# War fires promote short-term alien plant colonization in eastern Ukraine’s pine forests

**DOI:** 10.1038/s41598-026-66859-6

**Published:** 2026-09-03

**Authors:** Maksym Matsala, Roman Zadorozhniuk, Olena Miskova, Heorhii Bondarenko, Andrii Bilous

**Affiliations:** 1 Southern Swedish Forest Research Centre, Swedish University of Agricultural Sciences, Alnarp, Sweden; 2 National University of Life and Environmental Sciences of Ukraine, Kyiv, Ukraine; 3 M.G. Kholodny Institute of Botany, Kyiv, Ukraine; 4 V. N. Karazin Kharkiv National University, Kharkiv, Ukraine; 5 Sumy National Agrarian University, Sumy, Ukraine; 6 Hochschule Weihenstephan-Triesdorf, Weihenstephan-Triesdorf University of Applied Sciences, Freising, Germany

**Keywords:** Unmanned aerial vehicle, Drone, Forest reorganization, Plant invasions, Successional trajectory, Ecology, Ecology, Plant sciences

## Abstract

Russian full-scale military invasion has driven multiple ecosystem disturbances in the arid zone of eastern Ukraine, including fires of varying severity. Local forests, largely dominated by Scots pine (*Pinus sylvestris* L.), were planted as uniform monocultures in a region where low-density woodlands had historically prevailed. However, little is known about how military-related fires affect successional trajectories in disturbed pine forests. We conducted fully digital surveys of vascular plant composition using under-canopy imagery collected by an unmanned aerial vehicle. Transects were established in 29 pine stands with low to medium soil moisture content that had burned three years before the survey. We constructed linear models to examine short-term effects of fire severity on the proportion of alien flora, vascular plant diversity, and the success of tree regeneration. In line with our expectations, higher levels of stand damage were positively correlated with an increasing proportion of non-native plant species. However, this pattern was largely driven by Canadian horseweed (*Erigeron canadensis* L.), a widespread ruderal species that commonly appears as a short-term colonizer of disturbed forest sites. By contrast, we found no significant effects of burn severity on tree regeneration, which was poor across most transects, or on overall vascular plant diversity. We therefore discuss how war-altered disturbance regimes, including the potential for repeated fires, may interact with short-term ruderal colonization in artificial pine forests, particularly in relation to landscape-level fire risks and future successional pathways.

**Supplementary Information:**

The online version contains supplementary material available at https://doi.org/10.1038/s41598-026-66859-6.

## Introduction

Russian war in Ukraine has caused severe degradation of terrestrial environments, with likely long-term direct and indirect negative effects. Large-scale tree mortality due to fires and shelling^1^, pollution from drone optical fibers, unexploded ordnance and landmines, soil disturbance due to fortification construction^2^ and the abandonment of agricultural land^3^ are among the widely reported impacts. More unconventional impacts include ecosystem reorganization after the destruction of the Kakhovka Dam^4,5^, or cascading increases in landscape fire risk due to uncontrolled vegetation regrowth in mined landscape mosaics^6^. Many forests have been affected in the arid zones of eastern and southern Ukraine, where poor soils historically did not support canopy-closed tree habitats^7^. Consequently, prolonged warfare may cause these sandy landscapes to become bare once again, ultimately leading to deforestation and desertification. While the direct impacts of war are often mapped and reported, reliable studies on likely successional pathways in the post-conflict period are still lacking.

Among war-affected regions in Ukraine, the eastern part is particularly relevant for active investigations of ecosystem development in forested landscapes. Its plantations, woodlands, and canopy-closed natural groves represent a clear gradient of site fertility, from sandy drylands to river valleys, and are largely controlled by the Ukrainian Armed Forces, which enables field visits. Moreover, this area has experienced a variety of pyrogenic impacts, including single fires during the leaf-on season in 2022 amid active frontline clashes around the town of Izium in Kharkivska oblast, re-burned stands after wildfires in the dry season of 2024, and the completely devastated landscape of continuous warfare in Serebrianskyi Forest in Luhanska oblast^1^. In the fifth year of the full-scale military invasion, comprehensive field studies on vegetation conditions and ecosystem dynamics are still lacking in this region.

Repeated direct military impacts, including warm-season fires and year-round mechanical damage, can accelerate successional pathways towards forest reorganization in eastern Ukraine. Before large-scale artificial afforestation in the mid-1900s, this region had undergone several waves of deforestation, often linked to overgrazing in semi-open woodlands. These mosaics of sparse trees and shrubs among dry grasslands were historically typical of local shifting sands^7^. Wildfires had repeatedly damaged pine monocultures before 2022, while tree regeneration, both natural and artificial, often failed under drought conditions and competition with herbaceous species. However, higher-severity fires may be more favorable for successful tree recovery, as suggested by studies from another abandoned pine landscape, the Chornobyl Exclusion Zone^8,9^. Such phenomenon was shaped by broadleaved colonizers and supported by fire consumption of thick litter layers or damage to root biomass of herbaceous species.

Pre-war studies reported short-term colonization of burned sites in eastern Ukraine by alien flora, such as Canadian horseweed (*Erigeron canadensis* L.)^10^. This ruderal species was typically replaced later by forest specialists or removed during salvage logging and site preparation for planting. However, studies on how such alien plants may persist under expanding canopy openings and repeated fire impacts are lacking. For example, multiple sites in war-affected Ukraine have experienced repeated burning because of continuous fighting or wildfires spreading through areas affected by earlier combat^1^. Unlike many natural wildfires, these military-related fires are often driven by dense, repeated ignitions from shelling rather than by particularly favorable fire weather. They may therefore produce patchy burn patterns without consuming large continuous areas, leaving disturbed sites surrounded by relatively untouched, mature trees. At the same time, these highly flammable elements of the managed landscape typically remain uncleared of both landmines and the ground vegetation rapidly regrowing within newly formed canopy gaps.

Importantly, ongoing warfare and proximity to the frontline severely limit options for field data collection in affected forests. Most studies in Ukraine^1,11^and other countries (e.g^12,13^.,) have therefore relied on satellite data to map disturbances and post-conflict vegetation development. However, the use of even high-resolution satellite images has several limitations for ecological monitoring. First, their spatial resolution does not allow adequate delineation of understory flora or assessment of tree regeneration at the initial stage. Second, satellite data are often unsuitable for direct mapping of vegetation structure, particularly its vertical component. The development of vegetation layering, understory regrowth, and delayed crown mortality could be captured by airborne laser scanning (lidar) campaigns, but such missions are practically impossible in active war zones, as is currently (beginning of 2026) the case in Ukraine.

Close-range remote sensing can offer a solution for monitoring vegetation changes in forests where landmines and other unexploded ordnance create unconventional risks for ground observations. Although both active techniques, such as laser scanning (or lidar), and passive techniques, such as stereophotogrammetry, using data acquired by unmanned aerial vehicles are now widespread (e.g.,^14,15^), most studies focus on automated above-canopy flights. Below-canopy drone applications remain limited and mostly address the modelling of vegetation structure^16^ or biomass^17^, rather than the diversity of ground flora or natural tree regeneration. Importantly, lightweight drones have been shown to operate even in complex vegetated environments^18^, although manual control or geolocation correction is often required because of positioning drift during automated below-canopy flights.

We conducted our study in the pine-dominated forests of eastern Ukraine, a typical forest type near the contemporary frontline with Russia as of autumn 2025. This area was selected because it represented a sufficiently long early-successional period (three years) after the first fighting and subsequent fires in 2022. Importantly, the study area was accessible for data collection because it was located at a reasonable distance from the active warfare zone and was fully controlled by Ukrainian forces. We collected data on understory flora using below-canopy drone flights and tested three hypotheses. First, we assumed that war-related disturbances with increasing damage severity would promote the occurrence of non-native flora at the early stage of succession (H1). Second, we expected that higher-severity fires would favor regeneration of tree species (both conifers and early seral colonizers) in these dry pine landscapes (H2). Finally, we hypothesized that the gradient of damage severity would be negatively correlated with the diversity of vascular plant species, assuming the dominance of post-fire colonizers (H3).

## Results

### Alien species, tree regeneration, and vascular plant diversity

 In general, dry Scots pine forests studied three years after military fires (2022) were characterized by low vascular plant species diversity, the high ruderalization of ground vegetation, and poor tree regeneration (see Supplementary Materials). On average, around 23% of ground flora could be attributed to alien plant species (Table S1). The most common alien species was Canadian horseweed (*Erigeron canadensis* L.), with prickly lettuce (*Lactuca serriola* L.), common sowthistle (*Sonchus oleraceus* L.), and downy brome (*Bromus tectorum* L.) also present at less extent.

 Tree regeneration was generally scarce and spatially uneven (Table 1). The bootstrap-estimated mean number of tree regeneration per transect was 3.27 ± 8.66, reflecting a highly skewed distribution where a few transects with abundant regeneration drove the mean upward. Assuming that all photos were obtained from the flight altitude of 2 m (coverage of 6 m² per photo), the estimated density of tree regeneration was 681 ± 1779 individuals per ha (median: 212 individuals per ha). Because of vegetative propagation of common oak and silver birch, a single photo could contain up to 50 individuals of tree regeneration, representing a very small and locally dense regeneration patch rather than widespread recovery. However, such cases were exceptional: based on bootstrap resampling, 88.4% (or 26% of transects) of photos contained no tree regeneration at all. Among photos with recorded regeneration, the majority (9.6% of all photos) contained from 1 to 5 individuals of tree regeneration, while photos with 6–10 and 11–50 individuals each accounted for 1.0% of all sampled photos (Table S2).

**Table 1:** Summary characteristics of studied flora.

Parameter	Statistics at transect level				
	Min	25%-quantile	Median	75%-quantile	Max
Proportion of native flora	13.9%	65.5%	82.0%	93.9%	99.9%
Number of new trees	0	0	1	2	12 (47*)
Shannon diversity	0.36	0.66	0.91	1.11	1.72
Simpson evenness	0.27	0.42	0.57	0.65	0.86
Number of plant species (averaged)	3	6	7	10	15

*This plot was not used in modeling the number of new tree individuals.

Tree species richness per transect averaged 0.67 ± 0.75 species, indicating that where regeneration did occur, it was typically represented by a single species. We recorded tree regeneration on 24 transects represented by 11 woody species in total. The most frequently encountered species was common aspen (*Populus tremula* L.) present on 15 transects (62.5% of transects with regeneration), common oak recorded on 9 transects (37.5%), and Scots pine occurred on 4 transects (16.7%) but with only 9 individuals total. Other tree regeneration was represented by silver birch *(Betula pendula* Roth.), hybrid poplar (*Populus × canescens* (Aiton) Sm.), wild pear (*Pyrus communis* L.), black locust (*Robinia pseudoacacia* L.), and wych elm (*Ulmus glabra* Huds.), each recorded on two transects. Tatar maple (*Acer tataricum* L.), black poplar (*Populus nigra* L.), and willow (*Salix* spp.) were each found on a single transect only.

Average value of Shannon diversity was index 0.94 (for all vascular plants), which illustrated a mixture of species groups such as ruderals (e.g., great celandine, *Chelidonium majus* L.), forest-associated (e.g., angular Solomon’s seal, *Polygonatum odoratum* (Mill.) Druce), psammophilous (e.g., bushgrass, *Calamagrostis epigejos* (L.) Roth.) and those associated with both meadows and steppe remnants (e.g., spiked speedwell, *Veronica spicata* L.).

### Impact of fire severity on ground vegetation

Among our continuous proxies of fire severity (dNBR and average flame scorch length), both significantly impacted predictions of our response variables. However, dNBR was more flexible for GLMM convergence. Regarding the categorical predictor of relative burned patch size, it was significant only for some variables and when class of burned area was ‘large’. This predictor was not significant if combined with dNBR or flame scorch length. Acknowledging the high correlation between predictors (see Fig. S1 in Supplementary Materials), we assumed that dNBR was good enough as sole fire severity proxy.

**Fig. 1:**
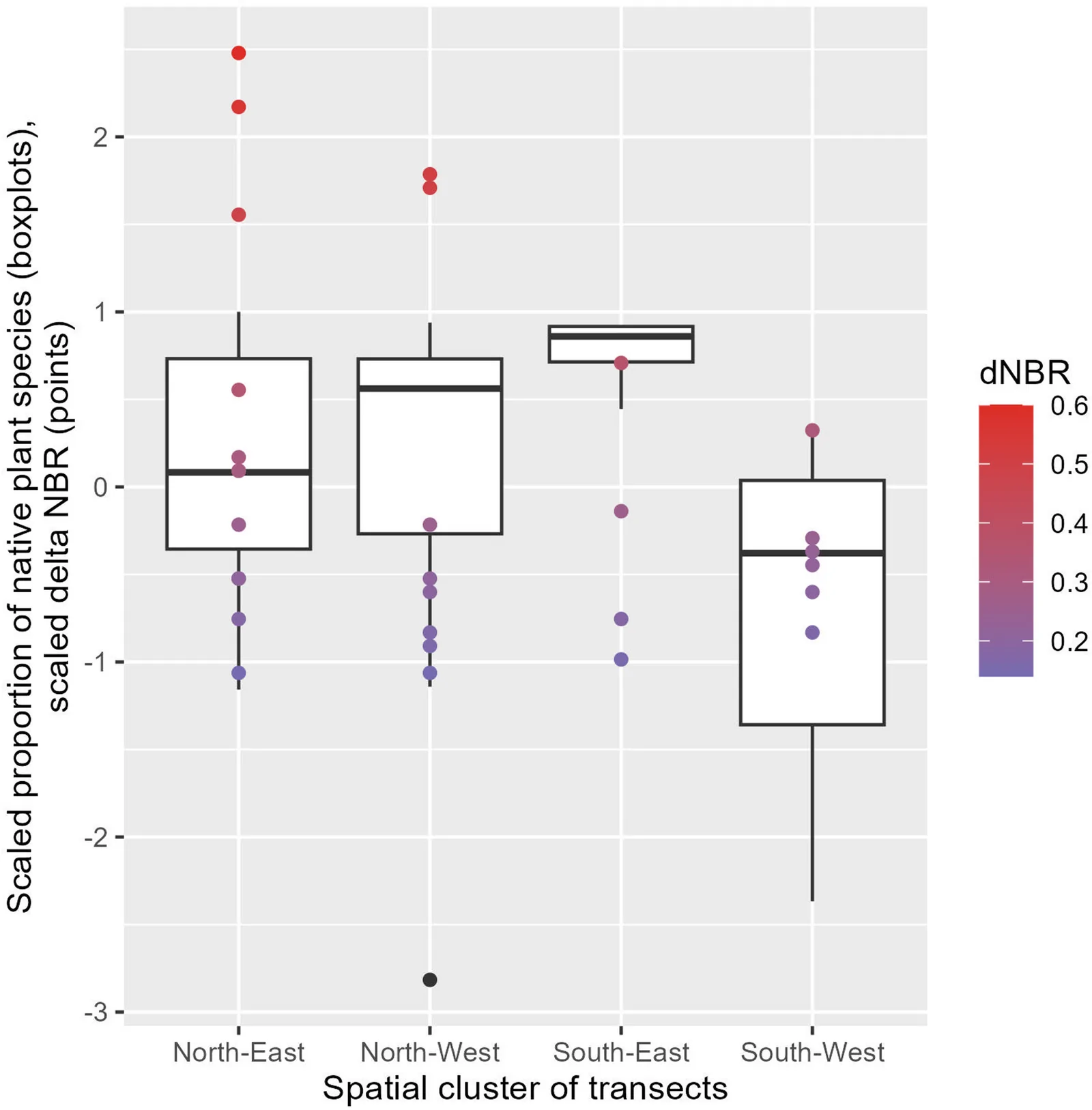
Variation of scaled proportion of native flora (boxplots) and respective dNBR (colored dots) respective to their spatial clusters (used as random effects in GLMMs).

**Fig. 2:**
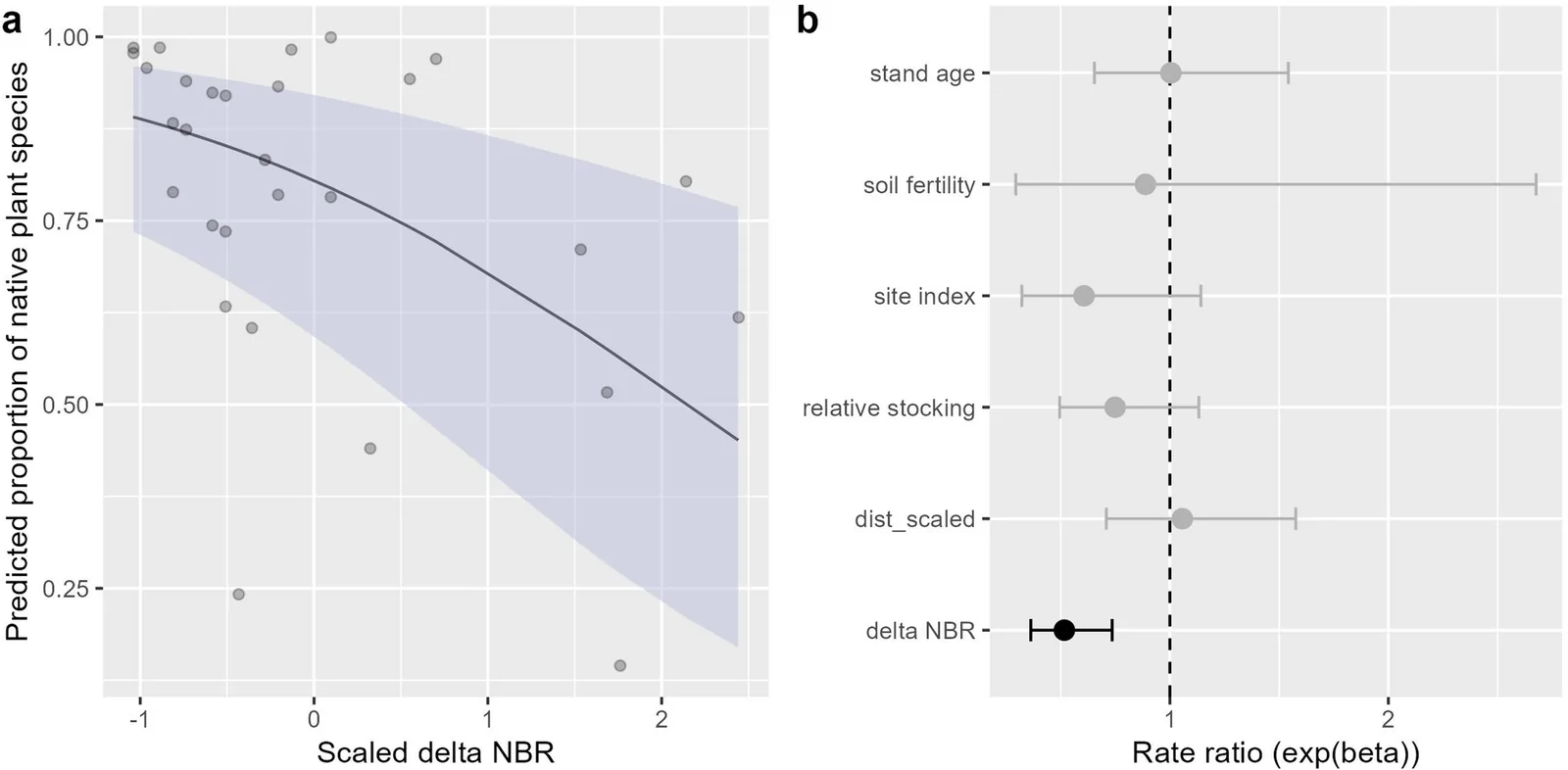
Linear effect of dNBR (scaled, **a**) and beta ratios for model coefficients and their standard deviations (**b**) in GLMM predicting the proportion of native flora in the ground vegetation (% of individuals).

We revealed statistically significant negative impact of fire severity (expressed as dNBR) on the proportion of native plant species three years after military fires in 2022 (−0.66 ± 0.18, marginal *R*^*2*^ = 0.53, Table 2). Only for this response variable, random effects (spatial cluster of transects) could explain a substantial portion of variance (0.46 ± 0.68). Given the observed pattern of correlation between dNBR, native plant species proportion, and spatial allocation of transects (Fig. 1), we also constructed an alternative model with spatial clusters used as a fixed effect (four discrete classes). In this way, we revealed their significance as also a linear predictor, while the effect of dNBR persisted as a negative and significant variable (Fig. 2). This finding supports our hypothesis (H1) about positive impact of damage severity on occurrence of alien plant species.

**Table 2:** Linear effects in the models.

Response variable for GLMM	Linear effect, estimate ± SD, *p*-value						
	Intercept	dNBR	Site index	Fertility class	Stand age	Relative stocking	Distance to firebreak
Proportion of native flora	− 0.28 ± 0.98, 0.79	− 0.66 ± 0.18, < 0.01***	0.50 ± 0.32, 0.12	− 0.12 ± 0.56, 0.83	0.01 ± 0.22, 0.98	− 0.29 ± 0.21, 0.17	0.05 ± 0.20, 0.79
Number of new trees	0.49 ± 0.98, 0.62	0.13 ± 0.14, 0.34	− 0.01 ± 0.34, 0.98	− 0.32 ± 0.63, 0.61	0.50 ± 0.23, 0.03*	− 0.13 ± 0.28, 0.66	0.11 ± 0.26, 0.67
Shannon diversity	1.78 ± 0.34, < 0.01***	0.00 ± 0.06, 0.99	− 0.27 ± 0.12, 0.04*	0.13 ± 0.20, 0.51	− 0.01 ± 0.08, 0.90	− 0.06 ± 0.07, 0.43	0.04 ± 0.07, 0.56
Simpson evenness	0.18 ± 0.15, 0.22	− 0.02 ± 0.03, 0.55	0.11 ± 0.05, 0.04*	− 0.03 ± 0.09, 0.71	− 0.01 ± 0.03, 0.80	0.01 ± 0.03, 0.92	− 0.02 ± 0.03, 0.53
Number of species (averaged)	13.76 ± 2.64, < 0.01***	− 0.57 ± 0.49, 0.25	− 1.99 ± 0.93, 0.03*	2.26 ± 1.52, 0.14	− 0.30 ± 0.60, 0.62	− 1.05 ± 0.52, 0.06	0.24 ± 0.52, 0.64

**Fig. 3:**
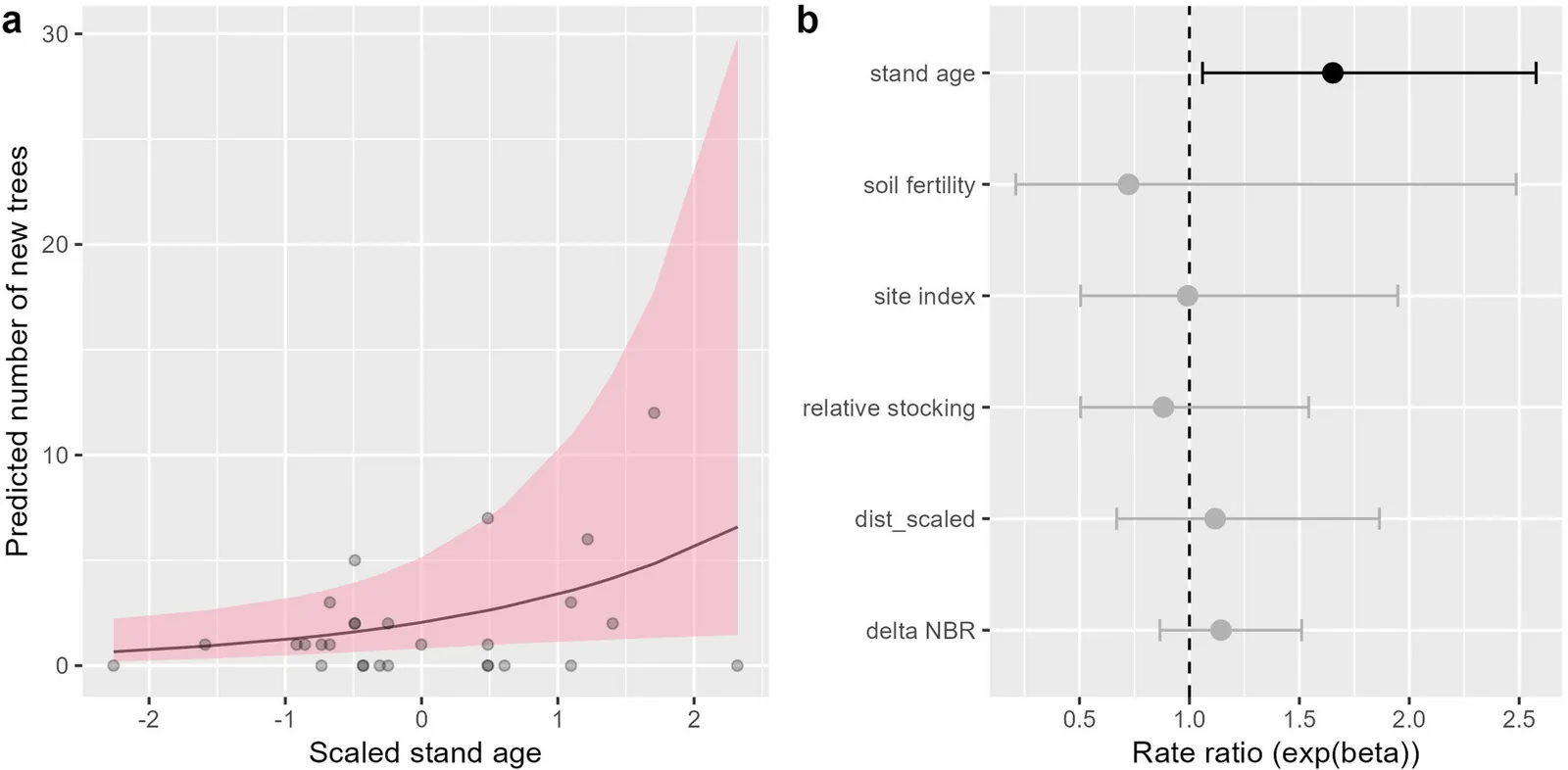
Linear effect of stand age (scaled, **a**) and beta ratios for model coefficients and their standard deviations (**b**) in GLMM predicting the number of new trees.

We did not uncover potential impact of fire severity on combined tree regeneration, thus rendering our second hypothesis (H2) not supported (Fig. 3). With marginal *R*^*2*^ = 0.33, this model revealed only significantly positive impact of stand age (0.50 ± 0.22) on the number of new tree individuals.

**Fig. 4:**
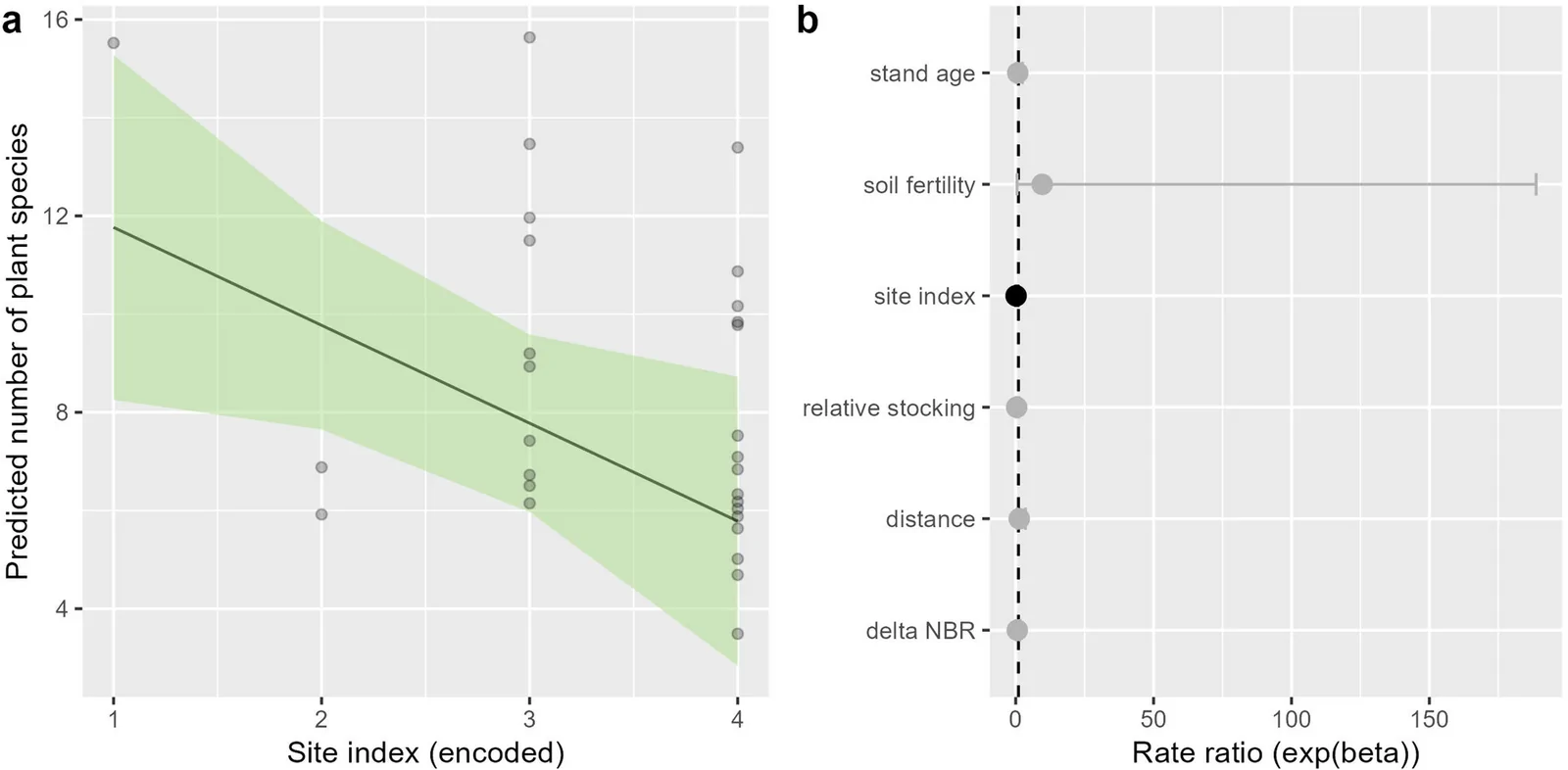
Linear effects of site index (encoded, **a**); and beta ratios for model coefficients and their standard deviations (**c**) in GLMM predicting the averaged number of vascular plant species per transect.

We revealed no significant impact of fire severity (both proxies) on Shannon diversity (marginal *R*^*2*^ = 0.27), Simpson evenness (*R*^*2*^ = 0.23), or averaged number of vascular plant species (*R*^*2*^ = 0.35). Site index had a negative effect on postfire species diversity (−0.27 ± 0.12, Fig. S2) and, respectively, a positive effect on Simpson evenness (0.11 ± 0.05, Fig. S3). GLMM for number of plant species could also explain substantial portion of variance with a statistically significant predictor of site index (negative effect, −1.99 ± 0.93, Fig. 4). This effect was still significant for all three response variables if removing three transects (~ 10% of data) containing the highest values of site index (3 or 4). Therefore, our findings could not also support assumptions for our third hypothesis of negative impact of fire severity (H3). Distance to firebreak, relative stocking, and fertility class were not significant in any models.

## Discussion and conclusions

### Alien flora in postfire forest communities

The data supported our expectation of a positive link between the presence of alien plant species and fire severity (H1). This finding is in line with studies of postfire pine forests in other biomes, for example in the Mediterranean region^19,20^. However, the non-native flora was largely represented by the short-lived Canadian horseweed. This invasive plant species has already become part of successional processes across a wide longitudinal gradient in Ukraine. Previous studies reported the dominance of Canadian horseweed during the first few years after wildfire both in our study region^10^ and in pine forests of northern Ukraine^9,21^. In all cases, this species was reported to decline in the third or fourth year of succession following wildfires, being rapidly replaced by meadow and then forest specialists.

We assume that the high presence of Canadian horseweed and other alien disturbance-adapted plants may drive a fire problem in the near future. In studied pine forests the high abundance of such plant species suggests increased availability of fine vegetation fuels even on the most severely disturbed sites. Under conditions of moisture deficit, this can increase early spring fire risk^22^, thereby sustaining horizontal fuel continuity between forested and non-forested parts of the landscape. Even that ruderals are usually quickly replaced within first years of postfire succession^10^, warfare in eastern Ukraine facilitates multiple reburns^1^. Such a novel fire regime may maintain the presence of ruderal species, including alien species such as Canadian horseweed, at a relatively stable level for a prolonged period, potentially up to a decade.

As our study covered the short-term outcomes of military fire disturbances, we did not expect to find many alien tree species in the ground stratum. These species may colonize open grasslands over time, as suggested by the most comprehensive study of vegetation succession in the region^23^. This study also identifies artificial plantations as a potential source for the spread of alien tree species such as Siberian elm (*Ulmus pumila* L.) and box elder (*Acer negundo* L.). Such experiments with the acclimatization of “exotic” tree species have continued since Soviet times and attempted to address the problem of climatic adaptation, while issues related to biodiversity and functional traits were largely ignored. Future implications of this process remain uncertain: alien tree species may rapidly recolonize from wetter patches where they are more likely to avoid fire damage. Meanwhile, the stochastic nature of warfare means that any forest, including those with low fire susceptibility, can be eradicated under continuous shelling.

### Tree regeneration after military fires

Our assumption that more severe fire impacts would promote tree regeneration (H2), both seeders (such as Scots pine) and broadleaved colonizers, was not supported by the empirical data. We expected that greater damage to tree canopies, by increasing light availability for new trees, and stronger reduction of ground-layer plant biomass, by reducing seed sources of competing herbs and increasing available space and nutrients, could facilitate the early recovery of woody vegetation. For example, a higher abundance of early-seral tree colonizers was recorded on the most severely disturbed sites after the 2020 wildfires in the Chornobyl Exclusion Zone^8,9^. A similar pattern for pine forests in northern Ukraine was also observed by Gumeniuk^24^. In our study, the high abundance of untouched mature pine trees surrounding relatively small, burned patches were not helpful in majority of cases. Importantly, above mentioned studies described forests growing in Polissia, which receives substantially higher annual precipitation than eastern Ukraine, approximately 550–700 mm compared with 350–500 mm^25^. In contrast, our transects were established mainly in dry pine forests. Therefore, soil water deficit may modulate tree regeneration success more strongly than the severity of the most recent disturbance.

Our model revealed a significant positive effect of stand age on postfire tree regeneration. We relate this finding to two potential mechanisms. The first is more straightforward and reflects dependence on available seed sources, as described, for example, by Shive et al.^26^, in dry forests of California, USA. As we mentioned above, military fires are often patchy and do not necessarily consume large areas, being driven by dense ignition occurrence under shelling rather than by favorable fire weather. Thus, disturbed sites may remain surrounded by intact trees that are old enough to provide seeds. The second mechanism may be related to disturbance legacies: older trees are more likely to persist after even severe wildfire in the form of large deadwood^27^. These structures may provide micro-shelters for new trees, especially under arid conditions such as those in our study area.

### Diversity of vascular plants and fire severity

General expectations that fire decreases plant species diversity several years after disturbance were not supported by our data (H3). We expected that increased light availability after canopy damage could shift ground-flora composition along the fire-severity gradient, mainly through greater dominance of ruderal species, as reported for northern Ukraine^28^. This pattern did not occur. Moreover, forest damage was not correlated with postfire evenness of vascular ground plants. These results suggest that, under the consistently dry conditions represented by our sites, understory vegetation structure may be constrained more by soil water availability than by canopy damage alone. This contrasts with the climatically similar but edaphically different landscapes of Khortytsia Island, where species evenness increased after the destruction of the Kakhovka Dam, partly due to ruderal colonization^4^. In that case, altered moisture availability was an important driver of vegetation structure because water levels changed after the disappearance of the Kakhovka Reservoir.

In our study, site productivity, expressed as Scots pine site index, was negatively correlated with vascular plant species richness but positively correlated with evenness. However, this relationship should not be interpreted simply as a direct effect of soil fertility on ground vegetation. Site index primarily reflects the potential dominant height and stem productivity of trees, whereas edaphic suitability for herbaceous vegetation was represented more directly by the soil fertility class, which was not a significant predictor in our models. Scots pine productivity is known to be constrained on very oligotrophic, dry, or excessively wet sites, while higher growth can occur under more mesic and moderately fertile conditions (e.g.,^29,30^). For example, one site with the most species richness (*n* = 15) represented dry soil conditions yet very low relative stocking of trees (50%), which could indicate more space and light for development of herbaceous layer. However, this observation does not explain why sites with ‘medium’ stand productivity (level 2 on Fig. 4) on average had more plant species than the most productive stands. In our case, however, the nonsignificant effect of soil fertility class and uniform moisture conditions suggest that the positive association between site index and species richness was probably not driven by soil properties alone.

### Limitations and future perspectives

Our study encompassed the most typical type of managed pine forest in the region: monocultures growing on dry soils. Importantly, other tree communities still persist in the war-affected areas of eastern Ukraine after four years of war. These include, for example, floodplain black alder (*Alnus glutinosa* (L.) Gaertn.) forests in swamps and common oak (*Quercus robur* L.) plantations, both associated with fertile soils along the Siverskyi Donets River. Some of these ecosystems have experienced damage of varying severity, but monitoring changes in their structure and composition is practically impossible because of landmines and canopy occlusion in remote sensing data. Therefore, it remains uncertain whether these military disturbances resulted in fire impacts, and which vegetation stratum, tree canopies or understory plants, was more strongly altered in these other forest types.

We also did not cover another important set of local pine landscapes, many of which have experienced multiple reburns since 2022. These include woodlands that resulted from largely failed regeneration efforts after the 1997 megafire^6^, patches of natural pine forests^7^, and even an endemic ecotype of “chalky” pine (*Pinus sylvestris* var. *cretacea* Kalenich.) growing in a designated nature reserve. Many of these unique dry coniferous ecosystems are so severely disturbed that they are unlikely to regenerate naturally in the foreseeable future. Less severely burned sites, however, may be crucial for understanding vegetation successional pathways in habitats that have historically been natural for this region of eastern Ukraine.

This study focused on the short-term stage of successional trajectories. However, there is no guarantee that future observations of vegetation dynamics under the same conditions, namely single fires and no new natural or human disturbances for at least a decade, will ever be feasible. Even if the war ends in peace or a frozen armed conflict, landmines will hinder effective land management across the entire region. Fencing “dangerous” forests will likely lead to the accumulation of vegetation fuels, as artificial barriers may prevent grazing by both domestic and wild animals. These highly continuous fuel mosaics could support fire regimes with very short return intervals, thereby producing a completely different trajectory of postfire succession than one described in this study.

## Methods and materials

### Study area

**Fig. 5:**
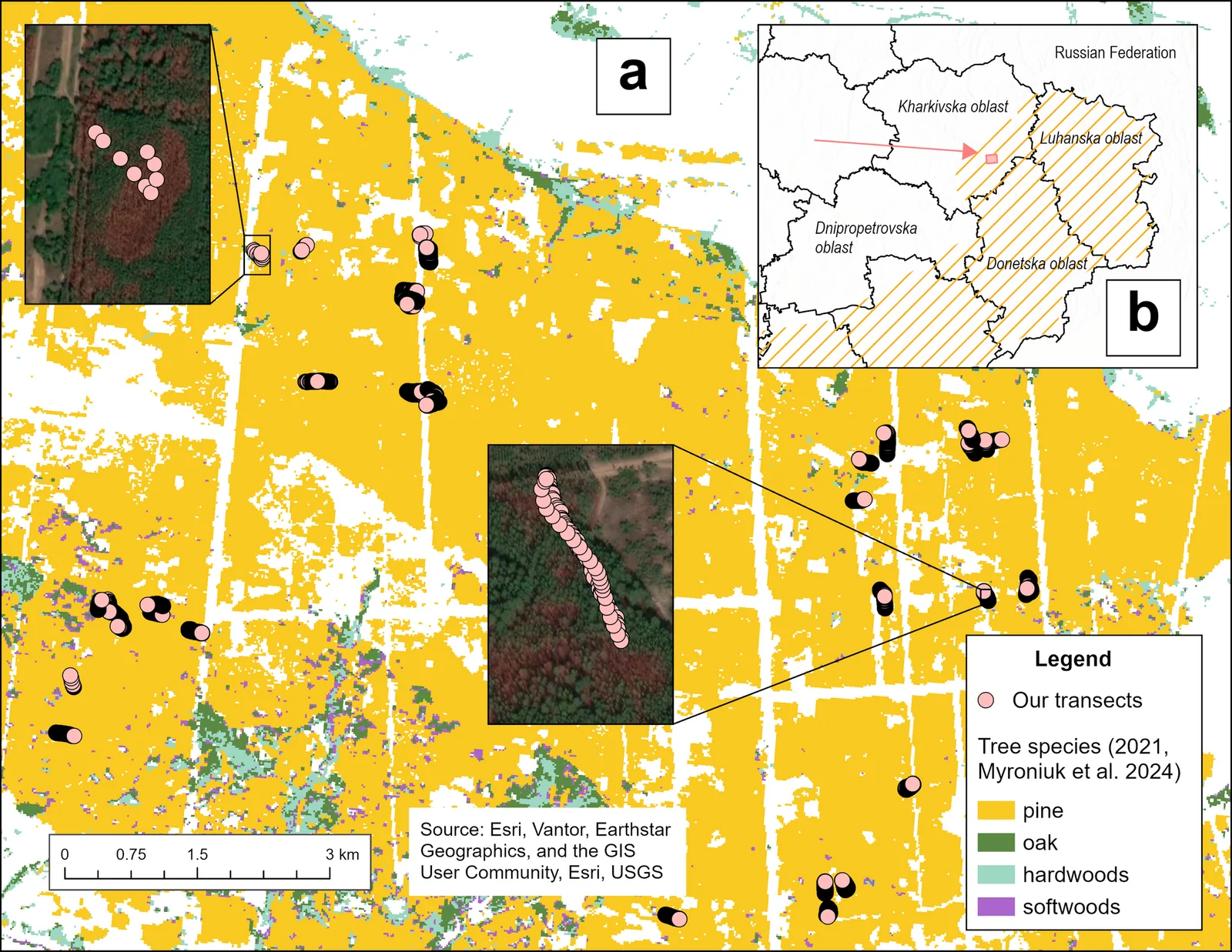
Locations of studied forests with established transects (**a**) over the map of tree species (as of 2021, by Myroniuk et al.^1^) and with two examples of very high-resolution satellite imagery of disturbed forest cover (map insets). The general location of landscape in eastern Ukraine (**b**) is marked with pink arrow, while hatched orange area depicts zone of active warfare since 2022. Background imagery: Esri (for ArcGIS Pro users and visualization only).

We collected data in forests around the town Izium, Kharkivska oblast (eastern Ukraine), 50 km away from the contemporary active frontline between Russian and Ukrainian forces (Fig. 5). The landscape is represented by managed Scots pine-dominated forests on the flat terrain, with total pre-war (2021) area of tree massifs ca. 60,000 ha. Soils are dry, sandy, and characterized by rather low nutrient content. Annual precipitation is 500–540 mm; average temperatures range from − 6.6 °C (January) to + 21.5 °C (July).

The studied forests had been a zone of active warfare in summer-autumn 2022 and recently exhibited multiple fires of different severity^6^ (Myroniuk et al., 2025). While there is no reported evidence on fire return interval in the region, last large wildfire burned hundreds of hectares around 20 years ago. Since autumn 2022, Izium forests have been under Ukrainian control and did not experience active warfare. Severe wildfires occurred in this area in May-June 2024 due to exceptional drought. We therefore established our transects (*n* = 29, one per pine-dominated stand) in forest patches which have not burned more than once since 2022.

### Field data

We used a fully digital framework to collect data on vascular plants in forests three years after fire (September 2025). This framework applied unmanned surveys of ground vegetation using drone DJI Phantom 4 Pro, equipped with default RGB camera. We launched this unmanned aerial vehicle (UAV) under tree canopies from a safe location (e.g., forest roads), thus avoiding risks related to landmines and other unexploded ordnance. We established transects from forest roads and firebreaks, which provided the only safely accessible entry points into the burned stands. We designed spatial locations of transects following two main criteria. First, transects were placed to remain within a single forest stand as delineated in the stand-wise forest management data, ensuring homogeneity of stand age, species composition and site conditions along its length. This was achieved for 27 of the 29 transects; the two remaining transects crossed two and three stands respectively. Second, transects were distributed across the assumed fire severity gradient to ensure representative sampling. Where a forest stand extended along the road but had a limited area, two flights were conducted and combined into a single transect. The median nearest-neighbor distance between transect line geometries was 155 m (mean ± standard deviation = 267 ± 334 m, range 2–1661 m). In two cases, transects started from approximately the same point on a forest road and extended in opposite directions into different forest stands. Consequently, the transects were only 2–36 m apart at their starting points but diverged to 209–448 m at their endpoints.

**Fig. 6:**
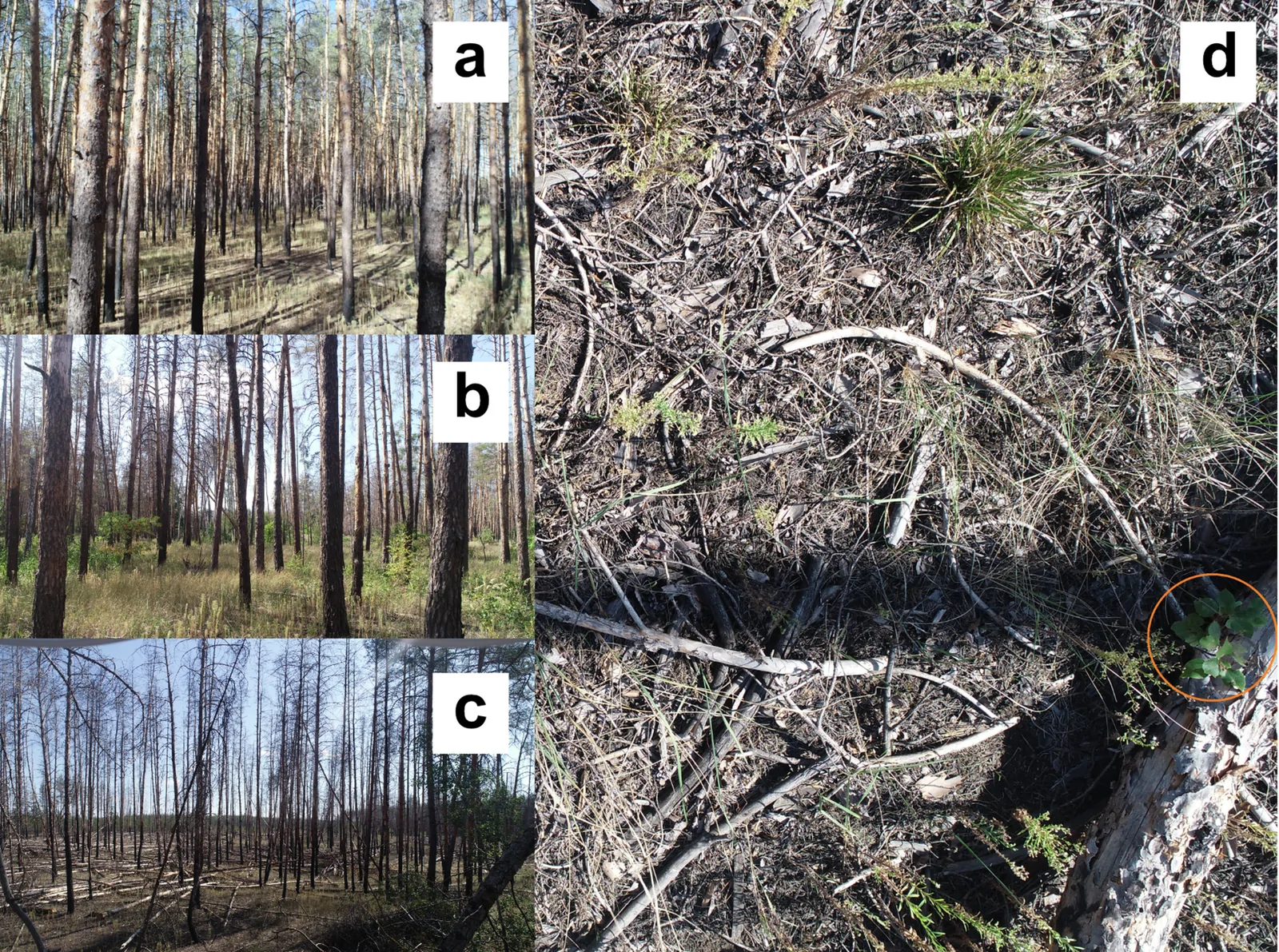
Examples of transects established in forests damaged with fires of low (**a**), medium (**b**), and high (**c**) severity. Horizontal (nadir) view-photograph shows example of how ground vegetation was recorded (**d**), with tree regeneration (common aspen) marked by orange circle.

Due to hardware limitations of the DJI Phantom 4 Pro, particularly restrictions on flight speed in automated scanning modes, all surveys were conducted manually. We operated UAV at a low and approximately constant speed (~ 2 km per hour), corresponding to up to 5% of its maximum flight speed, to ensure stable image acquisition along transects. We utilized a fixed time interval of 5 s between capturing photos along the transect. The UAV was briefly hovered every third interval to acquire a nadir photograph before continuing along the transect. Supplementary horizontal (oblique) photographs beneath the canopy were obtained during manual navigation of the UAV along the transect and served only to assist manual navigation beneath the canopy (Fig. 6). These oblique photographs were not used for vegetation cover estimation. All photos were captured at altitudes ranging between 1 and 2 m above the ground. We achieved a different length of transects (ranging 90–360 m, mean = 184.0 ± 72.2 m) and thus different number of nadir photographs per transect (ranging 8–63, mean ± standard deviation = 22.6 ± 16.6).

### Processing of field photos

We visually estimated photographs where we determined species and their percentage cover independently by two co-authors of this study and then species lists were cross-validated. To ensure comparability among transects with different sampling efforts, we applied a bootstrapping technique. We calculated our response variables (e.g., proportion of native flora or maximum number of vascular plant species) per randomly selected 8 photographs per transect with resampling. This averaging process also included photos without observed herbs or new tree individuals. We repeated this process 500 times, and final resulting values then were averaged per transect. For example, the list for the transect with the highest number of photographs (*n* = 63) contained 26 species of vascular plants, but on average (per 8 photographs) we recorded 15 species.

From plant species lists we calculated response variables for further modelling: proportion of native flora (% of coverage), number of new tree individuals, Shannon diversity index, Simpson evenness index, number of vascular plant species per transect (averaged).

### Ancillary data

**Fig. 7:**
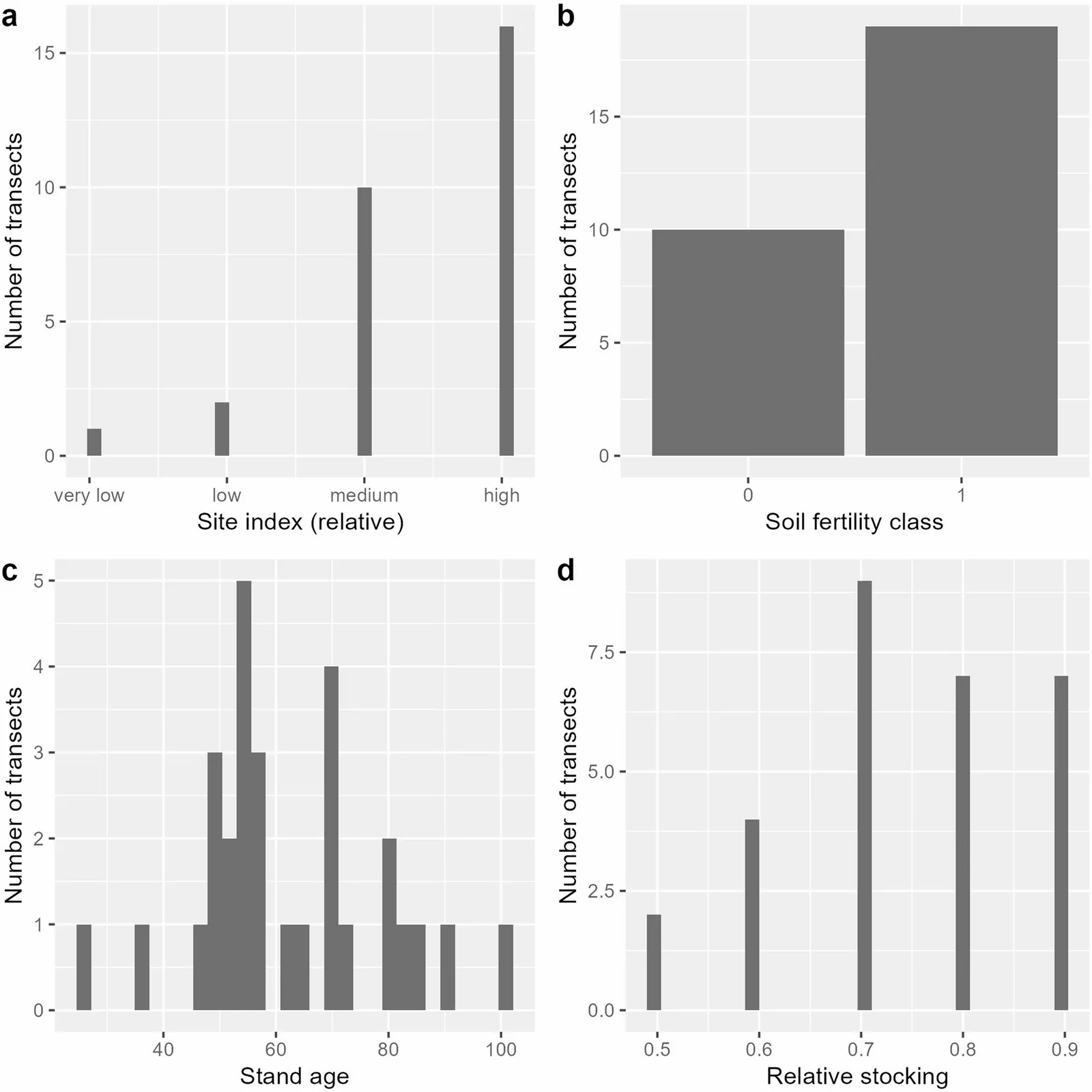
Distributions of predictors used in the models: encoded site index (**a**); soil fertility class (**b**); stand age (**c**); relative stocking (**d**).

We used stand-wise inventory data from 10-year forest management and planning data available for Izium state forests and created before the full-scale invasion by Russia. We georeferenced each transect and matched their locations with polygons containing attribute information about forests. We have been able to extract site index (encoded), fertility class (A or B), stand age, and relative stocking (Fig. 7). The site index was encoded to a discrete ascending scale according to Ukrainian forest inventory practices, with higher numbers reflecting higher potential dominant heights of Scots pine trees at age 100 years. Only three transects represented ‘low’ or ‘very low’ site index, while most pine stands were growing with a good productivity (Fig. 7b). Fertility class A represented poor sandy soils, while class B was closer to mesic soils in terms of nutrient content. We didn’t split our data by soil moisture class because almost all transects were established within homogeneous, dry conditions. Relative stocking ranged from 0 to 1, with ‘fully stocked’ (value 1) forests representing as much of tree basal area as specific ‘perfect’ conditions for a given combination of stand age, mean diameter and height. Additionally, we calculated the distance (in m) to the closest firebreak using available high-resolution satellite imagery in background maps using ArcGIS Pro software. We used firebreaks as a proxy of plant diversity source, 100 m-wide strips designed to split blocks of managed forest and prevent fire spread. Due to lack of resources at state forest enterprises, these firebreaks used to be not thoroughly cleaned of vegetation. In this way, firebreaks could support plant migrations (including early seral tree colonizers and alien plants) in a landscape dominated by managed Scots pine stands. For the same reason, we did not include distance to closest untouched pine stand that could produce seeds, as all our burned patches with transects were surrounded by such.

**Fig. 8:**
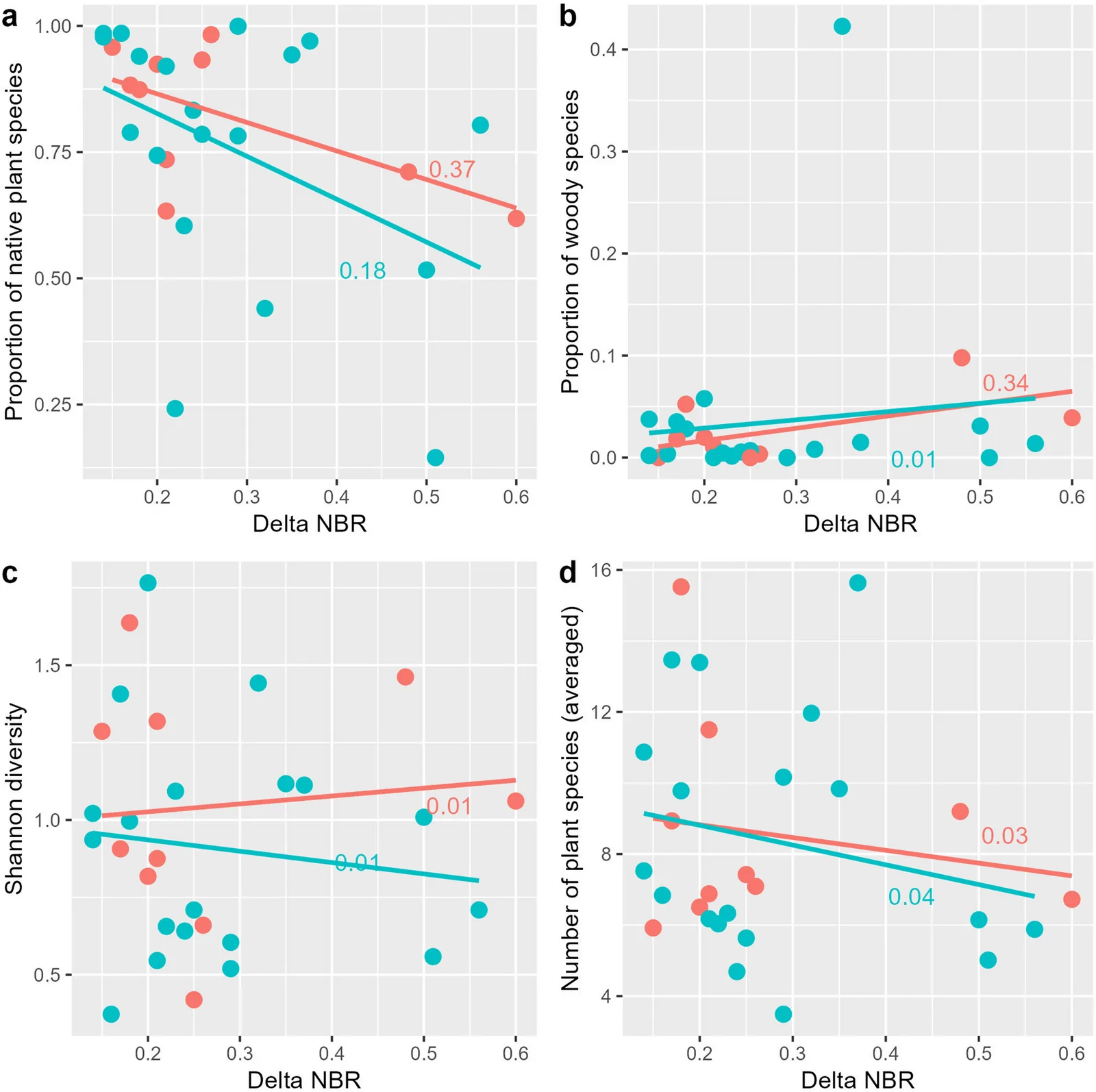
Dependencies between dNBR and response variables: proportion of native flora (**a**); proportion of tree regeneration among vascular plants (**b**); Shannon diversity (**c**); number of plant species per transect (averaged, **d**). Red dots show less fertile plots (class A) and blue dots represent more fertile plots (class B). Lines of respective colors show linear trends, with numbers reflecting *R*^*2*^ for dNBR as a single predictor.

We calculated three metrics to illustrate the impact of fires on ground vegetation. The first variable was delta Normalized Burn Ratio (dNBR^31^), a common and well-validated proxy of fire severity and damage to the forest cover (Fig. 8)^6^. Values of this metric typically ranged from 0.1 (low severity, likely only damage to ground vegetation) to 0.6 (very high severity, likely crown fire and destruction of tree layer). We calculated this spectral difference using cloud-free mosaics of Sentinel-2 satellite imagery (20 m spatial resolution) between August 2021 and August 2025 for the region of each transect. We used longer period (instead of comparing 2021 and 2022) due to postponed tree mortality (with stems falling observed in 2024) on some sites burned by low-severity fires, which allowed us to better delineate absolute dNBR over transect. However, these calculations also included certain spectral recovery which could occur for the last three years. Second, we manually examined mean flame scorch length (on the bark) on tree stems using our UAV during the field data collection. We visually approximated this parameter with a 0.5 m resolution. Third, we used dNBR maps to derive relative class of burned patch size: small, medium, or large (up to 18 ha for two largest patches). We did not calculate absolute value of burned area as some transects were in patches affected by low-severity fires with area unidentified at such spatial resolution (pixel size 20 × 20 m). In the further modelling we used only one variable (dNBR, flame scorch length, or class of patch size) as a proxy of fire severity.

### Models

We constructed a generalised linear mixed model (GLMM) for each response variable. Fixed effects included one proxy of fire severity (dNBR, relative size of burned patch, or scorch length), stand relative stocking, age, site index (encoded), and fertility class (Fig. 7). Random effects were designed to reveal the potential spatial structure in our data (Fig. 1). We assigned each transect to four ‘clusters’ located in relatively north-west, north-east, south-west, and south-east parts of Izium forests.

All predictors were scaled to be used in GLMMs. We used beta distribution to model the proportion of native flora (hypothesis H1), Poisson distribution to predict the number of tree regeneration (H2), and Gaussian distributions for GLMMs of Shannon diversity, Simpson evenness indices, and averaged number of plant species (H3). To adequately model tree regeneration, we removed one outlier plot (e.g., seen on Fig. 8b) with too many new trees (*n*= 47) from the training data set. We examined models (constructed in the R package {glmmTMB}^32^) for overdispersion in R package {DHARMa}^33^ and percentage of explained variance (in the form of marginal *R*-squared) using R package {performance}^34^.

## Supplementary Information

Below is the link to the electronic supplementary material.

Supplementary Material 1

## Data Availability

Summarized data on cover by species and tree regeneration specifically is available in Supplementary Materials. Authors can provide R code for models upon email request. The field data collection was carried out upon agreement with the Branch ‘Slobozhanskyi Forest Office’ of State Enterprise ‘Forests of Ukraine’ and located within Izium Forestry District. The UAV nadir photographs used in this study, together with a subset of oblique photographs and orthomosaic images, have been deposited in the Zenodo repository and are available at: https://doi.org/10.5281/zenodo.20832534.
