# Supplementary material for: War fires promote short-term alien plant colonization in eastern Ukraine’s pine forests
Source: Sci Rep. 2026 Sep 3;16:27652. doi: 10.1038/s41598-026-66859-6 (PMC13542255; doi:10.1038/s41598-026-66859-6)
Supplement: Supplementary file 1 [file 41598_2026_66859_MOESM1_ESM.pdf]

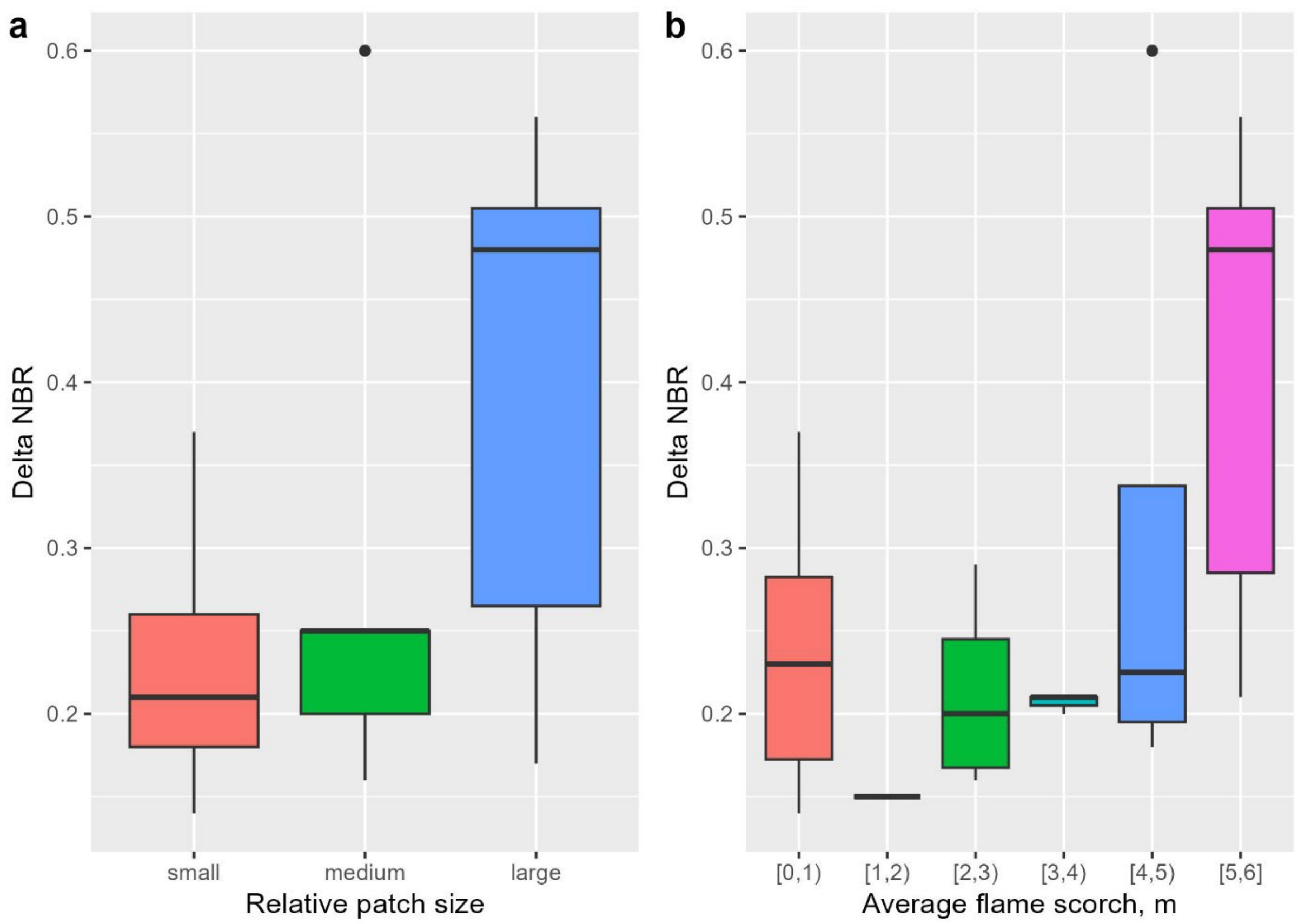

Figure S1. Distribution of relative burned patch sizes (a) and average flame scorch over bark (b) in relation to satellite-derived dNBR.

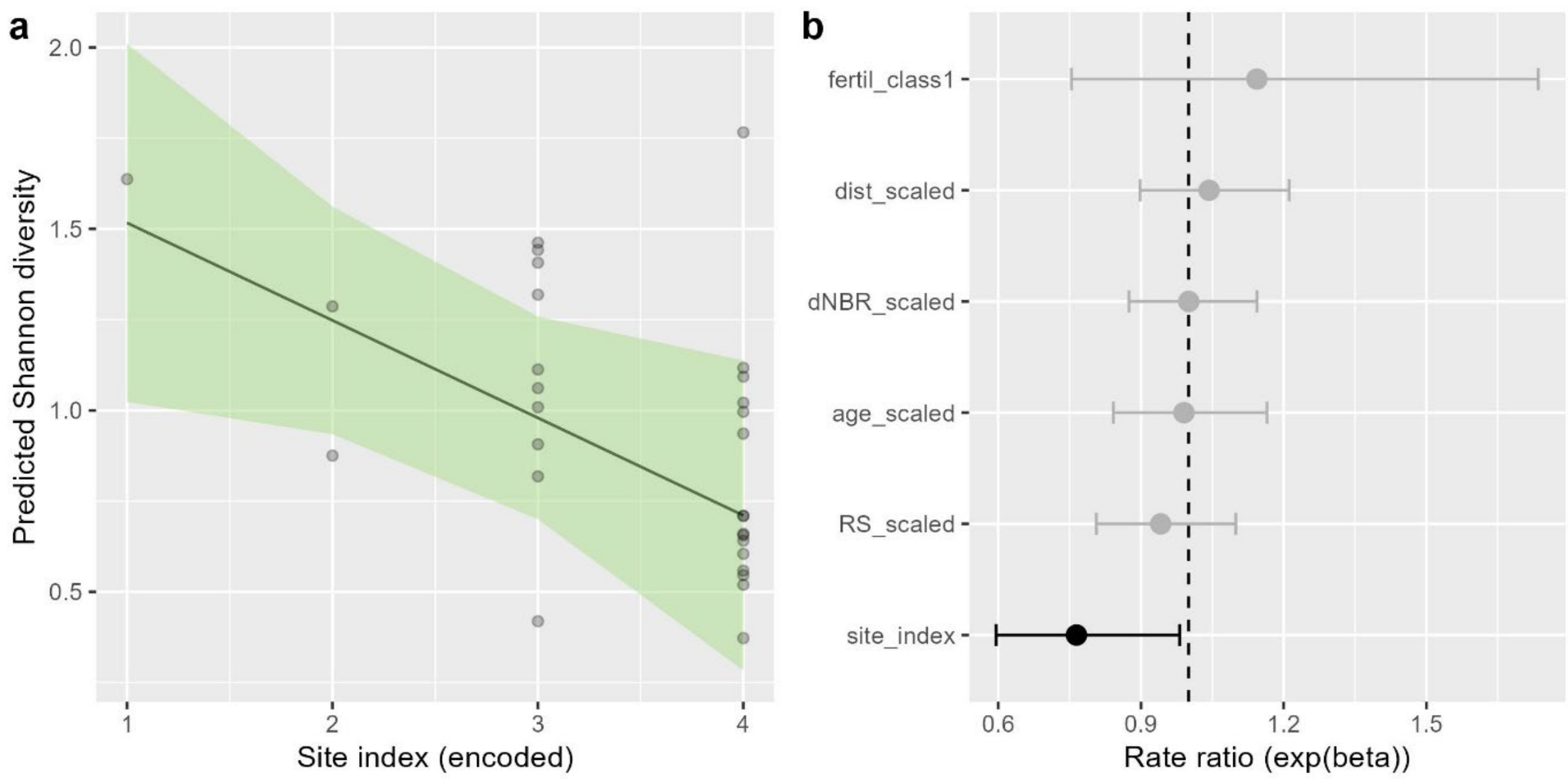

Figure S2. Effect of site index on predicted Shannon diversity (a) and beta-coefficients for all predictors in the model (b).

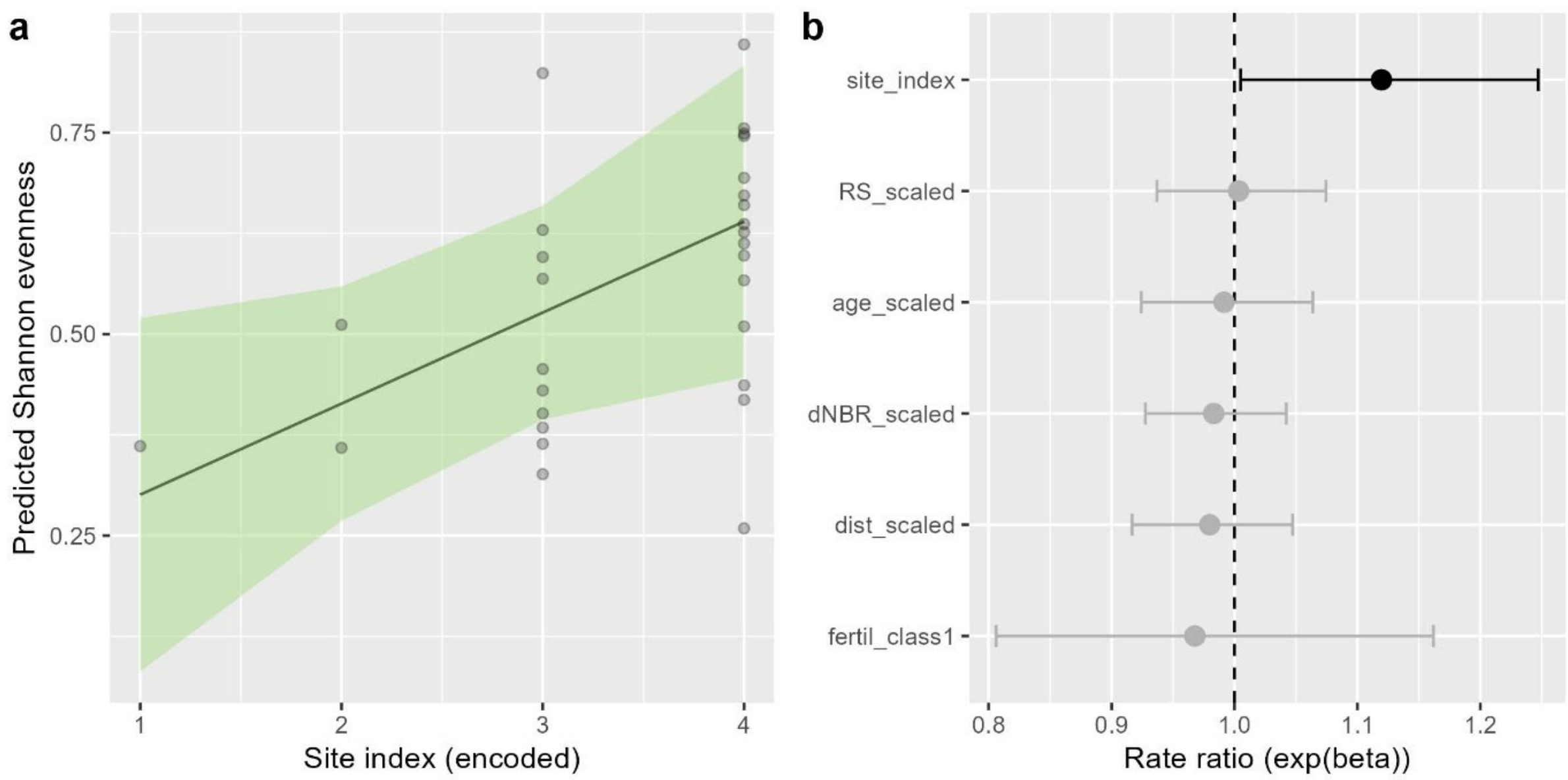

Figure S3. Effect of site index on predicted Shannon evenness (a) and beta-coefficients for all predictors in the model (b).

Table S1. List with vegetation cover by plant species detected on study transects.

Table S2. List with tree regeneration detected on study transects.

Plot_ID	Photo_ID	Species	coverage	native_alien	native_alien_mod	tree_shrub	family_name
Iz_10_1209	DJI_0637	Acer tataricum L.	0	n	1	1	Sapindaceae Juss.
Iz_10_1209	DJI_0637	Achillea millefolium L. s.l.	0	n	1	0	Asteraceae Bercht. & J.Presl
Iz_10_1209	DJI_0637	Allium oleraceum L.	0	n	1	0	Amaryllidaceae J.St.-Hil.
Iz_10_1209	DJI_0637	Ambrosia artemisiifolia L.	0	a	0	0	Asteraceae Bercht. & J.Presl
Iz_10_1209	DJI_0637	Amorpha fruticosa L.	0	a	0	0	Fabaceae Lindl.
Iz_10_1209	DJI_0637	Anthericum ramosum L.	0	n	1	0	Asparagaceae Juss.
Iz_10_1209	DJI_0637	Artemisia marschalliana Spreng.	0	n	1	0	Asteraceae Bercht. & J.Presl
Iz_10_1209	DJI_0637	Asparagus officinalis L.	0	n	1	0	Asparagaceae Juss.
Iz_10_1209	DJI_0637	Astragalus cicer L.	0	n	1	0	Fabaceae Lindl.
Iz_10_1209	DJI_0637	Bassia laniflora (S.G.Gmel.) A.J.Scott	0	n	1	0	Amaranthaceae Juss.
Iz_10_1209	DJI_0637	Berberis vulgaris L.	0	n	1	0	Berberidaceae Juss.
Iz_10_1209	DJI_0637	Betula pendula Roth.	0	n	1	1	Betulaceae Gray
Iz_10_1209	DJI_0637	Betula pendula Roth.	0	n	1	0	Poaceae Barnhart
Iz_10_1209	DJI_0637	Bromus inermis Leyss.	0		NA	NA	
Iz_10_1209	DJI_0637	Bromus squarrosus L.	0	a	0	0	Poaceae Barnhart
Iz_10_1209	DJI_0637	Bromus tectorum L.	0	a	0	0	Poaceae Barnhart
Iz_10_1209	DJI_0637	Calamagrostis epigejos (L.) Roth	2	n	1	0	Poaceae Barnhart
Iz_10_1209	DJI_0637	Carduus acanthoides L.	0	n	1	0	Asteraceae Bercht. & J.Presl
Iz_10_1209	DJI_0637	Carex sp.	0	n	1	0	Cyperaceae Juss.
Iz_10_1209	DJI_0637	Centaurea majorovii Dumbadze	0	n	1	0	Asteraceae Bercht. & J.Presl
Iz_10_1209	DJI_0637	Chamaecytisus ruthenicus (Fisch.ex Wol.) Klaskova	0	n	1	0	Fabaceae Lindl.
Iz_10_1209	DJI_0637	Chelidonium majus L.	0	n	1	0	Papaveraceae Juss.
Iz_10_1209	DJI_0637	Chenopodium betaceum Andrz.	0	n	1	0	Amaranthaceae Juss.
Iz_10_1209	DJI_0637	Chenopodium cf. album	0	n	1	0	Amaranthaceae Juss.
Iz_10_1209	DJI_0637	Chenopodium sp.	0		NA	0	Amaranthaceae Juss.
Iz_10_1209	DJI_0637	Chondrilla juncea L.	0	n	1	0	Asteraceae Bercht. & J.Presl
Iz_10_1209	DJI_0637	Cirsium vulgare (Savi) Ten.	0	n	1	0	Asteraceae Bercht. & J.Presl
Iz_10_1209	DJI_0637	Clinopodium acinos (L.) Kuntze	0	n	1	0	Lamiacea Lindl.
Iz_10_1209	DJI_0637	Convallaria majalis L.	0	n	1	0	Asparagaceae Juss.
Iz_10_1209	DJI_0637	Crataegus rhipidophylla Gand.	0	n	1	1	Rosaceae Juss.
Iz_10_1209	DJI_0637	Crepis foetida L.	0	n	1	0	Asteraceae Bercht. & J.Presl
Iz_10_1209	DJI_0637	Dactylis glomerata L.	0	n	1	0	Poaceae Barnhart
Iz_10_1209	DJI_0637	Dianthus campestris M.Bieb.	0	n	1	0	Caryophyllaceae Juss.
Iz_10_1209	DJI_0637	Digitaria sanguinalis (L.) Scop.	0	a	0	0	Poaceae Barnhart
Iz_10_1209	DJI_0637	Elymus repens (L.) Gould	0	n	1	0	Poaceae Barnhart
Iz_10_1209	DJI_0637	Epilobium tetragonum L.	0	n	1	0	Onagraceae Juss.
Iz_10_1209	DJI_0637	Eragrostis minor Host	0	a	0	0	Poaceae Barnhart
Iz_10_1209	DJI_0637	Erigeron canadensis L.	17	a	0	0	Asteraceae Bercht. & J.Presl
Iz_10_1209	DJI_0637	Euphorbia seguieriana Neck.	0	n	1	0	Euphorbiaceae Juss.
Iz_10_1209	DJI_0637	Fallopia dumetorum (L.) Holub	0	n	1	0	Polygonaceae Juss.
Iz_10_1209	DJI_0637	Festuca beckeri (Hack.) Trautv.	0	n	1	0	Poaceae Barnhart
Iz_10_1209	DJI_0637	Fragaria vesca L.	0	n	1	0	Rosaceae Juss.
Iz_10_1209	DJI_0637	Frangula alnus Mill.	0	n	1	1	Rhamnaceae Juss.
Iz_10_1209	DJI_0637	Fraxinus pennsylvanica Marshall	0	a	0	1	Oleaceae Hoffmanns. & Link
Iz_10_1209	DJI_0637	Galeopsis bifida Boenn.	0	n	1	0	Lamiacea Lindl.
Iz_10_1209	DJI_0637	Galium verum L.	0	n	1	0	Rubiaceae Juss.
Iz_10_1209	DJI_0637	Genista tinctoria L.	0	n	1	0	Fabaceae Lindl.
Iz_10_1209	DJI_0637	Geranium robertianum L.	0	n	1	0	Geraniaceae Juss.
Iz_10_1209	DJI_0637	Geranium sanquineum L.	0	n	1	0	Geraniaceae Juss.
Iz_10_1209	DJI_0637	Gypsophila paniculata L.	0	n	1	0	Caryophyllaceae Juss.
Iz_10_1209	DJI_0637	Helichrysum arenarium (L.) Moench	5	n	1	0	Asteraceae Bercht. & J.Presl
Iz_10_1209	DJI_0637	Herniaria polygama J.Gay	0	n	1	0	Caryophyllaceae Juss.
Iz_10_1209	DJI_0637	Hieracium robustum Fr.	0	n	1	0	Asteraceae Bercht. & J.Presl
Iz_10_1209	DJI_0637	Hieracium umbellatum L.	0	n	1	NA	Asteraceae Bercht. & J.Presl
Iz_10_1209	DJI_0637	Hylotelephium maximum (L.) Holub	0	n	1	0	Crassulaceae J.St.-Hil.
Iz_10_1209	DJI_0637	Jacobaea borysthenica (DC.) B.Nord. & Greuter	0	n	1	0	Asteraceae Bercht. & J.Presl
Iz_10_1209	DJI_0637	Jasione montana L.	0	n	1	0	Campanulaceae Juss.
Iz_10_1209	DJI_0637	Jurinea cyanoides (L.) Rchb.s.l.	0	n	1	0	Asteraceae Bercht. & J.Presl
Iz_10_1209	DJI_0637	Koeleria glauca (Spreng.) DC.	0	n	1	0	Poaceae Barnhart
Iz_10_1209	DJI_0637	Lactuca serriola L.	0	a	0	0	Asteraceae Bercht. & J.Presl
Iz_10_1209	DJI_0637	Linaria genistifolia (L.) Mill.	0	n	1	0	Plantaginaceae Juss.
Iz_10_1209	DJI_0637	Lomelosia argentea (L.) Greuter & Burdet	0	n	1	0	Caprifoliaceae Juss.
Iz_10_1209	DJI_0637	Melampyrum pratense L.	0	n	1	0	Orobanchaceae Vent.
Iz_10_1209	DJI_0637	Oenothera biennis L.	0	a	0	0	Onagraceae Juss.
Iz_10_1209	DJI_0637	Pentanema britannica (L.) D.Gut.Larr., Santos-Vicente, Anderb., E.Rico & M.M.Mart.Ort.	0	n	1	0	Asteraceae Bercht. & J.Presl
Iz_10_1209	DJI_0637	Peucedanum oreoselinum (L.) Moench	0	n	1	0	Apiaceae Lindl.
Iz_10_1209	DJI_0637	Peucedanum ruthenicum M.Bieb.	0	n	1	0	Apiaceae Lindl.
Iz_10_1209	DJI_0637	Pilosella echioides (Lumn.) F.W.Schultz & Sch.Bip.	0	n	1	0	Asteraceae Bercht. & J.Presl
Iz_10_1209	DJI_0637	Pilosella officinarum F. Schultz et Sch. Bip.	0	n	1	0	Asteraceae Bercht. & J.Presl
Iz_10_1209	DJI_0637	Pilosella sp.	0		NA	0	Asteraceae Bercht. & J.Presl
Iz_10_1209	DJI_0637	Pinus sylvestris L.	0	n	1	1	Pinaceae Spreng. ex F.Rudolphi
Iz_10_1209	DJI_0637	Poa annua L.	0	n	1	0	Poaceae Barnhart
Iz_10_1209	DJI_0637	Poa nemoralis L.	0	n	1	0	Poaceae Barnhart
Iz_10_1209	DJI_0637	Poa sp.	0		NA	0	Poaceae Barnhart
Iz_10_1209	DJI_0637	Polygonatum odoratum (Mill.) Druce	0	n	1	0	Asparagaceae Juss.
Iz_10_1209	DJI_0637	Polygonum arenarium Waldst. & Kit.	0	n	1	0	Polygonaceae Juss.
Iz_10_1209	DJI_0637	Populus nigra L.	0	n	1	1	Salicaceae Mirb.
Iz_10_1209	DJI_0637	Populus tremula L.	1	n	1	1	Salicaceae Mirb.
Iz_10_1209	DJI_0637	Populus Ã canescens (Aiton) Sm.	0	n	1	1	Salicaceae Mirb.
Iz_10_1209	DJI_0637	Potentilla argentea L.	0	n	1	0	Rosaceae Juss.
Iz_10_1209	DJI_0637	Psammophiliella muralis (L.) Ikonn.	0	n	1	0	Caryophyllaceae Juss.
Iz_10_1209	DJI_0637	Psephellus sumensis (Kalen.) Greuter	0	n	1	0	Asteraceae Bercht. & J.Presl
Iz_10_1209	DJI_0637	Pulsatilla pratensis (L.) Mill. s.l.	0	n	1	0	Ranunculaceae Juss.
Iz_10_1209	DJI_0637	Pyrus communis L.	0	a	0	1	Rosaceae Juss.
Iz_10_1209	DJI_0637	Quercus robur L.	0	n	1	1	Fagaceae Dumort.
Iz_10_1209	DJI_0637	Robinia pseudoacacia L.	0	a	0	1	Fabaceae Lindl.
Iz_10_1209	DJI_0637	Rosa sp.	0		NA	1	Rosaceae Juss.
Iz_10_1209	DJI_0637	Rubus idaeus L.	0	n	1	0	Rosaceae Juss.
Iz_10_1209	DJI_0637	Rumex acetosella L.	0	n	1	0	Polygonaceae Juss.
Iz_10_1209	DJI_0637	Rumex thyrsiflorus Fingerh.	0	n	1	0	Polygonaceae Juss.
Iz_10_1209	DJI_0637	Salix alba L.	0	n	1	1	Salicaceae Mirb.
Iz_10_1209	DJI_0637	Sambucus nigra L.	0	n	1	1	Viburnaceae Raf.
Iz_10_1209	DJI_0637	Secale sylvestre Host	0	n	1	0	Poaceae Barnhart
Iz_10_1209	DJI_0637	Sempervivum ruthenicum Schnittsp. & C.B. Lehm.	0	n	1	0	Crassulaceae J.St.-Hil.
Iz_10_1209	DJI_0637	Setaria viridis (L.) P. Beauv.	0	n	1	0	Poaceae Barnhart
Iz_10_1209	DJI_0637	Silene borysthenica (Gruner) Walters	0	n	1	0	Caryophyllaceae Juss.
Iz_10_1209	DJI_0637	Silene latifolia subsp. alba (Mill.) Greuter & Burdet	0	n	1	0	Caryophyllaceae Juss.

Iz_10_1209	DJI_0637	Solidago virgaurea L.	0	n	1	0	Asteraceae Bercht. & J.Presl
Iz_10_1209	DJI_0637	Sorbus aucuparia L.	0	n	1	1	Rosaceae Juss.
Iz_10_1209	DJI_0637	Stachys recta L.	0	n	1	0	Lamiacea Lindl.
Iz_10_1209	DJI_0637	Tanacetum vulgare L.	0	n	1	0	Asteraceae Bercht. & J.Presl
Iz_10_1209	DJI_0637	Thymus pallasianus Heinr.Braun	0	n	1	0	Lamiacea Lindl.
Iz_10_1209	DJI_0637	Tilia cordata Mill.	0	n	1	1	Malvaceae Juss.
Iz_10_1209	DJI_0637	Trifolium alpestre L.	0	n	1	0	Fabaceae Lindl.
Iz_10_1209	DJI_0637	Trifolium arvense L.	0	n	1	0	Fabaceae Lindl.
Iz_10_1209	DJI_0637	Ulmus glabra Huds.	0	n	1	1	Ulmaceae Mirb.
Iz_10_1209	DJI_0637	Verbascum densiflorum Bertol.	0	n	1	0	Scrophulariaceae Juss.
Iz_10_1209	DJI_0637	Verbascum sp.	0	n	1	0	Scrophulariaceae Juss.
Iz_10_1209	DJI_0637	Veronica austriaca L.	0	n	1	0	Plantaginaceae Juss.
Iz_10_1209	DJI_0637	Veronica incana L.	0	n	1	0	Plantaginaceae Juss.
Iz_10_1209	DJI_0637	Veronica verna L.	0	n	1	0	Plantaginaceae Juss.
Iz_10_1209	DJI_0637	Vincetoxicum hirundinaria Medik	0	n	1	0	Apocynaceae Juss.
Iz_10_1209	DJI_0643	Acer tataricum L.	0	n	1	1	Sapindaceae Juss.
Iz_10_1209	DJI_0643	Achillea millefolium L. s.l.	0	n	1	0	Asteraceae Bercht. & J.Presl
Iz_10_1209	DJI_0643	Allium oleraceum L.	0	n	1	0	Amaryllidaceae J.St.-Hil.
Iz_10_1209	DJI_0643	Ambrosia artemisiifolia L.	0	a	0	0	Asteraceae Bercht. & J.Presl
Iz_10_1209	DJI_0643	Amorpha fruticosa L.	0	a	0	0	Fabaceae Lindl.
Iz_10_1209	DJI_0643	Anthericum ramosum L.	0	n	1	0	Asparagaceae Juss.
Iz_10_1209	DJI_0643	Artemisia marschalliana Spreng.	0	n	1	0	Asteraceae Bercht. & J.Presl
Iz_10_1209	DJI_0643	Asparagus officinalis L.	0	n	1	0	Asparagaceae Juss.
Iz_10_1209	DJI_0643	Astragalus cicer L.	0	n	1	0	Fabaceae Lindl.
Iz_10_1209	DJI_0643	Bassia laniflora (S.G.Gmel.) A.J.Scott	0	n	1	0	Amaranthaceae Juss.
Iz_10_1209	DJI_0643	Berberis vulgaris L.	0	n	1	0	Berberidaceae Juss.
Iz_10_1209	DJI_0643	Betula pendula Roth.	0	n	1	1	Betulaceae Gray
Iz_10_1209	DJI_0643	Betula pendula Roth.	0	n	1	0	Poaceae Barnhart
Iz_10_1209	DJI_0643	Bromus inermis Leyss.	0		NA	NA	
Iz_10_1209	DJI_0643	Bromus squarrosus L.	0	a	0	0	Poaceae Barnhart
Iz_10_1209	DJI_0643	Bromus tectorum L.	0	a	0	0	Poaceae Barnhart
Iz_10_1209	DJI_0643	Calamagrostis epigejos (L.) Roth	25	n	1	0	Poaceae Barnhart
Iz_10_1209	DJI_0643	Carduus acanthoides L.	0	n	1	0	Asteraceae Bercht. & J.Presl
Iz_10_1209	DJI_0643	Carex sp.	0	n	1	0	Cyperaceae Juss.
Iz_10_1209	DJI_0643	Centaurea majorovii Dumbadze	0	n	1	0	Asteraceae Bercht. & J.Presl
Iz_10_1209	DJI_0643	Chamaecytisus ruthenicus (Fisch.ex Wol.) Klaskova	0	n	1	0	Fabaceae Lindl.
Iz_10_1209	DJI_0643	Chelidonium majus L.	0	n	1	0	Papaveraceae Juss.
Iz_10_1209	DJI_0643	Chenopodium betaceum Andrz.	0	n	1	0	Amaranthaceae Juss.
Iz_10_1209	DJI_0643	Chenopodium cf. album	0	n	1	0	Amaranthaceae Juss.
Iz_10_1209	DJI_0643	Chenopodium sp.	0		NA	0	Amaranthaceae Juss.
Iz_10_1209	DJI_0643	Chondrilla juncea L.	0	n	1	0	Asteraceae Bercht. & J.Presl
Iz_10_1209	DJI_0643	Cirsium vulgare (Savi) Ten.	0	n	1	0	Asteraceae Bercht. & J.Presl
Iz_10_1209	DJI_0643	Clinopodium acinos (L.) Kuntze	0	n	1	0	Lamiacea Lindl.
Iz_10_1209	DJI_0643	Convallaria majalis L.	0	n	1	0	Asparagaceae Juss.
Iz_10_1209	DJI_0643	Crataegus rhipidophylla Gand.	0	n	1	1	Rosaceae Juss.
Iz_10_1209	DJI_0643	Crepis foetida L.	0	n	1	0	Asteraceae Bercht. & J.Presl
Iz_10_1209	DJI_0643	Dactylis glomerata L.	0	n	1	0	Poaceae Barnhart
Iz_10_1209	DJI_0643	Dianthus campestris M.Bieb.	0	n	1	0	Caryophyllaceae Juss.
Iz_10_1209	DJI_0643	Digitaria sanguinalis (L.) Scop.	0	a	0	0	Poaceae Barnhart
Iz_10_1209	DJI_0643	Elymus repens (L.) Gould	0	n	1	0	Poaceae Barnhart
Iz_10_1209	DJI_0643	Epilobium tetragonum L.	0	n	1	0	Onagraceae Juss.
Iz_10_1209	DJI_0643	Eragrostis minor Host	0	a	0	0	Poaceae Barnhart
Iz_10_1209	DJI_0643	Erigeron canadensis L.	2	a	0	0	Asteraceae Bercht. & J.Presl
Iz_10_1209	DJI_0643	Euphorbia seguieriana Neck.	0	n	1	0	Euphorbiaceae Juss.
Iz_10_1209	DJI_0643	Fallopia dumetorum (L.) Holub	0	n	1	0	Polygonaceae Juss.
Iz_10_1209	DJI_0643	Festuca beckeri (Hack.) Trautv.	0	n	1	0	Poaceae Barnhart
Iz_10_1209	DJI_0643	Fragaria vesca L.	0	n	1	0	Rosaceae Juss.
Iz_10_1209	DJI_0643	Frangula alnus Mill.	0	n	1	1	Rhamnaceae Juss.
Iz_10_1209	DJI_0643	Fraxinus pennsylvanica Marshall	0	a	0	1	Oleaceae Hoffmanns. & Link
Iz_10_1209	DJI_0643	Galeopsis bifida Boenn.	0	n	1	0	Lamiacea Lindl.
Iz_10_1209	DJI_0643	Galium verum L.	0	n	1	0	Rubiaceae Juss.
Iz_10_1209	DJI_0643	Genista tinctoria L.	0	n	1	0	Fabaceae Lindl.
Iz_10_1209	DJI_0643	Geranium robertianum L.	0	n	1	0	Geraniaceae Juss.
Iz_10_1209	DJI_0643	Geranium sanquineum L.	0	n	1	0	Geraniaceae Juss.
Iz_10_1209	DJI_0643	Gypsophila paniculata L.	0	n	1	0	Caryophyllaceae Juss.
Iz_10_1209	DJI_0643	Helichrysum arenarium (L.) Moench	0	n	1	0	Asteraceae Bercht. & J.Presl
Iz_10_1209	DJI_0643	Herniaria polygama J.Gay	0	n	1	0	Caryophyllaceae Juss.
Iz_10_1209	DJI_0643	Hieracium robustum Fr.	0	n	1	0	Asteraceae Bercht. & J.Presl
Iz_10_1209	DJI_0643	Hieracium umbellatum L.	0	n	1	NA	Asteraceae Bercht. & J.Presl
Iz_10_1209	DJI_0643	Hylotelephium maximum (L.) Holub	0	n	1	0	Crassulaceae J.St.-Hil.
Iz_10_1209	DJI_0643	Jacobaea borysthenica (DC.) B.Nord. & Greuter	0	n	1	0	Asteraceae Bercht. & J.Presl
Iz_10_1209	DJI_0643	Jasione montana L.	0	n	1	0	Campanulaceae Juss.
Iz_10_1209	DJI_0643	Jurinea cyanoides (L.) Rchb.s.l.	0	n	1	0	Asteraceae Bercht. & J.Presl
Iz_10_1209	DJI_0643	Koeleria glauca (Spreng.) DC.	0	n	1	0	Poaceae Barnhart
Iz_10_1209	DJI_0643	Lactuca serriola L.	0	a	0	0	Asteraceae Bercht. & J.Presl
Iz_10_1209	DJI_0643	Linaria genistifolia (L.) Mill.	0	n	1	0	Plantaginaceae Juss.
Iz_10_1209	DJI_0643	Lomelosia argentea (L.) Greuter & Burdet	0	n	1	0	Caprifoliaceae Juss.
Iz_10_1209	DJI_0643	Melampyrum pratense L.	0	n	1	0	Orobanchaceae Vent.
Iz_10_1209	DJI_0643	Oenothera biennis L.	0	a	0	0	Onagraceae Juss.
Iz_10_1209	DJI_0643	Pentanema britannica (L.) D.Gut.Larr., Santos-Vicente, Anderb., E.Rico & M.M.Mart.Ort.	0	n	1	0	Asteraceae Bercht. & J.Presl
Iz_10_1209	DJI_0643	Peucedanum oreoselinum (L.) Moench	0	n	1	0	Apiaceae Lindl.
Iz_10_1209	DJI_0643	Peucedanum ruthenicum M.Bieb.	0	n	1	0	Apiaceae Lindl.
Iz_10_1209	DJI_0643	Pilosella echioides (Lumn.) F.W.Schultz & Sch.Bip.	0	n	1	0	Asteraceae Bercht. & J.Presl
Iz_10_1209	DJI_0643	Pilosella officinarum F. Schultz et Sch. Bip.	0	n	1	0	Asteraceae Bercht. & J.Presl
Iz_10_1209	DJI_0643	Pilosella sp.	0		NA	0	Asteraceae Bercht. & J.Presl
Iz_10_1209	DJI_0643	Pinus sylvestris L.	0	n	1	1	Pinaceae Spreng. ex F.Rudolphi
Iz_10_1209	DJI_0643	Poa annua L.	0	n	1	0	Poaceae Barnhart
Iz_10_1209	DJI_0643	Poa nemoralis L.	0	n	1	0	Poaceae Barnhart
Iz_10_1209	DJI_0643	Poa sp.	0		NA	0	Poaceae Barnhart
Iz_10_1209	DJI_0643	Polygonatum odoratum (Mill.) Druce	0	n	1	0	Asparagaceae Juss.
Iz_10_1209	DJI_0643	Polygonum arenarium Waldst. & Kit.	0	n	1	0	Polygonaceae Juss.
Iz_10_1209	DJI_0643	Populus nigra L.	0	n	1	1	Salicaceae Mirb.
Iz_10_1209	DJI_0643	Populus tremula L.	0	n	1	1	Salicaceae Mirb.
Iz_10_1209	DJI_0643	Populus Ã canescens (Aiton) Sm.	0	n	1	1	Salicaceae Mirb.
Iz_10_1209	DJI_0643	Potentilla argentea L.	0	n	1	0	Rosaceae Juss.
Iz_10_1209	DJI_0643	Psammophiliella muralis (L.) Ikonn.	0	n	1	0	Caryophyllaceae Juss.
Iz_10_1209	DJI_0643	Psephellus sumensis (Kalen.) Greuter	0	n	1	0	Asteraceae Bercht. & J.Presl
Iz_10_1209	DJI_0643	Pulsatilla pratensis (L.) Mill. s.l.	0	n	1	0	Ranunculaceae Juss.

Iz_10_1209	DJI_0643	Pyrus communis L.	0	a	0	1	Rosaceae Juss.
Iz_10_1209	DJI_0643	Quercus robur L.	0	n	1	1	Fagaceae Dumort.
Iz_10_1209	DJI_0643	Robinia pseudoacacia L.	0	a	0	1	Fabaceae Lindl.
Iz_10_1209	DJI_0643	Rosa sp.	0		NA	1	Rosaceae Juss.
Iz_10_1209	DJI_0643	Rubus idaeus L.	0	n	1	0	Rosaceae Juss.
Iz_10_1209	DJI_0643	Rumex acetosella L.	0	n	1	0	Polygonaceae Juss.
Iz_10_1209	DJI_0643	Rumex thyrsiflorus Fingerh.	0	n	1	0	Polygonaceae Juss.
Iz_10_1209	DJI_0643	Salix alba L.	0	n	1	1	Salicaceae Mirb.
Iz_10_1209	DJI_0643	Sambucus nigra L.	0	n	1	1	Viburnaceae Raf.
Iz_10_1209	DJI_0643	Secale sylvestre Host	0	n	1	0	Poaceae Barnhart
Iz_10_1209	DJI_0643	Sempervivum ruthenicum Schnittsp. & C.B. Lehm.	0	n	1	0	Crassulaceae J.St.-Hil.
Iz_10_1209	DJI_0643	Setaria viridis (L.) P. Beauv.	0	n	1	0	Poaceae Barnhart
Iz_10_1209	DJI_0643	Silene borysthenica (Gruner) Walters	0	n	1	0	Caryophyllaceae Juss.
Iz_10_1209	DJI_0643	Silene latifolia subsp. alba (Mill.) Greuter & Burdet	0	n	1	0	Caryophyllaceae Juss.
Iz_10_1209	DJI_0643	Solidago virgaurea L.	0	n	1	0	Asteraceae Bercht. & J.Presl
Iz_10_1209	DJI_0643	Sorbus aucuparia L.	0	n	1	1	Rosaceae Juss.
Iz_10_1209	DJI_0643	Stachys recta L.	0	n	1	0	Lamiacea Lindl.
Iz_10_1209	DJI_0643	Tanacetum vulgare L.	0	n	1	0	Asteraceae Bercht. & J.Presl
Iz_10_1209	DJI_0643	Thymus pallasianus Heinr.Braun	0	n	1	0	Lamiacea Lindl.
Iz_10_1209	DJI_0643	Tilia cordata Mill.	0	n	1	1	Malvaceae Juss.
Iz_10_1209	DJI_0643	Trifolium alpestre L.	0	n	1	0	Fabaceae Lindl.
Iz_10_1209	DJI_0643	Trifolium arvense L.	0	n	1	0	Fabaceae Lindl.
Iz_10_1209	DJI_0643	Ulmus glabra Huds.	0	n	1	1	Ulmaceae Mirb.
Iz_10_1209	DJI_0643	Verbascum densiflorum Bertol.	0	n	1	0	Scrophulariaceae Juss.
Iz_10_1209	DJI_0643	Verbascum sp.	0	n	1	0	Scrophulariaceae Juss.
Iz_10_1209	DJI_0643	Veronica austriaca L.	0	n	1	0	Plantaginaceae Juss.
Iz_10_1209	DJI_0643	Veronica incana L.	0	n	1	0	Plantaginaceae Juss.
Iz_10_1209	DJI_0643	Veronica verna L.	0	n	1	0	Plantaginaceae Juss.
Iz_10_1209	DJI_0643	Vincetoxicum hirundinaria Medik	0	n	1	0	Apocynaceae Juss.
Iz_10_1209	DJI_0649	Acer tataricum L.	0	n	1	1	Sapindaceae Juss.
Iz_10_1209	DJI_0649	Achillea millefolium L. s.l.	0	n	1	0	Asteraceae Bercht. & J.Presl
Iz_10_1209	DJI_0649	Allium oleraceum L.	0	n	1	0	Amaryllidaceae J.St.-Hil.
Iz_10_1209	DJI_0649	Ambrosia artemisiifolia L.	0	a	0	0	Asteraceae Bercht. & J.Presl
Iz_10_1209	DJI_0649	Amorpha fruticosa L.	0	a	0	0	Fabaceae Lindl.
Iz_10_1209	DJI_0649	Anthericum ramosum L.	0	n	1	0	Asparagaceae Juss.
Iz_10_1209	DJI_0649	Artemisia marschalliana Spreng.	0	n	1	0	Asteraceae Bercht. & J.Presl
Iz_10_1209	DJI_0649	Asparagus officinalis L.	0	n	1	0	Asparagaceae Juss.
Iz_10_1209	DJI_0649	Astragalus cicer L.	0	n	1	0	Fabaceae Lindl.
Iz_10_1209	DJI_0649	Bassia laniflora (S.G.Gmel.) A.J.Scott	0	n	1	0	Amaranthaceae Juss.
Iz_10_1209	DJI_0649	Berberis vulgaris L.	0	n	1	0	Berberidaceae Juss.
Iz_10_1209	DJI_0649	Betula pendula Roth.	0	n	1	1	Betulaceae Gray
Iz_10_1209	DJI_0649	Betula pendula Roth.	0	n	1	0	Poaceae Barnhart
Iz_10_1209	DJI_0649	Bromus inermis Leyss.	0		NA	NA	
Iz_10_1209	DJI_0649	Bromus squarrosus L.	0	a	0	0	Poaceae Barnhart
Iz_10_1209	DJI_0649	Bromus tectorum L.	0	a	0	0	Poaceae Barnhart
Iz_10_1209	DJI_0649	Calamagrostis epigejos (L.) Roth	15	n	1	0	Poaceae Barnhart
Iz_10_1209	DJI_0649	Carduus acanthoides L.	0	n	1	0	Asteraceae Bercht. & J.Presl
Iz_10_1209	DJI_0649	Carex sp.	0	n	1	0	Cyperaceae Juss.
Iz_10_1209	DJI_0649	Centaurea majorovii Dumbadze	0	n	1	0	Asteraceae Bercht. & J.Presl
Iz_10_1209	DJI_0649	Chamaecytisus ruthenicus (Fisch.ex Wol.) Klaskova	0	n	1	0	Fabaceae Lindl.
Iz_10_1209	DJI_0649	Chelidonium majus L.	0	n	1	0	Papaveraceae Juss.
Iz_10_1209	DJI_0649	Chenopodium betaceum Andrz.	0	n	1	0	Amaranthaceae Juss.
Iz_10_1209	DJI_0649	Chenopodium cf. album	0	n	1	0	Amaranthaceae Juss.
Iz_10_1209	DJI_0649	Chenopodium sp.	0		NA	0	Amaranthaceae Juss.
Iz_10_1209	DJI_0649	Chondrilla juncea L.	0	n	1	0	Asteraceae Bercht. & J.Presl
Iz_10_1209	DJI_0649	Cirsium vulgare (Savi) Ten.	0	n	1	0	Asteraceae Bercht. & J.Presl
Iz_10_1209	DJI_0649	Clinopodium acinos (L.) Kuntze	0	n	1	0	Lamiacea Lindl.
Iz_10_1209	DJI_0649	Convallaria majalis L.	0	n	1	0	Asparagaceae Juss.
Iz_10_1209	DJI_0649	Crataegus rhipidophylla Gand.	0	n	1	1	Rosaceae Juss.
Iz_10_1209	DJI_0649	Crepis foetida L.	0	n	1	0	Asteraceae Bercht. & J.Presl
Iz_10_1209	DJI_0649	Dactylis glomerata L.	0	n	1	0	Poaceae Barnhart
Iz_10_1209	DJI_0649	Dianthus campestris M.Bieb.	0	n	1	0	Caryophyllaceae Juss.
Iz_10_1209	DJI_0649	Digitaria sanguinalis (L.) Scop.	0	a	0	0	Poaceae Barnhart
Iz_10_1209	DJI_0649	Elymus repens (L.) Gould	0	n	1	0	Poaceae Barnhart
Iz_10_1209	DJI_0649	Epilobium tetragonum L.	0	n	1	0	Onagraceae Juss.
Iz_10_1209	DJI_0649	Eragrostis minor Host	0	a	0	0	Poaceae Barnhart
Iz_10_1209	DJI_0649	Erigeron canadensis L.	4	a	0	0	Asteraceae Bercht. & J.Presl
Iz_10_1209	DJI_0649	Euphorbia seguieriana Neck.	0	n	1	0	Euphorbiaceae Juss.
Iz_10_1209	DJI_0649	Fallopia dumetorum (L.) Holub	0	n	1	0	Polygonaceae Juss.
Iz_10_1209	DJI_0649	Festuca beckeri (Hack.) Trautv.	0	n	1	0	Poaceae Barnhart
Iz_10_1209	DJI_0649	Fragaria vesca L.	0	n	1	0	Rosaceae Juss.
Iz_10_1209	DJI_0649	Frangula alnus Mill.	0	n	1	1	Rhamnaceae Juss.
Iz_10_1209	DJI_0649	Fraxinus pennsylvanica Marshall	0	a	0	1	Oleaceae Hoffmanns. & Link
Iz_10_1209	DJI_0649	Galeopsis bifida Boenn.	0	n	1	0	Lamiacea Lindl.
Iz_10_1209	DJI_0649	Galium verum L.	0	n	1	0	Rubiaceae Juss.
Iz_10_1209	DJI_0649	Genista tinctoria L.	0	n	1	0	Fabaceae Lindl.
Iz_10_1209	DJI_0649	Geranium robertianum L.	0	n	1	0	Geraniaceae Juss.
Iz_10_1209	DJI_0649	Geranium sanquineum L.	0	n	1	0	Geraniaceae Juss.
Iz_10_1209	DJI_0649	Gypsophila paniculata L.	0	n	1	0	Caryophyllaceae Juss.
Iz_10_1209	DJI_0649	Helichrysum arenarium (L.) Moench	0	n	1	0	Asteraceae Bercht. & J.Presl
Iz_10_1209	DJI_0649	Herniaria polygama J.Gay	0	n	1	0	Caryophyllaceae Juss.
Iz_10_1209	DJI_0649	Hieracium robustum Fr.	0	n	1	0	Asteraceae Bercht. & J.Presl
Iz_10_1209	DJI_0649	Hieracium umbellatum L.	0	n	1	NA	Asteraceae Bercht. & J.Presl
Iz_10_1209	DJI_0649	Hylotelephium maximum (L.) Holub	0	n	1	0	Crassulaceae J.St.-Hil.
Iz_10_1209	DJI_0649	Jacobaea borysthenica (DC.) B.Nord. & Greuter	0	n	1	0	Asteraceae Bercht. & J.Presl
Iz_10_1209	DJI_0649	Jasione montana L.	0	n	1	0	Campanulaceae Juss.
Iz_10_1209	DJI_0649	Jurinea cyanoides (L.) Rchb.s.l.	0	n	1	0	Asteraceae Bercht. & J.Presl
Iz_10_1209	DJI_0649	Koeleria glauca (Spreng.) DC.	0	n	1	0	Poaceae Barnhart
Iz_10_1209	DJI_0649	Lactuca serriola L.	0	a	0	0	Asteraceae Bercht. & J.Presl
Iz_10_1209	DJI_0649	Linaria genistifolia (L.) Mill.	0	n	1	0	Plantaginaceae Juss.
Iz_10_1209	DJI_0649	Lomelosia argentea (L.) Greuter & Burdet	0	n	1	0	Caprifoliaceae Juss.
Iz_10_1209	DJI_0649	Melampyrum pratense L.	0	n	1	0	Orobanchaceae Vent.
Iz_10_1209	DJI_0649	Oenothera biennis L.	0	a	0	0	Onagraceae Juss.
Iz_10_1209	DJI_0649	Pentanema britannica (L.) D.Gut.Larr., Santos-Vicente, Anderb., E.Rico & M.M.Mart.Ort.	0	n	1	0	Asteraceae Bercht. & J.Presl
Iz_10_1209	DJI_0649	Peucedanum oreoselinum (L.) Moench	0	n	1	0	Apiaceae Lindl.
Iz_10_1209	DJI_0649	Peucedanum ruthenicum M.Bieb.	0	n	1	0	Apiaceae Lindl.
Iz_10_1209	DJI_0649	Pilosella echioides (Lumn.) F.W.Schultz & Sch.Bip.	0	n	1	0	Asteraceae Bercht. & J.Presl
Iz_10_1209	DJI_0649	Pilosella officinarum F. Schultz et Sch. Bip.	0	n	1	0	Asteraceae Bercht. & J.Presl

Iz_10_1209	DJI_0649	Pilosella sp.	0		NA	0	Asteraceae Bercht. & J.Presl
Iz_10_1209	DJI_0649	Pinus sylvestris L.	0	n	1	1	Pinaceae Spreng. ex F.Rudolphi
Iz_10_1209	DJI_0649	Poa annua L.	0	n	1	0	Poaceae Barnhart
Iz_10_1209	DJI_0649	Poa nemoralis L.	0	n	1	0	Poaceae Barnhart
Iz_10_1209	DJI_0649	Poa sp.	0		NA	0	Poaceae Barnhart
Iz_10_1209	DJI_0649	Polygonatum odoratum (Mill.) Druce	0	n	1	0	Asparagaceae Juss.
Iz_10_1209	DJI_0649	Polygonum arenarium Waldst. & Kit.	0	n	1	0	Polygonaceae Juss.
Iz_10_1209	DJI_0649	Populus nigra L.	0	n	1	1	Salicaceae Mirb.
Iz_10_1209	DJI_0649	Populus tremula L.	0	n	1	1	Salicaceae Mirb.
Iz_10_1209	DJI_0649	Populus Ã canescens (Aiton) Sm.	0	n	1	1	Salicaceae Mirb.
Iz_10_1209	DJI_0649	Potentilla argentea L.	0	n	1	0	Rosaceae Juss.
Iz_10_1209	DJI_0649	Psammophiliella muralis (L.) Ikonn.	0	n	1	0	Caryophyllaceae Juss.
Iz_10_1209	DJI_0649	Psephellus sumensis (Kalen.) Greuter	0	n	1	0	Asteraceae Bercht. & J.Presl
Iz_10_1209	DJI_0649	Pulsatilla pratensis (L.) Mill. s.l.	0	n	1	0	Ranunculaceae Juss.
Iz_10_1209	DJI_0649	Pyrus communis L.	0	a	0	1	Rosaceae Juss.
Iz_10_1209	DJI_0649	Quercus robur L.	0	n	1	1	Fagaceae Dumort.
Iz_10_1209	DJI_0649	Robinia pseudoacacia L.	0	a	0	1	Fabaceae Lindl.
Iz_10_1209	DJI_0649	Rosa sp.	0		NA	1	Rosaceae Juss.
Iz_10_1209	DJI_0649	Rubus idaeus L.	0	n	1	0	Rosaceae Juss.
Iz_10_1209	DJI_0649	Rumex acetosella L.	0	n	1	0	Polygonaceae Juss.
Iz_10_1209	DJI_0649	Rumex thyrsiflorus Fingerh.	0	n	1	0	Polygonaceae Juss.
Iz_10_1209	DJI_0649	Salix alba L.	0	n	1	1	Salicaceae Mirb.
Iz_10_1209	DJI_0649	Sambucus nigra L.	0	n	1	1	Viburnaceae Raf.
Iz_10_1209	DJI_0649	Secale sylvestre Host	0	n	1	0	Poaceae Barnhart
Iz_10_1209	DJI_0649	Sempervivum ruthenicum Schnittsp. & C.B. Lehm.	0	n	1	0	Crassulaceae J.St.-Hil.
Iz_10_1209	DJI_0649	Setaria viridis (L.) P. Beauv.	0	n	1	0	Poaceae Barnhart
Iz_10_1209	DJI_0649	Silene borysthenica (Gruner) Walters	0	n	1	0	Caryophyllaceae Juss.
Iz_10_1209	DJI_0649	Silene latifolia subsp. alba (Mill.) Greuter & Burdet	0	n	1	0	Caryophyllaceae Juss.
Iz_10_1209	DJI_0649	Solidago virgaurea L.	0	n	1	0	Asteraceae Bercht. & J.Presl
Iz_10_1209	DJI_0649	Sorbus aucuparia L.	0	n	1	1	Rosaceae Juss.
Iz_10_1209	DJI_0649	Stachys recta L.	0	n	1	0	Lamiacea Lindl.
Iz_10_1209	DJI_0649	Tanacetum vulgare L.	0	n	1	0	Asteraceae Bercht. & J.Presl
Iz_10_1209	DJI_0649	Thymus pallasianus Heinr.Braun	0	n	1	0	Lamiacea Lindl.
Iz_10_1209	DJI_0649	Tilia cordata Mill.	0	n	1	1	Malvaceae Juss.
Iz_10_1209	DJI_0649	Trifolium alpestre L.	0	n	1	0	Fabaceae Lindl.
Iz_10_1209	DJI_0649	Trifolium arvense L.	0	n	1	0	Fabaceae Lindl.
Iz_10_1209	DJI_0649	Ulmus glabra Huds.	0	n	1	1	Ulmaceae Mirb.
Iz_10_1209	DJI_0649	Verbascum densiflorum Bertol.	0	n	1	0	Scrophulariaceae Juss.
Iz_10_1209	DJI_0649	Verbascum sp.	0	n	1	0	Scrophulariaceae Juss.
Iz_10_1209	DJI_0649	Veronica austriaca L.	0	n	1	0	Plantaginaceae Juss.
Iz_10_1209	DJI_0649	Veronica incana L.	0	n	1	0	Plantaginaceae Juss.
Iz_10_1209	DJI_0649	Veronica verna L.	0	n	1	0	Plantaginaceae Juss.
Iz_10_1209	DJI_0649	Vincetoxicum hirundinaria Medik	0	n	1	0	Apocynaceae Juss.
Iz_10_1209	DJI_0654	Acer tataricum L.	0	n	1	1	Sapindaceae Juss.
Iz_10_1209	DJI_0654	Achillea millefolium L. s.l.	0	n	1	0	Asteraceae Bercht. & J.Presl
Iz_10_1209	DJI_0654	Allium oleraceum L.	0	n	1	0	Amaryllidaceae J.St.-Hil.
Iz_10_1209	DJI_0654	Ambrosia artemisiifolia L.	0	a	0	0	Asteraceae Bercht. & J.Presl
Iz_10_1209	DJI_0654	Amorpha fruticosa L.	0	a	0	0	Fabaceae Lindl.
Iz_10_1209	DJI_0654	Anthericum ramosum L.	0	n	1	0	Asparagaceae Juss.
Iz_10_1209	DJI_0654	Artemisia marschalliana Spreng.	0	n	1	0	Asteraceae Bercht. & J.Presl
Iz_10_1209	DJI_0654	Asparagus officinalis L.	0	n	1	0	Asparagaceae Juss.
Iz_10_1209	DJI_0654	Astragalus cicer L.	0	n	1	0	Fabaceae Lindl.
Iz_10_1209	DJI_0654	Bassia laniflora (S.G.Gmel.) A.J.Scott	0	n	1	0	Amaranthaceae Juss.
Iz_10_1209	DJI_0654	Berberis vulgaris L.	0	n	1	0	Berberidaceae Juss.
Iz_10_1209	DJI_0654	Betula pendula Roth.	0	n	1	1	Betulaceae Gray
Iz_10_1209	DJI_0654	Betula pendula Roth.	0	n	1	0	Poaceae Barnhart
Iz_10_1209	DJI_0654	Bromus inermis Leyss.	0		NA	NA	
Iz_10_1209	DJI_0654	Bromus squarrosus L.	0	a	0	0	Poaceae Barnhart
Iz_10_1209	DJI_0654	Bromus tectorum L.	0	a	0	0	Poaceae Barnhart
Iz_10_1209	DJI_0654	Calamagrostis epigejos (L.) Roth	80	n	1	0	Poaceae Barnhart
Iz_10_1209	DJI_0654	Carduus acanthoides L.	0	n	1	0	Asteraceae Bercht. & J.Presl
Iz_10_1209	DJI_0654	Carex sp.	0	n	1	0	Cyperaceae Juss.
Iz_10_1209	DJI_0654	Centaurea majorovii Dumbadze	0	n	1	0	Asteraceae Bercht. & J.Presl
Iz_10_1209	DJI_0654	Chamaecytisus ruthenicus (Fisch.ex Wol.) Klaskova	0	n	1	0	Fabaceae Lindl.
Iz_10_1209	DJI_0654	Chelidonium majus L.	0	n	1	0	Papaveraceae Juss.
Iz_10_1209	DJI_0654	Chenopodium betaceum Andrz.	0	n	1	0	Amaranthaceae Juss.
Iz_10_1209	DJI_0654	Chenopodium cf. album	0	n	1	0	Amaranthaceae Juss.
Iz_10_1209	DJI_0654	Chenopodium sp.	0		NA	0	Amaranthaceae Juss.
Iz_10_1209	DJI_0654	Chondrilla juncea L.	0	n	1	0	Asteraceae Bercht. & J.Presl
Iz_10_1209	DJI_0654	Cirsium vulgare (Savi) Ten.	0	n	1	0	Asteraceae Bercht. & J.Presl
Iz_10_1209	DJI_0654	Clinopodium acinos (L.) Kuntze	0	n	1	0	Lamiacea Lindl.
Iz_10_1209	DJI_0654	Convallaria majalis L.	0	n	1	0	Asparagaceae Juss.
Iz_10_1209	DJI_0654	Crataegus rhipidophylla Gand.	0	n	1	1	Rosaceae Juss.
Iz_10_1209	DJI_0654	Crepis foetida L.	0	n	1	0	Asteraceae Bercht. & J.Presl
Iz_10_1209	DJI_0654	Dactylis glomerata L.	0	n	1	0	Poaceae Barnhart
Iz_10_1209	DJI_0654	Dianthus campestris M.Bieb.	0	n	1	0	Caryophyllaceae Juss.
Iz_10_1209	DJI_0654	Digitaria sanguinalis (L.) Scop.	0	a	0	0	Poaceae Barnhart
Iz_10_1209	DJI_0654	Elymus repens (L.) Gould	0	n	1	0	Poaceae Barnhart
Iz_10_1209	DJI_0654	Epilobium tetragonum L.	0	n	1	0	Onagraceae Juss.
Iz_10_1209	DJI_0654	Eragrostis minor Host	0	a	0	0	Poaceae Barnhart
Iz_10_1209	DJI_0654	Erigeron canadensis L.	0	a	0	0	Asteraceae Bercht. & J.Presl
Iz_10_1209	DJI_0654	Euphorbia seguieriana Neck.	0	n	1	0	Euphorbiaceae Juss.
Iz_10_1209	DJI_0654	Fallopia dumetorum (L.) Holub	0	n	1	0	Polygonaceae Juss.
Iz_10_1209	DJI_0654	Festuca beckeri (Hack.) Trautv.	0	n	1	0	Poaceae Barnhart
Iz_10_1209	DJI_0654	Fragaria vesca L.	0	n	1	0	Rosaceae Juss.
Iz_10_1209	DJI_0654	Frangula alnus Mill.	0	n	1	1	Rhamnaceae Juss.
Iz_10_1209	DJI_0654	Fraxinus pennsylvanica Marshall	0	a	0	1	Oleaceae Hoffmanns. & Link
Iz_10_1209	DJI_0654	Galeopsis bifida Boenn.	0	n	1	0	Lamiacea Lindl.
Iz_10_1209	DJI_0654	Galium verum L.	0	n	1	0	Rubiaceae Juss.
Iz_10_1209	DJI_0654	Genista tinctoria L.	0	n	1	0	Fabaceae Lindl.
Iz_10_1209	DJI_0654	Geranium robertianum L.	0	n	1	0	Geraniaceae Juss.
Iz_10_1209	DJI_0654	Geranium sanquineum L.	0	n	1	0	Geraniaceae Juss.
Iz_10_1209	DJI_0654	Gypsophila paniculata L.	0	n	1	0	Caryophyllaceae Juss.
Iz_10_1209	DJI_0654	Helichrysum arenarium (L.) Moench	0	n	1	0	Asteraceae Bercht. & J.Presl
Iz_10_1209	DJI_0654	Herniaria polygama J.Gay	0	n	1	0	Caryophyllaceae Juss.
Iz_10_1209	DJI_0654	Hieracium robustum Fr.	0	n	1	0	Asteraceae Bercht. & J.Presl
Iz_10_1209	DJI_0654	Hieracium umbellatum L.	0	n	1	NA	Asteraceae Bercht. & J.Presl
Iz_10_1209	DJI_0654	Hylotelephium maximum (L.) Holub	0	n	1	0	Crassulaceae J.St.-Hil.

Iz_10_1209	DJI_0654	Jacobaea borysthenica (DC.) B.Nord. & Greuter	0	n	1	0	Asteraceae Bercht. & J.Presl
Iz_10_1209	DJI_0654	Jasione montana L.	0	n	1	0	Campanulaceae Juss.
Iz_10_1209	DJI_0654	Jurinea cyanoides (L.) Rchb.s.l.	0	n	1	0	Asteraceae Bercht. & J.Presl
Iz_10_1209	DJI_0654	Koeleria glauca (Spreng.) DC.	0	n	1	0	Poaceae Barnhart
Iz_10_1209	DJI_0654	Lactuca serriola L.	0	a	0	0	Asteraceae Bercht. & J.Presl
Iz_10_1209	DJI_0654	Linaria genistifolia (L.) Mill.	0	n	1	0	Plantaginaceae Juss.
Iz_10_1209	DJI_0654	Lomelosia argentea (L.) Greuter & Burdet	0	n	1	0	Caprifoliaceae Juss.
Iz_10_1209	DJI_0654	Melampyrum pratense L.	0	n	1	0	Orobanchaceae Vent.
Iz_10_1209	DJI_0654	Oenothera biennis L.	0	a	0	0	Onagraceae Juss.
Iz_10_1209	DJI_0654	Pentanema britannica (L.) D.Gut.Larr., Santos-Vicente, Anderb., E.Rico & M.M.Mart.Ort.	0	n	1	0	Asteraceae Bercht. & J.Presl
Iz_10_1209	DJI_0654	Peucedanum oreoselinum (L.) Moench	0	n	1	0	Apiaceae Lindl.
Iz_10_1209	DJI_0654	Peucedanum ruthenicum M.Bieb.	0	n	1	0	Apiaceae Lindl.
Iz_10_1209	DJI_0654	Pilosella echioides (Lumn.) F.W.Schultz & Sch.Bip.	0	n	1	0	Asteraceae Bercht. & J.Presl
Iz_10_1209	DJI_0654	Pilosella officinarum F. Schultz et Sch. Bip.	0	n	1	0	Asteraceae Bercht. & J.Presl
Iz_10_1209	DJI_0654	Pilosella sp.	0		NA	0	Asteraceae Bercht. & J.Presl
Iz_10_1209	DJI_0654	Pinus sylvestris L.	0	n	1	1	Pinaceae Spreng. ex F.Rudolphi
Iz_10_1209	DJI_0654	Poa annua L.	0	n	1	0	Poaceae Barnhart
Iz_10_1209	DJI_0654	Poa nemoralis L.	0	n	1	0	Poaceae Barnhart
Iz_10_1209	DJI_0654	Poa sp.	0		NA	0	Poaceae Barnhart
Iz_10_1209	DJI_0654	Polygonatum odoratum (Mill.) Druce	0	n	1	0	Asparagaceae Juss.
Iz_10_1209	DJI_0654	Polygonum arenarium Waldst. & Kit.	0	n	1	0	Polygonaceae Juss.
Iz_10_1209	DJI_0654	Populus nigra L.	0	n	1	1	Salicaceae Mirb.
Iz_10_1209	DJI_0654	Populus tremula L.	0	n	1	1	Salicaceae Mirb.
Iz_10_1209	DJI_0654	Populus Ã canescens (Aiton) Sm.	0	n	1	1	Salicaceae Mirb.
Iz_10_1209	DJI_0654	Potentilla argentea L.	0	n	1	0	Rosaceae Juss.
Iz_10_1209	DJI_0654	Psammophiliella muralis (L.) Ikonn.	0	n	1	0	Caryophyllaceae Juss.
Iz_10_1209	DJI_0654	Psephellus sumensis (Kalen.) Greuter	0	n	1	0	Asteraceae Bercht. & J.Presl
Iz_10_1209	DJI_0654	Pulsatilla pratensis (L.) Mill. s.l.	0	n	1	0	Ranunculaceae Juss.
Iz_10_1209	DJI_0654	Pyrus communis L.	0	a	0	1	Rosaceae Juss.
Iz_10_1209	DJI_0654	Quercus robur L.	0	n	1	1	Fagaceae Dumort.
Iz_10_1209	DJI_0654	Robinia pseudoacacia L.	0	a	0	1	Fabaceae Lindl.
Iz_10_1209	DJI_0654	Rosa sp.	0		NA	1	Rosaceae Juss.
Iz_10_1209	DJI_0654	Rubus idaeus L.	0	n	1	0	Rosaceae Juss.
Iz_10_1209	DJI_0654	Rumex acetosella L.	0	n	1	0	Polygonaceae Juss.
Iz_10_1209	DJI_0654	Rumex thyrsiflorus Fingerh.	0	n	1	0	Polygonaceae Juss.
Iz_10_1209	DJI_0654	Salix alba L.	0	n	1	1	Salicaceae Mirb.
Iz_10_1209	DJI_0654	Sambucus nigra L.	0	n	1	1	Viburnaceae Raf.
Iz_10_1209	DJI_0654	Secale sylvestre Host	0	n	1	0	Poaceae Barnhart
Iz_10_1209	DJI_0654	Sempervivum ruthenicum Schnittsp. & C.B. Lehm.	0	n	1	0	Crassulaceae J.St.-Hil.
Iz_10_1209	DJI_0654	Setaria viridis (L.) P. Beauv.	0	n	1	0	Poaceae Barnhart
Iz_10_1209	DJI_0654	Silene borysthenica (Gruner) Walters	0	n	1	0	Caryophyllaceae Juss.
Iz_10_1209	DJI_0654	Silene latifolia subsp. alba (Mill.) Greuter & Burdet	0	n	1	0	Caryophyllaceae Juss.
Iz_10_1209	DJI_0654	Solidago virgaurea L.	0	n	1	0	Asteraceae Bercht. & J.Presl
Iz_10_1209	DJI_0654	Sorbus aucuparia L.	0	n	1	1	Rosaceae Juss.
Iz_10_1209	DJI_0654	Stachys recta L.	0	n	1	0	Lamiacea Lindl.
Iz_10_1209	DJI_0654	Tanacetum vulgare L.	0	n	1	0	Asteraceae Bercht. & J.Presl
Iz_10_1209	DJI_0654	Thymus pallasianus Heinr.Braun	0	n	1	0	Lamiacea Lindl.
Iz_10_1209	DJI_0654	Tilia cordata Mill.	0	n	1	1	Malvaceae Juss.
Iz_10_1209	DJI_0654	Trifolium alpestre L.	0	n	1	0	Fabaceae Lindl.
Iz_10_1209	DJI_0654	Trifolium arvense L.	0	n	1	0	Fabaceae Lindl.
Iz_10_1209	DJI_0654	Ulmus glabra Huds.	0	n	1	1	Ulmaceae Mirb.
Iz_10_1209	DJI_0654	Verbascum densiflorum Bertol.	0	n	1	0	Scrophulariaceae Juss.
Iz_10_1209	DJI_0654	Verbascum sp.	0	n	1	0	Scrophulariaceae Juss.
Iz_10_1209	DJI_0654	Veronica austriaca L.	0	n	1	0	Plantaginaceae Juss.
Iz_10_1209	DJI_0654	Veronica incana L.	0	n	1	0	Plantaginaceae Juss.
Iz_10_1209	DJI_0654	Veronica verna L.	0	n	1	0	Plantaginaceae Juss.
Iz_10_1209	DJI_0654	Vincetoxicum hirundinaria Medik	0	n	1	0	Apocynaceae Juss.
Iz_10_1209	DJI_0658	Acer tataricum L.	0	n	1	1	Sapindaceae Juss.
Iz_10_1209	DJI_0658	Achillea millefolium L. s.l.	0	n	1	0	Asteraceae Bercht. & J.Presl
Iz_10_1209	DJI_0658	Allium oleraceum L.	0	n	1	0	Amaryllidaceae J.St.-Hil.
Iz_10_1209	DJI_0658	Ambrosia artemisiifolia L.	0	a	0	0	Asteraceae Bercht. & J.Presl
Iz_10_1209	DJI_0658	Amorpha fruticosa L.	0	a	0	0	Fabaceae Lindl.
Iz_10_1209	DJI_0658	Anthericum ramosum L.	0	n	1	0	Asparagaceae Juss.
Iz_10_1209	DJI_0658	Artemisia marschalliana Spreng.	0	n	1	0	Asteraceae Bercht. & J.Presl
Iz_10_1209	DJI_0658	Asparagus officinalis L.	0	n	1	0	Asparagaceae Juss.
Iz_10_1209	DJI_0658	Astragalus cicer L.	0	n	1	0	Fabaceae Lindl.
Iz_10_1209	DJI_0658	Bassia laniflora (S.G.Gmel.) A.J.Scott	0	n	1	0	Amaranthaceae Juss.
Iz_10_1209	DJI_0658	Berberis vulgaris L.	0	n	1	0	Berberidaceae Juss.
Iz_10_1209	DJI_0658	Betula pendula Roth.	0	n	1	1	Betulaceae Gray
Iz_10_1209	DJI_0658	Betula pendula Roth.	0	n	1	0	Poaceae Barnhart
Iz_10_1209	DJI_0658	Bromus inermis Leyss.	0		NA	NA	
Iz_10_1209	DJI_0658	Bromus squarrosus L.	0	a	0	0	Poaceae Barnhart
Iz_10_1209	DJI_0658	Bromus tectorum L.	0	a	0	0	Poaceae Barnhart
Iz_10_1209	DJI_0658	Calamagrostis epigejos (L.) Roth	10	n	1	0	Poaceae Barnhart
Iz_10_1209	DJI_0658	Carduus acanthoides L.	0	n	1	0	Asteraceae Bercht. & J.Presl
Iz_10_1209	DJI_0658	Carex sp.	0	n	1	0	Cyperaceae Juss.
Iz_10_1209	DJI_0658	Centaurea majorovii Dumbadze	0	n	1	0	Asteraceae Bercht. & J.Presl
Iz_10_1209	DJI_0658	Chamaecytisus ruthenicus (Fisch.ex Wol.) Klaskova	0	n	1	0	Fabaceae Lindl.
Iz_10_1209	DJI_0658	Chelidonium majus L.	0	n	1	0	Papaveraceae Juss.
Iz_10_1209	DJI_0658	Chenopodium betaceum Andrz.	0	n	1	0	Amaranthaceae Juss.
Iz_10_1209	DJI_0658	Chenopodium cf. album	0	n	1	0	Amaranthaceae Juss.
Iz_10_1209	DJI_0658	Chenopodium sp.	0		NA	0	Amaranthaceae Juss.
Iz_10_1209	DJI_0658	Chondrilla juncea L.	0	n	1	0	Asteraceae Bercht. & J.Presl
Iz_10_1209	DJI_0658	Cirsium vulgare (Savi) Ten.	0	n	1	0	Asteraceae Bercht. & J.Presl
Iz_10_1209	DJI_0658	Clinopodium acinos (L.) Kuntze	0	n	1	0	Lamiacea Lindl.
Iz_10_1209	DJI_0658	Convallaria majalis L.	0	n	1	0	Asparagaceae Juss.
Iz_10_1209	DJI_0658	Crataegus rhipidophylla Gand.	0	n	1	1	Rosaceae Juss.
Iz_10_1209	DJI_0658	Crepis foetida L.	0	n	1	0	Asteraceae Bercht. & J.Presl
Iz_10_1209	DJI_0658	Dactylis glomerata L.	0	n	1	0	Poaceae Barnhart
Iz_10_1209	DJI_0658	Dianthus campestris M.Bieb.	0	n	1	0	Caryophyllaceae Juss.
Iz_10_1209	DJI_0658	Digitaria sanguinalis (L.) Scop.	0	a	0	0	Poaceae Barnhart
Iz_10_1209	DJI_0658	Elymus repens (L.) Gould	0	n	1	0	Poaceae Barnhart
Iz_10_1209	DJI_0658	Epilobium tetragonum L.	0	n	1	0	Onagraceae Juss.
Iz_10_1209	DJI_0658	Eragrostis minor Host	0	a	0	0	Poaceae Barnhart
Iz_10_1209	DJI_0658	Erigeron canadensis L.	10	a	0	0	Asteraceae Bercht. & J.Presl
Iz_10_1209	DJI_0658	Euphorbia seguieriana Neck.	0	n	1	0	Euphorbiaceae Juss.
Iz_10_1209	DJI_0658	Fallopia dumetorum (L.) Holub	0	n	1	0	Polygonaceae Juss.
Iz_10_1209	DJI_0658	Festuca beckeri (Hack.) Trautv.	0	n	1	0	Poaceae Barnhart

Iz_10_1209	DJI_0658	Fragaria vesca L.	0	n	1	0	Rosaceae Juss.
Iz_10_1209	DJI_0658	Frangula alnus Mill.	0	n	1	1	Rhamnaceae Juss.
Iz_10_1209	DJI_0658	Fraxinus pennsylvanica Marshall	0	a	0	1	Oleaceae Hoffmanns. & Link
Iz_10_1209	DJI_0658	Galeopsis bifida Boenn.	0	n	1	0	Lamiacea Lindl.
Iz_10_1209	DJI_0658	Galium verum L.	0	n	1	0	Rubiaceae Juss.
Iz_10_1209	DJI_0658	Genista tinctoria L.	0	n	1	0	Fabaceae Lindl.
Iz_10_1209	DJI_0658	Geranium robertianum L.	0	n	1	0	Geraniaceae Juss.
Iz_10_1209	DJI_0658	Geranium sanquineum L.	0	n	1	0	Geraniaceae Juss.
Iz_10_1209	DJI_0658	Gypsophila paniculata L.	0	n	1	0	Caryophyllaceae Juss.
Iz_10_1209	DJI_0658	Helichrysum arenarium (L.) Moench	0	n	1	0	Asteraceae Bercht. & J.Presl
Iz_10_1209	DJI_0658	Herniaria polygama J.Gay	0	n	1	0	Caryophyllaceae Juss.
Iz_10_1209	DJI_0658	Hieracium robustum Fr.	0	n	1	0	Asteraceae Bercht. & J.Presl
Iz_10_1209	DJI_0658	Hieracium umbellatum L.	0	n	1	NA	Asteraceae Bercht. & J.Presl
Iz_10_1209	DJI_0658	Hylotelephium maximum (L.) Holub	0	n	1	0	Crassulaceae J.St.-Hil.
Iz_10_1209	DJI_0658	Jacobaea borysthenica (DC.) B.Nord. & Greuter	0	n	1	0	Asteraceae Bercht. & J.Presl
Iz_10_1209	DJI_0658	Jasione montana L.	0	n	1	0	Campanulaceae Juss.
Iz_10_1209	DJI_0658	Jurinea cyanoides (L.) Rchb.s.l.	0	n	1	0	Asteraceae Bercht. & J.Presl
Iz_10_1209	DJI_0658	Koeleria glauca (Spreng.) DC.	0	n	1	0	Poaceae Barnhart
Iz_10_1209	DJI_0658	Lactuca serriola L.	0	a	0	0	Asteraceae Bercht. & J.Presl
Iz_10_1209	DJI_0658	Linaria genistifolia (L.) Mill.	0	n	1	0	Plantaginaceae Juss.
Iz_10_1209	DJI_0658	Lomelosia argentea (L.) Greuter & Burdet	0	n	1	0	Caprifoliaceae Juss.
Iz_10_1209	DJI_0658	Melampyrum pratense L.	0	n	1	0	Orobanchaceae Vent.
Iz_10_1209	DJI_0658	Oenothera biennis L.	0	a	0	0	Onagraceae Juss.
Iz_10_1209	DJI_0658	Pentanema britannica (L.) D.Gut.Larr., Santos-Vicente, Anderb., E.Rico & M.M.Mart.Ort.	0	n	1	0	Asteraceae Bercht. & J.Presl
Iz_10_1209	DJI_0658	Peucedanum oreoselinum (L.) Moench	0	n	1	0	Apiaceae Lindl.
Iz_10_1209	DJI_0658	Peucedanum ruthenicum M.Bieb.	0	n	1	0	Apiaceae Lindl.
Iz_10_1209	DJI_0658	Pilosella echioides (Lumn.) F.W.Schultz & Sch.Bip.	0	n	1	0	Asteraceae Bercht. & J.Presl
Iz_10_1209	DJI_0658	Pilosella officinarum F. Schultz et Sch. Bip.	1	n	1	0	Asteraceae Bercht. & J.Presl
Iz_10_1209	DJI_0658	Pilosella sp.	0		NA	0	Asteraceae Bercht. & J.Presl
Iz_10_1209	DJI_0658	Pinus sylvestris L.	0	n	1	1	Pinaceae Spreng. ex F.Rudolphi
Iz_10_1209	DJI_0658	Poa annua L.	0	n	1	0	Poaceae Barnhart
Iz_10_1209	DJI_0658	Poa nemoralis L.	0	n	1	0	Poaceae Barnhart
Iz_10_1209	DJI_0658	Poa sp.	0		NA	0	Poaceae Barnhart
Iz_10_1209	DJI_0658	Polygonatum odoratum (Mill.) Druce	0	n	1	0	Asparagaceae Juss.
Iz_10_1209	DJI_0658	Polygonum arenarium Waldst. & Kit.	0	n	1	0	Polygonaceae Juss.
Iz_10_1209	DJI_0658	Populus nigra L.	0	n	1	1	Salicaceae Mirb.
Iz_10_1209	DJI_0658	Populus tremula L.	1	n	1	1	Salicaceae Mirb.
Iz_10_1209	DJI_0658	Populus Ã canescens (Aiton) Sm.	0	n	1	1	Salicaceae Mirb.
Iz_10_1209	DJI_0658	Potentilla argentea L.	0	n	1	0	Rosaceae Juss.
Iz_10_1209	DJI_0658	Psammophiliella muralis (L.) Ikonn.	0	n	1	0	Caryophyllaceae Juss.
Iz_10_1209	DJI_0658	Psephellus sumensis (Kalen.) Greuter	0	n	1	0	Asteraceae Bercht. & J.Presl
Iz_10_1209	DJI_0658	Pulsatilla pratensis (L.) Mill. s.l.	0	n	1	0	Ranunculaceae Juss.
Iz_10_1209	DJI_0658	Pyrus communis L.	0	a	0	1	Rosaceae Juss.
Iz_10_1209	DJI_0658	Quercus robur L.	0	n	1	1	Fagaceae Dumort.
Iz_10_1209	DJI_0658	Robinia pseudoacacia L.	0	a	0	1	Fabaceae Lindl.
Iz_10_1209	DJI_0658	Rosa sp.	0		NA	1	Rosaceae Juss.
Iz_10_1209	DJI_0658	Rubus idaeus L.	0	n	1	0	Rosaceae Juss.
Iz_10_1209	DJI_0658	Rumex acetosella L.	0	n	1	0	Polygonaceae Juss.
Iz_10_1209	DJI_0658	Rumex thyrsiflorus Fingerh.	0	n	1	0	Polygonaceae Juss.
Iz_10_1209	DJI_0658	Salix alba L.	0	n	1	1	Salicaceae Mirb.
Iz_10_1209	DJI_0658	Sambucus nigra L.	0	n	1	1	Viburnaceae Raf.
Iz_10_1209	DJI_0658	Secale sylvestre Host	0	n	1	0	Poaceae Barnhart
Iz_10_1209	DJI_0658	Sempervivum ruthenicum Schnittsp. & C.B. Lehm.	0	n	1	0	Crassulaceae J.St.-Hil.
Iz_10_1209	DJI_0658	Setaria viridis (L.) P. Beauv.	0	n	1	0	Poaceae Barnhart
Iz_10_1209	DJI_0658	Silene borysthenica (Gruner) Walters	0	n	1	0	Caryophyllaceae Juss.
Iz_10_1209	DJI_0658	Silene latifolia subsp. alba (Mill.) Greuter & Burdet	0	n	1	0	Caryophyllaceae Juss.
Iz_10_1209	DJI_0658	Solidago virgaurea L.	0	n	1	0	Asteraceae Bercht. & J.Presl
Iz_10_1209	DJI_0658	Sorbus aucuparia L.	0	n	1	1	Rosaceae Juss.
Iz_10_1209	DJI_0658	Stachys recta L.	0	n	1	0	Lamiacea Lindl.
Iz_10_1209	DJI_0658	Tanacetum vulgare L.	0	n	1	0	Asteraceae Bercht. & J.Presl
Iz_10_1209	DJI_0658	Thymus pallasianus Heinr.Braun	0	n	1	0	Lamiacea Lindl.
Iz_10_1209	DJI_0658	Tilia cordata Mill.	0	n	1	1	Malvaceae Juss.
Iz_10_1209	DJI_0658	Trifolium alpestre L.	0	n	1	0	Fabaceae Lindl.
Iz_10_1209	DJI_0658	Trifolium arvense L.	0	n	1	0	Fabaceae Lindl.
Iz_10_1209	DJI_0658	Ulmus glabra Huds.	0	n	1	1	Ulmaceae Mirb.
Iz_10_1209	DJI_0658	Verbascum densiflorum Bertol.	0	n	1	0	Scrophulariaceae Juss.
Iz_10_1209	DJI_0658	Verbascum sp.	0	n	1	0	Scrophulariaceae Juss.
Iz_10_1209	DJI_0658	Veronica austriaca L.	0	n	1	0	Plantaginaceae Juss.
Iz_10_1209	DJI_0658	Veronica incana L.	0	n	1	0	Plantaginaceae Juss.
Iz_10_1209	DJI_0658	Veronica verna L.	0	n	1	0	Plantaginaceae Juss.
Iz_10_1209	DJI_0658	Vincetoxicum hirundinaria Medik	0	n	1	0	Apocynaceae Juss.
Iz_10_1209	DJI_0662	Acer tataricum L.	0	n	1	1	Sapindaceae Juss.
Iz_10_1209	DJI_0662	Achillea millefolium L. s.l.	0	n	1	0	Asteraceae Bercht. & J.Presl
Iz_10_1209	DJI_0662	Allium oleraceum L.	0	n	1	0	Amaryllidaceae J.St.-Hil.
Iz_10_1209	DJI_0662	Ambrosia artemisiifolia L.	0	a	0	0	Asteraceae Bercht. & J.Presl
Iz_10_1209	DJI_0662	Amorpha fruticosa L.	0	a	0	0	Fabaceae Lindl.
Iz_10_1209	DJI_0662	Anthericum ramosum L.	0	n	1	0	Asparagaceae Juss.
Iz_10_1209	DJI_0662	Artemisia marschalliana Spreng.	0	n	1	0	Asteraceae Bercht. & J.Presl
Iz_10_1209	DJI_0662	Asparagus officinalis L.	0	n	1	0	Asparagaceae Juss.
Iz_10_1209	DJI_0662	Astragalus cicer L.	0	n	1	0	Fabaceae Lindl.
Iz_10_1209	DJI_0662	Bassia laniflora (S.G.Gmel.) A.J.Scott	0	n	1	0	Amaranthaceae Juss.
Iz_10_1209	DJI_0662	Berberis vulgaris L.	0	n	1	0	Berberidaceae Juss.
Iz_10_1209	DJI_0662	Betula pendula Roth.	0	n	1	1	Betulaceae Gray
Iz_10_1209	DJI_0662	Betula pendula Roth.	0	n	1	0	Poaceae Barnhart
Iz_10_1209	DJI_0662	Bromus inermis Leyss.	0		NA	NA	
Iz_10_1209	DJI_0662	Bromus squarrosus L.	0	a	0	0	Poaceae Barnhart
Iz_10_1209	DJI_0662	Bromus tectorum L.	0	a	0	0	Poaceae Barnhart
Iz_10_1209	DJI_0662	Calamagrostis epigejos (L.) Roth	1.5	n	1	0	Poaceae Barnhart
Iz_10_1209	DJI_0662	Carduus acanthoides L.	0	n	1	0	Asteraceae Bercht. & J.Presl
Iz_10_1209	DJI_0662	Carex sp.	0	n	1	0	Cyperaceae Juss.
Iz_10_1209	DJI_0662	Centaurea majorovii Dumbadze	0	n	1	0	Asteraceae Bercht. & J.Presl
Iz_10_1209	DJI_0662	Chamaecytisus ruthenicus (Fisch.ex Wol.) Klaskova	0	n	1	0	Fabaceae Lindl.
Iz_10_1209	DJI_0662	Chelidonium majus L.	0	n	1	0	Papaveraceae Juss.
Iz_10_1209	DJI_0662	Chenopodium betaceum Andrz.	0	n	1	0	Amaranthaceae Juss.
Iz_10_1209	DJI_0662	Chenopodium cf. album	0	n	1	0	Amaranthaceae Juss.
Iz_10_1209	DJI_0662	Chenopodium sp.	0		NA	0	Amaranthaceae Juss.
Iz_10_1209	DJI_0662	Chondrilla juncea L.	0	n	1	0	Asteraceae Bercht. & J.Presl
Iz_10_1209	DJI_0662	Cirsium vulgare (Savi) Ten.	0	n	1	0	Asteraceae Bercht. & J.Presl

Iz_10_1209	DJI_0662	Clinopodium acinos (L.) Kuntze	0	n	1	0	Lamiacea Lindl.
Iz_10_1209	DJI_0662	Convallaria majalis L.	0	n	1	0	Asparagaceae Juss.
Iz_10_1209	DJI_0662	Crataegus rhipidophylla Gand.	0	n	1	1	Rosaceae Juss.
Iz_10_1209	DJI_0662	Crepis foetida L.	0	n	1	0	Asteraceae Bercht. & J.Presl
Iz_10_1209	DJI_0662	Dactylis glomerata L.	0	n	1	0	Poaceae Barnhart
Iz_10_1209	DJI_0662	Dianthus campestris M.Bieb.	0	n	1	0	Caryophyllaceae Juss.
Iz_10_1209	DJI_0662	Digitaria sanguinalis (L.) Scop.	0	a	0	0	Poaceae Barnhart
Iz_10_1209	DJI_0662	Elymus repens (L.) Gould	0	n	1	0	Poaceae Barnhart
Iz_10_1209	DJI_0662	Epilobium tetragonum L.	0	n	1	0	Onagraceae Juss.
Iz_10_1209	DJI_0662	Eragrostis minor Host	0	a	0	0	Poaceae Barnhart
Iz_10_1209	DJI_0662	Erigeron canadensis L.	6	a	0	0	Asteraceae Bercht. & J.Presl
Iz_10_1209	DJI_0662	Euphorbia seguieriana Neck.	0	n	1	0	Euphorbiaceae Juss.
Iz_10_1209	DJI_0662	Fallopia dumetorum (L.) Holub	0	n	1	0	Polygonaceae Juss.
Iz_10_1209	DJI_0662	Festuca beckeri (Hack.) Trautv.	0	n	1	0	Poaceae Barnhart
Iz_10_1209	DJI_0662	Fragaria vesca L.	0	n	1	0	Rosaceae Juss.
Iz_10_1209	DJI_0662	Frangula alnus Mill.	0	n	1	1	Rhamnaceae Juss.
Iz_10_1209	DJI_0662	Fraxinus pennsylvanica Marshall	0	a	0	1	Oleaceae Hoffmanns. & Link
Iz_10_1209	DJI_0662	Galeopsis bifida Boenn.	0	n	1	0	Lamiacea Lindl.
Iz_10_1209	DJI_0662	Galium verum L.	0	n	1	0	Rubiaceae Juss.
Iz_10_1209	DJI_0662	Genista tinctoria L.	0	n	1	0	Fabaceae Lindl.
Iz_10_1209	DJI_0662	Geranium robertianum L.	0	n	1	0	Geraniaceae Juss.
Iz_10_1209	DJI_0662	Geranium sanquineum L.	0	n	1	0	Geraniaceae Juss.
Iz_10_1209	DJI_0662	Gypsophila paniculata L.	1	n	1	0	Caryophyllaceae Juss.
Iz_10_1209	DJI_0662	Helichrysum arenarium (L.) Moench	0	n	1	0	Asteraceae Bercht. & J.Presl
Iz_10_1209	DJI_0662	Herniaria polygama J.Gay	0	n	1	0	Caryophyllaceae Juss.
Iz_10_1209	DJI_0662	Hieracium robustum Fr.	0	n	1	0	Asteraceae Bercht. & J.Presl
Iz_10_1209	DJI_0662	Hieracium umbellatum L.	0	n	1	NA	Asteraceae Bercht. & J.Presl
Iz_10_1209	DJI_0662	Hylotelephium maximum (L.) Holub	0	n	1	0	Crassulaceae J.St.-Hil.
Iz_10_1209	DJI_0662	Jacobaea borysthenica (DC.) B.Nord. & Greuter	0	n	1	0	Asteraceae Bercht. & J.Presl
Iz_10_1209	DJI_0662	Jasione montana L.	0	n	1	0	Campanulaceae Juss.
Iz_10_1209	DJI_0662	Jurinea cyanoides (L.) Rchb.s.l.	0	n	1	0	Asteraceae Bercht. & J.Presl
Iz_10_1209	DJI_0662	Koeleria glauca (Spreng.) DC.	0	n	1	0	Poaceae Barnhart
Iz_10_1209	DJI_0662	Lactuca serriola L.	0	a	0	0	Asteraceae Bercht. & J.Presl
Iz_10_1209	DJI_0662	Linaria genistifolia (L.) Mill.	0	n	1	0	Plantaginaceae Juss.
Iz_10_1209	DJI_0662	Lomelosia argentea (L.) Greuter & Burdet	0	n	1	0	Caprifoliaceae Juss.
Iz_10_1209	DJI_0662	Melampyrum pratense L.	0	n	1	0	Orobanchaceae Vent.
Iz_10_1209	DJI_0662	Oenothera biennis L.	0	a	0	0	Onagraceae Juss.
Iz_10_1209	DJI_0662	Pentanema britannica (L.) D.Gut.Larr., Santos-Vicente, Anderb., E.Rico & M.M.Mart.Ort.	0	n	1	0	Asteraceae Bercht. & J.Presl
Iz_10_1209	DJI_0662	Peucedanum oreoselinum (L.) Moench	0	n	1	0	Apiaceae Lindl.
Iz_10_1209	DJI_0662	Peucedanum ruthenicum M.Bieb.	0	n	1	0	Apiaceae Lindl.
Iz_10_1209	DJI_0662	Pilosella echioides (Lumn.) F.W.Schultz & Sch.Bip.	0	n	1	0	Asteraceae Bercht. & J.Presl
Iz_10_1209	DJI_0662	Pilosella officinarum F. Schultz et Sch. Bip.	0	n	1	0	Asteraceae Bercht. & J.Presl
Iz_10_1209	DJI_0662	Pilosella sp.	0		NA	0	Asteraceae Bercht. & J.Presl
Iz_10_1209	DJI_0662	Pinus sylvestris L.	0	n	1	1	Pinaceae Spreng. ex F.Rudolphi
Iz_10_1209	DJI_0662	Poa annua L.	0	n	1	0	Poaceae Barnhart
Iz_10_1209	DJI_0662	Poa nemoralis L.	0	n	1	0	Poaceae Barnhart
Iz_10_1209	DJI_0662	Poa sp.	0		NA	0	Poaceae Barnhart
Iz_10_1209	DJI_0662	Polygonatum odoratum (Mill.) Druce	0	n	1	0	Asparagaceae Juss.
Iz_10_1209	DJI_0662	Polygonum arenarium Waldst. & Kit.	0	n	1	0	Polygonaceae Juss.
Iz_10_1209	DJI_0662	Populus nigra L.	0	n	1	1	Salicaceae Mirb.
Iz_10_1209	DJI_0662	Populus tremula L.	0	n	1	1	Salicaceae Mirb.
Iz_10_1209	DJI_0662	Populus Ã canescens (Aiton) Sm.	0	n	1	1	Salicaceae Mirb.
Iz_10_1209	DJI_0662	Potentilla argentea L.	0	n	1	0	Rosaceae Juss.
Iz_10_1209	DJI_0662	Psammophiliella muralis (L.) Ikonn.	0	n	1	0	Caryophyllaceae Juss.
Iz_10_1209	DJI_0662	Psephellus sumensis (Kalen.) Greuter	0	n	1	0	Asteraceae Bercht. & J.Presl
Iz_10_1209	DJI_0662	Pulsatilla pratensis (L.) Mill. s.l.	0	n	1	0	Ranunculaceae Juss.
Iz_10_1209	DJI_0662	Pyrus communis L.	0	a	0	1	Rosaceae Juss.
Iz_10_1209	DJI_0662	Quercus robur L.	0	n	1	1	Fagaceae Dumort.
Iz_10_1209	DJI_0662	Robinia pseudoacacia L.	0	a	0	1	Fabaceae Lindl.
Iz_10_1209	DJI_0662	Rosa sp.	0		NA	1	Rosaceae Juss.
Iz_10_1209	DJI_0662	Rubus idaeus L.	0	n	1	0	Rosaceae Juss.
Iz_10_1209	DJI_0662	Rumex acetosella L.	2	n	1	0	Polygonaceae Juss.
Iz_10_1209	DJI_0662	Rumex thyrsiflorus Fingerh.	0	n	1	0	Polygonaceae Juss.
Iz_10_1209	DJI_0662	Salix alba L.	0	n	1	1	Salicaceae Mirb.
Iz_10_1209	DJI_0662	Sambucus nigra L.	0	n	1	1	Viburnaceae Raf.
Iz_10_1209	DJI_0662	Secale sylvestre Host	0	n	1	0	Poaceae Barnhart
Iz_10_1209	DJI_0662	Sempervivum ruthenicum Schnittsp. & C.B. Lehm.	0	n	1	0	Crassulaceae J.St.-Hil.
Iz_10_1209	DJI_0662	Setaria viridis (L.) P. Beauv.	0	n	1	0	Poaceae Barnhart
Iz_10_1209	DJI_0662	Silene borysthenica (Gruner) Walters	0	n	1	0	Caryophyllaceae Juss.
Iz_10_1209	DJI_0662	Silene latifolia subsp. alba (Mill.) Greuter & Burdet	0	n	1	0	Caryophyllaceae Juss.
Iz_10_1209	DJI_0662	Solidago virgaurea L.	0	n	1	0	Asteraceae Bercht. & J.Presl
Iz_10_1209	DJI_0662	Sorbus aucuparia L.	0	n	1	1	Rosaceae Juss.
Iz_10_1209	DJI_0662	Stachys recta L.	0	n	1	0	Lamiacea Lindl.
Iz_10_1209	DJI_0662	Tanacetum vulgare L.	0	n	1	0	Asteraceae Bercht. & J.Presl
Iz_10_1209	DJI_0662	Thymus pallasianus Heinr.Braun	0	n	1	0	Lamiacea Lindl.
Iz_10_1209	DJI_0662	Tilia cordata Mill.	0	n	1	1	Malvaceae Juss.
Iz_10_1209	DJI_0662	Trifolium alpestre L.	0	n	1	0	Fabaceae Lindl.
Iz_10_1209	DJI_0662	Trifolium arvense L.	0	n	1	0	Fabaceae Lindl.
Iz_10_1209	DJI_0662	Ulmus glabra Huds.	0	n	1	1	Ulmaceae Mirb.
Iz_10_1209	DJI_0662	Verbascum densiflorum Bertol.	0	n	1	0	Scrophulariaceae Juss.
Iz_10_1209	DJI_0662	Verbascum sp.	0	n	1	0	Scrophulariaceae Juss.
Iz_10_1209	DJI_0662	Veronica austriaca L.	0	n	1	0	Plantaginaceae Juss.
Iz_10_1209	DJI_0662	Veronica incana L.	0	n	1	0	Plantaginaceae Juss.
Iz_10_1209	DJI_0662	Veronica verna L.	0	n	1	0	Plantaginaceae Juss.
Iz_10_1209	DJI_0662	Vincetoxicum hirundinaria Medik	0	n	1	0	Apocynaceae Juss.
Iz_10_1209	DJI_0666	Acer tataricum L.	0	n	1	1	Sapindaceae Juss.
Iz_10_1209	DJI_0666	Achillea millefolium L. s.l.	0	n	1	0	Asteraceae Bercht. & J.Presl
Iz_10_1209	DJI_0666	Allium oleraceum L.	0	n	1	0	Amaryllidaceae J.St.-Hil.
Iz_10_1209	DJI_0666	Ambrosia artemisiifolia L.	0	a	0	0	Asteraceae Bercht. & J.Presl
Iz_10_1209	DJI_0666	Amorpha fruticosa L.	0	a	0	0	Fabaceae Lindl.
Iz_10_1209	DJI_0666	Anthericum ramosum L.	0	n	1	0	Asparagaceae Juss.
Iz_10_1209	DJI_0666	Artemisia marschalliana Spreng.	0	n	1	0	Asteraceae Bercht. & J.Presl
Iz_10_1209	DJI_0666	Asparagus officinalis L.	0	n	1	0	Asparagaceae Juss.
Iz_10_1209	DJI_0666	Astragalus cicer L.	0	n	1	0	Fabaceae Lindl.
Iz_10_1209	DJI_0666	Bassia laniflora (S.G.Gmel.) A.J.Scott	0	n	1	0	Amaranthaceae Juss.
Iz_10_1209	DJI_0666	Berberis vulgaris L.	0	n	1	0	Berberidaceae Juss.
Iz_10_1209	DJI_0666	Betula pendula Roth.	0	n	1	1	Betulaceae Gray
Iz_10_1209	DJI_0666	Betula pendula Roth.	0	n	1	0	Poaceae Barnhart

Iz_10_1209	DJI_0666	Bromus inermis Leyss.	0		NA	NA	
Iz_10_1209	DJI_0666	Bromus squarrosus L.	0	a	0	0	Poaceae Barnhart
Iz_10_1209	DJI_0666	Bromus tectorum L.	0	a	0	0	Poaceae Barnhart
Iz_10_1209	DJI_0666	Calamagrostis epigejos (L.) Roth	25	n	1	0	Poaceae Barnhart
Iz_10_1209	DJI_0666	Carduus acanthoides L.	0	n	1	0	Asteraceae Bercht. & J.Presl
Iz_10_1209	DJI_0666	Carex sp.	0	n	1	0	Cyperaceae Juss.
Iz_10_1209	DJI_0666	Centaurea majorovii Dumbadze	0	n	1	0	Asteraceae Bercht. & J.Presl
Iz_10_1209	DJI_0666	Chamaecytisus ruthenicus (Fisch.ex Wol.) Klaskova	0	n	1	0	Fabaceae Lindl.
Iz_10_1209	DJI_0666	Chelidonium majus L.	0	n	1	0	Papaveraceae Juss.
Iz_10_1209	DJI_0666	Chenopodium betaceum Andrz.	0	n	1	0	Amaranthaceae Juss.
Iz_10_1209	DJI_0666	Chenopodium cf. album	0	n	1	0	Amaranthaceae Juss.
Iz_10_1209	DJI_0666	Chenopodium sp.	0		NA	0	Amaranthaceae Juss.
Iz_10_1209	DJI_0666	Chondrilla juncea L.	0	n	1	0	Asteraceae Bercht. & J.Presl
Iz_10_1209	DJI_0666	Cirsium vulgare (Savi) Ten.	0	n	1	0	Asteraceae Bercht. & J.Presl
Iz_10_1209	DJI_0666	Clinopodium acinos (L.) Kuntze	0	n	1	0	Lamiacea Lindl.
Iz_10_1209	DJI_0666	Convallaria majalis L.	0	n	1	0	Asparagaceae Juss.
Iz_10_1209	DJI_0666	Crataegus rhipidophylla Gand.	0	n	1	1	Rosaceae Juss.
Iz_10_1209	DJI_0666	Crepis foetida L.	0	n	1	0	Asteraceae Bercht. & J.Presl
Iz_10_1209	DJI_0666	Dactylis glomerata L.	0	n	1	0	Poaceae Barnhart
Iz_10_1209	DJI_0666	Dianthus campestris M.Bieb.	0	n	1	0	Caryophyllaceae Juss.
Iz_10_1209	DJI_0666	Digitaria sanguinalis (L.) Scop.	0	a	0	0	Poaceae Barnhart
Iz_10_1209	DJI_0666	Elymus repens (L.) Gould	0	n	1	0	Poaceae Barnhart
Iz_10_1209	DJI_0666	Epilobium tetragonum L.	0	n	1	0	Onagraceae Juss.
Iz_10_1209	DJI_0666	Eragrostis minor Host	0	a	0	0	Poaceae Barnhart
Iz_10_1209	DJI_0666	Erigeron canadensis L.	0.5	a	0	0	Asteraceae Bercht. & J.Presl
Iz_10_1209	DJI_0666	Euphorbia seguieriana Neck.	0	n	1	0	Euphorbiaceae Juss.
Iz_10_1209	DJI_0666	Fallopia dumetorum (L.) Holub	0	n	1	0	Polygonaceae Juss.
Iz_10_1209	DJI_0666	Festuca beckeri (Hack.) Trautv.	0	n	1	0	Poaceae Barnhart
Iz_10_1209	DJI_0666	Fragaria vesca L.	0	n	1	0	Rosaceae Juss.
Iz_10_1209	DJI_0666	Frangula alnus Mill.	0	n	1	1	Rhamnaceae Juss.
Iz_10_1209	DJI_0666	Fraxinus pennsylvanica Marshall	0	a	0	1	Oleaceae Hoffmanns. & Link
Iz_10_1209	DJI_0666	Galeopsis bifida Boenn.	0	n	1	0	Lamiacea Lindl.
Iz_10_1209	DJI_0666	Galium verum L.	0	n	1	0	Rubiaceae Juss.
Iz_10_1209	DJI_0666	Genista tinctoria L.	0	n	1	0	Fabaceae Lindl.
Iz_10_1209	DJI_0666	Geranium robertianum L.	0	n	1	0	Geraniaceae Juss.
Iz_10_1209	DJI_0666	Geranium sanquineum L.	0	n	1	0	Geraniaceae Juss.
Iz_10_1209	DJI_0666	Gypsophila paniculata L.	0	n	1	0	Caryophyllaceae Juss.
Iz_10_1209	DJI_0666	Helichrysum arenarium (L.) Moench	0	n	1	0	Asteraceae Bercht. & J.Presl
Iz_10_1209	DJI_0666	Herniaria polygama J.Gay	0	n	1	0	Caryophyllaceae Juss.
Iz_10_1209	DJI_0666	Hieracium robustum Fr.	0	n	1	0	Asteraceae Bercht. & J.Presl
Iz_10_1209	DJI_0666	Hieracium umbellatum L.	0	n	1	NA	Asteraceae Bercht. & J.Presl
Iz_10_1209	DJI_0666	Hylotelephium maximum (L.) Holub	0	n	1	0	Crassulaceae J.St.-Hil.
Iz_10_1209	DJI_0666	Jacobaea borysthenica (DC.) B.Nord. & Greuter	0	n	1	0	Asteraceae Bercht. & J.Presl
Iz_10_1209	DJI_0666	Jasione montana L.	0	n	1	0	Campanulaceae Juss.
Iz_10_1209	DJI_0666	Jurinea cyanoides (L.) Rchb.s.l.	0	n	1	0	Asteraceae Bercht. & J.Presl
Iz_10_1209	DJI_0666	Koeleria glauca (Spreng.) DC.	0	n	1	0	Poaceae Barnhart
Iz_10_1209	DJI_0666	Lactuca serriola L.	0	a	0	0	Asteraceae Bercht. & J.Presl
Iz_10_1209	DJI_0666	Linaria genistifolia (L.) Mill.	0	n	1	0	Plantaginaceae Juss.
Iz_10_1209	DJI_0666	Lomelosia argentea (L.) Greuter & Burdet	0	n	1	0	Caprifoliaceae Juss.
Iz_10_1209	DJI_0666	Melampyrum pratense L.	0	n	1	0	Orobanchaceae Vent.
Iz_10_1209	DJI_0666	Oenothera biennis L.	0	a	0	0	Onagraceae Juss.
Iz_10_1209	DJI_0666	Pentanema britannica (L.) D.Gut.Larr., Santos-Vicente, Anderb., E.Rico & M.M.Mart.Ort.	0	n	1	0	Asteraceae Bercht. & J.Presl
Iz_10_1209	DJI_0666	Peucedanum oreoselinum (L.) Moench	0	n	1	0	Apiaceae Lindl.
Iz_10_1209	DJI_0666	Peucedanum ruthenicum M.Bieb.	0	n	1	0	Apiaceae Lindl.
Iz_10_1209	DJI_0666	Pilosella echioides (Lumn.) F.W.Schultz & Sch.Bip.	0	n	1	0	Asteraceae Bercht. & J.Presl
Iz_10_1209	DJI_0666	Pilosella officinarum F. Schultz et Sch. Bip.	0	n	1	0	Asteraceae Bercht. & J.Presl
Iz_10_1209	DJI_0666	Pilosella sp.	0		NA	0	Asteraceae Bercht. & J.Presl
Iz_10_1209	DJI_0666	Pinus sylvestris L.	0	n	1	1	Pinaceae Spreng. ex F.Rudolphi
Iz_10_1209	DJI_0666	Poa annua L.	0	n	1	0	Poaceae Barnhart
Iz_10_1209	DJI_0666	Poa nemoralis L.	0	n	1	0	Poaceae Barnhart
Iz_10_1209	DJI_0666	Poa sp.	0		NA	0	Poaceae Barnhart
Iz_10_1209	DJI_0666	Polygonatum odoratum (Mill.) Druce	0	n	1	0	Asparagaceae Juss.
Iz_10_1209	DJI_0666	Polygonum arenarium Waldst. & Kit.	0	n	1	0	Polygonaceae Juss.
Iz_10_1209	DJI_0666	Populus nigra L.	0	n	1	1	Salicaceae Mirb.
Iz_10_1209	DJI_0666	Populus tremula L.	0	n	1	1	Salicaceae Mirb.
Iz_10_1209	DJI_0666	Populus Ã canescens (Aiton) Sm.	0	n	1	1	Salicaceae Mirb.
Iz_10_1209	DJI_0666	Potentilla argentea L.	0	n	1	0	Rosaceae Juss.
Iz_10_1209	DJI_0666	Psammophiliella muralis (L.) Ikonn.	0	n	1	0	Caryophyllaceae Juss.
Iz_10_1209	DJI_0666	Psephellus sumensis (Kalen.) Greuter	1	n	1	0	Asteraceae Bercht. & J.Presl
Iz_10_1209	DJI_0666	Pulsatilla pratensis (L.) Mill. s.l.	0	n	1	0	Ranunculaceae Juss.
Iz_10_1209	DJI_0666	Pyrus communis L.	0	a	0	1	Rosaceae Juss.
Iz_10_1209	DJI_0666	Quercus robur L.	0	n	1	1	Fagaceae Dumort.
Iz_10_1209	DJI_0666	Robinia pseudoacacia L.	0	a	0	1	Fabaceae Lindl.
Iz_10_1209	DJI_0666	Rosa sp.	0		NA	1	Rosaceae Juss.
Iz_10_1209	DJI_0666	Rubus idaeus L.	0	n	1	0	Rosaceae Juss.
Iz_10_1209	DJI_0666	Rumex acetosella L.	0	n	1	0	Polygonaceae Juss.
Iz_10_1209	DJI_0666	Rumex thyrsiflorus Fingerh.	0	n	1	0	Polygonaceae Juss.
Iz_10_1209	DJI_0666	Salix alba L.	0	n	1	1	Salicaceae Mirb.
Iz_10_1209	DJI_0666	Sambucus nigra L.	0	n	1	1	Viburnaceae Raf.
Iz_10_1209	DJI_0666	Secale sylvestre Host	0	n	1	0	Poaceae Barnhart
Iz_10_1209	DJI_0666	Sempervivum ruthenicum Schnittsp. & C.B. Lehm.	0	n	1	0	Crassulaceae J.St.-Hil.
Iz_10_1209	DJI_0666	Setaria viridis (L.) P. Beauv.	0	n	1	0	Poaceae Barnhart
Iz_10_1209	DJI_0666	Silene borysthenica (Gruner) Walters	0	n	1	0	Caryophyllaceae Juss.
Iz_10_1209	DJI_0666	Silene latifolia subsp. alba (Mill.) Greuter & Burdet	0	n	1	0	Caryophyllaceae Juss.
Iz_10_1209	DJI_0666	Solidago virgaurea L.	0	n	1	0	Asteraceae Bercht. & J.Presl
Iz_10_1209	DJI_0666	Sorbus aucuparia L.	0	n	1	1	Rosaceae Juss.
Iz_10_1209	DJI_0666	Stachys recta L.	0	n	1	0	Lamiacea Lindl.
Iz_10_1209	DJI_0666	Tanacetum vulgare L.	0	n	1	0	Asteraceae Bercht. & J.Presl
Iz_10_1209	DJI_0666	Thymus pallasianus Heinr.Braun	0	n	1	0	Lamiacea Lindl.
Iz_10_1209	DJI_0666	Tilia cordata Mill.	0	n	1	1	Malvaceae Juss.
Iz_10_1209	DJI_0666	Trifolium alpestre L.	0	n	1	0	Fabaceae Lindl.
Iz_10_1209	DJI_0666	Trifolium arvense L.	0	n	1	0	Fabaceae Lindl.
Iz_10_1209	DJI_0666	Ulmus glabra Huds.	0	n	1	1	Ulmaceae Mirb.
Iz_10_1209	DJI_0666	Verbascum densiflorum Bertol.	0	n	1	0	Scrophulariaceae Juss.
Iz_10_1209	DJI_0666	Verbascum sp.	0	n	1	0	Scrophulariaceae Juss.
Iz_10_1209	DJI_0666	Veronica austriaca L.	0	n	1	0	Plantaginaceae Juss.
Iz_10_1209	DJI_0666	Veronica incana L.	0	n	1	0	Plantaginaceae Juss.
Iz_10_1209	DJI_0666	Veronica verna L.	0	n	1	0	Plantaginaceae Juss.

Iz_10_1209	DJI_0666	Vincetoxicum hirundinaria Medik	0	n	1	0	Apocynaceae Juss.
Iz_10_1209	DJI_0669	Acer tataricum L.	0	n	1	1	Sapindaceae Juss.
Iz_10_1209	DJI_0669	Achillea millefolium L. s.l.	0	n	1	0	Asteraceae Bercht. & J.Presl
Iz_10_1209	DJI_0669	Allium oleraceum L.	0	n	1	0	Amaryllidaceae J.St.-Hil.
Iz_10_1209	DJI_0669	Ambrosia artemisiifolia L.	0	a	0	0	Asteraceae Bercht. & J.Presl
Iz_10_1209	DJI_0669	Amorpha fruticosa L.	0	a	0	0	Fabaceae Lindl.
Iz_10_1209	DJI_0669	Anthericum ramosum L.	0	n	1	0	Asparagaceae Juss.
Iz_10_1209	DJI_0669	Artemisia marschalliana Spreng.	0	n	1	0	Asteraceae Bercht. & J.Presl
Iz_10_1209	DJI_0669	Asparagus officinalis L.	0	n	1	0	Asparagaceae Juss.
Iz_10_1209	DJI_0669	Astragalus cicer L.	0	n	1	0	Fabaceae Lindl.
Iz_10_1209	DJI_0669	Bassia laniflora (S.G.Gmel.) A.J.Scott	0	n	1	0	Amaranthaceae Juss.
Iz_10_1209	DJI_0669	Berberis vulgaris L.	0	n	1	0	Berberidaceae Juss.
Iz_10_1209	DJI_0669	Betula pendula Roth.	0	n	1	1	Betulaceae Gray
Iz_10_1209	DJI_0669	Betula pendula Roth.	0	n	1	0	Poaceae Barnhart
Iz_10_1209	DJI_0669	Bromus inermis Leyss.	0		NA	NA	
Iz_10_1209	DJI_0669	Bromus squarrosus L.	0	a	0	0	Poaceae Barnhart
Iz_10_1209	DJI_0669	Bromus tectorum L.	0	a	0	0	Poaceae Barnhart
Iz_10_1209	DJI_0669	Calamagrostis epigejos (L.) Roth	10	n	1	0	Poaceae Barnhart
Iz_10_1209	DJI_0669	Carduus acanthoides L.	0	n	1	0	Asteraceae Bercht. & J.Presl
Iz_10_1209	DJI_0669	Carex sp.	0	n	1	0	Cyperaceae Juss.
Iz_10_1209	DJI_0669	Centaurea majorovii Dumbadze	0	n	1	0	Asteraceae Bercht. & J.Presl
Iz_10_1209	DJI_0669	Chamaecytisus ruthenicus (Fisch.ex Wol.) Klaskova	0	n	1	0	Fabaceae Lindl.
Iz_10_1209	DJI_0669	Chelidonium majus L.	0	n	1	0	Papaveraceae Juss.
Iz_10_1209	DJI_0669	Chenopodium betaceum Andrz.	0	n	1	0	Amaranthaceae Juss.
Iz_10_1209	DJI_0669	Chenopodium cf. album	0	n	1	0	Amaranthaceae Juss.
Iz_10_1209	DJI_0669	Chenopodium sp.	0		NA	0	Amaranthaceae Juss.
Iz_10_1209	DJI_0669	Chondrilla juncea L.	0	n	1	0	Asteraceae Bercht. & J.Presl
Iz_10_1209	DJI_0669	Cirsium vulgare (Savi) Ten.	0	n	1	0	Asteraceae Bercht. & J.Presl
Iz_10_1209	DJI_0669	Clinopodium acinos (L.) Kuntze	0	n	1	0	Lamiacea Lindl.
Iz_10_1209	DJI_0669	Convallaria majalis L.	0	n	1	0	Asparagaceae Juss.
Iz_10_1209	DJI_0669	Crataegus rhipidophylla Gand.	0	n	1	1	Rosaceae Juss.
Iz_10_1209	DJI_0669	Crepis foetida L.	0	n	1	0	Asteraceae Bercht. & J.Presl
Iz_10_1209	DJI_0669	Dactylis glomerata L.	0	n	1	0	Poaceae Barnhart
Iz_10_1209	DJI_0669	Dianthus campestris M.Bieb.	0	n	1	0	Caryophyllaceae Juss.
Iz_10_1209	DJI_0669	Digitaria sanguinalis (L.) Scop.	0	a	0	0	Poaceae Barnhart
Iz_10_1209	DJI_0669	Elymus repens (L.) Gould	0	n	1	0	Poaceae Barnhart
Iz_10_1209	DJI_0669	Epilobium tetragonum L.	0	n	1	0	Onagraceae Juss.
Iz_10_1209	DJI_0669	Eragrostis minor Host	0	a	0	0	Poaceae Barnhart
Iz_10_1209	DJI_0669	Erigeron canadensis L.	15	a	0	0	Asteraceae Bercht. & J.Presl
Iz_10_1209	DJI_0669	Euphorbia seguieriana Neck.	0	n	1	0	Euphorbiaceae Juss.
Iz_10_1209	DJI_0669	Fallopia dumetorum (L.) Holub	0	n	1	0	Polygonaceae Juss.
Iz_10_1209	DJI_0669	Festuca beckeri (Hack.) Trautv.	0	n	1	0	Poaceae Barnhart
Iz_10_1209	DJI_0669	Fragaria vesca L.	0	n	1	0	Rosaceae Juss.
Iz_10_1209	DJI_0669	Frangula alnus Mill.	0	n	1	1	Rhamnaceae Juss.
Iz_10_1209	DJI_0669	Fraxinus pennsylvanica Marshall	0	a	0	1	Oleaceae Hoffmanns. & Link
Iz_10_1209	DJI_0669	Galeopsis bifida Boenn.	0	n	1	0	Lamiacea Lindl.
Iz_10_1209	DJI_0669	Galium verum L.	0	n	1	0	Rubiaceae Juss.
Iz_10_1209	DJI_0669	Genista tinctoria L.	0	n	1	0	Fabaceae Lindl.
Iz_10_1209	DJI_0669	Geranium robertianum L.	0	n	1	0	Geraniaceae Juss.
Iz_10_1209	DJI_0669	Geranium sanquineum L.	0	n	1	0	Geraniaceae Juss.
Iz_10_1209	DJI_0669	Gypsophila paniculata L.	0	n	1	0	Caryophyllaceae Juss.
Iz_10_1209	DJI_0669	Helichrysum arenarium (L.) Moench	0	n	1	0	Asteraceae Bercht. & J.Presl
Iz_10_1209	DJI_0669	Herniaria polygama J.Gay	0	n	1	0	Caryophyllaceae Juss.
Iz_10_1209	DJI_0669	Hieracium robustum Fr.	0	n	1	0	Asteraceae Bercht. & J.Presl
Iz_10_1209	DJI_0669	Hieracium umbellatum L.	0	n	1	NA	Asteraceae Bercht. & J.Presl
Iz_10_1209	DJI_0669	Hylotelephium maximum (L.) Holub	0	n	1	0	Crassulaceae J.St.-Hil.
Iz_10_1209	DJI_0669	Jacobaea borysthenica (DC.) B.Nord. & Greuter	0	n	1	0	Asteraceae Bercht. & J.Presl
Iz_10_1209	DJI_0669	Jasione montana L.	0	n	1	0	Campanulaceae Juss.
Iz_10_1209	DJI_0669	Jurinea cyanoides (L.) Rchb.s.l.	0	n	1	0	Asteraceae Bercht. & J.Presl
Iz_10_1209	DJI_0669	Koeleria glauca (Spreng.) DC.	0	n	1	0	Poaceae Barnhart
Iz_10_1209	DJI_0669	Lactuca serriola L.	0	a	0	0	Asteraceae Bercht. & J.Presl
Iz_10_1209	DJI_0669	Linaria genistifolia (L.) Mill.	0	n	1	0	Plantaginaceae Juss.
Iz_10_1209	DJI_0669	Lomelosia argentea (L.) Greuter & Burdet	0	n	1	0	Caprifoliaceae Juss.
Iz_10_1209	DJI_0669	Melampyrum pratense L.	0	n	1	0	Orobanchaceae Vent.
Iz_10_1209	DJI_0669	Oenothera biennis L.	0	a	0	0	Onagraceae Juss.
Iz_10_1209	DJI_0669	Pentanema britannica (L.) D.Gut.Larr., Santos-Vicente, Anderb., E.Rico & M.M.Mart.Ort.	0	n	1	0	Asteraceae Bercht. & J.Presl
Iz_10_1209	DJI_0669	Peucedanum oreoselinum (L.) Moench	0	n	1	0	Apiaceae Lindl.
Iz_10_1209	DJI_0669	Peucedanum ruthenicum M.Bieb.	0	n	1	0	Apiaceae Lindl.
Iz_10_1209	DJI_0669	Pilosella echioides (Lumn.) F.W.Schultz & Sch.Bip.	0	n	1	0	Asteraceae Bercht. & J.Presl
Iz_10_1209	DJI_0669	Pilosella officinarum F. Schultz et Sch. Bip.	0	n	1	0	Asteraceae Bercht. & J.Presl
Iz_10_1209	DJI_0669	Pilosella sp.	0		NA	0	Asteraceae Bercht. & J.Presl
Iz_10_1209	DJI_0669	Pinus sylvestris L.	0	n	1	1	Pinaceae Spreng. ex F.Rudolphi
Iz_10_1209	DJI_0669	Poa annua L.	0	n	1	0	Poaceae Barnhart
Iz_10_1209	DJI_0669	Poa nemoralis L.	0	n	1	0	Poaceae Barnhart
Iz_10_1209	DJI_0669	Poa sp.	0		NA	0	Poaceae Barnhart
Iz_10_1209	DJI_0669	Polygonatum odoratum (Mill.) Druce	0	n	1	0	Asparagaceae Juss.
Iz_10_1209	DJI_0669	Polygonum arenarium Waldst. & Kit.	0	n	1	0	Polygonaceae Juss.
Iz_10_1209	DJI_0669	Populus nigra L.	0	n	1	1	Salicaceae Mirb.
Iz_10_1209	DJI_0669	Populus tremula L.	0	n	1	1	Salicaceae Mirb.
Iz_10_1209	DJI_0669	Populus Ã canescens (Aiton) Sm.	0	n	1	1	Salicaceae Mirb.
Iz_10_1209	DJI_0669	Potentilla argentea L.	0	n	1	0	Rosaceae Juss.
Iz_10_1209	DJI_0669	Psammophiliella muralis (L.) Ikonn.	0	n	1	0	Caryophyllaceae Juss.
Iz_10_1209	DJI_0669	Psephellus sumensis (Kalen.) Greuter	0	n	1	0	Asteraceae Bercht. & J.Presl
Iz_10_1209	DJI_0669	Pulsatilla pratensis (L.) Mill. s.l.	0	n	1	0	Ranunculaceae Juss.
Iz_10_1209	DJI_0669	Pyrus communis L.	0	a	0	1	Rosaceae Juss.
Iz_10_1209	DJI_0669	Quercus robur L.	0	n	1	1	Fagaceae Dumort.
Iz_10_1209	DJI_0669	Robinia pseudoacacia L.	0	a	0	1	Fabaceae Lindl.
Iz_10_1209	DJI_0669	Rosa sp.	0		NA	1	Rosaceae Juss.
Iz_10_1209	DJI_0669	Rubus idaeus L.	0	n	1	0	Rosaceae Juss.
Iz_10_1209	DJI_0669	Rumex acetosella L.	0	n	1	0	Polygonaceae Juss.
Iz_10_1209	DJI_0669	Rumex thyrsiflorus Fingerh.	0	n	1	0	Polygonaceae Juss.
Iz_10_1209	DJI_0669	Salix alba L.	0	n	1	1	Salicaceae Mirb.
Iz_10_1209	DJI_0669	Sambucus nigra L.	0	n	1	1	Viburnaceae Raf.
Iz_10_1209	DJI_0669	Secale sylvestre Host	0	n	1	0	Poaceae Barnhart
Iz_10_1209	DJI_0669	Sempervivum ruthenicum Schnittsp. & C.B. Lehm.	0	n	1	0	Crassulaceae J.St.-Hil.
Iz_10_1209	DJI_0669	Setaria viridis (L.) P. Beauv.	0	n	1	0	Poaceae Barnhart
Iz_10_1209	DJI_0669	Silene borysthenica (Gruner) Walters	0	n	1	0	Caryophyllaceae Juss.
Iz_10_1209	DJI_0669	Silene latifolia subsp. alba (Mill.) Greuter & Burdet	0	n	1	0	Caryophyllaceae Juss.

Iz_10_1209	DJI_0669	Solidago virgaurea L.	0	n	1	0	Asteraceae Bercht. & J.Presl
Iz_10_1209	DJI_0669	Sorbus aucuparia L.	0	n	1	1	Rosaceae Juss.
Iz_10_1209	DJI_0669	Stachys recta L.	0	n	1	0	Lamiacea Lindl.
Iz_10_1209	DJI_0669	Tanacetum vulgare L.	0	n	1	0	Asteraceae Bercht. & J.Presl
Iz_10_1209	DJI_0669	Thymus pallasianus Heinr.Braun	0	n	1	0	Lamiacea Lindl.
Iz_10_1209	DJI_0669	Tilia cordata Mill.	0	n	1	1	Malvaceae Juss.
Iz_10_1209	DJI_0669	Trifolium alpestre L.	0	n	1	0	Fabaceae Lindl.
Iz_10_1209	DJI_0669	Trifolium arvense L.	0	n	1	0	Fabaceae Lindl.
Iz_10_1209	DJI_0669	Ulmus glabra Huds.	0	n	1	1	Ulmaceae Mirb.
Iz_10_1209	DJI_0669	Verbascum densiflorum Bertol.	0	n	1	0	Scrophulariaceae Juss.
Iz_10_1209	DJI_0669	Verbascum sp.	0	n	1	0	Scrophulariaceae Juss.
Iz_10_1209	DJI_0669	Veronica austriaca L.	0	n	1	0	Plantaginaceae Juss.
Iz_10_1209	DJI_0669	Veronica incana L.	0	n	1	0	Plantaginaceae Juss.
Iz_10_1209	DJI_0669	Veronica verna L.	0	n	1	0	Plantaginaceae Juss.
Iz_10_1209	DJI_0669	Vincetoxicum hirundinaria Medik	0	n	1	0	Apocynaceae Juss.
Iz_10_1209	DJI_0672	Acer tataricum L.	0	n	1	1	Sapindaceae Juss.
Iz_10_1209	DJI_0672	Achillea millefolium L. s.l.	0	n	1	0	Asteraceae Bercht. & J.Presl
Iz_10_1209	DJI_0672	Allium oleraceum L.	0	n	1	0	Amaryllidaceae J.St.-Hil.
Iz_10_1209	DJI_0672	Ambrosia artemisiifolia L.	0	a	0	0	Asteraceae Bercht. & J.Presl
Iz_10_1209	DJI_0672	Amorpha fruticosa L.	0	a	0	0	Fabaceae Lindl.
Iz_10_1209	DJI_0672	Anthericum ramosum L.	0	n	1	0	Asparagaceae Juss.
Iz_10_1209	DJI_0672	Artemisia marschalliana Spreng.	0	n	1	0	Asteraceae Bercht. & J.Presl
Iz_10_1209	DJI_0672	Asparagus officinalis L.	0	n	1	0	Asparagaceae Juss.
Iz_10_1209	DJI_0672	Astragalus cicer L.	0	n	1	0	Fabaceae Lindl.
Iz_10_1209	DJI_0672	Bassia laniflora (S.G.Gmel.) A.J.Scott	0	n	1	0	Amaranthaceae Juss.
Iz_10_1209	DJI_0672	Berberis vulgaris L.	0	n	1	0	Berberidaceae Juss.
Iz_10_1209	DJI_0672	Betula pendula Roth.	0	n	1	1	Betulaceae Gray
Iz_10_1209	DJI_0672	Betula pendula Roth.	0	n	1	0	Poaceae Barnhart
Iz_10_1209	DJI_0672	Bromus inermis Leyss.	0		NA	NA	
Iz_10_1209	DJI_0672	Bromus squarrosus L.	0	a	0	0	Poaceae Barnhart
Iz_10_1209	DJI_0672	Bromus tectorum L.	0	a	0	0	Poaceae Barnhart
Iz_10_1209	DJI_0672	Calamagrostis epigejos (L.) Roth	0.5	n	1	0	Poaceae Barnhart
Iz_10_1209	DJI_0672	Carduus acanthoides L.	0	n	1	0	Asteraceae Bercht. & J.Presl
Iz_10_1209	DJI_0672	Carex sp.	0	n	1	0	Cyperaceae Juss.
Iz_10_1209	DJI_0672	Centaurea majorovii Dumbadze	0	n	1	0	Asteraceae Bercht. & J.Presl
Iz_10_1209	DJI_0672	Chamaecytisus ruthenicus (Fisch.ex Wol.) Klaskova	0	n	1	0	Fabaceae Lindl.
Iz_10_1209	DJI_0672	Chelidonium majus L.	0	n	1	0	Papaveraceae Juss.
Iz_10_1209	DJI_0672	Chenopodium betaceum Andrz.	0	n	1	0	Amaranthaceae Juss.
Iz_10_1209	DJI_0672	Chenopodium cf. album	0	n	1	0	Amaranthaceae Juss.
Iz_10_1209	DJI_0672	Chenopodium sp.	0		NA	0	Amaranthaceae Juss.
Iz_10_1209	DJI_0672	Chondrilla juncea L.	0	n	1	0	Asteraceae Bercht. & J.Presl
Iz_10_1209	DJI_0672	Cirsium vulgare (Savi) Ten.	0	n	1	0	Asteraceae Bercht. & J.Presl
Iz_10_1209	DJI_0672	Clinopodium acinos (L.) Kuntze	0	n	1	0	Lamiacea Lindl.
Iz_10_1209	DJI_0672	Convallaria majalis L.	0	n	1	0	Asparagaceae Juss.
Iz_10_1209	DJI_0672	Crataegus rhipidophylla Gand.	0	n	1	1	Rosaceae Juss.
Iz_10_1209	DJI_0672	Crepis foetida L.	0	n	1	0	Asteraceae Bercht. & J.Presl
Iz_10_1209	DJI_0672	Dactylis glomerata L.	0	n	1	0	Poaceae Barnhart
Iz_10_1209	DJI_0672	Dianthus campestris M.Bieb.	0	n	1	0	Caryophyllaceae Juss.
Iz_10_1209	DJI_0672	Digitaria sanguinalis (L.) Scop.	0	a	0	0	Poaceae Barnhart
Iz_10_1209	DJI_0672	Elymus repens (L.) Gould	0	n	1	0	Poaceae Barnhart
Iz_10_1209	DJI_0672	Epilobium tetragonum L.	0	n	1	0	Onagraceae Juss.
Iz_10_1209	DJI_0672	Eragrostis minor Host	0	a	0	0	Poaceae Barnhart
Iz_10_1209	DJI_0672	Erigeron canadensis L.	25	a	0	0	Asteraceae Bercht. & J.Presl
Iz_10_1209	DJI_0672	Euphorbia seguieriana Neck.	0	n	1	0	Euphorbiaceae Juss.
Iz_10_1209	DJI_0672	Fallopia dumetorum (L.) Holub	0	n	1	0	Polygonaceae Juss.
Iz_10_1209	DJI_0672	Festuca beckeri (Hack.) Trautv.	0	n	1	0	Poaceae Barnhart
Iz_10_1209	DJI_0672	Fragaria vesca L.	0	n	1	0	Rosaceae Juss.
Iz_10_1209	DJI_0672	Frangula alnus Mill.	0	n	1	1	Rhamnaceae Juss.
Iz_10_1209	DJI_0672	Fraxinus pennsylvanica Marshall	0	a	0	1	Oleaceae Hoffmanns. & Link
Iz_10_1209	DJI_0672	Galeopsis bifida Boenn.	0	n	1	0	Lamiacea Lindl.
Iz_10_1209	DJI_0672	Galium verum L.	0	n	1	0	Rubiaceae Juss.
Iz_10_1209	DJI_0672	Genista tinctoria L.	0	n	1	0	Fabaceae Lindl.
Iz_10_1209	DJI_0672	Geranium robertianum L.	0	n	1	0	Geraniaceae Juss.
Iz_10_1209	DJI_0672	Geranium sanquineum L.	0	n	1	0	Geraniaceae Juss.
Iz_10_1209	DJI_0672	Gypsophila paniculata L.	0	n	1	0	Caryophyllaceae Juss.
Iz_10_1209	DJI_0672	Helichrysum arenarium (L.) Moench	0	n	1	0	Asteraceae Bercht. & J.Presl
Iz_10_1209	DJI_0672	Herniaria polygama J.Gay	0	n	1	0	Caryophyllaceae Juss.
Iz_10_1209	DJI_0672	Hieracium robustum Fr.	0	n	1	0	Asteraceae Bercht. & J.Presl
Iz_10_1209	DJI_0672	Hieracium umbellatum L.	0	n	1	NA	Asteraceae Bercht. & J.Presl
Iz_10_1209	DJI_0672	Hylotelephium maximum (L.) Holub	0	n	1	0	Crassulaceae J.St.-Hil.
Iz_10_1209	DJI_0672	Jacobaea borysthenica (DC.) B.Nord. & Greuter	0	n	1	0	Asteraceae Bercht. & J.Presl
Iz_10_1209	DJI_0672	Jasione montana L.	0	n	1	0	Campanulaceae Juss.
Iz_10_1209	DJI_0672	Jurinea cyanoides (L.) Rchb.s.l.	0	n	1	0	Asteraceae Bercht. & J.Presl
Iz_10_1209	DJI_0672	Koeleria glauca (Spreng.) DC.	0	n	1	0	Poaceae Barnhart
Iz_10_1209	DJI_0672	Lactuca serriola L.	0	a	0	0	Asteraceae Bercht. & J.Presl
Iz_10_1209	DJI_0672	Linaria genistifolia (L.) Mill.	0	n	1	0	Plantaginaceae Juss.
Iz_10_1209	DJI_0672	Lomelosia argentea (L.) Greuter & Burdet	0	n	1	0	Caprifoliaceae Juss.
Iz_10_1209	DJI_0672	Melampyrum pratense L.	0	n	1	0	Orobanchaceae Vent.
Iz_10_1209	DJI_0672	Oenothera biennis L.	0	a	0	0	Onagraceae Juss.
Iz_10_1209	DJI_0672	Pentanema britannica (L.) D.Gut.Larr., Santos-Vicente, Anderb., E.Rico & M.M.Mart.Ort.	0	n	1	0	Asteraceae Bercht. & J.Presl
Iz_10_1209	DJI_0672	Peucedanum oreoselinum (L.) Moench	0	n	1	0	Apiaceae Lindl.
Iz_10_1209	DJI_0672	Peucedanum ruthenicum M.Bieb.	0	n	1	0	Apiaceae Lindl.
Iz_10_1209	DJI_0672	Pilosella echioides (Lumn.) F.W.Schultz & Sch.Bip.	0	n	1	0	Asteraceae Bercht. & J.Presl
Iz_10_1209	DJI_0672	Pilosella officinarum F. Schultz et Sch. Bip.	0	n	1	0	Asteraceae Bercht. & J.Presl
Iz_10_1209	DJI_0672	Pilosella sp.	0		NA	0	Asteraceae Bercht. & J.Presl
Iz_10_1209	DJI_0672	Pinus sylvestris L.	0	n	1	1	Pinaceae Spreng. ex F.Rudolphi
Iz_10_1209	DJI_0672	Poa annua L.	0	n	1	0	Poaceae Barnhart
Iz_10_1209	DJI_0672	Poa nemoralis L.	0	n	1	0	Poaceae Barnhart
Iz_10_1209	DJI_0672	Poa sp.	0		NA	0	Poaceae Barnhart
Iz_10_1209	DJI_0672	Polygonatum odoratum (Mill.) Druce	0	n	1	0	Asparagaceae Juss.
Iz_10_1209	DJI_0672	Polygonum arenarium Waldst. & Kit.	0	n	1	0	Polygonaceae Juss.
Iz_10_1209	DJI_0672	Populus nigra L.	0	n	1	1	Salicaceae Mirb.
Iz_10_1209	DJI_0672	Populus tremula L.	0.5	n	1	1	Salicaceae Mirb.
Iz_10_1209	DJI_0672	Populus Ã canescens (Aiton) Sm.	0	n	1	1	Salicaceae Mirb.
Iz_10_1209	DJI_0672	Potentilla argentea L.	0	n	1	0	Rosaceae Juss.
Iz_10_1209	DJI_0672	Psammophiliella muralis (L.) Ikonn.	0	n	1	0	Caryophyllaceae Juss.
Iz_10_1209	DJI_0672	Psephellus sumensis (Kalen.) Greuter	0	n	1	0	Asteraceae Bercht. & J.Presl
Iz_10_1209	DJI_0672	Pulsatilla pratensis (L.) Mill. s.l.	0	n	1	0	Ranunculaceae Juss.

Iz_10_1209	DJI_0672	Pyrus communis L.	0	a	0	1	Rosaceae Juss.
Iz_10_1209	DJI_0672	Quercus robur L.	0	n	1	1	Fagaceae Dumort.
Iz_10_1209	DJI_0672	Robinia pseudoacacia L.	0	a	0	1	Fabaceae Lindl.
Iz_10_1209	DJI_0672	Rosa sp.	0		NA	1	Rosaceae Juss.
Iz_10_1209	DJI_0672	Rubus idaeus L.	0	n	1	0	Rosaceae Juss.
Iz_10_1209	DJI_0672	Rumex acetosella L.	0	n	1	0	Polygonaceae Juss.
Iz_10_1209	DJI_0672	Rumex thyrsiflorus Fingerh.	0	n	1	0	Polygonaceae Juss.
Iz_10_1209	DJI_0672	Salix alba L.	0	n	1	1	Salicaceae Mirb.
Iz_10_1209	DJI_0672	Sambucus nigra L.	0	n	1	1	Viburnaceae Raf.
Iz_10_1209	DJI_0672	Secale sylvestre Host	0	n	1	0	Poaceae Barnhart
Iz_10_1209	DJI_0672	Sempervivum ruthenicum Schnittsp. & C.B. Lehm.	0	n	1	0	Crassulaceae J.St.-Hil.
Iz_10_1209	DJI_0672	Setaria viridis (L.) P. Beauv.	0	n	1	0	Poaceae Barnhart
Iz_10_1209	DJI_0672	Silene borysthenica (Gruner) Walters	0	n	1	0	Caryophyllaceae Juss.
Iz_10_1209	DJI_0672	Silene latifolia subsp. alba (Mill.) Greuter & Burdet	0	n	1	0	Caryophyllaceae Juss.
Iz_10_1209	DJI_0672	Solidago virgaurea L.	0	n	1	0	Asteraceae Bercht. & J.Presl
Iz_10_1209	DJI_0672	Sorbus aucuparia L.	0	n	1	1	Rosaceae Juss.
Iz_10_1209	DJI_0672	Stachys recta L.	0	n	1	0	Lamiacea Lindl.
Iz_10_1209	DJI_0672	Tanacetum vulgare L.	0	n	1	0	Asteraceae Bercht. & J.Presl
Iz_10_1209	DJI_0672	Thymus pallasianus Heinr.Braun	0	n	1	0	Lamiacea Lindl.
Iz_10_1209	DJI_0672	Tilia cordata Mill.	0	n	1	1	Malvaceae Juss.
Iz_10_1209	DJI_0672	Trifolium alpestre L.	0	n	1	0	Fabaceae Lindl.
Iz_10_1209	DJI_0672	Trifolium arvense L.	0	n	1	0	Fabaceae Lindl.
Iz_10_1209	DJI_0672	Ulmus glabra Huds.	0	n	1	1	Ulmaceae Mirb.
Iz_10_1209	DJI_0672	Verbascum densiflorum Bertol.	0	n	1	0	Scrophulariaceae Juss.
Iz_10_1209	DJI_0672	Verbascum sp.	0	n	1	0	Scrophulariaceae Juss.
Iz_10_1209	DJI_0672	Veronica austriaca L.	0	n	1	0	Plantaginaceae Juss.
Iz_10_1209	DJI_0672	Veronica incana L.	0	n	1	0	Plantaginaceae Juss.
Iz_10_1209	DJI_0672	Veronica verna L.	0	n	1	0	Plantaginaceae Juss.
Iz_10_1209	DJI_0672	Vincetoxicum hirundinaria Medik	0	n	1	0	Apocynaceae Juss.
Iz_10_1209	DJI_0675	Acer tataricum L.	0	n	1	1	Sapindaceae Juss.
Iz_10_1209	DJI_0675	Achillea millefolium L. s.l.	0	n	1	0	Asteraceae Bercht. & J.Presl
Iz_10_1209	DJI_0675	Allium oleraceum L.	0	n	1	0	Amaryllidaceae J.St.-Hil.
Iz_10_1209	DJI_0675	Ambrosia artemisiifolia L.	0	a	0	0	Asteraceae Bercht. & J.Presl
Iz_10_1209	DJI_0675	Amorpha fruticosa L.	0	a	0	0	Fabaceae Lindl.
Iz_10_1209	DJI_0675	Anthericum ramosum L.	0	n	1	0	Asparagaceae Juss.
Iz_10_1209	DJI_0675	Artemisia marschalliana Spreng.	0	n	1	0	Asteraceae Bercht. & J.Presl
Iz_10_1209	DJI_0675	Asparagus officinalis L.	0	n	1	0	Asparagaceae Juss.
Iz_10_1209	DJI_0675	Astragalus cicer L.	0	n	1	0	Fabaceae Lindl.
Iz_10_1209	DJI_0675	Bassia laniflora (S.G.Gmel.) A.J.Scott	0	n	1	0	Amaranthaceae Juss.
Iz_10_1209	DJI_0675	Berberis vulgaris L.	0	n	1	0	Berberidaceae Juss.
Iz_10_1209	DJI_0675	Betula pendula Roth.	0	n	1	1	Betulaceae Gray
Iz_10_1209	DJI_0675	Betula pendula Roth.	0	n	1	0	Poaceae Barnhart
Iz_10_1209	DJI_0675	Bromus inermis Leyss.	0		NA	NA	
Iz_10_1209	DJI_0675	Bromus squarrosus L.	0	a	0	0	Poaceae Barnhart
Iz_10_1209	DJI_0675	Bromus tectorum L.	0	a	0	0	Poaceae Barnhart
Iz_10_1209	DJI_0675	Calamagrostis epigejos (L.) Roth	5	n	1	0	Poaceae Barnhart
Iz_10_1209	DJI_0675	Carduus acanthoides L.	0	n	1	0	Asteraceae Bercht. & J.Presl
Iz_10_1209	DJI_0675	Carex sp.	0	n	1	0	Cyperaceae Juss.
Iz_10_1209	DJI_0675	Centaurea majorovii Dumbadze	0	n	1	0	Asteraceae Bercht. & J.Presl
Iz_10_1209	DJI_0675	Chamaecytisus ruthenicus (Fisch.ex Wol.) Klaskova	0	n	1	0	Fabaceae Lindl.
Iz_10_1209	DJI_0675	Chelidonium majus L.	0	n	1	0	Papaveraceae Juss.
Iz_10_1209	DJI_0675	Chenopodium betaceum Andrz.	0	n	1	0	Amaranthaceae Juss.
Iz_10_1209	DJI_0675	Chenopodium cf. album	0	n	1	0	Amaranthaceae Juss.
Iz_10_1209	DJI_0675	Chenopodium sp.	0		NA	0	Amaranthaceae Juss.
Iz_10_1209	DJI_0675	Chondrilla juncea L.	0	n	1	0	Asteraceae Bercht. & J.Presl
Iz_10_1209	DJI_0675	Cirsium vulgare (Savi) Ten.	0	n	1	0	Asteraceae Bercht. & J.Presl
Iz_10_1209	DJI_0675	Clinopodium acinos (L.) Kuntze	0	n	1	0	Lamiacea Lindl.
Iz_10_1209	DJI_0675	Convallaria majalis L.	0	n	1	0	Asparagaceae Juss.
Iz_10_1209	DJI_0675	Crataegus rhipidophylla Gand.	0	n	1	1	Rosaceae Juss.
Iz_10_1209	DJI_0675	Crepis foetida L.	0.5	n	1	0	Asteraceae Bercht. & J.Presl
Iz_10_1209	DJI_0675	Dactylis glomerata L.	0	n	1	0	Poaceae Barnhart
Iz_10_1209	DJI_0675	Dianthus campestris M.Bieb.	0	n	1	0	Caryophyllaceae Juss.
Iz_10_1209	DJI_0675	Digitaria sanguinalis (L.) Scop.	0	a	0	0	Poaceae Barnhart
Iz_10_1209	DJI_0675	Elymus repens (L.) Gould	0	n	1	0	Poaceae Barnhart
Iz_10_1209	DJI_0675	Epilobium tetragonum L.	0	n	1	0	Onagraceae Juss.
Iz_10_1209	DJI_0675	Eragrostis minor Host	0	a	0	0	Poaceae Barnhart
Iz_10_1209	DJI_0675	Erigeron canadensis L.	10	a	0	0	Asteraceae Bercht. & J.Presl
Iz_10_1209	DJI_0675	Euphorbia seguieriana Neck.	0	n	1	0	Euphorbiaceae Juss.
Iz_10_1209	DJI_0675	Fallopia dumetorum (L.) Holub	0	n	1	0	Polygonaceae Juss.
Iz_10_1209	DJI_0675	Festuca beckeri (Hack.) Trautv.	0	n	1	0	Poaceae Barnhart
Iz_10_1209	DJI_0675	Fragaria vesca L.	0	n	1	0	Rosaceae Juss.
Iz_10_1209	DJI_0675	Frangula alnus Mill.	0	n	1	1	Rhamnaceae Juss.
Iz_10_1209	DJI_0675	Fraxinus pennsylvanica Marshall	0	a	0	1	Oleaceae Hoffmanns. & Link
Iz_10_1209	DJI_0675	Galeopsis bifida Boenn.	0	n	1	0	Lamiacea Lindl.
Iz_10_1209	DJI_0675	Galium verum L.	0	n	1	0	Rubiaceae Juss.
Iz_10_1209	DJI_0675	Genista tinctoria L.	0	n	1	0	Fabaceae Lindl.
Iz_10_1209	DJI_0675	Geranium robertianum L.	0	n	1	0	Geraniaceae Juss.
Iz_10_1209	DJI_0675	Geranium sanquineum L.	0	n	1	0	Geraniaceae Juss.
Iz_10_1209	DJI_0675	Gypsophila paniculata L.	0	n	1	0	Caryophyllaceae Juss.
Iz_10_1209	DJI_0675	Helichrysum arenarium (L.) Moench	0	n	1	0	Asteraceae Bercht. & J.Presl
Iz_10_1209	DJI_0675	Herniaria polygama J.Gay	0	n	1	0	Caryophyllaceae Juss.
Iz_10_1209	DJI_0675	Hieracium robustum Fr.	0	n	1	0	Asteraceae Bercht. & J.Presl
Iz_10_1209	DJI_0675	Hieracium umbellatum L.	0	n	1	NA	Asteraceae Bercht. & J.Presl
Iz_10_1209	DJI_0675	Hylotelephium maximum (L.) Holub	0	n	1	0	Crassulaceae J.St.-Hil.
Iz_10_1209	DJI_0675	Jacobaea borysthenica (DC.) B.Nord. & Greuter	0	n	1	0	Asteraceae Bercht. & J.Presl
Iz_10_1209	DJI_0675	Jasione montana L.	0	n	1	0	Campanulaceae Juss.
Iz_10_1209	DJI_0675	Jurinea cyanoides (L.) Rchb.s.l.	0	n	1	0	Asteraceae Bercht. & J.Presl
Iz_10_1209	DJI_0675	Koeleria glauca (Spreng.) DC.	0	n	1	0	Poaceae Barnhart
Iz_10_1209	DJI_0675	Lactuca serriola L.	0	a	0	0	Asteraceae Bercht. & J.Presl
Iz_10_1209	DJI_0675	Linaria genistifolia (L.) Mill.	0	n	1	0	Plantaginaceae Juss.
Iz_10_1209	DJI_0675	Lomelosia argentea (L.) Greuter & Burdet	0	n	1	0	Caprifoliaceae Juss.
Iz_10_1209	DJI_0675	Melampyrum pratense L.	0	n	1	0	Orobanchaceae Vent.
Iz_10_1209	DJI_0675	Oenothera biennis L.	0	a	0	0	Onagraceae Juss.
Iz_10_1209	DJI_0675	Pentanema britannica (L.) D.Gut.Larr., Santos-Vicente, Anderb., E.Rico & M.M.Mart.Ort.	0	n	1	0	Asteraceae Bercht. & J.Presl
Iz_10_1209	DJI_0675	Peucedanum oreoselinum (L.) Moench	0	n	1	0	Apiaceae Lindl.
Iz_10_1209	DJI_0675	Peucedanum ruthenicum M.Bieb.	0	n	1	0	Apiaceae Lindl.
Iz_10_1209	DJI_0675	Pilosella echioides (Lumn.) F.W.Schultz & Sch.Bip.	0	n	1	0	Asteraceae Bercht. & J.Presl
Iz_10_1209	DJI_0675	Pilosella officinarum F. Schultz et Sch. Bip.	0	n	1	0	Asteraceae Bercht. & J.Presl

Iz_10_1209	DJI_0675	Pilosella sp.	0		NA	0	Asteraceae Bercht. & J.Presl
Iz_10_1209	DJI_0675	Pinus sylvestris L.	0	n	1	1	Pinaceae Spreng. ex F.Rudolphi
Iz_10_1209	DJI_0675	Poa annua L.	0	n	1	0	Poaceae Barnhart
Iz_10_1209	DJI_0675	Poa nemoralis L.	0	n	1	0	Poaceae Barnhart
Iz_10_1209	DJI_0675	Poa sp.	0		NA	0	Poaceae Barnhart
Iz_10_1209	DJI_0675	Polygonatum odoratum (Mill.) Druce	0	n	1	0	Asparagaceae Juss.
Iz_10_1209	DJI_0675	Polygonum arenarium Waldst. & Kit.	0	n	1	0	Polygonaceae Juss.
Iz_10_1209	DJI_0675	Populus nigra L.	0	n	1	1	Salicaceae Mirb.
Iz_10_1209	DJI_0675	Populus tremula L.	1	n	1	1	Salicaceae Mirb.
Iz_10_1209	DJI_0675	Populus Ã canescens (Aiton) Sm.	0	n	1	1	Salicaceae Mirb.
Iz_10_1209	DJI_0675	Potentilla argentea L.	0	n	1	0	Rosaceae Juss.
Iz_10_1209	DJI_0675	Psammophiliella muralis (L.) Ikonn.	0	n	1	0	Caryophyllaceae Juss.
Iz_10_1209	DJI_0675	Psephellus sumensis (Kalen.) Greuter	0	n	1	0	Asteraceae Bercht. & J.Presl
Iz_10_1209	DJI_0675	Pulsatilla pratensis (L.) Mill. s.l.	0	n	1	0	Ranunculaceae Juss.
Iz_10_1209	DJI_0675	Pyrus communis L.	0	a	0	1	Rosaceae Juss.
Iz_10_1209	DJI_0675	Quercus robur L.	0	n	1	1	Fagaceae Dumort.
Iz_10_1209	DJI_0675	Robinia pseudoacacia L.	0	a	0	1	Fabaceae Lindl.
Iz_10_1209	DJI_0675	Rosa sp.	0		NA	1	Rosaceae Juss.
Iz_10_1209	DJI_0675	Rubus idaeus L.	0	n	1	0	Rosaceae Juss.
Iz_10_1209	DJI_0675	Rumex acetosella L.	0	n	1	0	Polygonaceae Juss.
Iz_10_1209	DJI_0675	Rumex thyrsiflorus Fingerh.	0	n	1	0	Polygonaceae Juss.
Iz_10_1209	DJI_0675	Salix alba L.	0	n	1	1	Salicaceae Mirb.
Iz_10_1209	DJI_0675	Sambucus nigra L.	0	n	1	1	Viburnaceae Raf.
Iz_10_1209	DJI_0675	Secale sylvestre Host	0	n	1	0	Poaceae Barnhart
Iz_10_1209	DJI_0675	Sempervivum ruthenicum Schnittsp. & C.B. Lehm.	0	n	1	0	Crassulaceae J.St.-Hil.
Iz_10_1209	DJI_0675	Setaria viridis (L.) P. Beauv.	0	n	1	0	Poaceae Barnhart
Iz_10_1209	DJI_0675	Silene borysthenica (Gruner) Walters	0	n	1	0	Caryophyllaceae Juss.
Iz_10_1209	DJI_0675	Silene latifolia subsp. alba (Mill.) Greuter & Burdet	0	n	1	0	Caryophyllaceae Juss.
Iz_10_1209	DJI_0675	Solidago virgaurea L.	0	n	1	0	Asteraceae Bercht. & J.Presl
Iz_10_1209	DJI_0675	Sorbus aucuparia L.	0	n	1	1	Rosaceae Juss.
Iz_10_1209	DJI_0675	Stachys recta L.	0	n	1	0	Lamiacea Lindl.
Iz_10_1209	DJI_0675	Tanacetum vulgare L.	0	n	1	0	Asteraceae Bercht. & J.Presl
Iz_10_1209	DJI_0675	Thymus pallasianus Heinr.Braun	0	n	1	0	Lamiacea Lindl.
Iz_10_1209	DJI_0675	Tilia cordata Mill.	0	n	1	1	Malvaceae Juss.
Iz_10_1209	DJI_0675	Trifolium alpestre L.	0	n	1	0	Fabaceae Lindl.
Iz_10_1209	DJI_0675	Trifolium arvense L.	0	n	1	0	Fabaceae Lindl.
Iz_10_1209	DJI_0675	Ulmus glabra Huds.	0	n	1	1	Ulmaceae Mirb.
Iz_10_1209	DJI_0675	Verbascum densiflorum Bertol.	0	n	1	0	Scrophulariaceae Juss.
Iz_10_1209	DJI_0675	Verbascum sp.	0	n	1	0	Scrophulariaceae Juss.
Iz_10_1209	DJI_0675	Veronica austriaca L.	0	n	1	0	Plantaginaceae Juss.
Iz_10_1209	DJI_0675	Veronica incana L.	0	n	1	0	Plantaginaceae Juss.
Iz_10_1209	DJI_0675	Veronica verna L.	0	n	1	0	Plantaginaceae Juss.
Iz_10_1209	DJI_0675	Vincetoxicum hirundinaria Medik	0	n	1	0	Apocynaceae Juss.
Iz_10_1209	DJI_0678	Acer tataricum L.	0	n	1	1	Sapindaceae Juss.
Iz_10_1209	DJI_0678	Achillea millefolium L. s.l.	0	n	1	0	Asteraceae Bercht. & J.Presl
Iz_10_1209	DJI_0678	Allium oleraceum L.	0	n	1	0	Amaryllidaceae J.St.-Hil.
Iz_10_1209	DJI_0678	Ambrosia artemisiifolia L.	0	a	0	0	Asteraceae Bercht. & J.Presl
Iz_10_1209	DJI_0678	Amorpha fruticosa L.	0	a	0	0	Fabaceae Lindl.
Iz_10_1209	DJI_0678	Anthericum ramosum L.	0	n	1	0	Asparagaceae Juss.
Iz_10_1209	DJI_0678	Artemisia marschalliana Spreng.	0	n	1	0	Asteraceae Bercht. & J.Presl
Iz_10_1209	DJI_0678	Asparagus officinalis L.	0	n	1	0	Asparagaceae Juss.
Iz_10_1209	DJI_0678	Astragalus cicer L.	0	n	1	0	Fabaceae Lindl.
Iz_10_1209	DJI_0678	Bassia laniflora (S.G.Gmel.) A.J.Scott	0	n	1	0	Amaranthaceae Juss.
Iz_10_1209	DJI_0678	Berberis vulgaris L.	0	n	1	0	Berberidaceae Juss.
Iz_10_1209	DJI_0678	Betula pendula Roth.	0	n	1	1	Betulaceae Gray
Iz_10_1209	DJI_0678	Betula pendula Roth.	0	n	1	0	Poaceae Barnhart
Iz_10_1209	DJI_0678	Bromus inermis Leyss.	0		NA	NA	
Iz_10_1209	DJI_0678	Bromus squarrosus L.	0	a	0	0	Poaceae Barnhart
Iz_10_1209	DJI_0678	Bromus tectorum L.	0	a	0	0	Poaceae Barnhart
Iz_10_1209	DJI_0678	Calamagrostis epigejos (L.) Roth	0	n	1	0	Poaceae Barnhart
Iz_10_1209	DJI_0678	Carduus acanthoides L.	0	n	1	0	Asteraceae Bercht. & J.Presl
Iz_10_1209	DJI_0678	Carex sp.	0	n	1	0	Cyperaceae Juss.
Iz_10_1209	DJI_0678	Centaurea majorovii Dumbadze	0	n	1	0	Asteraceae Bercht. & J.Presl
Iz_10_1209	DJI_0678	Chamaecytisus ruthenicus (Fisch.ex Wol.) Klaskova	0	n	1	0	Fabaceae Lindl.
Iz_10_1209	DJI_0678	Chelidonium majus L.	0	n	1	0	Papaveraceae Juss.
Iz_10_1209	DJI_0678	Chenopodium betaceum Andrz.	0	n	1	0	Amaranthaceae Juss.
Iz_10_1209	DJI_0678	Chenopodium cf. album	0	n	1	0	Amaranthaceae Juss.
Iz_10_1209	DJI_0678	Chenopodium sp.	0		NA	0	Amaranthaceae Juss.
Iz_10_1209	DJI_0678	Chondrilla juncea L.	0	n	1	0	Asteraceae Bercht. & J.Presl
Iz_10_1209	DJI_0678	Cirsium vulgare (Savi) Ten.	0	n	1	0	Asteraceae Bercht. & J.Presl
Iz_10_1209	DJI_0678	Clinopodium acinos (L.) Kuntze	0	n	1	0	Lamiacea Lindl.
Iz_10_1209	DJI_0678	Convallaria majalis L.	0	n	1	0	Asparagaceae Juss.
Iz_10_1209	DJI_0678	Crataegus rhipidophylla Gand.	0	n	1	1	Rosaceae Juss.
Iz_10_1209	DJI_0678	Crepis foetida L.	0	n	1	0	Asteraceae Bercht. & J.Presl
Iz_10_1209	DJI_0678	Dactylis glomerata L.	0	n	1	0	Poaceae Barnhart
Iz_10_1209	DJI_0678	Dianthus campestris M.Bieb.	0	n	1	0	Caryophyllaceae Juss.
Iz_10_1209	DJI_0678	Digitaria sanguinalis (L.) Scop.	0	a	0	0	Poaceae Barnhart
Iz_10_1209	DJI_0678	Elymus repens (L.) Gould	0	n	1	0	Poaceae Barnhart
Iz_10_1209	DJI_0678	Epilobium tetragonum L.	0	n	1	0	Onagraceae Juss.
Iz_10_1209	DJI_0678	Eragrostis minor Host	0	a	0	0	Poaceae Barnhart
Iz_10_1209	DJI_0678	Erigeron canadensis L.	15	a	0	0	Asteraceae Bercht. & J.Presl
Iz_10_1209	DJI_0678	Euphorbia seguieriana Neck.	0	n	1	0	Euphorbiaceae Juss.
Iz_10_1209	DJI_0678	Fallopia dumetorum (L.) Holub	0	n	1	0	Polygonaceae Juss.
Iz_10_1209	DJI_0678	Festuca beckeri (Hack.) Trautv.	0	n	1	0	Poaceae Barnhart
Iz_10_1209	DJI_0678	Fragaria vesca L.	0	n	1	0	Rosaceae Juss.
Iz_10_1209	DJI_0678	Frangula alnus Mill.	0	n	1	1	Rhamnaceae Juss.
Iz_10_1209	DJI_0678	Fraxinus pennsylvanica Marshall	0	a	0	1	Oleaceae Hoffmanns. & Link
Iz_10_1209	DJI_0678	Galeopsis bifida Boenn.	0	n	1	0	Lamiacea Lindl.
Iz_10_1209	DJI_0678	Galium verum L.	0	n	1	0	Rubiaceae Juss.
Iz_10_1209	DJI_0678	Genista tinctoria L.	0	n	1	0	Fabaceae Lindl.
Iz_10_1209	DJI_0678	Geranium robertianum L.	0	n	1	0	Geraniaceae Juss.
Iz_10_1209	DJI_0678	Geranium sanquineum L.	0	n	1	0	Geraniaceae Juss.
Iz_10_1209	DJI_0678	Gypsophila paniculata L.	0	n	1	0	Caryophyllaceae Juss.
Iz_10_1209	DJI_0678	Helichrysum arenarium (L.) Moench	0	n	1	0	Asteraceae Bercht. & J.Presl
Iz_10_1209	DJI_0678	Herniaria polygama J.Gay	0	n	1	0	Caryophyllaceae Juss.
Iz_10_1209	DJI_0678	Hieracium robustum Fr.	0	n	1	0	Asteraceae Bercht. & J.Presl
Iz_10_1209	DJI_0678	Hieracium umbellatum L.	0	n	1	NA	Asteraceae Bercht. & J.Presl
Iz_10_1209	DJI_0678	Hylotelephium maximum (L.) Holub	0	n	1	0	Crassulaceae J.St.-Hil.

Iz_10_1209	DJI_0678	Jacobaea borysthenica (DC.) B.Nord. & Greuter	0	n	1	0	Asteraceae Bercht. & J.Presl
Iz_10_1209	DJI_0678	Jasione montana L.	0	n	1	0	Campanulaceae Juss.
Iz_10_1209	DJI_0678	Jurinea cyanoides (L.) Rchb.s.l.	0	n	1	0	Asteraceae Bercht. & J.Presl
Iz_10_1209	DJI_0678	Koeleria glauca (Spreng.) DC.	0	n	1	0	Poaceae Barnhart
Iz_10_1209	DJI_0678	Lactuca serriola L.	0	a	0	0	Asteraceae Bercht. & J.Presl
Iz_10_1209	DJI_0678	Linaria genistifolia (L.) Mill.	0	n	1	0	Plantaginaceae Juss.
Iz_10_1209	DJI_0678	Lomelosia argentea (L.) Greuter & Burdet	0	n	1	0	Caprifoliaceae Juss.
Iz_10_1209	DJI_0678	Melampyrum pratense L.	0	n	1	0	Orobanchaceae Vent.
Iz_10_1209	DJI_0678	Oenothera biennis L.	0	a	0	0	Onagraceae Juss.
Iz_10_1209	DJI_0678	Pentanema britannica (L.) D.Gut.Larr., Santos-Vicente, Anderb., E.Rico & M.M.Mart.Ort.	0	n	1	0	Asteraceae Bercht. & J.Presl
Iz_10_1209	DJI_0678	Peucedanum oreoselinum (L.) Moench	0	n	1	0	Apiaceae Lindl.
Iz_10_1209	DJI_0678	Peucedanum ruthenicum M.Bieb.	0	n	1	0	Apiaceae Lindl.
Iz_10_1209	DJI_0678	Pilosella echioides (Lumn.) F.W.Schultz & Sch.Bip.	0	n	1	0	Asteraceae Bercht. & J.Presl
Iz_10_1209	DJI_0678	Pilosella officinarum F. Schultz et Sch. Bip.	0.5	n	1	0	Asteraceae Bercht. & J.Presl
Iz_10_1209	DJI_0678	Pilosella sp.	0		NA	0	Asteraceae Bercht. & J.Presl
Iz_10_1209	DJI_0678	Pinus sylvestris L.	0	n	1	1	Pinaceae Spreng. ex F.Rudolphi
Iz_10_1209	DJI_0678	Poa annua L.	0	n	1	0	Poaceae Barnhart
Iz_10_1209	DJI_0678	Poa nemoralis L.	0	n	1	0	Poaceae Barnhart
Iz_10_1209	DJI_0678	Poa sp.	0		NA	0	Poaceae Barnhart
Iz_10_1209	DJI_0678	Polygonatum odoratum (Mill.) Druce	0	n	1	0	Asparagaceae Juss.
Iz_10_1209	DJI_0678	Polygonum arenarium Waldst. & Kit.	0	n	1	0	Polygonaceae Juss.
Iz_10_1209	DJI_0678	Populus nigra L.	0	n	1	1	Salicaceae Mirb.
Iz_10_1209	DJI_0678	Populus tremula L.	0	n	1	1	Salicaceae Mirb.
Iz_10_1209	DJI_0678	Populus Ã canescens (Aiton) Sm.	0	n	1	1	Salicaceae Mirb.
Iz_10_1209	DJI_0678	Potentilla argentea L.	0	n	1	0	Rosaceae Juss.
Iz_10_1209	DJI_0678	Psammophiliella muralis (L.) Ikonn.	0	n	1	0	Caryophyllaceae Juss.
Iz_10_1209	DJI_0678	Psephellus sumensis (Kalen.) Greuter	0	n	1	0	Asteraceae Bercht. & J.Presl
Iz_10_1209	DJI_0678	Pulsatilla pratensis (L.) Mill. s.l.	0	n	1	0	Ranunculaceae Juss.
Iz_10_1209	DJI_0678	Pyrus communis L.	0	a	0	1	Rosaceae Juss.
Iz_10_1209	DJI_0678	Quercus robur L.	0	n	1	1	Fagaceae Dumort.
Iz_10_1209	DJI_0678	Robinia pseudoacacia L.	0	a	0	1	Fabaceae Lindl.
Iz_10_1209	DJI_0678	Rosa sp.	0		NA	1	Rosaceae Juss.
Iz_10_1209	DJI_0678	Rubus idaeus L.	0	n	1	0	Rosaceae Juss.
Iz_10_1209	DJI_0678	Rumex acetosella L.	0	n	1	0	Polygonaceae Juss.
Iz_10_1209	DJI_0678	Rumex thyrsiflorus Fingerh.	0	n	1	0	Polygonaceae Juss.
Iz_10_1209	DJI_0678	Salix alba L.	0	n	1	1	Salicaceae Mirb.
Iz_10_1209	DJI_0678	Sambucus nigra L.	0	n	1	1	Viburnaceae Raf.
Iz_10_1209	DJI_0678	Secale sylvestre Host	0	n	1	0	Poaceae Barnhart
Iz_10_1209	DJI_0678	Sempervivum ruthenicum Schnittsp. & C.B. Lehm.	0	n	1	0	Crassulaceae J.St.-Hil.
Iz_10_1209	DJI_0678	Setaria viridis (L.) P. Beauv.	0	n	1	0	Poaceae Barnhart
Iz_10_1209	DJI_0678	Silene borysthenica (Gruner) Walters	0	n	1	0	Caryophyllaceae Juss.
Iz_10_1209	DJI_0678	Silene latifolia subsp. alba (Mill.) Greuter & Burdet	0	n	1	0	Caryophyllaceae Juss.
Iz_10_1209	DJI_0678	Solidago virgaurea L.	0	n	1	0	Asteraceae Bercht. & J.Presl
Iz_10_1209	DJI_0678	Sorbus aucuparia L.	0	n	1	1	Rosaceae Juss.
Iz_10_1209	DJI_0678	Stachys recta L.	0	n	1	0	Lamiacea Lindl.
Iz_10_1209	DJI_0678	Tanacetum vulgare L.	0	n	1	0	Asteraceae Bercht. & J.Presl
Iz_10_1209	DJI_0678	Thymus pallasianus Heinr.Braun	0	n	1	0	Lamiacea Lindl.
Iz_10_1209	DJI_0678	Tilia cordata Mill.	0	n	1	1	Malvaceae Juss.
Iz_10_1209	DJI_0678	Trifolium alpestre L.	0	n	1	0	Fabaceae Lindl.
Iz_10_1209	DJI_0678	Trifolium arvense L.	0	n	1	0	Fabaceae Lindl.
Iz_10_1209	DJI_0678	Ulmus glabra Huds.	0	n	1	1	Ulmaceae Mirb.
Iz_10_1209	DJI_0678	Verbascum densiflorum Bertol.	0	n	1	0	Scrophulariaceae Juss.
Iz_10_1209	DJI_0678	Verbascum sp.	0	n	1	0	Scrophulariaceae Juss.
Iz_10_1209	DJI_0678	Veronica austriaca L.	0	n	1	0	Plantaginaceae Juss.
Iz_10_1209	DJI_0678	Veronica incana L.	0	n	1	0	Plantaginaceae Juss.
Iz_10_1209	DJI_0678	Veronica verna L.	0	n	1	0	Plantaginaceae Juss.
Iz_10_1209	DJI_0678	Vincetoxicum hirundinaria Medik	0	n	1	0	Apocynaceae Juss.
Iz_10_1209	DJI_0681	Acer tataricum L.	0	n	1	1	Sapindaceae Juss.
Iz_10_1209	DJI_0681	Achillea millefolium L. s.l.	0	n	1	0	Asteraceae Bercht. & J.Presl
Iz_10_1209	DJI_0681	Allium oleraceum L.	0	n	1	0	Amaryllidaceae J.St.-Hil.
Iz_10_1209	DJI_0681	Ambrosia artemisiifolia L.	0	a	0	0	Asteraceae Bercht. & J.Presl
Iz_10_1209	DJI_0681	Amorpha fruticosa L.	0	a	0	0	Fabaceae Lindl.
Iz_10_1209	DJI_0681	Anthericum ramosum L.	0	n	1	0	Asparagaceae Juss.
Iz_10_1209	DJI_0681	Artemisia marschalliana Spreng.	0	n	1	0	Asteraceae Bercht. & J.Presl
Iz_10_1209	DJI_0681	Asparagus officinalis L.	0	n	1	0	Asparagaceae Juss.
Iz_10_1209	DJI_0681	Astragalus cicer L.	0	n	1	0	Fabaceae Lindl.
Iz_10_1209	DJI_0681	Bassia laniflora (S.G.Gmel.) A.J.Scott	0	n	1	0	Amaranthaceae Juss.
Iz_10_1209	DJI_0681	Berberis vulgaris L.	0	n	1	0	Berberidaceae Juss.
Iz_10_1209	DJI_0681	Betula pendula Roth.	0	n	1	1	Betulaceae Gray
Iz_10_1209	DJI_0681	Betula pendula Roth.	0	n	1	0	Poaceae Barnhart
Iz_10_1209	DJI_0681	Bromus inermis Leyss.	0		NA	NA	
Iz_10_1209	DJI_0681	Bromus squarrosus L.	0	a	0	0	Poaceae Barnhart
Iz_10_1209	DJI_0681	Bromus tectorum L.	0	a	0	0	Poaceae Barnhart
Iz_10_1209	DJI_0681	Calamagrostis epigejos (L.) Roth	1	n	1	0	Poaceae Barnhart
Iz_10_1209	DJI_0681	Carduus acanthoides L.	0	n	1	0	Asteraceae Bercht. & J.Presl
Iz_10_1209	DJI_0681	Carex sp.	0	n	1	0	Cyperaceae Juss.
Iz_10_1209	DJI_0681	Centaurea majorovii Dumbadze	0	n	1	0	Asteraceae Bercht. & J.Presl
Iz_10_1209	DJI_0681	Chamaecytisus ruthenicus (Fisch.ex Wol.) Klaskova	0	n	1	0	Fabaceae Lindl.
Iz_10_1209	DJI_0681	Chelidonium majus L.	0	n	1	0	Papaveraceae Juss.
Iz_10_1209	DJI_0681	Chenopodium betaceum Andrz.	0	n	1	0	Amaranthaceae Juss.
Iz_10_1209	DJI_0681	Chenopodium cf. album	0	n	1	0	Amaranthaceae Juss.
Iz_10_1209	DJI_0681	Chenopodium sp.	0		NA	0	Amaranthaceae Juss.
Iz_10_1209	DJI_0681	Chondrilla juncea L.	0	n	1	0	Asteraceae Bercht. & J.Presl
Iz_10_1209	DJI_0681	Cirsium vulgare (Savi) Ten.	0	n	1	0	Asteraceae Bercht. & J.Presl
Iz_10_1209	DJI_0681	Clinopodium acinos (L.) Kuntze	0	n	1	0	Lamiacea Lindl.
Iz_10_1209	DJI_0681	Convallaria majalis L.	0	n	1	0	Asparagaceae Juss.
Iz_10_1209	DJI_0681	Crataegus rhipidophylla Gand.	0	n	1	1	Rosaceae Juss.
Iz_10_1209	DJI_0681	Crepis foetida L.	0	n	1	0	Asteraceae Bercht. & J.Presl
Iz_10_1209	DJI_0681	Dactylis glomerata L.	0	n	1	0	Poaceae Barnhart
Iz_10_1209	DJI_0681	Dianthus campestris M.Bieb.	0	n	1	0	Caryophyllaceae Juss.
Iz_10_1209	DJI_0681	Digitaria sanguinalis (L.) Scop.	0	a	0	0	Poaceae Barnhart
Iz_10_1209	DJI_0681	Elymus repens (L.) Gould	0	n	1	0	Poaceae Barnhart
Iz_10_1209	DJI_0681	Epilobium tetragonum L.	0	n	1	0	Onagraceae Juss.
Iz_10_1209	DJI_0681	Eragrostis minor Host	0	a	0	0	Poaceae Barnhart
Iz_10_1209	DJI_0681	Erigeron canadensis L.	0.5	a	0	0	Asteraceae Bercht. & J.Presl
Iz_10_1209	DJI_0681	Euphorbia seguieriana Neck.	0	n	1	0	Euphorbiaceae Juss.
Iz_10_1209	DJI_0681	Fallopia dumetorum (L.) Holub	0	n	1	0	Polygonaceae Juss.
Iz_10_1209	DJI_0681	Festuca beckeri (Hack.) Trautv.	0	n	1	0	Poaceae Barnhart

Iz_10_1209	DJI_0681	Fragaria vesca L.	0	n	1	0	Rosaceae Juss.
Iz_10_1209	DJI_0681	Frangula alnus Mill.	0	n	1	1	Rhamnaceae Juss.
Iz_10_1209	DJI_0681	Fraxinus pennsylvanica Marshall	0	a	0	1	Oleaceae Hoffmanns. & Link
Iz_10_1209	DJI_0681	Galeopsis bifida Boenn.	0	n	1	0	Lamiacea Lindl.
Iz_10_1209	DJI_0681	Galium verum L.	0	n	1	0	Rubiaceae Juss.
Iz_10_1209	DJI_0681	Genista tinctoria L.	0	n	1	0	Fabaceae Lindl.
Iz_10_1209	DJI_0681	Geranium robertianum L.	0	n	1	0	Geraniaceae Juss.
Iz_10_1209	DJI_0681	Geranium sanquineum L.	0	n	1	0	Geraniaceae Juss.
Iz_10_1209	DJI_0681	Gypsophila paniculata L.	0	n	1	0	Caryophyllaceae Juss.
Iz_10_1209	DJI_0681	Helichrysum arenarium (L.) Moench	0	n	1	0	Asteraceae Bercht. & J.Presl
Iz_10_1209	DJI_0681	Herniaria polygama J.Gay	0	n	1	0	Caryophyllaceae Juss.
Iz_10_1209	DJI_0681	Hieracium robustum Fr.	0	n	1	0	Asteraceae Bercht. & J.Presl
Iz_10_1209	DJI_0681	Hieracium umbellatum L.	0	n	1	NA	Asteraceae Bercht. & J.Presl
Iz_10_1209	DJI_0681	Hylotelephium maximum (L.) Holub	0	n	1	0	Crassulaceae J.St.-Hil.
Iz_10_1209	DJI_0681	Jacobaea borysthenica (DC.) B.Nord. & Greuter	0	n	1	0	Asteraceae Bercht. & J.Presl
Iz_10_1209	DJI_0681	Jasione montana L.	0	n	1	0	Campanulaceae Juss.
Iz_10_1209	DJI_0681	Jurinea cyanoides (L.) Rchb.s.l.	0	n	1	0	Asteraceae Bercht. & J.Presl
Iz_10_1209	DJI_0681	Koeleria glauca (Spreng.) DC.	0	n	1	0	Poaceae Barnhart
Iz_10_1209	DJI_0681	Lactuca serriola L.	0	a	0	0	Asteraceae Bercht. & J.Presl
Iz_10_1209	DJI_0681	Linaria genistifolia (L.) Mill.	0	n	1	0	Plantaginaceae Juss.
Iz_10_1209	DJI_0681	Lomelosia argentea (L.) Greuter & Burdet	0	n	1	0	Caprifoliaceae Juss.
Iz_10_1209	DJI_0681	Melampyrum pratense L.	0	n	1	0	Orobanchaceae Vent.
Iz_10_1209	DJI_0681	Oenothera biennis L.	0	a	0	0	Onagraceae Juss.
Iz_10_1209	DJI_0681	Pentanema britannica (L.) D.Gut.Larr., Santos-Vicente, Anderb., E.Rico & M.M.Mart.Ort.	0	n	1	0	Asteraceae Bercht. & J.Presl
Iz_10_1209	DJI_0681	Peucedanum oreoselinum (L.) Moench	0	n	1	0	Apiaceae Lindl.
Iz_10_1209	DJI_0681	Peucedanum ruthenicum M.Bieb.	0	n	1	0	Apiaceae Lindl.
Iz_10_1209	DJI_0681	Pilosella echioides (Lumn.) F.W.Schultz & Sch.Bip.	0	n	1	0	Asteraceae Bercht. & J.Presl
Iz_10_1209	DJI_0681	Pilosella officinarum F. Schultz et Sch. Bip.	0	n	1	0	Asteraceae Bercht. & J.Presl
Iz_10_1209	DJI_0681	Pilosella sp.	0		NA	0	Asteraceae Bercht. & J.Presl
Iz_10_1209	DJI_0681	Pinus sylvestris L.	0	n	1	1	Pinaceae Spreng. ex F.Rudolphi
Iz_10_1209	DJI_0681	Poa annua L.	0	n	1	0	Poaceae Barnhart
Iz_10_1209	DJI_0681	Poa nemoralis L.	0	n	1	0	Poaceae Barnhart
Iz_10_1209	DJI_0681	Poa sp.	0		NA	0	Poaceae Barnhart
Iz_10_1209	DJI_0681	Polygonatum odoratum (Mill.) Druce	0	n	1	0	Asparagaceae Juss.
Iz_10_1209	DJI_0681	Polygonum arenarium Waldst. & Kit.	0	n	1	0	Polygonaceae Juss.
Iz_10_1209	DJI_0681	Populus nigra L.	0	n	1	1	Salicaceae Mirb.
Iz_10_1209	DJI_0681	Populus tremula L.	0	n	1	1	Salicaceae Mirb.
Iz_10_1209	DJI_0681	Populus Ã canescens (Aiton) Sm.	0	n	1	1	Salicaceae Mirb.
Iz_10_1209	DJI_0681	Potentilla argentea L.	0	n	1	0	Rosaceae Juss.
Iz_10_1209	DJI_0681	Psammophiliella muralis (L.) Ikonn.	0	n	1	0	Caryophyllaceae Juss.
Iz_10_1209	DJI_0681	Psephellus sumensis (Kalen.) Greuter	0	n	1	0	Asteraceae Bercht. & J.Presl
Iz_10_1209	DJI_0681	Pulsatilla pratensis (L.) Mill. s.l.	0	n	1	0	Ranunculaceae Juss.
Iz_10_1209	DJI_0681	Pyrus communis L.	0	a	0	1	Rosaceae Juss.
Iz_10_1209	DJI_0681	Quercus robur L.	0	n	1	1	Fagaceae Dumort.
Iz_10_1209	DJI_0681	Robinia pseudoacacia L.	0	a	0	1	Fabaceae Lindl.
Iz_10_1209	DJI_0681	Rosa sp.	0		NA	1	Rosaceae Juss.
Iz_10_1209	DJI_0681	Rubus idaeus L.	0	n	1	0	Rosaceae Juss.
Iz_10_1209	DJI_0681	Rumex acetosella L.	0	n	1	0	Polygonaceae Juss.
Iz_10_1209	DJI_0681	Rumex thyrsiflorus Fingerh.	0	n	1	0	Polygonaceae Juss.
Iz_10_1209	DJI_0681	Salix alba L.	0	n	1	1	Salicaceae Mirb.
Iz_10_1209	DJI_0681	Sambucus nigra L.	0	n	1	1	Viburnaceae Raf.
Iz_10_1209	DJI_0681	Secale sylvestre Host	0	n	1	0	Poaceae Barnhart
Iz_10_1209	DJI_0681	Sempervivum ruthenicum Schnittsp. & C.B. Lehm.	0	n	1	0	Crassulaceae J.St.-Hil.
Iz_10_1209	DJI_0681	Setaria viridis (L.) P. Beauv.	0	n	1	0	Poaceae Barnhart
Iz_10_1209	DJI_0681	Silene borysthenica (Gruner) Walters	0	n	1	0	Caryophyllaceae Juss.
Iz_10_1209	DJI_0681	Silene latifolia subsp. alba (Mill.) Greuter & Burdet	0	n	1	0	Caryophyllaceae Juss.
Iz_10_1209	DJI_0681	Solidago virgaurea L.	0	n	1	0	Asteraceae Bercht. & J.Presl
Iz_10_1209	DJI_0681	Sorbus aucuparia L.	0	n	1	1	Rosaceae Juss.
Iz_10_1209	DJI_0681	Stachys recta L.	0	n	1	0	Lamiacea Lindl.
Iz_10_1209	DJI_0681	Tanacetum vulgare L.	0	n	1	0	Asteraceae Bercht. & J.Presl
Iz_10_1209	DJI_0681	Thymus pallasianus Heinr.Braun	0	n	1	0	Lamiacea Lindl.
Iz_10_1209	DJI_0681	Tilia cordata Mill.	0	n	1	1	Malvaceae Juss.
Iz_10_1209	DJI_0681	Trifolium alpestre L.	0	n	1	0	Fabaceae Lindl.
Iz_10_1209	DJI_0681	Trifolium arvense L.	0	n	1	0	Fabaceae Lindl.
Iz_10_1209	DJI_0681	Ulmus glabra Huds.	0	n	1	1	Ulmaceae Mirb.
Iz_10_1209	DJI_0681	Verbascum densiflorum Bertol.	0	n	1	0	Scrophulariaceae Juss.
Iz_10_1209	DJI_0681	Verbascum sp.	0	n	1	0	Scrophulariaceae Juss.
Iz_10_1209	DJI_0681	Veronica austriaca L.	0	n	1	0	Plantaginaceae Juss.
Iz_10_1209	DJI_0681	Veronica incana L.	0	n	1	0	Plantaginaceae Juss.
Iz_10_1209	DJI_0681	Veronica verna L.	0	n	1	0	Plantaginaceae Juss.
Iz_10_1209	DJI_0681	Vincetoxicum hirundinaria Medik	0	n	1	0	Apocynaceae Juss.
Iz_10_1209	DJI_0683	Acer tataricum L.	0	n	1	1	Sapindaceae Juss.
Iz_10_1209	DJI_0683	Achillea millefolium L. s.l.	0	n	1	0	Asteraceae Bercht. & J.Presl
Iz_10_1209	DJI_0683	Allium oleraceum L.	0	n	1	0	Amaryllidaceae J.St.-Hil.
Iz_10_1209	DJI_0683	Ambrosia artemisiifolia L.	0	a	0	0	Asteraceae Bercht. & J.Presl
Iz_10_1209	DJI_0683	Amorpha fruticosa L.	0	a	0	0	Fabaceae Lindl.
Iz_10_1209	DJI_0683	Anthericum ramosum L.	0	n	1	0	Asparagaceae Juss.
Iz_10_1209	DJI_0683	Artemisia marschalliana Spreng.	0	n	1	0	Asteraceae Bercht. & J.Presl
Iz_10_1209	DJI_0683	Asparagus officinalis L.	0	n	1	0	Asparagaceae Juss.
Iz_10_1209	DJI_0683	Astragalus cicer L.	0	n	1	0	Fabaceae Lindl.
Iz_10_1209	DJI_0683	Bassia laniflora (S.G.Gmel.) A.J.Scott	0	n	1	0	Amaranthaceae Juss.
Iz_10_1209	DJI_0683	Berberis vulgaris L.	0	n	1	0	Berberidaceae Juss.
Iz_10_1209	DJI_0683	Betula pendula Roth.	0	n	1	1	Betulaceae Gray
Iz_10_1209	DJI_0683	Betula pendula Roth.	0	n	1	0	Poaceae Barnhart
Iz_10_1209	DJI_0683	Bromus inermis Leyss.	0		NA	NA	
Iz_10_1209	DJI_0683	Bromus squarrosus L.	0	a	0	0	Poaceae Barnhart
Iz_10_1209	DJI_0683	Bromus tectorum L.	0	a	0	0	Poaceae Barnhart
Iz_10_1209	DJI_0683	Calamagrostis epigejos (L.) Roth	0.2	n	1	0	Poaceae Barnhart
Iz_10_1209	DJI_0683	Carduus acanthoides L.	0	n	1	0	Asteraceae Bercht. & J.Presl
Iz_10_1209	DJI_0683	Carex sp.	0	n	1	0	Cyperaceae Juss.
Iz_10_1209	DJI_0683	Centaurea majorovii Dumbadze	0	n	1	0	Asteraceae Bercht. & J.Presl
Iz_10_1209	DJI_0683	Chamaecytisus ruthenicus (Fisch.ex Wol.) Klaskova	0	n	1	0	Fabaceae Lindl.
Iz_10_1209	DJI_0683	Chelidonium majus L.	0	n	1	0	Papaveraceae Juss.
Iz_10_1209	DJI_0683	Chenopodium betaceum Andrz.	0	n	1	0	Amaranthaceae Juss.
Iz_10_1209	DJI_0683	Chenopodium cf. album	0	n	1	0	Amaranthaceae Juss.
Iz_10_1209	DJI_0683	Chenopodium sp.	1		NA	0	Amaranthaceae Juss.
Iz_10_1209	DJI_0683	Chondrilla juncea L.	0	n	1	0	Asteraceae Bercht. & J.Presl
Iz_10_1209	DJI_0683	Cirsium vulgare (Savi) Ten.	0	n	1	0	Asteraceae Bercht. & J.Presl

Iz_10_1209	DJI_0683	Clinopodium acinos (L.) Kuntze	0	n	1	0	Lamiacea Lindl.
Iz_10_1209	DJI_0683	Convallaria majalis L.	0	n	1	0	Asparagaceae Juss.
Iz_10_1209	DJI_0683	Crataegus rhipidophylla Gand.	0	n	1	1	Rosaceae Juss.
Iz_10_1209	DJI_0683	Crepis foetida L.	0	n	1	0	Asteraceae Bercht. & J.Presl
Iz_10_1209	DJI_0683	Dactylis glomerata L.	0	n	1	0	Poaceae Barnhart
Iz_10_1209	DJI_0683	Dianthus campestris M.Bieb.	0	n	1	0	Caryophyllaceae Juss.
Iz_10_1209	DJI_0683	Digitaria sanguinalis (L.) Scop.	0	a	0	0	Poaceae Barnhart
Iz_10_1209	DJI_0683	Elymus repens (L.) Gould	0	n	1	0	Poaceae Barnhart
Iz_10_1209	DJI_0683	Epilobium tetragonum L.	0	n	1	0	Onagraceae Juss.
Iz_10_1209	DJI_0683	Eragrostis minor Host	0	a	0	0	Poaceae Barnhart
Iz_10_1209	DJI_0683	Erigeron canadensis L.	3	a	0	0	Asteraceae Bercht. & J.Presl
Iz_10_1209	DJI_0683	Euphorbia seguieriana Neck.	0	n	1	0	Euphorbiaceae Juss.
Iz_10_1209	DJI_0683	Fallopia dumetorum (L.) Holub	0	n	1	0	Polygonaceae Juss.
Iz_10_1209	DJI_0683	Festuca beckeri (Hack.) Trautv.	0	n	1	0	Poaceae Barnhart
Iz_10_1209	DJI_0683	Fragaria vesca L.	0	n	1	0	Rosaceae Juss.
Iz_10_1209	DJI_0683	Frangula alnus Mill.	0	n	1	1	Rhamnaceae Juss.
Iz_10_1209	DJI_0683	Fraxinus pennsylvanica Marshall	0	a	0	1	Oleaceae Hoffmanns. & Link
Iz_10_1209	DJI_0683	Galeopsis bifida Boenn.	0	n	1	0	Lamiacea Lindl.
Iz_10_1209	DJI_0683	Galium verum L.	0	n	1	0	Rubiaceae Juss.
Iz_10_1209	DJI_0683	Genista tinctoria L.	0	n	1	0	Fabaceae Lindl.
Iz_10_1209	DJI_0683	Geranium robertianum L.	0	n	1	0	Geraniaceae Juss.
Iz_10_1209	DJI_0683	Geranium sanquineum L.	0	n	1	0	Geraniaceae Juss.
Iz_10_1209	DJI_0683	Gypsophila paniculata L.	0	n	1	0	Caryophyllaceae Juss.
Iz_10_1209	DJI_0683	Helichrysum arenarium (L.) Moench	1	n	1	0	Asteraceae Bercht. & J.Presl
Iz_10_1209	DJI_0683	Herniaria polygama J.Gay	0	n	1	0	Caryophyllaceae Juss.
Iz_10_1209	DJI_0683	Hieracium robustum Fr.	0	n	1	0	Asteraceae Bercht. & J.Presl
Iz_10_1209	DJI_0683	Hieracium umbellatum L.	0	n	1	NA	Asteraceae Bercht. & J.Presl
Iz_10_1209	DJI_0683	Hylotelephium maximum (L.) Holub	0	n	1	0	Crassulaceae J.St.-Hil.
Iz_10_1209	DJI_0683	Jacobaea borysthenica (DC.) B.Nord. & Greuter	0	n	1	0	Asteraceae Bercht. & J.Presl
Iz_10_1209	DJI_0683	Jasione montana L.	0	n	1	0	Campanulaceae Juss.
Iz_10_1209	DJI_0683	Jurinea cyanoides (L.) Rchb.s.l.	0	n	1	0	Asteraceae Bercht. & J.Presl
Iz_10_1209	DJI_0683	Koeleria glauca (Spreng.) DC.	0	n	1	0	Poaceae Barnhart
Iz_10_1209	DJI_0683	Lactuca serriola L.	0	a	0	0	Asteraceae Bercht. & J.Presl
Iz_10_1209	DJI_0683	Linaria genistifolia (L.) Mill.	0	n	1	0	Plantaginaceae Juss.
Iz_10_1209	DJI_0683	Lomelosia argentea (L.) Greuter & Burdet	0	n	1	0	Caprifoliaceae Juss.
Iz_10_1209	DJI_0683	Melampyrum pratense L.	0	n	1	0	Orobanchaceae Vent.
Iz_10_1209	DJI_0683	Oenothera biennis L.	0	a	0	0	Onagraceae Juss.
Iz_10_1209	DJI_0683	Pentanema britannica (L.) D.Gut.Larr., Santos-Vicente, Anderb., E.Rico & M.M.Mart.Ort.	0	n	1	0	Asteraceae Bercht. & J.Presl
Iz_10_1209	DJI_0683	Peucedanum oreoselinum (L.) Moench	0	n	1	0	Apiaceae Lindl.
Iz_10_1209	DJI_0683	Peucedanum ruthenicum M.Bieb.	0	n	1	0	Apiaceae Lindl.
Iz_10_1209	DJI_0683	Pilosella echioides (Lumn.) F.W.Schultz & Sch.Bip.	0	n	1	0	Asteraceae Bercht. & J.Presl
Iz_10_1209	DJI_0683	Pilosella officinarum F. Schultz et Sch. Bip.	0	n	1	0	Asteraceae Bercht. & J.Presl
Iz_10_1209	DJI_0683	Pilosella sp.	0		NA	0	Asteraceae Bercht. & J.Presl
Iz_10_1209	DJI_0683	Pinus sylvestris L.	0	n	1	1	Pinaceae Spreng. ex F.Rudolphi
Iz_10_1209	DJI_0683	Poa annua L.	0	n	1	0	Poaceae Barnhart
Iz_10_1209	DJI_0683	Poa nemoralis L.	0	n	1	0	Poaceae Barnhart
Iz_10_1209	DJI_0683	Poa sp.	0		NA	0	Poaceae Barnhart
Iz_10_1209	DJI_0683	Polygonatum odoratum (Mill.) Druce	0	n	1	0	Asparagaceae Juss.
Iz_10_1209	DJI_0683	Polygonum arenarium Waldst. & Kit.	0	n	1	0	Polygonaceae Juss.
Iz_10_1209	DJI_0683	Populus nigra L.	0	n	1	1	Salicaceae Mirb.
Iz_10_1209	DJI_0683	Populus tremula L.	0	n	1	1	Salicaceae Mirb.
Iz_10_1209	DJI_0683	Populus Ã canescens (Aiton) Sm.	0	n	1	1	Salicaceae Mirb.
Iz_10_1209	DJI_0683	Potentilla argentea L.	0	n	1	0	Rosaceae Juss.
Iz_10_1209	DJI_0683	Psammophiliella muralis (L.) Ikonn.	0	n	1	0	Caryophyllaceae Juss.
Iz_10_1209	DJI_0683	Psephellus sumensis (Kalen.) Greuter	0	n	1	0	Asteraceae Bercht. & J.Presl
Iz_10_1209	DJI_0683	Pulsatilla pratensis (L.) Mill. s.l.	0	n	1	0	Ranunculaceae Juss.
Iz_10_1209	DJI_0683	Pyrus communis L.	0	a	0	1	Rosaceae Juss.
Iz_10_1209	DJI_0683	Quercus robur L.	0	n	1	1	Fagaceae Dumort.
Iz_10_1209	DJI_0683	Robinia pseudoacacia L.	0	a	0	1	Fabaceae Lindl.
Iz_10_1209	DJI_0683	Rosa sp.	0		NA	1	Rosaceae Juss.
Iz_10_1209	DJI_0683	Rubus idaeus L.	0	n	1	0	Rosaceae Juss.
Iz_10_1209	DJI_0683	Rumex acetosella L.	0	n	1	0	Polygonaceae Juss.
Iz_10_1209	DJI_0683	Rumex thyrsiflorus Fingerh.	0	n	1	0	Polygonaceae Juss.
Iz_10_1209	DJI_0683	Salix alba L.	0	n	1	1	Salicaceae Mirb.
Iz_10_1209	DJI_0683	Sambucus nigra L.	0	n	1	1	Viburnaceae Raf.
Iz_10_1209	DJI_0683	Secale sylvestre Host	0	n	1	0	Poaceae Barnhart
Iz_10_1209	DJI_0683	Sempervivum ruthenicum Schnittsp. & C.B. Lehm.	0	n	1	0	Crassulaceae J.St.-Hil.
Iz_10_1209	DJI_0683	Setaria viridis (L.) P. Beauv.	0	n	1	0	Poaceae Barnhart
Iz_10_1209	DJI_0683	Silene borysthenica (Gruner) Walters	0	n	1	0	Caryophyllaceae Juss.
Iz_10_1209	DJI_0683	Silene latifolia subsp. alba (Mill.) Greuter & Burdet	0	n	1	0	Caryophyllaceae Juss.
Iz_10_1209	DJI_0683	Solidago virgaurea L.	0	n	1	0	Asteraceae Bercht. & J.Presl
Iz_10_1209	DJI_0683	Sorbus aucuparia L.	0	n	1	1	Rosaceae Juss.
Iz_10_1209	DJI_0683	Stachys recta L.	0	n	1	0	Lamiacea Lindl.
Iz_10_1209	DJI_0683	Tanacetum vulgare L.	0	n	1	0	Asteraceae Bercht. & J.Presl
Iz_10_1209	DJI_0683	Thymus pallasianus Heinr.Braun	0	n	1	0	Lamiacea Lindl.
Iz_10_1209	DJI_0683	Tilia cordata Mill.	0	n	1	1	Malvaceae Juss.
Iz_10_1209	DJI_0683	Trifolium alpestre L.	0	n	1	0	Fabaceae Lindl.
Iz_10_1209	DJI_0683	Trifolium arvense L.	0	n	1	0	Fabaceae Lindl.
Iz_10_1209	DJI_0683	Ulmus glabra Huds.	0	n	1	1	Ulmaceae Mirb.
Iz_10_1209	DJI_0683	Verbascum densiflorum Bertol.	0	n	1	0	Scrophulariaceae Juss.
Iz_10_1209	DJI_0683	Verbascum sp.	0	n	1	0	Scrophulariaceae Juss.
Iz_10_1209	DJI_0683	Veronica austriaca L.	0	n	1	0	Plantaginaceae Juss.
Iz_10_1209	DJI_0683	Veronica incana L.	0	n	1	0	Plantaginaceae Juss.
Iz_10_1209	DJI_0683	Veronica verna L.	0	n	1	0	Plantaginaceae Juss.
Iz_10_1209	DJI_0683	Vincetoxicum hirundinaria Medik	0	n	1	0	Apocynaceae Juss.
Iz_10_1209	DJI_0687	Acer tataricum L.	0	n	1	1	Sapindaceae Juss.
Iz_10_1209	DJI_0687	Achillea millefolium L. s.l.	0	n	1	0	Asteraceae Bercht. & J.Presl
Iz_10_1209	DJI_0687	Allium oleraceum L.	0	n	1	0	Amaryllidaceae J.St.-Hil.
Iz_10_1209	DJI_0687	Ambrosia artemisiifolia L.	0	a	0	0	Asteraceae Bercht. & J.Presl
Iz_10_1209	DJI_0687	Amorpha fruticosa L.	0	a	0	0	Fabaceae Lindl.
Iz_10_1209	DJI_0687	Anthericum ramosum L.	0	n	1	0	Asparagaceae Juss.
Iz_10_1209	DJI_0687	Artemisia marschalliana Spreng.	0	n	1	0	Asteraceae Bercht. & J.Presl
Iz_10_1209	DJI_0687	Asparagus officinalis L.	0	n	1	0	Asparagaceae Juss.
Iz_10_1209	DJI_0687	Astragalus cicer L.	0	n	1	0	Fabaceae Lindl.
Iz_10_1209	DJI_0687	Bassia laniflora (S.G.Gmel.) A.J.Scott	0	n	1	0	Amaranthaceae Juss.
Iz_10_1209	DJI_0687	Berberis vulgaris L.	0	n	1	0	Berberidaceae Juss.
Iz_10_1209	DJI_0687	Betula pendula Roth.	0	n	1	1	Betulaceae Gray
Iz_10_1209	DJI_0687	Betula pendula Roth.	0	n	1	0	Poaceae Barnhart

Iz_10_1209	DJI_0687	Bromus inermis Leyss.	0		NA	NA	
Iz_10_1209	DJI_0687	Bromus squarrosus L.	0	a	0	0	Poaceae Barnhart
Iz_10_1209	DJI_0687	Bromus tectorum L.	0	a	0	0	Poaceae Barnhart
Iz_10_1209	DJI_0687	Calamagrostis epigejos (L.) Roth	15	n	1	0	Poaceae Barnhart
Iz_10_1209	DJI_0687	Carduus acanthoides L.	0	n	1	0	Asteraceae Bercht. & J.Presl
Iz_10_1209	DJI_0687	Carex sp.	0	n	1	0	Cyperaceae Juss.
Iz_10_1209	DJI_0687	Centaurea majorovii Dumbadze	0	n	1	0	Asteraceae Bercht. & J.Presl
Iz_10_1209	DJI_0687	Chamaecytisus ruthenicus (Fisch.ex Wol.) Klaskova	0	n	1	0	Fabaceae Lindl.
Iz_10_1209	DJI_0687	Chelidonium majus L.	0	n	1	0	Papaveraceae Juss.
Iz_10_1209	DJI_0687	Chenopodium betaceum Andrz.	0	n	1	0	Amaranthaceae Juss.
Iz_10_1209	DJI_0687	Chenopodium cf. album	0	n	1	0	Amaranthaceae Juss.
Iz_10_1209	DJI_0687	Chenopodium sp.	0		NA	0	Amaranthaceae Juss.
Iz_10_1209	DJI_0687	Chondrilla juncea L.	0	n	1	0	Asteraceae Bercht. & J.Presl
Iz_10_1209	DJI_0687	Cirsium vulgare (Savi) Ten.	0	n	1	0	Asteraceae Bercht. & J.Presl
Iz_10_1209	DJI_0687	Clinopodium acinos (L.) Kuntze	0	n	1	0	Lamiacea Lindl.
Iz_10_1209	DJI_0687	Convallaria majalis L.	0	n	1	0	Asparagaceae Juss.
Iz_10_1209	DJI_0687	Crataegus rhipidophylla Gand.	0	n	1	1	Rosaceae Juss.
Iz_10_1209	DJI_0687	Crepis foetida L.	0	n	1	0	Asteraceae Bercht. & J.Presl
Iz_10_1209	DJI_0687	Dactylis glomerata L.	0	n	1	0	Poaceae Barnhart
Iz_10_1209	DJI_0687	Dianthus campestris M.Bieb.	0	n	1	0	Caryophyllaceae Juss.
Iz_10_1209	DJI_0687	Digitaria sanguinalis (L.) Scop.	0	a	0	0	Poaceae Barnhart
Iz_10_1209	DJI_0687	Elymus repens (L.) Gould	0	n	1	0	Poaceae Barnhart
Iz_10_1209	DJI_0687	Epilobium tetragonum L.	0	n	1	0	Onagraceae Juss.
Iz_10_1209	DJI_0687	Eragrostis minor Host	0	a	0	0	Poaceae Barnhart
Iz_10_1209	DJI_0687	Erigeron canadensis L.	0.5	a	0	0	Asteraceae Bercht. & J.Presl
Iz_10_1209	DJI_0687	Euphorbia seguieriana Neck.	0	n	1	0	Euphorbiaceae Juss.
Iz_10_1209	DJI_0687	Fallopia dumetorum (L.) Holub	0	n	1	0	Polygonaceae Juss.
Iz_10_1209	DJI_0687	Festuca beckeri (Hack.) Trautv.	0	n	1	0	Poaceae Barnhart
Iz_10_1209	DJI_0687	Fragaria vesca L.	0	n	1	0	Rosaceae Juss.
Iz_10_1209	DJI_0687	Frangula alnus Mill.	0	n	1	1	Rhamnaceae Juss.
Iz_10_1209	DJI_0687	Fraxinus pennsylvanica Marshall	0	a	0	1	Oleaceae Hoffmanns. & Link
Iz_10_1209	DJI_0687	Galeopsis bifida Boenn.	0	n	1	0	Lamiacea Lindl.
Iz_10_1209	DJI_0687	Galium verum L.	0	n	1	0	Rubiaceae Juss.
Iz_10_1209	DJI_0687	Genista tinctoria L.	0	n	1	0	Fabaceae Lindl.
Iz_10_1209	DJI_0687	Geranium robertianum L.	0	n	1	0	Geraniaceae Juss.
Iz_10_1209	DJI_0687	Geranium sanquineum L.	0	n	1	0	Geraniaceae Juss.
Iz_10_1209	DJI_0687	Gypsophila paniculata L.	0	n	1	0	Caryophyllaceae Juss.
Iz_10_1209	DJI_0687	Helichrysum arenarium (L.) Moench	0	n	1	0	Asteraceae Bercht. & J.Presl
Iz_10_1209	DJI_0687	Herniaria polygama J.Gay	0	n	1	0	Caryophyllaceae Juss.
Iz_10_1209	DJI_0687	Hieracium robustum Fr.	0	n	1	0	Asteraceae Bercht. & J.Presl
Iz_10_1209	DJI_0687	Hieracium umbellatum L.	0	n	1	NA	Asteraceae Bercht. & J.Presl
Iz_10_1209	DJI_0687	Hylotelephium maximum (L.) Holub	0	n	1	0	Crassulaceae J.St.-Hil.
Iz_10_1209	DJI_0687	Jacobaea borysthenica (DC.) B.Nord. & Greuter	0	n	1	0	Asteraceae Bercht. & J.Presl
Iz_10_1209	DJI_0687	Jasione montana L.	0	n	1	0	Campanulaceae Juss.
Iz_10_1209	DJI_0687	Jurinea cyanoides (L.) Rchb.s.l.	0	n	1	0	Asteraceae Bercht. & J.Presl
Iz_10_1209	DJI_0687	Koeleria glauca (Spreng.) DC.	0	n	1	0	Poaceae Barnhart
Iz_10_1209	DJI_0687	Lactuca serriola L.	0	a	0	0	Asteraceae Bercht. & J.Presl
Iz_10_1209	DJI_0687	Linaria genistifolia (L.) Mill.	0	n	1	0	Plantaginaceae Juss.
Iz_10_1209	DJI_0687	Lomelosia argentea (L.) Greuter & Burdet	0	n	1	0	Caprifoliaceae Juss.
Iz_10_1209	DJI_0687	Melampyrum pratense L.	0	n	1	0	Orobanchaceae Vent.
Iz_10_1209	DJI_0687	Oenothera biennis L.	0	a	0	0	Onagraceae Juss.
Iz_10_1209	DJI_0687	Pentanema britannica (L.) D.Gut.Larr., Santos-Vicente, Anderb., E.Rico & M.M.Mart.Ort.	0	n	1	0	Asteraceae Bercht. & J.Presl
Iz_10_1209	DJI_0687	Peucedanum oreoselinum (L.) Moench	0	n	1	0	Apiaceae Lindl.
Iz_10_1209	DJI_0687	Peucedanum ruthenicum M.Bieb.	0	n	1	0	Apiaceae Lindl.
Iz_10_1209	DJI_0687	Pilosella echioides (Lumn.) F.W.Schultz & Sch.Bip.	0	n	1	0	Asteraceae Bercht. & J.Presl
Iz_10_1209	DJI_0687	Pilosella officinarum F. Schultz et Sch. Bip.	0	n	1	0	Asteraceae Bercht. & J.Presl
Iz_10_1209	DJI_0687	Pilosella sp.	0		NA	0	Asteraceae Bercht. & J.Presl
Iz_10_1209	DJI_0687	Pinus sylvestris L.	0	n	1	1	Pinaceae Spreng. ex F.Rudolphi
Iz_10_1209	DJI_0687	Poa annua L.	0	n	1	0	Poaceae Barnhart
Iz_10_1209	DJI_0687	Poa nemoralis L.	0	n	1	0	Poaceae Barnhart
Iz_10_1209	DJI_0687	Poa sp.	0		NA	0	Poaceae Barnhart
Iz_10_1209	DJI_0687	Polygonatum odoratum (Mill.) Druce	0	n	1	0	Asparagaceae Juss.
Iz_10_1209	DJI_0687	Polygonum arenarium Waldst. & Kit.	0	n	1	0	Polygonaceae Juss.
Iz_10_1209	DJI_0687	Populus nigra L.	0	n	1	1	Salicaceae Mirb.
Iz_10_1209	DJI_0687	Populus tremula L.	0	n	1	1	Salicaceae Mirb.
Iz_10_1209	DJI_0687	Populus Ã canescens (Aiton) Sm.	0	n	1	1	Salicaceae Mirb.
Iz_10_1209	DJI_0687	Potentilla argentea L.	0	n	1	0	Rosaceae Juss.
Iz_10_1209	DJI_0687	Psammophiliella muralis (L.) Ikonn.	0	n	1	0	Caryophyllaceae Juss.
Iz_10_1209	DJI_0687	Psephellus sumensis (Kalen.) Greuter	0	n	1	0	Asteraceae Bercht. & J.Presl
Iz_10_1209	DJI_0687	Pulsatilla pratensis (L.) Mill. s.l.	0	n	1	0	Ranunculaceae Juss.
Iz_10_1209	DJI_0687	Pyrus communis L.	0	a	0	1	Rosaceae Juss.
Iz_10_1209	DJI_0687	Quercus robur L.	0	n	1	1	Fagaceae Dumort.
Iz_10_1209	DJI_0687	Robinia pseudoacacia L.	0	a	0	1	Fabaceae Lindl.
Iz_10_1209	DJI_0687	Rosa sp.	0		NA	1	Rosaceae Juss.
Iz_10_1209	DJI_0687	Rubus idaeus L.	0	n	1	0	Rosaceae Juss.
Iz_10_1209	DJI_0687	Rumex acetosella L.	0	n	1	0	Polygonaceae Juss.
Iz_10_1209	DJI_0687	Rumex thyrsiflorus Fingerh.	0	n	1	0	Polygonaceae Juss.
Iz_10_1209	DJI_0687	Salix alba L.	0	n	1	1	Salicaceae Mirb.
Iz_10_1209	DJI_0687	Sambucus nigra L.	0	n	1	1	Viburnaceae Raf.
Iz_10_1209	DJI_0687	Secale sylvestre Host	0	n	1	0	Poaceae Barnhart
Iz_10_1209	DJI_0687	Sempervivum ruthenicum Schnittsp. & C.B. Lehm.	0	n	1	0	Crassulaceae J.St.-Hil.
Iz_10_1209	DJI_0687	Setaria viridis (L.) P. Beauv.	0	n	1	0	Poaceae Barnhart
Iz_10_1209	DJI_0687	Silene borysthenica (Gruner) Walters	0	n	1	0	Caryophyllaceae Juss.
Iz_10_1209	DJI_0687	Silene latifolia subsp. alba (Mill.) Greuter & Burdet	0	n	1	0	Caryophyllaceae Juss.
Iz_10_1209	DJI_0687	Solidago virgaurea L.	0	n	1	0	Asteraceae Bercht. & J.Presl
Iz_10_1209	DJI_0687	Sorbus aucuparia L.	0	n	1	1	Rosaceae Juss.
Iz_10_1209	DJI_0687	Stachys recta L.	0	n	1	0	Lamiacea Lindl.
Iz_10_1209	DJI_0687	Tanacetum vulgare L.	0	n	1	0	Asteraceae Bercht. & J.Presl
Iz_10_1209	DJI_0687	Thymus pallasianus Heinr.Braun	0	n	1	0	Lamiacea Lindl.
Iz_10_1209	DJI_0687	Tilia cordata Mill.	0	n	1	1	Malvaceae Juss.
Iz_10_1209	DJI_0687	Trifolium alpestre L.	0	n	1	0	Fabaceae Lindl.
Iz_10_1209	DJI_0687	Trifolium arvense L.	0	n	1	0	Fabaceae Lindl.
Iz_10_1209	DJI_0687	Ulmus glabra Huds.	0	n	1	1	Ulmaceae Mirb.
Iz_10_1209	DJI_0687	Verbascum densiflorum Bertol.	0	n	1	0	Scrophulariaceae Juss.
Iz_10_1209	DJI_0687	Verbascum sp.	0	n	1	0	Scrophulariaceae Juss.
Iz_10_1209	DJI_0687	Veronica austriaca L.	0	n	1	0	Plantaginaceae Juss.
Iz_10_1209	DJI_0687	Veronica incana L.	0	n	1	0	Plantaginaceae Juss.
Iz_10_1209	DJI_0687	Veronica verna L.	0	n	1	0	Plantaginaceae Juss.

Iz_10_1209	DJI_0687	Vincetoxicum hirundinaria Medik	0	n	1	0	Apocynaceae Juss.
Iz_10_1209	DJI_0691	Acer tataricum L.	0	n	1	1	Sapindaceae Juss.
Iz_10_1209	DJI_0691	Achillea millefolium L. s.l.	0	n	1	0	Asteraceae Bercht. & J.Presl
Iz_10_1209	DJI_0691	Allium oleraceum L.	0	n	1	0	Amaryllidaceae J.St.-Hil.
Iz_10_1209	DJI_0691	Ambrosia artemisiifolia L.	0	a	0	0	Asteraceae Bercht. & J.Presl
Iz_10_1209	DJI_0691	Amorpha fruticosa L.	0	a	0	0	Fabaceae Lindl.
Iz_10_1209	DJI_0691	Anthericum ramosum L.	0	n	1	0	Asparagaceae Juss.
Iz_10_1209	DJI_0691	Artemisia marschalliana Spreng.	0	n	1	0	Asteraceae Bercht. & J.Presl
Iz_10_1209	DJI_0691	Asparagus officinalis L.	0	n	1	0	Asparagaceae Juss.
Iz_10_1209	DJI_0691	Astragalus cicer L.	0	n	1	0	Fabaceae Lindl.
Iz_10_1209	DJI_0691	Bassia laniflora (S.G.Gmel.) A.J.Scott	0	n	1	0	Amaranthaceae Juss.
Iz_10_1209	DJI_0691	Berberis vulgaris L.	0	n	1	0	Berberidaceae Juss.
Iz_10_1209	DJI_0691	Betula pendula Roth.	0	n	1	1	Betulaceae Gray
Iz_10_1209	DJI_0691	Betula pendula Roth.	0	n	1	0	Poaceae Barnhart
Iz_10_1209	DJI_0691	Bromus inermis Leyss.	0		NA	NA	
Iz_10_1209	DJI_0691	Bromus squarrosus L.	0	a	0	0	Poaceae Barnhart
Iz_10_1209	DJI_0691	Bromus tectorum L.	0	a	0	0	Poaceae Barnhart
Iz_10_1209	DJI_0691	Calamagrostis epigejos (L.) Roth	10	n	1	0	Poaceae Barnhart
Iz_10_1209	DJI_0691	Carduus acanthoides L.	0	n	1	0	Asteraceae Bercht. & J.Presl
Iz_10_1209	DJI_0691	Carex sp.	0	n	1	0	Cyperaceae Juss.
Iz_10_1209	DJI_0691	Centaurea majorovii Dumbadze	0	n	1	0	Asteraceae Bercht. & J.Presl
Iz_10_1209	DJI_0691	Chamaecytisus ruthenicus (Fisch.ex Wol.) Klaskova	0	n	1	0	Fabaceae Lindl.
Iz_10_1209	DJI_0691	Chelidonium majus L.	0	n	1	0	Papaveraceae Juss.
Iz_10_1209	DJI_0691	Chenopodium betaceum Andrz.	0	n	1	0	Amaranthaceae Juss.
Iz_10_1209	DJI_0691	Chenopodium cf. album	0	n	1	0	Amaranthaceae Juss.
Iz_10_1209	DJI_0691	Chenopodium sp.	0		NA	0	Amaranthaceae Juss.
Iz_10_1209	DJI_0691	Chondrilla juncea L.	0	n	1	0	Asteraceae Bercht. & J.Presl
Iz_10_1209	DJI_0691	Cirsium vulgare (Savi) Ten.	0	n	1	0	Asteraceae Bercht. & J.Presl
Iz_10_1209	DJI_0691	Clinopodium acinos (L.) Kuntze	0	n	1	0	Lamiacea Lindl.
Iz_10_1209	DJI_0691	Convallaria majalis L.	0	n	1	0	Asparagaceae Juss.
Iz_10_1209	DJI_0691	Crataegus rhipidophylla Gand.	0	n	1	1	Rosaceae Juss.
Iz_10_1209	DJI_0691	Crepis foetida L.	0	n	1	0	Asteraceae Bercht. & J.Presl
Iz_10_1209	DJI_0691	Dactylis glomerata L.	0	n	1	0	Poaceae Barnhart
Iz_10_1209	DJI_0691	Dianthus campestris M.Bieb.	0	n	1	0	Caryophyllaceae Juss.
Iz_10_1209	DJI_0691	Digitaria sanguinalis (L.) Scop.	0	a	0	0	Poaceae Barnhart
Iz_10_1209	DJI_0691	Elymus repens (L.) Gould	0	n	1	0	Poaceae Barnhart
Iz_10_1209	DJI_0691	Epilobium tetragonum L.	0	n	1	0	Onagraceae Juss.
Iz_10_1209	DJI_0691	Eragrostis minor Host	0	a	0	0	Poaceae Barnhart
Iz_10_1209	DJI_0691	Erigeron canadensis L.	3	a	0	0	Asteraceae Bercht. & J.Presl
Iz_10_1209	DJI_0691	Euphorbia seguieriana Neck.	0	n	1	0	Euphorbiaceae Juss.
Iz_10_1209	DJI_0691	Fallopia dumetorum (L.) Holub	0	n	1	0	Polygonaceae Juss.
Iz_10_1209	DJI_0691	Festuca beckeri (Hack.) Trautv.	0	n	1	0	Poaceae Barnhart
Iz_10_1209	DJI_0691	Fragaria vesca L.	0	n	1	0	Rosaceae Juss.
Iz_10_1209	DJI_0691	Frangula alnus Mill.	0	n	1	1	Rhamnaceae Juss.
Iz_10_1209	DJI_0691	Fraxinus pennsylvanica Marshall	0	a	0	1	Oleaceae Hoffmanns. & Link
Iz_10_1209	DJI_0691	Galeopsis bifida Boenn.	0	n	1	0	Lamiacea Lindl.
Iz_10_1209	DJI_0691	Galium verum L.	0	n	1	0	Rubiaceae Juss.
Iz_10_1209	DJI_0691	Genista tinctoria L.	0	n	1	0	Fabaceae Lindl.
Iz_10_1209	DJI_0691	Geranium robertianum L.	0	n	1	0	Geraniaceae Juss.
Iz_10_1209	DJI_0691	Geranium sanquineum L.	0	n	1	0	Geraniaceae Juss.
Iz_10_1209	DJI_0691	Gypsophila paniculata L.	0	n	1	0	Caryophyllaceae Juss.
Iz_10_1209	DJI_0691	Helichrysum arenarium (L.) Moench	0	n	1	0	Asteraceae Bercht. & J.Presl
Iz_10_1209	DJI_0691	Herniaria polygama J.Gay	0	n	1	0	Caryophyllaceae Juss.
Iz_10_1209	DJI_0691	Hieracium robustum Fr.	0	n	1	0	Asteraceae Bercht. & J.Presl
Iz_10_1209	DJI_0691	Hieracium umbellatum L.	0	n	1	NA	Asteraceae Bercht. & J.Presl
Iz_10_1209	DJI_0691	Hylotelephium maximum (L.) Holub	0	n	1	0	Crassulaceae J.St.-Hil.
Iz_10_1209	DJI_0691	Jacobaea borysthenica (DC.) B.Nord. & Greuter	0	n	1	0	Asteraceae Bercht. & J.Presl
Iz_10_1209	DJI_0691	Jasione montana L.	0	n	1	0	Campanulaceae Juss.
Iz_10_1209	DJI_0691	Jurinea cyanoides (L.) Rchb.s.l.	0	n	1	0	Asteraceae Bercht. & J.Presl
Iz_10_1209	DJI_0691	Koeleria glauca (Spreng.) DC.	0	n	1	0	Poaceae Barnhart
Iz_10_1209	DJI_0691	Lactuca serriola L.	0	a	0	0	Asteraceae Bercht. & J.Presl
Iz_10_1209	DJI_0691	Linaria genistifolia (L.) Mill.	0	n	1	0	Plantaginaceae Juss.
Iz_10_1209	DJI_0691	Lomelosia argentea (L.) Greuter & Burdet	0	n	1	0	Caprifoliaceae Juss.
Iz_10_1209	DJI_0691	Melampyrum pratense L.	0	n	1	0	Orobanchaceae Vent.
Iz_10_1209	DJI_0691	Oenothera biennis L.	0	a	0	0	Onagraceae Juss.
Iz_10_1209	DJI_0691	Pentanema britannica (L.) D.Gut.Larr., Santos-Vicente, Anderb., E.Rico & M.M.Mart.Ort.	0	n	1	0	Asteraceae Bercht. & J.Presl
Iz_10_1209	DJI_0691	Peucedanum oreoselinum (L.) Moench	0	n	1	0	Apiaceae Lindl.
Iz_10_1209	DJI_0691	Peucedanum ruthenicum M.Bieb.	0	n	1	0	Apiaceae Lindl.
Iz_10_1209	DJI_0691	Pilosella echioides (Lumn.) F.W.Schultz & Sch.Bip.	0	n	1	0	Asteraceae Bercht. & J.Presl
Iz_10_1209	DJI_0691	Pilosella officinarum F. Schultz et Sch. Bip.	0	n	1	0	Asteraceae Bercht. & J.Presl
Iz_10_1209	DJI_0691	Pilosella sp.	0		NA	0	Asteraceae Bercht. & J.Presl
Iz_10_1209	DJI_0691	Pinus sylvestris L.	0	n	1	1	Pinaceae Spreng. ex F.Rudolphi
Iz_10_1209	DJI_0691	Poa annua L.	0	n	1	0	Poaceae Barnhart
Iz_10_1209	DJI_0691	Poa nemoralis L.	0	n	1	0	Poaceae Barnhart
Iz_10_1209	DJI_0691	Poa sp.	0		NA	0	Poaceae Barnhart
Iz_10_1209	DJI_0691	Polygonatum odoratum (Mill.) Druce	0	n	1	0	Asparagaceae Juss.
Iz_10_1209	DJI_0691	Polygonum arenarium Waldst. & Kit.	0	n	1	0	Polygonaceae Juss.
Iz_10_1209	DJI_0691	Populus nigra L.	0	n	1	1	Salicaceae Mirb.
Iz_10_1209	DJI_0691	Populus tremula L.	0	n	1	1	Salicaceae Mirb.
Iz_10_1209	DJI_0691	Populus Ã canescens (Aiton) Sm.	0	n	1	1	Salicaceae Mirb.
Iz_10_1209	DJI_0691	Potentilla argentea L.	0	n	1	0	Rosaceae Juss.
Iz_10_1209	DJI_0691	Psammophiliella muralis (L.) Ikonn.	0	n	1	0	Caryophyllaceae Juss.
Iz_10_1209	DJI_0691	Psephellus sumensis (Kalen.) Greuter	0	n	1	0	Asteraceae Bercht. & J.Presl
Iz_10_1209	DJI_0691	Pulsatilla pratensis (L.) Mill. s.l.	0	n	1	0	Ranunculaceae Juss.
Iz_10_1209	DJI_0691	Pyrus communis L.	0	a	0	1	Rosaceae Juss.
Iz_10_1209	DJI_0691	Quercus robur L.	0	n	1	1	Fagaceae Dumort.
Iz_10_1209	DJI_0691	Robinia pseudoacacia L.	0	a	0	1	Fabaceae Lindl.
Iz_10_1209	DJI_0691	Rosa sp.	0		NA	1	Rosaceae Juss.
Iz_10_1209	DJI_0691	Rubus idaeus L.	0	n	1	0	Rosaceae Juss.
Iz_10_1209	DJI_0691	Rumex acetosella L.	0	n	1	0	Polygonaceae Juss.
Iz_10_1209	DJI_0691	Rumex thyrsiflorus Fingerh.	0	n	1	0	Polygonaceae Juss.
Iz_10_1209	DJI_0691	Salix alba L.	0	n	1	1	Salicaceae Mirb.
Iz_10_1209	DJI_0691	Sambucus nigra L.	0	n	1	1	Viburnaceae Raf.
Iz_10_1209	DJI_0691	Secale sylvestre Host	0	n	1	0	Poaceae Barnhart
Iz_10_1209	DJI_0691	Sempervivum ruthenicum Schnittsp. & C.B. Lehm.	0	n	1	0	Crassulaceae J.St.-Hil.
Iz_10_1209	DJI_0691	Setaria viridis (L.) P. Beauv.	0	n	1	0	Poaceae Barnhart
Iz_10_1209	DJI_0691	Silene borysthenica (Gruner) Walters	0	n	1	0	Caryophyllaceae Juss.
Iz_10_1209	DJI_0691	Silene latifolia subsp. alba (Mill.) Greuter & Burdet	0	n	1	0	Caryophyllaceae Juss.

Iz_10_1209	DJI_0691	Solidago virgaurea L.	0	n	1	0	Asteraceae Bercht. & J.Presl
Iz_10_1209	DJI_0691	Sorbus aucuparia L.	0	n	1	1	Rosaceae Juss.
Iz_10_1209	DJI_0691	Stachys recta L.	0	n	1	0	Lamiacea Lindl.
Iz_10_1209	DJI_0691	Tanacetum vulgare L.	0	n	1	0	Asteraceae Bercht. & J.Presl
Iz_10_1209	DJI_0691	Thymus pallasianus Heinr.Braun	0	n	1	0	Lamiacea Lindl.
Iz_10_1209	DJI_0691	Tilia cordata Mill.	0	n	1	1	Malvaceae Juss.
Iz_10_1209	DJI_0691	Trifolium alpestre L.	0	n	1	0	Fabaceae Lindl.
Iz_10_1209	DJI_0691	Trifolium arvense L.	0	n	1	0	Fabaceae Lindl.
Iz_10_1209	DJI_0691	Ulmus glabra Huds.	0	n	1	1	Ulmaceae Mirb.
Iz_10_1209	DJI_0691	Verbascum densiflorum Bertol.	0	n	1	0	Scrophulariaceae Juss.
Iz_10_1209	DJI_0691	Verbascum sp.	1	n	1	0	Scrophulariaceae Juss.
Iz_10_1209	DJI_0691	Veronica austriaca L.	0	n	1	0	Plantaginaceae Juss.
Iz_10_1209	DJI_0691	Veronica incana L.	0	n	1	0	Plantaginaceae Juss.
Iz_10_1209	DJI_0691	Veronica verna L.	0	n	1	0	Plantaginaceae Juss.
Iz_10_1209	DJI_0691	Vincetoxicum hirundinaria Medik	0	n	1	0	Apocynaceae Juss.
Iz_11_1209	DJI_0719	Acer tataricum L.	0	n	1	1	Sapindaceae Juss.
Iz_11_1209	DJI_0719	Achillea millefolium L. s.l.	0	n	1	0	Asteraceae Bercht. & J.Presl
Iz_11_1209	DJI_0719	Allium oleraceum L.	0	n	1	0	Amaryllidaceae J.St.-Hil.
Iz_11_1209	DJI_0719	Ambrosia artemisiifolia L.	0	a	0	0	Asteraceae Bercht. & J.Presl
Iz_11_1209	DJI_0719	Amorpha fruticosa L.	0	a	0	0	Fabaceae Lindl.
Iz_11_1209	DJI_0719	Anthericum ramosum L.	0	n	1	0	Asparagaceae Juss.
Iz_11_1209	DJI_0719	Artemisia marschalliana Spreng.	0	n	1	0	Asteraceae Bercht. & J.Presl
Iz_11_1209	DJI_0719	Asparagus officinalis L.	0	n	1	0	Asparagaceae Juss.
Iz_11_1209	DJI_0719	Astragalus cicer L.	0	n	1	0	Fabaceae Lindl.
Iz_11_1209	DJI_0719	Bassia laniflora (S.G.Gmel.) A.J.Scott	0	n	1	0	Amaranthaceae Juss.
Iz_11_1209	DJI_0719	Berberis vulgaris L.	0	n	1	0	Berberidaceae Juss.
Iz_11_1209	DJI_0719	Betula pendula Roth.	0	n	1	1	Betulaceae Gray
Iz_11_1209	DJI_0719	Betula pendula Roth.	0	n	1	0	Poaceae Barnhart
Iz_11_1209	DJI_0719	Bromus inermis Leyss.	0		NA	NA	
Iz_11_1209	DJI_0719	Bromus squarrosus L.	0	a	0	0	Poaceae Barnhart
Iz_11_1209	DJI_0719	Bromus tectorum L.	0	a	0	0	Poaceae Barnhart
Iz_11_1209	DJI_0719	Calamagrostis epigejos (L.) Roth	0.2	n	1	0	Poaceae Barnhart
Iz_11_1209	DJI_0719	Carduus acanthoides L.	0	n	1	0	Asteraceae Bercht. & J.Presl
Iz_11_1209	DJI_0719	Carex sp.	0	n	1	0	Cyperaceae Juss.
Iz_11_1209	DJI_0719	Centaurea majorovii Dumbadze	0	n	1	0	Asteraceae Bercht. & J.Presl
Iz_11_1209	DJI_0719	Chamaecytisus ruthenicus (Fisch.ex Wol.) Klaskova	0	n	1	0	Fabaceae Lindl.
Iz_11_1209	DJI_0719	Chelidonium majus L.	0	n	1	0	Papaveraceae Juss.
Iz_11_1209	DJI_0719	Chenopodium betaceum Andrz.	0	n	1	0	Amaranthaceae Juss.
Iz_11_1209	DJI_0719	Chenopodium cf. album	0	n	1	0	Amaranthaceae Juss.
Iz_11_1209	DJI_0719	Chenopodium sp.	0		NA	0	Amaranthaceae Juss.
Iz_11_1209	DJI_0719	Chondrilla juncea L.	0	n	1	0	Asteraceae Bercht. & J.Presl
Iz_11_1209	DJI_0719	Cirsium vulgare (Savi) Ten.	0	n	1	0	Asteraceae Bercht. & J.Presl
Iz_11_1209	DJI_0719	Clinopodium acinos (L.) Kuntze	0	n	1	0	Lamiacea Lindl.
Iz_11_1209	DJI_0719	Convallaria majalis L.	0	n	1	0	Asparagaceae Juss.
Iz_11_1209	DJI_0719	Crataegus rhipidophylla Gand.	0	n	1	1	Rosaceae Juss.
Iz_11_1209	DJI_0719	Crepis foetida L.	0	n	1	0	Asteraceae Bercht. & J.Presl
Iz_11_1209	DJI_0719	Dactylis glomerata L.	0	n	1	0	Poaceae Barnhart
Iz_11_1209	DJI_0719	Dianthus campestris M.Bieb.	0	n	1	0	Caryophyllaceae Juss.
Iz_11_1209	DJI_0719	Digitaria sanguinalis (L.) Scop.	0	a	0	0	Poaceae Barnhart
Iz_11_1209	DJI_0719	Elymus repens (L.) Gould	0	n	1	0	Poaceae Barnhart
Iz_11_1209	DJI_0719	Epilobium tetragonum L.	0	n	1	0	Onagraceae Juss.
Iz_11_1209	DJI_0719	Eragrostis minor Host	0	a	0	0	Poaceae Barnhart
Iz_11_1209	DJI_0719	Erigeron canadensis L.	10	a	0	0	Asteraceae Bercht. & J.Presl
Iz_11_1209	DJI_0719	Euphorbia seguieriana Neck.	0	n	1	0	Euphorbiaceae Juss.
Iz_11_1209	DJI_0719	Euphorbia sp.	0		NA	NA	
Iz_11_1209	DJI_0719	Fallopia dumetorum (L.) Holub	0	n	1	0	Polygonaceae Juss.
Iz_11_1209	DJI_0719	Festuca beckeri (Hack.) Trautv.	0	n	1	0	Poaceae Barnhart
Iz_11_1209	DJI_0719	Fragaria vesca L.	0	n	1	0	Rosaceae Juss.
Iz_11_1209	DJI_0719	Frangula alnus Mill.	0	n	1	1	Rhamnaceae Juss.
Iz_11_1209	DJI_0719	Fraxinus pennsylvanica Marshall	0	a	0	1	Oleaceae Hoffmanns. & Link
Iz_11_1209	DJI_0719	Galeopsis bifida Boenn.	0	n	1	0	Lamiacea Lindl.
Iz_11_1209	DJI_0719	Galium verum L.	0	n	1	0	Rubiaceae Juss.
Iz_11_1209	DJI_0719	Genista tinctoria L.	0	n	1	0	Fabaceae Lindl.
Iz_11_1209	DJI_0719	Geranium robertianum L.	0	n	1	0	Geraniaceae Juss.
Iz_11_1209	DJI_0719	Geranium sanquineum L.	0	n	1	0	Geraniaceae Juss.
Iz_11_1209	DJI_0719	Gypsophila paniculata L.	0	n	1	0	Caryophyllaceae Juss.
Iz_11_1209	DJI_0719	Helichrysum arenarium (L.) Moench	0	n	1	0	Asteraceae Bercht. & J.Presl
Iz_11_1209	DJI_0719	Herniaria polygama J.Gay	0	n	1	0	Caryophyllaceae Juss.
Iz_11_1209	DJI_0719	Hieracium robustum Fr.	0	n	1	0	Asteraceae Bercht. & J.Presl
Iz_11_1209	DJI_0719	Hieracium umbellatum L.	0	n	1	NA	Asteraceae Bercht. & J.Presl
Iz_11_1209	DJI_0719	Hylotelephium maximum (L.) Holub	0	n	1	0	Crassulaceae J.St.-Hil.
Iz_11_1209	DJI_0719	Jacobaea borysthenica (DC.) B.Nord. & Greuter	0	n	1	0	Asteraceae Bercht. & J.Presl
Iz_11_1209	DJI_0719	Jasione montana L.	0	n	1	0	Campanulaceae Juss.
Iz_11_1209	DJI_0719	Jurinea cyanoides (L.) Rchb.s.l.	0	n	1	0	Asteraceae Bercht. & J.Presl
Iz_11_1209	DJI_0719	Koeleria glauca (Spreng.) DC.	0	n	1	0	Poaceae Barnhart
Iz_11_1209	DJI_0719	Lactuca serriola L.	0	a	0	0	Asteraceae Bercht. & J.Presl
Iz_11_1209	DJI_0719	Linaria genistifolia (L.) Mill.	0	n	1	0	Plantaginaceae Juss.
Iz_11_1209	DJI_0719	Lomelosia argentea (L.) Greuter & Burdet	0	n	1	0	Caprifoliaceae Juss.
Iz_11_1209	DJI_0719	Melampyrum pratense L.	0	n	1	0	Orobanchaceae Vent.
Iz_11_1209	DJI_0719	Oenothera biennis L.	0	a	0	0	Onagraceae Juss.
Iz_11_1209	DJI_0719	Pentanema britannica (L.) D.Gut.Larr., Santos-Vicente, Anderb., E.Rico & M.M.Mart.Ort.	0	n	1	0	Asteraceae Bercht. & J.Presl
Iz_11_1209	DJI_0719	Peucedanum oreoselinum (L.) Moench	0	n	1	0	Apiaceae Lindl.
Iz_11_1209	DJI_0719	Peucedanum ruthenicum M.Bieb.	0	n	1	0	Apiaceae Lindl.
Iz_11_1209	DJI_0719	Pilosella echioides (Lumn.) F.W.Schultz & Sch.Bip.	0	n	1	0	Asteraceae Bercht. & J.Presl
Iz_11_1209	DJI_0719	Pilosella officinarum F. Schultz et Sch. Bip.	0	n	1	0	Asteraceae Bercht. & J.Presl
Iz_11_1209	DJI_0719	Pilosella sp.	1		NA	0	Asteraceae Bercht. & J.Presl
Iz_11_1209	DJI_0719	Pinus sylvestris L.	0	n	1	1	Pinaceae Spreng. ex F.Rudolphi
Iz_11_1209	DJI_0719	Poa annua L.	0	n	1	0	Poaceae Barnhart
Iz_11_1209	DJI_0719	Poa nemoralis L.	0	n	1	0	Poaceae Barnhart
Iz_11_1209	DJI_0719	Poa sp.	0		NA	0	Poaceae Barnhart
Iz_11_1209	DJI_0719	Polygonatum odoratum (Mill.) Druce	0	n	1	0	Asparagaceae Juss.
Iz_11_1209	DJI_0719	Polygonum arenarium Waldst. & Kit.	0.5	n	1	0	Polygonaceae Juss.
Iz_11_1209	DJI_0719	Populus nigra L.	0	n	1	1	Salicaceae Mirb.
Iz_11_1209	DJI_0719	Populus tremula L.	0	n	1	1	Salicaceae Mirb.
Iz_11_1209	DJI_0719	Populus Ã canescens (Aiton) Sm.	0	n	1	1	Salicaceae Mirb.
Iz_11_1209	DJI_0719	Potentilla argentea L.	0	n	1	0	Rosaceae Juss.
Iz_11_1209	DJI_0719	Psammophiliella muralis (L.) Ikonn.	0	n	1	0	Caryophyllaceae Juss.
Iz_11_1209	DJI_0719	Psephellus sumensis (Kalen.) Greuter	0	n	1	0	Asteraceae Bercht. & J.Presl

Iz_11_1209	DJI_0719	Pulsatilla pratensis (L.) Mill. s.l.	0	n	1	0	Ranunculaceae Juss.
Iz_11_1209	DJI_0719	Pyrus communis L.	0	a	0	1	Rosaceae Juss.
Iz_11_1209	DJI_0719	Quercus robur L.	0	n	1	1	Fagaceae Dumort.
Iz_11_1209	DJI_0719	Robinia pseudoacacia L.	0	a	0	1	Fabaceae Lindl.
Iz_11_1209	DJI_0719	Rosa sp.	0		NA	1	Rosaceae Juss.
Iz_11_1209	DJI_0719	Rubus idaeus L.	0	n	1	0	Rosaceae Juss.
Iz_11_1209	DJI_0719	Rumex acetosella L.	0	n	1	0	Polygonaceae Juss.
Iz_11_1209	DJI_0719	Rumex thyrsiflorus Fingerh.	0	n	1	0	Polygonaceae Juss.
Iz_11_1209	DJI_0719	Salix alba L.	0	n	1	1	Salicaceae Mirb.
Iz_11_1209	DJI_0719	Sambucus nigra L.	0	n	1	1	Viburnaceae Raf.
Iz_11_1209	DJI_0719	Secale sylvestre Host	0	n	1	0	Poaceae Barnhart
Iz_11_1209	DJI_0719	Sempervivum ruthenicum Schnittsp. & C.B. Lehm.	0	n	1	0	Crassulaceae J.St.-Hil.
Iz_11_1209	DJI_0719	Setaria viridis (L.) P. Beauv.	0	n	1	0	Poaceae Barnhart
Iz_11_1209	DJI_0719	Silene borysthenica (Gruner) Walters	0	n	1	0	Caryophyllaceae Juss.
Iz_11_1209	DJI_0719	Silene latifolia subsp. alba (Mill.) Greuter & Burdet	0	n	1	0	Caryophyllaceae Juss.
Iz_11_1209	DJI_0719	Solidago virgaurea L.	0	n	1	0	Asteraceae Bercht. & J.Presl
Iz_11_1209	DJI_0719	Sorbus aucuparia L.	0	n	1	1	Rosaceae Juss.
Iz_11_1209	DJI_0719	Stachys recta L.	0	n	1	0	Lamiacea Lindl.
Iz_11_1209	DJI_0719	Tanacetum vulgare L.	0	n	1	0	Asteraceae Bercht. & J.Presl
Iz_11_1209	DJI_0719	Thymus pallasianus Heinr.Braun	0	n	1	0	Lamiacea Lindl.
Iz_11_1209	DJI_0719	Tilia cordata Mill.	0	n	1	1	Malvaceae Juss.
Iz_11_1209	DJI_0719	Trifolium alpestre L.	0	n	1	0	Fabaceae Lindl.
Iz_11_1209	DJI_0719	Trifolium arvense L.	0	n	1	0	Fabaceae Lindl.
Iz_11_1209	DJI_0719	Ulmus glabra Huds.	0	n	1	1	Ulmaceae Mirb.
Iz_11_1209	DJI_0719	Verbascum densiflorum Bertol.	0	n	1	0	Scrophulariaceae Juss.
Iz_11_1209	DJI_0719	Veronica austriaca L.	0	n	1	0	Plantaginaceae Juss.
Iz_11_1209	DJI_0719	Veronica incana L.	0	n	1	0	Plantaginaceae Juss.
Iz_11_1209	DJI_0719	Veronica verna L.	0	n	1	0	Plantaginaceae Juss.
Iz_11_1209	DJI_0719	Vincetoxicum hirundinaria Medik	0	n	1	0	Apocynaceae Juss.
Iz_11_1209	DJI_0722	Acer tataricum L.	0	n	1	1	Sapindaceae Juss.
Iz_11_1209	DJI_0722	Achillea millefolium L. s.l.	0	n	1	0	Asteraceae Bercht. & J.Presl
Iz_11_1209	DJI_0722	Allium oleraceum L.	0	n	1	0	Amaryllidaceae J.St.-Hil.
Iz_11_1209	DJI_0722	Ambrosia artemisiifolia L.	0	a	0	0	Asteraceae Bercht. & J.Presl
Iz_11_1209	DJI_0722	Amorpha fruticosa L.	0	a	0	0	Fabaceae Lindl.
Iz_11_1209	DJI_0722	Anthericum ramosum L.	0	n	1	0	Asparagaceae Juss.
Iz_11_1209	DJI_0722	Artemisia marschalliana Spreng.	0	n	1	0	Asteraceae Bercht. & J.Presl
Iz_11_1209	DJI_0722	Asparagus officinalis L.	0	n	1	0	Asparagaceae Juss.
Iz_11_1209	DJI_0722	Astragalus cicer L.	0	n	1	0	Fabaceae Lindl.
Iz_11_1209	DJI_0722	Bassia laniflora (S.G.Gmel.) A.J.Scott	0	n	1	0	Amaranthaceae Juss.
Iz_11_1209	DJI_0722	Berberis vulgaris L.	0	n	1	0	Berberidaceae Juss.
Iz_11_1209	DJI_0722	Betula pendula Roth.	0	n	1	1	Betulaceae Gray
Iz_11_1209	DJI_0722	Betula pendula Roth.	0	n	1	0	Poaceae Barnhart
Iz_11_1209	DJI_0722	Bromus inermis Leyss.	0		NA	NA	
Iz_11_1209	DJI_0722	Bromus squarrosus L.	0	a	0	0	Poaceae Barnhart
Iz_11_1209	DJI_0722	Bromus tectorum L.	0	a	0	0	Poaceae Barnhart
Iz_11_1209	DJI_0722	Calamagrostis epigejos (L.) Roth	2	n	1	0	Poaceae Barnhart
Iz_11_1209	DJI_0722	Carduus acanthoides L.	0	n	1	0	Asteraceae Bercht. & J.Presl
Iz_11_1209	DJI_0722	Carex sp.	0	n	1	0	Cyperaceae Juss.
Iz_11_1209	DJI_0722	Centaurea majorovii Dumbadze	0	n	1	0	Asteraceae Bercht. & J.Presl
Iz_11_1209	DJI_0722	Chamaecytisus ruthenicus (Fisch.ex Wol.) Klaskova	0	n	1	0	Fabaceae Lindl.
Iz_11_1209	DJI_0722	Chelidonium majus L.	0	n	1	0	Papaveraceae Juss.
Iz_11_1209	DJI_0722	Chenopodium betaceum Andrz.	0	n	1	0	Amaranthaceae Juss.
Iz_11_1209	DJI_0722	Chenopodium cf. album	0	n	1	0	Amaranthaceae Juss.
Iz_11_1209	DJI_0722	Chenopodium sp.	0		NA	0	Amaranthaceae Juss.
Iz_11_1209	DJI_0722	Chondrilla juncea L.	0	n	1	0	Asteraceae Bercht. & J.Presl
Iz_11_1209	DJI_0722	Cirsium vulgare (Savi) Ten.	0	n	1	0	Asteraceae Bercht. & J.Presl
Iz_11_1209	DJI_0722	Clinopodium acinos (L.) Kuntze	0	n	1	0	Lamiacea Lindl.
Iz_11_1209	DJI_0722	Convallaria majalis L.	0	n	1	0	Asparagaceae Juss.
Iz_11_1209	DJI_0722	Crataegus rhipidophylla Gand.	0	n	1	1	Rosaceae Juss.
Iz_11_1209	DJI_0722	Crepis foetida L.	0	n	1	0	Asteraceae Bercht. & J.Presl
Iz_11_1209	DJI_0722	Dactylis glomerata L.	0	n	1	0	Poaceae Barnhart
Iz_11_1209	DJI_0722	Dianthus campestris M.Bieb.	0	n	1	0	Caryophyllaceae Juss.
Iz_11_1209	DJI_0722	Digitaria sanguinalis (L.) Scop.	0	a	0	0	Poaceae Barnhart
Iz_11_1209	DJI_0722	Elymus repens (L.) Gould	0	n	1	0	Poaceae Barnhart
Iz_11_1209	DJI_0722	Epilobium tetragonum L.	0	n	1	0	Onagraceae Juss.
Iz_11_1209	DJI_0722	Eragrostis minor Host	0	a	0	0	Poaceae Barnhart
Iz_11_1209	DJI_0722	Erigeron canadensis L.	5	a	0	0	Asteraceae Bercht. & J.Presl
Iz_11_1209	DJI_0722	Euphorbia seguieriana Neck.	0	n	1	0	Euphorbiaceae Juss.
Iz_11_1209	DJI_0722	Euphorbia sp.	0		NA	NA	
Iz_11_1209	DJI_0722	Fallopia dumetorum (L.) Holub	0	n	1	0	Polygonaceae Juss.
Iz_11_1209	DJI_0722	Festuca beckeri (Hack.) Trautv.	0	n	1	0	Poaceae Barnhart
Iz_11_1209	DJI_0722	Fragaria vesca L.	0	n	1	0	Rosaceae Juss.
Iz_11_1209	DJI_0722	Frangula alnus Mill.	0	n	1	1	Rhamnaceae Juss.
Iz_11_1209	DJI_0722	Fraxinus pennsylvanica Marshall	0	a	0	1	Oleaceae Hoffmanns. & Link
Iz_11_1209	DJI_0722	Galeopsis bifida Boenn.	0	n	1	0	Lamiacea Lindl.
Iz_11_1209	DJI_0722	Galium verum L.	0	n	1	0	Rubiaceae Juss.
Iz_11_1209	DJI_0722	Genista tinctoria L.	0	n	1	0	Fabaceae Lindl.
Iz_11_1209	DJI_0722	Geranium robertianum L.	0	n	1	0	Geraniaceae Juss.
Iz_11_1209	DJI_0722	Geranium sanquineum L.	0	n	1	0	Geraniaceae Juss.
Iz_11_1209	DJI_0722	Gypsophila paniculata L.	0	n	1	0	Caryophyllaceae Juss.
Iz_11_1209	DJI_0722	Helichrysum arenarium (L.) Moench	0	n	1	0	Asteraceae Bercht. & J.Presl
Iz_11_1209	DJI_0722	Herniaria polygama J.Gay	0	n	1	0	Caryophyllaceae Juss.
Iz_11_1209	DJI_0722	Hieracium robustum Fr.	0	n	1	0	Asteraceae Bercht. & J.Presl
Iz_11_1209	DJI_0722	Hieracium umbellatum L.	0	n	1	NA	Asteraceae Bercht. & J.Presl
Iz_11_1209	DJI_0722	Hylotelephium maximum (L.) Holub	0	n	1	0	Crassulaceae J.St.-Hil.
Iz_11_1209	DJI_0722	Jacobaea borysthenica (DC.) B.Nord. & Greuter	0	n	1	0	Asteraceae Bercht. & J.Presl
Iz_11_1209	DJI_0722	Jasione montana L.	0	n	1	0	Campanulaceae Juss.
Iz_11_1209	DJI_0722	Jurinea cyanoides (L.) Rchb.s.l.	0	n	1	0	Asteraceae Bercht. & J.Presl
Iz_11_1209	DJI_0722	Koeleria glauca (Spreng.) DC.	0	n	1	0	Poaceae Barnhart
Iz_11_1209	DJI_0722	Lactuca serriola L.	0	a	0	0	Asteraceae Bercht. & J.Presl
Iz_11_1209	DJI_0722	Linaria genistifolia (L.) Mill.	0	n	1	0	Plantaginaceae Juss.
Iz_11_1209	DJI_0722	Lomelosia argentea (L.) Greuter & Burdet	0	n	1	0	Caprifoliaceae Juss.
Iz_11_1209	DJI_0722	Melampyrum pratense L.	0	n	1	0	Orobanchaceae Vent.
Iz_11_1209	DJI_0722	Oenothera biennis L.	0	a	0	0	Onagraceae Juss.
Iz_11_1209	DJI_0722	Pentanema britannica (L.) D.Gut.Larr., Santos-Vicente, Anderb., E.Rico & M.M.Mart.Ort.	0	n	1	0	Asteraceae Bercht. & J.Presl
Iz_11_1209	DJI_0722	Peucedanum oreoselinum (L.) Moench	0	n	1	0	Apiaceae Lindl.
Iz_11_1209	DJI_0722	Peucedanum ruthenicum M.Bieb.	0	n	1	0	Apiaceae Lindl.
Iz_11_1209	DJI_0722	Pilosella echioides (Lumn.) F.W.Schultz & Sch.Bip.	0	n	1	0	Asteraceae Bercht. & J.Presl

Iz_11_1209	DJI_0722	Pilosella officinarum F. Schultz et Sch. Bip.	0	n	1	0	Asteraceae Bercht. & J.Presl
Iz_11_1209	DJI_0722	Pilosella sp.	0		NA	0	Asteraceae Bercht. & J.Presl
Iz_11_1209	DJI_0722	Pinus sylvestris L.	0	n	1	1	Pinaceae Spreng. ex F.Rudolphi
Iz_11_1209	DJI_0722	Poa annua L.	0	n	1	0	Poaceae Barnhart
Iz_11_1209	DJI_0722	Poa nemoralis L.	0	n	1	0	Poaceae Barnhart
Iz_11_1209	DJI_0722	Poa sp.	0		NA	0	Poaceae Barnhart
Iz_11_1209	DJI_0722	Polygonatum odoratum (Mill.) Druce	0	n	1	0	Asparagaceae Juss.
Iz_11_1209	DJI_0722	Polygonum arenarium Waldst. & Kit.	0.5	n	1	0	Polygonaceae Juss.
Iz_11_1209	DJI_0722	Populus nigra L.	0	n	1	1	Salicaceae Mirb.
Iz_11_1209	DJI_0722	Populus tremula L.	0	n	1	1	Salicaceae Mirb.
Iz_11_1209	DJI_0722	Populus Ã canescens (Aiton) Sm.	0	n	1	1	Salicaceae Mirb.
Iz_11_1209	DJI_0722	Potentilla argentea L.	0	n	1	0	Rosaceae Juss.
Iz_11_1209	DJI_0722	Psammophiliella muralis (L.) Ikonn.	0	n	1	0	Caryophyllaceae Juss.
Iz_11_1209	DJI_0722	Psephellus sumensis (Kalen.) Greuter	0	n	1	0	Asteraceae Bercht. & J.Presl
Iz_11_1209	DJI_0722	Pulsatilla pratensis (L.) Mill. s.l.	0	n	1	0	Ranunculaceae Juss.
Iz_11_1209	DJI_0722	Pyrus communis L.	0	a	0	1	Rosaceae Juss.
Iz_11_1209	DJI_0722	Quercus robur L.	0	n	1	1	Fagaceae Dumort.
Iz_11_1209	DJI_0722	Robinia pseudoacacia L.	0	a	0	1	Fabaceae Lindl.
Iz_11_1209	DJI_0722	Rosa sp.	0		NA	1	Rosaceae Juss.
Iz_11_1209	DJI_0722	Rubus idaeus L.	0	n	1	0	Rosaceae Juss.
Iz_11_1209	DJI_0722	Rumex acetosella L.	0	n	1	0	Polygonaceae Juss.
Iz_11_1209	DJI_0722	Rumex thyrsiflorus Fingerh.	0	n	1	0	Polygonaceae Juss.
Iz_11_1209	DJI_0722	Salix alba L.	0	n	1	1	Salicaceae Mirb.
Iz_11_1209	DJI_0722	Sambucus nigra L.	0	n	1	1	Viburnaceae Raf.
Iz_11_1209	DJI_0722	Secale sylvestre Host	0	n	1	0	Poaceae Barnhart
Iz_11_1209	DJI_0722	Sempervivum ruthenicum Schnittsp. & C.B. Lehm.	0	n	1	0	Crassulaceae J.St.-Hil.
Iz_11_1209	DJI_0722	Setaria viridis (L.) P. Beauv.	0	n	1	0	Poaceae Barnhart
Iz_11_1209	DJI_0722	Silene borysthenica (Gruner) Walters	0	n	1	0	Caryophyllaceae Juss.
Iz_11_1209	DJI_0722	Silene latifolia subsp. alba (Mill.) Greuter & Burdet	0	n	1	0	Caryophyllaceae Juss.
Iz_11_1209	DJI_0722	Solidago virgaurea L.	0	n	1	0	Asteraceae Bercht. & J.Presl
Iz_11_1209	DJI_0722	Sorbus aucuparia L.	0	n	1	1	Rosaceae Juss.
Iz_11_1209	DJI_0722	Stachys recta L.	0	n	1	0	Lamiacea Lindl.
Iz_11_1209	DJI_0722	Tanacetum vulgare L.	0	n	1	0	Asteraceae Bercht. & J.Presl
Iz_11_1209	DJI_0722	Thymus pallasianus Heinr.Braun	0	n	1	0	Lamiacea Lindl.
Iz_11_1209	DJI_0722	Tilia cordata Mill.	0	n	1	1	Malvaceae Juss.
Iz_11_1209	DJI_0722	Trifolium alpestre L.	0	n	1	0	Fabaceae Lindl.
Iz_11_1209	DJI_0722	Trifolium arvense L.	0	n	1	0	Fabaceae Lindl.
Iz_11_1209	DJI_0722	Ulmus glabra Huds.	0	n	1	1	Ulmaceae Mirb.
Iz_11_1209	DJI_0722	Verbascum densiflorum Bertol.	0	n	1	0	Scrophulariaceae Juss.
Iz_11_1209	DJI_0722	Veronica austriaca L.	0	n	1	0	Plantaginaceae Juss.
Iz_11_1209	DJI_0722	Veronica incana L.	0	n	1	0	Plantaginaceae Juss.
Iz_11_1209	DJI_0722	Veronica verna L.	0	n	1	0	Plantaginaceae Juss.
Iz_11_1209	DJI_0722	Vincetoxicum hirundinaria Medik	0	n	1	0	Apocynaceae Juss.
Iz_11_1209	DJI_0725	Acer tataricum L.	0	n	1	1	Sapindaceae Juss.
Iz_11_1209	DJI_0725	Achillea millefolium L. s.l.	0	n	1	0	Asteraceae Bercht. & J.Presl
Iz_11_1209	DJI_0725	Allium oleraceum L.	0	n	1	0	Amaryllidaceae J.St.-Hil.
Iz_11_1209	DJI_0725	Ambrosia artemisiifolia L.	0	a	0	0	Asteraceae Bercht. & J.Presl
Iz_11_1209	DJI_0725	Amorpha fruticosa L.	0	a	0	0	Fabaceae Lindl.
Iz_11_1209	DJI_0725	Anthericum ramosum L.	0	n	1	0	Asparagaceae Juss.
Iz_11_1209	DJI_0725	Artemisia marschalliana Spreng.	0	n	1	0	Asteraceae Bercht. & J.Presl
Iz_11_1209	DJI_0725	Asparagus officinalis L.	0	n	1	0	Asparagaceae Juss.
Iz_11_1209	DJI_0725	Astragalus cicer L.	0	n	1	0	Fabaceae Lindl.
Iz_11_1209	DJI_0725	Bassia laniflora (S.G.Gmel.) A.J.Scott	0	n	1	0	Amaranthaceae Juss.
Iz_11_1209	DJI_0725	Berberis vulgaris L.	0	n	1	0	Berberidaceae Juss.
Iz_11_1209	DJI_0725	Betula pendula Roth.	0	n	1	1	Betulaceae Gray
Iz_11_1209	DJI_0725	Betula pendula Roth.	0	n	1	0	Poaceae Barnhart
Iz_11_1209	DJI_0725	Bromus inermis Leyss.	0		NA	NA	
Iz_11_1209	DJI_0725	Bromus squarrosus L.	0	a	0	0	Poaceae Barnhart
Iz_11_1209	DJI_0725	Bromus tectorum L.	0	a	0	0	Poaceae Barnhart
Iz_11_1209	DJI_0725	Calamagrostis epigejos (L.) Roth	0.5	n	1	0	Poaceae Barnhart
Iz_11_1209	DJI_0725	Carduus acanthoides L.	0	n	1	0	Asteraceae Bercht. & J.Presl
Iz_11_1209	DJI_0725	Carex sp.	0	n	1	0	Cyperaceae Juss.
Iz_11_1209	DJI_0725	Centaurea majorovii Dumbadze	0	n	1	0	Asteraceae Bercht. & J.Presl
Iz_11_1209	DJI_0725	Chamaecytisus ruthenicus (Fisch.ex Wol.) Klaskova	0	n	1	0	Fabaceae Lindl.
Iz_11_1209	DJI_0725	Chelidonium majus L.	1	n	1	0	Papaveraceae Juss.
Iz_11_1209	DJI_0725	Chenopodium betaceum Andrz.	0	n	1	0	Amaranthaceae Juss.
Iz_11_1209	DJI_0725	Chenopodium cf. album	0	n	1	0	Amaranthaceae Juss.
Iz_11_1209	DJI_0725	Chenopodium sp.	3		NA	0	Amaranthaceae Juss.
Iz_11_1209	DJI_0725	Chondrilla juncea L.	0	n	1	0	Asteraceae Bercht. & J.Presl
Iz_11_1209	DJI_0725	Cirsium vulgare (Savi) Ten.	0	n	1	0	Asteraceae Bercht. & J.Presl
Iz_11_1209	DJI_0725	Clinopodium acinos (L.) Kuntze	0	n	1	0	Lamiacea Lindl.
Iz_11_1209	DJI_0725	Convallaria majalis L.	0	n	1	0	Asparagaceae Juss.
Iz_11_1209	DJI_0725	Crataegus rhipidophylla Gand.	0	n	1	1	Rosaceae Juss.
Iz_11_1209	DJI_0725	Crepis foetida L.	0	n	1	0	Asteraceae Bercht. & J.Presl
Iz_11_1209	DJI_0725	Dactylis glomerata L.	0	n	1	0	Poaceae Barnhart
Iz_11_1209	DJI_0725	Dianthus campestris M.Bieb.	0	n	1	0	Caryophyllaceae Juss.
Iz_11_1209	DJI_0725	Digitaria sanguinalis (L.) Scop.	0	a	0	0	Poaceae Barnhart
Iz_11_1209	DJI_0725	Elymus repens (L.) Gould	0	n	1	0	Poaceae Barnhart
Iz_11_1209	DJI_0725	Epilobium tetragonum L.	0	n	1	0	Onagraceae Juss.
Iz_11_1209	DJI_0725	Eragrostis minor Host	0	a	0	0	Poaceae Barnhart
Iz_11_1209	DJI_0725	Erigeron canadensis L.	6	a	0	0	Asteraceae Bercht. & J.Presl
Iz_11_1209	DJI_0725	Euphorbia seguieriana Neck.	0	n	1	0	Euphorbiaceae Juss.
Iz_11_1209	DJI_0725	Euphorbia sp.	0		NA	NA	
Iz_11_1209	DJI_0725	Fallopia dumetorum (L.) Holub	0	n	1	0	Polygonaceae Juss.
Iz_11_1209	DJI_0725	Festuca beckeri (Hack.) Trautv.	0	n	1	0	Poaceae Barnhart
Iz_11_1209	DJI_0725	Fragaria vesca L.	0	n	1	0	Rosaceae Juss.
Iz_11_1209	DJI_0725	Frangula alnus Mill.	0	n	1	1	Rhamnaceae Juss.
Iz_11_1209	DJI_0725	Fraxinus pennsylvanica Marshall	0	a	0	1	Oleaceae Hoffmanns. & Link
Iz_11_1209	DJI_0725	Galeopsis bifida Boenn.	0	n	1	0	Lamiacea Lindl.
Iz_11_1209	DJI_0725	Galium verum L.	0	n	1	0	Rubiaceae Juss.
Iz_11_1209	DJI_0725	Genista tinctoria L.	0	n	1	0	Fabaceae Lindl.
Iz_11_1209	DJI_0725	Geranium robertianum L.	0	n	1	0	Geraniaceae Juss.
Iz_11_1209	DJI_0725	Geranium sanquineum L.	0	n	1	0	Geraniaceae Juss.
Iz_11_1209	DJI_0725	Gypsophila paniculata L.	0	n	1	0	Caryophyllaceae Juss.
Iz_11_1209	DJI_0725	Helichrysum arenarium (L.) Moench	0	n	1	0	Asteraceae Bercht. & J.Presl
Iz_11_1209	DJI_0725	Herniaria polygama J.Gay	0	n	1	0	Caryophyllaceae Juss.
Iz_11_1209	DJI_0725	Hieracium robustum Fr.	0	n	1	0	Asteraceae Bercht. & J.Presl
Iz_11_1209	DJI_0725	Hieracium umbellatum L.	0	n	1	NA	Asteraceae Bercht. & J.Presl

Iz_11_1209	DJI_0725	Hylotelephium maximum (L.) Holub	0	n	1	0	Crassulaceae J.St.-Hil.
Iz_11_1209	DJI_0725	Jacobaea borysthenica (DC.) B.Nord. & Greuter	0	n	1	0	Asteraceae Bercht. & J.Presl
Iz_11_1209	DJI_0725	Jasione montana L.	0	n	1	0	Campanulaceae Juss.
Iz_11_1209	DJI_0725	Jurinea cyanoides (L.) Rchb.s.l.	0	n	1	0	Asteraceae Bercht. & J.Presl
Iz_11_1209	DJI_0725	Koeleria glauca (Spreng.) DC.	0	n	1	0	Poaceae Barnhart
Iz_11_1209	DJI_0725	Lactuca serriola L.	0	a	0	0	Asteraceae Bercht. & J.Presl
Iz_11_1209	DJI_0725	Linaria genistifolia (L.) Mill.	0	n	1	0	Plantaginaceae Juss.
Iz_11_1209	DJI_0725	Lomelosia argentea (L.) Greuter & Burdet	0	n	1	0	Caprifoliaceae Juss.
Iz_11_1209	DJI_0725	Melampyrum pratense L.	0	n	1	0	Orobanchaceae Vent.
Iz_11_1209	DJI_0725	Oenothera biennis L.	0	a	0	0	Onagraceae Juss.
Iz_11_1209	DJI_0725	Pentanema britannica (L.) D.Gut.Larr., Santos-Vicente, Anderb., E.Rico & M.M.Mart.Ort.	0	n	1	0	Asteraceae Bercht. & J.Presl
Iz_11_1209	DJI_0725	Peucedanum oreoselinum (L.) Moench	0	n	1	0	Apiaceae Lindl.
Iz_11_1209	DJI_0725	Peucedanum ruthenicum M.Bieb.	0	n	1	0	Apiaceae Lindl.
Iz_11_1209	DJI_0725	Pilosella echioides (Lumn.) F.W.Schultz & Sch.Bip.	0	n	1	0	Asteraceae Bercht. & J.Presl
Iz_11_1209	DJI_0725	Pilosella officinarum F. Schultz et Sch. Bip.	0	n	1	0	Asteraceae Bercht. & J.Presl
Iz_11_1209	DJI_0725	Pilosella sp.	0		NA	0	Asteraceae Bercht. & J.Presl
Iz_11_1209	DJI_0725	Pinus sylvestris L.	0	n	1	1	Pinaceae Spreng. ex F.Rudolphi
Iz_11_1209	DJI_0725	Poa annua L.	0	n	1	0	Poaceae Barnhart
Iz_11_1209	DJI_0725	Poa nemoralis L.	0	n	1	0	Poaceae Barnhart
Iz_11_1209	DJI_0725	Poa sp.	0		NA	0	Poaceae Barnhart
Iz_11_1209	DJI_0725	Polygonatum odoratum (Mill.) Druce	0	n	1	0	Asparagaceae Juss.
Iz_11_1209	DJI_0725	Polygonum arenarium Waldst. & Kit.	0	n	1	0	Polygonaceae Juss.
Iz_11_1209	DJI_0725	Populus nigra L.	0	n	1	1	Salicaceae Mirb.
Iz_11_1209	DJI_0725	Populus tremula L.	0	n	1	1	Salicaceae Mirb.
Iz_11_1209	DJI_0725	Populus Ã canescens (Aiton) Sm.	0	n	1	1	Salicaceae Mirb.
Iz_11_1209	DJI_0725	Potentilla argentea L.	0	n	1	0	Rosaceae Juss.
Iz_11_1209	DJI_0725	Psammophiliella muralis (L.) Ikonn.	0	n	1	0	Caryophyllaceae Juss.
Iz_11_1209	DJI_0725	Psephellus sumensis (Kalen.) Greuter	0	n	1	0	Asteraceae Bercht. & J.Presl
Iz_11_1209	DJI_0725	Pulsatilla pratensis (L.) Mill. s.l.	0	n	1	0	Ranunculaceae Juss.
Iz_11_1209	DJI_0725	Pyrus communis L.	0	a	0	1	Rosaceae Juss.
Iz_11_1209	DJI_0725	Quercus robur L.	0	n	1	1	Fagaceae Dumort.
Iz_11_1209	DJI_0725	Robinia pseudoacacia L.	0	a	0	1	Fabaceae Lindl.
Iz_11_1209	DJI_0725	Rosa sp.	0		NA	1	Rosaceae Juss.
Iz_11_1209	DJI_0725	Rubus idaeus L.	0	n	1	0	Rosaceae Juss.
Iz_11_1209	DJI_0725	Rumex acetosella L.	0	n	1	0	Polygonaceae Juss.
Iz_11_1209	DJI_0725	Rumex thyrsiflorus Fingerh.	0	n	1	0	Polygonaceae Juss.
Iz_11_1209	DJI_0725	Salix alba L.	0	n	1	1	Salicaceae Mirb.
Iz_11_1209	DJI_0725	Sambucus nigra L.	0	n	1	1	Viburnaceae Raf.
Iz_11_1209	DJI_0725	Secale sylvestre Host	0	n	1	0	Poaceae Barnhart
Iz_11_1209	DJI_0725	Sempervivum ruthenicum Schnittsp. & C.B. Lehm.	0	n	1	0	Crassulaceae J.St.-Hil.
Iz_11_1209	DJI_0725	Setaria viridis (L.) P. Beauv.	0	n	1	0	Poaceae Barnhart
Iz_11_1209	DJI_0725	Silene borysthenica (Gruner) Walters	0	n	1	0	Caryophyllaceae Juss.
Iz_11_1209	DJI_0725	Silene latifolia subsp. alba (Mill.) Greuter & Burdet	0	n	1	0	Caryophyllaceae Juss.
Iz_11_1209	DJI_0725	Solidago virgaurea L.	0	n	1	0	Asteraceae Bercht. & J.Presl
Iz_11_1209	DJI_0725	Sorbus aucuparia L.	0	n	1	1	Rosaceae Juss.
Iz_11_1209	DJI_0725	Stachys recta L.	0	n	1	0	Lamiacea Lindl.
Iz_11_1209	DJI_0725	Tanacetum vulgare L.	0	n	1	0	Asteraceae Bercht. & J.Presl
Iz_11_1209	DJI_0725	Thymus pallasianus Heinr.Braun	0	n	1	0	Lamiacea Lindl.
Iz_11_1209	DJI_0725	Tilia cordata Mill.	0	n	1	1	Malvaceae Juss.
Iz_11_1209	DJI_0725	Trifolium alpestre L.	0	n	1	0	Fabaceae Lindl.
Iz_11_1209	DJI_0725	Trifolium arvense L.	0	n	1	0	Fabaceae Lindl.
Iz_11_1209	DJI_0725	Ulmus glabra Huds.	0	n	1	1	Ulmaceae Mirb.
Iz_11_1209	DJI_0725	Verbascum densiflorum Bertol.	0	n	1	0	Scrophulariaceae Juss.
Iz_11_1209	DJI_0725	Veronica austriaca L.	0	n	1	0	Plantaginaceae Juss.
Iz_11_1209	DJI_0725	Veronica incana L.	0	n	1	0	Plantaginaceae Juss.
Iz_11_1209	DJI_0725	Veronica verna L.	0	n	1	0	Plantaginaceae Juss.
Iz_11_1209	DJI_0725	Vincetoxicum hirundinaria Medik	0	n	1	0	Apocynaceae Juss.
Iz_11_1209	DJI_0728	Acer tataricum L.	0	n	1	1	Sapindaceae Juss.
Iz_11_1209	DJI_0728	Achillea millefolium L. s.l.	0	n	1	0	Asteraceae Bercht. & J.Presl
Iz_11_1209	DJI_0728	Allium oleraceum L.	0	n	1	0	Amaryllidaceae J.St.-Hil.
Iz_11_1209	DJI_0728	Ambrosia artemisiifolia L.	0	a	0	0	Asteraceae Bercht. & J.Presl
Iz_11_1209	DJI_0728	Amorpha fruticosa L.	0	a	0	0	Fabaceae Lindl.
Iz_11_1209	DJI_0728	Anthericum ramosum L.	0	n	1	0	Asparagaceae Juss.
Iz_11_1209	DJI_0728	Artemisia marschalliana Spreng.	0	n	1	0	Asteraceae Bercht. & J.Presl
Iz_11_1209	DJI_0728	Asparagus officinalis L.	0	n	1	0	Asparagaceae Juss.
Iz_11_1209	DJI_0728	Astragalus cicer L.	0	n	1	0	Fabaceae Lindl.
Iz_11_1209	DJI_0728	Bassia laniflora (S.G.Gmel.) A.J.Scott	0	n	1	0	Amaranthaceae Juss.
Iz_11_1209	DJI_0728	Berberis vulgaris L.	0	n	1	0	Berberidaceae Juss.
Iz_11_1209	DJI_0728	Betula pendula Roth.	0	n	1	1	Betulaceae Gray
Iz_11_1209	DJI_0728	Betula pendula Roth.	0	n	1	0	Poaceae Barnhart
Iz_11_1209	DJI_0728	Bromus inermis Leyss.	0		NA	NA	
Iz_11_1209	DJI_0728	Bromus squarrosus L.	0	a	0	0	Poaceae Barnhart
Iz_11_1209	DJI_0728	Bromus tectorum L.	0	a	0	0	Poaceae Barnhart
Iz_11_1209	DJI_0728	Calamagrostis epigejos (L.) Roth	3	n	1	0	Poaceae Barnhart
Iz_11_1209	DJI_0728	Carduus acanthoides L.	0	n	1	0	Asteraceae Bercht. & J.Presl
Iz_11_1209	DJI_0728	Carex sp.	8	n	1	0	Cyperaceae Juss.
Iz_11_1209	DJI_0728	Centaurea majorovii Dumbadze	0	n	1	0	Asteraceae Bercht. & J.Presl
Iz_11_1209	DJI_0728	Chamaecytisus ruthenicus (Fisch.ex Wol.) Klaskova	0	n	1	0	Fabaceae Lindl.
Iz_11_1209	DJI_0728	Chelidonium majus L.	1	n	1	0	Papaveraceae Juss.
Iz_11_1209	DJI_0728	Chenopodium betaceum Andrz.	0	n	1	0	Amaranthaceae Juss.
Iz_11_1209	DJI_0728	Chenopodium cf. album	0	n	1	0	Amaranthaceae Juss.
Iz_11_1209	DJI_0728	Chenopodium sp.	1		NA	0	Amaranthaceae Juss.
Iz_11_1209	DJI_0728	Chondrilla juncea L.	0	n	1	0	Asteraceae Bercht. & J.Presl
Iz_11_1209	DJI_0728	Cirsium vulgare (Savi) Ten.	0	n	1	0	Asteraceae Bercht. & J.Presl
Iz_11_1209	DJI_0728	Clinopodium acinos (L.) Kuntze	0	n	1	0	Lamiacea Lindl.
Iz_11_1209	DJI_0728	Convallaria majalis L.	0	n	1	0	Asparagaceae Juss.
Iz_11_1209	DJI_0728	Crataegus rhipidophylla Gand.	0	n	1	1	Rosaceae Juss.
Iz_11_1209	DJI_0728	Crepis foetida L.	0	n	1	0	Asteraceae Bercht. & J.Presl
Iz_11_1209	DJI_0728	Dactylis glomerata L.	0	n	1	0	Poaceae Barnhart
Iz_11_1209	DJI_0728	Dianthus campestris M.Bieb.	0	n	1	0	Caryophyllaceae Juss.
Iz_11_1209	DJI_0728	Digitaria sanguinalis (L.) Scop.	0	a	0	0	Poaceae Barnhart
Iz_11_1209	DJI_0728	Elymus repens (L.) Gould	0	n	1	0	Poaceae Barnhart
Iz_11_1209	DJI_0728	Epilobium tetragonum L.	0	n	1	0	Onagraceae Juss.
Iz_11_1209	DJI_0728	Eragrostis minor Host	0	a	0	0	Poaceae Barnhart
Iz_11_1209	DJI_0728	Erigeron canadensis L.	3	a	0	0	Asteraceae Bercht. & J.Presl
Iz_11_1209	DJI_0728	Euphorbia seguieriana Neck.	0	n	1	0	Euphorbiaceae Juss.
Iz_11_1209	DJI_0728	Euphorbia sp.	0		NA	NA	
Iz_11_1209	DJI_0728	Fallopia dumetorum (L.) Holub	0	n	1	0	Polygonaceae Juss.

Iz_11_1209	DJI_0728	Festuca beckeri (Hack.) Trautv.	0	n	1	0	Poaceae Barnhart
Iz_11_1209	DJI_0728	Fragaria vesca L.	0	n	1	0	Rosaceae Juss.
Iz_11_1209	DJI_0728	Frangula alnus Mill.	0	n	1	1	Rhamnaceae Juss.
Iz_11_1209	DJI_0728	Fraxinus pennsylvanica Marshall	0	a	0	1	Oleaceae Hoffmanns. & Link
Iz_11_1209	DJI_0728	Galeopsis bifida Boenn.	0	n	1	0	Lamiacea Lindl.
Iz_11_1209	DJI_0728	Galium verum L.	0	n	1	0	Rubiaceae Juss.
Iz_11_1209	DJI_0728	Genista tinctoria L.	0	n	1	0	Fabaceae Lindl.
Iz_11_1209	DJI_0728	Geranium robertianum L.	0	n	1	0	Geraniaceae Juss.
Iz_11_1209	DJI_0728	Geranium sanquineum L.	0	n	1	0	Geraniaceae Juss.
Iz_11_1209	DJI_0728	Gypsophila paniculata L.	0	n	1	0	Caryophyllaceae Juss.
Iz_11_1209	DJI_0728	Helichrysum arenarium (L.) Moench	0	n	1	0	Asteraceae Bercht. & J.Presl
Iz_11_1209	DJI_0728	Herniaria polygama J.Gay	0	n	1	0	Caryophyllaceae Juss.
Iz_11_1209	DJI_0728	Hieracium robustum Fr.	0	n	1	0	Asteraceae Bercht. & J.Presl
Iz_11_1209	DJI_0728	Hieracium umbellatum L.	0	n	1	NA	Asteraceae Bercht. & J.Presl
Iz_11_1209	DJI_0728	Hylotelephium maximum (L.) Holub	0	n	1	0	Crassulaceae J.St.-Hil.
Iz_11_1209	DJI_0728	Jacobaea borysthenica (DC.) B.Nord. & Greuter	0	n	1	0	Asteraceae Bercht. & J.Presl
Iz_11_1209	DJI_0728	Jasione montana L.	0	n	1	0	Campanulaceae Juss.
Iz_11_1209	DJI_0728	Jurinea cyanoides (L.) Rchb.s.l.	0	n	1	0	Asteraceae Bercht. & J.Presl
Iz_11_1209	DJI_0728	Koeleria glauca (Spreng.) DC.	0	n	1	0	Poaceae Barnhart
Iz_11_1209	DJI_0728	Lactuca serriola L.	0	a	0	0	Asteraceae Bercht. & J.Presl
Iz_11_1209	DJI_0728	Linaria genistifolia (L.) Mill.	0	n	1	0	Plantaginaceae Juss.
Iz_11_1209	DJI_0728	Lomelosia argentea (L.) Greuter & Burdet	0	n	1	0	Caprifoliaceae Juss.
Iz_11_1209	DJI_0728	Melampyrum pratense L.	0	n	1	0	Orobanchaceae Vent.
Iz_11_1209	DJI_0728	Oenothera biennis L.	0	a	0	0	Onagraceae Juss.
Iz_11_1209	DJI_0728	Pentanema britannica (L.) D.Gut.Larr., Santos-Vicente, Anderb., E.Rico & M.M.Mart.Ort.	0	n	1	0	Asteraceae Bercht. & J.Presl
Iz_11_1209	DJI_0728	Peucedanum oreoselinum (L.) Moench	0	n	1	0	Apiaceae Lindl.
Iz_11_1209	DJI_0728	Peucedanum ruthenicum M.Bieb.	0	n	1	0	Apiaceae Lindl.
Iz_11_1209	DJI_0728	Pilosella echioides (Lumn.) F.W.Schultz & Sch.Bip.	0	n	1	0	Asteraceae Bercht. & J.Presl
Iz_11_1209	DJI_0728	Pilosella officinarum F. Schultz et Sch. Bip.	0	n	1	0	Asteraceae Bercht. & J.Presl
Iz_11_1209	DJI_0728	Pilosella sp.	0		NA	0	Asteraceae Bercht. & J.Presl
Iz_11_1209	DJI_0728	Pinus sylvestris L.	0	n	1	1	Pinaceae Spreng. ex F.Rudolphi
Iz_11_1209	DJI_0728	Poa annua L.	0	n	1	0	Poaceae Barnhart
Iz_11_1209	DJI_0728	Poa nemoralis L.	0	n	1	0	Poaceae Barnhart
Iz_11_1209	DJI_0728	Poa sp.	0		NA	0	Poaceae Barnhart
Iz_11_1209	DJI_0728	Polygonatum odoratum (Mill.) Druce	0	n	1	0	Asparagaceae Juss.
Iz_11_1209	DJI_0728	Polygonum arenarium Waldst. & Kit.	0	n	1	0	Polygonaceae Juss.
Iz_11_1209	DJI_0728	Populus nigra L.	0	n	1	1	Salicaceae Mirb.
Iz_11_1209	DJI_0728	Populus tremula L.	0	n	1	1	Salicaceae Mirb.
Iz_11_1209	DJI_0728	Populus Ã canescens (Aiton) Sm.	0	n	1	1	Salicaceae Mirb.
Iz_11_1209	DJI_0728	Potentilla argentea L.	0	n	1	0	Rosaceae Juss.
Iz_11_1209	DJI_0728	Psammophiliella muralis (L.) Ikonn.	0	n	1	0	Caryophyllaceae Juss.
Iz_11_1209	DJI_0728	Psephellus sumensis (Kalen.) Greuter	0	n	1	0	Asteraceae Bercht. & J.Presl
Iz_11_1209	DJI_0728	Pulsatilla pratensis (L.) Mill. s.l.	0	n	1	0	Ranunculaceae Juss.
Iz_11_1209	DJI_0728	Pyrus communis L.	0	a	0	1	Rosaceae Juss.
Iz_11_1209	DJI_0728	Quercus robur L.	0	n	1	1	Fagaceae Dumort.
Iz_11_1209	DJI_0728	Robinia pseudoacacia L.	0	a	0	1	Fabaceae Lindl.
Iz_11_1209	DJI_0728	Rosa sp.	0		NA	1	Rosaceae Juss.
Iz_11_1209	DJI_0728	Rubus idaeus L.	0	n	1	0	Rosaceae Juss.
Iz_11_1209	DJI_0728	Rumex acetosella L.	0	n	1	0	Polygonaceae Juss.
Iz_11_1209	DJI_0728	Rumex thyrsiflorus Fingerh.	0	n	1	0	Polygonaceae Juss.
Iz_11_1209	DJI_0728	Salix alba L.	0	n	1	1	Salicaceae Mirb.
Iz_11_1209	DJI_0728	Sambucus nigra L.	0	n	1	1	Viburnaceae Raf.
Iz_11_1209	DJI_0728	Secale sylvestre Host	0	n	1	0	Poaceae Barnhart
Iz_11_1209	DJI_0728	Sempervivum ruthenicum Schnittsp. & C.B. Lehm.	1	n	1	0	Crassulaceae J.St.-Hil.
Iz_11_1209	DJI_0728	Setaria viridis (L.) P. Beauv.	0	n	1	0	Poaceae Barnhart
Iz_11_1209	DJI_0728	Silene borysthenica (Gruner) Walters	0	n	1	0	Caryophyllaceae Juss.
Iz_11_1209	DJI_0728	Silene latifolia subsp. alba (Mill.) Greuter & Burdet	0	n	1	0	Caryophyllaceae Juss.
Iz_11_1209	DJI_0728	Solidago virgaurea L.	0	n	1	0	Asteraceae Bercht. & J.Presl
Iz_11_1209	DJI_0728	Sorbus aucuparia L.	0	n	1	1	Rosaceae Juss.
Iz_11_1209	DJI_0728	Stachys recta L.	0	n	1	0	Lamiacea Lindl.
Iz_11_1209	DJI_0728	Tanacetum vulgare L.	0	n	1	0	Asteraceae Bercht. & J.Presl
Iz_11_1209	DJI_0728	Thymus pallasianus Heinr.Braun	0	n	1	0	Lamiacea Lindl.
Iz_11_1209	DJI_0728	Tilia cordata Mill.	0	n	1	1	Malvaceae Juss.
Iz_11_1209	DJI_0728	Trifolium alpestre L.	0	n	1	0	Fabaceae Lindl.
Iz_11_1209	DJI_0728	Trifolium arvense L.	0	n	1	0	Fabaceae Lindl.
Iz_11_1209	DJI_0728	Ulmus glabra Huds.	0	n	1	1	Ulmaceae Mirb.
Iz_11_1209	DJI_0728	Verbascum densiflorum Bertol.	0	n	1	0	Scrophulariaceae Juss.
Iz_11_1209	DJI_0728	Veronica austriaca L.	0	n	1	0	Plantaginaceae Juss.
Iz_11_1209	DJI_0728	Veronica incana L.	0	n	1	0	Plantaginaceae Juss.
Iz_11_1209	DJI_0728	Veronica verna L.	0	n	1	0	Plantaginaceae Juss.
Iz_11_1209	DJI_0728	Vincetoxicum hirundinaria Medik	0	n	1	0	Apocynaceae Juss.
Iz_11_1209	DJI_0733	Acer tataricum L.	0	n	1	1	Sapindaceae Juss.
Iz_11_1209	DJI_0733	Achillea millefolium L. s.l.	0	n	1	0	Asteraceae Bercht. & J.Presl
Iz_11_1209	DJI_0733	Allium oleraceum L.	0	n	1	0	Amaryllidaceae J.St.-Hil.
Iz_11_1209	DJI_0733	Ambrosia artemisiifolia L.	0	a	0	0	Asteraceae Bercht. & J.Presl
Iz_11_1209	DJI_0733	Amorpha fruticosa L.	0	a	0	0	Fabaceae Lindl.
Iz_11_1209	DJI_0733	Anthericum ramosum L.	0	n	1	0	Asparagaceae Juss.
Iz_11_1209	DJI_0733	Artemisia marschalliana Spreng.	0	n	1	0	Asteraceae Bercht. & J.Presl
Iz_11_1209	DJI_0733	Asparagus officinalis L.	0	n	1	0	Asparagaceae Juss.
Iz_11_1209	DJI_0733	Astragalus cicer L.	0	n	1	0	Fabaceae Lindl.
Iz_11_1209	DJI_0733	Bassia laniflora (S.G.Gmel.) A.J.Scott	0	n	1	0	Amaranthaceae Juss.
Iz_11_1209	DJI_0733	Berberis vulgaris L.	0	n	1	0	Berberidaceae Juss.
Iz_11_1209	DJI_0733	Betula pendula Roth.	0	n	1	1	Betulaceae Gray
Iz_11_1209	DJI_0733	Betula pendula Roth.	0	n	1	0	Poaceae Barnhart
Iz_11_1209	DJI_0733	Bromus inermis Leyss.	0		NA	NA	
Iz_11_1209	DJI_0733	Bromus squarrosus L.	0	a	0	0	Poaceae Barnhart
Iz_11_1209	DJI_0733	Bromus tectorum L.	0	a	0	0	Poaceae Barnhart
Iz_11_1209	DJI_0733	Calamagrostis epigejos (L.) Roth	0.5	n	1	0	Poaceae Barnhart
Iz_11_1209	DJI_0733	Carduus acanthoides L.	0	n	1	0	Asteraceae Bercht. & J.Presl
Iz_11_1209	DJI_0733	Carex sp.	0	n	1	0	Cyperaceae Juss.
Iz_11_1209	DJI_0733	Centaurea majorovii Dumbadze	0	n	1	0	Asteraceae Bercht. & J.Presl
Iz_11_1209	DJI_0733	Chamaecytisus ruthenicus (Fisch.ex Wol.) Klaskova	0	n	1	0	Fabaceae Lindl.
Iz_11_1209	DJI_0733	Chelidonium majus L.	0	n	1	0	Papaveraceae Juss.
Iz_11_1209	DJI_0733	Chenopodium betaceum Andrz.	0	n	1	0	Amaranthaceae Juss.
Iz_11_1209	DJI_0733	Chenopodium cf. album	0	n	1	0	Amaranthaceae Juss.
Iz_11_1209	DJI_0733	Chenopodium sp.	1		NA	0	Amaranthaceae Juss.
Iz_11_1209	DJI_0733	Chondrilla juncea L.	0	n	1	0	Asteraceae Bercht. & J.Presl
Iz_11_1209	DJI_0733	Cirsium vulgare (Savi) Ten.	0	n	1	0	Asteraceae Bercht. & J.Presl

Iz_11_1209	DJI_0733	Clinopodium acinos (L.) Kuntze	0	n	1	0	Lamiacea Lindl.
Iz_11_1209	DJI_0733	Convallaria majalis L.	0	n	1	0	Asparagaceae Juss.
Iz_11_1209	DJI_0733	Crataegus rhipidophylla Gand.	0	n	1	1	Rosaceae Juss.
Iz_11_1209	DJI_0733	Crepis foetida L.	0	n	1	0	Asteraceae Bercht. & J.Presl
Iz_11_1209	DJI_0733	Dactylis glomerata L.	0	n	1	0	Poaceae Barnhart
Iz_11_1209	DJI_0733	Dianthus campestris M.Bieb.	0	n	1	0	Caryophyllaceae Juss.
Iz_11_1209	DJI_0733	Digitaria sanguinalis (L.) Scop.	0	a	0	0	Poaceae Barnhart
Iz_11_1209	DJI_0733	Elymus repens (L.) Gould	0	n	1	0	Poaceae Barnhart
Iz_11_1209	DJI_0733	Epilobium tetragonum L.	0	n	1	0	Onagraceae Juss.
Iz_11_1209	DJI_0733	Eragrostis minor Host	0	a	0	0	Poaceae Barnhart
Iz_11_1209	DJI_0733	Erigeron canadensis L.	5	a	0	0	Asteraceae Bercht. & J.Presl
Iz_11_1209	DJI_0733	Euphorbia seguieriana Neck.	0	n	1	0	Euphorbiaceae Juss.
Iz_11_1209	DJI_0733	Euphorbia sp.	0		NA	NA	
Iz_11_1209	DJI_0733	Fallopia dumetorum (L.) Holub	0	n	1	0	Polygonaceae Juss.
Iz_11_1209	DJI_0733	Festuca beckeri (Hack.) Trautv.	0	n	1	0	Poaceae Barnhart
Iz_11_1209	DJI_0733	Fragaria vesca L.	0	n	1	0	Rosaceae Juss.
Iz_11_1209	DJI_0733	Frangula alnus Mill.	0	n	1	1	Rhamnaceae Juss.
Iz_11_1209	DJI_0733	Fraxinus pennsylvanica Marshall	0	a	0	1	Oleaceae Hoffmanns. & Link
Iz_11_1209	DJI_0733	Galeopsis bifida Boenn.	0	n	1	0	Lamiacea Lindl.
Iz_11_1209	DJI_0733	Galium verum L.	0	n	1	0	Rubiaceae Juss.
Iz_11_1209	DJI_0733	Genista tinctoria L.	0	n	1	0	Fabaceae Lindl.
Iz_11_1209	DJI_0733	Geranium robertianum L.	0	n	1	0	Geraniaceae Juss.
Iz_11_1209	DJI_0733	Geranium sanquineum L.	0	n	1	0	Geraniaceae Juss.
Iz_11_1209	DJI_0733	Gypsophila paniculata L.	0	n	1	0	Caryophyllaceae Juss.
Iz_11_1209	DJI_0733	Helichrysum arenarium (L.) Moench	0	n	1	0	Asteraceae Bercht. & J.Presl
Iz_11_1209	DJI_0733	Herniaria polygama J.Gay	0	n	1	0	Caryophyllaceae Juss.
Iz_11_1209	DJI_0733	Hieracium robustum Fr.	0	n	1	0	Asteraceae Bercht. & J.Presl
Iz_11_1209	DJI_0733	Hieracium umbellatum L.	0	n	1	NA	Asteraceae Bercht. & J.Presl
Iz_11_1209	DJI_0733	Hylotelephium maximum (L.) Holub	0	n	1	0	Crassulaceae J.St.-Hil.
Iz_11_1209	DJI_0733	Jacobaea borysthenica (DC.) B.Nord. & Greuter	0	n	1	0	Asteraceae Bercht. & J.Presl
Iz_11_1209	DJI_0733	Jasione montana L.	0	n	1	0	Campanulaceae Juss.
Iz_11_1209	DJI_0733	Jurinea cyanoides (L.) Rchb.s.l.	0	n	1	0	Asteraceae Bercht. & J.Presl
Iz_11_1209	DJI_0733	Koeleria glauca (Spreng.) DC.	0	n	1	0	Poaceae Barnhart
Iz_11_1209	DJI_0733	Lactuca serriola L.	0	a	0	0	Asteraceae Bercht. & J.Presl
Iz_11_1209	DJI_0733	Linaria genistifolia (L.) Mill.	0	n	1	0	Plantaginaceae Juss.
Iz_11_1209	DJI_0733	Lomelosia argentea (L.) Greuter & Burdet	0	n	1	0	Caprifoliaceae Juss.
Iz_11_1209	DJI_0733	Melampyrum pratense L.	0	n	1	0	Orobanchaceae Vent.
Iz_11_1209	DJI_0733	Oenothera biennis L.	0	a	0	0	Onagraceae Juss.
Iz_11_1209	DJI_0733	Pentanema britannica (L.) D.Gut.Larr., Santos-Vicente, Anderb., E.Rico & M.M.Mart.Ort.	0	n	1	0	Asteraceae Bercht. & J.Presl
Iz_11_1209	DJI_0733	Peucedanum oreoselinum (L.) Moench	0	n	1	0	Apiaceae Lindl.
Iz_11_1209	DJI_0733	Peucedanum ruthenicum M.Bieb.	0	n	1	0	Apiaceae Lindl.
Iz_11_1209	DJI_0733	Pilosella echioides (Lumn.) F.W.Schultz & Sch.Bip.	0	n	1	0	Asteraceae Bercht. & J.Presl
Iz_11_1209	DJI_0733	Pilosella officinarum F. Schultz et Sch. Bip.	0	n	1	0	Asteraceae Bercht. & J.Presl
Iz_11_1209	DJI_0733	Pilosella sp.	0		NA	0	Asteraceae Bercht. & J.Presl
Iz_11_1209	DJI_0733	Pinus sylvestris L.	0	n	1	1	Pinaceae Spreng. ex F.Rudolphi
Iz_11_1209	DJI_0733	Poa annua L.	0	n	1	0	Poaceae Barnhart
Iz_11_1209	DJI_0733	Poa nemoralis L.	0	n	1	0	Poaceae Barnhart
Iz_11_1209	DJI_0733	Poa sp.	0		NA	0	Poaceae Barnhart
Iz_11_1209	DJI_0733	Polygonatum odoratum (Mill.) Druce	0	n	1	0	Asparagaceae Juss.
Iz_11_1209	DJI_0733	Polygonum arenarium Waldst. & Kit.	0	n	1	0	Polygonaceae Juss.
Iz_11_1209	DJI_0733	Populus nigra L.	0	n	1	1	Salicaceae Mirb.
Iz_11_1209	DJI_0733	Populus tremula L.	0	n	1	1	Salicaceae Mirb.
Iz_11_1209	DJI_0733	Populus Ã canescens (Aiton) Sm.	0	n	1	1	Salicaceae Mirb.
Iz_11_1209	DJI_0733	Potentilla argentea L.	0	n	1	0	Rosaceae Juss.
Iz_11_1209	DJI_0733	Psammophiliella muralis (L.) Ikonn.	0	n	1	0	Caryophyllaceae Juss.
Iz_11_1209	DJI_0733	Psephellus sumensis (Kalen.) Greuter	0	n	1	0	Asteraceae Bercht. & J.Presl
Iz_11_1209	DJI_0733	Pulsatilla pratensis (L.) Mill. s.l.	0	n	1	0	Ranunculaceae Juss.
Iz_11_1209	DJI_0733	Pyrus communis L.	0	a	0	1	Rosaceae Juss.
Iz_11_1209	DJI_0733	Quercus robur L.	0	n	1	1	Fagaceae Dumort.
Iz_11_1209	DJI_0733	Robinia pseudoacacia L.	0	a	0	1	Fabaceae Lindl.
Iz_11_1209	DJI_0733	Rosa sp.	0		NA	1	Rosaceae Juss.
Iz_11_1209	DJI_0733	Rubus idaeus L.	0	n	1	0	Rosaceae Juss.
Iz_11_1209	DJI_0733	Rumex acetosella L.	0	n	1	0	Polygonaceae Juss.
Iz_11_1209	DJI_0733	Rumex thyrsiflorus Fingerh.	0	n	1	0	Polygonaceae Juss.
Iz_11_1209	DJI_0733	Salix alba L.	0	n	1	1	Salicaceae Mirb.
Iz_11_1209	DJI_0733	Sambucus nigra L.	0	n	1	1	Viburnaceae Raf.
Iz_11_1209	DJI_0733	Secale sylvestre Host	0	n	1	0	Poaceae Barnhart
Iz_11_1209	DJI_0733	Sempervivum ruthenicum Schnittsp. & C.B. Lehm.	0	n	1	0	Crassulaceae J.St.-Hil.
Iz_11_1209	DJI_0733	Setaria viridis (L.) P. Beauv.	0	n	1	0	Poaceae Barnhart
Iz_11_1209	DJI_0733	Silene borysthenica (Gruner) Walters	0	n	1	0	Caryophyllaceae Juss.
Iz_11_1209	DJI_0733	Silene latifolia subsp. alba (Mill.) Greuter & Burdet	0	n	1	0	Caryophyllaceae Juss.
Iz_11_1209	DJI_0733	Solidago virgaurea L.	0.2	n	1	0	Asteraceae Bercht. & J.Presl
Iz_11_1209	DJI_0733	Sorbus aucuparia L.	0	n	1	1	Rosaceae Juss.
Iz_11_1209	DJI_0733	Stachys recta L.	0	n	1	0	Lamiacea Lindl.
Iz_11_1209	DJI_0733	Tanacetum vulgare L.	0	n	1	0	Asteraceae Bercht. & J.Presl
Iz_11_1209	DJI_0733	Thymus pallasianus Heinr.Braun	0	n	1	0	Lamiacea Lindl.
Iz_11_1209	DJI_0733	Tilia cordata Mill.	0	n	1	1	Malvaceae Juss.
Iz_11_1209	DJI_0733	Trifolium alpestre L.	0	n	1	0	Fabaceae Lindl.
Iz_11_1209	DJI_0733	Trifolium arvense L.	0	n	1	0	Fabaceae Lindl.
Iz_11_1209	DJI_0733	Ulmus glabra Huds.	0	n	1	1	Ulmaceae Mirb.
Iz_11_1209	DJI_0733	Verbascum densiflorum Bertol.	0	n	1	0	Scrophulariaceae Juss.
Iz_11_1209	DJI_0733	Veronica austriaca L.	0	n	1	0	Plantaginaceae Juss.
Iz_11_1209	DJI_0733	Veronica incana L.	0	n	1	0	Plantaginaceae Juss.
Iz_11_1209	DJI_0733	Veronica verna L.	0	n	1	0	Plantaginaceae Juss.
Iz_11_1209	DJI_0733	Vincetoxicum hirundinaria Medik	0	n	1	0	Apocynaceae Juss.
Iz_11_1209	DJI_0737	Acer tataricum L.	0	n	1	1	Sapindaceae Juss.
Iz_11_1209	DJI_0737	Achillea millefolium L. s.l.	0	n	1	0	Asteraceae Bercht. & J.Presl
Iz_11_1209	DJI_0737	Allium oleraceum L.	0	n	1	0	Amaryllidaceae J.St.-Hil.
Iz_11_1209	DJI_0737	Ambrosia artemisiifolia L.	0	a	0	0	Asteraceae Bercht. & J.Presl
Iz_11_1209	DJI_0737	Amorpha fruticosa L.	0	a	0	0	Fabaceae Lindl.
Iz_11_1209	DJI_0737	Anthericum ramosum L.	0	n	1	0	Asparagaceae Juss.
Iz_11_1209	DJI_0737	Artemisia marschalliana Spreng.	0	n	1	0	Asteraceae Bercht. & J.Presl
Iz_11_1209	DJI_0737	Asparagus officinalis L.	0	n	1	0	Asparagaceae Juss.
Iz_11_1209	DJI_0737	Astragalus cicer L.	0	n	1	0	Fabaceae Lindl.
Iz_11_1209	DJI_0737	Bassia laniflora (S.G.Gmel.) A.J.Scott	0	n	1	0	Amaranthaceae Juss.
Iz_11_1209	DJI_0737	Berberis vulgaris L.	0	n	1	0	Berberidaceae Juss.
Iz_11_1209	DJI_0737	Betula pendula Roth.	0	n	1	1	Betulaceae Gray
Iz_11_1209	DJI_0737	Betula pendula Roth.	0	n	1	0	Poaceae Barnhart

Iz_11_1209	DJI_0737	Bromus inermis Leyss.	0		NA	NA	
Iz_11_1209	DJI_0737	Bromus squarrosus L.	0	a	0	0	Poaceae Barnhart
Iz_11_1209	DJI_0737	Bromus tectorum L.	0	a	0	0	Poaceae Barnhart
Iz_11_1209	DJI_0737	Calamagrostis epigejos (L.) Roth	15	n	1	0	Poaceae Barnhart
Iz_11_1209	DJI_0737	Carduus acanthoides L.	0	n	1	0	Asteraceae Bercht. & J.Presl
Iz_11_1209	DJI_0737	Carex sp.	2	n	1	0	Cyperaceae Juss.
Iz_11_1209	DJI_0737	Centaurea majorovii Dumbadze	0	n	1	0	Asteraceae Bercht. & J.Presl
Iz_11_1209	DJI_0737	Chamaecytisus ruthenicus (Fisch.ex Wol.) Klaskova	0	n	1	0	Fabaceae Lindl.
Iz_11_1209	DJI_0737	Chelidonium majus L.	0	n	1	0	Papaveraceae Juss.
Iz_11_1209	DJI_0737	Chenopodium betaceum Andrz.	0	n	1	0	Amaranthaceae Juss.
Iz_11_1209	DJI_0737	Chenopodium cf. album	0	n	1	0	Amaranthaceae Juss.
Iz_11_1209	DJI_0737	Chenopodium sp.	0		NA	0	Amaranthaceae Juss.
Iz_11_1209	DJI_0737	Chondrilla juncea L.	0	n	1	0	Asteraceae Bercht. & J.Presl
Iz_11_1209	DJI_0737	Cirsium vulgare (Savi) Ten.	0	n	1	0	Asteraceae Bercht. & J.Presl
Iz_11_1209	DJI_0737	Clinopodium acinos (L.) Kuntze	0	n	1	0	Lamiacea Lindl.
Iz_11_1209	DJI_0737	Convallaria majalis L.	0	n	1	0	Asparagaceae Juss.
Iz_11_1209	DJI_0737	Crataegus rhipidophylla Gand.	0	n	1	1	Rosaceae Juss.
Iz_11_1209	DJI_0737	Crepis foetida L.	0	n	1	0	Asteraceae Bercht. & J.Presl
Iz_11_1209	DJI_0737	Dactylis glomerata L.	0	n	1	0	Poaceae Barnhart
Iz_11_1209	DJI_0737	Dianthus campestris M.Bieb.	0	n	1	0	Caryophyllaceae Juss.
Iz_11_1209	DJI_0737	Digitaria sanguinalis (L.) Scop.	0	a	0	0	Poaceae Barnhart
Iz_11_1209	DJI_0737	Elymus repens (L.) Gould	0	n	1	0	Poaceae Barnhart
Iz_11_1209	DJI_0737	Epilobium tetragonum L.	0	n	1	0	Onagraceae Juss.
Iz_11_1209	DJI_0737	Eragrostis minor Host	0	a	0	0	Poaceae Barnhart
Iz_11_1209	DJI_0737	Erigeron canadensis L.	10	a	0	0	Asteraceae Bercht. & J.Presl
Iz_11_1209	DJI_0737	Euphorbia seguieriana Neck.	0	n	1	0	Euphorbiaceae Juss.
Iz_11_1209	DJI_0737	Euphorbia sp.	0		NA	NA	
Iz_11_1209	DJI_0737	Fallopia dumetorum (L.) Holub	0	n	1	0	Polygonaceae Juss.
Iz_11_1209	DJI_0737	Festuca beckeri (Hack.) Trautv.	0	n	1	0	Poaceae Barnhart
Iz_11_1209	DJI_0737	Fragaria vesca L.	0	n	1	0	Rosaceae Juss.
Iz_11_1209	DJI_0737	Frangula alnus Mill.	0	n	1	1	Rhamnaceae Juss.
Iz_11_1209	DJI_0737	Fraxinus pennsylvanica Marshall	0	a	0	1	Oleaceae Hoffmanns. & Link
Iz_11_1209	DJI_0737	Galeopsis bifida Boenn.	0	n	1	0	Lamiacea Lindl.
Iz_11_1209	DJI_0737	Galium verum L.	0	n	1	0	Rubiaceae Juss.
Iz_11_1209	DJI_0737	Genista tinctoria L.	0	n	1	0	Fabaceae Lindl.
Iz_11_1209	DJI_0737	Geranium robertianum L.	0	n	1	0	Geraniaceae Juss.
Iz_11_1209	DJI_0737	Geranium sanquineum L.	0	n	1	0	Geraniaceae Juss.
Iz_11_1209	DJI_0737	Gypsophila paniculata L.	0	n	1	0	Caryophyllaceae Juss.
Iz_11_1209	DJI_0737	Helichrysum arenarium (L.) Moench	0	n	1	0	Asteraceae Bercht. & J.Presl
Iz_11_1209	DJI_0737	Herniaria polygama J.Gay	0	n	1	0	Caryophyllaceae Juss.
Iz_11_1209	DJI_0737	Hieracium robustum Fr.	0	n	1	0	Asteraceae Bercht. & J.Presl
Iz_11_1209	DJI_0737	Hieracium umbellatum L.	0	n	1	NA	Asteraceae Bercht. & J.Presl
Iz_11_1209	DJI_0737	Hylotelephium maximum (L.) Holub	0	n	1	0	Crassulaceae J.St.-Hil.
Iz_11_1209	DJI_0737	Jacobaea borysthenica (DC.) B.Nord. & Greuter	0	n	1	0	Asteraceae Bercht. & J.Presl
Iz_11_1209	DJI_0737	Jasione montana L.	0	n	1	0	Campanulaceae Juss.
Iz_11_1209	DJI_0737	Jurinea cyanoides (L.) Rchb.s.l.	0	n	1	0	Asteraceae Bercht. & J.Presl
Iz_11_1209	DJI_0737	Koeleria glauca (Spreng.) DC.	0	n	1	0	Poaceae Barnhart
Iz_11_1209	DJI_0737	Lactuca serriola L.	0	a	0	0	Asteraceae Bercht. & J.Presl
Iz_11_1209	DJI_0737	Linaria genistifolia (L.) Mill.	0	n	1	0	Plantaginaceae Juss.
Iz_11_1209	DJI_0737	Lomelosia argentea (L.) Greuter & Burdet	0	n	1	0	Caprifoliaceae Juss.
Iz_11_1209	DJI_0737	Melampyrum pratense L.	0	n	1	0	Orobanchaceae Vent.
Iz_11_1209	DJI_0737	Oenothera biennis L.	0	a	0	0	Onagraceae Juss.
Iz_11_1209	DJI_0737	Pentanema britannica (L.) D.Gut.Larr., Santos-Vicente, Anderb., E.Rico & M.M.Mart.Ort.	0	n	1	0	Asteraceae Bercht. & J.Presl
Iz_11_1209	DJI_0737	Peucedanum oreoselinum (L.) Moench	0	n	1	0	Apiaceae Lindl.
Iz_11_1209	DJI_0737	Peucedanum ruthenicum M.Bieb.	0	n	1	0	Apiaceae Lindl.
Iz_11_1209	DJI_0737	Pilosella echioides (Lumn.) F.W.Schultz & Sch.Bip.	0	n	1	0	Asteraceae Bercht. & J.Presl
Iz_11_1209	DJI_0737	Pilosella officinarum F. Schultz et Sch. Bip.	0	n	1	0	Asteraceae Bercht. & J.Presl
Iz_11_1209	DJI_0737	Pilosella sp.	0		NA	0	Asteraceae Bercht. & J.Presl
Iz_11_1209	DJI_0737	Pinus sylvestris L.	0	n	1	1	Pinaceae Spreng. ex F.Rudolphi
Iz_11_1209	DJI_0737	Poa annua L.	0	n	1	0	Poaceae Barnhart
Iz_11_1209	DJI_0737	Poa nemoralis L.	0	n	1	0	Poaceae Barnhart
Iz_11_1209	DJI_0737	Poa sp.	0		NA	0	Poaceae Barnhart
Iz_11_1209	DJI_0737	Polygonatum odoratum (Mill.) Druce	0	n	1	0	Asparagaceae Juss.
Iz_11_1209	DJI_0737	Polygonum arenarium Waldst. & Kit.	0	n	1	0	Polygonaceae Juss.
Iz_11_1209	DJI_0737	Populus nigra L.	0	n	1	1	Salicaceae Mirb.
Iz_11_1209	DJI_0737	Populus tremula L.	0	n	1	1	Salicaceae Mirb.
Iz_11_1209	DJI_0737	Populus Ã canescens (Aiton) Sm.	0	n	1	1	Salicaceae Mirb.
Iz_11_1209	DJI_0737	Potentilla argentea L.	0	n	1	0	Rosaceae Juss.
Iz_11_1209	DJI_0737	Psammophiliella muralis (L.) Ikonn.	0	n	1	0	Caryophyllaceae Juss.
Iz_11_1209	DJI_0737	Psephellus sumensis (Kalen.) Greuter	0	n	1	0	Asteraceae Bercht. & J.Presl
Iz_11_1209	DJI_0737	Pulsatilla pratensis (L.) Mill. s.l.	0	n	1	0	Ranunculaceae Juss.
Iz_11_1209	DJI_0737	Pyrus communis L.	0	a	0	1	Rosaceae Juss.
Iz_11_1209	DJI_0737	Quercus robur L.	0	n	1	1	Fagaceae Dumort.
Iz_11_1209	DJI_0737	Robinia pseudoacacia L.	0	a	0	1	Fabaceae Lindl.
Iz_11_1209	DJI_0737	Rosa sp.	0		NA	1	Rosaceae Juss.
Iz_11_1209	DJI_0737	Rubus idaeus L.	0	n	1	0	Rosaceae Juss.
Iz_11_1209	DJI_0737	Rumex acetosella L.	0	n	1	0	Polygonaceae Juss.
Iz_11_1209	DJI_0737	Rumex thyrsiflorus Fingerh.	0	n	1	0	Polygonaceae Juss.
Iz_11_1209	DJI_0737	Salix alba L.	0	n	1	1	Salicaceae Mirb.
Iz_11_1209	DJI_0737	Sambucus nigra L.	0	n	1	1	Viburnaceae Raf.
Iz_11_1209	DJI_0737	Secale sylvestre Host	0	n	1	0	Poaceae Barnhart
Iz_11_1209	DJI_0737	Sempervivum ruthenicum Schnittsp. & C.B. Lehm.	0	n	1	0	Crassulaceae J.St.-Hil.
Iz_11_1209	DJI_0737	Setaria viridis (L.) P. Beauv.	0	n	1	0	Poaceae Barnhart
Iz_11_1209	DJI_0737	Silene borysthenica (Gruner) Walters	0	n	1	0	Caryophyllaceae Juss.
Iz_11_1209	DJI_0737	Silene latifolia subsp. alba (Mill.) Greuter & Burdet	0	n	1	0	Caryophyllaceae Juss.
Iz_11_1209	DJI_0737	Solidago virgaurea L.	0	n	1	0	Asteraceae Bercht. & J.Presl
Iz_11_1209	DJI_0737	Sorbus aucuparia L.	0	n	1	1	Rosaceae Juss.
Iz_11_1209	DJI_0737	Stachys recta L.	0	n	1	0	Lamiacea Lindl.
Iz_11_1209	DJI_0737	Tanacetum vulgare L.	0	n	1	0	Asteraceae Bercht. & J.Presl
Iz_11_1209	DJI_0737	Thymus pallasianus Heinr.Braun	0	n	1	0	Lamiacea Lindl.
Iz_11_1209	DJI_0737	Tilia cordata Mill.	0	n	1	1	Malvaceae Juss.
Iz_11_1209	DJI_0737	Trifolium alpestre L.	0	n	1	0	Fabaceae Lindl.
Iz_11_1209	DJI_0737	Trifolium arvense L.	0	n	1	0	Fabaceae Lindl.
Iz_11_1209	DJI_0737	Ulmus glabra Huds.	0	n	1	1	Ulmaceae Mirb.
Iz_11_1209	DJI_0737	Verbascum densiflorum Bertol.	0	n	1	0	Scrophulariaceae Juss.
Iz_11_1209	DJI_0737	Veronica austriaca L.	0	n	1	0	Plantaginaceae Juss.
Iz_11_1209	DJI_0737	Veronica incana L.	0	n	1	0	Plantaginaceae Juss.
Iz_11_1209	DJI_0737	Veronica verna L.	0	n	1	0	Plantaginaceae Juss.

Iz_11_1209	DJI_0737	Vincetoxicum hirundinaria Medik	0	n	1	0	Apocynaceae Juss.
Iz_11_1209	DJI_0740	Acer tataricum L.	0	n	1	1	Sapindaceae Juss.
Iz_11_1209	DJI_0740	Achillea millefolium L. s.l.	0	n	1	0	Asteraceae Bercht. & J.Presl
Iz_11_1209	DJI_0740	Allium oleraceum L.	0	n	1	0	Amaryllidaceae J.St.-Hil.
Iz_11_1209	DJI_0740	Ambrosia artemisiifolia L.	0	a	0	0	Asteraceae Bercht. & J.Presl
Iz_11_1209	DJI_0740	Amorpha fruticosa L.	0	a	0	0	Fabaceae Lindl.
Iz_11_1209	DJI_0740	Anthericum ramosum L.	0	n	1	0	Asparagaceae Juss.
Iz_11_1209	DJI_0740	Artemisia marschalliana Spreng.	0	n	1	0	Asteraceae Bercht. & J.Presl
Iz_11_1209	DJI_0740	Asparagus officinalis L.	0	n	1	0	Asparagaceae Juss.
Iz_11_1209	DJI_0740	Astragalus cicer L.	0	n	1	0	Fabaceae Lindl.
Iz_11_1209	DJI_0740	Bassia laniflora (S.G.Gmel.) A.J.Scott	0	n	1	0	Amaranthaceae Juss.
Iz_11_1209	DJI_0740	Berberis vulgaris L.	0	n	1	0	Berberidaceae Juss.
Iz_11_1209	DJI_0740	Betula pendula Roth.	0	n	1	1	Betulaceae Gray
Iz_11_1209	DJI_0740	Betula pendula Roth.	0	n	1	0	Poaceae Barnhart
Iz_11_1209	DJI_0740	Bromus inermis Leyss.	0		NA	NA	
Iz_11_1209	DJI_0740	Bromus squarrosus L.	0	a	0	0	Poaceae Barnhart
Iz_11_1209	DJI_0740	Bromus tectorum L.	0	a	0	0	Poaceae Barnhart
Iz_11_1209	DJI_0740	Calamagrostis epigejos (L.) Roth	50	n	1	0	Poaceae Barnhart
Iz_11_1209	DJI_0740	Carduus acanthoides L.	0	n	1	0	Asteraceae Bercht. & J.Presl
Iz_11_1209	DJI_0740	Carex sp.	0	n	1	0	Cyperaceae Juss.
Iz_11_1209	DJI_0740	Centaurea majorovii Dumbadze	0	n	1	0	Asteraceae Bercht. & J.Presl
Iz_11_1209	DJI_0740	Chamaecytisus ruthenicus (Fisch.ex Wol.) Klaskova	0	n	1	0	Fabaceae Lindl.
Iz_11_1209	DJI_0740	Chelidonium majus L.	0	n	1	0	Papaveraceae Juss.
Iz_11_1209	DJI_0740	Chenopodium betaceum Andrz.	0	n	1	0	Amaranthaceae Juss.
Iz_11_1209	DJI_0740	Chenopodium cf. album	0	n	1	0	Amaranthaceae Juss.
Iz_11_1209	DJI_0740	Chenopodium sp.	0		NA	0	Amaranthaceae Juss.
Iz_11_1209	DJI_0740	Chondrilla juncea L.	0	n	1	0	Asteraceae Bercht. & J.Presl
Iz_11_1209	DJI_0740	Cirsium vulgare (Savi) Ten.	0	n	1	0	Asteraceae Bercht. & J.Presl
Iz_11_1209	DJI_0740	Clinopodium acinos (L.) Kuntze	0	n	1	0	Lamiacea Lindl.
Iz_11_1209	DJI_0740	Convallaria majalis L.	0	n	1	0	Asparagaceae Juss.
Iz_11_1209	DJI_0740	Crataegus rhipidophylla Gand.	0	n	1	1	Rosaceae Juss.
Iz_11_1209	DJI_0740	Crepis foetida L.	0	n	1	0	Asteraceae Bercht. & J.Presl
Iz_11_1209	DJI_0740	Dactylis glomerata L.	0	n	1	0	Poaceae Barnhart
Iz_11_1209	DJI_0740	Dianthus campestris M.Bieb.	0	n	1	0	Caryophyllaceae Juss.
Iz_11_1209	DJI_0740	Digitaria sanguinalis (L.) Scop.	0	a	0	0	Poaceae Barnhart
Iz_11_1209	DJI_0740	Elymus repens (L.) Gould	0	n	1	0	Poaceae Barnhart
Iz_11_1209	DJI_0740	Epilobium tetragonum L.	0	n	1	0	Onagraceae Juss.
Iz_11_1209	DJI_0740	Eragrostis minor Host	0	a	0	0	Poaceae Barnhart
Iz_11_1209	DJI_0740	Erigeron canadensis L.	5	a	0	0	Asteraceae Bercht. & J.Presl
Iz_11_1209	DJI_0740	Euphorbia seguieriana Neck.	0	n	1	0	Euphorbiaceae Juss.
Iz_11_1209	DJI_0740	Euphorbia sp.	0		NA	NA	
Iz_11_1209	DJI_0740	Fallopia dumetorum (L.) Holub	0	n	1	0	Polygonaceae Juss.
Iz_11_1209	DJI_0740	Festuca beckeri (Hack.) Trautv.	0	n	1	0	Poaceae Barnhart
Iz_11_1209	DJI_0740	Fragaria vesca L.	0	n	1	0	Rosaceae Juss.
Iz_11_1209	DJI_0740	Frangula alnus Mill.	0	n	1	1	Rhamnaceae Juss.
Iz_11_1209	DJI_0740	Fraxinus pennsylvanica Marshall	0	a	0	1	Oleaceae Hoffmanns. & Link
Iz_11_1209	DJI_0740	Galeopsis bifida Boenn.	0	n	1	0	Lamiacea Lindl.
Iz_11_1209	DJI_0740	Galium verum L.	0	n	1	0	Rubiaceae Juss.
Iz_11_1209	DJI_0740	Genista tinctoria L.	0	n	1	0	Fabaceae Lindl.
Iz_11_1209	DJI_0740	Geranium robertianum L.	0	n	1	0	Geraniaceae Juss.
Iz_11_1209	DJI_0740	Geranium sanquineum L.	0	n	1	0	Geraniaceae Juss.
Iz_11_1209	DJI_0740	Gypsophila paniculata L.	0	n	1	0	Caryophyllaceae Juss.
Iz_11_1209	DJI_0740	Helichrysum arenarium (L.) Moench	0	n	1	0	Asteraceae Bercht. & J.Presl
Iz_11_1209	DJI_0740	Herniaria polygama J.Gay	0	n	1	0	Caryophyllaceae Juss.
Iz_11_1209	DJI_0740	Hieracium robustum Fr.	0	n	1	0	Asteraceae Bercht. & J.Presl
Iz_11_1209	DJI_0740	Hieracium umbellatum L.	0	n	1	NA	Asteraceae Bercht. & J.Presl
Iz_11_1209	DJI_0740	Hylotelephium maximum (L.) Holub	0	n	1	0	Crassulaceae J.St.-Hil.
Iz_11_1209	DJI_0740	Jacobaea borysthenica (DC.) B.Nord. & Greuter	0	n	1	0	Asteraceae Bercht. & J.Presl
Iz_11_1209	DJI_0740	Jasione montana L.	0	n	1	0	Campanulaceae Juss.
Iz_11_1209	DJI_0740	Jurinea cyanoides (L.) Rchb.s.l.	0	n	1	0	Asteraceae Bercht. & J.Presl
Iz_11_1209	DJI_0740	Koeleria glauca (Spreng.) DC.	0	n	1	0	Poaceae Barnhart
Iz_11_1209	DJI_0740	Lactuca serriola L.	0	a	0	0	Asteraceae Bercht. & J.Presl
Iz_11_1209	DJI_0740	Linaria genistifolia (L.) Mill.	0	n	1	0	Plantaginaceae Juss.
Iz_11_1209	DJI_0740	Lomelosia argentea (L.) Greuter & Burdet	0	n	1	0	Caprifoliaceae Juss.
Iz_11_1209	DJI_0740	Melampyrum pratense L.	0	n	1	0	Orobanchaceae Vent.
Iz_11_1209	DJI_0740	Oenothera biennis L.	0	a	0	0	Onagraceae Juss.
Iz_11_1209	DJI_0740	Pentanema britannica (L.) D.Gut.Larr., Santos-Vicente, Anderb., E.Rico & M.M.Mart.Ort.	0	n	1	0	Asteraceae Bercht. & J.Presl
Iz_11_1209	DJI_0740	Peucedanum oreoselinum (L.) Moench	0	n	1	0	Apiaceae Lindl.
Iz_11_1209	DJI_0740	Peucedanum ruthenicum M.Bieb.	0	n	1	0	Apiaceae Lindl.
Iz_11_1209	DJI_0740	Pilosella echioides (Lumn.) F.W.Schultz & Sch.Bip.	0	n	1	0	Asteraceae Bercht. & J.Presl
Iz_11_1209	DJI_0740	Pilosella officinarum F. Schultz et Sch. Bip.	0	n	1	0	Asteraceae Bercht. & J.Presl
Iz_11_1209	DJI_0740	Pilosella sp.	0		NA	0	Asteraceae Bercht. & J.Presl
Iz_11_1209	DJI_0740	Pinus sylvestris L.	0	n	1	1	Pinaceae Spreng. ex F.Rudolphi
Iz_11_1209	DJI_0740	Poa annua L.	0	n	1	0	Poaceae Barnhart
Iz_11_1209	DJI_0740	Poa nemoralis L.	0	n	1	0	Poaceae Barnhart
Iz_11_1209	DJI_0740	Poa sp.	0		NA	0	Poaceae Barnhart
Iz_11_1209	DJI_0740	Polygonatum odoratum (Mill.) Druce	0	n	1	0	Asparagaceae Juss.
Iz_11_1209	DJI_0740	Polygonum arenarium Waldst. & Kit.	0	n	1	0	Polygonaceae Juss.
Iz_11_1209	DJI_0740	Populus nigra L.	0	n	1	1	Salicaceae Mirb.
Iz_11_1209	DJI_0740	Populus tremula L.	0	n	1	1	Salicaceae Mirb.
Iz_11_1209	DJI_0740	Populus Ã canescens (Aiton) Sm.	0	n	1	1	Salicaceae Mirb.
Iz_11_1209	DJI_0740	Potentilla argentea L.	0	n	1	0	Rosaceae Juss.
Iz_11_1209	DJI_0740	Psammophiliella muralis (L.) Ikonn.	0	n	1	0	Caryophyllaceae Juss.
Iz_11_1209	DJI_0740	Psephellus sumensis (Kalen.) Greuter	0	n	1	0	Asteraceae Bercht. & J.Presl
Iz_11_1209	DJI_0740	Pulsatilla pratensis (L.) Mill. s.l.	0	n	1	0	Ranunculaceae Juss.
Iz_11_1209	DJI_0740	Pyrus communis L.	0	a	0	1	Rosaceae Juss.
Iz_11_1209	DJI_0740	Quercus robur L.	0	n	1	1	Fagaceae Dumort.
Iz_11_1209	DJI_0740	Robinia pseudoacacia L.	0	a	0	1	Fabaceae Lindl.
Iz_11_1209	DJI_0740	Rosa sp.	0		NA	1	Rosaceae Juss.
Iz_11_1209	DJI_0740	Rubus idaeus L.	0	n	1	0	Rosaceae Juss.
Iz_11_1209	DJI_0740	Rumex acetosella L.	0	n	1	0	Polygonaceae Juss.
Iz_11_1209	DJI_0740	Rumex thyrsiflorus Fingerh.	0	n	1	0	Polygonaceae Juss.
Iz_11_1209	DJI_0740	Salix alba L.	0	n	1	1	Salicaceae Mirb.
Iz_11_1209	DJI_0740	Sambucus nigra L.	0	n	1	1	Viburnaceae Raf.
Iz_11_1209	DJI_0740	Secale sylvestre Host	0	n	1	0	Poaceae Barnhart
Iz_11_1209	DJI_0740	Sempervivum ruthenicum Schnittsp. & C.B. Lehm.	0	n	1	0	Crassulaceae J.St.-Hil.
Iz_11_1209	DJI_0740	Setaria viridis (L.) P. Beauv.	0	n	1	0	Poaceae Barnhart
Iz_11_1209	DJI_0740	Silene borysthenica (Gruner) Walters	0	n	1	0	Caryophyllaceae Juss.

Iz_11_1209	DJI_0740	Silene latifolia subsp. alba (Mill.) Greuter & Burdet	0	n	1	0	Caryophyllaceae Juss.
Iz_11_1209	DJI_0740	Solidago virgaurea L.	0	n	1	0	Asteraceae Bercht. & J.Presl
Iz_11_1209	DJI_0740	Sorbus aucuparia L.	0	n	1	1	Rosaceae Juss.
Iz_11_1209	DJI_0740	Stachys recta L.	0	n	1	0	Lamiacea Lindl.
Iz_11_1209	DJI_0740	Tanacetum vulgare L.	0	n	1	0	Asteraceae Bercht. & J.Presl
Iz_11_1209	DJI_0740	Thymus pallasianus Heinr.Braun	0	n	1	0	Lamiacea Lindl.
Iz_11_1209	DJI_0740	Tilia cordata Mill.	0	n	1	1	Malvaceae Juss.
Iz_11_1209	DJI_0740	Trifolium alpestre L.	0	n	1	0	Fabaceae Lindl.
Iz_11_1209	DJI_0740	Trifolium arvense L.	0	n	1	0	Fabaceae Lindl.
Iz_11_1209	DJI_0740	Ulmus glabra Huds.	0	n	1	1	Ulmaceae Mirb.
Iz_11_1209	DJI_0740	Verbascum densiflorum Bertol.	0	n	1	0	Scrophulariaceae Juss.
Iz_11_1209	DJI_0740	Veronica austriaca L.	0	n	1	0	Plantaginaceae Juss.
Iz_11_1209	DJI_0740	Veronica incana L.	0	n	1	0	Plantaginaceae Juss.
Iz_11_1209	DJI_0740	Veronica verna L.	0	n	1	0	Plantaginaceae Juss.
Iz_11_1209	DJI_0740	Vincetoxicum hirundinaria Medik	0	n	1	0	Apocynaceae Juss.
Iz_11_1209	DJI_0743	Acer tataricum L.	0	n	1	1	Sapindaceae Juss.
Iz_11_1209	DJI_0743	Achillea millefolium L. s.l.	0	n	1	0	Asteraceae Bercht. & J.Presl
Iz_11_1209	DJI_0743	Allium oleraceum L.	0	n	1	0	Amaryllidaceae J.St.-Hil.
Iz_11_1209	DJI_0743	Ambrosia artemisiifolia L.	0	a	0	0	Asteraceae Bercht. & J.Presl
Iz_11_1209	DJI_0743	Amorpha fruticosa L.	0	a	0	0	Fabaceae Lindl.
Iz_11_1209	DJI_0743	Anthericum ramosum L.	0	n	1	0	Asparagaceae Juss.
Iz_11_1209	DJI_0743	Artemisia marschalliana Spreng.	0	n	1	0	Asteraceae Bercht. & J.Presl
Iz_11_1209	DJI_0743	Asparagus officinalis L.	0	n	1	0	Asparagaceae Juss.
Iz_11_1209	DJI_0743	Astragalus cicer L.	0	n	1	0	Fabaceae Lindl.
Iz_11_1209	DJI_0743	Bassia laniflora (S.G.Gmel.) A.J.Scott	0	n	1	0	Amaranthaceae Juss.
Iz_11_1209	DJI_0743	Berberis vulgaris L.	0	n	1	0	Berberidaceae Juss.
Iz_11_1209	DJI_0743	Betula pendula Roth.	0	n	1	1	Betulaceae Gray
Iz_11_1209	DJI_0743	Betula pendula Roth.	0	n	1	0	Poaceae Barnhart
Iz_11_1209	DJI_0743	Bromus inermis Leyss.	0		NA	NA	
Iz_11_1209	DJI_0743	Bromus squarrosus L.	0	a	0	0	Poaceae Barnhart
Iz_11_1209	DJI_0743	Bromus tectorum L.	0	a	0	0	Poaceae Barnhart
Iz_11_1209	DJI_0743	Calamagrostis epigejos (L.) Roth	0	n	1	0	Poaceae Barnhart
Iz_11_1209	DJI_0743	Carduus acanthoides L.	0	n	1	0	Asteraceae Bercht. & J.Presl
Iz_11_1209	DJI_0743	Carex sp.	0	n	1	0	Cyperaceae Juss.
Iz_11_1209	DJI_0743	Centaurea majorovii Dumbadze	0	n	1	0	Asteraceae Bercht. & J.Presl
Iz_11_1209	DJI_0743	Chamaecytisus ruthenicus (Fisch.ex Wol.) Klaskova	0	n	1	0	Fabaceae Lindl.
Iz_11_1209	DJI_0743	Chelidonium majus L.	0	n	1	0	Papaveraceae Juss.
Iz_11_1209	DJI_0743	Chenopodium betaceum Andrz.	0	n	1	0	Amaranthaceae Juss.
Iz_11_1209	DJI_0743	Chenopodium cf. album	0	n	1	0	Amaranthaceae Juss.
Iz_11_1209	DJI_0743	Chenopodium sp.	0		NA	0	Amaranthaceae Juss.
Iz_11_1209	DJI_0743	Chondrilla juncea L.	0	n	1	0	Asteraceae Bercht. & J.Presl
Iz_11_1209	DJI_0743	Cirsium vulgare (Savi) Ten.	0	n	1	0	Asteraceae Bercht. & J.Presl
Iz_11_1209	DJI_0743	Clinopodium acinos (L.) Kuntze	0	n	1	0	Lamiacea Lindl.
Iz_11_1209	DJI_0743	Convallaria majalis L.	0	n	1	0	Asparagaceae Juss.
Iz_11_1209	DJI_0743	Crataegus rhipidophylla Gand.	0	n	1	1	Rosaceae Juss.
Iz_11_1209	DJI_0743	Crepis foetida L.	0	n	1	0	Asteraceae Bercht. & J.Presl
Iz_11_1209	DJI_0743	Dactylis glomerata L.	0	n	1	0	Poaceae Barnhart
Iz_11_1209	DJI_0743	Dianthus campestris M.Bieb.	0	n	1	0	Caryophyllaceae Juss.
Iz_11_1209	DJI_0743	Digitaria sanguinalis (L.) Scop.	0	a	0	0	Poaceae Barnhart
Iz_11_1209	DJI_0743	Elymus repens (L.) Gould	0	n	1	0	Poaceae Barnhart
Iz_11_1209	DJI_0743	Epilobium tetragonum L.	0	n	1	0	Onagraceae Juss.
Iz_11_1209	DJI_0743	Eragrostis minor Host	0	a	0	0	Poaceae Barnhart
Iz_11_1209	DJI_0743	Erigeron canadensis L.	2	a	0	0	Asteraceae Bercht. & J.Presl
Iz_11_1209	DJI_0743	Euphorbia seguieriana Neck.	0	n	1	0	Euphorbiaceae Juss.
Iz_11_1209	DJI_0743	Euphorbia sp.	0		NA	NA	
Iz_11_1209	DJI_0743	Fallopia dumetorum (L.) Holub	0	n	1	0	Polygonaceae Juss.
Iz_11_1209	DJI_0743	Festuca beckeri (Hack.) Trautv.	0	n	1	0	Poaceae Barnhart
Iz_11_1209	DJI_0743	Fragaria vesca L.	0	n	1	0	Rosaceae Juss.
Iz_11_1209	DJI_0743	Frangula alnus Mill.	0	n	1	1	Rhamnaceae Juss.
Iz_11_1209	DJI_0743	Fraxinus pennsylvanica Marshall	0	a	0	1	Oleaceae Hoffmanns. & Link
Iz_11_1209	DJI_0743	Galeopsis bifida Boenn.	0	n	1	0	Lamiacea Lindl.
Iz_11_1209	DJI_0743	Galium verum L.	0	n	1	0	Rubiaceae Juss.
Iz_11_1209	DJI_0743	Genista tinctoria L.	0	n	1	0	Fabaceae Lindl.
Iz_11_1209	DJI_0743	Geranium robertianum L.	0	n	1	0	Geraniaceae Juss.
Iz_11_1209	DJI_0743	Geranium sanquineum L.	0	n	1	0	Geraniaceae Juss.
Iz_11_1209	DJI_0743	Gypsophila paniculata L.	0	n	1	0	Caryophyllaceae Juss.
Iz_11_1209	DJI_0743	Helichrysum arenarium (L.) Moench	0	n	1	0	Asteraceae Bercht. & J.Presl
Iz_11_1209	DJI_0743	Herniaria polygama J.Gay	0	n	1	0	Caryophyllaceae Juss.
Iz_11_1209	DJI_0743	Hieracium robustum Fr.	0	n	1	0	Asteraceae Bercht. & J.Presl
Iz_11_1209	DJI_0743	Hieracium umbellatum L.	0	n	1	NA	Asteraceae Bercht. & J.Presl
Iz_11_1209	DJI_0743	Hylotelephium maximum (L.) Holub	0	n	1	0	Crassulaceae J.St.-Hil.
Iz_11_1209	DJI_0743	Jacobaea borysthenica (DC.) B.Nord. & Greuter	0	n	1	0	Asteraceae Bercht. & J.Presl
Iz_11_1209	DJI_0743	Jasione montana L.	0	n	1	0	Campanulaceae Juss.
Iz_11_1209	DJI_0743	Jurinea cyanoides (L.) Rchb.s.l.	0	n	1	0	Asteraceae Bercht. & J.Presl
Iz_11_1209	DJI_0743	Koeleria glauca (Spreng.) DC.	0	n	1	0	Poaceae Barnhart
Iz_11_1209	DJI_0743	Lactuca serriola L.	0	a	0	0	Asteraceae Bercht. & J.Presl
Iz_11_1209	DJI_0743	Linaria genistifolia (L.) Mill.	0	n	1	0	Plantaginaceae Juss.
Iz_11_1209	DJI_0743	Lomelosia argentea (L.) Greuter & Burdet	0	n	1	0	Caprifoliaceae Juss.
Iz_11_1209	DJI_0743	Melampyrum pratense L.	0	n	1	0	Orobanchaceae Vent.
Iz_11_1209	DJI_0743	Oenothera biennis L.	0	a	0	0	Onagraceae Juss.
Iz_11_1209	DJI_0743	Pentanema britannica (L.) D.Gut.Larr., Santos-Vicente, Anderb., E.Rico & M.M.Mart.Ort.	0	n	1	0	Asteraceae Bercht. & J.Presl
Iz_11_1209	DJI_0743	Peucedanum oreoselinum (L.) Moench	0	n	1	0	Apiaceae Lindl.
Iz_11_1209	DJI_0743	Peucedanum ruthenicum M.Bieb.	0	n	1	0	Apiaceae Lindl.
Iz_11_1209	DJI_0743	Pilosella echioides (Lumn.) F.W.Schultz & Sch.Bip.	0	n	1	0	Asteraceae Bercht. & J.Presl
Iz_11_1209	DJI_0743	Pilosella officinarum F. Schultz et Sch. Bip.	0	n	1	0	Asteraceae Bercht. & J.Presl
Iz_11_1209	DJI_0743	Pilosella sp.	0		NA	0	Asteraceae Bercht. & J.Presl
Iz_11_1209	DJI_0743	Pinus sylvestris L.	0	n	1	1	Pinaceae Spreng. ex F.Rudolphi
Iz_11_1209	DJI_0743	Poa annua L.	0	n	1	0	Poaceae Barnhart
Iz_11_1209	DJI_0743	Poa nemoralis L.	0	n	1	0	Poaceae Barnhart
Iz_11_1209	DJI_0743	Poa sp.	0		NA	0	Poaceae Barnhart
Iz_11_1209	DJI_0743	Polygonatum odoratum (Mill.) Druce	0	n	1	0	Asparagaceae Juss.
Iz_11_1209	DJI_0743	Polygonum arenarium Waldst. & Kit.	0	n	1	0	Polygonaceae Juss.
Iz_11_1209	DJI_0743	Populus nigra L.	0	n	1	1	Salicaceae Mirb.
Iz_11_1209	DJI_0743	Populus tremula L.	0	n	1	1	Salicaceae Mirb.
Iz_11_1209	DJI_0743	Populus Ã canescens (Aiton) Sm.	0	n	1	1	Salicaceae Mirb.
Iz_11_1209	DJI_0743	Potentilla argentea L.	0	n	1	0	Rosaceae Juss.
Iz_11_1209	DJI_0743	Psammophiliella muralis (L.) Ikonn.	0	n	1	0	Caryophyllaceae Juss.
Iz_11_1209	DJI_0743	Psephellus sumensis (Kalen.) Greuter	0	n	1	0	Asteraceae Bercht. & J.Presl

Iz_11_1209	DJI_0743	Pulsatilla pratensis (L.) Mill. s.l.	0	n	1	0	Ranunculaceae Juss.
Iz_11_1209	DJI_0743	Pyrus communis L.	0	a	0	1	Rosaceae Juss.
Iz_11_1209	DJI_0743	Quercus robur L.	0	n	1	1	Fagaceae Dumort.
Iz_11_1209	DJI_0743	Robinia pseudoacacia L.	0	a	0	1	Fabaceae Lindl.
Iz_11_1209	DJI_0743	Rosa sp.	0		NA	1	Rosaceae Juss.
Iz_11_1209	DJI_0743	Rubus idaeus L.	0	n	1	0	Rosaceae Juss.
Iz_11_1209	DJI_0743	Rumex acetosella L.	0	n	1	0	Polygonaceae Juss.
Iz_11_1209	DJI_0743	Rumex thyrsiflorus Fingerh.	0	n	1	0	Polygonaceae Juss.
Iz_11_1209	DJI_0743	Salix alba L.	0	n	1	1	Salicaceae Mirb.
Iz_11_1209	DJI_0743	Sambucus nigra L.	0	n	1	1	Viburnaceae Raf.
Iz_11_1209	DJI_0743	Secale sylvestre Host	0	n	1	0	Poaceae Barnhart
Iz_11_1209	DJI_0743	Sempervivum ruthenicum Schnittsp. & C.B. Lehm.	0	n	1	0	Crassulaceae J.St.-Hil.
Iz_11_1209	DJI_0743	Setaria viridis (L.) P. Beauv.	0	n	1	0	Poaceae Barnhart
Iz_11_1209	DJI_0743	Silene borysthenica (Gruner) Walters	0	n	1	0	Caryophyllaceae Juss.
Iz_11_1209	DJI_0743	Silene latifolia subsp. alba (Mill.) Greuter & Burdet	0	n	1	0	Caryophyllaceae Juss.
Iz_11_1209	DJI_0743	Solidago virgaurea L.	0	n	1	0	Asteraceae Bercht. & J.Presl
Iz_11_1209	DJI_0743	Sorbus aucuparia L.	0	n	1	1	Rosaceae Juss.
Iz_11_1209	DJI_0743	Stachys recta L.	0	n	1	0	Lamiacea Lindl.
Iz_11_1209	DJI_0743	Tanacetum vulgare L.	0	n	1	0	Asteraceae Bercht. & J.Presl
Iz_11_1209	DJI_0743	Thymus pallasianus Heinr.Braun	0	n	1	0	Lamiacea Lindl.
Iz_11_1209	DJI_0743	Tilia cordata Mill.	0	n	1	1	Malvaceae Juss.
Iz_11_1209	DJI_0743	Trifolium alpestre L.	0	n	1	0	Fabaceae Lindl.
Iz_11_1209	DJI_0743	Trifolium arvense L.	0	n	1	0	Fabaceae Lindl.
Iz_11_1209	DJI_0743	Ulmus glabra Huds.	0	n	1	1	Ulmaceae Mirb.
Iz_11_1209	DJI_0743	Verbascum densiflorum Bertol.	0	n	1	0	Scrophulariaceae Juss.
Iz_11_1209	DJI_0743	Veronica austriaca L.	0	n	1	0	Plantaginaceae Juss.
Iz_11_1209	DJI_0743	Veronica incana L.	0	n	1	0	Plantaginaceae Juss.
Iz_11_1209	DJI_0743	Veronica verna L.	0	n	1	0	Plantaginaceae Juss.
Iz_11_1209	DJI_0743	Vincetoxicum hirundinaria Medik	0	n	1	0	Apocynaceae Juss.
Iz_11_1209	DJI_0746	Acer tataricum L.	0	n	1	1	Sapindaceae Juss.
Iz_11_1209	DJI_0746	Achillea millefolium L. s.l.	0	n	1	0	Asteraceae Bercht. & J.Presl
Iz_11_1209	DJI_0746	Allium oleraceum L.	0	n	1	0	Amaryllidaceae J.St.-Hil.
Iz_11_1209	DJI_0746	Ambrosia artemisiifolia L.	0	a	0	0	Asteraceae Bercht. & J.Presl
Iz_11_1209	DJI_0746	Amorpha fruticosa L.	0	a	0	0	Fabaceae Lindl.
Iz_11_1209	DJI_0746	Anthericum ramosum L.	0	n	1	0	Asparagaceae Juss.
Iz_11_1209	DJI_0746	Artemisia marschalliana Spreng.	0	n	1	0	Asteraceae Bercht. & J.Presl
Iz_11_1209	DJI_0746	Asparagus officinalis L.	0	n	1	0	Asparagaceae Juss.
Iz_11_1209	DJI_0746	Astragalus cicer L.	0	n	1	0	Fabaceae Lindl.
Iz_11_1209	DJI_0746	Bassia laniflora (S.G.Gmel.) A.J.Scott	0	n	1	0	Amaranthaceae Juss.
Iz_11_1209	DJI_0746	Berberis vulgaris L.	0	n	1	0	Berberidaceae Juss.
Iz_11_1209	DJI_0746	Betula pendula Roth.	0	n	1	1	Betulaceae Gray
Iz_11_1209	DJI_0746	Betula pendula Roth.	0	n	1	0	Poaceae Barnhart
Iz_11_1209	DJI_0746	Bromus inermis Leyss.	0		NA	NA	
Iz_11_1209	DJI_0746	Bromus squarrosus L.	0	a	0	0	Poaceae Barnhart
Iz_11_1209	DJI_0746	Bromus tectorum L.	0	a	0	0	Poaceae Barnhart
Iz_11_1209	DJI_0746	Calamagrostis epigejos (L.) Roth	2	n	1	0	Poaceae Barnhart
Iz_11_1209	DJI_0746	Carduus acanthoides L.	0	n	1	0	Asteraceae Bercht. & J.Presl
Iz_11_1209	DJI_0746	Carex sp.	0	n	1	0	Cyperaceae Juss.
Iz_11_1209	DJI_0746	Centaurea majorovii Dumbadze	0	n	1	0	Asteraceae Bercht. & J.Presl
Iz_11_1209	DJI_0746	Chamaecytisus ruthenicus (Fisch.ex Wol.) Klaskova	0	n	1	0	Fabaceae Lindl.
Iz_11_1209	DJI_0746	Chelidonium majus L.	0	n	1	0	Papaveraceae Juss.
Iz_11_1209	DJI_0746	Chenopodium betaceum Andrz.	0	n	1	0	Amaranthaceae Juss.
Iz_11_1209	DJI_0746	Chenopodium cf. album	0	n	1	0	Amaranthaceae Juss.
Iz_11_1209	DJI_0746	Chenopodium sp.	0		NA	0	Amaranthaceae Juss.
Iz_11_1209	DJI_0746	Chondrilla juncea L.	0	n	1	0	Asteraceae Bercht. & J.Presl
Iz_11_1209	DJI_0746	Cirsium vulgare (Savi) Ten.	0	n	1	0	Asteraceae Bercht. & J.Presl
Iz_11_1209	DJI_0746	Clinopodium acinos (L.) Kuntze	0	n	1	0	Lamiacea Lindl.
Iz_11_1209	DJI_0746	Convallaria majalis L.	0	n	1	0	Asparagaceae Juss.
Iz_11_1209	DJI_0746	Crataegus rhipidophylla Gand.	0	n	1	1	Rosaceae Juss.
Iz_11_1209	DJI_0746	Crepis foetida L.	0	n	1	0	Asteraceae Bercht. & J.Presl
Iz_11_1209	DJI_0746	Dactylis glomerata L.	0	n	1	0	Poaceae Barnhart
Iz_11_1209	DJI_0746	Dianthus campestris M.Bieb.	0	n	1	0	Caryophyllaceae Juss.
Iz_11_1209	DJI_0746	Digitaria sanguinalis (L.) Scop.	0	a	0	0	Poaceae Barnhart
Iz_11_1209	DJI_0746	Elymus repens (L.) Gould	0	n	1	0	Poaceae Barnhart
Iz_11_1209	DJI_0746	Epilobium tetragonum L.	0	n	1	0	Onagraceae Juss.
Iz_11_1209	DJI_0746	Eragrostis minor Host	0	a	0	0	Poaceae Barnhart
Iz_11_1209	DJI_0746	Erigeron canadensis L.	7	a	0	0	Asteraceae Bercht. & J.Presl
Iz_11_1209	DJI_0746	Euphorbia seguieriana Neck.	0	n	1	0	Euphorbiaceae Juss.
Iz_11_1209	DJI_0746	Euphorbia sp.	0		NA	NA	
Iz_11_1209	DJI_0746	Fallopia dumetorum (L.) Holub	0	n	1	0	Polygonaceae Juss.
Iz_11_1209	DJI_0746	Festuca beckeri (Hack.) Trautv.	0	n	1	0	Poaceae Barnhart
Iz_11_1209	DJI_0746	Fragaria vesca L.	0	n	1	0	Rosaceae Juss.
Iz_11_1209	DJI_0746	Frangula alnus Mill.	0	n	1	1	Rhamnaceae Juss.
Iz_11_1209	DJI_0746	Fraxinus pennsylvanica Marshall	0	a	0	1	Oleaceae Hoffmanns. & Link
Iz_11_1209	DJI_0746	Galeopsis bifida Boenn.	0	n	1	0	Lamiacea Lindl.
Iz_11_1209	DJI_0746	Galium verum L.	0	n	1	0	Rubiaceae Juss.
Iz_11_1209	DJI_0746	Genista tinctoria L.	0	n	1	0	Fabaceae Lindl.
Iz_11_1209	DJI_0746	Geranium robertianum L.	0	n	1	0	Geraniaceae Juss.
Iz_11_1209	DJI_0746	Geranium sanquineum L.	0	n	1	0	Geraniaceae Juss.
Iz_11_1209	DJI_0746	Gypsophila paniculata L.	0	n	1	0	Caryophyllaceae Juss.
Iz_11_1209	DJI_0746	Helichrysum arenarium (L.) Moench	0	n	1	0	Asteraceae Bercht. & J.Presl
Iz_11_1209	DJI_0746	Herniaria polygama J.Gay	0	n	1	0	Caryophyllaceae Juss.
Iz_11_1209	DJI_0746	Hieracium robustum Fr.	0	n	1	0	Asteraceae Bercht. & J.Presl
Iz_11_1209	DJI_0746	Hieracium umbellatum L.	0	n	1	NA	Asteraceae Bercht. & J.Presl
Iz_11_1209	DJI_0746	Hylotelephium maximum (L.) Holub	0	n	1	0	Crassulaceae J.St.-Hil.
Iz_11_1209	DJI_0746	Jacobaea borysthenica (DC.) B.Nord. & Greuter	0	n	1	0	Asteraceae Bercht. & J.Presl
Iz_11_1209	DJI_0746	Jasione montana L.	0	n	1	0	Campanulaceae Juss.
Iz_11_1209	DJI_0746	Jurinea cyanoides (L.) Rchb.s.l.	0	n	1	0	Asteraceae Bercht. & J.Presl
Iz_11_1209	DJI_0746	Koeleria glauca (Spreng.) DC.	0	n	1	0	Poaceae Barnhart
Iz_11_1209	DJI_0746	Lactuca serriola L.	0	a	0	0	Asteraceae Bercht. & J.Presl
Iz_11_1209	DJI_0746	Linaria genistifolia (L.) Mill.	0	n	1	0	Plantaginaceae Juss.
Iz_11_1209	DJI_0746	Lomelosia argentea (L.) Greuter & Burdet	0	n	1	0	Caprifoliaceae Juss.
Iz_11_1209	DJI_0746	Melampyrum pratense L.	0	n	1	0	Orobanchaceae Vent.
Iz_11_1209	DJI_0746	Oenothera biennis L.	0	a	0	0	Onagraceae Juss.
Iz_11_1209	DJI_0746	Pentanema britannica (L.) D.Gut.Larr., Santos-Vicente, Anderb., E.Rico & M.M.Mart.Ort.	0	n	1	0	Asteraceae Bercht. & J.Presl
Iz_11_1209	DJI_0746	Peucedanum oreoselinum (L.) Moench	0	n	1	0	Apiaceae Lindl.
Iz_11_1209	DJI_0746	Peucedanum ruthenicum M.Bieb.	0	n	1	0	Apiaceae Lindl.
Iz_11_1209	DJI_0746	Pilosella echioides (Lumn.) F.W.Schultz & Sch.Bip.	0	n	1	0	Asteraceae Bercht. & J.Presl

Iz_11_1209	DJI_0746	Pilosella officinarum F. Schultz et Sch. Bip.	0	n	1	0	Asteraceae Bercht. & J.Presl
Iz_11_1209	DJI_0746	Pilosella sp.	0		NA	0	Asteraceae Bercht. & J.Presl
Iz_11_1209	DJI_0746	Pinus sylvestris L.	0	n	1	1	Pinaceae Spreng. ex F.Rudolphi
Iz_11_1209	DJI_0746	Poa annua L.	0	n	1	0	Poaceae Barnhart
Iz_11_1209	DJI_0746	Poa nemoralis L.	0	n	1	0	Poaceae Barnhart
Iz_11_1209	DJI_0746	Poa sp.	0		NA	0	Poaceae Barnhart
Iz_11_1209	DJI_0746	Polygonatum odoratum (Mill.) Druce	0	n	1	0	Asparagaceae Juss.
Iz_11_1209	DJI_0746	Polygonum arenarium Waldst. & Kit.	0	n	1	0	Polygonaceae Juss.
Iz_11_1209	DJI_0746	Populus nigra L.	0	n	1	1	Salicaceae Mirb.
Iz_11_1209	DJI_0746	Populus tremula L.	0	n	1	1	Salicaceae Mirb.
Iz_11_1209	DJI_0746	Populus Ã canescens (Aiton) Sm.	0	n	1	1	Salicaceae Mirb.
Iz_11_1209	DJI_0746	Potentilla argentea L.	0	n	1	0	Rosaceae Juss.
Iz_11_1209	DJI_0746	Psammophiliella muralis (L.) Ikonn.	0	n	1	0	Caryophyllaceae Juss.
Iz_11_1209	DJI_0746	Psephellus sumensis (Kalen.) Greuter	0	n	1	0	Asteraceae Bercht. & J.Presl
Iz_11_1209	DJI_0746	Pulsatilla pratensis (L.) Mill. s.l.	0	n	1	0	Ranunculaceae Juss.
Iz_11_1209	DJI_0746	Pyrus communis L.	0	a	0	1	Rosaceae Juss.
Iz_11_1209	DJI_0746	Quercus robur L.	0	n	1	1	Fagaceae Dumort.
Iz_11_1209	DJI_0746	Robinia pseudoacacia L.	0	a	0	1	Fabaceae Lindl.
Iz_11_1209	DJI_0746	Rosa sp.	0		NA	1	Rosaceae Juss.
Iz_11_1209	DJI_0746	Rubus idaeus L.	0	n	1	0	Rosaceae Juss.
Iz_11_1209	DJI_0746	Rumex acetosella L.	10	n	1	0	Polygonaceae Juss.
Iz_11_1209	DJI_0746	Rumex thyrsiflorus Fingerh.	0	n	1	0	Polygonaceae Juss.
Iz_11_1209	DJI_0746	Salix alba L.	0	n	1	1	Salicaceae Mirb.
Iz_11_1209	DJI_0746	Sambucus nigra L.	0	n	1	1	Viburnaceae Raf.
Iz_11_1209	DJI_0746	Secale sylvestre Host	0	n	1	0	Poaceae Barnhart
Iz_11_1209	DJI_0746	Sempervivum ruthenicum Schnittsp. & C.B. Lehm.	0	n	1	0	Crassulaceae J.St.-Hil.
Iz_11_1209	DJI_0746	Setaria viridis (L.) P. Beauv.	0	n	1	0	Poaceae Barnhart
Iz_11_1209	DJI_0746	Silene borysthenica (Gruner) Walters	0	n	1	0	Caryophyllaceae Juss.
Iz_11_1209	DJI_0746	Silene latifolia subsp. alba (Mill.) Greuter & Burdet	0	n	1	0	Caryophyllaceae Juss.
Iz_11_1209	DJI_0746	Solidago virgaurea L.	0	n	1	0	Asteraceae Bercht. & J.Presl
Iz_11_1209	DJI_0746	Sorbus aucuparia L.	0	n	1	1	Rosaceae Juss.
Iz_11_1209	DJI_0746	Stachys recta L.	0	n	1	0	Lamiacea Lindl.
Iz_11_1209	DJI_0746	Tanacetum vulgare L.	0	n	1	0	Asteraceae Bercht. & J.Presl
Iz_11_1209	DJI_0746	Thymus pallasianus Heinr.Braun	0	n	1	0	Lamiacea Lindl.
Iz_11_1209	DJI_0746	Tilia cordata Mill.	0	n	1	1	Malvaceae Juss.
Iz_11_1209	DJI_0746	Trifolium alpestre L.	0	n	1	0	Fabaceae Lindl.
Iz_11_1209	DJI_0746	Trifolium arvense L.	0	n	1	0	Fabaceae Lindl.
Iz_11_1209	DJI_0746	Ulmus glabra Huds.	0	n	1	1	Ulmaceae Mirb.
Iz_11_1209	DJI_0746	Verbascum densiflorum Bertol.	0	n	1	0	Scrophulariaceae Juss.
Iz_11_1209	DJI_0746	Veronica austriaca L.	0	n	1	0	Plantaginaceae Juss.
Iz_11_1209	DJI_0746	Veronica incana L.	0	n	1	0	Plantaginaceae Juss.
Iz_11_1209	DJI_0746	Veronica verna L.	0	n	1	0	Plantaginaceae Juss.
Iz_11_1209	DJI_0746	Vincetoxicum hirundinaria Medik	0	n	1	0	Apocynaceae Juss.
Iz_11_1209	DJI_0749	Acer tataricum L.	0	n	1	1	Sapindaceae Juss.
Iz_11_1209	DJI_0749	Achillea millefolium L. s.l.	0	n	1	0	Asteraceae Bercht. & J.Presl
Iz_11_1209	DJI_0749	Allium oleraceum L.	0	n	1	0	Amaryllidaceae J.St.-Hil.
Iz_11_1209	DJI_0749	Ambrosia artemisiifolia L.	0	a	0	0	Asteraceae Bercht. & J.Presl
Iz_11_1209	DJI_0749	Amorpha fruticosa L.	0	a	0	0	Fabaceae Lindl.
Iz_11_1209	DJI_0749	Anthericum ramosum L.	0	n	1	0	Asparagaceae Juss.
Iz_11_1209	DJI_0749	Artemisia marschalliana Spreng.	0	n	1	0	Asteraceae Bercht. & J.Presl
Iz_11_1209	DJI_0749	Asparagus officinalis L.	0	n	1	0	Asparagaceae Juss.
Iz_11_1209	DJI_0749	Astragalus cicer L.	0	n	1	0	Fabaceae Lindl.
Iz_11_1209	DJI_0749	Bassia laniflora (S.G.Gmel.) A.J.Scott	0	n	1	0	Amaranthaceae Juss.
Iz_11_1209	DJI_0749	Berberis vulgaris L.	0	n	1	0	Berberidaceae Juss.
Iz_11_1209	DJI_0749	Betula pendula Roth.	0	n	1	1	Betulaceae Gray
Iz_11_1209	DJI_0749	Betula pendula Roth.	0	n	1	0	Poaceae Barnhart
Iz_11_1209	DJI_0749	Bromus inermis Leyss.	0		NA	NA	
Iz_11_1209	DJI_0749	Bromus squarrosus L.	0	a	0	0	Poaceae Barnhart
Iz_11_1209	DJI_0749	Bromus tectorum L.	0	a	0	0	Poaceae Barnhart
Iz_11_1209	DJI_0749	Calamagrostis epigejos (L.) Roth	2	n	1	0	Poaceae Barnhart
Iz_11_1209	DJI_0749	Carduus acanthoides L.	0	n	1	0	Asteraceae Bercht. & J.Presl
Iz_11_1209	DJI_0749	Carex sp.	0	n	1	0	Cyperaceae Juss.
Iz_11_1209	DJI_0749	Centaurea majorovii Dumbadze	0	n	1	0	Asteraceae Bercht. & J.Presl
Iz_11_1209	DJI_0749	Chamaecytisus ruthenicus (Fisch.ex Wol.) Klaskova	0	n	1	0	Fabaceae Lindl.
Iz_11_1209	DJI_0749	Chelidonium majus L.	0	n	1	0	Papaveraceae Juss.
Iz_11_1209	DJI_0749	Chenopodium betaceum Andrz.	0	n	1	0	Amaranthaceae Juss.
Iz_11_1209	DJI_0749	Chenopodium cf. album	0	n	1	0	Amaranthaceae Juss.
Iz_11_1209	DJI_0749	Chenopodium sp.	0		NA	0	Amaranthaceae Juss.
Iz_11_1209	DJI_0749	Chondrilla juncea L.	0	n	1	0	Asteraceae Bercht. & J.Presl
Iz_11_1209	DJI_0749	Cirsium vulgare (Savi) Ten.	0	n	1	0	Asteraceae Bercht. & J.Presl
Iz_11_1209	DJI_0749	Clinopodium acinos (L.) Kuntze	0	n	1	0	Lamiacea Lindl.
Iz_11_1209	DJI_0749	Convallaria majalis L.	0	n	1	0	Asparagaceae Juss.
Iz_11_1209	DJI_0749	Crataegus rhipidophylla Gand.	0	n	1	1	Rosaceae Juss.
Iz_11_1209	DJI_0749	Crepis foetida L.	0	n	1	0	Asteraceae Bercht. & J.Presl
Iz_11_1209	DJI_0749	Dactylis glomerata L.	0	n	1	0	Poaceae Barnhart
Iz_11_1209	DJI_0749	Dianthus campestris M.Bieb.	0	n	1	0	Caryophyllaceae Juss.
Iz_11_1209	DJI_0749	Digitaria sanguinalis (L.) Scop.	0	a	0	0	Poaceae Barnhart
Iz_11_1209	DJI_0749	Elymus repens (L.) Gould	0	n	1	0	Poaceae Barnhart
Iz_11_1209	DJI_0749	Epilobium tetragonum L.	0	n	1	0	Onagraceae Juss.
Iz_11_1209	DJI_0749	Eragrostis minor Host	0	a	0	0	Poaceae Barnhart
Iz_11_1209	DJI_0749	Erigeron canadensis L.	0	a	0	0	Asteraceae Bercht. & J.Presl
Iz_11_1209	DJI_0749	Euphorbia seguieriana Neck.	0	n	1	0	Euphorbiaceae Juss.
Iz_11_1209	DJI_0749	Euphorbia sp.	0		NA	NA	
Iz_11_1209	DJI_0749	Fallopia dumetorum (L.) Holub	0	n	1	0	Polygonaceae Juss.
Iz_11_1209	DJI_0749	Festuca beckeri (Hack.) Trautv.	1	n	1	0	Poaceae Barnhart
Iz_11_1209	DJI_0749	Fragaria vesca L.	0	n	1	0	Rosaceae Juss.
Iz_11_1209	DJI_0749	Frangula alnus Mill.	0	n	1	1	Rhamnaceae Juss.
Iz_11_1209	DJI_0749	Fraxinus pennsylvanica Marshall	0	a	0	1	Oleaceae Hoffmanns. & Link
Iz_11_1209	DJI_0749	Galeopsis bifida Boenn.	0	n	1	0	Lamiacea Lindl.
Iz_11_1209	DJI_0749	Galium verum L.	0	n	1	0	Rubiaceae Juss.
Iz_11_1209	DJI_0749	Genista tinctoria L.	0	n	1	0	Fabaceae Lindl.
Iz_11_1209	DJI_0749	Geranium robertianum L.	0	n	1	0	Geraniaceae Juss.
Iz_11_1209	DJI_0749	Geranium sanquineum L.	0	n	1	0	Geraniaceae Juss.
Iz_11_1209	DJI_0749	Gypsophila paniculata L.	0	n	1	0	Caryophyllaceae Juss.
Iz_11_1209	DJI_0749	Helichrysum arenarium (L.) Moench	0	n	1	0	Asteraceae Bercht. & J.Presl
Iz_11_1209	DJI_0749	Herniaria polygama J.Gay	0	n	1	0	Caryophyllaceae Juss.
Iz_11_1209	DJI_0749	Hieracium robustum Fr.	0	n	1	0	Asteraceae Bercht. & J.Presl
Iz_11_1209	DJI_0749	Hieracium umbellatum L.	0	n	1	NA	Asteraceae Bercht. & J.Presl

Iz_11_1209	DJI_0749	Hylotelephium maximum (L.) Holub	0	n	1	0	Crassulaceae J.St.-Hil.
Iz_11_1209	DJI_0749	Jacobaea borysthenica (DC.) B.Nord. & Greuter	0	n	1	0	Asteraceae Bercht. & J.Presl
Iz_11_1209	DJI_0749	Jasione montana L.	0	n	1	0	Campanulaceae Juss.
Iz_11_1209	DJI_0749	Jurinea cyanoides (L.) Rchb.s.l.	0	n	1	0	Asteraceae Bercht. & J.Presl
Iz_11_1209	DJI_0749	Koeleria glauca (Spreng.) DC.	0	n	1	0	Poaceae Barnhart
Iz_11_1209	DJI_0749	Lactuca serriola L.	0	a	0	0	Asteraceae Bercht. & J.Presl
Iz_11_1209	DJI_0749	Linaria genistifolia (L.) Mill.	0	n	1	0	Plantaginaceae Juss.
Iz_11_1209	DJI_0749	Lomelosia argentea (L.) Greuter & Burdet	0	n	1	0	Caprifoliaceae Juss.
Iz_11_1209	DJI_0749	Melampyrum pratense L.	0	n	1	0	Orobanchaceae Vent.
Iz_11_1209	DJI_0749	Oenothera biennis L.	0	a	0	0	Onagraceae Juss.
Iz_11_1209	DJI_0749	Pentanema britannica (L.) D.Gut.Larr., Santos-Vicente, Anderb., E.Rico & M.M.Mart.Ort.	0	n	1	0	Asteraceae Bercht. & J.Presl
Iz_11_1209	DJI_0749	Peucedanum oreoselinum (L.) Moench	0	n	1	0	Apiaceae Lindl.
Iz_11_1209	DJI_0749	Peucedanum ruthenicum M.Bieb.	0	n	1	0	Apiaceae Lindl.
Iz_11_1209	DJI_0749	Pilosella echioides (Lumn.) F.W.Schultz & Sch.Bip.	0	n	1	0	Asteraceae Bercht. & J.Presl
Iz_11_1209	DJI_0749	Pilosella officinarum F. Schultz et Sch. Bip.	0	n	1	0	Asteraceae Bercht. & J.Presl
Iz_11_1209	DJI_0749	Pilosella sp.	0		NA	0	Asteraceae Bercht. & J.Presl
Iz_11_1209	DJI_0749	Pinus sylvestris L.	0	n	1	1	Pinaceae Spreng. ex F.Rudolphi
Iz_11_1209	DJI_0749	Poa annua L.	0	n	1	0	Poaceae Barnhart
Iz_11_1209	DJI_0749	Poa nemoralis L.	0	n	1	0	Poaceae Barnhart
Iz_11_1209	DJI_0749	Poa sp.	0		NA	0	Poaceae Barnhart
Iz_11_1209	DJI_0749	Polygonatum odoratum (Mill.) Druce	0	n	1	0	Asparagaceae Juss.
Iz_11_1209	DJI_0749	Polygonum arenarium Waldst. & Kit.	0	n	1	0	Polygonaceae Juss.
Iz_11_1209	DJI_0749	Populus nigra L.	0	n	1	1	Salicaceae Mirb.
Iz_11_1209	DJI_0749	Populus tremula L.	0	n	1	1	Salicaceae Mirb.
Iz_11_1209	DJI_0749	Populus Ã canescens (Aiton) Sm.	0	n	1	1	Salicaceae Mirb.
Iz_11_1209	DJI_0749	Potentilla argentea L.	0	n	1	0	Rosaceae Juss.
Iz_11_1209	DJI_0749	Psammophiliella muralis (L.) Ikonn.	0	n	1	0	Caryophyllaceae Juss.
Iz_11_1209	DJI_0749	Psephellus sumensis (Kalen.) Greuter	0	n	1	0	Asteraceae Bercht. & J.Presl
Iz_11_1209	DJI_0749	Pulsatilla pratensis (L.) Mill. s.l.	0	n	1	0	Ranunculaceae Juss.
Iz_11_1209	DJI_0749	Pyrus communis L.	0	a	0	1	Rosaceae Juss.
Iz_11_1209	DJI_0749	Quercus robur L.	0	n	1	1	Fagaceae Dumort.
Iz_11_1209	DJI_0749	Robinia pseudoacacia L.	0	a	0	1	Fabaceae Lindl.
Iz_11_1209	DJI_0749	Rosa sp.	0		NA	1	Rosaceae Juss.
Iz_11_1209	DJI_0749	Rubus idaeus L.	0	n	1	0	Rosaceae Juss.
Iz_11_1209	DJI_0749	Rumex acetosella L.	7	n	1	0	Polygonaceae Juss.
Iz_11_1209	DJI_0749	Rumex thyrsiflorus Fingerh.	0	n	1	0	Polygonaceae Juss.
Iz_11_1209	DJI_0749	Salix alba L.	0	n	1	1	Salicaceae Mirb.
Iz_11_1209	DJI_0749	Sambucus nigra L.	0	n	1	1	Viburnaceae Raf.
Iz_11_1209	DJI_0749	Secale sylvestre Host	0	n	1	0	Poaceae Barnhart
Iz_11_1209	DJI_0749	Sempervivum ruthenicum Schnittsp. & C.B. Lehm.	0	n	1	0	Crassulaceae J.St.-Hil.
Iz_11_1209	DJI_0749	Setaria viridis (L.) P. Beauv.	0	n	1	0	Poaceae Barnhart
Iz_11_1209	DJI_0749	Silene borysthenica (Gruner) Walters	0	n	1	0	Caryophyllaceae Juss.
Iz_11_1209	DJI_0749	Silene latifolia subsp. alba (Mill.) Greuter & Burdet	0	n	1	0	Caryophyllaceae Juss.
Iz_11_1209	DJI_0749	Solidago virgaurea L.	0	n	1	0	Asteraceae Bercht. & J.Presl
Iz_11_1209	DJI_0749	Sorbus aucuparia L.	0	n	1	1	Rosaceae Juss.
Iz_11_1209	DJI_0749	Stachys recta L.	0	n	1	0	Lamiacea Lindl.
Iz_11_1209	DJI_0749	Tanacetum vulgare L.	0	n	1	0	Asteraceae Bercht. & J.Presl
Iz_11_1209	DJI_0749	Thymus pallasianus Heinr.Braun	0	n	1	0	Lamiacea Lindl.
Iz_11_1209	DJI_0749	Tilia cordata Mill.	0	n	1	1	Malvaceae Juss.
Iz_11_1209	DJI_0749	Trifolium alpestre L.	0	n	1	0	Fabaceae Lindl.
Iz_11_1209	DJI_0749	Trifolium arvense L.	0	n	1	0	Fabaceae Lindl.
Iz_11_1209	DJI_0749	Ulmus glabra Huds.	0	n	1	1	Ulmaceae Mirb.
Iz_11_1209	DJI_0749	Verbascum densiflorum Bertol.	0	n	1	0	Scrophulariaceae Juss.
Iz_11_1209	DJI_0749	Veronica austriaca L.	0	n	1	0	Plantaginaceae Juss.
Iz_11_1209	DJI_0749	Veronica incana L.	0	n	1	0	Plantaginaceae Juss.
Iz_11_1209	DJI_0749	Veronica verna L.	0	n	1	0	Plantaginaceae Juss.
Iz_11_1209	DJI_0749	Vincetoxicum hirundinaria Medik	0	n	1	0	Apocynaceae Juss.
Iz_11_1209	DJI_0751	Acer tataricum L.	0	n	1	1	Sapindaceae Juss.
Iz_11_1209	DJI_0751	Achillea millefolium L. s.l.	0	n	1	0	Asteraceae Bercht. & J.Presl
Iz_11_1209	DJI_0751	Allium oleraceum L.	0	n	1	0	Amaryllidaceae J.St.-Hil.
Iz_11_1209	DJI_0751	Ambrosia artemisiifolia L.	0	a	0	0	Asteraceae Bercht. & J.Presl
Iz_11_1209	DJI_0751	Amorpha fruticosa L.	0	a	0	0	Fabaceae Lindl.
Iz_11_1209	DJI_0751	Anthericum ramosum L.	0	n	1	0	Asparagaceae Juss.
Iz_11_1209	DJI_0751	Artemisia marschalliana Spreng.	0	n	1	0	Asteraceae Bercht. & J.Presl
Iz_11_1209	DJI_0751	Asparagus officinalis L.	0	n	1	0	Asparagaceae Juss.
Iz_11_1209	DJI_0751	Astragalus cicer L.	0	n	1	0	Fabaceae Lindl.
Iz_11_1209	DJI_0751	Bassia laniflora (S.G.Gmel.) A.J.Scott	0	n	1	0	Amaranthaceae Juss.
Iz_11_1209	DJI_0751	Berberis vulgaris L.	0	n	1	0	Berberidaceae Juss.
Iz_11_1209	DJI_0751	Betula pendula Roth.	0	n	1	1	Betulaceae Gray
Iz_11_1209	DJI_0751	Betula pendula Roth.	0	n	1	0	Poaceae Barnhart
Iz_11_1209	DJI_0751	Bromus inermis Leyss.	0		NA	NA	
Iz_11_1209	DJI_0751	Bromus squarrosus L.	0	a	0	0	Poaceae Barnhart
Iz_11_1209	DJI_0751	Bromus tectorum L.	0	a	0	0	Poaceae Barnhart
Iz_11_1209	DJI_0751	Calamagrostis epigejos (L.) Roth	0	n	1	0	Poaceae Barnhart
Iz_11_1209	DJI_0751	Carduus acanthoides L.	0	n	1	0	Asteraceae Bercht. & J.Presl
Iz_11_1209	DJI_0751	Carex sp.	0	n	1	0	Cyperaceae Juss.
Iz_11_1209	DJI_0751	Centaurea majorovii Dumbadze	0	n	1	0	Asteraceae Bercht. & J.Presl
Iz_11_1209	DJI_0751	Chamaecytisus ruthenicus (Fisch.ex Wol.) Klaskova	0	n	1	0	Fabaceae Lindl.
Iz_11_1209	DJI_0751	Chelidonium majus L.	0	n	1	0	Papaveraceae Juss.
Iz_11_1209	DJI_0751	Chenopodium betaceum Andrz.	0	n	1	0	Amaranthaceae Juss.
Iz_11_1209	DJI_0751	Chenopodium cf. album	0	n	1	0	Amaranthaceae Juss.
Iz_11_1209	DJI_0751	Chenopodium sp.	0		NA	0	Amaranthaceae Juss.
Iz_11_1209	DJI_0751	Chondrilla juncea L.	0	n	1	0	Asteraceae Bercht. & J.Presl
Iz_11_1209	DJI_0751	Cirsium vulgare (Savi) Ten.	0	n	1	0	Asteraceae Bercht. & J.Presl
Iz_11_1209	DJI_0751	Clinopodium acinos (L.) Kuntze	0	n	1	0	Lamiacea Lindl.
Iz_11_1209	DJI_0751	Convallaria majalis L.	0	n	1	0	Asparagaceae Juss.
Iz_11_1209	DJI_0751	Crataegus rhipidophylla Gand.	0	n	1	1	Rosaceae Juss.
Iz_11_1209	DJI_0751	Crepis foetida L.	0	n	1	0	Asteraceae Bercht. & J.Presl
Iz_11_1209	DJI_0751	Dactylis glomerata L.	0	n	1	0	Poaceae Barnhart
Iz_11_1209	DJI_0751	Dianthus campestris M.Bieb.	0	n	1	0	Caryophyllaceae Juss.
Iz_11_1209	DJI_0751	Digitaria sanguinalis (L.) Scop.	0	a	0	0	Poaceae Barnhart
Iz_11_1209	DJI_0751	Elymus repens (L.) Gould	0	n	1	0	Poaceae Barnhart
Iz_11_1209	DJI_0751	Epilobium tetragonum L.	0	n	1	0	Onagraceae Juss.
Iz_11_1209	DJI_0751	Eragrostis minor Host	0	a	0	0	Poaceae Barnhart
Iz_11_1209	DJI_0751	Erigeron canadensis L.	0	a	0	0	Asteraceae Bercht. & J.Presl
Iz_11_1209	DJI_0751	Euphorbia seguieriana Neck.	0	n	1	0	Euphorbiaceae Juss.
Iz_11_1209	DJI_0751	Euphorbia sp.	0		NA	NA	
Iz_11_1209	DJI_0751	Fallopia dumetorum (L.) Holub	0	n	1	0	Polygonaceae Juss.

Iz_11_1209	DJI_0751	Festuca beckeri (Hack.) Trautv.	0	n	1	0	Poaceae Barnhart
Iz_11_1209	DJI_0751	Fragaria vesca L.	0	n	1	0	Rosaceae Juss.
Iz_11_1209	DJI_0751	Frangula alnus Mill.	0	n	1	1	Rhamnaceae Juss.
Iz_11_1209	DJI_0751	Fraxinus pennsylvanica Marshall	0	a	0	1	Oleaceae Hoffmanns. & Link
Iz_11_1209	DJI_0751	Galeopsis bifida Boenn.	0	n	1	0	Lamiacea Lindl.
Iz_11_1209	DJI_0751	Galium verum L.	0	n	1	0	Rubiaceae Juss.
Iz_11_1209	DJI_0751	Genista tinctoria L.	0	n	1	0	Fabaceae Lindl.
Iz_11_1209	DJI_0751	Geranium robertianum L.	0	n	1	0	Geraniaceae Juss.
Iz_11_1209	DJI_0751	Geranium sanquineum L.	0	n	1	0	Geraniaceae Juss.
Iz_11_1209	DJI_0751	Gypsophila paniculata L.	0	n	1	0	Caryophyllaceae Juss.
Iz_11_1209	DJI_0751	Helichrysum arenarium (L.) Moench	0	n	1	0	Asteraceae Bercht. & J.Presl
Iz_11_1209	DJI_0751	Herniaria polygama J.Gay	0	n	1	0	Caryophyllaceae Juss.
Iz_11_1209	DJI_0751	Hieracium robustum Fr.	0	n	1	0	Asteraceae Bercht. & J.Presl
Iz_11_1209	DJI_0751	Hieracium umbellatum L.	0	n	1	NA	Asteraceae Bercht. & J.Presl
Iz_11_1209	DJI_0751	Hylotelephium maximum (L.) Holub	0	n	1	0	Crassulaceae J.St.-Hil.
Iz_11_1209	DJI_0751	Jacobaea borysthenica (DC.) B.Nord. & Greuter	0	n	1	0	Asteraceae Bercht. & J.Presl
Iz_11_1209	DJI_0751	Jasione montana L.	0	n	1	0	Campanulaceae Juss.
Iz_11_1209	DJI_0751	Jurinea cyanoides (L.) Rchb.s.l.	0	n	1	0	Asteraceae Bercht. & J.Presl
Iz_11_1209	DJI_0751	Koeleria glauca (Spreng.) DC.	0	n	1	0	Poaceae Barnhart
Iz_11_1209	DJI_0751	Lactuca serriola L.	0	a	0	0	Asteraceae Bercht. & J.Presl
Iz_11_1209	DJI_0751	Linaria genistifolia (L.) Mill.	0	n	1	0	Plantaginaceae Juss.
Iz_11_1209	DJI_0751	Lomelosia argentea (L.) Greuter & Burdet	0	n	1	0	Caprifoliaceae Juss.
Iz_11_1209	DJI_0751	Melampyrum pratense L.	0	n	1	0	Orobanchaceae Vent.
Iz_11_1209	DJI_0751	Oenothera biennis L.	0	a	0	0	Onagraceae Juss.
Iz_11_1209	DJI_0751	Pentanema britannica (L.) D.Gut.Larr., Santos-Vicente, Anderb., E.Rico & M.M.Mart.Ort.	0	n	1	0	Asteraceae Bercht. & J.Presl
Iz_11_1209	DJI_0751	Peucedanum oreoselinum (L.) Moench	0	n	1	0	Apiaceae Lindl.
Iz_11_1209	DJI_0751	Peucedanum ruthenicum M.Bieb.	0	n	1	0	Apiaceae Lindl.
Iz_11_1209	DJI_0751	Pilosella echioides (Lumn.) F.W.Schultz & Sch.Bip.	0	n	1	0	Asteraceae Bercht. & J.Presl
Iz_11_1209	DJI_0751	Pilosella officinarum F. Schultz et Sch. Bip.	0	n	1	0	Asteraceae Bercht. & J.Presl
Iz_11_1209	DJI_0751	Pilosella sp.	0		NA	0	Asteraceae Bercht. & J.Presl
Iz_11_1209	DJI_0751	Pinus sylvestris L.	0	n	1	1	Pinaceae Spreng. ex F.Rudolphi
Iz_11_1209	DJI_0751	Poa annua L.	0	n	1	0	Poaceae Barnhart
Iz_11_1209	DJI_0751	Poa nemoralis L.	0	n	1	0	Poaceae Barnhart
Iz_11_1209	DJI_0751	Poa sp.	0		NA	0	Poaceae Barnhart
Iz_11_1209	DJI_0751	Polygonatum odoratum (Mill.) Druce	0	n	1	0	Asparagaceae Juss.
Iz_11_1209	DJI_0751	Polygonum arenarium Waldst. & Kit.	0	n	1	0	Polygonaceae Juss.
Iz_11_1209	DJI_0751	Populus nigra L.	0	n	1	1	Salicaceae Mirb.
Iz_11_1209	DJI_0751	Populus tremula L.	0	n	1	1	Salicaceae Mirb.
Iz_11_1209	DJI_0751	Populus Ã canescens (Aiton) Sm.	0	n	1	1	Salicaceae Mirb.
Iz_11_1209	DJI_0751	Potentilla argentea L.	0	n	1	0	Rosaceae Juss.
Iz_11_1209	DJI_0751	Psammophiliella muralis (L.) Ikonn.	0	n	1	0	Caryophyllaceae Juss.
Iz_11_1209	DJI_0751	Psephellus sumensis (Kalen.) Greuter	0	n	1	0	Asteraceae Bercht. & J.Presl
Iz_11_1209	DJI_0751	Pulsatilla pratensis (L.) Mill. s.l.	0	n	1	0	Ranunculaceae Juss.
Iz_11_1209	DJI_0751	Pyrus communis L.	0	a	0	1	Rosaceae Juss.
Iz_11_1209	DJI_0751	Quercus robur L.	0	n	1	1	Fagaceae Dumort.
Iz_11_1209	DJI_0751	Robinia pseudoacacia L.	0	a	0	1	Fabaceae Lindl.
Iz_11_1209	DJI_0751	Rosa sp.	0		NA	1	Rosaceae Juss.
Iz_11_1209	DJI_0751	Rubus idaeus L.	0	n	1	0	Rosaceae Juss.
Iz_11_1209	DJI_0751	Rumex acetosella L.	5	n	1	0	Polygonaceae Juss.
Iz_11_1209	DJI_0751	Rumex thyrsiflorus Fingerh.	0	n	1	0	Polygonaceae Juss.
Iz_11_1209	DJI_0751	Salix alba L.	0	n	1	1	Salicaceae Mirb.
Iz_11_1209	DJI_0751	Sambucus nigra L.	0	n	1	1	Viburnaceae Raf.
Iz_11_1209	DJI_0751	Secale sylvestre Host	0	n	1	0	Poaceae Barnhart
Iz_11_1209	DJI_0751	Sempervivum ruthenicum Schnittsp. & C.B. Lehm.	0	n	1	0	Crassulaceae J.St.-Hil.
Iz_11_1209	DJI_0751	Setaria viridis (L.) P. Beauv.	0	n	1	0	Poaceae Barnhart
Iz_11_1209	DJI_0751	Silene borysthenica (Gruner) Walters	0	n	1	0	Caryophyllaceae Juss.
Iz_11_1209	DJI_0751	Silene latifolia subsp. alba (Mill.) Greuter & Burdet	0	n	1	0	Caryophyllaceae Juss.
Iz_11_1209	DJI_0751	Solidago virgaurea L.	0	n	1	0	Asteraceae Bercht. & J.Presl
Iz_11_1209	DJI_0751	Sorbus aucuparia L.	0	n	1	1	Rosaceae Juss.
Iz_11_1209	DJI_0751	Stachys recta L.	0	n	1	0	Lamiacea Lindl.
Iz_11_1209	DJI_0751	Tanacetum vulgare L.	0	n	1	0	Asteraceae Bercht. & J.Presl
Iz_11_1209	DJI_0751	Thymus pallasianus Heinr.Braun	0	n	1	0	Lamiacea Lindl.
Iz_11_1209	DJI_0751	Tilia cordata Mill.	0	n	1	1	Malvaceae Juss.
Iz_11_1209	DJI_0751	Trifolium alpestre L.	0	n	1	0	Fabaceae Lindl.
Iz_11_1209	DJI_0751	Trifolium arvense L.	0	n	1	0	Fabaceae Lindl.
Iz_11_1209	DJI_0751	Ulmus glabra Huds.	0	n	1	1	Ulmaceae Mirb.
Iz_11_1209	DJI_0751	Verbascum densiflorum Bertol.	0	n	1	0	Scrophulariaceae Juss.
Iz_11_1209	DJI_0751	Veronica austriaca L.	0	n	1	0	Plantaginaceae Juss.
Iz_11_1209	DJI_0751	Veronica incana L.	0	n	1	0	Plantaginaceae Juss.
Iz_11_1209	DJI_0751	Veronica verna L.	0	n	1	0	Plantaginaceae Juss.
Iz_11_1209	DJI_0751	Vincetoxicum hirundinaria Medik	0	n	1	0	Apocynaceae Juss.
Iz_11_1209	DJI_0757	Acer tataricum L.	0	n	1	1	Sapindaceae Juss.
Iz_11_1209	DJI_0757	Achillea millefolium L. s.l.	0	n	1	0	Asteraceae Bercht. & J.Presl
Iz_11_1209	DJI_0757	Allium oleraceum L.	0	n	1	0	Amaryllidaceae J.St.-Hil.
Iz_11_1209	DJI_0757	Ambrosia artemisiifolia L.	0	a	0	0	Asteraceae Bercht. & J.Presl
Iz_11_1209	DJI_0757	Amorpha fruticosa L.	0	a	0	0	Fabaceae Lindl.
Iz_11_1209	DJI_0757	Anthericum ramosum L.	0	n	1	0	Asparagaceae Juss.
Iz_11_1209	DJI_0757	Artemisia marschalliana Spreng.	0	n	1	0	Asteraceae Bercht. & J.Presl
Iz_11_1209	DJI_0757	Asparagus officinalis L.	0	n	1	0	Asparagaceae Juss.
Iz_11_1209	DJI_0757	Astragalus cicer L.	0	n	1	0	Fabaceae Lindl.
Iz_11_1209	DJI_0757	Bassia laniflora (S.G.Gmel.) A.J.Scott	0	n	1	0	Amaranthaceae Juss.
Iz_11_1209	DJI_0757	Berberis vulgaris L.	0	n	1	0	Berberidaceae Juss.
Iz_11_1209	DJI_0757	Betula pendula Roth.	0	n	1	1	Betulaceae Gray
Iz_11_1209	DJI_0757	Betula pendula Roth.	0	n	1	0	Poaceae Barnhart
Iz_11_1209	DJI_0757	Bromus inermis Leyss.	0		NA	NA	
Iz_11_1209	DJI_0757	Bromus squarrosus L.	0	a	0	0	Poaceae Barnhart
Iz_11_1209	DJI_0757	Bromus tectorum L.	0	a	0	0	Poaceae Barnhart
Iz_11_1209	DJI_0757	Calamagrostis epigejos (L.) Roth	40	n	1	0	Poaceae Barnhart
Iz_11_1209	DJI_0757	Carduus acanthoides L.	0	n	1	0	Asteraceae Bercht. & J.Presl
Iz_11_1209	DJI_0757	Carex sp.	0	n	1	0	Cyperaceae Juss.
Iz_11_1209	DJI_0757	Centaurea majorovii Dumbadze	0	n	1	0	Asteraceae Bercht. & J.Presl
Iz_11_1209	DJI_0757	Chamaecytisus ruthenicus (Fisch.ex Wol.) Klaskova	0	n	1	0	Fabaceae Lindl.
Iz_11_1209	DJI_0757	Chelidonium majus L.	0	n	1	0	Papaveraceae Juss.
Iz_11_1209	DJI_0757	Chenopodium betaceum Andrz.	0	n	1	0	Amaranthaceae Juss.
Iz_11_1209	DJI_0757	Chenopodium cf. album	0	n	1	0	Amaranthaceae Juss.
Iz_11_1209	DJI_0757	Chenopodium sp.	0		NA	0	Amaranthaceae Juss.
Iz_11_1209	DJI_0757	Chondrilla juncea L.	0	n	1	0	Asteraceae Bercht. & J.Presl
Iz_11_1209	DJI_0757	Cirsium vulgare (Savi) Ten.	0	n	1	0	Asteraceae Bercht. & J.Presl

Iz_11_1209	DJI_0757	Clinopodium acinos (L.) Kuntze	0	n	1	0	Lamiacea Lindl.
Iz_11_1209	DJI_0757	Convallaria majalis L.	0	n	1	0	Asparagaceae Juss.
Iz_11_1209	DJI_0757	Crataegus rhipidophylla Gand.	0	n	1	1	Rosaceae Juss.
Iz_11_1209	DJI_0757	Crepis foetida L.	0	n	1	0	Asteraceae Bercht. & J.Presl
Iz_11_1209	DJI_0757	Dactylis glomerata L.	0	n	1	0	Poaceae Barnhart
Iz_11_1209	DJI_0757	Dianthus campestris M.Bieb.	0	n	1	0	Caryophyllaceae Juss.
Iz_11_1209	DJI_0757	Digitaria sanguinalis (L.) Scop.	0	a	0	0	Poaceae Barnhart
Iz_11_1209	DJI_0757	Elymus repens (L.) Gould	0	n	1	0	Poaceae Barnhart
Iz_11_1209	DJI_0757	Epilobium tetragonum L.	0	n	1	0	Onagraceae Juss.
Iz_11_1209	DJI_0757	Eragrostis minor Host	0	a	0	0	Poaceae Barnhart
Iz_11_1209	DJI_0757	Erigeron canadensis L.	6	a	0	0	Asteraceae Bercht. & J.Presl
Iz_11_1209	DJI_0757	Euphorbia seguieriana Neck.	0	n	1	0	Euphorbiaceae Juss.
Iz_11_1209	DJI_0757	Euphorbia sp.	0		NA	NA	
Iz_11_1209	DJI_0757	Fallopia dumetorum (L.) Holub	0	n	1	0	Polygonaceae Juss.
Iz_11_1209	DJI_0757	Festuca beckeri (Hack.) Trautv.	0	n	1	0	Poaceae Barnhart
Iz_11_1209	DJI_0757	Fragaria vesca L.	0	n	1	0	Rosaceae Juss.
Iz_11_1209	DJI_0757	Frangula alnus Mill.	0	n	1	1	Rhamnaceae Juss.
Iz_11_1209	DJI_0757	Fraxinus pennsylvanica Marshall	0	a	0	1	Oleaceae Hoffmanns. & Link
Iz_11_1209	DJI_0757	Galeopsis bifida Boenn.	0	n	1	0	Lamiacea Lindl.
Iz_11_1209	DJI_0757	Galium verum L.	0	n	1	0	Rubiaceae Juss.
Iz_11_1209	DJI_0757	Genista tinctoria L.	0	n	1	0	Fabaceae Lindl.
Iz_11_1209	DJI_0757	Geranium robertianum L.	0	n	1	0	Geraniaceae Juss.
Iz_11_1209	DJI_0757	Geranium sanquineum L.	0	n	1	0	Geraniaceae Juss.
Iz_11_1209	DJI_0757	Gypsophila paniculata L.	0	n	1	0	Caryophyllaceae Juss.
Iz_11_1209	DJI_0757	Helichrysum arenarium (L.) Moench	0	n	1	0	Asteraceae Bercht. & J.Presl
Iz_11_1209	DJI_0757	Herniaria polygama J.Gay	0	n	1	0	Caryophyllaceae Juss.
Iz_11_1209	DJI_0757	Hieracium robustum Fr.	0	n	1	0	Asteraceae Bercht. & J.Presl
Iz_11_1209	DJI_0757	Hieracium umbellatum L.	0	n	1	NA	Asteraceae Bercht. & J.Presl
Iz_11_1209	DJI_0757	Hylotelephium maximum (L.) Holub	0	n	1	0	Crassulaceae J.St.-Hil.
Iz_11_1209	DJI_0757	Jacobaea borysthenica (DC.) B.Nord. & Greuter	0	n	1	0	Asteraceae Bercht. & J.Presl
Iz_11_1209	DJI_0757	Jasione montana L.	0	n	1	0	Campanulaceae Juss.
Iz_11_1209	DJI_0757	Jurinea cyanoides (L.) Rchb.s.l.	0	n	1	0	Asteraceae Bercht. & J.Presl
Iz_11_1209	DJI_0757	Koeleria glauca (Spreng.) DC.	0	n	1	0	Poaceae Barnhart
Iz_11_1209	DJI_0757	Lactuca serriola L.	0	a	0	0	Asteraceae Bercht. & J.Presl
Iz_11_1209	DJI_0757	Linaria genistifolia (L.) Mill.	0	n	1	0	Plantaginaceae Juss.
Iz_11_1209	DJI_0757	Lomelosia argentea (L.) Greuter & Burdet	0	n	1	0	Caprifoliaceae Juss.
Iz_11_1209	DJI_0757	Melampyrum pratense L.	0	n	1	0	Orobanchaceae Vent.
Iz_11_1209	DJI_0757	Oenothera biennis L.	0	a	0	0	Onagraceae Juss.
Iz_11_1209	DJI_0757	Pentanema britannica (L.) D.Gut.Larr., Santos-Vicente, Anderb., E.Rico & M.M.Mart.Ort.	0	n	1	0	Asteraceae Bercht. & J.Presl
Iz_11_1209	DJI_0757	Peucedanum oreoselinum (L.) Moench	0	n	1	0	Apiaceae Lindl.
Iz_11_1209	DJI_0757	Peucedanum ruthenicum M.Bieb.	0	n	1	0	Apiaceae Lindl.
Iz_11_1209	DJI_0757	Pilosella echioides (Lumn.) F.W.Schultz & Sch.Bip.	0	n	1	0	Asteraceae Bercht. & J.Presl
Iz_11_1209	DJI_0757	Pilosella officinarum F. Schultz et Sch. Bip.	0	n	1	0	Asteraceae Bercht. & J.Presl
Iz_11_1209	DJI_0757	Pilosella sp.	0		NA	0	Asteraceae Bercht. & J.Presl
Iz_11_1209	DJI_0757	Pinus sylvestris L.	0	n	1	1	Pinaceae Spreng. ex F.Rudolphi
Iz_11_1209	DJI_0757	Poa annua L.	0	n	1	0	Poaceae Barnhart
Iz_11_1209	DJI_0757	Poa nemoralis L.	0	n	1	0	Poaceae Barnhart
Iz_11_1209	DJI_0757	Poa sp.	0		NA	0	Poaceae Barnhart
Iz_11_1209	DJI_0757	Polygonatum odoratum (Mill.) Druce	0	n	1	0	Asparagaceae Juss.
Iz_11_1209	DJI_0757	Polygonum arenarium Waldst. & Kit.	0	n	1	0	Polygonaceae Juss.
Iz_11_1209	DJI_0757	Populus nigra L.	0	n	1	1	Salicaceae Mirb.
Iz_11_1209	DJI_0757	Populus tremula L.	4	n	1	1	Salicaceae Mirb.
Iz_11_1209	DJI_0757	Populus Ã canescens (Aiton) Sm.	0	n	1	1	Salicaceae Mirb.
Iz_11_1209	DJI_0757	Potentilla argentea L.	0	n	1	0	Rosaceae Juss.
Iz_11_1209	DJI_0757	Psammophiliella muralis (L.) Ikonn.	0	n	1	0	Caryophyllaceae Juss.
Iz_11_1209	DJI_0757	Psephellus sumensis (Kalen.) Greuter	0	n	1	0	Asteraceae Bercht. & J.Presl
Iz_11_1209	DJI_0757	Pulsatilla pratensis (L.) Mill. s.l.	0	n	1	0	Ranunculaceae Juss.
Iz_11_1209	DJI_0757	Pyrus communis L.	0	a	0	1	Rosaceae Juss.
Iz_11_1209	DJI_0757	Quercus robur L.	0	n	1	1	Fagaceae Dumort.
Iz_11_1209	DJI_0757	Robinia pseudoacacia L.	0	a	0	1	Fabaceae Lindl.
Iz_11_1209	DJI_0757	Rosa sp.	0		NA	1	Rosaceae Juss.
Iz_11_1209	DJI_0757	Rubus idaeus L.	0	n	1	0	Rosaceae Juss.
Iz_11_1209	DJI_0757	Rumex acetosella L.	3	n	1	0	Polygonaceae Juss.
Iz_11_1209	DJI_0757	Rumex thyrsiflorus Fingerh.	0	n	1	0	Polygonaceae Juss.
Iz_11_1209	DJI_0757	Salix alba L.	0	n	1	1	Salicaceae Mirb.
Iz_11_1209	DJI_0757	Sambucus nigra L.	0	n	1	1	Viburnaceae Raf.
Iz_11_1209	DJI_0757	Secale sylvestre Host	0	n	1	0	Poaceae Barnhart
Iz_11_1209	DJI_0757	Sempervivum ruthenicum Schnittsp. & C.B. Lehm.	0	n	1	0	Crassulaceae J.St.-Hil.
Iz_11_1209	DJI_0757	Setaria viridis (L.) P. Beauv.	0	n	1	0	Poaceae Barnhart
Iz_11_1209	DJI_0757	Silene borysthenica (Gruner) Walters	0	n	1	0	Caryophyllaceae Juss.
Iz_11_1209	DJI_0757	Silene latifolia subsp. alba (Mill.) Greuter & Burdet	0	n	1	0	Caryophyllaceae Juss.
Iz_11_1209	DJI_0757	Solidago virgaurea L.	0	n	1	0	Asteraceae Bercht. & J.Presl
Iz_11_1209	DJI_0757	Sorbus aucuparia L.	0	n	1	1	Rosaceae Juss.
Iz_11_1209	DJI_0757	Stachys recta L.	0	n	1	0	Lamiacea Lindl.
Iz_11_1209	DJI_0757	Tanacetum vulgare L.	0	n	1	0	Asteraceae Bercht. & J.Presl
Iz_11_1209	DJI_0757	Thymus pallasianus Heinr.Braun	0	n	1	0	Lamiacea Lindl.
Iz_11_1209	DJI_0757	Tilia cordata Mill.	0	n	1	1	Malvaceae Juss.
Iz_11_1209	DJI_0757	Trifolium alpestre L.	0	n	1	0	Fabaceae Lindl.
Iz_11_1209	DJI_0757	Trifolium arvense L.	0	n	1	0	Fabaceae Lindl.
Iz_11_1209	DJI_0757	Ulmus glabra Huds.	0	n	1	1	Ulmaceae Mirb.
Iz_11_1209	DJI_0757	Verbascum densiflorum Bertol.	0	n	1	0	Scrophulariaceae Juss.
Iz_11_1209	DJI_0757	Veronica austriaca L.	0	n	1	0	Plantaginaceae Juss.
Iz_11_1209	DJI_0757	Veronica incana L.	0	n	1	0	Plantaginaceae Juss.
Iz_11_1209	DJI_0757	Veronica verna L.	0	n	1	0	Plantaginaceae Juss.
Iz_11_1209	DJI_0757	Vincetoxicum hirundinaria Medik	0	n	1	0	Apocynaceae Juss.
Iz_11_1209	DJI_0761	Acer tataricum L.	0	n	1	1	Sapindaceae Juss.
Iz_11_1209	DJI_0761	Achillea millefolium L. s.l.	0	n	1	0	Asteraceae Bercht. & J.Presl
Iz_11_1209	DJI_0761	Allium oleraceum L.	0	n	1	0	Amaryllidaceae J.St.-Hil.
Iz_11_1209	DJI_0761	Ambrosia artemisiifolia L.	0	a	0	0	Asteraceae Bercht. & J.Presl
Iz_11_1209	DJI_0761	Amorpha fruticosa L.	0	a	0	0	Fabaceae Lindl.
Iz_11_1209	DJI_0761	Anthericum ramosum L.	0	n	1	0	Asparagaceae Juss.
Iz_11_1209	DJI_0761	Artemisia marschalliana Spreng.	0	n	1	0	Asteraceae Bercht. & J.Presl
Iz_11_1209	DJI_0761	Asparagus officinalis L.	0	n	1	0	Asparagaceae Juss.
Iz_11_1209	DJI_0761	Astragalus cicer L.	0	n	1	0	Fabaceae Lindl.
Iz_11_1209	DJI_0761	Bassia laniflora (S.G.Gmel.) A.J.Scott	0	n	1	0	Amaranthaceae Juss.
Iz_11_1209	DJI_0761	Berberis vulgaris L.	0	n	1	0	Berberidaceae Juss.
Iz_11_1209	DJI_0761	Betula pendula Roth.	0	n	1	1	Betulaceae Gray
Iz_11_1209	DJI_0761	Betula pendula Roth.	0	n	1	0	Poaceae Barnhart

Iz_11_1209	DJI_0761	Bromus inermis Leyss.	0		NA	NA	
Iz_11_1209	DJI_0761	Bromus squarrosus L.	0	a	0	0	Poaceae Barnhart
Iz_11_1209	DJI_0761	Bromus tectorum L.	0	a	0	0	Poaceae Barnhart
Iz_11_1209	DJI_0761	Calamagrostis epigejos (L.) Roth	70	n	1	0	Poaceae Barnhart
Iz_11_1209	DJI_0761	Carduus acanthoides L.	0	n	1	0	Asteraceae Bercht. & J.Presl
Iz_11_1209	DJI_0761	Carex sp.	0	n	1	0	Cyperaceae Juss.
Iz_11_1209	DJI_0761	Centaurea majorovii Dumbadze	0	n	1	0	Asteraceae Bercht. & J.Presl
Iz_11_1209	DJI_0761	Chamaecytisus ruthenicus (Fisch.ex Wol.) Klaskova	0	n	1	0	Fabaceae Lindl.
Iz_11_1209	DJI_0761	Chelidonium majus L.	0	n	1	0	Papaveraceae Juss.
Iz_11_1209	DJI_0761	Chenopodium betaceum Andrz.	0	n	1	0	Amaranthaceae Juss.
Iz_11_1209	DJI_0761	Chenopodium cf. album	0	n	1	0	Amaranthaceae Juss.
Iz_11_1209	DJI_0761	Chenopodium sp.	0		NA	0	Amaranthaceae Juss.
Iz_11_1209	DJI_0761	Chondrilla juncea L.	0	n	1	0	Asteraceae Bercht. & J.Presl
Iz_11_1209	DJI_0761	Cirsium vulgare (Savi) Ten.	0	n	1	0	Asteraceae Bercht. & J.Presl
Iz_11_1209	DJI_0761	Clinopodium acinos (L.) Kuntze	0	n	1	0	Lamiacea Lindl.
Iz_11_1209	DJI_0761	Convallaria majalis L.	0	n	1	0	Asparagaceae Juss.
Iz_11_1209	DJI_0761	Crataegus rhipidophylla Gand.	0	n	1	1	Rosaceae Juss.
Iz_11_1209	DJI_0761	Crepis foetida L.	0	n	1	0	Asteraceae Bercht. & J.Presl
Iz_11_1209	DJI_0761	Dactylis glomerata L.	0	n	1	0	Poaceae Barnhart
Iz_11_1209	DJI_0761	Dianthus campestris M.Bieb.	0	n	1	0	Caryophyllaceae Juss.
Iz_11_1209	DJI_0761	Digitaria sanguinalis (L.) Scop.	0	a	0	0	Poaceae Barnhart
Iz_11_1209	DJI_0761	Elymus repens (L.) Gould	0	n	1	0	Poaceae Barnhart
Iz_11_1209	DJI_0761	Epilobium tetragonum L.	0	n	1	0	Onagraceae Juss.
Iz_11_1209	DJI_0761	Eragrostis minor Host	0	a	0	0	Poaceae Barnhart
Iz_11_1209	DJI_0761	Erigeron canadensis L.	0	a	0	0	Asteraceae Bercht. & J.Presl
Iz_11_1209	DJI_0761	Euphorbia seguieriana Neck.	0	n	1	0	Euphorbiaceae Juss.
Iz_11_1209	DJI_0761	Euphorbia sp.	0		NA	NA	
Iz_11_1209	DJI_0761	Fallopia dumetorum (L.) Holub	0	n	1	0	Polygonaceae Juss.
Iz_11_1209	DJI_0761	Festuca beckeri (Hack.) Trautv.	0	n	1	0	Poaceae Barnhart
Iz_11_1209	DJI_0761	Fragaria vesca L.	0	n	1	0	Rosaceae Juss.
Iz_11_1209	DJI_0761	Frangula alnus Mill.	0	n	1	1	Rhamnaceae Juss.
Iz_11_1209	DJI_0761	Fraxinus pennsylvanica Marshall	0	a	0	1	Oleaceae Hoffmanns. & Link
Iz_11_1209	DJI_0761	Galeopsis bifida Boenn.	0	n	1	0	Lamiacea Lindl.
Iz_11_1209	DJI_0761	Galium verum L.	0	n	1	0	Rubiaceae Juss.
Iz_11_1209	DJI_0761	Genista tinctoria L.	0	n	1	0	Fabaceae Lindl.
Iz_11_1209	DJI_0761	Geranium robertianum L.	0	n	1	0	Geraniaceae Juss.
Iz_11_1209	DJI_0761	Geranium sanquineum L.	0	n	1	0	Geraniaceae Juss.
Iz_11_1209	DJI_0761	Gypsophila paniculata L.	0	n	1	0	Caryophyllaceae Juss.
Iz_11_1209	DJI_0761	Helichrysum arenarium (L.) Moench	0	n	1	0	Asteraceae Bercht. & J.Presl
Iz_11_1209	DJI_0761	Herniaria polygama J.Gay	0	n	1	0	Caryophyllaceae Juss.
Iz_11_1209	DJI_0761	Hieracium robustum Fr.	0	n	1	0	Asteraceae Bercht. & J.Presl
Iz_11_1209	DJI_0761	Hieracium umbellatum L.	0	n	1	NA	Asteraceae Bercht. & J.Presl
Iz_11_1209	DJI_0761	Hylotelephium maximum (L.) Holub	0	n	1	0	Crassulaceae J.St.-Hil.
Iz_11_1209	DJI_0761	Jacobaea borysthenica (DC.) B.Nord. & Greuter	0	n	1	0	Asteraceae Bercht. & J.Presl
Iz_11_1209	DJI_0761	Jasione montana L.	0	n	1	0	Campanulaceae Juss.
Iz_11_1209	DJI_0761	Jurinea cyanoides (L.) Rchb.s.l.	0	n	1	0	Asteraceae Bercht. & J.Presl
Iz_11_1209	DJI_0761	Koeleria glauca (Spreng.) DC.	0	n	1	0	Poaceae Barnhart
Iz_11_1209	DJI_0761	Lactuca serriola L.	0	a	0	0	Asteraceae Bercht. & J.Presl
Iz_11_1209	DJI_0761	Linaria genistifolia (L.) Mill.	0	n	1	0	Plantaginaceae Juss.
Iz_11_1209	DJI_0761	Lomelosia argentea (L.) Greuter & Burdet	0	n	1	0	Caprifoliaceae Juss.
Iz_11_1209	DJI_0761	Melampyrum pratense L.	0	n	1	0	Orobanchaceae Vent.
Iz_11_1209	DJI_0761	Oenothera biennis L.	0	a	0	0	Onagraceae Juss.
Iz_11_1209	DJI_0761	Pentanema britannica (L.) D.Gut.Larr., Santos-Vicente, Anderb., E.Rico & M.M.Mart.Ort.	0	n	1	0	Asteraceae Bercht. & J.Presl
Iz_11_1209	DJI_0761	Peucedanum oreoselinum (L.) Moench	0	n	1	0	Apiaceae Lindl.
Iz_11_1209	DJI_0761	Peucedanum ruthenicum M.Bieb.	0	n	1	0	Apiaceae Lindl.
Iz_11_1209	DJI_0761	Pilosella echioides (Lumn.) F.W.Schultz & Sch.Bip.	0	n	1	0	Asteraceae Bercht. & J.Presl
Iz_11_1209	DJI_0761	Pilosella officinarum F. Schultz et Sch. Bip.	0	n	1	0	Asteraceae Bercht. & J.Presl
Iz_11_1209	DJI_0761	Pilosella sp.	1		NA	0	Asteraceae Bercht. & J.Presl
Iz_11_1209	DJI_0761	Pinus sylvestris L.	0	n	1	1	Pinaceae Spreng. ex F.Rudolphi
Iz_11_1209	DJI_0761	Poa annua L.	0	n	1	0	Poaceae Barnhart
Iz_11_1209	DJI_0761	Poa nemoralis L.	0	n	1	0	Poaceae Barnhart
Iz_11_1209	DJI_0761	Poa sp.	0		NA	0	Poaceae Barnhart
Iz_11_1209	DJI_0761	Polygonatum odoratum (Mill.) Druce	0	n	1	0	Asparagaceae Juss.
Iz_11_1209	DJI_0761	Polygonum arenarium Waldst. & Kit.	0	n	1	0	Polygonaceae Juss.
Iz_11_1209	DJI_0761	Populus nigra L.	0	n	1	1	Salicaceae Mirb.
Iz_11_1209	DJI_0761	Populus tremula L.	0	n	1	1	Salicaceae Mirb.
Iz_11_1209	DJI_0761	Populus Ã canescens (Aiton) Sm.	0	n	1	1	Salicaceae Mirb.
Iz_11_1209	DJI_0761	Potentilla argentea L.	0	n	1	0	Rosaceae Juss.
Iz_11_1209	DJI_0761	Psammophiliella muralis (L.) Ikonn.	0	n	1	0	Caryophyllaceae Juss.
Iz_11_1209	DJI_0761	Psephellus sumensis (Kalen.) Greuter	0	n	1	0	Asteraceae Bercht. & J.Presl
Iz_11_1209	DJI_0761	Pulsatilla pratensis (L.) Mill. s.l.	0	n	1	0	Ranunculaceae Juss.
Iz_11_1209	DJI_0761	Pyrus communis L.	0	a	0	1	Rosaceae Juss.
Iz_11_1209	DJI_0761	Quercus robur L.	0	n	1	1	Fagaceae Dumort.
Iz_11_1209	DJI_0761	Robinia pseudoacacia L.	0	a	0	1	Fabaceae Lindl.
Iz_11_1209	DJI_0761	Rosa sp.	0		NA	1	Rosaceae Juss.
Iz_11_1209	DJI_0761	Rubus idaeus L.	0	n	1	0	Rosaceae Juss.
Iz_11_1209	DJI_0761	Rumex acetosella L.	0	n	1	0	Polygonaceae Juss.
Iz_11_1209	DJI_0761	Rumex thyrsiflorus Fingerh.	0	n	1	0	Polygonaceae Juss.
Iz_11_1209	DJI_0761	Salix alba L.	0	n	1	1	Salicaceae Mirb.
Iz_11_1209	DJI_0761	Sambucus nigra L.	0	n	1	1	Viburnaceae Raf.
Iz_11_1209	DJI_0761	Secale sylvestre Host	0	n	1	0	Poaceae Barnhart
Iz_11_1209	DJI_0761	Sempervivum ruthenicum Schnittsp. & C.B. Lehm.	0	n	1	0	Crassulaceae J.St.-Hil.
Iz_11_1209	DJI_0761	Setaria viridis (L.) P. Beauv.	0	n	1	0	Poaceae Barnhart
Iz_11_1209	DJI_0761	Silene borysthenica (Gruner) Walters	0	n	1	0	Caryophyllaceae Juss.
Iz_11_1209	DJI_0761	Silene latifolia subsp. alba (Mill.) Greuter & Burdet	0	n	1	0	Caryophyllaceae Juss.
Iz_11_1209	DJI_0761	Solidago virgaurea L.	0.5	n	1	0	Asteraceae Bercht. & J.Presl
Iz_11_1209	DJI_0761	Sorbus aucuparia L.	0	n	1	1	Rosaceae Juss.
Iz_11_1209	DJI_0761	Stachys recta L.	0	n	1	0	Lamiacea Lindl.
Iz_11_1209	DJI_0761	Tanacetum vulgare L.	0	n	1	0	Asteraceae Bercht. & J.Presl
Iz_11_1209	DJI_0761	Thymus pallasianus Heinr.Braun	0	n	1	0	Lamiacea Lindl.
Iz_11_1209	DJI_0761	Tilia cordata Mill.	0	n	1	1	Malvaceae Juss.
Iz_11_1209	DJI_0761	Trifolium alpestre L.	0	n	1	0	Fabaceae Lindl.
Iz_11_1209	DJI_0761	Trifolium arvense L.	0	n	1	0	Fabaceae Lindl.
Iz_11_1209	DJI_0761	Ulmus glabra Huds.	0	n	1	1	Ulmaceae Mirb.
Iz_11_1209	DJI_0761	Verbascum densiflorum Bertol.	0	n	1	0	Scrophulariaceae Juss.
Iz_11_1209	DJI_0761	Veronica austriaca L.	0	n	1	0	Plantaginaceae Juss.
Iz_11_1209	DJI_0761	Veronica incana L.	0	n	1	0	Plantaginaceae Juss.
Iz_11_1209	DJI_0761	Veronica verna L.	0	n	1	0	Plantaginaceae Juss.

Iz_11_1209	DJI_0761	Vincetoxicum hirundinaria Medik	0	n	1	0	Apocynaceae Juss.
Iz_11_1209	DJI_0765	Acer tataricum L.	0	n	1	1	Sapindaceae Juss.
Iz_11_1209	DJI_0765	Achillea millefolium L. s.l.	0	n	1	0	Asteraceae Bercht. & J.Presl
Iz_11_1209	DJI_0765	Allium oleraceum L.	0	n	1	0	Amaryllidaceae J.St.-Hil.
Iz_11_1209	DJI_0765	Ambrosia artemisiifolia L.	0	a	0	0	Asteraceae Bercht. & J.Presl
Iz_11_1209	DJI_0765	Amorpha fruticosa L.	0	a	0	0	Fabaceae Lindl.
Iz_11_1209	DJI_0765	Anthericum ramosum L.	0	n	1	0	Asparagaceae Juss.
Iz_11_1209	DJI_0765	Artemisia marschalliana Spreng.	0	n	1	0	Asteraceae Bercht. & J.Presl
Iz_11_1209	DJI_0765	Asparagus officinalis L.	0	n	1	0	Asparagaceae Juss.
Iz_11_1209	DJI_0765	Astragalus cicer L.	0	n	1	0	Fabaceae Lindl.
Iz_11_1209	DJI_0765	Bassia laniflora (S.G.Gmel.) A.J.Scott	0	n	1	0	Amaranthaceae Juss.
Iz_11_1209	DJI_0765	Berberis vulgaris L.	0	n	1	0	Berberidaceae Juss.
Iz_11_1209	DJI_0765	Betula pendula Roth.	0	n	1	1	Betulaceae Gray
Iz_11_1209	DJI_0765	Betula pendula Roth.	0	n	1	0	Poaceae Barnhart
Iz_11_1209	DJI_0765	Bromus inermis Leyss.	0		NA	NA	
Iz_11_1209	DJI_0765	Bromus squarrosus L.	0	a	0	0	Poaceae Barnhart
Iz_11_1209	DJI_0765	Bromus tectorum L.	0	a	0	0	Poaceae Barnhart
Iz_11_1209	DJI_0765	Calamagrostis epigejos (L.) Roth	20	n	1	0	Poaceae Barnhart
Iz_11_1209	DJI_0765	Carduus acanthoides L.	0	n	1	0	Asteraceae Bercht. & J.Presl
Iz_11_1209	DJI_0765	Carex sp.	0	n	1	0	Cyperaceae Juss.
Iz_11_1209	DJI_0765	Centaurea majorovii Dumbadze	0	n	1	0	Asteraceae Bercht. & J.Presl
Iz_11_1209	DJI_0765	Chamaecytisus ruthenicus (Fisch.ex Wol.) Klaskova	0	n	1	0	Fabaceae Lindl.
Iz_11_1209	DJI_0765	Chelidonium majus L.	0	n	1	0	Papaveraceae Juss.
Iz_11_1209	DJI_0765	Chenopodium betaceum Andrz.	0	n	1	0	Amaranthaceae Juss.
Iz_11_1209	DJI_0765	Chenopodium cf. album	0	n	1	0	Amaranthaceae Juss.
Iz_11_1209	DJI_0765	Chenopodium sp.	0		NA	0	Amaranthaceae Juss.
Iz_11_1209	DJI_0765	Chondrilla juncea L.	0	n	1	0	Asteraceae Bercht. & J.Presl
Iz_11_1209	DJI_0765	Cirsium vulgare (Savi) Ten.	0	n	1	0	Asteraceae Bercht. & J.Presl
Iz_11_1209	DJI_0765	Clinopodium acinos (L.) Kuntze	0	n	1	0	Lamiacea Lindl.
Iz_11_1209	DJI_0765	Convallaria majalis L.	0	n	1	0	Asparagaceae Juss.
Iz_11_1209	DJI_0765	Crataegus rhipidophylla Gand.	0	n	1	1	Rosaceae Juss.
Iz_11_1209	DJI_0765	Crepis foetida L.	0	n	1	0	Asteraceae Bercht. & J.Presl
Iz_11_1209	DJI_0765	Dactylis glomerata L.	0	n	1	0	Poaceae Barnhart
Iz_11_1209	DJI_0765	Dianthus campestris M.Bieb.	0	n	1	0	Caryophyllaceae Juss.
Iz_11_1209	DJI_0765	Digitaria sanguinalis (L.) Scop.	0	a	0	0	Poaceae Barnhart
Iz_11_1209	DJI_0765	Elymus repens (L.) Gould	0	n	1	0	Poaceae Barnhart
Iz_11_1209	DJI_0765	Epilobium tetragonum L.	0	n	1	0	Onagraceae Juss.
Iz_11_1209	DJI_0765	Eragrostis minor Host	0	a	0	0	Poaceae Barnhart
Iz_11_1209	DJI_0765	Erigeron canadensis L.	15	a	0	0	Asteraceae Bercht. & J.Presl
Iz_11_1209	DJI_0765	Euphorbia seguieriana Neck.	0	n	1	0	Euphorbiaceae Juss.
Iz_11_1209	DJI_0765	Euphorbia sp.	0		NA	NA	
Iz_11_1209	DJI_0765	Fallopia dumetorum (L.) Holub	0	n	1	0	Polygonaceae Juss.
Iz_11_1209	DJI_0765	Festuca beckeri (Hack.) Trautv.	0	n	1	0	Poaceae Barnhart
Iz_11_1209	DJI_0765	Fragaria vesca L.	0	n	1	0	Rosaceae Juss.
Iz_11_1209	DJI_0765	Frangula alnus Mill.	0	n	1	1	Rhamnaceae Juss.
Iz_11_1209	DJI_0765	Fraxinus pennsylvanica Marshall	0	a	0	1	Oleaceae Hoffmanns. & Link
Iz_11_1209	DJI_0765	Galeopsis bifida Boenn.	0	n	1	0	Lamiacea Lindl.
Iz_11_1209	DJI_0765	Galium verum L.	0	n	1	0	Rubiaceae Juss.
Iz_11_1209	DJI_0765	Genista tinctoria L.	0	n	1	0	Fabaceae Lindl.
Iz_11_1209	DJI_0765	Geranium robertianum L.	0	n	1	0	Geraniaceae Juss.
Iz_11_1209	DJI_0765	Geranium sanquineum L.	0	n	1	0	Geraniaceae Juss.
Iz_11_1209	DJI_0765	Gypsophila paniculata L.	0	n	1	0	Caryophyllaceae Juss.
Iz_11_1209	DJI_0765	Helichrysum arenarium (L.) Moench	0	n	1	0	Asteraceae Bercht. & J.Presl
Iz_11_1209	DJI_0765	Herniaria polygama J.Gay	0	n	1	0	Caryophyllaceae Juss.
Iz_11_1209	DJI_0765	Hieracium robustum Fr.	0	n	1	0	Asteraceae Bercht. & J.Presl
Iz_11_1209	DJI_0765	Hieracium umbellatum L.	0	n	1	NA	Asteraceae Bercht. & J.Presl
Iz_11_1209	DJI_0765	Hylotelephium maximum (L.) Holub	0	n	1	0	Crassulaceae J.St.-Hil.
Iz_11_1209	DJI_0765	Jacobaea borysthenica (DC.) B.Nord. & Greuter	0	n	1	0	Asteraceae Bercht. & J.Presl
Iz_11_1209	DJI_0765	Jasione montana L.	0	n	1	0	Campanulaceae Juss.
Iz_11_1209	DJI_0765	Jurinea cyanoides (L.) Rchb.s.l.	0	n	1	0	Asteraceae Bercht. & J.Presl
Iz_11_1209	DJI_0765	Koeleria glauca (Spreng.) DC.	0	n	1	0	Poaceae Barnhart
Iz_11_1209	DJI_0765	Lactuca serriola L.	0.2	a	0	0	Asteraceae Bercht. & J.Presl
Iz_11_1209	DJI_0765	Linaria genistifolia (L.) Mill.	0	n	1	0	Plantaginaceae Juss.
Iz_11_1209	DJI_0765	Lomelosia argentea (L.) Greuter & Burdet	0	n	1	0	Caprifoliaceae Juss.
Iz_11_1209	DJI_0765	Melampyrum pratense L.	0	n	1	0	Orobanchaceae Vent.
Iz_11_1209	DJI_0765	Oenothera biennis L.	0	a	0	0	Onagraceae Juss.
Iz_11_1209	DJI_0765	Pentanema britannica (L.) D.Gut.Larr., Santos-Vicente, Anderb., E.Rico & M.M.Mart.Ort.	0	n	1	0	Asteraceae Bercht. & J.Presl
Iz_11_1209	DJI_0765	Peucedanum oreoselinum (L.) Moench	0	n	1	0	Apiaceae Lindl.
Iz_11_1209	DJI_0765	Peucedanum ruthenicum M.Bieb.	0	n	1	0	Apiaceae Lindl.
Iz_11_1209	DJI_0765	Pilosella echioides (Lumn.) F.W.Schultz & Sch.Bip.	0	n	1	0	Asteraceae Bercht. & J.Presl
Iz_11_1209	DJI_0765	Pilosella officinarum F. Schultz et Sch. Bip.	0	n	1	0	Asteraceae Bercht. & J.Presl
Iz_11_1209	DJI_0765	Pilosella sp.	0		NA	0	Asteraceae Bercht. & J.Presl
Iz_11_1209	DJI_0765	Pinus sylvestris L.	0	n	1	1	Pinaceae Spreng. ex F.Rudolphi
Iz_11_1209	DJI_0765	Poa annua L.	0	n	1	0	Poaceae Barnhart
Iz_11_1209	DJI_0765	Poa nemoralis L.	0	n	1	0	Poaceae Barnhart
Iz_11_1209	DJI_0765	Poa sp.	0		NA	0	Poaceae Barnhart
Iz_11_1209	DJI_0765	Polygonatum odoratum (Mill.) Druce	0	n	1	0	Asparagaceae Juss.
Iz_11_1209	DJI_0765	Polygonum arenarium Waldst. & Kit.	0	n	1	0	Polygonaceae Juss.
Iz_11_1209	DJI_0765	Populus nigra L.	0	n	1	1	Salicaceae Mirb.
Iz_11_1209	DJI_0765	Populus tremula L.	0	n	1	1	Salicaceae Mirb.
Iz_11_1209	DJI_0765	Populus Ã canescens (Aiton) Sm.	0	n	1	1	Salicaceae Mirb.
Iz_11_1209	DJI_0765	Potentilla argentea L.	0	n	1	0	Rosaceae Juss.
Iz_11_1209	DJI_0765	Psammophiliella muralis (L.) Ikonn.	0	n	1	0	Caryophyllaceae Juss.
Iz_11_1209	DJI_0765	Psephellus sumensis (Kalen.) Greuter	0	n	1	0	Asteraceae Bercht. & J.Presl
Iz_11_1209	DJI_0765	Pulsatilla pratensis (L.) Mill. s.l.	0	n	1	0	Ranunculaceae Juss.
Iz_11_1209	DJI_0765	Pyrus communis L.	0	a	0	1	Rosaceae Juss.
Iz_11_1209	DJI_0765	Quercus robur L.	0	n	1	1	Fagaceae Dumort.
Iz_11_1209	DJI_0765	Robinia pseudoacacia L.	0	a	0	1	Fabaceae Lindl.
Iz_11_1209	DJI_0765	Rosa sp.	0		NA	1	Rosaceae Juss.
Iz_11_1209	DJI_0765	Rubus idaeus L.	0	n	1	0	Rosaceae Juss.
Iz_11_1209	DJI_0765	Rumex acetosella L.	0	n	1	0	Polygonaceae Juss.
Iz_11_1209	DJI_0765	Rumex thyrsiflorus Fingerh.	0	n	1	0	Polygonaceae Juss.
Iz_11_1209	DJI_0765	Salix alba L.	0	n	1	1	Salicaceae Mirb.
Iz_11_1209	DJI_0765	Sambucus nigra L.	0	n	1	1	Viburnaceae Raf.
Iz_11_1209	DJI_0765	Secale sylvestre Host	0	n	1	0	Poaceae Barnhart
Iz_11_1209	DJI_0765	Sempervivum ruthenicum Schnittsp. & C.B. Lehm.	0	n	1	0	Crassulaceae J.St.-Hil.
Iz_11_1209	DJI_0765	Setaria viridis (L.) P. Beauv.	0	n	1	0	Poaceae Barnhart
Iz_11_1209	DJI_0765	Silene borysthenica (Gruner) Walters	0	n	1	0	Caryophyllaceae Juss.

Iz_11_1209	DJI_0765	Silene latifolia subsp. alba (Mill.) Greuter & Burdet	0	n	1	0	Caryophyllaceae Juss.
Iz_11_1209	DJI_0765	Solidago virgaurea L.	0.5	n	1	0	Asteraceae Bercht. & J.Presl
Iz_11_1209	DJI_0765	Sorbus aucuparia L.	0	n	1	1	Rosaceae Juss.
Iz_11_1209	DJI_0765	Stachys recta L.	0	n	1	0	Lamiacea Lindl.
Iz_11_1209	DJI_0765	Tanacetum vulgare L.	0	n	1	0	Asteraceae Bercht. & J.Presl
Iz_11_1209	DJI_0765	Thymus pallasianus Heinr.Braun	0	n	1	0	Lamiacea Lindl.
Iz_11_1209	DJI_0765	Tilia cordata Mill.	0	n	1	1	Malvaceae Juss.
Iz_11_1209	DJI_0765	Trifolium alpestre L.	0	n	1	0	Fabaceae Lindl.
Iz_11_1209	DJI_0765	Trifolium arvense L.	0	n	1	0	Fabaceae Lindl.
Iz_11_1209	DJI_0765	Ulmus glabra Huds.	0	n	1	1	Ulmaceae Mirb.
Iz_11_1209	DJI_0765	Verbascum densiflorum Bertol.	0	n	1	0	Scrophulariaceae Juss.
Iz_11_1209	DJI_0765	Veronica austriaca L.	0	n	1	0	Plantaginaceae Juss.
Iz_11_1209	DJI_0765	Veronica incana L.	0	n	1	0	Plantaginaceae Juss.
Iz_11_1209	DJI_0765	Veronica verna L.	0	n	1	0	Plantaginaceae Juss.
Iz_11_1209	DJI_0765	Vincetoxicum hirundinaria Medik	0	n	1	0	Apocynaceae Juss.
Iz_11_1209	DJI_0771	Acer tataricum L.	0	n	1	1	Sapindaceae Juss.
Iz_11_1209	DJI_0771	Achillea millefolium L. s.l.	0	n	1	0	Asteraceae Bercht. & J.Presl
Iz_11_1209	DJI_0771	Allium oleraceum L.	0	n	1	0	Amaryllidaceae J.St.-Hil.
Iz_11_1209	DJI_0771	Ambrosia artemisiifolia L.	0	a	0	0	Asteraceae Bercht. & J.Presl
Iz_11_1209	DJI_0771	Amorpha fruticosa L.	0	a	0	0	Fabaceae Lindl.
Iz_11_1209	DJI_0771	Anthericum ramosum L.	0	n	1	0	Asparagaceae Juss.
Iz_11_1209	DJI_0771	Artemisia marschalliana Spreng.	0	n	1	0	Asteraceae Bercht. & J.Presl
Iz_11_1209	DJI_0771	Asparagus officinalis L.	0	n	1	0	Asparagaceae Juss.
Iz_11_1209	DJI_0771	Astragalus cicer L.	0	n	1	0	Fabaceae Lindl.
Iz_11_1209	DJI_0771	Bassia laniflora (S.G.Gmel.) A.J.Scott	0	n	1	0	Amaranthaceae Juss.
Iz_11_1209	DJI_0771	Berberis vulgaris L.	0	n	1	0	Berberidaceae Juss.
Iz_11_1209	DJI_0771	Betula pendula Roth.	0	n	1	1	Betulaceae Gray
Iz_11_1209	DJI_0771	Betula pendula Roth.	0	n	1	0	Poaceae Barnhart
Iz_11_1209	DJI_0771	Bromus inermis Leyss.	0		NA	NA	
Iz_11_1209	DJI_0771	Bromus squarrosus L.	0	a	0	0	Poaceae Barnhart
Iz_11_1209	DJI_0771	Bromus tectorum L.	0	a	0	0	Poaceae Barnhart
Iz_11_1209	DJI_0771	Calamagrostis epigejos (L.) Roth	2	n	1	0	Poaceae Barnhart
Iz_11_1209	DJI_0771	Carduus acanthoides L.	0	n	1	0	Asteraceae Bercht. & J.Presl
Iz_11_1209	DJI_0771	Carex sp.	0	n	1	0	Cyperaceae Juss.
Iz_11_1209	DJI_0771	Centaurea majorovii Dumbadze	0	n	1	0	Asteraceae Bercht. & J.Presl
Iz_11_1209	DJI_0771	Chamaecytisus ruthenicus (Fisch.ex Wol.) Klaskova	2	n	1	0	Fabaceae Lindl.
Iz_11_1209	DJI_0771	Chelidonium majus L.	0	n	1	0	Papaveraceae Juss.
Iz_11_1209	DJI_0771	Chenopodium betaceum Andrz.	0	n	1	0	Amaranthaceae Juss.
Iz_11_1209	DJI_0771	Chenopodium cf. album	0	n	1	0	Amaranthaceae Juss.
Iz_11_1209	DJI_0771	Chenopodium sp.	0		NA	0	Amaranthaceae Juss.
Iz_11_1209	DJI_0771	Chondrilla juncea L.	0	n	1	0	Asteraceae Bercht. & J.Presl
Iz_11_1209	DJI_0771	Cirsium vulgare (Savi) Ten.	0	n	1	0	Asteraceae Bercht. & J.Presl
Iz_11_1209	DJI_0771	Clinopodium acinos (L.) Kuntze	0	n	1	0	Lamiacea Lindl.
Iz_11_1209	DJI_0771	Convallaria majalis L.	0	n	1	0	Asparagaceae Juss.
Iz_11_1209	DJI_0771	Crataegus rhipidophylla Gand.	0	n	1	1	Rosaceae Juss.
Iz_11_1209	DJI_0771	Crepis foetida L.	0	n	1	0	Asteraceae Bercht. & J.Presl
Iz_11_1209	DJI_0771	Dactylis glomerata L.	0	n	1	0	Poaceae Barnhart
Iz_11_1209	DJI_0771	Dianthus campestris M.Bieb.	0	n	1	0	Caryophyllaceae Juss.
Iz_11_1209	DJI_0771	Digitaria sanguinalis (L.) Scop.	0	a	0	0	Poaceae Barnhart
Iz_11_1209	DJI_0771	Elymus repens (L.) Gould	0	n	1	0	Poaceae Barnhart
Iz_11_1209	DJI_0771	Epilobium tetragonum L.	0	n	1	0	Onagraceae Juss.
Iz_11_1209	DJI_0771	Eragrostis minor Host	0	a	0	0	Poaceae Barnhart
Iz_11_1209	DJI_0771	Erigeron canadensis L.	0	a	0	0	Asteraceae Bercht. & J.Presl
Iz_11_1209	DJI_0771	Euphorbia seguieriana Neck.	0	n	1	0	Euphorbiaceae Juss.
Iz_11_1209	DJI_0771	Euphorbia sp.	2		NA	NA	
Iz_11_1209	DJI_0771	Fallopia dumetorum (L.) Holub	0	n	1	0	Polygonaceae Juss.
Iz_11_1209	DJI_0771	Festuca beckeri (Hack.) Trautv.	0	n	1	0	Poaceae Barnhart
Iz_11_1209	DJI_0771	Fragaria vesca L.	0	n	1	0	Rosaceae Juss.
Iz_11_1209	DJI_0771	Frangula alnus Mill.	0	n	1	1	Rhamnaceae Juss.
Iz_11_1209	DJI_0771	Fraxinus pennsylvanica Marshall	0	a	0	1	Oleaceae Hoffmanns. & Link
Iz_11_1209	DJI_0771	Galeopsis bifida Boenn.	0	n	1	0	Lamiacea Lindl.
Iz_11_1209	DJI_0771	Galium verum L.	0	n	1	0	Rubiaceae Juss.
Iz_11_1209	DJI_0771	Genista tinctoria L.	0	n	1	0	Fabaceae Lindl.
Iz_11_1209	DJI_0771	Geranium robertianum L.	0	n	1	0	Geraniaceae Juss.
Iz_11_1209	DJI_0771	Geranium sanquineum L.	0	n	1	0	Geraniaceae Juss.
Iz_11_1209	DJI_0771	Gypsophila paniculata L.	0	n	1	0	Caryophyllaceae Juss.
Iz_11_1209	DJI_0771	Helichrysum arenarium (L.) Moench	6	n	1	0	Asteraceae Bercht. & J.Presl
Iz_11_1209	DJI_0771	Herniaria polygama J.Gay	0	n	1	0	Caryophyllaceae Juss.
Iz_11_1209	DJI_0771	Hieracium robustum Fr.	0	n	1	0	Asteraceae Bercht. & J.Presl
Iz_11_1209	DJI_0771	Hieracium umbellatum L.	0	n	1	NA	Asteraceae Bercht. & J.Presl
Iz_11_1209	DJI_0771	Hylotelephium maximum (L.) Holub	0	n	1	0	Crassulaceae J.St.-Hil.
Iz_11_1209	DJI_0771	Jacobaea borysthenica (DC.) B.Nord. & Greuter	0	n	1	0	Asteraceae Bercht. & J.Presl
Iz_11_1209	DJI_0771	Jasione montana L.	0	n	1	0	Campanulaceae Juss.
Iz_11_1209	DJI_0771	Jurinea cyanoides (L.) Rchb.s.l.	0	n	1	0	Asteraceae Bercht. & J.Presl
Iz_11_1209	DJI_0771	Koeleria glauca (Spreng.) DC.	4	n	1	0	Poaceae Barnhart
Iz_11_1209	DJI_0771	Lactuca serriola L.	0	a	0	0	Asteraceae Bercht. & J.Presl
Iz_11_1209	DJI_0771	Linaria genistifolia (L.) Mill.	0	n	1	0	Plantaginaceae Juss.
Iz_11_1209	DJI_0771	Lomelosia argentea (L.) Greuter & Burdet	0	n	1	0	Caprifoliaceae Juss.
Iz_11_1209	DJI_0771	Melampyrum pratense L.	0	n	1	0	Orobanchaceae Vent.
Iz_11_1209	DJI_0771	Oenothera biennis L.	0	a	0	0	Onagraceae Juss.
Iz_11_1209	DJI_0771	Pentanema britannica (L.) D.Gut.Larr., Santos-Vicente, Anderb., E.Rico & M.M.Mart.Ort.	0	n	1	0	Asteraceae Bercht. & J.Presl
Iz_11_1209	DJI_0771	Peucedanum oreoselinum (L.) Moench	0	n	1	0	Apiaceae Lindl.
Iz_11_1209	DJI_0771	Peucedanum ruthenicum M.Bieb.	0	n	1	0	Apiaceae Lindl.
Iz_11_1209	DJI_0771	Pilosella echioides (Lumn.) F.W.Schultz & Sch.Bip.	0	n	1	0	Asteraceae Bercht. & J.Presl
Iz_11_1209	DJI_0771	Pilosella officinarum F. Schultz et Sch. Bip.	0	n	1	0	Asteraceae Bercht. & J.Presl
Iz_11_1209	DJI_0771	Pilosella sp.	0		NA	0	Asteraceae Bercht. & J.Presl
Iz_11_1209	DJI_0771	Pinus sylvestris L.	0	n	1	1	Pinaceae Spreng. ex F.Rudolphi
Iz_11_1209	DJI_0771	Poa annua L.	0	n	1	0	Poaceae Barnhart
Iz_11_1209	DJI_0771	Poa nemoralis L.	0	n	1	0	Poaceae Barnhart
Iz_11_1209	DJI_0771	Poa sp.	1		NA	0	Poaceae Barnhart
Iz_11_1209	DJI_0771	Polygonatum odoratum (Mill.) Druce	0	n	1	0	Asparagaceae Juss.
Iz_11_1209	DJI_0771	Polygonum arenarium Waldst. & Kit.	0	n	1	0	Polygonaceae Juss.
Iz_11_1209	DJI_0771	Populus nigra L.	0	n	1	1	Salicaceae Mirb.
Iz_11_1209	DJI_0771	Populus tremula L.	0	n	1	1	Salicaceae Mirb.
Iz_11_1209	DJI_0771	Populus Ã canescens (Aiton) Sm.	0	n	1	1	Salicaceae Mirb.
Iz_11_1209	DJI_0771	Potentilla argentea L.	0	n	1	0	Rosaceae Juss.
Iz_11_1209	DJI_0771	Psammophiliella muralis (L.) Ikonn.	0	n	1	0	Caryophyllaceae Juss.
Iz_11_1209	DJI_0771	Psephellus sumensis (Kalen.) Greuter	0	n	1	0	Asteraceae Bercht. & J.Presl

Iz_11_1209	DJI_0771	Pulsatilla pratensis (L.) Mill. s.l.	0	n	1	0	Ranunculaceae Juss.
Iz_11_1209	DJI_0771	Pyrus communis L.	0	a	0	1	Rosaceae Juss.
Iz_11_1209	DJI_0771	Quercus robur L.	0	n	1	1	Fagaceae Dumort.
Iz_11_1209	DJI_0771	Robinia pseudoacacia L.	0	a	0	1	Fabaceae Lindl.
Iz_11_1209	DJI_0771	Rosa sp.	0		NA	1	Rosaceae Juss.
Iz_11_1209	DJI_0771	Rubus idaeus L.	0	n	1	0	Rosaceae Juss.
Iz_11_1209	DJI_0771	Rumex acetosella L.	2	n	1	0	Polygonaceae Juss.
Iz_11_1209	DJI_0771	Rumex thyrsiflorus Fingerh.	0	n	1	0	Polygonaceae Juss.
Iz_11_1209	DJI_0771	Salix alba L.	0	n	1	1	Salicaceae Mirb.
Iz_11_1209	DJI_0771	Sambucus nigra L.	0	n	1	1	Viburnaceae Raf.
Iz_11_1209	DJI_0771	Secale sylvestre Host	0	n	1	0	Poaceae Barnhart
Iz_11_1209	DJI_0771	Sempervivum ruthenicum Schnittsp. & C.B. Lehm.	0	n	1	0	Crassulaceae J.St.-Hil.
Iz_11_1209	DJI_0771	Setaria viridis (L.) P. Beauv.	0	n	1	0	Poaceae Barnhart
Iz_11_1209	DJI_0771	Silene borysthenica (Gruner) Walters	0	n	1	0	Caryophyllaceae Juss.
Iz_11_1209	DJI_0771	Silene latifolia subsp. alba (Mill.) Greuter & Burdet	0	n	1	0	Caryophyllaceae Juss.
Iz_11_1209	DJI_0771	Solidago virgaurea L.	1	n	1	0	Asteraceae Bercht. & J.Presl
Iz_11_1209	DJI_0771	Sorbus aucuparia L.	0	n	1	1	Rosaceae Juss.
Iz_11_1209	DJI_0771	Stachys recta L.	0	n	1	0	Lamiacea Lindl.
Iz_11_1209	DJI_0771	Tanacetum vulgare L.	0	n	1	0	Asteraceae Bercht. & J.Presl
Iz_11_1209	DJI_0771	Thymus pallasianus Heinr.Braun	0	n	1	0	Lamiacea Lindl.
Iz_11_1209	DJI_0771	Tilia cordata Mill.	0	n	1	1	Malvaceae Juss.
Iz_11_1209	DJI_0771	Trifolium alpestre L.	0	n	1	0	Fabaceae Lindl.
Iz_11_1209	DJI_0771	Trifolium arvense L.	0	n	1	0	Fabaceae Lindl.
Iz_11_1209	DJI_0771	Ulmus glabra Huds.	0	n	1	1	Ulmaceae Mirb.
Iz_11_1209	DJI_0771	Verbascum densiflorum Bertol.	0	n	1	0	Scrophulariaceae Juss.
Iz_11_1209	DJI_0771	Veronica austriaca L.	0	n	1	0	Plantaginaceae Juss.
Iz_11_1209	DJI_0771	Veronica incana L.	2	n	1	0	Plantaginaceae Juss.
Iz_11_1209	DJI_0771	Veronica verna L.	0	n	1	0	Plantaginaceae Juss.
Iz_11_1209	DJI_0771	Vincetoxicum hirundinaria Medik	0	n	1	0	Apocynaceae Juss.
Iz_12_1209	DJI_0785	Acer tataricum L.	0	n	1	1	Sapindaceae Juss.
Iz_12_1209	DJI_0785	Achillea millefolium L. s.l.	0	n	1	0	Asteraceae Bercht. & J.Presl
Iz_12_1209	DJI_0785	Allium oleraceum L.	0	n	1	0	Amaryllidaceae J.St.-Hil.
Iz_12_1209	DJI_0785	Ambrosia artemisiifolia L.	0	a	0	0	Asteraceae Bercht. & J.Presl
Iz_12_1209	DJI_0785	Amorpha fruticosa L.	0	a	0	0	Fabaceae Lindl.
Iz_12_1209	DJI_0785	Anthericum ramosum L.	0	n	1	0	Asparagaceae Juss.
Iz_12_1209	DJI_0785	Artemisia marschalliana Spreng.	0	n	1	0	Asteraceae Bercht. & J.Presl
Iz_12_1209	DJI_0785	Asparagus officinalis L.	0	n	1	0	Asparagaceae Juss.
Iz_12_1209	DJI_0785	Astragalus cicer L.	0	n	1	0	Fabaceae Lindl.
Iz_12_1209	DJI_0785	Bassia laniflora (S.G.Gmel.) A.J.Scott	0	n	1	0	Amaranthaceae Juss.
Iz_12_1209	DJI_0785	Berberis vulgaris L.	0	n	1	0	Berberidaceae Juss.
Iz_12_1209	DJI_0785	Betula pendula Roth.	0	n	1	1	Betulaceae Gray
Iz_12_1209	DJI_0785	Betula pendula Roth.	0	n	1	0	Poaceae Barnhart
Iz_12_1209	DJI_0785	Bromus inermis Leyss.	0		NA	NA	
Iz_12_1209	DJI_0785	Bromus squarrosus L.	0	a	0	0	Poaceae Barnhart
Iz_12_1209	DJI_0785	Bromus tectorum L.	0	a	0	0	Poaceae Barnhart
Iz_12_1209	DJI_0785	Calamagrostis epigejos (L.) Roth	1	n	1	0	Poaceae Barnhart
Iz_12_1209	DJI_0785	Carduus acanthoides L.	0	n	1	0	Asteraceae Bercht. & J.Presl
Iz_12_1209	DJI_0785	Carex sp.	0	n	1	0	Cyperaceae Juss.
Iz_12_1209	DJI_0785	Centaurea majorovii Dumbadze	0	n	1	0	Asteraceae Bercht. & J.Presl
Iz_12_1209	DJI_0785	Chamaecytisus ruthenicus (Fisch.ex Wol.) Klaskova	0	n	1	0	Fabaceae Lindl.
Iz_12_1209	DJI_0785	Chelidonium majus L.	0	n	1	0	Papaveraceae Juss.
Iz_12_1209	DJI_0785	Chenopodium betaceum Andrz.	0	n	1	0	Amaranthaceae Juss.
Iz_12_1209	DJI_0785	Chenopodium cf. album	0	n	1	0	Amaranthaceae Juss.
Iz_12_1209	DJI_0785	Chenopodium sp.	0		NA	0	Amaranthaceae Juss.
Iz_12_1209	DJI_0785	Chondrilla juncea L.	0	n	1	0	Asteraceae Bercht. & J.Presl
Iz_12_1209	DJI_0785	Cirsium vulgare (Savi) Ten.	0	n	1	0	Asteraceae Bercht. & J.Presl
Iz_12_1209	DJI_0785	Clinopodium acinos (L.) Kuntze	0	n	1	0	Lamiacea Lindl.
Iz_12_1209	DJI_0785	Convallaria majalis L.	0	n	1	0	Asparagaceae Juss.
Iz_12_1209	DJI_0785	Crataegus rhipidophylla Gand.	0	n	1	1	Rosaceae Juss.
Iz_12_1209	DJI_0785	Crepis foetida L.	0	n	1	0	Asteraceae Bercht. & J.Presl
Iz_12_1209	DJI_0785	Dactylis glomerata L.	0	n	1	0	Poaceae Barnhart
Iz_12_1209	DJI_0785	Dianthus campestris M.Bieb.	0	n	1	0	Caryophyllaceae Juss.
Iz_12_1209	DJI_0785	Digitaria sanguinalis (L.) Scop.	0	a	0	0	Poaceae Barnhart
Iz_12_1209	DJI_0785	Elymus repens (L.) Gould	0	n	1	0	Poaceae Barnhart
Iz_12_1209	DJI_0785	Epilobium tetragonum L.	0	n	1	0	Onagraceae Juss.
Iz_12_1209	DJI_0785	Eragrostis minor Host	0	a	0	0	Poaceae Barnhart
Iz_12_1209	DJI_0785	Erigeron canadensis L.	0	a	0	0	Asteraceae Bercht. & J.Presl
Iz_12_1209	DJI_0785	Euphorbia seguieriana Neck.	0	n	1	0	Euphorbiaceae Juss.
Iz_12_1209	DJI_0785	Fallopia dumetorum (L.) Holub	5	n	1	0	Polygonaceae Juss.
Iz_12_1209	DJI_0785	Festuca beckeri (Hack.) Trautv.	0	n	1	0	Poaceae Barnhart
Iz_12_1209	DJI_0785	Fragaria vesca L.	0	n	1	0	Rosaceae Juss.
Iz_12_1209	DJI_0785	Frangula alnus Mill.	0	n	1	1	Rhamnaceae Juss.
Iz_12_1209	DJI_0785	Fraxinus pennsylvanica Marshall	0	a	0	1	Oleaceae Hoffmanns. & Link
Iz_12_1209	DJI_0785	Galeopsis bifida Boenn.	0	n	1	0	Lamiacea Lindl.
Iz_12_1209	DJI_0785	Galium verum L.	0	n	1	0	Rubiaceae Juss.
Iz_12_1209	DJI_0785	Genista tinctoria L.	0	n	1	0	Fabaceae Lindl.
Iz_12_1209	DJI_0785	Geranium robertianum L.	0	n	1	0	Geraniaceae Juss.
Iz_12_1209	DJI_0785	Geranium sanquineum L.	0	n	1	0	Geraniaceae Juss.
Iz_12_1209	DJI_0785	Gypsophila paniculata L.	0	n	1	0	Caryophyllaceae Juss.
Iz_12_1209	DJI_0785	Helichrysum arenarium (L.) Moench	0	n	1	0	Asteraceae Bercht. & J.Presl
Iz_12_1209	DJI_0785	Herniaria polygama J.Gay	0	n	1	0	Caryophyllaceae Juss.
Iz_12_1209	DJI_0785	Hieracium robustum Fr.	0	n	1	0	Asteraceae Bercht. & J.Presl
Iz_12_1209	DJI_0785	Hieracium umbellatum L.	0	n	1	NA	Asteraceae Bercht. & J.Presl
Iz_12_1209	DJI_0785	Hylotelephium maximum (L.) Holub	0	n	1	0	Crassulaceae J.St.-Hil.
Iz_12_1209	DJI_0785	Jacobaea borysthenica (DC.) B.Nord. & Greuter	0	n	1	0	Asteraceae Bercht. & J.Presl
Iz_12_1209	DJI_0785	Jasione montana L.	0	n	1	0	Campanulaceae Juss.
Iz_12_1209	DJI_0785	Jurinea cyanoides (L.) Rchb.s.l.	0	n	1	0	Asteraceae Bercht. & J.Presl
Iz_12_1209	DJI_0785	Koeleria glauca (Spreng.) DC.	0	n	1	0	Poaceae Barnhart
Iz_12_1209	DJI_0785	Lactuca serriola L.	0	a	0	0	Asteraceae Bercht. & J.Presl
Iz_12_1209	DJI_0785	Linaria genistifolia (L.) Mill.	0	n	1	0	Plantaginaceae Juss.
Iz_12_1209	DJI_0785	Lomelosia argentea (L.) Greuter & Burdet	0	n	1	0	Caprifoliaceae Juss.
Iz_12_1209	DJI_0785	Melampyrum pratense L.	0	n	1	0	Orobanchaceae Vent.
Iz_12_1209	DJI_0785	Oenothera biennis L.	0	a	0	0	Onagraceae Juss.
Iz_12_1209	DJI_0785	Pentanema britannica (L.) D.Gut.Larr., Santos-Vicente, Anderb., E.Rico & M.M.Mart.Ort.	0	n	1	0	Asteraceae Bercht. & J.Presl
Iz_12_1209	DJI_0785	Peucedanum oreoselinum (L.) Moench	0	n	1	0	Apiaceae Lindl.
Iz_12_1209	DJI_0785	Peucedanum ruthenicum M.Bieb.	0	n	1	0	Apiaceae Lindl.
Iz_12_1209	DJI_0785	Pilosella echioides (Lumn.) F.W.Schultz & Sch.Bip.	0	n	1	0	Asteraceae Bercht. & J.Presl
Iz_12_1209	DJI_0785	Pilosella officinarum F. Schultz et Sch. Bip.	0	n	1	0	Asteraceae Bercht. & J.Presl

Iz_12_1209	DJI_0785	Pilosella sp.	0		NA	0	Asteraceae Bercht. & J.Presl
Iz_12_1209	DJI_0785	Pinus sylvestris L.	2	n	1	1	Pinaceae Spreng. ex F.Rudolphi
Iz_12_1209	DJI_0785	Poa annua L.	0	n	1	0	Poaceae Barnhart
Iz_12_1209	DJI_0785	Poa nemoralis L.	0	n	1	0	Poaceae Barnhart
Iz_12_1209	DJI_0785	Poa sp.	0		NA	0	Poaceae Barnhart
Iz_12_1209	DJI_0785	Polygonatum odoratum (Mill.) Druce	0	n	1	0	Asparagaceae Juss.
Iz_12_1209	DJI_0785	Polygonum arenarium Waldst. & Kit.	0	n	1	0	Polygonaceae Juss.
Iz_12_1209	DJI_0785	Populus nigra L.	0	n	1	1	Salicaceae Mirb.
Iz_12_1209	DJI_0785	Populus tremula L.	0	n	1	1	Salicaceae Mirb.
Iz_12_1209	DJI_0785	Populus Ã canescens (Aiton) Sm.	4	n	1	1	Salicaceae Mirb.
Iz_12_1209	DJI_0785	Potentilla argentea L.	0	n	1	0	Rosaceae Juss.
Iz_12_1209	DJI_0785	Psammophiliella muralis (L.) Ikonn.	0	n	1	0	Caryophyllaceae Juss.
Iz_12_1209	DJI_0785	Psephellus sumensis (Kalen.) Greuter	0	n	1	0	Asteraceae Bercht. & J.Presl
Iz_12_1209	DJI_0785	Pulsatilla pratensis (L.) Mill. s.l.	0	n	1	0	Ranunculaceae Juss.
Iz_12_1209	DJI_0785	Pyrus communis L.	0	a	0	1	Rosaceae Juss.
Iz_12_1209	DJI_0785	Quercus robur L.	3	n	1	1	Fagaceae Dumort.
Iz_12_1209	DJI_0785	Robinia pseudoacacia L.	0	a	0	1	Fabaceae Lindl.
Iz_12_1209	DJI_0785	Rosa sp.	0		NA	1	Rosaceae Juss.
Iz_12_1209	DJI_0785	Rubus idaeus L.	0	n	1	0	Rosaceae Juss.
Iz_12_1209	DJI_0785	Rumex acetosella L.	0	n	1	0	Polygonaceae Juss.
Iz_12_1209	DJI_0785	Rumex thyrsiflorus Fingerh.	0	n	1	0	Polygonaceae Juss.
Iz_12_1209	DJI_0785	Salix alba L.	0	n	1	1	Salicaceae Mirb.
Iz_12_1209	DJI_0785	Salix sp.	2		NA	1	
Iz_12_1209	DJI_0785	Sambucus nigra L.	0	n	1	1	Viburnaceae Raf.
Iz_12_1209	DJI_0785	Secale sylvestre Host	0	n	1	0	Poaceae Barnhart
Iz_12_1209	DJI_0785	Sempervivum ruthenicum Schnittsp. & C.B. Lehm.	0	n	1	0	Crassulaceae J.St.-Hil.
Iz_12_1209	DJI_0785	Setaria viridis (L.) P. Beauv.	0	n	1	0	Poaceae Barnhart
Iz_12_1209	DJI_0785	Silene borysthenica (Gruner) Walters	0	n	1	0	Caryophyllaceae Juss.
Iz_12_1209	DJI_0785	Silene latifolia subsp. alba (Mill.) Greuter & Burdet	0	n	1	0	Caryophyllaceae Juss.
Iz_12_1209	DJI_0785	Solidago virgaurea L.	0	n	1	0	Asteraceae Bercht. & J.Presl
Iz_12_1209	DJI_0785	Sorbus aucuparia L.	0	n	1	1	Rosaceae Juss.
Iz_12_1209	DJI_0785	Stachys recta L.	0	n	1	0	Lamiacea Lindl.
Iz_12_1209	DJI_0785	Tanacetum vulgare L.	0	n	1	0	Asteraceae Bercht. & J.Presl
Iz_12_1209	DJI_0785	Thymus pallasianus Heinr.Braun	0	n	1	0	Lamiacea Lindl.
Iz_12_1209	DJI_0785	Tilia cordata Mill.	0	n	1	1	Malvaceae Juss.
Iz_12_1209	DJI_0785	Trifolium alpestre L.	0	n	1	0	Fabaceae Lindl.
Iz_12_1209	DJI_0785	Trifolium arvense L.	0	n	1	0	Fabaceae Lindl.
Iz_12_1209	DJI_0785	Ulmus glabra Huds.	0	n	1	1	Ulmaceae Mirb.
Iz_12_1209	DJI_0785	Verbascum densiflorum Bertol.	0	n	1	0	Scrophulariaceae Juss.
Iz_12_1209	DJI_0785	Verbascum sp.	0	n	1	0	Scrophulariaceae Juss.
Iz_12_1209	DJI_0785	Veronica austriaca L.	0	n	1	0	Plantaginaceae Juss.
Iz_12_1209	DJI_0785	Veronica incana L.	0	n	1	0	Plantaginaceae Juss.
Iz_12_1209	DJI_0785	Veronica verna L.	0	n	1	0	Plantaginaceae Juss.
Iz_12_1209	DJI_0785	Vincetoxicum hirundinaria Medik	0	n	1	0	Apocynaceae Juss.
Iz_12_1209	DJI_0788	Acer tataricum L.	0	n	1	1	Sapindaceae Juss.
Iz_12_1209	DJI_0788	Achillea millefolium L. s.l.	0	n	1	0	Asteraceae Bercht. & J.Presl
Iz_12_1209	DJI_0788	Allium oleraceum L.	0	n	1	0	Amaryllidaceae J.St.-Hil.
Iz_12_1209	DJI_0788	Ambrosia artemisiifolia L.	0	a	0	0	Asteraceae Bercht. & J.Presl
Iz_12_1209	DJI_0788	Amorpha fruticosa L.	0	a	0	0	Fabaceae Lindl.
Iz_12_1209	DJI_0788	Anthericum ramosum L.	0	n	1	0	Asparagaceae Juss.
Iz_12_1209	DJI_0788	Artemisia marschalliana Spreng.	0	n	1	0	Asteraceae Bercht. & J.Presl
Iz_12_1209	DJI_0788	Asparagus officinalis L.	0	n	1	0	Asparagaceae Juss.
Iz_12_1209	DJI_0788	Astragalus cicer L.	0	n	1	0	Fabaceae Lindl.
Iz_12_1209	DJI_0788	Bassia laniflora (S.G.Gmel.) A.J.Scott	0	n	1	0	Amaranthaceae Juss.
Iz_12_1209	DJI_0788	Berberis vulgaris L.	0	n	1	0	Berberidaceae Juss.
Iz_12_1209	DJI_0788	Betula pendula Roth.	0	n	1	1	Betulaceae Gray
Iz_12_1209	DJI_0788	Betula pendula Roth.	0	n	1	0	Poaceae Barnhart
Iz_12_1209	DJI_0788	Bromus inermis Leyss.	0		NA	NA	
Iz_12_1209	DJI_0788	Bromus squarrosus L.	0	a	0	0	Poaceae Barnhart
Iz_12_1209	DJI_0788	Bromus tectorum L.	0	a	0	0	Poaceae Barnhart
Iz_12_1209	DJI_0788	Calamagrostis epigejos (L.) Roth	30	n	1	0	Poaceae Barnhart
Iz_12_1209	DJI_0788	Carduus acanthoides L.	0	n	1	0	Asteraceae Bercht. & J.Presl
Iz_12_1209	DJI_0788	Carex sp.	0	n	1	0	Cyperaceae Juss.
Iz_12_1209	DJI_0788	Centaurea majorovii Dumbadze	0	n	1	0	Asteraceae Bercht. & J.Presl
Iz_12_1209	DJI_0788	Chamaecytisus ruthenicus (Fisch.ex Wol.) Klaskova	0	n	1	0	Fabaceae Lindl.
Iz_12_1209	DJI_0788	Chelidonium majus L.	0	n	1	0	Papaveraceae Juss.
Iz_12_1209	DJI_0788	Chenopodium betaceum Andrz.	0	n	1	0	Amaranthaceae Juss.
Iz_12_1209	DJI_0788	Chenopodium cf. album	0	n	1	0	Amaranthaceae Juss.
Iz_12_1209	DJI_0788	Chenopodium sp.	0		NA	0	Amaranthaceae Juss.
Iz_12_1209	DJI_0788	Chondrilla juncea L.	0	n	1	0	Asteraceae Bercht. & J.Presl
Iz_12_1209	DJI_0788	Cirsium vulgare (Savi) Ten.	0	n	1	0	Asteraceae Bercht. & J.Presl
Iz_12_1209	DJI_0788	Clinopodium acinos (L.) Kuntze	0	n	1	0	Lamiacea Lindl.
Iz_12_1209	DJI_0788	Convallaria majalis L.	0	n	1	0	Asparagaceae Juss.
Iz_12_1209	DJI_0788	Crataegus rhipidophylla Gand.	0	n	1	1	Rosaceae Juss.
Iz_12_1209	DJI_0788	Crepis foetida L.	0	n	1	0	Asteraceae Bercht. & J.Presl
Iz_12_1209	DJI_0788	Dactylis glomerata L.	0	n	1	0	Poaceae Barnhart
Iz_12_1209	DJI_0788	Dianthus campestris M.Bieb.	0	n	1	0	Caryophyllaceae Juss.
Iz_12_1209	DJI_0788	Digitaria sanguinalis (L.) Scop.	0	a	0	0	Poaceae Barnhart
Iz_12_1209	DJI_0788	Elymus repens (L.) Gould	0	n	1	0	Poaceae Barnhart
Iz_12_1209	DJI_0788	Epilobium tetragonum L.	0	n	1	0	Onagraceae Juss.
Iz_12_1209	DJI_0788	Eragrostis minor Host	0	a	0	0	Poaceae Barnhart
Iz_12_1209	DJI_0788	Erigeron canadensis L.	7	a	0	0	Asteraceae Bercht. & J.Presl
Iz_12_1209	DJI_0788	Euphorbia seguieriana Neck.	0	n	1	0	Euphorbiaceae Juss.
Iz_12_1209	DJI_0788	Fallopia dumetorum (L.) Holub	0	n	1	0	Polygonaceae Juss.
Iz_12_1209	DJI_0788	Festuca beckeri (Hack.) Trautv.	0	n	1	0	Poaceae Barnhart
Iz_12_1209	DJI_0788	Fragaria vesca L.	0	n	1	0	Rosaceae Juss.
Iz_12_1209	DJI_0788	Frangula alnus Mill.	0	n	1	1	Rhamnaceae Juss.
Iz_12_1209	DJI_0788	Fraxinus pennsylvanica Marshall	0	a	0	1	Oleaceae Hoffmanns. & Link
Iz_12_1209	DJI_0788	Galeopsis bifida Boenn.	0	n	1	0	Lamiacea Lindl.
Iz_12_1209	DJI_0788	Galium verum L.	0	n	1	0	Rubiaceae Juss.
Iz_12_1209	DJI_0788	Genista tinctoria L.	0	n	1	0	Fabaceae Lindl.
Iz_12_1209	DJI_0788	Geranium robertianum L.	0	n	1	0	Geraniaceae Juss.
Iz_12_1209	DJI_0788	Geranium sanquineum L.	0	n	1	0	Geraniaceae Juss.
Iz_12_1209	DJI_0788	Gypsophila paniculata L.	0	n	1	0	Caryophyllaceae Juss.
Iz_12_1209	DJI_0788	Helichrysum arenarium (L.) Moench	0	n	1	0	Asteraceae Bercht. & J.Presl
Iz_12_1209	DJI_0788	Herniaria polygama J.Gay	0	n	1	0	Caryophyllaceae Juss.
Iz_12_1209	DJI_0788	Hieracium robustum Fr.	0	n	1	0	Asteraceae Bercht. & J.Presl
Iz_12_1209	DJI_0788	Hieracium umbellatum L.	0	n	1	NA	Asteraceae Bercht. & J.Presl

Iz_12_1209	DJI_0788	Hylotelephium maximum (L.) Holub	0	n	1	0	Crassulaceae J.St.-Hil.
Iz_12_1209	DJI_0788	Jacobaea borysthenica (DC.) B.Nord. & Greuter	0	n	1	0	Asteraceae Bercht. & J.Presl
Iz_12_1209	DJI_0788	Jasione montana L.	0	n	1	0	Campanulaceae Juss.
Iz_12_1209	DJI_0788	Jurinea cyanoides (L.) Rchb.s.l.	0	n	1	0	Asteraceae Bercht. & J.Presl
Iz_12_1209	DJI_0788	Koeleria glauca (Spreng.) DC.	0	n	1	0	Poaceae Barnhart
Iz_12_1209	DJI_0788	Lactuca serriola L.	0	a	0	0	Asteraceae Bercht. & J.Presl
Iz_12_1209	DJI_0788	Linaria genistifolia (L.) Mill.	0	n	1	0	Plantaginaceae Juss.
Iz_12_1209	DJI_0788	Lomelosia argentea (L.) Greuter & Burdet	0	n	1	0	Caprifoliaceae Juss.
Iz_12_1209	DJI_0788	Melampyrum pratense L.	0	n	1	0	Orobanchaceae Vent.
Iz_12_1209	DJI_0788	Oenothera biennis L.	0	a	0	0	Onagraceae Juss.
Iz_12_1209	DJI_0788	Pentanema britannica (L.) D.Gut.Larr., Santos-Vicente, Anderb., E.Rico & M.M.Mart.Ort.	0	n	1	0	Asteraceae Bercht. & J.Presl
Iz_12_1209	DJI_0788	Peucedanum oreoselinum (L.) Moench	0	n	1	0	Apiaceae Lindl.
Iz_12_1209	DJI_0788	Peucedanum ruthenicum M.Bieb.	0	n	1	0	Apiaceae Lindl.
Iz_12_1209	DJI_0788	Pilosella echioides (Lumn.) F.W.Schultz & Sch.Bip.	0	n	1	0	Asteraceae Bercht. & J.Presl
Iz_12_1209	DJI_0788	Pilosella officinarum F. Schultz et Sch. Bip.	0	n	1	0	Asteraceae Bercht. & J.Presl
Iz_12_1209	DJI_0788	Pilosella sp.	0		NA	0	Asteraceae Bercht. & J.Presl
Iz_12_1209	DJI_0788	Pinus sylvestris L.	0	n	1	1	Pinaceae Spreng. ex F.Rudolphi
Iz_12_1209	DJI_0788	Poa annua L.	0	n	1	0	Poaceae Barnhart
Iz_12_1209	DJI_0788	Poa nemoralis L.	0	n	1	0	Poaceae Barnhart
Iz_12_1209	DJI_0788	Poa sp.	0		NA	0	Poaceae Barnhart
Iz_12_1209	DJI_0788	Polygonatum odoratum (Mill.) Druce	0	n	1	0	Asparagaceae Juss.
Iz_12_1209	DJI_0788	Polygonum arenarium Waldst. & Kit.	0	n	1	0	Polygonaceae Juss.
Iz_12_1209	DJI_0788	Populus nigra L.	0	n	1	1	Salicaceae Mirb.
Iz_12_1209	DJI_0788	Populus tremula L.	0	n	1	1	Salicaceae Mirb.
Iz_12_1209	DJI_0788	Populus Ã canescens (Aiton) Sm.	0	n	1	1	Salicaceae Mirb.
Iz_12_1209	DJI_0788	Potentilla argentea L.	0	n	1	0	Rosaceae Juss.
Iz_12_1209	DJI_0788	Psammophiliella muralis (L.) Ikonn.	0	n	1	0	Caryophyllaceae Juss.
Iz_12_1209	DJI_0788	Psephellus sumensis (Kalen.) Greuter	0	n	1	0	Asteraceae Bercht. & J.Presl
Iz_12_1209	DJI_0788	Pulsatilla pratensis (L.) Mill. s.l.	0	n	1	0	Ranunculaceae Juss.
Iz_12_1209	DJI_0788	Pyrus communis L.	0	a	0	1	Rosaceae Juss.
Iz_12_1209	DJI_0788	Quercus robur L.	0	n	1	1	Fagaceae Dumort.
Iz_12_1209	DJI_0788	Robinia pseudoacacia L.	0	a	0	1	Fabaceae Lindl.
Iz_12_1209	DJI_0788	Rosa sp.	0		NA	1	Rosaceae Juss.
Iz_12_1209	DJI_0788	Rubus idaeus L.	0	n	1	0	Rosaceae Juss.
Iz_12_1209	DJI_0788	Rumex acetosella L.	0	n	1	0	Polygonaceae Juss.
Iz_12_1209	DJI_0788	Rumex thyrsiflorus Fingerh.	0	n	1	0	Polygonaceae Juss.
Iz_12_1209	DJI_0788	Salix alba L.	0	n	1	1	Salicaceae Mirb.
Iz_12_1209	DJI_0788	Salix sp.	0		NA	1	
Iz_12_1209	DJI_0788	Sambucus nigra L.	0	n	1	1	Viburnaceae Raf.
Iz_12_1209	DJI_0788	Secale sylvestre Host	0	n	1	0	Poaceae Barnhart
Iz_12_1209	DJI_0788	Sempervivum ruthenicum Schnittsp. & C.B. Lehm.	0	n	1	0	Crassulaceae J.St.-Hil.
Iz_12_1209	DJI_0788	Setaria viridis (L.) P. Beauv.	0	n	1	0	Poaceae Barnhart
Iz_12_1209	DJI_0788	Silene borysthenica (Gruner) Walters	0	n	1	0	Caryophyllaceae Juss.
Iz_12_1209	DJI_0788	Silene latifolia subsp. alba (Mill.) Greuter & Burdet	0	n	1	0	Caryophyllaceae Juss.
Iz_12_1209	DJI_0788	Solidago virgaurea L.	0	n	1	0	Asteraceae Bercht. & J.Presl
Iz_12_1209	DJI_0788	Sorbus aucuparia L.	2	n	1	1	Rosaceae Juss.
Iz_12_1209	DJI_0788	Stachys recta L.	0	n	1	0	Lamiacea Lindl.
Iz_12_1209	DJI_0788	Tanacetum vulgare L.	0	n	1	0	Asteraceae Bercht. & J.Presl
Iz_12_1209	DJI_0788	Thymus pallasianus Heinr.Braun	0	n	1	0	Lamiacea Lindl.
Iz_12_1209	DJI_0788	Tilia cordata Mill.	0	n	1	1	Malvaceae Juss.
Iz_12_1209	DJI_0788	Trifolium alpestre L.	0	n	1	0	Fabaceae Lindl.
Iz_12_1209	DJI_0788	Trifolium arvense L.	0	n	1	0	Fabaceae Lindl.
Iz_12_1209	DJI_0788	Ulmus glabra Huds.	0	n	1	1	Ulmaceae Mirb.
Iz_12_1209	DJI_0788	Verbascum densiflorum Bertol.	0	n	1	0	Scrophulariaceae Juss.
Iz_12_1209	DJI_0788	Verbascum sp.	0	n	1	0	Scrophulariaceae Juss.
Iz_12_1209	DJI_0788	Veronica austriaca L.	0	n	1	0	Plantaginaceae Juss.
Iz_12_1209	DJI_0788	Veronica incana L.	0	n	1	0	Plantaginaceae Juss.
Iz_12_1209	DJI_0788	Veronica verna L.	0	n	1	0	Plantaginaceae Juss.
Iz_12_1209	DJI_0788	Vincetoxicum hirundinaria Medik	0	n	1	0	Apocynaceae Juss.
Iz_12_1209	DJI_0791	Acer tataricum L.	0	n	1	1	Sapindaceae Juss.
Iz_12_1209	DJI_0791	Achillea millefolium L. s.l.	0	n	1	0	Asteraceae Bercht. & J.Presl
Iz_12_1209	DJI_0791	Allium oleraceum L.	0	n	1	0	Amaryllidaceae J.St.-Hil.
Iz_12_1209	DJI_0791	Ambrosia artemisiifolia L.	0	a	0	0	Asteraceae Bercht. & J.Presl
Iz_12_1209	DJI_0791	Amorpha fruticosa L.	0	a	0	0	Fabaceae Lindl.
Iz_12_1209	DJI_0791	Anthericum ramosum L.	0	n	1	0	Asparagaceae Juss.
Iz_12_1209	DJI_0791	Artemisia marschalliana Spreng.	0	n	1	0	Asteraceae Bercht. & J.Presl
Iz_12_1209	DJI_0791	Asparagus officinalis L.	0	n	1	0	Asparagaceae Juss.
Iz_12_1209	DJI_0791	Astragalus cicer L.	0	n	1	0	Fabaceae Lindl.
Iz_12_1209	DJI_0791	Bassia laniflora (S.G.Gmel.) A.J.Scott	0	n	1	0	Amaranthaceae Juss.
Iz_12_1209	DJI_0791	Berberis vulgaris L.	0	n	1	0	Berberidaceae Juss.
Iz_12_1209	DJI_0791	Betula pendula Roth.	0	n	1	1	Betulaceae Gray
Iz_12_1209	DJI_0791	Betula pendula Roth.	0	n	1	0	Poaceae Barnhart
Iz_12_1209	DJI_0791	Bromus inermis Leyss.	0		NA	NA	
Iz_12_1209	DJI_0791	Bromus squarrosus L.	0	a	0	0	Poaceae Barnhart
Iz_12_1209	DJI_0791	Bromus tectorum L.	0	a	0	0	Poaceae Barnhart
Iz_12_1209	DJI_0791	Calamagrostis epigejos (L.) Roth	75	n	1	0	Poaceae Barnhart
Iz_12_1209	DJI_0791	Carduus acanthoides L.	0	n	1	0	Asteraceae Bercht. & J.Presl
Iz_12_1209	DJI_0791	Carex sp.	0	n	1	0	Cyperaceae Juss.
Iz_12_1209	DJI_0791	Centaurea majorovii Dumbadze	0	n	1	0	Asteraceae Bercht. & J.Presl
Iz_12_1209	DJI_0791	Chamaecytisus ruthenicus (Fisch.ex Wol.) Klaskova	0	n	1	0	Fabaceae Lindl.
Iz_12_1209	DJI_0791	Chelidonium majus L.	0	n	1	0	Papaveraceae Juss.
Iz_12_1209	DJI_0791	Chenopodium betaceum Andrz.	0	n	1	0	Amaranthaceae Juss.
Iz_12_1209	DJI_0791	Chenopodium cf. album	0	n	1	0	Amaranthaceae Juss.
Iz_12_1209	DJI_0791	Chenopodium sp.	0		NA	0	Amaranthaceae Juss.
Iz_12_1209	DJI_0791	Chondrilla juncea L.	0	n	1	0	Asteraceae Bercht. & J.Presl
Iz_12_1209	DJI_0791	Cirsium vulgare (Savi) Ten.	0	n	1	0	Asteraceae Bercht. & J.Presl
Iz_12_1209	DJI_0791	Clinopodium acinos (L.) Kuntze	0	n	1	0	Lamiacea Lindl.
Iz_12_1209	DJI_0791	Convallaria majalis L.	0	n	1	0	Asparagaceae Juss.
Iz_12_1209	DJI_0791	Crataegus rhipidophylla Gand.	0	n	1	1	Rosaceae Juss.
Iz_12_1209	DJI_0791	Crepis foetida L.	0	n	1	0	Asteraceae Bercht. & J.Presl
Iz_12_1209	DJI_0791	Dactylis glomerata L.	0	n	1	0	Poaceae Barnhart
Iz_12_1209	DJI_0791	Dianthus campestris M.Bieb.	0	n	1	0	Caryophyllaceae Juss.
Iz_12_1209	DJI_0791	Digitaria sanguinalis (L.) Scop.	0	a	0	0	Poaceae Barnhart
Iz_12_1209	DJI_0791	Elymus repens (L.) Gould	0	n	1	0	Poaceae Barnhart
Iz_12_1209	DJI_0791	Epilobium tetragonum L.	0	n	1	0	Onagraceae Juss.
Iz_12_1209	DJI_0791	Eragrostis minor Host	0	a	0	0	Poaceae Barnhart
Iz_12_1209	DJI_0791	Erigeron canadensis L.	0	a	0	0	Asteraceae Bercht. & J.Presl
Iz_12_1209	DJI_0791	Euphorbia seguieriana Neck.	0	n	1	0	Euphorbiaceae Juss.

Iz_12_1209	DJI_0791	Fallopia dumetorum (L.) Holub	0	n	1	0	Polygonaceae Juss.
Iz_12_1209	DJI_0791	Festuca beckeri (Hack.) Trautv.	0	n	1	0	Poaceae Barnhart
Iz_12_1209	DJI_0791	Fragaria vesca L.	0	n	1	0	Rosaceae Juss.
Iz_12_1209	DJI_0791	Frangula alnus Mill.	0	n	1	1	Rhamnaceae Juss.
Iz_12_1209	DJI_0791	Fraxinus pennsylvanica Marshall	0	a	0	1	Oleaceae Hoffmanns. & Link
Iz_12_1209	DJI_0791	Galeopsis bifida Boenn.	0	n	1	0	Lamiacea Lindl.
Iz_12_1209	DJI_0791	Galium verum L.	0	n	1	0	Rubiaceae Juss.
Iz_12_1209	DJI_0791	Genista tinctoria L.	0	n	1	0	Fabaceae Lindl.
Iz_12_1209	DJI_0791	Geranium robertianum L.	0	n	1	0	Geraniaceae Juss.
Iz_12_1209	DJI_0791	Geranium sanquineum L.	0	n	1	0	Geraniaceae Juss.
Iz_12_1209	DJI_0791	Gypsophila paniculata L.	0	n	1	0	Caryophyllaceae Juss.
Iz_12_1209	DJI_0791	Helichrysum arenarium (L.) Moench	0	n	1	0	Asteraceae Bercht. & J.Presl
Iz_12_1209	DJI_0791	Herniaria polygama J.Gay	0	n	1	0	Caryophyllaceae Juss.
Iz_12_1209	DJI_0791	Hieracium robustum Fr.	0	n	1	0	Asteraceae Bercht. & J.Presl
Iz_12_1209	DJI_0791	Hieracium umbellatum L.	0	n	1	NA	Asteraceae Bercht. & J.Presl
Iz_12_1209	DJI_0791	Hylotelephium maximum (L.) Holub	0	n	1	0	Crassulaceae J.St.-Hil.
Iz_12_1209	DJI_0791	Jacobaea borysthenica (DC.) B.Nord. & Greuter	0	n	1	0	Asteraceae Bercht. & J.Presl
Iz_12_1209	DJI_0791	Jasione montana L.	0	n	1	0	Campanulaceae Juss.
Iz_12_1209	DJI_0791	Jurinea cyanoides (L.) Rchb.s.l.	0	n	1	0	Asteraceae Bercht. & J.Presl
Iz_12_1209	DJI_0791	Koeleria glauca (Spreng.) DC.	0	n	1	0	Poaceae Barnhart
Iz_12_1209	DJI_0791	Lactuca serriola L.	0	a	0	0	Asteraceae Bercht. & J.Presl
Iz_12_1209	DJI_0791	Linaria genistifolia (L.) Mill.	0	n	1	0	Plantaginaceae Juss.
Iz_12_1209	DJI_0791	Lomelosia argentea (L.) Greuter & Burdet	0	n	1	0	Caprifoliaceae Juss.
Iz_12_1209	DJI_0791	Melampyrum pratense L.	0	n	1	0	Orobanchaceae Vent.
Iz_12_1209	DJI_0791	Oenothera biennis L.	0	a	0	0	Onagraceae Juss.
Iz_12_1209	DJI_0791	Pentanema britannica (L.) D.Gut.Larr., Santos-Vicente, Anderb., E.Rico & M.M.Mart.Ort.	0	n	1	0	Asteraceae Bercht. & J.Presl
Iz_12_1209	DJI_0791	Peucedanum oreoselinum (L.) Moench	0	n	1	0	Apiaceae Lindl.
Iz_12_1209	DJI_0791	Peucedanum ruthenicum M.Bieb.	0	n	1	0	Apiaceae Lindl.
Iz_12_1209	DJI_0791	Pilosella echioides (Lumn.) F.W.Schultz & Sch.Bip.	0	n	1	0	Asteraceae Bercht. & J.Presl
Iz_12_1209	DJI_0791	Pilosella officinarum F. Schultz et Sch. Bip.	0	n	1	0	Asteraceae Bercht. & J.Presl
Iz_12_1209	DJI_0791	Pilosella sp.	0		NA	0	Asteraceae Bercht. & J.Presl
Iz_12_1209	DJI_0791	Pinus sylvestris L.	0	n	1	1	Pinaceae Spreng. ex F.Rudolphi
Iz_12_1209	DJI_0791	Poa annua L.	0	n	1	0	Poaceae Barnhart
Iz_12_1209	DJI_0791	Poa nemoralis L.	0	n	1	0	Poaceae Barnhart
Iz_12_1209	DJI_0791	Poa sp.	0		NA	0	Poaceae Barnhart
Iz_12_1209	DJI_0791	Polygonatum odoratum (Mill.) Druce	0	n	1	0	Asparagaceae Juss.
Iz_12_1209	DJI_0791	Polygonum arenarium Waldst. & Kit.	0	n	1	0	Polygonaceae Juss.
Iz_12_1209	DJI_0791	Populus nigra L.	0	n	1	1	Salicaceae Mirb.
Iz_12_1209	DJI_0791	Populus tremula L.	0	n	1	1	Salicaceae Mirb.
Iz_12_1209	DJI_0791	Populus Ã canescens (Aiton) Sm.	0	n	1	1	Salicaceae Mirb.
Iz_12_1209	DJI_0791	Potentilla argentea L.	0	n	1	0	Rosaceae Juss.
Iz_12_1209	DJI_0791	Psammophiliella muralis (L.) Ikonn.	0	n	1	0	Caryophyllaceae Juss.
Iz_12_1209	DJI_0791	Psephellus sumensis (Kalen.) Greuter	0	n	1	0	Asteraceae Bercht. & J.Presl
Iz_12_1209	DJI_0791	Pulsatilla pratensis (L.) Mill. s.l.	0	n	1	0	Ranunculaceae Juss.
Iz_12_1209	DJI_0791	Pyrus communis L.	0	a	0	1	Rosaceae Juss.
Iz_12_1209	DJI_0791	Quercus robur L.	0	n	1	1	Fagaceae Dumort.
Iz_12_1209	DJI_0791	Robinia pseudoacacia L.	0	a	0	1	Fabaceae Lindl.
Iz_12_1209	DJI_0791	Rosa sp.	0		NA	1	Rosaceae Juss.
Iz_12_1209	DJI_0791	Rubus idaeus L.	0	n	1	0	Rosaceae Juss.
Iz_12_1209	DJI_0791	Rumex acetosella L.	0	n	1	0	Polygonaceae Juss.
Iz_12_1209	DJI_0791	Rumex thyrsiflorus Fingerh.	0	n	1	0	Polygonaceae Juss.
Iz_12_1209	DJI_0791	Salix alba L.	0	n	1	1	Salicaceae Mirb.
Iz_12_1209	DJI_0791	Salix sp.	0		NA	1	
Iz_12_1209	DJI_0791	Sambucus nigra L.	0	n	1	1	Viburnaceae Raf.
Iz_12_1209	DJI_0791	Secale sylvestre Host	0	n	1	0	Poaceae Barnhart
Iz_12_1209	DJI_0791	Sempervivum ruthenicum Schnittsp. & C.B. Lehm.	0	n	1	0	Crassulaceae J.St.-Hil.
Iz_12_1209	DJI_0791	Setaria viridis (L.) P. Beauv.	0	n	1	0	Poaceae Barnhart
Iz_12_1209	DJI_0791	Silene borysthenica (Gruner) Walters	0	n	1	0	Caryophyllaceae Juss.
Iz_12_1209	DJI_0791	Silene latifolia subsp. alba (Mill.) Greuter & Burdet	0	n	1	0	Caryophyllaceae Juss.
Iz_12_1209	DJI_0791	Solidago virgaurea L.	0	n	1	0	Asteraceae Bercht. & J.Presl
Iz_12_1209	DJI_0791	Sorbus aucuparia L.	0	n	1	1	Rosaceae Juss.
Iz_12_1209	DJI_0791	Stachys recta L.	0	n	1	0	Lamiacea Lindl.
Iz_12_1209	DJI_0791	Tanacetum vulgare L.	0	n	1	0	Asteraceae Bercht. & J.Presl
Iz_12_1209	DJI_0791	Thymus pallasianus Heinr.Braun	0	n	1	0	Lamiacea Lindl.
Iz_12_1209	DJI_0791	Tilia cordata Mill.	0	n	1	1	Malvaceae Juss.
Iz_12_1209	DJI_0791	Trifolium alpestre L.	0	n	1	0	Fabaceae Lindl.
Iz_12_1209	DJI_0791	Trifolium arvense L.	0	n	1	0	Fabaceae Lindl.
Iz_12_1209	DJI_0791	Ulmus glabra Huds.	0	n	1	1	Ulmaceae Mirb.
Iz_12_1209	DJI_0791	Verbascum densiflorum Bertol.	0	n	1	0	Scrophulariaceae Juss.
Iz_12_1209	DJI_0791	Verbascum sp.	0	n	1	0	Scrophulariaceae Juss.
Iz_12_1209	DJI_0791	Veronica austriaca L.	0	n	1	0	Plantaginaceae Juss.
Iz_12_1209	DJI_0791	Veronica incana L.	0	n	1	0	Plantaginaceae Juss.
Iz_12_1209	DJI_0791	Veronica verna L.	0	n	1	0	Plantaginaceae Juss.
Iz_12_1209	DJI_0791	Vincetoxicum hirundinaria Medik	0	n	1	0	Apocynaceae Juss.
Iz_12_1209	DJI_0794	Acer tataricum L.	0	n	1	1	Sapindaceae Juss.
Iz_12_1209	DJI_0794	Achillea millefolium L. s.l.	0	n	1	0	Asteraceae Bercht. & J.Presl
Iz_12_1209	DJI_0794	Allium oleraceum L.	0	n	1	0	Amaryllidaceae J.St.-Hil.
Iz_12_1209	DJI_0794	Ambrosia artemisiifolia L.	0	a	0	0	Asteraceae Bercht. & J.Presl
Iz_12_1209	DJI_0794	Amorpha fruticosa L.	0	a	0	0	Fabaceae Lindl.
Iz_12_1209	DJI_0794	Anthericum ramosum L.	0	n	1	0	Asparagaceae Juss.
Iz_12_1209	DJI_0794	Artemisia marschalliana Spreng.	0	n	1	0	Asteraceae Bercht. & J.Presl
Iz_12_1209	DJI_0794	Asparagus officinalis L.	0	n	1	0	Asparagaceae Juss.
Iz_12_1209	DJI_0794	Astragalus cicer L.	0	n	1	0	Fabaceae Lindl.
Iz_12_1209	DJI_0794	Bassia laniflora (S.G.Gmel.) A.J.Scott	0	n	1	0	Amaranthaceae Juss.
Iz_12_1209	DJI_0794	Berberis vulgaris L.	0	n	1	0	Berberidaceae Juss.
Iz_12_1209	DJI_0794	Betula pendula Roth.	3	n	1	1	Betulaceae Gray
Iz_12_1209	DJI_0794	Betula pendula Roth.	3	n	1	0	Poaceae Barnhart
Iz_12_1209	DJI_0794	Bromus inermis Leyss.	0		NA	NA	
Iz_12_1209	DJI_0794	Bromus squarrosus L.	0	a	0	0	Poaceae Barnhart
Iz_12_1209	DJI_0794	Bromus tectorum L.	0	a	0	0	Poaceae Barnhart
Iz_12_1209	DJI_0794	Calamagrostis epigejos (L.) Roth	10	n	1	0	Poaceae Barnhart
Iz_12_1209	DJI_0794	Carduus acanthoides L.	0	n	1	0	Asteraceae Bercht. & J.Presl
Iz_12_1209	DJI_0794	Carex sp.	0	n	1	0	Cyperaceae Juss.
Iz_12_1209	DJI_0794	Centaurea majorovii Dumbadze	0	n	1	0	Asteraceae Bercht. & J.Presl
Iz_12_1209	DJI_0794	Chamaecytisus ruthenicus (Fisch.ex Wol.) Klaskova	0	n	1	0	Fabaceae Lindl.
Iz_12_1209	DJI_0794	Chelidonium majus L.	0	n	1	0	Papaveraceae Juss.
Iz_12_1209	DJI_0794	Chenopodium betaceum Andrz.	0	n	1	0	Amaranthaceae Juss.
Iz_12_1209	DJI_0794	Chenopodium cf. album	0	n	1	0	Amaranthaceae Juss.

Iz_12_1209	DJI_0794	Chenopodium sp.	0		NA	0	Amaranthaceae Juss.
Iz_12_1209	DJI_0794	Chondrilla juncea L.	0	n	1	0	Asteraceae Bercht. & J.Presl
Iz_12_1209	DJI_0794	Cirsium vulgare (Savi) Ten.	0	n	1	0	Asteraceae Bercht. & J.Presl
Iz_12_1209	DJI_0794	Clinopodium acinos (L.) Kuntze	0	n	1	0	Lamiacea Lindl.
Iz_12_1209	DJI_0794	Convallaria majalis L.	0	n	1	0	Asparagaceae Juss.
Iz_12_1209	DJI_0794	Crataegus rhipidophylla Gand.	0	n	1	1	Rosaceae Juss.
Iz_12_1209	DJI_0794	Crepis foetida L.	0	n	1	0	Asteraceae Bercht. & J.Presl
Iz_12_1209	DJI_0794	Dactylis glomerata L.	0	n	1	0	Poaceae Barnhart
Iz_12_1209	DJI_0794	Dianthus campestris M.Bieb.	0	n	1	0	Caryophyllaceae Juss.
Iz_12_1209	DJI_0794	Digitaria sanguinalis (L.) Scop.	0	a	0	0	Poaceae Barnhart
Iz_12_1209	DJI_0794	Elymus repens (L.) Gould	0	n	1	0	Poaceae Barnhart
Iz_12_1209	DJI_0794	Epilobium tetragonum L.	0	n	1	0	Onagraceae Juss.
Iz_12_1209	DJI_0794	Eragrostis minor Host	0	a	0	0	Poaceae Barnhart
Iz_12_1209	DJI_0794	Erigeron canadensis L.	10	a	0	0	Asteraceae Bercht. & J.Presl
Iz_12_1209	DJI_0794	Euphorbia seguieriana Neck.	0	n	1	0	Euphorbiaceae Juss.
Iz_12_1209	DJI_0794	Fallopia dumetorum (L.) Holub	2	n	1	0	Polygonaceae Juss.
Iz_12_1209	DJI_0794	Festuca beckeri (Hack.) Trautv.	0	n	1	0	Poaceae Barnhart
Iz_12_1209	DJI_0794	Fragaria vesca L.	0	n	1	0	Rosaceae Juss.
Iz_12_1209	DJI_0794	Frangula alnus Mill.	0	n	1	1	Rhamnaceae Juss.
Iz_12_1209	DJI_0794	Fraxinus pennsylvanica Marshall	0	a	0	1	Oleaceae Hoffmanns. & Link
Iz_12_1209	DJI_0794	Galeopsis bifida Boenn.	0	n	1	0	Lamiacea Lindl.
Iz_12_1209	DJI_0794	Galium verum L.	0	n	1	0	Rubiaceae Juss.
Iz_12_1209	DJI_0794	Genista tinctoria L.	0	n	1	0	Fabaceae Lindl.
Iz_12_1209	DJI_0794	Geranium robertianum L.	0	n	1	0	Geraniaceae Juss.
Iz_12_1209	DJI_0794	Geranium sanquineum L.	0	n	1	0	Geraniaceae Juss.
Iz_12_1209	DJI_0794	Gypsophila paniculata L.	0	n	1	0	Caryophyllaceae Juss.
Iz_12_1209	DJI_0794	Helichrysum arenarium (L.) Moench	0	n	1	0	Asteraceae Bercht. & J.Presl
Iz_12_1209	DJI_0794	Herniaria polygama J.Gay	0	n	1	0	Caryophyllaceae Juss.
Iz_12_1209	DJI_0794	Hieracium robustum Fr.	0	n	1	0	Asteraceae Bercht. & J.Presl
Iz_12_1209	DJI_0794	Hieracium umbellatum L.	0	n	1	NA	Asteraceae Bercht. & J.Presl
Iz_12_1209	DJI_0794	Hylotelephium maximum (L.) Holub	0	n	1	0	Crassulaceae J.St.-Hil.
Iz_12_1209	DJI_0794	Jacobaea borysthenica (DC.) B.Nord. & Greuter	0	n	1	0	Asteraceae Bercht. & J.Presl
Iz_12_1209	DJI_0794	Jasione montana L.	0	n	1	0	Campanulaceae Juss.
Iz_12_1209	DJI_0794	Jurinea cyanoides (L.) Rchb.s.l.	0	n	1	0	Asteraceae Bercht. & J.Presl
Iz_12_1209	DJI_0794	Koeleria glauca (Spreng.) DC.	0	n	1	0	Poaceae Barnhart
Iz_12_1209	DJI_0794	Lactuca serriola L.	0	a	0	0	Asteraceae Bercht. & J.Presl
Iz_12_1209	DJI_0794	Linaria genistifolia (L.) Mill.	0	n	1	0	Plantaginaceae Juss.
Iz_12_1209	DJI_0794	Lomelosia argentea (L.) Greuter & Burdet	0	n	1	0	Caprifoliaceae Juss.
Iz_12_1209	DJI_0794	Melampyrum pratense L.	0	n	1	0	Orobanchaceae Vent.
Iz_12_1209	DJI_0794	Oenothera biennis L.	0	a	0	0	Onagraceae Juss.
Iz_12_1209	DJI_0794	Pentanema britannica (L.) D.Gut.Larr., Santos-Vicente, Anderb., E.Rico & M.M.Mart.Ort.	0	n	1	0	Asteraceae Bercht. & J.Presl
Iz_12_1209	DJI_0794	Peucedanum oreoselinum (L.) Moench	0	n	1	0	Apiaceae Lindl.
Iz_12_1209	DJI_0794	Peucedanum ruthenicum M.Bieb.	0	n	1	0	Apiaceae Lindl.
Iz_12_1209	DJI_0794	Pilosella echioides (Lumn.) F.W.Schultz & Sch.Bip.	0	n	1	0	Asteraceae Bercht. & J.Presl
Iz_12_1209	DJI_0794	Pilosella officinarum F. Schultz et Sch. Bip.	0	n	1	0	Asteraceae Bercht. & J.Presl
Iz_12_1209	DJI_0794	Pilosella sp.	0		NA	0	Asteraceae Bercht. & J.Presl
Iz_12_1209	DJI_0794	Pinus sylvestris L.	0	n	1	1	Pinaceae Spreng. ex F.Rudolphi
Iz_12_1209	DJI_0794	Poa annua L.	0	n	1	0	Poaceae Barnhart
Iz_12_1209	DJI_0794	Poa nemoralis L.	0	n	1	0	Poaceae Barnhart
Iz_12_1209	DJI_0794	Poa sp.	0		NA	0	Poaceae Barnhart
Iz_12_1209	DJI_0794	Polygonatum odoratum (Mill.) Druce	0	n	1	0	Asparagaceae Juss.
Iz_12_1209	DJI_0794	Polygonum arenarium Waldst. & Kit.	0	n	1	0	Polygonaceae Juss.
Iz_12_1209	DJI_0794	Populus nigra L.	0	n	1	1	Salicaceae Mirb.
Iz_12_1209	DJI_0794	Populus tremula L.	0	n	1	1	Salicaceae Mirb.
Iz_12_1209	DJI_0794	Populus Ã canescens (Aiton) Sm.	0	n	1	1	Salicaceae Mirb.
Iz_12_1209	DJI_0794	Potentilla argentea L.	0	n	1	0	Rosaceae Juss.
Iz_12_1209	DJI_0794	Psammophiliella muralis (L.) Ikonn.	0	n	1	0	Caryophyllaceae Juss.
Iz_12_1209	DJI_0794	Psephellus sumensis (Kalen.) Greuter	0	n	1	0	Asteraceae Bercht. & J.Presl
Iz_12_1209	DJI_0794	Pulsatilla pratensis (L.) Mill. s.l.	0	n	1	0	Ranunculaceae Juss.
Iz_12_1209	DJI_0794	Pyrus communis L.	0	a	0	1	Rosaceae Juss.
Iz_12_1209	DJI_0794	Quercus robur L.	0	n	1	1	Fagaceae Dumort.
Iz_12_1209	DJI_0794	Robinia pseudoacacia L.	0	a	0	1	Fabaceae Lindl.
Iz_12_1209	DJI_0794	Rosa sp.	0		NA	1	Rosaceae Juss.
Iz_12_1209	DJI_0794	Rubus idaeus L.	0	n	1	0	Rosaceae Juss.
Iz_12_1209	DJI_0794	Rumex acetosella L.	0	n	1	0	Polygonaceae Juss.
Iz_12_1209	DJI_0794	Rumex thyrsiflorus Fingerh.	0	n	1	0	Polygonaceae Juss.
Iz_12_1209	DJI_0794	Salix alba L.	0	n	1	1	Salicaceae Mirb.
Iz_12_1209	DJI_0794	Salix sp.	0		NA	1	
Iz_12_1209	DJI_0794	Sambucus nigra L.	0	n	1	1	Viburnaceae Raf.
Iz_12_1209	DJI_0794	Secale sylvestre Host	0	n	1	0	Poaceae Barnhart
Iz_12_1209	DJI_0794	Sempervivum ruthenicum Schnittsp. & C.B. Lehm.	0	n	1	0	Crassulaceae J.St.-Hil.
Iz_12_1209	DJI_0794	Setaria viridis (L.) P. Beauv.	0	n	1	0	Poaceae Barnhart
Iz_12_1209	DJI_0794	Silene borysthenica (Gruner) Walters	0	n	1	0	Caryophyllaceae Juss.
Iz_12_1209	DJI_0794	Silene latifolia subsp. alba (Mill.) Greuter & Burdet	0	n	1	0	Caryophyllaceae Juss.
Iz_12_1209	DJI_0794	Solidago virgaurea L.	0	n	1	0	Asteraceae Bercht. & J.Presl
Iz_12_1209	DJI_0794	Sorbus aucuparia L.	0	n	1	1	Rosaceae Juss.
Iz_12_1209	DJI_0794	Stachys recta L.	0	n	1	0	Lamiacea Lindl.
Iz_12_1209	DJI_0794	Tanacetum vulgare L.	0	n	1	0	Asteraceae Bercht. & J.Presl
Iz_12_1209	DJI_0794	Thymus pallasianus Heinr.Braun	0	n	1	0	Lamiacea Lindl.
Iz_12_1209	DJI_0794	Tilia cordata Mill.	0	n	1	1	Malvaceae Juss.
Iz_12_1209	DJI_0794	Trifolium alpestre L.	0	n	1	0	Fabaceae Lindl.
Iz_12_1209	DJI_0794	Trifolium arvense L.	0	n	1	0	Fabaceae Lindl.
Iz_12_1209	DJI_0794	Ulmus glabra Huds.	0	n	1	1	Ulmaceae Mirb.
Iz_12_1209	DJI_0794	Verbascum densiflorum Bertol.	0	n	1	0	Scrophulariaceae Juss.
Iz_12_1209	DJI_0794	Verbascum sp.	2	n	1	0	Scrophulariaceae Juss.
Iz_12_1209	DJI_0794	Veronica austriaca L.	0	n	1	0	Plantaginaceae Juss.
Iz_12_1209	DJI_0794	Veronica incana L.	0	n	1	0	Plantaginaceae Juss.
Iz_12_1209	DJI_0794	Veronica verna L.	0	n	1	0	Plantaginaceae Juss.
Iz_12_1209	DJI_0794	Vincetoxicum hirundinaria Medik	0	n	1	0	Apocynaceae Juss.
Iz_12_1209	DJI_0798	Acer tataricum L.	0	n	1	1	Sapindaceae Juss.
Iz_12_1209	DJI_0798	Achillea millefolium L. s.l.	0	n	1	0	Asteraceae Bercht. & J.Presl
Iz_12_1209	DJI_0798	Allium oleraceum L.	0	n	1	0	Amaryllidaceae J.St.-Hil.
Iz_12_1209	DJI_0798	Ambrosia artemisiifolia L.	0	a	0	0	Asteraceae Bercht. & J.Presl
Iz_12_1209	DJI_0798	Amorpha fruticosa L.	0	a	0	0	Fabaceae Lindl.
Iz_12_1209	DJI_0798	Anthericum ramosum L.	0	n	1	0	Asparagaceae Juss.
Iz_12_1209	DJI_0798	Artemisia marschalliana Spreng.	0	n	1	0	Asteraceae Bercht. & J.Presl
Iz_12_1209	DJI_0798	Asparagus officinalis L.	0	n	1	0	Asparagaceae Juss.
Iz_12_1209	DJI_0798	Astragalus cicer L.	0	n	1	0	Fabaceae Lindl.

Iz_12_1209	DJI_0798	Bassia laniflora (S.G.Gmel.) A.J.Scott	0	n	1	0	Amaranthaceae Juss.
Iz_12_1209	DJI_0798	Berberis vulgaris L.	0	n	1	0	Berberidaceae Juss.
Iz_12_1209	DJI_0798	Betula pendula Roth.	15	n	1	1	Betulaceae Gray
Iz_12_1209	DJI_0798	Betula pendula Roth.	15	n	1	0	Poaceae Barnhart
Iz_12_1209	DJI_0798	Bromus inermis Leyss.	0		NA	NA	
Iz_12_1209	DJI_0798	Bromus squarrosus L.	0	a	0	0	Poaceae Barnhart
Iz_12_1209	DJI_0798	Bromus tectorum L.	0	a	0	0	Poaceae Barnhart
Iz_12_1209	DJI_0798	Calamagrostis epigejos (L.) Roth	1	n	1	0	Poaceae Barnhart
Iz_12_1209	DJI_0798	Carduus acanthoides L.	0	n	1	0	Asteraceae Bercht. & J.Presl
Iz_12_1209	DJI_0798	Carex sp.	0	n	1	0	Cyperaceae Juss.
Iz_12_1209	DJI_0798	Centaurea majorovii Dumbadze	0	n	1	0	Asteraceae Bercht. & J.Presl
Iz_12_1209	DJI_0798	Chamaecytisus ruthenicus (Fisch.ex Wol.) Klaskova	0	n	1	0	Fabaceae Lindl.
Iz_12_1209	DJI_0798	Chelidonium majus L.	0	n	1	0	Papaveraceae Juss.
Iz_12_1209	DJI_0798	Chenopodium betaceum Andrz.	0	n	1	0	Amaranthaceae Juss.
Iz_12_1209	DJI_0798	Chenopodium cf. album	0	n	1	0	Amaranthaceae Juss.
Iz_12_1209	DJI_0798	Chenopodium sp.	0		NA	0	Amaranthaceae Juss.
Iz_12_1209	DJI_0798	Chondrilla juncea L.	0	n	1	0	Asteraceae Bercht. & J.Presl
Iz_12_1209	DJI_0798	Cirsium vulgare (Savi) Ten.	0	n	1	0	Asteraceae Bercht. & J.Presl
Iz_12_1209	DJI_0798	Clinopodium acinos (L.) Kuntze	0	n	1	0	Lamiacea Lindl.
Iz_12_1209	DJI_0798	Convallaria majalis L.	0	n	1	0	Asparagaceae Juss.
Iz_12_1209	DJI_0798	Crataegus rhipidophylla Gand.	0	n	1	1	Rosaceae Juss.
Iz_12_1209	DJI_0798	Crepis foetida L.	0	n	1	0	Asteraceae Bercht. & J.Presl
Iz_12_1209	DJI_0798	Dactylis glomerata L.	0	n	1	0	Poaceae Barnhart
Iz_12_1209	DJI_0798	Dianthus campestris M.Bieb.	0	n	1	0	Caryophyllaceae Juss.
Iz_12_1209	DJI_0798	Digitaria sanguinalis (L.) Scop.	0	a	0	0	Poaceae Barnhart
Iz_12_1209	DJI_0798	Elymus repens (L.) Gould	0	n	1	0	Poaceae Barnhart
Iz_12_1209	DJI_0798	Epilobium tetragonum L.	0	n	1	0	Onagraceae Juss.
Iz_12_1209	DJI_0798	Eragrostis minor Host	0	a	0	0	Poaceae Barnhart
Iz_12_1209	DJI_0798	Erigeron canadensis L.	1	a	0	0	Asteraceae Bercht. & J.Presl
Iz_12_1209	DJI_0798	Euphorbia seguieriana Neck.	0	n	1	0	Euphorbiaceae Juss.
Iz_12_1209	DJI_0798	Fallopia dumetorum (L.) Holub	0	n	1	0	Polygonaceae Juss.
Iz_12_1209	DJI_0798	Festuca beckeri (Hack.) Trautv.	3	n	1	0	Poaceae Barnhart
Iz_12_1209	DJI_0798	Fragaria vesca L.	0	n	1	0	Rosaceae Juss.
Iz_12_1209	DJI_0798	Frangula alnus Mill.	0	n	1	1	Rhamnaceae Juss.
Iz_12_1209	DJI_0798	Fraxinus pennsylvanica Marshall	0	a	0	1	Oleaceae Hoffmanns. & Link
Iz_12_1209	DJI_0798	Galeopsis bifida Boenn.	0	n	1	0	Lamiacea Lindl.
Iz_12_1209	DJI_0798	Galium verum L.	0	n	1	0	Rubiaceae Juss.
Iz_12_1209	DJI_0798	Genista tinctoria L.	0	n	1	0	Fabaceae Lindl.
Iz_12_1209	DJI_0798	Geranium robertianum L.	0	n	1	0	Geraniaceae Juss.
Iz_12_1209	DJI_0798	Geranium sanquineum L.	0	n	1	0	Geraniaceae Juss.
Iz_12_1209	DJI_0798	Gypsophila paniculata L.	0	n	1	0	Caryophyllaceae Juss.
Iz_12_1209	DJI_0798	Helichrysum arenarium (L.) Moench	0	n	1	0	Asteraceae Bercht. & J.Presl
Iz_12_1209	DJI_0798	Herniaria polygama J.Gay	0	n	1	0	Caryophyllaceae Juss.
Iz_12_1209	DJI_0798	Hieracium robustum Fr.	0	n	1	0	Asteraceae Bercht. & J.Presl
Iz_12_1209	DJI_0798	Hieracium umbellatum L.	0	n	1	NA	Asteraceae Bercht. & J.Presl
Iz_12_1209	DJI_0798	Hylotelephium maximum (L.) Holub	0	n	1	0	Crassulaceae J.St.-Hil.
Iz_12_1209	DJI_0798	Jacobaea borysthenica (DC.) B.Nord. & Greuter	0	n	1	0	Asteraceae Bercht. & J.Presl
Iz_12_1209	DJI_0798	Jasione montana L.	0	n	1	0	Campanulaceae Juss.
Iz_12_1209	DJI_0798	Jurinea cyanoides (L.) Rchb.s.l.	0	n	1	0	Asteraceae Bercht. & J.Presl
Iz_12_1209	DJI_0798	Koeleria glauca (Spreng.) DC.	0	n	1	0	Poaceae Barnhart
Iz_12_1209	DJI_0798	Lactuca serriola L.	0	a	0	0	Asteraceae Bercht. & J.Presl
Iz_12_1209	DJI_0798	Linaria genistifolia (L.) Mill.	0	n	1	0	Plantaginaceae Juss.
Iz_12_1209	DJI_0798	Lomelosia argentea (L.) Greuter & Burdet	0	n	1	0	Caprifoliaceae Juss.
Iz_12_1209	DJI_0798	Melampyrum pratense L.	0	n	1	0	Orobanchaceae Vent.
Iz_12_1209	DJI_0798	Oenothera biennis L.	0	a	0	0	Onagraceae Juss.
Iz_12_1209	DJI_0798	Pentanema britannica (L.) D.Gut.Larr., Santos-Vicente, Anderb., E.Rico & M.M.Mart.Ort.	0	n	1	0	Asteraceae Bercht. & J.Presl
Iz_12_1209	DJI_0798	Peucedanum oreoselinum (L.) Moench	0	n	1	0	Apiaceae Lindl.
Iz_12_1209	DJI_0798	Peucedanum ruthenicum M.Bieb.	0	n	1	0	Apiaceae Lindl.
Iz_12_1209	DJI_0798	Pilosella echioides (Lumn.) F.W.Schultz & Sch.Bip.	0	n	1	0	Asteraceae Bercht. & J.Presl
Iz_12_1209	DJI_0798	Pilosella officinarum F. Schultz et Sch. Bip.	0	n	1	0	Asteraceae Bercht. & J.Presl
Iz_12_1209	DJI_0798	Pilosella sp.	0		NA	0	Asteraceae Bercht. & J.Presl
Iz_12_1209	DJI_0798	Pinus sylvestris L.	0	n	1	1	Pinaceae Spreng. ex F.Rudolphi
Iz_12_1209	DJI_0798	Poa annua L.	0	n	1	0	Poaceae Barnhart
Iz_12_1209	DJI_0798	Poa nemoralis L.	0	n	1	0	Poaceae Barnhart
Iz_12_1209	DJI_0798	Poa sp.	0		NA	0	Poaceae Barnhart
Iz_12_1209	DJI_0798	Polygonatum odoratum (Mill.) Druce	0	n	1	0	Asparagaceae Juss.
Iz_12_1209	DJI_0798	Polygonum arenarium Waldst. & Kit.	0	n	1	0	Polygonaceae Juss.
Iz_12_1209	DJI_0798	Populus nigra L.	0	n	1	1	Salicaceae Mirb.
Iz_12_1209	DJI_0798	Populus tremula L.	1	n	1	1	Salicaceae Mirb.
Iz_12_1209	DJI_0798	Populus Ã canescens (Aiton) Sm.	0	n	1	1	Salicaceae Mirb.
Iz_12_1209	DJI_0798	Potentilla argentea L.	0	n	1	0	Rosaceae Juss.
Iz_12_1209	DJI_0798	Psammophiliella muralis (L.) Ikonn.	0	n	1	0	Caryophyllaceae Juss.
Iz_12_1209	DJI_0798	Psephellus sumensis (Kalen.) Greuter	0	n	1	0	Asteraceae Bercht. & J.Presl
Iz_12_1209	DJI_0798	Pulsatilla pratensis (L.) Mill. s.l.	0	n	1	0	Ranunculaceae Juss.
Iz_12_1209	DJI_0798	Pyrus communis L.	0	a	0	1	Rosaceae Juss.
Iz_12_1209	DJI_0798	Quercus robur L.	0	n	1	1	Fagaceae Dumort.
Iz_12_1209	DJI_0798	Robinia pseudoacacia L.	0	a	0	1	Fabaceae Lindl.
Iz_12_1209	DJI_0798	Rosa sp.	0		NA	1	Rosaceae Juss.
Iz_12_1209	DJI_0798	Rubus idaeus L.	0.2	n	1	0	Rosaceae Juss.
Iz_12_1209	DJI_0798	Rumex acetosella L.	0	n	1	0	Polygonaceae Juss.
Iz_12_1209	DJI_0798	Rumex thyrsiflorus Fingerh.	0	n	1	0	Polygonaceae Juss.
Iz_12_1209	DJI_0798	Salix alba L.	0	n	1	1	Salicaceae Mirb.
Iz_12_1209	DJI_0798	Salix sp.	0		NA	1	
Iz_12_1209	DJI_0798	Sambucus nigra L.	0	n	1	1	Viburnaceae Raf.
Iz_12_1209	DJI_0798	Secale sylvestre Host	0	n	1	0	Poaceae Barnhart
Iz_12_1209	DJI_0798	Sempervivum ruthenicum Schnittsp. & C.B. Lehm.	0	n	1	0	Crassulaceae J.St.-Hil.
Iz_12_1209	DJI_0798	Setaria viridis (L.) P. Beauv.	0	n	1	0	Poaceae Barnhart
Iz_12_1209	DJI_0798	Silene borysthenica (Gruner) Walters	0	n	1	0	Caryophyllaceae Juss.
Iz_12_1209	DJI_0798	Silene latifolia subsp. alba (Mill.) Greuter & Burdet	0	n	1	0	Caryophyllaceae Juss.
Iz_12_1209	DJI_0798	Solidago virgaurea L.	0	n	1	0	Asteraceae Bercht. & J.Presl
Iz_12_1209	DJI_0798	Sorbus aucuparia L.	0	n	1	1	Rosaceae Juss.
Iz_12_1209	DJI_0798	Stachys recta L.	0	n	1	0	Lamiacea Lindl.
Iz_12_1209	DJI_0798	Tanacetum vulgare L.	0	n	1	0	Asteraceae Bercht. & J.Presl
Iz_12_1209	DJI_0798	Thymus pallasianus Heinr.Braun	0	n	1	0	Lamiacea Lindl.
Iz_12_1209	DJI_0798	Tilia cordata Mill.	0	n	1	1	Malvaceae Juss.
Iz_12_1209	DJI_0798	Trifolium alpestre L.	0	n	1	0	Fabaceae Lindl.
Iz_12_1209	DJI_0798	Trifolium arvense L.	0	n	1	0	Fabaceae Lindl.
Iz_12_1209	DJI_0798	Ulmus glabra Huds.	0	n	1	1	Ulmaceae Mirb.

Iz_12_1209	DJI_0798	Verbascum densiflorum Bertol.	0	n	1	0	Scrophulariaceae Juss.
Iz_12_1209	DJI_0798	Verbascum sp.	0	n	1	0	Scrophulariaceae Juss.
Iz_12_1209	DJI_0798	Veronica austriaca L.	0	n	1	0	Plantaginaceae Juss.
Iz_12_1209	DJI_0798	Veronica incana L.	0	n	1	0	Plantaginaceae Juss.
Iz_12_1209	DJI_0798	Veronica verna L.	0	n	1	0	Plantaginaceae Juss.
Iz_12_1209	DJI_0798	Vincetoxicum hirundinaria Medik	0	n	1	0	Apocynaceae Juss.
Iz_12_1209	DJI_0801	Acer tataricum L.	0	n	1	1	Sapindaceae Juss.
Iz_12_1209	DJI_0801	Achillea millefolium L. s.l.	0	n	1	0	Asteraceae Bercht. & J.Presl
Iz_12_1209	DJI_0801	Allium oleraceum L.	0	n	1	0	Amaryllidaceae J.St.-Hil.
Iz_12_1209	DJI_0801	Ambrosia artemisiifolia L.	0	a	0	0	Asteraceae Bercht. & J.Presl
Iz_12_1209	DJI_0801	Amorpha fruticosa L.	0	a	0	0	Fabaceae Lindl.
Iz_12_1209	DJI_0801	Anthericum ramosum L.	0	n	1	0	Asparagaceae Juss.
Iz_12_1209	DJI_0801	Artemisia marschalliana Spreng.	0	n	1	0	Asteraceae Bercht. & J.Presl
Iz_12_1209	DJI_0801	Asparagus officinalis L.	0	n	1	0	Asparagaceae Juss.
Iz_12_1209	DJI_0801	Astragalus cicer L.	0	n	1	0	Fabaceae Lindl.
Iz_12_1209	DJI_0801	Bassia laniflora (S.G.Gmel.) A.J.Scott	0	n	1	0	Amaranthaceae Juss.
Iz_12_1209	DJI_0801	Berberis vulgaris L.	0	n	1	0	Berberidaceae Juss.
Iz_12_1209	DJI_0801	Betula pendula Roth.	5	n	1	1	Betulaceae Gray
Iz_12_1209	DJI_0801	Betula pendula Roth.	5	n	1	0	Poaceae Barnhart
Iz_12_1209	DJI_0801	Bromus inermis Leyss.	0		NA	NA	
Iz_12_1209	DJI_0801	Bromus squarrosus L.	0	a	0	0	Poaceae Barnhart
Iz_12_1209	DJI_0801	Bromus tectorum L.	0	a	0	0	Poaceae Barnhart
Iz_12_1209	DJI_0801	Calamagrostis epigejos (L.) Roth	80	n	1	0	Poaceae Barnhart
Iz_12_1209	DJI_0801	Carduus acanthoides L.	0	n	1	0	Asteraceae Bercht. & J.Presl
Iz_12_1209	DJI_0801	Carex sp.	0	n	1	0	Cyperaceae Juss.
Iz_12_1209	DJI_0801	Centaurea majorovii Dumbadze	0	n	1	0	Asteraceae Bercht. & J.Presl
Iz_12_1209	DJI_0801	Chamaecytisus ruthenicus (Fisch.ex Wol.) Klaskova	0	n	1	0	Fabaceae Lindl.
Iz_12_1209	DJI_0801	Chelidonium majus L.	0	n	1	0	Papaveraceae Juss.
Iz_12_1209	DJI_0801	Chenopodium betaceum Andrz.	0	n	1	0	Amaranthaceae Juss.
Iz_12_1209	DJI_0801	Chenopodium cf. album	0	n	1	0	Amaranthaceae Juss.
Iz_12_1209	DJI_0801	Chenopodium sp.	0		NA	0	Amaranthaceae Juss.
Iz_12_1209	DJI_0801	Chondrilla juncea L.	0	n	1	0	Asteraceae Bercht. & J.Presl
Iz_12_1209	DJI_0801	Cirsium vulgare (Savi) Ten.	0	n	1	0	Asteraceae Bercht. & J.Presl
Iz_12_1209	DJI_0801	Clinopodium acinos (L.) Kuntze	0	n	1	0	Lamiacea Lindl.
Iz_12_1209	DJI_0801	Convallaria majalis L.	0	n	1	0	Asparagaceae Juss.
Iz_12_1209	DJI_0801	Crataegus rhipidophylla Gand.	0	n	1	1	Rosaceae Juss.
Iz_12_1209	DJI_0801	Crepis foetida L.	0	n	1	0	Asteraceae Bercht. & J.Presl
Iz_12_1209	DJI_0801	Dactylis glomerata L.	0	n	1	0	Poaceae Barnhart
Iz_12_1209	DJI_0801	Dianthus campestris M.Bieb.	0	n	1	0	Caryophyllaceae Juss.
Iz_12_1209	DJI_0801	Digitaria sanguinalis (L.) Scop.	0	a	0	0	Poaceae Barnhart
Iz_12_1209	DJI_0801	Elymus repens (L.) Gould	0	n	1	0	Poaceae Barnhart
Iz_12_1209	DJI_0801	Epilobium tetragonum L.	0	n	1	0	Onagraceae Juss.
Iz_12_1209	DJI_0801	Eragrostis minor Host	0	a	0	0	Poaceae Barnhart
Iz_12_1209	DJI_0801	Erigeron canadensis L.	0	a	0	0	Asteraceae Bercht. & J.Presl
Iz_12_1209	DJI_0801	Euphorbia seguieriana Neck.	0	n	1	0	Euphorbiaceae Juss.
Iz_12_1209	DJI_0801	Fallopia dumetorum (L.) Holub	0	n	1	0	Polygonaceae Juss.
Iz_12_1209	DJI_0801	Festuca beckeri (Hack.) Trautv.	0	n	1	0	Poaceae Barnhart
Iz_12_1209	DJI_0801	Fragaria vesca L.	0	n	1	0	Rosaceae Juss.
Iz_12_1209	DJI_0801	Frangula alnus Mill.	0	n	1	1	Rhamnaceae Juss.
Iz_12_1209	DJI_0801	Fraxinus pennsylvanica Marshall	0	a	0	1	Oleaceae Hoffmanns. & Link
Iz_12_1209	DJI_0801	Galeopsis bifida Boenn.	0	n	1	0	Lamiacea Lindl.
Iz_12_1209	DJI_0801	Galium verum L.	0	n	1	0	Rubiaceae Juss.
Iz_12_1209	DJI_0801	Genista tinctoria L.	0	n	1	0	Fabaceae Lindl.
Iz_12_1209	DJI_0801	Geranium robertianum L.	0	n	1	0	Geraniaceae Juss.
Iz_12_1209	DJI_0801	Geranium sanquineum L.	0	n	1	0	Geraniaceae Juss.
Iz_12_1209	DJI_0801	Gypsophila paniculata L.	0	n	1	0	Caryophyllaceae Juss.
Iz_12_1209	DJI_0801	Helichrysum arenarium (L.) Moench	0	n	1	0	Asteraceae Bercht. & J.Presl
Iz_12_1209	DJI_0801	Herniaria polygama J.Gay	0	n	1	0	Caryophyllaceae Juss.
Iz_12_1209	DJI_0801	Hieracium robustum Fr.	0	n	1	0	Asteraceae Bercht. & J.Presl
Iz_12_1209	DJI_0801	Hieracium umbellatum L.	0	n	1	NA	Asteraceae Bercht. & J.Presl
Iz_12_1209	DJI_0801	Hylotelephium maximum (L.) Holub	0	n	1	0	Crassulaceae J.St.-Hil.
Iz_12_1209	DJI_0801	Jacobaea borysthenica (DC.) B.Nord. & Greuter	0	n	1	0	Asteraceae Bercht. & J.Presl
Iz_12_1209	DJI_0801	Jasione montana L.	0	n	1	0	Campanulaceae Juss.
Iz_12_1209	DJI_0801	Jurinea cyanoides (L.) Rchb.s.l.	0	n	1	0	Asteraceae Bercht. & J.Presl
Iz_12_1209	DJI_0801	Koeleria glauca (Spreng.) DC.	0	n	1	0	Poaceae Barnhart
Iz_12_1209	DJI_0801	Lactuca serriola L.	0	a	0	0	Asteraceae Bercht. & J.Presl
Iz_12_1209	DJI_0801	Linaria genistifolia (L.) Mill.	0	n	1	0	Plantaginaceae Juss.
Iz_12_1209	DJI_0801	Lomelosia argentea (L.) Greuter & Burdet	0	n	1	0	Caprifoliaceae Juss.
Iz_12_1209	DJI_0801	Melampyrum pratense L.	0	n	1	0	Orobanchaceae Vent.
Iz_12_1209	DJI_0801	Oenothera biennis L.	0	a	0	0	Onagraceae Juss.
Iz_12_1209	DJI_0801	Pentanema britannica (L.) D.Gut.Larr., Santos-Vicente, Anderb., E.Rico & M.M.Mart.Ort.	0	n	1	0	Asteraceae Bercht. & J.Presl
Iz_12_1209	DJI_0801	Peucedanum oreoselinum (L.) Moench	0	n	1	0	Apiaceae Lindl.
Iz_12_1209	DJI_0801	Peucedanum ruthenicum M.Bieb.	0	n	1	0	Apiaceae Lindl.
Iz_12_1209	DJI_0801	Pilosella echioides (Lumn.) F.W.Schultz & Sch.Bip.	0	n	1	0	Asteraceae Bercht. & J.Presl
Iz_12_1209	DJI_0801	Pilosella officinarum F. Schultz et Sch. Bip.	0	n	1	0	Asteraceae Bercht. & J.Presl
Iz_12_1209	DJI_0801	Pilosella sp.	0		NA	0	Asteraceae Bercht. & J.Presl
Iz_12_1209	DJI_0801	Pinus sylvestris L.	0	n	1	1	Pinaceae Spreng. ex F.Rudolphi
Iz_12_1209	DJI_0801	Poa annua L.	0	n	1	0	Poaceae Barnhart
Iz_12_1209	DJI_0801	Poa nemoralis L.	0	n	1	0	Poaceae Barnhart
Iz_12_1209	DJI_0801	Poa sp.	0		NA	0	Poaceae Barnhart
Iz_12_1209	DJI_0801	Polygonatum odoratum (Mill.) Druce	0	n	1	0	Asparagaceae Juss.
Iz_12_1209	DJI_0801	Polygonum arenarium Waldst. & Kit.	0	n	1	0	Polygonaceae Juss.
Iz_12_1209	DJI_0801	Populus nigra L.	0	n	1	1	Salicaceae Mirb.
Iz_12_1209	DJI_0801	Populus tremula L.	0	n	1	1	Salicaceae Mirb.
Iz_12_1209	DJI_0801	Populus Ã canescens (Aiton) Sm.	0	n	1	1	Salicaceae Mirb.
Iz_12_1209	DJI_0801	Potentilla argentea L.	0	n	1	0	Rosaceae Juss.
Iz_12_1209	DJI_0801	Psammophiliella muralis (L.) Ikonn.	0	n	1	0	Caryophyllaceae Juss.
Iz_12_1209	DJI_0801	Psephellus sumensis (Kalen.) Greuter	0	n	1	0	Asteraceae Bercht. & J.Presl
Iz_12_1209	DJI_0801	Pulsatilla pratensis (L.) Mill. s.l.	0	n	1	0	Ranunculaceae Juss.
Iz_12_1209	DJI_0801	Pyrus communis L.	0	a	0	1	Rosaceae Juss.
Iz_12_1209	DJI_0801	Quercus robur L.	0	n	1	1	Fagaceae Dumort.
Iz_12_1209	DJI_0801	Robinia pseudoacacia L.	0	a	0	1	Fabaceae Lindl.
Iz_12_1209	DJI_0801	Rosa sp.	0		NA	1	Rosaceae Juss.
Iz_12_1209	DJI_0801	Rubus idaeus L.	0	n	1	0	Rosaceae Juss.
Iz_12_1209	DJI_0801	Rumex acetosella L.	0	n	1	0	Polygonaceae Juss.
Iz_12_1209	DJI_0801	Rumex thyrsiflorus Fingerh.	0	n	1	0	Polygonaceae Juss.
Iz_12_1209	DJI_0801	Salix alba L.	0	n	1	1	Salicaceae Mirb.
Iz_12_1209	DJI_0801	Salix sp.	0		NA	1	

Iz_12_1209	DJI_0801	Sambucus nigra L.	0	n	1	1	Viburnaceae Raf.
Iz_12_1209	DJI_0801	Secale sylvestre Host	0	n	1	0	Poaceae Barnhart
Iz_12_1209	DJI_0801	Sempervivum ruthenicum Schnittsp. & C.B. Lehm.	0	n	1	0	Crassulaceae J.St.-Hil.
Iz_12_1209	DJI_0801	Setaria viridis (L.) P. Beauv.	0	n	1	0	Poaceae Barnhart
Iz_12_1209	DJI_0801	Silene borysthenica (Gruner) Walters	0	n	1	0	Caryophyllaceae Juss.
Iz_12_1209	DJI_0801	Silene latifolia subsp. alba (Mill.) Greuter & Burdet	0	n	1	0	Caryophyllaceae Juss.
Iz_12_1209	DJI_0801	Solidago virgaurea L.	0	n	1	0	Asteraceae Bercht. & J.Presl
Iz_12_1209	DJI_0801	Sorbus aucuparia L.	0	n	1	1	Rosaceae Juss.
Iz_12_1209	DJI_0801	Stachys recta L.	0	n	1	0	Lamiacea Lindl.
Iz_12_1209	DJI_0801	Tanacetum vulgare L.	0	n	1	0	Asteraceae Bercht. & J.Presl
Iz_12_1209	DJI_0801	Thymus pallasianus Heinr.Braun	0	n	1	0	Lamiacea Lindl.
Iz_12_1209	DJI_0801	Tilia cordata Mill.	0	n	1	1	Malvaceae Juss.
Iz_12_1209	DJI_0801	Trifolium alpestre L.	0	n	1	0	Fabaceae Lindl.
Iz_12_1209	DJI_0801	Trifolium arvense L.	0	n	1	0	Fabaceae Lindl.
Iz_12_1209	DJI_0801	Ulmus glabra Huds.	0	n	1	1	Ulmaceae Mirb.
Iz_12_1209	DJI_0801	Verbascum densiflorum Bertol.	0	n	1	0	Scrophulariaceae Juss.
Iz_12_1209	DJI_0801	Verbascum sp.	0	n	1	0	Scrophulariaceae Juss.
Iz_12_1209	DJI_0801	Veronica austriaca L.	0	n	1	0	Plantaginaceae Juss.
Iz_12_1209	DJI_0801	Veronica incana L.	0	n	1	0	Plantaginaceae Juss.
Iz_12_1209	DJI_0801	Veronica verna L.	0	n	1	0	Plantaginaceae Juss.
Iz_12_1209	DJI_0801	Vincetoxicum hirundinaria Medik	2	n	1	0	Apocynaceae Juss.
Iz_12_1209	DJI_0805	Acer tataricum L.	0	n	1	1	Sapindaceae Juss.
Iz_12_1209	DJI_0805	Achillea millefolium L. s.l.	0	n	1	0	Asteraceae Bercht. & J.Presl
Iz_12_1209	DJI_0805	Allium oleraceum L.	0	n	1	0	Amaryllidaceae J.St.-Hil.
Iz_12_1209	DJI_0805	Ambrosia artemisiifolia L.	0	a	0	0	Asteraceae Bercht. & J.Presl
Iz_12_1209	DJI_0805	Amorpha fruticosa L.	0	a	0	0	Fabaceae Lindl.
Iz_12_1209	DJI_0805	Anthericum ramosum L.	0	n	1	0	Asparagaceae Juss.
Iz_12_1209	DJI_0805	Artemisia marschalliana Spreng.	0	n	1	0	Asteraceae Bercht. & J.Presl
Iz_12_1209	DJI_0805	Asparagus officinalis L.	0	n	1	0	Asparagaceae Juss.
Iz_12_1209	DJI_0805	Astragalus cicer L.	0	n	1	0	Fabaceae Lindl.
Iz_12_1209	DJI_0805	Bassia laniflora (S.G.Gmel.) A.J.Scott	0	n	1	0	Amaranthaceae Juss.
Iz_12_1209	DJI_0805	Berberis vulgaris L.	0	n	1	0	Berberidaceae Juss.
Iz_12_1209	DJI_0805	Betula pendula Roth.	2	n	1	1	Betulaceae Gray
Iz_12_1209	DJI_0805	Betula pendula Roth.	2	n	1	0	Poaceae Barnhart
Iz_12_1209	DJI_0805	Bromus inermis Leyss.	0		NA	NA	
Iz_12_1209	DJI_0805	Bromus squarrosus L.	0	a	0	0	Poaceae Barnhart
Iz_12_1209	DJI_0805	Bromus tectorum L.	0	a	0	0	Poaceae Barnhart
Iz_12_1209	DJI_0805	Calamagrostis epigejos (L.) Roth	10	n	1	0	Poaceae Barnhart
Iz_12_1209	DJI_0805	Carduus acanthoides L.	0	n	1	0	Asteraceae Bercht. & J.Presl
Iz_12_1209	DJI_0805	Carex sp.	0	n	1	0	Cyperaceae Juss.
Iz_12_1209	DJI_0805	Centaurea majorovii Dumbadze	0	n	1	0	Asteraceae Bercht. & J.Presl
Iz_12_1209	DJI_0805	Chamaecytisus ruthenicus (Fisch.ex Wol.) Klaskova	0	n	1	0	Fabaceae Lindl.
Iz_12_1209	DJI_0805	Chelidonium majus L.	0	n	1	0	Papaveraceae Juss.
Iz_12_1209	DJI_0805	Chenopodium betaceum Andrz.	0	n	1	0	Amaranthaceae Juss.
Iz_12_1209	DJI_0805	Chenopodium cf. album	0	n	1	0	Amaranthaceae Juss.
Iz_12_1209	DJI_0805	Chenopodium sp.	0		NA	0	Amaranthaceae Juss.
Iz_12_1209	DJI_0805	Chondrilla juncea L.	0	n	1	0	Asteraceae Bercht. & J.Presl
Iz_12_1209	DJI_0805	Cirsium vulgare (Savi) Ten.	0	n	1	0	Asteraceae Bercht. & J.Presl
Iz_12_1209	DJI_0805	Clinopodium acinos (L.) Kuntze	0	n	1	0	Lamiacea Lindl.
Iz_12_1209	DJI_0805	Convallaria majalis L.	0	n	1	0	Asparagaceae Juss.
Iz_12_1209	DJI_0805	Crataegus rhipidophylla Gand.	0	n	1	1	Rosaceae Juss.
Iz_12_1209	DJI_0805	Crepis foetida L.	0	n	1	0	Asteraceae Bercht. & J.Presl
Iz_12_1209	DJI_0805	Dactylis glomerata L.	0	n	1	0	Poaceae Barnhart
Iz_12_1209	DJI_0805	Dianthus campestris M.Bieb.	0	n	1	0	Caryophyllaceae Juss.
Iz_12_1209	DJI_0805	Digitaria sanguinalis (L.) Scop.	0	a	0	0	Poaceae Barnhart
Iz_12_1209	DJI_0805	Elymus repens (L.) Gould	0	n	1	0	Poaceae Barnhart
Iz_12_1209	DJI_0805	Epilobium tetragonum L.	0	n	1	0	Onagraceae Juss.
Iz_12_1209	DJI_0805	Eragrostis minor Host	0	a	0	0	Poaceae Barnhart
Iz_12_1209	DJI_0805	Erigeron canadensis L.	8	a	0	0	Asteraceae Bercht. & J.Presl
Iz_12_1209	DJI_0805	Euphorbia seguieriana Neck.	0	n	1	0	Euphorbiaceae Juss.
Iz_12_1209	DJI_0805	Fallopia dumetorum (L.) Holub	0	n	1	0	Polygonaceae Juss.
Iz_12_1209	DJI_0805	Festuca beckeri (Hack.) Trautv.	0	n	1	0	Poaceae Barnhart
Iz_12_1209	DJI_0805	Fragaria vesca L.	0	n	1	0	Rosaceae Juss.
Iz_12_1209	DJI_0805	Frangula alnus Mill.	0	n	1	1	Rhamnaceae Juss.
Iz_12_1209	DJI_0805	Fraxinus pennsylvanica Marshall	0	a	0	1	Oleaceae Hoffmanns. & Link
Iz_12_1209	DJI_0805	Galeopsis bifida Boenn.	0	n	1	0	Lamiacea Lindl.
Iz_12_1209	DJI_0805	Galium verum L.	0	n	1	0	Rubiaceae Juss.
Iz_12_1209	DJI_0805	Genista tinctoria L.	0	n	1	0	Fabaceae Lindl.
Iz_12_1209	DJI_0805	Geranium robertianum L.	0	n	1	0	Geraniaceae Juss.
Iz_12_1209	DJI_0805	Geranium sanquineum L.	0	n	1	0	Geraniaceae Juss.
Iz_12_1209	DJI_0805	Gypsophila paniculata L.	0	n	1	0	Caryophyllaceae Juss.
Iz_12_1209	DJI_0805	Helichrysum arenarium (L.) Moench	0	n	1	0	Asteraceae Bercht. & J.Presl
Iz_12_1209	DJI_0805	Herniaria polygama J.Gay	0	n	1	0	Caryophyllaceae Juss.
Iz_12_1209	DJI_0805	Hieracium robustum Fr.	0	n	1	0	Asteraceae Bercht. & J.Presl
Iz_12_1209	DJI_0805	Hieracium umbellatum L.	0	n	1	NA	Asteraceae Bercht. & J.Presl
Iz_12_1209	DJI_0805	Hylotelephium maximum (L.) Holub	0	n	1	0	Crassulaceae J.St.-Hil.
Iz_12_1209	DJI_0805	Jacobaea borysthenica (DC.) B.Nord. & Greuter	0	n	1	0	Asteraceae Bercht. & J.Presl
Iz_12_1209	DJI_0805	Jasione montana L.	0	n	1	0	Campanulaceae Juss.
Iz_12_1209	DJI_0805	Jurinea cyanoides (L.) Rchb.s.l.	0	n	1	0	Asteraceae Bercht. & J.Presl
Iz_12_1209	DJI_0805	Koeleria glauca (Spreng.) DC.	0	n	1	0	Poaceae Barnhart
Iz_12_1209	DJI_0805	Lactuca serriola L.	0	a	0	0	Asteraceae Bercht. & J.Presl
Iz_12_1209	DJI_0805	Linaria genistifolia (L.) Mill.	0	n	1	0	Plantaginaceae Juss.
Iz_12_1209	DJI_0805	Lomelosia argentea (L.) Greuter & Burdet	0	n	1	0	Caprifoliaceae Juss.
Iz_12_1209	DJI_0805	Melampyrum pratense L.	0	n	1	0	Orobanchaceae Vent.
Iz_12_1209	DJI_0805	Oenothera biennis L.	0	a	0	0	Onagraceae Juss.
Iz_12_1209	DJI_0805	Pentanema britannica (L.) D.Gut.Larr., Santos-Vicente, Anderb., E.Rico & M.M.Mart.Ort.	0	n	1	0	Asteraceae Bercht. & J.Presl
Iz_12_1209	DJI_0805	Peucedanum oreoselinum (L.) Moench	0	n	1	0	Apiaceae Lindl.
Iz_12_1209	DJI_0805	Peucedanum ruthenicum M.Bieb.	0	n	1	0	Apiaceae Lindl.
Iz_12_1209	DJI_0805	Pilosella echioides (Lumn.) F.W.Schultz & Sch.Bip.	0	n	1	0	Asteraceae Bercht. & J.Presl
Iz_12_1209	DJI_0805	Pilosella officinarum F. Schultz et Sch. Bip.	0	n	1	0	Asteraceae Bercht. & J.Presl
Iz_12_1209	DJI_0805	Pilosella sp.	0		NA	0	Asteraceae Bercht. & J.Presl
Iz_12_1209	DJI_0805	Pinus sylvestris L.	0	n	1	1	Pinaceae Spreng. ex F.Rudolphi
Iz_12_1209	DJI_0805	Poa annua L.	0	n	1	0	Poaceae Barnhart
Iz_12_1209	DJI_0805	Poa nemoralis L.	0	n	1	0	Poaceae Barnhart
Iz_12_1209	DJI_0805	Poa sp.	0		NA	0	Poaceae Barnhart
Iz_12_1209	DJI_0805	Polygonatum odoratum (Mill.) Druce	0	n	1	0	Asparagaceae Juss.
Iz_12_1209	DJI_0805	Polygonum arenarium Waldst. & Kit.	0	n	1	0	Polygonaceae Juss.
Iz_12_1209	DJI_0805	Populus nigra L.	0	n	1	1	Salicaceae Mirb.

Iz_12_1209	DJI_0805	Populus tremula L.	0	n	1	1	Salicaceae Mirb.
Iz_12_1209	DJI_0805	Populus Ã canescens (Aiton) Sm.	0	n	1	1	Salicaceae Mirb.
Iz_12_1209	DJI_0805	Potentilla argentea L.	0	n	1	0	Rosaceae Juss.
Iz_12_1209	DJI_0805	Psammophiliella muralis (L.) Ikonn.	0	n	1	0	Caryophyllaceae Juss.
Iz_12_1209	DJI_0805	Psephellus sumensis (Kalen.) Greuter	0	n	1	0	Asteraceae Bercht. & J.Presl
Iz_12_1209	DJI_0805	Pulsatilla pratensis (L.) Mill. s.l.	0	n	1	0	Ranunculaceae Juss.
Iz_12_1209	DJI_0805	Pyrus communis L.	0	a	0	1	Rosaceae Juss.
Iz_12_1209	DJI_0805	Quercus robur L.	0	n	1	1	Fagaceae Dumort.
Iz_12_1209	DJI_0805	Robinia pseudoacacia L.	0	a	0	1	Fabaceae Lindl.
Iz_12_1209	DJI_0805	Rosa sp.	0		NA	1	Rosaceae Juss.
Iz_12_1209	DJI_0805	Rubus idaeus L.	0	n	1	0	Rosaceae Juss.
Iz_12_1209	DJI_0805	Rumex acetosella L.	0	n	1	0	Polygonaceae Juss.
Iz_12_1209	DJI_0805	Rumex thyrsiflorus Fingerh.	0	n	1	0	Polygonaceae Juss.
Iz_12_1209	DJI_0805	Salix alba L.	0	n	1	1	Salicaceae Mirb.
Iz_12_1209	DJI_0805	Salix sp.	0		NA	1	
Iz_12_1209	DJI_0805	Sambucus nigra L.	0	n	1	1	Viburnaceae Raf.
Iz_12_1209	DJI_0805	Secale sylvestre Host	0	n	1	0	Poaceae Barnhart
Iz_12_1209	DJI_0805	Sempervivum ruthenicum Schnittsp. & C.B. Lehm.	0	n	1	0	Crassulaceae J.St.-Hil.
Iz_12_1209	DJI_0805	Setaria viridis (L.) P. Beauv.	0	n	1	0	Poaceae Barnhart
Iz_12_1209	DJI_0805	Silene borysthenica (Gruner) Walters	0	n	1	0	Caryophyllaceae Juss.
Iz_12_1209	DJI_0805	Silene latifolia subsp. alba (Mill.) Greuter & Burdet	0	n	1	0	Caryophyllaceae Juss.
Iz_12_1209	DJI_0805	Solidago virgaurea L.	0	n	1	0	Asteraceae Bercht. & J.Presl
Iz_12_1209	DJI_0805	Sorbus aucuparia L.	0	n	1	1	Rosaceae Juss.
Iz_12_1209	DJI_0805	Stachys recta L.	0	n	1	0	Lamiacea Lindl.
Iz_12_1209	DJI_0805	Tanacetum vulgare L.	0	n	1	0	Asteraceae Bercht. & J.Presl
Iz_12_1209	DJI_0805	Thymus pallasianus Heinr.Braun	0	n	1	0	Lamiacea Lindl.
Iz_12_1209	DJI_0805	Tilia cordata Mill.	0	n	1	1	Malvaceae Juss.
Iz_12_1209	DJI_0805	Trifolium alpestre L.	0	n	1	0	Fabaceae Lindl.
Iz_12_1209	DJI_0805	Trifolium arvense L.	0	n	1	0	Fabaceae Lindl.
Iz_12_1209	DJI_0805	Ulmus glabra Huds.	0	n	1	1	Ulmaceae Mirb.
Iz_12_1209	DJI_0805	Verbascum densiflorum Bertol.	0	n	1	0	Scrophulariaceae Juss.
Iz_12_1209	DJI_0805	Verbascum sp.	0	n	1	0	Scrophulariaceae Juss.
Iz_12_1209	DJI_0805	Veronica austriaca L.	0	n	1	0	Plantaginaceae Juss.
Iz_12_1209	DJI_0805	Veronica incana L.	0	n	1	0	Plantaginaceae Juss.
Iz_12_1209	DJI_0805	Veronica verna L.	0	n	1	0	Plantaginaceae Juss.
Iz_12_1209	DJI_0805	Vincetoxicum hirundinaria Medik	0	n	1	0	Apocynaceae Juss.
Iz_12_1209	DJI_0810	Acer tataricum L.	0	n	1	1	Sapindaceae Juss.
Iz_12_1209	DJI_0810	Achillea millefolium L. s.l.	0	n	1	0	Asteraceae Bercht. & J.Presl
Iz_12_1209	DJI_0810	Allium oleraceum L.	0	n	1	0	Amaryllidaceae J.St.-Hil.
Iz_12_1209	DJI_0810	Ambrosia artemisiifolia L.	0	a	0	0	Asteraceae Bercht. & J.Presl
Iz_12_1209	DJI_0810	Amorpha fruticosa L.	0	a	0	0	Fabaceae Lindl.
Iz_12_1209	DJI_0810	Anthericum ramosum L.	0	n	1	0	Asparagaceae Juss.
Iz_12_1209	DJI_0810	Artemisia marschalliana Spreng.	0	n	1	0	Asteraceae Bercht. & J.Presl
Iz_12_1209	DJI_0810	Asparagus officinalis L.	0	n	1	0	Asparagaceae Juss.
Iz_12_1209	DJI_0810	Astragalus cicer L.	0	n	1	0	Fabaceae Lindl.
Iz_12_1209	DJI_0810	Bassia laniflora (S.G.Gmel.) A.J.Scott	0	n	1	0	Amaranthaceae Juss.
Iz_12_1209	DJI_0810	Berberis vulgaris L.	0	n	1	0	Berberidaceae Juss.
Iz_12_1209	DJI_0810	Betula pendula Roth.	70	n	1	1	Betulaceae Gray
Iz_12_1209	DJI_0810	Betula pendula Roth.	70	n	1	0	Poaceae Barnhart
Iz_12_1209	DJI_0810	Bromus inermis Leyss.	0		NA	NA	
Iz_12_1209	DJI_0810	Bromus squarrosus L.	0	a	0	0	Poaceae Barnhart
Iz_12_1209	DJI_0810	Bromus tectorum L.	0	a	0	0	Poaceae Barnhart
Iz_12_1209	DJI_0810	Calamagrostis epigejos (L.) Roth	40	n	1	0	Poaceae Barnhart
Iz_12_1209	DJI_0810	Carduus acanthoides L.	0	n	1	0	Asteraceae Bercht. & J.Presl
Iz_12_1209	DJI_0810	Carex sp.	0	n	1	0	Cyperaceae Juss.
Iz_12_1209	DJI_0810	Centaurea majorovii Dumbadze	0	n	1	0	Asteraceae Bercht. & J.Presl
Iz_12_1209	DJI_0810	Chamaecytisus ruthenicus (Fisch.ex Wol.) Klaskova	0	n	1	0	Fabaceae Lindl.
Iz_12_1209	DJI_0810	Chelidonium majus L.	0	n	1	0	Papaveraceae Juss.
Iz_12_1209	DJI_0810	Chenopodium betaceum Andrz.	0	n	1	0	Amaranthaceae Juss.
Iz_12_1209	DJI_0810	Chenopodium cf. album	0	n	1	0	Amaranthaceae Juss.
Iz_12_1209	DJI_0810	Chenopodium sp.	0		NA	0	Amaranthaceae Juss.
Iz_12_1209	DJI_0810	Chondrilla juncea L.	0	n	1	0	Asteraceae Bercht. & J.Presl
Iz_12_1209	DJI_0810	Cirsium vulgare (Savi) Ten.	0	n	1	0	Asteraceae Bercht. & J.Presl
Iz_12_1209	DJI_0810	Clinopodium acinos (L.) Kuntze	0	n	1	0	Lamiacea Lindl.
Iz_12_1209	DJI_0810	Convallaria majalis L.	0	n	1	0	Asparagaceae Juss.
Iz_12_1209	DJI_0810	Crataegus rhipidophylla Gand.	0	n	1	1	Rosaceae Juss.
Iz_12_1209	DJI_0810	Crepis foetida L.	0	n	1	0	Asteraceae Bercht. & J.Presl
Iz_12_1209	DJI_0810	Dactylis glomerata L.	0	n	1	0	Poaceae Barnhart
Iz_12_1209	DJI_0810	Dianthus campestris M.Bieb.	0	n	1	0	Caryophyllaceae Juss.
Iz_12_1209	DJI_0810	Digitaria sanguinalis (L.) Scop.	0	a	0	0	Poaceae Barnhart
Iz_12_1209	DJI_0810	Elymus repens (L.) Gould	0	n	1	0	Poaceae Barnhart
Iz_12_1209	DJI_0810	Epilobium tetragonum L.	0	n	1	0	Onagraceae Juss.
Iz_12_1209	DJI_0810	Eragrostis minor Host	0	a	0	0	Poaceae Barnhart
Iz_12_1209	DJI_0810	Erigeron canadensis L.	0	a	0	0	Asteraceae Bercht. & J.Presl
Iz_12_1209	DJI_0810	Euphorbia seguieriana Neck.	0	n	1	0	Euphorbiaceae Juss.
Iz_12_1209	DJI_0810	Fallopia dumetorum (L.) Holub	0	n	1	0	Polygonaceae Juss.
Iz_12_1209	DJI_0810	Festuca beckeri (Hack.) Trautv.	0	n	1	0	Poaceae Barnhart
Iz_12_1209	DJI_0810	Fragaria vesca L.	0	n	1	0	Rosaceae Juss.
Iz_12_1209	DJI_0810	Frangula alnus Mill.	0	n	1	1	Rhamnaceae Juss.
Iz_12_1209	DJI_0810	Fraxinus pennsylvanica Marshall	0	a	0	1	Oleaceae Hoffmanns. & Link
Iz_12_1209	DJI_0810	Galeopsis bifida Boenn.	0	n	1	0	Lamiacea Lindl.
Iz_12_1209	DJI_0810	Galium verum L.	0	n	1	0	Rubiaceae Juss.
Iz_12_1209	DJI_0810	Genista tinctoria L.	0	n	1	0	Fabaceae Lindl.
Iz_12_1209	DJI_0810	Geranium robertianum L.	0	n	1	0	Geraniaceae Juss.
Iz_12_1209	DJI_0810	Geranium sanquineum L.	0	n	1	0	Geraniaceae Juss.
Iz_12_1209	DJI_0810	Gypsophila paniculata L.	0	n	1	0	Caryophyllaceae Juss.
Iz_12_1209	DJI_0810	Helichrysum arenarium (L.) Moench	0	n	1	0	Asteraceae Bercht. & J.Presl
Iz_12_1209	DJI_0810	Herniaria polygama J.Gay	0	n	1	0	Caryophyllaceae Juss.
Iz_12_1209	DJI_0810	Hieracium robustum Fr.	0	n	1	0	Asteraceae Bercht. & J.Presl
Iz_12_1209	DJI_0810	Hieracium umbellatum L.	0	n	1	NA	Asteraceae Bercht. & J.Presl
Iz_12_1209	DJI_0810	Hylotelephium maximum (L.) Holub	0	n	1	0	Crassulaceae J.St.-Hil.
Iz_12_1209	DJI_0810	Jacobaea borysthenica (DC.) B.Nord. & Greuter	0	n	1	0	Asteraceae Bercht. & J.Presl
Iz_12_1209	DJI_0810	Jasione montana L.	0	n	1	0	Campanulaceae Juss.
Iz_12_1209	DJI_0810	Jurinea cyanoides (L.) Rchb.s.l.	0	n	1	0	Asteraceae Bercht. & J.Presl
Iz_12_1209	DJI_0810	Koeleria glauca (Spreng.) DC.	0	n	1	0	Poaceae Barnhart
Iz_12_1209	DJI_0810	Lactuca serriola L.	0	a	0	0	Asteraceae Bercht. & J.Presl
Iz_12_1209	DJI_0810	Linaria genistifolia (L.) Mill.	0	n	1	0	Plantaginaceae Juss.
Iz_12_1209	DJI_0810	Lomelosia argentea (L.) Greuter & Burdet	0	n	1	0	Caprifoliaceae Juss.

Iz_12_1209	DJI_0810	Melampyrum pratense L.	0	n	1	0	Orobanchaceae Vent.
Iz_12_1209	DJI_0810	Oenothera biennis L.	0	a	0	0	Onagraceae Juss.
Iz_12_1209	DJI_0810	Pentanema britannica (L.) D.Gut.Larr., Santos-Vicente, Anderb., E.Rico & M.M.Mart.Ort.	0	n	1	0	Asteraceae Bercht. & J.Presl
Iz_12_1209	DJI_0810	Peucedanum oreoselinum (L.) Moench	0	n	1	0	Apiaceae Lindl.
Iz_12_1209	DJI_0810	Peucedanum ruthenicum M.Bieb.	0	n	1	0	Apiaceae Lindl.
Iz_12_1209	DJI_0810	Pilosella echioides (Lumn.) F.W.Schultz & Sch.Bip.	0	n	1	0	Asteraceae Bercht. & J.Presl
Iz_12_1209	DJI_0810	Pilosella officinarum F. Schultz et Sch. Bip.	0	n	1	0	Asteraceae Bercht. & J.Presl
Iz_12_1209	DJI_0810	Pilosella sp.	0		NA	0	Asteraceae Bercht. & J.Presl
Iz_12_1209	DJI_0810	Pinus sylvestris L.	0	n	1	1	Pinaceae Spreng. ex F.Rudolphi
Iz_12_1209	DJI_0810	Poa annua L.	0	n	1	0	Poaceae Barnhart
Iz_12_1209	DJI_0810	Poa nemoralis L.	0	n	1	0	Poaceae Barnhart
Iz_12_1209	DJI_0810	Poa sp.	0		NA	0	Poaceae Barnhart
Iz_12_1209	DJI_0810	Polygonatum odoratum (Mill.) Druce	0	n	1	0	Asparagaceae Juss.
Iz_12_1209	DJI_0810	Polygonum arenarium Waldst. & Kit.	0	n	1	0	Polygonaceae Juss.
Iz_12_1209	DJI_0810	Populus nigra L.	0	n	1	1	Salicaceae Mirb.
Iz_12_1209	DJI_0810	Populus tremula L.	0	n	1	1	Salicaceae Mirb.
Iz_12_1209	DJI_0810	Populus Ã canescens (Aiton) Sm.	0	n	1	1	Salicaceae Mirb.
Iz_12_1209	DJI_0810	Potentilla argentea L.	0	n	1	0	Rosaceae Juss.
Iz_12_1209	DJI_0810	Psammophiliella muralis (L.) Ikonn.	0	n	1	0	Caryophyllaceae Juss.
Iz_12_1209	DJI_0810	Psephellus sumensis (Kalen.) Greuter	0	n	1	0	Asteraceae Bercht. & J.Presl
Iz_12_1209	DJI_0810	Pulsatilla pratensis (L.) Mill. s.l.	0	n	1	0	Ranunculaceae Juss.
Iz_12_1209	DJI_0810	Pyrus communis L.	0	a	0	1	Rosaceae Juss.
Iz_12_1209	DJI_0810	Quercus robur L.	0	n	1	1	Fagaceae Dumort.
Iz_12_1209	DJI_0810	Robinia pseudoacacia L.	0	a	0	1	Fabaceae Lindl.
Iz_12_1209	DJI_0810	Rosa sp.	0		NA	1	Rosaceae Juss.
Iz_12_1209	DJI_0810	Rubus idaeus L.	0	n	1	0	Rosaceae Juss.
Iz_12_1209	DJI_0810	Rumex acetosella L.	0	n	1	0	Polygonaceae Juss.
Iz_12_1209	DJI_0810	Rumex thyrsiflorus Fingerh.	0	n	1	0	Polygonaceae Juss.
Iz_12_1209	DJI_0810	Salix alba L.	0	n	1	1	Salicaceae Mirb.
Iz_12_1209	DJI_0810	Salix sp.	0		NA	1	
Iz_12_1209	DJI_0810	Sambucus nigra L.	0	n	1	1	Viburnaceae Raf.
Iz_12_1209	DJI_0810	Secale sylvestre Host	0	n	1	0	Poaceae Barnhart
Iz_12_1209	DJI_0810	Sempervivum ruthenicum Schnittsp. & C.B. Lehm.	0	n	1	0	Crassulaceae J.St.-Hil.
Iz_12_1209	DJI_0810	Setaria viridis (L.) P. Beauv.	0	n	1	0	Poaceae Barnhart
Iz_12_1209	DJI_0810	Silene borysthenica (Gruner) Walters	0	n	1	0	Caryophyllaceae Juss.
Iz_12_1209	DJI_0810	Silene latifolia subsp. alba (Mill.) Greuter & Burdet	0	n	1	0	Caryophyllaceae Juss.
Iz_12_1209	DJI_0810	Solidago virgaurea L.	0	n	1	0	Asteraceae Bercht. & J.Presl
Iz_12_1209	DJI_0810	Sorbus aucuparia L.	0	n	1	1	Rosaceae Juss.
Iz_12_1209	DJI_0810	Stachys recta L.	0	n	1	0	Lamiacea Lindl.
Iz_12_1209	DJI_0810	Tanacetum vulgare L.	0	n	1	0	Asteraceae Bercht. & J.Presl
Iz_12_1209	DJI_0810	Thymus pallasianus Heinr.Braun	0	n	1	0	Lamiacea Lindl.
Iz_12_1209	DJI_0810	Tilia cordata Mill.	0	n	1	1	Malvaceae Juss.
Iz_12_1209	DJI_0810	Trifolium alpestre L.	0	n	1	0	Fabaceae Lindl.
Iz_12_1209	DJI_0810	Trifolium arvense L.	0	n	1	0	Fabaceae Lindl.
Iz_12_1209	DJI_0810	Ulmus glabra Huds.	0	n	1	1	Ulmaceae Mirb.
Iz_12_1209	DJI_0810	Verbascum densiflorum Bertol.	0	n	1	0	Scrophulariaceae Juss.
Iz_12_1209	DJI_0810	Verbascum sp.	0	n	1	0	Scrophulariaceae Juss.
Iz_12_1209	DJI_0810	Veronica austriaca L.	0	n	1	0	Plantaginaceae Juss.
Iz_12_1209	DJI_0810	Veronica incana L.	0	n	1	0	Plantaginaceae Juss.
Iz_12_1209	DJI_0810	Veronica verna L.	0	n	1	0	Plantaginaceae Juss.
Iz_12_1209	DJI_0810	Vincetoxicum hirundinaria Medik	0	n	1	0	Apocynaceae Juss.
Iz_12_1209	DJI_0814	Acer tataricum L.	0	n	1	1	Sapindaceae Juss.
Iz_12_1209	DJI_0814	Achillea millefolium L. s.l.	0	n	1	0	Asteraceae Bercht. & J.Presl
Iz_12_1209	DJI_0814	Allium oleraceum L.	0	n	1	0	Amaryllidaceae J.St.-Hil.
Iz_12_1209	DJI_0814	Ambrosia artemisiifolia L.	0	a	0	0	Asteraceae Bercht. & J.Presl
Iz_12_1209	DJI_0814	Amorpha fruticosa L.	0	a	0	0	Fabaceae Lindl.
Iz_12_1209	DJI_0814	Anthericum ramosum L.	0	n	1	0	Asparagaceae Juss.
Iz_12_1209	DJI_0814	Artemisia marschalliana Spreng.	0	n	1	0	Asteraceae Bercht. & J.Presl
Iz_12_1209	DJI_0814	Asparagus officinalis L.	0	n	1	0	Asparagaceae Juss.
Iz_12_1209	DJI_0814	Astragalus cicer L.	0	n	1	0	Fabaceae Lindl.
Iz_12_1209	DJI_0814	Bassia laniflora (S.G.Gmel.) A.J.Scott	0	n	1	0	Amaranthaceae Juss.
Iz_12_1209	DJI_0814	Berberis vulgaris L.	0	n	1	0	Berberidaceae Juss.
Iz_12_1209	DJI_0814	Betula pendula Roth.	70	n	1	1	Betulaceae Gray
Iz_12_1209	DJI_0814	Betula pendula Roth.	70	n	1	0	Poaceae Barnhart
Iz_12_1209	DJI_0814	Bromus inermis Leyss.	0		NA	NA	
Iz_12_1209	DJI_0814	Bromus squarrosus L.	0	a	0	0	Poaceae Barnhart
Iz_12_1209	DJI_0814	Bromus tectorum L.	0	a	0	0	Poaceae Barnhart
Iz_12_1209	DJI_0814	Calamagrostis epigejos (L.) Roth	30	n	1	0	Poaceae Barnhart
Iz_12_1209	DJI_0814	Carduus acanthoides L.	0	n	1	0	Asteraceae Bercht. & J.Presl
Iz_12_1209	DJI_0814	Carex sp.	0	n	1	0	Cyperaceae Juss.
Iz_12_1209	DJI_0814	Centaurea majorovii Dumbadze	0	n	1	0	Asteraceae Bercht. & J.Presl
Iz_12_1209	DJI_0814	Chamaecytisus ruthenicus (Fisch.ex Wol.) Klaskova	0	n	1	0	Fabaceae Lindl.
Iz_12_1209	DJI_0814	Chelidonium majus L.	0	n	1	0	Papaveraceae Juss.
Iz_12_1209	DJI_0814	Chenopodium betaceum Andrz.	0	n	1	0	Amaranthaceae Juss.
Iz_12_1209	DJI_0814	Chenopodium cf. album	0	n	1	0	Amaranthaceae Juss.
Iz_12_1209	DJI_0814	Chenopodium sp.	0		NA	0	Amaranthaceae Juss.
Iz_12_1209	DJI_0814	Chondrilla juncea L.	0	n	1	0	Asteraceae Bercht. & J.Presl
Iz_12_1209	DJI_0814	Cirsium vulgare (Savi) Ten.	0	n	1	0	Asteraceae Bercht. & J.Presl
Iz_12_1209	DJI_0814	Clinopodium acinos (L.) Kuntze	0	n	1	0	Lamiacea Lindl.
Iz_12_1209	DJI_0814	Convallaria majalis L.	0	n	1	0	Asparagaceae Juss.
Iz_12_1209	DJI_0814	Crataegus rhipidophylla Gand.	0	n	1	1	Rosaceae Juss.
Iz_12_1209	DJI_0814	Crepis foetida L.	0	n	1	0	Asteraceae Bercht. & J.Presl
Iz_12_1209	DJI_0814	Dactylis glomerata L.	0	n	1	0	Poaceae Barnhart
Iz_12_1209	DJI_0814	Dianthus campestris M.Bieb.	0	n	1	0	Caryophyllaceae Juss.
Iz_12_1209	DJI_0814	Digitaria sanguinalis (L.) Scop.	0	a	0	0	Poaceae Barnhart
Iz_12_1209	DJI_0814	Elymus repens (L.) Gould	0	n	1	0	Poaceae Barnhart
Iz_12_1209	DJI_0814	Epilobium tetragonum L.	0	n	1	0	Onagraceae Juss.
Iz_12_1209	DJI_0814	Eragrostis minor Host	0	a	0	0	Poaceae Barnhart
Iz_12_1209	DJI_0814	Erigeron canadensis L.	0	a	0	0	Asteraceae Bercht. & J.Presl
Iz_12_1209	DJI_0814	Euphorbia seguieriana Neck.	0	n	1	0	Euphorbiaceae Juss.
Iz_12_1209	DJI_0814	Fallopia dumetorum (L.) Holub	0	n	1	0	Polygonaceae Juss.
Iz_12_1209	DJI_0814	Festuca beckeri (Hack.) Trautv.	0	n	1	0	Poaceae Barnhart
Iz_12_1209	DJI_0814	Fragaria vesca L.	0	n	1	0	Rosaceae Juss.
Iz_12_1209	DJI_0814	Frangula alnus Mill.	0	n	1	1	Rhamnaceae Juss.
Iz_12_1209	DJI_0814	Fraxinus pennsylvanica Marshall	0	a	0	1	Oleaceae Hoffmanns. & Link
Iz_12_1209	DJI_0814	Galeopsis bifida Boenn.	0	n	1	0	Lamiacea Lindl.
Iz_12_1209	DJI_0814	Galium verum L.	0	n	1	0	Rubiaceae Juss.
Iz_12_1209	DJI_0814	Genista tinctoria L.	0	n	1	0	Fabaceae Lindl.

Iz_12_1209	DJI_0814	Geranium robertianum L.	0	n	1	0	Geraniaceae Juss.
Iz_12_1209	DJI_0814	Geranium sanquineum L.	0	n	1	0	Geraniaceae Juss.
Iz_12_1209	DJI_0814	Gypsophila paniculata L.	0	n	1	0	Caryophyllaceae Juss.
Iz_12_1209	DJI_0814	Helichrysum arenarium (L.) Moench	0	n	1	0	Asteraceae Bercht. & J.Presl
Iz_12_1209	DJI_0814	Herniaria polygama J.Gay	0	n	1	0	Caryophyllaceae Juss.
Iz_12_1209	DJI_0814	Hieracium robustum Fr.	0	n	1	0	Asteraceae Bercht. & J.Presl
Iz_12_1209	DJI_0814	Hieracium umbellatum L.	0	n	1	NA	Asteraceae Bercht. & J.Presl
Iz_12_1209	DJI_0814	Hylotelephium maximum (L.) Holub	0	n	1	0	Crassulaceae J.St.-Hil.
Iz_12_1209	DJI_0814	Jacobaea borysthenica (DC.) B.Nord. & Greuter	0	n	1	0	Asteraceae Bercht. & J.Presl
Iz_12_1209	DJI_0814	Jasione montana L.	0	n	1	0	Campanulaceae Juss.
Iz_12_1209	DJI_0814	Jurinea cyanoides (L.) Rchb.s.l.	0	n	1	0	Asteraceae Bercht. & J.Presl
Iz_12_1209	DJI_0814	Koeleria glauca (Spreng.) DC.	0	n	1	0	Poaceae Barnhart
Iz_12_1209	DJI_0814	Lactuca serriola L.	0	a	0	0	Asteraceae Bercht. & J.Presl
Iz_12_1209	DJI_0814	Linaria genistifolia (L.) Mill.	0	n	1	0	Plantaginaceae Juss.
Iz_12_1209	DJI_0814	Lomelosia argentea (L.) Greuter & Burdet	0	n	1	0	Caprifoliaceae Juss.
Iz_12_1209	DJI_0814	Melampyrum pratense L.	0	n	1	0	Orobanchaceae Vent.
Iz_12_1209	DJI_0814	Oenothera biennis L.	0	a	0	0	Onagraceae Juss.
Iz_12_1209	DJI_0814	Pentanema britannica (L.) D.Gut.Larr., Santos-Vicente, Anderb., E.Rico & M.M.Mart.Ort.	0	n	1	0	Asteraceae Bercht. & J.Presl
Iz_12_1209	DJI_0814	Peucedanum oreoselinum (L.) Moench	0	n	1	0	Apiaceae Lindl.
Iz_12_1209	DJI_0814	Peucedanum ruthenicum M.Bieb.	0	n	1	0	Apiaceae Lindl.
Iz_12_1209	DJI_0814	Pilosella echioides (Lumn.) F.W.Schultz & Sch.Bip.	0	n	1	0	Asteraceae Bercht. & J.Presl
Iz_12_1209	DJI_0814	Pilosella officinarum F. Schultz et Sch. Bip.	0	n	1	0	Asteraceae Bercht. & J.Presl
Iz_12_1209	DJI_0814	Pilosella sp.	0		NA	0	Asteraceae Bercht. & J.Presl
Iz_12_1209	DJI_0814	Pinus sylvestris L.	0	n	1	1	Pinaceae Spreng. ex F.Rudolphi
Iz_12_1209	DJI_0814	Poa annua L.	0	n	1	0	Poaceae Barnhart
Iz_12_1209	DJI_0814	Poa nemoralis L.	0	n	1	0	Poaceae Barnhart
Iz_12_1209	DJI_0814	Poa sp.	0		NA	0	Poaceae Barnhart
Iz_12_1209	DJI_0814	Polygonatum odoratum (Mill.) Druce	0	n	1	0	Asparagaceae Juss.
Iz_12_1209	DJI_0814	Polygonum arenarium Waldst. & Kit.	0	n	1	0	Polygonaceae Juss.
Iz_12_1209	DJI_0814	Populus nigra L.	0	n	1	1	Salicaceae Mirb.
Iz_12_1209	DJI_0814	Populus tremula L.	3	n	1	1	Salicaceae Mirb.
Iz_12_1209	DJI_0814	Populus Ã canescens (Aiton) Sm.	0	n	1	1	Salicaceae Mirb.
Iz_12_1209	DJI_0814	Potentilla argentea L.	0	n	1	0	Rosaceae Juss.
Iz_12_1209	DJI_0814	Psammophiliella muralis (L.) Ikonn.	0	n	1	0	Caryophyllaceae Juss.
Iz_12_1209	DJI_0814	Psephellus sumensis (Kalen.) Greuter	0	n	1	0	Asteraceae Bercht. & J.Presl
Iz_12_1209	DJI_0814	Pulsatilla pratensis (L.) Mill. s.l.	0	n	1	0	Ranunculaceae Juss.
Iz_12_1209	DJI_0814	Pyrus communis L.	0	a	0	1	Rosaceae Juss.
Iz_12_1209	DJI_0814	Quercus robur L.	0	n	1	1	Fagaceae Dumort.
Iz_12_1209	DJI_0814	Robinia pseudoacacia L.	0	a	0	1	Fabaceae Lindl.
Iz_12_1209	DJI_0814	Rosa sp.	0		NA	1	Rosaceae Juss.
Iz_12_1209	DJI_0814	Rubus idaeus L.	0	n	1	0	Rosaceae Juss.
Iz_12_1209	DJI_0814	Rumex acetosella L.	0	n	1	0	Polygonaceae Juss.
Iz_12_1209	DJI_0814	Rumex thyrsiflorus Fingerh.	0	n	1	0	Polygonaceae Juss.
Iz_12_1209	DJI_0814	Salix alba L.	0	n	1	1	Salicaceae Mirb.
Iz_12_1209	DJI_0814	Salix sp.	0		NA	1	
Iz_12_1209	DJI_0814	Sambucus nigra L.	0	n	1	1	Viburnaceae Raf.
Iz_12_1209	DJI_0814	Secale sylvestre Host	0	n	1	0	Poaceae Barnhart
Iz_12_1209	DJI_0814	Sempervivum ruthenicum Schnittsp. & C.B. Lehm.	0	n	1	0	Crassulaceae J.St.-Hil.
Iz_12_1209	DJI_0814	Setaria viridis (L.) P. Beauv.	0	n	1	0	Poaceae Barnhart
Iz_12_1209	DJI_0814	Silene borysthenica (Gruner) Walters	0	n	1	0	Caryophyllaceae Juss.
Iz_12_1209	DJI_0814	Silene latifolia subsp. alba (Mill.) Greuter & Burdet	0	n	1	0	Caryophyllaceae Juss.
Iz_12_1209	DJI_0814	Solidago virgaurea L.	0	n	1	0	Asteraceae Bercht. & J.Presl
Iz_12_1209	DJI_0814	Sorbus aucuparia L.	0	n	1	1	Rosaceae Juss.
Iz_12_1209	DJI_0814	Stachys recta L.	0	n	1	0	Lamiacea Lindl.
Iz_12_1209	DJI_0814	Tanacetum vulgare L.	0	n	1	0	Asteraceae Bercht. & J.Presl
Iz_12_1209	DJI_0814	Thymus pallasianus Heinr.Braun	0	n	1	0	Lamiacea Lindl.
Iz_12_1209	DJI_0814	Tilia cordata Mill.	0	n	1	1	Malvaceae Juss.
Iz_12_1209	DJI_0814	Trifolium alpestre L.	0	n	1	0	Fabaceae Lindl.
Iz_12_1209	DJI_0814	Trifolium arvense L.	0	n	1	0	Fabaceae Lindl.
Iz_12_1209	DJI_0814	Ulmus glabra Huds.	0	n	1	1	Ulmaceae Mirb.
Iz_12_1209	DJI_0814	Verbascum densiflorum Bertol.	0	n	1	0	Scrophulariaceae Juss.
Iz_12_1209	DJI_0814	Verbascum sp.	0	n	1	0	Scrophulariaceae Juss.
Iz_12_1209	DJI_0814	Veronica austriaca L.	0	n	1	0	Plantaginaceae Juss.
Iz_12_1209	DJI_0814	Veronica incana L.	0	n	1	0	Plantaginaceae Juss.
Iz_12_1209	DJI_0814	Veronica verna L.	0	n	1	0	Plantaginaceae Juss.
Iz_12_1209	DJI_0814	Vincetoxicum hirundinaria Medik	0	n	1	0	Apocynaceae Juss.
Iz_12_1209	DJI_0817	Acer tataricum L.	0	n	1	1	Sapindaceae Juss.
Iz_12_1209	DJI_0817	Achillea millefolium L. s.l.	0	n	1	0	Asteraceae Bercht. & J.Presl
Iz_12_1209	DJI_0817	Allium oleraceum L.	0	n	1	0	Amaryllidaceae J.St.-Hil.
Iz_12_1209	DJI_0817	Ambrosia artemisiifolia L.	0	a	0	0	Asteraceae Bercht. & J.Presl
Iz_12_1209	DJI_0817	Amorpha fruticosa L.	0	a	0	0	Fabaceae Lindl.
Iz_12_1209	DJI_0817	Anthericum ramosum L.	0	n	1	0	Asparagaceae Juss.
Iz_12_1209	DJI_0817	Artemisia marschalliana Spreng.	0	n	1	0	Asteraceae Bercht. & J.Presl
Iz_12_1209	DJI_0817	Asparagus officinalis L.	0	n	1	0	Asparagaceae Juss.
Iz_12_1209	DJI_0817	Astragalus cicer L.	0	n	1	0	Fabaceae Lindl.
Iz_12_1209	DJI_0817	Bassia laniflora (S.G.Gmel.) A.J.Scott	0	n	1	0	Amaranthaceae Juss.
Iz_12_1209	DJI_0817	Berberis vulgaris L.	0	n	1	0	Berberidaceae Juss.
Iz_12_1209	DJI_0817	Betula pendula Roth.	0	n	1	1	Betulaceae Gray
Iz_12_1209	DJI_0817	Betula pendula Roth.	0	n	1	0	Poaceae Barnhart
Iz_12_1209	DJI_0817	Bromus inermis Leyss.	0		NA	NA	
Iz_12_1209	DJI_0817	Bromus squarrosus L.	0	a	0	0	Poaceae Barnhart
Iz_12_1209	DJI_0817	Bromus tectorum L.	0	a	0	0	Poaceae Barnhart
Iz_12_1209	DJI_0817	Calamagrostis epigejos (L.) Roth	0	n	1	0	Poaceae Barnhart
Iz_12_1209	DJI_0817	Carduus acanthoides L.	0	n	1	0	Asteraceae Bercht. & J.Presl
Iz_12_1209	DJI_0817	Carex sp.	0	n	1	0	Cyperaceae Juss.
Iz_12_1209	DJI_0817	Centaurea majorovii Dumbadze	0	n	1	0	Asteraceae Bercht. & J.Presl
Iz_12_1209	DJI_0817	Chamaecytisus ruthenicus (Fisch.ex Wol.) Klaskova	0	n	1	0	Fabaceae Lindl.
Iz_12_1209	DJI_0817	Chelidonium majus L.	0	n	1	0	Papaveraceae Juss.
Iz_12_1209	DJI_0817	Chenopodium betaceum Andrz.	0	n	1	0	Amaranthaceae Juss.
Iz_12_1209	DJI_0817	Chenopodium cf. album	0	n	1	0	Amaranthaceae Juss.
Iz_12_1209	DJI_0817	Chenopodium sp.	0		NA	0	Amaranthaceae Juss.
Iz_12_1209	DJI_0817	Chondrilla juncea L.	0	n	1	0	Asteraceae Bercht. & J.Presl
Iz_12_1209	DJI_0817	Cirsium vulgare (Savi) Ten.	0	n	1	0	Asteraceae Bercht. & J.Presl
Iz_12_1209	DJI_0817	Clinopodium acinos (L.) Kuntze	0	n	1	0	Lamiacea Lindl.
Iz_12_1209	DJI_0817	Convallaria majalis L.	0	n	1	0	Asparagaceae Juss.
Iz_12_1209	DJI_0817	Crataegus rhipidophylla Gand.	0	n	1	1	Rosaceae Juss.
Iz_12_1209	DJI_0817	Crepis foetida L.	0	n	1	0	Asteraceae Bercht. & J.Presl
Iz_12_1209	DJI_0817	Dactylis glomerata L.	0	n	1	0	Poaceae Barnhart

Iz_12_1209	DJI_0817	Dianthus campestris M.Bieb.	0	n	1	0	Caryophyllaceae Juss.
Iz_12_1209	DJI_0817	Digitaria sanguinalis (L.) Scop.	0	a	0	0	Poaceae Barnhart
Iz_12_1209	DJI_0817	Elymus repens (L.) Gould	0	n	1	0	Poaceae Barnhart
Iz_12_1209	DJI_0817	Epilobium tetragonum L.	0	n	1	0	Onagraceae Juss.
Iz_12_1209	DJI_0817	Eragrostis minor Host	0	a	0	0	Poaceae Barnhart
Iz_12_1209	DJI_0817	Erigeron canadensis L.	10	a	0	0	Asteraceae Bercht. & J.Presl
Iz_12_1209	DJI_0817	Euphorbia seguieriana Neck.	0	n	1	0	Euphorbiaceae Juss.
Iz_12_1209	DJI_0817	Fallopia dumetorum (L.) Holub	0	n	1	0	Polygonaceae Juss.
Iz_12_1209	DJI_0817	Festuca beckeri (Hack.) Trautv.	0	n	1	0	Poaceae Barnhart
Iz_12_1209	DJI_0817	Fragaria vesca L.	0	n	1	0	Rosaceae Juss.
Iz_12_1209	DJI_0817	Frangula alnus Mill.	0	n	1	1	Rhamnaceae Juss.
Iz_12_1209	DJI_0817	Fraxinus pennsylvanica Marshall	0	a	0	1	Oleaceae Hoffmanns. & Link
Iz_12_1209	DJI_0817	Galeopsis bifida Boenn.	0	n	1	0	Lamiacea Lindl.
Iz_12_1209	DJI_0817	Galium verum L.	0	n	1	0	Rubiaceae Juss.
Iz_12_1209	DJI_0817	Genista tinctoria L.	0	n	1	0	Fabaceae Lindl.
Iz_12_1209	DJI_0817	Geranium robertianum L.	0	n	1	0	Geraniaceae Juss.
Iz_12_1209	DJI_0817	Geranium sanquineum L.	0	n	1	0	Geraniaceae Juss.
Iz_12_1209	DJI_0817	Gypsophila paniculata L.	0	n	1	0	Caryophyllaceae Juss.
Iz_12_1209	DJI_0817	Helichrysum arenarium (L.) Moench	0	n	1	0	Asteraceae Bercht. & J.Presl
Iz_12_1209	DJI_0817	Herniaria polygama J.Gay	0	n	1	0	Caryophyllaceae Juss.
Iz_12_1209	DJI_0817	Hieracium robustum Fr.	0	n	1	0	Asteraceae Bercht. & J.Presl
Iz_12_1209	DJI_0817	Hieracium umbellatum L.	0	n	1	NA	Asteraceae Bercht. & J.Presl
Iz_12_1209	DJI_0817	Hylotelephium maximum (L.) Holub	0	n	1	0	Crassulaceae J.St.-Hil.
Iz_12_1209	DJI_0817	Jacobaea borysthenica (DC.) B.Nord. & Greuter	0	n	1	0	Asteraceae Bercht. & J.Presl
Iz_12_1209	DJI_0817	Jasione montana L.	0	n	1	0	Campanulaceae Juss.
Iz_12_1209	DJI_0817	Jurinea cyanoides (L.) Rchb.s.l.	0	n	1	0	Asteraceae Bercht. & J.Presl
Iz_12_1209	DJI_0817	Koeleria glauca (Spreng.) DC.	0	n	1	0	Poaceae Barnhart
Iz_12_1209	DJI_0817	Lactuca serriola L.	0	a	0	0	Asteraceae Bercht. & J.Presl
Iz_12_1209	DJI_0817	Linaria genistifolia (L.) Mill.	0	n	1	0	Plantaginaceae Juss.
Iz_12_1209	DJI_0817	Lomelosia argentea (L.) Greuter & Burdet	0	n	1	0	Caprifoliaceae Juss.
Iz_12_1209	DJI_0817	Melampyrum pratense L.	0	n	1	0	Orobanchaceae Vent.
Iz_12_1209	DJI_0817	Oenothera biennis L.	0	a	0	0	Onagraceae Juss.
Iz_12_1209	DJI_0817	Pentanema britannica (L.) D.Gut.Larr., Santos-Vicente, Anderb., E.Rico & M.M.Mart.Ort.	0	n	1	0	Asteraceae Bercht. & J.Presl
Iz_12_1209	DJI_0817	Peucedanum oreoselinum (L.) Moench	0	n	1	0	Apiaceae Lindl.
Iz_12_1209	DJI_0817	Peucedanum ruthenicum M.Bieb.	0	n	1	0	Apiaceae Lindl.
Iz_12_1209	DJI_0817	Pilosella echioides (Lumn.) F.W.Schultz & Sch.Bip.	0	n	1	0	Asteraceae Bercht. & J.Presl
Iz_12_1209	DJI_0817	Pilosella officinarum F. Schultz et Sch. Bip.	0	n	1	0	Asteraceae Bercht. & J.Presl
Iz_12_1209	DJI_0817	Pilosella sp.	0		NA	0	Asteraceae Bercht. & J.Presl
Iz_12_1209	DJI_0817	Pinus sylvestris L.	0	n	1	1	Pinaceae Spreng. ex F.Rudolphi
Iz_12_1209	DJI_0817	Poa annua L.	0	n	1	0	Poaceae Barnhart
Iz_12_1209	DJI_0817	Poa nemoralis L.	0	n	1	0	Poaceae Barnhart
Iz_12_1209	DJI_0817	Poa sp.	0		NA	0	Poaceae Barnhart
Iz_12_1209	DJI_0817	Polygonatum odoratum (Mill.) Druce	0	n	1	0	Asparagaceae Juss.
Iz_12_1209	DJI_0817	Polygonum arenarium Waldst. & Kit.	0	n	1	0	Polygonaceae Juss.
Iz_12_1209	DJI_0817	Populus nigra L.	0	n	1	1	Salicaceae Mirb.
Iz_12_1209	DJI_0817	Populus tremula L.	0	n	1	1	Salicaceae Mirb.
Iz_12_1209	DJI_0817	Populus Ã canescens (Aiton) Sm.	0	n	1	1	Salicaceae Mirb.
Iz_12_1209	DJI_0817	Potentilla argentea L.	0	n	1	0	Rosaceae Juss.
Iz_12_1209	DJI_0817	Psammophiliella muralis (L.) Ikonn.	0	n	1	0	Caryophyllaceae Juss.
Iz_12_1209	DJI_0817	Psephellus sumensis (Kalen.) Greuter	0	n	1	0	Asteraceae Bercht. & J.Presl
Iz_12_1209	DJI_0817	Pulsatilla pratensis (L.) Mill. s.l.	0	n	1	0	Ranunculaceae Juss.
Iz_12_1209	DJI_0817	Pyrus communis L.	0	a	0	1	Rosaceae Juss.
Iz_12_1209	DJI_0817	Quercus robur L.	0	n	1	1	Fagaceae Dumort.
Iz_12_1209	DJI_0817	Robinia pseudoacacia L.	0	a	0	1	Fabaceae Lindl.
Iz_12_1209	DJI_0817	Rosa sp.	0		NA	1	Rosaceae Juss.
Iz_12_1209	DJI_0817	Rubus idaeus L.	0	n	1	0	Rosaceae Juss.
Iz_12_1209	DJI_0817	Rumex acetosella L.	0	n	1	0	Polygonaceae Juss.
Iz_12_1209	DJI_0817	Rumex thyrsiflorus Fingerh.	0	n	1	0	Polygonaceae Juss.
Iz_12_1209	DJI_0817	Salix alba L.	0	n	1	1	Salicaceae Mirb.
Iz_12_1209	DJI_0817	Salix sp.	0		NA	1	
Iz_12_1209	DJI_0817	Sambucus nigra L.	0	n	1	1	Viburnaceae Raf.
Iz_12_1209	DJI_0817	Secale sylvestre Host	0	n	1	0	Poaceae Barnhart
Iz_12_1209	DJI_0817	Sempervivum ruthenicum Schnittsp. & C.B. Lehm.	0	n	1	0	Crassulaceae J.St.-Hil.
Iz_12_1209	DJI_0817	Setaria viridis (L.) P. Beauv.	0	n	1	0	Poaceae Barnhart
Iz_12_1209	DJI_0817	Silene borysthenica (Gruner) Walters	0	n	1	0	Caryophyllaceae Juss.
Iz_12_1209	DJI_0817	Silene latifolia subsp. alba (Mill.) Greuter & Burdet	0	n	1	0	Caryophyllaceae Juss.
Iz_12_1209	DJI_0817	Solidago virgaurea L.	0	n	1	0	Asteraceae Bercht. & J.Presl
Iz_12_1209	DJI_0817	Sorbus aucuparia L.	0	n	1	1	Rosaceae Juss.
Iz_12_1209	DJI_0817	Stachys recta L.	0	n	1	0	Lamiacea Lindl.
Iz_12_1209	DJI_0817	Tanacetum vulgare L.	0	n	1	0	Asteraceae Bercht. & J.Presl
Iz_12_1209	DJI_0817	Thymus pallasianus Heinr.Braun	0	n	1	0	Lamiacea Lindl.
Iz_12_1209	DJI_0817	Tilia cordata Mill.	0	n	1	1	Malvaceae Juss.
Iz_12_1209	DJI_0817	Trifolium alpestre L.	0	n	1	0	Fabaceae Lindl.
Iz_12_1209	DJI_0817	Trifolium arvense L.	0	n	1	0	Fabaceae Lindl.
Iz_12_1209	DJI_0817	Ulmus glabra Huds.	0	n	1	1	Ulmaceae Mirb.
Iz_12_1209	DJI_0817	Verbascum densiflorum Bertol.	0	n	1	0	Scrophulariaceae Juss.
Iz_12_1209	DJI_0817	Verbascum sp.	0	n	1	0	Scrophulariaceae Juss.
Iz_12_1209	DJI_0817	Veronica austriaca L.	0	n	1	0	Plantaginaceae Juss.
Iz_12_1209	DJI_0817	Veronica incana L.	0	n	1	0	Plantaginaceae Juss.
Iz_12_1209	DJI_0817	Veronica verna L.	0	n	1	0	Plantaginaceae Juss.
Iz_12_1209	DJI_0817	Vincetoxicum hirundinaria Medik	0	n	1	0	Apocynaceae Juss.
Iz_12_1209	DJI_0825	Acer tataricum L.	0	n	1	1	Sapindaceae Juss.
Iz_12_1209	DJI_0825	Achillea millefolium L. s.l.	0	n	1	0	Asteraceae Bercht. & J.Presl
Iz_12_1209	DJI_0825	Allium oleraceum L.	0	n	1	0	Amaryllidaceae J.St.-Hil.
Iz_12_1209	DJI_0825	Ambrosia artemisiifolia L.	0	a	0	0	Asteraceae Bercht. & J.Presl
Iz_12_1209	DJI_0825	Amorpha fruticosa L.	0	a	0	0	Fabaceae Lindl.
Iz_12_1209	DJI_0825	Anthericum ramosum L.	0	n	1	0	Asparagaceae Juss.
Iz_12_1209	DJI_0825	Artemisia marschalliana Spreng.	0	n	1	0	Asteraceae Bercht. & J.Presl
Iz_12_1209	DJI_0825	Asparagus officinalis L.	0	n	1	0	Asparagaceae Juss.
Iz_12_1209	DJI_0825	Astragalus cicer L.	0	n	1	0	Fabaceae Lindl.
Iz_12_1209	DJI_0825	Bassia laniflora (S.G.Gmel.) A.J.Scott	0	n	1	0	Amaranthaceae Juss.
Iz_12_1209	DJI_0825	Berberis vulgaris L.	0	n	1	0	Berberidaceae Juss.
Iz_12_1209	DJI_0825	Betula pendula Roth.	0	n	1	1	Betulaceae Gray
Iz_12_1209	DJI_0825	Betula pendula Roth.	0	n	1	0	Poaceae Barnhart
Iz_12_1209	DJI_0825	Bromus inermis Leyss.	0		NA	NA	
Iz_12_1209	DJI_0825	Bromus squarrosus L.	0	a	0	0	Poaceae Barnhart
Iz_12_1209	DJI_0825	Bromus tectorum L.	0	a	0	0	Poaceae Barnhart
Iz_12_1209	DJI_0825	Calamagrostis epigejos (L.) Roth	20	n	1	0	Poaceae Barnhart

Iz_12_1209	DJI_0825	Carduus acanthoides L.	0	n	1	0	Asteraceae Bercht. & J.Presl
Iz_12_1209	DJI_0825	Carex sp.	0	n	1	0	Cyperaceae Juss.
Iz_12_1209	DJI_0825	Centaurea majorovii Dumbadze	0	n	1	0	Asteraceae Bercht. & J.Presl
Iz_12_1209	DJI_0825	Chamaecytisus ruthenicus (Fisch.ex Wol.) Klaskova	0	n	1	0	Fabaceae Lindl.
Iz_12_1209	DJI_0825	Chelidonium majus L.	0	n	1	0	Papaveraceae Juss.
Iz_12_1209	DJI_0825	Chenopodium betaceum Andrz.	0	n	1	0	Amaranthaceae Juss.
Iz_12_1209	DJI_0825	Chenopodium cf. album	0	n	1	0	Amaranthaceae Juss.
Iz_12_1209	DJI_0825	Chenopodium sp.	0		NA	0	Amaranthaceae Juss.
Iz_12_1209	DJI_0825	Chondrilla juncea L.	0	n	1	0	Asteraceae Bercht. & J.Presl
Iz_12_1209	DJI_0825	Cirsium vulgare (Savi) Ten.	0	n	1	0	Asteraceae Bercht. & J.Presl
Iz_12_1209	DJI_0825	Clinopodium acinos (L.) Kuntze	0	n	1	0	Lamiacea Lindl.
Iz_12_1209	DJI_0825	Convallaria majalis L.	0	n	1	0	Asparagaceae Juss.
Iz_12_1209	DJI_0825	Crataegus rhipidophylla Gand.	0	n	1	1	Rosaceae Juss.
Iz_12_1209	DJI_0825	Crepis foetida L.	0	n	1	0	Asteraceae Bercht. & J.Presl
Iz_12_1209	DJI_0825	Dactylis glomerata L.	0	n	1	0	Poaceae Barnhart
Iz_12_1209	DJI_0825	Dianthus campestris M.Bieb.	0	n	1	0	Caryophyllaceae Juss.
Iz_12_1209	DJI_0825	Digitaria sanguinalis (L.) Scop.	0	a	0	0	Poaceae Barnhart
Iz_12_1209	DJI_0825	Elymus repens (L.) Gould	0	n	1	0	Poaceae Barnhart
Iz_12_1209	DJI_0825	Epilobium tetragonum L.	0	n	1	0	Onagraceae Juss.
Iz_12_1209	DJI_0825	Eragrostis minor Host	0	a	0	0	Poaceae Barnhart
Iz_12_1209	DJI_0825	Erigeron canadensis L.	0	a	0	0	Asteraceae Bercht. & J.Presl
Iz_12_1209	DJI_0825	Euphorbia seguieriana Neck.	0	n	1	0	Euphorbiaceae Juss.
Iz_12_1209	DJI_0825	Fallopia dumetorum (L.) Holub	0	n	1	0	Polygonaceae Juss.
Iz_12_1209	DJI_0825	Festuca beckeri (Hack.) Trautv.	0	n	1	0	Poaceae Barnhart
Iz_12_1209	DJI_0825	Fragaria vesca L.	0	n	1	0	Rosaceae Juss.
Iz_12_1209	DJI_0825	Frangula alnus Mill.	0	n	1	1	Rhamnaceae Juss.
Iz_12_1209	DJI_0825	Fraxinus pennsylvanica Marshall	0	a	0	1	Oleaceae Hoffmanns. & Link
Iz_12_1209	DJI_0825	Galeopsis bifida Boenn.	0	n	1	0	Lamiacea Lindl.
Iz_12_1209	DJI_0825	Galium verum L.	0	n	1	0	Rubiaceae Juss.
Iz_12_1209	DJI_0825	Genista tinctoria L.	0	n	1	0	Fabaceae Lindl.
Iz_12_1209	DJI_0825	Geranium robertianum L.	0	n	1	0	Geraniaceae Juss.
Iz_12_1209	DJI_0825	Geranium sanquineum L.	0	n	1	0	Geraniaceae Juss.
Iz_12_1209	DJI_0825	Gypsophila paniculata L.	0	n	1	0	Caryophyllaceae Juss.
Iz_12_1209	DJI_0825	Helichrysum arenarium (L.) Moench	0	n	1	0	Asteraceae Bercht. & J.Presl
Iz_12_1209	DJI_0825	Herniaria polygama J.Gay	0	n	1	0	Caryophyllaceae Juss.
Iz_12_1209	DJI_0825	Hieracium robustum Fr.	0	n	1	0	Asteraceae Bercht. & J.Presl
Iz_12_1209	DJI_0825	Hieracium umbellatum L.	0	n	1	NA	Asteraceae Bercht. & J.Presl
Iz_12_1209	DJI_0825	Hylotelephium maximum (L.) Holub	0	n	1	0	Crassulaceae J.St.-Hil.
Iz_12_1209	DJI_0825	Jacobaea borysthenica (DC.) B.Nord. & Greuter	0	n	1	0	Asteraceae Bercht. & J.Presl
Iz_12_1209	DJI_0825	Jasione montana L.	0	n	1	0	Campanulaceae Juss.
Iz_12_1209	DJI_0825	Jurinea cyanoides (L.) Rchb.s.l.	0	n	1	0	Asteraceae Bercht. & J.Presl
Iz_12_1209	DJI_0825	Koeleria glauca (Spreng.) DC.	0	n	1	0	Poaceae Barnhart
Iz_12_1209	DJI_0825	Lactuca serriola L.	0	a	0	0	Asteraceae Bercht. & J.Presl
Iz_12_1209	DJI_0825	Linaria genistifolia (L.) Mill.	0	n	1	0	Plantaginaceae Juss.
Iz_12_1209	DJI_0825	Lomelosia argentea (L.) Greuter & Burdet	0	n	1	0	Caprifoliaceae Juss.
Iz_12_1209	DJI_0825	Melampyrum pratense L.	0	n	1	0	Orobanchaceae Vent.
Iz_12_1209	DJI_0825	Oenothera biennis L.	0	a	0	0	Onagraceae Juss.
Iz_12_1209	DJI_0825	Pentanema britannica (L.) D.Gut.Larr., Santos-Vicente, Anderb., E.Rico & M.M.Mart.Ort.	0	n	1	0	Asteraceae Bercht. & J.Presl
Iz_12_1209	DJI_0825	Peucedanum oreoselinum (L.) Moench	0	n	1	0	Apiaceae Lindl.
Iz_12_1209	DJI_0825	Peucedanum ruthenicum M.Bieb.	0	n	1	0	Apiaceae Lindl.
Iz_12_1209	DJI_0825	Pilosella echioides (Lumn.) F.W.Schultz & Sch.Bip.	0	n	1	0	Asteraceae Bercht. & J.Presl
Iz_12_1209	DJI_0825	Pilosella officinarum F. Schultz et Sch. Bip.	0	n	1	0	Asteraceae Bercht. & J.Presl
Iz_12_1209	DJI_0825	Pilosella sp.	0		NA	0	Asteraceae Bercht. & J.Presl
Iz_12_1209	DJI_0825	Pinus sylvestris L.	0	n	1	1	Pinaceae Spreng. ex F.Rudolphi
Iz_12_1209	DJI_0825	Poa annua L.	0	n	1	0	Poaceae Barnhart
Iz_12_1209	DJI_0825	Poa nemoralis L.	0	n	1	0	Poaceae Barnhart
Iz_12_1209	DJI_0825	Poa sp.	0		NA	0	Poaceae Barnhart
Iz_12_1209	DJI_0825	Polygonatum odoratum (Mill.) Druce	0	n	1	0	Asparagaceae Juss.
Iz_12_1209	DJI_0825	Polygonum arenarium Waldst. & Kit.	0	n	1	0	Polygonaceae Juss.
Iz_12_1209	DJI_0825	Populus nigra L.	0	n	1	1	Salicaceae Mirb.
Iz_12_1209	DJI_0825	Populus tremula L.	0	n	1	1	Salicaceae Mirb.
Iz_12_1209	DJI_0825	Populus Ã canescens (Aiton) Sm.	0	n	1	1	Salicaceae Mirb.
Iz_12_1209	DJI_0825	Potentilla argentea L.	0	n	1	0	Rosaceae Juss.
Iz_12_1209	DJI_0825	Psammophiliella muralis (L.) Ikonn.	0	n	1	0	Caryophyllaceae Juss.
Iz_12_1209	DJI_0825	Psephellus sumensis (Kalen.) Greuter	0	n	1	0	Asteraceae Bercht. & J.Presl
Iz_12_1209	DJI_0825	Pulsatilla pratensis (L.) Mill. s.l.	0	n	1	0	Ranunculaceae Juss.
Iz_12_1209	DJI_0825	Pyrus communis L.	0	a	0	1	Rosaceae Juss.
Iz_12_1209	DJI_0825	Quercus robur L.	0	n	1	1	Fagaceae Dumort.
Iz_12_1209	DJI_0825	Robinia pseudoacacia L.	0	a	0	1	Fabaceae Lindl.
Iz_12_1209	DJI_0825	Rosa sp.	0		NA	1	Rosaceae Juss.
Iz_12_1209	DJI_0825	Rubus idaeus L.	0	n	1	0	Rosaceae Juss.
Iz_12_1209	DJI_0825	Rumex acetosella L.	0	n	1	0	Polygonaceae Juss.
Iz_12_1209	DJI_0825	Rumex thyrsiflorus Fingerh.	0	n	1	0	Polygonaceae Juss.
Iz_12_1209	DJI_0825	Salix alba L.	0	n	1	1	Salicaceae Mirb.
Iz_12_1209	DJI_0825	Salix sp.	0		NA	1	
Iz_12_1209	DJI_0825	Sambucus nigra L.	0	n	1	1	Viburnaceae Raf.
Iz_12_1209	DJI_0825	Secale sylvestre Host	0	n	1	0	Poaceae Barnhart
Iz_12_1209	DJI_0825	Sempervivum ruthenicum Schnittsp. & C.B. Lehm.	0	n	1	0	Crassulaceae J.St.-Hil.
Iz_12_1209	DJI_0825	Setaria viridis (L.) P. Beauv.	0	n	1	0	Poaceae Barnhart
Iz_12_1209	DJI_0825	Silene borysthenica (Gruner) Walters	0	n	1	0	Caryophyllaceae Juss.
Iz_12_1209	DJI_0825	Silene latifolia subsp. alba (Mill.) Greuter & Burdet	0	n	1	0	Caryophyllaceae Juss.
Iz_12_1209	DJI_0825	Solidago virgaurea L.	0	n	1	0	Asteraceae Bercht. & J.Presl
Iz_12_1209	DJI_0825	Sorbus aucuparia L.	0	n	1	1	Rosaceae Juss.
Iz_12_1209	DJI_0825	Stachys recta L.	0	n	1	0	Lamiacea Lindl.
Iz_12_1209	DJI_0825	Tanacetum vulgare L.	0	n	1	0	Asteraceae Bercht. & J.Presl
Iz_12_1209	DJI_0825	Thymus pallasianus Heinr.Braun	0	n	1	0	Lamiacea Lindl.
Iz_12_1209	DJI_0825	Tilia cordata Mill.	0	n	1	1	Malvaceae Juss.
Iz_12_1209	DJI_0825	Trifolium alpestre L.	0	n	1	0	Fabaceae Lindl.
Iz_12_1209	DJI_0825	Trifolium arvense L.	0	n	1	0	Fabaceae Lindl.
Iz_12_1209	DJI_0825	Ulmus glabra Huds.	0	n	1	1	Ulmaceae Mirb.
Iz_12_1209	DJI_0825	Verbascum densiflorum Bertol.	0	n	1	0	Scrophulariaceae Juss.
Iz_12_1209	DJI_0825	Verbascum sp.	0	n	1	0	Scrophulariaceae Juss.
Iz_12_1209	DJI_0825	Veronica austriaca L.	0	n	1	0	Plantaginaceae Juss.
Iz_12_1209	DJI_0825	Veronica incana L.	0	n	1	0	Plantaginaceae Juss.
Iz_12_1209	DJI_0825	Veronica verna L.	0	n	1	0	Plantaginaceae Juss.
Iz_12_1209	DJI_0825	Vincetoxicum hirundinaria Medik	0	n	1	0	Apocynaceae Juss.
Iz_12_1209	DJI_0828	Acer tataricum L.	0	n	1	1	Sapindaceae Juss.
Iz_12_1209	DJI_0828	Achillea millefolium L. s.l.	0	n	1	0	Asteraceae Bercht. & J.Presl

Iz_12_1209	DJI_0828	Allium oleraceum L.	0	n	1	0	Amaryllidaceae J.St.-Hil.
Iz_12_1209	DJI_0828	Ambrosia artemisiifolia L.	0	a	0	0	Asteraceae Bercht. & J.Presl
Iz_12_1209	DJI_0828	Amorpha fruticosa L.	0	a	0	0	Fabaceae Lindl.
Iz_12_1209	DJI_0828	Anthericum ramosum L.	0	n	1	0	Asparagaceae Juss.
Iz_12_1209	DJI_0828	Artemisia marschalliana Spreng.	0	n	1	0	Asteraceae Bercht. & J.Presl
Iz_12_1209	DJI_0828	Asparagus officinalis L.	0	n	1	0	Asparagaceae Juss.
Iz_12_1209	DJI_0828	Astragalus cicer L.	0	n	1	0	Fabaceae Lindl.
Iz_12_1209	DJI_0828	Bassia laniflora (S.G.Gmel.) A.J.Scott	0	n	1	0	Amaranthaceae Juss.
Iz_12_1209	DJI_0828	Berberis vulgaris L.	0	n	1	0	Berberidaceae Juss.
Iz_12_1209	DJI_0828	Betula pendula Roth.	0	n	1	1	Betulaceae Gray
Iz_12_1209	DJI_0828	Betula pendula Roth.	0	n	1	0	Poaceae Barnhart
Iz_12_1209	DJI_0828	Bromus inermis Leyss.	0		NA	NA	
Iz_12_1209	DJI_0828	Bromus squarrosus L.	0	a	0	0	Poaceae Barnhart
Iz_12_1209	DJI_0828	Bromus tectorum L.	0	a	0	0	Poaceae Barnhart
Iz_12_1209	DJI_0828	Calamagrostis epigejos (L.) Roth	6	n	1	0	Poaceae Barnhart
Iz_12_1209	DJI_0828	Carduus acanthoides L.	0	n	1	0	Asteraceae Bercht. & J.Presl
Iz_12_1209	DJI_0828	Carex sp.	10	n	1	0	Cyperaceae Juss.
Iz_12_1209	DJI_0828	Centaurea majorovii Dumbadze	0	n	1	0	Asteraceae Bercht. & J.Presl
Iz_12_1209	DJI_0828	Chamaecytisus ruthenicus (Fisch.ex Wol.) Klaskova	0	n	1	0	Fabaceae Lindl.
Iz_12_1209	DJI_0828	Chelidonium majus L.	0	n	1	0	Papaveraceae Juss.
Iz_12_1209	DJI_0828	Chenopodium betaceum Andrz.	0	n	1	0	Amaranthaceae Juss.
Iz_12_1209	DJI_0828	Chenopodium cf. album	0	n	1	0	Amaranthaceae Juss.
Iz_12_1209	DJI_0828	Chenopodium sp.	0		NA	0	Amaranthaceae Juss.
Iz_12_1209	DJI_0828	Chondrilla juncea L.	0	n	1	0	Asteraceae Bercht. & J.Presl
Iz_12_1209	DJI_0828	Cirsium vulgare (Savi) Ten.	0	n	1	0	Asteraceae Bercht. & J.Presl
Iz_12_1209	DJI_0828	Clinopodium acinos (L.) Kuntze	0	n	1	0	Lamiacea Lindl.
Iz_12_1209	DJI_0828	Convallaria majalis L.	0	n	1	0	Asparagaceae Juss.
Iz_12_1209	DJI_0828	Crataegus rhipidophylla Gand.	0	n	1	1	Rosaceae Juss.
Iz_12_1209	DJI_0828	Crepis foetida L.	0	n	1	0	Asteraceae Bercht. & J.Presl
Iz_12_1209	DJI_0828	Dactylis glomerata L.	0	n	1	0	Poaceae Barnhart
Iz_12_1209	DJI_0828	Dianthus campestris M.Bieb.	0	n	1	0	Caryophyllaceae Juss.
Iz_12_1209	DJI_0828	Digitaria sanguinalis (L.) Scop.	0	a	0	0	Poaceae Barnhart
Iz_12_1209	DJI_0828	Elymus repens (L.) Gould	0	n	1	0	Poaceae Barnhart
Iz_12_1209	DJI_0828	Epilobium tetragonum L.	0	n	1	0	Onagraceae Juss.
Iz_12_1209	DJI_0828	Eragrostis minor Host	0	a	0	0	Poaceae Barnhart
Iz_12_1209	DJI_0828	Erigeron canadensis L.	0	a	0	0	Asteraceae Bercht. & J.Presl
Iz_12_1209	DJI_0828	Euphorbia seguieriana Neck.	0	n	1	0	Euphorbiaceae Juss.
Iz_12_1209	DJI_0828	Fallopia dumetorum (L.) Holub	0	n	1	0	Polygonaceae Juss.
Iz_12_1209	DJI_0828	Festuca beckeri (Hack.) Trautv.	0	n	1	0	Poaceae Barnhart
Iz_12_1209	DJI_0828	Fragaria vesca L.	0	n	1	0	Rosaceae Juss.
Iz_12_1209	DJI_0828	Frangula alnus Mill.	0	n	1	1	Rhamnaceae Juss.
Iz_12_1209	DJI_0828	Fraxinus pennsylvanica Marshall	0	a	0	1	Oleaceae Hoffmanns. & Link
Iz_12_1209	DJI_0828	Galeopsis bifida Boenn.	0	n	1	0	Lamiacea Lindl.
Iz_12_1209	DJI_0828	Galium verum L.	0	n	1	0	Rubiaceae Juss.
Iz_12_1209	DJI_0828	Genista tinctoria L.	0	n	1	0	Fabaceae Lindl.
Iz_12_1209	DJI_0828	Geranium robertianum L.	0	n	1	0	Geraniaceae Juss.
Iz_12_1209	DJI_0828	Geranium sanquineum L.	0	n	1	0	Geraniaceae Juss.
Iz_12_1209	DJI_0828	Gypsophila paniculata L.	0	n	1	0	Caryophyllaceae Juss.
Iz_12_1209	DJI_0828	Helichrysum arenarium (L.) Moench	0	n	1	0	Asteraceae Bercht. & J.Presl
Iz_12_1209	DJI_0828	Herniaria polygama J.Gay	0	n	1	0	Caryophyllaceae Juss.
Iz_12_1209	DJI_0828	Hieracium robustum Fr.	0	n	1	0	Asteraceae Bercht. & J.Presl
Iz_12_1209	DJI_0828	Hieracium umbellatum L.	0	n	1	NA	Asteraceae Bercht. & J.Presl
Iz_12_1209	DJI_0828	Hylotelephium maximum (L.) Holub	0	n	1	0	Crassulaceae J.St.-Hil.
Iz_12_1209	DJI_0828	Jacobaea borysthenica (DC.) B.Nord. & Greuter	0	n	1	0	Asteraceae Bercht. & J.Presl
Iz_12_1209	DJI_0828	Jasione montana L.	0	n	1	0	Campanulaceae Juss.
Iz_12_1209	DJI_0828	Jurinea cyanoides (L.) Rchb.s.l.	0	n	1	0	Asteraceae Bercht. & J.Presl
Iz_12_1209	DJI_0828	Koeleria glauca (Spreng.) DC.	0	n	1	0	Poaceae Barnhart
Iz_12_1209	DJI_0828	Lactuca serriola L.	0	a	0	0	Asteraceae Bercht. & J.Presl
Iz_12_1209	DJI_0828	Linaria genistifolia (L.) Mill.	0	n	1	0	Plantaginaceae Juss.
Iz_12_1209	DJI_0828	Lomelosia argentea (L.) Greuter & Burdet	0	n	1	0	Caprifoliaceae Juss.
Iz_12_1209	DJI_0828	Melampyrum pratense L.	0	n	1	0	Orobanchaceae Vent.
Iz_12_1209	DJI_0828	Oenothera biennis L.	0	a	0	0	Onagraceae Juss.
Iz_12_1209	DJI_0828	Pentanema britannica (L.) D.Gut.Larr., Santos-Vicente, Anderb., E.Rico & M.M.Mart.Ort.	0	n	1	0	Asteraceae Bercht. & J.Presl
Iz_12_1209	DJI_0828	Peucedanum oreoselinum (L.) Moench	0	n	1	0	Apiaceae Lindl.
Iz_12_1209	DJI_0828	Peucedanum ruthenicum M.Bieb.	0	n	1	0	Apiaceae Lindl.
Iz_12_1209	DJI_0828	Pilosella echioides (Lumn.) F.W.Schultz & Sch.Bip.	0	n	1	0	Asteraceae Bercht. & J.Presl
Iz_12_1209	DJI_0828	Pilosella officinarum F. Schultz et Sch. Bip.	0.5	n	1	0	Asteraceae Bercht. & J.Presl
Iz_12_1209	DJI_0828	Pilosella sp.	0		NA	0	Asteraceae Bercht. & J.Presl
Iz_12_1209	DJI_0828	Pinus sylvestris L.	0	n	1	1	Pinaceae Spreng. ex F.Rudolphi
Iz_12_1209	DJI_0828	Poa annua L.	0	n	1	0	Poaceae Barnhart
Iz_12_1209	DJI_0828	Poa nemoralis L.	0	n	1	0	Poaceae Barnhart
Iz_12_1209	DJI_0828	Poa sp.	0		NA	0	Poaceae Barnhart
Iz_12_1209	DJI_0828	Polygonatum odoratum (Mill.) Druce	0	n	1	0	Asparagaceae Juss.
Iz_12_1209	DJI_0828	Polygonum arenarium Waldst. & Kit.	0	n	1	0	Polygonaceae Juss.
Iz_12_1209	DJI_0828	Populus nigra L.	0	n	1	1	Salicaceae Mirb.
Iz_12_1209	DJI_0828	Populus tremula L.	0	n	1	1	Salicaceae Mirb.
Iz_12_1209	DJI_0828	Populus Ã canescens (Aiton) Sm.	0	n	1	1	Salicaceae Mirb.
Iz_12_1209	DJI_0828	Potentilla argentea L.	0	n	1	0	Rosaceae Juss.
Iz_12_1209	DJI_0828	Psammophiliella muralis (L.) Ikonn.	0	n	1	0	Caryophyllaceae Juss.
Iz_12_1209	DJI_0828	Psephellus sumensis (Kalen.) Greuter	0	n	1	0	Asteraceae Bercht. & J.Presl
Iz_12_1209	DJI_0828	Pulsatilla pratensis (L.) Mill. s.l.	0	n	1	0	Ranunculaceae Juss.
Iz_12_1209	DJI_0828	Pyrus communis L.	0	a	0	1	Rosaceae Juss.
Iz_12_1209	DJI_0828	Quercus robur L.	0	n	1	1	Fagaceae Dumort.
Iz_12_1209	DJI_0828	Robinia pseudoacacia L.	0	a	0	1	Fabaceae Lindl.
Iz_12_1209	DJI_0828	Rosa sp.	0		NA	1	Rosaceae Juss.
Iz_12_1209	DJI_0828	Rubus idaeus L.	0	n	1	0	Rosaceae Juss.
Iz_12_1209	DJI_0828	Rumex acetosella L.	0	n	1	0	Polygonaceae Juss.
Iz_12_1209	DJI_0828	Rumex thyrsiflorus Fingerh.	0	n	1	0	Polygonaceae Juss.
Iz_12_1209	DJI_0828	Salix alba L.	0	n	1	1	Salicaceae Mirb.
Iz_12_1209	DJI_0828	Salix sp.	0		NA	1	
Iz_12_1209	DJI_0828	Sambucus nigra L.	0	n	1	1	Viburnaceae Raf.
Iz_12_1209	DJI_0828	Secale sylvestre Host	0	n	1	0	Poaceae Barnhart
Iz_12_1209	DJI_0828	Sempervivum ruthenicum Schnittsp. & C.B. Lehm.	0	n	1	0	Crassulaceae J.St.-Hil.
Iz_12_1209	DJI_0828	Setaria viridis (L.) P. Beauv.	0	n	1	0	Poaceae Barnhart
Iz_12_1209	DJI_0828	Silene borysthenica (Gruner) Walters	0	n	1	0	Caryophyllaceae Juss.
Iz_12_1209	DJI_0828	Silene latifolia subsp. alba (Mill.) Greuter & Burdet	0	n	1	0	Caryophyllaceae Juss.
Iz_12_1209	DJI_0828	Solidago virgaurea L.	0	n	1	0	Asteraceae Bercht. & J.Presl
Iz_12_1209	DJI_0828	Sorbus aucuparia L.	0	n	1	1	Rosaceae Juss.

Iz_12_1209	DJI_0828	Stachys recta L.	0	n	1	0	Lamiacea Lindl.
Iz_12_1209	DJI_0828	Tanacetum vulgare L.	0	n	1	0	Asteraceae Bercht. & J.Presl
Iz_12_1209	DJI_0828	Thymus pallasianus Heinr.Braun	0	n	1	0	Lamiacea Lindl.
Iz_12_1209	DJI_0828	Tilia cordata Mill.	0	n	1	1	Malvaceae Juss.
Iz_12_1209	DJI_0828	Trifolium alpestre L.	0	n	1	0	Fabaceae Lindl.
Iz_12_1209	DJI_0828	Trifolium arvense L.	0	n	1	0	Fabaceae Lindl.
Iz_12_1209	DJI_0828	Ulmus glabra Huds.	0	n	1	1	Ulmaceae Mirb.
Iz_12_1209	DJI_0828	Verbascum densiflorum Bertol.	0	n	1	0	Scrophulariaceae Juss.
Iz_12_1209	DJI_0828	Verbascum sp.	0	n	1	0	Scrophulariaceae Juss.
Iz_12_1209	DJI_0828	Veronica austriaca L.	0	n	1	0	Plantaginaceae Juss.
Iz_12_1209	DJI_0828	Veronica incana L.	0	n	1	0	Plantaginaceae Juss.
Iz_12_1209	DJI_0828	Veronica verna L.	0	n	1	0	Plantaginaceae Juss.
Iz_12_1209	DJI_0828	Vincetoxicum hirundinaria Medik	0	n	1	0	Apocynaceae Juss.
Iz_12_1209	DJI_0831	Acer tataricum L.	0	n	1	1	Sapindaceae Juss.
Iz_12_1209	DJI_0831	Achillea millefolium L. s.l.	0	n	1	0	Asteraceae Bercht. & J.Presl
Iz_12_1209	DJI_0831	Allium oleraceum L.	0	n	1	0	Amaryllidaceae J.St.-Hil.
Iz_12_1209	DJI_0831	Ambrosia artemisiifolia L.	0	a	0	0	Asteraceae Bercht. & J.Presl
Iz_12_1209	DJI_0831	Amorpha fruticosa L.	0	a	0	0	Fabaceae Lindl.
Iz_12_1209	DJI_0831	Anthericum ramosum L.	0	n	1	0	Asparagaceae Juss.
Iz_12_1209	DJI_0831	Artemisia marschalliana Spreng.	0	n	1	0	Asteraceae Bercht. & J.Presl
Iz_12_1209	DJI_0831	Asparagus officinalis L.	0	n	1	0	Asparagaceae Juss.
Iz_12_1209	DJI_0831	Astragalus cicer L.	0	n	1	0	Fabaceae Lindl.
Iz_12_1209	DJI_0831	Bassia laniflora (S.G.Gmel.) A.J.Scott	0	n	1	0	Amaranthaceae Juss.
Iz_12_1209	DJI_0831	Berberis vulgaris L.	0	n	1	0	Berberidaceae Juss.
Iz_12_1209	DJI_0831	Betula pendula Roth.	0	n	1	1	Betulaceae Gray
Iz_12_1209	DJI_0831	Betula pendula Roth.	0	n	1	0	Poaceae Barnhart
Iz_12_1209	DJI_0831	Bromus inermis Leyss.	0		NA	NA	
Iz_12_1209	DJI_0831	Bromus squarrosus L.	0	a	0	0	Poaceae Barnhart
Iz_12_1209	DJI_0831	Bromus tectorum L.	0	a	0	0	Poaceae Barnhart
Iz_12_1209	DJI_0831	Calamagrostis epigejos (L.) Roth	70	n	1	0	Poaceae Barnhart
Iz_12_1209	DJI_0831	Carduus acanthoides L.	0	n	1	0	Asteraceae Bercht. & J.Presl
Iz_12_1209	DJI_0831	Carex sp.	0	n	1	0	Cyperaceae Juss.
Iz_12_1209	DJI_0831	Centaurea majorovii Dumbadze	0	n	1	0	Asteraceae Bercht. & J.Presl
Iz_12_1209	DJI_0831	Chamaecytisus ruthenicus (Fisch.ex Wol.) Klaskova	0	n	1	0	Fabaceae Lindl.
Iz_12_1209	DJI_0831	Chelidonium majus L.	0	n	1	0	Papaveraceae Juss.
Iz_12_1209	DJI_0831	Chenopodium betaceum Andrz.	0	n	1	0	Amaranthaceae Juss.
Iz_12_1209	DJI_0831	Chenopodium cf. album	0	n	1	0	Amaranthaceae Juss.
Iz_12_1209	DJI_0831	Chenopodium sp.	0		NA	0	Amaranthaceae Juss.
Iz_12_1209	DJI_0831	Chondrilla juncea L.	0	n	1	0	Asteraceae Bercht. & J.Presl
Iz_12_1209	DJI_0831	Cirsium vulgare (Savi) Ten.	0	n	1	0	Asteraceae Bercht. & J.Presl
Iz_12_1209	DJI_0831	Clinopodium acinos (L.) Kuntze	0	n	1	0	Lamiacea Lindl.
Iz_12_1209	DJI_0831	Convallaria majalis L.	0	n	1	0	Asparagaceae Juss.
Iz_12_1209	DJI_0831	Crataegus rhipidophylla Gand.	0	n	1	1	Rosaceae Juss.
Iz_12_1209	DJI_0831	Crepis foetida L.	0	n	1	0	Asteraceae Bercht. & J.Presl
Iz_12_1209	DJI_0831	Dactylis glomerata L.	0	n	1	0	Poaceae Barnhart
Iz_12_1209	DJI_0831	Dianthus campestris M.Bieb.	0	n	1	0	Caryophyllaceae Juss.
Iz_12_1209	DJI_0831	Digitaria sanguinalis (L.) Scop.	0	a	0	0	Poaceae Barnhart
Iz_12_1209	DJI_0831	Elymus repens (L.) Gould	0	n	1	0	Poaceae Barnhart
Iz_12_1209	DJI_0831	Epilobium tetragonum L.	0	n	1	0	Onagraceae Juss.
Iz_12_1209	DJI_0831	Eragrostis minor Host	0	a	0	0	Poaceae Barnhart
Iz_12_1209	DJI_0831	Erigeron canadensis L.	0	a	0	0	Asteraceae Bercht. & J.Presl
Iz_12_1209	DJI_0831	Euphorbia seguieriana Neck.	0	n	1	0	Euphorbiaceae Juss.
Iz_12_1209	DJI_0831	Fallopia dumetorum (L.) Holub	0	n	1	0	Polygonaceae Juss.
Iz_12_1209	DJI_0831	Festuca beckeri (Hack.) Trautv.	0	n	1	0	Poaceae Barnhart
Iz_12_1209	DJI_0831	Fragaria vesca L.	0	n	1	0	Rosaceae Juss.
Iz_12_1209	DJI_0831	Frangula alnus Mill.	0	n	1	1	Rhamnaceae Juss.
Iz_12_1209	DJI_0831	Fraxinus pennsylvanica Marshall	0	a	0	1	Oleaceae Hoffmanns. & Link
Iz_12_1209	DJI_0831	Galeopsis bifida Boenn.	0	n	1	0	Lamiacea Lindl.
Iz_12_1209	DJI_0831	Galium verum L.	0	n	1	0	Rubiaceae Juss.
Iz_12_1209	DJI_0831	Genista tinctoria L.	0	n	1	0	Fabaceae Lindl.
Iz_12_1209	DJI_0831	Geranium robertianum L.	0	n	1	0	Geraniaceae Juss.
Iz_12_1209	DJI_0831	Geranium sanquineum L.	0	n	1	0	Geraniaceae Juss.
Iz_12_1209	DJI_0831	Gypsophila paniculata L.	0	n	1	0	Caryophyllaceae Juss.
Iz_12_1209	DJI_0831	Helichrysum arenarium (L.) Moench	0	n	1	0	Asteraceae Bercht. & J.Presl
Iz_12_1209	DJI_0831	Herniaria polygama J.Gay	0	n	1	0	Caryophyllaceae Juss.
Iz_12_1209	DJI_0831	Hieracium robustum Fr.	0	n	1	0	Asteraceae Bercht. & J.Presl
Iz_12_1209	DJI_0831	Hieracium umbellatum L.	0	n	1	NA	Asteraceae Bercht. & J.Presl
Iz_12_1209	DJI_0831	Hylotelephium maximum (L.) Holub	0	n	1	0	Crassulaceae J.St.-Hil.
Iz_12_1209	DJI_0831	Jacobaea borysthenica (DC.) B.Nord. & Greuter	0	n	1	0	Asteraceae Bercht. & J.Presl
Iz_12_1209	DJI_0831	Jasione montana L.	0	n	1	0	Campanulaceae Juss.
Iz_12_1209	DJI_0831	Jurinea cyanoides (L.) Rchb.s.l.	0	n	1	0	Asteraceae Bercht. & J.Presl
Iz_12_1209	DJI_0831	Koeleria glauca (Spreng.) DC.	0	n	1	0	Poaceae Barnhart
Iz_12_1209	DJI_0831	Lactuca serriola L.	0	a	0	0	Asteraceae Bercht. & J.Presl
Iz_12_1209	DJI_0831	Linaria genistifolia (L.) Mill.	0	n	1	0	Plantaginaceae Juss.
Iz_12_1209	DJI_0831	Lomelosia argentea (L.) Greuter & Burdet	0	n	1	0	Caprifoliaceae Juss.
Iz_12_1209	DJI_0831	Melampyrum pratense L.	0	n	1	0	Orobanchaceae Vent.
Iz_12_1209	DJI_0831	Oenothera biennis L.	0	a	0	0	Onagraceae Juss.
Iz_12_1209	DJI_0831	Pentanema britannica (L.) D.Gut.Larr., Santos-Vicente, Anderb., E.Rico & M.M.Mart.Ort.	0	n	1	0	Asteraceae Bercht. & J.Presl
Iz_12_1209	DJI_0831	Peucedanum oreoselinum (L.) Moench	0	n	1	0	Apiaceae Lindl.
Iz_12_1209	DJI_0831	Peucedanum ruthenicum M.Bieb.	0	n	1	0	Apiaceae Lindl.
Iz_12_1209	DJI_0831	Pilosella echioides (Lumn.) F.W.Schultz & Sch.Bip.	0	n	1	0	Asteraceae Bercht. & J.Presl
Iz_12_1209	DJI_0831	Pilosella officinarum F. Schultz et Sch. Bip.	0	n	1	0	Asteraceae Bercht. & J.Presl
Iz_12_1209	DJI_0831	Pilosella sp.	0		NA	0	Asteraceae Bercht. & J.Presl
Iz_12_1209	DJI_0831	Pinus sylvestris L.	0	n	1	1	Pinaceae Spreng. ex F.Rudolphi
Iz_12_1209	DJI_0831	Poa annua L.	0	n	1	0	Poaceae Barnhart
Iz_12_1209	DJI_0831	Poa nemoralis L.	0	n	1	0	Poaceae Barnhart
Iz_12_1209	DJI_0831	Poa sp.	0		NA	0	Poaceae Barnhart
Iz_12_1209	DJI_0831	Polygonatum odoratum (Mill.) Druce	0	n	1	0	Asparagaceae Juss.
Iz_12_1209	DJI_0831	Polygonum arenarium Waldst. & Kit.	0	n	1	0	Polygonaceae Juss.
Iz_12_1209	DJI_0831	Populus nigra L.	0	n	1	1	Salicaceae Mirb.
Iz_12_1209	DJI_0831	Populus tremula L.	15	n	1	1	Salicaceae Mirb.
Iz_12_1209	DJI_0831	Populus Ã canescens (Aiton) Sm.	0	n	1	1	Salicaceae Mirb.
Iz_12_1209	DJI_0831	Potentilla argentea L.	0	n	1	0	Rosaceae Juss.
Iz_12_1209	DJI_0831	Psammophiliella muralis (L.) Ikonn.	0	n	1	0	Caryophyllaceae Juss.
Iz_12_1209	DJI_0831	Psephellus sumensis (Kalen.) Greuter	0	n	1	0	Asteraceae Bercht. & J.Presl
Iz_12_1209	DJI_0831	Pulsatilla pratensis (L.) Mill. s.l.	0	n	1	0	Ranunculaceae Juss.
Iz_12_1209	DJI_0831	Pyrus communis L.	0	a	0	1	Rosaceae Juss.
Iz_12_1209	DJI_0831	Quercus robur L.	0	n	1	1	Fagaceae Dumort.

Iz_12_1209	DJI_0831	Robinia pseudoacacia L.	0	a	0	1	Fabaceae Lindl.
Iz_12_1209	DJI_0831	Rosa sp.	0		NA	1	Rosaceae Juss.
Iz_12_1209	DJI_0831	Rubus idaeus L.	0	n	1	0	Rosaceae Juss.
Iz_12_1209	DJI_0831	Rumex acetosella L.	0	n	1	0	Polygonaceae Juss.
Iz_12_1209	DJI_0831	Rumex thyrsiflorus Fingerh.	0	n	1	0	Polygonaceae Juss.
Iz_12_1209	DJI_0831	Salix alba L.	0	n	1	1	Salicaceae Mirb.
Iz_12_1209	DJI_0831	Salix sp.	0		NA	1	
Iz_12_1209	DJI_0831	Sambucus nigra L.	0	n	1	1	Viburnaceae Raf.
Iz_12_1209	DJI_0831	Secale sylvestre Host	0	n	1	0	Poaceae Barnhart
Iz_12_1209	DJI_0831	Sempervivum ruthenicum Schnittsp. & C.B. Lehm.	0	n	1	0	Crassulaceae J.St.-Hil.
Iz_12_1209	DJI_0831	Setaria viridis (L.) P. Beauv.	0	n	1	0	Poaceae Barnhart
Iz_12_1209	DJI_0831	Silene borysthenica (Gruner) Walters	0	n	1	0	Caryophyllaceae Juss.
Iz_12_1209	DJI_0831	Silene latifolia subsp. alba (Mill.) Greuter & Burdet	0	n	1	0	Caryophyllaceae Juss.
Iz_12_1209	DJI_0831	Solidago virgaurea L.	0	n	1	0	Asteraceae Bercht. & J.Presl
Iz_12_1209	DJI_0831	Sorbus aucuparia L.	0	n	1	1	Rosaceae Juss.
Iz_12_1209	DJI_0831	Stachys recta L.	0	n	1	0	Lamiacea Lindl.
Iz_12_1209	DJI_0831	Tanacetum vulgare L.	0	n	1	0	Asteraceae Bercht. & J.Presl
Iz_12_1209	DJI_0831	Thymus pallasianus Heinr.Braun	0	n	1	0	Lamiacea Lindl.
Iz_12_1209	DJI_0831	Tilia cordata Mill.	0	n	1	1	Malvaceae Juss.
Iz_12_1209	DJI_0831	Trifolium alpestre L.	0	n	1	0	Fabaceae Lindl.
Iz_12_1209	DJI_0831	Trifolium arvense L.	0	n	1	0	Fabaceae Lindl.
Iz_12_1209	DJI_0831	Ulmus glabra Huds.	0	n	1	1	Ulmaceae Mirb.
Iz_12_1209	DJI_0831	Verbascum densiflorum Bertol.	0	n	1	0	Scrophulariaceae Juss.
Iz_12_1209	DJI_0831	Verbascum sp.	0	n	1	0	Scrophulariaceae Juss.
Iz_12_1209	DJI_0831	Veronica austriaca L.	0	n	1	0	Plantaginaceae Juss.
Iz_12_1209	DJI_0831	Veronica incana L.	0	n	1	0	Plantaginaceae Juss.
Iz_12_1209	DJI_0831	Veronica verna L.	0	n	1	0	Plantaginaceae Juss.
Iz_12_1209	DJI_0831	Vincetoxicum hirundinaria Medik	0	n	1	0	Apocynaceae Juss.
Iz_13_1209	DJI_0863	Acer tataricum L.	0	n	1	1	Sapindaceae Juss.
Iz_13_1209	DJI_0863	Achillea millefolium L. s.l.	0	n	1	0	Asteraceae Bercht. & J.Presl
Iz_13_1209	DJI_0863	Allium oleraceum L.	0	n	1	0	Amaryllidaceae J.St.-Hil.
Iz_13_1209	DJI_0863	Ambrosia artemisiifolia L.	0	a	0	0	Asteraceae Bercht. & J.Presl
Iz_13_1209	DJI_0863	Amorpha fruticosa L.	0	a	0	0	Fabaceae Lindl.
Iz_13_1209	DJI_0863	Anthericum ramosum L.	0	n	1	0	Asparagaceae Juss.
Iz_13_1209	DJI_0863	Artemisia marschalliana Spreng.	0	n	1	0	Asteraceae Bercht. & J.Presl
Iz_13_1209	DJI_0863	Asparagus officinalis L.	0	n	1	0	Asparagaceae Juss.
Iz_13_1209	DJI_0863	Astragalus cicer L.	0	n	1	0	Fabaceae Lindl.
Iz_13_1209	DJI_0863	Bassia laniflora (S.G.Gmel.) A.J.Scott	0	n	1	0	Amaranthaceae Juss.
Iz_13_1209	DJI_0863	Berberis vulgaris L.	0	n	1	0	Berberidaceae Juss.
Iz_13_1209	DJI_0863	Betula pendula Roth.	0	n	1	1	Betulaceae Gray
Iz_13_1209	DJI_0863	Betula pendula Roth.	0	n	1	0	Poaceae Barnhart
Iz_13_1209	DJI_0863	Bromus inermis Leyss.	0		NA	NA	
Iz_13_1209	DJI_0863	Bromus squarrosus L.	0	a	0	0	Poaceae Barnhart
Iz_13_1209	DJI_0863	Bromus tectorum L.	0	a	0	0	Poaceae Barnhart
Iz_13_1209	DJI_0863	Calamagrostis epigejos (L.) Roth	8	n	1	0	Poaceae Barnhart
Iz_13_1209	DJI_0863	Carduus acanthoides L.	0	n	1	0	Asteraceae Bercht. & J.Presl
Iz_13_1209	DJI_0863	Carex sp.	0	n	1	0	Cyperaceae Juss.
Iz_13_1209	DJI_0863	Centaurea majorovii Dumbadze	0	n	1	0	Asteraceae Bercht. & J.Presl
Iz_13_1209	DJI_0863	Chamaecytisus ruthenicus (Fisch.ex Wol.) Klaskova	0	n	1	0	Fabaceae Lindl.
Iz_13_1209	DJI_0863	Chelidonium majus L.	0	n	1	0	Papaveraceae Juss.
Iz_13_1209	DJI_0863	Chenopodium betaceum Andrz.	0	n	1	0	Amaranthaceae Juss.
Iz_13_1209	DJI_0863	Chenopodium cf. album	0	n	1	0	Amaranthaceae Juss.
Iz_13_1209	DJI_0863	Chenopodium sp.	0		NA	0	Amaranthaceae Juss.
Iz_13_1209	DJI_0863	Chondrilla juncea L.	0	n	1	0	Asteraceae Bercht. & J.Presl
Iz_13_1209	DJI_0863	Cirsium vulgare (Savi) Ten.	0	n	1	0	Asteraceae Bercht. & J.Presl
Iz_13_1209	DJI_0863	Clinopodium acinos (L.) Kuntze	0	n	1	0	Lamiacea Lindl.
Iz_13_1209	DJI_0863	Convallaria majalis L.	0	n	1	0	Asparagaceae Juss.
Iz_13_1209	DJI_0863	Crataegus rhipidophylla Gand.	0	n	1	1	Rosaceae Juss.
Iz_13_1209	DJI_0863	Crepis foetida L.	0	n	1	0	Asteraceae Bercht. & J.Presl
Iz_13_1209	DJI_0863	Dactylis glomerata L.	0	n	1	0	Poaceae Barnhart
Iz_13_1209	DJI_0863	Dianthus campestris M.Bieb.	0	n	1	0	Caryophyllaceae Juss.
Iz_13_1209	DJI_0863	Digitaria sanguinalis (L.) Scop.	0	a	0	0	Poaceae Barnhart
Iz_13_1209	DJI_0863	Elymus repens (L.) Gould	0	n	1	0	Poaceae Barnhart
Iz_13_1209	DJI_0863	Epilobium tetragonum L.	0	n	1	0	Onagraceae Juss.
Iz_13_1209	DJI_0863	Eragrostis minor Host	0	a	0	0	Poaceae Barnhart
Iz_13_1209	DJI_0863	Erigeron canadensis L.	3	a	0	0	Asteraceae Bercht. & J.Presl
Iz_13_1209	DJI_0863	Euphorbia seguieriana Neck.	0	n	1	0	Euphorbiaceae Juss.
Iz_13_1209	DJI_0863	Fallopia dumetorum (L.) Holub	0	n	1	0	Polygonaceae Juss.
Iz_13_1209	DJI_0863	Festuca beckeri (Hack.) Trautv.	0	n	1	0	Poaceae Barnhart
Iz_13_1209	DJI_0863	Fragaria vesca L.	0	n	1	0	Rosaceae Juss.
Iz_13_1209	DJI_0863	Frangula alnus Mill.	0	n	1	1	Rhamnaceae Juss.
Iz_13_1209	DJI_0863	Fraxinus pennsylvanica Marshall	0	a	0	1	Oleaceae Hoffmanns. & Link
Iz_13_1209	DJI_0863	Galeopsis bifida Boenn.	0	n	1	0	Lamiacea Lindl.
Iz_13_1209	DJI_0863	Galium verum L.	0	n	1	0	Rubiaceae Juss.
Iz_13_1209	DJI_0863	Genista tinctoria L.	0	n	1	0	Fabaceae Lindl.
Iz_13_1209	DJI_0863	Geranium robertianum L.	0	n	1	0	Geraniaceae Juss.
Iz_13_1209	DJI_0863	Geranium sanquineum L.	0	n	1	0	Geraniaceae Juss.
Iz_13_1209	DJI_0863	Gypsophila paniculata L.	0	n	1	0	Caryophyllaceae Juss.
Iz_13_1209	DJI_0863	Helichrysum arenarium (L.) Moench	0	n	1	0	Asteraceae Bercht. & J.Presl
Iz_13_1209	DJI_0863	Herniaria polygama J.Gay	0	n	1	0	Caryophyllaceae Juss.
Iz_13_1209	DJI_0863	Hieracium robustum Fr.	0	n	1	0	Asteraceae Bercht. & J.Presl
Iz_13_1209	DJI_0863	Hieracium umbellatum L.	0	n	1	NA	Asteraceae Bercht. & J.Presl
Iz_13_1209	DJI_0863	Hylotelephium maximum (L.) Holub	4	n	1	0	Crassulaceae J.St.-Hil.
Iz_13_1209	DJI_0863	Jacobaea borysthenica (DC.) B.Nord. & Greuter	0	n	1	0	Asteraceae Bercht. & J.Presl
Iz_13_1209	DJI_0863	Jasione montana L.	0	n	1	0	Campanulaceae Juss.
Iz_13_1209	DJI_0863	Jurinea cyanoides (L.) Rchb.s.l.	0	n	1	0	Asteraceae Bercht. & J.Presl
Iz_13_1209	DJI_0863	Koeleria glauca (Spreng.) DC.	0	n	1	0	Poaceae Barnhart
Iz_13_1209	DJI_0863	Lactuca serriola L.	0	a	0	0	Asteraceae Bercht. & J.Presl
Iz_13_1209	DJI_0863	Linaria genistifolia (L.) Mill.	0	n	1	0	Plantaginaceae Juss.
Iz_13_1209	DJI_0863	Lomelosia argentea (L.) Greuter & Burdet	0	n	1	0	Caprifoliaceae Juss.
Iz_13_1209	DJI_0863	Melampyrum pratense L.	0	n	1	0	Orobanchaceae Vent.
Iz_13_1209	DJI_0863	Oenothera biennis L.	0	a	0	0	Onagraceae Juss.
Iz_13_1209	DJI_0863	Pentanema britannica (L.) D.Gut.Larr., Santos-Vicente, Anderb., E.Rico & M.M.Mart.Ort.	0	n	1	0	Asteraceae Bercht. & J.Presl
Iz_13_1209	DJI_0863	Peucedanum oreoselinum (L.) Moench	0	n	1	0	Apiaceae Lindl.
Iz_13_1209	DJI_0863	Peucedanum ruthenicum M.Bieb.	0	n	1	0	Apiaceae Lindl.
Iz_13_1209	DJI_0863	Pilosella echioides (Lumn.) F.W.Schultz & Sch.Bip.	0	n	1	0	Asteraceae Bercht. & J.Presl
Iz_13_1209	DJI_0863	Pilosella officinarum F. Schultz et Sch. Bip.	0	n	1	0	Asteraceae Bercht. & J.Presl
Iz_13_1209	DJI_0863	Pilosella sp.	0		NA	0	Asteraceae Bercht. & J.Presl

Iz_13_1209	DJI_0863	Pinus sylvestris L.	0	n	1	1	Pinaceae Spreng. ex F.Rudolphi
Iz_13_1209	DJI_0863	Poa annua L.	0	n	1	0	Poaceae Barnhart
Iz_13_1209	DJI_0863	Poa nemoralis L.	0	n	1	0	Poaceae Barnhart
Iz_13_1209	DJI_0863	Poa sp.	0		NA	0	Poaceae Barnhart
Iz_13_1209	DJI_0863	Polygonatum odoratum (Mill.) Druce	0	n	1	0	Asparagaceae Juss.
Iz_13_1209	DJI_0863	Polygonum arenarium Waldst. & Kit.	0	n	1	0	Polygonaceae Juss.
Iz_13_1209	DJI_0863	Populus nigra L.	0	n	1	1	Salicaceae Mirb.
Iz_13_1209	DJI_0863	Populus sp.	0	n	1	1	Salicaceae Mirb.
Iz_13_1209	DJI_0863	Populus tremula L.	0	n	1	1	Salicaceae Mirb.
Iz_13_1209	DJI_0863	Populus Ã canescens (Aiton) Sm.	0	n	1	1	Salicaceae Mirb.
Iz_13_1209	DJI_0863	Potentilla argentea L.	0	n	1	0	Rosaceae Juss.
Iz_13_1209	DJI_0863	Psammophiliella muralis (L.) Ikonn.	0	n	1	0	Caryophyllaceae Juss.
Iz_13_1209	DJI_0863	Psephellus sumensis (Kalen.) Greuter	0	n	1	0	Asteraceae Bercht. & J.Presl
Iz_13_1209	DJI_0863	Pulsatilla pratensis (L.) Mill. s.l.	0	n	1	0	Ranunculaceae Juss.
Iz_13_1209	DJI_0863	Pyrus communis L.	0	a	0	1	Rosaceae Juss.
Iz_13_1209	DJI_0863	Quercus robur L.	0	n	1	1	Fagaceae Dumort.
Iz_13_1209	DJI_0863	Robinia pseudoacacia L.	0	a	0	1	Fabaceae Lindl.
Iz_13_1209	DJI_0863	Rosa sp.	0		NA	1	Rosaceae Juss.
Iz_13_1209	DJI_0863	Rubus idaeus L.	0	n	1	0	Rosaceae Juss.
Iz_13_1209	DJI_0863	Rumex acetosella L.	0	n	1	0	Polygonaceae Juss.
Iz_13_1209	DJI_0863	Rumex thyrsiflorus Fingerh.	0	n	1	0	Polygonaceae Juss.
Iz_13_1209	DJI_0863	Salix alba L.	0	n	1	1	Salicaceae Mirb.
Iz_13_1209	DJI_0863	Sambucus nigra L.	0	n	1	1	Viburnaceae Raf.
Iz_13_1209	DJI_0863	Secale sylvestre Host	0	n	1	0	Poaceae Barnhart
Iz_13_1209	DJI_0863	Sempervivum ruthenicum Schnittsp. & C.B. Lehm.	0	n	1	0	Crassulaceae J.St.-Hil.
Iz_13_1209	DJI_0863	Setaria viridis (L.) P. Beauv.	0	n	1	0	Poaceae Barnhart
Iz_13_1209	DJI_0863	Silene borysthenica (Gruner) Walters	0	n	1	0	Caryophyllaceae Juss.
Iz_13_1209	DJI_0863	Silene latifolia subsp. alba (Mill.) Greuter & Burdet	0	n	1	0	Caryophyllaceae Juss.
Iz_13_1209	DJI_0863	Solidago virgaurea L.	0	n	1	0	Asteraceae Bercht. & J.Presl
Iz_13_1209	DJI_0863	Sorbus aucuparia L.	0	n	1	1	Rosaceae Juss.
Iz_13_1209	DJI_0863	Stachys recta L.	0	n	1	0	Lamiacea Lindl.
Iz_13_1209	DJI_0863	Tanacetum vulgare L.	0	n	1	0	Asteraceae Bercht. & J.Presl
Iz_13_1209	DJI_0863	Thymus pallasianus Heinr.Braun	0	n	1	0	Lamiacea Lindl.
Iz_13_1209	DJI_0863	Tilia cordata Mill.	0	n	1	1	Malvaceae Juss.
Iz_13_1209	DJI_0863	Trifolium alpestre L.	0	n	1	0	Fabaceae Lindl.
Iz_13_1209	DJI_0863	Trifolium arvense L.	0	n	1	0	Fabaceae Lindl.
Iz_13_1209	DJI_0863	Ulmus glabra Huds.	0	n	1	1	Ulmaceae Mirb.
Iz_13_1209	DJI_0863	Verbascum densiflorum Bertol.	0	n	1	0	Scrophulariaceae Juss.
Iz_13_1209	DJI_0863	Veronica austriaca L.	0	n	1	0	Plantaginaceae Juss.
Iz_13_1209	DJI_0863	Veronica incana L.	0	n	1	0	Plantaginaceae Juss.
Iz_13_1209	DJI_0863	Veronica verna L.	0	n	1	0	Plantaginaceae Juss.
Iz_13_1209	DJI_0863	Vincetoxicum hirundinaria Medik	0	n	1	0	Apocynaceae Juss.
Iz_13_1209	DJI_0869	Acer tataricum L.	0	n	1	1	Sapindaceae Juss.
Iz_13_1209	DJI_0869	Achillea millefolium L. s.l.	0	n	1	0	Asteraceae Bercht. & J.Presl
Iz_13_1209	DJI_0869	Allium oleraceum L.	0	n	1	0	Amaryllidaceae J.St.-Hil.
Iz_13_1209	DJI_0869	Ambrosia artemisiifolia L.	0	a	0	0	Asteraceae Bercht. & J.Presl
Iz_13_1209	DJI_0869	Amorpha fruticosa L.	0	a	0	0	Fabaceae Lindl.
Iz_13_1209	DJI_0869	Anthericum ramosum L.	0	n	1	0	Asparagaceae Juss.
Iz_13_1209	DJI_0869	Artemisia marschalliana Spreng.	0	n	1	0	Asteraceae Bercht. & J.Presl
Iz_13_1209	DJI_0869	Asparagus officinalis L.	0	n	1	0	Asparagaceae Juss.
Iz_13_1209	DJI_0869	Astragalus cicer L.	0	n	1	0	Fabaceae Lindl.
Iz_13_1209	DJI_0869	Bassia laniflora (S.G.Gmel.) A.J.Scott	0	n	1	0	Amaranthaceae Juss.
Iz_13_1209	DJI_0869	Berberis vulgaris L.	0	n	1	0	Berberidaceae Juss.
Iz_13_1209	DJI_0869	Betula pendula Roth.	0	n	1	1	Betulaceae Gray
Iz_13_1209	DJI_0869	Betula pendula Roth.	0	n	1	0	Poaceae Barnhart
Iz_13_1209	DJI_0869	Bromus inermis Leyss.	0		NA	NA	
Iz_13_1209	DJI_0869	Bromus squarrosus L.	0	a	0	0	Poaceae Barnhart
Iz_13_1209	DJI_0869	Bromus tectorum L.	0	a	0	0	Poaceae Barnhart
Iz_13_1209	DJI_0869	Calamagrostis epigejos (L.) Roth	25	n	1	0	Poaceae Barnhart
Iz_13_1209	DJI_0869	Carduus acanthoides L.	0	n	1	0	Asteraceae Bercht. & J.Presl
Iz_13_1209	DJI_0869	Carex sp.	0	n	1	0	Cyperaceae Juss.
Iz_13_1209	DJI_0869	Centaurea majorovii Dumbadze	0	n	1	0	Asteraceae Bercht. & J.Presl
Iz_13_1209	DJI_0869	Chamaecytisus ruthenicus (Fisch.ex Wol.) Klaskova	0	n	1	0	Fabaceae Lindl.
Iz_13_1209	DJI_0869	Chelidonium majus L.	0	n	1	0	Papaveraceae Juss.
Iz_13_1209	DJI_0869	Chenopodium betaceum Andrz.	0	n	1	0	Amaranthaceae Juss.
Iz_13_1209	DJI_0869	Chenopodium cf. album	0	n	1	0	Amaranthaceae Juss.
Iz_13_1209	DJI_0869	Chenopodium sp.	0		NA	0	Amaranthaceae Juss.
Iz_13_1209	DJI_0869	Chondrilla juncea L.	0	n	1	0	Asteraceae Bercht. & J.Presl
Iz_13_1209	DJI_0869	Cirsium vulgare (Savi) Ten.	0	n	1	0	Asteraceae Bercht. & J.Presl
Iz_13_1209	DJI_0869	Clinopodium acinos (L.) Kuntze	0	n	1	0	Lamiacea Lindl.
Iz_13_1209	DJI_0869	Convallaria majalis L.	0	n	1	0	Asparagaceae Juss.
Iz_13_1209	DJI_0869	Crataegus rhipidophylla Gand.	0	n	1	1	Rosaceae Juss.
Iz_13_1209	DJI_0869	Crepis foetida L.	0	n	1	0	Asteraceae Bercht. & J.Presl
Iz_13_1209	DJI_0869	Dactylis glomerata L.	0	n	1	0	Poaceae Barnhart
Iz_13_1209	DJI_0869	Dianthus campestris M.Bieb.	0	n	1	0	Caryophyllaceae Juss.
Iz_13_1209	DJI_0869	Digitaria sanguinalis (L.) Scop.	0	a	0	0	Poaceae Barnhart
Iz_13_1209	DJI_0869	Elymus repens (L.) Gould	0	n	1	0	Poaceae Barnhart
Iz_13_1209	DJI_0869	Epilobium tetragonum L.	0	n	1	0	Onagraceae Juss.
Iz_13_1209	DJI_0869	Eragrostis minor Host	0	a	0	0	Poaceae Barnhart
Iz_13_1209	DJI_0869	Erigeron canadensis L.	6	a	0	0	Asteraceae Bercht. & J.Presl
Iz_13_1209	DJI_0869	Euphorbia seguieriana Neck.	0	n	1	0	Euphorbiaceae Juss.
Iz_13_1209	DJI_0869	Fallopia dumetorum (L.) Holub	0	n	1	0	Polygonaceae Juss.
Iz_13_1209	DJI_0869	Festuca beckeri (Hack.) Trautv.	0	n	1	0	Poaceae Barnhart
Iz_13_1209	DJI_0869	Fragaria vesca L.	0	n	1	0	Rosaceae Juss.
Iz_13_1209	DJI_0869	Frangula alnus Mill.	0	n	1	1	Rhamnaceae Juss.
Iz_13_1209	DJI_0869	Fraxinus pennsylvanica Marshall	0	a	0	1	Oleaceae Hoffmanns. & Link
Iz_13_1209	DJI_0869	Galeopsis bifida Boenn.	0	n	1	0	Lamiacea Lindl.
Iz_13_1209	DJI_0869	Galium verum L.	0	n	1	0	Rubiaceae Juss.
Iz_13_1209	DJI_0869	Genista tinctoria L.	0	n	1	0	Fabaceae Lindl.
Iz_13_1209	DJI_0869	Geranium robertianum L.	0	n	1	0	Geraniaceae Juss.
Iz_13_1209	DJI_0869	Geranium sanquineum L.	0	n	1	0	Geraniaceae Juss.
Iz_13_1209	DJI_0869	Gypsophila paniculata L.	0	n	1	0	Caryophyllaceae Juss.
Iz_13_1209	DJI_0869	Helichrysum arenarium (L.) Moench	0	n	1	0	Asteraceae Bercht. & J.Presl
Iz_13_1209	DJI_0869	Herniaria polygama J.Gay	0	n	1	0	Caryophyllaceae Juss.
Iz_13_1209	DJI_0869	Hieracium robustum Fr.	0	n	1	0	Asteraceae Bercht. & J.Presl
Iz_13_1209	DJI_0869	Hieracium umbellatum L.	0	n	1	NA	Asteraceae Bercht. & J.Presl
Iz_13_1209	DJI_0869	Hylotelephium maximum (L.) Holub	0	n	1	0	Crassulaceae J.St.-Hil.
Iz_13_1209	DJI_0869	Jacobaea borysthenica (DC.) B.Nord. & Greuter	0	n	1	0	Asteraceae Bercht. & J.Presl

Iz_13_1209	DJI_0869	Jasione montana L.	0	n	1	0	Campanulaceae Juss.
Iz_13_1209	DJI_0869	Jurinea cyanoides (L.) Rchb.s.l.	0	n	1	0	Asteraceae Bercht. & J.Presl
Iz_13_1209	DJI_0869	Koeleria glauca (Spreng.) DC.	0	n	1	0	Poaceae Barnhart
Iz_13_1209	DJI_0869	Lactuca serriola L.	0	a	0	0	Asteraceae Bercht. & J.Presl
Iz_13_1209	DJI_0869	Linaria genistifolia (L.) Mill.	0	n	1	0	Plantaginaceae Juss.
Iz_13_1209	DJI_0869	Lomelosia argentea (L.) Greuter & Burdet	0	n	1	0	Caprifoliaceae Juss.
Iz_13_1209	DJI_0869	Melampyrum pratense L.	0	n	1	0	Orobanchaceae Vent.
Iz_13_1209	DJI_0869	Oenothera biennis L.	0	a	0	0	Onagraceae Juss.
Iz_13_1209	DJI_0869	Pentanema britannica (L.) D.Gut.Larr., Santos-Vicente, Anderb., E.Rico & M.M.Mart.Ort.	0	n	1	0	Asteraceae Bercht. & J.Presl
Iz_13_1209	DJI_0869	Peucedanum oreoselinum (L.) Moench	0	n	1	0	Apiaceae Lindl.
Iz_13_1209	DJI_0869	Peucedanum ruthenicum M.Bieb.	0	n	1	0	Apiaceae Lindl.
Iz_13_1209	DJI_0869	Pilosella echioides (Lumn.) F.W.Schultz & Sch.Bip.	0	n	1	0	Asteraceae Bercht. & J.Presl
Iz_13_1209	DJI_0869	Pilosella officinarum F. Schultz et Sch. Bip.	0	n	1	0	Asteraceae Bercht. & J.Presl
Iz_13_1209	DJI_0869	Pilosella sp.	0		NA	0	Asteraceae Bercht. & J.Presl
Iz_13_1209	DJI_0869	Pinus sylvestris L.	0	n	1	1	Pinaceae Spreng. ex F.Rudolphi
Iz_13_1209	DJI_0869	Poa annua L.	0	n	1	0	Poaceae Barnhart
Iz_13_1209	DJI_0869	Poa nemoralis L.	0	n	1	0	Poaceae Barnhart
Iz_13_1209	DJI_0869	Poa sp.	0		NA	0	Poaceae Barnhart
Iz_13_1209	DJI_0869	Polygonatum odoratum (Mill.) Druce	0	n	1	0	Asparagaceae Juss.
Iz_13_1209	DJI_0869	Polygonum arenarium Waldst. & Kit.	0	n	1	0	Polygonaceae Juss.
Iz_13_1209	DJI_0869	Populus nigra L.	0	n	1	1	Salicaceae Mirb.
Iz_13_1209	DJI_0869	Populus sp.	0	n	1	1	Salicaceae Mirb.
Iz_13_1209	DJI_0869	Populus tremula L.	0	n	1	1	Salicaceae Mirb.
Iz_13_1209	DJI_0869	Populus Ã canescens (Aiton) Sm.	0	n	1	1	Salicaceae Mirb.
Iz_13_1209	DJI_0869	Potentilla argentea L.	0	n	1	0	Rosaceae Juss.
Iz_13_1209	DJI_0869	Psammophiliella muralis (L.) Ikonn.	0	n	1	0	Caryophyllaceae Juss.
Iz_13_1209	DJI_0869	Psephellus sumensis (Kalen.) Greuter	0	n	1	0	Asteraceae Bercht. & J.Presl
Iz_13_1209	DJI_0869	Pulsatilla pratensis (L.) Mill. s.l.	0	n	1	0	Ranunculaceae Juss.
Iz_13_1209	DJI_0869	Pyrus communis L.	0	a	0	1	Rosaceae Juss.
Iz_13_1209	DJI_0869	Quercus robur L.	0	n	1	1	Fagaceae Dumort.
Iz_13_1209	DJI_0869	Robinia pseudoacacia L.	0	a	0	1	Fabaceae Lindl.
Iz_13_1209	DJI_0869	Rosa sp.	0		NA	1	Rosaceae Juss.
Iz_13_1209	DJI_0869	Rubus idaeus L.	0	n	1	0	Rosaceae Juss.
Iz_13_1209	DJI_0869	Rumex acetosella L.	0	n	1	0	Polygonaceae Juss.
Iz_13_1209	DJI_0869	Rumex thyrsiflorus Fingerh.	0	n	1	0	Polygonaceae Juss.
Iz_13_1209	DJI_0869	Salix alba L.	0	n	1	1	Salicaceae Mirb.
Iz_13_1209	DJI_0869	Sambucus nigra L.	0	n	1	1	Viburnaceae Raf.
Iz_13_1209	DJI_0869	Secale sylvestre Host	0	n	1	0	Poaceae Barnhart
Iz_13_1209	DJI_0869	Sempervivum ruthenicum Schnittsp. & C.B. Lehm.	0	n	1	0	Crassulaceae J.St.-Hil.
Iz_13_1209	DJI_0869	Setaria viridis (L.) P. Beauv.	0	n	1	0	Poaceae Barnhart
Iz_13_1209	DJI_0869	Silene borysthenica (Gruner) Walters	0	n	1	0	Caryophyllaceae Juss.
Iz_13_1209	DJI_0869	Silene latifolia subsp. alba (Mill.) Greuter & Burdet	0	n	1	0	Caryophyllaceae Juss.
Iz_13_1209	DJI_0869	Solidago virgaurea L.	0	n	1	0	Asteraceae Bercht. & J.Presl
Iz_13_1209	DJI_0869	Sorbus aucuparia L.	0	n	1	1	Rosaceae Juss.
Iz_13_1209	DJI_0869	Stachys recta L.	0	n	1	0	Lamiacea Lindl.
Iz_13_1209	DJI_0869	Tanacetum vulgare L.	0	n	1	0	Asteraceae Bercht. & J.Presl
Iz_13_1209	DJI_0869	Thymus pallasianus Heinr.Braun	0	n	1	0	Lamiacea Lindl.
Iz_13_1209	DJI_0869	Tilia cordata Mill.	0	n	1	1	Malvaceae Juss.
Iz_13_1209	DJI_0869	Trifolium alpestre L.	0	n	1	0	Fabaceae Lindl.
Iz_13_1209	DJI_0869	Trifolium arvense L.	0	n	1	0	Fabaceae Lindl.
Iz_13_1209	DJI_0869	Ulmus glabra Huds.	0	n	1	1	Ulmaceae Mirb.
Iz_13_1209	DJI_0869	Verbascum densiflorum Bertol.	0	n	1	0	Scrophulariaceae Juss.
Iz_13_1209	DJI_0869	Veronica austriaca L.	0	n	1	0	Plantaginaceae Juss.
Iz_13_1209	DJI_0869	Veronica incana L.	0	n	1	0	Plantaginaceae Juss.
Iz_13_1209	DJI_0869	Veronica verna L.	0	n	1	0	Plantaginaceae Juss.
Iz_13_1209	DJI_0869	Vincetoxicum hirundinaria Medik	0	n	1	0	Apocynaceae Juss.
Iz_13_1209	DJI_0873	Acer tataricum L.	0	n	1	1	Sapindaceae Juss.
Iz_13_1209	DJI_0873	Achillea millefolium L. s.l.	0	n	1	0	Asteraceae Bercht. & J.Presl
Iz_13_1209	DJI_0873	Allium oleraceum L.	0	n	1	0	Amaryllidaceae J.St.-Hil.
Iz_13_1209	DJI_0873	Ambrosia artemisiifolia L.	0	a	0	0	Asteraceae Bercht. & J.Presl
Iz_13_1209	DJI_0873	Amorpha fruticosa L.	0	a	0	0	Fabaceae Lindl.
Iz_13_1209	DJI_0873	Anthericum ramosum L.	0	n	1	0	Asparagaceae Juss.
Iz_13_1209	DJI_0873	Artemisia marschalliana Spreng.	0	n	1	0	Asteraceae Bercht. & J.Presl
Iz_13_1209	DJI_0873	Asparagus officinalis L.	0	n	1	0	Asparagaceae Juss.
Iz_13_1209	DJI_0873	Astragalus cicer L.	0	n	1	0	Fabaceae Lindl.
Iz_13_1209	DJI_0873	Bassia laniflora (S.G.Gmel.) A.J.Scott	0	n	1	0	Amaranthaceae Juss.
Iz_13_1209	DJI_0873	Berberis vulgaris L.	0	n	1	0	Berberidaceae Juss.
Iz_13_1209	DJI_0873	Betula pendula Roth.	0	n	1	1	Betulaceae Gray
Iz_13_1209	DJI_0873	Betula pendula Roth.	0	n	1	0	Poaceae Barnhart
Iz_13_1209	DJI_0873	Bromus inermis Leyss.	0		NA	NA	
Iz_13_1209	DJI_0873	Bromus squarrosus L.	0	a	0	0	Poaceae Barnhart
Iz_13_1209	DJI_0873	Bromus tectorum L.	0	a	0	0	Poaceae Barnhart
Iz_13_1209	DJI_0873	Calamagrostis epigejos (L.) Roth	2	n	1	0	Poaceae Barnhart
Iz_13_1209	DJI_0873	Carduus acanthoides L.	0	n	1	0	Asteraceae Bercht. & J.Presl
Iz_13_1209	DJI_0873	Carex sp.	0	n	1	0	Cyperaceae Juss.
Iz_13_1209	DJI_0873	Centaurea majorovii Dumbadze	0	n	1	0	Asteraceae Bercht. & J.Presl
Iz_13_1209	DJI_0873	Chamaecytisus ruthenicus (Fisch.ex Wol.) Klaskova	0	n	1	0	Fabaceae Lindl.
Iz_13_1209	DJI_0873	Chelidonium majus L.	0	n	1	0	Papaveraceae Juss.
Iz_13_1209	DJI_0873	Chenopodium betaceum Andrz.	0	n	1	0	Amaranthaceae Juss.
Iz_13_1209	DJI_0873	Chenopodium cf. album	0	n	1	0	Amaranthaceae Juss.
Iz_13_1209	DJI_0873	Chenopodium sp.	0		NA	0	Amaranthaceae Juss.
Iz_13_1209	DJI_0873	Chondrilla juncea L.	0	n	1	0	Asteraceae Bercht. & J.Presl
Iz_13_1209	DJI_0873	Cirsium vulgare (Savi) Ten.	0	n	1	0	Asteraceae Bercht. & J.Presl
Iz_13_1209	DJI_0873	Clinopodium acinos (L.) Kuntze	0	n	1	0	Lamiacea Lindl.
Iz_13_1209	DJI_0873	Convallaria majalis L.	0	n	1	0	Asparagaceae Juss.
Iz_13_1209	DJI_0873	Crataegus rhipidophylla Gand.	0	n	1	1	Rosaceae Juss.
Iz_13_1209	DJI_0873	Crepis foetida L.	0	n	1	0	Asteraceae Bercht. & J.Presl
Iz_13_1209	DJI_0873	Dactylis glomerata L.	0	n	1	0	Poaceae Barnhart
Iz_13_1209	DJI_0873	Dianthus campestris M.Bieb.	0	n	1	0	Caryophyllaceae Juss.
Iz_13_1209	DJI_0873	Digitaria sanguinalis (L.) Scop.	0	a	0	0	Poaceae Barnhart
Iz_13_1209	DJI_0873	Elymus repens (L.) Gould	0	n	1	0	Poaceae Barnhart
Iz_13_1209	DJI_0873	Epilobium tetragonum L.	0	n	1	0	Onagraceae Juss.
Iz_13_1209	DJI_0873	Eragrostis minor Host	0	a	0	0	Poaceae Barnhart
Iz_13_1209	DJI_0873	Erigeron canadensis L.	7	a	0	0	Asteraceae Bercht. & J.Presl
Iz_13_1209	DJI_0873	Euphorbia seguieriana Neck.	0	n	1	0	Euphorbiaceae Juss.
Iz_13_1209	DJI_0873	Fallopia dumetorum (L.) Holub	0	n	1	0	Polygonaceae Juss.
Iz_13_1209	DJI_0873	Festuca beckeri (Hack.) Trautv.	0	n	1	0	Poaceae Barnhart
Iz_13_1209	DJI_0873	Fragaria vesca L.	0	n	1	0	Rosaceae Juss.

Iz_13_1209	DJI_0873	Frangula alnus Mill.	0	n	1	1	Rhamnaceae Juss.
Iz_13_1209	DJI_0873	Fraxinus pennsylvanica Marshall	0	a	0	1	Oleaceae Hoffmanns. & Link
Iz_13_1209	DJI_0873	Galeopsis bifida Boenn.	0	n	1	0	Lamiacea Lindl.
Iz_13_1209	DJI_0873	Galium verum L.	0	n	1	0	Rubiaceae Juss.
Iz_13_1209	DJI_0873	Genista tinctoria L.	0	n	1	0	Fabaceae Lindl.
Iz_13_1209	DJI_0873	Geranium robertianum L.	0	n	1	0	Geraniaceae Juss.
Iz_13_1209	DJI_0873	Geranium sanquineum L.	0	n	1	0	Geraniaceae Juss.
Iz_13_1209	DJI_0873	Gypsophila paniculata L.	0	n	1	0	Caryophyllaceae Juss.
Iz_13_1209	DJI_0873	Helichrysum arenarium (L.) Moench	0	n	1	0	Asteraceae Bercht. & J.Presl
Iz_13_1209	DJI_0873	Herniaria polygama J.Gay	0	n	1	0	Caryophyllaceae Juss.
Iz_13_1209	DJI_0873	Hieracium robustum Fr.	0	n	1	0	Asteraceae Bercht. & J.Presl
Iz_13_1209	DJI_0873	Hieracium umbellatum L.	0	n	1	NA	Asteraceae Bercht. & J.Presl
Iz_13_1209	DJI_0873	Hylotelephium maximum (L.) Holub	0	n	1	0	Crassulaceae J.St.-Hil.
Iz_13_1209	DJI_0873	Jacobaea borysthenica (DC.) B.Nord. & Greuter	0	n	1	0	Asteraceae Bercht. & J.Presl
Iz_13_1209	DJI_0873	Jasione montana L.	0	n	1	0	Campanulaceae Juss.
Iz_13_1209	DJI_0873	Jurinea cyanoides (L.) Rchb.s.l.	0	n	1	0	Asteraceae Bercht. & J.Presl
Iz_13_1209	DJI_0873	Koeleria glauca (Spreng.) DC.	0	n	1	0	Poaceae Barnhart
Iz_13_1209	DJI_0873	Lactuca serriola L.	0	a	0	0	Asteraceae Bercht. & J.Presl
Iz_13_1209	DJI_0873	Linaria genistifolia (L.) Mill.	0	n	1	0	Plantaginaceae Juss.
Iz_13_1209	DJI_0873	Lomelosia argentea (L.) Greuter & Burdet	0	n	1	0	Caprifoliaceae Juss.
Iz_13_1209	DJI_0873	Melampyrum pratense L.	0	n	1	0	Orobanchaceae Vent.
Iz_13_1209	DJI_0873	Oenothera biennis L.	0	a	0	0	Onagraceae Juss.
Iz_13_1209	DJI_0873	Pentanema britannica (L.) D.Gut.Larr., Santos-Vicente, Anderb., E.Rico & M.M.Mart.Ort.	0	n	1	0	Asteraceae Bercht. & J.Presl
Iz_13_1209	DJI_0873	Peucedanum oreoselinum (L.) Moench	0	n	1	0	Apiaceae Lindl.
Iz_13_1209	DJI_0873	Peucedanum ruthenicum M.Bieb.	0	n	1	0	Apiaceae Lindl.
Iz_13_1209	DJI_0873	Pilosella echioides (Lumn.) F.W.Schultz & Sch.Bip.	0	n	1	0	Asteraceae Bercht. & J.Presl
Iz_13_1209	DJI_0873	Pilosella officinarum F. Schultz et Sch. Bip.	0	n	1	0	Asteraceae Bercht. & J.Presl
Iz_13_1209	DJI_0873	Pilosella sp.	0		NA	0	Asteraceae Bercht. & J.Presl
Iz_13_1209	DJI_0873	Pinus sylvestris L.	0	n	1	1	Pinaceae Spreng. ex F.Rudolphi
Iz_13_1209	DJI_0873	Poa annua L.	0	n	1	0	Poaceae Barnhart
Iz_13_1209	DJI_0873	Poa nemoralis L.	0	n	1	0	Poaceae Barnhart
Iz_13_1209	DJI_0873	Poa sp.	0		NA	0	Poaceae Barnhart
Iz_13_1209	DJI_0873	Polygonatum odoratum (Mill.) Druce	0	n	1	0	Asparagaceae Juss.
Iz_13_1209	DJI_0873	Polygonum arenarium Waldst. & Kit.	0	n	1	0	Polygonaceae Juss.
Iz_13_1209	DJI_0873	Populus nigra L.	0	n	1	1	Salicaceae Mirb.
Iz_13_1209	DJI_0873	Populus sp.	0	n	1	1	Salicaceae Mirb.
Iz_13_1209	DJI_0873	Populus tremula L.	0	n	1	1	Salicaceae Mirb.
Iz_13_1209	DJI_0873	Populus Ã canescens (Aiton) Sm.	0	n	1	1	Salicaceae Mirb.
Iz_13_1209	DJI_0873	Potentilla argentea L.	0	n	1	0	Rosaceae Juss.
Iz_13_1209	DJI_0873	Psammophiliella muralis (L.) Ikonn.	0	n	1	0	Caryophyllaceae Juss.
Iz_13_1209	DJI_0873	Psephellus sumensis (Kalen.) Greuter	0	n	1	0	Asteraceae Bercht. & J.Presl
Iz_13_1209	DJI_0873	Pulsatilla pratensis (L.) Mill. s.l.	0	n	1	0	Ranunculaceae Juss.
Iz_13_1209	DJI_0873	Pyrus communis L.	0	a	0	1	Rosaceae Juss.
Iz_13_1209	DJI_0873	Quercus robur L.	0	n	1	1	Fagaceae Dumort.
Iz_13_1209	DJI_0873	Robinia pseudoacacia L.	0	a	0	1	Fabaceae Lindl.
Iz_13_1209	DJI_0873	Rosa sp.	0		NA	1	Rosaceae Juss.
Iz_13_1209	DJI_0873	Rubus idaeus L.	0	n	1	0	Rosaceae Juss.
Iz_13_1209	DJI_0873	Rumex acetosella L.	0	n	1	0	Polygonaceae Juss.
Iz_13_1209	DJI_0873	Rumex thyrsiflorus Fingerh.	0	n	1	0	Polygonaceae Juss.
Iz_13_1209	DJI_0873	Salix alba L.	0	n	1	1	Salicaceae Mirb.
Iz_13_1209	DJI_0873	Sambucus nigra L.	0	n	1	1	Viburnaceae Raf.
Iz_13_1209	DJI_0873	Secale sylvestre Host	0	n	1	0	Poaceae Barnhart
Iz_13_1209	DJI_0873	Sempervivum ruthenicum Schnittsp. & C.B. Lehm.	0	n	1	0	Crassulaceae J.St.-Hil.
Iz_13_1209	DJI_0873	Setaria viridis (L.) P. Beauv.	0	n	1	0	Poaceae Barnhart
Iz_13_1209	DJI_0873	Silene borysthenica (Gruner) Walters	0	n	1	0	Caryophyllaceae Juss.
Iz_13_1209	DJI_0873	Silene latifolia subsp. alba (Mill.) Greuter & Burdet	0	n	1	0	Caryophyllaceae Juss.
Iz_13_1209	DJI_0873	Solidago virgaurea L.	0	n	1	0	Asteraceae Bercht. & J.Presl
Iz_13_1209	DJI_0873	Sorbus aucuparia L.	0	n	1	1	Rosaceae Juss.
Iz_13_1209	DJI_0873	Stachys recta L.	0	n	1	0	Lamiacea Lindl.
Iz_13_1209	DJI_0873	Tanacetum vulgare L.	0	n	1	0	Asteraceae Bercht. & J.Presl
Iz_13_1209	DJI_0873	Thymus pallasianus Heinr.Braun	0	n	1	0	Lamiacea Lindl.
Iz_13_1209	DJI_0873	Tilia cordata Mill.	0	n	1	1	Malvaceae Juss.
Iz_13_1209	DJI_0873	Trifolium alpestre L.	0	n	1	0	Fabaceae Lindl.
Iz_13_1209	DJI_0873	Trifolium arvense L.	0	n	1	0	Fabaceae Lindl.
Iz_13_1209	DJI_0873	Ulmus glabra Huds.	0	n	1	1	Ulmaceae Mirb.
Iz_13_1209	DJI_0873	Verbascum densiflorum Bertol.	0	n	1	0	Scrophulariaceae Juss.
Iz_13_1209	DJI_0873	Veronica austriaca L.	0	n	1	0	Plantaginaceae Juss.
Iz_13_1209	DJI_0873	Veronica incana L.	0	n	1	0	Plantaginaceae Juss.
Iz_13_1209	DJI_0873	Veronica verna L.	0	n	1	0	Plantaginaceae Juss.
Iz_13_1209	DJI_0873	Vincetoxicum hirundinaria Medik	0	n	1	0	Apocynaceae Juss.
Iz_13_1209	DJI_0875	Acer tataricum L.	0	n	1	1	Sapindaceae Juss.
Iz_13_1209	DJI_0875	Achillea millefolium L. s.l.	0	n	1	0	Asteraceae Bercht. & J.Presl
Iz_13_1209	DJI_0875	Allium oleraceum L.	0	n	1	0	Amaryllidaceae J.St.-Hil.
Iz_13_1209	DJI_0875	Ambrosia artemisiifolia L.	0	a	0	0	Asteraceae Bercht. & J.Presl
Iz_13_1209	DJI_0875	Amorpha fruticosa L.	0	a	0	0	Fabaceae Lindl.
Iz_13_1209	DJI_0875	Anthericum ramosum L.	0	n	1	0	Asparagaceae Juss.
Iz_13_1209	DJI_0875	Artemisia marschalliana Spreng.	0	n	1	0	Asteraceae Bercht. & J.Presl
Iz_13_1209	DJI_0875	Asparagus officinalis L.	0	n	1	0	Asparagaceae Juss.
Iz_13_1209	DJI_0875	Astragalus cicer L.	0	n	1	0	Fabaceae Lindl.
Iz_13_1209	DJI_0875	Bassia laniflora (S.G.Gmel.) A.J.Scott	0	n	1	0	Amaranthaceae Juss.
Iz_13_1209	DJI_0875	Berberis vulgaris L.	0	n	1	0	Berberidaceae Juss.
Iz_13_1209	DJI_0875	Betula pendula Roth.	0	n	1	1	Betulaceae Gray
Iz_13_1209	DJI_0875	Betula pendula Roth.	0	n	1	0	Poaceae Barnhart
Iz_13_1209	DJI_0875	Bromus inermis Leyss.	0		NA	NA	
Iz_13_1209	DJI_0875	Bromus squarrosus L.	0	a	0	0	Poaceae Barnhart
Iz_13_1209	DJI_0875	Bromus tectorum L.	0	a	0	0	Poaceae Barnhart
Iz_13_1209	DJI_0875	Calamagrostis epigejos (L.) Roth	5	n	1	0	Poaceae Barnhart
Iz_13_1209	DJI_0875	Carduus acanthoides L.	0	n	1	0	Asteraceae Bercht. & J.Presl
Iz_13_1209	DJI_0875	Carex sp.	0	n	1	0	Cyperaceae Juss.
Iz_13_1209	DJI_0875	Centaurea majorovii Dumbadze	0	n	1	0	Asteraceae Bercht. & J.Presl
Iz_13_1209	DJI_0875	Chamaecytisus ruthenicus (Fisch.ex Wol.) Klaskova	0	n	1	0	Fabaceae Lindl.
Iz_13_1209	DJI_0875	Chelidonium majus L.	0	n	1	0	Papaveraceae Juss.
Iz_13_1209	DJI_0875	Chenopodium betaceum Andrz.	0	n	1	0	Amaranthaceae Juss.
Iz_13_1209	DJI_0875	Chenopodium cf. album	0	n	1	0	Amaranthaceae Juss.
Iz_13_1209	DJI_0875	Chenopodium sp.	0		NA	0	Amaranthaceae Juss.
Iz_13_1209	DJI_0875	Chondrilla juncea L.	0	n	1	0	Asteraceae Bercht. & J.Presl
Iz_13_1209	DJI_0875	Cirsium vulgare (Savi) Ten.	0	n	1	0	Asteraceae Bercht. & J.Presl
Iz_13_1209	DJI_0875	Clinopodium acinos (L.) Kuntze	0	n	1	0	Lamiacea Lindl.

Iz_13_1209	DJI_0875	Convallaria majalis L.	0	n	1	0	Asparagaceae Juss.
Iz_13_1209	DJI_0875	Crataegus rhipidophylla Gand.	0	n	1	1	Rosaceae Juss.
Iz_13_1209	DJI_0875	Crepis foetida L.	0	n	1	0	Asteraceae Bercht. & J.Presl
Iz_13_1209	DJI_0875	Dactylis glomerata L.	0	n	1	0	Poaceae Barnhart
Iz_13_1209	DJI_0875	Dianthus campestris M.Bieb.	0	n	1	0	Caryophyllaceae Juss.
Iz_13_1209	DJI_0875	Digitaria sanguinalis (L.) Scop.	0	a	0	0	Poaceae Barnhart
Iz_13_1209	DJI_0875	Elymus repens (L.) Gould	0	n	1	0	Poaceae Barnhart
Iz_13_1209	DJI_0875	Epilobium tetragonum L.	0	n	1	0	Onagraceae Juss.
Iz_13_1209	DJI_0875	Eragrostis minor Host	0	a	0	0	Poaceae Barnhart
Iz_13_1209	DJI_0875	Erigeron canadensis L.	3	a	0	0	Asteraceae Bercht. & J.Presl
Iz_13_1209	DJI_0875	Euphorbia seguieriana Neck.	0	n	1	0	Euphorbiaceae Juss.
Iz_13_1209	DJI_0875	Fallopia dumetorum (L.) Holub	0	n	1	0	Polygonaceae Juss.
Iz_13_1209	DJI_0875	Festuca beckeri (Hack.) Trautv.	0	n	1	0	Poaceae Barnhart
Iz_13_1209	DJI_0875	Fragaria vesca L.	0	n	1	0	Rosaceae Juss.
Iz_13_1209	DJI_0875	Frangula alnus Mill.	0	n	1	1	Rhamnaceae Juss.
Iz_13_1209	DJI_0875	Fraxinus pennsylvanica Marshall	0	a	0	1	Oleaceae Hoffmanns. & Link
Iz_13_1209	DJI_0875	Galeopsis bifida Boenn.	0	n	1	0	Lamiacea Lindl.
Iz_13_1209	DJI_0875	Galium verum L.	0	n	1	0	Rubiaceae Juss.
Iz_13_1209	DJI_0875	Genista tinctoria L.	0	n	1	0	Fabaceae Lindl.
Iz_13_1209	DJI_0875	Geranium robertianum L.	0	n	1	0	Geraniaceae Juss.
Iz_13_1209	DJI_0875	Geranium sanquineum L.	0	n	1	0	Geraniaceae Juss.
Iz_13_1209	DJI_0875	Gypsophila paniculata L.	0	n	1	0	Caryophyllaceae Juss.
Iz_13_1209	DJI_0875	Helichrysum arenarium (L.) Moench	0	n	1	0	Asteraceae Bercht. & J.Presl
Iz_13_1209	DJI_0875	Herniaria polygama J.Gay	0	n	1	0	Caryophyllaceae Juss.
Iz_13_1209	DJI_0875	Hieracium robustum Fr.	0	n	1	0	Asteraceae Bercht. & J.Presl
Iz_13_1209	DJI_0875	Hieracium umbellatum L.	0	n	1	NA	Asteraceae Bercht. & J.Presl
Iz_13_1209	DJI_0875	Hylotelephium maximum (L.) Holub	0	n	1	0	Crassulaceae J.St.-Hil.
Iz_13_1209	DJI_0875	Jacobaea borysthenica (DC.) B.Nord. & Greuter	0	n	1	0	Asteraceae Bercht. & J.Presl
Iz_13_1209	DJI_0875	Jasione montana L.	0	n	1	0	Campanulaceae Juss.
Iz_13_1209	DJI_0875	Jurinea cyanoides (L.) Rchb.s.l.	0	n	1	0	Asteraceae Bercht. & J.Presl
Iz_13_1209	DJI_0875	Koeleria glauca (Spreng.) DC.	0	n	1	0	Poaceae Barnhart
Iz_13_1209	DJI_0875	Lactuca serriola L.	0	a	0	0	Asteraceae Bercht. & J.Presl
Iz_13_1209	DJI_0875	Linaria genistifolia (L.) Mill.	0	n	1	0	Plantaginaceae Juss.
Iz_13_1209	DJI_0875	Lomelosia argentea (L.) Greuter & Burdet	0	n	1	0	Caprifoliaceae Juss.
Iz_13_1209	DJI_0875	Melampyrum pratense L.	0	n	1	0	Orobanchaceae Vent.
Iz_13_1209	DJI_0875	Oenothera biennis L.	0	a	0	0	Onagraceae Juss.
Iz_13_1209	DJI_0875	Pentanema britannica (L.) D.Gut.Larr., Santos-Vicente, Anderb., E.Rico & M.M.Mart.Ort.	0	n	1	0	Asteraceae Bercht. & J.Presl
Iz_13_1209	DJI_0875	Peucedanum oreoselinum (L.) Moench	0	n	1	0	Apiaceae Lindl.
Iz_13_1209	DJI_0875	Peucedanum ruthenicum M.Bieb.	0	n	1	0	Apiaceae Lindl.
Iz_13_1209	DJI_0875	Pilosella echioides (Lumn.) F.W.Schultz & Sch.Bip.	0	n	1	0	Asteraceae Bercht. & J.Presl
Iz_13_1209	DJI_0875	Pilosella officinarum F. Schultz et Sch. Bip.	0	n	1	0	Asteraceae Bercht. & J.Presl
Iz_13_1209	DJI_0875	Pilosella sp.	0		NA	0	Asteraceae Bercht. & J.Presl
Iz_13_1209	DJI_0875	Pinus sylvestris L.	0	n	1	1	Pinaceae Spreng. ex F.Rudolphi
Iz_13_1209	DJI_0875	Poa annua L.	0	n	1	0	Poaceae Barnhart
Iz_13_1209	DJI_0875	Poa nemoralis L.	0	n	1	0	Poaceae Barnhart
Iz_13_1209	DJI_0875	Poa sp.	0		NA	0	Poaceae Barnhart
Iz_13_1209	DJI_0875	Polygonatum odoratum (Mill.) Druce	0	n	1	0	Asparagaceae Juss.
Iz_13_1209	DJI_0875	Polygonum arenarium Waldst. & Kit.	0	n	1	0	Polygonaceae Juss.
Iz_13_1209	DJI_0875	Populus nigra L.	0	n	1	1	Salicaceae Mirb.
Iz_13_1209	DJI_0875	Populus sp.	0	n	1	1	Salicaceae Mirb.
Iz_13_1209	DJI_0875	Populus tremula L.	0	n	1	1	Salicaceae Mirb.
Iz_13_1209	DJI_0875	Populus Ã canescens (Aiton) Sm.	0	n	1	1	Salicaceae Mirb.
Iz_13_1209	DJI_0875	Potentilla argentea L.	0	n	1	0	Rosaceae Juss.
Iz_13_1209	DJI_0875	Psammophiliella muralis (L.) Ikonn.	0	n	1	0	Caryophyllaceae Juss.
Iz_13_1209	DJI_0875	Psephellus sumensis (Kalen.) Greuter	0	n	1	0	Asteraceae Bercht. & J.Presl
Iz_13_1209	DJI_0875	Pulsatilla pratensis (L.) Mill. s.l.	0	n	1	0	Ranunculaceae Juss.
Iz_13_1209	DJI_0875	Pyrus communis L.	0	a	0	1	Rosaceae Juss.
Iz_13_1209	DJI_0875	Quercus robur L.	0	n	1	1	Fagaceae Dumort.
Iz_13_1209	DJI_0875	Robinia pseudoacacia L.	0	a	0	1	Fabaceae Lindl.
Iz_13_1209	DJI_0875	Rosa sp.	0		NA	1	Rosaceae Juss.
Iz_13_1209	DJI_0875	Rubus idaeus L.	0	n	1	0	Rosaceae Juss.
Iz_13_1209	DJI_0875	Rumex acetosella L.	0	n	1	0	Polygonaceae Juss.
Iz_13_1209	DJI_0875	Rumex thyrsiflorus Fingerh.	0	n	1	0	Polygonaceae Juss.
Iz_13_1209	DJI_0875	Salix alba L.	0	n	1	1	Salicaceae Mirb.
Iz_13_1209	DJI_0875	Sambucus nigra L.	0	n	1	1	Viburnaceae Raf.
Iz_13_1209	DJI_0875	Secale sylvestre Host	0	n	1	0	Poaceae Barnhart
Iz_13_1209	DJI_0875	Sempervivum ruthenicum Schnittsp. & C.B. Lehm.	0	n	1	0	Crassulaceae J.St.-Hil.
Iz_13_1209	DJI_0875	Setaria viridis (L.) P. Beauv.	0	n	1	0	Poaceae Barnhart
Iz_13_1209	DJI_0875	Silene borysthenica (Gruner) Walters	0	n	1	0	Caryophyllaceae Juss.
Iz_13_1209	DJI_0875	Silene latifolia subsp. alba (Mill.) Greuter & Burdet	0	n	1	0	Caryophyllaceae Juss.
Iz_13_1209	DJI_0875	Solidago virgaurea L.	0	n	1	0	Asteraceae Bercht. & J.Presl
Iz_13_1209	DJI_0875	Sorbus aucuparia L.	0	n	1	1	Rosaceae Juss.
Iz_13_1209	DJI_0875	Stachys recta L.	0	n	1	0	Lamiacea Lindl.
Iz_13_1209	DJI_0875	Tanacetum vulgare L.	0	n	1	0	Asteraceae Bercht. & J.Presl
Iz_13_1209	DJI_0875	Thymus pallasianus Heinr.Braun	0	n	1	0	Lamiacea Lindl.
Iz_13_1209	DJI_0875	Tilia cordata Mill.	0	n	1	1	Malvaceae Juss.
Iz_13_1209	DJI_0875	Trifolium alpestre L.	0	n	1	0	Fabaceae Lindl.
Iz_13_1209	DJI_0875	Trifolium arvense L.	0	n	1	0	Fabaceae Lindl.
Iz_13_1209	DJI_0875	Ulmus glabra Huds.	0	n	1	1	Ulmaceae Mirb.
Iz_13_1209	DJI_0875	Verbascum densiflorum Bertol.	0	n	1	0	Scrophulariaceae Juss.
Iz_13_1209	DJI_0875	Veronica austriaca L.	0	n	1	0	Plantaginaceae Juss.
Iz_13_1209	DJI_0875	Veronica incana L.	0	n	1	0	Plantaginaceae Juss.
Iz_13_1209	DJI_0875	Veronica verna L.	0	n	1	0	Plantaginaceae Juss.
Iz_13_1209	DJI_0875	Vincetoxicum hirundinaria Medik	0	n	1	0	Apocynaceae Juss.
Iz_13_1209	DJI_0878	Acer tataricum L.	0	n	1	1	Sapindaceae Juss.
Iz_13_1209	DJI_0878	Achillea millefolium L. s.l.	0	n	1	0	Asteraceae Bercht. & J.Presl
Iz_13_1209	DJI_0878	Allium oleraceum L.	0	n	1	0	Amaryllidaceae J.St.-Hil.
Iz_13_1209	DJI_0878	Ambrosia artemisiifolia L.	0	a	0	0	Asteraceae Bercht. & J.Presl
Iz_13_1209	DJI_0878	Amorpha fruticosa L.	0	a	0	0	Fabaceae Lindl.
Iz_13_1209	DJI_0878	Anthericum ramosum L.	0	n	1	0	Asparagaceae Juss.
Iz_13_1209	DJI_0878	Artemisia marschalliana Spreng.	0	n	1	0	Asteraceae Bercht. & J.Presl
Iz_13_1209	DJI_0878	Asparagus officinalis L.	0	n	1	0	Asparagaceae Juss.
Iz_13_1209	DJI_0878	Astragalus cicer L.	0	n	1	0	Fabaceae Lindl.
Iz_13_1209	DJI_0878	Bassia laniflora (S.G.Gmel.) A.J.Scott	0	n	1	0	Amaranthaceae Juss.
Iz_13_1209	DJI_0878	Berberis vulgaris L.	0	n	1	0	Berberidaceae Juss.
Iz_13_1209	DJI_0878	Betula pendula Roth.	0	n	1	1	Betulaceae Gray
Iz_13_1209	DJI_0878	Betula pendula Roth.	0	n	1	0	Poaceae Barnhart
Iz_13_1209	DJI_0878	Bromus inermis Leyss.	0		NA	NA	

Iz_13_1209	DJI_0878	Bromus squarrosus L.	0	a	0	0	Poaceae Barnhart
Iz_13_1209	DJI_0878	Bromus tectorum L.	0	a	0	0	Poaceae Barnhart
Iz_13_1209	DJI_0878	Calamagrostis epigejos (L.) Roth	2	n	1	0	Poaceae Barnhart
Iz_13_1209	DJI_0878	Carduus acanthoides L.	0	n	1	0	Asteraceae Bercht. & J.Presl
Iz_13_1209	DJI_0878	Carex sp.	0	n	1	0	Cyperaceae Juss.
Iz_13_1209	DJI_0878	Centaurea majorovii Dumbadze	0	n	1	0	Asteraceae Bercht. & J.Presl
Iz_13_1209	DJI_0878	Chamaecytisus ruthenicus (Fisch.ex Wol.) Klaskova	0	n	1	0	Fabaceae Lindl.
Iz_13_1209	DJI_0878	Chelidonium majus L.	0	n	1	0	Papaveraceae Juss.
Iz_13_1209	DJI_0878	Chenopodium betaceum Andrz.	0	n	1	0	Amaranthaceae Juss.
Iz_13_1209	DJI_0878	Chenopodium cf. album	0	n	1	0	Amaranthaceae Juss.
Iz_13_1209	DJI_0878	Chenopodium sp.	0		NA	0	Amaranthaceae Juss.
Iz_13_1209	DJI_0878	Chondrilla juncea L.	0	n	1	0	Asteraceae Bercht. & J.Presl
Iz_13_1209	DJI_0878	Cirsium vulgare (Savi) Ten.	0	n	1	0	Asteraceae Bercht. & J.Presl
Iz_13_1209	DJI_0878	Clinopodium acinos (L.) Kuntze	0	n	1	0	Lamiacea Lindl.
Iz_13_1209	DJI_0878	Convallaria majalis L.	0	n	1	0	Asparagaceae Juss.
Iz_13_1209	DJI_0878	Crataegus rhipidophylla Gand.	0	n	1	1	Rosaceae Juss.
Iz_13_1209	DJI_0878	Crepis foetida L.	0	n	1	0	Asteraceae Bercht. & J.Presl
Iz_13_1209	DJI_0878	Dactylis glomerata L.	0	n	1	0	Poaceae Barnhart
Iz_13_1209	DJI_0878	Dianthus campestris M.Bieb.	0	n	1	0	Caryophyllaceae Juss.
Iz_13_1209	DJI_0878	Digitaria sanguinalis (L.) Scop.	0	a	0	0	Poaceae Barnhart
Iz_13_1209	DJI_0878	Elymus repens (L.) Gould	0	n	1	0	Poaceae Barnhart
Iz_13_1209	DJI_0878	Epilobium tetragonum L.	0	n	1	0	Onagraceae Juss.
Iz_13_1209	DJI_0878	Eragrostis minor Host	0	a	0	0	Poaceae Barnhart
Iz_13_1209	DJI_0878	Erigeron canadensis L.	5	a	0	0	Asteraceae Bercht. & J.Presl
Iz_13_1209	DJI_0878	Euphorbia seguieriana Neck.	0	n	1	0	Euphorbiaceae Juss.
Iz_13_1209	DJI_0878	Fallopia dumetorum (L.) Holub	0	n	1	0	Polygonaceae Juss.
Iz_13_1209	DJI_0878	Festuca beckeri (Hack.) Trautv.	0	n	1	0	Poaceae Barnhart
Iz_13_1209	DJI_0878	Fragaria vesca L.	0	n	1	0	Rosaceae Juss.
Iz_13_1209	DJI_0878	Frangula alnus Mill.	0	n	1	1	Rhamnaceae Juss.
Iz_13_1209	DJI_0878	Fraxinus pennsylvanica Marshall	0	a	0	1	Oleaceae Hoffmanns. & Link
Iz_13_1209	DJI_0878	Galeopsis bifida Boenn.	0	n	1	0	Lamiacea Lindl.
Iz_13_1209	DJI_0878	Galium verum L.	0	n	1	0	Rubiaceae Juss.
Iz_13_1209	DJI_0878	Genista tinctoria L.	0	n	1	0	Fabaceae Lindl.
Iz_13_1209	DJI_0878	Geranium robertianum L.	0	n	1	0	Geraniaceae Juss.
Iz_13_1209	DJI_0878	Geranium sanquineum L.	0	n	1	0	Geraniaceae Juss.
Iz_13_1209	DJI_0878	Gypsophila paniculata L.	0	n	1	0	Caryophyllaceae Juss.
Iz_13_1209	DJI_0878	Helichrysum arenarium (L.) Moench	0	n	1	0	Asteraceae Bercht. & J.Presl
Iz_13_1209	DJI_0878	Herniaria polygama J.Gay	0	n	1	0	Caryophyllaceae Juss.
Iz_13_1209	DJI_0878	Hieracium robustum Fr.	0	n	1	0	Asteraceae Bercht. & J.Presl
Iz_13_1209	DJI_0878	Hieracium umbellatum L.	0	n	1	NA	Asteraceae Bercht. & J.Presl
Iz_13_1209	DJI_0878	Hylotelephium maximum (L.) Holub	0	n	1	0	Crassulaceae J.St.-Hil.
Iz_13_1209	DJI_0878	Jacobaea borysthenica (DC.) B.Nord. & Greuter	0	n	1	0	Asteraceae Bercht. & J.Presl
Iz_13_1209	DJI_0878	Jasione montana L.	0	n	1	0	Campanulaceae Juss.
Iz_13_1209	DJI_0878	Jurinea cyanoides (L.) Rchb.s.l.	0	n	1	0	Asteraceae Bercht. & J.Presl
Iz_13_1209	DJI_0878	Koeleria glauca (Spreng.) DC.	0	n	1	0	Poaceae Barnhart
Iz_13_1209	DJI_0878	Lactuca serriola L.	0	a	0	0	Asteraceae Bercht. & J.Presl
Iz_13_1209	DJI_0878	Linaria genistifolia (L.) Mill.	0	n	1	0	Plantaginaceae Juss.
Iz_13_1209	DJI_0878	Lomelosia argentea (L.) Greuter & Burdet	0	n	1	0	Caprifoliaceae Juss.
Iz_13_1209	DJI_0878	Melampyrum pratense L.	0	n	1	0	Orobanchaceae Vent.
Iz_13_1209	DJI_0878	Oenothera biennis L.	0	a	0	0	Onagraceae Juss.
Iz_13_1209	DJI_0878	Pentanema britannica (L.) D.Gut.Larr., Santos-Vicente, Anderb., E.Rico & M.M.Mart.Ort.	0	n	1	0	Asteraceae Bercht. & J.Presl
Iz_13_1209	DJI_0878	Peucedanum oreoselinum (L.) Moench	0	n	1	0	Apiaceae Lindl.
Iz_13_1209	DJI_0878	Peucedanum ruthenicum M.Bieb.	0	n	1	0	Apiaceae Lindl.
Iz_13_1209	DJI_0878	Pilosella echioides (Lumn.) F.W.Schultz & Sch.Bip.	0	n	1	0	Asteraceae Bercht. & J.Presl
Iz_13_1209	DJI_0878	Pilosella officinarum F. Schultz et Sch. Bip.	0	n	1	0	Asteraceae Bercht. & J.Presl
Iz_13_1209	DJI_0878	Pilosella sp.	0		NA	0	Asteraceae Bercht. & J.Presl
Iz_13_1209	DJI_0878	Pinus sylvestris L.	0	n	1	1	Pinaceae Spreng. ex F.Rudolphi
Iz_13_1209	DJI_0878	Poa annua L.	0	n	1	0	Poaceae Barnhart
Iz_13_1209	DJI_0878	Poa nemoralis L.	0	n	1	0	Poaceae Barnhart
Iz_13_1209	DJI_0878	Poa sp.	0		NA	0	Poaceae Barnhart
Iz_13_1209	DJI_0878	Polygonatum odoratum (Mill.) Druce	0	n	1	0	Asparagaceae Juss.
Iz_13_1209	DJI_0878	Polygonum arenarium Waldst. & Kit.	0	n	1	0	Polygonaceae Juss.
Iz_13_1209	DJI_0878	Populus nigra L.	0	n	1	1	Salicaceae Mirb.
Iz_13_1209	DJI_0878	Populus sp.	0	n	1	1	Salicaceae Mirb.
Iz_13_1209	DJI_0878	Populus tremula L.	0	n	1	1	Salicaceae Mirb.
Iz_13_1209	DJI_0878	Populus Ã canescens (Aiton) Sm.	0	n	1	1	Salicaceae Mirb.
Iz_13_1209	DJI_0878	Potentilla argentea L.	0	n	1	0	Rosaceae Juss.
Iz_13_1209	DJI_0878	Psammophiliella muralis (L.) Ikonn.	0	n	1	0	Caryophyllaceae Juss.
Iz_13_1209	DJI_0878	Psephellus sumensis (Kalen.) Greuter	0	n	1	0	Asteraceae Bercht. & J.Presl
Iz_13_1209	DJI_0878	Pulsatilla pratensis (L.) Mill. s.l.	0	n	1	0	Ranunculaceae Juss.
Iz_13_1209	DJI_0878	Pyrus communis L.	0	a	0	1	Rosaceae Juss.
Iz_13_1209	DJI_0878	Quercus robur L.	0	n	1	1	Fagaceae Dumort.
Iz_13_1209	DJI_0878	Robinia pseudoacacia L.	0	a	0	1	Fabaceae Lindl.
Iz_13_1209	DJI_0878	Rosa sp.	0		NA	1	Rosaceae Juss.
Iz_13_1209	DJI_0878	Rubus idaeus L.	0	n	1	0	Rosaceae Juss.
Iz_13_1209	DJI_0878	Rumex acetosella L.	0	n	1	0	Polygonaceae Juss.
Iz_13_1209	DJI_0878	Rumex thyrsiflorus Fingerh.	0	n	1	0	Polygonaceae Juss.
Iz_13_1209	DJI_0878	Salix alba L.	0	n	1	1	Salicaceae Mirb.
Iz_13_1209	DJI_0878	Sambucus nigra L.	0	n	1	1	Viburnaceae Raf.
Iz_13_1209	DJI_0878	Secale sylvestre Host	0	n	1	0	Poaceae Barnhart
Iz_13_1209	DJI_0878	Sempervivum ruthenicum Schnittsp. & C.B. Lehm.	0	n	1	0	Crassulaceae J.St.-Hil.
Iz_13_1209	DJI_0878	Setaria viridis (L.) P. Beauv.	0	n	1	0	Poaceae Barnhart
Iz_13_1209	DJI_0878	Silene borysthenica (Gruner) Walters	0	n	1	0	Caryophyllaceae Juss.
Iz_13_1209	DJI_0878	Silene latifolia subsp. alba (Mill.) Greuter & Burdet	0	n	1	0	Caryophyllaceae Juss.
Iz_13_1209	DJI_0878	Solidago virgaurea L.	0	n	1	0	Asteraceae Bercht. & J.Presl
Iz_13_1209	DJI_0878	Sorbus aucuparia L.	0	n	1	1	Rosaceae Juss.
Iz_13_1209	DJI_0878	Stachys recta L.	0	n	1	0	Lamiacea Lindl.
Iz_13_1209	DJI_0878	Tanacetum vulgare L.	0	n	1	0	Asteraceae Bercht. & J.Presl
Iz_13_1209	DJI_0878	Thymus pallasianus Heinr.Braun	0	n	1	0	Lamiacea Lindl.
Iz_13_1209	DJI_0878	Tilia cordata Mill.	0	n	1	1	Malvaceae Juss.
Iz_13_1209	DJI_0878	Trifolium alpestre L.	0	n	1	0	Fabaceae Lindl.
Iz_13_1209	DJI_0878	Trifolium arvense L.	0	n	1	0	Fabaceae Lindl.
Iz_13_1209	DJI_0878	Ulmus glabra Huds.	0	n	1	1	Ulmaceae Mirb.
Iz_13_1209	DJI_0878	Verbascum densiflorum Bertol.	0	n	1	0	Scrophulariaceae Juss.
Iz_13_1209	DJI_0878	Veronica austriaca L.	0	n	1	0	Plantaginaceae Juss.
Iz_13_1209	DJI_0878	Veronica incana L.	0	n	1	0	Plantaginaceae Juss.
Iz_13_1209	DJI_0878	Veronica verna L.	0	n	1	0	Plantaginaceae Juss.
Iz_13_1209	DJI_0878	Vincetoxicum hirundinaria Medik	0	n	1	0	Apocynaceae Juss.

Iz_13_1209	DJI_0883	Acer tataricum L.	0	n	1	1	Sapindaceae Juss.
Iz_13_1209	DJI_0883	Achillea millefolium L. s.l.	0	n	1	0	Asteraceae Bercht. & J.Presl
Iz_13_1209	DJI_0883	Allium oleraceum L.	0	n	1	0	Amaryllidaceae J.St.-Hil.
Iz_13_1209	DJI_0883	Ambrosia artemisiifolia L.	0	a	0	0	Asteraceae Bercht. & J.Presl
Iz_13_1209	DJI_0883	Amorpha fruticosa L.	0	a	0	0	Fabaceae Lindl.
Iz_13_1209	DJI_0883	Anthericum ramosum L.	0	n	1	0	Asparagaceae Juss.
Iz_13_1209	DJI_0883	Artemisia marschalliana Spreng.	0	n	1	0	Asteraceae Bercht. & J.Presl
Iz_13_1209	DJI_0883	Asparagus officinalis L.	0	n	1	0	Asparagaceae Juss.
Iz_13_1209	DJI_0883	Astragalus cicer L.	0	n	1	0	Fabaceae Lindl.
Iz_13_1209	DJI_0883	Bassia laniflora (S.G.Gmel.) A.J.Scott	0	n	1	0	Amaranthaceae Juss.
Iz_13_1209	DJI_0883	Berberis vulgaris L.	0	n	1	0	Berberidaceae Juss.
Iz_13_1209	DJI_0883	Betula pendula Roth.	0	n	1	1	Betulaceae Gray
Iz_13_1209	DJI_0883	Betula pendula Roth.	0	n	1	0	Poaceae Barnhart
Iz_13_1209	DJI_0883	Bromus inermis Leyss.	0		NA	NA	
Iz_13_1209	DJI_0883	Bromus squarrosus L.	0	a	0	0	Poaceae Barnhart
Iz_13_1209	DJI_0883	Bromus tectorum L.	0	a	0	0	Poaceae Barnhart
Iz_13_1209	DJI_0883	Calamagrostis epigejos (L.) Roth	2	n	1	0	Poaceae Barnhart
Iz_13_1209	DJI_0883	Carduus acanthoides L.	0	n	1	0	Asteraceae Bercht. & J.Presl
Iz_13_1209	DJI_0883	Carex sp.	0	n	1	0	Cyperaceae Juss.
Iz_13_1209	DJI_0883	Centaurea majorovii Dumbadze	0	n	1	0	Asteraceae Bercht. & J.Presl
Iz_13_1209	DJI_0883	Chamaecytisus ruthenicus (Fisch.ex Wol.) Klaskova	0	n	1	0	Fabaceae Lindl.
Iz_13_1209	DJI_0883	Chelidonium majus L.	0	n	1	0	Papaveraceae Juss.
Iz_13_1209	DJI_0883	Chenopodium betaceum Andrz.	0	n	1	0	Amaranthaceae Juss.
Iz_13_1209	DJI_0883	Chenopodium cf. album	0	n	1	0	Amaranthaceae Juss.
Iz_13_1209	DJI_0883	Chenopodium sp.	0		NA	0	Amaranthaceae Juss.
Iz_13_1209	DJI_0883	Chondrilla juncea L.	0	n	1	0	Asteraceae Bercht. & J.Presl
Iz_13_1209	DJI_0883	Cirsium vulgare (Savi) Ten.	0	n	1	0	Asteraceae Bercht. & J.Presl
Iz_13_1209	DJI_0883	Clinopodium acinos (L.) Kuntze	0	n	1	0	Lamiacea Lindl.
Iz_13_1209	DJI_0883	Convallaria majalis L.	0	n	1	0	Asparagaceae Juss.
Iz_13_1209	DJI_0883	Crataegus rhipidophylla Gand.	0	n	1	1	Rosaceae Juss.
Iz_13_1209	DJI_0883	Crepis foetida L.	0	n	1	0	Asteraceae Bercht. & J.Presl
Iz_13_1209	DJI_0883	Dactylis glomerata L.	0	n	1	0	Poaceae Barnhart
Iz_13_1209	DJI_0883	Dianthus campestris M.Bieb.	0	n	1	0	Caryophyllaceae Juss.
Iz_13_1209	DJI_0883	Digitaria sanguinalis (L.) Scop.	0	a	0	0	Poaceae Barnhart
Iz_13_1209	DJI_0883	Elymus repens (L.) Gould	0	n	1	0	Poaceae Barnhart
Iz_13_1209	DJI_0883	Epilobium tetragonum L.	0	n	1	0	Onagraceae Juss.
Iz_13_1209	DJI_0883	Eragrostis minor Host	0	a	0	0	Poaceae Barnhart
Iz_13_1209	DJI_0883	Erigeron canadensis L.	2	a	0	0	Asteraceae Bercht. & J.Presl
Iz_13_1209	DJI_0883	Euphorbia seguieriana Neck.	0	n	1	0	Euphorbiaceae Juss.
Iz_13_1209	DJI_0883	Fallopia dumetorum (L.) Holub	0	n	1	0	Polygonaceae Juss.
Iz_13_1209	DJI_0883	Festuca beckeri (Hack.) Trautv.	0	n	1	0	Poaceae Barnhart
Iz_13_1209	DJI_0883	Fragaria vesca L.	0	n	1	0	Rosaceae Juss.
Iz_13_1209	DJI_0883	Frangula alnus Mill.	0	n	1	1	Rhamnaceae Juss.
Iz_13_1209	DJI_0883	Fraxinus pennsylvanica Marshall	0	a	0	1	Oleaceae Hoffmanns. & Link
Iz_13_1209	DJI_0883	Galeopsis bifida Boenn.	0	n	1	0	Lamiacea Lindl.
Iz_13_1209	DJI_0883	Galium verum L.	0	n	1	0	Rubiaceae Juss.
Iz_13_1209	DJI_0883	Genista tinctoria L.	0	n	1	0	Fabaceae Lindl.
Iz_13_1209	DJI_0883	Geranium robertianum L.	0	n	1	0	Geraniaceae Juss.
Iz_13_1209	DJI_0883	Geranium sanquineum L.	0	n	1	0	Geraniaceae Juss.
Iz_13_1209	DJI_0883	Gypsophila paniculata L.	0	n	1	0	Caryophyllaceae Juss.
Iz_13_1209	DJI_0883	Helichrysum arenarium (L.) Moench	0	n	1	0	Asteraceae Bercht. & J.Presl
Iz_13_1209	DJI_0883	Herniaria polygama J.Gay	0	n	1	0	Caryophyllaceae Juss.
Iz_13_1209	DJI_0883	Hieracium robustum Fr.	0	n	1	0	Asteraceae Bercht. & J.Presl
Iz_13_1209	DJI_0883	Hieracium umbellatum L.	0	n	1	NA	Asteraceae Bercht. & J.Presl
Iz_13_1209	DJI_0883	Hylotelephium maximum (L.) Holub	0	n	1	0	Crassulaceae J.St.-Hil.
Iz_13_1209	DJI_0883	Jacobaea borysthenica (DC.) B.Nord. & Greuter	0	n	1	0	Asteraceae Bercht. & J.Presl
Iz_13_1209	DJI_0883	Jasione montana L.	0	n	1	0	Campanulaceae Juss.
Iz_13_1209	DJI_0883	Jurinea cyanoides (L.) Rchb.s.l.	0	n	1	0	Asteraceae Bercht. & J.Presl
Iz_13_1209	DJI_0883	Koeleria glauca (Spreng.) DC.	0	n	1	0	Poaceae Barnhart
Iz_13_1209	DJI_0883	Lactuca serriola L.	0	a	0	0	Asteraceae Bercht. & J.Presl
Iz_13_1209	DJI_0883	Linaria genistifolia (L.) Mill.	0	n	1	0	Plantaginaceae Juss.
Iz_13_1209	DJI_0883	Lomelosia argentea (L.) Greuter & Burdet	0	n	1	0	Caprifoliaceae Juss.
Iz_13_1209	DJI_0883	Melampyrum pratense L.	0	n	1	0	Orobanchaceae Vent.
Iz_13_1209	DJI_0883	Oenothera biennis L.	0	a	0	0	Onagraceae Juss.
Iz_13_1209	DJI_0883	Pentanema britannica (L.) D.Gut.Larr., Santos-Vicente, Anderb., E.Rico & M.M.Mart.Ort.	0	n	1	0	Asteraceae Bercht. & J.Presl
Iz_13_1209	DJI_0883	Peucedanum oreoselinum (L.) Moench	0	n	1	0	Apiaceae Lindl.
Iz_13_1209	DJI_0883	Peucedanum ruthenicum M.Bieb.	0	n	1	0	Apiaceae Lindl.
Iz_13_1209	DJI_0883	Pilosella echioides (Lumn.) F.W.Schultz & Sch.Bip.	0	n	1	0	Asteraceae Bercht. & J.Presl
Iz_13_1209	DJI_0883	Pilosella officinarum F. Schultz et Sch. Bip.	0	n	1	0	Asteraceae Bercht. & J.Presl
Iz_13_1209	DJI_0883	Pilosella sp.	0		NA	0	Asteraceae Bercht. & J.Presl
Iz_13_1209	DJI_0883	Pinus sylvestris L.	0	n	1	1	Pinaceae Spreng. ex F.Rudolphi
Iz_13_1209	DJI_0883	Poa annua L.	0	n	1	0	Poaceae Barnhart
Iz_13_1209	DJI_0883	Poa nemoralis L.	0	n	1	0	Poaceae Barnhart
Iz_13_1209	DJI_0883	Poa sp.	0		NA	0	Poaceae Barnhart
Iz_13_1209	DJI_0883	Polygonatum odoratum (Mill.) Druce	0	n	1	0	Asparagaceae Juss.
Iz_13_1209	DJI_0883	Polygonum arenarium Waldst. & Kit.	0	n	1	0	Polygonaceae Juss.
Iz_13_1209	DJI_0883	Populus nigra L.	0	n	1	1	Salicaceae Mirb.
Iz_13_1209	DJI_0883	Populus sp.	0	n	1	1	Salicaceae Mirb.
Iz_13_1209	DJI_0883	Populus tremula L.	0	n	1	1	Salicaceae Mirb.
Iz_13_1209	DJI_0883	Populus Ã canescens (Aiton) Sm.	0	n	1	1	Salicaceae Mirb.
Iz_13_1209	DJI_0883	Potentilla argentea L.	0	n	1	0	Rosaceae Juss.
Iz_13_1209	DJI_0883	Psammophiliella muralis (L.) Ikonn.	0	n	1	0	Caryophyllaceae Juss.
Iz_13_1209	DJI_0883	Psephellus sumensis (Kalen.) Greuter	0	n	1	0	Asteraceae Bercht. & J.Presl
Iz_13_1209	DJI_0883	Pulsatilla pratensis (L.) Mill. s.l.	0	n	1	0	Ranunculaceae Juss.
Iz_13_1209	DJI_0883	Pyrus communis L.	0	a	0	1	Rosaceae Juss.
Iz_13_1209	DJI_0883	Quercus robur L.	0	n	1	1	Fagaceae Dumort.
Iz_13_1209	DJI_0883	Robinia pseudoacacia L.	0	a	0	1	Fabaceae Lindl.
Iz_13_1209	DJI_0883	Rosa sp.	0		NA	1	Rosaceae Juss.
Iz_13_1209	DJI_0883	Rubus idaeus L.	0	n	1	0	Rosaceae Juss.
Iz_13_1209	DJI_0883	Rumex acetosella L.	0	n	1	0	Polygonaceae Juss.
Iz_13_1209	DJI_0883	Rumex thyrsiflorus Fingerh.	0	n	1	0	Polygonaceae Juss.
Iz_13_1209	DJI_0883	Salix alba L.	0	n	1	1	Salicaceae Mirb.
Iz_13_1209	DJI_0883	Sambucus nigra L.	0	n	1	1	Viburnaceae Raf.
Iz_13_1209	DJI_0883	Secale sylvestre Host	0	n	1	0	Poaceae Barnhart
Iz_13_1209	DJI_0883	Sempervivum ruthenicum Schnittsp. & C.B. Lehm.	0	n	1	0	Crassulaceae J.St.-Hil.
Iz_13_1209	DJI_0883	Setaria viridis (L.) P. Beauv.	0	n	1	0	Poaceae Barnhart
Iz_13_1209	DJI_0883	Silene borysthenica (Gruner) Walters	0	n	1	0	Caryophyllaceae Juss.
Iz_13_1209	DJI_0883	Silene latifolia subsp. alba (Mill.) Greuter & Burdet	0	n	1	0	Caryophyllaceae Juss.

Iz_13_1209	DJI_0883	Solidago virgaurea L.	0	n	1	0	Asteraceae Bercht. & J.Presl
Iz_13_1209	DJI_0883	Sorbus aucuparia L.	0	n	1	1	Rosaceae Juss.
Iz_13_1209	DJI_0883	Stachys recta L.	0	n	1	0	Lamiacea Lindl.
Iz_13_1209	DJI_0883	Tanacetum vulgare L.	0	n	1	0	Asteraceae Bercht. & J.Presl
Iz_13_1209	DJI_0883	Thymus pallasianus Heinr.Braun	0	n	1	0	Lamiacea Lindl.
Iz_13_1209	DJI_0883	Tilia cordata Mill.	0	n	1	1	Malvaceae Juss.
Iz_13_1209	DJI_0883	Trifolium alpestre L.	0	n	1	0	Fabaceae Lindl.
Iz_13_1209	DJI_0883	Trifolium arvense L.	0	n	1	0	Fabaceae Lindl.
Iz_13_1209	DJI_0883	Ulmus glabra Huds.	0	n	1	1	Ulmaceae Mirb.
Iz_13_1209	DJI_0883	Verbascum densiflorum Bertol.	0	n	1	0	Scrophulariaceae Juss.
Iz_13_1209	DJI_0883	Veronica austriaca L.	0	n	1	0	Plantaginaceae Juss.
Iz_13_1209	DJI_0883	Veronica incana L.	0	n	1	0	Plantaginaceae Juss.
Iz_13_1209	DJI_0883	Veronica verna L.	0	n	1	0	Plantaginaceae Juss.
Iz_13_1209	DJI_0883	Vincetoxicum hirundinaria Medik	0	n	1	0	Apocynaceae Juss.
Iz_13_1209	DJI_0887	Acer tataricum L.	0	n	1	1	Sapindaceae Juss.
Iz_13_1209	DJI_0887	Achillea millefolium L. s.l.	0	n	1	0	Asteraceae Bercht. & J.Presl
Iz_13_1209	DJI_0887	Allium oleraceum L.	0	n	1	0	Amaryllidaceae J.St.-Hil.
Iz_13_1209	DJI_0887	Ambrosia artemisiifolia L.	0	a	0	0	Asteraceae Bercht. & J.Presl
Iz_13_1209	DJI_0887	Amorpha fruticosa L.	0	a	0	0	Fabaceae Lindl.
Iz_13_1209	DJI_0887	Anthericum ramosum L.	0	n	1	0	Asparagaceae Juss.
Iz_13_1209	DJI_0887	Artemisia marschalliana Spreng.	0	n	1	0	Asteraceae Bercht. & J.Presl
Iz_13_1209	DJI_0887	Asparagus officinalis L.	0	n	1	0	Asparagaceae Juss.
Iz_13_1209	DJI_0887	Astragalus cicer L.	0	n	1	0	Fabaceae Lindl.
Iz_13_1209	DJI_0887	Bassia laniflora (S.G.Gmel.) A.J.Scott	0	n	1	0	Amaranthaceae Juss.
Iz_13_1209	DJI_0887	Berberis vulgaris L.	0	n	1	0	Berberidaceae Juss.
Iz_13_1209	DJI_0887	Betula pendula Roth.	0	n	1	1	Betulaceae Gray
Iz_13_1209	DJI_0887	Betula pendula Roth.	0	n	1	0	Poaceae Barnhart
Iz_13_1209	DJI_0887	Bromus inermis Leyss.	0		NA	NA	
Iz_13_1209	DJI_0887	Bromus squarrosus L.	0	a	0	0	Poaceae Barnhart
Iz_13_1209	DJI_0887	Bromus tectorum L.	0	a	0	0	Poaceae Barnhart
Iz_13_1209	DJI_0887	Calamagrostis epigejos (L.) Roth	1	n	1	0	Poaceae Barnhart
Iz_13_1209	DJI_0887	Carduus acanthoides L.	0	n	1	0	Asteraceae Bercht. & J.Presl
Iz_13_1209	DJI_0887	Carex sp.	0	n	1	0	Cyperaceae Juss.
Iz_13_1209	DJI_0887	Centaurea majorovii Dumbadze	0	n	1	0	Asteraceae Bercht. & J.Presl
Iz_13_1209	DJI_0887	Chamaecytisus ruthenicus (Fisch.ex Wol.) Klaskova	0	n	1	0	Fabaceae Lindl.
Iz_13_1209	DJI_0887	Chelidonium majus L.	0	n	1	0	Papaveraceae Juss.
Iz_13_1209	DJI_0887	Chenopodium betaceum Andrz.	0	n	1	0	Amaranthaceae Juss.
Iz_13_1209	DJI_0887	Chenopodium cf. album	0	n	1	0	Amaranthaceae Juss.
Iz_13_1209	DJI_0887	Chenopodium sp.	0		NA	0	Amaranthaceae Juss.
Iz_13_1209	DJI_0887	Chondrilla juncea L.	0	n	1	0	Asteraceae Bercht. & J.Presl
Iz_13_1209	DJI_0887	Cirsium vulgare (Savi) Ten.	0	n	1	0	Asteraceae Bercht. & J.Presl
Iz_13_1209	DJI_0887	Clinopodium acinos (L.) Kuntze	0	n	1	0	Lamiacea Lindl.
Iz_13_1209	DJI_0887	Convallaria majalis L.	0	n	1	0	Asparagaceae Juss.
Iz_13_1209	DJI_0887	Crataegus rhipidophylla Gand.	0	n	1	1	Rosaceae Juss.
Iz_13_1209	DJI_0887	Crepis foetida L.	0	n	1	0	Asteraceae Bercht. & J.Presl
Iz_13_1209	DJI_0887	Dactylis glomerata L.	0	n	1	0	Poaceae Barnhart
Iz_13_1209	DJI_0887	Dianthus campestris M.Bieb.	0	n	1	0	Caryophyllaceae Juss.
Iz_13_1209	DJI_0887	Digitaria sanguinalis (L.) Scop.	0	a	0	0	Poaceae Barnhart
Iz_13_1209	DJI_0887	Elymus repens (L.) Gould	0	n	1	0	Poaceae Barnhart
Iz_13_1209	DJI_0887	Epilobium tetragonum L.	0	n	1	0	Onagraceae Juss.
Iz_13_1209	DJI_0887	Eragrostis minor Host	0	a	0	0	Poaceae Barnhart
Iz_13_1209	DJI_0887	Erigeron canadensis L.	2	a	0	0	Asteraceae Bercht. & J.Presl
Iz_13_1209	DJI_0887	Euphorbia seguieriana Neck.	0	n	1	0	Euphorbiaceae Juss.
Iz_13_1209	DJI_0887	Fallopia dumetorum (L.) Holub	0	n	1	0	Polygonaceae Juss.
Iz_13_1209	DJI_0887	Festuca beckeri (Hack.) Trautv.	0	n	1	0	Poaceae Barnhart
Iz_13_1209	DJI_0887	Fragaria vesca L.	0	n	1	0	Rosaceae Juss.
Iz_13_1209	DJI_0887	Frangula alnus Mill.	0	n	1	1	Rhamnaceae Juss.
Iz_13_1209	DJI_0887	Fraxinus pennsylvanica Marshall	0	a	0	1	Oleaceae Hoffmanns. & Link
Iz_13_1209	DJI_0887	Galeopsis bifida Boenn.	0	n	1	0	Lamiacea Lindl.
Iz_13_1209	DJI_0887	Galium verum L.	0	n	1	0	Rubiaceae Juss.
Iz_13_1209	DJI_0887	Genista tinctoria L.	0	n	1	0	Fabaceae Lindl.
Iz_13_1209	DJI_0887	Geranium robertianum L.	0	n	1	0	Geraniaceae Juss.
Iz_13_1209	DJI_0887	Geranium sanquineum L.	0	n	1	0	Geraniaceae Juss.
Iz_13_1209	DJI_0887	Gypsophila paniculata L.	0	n	1	0	Caryophyllaceae Juss.
Iz_13_1209	DJI_0887	Helichrysum arenarium (L.) Moench	0	n	1	0	Asteraceae Bercht. & J.Presl
Iz_13_1209	DJI_0887	Herniaria polygama J.Gay	0	n	1	0	Caryophyllaceae Juss.
Iz_13_1209	DJI_0887	Hieracium robustum Fr.	0	n	1	0	Asteraceae Bercht. & J.Presl
Iz_13_1209	DJI_0887	Hieracium umbellatum L.	0	n	1	NA	Asteraceae Bercht. & J.Presl
Iz_13_1209	DJI_0887	Hylotelephium maximum (L.) Holub	0	n	1	0	Crassulaceae J.St.-Hil.
Iz_13_1209	DJI_0887	Jacobaea borysthenica (DC.) B.Nord. & Greuter	0	n	1	0	Asteraceae Bercht. & J.Presl
Iz_13_1209	DJI_0887	Jasione montana L.	0	n	1	0	Campanulaceae Juss.
Iz_13_1209	DJI_0887	Jurinea cyanoides (L.) Rchb.s.l.	0	n	1	0	Asteraceae Bercht. & J.Presl
Iz_13_1209	DJI_0887	Koeleria glauca (Spreng.) DC.	0	n	1	0	Poaceae Barnhart
Iz_13_1209	DJI_0887	Lactuca serriola L.	0	a	0	0	Asteraceae Bercht. & J.Presl
Iz_13_1209	DJI_0887	Linaria genistifolia (L.) Mill.	0	n	1	0	Plantaginaceae Juss.
Iz_13_1209	DJI_0887	Lomelosia argentea (L.) Greuter & Burdet	0	n	1	0	Caprifoliaceae Juss.
Iz_13_1209	DJI_0887	Melampyrum pratense L.	0	n	1	0	Orobanchaceae Vent.
Iz_13_1209	DJI_0887	Oenothera biennis L.	0	a	0	0	Onagraceae Juss.
Iz_13_1209	DJI_0887	Pentanema britannica (L.) D.Gut.Larr., Santos-Vicente, Anderb., E.Rico & M.M.Mart.Ort.	0	n	1	0	Asteraceae Bercht. & J.Presl
Iz_13_1209	DJI_0887	Peucedanum oreoselinum (L.) Moench	0	n	1	0	Apiaceae Lindl.
Iz_13_1209	DJI_0887	Peucedanum ruthenicum M.Bieb.	0	n	1	0	Apiaceae Lindl.
Iz_13_1209	DJI_0887	Pilosella echioides (Lumn.) F.W.Schultz & Sch.Bip.	0	n	1	0	Asteraceae Bercht. & J.Presl
Iz_13_1209	DJI_0887	Pilosella officinarum F. Schultz et Sch. Bip.	0	n	1	0	Asteraceae Bercht. & J.Presl
Iz_13_1209	DJI_0887	Pilosella sp.	0		NA	0	Asteraceae Bercht. & J.Presl
Iz_13_1209	DJI_0887	Pinus sylvestris L.	0	n	1	1	Pinaceae Spreng. ex F.Rudolphi
Iz_13_1209	DJI_0887	Poa annua L.	0	n	1	0	Poaceae Barnhart
Iz_13_1209	DJI_0887	Poa nemoralis L.	0	n	1	0	Poaceae Barnhart
Iz_13_1209	DJI_0887	Poa sp.	0		NA	0	Poaceae Barnhart
Iz_13_1209	DJI_0887	Polygonatum odoratum (Mill.) Druce	0	n	1	0	Asparagaceae Juss.
Iz_13_1209	DJI_0887	Polygonum arenarium Waldst. & Kit.	0	n	1	0	Polygonaceae Juss.
Iz_13_1209	DJI_0887	Populus nigra L.	0	n	1	1	Salicaceae Mirb.
Iz_13_1209	DJI_0887	Populus sp.	0	n	1	1	Salicaceae Mirb.
Iz_13_1209	DJI_0887	Populus tremula L.	0	n	1	1	Salicaceae Mirb.
Iz_13_1209	DJI_0887	Populus Ã canescens (Aiton) Sm.	0	n	1	1	Salicaceae Mirb.
Iz_13_1209	DJI_0887	Potentilla argentea L.	0	n	1	0	Rosaceae Juss.
Iz_13_1209	DJI_0887	Psammophiliella muralis (L.) Ikonn.	0	n	1	0	Caryophyllaceae Juss.
Iz_13_1209	DJI_0887	Psephellus sumensis (Kalen.) Greuter	0	n	1	0	Asteraceae Bercht. & J.Presl
Iz_13_1209	DJI_0887	Pulsatilla pratensis (L.) Mill. s.l.	0	n	1	0	Ranunculaceae Juss.

Iz_13_1209	DJI_0887	Pyrus communis L.	0	a	0	1	Rosaceae Juss.
Iz_13_1209	DJI_0887	Quercus robur L.	0	n	1	1	Fagaceae Dumort.
Iz_13_1209	DJI_0887	Robinia pseudoacacia L.	0	a	0	1	Fabaceae Lindl.
Iz_13_1209	DJI_0887	Rosa sp.	0		NA	1	Rosaceae Juss.
Iz_13_1209	DJI_0887	Rubus idaeus L.	0	n	1	0	Rosaceae Juss.
Iz_13_1209	DJI_0887	Rumex acetosella L.	0	n	1	0	Polygonaceae Juss.
Iz_13_1209	DJI_0887	Rumex thyrsiflorus Fingerh.	0	n	1	0	Polygonaceae Juss.
Iz_13_1209	DJI_0887	Salix alba L.	0	n	1	1	Salicaceae Mirb.
Iz_13_1209	DJI_0887	Sambucus nigra L.	0	n	1	1	Viburnaceae Raf.
Iz_13_1209	DJI_0887	Secale sylvestre Host	0	n	1	0	Poaceae Barnhart
Iz_13_1209	DJI_0887	Sempervivum ruthenicum Schnittsp. & C.B. Lehm.	0	n	1	0	Crassulaceae J.St.-Hil.
Iz_13_1209	DJI_0887	Setaria viridis (L.) P. Beauv.	0	n	1	0	Poaceae Barnhart
Iz_13_1209	DJI_0887	Silene borysthenica (Gruner) Walters	0	n	1	0	Caryophyllaceae Juss.
Iz_13_1209	DJI_0887	Silene latifolia subsp. alba (Mill.) Greuter & Burdet	0	n	1	0	Caryophyllaceae Juss.
Iz_13_1209	DJI_0887	Solidago virgaurea L.	0	n	1	0	Asteraceae Bercht. & J.Presl
Iz_13_1209	DJI_0887	Sorbus aucuparia L.	0	n	1	1	Rosaceae Juss.
Iz_13_1209	DJI_0887	Stachys recta L.	0	n	1	0	Lamiacea Lindl.
Iz_13_1209	DJI_0887	Tanacetum vulgare L.	0	n	1	0	Asteraceae Bercht. & J.Presl
Iz_13_1209	DJI_0887	Thymus pallasianus Heinr.Braun	0	n	1	0	Lamiacea Lindl.
Iz_13_1209	DJI_0887	Tilia cordata Mill.	0	n	1	1	Malvaceae Juss.
Iz_13_1209	DJI_0887	Trifolium alpestre L.	0	n	1	0	Fabaceae Lindl.
Iz_13_1209	DJI_0887	Trifolium arvense L.	0	n	1	0	Fabaceae Lindl.
Iz_13_1209	DJI_0887	Ulmus glabra Huds.	0	n	1	1	Ulmaceae Mirb.
Iz_13_1209	DJI_0887	Verbascum densiflorum Bertol.	0	n	1	0	Scrophulariaceae Juss.
Iz_13_1209	DJI_0887	Veronica austriaca L.	0	n	1	0	Plantaginaceae Juss.
Iz_13_1209	DJI_0887	Veronica incana L.	0	n	1	0	Plantaginaceae Juss.
Iz_13_1209	DJI_0887	Veronica verna L.	0	n	1	0	Plantaginaceae Juss.
Iz_13_1209	DJI_0887	Vincetoxicum hirundinaria Medik	0	n	1	0	Apocynaceae Juss.
Iz_13_1209	DJI_0890	Acer tataricum L.	0	n	1	1	Sapindaceae Juss.
Iz_13_1209	DJI_0890	Achillea millefolium L. s.l.	0	n	1	0	Asteraceae Bercht. & J.Presl
Iz_13_1209	DJI_0890	Allium oleraceum L.	0	n	1	0	Amaryllidaceae J.St.-Hil.
Iz_13_1209	DJI_0890	Ambrosia artemisiifolia L.	0	a	0	0	Asteraceae Bercht. & J.Presl
Iz_13_1209	DJI_0890	Amorpha fruticosa L.	0	a	0	0	Fabaceae Lindl.
Iz_13_1209	DJI_0890	Anthericum ramosum L.	0	n	1	0	Asparagaceae Juss.
Iz_13_1209	DJI_0890	Artemisia marschalliana Spreng.	0	n	1	0	Asteraceae Bercht. & J.Presl
Iz_13_1209	DJI_0890	Asparagus officinalis L.	0	n	1	0	Asparagaceae Juss.
Iz_13_1209	DJI_0890	Astragalus cicer L.	0	n	1	0	Fabaceae Lindl.
Iz_13_1209	DJI_0890	Bassia laniflora (S.G.Gmel.) A.J.Scott	0	n	1	0	Amaranthaceae Juss.
Iz_13_1209	DJI_0890	Berberis vulgaris L.	0	n	1	0	Berberidaceae Juss.
Iz_13_1209	DJI_0890	Betula pendula Roth.	0	n	1	1	Betulaceae Gray
Iz_13_1209	DJI_0890	Betula pendula Roth.	0	n	1	0	Poaceae Barnhart
Iz_13_1209	DJI_0890	Bromus inermis Leyss.	0		NA	NA	
Iz_13_1209	DJI_0890	Bromus squarrosus L.	0	a	0	0	Poaceae Barnhart
Iz_13_1209	DJI_0890	Bromus tectorum L.	0	a	0	0	Poaceae Barnhart
Iz_13_1209	DJI_0890	Calamagrostis epigejos (L.) Roth	3	n	1	0	Poaceae Barnhart
Iz_13_1209	DJI_0890	Carduus acanthoides L.	0	n	1	0	Asteraceae Bercht. & J.Presl
Iz_13_1209	DJI_0890	Carex sp.	0	n	1	0	Cyperaceae Juss.
Iz_13_1209	DJI_0890	Centaurea majorovii Dumbadze	0	n	1	0	Asteraceae Bercht. & J.Presl
Iz_13_1209	DJI_0890	Chamaecytisus ruthenicus (Fisch.ex Wol.) Klaskova	0	n	1	0	Fabaceae Lindl.
Iz_13_1209	DJI_0890	Chelidonium majus L.	0	n	1	0	Papaveraceae Juss.
Iz_13_1209	DJI_0890	Chenopodium betaceum Andrz.	0	n	1	0	Amaranthaceae Juss.
Iz_13_1209	DJI_0890	Chenopodium cf. album	0	n	1	0	Amaranthaceae Juss.
Iz_13_1209	DJI_0890	Chenopodium sp.	0		NA	0	Amaranthaceae Juss.
Iz_13_1209	DJI_0890	Chondrilla juncea L.	0	n	1	0	Asteraceae Bercht. & J.Presl
Iz_13_1209	DJI_0890	Cirsium vulgare (Savi) Ten.	0	n	1	0	Asteraceae Bercht. & J.Presl
Iz_13_1209	DJI_0890	Clinopodium acinos (L.) Kuntze	0	n	1	0	Lamiacea Lindl.
Iz_13_1209	DJI_0890	Convallaria majalis L.	0	n	1	0	Asparagaceae Juss.
Iz_13_1209	DJI_0890	Crataegus rhipidophylla Gand.	0	n	1	1	Rosaceae Juss.
Iz_13_1209	DJI_0890	Crepis foetida L.	0	n	1	0	Asteraceae Bercht. & J.Presl
Iz_13_1209	DJI_0890	Dactylis glomerata L.	0	n	1	0	Poaceae Barnhart
Iz_13_1209	DJI_0890	Dianthus campestris M.Bieb.	0	n	1	0	Caryophyllaceae Juss.
Iz_13_1209	DJI_0890	Digitaria sanguinalis (L.) Scop.	0	a	0	0	Poaceae Barnhart
Iz_13_1209	DJI_0890	Elymus repens (L.) Gould	0	n	1	0	Poaceae Barnhart
Iz_13_1209	DJI_0890	Epilobium tetragonum L.	0	n	1	0	Onagraceae Juss.
Iz_13_1209	DJI_0890	Eragrostis minor Host	0	a	0	0	Poaceae Barnhart
Iz_13_1209	DJI_0890	Erigeron canadensis L.	5	a	0	0	Asteraceae Bercht. & J.Presl
Iz_13_1209	DJI_0890	Euphorbia seguieriana Neck.	0	n	1	0	Euphorbiaceae Juss.
Iz_13_1209	DJI_0890	Fallopia dumetorum (L.) Holub	0	n	1	0	Polygonaceae Juss.
Iz_13_1209	DJI_0890	Festuca beckeri (Hack.) Trautv.	0	n	1	0	Poaceae Barnhart
Iz_13_1209	DJI_0890	Fragaria vesca L.	0	n	1	0	Rosaceae Juss.
Iz_13_1209	DJI_0890	Frangula alnus Mill.	0	n	1	1	Rhamnaceae Juss.
Iz_13_1209	DJI_0890	Fraxinus pennsylvanica Marshall	0	a	0	1	Oleaceae Hoffmanns. & Link
Iz_13_1209	DJI_0890	Galeopsis bifida Boenn.	0	n	1	0	Lamiacea Lindl.
Iz_13_1209	DJI_0890	Galium verum L.	0	n	1	0	Rubiaceae Juss.
Iz_13_1209	DJI_0890	Genista tinctoria L.	0	n	1	0	Fabaceae Lindl.
Iz_13_1209	DJI_0890	Geranium robertianum L.	0	n	1	0	Geraniaceae Juss.
Iz_13_1209	DJI_0890	Geranium sanquineum L.	0	n	1	0	Geraniaceae Juss.
Iz_13_1209	DJI_0890	Gypsophila paniculata L.	0	n	1	0	Caryophyllaceae Juss.
Iz_13_1209	DJI_0890	Helichrysum arenarium (L.) Moench	0	n	1	0	Asteraceae Bercht. & J.Presl
Iz_13_1209	DJI_0890	Herniaria polygama J.Gay	0	n	1	0	Caryophyllaceae Juss.
Iz_13_1209	DJI_0890	Hieracium robustum Fr.	0	n	1	0	Asteraceae Bercht. & J.Presl
Iz_13_1209	DJI_0890	Hieracium umbellatum L.	0	n	1	NA	Asteraceae Bercht. & J.Presl
Iz_13_1209	DJI_0890	Hylotelephium maximum (L.) Holub	0	n	1	0	Crassulaceae J.St.-Hil.
Iz_13_1209	DJI_0890	Jacobaea borysthenica (DC.) B.Nord. & Greuter	0	n	1	0	Asteraceae Bercht. & J.Presl
Iz_13_1209	DJI_0890	Jasione montana L.	0	n	1	0	Campanulaceae Juss.
Iz_13_1209	DJI_0890	Jurinea cyanoides (L.) Rchb.s.l.	0	n	1	0	Asteraceae Bercht. & J.Presl
Iz_13_1209	DJI_0890	Koeleria glauca (Spreng.) DC.	0	n	1	0	Poaceae Barnhart
Iz_13_1209	DJI_0890	Lactuca serriola L.	0	a	0	0	Asteraceae Bercht. & J.Presl
Iz_13_1209	DJI_0890	Linaria genistifolia (L.) Mill.	0	n	1	0	Plantaginaceae Juss.
Iz_13_1209	DJI_0890	Lomelosia argentea (L.) Greuter & Burdet	0	n	1	0	Caprifoliaceae Juss.
Iz_13_1209	DJI_0890	Melampyrum pratense L.	0	n	1	0	Orobanchaceae Vent.
Iz_13_1209	DJI_0890	Oenothera biennis L.	0	a	0	0	Onagraceae Juss.
Iz_13_1209	DJI_0890	Pentanema britannica (L.) D.Gut.Larr., Santos-Vicente, Anderb., E.Rico & M.M.Mart.Ort.	0	n	1	0	Asteraceae Bercht. & J.Presl
Iz_13_1209	DJI_0890	Peucedanum oreoselinum (L.) Moench	0	n	1	0	Apiaceae Lindl.
Iz_13_1209	DJI_0890	Peucedanum ruthenicum M.Bieb.	0	n	1	0	Apiaceae Lindl.
Iz_13_1209	DJI_0890	Pilosella echioides (Lumn.) F.W.Schultz & Sch.Bip.	0	n	1	0	Asteraceae Bercht. & J.Presl
Iz_13_1209	DJI_0890	Pilosella officinarum F. Schultz et Sch. Bip.	0	n	1	0	Asteraceae Bercht. & J.Presl
Iz_13_1209	DJI_0890	Pilosella sp.	0		NA	0	Asteraceae Bercht. & J.Presl

Iz_13_1209	DJI_0890	Pinus sylvestris L.	0	n	1	1	Pinaceae Spreng. ex F.Rudolphi
Iz_13_1209	DJI_0890	Poa annua L.	0	n	1	0	Poaceae Barnhart
Iz_13_1209	DJI_0890	Poa nemoralis L.	0	n	1	0	Poaceae Barnhart
Iz_13_1209	DJI_0890	Poa sp.	0		NA	0	Poaceae Barnhart
Iz_13_1209	DJI_0890	Polygonatum odoratum (Mill.) Druce	0	n	1	0	Asparagaceae Juss.
Iz_13_1209	DJI_0890	Polygonum arenarium Waldst. & Kit.	0	n	1	0	Polygonaceae Juss.
Iz_13_1209	DJI_0890	Populus nigra L.	0	n	1	1	Salicaceae Mirb.
Iz_13_1209	DJI_0890	Populus sp.	0	n	1	1	Salicaceae Mirb.
Iz_13_1209	DJI_0890	Populus tremula L.	0	n	1	1	Salicaceae Mirb.
Iz_13_1209	DJI_0890	Populus Ã canescens (Aiton) Sm.	0	n	1	1	Salicaceae Mirb.
Iz_13_1209	DJI_0890	Potentilla argentea L.	0	n	1	0	Rosaceae Juss.
Iz_13_1209	DJI_0890	Psammophiliella muralis (L.) Ikonn.	0	n	1	0	Caryophyllaceae Juss.
Iz_13_1209	DJI_0890	Psephellus sumensis (Kalen.) Greuter	0	n	1	0	Asteraceae Bercht. & J.Presl
Iz_13_1209	DJI_0890	Pulsatilla pratensis (L.) Mill. s.l.	0	n	1	0	Ranunculaceae Juss.
Iz_13_1209	DJI_0890	Pyrus communis L.	0	a	0	1	Rosaceae Juss.
Iz_13_1209	DJI_0890	Quercus robur L.	0	n	1	1	Fagaceae Dumort.
Iz_13_1209	DJI_0890	Robinia pseudoacacia L.	0	a	0	1	Fabaceae Lindl.
Iz_13_1209	DJI_0890	Rosa sp.	0		NA	1	Rosaceae Juss.
Iz_13_1209	DJI_0890	Rubus idaeus L.	0	n	1	0	Rosaceae Juss.
Iz_13_1209	DJI_0890	Rumex acetosella L.	0	n	1	0	Polygonaceae Juss.
Iz_13_1209	DJI_0890	Rumex thyrsiflorus Fingerh.	0	n	1	0	Polygonaceae Juss.
Iz_13_1209	DJI_0890	Salix alba L.	0	n	1	1	Salicaceae Mirb.
Iz_13_1209	DJI_0890	Sambucus nigra L.	0	n	1	1	Viburnaceae Raf.
Iz_13_1209	DJI_0890	Secale sylvestre Host	0	n	1	0	Poaceae Barnhart
Iz_13_1209	DJI_0890	Sempervivum ruthenicum Schnittsp. & C.B. Lehm.	0	n	1	0	Crassulaceae J.St.-Hil.
Iz_13_1209	DJI_0890	Setaria viridis (L.) P. Beauv.	0	n	1	0	Poaceae Barnhart
Iz_13_1209	DJI_0890	Silene borysthenica (Gruner) Walters	0	n	1	0	Caryophyllaceae Juss.
Iz_13_1209	DJI_0890	Silene latifolia subsp. alba (Mill.) Greuter & Burdet	0	n	1	0	Caryophyllaceae Juss.
Iz_13_1209	DJI_0890	Solidago virgaurea L.	0	n	1	0	Asteraceae Bercht. & J.Presl
Iz_13_1209	DJI_0890	Sorbus aucuparia L.	0	n	1	1	Rosaceae Juss.
Iz_13_1209	DJI_0890	Stachys recta L.	0	n	1	0	Lamiacea Lindl.
Iz_13_1209	DJI_0890	Tanacetum vulgare L.	0	n	1	0	Asteraceae Bercht. & J.Presl
Iz_13_1209	DJI_0890	Thymus pallasianus Heinr.Braun	0	n	1	0	Lamiacea Lindl.
Iz_13_1209	DJI_0890	Tilia cordata Mill.	0	n	1	1	Malvaceae Juss.
Iz_13_1209	DJI_0890	Trifolium alpestre L.	0	n	1	0	Fabaceae Lindl.
Iz_13_1209	DJI_0890	Trifolium arvense L.	0	n	1	0	Fabaceae Lindl.
Iz_13_1209	DJI_0890	Ulmus glabra Huds.	0	n	1	1	Ulmaceae Mirb.
Iz_13_1209	DJI_0890	Verbascum densiflorum Bertol.	0	n	1	0	Scrophulariaceae Juss.
Iz_13_1209	DJI_0890	Veronica austriaca L.	0	n	1	0	Plantaginaceae Juss.
Iz_13_1209	DJI_0890	Veronica incana L.	0	n	1	0	Plantaginaceae Juss.
Iz_13_1209	DJI_0890	Veronica verna L.	0	n	1	0	Plantaginaceae Juss.
Iz_13_1209	DJI_0890	Vincetoxicum hirundinaria Medik	0	n	1	0	Apocynaceae Juss.
Iz_13_1209	DJI_0893	Acer tataricum L.	0	n	1	1	Sapindaceae Juss.
Iz_13_1209	DJI_0893	Achillea millefolium L. s.l.	0	n	1	0	Asteraceae Bercht. & J.Presl
Iz_13_1209	DJI_0893	Allium oleraceum L.	0	n	1	0	Amaryllidaceae J.St.-Hil.
Iz_13_1209	DJI_0893	Ambrosia artemisiifolia L.	0	a	0	0	Asteraceae Bercht. & J.Presl
Iz_13_1209	DJI_0893	Amorpha fruticosa L.	0	a	0	0	Fabaceae Lindl.
Iz_13_1209	DJI_0893	Anthericum ramosum L.	0	n	1	0	Asparagaceae Juss.
Iz_13_1209	DJI_0893	Artemisia marschalliana Spreng.	0	n	1	0	Asteraceae Bercht. & J.Presl
Iz_13_1209	DJI_0893	Asparagus officinalis L.	0	n	1	0	Asparagaceae Juss.
Iz_13_1209	DJI_0893	Astragalus cicer L.	0	n	1	0	Fabaceae Lindl.
Iz_13_1209	DJI_0893	Bassia laniflora (S.G.Gmel.) A.J.Scott	0	n	1	0	Amaranthaceae Juss.
Iz_13_1209	DJI_0893	Berberis vulgaris L.	0	n	1	0	Berberidaceae Juss.
Iz_13_1209	DJI_0893	Betula pendula Roth.	0	n	1	1	Betulaceae Gray
Iz_13_1209	DJI_0893	Betula pendula Roth.	0	n	1	0	Poaceae Barnhart
Iz_13_1209	DJI_0893	Bromus inermis Leyss.	0		NA	NA	
Iz_13_1209	DJI_0893	Bromus squarrosus L.	0	a	0	0	Poaceae Barnhart
Iz_13_1209	DJI_0893	Bromus tectorum L.	0	a	0	0	Poaceae Barnhart
Iz_13_1209	DJI_0893	Calamagrostis epigejos (L.) Roth	0	n	1	0	Poaceae Barnhart
Iz_13_1209	DJI_0893	Carduus acanthoides L.	0	n	1	0	Asteraceae Bercht. & J.Presl
Iz_13_1209	DJI_0893	Carex sp.	0	n	1	0	Cyperaceae Juss.
Iz_13_1209	DJI_0893	Centaurea majorovii Dumbadze	0	n	1	0	Asteraceae Bercht. & J.Presl
Iz_13_1209	DJI_0893	Chamaecytisus ruthenicus (Fisch.ex Wol.) Klaskova	0	n	1	0	Fabaceae Lindl.
Iz_13_1209	DJI_0893	Chelidonium majus L.	0	n	1	0	Papaveraceae Juss.
Iz_13_1209	DJI_0893	Chenopodium betaceum Andrz.	0	n	1	0	Amaranthaceae Juss.
Iz_13_1209	DJI_0893	Chenopodium cf. album	0	n	1	0	Amaranthaceae Juss.
Iz_13_1209	DJI_0893	Chenopodium sp.	0		NA	0	Amaranthaceae Juss.
Iz_13_1209	DJI_0893	Chondrilla juncea L.	0	n	1	0	Asteraceae Bercht. & J.Presl
Iz_13_1209	DJI_0893	Cirsium vulgare (Savi) Ten.	0	n	1	0	Asteraceae Bercht. & J.Presl
Iz_13_1209	DJI_0893	Clinopodium acinos (L.) Kuntze	0	n	1	0	Lamiacea Lindl.
Iz_13_1209	DJI_0893	Convallaria majalis L.	0	n	1	0	Asparagaceae Juss.
Iz_13_1209	DJI_0893	Crataegus rhipidophylla Gand.	0	n	1	1	Rosaceae Juss.
Iz_13_1209	DJI_0893	Crepis foetida L.	0	n	1	0	Asteraceae Bercht. & J.Presl
Iz_13_1209	DJI_0893	Dactylis glomerata L.	0	n	1	0	Poaceae Barnhart
Iz_13_1209	DJI_0893	Dianthus campestris M.Bieb.	0	n	1	0	Caryophyllaceae Juss.
Iz_13_1209	DJI_0893	Digitaria sanguinalis (L.) Scop.	0	a	0	0	Poaceae Barnhart
Iz_13_1209	DJI_0893	Elymus repens (L.) Gould	0	n	1	0	Poaceae Barnhart
Iz_13_1209	DJI_0893	Epilobium tetragonum L.	0	n	1	0	Onagraceae Juss.
Iz_13_1209	DJI_0893	Eragrostis minor Host	0	a	0	0	Poaceae Barnhart
Iz_13_1209	DJI_0893	Erigeron canadensis L.	2	a	0	0	Asteraceae Bercht. & J.Presl
Iz_13_1209	DJI_0893	Euphorbia seguieriana Neck.	0	n	1	0	Euphorbiaceae Juss.
Iz_13_1209	DJI_0893	Fallopia dumetorum (L.) Holub	0	n	1	0	Polygonaceae Juss.
Iz_13_1209	DJI_0893	Festuca beckeri (Hack.) Trautv.	0	n	1	0	Poaceae Barnhart
Iz_13_1209	DJI_0893	Fragaria vesca L.	0	n	1	0	Rosaceae Juss.
Iz_13_1209	DJI_0893	Frangula alnus Mill.	0	n	1	1	Rhamnaceae Juss.
Iz_13_1209	DJI_0893	Fraxinus pennsylvanica Marshall	0	a	0	1	Oleaceae Hoffmanns. & Link
Iz_13_1209	DJI_0893	Galeopsis bifida Boenn.	0	n	1	0	Lamiacea Lindl.
Iz_13_1209	DJI_0893	Galium verum L.	0	n	1	0	Rubiaceae Juss.
Iz_13_1209	DJI_0893	Genista tinctoria L.	0	n	1	0	Fabaceae Lindl.
Iz_13_1209	DJI_0893	Geranium robertianum L.	0	n	1	0	Geraniaceae Juss.
Iz_13_1209	DJI_0893	Geranium sanquineum L.	0	n	1	0	Geraniaceae Juss.
Iz_13_1209	DJI_0893	Gypsophila paniculata L.	0	n	1	0	Caryophyllaceae Juss.
Iz_13_1209	DJI_0893	Helichrysum arenarium (L.) Moench	0	n	1	0	Asteraceae Bercht. & J.Presl
Iz_13_1209	DJI_0893	Herniaria polygama J.Gay	0	n	1	0	Caryophyllaceae Juss.
Iz_13_1209	DJI_0893	Hieracium robustum Fr.	0	n	1	0	Asteraceae Bercht. & J.Presl
Iz_13_1209	DJI_0893	Hieracium umbellatum L.	0	n	1	NA	Asteraceae Bercht. & J.Presl
Iz_13_1209	DJI_0893	Hylotelephium maximum (L.) Holub	0	n	1	0	Crassulaceae J.St.-Hil.
Iz_13_1209	DJI_0893	Jacobaea borysthenica (DC.) B.Nord. & Greuter	0	n	1	0	Asteraceae Bercht. & J.Presl

Iz_13_1209	DJI_0893	Jasione montana L.	0	n	1	0	Campanulaceae Juss.
Iz_13_1209	DJI_0893	Jurinea cyanoides (L.) Rchb.s.l.	0	n	1	0	Asteraceae Bercht. & J.Presl
Iz_13_1209	DJI_0893	Koeleria glauca (Spreng.) DC.	0	n	1	0	Poaceae Barnhart
Iz_13_1209	DJI_0893	Lactuca serriola L.	0	a	0	0	Asteraceae Bercht. & J.Presl
Iz_13_1209	DJI_0893	Linaria genistifolia (L.) Mill.	0	n	1	0	Plantaginaceae Juss.
Iz_13_1209	DJI_0893	Lomelosia argentea (L.) Greuter & Burdet	0	n	1	0	Caprifoliaceae Juss.
Iz_13_1209	DJI_0893	Melampyrum pratense L.	0	n	1	0	Orobanchaceae Vent.
Iz_13_1209	DJI_0893	Oenothera biennis L.	0	a	0	0	Onagraceae Juss.
Iz_13_1209	DJI_0893	Pentanema britannica (L.) D.Gut.Larr., Santos-Vicente, Anderb., E.Rico & M.M.Mart.Ort.	0	n	1	0	Asteraceae Bercht. & J.Presl
Iz_13_1209	DJI_0893	Peucedanum oreoselinum (L.) Moench	0	n	1	0	Apiaceae Lindl.
Iz_13_1209	DJI_0893	Peucedanum ruthenicum M.Bieb.	0	n	1	0	Apiaceae Lindl.
Iz_13_1209	DJI_0893	Pilosella echioides (Lumn.) F.W.Schultz & Sch.Bip.	0	n	1	0	Asteraceae Bercht. & J.Presl
Iz_13_1209	DJI_0893	Pilosella officinarum F. Schultz et Sch. Bip.	0	n	1	0	Asteraceae Bercht. & J.Presl
Iz_13_1209	DJI_0893	Pilosella sp.	0		NA	0	Asteraceae Bercht. & J.Presl
Iz_13_1209	DJI_0893	Pinus sylvestris L.	0	n	1	1	Pinaceae Spreng. ex F.Rudolphi
Iz_13_1209	DJI_0893	Poa annua L.	0	n	1	0	Poaceae Barnhart
Iz_13_1209	DJI_0893	Poa nemoralis L.	0	n	1	0	Poaceae Barnhart
Iz_13_1209	DJI_0893	Poa sp.	0		NA	0	Poaceae Barnhart
Iz_13_1209	DJI_0893	Polygonatum odoratum (Mill.) Druce	0	n	1	0	Asparagaceae Juss.
Iz_13_1209	DJI_0893	Polygonum arenarium Waldst. & Kit.	0	n	1	0	Polygonaceae Juss.
Iz_13_1209	DJI_0893	Populus nigra L.	0	n	1	1	Salicaceae Mirb.
Iz_13_1209	DJI_0893	Populus sp.	0	n	1	1	Salicaceae Mirb.
Iz_13_1209	DJI_0893	Populus tremula L.	0	n	1	1	Salicaceae Mirb.
Iz_13_1209	DJI_0893	Populus Ã canescens (Aiton) Sm.	0	n	1	1	Salicaceae Mirb.
Iz_13_1209	DJI_0893	Potentilla argentea L.	0	n	1	0	Rosaceae Juss.
Iz_13_1209	DJI_0893	Psammophiliella muralis (L.) Ikonn.	0	n	1	0	Caryophyllaceae Juss.
Iz_13_1209	DJI_0893	Psephellus sumensis (Kalen.) Greuter	0	n	1	0	Asteraceae Bercht. & J.Presl
Iz_13_1209	DJI_0893	Pulsatilla pratensis (L.) Mill. s.l.	0	n	1	0	Ranunculaceae Juss.
Iz_13_1209	DJI_0893	Pyrus communis L.	0	a	0	1	Rosaceae Juss.
Iz_13_1209	DJI_0893	Quercus robur L.	0	n	1	1	Fagaceae Dumort.
Iz_13_1209	DJI_0893	Robinia pseudoacacia L.	0	a	0	1	Fabaceae Lindl.
Iz_13_1209	DJI_0893	Rosa sp.	0		NA	1	Rosaceae Juss.
Iz_13_1209	DJI_0893	Rubus idaeus L.	0	n	1	0	Rosaceae Juss.
Iz_13_1209	DJI_0893	Rumex acetosella L.	0	n	1	0	Polygonaceae Juss.
Iz_13_1209	DJI_0893	Rumex thyrsiflorus Fingerh.	0	n	1	0	Polygonaceae Juss.
Iz_13_1209	DJI_0893	Salix alba L.	0	n	1	1	Salicaceae Mirb.
Iz_13_1209	DJI_0893	Sambucus nigra L.	0	n	1	1	Viburnaceae Raf.
Iz_13_1209	DJI_0893	Secale sylvestre Host	0	n	1	0	Poaceae Barnhart
Iz_13_1209	DJI_0893	Sempervivum ruthenicum Schnittsp. & C.B. Lehm.	0	n	1	0	Crassulaceae J.St.-Hil.
Iz_13_1209	DJI_0893	Setaria viridis (L.) P. Beauv.	0	n	1	0	Poaceae Barnhart
Iz_13_1209	DJI_0893	Silene borysthenica (Gruner) Walters	0	n	1	0	Caryophyllaceae Juss.
Iz_13_1209	DJI_0893	Silene latifolia subsp. alba (Mill.) Greuter & Burdet	0	n	1	0	Caryophyllaceae Juss.
Iz_13_1209	DJI_0893	Solidago virgaurea L.	0	n	1	0	Asteraceae Bercht. & J.Presl
Iz_13_1209	DJI_0893	Sorbus aucuparia L.	0	n	1	1	Rosaceae Juss.
Iz_13_1209	DJI_0893	Stachys recta L.	0	n	1	0	Lamiacea Lindl.
Iz_13_1209	DJI_0893	Tanacetum vulgare L.	0	n	1	0	Asteraceae Bercht. & J.Presl
Iz_13_1209	DJI_0893	Thymus pallasianus Heinr.Braun	0	n	1	0	Lamiacea Lindl.
Iz_13_1209	DJI_0893	Tilia cordata Mill.	0	n	1	1	Malvaceae Juss.
Iz_13_1209	DJI_0893	Trifolium alpestre L.	0	n	1	0	Fabaceae Lindl.
Iz_13_1209	DJI_0893	Trifolium arvense L.	0	n	1	0	Fabaceae Lindl.
Iz_13_1209	DJI_0893	Ulmus glabra Huds.	0	n	1	1	Ulmaceae Mirb.
Iz_13_1209	DJI_0893	Verbascum densiflorum Bertol.	0	n	1	0	Scrophulariaceae Juss.
Iz_13_1209	DJI_0893	Veronica austriaca L.	0	n	1	0	Plantaginaceae Juss.
Iz_13_1209	DJI_0893	Veronica incana L.	0	n	1	0	Plantaginaceae Juss.
Iz_13_1209	DJI_0893	Veronica verna L.	0	n	1	0	Plantaginaceae Juss.
Iz_13_1209	DJI_0893	Vincetoxicum hirundinaria Medik	0	n	1	0	Apocynaceae Juss.
Iz_13_1209	DJI_0897	Acer tataricum L.	0	n	1	1	Sapindaceae Juss.
Iz_13_1209	DJI_0897	Achillea millefolium L. s.l.	0	n	1	0	Asteraceae Bercht. & J.Presl
Iz_13_1209	DJI_0897	Allium oleraceum L.	0	n	1	0	Amaryllidaceae J.St.-Hil.
Iz_13_1209	DJI_0897	Ambrosia artemisiifolia L.	0	a	0	0	Asteraceae Bercht. & J.Presl
Iz_13_1209	DJI_0897	Amorpha fruticosa L.	0	a	0	0	Fabaceae Lindl.
Iz_13_1209	DJI_0897	Anthericum ramosum L.	0	n	1	0	Asparagaceae Juss.
Iz_13_1209	DJI_0897	Artemisia marschalliana Spreng.	0	n	1	0	Asteraceae Bercht. & J.Presl
Iz_13_1209	DJI_0897	Asparagus officinalis L.	0	n	1	0	Asparagaceae Juss.
Iz_13_1209	DJI_0897	Astragalus cicer L.	0	n	1	0	Fabaceae Lindl.
Iz_13_1209	DJI_0897	Bassia laniflora (S.G.Gmel.) A.J.Scott	0	n	1	0	Amaranthaceae Juss.
Iz_13_1209	DJI_0897	Berberis vulgaris L.	0	n	1	0	Berberidaceae Juss.
Iz_13_1209	DJI_0897	Betula pendula Roth.	0	n	1	1	Betulaceae Gray
Iz_13_1209	DJI_0897	Betula pendula Roth.	0	n	1	0	Poaceae Barnhart
Iz_13_1209	DJI_0897	Bromus inermis Leyss.	0		NA	NA	
Iz_13_1209	DJI_0897	Bromus squarrosus L.	0	a	0	0	Poaceae Barnhart
Iz_13_1209	DJI_0897	Bromus tectorum L.	0	a	0	0	Poaceae Barnhart
Iz_13_1209	DJI_0897	Calamagrostis epigejos (L.) Roth	22	n	1	0	Poaceae Barnhart
Iz_13_1209	DJI_0897	Carduus acanthoides L.	0	n	1	0	Asteraceae Bercht. & J.Presl
Iz_13_1209	DJI_0897	Carex sp.	0	n	1	0	Cyperaceae Juss.
Iz_13_1209	DJI_0897	Centaurea majorovii Dumbadze	0	n	1	0	Asteraceae Bercht. & J.Presl
Iz_13_1209	DJI_0897	Chamaecytisus ruthenicus (Fisch.ex Wol.) Klaskova	0	n	1	0	Fabaceae Lindl.
Iz_13_1209	DJI_0897	Chelidonium majus L.	0	n	1	0	Papaveraceae Juss.
Iz_13_1209	DJI_0897	Chenopodium betaceum Andrz.	0	n	1	0	Amaranthaceae Juss.
Iz_13_1209	DJI_0897	Chenopodium cf. album	0	n	1	0	Amaranthaceae Juss.
Iz_13_1209	DJI_0897	Chenopodium sp.	0		NA	0	Amaranthaceae Juss.
Iz_13_1209	DJI_0897	Chondrilla juncea L.	0	n	1	0	Asteraceae Bercht. & J.Presl
Iz_13_1209	DJI_0897	Cirsium vulgare (Savi) Ten.	0	n	1	0	Asteraceae Bercht. & J.Presl
Iz_13_1209	DJI_0897	Clinopodium acinos (L.) Kuntze	0	n	1	0	Lamiacea Lindl.
Iz_13_1209	DJI_0897	Convallaria majalis L.	0	n	1	0	Asparagaceae Juss.
Iz_13_1209	DJI_0897	Crataegus rhipidophylla Gand.	0	n	1	1	Rosaceae Juss.
Iz_13_1209	DJI_0897	Crepis foetida L.	0	n	1	0	Asteraceae Bercht. & J.Presl
Iz_13_1209	DJI_0897	Dactylis glomerata L.	0	n	1	0	Poaceae Barnhart
Iz_13_1209	DJI_0897	Dianthus campestris M.Bieb.	0	n	1	0	Caryophyllaceae Juss.
Iz_13_1209	DJI_0897	Digitaria sanguinalis (L.) Scop.	0	a	0	0	Poaceae Barnhart
Iz_13_1209	DJI_0897	Elymus repens (L.) Gould	0	n	1	0	Poaceae Barnhart
Iz_13_1209	DJI_0897	Epilobium tetragonum L.	0	n	1	0	Onagraceae Juss.
Iz_13_1209	DJI_0897	Eragrostis minor Host	0	a	0	0	Poaceae Barnhart
Iz_13_1209	DJI_0897	Erigeron canadensis L.	10	a	0	0	Asteraceae Bercht. & J.Presl
Iz_13_1209	DJI_0897	Euphorbia seguieriana Neck.	0	n	1	0	Euphorbiaceae Juss.
Iz_13_1209	DJI_0897	Fallopia dumetorum (L.) Holub	0	n	1	0	Polygonaceae Juss.
Iz_13_1209	DJI_0897	Festuca beckeri (Hack.) Trautv.	0	n	1	0	Poaceae Barnhart
Iz_13_1209	DJI_0897	Fragaria vesca L.	0	n	1	0	Rosaceae Juss.

Iz_13_1209	DJI_0897	Frangula alnus Mill.	0	n	1	1	Rhamnaceae Juss.
Iz_13_1209	DJI_0897	Fraxinus pennsylvanica Marshall	0	a	0	1	Oleaceae Hoffmanns. & Link
Iz_13_1209	DJI_0897	Galeopsis bifida Boenn.	0	n	1	0	Lamiacea Lindl.
Iz_13_1209	DJI_0897	Galium verum L.	0	n	1	0	Rubiaceae Juss.
Iz_13_1209	DJI_0897	Genista tinctoria L.	0	n	1	0	Fabaceae Lindl.
Iz_13_1209	DJI_0897	Geranium robertianum L.	0	n	1	0	Geraniaceae Juss.
Iz_13_1209	DJI_0897	Geranium sanquineum L.	0	n	1	0	Geraniaceae Juss.
Iz_13_1209	DJI_0897	Gypsophila paniculata L.	0	n	1	0	Caryophyllaceae Juss.
Iz_13_1209	DJI_0897	Helichrysum arenarium (L.) Moench	0	n	1	0	Asteraceae Bercht. & J.Presl
Iz_13_1209	DJI_0897	Herniaria polygama J.Gay	0	n	1	0	Caryophyllaceae Juss.
Iz_13_1209	DJI_0897	Hieracium robustum Fr.	0	n	1	0	Asteraceae Bercht. & J.Presl
Iz_13_1209	DJI_0897	Hieracium umbellatum L.	0	n	1	NA	Asteraceae Bercht. & J.Presl
Iz_13_1209	DJI_0897	Hylotelephium maximum (L.) Holub	0	n	1	0	Crassulaceae J.St.-Hil.
Iz_13_1209	DJI_0897	Jacobaea borysthenica (DC.) B.Nord. & Greuter	0	n	1	0	Asteraceae Bercht. & J.Presl
Iz_13_1209	DJI_0897	Jasione montana L.	0	n	1	0	Campanulaceae Juss.
Iz_13_1209	DJI_0897	Jurinea cyanoides (L.) Rchb.s.l.	0	n	1	0	Asteraceae Bercht. & J.Presl
Iz_13_1209	DJI_0897	Koeleria glauca (Spreng.) DC.	0	n	1	0	Poaceae Barnhart
Iz_13_1209	DJI_0897	Lactuca serriola L.	0	a	0	0	Asteraceae Bercht. & J.Presl
Iz_13_1209	DJI_0897	Linaria genistifolia (L.) Mill.	0	n	1	0	Plantaginaceae Juss.
Iz_13_1209	DJI_0897	Lomelosia argentea (L.) Greuter & Burdet	0	n	1	0	Caprifoliaceae Juss.
Iz_13_1209	DJI_0897	Melampyrum pratense L.	0	n	1	0	Orobanchaceae Vent.
Iz_13_1209	DJI_0897	Oenothera biennis L.	0	a	0	0	Onagraceae Juss.
Iz_13_1209	DJI_0897	Pentanema britannica (L.) D.Gut.Larr., Santos-Vicente, Anderb., E.Rico & M.M.Mart.Ort.	0	n	1	0	Asteraceae Bercht. & J.Presl
Iz_13_1209	DJI_0897	Peucedanum oreoselinum (L.) Moench	0	n	1	0	Apiaceae Lindl.
Iz_13_1209	DJI_0897	Peucedanum ruthenicum M.Bieb.	0	n	1	0	Apiaceae Lindl.
Iz_13_1209	DJI_0897	Pilosella echioides (Lumn.) F.W.Schultz & Sch.Bip.	0	n	1	0	Asteraceae Bercht. & J.Presl
Iz_13_1209	DJI_0897	Pilosella officinarum F. Schultz et Sch. Bip.	0	n	1	0	Asteraceae Bercht. & J.Presl
Iz_13_1209	DJI_0897	Pilosella sp.	0		NA	0	Asteraceae Bercht. & J.Presl
Iz_13_1209	DJI_0897	Pinus sylvestris L.	0	n	1	1	Pinaceae Spreng. ex F.Rudolphi
Iz_13_1209	DJI_0897	Poa annua L.	0	n	1	0	Poaceae Barnhart
Iz_13_1209	DJI_0897	Poa nemoralis L.	0	n	1	0	Poaceae Barnhart
Iz_13_1209	DJI_0897	Poa sp.	0		NA	0	Poaceae Barnhart
Iz_13_1209	DJI_0897	Polygonatum odoratum (Mill.) Druce	0	n	1	0	Asparagaceae Juss.
Iz_13_1209	DJI_0897	Polygonum arenarium Waldst. & Kit.	0	n	1	0	Polygonaceae Juss.
Iz_13_1209	DJI_0897	Populus nigra L.	0	n	1	1	Salicaceae Mirb.
Iz_13_1209	DJI_0897	Populus sp.	0	n	1	1	Salicaceae Mirb.
Iz_13_1209	DJI_0897	Populus tremula L.	0	n	1	1	Salicaceae Mirb.
Iz_13_1209	DJI_0897	Populus Ã canescens (Aiton) Sm.	0	n	1	1	Salicaceae Mirb.
Iz_13_1209	DJI_0897	Potentilla argentea L.	0	n	1	0	Rosaceae Juss.
Iz_13_1209	DJI_0897	Psammophiliella muralis (L.) Ikonn.	0	n	1	0	Caryophyllaceae Juss.
Iz_13_1209	DJI_0897	Psephellus sumensis (Kalen.) Greuter	0	n	1	0	Asteraceae Bercht. & J.Presl
Iz_13_1209	DJI_0897	Pulsatilla pratensis (L.) Mill. s.l.	0	n	1	0	Ranunculaceae Juss.
Iz_13_1209	DJI_0897	Pyrus communis L.	0	a	0	1	Rosaceae Juss.
Iz_13_1209	DJI_0897	Quercus robur L.	0	n	1	1	Fagaceae Dumort.
Iz_13_1209	DJI_0897	Robinia pseudoacacia L.	0	a	0	1	Fabaceae Lindl.
Iz_13_1209	DJI_0897	Rosa sp.	0		NA	1	Rosaceae Juss.
Iz_13_1209	DJI_0897	Rubus idaeus L.	0	n	1	0	Rosaceae Juss.
Iz_13_1209	DJI_0897	Rumex acetosella L.	0	n	1	0	Polygonaceae Juss.
Iz_13_1209	DJI_0897	Rumex thyrsiflorus Fingerh.	0	n	1	0	Polygonaceae Juss.
Iz_13_1209	DJI_0897	Salix alba L.	0	n	1	1	Salicaceae Mirb.
Iz_13_1209	DJI_0897	Sambucus nigra L.	0	n	1	1	Viburnaceae Raf.
Iz_13_1209	DJI_0897	Secale sylvestre Host	0	n	1	0	Poaceae Barnhart
Iz_13_1209	DJI_0897	Sempervivum ruthenicum Schnittsp. & C.B. Lehm.	0	n	1	0	Crassulaceae J.St.-Hil.
Iz_13_1209	DJI_0897	Setaria viridis (L.) P. Beauv.	0	n	1	0	Poaceae Barnhart
Iz_13_1209	DJI_0897	Silene borysthenica (Gruner) Walters	0	n	1	0	Caryophyllaceae Juss.
Iz_13_1209	DJI_0897	Silene latifolia subsp. alba (Mill.) Greuter & Burdet	0	n	1	0	Caryophyllaceae Juss.
Iz_13_1209	DJI_0897	Solidago virgaurea L.	0	n	1	0	Asteraceae Bercht. & J.Presl
Iz_13_1209	DJI_0897	Sorbus aucuparia L.	0	n	1	1	Rosaceae Juss.
Iz_13_1209	DJI_0897	Stachys recta L.	0	n	1	0	Lamiacea Lindl.
Iz_13_1209	DJI_0897	Tanacetum vulgare L.	0	n	1	0	Asteraceae Bercht. & J.Presl
Iz_13_1209	DJI_0897	Thymus pallasianus Heinr.Braun	0	n	1	0	Lamiacea Lindl.
Iz_13_1209	DJI_0897	Tilia cordata Mill.	0	n	1	1	Malvaceae Juss.
Iz_13_1209	DJI_0897	Trifolium alpestre L.	0	n	1	0	Fabaceae Lindl.
Iz_13_1209	DJI_0897	Trifolium arvense L.	0	n	1	0	Fabaceae Lindl.
Iz_13_1209	DJI_0897	Ulmus glabra Huds.	0	n	1	1	Ulmaceae Mirb.
Iz_13_1209	DJI_0897	Verbascum densiflorum Bertol.	0	n	1	0	Scrophulariaceae Juss.
Iz_13_1209	DJI_0897	Veronica austriaca L.	0	n	1	0	Plantaginaceae Juss.
Iz_13_1209	DJI_0897	Veronica incana L.	0	n	1	0	Plantaginaceae Juss.
Iz_13_1209	DJI_0897	Veronica verna L.	0	n	1	0	Plantaginaceae Juss.
Iz_13_1209	DJI_0897	Vincetoxicum hirundinaria Medik	0	n	1	0	Apocynaceae Juss.
Iz_13_1209	DJI_0901	Acer tataricum L.	0	n	1	1	Sapindaceae Juss.
Iz_13_1209	DJI_0901	Achillea millefolium L. s.l.	0	n	1	0	Asteraceae Bercht. & J.Presl
Iz_13_1209	DJI_0901	Allium oleraceum L.	0	n	1	0	Amaryllidaceae J.St.-Hil.
Iz_13_1209	DJI_0901	Ambrosia artemisiifolia L.	0	a	0	0	Asteraceae Bercht. & J.Presl
Iz_13_1209	DJI_0901	Amorpha fruticosa L.	0	a	0	0	Fabaceae Lindl.
Iz_13_1209	DJI_0901	Anthericum ramosum L.	0	n	1	0	Asparagaceae Juss.
Iz_13_1209	DJI_0901	Artemisia marschalliana Spreng.	0	n	1	0	Asteraceae Bercht. & J.Presl
Iz_13_1209	DJI_0901	Asparagus officinalis L.	0	n	1	0	Asparagaceae Juss.
Iz_13_1209	DJI_0901	Astragalus cicer L.	0	n	1	0	Fabaceae Lindl.
Iz_13_1209	DJI_0901	Bassia laniflora (S.G.Gmel.) A.J.Scott	0	n	1	0	Amaranthaceae Juss.
Iz_13_1209	DJI_0901	Berberis vulgaris L.	0	n	1	0	Berberidaceae Juss.
Iz_13_1209	DJI_0901	Betula pendula Roth.	0	n	1	1	Betulaceae Gray
Iz_13_1209	DJI_0901	Betula pendula Roth.	0	n	1	0	Poaceae Barnhart
Iz_13_1209	DJI_0901	Bromus inermis Leyss.	0		NA	NA	
Iz_13_1209	DJI_0901	Bromus squarrosus L.	0	a	0	0	Poaceae Barnhart
Iz_13_1209	DJI_0901	Bromus tectorum L.	0	a	0	0	Poaceae Barnhart
Iz_13_1209	DJI_0901	Calamagrostis epigejos (L.) Roth	3	n	1	0	Poaceae Barnhart
Iz_13_1209	DJI_0901	Carduus acanthoides L.	0	n	1	0	Asteraceae Bercht. & J.Presl
Iz_13_1209	DJI_0901	Carex sp.	0	n	1	0	Cyperaceae Juss.
Iz_13_1209	DJI_0901	Centaurea majorovii Dumbadze	0	n	1	0	Asteraceae Bercht. & J.Presl
Iz_13_1209	DJI_0901	Chamaecytisus ruthenicus (Fisch.ex Wol.) Klaskova	0	n	1	0	Fabaceae Lindl.
Iz_13_1209	DJI_0901	Chelidonium majus L.	0	n	1	0	Papaveraceae Juss.
Iz_13_1209	DJI_0901	Chenopodium betaceum Andrz.	0	n	1	0	Amaranthaceae Juss.
Iz_13_1209	DJI_0901	Chenopodium cf. album	0	n	1	0	Amaranthaceae Juss.
Iz_13_1209	DJI_0901	Chenopodium sp.	0		NA	0	Amaranthaceae Juss.
Iz_13_1209	DJI_0901	Chondrilla juncea L.	0	n	1	0	Asteraceae Bercht. & J.Presl
Iz_13_1209	DJI_0901	Cirsium vulgare (Savi) Ten.	0	n	1	0	Asteraceae Bercht. & J.Presl
Iz_13_1209	DJI_0901	Clinopodium acinos (L.) Kuntze	0	n	1	0	Lamiacea Lindl.

Iz_13_1209	DJI_0901	Convallaria majalis L.	0	n	1	0	Asparagaceae Juss.
Iz_13_1209	DJI_0901	Crataegus rhipidophylla Gand.	0	n	1	1	Rosaceae Juss.
Iz_13_1209	DJI_0901	Crepis foetida L.	0	n	1	0	Asteraceae Bercht. & J.Presl
Iz_13_1209	DJI_0901	Dactylis glomerata L.	0	n	1	0	Poaceae Barnhart
Iz_13_1209	DJI_0901	Dianthus campestris M.Bieb.	0	n	1	0	Caryophyllaceae Juss.
Iz_13_1209	DJI_0901	Digitaria sanguinalis (L.) Scop.	0	a	0	0	Poaceae Barnhart
Iz_13_1209	DJI_0901	Elymus repens (L.) Gould	0	n	1	0	Poaceae Barnhart
Iz_13_1209	DJI_0901	Epilobium tetragonum L.	0	n	1	0	Onagraceae Juss.
Iz_13_1209	DJI_0901	Eragrostis minor Host	0	a	0	0	Poaceae Barnhart
Iz_13_1209	DJI_0901	Erigeron canadensis L.	5	a	0	0	Asteraceae Bercht. & J.Presl
Iz_13_1209	DJI_0901	Euphorbia seguieriana Neck.	0	n	1	0	Euphorbiaceae Juss.
Iz_13_1209	DJI_0901	Fallopia dumetorum (L.) Holub	0	n	1	0	Polygonaceae Juss.
Iz_13_1209	DJI_0901	Festuca beckeri (Hack.) Trautv.	0	n	1	0	Poaceae Barnhart
Iz_13_1209	DJI_0901	Fragaria vesca L.	0	n	1	0	Rosaceae Juss.
Iz_13_1209	DJI_0901	Frangula alnus Mill.	0	n	1	1	Rhamnaceae Juss.
Iz_13_1209	DJI_0901	Fraxinus pennsylvanica Marshall	0	a	0	1	Oleaceae Hoffmanns. & Link
Iz_13_1209	DJI_0901	Galeopsis bifida Boenn.	0	n	1	0	Lamiacea Lindl.
Iz_13_1209	DJI_0901	Galium verum L.	0	n	1	0	Rubiaceae Juss.
Iz_13_1209	DJI_0901	Genista tinctoria L.	0	n	1	0	Fabaceae Lindl.
Iz_13_1209	DJI_0901	Geranium robertianum L.	0	n	1	0	Geraniaceae Juss.
Iz_13_1209	DJI_0901	Geranium sanquineum L.	0	n	1	0	Geraniaceae Juss.
Iz_13_1209	DJI_0901	Gypsophila paniculata L.	0	n	1	0	Caryophyllaceae Juss.
Iz_13_1209	DJI_0901	Helichrysum arenarium (L.) Moench	0	n	1	0	Asteraceae Bercht. & J.Presl
Iz_13_1209	DJI_0901	Herniaria polygama J.Gay	0	n	1	0	Caryophyllaceae Juss.
Iz_13_1209	DJI_0901	Hieracium robustum Fr.	0	n	1	0	Asteraceae Bercht. & J.Presl
Iz_13_1209	DJI_0901	Hieracium umbellatum L.	0	n	1	NA	Asteraceae Bercht. & J.Presl
Iz_13_1209	DJI_0901	Hylotelephium maximum (L.) Holub	0	n	1	0	Crassulaceae J.St.-Hil.
Iz_13_1209	DJI_0901	Jacobaea borysthenica (DC.) B.Nord. & Greuter	0	n	1	0	Asteraceae Bercht. & J.Presl
Iz_13_1209	DJI_0901	Jasione montana L.	0	n	1	0	Campanulaceae Juss.
Iz_13_1209	DJI_0901	Jurinea cyanoides (L.) Rchb.s.l.	0	n	1	0	Asteraceae Bercht. & J.Presl
Iz_13_1209	DJI_0901	Koeleria glauca (Spreng.) DC.	0	n	1	0	Poaceae Barnhart
Iz_13_1209	DJI_0901	Lactuca serriola L.	0	a	0	0	Asteraceae Bercht. & J.Presl
Iz_13_1209	DJI_0901	Linaria genistifolia (L.) Mill.	0	n	1	0	Plantaginaceae Juss.
Iz_13_1209	DJI_0901	Lomelosia argentea (L.) Greuter & Burdet	0	n	1	0	Caprifoliaceae Juss.
Iz_13_1209	DJI_0901	Melampyrum pratense L.	0	n	1	0	Orobanchaceae Vent.
Iz_13_1209	DJI_0901	Oenothera biennis L.	0	a	0	0	Onagraceae Juss.
Iz_13_1209	DJI_0901	Pentanema britannica (L.) D.Gut.Larr., Santos-Vicente, Anderb., E.Rico & M.M.Mart.Ort.	0	n	1	0	Asteraceae Bercht. & J.Presl
Iz_13_1209	DJI_0901	Peucedanum oreoselinum (L.) Moench	0	n	1	0	Apiaceae Lindl.
Iz_13_1209	DJI_0901	Peucedanum ruthenicum M.Bieb.	0	n	1	0	Apiaceae Lindl.
Iz_13_1209	DJI_0901	Pilosella echioides (Lumn.) F.W.Schultz & Sch.Bip.	0	n	1	0	Asteraceae Bercht. & J.Presl
Iz_13_1209	DJI_0901	Pilosella officinarum F. Schultz et Sch. Bip.	0	n	1	0	Asteraceae Bercht. & J.Presl
Iz_13_1209	DJI_0901	Pilosella sp.	0		NA	0	Asteraceae Bercht. & J.Presl
Iz_13_1209	DJI_0901	Pinus sylvestris L.	0	n	1	1	Pinaceae Spreng. ex F.Rudolphi
Iz_13_1209	DJI_0901	Poa annua L.	0	n	1	0	Poaceae Barnhart
Iz_13_1209	DJI_0901	Poa nemoralis L.	0	n	1	0	Poaceae Barnhart
Iz_13_1209	DJI_0901	Poa sp.	0		NA	0	Poaceae Barnhart
Iz_13_1209	DJI_0901	Polygonatum odoratum (Mill.) Druce	0	n	1	0	Asparagaceae Juss.
Iz_13_1209	DJI_0901	Polygonum arenarium Waldst. & Kit.	0	n	1	0	Polygonaceae Juss.
Iz_13_1209	DJI_0901	Populus nigra L.	0	n	1	1	Salicaceae Mirb.
Iz_13_1209	DJI_0901	Populus sp.	0	n	1	1	Salicaceae Mirb.
Iz_13_1209	DJI_0901	Populus tremula L.	0	n	1	1	Salicaceae Mirb.
Iz_13_1209	DJI_0901	Populus Ã canescens (Aiton) Sm.	0	n	1	1	Salicaceae Mirb.
Iz_13_1209	DJI_0901	Potentilla argentea L.	0	n	1	0	Rosaceae Juss.
Iz_13_1209	DJI_0901	Psammophiliella muralis (L.) Ikonn.	0	n	1	0	Caryophyllaceae Juss.
Iz_13_1209	DJI_0901	Psephellus sumensis (Kalen.) Greuter	0	n	1	0	Asteraceae Bercht. & J.Presl
Iz_13_1209	DJI_0901	Pulsatilla pratensis (L.) Mill. s.l.	0	n	1	0	Ranunculaceae Juss.
Iz_13_1209	DJI_0901	Pyrus communis L.	0	a	0	1	Rosaceae Juss.
Iz_13_1209	DJI_0901	Quercus robur L.	0	n	1	1	Fagaceae Dumort.
Iz_13_1209	DJI_0901	Robinia pseudoacacia L.	0	a	0	1	Fabaceae Lindl.
Iz_13_1209	DJI_0901	Rosa sp.	0		NA	1	Rosaceae Juss.
Iz_13_1209	DJI_0901	Rubus idaeus L.	0	n	1	0	Rosaceae Juss.
Iz_13_1209	DJI_0901	Rumex acetosella L.	0	n	1	0	Polygonaceae Juss.
Iz_13_1209	DJI_0901	Rumex thyrsiflorus Fingerh.	0	n	1	0	Polygonaceae Juss.
Iz_13_1209	DJI_0901	Salix alba L.	0	n	1	1	Salicaceae Mirb.
Iz_13_1209	DJI_0901	Sambucus nigra L.	0	n	1	1	Viburnaceae Raf.
Iz_13_1209	DJI_0901	Secale sylvestre Host	0	n	1	0	Poaceae Barnhart
Iz_13_1209	DJI_0901	Sempervivum ruthenicum Schnittsp. & C.B. Lehm.	0	n	1	0	Crassulaceae J.St.-Hil.
Iz_13_1209	DJI_0901	Setaria viridis (L.) P. Beauv.	0	n	1	0	Poaceae Barnhart
Iz_13_1209	DJI_0901	Silene borysthenica (Gruner) Walters	0	n	1	0	Caryophyllaceae Juss.
Iz_13_1209	DJI_0901	Silene latifolia subsp. alba (Mill.) Greuter & Burdet	0	n	1	0	Caryophyllaceae Juss.
Iz_13_1209	DJI_0901	Solidago virgaurea L.	0	n	1	0	Asteraceae Bercht. & J.Presl
Iz_13_1209	DJI_0901	Sorbus aucuparia L.	0	n	1	1	Rosaceae Juss.
Iz_13_1209	DJI_0901	Stachys recta L.	0	n	1	0	Lamiacea Lindl.
Iz_13_1209	DJI_0901	Tanacetum vulgare L.	0	n	1	0	Asteraceae Bercht. & J.Presl
Iz_13_1209	DJI_0901	Thymus pallasianus Heinr.Braun	0	n	1	0	Lamiacea Lindl.
Iz_13_1209	DJI_0901	Tilia cordata Mill.	0	n	1	1	Malvaceae Juss.
Iz_13_1209	DJI_0901	Trifolium alpestre L.	0	n	1	0	Fabaceae Lindl.
Iz_13_1209	DJI_0901	Trifolium arvense L.	0	n	1	0	Fabaceae Lindl.
Iz_13_1209	DJI_0901	Ulmus glabra Huds.	0	n	1	1	Ulmaceae Mirb.
Iz_13_1209	DJI_0901	Verbascum densiflorum Bertol.	0	n	1	0	Scrophulariaceae Juss.
Iz_13_1209	DJI_0901	Veronica austriaca L.	0	n	1	0	Plantaginaceae Juss.
Iz_13_1209	DJI_0901	Veronica incana L.	0	n	1	0	Plantaginaceae Juss.
Iz_13_1209	DJI_0901	Veronica verna L.	0	n	1	0	Plantaginaceae Juss.
Iz_13_1209	DJI_0901	Vincetoxicum hirundinaria Medik	0	n	1	0	Apocynaceae Juss.
Iz_13_1209	DJI_0905	Acer tataricum L.	0	n	1	1	Sapindaceae Juss.
Iz_13_1209	DJI_0905	Achillea millefolium L. s.l.	0	n	1	0	Asteraceae Bercht. & J.Presl
Iz_13_1209	DJI_0905	Allium oleraceum L.	0	n	1	0	Amaryllidaceae J.St.-Hil.
Iz_13_1209	DJI_0905	Ambrosia artemisiifolia L.	0	a	0	0	Asteraceae Bercht. & J.Presl
Iz_13_1209	DJI_0905	Amorpha fruticosa L.	0	a	0	0	Fabaceae Lindl.
Iz_13_1209	DJI_0905	Anthericum ramosum L.	0	n	1	0	Asparagaceae Juss.
Iz_13_1209	DJI_0905	Artemisia marschalliana Spreng.	0	n	1	0	Asteraceae Bercht. & J.Presl
Iz_13_1209	DJI_0905	Asparagus officinalis L.	0	n	1	0	Asparagaceae Juss.
Iz_13_1209	DJI_0905	Astragalus cicer L.	0	n	1	0	Fabaceae Lindl.
Iz_13_1209	DJI_0905	Bassia laniflora (S.G.Gmel.) A.J.Scott	0	n	1	0	Amaranthaceae Juss.
Iz_13_1209	DJI_0905	Berberis vulgaris L.	0	n	1	0	Berberidaceae Juss.
Iz_13_1209	DJI_0905	Betula pendula Roth.	0	n	1	1	Betulaceae Gray
Iz_13_1209	DJI_0905	Betula pendula Roth.	0	n	1	0	Poaceae Barnhart
Iz_13_1209	DJI_0905	Bromus inermis Leyss.	0		NA	NA	

Iz_13_1209	DJI_0905	Bromus squarrosus L.	0	a	0	0	Poaceae Barnhart
Iz_13_1209	DJI_0905	Bromus tectorum L.	0	a	0	0	Poaceae Barnhart
Iz_13_1209	DJI_0905	Calamagrostis epigejos (L.) Roth	20	n	1	0	Poaceae Barnhart
Iz_13_1209	DJI_0905	Carduus acanthoides L.	0	n	1	0	Asteraceae Bercht. & J.Presl
Iz_13_1209	DJI_0905	Carex sp.	0	n	1	0	Cyperaceae Juss.
Iz_13_1209	DJI_0905	Centaurea majorovii Dumbadze	0	n	1	0	Asteraceae Bercht. & J.Presl
Iz_13_1209	DJI_0905	Chamaecytisus ruthenicus (Fisch.ex Wol.) Klaskova	0	n	1	0	Fabaceae Lindl.
Iz_13_1209	DJI_0905	Chelidonium majus L.	0	n	1	0	Papaveraceae Juss.
Iz_13_1209	DJI_0905	Chenopodium betaceum Andrz.	0	n	1	0	Amaranthaceae Juss.
Iz_13_1209	DJI_0905	Chenopodium cf. album	0	n	1	0	Amaranthaceae Juss.
Iz_13_1209	DJI_0905	Chenopodium sp.	0		NA	0	Amaranthaceae Juss.
Iz_13_1209	DJI_0905	Chondrilla juncea L.	0	n	1	0	Asteraceae Bercht. & J.Presl
Iz_13_1209	DJI_0905	Cirsium vulgare (Savi) Ten.	0	n	1	0	Asteraceae Bercht. & J.Presl
Iz_13_1209	DJI_0905	Clinopodium acinos (L.) Kuntze	0	n	1	0	Lamiacea Lindl.
Iz_13_1209	DJI_0905	Convallaria majalis L.	0	n	1	0	Asparagaceae Juss.
Iz_13_1209	DJI_0905	Crataegus rhipidophylla Gand.	0	n	1	1	Rosaceae Juss.
Iz_13_1209	DJI_0905	Crepis foetida L.	0	n	1	0	Asteraceae Bercht. & J.Presl
Iz_13_1209	DJI_0905	Dactylis glomerata L.	0	n	1	0	Poaceae Barnhart
Iz_13_1209	DJI_0905	Dianthus campestris M.Bieb.	0	n	1	0	Caryophyllaceae Juss.
Iz_13_1209	DJI_0905	Digitaria sanguinalis (L.) Scop.	0	a	0	0	Poaceae Barnhart
Iz_13_1209	DJI_0905	Elymus repens (L.) Gould	0	n	1	0	Poaceae Barnhart
Iz_13_1209	DJI_0905	Epilobium tetragonum L.	0	n	1	0	Onagraceae Juss.
Iz_13_1209	DJI_0905	Eragrostis minor Host	0	a	0	0	Poaceae Barnhart
Iz_13_1209	DJI_0905	Erigeron canadensis L.	5	a	0	0	Asteraceae Bercht. & J.Presl
Iz_13_1209	DJI_0905	Euphorbia seguieriana Neck.	0	n	1	0	Euphorbiaceae Juss.
Iz_13_1209	DJI_0905	Fallopia dumetorum (L.) Holub	0	n	1	0	Polygonaceae Juss.
Iz_13_1209	DJI_0905	Festuca beckeri (Hack.) Trautv.	0	n	1	0	Poaceae Barnhart
Iz_13_1209	DJI_0905	Fragaria vesca L.	0	n	1	0	Rosaceae Juss.
Iz_13_1209	DJI_0905	Frangula alnus Mill.	0	n	1	1	Rhamnaceae Juss.
Iz_13_1209	DJI_0905	Fraxinus pennsylvanica Marshall	0	a	0	1	Oleaceae Hoffmanns. & Link
Iz_13_1209	DJI_0905	Galeopsis bifida Boenn.	0	n	1	0	Lamiacea Lindl.
Iz_13_1209	DJI_0905	Galium verum L.	0	n	1	0	Rubiaceae Juss.
Iz_13_1209	DJI_0905	Genista tinctoria L.	0	n	1	0	Fabaceae Lindl.
Iz_13_1209	DJI_0905	Geranium robertianum L.	0	n	1	0	Geraniaceae Juss.
Iz_13_1209	DJI_0905	Geranium sanquineum L.	0	n	1	0	Geraniaceae Juss.
Iz_13_1209	DJI_0905	Gypsophila paniculata L.	0	n	1	0	Caryophyllaceae Juss.
Iz_13_1209	DJI_0905	Helichrysum arenarium (L.) Moench	0	n	1	0	Asteraceae Bercht. & J.Presl
Iz_13_1209	DJI_0905	Herniaria polygama J.Gay	0	n	1	0	Caryophyllaceae Juss.
Iz_13_1209	DJI_0905	Hieracium robustum Fr.	0	n	1	0	Asteraceae Bercht. & J.Presl
Iz_13_1209	DJI_0905	Hieracium umbellatum L.	0	n	1	NA	Asteraceae Bercht. & J.Presl
Iz_13_1209	DJI_0905	Hylotelephium maximum (L.) Holub	0	n	1	0	Crassulaceae J.St.-Hil.
Iz_13_1209	DJI_0905	Jacobaea borysthenica (DC.) B.Nord. & Greuter	0	n	1	0	Asteraceae Bercht. & J.Presl
Iz_13_1209	DJI_0905	Jasione montana L.	0	n	1	0	Campanulaceae Juss.
Iz_13_1209	DJI_0905	Jurinea cyanoides (L.) Rchb.s.l.	0	n	1	0	Asteraceae Bercht. & J.Presl
Iz_13_1209	DJI_0905	Koeleria glauca (Spreng.) DC.	0	n	1	0	Poaceae Barnhart
Iz_13_1209	DJI_0905	Lactuca serriola L.	0	a	0	0	Asteraceae Bercht. & J.Presl
Iz_13_1209	DJI_0905	Linaria genistifolia (L.) Mill.	0	n	1	0	Plantaginaceae Juss.
Iz_13_1209	DJI_0905	Lomelosia argentea (L.) Greuter & Burdet	0	n	1	0	Caprifoliaceae Juss.
Iz_13_1209	DJI_0905	Melampyrum pratense L.	0	n	1	0	Orobanchaceae Vent.
Iz_13_1209	DJI_0905	Oenothera biennis L.	0	a	0	0	Onagraceae Juss.
Iz_13_1209	DJI_0905	Pentanema britannica (L.) D.Gut.Larr., Santos-Vicente, Anderb., E.Rico & M.M.Mart.Ort.	0	n	1	0	Asteraceae Bercht. & J.Presl
Iz_13_1209	DJI_0905	Peucedanum oreoselinum (L.) Moench	0	n	1	0	Apiaceae Lindl.
Iz_13_1209	DJI_0905	Peucedanum ruthenicum M.Bieb.	0	n	1	0	Apiaceae Lindl.
Iz_13_1209	DJI_0905	Pilosella echioides (Lumn.) F.W.Schultz & Sch.Bip.	0	n	1	0	Asteraceae Bercht. & J.Presl
Iz_13_1209	DJI_0905	Pilosella officinarum F. Schultz et Sch. Bip.	0	n	1	0	Asteraceae Bercht. & J.Presl
Iz_13_1209	DJI_0905	Pilosella sp.	0		NA	0	Asteraceae Bercht. & J.Presl
Iz_13_1209	DJI_0905	Pinus sylvestris L.	0	n	1	1	Pinaceae Spreng. ex F.Rudolphi
Iz_13_1209	DJI_0905	Poa annua L.	0	n	1	0	Poaceae Barnhart
Iz_13_1209	DJI_0905	Poa nemoralis L.	0	n	1	0	Poaceae Barnhart
Iz_13_1209	DJI_0905	Poa sp.	0		NA	0	Poaceae Barnhart
Iz_13_1209	DJI_0905	Polygonatum odoratum (Mill.) Druce	0	n	1	0	Asparagaceae Juss.
Iz_13_1209	DJI_0905	Polygonum arenarium Waldst. & Kit.	0	n	1	0	Polygonaceae Juss.
Iz_13_1209	DJI_0905	Populus nigra L.	0	n	1	1	Salicaceae Mirb.
Iz_13_1209	DJI_0905	Populus sp.	0	n	1	1	Salicaceae Mirb.
Iz_13_1209	DJI_0905	Populus tremula L.	0	n	1	1	Salicaceae Mirb.
Iz_13_1209	DJI_0905	Populus Ã canescens (Aiton) Sm.	0	n	1	1	Salicaceae Mirb.
Iz_13_1209	DJI_0905	Potentilla argentea L.	0	n	1	0	Rosaceae Juss.
Iz_13_1209	DJI_0905	Psammophiliella muralis (L.) Ikonn.	0	n	1	0	Caryophyllaceae Juss.
Iz_13_1209	DJI_0905	Psephellus sumensis (Kalen.) Greuter	0	n	1	0	Asteraceae Bercht. & J.Presl
Iz_13_1209	DJI_0905	Pulsatilla pratensis (L.) Mill. s.l.	0	n	1	0	Ranunculaceae Juss.
Iz_13_1209	DJI_0905	Pyrus communis L.	0	a	0	1	Rosaceae Juss.
Iz_13_1209	DJI_0905	Quercus robur L.	0	n	1	1	Fagaceae Dumort.
Iz_13_1209	DJI_0905	Robinia pseudoacacia L.	0	a	0	1	Fabaceae Lindl.
Iz_13_1209	DJI_0905	Rosa sp.	0		NA	1	Rosaceae Juss.
Iz_13_1209	DJI_0905	Rubus idaeus L.	0	n	1	0	Rosaceae Juss.
Iz_13_1209	DJI_0905	Rumex acetosella L.	0	n	1	0	Polygonaceae Juss.
Iz_13_1209	DJI_0905	Rumex thyrsiflorus Fingerh.	0	n	1	0	Polygonaceae Juss.
Iz_13_1209	DJI_0905	Salix alba L.	0	n	1	1	Salicaceae Mirb.
Iz_13_1209	DJI_0905	Sambucus nigra L.	0	n	1	1	Viburnaceae Raf.
Iz_13_1209	DJI_0905	Secale sylvestre Host	0	n	1	0	Poaceae Barnhart
Iz_13_1209	DJI_0905	Sempervivum ruthenicum Schnittsp. & C.B. Lehm.	0	n	1	0	Crassulaceae J.St.-Hil.
Iz_13_1209	DJI_0905	Setaria viridis (L.) P. Beauv.	0	n	1	0	Poaceae Barnhart
Iz_13_1209	DJI_0905	Silene borysthenica (Gruner) Walters	0	n	1	0	Caryophyllaceae Juss.
Iz_13_1209	DJI_0905	Silene latifolia subsp. alba (Mill.) Greuter & Burdet	0	n	1	0	Caryophyllaceae Juss.
Iz_13_1209	DJI_0905	Solidago virgaurea L.	0	n	1	0	Asteraceae Bercht. & J.Presl
Iz_13_1209	DJI_0905	Sorbus aucuparia L.	0	n	1	1	Rosaceae Juss.
Iz_13_1209	DJI_0905	Stachys recta L.	0	n	1	0	Lamiacea Lindl.
Iz_13_1209	DJI_0905	Tanacetum vulgare L.	0	n	1	0	Asteraceae Bercht. & J.Presl
Iz_13_1209	DJI_0905	Thymus pallasianus Heinr.Braun	0	n	1	0	Lamiacea Lindl.
Iz_13_1209	DJI_0905	Tilia cordata Mill.	0	n	1	1	Malvaceae Juss.
Iz_13_1209	DJI_0905	Trifolium alpestre L.	0	n	1	0	Fabaceae Lindl.
Iz_13_1209	DJI_0905	Trifolium arvense L.	0	n	1	0	Fabaceae Lindl.
Iz_13_1209	DJI_0905	Ulmus glabra Huds.	0	n	1	1	Ulmaceae Mirb.
Iz_13_1209	DJI_0905	Verbascum densiflorum Bertol.	0	n	1	0	Scrophulariaceae Juss.
Iz_13_1209	DJI_0905	Veronica austriaca L.	0	n	1	0	Plantaginaceae Juss.
Iz_13_1209	DJI_0905	Veronica incana L.	0	n	1	0	Plantaginaceae Juss.
Iz_13_1209	DJI_0905	Veronica verna L.	0	n	1	0	Plantaginaceae Juss.
Iz_13_1209	DJI_0905	Vincetoxicum hirundinaria Medik	0	n	1	0	Apocynaceae Juss.

Iz_13_1209	DJI_0908	Acer tataricum L.	0	n	1	1	Sapindaceae Juss.
Iz_13_1209	DJI_0908	Achillea millefolium L. s.l.	0	n	1	0	Asteraceae Bercht. & J.Presl
Iz_13_1209	DJI_0908	Allium oleraceum L.	0	n	1	0	Amaryllidaceae J.St.-Hil.
Iz_13_1209	DJI_0908	Ambrosia artemisiifolia L.	0	a	0	0	Asteraceae Bercht. & J.Presl
Iz_13_1209	DJI_0908	Amorpha fruticosa L.	0	a	0	0	Fabaceae Lindl.
Iz_13_1209	DJI_0908	Anthericum ramosum L.	0	n	1	0	Asparagaceae Juss.
Iz_13_1209	DJI_0908	Artemisia marschalliana Spreng.	0	n	1	0	Asteraceae Bercht. & J.Presl
Iz_13_1209	DJI_0908	Asparagus officinalis L.	0	n	1	0	Asparagaceae Juss.
Iz_13_1209	DJI_0908	Astragalus cicer L.	0	n	1	0	Fabaceae Lindl.
Iz_13_1209	DJI_0908	Bassia laniflora (S.G.Gmel.) A.J.Scott	0	n	1	0	Amaranthaceae Juss.
Iz_13_1209	DJI_0908	Berberis vulgaris L.	0	n	1	0	Berberidaceae Juss.
Iz_13_1209	DJI_0908	Betula pendula Roth.	0	n	1	1	Betulaceae Gray
Iz_13_1209	DJI_0908	Betula pendula Roth.	0	n	1	0	Poaceae Barnhart
Iz_13_1209	DJI_0908	Bromus inermis Leyss.	0		NA	NA	
Iz_13_1209	DJI_0908	Bromus squarrosus L.	0	a	0	0	Poaceae Barnhart
Iz_13_1209	DJI_0908	Bromus tectorum L.	0	a	0	0	Poaceae Barnhart
Iz_13_1209	DJI_0908	Calamagrostis epigejos (L.) Roth	80	n	1	0	Poaceae Barnhart
Iz_13_1209	DJI_0908	Carduus acanthoides L.	0	n	1	0	Asteraceae Bercht. & J.Presl
Iz_13_1209	DJI_0908	Carex sp.	0	n	1	0	Cyperaceae Juss.
Iz_13_1209	DJI_0908	Centaurea majorovii Dumbadze	0	n	1	0	Asteraceae Bercht. & J.Presl
Iz_13_1209	DJI_0908	Chamaecytisus ruthenicus (Fisch.ex Wol.) Klaskova	0	n	1	0	Fabaceae Lindl.
Iz_13_1209	DJI_0908	Chelidonium majus L.	0	n	1	0	Papaveraceae Juss.
Iz_13_1209	DJI_0908	Chenopodium betaceum Andrz.	0	n	1	0	Amaranthaceae Juss.
Iz_13_1209	DJI_0908	Chenopodium cf. album	0	n	1	0	Amaranthaceae Juss.
Iz_13_1209	DJI_0908	Chenopodium sp.	0		NA	0	Amaranthaceae Juss.
Iz_13_1209	DJI_0908	Chondrilla juncea L.	0	n	1	0	Asteraceae Bercht. & J.Presl
Iz_13_1209	DJI_0908	Cirsium vulgare (Savi) Ten.	0	n	1	0	Asteraceae Bercht. & J.Presl
Iz_13_1209	DJI_0908	Clinopodium acinos (L.) Kuntze	0	n	1	0	Lamiacea Lindl.
Iz_13_1209	DJI_0908	Convallaria majalis L.	0	n	1	0	Asparagaceae Juss.
Iz_13_1209	DJI_0908	Crataegus rhipidophylla Gand.	0	n	1	1	Rosaceae Juss.
Iz_13_1209	DJI_0908	Crepis foetida L.	0	n	1	0	Asteraceae Bercht. & J.Presl
Iz_13_1209	DJI_0908	Dactylis glomerata L.	0	n	1	0	Poaceae Barnhart
Iz_13_1209	DJI_0908	Dianthus campestris M.Bieb.	0	n	1	0	Caryophyllaceae Juss.
Iz_13_1209	DJI_0908	Digitaria sanguinalis (L.) Scop.	0	a	0	0	Poaceae Barnhart
Iz_13_1209	DJI_0908	Elymus repens (L.) Gould	0	n	1	0	Poaceae Barnhart
Iz_13_1209	DJI_0908	Epilobium tetragonum L.	0	n	1	0	Onagraceae Juss.
Iz_13_1209	DJI_0908	Eragrostis minor Host	0	a	0	0	Poaceae Barnhart
Iz_13_1209	DJI_0908	Erigeron canadensis L.	1	a	0	0	Asteraceae Bercht. & J.Presl
Iz_13_1209	DJI_0908	Euphorbia seguieriana Neck.	0	n	1	0	Euphorbiaceae Juss.
Iz_13_1209	DJI_0908	Fallopia dumetorum (L.) Holub	0	n	1	0	Polygonaceae Juss.
Iz_13_1209	DJI_0908	Festuca beckeri (Hack.) Trautv.	0	n	1	0	Poaceae Barnhart
Iz_13_1209	DJI_0908	Fragaria vesca L.	0	n	1	0	Rosaceae Juss.
Iz_13_1209	DJI_0908	Frangula alnus Mill.	0	n	1	1	Rhamnaceae Juss.
Iz_13_1209	DJI_0908	Fraxinus pennsylvanica Marshall	0	a	0	1	Oleaceae Hoffmanns. & Link
Iz_13_1209	DJI_0908	Galeopsis bifida Boenn.	0	n	1	0	Lamiacea Lindl.
Iz_13_1209	DJI_0908	Galium verum L.	0	n	1	0	Rubiaceae Juss.
Iz_13_1209	DJI_0908	Genista tinctoria L.	0	n	1	0	Fabaceae Lindl.
Iz_13_1209	DJI_0908	Geranium robertianum L.	0	n	1	0	Geraniaceae Juss.
Iz_13_1209	DJI_0908	Geranium sanquineum L.	0	n	1	0	Geraniaceae Juss.
Iz_13_1209	DJI_0908	Gypsophila paniculata L.	0	n	1	0	Caryophyllaceae Juss.
Iz_13_1209	DJI_0908	Helichrysum arenarium (L.) Moench	0	n	1	0	Asteraceae Bercht. & J.Presl
Iz_13_1209	DJI_0908	Herniaria polygama J.Gay	0	n	1	0	Caryophyllaceae Juss.
Iz_13_1209	DJI_0908	Hieracium robustum Fr.	0	n	1	0	Asteraceae Bercht. & J.Presl
Iz_13_1209	DJI_0908	Hieracium umbellatum L.	0	n	1	NA	Asteraceae Bercht. & J.Presl
Iz_13_1209	DJI_0908	Hylotelephium maximum (L.) Holub	0	n	1	0	Crassulaceae J.St.-Hil.
Iz_13_1209	DJI_0908	Jacobaea borysthenica (DC.) B.Nord. & Greuter	0	n	1	0	Asteraceae Bercht. & J.Presl
Iz_13_1209	DJI_0908	Jasione montana L.	0	n	1	0	Campanulaceae Juss.
Iz_13_1209	DJI_0908	Jurinea cyanoides (L.) Rchb.s.l.	0	n	1	0	Asteraceae Bercht. & J.Presl
Iz_13_1209	DJI_0908	Koeleria glauca (Spreng.) DC.	0	n	1	0	Poaceae Barnhart
Iz_13_1209	DJI_0908	Lactuca serriola L.	0	a	0	0	Asteraceae Bercht. & J.Presl
Iz_13_1209	DJI_0908	Linaria genistifolia (L.) Mill.	0	n	1	0	Plantaginaceae Juss.
Iz_13_1209	DJI_0908	Lomelosia argentea (L.) Greuter & Burdet	0	n	1	0	Caprifoliaceae Juss.
Iz_13_1209	DJI_0908	Melampyrum pratense L.	0	n	1	0	Orobanchaceae Vent.
Iz_13_1209	DJI_0908	Oenothera biennis L.	0	a	0	0	Onagraceae Juss.
Iz_13_1209	DJI_0908	Pentanema britannica (L.) D.Gut.Larr., Santos-Vicente, Anderb., E.Rico & M.M.Mart.Ort.	0	n	1	0	Asteraceae Bercht. & J.Presl
Iz_13_1209	DJI_0908	Peucedanum oreoselinum (L.) Moench	0	n	1	0	Apiaceae Lindl.
Iz_13_1209	DJI_0908	Peucedanum ruthenicum M.Bieb.	0	n	1	0	Apiaceae Lindl.
Iz_13_1209	DJI_0908	Pilosella echioides (Lumn.) F.W.Schultz & Sch.Bip.	0	n	1	0	Asteraceae Bercht. & J.Presl
Iz_13_1209	DJI_0908	Pilosella officinarum F. Schultz et Sch. Bip.	0	n	1	0	Asteraceae Bercht. & J.Presl
Iz_13_1209	DJI_0908	Pilosella sp.	0		NA	0	Asteraceae Bercht. & J.Presl
Iz_13_1209	DJI_0908	Pinus sylvestris L.	0	n	1	1	Pinaceae Spreng. ex F.Rudolphi
Iz_13_1209	DJI_0908	Poa annua L.	0	n	1	0	Poaceae Barnhart
Iz_13_1209	DJI_0908	Poa nemoralis L.	0	n	1	0	Poaceae Barnhart
Iz_13_1209	DJI_0908	Poa sp.	0		NA	0	Poaceae Barnhart
Iz_13_1209	DJI_0908	Polygonatum odoratum (Mill.) Druce	0	n	1	0	Asparagaceae Juss.
Iz_13_1209	DJI_0908	Polygonum arenarium Waldst. & Kit.	0	n	1	0	Polygonaceae Juss.
Iz_13_1209	DJI_0908	Populus nigra L.	0	n	1	1	Salicaceae Mirb.
Iz_13_1209	DJI_0908	Populus sp.	0	n	1	1	Salicaceae Mirb.
Iz_13_1209	DJI_0908	Populus tremula L.	15	n	1	1	Salicaceae Mirb.
Iz_13_1209	DJI_0908	Populus Ã canescens (Aiton) Sm.	0	n	1	1	Salicaceae Mirb.
Iz_13_1209	DJI_0908	Potentilla argentea L.	0	n	1	0	Rosaceae Juss.
Iz_13_1209	DJI_0908	Psammophiliella muralis (L.) Ikonn.	0	n	1	0	Caryophyllaceae Juss.
Iz_13_1209	DJI_0908	Psephellus sumensis (Kalen.) Greuter	0	n	1	0	Asteraceae Bercht. & J.Presl
Iz_13_1209	DJI_0908	Pulsatilla pratensis (L.) Mill. s.l.	0	n	1	0	Ranunculaceae Juss.
Iz_13_1209	DJI_0908	Pyrus communis L.	0	a	0	1	Rosaceae Juss.
Iz_13_1209	DJI_0908	Quercus robur L.	0	n	1	1	Fagaceae Dumort.
Iz_13_1209	DJI_0908	Robinia pseudoacacia L.	0	a	0	1	Fabaceae Lindl.
Iz_13_1209	DJI_0908	Rosa sp.	0		NA	1	Rosaceae Juss.
Iz_13_1209	DJI_0908	Rubus idaeus L.	0	n	1	0	Rosaceae Juss.
Iz_13_1209	DJI_0908	Rumex acetosella L.	0	n	1	0	Polygonaceae Juss.
Iz_13_1209	DJI_0908	Rumex thyrsiflorus Fingerh.	0	n	1	0	Polygonaceae Juss.
Iz_13_1209	DJI_0908	Salix alba L.	0	n	1	1	Salicaceae Mirb.
Iz_13_1209	DJI_0908	Sambucus nigra L.	0	n	1	1	Viburnaceae Raf.
Iz_13_1209	DJI_0908	Secale sylvestre Host	0	n	1	0	Poaceae Barnhart
Iz_13_1209	DJI_0908	Sempervivum ruthenicum Schnittsp. & C.B. Lehm.	0	n	1	0	Crassulaceae J.St.-Hil.
Iz_13_1209	DJI_0908	Setaria viridis (L.) P. Beauv.	0	n	1	0	Poaceae Barnhart
Iz_13_1209	DJI_0908	Silene borysthenica (Gruner) Walters	0	n	1	0	Caryophyllaceae Juss.
Iz_13_1209	DJI_0908	Silene latifolia subsp. alba (Mill.) Greuter & Burdet	0	n	1	0	Caryophyllaceae Juss.

Iz_13_1209	DJI_0908	Solidago virgaurea L.	0	n	1	0	Asteraceae Bercht. & J.Presl
Iz_13_1209	DJI_0908	Sorbus aucuparia L.	0	n	1	1	Rosaceae Juss.
Iz_13_1209	DJI_0908	Stachys recta L.	0	n	1	0	Lamiacea Lindl.
Iz_13_1209	DJI_0908	Tanacetum vulgare L.	0	n	1	0	Asteraceae Bercht. & J.Presl
Iz_13_1209	DJI_0908	Thymus pallasianus Heinr.Braun	0	n	1	0	Lamiacea Lindl.
Iz_13_1209	DJI_0908	Tilia cordata Mill.	0	n	1	1	Malvaceae Juss.
Iz_13_1209	DJI_0908	Trifolium alpestre L.	0	n	1	0	Fabaceae Lindl.
Iz_13_1209	DJI_0908	Trifolium arvense L.	0	n	1	0	Fabaceae Lindl.
Iz_13_1209	DJI_0908	Ulmus glabra Huds.	0	n	1	1	Ulmaceae Mirb.
Iz_13_1209	DJI_0908	Verbascum densiflorum Bertol.	0	n	1	0	Scrophulariaceae Juss.
Iz_13_1209	DJI_0908	Veronica austriaca L.	0	n	1	0	Plantaginaceae Juss.
Iz_13_1209	DJI_0908	Veronica incana L.	0	n	1	0	Plantaginaceae Juss.
Iz_13_1209	DJI_0908	Veronica verna L.	0	n	1	0	Plantaginaceae Juss.
Iz_13_1209	DJI_0908	Vincetoxicum hirundinaria Medik	0	n	1	0	Apocynaceae Juss.
Iz_13_1209	DJI_0913	Acer tataricum L.	0	n	1	1	Sapindaceae Juss.
Iz_13_1209	DJI_0913	Achillea millefolium L. s.l.	0	n	1	0	Asteraceae Bercht. & J.Presl
Iz_13_1209	DJI_0913	Allium oleraceum L.	0	n	1	0	Amaryllidaceae J.St.-Hil.
Iz_13_1209	DJI_0913	Ambrosia artemisiifolia L.	0	a	0	0	Asteraceae Bercht. & J.Presl
Iz_13_1209	DJI_0913	Amorpha fruticosa L.	0	a	0	0	Fabaceae Lindl.
Iz_13_1209	DJI_0913	Anthericum ramosum L.	0	n	1	0	Asparagaceae Juss.
Iz_13_1209	DJI_0913	Artemisia marschalliana Spreng.	0	n	1	0	Asteraceae Bercht. & J.Presl
Iz_13_1209	DJI_0913	Asparagus officinalis L.	0	n	1	0	Asparagaceae Juss.
Iz_13_1209	DJI_0913	Astragalus cicer L.	0	n	1	0	Fabaceae Lindl.
Iz_13_1209	DJI_0913	Bassia laniflora (S.G.Gmel.) A.J.Scott	0	n	1	0	Amaranthaceae Juss.
Iz_13_1209	DJI_0913	Berberis vulgaris L.	0	n	1	0	Berberidaceae Juss.
Iz_13_1209	DJI_0913	Betula pendula Roth.	0	n	1	1	Betulaceae Gray
Iz_13_1209	DJI_0913	Betula pendula Roth.	0	n	1	0	Poaceae Barnhart
Iz_13_1209	DJI_0913	Bromus inermis Leyss.	0		NA	NA	
Iz_13_1209	DJI_0913	Bromus squarrosus L.	0	a	0	0	Poaceae Barnhart
Iz_13_1209	DJI_0913	Bromus tectorum L.	0	a	0	0	Poaceae Barnhart
Iz_13_1209	DJI_0913	Calamagrostis epigejos (L.) Roth	80	n	1	0	Poaceae Barnhart
Iz_13_1209	DJI_0913	Carduus acanthoides L.	0	n	1	0	Asteraceae Bercht. & J.Presl
Iz_13_1209	DJI_0913	Carex sp.	0	n	1	0	Cyperaceae Juss.
Iz_13_1209	DJI_0913	Centaurea majorovii Dumbadze	0	n	1	0	Asteraceae Bercht. & J.Presl
Iz_13_1209	DJI_0913	Chamaecytisus ruthenicus (Fisch.ex Wol.) Klaskova	0	n	1	0	Fabaceae Lindl.
Iz_13_1209	DJI_0913	Chelidonium majus L.	0	n	1	0	Papaveraceae Juss.
Iz_13_1209	DJI_0913	Chenopodium betaceum Andrz.	0	n	1	0	Amaranthaceae Juss.
Iz_13_1209	DJI_0913	Chenopodium cf. album	0	n	1	0	Amaranthaceae Juss.
Iz_13_1209	DJI_0913	Chenopodium sp.	0		NA	0	Amaranthaceae Juss.
Iz_13_1209	DJI_0913	Chondrilla juncea L.	0	n	1	0	Asteraceae Bercht. & J.Presl
Iz_13_1209	DJI_0913	Cirsium vulgare (Savi) Ten.	0	n	1	0	Asteraceae Bercht. & J.Presl
Iz_13_1209	DJI_0913	Clinopodium acinos (L.) Kuntze	0	n	1	0	Lamiacea Lindl.
Iz_13_1209	DJI_0913	Convallaria majalis L.	0	n	1	0	Asparagaceae Juss.
Iz_13_1209	DJI_0913	Crataegus rhipidophylla Gand.	0	n	1	1	Rosaceae Juss.
Iz_13_1209	DJI_0913	Crepis foetida L.	0	n	1	0	Asteraceae Bercht. & J.Presl
Iz_13_1209	DJI_0913	Dactylis glomerata L.	0	n	1	0	Poaceae Barnhart
Iz_13_1209	DJI_0913	Dianthus campestris M.Bieb.	0	n	1	0	Caryophyllaceae Juss.
Iz_13_1209	DJI_0913	Digitaria sanguinalis (L.) Scop.	0	a	0	0	Poaceae Barnhart
Iz_13_1209	DJI_0913	Elymus repens (L.) Gould	0	n	1	0	Poaceae Barnhart
Iz_13_1209	DJI_0913	Epilobium tetragonum L.	0	n	1	0	Onagraceae Juss.
Iz_13_1209	DJI_0913	Eragrostis minor Host	0	a	0	0	Poaceae Barnhart
Iz_13_1209	DJI_0913	Erigeron canadensis L.	1	a	0	0	Asteraceae Bercht. & J.Presl
Iz_13_1209	DJI_0913	Euphorbia seguieriana Neck.	0	n	1	0	Euphorbiaceae Juss.
Iz_13_1209	DJI_0913	Fallopia dumetorum (L.) Holub	0	n	1	0	Polygonaceae Juss.
Iz_13_1209	DJI_0913	Festuca beckeri (Hack.) Trautv.	0	n	1	0	Poaceae Barnhart
Iz_13_1209	DJI_0913	Fragaria vesca L.	0	n	1	0	Rosaceae Juss.
Iz_13_1209	DJI_0913	Frangula alnus Mill.	0	n	1	1	Rhamnaceae Juss.
Iz_13_1209	DJI_0913	Fraxinus pennsylvanica Marshall	0	a	0	1	Oleaceae Hoffmanns. & Link
Iz_13_1209	DJI_0913	Galeopsis bifida Boenn.	0	n	1	0	Lamiacea Lindl.
Iz_13_1209	DJI_0913	Galium verum L.	0	n	1	0	Rubiaceae Juss.
Iz_13_1209	DJI_0913	Genista tinctoria L.	0	n	1	0	Fabaceae Lindl.
Iz_13_1209	DJI_0913	Geranium robertianum L.	0	n	1	0	Geraniaceae Juss.
Iz_13_1209	DJI_0913	Geranium sanquineum L.	0	n	1	0	Geraniaceae Juss.
Iz_13_1209	DJI_0913	Gypsophila paniculata L.	0	n	1	0	Caryophyllaceae Juss.
Iz_13_1209	DJI_0913	Helichrysum arenarium (L.) Moench	0	n	1	0	Asteraceae Bercht. & J.Presl
Iz_13_1209	DJI_0913	Herniaria polygama J.Gay	0	n	1	0	Caryophyllaceae Juss.
Iz_13_1209	DJI_0913	Hieracium robustum Fr.	0	n	1	0	Asteraceae Bercht. & J.Presl
Iz_13_1209	DJI_0913	Hieracium umbellatum L.	0	n	1	NA	Asteraceae Bercht. & J.Presl
Iz_13_1209	DJI_0913	Hylotelephium maximum (L.) Holub	0	n	1	0	Crassulaceae J.St.-Hil.
Iz_13_1209	DJI_0913	Jacobaea borysthenica (DC.) B.Nord. & Greuter	0	n	1	0	Asteraceae Bercht. & J.Presl
Iz_13_1209	DJI_0913	Jasione montana L.	0	n	1	0	Campanulaceae Juss.
Iz_13_1209	DJI_0913	Jurinea cyanoides (L.) Rchb.s.l.	0	n	1	0	Asteraceae Bercht. & J.Presl
Iz_13_1209	DJI_0913	Koeleria glauca (Spreng.) DC.	0	n	1	0	Poaceae Barnhart
Iz_13_1209	DJI_0913	Lactuca serriola L.	0	a	0	0	Asteraceae Bercht. & J.Presl
Iz_13_1209	DJI_0913	Linaria genistifolia (L.) Mill.	0	n	1	0	Plantaginaceae Juss.
Iz_13_1209	DJI_0913	Lomelosia argentea (L.) Greuter & Burdet	0	n	1	0	Caprifoliaceae Juss.
Iz_13_1209	DJI_0913	Melampyrum pratense L.	0	n	1	0	Orobanchaceae Vent.
Iz_13_1209	DJI_0913	Oenothera biennis L.	0	a	0	0	Onagraceae Juss.
Iz_13_1209	DJI_0913	Pentanema britannica (L.) D.Gut.Larr., Santos-Vicente, Anderb., E.Rico & M.M.Mart.Ort.	0	n	1	0	Asteraceae Bercht. & J.Presl
Iz_13_1209	DJI_0913	Peucedanum oreoselinum (L.) Moench	0	n	1	0	Apiaceae Lindl.
Iz_13_1209	DJI_0913	Peucedanum ruthenicum M.Bieb.	0	n	1	0	Apiaceae Lindl.
Iz_13_1209	DJI_0913	Pilosella echioides (Lumn.) F.W.Schultz & Sch.Bip.	0	n	1	0	Asteraceae Bercht. & J.Presl
Iz_13_1209	DJI_0913	Pilosella officinarum F. Schultz et Sch. Bip.	0	n	1	0	Asteraceae Bercht. & J.Presl
Iz_13_1209	DJI_0913	Pilosella sp.	0		NA	0	Asteraceae Bercht. & J.Presl
Iz_13_1209	DJI_0913	Pinus sylvestris L.	0	n	1	1	Pinaceae Spreng. ex F.Rudolphi
Iz_13_1209	DJI_0913	Poa annua L.	0	n	1	0	Poaceae Barnhart
Iz_13_1209	DJI_0913	Poa nemoralis L.	0	n	1	0	Poaceae Barnhart
Iz_13_1209	DJI_0913	Poa sp.	0		NA	0	Poaceae Barnhart
Iz_13_1209	DJI_0913	Polygonatum odoratum (Mill.) Druce	0	n	1	0	Asparagaceae Juss.
Iz_13_1209	DJI_0913	Polygonum arenarium Waldst. & Kit.	0	n	1	0	Polygonaceae Juss.
Iz_13_1209	DJI_0913	Populus nigra L.	0	n	1	1	Salicaceae Mirb.
Iz_13_1209	DJI_0913	Populus sp.	0	n	1	1	Salicaceae Mirb.
Iz_13_1209	DJI_0913	Populus tremula L.	3	n	1	1	Salicaceae Mirb.
Iz_13_1209	DJI_0913	Populus Ã canescens (Aiton) Sm.	0	n	1	1	Salicaceae Mirb.
Iz_13_1209	DJI_0913	Potentilla argentea L.	0	n	1	0	Rosaceae Juss.
Iz_13_1209	DJI_0913	Psammophiliella muralis (L.) Ikonn.	0	n	1	0	Caryophyllaceae Juss.
Iz_13_1209	DJI_0913	Psephellus sumensis (Kalen.) Greuter	0	n	1	0	Asteraceae Bercht. & J.Presl
Iz_13_1209	DJI_0913	Pulsatilla pratensis (L.) Mill. s.l.	0	n	1	0	Ranunculaceae Juss.

Iz_13_1209	DJI_0913	Pyrus communis L.	0	a	0	1	Rosaceae Juss.
Iz_13_1209	DJI_0913	Quercus robur L.	0	n	1	1	Fagaceae Dumort.
Iz_13_1209	DJI_0913	Robinia pseudoacacia L.	0	a	0	1	Fabaceae Lindl.
Iz_13_1209	DJI_0913	Rosa sp.	0		NA	1	Rosaceae Juss.
Iz_13_1209	DJI_0913	Rubus idaeus L.	0	n	1	0	Rosaceae Juss.
Iz_13_1209	DJI_0913	Rumex acetosella L.	0	n	1	0	Polygonaceae Juss.
Iz_13_1209	DJI_0913	Rumex thyrsiflorus Fingerh.	0	n	1	0	Polygonaceae Juss.
Iz_13_1209	DJI_0913	Salix alba L.	0	n	1	1	Salicaceae Mirb.
Iz_13_1209	DJI_0913	Sambucus nigra L.	0	n	1	1	Viburnaceae Raf.
Iz_13_1209	DJI_0913	Secale sylvestre Host	0	n	1	0	Poaceae Barnhart
Iz_13_1209	DJI_0913	Sempervivum ruthenicum Schnittsp. & C.B. Lehm.	0	n	1	0	Crassulaceae J.St.-Hil.
Iz_13_1209	DJI_0913	Setaria viridis (L.) P. Beauv.	0	n	1	0	Poaceae Barnhart
Iz_13_1209	DJI_0913	Silene borysthenica (Gruner) Walters	0	n	1	0	Caryophyllaceae Juss.
Iz_13_1209	DJI_0913	Silene latifolia subsp. alba (Mill.) Greuter & Burdet	0	n	1	0	Caryophyllaceae Juss.
Iz_13_1209	DJI_0913	Solidago virgaurea L.	0	n	1	0	Asteraceae Bercht. & J.Presl
Iz_13_1209	DJI_0913	Sorbus aucuparia L.	0	n	1	1	Rosaceae Juss.
Iz_13_1209	DJI_0913	Stachys recta L.	0	n	1	0	Lamiacea Lindl.
Iz_13_1209	DJI_0913	Tanacetum vulgare L.	0	n	1	0	Asteraceae Bercht. & J.Presl
Iz_13_1209	DJI_0913	Thymus pallasianus Heinr.Braun	0	n	1	0	Lamiacea Lindl.
Iz_13_1209	DJI_0913	Tilia cordata Mill.	0	n	1	1	Malvaceae Juss.
Iz_13_1209	DJI_0913	Trifolium alpestre L.	0	n	1	0	Fabaceae Lindl.
Iz_13_1209	DJI_0913	Trifolium arvense L.	0	n	1	0	Fabaceae Lindl.
Iz_13_1209	DJI_0913	Ulmus glabra Huds.	0	n	1	1	Ulmaceae Mirb.
Iz_13_1209	DJI_0913	Verbascum densiflorum Bertol.	0	n	1	0	Scrophulariaceae Juss.
Iz_13_1209	DJI_0913	Veronica austriaca L.	0	n	1	0	Plantaginaceae Juss.
Iz_13_1209	DJI_0913	Veronica incana L.	0	n	1	0	Plantaginaceae Juss.
Iz_13_1209	DJI_0913	Veronica verna L.	0	n	1	0	Plantaginaceae Juss.
Iz_13_1209	DJI_0913	Vincetoxicum hirundinaria Medik	0	n	1	0	Apocynaceae Juss.
Iz_14_1209	DJI_0939	Acer tataricum L.	0	n	1	1	Sapindaceae Juss.
Iz_14_1209	DJI_0939	Achillea millefolium L. s.l.	0	n	1	0	Asteraceae Bercht. & J.Presl
Iz_14_1209	DJI_0939	Allium oleraceum L.	0	n	1	0	Amaryllidaceae J.St.-Hil.
Iz_14_1209	DJI_0939	Ambrosia artemisiifolia L.	0	a	0	0	Asteraceae Bercht. & J.Presl
Iz_14_1209	DJI_0939	Amorpha fruticosa L.	0	a	0	0	Fabaceae Lindl.
Iz_14_1209	DJI_0939	Anthericum ramosum L.	0	n	1	0	Asparagaceae Juss.
Iz_14_1209	DJI_0939	Artemisia marschalliana Spreng.	0	n	1	0	Asteraceae Bercht. & J.Presl
Iz_14_1209	DJI_0939	Asparagus officinalis L.	0	n	1	0	Asparagaceae Juss.
Iz_14_1209	DJI_0939	Astragalus cicer L.	0	n	1	0	Fabaceae Lindl.
Iz_14_1209	DJI_0939	Bassia laniflora (S.G.Gmel.) A.J.Scott	0	n	1	0	Amaranthaceae Juss.
Iz_14_1209	DJI_0939	Berberis vulgaris L.	0	n	1	0	Berberidaceae Juss.
Iz_14_1209	DJI_0939	Betula pendula Roth.	0	n	1	1	Betulaceae Gray
Iz_14_1209	DJI_0939	Betula pendula Roth.	0	n	1	0	Poaceae Barnhart
Iz_14_1209	DJI_0939	Bromus inermis Leyss.	0		NA	NA	
Iz_14_1209	DJI_0939	Bromus squarrosus L.	0	a	0	0	Poaceae Barnhart
Iz_14_1209	DJI_0939	Bromus tectorum L.	0	a	0	0	Poaceae Barnhart
Iz_14_1209	DJI_0939	Calamagrostis epigejos (L.) Roth	45	n	1	0	Poaceae Barnhart
Iz_14_1209	DJI_0939	Carduus acanthoides L.	0	n	1	0	Asteraceae Bercht. & J.Presl
Iz_14_1209	DJI_0939	Carex sp.	0	n	1	0	Cyperaceae Juss.
Iz_14_1209	DJI_0939	Centaurea majorovii Dumbadze	0	n	1	0	Asteraceae Bercht. & J.Presl
Iz_14_1209	DJI_0939	Chamaecytisus ruthenicus (Fisch.ex Wol.) Klaskova	0	n	1	0	Fabaceae Lindl.
Iz_14_1209	DJI_0939	Chelidonium majus L.	0	n	1	0	Papaveraceae Juss.
Iz_14_1209	DJI_0939	Chenopodium betaceum Andrz.	0	n	1	0	Amaranthaceae Juss.
Iz_14_1209	DJI_0939	Chenopodium cf. album	0	n	1	0	Amaranthaceae Juss.
Iz_14_1209	DJI_0939	Chenopodium sp.	0.2		NA	0	Amaranthaceae Juss.
Iz_14_1209	DJI_0939	Chondrilla juncea L.	0	n	1	0	Asteraceae Bercht. & J.Presl
Iz_14_1209	DJI_0939	Cirsium vulgare (Savi) Ten.	0	n	1	0	Asteraceae Bercht. & J.Presl
Iz_14_1209	DJI_0939	Clinopodium acinos (L.) Kuntze	0	n	1	0	Lamiacea Lindl.
Iz_14_1209	DJI_0939	Convallaria majalis L.	0	n	1	0	Asparagaceae Juss.
Iz_14_1209	DJI_0939	Crataegus rhipidophylla Gand.	0	n	1	1	Rosaceae Juss.
Iz_14_1209	DJI_0939	Crepis foetida L.	0	n	1	0	Asteraceae Bercht. & J.Presl
Iz_14_1209	DJI_0939	Dactylis glomerata L.	0	n	1	0	Poaceae Barnhart
Iz_14_1209	DJI_0939	Dianthus campestris M.Bieb.	0	n	1	0	Caryophyllaceae Juss.
Iz_14_1209	DJI_0939	Digitaria sanguinalis (L.) Scop.	0	a	0	0	Poaceae Barnhart
Iz_14_1209	DJI_0939	Elymus repens (L.) Gould	0	n	1	0	Poaceae Barnhart
Iz_14_1209	DJI_0939	Epilobium tetragonum L.	0	n	1	0	Onagraceae Juss.
Iz_14_1209	DJI_0939	Eragrostis minor Host	0	a	0	0	Poaceae Barnhart
Iz_14_1209	DJI_0939	Erigeron annuus (L.) Desf.	0	a	0	0	Asteraceae Bercht. & J.Presl
Iz_14_1209	DJI_0939	Erigeron canadensis L.	0	a	0	0	Asteraceae Bercht. & J.Presl
Iz_14_1209	DJI_0939	Euphorbia seguieriana Neck.	0	n	1	0	Euphorbiaceae Juss.
Iz_14_1209	DJI_0939	Euphorbia sp.	0		NA	NA	
Iz_14_1209	DJI_0939	Fallopia dumetorum (L.) Holub	0.2	n	1	0	Polygonaceae Juss.
Iz_14_1209	DJI_0939	Festuca beckeri (Hack.) Trautv.	0	n	1	0	Poaceae Barnhart
Iz_14_1209	DJI_0939	Fragaria vesca L.	0	n	1	0	Rosaceae Juss.
Iz_14_1209	DJI_0939	Frangula alnus Mill.	0	n	1	1	Rhamnaceae Juss.
Iz_14_1209	DJI_0939	Fraxinus pennsylvanica Marshall	0	a	0	1	Oleaceae Hoffmanns. & Link
Iz_14_1209	DJI_0939	Galeopsis bifida Boenn.	0	n	1	0	Lamiacea Lindl.
Iz_14_1209	DJI_0939	Galium verum L.	0	n	1	0	Rubiaceae Juss.
Iz_14_1209	DJI_0939	Genista tinctoria L.	0	n	1	0	Fabaceae Lindl.
Iz_14_1209	DJI_0939	Geranium robertianum L.	0	n	1	0	Geraniaceae Juss.
Iz_14_1209	DJI_0939	Geranium sanquineum L.	0	n	1	0	Geraniaceae Juss.
Iz_14_1209	DJI_0939	Gypsophila paniculata L.	10	n	1	0	Caryophyllaceae Juss.
Iz_14_1209	DJI_0939	Helichrysum arenarium (L.) Moench	0	n	1	0	Asteraceae Bercht. & J.Presl
Iz_14_1209	DJI_0939	Herniaria polygama J.Gay	0	n	1	0	Caryophyllaceae Juss.
Iz_14_1209	DJI_0939	Hieracium robustum Fr.	0	n	1	0	Asteraceae Bercht. & J.Presl
Iz_14_1209	DJI_0939	Hieracium umbellatum L.	0	n	1	NA	Asteraceae Bercht. & J.Presl
Iz_14_1209	DJI_0939	Hylotelephium maximum (L.) Holub	0	n	1	0	Crassulaceae J.St.-Hil.
Iz_14_1209	DJI_0939	Jacobaea borysthenica (DC.) B.Nord. & Greuter	0	n	1	0	Asteraceae Bercht. & J.Presl
Iz_14_1209	DJI_0939	Jasione montana L.	0	n	1	0	Campanulaceae Juss.
Iz_14_1209	DJI_0939	Jurinea cyanoides (L.) Rchb.s.l.	0	n	1	0	Asteraceae Bercht. & J.Presl
Iz_14_1209	DJI_0939	Koeleria glauca (Spreng.) DC.	0	n	1	0	Poaceae Barnhart
Iz_14_1209	DJI_0939	Lactuca serriola L.	0	a	0	0	Asteraceae Bercht. & J.Presl
Iz_14_1209	DJI_0939	Linaria genistifolia (L.) Mill.	0	n	1	0	Plantaginaceae Juss.
Iz_14_1209	DJI_0939	Lomelosia argentea (L.) Greuter & Burdet	0	n	1	0	Caprifoliaceae Juss.
Iz_14_1209	DJI_0939	Melampyrum pratense L.	0	n	1	0	Orobanchaceae Vent.
Iz_14_1209	DJI_0939	Oenothera biennis L.	0	a	0	0	Onagraceae Juss.
Iz_14_1209	DJI_0939	Pentanema britannica (L.) D.Gut.Larr., Santos-Vicente, Anderb., E.Rico & M.M.Mart.Ort.	0	n	1	0	Asteraceae Bercht. & J.Presl
Iz_14_1209	DJI_0939	Peucedanum oreoselinum (L.) Moench	0	n	1	0	Apiaceae Lindl.
Iz_14_1209	DJI_0939	Peucedanum ruthenicum M.Bieb.	0	n	1	0	Apiaceae Lindl.
Iz_14_1209	DJI_0939	Pilosella echioides (Lumn.) F.W.Schultz & Sch.Bip.	0	n	1	0	Asteraceae Bercht. & J.Presl

Iz_14_1209	DJI_0939	Pilosella officinarum F. Schultz et Sch. Bip.	0	n	1	0	Asteraceae Bercht. & J.Presl
Iz_14_1209	DJI_0939	Pilosella sp.	0		NA	0	Asteraceae Bercht. & J.Presl
Iz_14_1209	DJI_0939	Pinus sylvestris L.	0	n	1	1	Pinaceae Spreng. ex F.Rudolphi
Iz_14_1209	DJI_0939	Poa annua L.	0	n	1	0	Poaceae Barnhart
Iz_14_1209	DJI_0939	Poa nemoralis L.	0	n	1	0	Poaceae Barnhart
Iz_14_1209	DJI_0939	Poa sp.	0		NA	0	Poaceae Barnhart
Iz_14_1209	DJI_0939	Polygonatum odoratum (Mill.) Druce	0	n	1	0	Asparagaceae Juss.
Iz_14_1209	DJI_0939	Polygonum arenarium Waldst. & Kit.	0	n	1	0	Polygonaceae Juss.
Iz_14_1209	DJI_0939	Populus nigra L.	0	n	1	1	Salicaceae Mirb.
Iz_14_1209	DJI_0939	Populus tremula L.	0	n	1	1	Salicaceae Mirb.
Iz_14_1209	DJI_0939	Populus Ã canescens (Aiton) Sm.	0	n	1	1	Salicaceae Mirb.
Iz_14_1209	DJI_0939	Potentilla argentea L.	0	n	1	0	Rosaceae Juss.
Iz_14_1209	DJI_0939	Psammophiliella muralis (L.) Ikonn.	0	n	1	0	Caryophyllaceae Juss.
Iz_14_1209	DJI_0939	Psephellus sumensis (Kalen.) Greuter	0	n	1	0	Asteraceae Bercht. & J.Presl
Iz_14_1209	DJI_0939	Pulsatilla pratensis (L.) Mill. s.l.	0	n	1	0	Ranunculaceae Juss.
Iz_14_1209	DJI_0939	Pyrus communis L.	0	a	0	1	Rosaceae Juss.
Iz_14_1209	DJI_0939	Quercus robur L.	0	n	1	1	Fagaceae Dumort.
Iz_14_1209	DJI_0939	Robinia pseudoacacia L.	0	a	0	1	Fabaceae Lindl.
Iz_14_1209	DJI_0939	Rosa sp.	0		NA	1	Rosaceae Juss.
Iz_14_1209	DJI_0939	Rubus idaeus L.	0	n	1	0	Rosaceae Juss.
Iz_14_1209	DJI_0939	Rumex acetosella L.	0	n	1	0	Polygonaceae Juss.
Iz_14_1209	DJI_0939	Rumex thyrsiflorus Fingerh.	0	n	1	0	Polygonaceae Juss.
Iz_14_1209	DJI_0939	Salix alba L.	0	n	1	1	Salicaceae Mirb.
Iz_14_1209	DJI_0939	Sambucus nigra L.	0	n	1	1	Viburnaceae Raf.
Iz_14_1209	DJI_0939	Secale sylvestre Host	0	n	1	0	Poaceae Barnhart
Iz_14_1209	DJI_0939	Sempervivum ruthenicum Schnittsp. & C.B. Lehm.	0	n	1	0	Crassulaceae J.St.-Hil.
Iz_14_1209	DJI_0939	Setaria viridis (L.) P. Beauv.	0	n	1	0	Poaceae Barnhart
Iz_14_1209	DJI_0939	Silene borysthenica (Gruner) Walters	0	n	1	0	Caryophyllaceae Juss.
Iz_14_1209	DJI_0939	Silene latifolia subsp. alba (Mill.) Greuter & Burdet	0	n	1	0	Caryophyllaceae Juss.
Iz_14_1209	DJI_0939	Solidago virgaurea L.	0	n	1	0	Asteraceae Bercht. & J.Presl
Iz_14_1209	DJI_0939	Sorbus aucuparia L.	0	n	1	1	Rosaceae Juss.
Iz_14_1209	DJI_0939	Stachys recta L.	0	n	1	0	Lamiacea Lindl.
Iz_14_1209	DJI_0939	Tanacetum vulgare L.	0	n	1	0	Asteraceae Bercht. & J.Presl
Iz_14_1209	DJI_0939	Thymus pallasianus Heinr.Braun	0	n	1	0	Lamiacea Lindl.
Iz_14_1209	DJI_0939	Tilia cordata Mill.	0	n	1	1	Malvaceae Juss.
Iz_14_1209	DJI_0939	Trifolium alpestre L.	0	n	1	0	Fabaceae Lindl.
Iz_14_1209	DJI_0939	Trifolium arvense L.	0	n	1	0	Fabaceae Lindl.
Iz_14_1209	DJI_0939	Ulmus glabra Huds.	0	n	1	1	Ulmaceae Mirb.
Iz_14_1209	DJI_0939	Verbascum densiflorum Bertol.	0	n	1	0	Scrophulariaceae Juss.
Iz_14_1209	DJI_0939	Veronica austriaca L.	0	n	1	0	Plantaginaceae Juss.
Iz_14_1209	DJI_0939	Veronica incana L.	0	n	1	0	Plantaginaceae Juss.
Iz_14_1209	DJI_0939	Veronica verna L.	0	n	1	0	Plantaginaceae Juss.
Iz_14_1209	DJI_0939	Vincetoxicum hirundinaria Medik	0	n	1	0	Apocynaceae Juss.
Iz_14_1209	DJI_0942	Acer tataricum L.	0	n	1	1	Sapindaceae Juss.
Iz_14_1209	DJI_0942	Achillea millefolium L. s.l.	0	n	1	0	Asteraceae Bercht. & J.Presl
Iz_14_1209	DJI_0942	Allium oleraceum L.	0	n	1	0	Amaryllidaceae J.St.-Hil.
Iz_14_1209	DJI_0942	Ambrosia artemisiifolia L.	0	a	0	0	Asteraceae Bercht. & J.Presl
Iz_14_1209	DJI_0942	Amorpha fruticosa L.	0	a	0	0	Fabaceae Lindl.
Iz_14_1209	DJI_0942	Anthericum ramosum L.	0	n	1	0	Asparagaceae Juss.
Iz_14_1209	DJI_0942	Artemisia marschalliana Spreng.	0	n	1	0	Asteraceae Bercht. & J.Presl
Iz_14_1209	DJI_0942	Asparagus officinalis L.	0	n	1	0	Asparagaceae Juss.
Iz_14_1209	DJI_0942	Astragalus cicer L.	0	n	1	0	Fabaceae Lindl.
Iz_14_1209	DJI_0942	Bassia laniflora (S.G.Gmel.) A.J.Scott	0	n	1	0	Amaranthaceae Juss.
Iz_14_1209	DJI_0942	Berberis vulgaris L.	0	n	1	0	Berberidaceae Juss.
Iz_14_1209	DJI_0942	Betula pendula Roth.	0	n	1	1	Betulaceae Gray
Iz_14_1209	DJI_0942	Betula pendula Roth.	0	n	1	0	Poaceae Barnhart
Iz_14_1209	DJI_0942	Bromus inermis Leyss.	0		NA	NA	
Iz_14_1209	DJI_0942	Bromus squarrosus L.	0	a	0	0	Poaceae Barnhart
Iz_14_1209	DJI_0942	Bromus tectorum L.	0	a	0	0	Poaceae Barnhart
Iz_14_1209	DJI_0942	Calamagrostis epigejos (L.) Roth	8	n	1	0	Poaceae Barnhart
Iz_14_1209	DJI_0942	Carduus acanthoides L.	0	n	1	0	Asteraceae Bercht. & J.Presl
Iz_14_1209	DJI_0942	Carex sp.	0	n	1	0	Cyperaceae Juss.
Iz_14_1209	DJI_0942	Centaurea majorovii Dumbadze	0	n	1	0	Asteraceae Bercht. & J.Presl
Iz_14_1209	DJI_0942	Chamaecytisus ruthenicus (Fisch.ex Wol.) Klaskova	0	n	1	0	Fabaceae Lindl.
Iz_14_1209	DJI_0942	Chelidonium majus L.	10	n	1	0	Papaveraceae Juss.
Iz_14_1209	DJI_0942	Chenopodium betaceum Andrz.	0	n	1	0	Amaranthaceae Juss.
Iz_14_1209	DJI_0942	Chenopodium cf. album	0	n	1	0	Amaranthaceae Juss.
Iz_14_1209	DJI_0942	Chenopodium sp.	0		NA	0	Amaranthaceae Juss.
Iz_14_1209	DJI_0942	Chondrilla juncea L.	0	n	1	0	Asteraceae Bercht. & J.Presl
Iz_14_1209	DJI_0942	Cirsium vulgare (Savi) Ten.	0	n	1	0	Asteraceae Bercht. & J.Presl
Iz_14_1209	DJI_0942	Clinopodium acinos (L.) Kuntze	0	n	1	0	Lamiacea Lindl.
Iz_14_1209	DJI_0942	Convallaria majalis L.	0	n	1	0	Asparagaceae Juss.
Iz_14_1209	DJI_0942	Crataegus rhipidophylla Gand.	0	n	1	1	Rosaceae Juss.
Iz_14_1209	DJI_0942	Crepis foetida L.	0	n	1	0	Asteraceae Bercht. & J.Presl
Iz_14_1209	DJI_0942	Dactylis glomerata L.	0	n	1	0	Poaceae Barnhart
Iz_14_1209	DJI_0942	Dianthus campestris M.Bieb.	0	n	1	0	Caryophyllaceae Juss.
Iz_14_1209	DJI_0942	Digitaria sanguinalis (L.) Scop.	0	a	0	0	Poaceae Barnhart
Iz_14_1209	DJI_0942	Elymus repens (L.) Gould	0	n	1	0	Poaceae Barnhart
Iz_14_1209	DJI_0942	Epilobium tetragonum L.	0	n	1	0	Onagraceae Juss.
Iz_14_1209	DJI_0942	Eragrostis minor Host	0	a	0	0	Poaceae Barnhart
Iz_14_1209	DJI_0942	Erigeron annuus (L.) Desf.	0	a	0	0	Asteraceae Bercht. & J.Presl
Iz_14_1209	DJI_0942	Erigeron canadensis L.	0	a	0	0	Asteraceae Bercht. & J.Presl
Iz_14_1209	DJI_0942	Euphorbia seguieriana Neck.	0	n	1	0	Euphorbiaceae Juss.
Iz_14_1209	DJI_0942	Euphorbia sp.	0.2		NA	NA	
Iz_14_1209	DJI_0942	Fallopia dumetorum (L.) Holub	0	n	1	0	Polygonaceae Juss.
Iz_14_1209	DJI_0942	Festuca beckeri (Hack.) Trautv.	0	n	1	0	Poaceae Barnhart
Iz_14_1209	DJI_0942	Fragaria vesca L.	0	n	1	0	Rosaceae Juss.
Iz_14_1209	DJI_0942	Frangula alnus Mill.	0	n	1	1	Rhamnaceae Juss.
Iz_14_1209	DJI_0942	Fraxinus pennsylvanica Marshall	0	a	0	1	Oleaceae Hoffmanns. & Link
Iz_14_1209	DJI_0942	Galeopsis bifida Boenn.	0	n	1	0	Lamiacea Lindl.
Iz_14_1209	DJI_0942	Galium verum L.	4	n	1	0	Rubiaceae Juss.
Iz_14_1209	DJI_0942	Genista tinctoria L.	0	n	1	0	Fabaceae Lindl.
Iz_14_1209	DJI_0942	Geranium robertianum L.	0	n	1	0	Geraniaceae Juss.
Iz_14_1209	DJI_0942	Geranium sanquineum L.	0	n	1	0	Geraniaceae Juss.
Iz_14_1209	DJI_0942	Gypsophila paniculata L.	0	n	1	0	Caryophyllaceae Juss.
Iz_14_1209	DJI_0942	Helichrysum arenarium (L.) Moench	0	n	1	0	Asteraceae Bercht. & J.Presl
Iz_14_1209	DJI_0942	Herniaria polygama J.Gay	0	n	1	0	Caryophyllaceae Juss.
Iz_14_1209	DJI_0942	Hieracium robustum Fr.	0	n	1	0	Asteraceae Bercht. & J.Presl

Iz_14_1209	DJI_0942	Hieracium umbellatum L.	0	n	1	NA	Asteraceae Bercht. & J.Presl
Iz_14_1209	DJI_0942	Hylotelephium maximum (L.) Holub	0	n	1	0	Crassulaceae J.St.-Hil.
Iz_14_1209	DJI_0942	Jacobaea borysthenica (DC.) B.Nord. & Greuter	0	n	1	0	Asteraceae Bercht. & J.Presl
Iz_14_1209	DJI_0942	Jasione montana L.	0	n	1	0	Campanulaceae Juss.
Iz_14_1209	DJI_0942	Jurinea cyanoides (L.) Rchb.s.l.	0	n	1	0	Asteraceae Bercht. & J.Presl
Iz_14_1209	DJI_0942	Koeleria glauca (Spreng.) DC.	0	n	1	0	Poaceae Barnhart
Iz_14_1209	DJI_0942	Lactuca serriola L.	0	a	0	0	Asteraceae Bercht. & J.Presl
Iz_14_1209	DJI_0942	Linaria genistifolia (L.) Mill.	0	n	1	0	Plantaginaceae Juss.
Iz_14_1209	DJI_0942	Lomelosia argentea (L.) Greuter & Burdet	0	n	1	0	Caprifoliaceae Juss.
Iz_14_1209	DJI_0942	Melampyrum pratense L.	0	n	1	0	Orobanchaceae Vent.
Iz_14_1209	DJI_0942	Oenothera biennis L.	0	a	0	0	Onagraceae Juss.
Iz_14_1209	DJI_0942	Pentanema britannica (L.) D.Gut.Larr., Santos-Vicente, Anderb., E.Rico & M.M.Mart.Ort.	0	n	1	0	Asteraceae Bercht. & J.Presl
Iz_14_1209	DJI_0942	Peucedanum oreoselinum (L.) Moench	5	n	1	0	Apiaceae Lindl.
Iz_14_1209	DJI_0942	Peucedanum ruthenicum M.Bieb.	0	n	1	0	Apiaceae Lindl.
Iz_14_1209	DJI_0942	Pilosella echioides (Lumn.) F.W.Schultz & Sch.Bip.	0	n	1	0	Asteraceae Bercht. & J.Presl
Iz_14_1209	DJI_0942	Pilosella officinarum F. Schultz et Sch. Bip.	0	n	1	0	Asteraceae Bercht. & J.Presl
Iz_14_1209	DJI_0942	Pilosella sp.	0		NA	0	Asteraceae Bercht. & J.Presl
Iz_14_1209	DJI_0942	Pinus sylvestris L.	0	n	1	1	Pinaceae Spreng. ex F.Rudolphi
Iz_14_1209	DJI_0942	Poa annua L.	0	n	1	0	Poaceae Barnhart
Iz_14_1209	DJI_0942	Poa nemoralis L.	0	n	1	0	Poaceae Barnhart
Iz_14_1209	DJI_0942	Poa sp.	1		NA	0	Poaceae Barnhart
Iz_14_1209	DJI_0942	Polygonatum odoratum (Mill.) Druce	0	n	1	0	Asparagaceae Juss.
Iz_14_1209	DJI_0942	Polygonum arenarium Waldst. & Kit.	0	n	1	0	Polygonaceae Juss.
Iz_14_1209	DJI_0942	Populus nigra L.	0	n	1	1	Salicaceae Mirb.
Iz_14_1209	DJI_0942	Populus tremula L.	0	n	1	1	Salicaceae Mirb.
Iz_14_1209	DJI_0942	Populus Ã canescens (Aiton) Sm.	0	n	1	1	Salicaceae Mirb.
Iz_14_1209	DJI_0942	Potentilla argentea L.	0	n	1	0	Rosaceae Juss.
Iz_14_1209	DJI_0942	Psammophiliella muralis (L.) Ikonn.	0	n	1	0	Caryophyllaceae Juss.
Iz_14_1209	DJI_0942	Psephellus sumensis (Kalen.) Greuter	4	n	1	0	Asteraceae Bercht. & J.Presl
Iz_14_1209	DJI_0942	Pulsatilla pratensis (L.) Mill. s.l.	0	n	1	0	Ranunculaceae Juss.
Iz_14_1209	DJI_0942	Pyrus communis L.	0	a	0	1	Rosaceae Juss.
Iz_14_1209	DJI_0942	Quercus robur L.	0	n	1	1	Fagaceae Dumort.
Iz_14_1209	DJI_0942	Robinia pseudoacacia L.	0	a	0	1	Fabaceae Lindl.
Iz_14_1209	DJI_0942	Rosa sp.	0		NA	1	Rosaceae Juss.
Iz_14_1209	DJI_0942	Rubus idaeus L.	0	n	1	0	Rosaceae Juss.
Iz_14_1209	DJI_0942	Rumex acetosella L.	0	n	1	0	Polygonaceae Juss.
Iz_14_1209	DJI_0942	Rumex thyrsiflorus Fingerh.	0	n	1	0	Polygonaceae Juss.
Iz_14_1209	DJI_0942	Salix alba L.	0	n	1	1	Salicaceae Mirb.
Iz_14_1209	DJI_0942	Sambucus nigra L.	0	n	1	1	Viburnaceae Raf.
Iz_14_1209	DJI_0942	Secale sylvestre Host	0	n	1	0	Poaceae Barnhart
Iz_14_1209	DJI_0942	Sempervivum ruthenicum Schnittsp. & C.B. Lehm.	0	n	1	0	Crassulaceae J.St.-Hil.
Iz_14_1209	DJI_0942	Setaria viridis (L.) P. Beauv.	0	n	1	0	Poaceae Barnhart
Iz_14_1209	DJI_0942	Silene borysthenica (Gruner) Walters	0	n	1	0	Caryophyllaceae Juss.
Iz_14_1209	DJI_0942	Silene latifolia subsp. alba (Mill.) Greuter & Burdet	0	n	1	0	Caryophyllaceae Juss.
Iz_14_1209	DJI_0942	Solidago virgaurea L.	0	n	1	0	Asteraceae Bercht. & J.Presl
Iz_14_1209	DJI_0942	Sorbus aucuparia L.	0	n	1	1	Rosaceae Juss.
Iz_14_1209	DJI_0942	Stachys recta L.	0	n	1	0	Lamiacea Lindl.
Iz_14_1209	DJI_0942	Tanacetum vulgare L.	0	n	1	0	Asteraceae Bercht. & J.Presl
Iz_14_1209	DJI_0942	Thymus pallasianus Heinr.Braun	0	n	1	0	Lamiacea Lindl.
Iz_14_1209	DJI_0942	Tilia cordata Mill.	0	n	1	1	Malvaceae Juss.
Iz_14_1209	DJI_0942	Trifolium alpestre L.	0	n	1	0	Fabaceae Lindl.
Iz_14_1209	DJI_0942	Trifolium arvense L.	0	n	1	0	Fabaceae Lindl.
Iz_14_1209	DJI_0942	Ulmus glabra Huds.	0	n	1	1	Ulmaceae Mirb.
Iz_14_1209	DJI_0942	Verbascum densiflorum Bertol.	0	n	1	0	Scrophulariaceae Juss.
Iz_14_1209	DJI_0942	Veronica austriaca L.	0	n	1	0	Plantaginaceae Juss.
Iz_14_1209	DJI_0942	Veronica incana L.	0	n	1	0	Plantaginaceae Juss.
Iz_14_1209	DJI_0942	Veronica verna L.	0	n	1	0	Plantaginaceae Juss.
Iz_14_1209	DJI_0942	Vincetoxicum hirundinaria Medik	0	n	1	0	Apocynaceae Juss.
Iz_14_1209	DJI_0950	Acer tataricum L.	0	n	1	1	Sapindaceae Juss.
Iz_14_1209	DJI_0950	Achillea millefolium L. s.l.	0	n	1	0	Asteraceae Bercht. & J.Presl
Iz_14_1209	DJI_0950	Allium oleraceum L.	0	n	1	0	Amaryllidaceae J.St.-Hil.
Iz_14_1209	DJI_0950	Ambrosia artemisiifolia L.	0	a	0	0	Asteraceae Bercht. & J.Presl
Iz_14_1209	DJI_0950	Amorpha fruticosa L.	0	a	0	0	Fabaceae Lindl.
Iz_14_1209	DJI_0950	Anthericum ramosum L.	0	n	1	0	Asparagaceae Juss.
Iz_14_1209	DJI_0950	Artemisia marschalliana Spreng.	0	n	1	0	Asteraceae Bercht. & J.Presl
Iz_14_1209	DJI_0950	Asparagus officinalis L.	0	n	1	0	Asparagaceae Juss.
Iz_14_1209	DJI_0950	Astragalus cicer L.	0	n	1	0	Fabaceae Lindl.
Iz_14_1209	DJI_0950	Bassia laniflora (S.G.Gmel.) A.J.Scott	0	n	1	0	Amaranthaceae Juss.
Iz_14_1209	DJI_0950	Berberis vulgaris L.	0	n	1	0	Berberidaceae Juss.
Iz_14_1209	DJI_0950	Betula pendula Roth.	0	n	1	1	Betulaceae Gray
Iz_14_1209	DJI_0950	Betula pendula Roth.	0	n	1	0	Poaceae Barnhart
Iz_14_1209	DJI_0950	Bromus inermis Leyss.	0		NA	NA	
Iz_14_1209	DJI_0950	Bromus squarrosus L.	0	a	0	0	Poaceae Barnhart
Iz_14_1209	DJI_0950	Bromus tectorum L.	0	a	0	0	Poaceae Barnhart
Iz_14_1209	DJI_0950	Calamagrostis epigejos (L.) Roth	35	n	1	0	Poaceae Barnhart
Iz_14_1209	DJI_0950	Carduus acanthoides L.	0	n	1	0	Asteraceae Bercht. & J.Presl
Iz_14_1209	DJI_0950	Carex sp.	0	n	1	0	Cyperaceae Juss.
Iz_14_1209	DJI_0950	Centaurea majorovii Dumbadze	0	n	1	0	Asteraceae Bercht. & J.Presl
Iz_14_1209	DJI_0950	Chamaecytisus ruthenicus (Fisch.ex Wol.) Klaskova	0	n	1	0	Fabaceae Lindl.
Iz_14_1209	DJI_0950	Chelidonium majus L.	0.1	n	1	0	Papaveraceae Juss.
Iz_14_1209	DJI_0950	Chenopodium betaceum Andrz.	0	n	1	0	Amaranthaceae Juss.
Iz_14_1209	DJI_0950	Chenopodium cf. album	0	n	1	0	Amaranthaceae Juss.
Iz_14_1209	DJI_0950	Chenopodium sp.	0		NA	0	Amaranthaceae Juss.
Iz_14_1209	DJI_0950	Chondrilla juncea L.	0	n	1	0	Asteraceae Bercht. & J.Presl
Iz_14_1209	DJI_0950	Cirsium vulgare (Savi) Ten.	0	n	1	0	Asteraceae Bercht. & J.Presl
Iz_14_1209	DJI_0950	Clinopodium acinos (L.) Kuntze	0	n	1	0	Lamiacea Lindl.
Iz_14_1209	DJI_0950	Convallaria majalis L.	0	n	1	0	Asparagaceae Juss.
Iz_14_1209	DJI_0950	Crataegus rhipidophylla Gand.	0	n	1	1	Rosaceae Juss.
Iz_14_1209	DJI_0950	Crepis foetida L.	0	n	1	0	Asteraceae Bercht. & J.Presl
Iz_14_1209	DJI_0950	Dactylis glomerata L.	0	n	1	0	Poaceae Barnhart
Iz_14_1209	DJI_0950	Dianthus campestris M.Bieb.	0	n	1	0	Caryophyllaceae Juss.
Iz_14_1209	DJI_0950	Digitaria sanguinalis (L.) Scop.	0	a	0	0	Poaceae Barnhart
Iz_14_1209	DJI_0950	Elymus repens (L.) Gould	0	n	1	0	Poaceae Barnhart
Iz_14_1209	DJI_0950	Epilobium tetragonum L.	0	n	1	0	Onagraceae Juss.
Iz_14_1209	DJI_0950	Eragrostis minor Host	0	a	0	0	Poaceae Barnhart
Iz_14_1209	DJI_0950	Erigeron annuus (L.) Desf.	0	a	0	0	Asteraceae Bercht. & J.Presl
Iz_14_1209	DJI_0950	Erigeron canadensis L.	0	a	0	0	Asteraceae Bercht. & J.Presl
Iz_14_1209	DJI_0950	Euphorbia seguieriana Neck.	0	n	1	0	Euphorbiaceae Juss.

Iz_14_1209	DJI_0950	Euphorbia sp.	0		NA	NA	
Iz_14_1209	DJI_0950	Fallopia dumetorum (L.) Holub	2	n	1	0	Polygonaceae Juss.
Iz_14_1209	DJI_0950	Festuca beckeri (Hack.) Trautv.	0	n	1	0	Poaceae Barnhart
Iz_14_1209	DJI_0950	Fragaria vesca L.	0	n	1	0	Rosaceae Juss.
Iz_14_1209	DJI_0950	Frangula alnus Mill.	0	n	1	1	Rhamnaceae Juss.
Iz_14_1209	DJI_0950	Fraxinus pennsylvanica Marshall	0	a	0	1	Oleaceae Hoffmanns. & Link
Iz_14_1209	DJI_0950	Galeopsis bifida Boenn.	0	n	1	0	Lamiacea Lindl.
Iz_14_1209	DJI_0950	Galium verum L.	0	n	1	0	Rubiaceae Juss.
Iz_14_1209	DJI_0950	Genista tinctoria L.	0	n	1	0	Fabaceae Lindl.
Iz_14_1209	DJI_0950	Geranium robertianum L.	0	n	1	0	Geraniaceae Juss.
Iz_14_1209	DJI_0950	Geranium sanquineum L.	0	n	1	0	Geraniaceae Juss.
Iz_14_1209	DJI_0950	Gypsophila paniculata L.	0	n	1	0	Caryophyllaceae Juss.
Iz_14_1209	DJI_0950	Helichrysum arenarium (L.) Moench	0	n	1	0	Asteraceae Bercht. & J.Presl
Iz_14_1209	DJI_0950	Herniaria polygama J.Gay	0	n	1	0	Caryophyllaceae Juss.
Iz_14_1209	DJI_0950	Hieracium robustum Fr.	0	n	1	0	Asteraceae Bercht. & J.Presl
Iz_14_1209	DJI_0950	Hieracium umbellatum L.	0	n	1	NA	Asteraceae Bercht. & J.Presl
Iz_14_1209	DJI_0950	Hylotelephium maximum (L.) Holub	0	n	1	0	Crassulaceae J.St.-Hil.
Iz_14_1209	DJI_0950	Jacobaea borysthenica (DC.) B.Nord. & Greuter	0	n	1	0	Asteraceae Bercht. & J.Presl
Iz_14_1209	DJI_0950	Jasione montana L.	0	n	1	0	Campanulaceae Juss.
Iz_14_1209	DJI_0950	Jurinea cyanoides (L.) Rchb.s.l.	0	n	1	0	Asteraceae Bercht. & J.Presl
Iz_14_1209	DJI_0950	Koeleria glauca (Spreng.) DC.	0	n	1	0	Poaceae Barnhart
Iz_14_1209	DJI_0950	Lactuca serriola L.	0	a	0	0	Asteraceae Bercht. & J.Presl
Iz_14_1209	DJI_0950	Linaria genistifolia (L.) Mill.	0	n	1	0	Plantaginaceae Juss.
Iz_14_1209	DJI_0950	Lomelosia argentea (L.) Greuter & Burdet	0	n	1	0	Caprifoliaceae Juss.
Iz_14_1209	DJI_0950	Melampyrum pratense L.	0	n	1	0	Orobanchaceae Vent.
Iz_14_1209	DJI_0950	Oenothera biennis L.	0	a	0	0	Onagraceae Juss.
Iz_14_1209	DJI_0950	Pentanema britannica (L.) D.Gut.Larr., Santos-Vicente, Anderb., E.Rico & M.M.Mart.Ort.	0	n	1	0	Asteraceae Bercht. & J.Presl
Iz_14_1209	DJI_0950	Peucedanum oreoselinum (L.) Moench	0	n	1	0	Apiaceae Lindl.
Iz_14_1209	DJI_0950	Peucedanum ruthenicum M.Bieb.	0	n	1	0	Apiaceae Lindl.
Iz_14_1209	DJI_0950	Pilosella echioides (Lumn.) F.W.Schultz & Sch.Bip.	0	n	1	0	Asteraceae Bercht. & J.Presl
Iz_14_1209	DJI_0950	Pilosella officinarum F. Schultz et Sch. Bip.	0	n	1	0	Asteraceae Bercht. & J.Presl
Iz_14_1209	DJI_0950	Pilosella sp.	0		NA	0	Asteraceae Bercht. & J.Presl
Iz_14_1209	DJI_0950	Pinus sylvestris L.	0	n	1	1	Pinaceae Spreng. ex F.Rudolphi
Iz_14_1209	DJI_0950	Poa annua L.	0	n	1	0	Poaceae Barnhart
Iz_14_1209	DJI_0950	Poa nemoralis L.	0	n	1	0	Poaceae Barnhart
Iz_14_1209	DJI_0950	Poa sp.	0		NA	0	Poaceae Barnhart
Iz_14_1209	DJI_0950	Polygonatum odoratum (Mill.) Druce	0	n	1	0	Asparagaceae Juss.
Iz_14_1209	DJI_0950	Polygonum arenarium Waldst. & Kit.	0	n	1	0	Polygonaceae Juss.
Iz_14_1209	DJI_0950	Populus nigra L.	0	n	1	1	Salicaceae Mirb.
Iz_14_1209	DJI_0950	Populus tremula L.	0	n	1	1	Salicaceae Mirb.
Iz_14_1209	DJI_0950	Populus Ã canescens (Aiton) Sm.	0	n	1	1	Salicaceae Mirb.
Iz_14_1209	DJI_0950	Potentilla argentea L.	0	n	1	0	Rosaceae Juss.
Iz_14_1209	DJI_0950	Psammophiliella muralis (L.) Ikonn.	0	n	1	0	Caryophyllaceae Juss.
Iz_14_1209	DJI_0950	Psephellus sumensis (Kalen.) Greuter	0	n	1	0	Asteraceae Bercht. & J.Presl
Iz_14_1209	DJI_0950	Pulsatilla pratensis (L.) Mill. s.l.	0	n	1	0	Ranunculaceae Juss.
Iz_14_1209	DJI_0950	Pyrus communis L.	0	a	0	1	Rosaceae Juss.
Iz_14_1209	DJI_0950	Quercus robur L.	0	n	1	1	Fagaceae Dumort.
Iz_14_1209	DJI_0950	Robinia pseudoacacia L.	0	a	0	1	Fabaceae Lindl.
Iz_14_1209	DJI_0950	Rosa sp.	0		NA	1	Rosaceae Juss.
Iz_14_1209	DJI_0950	Rubus idaeus L.	0	n	1	0	Rosaceae Juss.
Iz_14_1209	DJI_0950	Rumex acetosella L.	0	n	1	0	Polygonaceae Juss.
Iz_14_1209	DJI_0950	Rumex thyrsiflorus Fingerh.	0	n	1	0	Polygonaceae Juss.
Iz_14_1209	DJI_0950	Salix alba L.	0	n	1	1	Salicaceae Mirb.
Iz_14_1209	DJI_0950	Sambucus nigra L.	0	n	1	1	Viburnaceae Raf.
Iz_14_1209	DJI_0950	Secale sylvestre Host	0	n	1	0	Poaceae Barnhart
Iz_14_1209	DJI_0950	Sempervivum ruthenicum Schnittsp. & C.B. Lehm.	5	n	1	0	Crassulaceae J.St.-Hil.
Iz_14_1209	DJI_0950	Setaria viridis (L.) P. Beauv.	0	n	1	0	Poaceae Barnhart
Iz_14_1209	DJI_0950	Silene borysthenica (Gruner) Walters	0	n	1	0	Caryophyllaceae Juss.
Iz_14_1209	DJI_0950	Silene latifolia subsp. alba (Mill.) Greuter & Burdet	0	n	1	0	Caryophyllaceae Juss.
Iz_14_1209	DJI_0950	Solidago virgaurea L.	0	n	1	0	Asteraceae Bercht. & J.Presl
Iz_14_1209	DJI_0950	Sorbus aucuparia L.	0	n	1	1	Rosaceae Juss.
Iz_14_1209	DJI_0950	Stachys recta L.	0	n	1	0	Lamiacea Lindl.
Iz_14_1209	DJI_0950	Tanacetum vulgare L.	0	n	1	0	Asteraceae Bercht. & J.Presl
Iz_14_1209	DJI_0950	Thymus pallasianus Heinr.Braun	0	n	1	0	Lamiacea Lindl.
Iz_14_1209	DJI_0950	Tilia cordata Mill.	0	n	1	1	Malvaceae Juss.
Iz_14_1209	DJI_0950	Trifolium alpestre L.	0	n	1	0	Fabaceae Lindl.
Iz_14_1209	DJI_0950	Trifolium arvense L.	0	n	1	0	Fabaceae Lindl.
Iz_14_1209	DJI_0950	Ulmus glabra Huds.	0	n	1	1	Ulmaceae Mirb.
Iz_14_1209	DJI_0950	Verbascum densiflorum Bertol.	0	n	1	0	Scrophulariaceae Juss.
Iz_14_1209	DJI_0950	Veronica austriaca L.	0	n	1	0	Plantaginaceae Juss.
Iz_14_1209	DJI_0950	Veronica incana L.	0	n	1	0	Plantaginaceae Juss.
Iz_14_1209	DJI_0950	Veronica verna L.	0	n	1	0	Plantaginaceae Juss.
Iz_14_1209	DJI_0950	Vincetoxicum hirundinaria Medik	0	n	1	0	Apocynaceae Juss.
Iz_14_1209	DJI_0953	Acer tataricum L.	0	n	1	1	Sapindaceae Juss.
Iz_14_1209	DJI_0953	Achillea millefolium L. s.l.	0	n	1	0	Asteraceae Bercht. & J.Presl
Iz_14_1209	DJI_0953	Allium oleraceum L.	0	n	1	0	Amaryllidaceae J.St.-Hil.
Iz_14_1209	DJI_0953	Ambrosia artemisiifolia L.	0	a	0	0	Asteraceae Bercht. & J.Presl
Iz_14_1209	DJI_0953	Amorpha fruticosa L.	0	a	0	0	Fabaceae Lindl.
Iz_14_1209	DJI_0953	Anthericum ramosum L.	0	n	1	0	Asparagaceae Juss.
Iz_14_1209	DJI_0953	Artemisia marschalliana Spreng.	0	n	1	0	Asteraceae Bercht. & J.Presl
Iz_14_1209	DJI_0953	Asparagus officinalis L.	0	n	1	0	Asparagaceae Juss.
Iz_14_1209	DJI_0953	Astragalus cicer L.	0	n	1	0	Fabaceae Lindl.
Iz_14_1209	DJI_0953	Bassia laniflora (S.G.Gmel.) A.J.Scott	0	n	1	0	Amaranthaceae Juss.
Iz_14_1209	DJI_0953	Berberis vulgaris L.	0	n	1	0	Berberidaceae Juss.
Iz_14_1209	DJI_0953	Betula pendula Roth.	0	n	1	1	Betulaceae Gray
Iz_14_1209	DJI_0953	Betula pendula Roth.	0	n	1	0	Poaceae Barnhart
Iz_14_1209	DJI_0953	Bromus inermis Leyss.	0		NA	NA	
Iz_14_1209	DJI_0953	Bromus squarrosus L.	0	a	0	0	Poaceae Barnhart
Iz_14_1209	DJI_0953	Bromus tectorum L.	0	a	0	0	Poaceae Barnhart
Iz_14_1209	DJI_0953	Calamagrostis epigejos (L.) Roth	20	n	1	0	Poaceae Barnhart
Iz_14_1209	DJI_0953	Carduus acanthoides L.	0	n	1	0	Asteraceae Bercht. & J.Presl
Iz_14_1209	DJI_0953	Carex sp.	0	n	1	0	Cyperaceae Juss.
Iz_14_1209	DJI_0953	Centaurea majorovii Dumbadze	0	n	1	0	Asteraceae Bercht. & J.Presl
Iz_14_1209	DJI_0953	Chamaecytisus ruthenicus (Fisch.ex Wol.) Klaskova	0	n	1	0	Fabaceae Lindl.
Iz_14_1209	DJI_0953	Chelidonium majus L.	2	n	1	0	Papaveraceae Juss.
Iz_14_1209	DJI_0953	Chenopodium betaceum Andrz.	0	n	1	0	Amaranthaceae Juss.
Iz_14_1209	DJI_0953	Chenopodium cf. album	0	n	1	0	Amaranthaceae Juss.
Iz_14_1209	DJI_0953	Chenopodium sp.	0.2		NA	0	Amaranthaceae Juss.

Iz_14_1209	DJI_0953	Chondrilla juncea L.	0	n	1	0	Asteraceae Bercht. & J.Presl
Iz_14_1209	DJI_0953	Cirsium vulgare (Savi) Ten.	0	n	1	0	Asteraceae Bercht. & J.Presl
Iz_14_1209	DJI_0953	Clinopodium acinos (L.) Kuntze	0	n	1	0	Lamiacea Lindl.
Iz_14_1209	DJI_0953	Convallaria majalis L.	0	n	1	0	Asparagaceae Juss.
Iz_14_1209	DJI_0953	Crataegus rhipidophylla Gand.	0	n	1	1	Rosaceae Juss.
Iz_14_1209	DJI_0953	Crepis foetida L.	0	n	1	0	Asteraceae Bercht. & J.Presl
Iz_14_1209	DJI_0953	Dactylis glomerata L.	0	n	1	0	Poaceae Barnhart
Iz_14_1209	DJI_0953	Dianthus campestris M.Bieb.	0	n	1	0	Caryophyllaceae Juss.
Iz_14_1209	DJI_0953	Digitaria sanguinalis (L.) Scop.	0	a	0	0	Poaceae Barnhart
Iz_14_1209	DJI_0953	Elymus repens (L.) Gould	0	n	1	0	Poaceae Barnhart
Iz_14_1209	DJI_0953	Epilobium tetragonum L.	0	n	1	0	Onagraceae Juss.
Iz_14_1209	DJI_0953	Eragrostis minor Host	0	a	0	0	Poaceae Barnhart
Iz_14_1209	DJI_0953	Erigeron annuus (L.) Desf.	0.2	a	0	0	Asteraceae Bercht. & J.Presl
Iz_14_1209	DJI_0953	Erigeron canadensis L.	2	a	0	0	Asteraceae Bercht. & J.Presl
Iz_14_1209	DJI_0953	Euphorbia seguieriana Neck.	0	n	1	0	Euphorbiaceae Juss.
Iz_14_1209	DJI_0953	Euphorbia sp.	0		NA	NA	
Iz_14_1209	DJI_0953	Fallopia dumetorum (L.) Holub	15	n	1	0	Polygonaceae Juss.
Iz_14_1209	DJI_0953	Festuca beckeri (Hack.) Trautv.	0	n	1	0	Poaceae Barnhart
Iz_14_1209	DJI_0953	Fragaria vesca L.	0	n	1	0	Rosaceae Juss.
Iz_14_1209	DJI_0953	Frangula alnus Mill.	0	n	1	1	Rhamnaceae Juss.
Iz_14_1209	DJI_0953	Fraxinus pennsylvanica Marshall	0	a	0	1	Oleaceae Hoffmanns. & Link
Iz_14_1209	DJI_0953	Galeopsis bifida Boenn.	0	n	1	0	Lamiacea Lindl.
Iz_14_1209	DJI_0953	Galium verum L.	0	n	1	0	Rubiaceae Juss.
Iz_14_1209	DJI_0953	Genista tinctoria L.	0	n	1	0	Fabaceae Lindl.
Iz_14_1209	DJI_0953	Geranium robertianum L.	0	n	1	0	Geraniaceae Juss.
Iz_14_1209	DJI_0953	Geranium sanquineum L.	0	n	1	0	Geraniaceae Juss.
Iz_14_1209	DJI_0953	Gypsophila paniculata L.	0	n	1	0	Caryophyllaceae Juss.
Iz_14_1209	DJI_0953	Helichrysum arenarium (L.) Moench	0	n	1	0	Asteraceae Bercht. & J.Presl
Iz_14_1209	DJI_0953	Herniaria polygama J.Gay	0	n	1	0	Caryophyllaceae Juss.
Iz_14_1209	DJI_0953	Hieracium robustum Fr.	1	n	1	0	Asteraceae Bercht. & J.Presl
Iz_14_1209	DJI_0953	Hieracium umbellatum L.	0	n	1	NA	Asteraceae Bercht. & J.Presl
Iz_14_1209	DJI_0953	Hylotelephium maximum (L.) Holub	0	n	1	0	Crassulaceae J.St.-Hil.
Iz_14_1209	DJI_0953	Jacobaea borysthenica (DC.) B.Nord. & Greuter	0	n	1	0	Asteraceae Bercht. & J.Presl
Iz_14_1209	DJI_0953	Jasione montana L.	0	n	1	0	Campanulaceae Juss.
Iz_14_1209	DJI_0953	Jurinea cyanoides (L.) Rchb.s.l.	0	n	1	0	Asteraceae Bercht. & J.Presl
Iz_14_1209	DJI_0953	Koeleria glauca (Spreng.) DC.	0	n	1	0	Poaceae Barnhart
Iz_14_1209	DJI_0953	Lactuca serriola L.	0	a	0	0	Asteraceae Bercht. & J.Presl
Iz_14_1209	DJI_0953	Linaria genistifolia (L.) Mill.	0	n	1	0	Plantaginaceae Juss.
Iz_14_1209	DJI_0953	Lomelosia argentea (L.) Greuter & Burdet	0	n	1	0	Caprifoliaceae Juss.
Iz_14_1209	DJI_0953	Melampyrum pratense L.	0	n	1	0	Orobanchaceae Vent.
Iz_14_1209	DJI_0953	Oenothera biennis L.	0	a	0	0	Onagraceae Juss.
Iz_14_1209	DJI_0953	Pentanema britannica (L.) D.Gut.Larr., Santos-Vicente, Anderb., E.Rico & M.M.Mart.Ort.	0	n	1	0	Asteraceae Bercht. & J.Presl
Iz_14_1209	DJI_0953	Peucedanum oreoselinum (L.) Moench	1	n	1	0	Apiaceae Lindl.
Iz_14_1209	DJI_0953	Peucedanum ruthenicum M.Bieb.	0	n	1	0	Apiaceae Lindl.
Iz_14_1209	DJI_0953	Pilosella echioides (Lumn.) F.W.Schultz & Sch.Bip.	0	n	1	0	Asteraceae Bercht. & J.Presl
Iz_14_1209	DJI_0953	Pilosella officinarum F. Schultz et Sch. Bip.	0	n	1	0	Asteraceae Bercht. & J.Presl
Iz_14_1209	DJI_0953	Pilosella sp.	0		NA	0	Asteraceae Bercht. & J.Presl
Iz_14_1209	DJI_0953	Pinus sylvestris L.	0	n	1	1	Pinaceae Spreng. ex F.Rudolphi
Iz_14_1209	DJI_0953	Poa annua L.	0	n	1	0	Poaceae Barnhart
Iz_14_1209	DJI_0953	Poa nemoralis L.	0	n	1	0	Poaceae Barnhart
Iz_14_1209	DJI_0953	Poa sp.	0		NA	0	Poaceae Barnhart
Iz_14_1209	DJI_0953	Polygonatum odoratum (Mill.) Druce	0	n	1	0	Asparagaceae Juss.
Iz_14_1209	DJI_0953	Polygonum arenarium Waldst. & Kit.	0	n	1	0	Polygonaceae Juss.
Iz_14_1209	DJI_0953	Populus nigra L.	1	n	1	1	Salicaceae Mirb.
Iz_14_1209	DJI_0953	Populus tremula L.	0	n	1	1	Salicaceae Mirb.
Iz_14_1209	DJI_0953	Populus Ã canescens (Aiton) Sm.	0	n	1	1	Salicaceae Mirb.
Iz_14_1209	DJI_0953	Potentilla argentea L.	0	n	1	0	Rosaceae Juss.
Iz_14_1209	DJI_0953	Psammophiliella muralis (L.) Ikonn.	0	n	1	0	Caryophyllaceae Juss.
Iz_14_1209	DJI_0953	Psephellus sumensis (Kalen.) Greuter	0	n	1	0	Asteraceae Bercht. & J.Presl
Iz_14_1209	DJI_0953	Pulsatilla pratensis (L.) Mill. s.l.	0	n	1	0	Ranunculaceae Juss.
Iz_14_1209	DJI_0953	Pyrus communis L.	0	a	0	1	Rosaceae Juss.
Iz_14_1209	DJI_0953	Quercus robur L.	2	n	1	1	Fagaceae Dumort.
Iz_14_1209	DJI_0953	Robinia pseudoacacia L.	0	a	0	1	Fabaceae Lindl.
Iz_14_1209	DJI_0953	Rosa sp.	0		NA	1	Rosaceae Juss.
Iz_14_1209	DJI_0953	Rubus idaeus L.	0	n	1	0	Rosaceae Juss.
Iz_14_1209	DJI_0953	Rumex acetosella L.	0	n	1	0	Polygonaceae Juss.
Iz_14_1209	DJI_0953	Rumex thyrsiflorus Fingerh.	0	n	1	0	Polygonaceae Juss.
Iz_14_1209	DJI_0953	Salix alba L.	0	n	1	1	Salicaceae Mirb.
Iz_14_1209	DJI_0953	Sambucus nigra L.	0	n	1	1	Viburnaceae Raf.
Iz_14_1209	DJI_0953	Secale sylvestre Host	0	n	1	0	Poaceae Barnhart
Iz_14_1209	DJI_0953	Sempervivum ruthenicum Schnittsp. & C.B. Lehm.	0	n	1	0	Crassulaceae J.St.-Hil.
Iz_14_1209	DJI_0953	Setaria viridis (L.) P. Beauv.	0	n	1	0	Poaceae Barnhart
Iz_14_1209	DJI_0953	Silene borysthenica (Gruner) Walters	0	n	1	0	Caryophyllaceae Juss.
Iz_14_1209	DJI_0953	Silene latifolia subsp. alba (Mill.) Greuter & Burdet	0	n	1	0	Caryophyllaceae Juss.
Iz_14_1209	DJI_0953	Solidago virgaurea L.	0	n	1	0	Asteraceae Bercht. & J.Presl
Iz_14_1209	DJI_0953	Sorbus aucuparia L.	0	n	1	1	Rosaceae Juss.
Iz_14_1209	DJI_0953	Stachys recta L.	0	n	1	0	Lamiacea Lindl.
Iz_14_1209	DJI_0953	Tanacetum vulgare L.	0	n	1	0	Asteraceae Bercht. & J.Presl
Iz_14_1209	DJI_0953	Thymus pallasianus Heinr.Braun	0	n	1	0	Lamiacea Lindl.
Iz_14_1209	DJI_0953	Tilia cordata Mill.	0	n	1	1	Malvaceae Juss.
Iz_14_1209	DJI_0953	Trifolium alpestre L.	0	n	1	0	Fabaceae Lindl.
Iz_14_1209	DJI_0953	Trifolium arvense L.	0	n	1	0	Fabaceae Lindl.
Iz_14_1209	DJI_0953	Ulmus glabra Huds.	0	n	1	1	Ulmaceae Mirb.
Iz_14_1209	DJI_0953	Verbascum densiflorum Bertol.	0	n	1	0	Scrophulariaceae Juss.
Iz_14_1209	DJI_0953	Veronica austriaca L.	0	n	1	0	Plantaginaceae Juss.
Iz_14_1209	DJI_0953	Veronica incana L.	0	n	1	0	Plantaginaceae Juss.
Iz_14_1209	DJI_0953	Veronica verna L.	0	n	1	0	Plantaginaceae Juss.
Iz_14_1209	DJI_0953	Vincetoxicum hirundinaria Medik	0	n	1	0	Apocynaceae Juss.
Iz_14_1209	DJI_0956	Acer tataricum L.	0	n	1	1	Sapindaceae Juss.
Iz_14_1209	DJI_0956	Achillea millefolium L. s.l.	0	n	1	0	Asteraceae Bercht. & J.Presl
Iz_14_1209	DJI_0956	Allium oleraceum L.	0	n	1	0	Amaryllidaceae J.St.-Hil.
Iz_14_1209	DJI_0956	Ambrosia artemisiifolia L.	0	a	0	0	Asteraceae Bercht. & J.Presl
Iz_14_1209	DJI_0956	Amorpha fruticosa L.	0	a	0	0	Fabaceae Lindl.
Iz_14_1209	DJI_0956	Anthericum ramosum L.	0	n	1	0	Asparagaceae Juss.
Iz_14_1209	DJI_0956	Artemisia marschalliana Spreng.	0	n	1	0	Asteraceae Bercht. & J.Presl
Iz_14_1209	DJI_0956	Asparagus officinalis L.	0	n	1	0	Asparagaceae Juss.
Iz_14_1209	DJI_0956	Astragalus cicer L.	0	n	1	0	Fabaceae Lindl.
Iz_14_1209	DJI_0956	Bassia laniflora (S.G.Gmel.) A.J.Scott	0	n	1	0	Amaranthaceae Juss.

Iz_14_1209	DJI_0956	Berberis vulgaris L.	0	n	1	0	Berberidaceae Juss.
Iz_14_1209	DJI_0956	Betula pendula Roth.	0	n	1	1	Betulaceae Gray
Iz_14_1209	DJI_0956	Betula pendula Roth.	0	n	1	0	Poaceae Barnhart
Iz_14_1209	DJI_0956	Bromus inermis Leyss.	0		NA	NA	
Iz_14_1209	DJI_0956	Bromus squarrosus L.	0	a	0	0	Poaceae Barnhart
Iz_14_1209	DJI_0956	Bromus tectorum L.	0	a	0	0	Poaceae Barnhart
Iz_14_1209	DJI_0956	Calamagrostis epigejos (L.) Roth	50	n	1	0	Poaceae Barnhart
Iz_14_1209	DJI_0956	Carduus acanthoides L.	0	n	1	0	Asteraceae Bercht. & J.Presl
Iz_14_1209	DJI_0956	Carex sp.	0	n	1	0	Cyperaceae Juss.
Iz_14_1209	DJI_0956	Centaurea majorovii Dumbadze	0	n	1	0	Asteraceae Bercht. & J.Presl
Iz_14_1209	DJI_0956	Chamaecytisus ruthenicus (Fisch.ex Wol.) Klaskova	0	n	1	0	Fabaceae Lindl.
Iz_14_1209	DJI_0956	Chelidonium majus L.	0.5	n	1	0	Papaveraceae Juss.
Iz_14_1209	DJI_0956	Chenopodium betaceum Andrz.	0	n	1	0	Amaranthaceae Juss.
Iz_14_1209	DJI_0956	Chenopodium cf. album	0	n	1	0	Amaranthaceae Juss.
Iz_14_1209	DJI_0956	Chenopodium sp.	0		NA	0	Amaranthaceae Juss.
Iz_14_1209	DJI_0956	Chondrilla juncea L.	0	n	1	0	Asteraceae Bercht. & J.Presl
Iz_14_1209	DJI_0956	Cirsium vulgare (Savi) Ten.	0	n	1	0	Asteraceae Bercht. & J.Presl
Iz_14_1209	DJI_0956	Clinopodium acinos (L.) Kuntze	0	n	1	0	Lamiacea Lindl.
Iz_14_1209	DJI_0956	Convallaria majalis L.	0	n	1	0	Asparagaceae Juss.
Iz_14_1209	DJI_0956	Crataegus rhipidophylla Gand.	0	n	1	1	Rosaceae Juss.
Iz_14_1209	DJI_0956	Crepis foetida L.	0	n	1	0	Asteraceae Bercht. & J.Presl
Iz_14_1209	DJI_0956	Dactylis glomerata L.	0	n	1	0	Poaceae Barnhart
Iz_14_1209	DJI_0956	Dianthus campestris M.Bieb.	0	n	1	0	Caryophyllaceae Juss.
Iz_14_1209	DJI_0956	Digitaria sanguinalis (L.) Scop.	0	a	0	0	Poaceae Barnhart
Iz_14_1209	DJI_0956	Elymus repens (L.) Gould	0	n	1	0	Poaceae Barnhart
Iz_14_1209	DJI_0956	Epilobium tetragonum L.	0	n	1	0	Onagraceae Juss.
Iz_14_1209	DJI_0956	Eragrostis minor Host	0	a	0	0	Poaceae Barnhart
Iz_14_1209	DJI_0956	Erigeron annuus (L.) Desf.	0	a	0	0	Asteraceae Bercht. & J.Presl
Iz_14_1209	DJI_0956	Erigeron canadensis L.	0	a	0	0	Asteraceae Bercht. & J.Presl
Iz_14_1209	DJI_0956	Euphorbia seguieriana Neck.	0	n	1	0	Euphorbiaceae Juss.
Iz_14_1209	DJI_0956	Euphorbia sp.	0		NA	NA	
Iz_14_1209	DJI_0956	Fallopia dumetorum (L.) Holub	10	n	1	0	Polygonaceae Juss.
Iz_14_1209	DJI_0956	Festuca beckeri (Hack.) Trautv.	0	n	1	0	Poaceae Barnhart
Iz_14_1209	DJI_0956	Fragaria vesca L.	0	n	1	0	Rosaceae Juss.
Iz_14_1209	DJI_0956	Frangula alnus Mill.	0	n	1	1	Rhamnaceae Juss.
Iz_14_1209	DJI_0956	Fraxinus pennsylvanica Marshall	0	a	0	1	Oleaceae Hoffmanns. & Link
Iz_14_1209	DJI_0956	Galeopsis bifida Boenn.	0	n	1	0	Lamiacea Lindl.
Iz_14_1209	DJI_0956	Galium verum L.	1	n	1	0	Rubiaceae Juss.
Iz_14_1209	DJI_0956	Genista tinctoria L.	0	n	1	0	Fabaceae Lindl.
Iz_14_1209	DJI_0956	Geranium robertianum L.	0	n	1	0	Geraniaceae Juss.
Iz_14_1209	DJI_0956	Geranium sanquineum L.	0	n	1	0	Geraniaceae Juss.
Iz_14_1209	DJI_0956	Gypsophila paniculata L.	0	n	1	0	Caryophyllaceae Juss.
Iz_14_1209	DJI_0956	Helichrysum arenarium (L.) Moench	0	n	1	0	Asteraceae Bercht. & J.Presl
Iz_14_1209	DJI_0956	Herniaria polygama J.Gay	0	n	1	0	Caryophyllaceae Juss.
Iz_14_1209	DJI_0956	Hieracium robustum Fr.	0	n	1	0	Asteraceae Bercht. & J.Presl
Iz_14_1209	DJI_0956	Hieracium umbellatum L.	0	n	1	NA	Asteraceae Bercht. & J.Presl
Iz_14_1209	DJI_0956	Hylotelephium maximum (L.) Holub	0	n	1	0	Crassulaceae J.St.-Hil.
Iz_14_1209	DJI_0956	Jacobaea borysthenica (DC.) B.Nord. & Greuter	0	n	1	0	Asteraceae Bercht. & J.Presl
Iz_14_1209	DJI_0956	Jasione montana L.	0	n	1	0	Campanulaceae Juss.
Iz_14_1209	DJI_0956	Jurinea cyanoides (L.) Rchb.s.l.	0	n	1	0	Asteraceae Bercht. & J.Presl
Iz_14_1209	DJI_0956	Koeleria glauca (Spreng.) DC.	0	n	1	0	Poaceae Barnhart
Iz_14_1209	DJI_0956	Lactuca serriola L.	0	a	0	0	Asteraceae Bercht. & J.Presl
Iz_14_1209	DJI_0956	Linaria genistifolia (L.) Mill.	0	n	1	0	Plantaginaceae Juss.
Iz_14_1209	DJI_0956	Lomelosia argentea (L.) Greuter & Burdet	0	n	1	0	Caprifoliaceae Juss.
Iz_14_1209	DJI_0956	Melampyrum pratense L.	0	n	1	0	Orobanchaceae Vent.
Iz_14_1209	DJI_0956	Oenothera biennis L.	0	a	0	0	Onagraceae Juss.
Iz_14_1209	DJI_0956	Pentanema britannica (L.) D.Gut.Larr., Santos-Vicente, Anderb., E.Rico & M.M.Mart.Ort.	0	n	1	0	Asteraceae Bercht. & J.Presl
Iz_14_1209	DJI_0956	Peucedanum oreoselinum (L.) Moench	0	n	1	0	Apiaceae Lindl.
Iz_14_1209	DJI_0956	Peucedanum ruthenicum M.Bieb.	0	n	1	0	Apiaceae Lindl.
Iz_14_1209	DJI_0956	Pilosella echioides (Lumn.) F.W.Schultz & Sch.Bip.	0	n	1	0	Asteraceae Bercht. & J.Presl
Iz_14_1209	DJI_0956	Pilosella officinarum F. Schultz et Sch. Bip.	0	n	1	0	Asteraceae Bercht. & J.Presl
Iz_14_1209	DJI_0956	Pilosella sp.	0		NA	0	Asteraceae Bercht. & J.Presl
Iz_14_1209	DJI_0956	Pinus sylvestris L.	0	n	1	1	Pinaceae Spreng. ex F.Rudolphi
Iz_14_1209	DJI_0956	Poa annua L.	0	n	1	0	Poaceae Barnhart
Iz_14_1209	DJI_0956	Poa nemoralis L.	0	n	1	0	Poaceae Barnhart
Iz_14_1209	DJI_0956	Poa sp.	0		NA	0	Poaceae Barnhart
Iz_14_1209	DJI_0956	Polygonatum odoratum (Mill.) Druce	0	n	1	0	Asparagaceae Juss.
Iz_14_1209	DJI_0956	Polygonum arenarium Waldst. & Kit.	0	n	1	0	Polygonaceae Juss.
Iz_14_1209	DJI_0956	Populus nigra L.	0	n	1	1	Salicaceae Mirb.
Iz_14_1209	DJI_0956	Populus tremula L.	0	n	1	1	Salicaceae Mirb.
Iz_14_1209	DJI_0956	Populus Ã canescens (Aiton) Sm.	0	n	1	1	Salicaceae Mirb.
Iz_14_1209	DJI_0956	Potentilla argentea L.	0	n	1	0	Rosaceae Juss.
Iz_14_1209	DJI_0956	Psammophiliella muralis (L.) Ikonn.	0	n	1	0	Caryophyllaceae Juss.
Iz_14_1209	DJI_0956	Psephellus sumensis (Kalen.) Greuter	0	n	1	0	Asteraceae Bercht. & J.Presl
Iz_14_1209	DJI_0956	Pulsatilla pratensis (L.) Mill. s.l.	0	n	1	0	Ranunculaceae Juss.
Iz_14_1209	DJI_0956	Pyrus communis L.	0	a	0	1	Rosaceae Juss.
Iz_14_1209	DJI_0956	Quercus robur L.	5	n	1	1	Fagaceae Dumort.
Iz_14_1209	DJI_0956	Robinia pseudoacacia L.	0	a	0	1	Fabaceae Lindl.
Iz_14_1209	DJI_0956	Rosa sp.	0		NA	1	Rosaceae Juss.
Iz_14_1209	DJI_0956	Rubus idaeus L.	0	n	1	0	Rosaceae Juss.
Iz_14_1209	DJI_0956	Rumex acetosella L.	0	n	1	0	Polygonaceae Juss.
Iz_14_1209	DJI_0956	Rumex thyrsiflorus Fingerh.	0	n	1	0	Polygonaceae Juss.
Iz_14_1209	DJI_0956	Salix alba L.	0	n	1	1	Salicaceae Mirb.
Iz_14_1209	DJI_0956	Sambucus nigra L.	0	n	1	1	Viburnaceae Raf.
Iz_14_1209	DJI_0956	Secale sylvestre Host	0	n	1	0	Poaceae Barnhart
Iz_14_1209	DJI_0956	Sempervivum ruthenicum Schnittsp. & C.B. Lehm.	0	n	1	0	Crassulaceae J.St.-Hil.
Iz_14_1209	DJI_0956	Setaria viridis (L.) P. Beauv.	0	n	1	0	Poaceae Barnhart
Iz_14_1209	DJI_0956	Silene borysthenica (Gruner) Walters	0	n	1	0	Caryophyllaceae Juss.
Iz_14_1209	DJI_0956	Silene latifolia subsp. alba (Mill.) Greuter & Burdet	0	n	1	0	Caryophyllaceae Juss.
Iz_14_1209	DJI_0956	Solidago virgaurea L.	0	n	1	0	Asteraceae Bercht. & J.Presl
Iz_14_1209	DJI_0956	Sorbus aucuparia L.	0	n	1	1	Rosaceae Juss.
Iz_14_1209	DJI_0956	Stachys recta L.	0	n	1	0	Lamiacea Lindl.
Iz_14_1209	DJI_0956	Tanacetum vulgare L.	0	n	1	0	Asteraceae Bercht. & J.Presl
Iz_14_1209	DJI_0956	Thymus pallasianus Heinr.Braun	0	n	1	0	Lamiacea Lindl.
Iz_14_1209	DJI_0956	Tilia cordata Mill.	0	n	1	1	Malvaceae Juss.
Iz_14_1209	DJI_0956	Trifolium alpestre L.	0	n	1	0	Fabaceae Lindl.
Iz_14_1209	DJI_0956	Trifolium arvense L.	0	n	1	0	Fabaceae Lindl.
Iz_14_1209	DJI_0956	Ulmus glabra Huds.	0	n	1	1	Ulmaceae Mirb.

Iz_14_1209	DJI_0956	Verbascum densiflorum Bertol.	0	n	1	0	Scrophulariaceae Juss.
Iz_14_1209	DJI_0956	Veronica austriaca L.	0	n	1	0	Plantaginaceae Juss.
Iz_14_1209	DJI_0956	Veronica incana L.	0	n	1	0	Plantaginaceae Juss.
Iz_14_1209	DJI_0956	Veronica verna L.	0	n	1	0	Plantaginaceae Juss.
Iz_14_1209	DJI_0956	Vincetoxicum hirundinaria Medik	0	n	1	0	Apocynaceae Juss.
Iz_14_1209	DJI_0960	Acer tataricum L.	0	n	1	1	Sapindaceae Juss.
Iz_14_1209	DJI_0960	Achillea millefolium L. s.l.	0	n	1	0	Asteraceae Bercht. & J.Presl
Iz_14_1209	DJI_0960	Allium oleraceum L.	0	n	1	0	Amaryllidaceae J.St.-Hil.
Iz_14_1209	DJI_0960	Ambrosia artemisiifolia L.	0	a	0	0	Asteraceae Bercht. & J.Presl
Iz_14_1209	DJI_0960	Amorpha fruticosa L.	0	a	0	0	Fabaceae Lindl.
Iz_14_1209	DJI_0960	Anthericum ramosum L.	0	n	1	0	Asparagaceae Juss.
Iz_14_1209	DJI_0960	Artemisia marschalliana Spreng.	0	n	1	0	Asteraceae Bercht. & J.Presl
Iz_14_1209	DJI_0960	Asparagus officinalis L.	0	n	1	0	Asparagaceae Juss.
Iz_14_1209	DJI_0960	Astragalus cicer L.	0	n	1	0	Fabaceae Lindl.
Iz_14_1209	DJI_0960	Bassia laniflora (S.G.Gmel.) A.J.Scott	0	n	1	0	Amaranthaceae Juss.
Iz_14_1209	DJI_0960	Berberis vulgaris L.	0	n	1	0	Berberidaceae Juss.
Iz_14_1209	DJI_0960	Betula pendula Roth.	0	n	1	1	Betulaceae Gray
Iz_14_1209	DJI_0960	Betula pendula Roth.	0	n	1	0	Poaceae Barnhart
Iz_14_1209	DJI_0960	Bromus inermis Leyss.	0		NA	NA	
Iz_14_1209	DJI_0960	Bromus squarrosus L.	0	a	0	0	Poaceae Barnhart
Iz_14_1209	DJI_0960	Bromus tectorum L.	0	a	0	0	Poaceae Barnhart
Iz_14_1209	DJI_0960	Calamagrostis epigejos (L.) Roth	45	n	1	0	Poaceae Barnhart
Iz_14_1209	DJI_0960	Carduus acanthoides L.	0	n	1	0	Asteraceae Bercht. & J.Presl
Iz_14_1209	DJI_0960	Carex sp.	0	n	1	0	Cyperaceae Juss.
Iz_14_1209	DJI_0960	Centaurea majorovii Dumbadze	0	n	1	0	Asteraceae Bercht. & J.Presl
Iz_14_1209	DJI_0960	Chamaecytisus ruthenicus (Fisch.ex Wol.) Klaskova	0	n	1	0	Fabaceae Lindl.
Iz_14_1209	DJI_0960	Chelidonium majus L.	1	n	1	0	Papaveraceae Juss.
Iz_14_1209	DJI_0960	Chenopodium betaceum Andrz.	0	n	1	0	Amaranthaceae Juss.
Iz_14_1209	DJI_0960	Chenopodium cf. album	1	n	1	0	Amaranthaceae Juss.
Iz_14_1209	DJI_0960	Chenopodium sp.	0		NA	0	Amaranthaceae Juss.
Iz_14_1209	DJI_0960	Chondrilla juncea L.	0	n	1	0	Asteraceae Bercht. & J.Presl
Iz_14_1209	DJI_0960	Cirsium vulgare (Savi) Ten.	0	n	1	0	Asteraceae Bercht. & J.Presl
Iz_14_1209	DJI_0960	Clinopodium acinos (L.) Kuntze	0	n	1	0	Lamiacea Lindl.
Iz_14_1209	DJI_0960	Convallaria majalis L.	0	n	1	0	Asparagaceae Juss.
Iz_14_1209	DJI_0960	Crataegus rhipidophylla Gand.	0	n	1	1	Rosaceae Juss.
Iz_14_1209	DJI_0960	Crepis foetida L.	0	n	1	0	Asteraceae Bercht. & J.Presl
Iz_14_1209	DJI_0960	Dactylis glomerata L.	0	n	1	0	Poaceae Barnhart
Iz_14_1209	DJI_0960	Dianthus campestris M.Bieb.	0	n	1	0	Caryophyllaceae Juss.
Iz_14_1209	DJI_0960	Digitaria sanguinalis (L.) Scop.	0	a	0	0	Poaceae Barnhart
Iz_14_1209	DJI_0960	Elymus repens (L.) Gould	0	n	1	0	Poaceae Barnhart
Iz_14_1209	DJI_0960	Epilobium tetragonum L.	0	n	1	0	Onagraceae Juss.
Iz_14_1209	DJI_0960	Eragrostis minor Host	0	a	0	0	Poaceae Barnhart
Iz_14_1209	DJI_0960	Erigeron annuus (L.) Desf.	0	a	0	0	Asteraceae Bercht. & J.Presl
Iz_14_1209	DJI_0960	Erigeron canadensis L.	0	a	0	0	Asteraceae Bercht. & J.Presl
Iz_14_1209	DJI_0960	Euphorbia seguieriana Neck.	0	n	1	0	Euphorbiaceae Juss.
Iz_14_1209	DJI_0960	Euphorbia sp.	0		NA	NA	
Iz_14_1209	DJI_0960	Fallopia dumetorum (L.) Holub	0	n	1	0	Polygonaceae Juss.
Iz_14_1209	DJI_0960	Festuca beckeri (Hack.) Trautv.	0	n	1	0	Poaceae Barnhart
Iz_14_1209	DJI_0960	Fragaria vesca L.	0	n	1	0	Rosaceae Juss.
Iz_14_1209	DJI_0960	Frangula alnus Mill.	0	n	1	1	Rhamnaceae Juss.
Iz_14_1209	DJI_0960	Fraxinus pennsylvanica Marshall	0	a	0	1	Oleaceae Hoffmanns. & Link
Iz_14_1209	DJI_0960	Galeopsis bifida Boenn.	0	n	1	0	Lamiacea Lindl.
Iz_14_1209	DJI_0960	Galium verum L.	0	n	1	0	Rubiaceae Juss.
Iz_14_1209	DJI_0960	Genista tinctoria L.	0	n	1	0	Fabaceae Lindl.
Iz_14_1209	DJI_0960	Geranium robertianum L.	0	n	1	0	Geraniaceae Juss.
Iz_14_1209	DJI_0960	Geranium sanquineum L.	0	n	1	0	Geraniaceae Juss.
Iz_14_1209	DJI_0960	Gypsophila paniculata L.	0	n	1	0	Caryophyllaceae Juss.
Iz_14_1209	DJI_0960	Helichrysum arenarium (L.) Moench	0	n	1	0	Asteraceae Bercht. & J.Presl
Iz_14_1209	DJI_0960	Herniaria polygama J.Gay	0	n	1	0	Caryophyllaceae Juss.
Iz_14_1209	DJI_0960	Hieracium robustum Fr.	0	n	1	0	Asteraceae Bercht. & J.Presl
Iz_14_1209	DJI_0960	Hieracium umbellatum L.	2	n	1	NA	Asteraceae Bercht. & J.Presl
Iz_14_1209	DJI_0960	Hylotelephium maximum (L.) Holub	0	n	1	0	Crassulaceae J.St.-Hil.
Iz_14_1209	DJI_0960	Jacobaea borysthenica (DC.) B.Nord. & Greuter	0	n	1	0	Asteraceae Bercht. & J.Presl
Iz_14_1209	DJI_0960	Jasione montana L.	0	n	1	0	Campanulaceae Juss.
Iz_14_1209	DJI_0960	Jurinea cyanoides (L.) Rchb.s.l.	0	n	1	0	Asteraceae Bercht. & J.Presl
Iz_14_1209	DJI_0960	Koeleria glauca (Spreng.) DC.	0	n	1	0	Poaceae Barnhart
Iz_14_1209	DJI_0960	Lactuca serriola L.	0	a	0	0	Asteraceae Bercht. & J.Presl
Iz_14_1209	DJI_0960	Linaria genistifolia (L.) Mill.	0	n	1	0	Plantaginaceae Juss.
Iz_14_1209	DJI_0960	Lomelosia argentea (L.) Greuter & Burdet	0	n	1	0	Caprifoliaceae Juss.
Iz_14_1209	DJI_0960	Melampyrum pratense L.	0	n	1	0	Orobanchaceae Vent.
Iz_14_1209	DJI_0960	Oenothera biennis L.	0	a	0	0	Onagraceae Juss.
Iz_14_1209	DJI_0960	Pentanema britannica (L.) D.Gut.Larr., Santos-Vicente, Anderb., E.Rico & M.M.Mart.Ort.	0	n	1	0	Asteraceae Bercht. & J.Presl
Iz_14_1209	DJI_0960	Peucedanum oreoselinum (L.) Moench	8	n	1	0	Apiaceae Lindl.
Iz_14_1209	DJI_0960	Peucedanum ruthenicum M.Bieb.	0	n	1	0	Apiaceae Lindl.
Iz_14_1209	DJI_0960	Pilosella echioides (Lumn.) F.W.Schultz & Sch.Bip.	0	n	1	0	Asteraceae Bercht. & J.Presl
Iz_14_1209	DJI_0960	Pilosella officinarum F. Schultz et Sch. Bip.	0	n	1	0	Asteraceae Bercht. & J.Presl
Iz_14_1209	DJI_0960	Pilosella sp.	0		NA	0	Asteraceae Bercht. & J.Presl
Iz_14_1209	DJI_0960	Pinus sylvestris L.	0	n	1	1	Pinaceae Spreng. ex F.Rudolphi
Iz_14_1209	DJI_0960	Poa annua L.	0	n	1	0	Poaceae Barnhart
Iz_14_1209	DJI_0960	Poa nemoralis L.	0	n	1	0	Poaceae Barnhart
Iz_14_1209	DJI_0960	Poa sp.	0		NA	0	Poaceae Barnhart
Iz_14_1209	DJI_0960	Polygonatum odoratum (Mill.) Druce	0	n	1	0	Asparagaceae Juss.
Iz_14_1209	DJI_0960	Polygonum arenarium Waldst. & Kit.	0	n	1	0	Polygonaceae Juss.
Iz_14_1209	DJI_0960	Populus nigra L.	0	n	1	1	Salicaceae Mirb.
Iz_14_1209	DJI_0960	Populus tremula L.	0	n	1	1	Salicaceae Mirb.
Iz_14_1209	DJI_0960	Populus Ã canescens (Aiton) Sm.	0	n	1	1	Salicaceae Mirb.
Iz_14_1209	DJI_0960	Potentilla argentea L.	0	n	1	0	Rosaceae Juss.
Iz_14_1209	DJI_0960	Psammophiliella muralis (L.) Ikonn.	0	n	1	0	Caryophyllaceae Juss.
Iz_14_1209	DJI_0960	Psephellus sumensis (Kalen.) Greuter	0	n	1	0	Asteraceae Bercht. & J.Presl
Iz_14_1209	DJI_0960	Pulsatilla pratensis (L.) Mill. s.l.	0	n	1	0	Ranunculaceae Juss.
Iz_14_1209	DJI_0960	Pyrus communis L.	0	a	0	1	Rosaceae Juss.
Iz_14_1209	DJI_0960	Quercus robur L.	0	n	1	1	Fagaceae Dumort.
Iz_14_1209	DJI_0960	Robinia pseudoacacia L.	0	a	0	1	Fabaceae Lindl.
Iz_14_1209	DJI_0960	Rosa sp.	0		NA	1	Rosaceae Juss.
Iz_14_1209	DJI_0960	Rubus idaeus L.	0	n	1	0	Rosaceae Juss.
Iz_14_1209	DJI_0960	Rumex acetosella L.	0	n	1	0	Polygonaceae Juss.
Iz_14_1209	DJI_0960	Rumex thyrsiflorus Fingerh.	0	n	1	0	Polygonaceae Juss.
Iz_14_1209	DJI_0960	Salix alba L.	0	n	1	1	Salicaceae Mirb.

Iz_14_1209	DJI_0960	Sambucus nigra L.	0	n	1	1	Viburnaceae Raf.
Iz_14_1209	DJI_0960	Secale sylvestre Host	0	n	1	0	Poaceae Barnhart
Iz_14_1209	DJI_0960	Sempervivum ruthenicum Schnittsp. & C.B. Lehm.	1.5	n	1	0	Crassulaceae J.St.-Hil.
Iz_14_1209	DJI_0960	Setaria viridis (L.) P. Beauv.	0	n	1	0	Poaceae Barnhart
Iz_14_1209	DJI_0960	Silene borysthenica (Gruner) Walters	0	n	1	0	Caryophyllaceae Juss.
Iz_14_1209	DJI_0960	Silene latifolia subsp. alba (Mill.) Greuter & Burdet	0	n	1	0	Caryophyllaceae Juss.
Iz_14_1209	DJI_0960	Solidago virgaurea L.	0	n	1	0	Asteraceae Bercht. & J.Presl
Iz_14_1209	DJI_0960	Sorbus aucuparia L.	0	n	1	1	Rosaceae Juss.
Iz_14_1209	DJI_0960	Stachys recta L.	0	n	1	0	Lamiacea Lindl.
Iz_14_1209	DJI_0960	Tanacetum vulgare L.	0	n	1	0	Asteraceae Bercht. & J.Presl
Iz_14_1209	DJI_0960	Thymus pallasianus Heinr.Braun	0	n	1	0	Lamiacea Lindl.
Iz_14_1209	DJI_0960	Tilia cordata Mill.	0	n	1	1	Malvaceae Juss.
Iz_14_1209	DJI_0960	Trifolium alpestre L.	0	n	1	0	Fabaceae Lindl.
Iz_14_1209	DJI_0960	Trifolium arvense L.	0	n	1	0	Fabaceae Lindl.
Iz_14_1209	DJI_0960	Ulmus glabra Huds.	0	n	1	1	Ulmaceae Mirb.
Iz_14_1209	DJI_0960	Verbascum densiflorum Bertol.	0	n	1	0	Scrophulariaceae Juss.
Iz_14_1209	DJI_0960	Veronica austriaca L.	0	n	1	0	Plantaginaceae Juss.
Iz_14_1209	DJI_0960	Veronica incana L.	0	n	1	0	Plantaginaceae Juss.
Iz_14_1209	DJI_0960	Veronica verna L.	0	n	1	0	Plantaginaceae Juss.
Iz_14_1209	DJI_0960	Vincetoxicum hirundinaria Medik	0	n	1	0	Apocynaceae Juss.
Iz_14_1209	DJI_0966	Acer tataricum L.	0	n	1	1	Sapindaceae Juss.
Iz_14_1209	DJI_0966	Achillea millefolium L. s.l.	0	n	1	0	Asteraceae Bercht. & J.Presl
Iz_14_1209	DJI_0966	Allium oleraceum L.	0	n	1	0	Amaryllidaceae J.St.-Hil.
Iz_14_1209	DJI_0966	Ambrosia artemisiifolia L.	0	a	0	0	Asteraceae Bercht. & J.Presl
Iz_14_1209	DJI_0966	Amorpha fruticosa L.	0	a	0	0	Fabaceae Lindl.
Iz_14_1209	DJI_0966	Anthericum ramosum L.	0	n	1	0	Asparagaceae Juss.
Iz_14_1209	DJI_0966	Artemisia marschalliana Spreng.	0	n	1	0	Asteraceae Bercht. & J.Presl
Iz_14_1209	DJI_0966	Asparagus officinalis L.	0	n	1	0	Asparagaceae Juss.
Iz_14_1209	DJI_0966	Astragalus cicer L.	0	n	1	0	Fabaceae Lindl.
Iz_14_1209	DJI_0966	Bassia laniflora (S.G.Gmel.) A.J.Scott	0	n	1	0	Amaranthaceae Juss.
Iz_14_1209	DJI_0966	Berberis vulgaris L.	0	n	1	0	Berberidaceae Juss.
Iz_14_1209	DJI_0966	Betula pendula Roth.	0	n	1	1	Betulaceae Gray
Iz_14_1209	DJI_0966	Betula pendula Roth.	0	n	1	0	Poaceae Barnhart
Iz_14_1209	DJI_0966	Bromus inermis Leyss.	0		NA	NA	
Iz_14_1209	DJI_0966	Bromus squarrosus L.	0	a	0	0	Poaceae Barnhart
Iz_14_1209	DJI_0966	Bromus tectorum L.	0	a	0	0	Poaceae Barnhart
Iz_14_1209	DJI_0966	Calamagrostis epigejos (L.) Roth	40	n	1	0	Poaceae Barnhart
Iz_14_1209	DJI_0966	Carduus acanthoides L.	0	n	1	0	Asteraceae Bercht. & J.Presl
Iz_14_1209	DJI_0966	Carex sp.	0	n	1	0	Cyperaceae Juss.
Iz_14_1209	DJI_0966	Centaurea majorovii Dumbadze	0	n	1	0	Asteraceae Bercht. & J.Presl
Iz_14_1209	DJI_0966	Chamaecytisus ruthenicus (Fisch.ex Wol.) Klaskova	0	n	1	0	Fabaceae Lindl.
Iz_14_1209	DJI_0966	Chelidonium majus L.	0	n	1	0	Papaveraceae Juss.
Iz_14_1209	DJI_0966	Chenopodium betaceum Andrz.	0	n	1	0	Amaranthaceae Juss.
Iz_14_1209	DJI_0966	Chenopodium cf. album	3	n	1	0	Amaranthaceae Juss.
Iz_14_1209	DJI_0966	Chenopodium sp.	0		NA	0	Amaranthaceae Juss.
Iz_14_1209	DJI_0966	Chondrilla juncea L.	0	n	1	0	Asteraceae Bercht. & J.Presl
Iz_14_1209	DJI_0966	Cirsium vulgare (Savi) Ten.	0	n	1	0	Asteraceae Bercht. & J.Presl
Iz_14_1209	DJI_0966	Clinopodium acinos (L.) Kuntze	0	n	1	0	Lamiacea Lindl.
Iz_14_1209	DJI_0966	Convallaria majalis L.	0	n	1	0	Asparagaceae Juss.
Iz_14_1209	DJI_0966	Crataegus rhipidophylla Gand.	0	n	1	1	Rosaceae Juss.
Iz_14_1209	DJI_0966	Crepis foetida L.	0	n	1	0	Asteraceae Bercht. & J.Presl
Iz_14_1209	DJI_0966	Dactylis glomerata L.	0	n	1	0	Poaceae Barnhart
Iz_14_1209	DJI_0966	Dianthus campestris M.Bieb.	0	n	1	0	Caryophyllaceae Juss.
Iz_14_1209	DJI_0966	Digitaria sanguinalis (L.) Scop.	0	a	0	0	Poaceae Barnhart
Iz_14_1209	DJI_0966	Elymus repens (L.) Gould	0	n	1	0	Poaceae Barnhart
Iz_14_1209	DJI_0966	Epilobium tetragonum L.	0	n	1	0	Onagraceae Juss.
Iz_14_1209	DJI_0966	Eragrostis minor Host	0	a	0	0	Poaceae Barnhart
Iz_14_1209	DJI_0966	Erigeron annuus (L.) Desf.	0	a	0	0	Asteraceae Bercht. & J.Presl
Iz_14_1209	DJI_0966	Erigeron canadensis L.	0	a	0	0	Asteraceae Bercht. & J.Presl
Iz_14_1209	DJI_0966	Euphorbia seguieriana Neck.	0	n	1	0	Euphorbiaceae Juss.
Iz_14_1209	DJI_0966	Euphorbia sp.	0		NA	NA	
Iz_14_1209	DJI_0966	Fallopia dumetorum (L.) Holub	1	n	1	0	Polygonaceae Juss.
Iz_14_1209	DJI_0966	Festuca beckeri (Hack.) Trautv.	0	n	1	0	Poaceae Barnhart
Iz_14_1209	DJI_0966	Fragaria vesca L.	0	n	1	0	Rosaceae Juss.
Iz_14_1209	DJI_0966	Frangula alnus Mill.	0	n	1	1	Rhamnaceae Juss.
Iz_14_1209	DJI_0966	Fraxinus pennsylvanica Marshall	0	a	0	1	Oleaceae Hoffmanns. & Link
Iz_14_1209	DJI_0966	Galeopsis bifida Boenn.	0	n	1	0	Lamiacea Lindl.
Iz_14_1209	DJI_0966	Galium verum L.	0	n	1	0	Rubiaceae Juss.
Iz_14_1209	DJI_0966	Genista tinctoria L.	0	n	1	0	Fabaceae Lindl.
Iz_14_1209	DJI_0966	Geranium robertianum L.	0	n	1	0	Geraniaceae Juss.
Iz_14_1209	DJI_0966	Geranium sanquineum L.	0	n	1	0	Geraniaceae Juss.
Iz_14_1209	DJI_0966	Gypsophila paniculata L.	0	n	1	0	Caryophyllaceae Juss.
Iz_14_1209	DJI_0966	Helichrysum arenarium (L.) Moench	0	n	1	0	Asteraceae Bercht. & J.Presl
Iz_14_1209	DJI_0966	Herniaria polygama J.Gay	0	n	1	0	Caryophyllaceae Juss.
Iz_14_1209	DJI_0966	Hieracium robustum Fr.	0	n	1	0	Asteraceae Bercht. & J.Presl
Iz_14_1209	DJI_0966	Hieracium umbellatum L.	0	n	1	NA	Asteraceae Bercht. & J.Presl
Iz_14_1209	DJI_0966	Hylotelephium maximum (L.) Holub	0	n	1	0	Crassulaceae J.St.-Hil.
Iz_14_1209	DJI_0966	Jacobaea borysthenica (DC.) B.Nord. & Greuter	0	n	1	0	Asteraceae Bercht. & J.Presl
Iz_14_1209	DJI_0966	Jasione montana L.	0	n	1	0	Campanulaceae Juss.
Iz_14_1209	DJI_0966	Jurinea cyanoides (L.) Rchb.s.l.	0	n	1	0	Asteraceae Bercht. & J.Presl
Iz_14_1209	DJI_0966	Koeleria glauca (Spreng.) DC.	0	n	1	0	Poaceae Barnhart
Iz_14_1209	DJI_0966	Lactuca serriola L.	0	a	0	0	Asteraceae Bercht. & J.Presl
Iz_14_1209	DJI_0966	Linaria genistifolia (L.) Mill.	0	n	1	0	Plantaginaceae Juss.
Iz_14_1209	DJI_0966	Lomelosia argentea (L.) Greuter & Burdet	0	n	1	0	Caprifoliaceae Juss.
Iz_14_1209	DJI_0966	Melampyrum pratense L.	0	n	1	0	Orobanchaceae Vent.
Iz_14_1209	DJI_0966	Oenothera biennis L.	0	a	0	0	Onagraceae Juss.
Iz_14_1209	DJI_0966	Pentanema britannica (L.) D.Gut.Larr., Santos-Vicente, Anderb., E.Rico & M.M.Mart.Ort.	0	n	1	0	Asteraceae Bercht. & J.Presl
Iz_14_1209	DJI_0966	Peucedanum oreoselinum (L.) Moench	10	n	1	0	Apiaceae Lindl.
Iz_14_1209	DJI_0966	Peucedanum ruthenicum M.Bieb.	0	n	1	0	Apiaceae Lindl.
Iz_14_1209	DJI_0966	Pilosella echioides (Lumn.) F.W.Schultz & Sch.Bip.	0	n	1	0	Asteraceae Bercht. & J.Presl
Iz_14_1209	DJI_0966	Pilosella officinarum F. Schultz et Sch. Bip.	0	n	1	0	Asteraceae Bercht. & J.Presl
Iz_14_1209	DJI_0966	Pilosella sp.	0		NA	0	Asteraceae Bercht. & J.Presl
Iz_14_1209	DJI_0966	Pinus sylvestris L.	0	n	1	1	Pinaceae Spreng. ex F.Rudolphi
Iz_14_1209	DJI_0966	Poa annua L.	0	n	1	0	Poaceae Barnhart
Iz_14_1209	DJI_0966	Poa nemoralis L.	0	n	1	0	Poaceae Barnhart
Iz_14_1209	DJI_0966	Poa sp.	0		NA	0	Poaceae Barnhart
Iz_14_1209	DJI_0966	Polygonatum odoratum (Mill.) Druce	0	n	1	0	Asparagaceae Juss.
Iz_14_1209	DJI_0966	Polygonum arenarium Waldst. & Kit.	0	n	1	0	Polygonaceae Juss.

Iz_14_1209	DJI_0966	Populus nigra L.	0	n	1	1	Salicaceae Mirb.
Iz_14_1209	DJI_0966	Populus tremula L.	0	n	1	1	Salicaceae Mirb.
Iz_14_1209	DJI_0966	Populus Ã canescens (Aiton) Sm.	0	n	1	1	Salicaceae Mirb.
Iz_14_1209	DJI_0966	Potentilla argentea L.	0	n	1	0	Rosaceae Juss.
Iz_14_1209	DJI_0966	Psammophiliella muralis (L.) Ikonn.	0	n	1	0	Caryophyllaceae Juss.
Iz_14_1209	DJI_0966	Psephellus sumensis (Kalen.) Greuter	1	n	1	0	Asteraceae Bercht. & J.Presl
Iz_14_1209	DJI_0966	Pulsatilla pratensis (L.) Mill. s.l.	0	n	1	0	Ranunculaceae Juss.
Iz_14_1209	DJI_0966	Pyrus communis L.	0	a	0	1	Rosaceae Juss.
Iz_14_1209	DJI_0966	Quercus robur L.	0	n	1	1	Fagaceae Dumort.
Iz_14_1209	DJI_0966	Robinia pseudoacacia L.	0	a	0	1	Fabaceae Lindl.
Iz_14_1209	DJI_0966	Rosa sp.	0		NA	1	Rosaceae Juss.
Iz_14_1209	DJI_0966	Rubus idaeus L.	0	n	1	0	Rosaceae Juss.
Iz_14_1209	DJI_0966	Rumex acetosella L.	0	n	1	0	Polygonaceae Juss.
Iz_14_1209	DJI_0966	Rumex thyrsiflorus Fingerh.	0	n	1	0	Polygonaceae Juss.
Iz_14_1209	DJI_0966	Salix alba L.	0	n	1	1	Salicaceae Mirb.
Iz_14_1209	DJI_0966	Sambucus nigra L.	0	n	1	1	Viburnaceae Raf.
Iz_14_1209	DJI_0966	Secale sylvestre Host	0	n	1	0	Poaceae Barnhart
Iz_14_1209	DJI_0966	Sempervivum ruthenicum Schnittsp. & C.B. Lehm.	0.5	n	1	0	Crassulaceae J.St.-Hil.
Iz_14_1209	DJI_0966	Setaria viridis (L.) P. Beauv.	0	n	1	0	Poaceae Barnhart
Iz_14_1209	DJI_0966	Silene borysthenica (Gruner) Walters	0	n	1	0	Caryophyllaceae Juss.
Iz_14_1209	DJI_0966	Silene latifolia subsp. alba (Mill.) Greuter & Burdet	0	n	1	0	Caryophyllaceae Juss.
Iz_14_1209	DJI_0966	Solidago virgaurea L.	0	n	1	0	Asteraceae Bercht. & J.Presl
Iz_14_1209	DJI_0966	Sorbus aucuparia L.	0	n	1	1	Rosaceae Juss.
Iz_14_1209	DJI_0966	Stachys recta L.	0	n	1	0	Lamiacea Lindl.
Iz_14_1209	DJI_0966	Tanacetum vulgare L.	0	n	1	0	Asteraceae Bercht. & J.Presl
Iz_14_1209	DJI_0966	Thymus pallasianus Heinr.Braun	0	n	1	0	Lamiacea Lindl.
Iz_14_1209	DJI_0966	Tilia cordata Mill.	0	n	1	1	Malvaceae Juss.
Iz_14_1209	DJI_0966	Trifolium alpestre L.	0	n	1	0	Fabaceae Lindl.
Iz_14_1209	DJI_0966	Trifolium arvense L.	0	n	1	0	Fabaceae Lindl.
Iz_14_1209	DJI_0966	Ulmus glabra Huds.	0	n	1	1	Ulmaceae Mirb.
Iz_14_1209	DJI_0966	Verbascum densiflorum Bertol.	0	n	1	0	Scrophulariaceae Juss.
Iz_14_1209	DJI_0966	Veronica austriaca L.	0	n	1	0	Plantaginaceae Juss.
Iz_14_1209	DJI_0966	Veronica incana L.	0	n	1	0	Plantaginaceae Juss.
Iz_14_1209	DJI_0966	Veronica verna L.	0	n	1	0	Plantaginaceae Juss.
Iz_14_1209	DJI_0966	Vincetoxicum hirundinaria Medik	0	n	1	0	Apocynaceae Juss.
Iz_14_1209	DJI_0972	Acer tataricum L.	0	n	1	1	Sapindaceae Juss.
Iz_14_1209	DJI_0972	Achillea millefolium L. s.l.	0	n	1	0	Asteraceae Bercht. & J.Presl
Iz_14_1209	DJI_0972	Allium oleraceum L.	0	n	1	0	Amaryllidaceae J.St.-Hil.
Iz_14_1209	DJI_0972	Ambrosia artemisiifolia L.	0	a	0	0	Asteraceae Bercht. & J.Presl
Iz_14_1209	DJI_0972	Amorpha fruticosa L.	0	a	0	0	Fabaceae Lindl.
Iz_14_1209	DJI_0972	Anthericum ramosum L.	0	n	1	0	Asparagaceae Juss.
Iz_14_1209	DJI_0972	Artemisia marschalliana Spreng.	0	n	1	0	Asteraceae Bercht. & J.Presl
Iz_14_1209	DJI_0972	Asparagus officinalis L.	0	n	1	0	Asparagaceae Juss.
Iz_14_1209	DJI_0972	Astragalus cicer L.	0	n	1	0	Fabaceae Lindl.
Iz_14_1209	DJI_0972	Bassia laniflora (S.G.Gmel.) A.J.Scott	0	n	1	0	Amaranthaceae Juss.
Iz_14_1209	DJI_0972	Berberis vulgaris L.	0	n	1	0	Berberidaceae Juss.
Iz_14_1209	DJI_0972	Betula pendula Roth.	0	n	1	1	Betulaceae Gray
Iz_14_1209	DJI_0972	Betula pendula Roth.	0	n	1	0	Poaceae Barnhart
Iz_14_1209	DJI_0972	Bromus inermis Leyss.	0		NA	NA	
Iz_14_1209	DJI_0972	Bromus squarrosus L.	0	a	0	0	Poaceae Barnhart
Iz_14_1209	DJI_0972	Bromus tectorum L.	0	a	0	0	Poaceae Barnhart
Iz_14_1209	DJI_0972	Calamagrostis epigejos (L.) Roth	10	n	1	0	Poaceae Barnhart
Iz_14_1209	DJI_0972	Carduus acanthoides L.	0	n	1	0	Asteraceae Bercht. & J.Presl
Iz_14_1209	DJI_0972	Carex sp.	3	n	1	0	Cyperaceae Juss.
Iz_14_1209	DJI_0972	Centaurea majorovii Dumbadze	0	n	1	0	Asteraceae Bercht. & J.Presl
Iz_14_1209	DJI_0972	Chamaecytisus ruthenicus (Fisch.ex Wol.) Klaskova	0	n	1	0	Fabaceae Lindl.
Iz_14_1209	DJI_0972	Chelidonium majus L.	3	n	1	0	Papaveraceae Juss.
Iz_14_1209	DJI_0972	Chenopodium betaceum Andrz.	0	n	1	0	Amaranthaceae Juss.
Iz_14_1209	DJI_0972	Chenopodium cf. album	0	n	1	0	Amaranthaceae Juss.
Iz_14_1209	DJI_0972	Chenopodium sp.	2		NA	0	Amaranthaceae Juss.
Iz_14_1209	DJI_0972	Chondrilla juncea L.	0	n	1	0	Asteraceae Bercht. & J.Presl
Iz_14_1209	DJI_0972	Cirsium vulgare (Savi) Ten.	0	n	1	0	Asteraceae Bercht. & J.Presl
Iz_14_1209	DJI_0972	Clinopodium acinos (L.) Kuntze	0	n	1	0	Lamiacea Lindl.
Iz_14_1209	DJI_0972	Convallaria majalis L.	0	n	1	0	Asparagaceae Juss.
Iz_14_1209	DJI_0972	Crataegus rhipidophylla Gand.	0	n	1	1	Rosaceae Juss.
Iz_14_1209	DJI_0972	Crepis foetida L.	0	n	1	0	Asteraceae Bercht. & J.Presl
Iz_14_1209	DJI_0972	Dactylis glomerata L.	0	n	1	0	Poaceae Barnhart
Iz_14_1209	DJI_0972	Dianthus campestris M.Bieb.	0	n	1	0	Caryophyllaceae Juss.
Iz_14_1209	DJI_0972	Digitaria sanguinalis (L.) Scop.	0	a	0	0	Poaceae Barnhart
Iz_14_1209	DJI_0972	Elymus repens (L.) Gould	0	n	1	0	Poaceae Barnhart
Iz_14_1209	DJI_0972	Epilobium tetragonum L.	0	n	1	0	Onagraceae Juss.
Iz_14_1209	DJI_0972	Eragrostis minor Host	0	a	0	0	Poaceae Barnhart
Iz_14_1209	DJI_0972	Erigeron annuus (L.) Desf.	0	a	0	0	Asteraceae Bercht. & J.Presl
Iz_14_1209	DJI_0972	Erigeron canadensis L.	4	a	0	0	Asteraceae Bercht. & J.Presl
Iz_14_1209	DJI_0972	Euphorbia seguieriana Neck.	0	n	1	0	Euphorbiaceae Juss.
Iz_14_1209	DJI_0972	Euphorbia sp.	0		NA	NA	
Iz_14_1209	DJI_0972	Fallopia dumetorum (L.) Holub	0	n	1	0	Polygonaceae Juss.
Iz_14_1209	DJI_0972	Festuca beckeri (Hack.) Trautv.	0	n	1	0	Poaceae Barnhart
Iz_14_1209	DJI_0972	Fragaria vesca L.	0	n	1	0	Rosaceae Juss.
Iz_14_1209	DJI_0972	Frangula alnus Mill.	0	n	1	1	Rhamnaceae Juss.
Iz_14_1209	DJI_0972	Fraxinus pennsylvanica Marshall	0	a	0	1	Oleaceae Hoffmanns. & Link
Iz_14_1209	DJI_0972	Galeopsis bifida Boenn.	0	n	1	0	Lamiacea Lindl.
Iz_14_1209	DJI_0972	Galium verum L.	0	n	1	0	Rubiaceae Juss.
Iz_14_1209	DJI_0972	Genista tinctoria L.	0	n	1	0	Fabaceae Lindl.
Iz_14_1209	DJI_0972	Geranium robertianum L.	0	n	1	0	Geraniaceae Juss.
Iz_14_1209	DJI_0972	Geranium sanquineum L.	0	n	1	0	Geraniaceae Juss.
Iz_14_1209	DJI_0972	Gypsophila paniculata L.	0	n	1	0	Caryophyllaceae Juss.
Iz_14_1209	DJI_0972	Helichrysum arenarium (L.) Moench	0	n	1	0	Asteraceae Bercht. & J.Presl
Iz_14_1209	DJI_0972	Herniaria polygama J.Gay	0	n	1	0	Caryophyllaceae Juss.
Iz_14_1209	DJI_0972	Hieracium robustum Fr.	0	n	1	0	Asteraceae Bercht. & J.Presl
Iz_14_1209	DJI_0972	Hieracium umbellatum L.	0	n	1	NA	Asteraceae Bercht. & J.Presl
Iz_14_1209	DJI_0972	Hylotelephium maximum (L.) Holub	0	n	1	0	Crassulaceae J.St.-Hil.
Iz_14_1209	DJI_0972	Jacobaea borysthenica (DC.) B.Nord. & Greuter	0	n	1	0	Asteraceae Bercht. & J.Presl
Iz_14_1209	DJI_0972	Jasione montana L.	0	n	1	0	Campanulaceae Juss.
Iz_14_1209	DJI_0972	Jurinea cyanoides (L.) Rchb.s.l.	0	n	1	0	Asteraceae Bercht. & J.Presl
Iz_14_1209	DJI_0972	Koeleria glauca (Spreng.) DC.	0	n	1	0	Poaceae Barnhart
Iz_14_1209	DJI_0972	Lactuca serriola L.	0	a	0	0	Asteraceae Bercht. & J.Presl
Iz_14_1209	DJI_0972	Linaria genistifolia (L.) Mill.	0	n	1	0	Plantaginaceae Juss.

Iz_14_1209	DJI_0972	Lomelosia argentea (L.) Greuter & Burdet	0	n	1	0	Caprifoliaceae Juss.
Iz_14_1209	DJI_0972	Melampyrum pratense L.	0	n	1	0	Orobanchaceae Vent.
Iz_14_1209	DJI_0972	Oenothera biennis L.	0	a	0	0	Onagraceae Juss.
Iz_14_1209	DJI_0972	Pentanema britannica (L.) D.Gut.Larr., Santos-Vicente, Anderb., E.Rico & M.M.Mart.Ort.	0	n	1	0	Asteraceae Bercht. & J.Presl
Iz_14_1209	DJI_0972	Peucedanum oreoselinum (L.) Moench	3	n	1	0	Apiaceae Lindl.
Iz_14_1209	DJI_0972	Peucedanum ruthenicum M.Bieb.	0	n	1	0	Apiaceae Lindl.
Iz_14_1209	DJI_0972	Pilosella echioides (Lumn.) F.W.Schultz & Sch.Bip.	0	n	1	0	Asteraceae Bercht. & J.Presl
Iz_14_1209	DJI_0972	Pilosella officinarum F. Schultz et Sch. Bip.	0	n	1	0	Asteraceae Bercht. & J.Presl
Iz_14_1209	DJI_0972	Pilosella sp.	0		NA	0	Asteraceae Bercht. & J.Presl
Iz_14_1209	DJI_0972	Pinus sylvestris L.	0	n	1	1	Pinaceae Spreng. ex F.Rudolphi
Iz_14_1209	DJI_0972	Poa annua L.	0	n	1	0	Poaceae Barnhart
Iz_14_1209	DJI_0972	Poa nemoralis L.	0	n	1	0	Poaceae Barnhart
Iz_14_1209	DJI_0972	Poa sp.	0		NA	0	Poaceae Barnhart
Iz_14_1209	DJI_0972	Polygonatum odoratum (Mill.) Druce	1	n	1	0	Asparagaceae Juss.
Iz_14_1209	DJI_0972	Polygonum arenarium Waldst. & Kit.	0	n	1	0	Polygonaceae Juss.
Iz_14_1209	DJI_0972	Populus nigra L.	0	n	1	1	Salicaceae Mirb.
Iz_14_1209	DJI_0972	Populus tremula L.	0	n	1	1	Salicaceae Mirb.
Iz_14_1209	DJI_0972	Populus Ã canescens (Aiton) Sm.	0	n	1	1	Salicaceae Mirb.
Iz_14_1209	DJI_0972	Potentilla argentea L.	0	n	1	0	Rosaceae Juss.
Iz_14_1209	DJI_0972	Psammophiliella muralis (L.) Ikonn.	0	n	1	0	Caryophyllaceae Juss.
Iz_14_1209	DJI_0972	Psephellus sumensis (Kalen.) Greuter	2	n	1	0	Asteraceae Bercht. & J.Presl
Iz_14_1209	DJI_0972	Pulsatilla pratensis (L.) Mill. s.l.	0	n	1	0	Ranunculaceae Juss.
Iz_14_1209	DJI_0972	Pyrus communis L.	0	a	0	1	Rosaceae Juss.
Iz_14_1209	DJI_0972	Quercus robur L.	0	n	1	1	Fagaceae Dumort.
Iz_14_1209	DJI_0972	Robinia pseudoacacia L.	0	a	0	1	Fabaceae Lindl.
Iz_14_1209	DJI_0972	Rosa sp.	0		NA	1	Rosaceae Juss.
Iz_14_1209	DJI_0972	Rubus idaeus L.	0	n	1	0	Rosaceae Juss.
Iz_14_1209	DJI_0972	Rumex acetosella L.	0	n	1	0	Polygonaceae Juss.
Iz_14_1209	DJI_0972	Rumex thyrsiflorus Fingerh.	0	n	1	0	Polygonaceae Juss.
Iz_14_1209	DJI_0972	Salix alba L.	0	n	1	1	Salicaceae Mirb.
Iz_14_1209	DJI_0972	Sambucus nigra L.	0	n	1	1	Viburnaceae Raf.
Iz_14_1209	DJI_0972	Secale sylvestre Host	0	n	1	0	Poaceae Barnhart
Iz_14_1209	DJI_0972	Sempervivum ruthenicum Schnittsp. & C.B. Lehm.	0	n	1	0	Crassulaceae J.St.-Hil.
Iz_14_1209	DJI_0972	Setaria viridis (L.) P. Beauv.	0	n	1	0	Poaceae Barnhart
Iz_14_1209	DJI_0972	Silene borysthenica (Gruner) Walters	0	n	1	0	Caryophyllaceae Juss.
Iz_14_1209	DJI_0972	Silene latifolia subsp. alba (Mill.) Greuter & Burdet	0	n	1	0	Caryophyllaceae Juss.
Iz_14_1209	DJI_0972	Solidago virgaurea L.	0	n	1	0	Asteraceae Bercht. & J.Presl
Iz_14_1209	DJI_0972	Sorbus aucuparia L.	0	n	1	1	Rosaceae Juss.
Iz_14_1209	DJI_0972	Stachys recta L.	0	n	1	0	Lamiacea Lindl.
Iz_14_1209	DJI_0972	Tanacetum vulgare L.	0	n	1	0	Asteraceae Bercht. & J.Presl
Iz_14_1209	DJI_0972	Thymus pallasianus Heinr.Braun	0	n	1	0	Lamiacea Lindl.
Iz_14_1209	DJI_0972	Tilia cordata Mill.	0	n	1	1	Malvaceae Juss.
Iz_14_1209	DJI_0972	Trifolium alpestre L.	0	n	1	0	Fabaceae Lindl.
Iz_14_1209	DJI_0972	Trifolium arvense L.	0	n	1	0	Fabaceae Lindl.
Iz_14_1209	DJI_0972	Ulmus glabra Huds.	1	n	1	1	Ulmaceae Mirb.
Iz_14_1209	DJI_0972	Verbascum densiflorum Bertol.	0	n	1	0	Scrophulariaceae Juss.
Iz_14_1209	DJI_0972	Veronica austriaca L.	0	n	1	0	Plantaginaceae Juss.
Iz_14_1209	DJI_0972	Veronica incana L.	0	n	1	0	Plantaginaceae Juss.
Iz_14_1209	DJI_0972	Veronica verna L.	0	n	1	0	Plantaginaceae Juss.
Iz_14_1209	DJI_0972	Vincetoxicum hirundinaria Medik	0	n	1	0	Apocynaceae Juss.
Iz_14_1209	DJI_0976	Acer tataricum L.	0	n	1	1	Sapindaceae Juss.
Iz_14_1209	DJI_0976	Achillea millefolium L. s.l.	0	n	1	0	Asteraceae Bercht. & J.Presl
Iz_14_1209	DJI_0976	Allium oleraceum L.	0	n	1	0	Amaryllidaceae J.St.-Hil.
Iz_14_1209	DJI_0976	Ambrosia artemisiifolia L.	0	a	0	0	Asteraceae Bercht. & J.Presl
Iz_14_1209	DJI_0976	Amorpha fruticosa L.	0	a	0	0	Fabaceae Lindl.
Iz_14_1209	DJI_0976	Anthericum ramosum L.	0	n	1	0	Asparagaceae Juss.
Iz_14_1209	DJI_0976	Artemisia marschalliana Spreng.	0	n	1	0	Asteraceae Bercht. & J.Presl
Iz_14_1209	DJI_0976	Asparagus officinalis L.	0	n	1	0	Asparagaceae Juss.
Iz_14_1209	DJI_0976	Astragalus cicer L.	0	n	1	0	Fabaceae Lindl.
Iz_14_1209	DJI_0976	Bassia laniflora (S.G.Gmel.) A.J.Scott	0	n	1	0	Amaranthaceae Juss.
Iz_14_1209	DJI_0976	Berberis vulgaris L.	0	n	1	0	Berberidaceae Juss.
Iz_14_1209	DJI_0976	Betula pendula Roth.	0	n	1	1	Betulaceae Gray
Iz_14_1209	DJI_0976	Betula pendula Roth.	0	n	1	0	Poaceae Barnhart
Iz_14_1209	DJI_0976	Bromus inermis Leyss.	0		NA	NA	
Iz_14_1209	DJI_0976	Bromus squarrosus L.	0	a	0	0	Poaceae Barnhart
Iz_14_1209	DJI_0976	Bromus tectorum L.	0	a	0	0	Poaceae Barnhart
Iz_14_1209	DJI_0976	Calamagrostis epigejos (L.) Roth	60	n	1	0	Poaceae Barnhart
Iz_14_1209	DJI_0976	Carduus acanthoides L.	0	n	1	0	Asteraceae Bercht. & J.Presl
Iz_14_1209	DJI_0976	Carex sp.	0	n	1	0	Cyperaceae Juss.
Iz_14_1209	DJI_0976	Centaurea majorovii Dumbadze	0	n	1	0	Asteraceae Bercht. & J.Presl
Iz_14_1209	DJI_0976	Chamaecytisus ruthenicus (Fisch.ex Wol.) Klaskova	0	n	1	0	Fabaceae Lindl.
Iz_14_1209	DJI_0976	Chelidonium majus L.	0	n	1	0	Papaveraceae Juss.
Iz_14_1209	DJI_0976	Chenopodium betaceum Andrz.	0	n	1	0	Amaranthaceae Juss.
Iz_14_1209	DJI_0976	Chenopodium cf. album	0	n	1	0	Amaranthaceae Juss.
Iz_14_1209	DJI_0976	Chenopodium sp.	0		NA	0	Amaranthaceae Juss.
Iz_14_1209	DJI_0976	Chondrilla juncea L.	0	n	1	0	Asteraceae Bercht. & J.Presl
Iz_14_1209	DJI_0976	Cirsium vulgare (Savi) Ten.	0	n	1	0	Asteraceae Bercht. & J.Presl
Iz_14_1209	DJI_0976	Clinopodium acinos (L.) Kuntze	0	n	1	0	Lamiacea Lindl.
Iz_14_1209	DJI_0976	Convallaria majalis L.	0	n	1	0	Asparagaceae Juss.
Iz_14_1209	DJI_0976	Crataegus rhipidophylla Gand.	0	n	1	1	Rosaceae Juss.
Iz_14_1209	DJI_0976	Crepis foetida L.	0	n	1	0	Asteraceae Bercht. & J.Presl
Iz_14_1209	DJI_0976	Dactylis glomerata L.	0	n	1	0	Poaceae Barnhart
Iz_14_1209	DJI_0976	Dianthus campestris M.Bieb.	0	n	1	0	Caryophyllaceae Juss.
Iz_14_1209	DJI_0976	Digitaria sanguinalis (L.) Scop.	0	a	0	0	Poaceae Barnhart
Iz_14_1209	DJI_0976	Elymus repens (L.) Gould	0	n	1	0	Poaceae Barnhart
Iz_14_1209	DJI_0976	Epilobium tetragonum L.	0	n	1	0	Onagraceae Juss.
Iz_14_1209	DJI_0976	Eragrostis minor Host	0	a	0	0	Poaceae Barnhart
Iz_14_1209	DJI_0976	Erigeron annuus (L.) Desf.	0	a	0	0	Asteraceae Bercht. & J.Presl
Iz_14_1209	DJI_0976	Erigeron canadensis L.	0	a	0	0	Asteraceae Bercht. & J.Presl
Iz_14_1209	DJI_0976	Euphorbia seguieriana Neck.	0	n	1	0	Euphorbiaceae Juss.
Iz_14_1209	DJI_0976	Euphorbia sp.	0		NA	NA	
Iz_14_1209	DJI_0976	Fallopia dumetorum (L.) Holub	3	n	1	0	Polygonaceae Juss.
Iz_14_1209	DJI_0976	Festuca beckeri (Hack.) Trautv.	0	n	1	0	Poaceae Barnhart
Iz_14_1209	DJI_0976	Fragaria vesca L.	0	n	1	0	Rosaceae Juss.
Iz_14_1209	DJI_0976	Frangula alnus Mill.	0	n	1	1	Rhamnaceae Juss.
Iz_14_1209	DJI_0976	Fraxinus pennsylvanica Marshall	0	a	0	1	Oleaceae Hoffmanns. & Link
Iz_14_1209	DJI_0976	Galeopsis bifida Boenn.	0	n	1	0	Lamiacea Lindl.
Iz_14_1209	DJI_0976	Galium verum L.	0	n	1	0	Rubiaceae Juss.

Iz_14_1209	DJI_0976	Genista tinctoria L.	0	n	1	0	Fabaceae Lindl.
Iz_14_1209	DJI_0976	Geranium robertianum L.	0	n	1	0	Geraniaceae Juss.
Iz_14_1209	DJI_0976	Geranium sanquineum L.	0	n	1	0	Geraniaceae Juss.
Iz_14_1209	DJI_0976	Gypsophila paniculata L.	0	n	1	0	Caryophyllaceae Juss.
Iz_14_1209	DJI_0976	Helichrysum arenarium (L.) Moench	0	n	1	0	Asteraceae Bercht. & J.Presl
Iz_14_1209	DJI_0976	Herniaria polygama J.Gay	0	n	1	0	Caryophyllaceae Juss.
Iz_14_1209	DJI_0976	Hieracium robustum Fr.	0	n	1	0	Asteraceae Bercht. & J.Presl
Iz_14_1209	DJI_0976	Hieracium umbellatum L.	0	n	1	NA	Asteraceae Bercht. & J.Presl
Iz_14_1209	DJI_0976	Hylotelephium maximum (L.) Holub	0	n	1	0	Crassulaceae J.St.-Hil.
Iz_14_1209	DJI_0976	Jacobaea borysthenica (DC.) B.Nord. & Greuter	0	n	1	0	Asteraceae Bercht. & J.Presl
Iz_14_1209	DJI_0976	Jasione montana L.	0	n	1	0	Campanulaceae Juss.
Iz_14_1209	DJI_0976	Jurinea cyanoides (L.) Rchb.s.l.	0	n	1	0	Asteraceae Bercht. & J.Presl
Iz_14_1209	DJI_0976	Koeleria glauca (Spreng.) DC.	0	n	1	0	Poaceae Barnhart
Iz_14_1209	DJI_0976	Lactuca serriola L.	0	a	0	0	Asteraceae Bercht. & J.Presl
Iz_14_1209	DJI_0976	Linaria genistifolia (L.) Mill.	0	n	1	0	Plantaginaceae Juss.
Iz_14_1209	DJI_0976	Lomelosia argentea (L.) Greuter & Burdet	0	n	1	0	Caprifoliaceae Juss.
Iz_14_1209	DJI_0976	Melampyrum pratense L.	0	n	1	0	Orobanchaceae Vent.
Iz_14_1209	DJI_0976	Oenothera biennis L.	0	a	0	0	Onagraceae Juss.
Iz_14_1209	DJI_0976	Pentanema britannica (L.) D.Gut.Larr., Santos-Vicente, Anderb., E.Rico & M.M.Mart.Ort.	0	n	1	0	Asteraceae Bercht. & J.Presl
Iz_14_1209	DJI_0976	Peucedanum oreoselinum (L.) Moench	0	n	1	0	Apiaceae Lindl.
Iz_14_1209	DJI_0976	Peucedanum ruthenicum M.Bieb.	0	n	1	0	Apiaceae Lindl.
Iz_14_1209	DJI_0976	Pilosella echioides (Lumn.) F.W.Schultz & Sch.Bip.	0	n	1	0	Asteraceae Bercht. & J.Presl
Iz_14_1209	DJI_0976	Pilosella officinarum F. Schultz et Sch. Bip.	0	n	1	0	Asteraceae Bercht. & J.Presl
Iz_14_1209	DJI_0976	Pilosella sp.	0		NA	0	Asteraceae Bercht. & J.Presl
Iz_14_1209	DJI_0976	Pinus sylvestris L.	0	n	1	1	Pinaceae Spreng. ex F.Rudolphi
Iz_14_1209	DJI_0976	Poa annua L.	0	n	1	0	Poaceae Barnhart
Iz_14_1209	DJI_0976	Poa nemoralis L.	0	n	1	0	Poaceae Barnhart
Iz_14_1209	DJI_0976	Poa sp.	0		NA	0	Poaceae Barnhart
Iz_14_1209	DJI_0976	Polygonatum odoratum (Mill.) Druce	0	n	1	0	Asparagaceae Juss.
Iz_14_1209	DJI_0976	Polygonum arenarium Waldst. & Kit.	0	n	1	0	Polygonaceae Juss.
Iz_14_1209	DJI_0976	Populus nigra L.	0	n	1	1	Salicaceae Mirb.
Iz_14_1209	DJI_0976	Populus tremula L.	0	n	1	1	Salicaceae Mirb.
Iz_14_1209	DJI_0976	Populus Ã canescens (Aiton) Sm.	0	n	1	1	Salicaceae Mirb.
Iz_14_1209	DJI_0976	Potentilla argentea L.	0	n	1	0	Rosaceae Juss.
Iz_14_1209	DJI_0976	Psammophiliella muralis (L.) Ikonn.	0	n	1	0	Caryophyllaceae Juss.
Iz_14_1209	DJI_0976	Psephellus sumensis (Kalen.) Greuter	0	n	1	0	Asteraceae Bercht. & J.Presl
Iz_14_1209	DJI_0976	Pulsatilla pratensis (L.) Mill. s.l.	0	n	1	0	Ranunculaceae Juss.
Iz_14_1209	DJI_0976	Pyrus communis L.	0	a	0	1	Rosaceae Juss.
Iz_14_1209	DJI_0976	Quercus robur L.	0	n	1	1	Fagaceae Dumort.
Iz_14_1209	DJI_0976	Robinia pseudoacacia L.	0	a	0	1	Fabaceae Lindl.
Iz_14_1209	DJI_0976	Rosa sp.	0		NA	1	Rosaceae Juss.
Iz_14_1209	DJI_0976	Rubus idaeus L.	0	n	1	0	Rosaceae Juss.
Iz_14_1209	DJI_0976	Rumex acetosella L.	0	n	1	0	Polygonaceae Juss.
Iz_14_1209	DJI_0976	Rumex thyrsiflorus Fingerh.	0	n	1	0	Polygonaceae Juss.
Iz_14_1209	DJI_0976	Salix alba L.	0	n	1	1	Salicaceae Mirb.
Iz_14_1209	DJI_0976	Sambucus nigra L.	0	n	1	1	Viburnaceae Raf.
Iz_14_1209	DJI_0976	Secale sylvestre Host	0	n	1	0	Poaceae Barnhart
Iz_14_1209	DJI_0976	Sempervivum ruthenicum Schnittsp. & C.B. Lehm.	0	n	1	0	Crassulaceae J.St.-Hil.
Iz_14_1209	DJI_0976	Setaria viridis (L.) P. Beauv.	0	n	1	0	Poaceae Barnhart
Iz_14_1209	DJI_0976	Silene borysthenica (Gruner) Walters	0	n	1	0	Caryophyllaceae Juss.
Iz_14_1209	DJI_0976	Silene latifolia subsp. alba (Mill.) Greuter & Burdet	0	n	1	0	Caryophyllaceae Juss.
Iz_14_1209	DJI_0976	Solidago virgaurea L.	0	n	1	0	Asteraceae Bercht. & J.Presl
Iz_14_1209	DJI_0976	Sorbus aucuparia L.	0	n	1	1	Rosaceae Juss.
Iz_14_1209	DJI_0976	Stachys recta L.	0	n	1	0	Lamiacea Lindl.
Iz_14_1209	DJI_0976	Tanacetum vulgare L.	0	n	1	0	Asteraceae Bercht. & J.Presl
Iz_14_1209	DJI_0976	Thymus pallasianus Heinr.Braun	0	n	1	0	Lamiacea Lindl.
Iz_14_1209	DJI_0976	Tilia cordata Mill.	0	n	1	1	Malvaceae Juss.
Iz_14_1209	DJI_0976	Trifolium alpestre L.	0	n	1	0	Fabaceae Lindl.
Iz_14_1209	DJI_0976	Trifolium arvense L.	0	n	1	0	Fabaceae Lindl.
Iz_14_1209	DJI_0976	Ulmus glabra Huds.	0	n	1	1	Ulmaceae Mirb.
Iz_14_1209	DJI_0976	Verbascum densiflorum Bertol.	0	n	1	0	Scrophulariaceae Juss.
Iz_14_1209	DJI_0976	Veronica austriaca L.	0	n	1	0	Plantaginaceae Juss.
Iz_14_1209	DJI_0976	Veronica incana L.	0	n	1	0	Plantaginaceae Juss.
Iz_14_1209	DJI_0976	Veronica verna L.	0	n	1	0	Plantaginaceae Juss.
Iz_14_1209	DJI_0976	Vincetoxicum hirundinaria Medik	0	n	1	0	Apocynaceae Juss.
Iz_14_1209	DJI_0979	Acer tataricum L.	0	n	1	1	Sapindaceae Juss.
Iz_14_1209	DJI_0979	Achillea millefolium L. s.l.	0	n	1	0	Asteraceae Bercht. & J.Presl
Iz_14_1209	DJI_0979	Allium oleraceum L.	0	n	1	0	Amaryllidaceae J.St.-Hil.
Iz_14_1209	DJI_0979	Ambrosia artemisiifolia L.	0	a	0	0	Asteraceae Bercht. & J.Presl
Iz_14_1209	DJI_0979	Amorpha fruticosa L.	0	a	0	0	Fabaceae Lindl.
Iz_14_1209	DJI_0979	Anthericum ramosum L.	0	n	1	0	Asparagaceae Juss.
Iz_14_1209	DJI_0979	Artemisia marschalliana Spreng.	0	n	1	0	Asteraceae Bercht. & J.Presl
Iz_14_1209	DJI_0979	Asparagus officinalis L.	0	n	1	0	Asparagaceae Juss.
Iz_14_1209	DJI_0979	Astragalus cicer L.	0	n	1	0	Fabaceae Lindl.
Iz_14_1209	DJI_0979	Bassia laniflora (S.G.Gmel.) A.J.Scott	0	n	1	0	Amaranthaceae Juss.
Iz_14_1209	DJI_0979	Berberis vulgaris L.	0	n	1	0	Berberidaceae Juss.
Iz_14_1209	DJI_0979	Betula pendula Roth.	0	n	1	1	Betulaceae Gray
Iz_14_1209	DJI_0979	Betula pendula Roth.	0	n	1	0	Poaceae Barnhart
Iz_14_1209	DJI_0979	Bromus inermis Leyss.	0		NA	NA	
Iz_14_1209	DJI_0979	Bromus squarrosus L.	0	a	0	0	Poaceae Barnhart
Iz_14_1209	DJI_0979	Bromus tectorum L.	0	a	0	0	Poaceae Barnhart
Iz_14_1209	DJI_0979	Calamagrostis epigejos (L.) Roth	50	n	1	0	Poaceae Barnhart
Iz_14_1209	DJI_0979	Carduus acanthoides L.	0	n	1	0	Asteraceae Bercht. & J.Presl
Iz_14_1209	DJI_0979	Carex sp.	0	n	1	0	Cyperaceae Juss.
Iz_14_1209	DJI_0979	Centaurea majorovii Dumbadze	0	n	1	0	Asteraceae Bercht. & J.Presl
Iz_14_1209	DJI_0979	Chamaecytisus ruthenicus (Fisch.ex Wol.) Klaskova	0	n	1	0	Fabaceae Lindl.
Iz_14_1209	DJI_0979	Chelidonium majus L.	2	n	1	0	Papaveraceae Juss.
Iz_14_1209	DJI_0979	Chenopodium betaceum Andrz.	0	n	1	0	Amaranthaceae Juss.
Iz_14_1209	DJI_0979	Chenopodium cf. album	0	n	1	0	Amaranthaceae Juss.
Iz_14_1209	DJI_0979	Chenopodium sp.	3		NA	0	Amaranthaceae Juss.
Iz_14_1209	DJI_0979	Chondrilla juncea L.	0	n	1	0	Asteraceae Bercht. & J.Presl
Iz_14_1209	DJI_0979	Cirsium vulgare (Savi) Ten.	0	n	1	0	Asteraceae Bercht. & J.Presl
Iz_14_1209	DJI_0979	Clinopodium acinos (L.) Kuntze	0	n	1	0	Lamiacea Lindl.
Iz_14_1209	DJI_0979	Convallaria majalis L.	0	n	1	0	Asparagaceae Juss.
Iz_14_1209	DJI_0979	Crataegus rhipidophylla Gand.	0	n	1	1	Rosaceae Juss.
Iz_14_1209	DJI_0979	Crepis foetida L.	0	n	1	0	Asteraceae Bercht. & J.Presl
Iz_14_1209	DJI_0979	Dactylis glomerata L.	0	n	1	0	Poaceae Barnhart
Iz_14_1209	DJI_0979	Dianthus campestris M.Bieb.	0	n	1	0	Caryophyllaceae Juss.

Iz_14_1209	DJI_0979	Digitaria sanguinalis (L.) Scop.	0	a	0	0	Poaceae Barnhart
Iz_14_1209	DJI_0979	Elymus repens (L.) Gould	0	n	1	0	Poaceae Barnhart
Iz_14_1209	DJI_0979	Epilobium tetragonum L.	0	n	1	0	Onagraceae Juss.
Iz_14_1209	DJI_0979	Eragrostis minor Host	0	a	0	0	Poaceae Barnhart
Iz_14_1209	DJI_0979	Erigeron annuus (L.) Desf.	0	a	0	0	Asteraceae Bercht. & J.Presl
Iz_14_1209	DJI_0979	Erigeron canadensis L.	5	a	0	0	Asteraceae Bercht. & J.Presl
Iz_14_1209	DJI_0979	Euphorbia seguieriana Neck.	0	n	1	0	Euphorbiaceae Juss.
Iz_14_1209	DJI_0979	Euphorbia sp.	0		NA	NA	
Iz_14_1209	DJI_0979	Fallopia dumetorum (L.) Holub	0	n	1	0	Polygonaceae Juss.
Iz_14_1209	DJI_0979	Festuca beckeri (Hack.) Trautv.	0	n	1	0	Poaceae Barnhart
Iz_14_1209	DJI_0979	Fragaria vesca L.	0	n	1	0	Rosaceae Juss.
Iz_14_1209	DJI_0979	Frangula alnus Mill.	0	n	1	1	Rhamnaceae Juss.
Iz_14_1209	DJI_0979	Fraxinus pennsylvanica Marshall	0	a	0	1	Oleaceae Hoffmanns. & Link
Iz_14_1209	DJI_0979	Galeopsis bifida Boenn.	0	n	1	0	Lamiacea Lindl.
Iz_14_1209	DJI_0979	Galium verum L.	0	n	1	0	Rubiaceae Juss.
Iz_14_1209	DJI_0979	Genista tinctoria L.	0	n	1	0	Fabaceae Lindl.
Iz_14_1209	DJI_0979	Geranium robertianum L.	0	n	1	0	Geraniaceae Juss.
Iz_14_1209	DJI_0979	Geranium sanquineum L.	0	n	1	0	Geraniaceae Juss.
Iz_14_1209	DJI_0979	Gypsophila paniculata L.	0	n	1	0	Caryophyllaceae Juss.
Iz_14_1209	DJI_0979	Helichrysum arenarium (L.) Moench	0	n	1	0	Asteraceae Bercht. & J.Presl
Iz_14_1209	DJI_0979	Herniaria polygama J.Gay	0	n	1	0	Caryophyllaceae Juss.
Iz_14_1209	DJI_0979	Hieracium robustum Fr.	0	n	1	0	Asteraceae Bercht. & J.Presl
Iz_14_1209	DJI_0979	Hieracium umbellatum L.	0	n	1	NA	Asteraceae Bercht. & J.Presl
Iz_14_1209	DJI_0979	Hylotelephium maximum (L.) Holub	0	n	1	0	Crassulaceae J.St.-Hil.
Iz_14_1209	DJI_0979	Jacobaea borysthenica (DC.) B.Nord. & Greuter	0	n	1	0	Asteraceae Bercht. & J.Presl
Iz_14_1209	DJI_0979	Jasione montana L.	0	n	1	0	Campanulaceae Juss.
Iz_14_1209	DJI_0979	Jurinea cyanoides (L.) Rchb.s.l.	0	n	1	0	Asteraceae Bercht. & J.Presl
Iz_14_1209	DJI_0979	Koeleria glauca (Spreng.) DC.	0	n	1	0	Poaceae Barnhart
Iz_14_1209	DJI_0979	Lactuca serriola L.	0	a	0	0	Asteraceae Bercht. & J.Presl
Iz_14_1209	DJI_0979	Linaria genistifolia (L.) Mill.	0	n	1	0	Plantaginaceae Juss.
Iz_14_1209	DJI_0979	Lomelosia argentea (L.) Greuter & Burdet	0	n	1	0	Caprifoliaceae Juss.
Iz_14_1209	DJI_0979	Melampyrum pratense L.	0	n	1	0	Orobanchaceae Vent.
Iz_14_1209	DJI_0979	Oenothera biennis L.	0	a	0	0	Onagraceae Juss.
Iz_14_1209	DJI_0979	Pentanema britannica (L.) D.Gut.Larr., Santos-Vicente, Anderb., E.Rico & M.M.Mart.Ort.	0	n	1	0	Asteraceae Bercht. & J.Presl
Iz_14_1209	DJI_0979	Peucedanum oreoselinum (L.) Moench	0	n	1	0	Apiaceae Lindl.
Iz_14_1209	DJI_0979	Peucedanum ruthenicum M.Bieb.	0	n	1	0	Apiaceae Lindl.
Iz_14_1209	DJI_0979	Pilosella echioides (Lumn.) F.W.Schultz & Sch.Bip.	0	n	1	0	Asteraceae Bercht. & J.Presl
Iz_14_1209	DJI_0979	Pilosella officinarum F. Schultz et Sch. Bip.	0	n	1	0	Asteraceae Bercht. & J.Presl
Iz_14_1209	DJI_0979	Pilosella sp.	0		NA	0	Asteraceae Bercht. & J.Presl
Iz_14_1209	DJI_0979	Pinus sylvestris L.	0	n	1	1	Pinaceae Spreng. ex F.Rudolphi
Iz_14_1209	DJI_0979	Poa annua L.	0	n	1	0	Poaceae Barnhart
Iz_14_1209	DJI_0979	Poa nemoralis L.	0	n	1	0	Poaceae Barnhart
Iz_14_1209	DJI_0979	Poa sp.	0		NA	0	Poaceae Barnhart
Iz_14_1209	DJI_0979	Polygonatum odoratum (Mill.) Druce	0	n	1	0	Asparagaceae Juss.
Iz_14_1209	DJI_0979	Polygonum arenarium Waldst. & Kit.	0	n	1	0	Polygonaceae Juss.
Iz_14_1209	DJI_0979	Populus nigra L.	0	n	1	1	Salicaceae Mirb.
Iz_14_1209	DJI_0979	Populus tremula L.	0	n	1	1	Salicaceae Mirb.
Iz_14_1209	DJI_0979	Populus Ã canescens (Aiton) Sm.	0	n	1	1	Salicaceae Mirb.
Iz_14_1209	DJI_0979	Potentilla argentea L.	0	n	1	0	Rosaceae Juss.
Iz_14_1209	DJI_0979	Psammophiliella muralis (L.) Ikonn.	0	n	1	0	Caryophyllaceae Juss.
Iz_14_1209	DJI_0979	Psephellus sumensis (Kalen.) Greuter	0	n	1	0	Asteraceae Bercht. & J.Presl
Iz_14_1209	DJI_0979	Pulsatilla pratensis (L.) Mill. s.l.	0	n	1	0	Ranunculaceae Juss.
Iz_14_1209	DJI_0979	Pyrus communis L.	0	a	0	1	Rosaceae Juss.
Iz_14_1209	DJI_0979	Quercus robur L.	0	n	1	1	Fagaceae Dumort.
Iz_14_1209	DJI_0979	Robinia pseudoacacia L.	0	a	0	1	Fabaceae Lindl.
Iz_14_1209	DJI_0979	Rosa sp.	0		NA	1	Rosaceae Juss.
Iz_14_1209	DJI_0979	Rubus idaeus L.	0	n	1	0	Rosaceae Juss.
Iz_14_1209	DJI_0979	Rumex acetosella L.	0	n	1	0	Polygonaceae Juss.
Iz_14_1209	DJI_0979	Rumex thyrsiflorus Fingerh.	0	n	1	0	Polygonaceae Juss.
Iz_14_1209	DJI_0979	Salix alba L.	0	n	1	1	Salicaceae Mirb.
Iz_14_1209	DJI_0979	Sambucus nigra L.	0	n	1	1	Viburnaceae Raf.
Iz_14_1209	DJI_0979	Secale sylvestre Host	0	n	1	0	Poaceae Barnhart
Iz_14_1209	DJI_0979	Sempervivum ruthenicum Schnittsp. & C.B. Lehm.	0	n	1	0	Crassulaceae J.St.-Hil.
Iz_14_1209	DJI_0979	Setaria viridis (L.) P. Beauv.	0	n	1	0	Poaceae Barnhart
Iz_14_1209	DJI_0979	Silene borysthenica (Gruner) Walters	0	n	1	0	Caryophyllaceae Juss.
Iz_14_1209	DJI_0979	Silene latifolia subsp. alba (Mill.) Greuter & Burdet	0	n	1	0	Caryophyllaceae Juss.
Iz_14_1209	DJI_0979	Solidago virgaurea L.	0	n	1	0	Asteraceae Bercht. & J.Presl
Iz_14_1209	DJI_0979	Sorbus aucuparia L.	0	n	1	1	Rosaceae Juss.
Iz_14_1209	DJI_0979	Stachys recta L.	0	n	1	0	Lamiacea Lindl.
Iz_14_1209	DJI_0979	Tanacetum vulgare L.	0	n	1	0	Asteraceae Bercht. & J.Presl
Iz_14_1209	DJI_0979	Thymus pallasianus Heinr.Braun	0	n	1	0	Lamiacea Lindl.
Iz_14_1209	DJI_0979	Tilia cordata Mill.	0	n	1	1	Malvaceae Juss.
Iz_14_1209	DJI_0979	Trifolium alpestre L.	0	n	1	0	Fabaceae Lindl.
Iz_14_1209	DJI_0979	Trifolium arvense L.	0	n	1	0	Fabaceae Lindl.
Iz_14_1209	DJI_0979	Ulmus glabra Huds.	0	n	1	1	Ulmaceae Mirb.
Iz_14_1209	DJI_0979	Verbascum densiflorum Bertol.	0	n	1	0	Scrophulariaceae Juss.
Iz_14_1209	DJI_0979	Veronica austriaca L.	0	n	1	0	Plantaginaceae Juss.
Iz_14_1209	DJI_0979	Veronica incana L.	0	n	1	0	Plantaginaceae Juss.
Iz_14_1209	DJI_0979	Veronica verna L.	0	n	1	0	Plantaginaceae Juss.
Iz_14_1209	DJI_0979	Vincetoxicum hirundinaria Medik	0	n	1	0	Apocynaceae Juss.
Iz_14_1209	DJI_0982	Acer tataricum L.	0	n	1	1	Sapindaceae Juss.
Iz_14_1209	DJI_0982	Achillea millefolium L. s.l.	0	n	1	0	Asteraceae Bercht. & J.Presl
Iz_14_1209	DJI_0982	Allium oleraceum L.	0	n	1	0	Amaryllidaceae J.St.-Hil.
Iz_14_1209	DJI_0982	Ambrosia artemisiifolia L.	0	a	0	0	Asteraceae Bercht. & J.Presl
Iz_14_1209	DJI_0982	Amorpha fruticosa L.	0	a	0	0	Fabaceae Lindl.
Iz_14_1209	DJI_0982	Anthericum ramosum L.	0	n	1	0	Asparagaceae Juss.
Iz_14_1209	DJI_0982	Artemisia marschalliana Spreng.	0	n	1	0	Asteraceae Bercht. & J.Presl
Iz_14_1209	DJI_0982	Asparagus officinalis L.	0	n	1	0	Asparagaceae Juss.
Iz_14_1209	DJI_0982	Astragalus cicer L.	0	n	1	0	Fabaceae Lindl.
Iz_14_1209	DJI_0982	Bassia laniflora (S.G.Gmel.) A.J.Scott	0	n	1	0	Amaranthaceae Juss.
Iz_14_1209	DJI_0982	Berberis vulgaris L.	0	n	1	0	Berberidaceae Juss.
Iz_14_1209	DJI_0982	Betula pendula Roth.	0	n	1	1	Betulaceae Gray
Iz_14_1209	DJI_0982	Betula pendula Roth.	0	n	1	0	Poaceae Barnhart
Iz_14_1209	DJI_0982	Bromus inermis Leyss.	0		NA	NA	
Iz_14_1209	DJI_0982	Bromus squarrosus L.	0	a	0	0	Poaceae Barnhart
Iz_14_1209	DJI_0982	Bromus tectorum L.	0	a	0	0	Poaceae Barnhart
Iz_14_1209	DJI_0982	Calamagrostis epigejos (L.) Roth	85	n	1	0	Poaceae Barnhart
Iz_14_1209	DJI_0982	Carduus acanthoides L.	0	n	1	0	Asteraceae Bercht. & J.Presl

Iz_14_1209	DJI_0982	Carex sp.	0	n	1	0	Cyperaceae Juss.
Iz_14_1209	DJI_0982	Centaurea majorovii Dumbadze	0	n	1	0	Asteraceae Bercht. & J.Presl
Iz_14_1209	DJI_0982	Chamaecytisus ruthenicus (Fisch.ex Wol.) Klaskova	0	n	1	0	Fabaceae Lindl.
Iz_14_1209	DJI_0982	Chelidonium majus L.	0	n	1	0	Papaveraceae Juss.
Iz_14_1209	DJI_0982	Chenopodium betaceum Andrz.	0	n	1	0	Amaranthaceae Juss.
Iz_14_1209	DJI_0982	Chenopodium cf. album	0	n	1	0	Amaranthaceae Juss.
Iz_14_1209	DJI_0982	Chenopodium sp.	0		NA	0	Amaranthaceae Juss.
Iz_14_1209	DJI_0982	Chondrilla juncea L.	0	n	1	0	Asteraceae Bercht. & J.Presl
Iz_14_1209	DJI_0982	Cirsium vulgare (Savi) Ten.	0	n	1	0	Asteraceae Bercht. & J.Presl
Iz_14_1209	DJI_0982	Clinopodium acinos (L.) Kuntze	0	n	1	0	Lamiacea Lindl.
Iz_14_1209	DJI_0982	Convallaria majalis L.	0	n	1	0	Asparagaceae Juss.
Iz_14_1209	DJI_0982	Crataegus rhipidophylla Gand.	0	n	1	1	Rosaceae Juss.
Iz_14_1209	DJI_0982	Crepis foetida L.	0	n	1	0	Asteraceae Bercht. & J.Presl
Iz_14_1209	DJI_0982	Dactylis glomerata L.	0	n	1	0	Poaceae Barnhart
Iz_14_1209	DJI_0982	Dianthus campestris M.Bieb.	0	n	1	0	Caryophyllaceae Juss.
Iz_14_1209	DJI_0982	Digitaria sanguinalis (L.) Scop.	0	a	0	0	Poaceae Barnhart
Iz_14_1209	DJI_0982	Elymus repens (L.) Gould	0	n	1	0	Poaceae Barnhart
Iz_14_1209	DJI_0982	Epilobium tetragonum L.	0	n	1	0	Onagraceae Juss.
Iz_14_1209	DJI_0982	Eragrostis minor Host	0	a	0	0	Poaceae Barnhart
Iz_14_1209	DJI_0982	Erigeron annuus (L.) Desf.	0	a	0	0	Asteraceae Bercht. & J.Presl
Iz_14_1209	DJI_0982	Erigeron canadensis L.	0	a	0	0	Asteraceae Bercht. & J.Presl
Iz_14_1209	DJI_0982	Euphorbia seguieriana Neck.	0	n	1	0	Euphorbiaceae Juss.
Iz_14_1209	DJI_0982	Euphorbia sp.	0		NA	NA	
Iz_14_1209	DJI_0982	Fallopia dumetorum (L.) Holub	5	n	1	0	Polygonaceae Juss.
Iz_14_1209	DJI_0982	Festuca beckeri (Hack.) Trautv.	0	n	1	0	Poaceae Barnhart
Iz_14_1209	DJI_0982	Fragaria vesca L.	0	n	1	0	Rosaceae Juss.
Iz_14_1209	DJI_0982	Frangula alnus Mill.	0	n	1	1	Rhamnaceae Juss.
Iz_14_1209	DJI_0982	Fraxinus pennsylvanica Marshall	0	a	0	1	Oleaceae Hoffmanns. & Link
Iz_14_1209	DJI_0982	Galeopsis bifida Boenn.	0	n	1	0	Lamiacea Lindl.
Iz_14_1209	DJI_0982	Galium verum L.	0	n	1	0	Rubiaceae Juss.
Iz_14_1209	DJI_0982	Genista tinctoria L.	0	n	1	0	Fabaceae Lindl.
Iz_14_1209	DJI_0982	Geranium robertianum L.	0	n	1	0	Geraniaceae Juss.
Iz_14_1209	DJI_0982	Geranium sanquineum L.	0	n	1	0	Geraniaceae Juss.
Iz_14_1209	DJI_0982	Gypsophila paniculata L.	0	n	1	0	Caryophyllaceae Juss.
Iz_14_1209	DJI_0982	Helichrysum arenarium (L.) Moench	0	n	1	0	Asteraceae Bercht. & J.Presl
Iz_14_1209	DJI_0982	Herniaria polygama J.Gay	0	n	1	0	Caryophyllaceae Juss.
Iz_14_1209	DJI_0982	Hieracium robustum Fr.	0	n	1	0	Asteraceae Bercht. & J.Presl
Iz_14_1209	DJI_0982	Hieracium umbellatum L.	0	n	1	NA	Asteraceae Bercht. & J.Presl
Iz_14_1209	DJI_0982	Hylotelephium maximum (L.) Holub	0	n	1	0	Crassulaceae J.St.-Hil.
Iz_14_1209	DJI_0982	Jacobaea borysthenica (DC.) B.Nord. & Greuter	0	n	1	0	Asteraceae Bercht. & J.Presl
Iz_14_1209	DJI_0982	Jasione montana L.	0	n	1	0	Campanulaceae Juss.
Iz_14_1209	DJI_0982	Jurinea cyanoides (L.) Rchb.s.l.	0	n	1	0	Asteraceae Bercht. & J.Presl
Iz_14_1209	DJI_0982	Koeleria glauca (Spreng.) DC.	0	n	1	0	Poaceae Barnhart
Iz_14_1209	DJI_0982	Lactuca serriola L.	0	a	0	0	Asteraceae Bercht. & J.Presl
Iz_14_1209	DJI_0982	Linaria genistifolia (L.) Mill.	0	n	1	0	Plantaginaceae Juss.
Iz_14_1209	DJI_0982	Lomelosia argentea (L.) Greuter & Burdet	0	n	1	0	Caprifoliaceae Juss.
Iz_14_1209	DJI_0982	Melampyrum pratense L.	0	n	1	0	Orobanchaceae Vent.
Iz_14_1209	DJI_0982	Oenothera biennis L.	0	a	0	0	Onagraceae Juss.
Iz_14_1209	DJI_0982	Pentanema britannica (L.) D.Gut.Larr., Santos-Vicente, Anderb., E.Rico & M.M.Mart.Ort.	0	n	1	0	Asteraceae Bercht. & J.Presl
Iz_14_1209	DJI_0982	Peucedanum oreoselinum (L.) Moench	0	n	1	0	Apiaceae Lindl.
Iz_14_1209	DJI_0982	Peucedanum ruthenicum M.Bieb.	0	n	1	0	Apiaceae Lindl.
Iz_14_1209	DJI_0982	Pilosella echioides (Lumn.) F.W.Schultz & Sch.Bip.	0	n	1	0	Asteraceae Bercht. & J.Presl
Iz_14_1209	DJI_0982	Pilosella officinarum F. Schultz et Sch. Bip.	0	n	1	0	Asteraceae Bercht. & J.Presl
Iz_14_1209	DJI_0982	Pilosella sp.	0		NA	0	Asteraceae Bercht. & J.Presl
Iz_14_1209	DJI_0982	Pinus sylvestris L.	0	n	1	1	Pinaceae Spreng. ex F.Rudolphi
Iz_14_1209	DJI_0982	Poa annua L.	0	n	1	0	Poaceae Barnhart
Iz_14_1209	DJI_0982	Poa nemoralis L.	0	n	1	0	Poaceae Barnhart
Iz_14_1209	DJI_0982	Poa sp.	0		NA	0	Poaceae Barnhart
Iz_14_1209	DJI_0982	Polygonatum odoratum (Mill.) Druce	0	n	1	0	Asparagaceae Juss.
Iz_14_1209	DJI_0982	Polygonum arenarium Waldst. & Kit.	0	n	1	0	Polygonaceae Juss.
Iz_14_1209	DJI_0982	Populus nigra L.	0	n	1	1	Salicaceae Mirb.
Iz_14_1209	DJI_0982	Populus tremula L.	0	n	1	1	Salicaceae Mirb.
Iz_14_1209	DJI_0982	Populus Ã canescens (Aiton) Sm.	0	n	1	1	Salicaceae Mirb.
Iz_14_1209	DJI_0982	Potentilla argentea L.	0	n	1	0	Rosaceae Juss.
Iz_14_1209	DJI_0982	Psammophiliella muralis (L.) Ikonn.	0	n	1	0	Caryophyllaceae Juss.
Iz_14_1209	DJI_0982	Psephellus sumensis (Kalen.) Greuter	0	n	1	0	Asteraceae Bercht. & J.Presl
Iz_14_1209	DJI_0982	Pulsatilla pratensis (L.) Mill. s.l.	0	n	1	0	Ranunculaceae Juss.
Iz_14_1209	DJI_0982	Pyrus communis L.	0	a	0	1	Rosaceae Juss.
Iz_14_1209	DJI_0982	Quercus robur L.	0	n	1	1	Fagaceae Dumort.
Iz_14_1209	DJI_0982	Robinia pseudoacacia L.	0	a	0	1	Fabaceae Lindl.
Iz_14_1209	DJI_0982	Rosa sp.	0		NA	1	Rosaceae Juss.
Iz_14_1209	DJI_0982	Rubus idaeus L.	0	n	1	0	Rosaceae Juss.
Iz_14_1209	DJI_0982	Rumex acetosella L.	0	n	1	0	Polygonaceae Juss.
Iz_14_1209	DJI_0982	Rumex thyrsiflorus Fingerh.	0	n	1	0	Polygonaceae Juss.
Iz_14_1209	DJI_0982	Salix alba L.	0	n	1	1	Salicaceae Mirb.
Iz_14_1209	DJI_0982	Sambucus nigra L.	0	n	1	1	Viburnaceae Raf.
Iz_14_1209	DJI_0982	Secale sylvestre Host	0	n	1	0	Poaceae Barnhart
Iz_14_1209	DJI_0982	Sempervivum ruthenicum Schnittsp. & C.B. Lehm.	0	n	1	0	Crassulaceae J.St.-Hil.
Iz_14_1209	DJI_0982	Setaria viridis (L.) P. Beauv.	0	n	1	0	Poaceae Barnhart
Iz_14_1209	DJI_0982	Silene borysthenica (Gruner) Walters	0	n	1	0	Caryophyllaceae Juss.
Iz_14_1209	DJI_0982	Silene latifolia subsp. alba (Mill.) Greuter & Burdet	0	n	1	0	Caryophyllaceae Juss.
Iz_14_1209	DJI_0982	Solidago virgaurea L.	0	n	1	0	Asteraceae Bercht. & J.Presl
Iz_14_1209	DJI_0982	Sorbus aucuparia L.	0	n	1	1	Rosaceae Juss.
Iz_14_1209	DJI_0982	Stachys recta L.	0	n	1	0	Lamiacea Lindl.
Iz_14_1209	DJI_0982	Tanacetum vulgare L.	0	n	1	0	Asteraceae Bercht. & J.Presl
Iz_14_1209	DJI_0982	Thymus pallasianus Heinr.Braun	0	n	1	0	Lamiacea Lindl.
Iz_14_1209	DJI_0982	Tilia cordata Mill.	0	n	1	1	Malvaceae Juss.
Iz_14_1209	DJI_0982	Trifolium alpestre L.	0	n	1	0	Fabaceae Lindl.
Iz_14_1209	DJI_0982	Trifolium arvense L.	0	n	1	0	Fabaceae Lindl.
Iz_14_1209	DJI_0982	Ulmus glabra Huds.	0	n	1	1	Ulmaceae Mirb.
Iz_14_1209	DJI_0982	Verbascum densiflorum Bertol.	0	n	1	0	Scrophulariaceae Juss.
Iz_14_1209	DJI_0982	Veronica austriaca L.	0	n	1	0	Plantaginaceae Juss.
Iz_14_1209	DJI_0982	Veronica incana L.	0	n	1	0	Plantaginaceae Juss.
Iz_14_1209	DJI_0982	Veronica verna L.	0	n	1	0	Plantaginaceae Juss.
Iz_14_1209	DJI_0982	Vincetoxicum hirundinaria Medik	0	n	1	0	Apocynaceae Juss.
Iz_15_1209	DJI_0046	Acer tataricum L.	0	n	1	1	Sapindaceae Juss.
Iz_15_1209	DJI_0046	Achillea millefolium L. s.l.	0	n	1	0	Asteraceae Bercht. & J.Presl
Iz_15_1209	DJI_0046	Allium oleraceum L.	0	n	1	0	Amaryllidaceae J.St.-Hil.

Iz_15_1209	DJI_0046	Ambrosia artemisiifolia L.	0	a	0	0	Asteraceae Bercht. & J.Presl
Iz_15_1209	DJI_0046	Amorpha fruticosa L.	0	a	0	0	Fabaceae Lindl.
Iz_15_1209	DJI_0046	Anthericum ramosum L.	0	n	1	0	Asparagaceae Juss.
Iz_15_1209	DJI_0046	Artemisia marschalliana Spreng.	0	n	1	0	Asteraceae Bercht. & J.Presl
Iz_15_1209	DJI_0046	Asparagus officinalis L.	0	n	1	0	Asparagaceae Juss.
Iz_15_1209	DJI_0046	Astragalus cicer L.	0	n	1	0	Fabaceae Lindl.
Iz_15_1209	DJI_0046	Bassia laniflora (S.G.Gmel.) A.J.Scott	0	n	1	0	Amaranthaceae Juss.
Iz_15_1209	DJI_0046	Berberis vulgaris L.	0	n	1	0	Berberidaceae Juss.
Iz_15_1209	DJI_0046	Betula pendula Roth.	0	n	1	1	Betulaceae Gray
Iz_15_1209	DJI_0046	Betula pendula Roth.	0	n	1	0	Poaceae Barnhart
Iz_15_1209	DJI_0046	Bromus inermis Leyss.	0		NA	NA	
Iz_15_1209	DJI_0046	Bromus squarrosus L.	0	a	0	0	Poaceae Barnhart
Iz_15_1209	DJI_0046	Bromus tectorum L.	0	a	0	0	Poaceae Barnhart
Iz_15_1209	DJI_0046	Calamagrostis epigejos (L.) Roth	5	n	1	0	Poaceae Barnhart
Iz_15_1209	DJI_0046	Carduus acanthoides L.	0	n	1	0	Asteraceae Bercht. & J.Presl
Iz_15_1209	DJI_0046	Carex sp.	0	n	1	0	Cyperaceae Juss.
Iz_15_1209	DJI_0046	Centaurea majorovii Dumbadze	0	n	1	0	Asteraceae Bercht. & J.Presl
Iz_15_1209	DJI_0046	Chamaecytisus ruthenicus (Fisch.ex Wol.) Klaskova	0	n	1	0	Fabaceae Lindl.
Iz_15_1209	DJI_0046	Chelidonium majus L.	0	n	1	0	Papaveraceae Juss.
Iz_15_1209	DJI_0046	Chenopodium betaceum Andrz.	0.5	n	1	0	Amaranthaceae Juss.
Iz_15_1209	DJI_0046	Chenopodium cf. album	0	n	1	0	Amaranthaceae Juss.
Iz_15_1209	DJI_0046	Chenopodium sp.	0		NA	0	Amaranthaceae Juss.
Iz_15_1209	DJI_0046	Chondrilla juncea L.	0	n	1	0	Asteraceae Bercht. & J.Presl
Iz_15_1209	DJI_0046	Cirsium vulgare (Savi) Ten.	0	n	1	0	Asteraceae Bercht. & J.Presl
Iz_15_1209	DJI_0046	Clinopodium acinos (L.) Kuntze	0	n	1	0	Lamiacea Lindl.
Iz_15_1209	DJI_0046	Convallaria majalis L.	0	n	1	0	Asparagaceae Juss.
Iz_15_1209	DJI_0046	Crataegus rhipidophylla Gand.	0	n	1	1	Rosaceae Juss.
Iz_15_1209	DJI_0046	Crepis foetida L.	0	n	1	0	Asteraceae Bercht. & J.Presl
Iz_15_1209	DJI_0046	Dactylis glomerata L.	0	n	1	0	Poaceae Barnhart
Iz_15_1209	DJI_0046	Dianthus campestris M.Bieb.	0	n	1	0	Caryophyllaceae Juss.
Iz_15_1209	DJI_0046	Digitaria sanguinalis (L.) Scop.	0	a	0	0	Poaceae Barnhart
Iz_15_1209	DJI_0046	Elymus repens (L.) Gould	0	n	1	0	Poaceae Barnhart
Iz_15_1209	DJI_0046	Epilobium tetragonum L.	0	n	1	0	Onagraceae Juss.
Iz_15_1209	DJI_0046	Eragrostis minor Host	0	a	0	0	Poaceae Barnhart
Iz_15_1209	DJI_0046	Erigeron canadensis L.	0	a	0	0	Asteraceae Bercht. & J.Presl
Iz_15_1209	DJI_0046	Euphorbia seguieriana Neck.	0	n	1	0	Euphorbiaceae Juss.
Iz_15_1209	DJI_0046	Fallopia dumetorum (L.) Holub	2	n	1	0	Polygonaceae Juss.
Iz_15_1209	DJI_0046	Festuca beckeri (Hack.) Trautv.	0	n	1	0	Poaceae Barnhart
Iz_15_1209	DJI_0046	Fragaria vesca L.	0	n	1	0	Rosaceae Juss.
Iz_15_1209	DJI_0046	Frangula alnus Mill.	0	n	1	1	Rhamnaceae Juss.
Iz_15_1209	DJI_0046	Fraxinus pennsylvanica Marshall	0	a	0	1	Oleaceae Hoffmanns. & Link
Iz_15_1209	DJI_0046	Galeopsis bifida Boenn.	0	n	1	0	Lamiacea Lindl.
Iz_15_1209	DJI_0046	Galium verum L.	0	n	1	0	Rubiaceae Juss.
Iz_15_1209	DJI_0046	Genista tinctoria L.	0	n	1	0	Fabaceae Lindl.
Iz_15_1209	DJI_0046	Geranium robertianum L.	0	n	1	0	Geraniaceae Juss.
Iz_15_1209	DJI_0046	Geranium sanquineum L.	0	n	1	0	Geraniaceae Juss.
Iz_15_1209	DJI_0046	Gypsophila paniculata L.	0	n	1	0	Caryophyllaceae Juss.
Iz_15_1209	DJI_0046	Helichrysum arenarium (L.) Moench	0	n	1	0	Asteraceae Bercht. & J.Presl
Iz_15_1209	DJI_0046	Herniaria polygama J.Gay	0	n	1	0	Caryophyllaceae Juss.
Iz_15_1209	DJI_0046	Hieracium robustum Fr.	0	n	1	0	Asteraceae Bercht. & J.Presl
Iz_15_1209	DJI_0046	Hieracium umbellatum L.	0	n	1	NA	Asteraceae Bercht. & J.Presl
Iz_15_1209	DJI_0046	Hylotelephium maximum (L.) Holub	0	n	1	0	Crassulaceae J.St.-Hil.
Iz_15_1209	DJI_0046	Jacobaea borysthenica (DC.) B.Nord. & Greuter	0	n	1	0	Asteraceae Bercht. & J.Presl
Iz_15_1209	DJI_0046	Jasione montana L.	0	n	1	0	Campanulaceae Juss.
Iz_15_1209	DJI_0046	Jurinea cyanoides (L.) Rchb.s.l.	0	n	1	0	Asteraceae Bercht. & J.Presl
Iz_15_1209	DJI_0046	Koeleria glauca (Spreng.) DC.	0	n	1	0	Poaceae Barnhart
Iz_15_1209	DJI_0046	Lactuca serriola L.	2	a	0	0	Asteraceae Bercht. & J.Presl
Iz_15_1209	DJI_0046	Linaria genistifolia (L.) Mill.	0	n	1	0	Plantaginaceae Juss.
Iz_15_1209	DJI_0046	Lomelosia argentea (L.) Greuter & Burdet	0	n	1	0	Caprifoliaceae Juss.
Iz_15_1209	DJI_0046	Melampyrum pratense L.	0	n	1	0	Orobanchaceae Vent.
Iz_15_1209	DJI_0046	Oenothera biennis L.	0	a	0	0	Onagraceae Juss.
Iz_15_1209	DJI_0046	Pentanema britannica (L.) D.Gut.Larr., Santos-Vicente, Anderb., E.Rico & M.M.Mart.Ort.	0	n	1	0	Asteraceae Bercht. & J.Presl
Iz_15_1209	DJI_0046	Peucedanum oreoselinum (L.) Moench	3	n	1	0	Apiaceae Lindl.
Iz_15_1209	DJI_0046	Peucedanum ruthenicum M.Bieb.	0	n	1	0	Apiaceae Lindl.
Iz_15_1209	DJI_0046	Pilosella echioides (Lumn.) F.W.Schultz & Sch.Bip.	0	n	1	0	Asteraceae Bercht. & J.Presl
Iz_15_1209	DJI_0046	Pilosella officinarum F. Schultz et Sch. Bip.	0	n	1	0	Asteraceae Bercht. & J.Presl
Iz_15_1209	DJI_0046	Pilosella sp.	0		NA	0	Asteraceae Bercht. & J.Presl
Iz_15_1209	DJI_0046	Pinus sylvestris L.	0	n	1	1	Pinaceae Spreng. ex F.Rudolphi
Iz_15_1209	DJI_0046	Poa annua L.	0	n	1	0	Poaceae Barnhart
Iz_15_1209	DJI_0046	Poa nemoralis L.	0	n	1	0	Poaceae Barnhart
Iz_15_1209	DJI_0046	Poa sp.	0		NA	0	Poaceae Barnhart
Iz_15_1209	DJI_0046	Polygonatum odoratum (Mill.) Druce	0	n	1	0	Asparagaceae Juss.
Iz_15_1209	DJI_0046	Polygonum arenarium Waldst. & Kit.	0	n	1	0	Polygonaceae Juss.
Iz_15_1209	DJI_0046	Populus nigra L.	0	n	1	1	Salicaceae Mirb.
Iz_15_1209	DJI_0046	Populus tremula L.	0	n	1	1	Salicaceae Mirb.
Iz_15_1209	DJI_0046	Populus Ã canescens (Aiton) Sm.	0	n	1	1	Salicaceae Mirb.
Iz_15_1209	DJI_0046	Potentilla argentea L.	0	n	1	0	Rosaceae Juss.
Iz_15_1209	DJI_0046	Psammophiliella muralis (L.) Ikonn.	0	n	1	0	Caryophyllaceae Juss.
Iz_15_1209	DJI_0046	Psephellus sumensis (Kalen.) Greuter	0	n	1	0	Asteraceae Bercht. & J.Presl
Iz_15_1209	DJI_0046	Pulsatilla pratensis (L.) Mill. s.l.	0	n	1	0	Ranunculaceae Juss.
Iz_15_1209	DJI_0046	Pyrus communis L.	0	a	0	1	Rosaceae Juss.
Iz_15_1209	DJI_0046	Quercus robur L.	0	n	1	1	Fagaceae Dumort.
Iz_15_1209	DJI_0046	Robinia pseudoacacia L.	0	a	0	1	Fabaceae Lindl.
Iz_15_1209	DJI_0046	Rosa sp.	0		NA	1	Rosaceae Juss.
Iz_15_1209	DJI_0046	Rubus idaeus L.	0	n	1	0	Rosaceae Juss.
Iz_15_1209	DJI_0046	Rumex acetosella L.	0	n	1	0	Polygonaceae Juss.
Iz_15_1209	DJI_0046	Rumex thyrsiflorus Fingerh.	0	n	1	0	Polygonaceae Juss.
Iz_15_1209	DJI_0046	Salix alba L.	0	n	1	1	Salicaceae Mirb.
Iz_15_1209	DJI_0046	Sambucus nigra L.	0	n	1	1	Viburnaceae Raf.
Iz_15_1209	DJI_0046	Secale sylvestre Host	0	n	1	0	Poaceae Barnhart
Iz_15_1209	DJI_0046	Sempervivum ruthenicum Schnittsp. & C.B. Lehm.	0	n	1	0	Crassulaceae J.St.-Hil.
Iz_15_1209	DJI_0046	Setaria viridis (L.) P. Beauv.	0	n	1	0	Poaceae Barnhart
Iz_15_1209	DJI_0046	Silene borysthenica (Gruner) Walters	0	n	1	0	Caryophyllaceae Juss.
Iz_15_1209	DJI_0046	Silene latifolia subsp. alba (Mill.) Greuter & Burdet	0	n	1	0	Caryophyllaceae Juss.
Iz_15_1209	DJI_0046	Solidago virgaurea L.	0	n	1	0	Asteraceae Bercht. & J.Presl
Iz_15_1209	DJI_0046	Sorbus aucuparia L.	0	n	1	1	Rosaceae Juss.
Iz_15_1209	DJI_0046	Stachys recta L.	0	n	1	0	Lamiacea Lindl.
Iz_15_1209	DJI_0046	Tanacetum vulgare L.	0	n	1	0	Asteraceae Bercht. & J.Presl

Iz_15_1209	DJI_0046	Thymus pallasianus Heinr.Braun	0	n	1	0	Lamiacea Lindl.
Iz_15_1209	DJI_0046	Tilia cordata Mill.	0	n	1	1	Malvaceae Juss.
Iz_15_1209	DJI_0046	Trifolium alpestre L.	0	n	1	0	Fabaceae Lindl.
Iz_15_1209	DJI_0046	Trifolium arvense L.	0	n	1	0	Fabaceae Lindl.
Iz_15_1209	DJI_0046	Ulmus glabra Huds.	0	n	1	1	Ulmaceae Mirb.
Iz_15_1209	DJI_0046	Verbascum densiflorum Bertol.	0	n	1	0	Scrophulariaceae Juss.
Iz_15_1209	DJI_0046	Veronica austriaca L.	0	n	1	0	Plantaginaceae Juss.
Iz_15_1209	DJI_0046	Veronica incana L.	0	n	1	0	Plantaginaceae Juss.
Iz_15_1209	DJI_0046	Veronica verna L.	0	n	1	0	Plantaginaceae Juss.
Iz_15_1209	DJI_0046	Vincetoxicum hirundinaria Medik	0	n	1	0	Apocynaceae Juss.
Iz_15_1209	DJI_0050	Acer tataricum L.	0	n	1	1	Sapindaceae Juss.
Iz_15_1209	DJI_0050	Achillea millefolium L. s.l.	0	n	1	0	Asteraceae Bercht. & J.Presl
Iz_15_1209	DJI_0050	Allium oleraceum L.	0	n	1	0	Amaryllidaceae J.St.-Hil.
Iz_15_1209	DJI_0050	Ambrosia artemisiifolia L.	0	a	0	0	Asteraceae Bercht. & J.Presl
Iz_15_1209	DJI_0050	Amorpha fruticosa L.	0	a	0	0	Fabaceae Lindl.
Iz_15_1209	DJI_0050	Anthericum ramosum L.	0	n	1	0	Asparagaceae Juss.
Iz_15_1209	DJI_0050	Artemisia marschalliana Spreng.	0	n	1	0	Asteraceae Bercht. & J.Presl
Iz_15_1209	DJI_0050	Asparagus officinalis L.	0	n	1	0	Asparagaceae Juss.
Iz_15_1209	DJI_0050	Astragalus cicer L.	0	n	1	0	Fabaceae Lindl.
Iz_15_1209	DJI_0050	Bassia laniflora (S.G.Gmel.) A.J.Scott	0	n	1	0	Amaranthaceae Juss.
Iz_15_1209	DJI_0050	Berberis vulgaris L.	0	n	1	0	Berberidaceae Juss.
Iz_15_1209	DJI_0050	Betula pendula Roth.	0	n	1	1	Betulaceae Gray
Iz_15_1209	DJI_0050	Betula pendula Roth.	0	n	1	0	Poaceae Barnhart
Iz_15_1209	DJI_0050	Bromus inermis Leyss.	0		NA	NA	
Iz_15_1209	DJI_0050	Bromus squarrosus L.	0	a	0	0	Poaceae Barnhart
Iz_15_1209	DJI_0050	Bromus tectorum L.	0	a	0	0	Poaceae Barnhart
Iz_15_1209	DJI_0050	Calamagrostis epigejos (L.) Roth	10	n	1	0	Poaceae Barnhart
Iz_15_1209	DJI_0050	Carduus acanthoides L.	0	n	1	0	Asteraceae Bercht. & J.Presl
Iz_15_1209	DJI_0050	Carex sp.	0	n	1	0	Cyperaceae Juss.
Iz_15_1209	DJI_0050	Centaurea majorovii Dumbadze	0	n	1	0	Asteraceae Bercht. & J.Presl
Iz_15_1209	DJI_0050	Chamaecytisus ruthenicus (Fisch.ex Wol.) Klaskova	0	n	1	0	Fabaceae Lindl.
Iz_15_1209	DJI_0050	Chelidonium majus L.	0	n	1	0	Papaveraceae Juss.
Iz_15_1209	DJI_0050	Chenopodium betaceum Andrz.	0	n	1	0	Amaranthaceae Juss.
Iz_15_1209	DJI_0050	Chenopodium cf. album	0	n	1	0	Amaranthaceae Juss.
Iz_15_1209	DJI_0050	Chenopodium sp.	0		NA	0	Amaranthaceae Juss.
Iz_15_1209	DJI_0050	Chondrilla juncea L.	0	n	1	0	Asteraceae Bercht. & J.Presl
Iz_15_1209	DJI_0050	Cirsium vulgare (Savi) Ten.	0	n	1	0	Asteraceae Bercht. & J.Presl
Iz_15_1209	DJI_0050	Clinopodium acinos (L.) Kuntze	0	n	1	0	Lamiacea Lindl.
Iz_15_1209	DJI_0050	Convallaria majalis L.	0	n	1	0	Asparagaceae Juss.
Iz_15_1209	DJI_0050	Crataegus rhipidophylla Gand.	0	n	1	1	Rosaceae Juss.
Iz_15_1209	DJI_0050	Crepis foetida L.	0	n	1	0	Asteraceae Bercht. & J.Presl
Iz_15_1209	DJI_0050	Dactylis glomerata L.	0	n	1	0	Poaceae Barnhart
Iz_15_1209	DJI_0050	Dianthus campestris M.Bieb.	0	n	1	0	Caryophyllaceae Juss.
Iz_15_1209	DJI_0050	Digitaria sanguinalis (L.) Scop.	0	a	0	0	Poaceae Barnhart
Iz_15_1209	DJI_0050	Elymus repens (L.) Gould	0	n	1	0	Poaceae Barnhart
Iz_15_1209	DJI_0050	Epilobium tetragonum L.	0	n	1	0	Onagraceae Juss.
Iz_15_1209	DJI_0050	Eragrostis minor Host	0	a	0	0	Poaceae Barnhart
Iz_15_1209	DJI_0050	Erigeron canadensis L.	0	a	0	0	Asteraceae Bercht. & J.Presl
Iz_15_1209	DJI_0050	Euphorbia seguieriana Neck.	0	n	1	0	Euphorbiaceae Juss.
Iz_15_1209	DJI_0050	Fallopia dumetorum (L.) Holub	0	n	1	0	Polygonaceae Juss.
Iz_15_1209	DJI_0050	Festuca beckeri (Hack.) Trautv.	0	n	1	0	Poaceae Barnhart
Iz_15_1209	DJI_0050	Fragaria vesca L.	0	n	1	0	Rosaceae Juss.
Iz_15_1209	DJI_0050	Frangula alnus Mill.	0	n	1	1	Rhamnaceae Juss.
Iz_15_1209	DJI_0050	Fraxinus pennsylvanica Marshall	0	a	0	1	Oleaceae Hoffmanns. & Link
Iz_15_1209	DJI_0050	Galeopsis bifida Boenn.	0	n	1	0	Lamiacea Lindl.
Iz_15_1209	DJI_0050	Galium verum L.	0	n	1	0	Rubiaceae Juss.
Iz_15_1209	DJI_0050	Genista tinctoria L.	0	n	1	0	Fabaceae Lindl.
Iz_15_1209	DJI_0050	Geranium robertianum L.	0	n	1	0	Geraniaceae Juss.
Iz_15_1209	DJI_0050	Geranium sanquineum L.	0	n	1	0	Geraniaceae Juss.
Iz_15_1209	DJI_0050	Gypsophila paniculata L.	0	n	1	0	Caryophyllaceae Juss.
Iz_15_1209	DJI_0050	Helichrysum arenarium (L.) Moench	0	n	1	0	Asteraceae Bercht. & J.Presl
Iz_15_1209	DJI_0050	Herniaria polygama J.Gay	0	n	1	0	Caryophyllaceae Juss.
Iz_15_1209	DJI_0050	Hieracium robustum Fr.	0	n	1	0	Asteraceae Bercht. & J.Presl
Iz_15_1209	DJI_0050	Hieracium umbellatum L.	0	n	1	NA	Asteraceae Bercht. & J.Presl
Iz_15_1209	DJI_0050	Hylotelephium maximum (L.) Holub	0	n	1	0	Crassulaceae J.St.-Hil.
Iz_15_1209	DJI_0050	Jacobaea borysthenica (DC.) B.Nord. & Greuter	0	n	1	0	Asteraceae Bercht. & J.Presl
Iz_15_1209	DJI_0050	Jasione montana L.	0	n	1	0	Campanulaceae Juss.
Iz_15_1209	DJI_0050	Jurinea cyanoides (L.) Rchb.s.l.	0	n	1	0	Asteraceae Bercht. & J.Presl
Iz_15_1209	DJI_0050	Koeleria glauca (Spreng.) DC.	0	n	1	0	Poaceae Barnhart
Iz_15_1209	DJI_0050	Lactuca serriola L.	0	a	0	0	Asteraceae Bercht. & J.Presl
Iz_15_1209	DJI_0050	Linaria genistifolia (L.) Mill.	0	n	1	0	Plantaginaceae Juss.
Iz_15_1209	DJI_0050	Lomelosia argentea (L.) Greuter & Burdet	0	n	1	0	Caprifoliaceae Juss.
Iz_15_1209	DJI_0050	Melampyrum pratense L.	0	n	1	0	Orobanchaceae Vent.
Iz_15_1209	DJI_0050	Oenothera biennis L.	0	a	0	0	Onagraceae Juss.
Iz_15_1209	DJI_0050	Pentanema britannica (L.) D.Gut.Larr., Santos-Vicente, Anderb., E.Rico & M.M.Mart.Ort.	0	n	1	0	Asteraceae Bercht. & J.Presl
Iz_15_1209	DJI_0050	Peucedanum oreoselinum (L.) Moench	0	n	1	0	Apiaceae Lindl.
Iz_15_1209	DJI_0050	Peucedanum ruthenicum M.Bieb.	0	n	1	0	Apiaceae Lindl.
Iz_15_1209	DJI_0050	Pilosella echioides (Lumn.) F.W.Schultz & Sch.Bip.	0	n	1	0	Asteraceae Bercht. & J.Presl
Iz_15_1209	DJI_0050	Pilosella officinarum F. Schultz et Sch. Bip.	0	n	1	0	Asteraceae Bercht. & J.Presl
Iz_15_1209	DJI_0050	Pilosella sp.	0		NA	0	Asteraceae Bercht. & J.Presl
Iz_15_1209	DJI_0050	Pinus sylvestris L.	0	n	1	1	Pinaceae Spreng. ex F.Rudolphi
Iz_15_1209	DJI_0050	Poa annua L.	0	n	1	0	Poaceae Barnhart
Iz_15_1209	DJI_0050	Poa nemoralis L.	0	n	1	0	Poaceae Barnhart
Iz_15_1209	DJI_0050	Poa sp.	0		NA	0	Poaceae Barnhart
Iz_15_1209	DJI_0050	Polygonatum odoratum (Mill.) Druce	0	n	1	0	Asparagaceae Juss.
Iz_15_1209	DJI_0050	Polygonum arenarium Waldst. & Kit.	0	n	1	0	Polygonaceae Juss.
Iz_15_1209	DJI_0050	Populus nigra L.	0	n	1	1	Salicaceae Mirb.
Iz_15_1209	DJI_0050	Populus tremula L.	0	n	1	1	Salicaceae Mirb.
Iz_15_1209	DJI_0050	Populus Ã canescens (Aiton) Sm.	0	n	1	1	Salicaceae Mirb.
Iz_15_1209	DJI_0050	Potentilla argentea L.	0	n	1	0	Rosaceae Juss.
Iz_15_1209	DJI_0050	Psammophiliella muralis (L.) Ikonn.	0	n	1	0	Caryophyllaceae Juss.
Iz_15_1209	DJI_0050	Psephellus sumensis (Kalen.) Greuter	0	n	1	0	Asteraceae Bercht. & J.Presl
Iz_15_1209	DJI_0050	Pulsatilla pratensis (L.) Mill. s.l.	0	n	1	0	Ranunculaceae Juss.
Iz_15_1209	DJI_0050	Pyrus communis L.	0	a	0	1	Rosaceae Juss.
Iz_15_1209	DJI_0050	Quercus robur L.	0	n	1	1	Fagaceae Dumort.
Iz_15_1209	DJI_0050	Robinia pseudoacacia L.	0	a	0	1	Fabaceae Lindl.
Iz_15_1209	DJI_0050	Rosa sp.	0		NA	1	Rosaceae Juss.
Iz_15_1209	DJI_0050	Rubus idaeus L.	0	n	1	0	Rosaceae Juss.

Iz_15_1209	DJI_0050	Rumex acetosella L.	0	n	1	0	Polygonaceae Juss.
Iz_15_1209	DJI_0050	Rumex thyrsiflorus Fingerh.	0	n	1	0	Polygonaceae Juss.
Iz_15_1209	DJI_0050	Salix alba L.	0	n	1	1	Salicaceae Mirb.
Iz_15_1209	DJI_0050	Sambucus nigra L.	0	n	1	1	Viburnaceae Raf.
Iz_15_1209	DJI_0050	Secale sylvestre Host	0	n	1	0	Poaceae Barnhart
Iz_15_1209	DJI_0050	Sempervivum ruthenicum Schnittsp. & C.B. Lehm.	0	n	1	0	Crassulaceae J.St.-Hil.
Iz_15_1209	DJI_0050	Setaria viridis (L.) P. Beauv.	0	n	1	0	Poaceae Barnhart
Iz_15_1209	DJI_0050	Silene borysthenica (Gruner) Walters	0	n	1	0	Caryophyllaceae Juss.
Iz_15_1209	DJI_0050	Silene latifolia subsp. alba (Mill.) Greuter & Burdet	0	n	1	0	Caryophyllaceae Juss.
Iz_15_1209	DJI_0050	Solidago virgaurea L.	0	n	1	0	Asteraceae Bercht. & J.Presl
Iz_15_1209	DJI_0050	Sorbus aucuparia L.	0	n	1	1	Rosaceae Juss.
Iz_15_1209	DJI_0050	Stachys recta L.	0	n	1	0	Lamiacea Lindl.
Iz_15_1209	DJI_0050	Tanacetum vulgare L.	0	n	1	0	Asteraceae Bercht. & J.Presl
Iz_15_1209	DJI_0050	Thymus pallasianus Heinr.Braun	0	n	1	0	Lamiacea Lindl.
Iz_15_1209	DJI_0050	Tilia cordata Mill.	0	n	1	1	Malvaceae Juss.
Iz_15_1209	DJI_0050	Trifolium alpestre L.	0	n	1	0	Fabaceae Lindl.
Iz_15_1209	DJI_0050	Trifolium arvense L.	0	n	1	0	Fabaceae Lindl.
Iz_15_1209	DJI_0050	Ulmus glabra Huds.	0	n	1	1	Ulmaceae Mirb.
Iz_15_1209	DJI_0050	Verbascum densiflorum Bertol.	0	n	1	0	Scrophulariaceae Juss.
Iz_15_1209	DJI_0050	Veronica austriaca L.	0	n	1	0	Plantaginaceae Juss.
Iz_15_1209	DJI_0050	Veronica incana L.	0	n	1	0	Plantaginaceae Juss.
Iz_15_1209	DJI_0050	Veronica verna L.	0	n	1	0	Plantaginaceae Juss.
Iz_15_1209	DJI_0050	Vincetoxicum hirundinaria Medik	0	n	1	0	Apocynaceae Juss.
Iz_15_1209	DJI_0053	Acer tataricum L.	0	n	1	1	Sapindaceae Juss.
Iz_15_1209	DJI_0053	Achillea millefolium L. s.l.	0	n	1	0	Asteraceae Bercht. & J.Presl
Iz_15_1209	DJI_0053	Allium oleraceum L.	0	n	1	0	Amaryllidaceae J.St.-Hil.
Iz_15_1209	DJI_0053	Ambrosia artemisiifolia L.	0	a	0	0	Asteraceae Bercht. & J.Presl
Iz_15_1209	DJI_0053	Amorpha fruticosa L.	0	a	0	0	Fabaceae Lindl.
Iz_15_1209	DJI_0053	Anthericum ramosum L.	0	n	1	0	Asparagaceae Juss.
Iz_15_1209	DJI_0053	Artemisia marschalliana Spreng.	0	n	1	0	Asteraceae Bercht. & J.Presl
Iz_15_1209	DJI_0053	Asparagus officinalis L.	0	n	1	0	Asparagaceae Juss.
Iz_15_1209	DJI_0053	Astragalus cicer L.	0	n	1	0	Fabaceae Lindl.
Iz_15_1209	DJI_0053	Bassia laniflora (S.G.Gmel.) A.J.Scott	0	n	1	0	Amaranthaceae Juss.
Iz_15_1209	DJI_0053	Berberis vulgaris L.	0	n	1	0	Berberidaceae Juss.
Iz_15_1209	DJI_0053	Betula pendula Roth.	0	n	1	1	Betulaceae Gray
Iz_15_1209	DJI_0053	Betula pendula Roth.	0	n	1	0	Poaceae Barnhart
Iz_15_1209	DJI_0053	Bromus inermis Leyss.	0		NA	NA	
Iz_15_1209	DJI_0053	Bromus squarrosus L.	0	a	0	0	Poaceae Barnhart
Iz_15_1209	DJI_0053	Bromus tectorum L.	0	a	0	0	Poaceae Barnhart
Iz_15_1209	DJI_0053	Calamagrostis epigejos (L.) Roth	3	n	1	0	Poaceae Barnhart
Iz_15_1209	DJI_0053	Carduus acanthoides L.	0	n	1	0	Asteraceae Bercht. & J.Presl
Iz_15_1209	DJI_0053	Carex sp.	0	n	1	0	Cyperaceae Juss.
Iz_15_1209	DJI_0053	Centaurea majorovii Dumbadze	0	n	1	0	Asteraceae Bercht. & J.Presl
Iz_15_1209	DJI_0053	Chamaecytisus ruthenicus (Fisch.ex Wol.) Klaskova	0	n	1	0	Fabaceae Lindl.
Iz_15_1209	DJI_0053	Chelidonium majus L.	0	n	1	0	Papaveraceae Juss.
Iz_15_1209	DJI_0053	Chenopodium betaceum Andrz.	0	n	1	0	Amaranthaceae Juss.
Iz_15_1209	DJI_0053	Chenopodium cf. album	0	n	1	0	Amaranthaceae Juss.
Iz_15_1209	DJI_0053	Chenopodium sp.	0		NA	0	Amaranthaceae Juss.
Iz_15_1209	DJI_0053	Chondrilla juncea L.	0	n	1	0	Asteraceae Bercht. & J.Presl
Iz_15_1209	DJI_0053	Cirsium vulgare (Savi) Ten.	0	n	1	0	Asteraceae Bercht. & J.Presl
Iz_15_1209	DJI_0053	Clinopodium acinos (L.) Kuntze	0	n	1	0	Lamiacea Lindl.
Iz_15_1209	DJI_0053	Convallaria majalis L.	0	n	1	0	Asparagaceae Juss.
Iz_15_1209	DJI_0053	Crataegus rhipidophylla Gand.	0	n	1	1	Rosaceae Juss.
Iz_15_1209	DJI_0053	Crepis foetida L.	0	n	1	0	Asteraceae Bercht. & J.Presl
Iz_15_1209	DJI_0053	Dactylis glomerata L.	0	n	1	0	Poaceae Barnhart
Iz_15_1209	DJI_0053	Dianthus campestris M.Bieb.	0	n	1	0	Caryophyllaceae Juss.
Iz_15_1209	DJI_0053	Digitaria sanguinalis (L.) Scop.	0	a	0	0	Poaceae Barnhart
Iz_15_1209	DJI_0053	Elymus repens (L.) Gould	0	n	1	0	Poaceae Barnhart
Iz_15_1209	DJI_0053	Epilobium tetragonum L.	0	n	1	0	Onagraceae Juss.
Iz_15_1209	DJI_0053	Eragrostis minor Host	0	a	0	0	Poaceae Barnhart
Iz_15_1209	DJI_0053	Erigeron canadensis L.	0	a	0	0	Asteraceae Bercht. & J.Presl
Iz_15_1209	DJI_0053	Euphorbia seguieriana Neck.	0	n	1	0	Euphorbiaceae Juss.
Iz_15_1209	DJI_0053	Fallopia dumetorum (L.) Holub	0	n	1	0	Polygonaceae Juss.
Iz_15_1209	DJI_0053	Festuca beckeri (Hack.) Trautv.	0	n	1	0	Poaceae Barnhart
Iz_15_1209	DJI_0053	Fragaria vesca L.	0	n	1	0	Rosaceae Juss.
Iz_15_1209	DJI_0053	Frangula alnus Mill.	0	n	1	1	Rhamnaceae Juss.
Iz_15_1209	DJI_0053	Fraxinus pennsylvanica Marshall	0	a	0	1	Oleaceae Hoffmanns. & Link
Iz_15_1209	DJI_0053	Galeopsis bifida Boenn.	0	n	1	0	Lamiacea Lindl.
Iz_15_1209	DJI_0053	Galium verum L.	0	n	1	0	Rubiaceae Juss.
Iz_15_1209	DJI_0053	Genista tinctoria L.	0	n	1	0	Fabaceae Lindl.
Iz_15_1209	DJI_0053	Geranium robertianum L.	0	n	1	0	Geraniaceae Juss.
Iz_15_1209	DJI_0053	Geranium sanquineum L.	0	n	1	0	Geraniaceae Juss.
Iz_15_1209	DJI_0053	Gypsophila paniculata L.	0	n	1	0	Caryophyllaceae Juss.
Iz_15_1209	DJI_0053	Helichrysum arenarium (L.) Moench	0	n	1	0	Asteraceae Bercht. & J.Presl
Iz_15_1209	DJI_0053	Herniaria polygama J.Gay	0	n	1	0	Caryophyllaceae Juss.
Iz_15_1209	DJI_0053	Hieracium robustum Fr.	0	n	1	0	Asteraceae Bercht. & J.Presl
Iz_15_1209	DJI_0053	Hieracium umbellatum L.	0	n	1	NA	Asteraceae Bercht. & J.Presl
Iz_15_1209	DJI_0053	Hylotelephium maximum (L.) Holub	0	n	1	0	Crassulaceae J.St.-Hil.
Iz_15_1209	DJI_0053	Jacobaea borysthenica (DC.) B.Nord. & Greuter	0	n	1	0	Asteraceae Bercht. & J.Presl
Iz_15_1209	DJI_0053	Jasione montana L.	0	n	1	0	Campanulaceae Juss.
Iz_15_1209	DJI_0053	Jurinea cyanoides (L.) Rchb.s.l.	0	n	1	0	Asteraceae Bercht. & J.Presl
Iz_15_1209	DJI_0053	Koeleria glauca (Spreng.) DC.	0	n	1	0	Poaceae Barnhart
Iz_15_1209	DJI_0053	Lactuca serriola L.	0	a	0	0	Asteraceae Bercht. & J.Presl
Iz_15_1209	DJI_0053	Linaria genistifolia (L.) Mill.	0	n	1	0	Plantaginaceae Juss.
Iz_15_1209	DJI_0053	Lomelosia argentea (L.) Greuter & Burdet	0	n	1	0	Caprifoliaceae Juss.
Iz_15_1209	DJI_0053	Melampyrum pratense L.	0	n	1	0	Orobanchaceae Vent.
Iz_15_1209	DJI_0053	Oenothera biennis L.	0	a	0	0	Onagraceae Juss.
Iz_15_1209	DJI_0053	Pentanema britannica (L.) D.Gut.Larr., Santos-Vicente, Anderb., E.Rico & M.M.Mart.Ort.	0	n	1	0	Asteraceae Bercht. & J.Presl
Iz_15_1209	DJI_0053	Peucedanum oreoselinum (L.) Moench	3	n	1	0	Apiaceae Lindl.
Iz_15_1209	DJI_0053	Peucedanum ruthenicum M.Bieb.	0	n	1	0	Apiaceae Lindl.
Iz_15_1209	DJI_0053	Pilosella echioides (Lumn.) F.W.Schultz & Sch.Bip.	0	n	1	0	Asteraceae Bercht. & J.Presl
Iz_15_1209	DJI_0053	Pilosella officinarum F. Schultz et Sch. Bip.	0	n	1	0	Asteraceae Bercht. & J.Presl
Iz_15_1209	DJI_0053	Pilosella sp.	0		NA	0	Asteraceae Bercht. & J.Presl
Iz_15_1209	DJI_0053	Pinus sylvestris L.	0	n	1	1	Pinaceae Spreng. ex F.Rudolphi
Iz_15_1209	DJI_0053	Poa annua L.	0	n	1	0	Poaceae Barnhart
Iz_15_1209	DJI_0053	Poa nemoralis L.	0	n	1	0	Poaceae Barnhart
Iz_15_1209	DJI_0053	Poa sp.	0		NA	0	Poaceae Barnhart
Iz_15_1209	DJI_0053	Polygonatum odoratum (Mill.) Druce	0.1	n	1	0	Asparagaceae Juss.

Iz_15_1209	DJI_0053	Polygonum arenarium Waldst. & Kit.	0	n	1	0	Polygonaceae Juss.
Iz_15_1209	DJI_0053	Populus nigra L.	0	n	1	1	Salicaceae Mirb.
Iz_15_1209	DJI_0053	Populus tremula L.	0	n	1	1	Salicaceae Mirb.
Iz_15_1209	DJI_0053	Populus Ã canescens (Aiton) Sm.	0	n	1	1	Salicaceae Mirb.
Iz_15_1209	DJI_0053	Potentilla argentea L.	0	n	1	0	Rosaceae Juss.
Iz_15_1209	DJI_0053	Psammophiliella muralis (L.) Ikonn.	0	n	1	0	Caryophyllaceae Juss.
Iz_15_1209	DJI_0053	Psephellus sumensis (Kalen.) Greuter	0	n	1	0	Asteraceae Bercht. & J.Presl
Iz_15_1209	DJI_0053	Pulsatilla pratensis (L.) Mill. s.l.	0	n	1	0	Ranunculaceae Juss.
Iz_15_1209	DJI_0053	Pyrus communis L.	0	a	0	1	Rosaceae Juss.
Iz_15_1209	DJI_0053	Quercus robur L.	0	n	1	1	Fagaceae Dumort.
Iz_15_1209	DJI_0053	Robinia pseudoacacia L.	0	a	0	1	Fabaceae Lindl.
Iz_15_1209	DJI_0053	Rosa sp.	0		NA	1	Rosaceae Juss.
Iz_15_1209	DJI_0053	Rubus idaeus L.	0	n	1	0	Rosaceae Juss.
Iz_15_1209	DJI_0053	Rumex acetosella L.	0	n	1	0	Polygonaceae Juss.
Iz_15_1209	DJI_0053	Rumex thyrsiflorus Fingerh.	0	n	1	0	Polygonaceae Juss.
Iz_15_1209	DJI_0053	Salix alba L.	0	n	1	1	Salicaceae Mirb.
Iz_15_1209	DJI_0053	Sambucus nigra L.	0	n	1	1	Viburnaceae Raf.
Iz_15_1209	DJI_0053	Secale sylvestre Host	0	n	1	0	Poaceae Barnhart
Iz_15_1209	DJI_0053	Sempervivum ruthenicum Schnittsp. & C.B. Lehm.	0	n	1	0	Crassulaceae J.St.-Hil.
Iz_15_1209	DJI_0053	Setaria viridis (L.) P. Beauv.	0	n	1	0	Poaceae Barnhart
Iz_15_1209	DJI_0053	Silene borysthenica (Gruner) Walters	0	n	1	0	Caryophyllaceae Juss.
Iz_15_1209	DJI_0053	Silene latifolia subsp. alba (Mill.) Greuter & Burdet	0	n	1	0	Caryophyllaceae Juss.
Iz_15_1209	DJI_0053	Solidago virgaurea L.	0	n	1	0	Asteraceae Bercht. & J.Presl
Iz_15_1209	DJI_0053	Sorbus aucuparia L.	0	n	1	1	Rosaceae Juss.
Iz_15_1209	DJI_0053	Stachys recta L.	0	n	1	0	Lamiacea Lindl.
Iz_15_1209	DJI_0053	Tanacetum vulgare L.	0	n	1	0	Asteraceae Bercht. & J.Presl
Iz_15_1209	DJI_0053	Thymus pallasianus Heinr.Braun	0	n	1	0	Lamiacea Lindl.
Iz_15_1209	DJI_0053	Tilia cordata Mill.	0	n	1	1	Malvaceae Juss.
Iz_15_1209	DJI_0053	Trifolium alpestre L.	0	n	1	0	Fabaceae Lindl.
Iz_15_1209	DJI_0053	Trifolium arvense L.	0	n	1	0	Fabaceae Lindl.
Iz_15_1209	DJI_0053	Ulmus glabra Huds.	0	n	1	1	Ulmaceae Mirb.
Iz_15_1209	DJI_0053	Verbascum densiflorum Bertol.	0	n	1	0	Scrophulariaceae Juss.
Iz_15_1209	DJI_0053	Veronica austriaca L.	0	n	1	0	Plantaginaceae Juss.
Iz_15_1209	DJI_0053	Veronica incana L.	0	n	1	0	Plantaginaceae Juss.
Iz_15_1209	DJI_0053	Veronica verna L.	0	n	1	0	Plantaginaceae Juss.
Iz_15_1209	DJI_0053	Vincetoxicum hirundinaria Medik	0	n	1	0	Apocynaceae Juss.
Iz_15_1209	DJI_0056	Acer tataricum L.	0	n	1	1	Sapindaceae Juss.
Iz_15_1209	DJI_0056	Achillea millefolium L. s.l.	0	n	1	0	Asteraceae Bercht. & J.Presl
Iz_15_1209	DJI_0056	Allium oleraceum L.	0	n	1	0	Amaryllidaceae J.St.-Hil.
Iz_15_1209	DJI_0056	Ambrosia artemisiifolia L.	0	a	0	0	Asteraceae Bercht. & J.Presl
Iz_15_1209	DJI_0056	Amorpha fruticosa L.	0	a	0	0	Fabaceae Lindl.
Iz_15_1209	DJI_0056	Anthericum ramosum L.	0	n	1	0	Asparagaceae Juss.
Iz_15_1209	DJI_0056	Artemisia marschalliana Spreng.	0	n	1	0	Asteraceae Bercht. & J.Presl
Iz_15_1209	DJI_0056	Asparagus officinalis L.	0	n	1	0	Asparagaceae Juss.
Iz_15_1209	DJI_0056	Astragalus cicer L.	0	n	1	0	Fabaceae Lindl.
Iz_15_1209	DJI_0056	Bassia laniflora (S.G.Gmel.) A.J.Scott	0	n	1	0	Amaranthaceae Juss.
Iz_15_1209	DJI_0056	Berberis vulgaris L.	0	n	1	0	Berberidaceae Juss.
Iz_15_1209	DJI_0056	Betula pendula Roth.	0	n	1	1	Betulaceae Gray
Iz_15_1209	DJI_0056	Betula pendula Roth.	0	n	1	0	Poaceae Barnhart
Iz_15_1209	DJI_0056	Bromus inermis Leyss.	0		NA	NA	
Iz_15_1209	DJI_0056	Bromus squarrosus L.	0	a	0	0	Poaceae Barnhart
Iz_15_1209	DJI_0056	Bromus tectorum L.	0	a	0	0	Poaceae Barnhart
Iz_15_1209	DJI_0056	Calamagrostis epigejos (L.) Roth	0	n	1	0	Poaceae Barnhart
Iz_15_1209	DJI_0056	Carduus acanthoides L.	0	n	1	0	Asteraceae Bercht. & J.Presl
Iz_15_1209	DJI_0056	Carex sp.	0	n	1	0	Cyperaceae Juss.
Iz_15_1209	DJI_0056	Centaurea majorovii Dumbadze	0	n	1	0	Asteraceae Bercht. & J.Presl
Iz_15_1209	DJI_0056	Chamaecytisus ruthenicus (Fisch.ex Wol.) Klaskova	0	n	1	0	Fabaceae Lindl.
Iz_15_1209	DJI_0056	Chelidonium majus L.	0	n	1	0	Papaveraceae Juss.
Iz_15_1209	DJI_0056	Chenopodium betaceum Andrz.	0	n	1	0	Amaranthaceae Juss.
Iz_15_1209	DJI_0056	Chenopodium cf. album	0	n	1	0	Amaranthaceae Juss.
Iz_15_1209	DJI_0056	Chenopodium sp.	0		NA	0	Amaranthaceae Juss.
Iz_15_1209	DJI_0056	Chondrilla juncea L.	0	n	1	0	Asteraceae Bercht. & J.Presl
Iz_15_1209	DJI_0056	Cirsium vulgare (Savi) Ten.	0	n	1	0	Asteraceae Bercht. & J.Presl
Iz_15_1209	DJI_0056	Clinopodium acinos (L.) Kuntze	0	n	1	0	Lamiacea Lindl.
Iz_15_1209	DJI_0056	Convallaria majalis L.	0	n	1	0	Asparagaceae Juss.
Iz_15_1209	DJI_0056	Crataegus rhipidophylla Gand.	0	n	1	1	Rosaceae Juss.
Iz_15_1209	DJI_0056	Crepis foetida L.	0	n	1	0	Asteraceae Bercht. & J.Presl
Iz_15_1209	DJI_0056	Dactylis glomerata L.	0	n	1	0	Poaceae Barnhart
Iz_15_1209	DJI_0056	Dianthus campestris M.Bieb.	0	n	1	0	Caryophyllaceae Juss.
Iz_15_1209	DJI_0056	Digitaria sanguinalis (L.) Scop.	0	a	0	0	Poaceae Barnhart
Iz_15_1209	DJI_0056	Elymus repens (L.) Gould	0	n	1	0	Poaceae Barnhart
Iz_15_1209	DJI_0056	Epilobium tetragonum L.	0	n	1	0	Onagraceae Juss.
Iz_15_1209	DJI_0056	Eragrostis minor Host	0	a	0	0	Poaceae Barnhart
Iz_15_1209	DJI_0056	Erigeron canadensis L.	0	a	0	0	Asteraceae Bercht. & J.Presl
Iz_15_1209	DJI_0056	Euphorbia seguieriana Neck.	0	n	1	0	Euphorbiaceae Juss.
Iz_15_1209	DJI_0056	Fallopia dumetorum (L.) Holub	0	n	1	0	Polygonaceae Juss.
Iz_15_1209	DJI_0056	Festuca beckeri (Hack.) Trautv.	0	n	1	0	Poaceae Barnhart
Iz_15_1209	DJI_0056	Fragaria vesca L.	0	n	1	0	Rosaceae Juss.
Iz_15_1209	DJI_0056	Frangula alnus Mill.	0	n	1	1	Rhamnaceae Juss.
Iz_15_1209	DJI_0056	Fraxinus pennsylvanica Marshall	0	a	0	1	Oleaceae Hoffmanns. & Link
Iz_15_1209	DJI_0056	Galeopsis bifida Boenn.	0	n	1	0	Lamiacea Lindl.
Iz_15_1209	DJI_0056	Galium verum L.	0	n	1	0	Rubiaceae Juss.
Iz_15_1209	DJI_0056	Genista tinctoria L.	0	n	1	0	Fabaceae Lindl.
Iz_15_1209	DJI_0056	Geranium robertianum L.	0	n	1	0	Geraniaceae Juss.
Iz_15_1209	DJI_0056	Geranium sanquineum L.	0	n	1	0	Geraniaceae Juss.
Iz_15_1209	DJI_0056	Gypsophila paniculata L.	0	n	1	0	Caryophyllaceae Juss.
Iz_15_1209	DJI_0056	Helichrysum arenarium (L.) Moench	0	n	1	0	Asteraceae Bercht. & J.Presl
Iz_15_1209	DJI_0056	Herniaria polygama J.Gay	0	n	1	0	Caryophyllaceae Juss.
Iz_15_1209	DJI_0056	Hieracium robustum Fr.	0	n	1	0	Asteraceae Bercht. & J.Presl
Iz_15_1209	DJI_0056	Hieracium umbellatum L.	0	n	1	NA	Asteraceae Bercht. & J.Presl
Iz_15_1209	DJI_0056	Hylotelephium maximum (L.) Holub	0	n	1	0	Crassulaceae J.St.-Hil.
Iz_15_1209	DJI_0056	Jacobaea borysthenica (DC.) B.Nord. & Greuter	0	n	1	0	Asteraceae Bercht. & J.Presl
Iz_15_1209	DJI_0056	Jasione montana L.	0	n	1	0	Campanulaceae Juss.
Iz_15_1209	DJI_0056	Jurinea cyanoides (L.) Rchb.s.l.	0	n	1	0	Asteraceae Bercht. & J.Presl
Iz_15_1209	DJI_0056	Koeleria glauca (Spreng.) DC.	0	n	1	0	Poaceae Barnhart
Iz_15_1209	DJI_0056	Lactuca serriola L.	0	a	0	0	Asteraceae Bercht. & J.Presl
Iz_15_1209	DJI_0056	Linaria genistifolia (L.) Mill.	0	n	1	0	Plantaginaceae Juss.
Iz_15_1209	DJI_0056	Lomelosia argentea (L.) Greuter & Burdet	0	n	1	0	Caprifoliaceae Juss.

Iz_15_1209	DJI_0056	Melampyrum pratense L.	0	n	1	0	Orobanchaceae Vent.
Iz_15_1209	DJI_0056	Oenothera biennis L.	0	a	0	0	Onagraceae Juss.
Iz_15_1209	DJI_0056	Pentanema britannica (L.) D.Gut.Larr., Santos-Vicente, Anderb., E.Rico & M.M.Mart.Ort.	0	n	1	0	Asteraceae Bercht. & J.Presl
Iz_15_1209	DJI_0056	Peucedanum oreoselinum (L.) Moench	1	n	1	0	Apiaceae Lindl.
Iz_15_1209	DJI_0056	Peucedanum ruthenicum M.Bieb.	0	n	1	0	Apiaceae Lindl.
Iz_15_1209	DJI_0056	Pilosella echioides (Lumn.) F.W.Schultz & Sch.Bip.	0	n	1	0	Asteraceae Bercht. & J.Presl
Iz_15_1209	DJI_0056	Pilosella officinarum F. Schultz et Sch. Bip.	0	n	1	0	Asteraceae Bercht. & J.Presl
Iz_15_1209	DJI_0056	Pilosella sp.	0		NA	0	Asteraceae Bercht. & J.Presl
Iz_15_1209	DJI_0056	Pinus sylvestris L.	0	n	1	1	Pinaceae Spreng. ex F.Rudolphi
Iz_15_1209	DJI_0056	Poa annua L.	0	n	1	0	Poaceae Barnhart
Iz_15_1209	DJI_0056	Poa nemoralis L.	0	n	1	0	Poaceae Barnhart
Iz_15_1209	DJI_0056	Poa sp.	0		NA	0	Poaceae Barnhart
Iz_15_1209	DJI_0056	Polygonatum odoratum (Mill.) Druce	0	n	1	0	Asparagaceae Juss.
Iz_15_1209	DJI_0056	Polygonum arenarium Waldst. & Kit.	0	n	1	0	Polygonaceae Juss.
Iz_15_1209	DJI_0056	Populus nigra L.	0	n	1	1	Salicaceae Mirb.
Iz_15_1209	DJI_0056	Populus tremula L.	0	n	1	1	Salicaceae Mirb.
Iz_15_1209	DJI_0056	Populus Ã canescens (Aiton) Sm.	0	n	1	1	Salicaceae Mirb.
Iz_15_1209	DJI_0056	Potentilla argentea L.	0	n	1	0	Rosaceae Juss.
Iz_15_1209	DJI_0056	Psammophiliella muralis (L.) Ikonn.	0	n	1	0	Caryophyllaceae Juss.
Iz_15_1209	DJI_0056	Psephellus sumensis (Kalen.) Greuter	0	n	1	0	Asteraceae Bercht. & J.Presl
Iz_15_1209	DJI_0056	Pulsatilla pratensis (L.) Mill. s.l.	0	n	1	0	Ranunculaceae Juss.
Iz_15_1209	DJI_0056	Pyrus communis L.	0	a	0	1	Rosaceae Juss.
Iz_15_1209	DJI_0056	Quercus robur L.	0	n	1	1	Fagaceae Dumort.
Iz_15_1209	DJI_0056	Robinia pseudoacacia L.	0	a	0	1	Fabaceae Lindl.
Iz_15_1209	DJI_0056	Rosa sp.	0		NA	1	Rosaceae Juss.
Iz_15_1209	DJI_0056	Rubus idaeus L.	0	n	1	0	Rosaceae Juss.
Iz_15_1209	DJI_0056	Rumex acetosella L.	0	n	1	0	Polygonaceae Juss.
Iz_15_1209	DJI_0056	Rumex thyrsiflorus Fingerh.	0	n	1	0	Polygonaceae Juss.
Iz_15_1209	DJI_0056	Salix alba L.	0	n	1	1	Salicaceae Mirb.
Iz_15_1209	DJI_0056	Sambucus nigra L.	0	n	1	1	Viburnaceae Raf.
Iz_15_1209	DJI_0056	Secale sylvestre Host	0	n	1	0	Poaceae Barnhart
Iz_15_1209	DJI_0056	Sempervivum ruthenicum Schnittsp. & C.B. Lehm.	0	n	1	0	Crassulaceae J.St.-Hil.
Iz_15_1209	DJI_0056	Setaria viridis (L.) P. Beauv.	0	n	1	0	Poaceae Barnhart
Iz_15_1209	DJI_0056	Silene borysthenica (Gruner) Walters	0	n	1	0	Caryophyllaceae Juss.
Iz_15_1209	DJI_0056	Silene latifolia subsp. alba (Mill.) Greuter & Burdet	0	n	1	0	Caryophyllaceae Juss.
Iz_15_1209	DJI_0056	Solidago virgaurea L.	0	n	1	0	Asteraceae Bercht. & J.Presl
Iz_15_1209	DJI_0056	Sorbus aucuparia L.	0	n	1	1	Rosaceae Juss.
Iz_15_1209	DJI_0056	Stachys recta L.	0	n	1	0	Lamiacea Lindl.
Iz_15_1209	DJI_0056	Tanacetum vulgare L.	0	n	1	0	Asteraceae Bercht. & J.Presl
Iz_15_1209	DJI_0056	Thymus pallasianus Heinr.Braun	0	n	1	0	Lamiacea Lindl.
Iz_15_1209	DJI_0056	Tilia cordata Mill.	0	n	1	1	Malvaceae Juss.
Iz_15_1209	DJI_0056	Trifolium alpestre L.	0	n	1	0	Fabaceae Lindl.
Iz_15_1209	DJI_0056	Trifolium arvense L.	0	n	1	0	Fabaceae Lindl.
Iz_15_1209	DJI_0056	Ulmus glabra Huds.	0	n	1	1	Ulmaceae Mirb.
Iz_15_1209	DJI_0056	Verbascum densiflorum Bertol.	0	n	1	0	Scrophulariaceae Juss.
Iz_15_1209	DJI_0056	Veronica austriaca L.	0	n	1	0	Plantaginaceae Juss.
Iz_15_1209	DJI_0056	Veronica incana L.	0	n	1	0	Plantaginaceae Juss.
Iz_15_1209	DJI_0056	Veronica verna L.	0	n	1	0	Plantaginaceae Juss.
Iz_15_1209	DJI_0056	Vincetoxicum hirundinaria Medik	0	n	1	0	Apocynaceae Juss.
Iz_15_1209	DJI_0065	Acer tataricum L.	0	n	1	1	Sapindaceae Juss.
Iz_15_1209	DJI_0065	Achillea millefolium L. s.l.	0	n	1	0	Asteraceae Bercht. & J.Presl
Iz_15_1209	DJI_0065	Allium oleraceum L.	0	n	1	0	Amaryllidaceae J.St.-Hil.
Iz_15_1209	DJI_0065	Ambrosia artemisiifolia L.	0	a	0	0	Asteraceae Bercht. & J.Presl
Iz_15_1209	DJI_0065	Amorpha fruticosa L.	0	a	0	0	Fabaceae Lindl.
Iz_15_1209	DJI_0065	Anthericum ramosum L.	0	n	1	0	Asparagaceae Juss.
Iz_15_1209	DJI_0065	Artemisia marschalliana Spreng.	0	n	1	0	Asteraceae Bercht. & J.Presl
Iz_15_1209	DJI_0065	Asparagus officinalis L.	0	n	1	0	Asparagaceae Juss.
Iz_15_1209	DJI_0065	Astragalus cicer L.	0	n	1	0	Fabaceae Lindl.
Iz_15_1209	DJI_0065	Bassia laniflora (S.G.Gmel.) A.J.Scott	0	n	1	0	Amaranthaceae Juss.
Iz_15_1209	DJI_0065	Berberis vulgaris L.	0	n	1	0	Berberidaceae Juss.
Iz_15_1209	DJI_0065	Betula pendula Roth.	0	n	1	1	Betulaceae Gray
Iz_15_1209	DJI_0065	Betula pendula Roth.	0	n	1	0	Poaceae Barnhart
Iz_15_1209	DJI_0065	Bromus inermis Leyss.	0		NA	NA	
Iz_15_1209	DJI_0065	Bromus squarrosus L.	0	a	0	0	Poaceae Barnhart
Iz_15_1209	DJI_0065	Bromus tectorum L.	0	a	0	0	Poaceae Barnhart
Iz_15_1209	DJI_0065	Calamagrostis epigejos (L.) Roth	0	n	1	0	Poaceae Barnhart
Iz_15_1209	DJI_0065	Carduus acanthoides L.	0	n	1	0	Asteraceae Bercht. & J.Presl
Iz_15_1209	DJI_0065	Carex sp.	0	n	1	0	Cyperaceae Juss.
Iz_15_1209	DJI_0065	Centaurea majorovii Dumbadze	0	n	1	0	Asteraceae Bercht. & J.Presl
Iz_15_1209	DJI_0065	Chamaecytisus ruthenicus (Fisch.ex Wol.) Klaskova	0	n	1	0	Fabaceae Lindl.
Iz_15_1209	DJI_0065	Chelidonium majus L.	0	n	1	0	Papaveraceae Juss.
Iz_15_1209	DJI_0065	Chenopodium betaceum Andrz.	0	n	1	0	Amaranthaceae Juss.
Iz_15_1209	DJI_0065	Chenopodium cf. album	0	n	1	0	Amaranthaceae Juss.
Iz_15_1209	DJI_0065	Chenopodium sp.	0		NA	0	Amaranthaceae Juss.
Iz_15_1209	DJI_0065	Chondrilla juncea L.	0	n	1	0	Asteraceae Bercht. & J.Presl
Iz_15_1209	DJI_0065	Cirsium vulgare (Savi) Ten.	0	n	1	0	Asteraceae Bercht. & J.Presl
Iz_15_1209	DJI_0065	Clinopodium acinos (L.) Kuntze	0	n	1	0	Lamiacea Lindl.
Iz_15_1209	DJI_0065	Convallaria majalis L.	0	n	1	0	Asparagaceae Juss.
Iz_15_1209	DJI_0065	Crataegus rhipidophylla Gand.	0	n	1	1	Rosaceae Juss.
Iz_15_1209	DJI_0065	Crepis foetida L.	0	n	1	0	Asteraceae Bercht. & J.Presl
Iz_15_1209	DJI_0065	Dactylis glomerata L.	0	n	1	0	Poaceae Barnhart
Iz_15_1209	DJI_0065	Dianthus campestris M.Bieb.	0	n	1	0	Caryophyllaceae Juss.
Iz_15_1209	DJI_0065	Digitaria sanguinalis (L.) Scop.	0	a	0	0	Poaceae Barnhart
Iz_15_1209	DJI_0065	Elymus repens (L.) Gould	0	n	1	0	Poaceae Barnhart
Iz_15_1209	DJI_0065	Epilobium tetragonum L.	0	n	1	0	Onagraceae Juss.
Iz_15_1209	DJI_0065	Eragrostis minor Host	0	a	0	0	Poaceae Barnhart
Iz_15_1209	DJI_0065	Erigeron canadensis L.	0	a	0	0	Asteraceae Bercht. & J.Presl
Iz_15_1209	DJI_0065	Euphorbia seguieriana Neck.	0	n	1	0	Euphorbiaceae Juss.
Iz_15_1209	DJI_0065	Fallopia dumetorum (L.) Holub	0	n	1	0	Polygonaceae Juss.
Iz_15_1209	DJI_0065	Festuca beckeri (Hack.) Trautv.	0	n	1	0	Poaceae Barnhart
Iz_15_1209	DJI_0065	Fragaria vesca L.	0	n	1	0	Rosaceae Juss.
Iz_15_1209	DJI_0065	Frangula alnus Mill.	0	n	1	1	Rhamnaceae Juss.
Iz_15_1209	DJI_0065	Fraxinus pennsylvanica Marshall	0	a	0	1	Oleaceae Hoffmanns. & Link
Iz_15_1209	DJI_0065	Galeopsis bifida Boenn.	0	n	1	0	Lamiacea Lindl.
Iz_15_1209	DJI_0065	Galium verum L.	0	n	1	0	Rubiaceae Juss.
Iz_15_1209	DJI_0065	Genista tinctoria L.	0	n	1	0	Fabaceae Lindl.
Iz_15_1209	DJI_0065	Geranium robertianum L.	0	n	1	0	Geraniaceae Juss.
Iz_15_1209	DJI_0065	Geranium sanquineum L.	0	n	1	0	Geraniaceae Juss.

Iz_15_1209	DJI_0065	Gypsophila paniculata L.	0	n	1	0	Caryophyllaceae Juss.
Iz_15_1209	DJI_0065	Helichrysum arenarium (L.) Moench	0	n	1	0	Asteraceae Bercht. & J.Presl
Iz_15_1209	DJI_0065	Herniaria polygama J.Gay	0	n	1	0	Caryophyllaceae Juss.
Iz_15_1209	DJI_0065	Hieracium robustum Fr.	0	n	1	0	Asteraceae Bercht. & J.Presl
Iz_15_1209	DJI_0065	Hieracium umbellatum L.	0	n	1	NA	Asteraceae Bercht. & J.Presl
Iz_15_1209	DJI_0065	Hylotelephium maximum (L.) Holub	0	n	1	0	Crassulaceae J.St.-Hil.
Iz_15_1209	DJI_0065	Jacobaea borysthenica (DC.) B.Nord. & Greuter	0	n	1	0	Asteraceae Bercht. & J.Presl
Iz_15_1209	DJI_0065	Jasione montana L.	0	n	1	0	Campanulaceae Juss.
Iz_15_1209	DJI_0065	Jurinea cyanoides (L.) Rchb.s.l.	0	n	1	0	Asteraceae Bercht. & J.Presl
Iz_15_1209	DJI_0065	Koeleria glauca (Spreng.) DC.	0	n	1	0	Poaceae Barnhart
Iz_15_1209	DJI_0065	Lactuca serriola L.	0	a	0	0	Asteraceae Bercht. & J.Presl
Iz_15_1209	DJI_0065	Linaria genistifolia (L.) Mill.	0	n	1	0	Plantaginaceae Juss.
Iz_15_1209	DJI_0065	Lomelosia argentea (L.) Greuter & Burdet	0	n	1	0	Caprifoliaceae Juss.
Iz_15_1209	DJI_0065	Melampyrum pratense L.	0	n	1	0	Orobanchaceae Vent.
Iz_15_1209	DJI_0065	Oenothera biennis L.	0	a	0	0	Onagraceae Juss.
Iz_15_1209	DJI_0065	Pentanema britannica (L.) D.Gut.Larr., Santos-Vicente, Anderb., E.Rico & M.M.Mart.Ort.	0	n	1	0	Asteraceae Bercht. & J.Presl
Iz_15_1209	DJI_0065	Peucedanum oreoselinum (L.) Moench	0.5	n	1	0	Apiaceae Lindl.
Iz_15_1209	DJI_0065	Peucedanum ruthenicum M.Bieb.	0	n	1	0	Apiaceae Lindl.
Iz_15_1209	DJI_0065	Pilosella echioides (Lumn.) F.W.Schultz & Sch.Bip.	0	n	1	0	Asteraceae Bercht. & J.Presl
Iz_15_1209	DJI_0065	Pilosella officinarum F. Schultz et Sch. Bip.	0	n	1	0	Asteraceae Bercht. & J.Presl
Iz_15_1209	DJI_0065	Pilosella sp.	0		NA	0	Asteraceae Bercht. & J.Presl
Iz_15_1209	DJI_0065	Pinus sylvestris L.	0	n	1	1	Pinaceae Spreng. ex F.Rudolphi
Iz_15_1209	DJI_0065	Poa annua L.	0	n	1	0	Poaceae Barnhart
Iz_15_1209	DJI_0065	Poa nemoralis L.	0	n	1	0	Poaceae Barnhart
Iz_15_1209	DJI_0065	Poa sp.	0		NA	0	Poaceae Barnhart
Iz_15_1209	DJI_0065	Polygonatum odoratum (Mill.) Druce	0	n	1	0	Asparagaceae Juss.
Iz_15_1209	DJI_0065	Polygonum arenarium Waldst. & Kit.	0	n	1	0	Polygonaceae Juss.
Iz_15_1209	DJI_0065	Populus nigra L.	0	n	1	1	Salicaceae Mirb.
Iz_15_1209	DJI_0065	Populus tremula L.	0	n	1	1	Salicaceae Mirb.
Iz_15_1209	DJI_0065	Populus Ã canescens (Aiton) Sm.	0	n	1	1	Salicaceae Mirb.
Iz_15_1209	DJI_0065	Potentilla argentea L.	0	n	1	0	Rosaceae Juss.
Iz_15_1209	DJI_0065	Psammophiliella muralis (L.) Ikonn.	0	n	1	0	Caryophyllaceae Juss.
Iz_15_1209	DJI_0065	Psephellus sumensis (Kalen.) Greuter	0	n	1	0	Asteraceae Bercht. & J.Presl
Iz_15_1209	DJI_0065	Pulsatilla pratensis (L.) Mill. s.l.	0	n	1	0	Ranunculaceae Juss.
Iz_15_1209	DJI_0065	Pyrus communis L.	0	a	0	1	Rosaceae Juss.
Iz_15_1209	DJI_0065	Quercus robur L.	0	n	1	1	Fagaceae Dumort.
Iz_15_1209	DJI_0065	Robinia pseudoacacia L.	0	a	0	1	Fabaceae Lindl.
Iz_15_1209	DJI_0065	Rosa sp.	0		NA	1	Rosaceae Juss.
Iz_15_1209	DJI_0065	Rubus idaeus L.	0	n	1	0	Rosaceae Juss.
Iz_15_1209	DJI_0065	Rumex acetosella L.	0	n	1	0	Polygonaceae Juss.
Iz_15_1209	DJI_0065	Rumex thyrsiflorus Fingerh.	0	n	1	0	Polygonaceae Juss.
Iz_15_1209	DJI_0065	Salix alba L.	0	n	1	1	Salicaceae Mirb.
Iz_15_1209	DJI_0065	Sambucus nigra L.	0	n	1	1	Viburnaceae Raf.
Iz_15_1209	DJI_0065	Secale sylvestre Host	0	n	1	0	Poaceae Barnhart
Iz_15_1209	DJI_0065	Sempervivum ruthenicum Schnittsp. & C.B. Lehm.	0	n	1	0	Crassulaceae J.St.-Hil.
Iz_15_1209	DJI_0065	Setaria viridis (L.) P. Beauv.	0	n	1	0	Poaceae Barnhart
Iz_15_1209	DJI_0065	Silene borysthenica (Gruner) Walters	0	n	1	0	Caryophyllaceae Juss.
Iz_15_1209	DJI_0065	Silene latifolia subsp. alba (Mill.) Greuter & Burdet	0	n	1	0	Caryophyllaceae Juss.
Iz_15_1209	DJI_0065	Solidago virgaurea L.	0	n	1	0	Asteraceae Bercht. & J.Presl
Iz_15_1209	DJI_0065	Sorbus aucuparia L.	0	n	1	1	Rosaceae Juss.
Iz_15_1209	DJI_0065	Stachys recta L.	0	n	1	0	Lamiacea Lindl.
Iz_15_1209	DJI_0065	Tanacetum vulgare L.	0	n	1	0	Asteraceae Bercht. & J.Presl
Iz_15_1209	DJI_0065	Thymus pallasianus Heinr.Braun	0	n	1	0	Lamiacea Lindl.
Iz_15_1209	DJI_0065	Tilia cordata Mill.	0	n	1	1	Malvaceae Juss.
Iz_15_1209	DJI_0065	Trifolium alpestre L.	0	n	1	0	Fabaceae Lindl.
Iz_15_1209	DJI_0065	Trifolium arvense L.	0	n	1	0	Fabaceae Lindl.
Iz_15_1209	DJI_0065	Ulmus glabra Huds.	0	n	1	1	Ulmaceae Mirb.
Iz_15_1209	DJI_0065	Verbascum densiflorum Bertol.	0	n	1	0	Scrophulariaceae Juss.
Iz_15_1209	DJI_0065	Veronica austriaca L.	0	n	1	0	Plantaginaceae Juss.
Iz_15_1209	DJI_0065	Veronica incana L.	0	n	1	0	Plantaginaceae Juss.
Iz_15_1209	DJI_0065	Veronica verna L.	0	n	1	0	Plantaginaceae Juss.
Iz_15_1209	DJI_0065	Vincetoxicum hirundinaria Medik	0	n	1	0	Apocynaceae Juss.
Iz_15_1209	DJI_0069	Acer tataricum L.	0	n	1	1	Sapindaceae Juss.
Iz_15_1209	DJI_0069	Achillea millefolium L. s.l.	0	n	1	0	Asteraceae Bercht. & J.Presl
Iz_15_1209	DJI_0069	Allium oleraceum L.	0	n	1	0	Amaryllidaceae J.St.-Hil.
Iz_15_1209	DJI_0069	Ambrosia artemisiifolia L.	0	a	0	0	Asteraceae Bercht. & J.Presl
Iz_15_1209	DJI_0069	Amorpha fruticosa L.	0	a	0	0	Fabaceae Lindl.
Iz_15_1209	DJI_0069	Anthericum ramosum L.	0	n	1	0	Asparagaceae Juss.
Iz_15_1209	DJI_0069	Artemisia marschalliana Spreng.	0	n	1	0	Asteraceae Bercht. & J.Presl
Iz_15_1209	DJI_0069	Asparagus officinalis L.	0	n	1	0	Asparagaceae Juss.
Iz_15_1209	DJI_0069	Astragalus cicer L.	0	n	1	0	Fabaceae Lindl.
Iz_15_1209	DJI_0069	Bassia laniflora (S.G.Gmel.) A.J.Scott	0	n	1	0	Amaranthaceae Juss.
Iz_15_1209	DJI_0069	Berberis vulgaris L.	0	n	1	0	Berberidaceae Juss.
Iz_15_1209	DJI_0069	Betula pendula Roth.	0	n	1	1	Betulaceae Gray
Iz_15_1209	DJI_0069	Betula pendula Roth.	0	n	1	0	Poaceae Barnhart
Iz_15_1209	DJI_0069	Bromus inermis Leyss.	0		NA	NA	
Iz_15_1209	DJI_0069	Bromus squarrosus L.	0	a	0	0	Poaceae Barnhart
Iz_15_1209	DJI_0069	Bromus tectorum L.	0	a	0	0	Poaceae Barnhart
Iz_15_1209	DJI_0069	Calamagrostis epigejos (L.) Roth	0.1	n	1	0	Poaceae Barnhart
Iz_15_1209	DJI_0069	Carduus acanthoides L.	0	n	1	0	Asteraceae Bercht. & J.Presl
Iz_15_1209	DJI_0069	Carex sp.	0	n	1	0	Cyperaceae Juss.
Iz_15_1209	DJI_0069	Centaurea majorovii Dumbadze	0	n	1	0	Asteraceae Bercht. & J.Presl
Iz_15_1209	DJI_0069	Chamaecytisus ruthenicus (Fisch.ex Wol.) Klaskova	0	n	1	0	Fabaceae Lindl.
Iz_15_1209	DJI_0069	Chelidonium majus L.	0	n	1	0	Papaveraceae Juss.
Iz_15_1209	DJI_0069	Chenopodium betaceum Andrz.	0	n	1	0	Amaranthaceae Juss.
Iz_15_1209	DJI_0069	Chenopodium cf. album	0	n	1	0	Amaranthaceae Juss.
Iz_15_1209	DJI_0069	Chenopodium sp.	0		NA	0	Amaranthaceae Juss.
Iz_15_1209	DJI_0069	Chondrilla juncea L.	0	n	1	0	Asteraceae Bercht. & J.Presl
Iz_15_1209	DJI_0069	Cirsium vulgare (Savi) Ten.	0	n	1	0	Asteraceae Bercht. & J.Presl
Iz_15_1209	DJI_0069	Clinopodium acinos (L.) Kuntze	0	n	1	0	Lamiacea Lindl.
Iz_15_1209	DJI_0069	Convallaria majalis L.	0	n	1	0	Asparagaceae Juss.
Iz_15_1209	DJI_0069	Crataegus rhipidophylla Gand.	0	n	1	1	Rosaceae Juss.
Iz_15_1209	DJI_0069	Crepis foetida L.	0	n	1	0	Asteraceae Bercht. & J.Presl
Iz_15_1209	DJI_0069	Dactylis glomerata L.	0	n	1	0	Poaceae Barnhart
Iz_15_1209	DJI_0069	Dianthus campestris M.Bieb.	0	n	1	0	Caryophyllaceae Juss.
Iz_15_1209	DJI_0069	Digitaria sanguinalis (L.) Scop.	0	a	0	0	Poaceae Barnhart
Iz_15_1209	DJI_0069	Elymus repens (L.) Gould	0	n	1	0	Poaceae Barnhart
Iz_15_1209	DJI_0069	Epilobium tetragonum L.	0	n	1	0	Onagraceae Juss.

Iz_15_1209	DJI_0069	Eragrostis minor Host	0	a	0	0	Poaceae Barnhart
Iz_15_1209	DJI_0069	Erigeron canadensis L.	0	a	0	0	Asteraceae Bercht. & J.Presl
Iz_15_1209	DJI_0069	Euphorbia seguieriana Neck.	0	n	1	0	Euphorbiaceae Juss.
Iz_15_1209	DJI_0069	Fallopia dumetorum (L.) Holub	0	n	1	0	Polygonaceae Juss.
Iz_15_1209	DJI_0069	Festuca beckeri (Hack.) Trautv.	0	n	1	0	Poaceae Barnhart
Iz_15_1209	DJI_0069	Fragaria vesca L.	0	n	1	0	Rosaceae Juss.
Iz_15_1209	DJI_0069	Frangula alnus Mill.	0	n	1	1	Rhamnaceae Juss.
Iz_15_1209	DJI_0069	Fraxinus pennsylvanica Marshall	0	a	0	1	Oleaceae Hoffmanns. & Link
Iz_15_1209	DJI_0069	Galeopsis bifida Boenn.	0	n	1	0	Lamiacea Lindl.
Iz_15_1209	DJI_0069	Galium verum L.	0	n	1	0	Rubiaceae Juss.
Iz_15_1209	DJI_0069	Genista tinctoria L.	0	n	1	0	Fabaceae Lindl.
Iz_15_1209	DJI_0069	Geranium robertianum L.	0	n	1	0	Geraniaceae Juss.
Iz_15_1209	DJI_0069	Geranium sanquineum L.	0	n	1	0	Geraniaceae Juss.
Iz_15_1209	DJI_0069	Gypsophila paniculata L.	0	n	1	0	Caryophyllaceae Juss.
Iz_15_1209	DJI_0069	Helichrysum arenarium (L.) Moench	0	n	1	0	Asteraceae Bercht. & J.Presl
Iz_15_1209	DJI_0069	Herniaria polygama J.Gay	0	n	1	0	Caryophyllaceae Juss.
Iz_15_1209	DJI_0069	Hieracium robustum Fr.	0	n	1	0	Asteraceae Bercht. & J.Presl
Iz_15_1209	DJI_0069	Hieracium umbellatum L.	0	n	1	NA	Asteraceae Bercht. & J.Presl
Iz_15_1209	DJI_0069	Hylotelephium maximum (L.) Holub	0	n	1	0	Crassulaceae J.St.-Hil.
Iz_15_1209	DJI_0069	Jacobaea borysthenica (DC.) B.Nord. & Greuter	0	n	1	0	Asteraceae Bercht. & J.Presl
Iz_15_1209	DJI_0069	Jasione montana L.	0	n	1	0	Campanulaceae Juss.
Iz_15_1209	DJI_0069	Jurinea cyanoides (L.) Rchb.s.l.	0	n	1	0	Asteraceae Bercht. & J.Presl
Iz_15_1209	DJI_0069	Koeleria glauca (Spreng.) DC.	0	n	1	0	Poaceae Barnhart
Iz_15_1209	DJI_0069	Lactuca serriola L.	0	a	0	0	Asteraceae Bercht. & J.Presl
Iz_15_1209	DJI_0069	Linaria genistifolia (L.) Mill.	0	n	1	0	Plantaginaceae Juss.
Iz_15_1209	DJI_0069	Lomelosia argentea (L.) Greuter & Burdet	0	n	1	0	Caprifoliaceae Juss.
Iz_15_1209	DJI_0069	Melampyrum pratense L.	0	n	1	0	Orobanchaceae Vent.
Iz_15_1209	DJI_0069	Oenothera biennis L.	0	a	0	0	Onagraceae Juss.
Iz_15_1209	DJI_0069	Pentanema britannica (L.) D.Gut.Larr., Santos-Vicente, Anderb., E.Rico & M.M.Mart.Ort.	0	n	1	0	Asteraceae Bercht. & J.Presl
Iz_15_1209	DJI_0069	Peucedanum oreoselinum (L.) Moench	0	n	1	0	Apiaceae Lindl.
Iz_15_1209	DJI_0069	Peucedanum ruthenicum M.Bieb.	0	n	1	0	Apiaceae Lindl.
Iz_15_1209	DJI_0069	Pilosella echioides (Lumn.) F.W.Schultz & Sch.Bip.	0	n	1	0	Asteraceae Bercht. & J.Presl
Iz_15_1209	DJI_0069	Pilosella officinarum F. Schultz et Sch. Bip.	0	n	1	0	Asteraceae Bercht. & J.Presl
Iz_15_1209	DJI_0069	Pilosella sp.	0		NA	0	Asteraceae Bercht. & J.Presl
Iz_15_1209	DJI_0069	Pinus sylvestris L.	0	n	1	1	Pinaceae Spreng. ex F.Rudolphi
Iz_15_1209	DJI_0069	Poa annua L.	0	n	1	0	Poaceae Barnhart
Iz_15_1209	DJI_0069	Poa nemoralis L.	0	n	1	0	Poaceae Barnhart
Iz_15_1209	DJI_0069	Poa sp.	0		NA	0	Poaceae Barnhart
Iz_15_1209	DJI_0069	Polygonatum odoratum (Mill.) Druce	0	n	1	0	Asparagaceae Juss.
Iz_15_1209	DJI_0069	Polygonum arenarium Waldst. & Kit.	0	n	1	0	Polygonaceae Juss.
Iz_15_1209	DJI_0069	Populus nigra L.	0	n	1	1	Salicaceae Mirb.
Iz_15_1209	DJI_0069	Populus tremula L.	0	n	1	1	Salicaceae Mirb.
Iz_15_1209	DJI_0069	Populus Ã canescens (Aiton) Sm.	0	n	1	1	Salicaceae Mirb.
Iz_15_1209	DJI_0069	Potentilla argentea L.	0	n	1	0	Rosaceae Juss.
Iz_15_1209	DJI_0069	Psammophiliella muralis (L.) Ikonn.	0	n	1	0	Caryophyllaceae Juss.
Iz_15_1209	DJI_0069	Psephellus sumensis (Kalen.) Greuter	0	n	1	0	Asteraceae Bercht. & J.Presl
Iz_15_1209	DJI_0069	Pulsatilla pratensis (L.) Mill. s.l.	0	n	1	0	Ranunculaceae Juss.
Iz_15_1209	DJI_0069	Pyrus communis L.	0	a	0	1	Rosaceae Juss.
Iz_15_1209	DJI_0069	Quercus robur L.	0	n	1	1	Fagaceae Dumort.
Iz_15_1209	DJI_0069	Robinia pseudoacacia L.	0	a	0	1	Fabaceae Lindl.
Iz_15_1209	DJI_0069	Rosa sp.	0		NA	1	Rosaceae Juss.
Iz_15_1209	DJI_0069	Rubus idaeus L.	0	n	1	0	Rosaceae Juss.
Iz_15_1209	DJI_0069	Rumex acetosella L.	0	n	1	0	Polygonaceae Juss.
Iz_15_1209	DJI_0069	Rumex thyrsiflorus Fingerh.	0	n	1	0	Polygonaceae Juss.
Iz_15_1209	DJI_0069	Salix alba L.	0	n	1	1	Salicaceae Mirb.
Iz_15_1209	DJI_0069	Sambucus nigra L.	0	n	1	1	Viburnaceae Raf.
Iz_15_1209	DJI_0069	Secale sylvestre Host	0	n	1	0	Poaceae Barnhart
Iz_15_1209	DJI_0069	Sempervivum ruthenicum Schnittsp. & C.B. Lehm.	0	n	1	0	Crassulaceae J.St.-Hil.
Iz_15_1209	DJI_0069	Setaria viridis (L.) P. Beauv.	0	n	1	0	Poaceae Barnhart
Iz_15_1209	DJI_0069	Silene borysthenica (Gruner) Walters	0	n	1	0	Caryophyllaceae Juss.
Iz_15_1209	DJI_0069	Silene latifolia subsp. alba (Mill.) Greuter & Burdet	0	n	1	0	Caryophyllaceae Juss.
Iz_15_1209	DJI_0069	Solidago virgaurea L.	0	n	1	0	Asteraceae Bercht. & J.Presl
Iz_15_1209	DJI_0069	Sorbus aucuparia L.	0	n	1	1	Rosaceae Juss.
Iz_15_1209	DJI_0069	Stachys recta L.	0	n	1	0	Lamiacea Lindl.
Iz_15_1209	DJI_0069	Tanacetum vulgare L.	0	n	1	0	Asteraceae Bercht. & J.Presl
Iz_15_1209	DJI_0069	Thymus pallasianus Heinr.Braun	0	n	1	0	Lamiacea Lindl.
Iz_15_1209	DJI_0069	Tilia cordata Mill.	0	n	1	1	Malvaceae Juss.
Iz_15_1209	DJI_0069	Trifolium alpestre L.	0	n	1	0	Fabaceae Lindl.
Iz_15_1209	DJI_0069	Trifolium arvense L.	0	n	1	0	Fabaceae Lindl.
Iz_15_1209	DJI_0069	Ulmus glabra Huds.	0	n	1	1	Ulmaceae Mirb.
Iz_15_1209	DJI_0069	Verbascum densiflorum Bertol.	0	n	1	0	Scrophulariaceae Juss.
Iz_15_1209	DJI_0069	Veronica austriaca L.	0	n	1	0	Plantaginaceae Juss.
Iz_15_1209	DJI_0069	Veronica incana L.	0	n	1	0	Plantaginaceae Juss.
Iz_15_1209	DJI_0069	Veronica verna L.	0	n	1	0	Plantaginaceae Juss.
Iz_15_1209	DJI_0069	Vincetoxicum hirundinaria Medik	0	n	1	0	Apocynaceae Juss.
Iz_15_1209	DJI_0072	Acer tataricum L.	0	n	1	1	Sapindaceae Juss.
Iz_15_1209	DJI_0072	Achillea millefolium L. s.l.	0	n	1	0	Asteraceae Bercht. & J.Presl
Iz_15_1209	DJI_0072	Allium oleraceum L.	0	n	1	0	Amaryllidaceae J.St.-Hil.
Iz_15_1209	DJI_0072	Ambrosia artemisiifolia L.	0	a	0	0	Asteraceae Bercht. & J.Presl
Iz_15_1209	DJI_0072	Amorpha fruticosa L.	0	a	0	0	Fabaceae Lindl.
Iz_15_1209	DJI_0072	Anthericum ramosum L.	0	n	1	0	Asparagaceae Juss.
Iz_15_1209	DJI_0072	Artemisia marschalliana Spreng.	0	n	1	0	Asteraceae Bercht. & J.Presl
Iz_15_1209	DJI_0072	Asparagus officinalis L.	0	n	1	0	Asparagaceae Juss.
Iz_15_1209	DJI_0072	Astragalus cicer L.	0	n	1	0	Fabaceae Lindl.
Iz_15_1209	DJI_0072	Bassia laniflora (S.G.Gmel.) A.J.Scott	0	n	1	0	Amaranthaceae Juss.
Iz_15_1209	DJI_0072	Berberis vulgaris L.	0	n	1	0	Berberidaceae Juss.
Iz_15_1209	DJI_0072	Betula pendula Roth.	0	n	1	1	Betulaceae Gray
Iz_15_1209	DJI_0072	Betula pendula Roth.	0	n	1	0	Poaceae Barnhart
Iz_15_1209	DJI_0072	Bromus inermis Leyss.	0		NA	NA	
Iz_15_1209	DJI_0072	Bromus squarrosus L.	0	a	0	0	Poaceae Barnhart
Iz_15_1209	DJI_0072	Bromus tectorum L.	0	a	0	0	Poaceae Barnhart
Iz_15_1209	DJI_0072	Calamagrostis epigejos (L.) Roth	0.1	n	1	0	Poaceae Barnhart
Iz_15_1209	DJI_0072	Carduus acanthoides L.	0	n	1	0	Asteraceae Bercht. & J.Presl
Iz_15_1209	DJI_0072	Carex sp.	2	n	1	0	Cyperaceae Juss.
Iz_15_1209	DJI_0072	Centaurea majorovii Dumbadze	0	n	1	0	Asteraceae Bercht. & J.Presl
Iz_15_1209	DJI_0072	Chamaecytisus ruthenicus (Fisch.ex Wol.) Klaskova	0	n	1	0	Fabaceae Lindl.
Iz_15_1209	DJI_0072	Chelidonium majus L.	0	n	1	0	Papaveraceae Juss.
Iz_15_1209	DJI_0072	Chenopodium betaceum Andrz.	0	n	1	0	Amaranthaceae Juss.

Iz_15_1209	DJI_0072	Chenopodium cf. album	0	n	1	0	Amaranthaceae Juss.
Iz_15_1209	DJI_0072	Chenopodium sp.	0		NA	0	Amaranthaceae Juss.
Iz_15_1209	DJI_0072	Chondrilla juncea L.	0	n	1	0	Asteraceae Bercht. & J.Presl
Iz_15_1209	DJI_0072	Cirsium vulgare (Savi) Ten.	0	n	1	0	Asteraceae Bercht. & J.Presl
Iz_15_1209	DJI_0072	Clinopodium acinos (L.) Kuntze	0	n	1	0	Lamiacea Lindl.
Iz_15_1209	DJI_0072	Convallaria majalis L.	0	n	1	0	Asparagaceae Juss.
Iz_15_1209	DJI_0072	Crataegus rhipidophylla Gand.	0	n	1	1	Rosaceae Juss.
Iz_15_1209	DJI_0072	Crepis foetida L.	0	n	1	0	Asteraceae Bercht. & J.Presl
Iz_15_1209	DJI_0072	Dactylis glomerata L.	0	n	1	0	Poaceae Barnhart
Iz_15_1209	DJI_0072	Dianthus campestris M.Bieb.	0	n	1	0	Caryophyllaceae Juss.
Iz_15_1209	DJI_0072	Digitaria sanguinalis (L.) Scop.	0	a	0	0	Poaceae Barnhart
Iz_15_1209	DJI_0072	Elymus repens (L.) Gould	0	n	1	0	Poaceae Barnhart
Iz_15_1209	DJI_0072	Epilobium tetragonum L.	0	n	1	0	Onagraceae Juss.
Iz_15_1209	DJI_0072	Eragrostis minor Host	0	a	0	0	Poaceae Barnhart
Iz_15_1209	DJI_0072	Erigeron canadensis L.	0	a	0	0	Asteraceae Bercht. & J.Presl
Iz_15_1209	DJI_0072	Euphorbia seguieriana Neck.	0	n	1	0	Euphorbiaceae Juss.
Iz_15_1209	DJI_0072	Fallopia dumetorum (L.) Holub	0	n	1	0	Polygonaceae Juss.
Iz_15_1209	DJI_0072	Festuca beckeri (Hack.) Trautv.	0	n	1	0	Poaceae Barnhart
Iz_15_1209	DJI_0072	Fragaria vesca L.	0	n	1	0	Rosaceae Juss.
Iz_15_1209	DJI_0072	Frangula alnus Mill.	0	n	1	1	Rhamnaceae Juss.
Iz_15_1209	DJI_0072	Fraxinus pennsylvanica Marshall	0	a	0	1	Oleaceae Hoffmanns. & Link
Iz_15_1209	DJI_0072	Galeopsis bifida Boenn.	0	n	1	0	Lamiacea Lindl.
Iz_15_1209	DJI_0072	Galium verum L.	0	n	1	0	Rubiaceae Juss.
Iz_15_1209	DJI_0072	Genista tinctoria L.	0	n	1	0	Fabaceae Lindl.
Iz_15_1209	DJI_0072	Geranium robertianum L.	0	n	1	0	Geraniaceae Juss.
Iz_15_1209	DJI_0072	Geranium sanquineum L.	0	n	1	0	Geraniaceae Juss.
Iz_15_1209	DJI_0072	Gypsophila paniculata L.	0	n	1	0	Caryophyllaceae Juss.
Iz_15_1209	DJI_0072	Helichrysum arenarium (L.) Moench	0	n	1	0	Asteraceae Bercht. & J.Presl
Iz_15_1209	DJI_0072	Herniaria polygama J.Gay	0	n	1	0	Caryophyllaceae Juss.
Iz_15_1209	DJI_0072	Hieracium robustum Fr.	0	n	1	0	Asteraceae Bercht. & J.Presl
Iz_15_1209	DJI_0072	Hieracium umbellatum L.	0	n	1	NA	Asteraceae Bercht. & J.Presl
Iz_15_1209	DJI_0072	Hylotelephium maximum (L.) Holub	0	n	1	0	Crassulaceae J.St.-Hil.
Iz_15_1209	DJI_0072	Jacobaea borysthenica (DC.) B.Nord. & Greuter	0	n	1	0	Asteraceae Bercht. & J.Presl
Iz_15_1209	DJI_0072	Jasione montana L.	0	n	1	0	Campanulaceae Juss.
Iz_15_1209	DJI_0072	Jurinea cyanoides (L.) Rchb.s.l.	0	n	1	0	Asteraceae Bercht. & J.Presl
Iz_15_1209	DJI_0072	Koeleria glauca (Spreng.) DC.	0	n	1	0	Poaceae Barnhart
Iz_15_1209	DJI_0072	Lactuca serriola L.	0	a	0	0	Asteraceae Bercht. & J.Presl
Iz_15_1209	DJI_0072	Linaria genistifolia (L.) Mill.	0	n	1	0	Plantaginaceae Juss.
Iz_15_1209	DJI_0072	Lomelosia argentea (L.) Greuter & Burdet	0	n	1	0	Caprifoliaceae Juss.
Iz_15_1209	DJI_0072	Melampyrum pratense L.	0	n	1	0	Orobanchaceae Vent.
Iz_15_1209	DJI_0072	Oenothera biennis L.	0	a	0	0	Onagraceae Juss.
Iz_15_1209	DJI_0072	Pentanema britannica (L.) D.Gut.Larr., Santos-Vicente, Anderb., E.Rico & M.M.Mart.Ort.	0	n	1	0	Asteraceae Bercht. & J.Presl
Iz_15_1209	DJI_0072	Peucedanum oreoselinum (L.) Moench	0	n	1	0	Apiaceae Lindl.
Iz_15_1209	DJI_0072	Peucedanum ruthenicum M.Bieb.	0	n	1	0	Apiaceae Lindl.
Iz_15_1209	DJI_0072	Pilosella echioides (Lumn.) F.W.Schultz & Sch.Bip.	0	n	1	0	Asteraceae Bercht. & J.Presl
Iz_15_1209	DJI_0072	Pilosella officinarum F. Schultz et Sch. Bip.	0	n	1	0	Asteraceae Bercht. & J.Presl
Iz_15_1209	DJI_0072	Pilosella sp.	0		NA	0	Asteraceae Bercht. & J.Presl
Iz_15_1209	DJI_0072	Pinus sylvestris L.	0	n	1	1	Pinaceae Spreng. ex F.Rudolphi
Iz_15_1209	DJI_0072	Poa annua L.	0	n	1	0	Poaceae Barnhart
Iz_15_1209	DJI_0072	Poa nemoralis L.	0	n	1	0	Poaceae Barnhart
Iz_15_1209	DJI_0072	Poa sp.	0		NA	0	Poaceae Barnhart
Iz_15_1209	DJI_0072	Polygonatum odoratum (Mill.) Druce	0	n	1	0	Asparagaceae Juss.
Iz_15_1209	DJI_0072	Polygonum arenarium Waldst. & Kit.	0	n	1	0	Polygonaceae Juss.
Iz_15_1209	DJI_0072	Populus nigra L.	0	n	1	1	Salicaceae Mirb.
Iz_15_1209	DJI_0072	Populus tremula L.	0	n	1	1	Salicaceae Mirb.
Iz_15_1209	DJI_0072	Populus Ã canescens (Aiton) Sm.	0	n	1	1	Salicaceae Mirb.
Iz_15_1209	DJI_0072	Potentilla argentea L.	0	n	1	0	Rosaceae Juss.
Iz_15_1209	DJI_0072	Psammophiliella muralis (L.) Ikonn.	0	n	1	0	Caryophyllaceae Juss.
Iz_15_1209	DJI_0072	Psephellus sumensis (Kalen.) Greuter	0	n	1	0	Asteraceae Bercht. & J.Presl
Iz_15_1209	DJI_0072	Pulsatilla pratensis (L.) Mill. s.l.	0	n	1	0	Ranunculaceae Juss.
Iz_15_1209	DJI_0072	Pyrus communis L.	0	a	0	1	Rosaceae Juss.
Iz_15_1209	DJI_0072	Quercus robur L.	0	n	1	1	Fagaceae Dumort.
Iz_15_1209	DJI_0072	Robinia pseudoacacia L.	0	a	0	1	Fabaceae Lindl.
Iz_15_1209	DJI_0072	Rosa sp.	0		NA	1	Rosaceae Juss.
Iz_15_1209	DJI_0072	Rubus idaeus L.	0	n	1	0	Rosaceae Juss.
Iz_15_1209	DJI_0072	Rumex acetosella L.	0	n	1	0	Polygonaceae Juss.
Iz_15_1209	DJI_0072	Rumex thyrsiflorus Fingerh.	0	n	1	0	Polygonaceae Juss.
Iz_15_1209	DJI_0072	Salix alba L.	0	n	1	1	Salicaceae Mirb.
Iz_15_1209	DJI_0072	Sambucus nigra L.	0	n	1	1	Viburnaceae Raf.
Iz_15_1209	DJI_0072	Secale sylvestre Host	0	n	1	0	Poaceae Barnhart
Iz_15_1209	DJI_0072	Sempervivum ruthenicum Schnittsp. & C.B. Lehm.	0	n	1	0	Crassulaceae J.St.-Hil.
Iz_15_1209	DJI_0072	Setaria viridis (L.) P. Beauv.	0	n	1	0	Poaceae Barnhart
Iz_15_1209	DJI_0072	Silene borysthenica (Gruner) Walters	0	n	1	0	Caryophyllaceae Juss.
Iz_15_1209	DJI_0072	Silene latifolia subsp. alba (Mill.) Greuter & Burdet	0	n	1	0	Caryophyllaceae Juss.
Iz_15_1209	DJI_0072	Solidago virgaurea L.	0	n	1	0	Asteraceae Bercht. & J.Presl
Iz_15_1209	DJI_0072	Sorbus aucuparia L.	0	n	1	1	Rosaceae Juss.
Iz_15_1209	DJI_0072	Stachys recta L.	0	n	1	0	Lamiacea Lindl.
Iz_15_1209	DJI_0072	Tanacetum vulgare L.	0	n	1	0	Asteraceae Bercht. & J.Presl
Iz_15_1209	DJI_0072	Thymus pallasianus Heinr.Braun	0	n	1	0	Lamiacea Lindl.
Iz_15_1209	DJI_0072	Tilia cordata Mill.	0	n	1	1	Malvaceae Juss.
Iz_15_1209	DJI_0072	Trifolium alpestre L.	0	n	1	0	Fabaceae Lindl.
Iz_15_1209	DJI_0072	Trifolium arvense L.	0	n	1	0	Fabaceae Lindl.
Iz_15_1209	DJI_0072	Ulmus glabra Huds.	0	n	1	1	Ulmaceae Mirb.
Iz_15_1209	DJI_0072	Verbascum densiflorum Bertol.	0	n	1	0	Scrophulariaceae Juss.
Iz_15_1209	DJI_0072	Veronica austriaca L.	0	n	1	0	Plantaginaceae Juss.
Iz_15_1209	DJI_0072	Veronica incana L.	0	n	1	0	Plantaginaceae Juss.
Iz_15_1209	DJI_0072	Veronica verna L.	0	n	1	0	Plantaginaceae Juss.
Iz_15_1209	DJI_0072	Vincetoxicum hirundinaria Medik	0	n	1	0	Apocynaceae Juss.
Iz_15_1209	DJI_0077	Acer tataricum L.	0	n	1	1	Sapindaceae Juss.
Iz_15_1209	DJI_0077	Achillea millefolium L. s.l.	0	n	1	0	Asteraceae Bercht. & J.Presl
Iz_15_1209	DJI_0077	Allium oleraceum L.	0	n	1	0	Amaryllidaceae J.St.-Hil.
Iz_15_1209	DJI_0077	Ambrosia artemisiifolia L.	0	a	0	0	Asteraceae Bercht. & J.Presl
Iz_15_1209	DJI_0077	Amorpha fruticosa L.	0	a	0	0	Fabaceae Lindl.
Iz_15_1209	DJI_0077	Anthericum ramosum L.	0	n	1	0	Asparagaceae Juss.
Iz_15_1209	DJI_0077	Artemisia marschalliana Spreng.	0	n	1	0	Asteraceae Bercht. & J.Presl
Iz_15_1209	DJI_0077	Asparagus officinalis L.	0	n	1	0	Asparagaceae Juss.
Iz_15_1209	DJI_0077	Astragalus cicer L.	0	n	1	0	Fabaceae Lindl.
Iz_15_1209	DJI_0077	Bassia laniflora (S.G.Gmel.) A.J.Scott	0	n	1	0	Amaranthaceae Juss.

Iz_15_1209	DJI_0077	Berberis vulgaris L.	0	n	1	0	Berberidaceae Juss.
Iz_15_1209	DJI_0077	Betula pendula Roth.	0	n	1	1	Betulaceae Gray
Iz_15_1209	DJI_0077	Betula pendula Roth.	0	n	1	0	Poaceae Barnhart
Iz_15_1209	DJI_0077	Bromus inermis Leyss.	0		NA	NA	
Iz_15_1209	DJI_0077	Bromus squarrosus L.	0	a	0	0	Poaceae Barnhart
Iz_15_1209	DJI_0077	Bromus tectorum L.	0	a	0	0	Poaceae Barnhart
Iz_15_1209	DJI_0077	Calamagrostis epigejos (L.) Roth	0.1	n	1	0	Poaceae Barnhart
Iz_15_1209	DJI_0077	Carduus acanthoides L.	0	n	1	0	Asteraceae Bercht. & J.Presl
Iz_15_1209	DJI_0077	Carex sp.	0	n	1	0	Cyperaceae Juss.
Iz_15_1209	DJI_0077	Centaurea majorovii Dumbadze	0	n	1	0	Asteraceae Bercht. & J.Presl
Iz_15_1209	DJI_0077	Chamaecytisus ruthenicus (Fisch.ex Wol.) Klaskova	0	n	1	0	Fabaceae Lindl.
Iz_15_1209	DJI_0077	Chelidonium majus L.	0	n	1	0	Papaveraceae Juss.
Iz_15_1209	DJI_0077	Chenopodium betaceum Andrz.	0	n	1	0	Amaranthaceae Juss.
Iz_15_1209	DJI_0077	Chenopodium cf. album	0	n	1	0	Amaranthaceae Juss.
Iz_15_1209	DJI_0077	Chenopodium sp.	0		NA	0	Amaranthaceae Juss.
Iz_15_1209	DJI_0077	Chondrilla juncea L.	0	n	1	0	Asteraceae Bercht. & J.Presl
Iz_15_1209	DJI_0077	Cirsium vulgare (Savi) Ten.	0	n	1	0	Asteraceae Bercht. & J.Presl
Iz_15_1209	DJI_0077	Clinopodium acinos (L.) Kuntze	0	n	1	0	Lamiacea Lindl.
Iz_15_1209	DJI_0077	Convallaria majalis L.	0	n	1	0	Asparagaceae Juss.
Iz_15_1209	DJI_0077	Crataegus rhipidophylla Gand.	0	n	1	1	Rosaceae Juss.
Iz_15_1209	DJI_0077	Crepis foetida L.	0	n	1	0	Asteraceae Bercht. & J.Presl
Iz_15_1209	DJI_0077	Dactylis glomerata L.	0	n	1	0	Poaceae Barnhart
Iz_15_1209	DJI_0077	Dianthus campestris M.Bieb.	0	n	1	0	Caryophyllaceae Juss.
Iz_15_1209	DJI_0077	Digitaria sanguinalis (L.) Scop.	0	a	0	0	Poaceae Barnhart
Iz_15_1209	DJI_0077	Elymus repens (L.) Gould	0	n	1	0	Poaceae Barnhart
Iz_15_1209	DJI_0077	Epilobium tetragonum L.	0	n	1	0	Onagraceae Juss.
Iz_15_1209	DJI_0077	Eragrostis minor Host	0	a	0	0	Poaceae Barnhart
Iz_15_1209	DJI_0077	Erigeron canadensis L.	0	a	0	0	Asteraceae Bercht. & J.Presl
Iz_15_1209	DJI_0077	Euphorbia seguieriana Neck.	0	n	1	0	Euphorbiaceae Juss.
Iz_15_1209	DJI_0077	Fallopia dumetorum (L.) Holub	0	n	1	0	Polygonaceae Juss.
Iz_15_1209	DJI_0077	Festuca beckeri (Hack.) Trautv.	0	n	1	0	Poaceae Barnhart
Iz_15_1209	DJI_0077	Fragaria vesca L.	0	n	1	0	Rosaceae Juss.
Iz_15_1209	DJI_0077	Frangula alnus Mill.	0	n	1	1	Rhamnaceae Juss.
Iz_15_1209	DJI_0077	Fraxinus pennsylvanica Marshall	0	a	0	1	Oleaceae Hoffmanns. & Link
Iz_15_1209	DJI_0077	Galeopsis bifida Boenn.	0	n	1	0	Lamiacea Lindl.
Iz_15_1209	DJI_0077	Galium verum L.	0	n	1	0	Rubiaceae Juss.
Iz_15_1209	DJI_0077	Genista tinctoria L.	0	n	1	0	Fabaceae Lindl.
Iz_15_1209	DJI_0077	Geranium robertianum L.	0	n	1	0	Geraniaceae Juss.
Iz_15_1209	DJI_0077	Geranium sanquineum L.	0	n	1	0	Geraniaceae Juss.
Iz_15_1209	DJI_0077	Gypsophila paniculata L.	0	n	1	0	Caryophyllaceae Juss.
Iz_15_1209	DJI_0077	Helichrysum arenarium (L.) Moench	0	n	1	0	Asteraceae Bercht. & J.Presl
Iz_15_1209	DJI_0077	Herniaria polygama J.Gay	0	n	1	0	Caryophyllaceae Juss.
Iz_15_1209	DJI_0077	Hieracium robustum Fr.	0	n	1	0	Asteraceae Bercht. & J.Presl
Iz_15_1209	DJI_0077	Hieracium umbellatum L.	0	n	1	NA	Asteraceae Bercht. & J.Presl
Iz_15_1209	DJI_0077	Hylotelephium maximum (L.) Holub	0	n	1	0	Crassulaceae J.St.-Hil.
Iz_15_1209	DJI_0077	Jacobaea borysthenica (DC.) B.Nord. & Greuter	0	n	1	0	Asteraceae Bercht. & J.Presl
Iz_15_1209	DJI_0077	Jasione montana L.	0	n	1	0	Campanulaceae Juss.
Iz_15_1209	DJI_0077	Jurinea cyanoides (L.) Rchb.s.l.	0	n	1	0	Asteraceae Bercht. & J.Presl
Iz_15_1209	DJI_0077	Koeleria glauca (Spreng.) DC.	0	n	1	0	Poaceae Barnhart
Iz_15_1209	DJI_0077	Lactuca serriola L.	0	a	0	0	Asteraceae Bercht. & J.Presl
Iz_15_1209	DJI_0077	Linaria genistifolia (L.) Mill.	0	n	1	0	Plantaginaceae Juss.
Iz_15_1209	DJI_0077	Lomelosia argentea (L.) Greuter & Burdet	0	n	1	0	Caprifoliaceae Juss.
Iz_15_1209	DJI_0077	Melampyrum pratense L.	0	n	1	0	Orobanchaceae Vent.
Iz_15_1209	DJI_0077	Oenothera biennis L.	0	a	0	0	Onagraceae Juss.
Iz_15_1209	DJI_0077	Pentanema britannica (L.) D.Gut.Larr., Santos-Vicente, Anderb., E.Rico & M.M.Mart.Ort.	0	n	1	0	Asteraceae Bercht. & J.Presl
Iz_15_1209	DJI_0077	Peucedanum oreoselinum (L.) Moench	0	n	1	0	Apiaceae Lindl.
Iz_15_1209	DJI_0077	Peucedanum ruthenicum M.Bieb.	0	n	1	0	Apiaceae Lindl.
Iz_15_1209	DJI_0077	Pilosella echioides (Lumn.) F.W.Schultz & Sch.Bip.	0	n	1	0	Asteraceae Bercht. & J.Presl
Iz_15_1209	DJI_0077	Pilosella officinarum F. Schultz et Sch. Bip.	0	n	1	0	Asteraceae Bercht. & J.Presl
Iz_15_1209	DJI_0077	Pilosella sp.	0		NA	0	Asteraceae Bercht. & J.Presl
Iz_15_1209	DJI_0077	Pinus sylvestris L.	0	n	1	1	Pinaceae Spreng. ex F.Rudolphi
Iz_15_1209	DJI_0077	Poa annua L.	0	n	1	0	Poaceae Barnhart
Iz_15_1209	DJI_0077	Poa nemoralis L.	0	n	1	0	Poaceae Barnhart
Iz_15_1209	DJI_0077	Poa sp.	0		NA	0	Poaceae Barnhart
Iz_15_1209	DJI_0077	Polygonatum odoratum (Mill.) Druce	1	n	1	0	Asparagaceae Juss.
Iz_15_1209	DJI_0077	Polygonum arenarium Waldst. & Kit.	0	n	1	0	Polygonaceae Juss.
Iz_15_1209	DJI_0077	Populus nigra L.	0	n	1	1	Salicaceae Mirb.
Iz_15_1209	DJI_0077	Populus tremula L.	0	n	1	1	Salicaceae Mirb.
Iz_15_1209	DJI_0077	Populus Ã canescens (Aiton) Sm.	0	n	1	1	Salicaceae Mirb.
Iz_15_1209	DJI_0077	Potentilla argentea L.	0	n	1	0	Rosaceae Juss.
Iz_15_1209	DJI_0077	Psammophiliella muralis (L.) Ikonn.	0	n	1	0	Caryophyllaceae Juss.
Iz_15_1209	DJI_0077	Psephellus sumensis (Kalen.) Greuter	0	n	1	0	Asteraceae Bercht. & J.Presl
Iz_15_1209	DJI_0077	Pulsatilla pratensis (L.) Mill. s.l.	0	n	1	0	Ranunculaceae Juss.
Iz_15_1209	DJI_0077	Pyrus communis L.	0	a	0	1	Rosaceae Juss.
Iz_15_1209	DJI_0077	Quercus robur L.	0	n	1	1	Fagaceae Dumort.
Iz_15_1209	DJI_0077	Robinia pseudoacacia L.	0	a	0	1	Fabaceae Lindl.
Iz_15_1209	DJI_0077	Rosa sp.	0		NA	1	Rosaceae Juss.
Iz_15_1209	DJI_0077	Rubus idaeus L.	0	n	1	0	Rosaceae Juss.
Iz_15_1209	DJI_0077	Rumex acetosella L.	0	n	1	0	Polygonaceae Juss.
Iz_15_1209	DJI_0077	Rumex thyrsiflorus Fingerh.	0	n	1	0	Polygonaceae Juss.
Iz_15_1209	DJI_0077	Salix alba L.	0	n	1	1	Salicaceae Mirb.
Iz_15_1209	DJI_0077	Sambucus nigra L.	0	n	1	1	Viburnaceae Raf.
Iz_15_1209	DJI_0077	Secale sylvestre Host	0	n	1	0	Poaceae Barnhart
Iz_15_1209	DJI_0077	Sempervivum ruthenicum Schnittsp. & C.B. Lehm.	0	n	1	0	Crassulaceae J.St.-Hil.
Iz_15_1209	DJI_0077	Setaria viridis (L.) P. Beauv.	0	n	1	0	Poaceae Barnhart
Iz_15_1209	DJI_0077	Silene borysthenica (Gruner) Walters	0	n	1	0	Caryophyllaceae Juss.
Iz_15_1209	DJI_0077	Silene latifolia subsp. alba (Mill.) Greuter & Burdet	0	n	1	0	Caryophyllaceae Juss.
Iz_15_1209	DJI_0077	Solidago virgaurea L.	0	n	1	0	Asteraceae Bercht. & J.Presl
Iz_15_1209	DJI_0077	Sorbus aucuparia L.	0	n	1	1	Rosaceae Juss.
Iz_15_1209	DJI_0077	Stachys recta L.	0	n	1	0	Lamiacea Lindl.
Iz_15_1209	DJI_0077	Tanacetum vulgare L.	0	n	1	0	Asteraceae Bercht. & J.Presl
Iz_15_1209	DJI_0077	Thymus pallasianus Heinr.Braun	0	n	1	0	Lamiacea Lindl.
Iz_15_1209	DJI_0077	Tilia cordata Mill.	0	n	1	1	Malvaceae Juss.
Iz_15_1209	DJI_0077	Trifolium alpestre L.	0	n	1	0	Fabaceae Lindl.
Iz_15_1209	DJI_0077	Trifolium arvense L.	0	n	1	0	Fabaceae Lindl.
Iz_15_1209	DJI_0077	Ulmus glabra Huds.	0	n	1	1	Ulmaceae Mirb.
Iz_15_1209	DJI_0077	Verbascum densiflorum Bertol.	0	n	1	0	Scrophulariaceae Juss.
Iz_15_1209	DJI_0077	Veronica austriaca L.	0	n	1	0	Plantaginaceae Juss.

Iz_15_1209	DJI_0077	Veronica incana L.	0	n	1	0	Plantaginaceae Juss.
Iz_15_1209	DJI_0077	Veronica verna L.	0	n	1	0	Plantaginaceae Juss.
Iz_15_1209	DJI_0077	Vincetoxicum hirundinaria Medik	0	n	1	0	Apocynaceae Juss.
Iz_15_1209	DJI_0080	Acer tataricum L.	0	n	1	1	Sapindaceae Juss.
Iz_15_1209	DJI_0080	Achillea millefolium L. s.l.	0	n	1	0	Asteraceae Bercht. & J.Presl
Iz_15_1209	DJI_0080	Allium oleraceum L.	0	n	1	0	Amaryllidaceae J.St.-Hil.
Iz_15_1209	DJI_0080	Ambrosia artemisiifolia L.	0	a	0	0	Asteraceae Bercht. & J.Presl
Iz_15_1209	DJI_0080	Amorpha fruticosa L.	0	a	0	0	Fabaceae Lindl.
Iz_15_1209	DJI_0080	Anthericum ramosum L.	0	n	1	0	Asparagaceae Juss.
Iz_15_1209	DJI_0080	Artemisia marschalliana Spreng.	0	n	1	0	Asteraceae Bercht. & J.Presl
Iz_15_1209	DJI_0080	Asparagus officinalis L.	0	n	1	0	Asparagaceae Juss.
Iz_15_1209	DJI_0080	Astragalus cicer L.	0	n	1	0	Fabaceae Lindl.
Iz_15_1209	DJI_0080	Bassia laniflora (S.G.Gmel.) A.J.Scott	0	n	1	0	Amaranthaceae Juss.
Iz_15_1209	DJI_0080	Berberis vulgaris L.	0	n	1	0	Berberidaceae Juss.
Iz_15_1209	DJI_0080	Betula pendula Roth.	0	n	1	1	Betulaceae Gray
Iz_15_1209	DJI_0080	Betula pendula Roth.	0	n	1	0	Poaceae Barnhart
Iz_15_1209	DJI_0080	Bromus inermis Leyss.	0		NA	NA	
Iz_15_1209	DJI_0080	Bromus squarrosus L.	0	a	0	0	Poaceae Barnhart
Iz_15_1209	DJI_0080	Bromus tectorum L.	0	a	0	0	Poaceae Barnhart
Iz_15_1209	DJI_0080	Calamagrostis epigejos (L.) Roth	0.1	n	1	0	Poaceae Barnhart
Iz_15_1209	DJI_0080	Carduus acanthoides L.	0	n	1	0	Asteraceae Bercht. & J.Presl
Iz_15_1209	DJI_0080	Carex sp.	0	n	1	0	Cyperaceae Juss.
Iz_15_1209	DJI_0080	Centaurea majorovii Dumbadze	0	n	1	0	Asteraceae Bercht. & J.Presl
Iz_15_1209	DJI_0080	Chamaecytisus ruthenicus (Fisch.ex Wol.) Klaskova	0	n	1	0	Fabaceae Lindl.
Iz_15_1209	DJI_0080	Chelidonium majus L.	0	n	1	0	Papaveraceae Juss.
Iz_15_1209	DJI_0080	Chenopodium betaceum Andrz.	0	n	1	0	Amaranthaceae Juss.
Iz_15_1209	DJI_0080	Chenopodium cf. album	0	n	1	0	Amaranthaceae Juss.
Iz_15_1209	DJI_0080	Chenopodium sp.	0		NA	0	Amaranthaceae Juss.
Iz_15_1209	DJI_0080	Chondrilla juncea L.	0	n	1	0	Asteraceae Bercht. & J.Presl
Iz_15_1209	DJI_0080	Cirsium vulgare (Savi) Ten.	0	n	1	0	Asteraceae Bercht. & J.Presl
Iz_15_1209	DJI_0080	Clinopodium acinos (L.) Kuntze	0	n	1	0	Lamiacea Lindl.
Iz_15_1209	DJI_0080	Convallaria majalis L.	0	n	1	0	Asparagaceae Juss.
Iz_15_1209	DJI_0080	Crataegus rhipidophylla Gand.	0	n	1	1	Rosaceae Juss.
Iz_15_1209	DJI_0080	Crepis foetida L.	0	n	1	0	Asteraceae Bercht. & J.Presl
Iz_15_1209	DJI_0080	Dactylis glomerata L.	0	n	1	0	Poaceae Barnhart
Iz_15_1209	DJI_0080	Dianthus campestris M.Bieb.	0	n	1	0	Caryophyllaceae Juss.
Iz_15_1209	DJI_0080	Digitaria sanguinalis (L.) Scop.	0	a	0	0	Poaceae Barnhart
Iz_15_1209	DJI_0080	Elymus repens (L.) Gould	0	n	1	0	Poaceae Barnhart
Iz_15_1209	DJI_0080	Epilobium tetragonum L.	0	n	1	0	Onagraceae Juss.
Iz_15_1209	DJI_0080	Eragrostis minor Host	0	a	0	0	Poaceae Barnhart
Iz_15_1209	DJI_0080	Erigeron canadensis L.	0	a	0	0	Asteraceae Bercht. & J.Presl
Iz_15_1209	DJI_0080	Euphorbia seguieriana Neck.	0	n	1	0	Euphorbiaceae Juss.
Iz_15_1209	DJI_0080	Fallopia dumetorum (L.) Holub	0	n	1	0	Polygonaceae Juss.
Iz_15_1209	DJI_0080	Festuca beckeri (Hack.) Trautv.	0	n	1	0	Poaceae Barnhart
Iz_15_1209	DJI_0080	Fragaria vesca L.	0	n	1	0	Rosaceae Juss.
Iz_15_1209	DJI_0080	Frangula alnus Mill.	0	n	1	1	Rhamnaceae Juss.
Iz_15_1209	DJI_0080	Fraxinus pennsylvanica Marshall	0	a	0	1	Oleaceae Hoffmanns. & Link
Iz_15_1209	DJI_0080	Galeopsis bifida Boenn.	0	n	1	0	Lamiacea Lindl.
Iz_15_1209	DJI_0080	Galium verum L.	0	n	1	0	Rubiaceae Juss.
Iz_15_1209	DJI_0080	Genista tinctoria L.	0	n	1	0	Fabaceae Lindl.
Iz_15_1209	DJI_0080	Geranium robertianum L.	0	n	1	0	Geraniaceae Juss.
Iz_15_1209	DJI_0080	Geranium sanquineum L.	0	n	1	0	Geraniaceae Juss.
Iz_15_1209	DJI_0080	Gypsophila paniculata L.	0	n	1	0	Caryophyllaceae Juss.
Iz_15_1209	DJI_0080	Helichrysum arenarium (L.) Moench	0	n	1	0	Asteraceae Bercht. & J.Presl
Iz_15_1209	DJI_0080	Herniaria polygama J.Gay	0	n	1	0	Caryophyllaceae Juss.
Iz_15_1209	DJI_0080	Hieracium robustum Fr.	0	n	1	0	Asteraceae Bercht. & J.Presl
Iz_15_1209	DJI_0080	Hieracium umbellatum L.	0	n	1	NA	Asteraceae Bercht. & J.Presl
Iz_15_1209	DJI_0080	Hylotelephium maximum (L.) Holub	0	n	1	0	Crassulaceae J.St.-Hil.
Iz_15_1209	DJI_0080	Jacobaea borysthenica (DC.) B.Nord. & Greuter	0	n	1	0	Asteraceae Bercht. & J.Presl
Iz_15_1209	DJI_0080	Jasione montana L.	0	n	1	0	Campanulaceae Juss.
Iz_15_1209	DJI_0080	Jurinea cyanoides (L.) Rchb.s.l.	0	n	1	0	Asteraceae Bercht. & J.Presl
Iz_15_1209	DJI_0080	Koeleria glauca (Spreng.) DC.	0	n	1	0	Poaceae Barnhart
Iz_15_1209	DJI_0080	Lactuca serriola L.	0	a	0	0	Asteraceae Bercht. & J.Presl
Iz_15_1209	DJI_0080	Linaria genistifolia (L.) Mill.	0	n	1	0	Plantaginaceae Juss.
Iz_15_1209	DJI_0080	Lomelosia argentea (L.) Greuter & Burdet	0	n	1	0	Caprifoliaceae Juss.
Iz_15_1209	DJI_0080	Melampyrum pratense L.	0	n	1	0	Orobanchaceae Vent.
Iz_15_1209	DJI_0080	Oenothera biennis L.	0	a	0	0	Onagraceae Juss.
Iz_15_1209	DJI_0080	Pentanema britannica (L.) D.Gut.Larr., Santos-Vicente, Anderb., E.Rico & M.M.Mart.Ort.	0	n	1	0	Asteraceae Bercht. & J.Presl
Iz_15_1209	DJI_0080	Peucedanum oreoselinum (L.) Moench	0	n	1	0	Apiaceae Lindl.
Iz_15_1209	DJI_0080	Peucedanum ruthenicum M.Bieb.	0	n	1	0	Apiaceae Lindl.
Iz_15_1209	DJI_0080	Pilosella echioides (Lumn.) F.W.Schultz & Sch.Bip.	0	n	1	0	Asteraceae Bercht. & J.Presl
Iz_15_1209	DJI_0080	Pilosella officinarum F. Schultz et Sch. Bip.	0	n	1	0	Asteraceae Bercht. & J.Presl
Iz_15_1209	DJI_0080	Pilosella sp.	0		NA	0	Asteraceae Bercht. & J.Presl
Iz_15_1209	DJI_0080	Pinus sylvestris L.	0	n	1	1	Pinaceae Spreng. ex F.Rudolphi
Iz_15_1209	DJI_0080	Poa annua L.	0	n	1	0	Poaceae Barnhart
Iz_15_1209	DJI_0080	Poa nemoralis L.	0	n	1	0	Poaceae Barnhart
Iz_15_1209	DJI_0080	Poa sp.	0		NA	0	Poaceae Barnhart
Iz_15_1209	DJI_0080	Polygonatum odoratum (Mill.) Druce	1	n	1	0	Asparagaceae Juss.
Iz_15_1209	DJI_0080	Polygonum arenarium Waldst. & Kit.	0	n	1	0	Polygonaceae Juss.
Iz_15_1209	DJI_0080	Populus nigra L.	0	n	1	1	Salicaceae Mirb.
Iz_15_1209	DJI_0080	Populus tremula L.	0	n	1	1	Salicaceae Mirb.
Iz_15_1209	DJI_0080	Populus Ã canescens (Aiton) Sm.	0	n	1	1	Salicaceae Mirb.
Iz_15_1209	DJI_0080	Potentilla argentea L.	0	n	1	0	Rosaceae Juss.
Iz_15_1209	DJI_0080	Psammophiliella muralis (L.) Ikonn.	0	n	1	0	Caryophyllaceae Juss.
Iz_15_1209	DJI_0080	Psephellus sumensis (Kalen.) Greuter	0	n	1	0	Asteraceae Bercht. & J.Presl
Iz_15_1209	DJI_0080	Pulsatilla pratensis (L.) Mill. s.l.	0	n	1	0	Ranunculaceae Juss.
Iz_15_1209	DJI_0080	Pyrus communis L.	0	a	0	1	Rosaceae Juss.
Iz_15_1209	DJI_0080	Quercus robur L.	0	n	1	1	Fagaceae Dumort.
Iz_15_1209	DJI_0080	Robinia pseudoacacia L.	0	a	0	1	Fabaceae Lindl.
Iz_15_1209	DJI_0080	Rosa sp.	0		NA	1	Rosaceae Juss.
Iz_15_1209	DJI_0080	Rubus idaeus L.	0	n	1	0	Rosaceae Juss.
Iz_15_1209	DJI_0080	Rumex acetosella L.	0	n	1	0	Polygonaceae Juss.
Iz_15_1209	DJI_0080	Rumex thyrsiflorus Fingerh.	0	n	1	0	Polygonaceae Juss.
Iz_15_1209	DJI_0080	Salix alba L.	0	n	1	1	Salicaceae Mirb.
Iz_15_1209	DJI_0080	Sambucus nigra L.	0	n	1	1	Viburnaceae Raf.
Iz_15_1209	DJI_0080	Secale sylvestre Host	0	n	1	0	Poaceae Barnhart
Iz_15_1209	DJI_0080	Sempervivum ruthenicum Schnittsp. & C.B. Lehm.	0	n	1	0	Crassulaceae J.St.-Hil.
Iz_15_1209	DJI_0080	Setaria viridis (L.) P. Beauv.	0	n	1	0	Poaceae Barnhart

Iz_15_1209	DJI_0080	Silene borysthenica (Gruner) Walters	0	n	1	0	Caryophyllaceae Juss.
Iz_15_1209	DJI_0080	Silene latifolia subsp. alba (Mill.) Greuter & Burdet	0	n	1	0	Caryophyllaceae Juss.
Iz_15_1209	DJI_0080	Solidago virgaurea L.	0	n	1	0	Asteraceae Bercht. & J.Presl
Iz_15_1209	DJI_0080	Sorbus aucuparia L.	0	n	1	1	Rosaceae Juss.
Iz_15_1209	DJI_0080	Stachys recta L.	0	n	1	0	Lamiacea Lindl.
Iz_15_1209	DJI_0080	Tanacetum vulgare L.	0	n	1	0	Asteraceae Bercht. & J.Presl
Iz_15_1209	DJI_0080	Thymus pallasianus Heinr.Braun	0	n	1	0	Lamiacea Lindl.
Iz_15_1209	DJI_0080	Tilia cordata Mill.	0	n	1	1	Malvaceae Juss.
Iz_15_1209	DJI_0080	Trifolium alpestre L.	0	n	1	0	Fabaceae Lindl.
Iz_15_1209	DJI_0080	Trifolium arvense L.	0	n	1	0	Fabaceae Lindl.
Iz_15_1209	DJI_0080	Ulmus glabra Huds.	0	n	1	1	Ulmaceae Mirb.
Iz_15_1209	DJI_0080	Verbascum densiflorum Bertol.	0	n	1	0	Scrophulariaceae Juss.
Iz_15_1209	DJI_0080	Veronica austriaca L.	0	n	1	0	Plantaginaceae Juss.
Iz_15_1209	DJI_0080	Veronica incana L.	0	n	1	0	Plantaginaceae Juss.
Iz_15_1209	DJI_0080	Veronica verna L.	0	n	1	0	Plantaginaceae Juss.
Iz_15_1209	DJI_0080	Vincetoxicum hirundinaria Medik	0	n	1	0	Apocynaceae Juss.
Iz_15_1209	DJI_0083	Acer tataricum L.	0	n	1	1	Sapindaceae Juss.
Iz_15_1209	DJI_0083	Achillea millefolium L. s.l.	0	n	1	0	Asteraceae Bercht. & J.Presl
Iz_15_1209	DJI_0083	Allium oleraceum L.	0	n	1	0	Amaryllidaceae J.St.-Hil.
Iz_15_1209	DJI_0083	Ambrosia artemisiifolia L.	0	a	0	0	Asteraceae Bercht. & J.Presl
Iz_15_1209	DJI_0083	Amorpha fruticosa L.	0	a	0	0	Fabaceae Lindl.
Iz_15_1209	DJI_0083	Anthericum ramosum L.	0	n	1	0	Asparagaceae Juss.
Iz_15_1209	DJI_0083	Artemisia marschalliana Spreng.	0	n	1	0	Asteraceae Bercht. & J.Presl
Iz_15_1209	DJI_0083	Asparagus officinalis L.	0	n	1	0	Asparagaceae Juss.
Iz_15_1209	DJI_0083	Astragalus cicer L.	0	n	1	0	Fabaceae Lindl.
Iz_15_1209	DJI_0083	Bassia laniflora (S.G.Gmel.) A.J.Scott	0	n	1	0	Amaranthaceae Juss.
Iz_15_1209	DJI_0083	Berberis vulgaris L.	0	n	1	0	Berberidaceae Juss.
Iz_15_1209	DJI_0083	Betula pendula Roth.	0	n	1	1	Betulaceae Gray
Iz_15_1209	DJI_0083	Betula pendula Roth.	0	n	1	0	Poaceae Barnhart
Iz_15_1209	DJI_0083	Bromus inermis Leyss.	0		NA	NA	
Iz_15_1209	DJI_0083	Bromus squarrosus L.	0	a	0	0	Poaceae Barnhart
Iz_15_1209	DJI_0083	Bromus tectorum L.	0	a	0	0	Poaceae Barnhart
Iz_15_1209	DJI_0083	Calamagrostis epigejos (L.) Roth	1	n	1	0	Poaceae Barnhart
Iz_15_1209	DJI_0083	Carduus acanthoides L.	0	n	1	0	Asteraceae Bercht. & J.Presl
Iz_15_1209	DJI_0083	Carex sp.	3	n	1	0	Cyperaceae Juss.
Iz_15_1209	DJI_0083	Centaurea majorovii Dumbadze	0	n	1	0	Asteraceae Bercht. & J.Presl
Iz_15_1209	DJI_0083	Chamaecytisus ruthenicus (Fisch.ex Wol.) Klaskova	0	n	1	0	Fabaceae Lindl.
Iz_15_1209	DJI_0083	Chelidonium majus L.	0.1	n	1	0	Papaveraceae Juss.
Iz_15_1209	DJI_0083	Chenopodium betaceum Andrz.	0	n	1	0	Amaranthaceae Juss.
Iz_15_1209	DJI_0083	Chenopodium cf. album	0	n	1	0	Amaranthaceae Juss.
Iz_15_1209	DJI_0083	Chenopodium sp.	0		NA	0	Amaranthaceae Juss.
Iz_15_1209	DJI_0083	Chondrilla juncea L.	0	n	1	0	Asteraceae Bercht. & J.Presl
Iz_15_1209	DJI_0083	Cirsium vulgare (Savi) Ten.	0	n	1	0	Asteraceae Bercht. & J.Presl
Iz_15_1209	DJI_0083	Clinopodium acinos (L.) Kuntze	0	n	1	0	Lamiacea Lindl.
Iz_15_1209	DJI_0083	Convallaria majalis L.	0	n	1	0	Asparagaceae Juss.
Iz_15_1209	DJI_0083	Crataegus rhipidophylla Gand.	0	n	1	1	Rosaceae Juss.
Iz_15_1209	DJI_0083	Crepis foetida L.	0	n	1	0	Asteraceae Bercht. & J.Presl
Iz_15_1209	DJI_0083	Dactylis glomerata L.	0	n	1	0	Poaceae Barnhart
Iz_15_1209	DJI_0083	Dianthus campestris M.Bieb.	0	n	1	0	Caryophyllaceae Juss.
Iz_15_1209	DJI_0083	Digitaria sanguinalis (L.) Scop.	0	a	0	0	Poaceae Barnhart
Iz_15_1209	DJI_0083	Elymus repens (L.) Gould	0	n	1	0	Poaceae Barnhart
Iz_15_1209	DJI_0083	Epilobium tetragonum L.	0	n	1	0	Onagraceae Juss.
Iz_15_1209	DJI_0083	Eragrostis minor Host	0	a	0	0	Poaceae Barnhart
Iz_15_1209	DJI_0083	Erigeron canadensis L.	0	a	0	0	Asteraceae Bercht. & J.Presl
Iz_15_1209	DJI_0083	Euphorbia seguieriana Neck.	0	n	1	0	Euphorbiaceae Juss.
Iz_15_1209	DJI_0083	Fallopia dumetorum (L.) Holub	0	n	1	0	Polygonaceae Juss.
Iz_15_1209	DJI_0083	Festuca beckeri (Hack.) Trautv.	0	n	1	0	Poaceae Barnhart
Iz_15_1209	DJI_0083	Fragaria vesca L.	0	n	1	0	Rosaceae Juss.
Iz_15_1209	DJI_0083	Frangula alnus Mill.	0	n	1	1	Rhamnaceae Juss.
Iz_15_1209	DJI_0083	Fraxinus pennsylvanica Marshall	0	a	0	1	Oleaceae Hoffmanns. & Link
Iz_15_1209	DJI_0083	Galeopsis bifida Boenn.	0	n	1	0	Lamiacea Lindl.
Iz_15_1209	DJI_0083	Galium verum L.	0	n	1	0	Rubiaceae Juss.
Iz_15_1209	DJI_0083	Genista tinctoria L.	0	n	1	0	Fabaceae Lindl.
Iz_15_1209	DJI_0083	Geranium robertianum L.	0	n	1	0	Geraniaceae Juss.
Iz_15_1209	DJI_0083	Geranium sanquineum L.	0	n	1	0	Geraniaceae Juss.
Iz_15_1209	DJI_0083	Gypsophila paniculata L.	0	n	1	0	Caryophyllaceae Juss.
Iz_15_1209	DJI_0083	Helichrysum arenarium (L.) Moench	0	n	1	0	Asteraceae Bercht. & J.Presl
Iz_15_1209	DJI_0083	Herniaria polygama J.Gay	0	n	1	0	Caryophyllaceae Juss.
Iz_15_1209	DJI_0083	Hieracium robustum Fr.	0	n	1	0	Asteraceae Bercht. & J.Presl
Iz_15_1209	DJI_0083	Hieracium umbellatum L.	0	n	1	NA	Asteraceae Bercht. & J.Presl
Iz_15_1209	DJI_0083	Hylotelephium maximum (L.) Holub	0	n	1	0	Crassulaceae J.St.-Hil.
Iz_15_1209	DJI_0083	Jacobaea borysthenica (DC.) B.Nord. & Greuter	0	n	1	0	Asteraceae Bercht. & J.Presl
Iz_15_1209	DJI_0083	Jasione montana L.	0	n	1	0	Campanulaceae Juss.
Iz_15_1209	DJI_0083	Jurinea cyanoides (L.) Rchb.s.l.	0	n	1	0	Asteraceae Bercht. & J.Presl
Iz_15_1209	DJI_0083	Koeleria glauca (Spreng.) DC.	0	n	1	0	Poaceae Barnhart
Iz_15_1209	DJI_0083	Lactuca serriola L.	0	a	0	0	Asteraceae Bercht. & J.Presl
Iz_15_1209	DJI_0083	Linaria genistifolia (L.) Mill.	0	n	1	0	Plantaginaceae Juss.
Iz_15_1209	DJI_0083	Lomelosia argentea (L.) Greuter & Burdet	0	n	1	0	Caprifoliaceae Juss.
Iz_15_1209	DJI_0083	Melampyrum pratense L.	0	n	1	0	Orobanchaceae Vent.
Iz_15_1209	DJI_0083	Oenothera biennis L.	0	a	0	0	Onagraceae Juss.
Iz_15_1209	DJI_0083	Pentanema britannica (L.) D.Gut.Larr., Santos-Vicente, Anderb., E.Rico & M.M.Mart.Ort.	0	n	1	0	Asteraceae Bercht. & J.Presl
Iz_15_1209	DJI_0083	Peucedanum oreoselinum (L.) Moench	0	n	1	0	Apiaceae Lindl.
Iz_15_1209	DJI_0083	Peucedanum ruthenicum M.Bieb.	0	n	1	0	Apiaceae Lindl.
Iz_15_1209	DJI_0083	Pilosella echioides (Lumn.) F.W.Schultz & Sch.Bip.	0	n	1	0	Asteraceae Bercht. & J.Presl
Iz_15_1209	DJI_0083	Pilosella officinarum F. Schultz et Sch. Bip.	0	n	1	0	Asteraceae Bercht. & J.Presl
Iz_15_1209	DJI_0083	Pilosella sp.	0		NA	0	Asteraceae Bercht. & J.Presl
Iz_15_1209	DJI_0083	Pinus sylvestris L.	0	n	1	1	Pinaceae Spreng. ex F.Rudolphi
Iz_15_1209	DJI_0083	Poa annua L.	0	n	1	0	Poaceae Barnhart
Iz_15_1209	DJI_0083	Poa nemoralis L.	0	n	1	0	Poaceae Barnhart
Iz_15_1209	DJI_0083	Poa sp.	0		NA	0	Poaceae Barnhart
Iz_15_1209	DJI_0083	Polygonatum odoratum (Mill.) Druce	0	n	1	0	Asparagaceae Juss.
Iz_15_1209	DJI_0083	Polygonum arenarium Waldst. & Kit.	0	n	1	0	Polygonaceae Juss.
Iz_15_1209	DJI_0083	Populus nigra L.	0	n	1	1	Salicaceae Mirb.
Iz_15_1209	DJI_0083	Populus tremula L.	0	n	1	1	Salicaceae Mirb.
Iz_15_1209	DJI_0083	Populus Ã canescens (Aiton) Sm.	0	n	1	1	Salicaceae Mirb.
Iz_15_1209	DJI_0083	Potentilla argentea L.	0	n	1	0	Rosaceae Juss.
Iz_15_1209	DJI_0083	Psammophiliella muralis (L.) Ikonn.	0	n	1	0	Caryophyllaceae Juss.
Iz_15_1209	DJI_0083	Psephellus sumensis (Kalen.) Greuter	0	n	1	0	Asteraceae Bercht. & J.Presl

Iz_15_1209	DJI_0083	Pulsatilla pratensis (L.) Mill. s.l.	0	n	1	0	Ranunculaceae Juss.
Iz_15_1209	DJI_0083	Pyrus communis L.	0	a	0	1	Rosaceae Juss.
Iz_15_1209	DJI_0083	Quercus robur L.	0	n	1	1	Fagaceae Dumort.
Iz_15_1209	DJI_0083	Robinia pseudoacacia L.	0	a	0	1	Fabaceae Lindl.
Iz_15_1209	DJI_0083	Rosa sp.	0		NA	1	Rosaceae Juss.
Iz_15_1209	DJI_0083	Rubus idaeus L.	0	n	1	0	Rosaceae Juss.
Iz_15_1209	DJI_0083	Rumex acetosella L.	0	n	1	0	Polygonaceae Juss.
Iz_15_1209	DJI_0083	Rumex thyrsiflorus Fingerh.	0	n	1	0	Polygonaceae Juss.
Iz_15_1209	DJI_0083	Salix alba L.	0	n	1	1	Salicaceae Mirb.
Iz_15_1209	DJI_0083	Sambucus nigra L.	0	n	1	1	Viburnaceae Raf.
Iz_15_1209	DJI_0083	Secale sylvestre Host	0	n	1	0	Poaceae Barnhart
Iz_15_1209	DJI_0083	Sempervivum ruthenicum Schnittsp. & C.B. Lehm.	0	n	1	0	Crassulaceae J.St.-Hil.
Iz_15_1209	DJI_0083	Setaria viridis (L.) P. Beauv.	0	n	1	0	Poaceae Barnhart
Iz_15_1209	DJI_0083	Silene borysthenica (Gruner) Walters	0	n	1	0	Caryophyllaceae Juss.
Iz_15_1209	DJI_0083	Silene latifolia subsp. alba (Mill.) Greuter & Burdet	0	n	1	0	Caryophyllaceae Juss.
Iz_15_1209	DJI_0083	Solidago virgaurea L.	0	n	1	0	Asteraceae Bercht. & J.Presl
Iz_15_1209	DJI_0083	Sorbus aucuparia L.	0	n	1	1	Rosaceae Juss.
Iz_15_1209	DJI_0083	Stachys recta L.	0	n	1	0	Lamiacea Lindl.
Iz_15_1209	DJI_0083	Tanacetum vulgare L.	0	n	1	0	Asteraceae Bercht. & J.Presl
Iz_15_1209	DJI_0083	Thymus pallasianus Heinr.Braun	0	n	1	0	Lamiacea Lindl.
Iz_15_1209	DJI_0083	Tilia cordata Mill.	0	n	1	1	Malvaceae Juss.
Iz_15_1209	DJI_0083	Trifolium alpestre L.	0	n	1	0	Fabaceae Lindl.
Iz_15_1209	DJI_0083	Trifolium arvense L.	0	n	1	0	Fabaceae Lindl.
Iz_15_1209	DJI_0083	Ulmus glabra Huds.	0	n	1	1	Ulmaceae Mirb.
Iz_15_1209	DJI_0083	Verbascum densiflorum Bertol.	0	n	1	0	Scrophulariaceae Juss.
Iz_15_1209	DJI_0083	Veronica austriaca L.	0	n	1	0	Plantaginaceae Juss.
Iz_15_1209	DJI_0083	Veronica incana L.	0	n	1	0	Plantaginaceae Juss.
Iz_15_1209	DJI_0083	Veronica verna L.	0	n	1	0	Plantaginaceae Juss.
Iz_15_1209	DJI_0083	Vincetoxicum hirundinaria Medik	0	n	1	0	Apocynaceae Juss.
Iz_16_1209	DJI_0103	Acer tataricum L.	0	n	1	1	Sapindaceae Juss.
Iz_16_1209	DJI_0103	Achillea millefolium L. s.l.	0	n	1	0	Asteraceae Bercht. & J.Presl
Iz_16_1209	DJI_0103	Allium oleraceum L.	0	n	1	0	Amaryllidaceae J.St.-Hil.
Iz_16_1209	DJI_0103	Ambrosia artemisiifolia L.	0	a	0	0	Asteraceae Bercht. & J.Presl
Iz_16_1209	DJI_0103	Amorpha fruticosa L.	0	a	0	0	Fabaceae Lindl.
Iz_16_1209	DJI_0103	Anthericum ramosum L.	0	n	1	0	Asparagaceae Juss.
Iz_16_1209	DJI_0103	Anthriscus sylvestris (L.) Hoffm.	0		NA	NA	
Iz_16_1209	DJI_0103	Artemisia marschalliana Spreng.	0	n	1	0	Asteraceae Bercht. & J.Presl
Iz_16_1209	DJI_0103	Asparagus officinalis L.	0	n	1	0	Asparagaceae Juss.
Iz_16_1209	DJI_0103	Astragalus cicer L.	0	n	1	0	Fabaceae Lindl.
Iz_16_1209	DJI_0103	Bassia laniflora (S.G.Gmel.) A.J.Scott	0	n	1	0	Amaranthaceae Juss.
Iz_16_1209	DJI_0103	Berberis vulgaris L.	0	n	1	0	Berberidaceae Juss.
Iz_16_1209	DJI_0103	Betula pendula Roth.	0	n	1	1	Betulaceae Gray
Iz_16_1209	DJI_0103	Betula pendula Roth.	0	n	1	0	Poaceae Barnhart
Iz_16_1209	DJI_0103	Bromus inermis Leyss.	0		NA	NA	
Iz_16_1209	DJI_0103	Bromus squarrosus L.	0	a	0	0	Poaceae Barnhart
Iz_16_1209	DJI_0103	Bromus tectorum L.	0	a	0	0	Poaceae Barnhart
Iz_16_1209	DJI_0103	Calamagrostis epigejos (L.) Roth	8	n	1	0	Poaceae Barnhart
Iz_16_1209	DJI_0103	Carduus acanthoides L.	0	n	1	0	Asteraceae Bercht. & J.Presl
Iz_16_1209	DJI_0103	Carex sp.	0	n	1	0	Cyperaceae Juss.
Iz_16_1209	DJI_0103	Centaurea majorovii Dumbadze	0	n	1	0	Asteraceae Bercht. & J.Presl
Iz_16_1209	DJI_0103	Cf. Gypsophila paniculata L.	0		NA	0	
Iz_16_1209	DJI_0103	Chamaecytisus ruthenicus (Fisch.ex Wol.) Klaskova	1.5	n	1	0	Fabaceae Lindl.
Iz_16_1209	DJI_0103	Chelidonium majus L.	0	n	1	0	Papaveraceae Juss.
Iz_16_1209	DJI_0103	Chenopodium betaceum Andrz.	0	n	1	0	Amaranthaceae Juss.
Iz_16_1209	DJI_0103	Chenopodium cf. album	0	n	1	0	Amaranthaceae Juss.
Iz_16_1209	DJI_0103	Chenopodium sp.	0.5		NA	0	Amaranthaceae Juss.
Iz_16_1209	DJI_0103	Chondrilla juncea L.	0	n	1	0	Asteraceae Bercht. & J.Presl
Iz_16_1209	DJI_0103	Cirsium vulgare (Savi) Ten.	0	n	1	0	Asteraceae Bercht. & J.Presl
Iz_16_1209	DJI_0103	Clinopodium acinos (L.) Kuntze	0	n	1	0	Lamiacea Lindl.
Iz_16_1209	DJI_0103	Convallaria majalis L.	0	n	1	0	Asparagaceae Juss.
Iz_16_1209	DJI_0103	Crataegus rhipidophylla Gand.	0	n	1	1	Rosaceae Juss.
Iz_16_1209	DJI_0103	Crepis foetida L.	0	n	1	0	Asteraceae Bercht. & J.Presl
Iz_16_1209	DJI_0103	Dactylis glomerata L.	0	n	1	0	Poaceae Barnhart
Iz_16_1209	DJI_0103	Dianthus campestris M.Bieb.	0	n	1	0	Caryophyllaceae Juss.
Iz_16_1209	DJI_0103	Digitaria sanguinalis (L.) Scop.	0	a	0	0	Poaceae Barnhart
Iz_16_1209	DJI_0103	Elymus repens (L.) Gould	0	n	1	0	Poaceae Barnhart
Iz_16_1209	DJI_0103	Epilobium tetragonum L.	0	n	1	0	Onagraceae Juss.
Iz_16_1209	DJI_0103	Eragrostis minor Host	0	a	0	0	Poaceae Barnhart
Iz_16_1209	DJI_0103	Erigeron canadensis L.	2	a	0	0	Asteraceae Bercht. & J.Presl
Iz_16_1209	DJI_0103	Euphorbia seguieriana Neck.	0	n	1	0	Euphorbiaceae Juss.
Iz_16_1209	DJI_0103	Euphorbia sp.	0		NA	NA	
Iz_16_1209	DJI_0103	Fallopia dumetorum (L.) Holub	0	n	1	0	Polygonaceae Juss.
Iz_16_1209	DJI_0103	Festuca beckeri (Hack.) Trautv.	0	n	1	0	Poaceae Barnhart
Iz_16_1209	DJI_0103	Fragaria vesca L.	0	n	1	0	Rosaceae Juss.
Iz_16_1209	DJI_0103	Frangula alnus Mill.	0	n	1	1	Rhamnaceae Juss.
Iz_16_1209	DJI_0103	Fraxinus pennsylvanica Marshall	0	a	0	1	Oleaceae Hoffmanns. & Link
Iz_16_1209	DJI_0103	Galeopsis bifida Boenn.	0	n	1	0	Lamiacea Lindl.
Iz_16_1209	DJI_0103	Galium verum L.	0	n	1	0	Rubiaceae Juss.
Iz_16_1209	DJI_0103	Genista tinctoria L.	0	n	1	0	Fabaceae Lindl.
Iz_16_1209	DJI_0103	Geranium robertianum L.	0	n	1	0	Geraniaceae Juss.
Iz_16_1209	DJI_0103	Geranium sanquineum L.	0	n	1	0	Geraniaceae Juss.
Iz_16_1209	DJI_0103	Gypsophila paniculata L.	0	n	1	0	Caryophyllaceae Juss.
Iz_16_1209	DJI_0103	Helichrysum arenarium (L.) Moench	0	n	1	0	Asteraceae Bercht. & J.Presl
Iz_16_1209	DJI_0103	Herniaria polygama J.Gay	0	n	1	0	Caryophyllaceae Juss.
Iz_16_1209	DJI_0103	Hieracium robustum Fr.	0	n	1	0	Asteraceae Bercht. & J.Presl
Iz_16_1209	DJI_0103	Hieracium umbellatum L.	0	n	1	NA	Asteraceae Bercht. & J.Presl
Iz_16_1209	DJI_0103	Hylotelephium maximum (L.) Holub	0	n	1	0	Crassulaceae J.St.-Hil.
Iz_16_1209	DJI_0103	Jacobaea borysthenica (DC.) B.Nord. & Greuter	0	n	1	0	Asteraceae Bercht. & J.Presl
Iz_16_1209	DJI_0103	Jasione montana L.	0	n	1	0	Campanulaceae Juss.
Iz_16_1209	DJI_0103	Jurinea cyanoides (L.) Rchb.s.l.	0	n	1	0	Asteraceae Bercht. & J.Presl
Iz_16_1209	DJI_0103	Koeleria glauca (Spreng.) DC.	0	n	1	0	Poaceae Barnhart
Iz_16_1209	DJI_0103	Lactuca serriola L.	0	a	0	0	Asteraceae Bercht. & J.Presl
Iz_16_1209	DJI_0103	Linaria genistifolia (L.) Mill.	0	n	1	0	Plantaginaceae Juss.
Iz_16_1209	DJI_0103	Lomelosia argentea (L.) Greuter & Burdet	0	n	1	0	Caprifoliaceae Juss.
Iz_16_1209	DJI_0103	Melampyrum pratense L.	0	n	1	0	Orobanchaceae Vent.
Iz_16_1209	DJI_0103	Oenothera biennis L.	0	a	0	0	Onagraceae Juss.
Iz_16_1209	DJI_0103	Pentanema britannica (L.) D.Gut.Larr., Santos-Vicente, Anderb., E.Rico & M.M.Mart.Ort.	0	n	1	0	Asteraceae Bercht. & J.Presl
Iz_16_1209	DJI_0103	Peucedanum oreoselinum (L.) Moench	1	n	1	0	Apiaceae Lindl.

Iz_16_1209	DJI_0103	Peucedanum ruthenicum M.Bieb.	0	n	1	0	Apiaceae Lindl.
Iz_16_1209	DJI_0103	Pilosella echioides (Lumn.) F.W.Schultz & Sch.Bip.	0	n	1	0	Asteraceae Bercht. & J.Presl
Iz_16_1209	DJI_0103	Pilosella officinarum F. Schultz et Sch. Bip.	0	n	1	0	Asteraceae Bercht. & J.Presl
Iz_16_1209	DJI_0103	Pilosella sp.	0		NA	0	Asteraceae Bercht. & J.Presl
Iz_16_1209	DJI_0103	Pinus sylvestris L.	0	n	1	1	Pinaceae Spreng. ex F.Rudolphi
Iz_16_1209	DJI_0103	Poa annua L.	0	n	1	0	Poaceae Barnhart
Iz_16_1209	DJI_0103	Poa nemoralis L.	0	n	1	0	Poaceae Barnhart
Iz_16_1209	DJI_0103	Poa sp.	0		NA	0	Poaceae Barnhart
Iz_16_1209	DJI_0103	Polygonatum odoratum (Mill.) Druce	2	n	1	0	Asparagaceae Juss.
Iz_16_1209	DJI_0103	Polygonum arenarium Waldst. & Kit.	0	n	1	0	Polygonaceae Juss.
Iz_16_1209	DJI_0103	Populus nigra L.	0	n	1	1	Salicaceae Mirb.
Iz_16_1209	DJI_0103	Populus tremula L.	0	n	1	1	Salicaceae Mirb.
Iz_16_1209	DJI_0103	Populus Ã canescens (Aiton) Sm.	0	n	1	1	Salicaceae Mirb.
Iz_16_1209	DJI_0103	Potentilla argentea L.	0	n	1	0	Rosaceae Juss.
Iz_16_1209	DJI_0103	Psammophiliella muralis (L.) Ikonn.	0	n	1	0	Caryophyllaceae Juss.
Iz_16_1209	DJI_0103	Psephellus sumensis (Kalen.) Greuter	0	n	1	0	Asteraceae Bercht. & J.Presl
Iz_16_1209	DJI_0103	Pulsatilla pratensis (L.) Mill. s.l.	0	n	1	0	Ranunculaceae Juss.
Iz_16_1209	DJI_0103	Pyrus communis L.	0	a	0	1	Rosaceae Juss.
Iz_16_1209	DJI_0103	Quercus robur L.	0	n	1	1	Fagaceae Dumort.
Iz_16_1209	DJI_0103	Robinia pseudoacacia L.	0	a	0	1	Fabaceae Lindl.
Iz_16_1209	DJI_0103	Rosa sp.	0		NA	1	Rosaceae Juss.
Iz_16_1209	DJI_0103	Rubus idaeus L.	0	n	1	0	Rosaceae Juss.
Iz_16_1209	DJI_0103	Rumex acetosella L.	0	n	1	0	Polygonaceae Juss.
Iz_16_1209	DJI_0103	Rumex thyrsiflorus Fingerh.	0	n	1	0	Polygonaceae Juss.
Iz_16_1209	DJI_0103	Salix alba L.	0	n	1	1	Salicaceae Mirb.
Iz_16_1209	DJI_0103	Sambucus nigra L.	0	n	1	1	Viburnaceae Raf.
Iz_16_1209	DJI_0103	Secale sylvestre Host	0	n	1	0	Poaceae Barnhart
Iz_16_1209	DJI_0103	Sempervivum ruthenicum Schnittsp. & C.B. Lehm.	0	n	1	0	Crassulaceae J.St.-Hil.
Iz_16_1209	DJI_0103	Setaria viridis (L.) P. Beauv.	0	n	1	0	Poaceae Barnhart
Iz_16_1209	DJI_0103	Silene borysthenica (Gruner) Walters	0	n	1	0	Caryophyllaceae Juss.
Iz_16_1209	DJI_0103	Silene latifolia subsp. alba (Mill.) Greuter & Burdet	0	n	1	0	Caryophyllaceae Juss.
Iz_16_1209	DJI_0103	Solidago virgaurea L.	0	n	1	0	Asteraceae Bercht. & J.Presl
Iz_16_1209	DJI_0103	Sorbus aucuparia L.	0	n	1	1	Rosaceae Juss.
Iz_16_1209	DJI_0103	Stachys recta L.	0	n	1	0	Lamiacea Lindl.
Iz_16_1209	DJI_0103	Tanacetum vulgare L.	0	n	1	0	Asteraceae Bercht. & J.Presl
Iz_16_1209	DJI_0103	Thymus pallasianus Heinr.Braun	0	n	1	0	Lamiacea Lindl.
Iz_16_1209	DJI_0103	Tilia cordata Mill.	0	n	1	1	Malvaceae Juss.
Iz_16_1209	DJI_0103	Trifolium alpestre L.	0	n	1	0	Fabaceae Lindl.
Iz_16_1209	DJI_0103	Trifolium arvense L.	0	n	1	0	Fabaceae Lindl.
Iz_16_1209	DJI_0103	Ulmus glabra Huds.	0	n	1	1	Ulmaceae Mirb.
Iz_16_1209	DJI_0103	Verbascum densiflorum Bertol.	0	n	1	0	Scrophulariaceae Juss.
Iz_16_1209	DJI_0103	Veronica austriaca L.	0	n	1	0	Plantaginaceae Juss.
Iz_16_1209	DJI_0103	Veronica incana L.	0	n	1	0	Plantaginaceae Juss.
Iz_16_1209	DJI_0103	Veronica verna L.	0	n	1	0	Plantaginaceae Juss.
Iz_16_1209	DJI_0103	Vincetoxicum hirundinaria Medik	0	n	1	0	Apocynaceae Juss.
Iz_16_1209	DJI_0106	Acer tataricum L.	0	n	1	1	Sapindaceae Juss.
Iz_16_1209	DJI_0106	Achillea millefolium L. s.l.	0	n	1	0	Asteraceae Bercht. & J.Presl
Iz_16_1209	DJI_0106	Allium oleraceum L.	0	n	1	0	Amaryllidaceae J.St.-Hil.
Iz_16_1209	DJI_0106	Ambrosia artemisiifolia L.	0	a	0	0	Asteraceae Bercht. & J.Presl
Iz_16_1209	DJI_0106	Amorpha fruticosa L.	0	a	0	0	Fabaceae Lindl.
Iz_16_1209	DJI_0106	Anthericum ramosum L.	0	n	1	0	Asparagaceae Juss.
Iz_16_1209	DJI_0106	Anthriscus sylvestris (L.) Hoffm.	0.5		NA	NA	
Iz_16_1209	DJI_0106	Artemisia marschalliana Spreng.	0	n	1	0	Asteraceae Bercht. & J.Presl
Iz_16_1209	DJI_0106	Asparagus officinalis L.	0	n	1	0	Asparagaceae Juss.
Iz_16_1209	DJI_0106	Astragalus cicer L.	0	n	1	0	Fabaceae Lindl.
Iz_16_1209	DJI_0106	Bassia laniflora (S.G.Gmel.) A.J.Scott	0	n	1	0	Amaranthaceae Juss.
Iz_16_1209	DJI_0106	Berberis vulgaris L.	0	n	1	0	Berberidaceae Juss.
Iz_16_1209	DJI_0106	Betula pendula Roth.	0	n	1	1	Betulaceae Gray
Iz_16_1209	DJI_0106	Betula pendula Roth.	0	n	1	0	Poaceae Barnhart
Iz_16_1209	DJI_0106	Bromus inermis Leyss.	0		NA	NA	
Iz_16_1209	DJI_0106	Bromus squarrosus L.	0	a	0	0	Poaceae Barnhart
Iz_16_1209	DJI_0106	Bromus tectorum L.	0	a	0	0	Poaceae Barnhart
Iz_16_1209	DJI_0106	Calamagrostis epigejos (L.) Roth	0.5	n	1	0	Poaceae Barnhart
Iz_16_1209	DJI_0106	Carduus acanthoides L.	0	n	1	0	Asteraceae Bercht. & J.Presl
Iz_16_1209	DJI_0106	Carex sp.	0	n	1	0	Cyperaceae Juss.
Iz_16_1209	DJI_0106	Centaurea majorovii Dumbadze	0	n	1	0	Asteraceae Bercht. & J.Presl
Iz_16_1209	DJI_0106	Cf. Gypsophila paniculata L.	0		NA	0	
Iz_16_1209	DJI_0106	Chamaecytisus ruthenicus (Fisch.ex Wol.) Klaskova	0	n	1	0	Fabaceae Lindl.
Iz_16_1209	DJI_0106	Chelidonium majus L.	0	n	1	0	Papaveraceae Juss.
Iz_16_1209	DJI_0106	Chenopodium betaceum Andrz.	0	n	1	0	Amaranthaceae Juss.
Iz_16_1209	DJI_0106	Chenopodium cf. album	0	n	1	0	Amaranthaceae Juss.
Iz_16_1209	DJI_0106	Chenopodium sp.	0		NA	0	Amaranthaceae Juss.
Iz_16_1209	DJI_0106	Chondrilla juncea L.	0	n	1	0	Asteraceae Bercht. & J.Presl
Iz_16_1209	DJI_0106	Cirsium vulgare (Savi) Ten.	0	n	1	0	Asteraceae Bercht. & J.Presl
Iz_16_1209	DJI_0106	Clinopodium acinos (L.) Kuntze	0	n	1	0	Lamiacea Lindl.
Iz_16_1209	DJI_0106	Convallaria majalis L.	0	n	1	0	Asparagaceae Juss.
Iz_16_1209	DJI_0106	Crataegus rhipidophylla Gand.	0	n	1	1	Rosaceae Juss.
Iz_16_1209	DJI_0106	Crepis foetida L.	0	n	1	0	Asteraceae Bercht. & J.Presl
Iz_16_1209	DJI_0106	Dactylis glomerata L.	0	n	1	0	Poaceae Barnhart
Iz_16_1209	DJI_0106	Dianthus campestris M.Bieb.	0	n	1	0	Caryophyllaceae Juss.
Iz_16_1209	DJI_0106	Digitaria sanguinalis (L.) Scop.	0	a	0	0	Poaceae Barnhart
Iz_16_1209	DJI_0106	Elymus repens (L.) Gould	0	n	1	0	Poaceae Barnhart
Iz_16_1209	DJI_0106	Epilobium tetragonum L.	0	n	1	0	Onagraceae Juss.
Iz_16_1209	DJI_0106	Eragrostis minor Host	0	a	0	0	Poaceae Barnhart
Iz_16_1209	DJI_0106	Erigeron canadensis L.	0	a	0	0	Asteraceae Bercht. & J.Presl
Iz_16_1209	DJI_0106	Euphorbia seguieriana Neck.	0	n	1	0	Euphorbiaceae Juss.
Iz_16_1209	DJI_0106	Euphorbia sp.	0		NA	NA	
Iz_16_1209	DJI_0106	Fallopia dumetorum (L.) Holub	0	n	1	0	Polygonaceae Juss.
Iz_16_1209	DJI_0106	Festuca beckeri (Hack.) Trautv.	0	n	1	0	Poaceae Barnhart
Iz_16_1209	DJI_0106	Fragaria vesca L.	0	n	1	0	Rosaceae Juss.
Iz_16_1209	DJI_0106	Frangula alnus Mill.	0	n	1	1	Rhamnaceae Juss.
Iz_16_1209	DJI_0106	Fraxinus pennsylvanica Marshall	0	a	0	1	Oleaceae Hoffmanns. & Link
Iz_16_1209	DJI_0106	Galeopsis bifida Boenn.	0	n	1	0	Lamiacea Lindl.
Iz_16_1209	DJI_0106	Galium verum L.	0	n	1	0	Rubiaceae Juss.
Iz_16_1209	DJI_0106	Genista tinctoria L.	0	n	1	0	Fabaceae Lindl.
Iz_16_1209	DJI_0106	Geranium robertianum L.	0	n	1	0	Geraniaceae Juss.
Iz_16_1209	DJI_0106	Geranium sanquineum L.	0	n	1	0	Geraniaceae Juss.
Iz_16_1209	DJI_0106	Gypsophila paniculata L.	0	n	1	0	Caryophyllaceae Juss.

Iz_16_1209	DJI_0106	Helichrysum arenarium (L.) Moench	0	n	1	0	Asteraceae Bercht. & J.Presl
Iz_16_1209	DJI_0106	Herniaria polygama J.Gay	0	n	1	0	Caryophyllaceae Juss.
Iz_16_1209	DJI_0106	Hieracium robustum Fr.	0	n	1	0	Asteraceae Bercht. & J.Presl
Iz_16_1209	DJI_0106	Hieracium umbellatum L.	0	n	1	NA	Asteraceae Bercht. & J.Presl
Iz_16_1209	DJI_0106	Hylotelephium maximum (L.) Holub	0	n	1	0	Crassulaceae J.St.-Hil.
Iz_16_1209	DJI_0106	Jacobaea borysthenica (DC.) B.Nord. & Greuter	0	n	1	0	Asteraceae Bercht. & J.Presl
Iz_16_1209	DJI_0106	Jasione montana L.	0	n	1	0	Campanulaceae Juss.
Iz_16_1209	DJI_0106	Jurinea cyanoides (L.) Rchb.s.l.	0	n	1	0	Asteraceae Bercht. & J.Presl
Iz_16_1209	DJI_0106	Koeleria glauca (Spreng.) DC.	0	n	1	0	Poaceae Barnhart
Iz_16_1209	DJI_0106	Lactuca serriola L.	0	a	0	0	Asteraceae Bercht. & J.Presl
Iz_16_1209	DJI_0106	Linaria genistifolia (L.) Mill.	0	n	1	0	Plantaginaceae Juss.
Iz_16_1209	DJI_0106	Lomelosia argentea (L.) Greuter & Burdet	0	n	1	0	Caprifoliaceae Juss.
Iz_16_1209	DJI_0106	Melampyrum pratense L.	0	n	1	0	Orobanchaceae Vent.
Iz_16_1209	DJI_0106	Oenothera biennis L.	0	a	0	0	Onagraceae Juss.
Iz_16_1209	DJI_0106	Pentanema britannica (L.) D.Gut.Larr., Santos-Vicente, Anderb., E.Rico & M.M.Mart.Ort.	0	n	1	0	Asteraceae Bercht. & J.Presl
Iz_16_1209	DJI_0106	Peucedanum oreoselinum (L.) Moench	0.5	n	1	0	Apiaceae Lindl.
Iz_16_1209	DJI_0106	Peucedanum ruthenicum M.Bieb.	0	n	1	0	Apiaceae Lindl.
Iz_16_1209	DJI_0106	Pilosella echioides (Lumn.) F.W.Schultz & Sch.Bip.	0	n	1	0	Asteraceae Bercht. & J.Presl
Iz_16_1209	DJI_0106	Pilosella officinarum F. Schultz et Sch. Bip.	0	n	1	0	Asteraceae Bercht. & J.Presl
Iz_16_1209	DJI_0106	Pilosella sp.	0		NA	0	Asteraceae Bercht. & J.Presl
Iz_16_1209	DJI_0106	Pinus sylvestris L.	0	n	1	1	Pinaceae Spreng. ex F.Rudolphi
Iz_16_1209	DJI_0106	Poa annua L.	0	n	1	0	Poaceae Barnhart
Iz_16_1209	DJI_0106	Poa nemoralis L.	0	n	1	0	Poaceae Barnhart
Iz_16_1209	DJI_0106	Poa sp.	0		NA	0	Poaceae Barnhart
Iz_16_1209	DJI_0106	Polygonatum odoratum (Mill.) Druce	0	n	1	0	Asparagaceae Juss.
Iz_16_1209	DJI_0106	Polygonum arenarium Waldst. & Kit.	0	n	1	0	Polygonaceae Juss.
Iz_16_1209	DJI_0106	Populus nigra L.	0	n	1	1	Salicaceae Mirb.
Iz_16_1209	DJI_0106	Populus tremula L.	0	n	1	1	Salicaceae Mirb.
Iz_16_1209	DJI_0106	Populus Ã canescens (Aiton) Sm.	0	n	1	1	Salicaceae Mirb.
Iz_16_1209	DJI_0106	Potentilla argentea L.	0	n	1	0	Rosaceae Juss.
Iz_16_1209	DJI_0106	Psammophiliella muralis (L.) Ikonn.	0	n	1	0	Caryophyllaceae Juss.
Iz_16_1209	DJI_0106	Psephellus sumensis (Kalen.) Greuter	0	n	1	0	Asteraceae Bercht. & J.Presl
Iz_16_1209	DJI_0106	Pulsatilla pratensis (L.) Mill. s.l.	0	n	1	0	Ranunculaceae Juss.
Iz_16_1209	DJI_0106	Pyrus communis L.	0	a	0	1	Rosaceae Juss.
Iz_16_1209	DJI_0106	Quercus robur L.	0.5	n	1	1	Fagaceae Dumort.
Iz_16_1209	DJI_0106	Robinia pseudoacacia L.	10	a	0	1	Fabaceae Lindl.
Iz_16_1209	DJI_0106	Rosa sp.	0		NA	1	Rosaceae Juss.
Iz_16_1209	DJI_0106	Rubus idaeus L.	0	n	1	0	Rosaceae Juss.
Iz_16_1209	DJI_0106	Rumex acetosella L.	0	n	1	0	Polygonaceae Juss.
Iz_16_1209	DJI_0106	Rumex thyrsiflorus Fingerh.	0	n	1	0	Polygonaceae Juss.
Iz_16_1209	DJI_0106	Salix alba L.	0	n	1	1	Salicaceae Mirb.
Iz_16_1209	DJI_0106	Sambucus nigra L.	0	n	1	1	Viburnaceae Raf.
Iz_16_1209	DJI_0106	Secale sylvestre Host	0	n	1	0	Poaceae Barnhart
Iz_16_1209	DJI_0106	Sempervivum ruthenicum Schnittsp. & C.B. Lehm.	0	n	1	0	Crassulaceae J.St.-Hil.
Iz_16_1209	DJI_0106	Setaria viridis (L.) P. Beauv.	0	n	1	0	Poaceae Barnhart
Iz_16_1209	DJI_0106	Silene borysthenica (Gruner) Walters	0	n	1	0	Caryophyllaceae Juss.
Iz_16_1209	DJI_0106	Silene latifolia subsp. alba (Mill.) Greuter & Burdet	0	n	1	0	Caryophyllaceae Juss.
Iz_16_1209	DJI_0106	Solidago virgaurea L.	0	n	1	0	Asteraceae Bercht. & J.Presl
Iz_16_1209	DJI_0106	Sorbus aucuparia L.	0	n	1	1	Rosaceae Juss.
Iz_16_1209	DJI_0106	Stachys recta L.	0	n	1	0	Lamiacea Lindl.
Iz_16_1209	DJI_0106	Tanacetum vulgare L.	0	n	1	0	Asteraceae Bercht. & J.Presl
Iz_16_1209	DJI_0106	Thymus pallasianus Heinr.Braun	0	n	1	0	Lamiacea Lindl.
Iz_16_1209	DJI_0106	Tilia cordata Mill.	0	n	1	1	Malvaceae Juss.
Iz_16_1209	DJI_0106	Trifolium alpestre L.	0	n	1	0	Fabaceae Lindl.
Iz_16_1209	DJI_0106	Trifolium arvense L.	0	n	1	0	Fabaceae Lindl.
Iz_16_1209	DJI_0106	Ulmus glabra Huds.	0	n	1	1	Ulmaceae Mirb.
Iz_16_1209	DJI_0106	Verbascum densiflorum Bertol.	0	n	1	0	Scrophulariaceae Juss.
Iz_16_1209	DJI_0106	Veronica austriaca L.	0	n	1	0	Plantaginaceae Juss.
Iz_16_1209	DJI_0106	Veronica incana L.	0.3	n	1	0	Plantaginaceae Juss.
Iz_16_1209	DJI_0106	Veronica verna L.	0	n	1	0	Plantaginaceae Juss.
Iz_16_1209	DJI_0106	Vincetoxicum hirundinaria Medik	0	n	1	0	Apocynaceae Juss.
Iz_16_1209	DJI_0109	Acer tataricum L.	0	n	1	1	Sapindaceae Juss.
Iz_16_1209	DJI_0109	Achillea millefolium L. s.l.	0	n	1	0	Asteraceae Bercht. & J.Presl
Iz_16_1209	DJI_0109	Allium oleraceum L.	0	n	1	0	Amaryllidaceae J.St.-Hil.
Iz_16_1209	DJI_0109	Ambrosia artemisiifolia L.	0	a	0	0	Asteraceae Bercht. & J.Presl
Iz_16_1209	DJI_0109	Amorpha fruticosa L.	0	a	0	0	Fabaceae Lindl.
Iz_16_1209	DJI_0109	Anthericum ramosum L.	0	n	1	0	Asparagaceae Juss.
Iz_16_1209	DJI_0109	Anthriscus sylvestris (L.) Hoffm.	0		NA	NA	
Iz_16_1209	DJI_0109	Artemisia marschalliana Spreng.	0	n	1	0	Asteraceae Bercht. & J.Presl
Iz_16_1209	DJI_0109	Asparagus officinalis L.	0	n	1	0	Asparagaceae Juss.
Iz_16_1209	DJI_0109	Astragalus cicer L.	0	n	1	0	Fabaceae Lindl.
Iz_16_1209	DJI_0109	Bassia laniflora (S.G.Gmel.) A.J.Scott	0	n	1	0	Amaranthaceae Juss.
Iz_16_1209	DJI_0109	Berberis vulgaris L.	0	n	1	0	Berberidaceae Juss.
Iz_16_1209	DJI_0109	Betula pendula Roth.	0	n	1	1	Betulaceae Gray
Iz_16_1209	DJI_0109	Betula pendula Roth.	0	n	1	0	Poaceae Barnhart
Iz_16_1209	DJI_0109	Bromus inermis Leyss.	0		NA	NA	
Iz_16_1209	DJI_0109	Bromus squarrosus L.	0	a	0	0	Poaceae Barnhart
Iz_16_1209	DJI_0109	Bromus tectorum L.	0	a	0	0	Poaceae Barnhart
Iz_16_1209	DJI_0109	Calamagrostis epigejos (L.) Roth	0.5	n	1	0	Poaceae Barnhart
Iz_16_1209	DJI_0109	Carduus acanthoides L.	0	n	1	0	Asteraceae Bercht. & J.Presl
Iz_16_1209	DJI_0109	Carex sp.	0	n	1	0	Cyperaceae Juss.
Iz_16_1209	DJI_0109	Centaurea majorovii Dumbadze	0	n	1	0	Asteraceae Bercht. & J.Presl
Iz_16_1209	DJI_0109	Cf. Gypsophila paniculata L.	0		NA	0	
Iz_16_1209	DJI_0109	Chamaecytisus ruthenicus (Fisch.ex Wol.) Klaskova	0	n	1	0	Fabaceae Lindl.
Iz_16_1209	DJI_0109	Chelidonium majus L.	0	n	1	0	Papaveraceae Juss.
Iz_16_1209	DJI_0109	Chenopodium betaceum Andrz.	0	n	1	0	Amaranthaceae Juss.
Iz_16_1209	DJI_0109	Chenopodium cf. album	1.5	n	1	0	Amaranthaceae Juss.
Iz_16_1209	DJI_0109	Chenopodium sp.	0		NA	0	Amaranthaceae Juss.
Iz_16_1209	DJI_0109	Chondrilla juncea L.	0	n	1	0	Asteraceae Bercht. & J.Presl
Iz_16_1209	DJI_0109	Cirsium vulgare (Savi) Ten.	0	n	1	0	Asteraceae Bercht. & J.Presl
Iz_16_1209	DJI_0109	Clinopodium acinos (L.) Kuntze	0	n	1	0	Lamiacea Lindl.
Iz_16_1209	DJI_0109	Convallaria majalis L.	0	n	1	0	Asparagaceae Juss.
Iz_16_1209	DJI_0109	Crataegus rhipidophylla Gand.	0	n	1	1	Rosaceae Juss.
Iz_16_1209	DJI_0109	Crepis foetida L.	0	n	1	0	Asteraceae Bercht. & J.Presl
Iz_16_1209	DJI_0109	Dactylis glomerata L.	0	n	1	0	Poaceae Barnhart
Iz_16_1209	DJI_0109	Dianthus campestris M.Bieb.	0	n	1	0	Caryophyllaceae Juss.
Iz_16_1209	DJI_0109	Digitaria sanguinalis (L.) Scop.	0	a	0	0	Poaceae Barnhart
Iz_16_1209	DJI_0109	Elymus repens (L.) Gould	0	n	1	0	Poaceae Barnhart

Iz_16_1209	DJI_0109	Epilobium tetragonum L.	0	n	1	0	Onagraceae Juss.
Iz_16_1209	DJI_0109	Eragrostis minor Host	0	a	0	0	Poaceae Barnhart
Iz_16_1209	DJI_0109	Erigeron canadensis L.	0	a	0	0	Asteraceae Bercht. & J.Presl
Iz_16_1209	DJI_0109	Euphorbia seguieriana Neck.	0	n	1	0	Euphorbiaceae Juss.
Iz_16_1209	DJI_0109	Euphorbia sp.	0		NA	NA	
Iz_16_1209	DJI_0109	Fallopia dumetorum (L.) Holub	0	n	1	0	Polygonaceae Juss.
Iz_16_1209	DJI_0109	Festuca beckeri (Hack.) Trautv.	0	n	1	0	Poaceae Barnhart
Iz_16_1209	DJI_0109	Fragaria vesca L.	0	n	1	0	Rosaceae Juss.
Iz_16_1209	DJI_0109	Frangula alnus Mill.	0	n	1	1	Rhamnaceae Juss.
Iz_16_1209	DJI_0109	Fraxinus pennsylvanica Marshall	0	a	0	1	Oleaceae Hoffmanns. & Link
Iz_16_1209	DJI_0109	Galeopsis bifida Boenn.	0	n	1	0	Lamiacea Lindl.
Iz_16_1209	DJI_0109	Galium verum L.	0	n	1	0	Rubiaceae Juss.
Iz_16_1209	DJI_0109	Genista tinctoria L.	0	n	1	0	Fabaceae Lindl.
Iz_16_1209	DJI_0109	Geranium robertianum L.	0	n	1	0	Geraniaceae Juss.
Iz_16_1209	DJI_0109	Geranium sanquineum L.	0	n	1	0	Geraniaceae Juss.
Iz_16_1209	DJI_0109	Gypsophila paniculata L.	0	n	1	0	Caryophyllaceae Juss.
Iz_16_1209	DJI_0109	Helichrysum arenarium (L.) Moench	0	n	1	0	Asteraceae Bercht. & J.Presl
Iz_16_1209	DJI_0109	Herniaria polygama J.Gay	0	n	1	0	Caryophyllaceae Juss.
Iz_16_1209	DJI_0109	Hieracium robustum Fr.	0	n	1	0	Asteraceae Bercht. & J.Presl
Iz_16_1209	DJI_0109	Hieracium umbellatum L.	0	n	1	NA	Asteraceae Bercht. & J.Presl
Iz_16_1209	DJI_0109	Hylotelephium maximum (L.) Holub	0	n	1	0	Crassulaceae J.St.-Hil.
Iz_16_1209	DJI_0109	Jacobaea borysthenica (DC.) B.Nord. & Greuter	0	n	1	0	Asteraceae Bercht. & J.Presl
Iz_16_1209	DJI_0109	Jasione montana L.	0	n	1	0	Campanulaceae Juss.
Iz_16_1209	DJI_0109	Jurinea cyanoides (L.) Rchb.s.l.	0	n	1	0	Asteraceae Bercht. & J.Presl
Iz_16_1209	DJI_0109	Koeleria glauca (Spreng.) DC.	0	n	1	0	Poaceae Barnhart
Iz_16_1209	DJI_0109	Lactuca serriola L.	0	a	0	0	Asteraceae Bercht. & J.Presl
Iz_16_1209	DJI_0109	Linaria genistifolia (L.) Mill.	0	n	1	0	Plantaginaceae Juss.
Iz_16_1209	DJI_0109	Lomelosia argentea (L.) Greuter & Burdet	0	n	1	0	Caprifoliaceae Juss.
Iz_16_1209	DJI_0109	Melampyrum pratense L.	0	n	1	0	Orobanchaceae Vent.
Iz_16_1209	DJI_0109	Oenothera biennis L.	0	a	0	0	Onagraceae Juss.
Iz_16_1209	DJI_0109	Pentanema britannica (L.) D.Gut.Larr., Santos-Vicente, Anderb., E.Rico & M.M.Mart.Ort.	0	n	1	0	Asteraceae Bercht. & J.Presl
Iz_16_1209	DJI_0109	Peucedanum oreoselinum (L.) Moench	0	n	1	0	Apiaceae Lindl.
Iz_16_1209	DJI_0109	Peucedanum ruthenicum M.Bieb.	0	n	1	0	Apiaceae Lindl.
Iz_16_1209	DJI_0109	Pilosella echioides (Lumn.) F.W.Schultz & Sch.Bip.	0	n	1	0	Asteraceae Bercht. & J.Presl
Iz_16_1209	DJI_0109	Pilosella officinarum F. Schultz et Sch. Bip.	0	n	1	0	Asteraceae Bercht. & J.Presl
Iz_16_1209	DJI_0109	Pilosella sp.	0		NA	0	Asteraceae Bercht. & J.Presl
Iz_16_1209	DJI_0109	Pinus sylvestris L.	0	n	1	1	Pinaceae Spreng. ex F.Rudolphi
Iz_16_1209	DJI_0109	Poa annua L.	0	n	1	0	Poaceae Barnhart
Iz_16_1209	DJI_0109	Poa nemoralis L.	0	n	1	0	Poaceae Barnhart
Iz_16_1209	DJI_0109	Poa sp.	0		NA	0	Poaceae Barnhart
Iz_16_1209	DJI_0109	Polygonatum odoratum (Mill.) Druce	0	n	1	0	Asparagaceae Juss.
Iz_16_1209	DJI_0109	Polygonum arenarium Waldst. & Kit.	0	n	1	0	Polygonaceae Juss.
Iz_16_1209	DJI_0109	Populus nigra L.	0	n	1	1	Salicaceae Mirb.
Iz_16_1209	DJI_0109	Populus tremula L.	0	n	1	1	Salicaceae Mirb.
Iz_16_1209	DJI_0109	Populus Ã canescens (Aiton) Sm.	0	n	1	1	Salicaceae Mirb.
Iz_16_1209	DJI_0109	Potentilla argentea L.	0	n	1	0	Rosaceae Juss.
Iz_16_1209	DJI_0109	Psammophiliella muralis (L.) Ikonn.	0	n	1	0	Caryophyllaceae Juss.
Iz_16_1209	DJI_0109	Psephellus sumensis (Kalen.) Greuter	0	n	1	0	Asteraceae Bercht. & J.Presl
Iz_16_1209	DJI_0109	Pulsatilla pratensis (L.) Mill. s.l.	0	n	1	0	Ranunculaceae Juss.
Iz_16_1209	DJI_0109	Pyrus communis L.	0	a	0	1	Rosaceae Juss.
Iz_16_1209	DJI_0109	Quercus robur L.	0	n	1	1	Fagaceae Dumort.
Iz_16_1209	DJI_0109	Robinia pseudoacacia L.	0	a	0	1	Fabaceae Lindl.
Iz_16_1209	DJI_0109	Rosa sp.	0		NA	1	Rosaceae Juss.
Iz_16_1209	DJI_0109	Rubus idaeus L.	0	n	1	0	Rosaceae Juss.
Iz_16_1209	DJI_0109	Rumex acetosella L.	0	n	1	0	Polygonaceae Juss.
Iz_16_1209	DJI_0109	Rumex thyrsiflorus Fingerh.	0	n	1	0	Polygonaceae Juss.
Iz_16_1209	DJI_0109	Salix alba L.	0	n	1	1	Salicaceae Mirb.
Iz_16_1209	DJI_0109	Sambucus nigra L.	0	n	1	1	Viburnaceae Raf.
Iz_16_1209	DJI_0109	Secale sylvestre Host	0	n	1	0	Poaceae Barnhart
Iz_16_1209	DJI_0109	Sempervivum ruthenicum Schnittsp. & C.B. Lehm.	0	n	1	0	Crassulaceae J.St.-Hil.
Iz_16_1209	DJI_0109	Setaria viridis (L.) P. Beauv.	0	n	1	0	Poaceae Barnhart
Iz_16_1209	DJI_0109	Silene borysthenica (Gruner) Walters	0	n	1	0	Caryophyllaceae Juss.
Iz_16_1209	DJI_0109	Silene latifolia subsp. alba (Mill.) Greuter & Burdet	0	n	1	0	Caryophyllaceae Juss.
Iz_16_1209	DJI_0109	Solidago virgaurea L.	0	n	1	0	Asteraceae Bercht. & J.Presl
Iz_16_1209	DJI_0109	Sorbus aucuparia L.	0	n	1	1	Rosaceae Juss.
Iz_16_1209	DJI_0109	Stachys recta L.	0	n	1	0	Lamiacea Lindl.
Iz_16_1209	DJI_0109	Tanacetum vulgare L.	0	n	1	0	Asteraceae Bercht. & J.Presl
Iz_16_1209	DJI_0109	Thymus pallasianus Heinr.Braun	0	n	1	0	Lamiacea Lindl.
Iz_16_1209	DJI_0109	Tilia cordata Mill.	0	n	1	1	Malvaceae Juss.
Iz_16_1209	DJI_0109	Trifolium alpestre L.	0	n	1	0	Fabaceae Lindl.
Iz_16_1209	DJI_0109	Trifolium arvense L.	0	n	1	0	Fabaceae Lindl.
Iz_16_1209	DJI_0109	Ulmus glabra Huds.	0	n	1	1	Ulmaceae Mirb.
Iz_16_1209	DJI_0109	Verbascum densiflorum Bertol.	0	n	1	0	Scrophulariaceae Juss.
Iz_16_1209	DJI_0109	Veronica austriaca L.	0	n	1	0	Plantaginaceae Juss.
Iz_16_1209	DJI_0109	Veronica incana L.	0	n	1	0	Plantaginaceae Juss.
Iz_16_1209	DJI_0109	Veronica verna L.	0	n	1	0	Plantaginaceae Juss.
Iz_16_1209	DJI_0109	Vincetoxicum hirundinaria Medik	0	n	1	0	Apocynaceae Juss.
Iz_16_1209	DJI_0114	Acer tataricum L.	0	n	1	1	Sapindaceae Juss.
Iz_16_1209	DJI_0114	Achillea millefolium L. s.l.	0	n	1	0	Asteraceae Bercht. & J.Presl
Iz_16_1209	DJI_0114	Allium oleraceum L.	0	n	1	0	Amaryllidaceae J.St.-Hil.
Iz_16_1209	DJI_0114	Ambrosia artemisiifolia L.	0	a	0	0	Asteraceae Bercht. & J.Presl
Iz_16_1209	DJI_0114	Amorpha fruticosa L.	0	a	0	0	Fabaceae Lindl.
Iz_16_1209	DJI_0114	Anthericum ramosum L.	0	n	1	0	Asparagaceae Juss.
Iz_16_1209	DJI_0114	Anthriscus sylvestris (L.) Hoffm.	0		NA	NA	
Iz_16_1209	DJI_0114	Artemisia marschalliana Spreng.	0	n	1	0	Asteraceae Bercht. & J.Presl
Iz_16_1209	DJI_0114	Asparagus officinalis L.	0	n	1	0	Asparagaceae Juss.
Iz_16_1209	DJI_0114	Astragalus cicer L.	0	n	1	0	Fabaceae Lindl.
Iz_16_1209	DJI_0114	Bassia laniflora (S.G.Gmel.) A.J.Scott	0	n	1	0	Amaranthaceae Juss.
Iz_16_1209	DJI_0114	Berberis vulgaris L.	0	n	1	0	Berberidaceae Juss.
Iz_16_1209	DJI_0114	Betula pendula Roth.	0	n	1	1	Betulaceae Gray
Iz_16_1209	DJI_0114	Betula pendula Roth.	0	n	1	0	Poaceae Barnhart
Iz_16_1209	DJI_0114	Bromus inermis Leyss.	0		NA	NA	
Iz_16_1209	DJI_0114	Bromus squarrosus L.	0	a	0	0	Poaceae Barnhart
Iz_16_1209	DJI_0114	Bromus tectorum L.	0	a	0	0	Poaceae Barnhart
Iz_16_1209	DJI_0114	Calamagrostis epigejos (L.) Roth	0	n	1	0	Poaceae Barnhart
Iz_16_1209	DJI_0114	Carduus acanthoides L.	0	n	1	0	Asteraceae Bercht. & J.Presl
Iz_16_1209	DJI_0114	Carex sp.	0	n	1	0	Cyperaceae Juss.
Iz_16_1209	DJI_0114	Centaurea majorovii Dumbadze	0	n	1	0	Asteraceae Bercht. & J.Presl

Iz_16_1209	DJI_0114	Cf. Gypsophila paniculata L.	0		NA	0	
Iz_16_1209	DJI_0114	Chamaecytisus ruthenicus (Fisch.ex Wol.) Klaskova	0	n	1	0	Fabaceae Lindl.
Iz_16_1209	DJI_0114	Chelidonium majus L.	0	n	1	0	Papaveraceae Juss.
Iz_16_1209	DJI_0114	Chenopodium betaceum Andrz.	0	n	1	0	Amaranthaceae Juss.
Iz_16_1209	DJI_0114	Chenopodium cf. album	0	n	1	0	Amaranthaceae Juss.
Iz_16_1209	DJI_0114	Chenopodium sp.	0		NA	0	Amaranthaceae Juss.
Iz_16_1209	DJI_0114	Chondrilla juncea L.	0	n	1	0	Asteraceae Bercht. & J.Presl
Iz_16_1209	DJI_0114	Cirsium vulgare (Savi) Ten.	0	n	1	0	Asteraceae Bercht. & J.Presl
Iz_16_1209	DJI_0114	Clinopodium acinos (L.) Kuntze	0	n	1	0	Lamiacea Lindl.
Iz_16_1209	DJI_0114	Convallaria majalis L.	0	n	1	0	Asparagaceae Juss.
Iz_16_1209	DJI_0114	Crataegus rhipidophylla Gand.	0	n	1	1	Rosaceae Juss.
Iz_16_1209	DJI_0114	Crepis foetida L.	0	n	1	0	Asteraceae Bercht. & J.Presl
Iz_16_1209	DJI_0114	Dactylis glomerata L.	0	n	1	0	Poaceae Barnhart
Iz_16_1209	DJI_0114	Dianthus campestris M.Bieb.	0	n	1	0	Caryophyllaceae Juss.
Iz_16_1209	DJI_0114	Digitaria sanguinalis (L.) Scop.	0	a	0	0	Poaceae Barnhart
Iz_16_1209	DJI_0114	Elymus repens (L.) Gould	0	n	1	0	Poaceae Barnhart
Iz_16_1209	DJI_0114	Epilobium tetragonum L.	0	n	1	0	Onagraceae Juss.
Iz_16_1209	DJI_0114	Eragrostis minor Host	0	a	0	0	Poaceae Barnhart
Iz_16_1209	DJI_0114	Erigeron canadensis L.	0.2	a	0	0	Asteraceae Bercht. & J.Presl
Iz_16_1209	DJI_0114	Euphorbia seguieriana Neck.	0	n	1	0	Euphorbiaceae Juss.
Iz_16_1209	DJI_0114	Euphorbia sp.	0		NA	NA	
Iz_16_1209	DJI_0114	Fallopia dumetorum (L.) Holub	0	n	1	0	Polygonaceae Juss.
Iz_16_1209	DJI_0114	Festuca beckeri (Hack.) Trautv.	0	n	1	0	Poaceae Barnhart
Iz_16_1209	DJI_0114	Fragaria vesca L.	0	n	1	0	Rosaceae Juss.
Iz_16_1209	DJI_0114	Frangula alnus Mill.	0	n	1	1	Rhamnaceae Juss.
Iz_16_1209	DJI_0114	Fraxinus pennsylvanica Marshall	0	a	0	1	Oleaceae Hoffmanns. & Link
Iz_16_1209	DJI_0114	Galeopsis bifida Boenn.	0	n	1	0	Lamiacea Lindl.
Iz_16_1209	DJI_0114	Galium verum L.	0	n	1	0	Rubiaceae Juss.
Iz_16_1209	DJI_0114	Genista tinctoria L.	0	n	1	0	Fabaceae Lindl.
Iz_16_1209	DJI_0114	Geranium robertianum L.	0	n	1	0	Geraniaceae Juss.
Iz_16_1209	DJI_0114	Geranium sanquineum L.	0	n	1	0	Geraniaceae Juss.
Iz_16_1209	DJI_0114	Gypsophila paniculata L.	0	n	1	0	Caryophyllaceae Juss.
Iz_16_1209	DJI_0114	Helichrysum arenarium (L.) Moench	0	n	1	0	Asteraceae Bercht. & J.Presl
Iz_16_1209	DJI_0114	Herniaria polygama J.Gay	0	n	1	0	Caryophyllaceae Juss.
Iz_16_1209	DJI_0114	Hieracium robustum Fr.	0	n	1	0	Asteraceae Bercht. & J.Presl
Iz_16_1209	DJI_0114	Hieracium umbellatum L.	0	n	1	NA	Asteraceae Bercht. & J.Presl
Iz_16_1209	DJI_0114	Hylotelephium maximum (L.) Holub	0	n	1	0	Crassulaceae J.St.-Hil.
Iz_16_1209	DJI_0114	Jacobaea borysthenica (DC.) B.Nord. & Greuter	0	n	1	0	Asteraceae Bercht. & J.Presl
Iz_16_1209	DJI_0114	Jasione montana L.	0	n	1	0	Campanulaceae Juss.
Iz_16_1209	DJI_0114	Jurinea cyanoides (L.) Rchb.s.l.	0	n	1	0	Asteraceae Bercht. & J.Presl
Iz_16_1209	DJI_0114	Koeleria glauca (Spreng.) DC.	0	n	1	0	Poaceae Barnhart
Iz_16_1209	DJI_0114	Lactuca serriola L.	0	a	0	0	Asteraceae Bercht. & J.Presl
Iz_16_1209	DJI_0114	Linaria genistifolia (L.) Mill.	0	n	1	0	Plantaginaceae Juss.
Iz_16_1209	DJI_0114	Lomelosia argentea (L.) Greuter & Burdet	0	n	1	0	Caprifoliaceae Juss.
Iz_16_1209	DJI_0114	Melampyrum pratense L.	0	n	1	0	Orobanchaceae Vent.
Iz_16_1209	DJI_0114	Oenothera biennis L.	0	a	0	0	Onagraceae Juss.
Iz_16_1209	DJI_0114	Pentanema britannica (L.) D.Gut.Larr., Santos-Vicente, Anderb., E.Rico & M.M.Mart.Ort.	0	n	1	0	Asteraceae Bercht. & J.Presl
Iz_16_1209	DJI_0114	Peucedanum oreoselinum (L.) Moench	0	n	1	0	Apiaceae Lindl.
Iz_16_1209	DJI_0114	Peucedanum ruthenicum M.Bieb.	0	n	1	0	Apiaceae Lindl.
Iz_16_1209	DJI_0114	Pilosella echioides (Lumn.) F.W.Schultz & Sch.Bip.	0	n	1	0	Asteraceae Bercht. & J.Presl
Iz_16_1209	DJI_0114	Pilosella officinarum F. Schultz et Sch. Bip.	0	n	1	0	Asteraceae Bercht. & J.Presl
Iz_16_1209	DJI_0114	Pilosella sp.	0		NA	0	Asteraceae Bercht. & J.Presl
Iz_16_1209	DJI_0114	Pinus sylvestris L.	0	n	1	1	Pinaceae Spreng. ex F.Rudolphi
Iz_16_1209	DJI_0114	Poa annua L.	0	n	1	0	Poaceae Barnhart
Iz_16_1209	DJI_0114	Poa nemoralis L.	0	n	1	0	Poaceae Barnhart
Iz_16_1209	DJI_0114	Poa sp.	0		NA	0	Poaceae Barnhart
Iz_16_1209	DJI_0114	Polygonatum odoratum (Mill.) Druce	0	n	1	0	Asparagaceae Juss.
Iz_16_1209	DJI_0114	Polygonum arenarium Waldst. & Kit.	0	n	1	0	Polygonaceae Juss.
Iz_16_1209	DJI_0114	Populus nigra L.	0	n	1	1	Salicaceae Mirb.
Iz_16_1209	DJI_0114	Populus tremula L.	0	n	1	1	Salicaceae Mirb.
Iz_16_1209	DJI_0114	Populus Ã canescens (Aiton) Sm.	0	n	1	1	Salicaceae Mirb.
Iz_16_1209	DJI_0114	Potentilla argentea L.	0	n	1	0	Rosaceae Juss.
Iz_16_1209	DJI_0114	Psammophiliella muralis (L.) Ikonn.	0	n	1	0	Caryophyllaceae Juss.
Iz_16_1209	DJI_0114	Psephellus sumensis (Kalen.) Greuter	0	n	1	0	Asteraceae Bercht. & J.Presl
Iz_16_1209	DJI_0114	Pulsatilla pratensis (L.) Mill. s.l.	0	n	1	0	Ranunculaceae Juss.
Iz_16_1209	DJI_0114	Pyrus communis L.	0	a	0	1	Rosaceae Juss.
Iz_16_1209	DJI_0114	Quercus robur L.	0	n	1	1	Fagaceae Dumort.
Iz_16_1209	DJI_0114	Robinia pseudoacacia L.	0	a	0	1	Fabaceae Lindl.
Iz_16_1209	DJI_0114	Rosa sp.	0		NA	1	Rosaceae Juss.
Iz_16_1209	DJI_0114	Rubus idaeus L.	0	n	1	0	Rosaceae Juss.
Iz_16_1209	DJI_0114	Rumex acetosella L.	0	n	1	0	Polygonaceae Juss.
Iz_16_1209	DJI_0114	Rumex thyrsiflorus Fingerh.	0	n	1	0	Polygonaceae Juss.
Iz_16_1209	DJI_0114	Salix alba L.	0	n	1	1	Salicaceae Mirb.
Iz_16_1209	DJI_0114	Sambucus nigra L.	0	n	1	1	Viburnaceae Raf.
Iz_16_1209	DJI_0114	Secale sylvestre Host	0	n	1	0	Poaceae Barnhart
Iz_16_1209	DJI_0114	Sempervivum ruthenicum Schnittsp. & C.B. Lehm.	0	n	1	0	Crassulaceae J.St.-Hil.
Iz_16_1209	DJI_0114	Setaria viridis (L.) P. Beauv.	0	n	1	0	Poaceae Barnhart
Iz_16_1209	DJI_0114	Silene borysthenica (Gruner) Walters	0	n	1	0	Caryophyllaceae Juss.
Iz_16_1209	DJI_0114	Silene latifolia subsp. alba (Mill.) Greuter & Burdet	0	n	1	0	Caryophyllaceae Juss.
Iz_16_1209	DJI_0114	Solidago virgaurea L.	0	n	1	0	Asteraceae Bercht. & J.Presl
Iz_16_1209	DJI_0114	Sorbus aucuparia L.	0	n	1	1	Rosaceae Juss.
Iz_16_1209	DJI_0114	Stachys recta L.	0	n	1	0	Lamiacea Lindl.
Iz_16_1209	DJI_0114	Tanacetum vulgare L.	0	n	1	0	Asteraceae Bercht. & J.Presl
Iz_16_1209	DJI_0114	Thymus pallasianus Heinr.Braun	0	n	1	0	Lamiacea Lindl.
Iz_16_1209	DJI_0114	Tilia cordata Mill.	0	n	1	1	Malvaceae Juss.
Iz_16_1209	DJI_0114	Trifolium alpestre L.	0	n	1	0	Fabaceae Lindl.
Iz_16_1209	DJI_0114	Trifolium arvense L.	0	n	1	0	Fabaceae Lindl.
Iz_16_1209	DJI_0114	Ulmus glabra Huds.	0	n	1	1	Ulmaceae Mirb.
Iz_16_1209	DJI_0114	Verbascum densiflorum Bertol.	0	n	1	0	Scrophulariaceae Juss.
Iz_16_1209	DJI_0114	Veronica austriaca L.	0	n	1	0	Plantaginaceae Juss.
Iz_16_1209	DJI_0114	Veronica incana L.	0	n	1	0	Plantaginaceae Juss.
Iz_16_1209	DJI_0114	Veronica verna L.	0	n	1	0	Plantaginaceae Juss.
Iz_16_1209	DJI_0114	Vincetoxicum hirundinaria Medik	0	n	1	0	Apocynaceae Juss.
Iz_16_1209	DJI_0119	Acer tataricum L.	0	n	1	1	Sapindaceae Juss.
Iz_16_1209	DJI_0119	Achillea millefolium L. s.l.	0	n	1	0	Asteraceae Bercht. & J.Presl
Iz_16_1209	DJI_0119	Allium oleraceum L.	0	n	1	0	Amaryllidaceae J.St.-Hil.
Iz_16_1209	DJI_0119	Ambrosia artemisiifolia L.	0	a	0	0	Asteraceae Bercht. & J.Presl
Iz_16_1209	DJI_0119	Amorpha fruticosa L.	0	a	0	0	Fabaceae Lindl.

Iz_16_1209	DJI_0119	Anthericum ramosum L.	0	n	1	0	Asparagaceae Juss.
Iz_16_1209	DJI_0119	Anthriscus sylvestris (L.) Hoffm.	0		NA	NA	
Iz_16_1209	DJI_0119	Artemisia marschalliana Spreng.	0	n	1	0	Asteraceae Bercht. & J.Presl
Iz_16_1209	DJI_0119	Asparagus officinalis L.	0	n	1	0	Asparagaceae Juss.
Iz_16_1209	DJI_0119	Astragalus cicer L.	0	n	1	0	Fabaceae Lindl.
Iz_16_1209	DJI_0119	Bassia laniflora (S.G.Gmel.) A.J.Scott	0	n	1	0	Amaranthaceae Juss.
Iz_16_1209	DJI_0119	Berberis vulgaris L.	0	n	1	0	Berberidaceae Juss.
Iz_16_1209	DJI_0119	Betula pendula Roth.	0	n	1	1	Betulaceae Gray
Iz_16_1209	DJI_0119	Betula pendula Roth.	0	n	1	0	Poaceae Barnhart
Iz_16_1209	DJI_0119	Bromus inermis Leyss.	0		NA	NA	
Iz_16_1209	DJI_0119	Bromus squarrosus L.	0	a	0	0	Poaceae Barnhart
Iz_16_1209	DJI_0119	Bromus tectorum L.	0	a	0	0	Poaceae Barnhart
Iz_16_1209	DJI_0119	Calamagrostis epigejos (L.) Roth	2	n	1	0	Poaceae Barnhart
Iz_16_1209	DJI_0119	Carduus acanthoides L.	0	n	1	0	Asteraceae Bercht. & J.Presl
Iz_16_1209	DJI_0119	Carex sp.	0	n	1	0	Cyperaceae Juss.
Iz_16_1209	DJI_0119	Centaurea majorovii Dumbadze	0	n	1	0	Asteraceae Bercht. & J.Presl
Iz_16_1209	DJI_0119	Cf. Gypsophila paniculata L.	0		NA	0	
Iz_16_1209	DJI_0119	Chamaecytisus ruthenicus (Fisch.ex Wol.) Klaskova	0	n	1	0	Fabaceae Lindl.
Iz_16_1209	DJI_0119	Chelidonium majus L.	0	n	1	0	Papaveraceae Juss.
Iz_16_1209	DJI_0119	Chenopodium betaceum Andrz.	0	n	1	0	Amaranthaceae Juss.
Iz_16_1209	DJI_0119	Chenopodium cf. album	0.5	n	1	0	Amaranthaceae Juss.
Iz_16_1209	DJI_0119	Chenopodium sp.	0		NA	0	Amaranthaceae Juss.
Iz_16_1209	DJI_0119	Chondrilla juncea L.	0	n	1	0	Asteraceae Bercht. & J.Presl
Iz_16_1209	DJI_0119	Cirsium vulgare (Savi) Ten.	0	n	1	0	Asteraceae Bercht. & J.Presl
Iz_16_1209	DJI_0119	Clinopodium acinos (L.) Kuntze	0	n	1	0	Lamiacea Lindl.
Iz_16_1209	DJI_0119	Convallaria majalis L.	0	n	1	0	Asparagaceae Juss.
Iz_16_1209	DJI_0119	Crataegus rhipidophylla Gand.	0	n	1	1	Rosaceae Juss.
Iz_16_1209	DJI_0119	Crepis foetida L.	0	n	1	0	Asteraceae Bercht. & J.Presl
Iz_16_1209	DJI_0119	Dactylis glomerata L.	0	n	1	0	Poaceae Barnhart
Iz_16_1209	DJI_0119	Dianthus campestris M.Bieb.	0	n	1	0	Caryophyllaceae Juss.
Iz_16_1209	DJI_0119	Digitaria sanguinalis (L.) Scop.	0	a	0	0	Poaceae Barnhart
Iz_16_1209	DJI_0119	Elymus repens (L.) Gould	0	n	1	0	Poaceae Barnhart
Iz_16_1209	DJI_0119	Epilobium tetragonum L.	0	n	1	0	Onagraceae Juss.
Iz_16_1209	DJI_0119	Eragrostis minor Host	0	a	0	0	Poaceae Barnhart
Iz_16_1209	DJI_0119	Erigeron canadensis L.	5	a	0	0	Asteraceae Bercht. & J.Presl
Iz_16_1209	DJI_0119	Euphorbia seguieriana Neck.	0	n	1	0	Euphorbiaceae Juss.
Iz_16_1209	DJI_0119	Euphorbia sp.	0		NA	NA	
Iz_16_1209	DJI_0119	Fallopia dumetorum (L.) Holub	0	n	1	0	Polygonaceae Juss.
Iz_16_1209	DJI_0119	Festuca beckeri (Hack.) Trautv.	0	n	1	0	Poaceae Barnhart
Iz_16_1209	DJI_0119	Fragaria vesca L.	0	n	1	0	Rosaceae Juss.
Iz_16_1209	DJI_0119	Frangula alnus Mill.	0	n	1	1	Rhamnaceae Juss.
Iz_16_1209	DJI_0119	Fraxinus pennsylvanica Marshall	0	a	0	1	Oleaceae Hoffmanns. & Link
Iz_16_1209	DJI_0119	Galeopsis bifida Boenn.	0	n	1	0	Lamiacea Lindl.
Iz_16_1209	DJI_0119	Galium verum L.	0	n	1	0	Rubiaceae Juss.
Iz_16_1209	DJI_0119	Genista tinctoria L.	0	n	1	0	Fabaceae Lindl.
Iz_16_1209	DJI_0119	Geranium robertianum L.	0	n	1	0	Geraniaceae Juss.
Iz_16_1209	DJI_0119	Geranium sanquineum L.	0	n	1	0	Geraniaceae Juss.
Iz_16_1209	DJI_0119	Gypsophila paniculata L.	0	n	1	0	Caryophyllaceae Juss.
Iz_16_1209	DJI_0119	Helichrysum arenarium (L.) Moench	0	n	1	0	Asteraceae Bercht. & J.Presl
Iz_16_1209	DJI_0119	Herniaria polygama J.Gay	0	n	1	0	Caryophyllaceae Juss.
Iz_16_1209	DJI_0119	Hieracium robustum Fr.	0	n	1	0	Asteraceae Bercht. & J.Presl
Iz_16_1209	DJI_0119	Hieracium umbellatum L.	2	n	1	NA	Asteraceae Bercht. & J.Presl
Iz_16_1209	DJI_0119	Hylotelephium maximum (L.) Holub	0	n	1	0	Crassulaceae J.St.-Hil.
Iz_16_1209	DJI_0119	Jacobaea borysthenica (DC.) B.Nord. & Greuter	0	n	1	0	Asteraceae Bercht. & J.Presl
Iz_16_1209	DJI_0119	Jasione montana L.	0	n	1	0	Campanulaceae Juss.
Iz_16_1209	DJI_0119	Jurinea cyanoides (L.) Rchb.s.l.	0	n	1	0	Asteraceae Bercht. & J.Presl
Iz_16_1209	DJI_0119	Koeleria glauca (Spreng.) DC.	0	n	1	0	Poaceae Barnhart
Iz_16_1209	DJI_0119	Lactuca serriola L.	0	a	0	0	Asteraceae Bercht. & J.Presl
Iz_16_1209	DJI_0119	Linaria genistifolia (L.) Mill.	0	n	1	0	Plantaginaceae Juss.
Iz_16_1209	DJI_0119	Lomelosia argentea (L.) Greuter & Burdet	0	n	1	0	Caprifoliaceae Juss.
Iz_16_1209	DJI_0119	Melampyrum pratense L.	0	n	1	0	Orobanchaceae Vent.
Iz_16_1209	DJI_0119	Oenothera biennis L.	3	a	0	0	Onagraceae Juss.
Iz_16_1209	DJI_0119	Pentanema britannica (L.) D.Gut.Larr., Santos-Vicente, Anderb., E.Rico & M.M.Mart.Ort.	0	n	1	0	Asteraceae Bercht. & J.Presl
Iz_16_1209	DJI_0119	Peucedanum oreoselinum (L.) Moench	0	n	1	0	Apiaceae Lindl.
Iz_16_1209	DJI_0119	Peucedanum ruthenicum M.Bieb.	0	n	1	0	Apiaceae Lindl.
Iz_16_1209	DJI_0119	Pilosella echioides (Lumn.) F.W.Schultz & Sch.Bip.	0	n	1	0	Asteraceae Bercht. & J.Presl
Iz_16_1209	DJI_0119	Pilosella officinarum F. Schultz et Sch. Bip.	0	n	1	0	Asteraceae Bercht. & J.Presl
Iz_16_1209	DJI_0119	Pilosella sp.	0		NA	0	Asteraceae Bercht. & J.Presl
Iz_16_1209	DJI_0119	Pinus sylvestris L.	0	n	1	1	Pinaceae Spreng. ex F.Rudolphi
Iz_16_1209	DJI_0119	Poa annua L.	0	n	1	0	Poaceae Barnhart
Iz_16_1209	DJI_0119	Poa nemoralis L.	0	n	1	0	Poaceae Barnhart
Iz_16_1209	DJI_0119	Poa sp.	0		NA	0	Poaceae Barnhart
Iz_16_1209	DJI_0119	Polygonatum odoratum (Mill.) Druce	0	n	1	0	Asparagaceae Juss.
Iz_16_1209	DJI_0119	Polygonum arenarium Waldst. & Kit.	0	n	1	0	Polygonaceae Juss.
Iz_16_1209	DJI_0119	Populus nigra L.	0	n	1	1	Salicaceae Mirb.
Iz_16_1209	DJI_0119	Populus tremula L.	0	n	1	1	Salicaceae Mirb.
Iz_16_1209	DJI_0119	Populus Ã canescens (Aiton) Sm.	0	n	1	1	Salicaceae Mirb.
Iz_16_1209	DJI_0119	Potentilla argentea L.	0	n	1	0	Rosaceae Juss.
Iz_16_1209	DJI_0119	Psammophiliella muralis (L.) Ikonn.	0	n	1	0	Caryophyllaceae Juss.
Iz_16_1209	DJI_0119	Psephellus sumensis (Kalen.) Greuter	0	n	1	0	Asteraceae Bercht. & J.Presl
Iz_16_1209	DJI_0119	Pulsatilla pratensis (L.) Mill. s.l.	0	n	1	0	Ranunculaceae Juss.
Iz_16_1209	DJI_0119	Pyrus communis L.	0	a	0	1	Rosaceae Juss.
Iz_16_1209	DJI_0119	Quercus robur L.	0	n	1	1	Fagaceae Dumort.
Iz_16_1209	DJI_0119	Robinia pseudoacacia L.	0	a	0	1	Fabaceae Lindl.
Iz_16_1209	DJI_0119	Rosa sp.	0		NA	1	Rosaceae Juss.
Iz_16_1209	DJI_0119	Rubus idaeus L.	0	n	1	0	Rosaceae Juss.
Iz_16_1209	DJI_0119	Rumex acetosella L.	0	n	1	0	Polygonaceae Juss.
Iz_16_1209	DJI_0119	Rumex thyrsiflorus Fingerh.	0	n	1	0	Polygonaceae Juss.
Iz_16_1209	DJI_0119	Salix alba L.	0	n	1	1	Salicaceae Mirb.
Iz_16_1209	DJI_0119	Sambucus nigra L.	0	n	1	1	Viburnaceae Raf.
Iz_16_1209	DJI_0119	Secale sylvestre Host	0	n	1	0	Poaceae Barnhart
Iz_16_1209	DJI_0119	Sempervivum ruthenicum Schnittsp. & C.B. Lehm.	0	n	1	0	Crassulaceae J.St.-Hil.
Iz_16_1209	DJI_0119	Setaria viridis (L.) P. Beauv.	0	n	1	0	Poaceae Barnhart
Iz_16_1209	DJI_0119	Silene borysthenica (Gruner) Walters	0	n	1	0	Caryophyllaceae Juss.
Iz_16_1209	DJI_0119	Silene latifolia subsp. alba (Mill.) Greuter & Burdet	0	n	1	0	Caryophyllaceae Juss.
Iz_16_1209	DJI_0119	Solidago virgaurea L.	2	n	1	0	Asteraceae Bercht. & J.Presl
Iz_16_1209	DJI_0119	Sorbus aucuparia L.	0	n	1	1	Rosaceae Juss.
Iz_16_1209	DJI_0119	Stachys recta L.	5	n	1	0	Lamiacea Lindl.

Iz_16_1209	DJI_0119	Tanacetum vulgare L.	0	n	1	0	Asteraceae Bercht. & J.Presl
Iz_16_1209	DJI_0119	Thymus pallasianus Heinr.Braun	0	n	1	0	Lamiacea Lindl.
Iz_16_1209	DJI_0119	Tilia cordata Mill.	0	n	1	1	Malvaceae Juss.
Iz_16_1209	DJI_0119	Trifolium alpestre L.	0	n	1	0	Fabaceae Lindl.
Iz_16_1209	DJI_0119	Trifolium arvense L.	0	n	1	0	Fabaceae Lindl.
Iz_16_1209	DJI_0119	Ulmus glabra Huds.	0	n	1	1	Ulmaceae Mirb.
Iz_16_1209	DJI_0119	Verbascum densiflorum Bertol.	0	n	1	0	Scrophulariaceae Juss.
Iz_16_1209	DJI_0119	Veronica austriaca L.	0	n	1	0	Plantaginaceae Juss.
Iz_16_1209	DJI_0119	Veronica incana L.	0	n	1	0	Plantaginaceae Juss.
Iz_16_1209	DJI_0119	Veronica verna L.	0	n	1	0	Plantaginaceae Juss.
Iz_16_1209	DJI_0119	Vincetoxicum hirundinaria Medik	0	n	1	0	Apocynaceae Juss.
Iz_16_1209	DJI_0122	Acer tataricum L.	0	n	1	1	Sapindaceae Juss.
Iz_16_1209	DJI_0122	Achillea millefolium L. s.l.	0	n	1	0	Asteraceae Bercht. & J.Presl
Iz_16_1209	DJI_0122	Allium oleraceum L.	0	n	1	0	Amaryllidaceae J.St.-Hil.
Iz_16_1209	DJI_0122	Ambrosia artemisiifolia L.	0	a	0	0	Asteraceae Bercht. & J.Presl
Iz_16_1209	DJI_0122	Amorpha fruticosa L.	0	a	0	0	Fabaceae Lindl.
Iz_16_1209	DJI_0122	Anthericum ramosum L.	0	n	1	0	Asparagaceae Juss.
Iz_16_1209	DJI_0122	Anthriscus sylvestris (L.) Hoffm.	0		NA	NA	
Iz_16_1209	DJI_0122	Artemisia marschalliana Spreng.	0	n	1	0	Asteraceae Bercht. & J.Presl
Iz_16_1209	DJI_0122	Asparagus officinalis L.	0	n	1	0	Asparagaceae Juss.
Iz_16_1209	DJI_0122	Astragalus cicer L.	0	n	1	0	Fabaceae Lindl.
Iz_16_1209	DJI_0122	Bassia laniflora (S.G.Gmel.) A.J.Scott	0	n	1	0	Amaranthaceae Juss.
Iz_16_1209	DJI_0122	Berberis vulgaris L.	0	n	1	0	Berberidaceae Juss.
Iz_16_1209	DJI_0122	Betula pendula Roth.	0	n	1	1	Betulaceae Gray
Iz_16_1209	DJI_0122	Betula pendula Roth.	0	n	1	0	Poaceae Barnhart
Iz_16_1209	DJI_0122	Bromus inermis Leyss.	0		NA	NA	
Iz_16_1209	DJI_0122	Bromus squarrosus L.	0	a	0	0	Poaceae Barnhart
Iz_16_1209	DJI_0122	Bromus tectorum L.	0	a	0	0	Poaceae Barnhart
Iz_16_1209	DJI_0122	Calamagrostis epigejos (L.) Roth	0.1	n	1	0	Poaceae Barnhart
Iz_16_1209	DJI_0122	Carduus acanthoides L.	0	n	1	0	Asteraceae Bercht. & J.Presl
Iz_16_1209	DJI_0122	Carex sp.	0	n	1	0	Cyperaceae Juss.
Iz_16_1209	DJI_0122	Centaurea majorovii Dumbadze	0	n	1	0	Asteraceae Bercht. & J.Presl
Iz_16_1209	DJI_0122	Cf. Gypsophila paniculata L.	0		NA	0	
Iz_16_1209	DJI_0122	Chamaecytisus ruthenicus (Fisch.ex Wol.) Klaskova	0	n	1	0	Fabaceae Lindl.
Iz_16_1209	DJI_0122	Chelidonium majus L.	0	n	1	0	Papaveraceae Juss.
Iz_16_1209	DJI_0122	Chenopodium betaceum Andrz.	0	n	1	0	Amaranthaceae Juss.
Iz_16_1209	DJI_0122	Chenopodium cf. album	0	n	1	0	Amaranthaceae Juss.
Iz_16_1209	DJI_0122	Chenopodium sp.	0		NA	0	Amaranthaceae Juss.
Iz_16_1209	DJI_0122	Chondrilla juncea L.	0	n	1	0	Asteraceae Bercht. & J.Presl
Iz_16_1209	DJI_0122	Cirsium vulgare (Savi) Ten.	0	n	1	0	Asteraceae Bercht. & J.Presl
Iz_16_1209	DJI_0122	Clinopodium acinos (L.) Kuntze	0	n	1	0	Lamiacea Lindl.
Iz_16_1209	DJI_0122	Convallaria majalis L.	0	n	1	0	Asparagaceae Juss.
Iz_16_1209	DJI_0122	Crataegus rhipidophylla Gand.	0	n	1	1	Rosaceae Juss.
Iz_16_1209	DJI_0122	Crepis foetida L.	0	n	1	0	Asteraceae Bercht. & J.Presl
Iz_16_1209	DJI_0122	Dactylis glomerata L.	0	n	1	0	Poaceae Barnhart
Iz_16_1209	DJI_0122	Dianthus campestris M.Bieb.	0	n	1	0	Caryophyllaceae Juss.
Iz_16_1209	DJI_0122	Digitaria sanguinalis (L.) Scop.	0	a	0	0	Poaceae Barnhart
Iz_16_1209	DJI_0122	Elymus repens (L.) Gould	0	n	1	0	Poaceae Barnhart
Iz_16_1209	DJI_0122	Epilobium tetragonum L.	0	n	1	0	Onagraceae Juss.
Iz_16_1209	DJI_0122	Eragrostis minor Host	0	a	0	0	Poaceae Barnhart
Iz_16_1209	DJI_0122	Erigeron canadensis L.	2	a	0	0	Asteraceae Bercht. & J.Presl
Iz_16_1209	DJI_0122	Euphorbia seguieriana Neck.	0	n	1	0	Euphorbiaceae Juss.
Iz_16_1209	DJI_0122	Euphorbia sp.	0		NA	NA	
Iz_16_1209	DJI_0122	Fallopia dumetorum (L.) Holub	0	n	1	0	Polygonaceae Juss.
Iz_16_1209	DJI_0122	Festuca beckeri (Hack.) Trautv.	0	n	1	0	Poaceae Barnhart
Iz_16_1209	DJI_0122	Fragaria vesca L.	0	n	1	0	Rosaceae Juss.
Iz_16_1209	DJI_0122	Frangula alnus Mill.	0	n	1	1	Rhamnaceae Juss.
Iz_16_1209	DJI_0122	Fraxinus pennsylvanica Marshall	0	a	0	1	Oleaceae Hoffmanns. & Link
Iz_16_1209	DJI_0122	Galeopsis bifida Boenn.	0	n	1	0	Lamiacea Lindl.
Iz_16_1209	DJI_0122	Galium verum L.	0	n	1	0	Rubiaceae Juss.
Iz_16_1209	DJI_0122	Genista tinctoria L.	0	n	1	0	Fabaceae Lindl.
Iz_16_1209	DJI_0122	Geranium robertianum L.	0	n	1	0	Geraniaceae Juss.
Iz_16_1209	DJI_0122	Geranium sanquineum L.	0	n	1	0	Geraniaceae Juss.
Iz_16_1209	DJI_0122	Gypsophila paniculata L.	0	n	1	0	Caryophyllaceae Juss.
Iz_16_1209	DJI_0122	Helichrysum arenarium (L.) Moench	0	n	1	0	Asteraceae Bercht. & J.Presl
Iz_16_1209	DJI_0122	Herniaria polygama J.Gay	0	n	1	0	Caryophyllaceae Juss.
Iz_16_1209	DJI_0122	Hieracium robustum Fr.	0	n	1	0	Asteraceae Bercht. & J.Presl
Iz_16_1209	DJI_0122	Hieracium umbellatum L.	0	n	1	NA	Asteraceae Bercht. & J.Presl
Iz_16_1209	DJI_0122	Hylotelephium maximum (L.) Holub	0	n	1	0	Crassulaceae J.St.-Hil.
Iz_16_1209	DJI_0122	Jacobaea borysthenica (DC.) B.Nord. & Greuter	0	n	1	0	Asteraceae Bercht. & J.Presl
Iz_16_1209	DJI_0122	Jasione montana L.	0	n	1	0	Campanulaceae Juss.
Iz_16_1209	DJI_0122	Jurinea cyanoides (L.) Rchb.s.l.	0	n	1	0	Asteraceae Bercht. & J.Presl
Iz_16_1209	DJI_0122	Koeleria glauca (Spreng.) DC.	0	n	1	0	Poaceae Barnhart
Iz_16_1209	DJI_0122	Lactuca serriola L.	0	a	0	0	Asteraceae Bercht. & J.Presl
Iz_16_1209	DJI_0122	Linaria genistifolia (L.) Mill.	0	n	1	0	Plantaginaceae Juss.
Iz_16_1209	DJI_0122	Lomelosia argentea (L.) Greuter & Burdet	0	n	1	0	Caprifoliaceae Juss.
Iz_16_1209	DJI_0122	Melampyrum pratense L.	0	n	1	0	Orobanchaceae Vent.
Iz_16_1209	DJI_0122	Oenothera biennis L.	0	a	0	0	Onagraceae Juss.
Iz_16_1209	DJI_0122	Pentanema britannica (L.) D.Gut.Larr., Santos-Vicente, Anderb., E.Rico & M.M.Mart.Ort.	0	n	1	0	Asteraceae Bercht. & J.Presl
Iz_16_1209	DJI_0122	Peucedanum oreoselinum (L.) Moench	0	n	1	0	Apiaceae Lindl.
Iz_16_1209	DJI_0122	Peucedanum ruthenicum M.Bieb.	0	n	1	0	Apiaceae Lindl.
Iz_16_1209	DJI_0122	Pilosella echioides (Lumn.) F.W.Schultz & Sch.Bip.	0	n	1	0	Asteraceae Bercht. & J.Presl
Iz_16_1209	DJI_0122	Pilosella officinarum F. Schultz et Sch. Bip.	0	n	1	0	Asteraceae Bercht. & J.Presl
Iz_16_1209	DJI_0122	Pilosella sp.	0		NA	0	Asteraceae Bercht. & J.Presl
Iz_16_1209	DJI_0122	Pinus sylvestris L.	0	n	1	1	Pinaceae Spreng. ex F.Rudolphi
Iz_16_1209	DJI_0122	Poa annua L.	0	n	1	0	Poaceae Barnhart
Iz_16_1209	DJI_0122	Poa nemoralis L.	0	n	1	0	Poaceae Barnhart
Iz_16_1209	DJI_0122	Poa sp.	0		NA	0	Poaceae Barnhart
Iz_16_1209	DJI_0122	Polygonatum odoratum (Mill.) Druce	0.5	n	1	0	Asparagaceae Juss.
Iz_16_1209	DJI_0122	Polygonum arenarium Waldst. & Kit.	0	n	1	0	Polygonaceae Juss.
Iz_16_1209	DJI_0122	Populus nigra L.	0	n	1	1	Salicaceae Mirb.
Iz_16_1209	DJI_0122	Populus tremula L.	0	n	1	1	Salicaceae Mirb.
Iz_16_1209	DJI_0122	Populus Ã canescens (Aiton) Sm.	0	n	1	1	Salicaceae Mirb.
Iz_16_1209	DJI_0122	Potentilla argentea L.	0	n	1	0	Rosaceae Juss.
Iz_16_1209	DJI_0122	Psammophiliella muralis (L.) Ikonn.	0	n	1	0	Caryophyllaceae Juss.
Iz_16_1209	DJI_0122	Psephellus sumensis (Kalen.) Greuter	1	n	1	0	Asteraceae Bercht. & J.Presl
Iz_16_1209	DJI_0122	Pulsatilla pratensis (L.) Mill. s.l.	0	n	1	0	Ranunculaceae Juss.
Iz_16_1209	DJI_0122	Pyrus communis L.	0	a	0	1	Rosaceae Juss.

Iz_16_1209	DJI_0122	Quercus robur L.	0	n	1	1	Fagaceae Dumort.
Iz_16_1209	DJI_0122	Robinia pseudoacacia L.	0	a	0	1	Fabaceae Lindl.
Iz_16_1209	DJI_0122	Rosa sp.	0		NA	1	Rosaceae Juss.
Iz_16_1209	DJI_0122	Rubus idaeus L.	0	n	1	0	Rosaceae Juss.
Iz_16_1209	DJI_0122	Rumex acetosella L.	0	n	1	0	Polygonaceae Juss.
Iz_16_1209	DJI_0122	Rumex thyrsiflorus Fingerh.	0	n	1	0	Polygonaceae Juss.
Iz_16_1209	DJI_0122	Salix alba L.	0	n	1	1	Salicaceae Mirb.
Iz_16_1209	DJI_0122	Sambucus nigra L.	0	n	1	1	Viburnaceae Raf.
Iz_16_1209	DJI_0122	Secale sylvestre Host	0	n	1	0	Poaceae Barnhart
Iz_16_1209	DJI_0122	Sempervivum ruthenicum Schnittsp. & C.B. Lehm.	0	n	1	0	Crassulaceae J.St.-Hil.
Iz_16_1209	DJI_0122	Setaria viridis (L.) P. Beauv.	0	n	1	0	Poaceae Barnhart
Iz_16_1209	DJI_0122	Silene borysthenica (Gruner) Walters	0	n	1	0	Caryophyllaceae Juss.
Iz_16_1209	DJI_0122	Silene latifolia subsp. alba (Mill.) Greuter & Burdet	0	n	1	0	Caryophyllaceae Juss.
Iz_16_1209	DJI_0122	Solidago virgaurea L.	0	n	1	0	Asteraceae Bercht. & J.Presl
Iz_16_1209	DJI_0122	Sorbus aucuparia L.	0	n	1	1	Rosaceae Juss.
Iz_16_1209	DJI_0122	Stachys recta L.	0	n	1	0	Lamiacea Lindl.
Iz_16_1209	DJI_0122	Tanacetum vulgare L.	0	n	1	0	Asteraceae Bercht. & J.Presl
Iz_16_1209	DJI_0122	Thymus pallasianus Heinr.Braun	0	n	1	0	Lamiacea Lindl.
Iz_16_1209	DJI_0122	Tilia cordata Mill.	0	n	1	1	Malvaceae Juss.
Iz_16_1209	DJI_0122	Trifolium alpestre L.	0	n	1	0	Fabaceae Lindl.
Iz_16_1209	DJI_0122	Trifolium arvense L.	0	n	1	0	Fabaceae Lindl.
Iz_16_1209	DJI_0122	Ulmus glabra Huds.	0	n	1	1	Ulmaceae Mirb.
Iz_16_1209	DJI_0122	Verbascum densiflorum Bertol.	0	n	1	0	Scrophulariaceae Juss.
Iz_16_1209	DJI_0122	Veronica austriaca L.	0	n	1	0	Plantaginaceae Juss.
Iz_16_1209	DJI_0122	Veronica incana L.	0	n	1	0	Plantaginaceae Juss.
Iz_16_1209	DJI_0122	Veronica verna L.	0	n	1	0	Plantaginaceae Juss.
Iz_16_1209	DJI_0122	Vincetoxicum hirundinaria Medik	0	n	1	0	Apocynaceae Juss.
Iz_16_1209	DJI_0127	Acer tataricum L.	0	n	1	1	Sapindaceae Juss.
Iz_16_1209	DJI_0127	Achillea millefolium L. s.l.	0	n	1	0	Asteraceae Bercht. & J.Presl
Iz_16_1209	DJI_0127	Allium oleraceum L.	0	n	1	0	Amaryllidaceae J.St.-Hil.
Iz_16_1209	DJI_0127	Ambrosia artemisiifolia L.	0	a	0	0	Asteraceae Bercht. & J.Presl
Iz_16_1209	DJI_0127	Amorpha fruticosa L.	0	a	0	0	Fabaceae Lindl.
Iz_16_1209	DJI_0127	Anthericum ramosum L.	0	n	1	0	Asparagaceae Juss.
Iz_16_1209	DJI_0127	Anthriscus sylvestris (L.) Hoffm.	0		NA	NA	
Iz_16_1209	DJI_0127	Artemisia marschalliana Spreng.	0	n	1	0	Asteraceae Bercht. & J.Presl
Iz_16_1209	DJI_0127	Asparagus officinalis L.	0	n	1	0	Asparagaceae Juss.
Iz_16_1209	DJI_0127	Astragalus cicer L.	0	n	1	0	Fabaceae Lindl.
Iz_16_1209	DJI_0127	Bassia laniflora (S.G.Gmel.) A.J.Scott	0	n	1	0	Amaranthaceae Juss.
Iz_16_1209	DJI_0127	Berberis vulgaris L.	0	n	1	0	Berberidaceae Juss.
Iz_16_1209	DJI_0127	Betula pendula Roth.	0	n	1	1	Betulaceae Gray
Iz_16_1209	DJI_0127	Betula pendula Roth.	0	n	1	0	Poaceae Barnhart
Iz_16_1209	DJI_0127	Bromus inermis Leyss.	0		NA	NA	
Iz_16_1209	DJI_0127	Bromus squarrosus L.	0	a	0	0	Poaceae Barnhart
Iz_16_1209	DJI_0127	Bromus tectorum L.	0	a	0	0	Poaceae Barnhart
Iz_16_1209	DJI_0127	Calamagrostis epigejos (L.) Roth	0.5	n	1	0	Poaceae Barnhart
Iz_16_1209	DJI_0127	Carduus acanthoides L.	0	n	1	0	Asteraceae Bercht. & J.Presl
Iz_16_1209	DJI_0127	Carex sp.	0	n	1	0	Cyperaceae Juss.
Iz_16_1209	DJI_0127	Centaurea majorovii Dumbadze	0	n	1	0	Asteraceae Bercht. & J.Presl
Iz_16_1209	DJI_0127	Cf. Gypsophila paniculata L.	0		NA	0	
Iz_16_1209	DJI_0127	Chamaecytisus ruthenicus (Fisch.ex Wol.) Klaskova	0	n	1	0	Fabaceae Lindl.
Iz_16_1209	DJI_0127	Chelidonium majus L.	0	n	1	0	Papaveraceae Juss.
Iz_16_1209	DJI_0127	Chenopodium betaceum Andrz.	0	n	1	0	Amaranthaceae Juss.
Iz_16_1209	DJI_0127	Chenopodium cf. album	0	n	1	0	Amaranthaceae Juss.
Iz_16_1209	DJI_0127	Chenopodium sp.	0		NA	0	Amaranthaceae Juss.
Iz_16_1209	DJI_0127	Chondrilla juncea L.	0	n	1	0	Asteraceae Bercht. & J.Presl
Iz_16_1209	DJI_0127	Cirsium vulgare (Savi) Ten.	0	n	1	0	Asteraceae Bercht. & J.Presl
Iz_16_1209	DJI_0127	Clinopodium acinos (L.) Kuntze	0	n	1	0	Lamiacea Lindl.
Iz_16_1209	DJI_0127	Convallaria majalis L.	0	n	1	0	Asparagaceae Juss.
Iz_16_1209	DJI_0127	Crataegus rhipidophylla Gand.	0	n	1	1	Rosaceae Juss.
Iz_16_1209	DJI_0127	Crepis foetida L.	0	n	1	0	Asteraceae Bercht. & J.Presl
Iz_16_1209	DJI_0127	Dactylis glomerata L.	0	n	1	0	Poaceae Barnhart
Iz_16_1209	DJI_0127	Dianthus campestris M.Bieb.	0	n	1	0	Caryophyllaceae Juss.
Iz_16_1209	DJI_0127	Digitaria sanguinalis (L.) Scop.	0	a	0	0	Poaceae Barnhart
Iz_16_1209	DJI_0127	Elymus repens (L.) Gould	0	n	1	0	Poaceae Barnhart
Iz_16_1209	DJI_0127	Epilobium tetragonum L.	0	n	1	0	Onagraceae Juss.
Iz_16_1209	DJI_0127	Eragrostis minor Host	0	a	0	0	Poaceae Barnhart
Iz_16_1209	DJI_0127	Erigeron canadensis L.	0	a	0	0	Asteraceae Bercht. & J.Presl
Iz_16_1209	DJI_0127	Euphorbia seguieriana Neck.	0	n	1	0	Euphorbiaceae Juss.
Iz_16_1209	DJI_0127	Euphorbia sp.	0		NA	NA	
Iz_16_1209	DJI_0127	Fallopia dumetorum (L.) Holub	0	n	1	0	Polygonaceae Juss.
Iz_16_1209	DJI_0127	Festuca beckeri (Hack.) Trautv.	0	n	1	0	Poaceae Barnhart
Iz_16_1209	DJI_0127	Fragaria vesca L.	0	n	1	0	Rosaceae Juss.
Iz_16_1209	DJI_0127	Frangula alnus Mill.	0	n	1	1	Rhamnaceae Juss.
Iz_16_1209	DJI_0127	Fraxinus pennsylvanica Marshall	0	a	0	1	Oleaceae Hoffmanns. & Link
Iz_16_1209	DJI_0127	Galeopsis bifida Boenn.	0	n	1	0	Lamiacea Lindl.
Iz_16_1209	DJI_0127	Galium verum L.	0	n	1	0	Rubiaceae Juss.
Iz_16_1209	DJI_0127	Genista tinctoria L.	0	n	1	0	Fabaceae Lindl.
Iz_16_1209	DJI_0127	Geranium robertianum L.	0	n	1	0	Geraniaceae Juss.
Iz_16_1209	DJI_0127	Geranium sanquineum L.	0	n	1	0	Geraniaceae Juss.
Iz_16_1209	DJI_0127	Gypsophila paniculata L.	0	n	1	0	Caryophyllaceae Juss.
Iz_16_1209	DJI_0127	Helichrysum arenarium (L.) Moench	0	n	1	0	Asteraceae Bercht. & J.Presl
Iz_16_1209	DJI_0127	Herniaria polygama J.Gay	0	n	1	0	Caryophyllaceae Juss.
Iz_16_1209	DJI_0127	Hieracium robustum Fr.	0	n	1	0	Asteraceae Bercht. & J.Presl
Iz_16_1209	DJI_0127	Hieracium umbellatum L.	0	n	1	NA	Asteraceae Bercht. & J.Presl
Iz_16_1209	DJI_0127	Hylotelephium maximum (L.) Holub	0	n	1	0	Crassulaceae J.St.-Hil.
Iz_16_1209	DJI_0127	Jacobaea borysthenica (DC.) B.Nord. & Greuter	0	n	1	0	Asteraceae Bercht. & J.Presl
Iz_16_1209	DJI_0127	Jasione montana L.	0	n	1	0	Campanulaceae Juss.
Iz_16_1209	DJI_0127	Jurinea cyanoides (L.) Rchb.s.l.	0	n	1	0	Asteraceae Bercht. & J.Presl
Iz_16_1209	DJI_0127	Koeleria glauca (Spreng.) DC.	0	n	1	0	Poaceae Barnhart
Iz_16_1209	DJI_0127	Lactuca serriola L.	0	a	0	0	Asteraceae Bercht. & J.Presl
Iz_16_1209	DJI_0127	Linaria genistifolia (L.) Mill.	0	n	1	0	Plantaginaceae Juss.
Iz_16_1209	DJI_0127	Lomelosia argentea (L.) Greuter & Burdet	0	n	1	0	Caprifoliaceae Juss.
Iz_16_1209	DJI_0127	Melampyrum pratense L.	0	n	1	0	Orobanchaceae Vent.
Iz_16_1209	DJI_0127	Oenothera biennis L.	0	a	0	0	Onagraceae Juss.
Iz_16_1209	DJI_0127	Pentanema britannica (L.) D.Gut.Larr., Santos-Vicente, Anderb., E.Rico & M.M.Mart.Ort.	0	n	1	0	Asteraceae Bercht. & J.Presl
Iz_16_1209	DJI_0127	Peucedanum oreoselinum (L.) Moench	0	n	1	0	Apiaceae Lindl.
Iz_16_1209	DJI_0127	Peucedanum ruthenicum M.Bieb.	0	n	1	0	Apiaceae Lindl.
Iz_16_1209	DJI_0127	Pilosella echioides (Lumn.) F.W.Schultz & Sch.Bip.	0	n	1	0	Asteraceae Bercht. & J.Presl

Iz_16_1209	DJI_0127	Pilosella officinarum F. Schultz et Sch. Bip.	0	n	1	0	Asteraceae Bercht. & J.Presl
Iz_16_1209	DJI_0127	Pilosella sp.	0		NA	0	Asteraceae Bercht. & J.Presl
Iz_16_1209	DJI_0127	Pinus sylvestris L.	0	n	1	1	Pinaceae Spreng. ex F.Rudolphi
Iz_16_1209	DJI_0127	Poa annua L.	0	n	1	0	Poaceae Barnhart
Iz_16_1209	DJI_0127	Poa nemoralis L.	0	n	1	0	Poaceae Barnhart
Iz_16_1209	DJI_0127	Poa sp.	0		NA	0	Poaceae Barnhart
Iz_16_1209	DJI_0127	Polygonatum odoratum (Mill.) Druce	0	n	1	0	Asparagaceae Juss.
Iz_16_1209	DJI_0127	Polygonum arenarium Waldst. & Kit.	0	n	1	0	Polygonaceae Juss.
Iz_16_1209	DJI_0127	Populus nigra L.	0	n	1	1	Salicaceae Mirb.
Iz_16_1209	DJI_0127	Populus tremula L.	0	n	1	1	Salicaceae Mirb.
Iz_16_1209	DJI_0127	Populus Ã canescens (Aiton) Sm.	0	n	1	1	Salicaceae Mirb.
Iz_16_1209	DJI_0127	Potentilla argentea L.	0	n	1	0	Rosaceae Juss.
Iz_16_1209	DJI_0127	Psammophiliella muralis (L.) Ikonn.	0	n	1	0	Caryophyllaceae Juss.
Iz_16_1209	DJI_0127	Psephellus sumensis (Kalen.) Greuter	0	n	1	0	Asteraceae Bercht. & J.Presl
Iz_16_1209	DJI_0127	Pulsatilla pratensis (L.) Mill. s.l.	0	n	1	0	Ranunculaceae Juss.
Iz_16_1209	DJI_0127	Pyrus communis L.	0	a	0	1	Rosaceae Juss.
Iz_16_1209	DJI_0127	Quercus robur L.	0	n	1	1	Fagaceae Dumort.
Iz_16_1209	DJI_0127	Robinia pseudoacacia L.	0	a	0	1	Fabaceae Lindl.
Iz_16_1209	DJI_0127	Rosa sp.	0		NA	1	Rosaceae Juss.
Iz_16_1209	DJI_0127	Rubus idaeus L.	0	n	1	0	Rosaceae Juss.
Iz_16_1209	DJI_0127	Rumex acetosella L.	0	n	1	0	Polygonaceae Juss.
Iz_16_1209	DJI_0127	Rumex thyrsiflorus Fingerh.	0	n	1	0	Polygonaceae Juss.
Iz_16_1209	DJI_0127	Salix alba L.	0	n	1	1	Salicaceae Mirb.
Iz_16_1209	DJI_0127	Sambucus nigra L.	0	n	1	1	Viburnaceae Raf.
Iz_16_1209	DJI_0127	Secale sylvestre Host	0	n	1	0	Poaceae Barnhart
Iz_16_1209	DJI_0127	Sempervivum ruthenicum Schnittsp. & C.B. Lehm.	0	n	1	0	Crassulaceae J.St.-Hil.
Iz_16_1209	DJI_0127	Setaria viridis (L.) P. Beauv.	0	n	1	0	Poaceae Barnhart
Iz_16_1209	DJI_0127	Silene borysthenica (Gruner) Walters	0	n	1	0	Caryophyllaceae Juss.
Iz_16_1209	DJI_0127	Silene latifolia subsp. alba (Mill.) Greuter & Burdet	0	n	1	0	Caryophyllaceae Juss.
Iz_16_1209	DJI_0127	Solidago virgaurea L.	0	n	1	0	Asteraceae Bercht. & J.Presl
Iz_16_1209	DJI_0127	Sorbus aucuparia L.	0	n	1	1	Rosaceae Juss.
Iz_16_1209	DJI_0127	Stachys recta L.	0	n	1	0	Lamiacea Lindl.
Iz_16_1209	DJI_0127	Tanacetum vulgare L.	0	n	1	0	Asteraceae Bercht. & J.Presl
Iz_16_1209	DJI_0127	Thymus pallasianus Heinr.Braun	0	n	1	0	Lamiacea Lindl.
Iz_16_1209	DJI_0127	Tilia cordata Mill.	0	n	1	1	Malvaceae Juss.
Iz_16_1209	DJI_0127	Trifolium alpestre L.	0	n	1	0	Fabaceae Lindl.
Iz_16_1209	DJI_0127	Trifolium arvense L.	0	n	1	0	Fabaceae Lindl.
Iz_16_1209	DJI_0127	Ulmus glabra Huds.	0	n	1	1	Ulmaceae Mirb.
Iz_16_1209	DJI_0127	Verbascum densiflorum Bertol.	0	n	1	0	Scrophulariaceae Juss.
Iz_16_1209	DJI_0127	Veronica austriaca L.	0	n	1	0	Plantaginaceae Juss.
Iz_16_1209	DJI_0127	Veronica incana L.	0	n	1	0	Plantaginaceae Juss.
Iz_16_1209	DJI_0127	Veronica verna L.	0	n	1	0	Plantaginaceae Juss.
Iz_16_1209	DJI_0127	Vincetoxicum hirundinaria Medik	0	n	1	0	Apocynaceae Juss.
Iz_16_1209	DJI_0130	Acer tataricum L.	0	n	1	1	Sapindaceae Juss.
Iz_16_1209	DJI_0130	Achillea millefolium L. s.l.	0	n	1	0	Asteraceae Bercht. & J.Presl
Iz_16_1209	DJI_0130	Allium oleraceum L.	0	n	1	0	Amaryllidaceae J.St.-Hil.
Iz_16_1209	DJI_0130	Ambrosia artemisiifolia L.	0	a	0	0	Asteraceae Bercht. & J.Presl
Iz_16_1209	DJI_0130	Amorpha fruticosa L.	0	a	0	0	Fabaceae Lindl.
Iz_16_1209	DJI_0130	Anthericum ramosum L.	0	n	1	0	Asparagaceae Juss.
Iz_16_1209	DJI_0130	Anthriscus sylvestris (L.) Hoffm.	0		NA	NA	
Iz_16_1209	DJI_0130	Artemisia marschalliana Spreng.	2	n	1	0	Asteraceae Bercht. & J.Presl
Iz_16_1209	DJI_0130	Asparagus officinalis L.	0	n	1	0	Asparagaceae Juss.
Iz_16_1209	DJI_0130	Astragalus cicer L.	0	n	1	0	Fabaceae Lindl.
Iz_16_1209	DJI_0130	Bassia laniflora (S.G.Gmel.) A.J.Scott	0	n	1	0	Amaranthaceae Juss.
Iz_16_1209	DJI_0130	Berberis vulgaris L.	0	n	1	0	Berberidaceae Juss.
Iz_16_1209	DJI_0130	Betula pendula Roth.	0	n	1	1	Betulaceae Gray
Iz_16_1209	DJI_0130	Betula pendula Roth.	0	n	1	0	Poaceae Barnhart
Iz_16_1209	DJI_0130	Bromus inermis Leyss.	0		NA	NA	
Iz_16_1209	DJI_0130	Bromus squarrosus L.	0	a	0	0	Poaceae Barnhart
Iz_16_1209	DJI_0130	Bromus tectorum L.	0	a	0	0	Poaceae Barnhart
Iz_16_1209	DJI_0130	Calamagrostis epigejos (L.) Roth	1	n	1	0	Poaceae Barnhart
Iz_16_1209	DJI_0130	Carduus acanthoides L.	0	n	1	0	Asteraceae Bercht. & J.Presl
Iz_16_1209	DJI_0130	Carex sp.	18	n	1	0	Cyperaceae Juss.
Iz_16_1209	DJI_0130	Centaurea majorovii Dumbadze	0	n	1	0	Asteraceae Bercht. & J.Presl
Iz_16_1209	DJI_0130	Cf. Gypsophila paniculata L.	0		NA	0	
Iz_16_1209	DJI_0130	Chamaecytisus ruthenicus (Fisch.ex Wol.) Klaskova	0	n	1	0	Fabaceae Lindl.
Iz_16_1209	DJI_0130	Chelidonium majus L.	0	n	1	0	Papaveraceae Juss.
Iz_16_1209	DJI_0130	Chenopodium betaceum Andrz.	0	n	1	0	Amaranthaceae Juss.
Iz_16_1209	DJI_0130	Chenopodium cf. album	0	n	1	0	Amaranthaceae Juss.
Iz_16_1209	DJI_0130	Chenopodium sp.	0		NA	0	Amaranthaceae Juss.
Iz_16_1209	DJI_0130	Chondrilla juncea L.	0	n	1	0	Asteraceae Bercht. & J.Presl
Iz_16_1209	DJI_0130	Cirsium vulgare (Savi) Ten.	0	n	1	0	Asteraceae Bercht. & J.Presl
Iz_16_1209	DJI_0130	Clinopodium acinos (L.) Kuntze	0	n	1	0	Lamiacea Lindl.
Iz_16_1209	DJI_0130	Convallaria majalis L.	0	n	1	0	Asparagaceae Juss.
Iz_16_1209	DJI_0130	Crataegus rhipidophylla Gand.	0	n	1	1	Rosaceae Juss.
Iz_16_1209	DJI_0130	Crepis foetida L.	0	n	1	0	Asteraceae Bercht. & J.Presl
Iz_16_1209	DJI_0130	Dactylis glomerata L.	0	n	1	0	Poaceae Barnhart
Iz_16_1209	DJI_0130	Dianthus campestris M.Bieb.	0	n	1	0	Caryophyllaceae Juss.
Iz_16_1209	DJI_0130	Digitaria sanguinalis (L.) Scop.	0	a	0	0	Poaceae Barnhart
Iz_16_1209	DJI_0130	Elymus repens (L.) Gould	0	n	1	0	Poaceae Barnhart
Iz_16_1209	DJI_0130	Epilobium tetragonum L.	0	n	1	0	Onagraceae Juss.
Iz_16_1209	DJI_0130	Eragrostis minor Host	0	a	0	0	Poaceae Barnhart
Iz_16_1209	DJI_0130	Erigeron canadensis L.	0	a	0	0	Asteraceae Bercht. & J.Presl
Iz_16_1209	DJI_0130	Euphorbia seguieriana Neck.	0	n	1	0	Euphorbiaceae Juss.
Iz_16_1209	DJI_0130	Euphorbia sp.	0		NA	NA	
Iz_16_1209	DJI_0130	Fallopia dumetorum (L.) Holub	0	n	1	0	Polygonaceae Juss.
Iz_16_1209	DJI_0130	Festuca beckeri (Hack.) Trautv.	0	n	1	0	Poaceae Barnhart
Iz_16_1209	DJI_0130	Fragaria vesca L.	0	n	1	0	Rosaceae Juss.
Iz_16_1209	DJI_0130	Frangula alnus Mill.	0	n	1	1	Rhamnaceae Juss.
Iz_16_1209	DJI_0130	Fraxinus pennsylvanica Marshall	0	a	0	1	Oleaceae Hoffmanns. & Link
Iz_16_1209	DJI_0130	Galeopsis bifida Boenn.	0	n	1	0	Lamiacea Lindl.
Iz_16_1209	DJI_0130	Galium verum L.	2	n	1	0	Rubiaceae Juss.
Iz_16_1209	DJI_0130	Genista tinctoria L.	0	n	1	0	Fabaceae Lindl.
Iz_16_1209	DJI_0130	Geranium robertianum L.	0	n	1	0	Geraniaceae Juss.
Iz_16_1209	DJI_0130	Geranium sanquineum L.	0	n	1	0	Geraniaceae Juss.
Iz_16_1209	DJI_0130	Gypsophila paniculata L.	0	n	1	0	Caryophyllaceae Juss.
Iz_16_1209	DJI_0130	Helichrysum arenarium (L.) Moench	0	n	1	0	Asteraceae Bercht. & J.Presl
Iz_16_1209	DJI_0130	Herniaria polygama J.Gay	0	n	1	0	Caryophyllaceae Juss.

Iz_16_1209	DJI_0130	Hieracium robustum Fr.	0	n	1	0	Asteraceae Bercht. & J.Presl
Iz_16_1209	DJI_0130	Hieracium umbellatum L.	0	n	1	NA	Asteraceae Bercht. & J.Presl
Iz_16_1209	DJI_0130	Hylotelephium maximum (L.) Holub	0	n	1	0	Crassulaceae J.St.-Hil.
Iz_16_1209	DJI_0130	Jacobaea borysthenica (DC.) B.Nord. & Greuter	0	n	1	0	Asteraceae Bercht. & J.Presl
Iz_16_1209	DJI_0130	Jasione montana L.	0	n	1	0	Campanulaceae Juss.
Iz_16_1209	DJI_0130	Jurinea cyanoides (L.) Rchb.s.l.	0	n	1	0	Asteraceae Bercht. & J.Presl
Iz_16_1209	DJI_0130	Koeleria glauca (Spreng.) DC.	0	n	1	0	Poaceae Barnhart
Iz_16_1209	DJI_0130	Lactuca serriola L.	0	a	0	0	Asteraceae Bercht. & J.Presl
Iz_16_1209	DJI_0130	Linaria genistifolia (L.) Mill.	0	n	1	0	Plantaginaceae Juss.
Iz_16_1209	DJI_0130	Lomelosia argentea (L.) Greuter & Burdet	0	n	1	0	Caprifoliaceae Juss.
Iz_16_1209	DJI_0130	Melampyrum pratense L.	0	n	1	0	Orobanchaceae Vent.
Iz_16_1209	DJI_0130	Oenothera biennis L.	0	a	0	0	Onagraceae Juss.
Iz_16_1209	DJI_0130	Pentanema britannica (L.) D.Gut.Larr., Santos-Vicente, Anderb., E.Rico & M.M.Mart.Ort.	0	n	1	0	Asteraceae Bercht. & J.Presl
Iz_16_1209	DJI_0130	Peucedanum oreoselinum (L.) Moench	0	n	1	0	Apiaceae Lindl.
Iz_16_1209	DJI_0130	Peucedanum ruthenicum M.Bieb.	0	n	1	0	Apiaceae Lindl.
Iz_16_1209	DJI_0130	Pilosella echioides (Lumn.) F.W.Schultz & Sch.Bip.	0	n	1	0	Asteraceae Bercht. & J.Presl
Iz_16_1209	DJI_0130	Pilosella officinarum F. Schultz et Sch. Bip.	0	n	1	0	Asteraceae Bercht. & J.Presl
Iz_16_1209	DJI_0130	Pilosella sp.	0		NA	0	Asteraceae Bercht. & J.Presl
Iz_16_1209	DJI_0130	Pinus sylvestris L.	0	n	1	1	Pinaceae Spreng. ex F.Rudolphi
Iz_16_1209	DJI_0130	Poa annua L.	0	n	1	0	Poaceae Barnhart
Iz_16_1209	DJI_0130	Poa nemoralis L.	0	n	1	0	Poaceae Barnhart
Iz_16_1209	DJI_0130	Poa sp.	0		NA	0	Poaceae Barnhart
Iz_16_1209	DJI_0130	Polygonatum odoratum (Mill.) Druce	8	n	1	0	Asparagaceae Juss.
Iz_16_1209	DJI_0130	Polygonum arenarium Waldst. & Kit.	0	n	1	0	Polygonaceae Juss.
Iz_16_1209	DJI_0130	Populus nigra L.	0	n	1	1	Salicaceae Mirb.
Iz_16_1209	DJI_0130	Populus tremula L.	0	n	1	1	Salicaceae Mirb.
Iz_16_1209	DJI_0130	Populus Ã canescens (Aiton) Sm.	0	n	1	1	Salicaceae Mirb.
Iz_16_1209	DJI_0130	Potentilla argentea L.	0	n	1	0	Rosaceae Juss.
Iz_16_1209	DJI_0130	Psammophiliella muralis (L.) Ikonn.	0	n	1	0	Caryophyllaceae Juss.
Iz_16_1209	DJI_0130	Psephellus sumensis (Kalen.) Greuter	5	n	1	0	Asteraceae Bercht. & J.Presl
Iz_16_1209	DJI_0130	Pulsatilla pratensis (L.) Mill. s.l.	0	n	1	0	Ranunculaceae Juss.
Iz_16_1209	DJI_0130	Pyrus communis L.	0	a	0	1	Rosaceae Juss.
Iz_16_1209	DJI_0130	Quercus robur L.	0	n	1	1	Fagaceae Dumort.
Iz_16_1209	DJI_0130	Robinia pseudoacacia L.	0	a	0	1	Fabaceae Lindl.
Iz_16_1209	DJI_0130	Rosa sp.	0		NA	1	Rosaceae Juss.
Iz_16_1209	DJI_0130	Rubus idaeus L.	0	n	1	0	Rosaceae Juss.
Iz_16_1209	DJI_0130	Rumex acetosella L.	0	n	1	0	Polygonaceae Juss.
Iz_16_1209	DJI_0130	Rumex thyrsiflorus Fingerh.	0	n	1	0	Polygonaceae Juss.
Iz_16_1209	DJI_0130	Salix alba L.	0	n	1	1	Salicaceae Mirb.
Iz_16_1209	DJI_0130	Sambucus nigra L.	0	n	1	1	Viburnaceae Raf.
Iz_16_1209	DJI_0130	Secale sylvestre Host	0	n	1	0	Poaceae Barnhart
Iz_16_1209	DJI_0130	Sempervivum ruthenicum Schnittsp. & C.B. Lehm.	0	n	1	0	Crassulaceae J.St.-Hil.
Iz_16_1209	DJI_0130	Setaria viridis (L.) P. Beauv.	0	n	1	0	Poaceae Barnhart
Iz_16_1209	DJI_0130	Silene borysthenica (Gruner) Walters	0	n	1	0	Caryophyllaceae Juss.
Iz_16_1209	DJI_0130	Silene latifolia subsp. alba (Mill.) Greuter & Burdet	0	n	1	0	Caryophyllaceae Juss.
Iz_16_1209	DJI_0130	Solidago virgaurea L.	0	n	1	0	Asteraceae Bercht. & J.Presl
Iz_16_1209	DJI_0130	Sorbus aucuparia L.	0	n	1	1	Rosaceae Juss.
Iz_16_1209	DJI_0130	Stachys recta L.	0	n	1	0	Lamiacea Lindl.
Iz_16_1209	DJI_0130	Tanacetum vulgare L.	0	n	1	0	Asteraceae Bercht. & J.Presl
Iz_16_1209	DJI_0130	Thymus pallasianus Heinr.Braun	0	n	1	0	Lamiacea Lindl.
Iz_16_1209	DJI_0130	Tilia cordata Mill.	0	n	1	1	Malvaceae Juss.
Iz_16_1209	DJI_0130	Trifolium alpestre L.	0	n	1	0	Fabaceae Lindl.
Iz_16_1209	DJI_0130	Trifolium arvense L.	0	n	1	0	Fabaceae Lindl.
Iz_16_1209	DJI_0130	Ulmus glabra Huds.	0	n	1	1	Ulmaceae Mirb.
Iz_16_1209	DJI_0130	Verbascum densiflorum Bertol.	0	n	1	0	Scrophulariaceae Juss.
Iz_16_1209	DJI_0130	Veronica austriaca L.	0	n	1	0	Plantaginaceae Juss.
Iz_16_1209	DJI_0130	Veronica incana L.	0	n	1	0	Plantaginaceae Juss.
Iz_16_1209	DJI_0130	Veronica verna L.	0	n	1	0	Plantaginaceae Juss.
Iz_16_1209	DJI_0130	Vincetoxicum hirundinaria Medik	0	n	1	0	Apocynaceae Juss.
Iz_16_1209	DJI_0135	Acer tataricum L.	0	n	1	1	Sapindaceae Juss.
Iz_16_1209	DJI_0135	Achillea millefolium L. s.l.	0	n	1	0	Asteraceae Bercht. & J.Presl
Iz_16_1209	DJI_0135	Allium oleraceum L.	0	n	1	0	Amaryllidaceae J.St.-Hil.
Iz_16_1209	DJI_0135	Ambrosia artemisiifolia L.	0	a	0	0	Asteraceae Bercht. & J.Presl
Iz_16_1209	DJI_0135	Amorpha fruticosa L.	0	a	0	0	Fabaceae Lindl.
Iz_16_1209	DJI_0135	Anthericum ramosum L.	0	n	1	0	Asparagaceae Juss.
Iz_16_1209	DJI_0135	Anthriscus sylvestris (L.) Hoffm.	0		NA	NA	
Iz_16_1209	DJI_0135	Artemisia marschalliana Spreng.	0	n	1	0	Asteraceae Bercht. & J.Presl
Iz_16_1209	DJI_0135	Asparagus officinalis L.	0	n	1	0	Asparagaceae Juss.
Iz_16_1209	DJI_0135	Astragalus cicer L.	0	n	1	0	Fabaceae Lindl.
Iz_16_1209	DJI_0135	Bassia laniflora (S.G.Gmel.) A.J.Scott	0	n	1	0	Amaranthaceae Juss.
Iz_16_1209	DJI_0135	Berberis vulgaris L.	0	n	1	0	Berberidaceae Juss.
Iz_16_1209	DJI_0135	Betula pendula Roth.	0	n	1	1	Betulaceae Gray
Iz_16_1209	DJI_0135	Betula pendula Roth.	0	n	1	0	Poaceae Barnhart
Iz_16_1209	DJI_0135	Bromus inermis Leyss.	0		NA	NA	
Iz_16_1209	DJI_0135	Bromus squarrosus L.	0	a	0	0	Poaceae Barnhart
Iz_16_1209	DJI_0135	Bromus tectorum L.	0	a	0	0	Poaceae Barnhart
Iz_16_1209	DJI_0135	Calamagrostis epigejos (L.) Roth	15	n	1	0	Poaceae Barnhart
Iz_16_1209	DJI_0135	Carduus acanthoides L.	0	n	1	0	Asteraceae Bercht. & J.Presl
Iz_16_1209	DJI_0135	Carex sp.	0	n	1	0	Cyperaceae Juss.
Iz_16_1209	DJI_0135	Centaurea majorovii Dumbadze	0	n	1	0	Asteraceae Bercht. & J.Presl
Iz_16_1209	DJI_0135	Cf. Gypsophila paniculata L.	0		NA	0	
Iz_16_1209	DJI_0135	Chamaecytisus ruthenicus (Fisch.ex Wol.) Klaskova	0	n	1	0	Fabaceae Lindl.
Iz_16_1209	DJI_0135	Chelidonium majus L.	0	n	1	0	Papaveraceae Juss.
Iz_16_1209	DJI_0135	Chenopodium betaceum Andrz.	0	n	1	0	Amaranthaceae Juss.
Iz_16_1209	DJI_0135	Chenopodium cf. album	0	n	1	0	Amaranthaceae Juss.
Iz_16_1209	DJI_0135	Chenopodium sp.	0.5		NA	0	Amaranthaceae Juss.
Iz_16_1209	DJI_0135	Chondrilla juncea L.	0	n	1	0	Asteraceae Bercht. & J.Presl
Iz_16_1209	DJI_0135	Cirsium vulgare (Savi) Ten.	0	n	1	0	Asteraceae Bercht. & J.Presl
Iz_16_1209	DJI_0135	Clinopodium acinos (L.) Kuntze	0	n	1	0	Lamiacea Lindl.
Iz_16_1209	DJI_0135	Convallaria majalis L.	0	n	1	0	Asparagaceae Juss.
Iz_16_1209	DJI_0135	Crataegus rhipidophylla Gand.	0	n	1	1	Rosaceae Juss.
Iz_16_1209	DJI_0135	Crepis foetida L.	0	n	1	0	Asteraceae Bercht. & J.Presl
Iz_16_1209	DJI_0135	Dactylis glomerata L.	0	n	1	0	Poaceae Barnhart
Iz_16_1209	DJI_0135	Dianthus campestris M.Bieb.	0	n	1	0	Caryophyllaceae Juss.
Iz_16_1209	DJI_0135	Digitaria sanguinalis (L.) Scop.	0	a	0	0	Poaceae Barnhart
Iz_16_1209	DJI_0135	Elymus repens (L.) Gould	0	n	1	0	Poaceae Barnhart
Iz_16_1209	DJI_0135	Epilobium tetragonum L.	0	n	1	0	Onagraceae Juss.
Iz_16_1209	DJI_0135	Eragrostis minor Host	0	a	0	0	Poaceae Barnhart

Iz_16_1209	DJI_0135	Erigeron canadensis L.	0	a	0	0	Asteraceae Bercht. & J.Presl
Iz_16_1209	DJI_0135	Euphorbia seguieriana Neck.	0	n	1	0	Euphorbiaceae Juss.
Iz_16_1209	DJI_0135	Euphorbia sp.	0		NA	NA	
Iz_16_1209	DJI_0135	Fallopia dumetorum (L.) Holub	4	n	1	0	Polygonaceae Juss.
Iz_16_1209	DJI_0135	Festuca beckeri (Hack.) Trautv.	0	n	1	0	Poaceae Barnhart
Iz_16_1209	DJI_0135	Fragaria vesca L.	0	n	1	0	Rosaceae Juss.
Iz_16_1209	DJI_0135	Frangula alnus Mill.	0	n	1	1	Rhamnaceae Juss.
Iz_16_1209	DJI_0135	Fraxinus pennsylvanica Marshall	0	a	0	1	Oleaceae Hoffmanns. & Link
Iz_16_1209	DJI_0135	Galeopsis bifida Boenn.	0	n	1	0	Lamiacea Lindl.
Iz_16_1209	DJI_0135	Galium verum L.	0	n	1	0	Rubiaceae Juss.
Iz_16_1209	DJI_0135	Genista tinctoria L.	0	n	1	0	Fabaceae Lindl.
Iz_16_1209	DJI_0135	Geranium robertianum L.	0	n	1	0	Geraniaceae Juss.
Iz_16_1209	DJI_0135	Geranium sanquineum L.	0	n	1	0	Geraniaceae Juss.
Iz_16_1209	DJI_0135	Gypsophila paniculata L.	0	n	1	0	Caryophyllaceae Juss.
Iz_16_1209	DJI_0135	Helichrysum arenarium (L.) Moench	0	n	1	0	Asteraceae Bercht. & J.Presl
Iz_16_1209	DJI_0135	Herniaria polygama J.Gay	0	n	1	0	Caryophyllaceae Juss.
Iz_16_1209	DJI_0135	Hieracium robustum Fr.	0	n	1	0	Asteraceae Bercht. & J.Presl
Iz_16_1209	DJI_0135	Hieracium umbellatum L.	0	n	1	NA	Asteraceae Bercht. & J.Presl
Iz_16_1209	DJI_0135	Hylotelephium maximum (L.) Holub	0	n	1	0	Crassulaceae J.St.-Hil.
Iz_16_1209	DJI_0135	Jacobaea borysthenica (DC.) B.Nord. & Greuter	0	n	1	0	Asteraceae Bercht. & J.Presl
Iz_16_1209	DJI_0135	Jasione montana L.	0	n	1	0	Campanulaceae Juss.
Iz_16_1209	DJI_0135	Jurinea cyanoides (L.) Rchb.s.l.	0	n	1	0	Asteraceae Bercht. & J.Presl
Iz_16_1209	DJI_0135	Koeleria glauca (Spreng.) DC.	0	n	1	0	Poaceae Barnhart
Iz_16_1209	DJI_0135	Lactuca serriola L.	0	a	0	0	Asteraceae Bercht. & J.Presl
Iz_16_1209	DJI_0135	Linaria genistifolia (L.) Mill.	0	n	1	0	Plantaginaceae Juss.
Iz_16_1209	DJI_0135	Lomelosia argentea (L.) Greuter & Burdet	0	n	1	0	Caprifoliaceae Juss.
Iz_16_1209	DJI_0135	Melampyrum pratense L.	0	n	1	0	Orobanchaceae Vent.
Iz_16_1209	DJI_0135	Oenothera biennis L.	0	a	0	0	Onagraceae Juss.
Iz_16_1209	DJI_0135	Pentanema britannica (L.) D.Gut.Larr., Santos-Vicente, Anderb., E.Rico & M.M.Mart.Ort.	0	n	1	0	Asteraceae Bercht. & J.Presl
Iz_16_1209	DJI_0135	Peucedanum oreoselinum (L.) Moench	2	n	1	0	Apiaceae Lindl.
Iz_16_1209	DJI_0135	Peucedanum ruthenicum M.Bieb.	1	n	1	0	Apiaceae Lindl.
Iz_16_1209	DJI_0135	Pilosella echioides (Lumn.) F.W.Schultz & Sch.Bip.	0	n	1	0	Asteraceae Bercht. & J.Presl
Iz_16_1209	DJI_0135	Pilosella officinarum F. Schultz et Sch. Bip.	0	n	1	0	Asteraceae Bercht. & J.Presl
Iz_16_1209	DJI_0135	Pilosella sp.	0		NA	0	Asteraceae Bercht. & J.Presl
Iz_16_1209	DJI_0135	Pinus sylvestris L.	0	n	1	1	Pinaceae Spreng. ex F.Rudolphi
Iz_16_1209	DJI_0135	Poa annua L.	0	n	1	0	Poaceae Barnhart
Iz_16_1209	DJI_0135	Poa nemoralis L.	0	n	1	0	Poaceae Barnhart
Iz_16_1209	DJI_0135	Poa sp.	0		NA	0	Poaceae Barnhart
Iz_16_1209	DJI_0135	Polygonatum odoratum (Mill.) Druce	0.5	n	1	0	Asparagaceae Juss.
Iz_16_1209	DJI_0135	Polygonum arenarium Waldst. & Kit.	0	n	1	0	Polygonaceae Juss.
Iz_16_1209	DJI_0135	Populus nigra L.	0	n	1	1	Salicaceae Mirb.
Iz_16_1209	DJI_0135	Populus tremula L.	0	n	1	1	Salicaceae Mirb.
Iz_16_1209	DJI_0135	Populus Ã canescens (Aiton) Sm.	0	n	1	1	Salicaceae Mirb.
Iz_16_1209	DJI_0135	Potentilla argentea L.	0	n	1	0	Rosaceae Juss.
Iz_16_1209	DJI_0135	Psammophiliella muralis (L.) Ikonn.	0	n	1	0	Caryophyllaceae Juss.
Iz_16_1209	DJI_0135	Psephellus sumensis (Kalen.) Greuter	0.5	n	1	0	Asteraceae Bercht. & J.Presl
Iz_16_1209	DJI_0135	Pulsatilla pratensis (L.) Mill. s.l.	0	n	1	0	Ranunculaceae Juss.
Iz_16_1209	DJI_0135	Pyrus communis L.	0	a	0	1	Rosaceae Juss.
Iz_16_1209	DJI_0135	Quercus robur L.	0	n	1	1	Fagaceae Dumort.
Iz_16_1209	DJI_0135	Robinia pseudoacacia L.	0	a	0	1	Fabaceae Lindl.
Iz_16_1209	DJI_0135	Rosa sp.	0		NA	1	Rosaceae Juss.
Iz_16_1209	DJI_0135	Rubus idaeus L.	0	n	1	0	Rosaceae Juss.
Iz_16_1209	DJI_0135	Rumex acetosella L.	0	n	1	0	Polygonaceae Juss.
Iz_16_1209	DJI_0135	Rumex thyrsiflorus Fingerh.	0	n	1	0	Polygonaceae Juss.
Iz_16_1209	DJI_0135	Salix alba L.	0	n	1	1	Salicaceae Mirb.
Iz_16_1209	DJI_0135	Sambucus nigra L.	0	n	1	1	Viburnaceae Raf.
Iz_16_1209	DJI_0135	Secale sylvestre Host	0	n	1	0	Poaceae Barnhart
Iz_16_1209	DJI_0135	Sempervivum ruthenicum Schnittsp. & C.B. Lehm.	0	n	1	0	Crassulaceae J.St.-Hil.
Iz_16_1209	DJI_0135	Setaria viridis (L.) P. Beauv.	0	n	1	0	Poaceae Barnhart
Iz_16_1209	DJI_0135	Silene borysthenica (Gruner) Walters	0	n	1	0	Caryophyllaceae Juss.
Iz_16_1209	DJI_0135	Silene latifolia subsp. alba (Mill.) Greuter & Burdet	0	n	1	0	Caryophyllaceae Juss.
Iz_16_1209	DJI_0135	Solidago virgaurea L.	0	n	1	0	Asteraceae Bercht. & J.Presl
Iz_16_1209	DJI_0135	Sorbus aucuparia L.	0	n	1	1	Rosaceae Juss.
Iz_16_1209	DJI_0135	Stachys recta L.	0	n	1	0	Lamiacea Lindl.
Iz_16_1209	DJI_0135	Tanacetum vulgare L.	0	n	1	0	Asteraceae Bercht. & J.Presl
Iz_16_1209	DJI_0135	Thymus pallasianus Heinr.Braun	0	n	1	0	Lamiacea Lindl.
Iz_16_1209	DJI_0135	Tilia cordata Mill.	0	n	1	1	Malvaceae Juss.
Iz_16_1209	DJI_0135	Trifolium alpestre L.	0	n	1	0	Fabaceae Lindl.
Iz_16_1209	DJI_0135	Trifolium arvense L.	0	n	1	0	Fabaceae Lindl.
Iz_16_1209	DJI_0135	Ulmus glabra Huds.	0	n	1	1	Ulmaceae Mirb.
Iz_16_1209	DJI_0135	Verbascum densiflorum Bertol.	0	n	1	0	Scrophulariaceae Juss.
Iz_16_1209	DJI_0135	Veronica austriaca L.	0	n	1	0	Plantaginaceae Juss.
Iz_16_1209	DJI_0135	Veronica incana L.	0	n	1	0	Plantaginaceae Juss.
Iz_16_1209	DJI_0135	Veronica verna L.	0	n	1	0	Plantaginaceae Juss.
Iz_16_1209	DJI_0135	Vincetoxicum hirundinaria Medik	0	n	1	0	Apocynaceae Juss.
Iz_16_1209	DJI_0146	Acer tataricum L.	0	n	1	1	Sapindaceae Juss.
Iz_16_1209	DJI_0146	Achillea millefolium L. s.l.	0	n	1	0	Asteraceae Bercht. & J.Presl
Iz_16_1209	DJI_0146	Allium oleraceum L.	0	n	1	0	Amaryllidaceae J.St.-Hil.
Iz_16_1209	DJI_0146	Ambrosia artemisiifolia L.	0	a	0	0	Asteraceae Bercht. & J.Presl
Iz_16_1209	DJI_0146	Amorpha fruticosa L.	0	a	0	0	Fabaceae Lindl.
Iz_16_1209	DJI_0146	Anthericum ramosum L.	0	n	1	0	Asparagaceae Juss.
Iz_16_1209	DJI_0146	Anthriscus sylvestris (L.) Hoffm.	0		NA	NA	
Iz_16_1209	DJI_0146	Artemisia marschalliana Spreng.	0	n	1	0	Asteraceae Bercht. & J.Presl
Iz_16_1209	DJI_0146	Asparagus officinalis L.	0	n	1	0	Asparagaceae Juss.
Iz_16_1209	DJI_0146	Astragalus cicer L.	0	n	1	0	Fabaceae Lindl.
Iz_16_1209	DJI_0146	Bassia laniflora (S.G.Gmel.) A.J.Scott	0	n	1	0	Amaranthaceae Juss.
Iz_16_1209	DJI_0146	Berberis vulgaris L.	0	n	1	0	Berberidaceae Juss.
Iz_16_1209	DJI_0146	Betula pendula Roth.	0	n	1	1	Betulaceae Gray
Iz_16_1209	DJI_0146	Betula pendula Roth.	0	n	1	0	Poaceae Barnhart
Iz_16_1209	DJI_0146	Bromus inermis Leyss.	0		NA	NA	
Iz_16_1209	DJI_0146	Bromus squarrosus L.	0	a	0	0	Poaceae Barnhart
Iz_16_1209	DJI_0146	Bromus tectorum L.	0	a	0	0	Poaceae Barnhart
Iz_16_1209	DJI_0146	Calamagrostis epigejos (L.) Roth	30	n	1	0	Poaceae Barnhart
Iz_16_1209	DJI_0146	Carduus acanthoides L.	0	n	1	0	Asteraceae Bercht. & J.Presl
Iz_16_1209	DJI_0146	Carex sp.	0	n	1	0	Cyperaceae Juss.
Iz_16_1209	DJI_0146	Centaurea majorovii Dumbadze	0	n	1	0	Asteraceae Bercht. & J.Presl
Iz_16_1209	DJI_0146	Cf. Gypsophila paniculata L.	0		NA	0	
Iz_16_1209	DJI_0146	Chamaecytisus ruthenicus (Fisch.ex Wol.) Klaskova	0	n	1	0	Fabaceae Lindl.

Iz_16_1209	DJI_0146	Chelidonium majus L.	0	n	1	0	Papaveraceae Juss.
Iz_16_1209	DJI_0146	Chenopodium betaceum Andrz.	0	n	1	0	Amaranthaceae Juss.
Iz_16_1209	DJI_0146	Chenopodium cf. album	0	n	1	0	Amaranthaceae Juss.
Iz_16_1209	DJI_0146	Chenopodium sp.	0		NA	0	Amaranthaceae Juss.
Iz_16_1209	DJI_0146	Chondrilla juncea L.	0	n	1	0	Asteraceae Bercht. & J.Presl
Iz_16_1209	DJI_0146	Cirsium vulgare (Savi) Ten.	0	n	1	0	Asteraceae Bercht. & J.Presl
Iz_16_1209	DJI_0146	Clinopodium acinos (L.) Kuntze	0	n	1	0	Lamiacea Lindl.
Iz_16_1209	DJI_0146	Convallaria majalis L.	0	n	1	0	Asparagaceae Juss.
Iz_16_1209	DJI_0146	Crataegus rhipidophylla Gand.	0	n	1	1	Rosaceae Juss.
Iz_16_1209	DJI_0146	Crepis foetida L.	0	n	1	0	Asteraceae Bercht. & J.Presl
Iz_16_1209	DJI_0146	Dactylis glomerata L.	0	n	1	0	Poaceae Barnhart
Iz_16_1209	DJI_0146	Dianthus campestris M.Bieb.	0	n	1	0	Caryophyllaceae Juss.
Iz_16_1209	DJI_0146	Digitaria sanguinalis (L.) Scop.	0	a	0	0	Poaceae Barnhart
Iz_16_1209	DJI_0146	Elymus repens (L.) Gould	0	n	1	0	Poaceae Barnhart
Iz_16_1209	DJI_0146	Epilobium tetragonum L.	0	n	1	0	Onagraceae Juss.
Iz_16_1209	DJI_0146	Eragrostis minor Host	0	a	0	0	Poaceae Barnhart
Iz_16_1209	DJI_0146	Erigeron canadensis L.	0	a	0	0	Asteraceae Bercht. & J.Presl
Iz_16_1209	DJI_0146	Euphorbia seguieriana Neck.	0	n	1	0	Euphorbiaceae Juss.
Iz_16_1209	DJI_0146	Euphorbia sp.	0		NA	NA	
Iz_16_1209	DJI_0146	Fallopia dumetorum (L.) Holub	0	n	1	0	Polygonaceae Juss.
Iz_16_1209	DJI_0146	Festuca beckeri (Hack.) Trautv.	0	n	1	0	Poaceae Barnhart
Iz_16_1209	DJI_0146	Fragaria vesca L.	0	n	1	0	Rosaceae Juss.
Iz_16_1209	DJI_0146	Frangula alnus Mill.	0	n	1	1	Rhamnaceae Juss.
Iz_16_1209	DJI_0146	Fraxinus pennsylvanica Marshall	0	a	0	1	Oleaceae Hoffmanns. & Link
Iz_16_1209	DJI_0146	Galeopsis bifida Boenn.	0	n	1	0	Lamiacea Lindl.
Iz_16_1209	DJI_0146	Galium verum L.	0	n	1	0	Rubiaceae Juss.
Iz_16_1209	DJI_0146	Genista tinctoria L.	0	n	1	0	Fabaceae Lindl.
Iz_16_1209	DJI_0146	Geranium robertianum L.	0	n	1	0	Geraniaceae Juss.
Iz_16_1209	DJI_0146	Geranium sanquineum L.	0	n	1	0	Geraniaceae Juss.
Iz_16_1209	DJI_0146	Gypsophila paniculata L.	0	n	1	0	Caryophyllaceae Juss.
Iz_16_1209	DJI_0146	Helichrysum arenarium (L.) Moench	0	n	1	0	Asteraceae Bercht. & J.Presl
Iz_16_1209	DJI_0146	Herniaria polygama J.Gay	0	n	1	0	Caryophyllaceae Juss.
Iz_16_1209	DJI_0146	Hieracium robustum Fr.	0	n	1	0	Asteraceae Bercht. & J.Presl
Iz_16_1209	DJI_0146	Hieracium umbellatum L.	0	n	1	NA	Asteraceae Bercht. & J.Presl
Iz_16_1209	DJI_0146	Hylotelephium maximum (L.) Holub	0	n	1	0	Crassulaceae J.St.-Hil.
Iz_16_1209	DJI_0146	Jacobaea borysthenica (DC.) B.Nord. & Greuter	0	n	1	0	Asteraceae Bercht. & J.Presl
Iz_16_1209	DJI_0146	Jasione montana L.	0	n	1	0	Campanulaceae Juss.
Iz_16_1209	DJI_0146	Jurinea cyanoides (L.) Rchb.s.l.	0	n	1	0	Asteraceae Bercht. & J.Presl
Iz_16_1209	DJI_0146	Koeleria glauca (Spreng.) DC.	0	n	1	0	Poaceae Barnhart
Iz_16_1209	DJI_0146	Lactuca serriola L.	0.2	a	0	0	Asteraceae Bercht. & J.Presl
Iz_16_1209	DJI_0146	Linaria genistifolia (L.) Mill.	0	n	1	0	Plantaginaceae Juss.
Iz_16_1209	DJI_0146	Lomelosia argentea (L.) Greuter & Burdet	0	n	1	0	Caprifoliaceae Juss.
Iz_16_1209	DJI_0146	Melampyrum pratense L.	0	n	1	0	Orobanchaceae Vent.
Iz_16_1209	DJI_0146	Oenothera biennis L.	0	a	0	0	Onagraceae Juss.
Iz_16_1209	DJI_0146	Pentanema britannica (L.) D.Gut.Larr., Santos-Vicente, Anderb., E.Rico & M.M.Mart.Ort.	0	n	1	0	Asteraceae Bercht. & J.Presl
Iz_16_1209	DJI_0146	Peucedanum oreoselinum (L.) Moench	1	n	1	0	Apiaceae Lindl.
Iz_16_1209	DJI_0146	Peucedanum ruthenicum M.Bieb.	0	n	1	0	Apiaceae Lindl.
Iz_16_1209	DJI_0146	Pilosella echioides (Lumn.) F.W.Schultz & Sch.Bip.	0	n	1	0	Asteraceae Bercht. & J.Presl
Iz_16_1209	DJI_0146	Pilosella officinarum F. Schultz et Sch. Bip.	0	n	1	0	Asteraceae Bercht. & J.Presl
Iz_16_1209	DJI_0146	Pilosella sp.	0		NA	0	Asteraceae Bercht. & J.Presl
Iz_16_1209	DJI_0146	Pinus sylvestris L.	0	n	1	1	Pinaceae Spreng. ex F.Rudolphi
Iz_16_1209	DJI_0146	Poa annua L.	0	n	1	0	Poaceae Barnhart
Iz_16_1209	DJI_0146	Poa nemoralis L.	0	n	1	0	Poaceae Barnhart
Iz_16_1209	DJI_0146	Poa sp.	0		NA	0	Poaceae Barnhart
Iz_16_1209	DJI_0146	Polygonatum odoratum (Mill.) Druce	0	n	1	0	Asparagaceae Juss.
Iz_16_1209	DJI_0146	Polygonum arenarium Waldst. & Kit.	0	n	1	0	Polygonaceae Juss.
Iz_16_1209	DJI_0146	Populus nigra L.	0	n	1	1	Salicaceae Mirb.
Iz_16_1209	DJI_0146	Populus tremula L.	0	n	1	1	Salicaceae Mirb.
Iz_16_1209	DJI_0146	Populus Ã canescens (Aiton) Sm.	0	n	1	1	Salicaceae Mirb.
Iz_16_1209	DJI_0146	Potentilla argentea L.	0	n	1	0	Rosaceae Juss.
Iz_16_1209	DJI_0146	Psammophiliella muralis (L.) Ikonn.	0	n	1	0	Caryophyllaceae Juss.
Iz_16_1209	DJI_0146	Psephellus sumensis (Kalen.) Greuter	0	n	1	0	Asteraceae Bercht. & J.Presl
Iz_16_1209	DJI_0146	Pulsatilla pratensis (L.) Mill. s.l.	0	n	1	0	Ranunculaceae Juss.
Iz_16_1209	DJI_0146	Pyrus communis L.	0	a	0	1	Rosaceae Juss.
Iz_16_1209	DJI_0146	Quercus robur L.	0	n	1	1	Fagaceae Dumort.
Iz_16_1209	DJI_0146	Robinia pseudoacacia L.	0	a	0	1	Fabaceae Lindl.
Iz_16_1209	DJI_0146	Rosa sp.	0		NA	1	Rosaceae Juss.
Iz_16_1209	DJI_0146	Rubus idaeus L.	0	n	1	0	Rosaceae Juss.
Iz_16_1209	DJI_0146	Rumex acetosella L.	0	n	1	0	Polygonaceae Juss.
Iz_16_1209	DJI_0146	Rumex thyrsiflorus Fingerh.	0	n	1	0	Polygonaceae Juss.
Iz_16_1209	DJI_0146	Salix alba L.	0	n	1	1	Salicaceae Mirb.
Iz_16_1209	DJI_0146	Sambucus nigra L.	0	n	1	1	Viburnaceae Raf.
Iz_16_1209	DJI_0146	Secale sylvestre Host	0	n	1	0	Poaceae Barnhart
Iz_16_1209	DJI_0146	Sempervivum ruthenicum Schnittsp. & C.B. Lehm.	0	n	1	0	Crassulaceae J.St.-Hil.
Iz_16_1209	DJI_0146	Setaria viridis (L.) P. Beauv.	0	n	1	0	Poaceae Barnhart
Iz_16_1209	DJI_0146	Silene borysthenica (Gruner) Walters	0	n	1	0	Caryophyllaceae Juss.
Iz_16_1209	DJI_0146	Silene latifolia subsp. alba (Mill.) Greuter & Burdet	0	n	1	0	Caryophyllaceae Juss.
Iz_16_1209	DJI_0146	Solidago virgaurea L.	0	n	1	0	Asteraceae Bercht. & J.Presl
Iz_16_1209	DJI_0146	Sorbus aucuparia L.	0	n	1	1	Rosaceae Juss.
Iz_16_1209	DJI_0146	Stachys recta L.	0	n	1	0	Lamiacea Lindl.
Iz_16_1209	DJI_0146	Tanacetum vulgare L.	0	n	1	0	Asteraceae Bercht. & J.Presl
Iz_16_1209	DJI_0146	Thymus pallasianus Heinr.Braun	0	n	1	0	Lamiacea Lindl.
Iz_16_1209	DJI_0146	Tilia cordata Mill.	0	n	1	1	Malvaceae Juss.
Iz_16_1209	DJI_0146	Trifolium alpestre L.	0	n	1	0	Fabaceae Lindl.
Iz_16_1209	DJI_0146	Trifolium arvense L.	0	n	1	0	Fabaceae Lindl.
Iz_16_1209	DJI_0146	Ulmus glabra Huds.	0	n	1	1	Ulmaceae Mirb.
Iz_16_1209	DJI_0146	Verbascum densiflorum Bertol.	0	n	1	0	Scrophulariaceae Juss.
Iz_16_1209	DJI_0146	Veronica austriaca L.	0	n	1	0	Plantaginaceae Juss.
Iz_16_1209	DJI_0146	Veronica incana L.	0	n	1	0	Plantaginaceae Juss.
Iz_16_1209	DJI_0146	Veronica verna L.	0	n	1	0	Plantaginaceae Juss.
Iz_16_1209	DJI_0146	Vincetoxicum hirundinaria Medik	0	n	1	0	Apocynaceae Juss.
Iz_16_1209	DJI_0150	Acer tataricum L.	0	n	1	1	Sapindaceae Juss.
Iz_16_1209	DJI_0150	Achillea millefolium L. s.l.	0	n	1	0	Asteraceae Bercht. & J.Presl
Iz_16_1209	DJI_0150	Allium oleraceum L.	0	n	1	0	Amaryllidaceae J.St.-Hil.
Iz_16_1209	DJI_0150	Ambrosia artemisiifolia L.	0	a	0	0	Asteraceae Bercht. & J.Presl
Iz_16_1209	DJI_0150	Amorpha fruticosa L.	0	a	0	0	Fabaceae Lindl.
Iz_16_1209	DJI_0150	Anthericum ramosum L.	0	n	1	0	Asparagaceae Juss.
Iz_16_1209	DJI_0150	Anthriscus sylvestris (L.) Hoffm.	0		NA	NA	

Iz_16_1209	DJI_0150	Artemisia marschalliana Spreng.	0	n	1	0	Asteraceae Bercht. & J.Presl
Iz_16_1209	DJI_0150	Asparagus officinalis L.	0	n	1	0	Asparagaceae Juss.
Iz_16_1209	DJI_0150	Astragalus cicer L.	0	n	1	0	Fabaceae Lindl.
Iz_16_1209	DJI_0150	Bassia laniflora (S.G.Gmel.) A.J.Scott	0	n	1	0	Amaranthaceae Juss.
Iz_16_1209	DJI_0150	Berberis vulgaris L.	0	n	1	0	Berberidaceae Juss.
Iz_16_1209	DJI_0150	Betula pendula Roth.	0	n	1	1	Betulaceae Gray
Iz_16_1209	DJI_0150	Betula pendula Roth.	0	n	1	0	Poaceae Barnhart
Iz_16_1209	DJI_0150	Bromus inermis Leyss.	0		NA	NA	
Iz_16_1209	DJI_0150	Bromus squarrosus L.	0	a	0	0	Poaceae Barnhart
Iz_16_1209	DJI_0150	Bromus tectorum L.	0	a	0	0	Poaceae Barnhart
Iz_16_1209	DJI_0150	Calamagrostis epigejos (L.) Roth	35	n	1	0	Poaceae Barnhart
Iz_16_1209	DJI_0150	Carduus acanthoides L.	0	n	1	0	Asteraceae Bercht. & J.Presl
Iz_16_1209	DJI_0150	Carex sp.	0	n	1	0	Cyperaceae Juss.
Iz_16_1209	DJI_0150	Centaurea majorovii Dumbadze	0	n	1	0	Asteraceae Bercht. & J.Presl
Iz_16_1209	DJI_0150	Cf. Gypsophila paniculata L.	0		NA	0	
Iz_16_1209	DJI_0150	Chamaecytisus ruthenicus (Fisch.ex Wol.) Klaskova	0	n	1	0	Fabaceae Lindl.
Iz_16_1209	DJI_0150	Chelidonium majus L.	0	n	1	0	Papaveraceae Juss.
Iz_16_1209	DJI_0150	Chenopodium betaceum Andrz.	0	n	1	0	Amaranthaceae Juss.
Iz_16_1209	DJI_0150	Chenopodium cf. album	0	n	1	0	Amaranthaceae Juss.
Iz_16_1209	DJI_0150	Chenopodium sp.	0		NA	0	Amaranthaceae Juss.
Iz_16_1209	DJI_0150	Chondrilla juncea L.	0	n	1	0	Asteraceae Bercht. & J.Presl
Iz_16_1209	DJI_0150	Cirsium vulgare (Savi) Ten.	0	n	1	0	Asteraceae Bercht. & J.Presl
Iz_16_1209	DJI_0150	Clinopodium acinos (L.) Kuntze	0	n	1	0	Lamiacea Lindl.
Iz_16_1209	DJI_0150	Convallaria majalis L.	0	n	1	0	Asparagaceae Juss.
Iz_16_1209	DJI_0150	Crataegus rhipidophylla Gand.	0	n	1	1	Rosaceae Juss.
Iz_16_1209	DJI_0150	Crepis foetida L.	0	n	1	0	Asteraceae Bercht. & J.Presl
Iz_16_1209	DJI_0150	Dactylis glomerata L.	0	n	1	0	Poaceae Barnhart
Iz_16_1209	DJI_0150	Dianthus campestris M.Bieb.	0	n	1	0	Caryophyllaceae Juss.
Iz_16_1209	DJI_0150	Digitaria sanguinalis (L.) Scop.	0	a	0	0	Poaceae Barnhart
Iz_16_1209	DJI_0150	Elymus repens (L.) Gould	0	n	1	0	Poaceae Barnhart
Iz_16_1209	DJI_0150	Epilobium tetragonum L.	0	n	1	0	Onagraceae Juss.
Iz_16_1209	DJI_0150	Eragrostis minor Host	0	a	0	0	Poaceae Barnhart
Iz_16_1209	DJI_0150	Erigeron canadensis L.	0	a	0	0	Asteraceae Bercht. & J.Presl
Iz_16_1209	DJI_0150	Euphorbia seguieriana Neck.	0	n	1	0	Euphorbiaceae Juss.
Iz_16_1209	DJI_0150	Euphorbia sp.	2		NA	NA	
Iz_16_1209	DJI_0150	Fallopia dumetorum (L.) Holub	0	n	1	0	Polygonaceae Juss.
Iz_16_1209	DJI_0150	Festuca beckeri (Hack.) Trautv.	0	n	1	0	Poaceae Barnhart
Iz_16_1209	DJI_0150	Fragaria vesca L.	0	n	1	0	Rosaceae Juss.
Iz_16_1209	DJI_0150	Frangula alnus Mill.	0	n	1	1	Rhamnaceae Juss.
Iz_16_1209	DJI_0150	Fraxinus pennsylvanica Marshall	0	a	0	1	Oleaceae Hoffmanns. & Link
Iz_16_1209	DJI_0150	Galeopsis bifida Boenn.	0	n	1	0	Lamiacea Lindl.
Iz_16_1209	DJI_0150	Galium verum L.	0	n	1	0	Rubiaceae Juss.
Iz_16_1209	DJI_0150	Genista tinctoria L.	0	n	1	0	Fabaceae Lindl.
Iz_16_1209	DJI_0150	Geranium robertianum L.	0	n	1	0	Geraniaceae Juss.
Iz_16_1209	DJI_0150	Geranium sanquineum L.	0	n	1	0	Geraniaceae Juss.
Iz_16_1209	DJI_0150	Gypsophila paniculata L.	0	n	1	0	Caryophyllaceae Juss.
Iz_16_1209	DJI_0150	Helichrysum arenarium (L.) Moench	0	n	1	0	Asteraceae Bercht. & J.Presl
Iz_16_1209	DJI_0150	Herniaria polygama J.Gay	0	n	1	0	Caryophyllaceae Juss.
Iz_16_1209	DJI_0150	Hieracium robustum Fr.	0	n	1	0	Asteraceae Bercht. & J.Presl
Iz_16_1209	DJI_0150	Hieracium umbellatum L.	0	n	1	NA	Asteraceae Bercht. & J.Presl
Iz_16_1209	DJI_0150	Hylotelephium maximum (L.) Holub	0	n	1	0	Crassulaceae J.St.-Hil.
Iz_16_1209	DJI_0150	Jacobaea borysthenica (DC.) B.Nord. & Greuter	0	n	1	0	Asteraceae Bercht. & J.Presl
Iz_16_1209	DJI_0150	Jasione montana L.	0	n	1	0	Campanulaceae Juss.
Iz_16_1209	DJI_0150	Jurinea cyanoides (L.) Rchb.s.l.	0	n	1	0	Asteraceae Bercht. & J.Presl
Iz_16_1209	DJI_0150	Koeleria glauca (Spreng.) DC.	0	n	1	0	Poaceae Barnhart
Iz_16_1209	DJI_0150	Lactuca serriola L.	0	a	0	0	Asteraceae Bercht. & J.Presl
Iz_16_1209	DJI_0150	Linaria genistifolia (L.) Mill.	0	n	1	0	Plantaginaceae Juss.
Iz_16_1209	DJI_0150	Lomelosia argentea (L.) Greuter & Burdet	0	n	1	0	Caprifoliaceae Juss.
Iz_16_1209	DJI_0150	Melampyrum pratense L.	0	n	1	0	Orobanchaceae Vent.
Iz_16_1209	DJI_0150	Oenothera biennis L.	0	a	0	0	Onagraceae Juss.
Iz_16_1209	DJI_0150	Pentanema britannica (L.) D.Gut.Larr., Santos-Vicente, Anderb., E.Rico & M.M.Mart.Ort.	0	n	1	0	Asteraceae Bercht. & J.Presl
Iz_16_1209	DJI_0150	Peucedanum oreoselinum (L.) Moench	0	n	1	0	Apiaceae Lindl.
Iz_16_1209	DJI_0150	Peucedanum ruthenicum M.Bieb.	0	n	1	0	Apiaceae Lindl.
Iz_16_1209	DJI_0150	Pilosella echioides (Lumn.) F.W.Schultz & Sch.Bip.	0	n	1	0	Asteraceae Bercht. & J.Presl
Iz_16_1209	DJI_0150	Pilosella officinarum F. Schultz et Sch. Bip.	0	n	1	0	Asteraceae Bercht. & J.Presl
Iz_16_1209	DJI_0150	Pilosella sp.	0		NA	0	Asteraceae Bercht. & J.Presl
Iz_16_1209	DJI_0150	Pinus sylvestris L.	0	n	1	1	Pinaceae Spreng. ex F.Rudolphi
Iz_16_1209	DJI_0150	Poa annua L.	0	n	1	0	Poaceae Barnhart
Iz_16_1209	DJI_0150	Poa nemoralis L.	0	n	1	0	Poaceae Barnhart
Iz_16_1209	DJI_0150	Poa sp.	0		NA	0	Poaceae Barnhart
Iz_16_1209	DJI_0150	Polygonatum odoratum (Mill.) Druce	0	n	1	0	Asparagaceae Juss.
Iz_16_1209	DJI_0150	Polygonum arenarium Waldst. & Kit.	0	n	1	0	Polygonaceae Juss.
Iz_16_1209	DJI_0150	Populus nigra L.	0	n	1	1	Salicaceae Mirb.
Iz_16_1209	DJI_0150	Populus tremula L.	0	n	1	1	Salicaceae Mirb.
Iz_16_1209	DJI_0150	Populus Ã canescens (Aiton) Sm.	0	n	1	1	Salicaceae Mirb.
Iz_16_1209	DJI_0150	Potentilla argentea L.	0	n	1	0	Rosaceae Juss.
Iz_16_1209	DJI_0150	Psammophiliella muralis (L.) Ikonn.	0	n	1	0	Caryophyllaceae Juss.
Iz_16_1209	DJI_0150	Psephellus sumensis (Kalen.) Greuter	1	n	1	0	Asteraceae Bercht. & J.Presl
Iz_16_1209	DJI_0150	Pulsatilla pratensis (L.) Mill. s.l.	0	n	1	0	Ranunculaceae Juss.
Iz_16_1209	DJI_0150	Pyrus communis L.	0	a	0	1	Rosaceae Juss.
Iz_16_1209	DJI_0150	Quercus robur L.	0	n	1	1	Fagaceae Dumort.
Iz_16_1209	DJI_0150	Robinia pseudoacacia L.	0	a	0	1	Fabaceae Lindl.
Iz_16_1209	DJI_0150	Rosa sp.	0		NA	1	Rosaceae Juss.
Iz_16_1209	DJI_0150	Rubus idaeus L.	0	n	1	0	Rosaceae Juss.
Iz_16_1209	DJI_0150	Rumex acetosella L.	0	n	1	0	Polygonaceae Juss.
Iz_16_1209	DJI_0150	Rumex thyrsiflorus Fingerh.	0	n	1	0	Polygonaceae Juss.
Iz_16_1209	DJI_0150	Salix alba L.	0	n	1	1	Salicaceae Mirb.
Iz_16_1209	DJI_0150	Sambucus nigra L.	0	n	1	1	Viburnaceae Raf.
Iz_16_1209	DJI_0150	Secale sylvestre Host	0	n	1	0	Poaceae Barnhart
Iz_16_1209	DJI_0150	Sempervivum ruthenicum Schnittsp. & C.B. Lehm.	0	n	1	0	Crassulaceae J.St.-Hil.
Iz_16_1209	DJI_0150	Setaria viridis (L.) P. Beauv.	0	n	1	0	Poaceae Barnhart
Iz_16_1209	DJI_0150	Silene borysthenica (Gruner) Walters	0	n	1	0	Caryophyllaceae Juss.
Iz_16_1209	DJI_0150	Silene latifolia subsp. alba (Mill.) Greuter & Burdet	0	n	1	0	Caryophyllaceae Juss.
Iz_16_1209	DJI_0150	Solidago virgaurea L.	0	n	1	0	Asteraceae Bercht. & J.Presl
Iz_16_1209	DJI_0150	Sorbus aucuparia L.	0	n	1	1	Rosaceae Juss.
Iz_16_1209	DJI_0150	Stachys recta L.	0	n	1	0	Lamiacea Lindl.
Iz_16_1209	DJI_0150	Tanacetum vulgare L.	0	n	1	0	Asteraceae Bercht. & J.Presl
Iz_16_1209	DJI_0150	Thymus pallasianus Heinr.Braun	0	n	1	0	Lamiacea Lindl.

Iz_16_1209	DJI_0150	Tilia cordata Mill.	0	n	1	1	Malvaceae Juss.
Iz_16_1209	DJI_0150	Trifolium alpestre L.	0	n	1	0	Fabaceae Lindl.
Iz_16_1209	DJI_0150	Trifolium arvense L.	0	n	1	0	Fabaceae Lindl.
Iz_16_1209	DJI_0150	Ulmus glabra Huds.	0	n	1	1	Ulmaceae Mirb.
Iz_16_1209	DJI_0150	Verbascum densiflorum Bertol.	0	n	1	0	Scrophulariaceae Juss.
Iz_16_1209	DJI_0150	Veronica austriaca L.	0	n	1	0	Plantaginaceae Juss.
Iz_16_1209	DJI_0150	Veronica incana L.	0	n	1	0	Plantaginaceae Juss.
Iz_16_1209	DJI_0150	Veronica verna L.	0	n	1	0	Plantaginaceae Juss.
Iz_16_1209	DJI_0150	Vincetoxicum hirundinaria Medik	0	n	1	0	Apocynaceae Juss.
Iz_16_1209	DJI_0157	Acer tataricum L.	0	n	1	1	Sapindaceae Juss.
Iz_16_1209	DJI_0157	Achillea millefolium L. s.l.	0	n	1	0	Asteraceae Bercht. & J.Presl
Iz_16_1209	DJI_0157	Allium oleraceum L.	0	n	1	0	Amaryllidaceae J.St.-Hil.
Iz_16_1209	DJI_0157	Ambrosia artemisiifolia L.	0	a	0	0	Asteraceae Bercht. & J.Presl
Iz_16_1209	DJI_0157	Amorpha fruticosa L.	0	a	0	0	Fabaceae Lindl.
Iz_16_1209	DJI_0157	Anthericum ramosum L.	0	n	1	0	Asparagaceae Juss.
Iz_16_1209	DJI_0157	Anthriscus sylvestris (L.) Hoffm.	0		NA	NA	
Iz_16_1209	DJI_0157	Artemisia marschalliana Spreng.	0	n	1	0	Asteraceae Bercht. & J.Presl
Iz_16_1209	DJI_0157	Asparagus officinalis L.	0	n	1	0	Asparagaceae Juss.
Iz_16_1209	DJI_0157	Astragalus cicer L.	0	n	1	0	Fabaceae Lindl.
Iz_16_1209	DJI_0157	Bassia laniflora (S.G.Gmel.) A.J.Scott	0	n	1	0	Amaranthaceae Juss.
Iz_16_1209	DJI_0157	Berberis vulgaris L.	0	n	1	0	Berberidaceae Juss.
Iz_16_1209	DJI_0157	Betula pendula Roth.	0	n	1	1	Betulaceae Gray
Iz_16_1209	DJI_0157	Betula pendula Roth.	0	n	1	0	Poaceae Barnhart
Iz_16_1209	DJI_0157	Bromus inermis Leyss.	0		NA	NA	
Iz_16_1209	DJI_0157	Bromus squarrosus L.	0	a	0	0	Poaceae Barnhart
Iz_16_1209	DJI_0157	Bromus tectorum L.	0	a	0	0	Poaceae Barnhart
Iz_16_1209	DJI_0157	Calamagrostis epigejos (L.) Roth	35	n	1	0	Poaceae Barnhart
Iz_16_1209	DJI_0157	Carduus acanthoides L.	0	n	1	0	Asteraceae Bercht. & J.Presl
Iz_16_1209	DJI_0157	Carex sp.	0	n	1	0	Cyperaceae Juss.
Iz_16_1209	DJI_0157	Centaurea majorovii Dumbadze	0	n	1	0	Asteraceae Bercht. & J.Presl
Iz_16_1209	DJI_0157	Cf. Gypsophila paniculata L.	0		NA	0	
Iz_16_1209	DJI_0157	Chamaecytisus ruthenicus (Fisch.ex Wol.) Klaskova	2	n	1	0	Fabaceae Lindl.
Iz_16_1209	DJI_0157	Chelidonium majus L.	0	n	1	0	Papaveraceae Juss.
Iz_16_1209	DJI_0157	Chenopodium betaceum Andrz.	0	n	1	0	Amaranthaceae Juss.
Iz_16_1209	DJI_0157	Chenopodium cf. album	0	n	1	0	Amaranthaceae Juss.
Iz_16_1209	DJI_0157	Chenopodium sp.	0		NA	0	Amaranthaceae Juss.
Iz_16_1209	DJI_0157	Chondrilla juncea L.	0	n	1	0	Asteraceae Bercht. & J.Presl
Iz_16_1209	DJI_0157	Cirsium vulgare (Savi) Ten.	0	n	1	0	Asteraceae Bercht. & J.Presl
Iz_16_1209	DJI_0157	Clinopodium acinos (L.) Kuntze	0	n	1	0	Lamiacea Lindl.
Iz_16_1209	DJI_0157	Convallaria majalis L.	0	n	1	0	Asparagaceae Juss.
Iz_16_1209	DJI_0157	Crataegus rhipidophylla Gand.	0	n	1	1	Rosaceae Juss.
Iz_16_1209	DJI_0157	Crepis foetida L.	0	n	1	0	Asteraceae Bercht. & J.Presl
Iz_16_1209	DJI_0157	Dactylis glomerata L.	0	n	1	0	Poaceae Barnhart
Iz_16_1209	DJI_0157	Dianthus campestris M.Bieb.	0	n	1	0	Caryophyllaceae Juss.
Iz_16_1209	DJI_0157	Digitaria sanguinalis (L.) Scop.	0	a	0	0	Poaceae Barnhart
Iz_16_1209	DJI_0157	Elymus repens (L.) Gould	0	n	1	0	Poaceae Barnhart
Iz_16_1209	DJI_0157	Epilobium tetragonum L.	0	n	1	0	Onagraceae Juss.
Iz_16_1209	DJI_0157	Eragrostis minor Host	0	a	0	0	Poaceae Barnhart
Iz_16_1209	DJI_0157	Erigeron canadensis L.	0.5	a	0	0	Asteraceae Bercht. & J.Presl
Iz_16_1209	DJI_0157	Euphorbia seguieriana Neck.	0	n	1	0	Euphorbiaceae Juss.
Iz_16_1209	DJI_0157	Euphorbia sp.	0		NA	NA	
Iz_16_1209	DJI_0157	Fallopia dumetorum (L.) Holub	4	n	1	0	Polygonaceae Juss.
Iz_16_1209	DJI_0157	Festuca beckeri (Hack.) Trautv.	0	n	1	0	Poaceae Barnhart
Iz_16_1209	DJI_0157	Fragaria vesca L.	0	n	1	0	Rosaceae Juss.
Iz_16_1209	DJI_0157	Frangula alnus Mill.	0	n	1	1	Rhamnaceae Juss.
Iz_16_1209	DJI_0157	Fraxinus pennsylvanica Marshall	0	a	0	1	Oleaceae Hoffmanns. & Link
Iz_16_1209	DJI_0157	Galeopsis bifida Boenn.	0	n	1	0	Lamiacea Lindl.
Iz_16_1209	DJI_0157	Galium verum L.	0	n	1	0	Rubiaceae Juss.
Iz_16_1209	DJI_0157	Genista tinctoria L.	0	n	1	0	Fabaceae Lindl.
Iz_16_1209	DJI_0157	Geranium robertianum L.	0	n	1	0	Geraniaceae Juss.
Iz_16_1209	DJI_0157	Geranium sanquineum L.	0	n	1	0	Geraniaceae Juss.
Iz_16_1209	DJI_0157	Gypsophila paniculata L.	2	n	1	0	Caryophyllaceae Juss.
Iz_16_1209	DJI_0157	Helichrysum arenarium (L.) Moench	0	n	1	0	Asteraceae Bercht. & J.Presl
Iz_16_1209	DJI_0157	Herniaria polygama J.Gay	0	n	1	0	Caryophyllaceae Juss.
Iz_16_1209	DJI_0157	Hieracium robustum Fr.	0	n	1	0	Asteraceae Bercht. & J.Presl
Iz_16_1209	DJI_0157	Hieracium umbellatum L.	0	n	1	NA	Asteraceae Bercht. & J.Presl
Iz_16_1209	DJI_0157	Hylotelephium maximum (L.) Holub	0	n	1	0	Crassulaceae J.St.-Hil.
Iz_16_1209	DJI_0157	Jacobaea borysthenica (DC.) B.Nord. & Greuter	0	n	1	0	Asteraceae Bercht. & J.Presl
Iz_16_1209	DJI_0157	Jasione montana L.	0	n	1	0	Campanulaceae Juss.
Iz_16_1209	DJI_0157	Jurinea cyanoides (L.) Rchb.s.l.	0	n	1	0	Asteraceae Bercht. & J.Presl
Iz_16_1209	DJI_0157	Koeleria glauca (Spreng.) DC.	0	n	1	0	Poaceae Barnhart
Iz_16_1209	DJI_0157	Lactuca serriola L.	0	a	0	0	Asteraceae Bercht. & J.Presl
Iz_16_1209	DJI_0157	Linaria genistifolia (L.) Mill.	0	n	1	0	Plantaginaceae Juss.
Iz_16_1209	DJI_0157	Lomelosia argentea (L.) Greuter & Burdet	0	n	1	0	Caprifoliaceae Juss.
Iz_16_1209	DJI_0157	Melampyrum pratense L.	0	n	1	0	Orobanchaceae Vent.
Iz_16_1209	DJI_0157	Oenothera biennis L.	0	a	0	0	Onagraceae Juss.
Iz_16_1209	DJI_0157	Pentanema britannica (L.) D.Gut.Larr., Santos-Vicente, Anderb., E.Rico & M.M.Mart.Ort.	0	n	1	0	Asteraceae Bercht. & J.Presl
Iz_16_1209	DJI_0157	Peucedanum oreoselinum (L.) Moench	0	n	1	0	Apiaceae Lindl.
Iz_16_1209	DJI_0157	Peucedanum ruthenicum M.Bieb.	0	n	1	0	Apiaceae Lindl.
Iz_16_1209	DJI_0157	Pilosella echioides (Lumn.) F.W.Schultz & Sch.Bip.	0	n	1	0	Asteraceae Bercht. & J.Presl
Iz_16_1209	DJI_0157	Pilosella officinarum F. Schultz et Sch. Bip.	0	n	1	0	Asteraceae Bercht. & J.Presl
Iz_16_1209	DJI_0157	Pilosella sp.	0		NA	0	Asteraceae Bercht. & J.Presl
Iz_16_1209	DJI_0157	Pinus sylvestris L.	0	n	1	1	Pinaceae Spreng. ex F.Rudolphi
Iz_16_1209	DJI_0157	Poa annua L.	0	n	1	0	Poaceae Barnhart
Iz_16_1209	DJI_0157	Poa nemoralis L.	0	n	1	0	Poaceae Barnhart
Iz_16_1209	DJI_0157	Poa sp.	0		NA	0	Poaceae Barnhart
Iz_16_1209	DJI_0157	Polygonatum odoratum (Mill.) Druce	0.2	n	1	0	Asparagaceae Juss.
Iz_16_1209	DJI_0157	Polygonum arenarium Waldst. & Kit.	0	n	1	0	Polygonaceae Juss.
Iz_16_1209	DJI_0157	Populus nigra L.	0	n	1	1	Salicaceae Mirb.
Iz_16_1209	DJI_0157	Populus tremula L.	0	n	1	1	Salicaceae Mirb.
Iz_16_1209	DJI_0157	Populus Ã canescens (Aiton) Sm.	0	n	1	1	Salicaceae Mirb.
Iz_16_1209	DJI_0157	Potentilla argentea L.	0	n	1	0	Rosaceae Juss.
Iz_16_1209	DJI_0157	Psammophiliella muralis (L.) Ikonn.	0	n	1	0	Caryophyllaceae Juss.
Iz_16_1209	DJI_0157	Psephellus sumensis (Kalen.) Greuter	0	n	1	0	Asteraceae Bercht. & J.Presl
Iz_16_1209	DJI_0157	Pulsatilla pratensis (L.) Mill. s.l.	0	n	1	0	Ranunculaceae Juss.
Iz_16_1209	DJI_0157	Pyrus communis L.	0	a	0	1	Rosaceae Juss.
Iz_16_1209	DJI_0157	Quercus robur L.	0	n	1	1	Fagaceae Dumort.
Iz_16_1209	DJI_0157	Robinia pseudoacacia L.	0	a	0	1	Fabaceae Lindl.

Iz_16_1209	DJI_0157	Rosa sp.	0		NA	1	Rosaceae Juss.
Iz_16_1209	DJI_0157	Rubus idaeus L.	0	n	1	0	Rosaceae Juss.
Iz_16_1209	DJI_0157	Rumex acetosella L.	0	n	1	0	Polygonaceae Juss.
Iz_16_1209	DJI_0157	Rumex thyrsiflorus Fingerh.	0	n	1	0	Polygonaceae Juss.
Iz_16_1209	DJI_0157	Salix alba L.	0	n	1	1	Salicaceae Mirb.
Iz_16_1209	DJI_0157	Sambucus nigra L.	0	n	1	1	Viburnaceae Raf.
Iz_16_1209	DJI_0157	Secale sylvestre Host	0	n	1	0	Poaceae Barnhart
Iz_16_1209	DJI_0157	Sempervivum ruthenicum Schnittsp. & C.B. Lehm.	0	n	1	0	Crassulaceae J.St.-Hil.
Iz_16_1209	DJI_0157	Setaria viridis (L.) P. Beauv.	0	n	1	0	Poaceae Barnhart
Iz_16_1209	DJI_0157	Silene borysthenica (Gruner) Walters	0	n	1	0	Caryophyllaceae Juss.
Iz_16_1209	DJI_0157	Silene latifolia subsp. alba (Mill.) Greuter & Burdet	0	n	1	0	Caryophyllaceae Juss.
Iz_16_1209	DJI_0157	Solidago virgaurea L.	0	n	1	0	Asteraceae Bercht. & J.Presl
Iz_16_1209	DJI_0157	Sorbus aucuparia L.	0	n	1	1	Rosaceae Juss.
Iz_16_1209	DJI_0157	Stachys recta L.	0	n	1	0	Lamiacea Lindl.
Iz_16_1209	DJI_0157	Tanacetum vulgare L.	0	n	1	0	Asteraceae Bercht. & J.Presl
Iz_16_1209	DJI_0157	Thymus pallasianus Heinr.Braun	0	n	1	0	Lamiacea Lindl.
Iz_16_1209	DJI_0157	Tilia cordata Mill.	0	n	1	1	Malvaceae Juss.
Iz_16_1209	DJI_0157	Trifolium alpestre L.	0	n	1	0	Fabaceae Lindl.
Iz_16_1209	DJI_0157	Trifolium arvense L.	0	n	1	0	Fabaceae Lindl.
Iz_16_1209	DJI_0157	Ulmus glabra Huds.	0	n	1	1	Ulmaceae Mirb.
Iz_16_1209	DJI_0157	Verbascum densiflorum Bertol.	0	n	1	0	Scrophulariaceae Juss.
Iz_16_1209	DJI_0157	Veronica austriaca L.	0	n	1	0	Plantaginaceae Juss.
Iz_16_1209	DJI_0157	Veronica incana L.	0	n	1	0	Plantaginaceae Juss.
Iz_16_1209	DJI_0157	Veronica verna L.	0	n	1	0	Plantaginaceae Juss.
Iz_16_1209	DJI_0157	Vincetoxicum hirundinaria Medik	0	n	1	0	Apocynaceae Juss.
Iz_1_1009	DJI_0212	Acer tataricum L.	0	n	1	1	Sapindaceae Juss.
Iz_1_1009	DJI_0212	Achillea millefolium L. s.l.	0	n	1	0	Asteraceae Bercht. & J.Presl
Iz_1_1009	DJI_0212	Allium oleraceum L.	0	n	1	0	Amaryllidaceae J.St.-Hil.
Iz_1_1009	DJI_0212	Ambrosia artemisiifolia L.	0	a	0	0	Asteraceae Bercht. & J.Presl
Iz_1_1009	DJI_0212	Amorpha fruticosa L.	0	a	0	0	Fabaceae Lindl.
Iz_1_1009	DJI_0212	Anthericum ramosum L.	0	n	1	0	Asparagaceae Juss.
Iz_1_1009	DJI_0212	Artemisia marschalliana Spreng.	0	n	1	0	Asteraceae Bercht. & J.Presl
Iz_1_1009	DJI_0212	Asparagus officinalis L.	0	n	1	0	Asparagaceae Juss.
Iz_1_1009	DJI_0212	Astragalus cicer L.	0	n	1	0	Fabaceae Lindl.
Iz_1_1009	DJI_0212	Bassia laniflora (S.G.Gmel.) A.J.Scott	0	n	1	0	Amaranthaceae Juss.
Iz_1_1009	DJI_0212	Berberis vulgaris L.	0	n	1	0	Berberidaceae Juss.
Iz_1_1009	DJI_0212	Betula pendula Roth.	0	n	1	1	Betulaceae Gray
Iz_1_1009	DJI_0212	Betula pendula Roth.	0	n	1	0	Poaceae Barnhart
Iz_1_1009	DJI_0212	Bromus inermis Leyss.	0		NA	NA	
Iz_1_1009	DJI_0212	Bromus squarrosus L.	0	a	0	0	Poaceae Barnhart
Iz_1_1009	DJI_0212	Bromus tectorum L.	0	a	0	0	Poaceae Barnhart
Iz_1_1009	DJI_0212	Calamagrostis epigejos (L.) Roth	0.1	n	1	0	Poaceae Barnhart
Iz_1_1009	DJI_0212	Carduus acanthoides L.	0	n	1	0	Asteraceae Bercht. & J.Presl
Iz_1_1009	DJI_0212	Carex sp.	0	n	1	0	Cyperaceae Juss.
Iz_1_1009	DJI_0212	Centaurea majorovii Dumbadze	0	n	1	0	Asteraceae Bercht. & J.Presl
Iz_1_1009	DJI_0212	Chamaecytisus ruthenicus (Fisch.ex Wol.) Klaskova	0	n	1	0	Fabaceae Lindl.
Iz_1_1009	DJI_0212	Chelidonium majus L.	0	n	1	0	Papaveraceae Juss.
Iz_1_1009	DJI_0212	Chenopodium betaceum Andrz.	0	n	1	0	Amaranthaceae Juss.
Iz_1_1009	DJI_0212	Chenopodium cf. album	0	n	1	0	Amaranthaceae Juss.
Iz_1_1009	DJI_0212	Chenopodium sp.	0		NA	0	Amaranthaceae Juss.
Iz_1_1009	DJI_0212	Chondrilla juncea L.	0	n	1	0	Asteraceae Bercht. & J.Presl
Iz_1_1009	DJI_0212	Cirsium vulgare (Savi) Ten.	0	n	1	0	Asteraceae Bercht. & J.Presl
Iz_1_1009	DJI_0212	Clinopodium acinos (L.) Kuntze	0	n	1	0	Lamiacea Lindl.
Iz_1_1009	DJI_0212	Convallaria majalis L.	0	n	1	0	Asparagaceae Juss.
Iz_1_1009	DJI_0212	Crataegus rhipidophylla Gand.	0	n	1	1	Rosaceae Juss.
Iz_1_1009	DJI_0212	Crepis foetida L.	0	n	1	0	Asteraceae Bercht. & J.Presl
Iz_1_1009	DJI_0212	Dactylis glomerata L.	0	n	1	0	Poaceae Barnhart
Iz_1_1009	DJI_0212	Dianthus campestris M.Bieb.	0	n	1	0	Caryophyllaceae Juss.
Iz_1_1009	DJI_0212	Digitaria sanguinalis (L.) Scop.	0	a	0	0	Poaceae Barnhart
Iz_1_1009	DJI_0212	Elymus repens (L.) Gould	0	n	1	0	Poaceae Barnhart
Iz_1_1009	DJI_0212	Epilobium tetragonum L.	0	n	1	0	Onagraceae Juss.
Iz_1_1009	DJI_0212	Eragrostis minor Host	0	a	0	0	Poaceae Barnhart
Iz_1_1009	DJI_0212	Erigeron canadensis L.	0	a	0	0	Asteraceae Bercht. & J.Presl
Iz_1_1009	DJI_0212	Euphorbia seguieriana Neck.	0	n	1	0	Euphorbiaceae Juss.
Iz_1_1009	DJI_0212	Fallopia dumetorum (L.) Holub	0	n	1	0	Polygonaceae Juss.
Iz_1_1009	DJI_0212	Festuca beckeri (Hack.) Trautv.	0	n	1	0	Poaceae Barnhart
Iz_1_1009	DJI_0212	Fragaria vesca L.	0	n	1	0	Rosaceae Juss.
Iz_1_1009	DJI_0212	Frangula alnus Mill.	0	n	1	1	Rhamnaceae Juss.
Iz_1_1009	DJI_0212	Fraxinus pennsylvanica Marshall	0	a	0	1	Oleaceae Hoffmanns. & Link
Iz_1_1009	DJI_0212	Galeopsis bifida Boenn.	0	n	1	0	Lamiacea Lindl.
Iz_1_1009	DJI_0212	Galium verum L.	0	n	1	0	Rubiaceae Juss.
Iz_1_1009	DJI_0212	Genista tinctoria L.	0	n	1	0	Fabaceae Lindl.
Iz_1_1009	DJI_0212	Geranium robertianum L.	0	n	1	0	Geraniaceae Juss.
Iz_1_1009	DJI_0212	Geranium sanquineum L.	0	n	1	0	Geraniaceae Juss.
Iz_1_1009	DJI_0212	Gypsophila paniculata L.	0	n	1	0	Caryophyllaceae Juss.
Iz_1_1009	DJI_0212	Helichrysum arenarium (L.) Moench	0	n	1	0	Asteraceae Bercht. & J.Presl
Iz_1_1009	DJI_0212	Herniaria polygama J.Gay	0	n	1	0	Caryophyllaceae Juss.
Iz_1_1009	DJI_0212	Hieracium robustum Fr.	0	n	1	0	Asteraceae Bercht. & J.Presl
Iz_1_1009	DJI_0212	Hieracium umbellatum L.	0	n	1	NA	Asteraceae Bercht. & J.Presl
Iz_1_1009	DJI_0212	Hylotelephium maximum (L.) Holub	0	n	1	0	Crassulaceae J.St.-Hil.
Iz_1_1009	DJI_0212	Jacobaea borysthenica (DC.) B.Nord. & Greuter	0	n	1	0	Asteraceae Bercht. & J.Presl
Iz_1_1009	DJI_0212	Jasione montana L.	0	n	1	0	Campanulaceae Juss.
Iz_1_1009	DJI_0212	Jurinea cyanoides (L.) Rchb.s.l.	0	n	1	0	Asteraceae Bercht. & J.Presl
Iz_1_1009	DJI_0212	Koeleria glauca (Spreng.) DC.	0	n	1	0	Poaceae Barnhart
Iz_1_1009	DJI_0212	Lactuca serriola L.	0	a	0	0	Asteraceae Bercht. & J.Presl
Iz_1_1009	DJI_0212	Linaria genistifolia (L.) Mill.	0	n	1	0	Plantaginaceae Juss.
Iz_1_1009	DJI_0212	Lomelosia argentea (L.) Greuter & Burdet	0	n	1	0	Caprifoliaceae Juss.
Iz_1_1009	DJI_0212	Melampyrum pratense L.	0	n	1	0	Orobanchaceae Vent.
Iz_1_1009	DJI_0212	Oenothera biennis L.	0	a	0	0	Onagraceae Juss.
Iz_1_1009	DJI_0212	Pentanema britannica (L.) D.Gut.Larr., Santos-Vicente, Anderb., E.Rico & M.M.Mart.Ort.	0	n	1	0	Asteraceae Bercht. & J.Presl
Iz_1_1009	DJI_0212	Peucedanum oreoselinum (L.) Moench	0	n	1	0	Apiaceae Lindl.
Iz_1_1009	DJI_0212	Peucedanum ruthenicum M.Bieb.	0	n	1	0	Apiaceae Lindl.
Iz_1_1009	DJI_0212	Pilosella echioides (Lumn.) F.W.Schultz & Sch.Bip.	0	n	1	0	Asteraceae Bercht. & J.Presl
Iz_1_1009	DJI_0212	Pilosella officinarum F. Schultz et Sch. Bip.	0	n	1	0	Asteraceae Bercht. & J.Presl
Iz_1_1009	DJI_0212	Pilosella sp.	0		NA	0	Asteraceae Bercht. & J.Presl
Iz_1_1009	DJI_0212	Pinus sylvestris L.	0	n	1	1	Pinaceae Spreng. ex F.Rudolphi
Iz_1_1009	DJI_0212	Poa annua L.	0	n	1	0	Poaceae Barnhart
Iz_1_1009	DJI_0212	Poa nemoralis L.	0	n	1	0	Poaceae Barnhart

Iz_1_1009	DJI_0212	Poa sp.	0		NA	0	Poaceae Barnhart
Iz_1_1009	DJI_0212	Polygonatum odoratum (Mill.) Druce	0	n	1	0	Asparagaceae Juss.
Iz_1_1009	DJI_0212	Polygonum arenarium Waldst. & Kit.	0	n	1	0	Polygonaceae Juss.
Iz_1_1009	DJI_0212	Populus nigra L.	0	n	1	1	Salicaceae Mirb.
Iz_1_1009	DJI_0212	Populus tremula L.	0	n	1	1	Salicaceae Mirb.
Iz_1_1009	DJI_0212	Populus Ã canescens (Aiton) Sm.	0	n	1	1	Salicaceae Mirb.
Iz_1_1009	DJI_0212	Potentilla argentea L.	0	n	1	0	Rosaceae Juss.
Iz_1_1009	DJI_0212	Psammophiliella muralis (L.) Ikonn.	0	n	1	0	Caryophyllaceae Juss.
Iz_1_1009	DJI_0212	Psephellus sumensis (Kalen.) Greuter	0	n	1	0	Asteraceae Bercht. & J.Presl
Iz_1_1009	DJI_0212	Pulsatilla pratensis (L.) Mill. s.l.	0	n	1	0	Ranunculaceae Juss.
Iz_1_1009	DJI_0212	Pyrus communis L.	0	a	0	1	Rosaceae Juss.
Iz_1_1009	DJI_0212	Quercus robur L.	0	n	1	1	Fagaceae Dumort.
Iz_1_1009	DJI_0212	Robinia pseudoacacia L.	0	a	0	1	Fabaceae Lindl.
Iz_1_1009	DJI_0212	Rosa sp.	0		NA	1	Rosaceae Juss.
Iz_1_1009	DJI_0212	Rubus idaeus L.	0	n	1	0	Rosaceae Juss.
Iz_1_1009	DJI_0212	Rumex acetosella L.	0	n	1	0	Polygonaceae Juss.
Iz_1_1009	DJI_0212	Rumex thyrsiflorus Fingerh.	0	n	1	0	Polygonaceae Juss.
Iz_1_1009	DJI_0212	Salix alba L.	0	n	1	1	Salicaceae Mirb.
Iz_1_1009	DJI_0212	Sambucus nigra L.	0	n	1	1	Viburnaceae Raf.
Iz_1_1009	DJI_0212	Secale sylvestre Host	0	n	1	0	Poaceae Barnhart
Iz_1_1009	DJI_0212	Sempervivum ruthenicum Schnittsp. & C.B. Lehm.	0	n	1	0	Crassulaceae J.St.-Hil.
Iz_1_1009	DJI_0212	Setaria viridis (L.) P. Beauv.	0	n	1	0	Poaceae Barnhart
Iz_1_1009	DJI_0212	Silene borysthenica (Gruner) Walters	0	n	1	0	Caryophyllaceae Juss.
Iz_1_1009	DJI_0212	Silene latifolia subsp. alba (Mill.) Greuter & Burdet	0	n	1	0	Caryophyllaceae Juss.
Iz_1_1009	DJI_0212	Solidago virgaurea L.	0	n	1	0	Asteraceae Bercht. & J.Presl
Iz_1_1009	DJI_0212	Sorbus aucuparia L.	0	n	1	1	Rosaceae Juss.
Iz_1_1009	DJI_0212	Stachys recta L.	0	n	1	0	Lamiacea Lindl.
Iz_1_1009	DJI_0212	Tanacetum vulgare L.	0	n	1	0	Asteraceae Bercht. & J.Presl
Iz_1_1009	DJI_0212	Thymus pallasianus Heinr.Braun	0	n	1	0	Lamiacea Lindl.
Iz_1_1009	DJI_0212	Tilia cordata Mill.	0	n	1	1	Malvaceae Juss.
Iz_1_1009	DJI_0212	Trifolium alpestre L.	0	n	1	0	Fabaceae Lindl.
Iz_1_1009	DJI_0212	Trifolium arvense L.	0	n	1	0	Fabaceae Lindl.
Iz_1_1009	DJI_0212	Ulmus glabra Huds.	0	n	1	1	Ulmaceae Mirb.
Iz_1_1009	DJI_0212	Verbascum densiflorum Bertol.	0	n	1	0	Scrophulariaceae Juss.
Iz_1_1009	DJI_0212	Veronica austriaca L.	0	n	1	0	Plantaginaceae Juss.
Iz_1_1009	DJI_0212	Veronica incana L.	0	n	1	0	Plantaginaceae Juss.
Iz_1_1009	DJI_0212	Veronica verna L.	0	n	1	0	Plantaginaceae Juss.
Iz_1_1009	DJI_0212	Vincetoxicum hirundinaria Medik	0	n	1	0	Apocynaceae Juss.
Iz_1_1009	DJI_0213	Acer tataricum L.	0	n	1	1	Sapindaceae Juss.
Iz_1_1009	DJI_0213	Achillea millefolium L. s.l.	0	n	1	0	Asteraceae Bercht. & J.Presl
Iz_1_1009	DJI_0213	Allium oleraceum L.	0	n	1	0	Amaryllidaceae J.St.-Hil.
Iz_1_1009	DJI_0213	Ambrosia artemisiifolia L.	0	a	0	0	Asteraceae Bercht. & J.Presl
Iz_1_1009	DJI_0213	Amorpha fruticosa L.	0	a	0	0	Fabaceae Lindl.
Iz_1_1009	DJI_0213	Anthericum ramosum L.	0	n	1	0	Asparagaceae Juss.
Iz_1_1009	DJI_0213	Artemisia marschalliana Spreng.	0	n	1	0	Asteraceae Bercht. & J.Presl
Iz_1_1009	DJI_0213	Asparagus officinalis L.	0	n	1	0	Asparagaceae Juss.
Iz_1_1009	DJI_0213	Astragalus cicer L.	0	n	1	0	Fabaceae Lindl.
Iz_1_1009	DJI_0213	Bassia laniflora (S.G.Gmel.) A.J.Scott	0	n	1	0	Amaranthaceae Juss.
Iz_1_1009	DJI_0213	Berberis vulgaris L.	0	n	1	0	Berberidaceae Juss.
Iz_1_1009	DJI_0213	Betula pendula Roth.	0	n	1	1	Betulaceae Gray
Iz_1_1009	DJI_0213	Betula pendula Roth.	0	n	1	0	Poaceae Barnhart
Iz_1_1009	DJI_0213	Bromus inermis Leyss.	0		NA	NA	
Iz_1_1009	DJI_0213	Bromus squarrosus L.	0	a	0	0	Poaceae Barnhart
Iz_1_1009	DJI_0213	Bromus tectorum L.	0	a	0	0	Poaceae Barnhart
Iz_1_1009	DJI_0213	Calamagrostis epigejos (L.) Roth	0.2	n	1	0	Poaceae Barnhart
Iz_1_1009	DJI_0213	Carduus acanthoides L.	0	n	1	0	Asteraceae Bercht. & J.Presl
Iz_1_1009	DJI_0213	Carex sp.	0	n	1	0	Cyperaceae Juss.
Iz_1_1009	DJI_0213	Centaurea majorovii Dumbadze	0	n	1	0	Asteraceae Bercht. & J.Presl
Iz_1_1009	DJI_0213	Chamaecytisus ruthenicus (Fisch.ex Wol.) Klaskova	0	n	1	0	Fabaceae Lindl.
Iz_1_1009	DJI_0213	Chelidonium majus L.	0	n	1	0	Papaveraceae Juss.
Iz_1_1009	DJI_0213	Chenopodium betaceum Andrz.	0	n	1	0	Amaranthaceae Juss.
Iz_1_1009	DJI_0213	Chenopodium cf. album	0	n	1	0	Amaranthaceae Juss.
Iz_1_1009	DJI_0213	Chenopodium sp.	0		NA	0	Amaranthaceae Juss.
Iz_1_1009	DJI_0213	Chondrilla juncea L.	0	n	1	0	Asteraceae Bercht. & J.Presl
Iz_1_1009	DJI_0213	Cirsium vulgare (Savi) Ten.	0	n	1	0	Asteraceae Bercht. & J.Presl
Iz_1_1009	DJI_0213	Clinopodium acinos (L.) Kuntze	0	n	1	0	Lamiacea Lindl.
Iz_1_1009	DJI_0213	Convallaria majalis L.	0	n	1	0	Asparagaceae Juss.
Iz_1_1009	DJI_0213	Crataegus rhipidophylla Gand.	0	n	1	1	Rosaceae Juss.
Iz_1_1009	DJI_0213	Crepis foetida L.	0	n	1	0	Asteraceae Bercht. & J.Presl
Iz_1_1009	DJI_0213	Dactylis glomerata L.	0	n	1	0	Poaceae Barnhart
Iz_1_1009	DJI_0213	Dianthus campestris M.Bieb.	0	n	1	0	Caryophyllaceae Juss.
Iz_1_1009	DJI_0213	Digitaria sanguinalis (L.) Scop.	0	a	0	0	Poaceae Barnhart
Iz_1_1009	DJI_0213	Elymus repens (L.) Gould	0	n	1	0	Poaceae Barnhart
Iz_1_1009	DJI_0213	Epilobium tetragonum L.	0	n	1	0	Onagraceae Juss.
Iz_1_1009	DJI_0213	Eragrostis minor Host	0	a	0	0	Poaceae Barnhart
Iz_1_1009	DJI_0213	Erigeron canadensis L.	0	a	0	0	Asteraceae Bercht. & J.Presl
Iz_1_1009	DJI_0213	Euphorbia seguieriana Neck.	0	n	1	0	Euphorbiaceae Juss.
Iz_1_1009	DJI_0213	Fallopia dumetorum (L.) Holub	0.1	n	1	0	Polygonaceae Juss.
Iz_1_1009	DJI_0213	Festuca beckeri (Hack.) Trautv.	0	n	1	0	Poaceae Barnhart
Iz_1_1009	DJI_0213	Fragaria vesca L.	0	n	1	0	Rosaceae Juss.
Iz_1_1009	DJI_0213	Frangula alnus Mill.	0	n	1	1	Rhamnaceae Juss.
Iz_1_1009	DJI_0213	Fraxinus pennsylvanica Marshall	0	a	0	1	Oleaceae Hoffmanns. & Link
Iz_1_1009	DJI_0213	Galeopsis bifida Boenn.	0	n	1	0	Lamiacea Lindl.
Iz_1_1009	DJI_0213	Galium verum L.	0	n	1	0	Rubiaceae Juss.
Iz_1_1009	DJI_0213	Genista tinctoria L.	0	n	1	0	Fabaceae Lindl.
Iz_1_1009	DJI_0213	Geranium robertianum L.	0	n	1	0	Geraniaceae Juss.
Iz_1_1009	DJI_0213	Geranium sanquineum L.	0	n	1	0	Geraniaceae Juss.
Iz_1_1009	DJI_0213	Gypsophila paniculata L.	0	n	1	0	Caryophyllaceae Juss.
Iz_1_1009	DJI_0213	Helichrysum arenarium (L.) Moench	0	n	1	0	Asteraceae Bercht. & J.Presl
Iz_1_1009	DJI_0213	Herniaria polygama J.Gay	0	n	1	0	Caryophyllaceae Juss.
Iz_1_1009	DJI_0213	Hieracium robustum Fr.	0	n	1	0	Asteraceae Bercht. & J.Presl
Iz_1_1009	DJI_0213	Hieracium umbellatum L.	0	n	1	NA	Asteraceae Bercht. & J.Presl
Iz_1_1009	DJI_0213	Hylotelephium maximum (L.) Holub	0	n	1	0	Crassulaceae J.St.-Hil.
Iz_1_1009	DJI_0213	Jacobaea borysthenica (DC.) B.Nord. & Greuter	0	n	1	0	Asteraceae Bercht. & J.Presl
Iz_1_1009	DJI_0213	Jasione montana L.	0	n	1	0	Campanulaceae Juss.
Iz_1_1009	DJI_0213	Jurinea cyanoides (L.) Rchb.s.l.	0	n	1	0	Asteraceae Bercht. & J.Presl
Iz_1_1009	DJI_0213	Koeleria glauca (Spreng.) DC.	0	n	1	0	Poaceae Barnhart
Iz_1_1009	DJI_0213	Lactuca serriola L.	0	a	0	0	Asteraceae Bercht. & J.Presl

Iz_1_1009	DJI_0213	Linaria genistifolia (L.) Mill.	0	n	1	0	Plantaginaceae Juss.
Iz_1_1009	DJI_0213	Lomelosia argentea (L.) Greuter & Burdet	0	n	1	0	Caprifoliaceae Juss.
Iz_1_1009	DJI_0213	Melampyrum pratense L.	0	n	1	0	Orobanchaceae Vent.
Iz_1_1009	DJI_0213	Oenothera biennis L.	0	a	0	0	Onagraceae Juss.
Iz_1_1009	DJI_0213	Pentanema britannica (L.) D.Gut.Larr., Santos-Vicente, Anderb., E.Rico & M.M.Mart.Ort.	0	n	1	0	Asteraceae Bercht. & J.Presl
Iz_1_1009	DJI_0213	Peucedanum oreoselinum (L.) Moench	0	n	1	0	Apiaceae Lindl.
Iz_1_1009	DJI_0213	Peucedanum ruthenicum M.Bieb.	0	n	1	0	Apiaceae Lindl.
Iz_1_1009	DJI_0213	Pilosella echioides (Lumn.) F.W.Schultz & Sch.Bip.	0	n	1	0	Asteraceae Bercht. & J.Presl
Iz_1_1009	DJI_0213	Pilosella officinarum F. Schultz et Sch. Bip.	0	n	1	0	Asteraceae Bercht. & J.Presl
Iz_1_1009	DJI_0213	Pilosella sp.	0		NA	0	Asteraceae Bercht. & J.Presl
Iz_1_1009	DJI_0213	Pinus sylvestris L.	0	n	1	1	Pinaceae Spreng. ex F.Rudolphi
Iz_1_1009	DJI_0213	Poa annua L.	0	n	1	0	Poaceae Barnhart
Iz_1_1009	DJI_0213	Poa nemoralis L.	0	n	1	0	Poaceae Barnhart
Iz_1_1009	DJI_0213	Poa sp.	0		NA	0	Poaceae Barnhart
Iz_1_1009	DJI_0213	Polygonatum odoratum (Mill.) Druce	0	n	1	0	Asparagaceae Juss.
Iz_1_1009	DJI_0213	Polygonum arenarium Waldst. & Kit.	0	n	1	0	Polygonaceae Juss.
Iz_1_1009	DJI_0213	Populus nigra L.	0	n	1	1	Salicaceae Mirb.
Iz_1_1009	DJI_0213	Populus tremula L.	0	n	1	1	Salicaceae Mirb.
Iz_1_1009	DJI_0213	Populus Ã canescens (Aiton) Sm.	0	n	1	1	Salicaceae Mirb.
Iz_1_1009	DJI_0213	Potentilla argentea L.	0	n	1	0	Rosaceae Juss.
Iz_1_1009	DJI_0213	Psammophiliella muralis (L.) Ikonn.	0	n	1	0	Caryophyllaceae Juss.
Iz_1_1009	DJI_0213	Psephellus sumensis (Kalen.) Greuter	0	n	1	0	Asteraceae Bercht. & J.Presl
Iz_1_1009	DJI_0213	Pulsatilla pratensis (L.) Mill. s.l.	0	n	1	0	Ranunculaceae Juss.
Iz_1_1009	DJI_0213	Pyrus communis L.	0	a	0	1	Rosaceae Juss.
Iz_1_1009	DJI_0213	Quercus robur L.	0	n	1	1	Fagaceae Dumort.
Iz_1_1009	DJI_0213	Robinia pseudoacacia L.	0	a	0	1	Fabaceae Lindl.
Iz_1_1009	DJI_0213	Rosa sp.	0		NA	1	Rosaceae Juss.
Iz_1_1009	DJI_0213	Rubus idaeus L.	0	n	1	0	Rosaceae Juss.
Iz_1_1009	DJI_0213	Rumex acetosella L.	0	n	1	0	Polygonaceae Juss.
Iz_1_1009	DJI_0213	Rumex thyrsiflorus Fingerh.	0	n	1	0	Polygonaceae Juss.
Iz_1_1009	DJI_0213	Salix alba L.	0	n	1	1	Salicaceae Mirb.
Iz_1_1009	DJI_0213	Sambucus nigra L.	0	n	1	1	Viburnaceae Raf.
Iz_1_1009	DJI_0213	Secale sylvestre Host	0	n	1	0	Poaceae Barnhart
Iz_1_1009	DJI_0213	Sempervivum ruthenicum Schnittsp. & C.B. Lehm.	0	n	1	0	Crassulaceae J.St.-Hil.
Iz_1_1009	DJI_0213	Setaria viridis (L.) P. Beauv.	0	n	1	0	Poaceae Barnhart
Iz_1_1009	DJI_0213	Silene borysthenica (Gruner) Walters	0	n	1	0	Caryophyllaceae Juss.
Iz_1_1009	DJI_0213	Silene latifolia subsp. alba (Mill.) Greuter & Burdet	0	n	1	0	Caryophyllaceae Juss.
Iz_1_1009	DJI_0213	Solidago virgaurea L.	0.2	n	1	0	Asteraceae Bercht. & J.Presl
Iz_1_1009	DJI_0213	Sorbus aucuparia L.	0	n	1	1	Rosaceae Juss.
Iz_1_1009	DJI_0213	Stachys recta L.	0	n	1	0	Lamiacea Lindl.
Iz_1_1009	DJI_0213	Tanacetum vulgare L.	0	n	1	0	Asteraceae Bercht. & J.Presl
Iz_1_1009	DJI_0213	Thymus pallasianus Heinr.Braun	0	n	1	0	Lamiacea Lindl.
Iz_1_1009	DJI_0213	Tilia cordata Mill.	0	n	1	1	Malvaceae Juss.
Iz_1_1009	DJI_0213	Trifolium alpestre L.	0	n	1	0	Fabaceae Lindl.
Iz_1_1009	DJI_0213	Trifolium arvense L.	0	n	1	0	Fabaceae Lindl.
Iz_1_1009	DJI_0213	Ulmus glabra Huds.	0	n	1	1	Ulmaceae Mirb.
Iz_1_1009	DJI_0213	Verbascum densiflorum Bertol.	0	n	1	0	Scrophulariaceae Juss.
Iz_1_1009	DJI_0213	Veronica austriaca L.	0	n	1	0	Plantaginaceae Juss.
Iz_1_1009	DJI_0213	Veronica incana L.	0	n	1	0	Plantaginaceae Juss.
Iz_1_1009	DJI_0213	Veronica verna L.	0	n	1	0	Plantaginaceae Juss.
Iz_1_1009	DJI_0213	Vincetoxicum hirundinaria Medik	0	n	1	0	Apocynaceae Juss.
Iz_1_1009	DJI_0215	Acer tataricum L.	0	n	1	1	Sapindaceae Juss.
Iz_1_1009	DJI_0215	Achillea millefolium L. s.l.	0	n	1	0	Asteraceae Bercht. & J.Presl
Iz_1_1009	DJI_0215	Allium oleraceum L.	0	n	1	0	Amaryllidaceae J.St.-Hil.
Iz_1_1009	DJI_0215	Ambrosia artemisiifolia L.	0	a	0	0	Asteraceae Bercht. & J.Presl
Iz_1_1009	DJI_0215	Amorpha fruticosa L.	0	a	0	0	Fabaceae Lindl.
Iz_1_1009	DJI_0215	Anthericum ramosum L.	0	n	1	0	Asparagaceae Juss.
Iz_1_1009	DJI_0215	Artemisia marschalliana Spreng.	0	n	1	0	Asteraceae Bercht. & J.Presl
Iz_1_1009	DJI_0215	Asparagus officinalis L.	0	n	1	0	Asparagaceae Juss.
Iz_1_1009	DJI_0215	Astragalus cicer L.	0	n	1	0	Fabaceae Lindl.
Iz_1_1009	DJI_0215	Bassia laniflora (S.G.Gmel.) A.J.Scott	0	n	1	0	Amaranthaceae Juss.
Iz_1_1009	DJI_0215	Berberis vulgaris L.	0	n	1	0	Berberidaceae Juss.
Iz_1_1009	DJI_0215	Betula pendula Roth.	0	n	1	1	Betulaceae Gray
Iz_1_1009	DJI_0215	Betula pendula Roth.	0	n	1	0	Poaceae Barnhart
Iz_1_1009	DJI_0215	Bromus inermis Leyss.	0		NA	NA	
Iz_1_1009	DJI_0215	Bromus squarrosus L.	0	a	0	0	Poaceae Barnhart
Iz_1_1009	DJI_0215	Bromus tectorum L.	0	a	0	0	Poaceae Barnhart
Iz_1_1009	DJI_0215	Calamagrostis epigejos (L.) Roth	3	n	1	0	Poaceae Barnhart
Iz_1_1009	DJI_0215	Carduus acanthoides L.	0	n	1	0	Asteraceae Bercht. & J.Presl
Iz_1_1009	DJI_0215	Carex sp.	0	n	1	0	Cyperaceae Juss.
Iz_1_1009	DJI_0215	Centaurea majorovii Dumbadze	0	n	1	0	Asteraceae Bercht. & J.Presl
Iz_1_1009	DJI_0215	Chamaecytisus ruthenicus (Fisch.ex Wol.) Klaskova	0	n	1	0	Fabaceae Lindl.
Iz_1_1009	DJI_0215	Chelidonium majus L.	0	n	1	0	Papaveraceae Juss.
Iz_1_1009	DJI_0215	Chenopodium betaceum Andrz.	0	n	1	0	Amaranthaceae Juss.
Iz_1_1009	DJI_0215	Chenopodium cf. album	0	n	1	0	Amaranthaceae Juss.
Iz_1_1009	DJI_0215	Chenopodium sp.	0		NA	0	Amaranthaceae Juss.
Iz_1_1009	DJI_0215	Chondrilla juncea L.	0	n	1	0	Asteraceae Bercht. & J.Presl
Iz_1_1009	DJI_0215	Cirsium vulgare (Savi) Ten.	0	n	1	0	Asteraceae Bercht. & J.Presl
Iz_1_1009	DJI_0215	Clinopodium acinos (L.) Kuntze	0	n	1	0	Lamiacea Lindl.
Iz_1_1009	DJI_0215	Convallaria majalis L.	0	n	1	0	Asparagaceae Juss.
Iz_1_1009	DJI_0215	Crataegus rhipidophylla Gand.	0	n	1	1	Rosaceae Juss.
Iz_1_1009	DJI_0215	Crepis foetida L.	0	n	1	0	Asteraceae Bercht. & J.Presl
Iz_1_1009	DJI_0215	Dactylis glomerata L.	0	n	1	0	Poaceae Barnhart
Iz_1_1009	DJI_0215	Dianthus campestris M.Bieb.	0	n	1	0	Caryophyllaceae Juss.
Iz_1_1009	DJI_0215	Digitaria sanguinalis (L.) Scop.	0	a	0	0	Poaceae Barnhart
Iz_1_1009	DJI_0215	Elymus repens (L.) Gould	0	n	1	0	Poaceae Barnhart
Iz_1_1009	DJI_0215	Epilobium tetragonum L.	0	n	1	0	Onagraceae Juss.
Iz_1_1009	DJI_0215	Eragrostis minor Host	0	a	0	0	Poaceae Barnhart
Iz_1_1009	DJI_0215	Erigeron canadensis L.	0	a	0	0	Asteraceae Bercht. & J.Presl
Iz_1_1009	DJI_0215	Euphorbia seguieriana Neck.	0	n	1	0	Euphorbiaceae Juss.
Iz_1_1009	DJI_0215	Fallopia dumetorum (L.) Holub	0	n	1	0	Polygonaceae Juss.
Iz_1_1009	DJI_0215	Festuca beckeri (Hack.) Trautv.	0	n	1	0	Poaceae Barnhart
Iz_1_1009	DJI_0215	Fragaria vesca L.	0	n	1	0	Rosaceae Juss.
Iz_1_1009	DJI_0215	Frangula alnus Mill.	0	n	1	1	Rhamnaceae Juss.
Iz_1_1009	DJI_0215	Fraxinus pennsylvanica Marshall	0	a	0	1	Oleaceae Hoffmanns. & Link
Iz_1_1009	DJI_0215	Galeopsis bifida Boenn.	0	n	1	0	Lamiacea Lindl.
Iz_1_1009	DJI_0215	Galium verum L.	0	n	1	0	Rubiaceae Juss.
Iz_1_1009	DJI_0215	Genista tinctoria L.	0	n	1	0	Fabaceae Lindl.

Iz_1_1009	DJI_0215	Geranium robertianum L.	0	n	1	0	Geraniaceae Juss.
Iz_1_1009	DJI_0215	Geranium sanquineum L.	0	n	1	0	Geraniaceae Juss.
Iz_1_1009	DJI_0215	Gypsophila paniculata L.	0	n	1	0	Caryophyllaceae Juss.
Iz_1_1009	DJI_0215	Helichrysum arenarium (L.) Moench	0	n	1	0	Asteraceae Bercht. & J.Presl
Iz_1_1009	DJI_0215	Herniaria polygama J.Gay	0	n	1	0	Caryophyllaceae Juss.
Iz_1_1009	DJI_0215	Hieracium robustum Fr.	0	n	1	0	Asteraceae Bercht. & J.Presl
Iz_1_1009	DJI_0215	Hieracium umbellatum L.	0	n	1	NA	Asteraceae Bercht. & J.Presl
Iz_1_1009	DJI_0215	Hylotelephium maximum (L.) Holub	0	n	1	0	Crassulaceae J.St.-Hil.
Iz_1_1009	DJI_0215	Jacobaea borysthenica (DC.) B.Nord. & Greuter	0	n	1	0	Asteraceae Bercht. & J.Presl
Iz_1_1009	DJI_0215	Jasione montana L.	0	n	1	0	Campanulaceae Juss.
Iz_1_1009	DJI_0215	Jurinea cyanoides (L.) Rchb.s.l.	0	n	1	0	Asteraceae Bercht. & J.Presl
Iz_1_1009	DJI_0215	Koeleria glauca (Spreng.) DC.	0	n	1	0	Poaceae Barnhart
Iz_1_1009	DJI_0215	Lactuca serriola L.	0	a	0	0	Asteraceae Bercht. & J.Presl
Iz_1_1009	DJI_0215	Linaria genistifolia (L.) Mill.	0	n	1	0	Plantaginaceae Juss.
Iz_1_1009	DJI_0215	Lomelosia argentea (L.) Greuter & Burdet	0	n	1	0	Caprifoliaceae Juss.
Iz_1_1009	DJI_0215	Melampyrum pratense L.	0	n	1	0	Orobanchaceae Vent.
Iz_1_1009	DJI_0215	Oenothera biennis L.	0	a	0	0	Onagraceae Juss.
Iz_1_1009	DJI_0215	Pentanema britannica (L.) D.Gut.Larr., Santos-Vicente, Anderb., E.Rico & M.M.Mart.Ort.	0	n	1	0	Asteraceae Bercht. & J.Presl
Iz_1_1009	DJI_0215	Peucedanum oreoselinum (L.) Moench	0	n	1	0	Apiaceae Lindl.
Iz_1_1009	DJI_0215	Peucedanum ruthenicum M.Bieb.	0	n	1	0	Apiaceae Lindl.
Iz_1_1009	DJI_0215	Pilosella echioides (Lumn.) F.W.Schultz & Sch.Bip.	0	n	1	0	Asteraceae Bercht. & J.Presl
Iz_1_1009	DJI_0215	Pilosella officinarum F. Schultz et Sch. Bip.	0	n	1	0	Asteraceae Bercht. & J.Presl
Iz_1_1009	DJI_0215	Pilosella sp.	0		NA	0	Asteraceae Bercht. & J.Presl
Iz_1_1009	DJI_0215	Pinus sylvestris L.	0	n	1	1	Pinaceae Spreng. ex F.Rudolphi
Iz_1_1009	DJI_0215	Poa annua L.	0	n	1	0	Poaceae Barnhart
Iz_1_1009	DJI_0215	Poa nemoralis L.	0	n	1	0	Poaceae Barnhart
Iz_1_1009	DJI_0215	Poa sp.	0		NA	0	Poaceae Barnhart
Iz_1_1009	DJI_0215	Polygonatum odoratum (Mill.) Druce	0	n	1	0	Asparagaceae Juss.
Iz_1_1009	DJI_0215	Polygonum arenarium Waldst. & Kit.	0	n	1	0	Polygonaceae Juss.
Iz_1_1009	DJI_0215	Populus nigra L.	0	n	1	1	Salicaceae Mirb.
Iz_1_1009	DJI_0215	Populus tremula L.	0	n	1	1	Salicaceae Mirb.
Iz_1_1009	DJI_0215	Populus Ã canescens (Aiton) Sm.	0	n	1	1	Salicaceae Mirb.
Iz_1_1009	DJI_0215	Potentilla argentea L.	0	n	1	0	Rosaceae Juss.
Iz_1_1009	DJI_0215	Psammophiliella muralis (L.) Ikonn.	0	n	1	0	Caryophyllaceae Juss.
Iz_1_1009	DJI_0215	Psephellus sumensis (Kalen.) Greuter	0	n	1	0	Asteraceae Bercht. & J.Presl
Iz_1_1009	DJI_0215	Pulsatilla pratensis (L.) Mill. s.l.	0	n	1	0	Ranunculaceae Juss.
Iz_1_1009	DJI_0215	Pyrus communis L.	0	a	0	1	Rosaceae Juss.
Iz_1_1009	DJI_0215	Quercus robur L.	0	n	1	1	Fagaceae Dumort.
Iz_1_1009	DJI_0215	Robinia pseudoacacia L.	0	a	0	1	Fabaceae Lindl.
Iz_1_1009	DJI_0215	Rosa sp.	0		NA	1	Rosaceae Juss.
Iz_1_1009	DJI_0215	Rubus idaeus L.	0	n	1	0	Rosaceae Juss.
Iz_1_1009	DJI_0215	Rumex acetosella L.	0	n	1	0	Polygonaceae Juss.
Iz_1_1009	DJI_0215	Rumex thyrsiflorus Fingerh.	0	n	1	0	Polygonaceae Juss.
Iz_1_1009	DJI_0215	Salix alba L.	0	n	1	1	Salicaceae Mirb.
Iz_1_1009	DJI_0215	Sambucus nigra L.	0	n	1	1	Viburnaceae Raf.
Iz_1_1009	DJI_0215	Secale sylvestre Host	0	n	1	0	Poaceae Barnhart
Iz_1_1009	DJI_0215	Sempervivum ruthenicum Schnittsp. & C.B. Lehm.	0	n	1	0	Crassulaceae J.St.-Hil.
Iz_1_1009	DJI_0215	Setaria viridis (L.) P. Beauv.	0	n	1	0	Poaceae Barnhart
Iz_1_1009	DJI_0215	Silene borysthenica (Gruner) Walters	0	n	1	0	Caryophyllaceae Juss.
Iz_1_1009	DJI_0215	Silene latifolia subsp. alba (Mill.) Greuter & Burdet	0	n	1	0	Caryophyllaceae Juss.
Iz_1_1009	DJI_0215	Solidago virgaurea L.	5	n	1	0	Asteraceae Bercht. & J.Presl
Iz_1_1009	DJI_0215	Sorbus aucuparia L.	0	n	1	1	Rosaceae Juss.
Iz_1_1009	DJI_0215	Stachys recta L.	0	n	1	0	Lamiacea Lindl.
Iz_1_1009	DJI_0215	Tanacetum vulgare L.	0	n	1	0	Asteraceae Bercht. & J.Presl
Iz_1_1009	DJI_0215	Thymus pallasianus Heinr.Braun	0	n	1	0	Lamiacea Lindl.
Iz_1_1009	DJI_0215	Tilia cordata Mill.	0	n	1	1	Malvaceae Juss.
Iz_1_1009	DJI_0215	Trifolium alpestre L.	0	n	1	0	Fabaceae Lindl.
Iz_1_1009	DJI_0215	Trifolium arvense L.	0	n	1	0	Fabaceae Lindl.
Iz_1_1009	DJI_0215	Ulmus glabra Huds.	0	n	1	1	Ulmaceae Mirb.
Iz_1_1009	DJI_0215	Verbascum densiflorum Bertol.	0	n	1	0	Scrophulariaceae Juss.
Iz_1_1009	DJI_0215	Veronica austriaca L.	0	n	1	0	Plantaginaceae Juss.
Iz_1_1009	DJI_0215	Veronica incana L.	0	n	1	0	Plantaginaceae Juss.
Iz_1_1009	DJI_0215	Veronica verna L.	0	n	1	0	Plantaginaceae Juss.
Iz_1_1009	DJI_0215	Vincetoxicum hirundinaria Medik	0	n	1	0	Apocynaceae Juss.
Iz_1_1009	DJI_0217	Acer tataricum L.	0	n	1	1	Sapindaceae Juss.
Iz_1_1009	DJI_0217	Achillea millefolium L. s.l.	0	n	1	0	Asteraceae Bercht. & J.Presl
Iz_1_1009	DJI_0217	Allium oleraceum L.	0	n	1	0	Amaryllidaceae J.St.-Hil.
Iz_1_1009	DJI_0217	Ambrosia artemisiifolia L.	0	a	0	0	Asteraceae Bercht. & J.Presl
Iz_1_1009	DJI_0217	Amorpha fruticosa L.	0	a	0	0	Fabaceae Lindl.
Iz_1_1009	DJI_0217	Anthericum ramosum L.	0	n	1	0	Asparagaceae Juss.
Iz_1_1009	DJI_0217	Artemisia marschalliana Spreng.	0	n	1	0	Asteraceae Bercht. & J.Presl
Iz_1_1009	DJI_0217	Asparagus officinalis L.	0	n	1	0	Asparagaceae Juss.
Iz_1_1009	DJI_0217	Astragalus cicer L.	0	n	1	0	Fabaceae Lindl.
Iz_1_1009	DJI_0217	Bassia laniflora (S.G.Gmel.) A.J.Scott	0	n	1	0	Amaranthaceae Juss.
Iz_1_1009	DJI_0217	Berberis vulgaris L.	0	n	1	0	Berberidaceae Juss.
Iz_1_1009	DJI_0217	Betula pendula Roth.	0	n	1	1	Betulaceae Gray
Iz_1_1009	DJI_0217	Betula pendula Roth.	0	n	1	0	Poaceae Barnhart
Iz_1_1009	DJI_0217	Bromus inermis Leyss.	0		NA	NA	
Iz_1_1009	DJI_0217	Bromus squarrosus L.	0	a	0	0	Poaceae Barnhart
Iz_1_1009	DJI_0217	Bromus tectorum L.	0	a	0	0	Poaceae Barnhart
Iz_1_1009	DJI_0217	Calamagrostis epigejos (L.) Roth	80	n	1	0	Poaceae Barnhart
Iz_1_1009	DJI_0217	Carduus acanthoides L.	0	n	1	0	Asteraceae Bercht. & J.Presl
Iz_1_1009	DJI_0217	Carex sp.	0	n	1	0	Cyperaceae Juss.
Iz_1_1009	DJI_0217	Centaurea majorovii Dumbadze	0	n	1	0	Asteraceae Bercht. & J.Presl
Iz_1_1009	DJI_0217	Chamaecytisus ruthenicus (Fisch.ex Wol.) Klaskova	0	n	1	0	Fabaceae Lindl.
Iz_1_1009	DJI_0217	Chelidonium majus L.	0	n	1	0	Papaveraceae Juss.
Iz_1_1009	DJI_0217	Chenopodium betaceum Andrz.	0	n	1	0	Amaranthaceae Juss.
Iz_1_1009	DJI_0217	Chenopodium cf. album	0	n	1	0	Amaranthaceae Juss.
Iz_1_1009	DJI_0217	Chenopodium sp.	0		NA	0	Amaranthaceae Juss.
Iz_1_1009	DJI_0217	Chondrilla juncea L.	0	n	1	0	Asteraceae Bercht. & J.Presl
Iz_1_1009	DJI_0217	Cirsium vulgare (Savi) Ten.	0	n	1	0	Asteraceae Bercht. & J.Presl
Iz_1_1009	DJI_0217	Clinopodium acinos (L.) Kuntze	0	n	1	0	Lamiacea Lindl.
Iz_1_1009	DJI_0217	Convallaria majalis L.	0	n	1	0	Asparagaceae Juss.
Iz_1_1009	DJI_0217	Crataegus rhipidophylla Gand.	0	n	1	1	Rosaceae Juss.
Iz_1_1009	DJI_0217	Crepis foetida L.	0	n	1	0	Asteraceae Bercht. & J.Presl
Iz_1_1009	DJI_0217	Dactylis glomerata L.	0	n	1	0	Poaceae Barnhart
Iz_1_1009	DJI_0217	Dianthus campestris M.Bieb.	0	n	1	0	Caryophyllaceae Juss.
Iz_1_1009	DJI_0217	Digitaria sanguinalis (L.) Scop.	0	a	0	0	Poaceae Barnhart

Iz_1_1009	DJI_0217	Elymus repens (L.) Gould	0	n	1	0	Poaceae Barnhart
Iz_1_1009	DJI_0217	Epilobium tetragonum L.	0	n	1	0	Onagraceae Juss.
Iz_1_1009	DJI_0217	Eragrostis minor Host	0	a	0	0	Poaceae Barnhart
Iz_1_1009	DJI_0217	Erigeron canadensis L.	0	a	0	0	Asteraceae Bercht. & J.Presl
Iz_1_1009	DJI_0217	Euphorbia seguieriana Neck.	0	n	1	0	Euphorbiaceae Juss.
Iz_1_1009	DJI_0217	Fallopia dumetorum (L.) Holub	0	n	1	0	Polygonaceae Juss.
Iz_1_1009	DJI_0217	Festuca beckeri (Hack.) Trautv.	0	n	1	0	Poaceae Barnhart
Iz_1_1009	DJI_0217	Fragaria vesca L.	0	n	1	0	Rosaceae Juss.
Iz_1_1009	DJI_0217	Frangula alnus Mill.	0	n	1	1	Rhamnaceae Juss.
Iz_1_1009	DJI_0217	Fraxinus pennsylvanica Marshall	0	a	0	1	Oleaceae Hoffmanns. & Link
Iz_1_1009	DJI_0217	Galeopsis bifida Boenn.	0	n	1	0	Lamiacea Lindl.
Iz_1_1009	DJI_0217	Galium verum L.	0	n	1	0	Rubiaceae Juss.
Iz_1_1009	DJI_0217	Genista tinctoria L.	0	n	1	0	Fabaceae Lindl.
Iz_1_1009	DJI_0217	Geranium robertianum L.	0	n	1	0	Geraniaceae Juss.
Iz_1_1009	DJI_0217	Geranium sanquineum L.	0	n	1	0	Geraniaceae Juss.
Iz_1_1009	DJI_0217	Gypsophila paniculata L.	0	n	1	0	Caryophyllaceae Juss.
Iz_1_1009	DJI_0217	Helichrysum arenarium (L.) Moench	0	n	1	0	Asteraceae Bercht. & J.Presl
Iz_1_1009	DJI_0217	Herniaria polygama J.Gay	0	n	1	0	Caryophyllaceae Juss.
Iz_1_1009	DJI_0217	Hieracium robustum Fr.	0	n	1	0	Asteraceae Bercht. & J.Presl
Iz_1_1009	DJI_0217	Hieracium umbellatum L.	0	n	1	NA	Asteraceae Bercht. & J.Presl
Iz_1_1009	DJI_0217	Hylotelephium maximum (L.) Holub	0	n	1	0	Crassulaceae J.St.-Hil.
Iz_1_1009	DJI_0217	Jacobaea borysthenica (DC.) B.Nord. & Greuter	0	n	1	0	Asteraceae Bercht. & J.Presl
Iz_1_1009	DJI_0217	Jasione montana L.	0	n	1	0	Campanulaceae Juss.
Iz_1_1009	DJI_0217	Jurinea cyanoides (L.) Rchb.s.l.	0	n	1	0	Asteraceae Bercht. & J.Presl
Iz_1_1009	DJI_0217	Koeleria glauca (Spreng.) DC.	0	n	1	0	Poaceae Barnhart
Iz_1_1009	DJI_0217	Lactuca serriola L.	0	a	0	0	Asteraceae Bercht. & J.Presl
Iz_1_1009	DJI_0217	Linaria genistifolia (L.) Mill.	0	n	1	0	Plantaginaceae Juss.
Iz_1_1009	DJI_0217	Lomelosia argentea (L.) Greuter & Burdet	0	n	1	0	Caprifoliaceae Juss.
Iz_1_1009	DJI_0217	Melampyrum pratense L.	0	n	1	0	Orobanchaceae Vent.
Iz_1_1009	DJI_0217	Oenothera biennis L.	0	a	0	0	Onagraceae Juss.
Iz_1_1009	DJI_0217	Pentanema britannica (L.) D.Gut.Larr., Santos-Vicente, Anderb., E.Rico & M.M.Mart.Ort.	0	n	1	0	Asteraceae Bercht. & J.Presl
Iz_1_1009	DJI_0217	Peucedanum oreoselinum (L.) Moench	0	n	1	0	Apiaceae Lindl.
Iz_1_1009	DJI_0217	Peucedanum ruthenicum M.Bieb.	0	n	1	0	Apiaceae Lindl.
Iz_1_1009	DJI_0217	Pilosella echioides (Lumn.) F.W.Schultz & Sch.Bip.	0	n	1	0	Asteraceae Bercht. & J.Presl
Iz_1_1009	DJI_0217	Pilosella officinarum F. Schultz et Sch. Bip.	0	n	1	0	Asteraceae Bercht. & J.Presl
Iz_1_1009	DJI_0217	Pilosella sp.	0		NA	0	Asteraceae Bercht. & J.Presl
Iz_1_1009	DJI_0217	Pinus sylvestris L.	0	n	1	1	Pinaceae Spreng. ex F.Rudolphi
Iz_1_1009	DJI_0217	Poa annua L.	0	n	1	0	Poaceae Barnhart
Iz_1_1009	DJI_0217	Poa nemoralis L.	0	n	1	0	Poaceae Barnhart
Iz_1_1009	DJI_0217	Poa sp.	0		NA	0	Poaceae Barnhart
Iz_1_1009	DJI_0217	Polygonatum odoratum (Mill.) Druce	0	n	1	0	Asparagaceae Juss.
Iz_1_1009	DJI_0217	Polygonum arenarium Waldst. & Kit.	0	n	1	0	Polygonaceae Juss.
Iz_1_1009	DJI_0217	Populus nigra L.	0	n	1	1	Salicaceae Mirb.
Iz_1_1009	DJI_0217	Populus tremula L.	0	n	1	1	Salicaceae Mirb.
Iz_1_1009	DJI_0217	Populus Ã canescens (Aiton) Sm.	0	n	1	1	Salicaceae Mirb.
Iz_1_1009	DJI_0217	Potentilla argentea L.	0	n	1	0	Rosaceae Juss.
Iz_1_1009	DJI_0217	Psammophiliella muralis (L.) Ikonn.	0	n	1	0	Caryophyllaceae Juss.
Iz_1_1009	DJI_0217	Psephellus sumensis (Kalen.) Greuter	0	n	1	0	Asteraceae Bercht. & J.Presl
Iz_1_1009	DJI_0217	Pulsatilla pratensis (L.) Mill. s.l.	0	n	1	0	Ranunculaceae Juss.
Iz_1_1009	DJI_0217	Pyrus communis L.	0	a	0	1	Rosaceae Juss.
Iz_1_1009	DJI_0217	Quercus robur L.	0	n	1	1	Fagaceae Dumort.
Iz_1_1009	DJI_0217	Robinia pseudoacacia L.	0	a	0	1	Fabaceae Lindl.
Iz_1_1009	DJI_0217	Rosa sp.	0		NA	1	Rosaceae Juss.
Iz_1_1009	DJI_0217	Rubus idaeus L.	0	n	1	0	Rosaceae Juss.
Iz_1_1009	DJI_0217	Rumex acetosella L.	0	n	1	0	Polygonaceae Juss.
Iz_1_1009	DJI_0217	Rumex thyrsiflorus Fingerh.	0	n	1	0	Polygonaceae Juss.
Iz_1_1009	DJI_0217	Salix alba L.	0	n	1	1	Salicaceae Mirb.
Iz_1_1009	DJI_0217	Sambucus nigra L.	0	n	1	1	Viburnaceae Raf.
Iz_1_1009	DJI_0217	Secale sylvestre Host	0	n	1	0	Poaceae Barnhart
Iz_1_1009	DJI_0217	Sempervivum ruthenicum Schnittsp. & C.B. Lehm.	0	n	1	0	Crassulaceae J.St.-Hil.
Iz_1_1009	DJI_0217	Setaria viridis (L.) P. Beauv.	0	n	1	0	Poaceae Barnhart
Iz_1_1009	DJI_0217	Silene borysthenica (Gruner) Walters	0	n	1	0	Caryophyllaceae Juss.
Iz_1_1009	DJI_0217	Silene latifolia subsp. alba (Mill.) Greuter & Burdet	0	n	1	0	Caryophyllaceae Juss.
Iz_1_1009	DJI_0217	Solidago virgaurea L.	0	n	1	0	Asteraceae Bercht. & J.Presl
Iz_1_1009	DJI_0217	Sorbus aucuparia L.	0	n	1	1	Rosaceae Juss.
Iz_1_1009	DJI_0217	Stachys recta L.	0	n	1	0	Lamiacea Lindl.
Iz_1_1009	DJI_0217	Tanacetum vulgare L.	0	n	1	0	Asteraceae Bercht. & J.Presl
Iz_1_1009	DJI_0217	Thymus pallasianus Heinr.Braun	0	n	1	0	Lamiacea Lindl.
Iz_1_1009	DJI_0217	Tilia cordata Mill.	0	n	1	1	Malvaceae Juss.
Iz_1_1009	DJI_0217	Trifolium alpestre L.	0	n	1	0	Fabaceae Lindl.
Iz_1_1009	DJI_0217	Trifolium arvense L.	0	n	1	0	Fabaceae Lindl.
Iz_1_1009	DJI_0217	Ulmus glabra Huds.	0	n	1	1	Ulmaceae Mirb.
Iz_1_1009	DJI_0217	Verbascum densiflorum Bertol.	0	n	1	0	Scrophulariaceae Juss.
Iz_1_1009	DJI_0217	Veronica austriaca L.	0	n	1	0	Plantaginaceae Juss.
Iz_1_1009	DJI_0217	Veronica incana L.	0	n	1	0	Plantaginaceae Juss.
Iz_1_1009	DJI_0217	Veronica verna L.	0	n	1	0	Plantaginaceae Juss.
Iz_1_1009	DJI_0217	Vincetoxicum hirundinaria Medik	0	n	1	0	Apocynaceae Juss.
Iz_1_1009	DJI_0218	Acer tataricum L.	0	n	1	1	Sapindaceae Juss.
Iz_1_1009	DJI_0218	Achillea millefolium L. s.l.	0	n	1	0	Asteraceae Bercht. & J.Presl
Iz_1_1009	DJI_0218	Allium oleraceum L.	0	n	1	0	Amaryllidaceae J.St.-Hil.
Iz_1_1009	DJI_0218	Ambrosia artemisiifolia L.	0	a	0	0	Asteraceae Bercht. & J.Presl
Iz_1_1009	DJI_0218	Amorpha fruticosa L.	0	a	0	0	Fabaceae Lindl.
Iz_1_1009	DJI_0218	Anthericum ramosum L.	0	n	1	0	Asparagaceae Juss.
Iz_1_1009	DJI_0218	Artemisia marschalliana Spreng.	0	n	1	0	Asteraceae Bercht. & J.Presl
Iz_1_1009	DJI_0218	Asparagus officinalis L.	0	n	1	0	Asparagaceae Juss.
Iz_1_1009	DJI_0218	Astragalus cicer L.	0	n	1	0	Fabaceae Lindl.
Iz_1_1009	DJI_0218	Bassia laniflora (S.G.Gmel.) A.J.Scott	0	n	1	0	Amaranthaceae Juss.
Iz_1_1009	DJI_0218	Berberis vulgaris L.	0	n	1	0	Berberidaceae Juss.
Iz_1_1009	DJI_0218	Betula pendula Roth.	0	n	1	1	Betulaceae Gray
Iz_1_1009	DJI_0218	Betula pendula Roth.	0	n	1	0	Poaceae Barnhart
Iz_1_1009	DJI_0218	Bromus inermis Leyss.	0		NA	NA	
Iz_1_1009	DJI_0218	Bromus squarrosus L.	0	a	0	0	Poaceae Barnhart
Iz_1_1009	DJI_0218	Bromus tectorum L.	0	a	0	0	Poaceae Barnhart
Iz_1_1009	DJI_0218	Calamagrostis epigejos (L.) Roth	70	n	1	0	Poaceae Barnhart
Iz_1_1009	DJI_0218	Carduus acanthoides L.	0	n	1	0	Asteraceae Bercht. & J.Presl
Iz_1_1009	DJI_0218	Carex sp.	0	n	1	0	Cyperaceae Juss.
Iz_1_1009	DJI_0218	Centaurea majorovii Dumbadze	0	n	1	0	Asteraceae Bercht. & J.Presl
Iz_1_1009	DJI_0218	Chamaecytisus ruthenicus (Fisch.ex Wol.) Klaskova	0	n	1	0	Fabaceae Lindl.

Iz_1_1009	DJI_0218	Chelidonium majus L.	0	n	1	0	Papaveraceae Juss.
Iz_1_1009	DJI_0218	Chenopodium betaceum Andrz.	0	n	1	0	Amaranthaceae Juss.
Iz_1_1009	DJI_0218	Chenopodium cf. album	2	n	1	0	Amaranthaceae Juss.
Iz_1_1009	DJI_0218	Chenopodium sp.	0		NA	0	Amaranthaceae Juss.
Iz_1_1009	DJI_0218	Chondrilla juncea L.	2	n	1	0	Asteraceae Bercht. & J.Presl
Iz_1_1009	DJI_0218	Cirsium vulgare (Savi) Ten.	0	n	1	0	Asteraceae Bercht. & J.Presl
Iz_1_1009	DJI_0218	Clinopodium acinos (L.) Kuntze	0	n	1	0	Lamiacea Lindl.
Iz_1_1009	DJI_0218	Convallaria majalis L.	0	n	1	0	Asparagaceae Juss.
Iz_1_1009	DJI_0218	Crataegus rhipidophylla Gand.	0	n	1	1	Rosaceae Juss.
Iz_1_1009	DJI_0218	Crepis foetida L.	0	n	1	0	Asteraceae Bercht. & J.Presl
Iz_1_1009	DJI_0218	Dactylis glomerata L.	0	n	1	0	Poaceae Barnhart
Iz_1_1009	DJI_0218	Dianthus campestris M.Bieb.	0	n	1	0	Caryophyllaceae Juss.
Iz_1_1009	DJI_0218	Digitaria sanguinalis (L.) Scop.	0	a	0	0	Poaceae Barnhart
Iz_1_1009	DJI_0218	Elymus repens (L.) Gould	0	n	1	0	Poaceae Barnhart
Iz_1_1009	DJI_0218	Epilobium tetragonum L.	0	n	1	0	Onagraceae Juss.
Iz_1_1009	DJI_0218	Eragrostis minor Host	0	a	0	0	Poaceae Barnhart
Iz_1_1009	DJI_0218	Erigeron canadensis L.	0	a	0	0	Asteraceae Bercht. & J.Presl
Iz_1_1009	DJI_0218	Euphorbia seguieriana Neck.	0	n	1	0	Euphorbiaceae Juss.
Iz_1_1009	DJI_0218	Fallopia dumetorum (L.) Holub	0	n	1	0	Polygonaceae Juss.
Iz_1_1009	DJI_0218	Festuca beckeri (Hack.) Trautv.	0	n	1	0	Poaceae Barnhart
Iz_1_1009	DJI_0218	Fragaria vesca L.	0	n	1	0	Rosaceae Juss.
Iz_1_1009	DJI_0218	Frangula alnus Mill.	0	n	1	1	Rhamnaceae Juss.
Iz_1_1009	DJI_0218	Fraxinus pennsylvanica Marshall	0	a	0	1	Oleaceae Hoffmanns. & Link
Iz_1_1009	DJI_0218	Galeopsis bifida Boenn.	0	n	1	0	Lamiacea Lindl.
Iz_1_1009	DJI_0218	Galium verum L.	0	n	1	0	Rubiaceae Juss.
Iz_1_1009	DJI_0218	Genista tinctoria L.	0	n	1	0	Fabaceae Lindl.
Iz_1_1009	DJI_0218	Geranium robertianum L.	0	n	1	0	Geraniaceae Juss.
Iz_1_1009	DJI_0218	Geranium sanquineum L.	0	n	1	0	Geraniaceae Juss.
Iz_1_1009	DJI_0218	Gypsophila paniculata L.	0	n	1	0	Caryophyllaceae Juss.
Iz_1_1009	DJI_0218	Helichrysum arenarium (L.) Moench	0	n	1	0	Asteraceae Bercht. & J.Presl
Iz_1_1009	DJI_0218	Herniaria polygama J.Gay	0	n	1	0	Caryophyllaceae Juss.
Iz_1_1009	DJI_0218	Hieracium robustum Fr.	0	n	1	0	Asteraceae Bercht. & J.Presl
Iz_1_1009	DJI_0218	Hieracium umbellatum L.	0	n	1	NA	Asteraceae Bercht. & J.Presl
Iz_1_1009	DJI_0218	Hylotelephium maximum (L.) Holub	0	n	1	0	Crassulaceae J.St.-Hil.
Iz_1_1009	DJI_0218	Jacobaea borysthenica (DC.) B.Nord. & Greuter	0	n	1	0	Asteraceae Bercht. & J.Presl
Iz_1_1009	DJI_0218	Jasione montana L.	0	n	1	0	Campanulaceae Juss.
Iz_1_1009	DJI_0218	Jurinea cyanoides (L.) Rchb.s.l.	0	n	1	0	Asteraceae Bercht. & J.Presl
Iz_1_1009	DJI_0218	Koeleria glauca (Spreng.) DC.	0	n	1	0	Poaceae Barnhart
Iz_1_1009	DJI_0218	Lactuca serriola L.	0	a	0	0	Asteraceae Bercht. & J.Presl
Iz_1_1009	DJI_0218	Linaria genistifolia (L.) Mill.	0	n	1	0	Plantaginaceae Juss.
Iz_1_1009	DJI_0218	Lomelosia argentea (L.) Greuter & Burdet	0	n	1	0	Caprifoliaceae Juss.
Iz_1_1009	DJI_0218	Melampyrum pratense L.	0	n	1	0	Orobanchaceae Vent.
Iz_1_1009	DJI_0218	Oenothera biennis L.	0	a	0	0	Onagraceae Juss.
Iz_1_1009	DJI_0218	Pentanema britannica (L.) D.Gut.Larr., Santos-Vicente, Anderb., E.Rico & M.M.Mart.Ort.	0	n	1	0	Asteraceae Bercht. & J.Presl
Iz_1_1009	DJI_0218	Peucedanum oreoselinum (L.) Moench	0	n	1	0	Apiaceae Lindl.
Iz_1_1009	DJI_0218	Peucedanum ruthenicum M.Bieb.	0	n	1	0	Apiaceae Lindl.
Iz_1_1009	DJI_0218	Pilosella echioides (Lumn.) F.W.Schultz & Sch.Bip.	0	n	1	0	Asteraceae Bercht. & J.Presl
Iz_1_1009	DJI_0218	Pilosella officinarum F. Schultz et Sch. Bip.	0	n	1	0	Asteraceae Bercht. & J.Presl
Iz_1_1009	DJI_0218	Pilosella sp.	0		NA	0	Asteraceae Bercht. & J.Presl
Iz_1_1009	DJI_0218	Pinus sylvestris L.	0	n	1	1	Pinaceae Spreng. ex F.Rudolphi
Iz_1_1009	DJI_0218	Poa annua L.	0	n	1	0	Poaceae Barnhart
Iz_1_1009	DJI_0218	Poa nemoralis L.	0	n	1	0	Poaceae Barnhart
Iz_1_1009	DJI_0218	Poa sp.	0		NA	0	Poaceae Barnhart
Iz_1_1009	DJI_0218	Polygonatum odoratum (Mill.) Druce	0	n	1	0	Asparagaceae Juss.
Iz_1_1009	DJI_0218	Polygonum arenarium Waldst. & Kit.	0	n	1	0	Polygonaceae Juss.
Iz_1_1009	DJI_0218	Populus nigra L.	0	n	1	1	Salicaceae Mirb.
Iz_1_1009	DJI_0218	Populus tremula L.	0	n	1	1	Salicaceae Mirb.
Iz_1_1009	DJI_0218	Populus Ã canescens (Aiton) Sm.	0	n	1	1	Salicaceae Mirb.
Iz_1_1009	DJI_0218	Potentilla argentea L.	0	n	1	0	Rosaceae Juss.
Iz_1_1009	DJI_0218	Psammophiliella muralis (L.) Ikonn.	0	n	1	0	Caryophyllaceae Juss.
Iz_1_1009	DJI_0218	Psephellus sumensis (Kalen.) Greuter	0	n	1	0	Asteraceae Bercht. & J.Presl
Iz_1_1009	DJI_0218	Pulsatilla pratensis (L.) Mill. s.l.	0	n	1	0	Ranunculaceae Juss.
Iz_1_1009	DJI_0218	Pyrus communis L.	0	a	0	1	Rosaceae Juss.
Iz_1_1009	DJI_0218	Quercus robur L.	0	n	1	1	Fagaceae Dumort.
Iz_1_1009	DJI_0218	Robinia pseudoacacia L.	0	a	0	1	Fabaceae Lindl.
Iz_1_1009	DJI_0218	Rosa sp.	0		NA	1	Rosaceae Juss.
Iz_1_1009	DJI_0218	Rubus idaeus L.	0	n	1	0	Rosaceae Juss.
Iz_1_1009	DJI_0218	Rumex acetosella L.	0	n	1	0	Polygonaceae Juss.
Iz_1_1009	DJI_0218	Rumex thyrsiflorus Fingerh.	0	n	1	0	Polygonaceae Juss.
Iz_1_1009	DJI_0218	Salix alba L.	0	n	1	1	Salicaceae Mirb.
Iz_1_1009	DJI_0218	Sambucus nigra L.	0	n	1	1	Viburnaceae Raf.
Iz_1_1009	DJI_0218	Secale sylvestre Host	0	n	1	0	Poaceae Barnhart
Iz_1_1009	DJI_0218	Sempervivum ruthenicum Schnittsp. & C.B. Lehm.	0	n	1	0	Crassulaceae J.St.-Hil.
Iz_1_1009	DJI_0218	Setaria viridis (L.) P. Beauv.	0	n	1	0	Poaceae Barnhart
Iz_1_1009	DJI_0218	Silene borysthenica (Gruner) Walters	0	n	1	0	Caryophyllaceae Juss.
Iz_1_1009	DJI_0218	Silene latifolia subsp. alba (Mill.) Greuter & Burdet	0	n	1	0	Caryophyllaceae Juss.
Iz_1_1009	DJI_0218	Solidago virgaurea L.	0	n	1	0	Asteraceae Bercht. & J.Presl
Iz_1_1009	DJI_0218	Sorbus aucuparia L.	0	n	1	1	Rosaceae Juss.
Iz_1_1009	DJI_0218	Stachys recta L.	0	n	1	0	Lamiacea Lindl.
Iz_1_1009	DJI_0218	Tanacetum vulgare L.	0	n	1	0	Asteraceae Bercht. & J.Presl
Iz_1_1009	DJI_0218	Thymus pallasianus Heinr.Braun	0	n	1	0	Lamiacea Lindl.
Iz_1_1009	DJI_0218	Tilia cordata Mill.	0	n	1	1	Malvaceae Juss.
Iz_1_1009	DJI_0218	Trifolium alpestre L.	0	n	1	0	Fabaceae Lindl.
Iz_1_1009	DJI_0218	Trifolium arvense L.	0	n	1	0	Fabaceae Lindl.
Iz_1_1009	DJI_0218	Ulmus glabra Huds.	0	n	1	1	Ulmaceae Mirb.
Iz_1_1009	DJI_0218	Verbascum densiflorum Bertol.	0	n	1	0	Scrophulariaceae Juss.
Iz_1_1009	DJI_0218	Veronica austriaca L.	0	n	1	0	Plantaginaceae Juss.
Iz_1_1009	DJI_0218	Veronica incana L.	0	n	1	0	Plantaginaceae Juss.
Iz_1_1009	DJI_0218	Veronica verna L.	0	n	1	0	Plantaginaceae Juss.
Iz_1_1009	DJI_0218	Vincetoxicum hirundinaria Medik	0	n	1	0	Apocynaceae Juss.
Iz_1_1009	DJI_0220	Acer tataricum L.	0	n	1	1	Sapindaceae Juss.
Iz_1_1009	DJI_0220	Achillea millefolium L. s.l.	0	n	1	0	Asteraceae Bercht. & J.Presl
Iz_1_1009	DJI_0220	Allium oleraceum L.	0	n	1	0	Amaryllidaceae J.St.-Hil.
Iz_1_1009	DJI_0220	Ambrosia artemisiifolia L.	0	a	0	0	Asteraceae Bercht. & J.Presl
Iz_1_1009	DJI_0220	Amorpha fruticosa L.	0	a	0	0	Fabaceae Lindl.
Iz_1_1009	DJI_0220	Anthericum ramosum L.	0	n	1	0	Asparagaceae Juss.
Iz_1_1009	DJI_0220	Artemisia marschalliana Spreng.	0	n	1	0	Asteraceae Bercht. & J.Presl
Iz_1_1009	DJI_0220	Asparagus officinalis L.	0	n	1	0	Asparagaceae Juss.

Iz_1_1009	DJI_0220	Astragalus cicer L.	0	n	1	0	Fabaceae Lindl.
Iz_1_1009	DJI_0220	Bassia laniflora (S.G.Gmel.) A.J.Scott	0	n	1	0	Amaranthaceae Juss.
Iz_1_1009	DJI_0220	Berberis vulgaris L.	0	n	1	0	Berberidaceae Juss.
Iz_1_1009	DJI_0220	Betula pendula Roth.	0	n	1	1	Betulaceae Gray
Iz_1_1009	DJI_0220	Betula pendula Roth.	0	n	1	0	Poaceae Barnhart
Iz_1_1009	DJI_0220	Bromus inermis Leyss.	0		NA	NA	
Iz_1_1009	DJI_0220	Bromus squarrosus L.	0	a	0	0	Poaceae Barnhart
Iz_1_1009	DJI_0220	Bromus tectorum L.	0	a	0	0	Poaceae Barnhart
Iz_1_1009	DJI_0220	Calamagrostis epigejos (L.) Roth	50	n	1	0	Poaceae Barnhart
Iz_1_1009	DJI_0220	Carduus acanthoides L.	0	n	1	0	Asteraceae Bercht. & J.Presl
Iz_1_1009	DJI_0220	Carex sp.	0	n	1	0	Cyperaceae Juss.
Iz_1_1009	DJI_0220	Centaurea majorovii Dumbadze	0	n	1	0	Asteraceae Bercht. & J.Presl
Iz_1_1009	DJI_0220	Chamaecytisus ruthenicus (Fisch.ex Wol.) Klaskova	0	n	1	0	Fabaceae Lindl.
Iz_1_1009	DJI_0220	Chelidonium majus L.	0	n	1	0	Papaveraceae Juss.
Iz_1_1009	DJI_0220	Chenopodium betaceum Andrz.	0	n	1	0	Amaranthaceae Juss.
Iz_1_1009	DJI_0220	Chenopodium cf. album	2	n	1	0	Amaranthaceae Juss.
Iz_1_1009	DJI_0220	Chenopodium sp.	0		NA	0	Amaranthaceae Juss.
Iz_1_1009	DJI_0220	Chondrilla juncea L.	2	n	1	0	Asteraceae Bercht. & J.Presl
Iz_1_1009	DJI_0220	Cirsium vulgare (Savi) Ten.	0	n	1	0	Asteraceae Bercht. & J.Presl
Iz_1_1009	DJI_0220	Clinopodium acinos (L.) Kuntze	0	n	1	0	Lamiacea Lindl.
Iz_1_1009	DJI_0220	Convallaria majalis L.	0	n	1	0	Asparagaceae Juss.
Iz_1_1009	DJI_0220	Crataegus rhipidophylla Gand.	0	n	1	1	Rosaceae Juss.
Iz_1_1009	DJI_0220	Crepis foetida L.	0	n	1	0	Asteraceae Bercht. & J.Presl
Iz_1_1009	DJI_0220	Dactylis glomerata L.	0	n	1	0	Poaceae Barnhart
Iz_1_1009	DJI_0220	Dianthus campestris M.Bieb.	0	n	1	0	Caryophyllaceae Juss.
Iz_1_1009	DJI_0220	Digitaria sanguinalis (L.) Scop.	0	a	0	0	Poaceae Barnhart
Iz_1_1009	DJI_0220	Elymus repens (L.) Gould	0	n	1	0	Poaceae Barnhart
Iz_1_1009	DJI_0220	Epilobium tetragonum L.	0	n	1	0	Onagraceae Juss.
Iz_1_1009	DJI_0220	Eragrostis minor Host	0	a	0	0	Poaceae Barnhart
Iz_1_1009	DJI_0220	Erigeron canadensis L.	10	a	0	0	Asteraceae Bercht. & J.Presl
Iz_1_1009	DJI_0220	Euphorbia seguieriana Neck.	0	n	1	0	Euphorbiaceae Juss.
Iz_1_1009	DJI_0220	Fallopia dumetorum (L.) Holub	0	n	1	0	Polygonaceae Juss.
Iz_1_1009	DJI_0220	Festuca beckeri (Hack.) Trautv.	0	n	1	0	Poaceae Barnhart
Iz_1_1009	DJI_0220	Fragaria vesca L.	0	n	1	0	Rosaceae Juss.
Iz_1_1009	DJI_0220	Frangula alnus Mill.	0	n	1	1	Rhamnaceae Juss.
Iz_1_1009	DJI_0220	Fraxinus pennsylvanica Marshall	0	a	0	1	Oleaceae Hoffmanns. & Link
Iz_1_1009	DJI_0220	Galeopsis bifida Boenn.	0	n	1	0	Lamiacea Lindl.
Iz_1_1009	DJI_0220	Galium verum L.	0	n	1	0	Rubiaceae Juss.
Iz_1_1009	DJI_0220	Genista tinctoria L.	0	n	1	0	Fabaceae Lindl.
Iz_1_1009	DJI_0220	Geranium robertianum L.	0	n	1	0	Geraniaceae Juss.
Iz_1_1009	DJI_0220	Geranium sanquineum L.	0	n	1	0	Geraniaceae Juss.
Iz_1_1009	DJI_0220	Gypsophila paniculata L.	0	n	1	0	Caryophyllaceae Juss.
Iz_1_1009	DJI_0220	Helichrysum arenarium (L.) Moench	0	n	1	0	Asteraceae Bercht. & J.Presl
Iz_1_1009	DJI_0220	Herniaria polygama J.Gay	0	n	1	0	Caryophyllaceae Juss.
Iz_1_1009	DJI_0220	Hieracium robustum Fr.	0	n	1	0	Asteraceae Bercht. & J.Presl
Iz_1_1009	DJI_0220	Hieracium umbellatum L.	0	n	1	NA	Asteraceae Bercht. & J.Presl
Iz_1_1009	DJI_0220	Hylotelephium maximum (L.) Holub	0	n	1	0	Crassulaceae J.St.-Hil.
Iz_1_1009	DJI_0220	Jacobaea borysthenica (DC.) B.Nord. & Greuter	0	n	1	0	Asteraceae Bercht. & J.Presl
Iz_1_1009	DJI_0220	Jasione montana L.	0	n	1	0	Campanulaceae Juss.
Iz_1_1009	DJI_0220	Jurinea cyanoides (L.) Rchb.s.l.	0	n	1	0	Asteraceae Bercht. & J.Presl
Iz_1_1009	DJI_0220	Koeleria glauca (Spreng.) DC.	0	n	1	0	Poaceae Barnhart
Iz_1_1009	DJI_0220	Lactuca serriola L.	0	a	0	0	Asteraceae Bercht. & J.Presl
Iz_1_1009	DJI_0220	Linaria genistifolia (L.) Mill.	0	n	1	0	Plantaginaceae Juss.
Iz_1_1009	DJI_0220	Lomelosia argentea (L.) Greuter & Burdet	0	n	1	0	Caprifoliaceae Juss.
Iz_1_1009	DJI_0220	Melampyrum pratense L.	0	n	1	0	Orobanchaceae Vent.
Iz_1_1009	DJI_0220	Oenothera biennis L.	0	a	0	0	Onagraceae Juss.
Iz_1_1009	DJI_0220	Pentanema britannica (L.) D.Gut.Larr., Santos-Vicente, Anderb., E.Rico & M.M.Mart.Ort.	0	n	1	0	Asteraceae Bercht. & J.Presl
Iz_1_1009	DJI_0220	Peucedanum oreoselinum (L.) Moench	0	n	1	0	Apiaceae Lindl.
Iz_1_1009	DJI_0220	Peucedanum ruthenicum M.Bieb.	0	n	1	0	Apiaceae Lindl.
Iz_1_1009	DJI_0220	Pilosella echioides (Lumn.) F.W.Schultz & Sch.Bip.	0	n	1	0	Asteraceae Bercht. & J.Presl
Iz_1_1009	DJI_0220	Pilosella officinarum F. Schultz et Sch. Bip.	0	n	1	0	Asteraceae Bercht. & J.Presl
Iz_1_1009	DJI_0220	Pilosella sp.	0		NA	0	Asteraceae Bercht. & J.Presl
Iz_1_1009	DJI_0220	Pinus sylvestris L.	0	n	1	1	Pinaceae Spreng. ex F.Rudolphi
Iz_1_1009	DJI_0220	Poa annua L.	0	n	1	0	Poaceae Barnhart
Iz_1_1009	DJI_0220	Poa nemoralis L.	0	n	1	0	Poaceae Barnhart
Iz_1_1009	DJI_0220	Poa sp.	0		NA	0	Poaceae Barnhart
Iz_1_1009	DJI_0220	Polygonatum odoratum (Mill.) Druce	0	n	1	0	Asparagaceae Juss.
Iz_1_1009	DJI_0220	Polygonum arenarium Waldst. & Kit.	0	n	1	0	Polygonaceae Juss.
Iz_1_1009	DJI_0220	Populus nigra L.	0	n	1	1	Salicaceae Mirb.
Iz_1_1009	DJI_0220	Populus tremula L.	0	n	1	1	Salicaceae Mirb.
Iz_1_1009	DJI_0220	Populus Ã canescens (Aiton) Sm.	0	n	1	1	Salicaceae Mirb.
Iz_1_1009	DJI_0220	Potentilla argentea L.	0	n	1	0	Rosaceae Juss.
Iz_1_1009	DJI_0220	Psammophiliella muralis (L.) Ikonn.	0	n	1	0	Caryophyllaceae Juss.
Iz_1_1009	DJI_0220	Psephellus sumensis (Kalen.) Greuter	0	n	1	0	Asteraceae Bercht. & J.Presl
Iz_1_1009	DJI_0220	Pulsatilla pratensis (L.) Mill. s.l.	0	n	1	0	Ranunculaceae Juss.
Iz_1_1009	DJI_0220	Pyrus communis L.	0	a	0	1	Rosaceae Juss.
Iz_1_1009	DJI_0220	Quercus robur L.	0	n	1	1	Fagaceae Dumort.
Iz_1_1009	DJI_0220	Robinia pseudoacacia L.	0	a	0	1	Fabaceae Lindl.
Iz_1_1009	DJI_0220	Rosa sp.	0		NA	1	Rosaceae Juss.
Iz_1_1009	DJI_0220	Rubus idaeus L.	0	n	1	0	Rosaceae Juss.
Iz_1_1009	DJI_0220	Rumex acetosella L.	0	n	1	0	Polygonaceae Juss.
Iz_1_1009	DJI_0220	Rumex thyrsiflorus Fingerh.	0	n	1	0	Polygonaceae Juss.
Iz_1_1009	DJI_0220	Salix alba L.	0	n	1	1	Salicaceae Mirb.
Iz_1_1009	DJI_0220	Sambucus nigra L.	0	n	1	1	Viburnaceae Raf.
Iz_1_1009	DJI_0220	Secale sylvestre Host	0	n	1	0	Poaceae Barnhart
Iz_1_1009	DJI_0220	Sempervivum ruthenicum Schnittsp. & C.B. Lehm.	0	n	1	0	Crassulaceae J.St.-Hil.
Iz_1_1009	DJI_0220	Setaria viridis (L.) P. Beauv.	0	n	1	0	Poaceae Barnhart
Iz_1_1009	DJI_0220	Silene borysthenica (Gruner) Walters	0	n	1	0	Caryophyllaceae Juss.
Iz_1_1009	DJI_0220	Silene latifolia subsp. alba (Mill.) Greuter & Burdet	0	n	1	0	Caryophyllaceae Juss.
Iz_1_1009	DJI_0220	Solidago virgaurea L.	2	n	1	0	Asteraceae Bercht. & J.Presl
Iz_1_1009	DJI_0220	Sorbus aucuparia L.	0	n	1	1	Rosaceae Juss.
Iz_1_1009	DJI_0220	Stachys recta L.	0	n	1	0	Lamiacea Lindl.
Iz_1_1009	DJI_0220	Tanacetum vulgare L.	0	n	1	0	Asteraceae Bercht. & J.Presl
Iz_1_1009	DJI_0220	Thymus pallasianus Heinr.Braun	0	n	1	0	Lamiacea Lindl.
Iz_1_1009	DJI_0220	Tilia cordata Mill.	0	n	1	1	Malvaceae Juss.
Iz_1_1009	DJI_0220	Trifolium alpestre L.	0	n	1	0	Fabaceae Lindl.
Iz_1_1009	DJI_0220	Trifolium arvense L.	0	n	1	0	Fabaceae Lindl.
Iz_1_1009	DJI_0220	Ulmus glabra Huds.	0	n	1	1	Ulmaceae Mirb.

Iz_1_1009	DJI_0220	Verbascum densiflorum Bertol.	0	n	1	0	Scrophulariaceae Juss.
Iz_1_1009	DJI_0220	Veronica austriaca L.	0	n	1	0	Plantaginaceae Juss.
Iz_1_1009	DJI_0220	Veronica incana L.	0	n	1	0	Plantaginaceae Juss.
Iz_1_1009	DJI_0220	Veronica verna L.	0	n	1	0	Plantaginaceae Juss.
Iz_1_1009	DJI_0220	Vincetoxicum hirundinaria Medik	0	n	1	0	Apocynaceae Juss.
Iz_1_1009	DJI_0229	Acer tataricum L.	0	n	1	1	Sapindaceae Juss.
Iz_1_1009	DJI_0229	Achillea millefolium L. s.l.	0	n	1	0	Asteraceae Bercht. & J.Presl
Iz_1_1009	DJI_0229	Allium oleraceum L.	0	n	1	0	Amaryllidaceae J.St.-Hil.
Iz_1_1009	DJI_0229	Ambrosia artemisiifolia L.	0	a	0	0	Asteraceae Bercht. & J.Presl
Iz_1_1009	DJI_0229	Amorpha fruticosa L.	0	a	0	0	Fabaceae Lindl.
Iz_1_1009	DJI_0229	Anthericum ramosum L.	0	n	1	0	Asparagaceae Juss.
Iz_1_1009	DJI_0229	Artemisia marschalliana Spreng.	0	n	1	0	Asteraceae Bercht. & J.Presl
Iz_1_1009	DJI_0229	Asparagus officinalis L.	0	n	1	0	Asparagaceae Juss.
Iz_1_1009	DJI_0229	Astragalus cicer L.	0	n	1	0	Fabaceae Lindl.
Iz_1_1009	DJI_0229	Bassia laniflora (S.G.Gmel.) A.J.Scott	0	n	1	0	Amaranthaceae Juss.
Iz_1_1009	DJI_0229	Berberis vulgaris L.	0	n	1	0	Berberidaceae Juss.
Iz_1_1009	DJI_0229	Betula pendula Roth.	0	n	1	1	Betulaceae Gray
Iz_1_1009	DJI_0229	Betula pendula Roth.	0	n	1	0	Poaceae Barnhart
Iz_1_1009	DJI_0229	Bromus inermis Leyss.	0		NA	NA	
Iz_1_1009	DJI_0229	Bromus squarrosus L.	0	a	0	0	Poaceae Barnhart
Iz_1_1009	DJI_0229	Bromus tectorum L.	0	a	0	0	Poaceae Barnhart
Iz_1_1009	DJI_0229	Calamagrostis epigejos (L.) Roth	45	n	1	0	Poaceae Barnhart
Iz_1_1009	DJI_0229	Carduus acanthoides L.	0	n	1	0	Asteraceae Bercht. & J.Presl
Iz_1_1009	DJI_0229	Carex sp.	0	n	1	0	Cyperaceae Juss.
Iz_1_1009	DJI_0229	Centaurea majorovii Dumbadze	0	n	1	0	Asteraceae Bercht. & J.Presl
Iz_1_1009	DJI_0229	Chamaecytisus ruthenicus (Fisch.ex Wol.) Klaskova	0	n	1	0	Fabaceae Lindl.
Iz_1_1009	DJI_0229	Chelidonium majus L.	0	n	1	0	Papaveraceae Juss.
Iz_1_1009	DJI_0229	Chenopodium betaceum Andrz.	0	n	1	0	Amaranthaceae Juss.
Iz_1_1009	DJI_0229	Chenopodium cf. album	0	n	1	0	Amaranthaceae Juss.
Iz_1_1009	DJI_0229	Chenopodium sp.	0		NA	0	Amaranthaceae Juss.
Iz_1_1009	DJI_0229	Chondrilla juncea L.	0	n	1	0	Asteraceae Bercht. & J.Presl
Iz_1_1009	DJI_0229	Cirsium vulgare (Savi) Ten.	0	n	1	0	Asteraceae Bercht. & J.Presl
Iz_1_1009	DJI_0229	Clinopodium acinos (L.) Kuntze	0	n	1	0	Lamiacea Lindl.
Iz_1_1009	DJI_0229	Convallaria majalis L.	0	n	1	0	Asparagaceae Juss.
Iz_1_1009	DJI_0229	Crataegus rhipidophylla Gand.	0	n	1	1	Rosaceae Juss.
Iz_1_1009	DJI_0229	Crepis foetida L.	0	n	1	0	Asteraceae Bercht. & J.Presl
Iz_1_1009	DJI_0229	Dactylis glomerata L.	0	n	1	0	Poaceae Barnhart
Iz_1_1009	DJI_0229	Dianthus campestris M.Bieb.	0	n	1	0	Caryophyllaceae Juss.
Iz_1_1009	DJI_0229	Digitaria sanguinalis (L.) Scop.	0	a	0	0	Poaceae Barnhart
Iz_1_1009	DJI_0229	Elymus repens (L.) Gould	0	n	1	0	Poaceae Barnhart
Iz_1_1009	DJI_0229	Epilobium tetragonum L.	0	n	1	0	Onagraceae Juss.
Iz_1_1009	DJI_0229	Eragrostis minor Host	0	a	0	0	Poaceae Barnhart
Iz_1_1009	DJI_0229	Erigeron canadensis L.	7	a	0	0	Asteraceae Bercht. & J.Presl
Iz_1_1009	DJI_0229	Euphorbia seguieriana Neck.	0	n	1	0	Euphorbiaceae Juss.
Iz_1_1009	DJI_0229	Fallopia dumetorum (L.) Holub	0	n	1	0	Polygonaceae Juss.
Iz_1_1009	DJI_0229	Festuca beckeri (Hack.) Trautv.	0	n	1	0	Poaceae Barnhart
Iz_1_1009	DJI_0229	Fragaria vesca L.	0	n	1	0	Rosaceae Juss.
Iz_1_1009	DJI_0229	Frangula alnus Mill.	0	n	1	1	Rhamnaceae Juss.
Iz_1_1009	DJI_0229	Fraxinus pennsylvanica Marshall	0	a	0	1	Oleaceae Hoffmanns. & Link
Iz_1_1009	DJI_0229	Galeopsis bifida Boenn.	0	n	1	0	Lamiacea Lindl.
Iz_1_1009	DJI_0229	Galium verum L.	0	n	1	0	Rubiaceae Juss.
Iz_1_1009	DJI_0229	Genista tinctoria L.	0	n	1	0	Fabaceae Lindl.
Iz_1_1009	DJI_0229	Geranium robertianum L.	0	n	1	0	Geraniaceae Juss.
Iz_1_1009	DJI_0229	Geranium sanquineum L.	0	n	1	0	Geraniaceae Juss.
Iz_1_1009	DJI_0229	Gypsophila paniculata L.	0	n	1	0	Caryophyllaceae Juss.
Iz_1_1009	DJI_0229	Helichrysum arenarium (L.) Moench	0	n	1	0	Asteraceae Bercht. & J.Presl
Iz_1_1009	DJI_0229	Herniaria polygama J.Gay	0	n	1	0	Caryophyllaceae Juss.
Iz_1_1009	DJI_0229	Hieracium robustum Fr.	0	n	1	0	Asteraceae Bercht. & J.Presl
Iz_1_1009	DJI_0229	Hieracium umbellatum L.	0	n	1	NA	Asteraceae Bercht. & J.Presl
Iz_1_1009	DJI_0229	Hylotelephium maximum (L.) Holub	0	n	1	0	Crassulaceae J.St.-Hil.
Iz_1_1009	DJI_0229	Jacobaea borysthenica (DC.) B.Nord. & Greuter	0	n	1	0	Asteraceae Bercht. & J.Presl
Iz_1_1009	DJI_0229	Jasione montana L.	0	n	1	0	Campanulaceae Juss.
Iz_1_1009	DJI_0229	Jurinea cyanoides (L.) Rchb.s.l.	0	n	1	0	Asteraceae Bercht. & J.Presl
Iz_1_1009	DJI_0229	Koeleria glauca (Spreng.) DC.	0	n	1	0	Poaceae Barnhart
Iz_1_1009	DJI_0229	Lactuca serriola L.	0	a	0	0	Asteraceae Bercht. & J.Presl
Iz_1_1009	DJI_0229	Linaria genistifolia (L.) Mill.	0	n	1	0	Plantaginaceae Juss.
Iz_1_1009	DJI_0229	Lomelosia argentea (L.) Greuter & Burdet	0	n	1	0	Caprifoliaceae Juss.
Iz_1_1009	DJI_0229	Melampyrum pratense L.	0	n	1	0	Orobanchaceae Vent.
Iz_1_1009	DJI_0229	Oenothera biennis L.	0	a	0	0	Onagraceae Juss.
Iz_1_1009	DJI_0229	Pentanema britannica (L.) D.Gut.Larr., Santos-Vicente, Anderb., E.Rico & M.M.Mart.Ort.	0	n	1	0	Asteraceae Bercht. & J.Presl
Iz_1_1009	DJI_0229	Peucedanum oreoselinum (L.) Moench	0	n	1	0	Apiaceae Lindl.
Iz_1_1009	DJI_0229	Peucedanum ruthenicum M.Bieb.	0	n	1	0	Apiaceae Lindl.
Iz_1_1009	DJI_0229	Pilosella echioides (Lumn.) F.W.Schultz & Sch.Bip.	0	n	1	0	Asteraceae Bercht. & J.Presl
Iz_1_1009	DJI_0229	Pilosella officinarum F. Schultz et Sch. Bip.	0	n	1	0	Asteraceae Bercht. & J.Presl
Iz_1_1009	DJI_0229	Pilosella sp.	0		NA	0	Asteraceae Bercht. & J.Presl
Iz_1_1009	DJI_0229	Pinus sylvestris L.	0	n	1	1	Pinaceae Spreng. ex F.Rudolphi
Iz_1_1009	DJI_0229	Poa annua L.	0	n	1	0	Poaceae Barnhart
Iz_1_1009	DJI_0229	Poa nemoralis L.	0	n	1	0	Poaceae Barnhart
Iz_1_1009	DJI_0229	Poa sp.	0		NA	0	Poaceae Barnhart
Iz_1_1009	DJI_0229	Polygonatum odoratum (Mill.) Druce	0	n	1	0	Asparagaceae Juss.
Iz_1_1009	DJI_0229	Polygonum arenarium Waldst. & Kit.	0	n	1	0	Polygonaceae Juss.
Iz_1_1009	DJI_0229	Populus nigra L.	0	n	1	1	Salicaceae Mirb.
Iz_1_1009	DJI_0229	Populus tremula L.	0	n	1	1	Salicaceae Mirb.
Iz_1_1009	DJI_0229	Populus Ã canescens (Aiton) Sm.	0	n	1	1	Salicaceae Mirb.
Iz_1_1009	DJI_0229	Potentilla argentea L.	0	n	1	0	Rosaceae Juss.
Iz_1_1009	DJI_0229	Psammophiliella muralis (L.) Ikonn.	0	n	1	0	Caryophyllaceae Juss.
Iz_1_1009	DJI_0229	Psephellus sumensis (Kalen.) Greuter	0	n	1	0	Asteraceae Bercht. & J.Presl
Iz_1_1009	DJI_0229	Pulsatilla pratensis (L.) Mill. s.l.	0	n	1	0	Ranunculaceae Juss.
Iz_1_1009	DJI_0229	Pyrus communis L.	0	a	0	1	Rosaceae Juss.
Iz_1_1009	DJI_0229	Quercus robur L.	10	n	1	1	Fagaceae Dumort.
Iz_1_1009	DJI_0229	Robinia pseudoacacia L.	0	a	0	1	Fabaceae Lindl.
Iz_1_1009	DJI_0229	Rosa sp.	0		NA	1	Rosaceae Juss.
Iz_1_1009	DJI_0229	Rubus idaeus L.	0	n	1	0	Rosaceae Juss.
Iz_1_1009	DJI_0229	Rumex acetosella L.	2	n	1	0	Polygonaceae Juss.
Iz_1_1009	DJI_0229	Rumex thyrsiflorus Fingerh.	0	n	1	0	Polygonaceae Juss.
Iz_1_1009	DJI_0229	Salix alba L.	0	n	1	1	Salicaceae Mirb.
Iz_1_1009	DJI_0229	Sambucus nigra L.	0	n	1	1	Viburnaceae Raf.
Iz_1_1009	DJI_0229	Secale sylvestre Host	0	n	1	0	Poaceae Barnhart

Iz_1_1009	DJI_0229	Sempervivum ruthenicum Schnittsp. & C.B. Lehm.	0	n	1	0	Crassulaceae J.St.-Hil.
Iz_1_1009	DJI_0229	Setaria viridis (L.) P. Beauv.	0	n	1	0	Poaceae Barnhart
Iz_1_1009	DJI_0229	Silene borysthenica (Gruner) Walters	0	n	1	0	Caryophyllaceae Juss.
Iz_1_1009	DJI_0229	Silene latifolia subsp. alba (Mill.) Greuter & Burdet	0	n	1	0	Caryophyllaceae Juss.
Iz_1_1009	DJI_0229	Solidago virgaurea L.	0	n	1	0	Asteraceae Bercht. & J.Presl
Iz_1_1009	DJI_0229	Sorbus aucuparia L.	0	n	1	1	Rosaceae Juss.
Iz_1_1009	DJI_0229	Stachys recta L.	0	n	1	0	Lamiacea Lindl.
Iz_1_1009	DJI_0229	Tanacetum vulgare L.	0	n	1	0	Asteraceae Bercht. & J.Presl
Iz_1_1009	DJI_0229	Thymus pallasianus Heinr.Braun	0	n	1	0	Lamiacea Lindl.
Iz_1_1009	DJI_0229	Tilia cordata Mill.	0	n	1	1	Malvaceae Juss.
Iz_1_1009	DJI_0229	Trifolium alpestre L.	0	n	1	0	Fabaceae Lindl.
Iz_1_1009	DJI_0229	Trifolium arvense L.	0	n	1	0	Fabaceae Lindl.
Iz_1_1009	DJI_0229	Ulmus glabra Huds.	0	n	1	1	Ulmaceae Mirb.
Iz_1_1009	DJI_0229	Verbascum densiflorum Bertol.	0	n	1	0	Scrophulariaceae Juss.
Iz_1_1009	DJI_0229	Veronica austriaca L.	0	n	1	0	Plantaginaceae Juss.
Iz_1_1009	DJI_0229	Veronica incana L.	0	n	1	0	Plantaginaceae Juss.
Iz_1_1009	DJI_0229	Veronica verna L.	0	n	1	0	Plantaginaceae Juss.
Iz_1_1009	DJI_0229	Vincetoxicum hirundinaria Medik	0	n	1	0	Apocynaceae Juss.
Iz_1_1009	DJI_0230	Acer tataricum L.	0	n	1	1	Sapindaceae Juss.
Iz_1_1009	DJI_0230	Achillea millefolium L. s.l.	0	n	1	0	Asteraceae Bercht. & J.Presl
Iz_1_1009	DJI_0230	Allium oleraceum L.	0	n	1	0	Amaryllidaceae J.St.-Hil.
Iz_1_1009	DJI_0230	Ambrosia artemisiifolia L.	0	a	0	0	Asteraceae Bercht. & J.Presl
Iz_1_1009	DJI_0230	Amorpha fruticosa L.	0	a	0	0	Fabaceae Lindl.
Iz_1_1009	DJI_0230	Anthericum ramosum L.	0	n	1	0	Asparagaceae Juss.
Iz_1_1009	DJI_0230	Artemisia marschalliana Spreng.	0	n	1	0	Asteraceae Bercht. & J.Presl
Iz_1_1009	DJI_0230	Asparagus officinalis L.	0	n	1	0	Asparagaceae Juss.
Iz_1_1009	DJI_0230	Astragalus cicer L.	0	n	1	0	Fabaceae Lindl.
Iz_1_1009	DJI_0230	Bassia laniflora (S.G.Gmel.) A.J.Scott	0	n	1	0	Amaranthaceae Juss.
Iz_1_1009	DJI_0230	Berberis vulgaris L.	0	n	1	0	Berberidaceae Juss.
Iz_1_1009	DJI_0230	Betula pendula Roth.	0	n	1	1	Betulaceae Gray
Iz_1_1009	DJI_0230	Betula pendula Roth.	0	n	1	0	Poaceae Barnhart
Iz_1_1009	DJI_0230	Bromus inermis Leyss.	0		NA	NA	
Iz_1_1009	DJI_0230	Bromus squarrosus L.	0	a	0	0	Poaceae Barnhart
Iz_1_1009	DJI_0230	Bromus tectorum L.	0	a	0	0	Poaceae Barnhart
Iz_1_1009	DJI_0230	Calamagrostis epigejos (L.) Roth	30	n	1	0	Poaceae Barnhart
Iz_1_1009	DJI_0230	Carduus acanthoides L.	0	n	1	0	Asteraceae Bercht. & J.Presl
Iz_1_1009	DJI_0230	Carex sp.	0	n	1	0	Cyperaceae Juss.
Iz_1_1009	DJI_0230	Centaurea majorovii Dumbadze	0	n	1	0	Asteraceae Bercht. & J.Presl
Iz_1_1009	DJI_0230	Chamaecytisus ruthenicus (Fisch.ex Wol.) Klaskova	0	n	1	0	Fabaceae Lindl.
Iz_1_1009	DJI_0230	Chelidonium majus L.	0	n	1	0	Papaveraceae Juss.
Iz_1_1009	DJI_0230	Chenopodium betaceum Andrz.	0	n	1	0	Amaranthaceae Juss.
Iz_1_1009	DJI_0230	Chenopodium cf. album	0	n	1	0	Amaranthaceae Juss.
Iz_1_1009	DJI_0230	Chenopodium sp.	0		NA	0	Amaranthaceae Juss.
Iz_1_1009	DJI_0230	Chondrilla juncea L.	0	n	1	0	Asteraceae Bercht. & J.Presl
Iz_1_1009	DJI_0230	Cirsium vulgare (Savi) Ten.	0	n	1	0	Asteraceae Bercht. & J.Presl
Iz_1_1009	DJI_0230	Clinopodium acinos (L.) Kuntze	0	n	1	0	Lamiacea Lindl.
Iz_1_1009	DJI_0230	Convallaria majalis L.	0	n	1	0	Asparagaceae Juss.
Iz_1_1009	DJI_0230	Crataegus rhipidophylla Gand.	0	n	1	1	Rosaceae Juss.
Iz_1_1009	DJI_0230	Crepis foetida L.	0	n	1	0	Asteraceae Bercht. & J.Presl
Iz_1_1009	DJI_0230	Dactylis glomerata L.	0	n	1	0	Poaceae Barnhart
Iz_1_1009	DJI_0230	Dianthus campestris M.Bieb.	0	n	1	0	Caryophyllaceae Juss.
Iz_1_1009	DJI_0230	Digitaria sanguinalis (L.) Scop.	0	a	0	0	Poaceae Barnhart
Iz_1_1009	DJI_0230	Elymus repens (L.) Gould	0	n	1	0	Poaceae Barnhart
Iz_1_1009	DJI_0230	Epilobium tetragonum L.	0	n	1	0	Onagraceae Juss.
Iz_1_1009	DJI_0230	Eragrostis minor Host	0	a	0	0	Poaceae Barnhart
Iz_1_1009	DJI_0230	Erigeron canadensis L.	2	a	0	0	Asteraceae Bercht. & J.Presl
Iz_1_1009	DJI_0230	Euphorbia seguieriana Neck.	0	n	1	0	Euphorbiaceae Juss.
Iz_1_1009	DJI_0230	Fallopia dumetorum (L.) Holub	0	n	1	0	Polygonaceae Juss.
Iz_1_1009	DJI_0230	Festuca beckeri (Hack.) Trautv.	0	n	1	0	Poaceae Barnhart
Iz_1_1009	DJI_0230	Fragaria vesca L.	0	n	1	0	Rosaceae Juss.
Iz_1_1009	DJI_0230	Frangula alnus Mill.	0	n	1	1	Rhamnaceae Juss.
Iz_1_1009	DJI_0230	Fraxinus pennsylvanica Marshall	0	a	0	1	Oleaceae Hoffmanns. & Link
Iz_1_1009	DJI_0230	Galeopsis bifida Boenn.	0	n	1	0	Lamiacea Lindl.
Iz_1_1009	DJI_0230	Galium verum L.	0	n	1	0	Rubiaceae Juss.
Iz_1_1009	DJI_0230	Genista tinctoria L.	0	n	1	0	Fabaceae Lindl.
Iz_1_1009	DJI_0230	Geranium robertianum L.	0	n	1	0	Geraniaceae Juss.
Iz_1_1009	DJI_0230	Geranium sanquineum L.	0	n	1	0	Geraniaceae Juss.
Iz_1_1009	DJI_0230	Gypsophila paniculata L.	0	n	1	0	Caryophyllaceae Juss.
Iz_1_1009	DJI_0230	Helichrysum arenarium (L.) Moench	0	n	1	0	Asteraceae Bercht. & J.Presl
Iz_1_1009	DJI_0230	Herniaria polygama J.Gay	0	n	1	0	Caryophyllaceae Juss.
Iz_1_1009	DJI_0230	Hieracium robustum Fr.	0	n	1	0	Asteraceae Bercht. & J.Presl
Iz_1_1009	DJI_0230	Hieracium umbellatum L.	0	n	1	NA	Asteraceae Bercht. & J.Presl
Iz_1_1009	DJI_0230	Hylotelephium maximum (L.) Holub	0	n	1	0	Crassulaceae J.St.-Hil.
Iz_1_1009	DJI_0230	Jacobaea borysthenica (DC.) B.Nord. & Greuter	0	n	1	0	Asteraceae Bercht. & J.Presl
Iz_1_1009	DJI_0230	Jasione montana L.	0	n	1	0	Campanulaceae Juss.
Iz_1_1009	DJI_0230	Jurinea cyanoides (L.) Rchb.s.l.	0	n	1	0	Asteraceae Bercht. & J.Presl
Iz_1_1009	DJI_0230	Koeleria glauca (Spreng.) DC.	0	n	1	0	Poaceae Barnhart
Iz_1_1009	DJI_0230	Lactuca serriola L.	0	a	0	0	Asteraceae Bercht. & J.Presl
Iz_1_1009	DJI_0230	Linaria genistifolia (L.) Mill.	0	n	1	0	Plantaginaceae Juss.
Iz_1_1009	DJI_0230	Lomelosia argentea (L.) Greuter & Burdet	0	n	1	0	Caprifoliaceae Juss.
Iz_1_1009	DJI_0230	Melampyrum pratense L.	0	n	1	0	Orobanchaceae Vent.
Iz_1_1009	DJI_0230	Oenothera biennis L.	0	a	0	0	Onagraceae Juss.
Iz_1_1009	DJI_0230	Pentanema britannica (L.) D.Gut.Larr., Santos-Vicente, Anderb., E.Rico & M.M.Mart.Ort.	0	n	1	0	Asteraceae Bercht. & J.Presl
Iz_1_1009	DJI_0230	Peucedanum oreoselinum (L.) Moench	0	n	1	0	Apiaceae Lindl.
Iz_1_1009	DJI_0230	Peucedanum ruthenicum M.Bieb.	0	n	1	0	Apiaceae Lindl.
Iz_1_1009	DJI_0230	Pilosella echioides (Lumn.) F.W.Schultz & Sch.Bip.	0	n	1	0	Asteraceae Bercht. & J.Presl
Iz_1_1009	DJI_0230	Pilosella officinarum F. Schultz et Sch. Bip.	0	n	1	0	Asteraceae Bercht. & J.Presl
Iz_1_1009	DJI_0230	Pilosella sp.	0		NA	0	Asteraceae Bercht. & J.Presl
Iz_1_1009	DJI_0230	Pinus sylvestris L.	0	n	1	1	Pinaceae Spreng. ex F.Rudolphi
Iz_1_1009	DJI_0230	Poa annua L.	0	n	1	0	Poaceae Barnhart
Iz_1_1009	DJI_0230	Poa nemoralis L.	0	n	1	0	Poaceae Barnhart
Iz_1_1009	DJI_0230	Poa sp.	0		NA	0	Poaceae Barnhart
Iz_1_1009	DJI_0230	Polygonatum odoratum (Mill.) Druce	0	n	1	0	Asparagaceae Juss.
Iz_1_1009	DJI_0230	Polygonum arenarium Waldst. & Kit.	0	n	1	0	Polygonaceae Juss.
Iz_1_1009	DJI_0230	Populus nigra L.	0	n	1	1	Salicaceae Mirb.
Iz_1_1009	DJI_0230	Populus tremula L.	0	n	1	1	Salicaceae Mirb.
Iz_1_1009	DJI_0230	Populus Ã canescens (Aiton) Sm.	0	n	1	1	Salicaceae Mirb.
Iz_1_1009	DJI_0230	Potentilla argentea L.	0	n	1	0	Rosaceae Juss.

Iz_1_1009	DJI_0230	Psammophiliella muralis (L.) Ikonn.	0	n	1	0	Caryophyllaceae Juss.
Iz_1_1009	DJI_0230	Psephellus sumensis (Kalen.) Greuter	0	n	1	0	Asteraceae Bercht. & J.Presl
Iz_1_1009	DJI_0230	Pulsatilla pratensis (L.) Mill. s.l.	0	n	1	0	Ranunculaceae Juss.
Iz_1_1009	DJI_0230	Pyrus communis L.	0	a	0	1	Rosaceae Juss.
Iz_1_1009	DJI_0230	Quercus robur L.	0	n	1	1	Fagaceae Dumort.
Iz_1_1009	DJI_0230	Robinia pseudoacacia L.	0	a	0	1	Fabaceae Lindl.
Iz_1_1009	DJI_0230	Rosa sp.	0		NA	1	Rosaceae Juss.
Iz_1_1009	DJI_0230	Rubus idaeus L.	0	n	1	0	Rosaceae Juss.
Iz_1_1009	DJI_0230	Rumex acetosella L.	0	n	1	0	Polygonaceae Juss.
Iz_1_1009	DJI_0230	Rumex thyrsiflorus Fingerh.	0	n	1	0	Polygonaceae Juss.
Iz_1_1009	DJI_0230	Salix alba L.	0	n	1	1	Salicaceae Mirb.
Iz_1_1009	DJI_0230	Sambucus nigra L.	0	n	1	1	Viburnaceae Raf.
Iz_1_1009	DJI_0230	Secale sylvestre Host	0	n	1	0	Poaceae Barnhart
Iz_1_1009	DJI_0230	Sempervivum ruthenicum Schnittsp. & C.B. Lehm.	0	n	1	0	Crassulaceae J.St.-Hil.
Iz_1_1009	DJI_0230	Setaria viridis (L.) P. Beauv.	0	n	1	0	Poaceae Barnhart
Iz_1_1009	DJI_0230	Silene borysthenica (Gruner) Walters	0	n	1	0	Caryophyllaceae Juss.
Iz_1_1009	DJI_0230	Silene latifolia subsp. alba (Mill.) Greuter & Burdet	0	n	1	0	Caryophyllaceae Juss.
Iz_1_1009	DJI_0230	Solidago virgaurea L.	0	n	1	0	Asteraceae Bercht. & J.Presl
Iz_1_1009	DJI_0230	Sorbus aucuparia L.	0	n	1	1	Rosaceae Juss.
Iz_1_1009	DJI_0230	Stachys recta L.	0	n	1	0	Lamiacea Lindl.
Iz_1_1009	DJI_0230	Tanacetum vulgare L.	0	n	1	0	Asteraceae Bercht. & J.Presl
Iz_1_1009	DJI_0230	Thymus pallasianus Heinr.Braun	0	n	1	0	Lamiacea Lindl.
Iz_1_1009	DJI_0230	Tilia cordata Mill.	0	n	1	1	Malvaceae Juss.
Iz_1_1009	DJI_0230	Trifolium alpestre L.	0	n	1	0	Fabaceae Lindl.
Iz_1_1009	DJI_0230	Trifolium arvense L.	0	n	1	0	Fabaceae Lindl.
Iz_1_1009	DJI_0230	Ulmus glabra Huds.	0	n	1	1	Ulmaceae Mirb.
Iz_1_1009	DJI_0230	Verbascum densiflorum Bertol.	0	n	1	0	Scrophulariaceae Juss.
Iz_1_1009	DJI_0230	Veronica austriaca L.	0	n	1	0	Plantaginaceae Juss.
Iz_1_1009	DJI_0230	Veronica incana L.	0	n	1	0	Plantaginaceae Juss.
Iz_1_1009	DJI_0230	Veronica verna L.	0	n	1	0	Plantaginaceae Juss.
Iz_1_1009	DJI_0230	Vincetoxicum hirundinaria Medik	0	n	1	0	Apocynaceae Juss.
Iz_1_1009	DJI_0231	Acer tataricum L.	0	n	1	1	Sapindaceae Juss.
Iz_1_1009	DJI_0231	Achillea millefolium L. s.l.	0	n	1	0	Asteraceae Bercht. & J.Presl
Iz_1_1009	DJI_0231	Allium oleraceum L.	0	n	1	0	Amaryllidaceae J.St.-Hil.
Iz_1_1009	DJI_0231	Ambrosia artemisiifolia L.	0	a	0	0	Asteraceae Bercht. & J.Presl
Iz_1_1009	DJI_0231	Amorpha fruticosa L.	0	a	0	0	Fabaceae Lindl.
Iz_1_1009	DJI_0231	Anthericum ramosum L.	0	n	1	0	Asparagaceae Juss.
Iz_1_1009	DJI_0231	Artemisia marschalliana Spreng.	0	n	1	0	Asteraceae Bercht. & J.Presl
Iz_1_1009	DJI_0231	Asparagus officinalis L.	0	n	1	0	Asparagaceae Juss.
Iz_1_1009	DJI_0231	Astragalus cicer L.	0	n	1	0	Fabaceae Lindl.
Iz_1_1009	DJI_0231	Bassia laniflora (S.G.Gmel.) A.J.Scott	0	n	1	0	Amaranthaceae Juss.
Iz_1_1009	DJI_0231	Berberis vulgaris L.	0	n	1	0	Berberidaceae Juss.
Iz_1_1009	DJI_0231	Betula pendula Roth.	0	n	1	1	Betulaceae Gray
Iz_1_1009	DJI_0231	Betula pendula Roth.	0	n	1	0	Poaceae Barnhart
Iz_1_1009	DJI_0231	Bromus inermis Leyss.	0		NA	NA	
Iz_1_1009	DJI_0231	Bromus squarrosus L.	0	a	0	0	Poaceae Barnhart
Iz_1_1009	DJI_0231	Bromus tectorum L.	0	a	0	0	Poaceae Barnhart
Iz_1_1009	DJI_0231	Calamagrostis epigejos (L.) Roth	60	n	1	0	Poaceae Barnhart
Iz_1_1009	DJI_0231	Carduus acanthoides L.	0	n	1	0	Asteraceae Bercht. & J.Presl
Iz_1_1009	DJI_0231	Carex sp.	0	n	1	0	Cyperaceae Juss.
Iz_1_1009	DJI_0231	Centaurea majorovii Dumbadze	0	n	1	0	Asteraceae Bercht. & J.Presl
Iz_1_1009	DJI_0231	Chamaecytisus ruthenicus (Fisch.ex Wol.) Klaskova	0	n	1	0	Fabaceae Lindl.
Iz_1_1009	DJI_0231	Chelidonium majus L.	0	n	1	0	Papaveraceae Juss.
Iz_1_1009	DJI_0231	Chenopodium betaceum Andrz.	0	n	1	0	Amaranthaceae Juss.
Iz_1_1009	DJI_0231	Chenopodium cf. album	0	n	1	0	Amaranthaceae Juss.
Iz_1_1009	DJI_0231	Chenopodium sp.	0		NA	0	Amaranthaceae Juss.
Iz_1_1009	DJI_0231	Chondrilla juncea L.	0	n	1	0	Asteraceae Bercht. & J.Presl
Iz_1_1009	DJI_0231	Cirsium vulgare (Savi) Ten.	0	n	1	0	Asteraceae Bercht. & J.Presl
Iz_1_1009	DJI_0231	Clinopodium acinos (L.) Kuntze	0	n	1	0	Lamiacea Lindl.
Iz_1_1009	DJI_0231	Convallaria majalis L.	0	n	1	0	Asparagaceae Juss.
Iz_1_1009	DJI_0231	Crataegus rhipidophylla Gand.	0	n	1	1	Rosaceae Juss.
Iz_1_1009	DJI_0231	Crepis foetida L.	0	n	1	0	Asteraceae Bercht. & J.Presl
Iz_1_1009	DJI_0231	Dactylis glomerata L.	0	n	1	0	Poaceae Barnhart
Iz_1_1009	DJI_0231	Dianthus campestris M.Bieb.	0	n	1	0	Caryophyllaceae Juss.
Iz_1_1009	DJI_0231	Digitaria sanguinalis (L.) Scop.	0	a	0	0	Poaceae Barnhart
Iz_1_1009	DJI_0231	Elymus repens (L.) Gould	0	n	1	0	Poaceae Barnhart
Iz_1_1009	DJI_0231	Epilobium tetragonum L.	0	n	1	0	Onagraceae Juss.
Iz_1_1009	DJI_0231	Eragrostis minor Host	0	a	0	0	Poaceae Barnhart
Iz_1_1009	DJI_0231	Erigeron canadensis L.	1	a	0	0	Asteraceae Bercht. & J.Presl
Iz_1_1009	DJI_0231	Euphorbia seguieriana Neck.	0	n	1	0	Euphorbiaceae Juss.
Iz_1_1009	DJI_0231	Fallopia dumetorum (L.) Holub	0	n	1	0	Polygonaceae Juss.
Iz_1_1009	DJI_0231	Festuca beckeri (Hack.) Trautv.	0	n	1	0	Poaceae Barnhart
Iz_1_1009	DJI_0231	Fragaria vesca L.	0	n	1	0	Rosaceae Juss.
Iz_1_1009	DJI_0231	Frangula alnus Mill.	0	n	1	1	Rhamnaceae Juss.
Iz_1_1009	DJI_0231	Fraxinus pennsylvanica Marshall	0	a	0	1	Oleaceae Hoffmanns. & Link
Iz_1_1009	DJI_0231	Galeopsis bifida Boenn.	0	n	1	0	Lamiacea Lindl.
Iz_1_1009	DJI_0231	Galium verum L.	0	n	1	0	Rubiaceae Juss.
Iz_1_1009	DJI_0231	Genista tinctoria L.	0	n	1	0	Fabaceae Lindl.
Iz_1_1009	DJI_0231	Geranium robertianum L.	0	n	1	0	Geraniaceae Juss.
Iz_1_1009	DJI_0231	Geranium sanquineum L.	0	n	1	0	Geraniaceae Juss.
Iz_1_1009	DJI_0231	Gypsophila paniculata L.	0	n	1	0	Caryophyllaceae Juss.
Iz_1_1009	DJI_0231	Helichrysum arenarium (L.) Moench	0	n	1	0	Asteraceae Bercht. & J.Presl
Iz_1_1009	DJI_0231	Herniaria polygama J.Gay	0	n	1	0	Caryophyllaceae Juss.
Iz_1_1009	DJI_0231	Hieracium robustum Fr.	0	n	1	0	Asteraceae Bercht. & J.Presl
Iz_1_1009	DJI_0231	Hieracium umbellatum L.	0	n	1	NA	Asteraceae Bercht. & J.Presl
Iz_1_1009	DJI_0231	Hylotelephium maximum (L.) Holub	0	n	1	0	Crassulaceae J.St.-Hil.
Iz_1_1009	DJI_0231	Jacobaea borysthenica (DC.) B.Nord. & Greuter	0	n	1	0	Asteraceae Bercht. & J.Presl
Iz_1_1009	DJI_0231	Jasione montana L.	0	n	1	0	Campanulaceae Juss.
Iz_1_1009	DJI_0231	Jurinea cyanoides (L.) Rchb.s.l.	0	n	1	0	Asteraceae Bercht. & J.Presl
Iz_1_1009	DJI_0231	Koeleria glauca (Spreng.) DC.	0	n	1	0	Poaceae Barnhart
Iz_1_1009	DJI_0231	Lactuca serriola L.	0	a	0	0	Asteraceae Bercht. & J.Presl
Iz_1_1009	DJI_0231	Linaria genistifolia (L.) Mill.	0	n	1	0	Plantaginaceae Juss.
Iz_1_1009	DJI_0231	Lomelosia argentea (L.) Greuter & Burdet	0	n	1	0	Caprifoliaceae Juss.
Iz_1_1009	DJI_0231	Melampyrum pratense L.	0	n	1	0	Orobanchaceae Vent.
Iz_1_1009	DJI_0231	Oenothera biennis L.	0	a	0	0	Onagraceae Juss.
Iz_1_1009	DJI_0231	Pentanema britannica (L.) D.Gut.Larr., Santos-Vicente, Anderb., E.Rico & M.M.Mart.Ort.	0	n	1	0	Asteraceae Bercht. & J.Presl
Iz_1_1009	DJI_0231	Peucedanum oreoselinum (L.) Moench	0	n	1	0	Apiaceae Lindl.
Iz_1_1009	DJI_0231	Peucedanum ruthenicum M.Bieb.	0	n	1	0	Apiaceae Lindl.

Iz_1_1009	DJI_0231	Pilosella echioides (Lumn.) F.W.Schultz & Sch.Bip.	0	n	1	0	Asteraceae Bercht. & J.Presl
Iz_1_1009	DJI_0231	Pilosella officinarum F. Schultz et Sch. Bip.	0	n	1	0	Asteraceae Bercht. & J.Presl
Iz_1_1009	DJI_0231	Pilosella sp.	0		NA	0	Asteraceae Bercht. & J.Presl
Iz_1_1009	DJI_0231	Pinus sylvestris L.	0	n	1	1	Pinaceae Spreng. ex F.Rudolphi
Iz_1_1009	DJI_0231	Poa annua L.	0	n	1	0	Poaceae Barnhart
Iz_1_1009	DJI_0231	Poa nemoralis L.	0	n	1	0	Poaceae Barnhart
Iz_1_1009	DJI_0231	Poa sp.	0		NA	0	Poaceae Barnhart
Iz_1_1009	DJI_0231	Polygonatum odoratum (Mill.) Druce	0	n	1	0	Asparagaceae Juss.
Iz_1_1009	DJI_0231	Polygonum arenarium Waldst. & Kit.	0	n	1	0	Polygonaceae Juss.
Iz_1_1009	DJI_0231	Populus nigra L.	0	n	1	1	Salicaceae Mirb.
Iz_1_1009	DJI_0231	Populus tremula L.	0	n	1	1	Salicaceae Mirb.
Iz_1_1009	DJI_0231	Populus Ã canescens (Aiton) Sm.	0	n	1	1	Salicaceae Mirb.
Iz_1_1009	DJI_0231	Potentilla argentea L.	0	n	1	0	Rosaceae Juss.
Iz_1_1009	DJI_0231	Psammophiliella muralis (L.) Ikonn.	0	n	1	0	Caryophyllaceae Juss.
Iz_1_1009	DJI_0231	Psephellus sumensis (Kalen.) Greuter	0	n	1	0	Asteraceae Bercht. & J.Presl
Iz_1_1009	DJI_0231	Pulsatilla pratensis (L.) Mill. s.l.	0	n	1	0	Ranunculaceae Juss.
Iz_1_1009	DJI_0231	Pyrus communis L.	0	a	0	1	Rosaceae Juss.
Iz_1_1009	DJI_0231	Quercus robur L.	0	n	1	1	Fagaceae Dumort.
Iz_1_1009	DJI_0231	Robinia pseudoacacia L.	0	a	0	1	Fabaceae Lindl.
Iz_1_1009	DJI_0231	Rosa sp.	0		NA	1	Rosaceae Juss.
Iz_1_1009	DJI_0231	Rubus idaeus L.	0	n	1	0	Rosaceae Juss.
Iz_1_1009	DJI_0231	Rumex acetosella L.	2	n	1	0	Polygonaceae Juss.
Iz_1_1009	DJI_0231	Rumex thyrsiflorus Fingerh.	0	n	1	0	Polygonaceae Juss.
Iz_1_1009	DJI_0231	Salix alba L.	0	n	1	1	Salicaceae Mirb.
Iz_1_1009	DJI_0231	Sambucus nigra L.	0	n	1	1	Viburnaceae Raf.
Iz_1_1009	DJI_0231	Secale sylvestre Host	0	n	1	0	Poaceae Barnhart
Iz_1_1009	DJI_0231	Sempervivum ruthenicum Schnittsp. & C.B. Lehm.	0	n	1	0	Crassulaceae J.St.-Hil.
Iz_1_1009	DJI_0231	Setaria viridis (L.) P. Beauv.	0	n	1	0	Poaceae Barnhart
Iz_1_1009	DJI_0231	Silene borysthenica (Gruner) Walters	0	n	1	0	Caryophyllaceae Juss.
Iz_1_1009	DJI_0231	Silene latifolia subsp. alba (Mill.) Greuter & Burdet	0	n	1	0	Caryophyllaceae Juss.
Iz_1_1009	DJI_0231	Solidago virgaurea L.	2	n	1	0	Asteraceae Bercht. & J.Presl
Iz_1_1009	DJI_0231	Sorbus aucuparia L.	0	n	1	1	Rosaceae Juss.
Iz_1_1009	DJI_0231	Stachys recta L.	0	n	1	0	Lamiacea Lindl.
Iz_1_1009	DJI_0231	Tanacetum vulgare L.	0	n	1	0	Asteraceae Bercht. & J.Presl
Iz_1_1009	DJI_0231	Thymus pallasianus Heinr.Braun	0	n	1	0	Lamiacea Lindl.
Iz_1_1009	DJI_0231	Tilia cordata Mill.	0	n	1	1	Malvaceae Juss.
Iz_1_1009	DJI_0231	Trifolium alpestre L.	0	n	1	0	Fabaceae Lindl.
Iz_1_1009	DJI_0231	Trifolium arvense L.	0	n	1	0	Fabaceae Lindl.
Iz_1_1009	DJI_0231	Ulmus glabra Huds.	0	n	1	1	Ulmaceae Mirb.
Iz_1_1009	DJI_0231	Verbascum densiflorum Bertol.	0	n	1	0	Scrophulariaceae Juss.
Iz_1_1009	DJI_0231	Veronica austriaca L.	0	n	1	0	Plantaginaceae Juss.
Iz_1_1009	DJI_0231	Veronica incana L.	0	n	1	0	Plantaginaceae Juss.
Iz_1_1009	DJI_0231	Veronica verna L.	0	n	1	0	Plantaginaceae Juss.
Iz_1_1009	DJI_0231	Vincetoxicum hirundinaria Medik	0	n	1	0	Apocynaceae Juss.
Iz_1_1009	DJI_0234	Acer tataricum L.	0	n	1	1	Sapindaceae Juss.
Iz_1_1009	DJI_0234	Achillea millefolium L. s.l.	0	n	1	0	Asteraceae Bercht. & J.Presl
Iz_1_1009	DJI_0234	Allium oleraceum L.	0	n	1	0	Amaryllidaceae J.St.-Hil.
Iz_1_1009	DJI_0234	Ambrosia artemisiifolia L.	0	a	0	0	Asteraceae Bercht. & J.Presl
Iz_1_1009	DJI_0234	Amorpha fruticosa L.	0	a	0	0	Fabaceae Lindl.
Iz_1_1009	DJI_0234	Anthericum ramosum L.	0	n	1	0	Asparagaceae Juss.
Iz_1_1009	DJI_0234	Artemisia marschalliana Spreng.	0	n	1	0	Asteraceae Bercht. & J.Presl
Iz_1_1009	DJI_0234	Asparagus officinalis L.	0	n	1	0	Asparagaceae Juss.
Iz_1_1009	DJI_0234	Astragalus cicer L.	0	n	1	0	Fabaceae Lindl.
Iz_1_1009	DJI_0234	Bassia laniflora (S.G.Gmel.) A.J.Scott	0	n	1	0	Amaranthaceae Juss.
Iz_1_1009	DJI_0234	Berberis vulgaris L.	0	n	1	0	Berberidaceae Juss.
Iz_1_1009	DJI_0234	Betula pendula Roth.	0	n	1	1	Betulaceae Gray
Iz_1_1009	DJI_0234	Betula pendula Roth.	0	n	1	0	Poaceae Barnhart
Iz_1_1009	DJI_0234	Bromus inermis Leyss.	0		NA	NA	
Iz_1_1009	DJI_0234	Bromus squarrosus L.	0	a	0	0	Poaceae Barnhart
Iz_1_1009	DJI_0234	Bromus tectorum L.	0	a	0	0	Poaceae Barnhart
Iz_1_1009	DJI_0234	Calamagrostis epigejos (L.) Roth	30	n	1	0	Poaceae Barnhart
Iz_1_1009	DJI_0234	Carduus acanthoides L.	0	n	1	0	Asteraceae Bercht. & J.Presl
Iz_1_1009	DJI_0234	Carex sp.	0	n	1	0	Cyperaceae Juss.
Iz_1_1009	DJI_0234	Centaurea majorovii Dumbadze	0	n	1	0	Asteraceae Bercht. & J.Presl
Iz_1_1009	DJI_0234	Chamaecytisus ruthenicus (Fisch.ex Wol.) Klaskova	0	n	1	0	Fabaceae Lindl.
Iz_1_1009	DJI_0234	Chelidonium majus L.	0	n	1	0	Papaveraceae Juss.
Iz_1_1009	DJI_0234	Chenopodium betaceum Andrz.	0	n	1	0	Amaranthaceae Juss.
Iz_1_1009	DJI_0234	Chenopodium cf. album	0	n	1	0	Amaranthaceae Juss.
Iz_1_1009	DJI_0234	Chenopodium sp.	0		NA	0	Amaranthaceae Juss.
Iz_1_1009	DJI_0234	Chondrilla juncea L.	0	n	1	0	Asteraceae Bercht. & J.Presl
Iz_1_1009	DJI_0234	Cirsium vulgare (Savi) Ten.	0	n	1	0	Asteraceae Bercht. & J.Presl
Iz_1_1009	DJI_0234	Clinopodium acinos (L.) Kuntze	0	n	1	0	Lamiacea Lindl.
Iz_1_1009	DJI_0234	Convallaria majalis L.	0	n	1	0	Asparagaceae Juss.
Iz_1_1009	DJI_0234	Crataegus rhipidophylla Gand.	0	n	1	1	Rosaceae Juss.
Iz_1_1009	DJI_0234	Crepis foetida L.	0	n	1	0	Asteraceae Bercht. & J.Presl
Iz_1_1009	DJI_0234	Dactylis glomerata L.	0	n	1	0	Poaceae Barnhart
Iz_1_1009	DJI_0234	Dianthus campestris M.Bieb.	0	n	1	0	Caryophyllaceae Juss.
Iz_1_1009	DJI_0234	Digitaria sanguinalis (L.) Scop.	0	a	0	0	Poaceae Barnhart
Iz_1_1009	DJI_0234	Elymus repens (L.) Gould	0	n	1	0	Poaceae Barnhart
Iz_1_1009	DJI_0234	Epilobium tetragonum L.	0	n	1	0	Onagraceae Juss.
Iz_1_1009	DJI_0234	Eragrostis minor Host	0	a	0	0	Poaceae Barnhart
Iz_1_1009	DJI_0234	Erigeron canadensis L.	5	a	0	0	Asteraceae Bercht. & J.Presl
Iz_1_1009	DJI_0234	Euphorbia seguieriana Neck.	0	n	1	0	Euphorbiaceae Juss.
Iz_1_1009	DJI_0234	Fallopia dumetorum (L.) Holub	0	n	1	0	Polygonaceae Juss.
Iz_1_1009	DJI_0234	Festuca beckeri (Hack.) Trautv.	0	n	1	0	Poaceae Barnhart
Iz_1_1009	DJI_0234	Fragaria vesca L.	0	n	1	0	Rosaceae Juss.
Iz_1_1009	DJI_0234	Frangula alnus Mill.	0	n	1	1	Rhamnaceae Juss.
Iz_1_1009	DJI_0234	Fraxinus pennsylvanica Marshall	0	a	0	1	Oleaceae Hoffmanns. & Link
Iz_1_1009	DJI_0234	Galeopsis bifida Boenn.	0	n	1	0	Lamiacea Lindl.
Iz_1_1009	DJI_0234	Galium verum L.	0	n	1	0	Rubiaceae Juss.
Iz_1_1009	DJI_0234	Genista tinctoria L.	0	n	1	0	Fabaceae Lindl.
Iz_1_1009	DJI_0234	Geranium robertianum L.	0	n	1	0	Geraniaceae Juss.
Iz_1_1009	DJI_0234	Geranium sanquineum L.	0	n	1	0	Geraniaceae Juss.
Iz_1_1009	DJI_0234	Gypsophila paniculata L.	0	n	1	0	Caryophyllaceae Juss.
Iz_1_1009	DJI_0234	Helichrysum arenarium (L.) Moench	0	n	1	0	Asteraceae Bercht. & J.Presl
Iz_1_1009	DJI_0234	Herniaria polygama J.Gay	0	n	1	0	Caryophyllaceae Juss.
Iz_1_1009	DJI_0234	Hieracium robustum Fr.	0	n	1	0	Asteraceae Bercht. & J.Presl
Iz_1_1009	DJI_0234	Hieracium umbellatum L.	0	n	1	NA	Asteraceae Bercht. & J.Presl

Iz_1_1009	DJI_0234	Hylotelephium maximum (L.) Holub	0	n	1	0	Crassulaceae J.St.-Hil.
Iz_1_1009	DJI_0234	Jacobaea borysthenica (DC.) B.Nord. & Greuter	0	n	1	0	Asteraceae Bercht. & J.Presl
Iz_1_1009	DJI_0234	Jasione montana L.	0	n	1	0	Campanulaceae Juss.
Iz_1_1009	DJI_0234	Jurinea cyanoides (L.) Rchb.s.l.	0	n	1	0	Asteraceae Bercht. & J.Presl
Iz_1_1009	DJI_0234	Koeleria glauca (Spreng.) DC.	0	n	1	0	Poaceae Barnhart
Iz_1_1009	DJI_0234	Lactuca serriola L.	0	a	0	0	Asteraceae Bercht. & J.Presl
Iz_1_1009	DJI_0234	Linaria genistifolia (L.) Mill.	0	n	1	0	Plantaginaceae Juss.
Iz_1_1009	DJI_0234	Lomelosia argentea (L.) Greuter & Burdet	0	n	1	0	Caprifoliaceae Juss.
Iz_1_1009	DJI_0234	Melampyrum pratense L.	0	n	1	0	Orobanchaceae Vent.
Iz_1_1009	DJI_0234	Oenothera biennis L.	0	a	0	0	Onagraceae Juss.
Iz_1_1009	DJI_0234	Pentanema britannica (L.) D.Gut.Larr., Santos-Vicente, Anderb., E.Rico & M.M.Mart.Ort.	0	n	1	0	Asteraceae Bercht. & J.Presl
Iz_1_1009	DJI_0234	Peucedanum oreoselinum (L.) Moench	0	n	1	0	Apiaceae Lindl.
Iz_1_1009	DJI_0234	Peucedanum ruthenicum M.Bieb.	0	n	1	0	Apiaceae Lindl.
Iz_1_1009	DJI_0234	Pilosella echioides (Lumn.) F.W.Schultz & Sch.Bip.	0	n	1	0	Asteraceae Bercht. & J.Presl
Iz_1_1009	DJI_0234	Pilosella officinarum F. Schultz et Sch. Bip.	0	n	1	0	Asteraceae Bercht. & J.Presl
Iz_1_1009	DJI_0234	Pilosella sp.	0		NA	0	Asteraceae Bercht. & J.Presl
Iz_1_1009	DJI_0234	Pinus sylvestris L.	0	n	1	1	Pinaceae Spreng. ex F.Rudolphi
Iz_1_1009	DJI_0234	Poa annua L.	0	n	1	0	Poaceae Barnhart
Iz_1_1009	DJI_0234	Poa nemoralis L.	0	n	1	0	Poaceae Barnhart
Iz_1_1009	DJI_0234	Poa sp.	0		NA	0	Poaceae Barnhart
Iz_1_1009	DJI_0234	Polygonatum odoratum (Mill.) Druce	0	n	1	0	Asparagaceae Juss.
Iz_1_1009	DJI_0234	Polygonum arenarium Waldst. & Kit.	0	n	1	0	Polygonaceae Juss.
Iz_1_1009	DJI_0234	Populus nigra L.	0	n	1	1	Salicaceae Mirb.
Iz_1_1009	DJI_0234	Populus tremula L.	0	n	1	1	Salicaceae Mirb.
Iz_1_1009	DJI_0234	Populus Ã canescens (Aiton) Sm.	0	n	1	1	Salicaceae Mirb.
Iz_1_1009	DJI_0234	Potentilla argentea L.	0	n	1	0	Rosaceae Juss.
Iz_1_1009	DJI_0234	Psammophiliella muralis (L.) Ikonn.	0	n	1	0	Caryophyllaceae Juss.
Iz_1_1009	DJI_0234	Psephellus sumensis (Kalen.) Greuter	0	n	1	0	Asteraceae Bercht. & J.Presl
Iz_1_1009	DJI_0234	Pulsatilla pratensis (L.) Mill. s.l.	0	n	1	0	Ranunculaceae Juss.
Iz_1_1009	DJI_0234	Pyrus communis L.	0	a	0	1	Rosaceae Juss.
Iz_1_1009	DJI_0234	Quercus robur L.	0	n	1	1	Fagaceae Dumort.
Iz_1_1009	DJI_0234	Robinia pseudoacacia L.	0	a	0	1	Fabaceae Lindl.
Iz_1_1009	DJI_0234	Rosa sp.	0		NA	1	Rosaceae Juss.
Iz_1_1009	DJI_0234	Rubus idaeus L.	0	n	1	0	Rosaceae Juss.
Iz_1_1009	DJI_0234	Rumex acetosella L.	5	n	1	0	Polygonaceae Juss.
Iz_1_1009	DJI_0234	Rumex thyrsiflorus Fingerh.	0	n	1	0	Polygonaceae Juss.
Iz_1_1009	DJI_0234	Salix alba L.	0	n	1	1	Salicaceae Mirb.
Iz_1_1009	DJI_0234	Sambucus nigra L.	0	n	1	1	Viburnaceae Raf.
Iz_1_1009	DJI_0234	Secale sylvestre Host	0	n	1	0	Poaceae Barnhart
Iz_1_1009	DJI_0234	Sempervivum ruthenicum Schnittsp. & C.B. Lehm.	0	n	1	0	Crassulaceae J.St.-Hil.
Iz_1_1009	DJI_0234	Setaria viridis (L.) P. Beauv.	0	n	1	0	Poaceae Barnhart
Iz_1_1009	DJI_0234	Silene borysthenica (Gruner) Walters	0	n	1	0	Caryophyllaceae Juss.
Iz_1_1009	DJI_0234	Silene latifolia subsp. alba (Mill.) Greuter & Burdet	0	n	1	0	Caryophyllaceae Juss.
Iz_1_1009	DJI_0234	Solidago virgaurea L.	1	n	1	0	Asteraceae Bercht. & J.Presl
Iz_1_1009	DJI_0234	Sorbus aucuparia L.	0	n	1	1	Rosaceae Juss.
Iz_1_1009	DJI_0234	Stachys recta L.	0	n	1	0	Lamiacea Lindl.
Iz_1_1009	DJI_0234	Tanacetum vulgare L.	0	n	1	0	Asteraceae Bercht. & J.Presl
Iz_1_1009	DJI_0234	Thymus pallasianus Heinr.Braun	0	n	1	0	Lamiacea Lindl.
Iz_1_1009	DJI_0234	Tilia cordata Mill.	0	n	1	1	Malvaceae Juss.
Iz_1_1009	DJI_0234	Trifolium alpestre L.	0	n	1	0	Fabaceae Lindl.
Iz_1_1009	DJI_0234	Trifolium arvense L.	0	n	1	0	Fabaceae Lindl.
Iz_1_1009	DJI_0234	Ulmus glabra Huds.	0	n	1	1	Ulmaceae Mirb.
Iz_1_1009	DJI_0234	Verbascum densiflorum Bertol.	0	n	1	0	Scrophulariaceae Juss.
Iz_1_1009	DJI_0234	Veronica austriaca L.	0	n	1	0	Plantaginaceae Juss.
Iz_1_1009	DJI_0234	Veronica incana L.	0	n	1	0	Plantaginaceae Juss.
Iz_1_1009	DJI_0234	Veronica verna L.	0	n	1	0	Plantaginaceae Juss.
Iz_1_1009	DJI_0234	Vincetoxicum hirundinaria Medik	0	n	1	0	Apocynaceae Juss.
Iz_1_1009	DJI_0238	Acer tataricum L.	0	n	1	1	Sapindaceae Juss.
Iz_1_1009	DJI_0238	Achillea millefolium L. s.l.	0	n	1	0	Asteraceae Bercht. & J.Presl
Iz_1_1009	DJI_0238	Allium oleraceum L.	0	n	1	0	Amaryllidaceae J.St.-Hil.
Iz_1_1009	DJI_0238	Ambrosia artemisiifolia L.	0	a	0	0	Asteraceae Bercht. & J.Presl
Iz_1_1009	DJI_0238	Amorpha fruticosa L.	0	a	0	0	Fabaceae Lindl.
Iz_1_1009	DJI_0238	Anthericum ramosum L.	0	n	1	0	Asparagaceae Juss.
Iz_1_1009	DJI_0238	Artemisia marschalliana Spreng.	0	n	1	0	Asteraceae Bercht. & J.Presl
Iz_1_1009	DJI_0238	Asparagus officinalis L.	0	n	1	0	Asparagaceae Juss.
Iz_1_1009	DJI_0238	Astragalus cicer L.	0	n	1	0	Fabaceae Lindl.
Iz_1_1009	DJI_0238	Bassia laniflora (S.G.Gmel.) A.J.Scott	0	n	1	0	Amaranthaceae Juss.
Iz_1_1009	DJI_0238	Berberis vulgaris L.	0	n	1	0	Berberidaceae Juss.
Iz_1_1009	DJI_0238	Betula pendula Roth.	0	n	1	1	Betulaceae Gray
Iz_1_1009	DJI_0238	Betula pendula Roth.	0	n	1	0	Poaceae Barnhart
Iz_1_1009	DJI_0238	Bromus inermis Leyss.	0		NA	NA	
Iz_1_1009	DJI_0238	Bromus squarrosus L.	0	a	0	0	Poaceae Barnhart
Iz_1_1009	DJI_0238	Bromus tectorum L.	0	a	0	0	Poaceae Barnhart
Iz_1_1009	DJI_0238	Calamagrostis epigejos (L.) Roth	2	n	1	0	Poaceae Barnhart
Iz_1_1009	DJI_0238	Carduus acanthoides L.	0	n	1	0	Asteraceae Bercht. & J.Presl
Iz_1_1009	DJI_0238	Carex sp.	0	n	1	0	Cyperaceae Juss.
Iz_1_1009	DJI_0238	Centaurea majorovii Dumbadze	0	n	1	0	Asteraceae Bercht. & J.Presl
Iz_1_1009	DJI_0238	Chamaecytisus ruthenicus (Fisch.ex Wol.) Klaskova	0	n	1	0	Fabaceae Lindl.
Iz_1_1009	DJI_0238	Chelidonium majus L.	0	n	1	0	Papaveraceae Juss.
Iz_1_1009	DJI_0238	Chenopodium betaceum Andrz.	0	n	1	0	Amaranthaceae Juss.
Iz_1_1009	DJI_0238	Chenopodium cf. album	0	n	1	0	Amaranthaceae Juss.
Iz_1_1009	DJI_0238	Chenopodium sp.	0		NA	0	Amaranthaceae Juss.
Iz_1_1009	DJI_0238	Chondrilla juncea L.	0	n	1	0	Asteraceae Bercht. & J.Presl
Iz_1_1009	DJI_0238	Cirsium vulgare (Savi) Ten.	0	n	1	0	Asteraceae Bercht. & J.Presl
Iz_1_1009	DJI_0238	Clinopodium acinos (L.) Kuntze	0	n	1	0	Lamiacea Lindl.
Iz_1_1009	DJI_0238	Convallaria majalis L.	0	n	1	0	Asparagaceae Juss.
Iz_1_1009	DJI_0238	Crataegus rhipidophylla Gand.	0	n	1	1	Rosaceae Juss.
Iz_1_1009	DJI_0238	Crepis foetida L.	0	n	1	0	Asteraceae Bercht. & J.Presl
Iz_1_1009	DJI_0238	Dactylis glomerata L.	0	n	1	0	Poaceae Barnhart
Iz_1_1009	DJI_0238	Dianthus campestris M.Bieb.	0	n	1	0	Caryophyllaceae Juss.
Iz_1_1009	DJI_0238	Digitaria sanguinalis (L.) Scop.	0	a	0	0	Poaceae Barnhart
Iz_1_1009	DJI_0238	Elymus repens (L.) Gould	0	n	1	0	Poaceae Barnhart
Iz_1_1009	DJI_0238	Epilobium tetragonum L.	0	n	1	0	Onagraceae Juss.
Iz_1_1009	DJI_0238	Eragrostis minor Host	0	a	0	0	Poaceae Barnhart
Iz_1_1009	DJI_0238	Erigeron canadensis L.	2	a	0	0	Asteraceae Bercht. & J.Presl
Iz_1_1009	DJI_0238	Euphorbia seguieriana Neck.	0	n	1	0	Euphorbiaceae Juss.
Iz_1_1009	DJI_0238	Fallopia dumetorum (L.) Holub	0	n	1	0	Polygonaceae Juss.
Iz_1_1009	DJI_0238	Festuca beckeri (Hack.) Trautv.	0	n	1	0	Poaceae Barnhart

Iz_1_1009	DJI_0238	Fragaria vesca L.	0	n	1	0	Rosaceae Juss.
Iz_1_1009	DJI_0238	Frangula alnus Mill.	0	n	1	1	Rhamnaceae Juss.
Iz_1_1009	DJI_0238	Fraxinus pennsylvanica Marshall	0	a	0	1	Oleaceae Hoffmanns. & Link
Iz_1_1009	DJI_0238	Galeopsis bifida Boenn.	0	n	1	0	Lamiacea Lindl.
Iz_1_1009	DJI_0238	Galium verum L.	0	n	1	0	Rubiaceae Juss.
Iz_1_1009	DJI_0238	Genista tinctoria L.	0	n	1	0	Fabaceae Lindl.
Iz_1_1009	DJI_0238	Geranium robertianum L.	0	n	1	0	Geraniaceae Juss.
Iz_1_1009	DJI_0238	Geranium sanquineum L.	0	n	1	0	Geraniaceae Juss.
Iz_1_1009	DJI_0238	Gypsophila paniculata L.	0	n	1	0	Caryophyllaceae Juss.
Iz_1_1009	DJI_0238	Helichrysum arenarium (L.) Moench	0	n	1	0	Asteraceae Bercht. & J.Presl
Iz_1_1009	DJI_0238	Herniaria polygama J.Gay	0	n	1	0	Caryophyllaceae Juss.
Iz_1_1009	DJI_0238	Hieracium robustum Fr.	0	n	1	0	Asteraceae Bercht. & J.Presl
Iz_1_1009	DJI_0238	Hieracium umbellatum L.	0	n	1	NA	Asteraceae Bercht. & J.Presl
Iz_1_1009	DJI_0238	Hylotelephium maximum (L.) Holub	0	n	1	0	Crassulaceae J.St.-Hil.
Iz_1_1009	DJI_0238	Jacobaea borysthenica (DC.) B.Nord. & Greuter	0	n	1	0	Asteraceae Bercht. & J.Presl
Iz_1_1009	DJI_0238	Jasione montana L.	0	n	1	0	Campanulaceae Juss.
Iz_1_1009	DJI_0238	Jurinea cyanoides (L.) Rchb.s.l.	0	n	1	0	Asteraceae Bercht. & J.Presl
Iz_1_1009	DJI_0238	Koeleria glauca (Spreng.) DC.	0	n	1	0	Poaceae Barnhart
Iz_1_1009	DJI_0238	Lactuca serriola L.	0	a	0	0	Asteraceae Bercht. & J.Presl
Iz_1_1009	DJI_0238	Linaria genistifolia (L.) Mill.	0	n	1	0	Plantaginaceae Juss.
Iz_1_1009	DJI_0238	Lomelosia argentea (L.) Greuter & Burdet	0	n	1	0	Caprifoliaceae Juss.
Iz_1_1009	DJI_0238	Melampyrum pratense L.	0	n	1	0	Orobanchaceae Vent.
Iz_1_1009	DJI_0238	Oenothera biennis L.	0	a	0	0	Onagraceae Juss.
Iz_1_1009	DJI_0238	Pentanema britannica (L.) D.Gut.Larr., Santos-Vicente, Anderb., E.Rico & M.M.Mart.Ort.	0	n	1	0	Asteraceae Bercht. & J.Presl
Iz_1_1009	DJI_0238	Peucedanum oreoselinum (L.) Moench	0	n	1	0	Apiaceae Lindl.
Iz_1_1009	DJI_0238	Peucedanum ruthenicum M.Bieb.	0	n	1	0	Apiaceae Lindl.
Iz_1_1009	DJI_0238	Pilosella echioides (Lumn.) F.W.Schultz & Sch.Bip.	0	n	1	0	Asteraceae Bercht. & J.Presl
Iz_1_1009	DJI_0238	Pilosella officinarum F. Schultz et Sch. Bip.	0	n	1	0	Asteraceae Bercht. & J.Presl
Iz_1_1009	DJI_0238	Pilosella sp.	0		NA	0	Asteraceae Bercht. & J.Presl
Iz_1_1009	DJI_0238	Pinus sylvestris L.	0	n	1	1	Pinaceae Spreng. ex F.Rudolphi
Iz_1_1009	DJI_0238	Poa annua L.	0	n	1	0	Poaceae Barnhart
Iz_1_1009	DJI_0238	Poa nemoralis L.	0	n	1	0	Poaceae Barnhart
Iz_1_1009	DJI_0238	Poa sp.	0		NA	0	Poaceae Barnhart
Iz_1_1009	DJI_0238	Polygonatum odoratum (Mill.) Druce	0	n	1	0	Asparagaceae Juss.
Iz_1_1009	DJI_0238	Polygonum arenarium Waldst. & Kit.	0	n	1	0	Polygonaceae Juss.
Iz_1_1009	DJI_0238	Populus nigra L.	0	n	1	1	Salicaceae Mirb.
Iz_1_1009	DJI_0238	Populus tremula L.	0	n	1	1	Salicaceae Mirb.
Iz_1_1009	DJI_0238	Populus Ã canescens (Aiton) Sm.	0	n	1	1	Salicaceae Mirb.
Iz_1_1009	DJI_0238	Potentilla argentea L.	0	n	1	0	Rosaceae Juss.
Iz_1_1009	DJI_0238	Psammophiliella muralis (L.) Ikonn.	0	n	1	0	Caryophyllaceae Juss.
Iz_1_1009	DJI_0238	Psephellus sumensis (Kalen.) Greuter	0	n	1	0	Asteraceae Bercht. & J.Presl
Iz_1_1009	DJI_0238	Pulsatilla pratensis (L.) Mill. s.l.	0	n	1	0	Ranunculaceae Juss.
Iz_1_1009	DJI_0238	Pyrus communis L.	0	a	0	1	Rosaceae Juss.
Iz_1_1009	DJI_0238	Quercus robur L.	0	n	1	1	Fagaceae Dumort.
Iz_1_1009	DJI_0238	Robinia pseudoacacia L.	0	a	0	1	Fabaceae Lindl.
Iz_1_1009	DJI_0238	Rosa sp.	0		NA	1	Rosaceae Juss.
Iz_1_1009	DJI_0238	Rubus idaeus L.	0	n	1	0	Rosaceae Juss.
Iz_1_1009	DJI_0238	Rumex acetosella L.	5	n	1	0	Polygonaceae Juss.
Iz_1_1009	DJI_0238	Rumex thyrsiflorus Fingerh.	0	n	1	0	Polygonaceae Juss.
Iz_1_1009	DJI_0238	Salix alba L.	0	n	1	1	Salicaceae Mirb.
Iz_1_1009	DJI_0238	Sambucus nigra L.	0	n	1	1	Viburnaceae Raf.
Iz_1_1009	DJI_0238	Secale sylvestre Host	0	n	1	0	Poaceae Barnhart
Iz_1_1009	DJI_0238	Sempervivum ruthenicum Schnittsp. & C.B. Lehm.	0	n	1	0	Crassulaceae J.St.-Hil.
Iz_1_1009	DJI_0238	Setaria viridis (L.) P. Beauv.	0	n	1	0	Poaceae Barnhart
Iz_1_1009	DJI_0238	Silene borysthenica (Gruner) Walters	0	n	1	0	Caryophyllaceae Juss.
Iz_1_1009	DJI_0238	Silene latifolia subsp. alba (Mill.) Greuter & Burdet	0	n	1	0	Caryophyllaceae Juss.
Iz_1_1009	DJI_0238	Solidago virgaurea L.	2	n	1	0	Asteraceae Bercht. & J.Presl
Iz_1_1009	DJI_0238	Sorbus aucuparia L.	0	n	1	1	Rosaceae Juss.
Iz_1_1009	DJI_0238	Stachys recta L.	0	n	1	0	Lamiacea Lindl.
Iz_1_1009	DJI_0238	Tanacetum vulgare L.	0	n	1	0	Asteraceae Bercht. & J.Presl
Iz_1_1009	DJI_0238	Thymus pallasianus Heinr.Braun	0	n	1	0	Lamiacea Lindl.
Iz_1_1009	DJI_0238	Tilia cordata Mill.	0	n	1	1	Malvaceae Juss.
Iz_1_1009	DJI_0238	Trifolium alpestre L.	0	n	1	0	Fabaceae Lindl.
Iz_1_1009	DJI_0238	Trifolium arvense L.	0	n	1	0	Fabaceae Lindl.
Iz_1_1009	DJI_0238	Ulmus glabra Huds.	0	n	1	1	Ulmaceae Mirb.
Iz_1_1009	DJI_0238	Verbascum densiflorum Bertol.	0	n	1	0	Scrophulariaceae Juss.
Iz_1_1009	DJI_0238	Veronica austriaca L.	0	n	1	0	Plantaginaceae Juss.
Iz_1_1009	DJI_0238	Veronica incana L.	0	n	1	0	Plantaginaceae Juss.
Iz_1_1009	DJI_0238	Veronica verna L.	0	n	1	0	Plantaginaceae Juss.
Iz_1_1009	DJI_0238	Vincetoxicum hirundinaria Medik	0	n	1	0	Apocynaceae Juss.
Iz_1_1009	DJI_0239	Acer tataricum L.	0	n	1	1	Sapindaceae Juss.
Iz_1_1009	DJI_0239	Achillea millefolium L. s.l.	0	n	1	0	Asteraceae Bercht. & J.Presl
Iz_1_1009	DJI_0239	Allium oleraceum L.	0	n	1	0	Amaryllidaceae J.St.-Hil.
Iz_1_1009	DJI_0239	Ambrosia artemisiifolia L.	0	a	0	0	Asteraceae Bercht. & J.Presl
Iz_1_1009	DJI_0239	Amorpha fruticosa L.	0	a	0	0	Fabaceae Lindl.
Iz_1_1009	DJI_0239	Anthericum ramosum L.	0	n	1	0	Asparagaceae Juss.
Iz_1_1009	DJI_0239	Artemisia marschalliana Spreng.	0	n	1	0	Asteraceae Bercht. & J.Presl
Iz_1_1009	DJI_0239	Asparagus officinalis L.	0	n	1	0	Asparagaceae Juss.
Iz_1_1009	DJI_0239	Astragalus cicer L.	0	n	1	0	Fabaceae Lindl.
Iz_1_1009	DJI_0239	Bassia laniflora (S.G.Gmel.) A.J.Scott	0	n	1	0	Amaranthaceae Juss.
Iz_1_1009	DJI_0239	Berberis vulgaris L.	0	n	1	0	Berberidaceae Juss.
Iz_1_1009	DJI_0239	Betula pendula Roth.	0	n	1	1	Betulaceae Gray
Iz_1_1009	DJI_0239	Betula pendula Roth.	0	n	1	0	Poaceae Barnhart
Iz_1_1009	DJI_0239	Bromus inermis Leyss.	0		NA	NA	
Iz_1_1009	DJI_0239	Bromus squarrosus L.	0	a	0	0	Poaceae Barnhart
Iz_1_1009	DJI_0239	Bromus tectorum L.	0	a	0	0	Poaceae Barnhart
Iz_1_1009	DJI_0239	Calamagrostis epigejos (L.) Roth	2	n	1	0	Poaceae Barnhart
Iz_1_1009	DJI_0239	Carduus acanthoides L.	0	n	1	0	Asteraceae Bercht. & J.Presl
Iz_1_1009	DJI_0239	Carex sp.	0	n	1	0	Cyperaceae Juss.
Iz_1_1009	DJI_0239	Centaurea majorovii Dumbadze	0	n	1	0	Asteraceae Bercht. & J.Presl
Iz_1_1009	DJI_0239	Chamaecytisus ruthenicus (Fisch.ex Wol.) Klaskova	0	n	1	0	Fabaceae Lindl.
Iz_1_1009	DJI_0239	Chelidonium majus L.	0	n	1	0	Papaveraceae Juss.
Iz_1_1009	DJI_0239	Chenopodium betaceum Andrz.	0	n	1	0	Amaranthaceae Juss.
Iz_1_1009	DJI_0239	Chenopodium cf. album	1	n	1	0	Amaranthaceae Juss.
Iz_1_1009	DJI_0239	Chenopodium sp.	0		NA	0	Amaranthaceae Juss.
Iz_1_1009	DJI_0239	Chondrilla juncea L.	1	n	1	0	Asteraceae Bercht. & J.Presl
Iz_1_1009	DJI_0239	Cirsium vulgare (Savi) Ten.	0	n	1	0	Asteraceae Bercht. & J.Presl
Iz_1_1009	DJI_0239	Clinopodium acinos (L.) Kuntze	0	n	1	0	Lamiacea Lindl.

Iz_1_1009	DJI_0239	Convallaria majalis L.	0	n	1	0	Asparagaceae Juss.
Iz_1_1009	DJI_0239	Crataegus rhipidophylla Gand.	0	n	1	1	Rosaceae Juss.
Iz_1_1009	DJI_0239	Crepis foetida L.	0	n	1	0	Asteraceae Bercht. & J.Presl
Iz_1_1009	DJI_0239	Dactylis glomerata L.	0	n	1	0	Poaceae Barnhart
Iz_1_1009	DJI_0239	Dianthus campestris M.Bieb.	0	n	1	0	Caryophyllaceae Juss.
Iz_1_1009	DJI_0239	Digitaria sanguinalis (L.) Scop.	0	a	0	0	Poaceae Barnhart
Iz_1_1009	DJI_0239	Elymus repens (L.) Gould	0	n	1	0	Poaceae Barnhart
Iz_1_1009	DJI_0239	Epilobium tetragonum L.	0	n	1	0	Onagraceae Juss.
Iz_1_1009	DJI_0239	Eragrostis minor Host	0	a	0	0	Poaceae Barnhart
Iz_1_1009	DJI_0239	Erigeron canadensis L.	3	a	0	0	Asteraceae Bercht. & J.Presl
Iz_1_1009	DJI_0239	Euphorbia seguieriana Neck.	0	n	1	0	Euphorbiaceae Juss.
Iz_1_1009	DJI_0239	Fallopia dumetorum (L.) Holub	0	n	1	0	Polygonaceae Juss.
Iz_1_1009	DJI_0239	Festuca beckeri (Hack.) Trautv.	0	n	1	0	Poaceae Barnhart
Iz_1_1009	DJI_0239	Fragaria vesca L.	0	n	1	0	Rosaceae Juss.
Iz_1_1009	DJI_0239	Frangula alnus Mill.	0	n	1	1	Rhamnaceae Juss.
Iz_1_1009	DJI_0239	Fraxinus pennsylvanica Marshall	0	a	0	1	Oleaceae Hoffmanns. & Link
Iz_1_1009	DJI_0239	Galeopsis bifida Boenn.	0	n	1	0	Lamiacea Lindl.
Iz_1_1009	DJI_0239	Galium verum L.	0	n	1	0	Rubiaceae Juss.
Iz_1_1009	DJI_0239	Genista tinctoria L.	0	n	1	0	Fabaceae Lindl.
Iz_1_1009	DJI_0239	Geranium robertianum L.	0	n	1	0	Geraniaceae Juss.
Iz_1_1009	DJI_0239	Geranium sanquineum L.	0	n	1	0	Geraniaceae Juss.
Iz_1_1009	DJI_0239	Gypsophila paniculata L.	0	n	1	0	Caryophyllaceae Juss.
Iz_1_1009	DJI_0239	Helichrysum arenarium (L.) Moench	0	n	1	0	Asteraceae Bercht. & J.Presl
Iz_1_1009	DJI_0239	Herniaria polygama J.Gay	0	n	1	0	Caryophyllaceae Juss.
Iz_1_1009	DJI_0239	Hieracium robustum Fr.	0	n	1	0	Asteraceae Bercht. & J.Presl
Iz_1_1009	DJI_0239	Hieracium umbellatum L.	0	n	1	NA	Asteraceae Bercht. & J.Presl
Iz_1_1009	DJI_0239	Hylotelephium maximum (L.) Holub	0	n	1	0	Crassulaceae J.St.-Hil.
Iz_1_1009	DJI_0239	Jacobaea borysthenica (DC.) B.Nord. & Greuter	0	n	1	0	Asteraceae Bercht. & J.Presl
Iz_1_1009	DJI_0239	Jasione montana L.	0	n	1	0	Campanulaceae Juss.
Iz_1_1009	DJI_0239	Jurinea cyanoides (L.) Rchb.s.l.	0	n	1	0	Asteraceae Bercht. & J.Presl
Iz_1_1009	DJI_0239	Koeleria glauca (Spreng.) DC.	0	n	1	0	Poaceae Barnhart
Iz_1_1009	DJI_0239	Lactuca serriola L.	0	a	0	0	Asteraceae Bercht. & J.Presl
Iz_1_1009	DJI_0239	Linaria genistifolia (L.) Mill.	0	n	1	0	Plantaginaceae Juss.
Iz_1_1009	DJI_0239	Lomelosia argentea (L.) Greuter & Burdet	0	n	1	0	Caprifoliaceae Juss.
Iz_1_1009	DJI_0239	Melampyrum pratense L.	0	n	1	0	Orobanchaceae Vent.
Iz_1_1009	DJI_0239	Oenothera biennis L.	0	a	0	0	Onagraceae Juss.
Iz_1_1009	DJI_0239	Pentanema britannica (L.) D.Gut.Larr., Santos-Vicente, Anderb., E.Rico & M.M.Mart.Ort.	0	n	1	0	Asteraceae Bercht. & J.Presl
Iz_1_1009	DJI_0239	Peucedanum oreoselinum (L.) Moench	0	n	1	0	Apiaceae Lindl.
Iz_1_1009	DJI_0239	Peucedanum ruthenicum M.Bieb.	0	n	1	0	Apiaceae Lindl.
Iz_1_1009	DJI_0239	Pilosella echioides (Lumn.) F.W.Schultz & Sch.Bip.	0	n	1	0	Asteraceae Bercht. & J.Presl
Iz_1_1009	DJI_0239	Pilosella officinarum F. Schultz et Sch. Bip.	0	n	1	0	Asteraceae Bercht. & J.Presl
Iz_1_1009	DJI_0239	Pilosella sp.	0		NA	0	Asteraceae Bercht. & J.Presl
Iz_1_1009	DJI_0239	Pinus sylvestris L.	0	n	1	1	Pinaceae Spreng. ex F.Rudolphi
Iz_1_1009	DJI_0239	Poa annua L.	0	n	1	0	Poaceae Barnhart
Iz_1_1009	DJI_0239	Poa nemoralis L.	0	n	1	0	Poaceae Barnhart
Iz_1_1009	DJI_0239	Poa sp.	0		NA	0	Poaceae Barnhart
Iz_1_1009	DJI_0239	Polygonatum odoratum (Mill.) Druce	0	n	1	0	Asparagaceae Juss.
Iz_1_1009	DJI_0239	Polygonum arenarium Waldst. & Kit.	0	n	1	0	Polygonaceae Juss.
Iz_1_1009	DJI_0239	Populus nigra L.	0	n	1	1	Salicaceae Mirb.
Iz_1_1009	DJI_0239	Populus tremula L.	0	n	1	1	Salicaceae Mirb.
Iz_1_1009	DJI_0239	Populus Ã canescens (Aiton) Sm.	0	n	1	1	Salicaceae Mirb.
Iz_1_1009	DJI_0239	Potentilla argentea L.	0	n	1	0	Rosaceae Juss.
Iz_1_1009	DJI_0239	Psammophiliella muralis (L.) Ikonn.	0	n	1	0	Caryophyllaceae Juss.
Iz_1_1009	DJI_0239	Psephellus sumensis (Kalen.) Greuter	0	n	1	0	Asteraceae Bercht. & J.Presl
Iz_1_1009	DJI_0239	Pulsatilla pratensis (L.) Mill. s.l.	0	n	1	0	Ranunculaceae Juss.
Iz_1_1009	DJI_0239	Pyrus communis L.	0	a	0	1	Rosaceae Juss.
Iz_1_1009	DJI_0239	Quercus robur L.	0	n	1	1	Fagaceae Dumort.
Iz_1_1009	DJI_0239	Robinia pseudoacacia L.	0	a	0	1	Fabaceae Lindl.
Iz_1_1009	DJI_0239	Rosa sp.	0		NA	1	Rosaceae Juss.
Iz_1_1009	DJI_0239	Rubus idaeus L.	0	n	1	0	Rosaceae Juss.
Iz_1_1009	DJI_0239	Rumex acetosella L.	5	n	1	0	Polygonaceae Juss.
Iz_1_1009	DJI_0239	Rumex thyrsiflorus Fingerh.	0	n	1	0	Polygonaceae Juss.
Iz_1_1009	DJI_0239	Salix alba L.	0	n	1	1	Salicaceae Mirb.
Iz_1_1009	DJI_0239	Sambucus nigra L.	0	n	1	1	Viburnaceae Raf.
Iz_1_1009	DJI_0239	Secale sylvestre Host	0	n	1	0	Poaceae Barnhart
Iz_1_1009	DJI_0239	Sempervivum ruthenicum Schnittsp. & C.B. Lehm.	0	n	1	0	Crassulaceae J.St.-Hil.
Iz_1_1009	DJI_0239	Setaria viridis (L.) P. Beauv.	0	n	1	0	Poaceae Barnhart
Iz_1_1009	DJI_0239	Silene borysthenica (Gruner) Walters	0	n	1	0	Caryophyllaceae Juss.
Iz_1_1009	DJI_0239	Silene latifolia subsp. alba (Mill.) Greuter & Burdet	0	n	1	0	Caryophyllaceae Juss.
Iz_1_1009	DJI_0239	Solidago virgaurea L.	2	n	1	0	Asteraceae Bercht. & J.Presl
Iz_1_1009	DJI_0239	Sorbus aucuparia L.	0	n	1	1	Rosaceae Juss.
Iz_1_1009	DJI_0239	Stachys recta L.	0	n	1	0	Lamiacea Lindl.
Iz_1_1009	DJI_0239	Tanacetum vulgare L.	0	n	1	0	Asteraceae Bercht. & J.Presl
Iz_1_1009	DJI_0239	Thymus pallasianus Heinr.Braun	0	n	1	0	Lamiacea Lindl.
Iz_1_1009	DJI_0239	Tilia cordata Mill.	0	n	1	1	Malvaceae Juss.
Iz_1_1009	DJI_0239	Trifolium alpestre L.	0	n	1	0	Fabaceae Lindl.
Iz_1_1009	DJI_0239	Trifolium arvense L.	0	n	1	0	Fabaceae Lindl.
Iz_1_1009	DJI_0239	Ulmus glabra Huds.	0	n	1	1	Ulmaceae Mirb.
Iz_1_1009	DJI_0239	Verbascum densiflorum Bertol.	0	n	1	0	Scrophulariaceae Juss.
Iz_1_1009	DJI_0239	Veronica austriaca L.	0	n	1	0	Plantaginaceae Juss.
Iz_1_1009	DJI_0239	Veronica incana L.	0	n	1	0	Plantaginaceae Juss.
Iz_1_1009	DJI_0239	Veronica verna L.	0	n	1	0	Plantaginaceae Juss.
Iz_1_1009	DJI_0239	Vincetoxicum hirundinaria Medik	0	n	1	0	Apocynaceae Juss.
Iz_1_1009	DJI_0242	Acer tataricum L.	0	n	1	1	Sapindaceae Juss.
Iz_1_1009	DJI_0242	Achillea millefolium L. s.l.	0	n	1	0	Asteraceae Bercht. & J.Presl
Iz_1_1009	DJI_0242	Allium oleraceum L.	0	n	1	0	Amaryllidaceae J.St.-Hil.
Iz_1_1009	DJI_0242	Ambrosia artemisiifolia L.	0	a	0	0	Asteraceae Bercht. & J.Presl
Iz_1_1009	DJI_0242	Amorpha fruticosa L.	0	a	0	0	Fabaceae Lindl.
Iz_1_1009	DJI_0242	Anthericum ramosum L.	0	n	1	0	Asparagaceae Juss.
Iz_1_1009	DJI_0242	Artemisia marschalliana Spreng.	0	n	1	0	Asteraceae Bercht. & J.Presl
Iz_1_1009	DJI_0242	Asparagus officinalis L.	0	n	1	0	Asparagaceae Juss.
Iz_1_1009	DJI_0242	Astragalus cicer L.	0	n	1	0	Fabaceae Lindl.
Iz_1_1009	DJI_0242	Bassia laniflora (S.G.Gmel.) A.J.Scott	0	n	1	0	Amaranthaceae Juss.
Iz_1_1009	DJI_0242	Berberis vulgaris L.	0	n	1	0	Berberidaceae Juss.
Iz_1_1009	DJI_0242	Betula pendula Roth.	0	n	1	1	Betulaceae Gray
Iz_1_1009	DJI_0242	Betula pendula Roth.	0	n	1	0	Poaceae Barnhart
Iz_1_1009	DJI_0242	Bromus inermis Leyss.	0		NA	NA	
Iz_1_1009	DJI_0242	Bromus squarrosus L.	0	a	0	0	Poaceae Barnhart

Iz_1_1009	DJI_0242	Bromus tectorum L.	0	a	0	0	Poaceae Barnhart
Iz_1_1009	DJI_0242	Calamagrostis epigejos (L.) Roth	0	n	1	0	Poaceae Barnhart
Iz_1_1009	DJI_0242	Carduus acanthoides L.	0	n	1	0	Asteraceae Bercht. & J.Presl
Iz_1_1009	DJI_0242	Carex sp.	0	n	1	0	Cyperaceae Juss.
Iz_1_1009	DJI_0242	Centaurea majorovii Dumbadze	0	n	1	0	Asteraceae Bercht. & J.Presl
Iz_1_1009	DJI_0242	Chamaecytisus ruthenicus (Fisch.ex Wol.) Klaskova	0	n	1	0	Fabaceae Lindl.
Iz_1_1009	DJI_0242	Chelidonium majus L.	0	n	1	0	Papaveraceae Juss.
Iz_1_1009	DJI_0242	Chenopodium betaceum Andrz.	0	n	1	0	Amaranthaceae Juss.
Iz_1_1009	DJI_0242	Chenopodium cf. album	0	n	1	0	Amaranthaceae Juss.
Iz_1_1009	DJI_0242	Chenopodium sp.	0		NA	0	Amaranthaceae Juss.
Iz_1_1009	DJI_0242	Chondrilla juncea L.	0	n	1	0	Asteraceae Bercht. & J.Presl
Iz_1_1009	DJI_0242	Cirsium vulgare (Savi) Ten.	0	n	1	0	Asteraceae Bercht. & J.Presl
Iz_1_1009	DJI_0242	Clinopodium acinos (L.) Kuntze	0	n	1	0	Lamiacea Lindl.
Iz_1_1009	DJI_0242	Convallaria majalis L.	0	n	1	0	Asparagaceae Juss.
Iz_1_1009	DJI_0242	Crataegus rhipidophylla Gand.	0	n	1	1	Rosaceae Juss.
Iz_1_1009	DJI_0242	Crepis foetida L.	0	n	1	0	Asteraceae Bercht. & J.Presl
Iz_1_1009	DJI_0242	Dactylis glomerata L.	0	n	1	0	Poaceae Barnhart
Iz_1_1009	DJI_0242	Dianthus campestris M.Bieb.	0	n	1	0	Caryophyllaceae Juss.
Iz_1_1009	DJI_0242	Digitaria sanguinalis (L.) Scop.	0	a	0	0	Poaceae Barnhart
Iz_1_1009	DJI_0242	Elymus repens (L.) Gould	0	n	1	0	Poaceae Barnhart
Iz_1_1009	DJI_0242	Epilobium tetragonum L.	0	n	1	0	Onagraceae Juss.
Iz_1_1009	DJI_0242	Eragrostis minor Host	0	a	0	0	Poaceae Barnhart
Iz_1_1009	DJI_0242	Erigeron canadensis L.	1	a	0	0	Asteraceae Bercht. & J.Presl
Iz_1_1009	DJI_0242	Euphorbia seguieriana Neck.	0	n	1	0	Euphorbiaceae Juss.
Iz_1_1009	DJI_0242	Fallopia dumetorum (L.) Holub	0	n	1	0	Polygonaceae Juss.
Iz_1_1009	DJI_0242	Festuca beckeri (Hack.) Trautv.	0	n	1	0	Poaceae Barnhart
Iz_1_1009	DJI_0242	Fragaria vesca L.	0	n	1	0	Rosaceae Juss.
Iz_1_1009	DJI_0242	Frangula alnus Mill.	0	n	1	1	Rhamnaceae Juss.
Iz_1_1009	DJI_0242	Fraxinus pennsylvanica Marshall	0	a	0	1	Oleaceae Hoffmanns. & Link
Iz_1_1009	DJI_0242	Galeopsis bifida Boenn.	0	n	1	0	Lamiacea Lindl.
Iz_1_1009	DJI_0242	Galium verum L.	0	n	1	0	Rubiaceae Juss.
Iz_1_1009	DJI_0242	Genista tinctoria L.	0	n	1	0	Fabaceae Lindl.
Iz_1_1009	DJI_0242	Geranium robertianum L.	0	n	1	0	Geraniaceae Juss.
Iz_1_1009	DJI_0242	Geranium sanquineum L.	0	n	1	0	Geraniaceae Juss.
Iz_1_1009	DJI_0242	Gypsophila paniculata L.	0	n	1	0	Caryophyllaceae Juss.
Iz_1_1009	DJI_0242	Helichrysum arenarium (L.) Moench	0	n	1	0	Asteraceae Bercht. & J.Presl
Iz_1_1009	DJI_0242	Herniaria polygama J.Gay	0	n	1	0	Caryophyllaceae Juss.
Iz_1_1009	DJI_0242	Hieracium robustum Fr.	0	n	1	0	Asteraceae Bercht. & J.Presl
Iz_1_1009	DJI_0242	Hieracium umbellatum L.	0	n	1	NA	Asteraceae Bercht. & J.Presl
Iz_1_1009	DJI_0242	Hylotelephium maximum (L.) Holub	0	n	1	0	Crassulaceae J.St.-Hil.
Iz_1_1009	DJI_0242	Jacobaea borysthenica (DC.) B.Nord. & Greuter	0	n	1	0	Asteraceae Bercht. & J.Presl
Iz_1_1009	DJI_0242	Jasione montana L.	0	n	1	0	Campanulaceae Juss.
Iz_1_1009	DJI_0242	Jurinea cyanoides (L.) Rchb.s.l.	0	n	1	0	Asteraceae Bercht. & J.Presl
Iz_1_1009	DJI_0242	Koeleria glauca (Spreng.) DC.	0	n	1	0	Poaceae Barnhart
Iz_1_1009	DJI_0242	Lactuca serriola L.	0	a	0	0	Asteraceae Bercht. & J.Presl
Iz_1_1009	DJI_0242	Linaria genistifolia (L.) Mill.	0	n	1	0	Plantaginaceae Juss.
Iz_1_1009	DJI_0242	Lomelosia argentea (L.) Greuter & Burdet	0	n	1	0	Caprifoliaceae Juss.
Iz_1_1009	DJI_0242	Melampyrum pratense L.	0	n	1	0	Orobanchaceae Vent.
Iz_1_1009	DJI_0242	Oenothera biennis L.	0	a	0	0	Onagraceae Juss.
Iz_1_1009	DJI_0242	Pentanema britannica (L.) D.Gut.Larr., Santos-Vicente, Anderb., E.Rico & M.M.Mart.Ort.	0	n	1	0	Asteraceae Bercht. & J.Presl
Iz_1_1009	DJI_0242	Peucedanum oreoselinum (L.) Moench	0	n	1	0	Apiaceae Lindl.
Iz_1_1009	DJI_0242	Peucedanum ruthenicum M.Bieb.	0	n	1	0	Apiaceae Lindl.
Iz_1_1009	DJI_0242	Pilosella echioides (Lumn.) F.W.Schultz & Sch.Bip.	0	n	1	0	Asteraceae Bercht. & J.Presl
Iz_1_1009	DJI_0242	Pilosella officinarum F. Schultz et Sch. Bip.	0	n	1	0	Asteraceae Bercht. & J.Presl
Iz_1_1009	DJI_0242	Pilosella sp.	0		NA	0	Asteraceae Bercht. & J.Presl
Iz_1_1009	DJI_0242	Pinus sylvestris L.	0	n	1	1	Pinaceae Spreng. ex F.Rudolphi
Iz_1_1009	DJI_0242	Poa annua L.	0	n	1	0	Poaceae Barnhart
Iz_1_1009	DJI_0242	Poa nemoralis L.	0	n	1	0	Poaceae Barnhart
Iz_1_1009	DJI_0242	Poa sp.	0		NA	0	Poaceae Barnhart
Iz_1_1009	DJI_0242	Polygonatum odoratum (Mill.) Druce	0	n	1	0	Asparagaceae Juss.
Iz_1_1009	DJI_0242	Polygonum arenarium Waldst. & Kit.	0	n	1	0	Polygonaceae Juss.
Iz_1_1009	DJI_0242	Populus nigra L.	0	n	1	1	Salicaceae Mirb.
Iz_1_1009	DJI_0242	Populus tremula L.	0	n	1	1	Salicaceae Mirb.
Iz_1_1009	DJI_0242	Populus Ã canescens (Aiton) Sm.	0	n	1	1	Salicaceae Mirb.
Iz_1_1009	DJI_0242	Potentilla argentea L.	0	n	1	0	Rosaceae Juss.
Iz_1_1009	DJI_0242	Psammophiliella muralis (L.) Ikonn.	0	n	1	0	Caryophyllaceae Juss.
Iz_1_1009	DJI_0242	Psephellus sumensis (Kalen.) Greuter	0	n	1	0	Asteraceae Bercht. & J.Presl
Iz_1_1009	DJI_0242	Pulsatilla pratensis (L.) Mill. s.l.	0	n	1	0	Ranunculaceae Juss.
Iz_1_1009	DJI_0242	Pyrus communis L.	0	a	0	1	Rosaceae Juss.
Iz_1_1009	DJI_0242	Quercus robur L.	0	n	1	1	Fagaceae Dumort.
Iz_1_1009	DJI_0242	Robinia pseudoacacia L.	0	a	0	1	Fabaceae Lindl.
Iz_1_1009	DJI_0242	Rosa sp.	0		NA	1	Rosaceae Juss.
Iz_1_1009	DJI_0242	Rubus idaeus L.	0	n	1	0	Rosaceae Juss.
Iz_1_1009	DJI_0242	Rumex acetosella L.	3	n	1	0	Polygonaceae Juss.
Iz_1_1009	DJI_0242	Rumex thyrsiflorus Fingerh.	0	n	1	0	Polygonaceae Juss.
Iz_1_1009	DJI_0242	Salix alba L.	0	n	1	1	Salicaceae Mirb.
Iz_1_1009	DJI_0242	Sambucus nigra L.	0	n	1	1	Viburnaceae Raf.
Iz_1_1009	DJI_0242	Secale sylvestre Host	0	n	1	0	Poaceae Barnhart
Iz_1_1009	DJI_0242	Sempervivum ruthenicum Schnittsp. & C.B. Lehm.	0	n	1	0	Crassulaceae J.St.-Hil.
Iz_1_1009	DJI_0242	Setaria viridis (L.) P. Beauv.	0	n	1	0	Poaceae Barnhart
Iz_1_1009	DJI_0242	Silene borysthenica (Gruner) Walters	0	n	1	0	Caryophyllaceae Juss.
Iz_1_1009	DJI_0242	Silene latifolia subsp. alba (Mill.) Greuter & Burdet	0	n	1	0	Caryophyllaceae Juss.
Iz_1_1009	DJI_0242	Solidago virgaurea L.	5	n	1	0	Asteraceae Bercht. & J.Presl
Iz_1_1009	DJI_0242	Sorbus aucuparia L.	0	n	1	1	Rosaceae Juss.
Iz_1_1009	DJI_0242	Stachys recta L.	0	n	1	0	Lamiacea Lindl.
Iz_1_1009	DJI_0242	Tanacetum vulgare L.	0	n	1	0	Asteraceae Bercht. & J.Presl
Iz_1_1009	DJI_0242	Thymus pallasianus Heinr.Braun	0	n	1	0	Lamiacea Lindl.
Iz_1_1009	DJI_0242	Tilia cordata Mill.	0	n	1	1	Malvaceae Juss.
Iz_1_1009	DJI_0242	Trifolium alpestre L.	0	n	1	0	Fabaceae Lindl.
Iz_1_1009	DJI_0242	Trifolium arvense L.	0	n	1	0	Fabaceae Lindl.
Iz_1_1009	DJI_0242	Ulmus glabra Huds.	0	n	1	1	Ulmaceae Mirb.
Iz_1_1009	DJI_0242	Verbascum densiflorum Bertol.	0	n	1	0	Scrophulariaceae Juss.
Iz_1_1009	DJI_0242	Veronica austriaca L.	0	n	1	0	Plantaginaceae Juss.
Iz_1_1009	DJI_0242	Veronica incana L.	0	n	1	0	Plantaginaceae Juss.
Iz_1_1009	DJI_0242	Veronica verna L.	0	n	1	0	Plantaginaceae Juss.
Iz_1_1009	DJI_0242	Vincetoxicum hirundinaria Medik	0	n	1	0	Apocynaceae Juss.
Iz_1_1009	DJI_0250	Acer tataricum L.	0	n	1	1	Sapindaceae Juss.
Iz_1_1009	DJI_0250	Achillea millefolium L. s.l.	0	n	1	0	Asteraceae Bercht. & J.Presl

Iz_1_1009	DJI_0250	Allium oleraceum L.	0	n	1	0	Amaryllidaceae J.St.-Hil.
Iz_1_1009	DJI_0250	Ambrosia artemisiifolia L.	0	a	0	0	Asteraceae Bercht. & J.Presl
Iz_1_1009	DJI_0250	Amorpha fruticosa L.	0	a	0	0	Fabaceae Lindl.
Iz_1_1009	DJI_0250	Anthericum ramosum L.	0	n	1	0	Asparagaceae Juss.
Iz_1_1009	DJI_0250	Artemisia marschalliana Spreng.	0	n	1	0	Asteraceae Bercht. & J.Presl
Iz_1_1009	DJI_0250	Asparagus officinalis L.	0	n	1	0	Asparagaceae Juss.
Iz_1_1009	DJI_0250	Astragalus cicer L.	0	n	1	0	Fabaceae Lindl.
Iz_1_1009	DJI_0250	Bassia laniflora (S.G.Gmel.) A.J.Scott	0	n	1	0	Amaranthaceae Juss.
Iz_1_1009	DJI_0250	Berberis vulgaris L.	0	n	1	0	Berberidaceae Juss.
Iz_1_1009	DJI_0250	Betula pendula Roth.	0	n	1	1	Betulaceae Gray
Iz_1_1009	DJI_0250	Betula pendula Roth.	0	n	1	0	Poaceae Barnhart
Iz_1_1009	DJI_0250	Bromus inermis Leyss.	0		NA	NA	
Iz_1_1009	DJI_0250	Bromus squarrosus L.	0	a	0	0	Poaceae Barnhart
Iz_1_1009	DJI_0250	Bromus tectorum L.	0	a	0	0	Poaceae Barnhart
Iz_1_1009	DJI_0250	Calamagrostis epigejos (L.) Roth	1	n	1	0	Poaceae Barnhart
Iz_1_1009	DJI_0250	Carduus acanthoides L.	0	n	1	0	Asteraceae Bercht. & J.Presl
Iz_1_1009	DJI_0250	Carex sp.	0	n	1	0	Cyperaceae Juss.
Iz_1_1009	DJI_0250	Centaurea majorovii Dumbadze	0	n	1	0	Asteraceae Bercht. & J.Presl
Iz_1_1009	DJI_0250	Chamaecytisus ruthenicus (Fisch.ex Wol.) Klaskova	0	n	1	0	Fabaceae Lindl.
Iz_1_1009	DJI_0250	Chelidonium majus L.	0	n	1	0	Papaveraceae Juss.
Iz_1_1009	DJI_0250	Chenopodium betaceum Andrz.	0	n	1	0	Amaranthaceae Juss.
Iz_1_1009	DJI_0250	Chenopodium cf. album	0	n	1	0	Amaranthaceae Juss.
Iz_1_1009	DJI_0250	Chenopodium sp.	0		NA	0	Amaranthaceae Juss.
Iz_1_1009	DJI_0250	Chondrilla juncea L.	0	n	1	0	Asteraceae Bercht. & J.Presl
Iz_1_1009	DJI_0250	Cirsium vulgare (Savi) Ten.	0	n	1	0	Asteraceae Bercht. & J.Presl
Iz_1_1009	DJI_0250	Clinopodium acinos (L.) Kuntze	0	n	1	0	Lamiacea Lindl.
Iz_1_1009	DJI_0250	Convallaria majalis L.	0	n	1	0	Asparagaceae Juss.
Iz_1_1009	DJI_0250	Crataegus rhipidophylla Gand.	0	n	1	1	Rosaceae Juss.
Iz_1_1009	DJI_0250	Crepis foetida L.	0	n	1	0	Asteraceae Bercht. & J.Presl
Iz_1_1009	DJI_0250	Dactylis glomerata L.	0	n	1	0	Poaceae Barnhart
Iz_1_1009	DJI_0250	Dianthus campestris M.Bieb.	0	n	1	0	Caryophyllaceae Juss.
Iz_1_1009	DJI_0250	Digitaria sanguinalis (L.) Scop.	0	a	0	0	Poaceae Barnhart
Iz_1_1009	DJI_0250	Elymus repens (L.) Gould	0	n	1	0	Poaceae Barnhart
Iz_1_1009	DJI_0250	Epilobium tetragonum L.	0	n	1	0	Onagraceae Juss.
Iz_1_1009	DJI_0250	Eragrostis minor Host	0	a	0	0	Poaceae Barnhart
Iz_1_1009	DJI_0250	Erigeron canadensis L.	2	a	0	0	Asteraceae Bercht. & J.Presl
Iz_1_1009	DJI_0250	Euphorbia seguieriana Neck.	0	n	1	0	Euphorbiaceae Juss.
Iz_1_1009	DJI_0250	Fallopia dumetorum (L.) Holub	0	n	1	0	Polygonaceae Juss.
Iz_1_1009	DJI_0250	Festuca beckeri (Hack.) Trautv.	0	n	1	0	Poaceae Barnhart
Iz_1_1009	DJI_0250	Fragaria vesca L.	0	n	1	0	Rosaceae Juss.
Iz_1_1009	DJI_0250	Frangula alnus Mill.	0	n	1	1	Rhamnaceae Juss.
Iz_1_1009	DJI_0250	Fraxinus pennsylvanica Marshall	0	a	0	1	Oleaceae Hoffmanns. & Link
Iz_1_1009	DJI_0250	Galeopsis bifida Boenn.	0	n	1	0	Lamiacea Lindl.
Iz_1_1009	DJI_0250	Galium verum L.	0	n	1	0	Rubiaceae Juss.
Iz_1_1009	DJI_0250	Genista tinctoria L.	0	n	1	0	Fabaceae Lindl.
Iz_1_1009	DJI_0250	Geranium robertianum L.	0	n	1	0	Geraniaceae Juss.
Iz_1_1009	DJI_0250	Geranium sanquineum L.	0	n	1	0	Geraniaceae Juss.
Iz_1_1009	DJI_0250	Gypsophila paniculata L.	0	n	1	0	Caryophyllaceae Juss.
Iz_1_1009	DJI_0250	Helichrysum arenarium (L.) Moench	0	n	1	0	Asteraceae Bercht. & J.Presl
Iz_1_1009	DJI_0250	Herniaria polygama J.Gay	0	n	1	0	Caryophyllaceae Juss.
Iz_1_1009	DJI_0250	Hieracium robustum Fr.	0	n	1	0	Asteraceae Bercht. & J.Presl
Iz_1_1009	DJI_0250	Hieracium umbellatum L.	0	n	1	NA	Asteraceae Bercht. & J.Presl
Iz_1_1009	DJI_0250	Hylotelephium maximum (L.) Holub	0	n	1	0	Crassulaceae J.St.-Hil.
Iz_1_1009	DJI_0250	Jacobaea borysthenica (DC.) B.Nord. & Greuter	0	n	1	0	Asteraceae Bercht. & J.Presl
Iz_1_1009	DJI_0250	Jasione montana L.	0	n	1	0	Campanulaceae Juss.
Iz_1_1009	DJI_0250	Jurinea cyanoides (L.) Rchb.s.l.	0	n	1	0	Asteraceae Bercht. & J.Presl
Iz_1_1009	DJI_0250	Koeleria glauca (Spreng.) DC.	0	n	1	0	Poaceae Barnhart
Iz_1_1009	DJI_0250	Lactuca serriola L.	0	a	0	0	Asteraceae Bercht. & J.Presl
Iz_1_1009	DJI_0250	Linaria genistifolia (L.) Mill.	0	n	1	0	Plantaginaceae Juss.
Iz_1_1009	DJI_0250	Lomelosia argentea (L.) Greuter & Burdet	0	n	1	0	Caprifoliaceae Juss.
Iz_1_1009	DJI_0250	Melampyrum pratense L.	0	n	1	0	Orobanchaceae Vent.
Iz_1_1009	DJI_0250	Oenothera biennis L.	0	a	0	0	Onagraceae Juss.
Iz_1_1009	DJI_0250	Pentanema britannica (L.) D.Gut.Larr., Santos-Vicente, Anderb., E.Rico & M.M.Mart.Ort.	0	n	1	0	Asteraceae Bercht. & J.Presl
Iz_1_1009	DJI_0250	Peucedanum oreoselinum (L.) Moench	0	n	1	0	Apiaceae Lindl.
Iz_1_1009	DJI_0250	Peucedanum ruthenicum M.Bieb.	0	n	1	0	Apiaceae Lindl.
Iz_1_1009	DJI_0250	Pilosella echioides (Lumn.) F.W.Schultz & Sch.Bip.	0	n	1	0	Asteraceae Bercht. & J.Presl
Iz_1_1009	DJI_0250	Pilosella officinarum F. Schultz et Sch. Bip.	0	n	1	0	Asteraceae Bercht. & J.Presl
Iz_1_1009	DJI_0250	Pilosella sp.	0		NA	0	Asteraceae Bercht. & J.Presl
Iz_1_1009	DJI_0250	Pinus sylvestris L.	0	n	1	1	Pinaceae Spreng. ex F.Rudolphi
Iz_1_1009	DJI_0250	Poa annua L.	0	n	1	0	Poaceae Barnhart
Iz_1_1009	DJI_0250	Poa nemoralis L.	0	n	1	0	Poaceae Barnhart
Iz_1_1009	DJI_0250	Poa sp.	0		NA	0	Poaceae Barnhart
Iz_1_1009	DJI_0250	Polygonatum odoratum (Mill.) Druce	0	n	1	0	Asparagaceae Juss.
Iz_1_1009	DJI_0250	Polygonum arenarium Waldst. & Kit.	0	n	1	0	Polygonaceae Juss.
Iz_1_1009	DJI_0250	Populus nigra L.	0	n	1	1	Salicaceae Mirb.
Iz_1_1009	DJI_0250	Populus tremula L.	0	n	1	1	Salicaceae Mirb.
Iz_1_1009	DJI_0250	Populus Ã canescens (Aiton) Sm.	0	n	1	1	Salicaceae Mirb.
Iz_1_1009	DJI_0250	Potentilla argentea L.	0	n	1	0	Rosaceae Juss.
Iz_1_1009	DJI_0250	Psammophiliella muralis (L.) Ikonn.	0	n	1	0	Caryophyllaceae Juss.
Iz_1_1009	DJI_0250	Psephellus sumensis (Kalen.) Greuter	0	n	1	0	Asteraceae Bercht. & J.Presl
Iz_1_1009	DJI_0250	Pulsatilla pratensis (L.) Mill. s.l.	0	n	1	0	Ranunculaceae Juss.
Iz_1_1009	DJI_0250	Pyrus communis L.	0	a	0	1	Rosaceae Juss.
Iz_1_1009	DJI_0250	Quercus robur L.	0	n	1	1	Fagaceae Dumort.
Iz_1_1009	DJI_0250	Robinia pseudoacacia L.	0	a	0	1	Fabaceae Lindl.
Iz_1_1009	DJI_0250	Rosa sp.	0		NA	1	Rosaceae Juss.
Iz_1_1009	DJI_0250	Rubus idaeus L.	0	n	1	0	Rosaceae Juss.
Iz_1_1009	DJI_0250	Rumex acetosella L.	0	n	1	0	Polygonaceae Juss.
Iz_1_1009	DJI_0250	Rumex thyrsiflorus Fingerh.	0	n	1	0	Polygonaceae Juss.
Iz_1_1009	DJI_0250	Salix alba L.	0	n	1	1	Salicaceae Mirb.
Iz_1_1009	DJI_0250	Sambucus nigra L.	0	n	1	1	Viburnaceae Raf.
Iz_1_1009	DJI_0250	Secale sylvestre Host	0	n	1	0	Poaceae Barnhart
Iz_1_1009	DJI_0250	Sempervivum ruthenicum Schnittsp. & C.B. Lehm.	0	n	1	0	Crassulaceae J.St.-Hil.
Iz_1_1009	DJI_0250	Setaria viridis (L.) P. Beauv.	0	n	1	0	Poaceae Barnhart
Iz_1_1009	DJI_0250	Silene borysthenica (Gruner) Walters	0	n	1	0	Caryophyllaceae Juss.
Iz_1_1009	DJI_0250	Silene latifolia subsp. alba (Mill.) Greuter & Burdet	0	n	1	0	Caryophyllaceae Juss.
Iz_1_1009	DJI_0250	Solidago virgaurea L.	2	n	1	0	Asteraceae Bercht. & J.Presl
Iz_1_1009	DJI_0250	Sorbus aucuparia L.	0	n	1	1	Rosaceae Juss.
Iz_1_1009	DJI_0250	Stachys recta L.	0	n	1	0	Lamiacea Lindl.

Iz_1_1009	DJI_0250	Tanacetum vulgare L.	0	n	1	0	Asteraceae Bercht. & J.Presl
Iz_1_1009	DJI_0250	Thymus pallasianus Heinr.Braun	0	n	1	0	Lamiacea Lindl.
Iz_1_1009	DJI_0250	Tilia cordata Mill.	0	n	1	1	Malvaceae Juss.
Iz_1_1009	DJI_0250	Trifolium alpestre L.	0	n	1	0	Fabaceae Lindl.
Iz_1_1009	DJI_0250	Trifolium arvense L.	0	n	1	0	Fabaceae Lindl.
Iz_1_1009	DJI_0250	Ulmus glabra Huds.	0	n	1	1	Ulmaceae Mirb.
Iz_1_1009	DJI_0250	Verbascum densiflorum Bertol.	0	n	1	0	Scrophulariaceae Juss.
Iz_1_1009	DJI_0250	Veronica austriaca L.	0	n	1	0	Plantaginaceae Juss.
Iz_1_1009	DJI_0250	Veronica incana L.	0	n	1	0	Plantaginaceae Juss.
Iz_1_1009	DJI_0250	Veronica verna L.	0	n	1	0	Plantaginaceae Juss.
Iz_1_1009	DJI_0250	Vincetoxicum hirundinaria Medik	0	n	1	0	Apocynaceae Juss.
Iz_1_1009	DJI_0256	Acer tataricum L.	0	n	1	1	Sapindaceae Juss.
Iz_1_1009	DJI_0256	Achillea millefolium L. s.l.	0	n	1	0	Asteraceae Bercht. & J.Presl
Iz_1_1009	DJI_0256	Allium oleraceum L.	0	n	1	0	Amaryllidaceae J.St.-Hil.
Iz_1_1009	DJI_0256	Ambrosia artemisiifolia L.	0	a	0	0	Asteraceae Bercht. & J.Presl
Iz_1_1009	DJI_0256	Amorpha fruticosa L.	0	a	0	0	Fabaceae Lindl.
Iz_1_1009	DJI_0256	Anthericum ramosum L.	0	n	1	0	Asparagaceae Juss.
Iz_1_1009	DJI_0256	Artemisia marschalliana Spreng.	0	n	1	0	Asteraceae Bercht. & J.Presl
Iz_1_1009	DJI_0256	Asparagus officinalis L.	0	n	1	0	Asparagaceae Juss.
Iz_1_1009	DJI_0256	Astragalus cicer L.	0	n	1	0	Fabaceae Lindl.
Iz_1_1009	DJI_0256	Bassia laniflora (S.G.Gmel.) A.J.Scott	0	n	1	0	Amaranthaceae Juss.
Iz_1_1009	DJI_0256	Berberis vulgaris L.	0	n	1	0	Berberidaceae Juss.
Iz_1_1009	DJI_0256	Betula pendula Roth.	0	n	1	1	Betulaceae Gray
Iz_1_1009	DJI_0256	Betula pendula Roth.	0	n	1	0	Poaceae Barnhart
Iz_1_1009	DJI_0256	Bromus inermis Leyss.	0		NA	NA	
Iz_1_1009	DJI_0256	Bromus squarrosus L.	0	a	0	0	Poaceae Barnhart
Iz_1_1009	DJI_0256	Bromus tectorum L.	0	a	0	0	Poaceae Barnhart
Iz_1_1009	DJI_0256	Calamagrostis epigejos (L.) Roth	3	n	1	0	Poaceae Barnhart
Iz_1_1009	DJI_0256	Carduus acanthoides L.	0	n	1	0	Asteraceae Bercht. & J.Presl
Iz_1_1009	DJI_0256	Carex sp.	0	n	1	0	Cyperaceae Juss.
Iz_1_1009	DJI_0256	Centaurea majorovii Dumbadze	0	n	1	0	Asteraceae Bercht. & J.Presl
Iz_1_1009	DJI_0256	Chamaecytisus ruthenicus (Fisch.ex Wol.) Klaskova	0	n	1	0	Fabaceae Lindl.
Iz_1_1009	DJI_0256	Chelidonium majus L.	0	n	1	0	Papaveraceae Juss.
Iz_1_1009	DJI_0256	Chenopodium betaceum Andrz.	0	n	1	0	Amaranthaceae Juss.
Iz_1_1009	DJI_0256	Chenopodium cf. album	0	n	1	0	Amaranthaceae Juss.
Iz_1_1009	DJI_0256	Chenopodium sp.	0		NA	0	Amaranthaceae Juss.
Iz_1_1009	DJI_0256	Chondrilla juncea L.	0	n	1	0	Asteraceae Bercht. & J.Presl
Iz_1_1009	DJI_0256	Cirsium vulgare (Savi) Ten.	0	n	1	0	Asteraceae Bercht. & J.Presl
Iz_1_1009	DJI_0256	Clinopodium acinos (L.) Kuntze	0	n	1	0	Lamiacea Lindl.
Iz_1_1009	DJI_0256	Convallaria majalis L.	0	n	1	0	Asparagaceae Juss.
Iz_1_1009	DJI_0256	Crataegus rhipidophylla Gand.	0	n	1	1	Rosaceae Juss.
Iz_1_1009	DJI_0256	Crepis foetida L.	0	n	1	0	Asteraceae Bercht. & J.Presl
Iz_1_1009	DJI_0256	Dactylis glomerata L.	0	n	1	0	Poaceae Barnhart
Iz_1_1009	DJI_0256	Dianthus campestris M.Bieb.	0	n	1	0	Caryophyllaceae Juss.
Iz_1_1009	DJI_0256	Digitaria sanguinalis (L.) Scop.	0	a	0	0	Poaceae Barnhart
Iz_1_1009	DJI_0256	Elymus repens (L.) Gould	0	n	1	0	Poaceae Barnhart
Iz_1_1009	DJI_0256	Epilobium tetragonum L.	0	n	1	0	Onagraceae Juss.
Iz_1_1009	DJI_0256	Eragrostis minor Host	0	a	0	0	Poaceae Barnhart
Iz_1_1009	DJI_0256	Erigeron canadensis L.	1	a	0	0	Asteraceae Bercht. & J.Presl
Iz_1_1009	DJI_0256	Euphorbia seguieriana Neck.	0	n	1	0	Euphorbiaceae Juss.
Iz_1_1009	DJI_0256	Fallopia dumetorum (L.) Holub	0	n	1	0	Polygonaceae Juss.
Iz_1_1009	DJI_0256	Festuca beckeri (Hack.) Trautv.	0	n	1	0	Poaceae Barnhart
Iz_1_1009	DJI_0256	Fragaria vesca L.	0	n	1	0	Rosaceae Juss.
Iz_1_1009	DJI_0256	Frangula alnus Mill.	0	n	1	1	Rhamnaceae Juss.
Iz_1_1009	DJI_0256	Fraxinus pennsylvanica Marshall	0	a	0	1	Oleaceae Hoffmanns. & Link
Iz_1_1009	DJI_0256	Galeopsis bifida Boenn.	0	n	1	0	Lamiacea Lindl.
Iz_1_1009	DJI_0256	Galium verum L.	0	n	1	0	Rubiaceae Juss.
Iz_1_1009	DJI_0256	Genista tinctoria L.	0	n	1	0	Fabaceae Lindl.
Iz_1_1009	DJI_0256	Geranium robertianum L.	0	n	1	0	Geraniaceae Juss.
Iz_1_1009	DJI_0256	Geranium sanquineum L.	0	n	1	0	Geraniaceae Juss.
Iz_1_1009	DJI_0256	Gypsophila paniculata L.	0	n	1	0	Caryophyllaceae Juss.
Iz_1_1009	DJI_0256	Helichrysum arenarium (L.) Moench	0	n	1	0	Asteraceae Bercht. & J.Presl
Iz_1_1009	DJI_0256	Herniaria polygama J.Gay	0	n	1	0	Caryophyllaceae Juss.
Iz_1_1009	DJI_0256	Hieracium robustum Fr.	0	n	1	0	Asteraceae Bercht. & J.Presl
Iz_1_1009	DJI_0256	Hieracium umbellatum L.	0	n	1	NA	Asteraceae Bercht. & J.Presl
Iz_1_1009	DJI_0256	Hylotelephium maximum (L.) Holub	0	n	1	0	Crassulaceae J.St.-Hil.
Iz_1_1009	DJI_0256	Jacobaea borysthenica (DC.) B.Nord. & Greuter	0	n	1	0	Asteraceae Bercht. & J.Presl
Iz_1_1009	DJI_0256	Jasione montana L.	0	n	1	0	Campanulaceae Juss.
Iz_1_1009	DJI_0256	Jurinea cyanoides (L.) Rchb.s.l.	0	n	1	0	Asteraceae Bercht. & J.Presl
Iz_1_1009	DJI_0256	Koeleria glauca (Spreng.) DC.	0	n	1	0	Poaceae Barnhart
Iz_1_1009	DJI_0256	Lactuca serriola L.	0	a	0	0	Asteraceae Bercht. & J.Presl
Iz_1_1009	DJI_0256	Linaria genistifolia (L.) Mill.	0	n	1	0	Plantaginaceae Juss.
Iz_1_1009	DJI_0256	Lomelosia argentea (L.) Greuter & Burdet	0	n	1	0	Caprifoliaceae Juss.
Iz_1_1009	DJI_0256	Melampyrum pratense L.	0	n	1	0	Orobanchaceae Vent.
Iz_1_1009	DJI_0256	Oenothera biennis L.	0	a	0	0	Onagraceae Juss.
Iz_1_1009	DJI_0256	Pentanema britannica (L.) D.Gut.Larr., Santos-Vicente, Anderb., E.Rico & M.M.Mart.Ort.	0	n	1	0	Asteraceae Bercht. & J.Presl
Iz_1_1009	DJI_0256	Peucedanum oreoselinum (L.) Moench	0	n	1	0	Apiaceae Lindl.
Iz_1_1009	DJI_0256	Peucedanum ruthenicum M.Bieb.	0	n	1	0	Apiaceae Lindl.
Iz_1_1009	DJI_0256	Pilosella echioides (Lumn.) F.W.Schultz & Sch.Bip.	0	n	1	0	Asteraceae Bercht. & J.Presl
Iz_1_1009	DJI_0256	Pilosella officinarum F. Schultz et Sch. Bip.	0	n	1	0	Asteraceae Bercht. & J.Presl
Iz_1_1009	DJI_0256	Pilosella sp.	0		NA	0	Asteraceae Bercht. & J.Presl
Iz_1_1009	DJI_0256	Pinus sylvestris L.	0	n	1	1	Pinaceae Spreng. ex F.Rudolphi
Iz_1_1009	DJI_0256	Poa annua L.	0	n	1	0	Poaceae Barnhart
Iz_1_1009	DJI_0256	Poa nemoralis L.	0	n	1	0	Poaceae Barnhart
Iz_1_1009	DJI_0256	Poa sp.	0		NA	0	Poaceae Barnhart
Iz_1_1009	DJI_0256	Polygonatum odoratum (Mill.) Druce	0	n	1	0	Asparagaceae Juss.
Iz_1_1009	DJI_0256	Polygonum arenarium Waldst. & Kit.	0	n	1	0	Polygonaceae Juss.
Iz_1_1009	DJI_0256	Populus nigra L.	0	n	1	1	Salicaceae Mirb.
Iz_1_1009	DJI_0256	Populus tremula L.	0	n	1	1	Salicaceae Mirb.
Iz_1_1009	DJI_0256	Populus Ã canescens (Aiton) Sm.	0	n	1	1	Salicaceae Mirb.
Iz_1_1009	DJI_0256	Potentilla argentea L.	0	n	1	0	Rosaceae Juss.
Iz_1_1009	DJI_0256	Psammophiliella muralis (L.) Ikonn.	0	n	1	0	Caryophyllaceae Juss.
Iz_1_1009	DJI_0256	Psephellus sumensis (Kalen.) Greuter	0	n	1	0	Asteraceae Bercht. & J.Presl
Iz_1_1009	DJI_0256	Pulsatilla pratensis (L.) Mill. s.l.	0	n	1	0	Ranunculaceae Juss.
Iz_1_1009	DJI_0256	Pyrus communis L.	0	a	0	1	Rosaceae Juss.
Iz_1_1009	DJI_0256	Quercus robur L.	0	n	1	1	Fagaceae Dumort.
Iz_1_1009	DJI_0256	Robinia pseudoacacia L.	0	a	0	1	Fabaceae Lindl.
Iz_1_1009	DJI_0256	Rosa sp.	0		NA	1	Rosaceae Juss.

Iz_1_1009	DJI_0256	Rubus idaeus L.	0	n	1	0	Rosaceae Juss.
Iz_1_1009	DJI_0256	Rumex acetosella L.	0	n	1	0	Polygonaceae Juss.
Iz_1_1009	DJI_0256	Rumex thyrsiflorus Fingerh.	0	n	1	0	Polygonaceae Juss.
Iz_1_1009	DJI_0256	Salix alba L.	0	n	1	1	Salicaceae Mirb.
Iz_1_1009	DJI_0256	Sambucus nigra L.	0	n	1	1	Viburnaceae Raf.
Iz_1_1009	DJI_0256	Secale sylvestre Host	0	n	1	0	Poaceae Barnhart
Iz_1_1009	DJI_0256	Sempervivum ruthenicum Schnittsp. & C.B. Lehm.	0	n	1	0	Crassulaceae J.St.-Hil.
Iz_1_1009	DJI_0256	Setaria viridis (L.) P. Beauv.	0	n	1	0	Poaceae Barnhart
Iz_1_1009	DJI_0256	Silene borysthenica (Gruner) Walters	0	n	1	0	Caryophyllaceae Juss.
Iz_1_1009	DJI_0256	Silene latifolia subsp. alba (Mill.) Greuter & Burdet	0	n	1	0	Caryophyllaceae Juss.
Iz_1_1009	DJI_0256	Solidago virgaurea L.	0	n	1	0	Asteraceae Bercht. & J.Presl
Iz_1_1009	DJI_0256	Sorbus aucuparia L.	0	n	1	1	Rosaceae Juss.
Iz_1_1009	DJI_0256	Stachys recta L.	0	n	1	0	Lamiacea Lindl.
Iz_1_1009	DJI_0256	Tanacetum vulgare L.	0	n	1	0	Asteraceae Bercht. & J.Presl
Iz_1_1009	DJI_0256	Thymus pallasianus Heinr.Braun	0	n	1	0	Lamiacea Lindl.
Iz_1_1009	DJI_0256	Tilia cordata Mill.	0	n	1	1	Malvaceae Juss.
Iz_1_1009	DJI_0256	Trifolium alpestre L.	0	n	1	0	Fabaceae Lindl.
Iz_1_1009	DJI_0256	Trifolium arvense L.	0	n	1	0	Fabaceae Lindl.
Iz_1_1009	DJI_0256	Ulmus glabra Huds.	0	n	1	1	Ulmaceae Mirb.
Iz_1_1009	DJI_0256	Verbascum densiflorum Bertol.	0	n	1	0	Scrophulariaceae Juss.
Iz_1_1009	DJI_0256	Veronica austriaca L.	0	n	1	0	Plantaginaceae Juss.
Iz_1_1009	DJI_0256	Veronica incana L.	0	n	1	0	Plantaginaceae Juss.
Iz_1_1009	DJI_0256	Veronica verna L.	0	n	1	0	Plantaginaceae Juss.
Iz_1_1009	DJI_0256	Vincetoxicum hirundinaria Medik	0	n	1	0	Apocynaceae Juss.
Iz_1_1109	DJI_0029	Acer tataricum L.	0	n	1	1	Sapindaceae Juss.
Iz_1_1109	DJI_0029	Achillea millefolium L. s.l.	0	n	1	0	Asteraceae Bercht. & J.Presl
Iz_1_1109	DJI_0029	Allium oleraceum L.	0	n	1	0	Amaryllidaceae J.St.-Hil.
Iz_1_1109	DJI_0029	Ambrosia artemisiifolia L.	0	a	0	0	Asteraceae Bercht. & J.Presl
Iz_1_1109	DJI_0029	Amorpha fruticosa L.	0	a	0	0	Fabaceae Lindl.
Iz_1_1109	DJI_0029	Anthericum ramosum L.	0	n	1	0	Asparagaceae Juss.
Iz_1_1109	DJI_0029	Artemisia marschalliana Spreng.	0	n	1	0	Asteraceae Bercht. & J.Presl
Iz_1_1109	DJI_0029	Asparagus officinalis L.	0	n	1	0	Asparagaceae Juss.
Iz_1_1109	DJI_0029	Astragalus cicer L.	0	n	1	0	Fabaceae Lindl.
Iz_1_1109	DJI_0029	Bassia laniflora (S.G.Gmel.) A.J.Scott	0	n	1	0	Amaranthaceae Juss.
Iz_1_1109	DJI_0029	Berberis vulgaris L.	0	n	1	0	Berberidaceae Juss.
Iz_1_1109	DJI_0029	Betula pendula Roth.	0	n	1	1	Betulaceae Gray
Iz_1_1109	DJI_0029	Betula pendula Roth.	0	n	1	0	Poaceae Barnhart
Iz_1_1109	DJI_0029	Bromus inermis Leyss.	0		NA	NA	
Iz_1_1109	DJI_0029	Bromus squarrosus L.	0	a	0	0	Poaceae Barnhart
Iz_1_1109	DJI_0029	Bromus tectorum L.	0	a	0	0	Poaceae Barnhart
Iz_1_1109	DJI_0029	Calamagrostis epigejos (L.) Roth	35	n	1	0	Poaceae Barnhart
Iz_1_1109	DJI_0029	Carduus acanthoides L.	0	n	1	0	Asteraceae Bercht. & J.Presl
Iz_1_1109	DJI_0029	Carex sp.	0	n	1	0	Cyperaceae Juss.
Iz_1_1109	DJI_0029	Centaurea majorovii Dumbadze	0	n	1	0	Asteraceae Bercht. & J.Presl
Iz_1_1109	DJI_0029	Chamaecytisus ruthenicus (Fisch.ex Wol.) Klaskova	0	n	1	0	Fabaceae Lindl.
Iz_1_1109	DJI_0029	Chelidonium majus L.	0	n	1	0	Papaveraceae Juss.
Iz_1_1109	DJI_0029	Chenopodium betaceum Andrz.	0	n	1	0	Amaranthaceae Juss.
Iz_1_1109	DJI_0029	Chenopodium cf. album	0	n	1	0	Amaranthaceae Juss.
Iz_1_1109	DJI_0029	Chenopodium sp.	0		NA	0	Amaranthaceae Juss.
Iz_1_1109	DJI_0029	Chondrilla juncea L.	0	n	1	0	Asteraceae Bercht. & J.Presl
Iz_1_1109	DJI_0029	Cirsium vulgare (Savi) Ten.	0	n	1	0	Asteraceae Bercht. & J.Presl
Iz_1_1109	DJI_0029	Clinopodium acinos (L.) Kuntze	0	n	1	0	Lamiacea Lindl.
Iz_1_1109	DJI_0029	Convallaria majalis L.	0	n	1	0	Asparagaceae Juss.
Iz_1_1109	DJI_0029	Crataegus rhipidophylla Gand.	0	n	1	1	Rosaceae Juss.
Iz_1_1109	DJI_0029	Crepis foetida L.	0	n	1	0	Asteraceae Bercht. & J.Presl
Iz_1_1109	DJI_0029	Dactylis glomerata L.	0	n	1	0	Poaceae Barnhart
Iz_1_1109	DJI_0029	Dianthus campestris M.Bieb.	0	n	1	0	Caryophyllaceae Juss.
Iz_1_1109	DJI_0029	Digitaria sanguinalis (L.) Scop.	0	a	0	0	Poaceae Barnhart
Iz_1_1109	DJI_0029	Elymus repens (L.) Gould	0	n	1	0	Poaceae Barnhart
Iz_1_1109	DJI_0029	Epilobium tetragonum L.	0	n	1	0	Onagraceae Juss.
Iz_1_1109	DJI_0029	Eragrostis minor Host	0	a	0	0	Poaceae Barnhart
Iz_1_1109	DJI_0029	Erigeron canadensis L.	0	a	0	0	Asteraceae Bercht. & J.Presl
Iz_1_1109	DJI_0029	Euphorbia seguieriana Neck.	0	n	1	0	Euphorbiaceae Juss.
Iz_1_1109	DJI_0029	Fallopia dumetorum (L.) Holub	0	n	1	0	Polygonaceae Juss.
Iz_1_1109	DJI_0029	Festuca beckeri (Hack.) Trautv.	0	n	1	0	Poaceae Barnhart
Iz_1_1109	DJI_0029	Fragaria vesca L.	0	n	1	0	Rosaceae Juss.
Iz_1_1109	DJI_0029	Frangula alnus Mill.	0	n	1	1	Rhamnaceae Juss.
Iz_1_1109	DJI_0029	Fraxinus pennsylvanica Marshall	0	a	0	1	Oleaceae Hoffmanns. & Link
Iz_1_1109	DJI_0029	Galeopsis bifida Boenn.	0	n	1	0	Lamiacea Lindl.
Iz_1_1109	DJI_0029	Galium verum L.	0	n	1	0	Rubiaceae Juss.
Iz_1_1109	DJI_0029	Genista tinctoria L.	0	n	1	0	Fabaceae Lindl.
Iz_1_1109	DJI_0029	Geranium robertianum L.	0	n	1	0	Geraniaceae Juss.
Iz_1_1109	DJI_0029	Geranium sanquineum L.	0	n	1	0	Geraniaceae Juss.
Iz_1_1109	DJI_0029	Gypsophila paniculata L.	0	n	1	0	Caryophyllaceae Juss.
Iz_1_1109	DJI_0029	Helichrysum arenarium (L.) Moench	0	n	1	0	Asteraceae Bercht. & J.Presl
Iz_1_1109	DJI_0029	Herniaria polygama J.Gay	0	n	1	0	Caryophyllaceae Juss.
Iz_1_1109	DJI_0029	Hieracium robustum Fr.	0	n	1	0	Asteraceae Bercht. & J.Presl
Iz_1_1109	DJI_0029	Hieracium umbellatum L.	0	n	1	NA	Asteraceae Bercht. & J.Presl
Iz_1_1109	DJI_0029	Hylotelephium maximum (L.) Holub	0.1	n	1	0	Crassulaceae J.St.-Hil.
Iz_1_1109	DJI_0029	Jacobaea borysthenica (DC.) B.Nord. & Greuter	0	n	1	0	Asteraceae Bercht. & J.Presl
Iz_1_1109	DJI_0029	Jasione montana L.	0	n	1	0	Campanulaceae Juss.
Iz_1_1109	DJI_0029	Jurinea cyanoides (L.) Rchb.s.l.	0	n	1	0	Asteraceae Bercht. & J.Presl
Iz_1_1109	DJI_0029	Koeleria glauca (Spreng.) DC.	0	n	1	0	Poaceae Barnhart
Iz_1_1109	DJI_0029	Lactuca serriola L.	0	a	0	0	Asteraceae Bercht. & J.Presl
Iz_1_1109	DJI_0029	Linaria genistifolia (L.) Mill.	0	n	1	0	Plantaginaceae Juss.
Iz_1_1109	DJI_0029	Lomelosia argentea (L.) Greuter & Burdet	0	n	1	0	Caprifoliaceae Juss.
Iz_1_1109	DJI_0029	Melampyrum pratense L.	0	n	1	0	Orobanchaceae Vent.
Iz_1_1109	DJI_0029	Oenothera biennis L.	0	a	0	0	Onagraceae Juss.
Iz_1_1109	DJI_0029	Pentanema britannica (L.) D.Gut.Larr., Santos-Vicente, Anderb., E.Rico & M.M.Mart.Ort.	0	n	1	0	Asteraceae Bercht. & J.Presl
Iz_1_1109	DJI_0029	Peucedanum oreoselinum (L.) Moench	0	n	1	0	Apiaceae Lindl.
Iz_1_1109	DJI_0029	Peucedanum ruthenicum M.Bieb.	0	n	1	0	Apiaceae Lindl.
Iz_1_1109	DJI_0029	Pilosella echioides (Lumn.) F.W.Schultz & Sch.Bip.	0	n	1	0	Asteraceae Bercht. & J.Presl
Iz_1_1109	DJI_0029	Pilosella officinarum F. Schultz et Sch. Bip.	0	n	1	0	Asteraceae Bercht. & J.Presl
Iz_1_1109	DJI_0029	Pilosella sp.	0		NA	0	Asteraceae Bercht. & J.Presl
Iz_1_1109	DJI_0029	Pinus sylvestris L.	0	n	1	1	Pinaceae Spreng. ex F.Rudolphi
Iz_1_1109	DJI_0029	Poa annua L.	0	n	1	0	Poaceae Barnhart
Iz_1_1109	DJI_0029	Poa nemoralis L.	0	n	1	0	Poaceae Barnhart
Iz_1_1109	DJI_0029	Poa sp.	0		NA	0	Poaceae Barnhart

Iz_1_1109	DJI_0029	Polygonatum odoratum (Mill.) Druce	0	n	1	0	Asparagaceae Juss.
Iz_1_1109	DJI_0029	Polygonum arenarium Waldst. & Kit.	0	n	1	0	Polygonaceae Juss.
Iz_1_1109	DJI_0029	Populus nigra L.	0	n	1	1	Salicaceae Mirb.
Iz_1_1109	DJI_0029	Populus sp.	0	n	1	1	Salicaceae Mirb.
Iz_1_1109	DJI_0029	Populus tremula L.	0	n	1	1	Salicaceae Mirb.
Iz_1_1109	DJI_0029	Populus Ã canescens (Aiton) Sm.	0	n	1	1	Salicaceae Mirb.
Iz_1_1109	DJI_0029	Potentilla argentea L.	0	n	1	0	Rosaceae Juss.
Iz_1_1109	DJI_0029	Psammophiliella muralis (L.) Ikonn.	0	n	1	0	Caryophyllaceae Juss.
Iz_1_1109	DJI_0029	Psephellus sumensis (Kalen.) Greuter	0	n	1	0	Asteraceae Bercht. & J.Presl
Iz_1_1109	DJI_0029	Pulsatilla pratensis (L.) Mill. s.l.	0	n	1	0	Ranunculaceae Juss.
Iz_1_1109	DJI_0029	Pyrus communis L.	0	a	0	1	Rosaceae Juss.
Iz_1_1109	DJI_0029	Quercus robur L.	0	n	1	1	Fagaceae Dumort.
Iz_1_1109	DJI_0029	Robinia pseudoacacia L.	0	a	0	1	Fabaceae Lindl.
Iz_1_1109	DJI_0029	Rosa sp.	0		NA	1	Rosaceae Juss.
Iz_1_1109	DJI_0029	Rubus idaeus L.	0	n	1	0	Rosaceae Juss.
Iz_1_1109	DJI_0029	Rumex acetosella L.	0	n	1	0	Polygonaceae Juss.
Iz_1_1109	DJI_0029	Rumex thyrsiflorus Fingerh.	0	n	1	0	Polygonaceae Juss.
Iz_1_1109	DJI_0029	Salix alba L.	0	n	1	1	Salicaceae Mirb.
Iz_1_1109	DJI_0029	Sambucus nigra L.	0	n	1	1	Viburnaceae Raf.
Iz_1_1109	DJI_0029	Secale sylvestre Host	0	n	1	0	Poaceae Barnhart
Iz_1_1109	DJI_0029	Sempervivum ruthenicum Schnittsp. & C.B. Lehm.	0	n	1	0	Crassulaceae J.St.-Hil.
Iz_1_1109	DJI_0029	Setaria viridis (L.) P. Beauv.	0	n	1	0	Poaceae Barnhart
Iz_1_1109	DJI_0029	Silene borysthenica (Gruner) Walters	0	n	1	0	Caryophyllaceae Juss.
Iz_1_1109	DJI_0029	Silene latifolia subsp. alba (Mill.) Greuter & Burdet	0	n	1	0	Caryophyllaceae Juss.
Iz_1_1109	DJI_0029	Silene nutans L.	0	n	1	0	Caryophyllaceae Juss.
Iz_1_1109	DJI_0029	Solanum nigrum L.	0		NA	NA	
Iz_1_1109	DJI_0029	Solidago virgaurea L.	0	n	1	0	Asteraceae Bercht. & J.Presl
Iz_1_1109	DJI_0029	Sorbus aucuparia L.	0	n	1	1	Rosaceae Juss.
Iz_1_1109	DJI_0029	Stachys recta L.	0	n	1	0	Lamiacea Lindl.
Iz_1_1109	DJI_0029	Tanacetum vulgare L.	0	n	1	0	Asteraceae Bercht. & J.Presl
Iz_1_1109	DJI_0029	Thymus pallasianus Heinr.Braun	0	n	1	0	Lamiacea Lindl.
Iz_1_1109	DJI_0029	Tilia cordata Mill.	0	n	1	1	Malvaceae Juss.
Iz_1_1109	DJI_0029	Trifolium alpestre L.	0	n	1	0	Fabaceae Lindl.
Iz_1_1109	DJI_0029	Trifolium arvense L.	0	n	1	0	Fabaceae Lindl.
Iz_1_1109	DJI_0029	Ulmus glabra Huds.	0	n	1	1	Ulmaceae Mirb.
Iz_1_1109	DJI_0029	Verbascum densiflorum Bertol.	0	n	1	0	Scrophulariaceae Juss.
Iz_1_1109	DJI_0029	Veronica austriaca L.	0	n	1	0	Plantaginaceae Juss.
Iz_1_1109	DJI_0029	Veronica incana L.	0	n	1	0	Plantaginaceae Juss.
Iz_1_1109	DJI_0029	Veronica verna L.	0	n	1	0	Plantaginaceae Juss.
Iz_1_1109	DJI_0029	Vincetoxicum hirundinaria Medik	0	n	1	0	Apocynaceae Juss.
Iz_1_1109	DJI_0032	Acer tataricum L.	0	n	1	1	Sapindaceae Juss.
Iz_1_1109	DJI_0032	Achillea millefolium L. s.l.	0	n	1	0	Asteraceae Bercht. & J.Presl
Iz_1_1109	DJI_0032	Allium oleraceum L.	0	n	1	0	Amaryllidaceae J.St.-Hil.
Iz_1_1109	DJI_0032	Ambrosia artemisiifolia L.	0	a	0	0	Asteraceae Bercht. & J.Presl
Iz_1_1109	DJI_0032	Amorpha fruticosa L.	0	a	0	0	Fabaceae Lindl.
Iz_1_1109	DJI_0032	Anthericum ramosum L.	0	n	1	0	Asparagaceae Juss.
Iz_1_1109	DJI_0032	Artemisia marschalliana Spreng.	0	n	1	0	Asteraceae Bercht. & J.Presl
Iz_1_1109	DJI_0032	Asparagus officinalis L.	0	n	1	0	Asparagaceae Juss.
Iz_1_1109	DJI_0032	Astragalus cicer L.	0	n	1	0	Fabaceae Lindl.
Iz_1_1109	DJI_0032	Bassia laniflora (S.G.Gmel.) A.J.Scott	0	n	1	0	Amaranthaceae Juss.
Iz_1_1109	DJI_0032	Berberis vulgaris L.	0	n	1	0	Berberidaceae Juss.
Iz_1_1109	DJI_0032	Betula pendula Roth.	0	n	1	1	Betulaceae Gray
Iz_1_1109	DJI_0032	Betula pendula Roth.	0	n	1	0	Poaceae Barnhart
Iz_1_1109	DJI_0032	Bromus inermis Leyss.	0		NA	NA	
Iz_1_1109	DJI_0032	Bromus squarrosus L.	0	a	0	0	Poaceae Barnhart
Iz_1_1109	DJI_0032	Bromus tectorum L.	0	a	0	0	Poaceae Barnhart
Iz_1_1109	DJI_0032	Calamagrostis epigejos (L.) Roth	0.5	n	1	0	Poaceae Barnhart
Iz_1_1109	DJI_0032	Carduus acanthoides L.	0	n	1	0	Asteraceae Bercht. & J.Presl
Iz_1_1109	DJI_0032	Carex sp.	0	n	1	0	Cyperaceae Juss.
Iz_1_1109	DJI_0032	Centaurea majorovii Dumbadze	0	n	1	0	Asteraceae Bercht. & J.Presl
Iz_1_1109	DJI_0032	Chamaecytisus ruthenicus (Fisch.ex Wol.) Klaskova	0	n	1	0	Fabaceae Lindl.
Iz_1_1109	DJI_0032	Chelidonium majus L.	0	n	1	0	Papaveraceae Juss.
Iz_1_1109	DJI_0032	Chenopodium betaceum Andrz.	0	n	1	0	Amaranthaceae Juss.
Iz_1_1109	DJI_0032	Chenopodium cf. album	0	n	1	0	Amaranthaceae Juss.
Iz_1_1109	DJI_0032	Chenopodium sp.	0		NA	0	Amaranthaceae Juss.
Iz_1_1109	DJI_0032	Chondrilla juncea L.	0	n	1	0	Asteraceae Bercht. & J.Presl
Iz_1_1109	DJI_0032	Cirsium vulgare (Savi) Ten.	0	n	1	0	Asteraceae Bercht. & J.Presl
Iz_1_1109	DJI_0032	Clinopodium acinos (L.) Kuntze	0	n	1	0	Lamiacea Lindl.
Iz_1_1109	DJI_0032	Convallaria majalis L.	0	n	1	0	Asparagaceae Juss.
Iz_1_1109	DJI_0032	Crataegus rhipidophylla Gand.	0	n	1	1	Rosaceae Juss.
Iz_1_1109	DJI_0032	Crepis foetida L.	0	n	1	0	Asteraceae Bercht. & J.Presl
Iz_1_1109	DJI_0032	Dactylis glomerata L.	0	n	1	0	Poaceae Barnhart
Iz_1_1109	DJI_0032	Dianthus campestris M.Bieb.	0	n	1	0	Caryophyllaceae Juss.
Iz_1_1109	DJI_0032	Digitaria sanguinalis (L.) Scop.	0	a	0	0	Poaceae Barnhart
Iz_1_1109	DJI_0032	Elymus repens (L.) Gould	0	n	1	0	Poaceae Barnhart
Iz_1_1109	DJI_0032	Epilobium tetragonum L.	0	n	1	0	Onagraceae Juss.
Iz_1_1109	DJI_0032	Eragrostis minor Host	0	a	0	0	Poaceae Barnhart
Iz_1_1109	DJI_0032	Erigeron canadensis L.	0	a	0	0	Asteraceae Bercht. & J.Presl
Iz_1_1109	DJI_0032	Euphorbia seguieriana Neck.	0	n	1	0	Euphorbiaceae Juss.
Iz_1_1109	DJI_0032	Fallopia dumetorum (L.) Holub	0	n	1	0	Polygonaceae Juss.
Iz_1_1109	DJI_0032	Festuca beckeri (Hack.) Trautv.	0	n	1	0	Poaceae Barnhart
Iz_1_1109	DJI_0032	Fragaria vesca L.	0	n	1	0	Rosaceae Juss.
Iz_1_1109	DJI_0032	Frangula alnus Mill.	0	n	1	1	Rhamnaceae Juss.
Iz_1_1109	DJI_0032	Fraxinus pennsylvanica Marshall	0	a	0	1	Oleaceae Hoffmanns. & Link
Iz_1_1109	DJI_0032	Galeopsis bifida Boenn.	0	n	1	0	Lamiacea Lindl.
Iz_1_1109	DJI_0032	Galium verum L.	0	n	1	0	Rubiaceae Juss.
Iz_1_1109	DJI_0032	Genista tinctoria L.	0	n	1	0	Fabaceae Lindl.
Iz_1_1109	DJI_0032	Geranium robertianum L.	0	n	1	0	Geraniaceae Juss.
Iz_1_1109	DJI_0032	Geranium sanquineum L.	0	n	1	0	Geraniaceae Juss.
Iz_1_1109	DJI_0032	Gypsophila paniculata L.	0	n	1	0	Caryophyllaceae Juss.
Iz_1_1109	DJI_0032	Helichrysum arenarium (L.) Moench	0	n	1	0	Asteraceae Bercht. & J.Presl
Iz_1_1109	DJI_0032	Herniaria polygama J.Gay	0	n	1	0	Caryophyllaceae Juss.
Iz_1_1109	DJI_0032	Hieracium robustum Fr.	0	n	1	0	Asteraceae Bercht. & J.Presl
Iz_1_1109	DJI_0032	Hieracium umbellatum L.	0	n	1	NA	Asteraceae Bercht. & J.Presl
Iz_1_1109	DJI_0032	Hylotelephium maximum (L.) Holub	0	n	1	0	Crassulaceae J.St.-Hil.
Iz_1_1109	DJI_0032	Jacobaea borysthenica (DC.) B.Nord. & Greuter	0	n	1	0	Asteraceae Bercht. & J.Presl
Iz_1_1109	DJI_0032	Jasione montana L.	0	n	1	0	Campanulaceae Juss.
Iz_1_1109	DJI_0032	Jurinea cyanoides (L.) Rchb.s.l.	0	n	1	0	Asteraceae Bercht. & J.Presl

Iz_1_1109	DJI_0032	Koeleria glauca (Spreng.) DC.	0	n	1	0	Poaceae Barnhart
Iz_1_1109	DJI_0032	Lactuca serriola L.	0	a	0	0	Asteraceae Bercht. & J.Presl
Iz_1_1109	DJI_0032	Linaria genistifolia (L.) Mill.	0	n	1	0	Plantaginaceae Juss.
Iz_1_1109	DJI_0032	Lomelosia argentea (L.) Greuter & Burdet	0	n	1	0	Caprifoliaceae Juss.
Iz_1_1109	DJI_0032	Melampyrum pratense L.	0	n	1	0	Orobanchaceae Vent.
Iz_1_1109	DJI_0032	Oenothera biennis L.	0	a	0	0	Onagraceae Juss.
Iz_1_1109	DJI_0032	Pentanema britannica (L.) D.Gut.Larr., Santos-Vicente, Anderb., E.Rico & M.M.Mart.Ort.	0	n	1	0	Asteraceae Bercht. & J.Presl
Iz_1_1109	DJI_0032	Peucedanum oreoselinum (L.) Moench	0	n	1	0	Apiaceae Lindl.
Iz_1_1109	DJI_0032	Peucedanum ruthenicum M.Bieb.	0	n	1	0	Apiaceae Lindl.
Iz_1_1109	DJI_0032	Pilosella echioides (Lumn.) F.W.Schultz & Sch.Bip.	0	n	1	0	Asteraceae Bercht. & J.Presl
Iz_1_1109	DJI_0032	Pilosella officinarum F. Schultz et Sch. Bip.	0	n	1	0	Asteraceae Bercht. & J.Presl
Iz_1_1109	DJI_0032	Pilosella sp.	0		NA	0	Asteraceae Bercht. & J.Presl
Iz_1_1109	DJI_0032	Pinus sylvestris L.	0	n	1	1	Pinaceae Spreng. ex F.Rudolphi
Iz_1_1109	DJI_0032	Poa annua L.	0	n	1	0	Poaceae Barnhart
Iz_1_1109	DJI_0032	Poa nemoralis L.	0	n	1	0	Poaceae Barnhart
Iz_1_1109	DJI_0032	Poa sp.	0		NA	0	Poaceae Barnhart
Iz_1_1109	DJI_0032	Polygonatum odoratum (Mill.) Druce	0	n	1	0	Asparagaceae Juss.
Iz_1_1109	DJI_0032	Polygonum arenarium Waldst. & Kit.	0	n	1	0	Polygonaceae Juss.
Iz_1_1109	DJI_0032	Populus nigra L.	0	n	1	1	Salicaceae Mirb.
Iz_1_1109	DJI_0032	Populus sp.	0	n	1	1	Salicaceae Mirb.
Iz_1_1109	DJI_0032	Populus tremula L.	0	n	1	1	Salicaceae Mirb.
Iz_1_1109	DJI_0032	Populus Ã canescens (Aiton) Sm.	0	n	1	1	Salicaceae Mirb.
Iz_1_1109	DJI_0032	Potentilla argentea L.	0	n	1	0	Rosaceae Juss.
Iz_1_1109	DJI_0032	Psammophiliella muralis (L.) Ikonn.	0	n	1	0	Caryophyllaceae Juss.
Iz_1_1109	DJI_0032	Psephellus sumensis (Kalen.) Greuter	0	n	1	0	Asteraceae Bercht. & J.Presl
Iz_1_1109	DJI_0032	Pulsatilla pratensis (L.) Mill. s.l.	0	n	1	0	Ranunculaceae Juss.
Iz_1_1109	DJI_0032	Pyrus communis L.	0	a	0	1	Rosaceae Juss.
Iz_1_1109	DJI_0032	Quercus robur L.	0	n	1	1	Fagaceae Dumort.
Iz_1_1109	DJI_0032	Robinia pseudoacacia L.	0	a	0	1	Fabaceae Lindl.
Iz_1_1109	DJI_0032	Rosa sp.	0		NA	1	Rosaceae Juss.
Iz_1_1109	DJI_0032	Rubus idaeus L.	0	n	1	0	Rosaceae Juss.
Iz_1_1109	DJI_0032	Rumex acetosella L.	1	n	1	0	Polygonaceae Juss.
Iz_1_1109	DJI_0032	Rumex thyrsiflorus Fingerh.	0	n	1	0	Polygonaceae Juss.
Iz_1_1109	DJI_0032	Salix alba L.	0	n	1	1	Salicaceae Mirb.
Iz_1_1109	DJI_0032	Sambucus nigra L.	0	n	1	1	Viburnaceae Raf.
Iz_1_1109	DJI_0032	Secale sylvestre Host	0	n	1	0	Poaceae Barnhart
Iz_1_1109	DJI_0032	Sempervivum ruthenicum Schnittsp. & C.B. Lehm.	0	n	1	0	Crassulaceae J.St.-Hil.
Iz_1_1109	DJI_0032	Setaria viridis (L.) P. Beauv.	0	n	1	0	Poaceae Barnhart
Iz_1_1109	DJI_0032	Silene borysthenica (Gruner) Walters	0	n	1	0	Caryophyllaceae Juss.
Iz_1_1109	DJI_0032	Silene latifolia subsp. alba (Mill.) Greuter & Burdet	0	n	1	0	Caryophyllaceae Juss.
Iz_1_1109	DJI_0032	Silene nutans L.	0	n	1	0	Caryophyllaceae Juss.
Iz_1_1109	DJI_0032	Solanum nigrum L.	0		NA	NA	
Iz_1_1109	DJI_0032	Solidago virgaurea L.	0	n	1	0	Asteraceae Bercht. & J.Presl
Iz_1_1109	DJI_0032	Sorbus aucuparia L.	0	n	1	1	Rosaceae Juss.
Iz_1_1109	DJI_0032	Stachys recta L.	0	n	1	0	Lamiacea Lindl.
Iz_1_1109	DJI_0032	Tanacetum vulgare L.	0	n	1	0	Asteraceae Bercht. & J.Presl
Iz_1_1109	DJI_0032	Thymus pallasianus Heinr.Braun	0	n	1	0	Lamiacea Lindl.
Iz_1_1109	DJI_0032	Tilia cordata Mill.	0	n	1	1	Malvaceae Juss.
Iz_1_1109	DJI_0032	Trifolium alpestre L.	0	n	1	0	Fabaceae Lindl.
Iz_1_1109	DJI_0032	Trifolium arvense L.	0	n	1	0	Fabaceae Lindl.
Iz_1_1109	DJI_0032	Ulmus glabra Huds.	0	n	1	1	Ulmaceae Mirb.
Iz_1_1109	DJI_0032	Verbascum densiflorum Bertol.	0	n	1	0	Scrophulariaceae Juss.
Iz_1_1109	DJI_0032	Veronica austriaca L.	0	n	1	0	Plantaginaceae Juss.
Iz_1_1109	DJI_0032	Veronica incana L.	0	n	1	0	Plantaginaceae Juss.
Iz_1_1109	DJI_0032	Veronica verna L.	0	n	1	0	Plantaginaceae Juss.
Iz_1_1109	DJI_0032	Vincetoxicum hirundinaria Medik	0	n	1	0	Apocynaceae Juss.
Iz_1_1109	DJI_0034	Acer tataricum L.	0	n	1	1	Sapindaceae Juss.
Iz_1_1109	DJI_0034	Achillea millefolium L. s.l.	0	n	1	0	Asteraceae Bercht. & J.Presl
Iz_1_1109	DJI_0034	Allium oleraceum L.	0	n	1	0	Amaryllidaceae J.St.-Hil.
Iz_1_1109	DJI_0034	Ambrosia artemisiifolia L.	0	a	0	0	Asteraceae Bercht. & J.Presl
Iz_1_1109	DJI_0034	Amorpha fruticosa L.	0	a	0	0	Fabaceae Lindl.
Iz_1_1109	DJI_0034	Anthericum ramosum L.	0	n	1	0	Asparagaceae Juss.
Iz_1_1109	DJI_0034	Artemisia marschalliana Spreng.	0	n	1	0	Asteraceae Bercht. & J.Presl
Iz_1_1109	DJI_0034	Asparagus officinalis L.	0	n	1	0	Asparagaceae Juss.
Iz_1_1109	DJI_0034	Astragalus cicer L.	0	n	1	0	Fabaceae Lindl.
Iz_1_1109	DJI_0034	Bassia laniflora (S.G.Gmel.) A.J.Scott	0	n	1	0	Amaranthaceae Juss.
Iz_1_1109	DJI_0034	Berberis vulgaris L.	0	n	1	0	Berberidaceae Juss.
Iz_1_1109	DJI_0034	Betula pendula Roth.	0	n	1	1	Betulaceae Gray
Iz_1_1109	DJI_0034	Betula pendula Roth.	0	n	1	0	Poaceae Barnhart
Iz_1_1109	DJI_0034	Bromus inermis Leyss.	0		NA	NA	
Iz_1_1109	DJI_0034	Bromus squarrosus L.	0	a	0	0	Poaceae Barnhart
Iz_1_1109	DJI_0034	Bromus tectorum L.	0	a	0	0	Poaceae Barnhart
Iz_1_1109	DJI_0034	Calamagrostis epigejos (L.) Roth	0	n	1	0	Poaceae Barnhart
Iz_1_1109	DJI_0034	Carduus acanthoides L.	0	n	1	0	Asteraceae Bercht. & J.Presl
Iz_1_1109	DJI_0034	Carex sp.	0	n	1	0	Cyperaceae Juss.
Iz_1_1109	DJI_0034	Centaurea majorovii Dumbadze	0	n	1	0	Asteraceae Bercht. & J.Presl
Iz_1_1109	DJI_0034	Chamaecytisus ruthenicus (Fisch.ex Wol.) Klaskova	0	n	1	0	Fabaceae Lindl.
Iz_1_1109	DJI_0034	Chelidonium majus L.	0	n	1	0	Papaveraceae Juss.
Iz_1_1109	DJI_0034	Chenopodium betaceum Andrz.	0	n	1	0	Amaranthaceae Juss.
Iz_1_1109	DJI_0034	Chenopodium cf. album	0	n	1	0	Amaranthaceae Juss.
Iz_1_1109	DJI_0034	Chenopodium sp.	0		NA	0	Amaranthaceae Juss.
Iz_1_1109	DJI_0034	Chondrilla juncea L.	0	n	1	0	Asteraceae Bercht. & J.Presl
Iz_1_1109	DJI_0034	Cirsium vulgare (Savi) Ten.	0	n	1	0	Asteraceae Bercht. & J.Presl
Iz_1_1109	DJI_0034	Clinopodium acinos (L.) Kuntze	0	n	1	0	Lamiacea Lindl.
Iz_1_1109	DJI_0034	Convallaria majalis L.	0	n	1	0	Asparagaceae Juss.
Iz_1_1109	DJI_0034	Crataegus rhipidophylla Gand.	0	n	1	1	Rosaceae Juss.
Iz_1_1109	DJI_0034	Crepis foetida L.	0	n	1	0	Asteraceae Bercht. & J.Presl
Iz_1_1109	DJI_0034	Dactylis glomerata L.	0	n	1	0	Poaceae Barnhart
Iz_1_1109	DJI_0034	Dianthus campestris M.Bieb.	0	n	1	0	Caryophyllaceae Juss.
Iz_1_1109	DJI_0034	Digitaria sanguinalis (L.) Scop.	0	a	0	0	Poaceae Barnhart
Iz_1_1109	DJI_0034	Elymus repens (L.) Gould	0	n	1	0	Poaceae Barnhart
Iz_1_1109	DJI_0034	Epilobium tetragonum L.	0	n	1	0	Onagraceae Juss.
Iz_1_1109	DJI_0034	Eragrostis minor Host	0	a	0	0	Poaceae Barnhart
Iz_1_1109	DJI_0034	Erigeron canadensis L.	0	a	0	0	Asteraceae Bercht. & J.Presl
Iz_1_1109	DJI_0034	Euphorbia seguieriana Neck.	0	n	1	0	Euphorbiaceae Juss.
Iz_1_1109	DJI_0034	Fallopia dumetorum (L.) Holub	0	n	1	0	Polygonaceae Juss.
Iz_1_1109	DJI_0034	Festuca beckeri (Hack.) Trautv.	0	n	1	0	Poaceae Barnhart
Iz_1_1109	DJI_0034	Fragaria vesca L.	0	n	1	0	Rosaceae Juss.

Iz_1_1109	DJI_0034	Frangula alnus Mill.	0	n	1	1	Rhamnaceae Juss.
Iz_1_1109	DJI_0034	Fraxinus pennsylvanica Marshall	0	a	0	1	Oleaceae Hoffmanns. & Link
Iz_1_1109	DJI_0034	Galeopsis bifida Boenn.	0	n	1	0	Lamiacea Lindl.
Iz_1_1109	DJI_0034	Galium verum L.	0	n	1	0	Rubiaceae Juss.
Iz_1_1109	DJI_0034	Genista tinctoria L.	0	n	1	0	Fabaceae Lindl.
Iz_1_1109	DJI_0034	Geranium robertianum L.	0	n	1	0	Geraniaceae Juss.
Iz_1_1109	DJI_0034	Geranium sanquineum L.	0	n	1	0	Geraniaceae Juss.
Iz_1_1109	DJI_0034	Gypsophila paniculata L.	0	n	1	0	Caryophyllaceae Juss.
Iz_1_1109	DJI_0034	Helichrysum arenarium (L.) Moench	0	n	1	0	Asteraceae Bercht. & J.Presl
Iz_1_1109	DJI_0034	Herniaria polygama J.Gay	0	n	1	0	Caryophyllaceae Juss.
Iz_1_1109	DJI_0034	Hieracium robustum Fr.	0	n	1	0	Asteraceae Bercht. & J.Presl
Iz_1_1109	DJI_0034	Hieracium umbellatum L.	0	n	1	NA	Asteraceae Bercht. & J.Presl
Iz_1_1109	DJI_0034	Hylotelephium maximum (L.) Holub	0	n	1	0	Crassulaceae J.St.-Hil.
Iz_1_1109	DJI_0034	Jacobaea borysthenica (DC.) B.Nord. & Greuter	0	n	1	0	Asteraceae Bercht. & J.Presl
Iz_1_1109	DJI_0034	Jasione montana L.	0	n	1	0	Campanulaceae Juss.
Iz_1_1109	DJI_0034	Jurinea cyanoides (L.) Rchb.s.l.	0	n	1	0	Asteraceae Bercht. & J.Presl
Iz_1_1109	DJI_0034	Koeleria glauca (Spreng.) DC.	0	n	1	0	Poaceae Barnhart
Iz_1_1109	DJI_0034	Lactuca serriola L.	0	a	0	0	Asteraceae Bercht. & J.Presl
Iz_1_1109	DJI_0034	Linaria genistifolia (L.) Mill.	0	n	1	0	Plantaginaceae Juss.
Iz_1_1109	DJI_0034	Lomelosia argentea (L.) Greuter & Burdet	0	n	1	0	Caprifoliaceae Juss.
Iz_1_1109	DJI_0034	Melampyrum pratense L.	0	n	1	0	Orobanchaceae Vent.
Iz_1_1109	DJI_0034	Oenothera biennis L.	0	a	0	0	Onagraceae Juss.
Iz_1_1109	DJI_0034	Pentanema britannica (L.) D.Gut.Larr., Santos-Vicente, Anderb., E.Rico & M.M.Mart.Ort.	0	n	1	0	Asteraceae Bercht. & J.Presl
Iz_1_1109	DJI_0034	Peucedanum oreoselinum (L.) Moench	0	n	1	0	Apiaceae Lindl.
Iz_1_1109	DJI_0034	Peucedanum ruthenicum M.Bieb.	0	n	1	0	Apiaceae Lindl.
Iz_1_1109	DJI_0034	Pilosella echioides (Lumn.) F.W.Schultz & Sch.Bip.	0	n	1	0	Asteraceae Bercht. & J.Presl
Iz_1_1109	DJI_0034	Pilosella officinarum F. Schultz et Sch. Bip.	0	n	1	0	Asteraceae Bercht. & J.Presl
Iz_1_1109	DJI_0034	Pilosella sp.	0		NA	0	Asteraceae Bercht. & J.Presl
Iz_1_1109	DJI_0034	Pinus sylvestris L.	0	n	1	1	Pinaceae Spreng. ex F.Rudolphi
Iz_1_1109	DJI_0034	Poa annua L.	0	n	1	0	Poaceae Barnhart
Iz_1_1109	DJI_0034	Poa nemoralis L.	0	n	1	0	Poaceae Barnhart
Iz_1_1109	DJI_0034	Poa sp.	0		NA	0	Poaceae Barnhart
Iz_1_1109	DJI_0034	Polygonatum odoratum (Mill.) Druce	0	n	1	0	Asparagaceae Juss.
Iz_1_1109	DJI_0034	Polygonum arenarium Waldst. & Kit.	0	n	1	0	Polygonaceae Juss.
Iz_1_1109	DJI_0034	Populus nigra L.	0	n	1	1	Salicaceae Mirb.
Iz_1_1109	DJI_0034	Populus sp.	0	n	1	1	Salicaceae Mirb.
Iz_1_1109	DJI_0034	Populus tremula L.	0	n	1	1	Salicaceae Mirb.
Iz_1_1109	DJI_0034	Populus Ã canescens (Aiton) Sm.	0	n	1	1	Salicaceae Mirb.
Iz_1_1109	DJI_0034	Potentilla argentea L.	0	n	1	0	Rosaceae Juss.
Iz_1_1109	DJI_0034	Psammophiliella muralis (L.) Ikonn.	0	n	1	0	Caryophyllaceae Juss.
Iz_1_1109	DJI_0034	Psephellus sumensis (Kalen.) Greuter	0	n	1	0	Asteraceae Bercht. & J.Presl
Iz_1_1109	DJI_0034	Pulsatilla pratensis (L.) Mill. s.l.	0	n	1	0	Ranunculaceae Juss.
Iz_1_1109	DJI_0034	Pyrus communis L.	0	a	0	1	Rosaceae Juss.
Iz_1_1109	DJI_0034	Quercus robur L.	0	n	1	1	Fagaceae Dumort.
Iz_1_1109	DJI_0034	Robinia pseudoacacia L.	0	a	0	1	Fabaceae Lindl.
Iz_1_1109	DJI_0034	Rosa sp.	0		NA	1	Rosaceae Juss.
Iz_1_1109	DJI_0034	Rubus idaeus L.	0	n	1	0	Rosaceae Juss.
Iz_1_1109	DJI_0034	Rumex acetosella L.	2	n	1	0	Polygonaceae Juss.
Iz_1_1109	DJI_0034	Rumex thyrsiflorus Fingerh.	0	n	1	0	Polygonaceae Juss.
Iz_1_1109	DJI_0034	Salix alba L.	0	n	1	1	Salicaceae Mirb.
Iz_1_1109	DJI_0034	Sambucus nigra L.	0	n	1	1	Viburnaceae Raf.
Iz_1_1109	DJI_0034	Secale sylvestre Host	0	n	1	0	Poaceae Barnhart
Iz_1_1109	DJI_0034	Sempervivum ruthenicum Schnittsp. & C.B. Lehm.	0	n	1	0	Crassulaceae J.St.-Hil.
Iz_1_1109	DJI_0034	Setaria viridis (L.) P. Beauv.	0	n	1	0	Poaceae Barnhart
Iz_1_1109	DJI_0034	Silene borysthenica (Gruner) Walters	0	n	1	0	Caryophyllaceae Juss.
Iz_1_1109	DJI_0034	Silene latifolia subsp. alba (Mill.) Greuter & Burdet	0	n	1	0	Caryophyllaceae Juss.
Iz_1_1109	DJI_0034	Silene nutans L.	0	n	1	0	Caryophyllaceae Juss.
Iz_1_1109	DJI_0034	Solanum nigrum L.	0		NA	NA	
Iz_1_1109	DJI_0034	Solidago virgaurea L.	0	n	1	0	Asteraceae Bercht. & J.Presl
Iz_1_1109	DJI_0034	Sorbus aucuparia L.	0	n	1	1	Rosaceae Juss.
Iz_1_1109	DJI_0034	Stachys recta L.	0	n	1	0	Lamiacea Lindl.
Iz_1_1109	DJI_0034	Tanacetum vulgare L.	0	n	1	0	Asteraceae Bercht. & J.Presl
Iz_1_1109	DJI_0034	Thymus pallasianus Heinr.Braun	0	n	1	0	Lamiacea Lindl.
Iz_1_1109	DJI_0034	Tilia cordata Mill.	0	n	1	1	Malvaceae Juss.
Iz_1_1109	DJI_0034	Trifolium alpestre L.	0	n	1	0	Fabaceae Lindl.
Iz_1_1109	DJI_0034	Trifolium arvense L.	0	n	1	0	Fabaceae Lindl.
Iz_1_1109	DJI_0034	Ulmus glabra Huds.	0	n	1	1	Ulmaceae Mirb.
Iz_1_1109	DJI_0034	Verbascum densiflorum Bertol.	0	n	1	0	Scrophulariaceae Juss.
Iz_1_1109	DJI_0034	Veronica austriaca L.	0	n	1	0	Plantaginaceae Juss.
Iz_1_1109	DJI_0034	Veronica incana L.	0	n	1	0	Plantaginaceae Juss.
Iz_1_1109	DJI_0034	Veronica verna L.	0	n	1	0	Plantaginaceae Juss.
Iz_1_1109	DJI_0034	Vincetoxicum hirundinaria Medik	0	n	1	0	Apocynaceae Juss.
Iz_1_1109	DJI_0037	Acer tataricum L.	0	n	1	1	Sapindaceae Juss.
Iz_1_1109	DJI_0037	Achillea millefolium L. s.l.	0	n	1	0	Asteraceae Bercht. & J.Presl
Iz_1_1109	DJI_0037	Allium oleraceum L.	0	n	1	0	Amaryllidaceae J.St.-Hil.
Iz_1_1109	DJI_0037	Ambrosia artemisiifolia L.	0	a	0	0	Asteraceae Bercht. & J.Presl
Iz_1_1109	DJI_0037	Amorpha fruticosa L.	0	a	0	0	Fabaceae Lindl.
Iz_1_1109	DJI_0037	Anthericum ramosum L.	0	n	1	0	Asparagaceae Juss.
Iz_1_1109	DJI_0037	Artemisia marschalliana Spreng.	0	n	1	0	Asteraceae Bercht. & J.Presl
Iz_1_1109	DJI_0037	Asparagus officinalis L.	0	n	1	0	Asparagaceae Juss.
Iz_1_1109	DJI_0037	Astragalus cicer L.	0	n	1	0	Fabaceae Lindl.
Iz_1_1109	DJI_0037	Bassia laniflora (S.G.Gmel.) A.J.Scott	0	n	1	0	Amaranthaceae Juss.
Iz_1_1109	DJI_0037	Berberis vulgaris L.	0	n	1	0	Berberidaceae Juss.
Iz_1_1109	DJI_0037	Betula pendula Roth.	0	n	1	1	Betulaceae Gray
Iz_1_1109	DJI_0037	Betula pendula Roth.	0	n	1	0	Poaceae Barnhart
Iz_1_1109	DJI_0037	Bromus inermis Leyss.	0		NA	NA	
Iz_1_1109	DJI_0037	Bromus squarrosus L.	0	a	0	0	Poaceae Barnhart
Iz_1_1109	DJI_0037	Bromus tectorum L.	0	a	0	0	Poaceae Barnhart
Iz_1_1109	DJI_0037	Calamagrostis epigejos (L.) Roth	0	n	1	0	Poaceae Barnhart
Iz_1_1109	DJI_0037	Carduus acanthoides L.	0	n	1	0	Asteraceae Bercht. & J.Presl
Iz_1_1109	DJI_0037	Carex sp.	0	n	1	0	Cyperaceae Juss.
Iz_1_1109	DJI_0037	Centaurea majorovii Dumbadze	0	n	1	0	Asteraceae Bercht. & J.Presl
Iz_1_1109	DJI_0037	Chamaecytisus ruthenicus (Fisch.ex Wol.) Klaskova	0	n	1	0	Fabaceae Lindl.
Iz_1_1109	DJI_0037	Chelidonium majus L.	0	n	1	0	Papaveraceae Juss.
Iz_1_1109	DJI_0037	Chenopodium betaceum Andrz.	0	n	1	0	Amaranthaceae Juss.
Iz_1_1109	DJI_0037	Chenopodium cf. album	0.1	n	1	0	Amaranthaceae Juss.
Iz_1_1109	DJI_0037	Chenopodium sp.	0		NA	0	Amaranthaceae Juss.
Iz_1_1109	DJI_0037	Chondrilla juncea L.	0	n	1	0	Asteraceae Bercht. & J.Presl

Iz_1_1109	DJI_0037	Cirsium vulgare (Savi) Ten.	0	n	1	0	Asteraceae Bercht. & J.Presl
Iz_1_1109	DJI_0037	Clinopodium acinos (L.) Kuntze	0	n	1	0	Lamiacea Lindl.
Iz_1_1109	DJI_0037	Convallaria majalis L.	0	n	1	0	Asparagaceae Juss.
Iz_1_1109	DJI_0037	Crataegus rhipidophylla Gand.	0	n	1	1	Rosaceae Juss.
Iz_1_1109	DJI_0037	Crepis foetida L.	0	n	1	0	Asteraceae Bercht. & J.Presl
Iz_1_1109	DJI_0037	Dactylis glomerata L.	0	n	1	0	Poaceae Barnhart
Iz_1_1109	DJI_0037	Dianthus campestris M.Bieb.	0	n	1	0	Caryophyllaceae Juss.
Iz_1_1109	DJI_0037	Digitaria sanguinalis (L.) Scop.	0	a	0	0	Poaceae Barnhart
Iz_1_1109	DJI_0037	Elymus repens (L.) Gould	0	n	1	0	Poaceae Barnhart
Iz_1_1109	DJI_0037	Epilobium tetragonum L.	0	n	1	0	Onagraceae Juss.
Iz_1_1109	DJI_0037	Eragrostis minor Host	0	a	0	0	Poaceae Barnhart
Iz_1_1109	DJI_0037	Erigeron canadensis L.	0	a	0	0	Asteraceae Bercht. & J.Presl
Iz_1_1109	DJI_0037	Euphorbia seguieriana Neck.	0	n	1	0	Euphorbiaceae Juss.
Iz_1_1109	DJI_0037	Fallopia dumetorum (L.) Holub	0	n	1	0	Polygonaceae Juss.
Iz_1_1109	DJI_0037	Festuca beckeri (Hack.) Trautv.	0	n	1	0	Poaceae Barnhart
Iz_1_1109	DJI_0037	Fragaria vesca L.	0	n	1	0	Rosaceae Juss.
Iz_1_1109	DJI_0037	Frangula alnus Mill.	0	n	1	1	Rhamnaceae Juss.
Iz_1_1109	DJI_0037	Fraxinus pennsylvanica Marshall	0	a	0	1	Oleaceae Hoffmanns. & Link
Iz_1_1109	DJI_0037	Galeopsis bifida Boenn.	0	n	1	0	Lamiacea Lindl.
Iz_1_1109	DJI_0037	Galium verum L.	0	n	1	0	Rubiaceae Juss.
Iz_1_1109	DJI_0037	Genista tinctoria L.	0	n	1	0	Fabaceae Lindl.
Iz_1_1109	DJI_0037	Geranium robertianum L.	0	n	1	0	Geraniaceae Juss.
Iz_1_1109	DJI_0037	Geranium sanquineum L.	0	n	1	0	Geraniaceae Juss.
Iz_1_1109	DJI_0037	Gypsophila paniculata L.	0	n	1	0	Caryophyllaceae Juss.
Iz_1_1109	DJI_0037	Helichrysum arenarium (L.) Moench	0	n	1	0	Asteraceae Bercht. & J.Presl
Iz_1_1109	DJI_0037	Herniaria polygama J.Gay	0	n	1	0	Caryophyllaceae Juss.
Iz_1_1109	DJI_0037	Hieracium robustum Fr.	0	n	1	0	Asteraceae Bercht. & J.Presl
Iz_1_1109	DJI_0037	Hieracium umbellatum L.	0	n	1	NA	Asteraceae Bercht. & J.Presl
Iz_1_1109	DJI_0037	Hylotelephium maximum (L.) Holub	0	n	1	0	Crassulaceae J.St.-Hil.
Iz_1_1109	DJI_0037	Jacobaea borysthenica (DC.) B.Nord. & Greuter	0	n	1	0	Asteraceae Bercht. & J.Presl
Iz_1_1109	DJI_0037	Jasione montana L.	0	n	1	0	Campanulaceae Juss.
Iz_1_1109	DJI_0037	Jurinea cyanoides (L.) Rchb.s.l.	0	n	1	0	Asteraceae Bercht. & J.Presl
Iz_1_1109	DJI_0037	Koeleria glauca (Spreng.) DC.	0	n	1	0	Poaceae Barnhart
Iz_1_1109	DJI_0037	Lactuca serriola L.	0	a	0	0	Asteraceae Bercht. & J.Presl
Iz_1_1109	DJI_0037	Linaria genistifolia (L.) Mill.	0	n	1	0	Plantaginaceae Juss.
Iz_1_1109	DJI_0037	Lomelosia argentea (L.) Greuter & Burdet	0	n	1	0	Caprifoliaceae Juss.
Iz_1_1109	DJI_0037	Melampyrum pratense L.	0	n	1	0	Orobanchaceae Vent.
Iz_1_1109	DJI_0037	Oenothera biennis L.	0	a	0	0	Onagraceae Juss.
Iz_1_1109	DJI_0037	Pentanema britannica (L.) D.Gut.Larr., Santos-Vicente, Anderb., E.Rico & M.M.Mart.Ort.	0	n	1	0	Asteraceae Bercht. & J.Presl
Iz_1_1109	DJI_0037	Peucedanum oreoselinum (L.) Moench	0	n	1	0	Apiaceae Lindl.
Iz_1_1109	DJI_0037	Peucedanum ruthenicum M.Bieb.	0	n	1	0	Apiaceae Lindl.
Iz_1_1109	DJI_0037	Pilosella echioides (Lumn.) F.W.Schultz & Sch.Bip.	0	n	1	0	Asteraceae Bercht. & J.Presl
Iz_1_1109	DJI_0037	Pilosella officinarum F. Schultz et Sch. Bip.	0	n	1	0	Asteraceae Bercht. & J.Presl
Iz_1_1109	DJI_0037	Pilosella sp.	0		NA	0	Asteraceae Bercht. & J.Presl
Iz_1_1109	DJI_0037	Pinus sylvestris L.	0	n	1	1	Pinaceae Spreng. ex F.Rudolphi
Iz_1_1109	DJI_0037	Poa annua L.	0	n	1	0	Poaceae Barnhart
Iz_1_1109	DJI_0037	Poa nemoralis L.	0	n	1	0	Poaceae Barnhart
Iz_1_1109	DJI_0037	Poa sp.	0		NA	0	Poaceae Barnhart
Iz_1_1109	DJI_0037	Polygonatum odoratum (Mill.) Druce	0	n	1	0	Asparagaceae Juss.
Iz_1_1109	DJI_0037	Polygonum arenarium Waldst. & Kit.	0	n	1	0	Polygonaceae Juss.
Iz_1_1109	DJI_0037	Populus nigra L.	0	n	1	1	Salicaceae Mirb.
Iz_1_1109	DJI_0037	Populus sp.	0	n	1	1	Salicaceae Mirb.
Iz_1_1109	DJI_0037	Populus tremula L.	0	n	1	1	Salicaceae Mirb.
Iz_1_1109	DJI_0037	Populus Ã canescens (Aiton) Sm.	0	n	1	1	Salicaceae Mirb.
Iz_1_1109	DJI_0037	Potentilla argentea L.	0	n	1	0	Rosaceae Juss.
Iz_1_1109	DJI_0037	Psammophiliella muralis (L.) Ikonn.	0	n	1	0	Caryophyllaceae Juss.
Iz_1_1109	DJI_0037	Psephellus sumensis (Kalen.) Greuter	0	n	1	0	Asteraceae Bercht. & J.Presl
Iz_1_1109	DJI_0037	Pulsatilla pratensis (L.) Mill. s.l.	0	n	1	0	Ranunculaceae Juss.
Iz_1_1109	DJI_0037	Pyrus communis L.	0	a	0	1	Rosaceae Juss.
Iz_1_1109	DJI_0037	Quercus robur L.	0	n	1	1	Fagaceae Dumort.
Iz_1_1109	DJI_0037	Robinia pseudoacacia L.	0	a	0	1	Fabaceae Lindl.
Iz_1_1109	DJI_0037	Rosa sp.	0		NA	1	Rosaceae Juss.
Iz_1_1109	DJI_0037	Rubus idaeus L.	0	n	1	0	Rosaceae Juss.
Iz_1_1109	DJI_0037	Rumex acetosella L.	0	n	1	0	Polygonaceae Juss.
Iz_1_1109	DJI_0037	Rumex thyrsiflorus Fingerh.	0	n	1	0	Polygonaceae Juss.
Iz_1_1109	DJI_0037	Salix alba L.	0	n	1	1	Salicaceae Mirb.
Iz_1_1109	DJI_0037	Sambucus nigra L.	0	n	1	1	Viburnaceae Raf.
Iz_1_1109	DJI_0037	Secale sylvestre Host	0	n	1	0	Poaceae Barnhart
Iz_1_1109	DJI_0037	Sempervivum ruthenicum Schnittsp. & C.B. Lehm.	0	n	1	0	Crassulaceae J.St.-Hil.
Iz_1_1109	DJI_0037	Setaria viridis (L.) P. Beauv.	0	n	1	0	Poaceae Barnhart
Iz_1_1109	DJI_0037	Silene borysthenica (Gruner) Walters	0	n	1	0	Caryophyllaceae Juss.
Iz_1_1109	DJI_0037	Silene latifolia subsp. alba (Mill.) Greuter & Burdet	0	n	1	0	Caryophyllaceae Juss.
Iz_1_1109	DJI_0037	Silene nutans L.	0	n	1	0	Caryophyllaceae Juss.
Iz_1_1109	DJI_0037	Solanum nigrum L.	0		NA	NA	
Iz_1_1109	DJI_0037	Solidago virgaurea L.	0	n	1	0	Asteraceae Bercht. & J.Presl
Iz_1_1109	DJI_0037	Sorbus aucuparia L.	0	n	1	1	Rosaceae Juss.
Iz_1_1109	DJI_0037	Stachys recta L.	0	n	1	0	Lamiacea Lindl.
Iz_1_1109	DJI_0037	Tanacetum vulgare L.	0	n	1	0	Asteraceae Bercht. & J.Presl
Iz_1_1109	DJI_0037	Thymus pallasianus Heinr.Braun	0	n	1	0	Lamiacea Lindl.
Iz_1_1109	DJI_0037	Tilia cordata Mill.	0	n	1	1	Malvaceae Juss.
Iz_1_1109	DJI_0037	Trifolium alpestre L.	0	n	1	0	Fabaceae Lindl.
Iz_1_1109	DJI_0037	Trifolium arvense L.	0	n	1	0	Fabaceae Lindl.
Iz_1_1109	DJI_0037	Ulmus glabra Huds.	0	n	1	1	Ulmaceae Mirb.
Iz_1_1109	DJI_0037	Verbascum densiflorum Bertol.	0	n	1	0	Scrophulariaceae Juss.
Iz_1_1109	DJI_0037	Veronica austriaca L.	0	n	1	0	Plantaginaceae Juss.
Iz_1_1109	DJI_0037	Veronica incana L.	0	n	1	0	Plantaginaceae Juss.
Iz_1_1109	DJI_0037	Veronica verna L.	0	n	1	0	Plantaginaceae Juss.
Iz_1_1109	DJI_0037	Vincetoxicum hirundinaria Medik	0	n	1	0	Apocynaceae Juss.
Iz_1_1109	DJI_0041	Acer tataricum L.	0	n	1	1	Sapindaceae Juss.
Iz_1_1109	DJI_0041	Achillea millefolium L. s.l.	0	n	1	0	Asteraceae Bercht. & J.Presl
Iz_1_1109	DJI_0041	Allium oleraceum L.	0	n	1	0	Amaryllidaceae J.St.-Hil.
Iz_1_1109	DJI_0041	Ambrosia artemisiifolia L.	0	a	0	0	Asteraceae Bercht. & J.Presl
Iz_1_1109	DJI_0041	Amorpha fruticosa L.	0	a	0	0	Fabaceae Lindl.
Iz_1_1109	DJI_0041	Anthericum ramosum L.	0	n	1	0	Asparagaceae Juss.
Iz_1_1109	DJI_0041	Artemisia marschalliana Spreng.	0	n	1	0	Asteraceae Bercht. & J.Presl
Iz_1_1109	DJI_0041	Asparagus officinalis L.	0	n	1	0	Asparagaceae Juss.
Iz_1_1109	DJI_0041	Astragalus cicer L.	0	n	1	0	Fabaceae Lindl.
Iz_1_1109	DJI_0041	Bassia laniflora (S.G.Gmel.) A.J.Scott	0	n	1	0	Amaranthaceae Juss.

Iz_1_1109	DJI_0041	Berberis vulgaris L.	0	n	1	0	Berberidaceae Juss.
Iz_1_1109	DJI_0041	Betula pendula Roth.	0	n	1	1	Betulaceae Gray
Iz_1_1109	DJI_0041	Betula pendula Roth.	0	n	1	0	Poaceae Barnhart
Iz_1_1109	DJI_0041	Bromus inermis Leyss.	0		NA	NA	
Iz_1_1109	DJI_0041	Bromus squarrosus L.	0	a	0	0	Poaceae Barnhart
Iz_1_1109	DJI_0041	Bromus tectorum L.	0	a	0	0	Poaceae Barnhart
Iz_1_1109	DJI_0041	Calamagrostis epigejos (L.) Roth	0	n	1	0	Poaceae Barnhart
Iz_1_1109	DJI_0041	Carduus acanthoides L.	0	n	1	0	Asteraceae Bercht. & J.Presl
Iz_1_1109	DJI_0041	Carex sp.	0	n	1	0	Cyperaceae Juss.
Iz_1_1109	DJI_0041	Centaurea majorovii Dumbadze	0	n	1	0	Asteraceae Bercht. & J.Presl
Iz_1_1109	DJI_0041	Chamaecytisus ruthenicus (Fisch.ex Wol.) Klaskova	0	n	1	0	Fabaceae Lindl.
Iz_1_1109	DJI_0041	Chelidonium majus L.	0	n	1	0	Papaveraceae Juss.
Iz_1_1109	DJI_0041	Chenopodium betaceum Andrz.	0	n	1	0	Amaranthaceae Juss.
Iz_1_1109	DJI_0041	Chenopodium cf. album	0.5	n	1	0	Amaranthaceae Juss.
Iz_1_1109	DJI_0041	Chenopodium sp.	0		NA	0	Amaranthaceae Juss.
Iz_1_1109	DJI_0041	Chondrilla juncea L.	0	n	1	0	Asteraceae Bercht. & J.Presl
Iz_1_1109	DJI_0041	Cirsium vulgare (Savi) Ten.	0	n	1	0	Asteraceae Bercht. & J.Presl
Iz_1_1109	DJI_0041	Clinopodium acinos (L.) Kuntze	0	n	1	0	Lamiacea Lindl.
Iz_1_1109	DJI_0041	Convallaria majalis L.	0	n	1	0	Asparagaceae Juss.
Iz_1_1109	DJI_0041	Crataegus rhipidophylla Gand.	0	n	1	1	Rosaceae Juss.
Iz_1_1109	DJI_0041	Crepis foetida L.	0	n	1	0	Asteraceae Bercht. & J.Presl
Iz_1_1109	DJI_0041	Dactylis glomerata L.	0	n	1	0	Poaceae Barnhart
Iz_1_1109	DJI_0041	Dianthus campestris M.Bieb.	0	n	1	0	Caryophyllaceae Juss.
Iz_1_1109	DJI_0041	Digitaria sanguinalis (L.) Scop.	0	a	0	0	Poaceae Barnhart
Iz_1_1109	DJI_0041	Elymus repens (L.) Gould	0	n	1	0	Poaceae Barnhart
Iz_1_1109	DJI_0041	Epilobium tetragonum L.	0	n	1	0	Onagraceae Juss.
Iz_1_1109	DJI_0041	Eragrostis minor Host	0	a	0	0	Poaceae Barnhart
Iz_1_1109	DJI_0041	Erigeron canadensis L.	0	a	0	0	Asteraceae Bercht. & J.Presl
Iz_1_1109	DJI_0041	Euphorbia seguieriana Neck.	0	n	1	0	Euphorbiaceae Juss.
Iz_1_1109	DJI_0041	Fallopia dumetorum (L.) Holub	0	n	1	0	Polygonaceae Juss.
Iz_1_1109	DJI_0041	Festuca beckeri (Hack.) Trautv.	0	n	1	0	Poaceae Barnhart
Iz_1_1109	DJI_0041	Fragaria vesca L.	0	n	1	0	Rosaceae Juss.
Iz_1_1109	DJI_0041	Frangula alnus Mill.	0	n	1	1	Rhamnaceae Juss.
Iz_1_1109	DJI_0041	Fraxinus pennsylvanica Marshall	0	a	0	1	Oleaceae Hoffmanns. & Link
Iz_1_1109	DJI_0041	Galeopsis bifida Boenn.	0	n	1	0	Lamiacea Lindl.
Iz_1_1109	DJI_0041	Galium verum L.	0	n	1	0	Rubiaceae Juss.
Iz_1_1109	DJI_0041	Genista tinctoria L.	0	n	1	0	Fabaceae Lindl.
Iz_1_1109	DJI_0041	Geranium robertianum L.	0	n	1	0	Geraniaceae Juss.
Iz_1_1109	DJI_0041	Geranium sanquineum L.	0	n	1	0	Geraniaceae Juss.
Iz_1_1109	DJI_0041	Gypsophila paniculata L.	0	n	1	0	Caryophyllaceae Juss.
Iz_1_1109	DJI_0041	Helichrysum arenarium (L.) Moench	0	n	1	0	Asteraceae Bercht. & J.Presl
Iz_1_1109	DJI_0041	Herniaria polygama J.Gay	0	n	1	0	Caryophyllaceae Juss.
Iz_1_1109	DJI_0041	Hieracium robustum Fr.	0	n	1	0	Asteraceae Bercht. & J.Presl
Iz_1_1109	DJI_0041	Hieracium umbellatum L.	0	n	1	NA	Asteraceae Bercht. & J.Presl
Iz_1_1109	DJI_0041	Hylotelephium maximum (L.) Holub	0	n	1	0	Crassulaceae J.St.-Hil.
Iz_1_1109	DJI_0041	Jacobaea borysthenica (DC.) B.Nord. & Greuter	0	n	1	0	Asteraceae Bercht. & J.Presl
Iz_1_1109	DJI_0041	Jasione montana L.	0	n	1	0	Campanulaceae Juss.
Iz_1_1109	DJI_0041	Jurinea cyanoides (L.) Rchb.s.l.	0	n	1	0	Asteraceae Bercht. & J.Presl
Iz_1_1109	DJI_0041	Koeleria glauca (Spreng.) DC.	0	n	1	0	Poaceae Barnhart
Iz_1_1109	DJI_0041	Lactuca serriola L.	0	a	0	0	Asteraceae Bercht. & J.Presl
Iz_1_1109	DJI_0041	Linaria genistifolia (L.) Mill.	0	n	1	0	Plantaginaceae Juss.
Iz_1_1109	DJI_0041	Lomelosia argentea (L.) Greuter & Burdet	0	n	1	0	Caprifoliaceae Juss.
Iz_1_1109	DJI_0041	Melampyrum pratense L.	0	n	1	0	Orobanchaceae Vent.
Iz_1_1109	DJI_0041	Oenothera biennis L.	0	a	0	0	Onagraceae Juss.
Iz_1_1109	DJI_0041	Pentanema britannica (L.) D.Gut.Larr., Santos-Vicente, Anderb., E.Rico & M.M.Mart.Ort.	0	n	1	0	Asteraceae Bercht. & J.Presl
Iz_1_1109	DJI_0041	Peucedanum oreoselinum (L.) Moench	0	n	1	0	Apiaceae Lindl.
Iz_1_1109	DJI_0041	Peucedanum ruthenicum M.Bieb.	0	n	1	0	Apiaceae Lindl.
Iz_1_1109	DJI_0041	Pilosella echioides (Lumn.) F.W.Schultz & Sch.Bip.	0	n	1	0	Asteraceae Bercht. & J.Presl
Iz_1_1109	DJI_0041	Pilosella officinarum F. Schultz et Sch. Bip.	0	n	1	0	Asteraceae Bercht. & J.Presl
Iz_1_1109	DJI_0041	Pilosella sp.	0		NA	0	Asteraceae Bercht. & J.Presl
Iz_1_1109	DJI_0041	Pinus sylvestris L.	0	n	1	1	Pinaceae Spreng. ex F.Rudolphi
Iz_1_1109	DJI_0041	Poa annua L.	0	n	1	0	Poaceae Barnhart
Iz_1_1109	DJI_0041	Poa nemoralis L.	0	n	1	0	Poaceae Barnhart
Iz_1_1109	DJI_0041	Poa sp.	0		NA	0	Poaceae Barnhart
Iz_1_1109	DJI_0041	Polygonatum odoratum (Mill.) Druce	0	n	1	0	Asparagaceae Juss.
Iz_1_1109	DJI_0041	Polygonum arenarium Waldst. & Kit.	0	n	1	0	Polygonaceae Juss.
Iz_1_1109	DJI_0041	Populus nigra L.	0	n	1	1	Salicaceae Mirb.
Iz_1_1109	DJI_0041	Populus sp.	0	n	1	1	Salicaceae Mirb.
Iz_1_1109	DJI_0041	Populus tremula L.	0	n	1	1	Salicaceae Mirb.
Iz_1_1109	DJI_0041	Populus Ã canescens (Aiton) Sm.	0	n	1	1	Salicaceae Mirb.
Iz_1_1109	DJI_0041	Potentilla argentea L.	0	n	1	0	Rosaceae Juss.
Iz_1_1109	DJI_0041	Psammophiliella muralis (L.) Ikonn.	0	n	1	0	Caryophyllaceae Juss.
Iz_1_1109	DJI_0041	Psephellus sumensis (Kalen.) Greuter	0	n	1	0	Asteraceae Bercht. & J.Presl
Iz_1_1109	DJI_0041	Pulsatilla pratensis (L.) Mill. s.l.	0	n	1	0	Ranunculaceae Juss.
Iz_1_1109	DJI_0041	Pyrus communis L.	0	a	0	1	Rosaceae Juss.
Iz_1_1109	DJI_0041	Quercus robur L.	0	n	1	1	Fagaceae Dumort.
Iz_1_1109	DJI_0041	Robinia pseudoacacia L.	0	a	0	1	Fabaceae Lindl.
Iz_1_1109	DJI_0041	Rosa sp.	0		NA	1	Rosaceae Juss.
Iz_1_1109	DJI_0041	Rubus idaeus L.	0	n	1	0	Rosaceae Juss.
Iz_1_1109	DJI_0041	Rumex acetosella L.	0	n	1	0	Polygonaceae Juss.
Iz_1_1109	DJI_0041	Rumex thyrsiflorus Fingerh.	0	n	1	0	Polygonaceae Juss.
Iz_1_1109	DJI_0041	Salix alba L.	0	n	1	1	Salicaceae Mirb.
Iz_1_1109	DJI_0041	Sambucus nigra L.	0	n	1	1	Viburnaceae Raf.
Iz_1_1109	DJI_0041	Secale sylvestre Host	0	n	1	0	Poaceae Barnhart
Iz_1_1109	DJI_0041	Sempervivum ruthenicum Schnittsp. & C.B. Lehm.	0	n	1	0	Crassulaceae J.St.-Hil.
Iz_1_1109	DJI_0041	Setaria viridis (L.) P. Beauv.	0	n	1	0	Poaceae Barnhart
Iz_1_1109	DJI_0041	Silene borysthenica (Gruner) Walters	0	n	1	0	Caryophyllaceae Juss.
Iz_1_1109	DJI_0041	Silene latifolia subsp. alba (Mill.) Greuter & Burdet	0	n	1	0	Caryophyllaceae Juss.
Iz_1_1109	DJI_0041	Silene nutans L.	0	n	1	0	Caryophyllaceae Juss.
Iz_1_1109	DJI_0041	Solanum nigrum L.	0		NA	NA	
Iz_1_1109	DJI_0041	Solidago virgaurea L.	0	n	1	0	Asteraceae Bercht. & J.Presl
Iz_1_1109	DJI_0041	Sorbus aucuparia L.	0	n	1	1	Rosaceae Juss.
Iz_1_1109	DJI_0041	Stachys recta L.	0	n	1	0	Lamiacea Lindl.
Iz_1_1109	DJI_0041	Tanacetum vulgare L.	0	n	1	0	Asteraceae Bercht. & J.Presl
Iz_1_1109	DJI_0041	Thymus pallasianus Heinr.Braun	0	n	1	0	Lamiacea Lindl.
Iz_1_1109	DJI_0041	Tilia cordata Mill.	0	n	1	1	Malvaceae Juss.
Iz_1_1109	DJI_0041	Trifolium alpestre L.	0	n	1	0	Fabaceae Lindl.
Iz_1_1109	DJI_0041	Trifolium arvense L.	0	n	1	0	Fabaceae Lindl.

Iz_1_1109	DJI_0041	Ulmus glabra Huds.	0	n	1	1	Ulmaceae Mirb.
Iz_1_1109	DJI_0041	Verbascum densiflorum Bertol.	0	n	1	0	Scrophulariaceae Juss.
Iz_1_1109	DJI_0041	Veronica austriaca L.	0	n	1	0	Plantaginaceae Juss.
Iz_1_1109	DJI_0041	Veronica incana L.	0	n	1	0	Plantaginaceae Juss.
Iz_1_1109	DJI_0041	Veronica verna L.	0	n	1	0	Plantaginaceae Juss.
Iz_1_1109	DJI_0041	Vincetoxicum hirundinaria Medik	0	n	1	0	Apocynaceae Juss.
Iz_1_1109	DJI_0044	Acer tataricum L.	0	n	1	1	Sapindaceae Juss.
Iz_1_1109	DJI_0044	Achillea millefolium L. s.l.	0	n	1	0	Asteraceae Bercht. & J.Presl
Iz_1_1109	DJI_0044	Allium oleraceum L.	0	n	1	0	Amaryllidaceae J.St.-Hil.
Iz_1_1109	DJI_0044	Ambrosia artemisiifolia L.	0	a	0	0	Asteraceae Bercht. & J.Presl
Iz_1_1109	DJI_0044	Amorpha fruticosa L.	0	a	0	0	Fabaceae Lindl.
Iz_1_1109	DJI_0044	Anthericum ramosum L.	0	n	1	0	Asparagaceae Juss.
Iz_1_1109	DJI_0044	Artemisia marschalliana Spreng.	0	n	1	0	Asteraceae Bercht. & J.Presl
Iz_1_1109	DJI_0044	Asparagus officinalis L.	0	n	1	0	Asparagaceae Juss.
Iz_1_1109	DJI_0044	Astragalus cicer L.	0	n	1	0	Fabaceae Lindl.
Iz_1_1109	DJI_0044	Bassia laniflora (S.G.Gmel.) A.J.Scott	0	n	1	0	Amaranthaceae Juss.
Iz_1_1109	DJI_0044	Berberis vulgaris L.	0	n	1	0	Berberidaceae Juss.
Iz_1_1109	DJI_0044	Betula pendula Roth.	0	n	1	1	Betulaceae Gray
Iz_1_1109	DJI_0044	Betula pendula Roth.	0	n	1	0	Poaceae Barnhart
Iz_1_1109	DJI_0044	Bromus inermis Leyss.	0		NA	NA	
Iz_1_1109	DJI_0044	Bromus squarrosus L.	0	a	0	0	Poaceae Barnhart
Iz_1_1109	DJI_0044	Bromus tectorum L.	0	a	0	0	Poaceae Barnhart
Iz_1_1109	DJI_0044	Calamagrostis epigejos (L.) Roth	0	n	1	0	Poaceae Barnhart
Iz_1_1109	DJI_0044	Carduus acanthoides L.	0	n	1	0	Asteraceae Bercht. & J.Presl
Iz_1_1109	DJI_0044	Carex sp.	0	n	1	0	Cyperaceae Juss.
Iz_1_1109	DJI_0044	Centaurea majorovii Dumbadze	0	n	1	0	Asteraceae Bercht. & J.Presl
Iz_1_1109	DJI_0044	Chamaecytisus ruthenicus (Fisch.ex Wol.) Klaskova	0	n	1	0	Fabaceae Lindl.
Iz_1_1109	DJI_0044	Chelidonium majus L.	0	n	1	0	Papaveraceae Juss.
Iz_1_1109	DJI_0044	Chenopodium betaceum Andrz.	0	n	1	0	Amaranthaceae Juss.
Iz_1_1109	DJI_0044	Chenopodium cf. album	0	n	1	0	Amaranthaceae Juss.
Iz_1_1109	DJI_0044	Chenopodium sp.	0		NA	0	Amaranthaceae Juss.
Iz_1_1109	DJI_0044	Chondrilla juncea L.	0	n	1	0	Asteraceae Bercht. & J.Presl
Iz_1_1109	DJI_0044	Cirsium vulgare (Savi) Ten.	0	n	1	0	Asteraceae Bercht. & J.Presl
Iz_1_1109	DJI_0044	Clinopodium acinos (L.) Kuntze	0	n	1	0	Lamiacea Lindl.
Iz_1_1109	DJI_0044	Convallaria majalis L.	0	n	1	0	Asparagaceae Juss.
Iz_1_1109	DJI_0044	Crataegus rhipidophylla Gand.	0	n	1	1	Rosaceae Juss.
Iz_1_1109	DJI_0044	Crepis foetida L.	0	n	1	0	Asteraceae Bercht. & J.Presl
Iz_1_1109	DJI_0044	Dactylis glomerata L.	0	n	1	0	Poaceae Barnhart
Iz_1_1109	DJI_0044	Dianthus campestris M.Bieb.	0	n	1	0	Caryophyllaceae Juss.
Iz_1_1109	DJI_0044	Digitaria sanguinalis (L.) Scop.	0	a	0	0	Poaceae Barnhart
Iz_1_1109	DJI_0044	Elymus repens (L.) Gould	0	n	1	0	Poaceae Barnhart
Iz_1_1109	DJI_0044	Epilobium tetragonum L.	0	n	1	0	Onagraceae Juss.
Iz_1_1109	DJI_0044	Eragrostis minor Host	0	a	0	0	Poaceae Barnhart
Iz_1_1109	DJI_0044	Erigeron canadensis L.	0.1	a	0	0	Asteraceae Bercht. & J.Presl
Iz_1_1109	DJI_0044	Euphorbia seguieriana Neck.	0	n	1	0	Euphorbiaceae Juss.
Iz_1_1109	DJI_0044	Fallopia dumetorum (L.) Holub	0	n	1	0	Polygonaceae Juss.
Iz_1_1109	DJI_0044	Festuca beckeri (Hack.) Trautv.	0	n	1	0	Poaceae Barnhart
Iz_1_1109	DJI_0044	Fragaria vesca L.	0	n	1	0	Rosaceae Juss.
Iz_1_1109	DJI_0044	Frangula alnus Mill.	0	n	1	1	Rhamnaceae Juss.
Iz_1_1109	DJI_0044	Fraxinus pennsylvanica Marshall	0	a	0	1	Oleaceae Hoffmanns. & Link
Iz_1_1109	DJI_0044	Galeopsis bifida Boenn.	0	n	1	0	Lamiacea Lindl.
Iz_1_1109	DJI_0044	Galium verum L.	0	n	1	0	Rubiaceae Juss.
Iz_1_1109	DJI_0044	Genista tinctoria L.	0	n	1	0	Fabaceae Lindl.
Iz_1_1109	DJI_0044	Geranium robertianum L.	0	n	1	0	Geraniaceae Juss.
Iz_1_1109	DJI_0044	Geranium sanquineum L.	0	n	1	0	Geraniaceae Juss.
Iz_1_1109	DJI_0044	Gypsophila paniculata L.	0	n	1	0	Caryophyllaceae Juss.
Iz_1_1109	DJI_0044	Helichrysum arenarium (L.) Moench	0	n	1	0	Asteraceae Bercht. & J.Presl
Iz_1_1109	DJI_0044	Herniaria polygama J.Gay	0	n	1	0	Caryophyllaceae Juss.
Iz_1_1109	DJI_0044	Hieracium robustum Fr.	0	n	1	0	Asteraceae Bercht. & J.Presl
Iz_1_1109	DJI_0044	Hieracium umbellatum L.	0	n	1	NA	Asteraceae Bercht. & J.Presl
Iz_1_1109	DJI_0044	Hylotelephium maximum (L.) Holub	0	n	1	0	Crassulaceae J.St.-Hil.
Iz_1_1109	DJI_0044	Jacobaea borysthenica (DC.) B.Nord. & Greuter	0	n	1	0	Asteraceae Bercht. & J.Presl
Iz_1_1109	DJI_0044	Jasione montana L.	0	n	1	0	Campanulaceae Juss.
Iz_1_1109	DJI_0044	Jurinea cyanoides (L.) Rchb.s.l.	0	n	1	0	Asteraceae Bercht. & J.Presl
Iz_1_1109	DJI_0044	Koeleria glauca (Spreng.) DC.	0	n	1	0	Poaceae Barnhart
Iz_1_1109	DJI_0044	Lactuca serriola L.	0	a	0	0	Asteraceae Bercht. & J.Presl
Iz_1_1109	DJI_0044	Linaria genistifolia (L.) Mill.	0	n	1	0	Plantaginaceae Juss.
Iz_1_1109	DJI_0044	Lomelosia argentea (L.) Greuter & Burdet	0	n	1	0	Caprifoliaceae Juss.
Iz_1_1109	DJI_0044	Melampyrum pratense L.	0	n	1	0	Orobanchaceae Vent.
Iz_1_1109	DJI_0044	Oenothera biennis L.	0	a	0	0	Onagraceae Juss.
Iz_1_1109	DJI_0044	Pentanema britannica (L.) D.Gut.Larr., Santos-Vicente, Anderb., E.Rico & M.M.Mart.Ort.	0	n	1	0	Asteraceae Bercht. & J.Presl
Iz_1_1109	DJI_0044	Peucedanum oreoselinum (L.) Moench	0	n	1	0	Apiaceae Lindl.
Iz_1_1109	DJI_0044	Peucedanum ruthenicum M.Bieb.	0	n	1	0	Apiaceae Lindl.
Iz_1_1109	DJI_0044	Pilosella echioides (Lumn.) F.W.Schultz & Sch.Bip.	0	n	1	0	Asteraceae Bercht. & J.Presl
Iz_1_1109	DJI_0044	Pilosella officinarum F. Schultz et Sch. Bip.	0	n	1	0	Asteraceae Bercht. & J.Presl
Iz_1_1109	DJI_0044	Pilosella sp.	0		NA	0	Asteraceae Bercht. & J.Presl
Iz_1_1109	DJI_0044	Pinus sylvestris L.	0	n	1	1	Pinaceae Spreng. ex F.Rudolphi
Iz_1_1109	DJI_0044	Poa annua L.	0	n	1	0	Poaceae Barnhart
Iz_1_1109	DJI_0044	Poa nemoralis L.	0	n	1	0	Poaceae Barnhart
Iz_1_1109	DJI_0044	Poa sp.	0		NA	0	Poaceae Barnhart
Iz_1_1109	DJI_0044	Polygonatum odoratum (Mill.) Druce	0	n	1	0	Asparagaceae Juss.
Iz_1_1109	DJI_0044	Polygonum arenarium Waldst. & Kit.	0	n	1	0	Polygonaceae Juss.
Iz_1_1109	DJI_0044	Populus nigra L.	0	n	1	1	Salicaceae Mirb.
Iz_1_1109	DJI_0044	Populus sp.	0	n	1	1	Salicaceae Mirb.
Iz_1_1109	DJI_0044	Populus tremula L.	0	n	1	1	Salicaceae Mirb.
Iz_1_1109	DJI_0044	Populus Ã canescens (Aiton) Sm.	0	n	1	1	Salicaceae Mirb.
Iz_1_1109	DJI_0044	Potentilla argentea L.	0	n	1	0	Rosaceae Juss.
Iz_1_1109	DJI_0044	Psammophiliella muralis (L.) Ikonn.	0	n	1	0	Caryophyllaceae Juss.
Iz_1_1109	DJI_0044	Psephellus sumensis (Kalen.) Greuter	0	n	1	0	Asteraceae Bercht. & J.Presl
Iz_1_1109	DJI_0044	Pulsatilla pratensis (L.) Mill. s.l.	0	n	1	0	Ranunculaceae Juss.
Iz_1_1109	DJI_0044	Pyrus communis L.	0	a	0	1	Rosaceae Juss.
Iz_1_1109	DJI_0044	Quercus robur L.	0	n	1	1	Fagaceae Dumort.
Iz_1_1109	DJI_0044	Robinia pseudoacacia L.	0	a	0	1	Fabaceae Lindl.
Iz_1_1109	DJI_0044	Rosa sp.	0		NA	1	Rosaceae Juss.
Iz_1_1109	DJI_0044	Rubus idaeus L.	0	n	1	0	Rosaceae Juss.
Iz_1_1109	DJI_0044	Rumex acetosella L.	0	n	1	0	Polygonaceae Juss.
Iz_1_1109	DJI_0044	Rumex thyrsiflorus Fingerh.	0	n	1	0	Polygonaceae Juss.
Iz_1_1109	DJI_0044	Salix alba L.	0	n	1	1	Salicaceae Mirb.

Iz_1_1109	DJI_0044	Sambucus nigra L.	0	n	1	1	Viburnaceae Raf.
Iz_1_1109	DJI_0044	Secale sylvestre Host	0	n	1	0	Poaceae Barnhart
Iz_1_1109	DJI_0044	Sempervivum ruthenicum Schnittsp. & C.B. Lehm.	0	n	1	0	Crassulaceae J.St.-Hil.
Iz_1_1109	DJI_0044	Setaria viridis (L.) P. Beauv.	0	n	1	0	Poaceae Barnhart
Iz_1_1109	DJI_0044	Silene borysthenica (Gruner) Walters	0	n	1	0	Caryophyllaceae Juss.
Iz_1_1109	DJI_0044	Silene latifolia subsp. alba (Mill.) Greuter & Burdet	0	n	1	0	Caryophyllaceae Juss.
Iz_1_1109	DJI_0044	Silene nutans L.	0	n	1	0	Caryophyllaceae Juss.
Iz_1_1109	DJI_0044	Solanum nigrum L.	0		NA	NA	
Iz_1_1109	DJI_0044	Solidago virgaurea L.	0	n	1	0	Asteraceae Bercht. & J.Presl
Iz_1_1109	DJI_0044	Sorbus aucuparia L.	0	n	1	1	Rosaceae Juss.
Iz_1_1109	DJI_0044	Stachys recta L.	0	n	1	0	Lamiacea Lindl.
Iz_1_1109	DJI_0044	Tanacetum vulgare L.	0	n	1	0	Asteraceae Bercht. & J.Presl
Iz_1_1109	DJI_0044	Thymus pallasianus Heinr.Braun	0	n	1	0	Lamiacea Lindl.
Iz_1_1109	DJI_0044	Tilia cordata Mill.	0	n	1	1	Malvaceae Juss.
Iz_1_1109	DJI_0044	Trifolium alpestre L.	0	n	1	0	Fabaceae Lindl.
Iz_1_1109	DJI_0044	Trifolium arvense L.	0	n	1	0	Fabaceae Lindl.
Iz_1_1109	DJI_0044	Ulmus glabra Huds.	0	n	1	1	Ulmaceae Mirb.
Iz_1_1109	DJI_0044	Verbascum densiflorum Bertol.	0.2	n	1	0	Scrophulariaceae Juss.
Iz_1_1109	DJI_0044	Veronica austriaca L.	0	n	1	0	Plantaginaceae Juss.
Iz_1_1109	DJI_0044	Veronica incana L.	0	n	1	0	Plantaginaceae Juss.
Iz_1_1109	DJI_0044	Veronica verna L.	0	n	1	0	Plantaginaceae Juss.
Iz_1_1109	DJI_0044	Vincetoxicum hirundinaria Medik	0	n	1	0	Apocynaceae Juss.
Iz_1_1109	DJI_0046	Acer tataricum L.	0	n	1	1	Sapindaceae Juss.
Iz_1_1109	DJI_0046	Achillea millefolium L. s.l.	0	n	1	0	Asteraceae Bercht. & J.Presl
Iz_1_1109	DJI_0046	Allium oleraceum L.	0	n	1	0	Amaryllidaceae J.St.-Hil.
Iz_1_1109	DJI_0046	Ambrosia artemisiifolia L.	0	a	0	0	Asteraceae Bercht. & J.Presl
Iz_1_1109	DJI_0046	Amorpha fruticosa L.	0	a	0	0	Fabaceae Lindl.
Iz_1_1109	DJI_0046	Anthericum ramosum L.	0	n	1	0	Asparagaceae Juss.
Iz_1_1109	DJI_0046	Artemisia marschalliana Spreng.	0	n	1	0	Asteraceae Bercht. & J.Presl
Iz_1_1109	DJI_0046	Asparagus officinalis L.	0	n	1	0	Asparagaceae Juss.
Iz_1_1109	DJI_0046	Astragalus cicer L.	0	n	1	0	Fabaceae Lindl.
Iz_1_1109	DJI_0046	Bassia laniflora (S.G.Gmel.) A.J.Scott	0	n	1	0	Amaranthaceae Juss.
Iz_1_1109	DJI_0046	Berberis vulgaris L.	0	n	1	0	Berberidaceae Juss.
Iz_1_1109	DJI_0046	Betula pendula Roth.	0	n	1	1	Betulaceae Gray
Iz_1_1109	DJI_0046	Betula pendula Roth.	0	n	1	0	Poaceae Barnhart
Iz_1_1109	DJI_0046	Bromus inermis Leyss.	0		NA	NA	
Iz_1_1109	DJI_0046	Bromus squarrosus L.	0	a	0	0	Poaceae Barnhart
Iz_1_1109	DJI_0046	Bromus tectorum L.	0	a	0	0	Poaceae Barnhart
Iz_1_1109	DJI_0046	Calamagrostis epigejos (L.) Roth	0	n	1	0	Poaceae Barnhart
Iz_1_1109	DJI_0046	Carduus acanthoides L.	0	n	1	0	Asteraceae Bercht. & J.Presl
Iz_1_1109	DJI_0046	Carex sp.	0	n	1	0	Cyperaceae Juss.
Iz_1_1109	DJI_0046	Centaurea majorovii Dumbadze	0	n	1	0	Asteraceae Bercht. & J.Presl
Iz_1_1109	DJI_0046	Chamaecytisus ruthenicus (Fisch.ex Wol.) Klaskova	0	n	1	0	Fabaceae Lindl.
Iz_1_1109	DJI_0046	Chelidonium majus L.	0	n	1	0	Papaveraceae Juss.
Iz_1_1109	DJI_0046	Chenopodium betaceum Andrz.	0	n	1	0	Amaranthaceae Juss.
Iz_1_1109	DJI_0046	Chenopodium cf. album	0.2	n	1	0	Amaranthaceae Juss.
Iz_1_1109	DJI_0046	Chenopodium sp.	0		NA	0	Amaranthaceae Juss.
Iz_1_1109	DJI_0046	Chondrilla juncea L.	0	n	1	0	Asteraceae Bercht. & J.Presl
Iz_1_1109	DJI_0046	Cirsium vulgare (Savi) Ten.	0	n	1	0	Asteraceae Bercht. & J.Presl
Iz_1_1109	DJI_0046	Clinopodium acinos (L.) Kuntze	0	n	1	0	Lamiacea Lindl.
Iz_1_1109	DJI_0046	Convallaria majalis L.	0	n	1	0	Asparagaceae Juss.
Iz_1_1109	DJI_0046	Crataegus rhipidophylla Gand.	0	n	1	1	Rosaceae Juss.
Iz_1_1109	DJI_0046	Crepis foetida L.	0	n	1	0	Asteraceae Bercht. & J.Presl
Iz_1_1109	DJI_0046	Dactylis glomerata L.	0	n	1	0	Poaceae Barnhart
Iz_1_1109	DJI_0046	Dianthus campestris M.Bieb.	0	n	1	0	Caryophyllaceae Juss.
Iz_1_1109	DJI_0046	Digitaria sanguinalis (L.) Scop.	0	a	0	0	Poaceae Barnhart
Iz_1_1109	DJI_0046	Elymus repens (L.) Gould	0	n	1	0	Poaceae Barnhart
Iz_1_1109	DJI_0046	Epilobium tetragonum L.	0	n	1	0	Onagraceae Juss.
Iz_1_1109	DJI_0046	Eragrostis minor Host	0	a	0	0	Poaceae Barnhart
Iz_1_1109	DJI_0046	Erigeron canadensis L.	0.1	a	0	0	Asteraceae Bercht. & J.Presl
Iz_1_1109	DJI_0046	Euphorbia seguieriana Neck.	0	n	1	0	Euphorbiaceae Juss.
Iz_1_1109	DJI_0046	Fallopia dumetorum (L.) Holub	0	n	1	0	Polygonaceae Juss.
Iz_1_1109	DJI_0046	Festuca beckeri (Hack.) Trautv.	0	n	1	0	Poaceae Barnhart
Iz_1_1109	DJI_0046	Fragaria vesca L.	0	n	1	0	Rosaceae Juss.
Iz_1_1109	DJI_0046	Frangula alnus Mill.	0	n	1	1	Rhamnaceae Juss.
Iz_1_1109	DJI_0046	Fraxinus pennsylvanica Marshall	0	a	0	1	Oleaceae Hoffmanns. & Link
Iz_1_1109	DJI_0046	Galeopsis bifida Boenn.	0	n	1	0	Lamiacea Lindl.
Iz_1_1109	DJI_0046	Galium verum L.	0	n	1	0	Rubiaceae Juss.
Iz_1_1109	DJI_0046	Genista tinctoria L.	0	n	1	0	Fabaceae Lindl.
Iz_1_1109	DJI_0046	Geranium robertianum L.	0	n	1	0	Geraniaceae Juss.
Iz_1_1109	DJI_0046	Geranium sanquineum L.	0	n	1	0	Geraniaceae Juss.
Iz_1_1109	DJI_0046	Gypsophila paniculata L.	0	n	1	0	Caryophyllaceae Juss.
Iz_1_1109	DJI_0046	Helichrysum arenarium (L.) Moench	0	n	1	0	Asteraceae Bercht. & J.Presl
Iz_1_1109	DJI_0046	Herniaria polygama J.Gay	0	n	1	0	Caryophyllaceae Juss.
Iz_1_1109	DJI_0046	Hieracium robustum Fr.	0	n	1	0	Asteraceae Bercht. & J.Presl
Iz_1_1109	DJI_0046	Hieracium umbellatum L.	0	n	1	NA	Asteraceae Bercht. & J.Presl
Iz_1_1109	DJI_0046	Hylotelephium maximum (L.) Holub	0	n	1	0	Crassulaceae J.St.-Hil.
Iz_1_1109	DJI_0046	Jacobaea borysthenica (DC.) B.Nord. & Greuter	0	n	1	0	Asteraceae Bercht. & J.Presl
Iz_1_1109	DJI_0046	Jasione montana L.	0	n	1	0	Campanulaceae Juss.
Iz_1_1109	DJI_0046	Jurinea cyanoides (L.) Rchb.s.l.	0	n	1	0	Asteraceae Bercht. & J.Presl
Iz_1_1109	DJI_0046	Koeleria glauca (Spreng.) DC.	0	n	1	0	Poaceae Barnhart
Iz_1_1109	DJI_0046	Lactuca serriola L.	0	a	0	0	Asteraceae Bercht. & J.Presl
Iz_1_1109	DJI_0046	Linaria genistifolia (L.) Mill.	0	n	1	0	Plantaginaceae Juss.
Iz_1_1109	DJI_0046	Lomelosia argentea (L.) Greuter & Burdet	0	n	1	0	Caprifoliaceae Juss.
Iz_1_1109	DJI_0046	Melampyrum pratense L.	0	n	1	0	Orobanchaceae Vent.
Iz_1_1109	DJI_0046	Oenothera biennis L.	0	a	0	0	Onagraceae Juss.
Iz_1_1109	DJI_0046	Pentanema britannica (L.) D.Gut.Larr., Santos-Vicente, Anderb., E.Rico & M.M.Mart.Ort.	0	n	1	0	Asteraceae Bercht. & J.Presl
Iz_1_1109	DJI_0046	Peucedanum oreoselinum (L.) Moench	0	n	1	0	Apiaceae Lindl.
Iz_1_1109	DJI_0046	Peucedanum ruthenicum M.Bieb.	0	n	1	0	Apiaceae Lindl.
Iz_1_1109	DJI_0046	Pilosella echioides (Lumn.) F.W.Schultz & Sch.Bip.	0	n	1	0	Asteraceae Bercht. & J.Presl
Iz_1_1109	DJI_0046	Pilosella officinarum F. Schultz et Sch. Bip.	0	n	1	0	Asteraceae Bercht. & J.Presl
Iz_1_1109	DJI_0046	Pilosella sp.	0		NA	0	Asteraceae Bercht. & J.Presl
Iz_1_1109	DJI_0046	Pinus sylvestris L.	0	n	1	1	Pinaceae Spreng. ex F.Rudolphi
Iz_1_1109	DJI_0046	Poa annua L.	0	n	1	0	Poaceae Barnhart
Iz_1_1109	DJI_0046	Poa nemoralis L.	0	n	1	0	Poaceae Barnhart
Iz_1_1109	DJI_0046	Poa sp.	0		NA	0	Poaceae Barnhart
Iz_1_1109	DJI_0046	Polygonatum odoratum (Mill.) Druce	0	n	1	0	Asparagaceae Juss.
Iz_1_1109	DJI_0046	Polygonum arenarium Waldst. & Kit.	0	n	1	0	Polygonaceae Juss.

Iz_1_1109	DJI_0046	Populus nigra L.	0	n	1	1	Salicaceae Mirb.
Iz_1_1109	DJI_0046	Populus sp.	0	n	1	1	Salicaceae Mirb.
Iz_1_1109	DJI_0046	Populus tremula L.	0	n	1	1	Salicaceae Mirb.
Iz_1_1109	DJI_0046	Populus Ã canescens (Aiton) Sm.	0	n	1	1	Salicaceae Mirb.
Iz_1_1109	DJI_0046	Potentilla argentea L.	0	n	1	0	Rosaceae Juss.
Iz_1_1109	DJI_0046	Psammophiliella muralis (L.) Ikonn.	0	n	1	0	Caryophyllaceae Juss.
Iz_1_1109	DJI_0046	Psephellus sumensis (Kalen.) Greuter	0	n	1	0	Asteraceae Bercht. & J.Presl
Iz_1_1109	DJI_0046	Pulsatilla pratensis (L.) Mill. s.l.	0	n	1	0	Ranunculaceae Juss.
Iz_1_1109	DJI_0046	Pyrus communis L.	0	a	0	1	Rosaceae Juss.
Iz_1_1109	DJI_0046	Quercus robur L.	0	n	1	1	Fagaceae Dumort.
Iz_1_1109	DJI_0046	Robinia pseudoacacia L.	0	a	0	1	Fabaceae Lindl.
Iz_1_1109	DJI_0046	Rosa sp.	0		NA	1	Rosaceae Juss.
Iz_1_1109	DJI_0046	Rubus idaeus L.	0	n	1	0	Rosaceae Juss.
Iz_1_1109	DJI_0046	Rumex acetosella L.	0	n	1	0	Polygonaceae Juss.
Iz_1_1109	DJI_0046	Rumex thyrsiflorus Fingerh.	0	n	1	0	Polygonaceae Juss.
Iz_1_1109	DJI_0046	Salix alba L.	0	n	1	1	Salicaceae Mirb.
Iz_1_1109	DJI_0046	Sambucus nigra L.	0	n	1	1	Viburnaceae Raf.
Iz_1_1109	DJI_0046	Secale sylvestre Host	0	n	1	0	Poaceae Barnhart
Iz_1_1109	DJI_0046	Sempervivum ruthenicum Schnittsp. & C.B. Lehm.	0	n	1	0	Crassulaceae J.St.-Hil.
Iz_1_1109	DJI_0046	Setaria viridis (L.) P. Beauv.	0	n	1	0	Poaceae Barnhart
Iz_1_1109	DJI_0046	Silene borysthenica (Gruner) Walters	0	n	1	0	Caryophyllaceae Juss.
Iz_1_1109	DJI_0046	Silene latifolia subsp. alba (Mill.) Greuter & Burdet	0	n	1	0	Caryophyllaceae Juss.
Iz_1_1109	DJI_0046	Silene nutans L.	0	n	1	0	Caryophyllaceae Juss.
Iz_1_1109	DJI_0046	Solanum nigrum L.	0		NA	NA	
Iz_1_1109	DJI_0046	Solidago virgaurea L.	0	n	1	0	Asteraceae Bercht. & J.Presl
Iz_1_1109	DJI_0046	Sorbus aucuparia L.	0	n	1	1	Rosaceae Juss.
Iz_1_1109	DJI_0046	Stachys recta L.	0	n	1	0	Lamiacea Lindl.
Iz_1_1109	DJI_0046	Tanacetum vulgare L.	0	n	1	0	Asteraceae Bercht. & J.Presl
Iz_1_1109	DJI_0046	Thymus pallasianus Heinr.Braun	0	n	1	0	Lamiacea Lindl.
Iz_1_1109	DJI_0046	Tilia cordata Mill.	0	n	1	1	Malvaceae Juss.
Iz_1_1109	DJI_0046	Trifolium alpestre L.	0	n	1	0	Fabaceae Lindl.
Iz_1_1109	DJI_0046	Trifolium arvense L.	0	n	1	0	Fabaceae Lindl.
Iz_1_1109	DJI_0046	Ulmus glabra Huds.	0	n	1	1	Ulmaceae Mirb.
Iz_1_1109	DJI_0046	Verbascum densiflorum Bertol.	0	n	1	0	Scrophulariaceae Juss.
Iz_1_1109	DJI_0046	Veronica austriaca L.	0	n	1	0	Plantaginaceae Juss.
Iz_1_1109	DJI_0046	Veronica incana L.	0	n	1	0	Plantaginaceae Juss.
Iz_1_1109	DJI_0046	Veronica verna L.	0	n	1	0	Plantaginaceae Juss.
Iz_1_1109	DJI_0046	Vincetoxicum hirundinaria Medik	0	n	1	0	Apocynaceae Juss.
Iz_1_1109	DJI_0048	Acer tataricum L.	0	n	1	1	Sapindaceae Juss.
Iz_1_1109	DJI_0048	Achillea millefolium L. s.l.	0	n	1	0	Asteraceae Bercht. & J.Presl
Iz_1_1109	DJI_0048	Allium oleraceum L.	0	n	1	0	Amaryllidaceae J.St.-Hil.
Iz_1_1109	DJI_0048	Ambrosia artemisiifolia L.	0	a	0	0	Asteraceae Bercht. & J.Presl
Iz_1_1109	DJI_0048	Amorpha fruticosa L.	0	a	0	0	Fabaceae Lindl.
Iz_1_1109	DJI_0048	Anthericum ramosum L.	0	n	1	0	Asparagaceae Juss.
Iz_1_1109	DJI_0048	Artemisia marschalliana Spreng.	0	n	1	0	Asteraceae Bercht. & J.Presl
Iz_1_1109	DJI_0048	Asparagus officinalis L.	0	n	1	0	Asparagaceae Juss.
Iz_1_1109	DJI_0048	Astragalus cicer L.	0	n	1	0	Fabaceae Lindl.
Iz_1_1109	DJI_0048	Bassia laniflora (S.G.Gmel.) A.J.Scott	0	n	1	0	Amaranthaceae Juss.
Iz_1_1109	DJI_0048	Berberis vulgaris L.	0	n	1	0	Berberidaceae Juss.
Iz_1_1109	DJI_0048	Betula pendula Roth.	0	n	1	1	Betulaceae Gray
Iz_1_1109	DJI_0048	Betula pendula Roth.	0	n	1	0	Poaceae Barnhart
Iz_1_1109	DJI_0048	Bromus inermis Leyss.	0		NA	NA	
Iz_1_1109	DJI_0048	Bromus squarrosus L.	0	a	0	0	Poaceae Barnhart
Iz_1_1109	DJI_0048	Bromus tectorum L.	0	a	0	0	Poaceae Barnhart
Iz_1_1109	DJI_0048	Calamagrostis epigejos (L.) Roth	0	n	1	0	Poaceae Barnhart
Iz_1_1109	DJI_0048	Carduus acanthoides L.	0	n	1	0	Asteraceae Bercht. & J.Presl
Iz_1_1109	DJI_0048	Carex sp.	0	n	1	0	Cyperaceae Juss.
Iz_1_1109	DJI_0048	Centaurea majorovii Dumbadze	0	n	1	0	Asteraceae Bercht. & J.Presl
Iz_1_1109	DJI_0048	Chamaecytisus ruthenicus (Fisch.ex Wol.) Klaskova	0	n	1	0	Fabaceae Lindl.
Iz_1_1109	DJI_0048	Chelidonium majus L.	0	n	1	0	Papaveraceae Juss.
Iz_1_1109	DJI_0048	Chenopodium betaceum Andrz.	0	n	1	0	Amaranthaceae Juss.
Iz_1_1109	DJI_0048	Chenopodium cf. album	0.5	n	1	0	Amaranthaceae Juss.
Iz_1_1109	DJI_0048	Chenopodium sp.	0		NA	0	Amaranthaceae Juss.
Iz_1_1109	DJI_0048	Chondrilla juncea L.	0	n	1	0	Asteraceae Bercht. & J.Presl
Iz_1_1109	DJI_0048	Cirsium vulgare (Savi) Ten.	0	n	1	0	Asteraceae Bercht. & J.Presl
Iz_1_1109	DJI_0048	Clinopodium acinos (L.) Kuntze	0	n	1	0	Lamiacea Lindl.
Iz_1_1109	DJI_0048	Convallaria majalis L.	0	n	1	0	Asparagaceae Juss.
Iz_1_1109	DJI_0048	Crataegus rhipidophylla Gand.	0	n	1	1	Rosaceae Juss.
Iz_1_1109	DJI_0048	Crepis foetida L.	0	n	1	0	Asteraceae Bercht. & J.Presl
Iz_1_1109	DJI_0048	Dactylis glomerata L.	0	n	1	0	Poaceae Barnhart
Iz_1_1109	DJI_0048	Dianthus campestris M.Bieb.	0	n	1	0	Caryophyllaceae Juss.
Iz_1_1109	DJI_0048	Digitaria sanguinalis (L.) Scop.	0	a	0	0	Poaceae Barnhart
Iz_1_1109	DJI_0048	Elymus repens (L.) Gould	0	n	1	0	Poaceae Barnhart
Iz_1_1109	DJI_0048	Epilobium tetragonum L.	0	n	1	0	Onagraceae Juss.
Iz_1_1109	DJI_0048	Eragrostis minor Host	0	a	0	0	Poaceae Barnhart
Iz_1_1109	DJI_0048	Erigeron canadensis L.	0.2	a	0	0	Asteraceae Bercht. & J.Presl
Iz_1_1109	DJI_0048	Euphorbia seguieriana Neck.	0	n	1	0	Euphorbiaceae Juss.
Iz_1_1109	DJI_0048	Fallopia dumetorum (L.) Holub	0	n	1	0	Polygonaceae Juss.
Iz_1_1109	DJI_0048	Festuca beckeri (Hack.) Trautv.	0	n	1	0	Poaceae Barnhart
Iz_1_1109	DJI_0048	Fragaria vesca L.	0	n	1	0	Rosaceae Juss.
Iz_1_1109	DJI_0048	Frangula alnus Mill.	0	n	1	1	Rhamnaceae Juss.
Iz_1_1109	DJI_0048	Fraxinus pennsylvanica Marshall	0	a	0	1	Oleaceae Hoffmanns. & Link
Iz_1_1109	DJI_0048	Galeopsis bifida Boenn.	0	n	1	0	Lamiacea Lindl.
Iz_1_1109	DJI_0048	Galium verum L.	0	n	1	0	Rubiaceae Juss.
Iz_1_1109	DJI_0048	Genista tinctoria L.	0	n	1	0	Fabaceae Lindl.
Iz_1_1109	DJI_0048	Geranium robertianum L.	0	n	1	0	Geraniaceae Juss.
Iz_1_1109	DJI_0048	Geranium sanquineum L.	0	n	1	0	Geraniaceae Juss.
Iz_1_1109	DJI_0048	Gypsophila paniculata L.	0	n	1	0	Caryophyllaceae Juss.
Iz_1_1109	DJI_0048	Helichrysum arenarium (L.) Moench	0	n	1	0	Asteraceae Bercht. & J.Presl
Iz_1_1109	DJI_0048	Herniaria polygama J.Gay	0	n	1	0	Caryophyllaceae Juss.
Iz_1_1109	DJI_0048	Hieracium robustum Fr.	0	n	1	0	Asteraceae Bercht. & J.Presl
Iz_1_1109	DJI_0048	Hieracium umbellatum L.	0	n	1	NA	Asteraceae Bercht. & J.Presl
Iz_1_1109	DJI_0048	Hylotelephium maximum (L.) Holub	0	n	1	0	Crassulaceae J.St.-Hil.
Iz_1_1109	DJI_0048	Jacobaea borysthenica (DC.) B.Nord. & Greuter	0	n	1	0	Asteraceae Bercht. & J.Presl
Iz_1_1109	DJI_0048	Jasione montana L.	0	n	1	0	Campanulaceae Juss.
Iz_1_1109	DJI_0048	Jurinea cyanoides (L.) Rchb.s.l.	0	n	1	0	Asteraceae Bercht. & J.Presl
Iz_1_1109	DJI_0048	Koeleria glauca (Spreng.) DC.	0	n	1	0	Poaceae Barnhart
Iz_1_1109	DJI_0048	Lactuca serriola L.	0	a	0	0	Asteraceae Bercht. & J.Presl

Iz_1_1109	DJI_0048	Linaria genistifolia (L.) Mill.	0	n	1	0	Plantaginaceae Juss.
Iz_1_1109	DJI_0048	Lomelosia argentea (L.) Greuter & Burdet	0	n	1	0	Caprifoliaceae Juss.
Iz_1_1109	DJI_0048	Melampyrum pratense L.	0	n	1	0	Orobanchaceae Vent.
Iz_1_1109	DJI_0048	Oenothera biennis L.	0	a	0	0	Onagraceae Juss.
Iz_1_1109	DJI_0048	Pentanema britannica (L.) D.Gut.Larr., Santos-Vicente, Anderb., E.Rico & M.M.Mart.Ort.	0	n	1	0	Asteraceae Bercht. & J.Presl
Iz_1_1109	DJI_0048	Peucedanum oreoselinum (L.) Moench	0	n	1	0	Apiaceae Lindl.
Iz_1_1109	DJI_0048	Peucedanum ruthenicum M.Bieb.	0	n	1	0	Apiaceae Lindl.
Iz_1_1109	DJI_0048	Pilosella echioides (Lumn.) F.W.Schultz & Sch.Bip.	0	n	1	0	Asteraceae Bercht. & J.Presl
Iz_1_1109	DJI_0048	Pilosella officinarum F. Schultz et Sch. Bip.	0	n	1	0	Asteraceae Bercht. & J.Presl
Iz_1_1109	DJI_0048	Pilosella sp.	0		NA	0	Asteraceae Bercht. & J.Presl
Iz_1_1109	DJI_0048	Pinus sylvestris L.	0	n	1	1	Pinaceae Spreng. ex F.Rudolphi
Iz_1_1109	DJI_0048	Poa annua L.	0	n	1	0	Poaceae Barnhart
Iz_1_1109	DJI_0048	Poa nemoralis L.	0	n	1	0	Poaceae Barnhart
Iz_1_1109	DJI_0048	Poa sp.	0		NA	0	Poaceae Barnhart
Iz_1_1109	DJI_0048	Polygonatum odoratum (Mill.) Druce	0	n	1	0	Asparagaceae Juss.
Iz_1_1109	DJI_0048	Polygonum arenarium Waldst. & Kit.	0	n	1	0	Polygonaceae Juss.
Iz_1_1109	DJI_0048	Populus nigra L.	0	n	1	1	Salicaceae Mirb.
Iz_1_1109	DJI_0048	Populus sp.	0	n	1	1	Salicaceae Mirb.
Iz_1_1109	DJI_0048	Populus tremula L.	0	n	1	1	Salicaceae Mirb.
Iz_1_1109	DJI_0048	Populus Ã canescens (Aiton) Sm.	0	n	1	1	Salicaceae Mirb.
Iz_1_1109	DJI_0048	Potentilla argentea L.	0	n	1	0	Rosaceae Juss.
Iz_1_1109	DJI_0048	Psammophiliella muralis (L.) Ikonn.	0	n	1	0	Caryophyllaceae Juss.
Iz_1_1109	DJI_0048	Psephellus sumensis (Kalen.) Greuter	0	n	1	0	Asteraceae Bercht. & J.Presl
Iz_1_1109	DJI_0048	Pulsatilla pratensis (L.) Mill. s.l.	0	n	1	0	Ranunculaceae Juss.
Iz_1_1109	DJI_0048	Pyrus communis L.	0	a	0	1	Rosaceae Juss.
Iz_1_1109	DJI_0048	Quercus robur L.	0	n	1	1	Fagaceae Dumort.
Iz_1_1109	DJI_0048	Robinia pseudoacacia L.	0	a	0	1	Fabaceae Lindl.
Iz_1_1109	DJI_0048	Rosa sp.	0		NA	1	Rosaceae Juss.
Iz_1_1109	DJI_0048	Rubus idaeus L.	0	n	1	0	Rosaceae Juss.
Iz_1_1109	DJI_0048	Rumex acetosella L.	0	n	1	0	Polygonaceae Juss.
Iz_1_1109	DJI_0048	Rumex thyrsiflorus Fingerh.	0	n	1	0	Polygonaceae Juss.
Iz_1_1109	DJI_0048	Salix alba L.	0	n	1	1	Salicaceae Mirb.
Iz_1_1109	DJI_0048	Sambucus nigra L.	0	n	1	1	Viburnaceae Raf.
Iz_1_1109	DJI_0048	Secale sylvestre Host	0	n	1	0	Poaceae Barnhart
Iz_1_1109	DJI_0048	Sempervivum ruthenicum Schnittsp. & C.B. Lehm.	0	n	1	0	Crassulaceae J.St.-Hil.
Iz_1_1109	DJI_0048	Setaria viridis (L.) P. Beauv.	0	n	1	0	Poaceae Barnhart
Iz_1_1109	DJI_0048	Silene borysthenica (Gruner) Walters	0	n	1	0	Caryophyllaceae Juss.
Iz_1_1109	DJI_0048	Silene latifolia subsp. alba (Mill.) Greuter & Burdet	0	n	1	0	Caryophyllaceae Juss.
Iz_1_1109	DJI_0048	Silene nutans L.	0	n	1	0	Caryophyllaceae Juss.
Iz_1_1109	DJI_0048	Solanum nigrum L.	0		NA	NA	
Iz_1_1109	DJI_0048	Solidago virgaurea L.	0	n	1	0	Asteraceae Bercht. & J.Presl
Iz_1_1109	DJI_0048	Sorbus aucuparia L.	0	n	1	1	Rosaceae Juss.
Iz_1_1109	DJI_0048	Stachys recta L.	0	n	1	0	Lamiacea Lindl.
Iz_1_1109	DJI_0048	Tanacetum vulgare L.	0	n	1	0	Asteraceae Bercht. & J.Presl
Iz_1_1109	DJI_0048	Thymus pallasianus Heinr.Braun	0	n	1	0	Lamiacea Lindl.
Iz_1_1109	DJI_0048	Tilia cordata Mill.	0	n	1	1	Malvaceae Juss.
Iz_1_1109	DJI_0048	Trifolium alpestre L.	0	n	1	0	Fabaceae Lindl.
Iz_1_1109	DJI_0048	Trifolium arvense L.	0	n	1	0	Fabaceae Lindl.
Iz_1_1109	DJI_0048	Ulmus glabra Huds.	0	n	1	1	Ulmaceae Mirb.
Iz_1_1109	DJI_0048	Verbascum densiflorum Bertol.	0	n	1	0	Scrophulariaceae Juss.
Iz_1_1109	DJI_0048	Veronica austriaca L.	0	n	1	0	Plantaginaceae Juss.
Iz_1_1109	DJI_0048	Veronica incana L.	0	n	1	0	Plantaginaceae Juss.
Iz_1_1109	DJI_0048	Veronica verna L.	0	n	1	0	Plantaginaceae Juss.
Iz_1_1109	DJI_0048	Vincetoxicum hirundinaria Medik	0	n	1	0	Apocynaceae Juss.
Iz_1_1109	DJI_0051	Acer tataricum L.	0	n	1	1	Sapindaceae Juss.
Iz_1_1109	DJI_0051	Achillea millefolium L. s.l.	0	n	1	0	Asteraceae Bercht. & J.Presl
Iz_1_1109	DJI_0051	Allium oleraceum L.	0	n	1	0	Amaryllidaceae J.St.-Hil.
Iz_1_1109	DJI_0051	Ambrosia artemisiifolia L.	0	a	0	0	Asteraceae Bercht. & J.Presl
Iz_1_1109	DJI_0051	Amorpha fruticosa L.	0	a	0	0	Fabaceae Lindl.
Iz_1_1109	DJI_0051	Anthericum ramosum L.	0	n	1	0	Asparagaceae Juss.
Iz_1_1109	DJI_0051	Artemisia marschalliana Spreng.	0	n	1	0	Asteraceae Bercht. & J.Presl
Iz_1_1109	DJI_0051	Asparagus officinalis L.	0	n	1	0	Asparagaceae Juss.
Iz_1_1109	DJI_0051	Astragalus cicer L.	0	n	1	0	Fabaceae Lindl.
Iz_1_1109	DJI_0051	Bassia laniflora (S.G.Gmel.) A.J.Scott	0	n	1	0	Amaranthaceae Juss.
Iz_1_1109	DJI_0051	Berberis vulgaris L.	0	n	1	0	Berberidaceae Juss.
Iz_1_1109	DJI_0051	Betula pendula Roth.	0	n	1	1	Betulaceae Gray
Iz_1_1109	DJI_0051	Betula pendula Roth.	0	n	1	0	Poaceae Barnhart
Iz_1_1109	DJI_0051	Bromus inermis Leyss.	0		NA	NA	
Iz_1_1109	DJI_0051	Bromus squarrosus L.	0	a	0	0	Poaceae Barnhart
Iz_1_1109	DJI_0051	Bromus tectorum L.	0	a	0	0	Poaceae Barnhart
Iz_1_1109	DJI_0051	Calamagrostis epigejos (L.) Roth	0.5	n	1	0	Poaceae Barnhart
Iz_1_1109	DJI_0051	Carduus acanthoides L.	0	n	1	0	Asteraceae Bercht. & J.Presl
Iz_1_1109	DJI_0051	Carex sp.	1	n	1	0	Cyperaceae Juss.
Iz_1_1109	DJI_0051	Centaurea majorovii Dumbadze	0	n	1	0	Asteraceae Bercht. & J.Presl
Iz_1_1109	DJI_0051	Chamaecytisus ruthenicus (Fisch.ex Wol.) Klaskova	0	n	1	0	Fabaceae Lindl.
Iz_1_1109	DJI_0051	Chelidonium majus L.	0	n	1	0	Papaveraceae Juss.
Iz_1_1109	DJI_0051	Chenopodium betaceum Andrz.	0	n	1	0	Amaranthaceae Juss.
Iz_1_1109	DJI_0051	Chenopodium cf. album	0	n	1	0	Amaranthaceae Juss.
Iz_1_1109	DJI_0051	Chenopodium sp.	0		NA	0	Amaranthaceae Juss.
Iz_1_1109	DJI_0051	Chondrilla juncea L.	0	n	1	0	Asteraceae Bercht. & J.Presl
Iz_1_1109	DJI_0051	Cirsium vulgare (Savi) Ten.	0	n	1	0	Asteraceae Bercht. & J.Presl
Iz_1_1109	DJI_0051	Clinopodium acinos (L.) Kuntze	0	n	1	0	Lamiacea Lindl.
Iz_1_1109	DJI_0051	Convallaria majalis L.	0	n	1	0	Asparagaceae Juss.
Iz_1_1109	DJI_0051	Crataegus rhipidophylla Gand.	0	n	1	1	Rosaceae Juss.
Iz_1_1109	DJI_0051	Crepis foetida L.	0	n	1	0	Asteraceae Bercht. & J.Presl
Iz_1_1109	DJI_0051	Dactylis glomerata L.	0	n	1	0	Poaceae Barnhart
Iz_1_1109	DJI_0051	Dianthus campestris M.Bieb.	0	n	1	0	Caryophyllaceae Juss.
Iz_1_1109	DJI_0051	Digitaria sanguinalis (L.) Scop.	0	a	0	0	Poaceae Barnhart
Iz_1_1109	DJI_0051	Elymus repens (L.) Gould	0	n	1	0	Poaceae Barnhart
Iz_1_1109	DJI_0051	Epilobium tetragonum L.	0	n	1	0	Onagraceae Juss.
Iz_1_1109	DJI_0051	Eragrostis minor Host	0	a	0	0	Poaceae Barnhart
Iz_1_1109	DJI_0051	Erigeron canadensis L.	0	a	0	0	Asteraceae Bercht. & J.Presl
Iz_1_1109	DJI_0051	Euphorbia seguieriana Neck.	0	n	1	0	Euphorbiaceae Juss.
Iz_1_1109	DJI_0051	Fallopia dumetorum (L.) Holub	0	n	1	0	Polygonaceae Juss.
Iz_1_1109	DJI_0051	Festuca beckeri (Hack.) Trautv.	0	n	1	0	Poaceae Barnhart
Iz_1_1109	DJI_0051	Fragaria vesca L.	0	n	1	0	Rosaceae Juss.
Iz_1_1109	DJI_0051	Frangula alnus Mill.	0	n	1	1	Rhamnaceae Juss.
Iz_1_1109	DJI_0051	Fraxinus pennsylvanica Marshall	0	a	0	1	Oleaceae Hoffmanns. & Link

Iz_1_1109	DJI_0051	Galeopsis bifida Boenn.	0	n	1	0	Lamiacea Lindl.
Iz_1_1109	DJI_0051	Galium verum L.	0	n	1	0	Rubiaceae Juss.
Iz_1_1109	DJI_0051	Genista tinctoria L.	0	n	1	0	Fabaceae Lindl.
Iz_1_1109	DJI_0051	Geranium robertianum L.	0	n	1	0	Geraniaceae Juss.
Iz_1_1109	DJI_0051	Geranium sanquineum L.	0	n	1	0	Geraniaceae Juss.
Iz_1_1109	DJI_0051	Gypsophila paniculata L.	0	n	1	0	Caryophyllaceae Juss.
Iz_1_1109	DJI_0051	Helichrysum arenarium (L.) Moench	0	n	1	0	Asteraceae Bercht. & J.Presl
Iz_1_1109	DJI_0051	Herniaria polygama J.Gay	0	n	1	0	Caryophyllaceae Juss.
Iz_1_1109	DJI_0051	Hieracium robustum Fr.	0	n	1	0	Asteraceae Bercht. & J.Presl
Iz_1_1109	DJI_0051	Hieracium umbellatum L.	0	n	1	NA	Asteraceae Bercht. & J.Presl
Iz_1_1109	DJI_0051	Hylotelephium maximum (L.) Holub	0	n	1	0	Crassulaceae J.St.-Hil.
Iz_1_1109	DJI_0051	Jacobaea borysthenica (DC.) B.Nord. & Greuter	0	n	1	0	Asteraceae Bercht. & J.Presl
Iz_1_1109	DJI_0051	Jasione montana L.	0	n	1	0	Campanulaceae Juss.
Iz_1_1109	DJI_0051	Jurinea cyanoides (L.) Rchb.s.l.	0	n	1	0	Asteraceae Bercht. & J.Presl
Iz_1_1109	DJI_0051	Koeleria glauca (Spreng.) DC.	0	n	1	0	Poaceae Barnhart
Iz_1_1109	DJI_0051	Lactuca serriola L.	0	a	0	0	Asteraceae Bercht. & J.Presl
Iz_1_1109	DJI_0051	Linaria genistifolia (L.) Mill.	0	n	1	0	Plantaginaceae Juss.
Iz_1_1109	DJI_0051	Lomelosia argentea (L.) Greuter & Burdet	0	n	1	0	Caprifoliaceae Juss.
Iz_1_1109	DJI_0051	Melampyrum pratense L.	0	n	1	0	Orobanchaceae Vent.
Iz_1_1109	DJI_0051	Oenothera biennis L.	0	a	0	0	Onagraceae Juss.
Iz_1_1109	DJI_0051	Pentanema britannica (L.) D.Gut.Larr., Santos-Vicente, Anderb., E.Rico & M.M.Mart.Ort.	0	n	1	0	Asteraceae Bercht. & J.Presl
Iz_1_1109	DJI_0051	Peucedanum oreoselinum (L.) Moench	0	n	1	0	Apiaceae Lindl.
Iz_1_1109	DJI_0051	Peucedanum ruthenicum M.Bieb.	0	n	1	0	Apiaceae Lindl.
Iz_1_1109	DJI_0051	Pilosella echioides (Lumn.) F.W.Schultz & Sch.Bip.	0	n	1	0	Asteraceae Bercht. & J.Presl
Iz_1_1109	DJI_0051	Pilosella officinarum F. Schultz et Sch. Bip.	0	n	1	0	Asteraceae Bercht. & J.Presl
Iz_1_1109	DJI_0051	Pilosella sp.	0		NA	0	Asteraceae Bercht. & J.Presl
Iz_1_1109	DJI_0051	Pinus sylvestris L.	0	n	1	1	Pinaceae Spreng. ex F.Rudolphi
Iz_1_1109	DJI_0051	Poa annua L.	0	n	1	0	Poaceae Barnhart
Iz_1_1109	DJI_0051	Poa nemoralis L.	0	n	1	0	Poaceae Barnhart
Iz_1_1109	DJI_0051	Poa sp.	0		NA	0	Poaceae Barnhart
Iz_1_1109	DJI_0051	Polygonatum odoratum (Mill.) Druce	0	n	1	0	Asparagaceae Juss.
Iz_1_1109	DJI_0051	Polygonum arenarium Waldst. & Kit.	0	n	1	0	Polygonaceae Juss.
Iz_1_1109	DJI_0051	Populus nigra L.	0	n	1	1	Salicaceae Mirb.
Iz_1_1109	DJI_0051	Populus sp.	0	n	1	1	Salicaceae Mirb.
Iz_1_1109	DJI_0051	Populus tremula L.	0	n	1	1	Salicaceae Mirb.
Iz_1_1109	DJI_0051	Populus Ã canescens (Aiton) Sm.	0	n	1	1	Salicaceae Mirb.
Iz_1_1109	DJI_0051	Potentilla argentea L.	0	n	1	0	Rosaceae Juss.
Iz_1_1109	DJI_0051	Psammophiliella muralis (L.) Ikonn.	0	n	1	0	Caryophyllaceae Juss.
Iz_1_1109	DJI_0051	Psephellus sumensis (Kalen.) Greuter	0	n	1	0	Asteraceae Bercht. & J.Presl
Iz_1_1109	DJI_0051	Pulsatilla pratensis (L.) Mill. s.l.	0	n	1	0	Ranunculaceae Juss.
Iz_1_1109	DJI_0051	Pyrus communis L.	0	a	0	1	Rosaceae Juss.
Iz_1_1109	DJI_0051	Quercus robur L.	0	n	1	1	Fagaceae Dumort.
Iz_1_1109	DJI_0051	Robinia pseudoacacia L.	0	a	0	1	Fabaceae Lindl.
Iz_1_1109	DJI_0051	Rosa sp.	0		NA	1	Rosaceae Juss.
Iz_1_1109	DJI_0051	Rubus idaeus L.	0	n	1	0	Rosaceae Juss.
Iz_1_1109	DJI_0051	Rumex acetosella L.	0	n	1	0	Polygonaceae Juss.
Iz_1_1109	DJI_0051	Rumex thyrsiflorus Fingerh.	0	n	1	0	Polygonaceae Juss.
Iz_1_1109	DJI_0051	Salix alba L.	0	n	1	1	Salicaceae Mirb.
Iz_1_1109	DJI_0051	Sambucus nigra L.	0	n	1	1	Viburnaceae Raf.
Iz_1_1109	DJI_0051	Secale sylvestre Host	0	n	1	0	Poaceae Barnhart
Iz_1_1109	DJI_0051	Sempervivum ruthenicum Schnittsp. & C.B. Lehm.	0	n	1	0	Crassulaceae J.St.-Hil.
Iz_1_1109	DJI_0051	Setaria viridis (L.) P. Beauv.	0	n	1	0	Poaceae Barnhart
Iz_1_1109	DJI_0051	Silene borysthenica (Gruner) Walters	0	n	1	0	Caryophyllaceae Juss.
Iz_1_1109	DJI_0051	Silene latifolia subsp. alba (Mill.) Greuter & Burdet	0	n	1	0	Caryophyllaceae Juss.
Iz_1_1109	DJI_0051	Silene nutans L.	0	n	1	0	Caryophyllaceae Juss.
Iz_1_1109	DJI_0051	Solanum nigrum L.	0		NA	NA	
Iz_1_1109	DJI_0051	Solidago virgaurea L.	0	n	1	0	Asteraceae Bercht. & J.Presl
Iz_1_1109	DJI_0051	Sorbus aucuparia L.	0	n	1	1	Rosaceae Juss.
Iz_1_1109	DJI_0051	Stachys recta L.	0	n	1	0	Lamiacea Lindl.
Iz_1_1109	DJI_0051	Tanacetum vulgare L.	0	n	1	0	Asteraceae Bercht. & J.Presl
Iz_1_1109	DJI_0051	Thymus pallasianus Heinr.Braun	0	n	1	0	Lamiacea Lindl.
Iz_1_1109	DJI_0051	Tilia cordata Mill.	0	n	1	1	Malvaceae Juss.
Iz_1_1109	DJI_0051	Trifolium alpestre L.	0	n	1	0	Fabaceae Lindl.
Iz_1_1109	DJI_0051	Trifolium arvense L.	0	n	1	0	Fabaceae Lindl.
Iz_1_1109	DJI_0051	Ulmus glabra Huds.	0	n	1	1	Ulmaceae Mirb.
Iz_1_1109	DJI_0051	Verbascum densiflorum Bertol.	0	n	1	0	Scrophulariaceae Juss.
Iz_1_1109	DJI_0051	Veronica austriaca L.	0	n	1	0	Plantaginaceae Juss.
Iz_1_1109	DJI_0051	Veronica incana L.	0	n	1	0	Plantaginaceae Juss.
Iz_1_1109	DJI_0051	Veronica verna L.	0	n	1	0	Plantaginaceae Juss.
Iz_1_1109	DJI_0051	Vincetoxicum hirundinaria Medik	0	n	1	0	Apocynaceae Juss.
Iz_1_1109	DJI_0055	Acer tataricum L.	0	n	1	1	Sapindaceae Juss.
Iz_1_1109	DJI_0055	Achillea millefolium L. s.l.	0	n	1	0	Asteraceae Bercht. & J.Presl
Iz_1_1109	DJI_0055	Allium oleraceum L.	0	n	1	0	Amaryllidaceae J.St.-Hil.
Iz_1_1109	DJI_0055	Ambrosia artemisiifolia L.	0	a	0	0	Asteraceae Bercht. & J.Presl
Iz_1_1109	DJI_0055	Amorpha fruticosa L.	0	a	0	0	Fabaceae Lindl.
Iz_1_1109	DJI_0055	Anthericum ramosum L.	0	n	1	0	Asparagaceae Juss.
Iz_1_1109	DJI_0055	Artemisia marschalliana Spreng.	0	n	1	0	Asteraceae Bercht. & J.Presl
Iz_1_1109	DJI_0055	Asparagus officinalis L.	0	n	1	0	Asparagaceae Juss.
Iz_1_1109	DJI_0055	Astragalus cicer L.	0	n	1	0	Fabaceae Lindl.
Iz_1_1109	DJI_0055	Bassia laniflora (S.G.Gmel.) A.J.Scott	0	n	1	0	Amaranthaceae Juss.
Iz_1_1109	DJI_0055	Berberis vulgaris L.	0	n	1	0	Berberidaceae Juss.
Iz_1_1109	DJI_0055	Betula pendula Roth.	0	n	1	1	Betulaceae Gray
Iz_1_1109	DJI_0055	Betula pendula Roth.	0	n	1	0	Poaceae Barnhart
Iz_1_1109	DJI_0055	Bromus inermis Leyss.	0		NA	NA	
Iz_1_1109	DJI_0055	Bromus squarrosus L.	0	a	0	0	Poaceae Barnhart
Iz_1_1109	DJI_0055	Bromus tectorum L.	0	a	0	0	Poaceae Barnhart
Iz_1_1109	DJI_0055	Calamagrostis epigejos (L.) Roth	35	n	1	0	Poaceae Barnhart
Iz_1_1109	DJI_0055	Carduus acanthoides L.	0	n	1	0	Asteraceae Bercht. & J.Presl
Iz_1_1109	DJI_0055	Carex sp.	0	n	1	0	Cyperaceae Juss.
Iz_1_1109	DJI_0055	Centaurea majorovii Dumbadze	0	n	1	0	Asteraceae Bercht. & J.Presl
Iz_1_1109	DJI_0055	Chamaecytisus ruthenicus (Fisch.ex Wol.) Klaskova	0	n	1	0	Fabaceae Lindl.
Iz_1_1109	DJI_0055	Chelidonium majus L.	0	n	1	0	Papaveraceae Juss.
Iz_1_1109	DJI_0055	Chenopodium betaceum Andrz.	0	n	1	0	Amaranthaceae Juss.
Iz_1_1109	DJI_0055	Chenopodium cf. album	0	n	1	0	Amaranthaceae Juss.
Iz_1_1109	DJI_0055	Chenopodium sp.	0		NA	0	Amaranthaceae Juss.
Iz_1_1109	DJI_0055	Chondrilla juncea L.	0	n	1	0	Asteraceae Bercht. & J.Presl
Iz_1_1109	DJI_0055	Cirsium vulgare (Savi) Ten.	0	n	1	0	Asteraceae Bercht. & J.Presl
Iz_1_1109	DJI_0055	Clinopodium acinos (L.) Kuntze	0	n	1	0	Lamiacea Lindl.

Iz_1_1109	DJI_0055	Convallaria majalis L.	0	n	1	0	Asparagaceae Juss.
Iz_1_1109	DJI_0055	Crataegus rhipidophylla Gand.	0	n	1	1	Rosaceae Juss.
Iz_1_1109	DJI_0055	Crepis foetida L.	0	n	1	0	Asteraceae Bercht. & J.Presl
Iz_1_1109	DJI_0055	Dactylis glomerata L.	0	n	1	0	Poaceae Barnhart
Iz_1_1109	DJI_0055	Dianthus campestris M.Bieb.	0	n	1	0	Caryophyllaceae Juss.
Iz_1_1109	DJI_0055	Digitaria sanguinalis (L.) Scop.	0	a	0	0	Poaceae Barnhart
Iz_1_1109	DJI_0055	Elymus repens (L.) Gould	0	n	1	0	Poaceae Barnhart
Iz_1_1109	DJI_0055	Epilobium tetragonum L.	0	n	1	0	Onagraceae Juss.
Iz_1_1109	DJI_0055	Eragrostis minor Host	0	a	0	0	Poaceae Barnhart
Iz_1_1109	DJI_0055	Erigeron canadensis L.	0.5	a	0	0	Asteraceae Bercht. & J.Presl
Iz_1_1109	DJI_0055	Euphorbia seguieriana Neck.	0	n	1	0	Euphorbiaceae Juss.
Iz_1_1109	DJI_0055	Fallopia dumetorum (L.) Holub	5	n	1	0	Polygonaceae Juss.
Iz_1_1109	DJI_0055	Festuca beckeri (Hack.) Trautv.	0	n	1	0	Poaceae Barnhart
Iz_1_1109	DJI_0055	Fragaria vesca L.	0	n	1	0	Rosaceae Juss.
Iz_1_1109	DJI_0055	Frangula alnus Mill.	0	n	1	1	Rhamnaceae Juss.
Iz_1_1109	DJI_0055	Fraxinus pennsylvanica Marshall	0	a	0	1	Oleaceae Hoffmanns. & Link
Iz_1_1109	DJI_0055	Galeopsis bifida Boenn.	0	n	1	0	Lamiacea Lindl.
Iz_1_1109	DJI_0055	Galium verum L.	0	n	1	0	Rubiaceae Juss.
Iz_1_1109	DJI_0055	Genista tinctoria L.	0	n	1	0	Fabaceae Lindl.
Iz_1_1109	DJI_0055	Geranium robertianum L.	0	n	1	0	Geraniaceae Juss.
Iz_1_1109	DJI_0055	Geranium sanquineum L.	0	n	1	0	Geraniaceae Juss.
Iz_1_1109	DJI_0055	Gypsophila paniculata L.	0	n	1	0	Caryophyllaceae Juss.
Iz_1_1109	DJI_0055	Helichrysum arenarium (L.) Moench	0	n	1	0	Asteraceae Bercht. & J.Presl
Iz_1_1109	DJI_0055	Herniaria polygama J.Gay	0	n	1	0	Caryophyllaceae Juss.
Iz_1_1109	DJI_0055	Hieracium robustum Fr.	0	n	1	0	Asteraceae Bercht. & J.Presl
Iz_1_1109	DJI_0055	Hieracium umbellatum L.	0	n	1	NA	Asteraceae Bercht. & J.Presl
Iz_1_1109	DJI_0055	Hylotelephium maximum (L.) Holub	0	n	1	0	Crassulaceae J.St.-Hil.
Iz_1_1109	DJI_0055	Jacobaea borysthenica (DC.) B.Nord. & Greuter	0	n	1	0	Asteraceae Bercht. & J.Presl
Iz_1_1109	DJI_0055	Jasione montana L.	0	n	1	0	Campanulaceae Juss.
Iz_1_1109	DJI_0055	Jurinea cyanoides (L.) Rchb.s.l.	0	n	1	0	Asteraceae Bercht. & J.Presl
Iz_1_1109	DJI_0055	Koeleria glauca (Spreng.) DC.	0	n	1	0	Poaceae Barnhart
Iz_1_1109	DJI_0055	Lactuca serriola L.	0	a	0	0	Asteraceae Bercht. & J.Presl
Iz_1_1109	DJI_0055	Linaria genistifolia (L.) Mill.	0	n	1	0	Plantaginaceae Juss.
Iz_1_1109	DJI_0055	Lomelosia argentea (L.) Greuter & Burdet	0	n	1	0	Caprifoliaceae Juss.
Iz_1_1109	DJI_0055	Melampyrum pratense L.	0	n	1	0	Orobanchaceae Vent.
Iz_1_1109	DJI_0055	Oenothera biennis L.	0	a	0	0	Onagraceae Juss.
Iz_1_1109	DJI_0055	Pentanema britannica (L.) D.Gut.Larr., Santos-Vicente, Anderb., E.Rico & M.M.Mart.Ort.	0	n	1	0	Asteraceae Bercht. & J.Presl
Iz_1_1109	DJI_0055	Peucedanum oreoselinum (L.) Moench	0	n	1	0	Apiaceae Lindl.
Iz_1_1109	DJI_0055	Peucedanum ruthenicum M.Bieb.	0	n	1	0	Apiaceae Lindl.
Iz_1_1109	DJI_0055	Pilosella echioides (Lumn.) F.W.Schultz & Sch.Bip.	0	n	1	0	Asteraceae Bercht. & J.Presl
Iz_1_1109	DJI_0055	Pilosella officinarum F. Schultz et Sch. Bip.	0	n	1	0	Asteraceae Bercht. & J.Presl
Iz_1_1109	DJI_0055	Pilosella sp.	0		NA	0	Asteraceae Bercht. & J.Presl
Iz_1_1109	DJI_0055	Pinus sylvestris L.	0	n	1	1	Pinaceae Spreng. ex F.Rudolphi
Iz_1_1109	DJI_0055	Poa annua L.	0	n	1	0	Poaceae Barnhart
Iz_1_1109	DJI_0055	Poa nemoralis L.	0	n	1	0	Poaceae Barnhart
Iz_1_1109	DJI_0055	Poa sp.	0		NA	0	Poaceae Barnhart
Iz_1_1109	DJI_0055	Polygonatum odoratum (Mill.) Druce	0	n	1	0	Asparagaceae Juss.
Iz_1_1109	DJI_0055	Polygonum arenarium Waldst. & Kit.	0	n	1	0	Polygonaceae Juss.
Iz_1_1109	DJI_0055	Populus nigra L.	0	n	1	1	Salicaceae Mirb.
Iz_1_1109	DJI_0055	Populus sp.	1	n	1	1	Salicaceae Mirb.
Iz_1_1109	DJI_0055	Populus tremula L.	0	n	1	1	Salicaceae Mirb.
Iz_1_1109	DJI_0055	Populus Ã canescens (Aiton) Sm.	0	n	1	1	Salicaceae Mirb.
Iz_1_1109	DJI_0055	Potentilla argentea L.	0	n	1	0	Rosaceae Juss.
Iz_1_1109	DJI_0055	Psammophiliella muralis (L.) Ikonn.	0	n	1	0	Caryophyllaceae Juss.
Iz_1_1109	DJI_0055	Psephellus sumensis (Kalen.) Greuter	0	n	1	0	Asteraceae Bercht. & J.Presl
Iz_1_1109	DJI_0055	Pulsatilla pratensis (L.) Mill. s.l.	0	n	1	0	Ranunculaceae Juss.
Iz_1_1109	DJI_0055	Pyrus communis L.	0	a	0	1	Rosaceae Juss.
Iz_1_1109	DJI_0055	Quercus robur L.	0	n	1	1	Fagaceae Dumort.
Iz_1_1109	DJI_0055	Robinia pseudoacacia L.	0	a	0	1	Fabaceae Lindl.
Iz_1_1109	DJI_0055	Rosa sp.	0		NA	1	Rosaceae Juss.
Iz_1_1109	DJI_0055	Rubus idaeus L.	0	n	1	0	Rosaceae Juss.
Iz_1_1109	DJI_0055	Rumex acetosella L.	0	n	1	0	Polygonaceae Juss.
Iz_1_1109	DJI_0055	Rumex thyrsiflorus Fingerh.	0	n	1	0	Polygonaceae Juss.
Iz_1_1109	DJI_0055	Salix alba L.	0	n	1	1	Salicaceae Mirb.
Iz_1_1109	DJI_0055	Sambucus nigra L.	0	n	1	1	Viburnaceae Raf.
Iz_1_1109	DJI_0055	Secale sylvestre Host	0	n	1	0	Poaceae Barnhart
Iz_1_1109	DJI_0055	Sempervivum ruthenicum Schnittsp. & C.B. Lehm.	0	n	1	0	Crassulaceae J.St.-Hil.
Iz_1_1109	DJI_0055	Setaria viridis (L.) P. Beauv.	0	n	1	0	Poaceae Barnhart
Iz_1_1109	DJI_0055	Silene borysthenica (Gruner) Walters	0	n	1	0	Caryophyllaceae Juss.
Iz_1_1109	DJI_0055	Silene latifolia subsp. alba (Mill.) Greuter & Burdet	0	n	1	0	Caryophyllaceae Juss.
Iz_1_1109	DJI_0055	Silene nutans L.	0	n	1	0	Caryophyllaceae Juss.
Iz_1_1109	DJI_0055	Solanum nigrum L.	0.2		NA	NA	
Iz_1_1109	DJI_0055	Solidago virgaurea L.	0	n	1	0	Asteraceae Bercht. & J.Presl
Iz_1_1109	DJI_0055	Sorbus aucuparia L.	0	n	1	1	Rosaceae Juss.
Iz_1_1109	DJI_0055	Stachys recta L.	0	n	1	0	Lamiacea Lindl.
Iz_1_1109	DJI_0055	Tanacetum vulgare L.	0	n	1	0	Asteraceae Bercht. & J.Presl
Iz_1_1109	DJI_0055	Thymus pallasianus Heinr.Braun	0	n	1	0	Lamiacea Lindl.
Iz_1_1109	DJI_0055	Tilia cordata Mill.	0	n	1	1	Malvaceae Juss.
Iz_1_1109	DJI_0055	Trifolium alpestre L.	0	n	1	0	Fabaceae Lindl.
Iz_1_1109	DJI_0055	Trifolium arvense L.	0	n	1	0	Fabaceae Lindl.
Iz_1_1109	DJI_0055	Ulmus glabra Huds.	0	n	1	1	Ulmaceae Mirb.
Iz_1_1109	DJI_0055	Verbascum densiflorum Bertol.	0	n	1	0	Scrophulariaceae Juss.
Iz_1_1109	DJI_0055	Veronica austriaca L.	0	n	1	0	Plantaginaceae Juss.
Iz_1_1109	DJI_0055	Veronica incana L.	0	n	1	0	Plantaginaceae Juss.
Iz_1_1109	DJI_0055	Veronica verna L.	0	n	1	0	Plantaginaceae Juss.
Iz_1_1109	DJI_0055	Vincetoxicum hirundinaria Medik	0	n	1	0	Apocynaceae Juss.
Iz_1_1109	DJI_0058	Acer tataricum L.	0	n	1	1	Sapindaceae Juss.
Iz_1_1109	DJI_0058	Achillea millefolium L. s.l.	0	n	1	0	Asteraceae Bercht. & J.Presl
Iz_1_1109	DJI_0058	Allium oleraceum L.	0	n	1	0	Amaryllidaceae J.St.-Hil.
Iz_1_1109	DJI_0058	Ambrosia artemisiifolia L.	0	a	0	0	Asteraceae Bercht. & J.Presl
Iz_1_1109	DJI_0058	Amorpha fruticosa L.	0	a	0	0	Fabaceae Lindl.
Iz_1_1109	DJI_0058	Anthericum ramosum L.	0	n	1	0	Asparagaceae Juss.
Iz_1_1109	DJI_0058	Artemisia marschalliana Spreng.	0	n	1	0	Asteraceae Bercht. & J.Presl
Iz_1_1109	DJI_0058	Asparagus officinalis L.	0	n	1	0	Asparagaceae Juss.
Iz_1_1109	DJI_0058	Astragalus cicer L.	0	n	1	0	Fabaceae Lindl.
Iz_1_1109	DJI_0058	Bassia laniflora (S.G.Gmel.) A.J.Scott	0	n	1	0	Amaranthaceae Juss.
Iz_1_1109	DJI_0058	Berberis vulgaris L.	0	n	1	0	Berberidaceae Juss.
Iz_1_1109	DJI_0058	Betula pendula Roth.	0	n	1	1	Betulaceae Gray

Iz_1_1109	DJI_0058	Betula pendula Roth.	0	n	1	0	Poaceae Barnhart
Iz_1_1109	DJI_0058	Bromus inermis Leyss.	0		NA	NA	
Iz_1_1109	DJI_0058	Bromus squarrosus L.	0	a	0	0	Poaceae Barnhart
Iz_1_1109	DJI_0058	Bromus tectorum L.	0	a	0	0	Poaceae Barnhart
Iz_1_1109	DJI_0058	Calamagrostis epigejos (L.) Roth	30	n	1	0	Poaceae Barnhart
Iz_1_1109	DJI_0058	Carduus acanthoides L.	0	n	1	0	Asteraceae Bercht. & J.Presl
Iz_1_1109	DJI_0058	Carex sp.	0	n	1	0	Cyperaceae Juss.
Iz_1_1109	DJI_0058	Centaurea majorovii Dumbadze	0	n	1	0	Asteraceae Bercht. & J.Presl
Iz_1_1109	DJI_0058	Chamaecytisus ruthenicus (Fisch.ex Wol.) Klaskova	0	n	1	0	Fabaceae Lindl.
Iz_1_1109	DJI_0058	Chelidonium majus L.	0	n	1	0	Papaveraceae Juss.
Iz_1_1109	DJI_0058	Chenopodium betaceum Andrz.	0	n	1	0	Amaranthaceae Juss.
Iz_1_1109	DJI_0058	Chenopodium cf. album	0	n	1	0	Amaranthaceae Juss.
Iz_1_1109	DJI_0058	Chenopodium sp.	0		NA	0	Amaranthaceae Juss.
Iz_1_1109	DJI_0058	Chondrilla juncea L.	0	n	1	0	Asteraceae Bercht. & J.Presl
Iz_1_1109	DJI_0058	Cirsium vulgare (Savi) Ten.	0	n	1	0	Asteraceae Bercht. & J.Presl
Iz_1_1109	DJI_0058	Clinopodium acinos (L.) Kuntze	0	n	1	0	Lamiacea Lindl.
Iz_1_1109	DJI_0058	Convallaria majalis L.	0	n	1	0	Asparagaceae Juss.
Iz_1_1109	DJI_0058	Crataegus rhipidophylla Gand.	0	n	1	1	Rosaceae Juss.
Iz_1_1109	DJI_0058	Crepis foetida L.	0	n	1	0	Asteraceae Bercht. & J.Presl
Iz_1_1109	DJI_0058	Dactylis glomerata L.	0	n	1	0	Poaceae Barnhart
Iz_1_1109	DJI_0058	Dianthus campestris M.Bieb.	0	n	1	0	Caryophyllaceae Juss.
Iz_1_1109	DJI_0058	Digitaria sanguinalis (L.) Scop.	0	a	0	0	Poaceae Barnhart
Iz_1_1109	DJI_0058	Elymus repens (L.) Gould	0	n	1	0	Poaceae Barnhart
Iz_1_1109	DJI_0058	Epilobium tetragonum L.	0	n	1	0	Onagraceae Juss.
Iz_1_1109	DJI_0058	Eragrostis minor Host	0	a	0	0	Poaceae Barnhart
Iz_1_1109	DJI_0058	Erigeron canadensis L.	0.1	a	0	0	Asteraceae Bercht. & J.Presl
Iz_1_1109	DJI_0058	Euphorbia seguieriana Neck.	0	n	1	0	Euphorbiaceae Juss.
Iz_1_1109	DJI_0058	Fallopia dumetorum (L.) Holub	5	n	1	0	Polygonaceae Juss.
Iz_1_1109	DJI_0058	Festuca beckeri (Hack.) Trautv.	0	n	1	0	Poaceae Barnhart
Iz_1_1109	DJI_0058	Fragaria vesca L.	0	n	1	0	Rosaceae Juss.
Iz_1_1109	DJI_0058	Frangula alnus Mill.	0	n	1	1	Rhamnaceae Juss.
Iz_1_1109	DJI_0058	Fraxinus pennsylvanica Marshall	0	a	0	1	Oleaceae Hoffmanns. & Link
Iz_1_1109	DJI_0058	Galeopsis bifida Boenn.	0	n	1	0	Lamiacea Lindl.
Iz_1_1109	DJI_0058	Galium verum L.	0	n	1	0	Rubiaceae Juss.
Iz_1_1109	DJI_0058	Genista tinctoria L.	0	n	1	0	Fabaceae Lindl.
Iz_1_1109	DJI_0058	Geranium robertianum L.	0	n	1	0	Geraniaceae Juss.
Iz_1_1109	DJI_0058	Geranium sanquineum L.	0	n	1	0	Geraniaceae Juss.
Iz_1_1109	DJI_0058	Gypsophila paniculata L.	0	n	1	0	Caryophyllaceae Juss.
Iz_1_1109	DJI_0058	Helichrysum arenarium (L.) Moench	0	n	1	0	Asteraceae Bercht. & J.Presl
Iz_1_1109	DJI_0058	Herniaria polygama J.Gay	0	n	1	0	Caryophyllaceae Juss.
Iz_1_1109	DJI_0058	Hieracium robustum Fr.	0	n	1	0	Asteraceae Bercht. & J.Presl
Iz_1_1109	DJI_0058	Hieracium umbellatum L.	0	n	1	NA	Asteraceae Bercht. & J.Presl
Iz_1_1109	DJI_0058	Hylotelephium maximum (L.) Holub	0	n	1	0	Crassulaceae J.St.-Hil.
Iz_1_1109	DJI_0058	Jacobaea borysthenica (DC.) B.Nord. & Greuter	0	n	1	0	Asteraceae Bercht. & J.Presl
Iz_1_1109	DJI_0058	Jasione montana L.	0	n	1	0	Campanulaceae Juss.
Iz_1_1109	DJI_0058	Jurinea cyanoides (L.) Rchb.s.l.	0	n	1	0	Asteraceae Bercht. & J.Presl
Iz_1_1109	DJI_0058	Koeleria glauca (Spreng.) DC.	0	n	1	0	Poaceae Barnhart
Iz_1_1109	DJI_0058	Lactuca serriola L.	0	a	0	0	Asteraceae Bercht. & J.Presl
Iz_1_1109	DJI_0058	Linaria genistifolia (L.) Mill.	0	n	1	0	Plantaginaceae Juss.
Iz_1_1109	DJI_0058	Lomelosia argentea (L.) Greuter & Burdet	0	n	1	0	Caprifoliaceae Juss.
Iz_1_1109	DJI_0058	Melampyrum pratense L.	0	n	1	0	Orobanchaceae Vent.
Iz_1_1109	DJI_0058	Oenothera biennis L.	0	a	0	0	Onagraceae Juss.
Iz_1_1109	DJI_0058	Pentanema britannica (L.) D.Gut.Larr., Santos-Vicente, Anderb., E.Rico & M.M.Mart.Ort.	0	n	1	0	Asteraceae Bercht. & J.Presl
Iz_1_1109	DJI_0058	Peucedanum oreoselinum (L.) Moench	0	n	1	0	Apiaceae Lindl.
Iz_1_1109	DJI_0058	Peucedanum ruthenicum M.Bieb.	0	n	1	0	Apiaceae Lindl.
Iz_1_1109	DJI_0058	Pilosella echioides (Lumn.) F.W.Schultz & Sch.Bip.	0	n	1	0	Asteraceae Bercht. & J.Presl
Iz_1_1109	DJI_0058	Pilosella officinarum F. Schultz et Sch. Bip.	0	n	1	0	Asteraceae Bercht. & J.Presl
Iz_1_1109	DJI_0058	Pilosella sp.	0		NA	0	Asteraceae Bercht. & J.Presl
Iz_1_1109	DJI_0058	Pinus sylvestris L.	0	n	1	1	Pinaceae Spreng. ex F.Rudolphi
Iz_1_1109	DJI_0058	Poa annua L.	0	n	1	0	Poaceae Barnhart
Iz_1_1109	DJI_0058	Poa nemoralis L.	0	n	1	0	Poaceae Barnhart
Iz_1_1109	DJI_0058	Poa sp.	0		NA	0	Poaceae Barnhart
Iz_1_1109	DJI_0058	Polygonatum odoratum (Mill.) Druce	0	n	1	0	Asparagaceae Juss.
Iz_1_1109	DJI_0058	Polygonum arenarium Waldst. & Kit.	0	n	1	0	Polygonaceae Juss.
Iz_1_1109	DJI_0058	Populus nigra L.	0	n	1	1	Salicaceae Mirb.
Iz_1_1109	DJI_0058	Populus sp.	0	n	1	1	Salicaceae Mirb.
Iz_1_1109	DJI_0058	Populus tremula L.	0	n	1	1	Salicaceae Mirb.
Iz_1_1109	DJI_0058	Populus Ã canescens (Aiton) Sm.	0	n	1	1	Salicaceae Mirb.
Iz_1_1109	DJI_0058	Potentilla argentea L.	0	n	1	0	Rosaceae Juss.
Iz_1_1109	DJI_0058	Psammophiliella muralis (L.) Ikonn.	0	n	1	0	Caryophyllaceae Juss.
Iz_1_1109	DJI_0058	Psephellus sumensis (Kalen.) Greuter	0	n	1	0	Asteraceae Bercht. & J.Presl
Iz_1_1109	DJI_0058	Pulsatilla pratensis (L.) Mill. s.l.	0	n	1	0	Ranunculaceae Juss.
Iz_1_1109	DJI_0058	Pyrus communis L.	0	a	0	1	Rosaceae Juss.
Iz_1_1109	DJI_0058	Quercus robur L.	0	n	1	1	Fagaceae Dumort.
Iz_1_1109	DJI_0058	Robinia pseudoacacia L.	0	a	0	1	Fabaceae Lindl.
Iz_1_1109	DJI_0058	Rosa sp.	0		NA	1	Rosaceae Juss.
Iz_1_1109	DJI_0058	Rubus idaeus L.	0	n	1	0	Rosaceae Juss.
Iz_1_1109	DJI_0058	Rumex acetosella L.	5	n	1	0	Polygonaceae Juss.
Iz_1_1109	DJI_0058	Rumex thyrsiflorus Fingerh.	0	n	1	0	Polygonaceae Juss.
Iz_1_1109	DJI_0058	Salix alba L.	0	n	1	1	Salicaceae Mirb.
Iz_1_1109	DJI_0058	Sambucus nigra L.	0	n	1	1	Viburnaceae Raf.
Iz_1_1109	DJI_0058	Secale sylvestre Host	0	n	1	0	Poaceae Barnhart
Iz_1_1109	DJI_0058	Sempervivum ruthenicum Schnittsp. & C.B. Lehm.	0	n	1	0	Crassulaceae J.St.-Hil.
Iz_1_1109	DJI_0058	Setaria viridis (L.) P. Beauv.	0	n	1	0	Poaceae Barnhart
Iz_1_1109	DJI_0058	Silene borysthenica (Gruner) Walters	0	n	1	0	Caryophyllaceae Juss.
Iz_1_1109	DJI_0058	Silene latifolia subsp. alba (Mill.) Greuter & Burdet	0	n	1	0	Caryophyllaceae Juss.
Iz_1_1109	DJI_0058	Silene nutans L.	0	n	1	0	Caryophyllaceae Juss.
Iz_1_1109	DJI_0058	Solanum nigrum L.	0		NA	NA	
Iz_1_1109	DJI_0058	Solidago virgaurea L.	0	n	1	0	Asteraceae Bercht. & J.Presl
Iz_1_1109	DJI_0058	Sorbus aucuparia L.	0	n	1	1	Rosaceae Juss.
Iz_1_1109	DJI_0058	Stachys recta L.	0	n	1	0	Lamiacea Lindl.
Iz_1_1109	DJI_0058	Tanacetum vulgare L.	0	n	1	0	Asteraceae Bercht. & J.Presl
Iz_1_1109	DJI_0058	Thymus pallasianus Heinr.Braun	0	n	1	0	Lamiacea Lindl.
Iz_1_1109	DJI_0058	Tilia cordata Mill.	0	n	1	1	Malvaceae Juss.
Iz_1_1109	DJI_0058	Trifolium alpestre L.	0	n	1	0	Fabaceae Lindl.
Iz_1_1109	DJI_0058	Trifolium arvense L.	0	n	1	0	Fabaceae Lindl.
Iz_1_1109	DJI_0058	Ulmus glabra Huds.	0	n	1	1	Ulmaceae Mirb.
Iz_1_1109	DJI_0058	Verbascum densiflorum Bertol.	0	n	1	0	Scrophulariaceae Juss.

Iz_1_1109	DJI_0058	Veronica austriaca L.	0	n	1	0	Plantaginaceae Juss.
Iz_1_1109	DJI_0058	Veronica incana L.	0	n	1	0	Plantaginaceae Juss.
Iz_1_1109	DJI_0058	Veronica verna L.	0	n	1	0	Plantaginaceae Juss.
Iz_1_1109	DJI_0058	Vincetoxicum hirundinaria Medik	0	n	1	0	Apocynaceae Juss.
Iz_1_1109	DJI_0060	Acer tataricum L.	0	n	1	1	Sapindaceae Juss.
Iz_1_1109	DJI_0060	Achillea millefolium L. s.l.	0	n	1	0	Asteraceae Bercht. & J.Presl
Iz_1_1109	DJI_0060	Allium oleraceum L.	0	n	1	0	Amaryllidaceae J.St.-Hil.
Iz_1_1109	DJI_0060	Ambrosia artemisiifolia L.	0	a	0	0	Asteraceae Bercht. & J.Presl
Iz_1_1109	DJI_0060	Amorpha fruticosa L.	0	a	0	0	Fabaceae Lindl.
Iz_1_1109	DJI_0060	Anthericum ramosum L.	0	n	1	0	Asparagaceae Juss.
Iz_1_1109	DJI_0060	Artemisia marschalliana Spreng.	0	n	1	0	Asteraceae Bercht. & J.Presl
Iz_1_1109	DJI_0060	Asparagus officinalis L.	0	n	1	0	Asparagaceae Juss.
Iz_1_1109	DJI_0060	Astragalus cicer L.	0	n	1	0	Fabaceae Lindl.
Iz_1_1109	DJI_0060	Bassia laniflora (S.G.Gmel.) A.J.Scott	0	n	1	0	Amaranthaceae Juss.
Iz_1_1109	DJI_0060	Berberis vulgaris L.	0	n	1	0	Berberidaceae Juss.
Iz_1_1109	DJI_0060	Betula pendula Roth.	0	n	1	1	Betulaceae Gray
Iz_1_1109	DJI_0060	Betula pendula Roth.	0	n	1	0	Poaceae Barnhart
Iz_1_1109	DJI_0060	Bromus inermis Leyss.	0		NA	NA	
Iz_1_1109	DJI_0060	Bromus squarrosus L.	0	a	0	0	Poaceae Barnhart
Iz_1_1109	DJI_0060	Bromus tectorum L.	0	a	0	0	Poaceae Barnhart
Iz_1_1109	DJI_0060	Calamagrostis epigejos (L.) Roth	70	n	1	0	Poaceae Barnhart
Iz_1_1109	DJI_0060	Carduus acanthoides L.	0	n	1	0	Asteraceae Bercht. & J.Presl
Iz_1_1109	DJI_0060	Carex sp.	0	n	1	0	Cyperaceae Juss.
Iz_1_1109	DJI_0060	Centaurea majorovii Dumbadze	0	n	1	0	Asteraceae Bercht. & J.Presl
Iz_1_1109	DJI_0060	Chamaecytisus ruthenicus (Fisch.ex Wol.) Klaskova	0	n	1	0	Fabaceae Lindl.
Iz_1_1109	DJI_0060	Chelidonium majus L.	0	n	1	0	Papaveraceae Juss.
Iz_1_1109	DJI_0060	Chenopodium betaceum Andrz.	0	n	1	0	Amaranthaceae Juss.
Iz_1_1109	DJI_0060	Chenopodium cf. album	0	n	1	0	Amaranthaceae Juss.
Iz_1_1109	DJI_0060	Chenopodium sp.	0		NA	0	Amaranthaceae Juss.
Iz_1_1109	DJI_0060	Chondrilla juncea L.	0	n	1	0	Asteraceae Bercht. & J.Presl
Iz_1_1109	DJI_0060	Cirsium vulgare (Savi) Ten.	0	n	1	0	Asteraceae Bercht. & J.Presl
Iz_1_1109	DJI_0060	Clinopodium acinos (L.) Kuntze	0	n	1	0	Lamiacea Lindl.
Iz_1_1109	DJI_0060	Convallaria majalis L.	0	n	1	0	Asparagaceae Juss.
Iz_1_1109	DJI_0060	Crataegus rhipidophylla Gand.	0	n	1	1	Rosaceae Juss.
Iz_1_1109	DJI_0060	Crepis foetida L.	0	n	1	0	Asteraceae Bercht. & J.Presl
Iz_1_1109	DJI_0060	Dactylis glomerata L.	0	n	1	0	Poaceae Barnhart
Iz_1_1109	DJI_0060	Dianthus campestris M.Bieb.	0	n	1	0	Caryophyllaceae Juss.
Iz_1_1109	DJI_0060	Digitaria sanguinalis (L.) Scop.	0	a	0	0	Poaceae Barnhart
Iz_1_1109	DJI_0060	Elymus repens (L.) Gould	0	n	1	0	Poaceae Barnhart
Iz_1_1109	DJI_0060	Epilobium tetragonum L.	0	n	1	0	Onagraceae Juss.
Iz_1_1109	DJI_0060	Eragrostis minor Host	0	a	0	0	Poaceae Barnhart
Iz_1_1109	DJI_0060	Erigeron canadensis L.	0	a	0	0	Asteraceae Bercht. & J.Presl
Iz_1_1109	DJI_0060	Euphorbia seguieriana Neck.	0	n	1	0	Euphorbiaceae Juss.
Iz_1_1109	DJI_0060	Fallopia dumetorum (L.) Holub	10	n	1	0	Polygonaceae Juss.
Iz_1_1109	DJI_0060	Festuca beckeri (Hack.) Trautv.	0	n	1	0	Poaceae Barnhart
Iz_1_1109	DJI_0060	Fragaria vesca L.	0	n	1	0	Rosaceae Juss.
Iz_1_1109	DJI_0060	Frangula alnus Mill.	0	n	1	1	Rhamnaceae Juss.
Iz_1_1109	DJI_0060	Fraxinus pennsylvanica Marshall	0	a	0	1	Oleaceae Hoffmanns. & Link
Iz_1_1109	DJI_0060	Galeopsis bifida Boenn.	0	n	1	0	Lamiacea Lindl.
Iz_1_1109	DJI_0060	Galium verum L.	0	n	1	0	Rubiaceae Juss.
Iz_1_1109	DJI_0060	Genista tinctoria L.	0	n	1	0	Fabaceae Lindl.
Iz_1_1109	DJI_0060	Geranium robertianum L.	0	n	1	0	Geraniaceae Juss.
Iz_1_1109	DJI_0060	Geranium sanquineum L.	0	n	1	0	Geraniaceae Juss.
Iz_1_1109	DJI_0060	Gypsophila paniculata L.	0	n	1	0	Caryophyllaceae Juss.
Iz_1_1109	DJI_0060	Helichrysum arenarium (L.) Moench	0	n	1	0	Asteraceae Bercht. & J.Presl
Iz_1_1109	DJI_0060	Herniaria polygama J.Gay	0	n	1	0	Caryophyllaceae Juss.
Iz_1_1109	DJI_0060	Hieracium robustum Fr.	0	n	1	0	Asteraceae Bercht. & J.Presl
Iz_1_1109	DJI_0060	Hieracium umbellatum L.	0	n	1	NA	Asteraceae Bercht. & J.Presl
Iz_1_1109	DJI_0060	Hylotelephium maximum (L.) Holub	0	n	1	0	Crassulaceae J.St.-Hil.
Iz_1_1109	DJI_0060	Jacobaea borysthenica (DC.) B.Nord. & Greuter	0	n	1	0	Asteraceae Bercht. & J.Presl
Iz_1_1109	DJI_0060	Jasione montana L.	0	n	1	0	Campanulaceae Juss.
Iz_1_1109	DJI_0060	Jurinea cyanoides (L.) Rchb.s.l.	0	n	1	0	Asteraceae Bercht. & J.Presl
Iz_1_1109	DJI_0060	Koeleria glauca (Spreng.) DC.	0	n	1	0	Poaceae Barnhart
Iz_1_1109	DJI_0060	Lactuca serriola L.	0	a	0	0	Asteraceae Bercht. & J.Presl
Iz_1_1109	DJI_0060	Linaria genistifolia (L.) Mill.	0	n	1	0	Plantaginaceae Juss.
Iz_1_1109	DJI_0060	Lomelosia argentea (L.) Greuter & Burdet	0	n	1	0	Caprifoliaceae Juss.
Iz_1_1109	DJI_0060	Melampyrum pratense L.	0	n	1	0	Orobanchaceae Vent.
Iz_1_1109	DJI_0060	Oenothera biennis L.	0	a	0	0	Onagraceae Juss.
Iz_1_1109	DJI_0060	Pentanema britannica (L.) D.Gut.Larr., Santos-Vicente, Anderb., E.Rico & M.M.Mart.Ort.	0	n	1	0	Asteraceae Bercht. & J.Presl
Iz_1_1109	DJI_0060	Peucedanum oreoselinum (L.) Moench	0	n	1	0	Apiaceae Lindl.
Iz_1_1109	DJI_0060	Peucedanum ruthenicum M.Bieb.	0	n	1	0	Apiaceae Lindl.
Iz_1_1109	DJI_0060	Pilosella echioides (Lumn.) F.W.Schultz & Sch.Bip.	0	n	1	0	Asteraceae Bercht. & J.Presl
Iz_1_1109	DJI_0060	Pilosella officinarum F. Schultz et Sch. Bip.	0	n	1	0	Asteraceae Bercht. & J.Presl
Iz_1_1109	DJI_0060	Pilosella sp.	0		NA	0	Asteraceae Bercht. & J.Presl
Iz_1_1109	DJI_0060	Pinus sylvestris L.	0	n	1	1	Pinaceae Spreng. ex F.Rudolphi
Iz_1_1109	DJI_0060	Poa annua L.	0	n	1	0	Poaceae Barnhart
Iz_1_1109	DJI_0060	Poa nemoralis L.	0	n	1	0	Poaceae Barnhart
Iz_1_1109	DJI_0060	Poa sp.	0		NA	0	Poaceae Barnhart
Iz_1_1109	DJI_0060	Polygonatum odoratum (Mill.) Druce	0	n	1	0	Asparagaceae Juss.
Iz_1_1109	DJI_0060	Polygonum arenarium Waldst. & Kit.	0	n	1	0	Polygonaceae Juss.
Iz_1_1109	DJI_0060	Populus nigra L.	0	n	1	1	Salicaceae Mirb.
Iz_1_1109	DJI_0060	Populus sp.	0	n	1	1	Salicaceae Mirb.
Iz_1_1109	DJI_0060	Populus tremula L.	0	n	1	1	Salicaceae Mirb.
Iz_1_1109	DJI_0060	Populus Ã canescens (Aiton) Sm.	0	n	1	1	Salicaceae Mirb.
Iz_1_1109	DJI_0060	Potentilla argentea L.	0	n	1	0	Rosaceae Juss.
Iz_1_1109	DJI_0060	Psammophiliella muralis (L.) Ikonn.	0	n	1	0	Caryophyllaceae Juss.
Iz_1_1109	DJI_0060	Psephellus sumensis (Kalen.) Greuter	0	n	1	0	Asteraceae Bercht. & J.Presl
Iz_1_1109	DJI_0060	Pulsatilla pratensis (L.) Mill. s.l.	0	n	1	0	Ranunculaceae Juss.
Iz_1_1109	DJI_0060	Pyrus communis L.	0	a	0	1	Rosaceae Juss.
Iz_1_1109	DJI_0060	Quercus robur L.	0	n	1	1	Fagaceae Dumort.
Iz_1_1109	DJI_0060	Robinia pseudoacacia L.	0	a	0	1	Fabaceae Lindl.
Iz_1_1109	DJI_0060	Rosa sp.	0		NA	1	Rosaceae Juss.
Iz_1_1109	DJI_0060	Rubus idaeus L.	0	n	1	0	Rosaceae Juss.
Iz_1_1109	DJI_0060	Rumex acetosella L.	0	n	1	0	Polygonaceae Juss.
Iz_1_1109	DJI_0060	Rumex thyrsiflorus Fingerh.	0	n	1	0	Polygonaceae Juss.
Iz_1_1109	DJI_0060	Salix alba L.	0	n	1	1	Salicaceae Mirb.
Iz_1_1109	DJI_0060	Sambucus nigra L.	0	n	1	1	Viburnaceae Raf.
Iz_1_1109	DJI_0060	Secale sylvestre Host	0	n	1	0	Poaceae Barnhart

Iz_1_1109	DJI_0060	Sempervivum ruthenicum Schnittsp. & C.B. Lehm.	0	n	1	0	Crassulaceae J.St.-Hil.
Iz_1_1109	DJI_0060	Setaria viridis (L.) P. Beauv.	0	n	1	0	Poaceae Barnhart
Iz_1_1109	DJI_0060	Silene borysthenica (Gruner) Walters	0	n	1	0	Caryophyllaceae Juss.
Iz_1_1109	DJI_0060	Silene latifolia subsp. alba (Mill.) Greuter & Burdet	0	n	1	0	Caryophyllaceae Juss.
Iz_1_1109	DJI_0060	Silene nutans L.	0	n	1	0	Caryophyllaceae Juss.
Iz_1_1109	DJI_0060	Solanum nigrum L.	0		NA	NA	
Iz_1_1109	DJI_0060	Solidago virgaurea L.	0	n	1	0	Asteraceae Bercht. & J.Presl
Iz_1_1109	DJI_0060	Sorbus aucuparia L.	0	n	1	1	Rosaceae Juss.
Iz_1_1109	DJI_0060	Stachys recta L.	0	n	1	0	Lamiacea Lindl.
Iz_1_1109	DJI_0060	Tanacetum vulgare L.	0	n	1	0	Asteraceae Bercht. & J.Presl
Iz_1_1109	DJI_0060	Thymus pallasianus Heinr.Braun	0	n	1	0	Lamiacea Lindl.
Iz_1_1109	DJI_0060	Tilia cordata Mill.	0	n	1	1	Malvaceae Juss.
Iz_1_1109	DJI_0060	Trifolium alpestre L.	0	n	1	0	Fabaceae Lindl.
Iz_1_1109	DJI_0060	Trifolium arvense L.	0	n	1	0	Fabaceae Lindl.
Iz_1_1109	DJI_0060	Ulmus glabra Huds.	0	n	1	1	Ulmaceae Mirb.
Iz_1_1109	DJI_0060	Verbascum densiflorum Bertol.	0	n	1	0	Scrophulariaceae Juss.
Iz_1_1109	DJI_0060	Veronica austriaca L.	0	n	1	0	Plantaginaceae Juss.
Iz_1_1109	DJI_0060	Veronica incana L.	0	n	1	0	Plantaginaceae Juss.
Iz_1_1109	DJI_0060	Veronica verna L.	0	n	1	0	Plantaginaceae Juss.
Iz_1_1109	DJI_0060	Vincetoxicum hirundinaria Medik	0	n	1	0	Apocynaceae Juss.
Iz_1_1109	DJI_0062	Acer tataricum L.	0	n	1	1	Sapindaceae Juss.
Iz_1_1109	DJI_0062	Achillea millefolium L. s.l.	0	n	1	0	Asteraceae Bercht. & J.Presl
Iz_1_1109	DJI_0062	Allium oleraceum L.	0	n	1	0	Amaryllidaceae J.St.-Hil.
Iz_1_1109	DJI_0062	Ambrosia artemisiifolia L.	0	a	0	0	Asteraceae Bercht. & J.Presl
Iz_1_1109	DJI_0062	Amorpha fruticosa L.	0	a	0	0	Fabaceae Lindl.
Iz_1_1109	DJI_0062	Anthericum ramosum L.	0	n	1	0	Asparagaceae Juss.
Iz_1_1109	DJI_0062	Artemisia marschalliana Spreng.	0	n	1	0	Asteraceae Bercht. & J.Presl
Iz_1_1109	DJI_0062	Asparagus officinalis L.	0	n	1	0	Asparagaceae Juss.
Iz_1_1109	DJI_0062	Astragalus cicer L.	0	n	1	0	Fabaceae Lindl.
Iz_1_1109	DJI_0062	Bassia laniflora (S.G.Gmel.) A.J.Scott	0	n	1	0	Amaranthaceae Juss.
Iz_1_1109	DJI_0062	Berberis vulgaris L.	0	n	1	0	Berberidaceae Juss.
Iz_1_1109	DJI_0062	Betula pendula Roth.	0	n	1	1	Betulaceae Gray
Iz_1_1109	DJI_0062	Betula pendula Roth.	0	n	1	0	Poaceae Barnhart
Iz_1_1109	DJI_0062	Bromus inermis Leyss.	0		NA	NA	
Iz_1_1109	DJI_0062	Bromus squarrosus L.	0	a	0	0	Poaceae Barnhart
Iz_1_1109	DJI_0062	Bromus tectorum L.	0	a	0	0	Poaceae Barnhart
Iz_1_1109	DJI_0062	Calamagrostis epigejos (L.) Roth	20	n	1	0	Poaceae Barnhart
Iz_1_1109	DJI_0062	Carduus acanthoides L.	0	n	1	0	Asteraceae Bercht. & J.Presl
Iz_1_1109	DJI_0062	Carex sp.	0	n	1	0	Cyperaceae Juss.
Iz_1_1109	DJI_0062	Centaurea majorovii Dumbadze	0	n	1	0	Asteraceae Bercht. & J.Presl
Iz_1_1109	DJI_0062	Chamaecytisus ruthenicus (Fisch.ex Wol.) Klaskova	0	n	1	0	Fabaceae Lindl.
Iz_1_1109	DJI_0062	Chelidonium majus L.	0.2	n	1	0	Papaveraceae Juss.
Iz_1_1109	DJI_0062	Chenopodium betaceum Andrz.	0	n	1	0	Amaranthaceae Juss.
Iz_1_1109	DJI_0062	Chenopodium cf. album	0	n	1	0	Amaranthaceae Juss.
Iz_1_1109	DJI_0062	Chenopodium sp.	0		NA	0	Amaranthaceae Juss.
Iz_1_1109	DJI_0062	Chondrilla juncea L.	0	n	1	0	Asteraceae Bercht. & J.Presl
Iz_1_1109	DJI_0062	Cirsium vulgare (Savi) Ten.	0	n	1	0	Asteraceae Bercht. & J.Presl
Iz_1_1109	DJI_0062	Clinopodium acinos (L.) Kuntze	0	n	1	0	Lamiacea Lindl.
Iz_1_1109	DJI_0062	Convallaria majalis L.	0	n	1	0	Asparagaceae Juss.
Iz_1_1109	DJI_0062	Crataegus rhipidophylla Gand.	0	n	1	1	Rosaceae Juss.
Iz_1_1109	DJI_0062	Crepis foetida L.	0	n	1	0	Asteraceae Bercht. & J.Presl
Iz_1_1109	DJI_0062	Dactylis glomerata L.	0	n	1	0	Poaceae Barnhart
Iz_1_1109	DJI_0062	Dianthus campestris M.Bieb.	0	n	1	0	Caryophyllaceae Juss.
Iz_1_1109	DJI_0062	Digitaria sanguinalis (L.) Scop.	0	a	0	0	Poaceae Barnhart
Iz_1_1109	DJI_0062	Elymus repens (L.) Gould	0	n	1	0	Poaceae Barnhart
Iz_1_1109	DJI_0062	Epilobium tetragonum L.	0	n	1	0	Onagraceae Juss.
Iz_1_1109	DJI_0062	Eragrostis minor Host	0	a	0	0	Poaceae Barnhart
Iz_1_1109	DJI_0062	Erigeron canadensis L.	0.1	a	0	0	Asteraceae Bercht. & J.Presl
Iz_1_1109	DJI_0062	Euphorbia seguieriana Neck.	0	n	1	0	Euphorbiaceae Juss.
Iz_1_1109	DJI_0062	Fallopia dumetorum (L.) Holub	0.5	n	1	0	Polygonaceae Juss.
Iz_1_1109	DJI_0062	Festuca beckeri (Hack.) Trautv.	0	n	1	0	Poaceae Barnhart
Iz_1_1109	DJI_0062	Fragaria vesca L.	0	n	1	0	Rosaceae Juss.
Iz_1_1109	DJI_0062	Frangula alnus Mill.	0	n	1	1	Rhamnaceae Juss.
Iz_1_1109	DJI_0062	Fraxinus pennsylvanica Marshall	0	a	0	1	Oleaceae Hoffmanns. & Link
Iz_1_1109	DJI_0062	Galeopsis bifida Boenn.	0	n	1	0	Lamiacea Lindl.
Iz_1_1109	DJI_0062	Galium verum L.	0	n	1	0	Rubiaceae Juss.
Iz_1_1109	DJI_0062	Genista tinctoria L.	0	n	1	0	Fabaceae Lindl.
Iz_1_1109	DJI_0062	Geranium robertianum L.	0	n	1	0	Geraniaceae Juss.
Iz_1_1109	DJI_0062	Geranium sanquineum L.	0	n	1	0	Geraniaceae Juss.
Iz_1_1109	DJI_0062	Gypsophila paniculata L.	0	n	1	0	Caryophyllaceae Juss.
Iz_1_1109	DJI_0062	Helichrysum arenarium (L.) Moench	0	n	1	0	Asteraceae Bercht. & J.Presl
Iz_1_1109	DJI_0062	Herniaria polygama J.Gay	0	n	1	0	Caryophyllaceae Juss.
Iz_1_1109	DJI_0062	Hieracium robustum Fr.	0	n	1	0	Asteraceae Bercht. & J.Presl
Iz_1_1109	DJI_0062	Hieracium umbellatum L.	0	n	1	NA	Asteraceae Bercht. & J.Presl
Iz_1_1109	DJI_0062	Hylotelephium maximum (L.) Holub	0	n	1	0	Crassulaceae J.St.-Hil.
Iz_1_1109	DJI_0062	Jacobaea borysthenica (DC.) B.Nord. & Greuter	0	n	1	0	Asteraceae Bercht. & J.Presl
Iz_1_1109	DJI_0062	Jasione montana L.	0	n	1	0	Campanulaceae Juss.
Iz_1_1109	DJI_0062	Jurinea cyanoides (L.) Rchb.s.l.	0	n	1	0	Asteraceae Bercht. & J.Presl
Iz_1_1109	DJI_0062	Koeleria glauca (Spreng.) DC.	0	n	1	0	Poaceae Barnhart
Iz_1_1109	DJI_0062	Lactuca serriola L.	0	a	0	0	Asteraceae Bercht. & J.Presl
Iz_1_1109	DJI_0062	Linaria genistifolia (L.) Mill.	0	n	1	0	Plantaginaceae Juss.
Iz_1_1109	DJI_0062	Lomelosia argentea (L.) Greuter & Burdet	0	n	1	0	Caprifoliaceae Juss.
Iz_1_1109	DJI_0062	Melampyrum pratense L.	0	n	1	0	Orobanchaceae Vent.
Iz_1_1109	DJI_0062	Oenothera biennis L.	0	a	0	0	Onagraceae Juss.
Iz_1_1109	DJI_0062	Pentanema britannica (L.) D.Gut.Larr., Santos-Vicente, Anderb., E.Rico & M.M.Mart.Ort.	0	n	1	0	Asteraceae Bercht. & J.Presl
Iz_1_1109	DJI_0062	Peucedanum oreoselinum (L.) Moench	0	n	1	0	Apiaceae Lindl.
Iz_1_1109	DJI_0062	Peucedanum ruthenicum M.Bieb.	0	n	1	0	Apiaceae Lindl.
Iz_1_1109	DJI_0062	Pilosella echioides (Lumn.) F.W.Schultz & Sch.Bip.	0	n	1	0	Asteraceae Bercht. & J.Presl
Iz_1_1109	DJI_0062	Pilosella officinarum F. Schultz et Sch. Bip.	0	n	1	0	Asteraceae Bercht. & J.Presl
Iz_1_1109	DJI_0062	Pilosella sp.	0		NA	0	Asteraceae Bercht. & J.Presl
Iz_1_1109	DJI_0062	Pinus sylvestris L.	0	n	1	1	Pinaceae Spreng. ex F.Rudolphi
Iz_1_1109	DJI_0062	Poa annua L.	0	n	1	0	Poaceae Barnhart
Iz_1_1109	DJI_0062	Poa nemoralis L.	0	n	1	0	Poaceae Barnhart
Iz_1_1109	DJI_0062	Poa sp.	0		NA	0	Poaceae Barnhart
Iz_1_1109	DJI_0062	Polygonatum odoratum (Mill.) Druce	0	n	1	0	Asparagaceae Juss.
Iz_1_1109	DJI_0062	Polygonum arenarium Waldst. & Kit.	0	n	1	0	Polygonaceae Juss.
Iz_1_1109	DJI_0062	Populus nigra L.	0	n	1	1	Salicaceae Mirb.
Iz_1_1109	DJI_0062	Populus sp.	0	n	1	1	Salicaceae Mirb.

Iz_1_1109	DJI_0062	Populus tremula L.	0	n	1	1	Salicaceae Mirb.
Iz_1_1109	DJI_0062	Populus Ã canescens (Aiton) Sm.	0	n	1	1	Salicaceae Mirb.
Iz_1_1109	DJI_0062	Potentilla argentea L.	0	n	1	0	Rosaceae Juss.
Iz_1_1109	DJI_0062	Psammophiliella muralis (L.) Ikonn.	0	n	1	0	Caryophyllaceae Juss.
Iz_1_1109	DJI_0062	Psephellus sumensis (Kalen.) Greuter	0	n	1	0	Asteraceae Bercht. & J.Presl
Iz_1_1109	DJI_0062	Pulsatilla pratensis (L.) Mill. s.l.	0	n	1	0	Ranunculaceae Juss.
Iz_1_1109	DJI_0062	Pyrus communis L.	0	a	0	1	Rosaceae Juss.
Iz_1_1109	DJI_0062	Quercus robur L.	0	n	1	1	Fagaceae Dumort.
Iz_1_1109	DJI_0062	Robinia pseudoacacia L.	0	a	0	1	Fabaceae Lindl.
Iz_1_1109	DJI_0062	Rosa sp.	0		NA	1	Rosaceae Juss.
Iz_1_1109	DJI_0062	Rubus idaeus L.	0	n	1	0	Rosaceae Juss.
Iz_1_1109	DJI_0062	Rumex acetosella L.	0	n	1	0	Polygonaceae Juss.
Iz_1_1109	DJI_0062	Rumex thyrsiflorus Fingerh.	0	n	1	0	Polygonaceae Juss.
Iz_1_1109	DJI_0062	Salix alba L.	0	n	1	1	Salicaceae Mirb.
Iz_1_1109	DJI_0062	Sambucus nigra L.	0	n	1	1	Viburnaceae Raf.
Iz_1_1109	DJI_0062	Secale sylvestre Host	0	n	1	0	Poaceae Barnhart
Iz_1_1109	DJI_0062	Sempervivum ruthenicum Schnittsp. & C.B. Lehm.	0	n	1	0	Crassulaceae J.St.-Hil.
Iz_1_1109	DJI_0062	Setaria viridis (L.) P. Beauv.	0	n	1	0	Poaceae Barnhart
Iz_1_1109	DJI_0062	Silene borysthenica (Gruner) Walters	0	n	1	0	Caryophyllaceae Juss.
Iz_1_1109	DJI_0062	Silene latifolia subsp. alba (Mill.) Greuter & Burdet	0	n	1	0	Caryophyllaceae Juss.
Iz_1_1109	DJI_0062	Silene nutans L.	0	n	1	0	Caryophyllaceae Juss.
Iz_1_1109	DJI_0062	Solanum nigrum L.	0		NA	NA	
Iz_1_1109	DJI_0062	Solidago virgaurea L.	0	n	1	0	Asteraceae Bercht. & J.Presl
Iz_1_1109	DJI_0062	Sorbus aucuparia L.	0	n	1	1	Rosaceae Juss.
Iz_1_1109	DJI_0062	Stachys recta L.	0	n	1	0	Lamiacea Lindl.
Iz_1_1109	DJI_0062	Tanacetum vulgare L.	0	n	1	0	Asteraceae Bercht. & J.Presl
Iz_1_1109	DJI_0062	Thymus pallasianus Heinr.Braun	0	n	1	0	Lamiacea Lindl.
Iz_1_1109	DJI_0062	Tilia cordata Mill.	0	n	1	1	Malvaceae Juss.
Iz_1_1109	DJI_0062	Trifolium alpestre L.	0	n	1	0	Fabaceae Lindl.
Iz_1_1109	DJI_0062	Trifolium arvense L.	0	n	1	0	Fabaceae Lindl.
Iz_1_1109	DJI_0062	Ulmus glabra Huds.	0	n	1	1	Ulmaceae Mirb.
Iz_1_1109	DJI_0062	Verbascum densiflorum Bertol.	0	n	1	0	Scrophulariaceae Juss.
Iz_1_1109	DJI_0062	Veronica austriaca L.	0	n	1	0	Plantaginaceae Juss.
Iz_1_1109	DJI_0062	Veronica incana L.	0	n	1	0	Plantaginaceae Juss.
Iz_1_1109	DJI_0062	Veronica verna L.	0	n	1	0	Plantaginaceae Juss.
Iz_1_1109	DJI_0062	Vincetoxicum hirundinaria Medik	0	n	1	0	Apocynaceae Juss.
Iz_1_1109	DJI_0064	Acer tataricum L.	0	n	1	1	Sapindaceae Juss.
Iz_1_1109	DJI_0064	Achillea millefolium L. s.l.	0	n	1	0	Asteraceae Bercht. & J.Presl
Iz_1_1109	DJI_0064	Allium oleraceum L.	0	n	1	0	Amaryllidaceae J.St.-Hil.
Iz_1_1109	DJI_0064	Ambrosia artemisiifolia L.	0	a	0	0	Asteraceae Bercht. & J.Presl
Iz_1_1109	DJI_0064	Amorpha fruticosa L.	0	a	0	0	Fabaceae Lindl.
Iz_1_1109	DJI_0064	Anthericum ramosum L.	0	n	1	0	Asparagaceae Juss.
Iz_1_1109	DJI_0064	Artemisia marschalliana Spreng.	0	n	1	0	Asteraceae Bercht. & J.Presl
Iz_1_1109	DJI_0064	Asparagus officinalis L.	0	n	1	0	Asparagaceae Juss.
Iz_1_1109	DJI_0064	Astragalus cicer L.	0	n	1	0	Fabaceae Lindl.
Iz_1_1109	DJI_0064	Bassia laniflora (S.G.Gmel.) A.J.Scott	0	n	1	0	Amaranthaceae Juss.
Iz_1_1109	DJI_0064	Berberis vulgaris L.	0	n	1	0	Berberidaceae Juss.
Iz_1_1109	DJI_0064	Betula pendula Roth.	0	n	1	1	Betulaceae Gray
Iz_1_1109	DJI_0064	Betula pendula Roth.	0	n	1	0	Poaceae Barnhart
Iz_1_1109	DJI_0064	Bromus inermis Leyss.	0		NA	NA	
Iz_1_1109	DJI_0064	Bromus squarrosus L.	0	a	0	0	Poaceae Barnhart
Iz_1_1109	DJI_0064	Bromus tectorum L.	0	a	0	0	Poaceae Barnhart
Iz_1_1109	DJI_0064	Calamagrostis epigejos (L.) Roth	10	n	1	0	Poaceae Barnhart
Iz_1_1109	DJI_0064	Carduus acanthoides L.	0	n	1	0	Asteraceae Bercht. & J.Presl
Iz_1_1109	DJI_0064	Carex sp.	0	n	1	0	Cyperaceae Juss.
Iz_1_1109	DJI_0064	Centaurea majorovii Dumbadze	0	n	1	0	Asteraceae Bercht. & J.Presl
Iz_1_1109	DJI_0064	Chamaecytisus ruthenicus (Fisch.ex Wol.) Klaskova	0	n	1	0	Fabaceae Lindl.
Iz_1_1109	DJI_0064	Chelidonium majus L.	0	n	1	0	Papaveraceae Juss.
Iz_1_1109	DJI_0064	Chenopodium betaceum Andrz.	0	n	1	0	Amaranthaceae Juss.
Iz_1_1109	DJI_0064	Chenopodium cf. album	0	n	1	0	Amaranthaceae Juss.
Iz_1_1109	DJI_0064	Chenopodium sp.	0		NA	0	Amaranthaceae Juss.
Iz_1_1109	DJI_0064	Chondrilla juncea L.	0	n	1	0	Asteraceae Bercht. & J.Presl
Iz_1_1109	DJI_0064	Cirsium vulgare (Savi) Ten.	0	n	1	0	Asteraceae Bercht. & J.Presl
Iz_1_1109	DJI_0064	Clinopodium acinos (L.) Kuntze	0	n	1	0	Lamiacea Lindl.
Iz_1_1109	DJI_0064	Convallaria majalis L.	0	n	1	0	Asparagaceae Juss.
Iz_1_1109	DJI_0064	Crataegus rhipidophylla Gand.	0	n	1	1	Rosaceae Juss.
Iz_1_1109	DJI_0064	Crepis foetida L.	0	n	1	0	Asteraceae Bercht. & J.Presl
Iz_1_1109	DJI_0064	Dactylis glomerata L.	0	n	1	0	Poaceae Barnhart
Iz_1_1109	DJI_0064	Dianthus campestris M.Bieb.	0	n	1	0	Caryophyllaceae Juss.
Iz_1_1109	DJI_0064	Digitaria sanguinalis (L.) Scop.	0	a	0	0	Poaceae Barnhart
Iz_1_1109	DJI_0064	Elymus repens (L.) Gould	0	n	1	0	Poaceae Barnhart
Iz_1_1109	DJI_0064	Epilobium tetragonum L.	0	n	1	0	Onagraceae Juss.
Iz_1_1109	DJI_0064	Eragrostis minor Host	0	a	0	0	Poaceae Barnhart
Iz_1_1109	DJI_0064	Erigeron canadensis L.	0.1	a	0	0	Asteraceae Bercht. & J.Presl
Iz_1_1109	DJI_0064	Euphorbia seguieriana Neck.	0	n	1	0	Euphorbiaceae Juss.
Iz_1_1109	DJI_0064	Fallopia dumetorum (L.) Holub	1	n	1	0	Polygonaceae Juss.
Iz_1_1109	DJI_0064	Festuca beckeri (Hack.) Trautv.	0	n	1	0	Poaceae Barnhart
Iz_1_1109	DJI_0064	Fragaria vesca L.	0	n	1	0	Rosaceae Juss.
Iz_1_1109	DJI_0064	Frangula alnus Mill.	0	n	1	1	Rhamnaceae Juss.
Iz_1_1109	DJI_0064	Fraxinus pennsylvanica Marshall	0	a	0	1	Oleaceae Hoffmanns. & Link
Iz_1_1109	DJI_0064	Galeopsis bifida Boenn.	0	n	1	0	Lamiacea Lindl.
Iz_1_1109	DJI_0064	Galium verum L.	0	n	1	0	Rubiaceae Juss.
Iz_1_1109	DJI_0064	Genista tinctoria L.	0	n	1	0	Fabaceae Lindl.
Iz_1_1109	DJI_0064	Geranium robertianum L.	0	n	1	0	Geraniaceae Juss.
Iz_1_1109	DJI_0064	Geranium sanquineum L.	0	n	1	0	Geraniaceae Juss.
Iz_1_1109	DJI_0064	Gypsophila paniculata L.	0	n	1	0	Caryophyllaceae Juss.
Iz_1_1109	DJI_0064	Helichrysum arenarium (L.) Moench	0	n	1	0	Asteraceae Bercht. & J.Presl
Iz_1_1109	DJI_0064	Herniaria polygama J.Gay	0	n	1	0	Caryophyllaceae Juss.
Iz_1_1109	DJI_0064	Hieracium robustum Fr.	0	n	1	0	Asteraceae Bercht. & J.Presl
Iz_1_1109	DJI_0064	Hieracium umbellatum L.	0	n	1	NA	Asteraceae Bercht. & J.Presl
Iz_1_1109	DJI_0064	Hylotelephium maximum (L.) Holub	0	n	1	0	Crassulaceae J.St.-Hil.
Iz_1_1109	DJI_0064	Jacobaea borysthenica (DC.) B.Nord. & Greuter	0	n	1	0	Asteraceae Bercht. & J.Presl
Iz_1_1109	DJI_0064	Jasione montana L.	0	n	1	0	Campanulaceae Juss.
Iz_1_1109	DJI_0064	Jurinea cyanoides (L.) Rchb.s.l.	0	n	1	0	Asteraceae Bercht. & J.Presl
Iz_1_1109	DJI_0064	Koeleria glauca (Spreng.) DC.	0	n	1	0	Poaceae Barnhart
Iz_1_1109	DJI_0064	Lactuca serriola L.	0	a	0	0	Asteraceae Bercht. & J.Presl
Iz_1_1109	DJI_0064	Linaria genistifolia (L.) Mill.	0	n	1	0	Plantaginaceae Juss.
Iz_1_1109	DJI_0064	Lomelosia argentea (L.) Greuter & Burdet	0	n	1	0	Caprifoliaceae Juss.

Iz_1_1109	DJI_0064	Melampyrum pratense L.	0	n	1	0	Orobanchaceae Vent.
Iz_1_1109	DJI_0064	Oenothera biennis L.	0	a	0	0	Onagraceae Juss.
Iz_1_1109	DJI_0064	Pentanema britannica (L.) D.Gut.Larr., Santos-Vicente, Anderb., E.Rico & M.M.Mart.Ort.	0	n	1	0	Asteraceae Bercht. & J.Presl
Iz_1_1109	DJI_0064	Peucedanum oreoselinum (L.) Moench	0	n	1	0	Apiaceae Lindl.
Iz_1_1109	DJI_0064	Peucedanum ruthenicum M.Bieb.	0	n	1	0	Apiaceae Lindl.
Iz_1_1109	DJI_0064	Pilosella echioides (Lumn.) F.W.Schultz & Sch.Bip.	0	n	1	0	Asteraceae Bercht. & J.Presl
Iz_1_1109	DJI_0064	Pilosella officinarum F. Schultz et Sch. Bip.	0	n	1	0	Asteraceae Bercht. & J.Presl
Iz_1_1109	DJI_0064	Pilosella sp.	0		NA	0	Asteraceae Bercht. & J.Presl
Iz_1_1109	DJI_0064	Pinus sylvestris L.	0	n	1	1	Pinaceae Spreng. ex F.Rudolphi
Iz_1_1109	DJI_0064	Poa annua L.	0	n	1	0	Poaceae Barnhart
Iz_1_1109	DJI_0064	Poa nemoralis L.	0	n	1	0	Poaceae Barnhart
Iz_1_1109	DJI_0064	Poa sp.	0		NA	0	Poaceae Barnhart
Iz_1_1109	DJI_0064	Polygonatum odoratum (Mill.) Druce	0	n	1	0	Asparagaceae Juss.
Iz_1_1109	DJI_0064	Polygonum arenarium Waldst. & Kit.	0	n	1	0	Polygonaceae Juss.
Iz_1_1109	DJI_0064	Populus nigra L.	0	n	1	1	Salicaceae Mirb.
Iz_1_1109	DJI_0064	Populus sp.	0	n	1	1	Salicaceae Mirb.
Iz_1_1109	DJI_0064	Populus tremula L.	0	n	1	1	Salicaceae Mirb.
Iz_1_1109	DJI_0064	Populus Ã canescens (Aiton) Sm.	0	n	1	1	Salicaceae Mirb.
Iz_1_1109	DJI_0064	Potentilla argentea L.	0	n	1	0	Rosaceae Juss.
Iz_1_1109	DJI_0064	Psammophiliella muralis (L.) Ikonn.	0	n	1	0	Caryophyllaceae Juss.
Iz_1_1109	DJI_0064	Psephellus sumensis (Kalen.) Greuter	0	n	1	0	Asteraceae Bercht. & J.Presl
Iz_1_1109	DJI_0064	Pulsatilla pratensis (L.) Mill. s.l.	0	n	1	0	Ranunculaceae Juss.
Iz_1_1109	DJI_0064	Pyrus communis L.	0	a	0	1	Rosaceae Juss.
Iz_1_1109	DJI_0064	Quercus robur L.	0	n	1	1	Fagaceae Dumort.
Iz_1_1109	DJI_0064	Robinia pseudoacacia L.	0	a	0	1	Fabaceae Lindl.
Iz_1_1109	DJI_0064	Rosa sp.	0		NA	1	Rosaceae Juss.
Iz_1_1109	DJI_0064	Rubus idaeus L.	0	n	1	0	Rosaceae Juss.
Iz_1_1109	DJI_0064	Rumex acetosella L.	0	n	1	0	Polygonaceae Juss.
Iz_1_1109	DJI_0064	Rumex thyrsiflorus Fingerh.	0	n	1	0	Polygonaceae Juss.
Iz_1_1109	DJI_0064	Salix alba L.	0	n	1	1	Salicaceae Mirb.
Iz_1_1109	DJI_0064	Sambucus nigra L.	0	n	1	1	Viburnaceae Raf.
Iz_1_1109	DJI_0064	Secale sylvestre Host	0	n	1	0	Poaceae Barnhart
Iz_1_1109	DJI_0064	Sempervivum ruthenicum Schnittsp. & C.B. Lehm.	0	n	1	0	Crassulaceae J.St.-Hil.
Iz_1_1109	DJI_0064	Setaria viridis (L.) P. Beauv.	0	n	1	0	Poaceae Barnhart
Iz_1_1109	DJI_0064	Silene borysthenica (Gruner) Walters	0	n	1	0	Caryophyllaceae Juss.
Iz_1_1109	DJI_0064	Silene latifolia subsp. alba (Mill.) Greuter & Burdet	0	n	1	0	Caryophyllaceae Juss.
Iz_1_1109	DJI_0064	Silene nutans L.	0	n	1	0	Caryophyllaceae Juss.
Iz_1_1109	DJI_0064	Solanum nigrum L.	0		NA	NA	
Iz_1_1109	DJI_0064	Solidago virgaurea L.	0	n	1	0	Asteraceae Bercht. & J.Presl
Iz_1_1109	DJI_0064	Sorbus aucuparia L.	0	n	1	1	Rosaceae Juss.
Iz_1_1109	DJI_0064	Stachys recta L.	0	n	1	0	Lamiacea Lindl.
Iz_1_1109	DJI_0064	Tanacetum vulgare L.	0	n	1	0	Asteraceae Bercht. & J.Presl
Iz_1_1109	DJI_0064	Thymus pallasianus Heinr.Braun	0	n	1	0	Lamiacea Lindl.
Iz_1_1109	DJI_0064	Tilia cordata Mill.	0	n	1	1	Malvaceae Juss.
Iz_1_1109	DJI_0064	Trifolium alpestre L.	0	n	1	0	Fabaceae Lindl.
Iz_1_1109	DJI_0064	Trifolium arvense L.	0	n	1	0	Fabaceae Lindl.
Iz_1_1109	DJI_0064	Ulmus glabra Huds.	0	n	1	1	Ulmaceae Mirb.
Iz_1_1109	DJI_0064	Verbascum densiflorum Bertol.	0	n	1	0	Scrophulariaceae Juss.
Iz_1_1109	DJI_0064	Veronica austriaca L.	0	n	1	0	Plantaginaceae Juss.
Iz_1_1109	DJI_0064	Veronica incana L.	0	n	1	0	Plantaginaceae Juss.
Iz_1_1109	DJI_0064	Veronica verna L.	0	n	1	0	Plantaginaceae Juss.
Iz_1_1109	DJI_0064	Vincetoxicum hirundinaria Medik	0	n	1	0	Apocynaceae Juss.
Iz_1_1109	DJI_0066	Acer tataricum L.	0	n	1	1	Sapindaceae Juss.
Iz_1_1109	DJI_0066	Achillea millefolium L. s.l.	0	n	1	0	Asteraceae Bercht. & J.Presl
Iz_1_1109	DJI_0066	Allium oleraceum L.	0	n	1	0	Amaryllidaceae J.St.-Hil.
Iz_1_1109	DJI_0066	Ambrosia artemisiifolia L.	0	a	0	0	Asteraceae Bercht. & J.Presl
Iz_1_1109	DJI_0066	Amorpha fruticosa L.	0	a	0	0	Fabaceae Lindl.
Iz_1_1109	DJI_0066	Anthericum ramosum L.	0	n	1	0	Asparagaceae Juss.
Iz_1_1109	DJI_0066	Artemisia marschalliana Spreng.	0	n	1	0	Asteraceae Bercht. & J.Presl
Iz_1_1109	DJI_0066	Asparagus officinalis L.	0	n	1	0	Asparagaceae Juss.
Iz_1_1109	DJI_0066	Astragalus cicer L.	0	n	1	0	Fabaceae Lindl.
Iz_1_1109	DJI_0066	Bassia laniflora (S.G.Gmel.) A.J.Scott	0	n	1	0	Amaranthaceae Juss.
Iz_1_1109	DJI_0066	Berberis vulgaris L.	0	n	1	0	Berberidaceae Juss.
Iz_1_1109	DJI_0066	Betula pendula Roth.	0	n	1	1	Betulaceae Gray
Iz_1_1109	DJI_0066	Betula pendula Roth.	0	n	1	0	Poaceae Barnhart
Iz_1_1109	DJI_0066	Bromus inermis Leyss.	0		NA	NA	
Iz_1_1109	DJI_0066	Bromus squarrosus L.	0	a	0	0	Poaceae Barnhart
Iz_1_1109	DJI_0066	Bromus tectorum L.	0	a	0	0	Poaceae Barnhart
Iz_1_1109	DJI_0066	Calamagrostis epigejos (L.) Roth	5	n	1	0	Poaceae Barnhart
Iz_1_1109	DJI_0066	Carduus acanthoides L.	0	n	1	0	Asteraceae Bercht. & J.Presl
Iz_1_1109	DJI_0066	Carex sp.	0	n	1	0	Cyperaceae Juss.
Iz_1_1109	DJI_0066	Centaurea majorovii Dumbadze	0	n	1	0	Asteraceae Bercht. & J.Presl
Iz_1_1109	DJI_0066	Chamaecytisus ruthenicus (Fisch.ex Wol.) Klaskova	0	n	1	0	Fabaceae Lindl.
Iz_1_1109	DJI_0066	Chelidonium majus L.	0	n	1	0	Papaveraceae Juss.
Iz_1_1109	DJI_0066	Chenopodium betaceum Andrz.	0	n	1	0	Amaranthaceae Juss.
Iz_1_1109	DJI_0066	Chenopodium cf. album	0	n	1	0	Amaranthaceae Juss.
Iz_1_1109	DJI_0066	Chenopodium sp.	0		NA	0	Amaranthaceae Juss.
Iz_1_1109	DJI_0066	Chondrilla juncea L.	0	n	1	0	Asteraceae Bercht. & J.Presl
Iz_1_1109	DJI_0066	Cirsium vulgare (Savi) Ten.	0	n	1	0	Asteraceae Bercht. & J.Presl
Iz_1_1109	DJI_0066	Clinopodium acinos (L.) Kuntze	0	n	1	0	Lamiacea Lindl.
Iz_1_1109	DJI_0066	Convallaria majalis L.	0	n	1	0	Asparagaceae Juss.
Iz_1_1109	DJI_0066	Crataegus rhipidophylla Gand.	0	n	1	1	Rosaceae Juss.
Iz_1_1109	DJI_0066	Crepis foetida L.	0	n	1	0	Asteraceae Bercht. & J.Presl
Iz_1_1109	DJI_0066	Dactylis glomerata L.	0	n	1	0	Poaceae Barnhart
Iz_1_1109	DJI_0066	Dianthus campestris M.Bieb.	0	n	1	0	Caryophyllaceae Juss.
Iz_1_1109	DJI_0066	Digitaria sanguinalis (L.) Scop.	0	a	0	0	Poaceae Barnhart
Iz_1_1109	DJI_0066	Elymus repens (L.) Gould	0	n	1	0	Poaceae Barnhart
Iz_1_1109	DJI_0066	Epilobium tetragonum L.	0	n	1	0	Onagraceae Juss.
Iz_1_1109	DJI_0066	Eragrostis minor Host	0	a	0	0	Poaceae Barnhart
Iz_1_1109	DJI_0066	Erigeron canadensis L.	0	a	0	0	Asteraceae Bercht. & J.Presl
Iz_1_1109	DJI_0066	Euphorbia seguieriana Neck.	0	n	1	0	Euphorbiaceae Juss.
Iz_1_1109	DJI_0066	Fallopia dumetorum (L.) Holub	0.3	n	1	0	Polygonaceae Juss.
Iz_1_1109	DJI_0066	Festuca beckeri (Hack.) Trautv.	0	n	1	0	Poaceae Barnhart
Iz_1_1109	DJI_0066	Fragaria vesca L.	0	n	1	0	Rosaceae Juss.
Iz_1_1109	DJI_0066	Frangula alnus Mill.	0	n	1	1	Rhamnaceae Juss.
Iz_1_1109	DJI_0066	Fraxinus pennsylvanica Marshall	0	a	0	1	Oleaceae Hoffmanns. & Link
Iz_1_1109	DJI_0066	Galeopsis bifida Boenn.	0	n	1	0	Lamiacea Lindl.
Iz_1_1109	DJI_0066	Galium verum L.	0	n	1	0	Rubiaceae Juss.

Iz_1_1109	DJI_0066	Genista tinctoria L.	0	n	1	0	Fabaceae Lindl.
Iz_1_1109	DJI_0066	Geranium robertianum L.	0	n	1	0	Geraniaceae Juss.
Iz_1_1109	DJI_0066	Geranium sanquineum L.	0	n	1	0	Geraniaceae Juss.
Iz_1_1109	DJI_0066	Gypsophila paniculata L.	0	n	1	0	Caryophyllaceae Juss.
Iz_1_1109	DJI_0066	Helichrysum arenarium (L.) Moench	0	n	1	0	Asteraceae Bercht. & J.Presl
Iz_1_1109	DJI_0066	Herniaria polygama J.Gay	0	n	1	0	Caryophyllaceae Juss.
Iz_1_1109	DJI_0066	Hieracium robustum Fr.	0	n	1	0	Asteraceae Bercht. & J.Presl
Iz_1_1109	DJI_0066	Hieracium umbellatum L.	0	n	1	NA	Asteraceae Bercht. & J.Presl
Iz_1_1109	DJI_0066	Hylotelephium maximum (L.) Holub	0	n	1	0	Crassulaceae J.St.-Hil.
Iz_1_1109	DJI_0066	Jacobaea borysthenica (DC.) B.Nord. & Greuter	0	n	1	0	Asteraceae Bercht. & J.Presl
Iz_1_1109	DJI_0066	Jasione montana L.	0	n	1	0	Campanulaceae Juss.
Iz_1_1109	DJI_0066	Jurinea cyanoides (L.) Rchb.s.l.	0	n	1	0	Asteraceae Bercht. & J.Presl
Iz_1_1109	DJI_0066	Koeleria glauca (Spreng.) DC.	0	n	1	0	Poaceae Barnhart
Iz_1_1109	DJI_0066	Lactuca serriola L.	0	a	0	0	Asteraceae Bercht. & J.Presl
Iz_1_1109	DJI_0066	Linaria genistifolia (L.) Mill.	0	n	1	0	Plantaginaceae Juss.
Iz_1_1109	DJI_0066	Lomelosia argentea (L.) Greuter & Burdet	0	n	1	0	Caprifoliaceae Juss.
Iz_1_1109	DJI_0066	Melampyrum pratense L.	0	n	1	0	Orobanchaceae Vent.
Iz_1_1109	DJI_0066	Oenothera biennis L.	0	a	0	0	Onagraceae Juss.
Iz_1_1109	DJI_0066	Pentanema britannica (L.) D.Gut.Larr., Santos-Vicente, Anderb., E.Rico & M.M.Mart.Ort.	0	n	1	0	Asteraceae Bercht. & J.Presl
Iz_1_1109	DJI_0066	Peucedanum oreoselinum (L.) Moench	0	n	1	0	Apiaceae Lindl.
Iz_1_1109	DJI_0066	Peucedanum ruthenicum M.Bieb.	0	n	1	0	Apiaceae Lindl.
Iz_1_1109	DJI_0066	Pilosella echioides (Lumn.) F.W.Schultz & Sch.Bip.	0	n	1	0	Asteraceae Bercht. & J.Presl
Iz_1_1109	DJI_0066	Pilosella officinarum F. Schultz et Sch. Bip.	0	n	1	0	Asteraceae Bercht. & J.Presl
Iz_1_1109	DJI_0066	Pilosella sp.	0		NA	0	Asteraceae Bercht. & J.Presl
Iz_1_1109	DJI_0066	Pinus sylvestris L.	0	n	1	1	Pinaceae Spreng. ex F.Rudolphi
Iz_1_1109	DJI_0066	Poa annua L.	0	n	1	0	Poaceae Barnhart
Iz_1_1109	DJI_0066	Poa nemoralis L.	0	n	1	0	Poaceae Barnhart
Iz_1_1109	DJI_0066	Poa sp.	0		NA	0	Poaceae Barnhart
Iz_1_1109	DJI_0066	Polygonatum odoratum (Mill.) Druce	0	n	1	0	Asparagaceae Juss.
Iz_1_1109	DJI_0066	Polygonum arenarium Waldst. & Kit.	0	n	1	0	Polygonaceae Juss.
Iz_1_1109	DJI_0066	Populus nigra L.	0	n	1	1	Salicaceae Mirb.
Iz_1_1109	DJI_0066	Populus sp.	0	n	1	1	Salicaceae Mirb.
Iz_1_1109	DJI_0066	Populus tremula L.	0	n	1	1	Salicaceae Mirb.
Iz_1_1109	DJI_0066	Populus Ã canescens (Aiton) Sm.	0	n	1	1	Salicaceae Mirb.
Iz_1_1109	DJI_0066	Potentilla argentea L.	0	n	1	0	Rosaceae Juss.
Iz_1_1109	DJI_0066	Psammophiliella muralis (L.) Ikonn.	0	n	1	0	Caryophyllaceae Juss.
Iz_1_1109	DJI_0066	Psephellus sumensis (Kalen.) Greuter	0	n	1	0	Asteraceae Bercht. & J.Presl
Iz_1_1109	DJI_0066	Pulsatilla pratensis (L.) Mill. s.l.	0	n	1	0	Ranunculaceae Juss.
Iz_1_1109	DJI_0066	Pyrus communis L.	0	a	0	1	Rosaceae Juss.
Iz_1_1109	DJI_0066	Quercus robur L.	0	n	1	1	Fagaceae Dumort.
Iz_1_1109	DJI_0066	Robinia pseudoacacia L.	0	a	0	1	Fabaceae Lindl.
Iz_1_1109	DJI_0066	Rosa sp.	0		NA	1	Rosaceae Juss.
Iz_1_1109	DJI_0066	Rubus idaeus L.	0	n	1	0	Rosaceae Juss.
Iz_1_1109	DJI_0066	Rumex acetosella L.	0.5	n	1	0	Polygonaceae Juss.
Iz_1_1109	DJI_0066	Rumex thyrsiflorus Fingerh.	0	n	1	0	Polygonaceae Juss.
Iz_1_1109	DJI_0066	Salix alba L.	0	n	1	1	Salicaceae Mirb.
Iz_1_1109	DJI_0066	Sambucus nigra L.	0	n	1	1	Viburnaceae Raf.
Iz_1_1109	DJI_0066	Secale sylvestre Host	0	n	1	0	Poaceae Barnhart
Iz_1_1109	DJI_0066	Sempervivum ruthenicum Schnittsp. & C.B. Lehm.	0	n	1	0	Crassulaceae J.St.-Hil.
Iz_1_1109	DJI_0066	Setaria viridis (L.) P. Beauv.	0	n	1	0	Poaceae Barnhart
Iz_1_1109	DJI_0066	Silene borysthenica (Gruner) Walters	0	n	1	0	Caryophyllaceae Juss.
Iz_1_1109	DJI_0066	Silene latifolia subsp. alba (Mill.) Greuter & Burdet	0	n	1	0	Caryophyllaceae Juss.
Iz_1_1109	DJI_0066	Silene nutans L.	0.5	n	1	0	Caryophyllaceae Juss.
Iz_1_1109	DJI_0066	Solanum nigrum L.	0		NA	NA	
Iz_1_1109	DJI_0066	Solidago virgaurea L.	0	n	1	0	Asteraceae Bercht. & J.Presl
Iz_1_1109	DJI_0066	Sorbus aucuparia L.	0	n	1	1	Rosaceae Juss.
Iz_1_1109	DJI_0066	Stachys recta L.	0	n	1	0	Lamiacea Lindl.
Iz_1_1109	DJI_0066	Tanacetum vulgare L.	0	n	1	0	Asteraceae Bercht. & J.Presl
Iz_1_1109	DJI_0066	Thymus pallasianus Heinr.Braun	0	n	1	0	Lamiacea Lindl.
Iz_1_1109	DJI_0066	Tilia cordata Mill.	0	n	1	1	Malvaceae Juss.
Iz_1_1109	DJI_0066	Trifolium alpestre L.	0	n	1	0	Fabaceae Lindl.
Iz_1_1109	DJI_0066	Trifolium arvense L.	0	n	1	0	Fabaceae Lindl.
Iz_1_1109	DJI_0066	Ulmus glabra Huds.	0	n	1	1	Ulmaceae Mirb.
Iz_1_1109	DJI_0066	Verbascum densiflorum Bertol.	0	n	1	0	Scrophulariaceae Juss.
Iz_1_1109	DJI_0066	Veronica austriaca L.	0	n	1	0	Plantaginaceae Juss.
Iz_1_1109	DJI_0066	Veronica incana L.	0	n	1	0	Plantaginaceae Juss.
Iz_1_1109	DJI_0066	Veronica verna L.	0	n	1	0	Plantaginaceae Juss.
Iz_1_1109	DJI_0066	Vincetoxicum hirundinaria Medik	0	n	1	0	Apocynaceae Juss.
Iz_1_1109	DJI_0068	Acer tataricum L.	0	n	1	1	Sapindaceae Juss.
Iz_1_1109	DJI_0068	Achillea millefolium L. s.l.	0	n	1	0	Asteraceae Bercht. & J.Presl
Iz_1_1109	DJI_0068	Allium oleraceum L.	0	n	1	0	Amaryllidaceae J.St.-Hil.
Iz_1_1109	DJI_0068	Ambrosia artemisiifolia L.	0	a	0	0	Asteraceae Bercht. & J.Presl
Iz_1_1109	DJI_0068	Amorpha fruticosa L.	0	a	0	0	Fabaceae Lindl.
Iz_1_1109	DJI_0068	Anthericum ramosum L.	0	n	1	0	Asparagaceae Juss.
Iz_1_1109	DJI_0068	Artemisia marschalliana Spreng.	0	n	1	0	Asteraceae Bercht. & J.Presl
Iz_1_1109	DJI_0068	Asparagus officinalis L.	0	n	1	0	Asparagaceae Juss.
Iz_1_1109	DJI_0068	Astragalus cicer L.	0	n	1	0	Fabaceae Lindl.
Iz_1_1109	DJI_0068	Bassia laniflora (S.G.Gmel.) A.J.Scott	0	n	1	0	Amaranthaceae Juss.
Iz_1_1109	DJI_0068	Berberis vulgaris L.	0	n	1	0	Berberidaceae Juss.
Iz_1_1109	DJI_0068	Betula pendula Roth.	0	n	1	1	Betulaceae Gray
Iz_1_1109	DJI_0068	Betula pendula Roth.	0	n	1	0	Poaceae Barnhart
Iz_1_1109	DJI_0068	Bromus inermis Leyss.	0		NA	NA	
Iz_1_1109	DJI_0068	Bromus squarrosus L.	0	a	0	0	Poaceae Barnhart
Iz_1_1109	DJI_0068	Bromus tectorum L.	0	a	0	0	Poaceae Barnhart
Iz_1_1109	DJI_0068	Calamagrostis epigejos (L.) Roth	10	n	1	0	Poaceae Barnhart
Iz_1_1109	DJI_0068	Carduus acanthoides L.	0	n	1	0	Asteraceae Bercht. & J.Presl
Iz_1_1109	DJI_0068	Carex sp.	0	n	1	0	Cyperaceae Juss.
Iz_1_1109	DJI_0068	Centaurea majorovii Dumbadze	0	n	1	0	Asteraceae Bercht. & J.Presl
Iz_1_1109	DJI_0068	Chamaecytisus ruthenicus (Fisch.ex Wol.) Klaskova	0	n	1	0	Fabaceae Lindl.
Iz_1_1109	DJI_0068	Chelidonium majus L.	0	n	1	0	Papaveraceae Juss.
Iz_1_1109	DJI_0068	Chenopodium betaceum Andrz.	0	n	1	0	Amaranthaceae Juss.
Iz_1_1109	DJI_0068	Chenopodium cf. album	0	n	1	0	Amaranthaceae Juss.
Iz_1_1109	DJI_0068	Chenopodium sp.	0		NA	0	Amaranthaceae Juss.
Iz_1_1109	DJI_0068	Chondrilla juncea L.	0	n	1	0	Asteraceae Bercht. & J.Presl
Iz_1_1109	DJI_0068	Cirsium vulgare (Savi) Ten.	0	n	1	0	Asteraceae Bercht. & J.Presl
Iz_1_1109	DJI_0068	Clinopodium acinos (L.) Kuntze	0	n	1	0	Lamiacea Lindl.
Iz_1_1109	DJI_0068	Convallaria majalis L.	0	n	1	0	Asparagaceae Juss.
Iz_1_1109	DJI_0068	Crataegus rhipidophylla Gand.	0	n	1	1	Rosaceae Juss.

Iz_1_1109	DJI_0068	Crepis foetida L.	0	n	1	0	Asteraceae Bercht. & J.Presl
Iz_1_1109	DJI_0068	Dactylis glomerata L.	0	n	1	0	Poaceae Barnhart
Iz_1_1109	DJI_0068	Dianthus campestris M.Bieb.	0	n	1	0	Caryophyllaceae Juss.
Iz_1_1109	DJI_0068	Digitaria sanguinalis (L.) Scop.	0	a	0	0	Poaceae Barnhart
Iz_1_1109	DJI_0068	Elymus repens (L.) Gould	0	n	1	0	Poaceae Barnhart
Iz_1_1109	DJI_0068	Epilobium tetragonum L.	0	n	1	0	Onagraceae Juss.
Iz_1_1109	DJI_0068	Eragrostis minor Host	0	a	0	0	Poaceae Barnhart
Iz_1_1109	DJI_0068	Erigeron canadensis L.	0.2	a	0	0	Asteraceae Bercht. & J.Presl
Iz_1_1109	DJI_0068	Euphorbia seguieriana Neck.	0	n	1	0	Euphorbiaceae Juss.
Iz_1_1109	DJI_0068	Fallopia dumetorum (L.) Holub	2	n	1	0	Polygonaceae Juss.
Iz_1_1109	DJI_0068	Festuca beckeri (Hack.) Trautv.	0	n	1	0	Poaceae Barnhart
Iz_1_1109	DJI_0068	Fragaria vesca L.	0	n	1	0	Rosaceae Juss.
Iz_1_1109	DJI_0068	Frangula alnus Mill.	0	n	1	1	Rhamnaceae Juss.
Iz_1_1109	DJI_0068	Fraxinus pennsylvanica Marshall	0	a	0	1	Oleaceae Hoffmanns. & Link
Iz_1_1109	DJI_0068	Galeopsis bifida Boenn.	0	n	1	0	Lamiacea Lindl.
Iz_1_1109	DJI_0068	Galium verum L.	0	n	1	0	Rubiaceae Juss.
Iz_1_1109	DJI_0068	Genista tinctoria L.	0	n	1	0	Fabaceae Lindl.
Iz_1_1109	DJI_0068	Geranium robertianum L.	0	n	1	0	Geraniaceae Juss.
Iz_1_1109	DJI_0068	Geranium sanquineum L.	0	n	1	0	Geraniaceae Juss.
Iz_1_1109	DJI_0068	Gypsophila paniculata L.	0	n	1	0	Caryophyllaceae Juss.
Iz_1_1109	DJI_0068	Helichrysum arenarium (L.) Moench	0	n	1	0	Asteraceae Bercht. & J.Presl
Iz_1_1109	DJI_0068	Herniaria polygama J.Gay	0	n	1	0	Caryophyllaceae Juss.
Iz_1_1109	DJI_0068	Hieracium robustum Fr.	0	n	1	0	Asteraceae Bercht. & J.Presl
Iz_1_1109	DJI_0068	Hieracium umbellatum L.	0	n	1	NA	Asteraceae Bercht. & J.Presl
Iz_1_1109	DJI_0068	Hylotelephium maximum (L.) Holub	0	n	1	0	Crassulaceae J.St.-Hil.
Iz_1_1109	DJI_0068	Jacobaea borysthenica (DC.) B.Nord. & Greuter	0	n	1	0	Asteraceae Bercht. & J.Presl
Iz_1_1109	DJI_0068	Jasione montana L.	0	n	1	0	Campanulaceae Juss.
Iz_1_1109	DJI_0068	Jurinea cyanoides (L.) Rchb.s.l.	0	n	1	0	Asteraceae Bercht. & J.Presl
Iz_1_1109	DJI_0068	Koeleria glauca (Spreng.) DC.	0	n	1	0	Poaceae Barnhart
Iz_1_1109	DJI_0068	Lactuca serriola L.	0	a	0	0	Asteraceae Bercht. & J.Presl
Iz_1_1109	DJI_0068	Linaria genistifolia (L.) Mill.	0	n	1	0	Plantaginaceae Juss.
Iz_1_1109	DJI_0068	Lomelosia argentea (L.) Greuter & Burdet	0	n	1	0	Caprifoliaceae Juss.
Iz_1_1109	DJI_0068	Melampyrum pratense L.	0	n	1	0	Orobanchaceae Vent.
Iz_1_1109	DJI_0068	Oenothera biennis L.	0	a	0	0	Onagraceae Juss.
Iz_1_1109	DJI_0068	Pentanema britannica (L.) D.Gut.Larr., Santos-Vicente, Anderb., E.Rico & M.M.Mart.Ort.	0	n	1	0	Asteraceae Bercht. & J.Presl
Iz_1_1109	DJI_0068	Peucedanum oreoselinum (L.) Moench	0	n	1	0	Apiaceae Lindl.
Iz_1_1109	DJI_0068	Peucedanum ruthenicum M.Bieb.	0	n	1	0	Apiaceae Lindl.
Iz_1_1109	DJI_0068	Pilosella echioides (Lumn.) F.W.Schultz & Sch.Bip.	0	n	1	0	Asteraceae Bercht. & J.Presl
Iz_1_1109	DJI_0068	Pilosella officinarum F. Schultz et Sch. Bip.	0	n	1	0	Asteraceae Bercht. & J.Presl
Iz_1_1109	DJI_0068	Pilosella sp.	0		NA	0	Asteraceae Bercht. & J.Presl
Iz_1_1109	DJI_0068	Pinus sylvestris L.	0	n	1	1	Pinaceae Spreng. ex F.Rudolphi
Iz_1_1109	DJI_0068	Poa annua L.	0	n	1	0	Poaceae Barnhart
Iz_1_1109	DJI_0068	Poa nemoralis L.	0	n	1	0	Poaceae Barnhart
Iz_1_1109	DJI_0068	Poa sp.	0		NA	0	Poaceae Barnhart
Iz_1_1109	DJI_0068	Polygonatum odoratum (Mill.) Druce	0	n	1	0	Asparagaceae Juss.
Iz_1_1109	DJI_0068	Polygonum arenarium Waldst. & Kit.	0	n	1	0	Polygonaceae Juss.
Iz_1_1109	DJI_0068	Populus nigra L.	0	n	1	1	Salicaceae Mirb.
Iz_1_1109	DJI_0068	Populus sp.	0	n	1	1	Salicaceae Mirb.
Iz_1_1109	DJI_0068	Populus tremula L.	0	n	1	1	Salicaceae Mirb.
Iz_1_1109	DJI_0068	Populus Ã canescens (Aiton) Sm.	0	n	1	1	Salicaceae Mirb.
Iz_1_1109	DJI_0068	Potentilla argentea L.	0	n	1	0	Rosaceae Juss.
Iz_1_1109	DJI_0068	Psammophiliella muralis (L.) Ikonn.	0	n	1	0	Caryophyllaceae Juss.
Iz_1_1109	DJI_0068	Psephellus sumensis (Kalen.) Greuter	0	n	1	0	Asteraceae Bercht. & J.Presl
Iz_1_1109	DJI_0068	Pulsatilla pratensis (L.) Mill. s.l.	0	n	1	0	Ranunculaceae Juss.
Iz_1_1109	DJI_0068	Pyrus communis L.	0	a	0	1	Rosaceae Juss.
Iz_1_1109	DJI_0068	Quercus robur L.	0	n	1	1	Fagaceae Dumort.
Iz_1_1109	DJI_0068	Robinia pseudoacacia L.	0	a	0	1	Fabaceae Lindl.
Iz_1_1109	DJI_0068	Rosa sp.	0		NA	1	Rosaceae Juss.
Iz_1_1109	DJI_0068	Rubus idaeus L.	0	n	1	0	Rosaceae Juss.
Iz_1_1109	DJI_0068	Rumex acetosella L.	0	n	1	0	Polygonaceae Juss.
Iz_1_1109	DJI_0068	Rumex thyrsiflorus Fingerh.	0	n	1	0	Polygonaceae Juss.
Iz_1_1109	DJI_0068	Salix alba L.	0	n	1	1	Salicaceae Mirb.
Iz_1_1109	DJI_0068	Sambucus nigra L.	0	n	1	1	Viburnaceae Raf.
Iz_1_1109	DJI_0068	Secale sylvestre Host	0	n	1	0	Poaceae Barnhart
Iz_1_1109	DJI_0068	Sempervivum ruthenicum Schnittsp. & C.B. Lehm.	0	n	1	0	Crassulaceae J.St.-Hil.
Iz_1_1109	DJI_0068	Setaria viridis (L.) P. Beauv.	0	n	1	0	Poaceae Barnhart
Iz_1_1109	DJI_0068	Silene borysthenica (Gruner) Walters	0	n	1	0	Caryophyllaceae Juss.
Iz_1_1109	DJI_0068	Silene latifolia subsp. alba (Mill.) Greuter & Burdet	0	n	1	0	Caryophyllaceae Juss.
Iz_1_1109	DJI_0068	Silene nutans L.	0	n	1	0	Caryophyllaceae Juss.
Iz_1_1109	DJI_0068	Solanum nigrum L.	0		NA	NA	
Iz_1_1109	DJI_0068	Solidago virgaurea L.	0	n	1	0	Asteraceae Bercht. & J.Presl
Iz_1_1109	DJI_0068	Sorbus aucuparia L.	0	n	1	1	Rosaceae Juss.
Iz_1_1109	DJI_0068	Stachys recta L.	0	n	1	0	Lamiacea Lindl.
Iz_1_1109	DJI_0068	Tanacetum vulgare L.	0	n	1	0	Asteraceae Bercht. & J.Presl
Iz_1_1109	DJI_0068	Thymus pallasianus Heinr.Braun	0	n	1	0	Lamiacea Lindl.
Iz_1_1109	DJI_0068	Tilia cordata Mill.	0	n	1	1	Malvaceae Juss.
Iz_1_1109	DJI_0068	Trifolium alpestre L.	0	n	1	0	Fabaceae Lindl.
Iz_1_1109	DJI_0068	Trifolium arvense L.	0	n	1	0	Fabaceae Lindl.
Iz_1_1109	DJI_0068	Ulmus glabra Huds.	0	n	1	1	Ulmaceae Mirb.
Iz_1_1109	DJI_0068	Verbascum densiflorum Bertol.	0	n	1	0	Scrophulariaceae Juss.
Iz_1_1109	DJI_0068	Veronica austriaca L.	0	n	1	0	Plantaginaceae Juss.
Iz_1_1109	DJI_0068	Veronica incana L.	0	n	1	0	Plantaginaceae Juss.
Iz_1_1109	DJI_0068	Veronica verna L.	0	n	1	0	Plantaginaceae Juss.
Iz_1_1109	DJI_0068	Vincetoxicum hirundinaria Medik	0	n	1	0	Apocynaceae Juss.
Iz_1_1109	DJI_0071	Acer tataricum L.	0	n	1	1	Sapindaceae Juss.
Iz_1_1109	DJI_0071	Achillea millefolium L. s.l.	0	n	1	0	Asteraceae Bercht. & J.Presl
Iz_1_1109	DJI_0071	Allium oleraceum L.	0	n	1	0	Amaryllidaceae J.St.-Hil.
Iz_1_1109	DJI_0071	Ambrosia artemisiifolia L.	0	a	0	0	Asteraceae Bercht. & J.Presl
Iz_1_1109	DJI_0071	Amorpha fruticosa L.	0	a	0	0	Fabaceae Lindl.
Iz_1_1109	DJI_0071	Anthericum ramosum L.	0	n	1	0	Asparagaceae Juss.
Iz_1_1109	DJI_0071	Artemisia marschalliana Spreng.	0	n	1	0	Asteraceae Bercht. & J.Presl
Iz_1_1109	DJI_0071	Asparagus officinalis L.	0	n	1	0	Asparagaceae Juss.
Iz_1_1109	DJI_0071	Astragalus cicer L.	0	n	1	0	Fabaceae Lindl.
Iz_1_1109	DJI_0071	Bassia laniflora (S.G.Gmel.) A.J.Scott	0	n	1	0	Amaranthaceae Juss.
Iz_1_1109	DJI_0071	Berberis vulgaris L.	0	n	1	0	Berberidaceae Juss.
Iz_1_1109	DJI_0071	Betula pendula Roth.	0	n	1	1	Betulaceae Gray
Iz_1_1109	DJI_0071	Betula pendula Roth.	0	n	1	0	Poaceae Barnhart
Iz_1_1109	DJI_0071	Bromus inermis Leyss.	0		NA	NA	

Iz_1_1109	DJI_0071	Bromus squarrosus L.	0	a	0	0	Poaceae Barnhart
Iz_1_1109	DJI_0071	Bromus tectorum L.	0	a	0	0	Poaceae Barnhart
Iz_1_1109	DJI_0071	Calamagrostis epigejos (L.) Roth	1	n	1	0	Poaceae Barnhart
Iz_1_1109	DJI_0071	Carduus acanthoides L.	0	n	1	0	Asteraceae Bercht. & J.Presl
Iz_1_1109	DJI_0071	Carex sp.	0	n	1	0	Cyperaceae Juss.
Iz_1_1109	DJI_0071	Centaurea majorovii Dumbadze	0	n	1	0	Asteraceae Bercht. & J.Presl
Iz_1_1109	DJI_0071	Chamaecytisus ruthenicus (Fisch.ex Wol.) Klaskova	0	n	1	0	Fabaceae Lindl.
Iz_1_1109	DJI_0071	Chelidonium majus L.	0	n	1	0	Papaveraceae Juss.
Iz_1_1109	DJI_0071	Chenopodium betaceum Andrz.	0	n	1	0	Amaranthaceae Juss.
Iz_1_1109	DJI_0071	Chenopodium cf. album	0	n	1	0	Amaranthaceae Juss.
Iz_1_1109	DJI_0071	Chenopodium sp.	0		NA	0	Amaranthaceae Juss.
Iz_1_1109	DJI_0071	Chondrilla juncea L.	0	n	1	0	Asteraceae Bercht. & J.Presl
Iz_1_1109	DJI_0071	Cirsium vulgare (Savi) Ten.	0	n	1	0	Asteraceae Bercht. & J.Presl
Iz_1_1109	DJI_0071	Clinopodium acinos (L.) Kuntze	0	n	1	0	Lamiacea Lindl.
Iz_1_1109	DJI_0071	Convallaria majalis L.	0	n	1	0	Asparagaceae Juss.
Iz_1_1109	DJI_0071	Crataegus rhipidophylla Gand.	0	n	1	1	Rosaceae Juss.
Iz_1_1109	DJI_0071	Crepis foetida L.	0	n	1	0	Asteraceae Bercht. & J.Presl
Iz_1_1109	DJI_0071	Dactylis glomerata L.	0	n	1	0	Poaceae Barnhart
Iz_1_1109	DJI_0071	Dianthus campestris M.Bieb.	0	n	1	0	Caryophyllaceae Juss.
Iz_1_1109	DJI_0071	Digitaria sanguinalis (L.) Scop.	0	a	0	0	Poaceae Barnhart
Iz_1_1109	DJI_0071	Elymus repens (L.) Gould	0	n	1	0	Poaceae Barnhart
Iz_1_1109	DJI_0071	Epilobium tetragonum L.	0	n	1	0	Onagraceae Juss.
Iz_1_1109	DJI_0071	Eragrostis minor Host	0	a	0	0	Poaceae Barnhart
Iz_1_1109	DJI_0071	Erigeron canadensis L.	0.2	a	0	0	Asteraceae Bercht. & J.Presl
Iz_1_1109	DJI_0071	Euphorbia seguieriana Neck.	0	n	1	0	Euphorbiaceae Juss.
Iz_1_1109	DJI_0071	Fallopia dumetorum (L.) Holub	0.5	n	1	0	Polygonaceae Juss.
Iz_1_1109	DJI_0071	Festuca beckeri (Hack.) Trautv.	0	n	1	0	Poaceae Barnhart
Iz_1_1109	DJI_0071	Fragaria vesca L.	0	n	1	0	Rosaceae Juss.
Iz_1_1109	DJI_0071	Frangula alnus Mill.	0	n	1	1	Rhamnaceae Juss.
Iz_1_1109	DJI_0071	Fraxinus pennsylvanica Marshall	0	a	0	1	Oleaceae Hoffmanns. & Link
Iz_1_1109	DJI_0071	Galeopsis bifida Boenn.	0	n	1	0	Lamiacea Lindl.
Iz_1_1109	DJI_0071	Galium verum L.	0	n	1	0	Rubiaceae Juss.
Iz_1_1109	DJI_0071	Genista tinctoria L.	0	n	1	0	Fabaceae Lindl.
Iz_1_1109	DJI_0071	Geranium robertianum L.	0	n	1	0	Geraniaceae Juss.
Iz_1_1109	DJI_0071	Geranium sanquineum L.	0	n	1	0	Geraniaceae Juss.
Iz_1_1109	DJI_0071	Gypsophila paniculata L.	0	n	1	0	Caryophyllaceae Juss.
Iz_1_1109	DJI_0071	Helichrysum arenarium (L.) Moench	0	n	1	0	Asteraceae Bercht. & J.Presl
Iz_1_1109	DJI_0071	Herniaria polygama J.Gay	0	n	1	0	Caryophyllaceae Juss.
Iz_1_1109	DJI_0071	Hieracium robustum Fr.	0	n	1	0	Asteraceae Bercht. & J.Presl
Iz_1_1109	DJI_0071	Hieracium umbellatum L.	0	n	1	NA	Asteraceae Bercht. & J.Presl
Iz_1_1109	DJI_0071	Hylotelephium maximum (L.) Holub	0	n	1	0	Crassulaceae J.St.-Hil.
Iz_1_1109	DJI_0071	Jacobaea borysthenica (DC.) B.Nord. & Greuter	0	n	1	0	Asteraceae Bercht. & J.Presl
Iz_1_1109	DJI_0071	Jasione montana L.	0	n	1	0	Campanulaceae Juss.
Iz_1_1109	DJI_0071	Jurinea cyanoides (L.) Rchb.s.l.	0	n	1	0	Asteraceae Bercht. & J.Presl
Iz_1_1109	DJI_0071	Koeleria glauca (Spreng.) DC.	0	n	1	0	Poaceae Barnhart
Iz_1_1109	DJI_0071	Lactuca serriola L.	0	a	0	0	Asteraceae Bercht. & J.Presl
Iz_1_1109	DJI_0071	Linaria genistifolia (L.) Mill.	0	n	1	0	Plantaginaceae Juss.
Iz_1_1109	DJI_0071	Lomelosia argentea (L.) Greuter & Burdet	0	n	1	0	Caprifoliaceae Juss.
Iz_1_1109	DJI_0071	Melampyrum pratense L.	0	n	1	0	Orobanchaceae Vent.
Iz_1_1109	DJI_0071	Oenothera biennis L.	0	a	0	0	Onagraceae Juss.
Iz_1_1109	DJI_0071	Pentanema britannica (L.) D.Gut.Larr., Santos-Vicente, Anderb., E.Rico & M.M.Mart.Ort.	0	n	1	0	Asteraceae Bercht. & J.Presl
Iz_1_1109	DJI_0071	Peucedanum oreoselinum (L.) Moench	0	n	1	0	Apiaceae Lindl.
Iz_1_1109	DJI_0071	Peucedanum ruthenicum M.Bieb.	0	n	1	0	Apiaceae Lindl.
Iz_1_1109	DJI_0071	Pilosella echioides (Lumn.) F.W.Schultz & Sch.Bip.	0	n	1	0	Asteraceae Bercht. & J.Presl
Iz_1_1109	DJI_0071	Pilosella officinarum F. Schultz et Sch. Bip.	0	n	1	0	Asteraceae Bercht. & J.Presl
Iz_1_1109	DJI_0071	Pilosella sp.	0		NA	0	Asteraceae Bercht. & J.Presl
Iz_1_1109	DJI_0071	Pinus sylvestris L.	0	n	1	1	Pinaceae Spreng. ex F.Rudolphi
Iz_1_1109	DJI_0071	Poa annua L.	0	n	1	0	Poaceae Barnhart
Iz_1_1109	DJI_0071	Poa nemoralis L.	0	n	1	0	Poaceae Barnhart
Iz_1_1109	DJI_0071	Poa sp.	0		NA	0	Poaceae Barnhart
Iz_1_1109	DJI_0071	Polygonatum odoratum (Mill.) Druce	0	n	1	0	Asparagaceae Juss.
Iz_1_1109	DJI_0071	Polygonum arenarium Waldst. & Kit.	0	n	1	0	Polygonaceae Juss.
Iz_1_1109	DJI_0071	Populus nigra L.	0	n	1	1	Salicaceae Mirb.
Iz_1_1109	DJI_0071	Populus sp.	0	n	1	1	Salicaceae Mirb.
Iz_1_1109	DJI_0071	Populus tremula L.	0	n	1	1	Salicaceae Mirb.
Iz_1_1109	DJI_0071	Populus Ã canescens (Aiton) Sm.	0	n	1	1	Salicaceae Mirb.
Iz_1_1109	DJI_0071	Potentilla argentea L.	0	n	1	0	Rosaceae Juss.
Iz_1_1109	DJI_0071	Psammophiliella muralis (L.) Ikonn.	0	n	1	0	Caryophyllaceae Juss.
Iz_1_1109	DJI_0071	Psephellus sumensis (Kalen.) Greuter	0	n	1	0	Asteraceae Bercht. & J.Presl
Iz_1_1109	DJI_0071	Pulsatilla pratensis (L.) Mill. s.l.	0	n	1	0	Ranunculaceae Juss.
Iz_1_1109	DJI_0071	Pyrus communis L.	0	a	0	1	Rosaceae Juss.
Iz_1_1109	DJI_0071	Quercus robur L.	0	n	1	1	Fagaceae Dumort.
Iz_1_1109	DJI_0071	Robinia pseudoacacia L.	0	a	0	1	Fabaceae Lindl.
Iz_1_1109	DJI_0071	Rosa sp.	0		NA	1	Rosaceae Juss.
Iz_1_1109	DJI_0071	Rubus idaeus L.	0	n	1	0	Rosaceae Juss.
Iz_1_1109	DJI_0071	Rumex acetosella L.	0	n	1	0	Polygonaceae Juss.
Iz_1_1109	DJI_0071	Rumex thyrsiflorus Fingerh.	0	n	1	0	Polygonaceae Juss.
Iz_1_1109	DJI_0071	Salix alba L.	0	n	1	1	Salicaceae Mirb.
Iz_1_1109	DJI_0071	Sambucus nigra L.	0	n	1	1	Viburnaceae Raf.
Iz_1_1109	DJI_0071	Secale sylvestre Host	0	n	1	0	Poaceae Barnhart
Iz_1_1109	DJI_0071	Sempervivum ruthenicum Schnittsp. & C.B. Lehm.	0	n	1	0	Crassulaceae J.St.-Hil.
Iz_1_1109	DJI_0071	Setaria viridis (L.) P. Beauv.	0	n	1	0	Poaceae Barnhart
Iz_1_1109	DJI_0071	Silene borysthenica (Gruner) Walters	0	n	1	0	Caryophyllaceae Juss.
Iz_1_1109	DJI_0071	Silene latifolia subsp. alba (Mill.) Greuter & Burdet	0	n	1	0	Caryophyllaceae Juss.
Iz_1_1109	DJI_0071	Silene nutans L.	0	n	1	0	Caryophyllaceae Juss.
Iz_1_1109	DJI_0071	Solanum nigrum L.	0		NA	NA	
Iz_1_1109	DJI_0071	Solidago virgaurea L.	0	n	1	0	Asteraceae Bercht. & J.Presl
Iz_1_1109	DJI_0071	Sorbus aucuparia L.	0	n	1	1	Rosaceae Juss.
Iz_1_1109	DJI_0071	Stachys recta L.	0	n	1	0	Lamiacea Lindl.
Iz_1_1109	DJI_0071	Tanacetum vulgare L.	0	n	1	0	Asteraceae Bercht. & J.Presl
Iz_1_1109	DJI_0071	Thymus pallasianus Heinr.Braun	0	n	1	0	Lamiacea Lindl.
Iz_1_1109	DJI_0071	Tilia cordata Mill.	0	n	1	1	Malvaceae Juss.
Iz_1_1109	DJI_0071	Trifolium alpestre L.	0	n	1	0	Fabaceae Lindl.
Iz_1_1109	DJI_0071	Trifolium arvense L.	0	n	1	0	Fabaceae Lindl.
Iz_1_1109	DJI_0071	Ulmus glabra Huds.	0	n	1	1	Ulmaceae Mirb.
Iz_1_1109	DJI_0071	Verbascum densiflorum Bertol.	0	n	1	0	Scrophulariaceae Juss.
Iz_1_1109	DJI_0071	Veronica austriaca L.	0	n	1	0	Plantaginaceae Juss.
Iz_1_1109	DJI_0071	Veronica incana L.	0	n	1	0	Plantaginaceae Juss.

Iz_1_1109	DJI_0071	Veronica verna L.	0	n	1	0	Plantaginaceae Juss.
Iz_1_1109	DJI_0071	Vincetoxicum hirundinaria Medik	0	n	1	0	Apocynaceae Juss.
Iz_1_1109	DJI_0074	Acer tataricum L.	0	n	1	1	Sapindaceae Juss.
Iz_1_1109	DJI_0074	Achillea millefolium L. s.l.	0	n	1	0	Asteraceae Bercht. & J.Presl
Iz_1_1109	DJI_0074	Allium oleraceum L.	0	n	1	0	Amaryllidaceae J.St.-Hil.
Iz_1_1109	DJI_0074	Ambrosia artemisiifolia L.	0	a	0	0	Asteraceae Bercht. & J.Presl
Iz_1_1109	DJI_0074	Amorpha fruticosa L.	0	a	0	0	Fabaceae Lindl.
Iz_1_1109	DJI_0074	Anthericum ramosum L.	0	n	1	0	Asparagaceae Juss.
Iz_1_1109	DJI_0074	Artemisia marschalliana Spreng.	0	n	1	0	Asteraceae Bercht. & J.Presl
Iz_1_1109	DJI_0074	Asparagus officinalis L.	0	n	1	0	Asparagaceae Juss.
Iz_1_1109	DJI_0074	Astragalus cicer L.	0	n	1	0	Fabaceae Lindl.
Iz_1_1109	DJI_0074	Bassia laniflora (S.G.Gmel.) A.J.Scott	0	n	1	0	Amaranthaceae Juss.
Iz_1_1109	DJI_0074	Berberis vulgaris L.	0	n	1	0	Berberidaceae Juss.
Iz_1_1109	DJI_0074	Betula pendula Roth.	0	n	1	1	Betulaceae Gray
Iz_1_1109	DJI_0074	Betula pendula Roth.	0	n	1	0	Poaceae Barnhart
Iz_1_1109	DJI_0074	Bromus inermis Leyss.	0		NA	NA	
Iz_1_1109	DJI_0074	Bromus squarrosus L.	0	a	0	0	Poaceae Barnhart
Iz_1_1109	DJI_0074	Bromus tectorum L.	0	a	0	0	Poaceae Barnhart
Iz_1_1109	DJI_0074	Calamagrostis epigejos (L.) Roth	0	n	1	0	Poaceae Barnhart
Iz_1_1109	DJI_0074	Carduus acanthoides L.	0	n	1	0	Asteraceae Bercht. & J.Presl
Iz_1_1109	DJI_0074	Carex sp.	0	n	1	0	Cyperaceae Juss.
Iz_1_1109	DJI_0074	Centaurea majorovii Dumbadze	0	n	1	0	Asteraceae Bercht. & J.Presl
Iz_1_1109	DJI_0074	Chamaecytisus ruthenicus (Fisch.ex Wol.) Klaskova	0	n	1	0	Fabaceae Lindl.
Iz_1_1109	DJI_0074	Chelidonium majus L.	0	n	1	0	Papaveraceae Juss.
Iz_1_1109	DJI_0074	Chenopodium betaceum Andrz.	0	n	1	0	Amaranthaceae Juss.
Iz_1_1109	DJI_0074	Chenopodium cf. album	0	n	1	0	Amaranthaceae Juss.
Iz_1_1109	DJI_0074	Chenopodium sp.	0		NA	0	Amaranthaceae Juss.
Iz_1_1109	DJI_0074	Chondrilla juncea L.	0	n	1	0	Asteraceae Bercht. & J.Presl
Iz_1_1109	DJI_0074	Cirsium vulgare (Savi) Ten.	0	n	1	0	Asteraceae Bercht. & J.Presl
Iz_1_1109	DJI_0074	Clinopodium acinos (L.) Kuntze	0	n	1	0	Lamiacea Lindl.
Iz_1_1109	DJI_0074	Convallaria majalis L.	0	n	1	0	Asparagaceae Juss.
Iz_1_1109	DJI_0074	Crataegus rhipidophylla Gand.	0	n	1	1	Rosaceae Juss.
Iz_1_1109	DJI_0074	Crepis foetida L.	0	n	1	0	Asteraceae Bercht. & J.Presl
Iz_1_1109	DJI_0074	Dactylis glomerata L.	0	n	1	0	Poaceae Barnhart
Iz_1_1109	DJI_0074	Dianthus campestris M.Bieb.	0	n	1	0	Caryophyllaceae Juss.
Iz_1_1109	DJI_0074	Digitaria sanguinalis (L.) Scop.	0	a	0	0	Poaceae Barnhart
Iz_1_1109	DJI_0074	Elymus repens (L.) Gould	0	n	1	0	Poaceae Barnhart
Iz_1_1109	DJI_0074	Epilobium tetragonum L.	0	n	1	0	Onagraceae Juss.
Iz_1_1109	DJI_0074	Eragrostis minor Host	0	a	0	0	Poaceae Barnhart
Iz_1_1109	DJI_0074	Erigeron canadensis L.	0	a	0	0	Asteraceae Bercht. & J.Presl
Iz_1_1109	DJI_0074	Euphorbia seguieriana Neck.	0	n	1	0	Euphorbiaceae Juss.
Iz_1_1109	DJI_0074	Fallopia dumetorum (L.) Holub	0	n	1	0	Polygonaceae Juss.
Iz_1_1109	DJI_0074	Festuca beckeri (Hack.) Trautv.	0	n	1	0	Poaceae Barnhart
Iz_1_1109	DJI_0074	Fragaria vesca L.	0	n	1	0	Rosaceae Juss.
Iz_1_1109	DJI_0074	Frangula alnus Mill.	0	n	1	1	Rhamnaceae Juss.
Iz_1_1109	DJI_0074	Fraxinus pennsylvanica Marshall	0	a	0	1	Oleaceae Hoffmanns. & Link
Iz_1_1109	DJI_0074	Galeopsis bifida Boenn.	0	n	1	0	Lamiacea Lindl.
Iz_1_1109	DJI_0074	Galium verum L.	0	n	1	0	Rubiaceae Juss.
Iz_1_1109	DJI_0074	Genista tinctoria L.	0	n	1	0	Fabaceae Lindl.
Iz_1_1109	DJI_0074	Geranium robertianum L.	0	n	1	0	Geraniaceae Juss.
Iz_1_1109	DJI_0074	Geranium sanquineum L.	0	n	1	0	Geraniaceae Juss.
Iz_1_1109	DJI_0074	Gypsophila paniculata L.	0	n	1	0	Caryophyllaceae Juss.
Iz_1_1109	DJI_0074	Helichrysum arenarium (L.) Moench	0	n	1	0	Asteraceae Bercht. & J.Presl
Iz_1_1109	DJI_0074	Herniaria polygama J.Gay	0	n	1	0	Caryophyllaceae Juss.
Iz_1_1109	DJI_0074	Hieracium robustum Fr.	0	n	1	0	Asteraceae Bercht. & J.Presl
Iz_1_1109	DJI_0074	Hieracium umbellatum L.	0	n	1	NA	Asteraceae Bercht. & J.Presl
Iz_1_1109	DJI_0074	Hylotelephium maximum (L.) Holub	0	n	1	0	Crassulaceae J.St.-Hil.
Iz_1_1109	DJI_0074	Jacobaea borysthenica (DC.) B.Nord. & Greuter	0	n	1	0	Asteraceae Bercht. & J.Presl
Iz_1_1109	DJI_0074	Jasione montana L.	0	n	1	0	Campanulaceae Juss.
Iz_1_1109	DJI_0074	Jurinea cyanoides (L.) Rchb.s.l.	0	n	1	0	Asteraceae Bercht. & J.Presl
Iz_1_1109	DJI_0074	Koeleria glauca (Spreng.) DC.	0	n	1	0	Poaceae Barnhart
Iz_1_1109	DJI_0074	Lactuca serriola L.	0.1	a	0	0	Asteraceae Bercht. & J.Presl
Iz_1_1109	DJI_0074	Linaria genistifolia (L.) Mill.	0	n	1	0	Plantaginaceae Juss.
Iz_1_1109	DJI_0074	Lomelosia argentea (L.) Greuter & Burdet	0	n	1	0	Caprifoliaceae Juss.
Iz_1_1109	DJI_0074	Melampyrum pratense L.	0	n	1	0	Orobanchaceae Vent.
Iz_1_1109	DJI_0074	Oenothera biennis L.	0	a	0	0	Onagraceae Juss.
Iz_1_1109	DJI_0074	Pentanema britannica (L.) D.Gut.Larr., Santos-Vicente, Anderb., E.Rico & M.M.Mart.Ort.	0	n	1	0	Asteraceae Bercht. & J.Presl
Iz_1_1109	DJI_0074	Peucedanum oreoselinum (L.) Moench	0	n	1	0	Apiaceae Lindl.
Iz_1_1109	DJI_0074	Peucedanum ruthenicum M.Bieb.	0	n	1	0	Apiaceae Lindl.
Iz_1_1109	DJI_0074	Pilosella echioides (Lumn.) F.W.Schultz & Sch.Bip.	0	n	1	0	Asteraceae Bercht. & J.Presl
Iz_1_1109	DJI_0074	Pilosella officinarum F. Schultz et Sch. Bip.	0	n	1	0	Asteraceae Bercht. & J.Presl
Iz_1_1109	DJI_0074	Pilosella sp.	0		NA	0	Asteraceae Bercht. & J.Presl
Iz_1_1109	DJI_0074	Pinus sylvestris L.	0	n	1	1	Pinaceae Spreng. ex F.Rudolphi
Iz_1_1109	DJI_0074	Poa annua L.	0	n	1	0	Poaceae Barnhart
Iz_1_1109	DJI_0074	Poa nemoralis L.	0	n	1	0	Poaceae Barnhart
Iz_1_1109	DJI_0074	Poa sp.	0		NA	0	Poaceae Barnhart
Iz_1_1109	DJI_0074	Polygonatum odoratum (Mill.) Druce	0	n	1	0	Asparagaceae Juss.
Iz_1_1109	DJI_0074	Polygonum arenarium Waldst. & Kit.	0	n	1	0	Polygonaceae Juss.
Iz_1_1109	DJI_0074	Populus nigra L.	0	n	1	1	Salicaceae Mirb.
Iz_1_1109	DJI_0074	Populus sp.	0	n	1	1	Salicaceae Mirb.
Iz_1_1109	DJI_0074	Populus tremula L.	0	n	1	1	Salicaceae Mirb.
Iz_1_1109	DJI_0074	Populus Ã canescens (Aiton) Sm.	0	n	1	1	Salicaceae Mirb.
Iz_1_1109	DJI_0074	Potentilla argentea L.	0	n	1	0	Rosaceae Juss.
Iz_1_1109	DJI_0074	Psammophiliella muralis (L.) Ikonn.	0	n	1	0	Caryophyllaceae Juss.
Iz_1_1109	DJI_0074	Psephellus sumensis (Kalen.) Greuter	0	n	1	0	Asteraceae Bercht. & J.Presl
Iz_1_1109	DJI_0074	Pulsatilla pratensis (L.) Mill. s.l.	0	n	1	0	Ranunculaceae Juss.
Iz_1_1109	DJI_0074	Pyrus communis L.	0	a	0	1	Rosaceae Juss.
Iz_1_1109	DJI_0074	Quercus robur L.	0	n	1	1	Fagaceae Dumort.
Iz_1_1109	DJI_0074	Robinia pseudoacacia L.	0	a	0	1	Fabaceae Lindl.
Iz_1_1109	DJI_0074	Rosa sp.	0		NA	1	Rosaceae Juss.
Iz_1_1109	DJI_0074	Rubus idaeus L.	0	n	1	0	Rosaceae Juss.
Iz_1_1109	DJI_0074	Rumex acetosella L.	0	n	1	0	Polygonaceae Juss.
Iz_1_1109	DJI_0074	Rumex thyrsiflorus Fingerh.	0	n	1	0	Polygonaceae Juss.
Iz_1_1109	DJI_0074	Salix alba L.	0	n	1	1	Salicaceae Mirb.
Iz_1_1109	DJI_0074	Sambucus nigra L.	0	n	1	1	Viburnaceae Raf.
Iz_1_1109	DJI_0074	Secale sylvestre Host	0	n	1	0	Poaceae Barnhart
Iz_1_1109	DJI_0074	Sempervivum ruthenicum Schnittsp. & C.B. Lehm.	0	n	1	0	Crassulaceae J.St.-Hil.
Iz_1_1109	DJI_0074	Setaria viridis (L.) P. Beauv.	0	n	1	0	Poaceae Barnhart

Iz_1_1109	DJI_0074	Silene borysthenica (Gruner) Walters	0	n	1	0	Caryophyllaceae Juss.
Iz_1_1109	DJI_0074	Silene latifolia subsp. alba (Mill.) Greuter & Burdet	0	n	1	0	Caryophyllaceae Juss.
Iz_1_1109	DJI_0074	Silene nutans L.	0	n	1	0	Caryophyllaceae Juss.
Iz_1_1109	DJI_0074	Solanum nigrum L.	0		NA	NA	
Iz_1_1109	DJI_0074	Solidago virgaurea L.	0	n	1	0	Asteraceae Bercht. & J.Presl
Iz_1_1109	DJI_0074	Sorbus aucuparia L.	0	n	1	1	Rosaceae Juss.
Iz_1_1109	DJI_0074	Stachys recta L.	0	n	1	0	Lamiacea Lindl.
Iz_1_1109	DJI_0074	Tanacetum vulgare L.	0	n	1	0	Asteraceae Bercht. & J.Presl
Iz_1_1109	DJI_0074	Thymus pallasianus Heinr.Braun	0	n	1	0	Lamiacea Lindl.
Iz_1_1109	DJI_0074	Tilia cordata Mill.	0	n	1	1	Malvaceae Juss.
Iz_1_1109	DJI_0074	Trifolium alpestre L.	0	n	1	0	Fabaceae Lindl.
Iz_1_1109	DJI_0074	Trifolium arvense L.	0	n	1	0	Fabaceae Lindl.
Iz_1_1109	DJI_0074	Ulmus glabra Huds.	0	n	1	1	Ulmaceae Mirb.
Iz_1_1109	DJI_0074	Verbascum densiflorum Bertol.	0	n	1	0	Scrophulariaceae Juss.
Iz_1_1109	DJI_0074	Veronica austriaca L.	0	n	1	0	Plantaginaceae Juss.
Iz_1_1109	DJI_0074	Veronica incana L.	0	n	1	0	Plantaginaceae Juss.
Iz_1_1109	DJI_0074	Veronica verna L.	0	n	1	0	Plantaginaceae Juss.
Iz_1_1109	DJI_0074	Vincetoxicum hirundinaria Medik	0	n	1	0	Apocynaceae Juss.
Iz_1_1109	DJI_0076	Acer tataricum L.	0	n	1	1	Sapindaceae Juss.
Iz_1_1109	DJI_0076	Achillea millefolium L. s.l.	0	n	1	0	Asteraceae Bercht. & J.Presl
Iz_1_1109	DJI_0076	Allium oleraceum L.	0	n	1	0	Amaryllidaceae J.St.-Hil.
Iz_1_1109	DJI_0076	Ambrosia artemisiifolia L.	0	a	0	0	Asteraceae Bercht. & J.Presl
Iz_1_1109	DJI_0076	Amorpha fruticosa L.	0	a	0	0	Fabaceae Lindl.
Iz_1_1109	DJI_0076	Anthericum ramosum L.	0	n	1	0	Asparagaceae Juss.
Iz_1_1109	DJI_0076	Artemisia marschalliana Spreng.	0	n	1	0	Asteraceae Bercht. & J.Presl
Iz_1_1109	DJI_0076	Asparagus officinalis L.	0	n	1	0	Asparagaceae Juss.
Iz_1_1109	DJI_0076	Astragalus cicer L.	0	n	1	0	Fabaceae Lindl.
Iz_1_1109	DJI_0076	Bassia laniflora (S.G.Gmel.) A.J.Scott	0	n	1	0	Amaranthaceae Juss.
Iz_1_1109	DJI_0076	Berberis vulgaris L.	0	n	1	0	Berberidaceae Juss.
Iz_1_1109	DJI_0076	Betula pendula Roth.	0	n	1	1	Betulaceae Gray
Iz_1_1109	DJI_0076	Betula pendula Roth.	0	n	1	0	Poaceae Barnhart
Iz_1_1109	DJI_0076	Bromus inermis Leyss.	0		NA	NA	
Iz_1_1109	DJI_0076	Bromus squarrosus L.	0	a	0	0	Poaceae Barnhart
Iz_1_1109	DJI_0076	Bromus tectorum L.	0	a	0	0	Poaceae Barnhart
Iz_1_1109	DJI_0076	Calamagrostis epigejos (L.) Roth	0	n	1	0	Poaceae Barnhart
Iz_1_1109	DJI_0076	Carduus acanthoides L.	0	n	1	0	Asteraceae Bercht. & J.Presl
Iz_1_1109	DJI_0076	Carex sp.	0	n	1	0	Cyperaceae Juss.
Iz_1_1109	DJI_0076	Centaurea majorovii Dumbadze	0	n	1	0	Asteraceae Bercht. & J.Presl
Iz_1_1109	DJI_0076	Chamaecytisus ruthenicus (Fisch.ex Wol.) Klaskova	0	n	1	0	Fabaceae Lindl.
Iz_1_1109	DJI_0076	Chelidonium majus L.	0	n	1	0	Papaveraceae Juss.
Iz_1_1109	DJI_0076	Chenopodium betaceum Andrz.	0.5	n	1	0	Amaranthaceae Juss.
Iz_1_1109	DJI_0076	Chenopodium cf. album	0	n	1	0	Amaranthaceae Juss.
Iz_1_1109	DJI_0076	Chenopodium sp.	0		NA	0	Amaranthaceae Juss.
Iz_1_1109	DJI_0076	Chondrilla juncea L.	0	n	1	0	Asteraceae Bercht. & J.Presl
Iz_1_1109	DJI_0076	Cirsium vulgare (Savi) Ten.	0	n	1	0	Asteraceae Bercht. & J.Presl
Iz_1_1109	DJI_0076	Clinopodium acinos (L.) Kuntze	0	n	1	0	Lamiacea Lindl.
Iz_1_1109	DJI_0076	Convallaria majalis L.	0	n	1	0	Asparagaceae Juss.
Iz_1_1109	DJI_0076	Crataegus rhipidophylla Gand.	0	n	1	1	Rosaceae Juss.
Iz_1_1109	DJI_0076	Crepis foetida L.	0	n	1	0	Asteraceae Bercht. & J.Presl
Iz_1_1109	DJI_0076	Dactylis glomerata L.	0	n	1	0	Poaceae Barnhart
Iz_1_1109	DJI_0076	Dianthus campestris M.Bieb.	0	n	1	0	Caryophyllaceae Juss.
Iz_1_1109	DJI_0076	Digitaria sanguinalis (L.) Scop.	0	a	0	0	Poaceae Barnhart
Iz_1_1109	DJI_0076	Elymus repens (L.) Gould	0	n	1	0	Poaceae Barnhart
Iz_1_1109	DJI_0076	Epilobium tetragonum L.	0	n	1	0	Onagraceae Juss.
Iz_1_1109	DJI_0076	Eragrostis minor Host	0	a	0	0	Poaceae Barnhart
Iz_1_1109	DJI_0076	Erigeron canadensis L.	0	a	0	0	Asteraceae Bercht. & J.Presl
Iz_1_1109	DJI_0076	Euphorbia seguieriana Neck.	0	n	1	0	Euphorbiaceae Juss.
Iz_1_1109	DJI_0076	Fallopia dumetorum (L.) Holub	0	n	1	0	Polygonaceae Juss.
Iz_1_1109	DJI_0076	Festuca beckeri (Hack.) Trautv.	0	n	1	0	Poaceae Barnhart
Iz_1_1109	DJI_0076	Fragaria vesca L.	0	n	1	0	Rosaceae Juss.
Iz_1_1109	DJI_0076	Frangula alnus Mill.	0	n	1	1	Rhamnaceae Juss.
Iz_1_1109	DJI_0076	Fraxinus pennsylvanica Marshall	0	a	0	1	Oleaceae Hoffmanns. & Link
Iz_1_1109	DJI_0076	Galeopsis bifida Boenn.	0	n	1	0	Lamiacea Lindl.
Iz_1_1109	DJI_0076	Galium verum L.	0	n	1	0	Rubiaceae Juss.
Iz_1_1109	DJI_0076	Genista tinctoria L.	0	n	1	0	Fabaceae Lindl.
Iz_1_1109	DJI_0076	Geranium robertianum L.	0	n	1	0	Geraniaceae Juss.
Iz_1_1109	DJI_0076	Geranium sanquineum L.	0	n	1	0	Geraniaceae Juss.
Iz_1_1109	DJI_0076	Gypsophila paniculata L.	0	n	1	0	Caryophyllaceae Juss.
Iz_1_1109	DJI_0076	Helichrysum arenarium (L.) Moench	0	n	1	0	Asteraceae Bercht. & J.Presl
Iz_1_1109	DJI_0076	Herniaria polygama J.Gay	0	n	1	0	Caryophyllaceae Juss.
Iz_1_1109	DJI_0076	Hieracium robustum Fr.	0	n	1	0	Asteraceae Bercht. & J.Presl
Iz_1_1109	DJI_0076	Hieracium umbellatum L.	0	n	1	NA	Asteraceae Bercht. & J.Presl
Iz_1_1109	DJI_0076	Hylotelephium maximum (L.) Holub	0	n	1	0	Crassulaceae J.St.-Hil.
Iz_1_1109	DJI_0076	Jacobaea borysthenica (DC.) B.Nord. & Greuter	0	n	1	0	Asteraceae Bercht. & J.Presl
Iz_1_1109	DJI_0076	Jasione montana L.	0	n	1	0	Campanulaceae Juss.
Iz_1_1109	DJI_0076	Jurinea cyanoides (L.) Rchb.s.l.	0	n	1	0	Asteraceae Bercht. & J.Presl
Iz_1_1109	DJI_0076	Koeleria glauca (Spreng.) DC.	0	n	1	0	Poaceae Barnhart
Iz_1_1109	DJI_0076	Lactuca serriola L.	0	a	0	0	Asteraceae Bercht. & J.Presl
Iz_1_1109	DJI_0076	Linaria genistifolia (L.) Mill.	0	n	1	0	Plantaginaceae Juss.
Iz_1_1109	DJI_0076	Lomelosia argentea (L.) Greuter & Burdet	0	n	1	0	Caprifoliaceae Juss.
Iz_1_1109	DJI_0076	Melampyrum pratense L.	0	n	1	0	Orobanchaceae Vent.
Iz_1_1109	DJI_0076	Oenothera biennis L.	0	a	0	0	Onagraceae Juss.
Iz_1_1109	DJI_0076	Pentanema britannica (L.) D.Gut.Larr., Santos-Vicente, Anderb., E.Rico & M.M.Mart.Ort.	0	n	1	0	Asteraceae Bercht. & J.Presl
Iz_1_1109	DJI_0076	Peucedanum oreoselinum (L.) Moench	0	n	1	0	Apiaceae Lindl.
Iz_1_1109	DJI_0076	Peucedanum ruthenicum M.Bieb.	0	n	1	0	Apiaceae Lindl.
Iz_1_1109	DJI_0076	Pilosella echioides (Lumn.) F.W.Schultz & Sch.Bip.	0	n	1	0	Asteraceae Bercht. & J.Presl
Iz_1_1109	DJI_0076	Pilosella officinarum F. Schultz et Sch. Bip.	0	n	1	0	Asteraceae Bercht. & J.Presl
Iz_1_1109	DJI_0076	Pilosella sp.	0		NA	0	Asteraceae Bercht. & J.Presl
Iz_1_1109	DJI_0076	Pinus sylvestris L.	0	n	1	1	Pinaceae Spreng. ex F.Rudolphi
Iz_1_1109	DJI_0076	Poa annua L.	0	n	1	0	Poaceae Barnhart
Iz_1_1109	DJI_0076	Poa nemoralis L.	0	n	1	0	Poaceae Barnhart
Iz_1_1109	DJI_0076	Poa sp.	0		NA	0	Poaceae Barnhart
Iz_1_1109	DJI_0076	Polygonatum odoratum (Mill.) Druce	0	n	1	0	Asparagaceae Juss.
Iz_1_1109	DJI_0076	Polygonum arenarium Waldst. & Kit.	0	n	1	0	Polygonaceae Juss.
Iz_1_1109	DJI_0076	Populus nigra L.	0	n	1	1	Salicaceae Mirb.
Iz_1_1109	DJI_0076	Populus sp.	0	n	1	1	Salicaceae Mirb.
Iz_1_1109	DJI_0076	Populus tremula L.	0	n	1	1	Salicaceae Mirb.
Iz_1_1109	DJI_0076	Populus Ã canescens (Aiton) Sm.	0	n	1	1	Salicaceae Mirb.

Iz_1_1109	DJI_0076	Potentilla argentea L.	0	n	1	0	Rosaceae Juss.
Iz_1_1109	DJI_0076	Psammophiliella muralis (L.) Ikonn.	0	n	1	0	Caryophyllaceae Juss.
Iz_1_1109	DJI_0076	Psephellus sumensis (Kalen.) Greuter	0	n	1	0	Asteraceae Bercht. & J.Presl
Iz_1_1109	DJI_0076	Pulsatilla pratensis (L.) Mill. s.l.	0	n	1	0	Ranunculaceae Juss.
Iz_1_1109	DJI_0076	Pyrus communis L.	0	a	0	1	Rosaceae Juss.
Iz_1_1109	DJI_0076	Quercus robur L.	0	n	1	1	Fagaceae Dumort.
Iz_1_1109	DJI_0076	Robinia pseudoacacia L.	0	a	0	1	Fabaceae Lindl.
Iz_1_1109	DJI_0076	Rosa sp.	0		NA	1	Rosaceae Juss.
Iz_1_1109	DJI_0076	Rubus idaeus L.	0	n	1	0	Rosaceae Juss.
Iz_1_1109	DJI_0076	Rumex acetosella L.	0	n	1	0	Polygonaceae Juss.
Iz_1_1109	DJI_0076	Rumex thyrsiflorus Fingerh.	0	n	1	0	Polygonaceae Juss.
Iz_1_1109	DJI_0076	Salix alba L.	0	n	1	1	Salicaceae Mirb.
Iz_1_1109	DJI_0076	Sambucus nigra L.	0	n	1	1	Viburnaceae Raf.
Iz_1_1109	DJI_0076	Secale sylvestre Host	0	n	1	0	Poaceae Barnhart
Iz_1_1109	DJI_0076	Sempervivum ruthenicum Schnittsp. & C.B. Lehm.	0	n	1	0	Crassulaceae J.St.-Hil.
Iz_1_1109	DJI_0076	Setaria viridis (L.) P. Beauv.	0	n	1	0	Poaceae Barnhart
Iz_1_1109	DJI_0076	Silene borysthenica (Gruner) Walters	0	n	1	0	Caryophyllaceae Juss.
Iz_1_1109	DJI_0076	Silene latifolia subsp. alba (Mill.) Greuter & Burdet	0	n	1	0	Caryophyllaceae Juss.
Iz_1_1109	DJI_0076	Silene nutans L.	0	n	1	0	Caryophyllaceae Juss.
Iz_1_1109	DJI_0076	Solanum nigrum L.	0		NA	NA	
Iz_1_1109	DJI_0076	Solidago virgaurea L.	0	n	1	0	Asteraceae Bercht. & J.Presl
Iz_1_1109	DJI_0076	Sorbus aucuparia L.	0	n	1	1	Rosaceae Juss.
Iz_1_1109	DJI_0076	Stachys recta L.	0	n	1	0	Lamiacea Lindl.
Iz_1_1109	DJI_0076	Tanacetum vulgare L.	0	n	1	0	Asteraceae Bercht. & J.Presl
Iz_1_1109	DJI_0076	Thymus pallasianus Heinr.Braun	0	n	1	0	Lamiacea Lindl.
Iz_1_1109	DJI_0076	Tilia cordata Mill.	0	n	1	1	Malvaceae Juss.
Iz_1_1109	DJI_0076	Trifolium alpestre L.	0	n	1	0	Fabaceae Lindl.
Iz_1_1109	DJI_0076	Trifolium arvense L.	0	n	1	0	Fabaceae Lindl.
Iz_1_1109	DJI_0076	Ulmus glabra Huds.	0	n	1	1	Ulmaceae Mirb.
Iz_1_1109	DJI_0076	Verbascum densiflorum Bertol.	0	n	1	0	Scrophulariaceae Juss.
Iz_1_1109	DJI_0076	Veronica austriaca L.	0	n	1	0	Plantaginaceae Juss.
Iz_1_1109	DJI_0076	Veronica incana L.	0	n	1	0	Plantaginaceae Juss.
Iz_1_1109	DJI_0076	Veronica verna L.	0	n	1	0	Plantaginaceae Juss.
Iz_1_1109	DJI_0076	Vincetoxicum hirundinaria Medik	0	n	1	0	Apocynaceae Juss.
Iz_1_1109	DJI_0080	Acer tataricum L.	0	n	1	1	Sapindaceae Juss.
Iz_1_1109	DJI_0080	Achillea millefolium L. s.l.	0	n	1	0	Asteraceae Bercht. & J.Presl
Iz_1_1109	DJI_0080	Allium oleraceum L.	0	n	1	0	Amaryllidaceae J.St.-Hil.
Iz_1_1109	DJI_0080	Ambrosia artemisiifolia L.	0	a	0	0	Asteraceae Bercht. & J.Presl
Iz_1_1109	DJI_0080	Amorpha fruticosa L.	0	a	0	0	Fabaceae Lindl.
Iz_1_1109	DJI_0080	Anthericum ramosum L.	0	n	1	0	Asparagaceae Juss.
Iz_1_1109	DJI_0080	Artemisia marschalliana Spreng.	0	n	1	0	Asteraceae Bercht. & J.Presl
Iz_1_1109	DJI_0080	Asparagus officinalis L.	0	n	1	0	Asparagaceae Juss.
Iz_1_1109	DJI_0080	Astragalus cicer L.	0	n	1	0	Fabaceae Lindl.
Iz_1_1109	DJI_0080	Bassia laniflora (S.G.Gmel.) A.J.Scott	0	n	1	0	Amaranthaceae Juss.
Iz_1_1109	DJI_0080	Berberis vulgaris L.	0	n	1	0	Berberidaceae Juss.
Iz_1_1109	DJI_0080	Betula pendula Roth.	0	n	1	1	Betulaceae Gray
Iz_1_1109	DJI_0080	Betula pendula Roth.	0	n	1	0	Poaceae Barnhart
Iz_1_1109	DJI_0080	Bromus inermis Leyss.	0		NA	NA	
Iz_1_1109	DJI_0080	Bromus squarrosus L.	0	a	0	0	Poaceae Barnhart
Iz_1_1109	DJI_0080	Bromus tectorum L.	0	a	0	0	Poaceae Barnhart
Iz_1_1109	DJI_0080	Calamagrostis epigejos (L.) Roth	0.5	n	1	0	Poaceae Barnhart
Iz_1_1109	DJI_0080	Carduus acanthoides L.	0	n	1	0	Asteraceae Bercht. & J.Presl
Iz_1_1109	DJI_0080	Carex sp.	0	n	1	0	Cyperaceae Juss.
Iz_1_1109	DJI_0080	Centaurea majorovii Dumbadze	0	n	1	0	Asteraceae Bercht. & J.Presl
Iz_1_1109	DJI_0080	Chamaecytisus ruthenicus (Fisch.ex Wol.) Klaskova	0	n	1	0	Fabaceae Lindl.
Iz_1_1109	DJI_0080	Chelidonium majus L.	0	n	1	0	Papaveraceae Juss.
Iz_1_1109	DJI_0080	Chenopodium betaceum Andrz.	0	n	1	0	Amaranthaceae Juss.
Iz_1_1109	DJI_0080	Chenopodium cf. album	0	n	1	0	Amaranthaceae Juss.
Iz_1_1109	DJI_0080	Chenopodium sp.	0		NA	0	Amaranthaceae Juss.
Iz_1_1109	DJI_0080	Chondrilla juncea L.	0	n	1	0	Asteraceae Bercht. & J.Presl
Iz_1_1109	DJI_0080	Cirsium vulgare (Savi) Ten.	0	n	1	0	Asteraceae Bercht. & J.Presl
Iz_1_1109	DJI_0080	Clinopodium acinos (L.) Kuntze	0	n	1	0	Lamiacea Lindl.
Iz_1_1109	DJI_0080	Convallaria majalis L.	0	n	1	0	Asparagaceae Juss.
Iz_1_1109	DJI_0080	Crataegus rhipidophylla Gand.	0	n	1	1	Rosaceae Juss.
Iz_1_1109	DJI_0080	Crepis foetida L.	0	n	1	0	Asteraceae Bercht. & J.Presl
Iz_1_1109	DJI_0080	Dactylis glomerata L.	0	n	1	0	Poaceae Barnhart
Iz_1_1109	DJI_0080	Dianthus campestris M.Bieb.	0	n	1	0	Caryophyllaceae Juss.
Iz_1_1109	DJI_0080	Digitaria sanguinalis (L.) Scop.	0	a	0	0	Poaceae Barnhart
Iz_1_1109	DJI_0080	Elymus repens (L.) Gould	0	n	1	0	Poaceae Barnhart
Iz_1_1109	DJI_0080	Epilobium tetragonum L.	0	n	1	0	Onagraceae Juss.
Iz_1_1109	DJI_0080	Eragrostis minor Host	0	a	0	0	Poaceae Barnhart
Iz_1_1109	DJI_0080	Erigeron canadensis L.	0	a	0	0	Asteraceae Bercht. & J.Presl
Iz_1_1109	DJI_0080	Euphorbia seguieriana Neck.	0	n	1	0	Euphorbiaceae Juss.
Iz_1_1109	DJI_0080	Fallopia dumetorum (L.) Holub	0	n	1	0	Polygonaceae Juss.
Iz_1_1109	DJI_0080	Festuca beckeri (Hack.) Trautv.	0	n	1	0	Poaceae Barnhart
Iz_1_1109	DJI_0080	Fragaria vesca L.	0	n	1	0	Rosaceae Juss.
Iz_1_1109	DJI_0080	Frangula alnus Mill.	0	n	1	1	Rhamnaceae Juss.
Iz_1_1109	DJI_0080	Fraxinus pennsylvanica Marshall	0	a	0	1	Oleaceae Hoffmanns. & Link
Iz_1_1109	DJI_0080	Galeopsis bifida Boenn.	0	n	1	0	Lamiacea Lindl.
Iz_1_1109	DJI_0080	Galium verum L.	0	n	1	0	Rubiaceae Juss.
Iz_1_1109	DJI_0080	Genista tinctoria L.	0	n	1	0	Fabaceae Lindl.
Iz_1_1109	DJI_0080	Geranium robertianum L.	0	n	1	0	Geraniaceae Juss.
Iz_1_1109	DJI_0080	Geranium sanquineum L.	0	n	1	0	Geraniaceae Juss.
Iz_1_1109	DJI_0080	Gypsophila paniculata L.	0	n	1	0	Caryophyllaceae Juss.
Iz_1_1109	DJI_0080	Helichrysum arenarium (L.) Moench	0	n	1	0	Asteraceae Bercht. & J.Presl
Iz_1_1109	DJI_0080	Herniaria polygama J.Gay	0	n	1	0	Caryophyllaceae Juss.
Iz_1_1109	DJI_0080	Hieracium robustum Fr.	0	n	1	0	Asteraceae Bercht. & J.Presl
Iz_1_1109	DJI_0080	Hieracium umbellatum L.	0	n	1	NA	Asteraceae Bercht. & J.Presl
Iz_1_1109	DJI_0080	Hylotelephium maximum (L.) Holub	0	n	1	0	Crassulaceae J.St.-Hil.
Iz_1_1109	DJI_0080	Jacobaea borysthenica (DC.) B.Nord. & Greuter	0	n	1	0	Asteraceae Bercht. & J.Presl
Iz_1_1109	DJI_0080	Jasione montana L.	0	n	1	0	Campanulaceae Juss.
Iz_1_1109	DJI_0080	Jurinea cyanoides (L.) Rchb.s.l.	0	n	1	0	Asteraceae Bercht. & J.Presl
Iz_1_1109	DJI_0080	Koeleria glauca (Spreng.) DC.	0	n	1	0	Poaceae Barnhart
Iz_1_1109	DJI_0080	Lactuca serriola L.	0	a	0	0	Asteraceae Bercht. & J.Presl
Iz_1_1109	DJI_0080	Linaria genistifolia (L.) Mill.	0	n	1	0	Plantaginaceae Juss.
Iz_1_1109	DJI_0080	Lomelosia argentea (L.) Greuter & Burdet	0	n	1	0	Caprifoliaceae Juss.
Iz_1_1109	DJI_0080	Melampyrum pratense L.	0	n	1	0	Orobanchaceae Vent.
Iz_1_1109	DJI_0080	Oenothera biennis L.	0	a	0	0	Onagraceae Juss.

Iz_1_1109	DJI_0080	Pentanema britannica (L.) D.Gut.Larr., Santos-Vicente, Anderb., E.Rico & M.M.Mart.Ort.	0	n	1	0	Asteraceae Bercht. & J.Presl
Iz_1_1109	DJI_0080	Peucedanum oreoselinum (L.) Moench	0	n	1	0	Apiaceae Lindl.
Iz_1_1109	DJI_0080	Peucedanum ruthenicum M.Bieb.	0	n	1	0	Apiaceae Lindl.
Iz_1_1109	DJI_0080	Pilosella echioides (Lumn.) F.W.Schultz & Sch.Bip.	0	n	1	0	Asteraceae Bercht. & J.Presl
Iz_1_1109	DJI_0080	Pilosella officinarum F. Schultz et Sch. Bip.	0	n	1	0	Asteraceae Bercht. & J.Presl
Iz_1_1109	DJI_0080	Pilosella sp.	0		NA	0	Asteraceae Bercht. & J.Presl
Iz_1_1109	DJI_0080	Pinus sylvestris L.	0	n	1	1	Pinaceae Spreng. ex F.Rudolphi
Iz_1_1109	DJI_0080	Poa annua L.	0	n	1	0	Poaceae Barnhart
Iz_1_1109	DJI_0080	Poa nemoralis L.	0	n	1	0	Poaceae Barnhart
Iz_1_1109	DJI_0080	Poa sp.	0		NA	0	Poaceae Barnhart
Iz_1_1109	DJI_0080	Polygonatum odoratum (Mill.) Druce	0	n	1	0	Asparagaceae Juss.
Iz_1_1109	DJI_0080	Polygonum arenarium Waldst. & Kit.	0	n	1	0	Polygonaceae Juss.
Iz_1_1109	DJI_0080	Populus nigra L.	0	n	1	1	Salicaceae Mirb.
Iz_1_1109	DJI_0080	Populus sp.	0	n	1	1	Salicaceae Mirb.
Iz_1_1109	DJI_0080	Populus tremula L.	0	n	1	1	Salicaceae Mirb.
Iz_1_1109	DJI_0080	Populus Ã canescens (Aiton) Sm.	0	n	1	1	Salicaceae Mirb.
Iz_1_1109	DJI_0080	Potentilla argentea L.	0	n	1	0	Rosaceae Juss.
Iz_1_1109	DJI_0080	Psammophiliella muralis (L.) Ikonn.	0	n	1	0	Caryophyllaceae Juss.
Iz_1_1109	DJI_0080	Psephellus sumensis (Kalen.) Greuter	0	n	1	0	Asteraceae Bercht. & J.Presl
Iz_1_1109	DJI_0080	Pulsatilla pratensis (L.) Mill. s.l.	0	n	1	0	Ranunculaceae Juss.
Iz_1_1109	DJI_0080	Pyrus communis L.	0	a	0	1	Rosaceae Juss.
Iz_1_1109	DJI_0080	Quercus robur L.	0	n	1	1	Fagaceae Dumort.
Iz_1_1109	DJI_0080	Robinia pseudoacacia L.	0	a	0	1	Fabaceae Lindl.
Iz_1_1109	DJI_0080	Rosa sp.	0		NA	1	Rosaceae Juss.
Iz_1_1109	DJI_0080	Rubus idaeus L.	0	n	1	0	Rosaceae Juss.
Iz_1_1109	DJI_0080	Rumex acetosella L.	0	n	1	0	Polygonaceae Juss.
Iz_1_1109	DJI_0080	Rumex thyrsiflorus Fingerh.	0	n	1	0	Polygonaceae Juss.
Iz_1_1109	DJI_0080	Salix alba L.	0	n	1	1	Salicaceae Mirb.
Iz_1_1109	DJI_0080	Sambucus nigra L.	0	n	1	1	Viburnaceae Raf.
Iz_1_1109	DJI_0080	Secale sylvestre Host	0	n	1	0	Poaceae Barnhart
Iz_1_1109	DJI_0080	Sempervivum ruthenicum Schnittsp. & C.B. Lehm.	0	n	1	0	Crassulaceae J.St.-Hil.
Iz_1_1109	DJI_0080	Setaria viridis (L.) P. Beauv.	0	n	1	0	Poaceae Barnhart
Iz_1_1109	DJI_0080	Silene borysthenica (Gruner) Walters	0	n	1	0	Caryophyllaceae Juss.
Iz_1_1109	DJI_0080	Silene latifolia subsp. alba (Mill.) Greuter & Burdet	0	n	1	0	Caryophyllaceae Juss.
Iz_1_1109	DJI_0080	Silene nutans L.	0	n	1	0	Caryophyllaceae Juss.
Iz_1_1109	DJI_0080	Solanum nigrum L.	0		NA	NA	
Iz_1_1109	DJI_0080	Solidago virgaurea L.	0	n	1	0	Asteraceae Bercht. & J.Presl
Iz_1_1109	DJI_0080	Sorbus aucuparia L.	0	n	1	1	Rosaceae Juss.
Iz_1_1109	DJI_0080	Stachys recta L.	0	n	1	0	Lamiacea Lindl.
Iz_1_1109	DJI_0080	Tanacetum vulgare L.	0	n	1	0	Asteraceae Bercht. & J.Presl
Iz_1_1109	DJI_0080	Thymus pallasianus Heinr.Braun	0	n	1	0	Lamiacea Lindl.
Iz_1_1109	DJI_0080	Tilia cordata Mill.	0	n	1	1	Malvaceae Juss.
Iz_1_1109	DJI_0080	Trifolium alpestre L.	0	n	1	0	Fabaceae Lindl.
Iz_1_1109	DJI_0080	Trifolium arvense L.	0	n	1	0	Fabaceae Lindl.
Iz_1_1109	DJI_0080	Ulmus glabra Huds.	0	n	1	1	Ulmaceae Mirb.
Iz_1_1109	DJI_0080	Verbascum densiflorum Bertol.	0	n	1	0	Scrophulariaceae Juss.
Iz_1_1109	DJI_0080	Veronica austriaca L.	0	n	1	0	Plantaginaceae Juss.
Iz_1_1109	DJI_0080	Veronica incana L.	0	n	1	0	Plantaginaceae Juss.
Iz_1_1109	DJI_0080	Veronica verna L.	0	n	1	0	Plantaginaceae Juss.
Iz_1_1109	DJI_0080	Vincetoxicum hirundinaria Medik	0	n	1	0	Apocynaceae Juss.
Iz_1_1109	DJI_0083	Acer tataricum L.	0	n	1	1	Sapindaceae Juss.
Iz_1_1109	DJI_0083	Achillea millefolium L. s.l.	0	n	1	0	Asteraceae Bercht. & J.Presl
Iz_1_1109	DJI_0083	Allium oleraceum L.	0	n	1	0	Amaryllidaceae J.St.-Hil.
Iz_1_1109	DJI_0083	Ambrosia artemisiifolia L.	0	a	0	0	Asteraceae Bercht. & J.Presl
Iz_1_1109	DJI_0083	Amorpha fruticosa L.	0	a	0	0	Fabaceae Lindl.
Iz_1_1109	DJI_0083	Anthericum ramosum L.	0	n	1	0	Asparagaceae Juss.
Iz_1_1109	DJI_0083	Artemisia marschalliana Spreng.	0	n	1	0	Asteraceae Bercht. & J.Presl
Iz_1_1109	DJI_0083	Asparagus officinalis L.	0	n	1	0	Asparagaceae Juss.
Iz_1_1109	DJI_0083	Astragalus cicer L.	0	n	1	0	Fabaceae Lindl.
Iz_1_1109	DJI_0083	Bassia laniflora (S.G.Gmel.) A.J.Scott	0	n	1	0	Amaranthaceae Juss.
Iz_1_1109	DJI_0083	Berberis vulgaris L.	0	n	1	0	Berberidaceae Juss.
Iz_1_1109	DJI_0083	Betula pendula Roth.	0	n	1	1	Betulaceae Gray
Iz_1_1109	DJI_0083	Betula pendula Roth.	0	n	1	0	Poaceae Barnhart
Iz_1_1109	DJI_0083	Bromus inermis Leyss.	0		NA	NA	
Iz_1_1109	DJI_0083	Bromus squarrosus L.	0	a	0	0	Poaceae Barnhart
Iz_1_1109	DJI_0083	Bromus tectorum L.	0	a	0	0	Poaceae Barnhart
Iz_1_1109	DJI_0083	Calamagrostis epigejos (L.) Roth	0.5	n	1	0	Poaceae Barnhart
Iz_1_1109	DJI_0083	Carduus acanthoides L.	0	n	1	0	Asteraceae Bercht. & J.Presl
Iz_1_1109	DJI_0083	Carex sp.	0	n	1	0	Cyperaceae Juss.
Iz_1_1109	DJI_0083	Centaurea majorovii Dumbadze	0	n	1	0	Asteraceae Bercht. & J.Presl
Iz_1_1109	DJI_0083	Chamaecytisus ruthenicus (Fisch.ex Wol.) Klaskova	0	n	1	0	Fabaceae Lindl.
Iz_1_1109	DJI_0083	Chelidonium majus L.	0	n	1	0	Papaveraceae Juss.
Iz_1_1109	DJI_0083	Chenopodium betaceum Andrz.	0	n	1	0	Amaranthaceae Juss.
Iz_1_1109	DJI_0083	Chenopodium cf. album	0	n	1	0	Amaranthaceae Juss.
Iz_1_1109	DJI_0083	Chenopodium sp.	0		NA	0	Amaranthaceae Juss.
Iz_1_1109	DJI_0083	Chondrilla juncea L.	0	n	1	0	Asteraceae Bercht. & J.Presl
Iz_1_1109	DJI_0083	Cirsium vulgare (Savi) Ten.	0	n	1	0	Asteraceae Bercht. & J.Presl
Iz_1_1109	DJI_0083	Clinopodium acinos (L.) Kuntze	0	n	1	0	Lamiacea Lindl.
Iz_1_1109	DJI_0083	Convallaria majalis L.	0	n	1	0	Asparagaceae Juss.
Iz_1_1109	DJI_0083	Crataegus rhipidophylla Gand.	0	n	1	1	Rosaceae Juss.
Iz_1_1109	DJI_0083	Crepis foetida L.	0	n	1	0	Asteraceae Bercht. & J.Presl
Iz_1_1109	DJI_0083	Dactylis glomerata L.	0	n	1	0	Poaceae Barnhart
Iz_1_1109	DJI_0083	Dianthus campestris M.Bieb.	0	n	1	0	Caryophyllaceae Juss.
Iz_1_1109	DJI_0083	Digitaria sanguinalis (L.) Scop.	0	a	0	0	Poaceae Barnhart
Iz_1_1109	DJI_0083	Elymus repens (L.) Gould	0	n	1	0	Poaceae Barnhart
Iz_1_1109	DJI_0083	Epilobium tetragonum L.	0	n	1	0	Onagraceae Juss.
Iz_1_1109	DJI_0083	Eragrostis minor Host	0	a	0	0	Poaceae Barnhart
Iz_1_1109	DJI_0083	Erigeron canadensis L.	0.2	a	0	0	Asteraceae Bercht. & J.Presl
Iz_1_1109	DJI_0083	Euphorbia seguieriana Neck.	0	n	1	0	Euphorbiaceae Juss.
Iz_1_1109	DJI_0083	Fallopia dumetorum (L.) Holub	0	n	1	0	Polygonaceae Juss.
Iz_1_1109	DJI_0083	Festuca beckeri (Hack.) Trautv.	0	n	1	0	Poaceae Barnhart
Iz_1_1109	DJI_0083	Fragaria vesca L.	0	n	1	0	Rosaceae Juss.
Iz_1_1109	DJI_0083	Frangula alnus Mill.	0	n	1	1	Rhamnaceae Juss.
Iz_1_1109	DJI_0083	Fraxinus pennsylvanica Marshall	0	a	0	1	Oleaceae Hoffmanns. & Link
Iz_1_1109	DJI_0083	Galeopsis bifida Boenn.	0	n	1	0	Lamiacea Lindl.
Iz_1_1109	DJI_0083	Galium verum L.	0	n	1	0	Rubiaceae Juss.
Iz_1_1109	DJI_0083	Genista tinctoria L.	0	n	1	0	Fabaceae Lindl.
Iz_1_1109	DJI_0083	Geranium robertianum L.	0	n	1	0	Geraniaceae Juss.

Iz_1_1109	DJI_0083	Geranium sanquineum L.	0	n	1	0	Geraniaceae Juss.
Iz_1_1109	DJI_0083	Gypsophila paniculata L.	0	n	1	0	Caryophyllaceae Juss.
Iz_1_1109	DJI_0083	Helichrysum arenarium (L.) Moench	0	n	1	0	Asteraceae Bercht. & J.Presl
Iz_1_1109	DJI_0083	Herniaria polygama J.Gay	0	n	1	0	Caryophyllaceae Juss.
Iz_1_1109	DJI_0083	Hieracium robustum Fr.	0	n	1	0	Asteraceae Bercht. & J.Presl
Iz_1_1109	DJI_0083	Hieracium umbellatum L.	0	n	1	NA	Asteraceae Bercht. & J.Presl
Iz_1_1109	DJI_0083	Hylotelephium maximum (L.) Holub	0	n	1	0	Crassulaceae J.St.-Hil.
Iz_1_1109	DJI_0083	Jacobaea borysthenica (DC.) B.Nord. & Greuter	0	n	1	0	Asteraceae Bercht. & J.Presl
Iz_1_1109	DJI_0083	Jasione montana L.	0	n	1	0	Campanulaceae Juss.
Iz_1_1109	DJI_0083	Jurinea cyanoides (L.) Rchb.s.l.	0	n	1	0	Asteraceae Bercht. & J.Presl
Iz_1_1109	DJI_0083	Koeleria glauca (Spreng.) DC.	0	n	1	0	Poaceae Barnhart
Iz_1_1109	DJI_0083	Lactuca serriola L.	0	a	0	0	Asteraceae Bercht. & J.Presl
Iz_1_1109	DJI_0083	Linaria genistifolia (L.) Mill.	0	n	1	0	Plantaginaceae Juss.
Iz_1_1109	DJI_0083	Lomelosia argentea (L.) Greuter & Burdet	0	n	1	0	Caprifoliaceae Juss.
Iz_1_1109	DJI_0083	Melampyrum pratense L.	0	n	1	0	Orobanchaceae Vent.
Iz_1_1109	DJI_0083	Oenothera biennis L.	0	a	0	0	Onagraceae Juss.
Iz_1_1109	DJI_0083	Pentanema britannica (L.) D.Gut.Larr., Santos-Vicente, Anderb., E.Rico & M.M.Mart.Ort.	0	n	1	0	Asteraceae Bercht. & J.Presl
Iz_1_1109	DJI_0083	Peucedanum oreoselinum (L.) Moench	0	n	1	0	Apiaceae Lindl.
Iz_1_1109	DJI_0083	Peucedanum ruthenicum M.Bieb.	0	n	1	0	Apiaceae Lindl.
Iz_1_1109	DJI_0083	Pilosella echioides (Lumn.) F.W.Schultz & Sch.Bip.	0	n	1	0	Asteraceae Bercht. & J.Presl
Iz_1_1109	DJI_0083	Pilosella officinarum F. Schultz et Sch. Bip.	0	n	1	0	Asteraceae Bercht. & J.Presl
Iz_1_1109	DJI_0083	Pilosella sp.	0		NA	0	Asteraceae Bercht. & J.Presl
Iz_1_1109	DJI_0083	Pinus sylvestris L.	0	n	1	1	Pinaceae Spreng. ex F.Rudolphi
Iz_1_1109	DJI_0083	Poa annua L.	0	n	1	0	Poaceae Barnhart
Iz_1_1109	DJI_0083	Poa nemoralis L.	0	n	1	0	Poaceae Barnhart
Iz_1_1109	DJI_0083	Poa sp.	0		NA	0	Poaceae Barnhart
Iz_1_1109	DJI_0083	Polygonatum odoratum (Mill.) Druce	0	n	1	0	Asparagaceae Juss.
Iz_1_1109	DJI_0083	Polygonum arenarium Waldst. & Kit.	0	n	1	0	Polygonaceae Juss.
Iz_1_1109	DJI_0083	Populus nigra L.	0	n	1	1	Salicaceae Mirb.
Iz_1_1109	DJI_0083	Populus sp.	0	n	1	1	Salicaceae Mirb.
Iz_1_1109	DJI_0083	Populus tremula L.	0	n	1	1	Salicaceae Mirb.
Iz_1_1109	DJI_0083	Populus Ã canescens (Aiton) Sm.	0	n	1	1	Salicaceae Mirb.
Iz_1_1109	DJI_0083	Potentilla argentea L.	0	n	1	0	Rosaceae Juss.
Iz_1_1109	DJI_0083	Psammophiliella muralis (L.) Ikonn.	0	n	1	0	Caryophyllaceae Juss.
Iz_1_1109	DJI_0083	Psephellus sumensis (Kalen.) Greuter	0	n	1	0	Asteraceae Bercht. & J.Presl
Iz_1_1109	DJI_0083	Pulsatilla pratensis (L.) Mill. s.l.	0	n	1	0	Ranunculaceae Juss.
Iz_1_1109	DJI_0083	Pyrus communis L.	0	a	0	1	Rosaceae Juss.
Iz_1_1109	DJI_0083	Quercus robur L.	0	n	1	1	Fagaceae Dumort.
Iz_1_1109	DJI_0083	Robinia pseudoacacia L.	0	a	0	1	Fabaceae Lindl.
Iz_1_1109	DJI_0083	Rosa sp.	0		NA	1	Rosaceae Juss.
Iz_1_1109	DJI_0083	Rubus idaeus L.	0	n	1	0	Rosaceae Juss.
Iz_1_1109	DJI_0083	Rumex acetosella L.	0	n	1	0	Polygonaceae Juss.
Iz_1_1109	DJI_0083	Rumex thyrsiflorus Fingerh.	0	n	1	0	Polygonaceae Juss.
Iz_1_1109	DJI_0083	Salix alba L.	0	n	1	1	Salicaceae Mirb.
Iz_1_1109	DJI_0083	Sambucus nigra L.	0	n	1	1	Viburnaceae Raf.
Iz_1_1109	DJI_0083	Secale sylvestre Host	0	n	1	0	Poaceae Barnhart
Iz_1_1109	DJI_0083	Sempervivum ruthenicum Schnittsp. & C.B. Lehm.	0	n	1	0	Crassulaceae J.St.-Hil.
Iz_1_1109	DJI_0083	Setaria viridis (L.) P. Beauv.	0	n	1	0	Poaceae Barnhart
Iz_1_1109	DJI_0083	Silene borysthenica (Gruner) Walters	0	n	1	0	Caryophyllaceae Juss.
Iz_1_1109	DJI_0083	Silene latifolia subsp. alba (Mill.) Greuter & Burdet	0	n	1	0	Caryophyllaceae Juss.
Iz_1_1109	DJI_0083	Silene nutans L.	0	n	1	0	Caryophyllaceae Juss.
Iz_1_1109	DJI_0083	Solanum nigrum L.	0		NA	NA	
Iz_1_1109	DJI_0083	Solidago virgaurea L.	0	n	1	0	Asteraceae Bercht. & J.Presl
Iz_1_1109	DJI_0083	Sorbus aucuparia L.	0	n	1	1	Rosaceae Juss.
Iz_1_1109	DJI_0083	Stachys recta L.	0	n	1	0	Lamiacea Lindl.
Iz_1_1109	DJI_0083	Tanacetum vulgare L.	0	n	1	0	Asteraceae Bercht. & J.Presl
Iz_1_1109	DJI_0083	Thymus pallasianus Heinr.Braun	0	n	1	0	Lamiacea Lindl.
Iz_1_1109	DJI_0083	Tilia cordata Mill.	0	n	1	1	Malvaceae Juss.
Iz_1_1109	DJI_0083	Trifolium alpestre L.	0	n	1	0	Fabaceae Lindl.
Iz_1_1109	DJI_0083	Trifolium arvense L.	0	n	1	0	Fabaceae Lindl.
Iz_1_1109	DJI_0083	Ulmus glabra Huds.	0	n	1	1	Ulmaceae Mirb.
Iz_1_1109	DJI_0083	Verbascum densiflorum Bertol.	0	n	1	0	Scrophulariaceae Juss.
Iz_1_1109	DJI_0083	Veronica austriaca L.	0	n	1	0	Plantaginaceae Juss.
Iz_1_1109	DJI_0083	Veronica incana L.	0	n	1	0	Plantaginaceae Juss.
Iz_1_1109	DJI_0083	Veronica verna L.	0	n	1	0	Plantaginaceae Juss.
Iz_1_1109	DJI_0083	Vincetoxicum hirundinaria Medik	0	n	1	0	Apocynaceae Juss.
Iz_1_1109	DJI_0090	Acer tataricum L.	0	n	1	1	Sapindaceae Juss.
Iz_1_1109	DJI_0090	Achillea millefolium L. s.l.	0	n	1	0	Asteraceae Bercht. & J.Presl
Iz_1_1109	DJI_0090	Allium oleraceum L.	0	n	1	0	Amaryllidaceae J.St.-Hil.
Iz_1_1109	DJI_0090	Ambrosia artemisiifolia L.	0	a	0	0	Asteraceae Bercht. & J.Presl
Iz_1_1109	DJI_0090	Amorpha fruticosa L.	0	a	0	0	Fabaceae Lindl.
Iz_1_1109	DJI_0090	Anthericum ramosum L.	0	n	1	0	Asparagaceae Juss.
Iz_1_1109	DJI_0090	Artemisia marschalliana Spreng.	0	n	1	0	Asteraceae Bercht. & J.Presl
Iz_1_1109	DJI_0090	Asparagus officinalis L.	0	n	1	0	Asparagaceae Juss.
Iz_1_1109	DJI_0090	Astragalus cicer L.	0	n	1	0	Fabaceae Lindl.
Iz_1_1109	DJI_0090	Bassia laniflora (S.G.Gmel.) A.J.Scott	0	n	1	0	Amaranthaceae Juss.
Iz_1_1109	DJI_0090	Berberis vulgaris L.	0	n	1	0	Berberidaceae Juss.
Iz_1_1109	DJI_0090	Betula pendula Roth.	0	n	1	1	Betulaceae Gray
Iz_1_1109	DJI_0090	Betula pendula Roth.	0	n	1	0	Poaceae Barnhart
Iz_1_1109	DJI_0090	Bromus inermis Leyss.	0		NA	NA	
Iz_1_1109	DJI_0090	Bromus squarrosus L.	0	a	0	0	Poaceae Barnhart
Iz_1_1109	DJI_0090	Bromus tectorum L.	0	a	0	0	Poaceae Barnhart
Iz_1_1109	DJI_0090	Calamagrostis epigejos (L.) Roth	0	n	1	0	Poaceae Barnhart
Iz_1_1109	DJI_0090	Carduus acanthoides L.	0	n	1	0	Asteraceae Bercht. & J.Presl
Iz_1_1109	DJI_0090	Carex sp.	0	n	1	0	Cyperaceae Juss.
Iz_1_1109	DJI_0090	Centaurea majorovii Dumbadze	0	n	1	0	Asteraceae Bercht. & J.Presl
Iz_1_1109	DJI_0090	Chamaecytisus ruthenicus (Fisch.ex Wol.) Klaskova	0	n	1	0	Fabaceae Lindl.
Iz_1_1109	DJI_0090	Chelidonium majus L.	0	n	1	0	Papaveraceae Juss.
Iz_1_1109	DJI_0090	Chenopodium betaceum Andrz.	0	n	1	0	Amaranthaceae Juss.
Iz_1_1109	DJI_0090	Chenopodium cf. album	0	n	1	0	Amaranthaceae Juss.
Iz_1_1109	DJI_0090	Chenopodium sp.	0		NA	0	Amaranthaceae Juss.
Iz_1_1109	DJI_0090	Chondrilla juncea L.	0	n	1	0	Asteraceae Bercht. & J.Presl
Iz_1_1109	DJI_0090	Cirsium vulgare (Savi) Ten.	0	n	1	0	Asteraceae Bercht. & J.Presl
Iz_1_1109	DJI_0090	Clinopodium acinos (L.) Kuntze	0	n	1	0	Lamiacea Lindl.
Iz_1_1109	DJI_0090	Convallaria majalis L.	0	n	1	0	Asparagaceae Juss.
Iz_1_1109	DJI_0090	Crataegus rhipidophylla Gand.	0	n	1	1	Rosaceae Juss.
Iz_1_1109	DJI_0090	Crepis foetida L.	0	n	1	0	Asteraceae Bercht. & J.Presl
Iz_1_1109	DJI_0090	Dactylis glomerata L.	0	n	1	0	Poaceae Barnhart

Iz_1_1109	DJI_0090	Dianthus campestris M.Bieb.	0	n	1	0	Caryophyllaceae Juss.
Iz_1_1109	DJI_0090	Digitaria sanguinalis (L.) Scop.	0	a	0	0	Poaceae Barnhart
Iz_1_1109	DJI_0090	Elymus repens (L.) Gould	0	n	1	0	Poaceae Barnhart
Iz_1_1109	DJI_0090	Epilobium tetragonum L.	0	n	1	0	Onagraceae Juss.
Iz_1_1109	DJI_0090	Eragrostis minor Host	0	a	0	0	Poaceae Barnhart
Iz_1_1109	DJI_0090	Erigeron canadensis L.	0.3	a	0	0	Asteraceae Bercht. & J.Presl
Iz_1_1109	DJI_0090	Euphorbia seguieriana Neck.	0	n	1	0	Euphorbiaceae Juss.
Iz_1_1109	DJI_0090	Fallopia dumetorum (L.) Holub	0	n	1	0	Polygonaceae Juss.
Iz_1_1109	DJI_0090	Festuca beckeri (Hack.) Trautv.	0	n	1	0	Poaceae Barnhart
Iz_1_1109	DJI_0090	Fragaria vesca L.	0	n	1	0	Rosaceae Juss.
Iz_1_1109	DJI_0090	Frangula alnus Mill.	0	n	1	1	Rhamnaceae Juss.
Iz_1_1109	DJI_0090	Fraxinus pennsylvanica Marshall	0	a	0	1	Oleaceae Hoffmanns. & Link
Iz_1_1109	DJI_0090	Galeopsis bifida Boenn.	0	n	1	0	Lamiacea Lindl.
Iz_1_1109	DJI_0090	Galium verum L.	0	n	1	0	Rubiaceae Juss.
Iz_1_1109	DJI_0090	Genista tinctoria L.	0	n	1	0	Fabaceae Lindl.
Iz_1_1109	DJI_0090	Geranium robertianum L.	0	n	1	0	Geraniaceae Juss.
Iz_1_1109	DJI_0090	Geranium sanquineum L.	0	n	1	0	Geraniaceae Juss.
Iz_1_1109	DJI_0090	Gypsophila paniculata L.	0	n	1	0	Caryophyllaceae Juss.
Iz_1_1109	DJI_0090	Helichrysum arenarium (L.) Moench	0	n	1	0	Asteraceae Bercht. & J.Presl
Iz_1_1109	DJI_0090	Herniaria polygama J.Gay	0	n	1	0	Caryophyllaceae Juss.
Iz_1_1109	DJI_0090	Hieracium robustum Fr.	0	n	1	0	Asteraceae Bercht. & J.Presl
Iz_1_1109	DJI_0090	Hieracium umbellatum L.	0	n	1	NA	Asteraceae Bercht. & J.Presl
Iz_1_1109	DJI_0090	Hylotelephium maximum (L.) Holub	0	n	1	0	Crassulaceae J.St.-Hil.
Iz_1_1109	DJI_0090	Jacobaea borysthenica (DC.) B.Nord. & Greuter	0	n	1	0	Asteraceae Bercht. & J.Presl
Iz_1_1109	DJI_0090	Jasione montana L.	0	n	1	0	Campanulaceae Juss.
Iz_1_1109	DJI_0090	Jurinea cyanoides (L.) Rchb.s.l.	0	n	1	0	Asteraceae Bercht. & J.Presl
Iz_1_1109	DJI_0090	Koeleria glauca (Spreng.) DC.	0	n	1	0	Poaceae Barnhart
Iz_1_1109	DJI_0090	Lactuca serriola L.	0	a	0	0	Asteraceae Bercht. & J.Presl
Iz_1_1109	DJI_0090	Linaria genistifolia (L.) Mill.	0	n	1	0	Plantaginaceae Juss.
Iz_1_1109	DJI_0090	Lomelosia argentea (L.) Greuter & Burdet	0	n	1	0	Caprifoliaceae Juss.
Iz_1_1109	DJI_0090	Melampyrum pratense L.	0	n	1	0	Orobanchaceae Vent.
Iz_1_1109	DJI_0090	Oenothera biennis L.	0	a	0	0	Onagraceae Juss.
Iz_1_1109	DJI_0090	Pentanema britannica (L.) D.Gut.Larr., Santos-Vicente, Anderb., E.Rico & M.M.Mart.Ort.	0	n	1	0	Asteraceae Bercht. & J.Presl
Iz_1_1109	DJI_0090	Peucedanum oreoselinum (L.) Moench	0	n	1	0	Apiaceae Lindl.
Iz_1_1109	DJI_0090	Peucedanum ruthenicum M.Bieb.	0	n	1	0	Apiaceae Lindl.
Iz_1_1109	DJI_0090	Pilosella echioides (Lumn.) F.W.Schultz & Sch.Bip.	0	n	1	0	Asteraceae Bercht. & J.Presl
Iz_1_1109	DJI_0090	Pilosella officinarum F. Schultz et Sch. Bip.	0	n	1	0	Asteraceae Bercht. & J.Presl
Iz_1_1109	DJI_0090	Pilosella sp.	0		NA	0	Asteraceae Bercht. & J.Presl
Iz_1_1109	DJI_0090	Pinus sylvestris L.	0	n	1	1	Pinaceae Spreng. ex F.Rudolphi
Iz_1_1109	DJI_0090	Poa annua L.	0	n	1	0	Poaceae Barnhart
Iz_1_1109	DJI_0090	Poa nemoralis L.	0	n	1	0	Poaceae Barnhart
Iz_1_1109	DJI_0090	Poa sp.	0		NA	0	Poaceae Barnhart
Iz_1_1109	DJI_0090	Polygonatum odoratum (Mill.) Druce	0	n	1	0	Asparagaceae Juss.
Iz_1_1109	DJI_0090	Polygonum arenarium Waldst. & Kit.	0	n	1	0	Polygonaceae Juss.
Iz_1_1109	DJI_0090	Populus nigra L.	0	n	1	1	Salicaceae Mirb.
Iz_1_1109	DJI_0090	Populus sp.	0	n	1	1	Salicaceae Mirb.
Iz_1_1109	DJI_0090	Populus tremula L.	0	n	1	1	Salicaceae Mirb.
Iz_1_1109	DJI_0090	Populus Ã canescens (Aiton) Sm.	0	n	1	1	Salicaceae Mirb.
Iz_1_1109	DJI_0090	Potentilla argentea L.	0	n	1	0	Rosaceae Juss.
Iz_1_1109	DJI_0090	Psammophiliella muralis (L.) Ikonn.	0	n	1	0	Caryophyllaceae Juss.
Iz_1_1109	DJI_0090	Psephellus sumensis (Kalen.) Greuter	0	n	1	0	Asteraceae Bercht. & J.Presl
Iz_1_1109	DJI_0090	Pulsatilla pratensis (L.) Mill. s.l.	0	n	1	0	Ranunculaceae Juss.
Iz_1_1109	DJI_0090	Pyrus communis L.	0	a	0	1	Rosaceae Juss.
Iz_1_1109	DJI_0090	Quercus robur L.	0	n	1	1	Fagaceae Dumort.
Iz_1_1109	DJI_0090	Robinia pseudoacacia L.	0	a	0	1	Fabaceae Lindl.
Iz_1_1109	DJI_0090	Rosa sp.	0		NA	1	Rosaceae Juss.
Iz_1_1109	DJI_0090	Rubus idaeus L.	0	n	1	0	Rosaceae Juss.
Iz_1_1109	DJI_0090	Rumex acetosella L.	0	n	1	0	Polygonaceae Juss.
Iz_1_1109	DJI_0090	Rumex thyrsiflorus Fingerh.	0	n	1	0	Polygonaceae Juss.
Iz_1_1109	DJI_0090	Salix alba L.	0	n	1	1	Salicaceae Mirb.
Iz_1_1109	DJI_0090	Sambucus nigra L.	0	n	1	1	Viburnaceae Raf.
Iz_1_1109	DJI_0090	Secale sylvestre Host	0	n	1	0	Poaceae Barnhart
Iz_1_1109	DJI_0090	Sempervivum ruthenicum Schnittsp. & C.B. Lehm.	0	n	1	0	Crassulaceae J.St.-Hil.
Iz_1_1109	DJI_0090	Setaria viridis (L.) P. Beauv.	0	n	1	0	Poaceae Barnhart
Iz_1_1109	DJI_0090	Silene borysthenica (Gruner) Walters	0	n	1	0	Caryophyllaceae Juss.
Iz_1_1109	DJI_0090	Silene latifolia subsp. alba (Mill.) Greuter & Burdet	0	n	1	0	Caryophyllaceae Juss.
Iz_1_1109	DJI_0090	Silene nutans L.	0	n	1	0	Caryophyllaceae Juss.
Iz_1_1109	DJI_0090	Solanum nigrum L.	0		NA	NA	
Iz_1_1109	DJI_0090	Solidago virgaurea L.	0	n	1	0	Asteraceae Bercht. & J.Presl
Iz_1_1109	DJI_0090	Sorbus aucuparia L.	0	n	1	1	Rosaceae Juss.
Iz_1_1109	DJI_0090	Stachys recta L.	0	n	1	0	Lamiacea Lindl.
Iz_1_1109	DJI_0090	Tanacetum vulgare L.	0	n	1	0	Asteraceae Bercht. & J.Presl
Iz_1_1109	DJI_0090	Thymus pallasianus Heinr.Braun	0	n	1	0	Lamiacea Lindl.
Iz_1_1109	DJI_0090	Tilia cordata Mill.	0	n	1	1	Malvaceae Juss.
Iz_1_1109	DJI_0090	Trifolium alpestre L.	0	n	1	0	Fabaceae Lindl.
Iz_1_1109	DJI_0090	Trifolium arvense L.	0	n	1	0	Fabaceae Lindl.
Iz_1_1109	DJI_0090	Ulmus glabra Huds.	0	n	1	1	Ulmaceae Mirb.
Iz_1_1109	DJI_0090	Verbascum densiflorum Bertol.	0	n	1	0	Scrophulariaceae Juss.
Iz_1_1109	DJI_0090	Veronica austriaca L.	0	n	1	0	Plantaginaceae Juss.
Iz_1_1109	DJI_0090	Veronica incana L.	0	n	1	0	Plantaginaceae Juss.
Iz_1_1109	DJI_0090	Veronica verna L.	0	n	1	0	Plantaginaceae Juss.
Iz_1_1109	DJI_0090	Vincetoxicum hirundinaria Medik	0	n	1	0	Apocynaceae Juss.
Iz_1_1109	DJI_0098	Acer tataricum L.	0	n	1	1	Sapindaceae Juss.
Iz_1_1109	DJI_0098	Achillea millefolium L. s.l.	0	n	1	0	Asteraceae Bercht. & J.Presl
Iz_1_1109	DJI_0098	Allium oleraceum L.	0	n	1	0	Amaryllidaceae J.St.-Hil.
Iz_1_1109	DJI_0098	Ambrosia artemisiifolia L.	0	a	0	0	Asteraceae Bercht. & J.Presl
Iz_1_1109	DJI_0098	Amorpha fruticosa L.	0	a	0	0	Fabaceae Lindl.
Iz_1_1109	DJI_0098	Anthericum ramosum L.	0	n	1	0	Asparagaceae Juss.
Iz_1_1109	DJI_0098	Artemisia marschalliana Spreng.	0	n	1	0	Asteraceae Bercht. & J.Presl
Iz_1_1109	DJI_0098	Asparagus officinalis L.	0	n	1	0	Asparagaceae Juss.
Iz_1_1109	DJI_0098	Astragalus cicer L.	0	n	1	0	Fabaceae Lindl.
Iz_1_1109	DJI_0098	Bassia laniflora (S.G.Gmel.) A.J.Scott	0	n	1	0	Amaranthaceae Juss.
Iz_1_1109	DJI_0098	Berberis vulgaris L.	0	n	1	0	Berberidaceae Juss.
Iz_1_1109	DJI_0098	Betula pendula Roth.	0	n	1	1	Betulaceae Gray
Iz_1_1109	DJI_0098	Betula pendula Roth.	0	n	1	0	Poaceae Barnhart
Iz_1_1109	DJI_0098	Bromus inermis Leyss.	0		NA	NA	
Iz_1_1109	DJI_0098	Bromus squarrosus L.	0	a	0	0	Poaceae Barnhart
Iz_1_1109	DJI_0098	Bromus tectorum L.	0	a	0	0	Poaceae Barnhart

Iz_1_1109	DJI_0098	Calamagrostis epigejos (L.) Roth	0	n	1	0	Poaceae Barnhart
Iz_1_1109	DJI_0098	Carduus acanthoides L.	0	n	1	0	Asteraceae Bercht. & J.Presl
Iz_1_1109	DJI_0098	Carex sp.	0	n	1	0	Cyperaceae Juss.
Iz_1_1109	DJI_0098	Centaurea majorovii Dumbadze	0	n	1	0	Asteraceae Bercht. & J.Presl
Iz_1_1109	DJI_0098	Chamaecytisus ruthenicus (Fisch.ex Wol.) Klaskova	0	n	1	0	Fabaceae Lindl.
Iz_1_1109	DJI_0098	Chelidonium majus L.	0	n	1	0	Papaveraceae Juss.
Iz_1_1109	DJI_0098	Chenopodium betaceum Andrz.	0	n	1	0	Amaranthaceae Juss.
Iz_1_1109	DJI_0098	Chenopodium cf. album	0	n	1	0	Amaranthaceae Juss.
Iz_1_1109	DJI_0098	Chenopodium sp.	0		NA	0	Amaranthaceae Juss.
Iz_1_1109	DJI_0098	Chondrilla juncea L.	0	n	1	0	Asteraceae Bercht. & J.Presl
Iz_1_1109	DJI_0098	Cirsium vulgare (Savi) Ten.	0	n	1	0	Asteraceae Bercht. & J.Presl
Iz_1_1109	DJI_0098	Clinopodium acinos (L.) Kuntze	0	n	1	0	Lamiacea Lindl.
Iz_1_1109	DJI_0098	Convallaria majalis L.	0	n	1	0	Asparagaceae Juss.
Iz_1_1109	DJI_0098	Crataegus rhipidophylla Gand.	0	n	1	1	Rosaceae Juss.
Iz_1_1109	DJI_0098	Crepis foetida L.	0	n	1	0	Asteraceae Bercht. & J.Presl
Iz_1_1109	DJI_0098	Dactylis glomerata L.	0	n	1	0	Poaceae Barnhart
Iz_1_1109	DJI_0098	Dianthus campestris M.Bieb.	0	n	1	0	Caryophyllaceae Juss.
Iz_1_1109	DJI_0098	Digitaria sanguinalis (L.) Scop.	0	a	0	0	Poaceae Barnhart
Iz_1_1109	DJI_0098	Elymus repens (L.) Gould	0	n	1	0	Poaceae Barnhart
Iz_1_1109	DJI_0098	Epilobium tetragonum L.	0	n	1	0	Onagraceae Juss.
Iz_1_1109	DJI_0098	Eragrostis minor Host	0	a	0	0	Poaceae Barnhart
Iz_1_1109	DJI_0098	Erigeron canadensis L.	0	a	0	0	Asteraceae Bercht. & J.Presl
Iz_1_1109	DJI_0098	Euphorbia seguieriana Neck.	0	n	1	0	Euphorbiaceae Juss.
Iz_1_1109	DJI_0098	Fallopia dumetorum (L.) Holub	0	n	1	0	Polygonaceae Juss.
Iz_1_1109	DJI_0098	Festuca beckeri (Hack.) Trautv.	0	n	1	0	Poaceae Barnhart
Iz_1_1109	DJI_0098	Fragaria vesca L.	0	n	1	0	Rosaceae Juss.
Iz_1_1109	DJI_0098	Frangula alnus Mill.	0	n	1	1	Rhamnaceae Juss.
Iz_1_1109	DJI_0098	Fraxinus pennsylvanica Marshall	0	a	0	1	Oleaceae Hoffmanns. & Link
Iz_1_1109	DJI_0098	Galeopsis bifida Boenn.	0	n	1	0	Lamiacea Lindl.
Iz_1_1109	DJI_0098	Galium verum L.	0	n	1	0	Rubiaceae Juss.
Iz_1_1109	DJI_0098	Genista tinctoria L.	0	n	1	0	Fabaceae Lindl.
Iz_1_1109	DJI_0098	Geranium robertianum L.	0	n	1	0	Geraniaceae Juss.
Iz_1_1109	DJI_0098	Geranium sanquineum L.	0	n	1	0	Geraniaceae Juss.
Iz_1_1109	DJI_0098	Gypsophila paniculata L.	0	n	1	0	Caryophyllaceae Juss.
Iz_1_1109	DJI_0098	Helichrysum arenarium (L.) Moench	0	n	1	0	Asteraceae Bercht. & J.Presl
Iz_1_1109	DJI_0098	Herniaria polygama J.Gay	0	n	1	0	Caryophyllaceae Juss.
Iz_1_1109	DJI_0098	Hieracium robustum Fr.	0	n	1	0	Asteraceae Bercht. & J.Presl
Iz_1_1109	DJI_0098	Hieracium umbellatum L.	0	n	1	NA	Asteraceae Bercht. & J.Presl
Iz_1_1109	DJI_0098	Hylotelephium maximum (L.) Holub	0	n	1	0	Crassulaceae J.St.-Hil.
Iz_1_1109	DJI_0098	Jacobaea borysthenica (DC.) B.Nord. & Greuter	0	n	1	0	Asteraceae Bercht. & J.Presl
Iz_1_1109	DJI_0098	Jasione montana L.	0	n	1	0	Campanulaceae Juss.
Iz_1_1109	DJI_0098	Jurinea cyanoides (L.) Rchb.s.l.	0	n	1	0	Asteraceae Bercht. & J.Presl
Iz_1_1109	DJI_0098	Koeleria glauca (Spreng.) DC.	0	n	1	0	Poaceae Barnhart
Iz_1_1109	DJI_0098	Lactuca serriola L.	0	a	0	0	Asteraceae Bercht. & J.Presl
Iz_1_1109	DJI_0098	Linaria genistifolia (L.) Mill.	0	n	1	0	Plantaginaceae Juss.
Iz_1_1109	DJI_0098	Lomelosia argentea (L.) Greuter & Burdet	0	n	1	0	Caprifoliaceae Juss.
Iz_1_1109	DJI_0098	Melampyrum pratense L.	0	n	1	0	Orobanchaceae Vent.
Iz_1_1109	DJI_0098	Oenothera biennis L.	0	a	0	0	Onagraceae Juss.
Iz_1_1109	DJI_0098	Pentanema britannica (L.) D.Gut.Larr., Santos-Vicente, Anderb., E.Rico & M.M.Mart.Ort.	0	n	1	0	Asteraceae Bercht. & J.Presl
Iz_1_1109	DJI_0098	Peucedanum oreoselinum (L.) Moench	0	n	1	0	Apiaceae Lindl.
Iz_1_1109	DJI_0098	Peucedanum ruthenicum M.Bieb.	0	n	1	0	Apiaceae Lindl.
Iz_1_1109	DJI_0098	Pilosella echioides (Lumn.) F.W.Schultz & Sch.Bip.	0	n	1	0	Asteraceae Bercht. & J.Presl
Iz_1_1109	DJI_0098	Pilosella officinarum F. Schultz et Sch. Bip.	0	n	1	0	Asteraceae Bercht. & J.Presl
Iz_1_1109	DJI_0098	Pilosella sp.	0		NA	0	Asteraceae Bercht. & J.Presl
Iz_1_1109	DJI_0098	Pinus sylvestris L.	0	n	1	1	Pinaceae Spreng. ex F.Rudolphi
Iz_1_1109	DJI_0098	Poa annua L.	0	n	1	0	Poaceae Barnhart
Iz_1_1109	DJI_0098	Poa nemoralis L.	0	n	1	0	Poaceae Barnhart
Iz_1_1109	DJI_0098	Poa sp.	0		NA	0	Poaceae Barnhart
Iz_1_1109	DJI_0098	Polygonatum odoratum (Mill.) Druce	0	n	1	0	Asparagaceae Juss.
Iz_1_1109	DJI_0098	Polygonum arenarium Waldst. & Kit.	0	n	1	0	Polygonaceae Juss.
Iz_1_1109	DJI_0098	Populus nigra L.	0	n	1	1	Salicaceae Mirb.
Iz_1_1109	DJI_0098	Populus sp.	0	n	1	1	Salicaceae Mirb.
Iz_1_1109	DJI_0098	Populus tremula L.	0	n	1	1	Salicaceae Mirb.
Iz_1_1109	DJI_0098	Populus Ã canescens (Aiton) Sm.	0	n	1	1	Salicaceae Mirb.
Iz_1_1109	DJI_0098	Potentilla argentea L.	0	n	1	0	Rosaceae Juss.
Iz_1_1109	DJI_0098	Psammophiliella muralis (L.) Ikonn.	0	n	1	0	Caryophyllaceae Juss.
Iz_1_1109	DJI_0098	Psephellus sumensis (Kalen.) Greuter	0	n	1	0	Asteraceae Bercht. & J.Presl
Iz_1_1109	DJI_0098	Pulsatilla pratensis (L.) Mill. s.l.	0	n	1	0	Ranunculaceae Juss.
Iz_1_1109	DJI_0098	Pyrus communis L.	0	a	0	1	Rosaceae Juss.
Iz_1_1109	DJI_0098	Quercus robur L.	0	n	1	1	Fagaceae Dumort.
Iz_1_1109	DJI_0098	Robinia pseudoacacia L.	0	a	0	1	Fabaceae Lindl.
Iz_1_1109	DJI_0098	Rosa sp.	0		NA	1	Rosaceae Juss.
Iz_1_1109	DJI_0098	Rubus idaeus L.	0	n	1	0	Rosaceae Juss.
Iz_1_1109	DJI_0098	Rumex acetosella L.	0	n	1	0	Polygonaceae Juss.
Iz_1_1109	DJI_0098	Rumex thyrsiflorus Fingerh.	0	n	1	0	Polygonaceae Juss.
Iz_1_1109	DJI_0098	Salix alba L.	0	n	1	1	Salicaceae Mirb.
Iz_1_1109	DJI_0098	Sambucus nigra L.	0	n	1	1	Viburnaceae Raf.
Iz_1_1109	DJI_0098	Secale sylvestre Host	0	n	1	0	Poaceae Barnhart
Iz_1_1109	DJI_0098	Sempervivum ruthenicum Schnittsp. & C.B. Lehm.	0	n	1	0	Crassulaceae J.St.-Hil.
Iz_1_1109	DJI_0098	Setaria viridis (L.) P. Beauv.	0	n	1	0	Poaceae Barnhart
Iz_1_1109	DJI_0098	Silene borysthenica (Gruner) Walters	0	n	1	0	Caryophyllaceae Juss.
Iz_1_1109	DJI_0098	Silene latifolia subsp. alba (Mill.) Greuter & Burdet	0	n	1	0	Caryophyllaceae Juss.
Iz_1_1109	DJI_0098	Silene nutans L.	0	n	1	0	Caryophyllaceae Juss.
Iz_1_1109	DJI_0098	Solanum nigrum L.	0		NA	NA	
Iz_1_1109	DJI_0098	Solidago virgaurea L.	0	n	1	0	Asteraceae Bercht. & J.Presl
Iz_1_1109	DJI_0098	Sorbus aucuparia L.	0	n	1	1	Rosaceae Juss.
Iz_1_1109	DJI_0098	Stachys recta L.	0	n	1	0	Lamiacea Lindl.
Iz_1_1109	DJI_0098	Tanacetum vulgare L.	0	n	1	0	Asteraceae Bercht. & J.Presl
Iz_1_1109	DJI_0098	Thymus pallasianus Heinr.Braun	0	n	1	0	Lamiacea Lindl.
Iz_1_1109	DJI_0098	Tilia cordata Mill.	0	n	1	1	Malvaceae Juss.
Iz_1_1109	DJI_0098	Trifolium alpestre L.	0	n	1	0	Fabaceae Lindl.
Iz_1_1109	DJI_0098	Trifolium arvense L.	0	n	1	0	Fabaceae Lindl.
Iz_1_1109	DJI_0098	Ulmus glabra Huds.	0	n	1	1	Ulmaceae Mirb.
Iz_1_1109	DJI_0098	Verbascum densiflorum Bertol.	0	n	1	0	Scrophulariaceae Juss.
Iz_1_1109	DJI_0098	Veronica austriaca L.	0	n	1	0	Plantaginaceae Juss.
Iz_1_1109	DJI_0098	Veronica incana L.	0	n	1	0	Plantaginaceae Juss.
Iz_1_1109	DJI_0098	Veronica verna L.	0	n	1	0	Plantaginaceae Juss.
Iz_1_1109	DJI_0098	Vincetoxicum hirundinaria Medik	0	n	1	0	Apocynaceae Juss.

Iz_1_1109	DJI_0102	Acer tataricum L.	0	n	1	1	Sapindaceae Juss.
Iz_1_1109	DJI_0102	Achillea millefolium L. s.l.	0	n	1	0	Asteraceae Bercht. & J.Presl
Iz_1_1109	DJI_0102	Allium oleraceum L.	0	n	1	0	Amaryllidaceae J.St.-Hil.
Iz_1_1109	DJI_0102	Ambrosia artemisiifolia L.	0	a	0	0	Asteraceae Bercht. & J.Presl
Iz_1_1109	DJI_0102	Amorpha fruticosa L.	0	a	0	0	Fabaceae Lindl.
Iz_1_1109	DJI_0102	Anthericum ramosum L.	0	n	1	0	Asparagaceae Juss.
Iz_1_1109	DJI_0102	Artemisia marschalliana Spreng.	0	n	1	0	Asteraceae Bercht. & J.Presl
Iz_1_1109	DJI_0102	Asparagus officinalis L.	0	n	1	0	Asparagaceae Juss.
Iz_1_1109	DJI_0102	Astragalus cicer L.	0	n	1	0	Fabaceae Lindl.
Iz_1_1109	DJI_0102	Bassia laniflora (S.G.Gmel.) A.J.Scott	0	n	1	0	Amaranthaceae Juss.
Iz_1_1109	DJI_0102	Berberis vulgaris L.	0	n	1	0	Berberidaceae Juss.
Iz_1_1109	DJI_0102	Betula pendula Roth.	0	n	1	1	Betulaceae Gray
Iz_1_1109	DJI_0102	Betula pendula Roth.	0	n	1	0	Poaceae Barnhart
Iz_1_1109	DJI_0102	Bromus inermis Leyss.	0		NA	NA	
Iz_1_1109	DJI_0102	Bromus squarrosus L.	0	a	0	0	Poaceae Barnhart
Iz_1_1109	DJI_0102	Bromus tectorum L.	0	a	0	0	Poaceae Barnhart
Iz_1_1109	DJI_0102	Calamagrostis epigejos (L.) Roth	2	n	1	0	Poaceae Barnhart
Iz_1_1109	DJI_0102	Carduus acanthoides L.	0	n	1	0	Asteraceae Bercht. & J.Presl
Iz_1_1109	DJI_0102	Carex sp.	0	n	1	0	Cyperaceae Juss.
Iz_1_1109	DJI_0102	Centaurea majorovii Dumbadze	0	n	1	0	Asteraceae Bercht. & J.Presl
Iz_1_1109	DJI_0102	Chamaecytisus ruthenicus (Fisch.ex Wol.) Klaskova	0	n	1	0	Fabaceae Lindl.
Iz_1_1109	DJI_0102	Chelidonium majus L.	0	n	1	0	Papaveraceae Juss.
Iz_1_1109	DJI_0102	Chenopodium betaceum Andrz.	0	n	1	0	Amaranthaceae Juss.
Iz_1_1109	DJI_0102	Chenopodium cf. album	0	n	1	0	Amaranthaceae Juss.
Iz_1_1109	DJI_0102	Chenopodium sp.	0		NA	0	Amaranthaceae Juss.
Iz_1_1109	DJI_0102	Chondrilla juncea L.	0	n	1	0	Asteraceae Bercht. & J.Presl
Iz_1_1109	DJI_0102	Cirsium vulgare (Savi) Ten.	0	n	1	0	Asteraceae Bercht. & J.Presl
Iz_1_1109	DJI_0102	Clinopodium acinos (L.) Kuntze	0	n	1	0	Lamiacea Lindl.
Iz_1_1109	DJI_0102	Convallaria majalis L.	0	n	1	0	Asparagaceae Juss.
Iz_1_1109	DJI_0102	Crataegus rhipidophylla Gand.	0	n	1	1	Rosaceae Juss.
Iz_1_1109	DJI_0102	Crepis foetida L.	0	n	1	0	Asteraceae Bercht. & J.Presl
Iz_1_1109	DJI_0102	Dactylis glomerata L.	0	n	1	0	Poaceae Barnhart
Iz_1_1109	DJI_0102	Dianthus campestris M.Bieb.	0	n	1	0	Caryophyllaceae Juss.
Iz_1_1109	DJI_0102	Digitaria sanguinalis (L.) Scop.	0	a	0	0	Poaceae Barnhart
Iz_1_1109	DJI_0102	Elymus repens (L.) Gould	0	n	1	0	Poaceae Barnhart
Iz_1_1109	DJI_0102	Epilobium tetragonum L.	0	n	1	0	Onagraceae Juss.
Iz_1_1109	DJI_0102	Eragrostis minor Host	0	a	0	0	Poaceae Barnhart
Iz_1_1109	DJI_0102	Erigeron canadensis L.	0.2	a	0	0	Asteraceae Bercht. & J.Presl
Iz_1_1109	DJI_0102	Euphorbia seguieriana Neck.	0	n	1	0	Euphorbiaceae Juss.
Iz_1_1109	DJI_0102	Fallopia dumetorum (L.) Holub	0.1	n	1	0	Polygonaceae Juss.
Iz_1_1109	DJI_0102	Festuca beckeri (Hack.) Trautv.	0	n	1	0	Poaceae Barnhart
Iz_1_1109	DJI_0102	Fragaria vesca L.	0	n	1	0	Rosaceae Juss.
Iz_1_1109	DJI_0102	Frangula alnus Mill.	0	n	1	1	Rhamnaceae Juss.
Iz_1_1109	DJI_0102	Fraxinus pennsylvanica Marshall	0	a	0	1	Oleaceae Hoffmanns. & Link
Iz_1_1109	DJI_0102	Galeopsis bifida Boenn.	0	n	1	0	Lamiacea Lindl.
Iz_1_1109	DJI_0102	Galium verum L.	0	n	1	0	Rubiaceae Juss.
Iz_1_1109	DJI_0102	Genista tinctoria L.	0	n	1	0	Fabaceae Lindl.
Iz_1_1109	DJI_0102	Geranium robertianum L.	0	n	1	0	Geraniaceae Juss.
Iz_1_1109	DJI_0102	Geranium sanquineum L.	0	n	1	0	Geraniaceae Juss.
Iz_1_1109	DJI_0102	Gypsophila paniculata L.	0	n	1	0	Caryophyllaceae Juss.
Iz_1_1109	DJI_0102	Helichrysum arenarium (L.) Moench	0	n	1	0	Asteraceae Bercht. & J.Presl
Iz_1_1109	DJI_0102	Herniaria polygama J.Gay	0	n	1	0	Caryophyllaceae Juss.
Iz_1_1109	DJI_0102	Hieracium robustum Fr.	0	n	1	0	Asteraceae Bercht. & J.Presl
Iz_1_1109	DJI_0102	Hieracium umbellatum L.	0	n	1	NA	Asteraceae Bercht. & J.Presl
Iz_1_1109	DJI_0102	Hylotelephium maximum (L.) Holub	0	n	1	0	Crassulaceae J.St.-Hil.
Iz_1_1109	DJI_0102	Jacobaea borysthenica (DC.) B.Nord. & Greuter	0	n	1	0	Asteraceae Bercht. & J.Presl
Iz_1_1109	DJI_0102	Jasione montana L.	0	n	1	0	Campanulaceae Juss.
Iz_1_1109	DJI_0102	Jurinea cyanoides (L.) Rchb.s.l.	0	n	1	0	Asteraceae Bercht. & J.Presl
Iz_1_1109	DJI_0102	Koeleria glauca (Spreng.) DC.	0	n	1	0	Poaceae Barnhart
Iz_1_1109	DJI_0102	Lactuca serriola L.	0	a	0	0	Asteraceae Bercht. & J.Presl
Iz_1_1109	DJI_0102	Linaria genistifolia (L.) Mill.	0	n	1	0	Plantaginaceae Juss.
Iz_1_1109	DJI_0102	Lomelosia argentea (L.) Greuter & Burdet	0	n	1	0	Caprifoliaceae Juss.
Iz_1_1109	DJI_0102	Melampyrum pratense L.	0	n	1	0	Orobanchaceae Vent.
Iz_1_1109	DJI_0102	Oenothera biennis L.	0	a	0	0	Onagraceae Juss.
Iz_1_1109	DJI_0102	Pentanema britannica (L.) D.Gut.Larr., Santos-Vicente, Anderb., E.Rico & M.M.Mart.Ort.	0	n	1	0	Asteraceae Bercht. & J.Presl
Iz_1_1109	DJI_0102	Peucedanum oreoselinum (L.) Moench	0	n	1	0	Apiaceae Lindl.
Iz_1_1109	DJI_0102	Peucedanum ruthenicum M.Bieb.	0	n	1	0	Apiaceae Lindl.
Iz_1_1109	DJI_0102	Pilosella echioides (Lumn.) F.W.Schultz & Sch.Bip.	0	n	1	0	Asteraceae Bercht. & J.Presl
Iz_1_1109	DJI_0102	Pilosella officinarum F. Schultz et Sch. Bip.	0	n	1	0	Asteraceae Bercht. & J.Presl
Iz_1_1109	DJI_0102	Pilosella sp.	0		NA	0	Asteraceae Bercht. & J.Presl
Iz_1_1109	DJI_0102	Pinus sylvestris L.	0	n	1	1	Pinaceae Spreng. ex F.Rudolphi
Iz_1_1109	DJI_0102	Poa annua L.	0	n	1	0	Poaceae Barnhart
Iz_1_1109	DJI_0102	Poa nemoralis L.	0	n	1	0	Poaceae Barnhart
Iz_1_1109	DJI_0102	Poa sp.	0		NA	0	Poaceae Barnhart
Iz_1_1109	DJI_0102	Polygonatum odoratum (Mill.) Druce	0	n	1	0	Asparagaceae Juss.
Iz_1_1109	DJI_0102	Polygonum arenarium Waldst. & Kit.	0	n	1	0	Polygonaceae Juss.
Iz_1_1109	DJI_0102	Populus nigra L.	0	n	1	1	Salicaceae Mirb.
Iz_1_1109	DJI_0102	Populus sp.	0	n	1	1	Salicaceae Mirb.
Iz_1_1109	DJI_0102	Populus tremula L.	0	n	1	1	Salicaceae Mirb.
Iz_1_1109	DJI_0102	Populus Ã canescens (Aiton) Sm.	0	n	1	1	Salicaceae Mirb.
Iz_1_1109	DJI_0102	Potentilla argentea L.	0	n	1	0	Rosaceae Juss.
Iz_1_1109	DJI_0102	Psammophiliella muralis (L.) Ikonn.	0	n	1	0	Caryophyllaceae Juss.
Iz_1_1109	DJI_0102	Psephellus sumensis (Kalen.) Greuter	0	n	1	0	Asteraceae Bercht. & J.Presl
Iz_1_1109	DJI_0102	Pulsatilla pratensis (L.) Mill. s.l.	0	n	1	0	Ranunculaceae Juss.
Iz_1_1109	DJI_0102	Pyrus communis L.	0	a	0	1	Rosaceae Juss.
Iz_1_1109	DJI_0102	Quercus robur L.	0	n	1	1	Fagaceae Dumort.
Iz_1_1109	DJI_0102	Robinia pseudoacacia L.	0	a	0	1	Fabaceae Lindl.
Iz_1_1109	DJI_0102	Rosa sp.	0		NA	1	Rosaceae Juss.
Iz_1_1109	DJI_0102	Rubus idaeus L.	0	n	1	0	Rosaceae Juss.
Iz_1_1109	DJI_0102	Rumex acetosella L.	0	n	1	0	Polygonaceae Juss.
Iz_1_1109	DJI_0102	Rumex thyrsiflorus Fingerh.	0	n	1	0	Polygonaceae Juss.
Iz_1_1109	DJI_0102	Salix alba L.	0	n	1	1	Salicaceae Mirb.
Iz_1_1109	DJI_0102	Sambucus nigra L.	0	n	1	1	Viburnaceae Raf.
Iz_1_1109	DJI_0102	Secale sylvestre Host	0	n	1	0	Poaceae Barnhart
Iz_1_1109	DJI_0102	Sempervivum ruthenicum Schnittsp. & C.B. Lehm.	0	n	1	0	Crassulaceae J.St.-Hil.
Iz_1_1109	DJI_0102	Setaria viridis (L.) P. Beauv.	0	n	1	0	Poaceae Barnhart
Iz_1_1109	DJI_0102	Silene borysthenica (Gruner) Walters	0	n	1	0	Caryophyllaceae Juss.
Iz_1_1109	DJI_0102	Silene latifolia subsp. alba (Mill.) Greuter & Burdet	0	n	1	0	Caryophyllaceae Juss.

Iz_1_1109	DJI_0102	Silene nutans L.	0	n	1	0	Caryophyllaceae Juss.
Iz_1_1109	DJI_0102	Solanum nigrum L.	0		NA	NA	
Iz_1_1109	DJI_0102	Solidago virgaurea L.	0	n	1	0	Asteraceae Bercht. & J.Presl
Iz_1_1109	DJI_0102	Sorbus aucuparia L.	0	n	1	1	Rosaceae Juss.
Iz_1_1109	DJI_0102	Stachys recta L.	0	n	1	0	Lamiacea Lindl.
Iz_1_1109	DJI_0102	Tanacetum vulgare L.	0	n	1	0	Asteraceae Bercht. & J.Presl
Iz_1_1109	DJI_0102	Thymus pallasianus Heinr.Braun	0	n	1	0	Lamiacea Lindl.
Iz_1_1109	DJI_0102	Tilia cordata Mill.	0	n	1	1	Malvaceae Juss.
Iz_1_1109	DJI_0102	Trifolium alpestre L.	0	n	1	0	Fabaceae Lindl.
Iz_1_1109	DJI_0102	Trifolium arvense L.	0	n	1	0	Fabaceae Lindl.
Iz_1_1109	DJI_0102	Ulmus glabra Huds.	0	n	1	1	Ulmaceae Mirb.
Iz_1_1109	DJI_0102	Verbascum densiflorum Bertol.	0	n	1	0	Scrophulariaceae Juss.
Iz_1_1109	DJI_0102	Veronica austriaca L.	0	n	1	0	Plantaginaceae Juss.
Iz_1_1109	DJI_0102	Veronica incana L.	0	n	1	0	Plantaginaceae Juss.
Iz_1_1109	DJI_0102	Veronica verna L.	0	n	1	0	Plantaginaceae Juss.
Iz_1_1109	DJI_0102	Vincetoxicum hirundinaria Medik	0	n	1	0	Apocynaceae Juss.
Iz_1_1209	DJI_0005	Acer tataricum L.	0	n	1	1	Sapindaceae Juss.
Iz_1_1209	DJI_0005	Achillea millefolium L. s.l.	0	n	1	0	Asteraceae Bercht. & J.Presl
Iz_1_1209	DJI_0005	Allium oleraceum L.	0	n	1	0	Amaryllidaceae J.St.-Hil.
Iz_1_1209	DJI_0005	Ambrosia artemisiifolia L.	0	a	0	0	Asteraceae Bercht. & J.Presl
Iz_1_1209	DJI_0005	Amorpha fruticosa L.	0	a	0	0	Fabaceae Lindl.
Iz_1_1209	DJI_0005	Anthericum ramosum L.	0	n	1	0	Asparagaceae Juss.
Iz_1_1209	DJI_0005	Artemisia marschalliana Spreng.	0	n	1	0	Asteraceae Bercht. & J.Presl
Iz_1_1209	DJI_0005	Asparagus officinalis L.	0	n	1	0	Asparagaceae Juss.
Iz_1_1209	DJI_0005	Astragalus cicer L.	0	n	1	0	Fabaceae Lindl.
Iz_1_1209	DJI_0005	Bassia laniflora (S.G.Gmel.) A.J.Scott	0	n	1	0	Amaranthaceae Juss.
Iz_1_1209	DJI_0005	Berberis vulgaris L.	0	n	1	0	Berberidaceae Juss.
Iz_1_1209	DJI_0005	Betula pendula Roth.	0	n	1	1	Betulaceae Gray
Iz_1_1209	DJI_0005	Betula pendula Roth.	0	n	1	0	Poaceae Barnhart
Iz_1_1209	DJI_0005	Bromus inermis Leyss.	0		NA	NA	
Iz_1_1209	DJI_0005	Bromus squarrosus L.	0	a	0	0	Poaceae Barnhart
Iz_1_1209	DJI_0005	Bromus tectorum L.	0	a	0	0	Poaceae Barnhart
Iz_1_1209	DJI_0005	Calamagrostis epigejos (L.) Roth	2	n	1	0	Poaceae Barnhart
Iz_1_1209	DJI_0005	Carduus acanthoides L.	0	n	1	0	Asteraceae Bercht. & J.Presl
Iz_1_1209	DJI_0005	Carex sp.	0	n	1	0	Cyperaceae Juss.
Iz_1_1209	DJI_0005	Centaurea majorovii Dumbadze	0	n	1	0	Asteraceae Bercht. & J.Presl
Iz_1_1209	DJI_0005	Chamaecytisus ruthenicus (Fisch.ex Wol.) Klaskova	0	n	1	0	Fabaceae Lindl.
Iz_1_1209	DJI_0005	Chelidonium majus L.	0	n	1	0	Papaveraceae Juss.
Iz_1_1209	DJI_0005	Chenopodium betaceum Andrz.	0	n	1	0	Amaranthaceae Juss.
Iz_1_1209	DJI_0005	Chenopodium cf. album	0	n	1	0	Amaranthaceae Juss.
Iz_1_1209	DJI_0005	Chenopodium sp.	0		NA	0	Amaranthaceae Juss.
Iz_1_1209	DJI_0005	Chondrilla juncea L.	0	n	1	0	Asteraceae Bercht. & J.Presl
Iz_1_1209	DJI_0005	Cirsium vulgare (Savi) Ten.	0	n	1	0	Asteraceae Bercht. & J.Presl
Iz_1_1209	DJI_0005	Clinopodium acinos (L.) Kuntze	0	n	1	0	Lamiacea Lindl.
Iz_1_1209	DJI_0005	Convallaria majalis L.	0	n	1	0	Asparagaceae Juss.
Iz_1_1209	DJI_0005	Crataegus rhipidophylla Gand.	0	n	1	1	Rosaceae Juss.
Iz_1_1209	DJI_0005	Crepis foetida L.	0	n	1	0	Asteraceae Bercht. & J.Presl
Iz_1_1209	DJI_0005	Dactylis glomerata L.	0	n	1	0	Poaceae Barnhart
Iz_1_1209	DJI_0005	Dianthus campestris M.Bieb.	0	n	1	0	Caryophyllaceae Juss.
Iz_1_1209	DJI_0005	Digitaria sanguinalis (L.) Scop.	0	a	0	0	Poaceae Barnhart
Iz_1_1209	DJI_0005	Elymus repens (L.) Gould	0	n	1	0	Poaceae Barnhart
Iz_1_1209	DJI_0005	Epilobium tetragonum L.	0	n	1	0	Onagraceae Juss.
Iz_1_1209	DJI_0005	Eragrostis minor Host	0	a	0	0	Poaceae Barnhart
Iz_1_1209	DJI_0005	Erigeron canadensis L.	0	a	0	0	Asteraceae Bercht. & J.Presl
Iz_1_1209	DJI_0005	Euphorbia seguieriana Neck.	0	n	1	0	Euphorbiaceae Juss.
Iz_1_1209	DJI_0005	Fallopia dumetorum (L.) Holub	0	n	1	0	Polygonaceae Juss.
Iz_1_1209	DJI_0005	Festuca beckeri (Hack.) Trautv.	0	n	1	0	Poaceae Barnhart
Iz_1_1209	DJI_0005	Fragaria vesca L.	0	n	1	0	Rosaceae Juss.
Iz_1_1209	DJI_0005	Frangula alnus Mill.	0	n	1	1	Rhamnaceae Juss.
Iz_1_1209	DJI_0005	Fraxinus pennsylvanica Marshall	0	a	0	1	Oleaceae Hoffmanns. & Link
Iz_1_1209	DJI_0005	Galeopsis bifida Boenn.	0	n	1	0	Lamiacea Lindl.
Iz_1_1209	DJI_0005	Galium verum L.	0	n	1	0	Rubiaceae Juss.
Iz_1_1209	DJI_0005	Genista tinctoria L.	0	n	1	0	Fabaceae Lindl.
Iz_1_1209	DJI_0005	Geranium robertianum L.	0	n	1	0	Geraniaceae Juss.
Iz_1_1209	DJI_0005	Geranium sanquineum L.	0	n	1	0	Geraniaceae Juss.
Iz_1_1209	DJI_0005	Gypsophila paniculata L.	0	n	1	0	Caryophyllaceae Juss.
Iz_1_1209	DJI_0005	Helichrysum arenarium (L.) Moench	0	n	1	0	Asteraceae Bercht. & J.Presl
Iz_1_1209	DJI_0005	Herniaria polygama J.Gay	0	n	1	0	Caryophyllaceae Juss.
Iz_1_1209	DJI_0005	Hieracium robustum Fr.	0	n	1	0	Asteraceae Bercht. & J.Presl
Iz_1_1209	DJI_0005	Hieracium umbellatum L.	0	n	1	NA	Asteraceae Bercht. & J.Presl
Iz_1_1209	DJI_0005	Hylotelephium maximum (L.) Holub	0	n	1	0	Crassulaceae J.St.-Hil.
Iz_1_1209	DJI_0005	Jacobaea borysthenica (DC.) B.Nord. & Greuter	0	n	1	0	Asteraceae Bercht. & J.Presl
Iz_1_1209	DJI_0005	Jasione montana L.	0	n	1	0	Campanulaceae Juss.
Iz_1_1209	DJI_0005	Jurinea cyanoides (L.) Rchb.s.l.	0	n	1	0	Asteraceae Bercht. & J.Presl
Iz_1_1209	DJI_0005	Koeleria glauca (Spreng.) DC.	0	n	1	0	Poaceae Barnhart
Iz_1_1209	DJI_0005	Lactuca serriola L.	0	a	0	0	Asteraceae Bercht. & J.Presl
Iz_1_1209	DJI_0005	Linaria genistifolia (L.) Mill.	0	n	1	0	Plantaginaceae Juss.
Iz_1_1209	DJI_0005	Lomelosia argentea (L.) Greuter & Burdet	0	n	1	0	Caprifoliaceae Juss.
Iz_1_1209	DJI_0005	Melampyrum pratense L.	0	n	1	0	Orobanchaceae Vent.
Iz_1_1209	DJI_0005	Oenothera biennis L.	0	a	0	0	Onagraceae Juss.
Iz_1_1209	DJI_0005	Pentanema britannica (L.) D.Gut.Larr., Santos-Vicente, Anderb., E.Rico & M.M.Mart.Ort.	0	n	1	0	Asteraceae Bercht. & J.Presl
Iz_1_1209	DJI_0005	Peucedanum oreoselinum (L.) Moench	0.5	n	1	0	Apiaceae Lindl.
Iz_1_1209	DJI_0005	Peucedanum ruthenicum M.Bieb.	0	n	1	0	Apiaceae Lindl.
Iz_1_1209	DJI_0005	Pilosella echioides (Lumn.) F.W.Schultz & Sch.Bip.	0	n	1	0	Asteraceae Bercht. & J.Presl
Iz_1_1209	DJI_0005	Pilosella officinarum F. Schultz et Sch. Bip.	0	n	1	0	Asteraceae Bercht. & J.Presl
Iz_1_1209	DJI_0005	Pilosella sp.	0		NA	0	Asteraceae Bercht. & J.Presl
Iz_1_1209	DJI_0005	Pinus sylvestris L.	0	n	1	1	Pinaceae Spreng. ex F.Rudolphi
Iz_1_1209	DJI_0005	Poa annua L.	0	n	1	0	Poaceae Barnhart
Iz_1_1209	DJI_0005	Poa nemoralis L.	0	n	1	0	Poaceae Barnhart
Iz_1_1209	DJI_0005	Poa sp.	0		NA	0	Poaceae Barnhart
Iz_1_1209	DJI_0005	Polygonatum odoratum (Mill.) Druce	0	n	1	0	Asparagaceae Juss.
Iz_1_1209	DJI_0005	Polygonum arenarium Waldst. & Kit.	0	n	1	0	Polygonaceae Juss.
Iz_1_1209	DJI_0005	Populus nigra L.	0	n	1	1	Salicaceae Mirb.
Iz_1_1209	DJI_0005	Populus tremula L.	0	n	1	1	Salicaceae Mirb.
Iz_1_1209	DJI_0005	Populus Ã canescens (Aiton) Sm.	0	n	1	1	Salicaceae Mirb.
Iz_1_1209	DJI_0005	Potentilla argentea L.	0	n	1	0	Rosaceae Juss.
Iz_1_1209	DJI_0005	Psammophiliella muralis (L.) Ikonn.	0	n	1	0	Caryophyllaceae Juss.
Iz_1_1209	DJI_0005	Psephellus sumensis (Kalen.) Greuter	0	n	1	0	Asteraceae Bercht. & J.Presl

Iz_1_1209	DJI_0005	Pulsatilla pratensis (L.) Mill. s.l.	0	n	1	0	Ranunculaceae Juss.
Iz_1_1209	DJI_0005	Pyrus communis L.	0	a	0	1	Rosaceae Juss.
Iz_1_1209	DJI_0005	Quercus robur L.	0	n	1	1	Fagaceae Dumort.
Iz_1_1209	DJI_0005	Robinia pseudoacacia L.	0	a	0	1	Fabaceae Lindl.
Iz_1_1209	DJI_0005	Rosa sp.	0		NA	1	Rosaceae Juss.
Iz_1_1209	DJI_0005	Rubus idaeus L.	0	n	1	0	Rosaceae Juss.
Iz_1_1209	DJI_0005	Rumex acetosella L.	0.5	n	1	0	Polygonaceae Juss.
Iz_1_1209	DJI_0005	Rumex thyrsiflorus Fingerh.	0	n	1	0	Polygonaceae Juss.
Iz_1_1209	DJI_0005	Salix alba L.	0	n	1	1	Salicaceae Mirb.
Iz_1_1209	DJI_0005	Sambucus nigra L.	0	n	1	1	Viburnaceae Raf.
Iz_1_1209	DJI_0005	Secale sylvestre Host	0	n	1	0	Poaceae Barnhart
Iz_1_1209	DJI_0005	Sempervivum ruthenicum Schnittsp. & C.B. Lehm.	0	n	1	0	Crassulaceae J.St.-Hil.
Iz_1_1209	DJI_0005	Setaria viridis (L.) P. Beauv.	0	n	1	0	Poaceae Barnhart
Iz_1_1209	DJI_0005	Silene borysthenica (Gruner) Walters	0	n	1	0	Caryophyllaceae Juss.
Iz_1_1209	DJI_0005	Silene latifolia subsp. alba (Mill.) Greuter & Burdet	0	n	1	0	Caryophyllaceae Juss.
Iz_1_1209	DJI_0005	Solidago virgaurea L.	0	n	1	0	Asteraceae Bercht. & J.Presl
Iz_1_1209	DJI_0005	Sorbus aucuparia L.	0	n	1	1	Rosaceae Juss.
Iz_1_1209	DJI_0005	Stachys recta L.	0	n	1	0	Lamiacea Lindl.
Iz_1_1209	DJI_0005	Tanacetum vulgare L.	0	n	1	0	Asteraceae Bercht. & J.Presl
Iz_1_1209	DJI_0005	Thymus pallasianus Heinr.Braun	0	n	1	0	Lamiacea Lindl.
Iz_1_1209	DJI_0005	Tilia cordata Mill.	0	n	1	1	Malvaceae Juss.
Iz_1_1209	DJI_0005	Trifolium alpestre L.	0	n	1	0	Fabaceae Lindl.
Iz_1_1209	DJI_0005	Trifolium arvense L.	0	n	1	0	Fabaceae Lindl.
Iz_1_1209	DJI_0005	Ulmus glabra Huds.	0	n	1	1	Ulmaceae Mirb.
Iz_1_1209	DJI_0005	Verbascum densiflorum Bertol.	0	n	1	0	Scrophulariaceae Juss.
Iz_1_1209	DJI_0005	Veronica austriaca L.	0	n	1	0	Plantaginaceae Juss.
Iz_1_1209	DJI_0005	Veronica incana L.	0	n	1	0	Plantaginaceae Juss.
Iz_1_1209	DJI_0005	Veronica verna L.	0	n	1	0	Plantaginaceae Juss.
Iz_1_1209	DJI_0005	Vincetoxicum hirundinaria Medik	0	n	1	0	Apocynaceae Juss.
Iz_1_1209	DJI_0009	Acer tataricum L.	0	n	1	1	Sapindaceae Juss.
Iz_1_1209	DJI_0009	Achillea millefolium L. s.l.	0	n	1	0	Asteraceae Bercht. & J.Presl
Iz_1_1209	DJI_0009	Allium oleraceum L.	0	n	1	0	Amaryllidaceae J.St.-Hil.
Iz_1_1209	DJI_0009	Ambrosia artemisiifolia L.	0	a	0	0	Asteraceae Bercht. & J.Presl
Iz_1_1209	DJI_0009	Amorpha fruticosa L.	0	a	0	0	Fabaceae Lindl.
Iz_1_1209	DJI_0009	Anthericum ramosum L.	0	n	1	0	Asparagaceae Juss.
Iz_1_1209	DJI_0009	Artemisia marschalliana Spreng.	0	n	1	0	Asteraceae Bercht. & J.Presl
Iz_1_1209	DJI_0009	Asparagus officinalis L.	0	n	1	0	Asparagaceae Juss.
Iz_1_1209	DJI_0009	Astragalus cicer L.	0	n	1	0	Fabaceae Lindl.
Iz_1_1209	DJI_0009	Bassia laniflora (S.G.Gmel.) A.J.Scott	0	n	1	0	Amaranthaceae Juss.
Iz_1_1209	DJI_0009	Berberis vulgaris L.	0	n	1	0	Berberidaceae Juss.
Iz_1_1209	DJI_0009	Betula pendula Roth.	0.1	n	1	1	Betulaceae Gray
Iz_1_1209	DJI_0009	Betula pendula Roth.	0.1	n	1	0	Poaceae Barnhart
Iz_1_1209	DJI_0009	Bromus inermis Leyss.	0		NA	NA	
Iz_1_1209	DJI_0009	Bromus squarrosus L.	0	a	0	0	Poaceae Barnhart
Iz_1_1209	DJI_0009	Bromus tectorum L.	0	a	0	0	Poaceae Barnhart
Iz_1_1209	DJI_0009	Calamagrostis epigejos (L.) Roth	10	n	1	0	Poaceae Barnhart
Iz_1_1209	DJI_0009	Carduus acanthoides L.	0	n	1	0	Asteraceae Bercht. & J.Presl
Iz_1_1209	DJI_0009	Carex sp.	0	n	1	0	Cyperaceae Juss.
Iz_1_1209	DJI_0009	Centaurea majorovii Dumbadze	0	n	1	0	Asteraceae Bercht. & J.Presl
Iz_1_1209	DJI_0009	Chamaecytisus ruthenicus (Fisch.ex Wol.) Klaskova	0	n	1	0	Fabaceae Lindl.
Iz_1_1209	DJI_0009	Chelidonium majus L.	0	n	1	0	Papaveraceae Juss.
Iz_1_1209	DJI_0009	Chenopodium betaceum Andrz.	0	n	1	0	Amaranthaceae Juss.
Iz_1_1209	DJI_0009	Chenopodium cf. album	0	n	1	0	Amaranthaceae Juss.
Iz_1_1209	DJI_0009	Chenopodium sp.	0		NA	0	Amaranthaceae Juss.
Iz_1_1209	DJI_0009	Chondrilla juncea L.	0	n	1	0	Asteraceae Bercht. & J.Presl
Iz_1_1209	DJI_0009	Cirsium vulgare (Savi) Ten.	0	n	1	0	Asteraceae Bercht. & J.Presl
Iz_1_1209	DJI_0009	Clinopodium acinos (L.) Kuntze	0	n	1	0	Lamiacea Lindl.
Iz_1_1209	DJI_0009	Convallaria majalis L.	0	n	1	0	Asparagaceae Juss.
Iz_1_1209	DJI_0009	Crataegus rhipidophylla Gand.	0	n	1	1	Rosaceae Juss.
Iz_1_1209	DJI_0009	Crepis foetida L.	0	n	1	0	Asteraceae Bercht. & J.Presl
Iz_1_1209	DJI_0009	Dactylis glomerata L.	0	n	1	0	Poaceae Barnhart
Iz_1_1209	DJI_0009	Dianthus campestris M.Bieb.	0	n	1	0	Caryophyllaceae Juss.
Iz_1_1209	DJI_0009	Digitaria sanguinalis (L.) Scop.	0	a	0	0	Poaceae Barnhart
Iz_1_1209	DJI_0009	Elymus repens (L.) Gould	0	n	1	0	Poaceae Barnhart
Iz_1_1209	DJI_0009	Epilobium tetragonum L.	0	n	1	0	Onagraceae Juss.
Iz_1_1209	DJI_0009	Eragrostis minor Host	0	a	0	0	Poaceae Barnhart
Iz_1_1209	DJI_0009	Erigeron canadensis L.	0	a	0	0	Asteraceae Bercht. & J.Presl
Iz_1_1209	DJI_0009	Euphorbia seguieriana Neck.	0	n	1	0	Euphorbiaceae Juss.
Iz_1_1209	DJI_0009	Fallopia dumetorum (L.) Holub	0	n	1	0	Polygonaceae Juss.
Iz_1_1209	DJI_0009	Festuca beckeri (Hack.) Trautv.	0	n	1	0	Poaceae Barnhart
Iz_1_1209	DJI_0009	Fragaria vesca L.	0	n	1	0	Rosaceae Juss.
Iz_1_1209	DJI_0009	Frangula alnus Mill.	0	n	1	1	Rhamnaceae Juss.
Iz_1_1209	DJI_0009	Fraxinus pennsylvanica Marshall	0	a	0	1	Oleaceae Hoffmanns. & Link
Iz_1_1209	DJI_0009	Galeopsis bifida Boenn.	0	n	1	0	Lamiacea Lindl.
Iz_1_1209	DJI_0009	Galium verum L.	0	n	1	0	Rubiaceae Juss.
Iz_1_1209	DJI_0009	Genista tinctoria L.	0	n	1	0	Fabaceae Lindl.
Iz_1_1209	DJI_0009	Geranium robertianum L.	0	n	1	0	Geraniaceae Juss.
Iz_1_1209	DJI_0009	Geranium sanquineum L.	0	n	1	0	Geraniaceae Juss.
Iz_1_1209	DJI_0009	Gypsophila paniculata L.	0	n	1	0	Caryophyllaceae Juss.
Iz_1_1209	DJI_0009	Helichrysum arenarium (L.) Moench	0	n	1	0	Asteraceae Bercht. & J.Presl
Iz_1_1209	DJI_0009	Herniaria polygama J.Gay	0	n	1	0	Caryophyllaceae Juss.
Iz_1_1209	DJI_0009	Hieracium robustum Fr.	0	n	1	0	Asteraceae Bercht. & J.Presl
Iz_1_1209	DJI_0009	Hieracium umbellatum L.	0	n	1	NA	Asteraceae Bercht. & J.Presl
Iz_1_1209	DJI_0009	Hylotelephium maximum (L.) Holub	0	n	1	0	Crassulaceae J.St.-Hil.
Iz_1_1209	DJI_0009	Jacobaea borysthenica (DC.) B.Nord. & Greuter	0	n	1	0	Asteraceae Bercht. & J.Presl
Iz_1_1209	DJI_0009	Jasione montana L.	0	n	1	0	Campanulaceae Juss.
Iz_1_1209	DJI_0009	Jurinea cyanoides (L.) Rchb.s.l.	0	n	1	0	Asteraceae Bercht. & J.Presl
Iz_1_1209	DJI_0009	Koeleria glauca (Spreng.) DC.	0	n	1	0	Poaceae Barnhart
Iz_1_1209	DJI_0009	Lactuca serriola L.	0	a	0	0	Asteraceae Bercht. & J.Presl
Iz_1_1209	DJI_0009	Linaria genistifolia (L.) Mill.	0	n	1	0	Plantaginaceae Juss.
Iz_1_1209	DJI_0009	Lomelosia argentea (L.) Greuter & Burdet	0	n	1	0	Caprifoliaceae Juss.
Iz_1_1209	DJI_0009	Melampyrum pratense L.	0	n	1	0	Orobanchaceae Vent.
Iz_1_1209	DJI_0009	Oenothera biennis L.	0	a	0	0	Onagraceae Juss.
Iz_1_1209	DJI_0009	Pentanema britannica (L.) D.Gut.Larr., Santos-Vicente, Anderb., E.Rico & M.M.Mart.Ort.	0	n	1	0	Asteraceae Bercht. & J.Presl
Iz_1_1209	DJI_0009	Peucedanum oreoselinum (L.) Moench	0	n	1	0	Apiaceae Lindl.
Iz_1_1209	DJI_0009	Peucedanum ruthenicum M.Bieb.	0	n	1	0	Apiaceae Lindl.
Iz_1_1209	DJI_0009	Pilosella echioides (Lumn.) F.W.Schultz & Sch.Bip.	0	n	1	0	Asteraceae Bercht. & J.Presl
Iz_1_1209	DJI_0009	Pilosella officinarum F. Schultz et Sch. Bip.	0	n	1	0	Asteraceae Bercht. & J.Presl

Iz_1_1209	DJI_0009	Pilosella sp.	0		NA	0	Asteraceae Bercht. & J.Presl
Iz_1_1209	DJI_0009	Pinus sylvestris L.	0	n	1	1	Pinaceae Spreng. ex F.Rudolphi
Iz_1_1209	DJI_0009	Poa annua L.	0	n	1	0	Poaceae Barnhart
Iz_1_1209	DJI_0009	Poa nemoralis L.	0	n	1	0	Poaceae Barnhart
Iz_1_1209	DJI_0009	Poa sp.	0		NA	0	Poaceae Barnhart
Iz_1_1209	DJI_0009	Polygonatum odoratum (Mill.) Druce	0	n	1	0	Asparagaceae Juss.
Iz_1_1209	DJI_0009	Polygonum arenarium Waldst. & Kit.	0	n	1	0	Polygonaceae Juss.
Iz_1_1209	DJI_0009	Populus nigra L.	0	n	1	1	Salicaceae Mirb.
Iz_1_1209	DJI_0009	Populus tremula L.	0	n	1	1	Salicaceae Mirb.
Iz_1_1209	DJI_0009	Populus Ã canescens (Aiton) Sm.	0	n	1	1	Salicaceae Mirb.
Iz_1_1209	DJI_0009	Potentilla argentea L.	0	n	1	0	Rosaceae Juss.
Iz_1_1209	DJI_0009	Psammophiliella muralis (L.) Ikonn.	0	n	1	0	Caryophyllaceae Juss.
Iz_1_1209	DJI_0009	Psephellus sumensis (Kalen.) Greuter	0	n	1	0	Asteraceae Bercht. & J.Presl
Iz_1_1209	DJI_0009	Pulsatilla pratensis (L.) Mill. s.l.	0	n	1	0	Ranunculaceae Juss.
Iz_1_1209	DJI_0009	Pyrus communis L.	0	a	0	1	Rosaceae Juss.
Iz_1_1209	DJI_0009	Quercus robur L.	0	n	1	1	Fagaceae Dumort.
Iz_1_1209	DJI_0009	Robinia pseudoacacia L.	0	a	0	1	Fabaceae Lindl.
Iz_1_1209	DJI_0009	Rosa sp.	0		NA	1	Rosaceae Juss.
Iz_1_1209	DJI_0009	Rubus idaeus L.	0	n	1	0	Rosaceae Juss.
Iz_1_1209	DJI_0009	Rumex acetosella L.	0	n	1	0	Polygonaceae Juss.
Iz_1_1209	DJI_0009	Rumex thyrsiflorus Fingerh.	0	n	1	0	Polygonaceae Juss.
Iz_1_1209	DJI_0009	Salix alba L.	0	n	1	1	Salicaceae Mirb.
Iz_1_1209	DJI_0009	Sambucus nigra L.	0	n	1	1	Viburnaceae Raf.
Iz_1_1209	DJI_0009	Secale sylvestre Host	0	n	1	0	Poaceae Barnhart
Iz_1_1209	DJI_0009	Sempervivum ruthenicum Schnittsp. & C.B. Lehm.	0	n	1	0	Crassulaceae J.St.-Hil.
Iz_1_1209	DJI_0009	Setaria viridis (L.) P. Beauv.	0	n	1	0	Poaceae Barnhart
Iz_1_1209	DJI_0009	Silene borysthenica (Gruner) Walters	0	n	1	0	Caryophyllaceae Juss.
Iz_1_1209	DJI_0009	Silene latifolia subsp. alba (Mill.) Greuter & Burdet	0	n	1	0	Caryophyllaceae Juss.
Iz_1_1209	DJI_0009	Solidago virgaurea L.	0	n	1	0	Asteraceae Bercht. & J.Presl
Iz_1_1209	DJI_0009	Sorbus aucuparia L.	0	n	1	1	Rosaceae Juss.
Iz_1_1209	DJI_0009	Stachys recta L.	0	n	1	0	Lamiacea Lindl.
Iz_1_1209	DJI_0009	Tanacetum vulgare L.	0	n	1	0	Asteraceae Bercht. & J.Presl
Iz_1_1209	DJI_0009	Thymus pallasianus Heinr.Braun	0	n	1	0	Lamiacea Lindl.
Iz_1_1209	DJI_0009	Tilia cordata Mill.	0	n	1	1	Malvaceae Juss.
Iz_1_1209	DJI_0009	Trifolium alpestre L.	0	n	1	0	Fabaceae Lindl.
Iz_1_1209	DJI_0009	Trifolium arvense L.	0	n	1	0	Fabaceae Lindl.
Iz_1_1209	DJI_0009	Ulmus glabra Huds.	0	n	1	1	Ulmaceae Mirb.
Iz_1_1209	DJI_0009	Verbascum densiflorum Bertol.	0	n	1	0	Scrophulariaceae Juss.
Iz_1_1209	DJI_0009	Veronica austriaca L.	0	n	1	0	Plantaginaceae Juss.
Iz_1_1209	DJI_0009	Veronica incana L.	0	n	1	0	Plantaginaceae Juss.
Iz_1_1209	DJI_0009	Veronica verna L.	0	n	1	0	Plantaginaceae Juss.
Iz_1_1209	DJI_0009	Vincetoxicum hirundinaria Medik	0	n	1	0	Apocynaceae Juss.
Iz_1_1209	DJI_0013	Acer tataricum L.	0	n	1	1	Sapindaceae Juss.
Iz_1_1209	DJI_0013	Achillea millefolium L. s.l.	0	n	1	0	Asteraceae Bercht. & J.Presl
Iz_1_1209	DJI_0013	Allium oleraceum L.	0	n	1	0	Amaryllidaceae J.St.-Hil.
Iz_1_1209	DJI_0013	Ambrosia artemisiifolia L.	0	a	0	0	Asteraceae Bercht. & J.Presl
Iz_1_1209	DJI_0013	Amorpha fruticosa L.	0	a	0	0	Fabaceae Lindl.
Iz_1_1209	DJI_0013	Anthericum ramosum L.	0	n	1	0	Asparagaceae Juss.
Iz_1_1209	DJI_0013	Artemisia marschalliana Spreng.	0	n	1	0	Asteraceae Bercht. & J.Presl
Iz_1_1209	DJI_0013	Asparagus officinalis L.	0	n	1	0	Asparagaceae Juss.
Iz_1_1209	DJI_0013	Astragalus cicer L.	0	n	1	0	Fabaceae Lindl.
Iz_1_1209	DJI_0013	Bassia laniflora (S.G.Gmel.) A.J.Scott	0	n	1	0	Amaranthaceae Juss.
Iz_1_1209	DJI_0013	Berberis vulgaris L.	0	n	1	0	Berberidaceae Juss.
Iz_1_1209	DJI_0013	Betula pendula Roth.	0	n	1	1	Betulaceae Gray
Iz_1_1209	DJI_0013	Betula pendula Roth.	0	n	1	0	Poaceae Barnhart
Iz_1_1209	DJI_0013	Bromus inermis Leyss.	0		NA	NA	
Iz_1_1209	DJI_0013	Bromus squarrosus L.	0	a	0	0	Poaceae Barnhart
Iz_1_1209	DJI_0013	Bromus tectorum L.	0	a	0	0	Poaceae Barnhart
Iz_1_1209	DJI_0013	Calamagrostis epigejos (L.) Roth	2	n	1	0	Poaceae Barnhart
Iz_1_1209	DJI_0013	Carduus acanthoides L.	0	n	1	0	Asteraceae Bercht. & J.Presl
Iz_1_1209	DJI_0013	Carex sp.	0	n	1	0	Cyperaceae Juss.
Iz_1_1209	DJI_0013	Centaurea majorovii Dumbadze	0	n	1	0	Asteraceae Bercht. & J.Presl
Iz_1_1209	DJI_0013	Chamaecytisus ruthenicus (Fisch.ex Wol.) Klaskova	0	n	1	0	Fabaceae Lindl.
Iz_1_1209	DJI_0013	Chelidonium majus L.	0.5	n	1	0	Papaveraceae Juss.
Iz_1_1209	DJI_0013	Chenopodium betaceum Andrz.	0	n	1	0	Amaranthaceae Juss.
Iz_1_1209	DJI_0013	Chenopodium cf. album	0	n	1	0	Amaranthaceae Juss.
Iz_1_1209	DJI_0013	Chenopodium sp.	0		NA	0	Amaranthaceae Juss.
Iz_1_1209	DJI_0013	Chondrilla juncea L.	0	n	1	0	Asteraceae Bercht. & J.Presl
Iz_1_1209	DJI_0013	Cirsium vulgare (Savi) Ten.	0	n	1	0	Asteraceae Bercht. & J.Presl
Iz_1_1209	DJI_0013	Clinopodium acinos (L.) Kuntze	0	n	1	0	Lamiacea Lindl.
Iz_1_1209	DJI_0013	Convallaria majalis L.	0	n	1	0	Asparagaceae Juss.
Iz_1_1209	DJI_0013	Crataegus rhipidophylla Gand.	0	n	1	1	Rosaceae Juss.
Iz_1_1209	DJI_0013	Crepis foetida L.	0	n	1	0	Asteraceae Bercht. & J.Presl
Iz_1_1209	DJI_0013	Dactylis glomerata L.	0	n	1	0	Poaceae Barnhart
Iz_1_1209	DJI_0013	Dianthus campestris M.Bieb.	0	n	1	0	Caryophyllaceae Juss.
Iz_1_1209	DJI_0013	Digitaria sanguinalis (L.) Scop.	0	a	0	0	Poaceae Barnhart
Iz_1_1209	DJI_0013	Elymus repens (L.) Gould	0	n	1	0	Poaceae Barnhart
Iz_1_1209	DJI_0013	Epilobium tetragonum L.	0	n	1	0	Onagraceae Juss.
Iz_1_1209	DJI_0013	Eragrostis minor Host	0	a	0	0	Poaceae Barnhart
Iz_1_1209	DJI_0013	Erigeron canadensis L.	0	a	0	0	Asteraceae Bercht. & J.Presl
Iz_1_1209	DJI_0013	Euphorbia seguieriana Neck.	0	n	1	0	Euphorbiaceae Juss.
Iz_1_1209	DJI_0013	Fallopia dumetorum (L.) Holub	0	n	1	0	Polygonaceae Juss.
Iz_1_1209	DJI_0013	Festuca beckeri (Hack.) Trautv.	0	n	1	0	Poaceae Barnhart
Iz_1_1209	DJI_0013	Fragaria vesca L.	0	n	1	0	Rosaceae Juss.
Iz_1_1209	DJI_0013	Frangula alnus Mill.	0	n	1	1	Rhamnaceae Juss.
Iz_1_1209	DJI_0013	Fraxinus pennsylvanica Marshall	0	a	0	1	Oleaceae Hoffmanns. & Link
Iz_1_1209	DJI_0013	Galeopsis bifida Boenn.	0	n	1	0	Lamiacea Lindl.
Iz_1_1209	DJI_0013	Galium verum L.	0	n	1	0	Rubiaceae Juss.
Iz_1_1209	DJI_0013	Genista tinctoria L.	0	n	1	0	Fabaceae Lindl.
Iz_1_1209	DJI_0013	Geranium robertianum L.	0	n	1	0	Geraniaceae Juss.
Iz_1_1209	DJI_0013	Geranium sanquineum L.	0	n	1	0	Geraniaceae Juss.
Iz_1_1209	DJI_0013	Gypsophila paniculata L.	0	n	1	0	Caryophyllaceae Juss.
Iz_1_1209	DJI_0013	Helichrysum arenarium (L.) Moench	0	n	1	0	Asteraceae Bercht. & J.Presl
Iz_1_1209	DJI_0013	Herniaria polygama J.Gay	0	n	1	0	Caryophyllaceae Juss.
Iz_1_1209	DJI_0013	Hieracium robustum Fr.	0	n	1	0	Asteraceae Bercht. & J.Presl
Iz_1_1209	DJI_0013	Hieracium umbellatum L.	0	n	1	NA	Asteraceae Bercht. & J.Presl
Iz_1_1209	DJI_0013	Hylotelephium maximum (L.) Holub	0	n	1	0	Crassulaceae J.St.-Hil.
Iz_1_1209	DJI_0013	Jacobaea borysthenica (DC.) B.Nord. & Greuter	0	n	1	0	Asteraceae Bercht. & J.Presl

Iz_1_1209	DJI_0013	Jasione montana L.	0	n	1	0	Campanulaceae Juss.
Iz_1_1209	DJI_0013	Jurinea cyanoides (L.) Rchb.s.l.	0	n	1	0	Asteraceae Bercht. & J.Presl
Iz_1_1209	DJI_0013	Koeleria glauca (Spreng.) DC.	0	n	1	0	Poaceae Barnhart
Iz_1_1209	DJI_0013	Lactuca serriola L.	0	a	0	0	Asteraceae Bercht. & J.Presl
Iz_1_1209	DJI_0013	Linaria genistifolia (L.) Mill.	0	n	1	0	Plantaginaceae Juss.
Iz_1_1209	DJI_0013	Lomelosia argentea (L.) Greuter & Burdet	0	n	1	0	Caprifoliaceae Juss.
Iz_1_1209	DJI_0013	Melampyrum pratense L.	0	n	1	0	Orobanchaceae Vent.
Iz_1_1209	DJI_0013	Oenothera biennis L.	0	a	0	0	Onagraceae Juss.
Iz_1_1209	DJI_0013	Pentanema britannica (L.) D.Gut.Larr., Santos-Vicente, Anderb., E.Rico & M.M.Mart.Ort.	0	n	1	0	Asteraceae Bercht. & J.Presl
Iz_1_1209	DJI_0013	Peucedanum oreoselinum (L.) Moench	0.5	n	1	0	Apiaceae Lindl.
Iz_1_1209	DJI_0013	Peucedanum ruthenicum M.Bieb.	0	n	1	0	Apiaceae Lindl.
Iz_1_1209	DJI_0013	Pilosella echioides (Lumn.) F.W.Schultz & Sch.Bip.	0	n	1	0	Asteraceae Bercht. & J.Presl
Iz_1_1209	DJI_0013	Pilosella officinarum F. Schultz et Sch. Bip.	0	n	1	0	Asteraceae Bercht. & J.Presl
Iz_1_1209	DJI_0013	Pilosella sp.	0		NA	0	Asteraceae Bercht. & J.Presl
Iz_1_1209	DJI_0013	Pinus sylvestris L.	0	n	1	1	Pinaceae Spreng. ex F.Rudolphi
Iz_1_1209	DJI_0013	Poa annua L.	0	n	1	0	Poaceae Barnhart
Iz_1_1209	DJI_0013	Poa nemoralis L.	0	n	1	0	Poaceae Barnhart
Iz_1_1209	DJI_0013	Poa sp.	0		NA	0	Poaceae Barnhart
Iz_1_1209	DJI_0013	Polygonatum odoratum (Mill.) Druce	0	n	1	0	Asparagaceae Juss.
Iz_1_1209	DJI_0013	Polygonum arenarium Waldst. & Kit.	0	n	1	0	Polygonaceae Juss.
Iz_1_1209	DJI_0013	Populus nigra L.	0	n	1	1	Salicaceae Mirb.
Iz_1_1209	DJI_0013	Populus tremula L.	0	n	1	1	Salicaceae Mirb.
Iz_1_1209	DJI_0013	Populus Ã canescens (Aiton) Sm.	0	n	1	1	Salicaceae Mirb.
Iz_1_1209	DJI_0013	Potentilla argentea L.	0	n	1	0	Rosaceae Juss.
Iz_1_1209	DJI_0013	Psammophiliella muralis (L.) Ikonn.	0	n	1	0	Caryophyllaceae Juss.
Iz_1_1209	DJI_0013	Psephellus sumensis (Kalen.) Greuter	0	n	1	0	Asteraceae Bercht. & J.Presl
Iz_1_1209	DJI_0013	Pulsatilla pratensis (L.) Mill. s.l.	0	n	1	0	Ranunculaceae Juss.
Iz_1_1209	DJI_0013	Pyrus communis L.	0	a	0	1	Rosaceae Juss.
Iz_1_1209	DJI_0013	Quercus robur L.	0	n	1	1	Fagaceae Dumort.
Iz_1_1209	DJI_0013	Robinia pseudoacacia L.	0	a	0	1	Fabaceae Lindl.
Iz_1_1209	DJI_0013	Rosa sp.	0		NA	1	Rosaceae Juss.
Iz_1_1209	DJI_0013	Rubus idaeus L.	0	n	1	0	Rosaceae Juss.
Iz_1_1209	DJI_0013	Rumex acetosella L.	0	n	1	0	Polygonaceae Juss.
Iz_1_1209	DJI_0013	Rumex thyrsiflorus Fingerh.	0	n	1	0	Polygonaceae Juss.
Iz_1_1209	DJI_0013	Salix alba L.	0	n	1	1	Salicaceae Mirb.
Iz_1_1209	DJI_0013	Sambucus nigra L.	0	n	1	1	Viburnaceae Raf.
Iz_1_1209	DJI_0013	Secale sylvestre Host	0	n	1	0	Poaceae Barnhart
Iz_1_1209	DJI_0013	Sempervivum ruthenicum Schnittsp. & C.B. Lehm.	0	n	1	0	Crassulaceae J.St.-Hil.
Iz_1_1209	DJI_0013	Setaria viridis (L.) P. Beauv.	0	n	1	0	Poaceae Barnhart
Iz_1_1209	DJI_0013	Silene borysthenica (Gruner) Walters	0	n	1	0	Caryophyllaceae Juss.
Iz_1_1209	DJI_0013	Silene latifolia subsp. alba (Mill.) Greuter & Burdet	0	n	1	0	Caryophyllaceae Juss.
Iz_1_1209	DJI_0013	Solidago virgaurea L.	0	n	1	0	Asteraceae Bercht. & J.Presl
Iz_1_1209	DJI_0013	Sorbus aucuparia L.	0	n	1	1	Rosaceae Juss.
Iz_1_1209	DJI_0013	Stachys recta L.	0	n	1	0	Lamiacea Lindl.
Iz_1_1209	DJI_0013	Tanacetum vulgare L.	0	n	1	0	Asteraceae Bercht. & J.Presl
Iz_1_1209	DJI_0013	Thymus pallasianus Heinr.Braun	0	n	1	0	Lamiacea Lindl.
Iz_1_1209	DJI_0013	Tilia cordata Mill.	0	n	1	1	Malvaceae Juss.
Iz_1_1209	DJI_0013	Trifolium alpestre L.	0	n	1	0	Fabaceae Lindl.
Iz_1_1209	DJI_0013	Trifolium arvense L.	0	n	1	0	Fabaceae Lindl.
Iz_1_1209	DJI_0013	Ulmus glabra Huds.	0	n	1	1	Ulmaceae Mirb.
Iz_1_1209	DJI_0013	Verbascum densiflorum Bertol.	0	n	1	0	Scrophulariaceae Juss.
Iz_1_1209	DJI_0013	Veronica austriaca L.	0	n	1	0	Plantaginaceae Juss.
Iz_1_1209	DJI_0013	Veronica incana L.	0	n	1	0	Plantaginaceae Juss.
Iz_1_1209	DJI_0013	Veronica verna L.	0	n	1	0	Plantaginaceae Juss.
Iz_1_1209	DJI_0013	Vincetoxicum hirundinaria Medik	0	n	1	0	Apocynaceae Juss.
Iz_1_1209	DJI_0016	Acer tataricum L.	0	n	1	1	Sapindaceae Juss.
Iz_1_1209	DJI_0016	Achillea millefolium L. s.l.	0	n	1	0	Asteraceae Bercht. & J.Presl
Iz_1_1209	DJI_0016	Allium oleraceum L.	0	n	1	0	Amaryllidaceae J.St.-Hil.
Iz_1_1209	DJI_0016	Ambrosia artemisiifolia L.	0	a	0	0	Asteraceae Bercht. & J.Presl
Iz_1_1209	DJI_0016	Amorpha fruticosa L.	0	a	0	0	Fabaceae Lindl.
Iz_1_1209	DJI_0016	Anthericum ramosum L.	0	n	1	0	Asparagaceae Juss.
Iz_1_1209	DJI_0016	Artemisia marschalliana Spreng.	0	n	1	0	Asteraceae Bercht. & J.Presl
Iz_1_1209	DJI_0016	Asparagus officinalis L.	0	n	1	0	Asparagaceae Juss.
Iz_1_1209	DJI_0016	Astragalus cicer L.	0	n	1	0	Fabaceae Lindl.
Iz_1_1209	DJI_0016	Bassia laniflora (S.G.Gmel.) A.J.Scott	0	n	1	0	Amaranthaceae Juss.
Iz_1_1209	DJI_0016	Berberis vulgaris L.	0	n	1	0	Berberidaceae Juss.
Iz_1_1209	DJI_0016	Betula pendula Roth.	0	n	1	1	Betulaceae Gray
Iz_1_1209	DJI_0016	Betula pendula Roth.	0	n	1	0	Poaceae Barnhart
Iz_1_1209	DJI_0016	Bromus inermis Leyss.	0		NA	NA	
Iz_1_1209	DJI_0016	Bromus squarrosus L.	0	a	0	0	Poaceae Barnhart
Iz_1_1209	DJI_0016	Bromus tectorum L.	0	a	0	0	Poaceae Barnhart
Iz_1_1209	DJI_0016	Calamagrostis epigejos (L.) Roth	2	n	1	0	Poaceae Barnhart
Iz_1_1209	DJI_0016	Carduus acanthoides L.	0	n	1	0	Asteraceae Bercht. & J.Presl
Iz_1_1209	DJI_0016	Carex sp.	0	n	1	0	Cyperaceae Juss.
Iz_1_1209	DJI_0016	Centaurea majorovii Dumbadze	0	n	1	0	Asteraceae Bercht. & J.Presl
Iz_1_1209	DJI_0016	Chamaecytisus ruthenicus (Fisch.ex Wol.) Klaskova	0	n	1	0	Fabaceae Lindl.
Iz_1_1209	DJI_0016	Chelidonium majus L.	0.1	n	1	0	Papaveraceae Juss.
Iz_1_1209	DJI_0016	Chenopodium betaceum Andrz.	0	n	1	0	Amaranthaceae Juss.
Iz_1_1209	DJI_0016	Chenopodium cf. album	0	n	1	0	Amaranthaceae Juss.
Iz_1_1209	DJI_0016	Chenopodium sp.	0		NA	0	Amaranthaceae Juss.
Iz_1_1209	DJI_0016	Chondrilla juncea L.	0	n	1	0	Asteraceae Bercht. & J.Presl
Iz_1_1209	DJI_0016	Cirsium vulgare (Savi) Ten.	0	n	1	0	Asteraceae Bercht. & J.Presl
Iz_1_1209	DJI_0016	Clinopodium acinos (L.) Kuntze	0	n	1	0	Lamiacea Lindl.
Iz_1_1209	DJI_0016	Convallaria majalis L.	0	n	1	0	Asparagaceae Juss.
Iz_1_1209	DJI_0016	Crataegus rhipidophylla Gand.	0	n	1	1	Rosaceae Juss.
Iz_1_1209	DJI_0016	Crepis foetida L.	0	n	1	0	Asteraceae Bercht. & J.Presl
Iz_1_1209	DJI_0016	Dactylis glomerata L.	0	n	1	0	Poaceae Barnhart
Iz_1_1209	DJI_0016	Dianthus campestris M.Bieb.	0	n	1	0	Caryophyllaceae Juss.
Iz_1_1209	DJI_0016	Digitaria sanguinalis (L.) Scop.	0	a	0	0	Poaceae Barnhart
Iz_1_1209	DJI_0016	Elymus repens (L.) Gould	0	n	1	0	Poaceae Barnhart
Iz_1_1209	DJI_0016	Epilobium tetragonum L.	0	n	1	0	Onagraceae Juss.
Iz_1_1209	DJI_0016	Eragrostis minor Host	0	a	0	0	Poaceae Barnhart
Iz_1_1209	DJI_0016	Erigeron canadensis L.	0	a	0	0	Asteraceae Bercht. & J.Presl
Iz_1_1209	DJI_0016	Euphorbia seguieriana Neck.	0	n	1	0	Euphorbiaceae Juss.
Iz_1_1209	DJI_0016	Fallopia dumetorum (L.) Holub	0	n	1	0	Polygonaceae Juss.
Iz_1_1209	DJI_0016	Festuca beckeri (Hack.) Trautv.	0	n	1	0	Poaceae Barnhart
Iz_1_1209	DJI_0016	Fragaria vesca L.	0	n	1	0	Rosaceae Juss.
Iz_1_1209	DJI_0016	Frangula alnus Mill.	0	n	1	1	Rhamnaceae Juss.

Iz_1_1209	DJI_0016	Fraxinus pennsylvanica Marshall	0	a	0	1	Oleaceae Hoffmanns. & Link
Iz_1_1209	DJI_0016	Galeopsis bifida Boenn.	0	n	1	0	Lamiacea Lindl.
Iz_1_1209	DJI_0016	Galium verum L.	0	n	1	0	Rubiaceae Juss.
Iz_1_1209	DJI_0016	Genista tinctoria L.	0	n	1	0	Fabaceae Lindl.
Iz_1_1209	DJI_0016	Geranium robertianum L.	0	n	1	0	Geraniaceae Juss.
Iz_1_1209	DJI_0016	Geranium sanquineum L.	0	n	1	0	Geraniaceae Juss.
Iz_1_1209	DJI_0016	Gypsophila paniculata L.	0	n	1	0	Caryophyllaceae Juss.
Iz_1_1209	DJI_0016	Helichrysum arenarium (L.) Moench	0	n	1	0	Asteraceae Bercht. & J.Presl
Iz_1_1209	DJI_0016	Herniaria polygama J.Gay	0	n	1	0	Caryophyllaceae Juss.
Iz_1_1209	DJI_0016	Hieracium robustum Fr.	0	n	1	0	Asteraceae Bercht. & J.Presl
Iz_1_1209	DJI_0016	Hieracium umbellatum L.	0	n	1	NA	Asteraceae Bercht. & J.Presl
Iz_1_1209	DJI_0016	Hylotelephium maximum (L.) Holub	0	n	1	0	Crassulaceae J.St.-Hil.
Iz_1_1209	DJI_0016	Jacobaea borysthenica (DC.) B.Nord. & Greuter	0	n	1	0	Asteraceae Bercht. & J.Presl
Iz_1_1209	DJI_0016	Jasione montana L.	0	n	1	0	Campanulaceae Juss.
Iz_1_1209	DJI_0016	Jurinea cyanoides (L.) Rchb.s.l.	0	n	1	0	Asteraceae Bercht. & J.Presl
Iz_1_1209	DJI_0016	Koeleria glauca (Spreng.) DC.	0	n	1	0	Poaceae Barnhart
Iz_1_1209	DJI_0016	Lactuca serriola L.	0	a	0	0	Asteraceae Bercht. & J.Presl
Iz_1_1209	DJI_0016	Linaria genistifolia (L.) Mill.	0	n	1	0	Plantaginaceae Juss.
Iz_1_1209	DJI_0016	Lomelosia argentea (L.) Greuter & Burdet	0	n	1	0	Caprifoliaceae Juss.
Iz_1_1209	DJI_0016	Melampyrum pratense L.	0	n	1	0	Orobanchaceae Vent.
Iz_1_1209	DJI_0016	Oenothera biennis L.	0	a	0	0	Onagraceae Juss.
Iz_1_1209	DJI_0016	Pentanema britannica (L.) D.Gut.Larr., Santos-Vicente, Anderb., E.Rico & M.M.Mart.Ort.	0	n	1	0	Asteraceae Bercht. & J.Presl
Iz_1_1209	DJI_0016	Peucedanum oreoselinum (L.) Moench	3	n	1	0	Apiaceae Lindl.
Iz_1_1209	DJI_0016	Peucedanum ruthenicum M.Bieb.	0	n	1	0	Apiaceae Lindl.
Iz_1_1209	DJI_0016	Pilosella echioides (Lumn.) F.W.Schultz & Sch.Bip.	0	n	1	0	Asteraceae Bercht. & J.Presl
Iz_1_1209	DJI_0016	Pilosella officinarum F. Schultz et Sch. Bip.	0	n	1	0	Asteraceae Bercht. & J.Presl
Iz_1_1209	DJI_0016	Pilosella sp.	0		NA	0	Asteraceae Bercht. & J.Presl
Iz_1_1209	DJI_0016	Pinus sylvestris L.	0	n	1	1	Pinaceae Spreng. ex F.Rudolphi
Iz_1_1209	DJI_0016	Poa annua L.	0	n	1	0	Poaceae Barnhart
Iz_1_1209	DJI_0016	Poa nemoralis L.	0	n	1	0	Poaceae Barnhart
Iz_1_1209	DJI_0016	Poa sp.	0		NA	0	Poaceae Barnhart
Iz_1_1209	DJI_0016	Polygonatum odoratum (Mill.) Druce	0	n	1	0	Asparagaceae Juss.
Iz_1_1209	DJI_0016	Polygonum arenarium Waldst. & Kit.	0	n	1	0	Polygonaceae Juss.
Iz_1_1209	DJI_0016	Populus nigra L.	0	n	1	1	Salicaceae Mirb.
Iz_1_1209	DJI_0016	Populus tremula L.	0	n	1	1	Salicaceae Mirb.
Iz_1_1209	DJI_0016	Populus Ã canescens (Aiton) Sm.	0	n	1	1	Salicaceae Mirb.
Iz_1_1209	DJI_0016	Potentilla argentea L.	0	n	1	0	Rosaceae Juss.
Iz_1_1209	DJI_0016	Psammophiliella muralis (L.) Ikonn.	0	n	1	0	Caryophyllaceae Juss.
Iz_1_1209	DJI_0016	Psephellus sumensis (Kalen.) Greuter	0	n	1	0	Asteraceae Bercht. & J.Presl
Iz_1_1209	DJI_0016	Pulsatilla pratensis (L.) Mill. s.l.	0	n	1	0	Ranunculaceae Juss.
Iz_1_1209	DJI_0016	Pyrus communis L.	0	a	0	1	Rosaceae Juss.
Iz_1_1209	DJI_0016	Quercus robur L.	0	n	1	1	Fagaceae Dumort.
Iz_1_1209	DJI_0016	Robinia pseudoacacia L.	0	a	0	1	Fabaceae Lindl.
Iz_1_1209	DJI_0016	Rosa sp.	0		NA	1	Rosaceae Juss.
Iz_1_1209	DJI_0016	Rubus idaeus L.	0	n	1	0	Rosaceae Juss.
Iz_1_1209	DJI_0016	Rumex acetosella L.	0	n	1	0	Polygonaceae Juss.
Iz_1_1209	DJI_0016	Rumex thyrsiflorus Fingerh.	0	n	1	0	Polygonaceae Juss.
Iz_1_1209	DJI_0016	Salix alba L.	0	n	1	1	Salicaceae Mirb.
Iz_1_1209	DJI_0016	Sambucus nigra L.	0	n	1	1	Viburnaceae Raf.
Iz_1_1209	DJI_0016	Secale sylvestre Host	0	n	1	0	Poaceae Barnhart
Iz_1_1209	DJI_0016	Sempervivum ruthenicum Schnittsp. & C.B. Lehm.	0	n	1	0	Crassulaceae J.St.-Hil.
Iz_1_1209	DJI_0016	Setaria viridis (L.) P. Beauv.	0	n	1	0	Poaceae Barnhart
Iz_1_1209	DJI_0016	Silene borysthenica (Gruner) Walters	0	n	1	0	Caryophyllaceae Juss.
Iz_1_1209	DJI_0016	Silene latifolia subsp. alba (Mill.) Greuter & Burdet	0	n	1	0	Caryophyllaceae Juss.
Iz_1_1209	DJI_0016	Solidago virgaurea L.	0	n	1	0	Asteraceae Bercht. & J.Presl
Iz_1_1209	DJI_0016	Sorbus aucuparia L.	0	n	1	1	Rosaceae Juss.
Iz_1_1209	DJI_0016	Stachys recta L.	0	n	1	0	Lamiacea Lindl.
Iz_1_1209	DJI_0016	Tanacetum vulgare L.	0	n	1	0	Asteraceae Bercht. & J.Presl
Iz_1_1209	DJI_0016	Thymus pallasianus Heinr.Braun	0	n	1	0	Lamiacea Lindl.
Iz_1_1209	DJI_0016	Tilia cordata Mill.	0	n	1	1	Malvaceae Juss.
Iz_1_1209	DJI_0016	Trifolium alpestre L.	0	n	1	0	Fabaceae Lindl.
Iz_1_1209	DJI_0016	Trifolium arvense L.	0	n	1	0	Fabaceae Lindl.
Iz_1_1209	DJI_0016	Ulmus glabra Huds.	0	n	1	1	Ulmaceae Mirb.
Iz_1_1209	DJI_0016	Verbascum densiflorum Bertol.	0	n	1	0	Scrophulariaceae Juss.
Iz_1_1209	DJI_0016	Veronica austriaca L.	0	n	1	0	Plantaginaceae Juss.
Iz_1_1209	DJI_0016	Veronica incana L.	0	n	1	0	Plantaginaceae Juss.
Iz_1_1209	DJI_0016	Veronica verna L.	0	n	1	0	Plantaginaceae Juss.
Iz_1_1209	DJI_0016	Vincetoxicum hirundinaria Medik	0	n	1	0	Apocynaceae Juss.
Iz_1_1209	DJI_0020	Acer tataricum L.	0	n	1	1	Sapindaceae Juss.
Iz_1_1209	DJI_0020	Achillea millefolium L. s.l.	0	n	1	0	Asteraceae Bercht. & J.Presl
Iz_1_1209	DJI_0020	Allium oleraceum L.	0	n	1	0	Amaryllidaceae J.St.-Hil.
Iz_1_1209	DJI_0020	Ambrosia artemisiifolia L.	0	a	0	0	Asteraceae Bercht. & J.Presl
Iz_1_1209	DJI_0020	Amorpha fruticosa L.	0	a	0	0	Fabaceae Lindl.
Iz_1_1209	DJI_0020	Anthericum ramosum L.	0	n	1	0	Asparagaceae Juss.
Iz_1_1209	DJI_0020	Artemisia marschalliana Spreng.	0	n	1	0	Asteraceae Bercht. & J.Presl
Iz_1_1209	DJI_0020	Asparagus officinalis L.	0	n	1	0	Asparagaceae Juss.
Iz_1_1209	DJI_0020	Astragalus cicer L.	0	n	1	0	Fabaceae Lindl.
Iz_1_1209	DJI_0020	Bassia laniflora (S.G.Gmel.) A.J.Scott	0	n	1	0	Amaranthaceae Juss.
Iz_1_1209	DJI_0020	Berberis vulgaris L.	0	n	1	0	Berberidaceae Juss.
Iz_1_1209	DJI_0020	Betula pendula Roth.	0	n	1	1	Betulaceae Gray
Iz_1_1209	DJI_0020	Betula pendula Roth.	0	n	1	0	Poaceae Barnhart
Iz_1_1209	DJI_0020	Bromus inermis Leyss.	0		NA	NA	
Iz_1_1209	DJI_0020	Bromus squarrosus L.	0	a	0	0	Poaceae Barnhart
Iz_1_1209	DJI_0020	Bromus tectorum L.	0	a	0	0	Poaceae Barnhart
Iz_1_1209	DJI_0020	Calamagrostis epigejos (L.) Roth	0.5	n	1	0	Poaceae Barnhart
Iz_1_1209	DJI_0020	Carduus acanthoides L.	0	n	1	0	Asteraceae Bercht. & J.Presl
Iz_1_1209	DJI_0020	Carex sp.	0	n	1	0	Cyperaceae Juss.
Iz_1_1209	DJI_0020	Centaurea majorovii Dumbadze	0	n	1	0	Asteraceae Bercht. & J.Presl
Iz_1_1209	DJI_0020	Chamaecytisus ruthenicus (Fisch.ex Wol.) Klaskova	0	n	1	0	Fabaceae Lindl.
Iz_1_1209	DJI_0020	Chelidonium majus L.	2	n	1	0	Papaveraceae Juss.
Iz_1_1209	DJI_0020	Chenopodium betaceum Andrz.	0	n	1	0	Amaranthaceae Juss.
Iz_1_1209	DJI_0020	Chenopodium cf. album	0	n	1	0	Amaranthaceae Juss.
Iz_1_1209	DJI_0020	Chenopodium sp.	0		NA	0	Amaranthaceae Juss.
Iz_1_1209	DJI_0020	Chondrilla juncea L.	0	n	1	0	Asteraceae Bercht. & J.Presl
Iz_1_1209	DJI_0020	Cirsium vulgare (Savi) Ten.	0	n	1	0	Asteraceae Bercht. & J.Presl
Iz_1_1209	DJI_0020	Clinopodium acinos (L.) Kuntze	0	n	1	0	Lamiacea Lindl.
Iz_1_1209	DJI_0020	Convallaria majalis L.	0	n	1	0	Asparagaceae Juss.
Iz_1_1209	DJI_0020	Crataegus rhipidophylla Gand.	0	n	1	1	Rosaceae Juss.

Iz_1_1209	DJI_0020	Crepis foetida L.	0	n	1	0	Asteraceae Bercht. & J.Presl
Iz_1_1209	DJI_0020	Dactylis glomerata L.	0	n	1	0	Poaceae Barnhart
Iz_1_1209	DJI_0020	Dianthus campestris M.Bieb.	0	n	1	0	Caryophyllaceae Juss.
Iz_1_1209	DJI_0020	Digitaria sanguinalis (L.) Scop.	0	a	0	0	Poaceae Barnhart
Iz_1_1209	DJI_0020	Elymus repens (L.) Gould	0	n	1	0	Poaceae Barnhart
Iz_1_1209	DJI_0020	Epilobium tetragonum L.	0	n	1	0	Onagraceae Juss.
Iz_1_1209	DJI_0020	Eragrostis minor Host	0	a	0	0	Poaceae Barnhart
Iz_1_1209	DJI_0020	Erigeron canadensis L.	0	a	0	0	Asteraceae Bercht. & J.Presl
Iz_1_1209	DJI_0020	Euphorbia seguieriana Neck.	0	n	1	0	Euphorbiaceae Juss.
Iz_1_1209	DJI_0020	Fallopia dumetorum (L.) Holub	0	n	1	0	Polygonaceae Juss.
Iz_1_1209	DJI_0020	Festuca beckeri (Hack.) Trautv.	0	n	1	0	Poaceae Barnhart
Iz_1_1209	DJI_0020	Fragaria vesca L.	0	n	1	0	Rosaceae Juss.
Iz_1_1209	DJI_0020	Frangula alnus Mill.	0	n	1	1	Rhamnaceae Juss.
Iz_1_1209	DJI_0020	Fraxinus pennsylvanica Marshall	0	a	0	1	Oleaceae Hoffmanns. & Link
Iz_1_1209	DJI_0020	Galeopsis bifida Boenn.	0	n	1	0	Lamiacea Lindl.
Iz_1_1209	DJI_0020	Galium verum L.	0	n	1	0	Rubiaceae Juss.
Iz_1_1209	DJI_0020	Genista tinctoria L.	0	n	1	0	Fabaceae Lindl.
Iz_1_1209	DJI_0020	Geranium robertianum L.	0.2	n	1	0	Geraniaceae Juss.
Iz_1_1209	DJI_0020	Geranium sanquineum L.	0	n	1	0	Geraniaceae Juss.
Iz_1_1209	DJI_0020	Gypsophila paniculata L.	0	n	1	0	Caryophyllaceae Juss.
Iz_1_1209	DJI_0020	Helichrysum arenarium (L.) Moench	0	n	1	0	Asteraceae Bercht. & J.Presl
Iz_1_1209	DJI_0020	Herniaria polygama J.Gay	0	n	1	0	Caryophyllaceae Juss.
Iz_1_1209	DJI_0020	Hieracium robustum Fr.	0	n	1	0	Asteraceae Bercht. & J.Presl
Iz_1_1209	DJI_0020	Hieracium umbellatum L.	0	n	1	NA	Asteraceae Bercht. & J.Presl
Iz_1_1209	DJI_0020	Hylotelephium maximum (L.) Holub	0	n	1	0	Crassulaceae J.St.-Hil.
Iz_1_1209	DJI_0020	Jacobaea borysthenica (DC.) B.Nord. & Greuter	0	n	1	0	Asteraceae Bercht. & J.Presl
Iz_1_1209	DJI_0020	Jasione montana L.	0	n	1	0	Campanulaceae Juss.
Iz_1_1209	DJI_0020	Jurinea cyanoides (L.) Rchb.s.l.	0	n	1	0	Asteraceae Bercht. & J.Presl
Iz_1_1209	DJI_0020	Koeleria glauca (Spreng.) DC.	0	n	1	0	Poaceae Barnhart
Iz_1_1209	DJI_0020	Lactuca serriola L.	0	a	0	0	Asteraceae Bercht. & J.Presl
Iz_1_1209	DJI_0020	Linaria genistifolia (L.) Mill.	0	n	1	0	Plantaginaceae Juss.
Iz_1_1209	DJI_0020	Lomelosia argentea (L.) Greuter & Burdet	0	n	1	0	Caprifoliaceae Juss.
Iz_1_1209	DJI_0020	Melampyrum pratense L.	0	n	1	0	Orobanchaceae Vent.
Iz_1_1209	DJI_0020	Oenothera biennis L.	0	a	0	0	Onagraceae Juss.
Iz_1_1209	DJI_0020	Pentanema britannica (L.) D.Gut.Larr., Santos-Vicente, Anderb., E.Rico & M.M.Mart.Ort.	0	n	1	0	Asteraceae Bercht. & J.Presl
Iz_1_1209	DJI_0020	Peucedanum oreoselinum (L.) Moench	0	n	1	0	Apiaceae Lindl.
Iz_1_1209	DJI_0020	Peucedanum ruthenicum M.Bieb.	0	n	1	0	Apiaceae Lindl.
Iz_1_1209	DJI_0020	Pilosella echioides (Lumn.) F.W.Schultz & Sch.Bip.	0	n	1	0	Asteraceae Bercht. & J.Presl
Iz_1_1209	DJI_0020	Pilosella officinarum F. Schultz et Sch. Bip.	0	n	1	0	Asteraceae Bercht. & J.Presl
Iz_1_1209	DJI_0020	Pilosella sp.	0		NA	0	Asteraceae Bercht. & J.Presl
Iz_1_1209	DJI_0020	Pinus sylvestris L.	0	n	1	1	Pinaceae Spreng. ex F.Rudolphi
Iz_1_1209	DJI_0020	Poa annua L.	0	n	1	0	Poaceae Barnhart
Iz_1_1209	DJI_0020	Poa nemoralis L.	0	n	1	0	Poaceae Barnhart
Iz_1_1209	DJI_0020	Poa sp.	0		NA	0	Poaceae Barnhart
Iz_1_1209	DJI_0020	Polygonatum odoratum (Mill.) Druce	0	n	1	0	Asparagaceae Juss.
Iz_1_1209	DJI_0020	Polygonum arenarium Waldst. & Kit.	0	n	1	0	Polygonaceae Juss.
Iz_1_1209	DJI_0020	Populus nigra L.	0	n	1	1	Salicaceae Mirb.
Iz_1_1209	DJI_0020	Populus tremula L.	0	n	1	1	Salicaceae Mirb.
Iz_1_1209	DJI_0020	Populus Ã canescens (Aiton) Sm.	0	n	1	1	Salicaceae Mirb.
Iz_1_1209	DJI_0020	Potentilla argentea L.	0	n	1	0	Rosaceae Juss.
Iz_1_1209	DJI_0020	Psammophiliella muralis (L.) Ikonn.	0	n	1	0	Caryophyllaceae Juss.
Iz_1_1209	DJI_0020	Psephellus sumensis (Kalen.) Greuter	0	n	1	0	Asteraceae Bercht. & J.Presl
Iz_1_1209	DJI_0020	Pulsatilla pratensis (L.) Mill. s.l.	0	n	1	0	Ranunculaceae Juss.
Iz_1_1209	DJI_0020	Pyrus communis L.	0	a	0	1	Rosaceae Juss.
Iz_1_1209	DJI_0020	Quercus robur L.	0	n	1	1	Fagaceae Dumort.
Iz_1_1209	DJI_0020	Robinia pseudoacacia L.	0	a	0	1	Fabaceae Lindl.
Iz_1_1209	DJI_0020	Rosa sp.	0		NA	1	Rosaceae Juss.
Iz_1_1209	DJI_0020	Rubus idaeus L.	0	n	1	0	Rosaceae Juss.
Iz_1_1209	DJI_0020	Rumex acetosella L.	0	n	1	0	Polygonaceae Juss.
Iz_1_1209	DJI_0020	Rumex thyrsiflorus Fingerh.	2	n	1	0	Polygonaceae Juss.
Iz_1_1209	DJI_0020	Salix alba L.	0	n	1	1	Salicaceae Mirb.
Iz_1_1209	DJI_0020	Sambucus nigra L.	0	n	1	1	Viburnaceae Raf.
Iz_1_1209	DJI_0020	Secale sylvestre Host	0	n	1	0	Poaceae Barnhart
Iz_1_1209	DJI_0020	Sempervivum ruthenicum Schnittsp. & C.B. Lehm.	0	n	1	0	Crassulaceae J.St.-Hil.
Iz_1_1209	DJI_0020	Setaria viridis (L.) P. Beauv.	0	n	1	0	Poaceae Barnhart
Iz_1_1209	DJI_0020	Silene borysthenica (Gruner) Walters	0	n	1	0	Caryophyllaceae Juss.
Iz_1_1209	DJI_0020	Silene latifolia subsp. alba (Mill.) Greuter & Burdet	0	n	1	0	Caryophyllaceae Juss.
Iz_1_1209	DJI_0020	Solidago virgaurea L.	0	n	1	0	Asteraceae Bercht. & J.Presl
Iz_1_1209	DJI_0020	Sorbus aucuparia L.	0	n	1	1	Rosaceae Juss.
Iz_1_1209	DJI_0020	Stachys recta L.	0	n	1	0	Lamiacea Lindl.
Iz_1_1209	DJI_0020	Tanacetum vulgare L.	0	n	1	0	Asteraceae Bercht. & J.Presl
Iz_1_1209	DJI_0020	Thymus pallasianus Heinr.Braun	0	n	1	0	Lamiacea Lindl.
Iz_1_1209	DJI_0020	Tilia cordata Mill.	0	n	1	1	Malvaceae Juss.
Iz_1_1209	DJI_0020	Trifolium alpestre L.	0	n	1	0	Fabaceae Lindl.
Iz_1_1209	DJI_0020	Trifolium arvense L.	0	n	1	0	Fabaceae Lindl.
Iz_1_1209	DJI_0020	Ulmus glabra Huds.	0	n	1	1	Ulmaceae Mirb.
Iz_1_1209	DJI_0020	Verbascum densiflorum Bertol.	0	n	1	0	Scrophulariaceae Juss.
Iz_1_1209	DJI_0020	Veronica austriaca L.	0	n	1	0	Plantaginaceae Juss.
Iz_1_1209	DJI_0020	Veronica incana L.	0	n	1	0	Plantaginaceae Juss.
Iz_1_1209	DJI_0020	Veronica verna L.	0	n	1	0	Plantaginaceae Juss.
Iz_1_1209	DJI_0020	Vincetoxicum hirundinaria Medik	0	n	1	0	Apocynaceae Juss.
Iz_1_1209	DJI_0023	Acer tataricum L.	0	n	1	1	Sapindaceae Juss.
Iz_1_1209	DJI_0023	Achillea millefolium L. s.l.	0	n	1	0	Asteraceae Bercht. & J.Presl
Iz_1_1209	DJI_0023	Allium oleraceum L.	0	n	1	0	Amaryllidaceae J.St.-Hil.
Iz_1_1209	DJI_0023	Ambrosia artemisiifolia L.	0	a	0	0	Asteraceae Bercht. & J.Presl
Iz_1_1209	DJI_0023	Amorpha fruticosa L.	0	a	0	0	Fabaceae Lindl.
Iz_1_1209	DJI_0023	Anthericum ramosum L.	0	n	1	0	Asparagaceae Juss.
Iz_1_1209	DJI_0023	Artemisia marschalliana Spreng.	0	n	1	0	Asteraceae Bercht. & J.Presl
Iz_1_1209	DJI_0023	Asparagus officinalis L.	0	n	1	0	Asparagaceae Juss.
Iz_1_1209	DJI_0023	Astragalus cicer L.	0	n	1	0	Fabaceae Lindl.
Iz_1_1209	DJI_0023	Bassia laniflora (S.G.Gmel.) A.J.Scott	0	n	1	0	Amaranthaceae Juss.
Iz_1_1209	DJI_0023	Berberis vulgaris L.	0	n	1	0	Berberidaceae Juss.
Iz_1_1209	DJI_0023	Betula pendula Roth.	0	n	1	1	Betulaceae Gray
Iz_1_1209	DJI_0023	Betula pendula Roth.	0	n	1	0	Poaceae Barnhart
Iz_1_1209	DJI_0023	Bromus inermis Leyss.	0		NA	NA	
Iz_1_1209	DJI_0023	Bromus squarrosus L.	0	a	0	0	Poaceae Barnhart
Iz_1_1209	DJI_0023	Bromus tectorum L.	0	a	0	0	Poaceae Barnhart
Iz_1_1209	DJI_0023	Calamagrostis epigejos (L.) Roth	25	n	1	0	Poaceae Barnhart

Iz_1_1209	DJI_0023	Carduus acanthoides L.	0	n	1	0	Asteraceae Bercht. & J.Presl
Iz_1_1209	DJI_0023	Carex sp.	0	n	1	0	Cyperaceae Juss.
Iz_1_1209	DJI_0023	Centaurea majorovii Dumbadze	0	n	1	0	Asteraceae Bercht. & J.Presl
Iz_1_1209	DJI_0023	Chamaecytisus ruthenicus (Fisch.ex Wol.) Klaskova	0	n	1	0	Fabaceae Lindl.
Iz_1_1209	DJI_0023	Chelidonium majus L.	0	n	1	0	Papaveraceae Juss.
Iz_1_1209	DJI_0023	Chenopodium betaceum Andrz.	0	n	1	0	Amaranthaceae Juss.
Iz_1_1209	DJI_0023	Chenopodium cf. album	0	n	1	0	Amaranthaceae Juss.
Iz_1_1209	DJI_0023	Chenopodium sp.	0		NA	0	Amaranthaceae Juss.
Iz_1_1209	DJI_0023	Chondrilla juncea L.	0	n	1	0	Asteraceae Bercht. & J.Presl
Iz_1_1209	DJI_0023	Cirsium vulgare (Savi) Ten.	0	n	1	0	Asteraceae Bercht. & J.Presl
Iz_1_1209	DJI_0023	Clinopodium acinos (L.) Kuntze	0	n	1	0	Lamiacea Lindl.
Iz_1_1209	DJI_0023	Convallaria majalis L.	0	n	1	0	Asparagaceae Juss.
Iz_1_1209	DJI_0023	Crataegus rhipidophylla Gand.	0	n	1	1	Rosaceae Juss.
Iz_1_1209	DJI_0023	Crepis foetida L.	0	n	1	0	Asteraceae Bercht. & J.Presl
Iz_1_1209	DJI_0023	Dactylis glomerata L.	0	n	1	0	Poaceae Barnhart
Iz_1_1209	DJI_0023	Dianthus campestris M.Bieb.	0	n	1	0	Caryophyllaceae Juss.
Iz_1_1209	DJI_0023	Digitaria sanguinalis (L.) Scop.	0	a	0	0	Poaceae Barnhart
Iz_1_1209	DJI_0023	Elymus repens (L.) Gould	0	n	1	0	Poaceae Barnhart
Iz_1_1209	DJI_0023	Epilobium tetragonum L.	0	n	1	0	Onagraceae Juss.
Iz_1_1209	DJI_0023	Eragrostis minor Host	0	a	0	0	Poaceae Barnhart
Iz_1_1209	DJI_0023	Erigeron canadensis L.	0.5	a	0	0	Asteraceae Bercht. & J.Presl
Iz_1_1209	DJI_0023	Euphorbia seguieriana Neck.	0	n	1	0	Euphorbiaceae Juss.
Iz_1_1209	DJI_0023	Fallopia dumetorum (L.) Holub	1	n	1	0	Polygonaceae Juss.
Iz_1_1209	DJI_0023	Festuca beckeri (Hack.) Trautv.	0	n	1	0	Poaceae Barnhart
Iz_1_1209	DJI_0023	Fragaria vesca L.	0	n	1	0	Rosaceae Juss.
Iz_1_1209	DJI_0023	Frangula alnus Mill.	0	n	1	1	Rhamnaceae Juss.
Iz_1_1209	DJI_0023	Fraxinus pennsylvanica Marshall	0	a	0	1	Oleaceae Hoffmanns. & Link
Iz_1_1209	DJI_0023	Galeopsis bifida Boenn.	0	n	1	0	Lamiacea Lindl.
Iz_1_1209	DJI_0023	Galium verum L.	0	n	1	0	Rubiaceae Juss.
Iz_1_1209	DJI_0023	Genista tinctoria L.	0	n	1	0	Fabaceae Lindl.
Iz_1_1209	DJI_0023	Geranium robertianum L.	0	n	1	0	Geraniaceae Juss.
Iz_1_1209	DJI_0023	Geranium sanquineum L.	0	n	1	0	Geraniaceae Juss.
Iz_1_1209	DJI_0023	Gypsophila paniculata L.	0	n	1	0	Caryophyllaceae Juss.
Iz_1_1209	DJI_0023	Helichrysum arenarium (L.) Moench	0	n	1	0	Asteraceae Bercht. & J.Presl
Iz_1_1209	DJI_0023	Herniaria polygama J.Gay	0	n	1	0	Caryophyllaceae Juss.
Iz_1_1209	DJI_0023	Hieracium robustum Fr.	0	n	1	0	Asteraceae Bercht. & J.Presl
Iz_1_1209	DJI_0023	Hieracium umbellatum L.	0	n	1	NA	Asteraceae Bercht. & J.Presl
Iz_1_1209	DJI_0023	Hylotelephium maximum (L.) Holub	0	n	1	0	Crassulaceae J.St.-Hil.
Iz_1_1209	DJI_0023	Jacobaea borysthenica (DC.) B.Nord. & Greuter	0	n	1	0	Asteraceae Bercht. & J.Presl
Iz_1_1209	DJI_0023	Jasione montana L.	0	n	1	0	Campanulaceae Juss.
Iz_1_1209	DJI_0023	Jurinea cyanoides (L.) Rchb.s.l.	0	n	1	0	Asteraceae Bercht. & J.Presl
Iz_1_1209	DJI_0023	Koeleria glauca (Spreng.) DC.	0	n	1	0	Poaceae Barnhart
Iz_1_1209	DJI_0023	Lactuca serriola L.	0	a	0	0	Asteraceae Bercht. & J.Presl
Iz_1_1209	DJI_0023	Linaria genistifolia (L.) Mill.	0	n	1	0	Plantaginaceae Juss.
Iz_1_1209	DJI_0023	Lomelosia argentea (L.) Greuter & Burdet	0	n	1	0	Caprifoliaceae Juss.
Iz_1_1209	DJI_0023	Melampyrum pratense L.	0	n	1	0	Orobanchaceae Vent.
Iz_1_1209	DJI_0023	Oenothera biennis L.	0	a	0	0	Onagraceae Juss.
Iz_1_1209	DJI_0023	Pentanema britannica (L.) D.Gut.Larr., Santos-Vicente, Anderb., E.Rico & M.M.Mart.Ort.	0	n	1	0	Asteraceae Bercht. & J.Presl
Iz_1_1209	DJI_0023	Peucedanum oreoselinum (L.) Moench	1	n	1	0	Apiaceae Lindl.
Iz_1_1209	DJI_0023	Peucedanum ruthenicum M.Bieb.	0	n	1	0	Apiaceae Lindl.
Iz_1_1209	DJI_0023	Pilosella echioides (Lumn.) F.W.Schultz & Sch.Bip.	0	n	1	0	Asteraceae Bercht. & J.Presl
Iz_1_1209	DJI_0023	Pilosella officinarum F. Schultz et Sch. Bip.	0	n	1	0	Asteraceae Bercht. & J.Presl
Iz_1_1209	DJI_0023	Pilosella sp.	0		NA	0	Asteraceae Bercht. & J.Presl
Iz_1_1209	DJI_0023	Pinus sylvestris L.	0	n	1	1	Pinaceae Spreng. ex F.Rudolphi
Iz_1_1209	DJI_0023	Poa annua L.	0	n	1	0	Poaceae Barnhart
Iz_1_1209	DJI_0023	Poa nemoralis L.	0	n	1	0	Poaceae Barnhart
Iz_1_1209	DJI_0023	Poa sp.	0		NA	0	Poaceae Barnhart
Iz_1_1209	DJI_0023	Polygonatum odoratum (Mill.) Druce	0	n	1	0	Asparagaceae Juss.
Iz_1_1209	DJI_0023	Polygonum arenarium Waldst. & Kit.	0	n	1	0	Polygonaceae Juss.
Iz_1_1209	DJI_0023	Populus nigra L.	0	n	1	1	Salicaceae Mirb.
Iz_1_1209	DJI_0023	Populus tremula L.	0	n	1	1	Salicaceae Mirb.
Iz_1_1209	DJI_0023	Populus Ã canescens (Aiton) Sm.	0	n	1	1	Salicaceae Mirb.
Iz_1_1209	DJI_0023	Potentilla argentea L.	0	n	1	0	Rosaceae Juss.
Iz_1_1209	DJI_0023	Psammophiliella muralis (L.) Ikonn.	0	n	1	0	Caryophyllaceae Juss.
Iz_1_1209	DJI_0023	Psephellus sumensis (Kalen.) Greuter	0	n	1	0	Asteraceae Bercht. & J.Presl
Iz_1_1209	DJI_0023	Pulsatilla pratensis (L.) Mill. s.l.	0	n	1	0	Ranunculaceae Juss.
Iz_1_1209	DJI_0023	Pyrus communis L.	0	a	0	1	Rosaceae Juss.
Iz_1_1209	DJI_0023	Quercus robur L.	0	n	1	1	Fagaceae Dumort.
Iz_1_1209	DJI_0023	Robinia pseudoacacia L.	0	a	0	1	Fabaceae Lindl.
Iz_1_1209	DJI_0023	Rosa sp.	0		NA	1	Rosaceae Juss.
Iz_1_1209	DJI_0023	Rubus idaeus L.	0	n	1	0	Rosaceae Juss.
Iz_1_1209	DJI_0023	Rumex acetosella L.	0	n	1	0	Polygonaceae Juss.
Iz_1_1209	DJI_0023	Rumex thyrsiflorus Fingerh.	0	n	1	0	Polygonaceae Juss.
Iz_1_1209	DJI_0023	Salix alba L.	0	n	1	1	Salicaceae Mirb.
Iz_1_1209	DJI_0023	Sambucus nigra L.	0	n	1	1	Viburnaceae Raf.
Iz_1_1209	DJI_0023	Secale sylvestre Host	0	n	1	0	Poaceae Barnhart
Iz_1_1209	DJI_0023	Sempervivum ruthenicum Schnittsp. & C.B. Lehm.	0	n	1	0	Crassulaceae J.St.-Hil.
Iz_1_1209	DJI_0023	Setaria viridis (L.) P. Beauv.	0	n	1	0	Poaceae Barnhart
Iz_1_1209	DJI_0023	Silene borysthenica (Gruner) Walters	0	n	1	0	Caryophyllaceae Juss.
Iz_1_1209	DJI_0023	Silene latifolia subsp. alba (Mill.) Greuter & Burdet	0	n	1	0	Caryophyllaceae Juss.
Iz_1_1209	DJI_0023	Solidago virgaurea L.	2	n	1	0	Asteraceae Bercht. & J.Presl
Iz_1_1209	DJI_0023	Sorbus aucuparia L.	0	n	1	1	Rosaceae Juss.
Iz_1_1209	DJI_0023	Stachys recta L.	0	n	1	0	Lamiacea Lindl.
Iz_1_1209	DJI_0023	Tanacetum vulgare L.	0	n	1	0	Asteraceae Bercht. & J.Presl
Iz_1_1209	DJI_0023	Thymus pallasianus Heinr.Braun	0	n	1	0	Lamiacea Lindl.
Iz_1_1209	DJI_0023	Tilia cordata Mill.	0	n	1	1	Malvaceae Juss.
Iz_1_1209	DJI_0023	Trifolium alpestre L.	0	n	1	0	Fabaceae Lindl.
Iz_1_1209	DJI_0023	Trifolium arvense L.	0	n	1	0	Fabaceae Lindl.
Iz_1_1209	DJI_0023	Ulmus glabra Huds.	0	n	1	1	Ulmaceae Mirb.
Iz_1_1209	DJI_0023	Verbascum densiflorum Bertol.	0	n	1	0	Scrophulariaceae Juss.
Iz_1_1209	DJI_0023	Veronica austriaca L.	0	n	1	0	Plantaginaceae Juss.
Iz_1_1209	DJI_0023	Veronica incana L.	0	n	1	0	Plantaginaceae Juss.
Iz_1_1209	DJI_0023	Veronica verna L.	0	n	1	0	Plantaginaceae Juss.
Iz_1_1209	DJI_0023	Vincetoxicum hirundinaria Medik	0	n	1	0	Apocynaceae Juss.
Iz_1_1209	DJI_0026	Acer tataricum L.	0	n	1	1	Sapindaceae Juss.
Iz_1_1209	DJI_0026	Achillea millefolium L. s.l.	0	n	1	0	Asteraceae Bercht. & J.Presl
Iz_1_1209	DJI_0026	Allium oleraceum L.	0	n	1	0	Amaryllidaceae J.St.-Hil.
Iz_1_1209	DJI_0026	Ambrosia artemisiifolia L.	0	a	0	0	Asteraceae Bercht. & J.Presl

Iz_1_1209	DJI_0026	Amorpha fruticosa L.	0	a	0	0	Fabaceae Lindl.
Iz_1_1209	DJI_0026	Anthericum ramosum L.	0	n	1	0	Asparagaceae Juss.
Iz_1_1209	DJI_0026	Artemisia marschalliana Spreng.	0	n	1	0	Asteraceae Bercht. & J.Presl
Iz_1_1209	DJI_0026	Asparagus officinalis L.	0	n	1	0	Asparagaceae Juss.
Iz_1_1209	DJI_0026	Astragalus cicer L.	0	n	1	0	Fabaceae Lindl.
Iz_1_1209	DJI_0026	Bassia laniflora (S.G.Gmel.) A.J.Scott	0	n	1	0	Amaranthaceae Juss.
Iz_1_1209	DJI_0026	Berberis vulgaris L.	0	n	1	0	Berberidaceae Juss.
Iz_1_1209	DJI_0026	Betula pendula Roth.	0	n	1	1	Betulaceae Gray
Iz_1_1209	DJI_0026	Betula pendula Roth.	0	n	1	0	Poaceae Barnhart
Iz_1_1209	DJI_0026	Bromus inermis Leyss.	0		NA	NA	
Iz_1_1209	DJI_0026	Bromus squarrosus L.	0	a	0	0	Poaceae Barnhart
Iz_1_1209	DJI_0026	Bromus tectorum L.	0	a	0	0	Poaceae Barnhart
Iz_1_1209	DJI_0026	Calamagrostis epigejos (L.) Roth	17	n	1	0	Poaceae Barnhart
Iz_1_1209	DJI_0026	Carduus acanthoides L.	0	n	1	0	Asteraceae Bercht. & J.Presl
Iz_1_1209	DJI_0026	Carex sp.	0	n	1	0	Cyperaceae Juss.
Iz_1_1209	DJI_0026	Centaurea majorovii Dumbadze	0	n	1	0	Asteraceae Bercht. & J.Presl
Iz_1_1209	DJI_0026	Chamaecytisus ruthenicus (Fisch.ex Wol.) Klaskova	0	n	1	0	Fabaceae Lindl.
Iz_1_1209	DJI_0026	Chelidonium majus L.	0	n	1	0	Papaveraceae Juss.
Iz_1_1209	DJI_0026	Chenopodium betaceum Andrz.	0	n	1	0	Amaranthaceae Juss.
Iz_1_1209	DJI_0026	Chenopodium cf. album	0	n	1	0	Amaranthaceae Juss.
Iz_1_1209	DJI_0026	Chenopodium sp.	0		NA	0	Amaranthaceae Juss.
Iz_1_1209	DJI_0026	Chondrilla juncea L.	0	n	1	0	Asteraceae Bercht. & J.Presl
Iz_1_1209	DJI_0026	Cirsium vulgare (Savi) Ten.	0	n	1	0	Asteraceae Bercht. & J.Presl
Iz_1_1209	DJI_0026	Clinopodium acinos (L.) Kuntze	0	n	1	0	Lamiacea Lindl.
Iz_1_1209	DJI_0026	Convallaria majalis L.	0	n	1	0	Asparagaceae Juss.
Iz_1_1209	DJI_0026	Crataegus rhipidophylla Gand.	0	n	1	1	Rosaceae Juss.
Iz_1_1209	DJI_0026	Crepis foetida L.	0	n	1	0	Asteraceae Bercht. & J.Presl
Iz_1_1209	DJI_0026	Dactylis glomerata L.	0	n	1	0	Poaceae Barnhart
Iz_1_1209	DJI_0026	Dianthus campestris M.Bieb.	0	n	1	0	Caryophyllaceae Juss.
Iz_1_1209	DJI_0026	Digitaria sanguinalis (L.) Scop.	0	a	0	0	Poaceae Barnhart
Iz_1_1209	DJI_0026	Elymus repens (L.) Gould	0	n	1	0	Poaceae Barnhart
Iz_1_1209	DJI_0026	Epilobium tetragonum L.	0	n	1	0	Onagraceae Juss.
Iz_1_1209	DJI_0026	Eragrostis minor Host	0	a	0	0	Poaceae Barnhart
Iz_1_1209	DJI_0026	Erigeron canadensis L.	0	a	0	0	Asteraceae Bercht. & J.Presl
Iz_1_1209	DJI_0026	Euphorbia seguieriana Neck.	0	n	1	0	Euphorbiaceae Juss.
Iz_1_1209	DJI_0026	Fallopia dumetorum (L.) Holub	1	n	1	0	Polygonaceae Juss.
Iz_1_1209	DJI_0026	Festuca beckeri (Hack.) Trautv.	0	n	1	0	Poaceae Barnhart
Iz_1_1209	DJI_0026	Fragaria vesca L.	0	n	1	0	Rosaceae Juss.
Iz_1_1209	DJI_0026	Frangula alnus Mill.	0	n	1	1	Rhamnaceae Juss.
Iz_1_1209	DJI_0026	Fraxinus pennsylvanica Marshall	0	a	0	1	Oleaceae Hoffmanns. & Link
Iz_1_1209	DJI_0026	Galeopsis bifida Boenn.	0	n	1	0	Lamiacea Lindl.
Iz_1_1209	DJI_0026	Galium verum L.	0	n	1	0	Rubiaceae Juss.
Iz_1_1209	DJI_0026	Genista tinctoria L.	0	n	1	0	Fabaceae Lindl.
Iz_1_1209	DJI_0026	Geranium robertianum L.	0	n	1	0	Geraniaceae Juss.
Iz_1_1209	DJI_0026	Geranium sanquineum L.	0	n	1	0	Geraniaceae Juss.
Iz_1_1209	DJI_0026	Gypsophila paniculata L.	0	n	1	0	Caryophyllaceae Juss.
Iz_1_1209	DJI_0026	Helichrysum arenarium (L.) Moench	0	n	1	0	Asteraceae Bercht. & J.Presl
Iz_1_1209	DJI_0026	Herniaria polygama J.Gay	0	n	1	0	Caryophyllaceae Juss.
Iz_1_1209	DJI_0026	Hieracium robustum Fr.	0	n	1	0	Asteraceae Bercht. & J.Presl
Iz_1_1209	DJI_0026	Hieracium umbellatum L.	0	n	1	NA	Asteraceae Bercht. & J.Presl
Iz_1_1209	DJI_0026	Hylotelephium maximum (L.) Holub	0	n	1	0	Crassulaceae J.St.-Hil.
Iz_1_1209	DJI_0026	Jacobaea borysthenica (DC.) B.Nord. & Greuter	0	n	1	0	Asteraceae Bercht. & J.Presl
Iz_1_1209	DJI_0026	Jasione montana L.	0	n	1	0	Campanulaceae Juss.
Iz_1_1209	DJI_0026	Jurinea cyanoides (L.) Rchb.s.l.	0	n	1	0	Asteraceae Bercht. & J.Presl
Iz_1_1209	DJI_0026	Koeleria glauca (Spreng.) DC.	0	n	1	0	Poaceae Barnhart
Iz_1_1209	DJI_0026	Lactuca serriola L.	0	a	0	0	Asteraceae Bercht. & J.Presl
Iz_1_1209	DJI_0026	Linaria genistifolia (L.) Mill.	0	n	1	0	Plantaginaceae Juss.
Iz_1_1209	DJI_0026	Lomelosia argentea (L.) Greuter & Burdet	0	n	1	0	Caprifoliaceae Juss.
Iz_1_1209	DJI_0026	Melampyrum pratense L.	0	n	1	0	Orobanchaceae Vent.
Iz_1_1209	DJI_0026	Oenothera biennis L.	0	a	0	0	Onagraceae Juss.
Iz_1_1209	DJI_0026	Pentanema britannica (L.) D.Gut.Larr., Santos-Vicente, Anderb., E.Rico & M.M.Mart.Ort.	0	n	1	0	Asteraceae Bercht. & J.Presl
Iz_1_1209	DJI_0026	Peucedanum oreoselinum (L.) Moench	0	n	1	0	Apiaceae Lindl.
Iz_1_1209	DJI_0026	Peucedanum ruthenicum M.Bieb.	0	n	1	0	Apiaceae Lindl.
Iz_1_1209	DJI_0026	Pilosella echioides (Lumn.) F.W.Schultz & Sch.Bip.	0	n	1	0	Asteraceae Bercht. & J.Presl
Iz_1_1209	DJI_0026	Pilosella officinarum F. Schultz et Sch. Bip.	0	n	1	0	Asteraceae Bercht. & J.Presl
Iz_1_1209	DJI_0026	Pilosella sp.	0		NA	0	Asteraceae Bercht. & J.Presl
Iz_1_1209	DJI_0026	Pinus sylvestris L.	0	n	1	1	Pinaceae Spreng. ex F.Rudolphi
Iz_1_1209	DJI_0026	Poa annua L.	0	n	1	0	Poaceae Barnhart
Iz_1_1209	DJI_0026	Poa nemoralis L.	0	n	1	0	Poaceae Barnhart
Iz_1_1209	DJI_0026	Poa sp.	0		NA	0	Poaceae Barnhart
Iz_1_1209	DJI_0026	Polygonatum odoratum (Mill.) Druce	0	n	1	0	Asparagaceae Juss.
Iz_1_1209	DJI_0026	Polygonum arenarium Waldst. & Kit.	0	n	1	0	Polygonaceae Juss.
Iz_1_1209	DJI_0026	Populus nigra L.	0	n	1	1	Salicaceae Mirb.
Iz_1_1209	DJI_0026	Populus tremula L.	0	n	1	1	Salicaceae Mirb.
Iz_1_1209	DJI_0026	Populus Ã canescens (Aiton) Sm.	0	n	1	1	Salicaceae Mirb.
Iz_1_1209	DJI_0026	Potentilla argentea L.	0	n	1	0	Rosaceae Juss.
Iz_1_1209	DJI_0026	Psammophiliella muralis (L.) Ikonn.	0	n	1	0	Caryophyllaceae Juss.
Iz_1_1209	DJI_0026	Psephellus sumensis (Kalen.) Greuter	0	n	1	0	Asteraceae Bercht. & J.Presl
Iz_1_1209	DJI_0026	Pulsatilla pratensis (L.) Mill. s.l.	0	n	1	0	Ranunculaceae Juss.
Iz_1_1209	DJI_0026	Pyrus communis L.	0	a	0	1	Rosaceae Juss.
Iz_1_1209	DJI_0026	Quercus robur L.	0	n	1	1	Fagaceae Dumort.
Iz_1_1209	DJI_0026	Robinia pseudoacacia L.	0	a	0	1	Fabaceae Lindl.
Iz_1_1209	DJI_0026	Rosa sp.	0		NA	1	Rosaceae Juss.
Iz_1_1209	DJI_0026	Rubus idaeus L.	0	n	1	0	Rosaceae Juss.
Iz_1_1209	DJI_0026	Rumex acetosella L.	0	n	1	0	Polygonaceae Juss.
Iz_1_1209	DJI_0026	Rumex thyrsiflorus Fingerh.	0	n	1	0	Polygonaceae Juss.
Iz_1_1209	DJI_0026	Salix alba L.	0	n	1	1	Salicaceae Mirb.
Iz_1_1209	DJI_0026	Sambucus nigra L.	0	n	1	1	Viburnaceae Raf.
Iz_1_1209	DJI_0026	Secale sylvestre Host	0	n	1	0	Poaceae Barnhart
Iz_1_1209	DJI_0026	Sempervivum ruthenicum Schnittsp. & C.B. Lehm.	0	n	1	0	Crassulaceae J.St.-Hil.
Iz_1_1209	DJI_0026	Setaria viridis (L.) P. Beauv.	0	n	1	0	Poaceae Barnhart
Iz_1_1209	DJI_0026	Silene borysthenica (Gruner) Walters	0	n	1	0	Caryophyllaceae Juss.
Iz_1_1209	DJI_0026	Silene latifolia subsp. alba (Mill.) Greuter & Burdet	0	n	1	0	Caryophyllaceae Juss.
Iz_1_1209	DJI_0026	Solidago virgaurea L.	0	n	1	0	Asteraceae Bercht. & J.Presl
Iz_1_1209	DJI_0026	Sorbus aucuparia L.	0	n	1	1	Rosaceae Juss.
Iz_1_1209	DJI_0026	Stachys recta L.	0	n	1	0	Lamiacea Lindl.
Iz_1_1209	DJI_0026	Tanacetum vulgare L.	0	n	1	0	Asteraceae Bercht. & J.Presl
Iz_1_1209	DJI_0026	Thymus pallasianus Heinr.Braun	0	n	1	0	Lamiacea Lindl.

Iz_1_1209	DJI_0026	Tilia cordata Mill.	0	n	1	1	Malvaceae Juss.
Iz_1_1209	DJI_0026	Trifolium alpestre L.	0	n	1	0	Fabaceae Lindl.
Iz_1_1209	DJI_0026	Trifolium arvense L.	0	n	1	0	Fabaceae Lindl.
Iz_1_1209	DJI_0026	Ulmus glabra Huds.	0	n	1	1	Ulmaceae Mirb.
Iz_1_1209	DJI_0026	Verbascum densiflorum Bertol.	0	n	1	0	Scrophulariaceae Juss.
Iz_1_1209	DJI_0026	Veronica austriaca L.	0	n	1	0	Plantaginaceae Juss.
Iz_1_1209	DJI_0026	Veronica incana L.	0	n	1	0	Plantaginaceae Juss.
Iz_1_1209	DJI_0026	Veronica verna L.	0	n	1	0	Plantaginaceae Juss.
Iz_1_1209	DJI_0026	Vincetoxicum hirundinaria Medik	0	n	1	0	Apocynaceae Juss.
Iz_1_1209	DJI_0029	Acer tataricum L.	0	n	1	1	Sapindaceae Juss.
Iz_1_1209	DJI_0029	Achillea millefolium L. s.l.	0	n	1	0	Asteraceae Bercht. & J.Presl
Iz_1_1209	DJI_0029	Allium oleraceum L.	0	n	1	0	Amaryllidaceae J.St.-Hil.
Iz_1_1209	DJI_0029	Ambrosia artemisiifolia L.	0	a	0	0	Asteraceae Bercht. & J.Presl
Iz_1_1209	DJI_0029	Amorpha fruticosa L.	0	a	0	0	Fabaceae Lindl.
Iz_1_1209	DJI_0029	Anthericum ramosum L.	0	n	1	0	Asparagaceae Juss.
Iz_1_1209	DJI_0029	Artemisia marschalliana Spreng.	0	n	1	0	Asteraceae Bercht. & J.Presl
Iz_1_1209	DJI_0029	Asparagus officinalis L.	0	n	1	0	Asparagaceae Juss.
Iz_1_1209	DJI_0029	Astragalus cicer L.	0	n	1	0	Fabaceae Lindl.
Iz_1_1209	DJI_0029	Bassia laniflora (S.G.Gmel.) A.J.Scott	0	n	1	0	Amaranthaceae Juss.
Iz_1_1209	DJI_0029	Berberis vulgaris L.	0	n	1	0	Berberidaceae Juss.
Iz_1_1209	DJI_0029	Betula pendula Roth.	0	n	1	1	Betulaceae Gray
Iz_1_1209	DJI_0029	Betula pendula Roth.	0	n	1	0	Poaceae Barnhart
Iz_1_1209	DJI_0029	Bromus inermis Leyss.	0		NA	NA	
Iz_1_1209	DJI_0029	Bromus squarrosus L.	0	a	0	0	Poaceae Barnhart
Iz_1_1209	DJI_0029	Bromus tectorum L.	0	a	0	0	Poaceae Barnhart
Iz_1_1209	DJI_0029	Calamagrostis epigejos (L.) Roth	6	n	1	0	Poaceae Barnhart
Iz_1_1209	DJI_0029	Carduus acanthoides L.	0	n	1	0	Asteraceae Bercht. & J.Presl
Iz_1_1209	DJI_0029	Carex sp.	0	n	1	0	Cyperaceae Juss.
Iz_1_1209	DJI_0029	Centaurea majorovii Dumbadze	0	n	1	0	Asteraceae Bercht. & J.Presl
Iz_1_1209	DJI_0029	Chamaecytisus ruthenicus (Fisch.ex Wol.) Klaskova	0	n	1	0	Fabaceae Lindl.
Iz_1_1209	DJI_0029	Chelidonium majus L.	0	n	1	0	Papaveraceae Juss.
Iz_1_1209	DJI_0029	Chenopodium betaceum Andrz.	0	n	1	0	Amaranthaceae Juss.
Iz_1_1209	DJI_0029	Chenopodium cf. album	0	n	1	0	Amaranthaceae Juss.
Iz_1_1209	DJI_0029	Chenopodium sp.	0		NA	0	Amaranthaceae Juss.
Iz_1_1209	DJI_0029	Chondrilla juncea L.	0	n	1	0	Asteraceae Bercht. & J.Presl
Iz_1_1209	DJI_0029	Cirsium vulgare (Savi) Ten.	0	n	1	0	Asteraceae Bercht. & J.Presl
Iz_1_1209	DJI_0029	Clinopodium acinos (L.) Kuntze	0	n	1	0	Lamiacea Lindl.
Iz_1_1209	DJI_0029	Convallaria majalis L.	0	n	1	0	Asparagaceae Juss.
Iz_1_1209	DJI_0029	Crataegus rhipidophylla Gand.	0	n	1	1	Rosaceae Juss.
Iz_1_1209	DJI_0029	Crepis foetida L.	0	n	1	0	Asteraceae Bercht. & J.Presl
Iz_1_1209	DJI_0029	Dactylis glomerata L.	0	n	1	0	Poaceae Barnhart
Iz_1_1209	DJI_0029	Dianthus campestris M.Bieb.	0	n	1	0	Caryophyllaceae Juss.
Iz_1_1209	DJI_0029	Digitaria sanguinalis (L.) Scop.	0	a	0	0	Poaceae Barnhart
Iz_1_1209	DJI_0029	Elymus repens (L.) Gould	0	n	1	0	Poaceae Barnhart
Iz_1_1209	DJI_0029	Epilobium tetragonum L.	0	n	1	0	Onagraceae Juss.
Iz_1_1209	DJI_0029	Eragrostis minor Host	0	a	0	0	Poaceae Barnhart
Iz_1_1209	DJI_0029	Erigeron canadensis L.	0	a	0	0	Asteraceae Bercht. & J.Presl
Iz_1_1209	DJI_0029	Euphorbia seguieriana Neck.	0	n	1	0	Euphorbiaceae Juss.
Iz_1_1209	DJI_0029	Fallopia dumetorum (L.) Holub	10	n	1	0	Polygonaceae Juss.
Iz_1_1209	DJI_0029	Festuca beckeri (Hack.) Trautv.	0	n	1	0	Poaceae Barnhart
Iz_1_1209	DJI_0029	Fragaria vesca L.	0	n	1	0	Rosaceae Juss.
Iz_1_1209	DJI_0029	Frangula alnus Mill.	0	n	1	1	Rhamnaceae Juss.
Iz_1_1209	DJI_0029	Fraxinus pennsylvanica Marshall	0	a	0	1	Oleaceae Hoffmanns. & Link
Iz_1_1209	DJI_0029	Galeopsis bifida Boenn.	0	n	1	0	Lamiacea Lindl.
Iz_1_1209	DJI_0029	Galium verum L.	0	n	1	0	Rubiaceae Juss.
Iz_1_1209	DJI_0029	Genista tinctoria L.	0	n	1	0	Fabaceae Lindl.
Iz_1_1209	DJI_0029	Geranium robertianum L.	0	n	1	0	Geraniaceae Juss.
Iz_1_1209	DJI_0029	Geranium sanquineum L.	0	n	1	0	Geraniaceae Juss.
Iz_1_1209	DJI_0029	Gypsophila paniculata L.	0	n	1	0	Caryophyllaceae Juss.
Iz_1_1209	DJI_0029	Helichrysum arenarium (L.) Moench	0	n	1	0	Asteraceae Bercht. & J.Presl
Iz_1_1209	DJI_0029	Herniaria polygama J.Gay	0	n	1	0	Caryophyllaceae Juss.
Iz_1_1209	DJI_0029	Hieracium robustum Fr.	0	n	1	0	Asteraceae Bercht. & J.Presl
Iz_1_1209	DJI_0029	Hieracium umbellatum L.	0	n	1	NA	Asteraceae Bercht. & J.Presl
Iz_1_1209	DJI_0029	Hylotelephium maximum (L.) Holub	0	n	1	0	Crassulaceae J.St.-Hil.
Iz_1_1209	DJI_0029	Jacobaea borysthenica (DC.) B.Nord. & Greuter	0	n	1	0	Asteraceae Bercht. & J.Presl
Iz_1_1209	DJI_0029	Jasione montana L.	0	n	1	0	Campanulaceae Juss.
Iz_1_1209	DJI_0029	Jurinea cyanoides (L.) Rchb.s.l.	0	n	1	0	Asteraceae Bercht. & J.Presl
Iz_1_1209	DJI_0029	Koeleria glauca (Spreng.) DC.	0	n	1	0	Poaceae Barnhart
Iz_1_1209	DJI_0029	Lactuca serriola L.	0	a	0	0	Asteraceae Bercht. & J.Presl
Iz_1_1209	DJI_0029	Linaria genistifolia (L.) Mill.	0	n	1	0	Plantaginaceae Juss.
Iz_1_1209	DJI_0029	Lomelosia argentea (L.) Greuter & Burdet	0	n	1	0	Caprifoliaceae Juss.
Iz_1_1209	DJI_0029	Melampyrum pratense L.	0	n	1	0	Orobanchaceae Vent.
Iz_1_1209	DJI_0029	Oenothera biennis L.	0	a	0	0	Onagraceae Juss.
Iz_1_1209	DJI_0029	Pentanema britannica (L.) D.Gut.Larr., Santos-Vicente, Anderb., E.Rico & M.M.Mart.Ort.	0	n	1	0	Asteraceae Bercht. & J.Presl
Iz_1_1209	DJI_0029	Peucedanum oreoselinum (L.) Moench	0	n	1	0	Apiaceae Lindl.
Iz_1_1209	DJI_0029	Peucedanum ruthenicum M.Bieb.	0	n	1	0	Apiaceae Lindl.
Iz_1_1209	DJI_0029	Pilosella echioides (Lumn.) F.W.Schultz & Sch.Bip.	0	n	1	0	Asteraceae Bercht. & J.Presl
Iz_1_1209	DJI_0029	Pilosella officinarum F. Schultz et Sch. Bip.	0	n	1	0	Asteraceae Bercht. & J.Presl
Iz_1_1209	DJI_0029	Pilosella sp.	0		NA	0	Asteraceae Bercht. & J.Presl
Iz_1_1209	DJI_0029	Pinus sylvestris L.	0	n	1	1	Pinaceae Spreng. ex F.Rudolphi
Iz_1_1209	DJI_0029	Poa annua L.	0	n	1	0	Poaceae Barnhart
Iz_1_1209	DJI_0029	Poa nemoralis L.	0	n	1	0	Poaceae Barnhart
Iz_1_1209	DJI_0029	Poa sp.	0		NA	0	Poaceae Barnhart
Iz_1_1209	DJI_0029	Polygonatum odoratum (Mill.) Druce	0	n	1	0	Asparagaceae Juss.
Iz_1_1209	DJI_0029	Polygonum arenarium Waldst. & Kit.	0	n	1	0	Polygonaceae Juss.
Iz_1_1209	DJI_0029	Populus nigra L.	0	n	1	1	Salicaceae Mirb.
Iz_1_1209	DJI_0029	Populus tremula L.	0	n	1	1	Salicaceae Mirb.
Iz_1_1209	DJI_0029	Populus Ã canescens (Aiton) Sm.	0	n	1	1	Salicaceae Mirb.
Iz_1_1209	DJI_0029	Potentilla argentea L.	0	n	1	0	Rosaceae Juss.
Iz_1_1209	DJI_0029	Psammophiliella muralis (L.) Ikonn.	0	n	1	0	Caryophyllaceae Juss.
Iz_1_1209	DJI_0029	Psephellus sumensis (Kalen.) Greuter	0	n	1	0	Asteraceae Bercht. & J.Presl
Iz_1_1209	DJI_0029	Pulsatilla pratensis (L.) Mill. s.l.	0	n	1	0	Ranunculaceae Juss.
Iz_1_1209	DJI_0029	Pyrus communis L.	0	a	0	1	Rosaceae Juss.
Iz_1_1209	DJI_0029	Quercus robur L.	0	n	1	1	Fagaceae Dumort.
Iz_1_1209	DJI_0029	Robinia pseudoacacia L.	0	a	0	1	Fabaceae Lindl.
Iz_1_1209	DJI_0029	Rosa sp.	0		NA	1	Rosaceae Juss.
Iz_1_1209	DJI_0029	Rubus idaeus L.	0	n	1	0	Rosaceae Juss.
Iz_1_1209	DJI_0029	Rumex acetosella L.	0	n	1	0	Polygonaceae Juss.

Iz_1_1209	DJI_0029	Rumex thyrsiflorus Fingerh.	0	n	1	0	Polygonaceae Juss.
Iz_1_1209	DJI_0029	Salix alba L.	0	n	1	1	Salicaceae Mirb.
Iz_1_1209	DJI_0029	Sambucus nigra L.	0	n	1	1	Viburnaceae Raf.
Iz_1_1209	DJI_0029	Secale sylvestre Host	0	n	1	0	Poaceae Barnhart
Iz_1_1209	DJI_0029	Sempervivum ruthenicum Schnittsp. & C.B. Lehm.	0	n	1	0	Crassulaceae J.St.-Hil.
Iz_1_1209	DJI_0029	Setaria viridis (L.) P. Beauv.	0	n	1	0	Poaceae Barnhart
Iz_1_1209	DJI_0029	Silene borysthenica (Gruner) Walters	0	n	1	0	Caryophyllaceae Juss.
Iz_1_1209	DJI_0029	Silene latifolia subsp. alba (Mill.) Greuter & Burdet	0	n	1	0	Caryophyllaceae Juss.
Iz_1_1209	DJI_0029	Solidago virgaurea L.	0	n	1	0	Asteraceae Bercht. & J.Presl
Iz_1_1209	DJI_0029	Sorbus aucuparia L.	0	n	1	1	Rosaceae Juss.
Iz_1_1209	DJI_0029	Stachys recta L.	0	n	1	0	Lamiacea Lindl.
Iz_1_1209	DJI_0029	Tanacetum vulgare L.	0	n	1	0	Asteraceae Bercht. & J.Presl
Iz_1_1209	DJI_0029	Thymus pallasianus Heinr.Braun	0	n	1	0	Lamiacea Lindl.
Iz_1_1209	DJI_0029	Tilia cordata Mill.	0	n	1	1	Malvaceae Juss.
Iz_1_1209	DJI_0029	Trifolium alpestre L.	0	n	1	0	Fabaceae Lindl.
Iz_1_1209	DJI_0029	Trifolium arvense L.	0	n	1	0	Fabaceae Lindl.
Iz_1_1209	DJI_0029	Ulmus glabra Huds.	0	n	1	1	Ulmaceae Mirb.
Iz_1_1209	DJI_0029	Verbascum densiflorum Bertol.	0	n	1	0	Scrophulariaceae Juss.
Iz_1_1209	DJI_0029	Veronica austriaca L.	0	n	1	0	Plantaginaceae Juss.
Iz_1_1209	DJI_0029	Veronica incana L.	0	n	1	0	Plantaginaceae Juss.
Iz_1_1209	DJI_0029	Veronica verna L.	0	n	1	0	Plantaginaceae Juss.
Iz_1_1209	DJI_0029	Vincetoxicum hirundinaria Medik	0	n	1	0	Apocynaceae Juss.
Iz_1_1209	DJI_0032	Acer tataricum L.	0	n	1	1	Sapindaceae Juss.
Iz_1_1209	DJI_0032	Achillea millefolium L. s.l.	0	n	1	0	Asteraceae Bercht. & J.Presl
Iz_1_1209	DJI_0032	Allium oleraceum L.	0	n	1	0	Amaryllidaceae J.St.-Hil.
Iz_1_1209	DJI_0032	Ambrosia artemisiifolia L.	0	a	0	0	Asteraceae Bercht. & J.Presl
Iz_1_1209	DJI_0032	Amorpha fruticosa L.	0	a	0	0	Fabaceae Lindl.
Iz_1_1209	DJI_0032	Anthericum ramosum L.	0	n	1	0	Asparagaceae Juss.
Iz_1_1209	DJI_0032	Artemisia marschalliana Spreng.	0	n	1	0	Asteraceae Bercht. & J.Presl
Iz_1_1209	DJI_0032	Asparagus officinalis L.	0	n	1	0	Asparagaceae Juss.
Iz_1_1209	DJI_0032	Astragalus cicer L.	0	n	1	0	Fabaceae Lindl.
Iz_1_1209	DJI_0032	Bassia laniflora (S.G.Gmel.) A.J.Scott	0	n	1	0	Amaranthaceae Juss.
Iz_1_1209	DJI_0032	Berberis vulgaris L.	0	n	1	0	Berberidaceae Juss.
Iz_1_1209	DJI_0032	Betula pendula Roth.	0	n	1	1	Betulaceae Gray
Iz_1_1209	DJI_0032	Betula pendula Roth.	0	n	1	0	Poaceae Barnhart
Iz_1_1209	DJI_0032	Bromus inermis Leyss.	0		NA	NA	
Iz_1_1209	DJI_0032	Bromus squarrosus L.	0	a	0	0	Poaceae Barnhart
Iz_1_1209	DJI_0032	Bromus tectorum L.	0	a	0	0	Poaceae Barnhart
Iz_1_1209	DJI_0032	Calamagrostis epigejos (L.) Roth	3	n	1	0	Poaceae Barnhart
Iz_1_1209	DJI_0032	Carduus acanthoides L.	0	n	1	0	Asteraceae Bercht. & J.Presl
Iz_1_1209	DJI_0032	Carex sp.	0	n	1	0	Cyperaceae Juss.
Iz_1_1209	DJI_0032	Centaurea majorovii Dumbadze	0	n	1	0	Asteraceae Bercht. & J.Presl
Iz_1_1209	DJI_0032	Chamaecytisus ruthenicus (Fisch.ex Wol.) Klaskova	0	n	1	0	Fabaceae Lindl.
Iz_1_1209	DJI_0032	Chelidonium majus L.	0	n	1	0	Papaveraceae Juss.
Iz_1_1209	DJI_0032	Chenopodium betaceum Andrz.	0	n	1	0	Amaranthaceae Juss.
Iz_1_1209	DJI_0032	Chenopodium cf. album	0	n	1	0	Amaranthaceae Juss.
Iz_1_1209	DJI_0032	Chenopodium sp.	0		NA	0	Amaranthaceae Juss.
Iz_1_1209	DJI_0032	Chondrilla juncea L.	0	n	1	0	Asteraceae Bercht. & J.Presl
Iz_1_1209	DJI_0032	Cirsium vulgare (Savi) Ten.	0	n	1	0	Asteraceae Bercht. & J.Presl
Iz_1_1209	DJI_0032	Clinopodium acinos (L.) Kuntze	0	n	1	0	Lamiacea Lindl.
Iz_1_1209	DJI_0032	Convallaria majalis L.	0	n	1	0	Asparagaceae Juss.
Iz_1_1209	DJI_0032	Crataegus rhipidophylla Gand.	0	n	1	1	Rosaceae Juss.
Iz_1_1209	DJI_0032	Crepis foetida L.	0	n	1	0	Asteraceae Bercht. & J.Presl
Iz_1_1209	DJI_0032	Dactylis glomerata L.	0	n	1	0	Poaceae Barnhart
Iz_1_1209	DJI_0032	Dianthus campestris M.Bieb.	0	n	1	0	Caryophyllaceae Juss.
Iz_1_1209	DJI_0032	Digitaria sanguinalis (L.) Scop.	0	a	0	0	Poaceae Barnhart
Iz_1_1209	DJI_0032	Elymus repens (L.) Gould	0	n	1	0	Poaceae Barnhart
Iz_1_1209	DJI_0032	Epilobium tetragonum L.	0	n	1	0	Onagraceae Juss.
Iz_1_1209	DJI_0032	Eragrostis minor Host	0	a	0	0	Poaceae Barnhart
Iz_1_1209	DJI_0032	Erigeron canadensis L.	1	a	0	0	Asteraceae Bercht. & J.Presl
Iz_1_1209	DJI_0032	Euphorbia seguieriana Neck.	0	n	1	0	Euphorbiaceae Juss.
Iz_1_1209	DJI_0032	Fallopia dumetorum (L.) Holub	2	n	1	0	Polygonaceae Juss.
Iz_1_1209	DJI_0032	Festuca beckeri (Hack.) Trautv.	0	n	1	0	Poaceae Barnhart
Iz_1_1209	DJI_0032	Fragaria vesca L.	0	n	1	0	Rosaceae Juss.
Iz_1_1209	DJI_0032	Frangula alnus Mill.	0	n	1	1	Rhamnaceae Juss.
Iz_1_1209	DJI_0032	Fraxinus pennsylvanica Marshall	0	a	0	1	Oleaceae Hoffmanns. & Link
Iz_1_1209	DJI_0032	Galeopsis bifida Boenn.	0	n	1	0	Lamiacea Lindl.
Iz_1_1209	DJI_0032	Galium verum L.	0	n	1	0	Rubiaceae Juss.
Iz_1_1209	DJI_0032	Genista tinctoria L.	0	n	1	0	Fabaceae Lindl.
Iz_1_1209	DJI_0032	Geranium robertianum L.	0	n	1	0	Geraniaceae Juss.
Iz_1_1209	DJI_0032	Geranium sanquineum L.	0	n	1	0	Geraniaceae Juss.
Iz_1_1209	DJI_0032	Gypsophila paniculata L.	0	n	1	0	Caryophyllaceae Juss.
Iz_1_1209	DJI_0032	Helichrysum arenarium (L.) Moench	0	n	1	0	Asteraceae Bercht. & J.Presl
Iz_1_1209	DJI_0032	Herniaria polygama J.Gay	0	n	1	0	Caryophyllaceae Juss.
Iz_1_1209	DJI_0032	Hieracium robustum Fr.	0	n	1	0	Asteraceae Bercht. & J.Presl
Iz_1_1209	DJI_0032	Hieracium umbellatum L.	0	n	1	NA	Asteraceae Bercht. & J.Presl
Iz_1_1209	DJI_0032	Hylotelephium maximum (L.) Holub	0	n	1	0	Crassulaceae J.St.-Hil.
Iz_1_1209	DJI_0032	Jacobaea borysthenica (DC.) B.Nord. & Greuter	0	n	1	0	Asteraceae Bercht. & J.Presl
Iz_1_1209	DJI_0032	Jasione montana L.	0	n	1	0	Campanulaceae Juss.
Iz_1_1209	DJI_0032	Jurinea cyanoides (L.) Rchb.s.l.	0	n	1	0	Asteraceae Bercht. & J.Presl
Iz_1_1209	DJI_0032	Koeleria glauca (Spreng.) DC.	0	n	1	0	Poaceae Barnhart
Iz_1_1209	DJI_0032	Lactuca serriola L.	0	a	0	0	Asteraceae Bercht. & J.Presl
Iz_1_1209	DJI_0032	Linaria genistifolia (L.) Mill.	0	n	1	0	Plantaginaceae Juss.
Iz_1_1209	DJI_0032	Lomelosia argentea (L.) Greuter & Burdet	0	n	1	0	Caprifoliaceae Juss.
Iz_1_1209	DJI_0032	Melampyrum pratense L.	0	n	1	0	Orobanchaceae Vent.
Iz_1_1209	DJI_0032	Oenothera biennis L.	0	a	0	0	Onagraceae Juss.
Iz_1_1209	DJI_0032	Pentanema britannica (L.) D.Gut.Larr., Santos-Vicente, Anderb., E.Rico & M.M.Mart.Ort.	0	n	1	0	Asteraceae Bercht. & J.Presl
Iz_1_1209	DJI_0032	Peucedanum oreoselinum (L.) Moench	1	n	1	0	Apiaceae Lindl.
Iz_1_1209	DJI_0032	Peucedanum ruthenicum M.Bieb.	0	n	1	0	Apiaceae Lindl.
Iz_1_1209	DJI_0032	Pilosella echioides (Lumn.) F.W.Schultz & Sch.Bip.	0	n	1	0	Asteraceae Bercht. & J.Presl
Iz_1_1209	DJI_0032	Pilosella officinarum F. Schultz et Sch. Bip.	0	n	1	0	Asteraceae Bercht. & J.Presl
Iz_1_1209	DJI_0032	Pilosella sp.	0		NA	0	Asteraceae Bercht. & J.Presl
Iz_1_1209	DJI_0032	Pinus sylvestris L.	0	n	1	1	Pinaceae Spreng. ex F.Rudolphi
Iz_1_1209	DJI_0032	Poa annua L.	0	n	1	0	Poaceae Barnhart
Iz_1_1209	DJI_0032	Poa nemoralis L.	0	n	1	0	Poaceae Barnhart
Iz_1_1209	DJI_0032	Poa sp.	0		NA	0	Poaceae Barnhart
Iz_1_1209	DJI_0032	Polygonatum odoratum (Mill.) Druce	0	n	1	0	Asparagaceae Juss.
Iz_1_1209	DJI_0032	Polygonum arenarium Waldst. & Kit.	0	n	1	0	Polygonaceae Juss.

Iz_1_1209	DJI_0032	Populus nigra L.	0	n	1	1	Salicaceae Mirb.
Iz_1_1209	DJI_0032	Populus tremula L.	0	n	1	1	Salicaceae Mirb.
Iz_1_1209	DJI_0032	Populus Ã canescens (Aiton) Sm.	0	n	1	1	Salicaceae Mirb.
Iz_1_1209	DJI_0032	Potentilla argentea L.	0	n	1	0	Rosaceae Juss.
Iz_1_1209	DJI_0032	Psammophiliella muralis (L.) Ikonn.	0	n	1	0	Caryophyllaceae Juss.
Iz_1_1209	DJI_0032	Psephellus sumensis (Kalen.) Greuter	0	n	1	0	Asteraceae Bercht. & J.Presl
Iz_1_1209	DJI_0032	Pulsatilla pratensis (L.) Mill. s.l.	0	n	1	0	Ranunculaceae Juss.
Iz_1_1209	DJI_0032	Pyrus communis L.	0	a	0	1	Rosaceae Juss.
Iz_1_1209	DJI_0032	Quercus robur L.	0	n	1	1	Fagaceae Dumort.
Iz_1_1209	DJI_0032	Robinia pseudoacacia L.	0	a	0	1	Fabaceae Lindl.
Iz_1_1209	DJI_0032	Rosa sp.	0		NA	1	Rosaceae Juss.
Iz_1_1209	DJI_0032	Rubus idaeus L.	0	n	1	0	Rosaceae Juss.
Iz_1_1209	DJI_0032	Rumex acetosella L.	0	n	1	0	Polygonaceae Juss.
Iz_1_1209	DJI_0032	Rumex thyrsiflorus Fingerh.	0	n	1	0	Polygonaceae Juss.
Iz_1_1209	DJI_0032	Salix alba L.	0	n	1	1	Salicaceae Mirb.
Iz_1_1209	DJI_0032	Sambucus nigra L.	0	n	1	1	Viburnaceae Raf.
Iz_1_1209	DJI_0032	Secale sylvestre Host	0	n	1	0	Poaceae Barnhart
Iz_1_1209	DJI_0032	Sempervivum ruthenicum Schnittsp. & C.B. Lehm.	0	n	1	0	Crassulaceae J.St.-Hil.
Iz_1_1209	DJI_0032	Setaria viridis (L.) P. Beauv.	0	n	1	0	Poaceae Barnhart
Iz_1_1209	DJI_0032	Silene borysthenica (Gruner) Walters	0	n	1	0	Caryophyllaceae Juss.
Iz_1_1209	DJI_0032	Silene latifolia subsp. alba (Mill.) Greuter & Burdet	0	n	1	0	Caryophyllaceae Juss.
Iz_1_1209	DJI_0032	Solidago virgaurea L.	0	n	1	0	Asteraceae Bercht. & J.Presl
Iz_1_1209	DJI_0032	Sorbus aucuparia L.	0	n	1	1	Rosaceae Juss.
Iz_1_1209	DJI_0032	Stachys recta L.	0	n	1	0	Lamiacea Lindl.
Iz_1_1209	DJI_0032	Tanacetum vulgare L.	0	n	1	0	Asteraceae Bercht. & J.Presl
Iz_1_1209	DJI_0032	Thymus pallasianus Heinr.Braun	0	n	1	0	Lamiacea Lindl.
Iz_1_1209	DJI_0032	Tilia cordata Mill.	0	n	1	1	Malvaceae Juss.
Iz_1_1209	DJI_0032	Trifolium alpestre L.	0	n	1	0	Fabaceae Lindl.
Iz_1_1209	DJI_0032	Trifolium arvense L.	0	n	1	0	Fabaceae Lindl.
Iz_1_1209	DJI_0032	Ulmus glabra Huds.	0	n	1	1	Ulmaceae Mirb.
Iz_1_1209	DJI_0032	Verbascum densiflorum Bertol.	0	n	1	0	Scrophulariaceae Juss.
Iz_1_1209	DJI_0032	Veronica austriaca L.	0	n	1	0	Plantaginaceae Juss.
Iz_1_1209	DJI_0032	Veronica incana L.	0	n	1	0	Plantaginaceae Juss.
Iz_1_1209	DJI_0032	Veronica verna L.	0	n	1	0	Plantaginaceae Juss.
Iz_1_1209	DJI_0032	Vincetoxicum hirundinaria Medik	0	n	1	0	Apocynaceae Juss.
Iz_1_1209	DJI_0035	Acer tataricum L.	0	n	1	1	Sapindaceae Juss.
Iz_1_1209	DJI_0035	Achillea millefolium L. s.l.	0	n	1	0	Asteraceae Bercht. & J.Presl
Iz_1_1209	DJI_0035	Allium oleraceum L.	0	n	1	0	Amaryllidaceae J.St.-Hil.
Iz_1_1209	DJI_0035	Ambrosia artemisiifolia L.	0	a	0	0	Asteraceae Bercht. & J.Presl
Iz_1_1209	DJI_0035	Amorpha fruticosa L.	0	a	0	0	Fabaceae Lindl.
Iz_1_1209	DJI_0035	Anthericum ramosum L.	0	n	1	0	Asparagaceae Juss.
Iz_1_1209	DJI_0035	Artemisia marschalliana Spreng.	0	n	1	0	Asteraceae Bercht. & J.Presl
Iz_1_1209	DJI_0035	Asparagus officinalis L.	0	n	1	0	Asparagaceae Juss.
Iz_1_1209	DJI_0035	Astragalus cicer L.	0	n	1	0	Fabaceae Lindl.
Iz_1_1209	DJI_0035	Bassia laniflora (S.G.Gmel.) A.J.Scott	0	n	1	0	Amaranthaceae Juss.
Iz_1_1209	DJI_0035	Berberis vulgaris L.	0	n	1	0	Berberidaceae Juss.
Iz_1_1209	DJI_0035	Betula pendula Roth.	0	n	1	1	Betulaceae Gray
Iz_1_1209	DJI_0035	Betula pendula Roth.	0	n	1	0	Poaceae Barnhart
Iz_1_1209	DJI_0035	Bromus inermis Leyss.	0		NA	NA	
Iz_1_1209	DJI_0035	Bromus squarrosus L.	0	a	0	0	Poaceae Barnhart
Iz_1_1209	DJI_0035	Bromus tectorum L.	0	a	0	0	Poaceae Barnhart
Iz_1_1209	DJI_0035	Calamagrostis epigejos (L.) Roth	3	n	1	0	Poaceae Barnhart
Iz_1_1209	DJI_0035	Carduus acanthoides L.	0	n	1	0	Asteraceae Bercht. & J.Presl
Iz_1_1209	DJI_0035	Carex sp.	0	n	1	0	Cyperaceae Juss.
Iz_1_1209	DJI_0035	Centaurea majorovii Dumbadze	0	n	1	0	Asteraceae Bercht. & J.Presl
Iz_1_1209	DJI_0035	Chamaecytisus ruthenicus (Fisch.ex Wol.) Klaskova	0	n	1	0	Fabaceae Lindl.
Iz_1_1209	DJI_0035	Chelidonium majus L.	0	n	1	0	Papaveraceae Juss.
Iz_1_1209	DJI_0035	Chenopodium betaceum Andrz.	0	n	1	0	Amaranthaceae Juss.
Iz_1_1209	DJI_0035	Chenopodium cf. album	0	n	1	0	Amaranthaceae Juss.
Iz_1_1209	DJI_0035	Chenopodium sp.	0		NA	0	Amaranthaceae Juss.
Iz_1_1209	DJI_0035	Chondrilla juncea L.	0	n	1	0	Asteraceae Bercht. & J.Presl
Iz_1_1209	DJI_0035	Cirsium vulgare (Savi) Ten.	0	n	1	0	Asteraceae Bercht. & J.Presl
Iz_1_1209	DJI_0035	Clinopodium acinos (L.) Kuntze	0	n	1	0	Lamiacea Lindl.
Iz_1_1209	DJI_0035	Convallaria majalis L.	0	n	1	0	Asparagaceae Juss.
Iz_1_1209	DJI_0035	Crataegus rhipidophylla Gand.	0	n	1	1	Rosaceae Juss.
Iz_1_1209	DJI_0035	Crepis foetida L.	0	n	1	0	Asteraceae Bercht. & J.Presl
Iz_1_1209	DJI_0035	Dactylis glomerata L.	0	n	1	0	Poaceae Barnhart
Iz_1_1209	DJI_0035	Dianthus campestris M.Bieb.	0	n	1	0	Caryophyllaceae Juss.
Iz_1_1209	DJI_0035	Digitaria sanguinalis (L.) Scop.	0	a	0	0	Poaceae Barnhart
Iz_1_1209	DJI_0035	Elymus repens (L.) Gould	0	n	1	0	Poaceae Barnhart
Iz_1_1209	DJI_0035	Epilobium tetragonum L.	0	n	1	0	Onagraceae Juss.
Iz_1_1209	DJI_0035	Eragrostis minor Host	0	a	0	0	Poaceae Barnhart
Iz_1_1209	DJI_0035	Erigeron canadensis L.	0	a	0	0	Asteraceae Bercht. & J.Presl
Iz_1_1209	DJI_0035	Euphorbia seguieriana Neck.	0	n	1	0	Euphorbiaceae Juss.
Iz_1_1209	DJI_0035	Fallopia dumetorum (L.) Holub	3	n	1	0	Polygonaceae Juss.
Iz_1_1209	DJI_0035	Festuca beckeri (Hack.) Trautv.	0	n	1	0	Poaceae Barnhart
Iz_1_1209	DJI_0035	Fragaria vesca L.	0	n	1	0	Rosaceae Juss.
Iz_1_1209	DJI_0035	Frangula alnus Mill.	0	n	1	1	Rhamnaceae Juss.
Iz_1_1209	DJI_0035	Fraxinus pennsylvanica Marshall	0	a	0	1	Oleaceae Hoffmanns. & Link
Iz_1_1209	DJI_0035	Galeopsis bifida Boenn.	0	n	1	0	Lamiacea Lindl.
Iz_1_1209	DJI_0035	Galium verum L.	0	n	1	0	Rubiaceae Juss.
Iz_1_1209	DJI_0035	Genista tinctoria L.	0	n	1	0	Fabaceae Lindl.
Iz_1_1209	DJI_0035	Geranium robertianum L.	0	n	1	0	Geraniaceae Juss.
Iz_1_1209	DJI_0035	Geranium sanquineum L.	0	n	1	0	Geraniaceae Juss.
Iz_1_1209	DJI_0035	Gypsophila paniculata L.	0	n	1	0	Caryophyllaceae Juss.
Iz_1_1209	DJI_0035	Helichrysum arenarium (L.) Moench	0	n	1	0	Asteraceae Bercht. & J.Presl
Iz_1_1209	DJI_0035	Herniaria polygama J.Gay	0	n	1	0	Caryophyllaceae Juss.
Iz_1_1209	DJI_0035	Hieracium robustum Fr.	0	n	1	0	Asteraceae Bercht. & J.Presl
Iz_1_1209	DJI_0035	Hieracium umbellatum L.	0	n	1	NA	Asteraceae Bercht. & J.Presl
Iz_1_1209	DJI_0035	Hylotelephium maximum (L.) Holub	0	n	1	0	Crassulaceae J.St.-Hil.
Iz_1_1209	DJI_0035	Jacobaea borysthenica (DC.) B.Nord. & Greuter	0	n	1	0	Asteraceae Bercht. & J.Presl
Iz_1_1209	DJI_0035	Jasione montana L.	0	n	1	0	Campanulaceae Juss.
Iz_1_1209	DJI_0035	Jurinea cyanoides (L.) Rchb.s.l.	0	n	1	0	Asteraceae Bercht. & J.Presl
Iz_1_1209	DJI_0035	Koeleria glauca (Spreng.) DC.	0	n	1	0	Poaceae Barnhart
Iz_1_1209	DJI_0035	Lactuca serriola L.	0	a	0	0	Asteraceae Bercht. & J.Presl
Iz_1_1209	DJI_0035	Linaria genistifolia (L.) Mill.	0	n	1	0	Plantaginaceae Juss.
Iz_1_1209	DJI_0035	Lomelosia argentea (L.) Greuter & Burdet	0	n	1	0	Caprifoliaceae Juss.
Iz_1_1209	DJI_0035	Melampyrum pratense L.	0	n	1	0	Orobanchaceae Vent.

Iz_1_1209	DJI_0035	Oenothera biennis L.	0	a	0	0	Onagraceae Juss.
Iz_1_1209	DJI_0035	Pentanema britannica (L.) D.Gut.Larr., Santos-Vicente, Anderb., E.Rico & M.M.Mart.Ort.	0	n	1	0	Asteraceae Bercht. & J.Presl
Iz_1_1209	DJI_0035	Peucedanum oreoselinum (L.) Moench	0	n	1	0	Apiaceae Lindl.
Iz_1_1209	DJI_0035	Peucedanum ruthenicum M.Bieb.	0	n	1	0	Apiaceae Lindl.
Iz_1_1209	DJI_0035	Pilosella echioides (Lumn.) F.W.Schultz & Sch.Bip.	0	n	1	0	Asteraceae Bercht. & J.Presl
Iz_1_1209	DJI_0035	Pilosella officinarum F. Schultz et Sch. Bip.	0	n	1	0	Asteraceae Bercht. & J.Presl
Iz_1_1209	DJI_0035	Pilosella sp.	0		NA	0	Asteraceae Bercht. & J.Presl
Iz_1_1209	DJI_0035	Pinus sylvestris L.	0	n	1	1	Pinaceae Spreng. ex F.Rudolphi
Iz_1_1209	DJI_0035	Poa annua L.	0	n	1	0	Poaceae Barnhart
Iz_1_1209	DJI_0035	Poa nemoralis L.	0	n	1	0	Poaceae Barnhart
Iz_1_1209	DJI_0035	Poa sp.	0		NA	0	Poaceae Barnhart
Iz_1_1209	DJI_0035	Polygonatum odoratum (Mill.) Druce	0	n	1	0	Asparagaceae Juss.
Iz_1_1209	DJI_0035	Polygonum arenarium Waldst. & Kit.	0	n	1	0	Polygonaceae Juss.
Iz_1_1209	DJI_0035	Populus nigra L.	0	n	1	1	Salicaceae Mirb.
Iz_1_1209	DJI_0035	Populus tremula L.	0	n	1	1	Salicaceae Mirb.
Iz_1_1209	DJI_0035	Populus Ã canescens (Aiton) Sm.	0	n	1	1	Salicaceae Mirb.
Iz_1_1209	DJI_0035	Potentilla argentea L.	0	n	1	0	Rosaceae Juss.
Iz_1_1209	DJI_0035	Psammophiliella muralis (L.) Ikonn.	0	n	1	0	Caryophyllaceae Juss.
Iz_1_1209	DJI_0035	Psephellus sumensis (Kalen.) Greuter	0	n	1	0	Asteraceae Bercht. & J.Presl
Iz_1_1209	DJI_0035	Pulsatilla pratensis (L.) Mill. s.l.	0	n	1	0	Ranunculaceae Juss.
Iz_1_1209	DJI_0035	Pyrus communis L.	0	a	0	1	Rosaceae Juss.
Iz_1_1209	DJI_0035	Quercus robur L.	0	n	1	1	Fagaceae Dumort.
Iz_1_1209	DJI_0035	Robinia pseudoacacia L.	0	a	0	1	Fabaceae Lindl.
Iz_1_1209	DJI_0035	Rosa sp.	0		NA	1	Rosaceae Juss.
Iz_1_1209	DJI_0035	Rubus idaeus L.	0	n	1	0	Rosaceae Juss.
Iz_1_1209	DJI_0035	Rumex acetosella L.	0	n	1	0	Polygonaceae Juss.
Iz_1_1209	DJI_0035	Rumex thyrsiflorus Fingerh.	0	n	1	0	Polygonaceae Juss.
Iz_1_1209	DJI_0035	Salix alba L.	0	n	1	1	Salicaceae Mirb.
Iz_1_1209	DJI_0035	Sambucus nigra L.	0	n	1	1	Viburnaceae Raf.
Iz_1_1209	DJI_0035	Secale sylvestre Host	0	n	1	0	Poaceae Barnhart
Iz_1_1209	DJI_0035	Sempervivum ruthenicum Schnittsp. & C.B. Lehm.	0	n	1	0	Crassulaceae J.St.-Hil.
Iz_1_1209	DJI_0035	Setaria viridis (L.) P. Beauv.	0	n	1	0	Poaceae Barnhart
Iz_1_1209	DJI_0035	Silene borysthenica (Gruner) Walters	0	n	1	0	Caryophyllaceae Juss.
Iz_1_1209	DJI_0035	Silene latifolia subsp. alba (Mill.) Greuter & Burdet	0	n	1	0	Caryophyllaceae Juss.
Iz_1_1209	DJI_0035	Solidago virgaurea L.	0	n	1	0	Asteraceae Bercht. & J.Presl
Iz_1_1209	DJI_0035	Sorbus aucuparia L.	0	n	1	1	Rosaceae Juss.
Iz_1_1209	DJI_0035	Stachys recta L.	0	n	1	0	Lamiacea Lindl.
Iz_1_1209	DJI_0035	Tanacetum vulgare L.	0	n	1	0	Asteraceae Bercht. & J.Presl
Iz_1_1209	DJI_0035	Thymus pallasianus Heinr.Braun	0	n	1	0	Lamiacea Lindl.
Iz_1_1209	DJI_0035	Tilia cordata Mill.	0	n	1	1	Malvaceae Juss.
Iz_1_1209	DJI_0035	Trifolium alpestre L.	0	n	1	0	Fabaceae Lindl.
Iz_1_1209	DJI_0035	Trifolium arvense L.	0	n	1	0	Fabaceae Lindl.
Iz_1_1209	DJI_0035	Ulmus glabra Huds.	0	n	1	1	Ulmaceae Mirb.
Iz_1_1209	DJI_0035	Verbascum densiflorum Bertol.	0	n	1	0	Scrophulariaceae Juss.
Iz_1_1209	DJI_0035	Veronica austriaca L.	0	n	1	0	Plantaginaceae Juss.
Iz_1_1209	DJI_0035	Veronica incana L.	0	n	1	0	Plantaginaceae Juss.
Iz_1_1209	DJI_0035	Veronica verna L.	0	n	1	0	Plantaginaceae Juss.
Iz_1_1209	DJI_0035	Vincetoxicum hirundinaria Medik	0	n	1	0	Apocynaceae Juss.
Iz_2_1009	DJI_0437	Acer tataricum L.	0	n	1	1	Sapindaceae Juss.
Iz_2_1009	DJI_0437	Achillea millefolium L. s.l.	0	n	1	0	Asteraceae Bercht. & J.Presl
Iz_2_1009	DJI_0437	Allium oleraceum L.	0	n	1	0	Amaryllidaceae J.St.-Hil.
Iz_2_1009	DJI_0437	Ambrosia artemisiifolia L.	0	a	0	0	Asteraceae Bercht. & J.Presl
Iz_2_1009	DJI_0437	Amorpha fruticosa L.	0	a	0	0	Fabaceae Lindl.
Iz_2_1009	DJI_0437	Anthericum ramosum L.	0	n	1	0	Asparagaceae Juss.
Iz_2_1009	DJI_0437	Artemisia marschalliana Spreng.	0	n	1	0	Asteraceae Bercht. & J.Presl
Iz_2_1009	DJI_0437	Asparagus officinalis L.	0	n	1	0	Asparagaceae Juss.
Iz_2_1009	DJI_0437	Astragalus cicer L.	0	n	1	0	Fabaceae Lindl.
Iz_2_1009	DJI_0437	Bassia laniflora (S.G.Gmel.) A.J.Scott	0	n	1	0	Amaranthaceae Juss.
Iz_2_1009	DJI_0437	Berberis vulgaris L.	0	n	1	0	Berberidaceae Juss.
Iz_2_1009	DJI_0437	Betula pendula Roth.	0	n	1	1	Betulaceae Gray
Iz_2_1009	DJI_0437	Betula pendula Roth.	0	n	1	0	Poaceae Barnhart
Iz_2_1009	DJI_0437	Bromus inermis Leyss.	0		NA	NA	
Iz_2_1009	DJI_0437	Bromus squarrosus L.	0	a	0	0	Poaceae Barnhart
Iz_2_1009	DJI_0437	Bromus tectorum L.	0	a	0	0	Poaceae Barnhart
Iz_2_1009	DJI_0437	Calamagrostis epigejos (L.) Roth	20	n	1	0	Poaceae Barnhart
Iz_2_1009	DJI_0437	Carduus acanthoides L.	0	n	1	0	Asteraceae Bercht. & J.Presl
Iz_2_1009	DJI_0437	Carex sp.	0	n	1	0	Cyperaceae Juss.
Iz_2_1009	DJI_0437	Centaurea majorovii Dumbadze	0	n	1	0	Asteraceae Bercht. & J.Presl
Iz_2_1009	DJI_0437	Cf. Solidago virgaurea	0		NA	NA	
Iz_2_1009	DJI_0437	Chamaecytisus ruthenicus (Fisch.ex Wol.) Klaskova	0	n	1	0	Fabaceae Lindl.
Iz_2_1009	DJI_0437	Chelidonium majus L.	0	n	1	0	Papaveraceae Juss.
Iz_2_1009	DJI_0437	Chenopodium betaceum Andrz.	0	n	1	0	Amaranthaceae Juss.
Iz_2_1009	DJI_0437	Chenopodium cf. album	0	n	1	0	Amaranthaceae Juss.
Iz_2_1009	DJI_0437	Chenopodium sp.	0		NA	0	Amaranthaceae Juss.
Iz_2_1009	DJI_0437	Chondrilla juncea L.	0	n	1	0	Asteraceae Bercht. & J.Presl
Iz_2_1009	DJI_0437	Cirsium vulgare (Savi) Ten.	0	n	1	0	Asteraceae Bercht. & J.Presl
Iz_2_1009	DJI_0437	Clinopodium acinos (L.) Kuntze	0	n	1	0	Lamiacea Lindl.
Iz_2_1009	DJI_0437	Convallaria majalis L.	0	n	1	0	Asparagaceae Juss.
Iz_2_1009	DJI_0437	Crataegus rhipidophylla Gand.	0	n	1	1	Rosaceae Juss.
Iz_2_1009	DJI_0437	Crepis foetida L.	0	n	1	0	Asteraceae Bercht. & J.Presl
Iz_2_1009	DJI_0437	Dactylis glomerata L.	0	n	1	0	Poaceae Barnhart
Iz_2_1009	DJI_0437	Dianthus campestris M.Bieb.	0	n	1	0	Caryophyllaceae Juss.
Iz_2_1009	DJI_0437	Digitaria sanguinalis (L.) Scop.	0	a	0	0	Poaceae Barnhart
Iz_2_1009	DJI_0437	Elymus repens (L.) Gould	0	n	1	0	Poaceae Barnhart
Iz_2_1009	DJI_0437	Epilobium tetragonum L.	0	n	1	0	Onagraceae Juss.
Iz_2_1009	DJI_0437	Eragrostis minor Host	0	a	0	0	Poaceae Barnhart
Iz_2_1009	DJI_0437	Erigeron canadensis L.	0	a	0	0	Asteraceae Bercht. & J.Presl
Iz_2_1009	DJI_0437	Euphorbia seguieriana Neck.	0	n	1	0	Euphorbiaceae Juss.
Iz_2_1009	DJI_0437	Fallopia dumetorum (L.) Holub	0	n	1	0	Polygonaceae Juss.
Iz_2_1009	DJI_0437	Festuca beckeri (Hack.) Trautv.	0	n	1	0	Poaceae Barnhart
Iz_2_1009	DJI_0437	Fragaria vesca L.	0	n	1	0	Rosaceae Juss.
Iz_2_1009	DJI_0437	Frangula alnus Mill.	0	n	1	1	Rhamnaceae Juss.
Iz_2_1009	DJI_0437	Fraxinus pennsylvanica Marshall	0	a	0	1	Oleaceae Hoffmanns. & Link
Iz_2_1009	DJI_0437	Galeopsis bifida Boenn.	0	n	1	0	Lamiacea Lindl.
Iz_2_1009	DJI_0437	Galium verum L.	0	n	1	0	Rubiaceae Juss.
Iz_2_1009	DJI_0437	Genista tinctoria L.	0	n	1	0	Fabaceae Lindl.
Iz_2_1009	DJI_0437	Geranium robertianum L.	0	n	1	0	Geraniaceae Juss.
Iz_2_1009	DJI_0437	Geranium sanquineum L.	0	n	1	0	Geraniaceae Juss.

Iz_2_1009	DJI_0437	Gypsophila paniculata L.	0	n	1	0	Caryophyllaceae Juss.
Iz_2_1009	DJI_0437	Helichrysum arenarium (L.) Moench	0	n	1	0	Asteraceae Bercht. & J.Presl
Iz_2_1009	DJI_0437	Herniaria polygama J.Gay	0	n	1	0	Caryophyllaceae Juss.
Iz_2_1009	DJI_0437	Hieracium robustum Fr.	0	n	1	0	Asteraceae Bercht. & J.Presl
Iz_2_1009	DJI_0437	Hieracium umbellatum L.	0	n	1	NA	Asteraceae Bercht. & J.Presl
Iz_2_1009	DJI_0437	Hylotelephium maximum (L.) Holub	0	n	1	0	Crassulaceae J.St.-Hil.
Iz_2_1009	DJI_0437	Jacobaea borysthenica (DC.) B.Nord. & Greuter	0	n	1	0	Asteraceae Bercht. & J.Presl
Iz_2_1009	DJI_0437	Jasione montana L.	0	n	1	0	Campanulaceae Juss.
Iz_2_1009	DJI_0437	Jurinea cyanoides (L.) Rchb.s.l.	0	n	1	0	Asteraceae Bercht. & J.Presl
Iz_2_1009	DJI_0437	Koeleria glauca (Spreng.) DC.	0	n	1	0	Poaceae Barnhart
Iz_2_1009	DJI_0437	Lactuca serriola L.	0	a	0	0	Asteraceae Bercht. & J.Presl
Iz_2_1009	DJI_0437	Linaria genistifolia (L.) Mill.	0	n	1	0	Plantaginaceae Juss.
Iz_2_1009	DJI_0437	Lomelosia argentea (L.) Greuter & Burdet	0	n	1	0	Caprifoliaceae Juss.
Iz_2_1009	DJI_0437	Melampyrum pratense L.	0	n	1	0	Orobanchaceae Vent.
Iz_2_1009	DJI_0437	Oenothera biennis L.	0	a	0	0	Onagraceae Juss.
Iz_2_1009	DJI_0437	Pentanema britannica (L.) D.Gut.Larr., Santos-Vicente, Anderb., E.Rico & M.M.Mart.Ort.	0	n	1	0	Asteraceae Bercht. & J.Presl
Iz_2_1009	DJI_0437	Peucedanum oreoselinum (L.) Moench	0	n	1	0	Apiaceae Lindl.
Iz_2_1009	DJI_0437	Peucedanum ruthenicum M.Bieb.	0	n	1	0	Apiaceae Lindl.
Iz_2_1009	DJI_0437	Pilosella echioides (Lumn.) F.W.Schultz & Sch.Bip.	0	n	1	0	Asteraceae Bercht. & J.Presl
Iz_2_1009	DJI_0437	Pilosella officinarum F. Schultz et Sch. Bip.	0	n	1	0	Asteraceae Bercht. & J.Presl
Iz_2_1009	DJI_0437	Pilosella sp.	0		NA	0	Asteraceae Bercht. & J.Presl
Iz_2_1009	DJI_0437	Pinus sylvestris L.	0	n	1	1	Pinaceae Spreng. ex F.Rudolphi
Iz_2_1009	DJI_0437	Poa annua L.	0	n	1	0	Poaceae Barnhart
Iz_2_1009	DJI_0437	Poa nemoralis L.	0	n	1	0	Poaceae Barnhart
Iz_2_1009	DJI_0437	Poa sp.	0		NA	0	Poaceae Barnhart
Iz_2_1009	DJI_0437	Polygonatum odoratum (Mill.) Druce	0	n	1	0	Asparagaceae Juss.
Iz_2_1009	DJI_0437	Polygonum arenarium Waldst. & Kit.	0	n	1	0	Polygonaceae Juss.
Iz_2_1009	DJI_0437	Populus nigra L.	0	n	1	1	Salicaceae Mirb.
Iz_2_1009	DJI_0437	Populus tremula L.	0	n	1	1	Salicaceae Mirb.
Iz_2_1009	DJI_0437	Populus Ã canescens (Aiton) Sm.	0	n	1	1	Salicaceae Mirb.
Iz_2_1009	DJI_0437	Potentilla argentea L.	0	n	1	0	Rosaceae Juss.
Iz_2_1009	DJI_0437	Psammophiliella muralis (L.) Ikonn.	0	n	1	0	Caryophyllaceae Juss.
Iz_2_1009	DJI_0437	Psephellus sumensis (Kalen.) Greuter	0	n	1	0	Asteraceae Bercht. & J.Presl
Iz_2_1009	DJI_0437	Pulsatilla pratensis (L.) Mill. s.l.	0	n	1	0	Ranunculaceae Juss.
Iz_2_1009	DJI_0437	Pyrus communis L.	0	a	0	1	Rosaceae Juss.
Iz_2_1009	DJI_0437	Quercus robur L.	0	n	1	1	Fagaceae Dumort.
Iz_2_1009	DJI_0437	Robinia pseudoacacia L.	0	a	0	1	Fabaceae Lindl.
Iz_2_1009	DJI_0437	Rosa sp.	0		NA	1	Rosaceae Juss.
Iz_2_1009	DJI_0437	Rubus idaeus L.	0	n	1	0	Rosaceae Juss.
Iz_2_1009	DJI_0437	Rumex acetosella L.	0	n	1	0	Polygonaceae Juss.
Iz_2_1009	DJI_0437	Rumex thyrsiflorus Fingerh.	0	n	1	0	Polygonaceae Juss.
Iz_2_1009	DJI_0437	Salix alba L.	0	n	1	1	Salicaceae Mirb.
Iz_2_1009	DJI_0437	Sambucus nigra L.	0	n	1	1	Viburnaceae Raf.
Iz_2_1009	DJI_0437	Secale sylvestre Host	0	n	1	0	Poaceae Barnhart
Iz_2_1009	DJI_0437	Sempervivum ruthenicum Schnittsp. & C.B. Lehm.	0	n	1	0	Crassulaceae J.St.-Hil.
Iz_2_1009	DJI_0437	Setaria viridis (L.) P. Beauv.	0	n	1	0	Poaceae Barnhart
Iz_2_1009	DJI_0437	Silene borysthenica (Gruner) Walters	0	n	1	0	Caryophyllaceae Juss.
Iz_2_1009	DJI_0437	Silene latifolia subsp. alba (Mill.) Greuter & Burdet	0	n	1	0	Caryophyllaceae Juss.
Iz_2_1009	DJI_0437	Solidago virgaurea L.	0	n	1	0	Asteraceae Bercht. & J.Presl
Iz_2_1009	DJI_0437	Sorbus aucuparia L.	0	n	1	1	Rosaceae Juss.
Iz_2_1009	DJI_0437	Stachys recta L.	0	n	1	0	Lamiacea Lindl.
Iz_2_1009	DJI_0437	Tanacetum vulgare L.	0	n	1	0	Asteraceae Bercht. & J.Presl
Iz_2_1009	DJI_0437	Thymus pallasianus Heinr.Braun	0	n	1	0	Lamiacea Lindl.
Iz_2_1009	DJI_0437	Tilia cordata Mill.	0	n	1	1	Malvaceae Juss.
Iz_2_1009	DJI_0437	Trifolium alpestre L.	0	n	1	0	Fabaceae Lindl.
Iz_2_1009	DJI_0437	Trifolium arvense L.	0	n	1	0	Fabaceae Lindl.
Iz_2_1009	DJI_0437	Ulmus glabra Huds.	0	n	1	1	Ulmaceae Mirb.
Iz_2_1009	DJI_0437	Verbascum densiflorum Bertol.	0	n	1	0	Scrophulariaceae Juss.
Iz_2_1009	DJI_0437	Veronica austriaca L.	0	n	1	0	Plantaginaceae Juss.
Iz_2_1009	DJI_0437	Veronica incana L.	0	n	1	0	Plantaginaceae Juss.
Iz_2_1009	DJI_0437	Veronica verna L.	0	n	1	0	Plantaginaceae Juss.
Iz_2_1009	DJI_0437	Vincetoxicum hirundinaria Medik	0	n	1	0	Apocynaceae Juss.
Iz_2_1009	DJI_0438	Acer tataricum L.	0	n	1	1	Sapindaceae Juss.
Iz_2_1009	DJI_0438	Achillea millefolium L. s.l.	0	n	1	0	Asteraceae Bercht. & J.Presl
Iz_2_1009	DJI_0438	Allium oleraceum L.	0	n	1	0	Amaryllidaceae J.St.-Hil.
Iz_2_1009	DJI_0438	Ambrosia artemisiifolia L.	0	a	0	0	Asteraceae Bercht. & J.Presl
Iz_2_1009	DJI_0438	Amorpha fruticosa L.	0	a	0	0	Fabaceae Lindl.
Iz_2_1009	DJI_0438	Anthericum ramosum L.	0	n	1	0	Asparagaceae Juss.
Iz_2_1009	DJI_0438	Artemisia marschalliana Spreng.	0	n	1	0	Asteraceae Bercht. & J.Presl
Iz_2_1009	DJI_0438	Asparagus officinalis L.	0	n	1	0	Asparagaceae Juss.
Iz_2_1009	DJI_0438	Astragalus cicer L.	0	n	1	0	Fabaceae Lindl.
Iz_2_1009	DJI_0438	Bassia laniflora (S.G.Gmel.) A.J.Scott	0	n	1	0	Amaranthaceae Juss.
Iz_2_1009	DJI_0438	Berberis vulgaris L.	0	n	1	0	Berberidaceae Juss.
Iz_2_1009	DJI_0438	Betula pendula Roth.	0	n	1	1	Betulaceae Gray
Iz_2_1009	DJI_0438	Betula pendula Roth.	0	n	1	0	Poaceae Barnhart
Iz_2_1009	DJI_0438	Bromus inermis Leyss.	0		NA	NA	
Iz_2_1009	DJI_0438	Bromus squarrosus L.	0	a	0	0	Poaceae Barnhart
Iz_2_1009	DJI_0438	Bromus tectorum L.	0	a	0	0	Poaceae Barnhart
Iz_2_1009	DJI_0438	Calamagrostis epigejos (L.) Roth	2	n	1	0	Poaceae Barnhart
Iz_2_1009	DJI_0438	Carduus acanthoides L.	0	n	1	0	Asteraceae Bercht. & J.Presl
Iz_2_1009	DJI_0438	Carex sp.	0	n	1	0	Cyperaceae Juss.
Iz_2_1009	DJI_0438	Centaurea majorovii Dumbadze	0	n	1	0	Asteraceae Bercht. & J.Presl
Iz_2_1009	DJI_0438	Cf. Solidago virgaurea	0		NA	NA	
Iz_2_1009	DJI_0438	Chamaecytisus ruthenicus (Fisch.ex Wol.) Klaskova	0	n	1	0	Fabaceae Lindl.
Iz_2_1009	DJI_0438	Chelidonium majus L.	0	n	1	0	Papaveraceae Juss.
Iz_2_1009	DJI_0438	Chenopodium betaceum Andrz.	0	n	1	0	Amaranthaceae Juss.
Iz_2_1009	DJI_0438	Chenopodium cf. album	0	n	1	0	Amaranthaceae Juss.
Iz_2_1009	DJI_0438	Chenopodium sp.	0		NA	0	Amaranthaceae Juss.
Iz_2_1009	DJI_0438	Chondrilla juncea L.	0	n	1	0	Asteraceae Bercht. & J.Presl
Iz_2_1009	DJI_0438	Cirsium vulgare (Savi) Ten.	0	n	1	0	Asteraceae Bercht. & J.Presl
Iz_2_1009	DJI_0438	Clinopodium acinos (L.) Kuntze	0	n	1	0	Lamiacea Lindl.
Iz_2_1009	DJI_0438	Convallaria majalis L.	0	n	1	0	Asparagaceae Juss.
Iz_2_1009	DJI_0438	Crataegus rhipidophylla Gand.	0	n	1	1	Rosaceae Juss.
Iz_2_1009	DJI_0438	Crepis foetida L.	0	n	1	0	Asteraceae Bercht. & J.Presl
Iz_2_1009	DJI_0438	Dactylis glomerata L.	0	n	1	0	Poaceae Barnhart
Iz_2_1009	DJI_0438	Dianthus campestris M.Bieb.	0	n	1	0	Caryophyllaceae Juss.
Iz_2_1009	DJI_0438	Digitaria sanguinalis (L.) Scop.	0	a	0	0	Poaceae Barnhart
Iz_2_1009	DJI_0438	Elymus repens (L.) Gould	0	n	1	0	Poaceae Barnhart

Iz_2_1009	DJI_0438	Epilobium tetragonum L.	0	n	1	0	Onagraceae Juss.
Iz_2_1009	DJI_0438	Eragrostis minor Host	0	a	0	0	Poaceae Barnhart
Iz_2_1009	DJI_0438	Erigeron canadensis L.	0.2	a	0	0	Asteraceae Bercht. & J.Presl
Iz_2_1009	DJI_0438	Euphorbia seguieriana Neck.	0	n	1	0	Euphorbiaceae Juss.
Iz_2_1009	DJI_0438	Fallopia dumetorum (L.) Holub	0	n	1	0	Polygonaceae Juss.
Iz_2_1009	DJI_0438	Festuca beckeri (Hack.) Trautv.	0	n	1	0	Poaceae Barnhart
Iz_2_1009	DJI_0438	Fragaria vesca L.	0	n	1	0	Rosaceae Juss.
Iz_2_1009	DJI_0438	Frangula alnus Mill.	0	n	1	1	Rhamnaceae Juss.
Iz_2_1009	DJI_0438	Fraxinus pennsylvanica Marshall	0	a	0	1	Oleaceae Hoffmanns. & Link
Iz_2_1009	DJI_0438	Galeopsis bifida Boenn.	0	n	1	0	Lamiacea Lindl.
Iz_2_1009	DJI_0438	Galium verum L.	0	n	1	0	Rubiaceae Juss.
Iz_2_1009	DJI_0438	Genista tinctoria L.	0	n	1	0	Fabaceae Lindl.
Iz_2_1009	DJI_0438	Geranium robertianum L.	0	n	1	0	Geraniaceae Juss.
Iz_2_1009	DJI_0438	Geranium sanquineum L.	0	n	1	0	Geraniaceae Juss.
Iz_2_1009	DJI_0438	Gypsophila paniculata L.	0	n	1	0	Caryophyllaceae Juss.
Iz_2_1009	DJI_0438	Helichrysum arenarium (L.) Moench	0	n	1	0	Asteraceae Bercht. & J.Presl
Iz_2_1009	DJI_0438	Herniaria polygama J.Gay	0	n	1	0	Caryophyllaceae Juss.
Iz_2_1009	DJI_0438	Hieracium robustum Fr.	0	n	1	0	Asteraceae Bercht. & J.Presl
Iz_2_1009	DJI_0438	Hieracium umbellatum L.	0	n	1	NA	Asteraceae Bercht. & J.Presl
Iz_2_1009	DJI_0438	Hylotelephium maximum (L.) Holub	0	n	1	0	Crassulaceae J.St.-Hil.
Iz_2_1009	DJI_0438	Jacobaea borysthenica (DC.) B.Nord. & Greuter	0	n	1	0	Asteraceae Bercht. & J.Presl
Iz_2_1009	DJI_0438	Jasione montana L.	0	n	1	0	Campanulaceae Juss.
Iz_2_1009	DJI_0438	Jurinea cyanoides (L.) Rchb.s.l.	0	n	1	0	Asteraceae Bercht. & J.Presl
Iz_2_1009	DJI_0438	Koeleria glauca (Spreng.) DC.	0	n	1	0	Poaceae Barnhart
Iz_2_1009	DJI_0438	Lactuca serriola L.	0	a	0	0	Asteraceae Bercht. & J.Presl
Iz_2_1009	DJI_0438	Linaria genistifolia (L.) Mill.	0	n	1	0	Plantaginaceae Juss.
Iz_2_1009	DJI_0438	Lomelosia argentea (L.) Greuter & Burdet	0	n	1	0	Caprifoliaceae Juss.
Iz_2_1009	DJI_0438	Melampyrum pratense L.	0	n	1	0	Orobanchaceae Vent.
Iz_2_1009	DJI_0438	Oenothera biennis L.	0	a	0	0	Onagraceae Juss.
Iz_2_1009	DJI_0438	Pentanema britannica (L.) D.Gut.Larr., Santos-Vicente, Anderb., E.Rico & M.M.Mart.Ort.	0	n	1	0	Asteraceae Bercht. & J.Presl
Iz_2_1009	DJI_0438	Peucedanum oreoselinum (L.) Moench	0	n	1	0	Apiaceae Lindl.
Iz_2_1009	DJI_0438	Peucedanum ruthenicum M.Bieb.	0	n	1	0	Apiaceae Lindl.
Iz_2_1009	DJI_0438	Pilosella echioides (Lumn.) F.W.Schultz & Sch.Bip.	0	n	1	0	Asteraceae Bercht. & J.Presl
Iz_2_1009	DJI_0438	Pilosella officinarum F. Schultz et Sch. Bip.	0	n	1	0	Asteraceae Bercht. & J.Presl
Iz_2_1009	DJI_0438	Pilosella sp.	0		NA	0	Asteraceae Bercht. & J.Presl
Iz_2_1009	DJI_0438	Pinus sylvestris L.	0	n	1	1	Pinaceae Spreng. ex F.Rudolphi
Iz_2_1009	DJI_0438	Poa annua L.	0	n	1	0	Poaceae Barnhart
Iz_2_1009	DJI_0438	Poa nemoralis L.	0	n	1	0	Poaceae Barnhart
Iz_2_1009	DJI_0438	Poa sp.	0		NA	0	Poaceae Barnhart
Iz_2_1009	DJI_0438	Polygonatum odoratum (Mill.) Druce	0	n	1	0	Asparagaceae Juss.
Iz_2_1009	DJI_0438	Polygonum arenarium Waldst. & Kit.	0	n	1	0	Polygonaceae Juss.
Iz_2_1009	DJI_0438	Populus nigra L.	0	n	1	1	Salicaceae Mirb.
Iz_2_1009	DJI_0438	Populus tremula L.	0	n	1	1	Salicaceae Mirb.
Iz_2_1009	DJI_0438	Populus Ã canescens (Aiton) Sm.	0	n	1	1	Salicaceae Mirb.
Iz_2_1009	DJI_0438	Potentilla argentea L.	0	n	1	0	Rosaceae Juss.
Iz_2_1009	DJI_0438	Psammophiliella muralis (L.) Ikonn.	0	n	1	0	Caryophyllaceae Juss.
Iz_2_1009	DJI_0438	Psephellus sumensis (Kalen.) Greuter	0	n	1	0	Asteraceae Bercht. & J.Presl
Iz_2_1009	DJI_0438	Pulsatilla pratensis (L.) Mill. s.l.	0	n	1	0	Ranunculaceae Juss.
Iz_2_1009	DJI_0438	Pyrus communis L.	0	a	0	1	Rosaceae Juss.
Iz_2_1009	DJI_0438	Quercus robur L.	0	n	1	1	Fagaceae Dumort.
Iz_2_1009	DJI_0438	Robinia pseudoacacia L.	0	a	0	1	Fabaceae Lindl.
Iz_2_1009	DJI_0438	Rosa sp.	0		NA	1	Rosaceae Juss.
Iz_2_1009	DJI_0438	Rubus idaeus L.	0	n	1	0	Rosaceae Juss.
Iz_2_1009	DJI_0438	Rumex acetosella L.	0	n	1	0	Polygonaceae Juss.
Iz_2_1009	DJI_0438	Rumex thyrsiflorus Fingerh.	0	n	1	0	Polygonaceae Juss.
Iz_2_1009	DJI_0438	Salix alba L.	0	n	1	1	Salicaceae Mirb.
Iz_2_1009	DJI_0438	Sambucus nigra L.	0	n	1	1	Viburnaceae Raf.
Iz_2_1009	DJI_0438	Secale sylvestre Host	0	n	1	0	Poaceae Barnhart
Iz_2_1009	DJI_0438	Sempervivum ruthenicum Schnittsp. & C.B. Lehm.	0	n	1	0	Crassulaceae J.St.-Hil.
Iz_2_1009	DJI_0438	Setaria viridis (L.) P. Beauv.	0	n	1	0	Poaceae Barnhart
Iz_2_1009	DJI_0438	Silene borysthenica (Gruner) Walters	0	n	1	0	Caryophyllaceae Juss.
Iz_2_1009	DJI_0438	Silene latifolia subsp. alba (Mill.) Greuter & Burdet	0	n	1	0	Caryophyllaceae Juss.
Iz_2_1009	DJI_0438	Solidago virgaurea L.	0	n	1	0	Asteraceae Bercht. & J.Presl
Iz_2_1009	DJI_0438	Sorbus aucuparia L.	0	n	1	1	Rosaceae Juss.
Iz_2_1009	DJI_0438	Stachys recta L.	0	n	1	0	Lamiacea Lindl.
Iz_2_1009	DJI_0438	Tanacetum vulgare L.	0	n	1	0	Asteraceae Bercht. & J.Presl
Iz_2_1009	DJI_0438	Thymus pallasianus Heinr.Braun	0	n	1	0	Lamiacea Lindl.
Iz_2_1009	DJI_0438	Tilia cordata Mill.	0	n	1	1	Malvaceae Juss.
Iz_2_1009	DJI_0438	Trifolium alpestre L.	0	n	1	0	Fabaceae Lindl.
Iz_2_1009	DJI_0438	Trifolium arvense L.	0	n	1	0	Fabaceae Lindl.
Iz_2_1009	DJI_0438	Ulmus glabra Huds.	0	n	1	1	Ulmaceae Mirb.
Iz_2_1009	DJI_0438	Verbascum densiflorum Bertol.	0	n	1	0	Scrophulariaceae Juss.
Iz_2_1009	DJI_0438	Veronica austriaca L.	0	n	1	0	Plantaginaceae Juss.
Iz_2_1009	DJI_0438	Veronica incana L.	0	n	1	0	Plantaginaceae Juss.
Iz_2_1009	DJI_0438	Veronica verna L.	0	n	1	0	Plantaginaceae Juss.
Iz_2_1009	DJI_0438	Vincetoxicum hirundinaria Medik	0	n	1	0	Apocynaceae Juss.
Iz_2_1009	DJI_0439	Acer tataricum L.	0	n	1	1	Sapindaceae Juss.
Iz_2_1009	DJI_0439	Achillea millefolium L. s.l.	0	n	1	0	Asteraceae Bercht. & J.Presl
Iz_2_1009	DJI_0439	Allium oleraceum L.	0	n	1	0	Amaryllidaceae J.St.-Hil.
Iz_2_1009	DJI_0439	Ambrosia artemisiifolia L.	0	a	0	0	Asteraceae Bercht. & J.Presl
Iz_2_1009	DJI_0439	Amorpha fruticosa L.	0	a	0	0	Fabaceae Lindl.
Iz_2_1009	DJI_0439	Anthericum ramosum L.	0	n	1	0	Asparagaceae Juss.
Iz_2_1009	DJI_0439	Artemisia marschalliana Spreng.	0	n	1	0	Asteraceae Bercht. & J.Presl
Iz_2_1009	DJI_0439	Asparagus officinalis L.	0	n	1	0	Asparagaceae Juss.
Iz_2_1009	DJI_0439	Astragalus cicer L.	0	n	1	0	Fabaceae Lindl.
Iz_2_1009	DJI_0439	Bassia laniflora (S.G.Gmel.) A.J.Scott	0	n	1	0	Amaranthaceae Juss.
Iz_2_1009	DJI_0439	Berberis vulgaris L.	0	n	1	0	Berberidaceae Juss.
Iz_2_1009	DJI_0439	Betula pendula Roth.	0	n	1	1	Betulaceae Gray
Iz_2_1009	DJI_0439	Betula pendula Roth.	0	n	1	0	Poaceae Barnhart
Iz_2_1009	DJI_0439	Bromus inermis Leyss.	0		NA	NA	
Iz_2_1009	DJI_0439	Bromus squarrosus L.	0	a	0	0	Poaceae Barnhart
Iz_2_1009	DJI_0439	Bromus tectorum L.	0	a	0	0	Poaceae Barnhart
Iz_2_1009	DJI_0439	Calamagrostis epigejos (L.) Roth	3	n	1	0	Poaceae Barnhart
Iz_2_1009	DJI_0439	Carduus acanthoides L.	0	n	1	0	Asteraceae Bercht. & J.Presl
Iz_2_1009	DJI_0439	Carex sp.	0	n	1	0	Cyperaceae Juss.
Iz_2_1009	DJI_0439	Centaurea majorovii Dumbadze	0	n	1	0	Asteraceae Bercht. & J.Presl
Iz_2_1009	DJI_0439	Cf. Solidago virgaurea	0		NA	NA	
Iz_2_1009	DJI_0439	Chamaecytisus ruthenicus (Fisch.ex Wol.) Klaskova	0	n	1	0	Fabaceae Lindl.

Iz_2_1009	DJI_0439	Chelidonium majus L.	0	n	1	0	Papaveraceae Juss.
Iz_2_1009	DJI_0439	Chenopodium betaceum Andrz.	0	n	1	0	Amaranthaceae Juss.
Iz_2_1009	DJI_0439	Chenopodium cf. album	0	n	1	0	Amaranthaceae Juss.
Iz_2_1009	DJI_0439	Chenopodium sp.	0		NA	0	Amaranthaceae Juss.
Iz_2_1009	DJI_0439	Chondrilla juncea L.	0	n	1	0	Asteraceae Bercht. & J.Presl
Iz_2_1009	DJI_0439	Cirsium vulgare (Savi) Ten.	0	n	1	0	Asteraceae Bercht. & J.Presl
Iz_2_1009	DJI_0439	Clinopodium acinos (L.) Kuntze	0	n	1	0	Lamiacea Lindl.
Iz_2_1009	DJI_0439	Convallaria majalis L.	0	n	1	0	Asparagaceae Juss.
Iz_2_1009	DJI_0439	Crataegus rhipidophylla Gand.	0	n	1	1	Rosaceae Juss.
Iz_2_1009	DJI_0439	Crepis foetida L.	0	n	1	0	Asteraceae Bercht. & J.Presl
Iz_2_1009	DJI_0439	Dactylis glomerata L.	0	n	1	0	Poaceae Barnhart
Iz_2_1009	DJI_0439	Dianthus campestris M.Bieb.	0	n	1	0	Caryophyllaceae Juss.
Iz_2_1009	DJI_0439	Digitaria sanguinalis (L.) Scop.	0	a	0	0	Poaceae Barnhart
Iz_2_1009	DJI_0439	Elymus repens (L.) Gould	0	n	1	0	Poaceae Barnhart
Iz_2_1009	DJI_0439	Epilobium tetragonum L.	0	n	1	0	Onagraceae Juss.
Iz_2_1009	DJI_0439	Eragrostis minor Host	0	a	0	0	Poaceae Barnhart
Iz_2_1009	DJI_0439	Erigeron canadensis L.	0	a	0	0	Asteraceae Bercht. & J.Presl
Iz_2_1009	DJI_0439	Euphorbia seguieriana Neck.	0	n	1	0	Euphorbiaceae Juss.
Iz_2_1009	DJI_0439	Fallopia dumetorum (L.) Holub	0	n	1	0	Polygonaceae Juss.
Iz_2_1009	DJI_0439	Festuca beckeri (Hack.) Trautv.	0	n	1	0	Poaceae Barnhart
Iz_2_1009	DJI_0439	Fragaria vesca L.	0	n	1	0	Rosaceae Juss.
Iz_2_1009	DJI_0439	Frangula alnus Mill.	0	n	1	1	Rhamnaceae Juss.
Iz_2_1009	DJI_0439	Fraxinus pennsylvanica Marshall	0	a	0	1	Oleaceae Hoffmanns. & Link
Iz_2_1009	DJI_0439	Galeopsis bifida Boenn.	0	n	1	0	Lamiacea Lindl.
Iz_2_1009	DJI_0439	Galium verum L.	0	n	1	0	Rubiaceae Juss.
Iz_2_1009	DJI_0439	Genista tinctoria L.	0	n	1	0	Fabaceae Lindl.
Iz_2_1009	DJI_0439	Geranium robertianum L.	0	n	1	0	Geraniaceae Juss.
Iz_2_1009	DJI_0439	Geranium sanquineum L.	0	n	1	0	Geraniaceae Juss.
Iz_2_1009	DJI_0439	Gypsophila paniculata L.	0	n	1	0	Caryophyllaceae Juss.
Iz_2_1009	DJI_0439	Helichrysum arenarium (L.) Moench	0	n	1	0	Asteraceae Bercht. & J.Presl
Iz_2_1009	DJI_0439	Herniaria polygama J.Gay	0	n	1	0	Caryophyllaceae Juss.
Iz_2_1009	DJI_0439	Hieracium robustum Fr.	0	n	1	0	Asteraceae Bercht. & J.Presl
Iz_2_1009	DJI_0439	Hieracium umbellatum L.	0	n	1	NA	Asteraceae Bercht. & J.Presl
Iz_2_1009	DJI_0439	Hylotelephium maximum (L.) Holub	0	n	1	0	Crassulaceae J.St.-Hil.
Iz_2_1009	DJI_0439	Jacobaea borysthenica (DC.) B.Nord. & Greuter	0	n	1	0	Asteraceae Bercht. & J.Presl
Iz_2_1009	DJI_0439	Jasione montana L.	0	n	1	0	Campanulaceae Juss.
Iz_2_1009	DJI_0439	Jurinea cyanoides (L.) Rchb.s.l.	0	n	1	0	Asteraceae Bercht. & J.Presl
Iz_2_1009	DJI_0439	Koeleria glauca (Spreng.) DC.	0	n	1	0	Poaceae Barnhart
Iz_2_1009	DJI_0439	Lactuca serriola L.	0	a	0	0	Asteraceae Bercht. & J.Presl
Iz_2_1009	DJI_0439	Linaria genistifolia (L.) Mill.	0	n	1	0	Plantaginaceae Juss.
Iz_2_1009	DJI_0439	Lomelosia argentea (L.) Greuter & Burdet	0	n	1	0	Caprifoliaceae Juss.
Iz_2_1009	DJI_0439	Melampyrum pratense L.	0	n	1	0	Orobanchaceae Vent.
Iz_2_1009	DJI_0439	Oenothera biennis L.	0	a	0	0	Onagraceae Juss.
Iz_2_1009	DJI_0439	Pentanema britannica (L.) D.Gut.Larr., Santos-Vicente, Anderb., E.Rico & M.M.Mart.Ort.	0	n	1	0	Asteraceae Bercht. & J.Presl
Iz_2_1009	DJI_0439	Peucedanum oreoselinum (L.) Moench	0	n	1	0	Apiaceae Lindl.
Iz_2_1009	DJI_0439	Peucedanum ruthenicum M.Bieb.	0	n	1	0	Apiaceae Lindl.
Iz_2_1009	DJI_0439	Pilosella echioides (Lumn.) F.W.Schultz & Sch.Bip.	0	n	1	0	Asteraceae Bercht. & J.Presl
Iz_2_1009	DJI_0439	Pilosella officinarum F. Schultz et Sch. Bip.	0	n	1	0	Asteraceae Bercht. & J.Presl
Iz_2_1009	DJI_0439	Pilosella sp.	0		NA	0	Asteraceae Bercht. & J.Presl
Iz_2_1009	DJI_0439	Pinus sylvestris L.	0	n	1	1	Pinaceae Spreng. ex F.Rudolphi
Iz_2_1009	DJI_0439	Poa annua L.	0	n	1	0	Poaceae Barnhart
Iz_2_1009	DJI_0439	Poa nemoralis L.	0	n	1	0	Poaceae Barnhart
Iz_2_1009	DJI_0439	Poa sp.	0		NA	0	Poaceae Barnhart
Iz_2_1009	DJI_0439	Polygonatum odoratum (Mill.) Druce	0	n	1	0	Asparagaceae Juss.
Iz_2_1009	DJI_0439	Polygonum arenarium Waldst. & Kit.	0	n	1	0	Polygonaceae Juss.
Iz_2_1009	DJI_0439	Populus nigra L.	0	n	1	1	Salicaceae Mirb.
Iz_2_1009	DJI_0439	Populus tremula L.	0	n	1	1	Salicaceae Mirb.
Iz_2_1009	DJI_0439	Populus Ã canescens (Aiton) Sm.	0	n	1	1	Salicaceae Mirb.
Iz_2_1009	DJI_0439	Potentilla argentea L.	0	n	1	0	Rosaceae Juss.
Iz_2_1009	DJI_0439	Psammophiliella muralis (L.) Ikonn.	0	n	1	0	Caryophyllaceae Juss.
Iz_2_1009	DJI_0439	Psephellus sumensis (Kalen.) Greuter	0	n	1	0	Asteraceae Bercht. & J.Presl
Iz_2_1009	DJI_0439	Pulsatilla pratensis (L.) Mill. s.l.	0	n	1	0	Ranunculaceae Juss.
Iz_2_1009	DJI_0439	Pyrus communis L.	0	a	0	1	Rosaceae Juss.
Iz_2_1009	DJI_0439	Quercus robur L.	0	n	1	1	Fagaceae Dumort.
Iz_2_1009	DJI_0439	Robinia pseudoacacia L.	0	a	0	1	Fabaceae Lindl.
Iz_2_1009	DJI_0439	Rosa sp.	0		NA	1	Rosaceae Juss.
Iz_2_1009	DJI_0439	Rubus idaeus L.	0	n	1	0	Rosaceae Juss.
Iz_2_1009	DJI_0439	Rumex acetosella L.	0	n	1	0	Polygonaceae Juss.
Iz_2_1009	DJI_0439	Rumex thyrsiflorus Fingerh.	0	n	1	0	Polygonaceae Juss.
Iz_2_1009	DJI_0439	Salix alba L.	0	n	1	1	Salicaceae Mirb.
Iz_2_1009	DJI_0439	Sambucus nigra L.	0	n	1	1	Viburnaceae Raf.
Iz_2_1009	DJI_0439	Secale sylvestre Host	0	n	1	0	Poaceae Barnhart
Iz_2_1009	DJI_0439	Sempervivum ruthenicum Schnittsp. & C.B. Lehm.	0	n	1	0	Crassulaceae J.St.-Hil.
Iz_2_1009	DJI_0439	Setaria viridis (L.) P. Beauv.	0	n	1	0	Poaceae Barnhart
Iz_2_1009	DJI_0439	Silene borysthenica (Gruner) Walters	0	n	1	0	Caryophyllaceae Juss.
Iz_2_1009	DJI_0439	Silene latifolia subsp. alba (Mill.) Greuter & Burdet	0	n	1	0	Caryophyllaceae Juss.
Iz_2_1009	DJI_0439	Solidago virgaurea L.	0.5	n	1	0	Asteraceae Bercht. & J.Presl
Iz_2_1009	DJI_0439	Sorbus aucuparia L.	0	n	1	1	Rosaceae Juss.
Iz_2_1009	DJI_0439	Stachys recta L.	0	n	1	0	Lamiacea Lindl.
Iz_2_1009	DJI_0439	Tanacetum vulgare L.	0	n	1	0	Asteraceae Bercht. & J.Presl
Iz_2_1009	DJI_0439	Thymus pallasianus Heinr.Braun	0	n	1	0	Lamiacea Lindl.
Iz_2_1009	DJI_0439	Tilia cordata Mill.	0	n	1	1	Malvaceae Juss.
Iz_2_1009	DJI_0439	Trifolium alpestre L.	0	n	1	0	Fabaceae Lindl.
Iz_2_1009	DJI_0439	Trifolium arvense L.	0	n	1	0	Fabaceae Lindl.
Iz_2_1009	DJI_0439	Ulmus glabra Huds.	0	n	1	1	Ulmaceae Mirb.
Iz_2_1009	DJI_0439	Verbascum densiflorum Bertol.	0	n	1	0	Scrophulariaceae Juss.
Iz_2_1009	DJI_0439	Veronica austriaca L.	0	n	1	0	Plantaginaceae Juss.
Iz_2_1009	DJI_0439	Veronica incana L.	0	n	1	0	Plantaginaceae Juss.
Iz_2_1009	DJI_0439	Veronica verna L.	0	n	1	0	Plantaginaceae Juss.
Iz_2_1009	DJI_0439	Vincetoxicum hirundinaria Medik	0	n	1	0	Apocynaceae Juss.
Iz_2_1009	DJI_0440	Acer tataricum L.	0	n	1	1	Sapindaceae Juss.
Iz_2_1009	DJI_0440	Achillea millefolium L. s.l.	0	n	1	0	Asteraceae Bercht. & J.Presl
Iz_2_1009	DJI_0440	Allium oleraceum L.	0	n	1	0	Amaryllidaceae J.St.-Hil.
Iz_2_1009	DJI_0440	Ambrosia artemisiifolia L.	0	a	0	0	Asteraceae Bercht. & J.Presl
Iz_2_1009	DJI_0440	Amorpha fruticosa L.	0	a	0	0	Fabaceae Lindl.
Iz_2_1009	DJI_0440	Anthericum ramosum L.	0	n	1	0	Asparagaceae Juss.
Iz_2_1009	DJI_0440	Artemisia marschalliana Spreng.	0	n	1	0	Asteraceae Bercht. & J.Presl
Iz_2_1009	DJI_0440	Asparagus officinalis L.	0	n	1	0	Asparagaceae Juss.

Iz_2_1009	DJI_0440	Astragalus cicer L.	0	n	1	0	Fabaceae Lindl.
Iz_2_1009	DJI_0440	Bassia laniflora (S.G.Gmel.) A.J.Scott	0	n	1	0	Amaranthaceae Juss.
Iz_2_1009	DJI_0440	Berberis vulgaris L.	0	n	1	0	Berberidaceae Juss.
Iz_2_1009	DJI_0440	Betula pendula Roth.	0	n	1	1	Betulaceae Gray
Iz_2_1009	DJI_0440	Betula pendula Roth.	0	n	1	0	Poaceae Barnhart
Iz_2_1009	DJI_0440	Bromus inermis Leyss.	0		NA	NA	
Iz_2_1009	DJI_0440	Bromus squarrosus L.	0	a	0	0	Poaceae Barnhart
Iz_2_1009	DJI_0440	Bromus tectorum L.	0	a	0	0	Poaceae Barnhart
Iz_2_1009	DJI_0440	Calamagrostis epigejos (L.) Roth	5	n	1	0	Poaceae Barnhart
Iz_2_1009	DJI_0440	Carduus acanthoides L.	0	n	1	0	Asteraceae Bercht. & J.Presl
Iz_2_1009	DJI_0440	Carex sp.	0	n	1	0	Cyperaceae Juss.
Iz_2_1009	DJI_0440	Centaurea majorovii Dumbadze	0	n	1	0	Asteraceae Bercht. & J.Presl
Iz_2_1009	DJI_0440	Cf. Solidago virgaurea	0		NA	NA	
Iz_2_1009	DJI_0440	Chamaecytisus ruthenicus (Fisch.ex Wol.) Klaskova	0	n	1	0	Fabaceae Lindl.
Iz_2_1009	DJI_0440	Chelidonium majus L.	0	n	1	0	Papaveraceae Juss.
Iz_2_1009	DJI_0440	Chenopodium betaceum Andrz.	0	n	1	0	Amaranthaceae Juss.
Iz_2_1009	DJI_0440	Chenopodium cf. album	0	n	1	0	Amaranthaceae Juss.
Iz_2_1009	DJI_0440	Chenopodium sp.	0		NA	0	Amaranthaceae Juss.
Iz_2_1009	DJI_0440	Chondrilla juncea L.	0	n	1	0	Asteraceae Bercht. & J.Presl
Iz_2_1009	DJI_0440	Cirsium vulgare (Savi) Ten.	0	n	1	0	Asteraceae Bercht. & J.Presl
Iz_2_1009	DJI_0440	Clinopodium acinos (L.) Kuntze	0	n	1	0	Lamiacea Lindl.
Iz_2_1009	DJI_0440	Convallaria majalis L.	0	n	1	0	Asparagaceae Juss.
Iz_2_1009	DJI_0440	Crataegus rhipidophylla Gand.	0	n	1	1	Rosaceae Juss.
Iz_2_1009	DJI_0440	Crepis foetida L.	0	n	1	0	Asteraceae Bercht. & J.Presl
Iz_2_1009	DJI_0440	Dactylis glomerata L.	0	n	1	0	Poaceae Barnhart
Iz_2_1009	DJI_0440	Dianthus campestris M.Bieb.	0	n	1	0	Caryophyllaceae Juss.
Iz_2_1009	DJI_0440	Digitaria sanguinalis (L.) Scop.	0	a	0	0	Poaceae Barnhart
Iz_2_1009	DJI_0440	Elymus repens (L.) Gould	0	n	1	0	Poaceae Barnhart
Iz_2_1009	DJI_0440	Epilobium tetragonum L.	0	n	1	0	Onagraceae Juss.
Iz_2_1009	DJI_0440	Eragrostis minor Host	0	a	0	0	Poaceae Barnhart
Iz_2_1009	DJI_0440	Erigeron canadensis L.	0	a	0	0	Asteraceae Bercht. & J.Presl
Iz_2_1009	DJI_0440	Euphorbia seguieriana Neck.	0	n	1	0	Euphorbiaceae Juss.
Iz_2_1009	DJI_0440	Fallopia dumetorum (L.) Holub	0	n	1	0	Polygonaceae Juss.
Iz_2_1009	DJI_0440	Festuca beckeri (Hack.) Trautv.	0	n	1	0	Poaceae Barnhart
Iz_2_1009	DJI_0440	Fragaria vesca L.	0	n	1	0	Rosaceae Juss.
Iz_2_1009	DJI_0440	Frangula alnus Mill.	0	n	1	1	Rhamnaceae Juss.
Iz_2_1009	DJI_0440	Fraxinus pennsylvanica Marshall	0	a	0	1	Oleaceae Hoffmanns. & Link
Iz_2_1009	DJI_0440	Galeopsis bifida Boenn.	0	n	1	0	Lamiacea Lindl.
Iz_2_1009	DJI_0440	Galium verum L.	0	n	1	0	Rubiaceae Juss.
Iz_2_1009	DJI_0440	Genista tinctoria L.	0	n	1	0	Fabaceae Lindl.
Iz_2_1009	DJI_0440	Geranium robertianum L.	0	n	1	0	Geraniaceae Juss.
Iz_2_1009	DJI_0440	Geranium sanquineum L.	0	n	1	0	Geraniaceae Juss.
Iz_2_1009	DJI_0440	Gypsophila paniculata L.	0	n	1	0	Caryophyllaceae Juss.
Iz_2_1009	DJI_0440	Helichrysum arenarium (L.) Moench	0	n	1	0	Asteraceae Bercht. & J.Presl
Iz_2_1009	DJI_0440	Herniaria polygama J.Gay	0	n	1	0	Caryophyllaceae Juss.
Iz_2_1009	DJI_0440	Hieracium robustum Fr.	0	n	1	0	Asteraceae Bercht. & J.Presl
Iz_2_1009	DJI_0440	Hieracium umbellatum L.	0	n	1	NA	Asteraceae Bercht. & J.Presl
Iz_2_1009	DJI_0440	Hylotelephium maximum (L.) Holub	0	n	1	0	Crassulaceae J.St.-Hil.
Iz_2_1009	DJI_0440	Jacobaea borysthenica (DC.) B.Nord. & Greuter	0	n	1	0	Asteraceae Bercht. & J.Presl
Iz_2_1009	DJI_0440	Jasione montana L.	0	n	1	0	Campanulaceae Juss.
Iz_2_1009	DJI_0440	Jurinea cyanoides (L.) Rchb.s.l.	0	n	1	0	Asteraceae Bercht. & J.Presl
Iz_2_1009	DJI_0440	Koeleria glauca (Spreng.) DC.	0	n	1	0	Poaceae Barnhart
Iz_2_1009	DJI_0440	Lactuca serriola L.	0	a	0	0	Asteraceae Bercht. & J.Presl
Iz_2_1009	DJI_0440	Linaria genistifolia (L.) Mill.	0	n	1	0	Plantaginaceae Juss.
Iz_2_1009	DJI_0440	Lomelosia argentea (L.) Greuter & Burdet	0	n	1	0	Caprifoliaceae Juss.
Iz_2_1009	DJI_0440	Melampyrum pratense L.	0	n	1	0	Orobanchaceae Vent.
Iz_2_1009	DJI_0440	Oenothera biennis L.	0	a	0	0	Onagraceae Juss.
Iz_2_1009	DJI_0440	Pentanema britannica (L.) D.Gut.Larr., Santos-Vicente, Anderb., E.Rico & M.M.Mart.Ort.	0	n	1	0	Asteraceae Bercht. & J.Presl
Iz_2_1009	DJI_0440	Peucedanum oreoselinum (L.) Moench	0	n	1	0	Apiaceae Lindl.
Iz_2_1009	DJI_0440	Peucedanum ruthenicum M.Bieb.	0	n	1	0	Apiaceae Lindl.
Iz_2_1009	DJI_0440	Pilosella echioides (Lumn.) F.W.Schultz & Sch.Bip.	0	n	1	0	Asteraceae Bercht. & J.Presl
Iz_2_1009	DJI_0440	Pilosella officinarum F. Schultz et Sch. Bip.	0	n	1	0	Asteraceae Bercht. & J.Presl
Iz_2_1009	DJI_0440	Pilosella sp.	0		NA	0	Asteraceae Bercht. & J.Presl
Iz_2_1009	DJI_0440	Pinus sylvestris L.	0	n	1	1	Pinaceae Spreng. ex F.Rudolphi
Iz_2_1009	DJI_0440	Poa annua L.	0	n	1	0	Poaceae Barnhart
Iz_2_1009	DJI_0440	Poa nemoralis L.	0	n	1	0	Poaceae Barnhart
Iz_2_1009	DJI_0440	Poa sp.	0		NA	0	Poaceae Barnhart
Iz_2_1009	DJI_0440	Polygonatum odoratum (Mill.) Druce	0	n	1	0	Asparagaceae Juss.
Iz_2_1009	DJI_0440	Polygonum arenarium Waldst. & Kit.	0	n	1	0	Polygonaceae Juss.
Iz_2_1009	DJI_0440	Populus nigra L.	0	n	1	1	Salicaceae Mirb.
Iz_2_1009	DJI_0440	Populus tremula L.	0	n	1	1	Salicaceae Mirb.
Iz_2_1009	DJI_0440	Populus Ã canescens (Aiton) Sm.	0	n	1	1	Salicaceae Mirb.
Iz_2_1009	DJI_0440	Potentilla argentea L.	0	n	1	0	Rosaceae Juss.
Iz_2_1009	DJI_0440	Psammophiliella muralis (L.) Ikonn.	0	n	1	0	Caryophyllaceae Juss.
Iz_2_1009	DJI_0440	Psephellus sumensis (Kalen.) Greuter	0	n	1	0	Asteraceae Bercht. & J.Presl
Iz_2_1009	DJI_0440	Pulsatilla pratensis (L.) Mill. s.l.	0	n	1	0	Ranunculaceae Juss.
Iz_2_1009	DJI_0440	Pyrus communis L.	0	a	0	1	Rosaceae Juss.
Iz_2_1009	DJI_0440	Quercus robur L.	0	n	1	1	Fagaceae Dumort.
Iz_2_1009	DJI_0440	Robinia pseudoacacia L.	0	a	0	1	Fabaceae Lindl.
Iz_2_1009	DJI_0440	Rosa sp.	0		NA	1	Rosaceae Juss.
Iz_2_1009	DJI_0440	Rubus idaeus L.	0	n	1	0	Rosaceae Juss.
Iz_2_1009	DJI_0440	Rumex acetosella L.	0	n	1	0	Polygonaceae Juss.
Iz_2_1009	DJI_0440	Rumex thyrsiflorus Fingerh.	0	n	1	0	Polygonaceae Juss.
Iz_2_1009	DJI_0440	Salix alba L.	0	n	1	1	Salicaceae Mirb.
Iz_2_1009	DJI_0440	Sambucus nigra L.	0	n	1	1	Viburnaceae Raf.
Iz_2_1009	DJI_0440	Secale sylvestre Host	0	n	1	0	Poaceae Barnhart
Iz_2_1009	DJI_0440	Sempervivum ruthenicum Schnittsp. & C.B. Lehm.	0	n	1	0	Crassulaceae J.St.-Hil.
Iz_2_1009	DJI_0440	Setaria viridis (L.) P. Beauv.	0	n	1	0	Poaceae Barnhart
Iz_2_1009	DJI_0440	Silene borysthenica (Gruner) Walters	0	n	1	0	Caryophyllaceae Juss.
Iz_2_1009	DJI_0440	Silene latifolia subsp. alba (Mill.) Greuter & Burdet	0	n	1	0	Caryophyllaceae Juss.
Iz_2_1009	DJI_0440	Solidago virgaurea L.	0	n	1	0	Asteraceae Bercht. & J.Presl
Iz_2_1009	DJI_0440	Sorbus aucuparia L.	0	n	1	1	Rosaceae Juss.
Iz_2_1009	DJI_0440	Stachys recta L.	0	n	1	0	Lamiacea Lindl.
Iz_2_1009	DJI_0440	Tanacetum vulgare L.	0	n	1	0	Asteraceae Bercht. & J.Presl
Iz_2_1009	DJI_0440	Thymus pallasianus Heinr.Braun	0	n	1	0	Lamiacea Lindl.
Iz_2_1009	DJI_0440	Tilia cordata Mill.	0	n	1	1	Malvaceae Juss.
Iz_2_1009	DJI_0440	Trifolium alpestre L.	0	n	1	0	Fabaceae Lindl.
Iz_2_1009	DJI_0440	Trifolium arvense L.	0	n	1	0	Fabaceae Lindl.

Iz_2_1009	DJI_0440	Ulmus glabra Huds.	0	n	1	1	Ulmaceae Mirb.
Iz_2_1009	DJI_0440	Verbascum densiflorum Bertol.	0	n	1	0	Scrophulariaceae Juss.
Iz_2_1009	DJI_0440	Veronica austriaca L.	0	n	1	0	Plantaginaceae Juss.
Iz_2_1009	DJI_0440	Veronica incana L.	0	n	1	0	Plantaginaceae Juss.
Iz_2_1009	DJI_0440	Veronica verna L.	0	n	1	0	Plantaginaceae Juss.
Iz_2_1009	DJI_0440	Vincetoxicum hirundinaria Medik	0	n	1	0	Apocynaceae Juss.
Iz_2_1009	DJI_0443	Acer tataricum L.	0	n	1	1	Sapindaceae Juss.
Iz_2_1009	DJI_0443	Achillea millefolium L. s.l.	0	n	1	0	Asteraceae Bercht. & J.Presl
Iz_2_1009	DJI_0443	Allium oleraceum L.	0	n	1	0	Amaryllidaceae J.St.-Hil.
Iz_2_1009	DJI_0443	Ambrosia artemisiifolia L.	0	a	0	0	Asteraceae Bercht. & J.Presl
Iz_2_1009	DJI_0443	Amorpha fruticosa L.	0	a	0	0	Fabaceae Lindl.
Iz_2_1009	DJI_0443	Anthericum ramosum L.	0	n	1	0	Asparagaceae Juss.
Iz_2_1009	DJI_0443	Artemisia marschalliana Spreng.	0	n	1	0	Asteraceae Bercht. & J.Presl
Iz_2_1009	DJI_0443	Asparagus officinalis L.	0	n	1	0	Asparagaceae Juss.
Iz_2_1009	DJI_0443	Astragalus cicer L.	0	n	1	0	Fabaceae Lindl.
Iz_2_1009	DJI_0443	Bassia laniflora (S.G.Gmel.) A.J.Scott	0	n	1	0	Amaranthaceae Juss.
Iz_2_1009	DJI_0443	Berberis vulgaris L.	0	n	1	0	Berberidaceae Juss.
Iz_2_1009	DJI_0443	Betula pendula Roth.	0	n	1	1	Betulaceae Gray
Iz_2_1009	DJI_0443	Betula pendula Roth.	0	n	1	0	Poaceae Barnhart
Iz_2_1009	DJI_0443	Bromus inermis Leyss.	0		NA	NA	
Iz_2_1009	DJI_0443	Bromus squarrosus L.	0	a	0	0	Poaceae Barnhart
Iz_2_1009	DJI_0443	Bromus tectorum L.	0	a	0	0	Poaceae Barnhart
Iz_2_1009	DJI_0443	Calamagrostis epigejos (L.) Roth	1	n	1	0	Poaceae Barnhart
Iz_2_1009	DJI_0443	Carduus acanthoides L.	0	n	1	0	Asteraceae Bercht. & J.Presl
Iz_2_1009	DJI_0443	Carex sp.	0	n	1	0	Cyperaceae Juss.
Iz_2_1009	DJI_0443	Centaurea majorovii Dumbadze	0	n	1	0	Asteraceae Bercht. & J.Presl
Iz_2_1009	DJI_0443	Cf. Solidago virgaurea	0		NA	NA	
Iz_2_1009	DJI_0443	Chamaecytisus ruthenicus (Fisch.ex Wol.) Klaskova	0	n	1	0	Fabaceae Lindl.
Iz_2_1009	DJI_0443	Chelidonium majus L.	0	n	1	0	Papaveraceae Juss.
Iz_2_1009	DJI_0443	Chenopodium betaceum Andrz.	0	n	1	0	Amaranthaceae Juss.
Iz_2_1009	DJI_0443	Chenopodium cf. album	0	n	1	0	Amaranthaceae Juss.
Iz_2_1009	DJI_0443	Chenopodium sp.	0		NA	0	Amaranthaceae Juss.
Iz_2_1009	DJI_0443	Chondrilla juncea L.	0	n	1	0	Asteraceae Bercht. & J.Presl
Iz_2_1009	DJI_0443	Cirsium vulgare (Savi) Ten.	0	n	1	0	Asteraceae Bercht. & J.Presl
Iz_2_1009	DJI_0443	Clinopodium acinos (L.) Kuntze	0	n	1	0	Lamiacea Lindl.
Iz_2_1009	DJI_0443	Convallaria majalis L.	0	n	1	0	Asparagaceae Juss.
Iz_2_1009	DJI_0443	Crataegus rhipidophylla Gand.	0	n	1	1	Rosaceae Juss.
Iz_2_1009	DJI_0443	Crepis foetida L.	0	n	1	0	Asteraceae Bercht. & J.Presl
Iz_2_1009	DJI_0443	Dactylis glomerata L.	0	n	1	0	Poaceae Barnhart
Iz_2_1009	DJI_0443	Dianthus campestris M.Bieb.	0	n	1	0	Caryophyllaceae Juss.
Iz_2_1009	DJI_0443	Digitaria sanguinalis (L.) Scop.	0	a	0	0	Poaceae Barnhart
Iz_2_1009	DJI_0443	Elymus repens (L.) Gould	0	n	1	0	Poaceae Barnhart
Iz_2_1009	DJI_0443	Epilobium tetragonum L.	0	n	1	0	Onagraceae Juss.
Iz_2_1009	DJI_0443	Eragrostis minor Host	0	a	0	0	Poaceae Barnhart
Iz_2_1009	DJI_0443	Erigeron canadensis L.	0	a	0	0	Asteraceae Bercht. & J.Presl
Iz_2_1009	DJI_0443	Euphorbia seguieriana Neck.	0	n	1	0	Euphorbiaceae Juss.
Iz_2_1009	DJI_0443	Fallopia dumetorum (L.) Holub	0	n	1	0	Polygonaceae Juss.
Iz_2_1009	DJI_0443	Festuca beckeri (Hack.) Trautv.	0	n	1	0	Poaceae Barnhart
Iz_2_1009	DJI_0443	Fragaria vesca L.	0	n	1	0	Rosaceae Juss.
Iz_2_1009	DJI_0443	Frangula alnus Mill.	0	n	1	1	Rhamnaceae Juss.
Iz_2_1009	DJI_0443	Fraxinus pennsylvanica Marshall	0	a	0	1	Oleaceae Hoffmanns. & Link
Iz_2_1009	DJI_0443	Galeopsis bifida Boenn.	0	n	1	0	Lamiacea Lindl.
Iz_2_1009	DJI_0443	Galium verum L.	0	n	1	0	Rubiaceae Juss.
Iz_2_1009	DJI_0443	Genista tinctoria L.	0	n	1	0	Fabaceae Lindl.
Iz_2_1009	DJI_0443	Geranium robertianum L.	1	n	1	0	Geraniaceae Juss.
Iz_2_1009	DJI_0443	Geranium sanquineum L.	0	n	1	0	Geraniaceae Juss.
Iz_2_1009	DJI_0443	Gypsophila paniculata L.	0	n	1	0	Caryophyllaceae Juss.
Iz_2_1009	DJI_0443	Helichrysum arenarium (L.) Moench	0	n	1	0	Asteraceae Bercht. & J.Presl
Iz_2_1009	DJI_0443	Herniaria polygama J.Gay	0	n	1	0	Caryophyllaceae Juss.
Iz_2_1009	DJI_0443	Hieracium robustum Fr.	0	n	1	0	Asteraceae Bercht. & J.Presl
Iz_2_1009	DJI_0443	Hieracium umbellatum L.	0	n	1	NA	Asteraceae Bercht. & J.Presl
Iz_2_1009	DJI_0443	Hylotelephium maximum (L.) Holub	0	n	1	0	Crassulaceae J.St.-Hil.
Iz_2_1009	DJI_0443	Jacobaea borysthenica (DC.) B.Nord. & Greuter	0	n	1	0	Asteraceae Bercht. & J.Presl
Iz_2_1009	DJI_0443	Jasione montana L.	0	n	1	0	Campanulaceae Juss.
Iz_2_1009	DJI_0443	Jurinea cyanoides (L.) Rchb.s.l.	0	n	1	0	Asteraceae Bercht. & J.Presl
Iz_2_1009	DJI_0443	Koeleria glauca (Spreng.) DC.	0	n	1	0	Poaceae Barnhart
Iz_2_1009	DJI_0443	Lactuca serriola L.	0	a	0	0	Asteraceae Bercht. & J.Presl
Iz_2_1009	DJI_0443	Linaria genistifolia (L.) Mill.	0	n	1	0	Plantaginaceae Juss.
Iz_2_1009	DJI_0443	Lomelosia argentea (L.) Greuter & Burdet	0	n	1	0	Caprifoliaceae Juss.
Iz_2_1009	DJI_0443	Melampyrum pratense L.	0	n	1	0	Orobanchaceae Vent.
Iz_2_1009	DJI_0443	Oenothera biennis L.	0	a	0	0	Onagraceae Juss.
Iz_2_1009	DJI_0443	Pentanema britannica (L.) D.Gut.Larr., Santos-Vicente, Anderb., E.Rico & M.M.Mart.Ort.	0	n	1	0	Asteraceae Bercht. & J.Presl
Iz_2_1009	DJI_0443	Peucedanum oreoselinum (L.) Moench	0	n	1	0	Apiaceae Lindl.
Iz_2_1009	DJI_0443	Peucedanum ruthenicum M.Bieb.	0	n	1	0	Apiaceae Lindl.
Iz_2_1009	DJI_0443	Pilosella echioides (Lumn.) F.W.Schultz & Sch.Bip.	0	n	1	0	Asteraceae Bercht. & J.Presl
Iz_2_1009	DJI_0443	Pilosella officinarum F. Schultz et Sch. Bip.	0	n	1	0	Asteraceae Bercht. & J.Presl
Iz_2_1009	DJI_0443	Pilosella sp.	0		NA	0	Asteraceae Bercht. & J.Presl
Iz_2_1009	DJI_0443	Pinus sylvestris L.	0	n	1	1	Pinaceae Spreng. ex F.Rudolphi
Iz_2_1009	DJI_0443	Poa annua L.	0	n	1	0	Poaceae Barnhart
Iz_2_1009	DJI_0443	Poa nemoralis L.	0	n	1	0	Poaceae Barnhart
Iz_2_1009	DJI_0443	Poa sp.	0		NA	0	Poaceae Barnhart
Iz_2_1009	DJI_0443	Polygonatum odoratum (Mill.) Druce	0	n	1	0	Asparagaceae Juss.
Iz_2_1009	DJI_0443	Polygonum arenarium Waldst. & Kit.	0	n	1	0	Polygonaceae Juss.
Iz_2_1009	DJI_0443	Populus nigra L.	0	n	1	1	Salicaceae Mirb.
Iz_2_1009	DJI_0443	Populus tremula L.	0	n	1	1	Salicaceae Mirb.
Iz_2_1009	DJI_0443	Populus Ã canescens (Aiton) Sm.	0	n	1	1	Salicaceae Mirb.
Iz_2_1009	DJI_0443	Potentilla argentea L.	0	n	1	0	Rosaceae Juss.
Iz_2_1009	DJI_0443	Psammophiliella muralis (L.) Ikonn.	0	n	1	0	Caryophyllaceae Juss.
Iz_2_1009	DJI_0443	Psephellus sumensis (Kalen.) Greuter	0	n	1	0	Asteraceae Bercht. & J.Presl
Iz_2_1009	DJI_0443	Pulsatilla pratensis (L.) Mill. s.l.	0	n	1	0	Ranunculaceae Juss.
Iz_2_1009	DJI_0443	Pyrus communis L.	0	a	0	1	Rosaceae Juss.
Iz_2_1009	DJI_0443	Quercus robur L.	0	n	1	1	Fagaceae Dumort.
Iz_2_1009	DJI_0443	Robinia pseudoacacia L.	0	a	0	1	Fabaceae Lindl.
Iz_2_1009	DJI_0443	Rosa sp.	0		NA	1	Rosaceae Juss.
Iz_2_1009	DJI_0443	Rubus idaeus L.	0	n	1	0	Rosaceae Juss.
Iz_2_1009	DJI_0443	Rumex acetosella L.	0	n	1	0	Polygonaceae Juss.
Iz_2_1009	DJI_0443	Rumex thyrsiflorus Fingerh.	0	n	1	0	Polygonaceae Juss.
Iz_2_1009	DJI_0443	Salix alba L.	0	n	1	1	Salicaceae Mirb.

Iz_2_1009	DJI_0443	Sambucus nigra L.	0	n	1	1	Viburnaceae Raf.
Iz_2_1009	DJI_0443	Secale sylvestre Host	0	n	1	0	Poaceae Barnhart
Iz_2_1009	DJI_0443	Sempervivum ruthenicum Schnittsp. & C.B. Lehm.	0	n	1	0	Crassulaceae J.St.-Hil.
Iz_2_1009	DJI_0443	Setaria viridis (L.) P. Beauv.	0	n	1	0	Poaceae Barnhart
Iz_2_1009	DJI_0443	Silene borysthenica (Gruner) Walters	0	n	1	0	Caryophyllaceae Juss.
Iz_2_1009	DJI_0443	Silene latifolia subsp. alba (Mill.) Greuter & Burdet	0	n	1	0	Caryophyllaceae Juss.
Iz_2_1009	DJI_0443	Solidago virgaurea L.	0	n	1	0	Asteraceae Bercht. & J.Presl
Iz_2_1009	DJI_0443	Sorbus aucuparia L.	0	n	1	1	Rosaceae Juss.
Iz_2_1009	DJI_0443	Stachys recta L.	0	n	1	0	Lamiacea Lindl.
Iz_2_1009	DJI_0443	Tanacetum vulgare L.	0	n	1	0	Asteraceae Bercht. & J.Presl
Iz_2_1009	DJI_0443	Thymus pallasianus Heinr.Braun	0	n	1	0	Lamiacea Lindl.
Iz_2_1009	DJI_0443	Tilia cordata Mill.	0	n	1	1	Malvaceae Juss.
Iz_2_1009	DJI_0443	Trifolium alpestre L.	0	n	1	0	Fabaceae Lindl.
Iz_2_1009	DJI_0443	Trifolium arvense L.	0	n	1	0	Fabaceae Lindl.
Iz_2_1009	DJI_0443	Ulmus glabra Huds.	0	n	1	1	Ulmaceae Mirb.
Iz_2_1009	DJI_0443	Verbascum densiflorum Bertol.	0	n	1	0	Scrophulariaceae Juss.
Iz_2_1009	DJI_0443	Veronica austriaca L.	0	n	1	0	Plantaginaceae Juss.
Iz_2_1009	DJI_0443	Veronica incana L.	0	n	1	0	Plantaginaceae Juss.
Iz_2_1009	DJI_0443	Veronica verna L.	0	n	1	0	Plantaginaceae Juss.
Iz_2_1009	DJI_0443	Vincetoxicum hirundinaria Medik	0	n	1	0	Apocynaceae Juss.
Iz_2_1009	DJI_0444	Acer tataricum L.	0	n	1	1	Sapindaceae Juss.
Iz_2_1009	DJI_0444	Achillea millefolium L. s.l.	0	n	1	0	Asteraceae Bercht. & J.Presl
Iz_2_1009	DJI_0444	Allium oleraceum L.	0	n	1	0	Amaryllidaceae J.St.-Hil.
Iz_2_1009	DJI_0444	Ambrosia artemisiifolia L.	0	a	0	0	Asteraceae Bercht. & J.Presl
Iz_2_1009	DJI_0444	Amorpha fruticosa L.	0	a	0	0	Fabaceae Lindl.
Iz_2_1009	DJI_0444	Anthericum ramosum L.	0	n	1	0	Asparagaceae Juss.
Iz_2_1009	DJI_0444	Artemisia marschalliana Spreng.	0	n	1	0	Asteraceae Bercht. & J.Presl
Iz_2_1009	DJI_0444	Asparagus officinalis L.	0	n	1	0	Asparagaceae Juss.
Iz_2_1009	DJI_0444	Astragalus cicer L.	0	n	1	0	Fabaceae Lindl.
Iz_2_1009	DJI_0444	Bassia laniflora (S.G.Gmel.) A.J.Scott	0	n	1	0	Amaranthaceae Juss.
Iz_2_1009	DJI_0444	Berberis vulgaris L.	0	n	1	0	Berberidaceae Juss.
Iz_2_1009	DJI_0444	Betula pendula Roth.	0	n	1	1	Betulaceae Gray
Iz_2_1009	DJI_0444	Betula pendula Roth.	0	n	1	0	Poaceae Barnhart
Iz_2_1009	DJI_0444	Bromus inermis Leyss.	0		NA	NA	
Iz_2_1009	DJI_0444	Bromus squarrosus L.	0	a	0	0	Poaceae Barnhart
Iz_2_1009	DJI_0444	Bromus tectorum L.	0	a	0	0	Poaceae Barnhart
Iz_2_1009	DJI_0444	Calamagrostis epigejos (L.) Roth	2	n	1	0	Poaceae Barnhart
Iz_2_1009	DJI_0444	Carduus acanthoides L.	0	n	1	0	Asteraceae Bercht. & J.Presl
Iz_2_1009	DJI_0444	Carex sp.	0	n	1	0	Cyperaceae Juss.
Iz_2_1009	DJI_0444	Centaurea majorovii Dumbadze	0	n	1	0	Asteraceae Bercht. & J.Presl
Iz_2_1009	DJI_0444	Cf. Solidago virgaurea	0		NA	NA	
Iz_2_1009	DJI_0444	Chamaecytisus ruthenicus (Fisch.ex Wol.) Klaskova	0	n	1	0	Fabaceae Lindl.
Iz_2_1009	DJI_0444	Chelidonium majus L.	0	n	1	0	Papaveraceae Juss.
Iz_2_1009	DJI_0444	Chenopodium betaceum Andrz.	0	n	1	0	Amaranthaceae Juss.
Iz_2_1009	DJI_0444	Chenopodium cf. album	0	n	1	0	Amaranthaceae Juss.
Iz_2_1009	DJI_0444	Chenopodium sp.	0		NA	0	Amaranthaceae Juss.
Iz_2_1009	DJI_0444	Chondrilla juncea L.	0	n	1	0	Asteraceae Bercht. & J.Presl
Iz_2_1009	DJI_0444	Cirsium vulgare (Savi) Ten.	0	n	1	0	Asteraceae Bercht. & J.Presl
Iz_2_1009	DJI_0444	Clinopodium acinos (L.) Kuntze	0	n	1	0	Lamiacea Lindl.
Iz_2_1009	DJI_0444	Convallaria majalis L.	0	n	1	0	Asparagaceae Juss.
Iz_2_1009	DJI_0444	Crataegus rhipidophylla Gand.	0	n	1	1	Rosaceae Juss.
Iz_2_1009	DJI_0444	Crepis foetida L.	0	n	1	0	Asteraceae Bercht. & J.Presl
Iz_2_1009	DJI_0444	Dactylis glomerata L.	0	n	1	0	Poaceae Barnhart
Iz_2_1009	DJI_0444	Dianthus campestris M.Bieb.	0	n	1	0	Caryophyllaceae Juss.
Iz_2_1009	DJI_0444	Digitaria sanguinalis (L.) Scop.	0	a	0	0	Poaceae Barnhart
Iz_2_1009	DJI_0444	Elymus repens (L.) Gould	0	n	1	0	Poaceae Barnhart
Iz_2_1009	DJI_0444	Epilobium tetragonum L.	0	n	1	0	Onagraceae Juss.
Iz_2_1009	DJI_0444	Eragrostis minor Host	0	a	0	0	Poaceae Barnhart
Iz_2_1009	DJI_0444	Erigeron canadensis L.	0	a	0	0	Asteraceae Bercht. & J.Presl
Iz_2_1009	DJI_0444	Euphorbia seguieriana Neck.	0	n	1	0	Euphorbiaceae Juss.
Iz_2_1009	DJI_0444	Fallopia dumetorum (L.) Holub	0	n	1	0	Polygonaceae Juss.
Iz_2_1009	DJI_0444	Festuca beckeri (Hack.) Trautv.	0	n	1	0	Poaceae Barnhart
Iz_2_1009	DJI_0444	Fragaria vesca L.	0	n	1	0	Rosaceae Juss.
Iz_2_1009	DJI_0444	Frangula alnus Mill.	0	n	1	1	Rhamnaceae Juss.
Iz_2_1009	DJI_0444	Fraxinus pennsylvanica Marshall	0	a	0	1	Oleaceae Hoffmanns. & Link
Iz_2_1009	DJI_0444	Galeopsis bifida Boenn.	0	n	1	0	Lamiacea Lindl.
Iz_2_1009	DJI_0444	Galium verum L.	0	n	1	0	Rubiaceae Juss.
Iz_2_1009	DJI_0444	Genista tinctoria L.	0	n	1	0	Fabaceae Lindl.
Iz_2_1009	DJI_0444	Geranium robertianum L.	1	n	1	0	Geraniaceae Juss.
Iz_2_1009	DJI_0444	Geranium sanquineum L.	0	n	1	0	Geraniaceae Juss.
Iz_2_1009	DJI_0444	Gypsophila paniculata L.	0	n	1	0	Caryophyllaceae Juss.
Iz_2_1009	DJI_0444	Helichrysum arenarium (L.) Moench	0	n	1	0	Asteraceae Bercht. & J.Presl
Iz_2_1009	DJI_0444	Herniaria polygama J.Gay	0	n	1	0	Caryophyllaceae Juss.
Iz_2_1009	DJI_0444	Hieracium robustum Fr.	0	n	1	0	Asteraceae Bercht. & J.Presl
Iz_2_1009	DJI_0444	Hieracium umbellatum L.	0	n	1	NA	Asteraceae Bercht. & J.Presl
Iz_2_1009	DJI_0444	Hylotelephium maximum (L.) Holub	0	n	1	0	Crassulaceae J.St.-Hil.
Iz_2_1009	DJI_0444	Jacobaea borysthenica (DC.) B.Nord. & Greuter	0	n	1	0	Asteraceae Bercht. & J.Presl
Iz_2_1009	DJI_0444	Jasione montana L.	0	n	1	0	Campanulaceae Juss.
Iz_2_1009	DJI_0444	Jurinea cyanoides (L.) Rchb.s.l.	0	n	1	0	Asteraceae Bercht. & J.Presl
Iz_2_1009	DJI_0444	Koeleria glauca (Spreng.) DC.	0	n	1	0	Poaceae Barnhart
Iz_2_1009	DJI_0444	Lactuca serriola L.	0	a	0	0	Asteraceae Bercht. & J.Presl
Iz_2_1009	DJI_0444	Linaria genistifolia (L.) Mill.	0	n	1	0	Plantaginaceae Juss.
Iz_2_1009	DJI_0444	Lomelosia argentea (L.) Greuter & Burdet	0	n	1	0	Caprifoliaceae Juss.
Iz_2_1009	DJI_0444	Melampyrum pratense L.	0	n	1	0	Orobanchaceae Vent.
Iz_2_1009	DJI_0444	Oenothera biennis L.	0	a	0	0	Onagraceae Juss.
Iz_2_1009	DJI_0444	Pentanema britannica (L.) D.Gut.Larr., Santos-Vicente, Anderb., E.Rico & M.M.Mart.Ort.	0	n	1	0	Asteraceae Bercht. & J.Presl
Iz_2_1009	DJI_0444	Peucedanum oreoselinum (L.) Moench	0	n	1	0	Apiaceae Lindl.
Iz_2_1009	DJI_0444	Peucedanum ruthenicum M.Bieb.	0	n	1	0	Apiaceae Lindl.
Iz_2_1009	DJI_0444	Pilosella echioides (Lumn.) F.W.Schultz & Sch.Bip.	0	n	1	0	Asteraceae Bercht. & J.Presl
Iz_2_1009	DJI_0444	Pilosella officinarum F. Schultz et Sch. Bip.	0	n	1	0	Asteraceae Bercht. & J.Presl
Iz_2_1009	DJI_0444	Pilosella sp.	0		NA	0	Asteraceae Bercht. & J.Presl
Iz_2_1009	DJI_0444	Pinus sylvestris L.	0	n	1	1	Pinaceae Spreng. ex F.Rudolphi
Iz_2_1009	DJI_0444	Poa annua L.	0	n	1	0	Poaceae Barnhart
Iz_2_1009	DJI_0444	Poa nemoralis L.	0	n	1	0	Poaceae Barnhart
Iz_2_1009	DJI_0444	Poa sp.	0		NA	0	Poaceae Barnhart
Iz_2_1009	DJI_0444	Polygonatum odoratum (Mill.) Druce	0	n	1	0	Asparagaceae Juss.
Iz_2_1009	DJI_0444	Polygonum arenarium Waldst. & Kit.	0	n	1	0	Polygonaceae Juss.
Iz_2_1009	DJI_0444	Populus nigra L.	0	n	1	1	Salicaceae Mirb.

Iz_2_1009	DJI_0444	Populus tremula L.	0	n	1	1	Salicaceae Mirb.
Iz_2_1009	DJI_0444	Populus Ã canescens (Aiton) Sm.	0	n	1	1	Salicaceae Mirb.
Iz_2_1009	DJI_0444	Potentilla argentea L.	0	n	1	0	Rosaceae Juss.
Iz_2_1009	DJI_0444	Psammophiliella muralis (L.) Ikonn.	0	n	1	0	Caryophyllaceae Juss.
Iz_2_1009	DJI_0444	Psephellus sumensis (Kalen.) Greuter	0	n	1	0	Asteraceae Bercht. & J.Presl
Iz_2_1009	DJI_0444	Pulsatilla pratensis (L.) Mill. s.l.	0	n	1	0	Ranunculaceae Juss.
Iz_2_1009	DJI_0444	Pyrus communis L.	0	a	0	1	Rosaceae Juss.
Iz_2_1009	DJI_0444	Quercus robur L.	0	n	1	1	Fagaceae Dumort.
Iz_2_1009	DJI_0444	Robinia pseudoacacia L.	0	a	0	1	Fabaceae Lindl.
Iz_2_1009	DJI_0444	Rosa sp.	0		NA	1	Rosaceae Juss.
Iz_2_1009	DJI_0444	Rubus idaeus L.	0	n	1	0	Rosaceae Juss.
Iz_2_1009	DJI_0444	Rumex acetosella L.	0	n	1	0	Polygonaceae Juss.
Iz_2_1009	DJI_0444	Rumex thyrsiflorus Fingerh.	0	n	1	0	Polygonaceae Juss.
Iz_2_1009	DJI_0444	Salix alba L.	0	n	1	1	Salicaceae Mirb.
Iz_2_1009	DJI_0444	Sambucus nigra L.	0	n	1	1	Viburnaceae Raf.
Iz_2_1009	DJI_0444	Secale sylvestre Host	0	n	1	0	Poaceae Barnhart
Iz_2_1009	DJI_0444	Sempervivum ruthenicum Schnittsp. & C.B. Lehm.	0	n	1	0	Crassulaceae J.St.-Hil.
Iz_2_1009	DJI_0444	Setaria viridis (L.) P. Beauv.	0	n	1	0	Poaceae Barnhart
Iz_2_1009	DJI_0444	Silene borysthenica (Gruner) Walters	0	n	1	0	Caryophyllaceae Juss.
Iz_2_1009	DJI_0444	Silene latifolia subsp. alba (Mill.) Greuter & Burdet	0	n	1	0	Caryophyllaceae Juss.
Iz_2_1009	DJI_0444	Solidago virgaurea L.	0	n	1	0	Asteraceae Bercht. & J.Presl
Iz_2_1009	DJI_0444	Sorbus aucuparia L.	0	n	1	1	Rosaceae Juss.
Iz_2_1009	DJI_0444	Stachys recta L.	0	n	1	0	Lamiacea Lindl.
Iz_2_1009	DJI_0444	Tanacetum vulgare L.	0	n	1	0	Asteraceae Bercht. & J.Presl
Iz_2_1009	DJI_0444	Thymus pallasianus Heinr.Braun	0	n	1	0	Lamiacea Lindl.
Iz_2_1009	DJI_0444	Tilia cordata Mill.	0	n	1	1	Malvaceae Juss.
Iz_2_1009	DJI_0444	Trifolium alpestre L.	0	n	1	0	Fabaceae Lindl.
Iz_2_1009	DJI_0444	Trifolium arvense L.	0	n	1	0	Fabaceae Lindl.
Iz_2_1009	DJI_0444	Ulmus glabra Huds.	0	n	1	1	Ulmaceae Mirb.
Iz_2_1009	DJI_0444	Verbascum densiflorum Bertol.	0	n	1	0	Scrophulariaceae Juss.
Iz_2_1009	DJI_0444	Veronica austriaca L.	0	n	1	0	Plantaginaceae Juss.
Iz_2_1009	DJI_0444	Veronica incana L.	0	n	1	0	Plantaginaceae Juss.
Iz_2_1009	DJI_0444	Veronica verna L.	0	n	1	0	Plantaginaceae Juss.
Iz_2_1009	DJI_0444	Vincetoxicum hirundinaria Medik	0	n	1	0	Apocynaceae Juss.
Iz_2_1009	DJI_0445	Acer tataricum L.	0	n	1	1	Sapindaceae Juss.
Iz_2_1009	DJI_0445	Achillea millefolium L. s.l.	0	n	1	0	Asteraceae Bercht. & J.Presl
Iz_2_1009	DJI_0445	Allium oleraceum L.	0	n	1	0	Amaryllidaceae J.St.-Hil.
Iz_2_1009	DJI_0445	Ambrosia artemisiifolia L.	0	a	0	0	Asteraceae Bercht. & J.Presl
Iz_2_1009	DJI_0445	Amorpha fruticosa L.	0	a	0	0	Fabaceae Lindl.
Iz_2_1009	DJI_0445	Anthericum ramosum L.	0	n	1	0	Asparagaceae Juss.
Iz_2_1009	DJI_0445	Artemisia marschalliana Spreng.	0	n	1	0	Asteraceae Bercht. & J.Presl
Iz_2_1009	DJI_0445	Asparagus officinalis L.	0	n	1	0	Asparagaceae Juss.
Iz_2_1009	DJI_0445	Astragalus cicer L.	0	n	1	0	Fabaceae Lindl.
Iz_2_1009	DJI_0445	Bassia laniflora (S.G.Gmel.) A.J.Scott	0	n	1	0	Amaranthaceae Juss.
Iz_2_1009	DJI_0445	Berberis vulgaris L.	0	n	1	0	Berberidaceae Juss.
Iz_2_1009	DJI_0445	Betula pendula Roth.	0	n	1	1	Betulaceae Gray
Iz_2_1009	DJI_0445	Betula pendula Roth.	0	n	1	0	Poaceae Barnhart
Iz_2_1009	DJI_0445	Bromus inermis Leyss.	0		NA	NA	
Iz_2_1009	DJI_0445	Bromus squarrosus L.	0	a	0	0	Poaceae Barnhart
Iz_2_1009	DJI_0445	Bromus tectorum L.	0	a	0	0	Poaceae Barnhart
Iz_2_1009	DJI_0445	Calamagrostis epigejos (L.) Roth	10	n	1	0	Poaceae Barnhart
Iz_2_1009	DJI_0445	Carduus acanthoides L.	0	n	1	0	Asteraceae Bercht. & J.Presl
Iz_2_1009	DJI_0445	Carex sp.	0	n	1	0	Cyperaceae Juss.
Iz_2_1009	DJI_0445	Centaurea majorovii Dumbadze	0	n	1	0	Asteraceae Bercht. & J.Presl
Iz_2_1009	DJI_0445	Cf. Solidago virgaurea	0		NA	NA	
Iz_2_1009	DJI_0445	Chamaecytisus ruthenicus (Fisch.ex Wol.) Klaskova	0	n	1	0	Fabaceae Lindl.
Iz_2_1009	DJI_0445	Chelidonium majus L.	0	n	1	0	Papaveraceae Juss.
Iz_2_1009	DJI_0445	Chenopodium betaceum Andrz.	0	n	1	0	Amaranthaceae Juss.
Iz_2_1009	DJI_0445	Chenopodium cf. album	0	n	1	0	Amaranthaceae Juss.
Iz_2_1009	DJI_0445	Chenopodium sp.	0		NA	0	Amaranthaceae Juss.
Iz_2_1009	DJI_0445	Chondrilla juncea L.	0	n	1	0	Asteraceae Bercht. & J.Presl
Iz_2_1009	DJI_0445	Cirsium vulgare (Savi) Ten.	0	n	1	0	Asteraceae Bercht. & J.Presl
Iz_2_1009	DJI_0445	Clinopodium acinos (L.) Kuntze	0	n	1	0	Lamiacea Lindl.
Iz_2_1009	DJI_0445	Convallaria majalis L.	0	n	1	0	Asparagaceae Juss.
Iz_2_1009	DJI_0445	Crataegus rhipidophylla Gand.	0	n	1	1	Rosaceae Juss.
Iz_2_1009	DJI_0445	Crepis foetida L.	0	n	1	0	Asteraceae Bercht. & J.Presl
Iz_2_1009	DJI_0445	Dactylis glomerata L.	0	n	1	0	Poaceae Barnhart
Iz_2_1009	DJI_0445	Dianthus campestris M.Bieb.	0	n	1	0	Caryophyllaceae Juss.
Iz_2_1009	DJI_0445	Digitaria sanguinalis (L.) Scop.	0	a	0	0	Poaceae Barnhart
Iz_2_1009	DJI_0445	Elymus repens (L.) Gould	0	n	1	0	Poaceae Barnhart
Iz_2_1009	DJI_0445	Epilobium tetragonum L.	0	n	1	0	Onagraceae Juss.
Iz_2_1009	DJI_0445	Eragrostis minor Host	0	a	0	0	Poaceae Barnhart
Iz_2_1009	DJI_0445	Erigeron canadensis L.	0.2	a	0	0	Asteraceae Bercht. & J.Presl
Iz_2_1009	DJI_0445	Euphorbia seguieriana Neck.	0	n	1	0	Euphorbiaceae Juss.
Iz_2_1009	DJI_0445	Fallopia dumetorum (L.) Holub	0	n	1	0	Polygonaceae Juss.
Iz_2_1009	DJI_0445	Festuca beckeri (Hack.) Trautv.	0	n	1	0	Poaceae Barnhart
Iz_2_1009	DJI_0445	Fragaria vesca L.	0	n	1	0	Rosaceae Juss.
Iz_2_1009	DJI_0445	Frangula alnus Mill.	0	n	1	1	Rhamnaceae Juss.
Iz_2_1009	DJI_0445	Fraxinus pennsylvanica Marshall	0	a	0	1	Oleaceae Hoffmanns. & Link
Iz_2_1009	DJI_0445	Galeopsis bifida Boenn.	0	n	1	0	Lamiacea Lindl.
Iz_2_1009	DJI_0445	Galium verum L.	0	n	1	0	Rubiaceae Juss.
Iz_2_1009	DJI_0445	Genista tinctoria L.	0	n	1	0	Fabaceae Lindl.
Iz_2_1009	DJI_0445	Geranium robertianum L.	1	n	1	0	Geraniaceae Juss.
Iz_2_1009	DJI_0445	Geranium sanquineum L.	0	n	1	0	Geraniaceae Juss.
Iz_2_1009	DJI_0445	Gypsophila paniculata L.	0	n	1	0	Caryophyllaceae Juss.
Iz_2_1009	DJI_0445	Helichrysum arenarium (L.) Moench	0	n	1	0	Asteraceae Bercht. & J.Presl
Iz_2_1009	DJI_0445	Herniaria polygama J.Gay	0	n	1	0	Caryophyllaceae Juss.
Iz_2_1009	DJI_0445	Hieracium robustum Fr.	0	n	1	0	Asteraceae Bercht. & J.Presl
Iz_2_1009	DJI_0445	Hieracium umbellatum L.	0	n	1	NA	Asteraceae Bercht. & J.Presl
Iz_2_1009	DJI_0445	Hylotelephium maximum (L.) Holub	0	n	1	0	Crassulaceae J.St.-Hil.
Iz_2_1009	DJI_0445	Jacobaea borysthenica (DC.) B.Nord. & Greuter	0	n	1	0	Asteraceae Bercht. & J.Presl
Iz_2_1009	DJI_0445	Jasione montana L.	0	n	1	0	Campanulaceae Juss.
Iz_2_1009	DJI_0445	Jurinea cyanoides (L.) Rchb.s.l.	0	n	1	0	Asteraceae Bercht. & J.Presl
Iz_2_1009	DJI_0445	Koeleria glauca (Spreng.) DC.	0	n	1	0	Poaceae Barnhart
Iz_2_1009	DJI_0445	Lactuca serriola L.	0	a	0	0	Asteraceae Bercht. & J.Presl
Iz_2_1009	DJI_0445	Linaria genistifolia (L.) Mill.	0	n	1	0	Plantaginaceae Juss.
Iz_2_1009	DJI_0445	Lomelosia argentea (L.) Greuter & Burdet	0	n	1	0	Caprifoliaceae Juss.
Iz_2_1009	DJI_0445	Melampyrum pratense L.	0	n	1	0	Orobanchaceae Vent.

Iz_2_1009	DJI_0445	Oenothera biennis L.	0	a	0	0	Onagraceae Juss.
Iz_2_1009	DJI_0445	Pentanema britannica (L.) D.Gut.Larr., Santos-Vicente, Anderb., E.Rico & M.M.Mart.Ort.	0	n	1	0	Asteraceae Bercht. & J.Presl
Iz_2_1009	DJI_0445	Peucedanum oreoselinum (L.) Moench	0	n	1	0	Apiaceae Lindl.
Iz_2_1009	DJI_0445	Peucedanum ruthenicum M.Bieb.	0	n	1	0	Apiaceae Lindl.
Iz_2_1009	DJI_0445	Pilosella echioides (Lumn.) F.W.Schultz & Sch.Bip.	0	n	1	0	Asteraceae Bercht. & J.Presl
Iz_2_1009	DJI_0445	Pilosella officinarum F. Schultz et Sch. Bip.	0	n	1	0	Asteraceae Bercht. & J.Presl
Iz_2_1009	DJI_0445	Pilosella sp.	0		NA	0	Asteraceae Bercht. & J.Presl
Iz_2_1009	DJI_0445	Pinus sylvestris L.	0	n	1	1	Pinaceae Spreng. ex F.Rudolphi
Iz_2_1009	DJI_0445	Poa annua L.	0	n	1	0	Poaceae Barnhart
Iz_2_1009	DJI_0445	Poa nemoralis L.	0	n	1	0	Poaceae Barnhart
Iz_2_1009	DJI_0445	Poa sp.	0		NA	0	Poaceae Barnhart
Iz_2_1009	DJI_0445	Polygonatum odoratum (Mill.) Druce	0	n	1	0	Asparagaceae Juss.
Iz_2_1009	DJI_0445	Polygonum arenarium Waldst. & Kit.	0	n	1	0	Polygonaceae Juss.
Iz_2_1009	DJI_0445	Populus nigra L.	0	n	1	1	Salicaceae Mirb.
Iz_2_1009	DJI_0445	Populus tremula L.	0	n	1	1	Salicaceae Mirb.
Iz_2_1009	DJI_0445	Populus Ã canescens (Aiton) Sm.	0	n	1	1	Salicaceae Mirb.
Iz_2_1009	DJI_0445	Potentilla argentea L.	0	n	1	0	Rosaceae Juss.
Iz_2_1009	DJI_0445	Psammophiliella muralis (L.) Ikonn.	0	n	1	0	Caryophyllaceae Juss.
Iz_2_1009	DJI_0445	Psephellus sumensis (Kalen.) Greuter	0	n	1	0	Asteraceae Bercht. & J.Presl
Iz_2_1009	DJI_0445	Pulsatilla pratensis (L.) Mill. s.l.	0	n	1	0	Ranunculaceae Juss.
Iz_2_1009	DJI_0445	Pyrus communis L.	0	a	0	1	Rosaceae Juss.
Iz_2_1009	DJI_0445	Quercus robur L.	0	n	1	1	Fagaceae Dumort.
Iz_2_1009	DJI_0445	Robinia pseudoacacia L.	0	a	0	1	Fabaceae Lindl.
Iz_2_1009	DJI_0445	Rosa sp.	0		NA	1	Rosaceae Juss.
Iz_2_1009	DJI_0445	Rubus idaeus L.	0	n	1	0	Rosaceae Juss.
Iz_2_1009	DJI_0445	Rumex acetosella L.	0	n	1	0	Polygonaceae Juss.
Iz_2_1009	DJI_0445	Rumex thyrsiflorus Fingerh.	0	n	1	0	Polygonaceae Juss.
Iz_2_1009	DJI_0445	Salix alba L.	0	n	1	1	Salicaceae Mirb.
Iz_2_1009	DJI_0445	Sambucus nigra L.	0	n	1	1	Viburnaceae Raf.
Iz_2_1009	DJI_0445	Secale sylvestre Host	0	n	1	0	Poaceae Barnhart
Iz_2_1009	DJI_0445	Sempervivum ruthenicum Schnittsp. & C.B. Lehm.	0	n	1	0	Crassulaceae J.St.-Hil.
Iz_2_1009	DJI_0445	Setaria viridis (L.) P. Beauv.	0	n	1	0	Poaceae Barnhart
Iz_2_1009	DJI_0445	Silene borysthenica (Gruner) Walters	0	n	1	0	Caryophyllaceae Juss.
Iz_2_1009	DJI_0445	Silene latifolia subsp. alba (Mill.) Greuter & Burdet	0	n	1	0	Caryophyllaceae Juss.
Iz_2_1009	DJI_0445	Solidago virgaurea L.	0	n	1	0	Asteraceae Bercht. & J.Presl
Iz_2_1009	DJI_0445	Sorbus aucuparia L.	0	n	1	1	Rosaceae Juss.
Iz_2_1009	DJI_0445	Stachys recta L.	0	n	1	0	Lamiacea Lindl.
Iz_2_1009	DJI_0445	Tanacetum vulgare L.	0	n	1	0	Asteraceae Bercht. & J.Presl
Iz_2_1009	DJI_0445	Thymus pallasianus Heinr.Braun	0	n	1	0	Lamiacea Lindl.
Iz_2_1009	DJI_0445	Tilia cordata Mill.	0	n	1	1	Malvaceae Juss.
Iz_2_1009	DJI_0445	Trifolium alpestre L.	0	n	1	0	Fabaceae Lindl.
Iz_2_1009	DJI_0445	Trifolium arvense L.	0	n	1	0	Fabaceae Lindl.
Iz_2_1009	DJI_0445	Ulmus glabra Huds.	0	n	1	1	Ulmaceae Mirb.
Iz_2_1009	DJI_0445	Verbascum densiflorum Bertol.	0	n	1	0	Scrophulariaceae Juss.
Iz_2_1009	DJI_0445	Veronica austriaca L.	0	n	1	0	Plantaginaceae Juss.
Iz_2_1009	DJI_0445	Veronica incana L.	0	n	1	0	Plantaginaceae Juss.
Iz_2_1009	DJI_0445	Veronica verna L.	0	n	1	0	Plantaginaceae Juss.
Iz_2_1009	DJI_0445	Vincetoxicum hirundinaria Medik	0	n	1	0	Apocynaceae Juss.
Iz_2_1009	DJI_0446	Acer tataricum L.	0	n	1	1	Sapindaceae Juss.
Iz_2_1009	DJI_0446	Achillea millefolium L. s.l.	0	n	1	0	Asteraceae Bercht. & J.Presl
Iz_2_1009	DJI_0446	Allium oleraceum L.	0	n	1	0	Amaryllidaceae J.St.-Hil.
Iz_2_1009	DJI_0446	Ambrosia artemisiifolia L.	0	a	0	0	Asteraceae Bercht. & J.Presl
Iz_2_1009	DJI_0446	Amorpha fruticosa L.	0	a	0	0	Fabaceae Lindl.
Iz_2_1009	DJI_0446	Anthericum ramosum L.	0	n	1	0	Asparagaceae Juss.
Iz_2_1009	DJI_0446	Artemisia marschalliana Spreng.	0	n	1	0	Asteraceae Bercht. & J.Presl
Iz_2_1009	DJI_0446	Asparagus officinalis L.	0	n	1	0	Asparagaceae Juss.
Iz_2_1009	DJI_0446	Astragalus cicer L.	0	n	1	0	Fabaceae Lindl.
Iz_2_1009	DJI_0446	Bassia laniflora (S.G.Gmel.) A.J.Scott	0	n	1	0	Amaranthaceae Juss.
Iz_2_1009	DJI_0446	Berberis vulgaris L.	0	n	1	0	Berberidaceae Juss.
Iz_2_1009	DJI_0446	Betula pendula Roth.	0	n	1	1	Betulaceae Gray
Iz_2_1009	DJI_0446	Betula pendula Roth.	0	n	1	0	Poaceae Barnhart
Iz_2_1009	DJI_0446	Bromus inermis Leyss.	0		NA	NA	
Iz_2_1009	DJI_0446	Bromus squarrosus L.	0	a	0	0	Poaceae Barnhart
Iz_2_1009	DJI_0446	Bromus tectorum L.	0	a	0	0	Poaceae Barnhart
Iz_2_1009	DJI_0446	Calamagrostis epigejos (L.) Roth	10	n	1	0	Poaceae Barnhart
Iz_2_1009	DJI_0446	Carduus acanthoides L.	0	n	1	0	Asteraceae Bercht. & J.Presl
Iz_2_1009	DJI_0446	Carex sp.	0	n	1	0	Cyperaceae Juss.
Iz_2_1009	DJI_0446	Centaurea majorovii Dumbadze	0	n	1	0	Asteraceae Bercht. & J.Presl
Iz_2_1009	DJI_0446	Cf. Solidago virgaurea	0		NA	NA	
Iz_2_1009	DJI_0446	Chamaecytisus ruthenicus (Fisch.ex Wol.) Klaskova	0	n	1	0	Fabaceae Lindl.
Iz_2_1009	DJI_0446	Chelidonium majus L.	0	n	1	0	Papaveraceae Juss.
Iz_2_1009	DJI_0446	Chenopodium betaceum Andrz.	0	n	1	0	Amaranthaceae Juss.
Iz_2_1009	DJI_0446	Chenopodium cf. album	0	n	1	0	Amaranthaceae Juss.
Iz_2_1009	DJI_0446	Chenopodium sp.	0		NA	0	Amaranthaceae Juss.
Iz_2_1009	DJI_0446	Chondrilla juncea L.	0	n	1	0	Asteraceae Bercht. & J.Presl
Iz_2_1009	DJI_0446	Cirsium vulgare (Savi) Ten.	0	n	1	0	Asteraceae Bercht. & J.Presl
Iz_2_1009	DJI_0446	Clinopodium acinos (L.) Kuntze	0	n	1	0	Lamiacea Lindl.
Iz_2_1009	DJI_0446	Convallaria majalis L.	0	n	1	0	Asparagaceae Juss.
Iz_2_1009	DJI_0446	Crataegus rhipidophylla Gand.	0	n	1	1	Rosaceae Juss.
Iz_2_1009	DJI_0446	Crepis foetida L.	0	n	1	0	Asteraceae Bercht. & J.Presl
Iz_2_1009	DJI_0446	Dactylis glomerata L.	0	n	1	0	Poaceae Barnhart
Iz_2_1009	DJI_0446	Dianthus campestris M.Bieb.	0	n	1	0	Caryophyllaceae Juss.
Iz_2_1009	DJI_0446	Digitaria sanguinalis (L.) Scop.	0	a	0	0	Poaceae Barnhart
Iz_2_1009	DJI_0446	Elymus repens (L.) Gould	0	n	1	0	Poaceae Barnhart
Iz_2_1009	DJI_0446	Epilobium tetragonum L.	0	n	1	0	Onagraceae Juss.
Iz_2_1009	DJI_0446	Eragrostis minor Host	0	a	0	0	Poaceae Barnhart
Iz_2_1009	DJI_0446	Erigeron canadensis L.	0.2	a	0	0	Asteraceae Bercht. & J.Presl
Iz_2_1009	DJI_0446	Euphorbia seguieriana Neck.	0	n	1	0	Euphorbiaceae Juss.
Iz_2_1009	DJI_0446	Fallopia dumetorum (L.) Holub	0	n	1	0	Polygonaceae Juss.
Iz_2_1009	DJI_0446	Festuca beckeri (Hack.) Trautv.	0	n	1	0	Poaceae Barnhart
Iz_2_1009	DJI_0446	Fragaria vesca L.	0	n	1	0	Rosaceae Juss.
Iz_2_1009	DJI_0446	Frangula alnus Mill.	0	n	1	1	Rhamnaceae Juss.
Iz_2_1009	DJI_0446	Fraxinus pennsylvanica Marshall	0	a	0	1	Oleaceae Hoffmanns. & Link
Iz_2_1009	DJI_0446	Galeopsis bifida Boenn.	0	n	1	0	Lamiacea Lindl.
Iz_2_1009	DJI_0446	Galium verum L.	0	n	1	0	Rubiaceae Juss.
Iz_2_1009	DJI_0446	Genista tinctoria L.	0	n	1	0	Fabaceae Lindl.
Iz_2_1009	DJI_0446	Geranium robertianum L.	0	n	1	0	Geraniaceae Juss.
Iz_2_1009	DJI_0446	Geranium sanquineum L.	0	n	1	0	Geraniaceae Juss.

Iz_2_1009	DJI_0446	Gypsophila paniculata L.	0	n	1	0	Caryophyllaceae Juss.
Iz_2_1009	DJI_0446	Helichrysum arenarium (L.) Moench	0	n	1	0	Asteraceae Bercht. & J.Presl
Iz_2_1009	DJI_0446	Herniaria polygama J.Gay	0	n	1	0	Caryophyllaceae Juss.
Iz_2_1009	DJI_0446	Hieracium robustum Fr.	0	n	1	0	Asteraceae Bercht. & J.Presl
Iz_2_1009	DJI_0446	Hieracium umbellatum L.	0	n	1	NA	Asteraceae Bercht. & J.Presl
Iz_2_1009	DJI_0446	Hylotelephium maximum (L.) Holub	0	n	1	0	Crassulaceae J.St.-Hil.
Iz_2_1009	DJI_0446	Jacobaea borysthenica (DC.) B.Nord. & Greuter	0	n	1	0	Asteraceae Bercht. & J.Presl
Iz_2_1009	DJI_0446	Jasione montana L.	0	n	1	0	Campanulaceae Juss.
Iz_2_1009	DJI_0446	Jurinea cyanoides (L.) Rchb.s.l.	0	n	1	0	Asteraceae Bercht. & J.Presl
Iz_2_1009	DJI_0446	Koeleria glauca (Spreng.) DC.	0	n	1	0	Poaceae Barnhart
Iz_2_1009	DJI_0446	Lactuca serriola L.	0	a	0	0	Asteraceae Bercht. & J.Presl
Iz_2_1009	DJI_0446	Linaria genistifolia (L.) Mill.	0	n	1	0	Plantaginaceae Juss.
Iz_2_1009	DJI_0446	Lomelosia argentea (L.) Greuter & Burdet	0	n	1	0	Caprifoliaceae Juss.
Iz_2_1009	DJI_0446	Melampyrum pratense L.	0	n	1	0	Orobanchaceae Vent.
Iz_2_1009	DJI_0446	Oenothera biennis L.	0	a	0	0	Onagraceae Juss.
Iz_2_1009	DJI_0446	Pentanema britannica (L.) D.Gut.Larr., Santos-Vicente, Anderb., E.Rico & M.M.Mart.Ort.	0	n	1	0	Asteraceae Bercht. & J.Presl
Iz_2_1009	DJI_0446	Peucedanum oreoselinum (L.) Moench	0.5	n	1	0	Apiaceae Lindl.
Iz_2_1009	DJI_0446	Peucedanum ruthenicum M.Bieb.	0	n	1	0	Apiaceae Lindl.
Iz_2_1009	DJI_0446	Pilosella echioides (Lumn.) F.W.Schultz & Sch.Bip.	0	n	1	0	Asteraceae Bercht. & J.Presl
Iz_2_1009	DJI_0446	Pilosella officinarum F. Schultz et Sch. Bip.	0	n	1	0	Asteraceae Bercht. & J.Presl
Iz_2_1009	DJI_0446	Pilosella sp.	0		NA	0	Asteraceae Bercht. & J.Presl
Iz_2_1009	DJI_0446	Pinus sylvestris L.	0	n	1	1	Pinaceae Spreng. ex F.Rudolphi
Iz_2_1009	DJI_0446	Poa annua L.	0	n	1	0	Poaceae Barnhart
Iz_2_1009	DJI_0446	Poa nemoralis L.	0	n	1	0	Poaceae Barnhart
Iz_2_1009	DJI_0446	Poa sp.	0		NA	0	Poaceae Barnhart
Iz_2_1009	DJI_0446	Polygonatum odoratum (Mill.) Druce	0	n	1	0	Asparagaceae Juss.
Iz_2_1009	DJI_0446	Polygonum arenarium Waldst. & Kit.	0	n	1	0	Polygonaceae Juss.
Iz_2_1009	DJI_0446	Populus nigra L.	0	n	1	1	Salicaceae Mirb.
Iz_2_1009	DJI_0446	Populus tremula L.	0	n	1	1	Salicaceae Mirb.
Iz_2_1009	DJI_0446	Populus Ã canescens (Aiton) Sm.	0	n	1	1	Salicaceae Mirb.
Iz_2_1009	DJI_0446	Potentilla argentea L.	0	n	1	0	Rosaceae Juss.
Iz_2_1009	DJI_0446	Psammophiliella muralis (L.) Ikonn.	0	n	1	0	Caryophyllaceae Juss.
Iz_2_1009	DJI_0446	Psephellus sumensis (Kalen.) Greuter	0	n	1	0	Asteraceae Bercht. & J.Presl
Iz_2_1009	DJI_0446	Pulsatilla pratensis (L.) Mill. s.l.	0	n	1	0	Ranunculaceae Juss.
Iz_2_1009	DJI_0446	Pyrus communis L.	0	a	0	1	Rosaceae Juss.
Iz_2_1009	DJI_0446	Quercus robur L.	0	n	1	1	Fagaceae Dumort.
Iz_2_1009	DJI_0446	Robinia pseudoacacia L.	0	a	0	1	Fabaceae Lindl.
Iz_2_1009	DJI_0446	Rosa sp.	0		NA	1	Rosaceae Juss.
Iz_2_1009	DJI_0446	Rubus idaeus L.	0	n	1	0	Rosaceae Juss.
Iz_2_1009	DJI_0446	Rumex acetosella L.	0	n	1	0	Polygonaceae Juss.
Iz_2_1009	DJI_0446	Rumex thyrsiflorus Fingerh.	0	n	1	0	Polygonaceae Juss.
Iz_2_1009	DJI_0446	Salix alba L.	0	n	1	1	Salicaceae Mirb.
Iz_2_1009	DJI_0446	Sambucus nigra L.	0	n	1	1	Viburnaceae Raf.
Iz_2_1009	DJI_0446	Secale sylvestre Host	0	n	1	0	Poaceae Barnhart
Iz_2_1009	DJI_0446	Sempervivum ruthenicum Schnittsp. & C.B. Lehm.	0	n	1	0	Crassulaceae J.St.-Hil.
Iz_2_1009	DJI_0446	Setaria viridis (L.) P. Beauv.	0	n	1	0	Poaceae Barnhart
Iz_2_1009	DJI_0446	Silene borysthenica (Gruner) Walters	0	n	1	0	Caryophyllaceae Juss.
Iz_2_1009	DJI_0446	Silene latifolia subsp. alba (Mill.) Greuter & Burdet	0	n	1	0	Caryophyllaceae Juss.
Iz_2_1009	DJI_0446	Solidago virgaurea L.	3	n	1	0	Asteraceae Bercht. & J.Presl
Iz_2_1009	DJI_0446	Sorbus aucuparia L.	0	n	1	1	Rosaceae Juss.
Iz_2_1009	DJI_0446	Stachys recta L.	0	n	1	0	Lamiacea Lindl.
Iz_2_1009	DJI_0446	Tanacetum vulgare L.	0	n	1	0	Asteraceae Bercht. & J.Presl
Iz_2_1009	DJI_0446	Thymus pallasianus Heinr.Braun	0	n	1	0	Lamiacea Lindl.
Iz_2_1009	DJI_0446	Tilia cordata Mill.	0	n	1	1	Malvaceae Juss.
Iz_2_1009	DJI_0446	Trifolium alpestre L.	0	n	1	0	Fabaceae Lindl.
Iz_2_1009	DJI_0446	Trifolium arvense L.	0	n	1	0	Fabaceae Lindl.
Iz_2_1009	DJI_0446	Ulmus glabra Huds.	0	n	1	1	Ulmaceae Mirb.
Iz_2_1009	DJI_0446	Verbascum densiflorum Bertol.	0	n	1	0	Scrophulariaceae Juss.
Iz_2_1009	DJI_0446	Veronica austriaca L.	0	n	1	0	Plantaginaceae Juss.
Iz_2_1009	DJI_0446	Veronica incana L.	0	n	1	0	Plantaginaceae Juss.
Iz_2_1009	DJI_0446	Veronica verna L.	0	n	1	0	Plantaginaceae Juss.
Iz_2_1009	DJI_0446	Vincetoxicum hirundinaria Medik	0	n	1	0	Apocynaceae Juss.
Iz_2_1009	DJI_0447	Acer tataricum L.	0	n	1	1	Sapindaceae Juss.
Iz_2_1009	DJI_0447	Achillea millefolium L. s.l.	0	n	1	0	Asteraceae Bercht. & J.Presl
Iz_2_1009	DJI_0447	Allium oleraceum L.	0	n	1	0	Amaryllidaceae J.St.-Hil.
Iz_2_1009	DJI_0447	Ambrosia artemisiifolia L.	0	a	0	0	Asteraceae Bercht. & J.Presl
Iz_2_1009	DJI_0447	Amorpha fruticosa L.	0	a	0	0	Fabaceae Lindl.
Iz_2_1009	DJI_0447	Anthericum ramosum L.	0	n	1	0	Asparagaceae Juss.
Iz_2_1009	DJI_0447	Artemisia marschalliana Spreng.	0	n	1	0	Asteraceae Bercht. & J.Presl
Iz_2_1009	DJI_0447	Asparagus officinalis L.	0	n	1	0	Asparagaceae Juss.
Iz_2_1009	DJI_0447	Astragalus cicer L.	0	n	1	0	Fabaceae Lindl.
Iz_2_1009	DJI_0447	Bassia laniflora (S.G.Gmel.) A.J.Scott	0	n	1	0	Amaranthaceae Juss.
Iz_2_1009	DJI_0447	Berberis vulgaris L.	0	n	1	0	Berberidaceae Juss.
Iz_2_1009	DJI_0447	Betula pendula Roth.	0	n	1	1	Betulaceae Gray
Iz_2_1009	DJI_0447	Betula pendula Roth.	0	n	1	0	Poaceae Barnhart
Iz_2_1009	DJI_0447	Bromus inermis Leyss.	0		NA	NA	
Iz_2_1009	DJI_0447	Bromus squarrosus L.	0	a	0	0	Poaceae Barnhart
Iz_2_1009	DJI_0447	Bromus tectorum L.	0	a	0	0	Poaceae Barnhart
Iz_2_1009	DJI_0447	Calamagrostis epigejos (L.) Roth	10	n	1	0	Poaceae Barnhart
Iz_2_1009	DJI_0447	Carduus acanthoides L.	0	n	1	0	Asteraceae Bercht. & J.Presl
Iz_2_1009	DJI_0447	Carex sp.	0	n	1	0	Cyperaceae Juss.
Iz_2_1009	DJI_0447	Centaurea majorovii Dumbadze	0	n	1	0	Asteraceae Bercht. & J.Presl
Iz_2_1009	DJI_0447	Cf. Solidago virgaurea	0		NA	NA	
Iz_2_1009	DJI_0447	Chamaecytisus ruthenicus (Fisch.ex Wol.) Klaskova	0	n	1	0	Fabaceae Lindl.
Iz_2_1009	DJI_0447	Chelidonium majus L.	0	n	1	0	Papaveraceae Juss.
Iz_2_1009	DJI_0447	Chenopodium betaceum Andrz.	0	n	1	0	Amaranthaceae Juss.
Iz_2_1009	DJI_0447	Chenopodium cf. album	0	n	1	0	Amaranthaceae Juss.
Iz_2_1009	DJI_0447	Chenopodium sp.	0		NA	0	Amaranthaceae Juss.
Iz_2_1009	DJI_0447	Chondrilla juncea L.	0	n	1	0	Asteraceae Bercht. & J.Presl
Iz_2_1009	DJI_0447	Cirsium vulgare (Savi) Ten.	0	n	1	0	Asteraceae Bercht. & J.Presl
Iz_2_1009	DJI_0447	Clinopodium acinos (L.) Kuntze	0	n	1	0	Lamiacea Lindl.
Iz_2_1009	DJI_0447	Convallaria majalis L.	0	n	1	0	Asparagaceae Juss.
Iz_2_1009	DJI_0447	Crataegus rhipidophylla Gand.	0	n	1	1	Rosaceae Juss.
Iz_2_1009	DJI_0447	Crepis foetida L.	0	n	1	0	Asteraceae Bercht. & J.Presl
Iz_2_1009	DJI_0447	Dactylis glomerata L.	0	n	1	0	Poaceae Barnhart
Iz_2_1009	DJI_0447	Dianthus campestris M.Bieb.	0	n	1	0	Caryophyllaceae Juss.
Iz_2_1009	DJI_0447	Digitaria sanguinalis (L.) Scop.	0	a	0	0	Poaceae Barnhart
Iz_2_1009	DJI_0447	Elymus repens (L.) Gould	0	n	1	0	Poaceae Barnhart

Iz_2_1009	DJI_0447	Epilobium tetragonum L.	0	n	1	0	Onagraceae Juss.
Iz_2_1009	DJI_0447	Eragrostis minor Host	0	a	0	0	Poaceae Barnhart
Iz_2_1009	DJI_0447	Erigeron canadensis L.	0	a	0	0	Asteraceae Bercht. & J.Presl
Iz_2_1009	DJI_0447	Euphorbia seguieriana Neck.	0	n	1	0	Euphorbiaceae Juss.
Iz_2_1009	DJI_0447	Fallopia dumetorum (L.) Holub	0	n	1	0	Polygonaceae Juss.
Iz_2_1009	DJI_0447	Festuca beckeri (Hack.) Trautv.	0	n	1	0	Poaceae Barnhart
Iz_2_1009	DJI_0447	Fragaria vesca L.	0	n	1	0	Rosaceae Juss.
Iz_2_1009	DJI_0447	Frangula alnus Mill.	0	n	1	1	Rhamnaceae Juss.
Iz_2_1009	DJI_0447	Fraxinus pennsylvanica Marshall	0	a	0	1	Oleaceae Hoffmanns. & Link
Iz_2_1009	DJI_0447	Galeopsis bifida Boenn.	0	n	1	0	Lamiacea Lindl.
Iz_2_1009	DJI_0447	Galium verum L.	0	n	1	0	Rubiaceae Juss.
Iz_2_1009	DJI_0447	Genista tinctoria L.	0	n	1	0	Fabaceae Lindl.
Iz_2_1009	DJI_0447	Geranium robertianum L.	1	n	1	0	Geraniaceae Juss.
Iz_2_1009	DJI_0447	Geranium sanquineum L.	0	n	1	0	Geraniaceae Juss.
Iz_2_1009	DJI_0447	Gypsophila paniculata L.	0	n	1	0	Caryophyllaceae Juss.
Iz_2_1009	DJI_0447	Helichrysum arenarium (L.) Moench	0	n	1	0	Asteraceae Bercht. & J.Presl
Iz_2_1009	DJI_0447	Herniaria polygama J.Gay	0	n	1	0	Caryophyllaceae Juss.
Iz_2_1009	DJI_0447	Hieracium robustum Fr.	0	n	1	0	Asteraceae Bercht. & J.Presl
Iz_2_1009	DJI_0447	Hieracium umbellatum L.	0	n	1	NA	Asteraceae Bercht. & J.Presl
Iz_2_1009	DJI_0447	Hylotelephium maximum (L.) Holub	0	n	1	0	Crassulaceae J.St.-Hil.
Iz_2_1009	DJI_0447	Jacobaea borysthenica (DC.) B.Nord. & Greuter	0	n	1	0	Asteraceae Bercht. & J.Presl
Iz_2_1009	DJI_0447	Jasione montana L.	0	n	1	0	Campanulaceae Juss.
Iz_2_1009	DJI_0447	Jurinea cyanoides (L.) Rchb.s.l.	0	n	1	0	Asteraceae Bercht. & J.Presl
Iz_2_1009	DJI_0447	Koeleria glauca (Spreng.) DC.	0	n	1	0	Poaceae Barnhart
Iz_2_1009	DJI_0447	Lactuca serriola L.	0	a	0	0	Asteraceae Bercht. & J.Presl
Iz_2_1009	DJI_0447	Linaria genistifolia (L.) Mill.	0	n	1	0	Plantaginaceae Juss.
Iz_2_1009	DJI_0447	Lomelosia argentea (L.) Greuter & Burdet	0	n	1	0	Caprifoliaceae Juss.
Iz_2_1009	DJI_0447	Melampyrum pratense L.	0	n	1	0	Orobanchaceae Vent.
Iz_2_1009	DJI_0447	Oenothera biennis L.	0	a	0	0	Onagraceae Juss.
Iz_2_1009	DJI_0447	Pentanema britannica (L.) D.Gut.Larr., Santos-Vicente, Anderb., E.Rico & M.M.Mart.Ort.	0	n	1	0	Asteraceae Bercht. & J.Presl
Iz_2_1009	DJI_0447	Peucedanum oreoselinum (L.) Moench	0	n	1	0	Apiaceae Lindl.
Iz_2_1009	DJI_0447	Peucedanum ruthenicum M.Bieb.	0	n	1	0	Apiaceae Lindl.
Iz_2_1009	DJI_0447	Pilosella echioides (Lumn.) F.W.Schultz & Sch.Bip.	0	n	1	0	Asteraceae Bercht. & J.Presl
Iz_2_1009	DJI_0447	Pilosella officinarum F. Schultz et Sch. Bip.	0	n	1	0	Asteraceae Bercht. & J.Presl
Iz_2_1009	DJI_0447	Pilosella sp.	0		NA	0	Asteraceae Bercht. & J.Presl
Iz_2_1009	DJI_0447	Pinus sylvestris L.	0	n	1	1	Pinaceae Spreng. ex F.Rudolphi
Iz_2_1009	DJI_0447	Poa annua L.	0	n	1	0	Poaceae Barnhart
Iz_2_1009	DJI_0447	Poa nemoralis L.	0	n	1	0	Poaceae Barnhart
Iz_2_1009	DJI_0447	Poa sp.	0		NA	0	Poaceae Barnhart
Iz_2_1009	DJI_0447	Polygonatum odoratum (Mill.) Druce	0	n	1	0	Asparagaceae Juss.
Iz_2_1009	DJI_0447	Polygonum arenarium Waldst. & Kit.	0	n	1	0	Polygonaceae Juss.
Iz_2_1009	DJI_0447	Populus nigra L.	0	n	1	1	Salicaceae Mirb.
Iz_2_1009	DJI_0447	Populus tremula L.	0	n	1	1	Salicaceae Mirb.
Iz_2_1009	DJI_0447	Populus Ã canescens (Aiton) Sm.	0	n	1	1	Salicaceae Mirb.
Iz_2_1009	DJI_0447	Potentilla argentea L.	0	n	1	0	Rosaceae Juss.
Iz_2_1009	DJI_0447	Psammophiliella muralis (L.) Ikonn.	0	n	1	0	Caryophyllaceae Juss.
Iz_2_1009	DJI_0447	Psephellus sumensis (Kalen.) Greuter	0	n	1	0	Asteraceae Bercht. & J.Presl
Iz_2_1009	DJI_0447	Pulsatilla pratensis (L.) Mill. s.l.	0	n	1	0	Ranunculaceae Juss.
Iz_2_1009	DJI_0447	Pyrus communis L.	0	a	0	1	Rosaceae Juss.
Iz_2_1009	DJI_0447	Quercus robur L.	0	n	1	1	Fagaceae Dumort.
Iz_2_1009	DJI_0447	Robinia pseudoacacia L.	0	a	0	1	Fabaceae Lindl.
Iz_2_1009	DJI_0447	Rosa sp.	0		NA	1	Rosaceae Juss.
Iz_2_1009	DJI_0447	Rubus idaeus L.	0	n	1	0	Rosaceae Juss.
Iz_2_1009	DJI_0447	Rumex acetosella L.	0	n	1	0	Polygonaceae Juss.
Iz_2_1009	DJI_0447	Rumex thyrsiflorus Fingerh.	0	n	1	0	Polygonaceae Juss.
Iz_2_1009	DJI_0447	Salix alba L.	0	n	1	1	Salicaceae Mirb.
Iz_2_1009	DJI_0447	Sambucus nigra L.	0	n	1	1	Viburnaceae Raf.
Iz_2_1009	DJI_0447	Secale sylvestre Host	0	n	1	0	Poaceae Barnhart
Iz_2_1009	DJI_0447	Sempervivum ruthenicum Schnittsp. & C.B. Lehm.	0	n	1	0	Crassulaceae J.St.-Hil.
Iz_2_1009	DJI_0447	Setaria viridis (L.) P. Beauv.	0	n	1	0	Poaceae Barnhart
Iz_2_1009	DJI_0447	Silene borysthenica (Gruner) Walters	0	n	1	0	Caryophyllaceae Juss.
Iz_2_1009	DJI_0447	Silene latifolia subsp. alba (Mill.) Greuter & Burdet	0	n	1	0	Caryophyllaceae Juss.
Iz_2_1009	DJI_0447	Solidago virgaurea L.	4	n	1	0	Asteraceae Bercht. & J.Presl
Iz_2_1009	DJI_0447	Sorbus aucuparia L.	0	n	1	1	Rosaceae Juss.
Iz_2_1009	DJI_0447	Stachys recta L.	0	n	1	0	Lamiacea Lindl.
Iz_2_1009	DJI_0447	Tanacetum vulgare L.	0	n	1	0	Asteraceae Bercht. & J.Presl
Iz_2_1009	DJI_0447	Thymus pallasianus Heinr.Braun	0	n	1	0	Lamiacea Lindl.
Iz_2_1009	DJI_0447	Tilia cordata Mill.	0	n	1	1	Malvaceae Juss.
Iz_2_1009	DJI_0447	Trifolium alpestre L.	0	n	1	0	Fabaceae Lindl.
Iz_2_1009	DJI_0447	Trifolium arvense L.	0	n	1	0	Fabaceae Lindl.
Iz_2_1009	DJI_0447	Ulmus glabra Huds.	0	n	1	1	Ulmaceae Mirb.
Iz_2_1009	DJI_0447	Verbascum densiflorum Bertol.	0	n	1	0	Scrophulariaceae Juss.
Iz_2_1009	DJI_0447	Veronica austriaca L.	0	n	1	0	Plantaginaceae Juss.
Iz_2_1009	DJI_0447	Veronica incana L.	0	n	1	0	Plantaginaceae Juss.
Iz_2_1009	DJI_0447	Veronica verna L.	0	n	1	0	Plantaginaceae Juss.
Iz_2_1009	DJI_0447	Vincetoxicum hirundinaria Medik	0	n	1	0	Apocynaceae Juss.
Iz_2_1009	DJI_0448	Acer tataricum L.	0	n	1	1	Sapindaceae Juss.
Iz_2_1009	DJI_0448	Achillea millefolium L. s.l.	0	n	1	0	Asteraceae Bercht. & J.Presl
Iz_2_1009	DJI_0448	Allium oleraceum L.	0	n	1	0	Amaryllidaceae J.St.-Hil.
Iz_2_1009	DJI_0448	Ambrosia artemisiifolia L.	0	a	0	0	Asteraceae Bercht. & J.Presl
Iz_2_1009	DJI_0448	Amorpha fruticosa L.	0	a	0	0	Fabaceae Lindl.
Iz_2_1009	DJI_0448	Anthericum ramosum L.	0	n	1	0	Asparagaceae Juss.
Iz_2_1009	DJI_0448	Artemisia marschalliana Spreng.	0	n	1	0	Asteraceae Bercht. & J.Presl
Iz_2_1009	DJI_0448	Asparagus officinalis L.	0	n	1	0	Asparagaceae Juss.
Iz_2_1009	DJI_0448	Astragalus cicer L.	0	n	1	0	Fabaceae Lindl.
Iz_2_1009	DJI_0448	Bassia laniflora (S.G.Gmel.) A.J.Scott	0	n	1	0	Amaranthaceae Juss.
Iz_2_1009	DJI_0448	Berberis vulgaris L.	0	n	1	0	Berberidaceae Juss.
Iz_2_1009	DJI_0448	Betula pendula Roth.	0	n	1	1	Betulaceae Gray
Iz_2_1009	DJI_0448	Betula pendula Roth.	0	n	1	0	Poaceae Barnhart
Iz_2_1009	DJI_0448	Bromus inermis Leyss.	0		NA	NA	
Iz_2_1009	DJI_0448	Bromus squarrosus L.	0	a	0	0	Poaceae Barnhart
Iz_2_1009	DJI_0448	Bromus tectorum L.	0	a	0	0	Poaceae Barnhart
Iz_2_1009	DJI_0448	Calamagrostis epigejos (L.) Roth	30	n	1	0	Poaceae Barnhart
Iz_2_1009	DJI_0448	Carduus acanthoides L.	0	n	1	0	Asteraceae Bercht. & J.Presl
Iz_2_1009	DJI_0448	Carex sp.	0	n	1	0	Cyperaceae Juss.
Iz_2_1009	DJI_0448	Centaurea majorovii Dumbadze	0	n	1	0	Asteraceae Bercht. & J.Presl
Iz_2_1009	DJI_0448	Cf. Solidago virgaurea	0		NA	NA	
Iz_2_1009	DJI_0448	Chamaecytisus ruthenicus (Fisch.ex Wol.) Klaskova	0	n	1	0	Fabaceae Lindl.

Iz_2_1009	DJI_0448	Chelidonium majus L.	0	n	1	0	Papaveraceae Juss.
Iz_2_1009	DJI_0448	Chenopodium betaceum Andrz.	0	n	1	0	Amaranthaceae Juss.
Iz_2_1009	DJI_0448	Chenopodium cf. album	0	n	1	0	Amaranthaceae Juss.
Iz_2_1009	DJI_0448	Chenopodium sp.	0		NA	0	Amaranthaceae Juss.
Iz_2_1009	DJI_0448	Chondrilla juncea L.	0	n	1	0	Asteraceae Bercht. & J.Presl
Iz_2_1009	DJI_0448	Cirsium vulgare (Savi) Ten.	0	n	1	0	Asteraceae Bercht. & J.Presl
Iz_2_1009	DJI_0448	Clinopodium acinos (L.) Kuntze	0	n	1	0	Lamiacea Lindl.
Iz_2_1009	DJI_0448	Convallaria majalis L.	0	n	1	0	Asparagaceae Juss.
Iz_2_1009	DJI_0448	Crataegus rhipidophylla Gand.	0	n	1	1	Rosaceae Juss.
Iz_2_1009	DJI_0448	Crepis foetida L.	0	n	1	0	Asteraceae Bercht. & J.Presl
Iz_2_1009	DJI_0448	Dactylis glomerata L.	0	n	1	0	Poaceae Barnhart
Iz_2_1009	DJI_0448	Dianthus campestris M.Bieb.	0	n	1	0	Caryophyllaceae Juss.
Iz_2_1009	DJI_0448	Digitaria sanguinalis (L.) Scop.	0	a	0	0	Poaceae Barnhart
Iz_2_1009	DJI_0448	Elymus repens (L.) Gould	0	n	1	0	Poaceae Barnhart
Iz_2_1009	DJI_0448	Epilobium tetragonum L.	0	n	1	0	Onagraceae Juss.
Iz_2_1009	DJI_0448	Eragrostis minor Host	0	a	0	0	Poaceae Barnhart
Iz_2_1009	DJI_0448	Erigeron canadensis L.	0	a	0	0	Asteraceae Bercht. & J.Presl
Iz_2_1009	DJI_0448	Euphorbia seguieriana Neck.	0	n	1	0	Euphorbiaceae Juss.
Iz_2_1009	DJI_0448	Fallopia dumetorum (L.) Holub	0	n	1	0	Polygonaceae Juss.
Iz_2_1009	DJI_0448	Festuca beckeri (Hack.) Trautv.	0	n	1	0	Poaceae Barnhart
Iz_2_1009	DJI_0448	Fragaria vesca L.	0	n	1	0	Rosaceae Juss.
Iz_2_1009	DJI_0448	Frangula alnus Mill.	0	n	1	1	Rhamnaceae Juss.
Iz_2_1009	DJI_0448	Fraxinus pennsylvanica Marshall	0	a	0	1	Oleaceae Hoffmanns. & Link
Iz_2_1009	DJI_0448	Galeopsis bifida Boenn.	0	n	1	0	Lamiacea Lindl.
Iz_2_1009	DJI_0448	Galium verum L.	0	n	1	0	Rubiaceae Juss.
Iz_2_1009	DJI_0448	Genista tinctoria L.	0	n	1	0	Fabaceae Lindl.
Iz_2_1009	DJI_0448	Geranium robertianum L.	0	n	1	0	Geraniaceae Juss.
Iz_2_1009	DJI_0448	Geranium sanquineum L.	0	n	1	0	Geraniaceae Juss.
Iz_2_1009	DJI_0448	Gypsophila paniculata L.	0	n	1	0	Caryophyllaceae Juss.
Iz_2_1009	DJI_0448	Helichrysum arenarium (L.) Moench	0	n	1	0	Asteraceae Bercht. & J.Presl
Iz_2_1009	DJI_0448	Herniaria polygama J.Gay	0	n	1	0	Caryophyllaceae Juss.
Iz_2_1009	DJI_0448	Hieracium robustum Fr.	0	n	1	0	Asteraceae Bercht. & J.Presl
Iz_2_1009	DJI_0448	Hieracium umbellatum L.	0	n	1	NA	Asteraceae Bercht. & J.Presl
Iz_2_1009	DJI_0448	Hylotelephium maximum (L.) Holub	0	n	1	0	Crassulaceae J.St.-Hil.
Iz_2_1009	DJI_0448	Jacobaea borysthenica (DC.) B.Nord. & Greuter	0	n	1	0	Asteraceae Bercht. & J.Presl
Iz_2_1009	DJI_0448	Jasione montana L.	0	n	1	0	Campanulaceae Juss.
Iz_2_1009	DJI_0448	Jurinea cyanoides (L.) Rchb.s.l.	0	n	1	0	Asteraceae Bercht. & J.Presl
Iz_2_1009	DJI_0448	Koeleria glauca (Spreng.) DC.	0	n	1	0	Poaceae Barnhart
Iz_2_1009	DJI_0448	Lactuca serriola L.	0	a	0	0	Asteraceae Bercht. & J.Presl
Iz_2_1009	DJI_0448	Linaria genistifolia (L.) Mill.	0	n	1	0	Plantaginaceae Juss.
Iz_2_1009	DJI_0448	Lomelosia argentea (L.) Greuter & Burdet	0	n	1	0	Caprifoliaceae Juss.
Iz_2_1009	DJI_0448	Melampyrum pratense L.	0	n	1	0	Orobanchaceae Vent.
Iz_2_1009	DJI_0448	Oenothera biennis L.	0	a	0	0	Onagraceae Juss.
Iz_2_1009	DJI_0448	Pentanema britannica (L.) D.Gut.Larr., Santos-Vicente, Anderb., E.Rico & M.M.Mart.Ort.	0	n	1	0	Asteraceae Bercht. & J.Presl
Iz_2_1009	DJI_0448	Peucedanum oreoselinum (L.) Moench	0	n	1	0	Apiaceae Lindl.
Iz_2_1009	DJI_0448	Peucedanum ruthenicum M.Bieb.	0	n	1	0	Apiaceae Lindl.
Iz_2_1009	DJI_0448	Pilosella echioides (Lumn.) F.W.Schultz & Sch.Bip.	0	n	1	0	Asteraceae Bercht. & J.Presl
Iz_2_1009	DJI_0448	Pilosella officinarum F. Schultz et Sch. Bip.	0	n	1	0	Asteraceae Bercht. & J.Presl
Iz_2_1009	DJI_0448	Pilosella sp.	0		NA	0	Asteraceae Bercht. & J.Presl
Iz_2_1009	DJI_0448	Pinus sylvestris L.	0	n	1	1	Pinaceae Spreng. ex F.Rudolphi
Iz_2_1009	DJI_0448	Poa annua L.	0	n	1	0	Poaceae Barnhart
Iz_2_1009	DJI_0448	Poa nemoralis L.	0	n	1	0	Poaceae Barnhart
Iz_2_1009	DJI_0448	Poa sp.	0		NA	0	Poaceae Barnhart
Iz_2_1009	DJI_0448	Polygonatum odoratum (Mill.) Druce	0	n	1	0	Asparagaceae Juss.
Iz_2_1009	DJI_0448	Polygonum arenarium Waldst. & Kit.	0	n	1	0	Polygonaceae Juss.
Iz_2_1009	DJI_0448	Populus nigra L.	0	n	1	1	Salicaceae Mirb.
Iz_2_1009	DJI_0448	Populus tremula L.	0	n	1	1	Salicaceae Mirb.
Iz_2_1009	DJI_0448	Populus Ã canescens (Aiton) Sm.	0	n	1	1	Salicaceae Mirb.
Iz_2_1009	DJI_0448	Potentilla argentea L.	0	n	1	0	Rosaceae Juss.
Iz_2_1009	DJI_0448	Psammophiliella muralis (L.) Ikonn.	0	n	1	0	Caryophyllaceae Juss.
Iz_2_1009	DJI_0448	Psephellus sumensis (Kalen.) Greuter	0	n	1	0	Asteraceae Bercht. & J.Presl
Iz_2_1009	DJI_0448	Pulsatilla pratensis (L.) Mill. s.l.	0	n	1	0	Ranunculaceae Juss.
Iz_2_1009	DJI_0448	Pyrus communis L.	0	a	0	1	Rosaceae Juss.
Iz_2_1009	DJI_0448	Quercus robur L.	0	n	1	1	Fagaceae Dumort.
Iz_2_1009	DJI_0448	Robinia pseudoacacia L.	0	a	0	1	Fabaceae Lindl.
Iz_2_1009	DJI_0448	Rosa sp.	0		NA	1	Rosaceae Juss.
Iz_2_1009	DJI_0448	Rubus idaeus L.	0	n	1	0	Rosaceae Juss.
Iz_2_1009	DJI_0448	Rumex acetosella L.	0	n	1	0	Polygonaceae Juss.
Iz_2_1009	DJI_0448	Rumex thyrsiflorus Fingerh.	0	n	1	0	Polygonaceae Juss.
Iz_2_1009	DJI_0448	Salix alba L.	0	n	1	1	Salicaceae Mirb.
Iz_2_1009	DJI_0448	Sambucus nigra L.	0	n	1	1	Viburnaceae Raf.
Iz_2_1009	DJI_0448	Secale sylvestre Host	0	n	1	0	Poaceae Barnhart
Iz_2_1009	DJI_0448	Sempervivum ruthenicum Schnittsp. & C.B. Lehm.	0	n	1	0	Crassulaceae J.St.-Hil.
Iz_2_1009	DJI_0448	Setaria viridis (L.) P. Beauv.	0	n	1	0	Poaceae Barnhart
Iz_2_1009	DJI_0448	Silene borysthenica (Gruner) Walters	0	n	1	0	Caryophyllaceae Juss.
Iz_2_1009	DJI_0448	Silene latifolia subsp. alba (Mill.) Greuter & Burdet	0	n	1	0	Caryophyllaceae Juss.
Iz_2_1009	DJI_0448	Solidago virgaurea L.	0	n	1	0	Asteraceae Bercht. & J.Presl
Iz_2_1009	DJI_0448	Sorbus aucuparia L.	0	n	1	1	Rosaceae Juss.
Iz_2_1009	DJI_0448	Stachys recta L.	0	n	1	0	Lamiacea Lindl.
Iz_2_1009	DJI_0448	Tanacetum vulgare L.	0	n	1	0	Asteraceae Bercht. & J.Presl
Iz_2_1009	DJI_0448	Thymus pallasianus Heinr.Braun	0	n	1	0	Lamiacea Lindl.
Iz_2_1009	DJI_0448	Tilia cordata Mill.	0	n	1	1	Malvaceae Juss.
Iz_2_1009	DJI_0448	Trifolium alpestre L.	0	n	1	0	Fabaceae Lindl.
Iz_2_1009	DJI_0448	Trifolium arvense L.	0	n	1	0	Fabaceae Lindl.
Iz_2_1009	DJI_0448	Ulmus glabra Huds.	0	n	1	1	Ulmaceae Mirb.
Iz_2_1009	DJI_0448	Verbascum densiflorum Bertol.	0	n	1	0	Scrophulariaceae Juss.
Iz_2_1009	DJI_0448	Veronica austriaca L.	0	n	1	0	Plantaginaceae Juss.
Iz_2_1009	DJI_0448	Veronica incana L.	0	n	1	0	Plantaginaceae Juss.
Iz_2_1009	DJI_0448	Veronica verna L.	0	n	1	0	Plantaginaceae Juss.
Iz_2_1009	DJI_0448	Vincetoxicum hirundinaria Medik	0	n	1	0	Apocynaceae Juss.
Iz_2_1009	DJI_0449	Acer tataricum L.	0	n	1	1	Sapindaceae Juss.
Iz_2_1009	DJI_0449	Achillea millefolium L. s.l.	0	n	1	0	Asteraceae Bercht. & J.Presl
Iz_2_1009	DJI_0449	Allium oleraceum L.	0	n	1	0	Amaryllidaceae J.St.-Hil.
Iz_2_1009	DJI_0449	Ambrosia artemisiifolia L.	0	a	0	0	Asteraceae Bercht. & J.Presl
Iz_2_1009	DJI_0449	Amorpha fruticosa L.	0	a	0	0	Fabaceae Lindl.
Iz_2_1009	DJI_0449	Anthericum ramosum L.	0	n	1	0	Asparagaceae Juss.
Iz_2_1009	DJI_0449	Artemisia marschalliana Spreng.	0	n	1	0	Asteraceae Bercht. & J.Presl
Iz_2_1009	DJI_0449	Asparagus officinalis L.	0	n	1	0	Asparagaceae Juss.

Iz_2_1009	DJI_0449	Astragalus cicer L.	0	n	1	0	Fabaceae Lindl.
Iz_2_1009	DJI_0449	Bassia laniflora (S.G.Gmel.) A.J.Scott	0	n	1	0	Amaranthaceae Juss.
Iz_2_1009	DJI_0449	Berberis vulgaris L.	0	n	1	0	Berberidaceae Juss.
Iz_2_1009	DJI_0449	Betula pendula Roth.	0	n	1	1	Betulaceae Gray
Iz_2_1009	DJI_0449	Betula pendula Roth.	0	n	1	0	Poaceae Barnhart
Iz_2_1009	DJI_0449	Bromus inermis Leyss.	0		NA	NA	
Iz_2_1009	DJI_0449	Bromus squarrosus L.	0	a	0	0	Poaceae Barnhart
Iz_2_1009	DJI_0449	Bromus tectorum L.	0	a	0	0	Poaceae Barnhart
Iz_2_1009	DJI_0449	Calamagrostis epigejos (L.) Roth	25	n	1	0	Poaceae Barnhart
Iz_2_1009	DJI_0449	Carduus acanthoides L.	0	n	1	0	Asteraceae Bercht. & J.Presl
Iz_2_1009	DJI_0449	Carex sp.	0	n	1	0	Cyperaceae Juss.
Iz_2_1009	DJI_0449	Centaurea majorovii Dumbadze	0	n	1	0	Asteraceae Bercht. & J.Presl
Iz_2_1009	DJI_0449	Cf. Solidago virgaurea	0		NA	NA	
Iz_2_1009	DJI_0449	Chamaecytisus ruthenicus (Fisch.ex Wol.) Klaskova	0	n	1	0	Fabaceae Lindl.
Iz_2_1009	DJI_0449	Chelidonium majus L.	0	n	1	0	Papaveraceae Juss.
Iz_2_1009	DJI_0449	Chenopodium betaceum Andrz.	0	n	1	0	Amaranthaceae Juss.
Iz_2_1009	DJI_0449	Chenopodium cf. album	0	n	1	0	Amaranthaceae Juss.
Iz_2_1009	DJI_0449	Chenopodium sp.	0		NA	0	Amaranthaceae Juss.
Iz_2_1009	DJI_0449	Chondrilla juncea L.	0	n	1	0	Asteraceae Bercht. & J.Presl
Iz_2_1009	DJI_0449	Cirsium vulgare (Savi) Ten.	0	n	1	0	Asteraceae Bercht. & J.Presl
Iz_2_1009	DJI_0449	Clinopodium acinos (L.) Kuntze	0	n	1	0	Lamiacea Lindl.
Iz_2_1009	DJI_0449	Convallaria majalis L.	0	n	1	0	Asparagaceae Juss.
Iz_2_1009	DJI_0449	Crataegus rhipidophylla Gand.	0	n	1	1	Rosaceae Juss.
Iz_2_1009	DJI_0449	Crepis foetida L.	0	n	1	0	Asteraceae Bercht. & J.Presl
Iz_2_1009	DJI_0449	Dactylis glomerata L.	0	n	1	0	Poaceae Barnhart
Iz_2_1009	DJI_0449	Dianthus campestris M.Bieb.	0	n	1	0	Caryophyllaceae Juss.
Iz_2_1009	DJI_0449	Digitaria sanguinalis (L.) Scop.	0	a	0	0	Poaceae Barnhart
Iz_2_1009	DJI_0449	Elymus repens (L.) Gould	0	n	1	0	Poaceae Barnhart
Iz_2_1009	DJI_0449	Epilobium tetragonum L.	0	n	1	0	Onagraceae Juss.
Iz_2_1009	DJI_0449	Eragrostis minor Host	0	a	0	0	Poaceae Barnhart
Iz_2_1009	DJI_0449	Erigeron canadensis L.	0	a	0	0	Asteraceae Bercht. & J.Presl
Iz_2_1009	DJI_0449	Euphorbia seguieriana Neck.	0	n	1	0	Euphorbiaceae Juss.
Iz_2_1009	DJI_0449	Fallopia dumetorum (L.) Holub	0	n	1	0	Polygonaceae Juss.
Iz_2_1009	DJI_0449	Festuca beckeri (Hack.) Trautv.	0	n	1	0	Poaceae Barnhart
Iz_2_1009	DJI_0449	Fragaria vesca L.	0	n	1	0	Rosaceae Juss.
Iz_2_1009	DJI_0449	Frangula alnus Mill.	0	n	1	1	Rhamnaceae Juss.
Iz_2_1009	DJI_0449	Fraxinus pennsylvanica Marshall	0	a	0	1	Oleaceae Hoffmanns. & Link
Iz_2_1009	DJI_0449	Galeopsis bifida Boenn.	0	n	1	0	Lamiacea Lindl.
Iz_2_1009	DJI_0449	Galium verum L.	0	n	1	0	Rubiaceae Juss.
Iz_2_1009	DJI_0449	Genista tinctoria L.	0	n	1	0	Fabaceae Lindl.
Iz_2_1009	DJI_0449	Geranium robertianum L.	0	n	1	0	Geraniaceae Juss.
Iz_2_1009	DJI_0449	Geranium sanquineum L.	0	n	1	0	Geraniaceae Juss.
Iz_2_1009	DJI_0449	Gypsophila paniculata L.	0	n	1	0	Caryophyllaceae Juss.
Iz_2_1009	DJI_0449	Helichrysum arenarium (L.) Moench	0	n	1	0	Asteraceae Bercht. & J.Presl
Iz_2_1009	DJI_0449	Herniaria polygama J.Gay	0	n	1	0	Caryophyllaceae Juss.
Iz_2_1009	DJI_0449	Hieracium robustum Fr.	0	n	1	0	Asteraceae Bercht. & J.Presl
Iz_2_1009	DJI_0449	Hieracium umbellatum L.	0	n	1	NA	Asteraceae Bercht. & J.Presl
Iz_2_1009	DJI_0449	Hylotelephium maximum (L.) Holub	0	n	1	0	Crassulaceae J.St.-Hil.
Iz_2_1009	DJI_0449	Jacobaea borysthenica (DC.) B.Nord. & Greuter	0	n	1	0	Asteraceae Bercht. & J.Presl
Iz_2_1009	DJI_0449	Jasione montana L.	0	n	1	0	Campanulaceae Juss.
Iz_2_1009	DJI_0449	Jurinea cyanoides (L.) Rchb.s.l.	0	n	1	0	Asteraceae Bercht. & J.Presl
Iz_2_1009	DJI_0449	Koeleria glauca (Spreng.) DC.	0	n	1	0	Poaceae Barnhart
Iz_2_1009	DJI_0449	Lactuca serriola L.	0	a	0	0	Asteraceae Bercht. & J.Presl
Iz_2_1009	DJI_0449	Linaria genistifolia (L.) Mill.	0	n	1	0	Plantaginaceae Juss.
Iz_2_1009	DJI_0449	Lomelosia argentea (L.) Greuter & Burdet	0	n	1	0	Caprifoliaceae Juss.
Iz_2_1009	DJI_0449	Melampyrum pratense L.	0	n	1	0	Orobanchaceae Vent.
Iz_2_1009	DJI_0449	Oenothera biennis L.	0	a	0	0	Onagraceae Juss.
Iz_2_1009	DJI_0449	Pentanema britannica (L.) D.Gut.Larr., Santos-Vicente, Anderb., E.Rico & M.M.Mart.Ort.	0	n	1	0	Asteraceae Bercht. & J.Presl
Iz_2_1009	DJI_0449	Peucedanum oreoselinum (L.) Moench	0	n	1	0	Apiaceae Lindl.
Iz_2_1009	DJI_0449	Peucedanum ruthenicum M.Bieb.	0	n	1	0	Apiaceae Lindl.
Iz_2_1009	DJI_0449	Pilosella echioides (Lumn.) F.W.Schultz & Sch.Bip.	0	n	1	0	Asteraceae Bercht. & J.Presl
Iz_2_1009	DJI_0449	Pilosella officinarum F. Schultz et Sch. Bip.	0	n	1	0	Asteraceae Bercht. & J.Presl
Iz_2_1009	DJI_0449	Pilosella sp.	0		NA	0	Asteraceae Bercht. & J.Presl
Iz_2_1009	DJI_0449	Pinus sylvestris L.	0	n	1	1	Pinaceae Spreng. ex F.Rudolphi
Iz_2_1009	DJI_0449	Poa annua L.	0	n	1	0	Poaceae Barnhart
Iz_2_1009	DJI_0449	Poa nemoralis L.	0	n	1	0	Poaceae Barnhart
Iz_2_1009	DJI_0449	Poa sp.	0		NA	0	Poaceae Barnhart
Iz_2_1009	DJI_0449	Polygonatum odoratum (Mill.) Druce	4	n	1	0	Asparagaceae Juss.
Iz_2_1009	DJI_0449	Polygonum arenarium Waldst. & Kit.	0	n	1	0	Polygonaceae Juss.
Iz_2_1009	DJI_0449	Populus nigra L.	0	n	1	1	Salicaceae Mirb.
Iz_2_1009	DJI_0449	Populus tremula L.	0	n	1	1	Salicaceae Mirb.
Iz_2_1009	DJI_0449	Populus Ã canescens (Aiton) Sm.	0	n	1	1	Salicaceae Mirb.
Iz_2_1009	DJI_0449	Potentilla argentea L.	0	n	1	0	Rosaceae Juss.
Iz_2_1009	DJI_0449	Psammophiliella muralis (L.) Ikonn.	0	n	1	0	Caryophyllaceae Juss.
Iz_2_1009	DJI_0449	Psephellus sumensis (Kalen.) Greuter	0	n	1	0	Asteraceae Bercht. & J.Presl
Iz_2_1009	DJI_0449	Pulsatilla pratensis (L.) Mill. s.l.	0	n	1	0	Ranunculaceae Juss.
Iz_2_1009	DJI_0449	Pyrus communis L.	0	a	0	1	Rosaceae Juss.
Iz_2_1009	DJI_0449	Quercus robur L.	0	n	1	1	Fagaceae Dumort.
Iz_2_1009	DJI_0449	Robinia pseudoacacia L.	0	a	0	1	Fabaceae Lindl.
Iz_2_1009	DJI_0449	Rosa sp.	0		NA	1	Rosaceae Juss.
Iz_2_1009	DJI_0449	Rubus idaeus L.	0	n	1	0	Rosaceae Juss.
Iz_2_1009	DJI_0449	Rumex acetosella L.	0	n	1	0	Polygonaceae Juss.
Iz_2_1009	DJI_0449	Rumex thyrsiflorus Fingerh.	0	n	1	0	Polygonaceae Juss.
Iz_2_1009	DJI_0449	Salix alba L.	0	n	1	1	Salicaceae Mirb.
Iz_2_1009	DJI_0449	Sambucus nigra L.	0	n	1	1	Viburnaceae Raf.
Iz_2_1009	DJI_0449	Secale sylvestre Host	0	n	1	0	Poaceae Barnhart
Iz_2_1009	DJI_0449	Sempervivum ruthenicum Schnittsp. & C.B. Lehm.	0	n	1	0	Crassulaceae J.St.-Hil.
Iz_2_1009	DJI_0449	Setaria viridis (L.) P. Beauv.	0	n	1	0	Poaceae Barnhart
Iz_2_1009	DJI_0449	Silene borysthenica (Gruner) Walters	0	n	1	0	Caryophyllaceae Juss.
Iz_2_1009	DJI_0449	Silene latifolia subsp. alba (Mill.) Greuter & Burdet	0	n	1	0	Caryophyllaceae Juss.
Iz_2_1009	DJI_0449	Solidago virgaurea L.	0	n	1	0	Asteraceae Bercht. & J.Presl
Iz_2_1009	DJI_0449	Sorbus aucuparia L.	0	n	1	1	Rosaceae Juss.
Iz_2_1009	DJI_0449	Stachys recta L.	0	n	1	0	Lamiacea Lindl.
Iz_2_1009	DJI_0449	Tanacetum vulgare L.	0	n	1	0	Asteraceae Bercht. & J.Presl
Iz_2_1009	DJI_0449	Thymus pallasianus Heinr.Braun	0	n	1	0	Lamiacea Lindl.
Iz_2_1009	DJI_0449	Tilia cordata Mill.	0	n	1	1	Malvaceae Juss.
Iz_2_1009	DJI_0449	Trifolium alpestre L.	0	n	1	0	Fabaceae Lindl.
Iz_2_1009	DJI_0449	Trifolium arvense L.	0	n	1	0	Fabaceae Lindl.

Iz_2_1009	DJI_0449	Ulmus glabra Huds.	0	n	1	1	Ulmaceae Mirb.
Iz_2_1009	DJI_0449	Verbascum densiflorum Bertol.	0	n	1	0	Scrophulariaceae Juss.
Iz_2_1009	DJI_0449	Veronica austriaca L.	0	n	1	0	Plantaginaceae Juss.
Iz_2_1009	DJI_0449	Veronica incana L.	0	n	1	0	Plantaginaceae Juss.
Iz_2_1009	DJI_0449	Veronica verna L.	0	n	1	0	Plantaginaceae Juss.
Iz_2_1009	DJI_0449	Vincetoxicum hirundinaria Medik	0	n	1	0	Apocynaceae Juss.
Iz_2_1009	DJI_0450	Acer tataricum L.	0	n	1	1	Sapindaceae Juss.
Iz_2_1009	DJI_0450	Achillea millefolium L. s.l.	0	n	1	0	Asteraceae Bercht. & J.Presl
Iz_2_1009	DJI_0450	Allium oleraceum L.	0	n	1	0	Amaryllidaceae J.St.-Hil.
Iz_2_1009	DJI_0450	Ambrosia artemisiifolia L.	0	a	0	0	Asteraceae Bercht. & J.Presl
Iz_2_1009	DJI_0450	Amorpha fruticosa L.	0	a	0	0	Fabaceae Lindl.
Iz_2_1009	DJI_0450	Anthericum ramosum L.	0	n	1	0	Asparagaceae Juss.
Iz_2_1009	DJI_0450	Artemisia marschalliana Spreng.	0	n	1	0	Asteraceae Bercht. & J.Presl
Iz_2_1009	DJI_0450	Asparagus officinalis L.	0	n	1	0	Asparagaceae Juss.
Iz_2_1009	DJI_0450	Astragalus cicer L.	0	n	1	0	Fabaceae Lindl.
Iz_2_1009	DJI_0450	Bassia laniflora (S.G.Gmel.) A.J.Scott	0	n	1	0	Amaranthaceae Juss.
Iz_2_1009	DJI_0450	Berberis vulgaris L.	0	n	1	0	Berberidaceae Juss.
Iz_2_1009	DJI_0450	Betula pendula Roth.	0	n	1	1	Betulaceae Gray
Iz_2_1009	DJI_0450	Betula pendula Roth.	0	n	1	0	Poaceae Barnhart
Iz_2_1009	DJI_0450	Bromus inermis Leyss.	0		NA	NA	
Iz_2_1009	DJI_0450	Bromus squarrosus L.	0	a	0	0	Poaceae Barnhart
Iz_2_1009	DJI_0450	Bromus tectorum L.	0	a	0	0	Poaceae Barnhart
Iz_2_1009	DJI_0450	Calamagrostis epigejos (L.) Roth	10	n	1	0	Poaceae Barnhart
Iz_2_1009	DJI_0450	Carduus acanthoides L.	0	n	1	0	Asteraceae Bercht. & J.Presl
Iz_2_1009	DJI_0450	Carex sp.	0	n	1	0	Cyperaceae Juss.
Iz_2_1009	DJI_0450	Centaurea majorovii Dumbadze	0	n	1	0	Asteraceae Bercht. & J.Presl
Iz_2_1009	DJI_0450	Cf. Solidago virgaurea	0		NA	NA	
Iz_2_1009	DJI_0450	Chamaecytisus ruthenicus (Fisch.ex Wol.) Klaskova	0	n	1	0	Fabaceae Lindl.
Iz_2_1009	DJI_0450	Chelidonium majus L.	0	n	1	0	Papaveraceae Juss.
Iz_2_1009	DJI_0450	Chenopodium betaceum Andrz.	0	n	1	0	Amaranthaceae Juss.
Iz_2_1009	DJI_0450	Chenopodium cf. album	0	n	1	0	Amaranthaceae Juss.
Iz_2_1009	DJI_0450	Chenopodium sp.	0		NA	0	Amaranthaceae Juss.
Iz_2_1009	DJI_0450	Chondrilla juncea L.	0	n	1	0	Asteraceae Bercht. & J.Presl
Iz_2_1009	DJI_0450	Cirsium vulgare (Savi) Ten.	0	n	1	0	Asteraceae Bercht. & J.Presl
Iz_2_1009	DJI_0450	Clinopodium acinos (L.) Kuntze	0	n	1	0	Lamiacea Lindl.
Iz_2_1009	DJI_0450	Convallaria majalis L.	0	n	1	0	Asparagaceae Juss.
Iz_2_1009	DJI_0450	Crataegus rhipidophylla Gand.	0	n	1	1	Rosaceae Juss.
Iz_2_1009	DJI_0450	Crepis foetida L.	0	n	1	0	Asteraceae Bercht. & J.Presl
Iz_2_1009	DJI_0450	Dactylis glomerata L.	0	n	1	0	Poaceae Barnhart
Iz_2_1009	DJI_0450	Dianthus campestris M.Bieb.	0	n	1	0	Caryophyllaceae Juss.
Iz_2_1009	DJI_0450	Digitaria sanguinalis (L.) Scop.	0	a	0	0	Poaceae Barnhart
Iz_2_1009	DJI_0450	Elymus repens (L.) Gould	0	n	1	0	Poaceae Barnhart
Iz_2_1009	DJI_0450	Epilobium tetragonum L.	0	n	1	0	Onagraceae Juss.
Iz_2_1009	DJI_0450	Eragrostis minor Host	0	a	0	0	Poaceae Barnhart
Iz_2_1009	DJI_0450	Erigeron canadensis L.	0.2	a	0	0	Asteraceae Bercht. & J.Presl
Iz_2_1009	DJI_0450	Euphorbia seguieriana Neck.	0	n	1	0	Euphorbiaceae Juss.
Iz_2_1009	DJI_0450	Fallopia dumetorum (L.) Holub	0	n	1	0	Polygonaceae Juss.
Iz_2_1009	DJI_0450	Festuca beckeri (Hack.) Trautv.	0	n	1	0	Poaceae Barnhart
Iz_2_1009	DJI_0450	Fragaria vesca L.	0	n	1	0	Rosaceae Juss.
Iz_2_1009	DJI_0450	Frangula alnus Mill.	0	n	1	1	Rhamnaceae Juss.
Iz_2_1009	DJI_0450	Fraxinus pennsylvanica Marshall	0	a	0	1	Oleaceae Hoffmanns. & Link
Iz_2_1009	DJI_0450	Galeopsis bifida Boenn.	0	n	1	0	Lamiacea Lindl.
Iz_2_1009	DJI_0450	Galium verum L.	0	n	1	0	Rubiaceae Juss.
Iz_2_1009	DJI_0450	Genista tinctoria L.	0	n	1	0	Fabaceae Lindl.
Iz_2_1009	DJI_0450	Geranium robertianum L.	0	n	1	0	Geraniaceae Juss.
Iz_2_1009	DJI_0450	Geranium sanquineum L.	0	n	1	0	Geraniaceae Juss.
Iz_2_1009	DJI_0450	Gypsophila paniculata L.	0	n	1	0	Caryophyllaceae Juss.
Iz_2_1009	DJI_0450	Helichrysum arenarium (L.) Moench	0	n	1	0	Asteraceae Bercht. & J.Presl
Iz_2_1009	DJI_0450	Herniaria polygama J.Gay	0	n	1	0	Caryophyllaceae Juss.
Iz_2_1009	DJI_0450	Hieracium robustum Fr.	0	n	1	0	Asteraceae Bercht. & J.Presl
Iz_2_1009	DJI_0450	Hieracium umbellatum L.	0	n	1	NA	Asteraceae Bercht. & J.Presl
Iz_2_1009	DJI_0450	Hylotelephium maximum (L.) Holub	0	n	1	0	Crassulaceae J.St.-Hil.
Iz_2_1009	DJI_0450	Jacobaea borysthenica (DC.) B.Nord. & Greuter	0	n	1	0	Asteraceae Bercht. & J.Presl
Iz_2_1009	DJI_0450	Jasione montana L.	0	n	1	0	Campanulaceae Juss.
Iz_2_1009	DJI_0450	Jurinea cyanoides (L.) Rchb.s.l.	0	n	1	0	Asteraceae Bercht. & J.Presl
Iz_2_1009	DJI_0450	Koeleria glauca (Spreng.) DC.	0	n	1	0	Poaceae Barnhart
Iz_2_1009	DJI_0450	Lactuca serriola L.	0	a	0	0	Asteraceae Bercht. & J.Presl
Iz_2_1009	DJI_0450	Linaria genistifolia (L.) Mill.	0	n	1	0	Plantaginaceae Juss.
Iz_2_1009	DJI_0450	Lomelosia argentea (L.) Greuter & Burdet	0	n	1	0	Caprifoliaceae Juss.
Iz_2_1009	DJI_0450	Melampyrum pratense L.	0	n	1	0	Orobanchaceae Vent.
Iz_2_1009	DJI_0450	Oenothera biennis L.	0	a	0	0	Onagraceae Juss.
Iz_2_1009	DJI_0450	Pentanema britannica (L.) D.Gut.Larr., Santos-Vicente, Anderb., E.Rico & M.M.Mart.Ort.	0	n	1	0	Asteraceae Bercht. & J.Presl
Iz_2_1009	DJI_0450	Peucedanum oreoselinum (L.) Moench	2	n	1	0	Apiaceae Lindl.
Iz_2_1009	DJI_0450	Peucedanum ruthenicum M.Bieb.	0	n	1	0	Apiaceae Lindl.
Iz_2_1009	DJI_0450	Pilosella echioides (Lumn.) F.W.Schultz & Sch.Bip.	0	n	1	0	Asteraceae Bercht. & J.Presl
Iz_2_1009	DJI_0450	Pilosella officinarum F. Schultz et Sch. Bip.	0	n	1	0	Asteraceae Bercht. & J.Presl
Iz_2_1009	DJI_0450	Pilosella sp.	0		NA	0	Asteraceae Bercht. & J.Presl
Iz_2_1009	DJI_0450	Pinus sylvestris L.	0	n	1	1	Pinaceae Spreng. ex F.Rudolphi
Iz_2_1009	DJI_0450	Poa annua L.	0	n	1	0	Poaceae Barnhart
Iz_2_1009	DJI_0450	Poa nemoralis L.	0	n	1	0	Poaceae Barnhart
Iz_2_1009	DJI_0450	Poa sp.	0		NA	0	Poaceae Barnhart
Iz_2_1009	DJI_0450	Polygonatum odoratum (Mill.) Druce	0	n	1	0	Asparagaceae Juss.
Iz_2_1009	DJI_0450	Polygonum arenarium Waldst. & Kit.	0	n	1	0	Polygonaceae Juss.
Iz_2_1009	DJI_0450	Populus nigra L.	0	n	1	1	Salicaceae Mirb.
Iz_2_1009	DJI_0450	Populus tremula L.	0	n	1	1	Salicaceae Mirb.
Iz_2_1009	DJI_0450	Populus Ã canescens (Aiton) Sm.	0	n	1	1	Salicaceae Mirb.
Iz_2_1009	DJI_0450	Potentilla argentea L.	0	n	1	0	Rosaceae Juss.
Iz_2_1009	DJI_0450	Psammophiliella muralis (L.) Ikonn.	0	n	1	0	Caryophyllaceae Juss.
Iz_2_1009	DJI_0450	Psephellus sumensis (Kalen.) Greuter	0	n	1	0	Asteraceae Bercht. & J.Presl
Iz_2_1009	DJI_0450	Pulsatilla pratensis (L.) Mill. s.l.	0	n	1	0	Ranunculaceae Juss.
Iz_2_1009	DJI_0450	Pyrus communis L.	0	a	0	1	Rosaceae Juss.
Iz_2_1009	DJI_0450	Quercus robur L.	0	n	1	1	Fagaceae Dumort.
Iz_2_1009	DJI_0450	Robinia pseudoacacia L.	0	a	0	1	Fabaceae Lindl.
Iz_2_1009	DJI_0450	Rosa sp.	0		NA	1	Rosaceae Juss.
Iz_2_1009	DJI_0450	Rubus idaeus L.	0	n	1	0	Rosaceae Juss.
Iz_2_1009	DJI_0450	Rumex acetosella L.	0	n	1	0	Polygonaceae Juss.
Iz_2_1009	DJI_0450	Rumex thyrsiflorus Fingerh.	0	n	1	0	Polygonaceae Juss.
Iz_2_1009	DJI_0450	Salix alba L.	0	n	1	1	Salicaceae Mirb.

Iz_2_1009	DJI_0450	Sambucus nigra L.	0	n	1	1	Viburnaceae Raf.
Iz_2_1009	DJI_0450	Secale sylvestre Host	0	n	1	0	Poaceae Barnhart
Iz_2_1009	DJI_0450	Sempervivum ruthenicum Schnittsp. & C.B. Lehm.	0	n	1	0	Crassulaceae J.St.-Hil.
Iz_2_1009	DJI_0450	Setaria viridis (L.) P. Beauv.	0	n	1	0	Poaceae Barnhart
Iz_2_1009	DJI_0450	Silene borysthenica (Gruner) Walters	0	n	1	0	Caryophyllaceae Juss.
Iz_2_1009	DJI_0450	Silene latifolia subsp. alba (Mill.) Greuter & Burdet	0	n	1	0	Caryophyllaceae Juss.
Iz_2_1009	DJI_0450	Solidago virgaurea L.	1	n	1	0	Asteraceae Bercht. & J.Presl
Iz_2_1009	DJI_0450	Sorbus aucuparia L.	0	n	1	1	Rosaceae Juss.
Iz_2_1009	DJI_0450	Stachys recta L.	0	n	1	0	Lamiacea Lindl.
Iz_2_1009	DJI_0450	Tanacetum vulgare L.	0	n	1	0	Asteraceae Bercht. & J.Presl
Iz_2_1009	DJI_0450	Thymus pallasianus Heinr.Braun	0	n	1	0	Lamiacea Lindl.
Iz_2_1009	DJI_0450	Tilia cordata Mill.	0	n	1	1	Malvaceae Juss.
Iz_2_1009	DJI_0450	Trifolium alpestre L.	0	n	1	0	Fabaceae Lindl.
Iz_2_1009	DJI_0450	Trifolium arvense L.	0	n	1	0	Fabaceae Lindl.
Iz_2_1009	DJI_0450	Ulmus glabra Huds.	0	n	1	1	Ulmaceae Mirb.
Iz_2_1009	DJI_0450	Verbascum densiflorum Bertol.	0	n	1	0	Scrophulariaceae Juss.
Iz_2_1009	DJI_0450	Veronica austriaca L.	0	n	1	0	Plantaginaceae Juss.
Iz_2_1009	DJI_0450	Veronica incana L.	0	n	1	0	Plantaginaceae Juss.
Iz_2_1009	DJI_0450	Veronica verna L.	0	n	1	0	Plantaginaceae Juss.
Iz_2_1009	DJI_0450	Vincetoxicum hirundinaria Medik	0	n	1	0	Apocynaceae Juss.
Iz_2_1009	DJI_0453	Acer tataricum L.	0	n	1	1	Sapindaceae Juss.
Iz_2_1009	DJI_0453	Achillea millefolium L. s.l.	0	n	1	0	Asteraceae Bercht. & J.Presl
Iz_2_1009	DJI_0453	Allium oleraceum L.	0	n	1	0	Amaryllidaceae J.St.-Hil.
Iz_2_1009	DJI_0453	Ambrosia artemisiifolia L.	0	a	0	0	Asteraceae Bercht. & J.Presl
Iz_2_1009	DJI_0453	Amorpha fruticosa L.	0	a	0	0	Fabaceae Lindl.
Iz_2_1009	DJI_0453	Anthericum ramosum L.	0	n	1	0	Asparagaceae Juss.
Iz_2_1009	DJI_0453	Artemisia marschalliana Spreng.	0	n	1	0	Asteraceae Bercht. & J.Presl
Iz_2_1009	DJI_0453	Asparagus officinalis L.	0	n	1	0	Asparagaceae Juss.
Iz_2_1009	DJI_0453	Astragalus cicer L.	0	n	1	0	Fabaceae Lindl.
Iz_2_1009	DJI_0453	Bassia laniflora (S.G.Gmel.) A.J.Scott	0	n	1	0	Amaranthaceae Juss.
Iz_2_1009	DJI_0453	Berberis vulgaris L.	0	n	1	0	Berberidaceae Juss.
Iz_2_1009	DJI_0453	Betula pendula Roth.	0	n	1	1	Betulaceae Gray
Iz_2_1009	DJI_0453	Betula pendula Roth.	0	n	1	0	Poaceae Barnhart
Iz_2_1009	DJI_0453	Bromus inermis Leyss.	0		NA	NA	
Iz_2_1009	DJI_0453	Bromus squarrosus L.	0	a	0	0	Poaceae Barnhart
Iz_2_1009	DJI_0453	Bromus tectorum L.	0	a	0	0	Poaceae Barnhart
Iz_2_1009	DJI_0453	Calamagrostis epigejos (L.) Roth	40	n	1	0	Poaceae Barnhart
Iz_2_1009	DJI_0453	Carduus acanthoides L.	0	n	1	0	Asteraceae Bercht. & J.Presl
Iz_2_1009	DJI_0453	Carex sp.	0	n	1	0	Cyperaceae Juss.
Iz_2_1009	DJI_0453	Centaurea majorovii Dumbadze	0	n	1	0	Asteraceae Bercht. & J.Presl
Iz_2_1009	DJI_0453	Cf. Solidago virgaurea	0		NA	NA	
Iz_2_1009	DJI_0453	Chamaecytisus ruthenicus (Fisch.ex Wol.) Klaskova	0	n	1	0	Fabaceae Lindl.
Iz_2_1009	DJI_0453	Chelidonium majus L.	0	n	1	0	Papaveraceae Juss.
Iz_2_1009	DJI_0453	Chenopodium betaceum Andrz.	0	n	1	0	Amaranthaceae Juss.
Iz_2_1009	DJI_0453	Chenopodium cf. album	0	n	1	0	Amaranthaceae Juss.
Iz_2_1009	DJI_0453	Chenopodium sp.	0		NA	0	Amaranthaceae Juss.
Iz_2_1009	DJI_0453	Chondrilla juncea L.	0	n	1	0	Asteraceae Bercht. & J.Presl
Iz_2_1009	DJI_0453	Cirsium vulgare (Savi) Ten.	0	n	1	0	Asteraceae Bercht. & J.Presl
Iz_2_1009	DJI_0453	Clinopodium acinos (L.) Kuntze	0	n	1	0	Lamiacea Lindl.
Iz_2_1009	DJI_0453	Convallaria majalis L.	0	n	1	0	Asparagaceae Juss.
Iz_2_1009	DJI_0453	Crataegus rhipidophylla Gand.	0	n	1	1	Rosaceae Juss.
Iz_2_1009	DJI_0453	Crepis foetida L.	0	n	1	0	Asteraceae Bercht. & J.Presl
Iz_2_1009	DJI_0453	Dactylis glomerata L.	0	n	1	0	Poaceae Barnhart
Iz_2_1009	DJI_0453	Dianthus campestris M.Bieb.	0	n	1	0	Caryophyllaceae Juss.
Iz_2_1009	DJI_0453	Digitaria sanguinalis (L.) Scop.	0	a	0	0	Poaceae Barnhart
Iz_2_1009	DJI_0453	Elymus repens (L.) Gould	0	n	1	0	Poaceae Barnhart
Iz_2_1009	DJI_0453	Epilobium tetragonum L.	0	n	1	0	Onagraceae Juss.
Iz_2_1009	DJI_0453	Eragrostis minor Host	0	a	0	0	Poaceae Barnhart
Iz_2_1009	DJI_0453	Erigeron canadensis L.	0	a	0	0	Asteraceae Bercht. & J.Presl
Iz_2_1009	DJI_0453	Euphorbia seguieriana Neck.	0	n	1	0	Euphorbiaceae Juss.
Iz_2_1009	DJI_0453	Fallopia dumetorum (L.) Holub	1	n	1	0	Polygonaceae Juss.
Iz_2_1009	DJI_0453	Festuca beckeri (Hack.) Trautv.	0	n	1	0	Poaceae Barnhart
Iz_2_1009	DJI_0453	Fragaria vesca L.	0	n	1	0	Rosaceae Juss.
Iz_2_1009	DJI_0453	Frangula alnus Mill.	0	n	1	1	Rhamnaceae Juss.
Iz_2_1009	DJI_0453	Fraxinus pennsylvanica Marshall	0	a	0	1	Oleaceae Hoffmanns. & Link
Iz_2_1009	DJI_0453	Galeopsis bifida Boenn.	0	n	1	0	Lamiacea Lindl.
Iz_2_1009	DJI_0453	Galium verum L.	0	n	1	0	Rubiaceae Juss.
Iz_2_1009	DJI_0453	Genista tinctoria L.	0	n	1	0	Fabaceae Lindl.
Iz_2_1009	DJI_0453	Geranium robertianum L.	1.5	n	1	0	Geraniaceae Juss.
Iz_2_1009	DJI_0453	Geranium sanquineum L.	0	n	1	0	Geraniaceae Juss.
Iz_2_1009	DJI_0453	Gypsophila paniculata L.	0	n	1	0	Caryophyllaceae Juss.
Iz_2_1009	DJI_0453	Helichrysum arenarium (L.) Moench	0	n	1	0	Asteraceae Bercht. & J.Presl
Iz_2_1009	DJI_0453	Herniaria polygama J.Gay	0	n	1	0	Caryophyllaceae Juss.
Iz_2_1009	DJI_0453	Hieracium robustum Fr.	0	n	1	0	Asteraceae Bercht. & J.Presl
Iz_2_1009	DJI_0453	Hieracium umbellatum L.	0	n	1	NA	Asteraceae Bercht. & J.Presl
Iz_2_1009	DJI_0453	Hylotelephium maximum (L.) Holub	0	n	1	0	Crassulaceae J.St.-Hil.
Iz_2_1009	DJI_0453	Jacobaea borysthenica (DC.) B.Nord. & Greuter	0	n	1	0	Asteraceae Bercht. & J.Presl
Iz_2_1009	DJI_0453	Jasione montana L.	0	n	1	0	Campanulaceae Juss.
Iz_2_1009	DJI_0453	Jurinea cyanoides (L.) Rchb.s.l.	0	n	1	0	Asteraceae Bercht. & J.Presl
Iz_2_1009	DJI_0453	Koeleria glauca (Spreng.) DC.	0	n	1	0	Poaceae Barnhart
Iz_2_1009	DJI_0453	Lactuca serriola L.	0	a	0	0	Asteraceae Bercht. & J.Presl
Iz_2_1009	DJI_0453	Linaria genistifolia (L.) Mill.	0	n	1	0	Plantaginaceae Juss.
Iz_2_1009	DJI_0453	Lomelosia argentea (L.) Greuter & Burdet	0	n	1	0	Caprifoliaceae Juss.
Iz_2_1009	DJI_0453	Melampyrum pratense L.	0	n	1	0	Orobanchaceae Vent.
Iz_2_1009	DJI_0453	Oenothera biennis L.	0	a	0	0	Onagraceae Juss.
Iz_2_1009	DJI_0453	Pentanema britannica (L.) D.Gut.Larr., Santos-Vicente, Anderb., E.Rico & M.M.Mart.Ort.	0	n	1	0	Asteraceae Bercht. & J.Presl
Iz_2_1009	DJI_0453	Peucedanum oreoselinum (L.) Moench	0	n	1	0	Apiaceae Lindl.
Iz_2_1009	DJI_0453	Peucedanum ruthenicum M.Bieb.	0	n	1	0	Apiaceae Lindl.
Iz_2_1009	DJI_0453	Pilosella echioides (Lumn.) F.W.Schultz & Sch.Bip.	0	n	1	0	Asteraceae Bercht. & J.Presl
Iz_2_1009	DJI_0453	Pilosella officinarum F. Schultz et Sch. Bip.	0	n	1	0	Asteraceae Bercht. & J.Presl
Iz_2_1009	DJI_0453	Pilosella sp.	0		NA	0	Asteraceae Bercht. & J.Presl
Iz_2_1009	DJI_0453	Pinus sylvestris L.	0	n	1	1	Pinaceae Spreng. ex F.Rudolphi
Iz_2_1009	DJI_0453	Poa annua L.	0	n	1	0	Poaceae Barnhart
Iz_2_1009	DJI_0453	Poa nemoralis L.	0	n	1	0	Poaceae Barnhart
Iz_2_1009	DJI_0453	Poa sp.	0		NA	0	Poaceae Barnhart
Iz_2_1009	DJI_0453	Polygonatum odoratum (Mill.) Druce	0	n	1	0	Asparagaceae Juss.
Iz_2_1009	DJI_0453	Polygonum arenarium Waldst. & Kit.	0	n	1	0	Polygonaceae Juss.
Iz_2_1009	DJI_0453	Populus nigra L.	0	n	1	1	Salicaceae Mirb.

Iz_2_1009	DJI_0453	Populus tremula L.	0	n	1	1	Salicaceae Mirb.
Iz_2_1009	DJI_0453	Populus Ã canescens (Aiton) Sm.	0	n	1	1	Salicaceae Mirb.
Iz_2_1009	DJI_0453	Potentilla argentea L.	0	n	1	0	Rosaceae Juss.
Iz_2_1009	DJI_0453	Psammophiliella muralis (L.) Ikonn.	0	n	1	0	Caryophyllaceae Juss.
Iz_2_1009	DJI_0453	Psephellus sumensis (Kalen.) Greuter	0	n	1	0	Asteraceae Bercht. & J.Presl
Iz_2_1009	DJI_0453	Pulsatilla pratensis (L.) Mill. s.l.	0	n	1	0	Ranunculaceae Juss.
Iz_2_1009	DJI_0453	Pyrus communis L.	0	a	0	1	Rosaceae Juss.
Iz_2_1009	DJI_0453	Quercus robur L.	0	n	1	1	Fagaceae Dumort.
Iz_2_1009	DJI_0453	Robinia pseudoacacia L.	0	a	0	1	Fabaceae Lindl.
Iz_2_1009	DJI_0453	Rosa sp.	0		NA	1	Rosaceae Juss.
Iz_2_1009	DJI_0453	Rubus idaeus L.	0	n	1	0	Rosaceae Juss.
Iz_2_1009	DJI_0453	Rumex acetosella L.	0	n	1	0	Polygonaceae Juss.
Iz_2_1009	DJI_0453	Rumex thyrsiflorus Fingerh.	0	n	1	0	Polygonaceae Juss.
Iz_2_1009	DJI_0453	Salix alba L.	0	n	1	1	Salicaceae Mirb.
Iz_2_1009	DJI_0453	Sambucus nigra L.	0	n	1	1	Viburnaceae Raf.
Iz_2_1009	DJI_0453	Secale sylvestre Host	0	n	1	0	Poaceae Barnhart
Iz_2_1009	DJI_0453	Sempervivum ruthenicum Schnittsp. & C.B. Lehm.	0	n	1	0	Crassulaceae J.St.-Hil.
Iz_2_1009	DJI_0453	Setaria viridis (L.) P. Beauv.	0	n	1	0	Poaceae Barnhart
Iz_2_1009	DJI_0453	Silene borysthenica (Gruner) Walters	0	n	1	0	Caryophyllaceae Juss.
Iz_2_1009	DJI_0453	Silene latifolia subsp. alba (Mill.) Greuter & Burdet	0	n	1	0	Caryophyllaceae Juss.
Iz_2_1009	DJI_0453	Solidago virgaurea L.	0.2	n	1	0	Asteraceae Bercht. & J.Presl
Iz_2_1009	DJI_0453	Sorbus aucuparia L.	0	n	1	1	Rosaceae Juss.
Iz_2_1009	DJI_0453	Stachys recta L.	0	n	1	0	Lamiacea Lindl.
Iz_2_1009	DJI_0453	Tanacetum vulgare L.	0	n	1	0	Asteraceae Bercht. & J.Presl
Iz_2_1009	DJI_0453	Thymus pallasianus Heinr.Braun	0	n	1	0	Lamiacea Lindl.
Iz_2_1009	DJI_0453	Tilia cordata Mill.	0	n	1	1	Malvaceae Juss.
Iz_2_1009	DJI_0453	Trifolium alpestre L.	0	n	1	0	Fabaceae Lindl.
Iz_2_1009	DJI_0453	Trifolium arvense L.	0	n	1	0	Fabaceae Lindl.
Iz_2_1009	DJI_0453	Ulmus glabra Huds.	0	n	1	1	Ulmaceae Mirb.
Iz_2_1009	DJI_0453	Verbascum densiflorum Bertol.	0	n	1	0	Scrophulariaceae Juss.
Iz_2_1009	DJI_0453	Veronica austriaca L.	0	n	1	0	Plantaginaceae Juss.
Iz_2_1009	DJI_0453	Veronica incana L.	0	n	1	0	Plantaginaceae Juss.
Iz_2_1009	DJI_0453	Veronica verna L.	0	n	1	0	Plantaginaceae Juss.
Iz_2_1009	DJI_0453	Vincetoxicum hirundinaria Medik	0	n	1	0	Apocynaceae Juss.
Iz_2_1009	DJI_0454	Acer tataricum L.	0	n	1	1	Sapindaceae Juss.
Iz_2_1009	DJI_0454	Achillea millefolium L. s.l.	0	n	1	0	Asteraceae Bercht. & J.Presl
Iz_2_1009	DJI_0454	Allium oleraceum L.	0	n	1	0	Amaryllidaceae J.St.-Hil.
Iz_2_1009	DJI_0454	Ambrosia artemisiifolia L.	0	a	0	0	Asteraceae Bercht. & J.Presl
Iz_2_1009	DJI_0454	Amorpha fruticosa L.	0	a	0	0	Fabaceae Lindl.
Iz_2_1009	DJI_0454	Anthericum ramosum L.	0	n	1	0	Asparagaceae Juss.
Iz_2_1009	DJI_0454	Artemisia marschalliana Spreng.	0	n	1	0	Asteraceae Bercht. & J.Presl
Iz_2_1009	DJI_0454	Asparagus officinalis L.	0	n	1	0	Asparagaceae Juss.
Iz_2_1009	DJI_0454	Astragalus cicer L.	0	n	1	0	Fabaceae Lindl.
Iz_2_1009	DJI_0454	Bassia laniflora (S.G.Gmel.) A.J.Scott	0	n	1	0	Amaranthaceae Juss.
Iz_2_1009	DJI_0454	Berberis vulgaris L.	0	n	1	0	Berberidaceae Juss.
Iz_2_1009	DJI_0454	Betula pendula Roth.	0	n	1	1	Betulaceae Gray
Iz_2_1009	DJI_0454	Betula pendula Roth.	0	n	1	0	Poaceae Barnhart
Iz_2_1009	DJI_0454	Bromus inermis Leyss.	0		NA	NA	
Iz_2_1009	DJI_0454	Bromus squarrosus L.	0	a	0	0	Poaceae Barnhart
Iz_2_1009	DJI_0454	Bromus tectorum L.	0	a	0	0	Poaceae Barnhart
Iz_2_1009	DJI_0454	Calamagrostis epigejos (L.) Roth	35	n	1	0	Poaceae Barnhart
Iz_2_1009	DJI_0454	Carduus acanthoides L.	0	n	1	0	Asteraceae Bercht. & J.Presl
Iz_2_1009	DJI_0454	Carex sp.	0	n	1	0	Cyperaceae Juss.
Iz_2_1009	DJI_0454	Centaurea majorovii Dumbadze	0	n	1	0	Asteraceae Bercht. & J.Presl
Iz_2_1009	DJI_0454	Cf. Solidago virgaurea	0		NA	NA	
Iz_2_1009	DJI_0454	Chamaecytisus ruthenicus (Fisch.ex Wol.) Klaskova	0	n	1	0	Fabaceae Lindl.
Iz_2_1009	DJI_0454	Chelidonium majus L.	0.2	n	1	0	Papaveraceae Juss.
Iz_2_1009	DJI_0454	Chenopodium betaceum Andrz.	0	n	1	0	Amaranthaceae Juss.
Iz_2_1009	DJI_0454	Chenopodium cf. album	0	n	1	0	Amaranthaceae Juss.
Iz_2_1009	DJI_0454	Chenopodium sp.	0		NA	0	Amaranthaceae Juss.
Iz_2_1009	DJI_0454	Chondrilla juncea L.	0	n	1	0	Asteraceae Bercht. & J.Presl
Iz_2_1009	DJI_0454	Cirsium vulgare (Savi) Ten.	0	n	1	0	Asteraceae Bercht. & J.Presl
Iz_2_1009	DJI_0454	Clinopodium acinos (L.) Kuntze	0	n	1	0	Lamiacea Lindl.
Iz_2_1009	DJI_0454	Convallaria majalis L.	0	n	1	0	Asparagaceae Juss.
Iz_2_1009	DJI_0454	Crataegus rhipidophylla Gand.	0	n	1	1	Rosaceae Juss.
Iz_2_1009	DJI_0454	Crepis foetida L.	0	n	1	0	Asteraceae Bercht. & J.Presl
Iz_2_1009	DJI_0454	Dactylis glomerata L.	0	n	1	0	Poaceae Barnhart
Iz_2_1009	DJI_0454	Dianthus campestris M.Bieb.	0	n	1	0	Caryophyllaceae Juss.
Iz_2_1009	DJI_0454	Digitaria sanguinalis (L.) Scop.	0	a	0	0	Poaceae Barnhart
Iz_2_1009	DJI_0454	Elymus repens (L.) Gould	0	n	1	0	Poaceae Barnhart
Iz_2_1009	DJI_0454	Epilobium tetragonum L.	0	n	1	0	Onagraceae Juss.
Iz_2_1009	DJI_0454	Eragrostis minor Host	0	a	0	0	Poaceae Barnhart
Iz_2_1009	DJI_0454	Erigeron canadensis L.	0	a	0	0	Asteraceae Bercht. & J.Presl
Iz_2_1009	DJI_0454	Euphorbia seguieriana Neck.	0	n	1	0	Euphorbiaceae Juss.
Iz_2_1009	DJI_0454	Fallopia dumetorum (L.) Holub	3	n	1	0	Polygonaceae Juss.
Iz_2_1009	DJI_0454	Festuca beckeri (Hack.) Trautv.	0	n	1	0	Poaceae Barnhart
Iz_2_1009	DJI_0454	Fragaria vesca L.	0	n	1	0	Rosaceae Juss.
Iz_2_1009	DJI_0454	Frangula alnus Mill.	0	n	1	1	Rhamnaceae Juss.
Iz_2_1009	DJI_0454	Fraxinus pennsylvanica Marshall	0	a	0	1	Oleaceae Hoffmanns. & Link
Iz_2_1009	DJI_0454	Galeopsis bifida Boenn.	0	n	1	0	Lamiacea Lindl.
Iz_2_1009	DJI_0454	Galium verum L.	0	n	1	0	Rubiaceae Juss.
Iz_2_1009	DJI_0454	Genista tinctoria L.	0	n	1	0	Fabaceae Lindl.
Iz_2_1009	DJI_0454	Geranium robertianum L.	0	n	1	0	Geraniaceae Juss.
Iz_2_1009	DJI_0454	Geranium sanquineum L.	0	n	1	0	Geraniaceae Juss.
Iz_2_1009	DJI_0454	Gypsophila paniculata L.	0	n	1	0	Caryophyllaceae Juss.
Iz_2_1009	DJI_0454	Helichrysum arenarium (L.) Moench	0	n	1	0	Asteraceae Bercht. & J.Presl
Iz_2_1009	DJI_0454	Herniaria polygama J.Gay	0	n	1	0	Caryophyllaceae Juss.
Iz_2_1009	DJI_0454	Hieracium robustum Fr.	0	n	1	0	Asteraceae Bercht. & J.Presl
Iz_2_1009	DJI_0454	Hieracium umbellatum L.	0	n	1	NA	Asteraceae Bercht. & J.Presl
Iz_2_1009	DJI_0454	Hylotelephium maximum (L.) Holub	0	n	1	0	Crassulaceae J.St.-Hil.
Iz_2_1009	DJI_0454	Jacobaea borysthenica (DC.) B.Nord. & Greuter	0	n	1	0	Asteraceae Bercht. & J.Presl
Iz_2_1009	DJI_0454	Jasione montana L.	0	n	1	0	Campanulaceae Juss.
Iz_2_1009	DJI_0454	Jurinea cyanoides (L.) Rchb.s.l.	0	n	1	0	Asteraceae Bercht. & J.Presl
Iz_2_1009	DJI_0454	Koeleria glauca (Spreng.) DC.	0	n	1	0	Poaceae Barnhart
Iz_2_1009	DJI_0454	Lactuca serriola L.	0	a	0	0	Asteraceae Bercht. & J.Presl
Iz_2_1009	DJI_0454	Linaria genistifolia (L.) Mill.	0	n	1	0	Plantaginaceae Juss.
Iz_2_1009	DJI_0454	Lomelosia argentea (L.) Greuter & Burdet	0	n	1	0	Caprifoliaceae Juss.
Iz_2_1009	DJI_0454	Melampyrum pratense L.	0	n	1	0	Orobanchaceae Vent.

Iz_2_1009	DJI_0454	Oenothera biennis L.	0	a	0	0	Onagraceae Juss.
Iz_2_1009	DJI_0454	Pentanema britannica (L.) D.Gut.Larr., Santos-Vicente, Anderb., E.Rico & M.M.Mart.Ort.	0	n	1	0	Asteraceae Bercht. & J.Presl
Iz_2_1009	DJI_0454	Peucedanum oreoselinum (L.) Moench	0	n	1	0	Apiaceae Lindl.
Iz_2_1009	DJI_0454	Peucedanum ruthenicum M.Bieb.	0	n	1	0	Apiaceae Lindl.
Iz_2_1009	DJI_0454	Pilosella echioides (Lumn.) F.W.Schultz & Sch.Bip.	0	n	1	0	Asteraceae Bercht. & J.Presl
Iz_2_1009	DJI_0454	Pilosella officinarum F. Schultz et Sch. Bip.	0	n	1	0	Asteraceae Bercht. & J.Presl
Iz_2_1009	DJI_0454	Pilosella sp.	0		NA	0	Asteraceae Bercht. & J.Presl
Iz_2_1009	DJI_0454	Pinus sylvestris L.	0	n	1	1	Pinaceae Spreng. ex F.Rudolphi
Iz_2_1009	DJI_0454	Poa annua L.	0	n	1	0	Poaceae Barnhart
Iz_2_1009	DJI_0454	Poa nemoralis L.	0	n	1	0	Poaceae Barnhart
Iz_2_1009	DJI_0454	Poa sp.	0		NA	0	Poaceae Barnhart
Iz_2_1009	DJI_0454	Polygonatum odoratum (Mill.) Druce	0	n	1	0	Asparagaceae Juss.
Iz_2_1009	DJI_0454	Polygonum arenarium Waldst. & Kit.	0	n	1	0	Polygonaceae Juss.
Iz_2_1009	DJI_0454	Populus nigra L.	0	n	1	1	Salicaceae Mirb.
Iz_2_1009	DJI_0454	Populus tremula L.	0	n	1	1	Salicaceae Mirb.
Iz_2_1009	DJI_0454	Populus Ã canescens (Aiton) Sm.	0	n	1	1	Salicaceae Mirb.
Iz_2_1009	DJI_0454	Potentilla argentea L.	0	n	1	0	Rosaceae Juss.
Iz_2_1009	DJI_0454	Psammophiliella muralis (L.) Ikonn.	0	n	1	0	Caryophyllaceae Juss.
Iz_2_1009	DJI_0454	Psephellus sumensis (Kalen.) Greuter	0	n	1	0	Asteraceae Bercht. & J.Presl
Iz_2_1009	DJI_0454	Pulsatilla pratensis (L.) Mill. s.l.	0	n	1	0	Ranunculaceae Juss.
Iz_2_1009	DJI_0454	Pyrus communis L.	0	a	0	1	Rosaceae Juss.
Iz_2_1009	DJI_0454	Quercus robur L.	0	n	1	1	Fagaceae Dumort.
Iz_2_1009	DJI_0454	Robinia pseudoacacia L.	0	a	0	1	Fabaceae Lindl.
Iz_2_1009	DJI_0454	Rosa sp.	0		NA	1	Rosaceae Juss.
Iz_2_1009	DJI_0454	Rubus idaeus L.	0	n	1	0	Rosaceae Juss.
Iz_2_1009	DJI_0454	Rumex acetosella L.	0	n	1	0	Polygonaceae Juss.
Iz_2_1009	DJI_0454	Rumex thyrsiflorus Fingerh.	0	n	1	0	Polygonaceae Juss.
Iz_2_1009	DJI_0454	Salix alba L.	0	n	1	1	Salicaceae Mirb.
Iz_2_1009	DJI_0454	Sambucus nigra L.	0	n	1	1	Viburnaceae Raf.
Iz_2_1009	DJI_0454	Secale sylvestre Host	0	n	1	0	Poaceae Barnhart
Iz_2_1009	DJI_0454	Sempervivum ruthenicum Schnittsp. & C.B. Lehm.	0	n	1	0	Crassulaceae J.St.-Hil.
Iz_2_1009	DJI_0454	Setaria viridis (L.) P. Beauv.	0	n	1	0	Poaceae Barnhart
Iz_2_1009	DJI_0454	Silene borysthenica (Gruner) Walters	0	n	1	0	Caryophyllaceae Juss.
Iz_2_1009	DJI_0454	Silene latifolia subsp. alba (Mill.) Greuter & Burdet	0	n	1	0	Caryophyllaceae Juss.
Iz_2_1009	DJI_0454	Solidago virgaurea L.	0	n	1	0	Asteraceae Bercht. & J.Presl
Iz_2_1009	DJI_0454	Sorbus aucuparia L.	0	n	1	1	Rosaceae Juss.
Iz_2_1009	DJI_0454	Stachys recta L.	0	n	1	0	Lamiacea Lindl.
Iz_2_1009	DJI_0454	Tanacetum vulgare L.	0	n	1	0	Asteraceae Bercht. & J.Presl
Iz_2_1009	DJI_0454	Thymus pallasianus Heinr.Braun	0	n	1	0	Lamiacea Lindl.
Iz_2_1009	DJI_0454	Tilia cordata Mill.	0	n	1	1	Malvaceae Juss.
Iz_2_1009	DJI_0454	Trifolium alpestre L.	0	n	1	0	Fabaceae Lindl.
Iz_2_1009	DJI_0454	Trifolium arvense L.	0	n	1	0	Fabaceae Lindl.
Iz_2_1009	DJI_0454	Ulmus glabra Huds.	0	n	1	1	Ulmaceae Mirb.
Iz_2_1009	DJI_0454	Verbascum densiflorum Bertol.	0	n	1	0	Scrophulariaceae Juss.
Iz_2_1009	DJI_0454	Veronica austriaca L.	0	n	1	0	Plantaginaceae Juss.
Iz_2_1009	DJI_0454	Veronica incana L.	0	n	1	0	Plantaginaceae Juss.
Iz_2_1009	DJI_0454	Veronica verna L.	0	n	1	0	Plantaginaceae Juss.
Iz_2_1009	DJI_0454	Vincetoxicum hirundinaria Medik	0	n	1	0	Apocynaceae Juss.
Iz_2_1009	DJI_0459	Acer tataricum L.	0	n	1	1	Sapindaceae Juss.
Iz_2_1009	DJI_0459	Achillea millefolium L. s.l.	0	n	1	0	Asteraceae Bercht. & J.Presl
Iz_2_1009	DJI_0459	Allium oleraceum L.	0	n	1	0	Amaryllidaceae J.St.-Hil.
Iz_2_1009	DJI_0459	Ambrosia artemisiifolia L.	0	a	0	0	Asteraceae Bercht. & J.Presl
Iz_2_1009	DJI_0459	Amorpha fruticosa L.	0	a	0	0	Fabaceae Lindl.
Iz_2_1009	DJI_0459	Anthericum ramosum L.	0	n	1	0	Asparagaceae Juss.
Iz_2_1009	DJI_0459	Artemisia marschalliana Spreng.	0	n	1	0	Asteraceae Bercht. & J.Presl
Iz_2_1009	DJI_0459	Asparagus officinalis L.	0	n	1	0	Asparagaceae Juss.
Iz_2_1009	DJI_0459	Astragalus cicer L.	0	n	1	0	Fabaceae Lindl.
Iz_2_1009	DJI_0459	Bassia laniflora (S.G.Gmel.) A.J.Scott	0	n	1	0	Amaranthaceae Juss.
Iz_2_1009	DJI_0459	Berberis vulgaris L.	0	n	1	0	Berberidaceae Juss.
Iz_2_1009	DJI_0459	Betula pendula Roth.	0	n	1	1	Betulaceae Gray
Iz_2_1009	DJI_0459	Betula pendula Roth.	0	n	1	0	Poaceae Barnhart
Iz_2_1009	DJI_0459	Bromus inermis Leyss.	0		NA	NA	
Iz_2_1009	DJI_0459	Bromus squarrosus L.	0	a	0	0	Poaceae Barnhart
Iz_2_1009	DJI_0459	Bromus tectorum L.	0	a	0	0	Poaceae Barnhart
Iz_2_1009	DJI_0459	Calamagrostis epigejos (L.) Roth	45	n	1	0	Poaceae Barnhart
Iz_2_1009	DJI_0459	Carduus acanthoides L.	0	n	1	0	Asteraceae Bercht. & J.Presl
Iz_2_1009	DJI_0459	Carex sp.	0	n	1	0	Cyperaceae Juss.
Iz_2_1009	DJI_0459	Centaurea majorovii Dumbadze	0	n	1	0	Asteraceae Bercht. & J.Presl
Iz_2_1009	DJI_0459	Cf. Solidago virgaurea	0		NA	NA	
Iz_2_1009	DJI_0459	Chamaecytisus ruthenicus (Fisch.ex Wol.) Klaskova	0	n	1	0	Fabaceae Lindl.
Iz_2_1009	DJI_0459	Chelidonium majus L.	0.5	n	1	0	Papaveraceae Juss.
Iz_2_1009	DJI_0459	Chenopodium betaceum Andrz.	0	n	1	0	Amaranthaceae Juss.
Iz_2_1009	DJI_0459	Chenopodium cf. album	0	n	1	0	Amaranthaceae Juss.
Iz_2_1009	DJI_0459	Chenopodium sp.	0		NA	0	Amaranthaceae Juss.
Iz_2_1009	DJI_0459	Chondrilla juncea L.	0	n	1	0	Asteraceae Bercht. & J.Presl
Iz_2_1009	DJI_0459	Cirsium vulgare (Savi) Ten.	0	n	1	0	Asteraceae Bercht. & J.Presl
Iz_2_1009	DJI_0459	Clinopodium acinos (L.) Kuntze	0	n	1	0	Lamiacea Lindl.
Iz_2_1009	DJI_0459	Convallaria majalis L.	0	n	1	0	Asparagaceae Juss.
Iz_2_1009	DJI_0459	Crataegus rhipidophylla Gand.	0	n	1	1	Rosaceae Juss.
Iz_2_1009	DJI_0459	Crepis foetida L.	0	n	1	0	Asteraceae Bercht. & J.Presl
Iz_2_1009	DJI_0459	Dactylis glomerata L.	0	n	1	0	Poaceae Barnhart
Iz_2_1009	DJI_0459	Dianthus campestris M.Bieb.	0	n	1	0	Caryophyllaceae Juss.
Iz_2_1009	DJI_0459	Digitaria sanguinalis (L.) Scop.	0	a	0	0	Poaceae Barnhart
Iz_2_1009	DJI_0459	Elymus repens (L.) Gould	0	n	1	0	Poaceae Barnhart
Iz_2_1009	DJI_0459	Epilobium tetragonum L.	0	n	1	0	Onagraceae Juss.
Iz_2_1009	DJI_0459	Eragrostis minor Host	0	a	0	0	Poaceae Barnhart
Iz_2_1009	DJI_0459	Erigeron canadensis L.	0	a	0	0	Asteraceae Bercht. & J.Presl
Iz_2_1009	DJI_0459	Euphorbia seguieriana Neck.	0	n	1	0	Euphorbiaceae Juss.
Iz_2_1009	DJI_0459	Fallopia dumetorum (L.) Holub	0	n	1	0	Polygonaceae Juss.
Iz_2_1009	DJI_0459	Festuca beckeri (Hack.) Trautv.	0	n	1	0	Poaceae Barnhart
Iz_2_1009	DJI_0459	Fragaria vesca L.	0	n	1	0	Rosaceae Juss.
Iz_2_1009	DJI_0459	Frangula alnus Mill.	0	n	1	1	Rhamnaceae Juss.
Iz_2_1009	DJI_0459	Fraxinus pennsylvanica Marshall	0	a	0	1	Oleaceae Hoffmanns. & Link
Iz_2_1009	DJI_0459	Galeopsis bifida Boenn.	0	n	1	0	Lamiacea Lindl.
Iz_2_1009	DJI_0459	Galium verum L.	0	n	1	0	Rubiaceae Juss.
Iz_2_1009	DJI_0459	Genista tinctoria L.	0	n	1	0	Fabaceae Lindl.
Iz_2_1009	DJI_0459	Geranium robertianum L.	2	n	1	0	Geraniaceae Juss.
Iz_2_1009	DJI_0459	Geranium sanquineum L.	0	n	1	0	Geraniaceae Juss.

Iz_2_1009	DJI_0459	Gypsophila paniculata L.	0	n	1	0	Caryophyllaceae Juss.
Iz_2_1009	DJI_0459	Helichrysum arenarium (L.) Moench	0	n	1	0	Asteraceae Bercht. & J.Presl
Iz_2_1009	DJI_0459	Herniaria polygama J.Gay	0	n	1	0	Caryophyllaceae Juss.
Iz_2_1009	DJI_0459	Hieracium robustum Fr.	0	n	1	0	Asteraceae Bercht. & J.Presl
Iz_2_1009	DJI_0459	Hieracium umbellatum L.	0	n	1	NA	Asteraceae Bercht. & J.Presl
Iz_2_1009	DJI_0459	Hylotelephium maximum (L.) Holub	0	n	1	0	Crassulaceae J.St.-Hil.
Iz_2_1009	DJI_0459	Jacobaea borysthenica (DC.) B.Nord. & Greuter	0	n	1	0	Asteraceae Bercht. & J.Presl
Iz_2_1009	DJI_0459	Jasione montana L.	0	n	1	0	Campanulaceae Juss.
Iz_2_1009	DJI_0459	Jurinea cyanoides (L.) Rchb.s.l.	0	n	1	0	Asteraceae Bercht. & J.Presl
Iz_2_1009	DJI_0459	Koeleria glauca (Spreng.) DC.	0	n	1	0	Poaceae Barnhart
Iz_2_1009	DJI_0459	Lactuca serriola L.	0	a	0	0	Asteraceae Bercht. & J.Presl
Iz_2_1009	DJI_0459	Linaria genistifolia (L.) Mill.	0	n	1	0	Plantaginaceae Juss.
Iz_2_1009	DJI_0459	Lomelosia argentea (L.) Greuter & Burdet	0	n	1	0	Caprifoliaceae Juss.
Iz_2_1009	DJI_0459	Melampyrum pratense L.	0	n	1	0	Orobanchaceae Vent.
Iz_2_1009	DJI_0459	Oenothera biennis L.	0	a	0	0	Onagraceae Juss.
Iz_2_1009	DJI_0459	Pentanema britannica (L.) D.Gut.Larr., Santos-Vicente, Anderb., E.Rico & M.M.Mart.Ort.	0	n	1	0	Asteraceae Bercht. & J.Presl
Iz_2_1009	DJI_0459	Peucedanum oreoselinum (L.) Moench	2	n	1	0	Apiaceae Lindl.
Iz_2_1009	DJI_0459	Peucedanum ruthenicum M.Bieb.	0	n	1	0	Apiaceae Lindl.
Iz_2_1009	DJI_0459	Pilosella echioides (Lumn.) F.W.Schultz & Sch.Bip.	0	n	1	0	Asteraceae Bercht. & J.Presl
Iz_2_1009	DJI_0459	Pilosella officinarum F. Schultz et Sch. Bip.	0	n	1	0	Asteraceae Bercht. & J.Presl
Iz_2_1009	DJI_0459	Pilosella sp.	0		NA	0	Asteraceae Bercht. & J.Presl
Iz_2_1009	DJI_0459	Pinus sylvestris L.	0	n	1	1	Pinaceae Spreng. ex F.Rudolphi
Iz_2_1009	DJI_0459	Poa annua L.	0	n	1	0	Poaceae Barnhart
Iz_2_1009	DJI_0459	Poa nemoralis L.	0	n	1	0	Poaceae Barnhart
Iz_2_1009	DJI_0459	Poa sp.	0		NA	0	Poaceae Barnhart
Iz_2_1009	DJI_0459	Polygonatum odoratum (Mill.) Druce	0	n	1	0	Asparagaceae Juss.
Iz_2_1009	DJI_0459	Polygonum arenarium Waldst. & Kit.	0	n	1	0	Polygonaceae Juss.
Iz_2_1009	DJI_0459	Populus nigra L.	0	n	1	1	Salicaceae Mirb.
Iz_2_1009	DJI_0459	Populus tremula L.	0	n	1	1	Salicaceae Mirb.
Iz_2_1009	DJI_0459	Populus Ã canescens (Aiton) Sm.	0	n	1	1	Salicaceae Mirb.
Iz_2_1009	DJI_0459	Potentilla argentea L.	0	n	1	0	Rosaceae Juss.
Iz_2_1009	DJI_0459	Psammophiliella muralis (L.) Ikonn.	0	n	1	0	Caryophyllaceae Juss.
Iz_2_1009	DJI_0459	Psephellus sumensis (Kalen.) Greuter	0	n	1	0	Asteraceae Bercht. & J.Presl
Iz_2_1009	DJI_0459	Pulsatilla pratensis (L.) Mill. s.l.	0	n	1	0	Ranunculaceae Juss.
Iz_2_1009	DJI_0459	Pyrus communis L.	0	a	0	1	Rosaceae Juss.
Iz_2_1009	DJI_0459	Quercus robur L.	0	n	1	1	Fagaceae Dumort.
Iz_2_1009	DJI_0459	Robinia pseudoacacia L.	0	a	0	1	Fabaceae Lindl.
Iz_2_1009	DJI_0459	Rosa sp.	0		NA	1	Rosaceae Juss.
Iz_2_1009	DJI_0459	Rubus idaeus L.	0	n	1	0	Rosaceae Juss.
Iz_2_1009	DJI_0459	Rumex acetosella L.	0	n	1	0	Polygonaceae Juss.
Iz_2_1009	DJI_0459	Rumex thyrsiflorus Fingerh.	0	n	1	0	Polygonaceae Juss.
Iz_2_1009	DJI_0459	Salix alba L.	0	n	1	1	Salicaceae Mirb.
Iz_2_1009	DJI_0459	Sambucus nigra L.	0	n	1	1	Viburnaceae Raf.
Iz_2_1009	DJI_0459	Secale sylvestre Host	0	n	1	0	Poaceae Barnhart
Iz_2_1009	DJI_0459	Sempervivum ruthenicum Schnittsp. & C.B. Lehm.	0	n	1	0	Crassulaceae J.St.-Hil.
Iz_2_1009	DJI_0459	Setaria viridis (L.) P. Beauv.	0	n	1	0	Poaceae Barnhart
Iz_2_1009	DJI_0459	Silene borysthenica (Gruner) Walters	0	n	1	0	Caryophyllaceae Juss.
Iz_2_1009	DJI_0459	Silene latifolia subsp. alba (Mill.) Greuter & Burdet	0	n	1	0	Caryophyllaceae Juss.
Iz_2_1009	DJI_0459	Solidago virgaurea L.	0	n	1	0	Asteraceae Bercht. & J.Presl
Iz_2_1009	DJI_0459	Sorbus aucuparia L.	0	n	1	1	Rosaceae Juss.
Iz_2_1009	DJI_0459	Stachys recta L.	0	n	1	0	Lamiacea Lindl.
Iz_2_1009	DJI_0459	Tanacetum vulgare L.	0	n	1	0	Asteraceae Bercht. & J.Presl
Iz_2_1009	DJI_0459	Thymus pallasianus Heinr.Braun	0	n	1	0	Lamiacea Lindl.
Iz_2_1009	DJI_0459	Tilia cordata Mill.	0	n	1	1	Malvaceae Juss.
Iz_2_1009	DJI_0459	Trifolium alpestre L.	0	n	1	0	Fabaceae Lindl.
Iz_2_1009	DJI_0459	Trifolium arvense L.	0	n	1	0	Fabaceae Lindl.
Iz_2_1009	DJI_0459	Ulmus glabra Huds.	0	n	1	1	Ulmaceae Mirb.
Iz_2_1009	DJI_0459	Verbascum densiflorum Bertol.	0	n	1	0	Scrophulariaceae Juss.
Iz_2_1009	DJI_0459	Veronica austriaca L.	0	n	1	0	Plantaginaceae Juss.
Iz_2_1009	DJI_0459	Veronica incana L.	0	n	1	0	Plantaginaceae Juss.
Iz_2_1009	DJI_0459	Veronica verna L.	0	n	1	0	Plantaginaceae Juss.
Iz_2_1009	DJI_0459	Vincetoxicum hirundinaria Medik	0	n	1	0	Apocynaceae Juss.
Iz_2_1009	DJI_0461	Acer tataricum L.	0	n	1	1	Sapindaceae Juss.
Iz_2_1009	DJI_0461	Achillea millefolium L. s.l.	0	n	1	0	Asteraceae Bercht. & J.Presl
Iz_2_1009	DJI_0461	Allium oleraceum L.	0	n	1	0	Amaryllidaceae J.St.-Hil.
Iz_2_1009	DJI_0461	Ambrosia artemisiifolia L.	0	a	0	0	Asteraceae Bercht. & J.Presl
Iz_2_1009	DJI_0461	Amorpha fruticosa L.	0	a	0	0	Fabaceae Lindl.
Iz_2_1009	DJI_0461	Anthericum ramosum L.	0	n	1	0	Asparagaceae Juss.
Iz_2_1009	DJI_0461	Artemisia marschalliana Spreng.	0	n	1	0	Asteraceae Bercht. & J.Presl
Iz_2_1009	DJI_0461	Asparagus officinalis L.	0	n	1	0	Asparagaceae Juss.
Iz_2_1009	DJI_0461	Astragalus cicer L.	0	n	1	0	Fabaceae Lindl.
Iz_2_1009	DJI_0461	Bassia laniflora (S.G.Gmel.) A.J.Scott	0	n	1	0	Amaranthaceae Juss.
Iz_2_1009	DJI_0461	Berberis vulgaris L.	0	n	1	0	Berberidaceae Juss.
Iz_2_1009	DJI_0461	Betula pendula Roth.	0	n	1	1	Betulaceae Gray
Iz_2_1009	DJI_0461	Betula pendula Roth.	0	n	1	0	Poaceae Barnhart
Iz_2_1009	DJI_0461	Bromus inermis Leyss.	0		NA	NA	
Iz_2_1009	DJI_0461	Bromus squarrosus L.	0	a	0	0	Poaceae Barnhart
Iz_2_1009	DJI_0461	Bromus tectorum L.	0	a	0	0	Poaceae Barnhart
Iz_2_1009	DJI_0461	Calamagrostis epigejos (L.) Roth	30	n	1	0	Poaceae Barnhart
Iz_2_1009	DJI_0461	Carduus acanthoides L.	0	n	1	0	Asteraceae Bercht. & J.Presl
Iz_2_1009	DJI_0461	Carex sp.	0	n	1	0	Cyperaceae Juss.
Iz_2_1009	DJI_0461	Centaurea majorovii Dumbadze	0	n	1	0	Asteraceae Bercht. & J.Presl
Iz_2_1009	DJI_0461	Cf. Solidago virgaurea	0		NA	NA	
Iz_2_1009	DJI_0461	Chamaecytisus ruthenicus (Fisch.ex Wol.) Klaskova	0	n	1	0	Fabaceae Lindl.
Iz_2_1009	DJI_0461	Chelidonium majus L.	0.5	n	1	0	Papaveraceae Juss.
Iz_2_1009	DJI_0461	Chenopodium betaceum Andrz.	0	n	1	0	Amaranthaceae Juss.
Iz_2_1009	DJI_0461	Chenopodium cf. album	0	n	1	0	Amaranthaceae Juss.
Iz_2_1009	DJI_0461	Chenopodium sp.	0		NA	0	Amaranthaceae Juss.
Iz_2_1009	DJI_0461	Chondrilla juncea L.	0	n	1	0	Asteraceae Bercht. & J.Presl
Iz_2_1009	DJI_0461	Cirsium vulgare (Savi) Ten.	0	n	1	0	Asteraceae Bercht. & J.Presl
Iz_2_1009	DJI_0461	Clinopodium acinos (L.) Kuntze	0	n	1	0	Lamiacea Lindl.
Iz_2_1009	DJI_0461	Convallaria majalis L.	0	n	1	0	Asparagaceae Juss.
Iz_2_1009	DJI_0461	Crataegus rhipidophylla Gand.	0	n	1	1	Rosaceae Juss.
Iz_2_1009	DJI_0461	Crepis foetida L.	0	n	1	0	Asteraceae Bercht. & J.Presl
Iz_2_1009	DJI_0461	Dactylis glomerata L.	0	n	1	0	Poaceae Barnhart
Iz_2_1009	DJI_0461	Dianthus campestris M.Bieb.	0	n	1	0	Caryophyllaceae Juss.
Iz_2_1009	DJI_0461	Digitaria sanguinalis (L.) Scop.	0	a	0	0	Poaceae Barnhart
Iz_2_1009	DJI_0461	Elymus repens (L.) Gould	0	n	1	0	Poaceae Barnhart

Iz_2_1009	DJI_0461	Epilobium tetragonum L.	0	n	1	0	Onagraceae Juss.
Iz_2_1009	DJI_0461	Eragrostis minor Host	0	a	0	0	Poaceae Barnhart
Iz_2_1009	DJI_0461	Erigeron canadensis L.	0	a	0	0	Asteraceae Bercht. & J.Presl
Iz_2_1009	DJI_0461	Euphorbia seguieriana Neck.	0	n	1	0	Euphorbiaceae Juss.
Iz_2_1009	DJI_0461	Fallopia dumetorum (L.) Holub	2	n	1	0	Polygonaceae Juss.
Iz_2_1009	DJI_0461	Festuca beckeri (Hack.) Trautv.	0	n	1	0	Poaceae Barnhart
Iz_2_1009	DJI_0461	Fragaria vesca L.	0	n	1	0	Rosaceae Juss.
Iz_2_1009	DJI_0461	Frangula alnus Mill.	0	n	1	1	Rhamnaceae Juss.
Iz_2_1009	DJI_0461	Fraxinus pennsylvanica Marshall	0	a	0	1	Oleaceae Hoffmanns. & Link
Iz_2_1009	DJI_0461	Galeopsis bifida Boenn.	0	n	1	0	Lamiacea Lindl.
Iz_2_1009	DJI_0461	Galium verum L.	0	n	1	0	Rubiaceae Juss.
Iz_2_1009	DJI_0461	Genista tinctoria L.	0	n	1	0	Fabaceae Lindl.
Iz_2_1009	DJI_0461	Geranium robertianum L.	0	n	1	0	Geraniaceae Juss.
Iz_2_1009	DJI_0461	Geranium sanquineum L.	0	n	1	0	Geraniaceae Juss.
Iz_2_1009	DJI_0461	Gypsophila paniculata L.	0	n	1	0	Caryophyllaceae Juss.
Iz_2_1009	DJI_0461	Helichrysum arenarium (L.) Moench	0	n	1	0	Asteraceae Bercht. & J.Presl
Iz_2_1009	DJI_0461	Herniaria polygama J.Gay	0	n	1	0	Caryophyllaceae Juss.
Iz_2_1009	DJI_0461	Hieracium robustum Fr.	0	n	1	0	Asteraceae Bercht. & J.Presl
Iz_2_1009	DJI_0461	Hieracium umbellatum L.	0	n	1	NA	Asteraceae Bercht. & J.Presl
Iz_2_1009	DJI_0461	Hylotelephium maximum (L.) Holub	0	n	1	0	Crassulaceae J.St.-Hil.
Iz_2_1009	DJI_0461	Jacobaea borysthenica (DC.) B.Nord. & Greuter	0	n	1	0	Asteraceae Bercht. & J.Presl
Iz_2_1009	DJI_0461	Jasione montana L.	0	n	1	0	Campanulaceae Juss.
Iz_2_1009	DJI_0461	Jurinea cyanoides (L.) Rchb.s.l.	0	n	1	0	Asteraceae Bercht. & J.Presl
Iz_2_1009	DJI_0461	Koeleria glauca (Spreng.) DC.	0	n	1	0	Poaceae Barnhart
Iz_2_1009	DJI_0461	Lactuca serriola L.	0	a	0	0	Asteraceae Bercht. & J.Presl
Iz_2_1009	DJI_0461	Linaria genistifolia (L.) Mill.	0	n	1	0	Plantaginaceae Juss.
Iz_2_1009	DJI_0461	Lomelosia argentea (L.) Greuter & Burdet	0	n	1	0	Caprifoliaceae Juss.
Iz_2_1009	DJI_0461	Melampyrum pratense L.	0	n	1	0	Orobanchaceae Vent.
Iz_2_1009	DJI_0461	Oenothera biennis L.	0	a	0	0	Onagraceae Juss.
Iz_2_1009	DJI_0461	Pentanema britannica (L.) D.Gut.Larr., Santos-Vicente, Anderb., E.Rico & M.M.Mart.Ort.	0	n	1	0	Asteraceae Bercht. & J.Presl
Iz_2_1009	DJI_0461	Peucedanum oreoselinum (L.) Moench	1	n	1	0	Apiaceae Lindl.
Iz_2_1009	DJI_0461	Peucedanum ruthenicum M.Bieb.	0	n	1	0	Apiaceae Lindl.
Iz_2_1009	DJI_0461	Pilosella echioides (Lumn.) F.W.Schultz & Sch.Bip.	0	n	1	0	Asteraceae Bercht. & J.Presl
Iz_2_1009	DJI_0461	Pilosella officinarum F. Schultz et Sch. Bip.	0	n	1	0	Asteraceae Bercht. & J.Presl
Iz_2_1009	DJI_0461	Pilosella sp.	0		NA	0	Asteraceae Bercht. & J.Presl
Iz_2_1009	DJI_0461	Pinus sylvestris L.	0	n	1	1	Pinaceae Spreng. ex F.Rudolphi
Iz_2_1009	DJI_0461	Poa annua L.	0	n	1	0	Poaceae Barnhart
Iz_2_1009	DJI_0461	Poa nemoralis L.	0	n	1	0	Poaceae Barnhart
Iz_2_1009	DJI_0461	Poa sp.	0		NA	0	Poaceae Barnhart
Iz_2_1009	DJI_0461	Polygonatum odoratum (Mill.) Druce	0	n	1	0	Asparagaceae Juss.
Iz_2_1009	DJI_0461	Polygonum arenarium Waldst. & Kit.	0	n	1	0	Polygonaceae Juss.
Iz_2_1009	DJI_0461	Populus nigra L.	0	n	1	1	Salicaceae Mirb.
Iz_2_1009	DJI_0461	Populus tremula L.	0	n	1	1	Salicaceae Mirb.
Iz_2_1009	DJI_0461	Populus Ã canescens (Aiton) Sm.	0	n	1	1	Salicaceae Mirb.
Iz_2_1009	DJI_0461	Potentilla argentea L.	0	n	1	0	Rosaceae Juss.
Iz_2_1009	DJI_0461	Psammophiliella muralis (L.) Ikonn.	0	n	1	0	Caryophyllaceae Juss.
Iz_2_1009	DJI_0461	Psephellus sumensis (Kalen.) Greuter	0	n	1	0	Asteraceae Bercht. & J.Presl
Iz_2_1009	DJI_0461	Pulsatilla pratensis (L.) Mill. s.l.	0	n	1	0	Ranunculaceae Juss.
Iz_2_1009	DJI_0461	Pyrus communis L.	0	a	0	1	Rosaceae Juss.
Iz_2_1009	DJI_0461	Quercus robur L.	0	n	1	1	Fagaceae Dumort.
Iz_2_1009	DJI_0461	Robinia pseudoacacia L.	0	a	0	1	Fabaceae Lindl.
Iz_2_1009	DJI_0461	Rosa sp.	0		NA	1	Rosaceae Juss.
Iz_2_1009	DJI_0461	Rubus idaeus L.	0	n	1	0	Rosaceae Juss.
Iz_2_1009	DJI_0461	Rumex acetosella L.	0	n	1	0	Polygonaceae Juss.
Iz_2_1009	DJI_0461	Rumex thyrsiflorus Fingerh.	0	n	1	0	Polygonaceae Juss.
Iz_2_1009	DJI_0461	Salix alba L.	0	n	1	1	Salicaceae Mirb.
Iz_2_1009	DJI_0461	Sambucus nigra L.	0	n	1	1	Viburnaceae Raf.
Iz_2_1009	DJI_0461	Secale sylvestre Host	0	n	1	0	Poaceae Barnhart
Iz_2_1009	DJI_0461	Sempervivum ruthenicum Schnittsp. & C.B. Lehm.	0	n	1	0	Crassulaceae J.St.-Hil.
Iz_2_1009	DJI_0461	Setaria viridis (L.) P. Beauv.	0	n	1	0	Poaceae Barnhart
Iz_2_1009	DJI_0461	Silene borysthenica (Gruner) Walters	0	n	1	0	Caryophyllaceae Juss.
Iz_2_1009	DJI_0461	Silene latifolia subsp. alba (Mill.) Greuter & Burdet	0	n	1	0	Caryophyllaceae Juss.
Iz_2_1009	DJI_0461	Solidago virgaurea L.	1	n	1	0	Asteraceae Bercht. & J.Presl
Iz_2_1009	DJI_0461	Sorbus aucuparia L.	0	n	1	1	Rosaceae Juss.
Iz_2_1009	DJI_0461	Stachys recta L.	0	n	1	0	Lamiacea Lindl.
Iz_2_1009	DJI_0461	Tanacetum vulgare L.	0	n	1	0	Asteraceae Bercht. & J.Presl
Iz_2_1009	DJI_0461	Thymus pallasianus Heinr.Braun	0	n	1	0	Lamiacea Lindl.
Iz_2_1009	DJI_0461	Tilia cordata Mill.	0	n	1	1	Malvaceae Juss.
Iz_2_1009	DJI_0461	Trifolium alpestre L.	0	n	1	0	Fabaceae Lindl.
Iz_2_1009	DJI_0461	Trifolium arvense L.	0	n	1	0	Fabaceae Lindl.
Iz_2_1009	DJI_0461	Ulmus glabra Huds.	0	n	1	1	Ulmaceae Mirb.
Iz_2_1009	DJI_0461	Verbascum densiflorum Bertol.	0	n	1	0	Scrophulariaceae Juss.
Iz_2_1009	DJI_0461	Veronica austriaca L.	0	n	1	0	Plantaginaceae Juss.
Iz_2_1009	DJI_0461	Veronica incana L.	0	n	1	0	Plantaginaceae Juss.
Iz_2_1009	DJI_0461	Veronica verna L.	0	n	1	0	Plantaginaceae Juss.
Iz_2_1009	DJI_0461	Vincetoxicum hirundinaria Medik	0	n	1	0	Apocynaceae Juss.
Iz_2_1009	DJI_0462	Acer tataricum L.	0	n	1	1	Sapindaceae Juss.
Iz_2_1009	DJI_0462	Achillea millefolium L. s.l.	0	n	1	0	Asteraceae Bercht. & J.Presl
Iz_2_1009	DJI_0462	Allium oleraceum L.	0	n	1	0	Amaryllidaceae J.St.-Hil.
Iz_2_1009	DJI_0462	Ambrosia artemisiifolia L.	0	a	0	0	Asteraceae Bercht. & J.Presl
Iz_2_1009	DJI_0462	Amorpha fruticosa L.	0	a	0	0	Fabaceae Lindl.
Iz_2_1009	DJI_0462	Anthericum ramosum L.	0	n	1	0	Asparagaceae Juss.
Iz_2_1009	DJI_0462	Artemisia marschalliana Spreng.	0	n	1	0	Asteraceae Bercht. & J.Presl
Iz_2_1009	DJI_0462	Asparagus officinalis L.	0	n	1	0	Asparagaceae Juss.
Iz_2_1009	DJI_0462	Astragalus cicer L.	0	n	1	0	Fabaceae Lindl.
Iz_2_1009	DJI_0462	Bassia laniflora (S.G.Gmel.) A.J.Scott	0	n	1	0	Amaranthaceae Juss.
Iz_2_1009	DJI_0462	Berberis vulgaris L.	0	n	1	0	Berberidaceae Juss.
Iz_2_1009	DJI_0462	Betula pendula Roth.	0	n	1	1	Betulaceae Gray
Iz_2_1009	DJI_0462	Betula pendula Roth.	0	n	1	0	Poaceae Barnhart
Iz_2_1009	DJI_0462	Bromus inermis Leyss.	0		NA	NA	
Iz_2_1009	DJI_0462	Bromus squarrosus L.	0	a	0	0	Poaceae Barnhart
Iz_2_1009	DJI_0462	Bromus tectorum L.	0	a	0	0	Poaceae Barnhart
Iz_2_1009	DJI_0462	Calamagrostis epigejos (L.) Roth	50	n	1	0	Poaceae Barnhart
Iz_2_1009	DJI_0462	Carduus acanthoides L.	0	n	1	0	Asteraceae Bercht. & J.Presl
Iz_2_1009	DJI_0462	Carex sp.	0	n	1	0	Cyperaceae Juss.
Iz_2_1009	DJI_0462	Centaurea majorovii Dumbadze	0	n	1	0	Asteraceae Bercht. & J.Presl
Iz_2_1009	DJI_0462	Cf. Solidago virgaurea	0		NA	NA	
Iz_2_1009	DJI_0462	Chamaecytisus ruthenicus (Fisch.ex Wol.) Klaskova	0	n	1	0	Fabaceae Lindl.

Iz_2_1009	DJI_0462	Chelidonium majus L.	3	n	1	0	Papaveraceae Juss.
Iz_2_1009	DJI_0462	Chenopodium betaceum Andrz.	0	n	1	0	Amaranthaceae Juss.
Iz_2_1009	DJI_0462	Chenopodium cf. album	0	n	1	0	Amaranthaceae Juss.
Iz_2_1009	DJI_0462	Chenopodium sp.	0		NA	0	Amaranthaceae Juss.
Iz_2_1009	DJI_0462	Chondrilla juncea L.	0	n	1	0	Asteraceae Bercht. & J.Presl
Iz_2_1009	DJI_0462	Cirsium vulgare (Savi) Ten.	0	n	1	0	Asteraceae Bercht. & J.Presl
Iz_2_1009	DJI_0462	Clinopodium acinos (L.) Kuntze	0	n	1	0	Lamiacea Lindl.
Iz_2_1009	DJI_0462	Convallaria majalis L.	0	n	1	0	Asparagaceae Juss.
Iz_2_1009	DJI_0462	Crataegus rhipidophylla Gand.	0	n	1	1	Rosaceae Juss.
Iz_2_1009	DJI_0462	Crepis foetida L.	0	n	1	0	Asteraceae Bercht. & J.Presl
Iz_2_1009	DJI_0462	Dactylis glomerata L.	0	n	1	0	Poaceae Barnhart
Iz_2_1009	DJI_0462	Dianthus campestris M.Bieb.	0	n	1	0	Caryophyllaceae Juss.
Iz_2_1009	DJI_0462	Digitaria sanguinalis (L.) Scop.	0	a	0	0	Poaceae Barnhart
Iz_2_1009	DJI_0462	Elymus repens (L.) Gould	0	n	1	0	Poaceae Barnhart
Iz_2_1009	DJI_0462	Epilobium tetragonum L.	0	n	1	0	Onagraceae Juss.
Iz_2_1009	DJI_0462	Eragrostis minor Host	0	a	0	0	Poaceae Barnhart
Iz_2_1009	DJI_0462	Erigeron canadensis L.	0	a	0	0	Asteraceae Bercht. & J.Presl
Iz_2_1009	DJI_0462	Euphorbia seguieriana Neck.	0	n	1	0	Euphorbiaceae Juss.
Iz_2_1009	DJI_0462	Fallopia dumetorum (L.) Holub	0	n	1	0	Polygonaceae Juss.
Iz_2_1009	DJI_0462	Festuca beckeri (Hack.) Trautv.	0	n	1	0	Poaceae Barnhart
Iz_2_1009	DJI_0462	Fragaria vesca L.	0	n	1	0	Rosaceae Juss.
Iz_2_1009	DJI_0462	Frangula alnus Mill.	0	n	1	1	Rhamnaceae Juss.
Iz_2_1009	DJI_0462	Fraxinus pennsylvanica Marshall	0	a	0	1	Oleaceae Hoffmanns. & Link
Iz_2_1009	DJI_0462	Galeopsis bifida Boenn.	0	n	1	0	Lamiacea Lindl.
Iz_2_1009	DJI_0462	Galium verum L.	0	n	1	0	Rubiaceae Juss.
Iz_2_1009	DJI_0462	Genista tinctoria L.	0	n	1	0	Fabaceae Lindl.
Iz_2_1009	DJI_0462	Geranium robertianum L.	4	n	1	0	Geraniaceae Juss.
Iz_2_1009	DJI_0462	Geranium sanquineum L.	0	n	1	0	Geraniaceae Juss.
Iz_2_1009	DJI_0462	Gypsophila paniculata L.	0	n	1	0	Caryophyllaceae Juss.
Iz_2_1009	DJI_0462	Helichrysum arenarium (L.) Moench	0	n	1	0	Asteraceae Bercht. & J.Presl
Iz_2_1009	DJI_0462	Herniaria polygama J.Gay	0	n	1	0	Caryophyllaceae Juss.
Iz_2_1009	DJI_0462	Hieracium robustum Fr.	0	n	1	0	Asteraceae Bercht. & J.Presl
Iz_2_1009	DJI_0462	Hieracium umbellatum L.	0	n	1	NA	Asteraceae Bercht. & J.Presl
Iz_2_1009	DJI_0462	Hylotelephium maximum (L.) Holub	0	n	1	0	Crassulaceae J.St.-Hil.
Iz_2_1009	DJI_0462	Jacobaea borysthenica (DC.) B.Nord. & Greuter	0	n	1	0	Asteraceae Bercht. & J.Presl
Iz_2_1009	DJI_0462	Jasione montana L.	0	n	1	0	Campanulaceae Juss.
Iz_2_1009	DJI_0462	Jurinea cyanoides (L.) Rchb.s.l.	0	n	1	0	Asteraceae Bercht. & J.Presl
Iz_2_1009	DJI_0462	Koeleria glauca (Spreng.) DC.	0	n	1	0	Poaceae Barnhart
Iz_2_1009	DJI_0462	Lactuca serriola L.	0	a	0	0	Asteraceae Bercht. & J.Presl
Iz_2_1009	DJI_0462	Linaria genistifolia (L.) Mill.	0	n	1	0	Plantaginaceae Juss.
Iz_2_1009	DJI_0462	Lomelosia argentea (L.) Greuter & Burdet	0	n	1	0	Caprifoliaceae Juss.
Iz_2_1009	DJI_0462	Melampyrum pratense L.	0	n	1	0	Orobanchaceae Vent.
Iz_2_1009	DJI_0462	Oenothera biennis L.	0	a	0	0	Onagraceae Juss.
Iz_2_1009	DJI_0462	Pentanema britannica (L.) D.Gut.Larr., Santos-Vicente, Anderb., E.Rico & M.M.Mart.Ort.	0	n	1	0	Asteraceae Bercht. & J.Presl
Iz_2_1009	DJI_0462	Peucedanum oreoselinum (L.) Moench	0	n	1	0	Apiaceae Lindl.
Iz_2_1009	DJI_0462	Peucedanum ruthenicum M.Bieb.	0	n	1	0	Apiaceae Lindl.
Iz_2_1009	DJI_0462	Pilosella echioides (Lumn.) F.W.Schultz & Sch.Bip.	0	n	1	0	Asteraceae Bercht. & J.Presl
Iz_2_1009	DJI_0462	Pilosella officinarum F. Schultz et Sch. Bip.	0	n	1	0	Asteraceae Bercht. & J.Presl
Iz_2_1009	DJI_0462	Pilosella sp.	0		NA	0	Asteraceae Bercht. & J.Presl
Iz_2_1009	DJI_0462	Pinus sylvestris L.	0	n	1	1	Pinaceae Spreng. ex F.Rudolphi
Iz_2_1009	DJI_0462	Poa annua L.	0	n	1	0	Poaceae Barnhart
Iz_2_1009	DJI_0462	Poa nemoralis L.	0	n	1	0	Poaceae Barnhart
Iz_2_1009	DJI_0462	Poa sp.	0		NA	0	Poaceae Barnhart
Iz_2_1009	DJI_0462	Polygonatum odoratum (Mill.) Druce	0	n	1	0	Asparagaceae Juss.
Iz_2_1009	DJI_0462	Polygonum arenarium Waldst. & Kit.	0	n	1	0	Polygonaceae Juss.
Iz_2_1009	DJI_0462	Populus nigra L.	0	n	1	1	Salicaceae Mirb.
Iz_2_1009	DJI_0462	Populus tremula L.	0	n	1	1	Salicaceae Mirb.
Iz_2_1009	DJI_0462	Populus Ã canescens (Aiton) Sm.	0	n	1	1	Salicaceae Mirb.
Iz_2_1009	DJI_0462	Potentilla argentea L.	0	n	1	0	Rosaceae Juss.
Iz_2_1009	DJI_0462	Psammophiliella muralis (L.) Ikonn.	0	n	1	0	Caryophyllaceae Juss.
Iz_2_1009	DJI_0462	Psephellus sumensis (Kalen.) Greuter	0	n	1	0	Asteraceae Bercht. & J.Presl
Iz_2_1009	DJI_0462	Pulsatilla pratensis (L.) Mill. s.l.	0	n	1	0	Ranunculaceae Juss.
Iz_2_1009	DJI_0462	Pyrus communis L.	0	a	0	1	Rosaceae Juss.
Iz_2_1009	DJI_0462	Quercus robur L.	0	n	1	1	Fagaceae Dumort.
Iz_2_1009	DJI_0462	Robinia pseudoacacia L.	0	a	0	1	Fabaceae Lindl.
Iz_2_1009	DJI_0462	Rosa sp.	0		NA	1	Rosaceae Juss.
Iz_2_1009	DJI_0462	Rubus idaeus L.	0	n	1	0	Rosaceae Juss.
Iz_2_1009	DJI_0462	Rumex acetosella L.	0	n	1	0	Polygonaceae Juss.
Iz_2_1009	DJI_0462	Rumex thyrsiflorus Fingerh.	0	n	1	0	Polygonaceae Juss.
Iz_2_1009	DJI_0462	Salix alba L.	0	n	1	1	Salicaceae Mirb.
Iz_2_1009	DJI_0462	Sambucus nigra L.	0	n	1	1	Viburnaceae Raf.
Iz_2_1009	DJI_0462	Secale sylvestre Host	0	n	1	0	Poaceae Barnhart
Iz_2_1009	DJI_0462	Sempervivum ruthenicum Schnittsp. & C.B. Lehm.	0	n	1	0	Crassulaceae J.St.-Hil.
Iz_2_1009	DJI_0462	Setaria viridis (L.) P. Beauv.	0	n	1	0	Poaceae Barnhart
Iz_2_1009	DJI_0462	Silene borysthenica (Gruner) Walters	0	n	1	0	Caryophyllaceae Juss.
Iz_2_1009	DJI_0462	Silene latifolia subsp. alba (Mill.) Greuter & Burdet	0	n	1	0	Caryophyllaceae Juss.
Iz_2_1009	DJI_0462	Solidago virgaurea L.	0	n	1	0	Asteraceae Bercht. & J.Presl
Iz_2_1009	DJI_0462	Sorbus aucuparia L.	0	n	1	1	Rosaceae Juss.
Iz_2_1009	DJI_0462	Stachys recta L.	0	n	1	0	Lamiacea Lindl.
Iz_2_1009	DJI_0462	Tanacetum vulgare L.	0	n	1	0	Asteraceae Bercht. & J.Presl
Iz_2_1009	DJI_0462	Thymus pallasianus Heinr.Braun	0	n	1	0	Lamiacea Lindl.
Iz_2_1009	DJI_0462	Tilia cordata Mill.	0	n	1	1	Malvaceae Juss.
Iz_2_1009	DJI_0462	Trifolium alpestre L.	0	n	1	0	Fabaceae Lindl.
Iz_2_1009	DJI_0462	Trifolium arvense L.	0	n	1	0	Fabaceae Lindl.
Iz_2_1009	DJI_0462	Ulmus glabra Huds.	0	n	1	1	Ulmaceae Mirb.
Iz_2_1009	DJI_0462	Verbascum densiflorum Bertol.	0	n	1	0	Scrophulariaceae Juss.
Iz_2_1009	DJI_0462	Veronica austriaca L.	0	n	1	0	Plantaginaceae Juss.
Iz_2_1009	DJI_0462	Veronica incana L.	0	n	1	0	Plantaginaceae Juss.
Iz_2_1009	DJI_0462	Veronica verna L.	0	n	1	0	Plantaginaceae Juss.
Iz_2_1009	DJI_0462	Vincetoxicum hirundinaria Medik	0	n	1	0	Apocynaceae Juss.
Iz_2_1009	DJI_0463	Acer tataricum L.	0	n	1	1	Sapindaceae Juss.
Iz_2_1009	DJI_0463	Achillea millefolium L. s.l.	0	n	1	0	Asteraceae Bercht. & J.Presl
Iz_2_1009	DJI_0463	Allium oleraceum L.	0	n	1	0	Amaryllidaceae J.St.-Hil.
Iz_2_1009	DJI_0463	Ambrosia artemisiifolia L.	0	a	0	0	Asteraceae Bercht. & J.Presl
Iz_2_1009	DJI_0463	Amorpha fruticosa L.	0	a	0	0	Fabaceae Lindl.
Iz_2_1009	DJI_0463	Anthericum ramosum L.	0	n	1	0	Asparagaceae Juss.
Iz_2_1009	DJI_0463	Artemisia marschalliana Spreng.	0	n	1	0	Asteraceae Bercht. & J.Presl
Iz_2_1009	DJI_0463	Asparagus officinalis L.	0	n	1	0	Asparagaceae Juss.

Iz_2_1009	DJI_0463	Astragalus cicer L.	0	n	1	0	Fabaceae Lindl.
Iz_2_1009	DJI_0463	Bassia laniflora (S.G.Gmel.) A.J.Scott	0	n	1	0	Amaranthaceae Juss.
Iz_2_1009	DJI_0463	Berberis vulgaris L.	0	n	1	0	Berberidaceae Juss.
Iz_2_1009	DJI_0463	Betula pendula Roth.	0	n	1	1	Betulaceae Gray
Iz_2_1009	DJI_0463	Betula pendula Roth.	0	n	1	0	Poaceae Barnhart
Iz_2_1009	DJI_0463	Bromus inermis Leyss.	0		NA	NA	
Iz_2_1009	DJI_0463	Bromus squarrosus L.	0	a	0	0	Poaceae Barnhart
Iz_2_1009	DJI_0463	Bromus tectorum L.	0	a	0	0	Poaceae Barnhart
Iz_2_1009	DJI_0463	Calamagrostis epigejos (L.) Roth	60	n	1	0	Poaceae Barnhart
Iz_2_1009	DJI_0463	Carduus acanthoides L.	0	n	1	0	Asteraceae Bercht. & J.Presl
Iz_2_1009	DJI_0463	Carex sp.	0	n	1	0	Cyperaceae Juss.
Iz_2_1009	DJI_0463	Centaurea majorovii Dumbadze	0	n	1	0	Asteraceae Bercht. & J.Presl
Iz_2_1009	DJI_0463	Cf. Solidago virgaurea	0		NA	NA	
Iz_2_1009	DJI_0463	Chamaecytisus ruthenicus (Fisch.ex Wol.) Klaskova	0	n	1	0	Fabaceae Lindl.
Iz_2_1009	DJI_0463	Chelidonium majus L.	3	n	1	0	Papaveraceae Juss.
Iz_2_1009	DJI_0463	Chenopodium betaceum Andrz.	0	n	1	0	Amaranthaceae Juss.
Iz_2_1009	DJI_0463	Chenopodium cf. album	0	n	1	0	Amaranthaceae Juss.
Iz_2_1009	DJI_0463	Chenopodium sp.	0		NA	0	Amaranthaceae Juss.
Iz_2_1009	DJI_0463	Chondrilla juncea L.	0	n	1	0	Asteraceae Bercht. & J.Presl
Iz_2_1009	DJI_0463	Cirsium vulgare (Savi) Ten.	0	n	1	0	Asteraceae Bercht. & J.Presl
Iz_2_1009	DJI_0463	Clinopodium acinos (L.) Kuntze	0	n	1	0	Lamiacea Lindl.
Iz_2_1009	DJI_0463	Convallaria majalis L.	0	n	1	0	Asparagaceae Juss.
Iz_2_1009	DJI_0463	Crataegus rhipidophylla Gand.	0	n	1	1	Rosaceae Juss.
Iz_2_1009	DJI_0463	Crepis foetida L.	0	n	1	0	Asteraceae Bercht. & J.Presl
Iz_2_1009	DJI_0463	Dactylis glomerata L.	0	n	1	0	Poaceae Barnhart
Iz_2_1009	DJI_0463	Dianthus campestris M.Bieb.	0	n	1	0	Caryophyllaceae Juss.
Iz_2_1009	DJI_0463	Digitaria sanguinalis (L.) Scop.	0	a	0	0	Poaceae Barnhart
Iz_2_1009	DJI_0463	Elymus repens (L.) Gould	0	n	1	0	Poaceae Barnhart
Iz_2_1009	DJI_0463	Epilobium tetragonum L.	0	n	1	0	Onagraceae Juss.
Iz_2_1009	DJI_0463	Eragrostis minor Host	0	a	0	0	Poaceae Barnhart
Iz_2_1009	DJI_0463	Erigeron canadensis L.	0	a	0	0	Asteraceae Bercht. & J.Presl
Iz_2_1009	DJI_0463	Euphorbia seguieriana Neck.	0	n	1	0	Euphorbiaceae Juss.
Iz_2_1009	DJI_0463	Fallopia dumetorum (L.) Holub	0	n	1	0	Polygonaceae Juss.
Iz_2_1009	DJI_0463	Festuca beckeri (Hack.) Trautv.	0	n	1	0	Poaceae Barnhart
Iz_2_1009	DJI_0463	Fragaria vesca L.	0	n	1	0	Rosaceae Juss.
Iz_2_1009	DJI_0463	Frangula alnus Mill.	0	n	1	1	Rhamnaceae Juss.
Iz_2_1009	DJI_0463	Fraxinus pennsylvanica Marshall	0	a	0	1	Oleaceae Hoffmanns. & Link
Iz_2_1009	DJI_0463	Galeopsis bifida Boenn.	0	n	1	0	Lamiacea Lindl.
Iz_2_1009	DJI_0463	Galium verum L.	0	n	1	0	Rubiaceae Juss.
Iz_2_1009	DJI_0463	Genista tinctoria L.	0	n	1	0	Fabaceae Lindl.
Iz_2_1009	DJI_0463	Geranium robertianum L.	5	n	1	0	Geraniaceae Juss.
Iz_2_1009	DJI_0463	Geranium sanquineum L.	0	n	1	0	Geraniaceae Juss.
Iz_2_1009	DJI_0463	Gypsophila paniculata L.	0	n	1	0	Caryophyllaceae Juss.
Iz_2_1009	DJI_0463	Helichrysum arenarium (L.) Moench	0	n	1	0	Asteraceae Bercht. & J.Presl
Iz_2_1009	DJI_0463	Herniaria polygama J.Gay	0	n	1	0	Caryophyllaceae Juss.
Iz_2_1009	DJI_0463	Hieracium robustum Fr.	0	n	1	0	Asteraceae Bercht. & J.Presl
Iz_2_1009	DJI_0463	Hieracium umbellatum L.	0	n	1	NA	Asteraceae Bercht. & J.Presl
Iz_2_1009	DJI_0463	Hylotelephium maximum (L.) Holub	0	n	1	0	Crassulaceae J.St.-Hil.
Iz_2_1009	DJI_0463	Jacobaea borysthenica (DC.) B.Nord. & Greuter	0	n	1	0	Asteraceae Bercht. & J.Presl
Iz_2_1009	DJI_0463	Jasione montana L.	0	n	1	0	Campanulaceae Juss.
Iz_2_1009	DJI_0463	Jurinea cyanoides (L.) Rchb.s.l.	0	n	1	0	Asteraceae Bercht. & J.Presl
Iz_2_1009	DJI_0463	Koeleria glauca (Spreng.) DC.	0	n	1	0	Poaceae Barnhart
Iz_2_1009	DJI_0463	Lactuca serriola L.	0	a	0	0	Asteraceae Bercht. & J.Presl
Iz_2_1009	DJI_0463	Linaria genistifolia (L.) Mill.	0	n	1	0	Plantaginaceae Juss.
Iz_2_1009	DJI_0463	Lomelosia argentea (L.) Greuter & Burdet	0	n	1	0	Caprifoliaceae Juss.
Iz_2_1009	DJI_0463	Melampyrum pratense L.	0	n	1	0	Orobanchaceae Vent.
Iz_2_1009	DJI_0463	Oenothera biennis L.	0	a	0	0	Onagraceae Juss.
Iz_2_1009	DJI_0463	Pentanema britannica (L.) D.Gut.Larr., Santos-Vicente, Anderb., E.Rico & M.M.Mart.Ort.	0	n	1	0	Asteraceae Bercht. & J.Presl
Iz_2_1009	DJI_0463	Peucedanum oreoselinum (L.) Moench	0	n	1	0	Apiaceae Lindl.
Iz_2_1009	DJI_0463	Peucedanum ruthenicum M.Bieb.	0	n	1	0	Apiaceae Lindl.
Iz_2_1009	DJI_0463	Pilosella echioides (Lumn.) F.W.Schultz & Sch.Bip.	0	n	1	0	Asteraceae Bercht. & J.Presl
Iz_2_1009	DJI_0463	Pilosella officinarum F. Schultz et Sch. Bip.	0	n	1	0	Asteraceae Bercht. & J.Presl
Iz_2_1009	DJI_0463	Pilosella sp.	0		NA	0	Asteraceae Bercht. & J.Presl
Iz_2_1009	DJI_0463	Pinus sylvestris L.	0	n	1	1	Pinaceae Spreng. ex F.Rudolphi
Iz_2_1009	DJI_0463	Poa annua L.	0	n	1	0	Poaceae Barnhart
Iz_2_1009	DJI_0463	Poa nemoralis L.	0	n	1	0	Poaceae Barnhart
Iz_2_1009	DJI_0463	Poa sp.	0		NA	0	Poaceae Barnhart
Iz_2_1009	DJI_0463	Polygonatum odoratum (Mill.) Druce	0	n	1	0	Asparagaceae Juss.
Iz_2_1009	DJI_0463	Polygonum arenarium Waldst. & Kit.	0	n	1	0	Polygonaceae Juss.
Iz_2_1009	DJI_0463	Populus nigra L.	0	n	1	1	Salicaceae Mirb.
Iz_2_1009	DJI_0463	Populus tremula L.	0	n	1	1	Salicaceae Mirb.
Iz_2_1009	DJI_0463	Populus Ã canescens (Aiton) Sm.	0	n	1	1	Salicaceae Mirb.
Iz_2_1009	DJI_0463	Potentilla argentea L.	0	n	1	0	Rosaceae Juss.
Iz_2_1009	DJI_0463	Psammophiliella muralis (L.) Ikonn.	0	n	1	0	Caryophyllaceae Juss.
Iz_2_1009	DJI_0463	Psephellus sumensis (Kalen.) Greuter	0	n	1	0	Asteraceae Bercht. & J.Presl
Iz_2_1009	DJI_0463	Pulsatilla pratensis (L.) Mill. s.l.	0	n	1	0	Ranunculaceae Juss.
Iz_2_1009	DJI_0463	Pyrus communis L.	0	a	0	1	Rosaceae Juss.
Iz_2_1009	DJI_0463	Quercus robur L.	0	n	1	1	Fagaceae Dumort.
Iz_2_1009	DJI_0463	Robinia pseudoacacia L.	0	a	0	1	Fabaceae Lindl.
Iz_2_1009	DJI_0463	Rosa sp.	0		NA	1	Rosaceae Juss.
Iz_2_1009	DJI_0463	Rubus idaeus L.	0	n	1	0	Rosaceae Juss.
Iz_2_1009	DJI_0463	Rumex acetosella L.	0	n	1	0	Polygonaceae Juss.
Iz_2_1009	DJI_0463	Rumex thyrsiflorus Fingerh.	0	n	1	0	Polygonaceae Juss.
Iz_2_1009	DJI_0463	Salix alba L.	0	n	1	1	Salicaceae Mirb.
Iz_2_1009	DJI_0463	Sambucus nigra L.	0	n	1	1	Viburnaceae Raf.
Iz_2_1009	DJI_0463	Secale sylvestre Host	0	n	1	0	Poaceae Barnhart
Iz_2_1009	DJI_0463	Sempervivum ruthenicum Schnittsp. & C.B. Lehm.	0	n	1	0	Crassulaceae J.St.-Hil.
Iz_2_1009	DJI_0463	Setaria viridis (L.) P. Beauv.	0	n	1	0	Poaceae Barnhart
Iz_2_1009	DJI_0463	Silene borysthenica (Gruner) Walters	0	n	1	0	Caryophyllaceae Juss.
Iz_2_1009	DJI_0463	Silene latifolia subsp. alba (Mill.) Greuter & Burdet	0	n	1	0	Caryophyllaceae Juss.
Iz_2_1009	DJI_0463	Solidago virgaurea L.	0	n	1	0	Asteraceae Bercht. & J.Presl
Iz_2_1009	DJI_0463	Sorbus aucuparia L.	0	n	1	1	Rosaceae Juss.
Iz_2_1009	DJI_0463	Stachys recta L.	0	n	1	0	Lamiacea Lindl.
Iz_2_1009	DJI_0463	Tanacetum vulgare L.	0	n	1	0	Asteraceae Bercht. & J.Presl
Iz_2_1009	DJI_0463	Thymus pallasianus Heinr.Braun	0	n	1	0	Lamiacea Lindl.
Iz_2_1009	DJI_0463	Tilia cordata Mill.	0	n	1	1	Malvaceae Juss.
Iz_2_1009	DJI_0463	Trifolium alpestre L.	0	n	1	0	Fabaceae Lindl.
Iz_2_1009	DJI_0463	Trifolium arvense L.	0	n	1	0	Fabaceae Lindl.

Iz_2_1009	DJI_0463	Ulmus glabra Huds.	0	n	1	1	Ulmaceae Mirb.
Iz_2_1009	DJI_0463	Verbascum densiflorum Bertol.	0	n	1	0	Scrophulariaceae Juss.
Iz_2_1009	DJI_0463	Veronica austriaca L.	0	n	1	0	Plantaginaceae Juss.
Iz_2_1009	DJI_0463	Veronica incana L.	0	n	1	0	Plantaginaceae Juss.
Iz_2_1009	DJI_0463	Veronica verna L.	0	n	1	0	Plantaginaceae Juss.
Iz_2_1009	DJI_0463	Vincetoxicum hirundinaria Medik	0	n	1	0	Apocynaceae Juss.
Iz_2_1009	DJI_0469	Acer tataricum L.	0	n	1	1	Sapindaceae Juss.
Iz_2_1009	DJI_0469	Achillea millefolium L. s.l.	0	n	1	0	Asteraceae Bercht. & J.Presl
Iz_2_1009	DJI_0469	Allium oleraceum L.	0	n	1	0	Amaryllidaceae J.St.-Hil.
Iz_2_1009	DJI_0469	Ambrosia artemisiifolia L.	0	a	0	0	Asteraceae Bercht. & J.Presl
Iz_2_1009	DJI_0469	Amorpha fruticosa L.	0	a	0	0	Fabaceae Lindl.
Iz_2_1009	DJI_0469	Anthericum ramosum L.	0	n	1	0	Asparagaceae Juss.
Iz_2_1009	DJI_0469	Artemisia marschalliana Spreng.	0	n	1	0	Asteraceae Bercht. & J.Presl
Iz_2_1009	DJI_0469	Asparagus officinalis L.	0	n	1	0	Asparagaceae Juss.
Iz_2_1009	DJI_0469	Astragalus cicer L.	0	n	1	0	Fabaceae Lindl.
Iz_2_1009	DJI_0469	Bassia laniflora (S.G.Gmel.) A.J.Scott	0	n	1	0	Amaranthaceae Juss.
Iz_2_1009	DJI_0469	Berberis vulgaris L.	0	n	1	0	Berberidaceae Juss.
Iz_2_1009	DJI_0469	Betula pendula Roth.	0	n	1	1	Betulaceae Gray
Iz_2_1009	DJI_0469	Betula pendula Roth.	0	n	1	0	Poaceae Barnhart
Iz_2_1009	DJI_0469	Bromus inermis Leyss.	0		NA	NA	
Iz_2_1009	DJI_0469	Bromus squarrosus L.	0	a	0	0	Poaceae Barnhart
Iz_2_1009	DJI_0469	Bromus tectorum L.	0	a	0	0	Poaceae Barnhart
Iz_2_1009	DJI_0469	Calamagrostis epigejos (L.) Roth	40	n	1	0	Poaceae Barnhart
Iz_2_1009	DJI_0469	Carduus acanthoides L.	0	n	1	0	Asteraceae Bercht. & J.Presl
Iz_2_1009	DJI_0469	Carex sp.	0	n	1	0	Cyperaceae Juss.
Iz_2_1009	DJI_0469	Centaurea majorovii Dumbadze	0	n	1	0	Asteraceae Bercht. & J.Presl
Iz_2_1009	DJI_0469	Cf. Solidago virgaurea	0		NA	NA	
Iz_2_1009	DJI_0469	Chamaecytisus ruthenicus (Fisch.ex Wol.) Klaskova	0	n	1	0	Fabaceae Lindl.
Iz_2_1009	DJI_0469	Chelidonium majus L.	0.5	n	1	0	Papaveraceae Juss.
Iz_2_1009	DJI_0469	Chenopodium betaceum Andrz.	0	n	1	0	Amaranthaceae Juss.
Iz_2_1009	DJI_0469	Chenopodium cf. album	0	n	1	0	Amaranthaceae Juss.
Iz_2_1009	DJI_0469	Chenopodium sp.	0		NA	0	Amaranthaceae Juss.
Iz_2_1009	DJI_0469	Chondrilla juncea L.	0	n	1	0	Asteraceae Bercht. & J.Presl
Iz_2_1009	DJI_0469	Cirsium vulgare (Savi) Ten.	0	n	1	0	Asteraceae Bercht. & J.Presl
Iz_2_1009	DJI_0469	Clinopodium acinos (L.) Kuntze	0	n	1	0	Lamiacea Lindl.
Iz_2_1009	DJI_0469	Convallaria majalis L.	0	n	1	0	Asparagaceae Juss.
Iz_2_1009	DJI_0469	Crataegus rhipidophylla Gand.	0	n	1	1	Rosaceae Juss.
Iz_2_1009	DJI_0469	Crepis foetida L.	0	n	1	0	Asteraceae Bercht. & J.Presl
Iz_2_1009	DJI_0469	Dactylis glomerata L.	0	n	1	0	Poaceae Barnhart
Iz_2_1009	DJI_0469	Dianthus campestris M.Bieb.	0	n	1	0	Caryophyllaceae Juss.
Iz_2_1009	DJI_0469	Digitaria sanguinalis (L.) Scop.	0	a	0	0	Poaceae Barnhart
Iz_2_1009	DJI_0469	Elymus repens (L.) Gould	0	n	1	0	Poaceae Barnhart
Iz_2_1009	DJI_0469	Epilobium tetragonum L.	0	n	1	0	Onagraceae Juss.
Iz_2_1009	DJI_0469	Eragrostis minor Host	0	a	0	0	Poaceae Barnhart
Iz_2_1009	DJI_0469	Erigeron canadensis L.	0	a	0	0	Asteraceae Bercht. & J.Presl
Iz_2_1009	DJI_0469	Euphorbia seguieriana Neck.	0	n	1	0	Euphorbiaceae Juss.
Iz_2_1009	DJI_0469	Fallopia dumetorum (L.) Holub	0	n	1	0	Polygonaceae Juss.
Iz_2_1009	DJI_0469	Festuca beckeri (Hack.) Trautv.	0	n	1	0	Poaceae Barnhart
Iz_2_1009	DJI_0469	Fragaria vesca L.	0	n	1	0	Rosaceae Juss.
Iz_2_1009	DJI_0469	Frangula alnus Mill.	0	n	1	1	Rhamnaceae Juss.
Iz_2_1009	DJI_0469	Fraxinus pennsylvanica Marshall	0	a	0	1	Oleaceae Hoffmanns. & Link
Iz_2_1009	DJI_0469	Galeopsis bifida Boenn.	0	n	1	0	Lamiacea Lindl.
Iz_2_1009	DJI_0469	Galium verum L.	0	n	1	0	Rubiaceae Juss.
Iz_2_1009	DJI_0469	Genista tinctoria L.	0	n	1	0	Fabaceae Lindl.
Iz_2_1009	DJI_0469	Geranium robertianum L.	1	n	1	0	Geraniaceae Juss.
Iz_2_1009	DJI_0469	Geranium sanquineum L.	0	n	1	0	Geraniaceae Juss.
Iz_2_1009	DJI_0469	Gypsophila paniculata L.	0	n	1	0	Caryophyllaceae Juss.
Iz_2_1009	DJI_0469	Helichrysum arenarium (L.) Moench	0	n	1	0	Asteraceae Bercht. & J.Presl
Iz_2_1009	DJI_0469	Herniaria polygama J.Gay	0	n	1	0	Caryophyllaceae Juss.
Iz_2_1009	DJI_0469	Hieracium robustum Fr.	0	n	1	0	Asteraceae Bercht. & J.Presl
Iz_2_1009	DJI_0469	Hieracium umbellatum L.	0	n	1	NA	Asteraceae Bercht. & J.Presl
Iz_2_1009	DJI_0469	Hylotelephium maximum (L.) Holub	0	n	1	0	Crassulaceae J.St.-Hil.
Iz_2_1009	DJI_0469	Jacobaea borysthenica (DC.) B.Nord. & Greuter	0	n	1	0	Asteraceae Bercht. & J.Presl
Iz_2_1009	DJI_0469	Jasione montana L.	0	n	1	0	Campanulaceae Juss.
Iz_2_1009	DJI_0469	Jurinea cyanoides (L.) Rchb.s.l.	0	n	1	0	Asteraceae Bercht. & J.Presl
Iz_2_1009	DJI_0469	Koeleria glauca (Spreng.) DC.	0	n	1	0	Poaceae Barnhart
Iz_2_1009	DJI_0469	Lactuca serriola L.	0	a	0	0	Asteraceae Bercht. & J.Presl
Iz_2_1009	DJI_0469	Linaria genistifolia (L.) Mill.	0	n	1	0	Plantaginaceae Juss.
Iz_2_1009	DJI_0469	Lomelosia argentea (L.) Greuter & Burdet	0	n	1	0	Caprifoliaceae Juss.
Iz_2_1009	DJI_0469	Melampyrum pratense L.	0	n	1	0	Orobanchaceae Vent.
Iz_2_1009	DJI_0469	Oenothera biennis L.	0	a	0	0	Onagraceae Juss.
Iz_2_1009	DJI_0469	Pentanema britannica (L.) D.Gut.Larr., Santos-Vicente, Anderb., E.Rico & M.M.Mart.Ort.	0	n	1	0	Asteraceae Bercht. & J.Presl
Iz_2_1009	DJI_0469	Peucedanum oreoselinum (L.) Moench	0	n	1	0	Apiaceae Lindl.
Iz_2_1009	DJI_0469	Peucedanum ruthenicum M.Bieb.	0	n	1	0	Apiaceae Lindl.
Iz_2_1009	DJI_0469	Pilosella echioides (Lumn.) F.W.Schultz & Sch.Bip.	0	n	1	0	Asteraceae Bercht. & J.Presl
Iz_2_1009	DJI_0469	Pilosella officinarum F. Schultz et Sch. Bip.	0	n	1	0	Asteraceae Bercht. & J.Presl
Iz_2_1009	DJI_0469	Pilosella sp.	0		NA	0	Asteraceae Bercht. & J.Presl
Iz_2_1009	DJI_0469	Pinus sylvestris L.	0	n	1	1	Pinaceae Spreng. ex F.Rudolphi
Iz_2_1009	DJI_0469	Poa annua L.	0	n	1	0	Poaceae Barnhart
Iz_2_1009	DJI_0469	Poa nemoralis L.	0	n	1	0	Poaceae Barnhart
Iz_2_1009	DJI_0469	Poa sp.	0		NA	0	Poaceae Barnhart
Iz_2_1009	DJI_0469	Polygonatum odoratum (Mill.) Druce	0	n	1	0	Asparagaceae Juss.
Iz_2_1009	DJI_0469	Polygonum arenarium Waldst. & Kit.	0	n	1	0	Polygonaceae Juss.
Iz_2_1009	DJI_0469	Populus nigra L.	0	n	1	1	Salicaceae Mirb.
Iz_2_1009	DJI_0469	Populus tremula L.	0	n	1	1	Salicaceae Mirb.
Iz_2_1009	DJI_0469	Populus Ã canescens (Aiton) Sm.	0	n	1	1	Salicaceae Mirb.
Iz_2_1009	DJI_0469	Potentilla argentea L.	0	n	1	0	Rosaceae Juss.
Iz_2_1009	DJI_0469	Psammophiliella muralis (L.) Ikonn.	0	n	1	0	Caryophyllaceae Juss.
Iz_2_1009	DJI_0469	Psephellus sumensis (Kalen.) Greuter	0	n	1	0	Asteraceae Bercht. & J.Presl
Iz_2_1009	DJI_0469	Pulsatilla pratensis (L.) Mill. s.l.	0	n	1	0	Ranunculaceae Juss.
Iz_2_1009	DJI_0469	Pyrus communis L.	0	a	0	1	Rosaceae Juss.
Iz_2_1009	DJI_0469	Quercus robur L.	0	n	1	1	Fagaceae Dumort.
Iz_2_1009	DJI_0469	Robinia pseudoacacia L.	0	a	0	1	Fabaceae Lindl.
Iz_2_1009	DJI_0469	Rosa sp.	0		NA	1	Rosaceae Juss.
Iz_2_1009	DJI_0469	Rubus idaeus L.	0	n	1	0	Rosaceae Juss.
Iz_2_1009	DJI_0469	Rumex acetosella L.	0	n	1	0	Polygonaceae Juss.
Iz_2_1009	DJI_0469	Rumex thyrsiflorus Fingerh.	0	n	1	0	Polygonaceae Juss.
Iz_2_1009	DJI_0469	Salix alba L.	0	n	1	1	Salicaceae Mirb.

Iz_2_1009	DJI_0469	Sambucus nigra L.	0	n	1	1	Viburnaceae Raf.
Iz_2_1009	DJI_0469	Secale sylvestre Host	0	n	1	0	Poaceae Barnhart
Iz_2_1009	DJI_0469	Sempervivum ruthenicum Schnittsp. & C.B. Lehm.	0	n	1	0	Crassulaceae J.St.-Hil.
Iz_2_1009	DJI_0469	Setaria viridis (L.) P. Beauv.	0	n	1	0	Poaceae Barnhart
Iz_2_1009	DJI_0469	Silene borysthenica (Gruner) Walters	0	n	1	0	Caryophyllaceae Juss.
Iz_2_1009	DJI_0469	Silene latifolia subsp. alba (Mill.) Greuter & Burdet	0	n	1	0	Caryophyllaceae Juss.
Iz_2_1009	DJI_0469	Solidago virgaurea L.	0	n	1	0	Asteraceae Bercht. & J.Presl
Iz_2_1009	DJI_0469	Sorbus aucuparia L.	0	n	1	1	Rosaceae Juss.
Iz_2_1009	DJI_0469	Stachys recta L.	0	n	1	0	Lamiacea Lindl.
Iz_2_1009	DJI_0469	Tanacetum vulgare L.	0	n	1	0	Asteraceae Bercht. & J.Presl
Iz_2_1009	DJI_0469	Thymus pallasianus Heinr.Braun	0	n	1	0	Lamiacea Lindl.
Iz_2_1009	DJI_0469	Tilia cordata Mill.	0	n	1	1	Malvaceae Juss.
Iz_2_1009	DJI_0469	Trifolium alpestre L.	0	n	1	0	Fabaceae Lindl.
Iz_2_1009	DJI_0469	Trifolium arvense L.	0	n	1	0	Fabaceae Lindl.
Iz_2_1009	DJI_0469	Ulmus glabra Huds.	0	n	1	1	Ulmaceae Mirb.
Iz_2_1009	DJI_0469	Verbascum densiflorum Bertol.	0	n	1	0	Scrophulariaceae Juss.
Iz_2_1009	DJI_0469	Veronica austriaca L.	0	n	1	0	Plantaginaceae Juss.
Iz_2_1009	DJI_0469	Veronica incana L.	0	n	1	0	Plantaginaceae Juss.
Iz_2_1009	DJI_0469	Veronica verna L.	0	n	1	0	Plantaginaceae Juss.
Iz_2_1009	DJI_0469	Vincetoxicum hirundinaria Medik	0	n	1	0	Apocynaceae Juss.
Iz_2_1009	DJI_0471	Acer tataricum L.	0	n	1	1	Sapindaceae Juss.
Iz_2_1009	DJI_0471	Achillea millefolium L. s.l.	0	n	1	0	Asteraceae Bercht. & J.Presl
Iz_2_1009	DJI_0471	Allium oleraceum L.	0	n	1	0	Amaryllidaceae J.St.-Hil.
Iz_2_1009	DJI_0471	Ambrosia artemisiifolia L.	0	a	0	0	Asteraceae Bercht. & J.Presl
Iz_2_1009	DJI_0471	Amorpha fruticosa L.	0	a	0	0	Fabaceae Lindl.
Iz_2_1009	DJI_0471	Anthericum ramosum L.	0	n	1	0	Asparagaceae Juss.
Iz_2_1009	DJI_0471	Artemisia marschalliana Spreng.	0	n	1	0	Asteraceae Bercht. & J.Presl
Iz_2_1009	DJI_0471	Asparagus officinalis L.	0	n	1	0	Asparagaceae Juss.
Iz_2_1009	DJI_0471	Astragalus cicer L.	0	n	1	0	Fabaceae Lindl.
Iz_2_1009	DJI_0471	Bassia laniflora (S.G.Gmel.) A.J.Scott	0	n	1	0	Amaranthaceae Juss.
Iz_2_1009	DJI_0471	Berberis vulgaris L.	0	n	1	0	Berberidaceae Juss.
Iz_2_1009	DJI_0471	Betula pendula Roth.	0	n	1	1	Betulaceae Gray
Iz_2_1009	DJI_0471	Betula pendula Roth.	0	n	1	0	Poaceae Barnhart
Iz_2_1009	DJI_0471	Bromus inermis Leyss.	0		NA	NA	
Iz_2_1009	DJI_0471	Bromus squarrosus L.	0	a	0	0	Poaceae Barnhart
Iz_2_1009	DJI_0471	Bromus tectorum L.	0	a	0	0	Poaceae Barnhart
Iz_2_1009	DJI_0471	Calamagrostis epigejos (L.) Roth	50	n	1	0	Poaceae Barnhart
Iz_2_1009	DJI_0471	Carduus acanthoides L.	0	n	1	0	Asteraceae Bercht. & J.Presl
Iz_2_1009	DJI_0471	Carex sp.	0	n	1	0	Cyperaceae Juss.
Iz_2_1009	DJI_0471	Centaurea majorovii Dumbadze	0	n	1	0	Asteraceae Bercht. & J.Presl
Iz_2_1009	DJI_0471	Cf. Solidago virgaurea	0		NA	NA	
Iz_2_1009	DJI_0471	Chamaecytisus ruthenicus (Fisch.ex Wol.) Klaskova	0	n	1	0	Fabaceae Lindl.
Iz_2_1009	DJI_0471	Chelidonium majus L.	0	n	1	0	Papaveraceae Juss.
Iz_2_1009	DJI_0471	Chenopodium betaceum Andrz.	0	n	1	0	Amaranthaceae Juss.
Iz_2_1009	DJI_0471	Chenopodium cf. album	0	n	1	0	Amaranthaceae Juss.
Iz_2_1009	DJI_0471	Chenopodium sp.	0		NA	0	Amaranthaceae Juss.
Iz_2_1009	DJI_0471	Chondrilla juncea L.	0	n	1	0	Asteraceae Bercht. & J.Presl
Iz_2_1009	DJI_0471	Cirsium vulgare (Savi) Ten.	0	n	1	0	Asteraceae Bercht. & J.Presl
Iz_2_1009	DJI_0471	Clinopodium acinos (L.) Kuntze	0	n	1	0	Lamiacea Lindl.
Iz_2_1009	DJI_0471	Convallaria majalis L.	0	n	1	0	Asparagaceae Juss.
Iz_2_1009	DJI_0471	Crataegus rhipidophylla Gand.	0	n	1	1	Rosaceae Juss.
Iz_2_1009	DJI_0471	Crepis foetida L.	0	n	1	0	Asteraceae Bercht. & J.Presl
Iz_2_1009	DJI_0471	Dactylis glomerata L.	0	n	1	0	Poaceae Barnhart
Iz_2_1009	DJI_0471	Dianthus campestris M.Bieb.	0	n	1	0	Caryophyllaceae Juss.
Iz_2_1009	DJI_0471	Digitaria sanguinalis (L.) Scop.	0	a	0	0	Poaceae Barnhart
Iz_2_1009	DJI_0471	Elymus repens (L.) Gould	0	n	1	0	Poaceae Barnhart
Iz_2_1009	DJI_0471	Epilobium tetragonum L.	0	n	1	0	Onagraceae Juss.
Iz_2_1009	DJI_0471	Eragrostis minor Host	0	a	0	0	Poaceae Barnhart
Iz_2_1009	DJI_0471	Erigeron canadensis L.	0	a	0	0	Asteraceae Bercht. & J.Presl
Iz_2_1009	DJI_0471	Euphorbia seguieriana Neck.	0	n	1	0	Euphorbiaceae Juss.
Iz_2_1009	DJI_0471	Fallopia dumetorum (L.) Holub	0	n	1	0	Polygonaceae Juss.
Iz_2_1009	DJI_0471	Festuca beckeri (Hack.) Trautv.	0	n	1	0	Poaceae Barnhart
Iz_2_1009	DJI_0471	Fragaria vesca L.	0	n	1	0	Rosaceae Juss.
Iz_2_1009	DJI_0471	Frangula alnus Mill.	0	n	1	1	Rhamnaceae Juss.
Iz_2_1009	DJI_0471	Fraxinus pennsylvanica Marshall	0	a	0	1	Oleaceae Hoffmanns. & Link
Iz_2_1009	DJI_0471	Galeopsis bifida Boenn.	0	n	1	0	Lamiacea Lindl.
Iz_2_1009	DJI_0471	Galium verum L.	0	n	1	0	Rubiaceae Juss.
Iz_2_1009	DJI_0471	Genista tinctoria L.	0	n	1	0	Fabaceae Lindl.
Iz_2_1009	DJI_0471	Geranium robertianum L.	0	n	1	0	Geraniaceae Juss.
Iz_2_1009	DJI_0471	Geranium sanquineum L.	0	n	1	0	Geraniaceae Juss.
Iz_2_1009	DJI_0471	Gypsophila paniculata L.	0	n	1	0	Caryophyllaceae Juss.
Iz_2_1009	DJI_0471	Helichrysum arenarium (L.) Moench	0	n	1	0	Asteraceae Bercht. & J.Presl
Iz_2_1009	DJI_0471	Herniaria polygama J.Gay	0	n	1	0	Caryophyllaceae Juss.
Iz_2_1009	DJI_0471	Hieracium robustum Fr.	0	n	1	0	Asteraceae Bercht. & J.Presl
Iz_2_1009	DJI_0471	Hieracium umbellatum L.	0	n	1	NA	Asteraceae Bercht. & J.Presl
Iz_2_1009	DJI_0471	Hylotelephium maximum (L.) Holub	0	n	1	0	Crassulaceae J.St.-Hil.
Iz_2_1009	DJI_0471	Jacobaea borysthenica (DC.) B.Nord. & Greuter	0	n	1	0	Asteraceae Bercht. & J.Presl
Iz_2_1009	DJI_0471	Jasione montana L.	0	n	1	0	Campanulaceae Juss.
Iz_2_1009	DJI_0471	Jurinea cyanoides (L.) Rchb.s.l.	0	n	1	0	Asteraceae Bercht. & J.Presl
Iz_2_1009	DJI_0471	Koeleria glauca (Spreng.) DC.	0	n	1	0	Poaceae Barnhart
Iz_2_1009	DJI_0471	Lactuca serriola L.	0	a	0	0	Asteraceae Bercht. & J.Presl
Iz_2_1009	DJI_0471	Linaria genistifolia (L.) Mill.	0	n	1	0	Plantaginaceae Juss.
Iz_2_1009	DJI_0471	Lomelosia argentea (L.) Greuter & Burdet	0	n	1	0	Caprifoliaceae Juss.
Iz_2_1009	DJI_0471	Melampyrum pratense L.	0	n	1	0	Orobanchaceae Vent.
Iz_2_1009	DJI_0471	Oenothera biennis L.	0	a	0	0	Onagraceae Juss.
Iz_2_1009	DJI_0471	Pentanema britannica (L.) D.Gut.Larr., Santos-Vicente, Anderb., E.Rico & M.M.Mart.Ort.	0	n	1	0	Asteraceae Bercht. & J.Presl
Iz_2_1009	DJI_0471	Peucedanum oreoselinum (L.) Moench	0	n	1	0	Apiaceae Lindl.
Iz_2_1009	DJI_0471	Peucedanum ruthenicum M.Bieb.	0	n	1	0	Apiaceae Lindl.
Iz_2_1009	DJI_0471	Pilosella echioides (Lumn.) F.W.Schultz & Sch.Bip.	0	n	1	0	Asteraceae Bercht. & J.Presl
Iz_2_1009	DJI_0471	Pilosella officinarum F. Schultz et Sch. Bip.	0	n	1	0	Asteraceae Bercht. & J.Presl
Iz_2_1009	DJI_0471	Pilosella sp.	0		NA	0	Asteraceae Bercht. & J.Presl
Iz_2_1009	DJI_0471	Pinus sylvestris L.	0	n	1	1	Pinaceae Spreng. ex F.Rudolphi
Iz_2_1009	DJI_0471	Poa annua L.	0	n	1	0	Poaceae Barnhart
Iz_2_1009	DJI_0471	Poa nemoralis L.	0	n	1	0	Poaceae Barnhart
Iz_2_1009	DJI_0471	Poa sp.	0		NA	0	Poaceae Barnhart
Iz_2_1009	DJI_0471	Polygonatum odoratum (Mill.) Druce	0	n	1	0	Asparagaceae Juss.
Iz_2_1009	DJI_0471	Polygonum arenarium Waldst. & Kit.	0	n	1	0	Polygonaceae Juss.
Iz_2_1009	DJI_0471	Populus nigra L.	0	n	1	1	Salicaceae Mirb.

Iz_2_1009	DJI_0471	Populus tremula L.	0	n	1	1	Salicaceae Mirb.
Iz_2_1009	DJI_0471	Populus Ã canescens (Aiton) Sm.	0	n	1	1	Salicaceae Mirb.
Iz_2_1009	DJI_0471	Potentilla argentea L.	0	n	1	0	Rosaceae Juss.
Iz_2_1009	DJI_0471	Psammophiliella muralis (L.) Ikonn.	0	n	1	0	Caryophyllaceae Juss.
Iz_2_1009	DJI_0471	Psephellus sumensis (Kalen.) Greuter	0	n	1	0	Asteraceae Bercht. & J.Presl
Iz_2_1009	DJI_0471	Pulsatilla pratensis (L.) Mill. s.l.	0	n	1	0	Ranunculaceae Juss.
Iz_2_1009	DJI_0471	Pyrus communis L.	0	a	0	1	Rosaceae Juss.
Iz_2_1009	DJI_0471	Quercus robur L.	0	n	1	1	Fagaceae Dumort.
Iz_2_1009	DJI_0471	Robinia pseudoacacia L.	0	a	0	1	Fabaceae Lindl.
Iz_2_1009	DJI_0471	Rosa sp.	0		NA	1	Rosaceae Juss.
Iz_2_1009	DJI_0471	Rubus idaeus L.	0	n	1	0	Rosaceae Juss.
Iz_2_1009	DJI_0471	Rumex acetosella L.	0	n	1	0	Polygonaceae Juss.
Iz_2_1009	DJI_0471	Rumex thyrsiflorus Fingerh.	0	n	1	0	Polygonaceae Juss.
Iz_2_1009	DJI_0471	Salix alba L.	0	n	1	1	Salicaceae Mirb.
Iz_2_1009	DJI_0471	Sambucus nigra L.	0	n	1	1	Viburnaceae Raf.
Iz_2_1009	DJI_0471	Secale sylvestre Host	0	n	1	0	Poaceae Barnhart
Iz_2_1009	DJI_0471	Sempervivum ruthenicum Schnittsp. & C.B. Lehm.	0	n	1	0	Crassulaceae J.St.-Hil.
Iz_2_1009	DJI_0471	Setaria viridis (L.) P. Beauv.	0	n	1	0	Poaceae Barnhart
Iz_2_1009	DJI_0471	Silene borysthenica (Gruner) Walters	0	n	1	0	Caryophyllaceae Juss.
Iz_2_1009	DJI_0471	Silene latifolia subsp. alba (Mill.) Greuter & Burdet	0	n	1	0	Caryophyllaceae Juss.
Iz_2_1009	DJI_0471	Solidago virgaurea L.	0	n	1	0	Asteraceae Bercht. & J.Presl
Iz_2_1009	DJI_0471	Sorbus aucuparia L.	0	n	1	1	Rosaceae Juss.
Iz_2_1009	DJI_0471	Stachys recta L.	0	n	1	0	Lamiacea Lindl.
Iz_2_1009	DJI_0471	Tanacetum vulgare L.	0	n	1	0	Asteraceae Bercht. & J.Presl
Iz_2_1009	DJI_0471	Thymus pallasianus Heinr.Braun	0	n	1	0	Lamiacea Lindl.
Iz_2_1009	DJI_0471	Tilia cordata Mill.	0	n	1	1	Malvaceae Juss.
Iz_2_1009	DJI_0471	Trifolium alpestre L.	0	n	1	0	Fabaceae Lindl.
Iz_2_1009	DJI_0471	Trifolium arvense L.	0	n	1	0	Fabaceae Lindl.
Iz_2_1009	DJI_0471	Ulmus glabra Huds.	0	n	1	1	Ulmaceae Mirb.
Iz_2_1009	DJI_0471	Verbascum densiflorum Bertol.	0	n	1	0	Scrophulariaceae Juss.
Iz_2_1009	DJI_0471	Veronica austriaca L.	0	n	1	0	Plantaginaceae Juss.
Iz_2_1009	DJI_0471	Veronica incana L.	0	n	1	0	Plantaginaceae Juss.
Iz_2_1009	DJI_0471	Veronica verna L.	0	n	1	0	Plantaginaceae Juss.
Iz_2_1009	DJI_0471	Vincetoxicum hirundinaria Medik	0	n	1	0	Apocynaceae Juss.
Iz_2_1009	DJI_0472	Acer tataricum L.	0	n	1	1	Sapindaceae Juss.
Iz_2_1009	DJI_0472	Achillea millefolium L. s.l.	0	n	1	0	Asteraceae Bercht. & J.Presl
Iz_2_1009	DJI_0472	Allium oleraceum L.	0	n	1	0	Amaryllidaceae J.St.-Hil.
Iz_2_1009	DJI_0472	Ambrosia artemisiifolia L.	0	a	0	0	Asteraceae Bercht. & J.Presl
Iz_2_1009	DJI_0472	Amorpha fruticosa L.	0	a	0	0	Fabaceae Lindl.
Iz_2_1009	DJI_0472	Anthericum ramosum L.	0	n	1	0	Asparagaceae Juss.
Iz_2_1009	DJI_0472	Artemisia marschalliana Spreng.	0	n	1	0	Asteraceae Bercht. & J.Presl
Iz_2_1009	DJI_0472	Asparagus officinalis L.	0	n	1	0	Asparagaceae Juss.
Iz_2_1009	DJI_0472	Astragalus cicer L.	0	n	1	0	Fabaceae Lindl.
Iz_2_1009	DJI_0472	Bassia laniflora (S.G.Gmel.) A.J.Scott	0	n	1	0	Amaranthaceae Juss.
Iz_2_1009	DJI_0472	Berberis vulgaris L.	0	n	1	0	Berberidaceae Juss.
Iz_2_1009	DJI_0472	Betula pendula Roth.	0	n	1	1	Betulaceae Gray
Iz_2_1009	DJI_0472	Betula pendula Roth.	0	n	1	0	Poaceae Barnhart
Iz_2_1009	DJI_0472	Bromus inermis Leyss.	0		NA	NA	
Iz_2_1009	DJI_0472	Bromus squarrosus L.	0	a	0	0	Poaceae Barnhart
Iz_2_1009	DJI_0472	Bromus tectorum L.	0	a	0	0	Poaceae Barnhart
Iz_2_1009	DJI_0472	Calamagrostis epigejos (L.) Roth	60	n	1	0	Poaceae Barnhart
Iz_2_1009	DJI_0472	Carduus acanthoides L.	0	n	1	0	Asteraceae Bercht. & J.Presl
Iz_2_1009	DJI_0472	Carex sp.	0	n	1	0	Cyperaceae Juss.
Iz_2_1009	DJI_0472	Centaurea majorovii Dumbadze	0	n	1	0	Asteraceae Bercht. & J.Presl
Iz_2_1009	DJI_0472	Cf. Solidago virgaurea	1		NA	NA	
Iz_2_1009	DJI_0472	Chamaecytisus ruthenicus (Fisch.ex Wol.) Klaskova	0	n	1	0	Fabaceae Lindl.
Iz_2_1009	DJI_0472	Chelidonium majus L.	0	n	1	0	Papaveraceae Juss.
Iz_2_1009	DJI_0472	Chenopodium betaceum Andrz.	0	n	1	0	Amaranthaceae Juss.
Iz_2_1009	DJI_0472	Chenopodium cf. album	0	n	1	0	Amaranthaceae Juss.
Iz_2_1009	DJI_0472	Chenopodium sp.	0		NA	0	Amaranthaceae Juss.
Iz_2_1009	DJI_0472	Chondrilla juncea L.	0	n	1	0	Asteraceae Bercht. & J.Presl
Iz_2_1009	DJI_0472	Cirsium vulgare (Savi) Ten.	0	n	1	0	Asteraceae Bercht. & J.Presl
Iz_2_1009	DJI_0472	Clinopodium acinos (L.) Kuntze	0	n	1	0	Lamiacea Lindl.
Iz_2_1009	DJI_0472	Convallaria majalis L.	0	n	1	0	Asparagaceae Juss.
Iz_2_1009	DJI_0472	Crataegus rhipidophylla Gand.	0	n	1	1	Rosaceae Juss.
Iz_2_1009	DJI_0472	Crepis foetida L.	0	n	1	0	Asteraceae Bercht. & J.Presl
Iz_2_1009	DJI_0472	Dactylis glomerata L.	0	n	1	0	Poaceae Barnhart
Iz_2_1009	DJI_0472	Dianthus campestris M.Bieb.	0	n	1	0	Caryophyllaceae Juss.
Iz_2_1009	DJI_0472	Digitaria sanguinalis (L.) Scop.	0	a	0	0	Poaceae Barnhart
Iz_2_1009	DJI_0472	Elymus repens (L.) Gould	0	n	1	0	Poaceae Barnhart
Iz_2_1009	DJI_0472	Epilobium tetragonum L.	0	n	1	0	Onagraceae Juss.
Iz_2_1009	DJI_0472	Eragrostis minor Host	0	a	0	0	Poaceae Barnhart
Iz_2_1009	DJI_0472	Erigeron canadensis L.	0	a	0	0	Asteraceae Bercht. & J.Presl
Iz_2_1009	DJI_0472	Euphorbia seguieriana Neck.	0	n	1	0	Euphorbiaceae Juss.
Iz_2_1009	DJI_0472	Fallopia dumetorum (L.) Holub	0	n	1	0	Polygonaceae Juss.
Iz_2_1009	DJI_0472	Festuca beckeri (Hack.) Trautv.	0	n	1	0	Poaceae Barnhart
Iz_2_1009	DJI_0472	Fragaria vesca L.	0	n	1	0	Rosaceae Juss.
Iz_2_1009	DJI_0472	Frangula alnus Mill.	0	n	1	1	Rhamnaceae Juss.
Iz_2_1009	DJI_0472	Fraxinus pennsylvanica Marshall	0	a	0	1	Oleaceae Hoffmanns. & Link
Iz_2_1009	DJI_0472	Galeopsis bifida Boenn.	0	n	1	0	Lamiacea Lindl.
Iz_2_1009	DJI_0472	Galium verum L.	0	n	1	0	Rubiaceae Juss.
Iz_2_1009	DJI_0472	Genista tinctoria L.	0	n	1	0	Fabaceae Lindl.
Iz_2_1009	DJI_0472	Geranium robertianum L.	0	n	1	0	Geraniaceae Juss.
Iz_2_1009	DJI_0472	Geranium sanquineum L.	0	n	1	0	Geraniaceae Juss.
Iz_2_1009	DJI_0472	Gypsophila paniculata L.	0	n	1	0	Caryophyllaceae Juss.
Iz_2_1009	DJI_0472	Helichrysum arenarium (L.) Moench	0	n	1	0	Asteraceae Bercht. & J.Presl
Iz_2_1009	DJI_0472	Herniaria polygama J.Gay	0	n	1	0	Caryophyllaceae Juss.
Iz_2_1009	DJI_0472	Hieracium robustum Fr.	0	n	1	0	Asteraceae Bercht. & J.Presl
Iz_2_1009	DJI_0472	Hieracium umbellatum L.	0	n	1	NA	Asteraceae Bercht. & J.Presl
Iz_2_1009	DJI_0472	Hylotelephium maximum (L.) Holub	0	n	1	0	Crassulaceae J.St.-Hil.
Iz_2_1009	DJI_0472	Jacobaea borysthenica (DC.) B.Nord. & Greuter	0	n	1	0	Asteraceae Bercht. & J.Presl
Iz_2_1009	DJI_0472	Jasione montana L.	0	n	1	0	Campanulaceae Juss.
Iz_2_1009	DJI_0472	Jurinea cyanoides (L.) Rchb.s.l.	0	n	1	0	Asteraceae Bercht. & J.Presl
Iz_2_1009	DJI_0472	Koeleria glauca (Spreng.) DC.	0	n	1	0	Poaceae Barnhart
Iz_2_1009	DJI_0472	Lactuca serriola L.	0	a	0	0	Asteraceae Bercht. & J.Presl
Iz_2_1009	DJI_0472	Linaria genistifolia (L.) Mill.	0	n	1	0	Plantaginaceae Juss.
Iz_2_1009	DJI_0472	Lomelosia argentea (L.) Greuter & Burdet	0	n	1	0	Caprifoliaceae Juss.
Iz_2_1009	DJI_0472	Melampyrum pratense L.	0	n	1	0	Orobanchaceae Vent.

Iz_2_1009	DJI_0472	Oenothera biennis L.	0	a	0	0	Onagraceae Juss.
Iz_2_1009	DJI_0472	Pentanema britannica (L.) D.Gut.Larr., Santos-Vicente, Anderb., E.Rico & M.M.Mart.Ort.	0	n	1	0	Asteraceae Bercht. & J.Presl
Iz_2_1009	DJI_0472	Peucedanum oreoselinum (L.) Moench	0	n	1	0	Apiaceae Lindl.
Iz_2_1009	DJI_0472	Peucedanum ruthenicum M.Bieb.	0	n	1	0	Apiaceae Lindl.
Iz_2_1009	DJI_0472	Pilosella echioides (Lumn.) F.W.Schultz & Sch.Bip.	0	n	1	0	Asteraceae Bercht. & J.Presl
Iz_2_1009	DJI_0472	Pilosella officinarum F. Schultz et Sch. Bip.	0	n	1	0	Asteraceae Bercht. & J.Presl
Iz_2_1009	DJI_0472	Pilosella sp.	0		NA	0	Asteraceae Bercht. & J.Presl
Iz_2_1009	DJI_0472	Pinus sylvestris L.	0	n	1	1	Pinaceae Spreng. ex F.Rudolphi
Iz_2_1009	DJI_0472	Poa annua L.	0	n	1	0	Poaceae Barnhart
Iz_2_1009	DJI_0472	Poa nemoralis L.	0	n	1	0	Poaceae Barnhart
Iz_2_1009	DJI_0472	Poa sp.	0		NA	0	Poaceae Barnhart
Iz_2_1009	DJI_0472	Polygonatum odoratum (Mill.) Druce	0	n	1	0	Asparagaceae Juss.
Iz_2_1009	DJI_0472	Polygonum arenarium Waldst. & Kit.	0	n	1	0	Polygonaceae Juss.
Iz_2_1009	DJI_0472	Populus nigra L.	0	n	1	1	Salicaceae Mirb.
Iz_2_1009	DJI_0472	Populus tremula L.	0	n	1	1	Salicaceae Mirb.
Iz_2_1009	DJI_0472	Populus Ã canescens (Aiton) Sm.	0	n	1	1	Salicaceae Mirb.
Iz_2_1009	DJI_0472	Potentilla argentea L.	0	n	1	0	Rosaceae Juss.
Iz_2_1009	DJI_0472	Psammophiliella muralis (L.) Ikonn.	0	n	1	0	Caryophyllaceae Juss.
Iz_2_1009	DJI_0472	Psephellus sumensis (Kalen.) Greuter	0	n	1	0	Asteraceae Bercht. & J.Presl
Iz_2_1009	DJI_0472	Pulsatilla pratensis (L.) Mill. s.l.	0	n	1	0	Ranunculaceae Juss.
Iz_2_1009	DJI_0472	Pyrus communis L.	0	a	0	1	Rosaceae Juss.
Iz_2_1009	DJI_0472	Quercus robur L.	0	n	1	1	Fagaceae Dumort.
Iz_2_1009	DJI_0472	Robinia pseudoacacia L.	0	a	0	1	Fabaceae Lindl.
Iz_2_1009	DJI_0472	Rosa sp.	0		NA	1	Rosaceae Juss.
Iz_2_1009	DJI_0472	Rubus idaeus L.	0	n	1	0	Rosaceae Juss.
Iz_2_1009	DJI_0472	Rumex acetosella L.	0	n	1	0	Polygonaceae Juss.
Iz_2_1009	DJI_0472	Rumex thyrsiflorus Fingerh.	0	n	1	0	Polygonaceae Juss.
Iz_2_1009	DJI_0472	Salix alba L.	0	n	1	1	Salicaceae Mirb.
Iz_2_1009	DJI_0472	Sambucus nigra L.	0	n	1	1	Viburnaceae Raf.
Iz_2_1009	DJI_0472	Secale sylvestre Host	0	n	1	0	Poaceae Barnhart
Iz_2_1009	DJI_0472	Sempervivum ruthenicum Schnittsp. & C.B. Lehm.	0	n	1	0	Crassulaceae J.St.-Hil.
Iz_2_1009	DJI_0472	Setaria viridis (L.) P. Beauv.	0	n	1	0	Poaceae Barnhart
Iz_2_1009	DJI_0472	Silene borysthenica (Gruner) Walters	0	n	1	0	Caryophyllaceae Juss.
Iz_2_1009	DJI_0472	Silene latifolia subsp. alba (Mill.) Greuter & Burdet	0	n	1	0	Caryophyllaceae Juss.
Iz_2_1009	DJI_0472	Solidago virgaurea L.	0	n	1	0	Asteraceae Bercht. & J.Presl
Iz_2_1009	DJI_0472	Sorbus aucuparia L.	0	n	1	1	Rosaceae Juss.
Iz_2_1009	DJI_0472	Stachys recta L.	0	n	1	0	Lamiacea Lindl.
Iz_2_1009	DJI_0472	Tanacetum vulgare L.	0	n	1	0	Asteraceae Bercht. & J.Presl
Iz_2_1009	DJI_0472	Thymus pallasianus Heinr.Braun	0	n	1	0	Lamiacea Lindl.
Iz_2_1009	DJI_0472	Tilia cordata Mill.	0	n	1	1	Malvaceae Juss.
Iz_2_1009	DJI_0472	Trifolium alpestre L.	0	n	1	0	Fabaceae Lindl.
Iz_2_1009	DJI_0472	Trifolium arvense L.	0	n	1	0	Fabaceae Lindl.
Iz_2_1009	DJI_0472	Ulmus glabra Huds.	0	n	1	1	Ulmaceae Mirb.
Iz_2_1009	DJI_0472	Verbascum densiflorum Bertol.	0	n	1	0	Scrophulariaceae Juss.
Iz_2_1009	DJI_0472	Veronica austriaca L.	0	n	1	0	Plantaginaceae Juss.
Iz_2_1009	DJI_0472	Veronica incana L.	0	n	1	0	Plantaginaceae Juss.
Iz_2_1009	DJI_0472	Veronica verna L.	0	n	1	0	Plantaginaceae Juss.
Iz_2_1009	DJI_0472	Vincetoxicum hirundinaria Medik	0	n	1	0	Apocynaceae Juss.
Iz_2_1009	DJI_0473	Acer tataricum L.	0	n	1	1	Sapindaceae Juss.
Iz_2_1009	DJI_0473	Achillea millefolium L. s.l.	0	n	1	0	Asteraceae Bercht. & J.Presl
Iz_2_1009	DJI_0473	Allium oleraceum L.	0	n	1	0	Amaryllidaceae J.St.-Hil.
Iz_2_1009	DJI_0473	Ambrosia artemisiifolia L.	0	a	0	0	Asteraceae Bercht. & J.Presl
Iz_2_1009	DJI_0473	Amorpha fruticosa L.	0	a	0	0	Fabaceae Lindl.
Iz_2_1009	DJI_0473	Anthericum ramosum L.	0	n	1	0	Asparagaceae Juss.
Iz_2_1009	DJI_0473	Artemisia marschalliana Spreng.	0	n	1	0	Asteraceae Bercht. & J.Presl
Iz_2_1009	DJI_0473	Asparagus officinalis L.	0	n	1	0	Asparagaceae Juss.
Iz_2_1009	DJI_0473	Astragalus cicer L.	0	n	1	0	Fabaceae Lindl.
Iz_2_1009	DJI_0473	Bassia laniflora (S.G.Gmel.) A.J.Scott	0	n	1	0	Amaranthaceae Juss.
Iz_2_1009	DJI_0473	Berberis vulgaris L.	0	n	1	0	Berberidaceae Juss.
Iz_2_1009	DJI_0473	Betula pendula Roth.	0	n	1	1	Betulaceae Gray
Iz_2_1009	DJI_0473	Betula pendula Roth.	0	n	1	0	Poaceae Barnhart
Iz_2_1009	DJI_0473	Bromus inermis Leyss.	0		NA	NA	
Iz_2_1009	DJI_0473	Bromus squarrosus L.	0	a	0	0	Poaceae Barnhart
Iz_2_1009	DJI_0473	Bromus tectorum L.	0	a	0	0	Poaceae Barnhart
Iz_2_1009	DJI_0473	Calamagrostis epigejos (L.) Roth	80	n	1	0	Poaceae Barnhart
Iz_2_1009	DJI_0473	Carduus acanthoides L.	0	n	1	0	Asteraceae Bercht. & J.Presl
Iz_2_1009	DJI_0473	Carex sp.	0	n	1	0	Cyperaceae Juss.
Iz_2_1009	DJI_0473	Centaurea majorovii Dumbadze	0	n	1	0	Asteraceae Bercht. & J.Presl
Iz_2_1009	DJI_0473	Cf. Solidago virgaurea	0		NA	NA	
Iz_2_1009	DJI_0473	Chamaecytisus ruthenicus (Fisch.ex Wol.) Klaskova	0	n	1	0	Fabaceae Lindl.
Iz_2_1009	DJI_0473	Chelidonium majus L.	0	n	1	0	Papaveraceae Juss.
Iz_2_1009	DJI_0473	Chenopodium betaceum Andrz.	0	n	1	0	Amaranthaceae Juss.
Iz_2_1009	DJI_0473	Chenopodium cf. album	0	n	1	0	Amaranthaceae Juss.
Iz_2_1009	DJI_0473	Chenopodium sp.	0		NA	0	Amaranthaceae Juss.
Iz_2_1009	DJI_0473	Chondrilla juncea L.	0	n	1	0	Asteraceae Bercht. & J.Presl
Iz_2_1009	DJI_0473	Cirsium vulgare (Savi) Ten.	0	n	1	0	Asteraceae Bercht. & J.Presl
Iz_2_1009	DJI_0473	Clinopodium acinos (L.) Kuntze	0	n	1	0	Lamiacea Lindl.
Iz_2_1009	DJI_0473	Convallaria majalis L.	0	n	1	0	Asparagaceae Juss.
Iz_2_1009	DJI_0473	Crataegus rhipidophylla Gand.	0	n	1	1	Rosaceae Juss.
Iz_2_1009	DJI_0473	Crepis foetida L.	0	n	1	0	Asteraceae Bercht. & J.Presl
Iz_2_1009	DJI_0473	Dactylis glomerata L.	0	n	1	0	Poaceae Barnhart
Iz_2_1009	DJI_0473	Dianthus campestris M.Bieb.	0	n	1	0	Caryophyllaceae Juss.
Iz_2_1009	DJI_0473	Digitaria sanguinalis (L.) Scop.	0	a	0	0	Poaceae Barnhart
Iz_2_1009	DJI_0473	Elymus repens (L.) Gould	0	n	1	0	Poaceae Barnhart
Iz_2_1009	DJI_0473	Epilobium tetragonum L.	0	n	1	0	Onagraceae Juss.
Iz_2_1009	DJI_0473	Eragrostis minor Host	0	a	0	0	Poaceae Barnhart
Iz_2_1009	DJI_0473	Erigeron canadensis L.	0	a	0	0	Asteraceae Bercht. & J.Presl
Iz_2_1009	DJI_0473	Euphorbia seguieriana Neck.	0	n	1	0	Euphorbiaceae Juss.
Iz_2_1009	DJI_0473	Fallopia dumetorum (L.) Holub	0	n	1	0	Polygonaceae Juss.
Iz_2_1009	DJI_0473	Festuca beckeri (Hack.) Trautv.	0	n	1	0	Poaceae Barnhart
Iz_2_1009	DJI_0473	Fragaria vesca L.	0	n	1	0	Rosaceae Juss.
Iz_2_1009	DJI_0473	Frangula alnus Mill.	0	n	1	1	Rhamnaceae Juss.
Iz_2_1009	DJI_0473	Fraxinus pennsylvanica Marshall	0	a	0	1	Oleaceae Hoffmanns. & Link
Iz_2_1009	DJI_0473	Galeopsis bifida Boenn.	0	n	1	0	Lamiacea Lindl.
Iz_2_1009	DJI_0473	Galium verum L.	0	n	1	0	Rubiaceae Juss.
Iz_2_1009	DJI_0473	Genista tinctoria L.	0	n	1	0	Fabaceae Lindl.
Iz_2_1009	DJI_0473	Geranium robertianum L.	0	n	1	0	Geraniaceae Juss.
Iz_2_1009	DJI_0473	Geranium sanquineum L.	0	n	1	0	Geraniaceae Juss.

Iz_2_1009	DJI_0473	Gypsophila paniculata L.	0	n	1	0	Caryophyllaceae Juss.
Iz_2_1009	DJI_0473	Helichrysum arenarium (L.) Moench	0	n	1	0	Asteraceae Bercht. & J.Presl
Iz_2_1009	DJI_0473	Herniaria polygama J.Gay	0	n	1	0	Caryophyllaceae Juss.
Iz_2_1009	DJI_0473	Hieracium robustum Fr.	0	n	1	0	Asteraceae Bercht. & J.Presl
Iz_2_1009	DJI_0473	Hieracium umbellatum L.	0	n	1	NA	Asteraceae Bercht. & J.Presl
Iz_2_1009	DJI_0473	Hylotelephium maximum (L.) Holub	0	n	1	0	Crassulaceae J.St.-Hil.
Iz_2_1009	DJI_0473	Jacobaea borysthenica (DC.) B.Nord. & Greuter	0	n	1	0	Asteraceae Bercht. & J.Presl
Iz_2_1009	DJI_0473	Jasione montana L.	0	n	1	0	Campanulaceae Juss.
Iz_2_1009	DJI_0473	Jurinea cyanoides (L.) Rchb.s.l.	0	n	1	0	Asteraceae Bercht. & J.Presl
Iz_2_1009	DJI_0473	Koeleria glauca (Spreng.) DC.	0	n	1	0	Poaceae Barnhart
Iz_2_1009	DJI_0473	Lactuca serriola L.	0	a	0	0	Asteraceae Bercht. & J.Presl
Iz_2_1009	DJI_0473	Linaria genistifolia (L.) Mill.	0	n	1	0	Plantaginaceae Juss.
Iz_2_1009	DJI_0473	Lomelosia argentea (L.) Greuter & Burdet	0	n	1	0	Caprifoliaceae Juss.
Iz_2_1009	DJI_0473	Melampyrum pratense L.	0	n	1	0	Orobanchaceae Vent.
Iz_2_1009	DJI_0473	Oenothera biennis L.	0	a	0	0	Onagraceae Juss.
Iz_2_1009	DJI_0473	Pentanema britannica (L.) D.Gut.Larr., Santos-Vicente, Anderb., E.Rico & M.M.Mart.Ort.	0	n	1	0	Asteraceae Bercht. & J.Presl
Iz_2_1009	DJI_0473	Peucedanum oreoselinum (L.) Moench	1	n	1	0	Apiaceae Lindl.
Iz_2_1009	DJI_0473	Peucedanum ruthenicum M.Bieb.	0	n	1	0	Apiaceae Lindl.
Iz_2_1009	DJI_0473	Pilosella echioides (Lumn.) F.W.Schultz & Sch.Bip.	0	n	1	0	Asteraceae Bercht. & J.Presl
Iz_2_1009	DJI_0473	Pilosella officinarum F. Schultz et Sch. Bip.	0	n	1	0	Asteraceae Bercht. & J.Presl
Iz_2_1009	DJI_0473	Pilosella sp.	0		NA	0	Asteraceae Bercht. & J.Presl
Iz_2_1009	DJI_0473	Pinus sylvestris L.	0	n	1	1	Pinaceae Spreng. ex F.Rudolphi
Iz_2_1009	DJI_0473	Poa annua L.	0	n	1	0	Poaceae Barnhart
Iz_2_1009	DJI_0473	Poa nemoralis L.	0	n	1	0	Poaceae Barnhart
Iz_2_1009	DJI_0473	Poa sp.	0		NA	0	Poaceae Barnhart
Iz_2_1009	DJI_0473	Polygonatum odoratum (Mill.) Druce	0	n	1	0	Asparagaceae Juss.
Iz_2_1009	DJI_0473	Polygonum arenarium Waldst. & Kit.	0	n	1	0	Polygonaceae Juss.
Iz_2_1009	DJI_0473	Populus nigra L.	0	n	1	1	Salicaceae Mirb.
Iz_2_1009	DJI_0473	Populus tremula L.	0	n	1	1	Salicaceae Mirb.
Iz_2_1009	DJI_0473	Populus Ã canescens (Aiton) Sm.	0	n	1	1	Salicaceae Mirb.
Iz_2_1009	DJI_0473	Potentilla argentea L.	0	n	1	0	Rosaceae Juss.
Iz_2_1009	DJI_0473	Psammophiliella muralis (L.) Ikonn.	0	n	1	0	Caryophyllaceae Juss.
Iz_2_1009	DJI_0473	Psephellus sumensis (Kalen.) Greuter	0	n	1	0	Asteraceae Bercht. & J.Presl
Iz_2_1009	DJI_0473	Pulsatilla pratensis (L.) Mill. s.l.	0	n	1	0	Ranunculaceae Juss.
Iz_2_1009	DJI_0473	Pyrus communis L.	0	a	0	1	Rosaceae Juss.
Iz_2_1009	DJI_0473	Quercus robur L.	0	n	1	1	Fagaceae Dumort.
Iz_2_1009	DJI_0473	Robinia pseudoacacia L.	0	a	0	1	Fabaceae Lindl.
Iz_2_1009	DJI_0473	Rosa sp.	0		NA	1	Rosaceae Juss.
Iz_2_1009	DJI_0473	Rubus idaeus L.	0	n	1	0	Rosaceae Juss.
Iz_2_1009	DJI_0473	Rumex acetosella L.	0	n	1	0	Polygonaceae Juss.
Iz_2_1009	DJI_0473	Rumex thyrsiflorus Fingerh.	0	n	1	0	Polygonaceae Juss.
Iz_2_1009	DJI_0473	Salix alba L.	0	n	1	1	Salicaceae Mirb.
Iz_2_1009	DJI_0473	Sambucus nigra L.	0	n	1	1	Viburnaceae Raf.
Iz_2_1009	DJI_0473	Secale sylvestre Host	0	n	1	0	Poaceae Barnhart
Iz_2_1009	DJI_0473	Sempervivum ruthenicum Schnittsp. & C.B. Lehm.	0	n	1	0	Crassulaceae J.St.-Hil.
Iz_2_1009	DJI_0473	Setaria viridis (L.) P. Beauv.	0	n	1	0	Poaceae Barnhart
Iz_2_1009	DJI_0473	Silene borysthenica (Gruner) Walters	0	n	1	0	Caryophyllaceae Juss.
Iz_2_1009	DJI_0473	Silene latifolia subsp. alba (Mill.) Greuter & Burdet	0	n	1	0	Caryophyllaceae Juss.
Iz_2_1009	DJI_0473	Solidago virgaurea L.	0	n	1	0	Asteraceae Bercht. & J.Presl
Iz_2_1009	DJI_0473	Sorbus aucuparia L.	0	n	1	1	Rosaceae Juss.
Iz_2_1009	DJI_0473	Stachys recta L.	0	n	1	0	Lamiacea Lindl.
Iz_2_1009	DJI_0473	Tanacetum vulgare L.	0	n	1	0	Asteraceae Bercht. & J.Presl
Iz_2_1009	DJI_0473	Thymus pallasianus Heinr.Braun	0	n	1	0	Lamiacea Lindl.
Iz_2_1009	DJI_0473	Tilia cordata Mill.	0	n	1	1	Malvaceae Juss.
Iz_2_1009	DJI_0473	Trifolium alpestre L.	0	n	1	0	Fabaceae Lindl.
Iz_2_1009	DJI_0473	Trifolium arvense L.	0	n	1	0	Fabaceae Lindl.
Iz_2_1009	DJI_0473	Ulmus glabra Huds.	0	n	1	1	Ulmaceae Mirb.
Iz_2_1009	DJI_0473	Verbascum densiflorum Bertol.	0	n	1	0	Scrophulariaceae Juss.
Iz_2_1009	DJI_0473	Veronica austriaca L.	0	n	1	0	Plantaginaceae Juss.
Iz_2_1009	DJI_0473	Veronica incana L.	0	n	1	0	Plantaginaceae Juss.
Iz_2_1009	DJI_0473	Veronica verna L.	0	n	1	0	Plantaginaceae Juss.
Iz_2_1009	DJI_0473	Vincetoxicum hirundinaria Medik	0	n	1	0	Apocynaceae Juss.
Iz_2_1009	DJI_0475	Acer tataricum L.	0	n	1	1	Sapindaceae Juss.
Iz_2_1009	DJI_0475	Achillea millefolium L. s.l.	0	n	1	0	Asteraceae Bercht. & J.Presl
Iz_2_1009	DJI_0475	Allium oleraceum L.	0	n	1	0	Amaryllidaceae J.St.-Hil.
Iz_2_1009	DJI_0475	Ambrosia artemisiifolia L.	0	a	0	0	Asteraceae Bercht. & J.Presl
Iz_2_1009	DJI_0475	Amorpha fruticosa L.	0	a	0	0	Fabaceae Lindl.
Iz_2_1009	DJI_0475	Anthericum ramosum L.	0	n	1	0	Asparagaceae Juss.
Iz_2_1009	DJI_0475	Artemisia marschalliana Spreng.	0	n	1	0	Asteraceae Bercht. & J.Presl
Iz_2_1009	DJI_0475	Asparagus officinalis L.	0	n	1	0	Asparagaceae Juss.
Iz_2_1009	DJI_0475	Astragalus cicer L.	0	n	1	0	Fabaceae Lindl.
Iz_2_1009	DJI_0475	Bassia laniflora (S.G.Gmel.) A.J.Scott	0	n	1	0	Amaranthaceae Juss.
Iz_2_1009	DJI_0475	Berberis vulgaris L.	0	n	1	0	Berberidaceae Juss.
Iz_2_1009	DJI_0475	Betula pendula Roth.	0	n	1	1	Betulaceae Gray
Iz_2_1009	DJI_0475	Betula pendula Roth.	0	n	1	0	Poaceae Barnhart
Iz_2_1009	DJI_0475	Bromus inermis Leyss.	0		NA	NA	
Iz_2_1009	DJI_0475	Bromus squarrosus L.	0	a	0	0	Poaceae Barnhart
Iz_2_1009	DJI_0475	Bromus tectorum L.	0	a	0	0	Poaceae Barnhart
Iz_2_1009	DJI_0475	Calamagrostis epigejos (L.) Roth	80	n	1	0	Poaceae Barnhart
Iz_2_1009	DJI_0475	Carduus acanthoides L.	0	n	1	0	Asteraceae Bercht. & J.Presl
Iz_2_1009	DJI_0475	Carex sp.	0	n	1	0	Cyperaceae Juss.
Iz_2_1009	DJI_0475	Centaurea majorovii Dumbadze	0	n	1	0	Asteraceae Bercht. & J.Presl
Iz_2_1009	DJI_0475	Cf. Solidago virgaurea	0		NA	NA	
Iz_2_1009	DJI_0475	Chamaecytisus ruthenicus (Fisch.ex Wol.) Klaskova	0	n	1	0	Fabaceae Lindl.
Iz_2_1009	DJI_0475	Chelidonium majus L.	0	n	1	0	Papaveraceae Juss.
Iz_2_1009	DJI_0475	Chenopodium betaceum Andrz.	0	n	1	0	Amaranthaceae Juss.
Iz_2_1009	DJI_0475	Chenopodium cf. album	0	n	1	0	Amaranthaceae Juss.
Iz_2_1009	DJI_0475	Chenopodium sp.	0		NA	0	Amaranthaceae Juss.
Iz_2_1009	DJI_0475	Chondrilla juncea L.	0	n	1	0	Asteraceae Bercht. & J.Presl
Iz_2_1009	DJI_0475	Cirsium vulgare (Savi) Ten.	0	n	1	0	Asteraceae Bercht. & J.Presl
Iz_2_1009	DJI_0475	Clinopodium acinos (L.) Kuntze	0	n	1	0	Lamiacea Lindl.
Iz_2_1009	DJI_0475	Convallaria majalis L.	0	n	1	0	Asparagaceae Juss.
Iz_2_1009	DJI_0475	Crataegus rhipidophylla Gand.	0	n	1	1	Rosaceae Juss.
Iz_2_1009	DJI_0475	Crepis foetida L.	0	n	1	0	Asteraceae Bercht. & J.Presl
Iz_2_1009	DJI_0475	Dactylis glomerata L.	0	n	1	0	Poaceae Barnhart
Iz_2_1009	DJI_0475	Dianthus campestris M.Bieb.	0	n	1	0	Caryophyllaceae Juss.
Iz_2_1009	DJI_0475	Digitaria sanguinalis (L.) Scop.	0	a	0	0	Poaceae Barnhart
Iz_2_1009	DJI_0475	Elymus repens (L.) Gould	0	n	1	0	Poaceae Barnhart

Iz_2_1009	DJI_0475	Epilobium tetragonum L.	0	n	1	0	Onagraceae Juss.
Iz_2_1009	DJI_0475	Eragrostis minor Host	0	a	0	0	Poaceae Barnhart
Iz_2_1009	DJI_0475	Erigeron canadensis L.	0	a	0	0	Asteraceae Bercht. & J.Presl
Iz_2_1009	DJI_0475	Euphorbia seguieriana Neck.	0	n	1	0	Euphorbiaceae Juss.
Iz_2_1009	DJI_0475	Fallopia dumetorum (L.) Holub	0	n	1	0	Polygonaceae Juss.
Iz_2_1009	DJI_0475	Festuca beckeri (Hack.) Trautv.	0	n	1	0	Poaceae Barnhart
Iz_2_1009	DJI_0475	Fragaria vesca L.	0	n	1	0	Rosaceae Juss.
Iz_2_1009	DJI_0475	Frangula alnus Mill.	0	n	1	1	Rhamnaceae Juss.
Iz_2_1009	DJI_0475	Fraxinus pennsylvanica Marshall	0	a	0	1	Oleaceae Hoffmanns. & Link
Iz_2_1009	DJI_0475	Galeopsis bifida Boenn.	0	n	1	0	Lamiacea Lindl.
Iz_2_1009	DJI_0475	Galium verum L.	0	n	1	0	Rubiaceae Juss.
Iz_2_1009	DJI_0475	Genista tinctoria L.	0	n	1	0	Fabaceae Lindl.
Iz_2_1009	DJI_0475	Geranium robertianum L.	0	n	1	0	Geraniaceae Juss.
Iz_2_1009	DJI_0475	Geranium sanquineum L.	0	n	1	0	Geraniaceae Juss.
Iz_2_1009	DJI_0475	Gypsophila paniculata L.	0	n	1	0	Caryophyllaceae Juss.
Iz_2_1009	DJI_0475	Helichrysum arenarium (L.) Moench	0	n	1	0	Asteraceae Bercht. & J.Presl
Iz_2_1009	DJI_0475	Herniaria polygama J.Gay	0	n	1	0	Caryophyllaceae Juss.
Iz_2_1009	DJI_0475	Hieracium robustum Fr.	0	n	1	0	Asteraceae Bercht. & J.Presl
Iz_2_1009	DJI_0475	Hieracium umbellatum L.	0	n	1	NA	Asteraceae Bercht. & J.Presl
Iz_2_1009	DJI_0475	Hylotelephium maximum (L.) Holub	0	n	1	0	Crassulaceae J.St.-Hil.
Iz_2_1009	DJI_0475	Jacobaea borysthenica (DC.) B.Nord. & Greuter	0	n	1	0	Asteraceae Bercht. & J.Presl
Iz_2_1009	DJI_0475	Jasione montana L.	0	n	1	0	Campanulaceae Juss.
Iz_2_1009	DJI_0475	Jurinea cyanoides (L.) Rchb.s.l.	0	n	1	0	Asteraceae Bercht. & J.Presl
Iz_2_1009	DJI_0475	Koeleria glauca (Spreng.) DC.	0	n	1	0	Poaceae Barnhart
Iz_2_1009	DJI_0475	Lactuca serriola L.	0	a	0	0	Asteraceae Bercht. & J.Presl
Iz_2_1009	DJI_0475	Linaria genistifolia (L.) Mill.	0	n	1	0	Plantaginaceae Juss.
Iz_2_1009	DJI_0475	Lomelosia argentea (L.) Greuter & Burdet	0	n	1	0	Caprifoliaceae Juss.
Iz_2_1009	DJI_0475	Melampyrum pratense L.	0	n	1	0	Orobanchaceae Vent.
Iz_2_1009	DJI_0475	Oenothera biennis L.	0	a	0	0	Onagraceae Juss.
Iz_2_1009	DJI_0475	Pentanema britannica (L.) D.Gut.Larr., Santos-Vicente, Anderb., E.Rico & M.M.Mart.Ort.	0	n	1	0	Asteraceae Bercht. & J.Presl
Iz_2_1009	DJI_0475	Peucedanum oreoselinum (L.) Moench	0	n	1	0	Apiaceae Lindl.
Iz_2_1009	DJI_0475	Peucedanum ruthenicum M.Bieb.	0	n	1	0	Apiaceae Lindl.
Iz_2_1009	DJI_0475	Pilosella echioides (Lumn.) F.W.Schultz & Sch.Bip.	0	n	1	0	Asteraceae Bercht. & J.Presl
Iz_2_1009	DJI_0475	Pilosella officinarum F. Schultz et Sch. Bip.	0	n	1	0	Asteraceae Bercht. & J.Presl
Iz_2_1009	DJI_0475	Pilosella sp.	0		NA	0	Asteraceae Bercht. & J.Presl
Iz_2_1009	DJI_0475	Pinus sylvestris L.	0	n	1	1	Pinaceae Spreng. ex F.Rudolphi
Iz_2_1009	DJI_0475	Poa annua L.	0	n	1	0	Poaceae Barnhart
Iz_2_1009	DJI_0475	Poa nemoralis L.	0	n	1	0	Poaceae Barnhart
Iz_2_1009	DJI_0475	Poa sp.	0		NA	0	Poaceae Barnhart
Iz_2_1009	DJI_0475	Polygonatum odoratum (Mill.) Druce	0	n	1	0	Asparagaceae Juss.
Iz_2_1009	DJI_0475	Polygonum arenarium Waldst. & Kit.	0	n	1	0	Polygonaceae Juss.
Iz_2_1009	DJI_0475	Populus nigra L.	0	n	1	1	Salicaceae Mirb.
Iz_2_1009	DJI_0475	Populus tremula L.	0	n	1	1	Salicaceae Mirb.
Iz_2_1009	DJI_0475	Populus Ã canescens (Aiton) Sm.	0	n	1	1	Salicaceae Mirb.
Iz_2_1009	DJI_0475	Potentilla argentea L.	0	n	1	0	Rosaceae Juss.
Iz_2_1009	DJI_0475	Psammophiliella muralis (L.) Ikonn.	0	n	1	0	Caryophyllaceae Juss.
Iz_2_1009	DJI_0475	Psephellus sumensis (Kalen.) Greuter	0	n	1	0	Asteraceae Bercht. & J.Presl
Iz_2_1009	DJI_0475	Pulsatilla pratensis (L.) Mill. s.l.	0	n	1	0	Ranunculaceae Juss.
Iz_2_1009	DJI_0475	Pyrus communis L.	0	a	0	1	Rosaceae Juss.
Iz_2_1009	DJI_0475	Quercus robur L.	0	n	1	1	Fagaceae Dumort.
Iz_2_1009	DJI_0475	Robinia pseudoacacia L.	0	a	0	1	Fabaceae Lindl.
Iz_2_1009	DJI_0475	Rosa sp.	0		NA	1	Rosaceae Juss.
Iz_2_1009	DJI_0475	Rubus idaeus L.	0	n	1	0	Rosaceae Juss.
Iz_2_1009	DJI_0475	Rumex acetosella L.	0	n	1	0	Polygonaceae Juss.
Iz_2_1009	DJI_0475	Rumex thyrsiflorus Fingerh.	0	n	1	0	Polygonaceae Juss.
Iz_2_1009	DJI_0475	Salix alba L.	0	n	1	1	Salicaceae Mirb.
Iz_2_1009	DJI_0475	Sambucus nigra L.	0	n	1	1	Viburnaceae Raf.
Iz_2_1009	DJI_0475	Secale sylvestre Host	0	n	1	0	Poaceae Barnhart
Iz_2_1009	DJI_0475	Sempervivum ruthenicum Schnittsp. & C.B. Lehm.	0	n	1	0	Crassulaceae J.St.-Hil.
Iz_2_1009	DJI_0475	Setaria viridis (L.) P. Beauv.	0	n	1	0	Poaceae Barnhart
Iz_2_1009	DJI_0475	Silene borysthenica (Gruner) Walters	0	n	1	0	Caryophyllaceae Juss.
Iz_2_1009	DJI_0475	Silene latifolia subsp. alba (Mill.) Greuter & Burdet	0	n	1	0	Caryophyllaceae Juss.
Iz_2_1009	DJI_0475	Solidago virgaurea L.	0	n	1	0	Asteraceae Bercht. & J.Presl
Iz_2_1009	DJI_0475	Sorbus aucuparia L.	0	n	1	1	Rosaceae Juss.
Iz_2_1009	DJI_0475	Stachys recta L.	0	n	1	0	Lamiacea Lindl.
Iz_2_1009	DJI_0475	Tanacetum vulgare L.	0	n	1	0	Asteraceae Bercht. & J.Presl
Iz_2_1009	DJI_0475	Thymus pallasianus Heinr.Braun	0	n	1	0	Lamiacea Lindl.
Iz_2_1009	DJI_0475	Tilia cordata Mill.	0	n	1	1	Malvaceae Juss.
Iz_2_1009	DJI_0475	Trifolium alpestre L.	0	n	1	0	Fabaceae Lindl.
Iz_2_1009	DJI_0475	Trifolium arvense L.	0	n	1	0	Fabaceae Lindl.
Iz_2_1009	DJI_0475	Ulmus glabra Huds.	0	n	1	1	Ulmaceae Mirb.
Iz_2_1009	DJI_0475	Verbascum densiflorum Bertol.	0	n	1	0	Scrophulariaceae Juss.
Iz_2_1009	DJI_0475	Veronica austriaca L.	0	n	1	0	Plantaginaceae Juss.
Iz_2_1009	DJI_0475	Veronica incana L.	0	n	1	0	Plantaginaceae Juss.
Iz_2_1009	DJI_0475	Veronica verna L.	0	n	1	0	Plantaginaceae Juss.
Iz_2_1009	DJI_0475	Vincetoxicum hirundinaria Medik	0	n	1	0	Apocynaceae Juss.
Iz_2_1009	DJI_0478	Acer tataricum L.	0	n	1	1	Sapindaceae Juss.
Iz_2_1009	DJI_0478	Achillea millefolium L. s.l.	0	n	1	0	Asteraceae Bercht. & J.Presl
Iz_2_1009	DJI_0478	Allium oleraceum L.	0	n	1	0	Amaryllidaceae J.St.-Hil.
Iz_2_1009	DJI_0478	Ambrosia artemisiifolia L.	0	a	0	0	Asteraceae Bercht. & J.Presl
Iz_2_1009	DJI_0478	Amorpha fruticosa L.	0	a	0	0	Fabaceae Lindl.
Iz_2_1009	DJI_0478	Anthericum ramosum L.	0	n	1	0	Asparagaceae Juss.
Iz_2_1009	DJI_0478	Artemisia marschalliana Spreng.	0	n	1	0	Asteraceae Bercht. & J.Presl
Iz_2_1009	DJI_0478	Asparagus officinalis L.	0	n	1	0	Asparagaceae Juss.
Iz_2_1009	DJI_0478	Astragalus cicer L.	0	n	1	0	Fabaceae Lindl.
Iz_2_1009	DJI_0478	Bassia laniflora (S.G.Gmel.) A.J.Scott	0	n	1	0	Amaranthaceae Juss.
Iz_2_1009	DJI_0478	Berberis vulgaris L.	0	n	1	0	Berberidaceae Juss.
Iz_2_1009	DJI_0478	Betula pendula Roth.	0	n	1	1	Betulaceae Gray
Iz_2_1009	DJI_0478	Betula pendula Roth.	0	n	1	0	Poaceae Barnhart
Iz_2_1009	DJI_0478	Bromus inermis Leyss.	0		NA	NA	
Iz_2_1009	DJI_0478	Bromus squarrosus L.	0	a	0	0	Poaceae Barnhart
Iz_2_1009	DJI_0478	Bromus tectorum L.	0	a	0	0	Poaceae Barnhart
Iz_2_1009	DJI_0478	Calamagrostis epigejos (L.) Roth	75	n	1	0	Poaceae Barnhart
Iz_2_1009	DJI_0478	Carduus acanthoides L.	0	n	1	0	Asteraceae Bercht. & J.Presl
Iz_2_1009	DJI_0478	Carex sp.	0	n	1	0	Cyperaceae Juss.
Iz_2_1009	DJI_0478	Centaurea majorovii Dumbadze	0	n	1	0	Asteraceae Bercht. & J.Presl
Iz_2_1009	DJI_0478	Cf. Solidago virgaurea	0		NA	NA	
Iz_2_1009	DJI_0478	Chamaecytisus ruthenicus (Fisch.ex Wol.) Klaskova	0	n	1	0	Fabaceae Lindl.

Iz_2_1009	DJI_0478	Chelidonium majus L.	0.5	n	1	0	Papaveraceae Juss.
Iz_2_1009	DJI_0478	Chenopodium betaceum Andrz.	0	n	1	0	Amaranthaceae Juss.
Iz_2_1009	DJI_0478	Chenopodium cf. album	0	n	1	0	Amaranthaceae Juss.
Iz_2_1009	DJI_0478	Chenopodium sp.	0		NA	0	Amaranthaceae Juss.
Iz_2_1009	DJI_0478	Chondrilla juncea L.	0	n	1	0	Asteraceae Bercht. & J.Presl
Iz_2_1009	DJI_0478	Cirsium vulgare (Savi) Ten.	0	n	1	0	Asteraceae Bercht. & J.Presl
Iz_2_1009	DJI_0478	Clinopodium acinos (L.) Kuntze	0	n	1	0	Lamiacea Lindl.
Iz_2_1009	DJI_0478	Convallaria majalis L.	0	n	1	0	Asparagaceae Juss.
Iz_2_1009	DJI_0478	Crataegus rhipidophylla Gand.	0	n	1	1	Rosaceae Juss.
Iz_2_1009	DJI_0478	Crepis foetida L.	0	n	1	0	Asteraceae Bercht. & J.Presl
Iz_2_1009	DJI_0478	Dactylis glomerata L.	0	n	1	0	Poaceae Barnhart
Iz_2_1009	DJI_0478	Dianthus campestris M.Bieb.	0	n	1	0	Caryophyllaceae Juss.
Iz_2_1009	DJI_0478	Digitaria sanguinalis (L.) Scop.	0	a	0	0	Poaceae Barnhart
Iz_2_1009	DJI_0478	Elymus repens (L.) Gould	0	n	1	0	Poaceae Barnhart
Iz_2_1009	DJI_0478	Epilobium tetragonum L.	0	n	1	0	Onagraceae Juss.
Iz_2_1009	DJI_0478	Eragrostis minor Host	0	a	0	0	Poaceae Barnhart
Iz_2_1009	DJI_0478	Erigeron canadensis L.	0	a	0	0	Asteraceae Bercht. & J.Presl
Iz_2_1009	DJI_0478	Euphorbia seguieriana Neck.	0	n	1	0	Euphorbiaceae Juss.
Iz_2_1009	DJI_0478	Fallopia dumetorum (L.) Holub	0	n	1	0	Polygonaceae Juss.
Iz_2_1009	DJI_0478	Festuca beckeri (Hack.) Trautv.	0	n	1	0	Poaceae Barnhart
Iz_2_1009	DJI_0478	Fragaria vesca L.	0	n	1	0	Rosaceae Juss.
Iz_2_1009	DJI_0478	Frangula alnus Mill.	0	n	1	1	Rhamnaceae Juss.
Iz_2_1009	DJI_0478	Fraxinus pennsylvanica Marshall	0	a	0	1	Oleaceae Hoffmanns. & Link
Iz_2_1009	DJI_0478	Galeopsis bifida Boenn.	0	n	1	0	Lamiacea Lindl.
Iz_2_1009	DJI_0478	Galium verum L.	0	n	1	0	Rubiaceae Juss.
Iz_2_1009	DJI_0478	Genista tinctoria L.	0	n	1	0	Fabaceae Lindl.
Iz_2_1009	DJI_0478	Geranium robertianum L.	3	n	1	0	Geraniaceae Juss.
Iz_2_1009	DJI_0478	Geranium sanquineum L.	0	n	1	0	Geraniaceae Juss.
Iz_2_1009	DJI_0478	Gypsophila paniculata L.	0	n	1	0	Caryophyllaceae Juss.
Iz_2_1009	DJI_0478	Helichrysum arenarium (L.) Moench	0	n	1	0	Asteraceae Bercht. & J.Presl
Iz_2_1009	DJI_0478	Herniaria polygama J.Gay	0	n	1	0	Caryophyllaceae Juss.
Iz_2_1009	DJI_0478	Hieracium robustum Fr.	0	n	1	0	Asteraceae Bercht. & J.Presl
Iz_2_1009	DJI_0478	Hieracium umbellatum L.	0	n	1	NA	Asteraceae Bercht. & J.Presl
Iz_2_1009	DJI_0478	Hylotelephium maximum (L.) Holub	0	n	1	0	Crassulaceae J.St.-Hil.
Iz_2_1009	DJI_0478	Jacobaea borysthenica (DC.) B.Nord. & Greuter	0	n	1	0	Asteraceae Bercht. & J.Presl
Iz_2_1009	DJI_0478	Jasione montana L.	0	n	1	0	Campanulaceae Juss.
Iz_2_1009	DJI_0478	Jurinea cyanoides (L.) Rchb.s.l.	0	n	1	0	Asteraceae Bercht. & J.Presl
Iz_2_1009	DJI_0478	Koeleria glauca (Spreng.) DC.	0	n	1	0	Poaceae Barnhart
Iz_2_1009	DJI_0478	Lactuca serriola L.	0	a	0	0	Asteraceae Bercht. & J.Presl
Iz_2_1009	DJI_0478	Linaria genistifolia (L.) Mill.	0	n	1	0	Plantaginaceae Juss.
Iz_2_1009	DJI_0478	Lomelosia argentea (L.) Greuter & Burdet	0	n	1	0	Caprifoliaceae Juss.
Iz_2_1009	DJI_0478	Melampyrum pratense L.	0	n	1	0	Orobanchaceae Vent.
Iz_2_1009	DJI_0478	Oenothera biennis L.	0	a	0	0	Onagraceae Juss.
Iz_2_1009	DJI_0478	Pentanema britannica (L.) D.Gut.Larr., Santos-Vicente, Anderb., E.Rico & M.M.Mart.Ort.	0	n	1	0	Asteraceae Bercht. & J.Presl
Iz_2_1009	DJI_0478	Peucedanum oreoselinum (L.) Moench	2	n	1	0	Apiaceae Lindl.
Iz_2_1009	DJI_0478	Peucedanum ruthenicum M.Bieb.	0	n	1	0	Apiaceae Lindl.
Iz_2_1009	DJI_0478	Pilosella echioides (Lumn.) F.W.Schultz & Sch.Bip.	0	n	1	0	Asteraceae Bercht. & J.Presl
Iz_2_1009	DJI_0478	Pilosella officinarum F. Schultz et Sch. Bip.	0	n	1	0	Asteraceae Bercht. & J.Presl
Iz_2_1009	DJI_0478	Pilosella sp.	0		NA	0	Asteraceae Bercht. & J.Presl
Iz_2_1009	DJI_0478	Pinus sylvestris L.	0	n	1	1	Pinaceae Spreng. ex F.Rudolphi
Iz_2_1009	DJI_0478	Poa annua L.	0	n	1	0	Poaceae Barnhart
Iz_2_1009	DJI_0478	Poa nemoralis L.	0	n	1	0	Poaceae Barnhart
Iz_2_1009	DJI_0478	Poa sp.	0		NA	0	Poaceae Barnhart
Iz_2_1009	DJI_0478	Polygonatum odoratum (Mill.) Druce	0	n	1	0	Asparagaceae Juss.
Iz_2_1009	DJI_0478	Polygonum arenarium Waldst. & Kit.	0	n	1	0	Polygonaceae Juss.
Iz_2_1009	DJI_0478	Populus nigra L.	0	n	1	1	Salicaceae Mirb.
Iz_2_1009	DJI_0478	Populus tremula L.	0	n	1	1	Salicaceae Mirb.
Iz_2_1009	DJI_0478	Populus Ã canescens (Aiton) Sm.	0	n	1	1	Salicaceae Mirb.
Iz_2_1009	DJI_0478	Potentilla argentea L.	0	n	1	0	Rosaceae Juss.
Iz_2_1009	DJI_0478	Psammophiliella muralis (L.) Ikonn.	0	n	1	0	Caryophyllaceae Juss.
Iz_2_1009	DJI_0478	Psephellus sumensis (Kalen.) Greuter	0	n	1	0	Asteraceae Bercht. & J.Presl
Iz_2_1009	DJI_0478	Pulsatilla pratensis (L.) Mill. s.l.	0	n	1	0	Ranunculaceae Juss.
Iz_2_1009	DJI_0478	Pyrus communis L.	0	a	0	1	Rosaceae Juss.
Iz_2_1009	DJI_0478	Quercus robur L.	0	n	1	1	Fagaceae Dumort.
Iz_2_1009	DJI_0478	Robinia pseudoacacia L.	0	a	0	1	Fabaceae Lindl.
Iz_2_1009	DJI_0478	Rosa sp.	0		NA	1	Rosaceae Juss.
Iz_2_1009	DJI_0478	Rubus idaeus L.	0	n	1	0	Rosaceae Juss.
Iz_2_1009	DJI_0478	Rumex acetosella L.	0	n	1	0	Polygonaceae Juss.
Iz_2_1009	DJI_0478	Rumex thyrsiflorus Fingerh.	0	n	1	0	Polygonaceae Juss.
Iz_2_1009	DJI_0478	Salix alba L.	0	n	1	1	Salicaceae Mirb.
Iz_2_1009	DJI_0478	Sambucus nigra L.	0	n	1	1	Viburnaceae Raf.
Iz_2_1009	DJI_0478	Secale sylvestre Host	0	n	1	0	Poaceae Barnhart
Iz_2_1009	DJI_0478	Sempervivum ruthenicum Schnittsp. & C.B. Lehm.	0	n	1	0	Crassulaceae J.St.-Hil.
Iz_2_1009	DJI_0478	Setaria viridis (L.) P. Beauv.	0	n	1	0	Poaceae Barnhart
Iz_2_1009	DJI_0478	Silene borysthenica (Gruner) Walters	0	n	1	0	Caryophyllaceae Juss.
Iz_2_1009	DJI_0478	Silene latifolia subsp. alba (Mill.) Greuter & Burdet	0	n	1	0	Caryophyllaceae Juss.
Iz_2_1009	DJI_0478	Solidago virgaurea L.	0	n	1	0	Asteraceae Bercht. & J.Presl
Iz_2_1009	DJI_0478	Sorbus aucuparia L.	0	n	1	1	Rosaceae Juss.
Iz_2_1009	DJI_0478	Stachys recta L.	0	n	1	0	Lamiacea Lindl.
Iz_2_1009	DJI_0478	Tanacetum vulgare L.	0	n	1	0	Asteraceae Bercht. & J.Presl
Iz_2_1009	DJI_0478	Thymus pallasianus Heinr.Braun	0	n	1	0	Lamiacea Lindl.
Iz_2_1009	DJI_0478	Tilia cordata Mill.	0	n	1	1	Malvaceae Juss.
Iz_2_1009	DJI_0478	Trifolium alpestre L.	0	n	1	0	Fabaceae Lindl.
Iz_2_1009	DJI_0478	Trifolium arvense L.	0	n	1	0	Fabaceae Lindl.
Iz_2_1009	DJI_0478	Ulmus glabra Huds.	0	n	1	1	Ulmaceae Mirb.
Iz_2_1009	DJI_0478	Verbascum densiflorum Bertol.	0	n	1	0	Scrophulariaceae Juss.
Iz_2_1009	DJI_0478	Veronica austriaca L.	0	n	1	0	Plantaginaceae Juss.
Iz_2_1009	DJI_0478	Veronica incana L.	0	n	1	0	Plantaginaceae Juss.
Iz_2_1009	DJI_0478	Veronica verna L.	0	n	1	0	Plantaginaceae Juss.
Iz_2_1009	DJI_0478	Vincetoxicum hirundinaria Medik	0	n	1	0	Apocynaceae Juss.
Iz_2_1009	DJI_0481	Acer tataricum L.	0	n	1	1	Sapindaceae Juss.
Iz_2_1009	DJI_0481	Achillea millefolium L. s.l.	0	n	1	0	Asteraceae Bercht. & J.Presl
Iz_2_1009	DJI_0481	Allium oleraceum L.	0	n	1	0	Amaryllidaceae J.St.-Hil.
Iz_2_1009	DJI_0481	Ambrosia artemisiifolia L.	0	a	0	0	Asteraceae Bercht. & J.Presl
Iz_2_1009	DJI_0481	Amorpha fruticosa L.	0	a	0	0	Fabaceae Lindl.
Iz_2_1009	DJI_0481	Anthericum ramosum L.	0	n	1	0	Asparagaceae Juss.
Iz_2_1009	DJI_0481	Artemisia marschalliana Spreng.	0	n	1	0	Asteraceae Bercht. & J.Presl
Iz_2_1009	DJI_0481	Asparagus officinalis L.	0	n	1	0	Asparagaceae Juss.

Iz_2_1009	DJI_0481	Astragalus cicer L.	0	n	1	0	Fabaceae Lindl.
Iz_2_1009	DJI_0481	Bassia laniflora (S.G.Gmel.) A.J.Scott	0	n	1	0	Amaranthaceae Juss.
Iz_2_1009	DJI_0481	Berberis vulgaris L.	0	n	1	0	Berberidaceae Juss.
Iz_2_1009	DJI_0481	Betula pendula Roth.	0	n	1	1	Betulaceae Gray
Iz_2_1009	DJI_0481	Betula pendula Roth.	0	n	1	0	Poaceae Barnhart
Iz_2_1009	DJI_0481	Bromus inermis Leyss.	0		NA	NA	
Iz_2_1009	DJI_0481	Bromus squarrosus L.	0	a	0	0	Poaceae Barnhart
Iz_2_1009	DJI_0481	Bromus tectorum L.	0	a	0	0	Poaceae Barnhart
Iz_2_1009	DJI_0481	Calamagrostis epigejos (L.) Roth	70	n	1	0	Poaceae Barnhart
Iz_2_1009	DJI_0481	Carduus acanthoides L.	0	n	1	0	Asteraceae Bercht. & J.Presl
Iz_2_1009	DJI_0481	Carex sp.	0	n	1	0	Cyperaceae Juss.
Iz_2_1009	DJI_0481	Centaurea majorovii Dumbadze	0	n	1	0	Asteraceae Bercht. & J.Presl
Iz_2_1009	DJI_0481	Cf. Solidago virgaurea	0		NA	NA	
Iz_2_1009	DJI_0481	Chamaecytisus ruthenicus (Fisch.ex Wol.) Klaskova	0	n	1	0	Fabaceae Lindl.
Iz_2_1009	DJI_0481	Chelidonium majus L.	5	n	1	0	Papaveraceae Juss.
Iz_2_1009	DJI_0481	Chenopodium betaceum Andrz.	0	n	1	0	Amaranthaceae Juss.
Iz_2_1009	DJI_0481	Chenopodium cf. album	0	n	1	0	Amaranthaceae Juss.
Iz_2_1009	DJI_0481	Chenopodium sp.	0		NA	0	Amaranthaceae Juss.
Iz_2_1009	DJI_0481	Chondrilla juncea L.	0	n	1	0	Asteraceae Bercht. & J.Presl
Iz_2_1009	DJI_0481	Cirsium vulgare (Savi) Ten.	0	n	1	0	Asteraceae Bercht. & J.Presl
Iz_2_1009	DJI_0481	Clinopodium acinos (L.) Kuntze	0	n	1	0	Lamiacea Lindl.
Iz_2_1009	DJI_0481	Convallaria majalis L.	0	n	1	0	Asparagaceae Juss.
Iz_2_1009	DJI_0481	Crataegus rhipidophylla Gand.	0	n	1	1	Rosaceae Juss.
Iz_2_1009	DJI_0481	Crepis foetida L.	0	n	1	0	Asteraceae Bercht. & J.Presl
Iz_2_1009	DJI_0481	Dactylis glomerata L.	0	n	1	0	Poaceae Barnhart
Iz_2_1009	DJI_0481	Dianthus campestris M.Bieb.	0	n	1	0	Caryophyllaceae Juss.
Iz_2_1009	DJI_0481	Digitaria sanguinalis (L.) Scop.	0	a	0	0	Poaceae Barnhart
Iz_2_1009	DJI_0481	Elymus repens (L.) Gould	0	n	1	0	Poaceae Barnhart
Iz_2_1009	DJI_0481	Epilobium tetragonum L.	0	n	1	0	Onagraceae Juss.
Iz_2_1009	DJI_0481	Eragrostis minor Host	0	a	0	0	Poaceae Barnhart
Iz_2_1009	DJI_0481	Erigeron canadensis L.	0	a	0	0	Asteraceae Bercht. & J.Presl
Iz_2_1009	DJI_0481	Euphorbia seguieriana Neck.	0	n	1	0	Euphorbiaceae Juss.
Iz_2_1009	DJI_0481	Fallopia dumetorum (L.) Holub	0	n	1	0	Polygonaceae Juss.
Iz_2_1009	DJI_0481	Festuca beckeri (Hack.) Trautv.	0	n	1	0	Poaceae Barnhart
Iz_2_1009	DJI_0481	Fragaria vesca L.	0	n	1	0	Rosaceae Juss.
Iz_2_1009	DJI_0481	Frangula alnus Mill.	0	n	1	1	Rhamnaceae Juss.
Iz_2_1009	DJI_0481	Fraxinus pennsylvanica Marshall	0	a	0	1	Oleaceae Hoffmanns. & Link
Iz_2_1009	DJI_0481	Galeopsis bifida Boenn.	0	n	1	0	Lamiacea Lindl.
Iz_2_1009	DJI_0481	Galium verum L.	0	n	1	0	Rubiaceae Juss.
Iz_2_1009	DJI_0481	Genista tinctoria L.	0	n	1	0	Fabaceae Lindl.
Iz_2_1009	DJI_0481	Geranium robertianum L.	0	n	1	0	Geraniaceae Juss.
Iz_2_1009	DJI_0481	Geranium sanquineum L.	0	n	1	0	Geraniaceae Juss.
Iz_2_1009	DJI_0481	Gypsophila paniculata L.	0	n	1	0	Caryophyllaceae Juss.
Iz_2_1009	DJI_0481	Helichrysum arenarium (L.) Moench	0	n	1	0	Asteraceae Bercht. & J.Presl
Iz_2_1009	DJI_0481	Herniaria polygama J.Gay	0	n	1	0	Caryophyllaceae Juss.
Iz_2_1009	DJI_0481	Hieracium robustum Fr.	0	n	1	0	Asteraceae Bercht. & J.Presl
Iz_2_1009	DJI_0481	Hieracium umbellatum L.	2	n	1	NA	Asteraceae Bercht. & J.Presl
Iz_2_1009	DJI_0481	Hylotelephium maximum (L.) Holub	0	n	1	0	Crassulaceae J.St.-Hil.
Iz_2_1009	DJI_0481	Jacobaea borysthenica (DC.) B.Nord. & Greuter	0	n	1	0	Asteraceae Bercht. & J.Presl
Iz_2_1009	DJI_0481	Jasione montana L.	0	n	1	0	Campanulaceae Juss.
Iz_2_1009	DJI_0481	Jurinea cyanoides (L.) Rchb.s.l.	0	n	1	0	Asteraceae Bercht. & J.Presl
Iz_2_1009	DJI_0481	Koeleria glauca (Spreng.) DC.	0	n	1	0	Poaceae Barnhart
Iz_2_1009	DJI_0481	Lactuca serriola L.	0	a	0	0	Asteraceae Bercht. & J.Presl
Iz_2_1009	DJI_0481	Linaria genistifolia (L.) Mill.	0	n	1	0	Plantaginaceae Juss.
Iz_2_1009	DJI_0481	Lomelosia argentea (L.) Greuter & Burdet	0	n	1	0	Caprifoliaceae Juss.
Iz_2_1009	DJI_0481	Melampyrum pratense L.	0	n	1	0	Orobanchaceae Vent.
Iz_2_1009	DJI_0481	Oenothera biennis L.	0	a	0	0	Onagraceae Juss.
Iz_2_1009	DJI_0481	Pentanema britannica (L.) D.Gut.Larr., Santos-Vicente, Anderb., E.Rico & M.M.Mart.Ort.	0	n	1	0	Asteraceae Bercht. & J.Presl
Iz_2_1009	DJI_0481	Peucedanum oreoselinum (L.) Moench	2	n	1	0	Apiaceae Lindl.
Iz_2_1009	DJI_0481	Peucedanum ruthenicum M.Bieb.	0	n	1	0	Apiaceae Lindl.
Iz_2_1009	DJI_0481	Pilosella echioides (Lumn.) F.W.Schultz & Sch.Bip.	0	n	1	0	Asteraceae Bercht. & J.Presl
Iz_2_1009	DJI_0481	Pilosella officinarum F. Schultz et Sch. Bip.	0	n	1	0	Asteraceae Bercht. & J.Presl
Iz_2_1009	DJI_0481	Pilosella sp.	0		NA	0	Asteraceae Bercht. & J.Presl
Iz_2_1009	DJI_0481	Pinus sylvestris L.	0	n	1	1	Pinaceae Spreng. ex F.Rudolphi
Iz_2_1009	DJI_0481	Poa annua L.	0	n	1	0	Poaceae Barnhart
Iz_2_1009	DJI_0481	Poa nemoralis L.	0	n	1	0	Poaceae Barnhart
Iz_2_1009	DJI_0481	Poa sp.	0		NA	0	Poaceae Barnhart
Iz_2_1009	DJI_0481	Polygonatum odoratum (Mill.) Druce	0	n	1	0	Asparagaceae Juss.
Iz_2_1009	DJI_0481	Polygonum arenarium Waldst. & Kit.	0	n	1	0	Polygonaceae Juss.
Iz_2_1009	DJI_0481	Populus nigra L.	0	n	1	1	Salicaceae Mirb.
Iz_2_1009	DJI_0481	Populus tremula L.	0	n	1	1	Salicaceae Mirb.
Iz_2_1009	DJI_0481	Populus Ã canescens (Aiton) Sm.	0	n	1	1	Salicaceae Mirb.
Iz_2_1009	DJI_0481	Potentilla argentea L.	0	n	1	0	Rosaceae Juss.
Iz_2_1009	DJI_0481	Psammophiliella muralis (L.) Ikonn.	0	n	1	0	Caryophyllaceae Juss.
Iz_2_1009	DJI_0481	Psephellus sumensis (Kalen.) Greuter	0	n	1	0	Asteraceae Bercht. & J.Presl
Iz_2_1009	DJI_0481	Pulsatilla pratensis (L.) Mill. s.l.	0	n	1	0	Ranunculaceae Juss.
Iz_2_1009	DJI_0481	Pyrus communis L.	0	a	0	1	Rosaceae Juss.
Iz_2_1009	DJI_0481	Quercus robur L.	1	n	1	1	Fagaceae Dumort.
Iz_2_1009	DJI_0481	Robinia pseudoacacia L.	0	a	0	1	Fabaceae Lindl.
Iz_2_1009	DJI_0481	Rosa sp.	0		NA	1	Rosaceae Juss.
Iz_2_1009	DJI_0481	Rubus idaeus L.	0	n	1	0	Rosaceae Juss.
Iz_2_1009	DJI_0481	Rumex acetosella L.	0	n	1	0	Polygonaceae Juss.
Iz_2_1009	DJI_0481	Rumex thyrsiflorus Fingerh.	0	n	1	0	Polygonaceae Juss.
Iz_2_1009	DJI_0481	Salix alba L.	0	n	1	1	Salicaceae Mirb.
Iz_2_1009	DJI_0481	Sambucus nigra L.	0	n	1	1	Viburnaceae Raf.
Iz_2_1009	DJI_0481	Secale sylvestre Host	0	n	1	0	Poaceae Barnhart
Iz_2_1009	DJI_0481	Sempervivum ruthenicum Schnittsp. & C.B. Lehm.	0	n	1	0	Crassulaceae J.St.-Hil.
Iz_2_1009	DJI_0481	Setaria viridis (L.) P. Beauv.	0	n	1	0	Poaceae Barnhart
Iz_2_1009	DJI_0481	Silene borysthenica (Gruner) Walters	0	n	1	0	Caryophyllaceae Juss.
Iz_2_1009	DJI_0481	Silene latifolia subsp. alba (Mill.) Greuter & Burdet	0	n	1	0	Caryophyllaceae Juss.
Iz_2_1009	DJI_0481	Solidago virgaurea L.	0	n	1	0	Asteraceae Bercht. & J.Presl
Iz_2_1009	DJI_0481	Sorbus aucuparia L.	0	n	1	1	Rosaceae Juss.
Iz_2_1009	DJI_0481	Stachys recta L.	0	n	1	0	Lamiacea Lindl.
Iz_2_1009	DJI_0481	Tanacetum vulgare L.	0	n	1	0	Asteraceae Bercht. & J.Presl
Iz_2_1009	DJI_0481	Thymus pallasianus Heinr.Braun	0	n	1	0	Lamiacea Lindl.
Iz_2_1009	DJI_0481	Tilia cordata Mill.	0	n	1	1	Malvaceae Juss.
Iz_2_1009	DJI_0481	Trifolium alpestre L.	0	n	1	0	Fabaceae Lindl.
Iz_2_1009	DJI_0481	Trifolium arvense L.	0	n	1	0	Fabaceae Lindl.

Iz_2_1009	DJI_0481	Ulmus glabra Huds.	0	n	1	1	Ulmaceae Mirb.
Iz_2_1009	DJI_0481	Verbascum densiflorum Bertol.	0	n	1	0	Scrophulariaceae Juss.
Iz_2_1009	DJI_0481	Veronica austriaca L.	0	n	1	0	Plantaginaceae Juss.
Iz_2_1009	DJI_0481	Veronica incana L.	0	n	1	0	Plantaginaceae Juss.
Iz_2_1009	DJI_0481	Veronica verna L.	0	n	1	0	Plantaginaceae Juss.
Iz_2_1009	DJI_0481	Vincetoxicum hirundinaria Medik	0	n	1	0	Apocynaceae Juss.
Iz_2_1009	DJI_0491	Acer tataricum L.	0	n	1	1	Sapindaceae Juss.
Iz_2_1009	DJI_0491	Achillea millefolium L. s.l.	0	n	1	0	Asteraceae Bercht. & J.Presl
Iz_2_1009	DJI_0491	Allium oleraceum L.	0	n	1	0	Amaryllidaceae J.St.-Hil.
Iz_2_1009	DJI_0491	Ambrosia artemisiifolia L.	0	a	0	0	Asteraceae Bercht. & J.Presl
Iz_2_1009	DJI_0491	Amorpha fruticosa L.	0	a	0	0	Fabaceae Lindl.
Iz_2_1009	DJI_0491	Anthericum ramosum L.	0	n	1	0	Asparagaceae Juss.
Iz_2_1009	DJI_0491	Artemisia marschalliana Spreng.	0	n	1	0	Asteraceae Bercht. & J.Presl
Iz_2_1009	DJI_0491	Asparagus officinalis L.	0	n	1	0	Asparagaceae Juss.
Iz_2_1009	DJI_0491	Astragalus cicer L.	0	n	1	0	Fabaceae Lindl.
Iz_2_1009	DJI_0491	Bassia laniflora (S.G.Gmel.) A.J.Scott	0	n	1	0	Amaranthaceae Juss.
Iz_2_1009	DJI_0491	Berberis vulgaris L.	0	n	1	0	Berberidaceae Juss.
Iz_2_1009	DJI_0491	Betula pendula Roth.	0	n	1	1	Betulaceae Gray
Iz_2_1009	DJI_0491	Betula pendula Roth.	0	n	1	0	Poaceae Barnhart
Iz_2_1009	DJI_0491	Bromus inermis Leyss.	0		NA	NA	
Iz_2_1009	DJI_0491	Bromus squarrosus L.	0	a	0	0	Poaceae Barnhart
Iz_2_1009	DJI_0491	Bromus tectorum L.	0	a	0	0	Poaceae Barnhart
Iz_2_1009	DJI_0491	Calamagrostis epigejos (L.) Roth	15	n	1	0	Poaceae Barnhart
Iz_2_1009	DJI_0491	Carduus acanthoides L.	0	n	1	0	Asteraceae Bercht. & J.Presl
Iz_2_1009	DJI_0491	Carex sp.	0	n	1	0	Cyperaceae Juss.
Iz_2_1009	DJI_0491	Centaurea majorovii Dumbadze	0	n	1	0	Asteraceae Bercht. & J.Presl
Iz_2_1009	DJI_0491	Cf. Solidago virgaurea	0		NA	NA	
Iz_2_1009	DJI_0491	Chamaecytisus ruthenicus (Fisch.ex Wol.) Klaskova	0	n	1	0	Fabaceae Lindl.
Iz_2_1009	DJI_0491	Chelidonium majus L.	0	n	1	0	Papaveraceae Juss.
Iz_2_1009	DJI_0491	Chenopodium betaceum Andrz.	0	n	1	0	Amaranthaceae Juss.
Iz_2_1009	DJI_0491	Chenopodium cf. album	0	n	1	0	Amaranthaceae Juss.
Iz_2_1009	DJI_0491	Chenopodium sp.	0		NA	0	Amaranthaceae Juss.
Iz_2_1009	DJI_0491	Chondrilla juncea L.	0	n	1	0	Asteraceae Bercht. & J.Presl
Iz_2_1009	DJI_0491	Cirsium vulgare (Savi) Ten.	0	n	1	0	Asteraceae Bercht. & J.Presl
Iz_2_1009	DJI_0491	Clinopodium acinos (L.) Kuntze	0	n	1	0	Lamiacea Lindl.
Iz_2_1009	DJI_0491	Convallaria majalis L.	0	n	1	0	Asparagaceae Juss.
Iz_2_1009	DJI_0491	Crataegus rhipidophylla Gand.	0	n	1	1	Rosaceae Juss.
Iz_2_1009	DJI_0491	Crepis foetida L.	0	n	1	0	Asteraceae Bercht. & J.Presl
Iz_2_1009	DJI_0491	Dactylis glomerata L.	0	n	1	0	Poaceae Barnhart
Iz_2_1009	DJI_0491	Dianthus campestris M.Bieb.	0	n	1	0	Caryophyllaceae Juss.
Iz_2_1009	DJI_0491	Digitaria sanguinalis (L.) Scop.	0	a	0	0	Poaceae Barnhart
Iz_2_1009	DJI_0491	Elymus repens (L.) Gould	0	n	1	0	Poaceae Barnhart
Iz_2_1009	DJI_0491	Epilobium tetragonum L.	0	n	1	0	Onagraceae Juss.
Iz_2_1009	DJI_0491	Eragrostis minor Host	0	a	0	0	Poaceae Barnhart
Iz_2_1009	DJI_0491	Erigeron canadensis L.	0	a	0	0	Asteraceae Bercht. & J.Presl
Iz_2_1009	DJI_0491	Euphorbia seguieriana Neck.	0	n	1	0	Euphorbiaceae Juss.
Iz_2_1009	DJI_0491	Fallopia dumetorum (L.) Holub	0	n	1	0	Polygonaceae Juss.
Iz_2_1009	DJI_0491	Festuca beckeri (Hack.) Trautv.	0	n	1	0	Poaceae Barnhart
Iz_2_1009	DJI_0491	Fragaria vesca L.	0	n	1	0	Rosaceae Juss.
Iz_2_1009	DJI_0491	Frangula alnus Mill.	0	n	1	1	Rhamnaceae Juss.
Iz_2_1009	DJI_0491	Fraxinus pennsylvanica Marshall	0	a	0	1	Oleaceae Hoffmanns. & Link
Iz_2_1009	DJI_0491	Galeopsis bifida Boenn.	0	n	1	0	Lamiacea Lindl.
Iz_2_1009	DJI_0491	Galium verum L.	0	n	1	0	Rubiaceae Juss.
Iz_2_1009	DJI_0491	Genista tinctoria L.	0	n	1	0	Fabaceae Lindl.
Iz_2_1009	DJI_0491	Geranium robertianum L.	0	n	1	0	Geraniaceae Juss.
Iz_2_1009	DJI_0491	Geranium sanquineum L.	0	n	1	0	Geraniaceae Juss.
Iz_2_1009	DJI_0491	Gypsophila paniculata L.	0	n	1	0	Caryophyllaceae Juss.
Iz_2_1009	DJI_0491	Helichrysum arenarium (L.) Moench	0	n	1	0	Asteraceae Bercht. & J.Presl
Iz_2_1009	DJI_0491	Herniaria polygama J.Gay	0	n	1	0	Caryophyllaceae Juss.
Iz_2_1009	DJI_0491	Hieracium robustum Fr.	0	n	1	0	Asteraceae Bercht. & J.Presl
Iz_2_1009	DJI_0491	Hieracium umbellatum L.	0	n	1	NA	Asteraceae Bercht. & J.Presl
Iz_2_1009	DJI_0491	Hylotelephium maximum (L.) Holub	0	n	1	0	Crassulaceae J.St.-Hil.
Iz_2_1009	DJI_0491	Jacobaea borysthenica (DC.) B.Nord. & Greuter	0	n	1	0	Asteraceae Bercht. & J.Presl
Iz_2_1009	DJI_0491	Jasione montana L.	0	n	1	0	Campanulaceae Juss.
Iz_2_1009	DJI_0491	Jurinea cyanoides (L.) Rchb.s.l.	0	n	1	0	Asteraceae Bercht. & J.Presl
Iz_2_1009	DJI_0491	Koeleria glauca (Spreng.) DC.	0	n	1	0	Poaceae Barnhart
Iz_2_1009	DJI_0491	Lactuca serriola L.	0	a	0	0	Asteraceae Bercht. & J.Presl
Iz_2_1009	DJI_0491	Linaria genistifolia (L.) Mill.	0	n	1	0	Plantaginaceae Juss.
Iz_2_1009	DJI_0491	Lomelosia argentea (L.) Greuter & Burdet	0	n	1	0	Caprifoliaceae Juss.
Iz_2_1009	DJI_0491	Melampyrum pratense L.	0	n	1	0	Orobanchaceae Vent.
Iz_2_1009	DJI_0491	Oenothera biennis L.	0	a	0	0	Onagraceae Juss.
Iz_2_1009	DJI_0491	Pentanema britannica (L.) D.Gut.Larr., Santos-Vicente, Anderb., E.Rico & M.M.Mart.Ort.	0	n	1	0	Asteraceae Bercht. & J.Presl
Iz_2_1009	DJI_0491	Peucedanum oreoselinum (L.) Moench	0	n	1	0	Apiaceae Lindl.
Iz_2_1009	DJI_0491	Peucedanum ruthenicum M.Bieb.	0	n	1	0	Apiaceae Lindl.
Iz_2_1009	DJI_0491	Pilosella echioides (Lumn.) F.W.Schultz & Sch.Bip.	0	n	1	0	Asteraceae Bercht. & J.Presl
Iz_2_1009	DJI_0491	Pilosella officinarum F. Schultz et Sch. Bip.	0	n	1	0	Asteraceae Bercht. & J.Presl
Iz_2_1009	DJI_0491	Pilosella sp.	0		NA	0	Asteraceae Bercht. & J.Presl
Iz_2_1009	DJI_0491	Pinus sylvestris L.	0	n	1	1	Pinaceae Spreng. ex F.Rudolphi
Iz_2_1009	DJI_0491	Poa annua L.	0	n	1	0	Poaceae Barnhart
Iz_2_1009	DJI_0491	Poa nemoralis L.	0	n	1	0	Poaceae Barnhart
Iz_2_1009	DJI_0491	Poa sp.	0		NA	0	Poaceae Barnhart
Iz_2_1009	DJI_0491	Polygonatum odoratum (Mill.) Druce	0	n	1	0	Asparagaceae Juss.
Iz_2_1009	DJI_0491	Polygonum arenarium Waldst. & Kit.	0	n	1	0	Polygonaceae Juss.
Iz_2_1009	DJI_0491	Populus nigra L.	0	n	1	1	Salicaceae Mirb.
Iz_2_1009	DJI_0491	Populus tremula L.	0	n	1	1	Salicaceae Mirb.
Iz_2_1009	DJI_0491	Populus Ã canescens (Aiton) Sm.	0	n	1	1	Salicaceae Mirb.
Iz_2_1009	DJI_0491	Potentilla argentea L.	0	n	1	0	Rosaceae Juss.
Iz_2_1009	DJI_0491	Psammophiliella muralis (L.) Ikonn.	0	n	1	0	Caryophyllaceae Juss.
Iz_2_1009	DJI_0491	Psephellus sumensis (Kalen.) Greuter	0	n	1	0	Asteraceae Bercht. & J.Presl
Iz_2_1009	DJI_0491	Pulsatilla pratensis (L.) Mill. s.l.	0	n	1	0	Ranunculaceae Juss.
Iz_2_1009	DJI_0491	Pyrus communis L.	0	a	0	1	Rosaceae Juss.
Iz_2_1009	DJI_0491	Quercus robur L.	0	n	1	1	Fagaceae Dumort.
Iz_2_1009	DJI_0491	Robinia pseudoacacia L.	0	a	0	1	Fabaceae Lindl.
Iz_2_1009	DJI_0491	Rosa sp.	0		NA	1	Rosaceae Juss.
Iz_2_1009	DJI_0491	Rubus idaeus L.	0	n	1	0	Rosaceae Juss.
Iz_2_1009	DJI_0491	Rumex acetosella L.	0	n	1	0	Polygonaceae Juss.
Iz_2_1009	DJI_0491	Rumex thyrsiflorus Fingerh.	0	n	1	0	Polygonaceae Juss.
Iz_2_1009	DJI_0491	Salix alba L.	0	n	1	1	Salicaceae Mirb.

Iz_2_1009	DJI_0491	Sambucus nigra L.	0	n	1	1	Viburnaceae Raf.
Iz_2_1009	DJI_0491	Secale sylvestre Host	0	n	1	0	Poaceae Barnhart
Iz_2_1009	DJI_0491	Sempervivum ruthenicum Schnittsp. & C.B. Lehm.	0	n	1	0	Crassulaceae J.St.-Hil.
Iz_2_1009	DJI_0491	Setaria viridis (L.) P. Beauv.	0	n	1	0	Poaceae Barnhart
Iz_2_1009	DJI_0491	Silene borysthenica (Gruner) Walters	0	n	1	0	Caryophyllaceae Juss.
Iz_2_1009	DJI_0491	Silene latifolia subsp. alba (Mill.) Greuter & Burdet	0	n	1	0	Caryophyllaceae Juss.
Iz_2_1009	DJI_0491	Solidago virgaurea L.	0	n	1	0	Asteraceae Bercht. & J.Presl
Iz_2_1009	DJI_0491	Sorbus aucuparia L.	0	n	1	1	Rosaceae Juss.
Iz_2_1009	DJI_0491	Stachys recta L.	0	n	1	0	Lamiacea Lindl.
Iz_2_1009	DJI_0491	Tanacetum vulgare L.	0	n	1	0	Asteraceae Bercht. & J.Presl
Iz_2_1009	DJI_0491	Thymus pallasianus Heinr.Braun	0	n	1	0	Lamiacea Lindl.
Iz_2_1009	DJI_0491	Tilia cordata Mill.	0	n	1	1	Malvaceae Juss.
Iz_2_1009	DJI_0491	Trifolium alpestre L.	0	n	1	0	Fabaceae Lindl.
Iz_2_1009	DJI_0491	Trifolium arvense L.	0	n	1	0	Fabaceae Lindl.
Iz_2_1009	DJI_0491	Ulmus glabra Huds.	0	n	1	1	Ulmaceae Mirb.
Iz_2_1009	DJI_0491	Verbascum densiflorum Bertol.	0	n	1	0	Scrophulariaceae Juss.
Iz_2_1009	DJI_0491	Veronica austriaca L.	0	n	1	0	Plantaginaceae Juss.
Iz_2_1009	DJI_0491	Veronica incana L.	0	n	1	0	Plantaginaceae Juss.
Iz_2_1009	DJI_0491	Veronica verna L.	0	n	1	0	Plantaginaceae Juss.
Iz_2_1009	DJI_0491	Vincetoxicum hirundinaria Medik	0	n	1	0	Apocynaceae Juss.
Iz_2_1009	DJI_0494	Acer tataricum L.	0	n	1	1	Sapindaceae Juss.
Iz_2_1009	DJI_0494	Achillea millefolium L. s.l.	0	n	1	0	Asteraceae Bercht. & J.Presl
Iz_2_1009	DJI_0494	Allium oleraceum L.	0	n	1	0	Amaryllidaceae J.St.-Hil.
Iz_2_1009	DJI_0494	Ambrosia artemisiifolia L.	0	a	0	0	Asteraceae Bercht. & J.Presl
Iz_2_1009	DJI_0494	Amorpha fruticosa L.	0	a	0	0	Fabaceae Lindl.
Iz_2_1009	DJI_0494	Anthericum ramosum L.	0	n	1	0	Asparagaceae Juss.
Iz_2_1009	DJI_0494	Artemisia marschalliana Spreng.	0	n	1	0	Asteraceae Bercht. & J.Presl
Iz_2_1009	DJI_0494	Asparagus officinalis L.	0	n	1	0	Asparagaceae Juss.
Iz_2_1009	DJI_0494	Astragalus cicer L.	0	n	1	0	Fabaceae Lindl.
Iz_2_1009	DJI_0494	Bassia laniflora (S.G.Gmel.) A.J.Scott	0	n	1	0	Amaranthaceae Juss.
Iz_2_1009	DJI_0494	Berberis vulgaris L.	0	n	1	0	Berberidaceae Juss.
Iz_2_1009	DJI_0494	Betula pendula Roth.	0	n	1	1	Betulaceae Gray
Iz_2_1009	DJI_0494	Betula pendula Roth.	0	n	1	0	Poaceae Barnhart
Iz_2_1009	DJI_0494	Bromus inermis Leyss.	0		NA	NA	
Iz_2_1009	DJI_0494	Bromus squarrosus L.	0	a	0	0	Poaceae Barnhart
Iz_2_1009	DJI_0494	Bromus tectorum L.	0	a	0	0	Poaceae Barnhart
Iz_2_1009	DJI_0494	Calamagrostis epigejos (L.) Roth	25	n	1	0	Poaceae Barnhart
Iz_2_1009	DJI_0494	Carduus acanthoides L.	0	n	1	0	Asteraceae Bercht. & J.Presl
Iz_2_1009	DJI_0494	Carex sp.	0	n	1	0	Cyperaceae Juss.
Iz_2_1009	DJI_0494	Centaurea majorovii Dumbadze	0	n	1	0	Asteraceae Bercht. & J.Presl
Iz_2_1009	DJI_0494	Cf. Solidago virgaurea	0		NA	NA	
Iz_2_1009	DJI_0494	Chamaecytisus ruthenicus (Fisch.ex Wol.) Klaskova	0	n	1	0	Fabaceae Lindl.
Iz_2_1009	DJI_0494	Chelidonium majus L.	0	n	1	0	Papaveraceae Juss.
Iz_2_1009	DJI_0494	Chenopodium betaceum Andrz.	0	n	1	0	Amaranthaceae Juss.
Iz_2_1009	DJI_0494	Chenopodium cf. album	0	n	1	0	Amaranthaceae Juss.
Iz_2_1009	DJI_0494	Chenopodium sp.	0		NA	0	Amaranthaceae Juss.
Iz_2_1009	DJI_0494	Chondrilla juncea L.	0	n	1	0	Asteraceae Bercht. & J.Presl
Iz_2_1009	DJI_0494	Cirsium vulgare (Savi) Ten.	0	n	1	0	Asteraceae Bercht. & J.Presl
Iz_2_1009	DJI_0494	Clinopodium acinos (L.) Kuntze	0	n	1	0	Lamiacea Lindl.
Iz_2_1009	DJI_0494	Convallaria majalis L.	0	n	1	0	Asparagaceae Juss.
Iz_2_1009	DJI_0494	Crataegus rhipidophylla Gand.	0	n	1	1	Rosaceae Juss.
Iz_2_1009	DJI_0494	Crepis foetida L.	0	n	1	0	Asteraceae Bercht. & J.Presl
Iz_2_1009	DJI_0494	Dactylis glomerata L.	0	n	1	0	Poaceae Barnhart
Iz_2_1009	DJI_0494	Dianthus campestris M.Bieb.	0	n	1	0	Caryophyllaceae Juss.
Iz_2_1009	DJI_0494	Digitaria sanguinalis (L.) Scop.	0	a	0	0	Poaceae Barnhart
Iz_2_1009	DJI_0494	Elymus repens (L.) Gould	0	n	1	0	Poaceae Barnhart
Iz_2_1009	DJI_0494	Epilobium tetragonum L.	0	n	1	0	Onagraceae Juss.
Iz_2_1009	DJI_0494	Eragrostis minor Host	0	a	0	0	Poaceae Barnhart
Iz_2_1009	DJI_0494	Erigeron canadensis L.	0	a	0	0	Asteraceae Bercht. & J.Presl
Iz_2_1009	DJI_0494	Euphorbia seguieriana Neck.	0	n	1	0	Euphorbiaceae Juss.
Iz_2_1009	DJI_0494	Fallopia dumetorum (L.) Holub	0	n	1	0	Polygonaceae Juss.
Iz_2_1009	DJI_0494	Festuca beckeri (Hack.) Trautv.	0	n	1	0	Poaceae Barnhart
Iz_2_1009	DJI_0494	Fragaria vesca L.	0	n	1	0	Rosaceae Juss.
Iz_2_1009	DJI_0494	Frangula alnus Mill.	0	n	1	1	Rhamnaceae Juss.
Iz_2_1009	DJI_0494	Fraxinus pennsylvanica Marshall	0	a	0	1	Oleaceae Hoffmanns. & Link
Iz_2_1009	DJI_0494	Galeopsis bifida Boenn.	0	n	1	0	Lamiacea Lindl.
Iz_2_1009	DJI_0494	Galium verum L.	0	n	1	0	Rubiaceae Juss.
Iz_2_1009	DJI_0494	Genista tinctoria L.	0	n	1	0	Fabaceae Lindl.
Iz_2_1009	DJI_0494	Geranium robertianum L.	1	n	1	0	Geraniaceae Juss.
Iz_2_1009	DJI_0494	Geranium sanquineum L.	0	n	1	0	Geraniaceae Juss.
Iz_2_1009	DJI_0494	Gypsophila paniculata L.	0	n	1	0	Caryophyllaceae Juss.
Iz_2_1009	DJI_0494	Helichrysum arenarium (L.) Moench	0	n	1	0	Asteraceae Bercht. & J.Presl
Iz_2_1009	DJI_0494	Herniaria polygama J.Gay	0	n	1	0	Caryophyllaceae Juss.
Iz_2_1009	DJI_0494	Hieracium robustum Fr.	0	n	1	0	Asteraceae Bercht. & J.Presl
Iz_2_1009	DJI_0494	Hieracium umbellatum L.	0	n	1	NA	Asteraceae Bercht. & J.Presl
Iz_2_1009	DJI_0494	Hylotelephium maximum (L.) Holub	0	n	1	0	Crassulaceae J.St.-Hil.
Iz_2_1009	DJI_0494	Jacobaea borysthenica (DC.) B.Nord. & Greuter	0	n	1	0	Asteraceae Bercht. & J.Presl
Iz_2_1009	DJI_0494	Jasione montana L.	0	n	1	0	Campanulaceae Juss.
Iz_2_1009	DJI_0494	Jurinea cyanoides (L.) Rchb.s.l.	0	n	1	0	Asteraceae Bercht. & J.Presl
Iz_2_1009	DJI_0494	Koeleria glauca (Spreng.) DC.	0	n	1	0	Poaceae Barnhart
Iz_2_1009	DJI_0494	Lactuca serriola L.	0	a	0	0	Asteraceae Bercht. & J.Presl
Iz_2_1009	DJI_0494	Linaria genistifolia (L.) Mill.	0	n	1	0	Plantaginaceae Juss.
Iz_2_1009	DJI_0494	Lomelosia argentea (L.) Greuter & Burdet	0	n	1	0	Caprifoliaceae Juss.
Iz_2_1009	DJI_0494	Melampyrum pratense L.	0	n	1	0	Orobanchaceae Vent.
Iz_2_1009	DJI_0494	Oenothera biennis L.	0	a	0	0	Onagraceae Juss.
Iz_2_1009	DJI_0494	Pentanema britannica (L.) D.Gut.Larr., Santos-Vicente, Anderb., E.Rico & M.M.Mart.Ort.	0	n	1	0	Asteraceae Bercht. & J.Presl
Iz_2_1009	DJI_0494	Peucedanum oreoselinum (L.) Moench	0	n	1	0	Apiaceae Lindl.
Iz_2_1009	DJI_0494	Peucedanum ruthenicum M.Bieb.	0	n	1	0	Apiaceae Lindl.
Iz_2_1009	DJI_0494	Pilosella echioides (Lumn.) F.W.Schultz & Sch.Bip.	0	n	1	0	Asteraceae Bercht. & J.Presl
Iz_2_1009	DJI_0494	Pilosella officinarum F. Schultz et Sch. Bip.	0	n	1	0	Asteraceae Bercht. & J.Presl
Iz_2_1009	DJI_0494	Pilosella sp.	0		NA	0	Asteraceae Bercht. & J.Presl
Iz_2_1009	DJI_0494	Pinus sylvestris L.	0	n	1	1	Pinaceae Spreng. ex F.Rudolphi
Iz_2_1009	DJI_0494	Poa annua L.	0	n	1	0	Poaceae Barnhart
Iz_2_1009	DJI_0494	Poa nemoralis L.	0	n	1	0	Poaceae Barnhart
Iz_2_1009	DJI_0494	Poa sp.	0		NA	0	Poaceae Barnhart
Iz_2_1009	DJI_0494	Polygonatum odoratum (Mill.) Druce	0	n	1	0	Asparagaceae Juss.
Iz_2_1009	DJI_0494	Polygonum arenarium Waldst. & Kit.	0	n	1	0	Polygonaceae Juss.
Iz_2_1009	DJI_0494	Populus nigra L.	0	n	1	1	Salicaceae Mirb.

Iz_2_1009	DJI_0494	Populus tremula L.	0	n	1	1	Salicaceae Mirb.
Iz_2_1009	DJI_0494	Populus Ã canescens (Aiton) Sm.	0	n	1	1	Salicaceae Mirb.
Iz_2_1009	DJI_0494	Potentilla argentea L.	0	n	1	0	Rosaceae Juss.
Iz_2_1009	DJI_0494	Psammophiliella muralis (L.) Ikonn.	0	n	1	0	Caryophyllaceae Juss.
Iz_2_1009	DJI_0494	Psephellus sumensis (Kalen.) Greuter	0	n	1	0	Asteraceae Bercht. & J.Presl
Iz_2_1009	DJI_0494	Pulsatilla pratensis (L.) Mill. s.l.	0	n	1	0	Ranunculaceae Juss.
Iz_2_1009	DJI_0494	Pyrus communis L.	0	a	0	1	Rosaceae Juss.
Iz_2_1009	DJI_0494	Quercus robur L.	0	n	1	1	Fagaceae Dumort.
Iz_2_1009	DJI_0494	Robinia pseudoacacia L.	0	a	0	1	Fabaceae Lindl.
Iz_2_1009	DJI_0494	Rosa sp.	0		NA	1	Rosaceae Juss.
Iz_2_1009	DJI_0494	Rubus idaeus L.	0	n	1	0	Rosaceae Juss.
Iz_2_1009	DJI_0494	Rumex acetosella L.	0	n	1	0	Polygonaceae Juss.
Iz_2_1009	DJI_0494	Rumex thyrsiflorus Fingerh.	0	n	1	0	Polygonaceae Juss.
Iz_2_1009	DJI_0494	Salix alba L.	0	n	1	1	Salicaceae Mirb.
Iz_2_1009	DJI_0494	Sambucus nigra L.	0	n	1	1	Viburnaceae Raf.
Iz_2_1009	DJI_0494	Secale sylvestre Host	0	n	1	0	Poaceae Barnhart
Iz_2_1009	DJI_0494	Sempervivum ruthenicum Schnittsp. & C.B. Lehm.	0	n	1	0	Crassulaceae J.St.-Hil.
Iz_2_1009	DJI_0494	Setaria viridis (L.) P. Beauv.	0	n	1	0	Poaceae Barnhart
Iz_2_1009	DJI_0494	Silene borysthenica (Gruner) Walters	0	n	1	0	Caryophyllaceae Juss.
Iz_2_1009	DJI_0494	Silene latifolia subsp. alba (Mill.) Greuter & Burdet	0	n	1	0	Caryophyllaceae Juss.
Iz_2_1009	DJI_0494	Solidago virgaurea L.	0	n	1	0	Asteraceae Bercht. & J.Presl
Iz_2_1009	DJI_0494	Sorbus aucuparia L.	0	n	1	1	Rosaceae Juss.
Iz_2_1009	DJI_0494	Stachys recta L.	0	n	1	0	Lamiacea Lindl.
Iz_2_1009	DJI_0494	Tanacetum vulgare L.	0	n	1	0	Asteraceae Bercht. & J.Presl
Iz_2_1009	DJI_0494	Thymus pallasianus Heinr.Braun	0	n	1	0	Lamiacea Lindl.
Iz_2_1009	DJI_0494	Tilia cordata Mill.	0	n	1	1	Malvaceae Juss.
Iz_2_1009	DJI_0494	Trifolium alpestre L.	0	n	1	0	Fabaceae Lindl.
Iz_2_1009	DJI_0494	Trifolium arvense L.	0	n	1	0	Fabaceae Lindl.
Iz_2_1009	DJI_0494	Ulmus glabra Huds.	0	n	1	1	Ulmaceae Mirb.
Iz_2_1009	DJI_0494	Verbascum densiflorum Bertol.	0	n	1	0	Scrophulariaceae Juss.
Iz_2_1009	DJI_0494	Veronica austriaca L.	0	n	1	0	Plantaginaceae Juss.
Iz_2_1009	DJI_0494	Veronica incana L.	0	n	1	0	Plantaginaceae Juss.
Iz_2_1009	DJI_0494	Veronica verna L.	0	n	1	0	Plantaginaceae Juss.
Iz_2_1009	DJI_0494	Vincetoxicum hirundinaria Medik	0	n	1	0	Apocynaceae Juss.
Iz_2_1009	DJI_0497	Acer tataricum L.	0	n	1	1	Sapindaceae Juss.
Iz_2_1009	DJI_0497	Achillea millefolium L. s.l.	0	n	1	0	Asteraceae Bercht. & J.Presl
Iz_2_1009	DJI_0497	Allium oleraceum L.	0	n	1	0	Amaryllidaceae J.St.-Hil.
Iz_2_1009	DJI_0497	Ambrosia artemisiifolia L.	0	a	0	0	Asteraceae Bercht. & J.Presl
Iz_2_1009	DJI_0497	Amorpha fruticosa L.	0	a	0	0	Fabaceae Lindl.
Iz_2_1009	DJI_0497	Anthericum ramosum L.	0	n	1	0	Asparagaceae Juss.
Iz_2_1009	DJI_0497	Artemisia marschalliana Spreng.	0	n	1	0	Asteraceae Bercht. & J.Presl
Iz_2_1009	DJI_0497	Asparagus officinalis L.	0	n	1	0	Asparagaceae Juss.
Iz_2_1009	DJI_0497	Astragalus cicer L.	0	n	1	0	Fabaceae Lindl.
Iz_2_1009	DJI_0497	Bassia laniflora (S.G.Gmel.) A.J.Scott	0	n	1	0	Amaranthaceae Juss.
Iz_2_1009	DJI_0497	Berberis vulgaris L.	0	n	1	0	Berberidaceae Juss.
Iz_2_1009	DJI_0497	Betula pendula Roth.	0	n	1	1	Betulaceae Gray
Iz_2_1009	DJI_0497	Betula pendula Roth.	0	n	1	0	Poaceae Barnhart
Iz_2_1009	DJI_0497	Bromus inermis Leyss.	0		NA	NA	
Iz_2_1009	DJI_0497	Bromus squarrosus L.	0	a	0	0	Poaceae Barnhart
Iz_2_1009	DJI_0497	Bromus tectorum L.	0	a	0	0	Poaceae Barnhart
Iz_2_1009	DJI_0497	Calamagrostis epigejos (L.) Roth	20	n	1	0	Poaceae Barnhart
Iz_2_1009	DJI_0497	Carduus acanthoides L.	0	n	1	0	Asteraceae Bercht. & J.Presl
Iz_2_1009	DJI_0497	Carex sp.	0	n	1	0	Cyperaceae Juss.
Iz_2_1009	DJI_0497	Centaurea majorovii Dumbadze	0	n	1	0	Asteraceae Bercht. & J.Presl
Iz_2_1009	DJI_0497	Cf. Solidago virgaurea	0		NA	NA	
Iz_2_1009	DJI_0497	Chamaecytisus ruthenicus (Fisch.ex Wol.) Klaskova	0	n	1	0	Fabaceae Lindl.
Iz_2_1009	DJI_0497	Chelidonium majus L.	0	n	1	0	Papaveraceae Juss.
Iz_2_1009	DJI_0497	Chenopodium betaceum Andrz.	0	n	1	0	Amaranthaceae Juss.
Iz_2_1009	DJI_0497	Chenopodium cf. album	0	n	1	0	Amaranthaceae Juss.
Iz_2_1009	DJI_0497	Chenopodium sp.	0		NA	0	Amaranthaceae Juss.
Iz_2_1009	DJI_0497	Chondrilla juncea L.	0	n	1	0	Asteraceae Bercht. & J.Presl
Iz_2_1009	DJI_0497	Cirsium vulgare (Savi) Ten.	0	n	1	0	Asteraceae Bercht. & J.Presl
Iz_2_1009	DJI_0497	Clinopodium acinos (L.) Kuntze	0	n	1	0	Lamiacea Lindl.
Iz_2_1009	DJI_0497	Convallaria majalis L.	0	n	1	0	Asparagaceae Juss.
Iz_2_1009	DJI_0497	Crataegus rhipidophylla Gand.	0	n	1	1	Rosaceae Juss.
Iz_2_1009	DJI_0497	Crepis foetida L.	0	n	1	0	Asteraceae Bercht. & J.Presl
Iz_2_1009	DJI_0497	Dactylis glomerata L.	0	n	1	0	Poaceae Barnhart
Iz_2_1009	DJI_0497	Dianthus campestris M.Bieb.	0	n	1	0	Caryophyllaceae Juss.
Iz_2_1009	DJI_0497	Digitaria sanguinalis (L.) Scop.	0	a	0	0	Poaceae Barnhart
Iz_2_1009	DJI_0497	Elymus repens (L.) Gould	0	n	1	0	Poaceae Barnhart
Iz_2_1009	DJI_0497	Epilobium tetragonum L.	0	n	1	0	Onagraceae Juss.
Iz_2_1009	DJI_0497	Eragrostis minor Host	0	a	0	0	Poaceae Barnhart
Iz_2_1009	DJI_0497	Erigeron canadensis L.	0	a	0	0	Asteraceae Bercht. & J.Presl
Iz_2_1009	DJI_0497	Euphorbia seguieriana Neck.	0	n	1	0	Euphorbiaceae Juss.
Iz_2_1009	DJI_0497	Fallopia dumetorum (L.) Holub	0	n	1	0	Polygonaceae Juss.
Iz_2_1009	DJI_0497	Festuca beckeri (Hack.) Trautv.	0	n	1	0	Poaceae Barnhart
Iz_2_1009	DJI_0497	Fragaria vesca L.	0	n	1	0	Rosaceae Juss.
Iz_2_1009	DJI_0497	Frangula alnus Mill.	0	n	1	1	Rhamnaceae Juss.
Iz_2_1009	DJI_0497	Fraxinus pennsylvanica Marshall	0	a	0	1	Oleaceae Hoffmanns. & Link
Iz_2_1009	DJI_0497	Galeopsis bifida Boenn.	0	n	1	0	Lamiacea Lindl.
Iz_2_1009	DJI_0497	Galium verum L.	0	n	1	0	Rubiaceae Juss.
Iz_2_1009	DJI_0497	Genista tinctoria L.	0	n	1	0	Fabaceae Lindl.
Iz_2_1009	DJI_0497	Geranium robertianum L.	1	n	1	0	Geraniaceae Juss.
Iz_2_1009	DJI_0497	Geranium sanquineum L.	0	n	1	0	Geraniaceae Juss.
Iz_2_1009	DJI_0497	Gypsophila paniculata L.	0	n	1	0	Caryophyllaceae Juss.
Iz_2_1009	DJI_0497	Helichrysum arenarium (L.) Moench	0	n	1	0	Asteraceae Bercht. & J.Presl
Iz_2_1009	DJI_0497	Herniaria polygama J.Gay	0	n	1	0	Caryophyllaceae Juss.
Iz_2_1009	DJI_0497	Hieracium robustum Fr.	0	n	1	0	Asteraceae Bercht. & J.Presl
Iz_2_1009	DJI_0497	Hieracium umbellatum L.	0	n	1	NA	Asteraceae Bercht. & J.Presl
Iz_2_1009	DJI_0497	Hylotelephium maximum (L.) Holub	0	n	1	0	Crassulaceae J.St.-Hil.
Iz_2_1009	DJI_0497	Jacobaea borysthenica (DC.) B.Nord. & Greuter	0	n	1	0	Asteraceae Bercht. & J.Presl
Iz_2_1009	DJI_0497	Jasione montana L.	0	n	1	0	Campanulaceae Juss.
Iz_2_1009	DJI_0497	Jurinea cyanoides (L.) Rchb.s.l.	0	n	1	0	Asteraceae Bercht. & J.Presl
Iz_2_1009	DJI_0497	Koeleria glauca (Spreng.) DC.	0	n	1	0	Poaceae Barnhart
Iz_2_1009	DJI_0497	Lactuca serriola L.	0	a	0	0	Asteraceae Bercht. & J.Presl
Iz_2_1009	DJI_0497	Linaria genistifolia (L.) Mill.	0	n	1	0	Plantaginaceae Juss.
Iz_2_1009	DJI_0497	Lomelosia argentea (L.) Greuter & Burdet	0	n	1	0	Caprifoliaceae Juss.
Iz_2_1009	DJI_0497	Melampyrum pratense L.	0	n	1	0	Orobanchaceae Vent.

Iz_2_1009	DJI_0497	Oenothera biennis L.	0	a	0	0	Onagraceae Juss.
Iz_2_1009	DJI_0497	Pentanema britannica (L.) D.Gut.Larr., Santos-Vicente, Anderb., E.Rico & M.M.Mart.Ort.	0	n	1	0	Asteraceae Bercht. & J.Presl
Iz_2_1009	DJI_0497	Peucedanum oreoselinum (L.) Moench	1	n	1	0	Apiaceae Lindl.
Iz_2_1009	DJI_0497	Peucedanum ruthenicum M.Bieb.	0	n	1	0	Apiaceae Lindl.
Iz_2_1009	DJI_0497	Pilosella echioides (Lumn.) F.W.Schultz & Sch.Bip.	0	n	1	0	Asteraceae Bercht. & J.Presl
Iz_2_1009	DJI_0497	Pilosella officinarum F. Schultz et Sch. Bip.	0	n	1	0	Asteraceae Bercht. & J.Presl
Iz_2_1009	DJI_0497	Pilosella sp.	0		NA	0	Asteraceae Bercht. & J.Presl
Iz_2_1009	DJI_0497	Pinus sylvestris L.	0	n	1	1	Pinaceae Spreng. ex F.Rudolphi
Iz_2_1009	DJI_0497	Poa annua L.	0	n	1	0	Poaceae Barnhart
Iz_2_1009	DJI_0497	Poa nemoralis L.	0	n	1	0	Poaceae Barnhart
Iz_2_1009	DJI_0497	Poa sp.	0		NA	0	Poaceae Barnhart
Iz_2_1009	DJI_0497	Polygonatum odoratum (Mill.) Druce	0	n	1	0	Asparagaceae Juss.
Iz_2_1009	DJI_0497	Polygonum arenarium Waldst. & Kit.	0	n	1	0	Polygonaceae Juss.
Iz_2_1009	DJI_0497	Populus nigra L.	0	n	1	1	Salicaceae Mirb.
Iz_2_1009	DJI_0497	Populus tremula L.	0	n	1	1	Salicaceae Mirb.
Iz_2_1009	DJI_0497	Populus Ã canescens (Aiton) Sm.	0	n	1	1	Salicaceae Mirb.
Iz_2_1009	DJI_0497	Potentilla argentea L.	0	n	1	0	Rosaceae Juss.
Iz_2_1009	DJI_0497	Psammophiliella muralis (L.) Ikonn.	0	n	1	0	Caryophyllaceae Juss.
Iz_2_1009	DJI_0497	Psephellus sumensis (Kalen.) Greuter	0	n	1	0	Asteraceae Bercht. & J.Presl
Iz_2_1009	DJI_0497	Pulsatilla pratensis (L.) Mill. s.l.	0	n	1	0	Ranunculaceae Juss.
Iz_2_1009	DJI_0497	Pyrus communis L.	0	a	0	1	Rosaceae Juss.
Iz_2_1009	DJI_0497	Quercus robur L.	0	n	1	1	Fagaceae Dumort.
Iz_2_1009	DJI_0497	Robinia pseudoacacia L.	0	a	0	1	Fabaceae Lindl.
Iz_2_1009	DJI_0497	Rosa sp.	0		NA	1	Rosaceae Juss.
Iz_2_1009	DJI_0497	Rubus idaeus L.	0	n	1	0	Rosaceae Juss.
Iz_2_1009	DJI_0497	Rumex acetosella L.	0	n	1	0	Polygonaceae Juss.
Iz_2_1009	DJI_0497	Rumex thyrsiflorus Fingerh.	0	n	1	0	Polygonaceae Juss.
Iz_2_1009	DJI_0497	Salix alba L.	0	n	1	1	Salicaceae Mirb.
Iz_2_1009	DJI_0497	Sambucus nigra L.	0	n	1	1	Viburnaceae Raf.
Iz_2_1009	DJI_0497	Secale sylvestre Host	0	n	1	0	Poaceae Barnhart
Iz_2_1009	DJI_0497	Sempervivum ruthenicum Schnittsp. & C.B. Lehm.	0	n	1	0	Crassulaceae J.St.-Hil.
Iz_2_1009	DJI_0497	Setaria viridis (L.) P. Beauv.	0	n	1	0	Poaceae Barnhart
Iz_2_1009	DJI_0497	Silene borysthenica (Gruner) Walters	0	n	1	0	Caryophyllaceae Juss.
Iz_2_1009	DJI_0497	Silene latifolia subsp. alba (Mill.) Greuter & Burdet	0	n	1	0	Caryophyllaceae Juss.
Iz_2_1009	DJI_0497	Solidago virgaurea L.	0	n	1	0	Asteraceae Bercht. & J.Presl
Iz_2_1009	DJI_0497	Sorbus aucuparia L.	0	n	1	1	Rosaceae Juss.
Iz_2_1009	DJI_0497	Stachys recta L.	0	n	1	0	Lamiacea Lindl.
Iz_2_1009	DJI_0497	Tanacetum vulgare L.	0	n	1	0	Asteraceae Bercht. & J.Presl
Iz_2_1009	DJI_0497	Thymus pallasianus Heinr.Braun	0	n	1	0	Lamiacea Lindl.
Iz_2_1009	DJI_0497	Tilia cordata Mill.	0	n	1	1	Malvaceae Juss.
Iz_2_1009	DJI_0497	Trifolium alpestre L.	0	n	1	0	Fabaceae Lindl.
Iz_2_1009	DJI_0497	Trifolium arvense L.	0	n	1	0	Fabaceae Lindl.
Iz_2_1009	DJI_0497	Ulmus glabra Huds.	0	n	1	1	Ulmaceae Mirb.
Iz_2_1009	DJI_0497	Verbascum densiflorum Bertol.	0	n	1	0	Scrophulariaceae Juss.
Iz_2_1009	DJI_0497	Veronica austriaca L.	0	n	1	0	Plantaginaceae Juss.
Iz_2_1009	DJI_0497	Veronica incana L.	0	n	1	0	Plantaginaceae Juss.
Iz_2_1009	DJI_0497	Veronica verna L.	0	n	1	0	Plantaginaceae Juss.
Iz_2_1009	DJI_0497	Vincetoxicum hirundinaria Medik	0	n	1	0	Apocynaceae Juss.
Iz_2_1009	DJI_0498	Acer tataricum L.	0	n	1	1	Sapindaceae Juss.
Iz_2_1009	DJI_0498	Achillea millefolium L. s.l.	0	n	1	0	Asteraceae Bercht. & J.Presl
Iz_2_1009	DJI_0498	Allium oleraceum L.	0	n	1	0	Amaryllidaceae J.St.-Hil.
Iz_2_1009	DJI_0498	Ambrosia artemisiifolia L.	0	a	0	0	Asteraceae Bercht. & J.Presl
Iz_2_1009	DJI_0498	Amorpha fruticosa L.	0	a	0	0	Fabaceae Lindl.
Iz_2_1009	DJI_0498	Anthericum ramosum L.	0	n	1	0	Asparagaceae Juss.
Iz_2_1009	DJI_0498	Artemisia marschalliana Spreng.	0	n	1	0	Asteraceae Bercht. & J.Presl
Iz_2_1009	DJI_0498	Asparagus officinalis L.	0	n	1	0	Asparagaceae Juss.
Iz_2_1009	DJI_0498	Astragalus cicer L.	0	n	1	0	Fabaceae Lindl.
Iz_2_1009	DJI_0498	Bassia laniflora (S.G.Gmel.) A.J.Scott	0	n	1	0	Amaranthaceae Juss.
Iz_2_1009	DJI_0498	Berberis vulgaris L.	0	n	1	0	Berberidaceae Juss.
Iz_2_1009	DJI_0498	Betula pendula Roth.	0	n	1	1	Betulaceae Gray
Iz_2_1009	DJI_0498	Betula pendula Roth.	0	n	1	0	Poaceae Barnhart
Iz_2_1009	DJI_0498	Bromus inermis Leyss.	0		NA	NA	
Iz_2_1009	DJI_0498	Bromus squarrosus L.	0	a	0	0	Poaceae Barnhart
Iz_2_1009	DJI_0498	Bromus tectorum L.	0	a	0	0	Poaceae Barnhart
Iz_2_1009	DJI_0498	Calamagrostis epigejos (L.) Roth	25	n	1	0	Poaceae Barnhart
Iz_2_1009	DJI_0498	Carduus acanthoides L.	0	n	1	0	Asteraceae Bercht. & J.Presl
Iz_2_1009	DJI_0498	Carex sp.	0	n	1	0	Cyperaceae Juss.
Iz_2_1009	DJI_0498	Centaurea majorovii Dumbadze	0	n	1	0	Asteraceae Bercht. & J.Presl
Iz_2_1009	DJI_0498	Cf. Solidago virgaurea	0		NA	NA	
Iz_2_1009	DJI_0498	Chamaecytisus ruthenicus (Fisch.ex Wol.) Klaskova	0	n	1	0	Fabaceae Lindl.
Iz_2_1009	DJI_0498	Chelidonium majus L.	0	n	1	0	Papaveraceae Juss.
Iz_2_1009	DJI_0498	Chenopodium betaceum Andrz.	0	n	1	0	Amaranthaceae Juss.
Iz_2_1009	DJI_0498	Chenopodium cf. album	0	n	1	0	Amaranthaceae Juss.
Iz_2_1009	DJI_0498	Chenopodium sp.	0		NA	0	Amaranthaceae Juss.
Iz_2_1009	DJI_0498	Chondrilla juncea L.	0	n	1	0	Asteraceae Bercht. & J.Presl
Iz_2_1009	DJI_0498	Cirsium vulgare (Savi) Ten.	0	n	1	0	Asteraceae Bercht. & J.Presl
Iz_2_1009	DJI_0498	Clinopodium acinos (L.) Kuntze	0	n	1	0	Lamiacea Lindl.
Iz_2_1009	DJI_0498	Convallaria majalis L.	0	n	1	0	Asparagaceae Juss.
Iz_2_1009	DJI_0498	Crataegus rhipidophylla Gand.	0	n	1	1	Rosaceae Juss.
Iz_2_1009	DJI_0498	Crepis foetida L.	0	n	1	0	Asteraceae Bercht. & J.Presl
Iz_2_1009	DJI_0498	Dactylis glomerata L.	0	n	1	0	Poaceae Barnhart
Iz_2_1009	DJI_0498	Dianthus campestris M.Bieb.	0	n	1	0	Caryophyllaceae Juss.
Iz_2_1009	DJI_0498	Digitaria sanguinalis (L.) Scop.	0	a	0	0	Poaceae Barnhart
Iz_2_1009	DJI_0498	Elymus repens (L.) Gould	0	n	1	0	Poaceae Barnhart
Iz_2_1009	DJI_0498	Epilobium tetragonum L.	0	n	1	0	Onagraceae Juss.
Iz_2_1009	DJI_0498	Eragrostis minor Host	0	a	0	0	Poaceae Barnhart
Iz_2_1009	DJI_0498	Erigeron canadensis L.	0	a	0	0	Asteraceae Bercht. & J.Presl
Iz_2_1009	DJI_0498	Euphorbia seguieriana Neck.	0	n	1	0	Euphorbiaceae Juss.
Iz_2_1009	DJI_0498	Fallopia dumetorum (L.) Holub	0	n	1	0	Polygonaceae Juss.
Iz_2_1009	DJI_0498	Festuca beckeri (Hack.) Trautv.	0	n	1	0	Poaceae Barnhart
Iz_2_1009	DJI_0498	Fragaria vesca L.	0	n	1	0	Rosaceae Juss.
Iz_2_1009	DJI_0498	Frangula alnus Mill.	0	n	1	1	Rhamnaceae Juss.
Iz_2_1009	DJI_0498	Fraxinus pennsylvanica Marshall	0	a	0	1	Oleaceae Hoffmanns. & Link
Iz_2_1009	DJI_0498	Galeopsis bifida Boenn.	0	n	1	0	Lamiacea Lindl.
Iz_2_1009	DJI_0498	Galium verum L.	0	n	1	0	Rubiaceae Juss.
Iz_2_1009	DJI_0498	Genista tinctoria L.	0	n	1	0	Fabaceae Lindl.
Iz_2_1009	DJI_0498	Geranium robertianum L.	1	n	1	0	Geraniaceae Juss.
Iz_2_1009	DJI_0498	Geranium sanquineum L.	0	n	1	0	Geraniaceae Juss.

Iz_2_1009	DJI_0498	Gypsophila paniculata L.	0	n	1	0	Caryophyllaceae Juss.
Iz_2_1009	DJI_0498	Helichrysum arenarium (L.) Moench	0	n	1	0	Asteraceae Bercht. & J.Presl
Iz_2_1009	DJI_0498	Herniaria polygama J.Gay	0	n	1	0	Caryophyllaceae Juss.
Iz_2_1009	DJI_0498	Hieracium robustum Fr.	0	n	1	0	Asteraceae Bercht. & J.Presl
Iz_2_1009	DJI_0498	Hieracium umbellatum L.	0	n	1	NA	Asteraceae Bercht. & J.Presl
Iz_2_1009	DJI_0498	Hylotelephium maximum (L.) Holub	0	n	1	0	Crassulaceae J.St.-Hil.
Iz_2_1009	DJI_0498	Jacobaea borysthenica (DC.) B.Nord. & Greuter	0	n	1	0	Asteraceae Bercht. & J.Presl
Iz_2_1009	DJI_0498	Jasione montana L.	0	n	1	0	Campanulaceae Juss.
Iz_2_1009	DJI_0498	Jurinea cyanoides (L.) Rchb.s.l.	0	n	1	0	Asteraceae Bercht. & J.Presl
Iz_2_1009	DJI_0498	Koeleria glauca (Spreng.) DC.	0	n	1	0	Poaceae Barnhart
Iz_2_1009	DJI_0498	Lactuca serriola L.	0	a	0	0	Asteraceae Bercht. & J.Presl
Iz_2_1009	DJI_0498	Linaria genistifolia (L.) Mill.	0	n	1	0	Plantaginaceae Juss.
Iz_2_1009	DJI_0498	Lomelosia argentea (L.) Greuter & Burdet	0	n	1	0	Caprifoliaceae Juss.
Iz_2_1009	DJI_0498	Melampyrum pratense L.	0	n	1	0	Orobanchaceae Vent.
Iz_2_1009	DJI_0498	Oenothera biennis L.	0	a	0	0	Onagraceae Juss.
Iz_2_1009	DJI_0498	Pentanema britannica (L.) D.Gut.Larr., Santos-Vicente, Anderb., E.Rico & M.M.Mart.Ort.	0	n	1	0	Asteraceae Bercht. & J.Presl
Iz_2_1009	DJI_0498	Peucedanum oreoselinum (L.) Moench	1	n	1	0	Apiaceae Lindl.
Iz_2_1009	DJI_0498	Peucedanum ruthenicum M.Bieb.	0	n	1	0	Apiaceae Lindl.
Iz_2_1009	DJI_0498	Pilosella echioides (Lumn.) F.W.Schultz & Sch.Bip.	0	n	1	0	Asteraceae Bercht. & J.Presl
Iz_2_1009	DJI_0498	Pilosella officinarum F. Schultz et Sch. Bip.	0	n	1	0	Asteraceae Bercht. & J.Presl
Iz_2_1009	DJI_0498	Pilosella sp.	0		NA	0	Asteraceae Bercht. & J.Presl
Iz_2_1009	DJI_0498	Pinus sylvestris L.	0	n	1	1	Pinaceae Spreng. ex F.Rudolphi
Iz_2_1009	DJI_0498	Poa annua L.	0	n	1	0	Poaceae Barnhart
Iz_2_1009	DJI_0498	Poa nemoralis L.	0	n	1	0	Poaceae Barnhart
Iz_2_1009	DJI_0498	Poa sp.	0		NA	0	Poaceae Barnhart
Iz_2_1009	DJI_0498	Polygonatum odoratum (Mill.) Druce	0	n	1	0	Asparagaceae Juss.
Iz_2_1009	DJI_0498	Polygonum arenarium Waldst. & Kit.	0	n	1	0	Polygonaceae Juss.
Iz_2_1009	DJI_0498	Populus nigra L.	0	n	1	1	Salicaceae Mirb.
Iz_2_1009	DJI_0498	Populus tremula L.	0	n	1	1	Salicaceae Mirb.
Iz_2_1009	DJI_0498	Populus Ã canescens (Aiton) Sm.	0	n	1	1	Salicaceae Mirb.
Iz_2_1009	DJI_0498	Potentilla argentea L.	0	n	1	0	Rosaceae Juss.
Iz_2_1009	DJI_0498	Psammophiliella muralis (L.) Ikonn.	0	n	1	0	Caryophyllaceae Juss.
Iz_2_1009	DJI_0498	Psephellus sumensis (Kalen.) Greuter	0	n	1	0	Asteraceae Bercht. & J.Presl
Iz_2_1009	DJI_0498	Pulsatilla pratensis (L.) Mill. s.l.	0	n	1	0	Ranunculaceae Juss.
Iz_2_1009	DJI_0498	Pyrus communis L.	0	a	0	1	Rosaceae Juss.
Iz_2_1009	DJI_0498	Quercus robur L.	3	n	1	1	Fagaceae Dumort.
Iz_2_1009	DJI_0498	Robinia pseudoacacia L.	0	a	0	1	Fabaceae Lindl.
Iz_2_1009	DJI_0498	Rosa sp.	0		NA	1	Rosaceae Juss.
Iz_2_1009	DJI_0498	Rubus idaeus L.	0	n	1	0	Rosaceae Juss.
Iz_2_1009	DJI_0498	Rumex acetosella L.	0	n	1	0	Polygonaceae Juss.
Iz_2_1009	DJI_0498	Rumex thyrsiflorus Fingerh.	0	n	1	0	Polygonaceae Juss.
Iz_2_1009	DJI_0498	Salix alba L.	0	n	1	1	Salicaceae Mirb.
Iz_2_1009	DJI_0498	Sambucus nigra L.	0	n	1	1	Viburnaceae Raf.
Iz_2_1009	DJI_0498	Secale sylvestre Host	0	n	1	0	Poaceae Barnhart
Iz_2_1009	DJI_0498	Sempervivum ruthenicum Schnittsp. & C.B. Lehm.	0	n	1	0	Crassulaceae J.St.-Hil.
Iz_2_1009	DJI_0498	Setaria viridis (L.) P. Beauv.	0	n	1	0	Poaceae Barnhart
Iz_2_1009	DJI_0498	Silene borysthenica (Gruner) Walters	0	n	1	0	Caryophyllaceae Juss.
Iz_2_1009	DJI_0498	Silene latifolia subsp. alba (Mill.) Greuter & Burdet	0	n	1	0	Caryophyllaceae Juss.
Iz_2_1009	DJI_0498	Solidago virgaurea L.	0	n	1	0	Asteraceae Bercht. & J.Presl
Iz_2_1009	DJI_0498	Sorbus aucuparia L.	0	n	1	1	Rosaceae Juss.
Iz_2_1009	DJI_0498	Stachys recta L.	0	n	1	0	Lamiacea Lindl.
Iz_2_1009	DJI_0498	Tanacetum vulgare L.	0	n	1	0	Asteraceae Bercht. & J.Presl
Iz_2_1009	DJI_0498	Thymus pallasianus Heinr.Braun	0	n	1	0	Lamiacea Lindl.
Iz_2_1009	DJI_0498	Tilia cordata Mill.	0	n	1	1	Malvaceae Juss.
Iz_2_1009	DJI_0498	Trifolium alpestre L.	0	n	1	0	Fabaceae Lindl.
Iz_2_1009	DJI_0498	Trifolium arvense L.	0	n	1	0	Fabaceae Lindl.
Iz_2_1009	DJI_0498	Ulmus glabra Huds.	0	n	1	1	Ulmaceae Mirb.
Iz_2_1009	DJI_0498	Verbascum densiflorum Bertol.	0	n	1	0	Scrophulariaceae Juss.
Iz_2_1009	DJI_0498	Veronica austriaca L.	0	n	1	0	Plantaginaceae Juss.
Iz_2_1009	DJI_0498	Veronica incana L.	0	n	1	0	Plantaginaceae Juss.
Iz_2_1009	DJI_0498	Veronica verna L.	0	n	1	0	Plantaginaceae Juss.
Iz_2_1009	DJI_0498	Vincetoxicum hirundinaria Medik	0	n	1	0	Apocynaceae Juss.
Iz_2_1009	DJI_0500	Acer tataricum L.	0	n	1	1	Sapindaceae Juss.
Iz_2_1009	DJI_0500	Achillea millefolium L. s.l.	0	n	1	0	Asteraceae Bercht. & J.Presl
Iz_2_1009	DJI_0500	Allium oleraceum L.	0	n	1	0	Amaryllidaceae J.St.-Hil.
Iz_2_1009	DJI_0500	Ambrosia artemisiifolia L.	0	a	0	0	Asteraceae Bercht. & J.Presl
Iz_2_1009	DJI_0500	Amorpha fruticosa L.	0	a	0	0	Fabaceae Lindl.
Iz_2_1009	DJI_0500	Anthericum ramosum L.	0	n	1	0	Asparagaceae Juss.
Iz_2_1009	DJI_0500	Artemisia marschalliana Spreng.	0	n	1	0	Asteraceae Bercht. & J.Presl
Iz_2_1009	DJI_0500	Asparagus officinalis L.	0	n	1	0	Asparagaceae Juss.
Iz_2_1009	DJI_0500	Astragalus cicer L.	0	n	1	0	Fabaceae Lindl.
Iz_2_1009	DJI_0500	Bassia laniflora (S.G.Gmel.) A.J.Scott	0	n	1	0	Amaranthaceae Juss.
Iz_2_1009	DJI_0500	Berberis vulgaris L.	0	n	1	0	Berberidaceae Juss.
Iz_2_1009	DJI_0500	Betula pendula Roth.	0	n	1	1	Betulaceae Gray
Iz_2_1009	DJI_0500	Betula pendula Roth.	0	n	1	0	Poaceae Barnhart
Iz_2_1009	DJI_0500	Bromus inermis Leyss.	0		NA	NA	
Iz_2_1009	DJI_0500	Bromus squarrosus L.	0	a	0	0	Poaceae Barnhart
Iz_2_1009	DJI_0500	Bromus tectorum L.	0	a	0	0	Poaceae Barnhart
Iz_2_1009	DJI_0500	Calamagrostis epigejos (L.) Roth	5	n	1	0	Poaceae Barnhart
Iz_2_1009	DJI_0500	Carduus acanthoides L.	0	n	1	0	Asteraceae Bercht. & J.Presl
Iz_2_1009	DJI_0500	Carex sp.	0	n	1	0	Cyperaceae Juss.
Iz_2_1009	DJI_0500	Centaurea majorovii Dumbadze	0	n	1	0	Asteraceae Bercht. & J.Presl
Iz_2_1009	DJI_0500	Cf. Solidago virgaurea	0		NA	NA	
Iz_2_1009	DJI_0500	Chamaecytisus ruthenicus (Fisch.ex Wol.) Klaskova	0	n	1	0	Fabaceae Lindl.
Iz_2_1009	DJI_0500	Chelidonium majus L.	0	n	1	0	Papaveraceae Juss.
Iz_2_1009	DJI_0500	Chenopodium betaceum Andrz.	0	n	1	0	Amaranthaceae Juss.
Iz_2_1009	DJI_0500	Chenopodium cf. album	0	n	1	0	Amaranthaceae Juss.
Iz_2_1009	DJI_0500	Chenopodium sp.	0		NA	0	Amaranthaceae Juss.
Iz_2_1009	DJI_0500	Chondrilla juncea L.	0	n	1	0	Asteraceae Bercht. & J.Presl
Iz_2_1009	DJI_0500	Cirsium vulgare (Savi) Ten.	0	n	1	0	Asteraceae Bercht. & J.Presl
Iz_2_1009	DJI_0500	Clinopodium acinos (L.) Kuntze	0	n	1	0	Lamiacea Lindl.
Iz_2_1009	DJI_0500	Convallaria majalis L.	0	n	1	0	Asparagaceae Juss.
Iz_2_1009	DJI_0500	Crataegus rhipidophylla Gand.	0	n	1	1	Rosaceae Juss.
Iz_2_1009	DJI_0500	Crepis foetida L.	0	n	1	0	Asteraceae Bercht. & J.Presl
Iz_2_1009	DJI_0500	Dactylis glomerata L.	0	n	1	0	Poaceae Barnhart
Iz_2_1009	DJI_0500	Dianthus campestris M.Bieb.	0	n	1	0	Caryophyllaceae Juss.
Iz_2_1009	DJI_0500	Digitaria sanguinalis (L.) Scop.	0	a	0	0	Poaceae Barnhart
Iz_2_1009	DJI_0500	Elymus repens (L.) Gould	0	n	1	0	Poaceae Barnhart

Iz_2_1009	DJI_0500	Epilobium tetragonum L.	0	n	1	0	Onagraceae Juss.
Iz_2_1009	DJI_0500	Eragrostis minor Host	0	a	0	0	Poaceae Barnhart
Iz_2_1009	DJI_0500	Erigeron canadensis L.	0	a	0	0	Asteraceae Bercht. & J.Presl
Iz_2_1009	DJI_0500	Euphorbia seguieriana Neck.	0	n	1	0	Euphorbiaceae Juss.
Iz_2_1009	DJI_0500	Fallopia dumetorum (L.) Holub	0	n	1	0	Polygonaceae Juss.
Iz_2_1009	DJI_0500	Festuca beckeri (Hack.) Trautv.	0	n	1	0	Poaceae Barnhart
Iz_2_1009	DJI_0500	Fragaria vesca L.	0	n	1	0	Rosaceae Juss.
Iz_2_1009	DJI_0500	Frangula alnus Mill.	0	n	1	1	Rhamnaceae Juss.
Iz_2_1009	DJI_0500	Fraxinus pennsylvanica Marshall	0	a	0	1	Oleaceae Hoffmanns. & Link
Iz_2_1009	DJI_0500	Galeopsis bifida Boenn.	0	n	1	0	Lamiacea Lindl.
Iz_2_1009	DJI_0500	Galium verum L.	0	n	1	0	Rubiaceae Juss.
Iz_2_1009	DJI_0500	Genista tinctoria L.	0	n	1	0	Fabaceae Lindl.
Iz_2_1009	DJI_0500	Geranium robertianum L.	0	n	1	0	Geraniaceae Juss.
Iz_2_1009	DJI_0500	Geranium sanquineum L.	0	n	1	0	Geraniaceae Juss.
Iz_2_1009	DJI_0500	Gypsophila paniculata L.	0	n	1	0	Caryophyllaceae Juss.
Iz_2_1009	DJI_0500	Helichrysum arenarium (L.) Moench	0	n	1	0	Asteraceae Bercht. & J.Presl
Iz_2_1009	DJI_0500	Herniaria polygama J.Gay	0	n	1	0	Caryophyllaceae Juss.
Iz_2_1009	DJI_0500	Hieracium robustum Fr.	0	n	1	0	Asteraceae Bercht. & J.Presl
Iz_2_1009	DJI_0500	Hieracium umbellatum L.	0	n	1	NA	Asteraceae Bercht. & J.Presl
Iz_2_1009	DJI_0500	Hylotelephium maximum (L.) Holub	0	n	1	0	Crassulaceae J.St.-Hil.
Iz_2_1009	DJI_0500	Jacobaea borysthenica (DC.) B.Nord. & Greuter	0	n	1	0	Asteraceae Bercht. & J.Presl
Iz_2_1009	DJI_0500	Jasione montana L.	0	n	1	0	Campanulaceae Juss.
Iz_2_1009	DJI_0500	Jurinea cyanoides (L.) Rchb.s.l.	0	n	1	0	Asteraceae Bercht. & J.Presl
Iz_2_1009	DJI_0500	Koeleria glauca (Spreng.) DC.	0	n	1	0	Poaceae Barnhart
Iz_2_1009	DJI_0500	Lactuca serriola L.	0	a	0	0	Asteraceae Bercht. & J.Presl
Iz_2_1009	DJI_0500	Linaria genistifolia (L.) Mill.	0	n	1	0	Plantaginaceae Juss.
Iz_2_1009	DJI_0500	Lomelosia argentea (L.) Greuter & Burdet	0	n	1	0	Caprifoliaceae Juss.
Iz_2_1009	DJI_0500	Melampyrum pratense L.	0	n	1	0	Orobanchaceae Vent.
Iz_2_1009	DJI_0500	Oenothera biennis L.	0	a	0	0	Onagraceae Juss.
Iz_2_1009	DJI_0500	Pentanema britannica (L.) D.Gut.Larr., Santos-Vicente, Anderb., E.Rico & M.M.Mart.Ort.	0	n	1	0	Asteraceae Bercht. & J.Presl
Iz_2_1009	DJI_0500	Peucedanum oreoselinum (L.) Moench	0	n	1	0	Apiaceae Lindl.
Iz_2_1009	DJI_0500	Peucedanum ruthenicum M.Bieb.	0	n	1	0	Apiaceae Lindl.
Iz_2_1009	DJI_0500	Pilosella echioides (Lumn.) F.W.Schultz & Sch.Bip.	0	n	1	0	Asteraceae Bercht. & J.Presl
Iz_2_1009	DJI_0500	Pilosella officinarum F. Schultz et Sch. Bip.	0	n	1	0	Asteraceae Bercht. & J.Presl
Iz_2_1009	DJI_0500	Pilosella sp.	0		NA	0	Asteraceae Bercht. & J.Presl
Iz_2_1009	DJI_0500	Pinus sylvestris L.	0	n	1	1	Pinaceae Spreng. ex F.Rudolphi
Iz_2_1009	DJI_0500	Poa annua L.	0	n	1	0	Poaceae Barnhart
Iz_2_1009	DJI_0500	Poa nemoralis L.	0	n	1	0	Poaceae Barnhart
Iz_2_1009	DJI_0500	Poa sp.	0		NA	0	Poaceae Barnhart
Iz_2_1009	DJI_0500	Polygonatum odoratum (Mill.) Druce	0	n	1	0	Asparagaceae Juss.
Iz_2_1009	DJI_0500	Polygonum arenarium Waldst. & Kit.	0	n	1	0	Polygonaceae Juss.
Iz_2_1009	DJI_0500	Populus nigra L.	0	n	1	1	Salicaceae Mirb.
Iz_2_1009	DJI_0500	Populus tremula L.	0	n	1	1	Salicaceae Mirb.
Iz_2_1009	DJI_0500	Populus Ã canescens (Aiton) Sm.	0	n	1	1	Salicaceae Mirb.
Iz_2_1009	DJI_0500	Potentilla argentea L.	0	n	1	0	Rosaceae Juss.
Iz_2_1009	DJI_0500	Psammophiliella muralis (L.) Ikonn.	0	n	1	0	Caryophyllaceae Juss.
Iz_2_1009	DJI_0500	Psephellus sumensis (Kalen.) Greuter	0	n	1	0	Asteraceae Bercht. & J.Presl
Iz_2_1009	DJI_0500	Pulsatilla pratensis (L.) Mill. s.l.	0	n	1	0	Ranunculaceae Juss.
Iz_2_1009	DJI_0500	Pyrus communis L.	0	a	0	1	Rosaceae Juss.
Iz_2_1009	DJI_0500	Quercus robur L.	0.5	n	1	1	Fagaceae Dumort.
Iz_2_1009	DJI_0500	Robinia pseudoacacia L.	0	a	0	1	Fabaceae Lindl.
Iz_2_1009	DJI_0500	Rosa sp.	0		NA	1	Rosaceae Juss.
Iz_2_1009	DJI_0500	Rubus idaeus L.	0	n	1	0	Rosaceae Juss.
Iz_2_1009	DJI_0500	Rumex acetosella L.	0	n	1	0	Polygonaceae Juss.
Iz_2_1009	DJI_0500	Rumex thyrsiflorus Fingerh.	0	n	1	0	Polygonaceae Juss.
Iz_2_1009	DJI_0500	Salix alba L.	0	n	1	1	Salicaceae Mirb.
Iz_2_1009	DJI_0500	Sambucus nigra L.	0	n	1	1	Viburnaceae Raf.
Iz_2_1009	DJI_0500	Secale sylvestre Host	0	n	1	0	Poaceae Barnhart
Iz_2_1009	DJI_0500	Sempervivum ruthenicum Schnittsp. & C.B. Lehm.	0	n	1	0	Crassulaceae J.St.-Hil.
Iz_2_1009	DJI_0500	Setaria viridis (L.) P. Beauv.	0	n	1	0	Poaceae Barnhart
Iz_2_1009	DJI_0500	Silene borysthenica (Gruner) Walters	0	n	1	0	Caryophyllaceae Juss.
Iz_2_1009	DJI_0500	Silene latifolia subsp. alba (Mill.) Greuter & Burdet	0	n	1	0	Caryophyllaceae Juss.
Iz_2_1009	DJI_0500	Solidago virgaurea L.	0	n	1	0	Asteraceae Bercht. & J.Presl
Iz_2_1009	DJI_0500	Sorbus aucuparia L.	0	n	1	1	Rosaceae Juss.
Iz_2_1009	DJI_0500	Stachys recta L.	0	n	1	0	Lamiacea Lindl.
Iz_2_1009	DJI_0500	Tanacetum vulgare L.	0	n	1	0	Asteraceae Bercht. & J.Presl
Iz_2_1009	DJI_0500	Thymus pallasianus Heinr.Braun	0	n	1	0	Lamiacea Lindl.
Iz_2_1009	DJI_0500	Tilia cordata Mill.	0	n	1	1	Malvaceae Juss.
Iz_2_1009	DJI_0500	Trifolium alpestre L.	0	n	1	0	Fabaceae Lindl.
Iz_2_1009	DJI_0500	Trifolium arvense L.	0	n	1	0	Fabaceae Lindl.
Iz_2_1009	DJI_0500	Ulmus glabra Huds.	0	n	1	1	Ulmaceae Mirb.
Iz_2_1009	DJI_0500	Verbascum densiflorum Bertol.	0	n	1	0	Scrophulariaceae Juss.
Iz_2_1009	DJI_0500	Veronica austriaca L.	0	n	1	0	Plantaginaceae Juss.
Iz_2_1009	DJI_0500	Veronica incana L.	0	n	1	0	Plantaginaceae Juss.
Iz_2_1009	DJI_0500	Veronica verna L.	0	n	1	0	Plantaginaceae Juss.
Iz_2_1009	DJI_0500	Vincetoxicum hirundinaria Medik	0	n	1	0	Apocynaceae Juss.
Iz_2_1009	DJI_0504	Acer tataricum L.	0	n	1	1	Sapindaceae Juss.
Iz_2_1009	DJI_0504	Achillea millefolium L. s.l.	0	n	1	0	Asteraceae Bercht. & J.Presl
Iz_2_1009	DJI_0504	Allium oleraceum L.	0	n	1	0	Amaryllidaceae J.St.-Hil.
Iz_2_1009	DJI_0504	Ambrosia artemisiifolia L.	0	a	0	0	Asteraceae Bercht. & J.Presl
Iz_2_1009	DJI_0504	Amorpha fruticosa L.	0	a	0	0	Fabaceae Lindl.
Iz_2_1009	DJI_0504	Anthericum ramosum L.	0	n	1	0	Asparagaceae Juss.
Iz_2_1009	DJI_0504	Artemisia marschalliana Spreng.	0	n	1	0	Asteraceae Bercht. & J.Presl
Iz_2_1009	DJI_0504	Asparagus officinalis L.	0	n	1	0	Asparagaceae Juss.
Iz_2_1009	DJI_0504	Astragalus cicer L.	0	n	1	0	Fabaceae Lindl.
Iz_2_1009	DJI_0504	Bassia laniflora (S.G.Gmel.) A.J.Scott	0	n	1	0	Amaranthaceae Juss.
Iz_2_1009	DJI_0504	Berberis vulgaris L.	0	n	1	0	Berberidaceae Juss.
Iz_2_1009	DJI_0504	Betula pendula Roth.	0	n	1	1	Betulaceae Gray
Iz_2_1009	DJI_0504	Betula pendula Roth.	0	n	1	0	Poaceae Barnhart
Iz_2_1009	DJI_0504	Bromus inermis Leyss.	0		NA	NA	
Iz_2_1009	DJI_0504	Bromus squarrosus L.	0	a	0	0	Poaceae Barnhart
Iz_2_1009	DJI_0504	Bromus tectorum L.	0	a	0	0	Poaceae Barnhart
Iz_2_1009	DJI_0504	Calamagrostis epigejos (L.) Roth	1	n	1	0	Poaceae Barnhart
Iz_2_1009	DJI_0504	Carduus acanthoides L.	0	n	1	0	Asteraceae Bercht. & J.Presl
Iz_2_1009	DJI_0504	Carex sp.	0	n	1	0	Cyperaceae Juss.
Iz_2_1009	DJI_0504	Centaurea majorovii Dumbadze	0	n	1	0	Asteraceae Bercht. & J.Presl
Iz_2_1009	DJI_0504	Cf. Solidago virgaurea	0		NA	NA	
Iz_2_1009	DJI_0504	Chamaecytisus ruthenicus (Fisch.ex Wol.) Klaskova	0	n	1	0	Fabaceae Lindl.

Iz_2_1009	DJI_0504	Chelidonium majus L.	0	n	1	0	Papaveraceae Juss.
Iz_2_1009	DJI_0504	Chenopodium betaceum Andrz.	0	n	1	0	Amaranthaceae Juss.
Iz_2_1009	DJI_0504	Chenopodium cf. album	0	n	1	0	Amaranthaceae Juss.
Iz_2_1009	DJI_0504	Chenopodium sp.	0		NA	0	Amaranthaceae Juss.
Iz_2_1009	DJI_0504	Chondrilla juncea L.	0	n	1	0	Asteraceae Bercht. & J.Presl
Iz_2_1009	DJI_0504	Cirsium vulgare (Savi) Ten.	0	n	1	0	Asteraceae Bercht. & J.Presl
Iz_2_1009	DJI_0504	Clinopodium acinos (L.) Kuntze	0	n	1	0	Lamiacea Lindl.
Iz_2_1009	DJI_0504	Convallaria majalis L.	0	n	1	0	Asparagaceae Juss.
Iz_2_1009	DJI_0504	Crataegus rhipidophylla Gand.	0	n	1	1	Rosaceae Juss.
Iz_2_1009	DJI_0504	Crepis foetida L.	0	n	1	0	Asteraceae Bercht. & J.Presl
Iz_2_1009	DJI_0504	Dactylis glomerata L.	0	n	1	0	Poaceae Barnhart
Iz_2_1009	DJI_0504	Dianthus campestris M.Bieb.	0	n	1	0	Caryophyllaceae Juss.
Iz_2_1009	DJI_0504	Digitaria sanguinalis (L.) Scop.	0	a	0	0	Poaceae Barnhart
Iz_2_1009	DJI_0504	Elymus repens (L.) Gould	0	n	1	0	Poaceae Barnhart
Iz_2_1009	DJI_0504	Epilobium tetragonum L.	0	n	1	0	Onagraceae Juss.
Iz_2_1009	DJI_0504	Eragrostis minor Host	0	a	0	0	Poaceae Barnhart
Iz_2_1009	DJI_0504	Erigeron canadensis L.	0	a	0	0	Asteraceae Bercht. & J.Presl
Iz_2_1009	DJI_0504	Euphorbia seguieriana Neck.	0	n	1	0	Euphorbiaceae Juss.
Iz_2_1009	DJI_0504	Fallopia dumetorum (L.) Holub	0	n	1	0	Polygonaceae Juss.
Iz_2_1009	DJI_0504	Festuca beckeri (Hack.) Trautv.	0	n	1	0	Poaceae Barnhart
Iz_2_1009	DJI_0504	Fragaria vesca L.	0	n	1	0	Rosaceae Juss.
Iz_2_1009	DJI_0504	Frangula alnus Mill.	0	n	1	1	Rhamnaceae Juss.
Iz_2_1009	DJI_0504	Fraxinus pennsylvanica Marshall	0	a	0	1	Oleaceae Hoffmanns. & Link
Iz_2_1009	DJI_0504	Galeopsis bifida Boenn.	0	n	1	0	Lamiacea Lindl.
Iz_2_1009	DJI_0504	Galium verum L.	0	n	1	0	Rubiaceae Juss.
Iz_2_1009	DJI_0504	Genista tinctoria L.	0	n	1	0	Fabaceae Lindl.
Iz_2_1009	DJI_0504	Geranium robertianum L.	0	n	1	0	Geraniaceae Juss.
Iz_2_1009	DJI_0504	Geranium sanquineum L.	0	n	1	0	Geraniaceae Juss.
Iz_2_1009	DJI_0504	Gypsophila paniculata L.	0	n	1	0	Caryophyllaceae Juss.
Iz_2_1009	DJI_0504	Helichrysum arenarium (L.) Moench	0	n	1	0	Asteraceae Bercht. & J.Presl
Iz_2_1009	DJI_0504	Herniaria polygama J.Gay	0	n	1	0	Caryophyllaceae Juss.
Iz_2_1009	DJI_0504	Hieracium robustum Fr.	0	n	1	0	Asteraceae Bercht. & J.Presl
Iz_2_1009	DJI_0504	Hieracium umbellatum L.	0	n	1	NA	Asteraceae Bercht. & J.Presl
Iz_2_1009	DJI_0504	Hylotelephium maximum (L.) Holub	0	n	1	0	Crassulaceae J.St.-Hil.
Iz_2_1009	DJI_0504	Jacobaea borysthenica (DC.) B.Nord. & Greuter	0	n	1	0	Asteraceae Bercht. & J.Presl
Iz_2_1009	DJI_0504	Jasione montana L.	0	n	1	0	Campanulaceae Juss.
Iz_2_1009	DJI_0504	Jurinea cyanoides (L.) Rchb.s.l.	0	n	1	0	Asteraceae Bercht. & J.Presl
Iz_2_1009	DJI_0504	Koeleria glauca (Spreng.) DC.	0	n	1	0	Poaceae Barnhart
Iz_2_1009	DJI_0504	Lactuca serriola L.	0	a	0	0	Asteraceae Bercht. & J.Presl
Iz_2_1009	DJI_0504	Linaria genistifolia (L.) Mill.	0	n	1	0	Plantaginaceae Juss.
Iz_2_1009	DJI_0504	Lomelosia argentea (L.) Greuter & Burdet	0	n	1	0	Caprifoliaceae Juss.
Iz_2_1009	DJI_0504	Melampyrum pratense L.	0	n	1	0	Orobanchaceae Vent.
Iz_2_1009	DJI_0504	Oenothera biennis L.	0	a	0	0	Onagraceae Juss.
Iz_2_1009	DJI_0504	Pentanema britannica (L.) D.Gut.Larr., Santos-Vicente, Anderb., E.Rico & M.M.Mart.Ort.	0	n	1	0	Asteraceae Bercht. & J.Presl
Iz_2_1009	DJI_0504	Peucedanum oreoselinum (L.) Moench	0	n	1	0	Apiaceae Lindl.
Iz_2_1009	DJI_0504	Peucedanum ruthenicum M.Bieb.	0	n	1	0	Apiaceae Lindl.
Iz_2_1009	DJI_0504	Pilosella echioides (Lumn.) F.W.Schultz & Sch.Bip.	0	n	1	0	Asteraceae Bercht. & J.Presl
Iz_2_1009	DJI_0504	Pilosella officinarum F. Schultz et Sch. Bip.	0	n	1	0	Asteraceae Bercht. & J.Presl
Iz_2_1009	DJI_0504	Pilosella sp.	0		NA	0	Asteraceae Bercht. & J.Presl
Iz_2_1009	DJI_0504	Pinus sylvestris L.	0	n	1	1	Pinaceae Spreng. ex F.Rudolphi
Iz_2_1009	DJI_0504	Poa annua L.	0	n	1	0	Poaceae Barnhart
Iz_2_1009	DJI_0504	Poa nemoralis L.	0	n	1	0	Poaceae Barnhart
Iz_2_1009	DJI_0504	Poa sp.	0		NA	0	Poaceae Barnhart
Iz_2_1009	DJI_0504	Polygonatum odoratum (Mill.) Druce	0	n	1	0	Asparagaceae Juss.
Iz_2_1009	DJI_0504	Polygonum arenarium Waldst. & Kit.	0	n	1	0	Polygonaceae Juss.
Iz_2_1009	DJI_0504	Populus nigra L.	0	n	1	1	Salicaceae Mirb.
Iz_2_1009	DJI_0504	Populus tremula L.	0	n	1	1	Salicaceae Mirb.
Iz_2_1009	DJI_0504	Populus Ã canescens (Aiton) Sm.	0	n	1	1	Salicaceae Mirb.
Iz_2_1009	DJI_0504	Potentilla argentea L.	0	n	1	0	Rosaceae Juss.
Iz_2_1009	DJI_0504	Psammophiliella muralis (L.) Ikonn.	0	n	1	0	Caryophyllaceae Juss.
Iz_2_1009	DJI_0504	Psephellus sumensis (Kalen.) Greuter	0	n	1	0	Asteraceae Bercht. & J.Presl
Iz_2_1009	DJI_0504	Pulsatilla pratensis (L.) Mill. s.l.	0	n	1	0	Ranunculaceae Juss.
Iz_2_1009	DJI_0504	Pyrus communis L.	0	a	0	1	Rosaceae Juss.
Iz_2_1009	DJI_0504	Quercus robur L.	2	n	1	1	Fagaceae Dumort.
Iz_2_1009	DJI_0504	Robinia pseudoacacia L.	0	a	0	1	Fabaceae Lindl.
Iz_2_1009	DJI_0504	Rosa sp.	0		NA	1	Rosaceae Juss.
Iz_2_1009	DJI_0504	Rubus idaeus L.	0	n	1	0	Rosaceae Juss.
Iz_2_1009	DJI_0504	Rumex acetosella L.	0	n	1	0	Polygonaceae Juss.
Iz_2_1009	DJI_0504	Rumex thyrsiflorus Fingerh.	0	n	1	0	Polygonaceae Juss.
Iz_2_1009	DJI_0504	Salix alba L.	0	n	1	1	Salicaceae Mirb.
Iz_2_1009	DJI_0504	Sambucus nigra L.	0	n	1	1	Viburnaceae Raf.
Iz_2_1009	DJI_0504	Secale sylvestre Host	0	n	1	0	Poaceae Barnhart
Iz_2_1009	DJI_0504	Sempervivum ruthenicum Schnittsp. & C.B. Lehm.	0	n	1	0	Crassulaceae J.St.-Hil.
Iz_2_1009	DJI_0504	Setaria viridis (L.) P. Beauv.	0	n	1	0	Poaceae Barnhart
Iz_2_1009	DJI_0504	Silene borysthenica (Gruner) Walters	0	n	1	0	Caryophyllaceae Juss.
Iz_2_1009	DJI_0504	Silene latifolia subsp. alba (Mill.) Greuter & Burdet	0	n	1	0	Caryophyllaceae Juss.
Iz_2_1009	DJI_0504	Solidago virgaurea L.	0	n	1	0	Asteraceae Bercht. & J.Presl
Iz_2_1009	DJI_0504	Sorbus aucuparia L.	0	n	1	1	Rosaceae Juss.
Iz_2_1009	DJI_0504	Stachys recta L.	0	n	1	0	Lamiacea Lindl.
Iz_2_1009	DJI_0504	Tanacetum vulgare L.	0	n	1	0	Asteraceae Bercht. & J.Presl
Iz_2_1009	DJI_0504	Thymus pallasianus Heinr.Braun	0	n	1	0	Lamiacea Lindl.
Iz_2_1009	DJI_0504	Tilia cordata Mill.	0	n	1	1	Malvaceae Juss.
Iz_2_1009	DJI_0504	Trifolium alpestre L.	0	n	1	0	Fabaceae Lindl.
Iz_2_1009	DJI_0504	Trifolium arvense L.	0	n	1	0	Fabaceae Lindl.
Iz_2_1009	DJI_0504	Ulmus glabra Huds.	0	n	1	1	Ulmaceae Mirb.
Iz_2_1009	DJI_0504	Verbascum densiflorum Bertol.	0	n	1	0	Scrophulariaceae Juss.
Iz_2_1009	DJI_0504	Veronica austriaca L.	0	n	1	0	Plantaginaceae Juss.
Iz_2_1009	DJI_0504	Veronica incana L.	0	n	1	0	Plantaginaceae Juss.
Iz_2_1009	DJI_0504	Veronica verna L.	0	n	1	0	Plantaginaceae Juss.
Iz_2_1009	DJI_0504	Vincetoxicum hirundinaria Medik	0	n	1	0	Apocynaceae Juss.
Iz_2_1009	DJI_0507	Acer tataricum L.	0	n	1	1	Sapindaceae Juss.
Iz_2_1009	DJI_0507	Achillea millefolium L. s.l.	0	n	1	0	Asteraceae Bercht. & J.Presl
Iz_2_1009	DJI_0507	Allium oleraceum L.	0	n	1	0	Amaryllidaceae J.St.-Hil.
Iz_2_1009	DJI_0507	Ambrosia artemisiifolia L.	0	a	0	0	Asteraceae Bercht. & J.Presl
Iz_2_1009	DJI_0507	Amorpha fruticosa L.	0	a	0	0	Fabaceae Lindl.
Iz_2_1009	DJI_0507	Anthericum ramosum L.	0	n	1	0	Asparagaceae Juss.
Iz_2_1009	DJI_0507	Artemisia marschalliana Spreng.	0	n	1	0	Asteraceae Bercht. & J.Presl
Iz_2_1009	DJI_0507	Asparagus officinalis L.	0	n	1	0	Asparagaceae Juss.

Iz_2_1009	DJI_0507	Astragalus cicer L.	0	n	1	0	Fabaceae Lindl.
Iz_2_1009	DJI_0507	Bassia laniflora (S.G.Gmel.) A.J.Scott	0	n	1	0	Amaranthaceae Juss.
Iz_2_1009	DJI_0507	Berberis vulgaris L.	0	n	1	0	Berberidaceae Juss.
Iz_2_1009	DJI_0507	Betula pendula Roth.	0	n	1	1	Betulaceae Gray
Iz_2_1009	DJI_0507	Betula pendula Roth.	0	n	1	0	Poaceae Barnhart
Iz_2_1009	DJI_0507	Bromus inermis Leyss.	0		NA	NA	
Iz_2_1009	DJI_0507	Bromus squarrosus L.	0	a	0	0	Poaceae Barnhart
Iz_2_1009	DJI_0507	Bromus tectorum L.	0	a	0	0	Poaceae Barnhart
Iz_2_1009	DJI_0507	Calamagrostis epigejos (L.) Roth	2	n	1	0	Poaceae Barnhart
Iz_2_1009	DJI_0507	Carduus acanthoides L.	0	n	1	0	Asteraceae Bercht. & J.Presl
Iz_2_1009	DJI_0507	Carex sp.	0	n	1	0	Cyperaceae Juss.
Iz_2_1009	DJI_0507	Centaurea majorovii Dumbadze	0	n	1	0	Asteraceae Bercht. & J.Presl
Iz_2_1009	DJI_0507	Cf. Solidago virgaurea	0		NA	NA	
Iz_2_1009	DJI_0507	Chamaecytisus ruthenicus (Fisch.ex Wol.) Klaskova	0	n	1	0	Fabaceae Lindl.
Iz_2_1009	DJI_0507	Chelidonium majus L.	0	n	1	0	Papaveraceae Juss.
Iz_2_1009	DJI_0507	Chenopodium betaceum Andrz.	0	n	1	0	Amaranthaceae Juss.
Iz_2_1009	DJI_0507	Chenopodium cf. album	0	n	1	0	Amaranthaceae Juss.
Iz_2_1009	DJI_0507	Chenopodium sp.	0		NA	0	Amaranthaceae Juss.
Iz_2_1009	DJI_0507	Chondrilla juncea L.	0	n	1	0	Asteraceae Bercht. & J.Presl
Iz_2_1009	DJI_0507	Cirsium vulgare (Savi) Ten.	0	n	1	0	Asteraceae Bercht. & J.Presl
Iz_2_1009	DJI_0507	Clinopodium acinos (L.) Kuntze	0	n	1	0	Lamiacea Lindl.
Iz_2_1009	DJI_0507	Convallaria majalis L.	0	n	1	0	Asparagaceae Juss.
Iz_2_1009	DJI_0507	Crataegus rhipidophylla Gand.	0	n	1	1	Rosaceae Juss.
Iz_2_1009	DJI_0507	Crepis foetida L.	0	n	1	0	Asteraceae Bercht. & J.Presl
Iz_2_1009	DJI_0507	Dactylis glomerata L.	0	n	1	0	Poaceae Barnhart
Iz_2_1009	DJI_0507	Dianthus campestris M.Bieb.	0	n	1	0	Caryophyllaceae Juss.
Iz_2_1009	DJI_0507	Digitaria sanguinalis (L.) Scop.	0	a	0	0	Poaceae Barnhart
Iz_2_1009	DJI_0507	Elymus repens (L.) Gould	0	n	1	0	Poaceae Barnhart
Iz_2_1009	DJI_0507	Epilobium tetragonum L.	0	n	1	0	Onagraceae Juss.
Iz_2_1009	DJI_0507	Eragrostis minor Host	0	a	0	0	Poaceae Barnhart
Iz_2_1009	DJI_0507	Erigeron canadensis L.	0	a	0	0	Asteraceae Bercht. & J.Presl
Iz_2_1009	DJI_0507	Euphorbia seguieriana Neck.	0	n	1	0	Euphorbiaceae Juss.
Iz_2_1009	DJI_0507	Fallopia dumetorum (L.) Holub	0	n	1	0	Polygonaceae Juss.
Iz_2_1009	DJI_0507	Festuca beckeri (Hack.) Trautv.	0	n	1	0	Poaceae Barnhart
Iz_2_1009	DJI_0507	Fragaria vesca L.	0	n	1	0	Rosaceae Juss.
Iz_2_1009	DJI_0507	Frangula alnus Mill.	0	n	1	1	Rhamnaceae Juss.
Iz_2_1009	DJI_0507	Fraxinus pennsylvanica Marshall	0	a	0	1	Oleaceae Hoffmanns. & Link
Iz_2_1009	DJI_0507	Galeopsis bifida Boenn.	0	n	1	0	Lamiacea Lindl.
Iz_2_1009	DJI_0507	Galium verum L.	0	n	1	0	Rubiaceae Juss.
Iz_2_1009	DJI_0507	Genista tinctoria L.	0	n	1	0	Fabaceae Lindl.
Iz_2_1009	DJI_0507	Geranium robertianum L.	0	n	1	0	Geraniaceae Juss.
Iz_2_1009	DJI_0507	Geranium sanquineum L.	0	n	1	0	Geraniaceae Juss.
Iz_2_1009	DJI_0507	Gypsophila paniculata L.	0	n	1	0	Caryophyllaceae Juss.
Iz_2_1009	DJI_0507	Helichrysum arenarium (L.) Moench	0	n	1	0	Asteraceae Bercht. & J.Presl
Iz_2_1009	DJI_0507	Herniaria polygama J.Gay	0	n	1	0	Caryophyllaceae Juss.
Iz_2_1009	DJI_0507	Hieracium robustum Fr.	0	n	1	0	Asteraceae Bercht. & J.Presl
Iz_2_1009	DJI_0507	Hieracium umbellatum L.	0	n	1	NA	Asteraceae Bercht. & J.Presl
Iz_2_1009	DJI_0507	Hylotelephium maximum (L.) Holub	0	n	1	0	Crassulaceae J.St.-Hil.
Iz_2_1009	DJI_0507	Jacobaea borysthenica (DC.) B.Nord. & Greuter	0	n	1	0	Asteraceae Bercht. & J.Presl
Iz_2_1009	DJI_0507	Jasione montana L.	0	n	1	0	Campanulaceae Juss.
Iz_2_1009	DJI_0507	Jurinea cyanoides (L.) Rchb.s.l.	0	n	1	0	Asteraceae Bercht. & J.Presl
Iz_2_1009	DJI_0507	Koeleria glauca (Spreng.) DC.	0	n	1	0	Poaceae Barnhart
Iz_2_1009	DJI_0507	Lactuca serriola L.	0	a	0	0	Asteraceae Bercht. & J.Presl
Iz_2_1009	DJI_0507	Linaria genistifolia (L.) Mill.	0	n	1	0	Plantaginaceae Juss.
Iz_2_1009	DJI_0507	Lomelosia argentea (L.) Greuter & Burdet	0	n	1	0	Caprifoliaceae Juss.
Iz_2_1009	DJI_0507	Melampyrum pratense L.	0	n	1	0	Orobanchaceae Vent.
Iz_2_1009	DJI_0507	Oenothera biennis L.	0	a	0	0	Onagraceae Juss.
Iz_2_1009	DJI_0507	Pentanema britannica (L.) D.Gut.Larr., Santos-Vicente, Anderb., E.Rico & M.M.Mart.Ort.	0	n	1	0	Asteraceae Bercht. & J.Presl
Iz_2_1009	DJI_0507	Peucedanum oreoselinum (L.) Moench	0	n	1	0	Apiaceae Lindl.
Iz_2_1009	DJI_0507	Peucedanum ruthenicum M.Bieb.	0	n	1	0	Apiaceae Lindl.
Iz_2_1009	DJI_0507	Pilosella echioides (Lumn.) F.W.Schultz & Sch.Bip.	0	n	1	0	Asteraceae Bercht. & J.Presl
Iz_2_1009	DJI_0507	Pilosella officinarum F. Schultz et Sch. Bip.	0	n	1	0	Asteraceae Bercht. & J.Presl
Iz_2_1009	DJI_0507	Pilosella sp.	0		NA	0	Asteraceae Bercht. & J.Presl
Iz_2_1009	DJI_0507	Pinus sylvestris L.	0	n	1	1	Pinaceae Spreng. ex F.Rudolphi
Iz_2_1009	DJI_0507	Poa annua L.	0	n	1	0	Poaceae Barnhart
Iz_2_1009	DJI_0507	Poa nemoralis L.	0	n	1	0	Poaceae Barnhart
Iz_2_1009	DJI_0507	Poa sp.	0		NA	0	Poaceae Barnhart
Iz_2_1009	DJI_0507	Polygonatum odoratum (Mill.) Druce	0	n	1	0	Asparagaceae Juss.
Iz_2_1009	DJI_0507	Polygonum arenarium Waldst. & Kit.	0	n	1	0	Polygonaceae Juss.
Iz_2_1009	DJI_0507	Populus nigra L.	0	n	1	1	Salicaceae Mirb.
Iz_2_1009	DJI_0507	Populus tremula L.	0	n	1	1	Salicaceae Mirb.
Iz_2_1009	DJI_0507	Populus Ã canescens (Aiton) Sm.	0	n	1	1	Salicaceae Mirb.
Iz_2_1009	DJI_0507	Potentilla argentea L.	0	n	1	0	Rosaceae Juss.
Iz_2_1009	DJI_0507	Psammophiliella muralis (L.) Ikonn.	0	n	1	0	Caryophyllaceae Juss.
Iz_2_1009	DJI_0507	Psephellus sumensis (Kalen.) Greuter	0	n	1	0	Asteraceae Bercht. & J.Presl
Iz_2_1009	DJI_0507	Pulsatilla pratensis (L.) Mill. s.l.	0	n	1	0	Ranunculaceae Juss.
Iz_2_1009	DJI_0507	Pyrus communis L.	0	a	0	1	Rosaceae Juss.
Iz_2_1009	DJI_0507	Quercus robur L.	0	n	1	1	Fagaceae Dumort.
Iz_2_1009	DJI_0507	Robinia pseudoacacia L.	0	a	0	1	Fabaceae Lindl.
Iz_2_1009	DJI_0507	Rosa sp.	0		NA	1	Rosaceae Juss.
Iz_2_1009	DJI_0507	Rubus idaeus L.	0	n	1	0	Rosaceae Juss.
Iz_2_1009	DJI_0507	Rumex acetosella L.	0	n	1	0	Polygonaceae Juss.
Iz_2_1009	DJI_0507	Rumex thyrsiflorus Fingerh.	0	n	1	0	Polygonaceae Juss.
Iz_2_1009	DJI_0507	Salix alba L.	0	n	1	1	Salicaceae Mirb.
Iz_2_1009	DJI_0507	Sambucus nigra L.	0	n	1	1	Viburnaceae Raf.
Iz_2_1009	DJI_0507	Secale sylvestre Host	0	n	1	0	Poaceae Barnhart
Iz_2_1009	DJI_0507	Sempervivum ruthenicum Schnittsp. & C.B. Lehm.	0	n	1	0	Crassulaceae J.St.-Hil.
Iz_2_1009	DJI_0507	Setaria viridis (L.) P. Beauv.	0	n	1	0	Poaceae Barnhart
Iz_2_1009	DJI_0507	Silene borysthenica (Gruner) Walters	0	n	1	0	Caryophyllaceae Juss.
Iz_2_1009	DJI_0507	Silene latifolia subsp. alba (Mill.) Greuter & Burdet	0	n	1	0	Caryophyllaceae Juss.
Iz_2_1009	DJI_0507	Solidago virgaurea L.	0	n	1	0	Asteraceae Bercht. & J.Presl
Iz_2_1009	DJI_0507	Sorbus aucuparia L.	0	n	1	1	Rosaceae Juss.
Iz_2_1009	DJI_0507	Stachys recta L.	0	n	1	0	Lamiacea Lindl.
Iz_2_1009	DJI_0507	Tanacetum vulgare L.	0	n	1	0	Asteraceae Bercht. & J.Presl
Iz_2_1009	DJI_0507	Thymus pallasianus Heinr.Braun	0	n	1	0	Lamiacea Lindl.
Iz_2_1009	DJI_0507	Tilia cordata Mill.	0	n	1	1	Malvaceae Juss.
Iz_2_1009	DJI_0507	Trifolium alpestre L.	0	n	1	0	Fabaceae Lindl.
Iz_2_1009	DJI_0507	Trifolium arvense L.	0	n	1	0	Fabaceae Lindl.

Iz_2_1009	DJI_0507	Ulmus glabra Huds.	0	n	1	1	Ulmaceae Mirb.
Iz_2_1009	DJI_0507	Verbascum densiflorum Bertol.	0	n	1	0	Scrophulariaceae Juss.
Iz_2_1009	DJI_0507	Veronica austriaca L.	0	n	1	0	Plantaginaceae Juss.
Iz_2_1009	DJI_0507	Veronica incana L.	0	n	1	0	Plantaginaceae Juss.
Iz_2_1009	DJI_0507	Veronica verna L.	0	n	1	0	Plantaginaceae Juss.
Iz_2_1009	DJI_0507	Vincetoxicum hirundinaria Medik	0	n	1	0	Apocynaceae Juss.
Iz_2_1009	DJI_0508	Acer tataricum L.	0	n	1	1	Sapindaceae Juss.
Iz_2_1009	DJI_0508	Achillea millefolium L. s.l.	0	n	1	0	Asteraceae Bercht. & J.Presl
Iz_2_1009	DJI_0508	Allium oleraceum L.	0	n	1	0	Amaryllidaceae J.St.-Hil.
Iz_2_1009	DJI_0508	Ambrosia artemisiifolia L.	0	a	0	0	Asteraceae Bercht. & J.Presl
Iz_2_1009	DJI_0508	Amorpha fruticosa L.	0	a	0	0	Fabaceae Lindl.
Iz_2_1009	DJI_0508	Anthericum ramosum L.	0	n	1	0	Asparagaceae Juss.
Iz_2_1009	DJI_0508	Artemisia marschalliana Spreng.	0	n	1	0	Asteraceae Bercht. & J.Presl
Iz_2_1009	DJI_0508	Asparagus officinalis L.	0	n	1	0	Asparagaceae Juss.
Iz_2_1009	DJI_0508	Astragalus cicer L.	0	n	1	0	Fabaceae Lindl.
Iz_2_1009	DJI_0508	Bassia laniflora (S.G.Gmel.) A.J.Scott	0	n	1	0	Amaranthaceae Juss.
Iz_2_1009	DJI_0508	Berberis vulgaris L.	0	n	1	0	Berberidaceae Juss.
Iz_2_1009	DJI_0508	Betula pendula Roth.	0	n	1	1	Betulaceae Gray
Iz_2_1009	DJI_0508	Betula pendula Roth.	0	n	1	0	Poaceae Barnhart
Iz_2_1009	DJI_0508	Bromus inermis Leyss.	0		NA	NA	
Iz_2_1009	DJI_0508	Bromus squarrosus L.	0	a	0	0	Poaceae Barnhart
Iz_2_1009	DJI_0508	Bromus tectorum L.	0	a	0	0	Poaceae Barnhart
Iz_2_1009	DJI_0508	Calamagrostis epigejos (L.) Roth	20	n	1	0	Poaceae Barnhart
Iz_2_1009	DJI_0508	Carduus acanthoides L.	0	n	1	0	Asteraceae Bercht. & J.Presl
Iz_2_1009	DJI_0508	Carex sp.	0	n	1	0	Cyperaceae Juss.
Iz_2_1009	DJI_0508	Centaurea majorovii Dumbadze	0	n	1	0	Asteraceae Bercht. & J.Presl
Iz_2_1009	DJI_0508	Cf. Solidago virgaurea	0		NA	NA	
Iz_2_1009	DJI_0508	Chamaecytisus ruthenicus (Fisch.ex Wol.) Klaskova	0	n	1	0	Fabaceae Lindl.
Iz_2_1009	DJI_0508	Chelidonium majus L.	0	n	1	0	Papaveraceae Juss.
Iz_2_1009	DJI_0508	Chenopodium betaceum Andrz.	0	n	1	0	Amaranthaceae Juss.
Iz_2_1009	DJI_0508	Chenopodium cf. album	0	n	1	0	Amaranthaceae Juss.
Iz_2_1009	DJI_0508	Chenopodium sp.	0		NA	0	Amaranthaceae Juss.
Iz_2_1009	DJI_0508	Chondrilla juncea L.	0	n	1	0	Asteraceae Bercht. & J.Presl
Iz_2_1009	DJI_0508	Cirsium vulgare (Savi) Ten.	0	n	1	0	Asteraceae Bercht. & J.Presl
Iz_2_1009	DJI_0508	Clinopodium acinos (L.) Kuntze	0	n	1	0	Lamiacea Lindl.
Iz_2_1009	DJI_0508	Convallaria majalis L.	0	n	1	0	Asparagaceae Juss.
Iz_2_1009	DJI_0508	Crataegus rhipidophylla Gand.	0	n	1	1	Rosaceae Juss.
Iz_2_1009	DJI_0508	Crepis foetida L.	0	n	1	0	Asteraceae Bercht. & J.Presl
Iz_2_1009	DJI_0508	Dactylis glomerata L.	0	n	1	0	Poaceae Barnhart
Iz_2_1009	DJI_0508	Dianthus campestris M.Bieb.	0	n	1	0	Caryophyllaceae Juss.
Iz_2_1009	DJI_0508	Digitaria sanguinalis (L.) Scop.	0	a	0	0	Poaceae Barnhart
Iz_2_1009	DJI_0508	Elymus repens (L.) Gould	0	n	1	0	Poaceae Barnhart
Iz_2_1009	DJI_0508	Epilobium tetragonum L.	0	n	1	0	Onagraceae Juss.
Iz_2_1009	DJI_0508	Eragrostis minor Host	0	a	0	0	Poaceae Barnhart
Iz_2_1009	DJI_0508	Erigeron canadensis L.	0	a	0	0	Asteraceae Bercht. & J.Presl
Iz_2_1009	DJI_0508	Euphorbia seguieriana Neck.	0	n	1	0	Euphorbiaceae Juss.
Iz_2_1009	DJI_0508	Fallopia dumetorum (L.) Holub	0	n	1	0	Polygonaceae Juss.
Iz_2_1009	DJI_0508	Festuca beckeri (Hack.) Trautv.	0	n	1	0	Poaceae Barnhart
Iz_2_1009	DJI_0508	Fragaria vesca L.	0	n	1	0	Rosaceae Juss.
Iz_2_1009	DJI_0508	Frangula alnus Mill.	0	n	1	1	Rhamnaceae Juss.
Iz_2_1009	DJI_0508	Fraxinus pennsylvanica Marshall	0	a	0	1	Oleaceae Hoffmanns. & Link
Iz_2_1009	DJI_0508	Galeopsis bifida Boenn.	0	n	1	0	Lamiacea Lindl.
Iz_2_1009	DJI_0508	Galium verum L.	0	n	1	0	Rubiaceae Juss.
Iz_2_1009	DJI_0508	Genista tinctoria L.	0	n	1	0	Fabaceae Lindl.
Iz_2_1009	DJI_0508	Geranium robertianum L.	0	n	1	0	Geraniaceae Juss.
Iz_2_1009	DJI_0508	Geranium sanquineum L.	0	n	1	0	Geraniaceae Juss.
Iz_2_1009	DJI_0508	Gypsophila paniculata L.	0	n	1	0	Caryophyllaceae Juss.
Iz_2_1009	DJI_0508	Helichrysum arenarium (L.) Moench	0	n	1	0	Asteraceae Bercht. & J.Presl
Iz_2_1009	DJI_0508	Herniaria polygama J.Gay	0	n	1	0	Caryophyllaceae Juss.
Iz_2_1009	DJI_0508	Hieracium robustum Fr.	0	n	1	0	Asteraceae Bercht. & J.Presl
Iz_2_1009	DJI_0508	Hieracium umbellatum L.	0	n	1	NA	Asteraceae Bercht. & J.Presl
Iz_2_1009	DJI_0508	Hylotelephium maximum (L.) Holub	0	n	1	0	Crassulaceae J.St.-Hil.
Iz_2_1009	DJI_0508	Jacobaea borysthenica (DC.) B.Nord. & Greuter	0	n	1	0	Asteraceae Bercht. & J.Presl
Iz_2_1009	DJI_0508	Jasione montana L.	0	n	1	0	Campanulaceae Juss.
Iz_2_1009	DJI_0508	Jurinea cyanoides (L.) Rchb.s.l.	0	n	1	0	Asteraceae Bercht. & J.Presl
Iz_2_1009	DJI_0508	Koeleria glauca (Spreng.) DC.	0	n	1	0	Poaceae Barnhart
Iz_2_1009	DJI_0508	Lactuca serriola L.	0	a	0	0	Asteraceae Bercht. & J.Presl
Iz_2_1009	DJI_0508	Linaria genistifolia (L.) Mill.	0	n	1	0	Plantaginaceae Juss.
Iz_2_1009	DJI_0508	Lomelosia argentea (L.) Greuter & Burdet	0	n	1	0	Caprifoliaceae Juss.
Iz_2_1009	DJI_0508	Melampyrum pratense L.	0	n	1	0	Orobanchaceae Vent.
Iz_2_1009	DJI_0508	Oenothera biennis L.	0	a	0	0	Onagraceae Juss.
Iz_2_1009	DJI_0508	Pentanema britannica (L.) D.Gut.Larr., Santos-Vicente, Anderb., E.Rico & M.M.Mart.Ort.	0	n	1	0	Asteraceae Bercht. & J.Presl
Iz_2_1009	DJI_0508	Peucedanum oreoselinum (L.) Moench	0	n	1	0	Apiaceae Lindl.
Iz_2_1009	DJI_0508	Peucedanum ruthenicum M.Bieb.	0	n	1	0	Apiaceae Lindl.
Iz_2_1009	DJI_0508	Pilosella echioides (Lumn.) F.W.Schultz & Sch.Bip.	0	n	1	0	Asteraceae Bercht. & J.Presl
Iz_2_1009	DJI_0508	Pilosella officinarum F. Schultz et Sch. Bip.	0	n	1	0	Asteraceae Bercht. & J.Presl
Iz_2_1009	DJI_0508	Pilosella sp.	0		NA	0	Asteraceae Bercht. & J.Presl
Iz_2_1009	DJI_0508	Pinus sylvestris L.	0	n	1	1	Pinaceae Spreng. ex F.Rudolphi
Iz_2_1009	DJI_0508	Poa annua L.	0	n	1	0	Poaceae Barnhart
Iz_2_1009	DJI_0508	Poa nemoralis L.	0	n	1	0	Poaceae Barnhart
Iz_2_1009	DJI_0508	Poa sp.	0		NA	0	Poaceae Barnhart
Iz_2_1009	DJI_0508	Polygonatum odoratum (Mill.) Druce	0	n	1	0	Asparagaceae Juss.
Iz_2_1009	DJI_0508	Polygonum arenarium Waldst. & Kit.	0	n	1	0	Polygonaceae Juss.
Iz_2_1009	DJI_0508	Populus nigra L.	0	n	1	1	Salicaceae Mirb.
Iz_2_1009	DJI_0508	Populus tremula L.	0	n	1	1	Salicaceae Mirb.
Iz_2_1009	DJI_0508	Populus Ã canescens (Aiton) Sm.	0	n	1	1	Salicaceae Mirb.
Iz_2_1009	DJI_0508	Potentilla argentea L.	0	n	1	0	Rosaceae Juss.
Iz_2_1009	DJI_0508	Psammophiliella muralis (L.) Ikonn.	0	n	1	0	Caryophyllaceae Juss.
Iz_2_1009	DJI_0508	Psephellus sumensis (Kalen.) Greuter	0	n	1	0	Asteraceae Bercht. & J.Presl
Iz_2_1009	DJI_0508	Pulsatilla pratensis (L.) Mill. s.l.	0	n	1	0	Ranunculaceae Juss.
Iz_2_1009	DJI_0508	Pyrus communis L.	0	a	0	1	Rosaceae Juss.
Iz_2_1009	DJI_0508	Quercus robur L.	0	n	1	1	Fagaceae Dumort.
Iz_2_1009	DJI_0508	Robinia pseudoacacia L.	0	a	0	1	Fabaceae Lindl.
Iz_2_1009	DJI_0508	Rosa sp.	0		NA	1	Rosaceae Juss.
Iz_2_1009	DJI_0508	Rubus idaeus L.	0	n	1	0	Rosaceae Juss.
Iz_2_1009	DJI_0508	Rumex acetosella L.	0	n	1	0	Polygonaceae Juss.
Iz_2_1009	DJI_0508	Rumex thyrsiflorus Fingerh.	0	n	1	0	Polygonaceae Juss.
Iz_2_1009	DJI_0508	Salix alba L.	0	n	1	1	Salicaceae Mirb.

Iz_2_1009	DJI_0508	Sambucus nigra L.	0	n	1	1	Viburnaceae Raf.
Iz_2_1009	DJI_0508	Secale sylvestre Host	0	n	1	0	Poaceae Barnhart
Iz_2_1009	DJI_0508	Sempervivum ruthenicum Schnittsp. & C.B. Lehm.	0	n	1	0	Crassulaceae J.St.-Hil.
Iz_2_1009	DJI_0508	Setaria viridis (L.) P. Beauv.	0	n	1	0	Poaceae Barnhart
Iz_2_1009	DJI_0508	Silene borysthenica (Gruner) Walters	0	n	1	0	Caryophyllaceae Juss.
Iz_2_1009	DJI_0508	Silene latifolia subsp. alba (Mill.) Greuter & Burdet	0	n	1	0	Caryophyllaceae Juss.
Iz_2_1009	DJI_0508	Solidago virgaurea L.	0	n	1	0	Asteraceae Bercht. & J.Presl
Iz_2_1009	DJI_0508	Sorbus aucuparia L.	0	n	1	1	Rosaceae Juss.
Iz_2_1009	DJI_0508	Stachys recta L.	0	n	1	0	Lamiacea Lindl.
Iz_2_1009	DJI_0508	Tanacetum vulgare L.	0	n	1	0	Asteraceae Bercht. & J.Presl
Iz_2_1009	DJI_0508	Thymus pallasianus Heinr.Braun	0	n	1	0	Lamiacea Lindl.
Iz_2_1009	DJI_0508	Tilia cordata Mill.	0	n	1	1	Malvaceae Juss.
Iz_2_1009	DJI_0508	Trifolium alpestre L.	0	n	1	0	Fabaceae Lindl.
Iz_2_1009	DJI_0508	Trifolium arvense L.	0	n	1	0	Fabaceae Lindl.
Iz_2_1009	DJI_0508	Ulmus glabra Huds.	0	n	1	1	Ulmaceae Mirb.
Iz_2_1009	DJI_0508	Verbascum densiflorum Bertol.	0	n	1	0	Scrophulariaceae Juss.
Iz_2_1009	DJI_0508	Veronica austriaca L.	0	n	1	0	Plantaginaceae Juss.
Iz_2_1009	DJI_0508	Veronica incana L.	0	n	1	0	Plantaginaceae Juss.
Iz_2_1009	DJI_0508	Veronica verna L.	0	n	1	0	Plantaginaceae Juss.
Iz_2_1009	DJI_0508	Vincetoxicum hirundinaria Medik	0	n	1	0	Apocynaceae Juss.
Iz_2_1009	DJI_0511	Acer tataricum L.	0	n	1	1	Sapindaceae Juss.
Iz_2_1009	DJI_0511	Achillea millefolium L. s.l.	0	n	1	0	Asteraceae Bercht. & J.Presl
Iz_2_1009	DJI_0511	Allium oleraceum L.	0	n	1	0	Amaryllidaceae J.St.-Hil.
Iz_2_1009	DJI_0511	Ambrosia artemisiifolia L.	0	a	0	0	Asteraceae Bercht. & J.Presl
Iz_2_1009	DJI_0511	Amorpha fruticosa L.	0	a	0	0	Fabaceae Lindl.
Iz_2_1009	DJI_0511	Anthericum ramosum L.	0	n	1	0	Asparagaceae Juss.
Iz_2_1009	DJI_0511	Artemisia marschalliana Spreng.	0	n	1	0	Asteraceae Bercht. & J.Presl
Iz_2_1009	DJI_0511	Asparagus officinalis L.	0	n	1	0	Asparagaceae Juss.
Iz_2_1009	DJI_0511	Astragalus cicer L.	0	n	1	0	Fabaceae Lindl.
Iz_2_1009	DJI_0511	Bassia laniflora (S.G.Gmel.) A.J.Scott	0	n	1	0	Amaranthaceae Juss.
Iz_2_1009	DJI_0511	Berberis vulgaris L.	0	n	1	0	Berberidaceae Juss.
Iz_2_1009	DJI_0511	Betula pendula Roth.	0	n	1	1	Betulaceae Gray
Iz_2_1009	DJI_0511	Betula pendula Roth.	0	n	1	0	Poaceae Barnhart
Iz_2_1009	DJI_0511	Bromus inermis Leyss.	0		NA	NA	
Iz_2_1009	DJI_0511	Bromus squarrosus L.	0	a	0	0	Poaceae Barnhart
Iz_2_1009	DJI_0511	Bromus tectorum L.	0	a	0	0	Poaceae Barnhart
Iz_2_1009	DJI_0511	Calamagrostis epigejos (L.) Roth	60	n	1	0	Poaceae Barnhart
Iz_2_1009	DJI_0511	Carduus acanthoides L.	0	n	1	0	Asteraceae Bercht. & J.Presl
Iz_2_1009	DJI_0511	Carex sp.	0	n	1	0	Cyperaceae Juss.
Iz_2_1009	DJI_0511	Centaurea majorovii Dumbadze	0	n	1	0	Asteraceae Bercht. & J.Presl
Iz_2_1009	DJI_0511	Cf. Solidago virgaurea	0		NA	NA	
Iz_2_1009	DJI_0511	Chamaecytisus ruthenicus (Fisch.ex Wol.) Klaskova	0	n	1	0	Fabaceae Lindl.
Iz_2_1009	DJI_0511	Chelidonium majus L.	0	n	1	0	Papaveraceae Juss.
Iz_2_1009	DJI_0511	Chenopodium betaceum Andrz.	0	n	1	0	Amaranthaceae Juss.
Iz_2_1009	DJI_0511	Chenopodium cf. album	0	n	1	0	Amaranthaceae Juss.
Iz_2_1009	DJI_0511	Chenopodium sp.	0		NA	0	Amaranthaceae Juss.
Iz_2_1009	DJI_0511	Chondrilla juncea L.	0	n	1	0	Asteraceae Bercht. & J.Presl
Iz_2_1009	DJI_0511	Cirsium vulgare (Savi) Ten.	0	n	1	0	Asteraceae Bercht. & J.Presl
Iz_2_1009	DJI_0511	Clinopodium acinos (L.) Kuntze	0	n	1	0	Lamiacea Lindl.
Iz_2_1009	DJI_0511	Convallaria majalis L.	0	n	1	0	Asparagaceae Juss.
Iz_2_1009	DJI_0511	Crataegus rhipidophylla Gand.	0	n	1	1	Rosaceae Juss.
Iz_2_1009	DJI_0511	Crepis foetida L.	0	n	1	0	Asteraceae Bercht. & J.Presl
Iz_2_1009	DJI_0511	Dactylis glomerata L.	0	n	1	0	Poaceae Barnhart
Iz_2_1009	DJI_0511	Dianthus campestris M.Bieb.	0	n	1	0	Caryophyllaceae Juss.
Iz_2_1009	DJI_0511	Digitaria sanguinalis (L.) Scop.	0	a	0	0	Poaceae Barnhart
Iz_2_1009	DJI_0511	Elymus repens (L.) Gould	0	n	1	0	Poaceae Barnhart
Iz_2_1009	DJI_0511	Epilobium tetragonum L.	0	n	1	0	Onagraceae Juss.
Iz_2_1009	DJI_0511	Eragrostis minor Host	0	a	0	0	Poaceae Barnhart
Iz_2_1009	DJI_0511	Erigeron canadensis L.	0	a	0	0	Asteraceae Bercht. & J.Presl
Iz_2_1009	DJI_0511	Euphorbia seguieriana Neck.	0	n	1	0	Euphorbiaceae Juss.
Iz_2_1009	DJI_0511	Fallopia dumetorum (L.) Holub	0	n	1	0	Polygonaceae Juss.
Iz_2_1009	DJI_0511	Festuca beckeri (Hack.) Trautv.	0	n	1	0	Poaceae Barnhart
Iz_2_1009	DJI_0511	Fragaria vesca L.	0	n	1	0	Rosaceae Juss.
Iz_2_1009	DJI_0511	Frangula alnus Mill.	0	n	1	1	Rhamnaceae Juss.
Iz_2_1009	DJI_0511	Fraxinus pennsylvanica Marshall	0	a	0	1	Oleaceae Hoffmanns. & Link
Iz_2_1009	DJI_0511	Galeopsis bifida Boenn.	0	n	1	0	Lamiacea Lindl.
Iz_2_1009	DJI_0511	Galium verum L.	0	n	1	0	Rubiaceae Juss.
Iz_2_1009	DJI_0511	Genista tinctoria L.	0	n	1	0	Fabaceae Lindl.
Iz_2_1009	DJI_0511	Geranium robertianum L.	0	n	1	0	Geraniaceae Juss.
Iz_2_1009	DJI_0511	Geranium sanquineum L.	0	n	1	0	Geraniaceae Juss.
Iz_2_1009	DJI_0511	Gypsophila paniculata L.	0	n	1	0	Caryophyllaceae Juss.
Iz_2_1009	DJI_0511	Helichrysum arenarium (L.) Moench	0	n	1	0	Asteraceae Bercht. & J.Presl
Iz_2_1009	DJI_0511	Herniaria polygama J.Gay	0	n	1	0	Caryophyllaceae Juss.
Iz_2_1009	DJI_0511	Hieracium robustum Fr.	0	n	1	0	Asteraceae Bercht. & J.Presl
Iz_2_1009	DJI_0511	Hieracium umbellatum L.	0	n	1	NA	Asteraceae Bercht. & J.Presl
Iz_2_1009	DJI_0511	Hylotelephium maximum (L.) Holub	0	n	1	0	Crassulaceae J.St.-Hil.
Iz_2_1009	DJI_0511	Jacobaea borysthenica (DC.) B.Nord. & Greuter	0	n	1	0	Asteraceae Bercht. & J.Presl
Iz_2_1009	DJI_0511	Jasione montana L.	0	n	1	0	Campanulaceae Juss.
Iz_2_1009	DJI_0511	Jurinea cyanoides (L.) Rchb.s.l.	0	n	1	0	Asteraceae Bercht. & J.Presl
Iz_2_1009	DJI_0511	Koeleria glauca (Spreng.) DC.	0	n	1	0	Poaceae Barnhart
Iz_2_1009	DJI_0511	Lactuca serriola L.	0	a	0	0	Asteraceae Bercht. & J.Presl
Iz_2_1009	DJI_0511	Linaria genistifolia (L.) Mill.	0	n	1	0	Plantaginaceae Juss.
Iz_2_1009	DJI_0511	Lomelosia argentea (L.) Greuter & Burdet	0	n	1	0	Caprifoliaceae Juss.
Iz_2_1009	DJI_0511	Melampyrum pratense L.	0	n	1	0	Orobanchaceae Vent.
Iz_2_1009	DJI_0511	Oenothera biennis L.	0	a	0	0	Onagraceae Juss.
Iz_2_1009	DJI_0511	Pentanema britannica (L.) D.Gut.Larr., Santos-Vicente, Anderb., E.Rico & M.M.Mart.Ort.	0	n	1	0	Asteraceae Bercht. & J.Presl
Iz_2_1009	DJI_0511	Peucedanum oreoselinum (L.) Moench	0	n	1	0	Apiaceae Lindl.
Iz_2_1009	DJI_0511	Peucedanum ruthenicum M.Bieb.	0	n	1	0	Apiaceae Lindl.
Iz_2_1009	DJI_0511	Pilosella echioides (Lumn.) F.W.Schultz & Sch.Bip.	0	n	1	0	Asteraceae Bercht. & J.Presl
Iz_2_1009	DJI_0511	Pilosella officinarum F. Schultz et Sch. Bip.	0	n	1	0	Asteraceae Bercht. & J.Presl
Iz_2_1009	DJI_0511	Pilosella sp.	0		NA	0	Asteraceae Bercht. & J.Presl
Iz_2_1009	DJI_0511	Pinus sylvestris L.	0	n	1	1	Pinaceae Spreng. ex F.Rudolphi
Iz_2_1009	DJI_0511	Poa annua L.	0	n	1	0	Poaceae Barnhart
Iz_2_1009	DJI_0511	Poa nemoralis L.	0	n	1	0	Poaceae Barnhart
Iz_2_1009	DJI_0511	Poa sp.	0		NA	0	Poaceae Barnhart
Iz_2_1009	DJI_0511	Polygonatum odoratum (Mill.) Druce	0	n	1	0	Asparagaceae Juss.
Iz_2_1009	DJI_0511	Polygonum arenarium Waldst. & Kit.	0	n	1	0	Polygonaceae Juss.
Iz_2_1009	DJI_0511	Populus nigra L.	0	n	1	1	Salicaceae Mirb.

Iz_2_1009	DJI_0511	Populus tremula L.	0	n	1	1	Salicaceae Mirb.
Iz_2_1009	DJI_0511	Populus Ã canescens (Aiton) Sm.	0	n	1	1	Salicaceae Mirb.
Iz_2_1009	DJI_0511	Potentilla argentea L.	0	n	1	0	Rosaceae Juss.
Iz_2_1009	DJI_0511	Psammophiliella muralis (L.) Ikonn.	0	n	1	0	Caryophyllaceae Juss.
Iz_2_1009	DJI_0511	Psephellus sumensis (Kalen.) Greuter	0	n	1	0	Asteraceae Bercht. & J.Presl
Iz_2_1009	DJI_0511	Pulsatilla pratensis (L.) Mill. s.l.	0	n	1	0	Ranunculaceae Juss.
Iz_2_1009	DJI_0511	Pyrus communis L.	0	a	0	1	Rosaceae Juss.
Iz_2_1009	DJI_0511	Quercus robur L.	0	n	1	1	Fagaceae Dumort.
Iz_2_1009	DJI_0511	Robinia pseudoacacia L.	0	a	0	1	Fabaceae Lindl.
Iz_2_1009	DJI_0511	Rosa sp.	0		NA	1	Rosaceae Juss.
Iz_2_1009	DJI_0511	Rubus idaeus L.	0	n	1	0	Rosaceae Juss.
Iz_2_1009	DJI_0511	Rumex acetosella L.	0	n	1	0	Polygonaceae Juss.
Iz_2_1009	DJI_0511	Rumex thyrsiflorus Fingerh.	0	n	1	0	Polygonaceae Juss.
Iz_2_1009	DJI_0511	Salix alba L.	0	n	1	1	Salicaceae Mirb.
Iz_2_1009	DJI_0511	Sambucus nigra L.	0	n	1	1	Viburnaceae Raf.
Iz_2_1009	DJI_0511	Secale sylvestre Host	0	n	1	0	Poaceae Barnhart
Iz_2_1009	DJI_0511	Sempervivum ruthenicum Schnittsp. & C.B. Lehm.	0	n	1	0	Crassulaceae J.St.-Hil.
Iz_2_1009	DJI_0511	Setaria viridis (L.) P. Beauv.	0	n	1	0	Poaceae Barnhart
Iz_2_1009	DJI_0511	Silene borysthenica (Gruner) Walters	0	n	1	0	Caryophyllaceae Juss.
Iz_2_1009	DJI_0511	Silene latifolia subsp. alba (Mill.) Greuter & Burdet	0	n	1	0	Caryophyllaceae Juss.
Iz_2_1009	DJI_0511	Solidago virgaurea L.	0	n	1	0	Asteraceae Bercht. & J.Presl
Iz_2_1009	DJI_0511	Sorbus aucuparia L.	0	n	1	1	Rosaceae Juss.
Iz_2_1009	DJI_0511	Stachys recta L.	0	n	1	0	Lamiacea Lindl.
Iz_2_1009	DJI_0511	Tanacetum vulgare L.	0	n	1	0	Asteraceae Bercht. & J.Presl
Iz_2_1009	DJI_0511	Thymus pallasianus Heinr.Braun	0	n	1	0	Lamiacea Lindl.
Iz_2_1009	DJI_0511	Tilia cordata Mill.	0	n	1	1	Malvaceae Juss.
Iz_2_1009	DJI_0511	Trifolium alpestre L.	0	n	1	0	Fabaceae Lindl.
Iz_2_1009	DJI_0511	Trifolium arvense L.	0	n	1	0	Fabaceae Lindl.
Iz_2_1009	DJI_0511	Ulmus glabra Huds.	0	n	1	1	Ulmaceae Mirb.
Iz_2_1009	DJI_0511	Verbascum densiflorum Bertol.	0	n	1	0	Scrophulariaceae Juss.
Iz_2_1009	DJI_0511	Veronica austriaca L.	0	n	1	0	Plantaginaceae Juss.
Iz_2_1009	DJI_0511	Veronica incana L.	0	n	1	0	Plantaginaceae Juss.
Iz_2_1009	DJI_0511	Veronica verna L.	0	n	1	0	Plantaginaceae Juss.
Iz_2_1009	DJI_0511	Vincetoxicum hirundinaria Medik	0	n	1	0	Apocynaceae Juss.
Iz_2_1009	DJI_0513	Acer tataricum L.	0	n	1	1	Sapindaceae Juss.
Iz_2_1009	DJI_0513	Achillea millefolium L. s.l.	0	n	1	0	Asteraceae Bercht. & J.Presl
Iz_2_1009	DJI_0513	Allium oleraceum L.	0	n	1	0	Amaryllidaceae J.St.-Hil.
Iz_2_1009	DJI_0513	Ambrosia artemisiifolia L.	0	a	0	0	Asteraceae Bercht. & J.Presl
Iz_2_1009	DJI_0513	Amorpha fruticosa L.	0	a	0	0	Fabaceae Lindl.
Iz_2_1009	DJI_0513	Anthericum ramosum L.	0	n	1	0	Asparagaceae Juss.
Iz_2_1009	DJI_0513	Artemisia marschalliana Spreng.	0	n	1	0	Asteraceae Bercht. & J.Presl
Iz_2_1009	DJI_0513	Asparagus officinalis L.	0	n	1	0	Asparagaceae Juss.
Iz_2_1009	DJI_0513	Astragalus cicer L.	0	n	1	0	Fabaceae Lindl.
Iz_2_1009	DJI_0513	Bassia laniflora (S.G.Gmel.) A.J.Scott	0	n	1	0	Amaranthaceae Juss.
Iz_2_1009	DJI_0513	Berberis vulgaris L.	0	n	1	0	Berberidaceae Juss.
Iz_2_1009	DJI_0513	Betula pendula Roth.	0	n	1	1	Betulaceae Gray
Iz_2_1009	DJI_0513	Betula pendula Roth.	0	n	1	0	Poaceae Barnhart
Iz_2_1009	DJI_0513	Bromus inermis Leyss.	0		NA	NA	
Iz_2_1009	DJI_0513	Bromus squarrosus L.	0	a	0	0	Poaceae Barnhart
Iz_2_1009	DJI_0513	Bromus tectorum L.	0	a	0	0	Poaceae Barnhart
Iz_2_1009	DJI_0513	Calamagrostis epigejos (L.) Roth	50	n	1	0	Poaceae Barnhart
Iz_2_1009	DJI_0513	Carduus acanthoides L.	0	n	1	0	Asteraceae Bercht. & J.Presl
Iz_2_1009	DJI_0513	Carex sp.	0	n	1	0	Cyperaceae Juss.
Iz_2_1009	DJI_0513	Centaurea majorovii Dumbadze	0	n	1	0	Asteraceae Bercht. & J.Presl
Iz_2_1009	DJI_0513	Cf. Solidago virgaurea	0		NA	NA	
Iz_2_1009	DJI_0513	Chamaecytisus ruthenicus (Fisch.ex Wol.) Klaskova	0	n	1	0	Fabaceae Lindl.
Iz_2_1009	DJI_0513	Chelidonium majus L.	0	n	1	0	Papaveraceae Juss.
Iz_2_1009	DJI_0513	Chenopodium betaceum Andrz.	0	n	1	0	Amaranthaceae Juss.
Iz_2_1009	DJI_0513	Chenopodium cf. album	0	n	1	0	Amaranthaceae Juss.
Iz_2_1009	DJI_0513	Chenopodium sp.	0		NA	0	Amaranthaceae Juss.
Iz_2_1009	DJI_0513	Chondrilla juncea L.	0	n	1	0	Asteraceae Bercht. & J.Presl
Iz_2_1009	DJI_0513	Cirsium vulgare (Savi) Ten.	0	n	1	0	Asteraceae Bercht. & J.Presl
Iz_2_1009	DJI_0513	Clinopodium acinos (L.) Kuntze	0	n	1	0	Lamiacea Lindl.
Iz_2_1009	DJI_0513	Convallaria majalis L.	0	n	1	0	Asparagaceae Juss.
Iz_2_1009	DJI_0513	Crataegus rhipidophylla Gand.	0	n	1	1	Rosaceae Juss.
Iz_2_1009	DJI_0513	Crepis foetida L.	0	n	1	0	Asteraceae Bercht. & J.Presl
Iz_2_1009	DJI_0513	Dactylis glomerata L.	0	n	1	0	Poaceae Barnhart
Iz_2_1009	DJI_0513	Dianthus campestris M.Bieb.	0	n	1	0	Caryophyllaceae Juss.
Iz_2_1009	DJI_0513	Digitaria sanguinalis (L.) Scop.	0	a	0	0	Poaceae Barnhart
Iz_2_1009	DJI_0513	Elymus repens (L.) Gould	0	n	1	0	Poaceae Barnhart
Iz_2_1009	DJI_0513	Epilobium tetragonum L.	0	n	1	0	Onagraceae Juss.
Iz_2_1009	DJI_0513	Eragrostis minor Host	0	a	0	0	Poaceae Barnhart
Iz_2_1009	DJI_0513	Erigeron canadensis L.	0	a	0	0	Asteraceae Bercht. & J.Presl
Iz_2_1009	DJI_0513	Euphorbia seguieriana Neck.	0	n	1	0	Euphorbiaceae Juss.
Iz_2_1009	DJI_0513	Fallopia dumetorum (L.) Holub	0	n	1	0	Polygonaceae Juss.
Iz_2_1009	DJI_0513	Festuca beckeri (Hack.) Trautv.	0	n	1	0	Poaceae Barnhart
Iz_2_1009	DJI_0513	Fragaria vesca L.	0	n	1	0	Rosaceae Juss.
Iz_2_1009	DJI_0513	Frangula alnus Mill.	0	n	1	1	Rhamnaceae Juss.
Iz_2_1009	DJI_0513	Fraxinus pennsylvanica Marshall	0	a	0	1	Oleaceae Hoffmanns. & Link
Iz_2_1009	DJI_0513	Galeopsis bifida Boenn.	0	n	1	0	Lamiacea Lindl.
Iz_2_1009	DJI_0513	Galium verum L.	0	n	1	0	Rubiaceae Juss.
Iz_2_1009	DJI_0513	Genista tinctoria L.	0	n	1	0	Fabaceae Lindl.
Iz_2_1009	DJI_0513	Geranium robertianum L.	0	n	1	0	Geraniaceae Juss.
Iz_2_1009	DJI_0513	Geranium sanquineum L.	0	n	1	0	Geraniaceae Juss.
Iz_2_1009	DJI_0513	Gypsophila paniculata L.	0	n	1	0	Caryophyllaceae Juss.
Iz_2_1009	DJI_0513	Helichrysum arenarium (L.) Moench	0	n	1	0	Asteraceae Bercht. & J.Presl
Iz_2_1009	DJI_0513	Herniaria polygama J.Gay	0	n	1	0	Caryophyllaceae Juss.
Iz_2_1009	DJI_0513	Hieracium robustum Fr.	0	n	1	0	Asteraceae Bercht. & J.Presl
Iz_2_1009	DJI_0513	Hieracium umbellatum L.	0	n	1	NA	Asteraceae Bercht. & J.Presl
Iz_2_1009	DJI_0513	Hylotelephium maximum (L.) Holub	0	n	1	0	Crassulaceae J.St.-Hil.
Iz_2_1009	DJI_0513	Jacobaea borysthenica (DC.) B.Nord. & Greuter	0	n	1	0	Asteraceae Bercht. & J.Presl
Iz_2_1009	DJI_0513	Jasione montana L.	0	n	1	0	Campanulaceae Juss.
Iz_2_1009	DJI_0513	Jurinea cyanoides (L.) Rchb.s.l.	0	n	1	0	Asteraceae Bercht. & J.Presl
Iz_2_1009	DJI_0513	Koeleria glauca (Spreng.) DC.	0	n	1	0	Poaceae Barnhart
Iz_2_1009	DJI_0513	Lactuca serriola L.	0	a	0	0	Asteraceae Bercht. & J.Presl
Iz_2_1009	DJI_0513	Linaria genistifolia (L.) Mill.	0	n	1	0	Plantaginaceae Juss.
Iz_2_1009	DJI_0513	Lomelosia argentea (L.) Greuter & Burdet	0	n	1	0	Caprifoliaceae Juss.
Iz_2_1009	DJI_0513	Melampyrum pratense L.	0	n	1	0	Orobanchaceae Vent.

Iz_2_1009	DJI_0513	Oenothera biennis L.	0	a	0	0	Onagraceae Juss.
Iz_2_1009	DJI_0513	Pentanema britannica (L.) D.Gut.Larr., Santos-Vicente, Anderb., E.Rico & M.M.Mart.Ort.	0	n	1	0	Asteraceae Bercht. & J.Presl
Iz_2_1009	DJI_0513	Peucedanum oreoselinum (L.) Moench	0	n	1	0	Apiaceae Lindl.
Iz_2_1009	DJI_0513	Peucedanum ruthenicum M.Bieb.	0	n	1	0	Apiaceae Lindl.
Iz_2_1009	DJI_0513	Pilosella echioides (Lumn.) F.W.Schultz & Sch.Bip.	0	n	1	0	Asteraceae Bercht. & J.Presl
Iz_2_1009	DJI_0513	Pilosella officinarum F. Schultz et Sch. Bip.	0	n	1	0	Asteraceae Bercht. & J.Presl
Iz_2_1009	DJI_0513	Pilosella sp.	0		NA	0	Asteraceae Bercht. & J.Presl
Iz_2_1009	DJI_0513	Pinus sylvestris L.	0	n	1	1	Pinaceae Spreng. ex F.Rudolphi
Iz_2_1009	DJI_0513	Poa annua L.	0	n	1	0	Poaceae Barnhart
Iz_2_1009	DJI_0513	Poa nemoralis L.	0	n	1	0	Poaceae Barnhart
Iz_2_1009	DJI_0513	Poa sp.	0		NA	0	Poaceae Barnhart
Iz_2_1009	DJI_0513	Polygonatum odoratum (Mill.) Druce	0	n	1	0	Asparagaceae Juss.
Iz_2_1009	DJI_0513	Polygonum arenarium Waldst. & Kit.	0	n	1	0	Polygonaceae Juss.
Iz_2_1009	DJI_0513	Populus nigra L.	0	n	1	1	Salicaceae Mirb.
Iz_2_1009	DJI_0513	Populus tremula L.	0	n	1	1	Salicaceae Mirb.
Iz_2_1009	DJI_0513	Populus Ã canescens (Aiton) Sm.	0	n	1	1	Salicaceae Mirb.
Iz_2_1009	DJI_0513	Potentilla argentea L.	0	n	1	0	Rosaceae Juss.
Iz_2_1009	DJI_0513	Psammophiliella muralis (L.) Ikonn.	0	n	1	0	Caryophyllaceae Juss.
Iz_2_1009	DJI_0513	Psephellus sumensis (Kalen.) Greuter	0	n	1	0	Asteraceae Bercht. & J.Presl
Iz_2_1009	DJI_0513	Pulsatilla pratensis (L.) Mill. s.l.	0	n	1	0	Ranunculaceae Juss.
Iz_2_1009	DJI_0513	Pyrus communis L.	0	a	0	1	Rosaceae Juss.
Iz_2_1009	DJI_0513	Quercus robur L.	0	n	1	1	Fagaceae Dumort.
Iz_2_1009	DJI_0513	Robinia pseudoacacia L.	0	a	0	1	Fabaceae Lindl.
Iz_2_1009	DJI_0513	Rosa sp.	0		NA	1	Rosaceae Juss.
Iz_2_1009	DJI_0513	Rubus idaeus L.	0	n	1	0	Rosaceae Juss.
Iz_2_1009	DJI_0513	Rumex acetosella L.	0	n	1	0	Polygonaceae Juss.
Iz_2_1009	DJI_0513	Rumex thyrsiflorus Fingerh.	0	n	1	0	Polygonaceae Juss.
Iz_2_1009	DJI_0513	Salix alba L.	0	n	1	1	Salicaceae Mirb.
Iz_2_1009	DJI_0513	Sambucus nigra L.	0	n	1	1	Viburnaceae Raf.
Iz_2_1009	DJI_0513	Secale sylvestre Host	0	n	1	0	Poaceae Barnhart
Iz_2_1009	DJI_0513	Sempervivum ruthenicum Schnittsp. & C.B. Lehm.	0	n	1	0	Crassulaceae J.St.-Hil.
Iz_2_1009	DJI_0513	Setaria viridis (L.) P. Beauv.	0	n	1	0	Poaceae Barnhart
Iz_2_1009	DJI_0513	Silene borysthenica (Gruner) Walters	0	n	1	0	Caryophyllaceae Juss.
Iz_2_1009	DJI_0513	Silene latifolia subsp. alba (Mill.) Greuter & Burdet	0	n	1	0	Caryophyllaceae Juss.
Iz_2_1009	DJI_0513	Solidago virgaurea L.	0	n	1	0	Asteraceae Bercht. & J.Presl
Iz_2_1009	DJI_0513	Sorbus aucuparia L.	0	n	1	1	Rosaceae Juss.
Iz_2_1009	DJI_0513	Stachys recta L.	0	n	1	0	Lamiacea Lindl.
Iz_2_1009	DJI_0513	Tanacetum vulgare L.	0	n	1	0	Asteraceae Bercht. & J.Presl
Iz_2_1009	DJI_0513	Thymus pallasianus Heinr.Braun	0	n	1	0	Lamiacea Lindl.
Iz_2_1009	DJI_0513	Tilia cordata Mill.	0	n	1	1	Malvaceae Juss.
Iz_2_1009	DJI_0513	Trifolium alpestre L.	0	n	1	0	Fabaceae Lindl.
Iz_2_1009	DJI_0513	Trifolium arvense L.	0	n	1	0	Fabaceae Lindl.
Iz_2_1009	DJI_0513	Ulmus glabra Huds.	0	n	1	1	Ulmaceae Mirb.
Iz_2_1009	DJI_0513	Verbascum densiflorum Bertol.	0	n	1	0	Scrophulariaceae Juss.
Iz_2_1009	DJI_0513	Veronica austriaca L.	0	n	1	0	Plantaginaceae Juss.
Iz_2_1009	DJI_0513	Veronica incana L.	0	n	1	0	Plantaginaceae Juss.
Iz_2_1009	DJI_0513	Veronica verna L.	0	n	1	0	Plantaginaceae Juss.
Iz_2_1009	DJI_0513	Vincetoxicum hirundinaria Medik	0	n	1	0	Apocynaceae Juss.
Iz_2_1009	DJI_0515	Acer tataricum L.	0	n	1	1	Sapindaceae Juss.
Iz_2_1009	DJI_0515	Achillea millefolium L. s.l.	0	n	1	0	Asteraceae Bercht. & J.Presl
Iz_2_1009	DJI_0515	Allium oleraceum L.	0	n	1	0	Amaryllidaceae J.St.-Hil.
Iz_2_1009	DJI_0515	Ambrosia artemisiifolia L.	0	a	0	0	Asteraceae Bercht. & J.Presl
Iz_2_1009	DJI_0515	Amorpha fruticosa L.	0	a	0	0	Fabaceae Lindl.
Iz_2_1009	DJI_0515	Anthericum ramosum L.	0	n	1	0	Asparagaceae Juss.
Iz_2_1009	DJI_0515	Artemisia marschalliana Spreng.	0	n	1	0	Asteraceae Bercht. & J.Presl
Iz_2_1009	DJI_0515	Asparagus officinalis L.	0	n	1	0	Asparagaceae Juss.
Iz_2_1009	DJI_0515	Astragalus cicer L.	0	n	1	0	Fabaceae Lindl.
Iz_2_1009	DJI_0515	Bassia laniflora (S.G.Gmel.) A.J.Scott	0	n	1	0	Amaranthaceae Juss.
Iz_2_1009	DJI_0515	Berberis vulgaris L.	0	n	1	0	Berberidaceae Juss.
Iz_2_1009	DJI_0515	Betula pendula Roth.	0	n	1	1	Betulaceae Gray
Iz_2_1009	DJI_0515	Betula pendula Roth.	0	n	1	0	Poaceae Barnhart
Iz_2_1009	DJI_0515	Bromus inermis Leyss.	0		NA	NA	
Iz_2_1009	DJI_0515	Bromus squarrosus L.	0	a	0	0	Poaceae Barnhart
Iz_2_1009	DJI_0515	Bromus tectorum L.	0	a	0	0	Poaceae Barnhart
Iz_2_1009	DJI_0515	Calamagrostis epigejos (L.) Roth	60	n	1	0	Poaceae Barnhart
Iz_2_1009	DJI_0515	Carduus acanthoides L.	0	n	1	0	Asteraceae Bercht. & J.Presl
Iz_2_1009	DJI_0515	Carex sp.	0	n	1	0	Cyperaceae Juss.
Iz_2_1009	DJI_0515	Centaurea majorovii Dumbadze	0	n	1	0	Asteraceae Bercht. & J.Presl
Iz_2_1009	DJI_0515	Cf. Solidago virgaurea	0		NA	NA	
Iz_2_1009	DJI_0515	Chamaecytisus ruthenicus (Fisch.ex Wol.) Klaskova	0	n	1	0	Fabaceae Lindl.
Iz_2_1009	DJI_0515	Chelidonium majus L.	0	n	1	0	Papaveraceae Juss.
Iz_2_1009	DJI_0515	Chenopodium betaceum Andrz.	0	n	1	0	Amaranthaceae Juss.
Iz_2_1009	DJI_0515	Chenopodium cf. album	0	n	1	0	Amaranthaceae Juss.
Iz_2_1009	DJI_0515	Chenopodium sp.	0		NA	0	Amaranthaceae Juss.
Iz_2_1009	DJI_0515	Chondrilla juncea L.	0	n	1	0	Asteraceae Bercht. & J.Presl
Iz_2_1009	DJI_0515	Cirsium vulgare (Savi) Ten.	0	n	1	0	Asteraceae Bercht. & J.Presl
Iz_2_1009	DJI_0515	Clinopodium acinos (L.) Kuntze	0	n	1	0	Lamiacea Lindl.
Iz_2_1009	DJI_0515	Convallaria majalis L.	0	n	1	0	Asparagaceae Juss.
Iz_2_1009	DJI_0515	Crataegus rhipidophylla Gand.	0	n	1	1	Rosaceae Juss.
Iz_2_1009	DJI_0515	Crepis foetida L.	0	n	1	0	Asteraceae Bercht. & J.Presl
Iz_2_1009	DJI_0515	Dactylis glomerata L.	0	n	1	0	Poaceae Barnhart
Iz_2_1009	DJI_0515	Dianthus campestris M.Bieb.	0	n	1	0	Caryophyllaceae Juss.
Iz_2_1009	DJI_0515	Digitaria sanguinalis (L.) Scop.	0	a	0	0	Poaceae Barnhart
Iz_2_1009	DJI_0515	Elymus repens (L.) Gould	0	n	1	0	Poaceae Barnhart
Iz_2_1009	DJI_0515	Epilobium tetragonum L.	0	n	1	0	Onagraceae Juss.
Iz_2_1009	DJI_0515	Eragrostis minor Host	0	a	0	0	Poaceae Barnhart
Iz_2_1009	DJI_0515	Erigeron canadensis L.	0	a	0	0	Asteraceae Bercht. & J.Presl
Iz_2_1009	DJI_0515	Euphorbia seguieriana Neck.	0	n	1	0	Euphorbiaceae Juss.
Iz_2_1009	DJI_0515	Fallopia dumetorum (L.) Holub	0	n	1	0	Polygonaceae Juss.
Iz_2_1009	DJI_0515	Festuca beckeri (Hack.) Trautv.	0	n	1	0	Poaceae Barnhart
Iz_2_1009	DJI_0515	Fragaria vesca L.	0	n	1	0	Rosaceae Juss.
Iz_2_1009	DJI_0515	Frangula alnus Mill.	0	n	1	1	Rhamnaceae Juss.
Iz_2_1009	DJI_0515	Fraxinus pennsylvanica Marshall	0	a	0	1	Oleaceae Hoffmanns. & Link
Iz_2_1009	DJI_0515	Galeopsis bifida Boenn.	0	n	1	0	Lamiacea Lindl.
Iz_2_1009	DJI_0515	Galium verum L.	0	n	1	0	Rubiaceae Juss.
Iz_2_1009	DJI_0515	Genista tinctoria L.	0	n	1	0	Fabaceae Lindl.
Iz_2_1009	DJI_0515	Geranium robertianum L.	0	n	1	0	Geraniaceae Juss.
Iz_2_1009	DJI_0515	Geranium sanquineum L.	0	n	1	0	Geraniaceae Juss.

Iz_2_1009	DJI_0515	Gypsophila paniculata L.	0	n	1	0	Caryophyllaceae Juss.
Iz_2_1009	DJI_0515	Helichrysum arenarium (L.) Moench	0	n	1	0	Asteraceae Bercht. & J.Presl
Iz_2_1009	DJI_0515	Herniaria polygama J.Gay	0	n	1	0	Caryophyllaceae Juss.
Iz_2_1009	DJI_0515	Hieracium robustum Fr.	0	n	1	0	Asteraceae Bercht. & J.Presl
Iz_2_1009	DJI_0515	Hieracium umbellatum L.	0	n	1	NA	Asteraceae Bercht. & J.Presl
Iz_2_1009	DJI_0515	Hylotelephium maximum (L.) Holub	0	n	1	0	Crassulaceae J.St.-Hil.
Iz_2_1009	DJI_0515	Jacobaea borysthenica (DC.) B.Nord. & Greuter	0	n	1	0	Asteraceae Bercht. & J.Presl
Iz_2_1009	DJI_0515	Jasione montana L.	0	n	1	0	Campanulaceae Juss.
Iz_2_1009	DJI_0515	Jurinea cyanoides (L.) Rchb.s.l.	0	n	1	0	Asteraceae Bercht. & J.Presl
Iz_2_1009	DJI_0515	Koeleria glauca (Spreng.) DC.	0	n	1	0	Poaceae Barnhart
Iz_2_1009	DJI_0515	Lactuca serriola L.	0	a	0	0	Asteraceae Bercht. & J.Presl
Iz_2_1009	DJI_0515	Linaria genistifolia (L.) Mill.	0	n	1	0	Plantaginaceae Juss.
Iz_2_1009	DJI_0515	Lomelosia argentea (L.) Greuter & Burdet	0	n	1	0	Caprifoliaceae Juss.
Iz_2_1009	DJI_0515	Melampyrum pratense L.	0	n	1	0	Orobanchaceae Vent.
Iz_2_1009	DJI_0515	Oenothera biennis L.	0	a	0	0	Onagraceae Juss.
Iz_2_1009	DJI_0515	Pentanema britannica (L.) D.Gut.Larr., Santos-Vicente, Anderb., E.Rico & M.M.Mart.Ort.	0	n	1	0	Asteraceae Bercht. & J.Presl
Iz_2_1009	DJI_0515	Peucedanum oreoselinum (L.) Moench	0	n	1	0	Apiaceae Lindl.
Iz_2_1009	DJI_0515	Peucedanum ruthenicum M.Bieb.	0	n	1	0	Apiaceae Lindl.
Iz_2_1009	DJI_0515	Pilosella echioides (Lumn.) F.W.Schultz & Sch.Bip.	0	n	1	0	Asteraceae Bercht. & J.Presl
Iz_2_1009	DJI_0515	Pilosella officinarum F. Schultz et Sch. Bip.	0	n	1	0	Asteraceae Bercht. & J.Presl
Iz_2_1009	DJI_0515	Pilosella sp.	0		NA	0	Asteraceae Bercht. & J.Presl
Iz_2_1009	DJI_0515	Pinus sylvestris L.	0	n	1	1	Pinaceae Spreng. ex F.Rudolphi
Iz_2_1009	DJI_0515	Poa annua L.	0	n	1	0	Poaceae Barnhart
Iz_2_1009	DJI_0515	Poa nemoralis L.	0	n	1	0	Poaceae Barnhart
Iz_2_1009	DJI_0515	Poa sp.	0		NA	0	Poaceae Barnhart
Iz_2_1009	DJI_0515	Polygonatum odoratum (Mill.) Druce	0	n	1	0	Asparagaceae Juss.
Iz_2_1009	DJI_0515	Polygonum arenarium Waldst. & Kit.	0	n	1	0	Polygonaceae Juss.
Iz_2_1009	DJI_0515	Populus nigra L.	0	n	1	1	Salicaceae Mirb.
Iz_2_1009	DJI_0515	Populus tremula L.	0	n	1	1	Salicaceae Mirb.
Iz_2_1009	DJI_0515	Populus Ã canescens (Aiton) Sm.	0	n	1	1	Salicaceae Mirb.
Iz_2_1009	DJI_0515	Potentilla argentea L.	0	n	1	0	Rosaceae Juss.
Iz_2_1009	DJI_0515	Psammophiliella muralis (L.) Ikonn.	0	n	1	0	Caryophyllaceae Juss.
Iz_2_1009	DJI_0515	Psephellus sumensis (Kalen.) Greuter	0	n	1	0	Asteraceae Bercht. & J.Presl
Iz_2_1009	DJI_0515	Pulsatilla pratensis (L.) Mill. s.l.	0	n	1	0	Ranunculaceae Juss.
Iz_2_1009	DJI_0515	Pyrus communis L.	0	a	0	1	Rosaceae Juss.
Iz_2_1009	DJI_0515	Quercus robur L.	0	n	1	1	Fagaceae Dumort.
Iz_2_1009	DJI_0515	Robinia pseudoacacia L.	0	a	0	1	Fabaceae Lindl.
Iz_2_1009	DJI_0515	Rosa sp.	0		NA	1	Rosaceae Juss.
Iz_2_1009	DJI_0515	Rubus idaeus L.	0	n	1	0	Rosaceae Juss.
Iz_2_1009	DJI_0515	Rumex acetosella L.	0	n	1	0	Polygonaceae Juss.
Iz_2_1009	DJI_0515	Rumex thyrsiflorus Fingerh.	0	n	1	0	Polygonaceae Juss.
Iz_2_1009	DJI_0515	Salix alba L.	0	n	1	1	Salicaceae Mirb.
Iz_2_1009	DJI_0515	Sambucus nigra L.	0	n	1	1	Viburnaceae Raf.
Iz_2_1009	DJI_0515	Secale sylvestre Host	0	n	1	0	Poaceae Barnhart
Iz_2_1009	DJI_0515	Sempervivum ruthenicum Schnittsp. & C.B. Lehm.	0	n	1	0	Crassulaceae J.St.-Hil.
Iz_2_1009	DJI_0515	Setaria viridis (L.) P. Beauv.	0	n	1	0	Poaceae Barnhart
Iz_2_1009	DJI_0515	Silene borysthenica (Gruner) Walters	0	n	1	0	Caryophyllaceae Juss.
Iz_2_1009	DJI_0515	Silene latifolia subsp. alba (Mill.) Greuter & Burdet	0	n	1	0	Caryophyllaceae Juss.
Iz_2_1009	DJI_0515	Solidago virgaurea L.	1	n	1	0	Asteraceae Bercht. & J.Presl
Iz_2_1009	DJI_0515	Sorbus aucuparia L.	0	n	1	1	Rosaceae Juss.
Iz_2_1009	DJI_0515	Stachys recta L.	0	n	1	0	Lamiacea Lindl.
Iz_2_1009	DJI_0515	Tanacetum vulgare L.	0	n	1	0	Asteraceae Bercht. & J.Presl
Iz_2_1009	DJI_0515	Thymus pallasianus Heinr.Braun	0	n	1	0	Lamiacea Lindl.
Iz_2_1009	DJI_0515	Tilia cordata Mill.	0	n	1	1	Malvaceae Juss.
Iz_2_1009	DJI_0515	Trifolium alpestre L.	0	n	1	0	Fabaceae Lindl.
Iz_2_1009	DJI_0515	Trifolium arvense L.	0	n	1	0	Fabaceae Lindl.
Iz_2_1009	DJI_0515	Ulmus glabra Huds.	0	n	1	1	Ulmaceae Mirb.
Iz_2_1009	DJI_0515	Verbascum densiflorum Bertol.	0	n	1	0	Scrophulariaceae Juss.
Iz_2_1009	DJI_0515	Veronica austriaca L.	0	n	1	0	Plantaginaceae Juss.
Iz_2_1009	DJI_0515	Veronica incana L.	0	n	1	0	Plantaginaceae Juss.
Iz_2_1009	DJI_0515	Veronica verna L.	0	n	1	0	Plantaginaceae Juss.
Iz_2_1009	DJI_0515	Vincetoxicum hirundinaria Medik	0	n	1	0	Apocynaceae Juss.
Iz_2_1009	DJI_0524	Acer tataricum L.	0	n	1	1	Sapindaceae Juss.
Iz_2_1009	DJI_0524	Achillea millefolium L. s.l.	0	n	1	0	Asteraceae Bercht. & J.Presl
Iz_2_1009	DJI_0524	Allium oleraceum L.	0	n	1	0	Amaryllidaceae J.St.-Hil.
Iz_2_1009	DJI_0524	Ambrosia artemisiifolia L.	0	a	0	0	Asteraceae Bercht. & J.Presl
Iz_2_1009	DJI_0524	Amorpha fruticosa L.	0	a	0	0	Fabaceae Lindl.
Iz_2_1009	DJI_0524	Anthericum ramosum L.	0	n	1	0	Asparagaceae Juss.
Iz_2_1009	DJI_0524	Artemisia marschalliana Spreng.	0	n	1	0	Asteraceae Bercht. & J.Presl
Iz_2_1009	DJI_0524	Asparagus officinalis L.	0	n	1	0	Asparagaceae Juss.
Iz_2_1009	DJI_0524	Astragalus cicer L.	0	n	1	0	Fabaceae Lindl.
Iz_2_1009	DJI_0524	Bassia laniflora (S.G.Gmel.) A.J.Scott	0	n	1	0	Amaranthaceae Juss.
Iz_2_1009	DJI_0524	Berberis vulgaris L.	0	n	1	0	Berberidaceae Juss.
Iz_2_1009	DJI_0524	Betula pendula Roth.	0	n	1	1	Betulaceae Gray
Iz_2_1009	DJI_0524	Betula pendula Roth.	0	n	1	0	Poaceae Barnhart
Iz_2_1009	DJI_0524	Bromus inermis Leyss.	0		NA	NA	
Iz_2_1009	DJI_0524	Bromus squarrosus L.	0	a	0	0	Poaceae Barnhart
Iz_2_1009	DJI_0524	Bromus tectorum L.	0	a	0	0	Poaceae Barnhart
Iz_2_1009	DJI_0524	Calamagrostis epigejos (L.) Roth	55	n	1	0	Poaceae Barnhart
Iz_2_1009	DJI_0524	Carduus acanthoides L.	0	n	1	0	Asteraceae Bercht. & J.Presl
Iz_2_1009	DJI_0524	Carex sp.	0	n	1	0	Cyperaceae Juss.
Iz_2_1009	DJI_0524	Centaurea majorovii Dumbadze	0	n	1	0	Asteraceae Bercht. & J.Presl
Iz_2_1009	DJI_0524	Cf. Solidago virgaurea	0		NA	NA	
Iz_2_1009	DJI_0524	Chamaecytisus ruthenicus (Fisch.ex Wol.) Klaskova	0	n	1	0	Fabaceae Lindl.
Iz_2_1009	DJI_0524	Chelidonium majus L.	0	n	1	0	Papaveraceae Juss.
Iz_2_1009	DJI_0524	Chenopodium betaceum Andrz.	0	n	1	0	Amaranthaceae Juss.
Iz_2_1009	DJI_0524	Chenopodium cf. album	0	n	1	0	Amaranthaceae Juss.
Iz_2_1009	DJI_0524	Chenopodium sp.	0		NA	0	Amaranthaceae Juss.
Iz_2_1009	DJI_0524	Chondrilla juncea L.	0	n	1	0	Asteraceae Bercht. & J.Presl
Iz_2_1009	DJI_0524	Cirsium vulgare (Savi) Ten.	0	n	1	0	Asteraceae Bercht. & J.Presl
Iz_2_1009	DJI_0524	Clinopodium acinos (L.) Kuntze	0	n	1	0	Lamiacea Lindl.
Iz_2_1009	DJI_0524	Convallaria majalis L.	0	n	1	0	Asparagaceae Juss.
Iz_2_1009	DJI_0524	Crataegus rhipidophylla Gand.	0	n	1	1	Rosaceae Juss.
Iz_2_1009	DJI_0524	Crepis foetida L.	0	n	1	0	Asteraceae Bercht. & J.Presl
Iz_2_1009	DJI_0524	Dactylis glomerata L.	0	n	1	0	Poaceae Barnhart
Iz_2_1009	DJI_0524	Dianthus campestris M.Bieb.	0	n	1	0	Caryophyllaceae Juss.
Iz_2_1009	DJI_0524	Digitaria sanguinalis (L.) Scop.	0	a	0	0	Poaceae Barnhart
Iz_2_1009	DJI_0524	Elymus repens (L.) Gould	0	n	1	0	Poaceae Barnhart

Iz_2_1009	DJI_0524	Epilobium tetragonum L.	0	n	1	0	Onagraceae Juss.
Iz_2_1009	DJI_0524	Eragrostis minor Host	0	a	0	0	Poaceae Barnhart
Iz_2_1009	DJI_0524	Erigeron canadensis L.	0	a	0	0	Asteraceae Bercht. & J.Presl
Iz_2_1009	DJI_0524	Euphorbia seguieriana Neck.	0	n	1	0	Euphorbiaceae Juss.
Iz_2_1009	DJI_0524	Fallopia dumetorum (L.) Holub	0	n	1	0	Polygonaceae Juss.
Iz_2_1009	DJI_0524	Festuca beckeri (Hack.) Trautv.	0	n	1	0	Poaceae Barnhart
Iz_2_1009	DJI_0524	Fragaria vesca L.	0	n	1	0	Rosaceae Juss.
Iz_2_1009	DJI_0524	Frangula alnus Mill.	0	n	1	1	Rhamnaceae Juss.
Iz_2_1009	DJI_0524	Fraxinus pennsylvanica Marshall	0	a	0	1	Oleaceae Hoffmanns. & Link
Iz_2_1009	DJI_0524	Galeopsis bifida Boenn.	0	n	1	0	Lamiacea Lindl.
Iz_2_1009	DJI_0524	Galium verum L.	0	n	1	0	Rubiaceae Juss.
Iz_2_1009	DJI_0524	Genista tinctoria L.	0	n	1	0	Fabaceae Lindl.
Iz_2_1009	DJI_0524	Geranium robertianum L.	0	n	1	0	Geraniaceae Juss.
Iz_2_1009	DJI_0524	Geranium sanquineum L.	0	n	1	0	Geraniaceae Juss.
Iz_2_1009	DJI_0524	Gypsophila paniculata L.	0	n	1	0	Caryophyllaceae Juss.
Iz_2_1009	DJI_0524	Helichrysum arenarium (L.) Moench	0	n	1	0	Asteraceae Bercht. & J.Presl
Iz_2_1009	DJI_0524	Herniaria polygama J.Gay	0	n	1	0	Caryophyllaceae Juss.
Iz_2_1009	DJI_0524	Hieracium robustum Fr.	0	n	1	0	Asteraceae Bercht. & J.Presl
Iz_2_1009	DJI_0524	Hieracium umbellatum L.	0	n	1	NA	Asteraceae Bercht. & J.Presl
Iz_2_1009	DJI_0524	Hylotelephium maximum (L.) Holub	0	n	1	0	Crassulaceae J.St.-Hil.
Iz_2_1009	DJI_0524	Jacobaea borysthenica (DC.) B.Nord. & Greuter	0	n	1	0	Asteraceae Bercht. & J.Presl
Iz_2_1009	DJI_0524	Jasione montana L.	0	n	1	0	Campanulaceae Juss.
Iz_2_1009	DJI_0524	Jurinea cyanoides (L.) Rchb.s.l.	0	n	1	0	Asteraceae Bercht. & J.Presl
Iz_2_1009	DJI_0524	Koeleria glauca (Spreng.) DC.	0	n	1	0	Poaceae Barnhart
Iz_2_1009	DJI_0524	Lactuca serriola L.	0	a	0	0	Asteraceae Bercht. & J.Presl
Iz_2_1009	DJI_0524	Linaria genistifolia (L.) Mill.	0	n	1	0	Plantaginaceae Juss.
Iz_2_1009	DJI_0524	Lomelosia argentea (L.) Greuter & Burdet	0	n	1	0	Caprifoliaceae Juss.
Iz_2_1009	DJI_0524	Melampyrum pratense L.	0	n	1	0	Orobanchaceae Vent.
Iz_2_1009	DJI_0524	Oenothera biennis L.	0	a	0	0	Onagraceae Juss.
Iz_2_1009	DJI_0524	Pentanema britannica (L.) D.Gut.Larr., Santos-Vicente, Anderb., E.Rico & M.M.Mart.Ort.	0	n	1	0	Asteraceae Bercht. & J.Presl
Iz_2_1009	DJI_0524	Peucedanum oreoselinum (L.) Moench	3	n	1	0	Apiaceae Lindl.
Iz_2_1009	DJI_0524	Peucedanum ruthenicum M.Bieb.	0	n	1	0	Apiaceae Lindl.
Iz_2_1009	DJI_0524	Pilosella echioides (Lumn.) F.W.Schultz & Sch.Bip.	0	n	1	0	Asteraceae Bercht. & J.Presl
Iz_2_1009	DJI_0524	Pilosella officinarum F. Schultz et Sch. Bip.	0	n	1	0	Asteraceae Bercht. & J.Presl
Iz_2_1009	DJI_0524	Pilosella sp.	0		NA	0	Asteraceae Bercht. & J.Presl
Iz_2_1009	DJI_0524	Pinus sylvestris L.	0	n	1	1	Pinaceae Spreng. ex F.Rudolphi
Iz_2_1009	DJI_0524	Poa annua L.	0	n	1	0	Poaceae Barnhart
Iz_2_1009	DJI_0524	Poa nemoralis L.	0	n	1	0	Poaceae Barnhart
Iz_2_1009	DJI_0524	Poa sp.	0		NA	0	Poaceae Barnhart
Iz_2_1009	DJI_0524	Polygonatum odoratum (Mill.) Druce	0	n	1	0	Asparagaceae Juss.
Iz_2_1009	DJI_0524	Polygonum arenarium Waldst. & Kit.	0	n	1	0	Polygonaceae Juss.
Iz_2_1009	DJI_0524	Populus nigra L.	0	n	1	1	Salicaceae Mirb.
Iz_2_1009	DJI_0524	Populus tremula L.	0	n	1	1	Salicaceae Mirb.
Iz_2_1009	DJI_0524	Populus Ã canescens (Aiton) Sm.	0	n	1	1	Salicaceae Mirb.
Iz_2_1009	DJI_0524	Potentilla argentea L.	0	n	1	0	Rosaceae Juss.
Iz_2_1009	DJI_0524	Psammophiliella muralis (L.) Ikonn.	0	n	1	0	Caryophyllaceae Juss.
Iz_2_1009	DJI_0524	Psephellus sumensis (Kalen.) Greuter	0	n	1	0	Asteraceae Bercht. & J.Presl
Iz_2_1009	DJI_0524	Pulsatilla pratensis (L.) Mill. s.l.	0	n	1	0	Ranunculaceae Juss.
Iz_2_1009	DJI_0524	Pyrus communis L.	0	a	0	1	Rosaceae Juss.
Iz_2_1009	DJI_0524	Quercus robur L.	0	n	1	1	Fagaceae Dumort.
Iz_2_1009	DJI_0524	Robinia pseudoacacia L.	0	a	0	1	Fabaceae Lindl.
Iz_2_1009	DJI_0524	Rosa sp.	0		NA	1	Rosaceae Juss.
Iz_2_1009	DJI_0524	Rubus idaeus L.	0	n	1	0	Rosaceae Juss.
Iz_2_1009	DJI_0524	Rumex acetosella L.	0	n	1	0	Polygonaceae Juss.
Iz_2_1009	DJI_0524	Rumex thyrsiflorus Fingerh.	0	n	1	0	Polygonaceae Juss.
Iz_2_1009	DJI_0524	Salix alba L.	0	n	1	1	Salicaceae Mirb.
Iz_2_1009	DJI_0524	Sambucus nigra L.	0	n	1	1	Viburnaceae Raf.
Iz_2_1009	DJI_0524	Secale sylvestre Host	0	n	1	0	Poaceae Barnhart
Iz_2_1009	DJI_0524	Sempervivum ruthenicum Schnittsp. & C.B. Lehm.	0	n	1	0	Crassulaceae J.St.-Hil.
Iz_2_1009	DJI_0524	Setaria viridis (L.) P. Beauv.	0	n	1	0	Poaceae Barnhart
Iz_2_1009	DJI_0524	Silene borysthenica (Gruner) Walters	0	n	1	0	Caryophyllaceae Juss.
Iz_2_1009	DJI_0524	Silene latifolia subsp. alba (Mill.) Greuter & Burdet	0	n	1	0	Caryophyllaceae Juss.
Iz_2_1009	DJI_0524	Solidago virgaurea L.	0	n	1	0	Asteraceae Bercht. & J.Presl
Iz_2_1009	DJI_0524	Sorbus aucuparia L.	0	n	1	1	Rosaceae Juss.
Iz_2_1009	DJI_0524	Stachys recta L.	0	n	1	0	Lamiacea Lindl.
Iz_2_1009	DJI_0524	Tanacetum vulgare L.	0	n	1	0	Asteraceae Bercht. & J.Presl
Iz_2_1009	DJI_0524	Thymus pallasianus Heinr.Braun	0	n	1	0	Lamiacea Lindl.
Iz_2_1009	DJI_0524	Tilia cordata Mill.	0	n	1	1	Malvaceae Juss.
Iz_2_1009	DJI_0524	Trifolium alpestre L.	0	n	1	0	Fabaceae Lindl.
Iz_2_1009	DJI_0524	Trifolium arvense L.	0	n	1	0	Fabaceae Lindl.
Iz_2_1009	DJI_0524	Ulmus glabra Huds.	0	n	1	1	Ulmaceae Mirb.
Iz_2_1009	DJI_0524	Verbascum densiflorum Bertol.	0	n	1	0	Scrophulariaceae Juss.
Iz_2_1009	DJI_0524	Veronica austriaca L.	0	n	1	0	Plantaginaceae Juss.
Iz_2_1009	DJI_0524	Veronica incana L.	0	n	1	0	Plantaginaceae Juss.
Iz_2_1009	DJI_0524	Veronica verna L.	0	n	1	0	Plantaginaceae Juss.
Iz_2_1009	DJI_0524	Vincetoxicum hirundinaria Medik	0	n	1	0	Apocynaceae Juss.
Iz_2_1009	DJI_0526	Acer tataricum L.	0	n	1	1	Sapindaceae Juss.
Iz_2_1009	DJI_0526	Achillea millefolium L. s.l.	0	n	1	0	Asteraceae Bercht. & J.Presl
Iz_2_1009	DJI_0526	Allium oleraceum L.	0	n	1	0	Amaryllidaceae J.St.-Hil.
Iz_2_1009	DJI_0526	Ambrosia artemisiifolia L.	0	a	0	0	Asteraceae Bercht. & J.Presl
Iz_2_1009	DJI_0526	Amorpha fruticosa L.	0	a	0	0	Fabaceae Lindl.
Iz_2_1009	DJI_0526	Anthericum ramosum L.	0	n	1	0	Asparagaceae Juss.
Iz_2_1009	DJI_0526	Artemisia marschalliana Spreng.	0	n	1	0	Asteraceae Bercht. & J.Presl
Iz_2_1009	DJI_0526	Asparagus officinalis L.	0	n	1	0	Asparagaceae Juss.
Iz_2_1009	DJI_0526	Astragalus cicer L.	0	n	1	0	Fabaceae Lindl.
Iz_2_1009	DJI_0526	Bassia laniflora (S.G.Gmel.) A.J.Scott	0	n	1	0	Amaranthaceae Juss.
Iz_2_1009	DJI_0526	Berberis vulgaris L.	0	n	1	0	Berberidaceae Juss.
Iz_2_1009	DJI_0526	Betula pendula Roth.	0	n	1	1	Betulaceae Gray
Iz_2_1009	DJI_0526	Betula pendula Roth.	0	n	1	0	Poaceae Barnhart
Iz_2_1009	DJI_0526	Bromus inermis Leyss.	0		NA	NA	
Iz_2_1009	DJI_0526	Bromus squarrosus L.	0	a	0	0	Poaceae Barnhart
Iz_2_1009	DJI_0526	Bromus tectorum L.	0	a	0	0	Poaceae Barnhart
Iz_2_1009	DJI_0526	Calamagrostis epigejos (L.) Roth	40	n	1	0	Poaceae Barnhart
Iz_2_1009	DJI_0526	Carduus acanthoides L.	0	n	1	0	Asteraceae Bercht. & J.Presl
Iz_2_1009	DJI_0526	Carex sp.	0	n	1	0	Cyperaceae Juss.
Iz_2_1009	DJI_0526	Centaurea majorovii Dumbadze	0	n	1	0	Asteraceae Bercht. & J.Presl
Iz_2_1009	DJI_0526	Cf. Solidago virgaurea	0		NA	NA	
Iz_2_1009	DJI_0526	Chamaecytisus ruthenicus (Fisch.ex Wol.) Klaskova	0	n	1	0	Fabaceae Lindl.

Iz_2_1009	DJI_0526	Chelidonium majus L.	0	n	1	0	Papaveraceae Juss.
Iz_2_1009	DJI_0526	Chenopodium betaceum Andrz.	0	n	1	0	Amaranthaceae Juss.
Iz_2_1009	DJI_0526	Chenopodium cf. album	0	n	1	0	Amaranthaceae Juss.
Iz_2_1009	DJI_0526	Chenopodium sp.	0		NA	0	Amaranthaceae Juss.
Iz_2_1009	DJI_0526	Chondrilla juncea L.	0	n	1	0	Asteraceae Bercht. & J.Presl
Iz_2_1009	DJI_0526	Cirsium vulgare (Savi) Ten.	0	n	1	0	Asteraceae Bercht. & J.Presl
Iz_2_1009	DJI_0526	Clinopodium acinos (L.) Kuntze	0	n	1	0	Lamiacea Lindl.
Iz_2_1009	DJI_0526	Convallaria majalis L.	0	n	1	0	Asparagaceae Juss.
Iz_2_1009	DJI_0526	Crataegus rhipidophylla Gand.	0	n	1	1	Rosaceae Juss.
Iz_2_1009	DJI_0526	Crepis foetida L.	0	n	1	0	Asteraceae Bercht. & J.Presl
Iz_2_1009	DJI_0526	Dactylis glomerata L.	0	n	1	0	Poaceae Barnhart
Iz_2_1009	DJI_0526	Dianthus campestris M.Bieb.	0	n	1	0	Caryophyllaceae Juss.
Iz_2_1009	DJI_0526	Digitaria sanguinalis (L.) Scop.	0	a	0	0	Poaceae Barnhart
Iz_2_1009	DJI_0526	Elymus repens (L.) Gould	0	n	1	0	Poaceae Barnhart
Iz_2_1009	DJI_0526	Epilobium tetragonum L.	0	n	1	0	Onagraceae Juss.
Iz_2_1009	DJI_0526	Eragrostis minor Host	0	a	0	0	Poaceae Barnhart
Iz_2_1009	DJI_0526	Erigeron canadensis L.	0	a	0	0	Asteraceae Bercht. & J.Presl
Iz_2_1009	DJI_0526	Euphorbia seguieriana Neck.	0	n	1	0	Euphorbiaceae Juss.
Iz_2_1009	DJI_0526	Fallopia dumetorum (L.) Holub	0	n	1	0	Polygonaceae Juss.
Iz_2_1009	DJI_0526	Festuca beckeri (Hack.) Trautv.	0	n	1	0	Poaceae Barnhart
Iz_2_1009	DJI_0526	Fragaria vesca L.	0	n	1	0	Rosaceae Juss.
Iz_2_1009	DJI_0526	Frangula alnus Mill.	0	n	1	1	Rhamnaceae Juss.
Iz_2_1009	DJI_0526	Fraxinus pennsylvanica Marshall	0	a	0	1	Oleaceae Hoffmanns. & Link
Iz_2_1009	DJI_0526	Galeopsis bifida Boenn.	0	n	1	0	Lamiacea Lindl.
Iz_2_1009	DJI_0526	Galium verum L.	0	n	1	0	Rubiaceae Juss.
Iz_2_1009	DJI_0526	Genista tinctoria L.	0	n	1	0	Fabaceae Lindl.
Iz_2_1009	DJI_0526	Geranium robertianum L.	0	n	1	0	Geraniaceae Juss.
Iz_2_1009	DJI_0526	Geranium sanquineum L.	0	n	1	0	Geraniaceae Juss.
Iz_2_1009	DJI_0526	Gypsophila paniculata L.	0	n	1	0	Caryophyllaceae Juss.
Iz_2_1009	DJI_0526	Helichrysum arenarium (L.) Moench	0	n	1	0	Asteraceae Bercht. & J.Presl
Iz_2_1009	DJI_0526	Herniaria polygama J.Gay	0	n	1	0	Caryophyllaceae Juss.
Iz_2_1009	DJI_0526	Hieracium robustum Fr.	0	n	1	0	Asteraceae Bercht. & J.Presl
Iz_2_1009	DJI_0526	Hieracium umbellatum L.	0	n	1	NA	Asteraceae Bercht. & J.Presl
Iz_2_1009	DJI_0526	Hylotelephium maximum (L.) Holub	0	n	1	0	Crassulaceae J.St.-Hil.
Iz_2_1009	DJI_0526	Jacobaea borysthenica (DC.) B.Nord. & Greuter	0	n	1	0	Asteraceae Bercht. & J.Presl
Iz_2_1009	DJI_0526	Jasione montana L.	0	n	1	0	Campanulaceae Juss.
Iz_2_1009	DJI_0526	Jurinea cyanoides (L.) Rchb.s.l.	0	n	1	0	Asteraceae Bercht. & J.Presl
Iz_2_1009	DJI_0526	Koeleria glauca (Spreng.) DC.	0	n	1	0	Poaceae Barnhart
Iz_2_1009	DJI_0526	Lactuca serriola L.	0	a	0	0	Asteraceae Bercht. & J.Presl
Iz_2_1009	DJI_0526	Linaria genistifolia (L.) Mill.	0	n	1	0	Plantaginaceae Juss.
Iz_2_1009	DJI_0526	Lomelosia argentea (L.) Greuter & Burdet	0	n	1	0	Caprifoliaceae Juss.
Iz_2_1009	DJI_0526	Melampyrum pratense L.	0	n	1	0	Orobanchaceae Vent.
Iz_2_1009	DJI_0526	Oenothera biennis L.	0	a	0	0	Onagraceae Juss.
Iz_2_1009	DJI_0526	Pentanema britannica (L.) D.Gut.Larr., Santos-Vicente, Anderb., E.Rico & M.M.Mart.Ort.	0	n	1	0	Asteraceae Bercht. & J.Presl
Iz_2_1009	DJI_0526	Peucedanum oreoselinum (L.) Moench	2	n	1	0	Apiaceae Lindl.
Iz_2_1009	DJI_0526	Peucedanum ruthenicum M.Bieb.	0	n	1	0	Apiaceae Lindl.
Iz_2_1009	DJI_0526	Pilosella echioides (Lumn.) F.W.Schultz & Sch.Bip.	0	n	1	0	Asteraceae Bercht. & J.Presl
Iz_2_1009	DJI_0526	Pilosella officinarum F. Schultz et Sch. Bip.	0	n	1	0	Asteraceae Bercht. & J.Presl
Iz_2_1009	DJI_0526	Pilosella sp.	0		NA	0	Asteraceae Bercht. & J.Presl
Iz_2_1009	DJI_0526	Pinus sylvestris L.	0	n	1	1	Pinaceae Spreng. ex F.Rudolphi
Iz_2_1009	DJI_0526	Poa annua L.	0	n	1	0	Poaceae Barnhart
Iz_2_1009	DJI_0526	Poa nemoralis L.	0	n	1	0	Poaceae Barnhart
Iz_2_1009	DJI_0526	Poa sp.	0		NA	0	Poaceae Barnhart
Iz_2_1009	DJI_0526	Polygonatum odoratum (Mill.) Druce	0	n	1	0	Asparagaceae Juss.
Iz_2_1009	DJI_0526	Polygonum arenarium Waldst. & Kit.	0	n	1	0	Polygonaceae Juss.
Iz_2_1009	DJI_0526	Populus nigra L.	0	n	1	1	Salicaceae Mirb.
Iz_2_1009	DJI_0526	Populus tremula L.	0	n	1	1	Salicaceae Mirb.
Iz_2_1009	DJI_0526	Populus Ã canescens (Aiton) Sm.	0	n	1	1	Salicaceae Mirb.
Iz_2_1009	DJI_0526	Potentilla argentea L.	0	n	1	0	Rosaceae Juss.
Iz_2_1009	DJI_0526	Psammophiliella muralis (L.) Ikonn.	0	n	1	0	Caryophyllaceae Juss.
Iz_2_1009	DJI_0526	Psephellus sumensis (Kalen.) Greuter	0	n	1	0	Asteraceae Bercht. & J.Presl
Iz_2_1009	DJI_0526	Pulsatilla pratensis (L.) Mill. s.l.	0	n	1	0	Ranunculaceae Juss.
Iz_2_1009	DJI_0526	Pyrus communis L.	0	a	0	1	Rosaceae Juss.
Iz_2_1009	DJI_0526	Quercus robur L.	0	n	1	1	Fagaceae Dumort.
Iz_2_1009	DJI_0526	Robinia pseudoacacia L.	0	a	0	1	Fabaceae Lindl.
Iz_2_1009	DJI_0526	Rosa sp.	0		NA	1	Rosaceae Juss.
Iz_2_1009	DJI_0526	Rubus idaeus L.	0	n	1	0	Rosaceae Juss.
Iz_2_1009	DJI_0526	Rumex acetosella L.	0	n	1	0	Polygonaceae Juss.
Iz_2_1009	DJI_0526	Rumex thyrsiflorus Fingerh.	0	n	1	0	Polygonaceae Juss.
Iz_2_1009	DJI_0526	Salix alba L.	0	n	1	1	Salicaceae Mirb.
Iz_2_1009	DJI_0526	Sambucus nigra L.	0	n	1	1	Viburnaceae Raf.
Iz_2_1009	DJI_0526	Secale sylvestre Host	0	n	1	0	Poaceae Barnhart
Iz_2_1009	DJI_0526	Sempervivum ruthenicum Schnittsp. & C.B. Lehm.	0	n	1	0	Crassulaceae J.St.-Hil.
Iz_2_1009	DJI_0526	Setaria viridis (L.) P. Beauv.	0	n	1	0	Poaceae Barnhart
Iz_2_1009	DJI_0526	Silene borysthenica (Gruner) Walters	0	n	1	0	Caryophyllaceae Juss.
Iz_2_1009	DJI_0526	Silene latifolia subsp. alba (Mill.) Greuter & Burdet	0	n	1	0	Caryophyllaceae Juss.
Iz_2_1009	DJI_0526	Solidago virgaurea L.	2	n	1	0	Asteraceae Bercht. & J.Presl
Iz_2_1009	DJI_0526	Sorbus aucuparia L.	0	n	1	1	Rosaceae Juss.
Iz_2_1009	DJI_0526	Stachys recta L.	0	n	1	0	Lamiacea Lindl.
Iz_2_1009	DJI_0526	Tanacetum vulgare L.	0	n	1	0	Asteraceae Bercht. & J.Presl
Iz_2_1009	DJI_0526	Thymus pallasianus Heinr.Braun	0	n	1	0	Lamiacea Lindl.
Iz_2_1009	DJI_0526	Tilia cordata Mill.	0	n	1	1	Malvaceae Juss.
Iz_2_1009	DJI_0526	Trifolium alpestre L.	0	n	1	0	Fabaceae Lindl.
Iz_2_1009	DJI_0526	Trifolium arvense L.	0	n	1	0	Fabaceae Lindl.
Iz_2_1009	DJI_0526	Ulmus glabra Huds.	0	n	1	1	Ulmaceae Mirb.
Iz_2_1009	DJI_0526	Verbascum densiflorum Bertol.	0	n	1	0	Scrophulariaceae Juss.
Iz_2_1009	DJI_0526	Veronica austriaca L.	0	n	1	0	Plantaginaceae Juss.
Iz_2_1009	DJI_0526	Veronica incana L.	0	n	1	0	Plantaginaceae Juss.
Iz_2_1009	DJI_0526	Veronica verna L.	0	n	1	0	Plantaginaceae Juss.
Iz_2_1009	DJI_0526	Vincetoxicum hirundinaria Medik	0	n	1	0	Apocynaceae Juss.
Iz_2_1009	DJI_0527	Acer tataricum L.	0	n	1	1	Sapindaceae Juss.
Iz_2_1009	DJI_0527	Achillea millefolium L. s.l.	0	n	1	0	Asteraceae Bercht. & J.Presl
Iz_2_1009	DJI_0527	Allium oleraceum L.	0	n	1	0	Amaryllidaceae J.St.-Hil.
Iz_2_1009	DJI_0527	Ambrosia artemisiifolia L.	0	a	0	0	Asteraceae Bercht. & J.Presl
Iz_2_1009	DJI_0527	Amorpha fruticosa L.	0	a	0	0	Fabaceae Lindl.
Iz_2_1009	DJI_0527	Anthericum ramosum L.	0	n	1	0	Asparagaceae Juss.
Iz_2_1009	DJI_0527	Artemisia marschalliana Spreng.	0	n	1	0	Asteraceae Bercht. & J.Presl
Iz_2_1009	DJI_0527	Asparagus officinalis L.	0	n	1	0	Asparagaceae Juss.

Iz_2_1009	DJI_0527	Astragalus cicer L.	0	n	1	0	Fabaceae Lindl.
Iz_2_1009	DJI_0527	Bassia laniflora (S.G.Gmel.) A.J.Scott	0	n	1	0	Amaranthaceae Juss.
Iz_2_1009	DJI_0527	Berberis vulgaris L.	0	n	1	0	Berberidaceae Juss.
Iz_2_1009	DJI_0527	Betula pendula Roth.	0	n	1	1	Betulaceae Gray
Iz_2_1009	DJI_0527	Betula pendula Roth.	0	n	1	0	Poaceae Barnhart
Iz_2_1009	DJI_0527	Bromus inermis Leyss.	0		NA	NA	
Iz_2_1009	DJI_0527	Bromus squarrosus L.	0	a	0	0	Poaceae Barnhart
Iz_2_1009	DJI_0527	Bromus tectorum L.	0	a	0	0	Poaceae Barnhart
Iz_2_1009	DJI_0527	Calamagrostis epigejos (L.) Roth	15	n	1	0	Poaceae Barnhart
Iz_2_1009	DJI_0527	Carduus acanthoides L.	0	n	1	0	Asteraceae Bercht. & J.Presl
Iz_2_1009	DJI_0527	Carex sp.	0	n	1	0	Cyperaceae Juss.
Iz_2_1009	DJI_0527	Centaurea majorovii Dumbadze	0	n	1	0	Asteraceae Bercht. & J.Presl
Iz_2_1009	DJI_0527	Cf. Solidago virgaurea	0		NA	NA	
Iz_2_1009	DJI_0527	Chamaecytisus ruthenicus (Fisch.ex Wol.) Klaskova	0	n	1	0	Fabaceae Lindl.
Iz_2_1009	DJI_0527	Chelidonium majus L.	0	n	1	0	Papaveraceae Juss.
Iz_2_1009	DJI_0527	Chenopodium betaceum Andrz.	0	n	1	0	Amaranthaceae Juss.
Iz_2_1009	DJI_0527	Chenopodium cf. album	1	n	1	0	Amaranthaceae Juss.
Iz_2_1009	DJI_0527	Chenopodium sp.	0		NA	0	Amaranthaceae Juss.
Iz_2_1009	DJI_0527	Chondrilla juncea L.	0	n	1	0	Asteraceae Bercht. & J.Presl
Iz_2_1009	DJI_0527	Cirsium vulgare (Savi) Ten.	0	n	1	0	Asteraceae Bercht. & J.Presl
Iz_2_1009	DJI_0527	Clinopodium acinos (L.) Kuntze	0	n	1	0	Lamiacea Lindl.
Iz_2_1009	DJI_0527	Convallaria majalis L.	0	n	1	0	Asparagaceae Juss.
Iz_2_1009	DJI_0527	Crataegus rhipidophylla Gand.	0	n	1	1	Rosaceae Juss.
Iz_2_1009	DJI_0527	Crepis foetida L.	0	n	1	0	Asteraceae Bercht. & J.Presl
Iz_2_1009	DJI_0527	Dactylis glomerata L.	0	n	1	0	Poaceae Barnhart
Iz_2_1009	DJI_0527	Dianthus campestris M.Bieb.	0	n	1	0	Caryophyllaceae Juss.
Iz_2_1009	DJI_0527	Digitaria sanguinalis (L.) Scop.	0	a	0	0	Poaceae Barnhart
Iz_2_1009	DJI_0527	Elymus repens (L.) Gould	0	n	1	0	Poaceae Barnhart
Iz_2_1009	DJI_0527	Epilobium tetragonum L.	0	n	1	0	Onagraceae Juss.
Iz_2_1009	DJI_0527	Eragrostis minor Host	0	a	0	0	Poaceae Barnhart
Iz_2_1009	DJI_0527	Erigeron canadensis L.	0	a	0	0	Asteraceae Bercht. & J.Presl
Iz_2_1009	DJI_0527	Euphorbia seguieriana Neck.	0	n	1	0	Euphorbiaceae Juss.
Iz_2_1009	DJI_0527	Fallopia dumetorum (L.) Holub	0	n	1	0	Polygonaceae Juss.
Iz_2_1009	DJI_0527	Festuca beckeri (Hack.) Trautv.	0	n	1	0	Poaceae Barnhart
Iz_2_1009	DJI_0527	Fragaria vesca L.	0	n	1	0	Rosaceae Juss.
Iz_2_1009	DJI_0527	Frangula alnus Mill.	0	n	1	1	Rhamnaceae Juss.
Iz_2_1009	DJI_0527	Fraxinus pennsylvanica Marshall	0	a	0	1	Oleaceae Hoffmanns. & Link
Iz_2_1009	DJI_0527	Galeopsis bifida Boenn.	0	n	1	0	Lamiacea Lindl.
Iz_2_1009	DJI_0527	Galium verum L.	0	n	1	0	Rubiaceae Juss.
Iz_2_1009	DJI_0527	Genista tinctoria L.	0	n	1	0	Fabaceae Lindl.
Iz_2_1009	DJI_0527	Geranium robertianum L.	0	n	1	0	Geraniaceae Juss.
Iz_2_1009	DJI_0527	Geranium sanquineum L.	0	n	1	0	Geraniaceae Juss.
Iz_2_1009	DJI_0527	Gypsophila paniculata L.	0	n	1	0	Caryophyllaceae Juss.
Iz_2_1009	DJI_0527	Helichrysum arenarium (L.) Moench	0	n	1	0	Asteraceae Bercht. & J.Presl
Iz_2_1009	DJI_0527	Herniaria polygama J.Gay	0	n	1	0	Caryophyllaceae Juss.
Iz_2_1009	DJI_0527	Hieracium robustum Fr.	0	n	1	0	Asteraceae Bercht. & J.Presl
Iz_2_1009	DJI_0527	Hieracium umbellatum L.	0	n	1	NA	Asteraceae Bercht. & J.Presl
Iz_2_1009	DJI_0527	Hylotelephium maximum (L.) Holub	0	n	1	0	Crassulaceae J.St.-Hil.
Iz_2_1009	DJI_0527	Jacobaea borysthenica (DC.) B.Nord. & Greuter	0	n	1	0	Asteraceae Bercht. & J.Presl
Iz_2_1009	DJI_0527	Jasione montana L.	0	n	1	0	Campanulaceae Juss.
Iz_2_1009	DJI_0527	Jurinea cyanoides (L.) Rchb.s.l.	0	n	1	0	Asteraceae Bercht. & J.Presl
Iz_2_1009	DJI_0527	Koeleria glauca (Spreng.) DC.	0	n	1	0	Poaceae Barnhart
Iz_2_1009	DJI_0527	Lactuca serriola L.	0	a	0	0	Asteraceae Bercht. & J.Presl
Iz_2_1009	DJI_0527	Linaria genistifolia (L.) Mill.	0	n	1	0	Plantaginaceae Juss.
Iz_2_1009	DJI_0527	Lomelosia argentea (L.) Greuter & Burdet	0	n	1	0	Caprifoliaceae Juss.
Iz_2_1009	DJI_0527	Melampyrum pratense L.	0	n	1	0	Orobanchaceae Vent.
Iz_2_1009	DJI_0527	Oenothera biennis L.	0	a	0	0	Onagraceae Juss.
Iz_2_1009	DJI_0527	Pentanema britannica (L.) D.Gut.Larr., Santos-Vicente, Anderb., E.Rico & M.M.Mart.Ort.	0	n	1	0	Asteraceae Bercht. & J.Presl
Iz_2_1009	DJI_0527	Peucedanum oreoselinum (L.) Moench	2	n	1	0	Apiaceae Lindl.
Iz_2_1009	DJI_0527	Peucedanum ruthenicum M.Bieb.	0	n	1	0	Apiaceae Lindl.
Iz_2_1009	DJI_0527	Pilosella echioides (Lumn.) F.W.Schultz & Sch.Bip.	0	n	1	0	Asteraceae Bercht. & J.Presl
Iz_2_1009	DJI_0527	Pilosella officinarum F. Schultz et Sch. Bip.	0	n	1	0	Asteraceae Bercht. & J.Presl
Iz_2_1009	DJI_0527	Pilosella sp.	0		NA	0	Asteraceae Bercht. & J.Presl
Iz_2_1009	DJI_0527	Pinus sylvestris L.	0	n	1	1	Pinaceae Spreng. ex F.Rudolphi
Iz_2_1009	DJI_0527	Poa annua L.	0	n	1	0	Poaceae Barnhart
Iz_2_1009	DJI_0527	Poa nemoralis L.	0	n	1	0	Poaceae Barnhart
Iz_2_1009	DJI_0527	Poa sp.	0		NA	0	Poaceae Barnhart
Iz_2_1009	DJI_0527	Polygonatum odoratum (Mill.) Druce	0	n	1	0	Asparagaceae Juss.
Iz_2_1009	DJI_0527	Polygonum arenarium Waldst. & Kit.	0	n	1	0	Polygonaceae Juss.
Iz_2_1009	DJI_0527	Populus nigra L.	0	n	1	1	Salicaceae Mirb.
Iz_2_1009	DJI_0527	Populus tremula L.	0	n	1	1	Salicaceae Mirb.
Iz_2_1009	DJI_0527	Populus Ã canescens (Aiton) Sm.	0	n	1	1	Salicaceae Mirb.
Iz_2_1009	DJI_0527	Potentilla argentea L.	0	n	1	0	Rosaceae Juss.
Iz_2_1009	DJI_0527	Psammophiliella muralis (L.) Ikonn.	0	n	1	0	Caryophyllaceae Juss.
Iz_2_1009	DJI_0527	Psephellus sumensis (Kalen.) Greuter	0	n	1	0	Asteraceae Bercht. & J.Presl
Iz_2_1009	DJI_0527	Pulsatilla pratensis (L.) Mill. s.l.	0	n	1	0	Ranunculaceae Juss.
Iz_2_1009	DJI_0527	Pyrus communis L.	0	a	0	1	Rosaceae Juss.
Iz_2_1009	DJI_0527	Quercus robur L.	0	n	1	1	Fagaceae Dumort.
Iz_2_1009	DJI_0527	Robinia pseudoacacia L.	0	a	0	1	Fabaceae Lindl.
Iz_2_1009	DJI_0527	Rosa sp.	0		NA	1	Rosaceae Juss.
Iz_2_1009	DJI_0527	Rubus idaeus L.	0	n	1	0	Rosaceae Juss.
Iz_2_1009	DJI_0527	Rumex acetosella L.	0	n	1	0	Polygonaceae Juss.
Iz_2_1009	DJI_0527	Rumex thyrsiflorus Fingerh.	0	n	1	0	Polygonaceae Juss.
Iz_2_1009	DJI_0527	Salix alba L.	0	n	1	1	Salicaceae Mirb.
Iz_2_1009	DJI_0527	Sambucus nigra L.	0	n	1	1	Viburnaceae Raf.
Iz_2_1009	DJI_0527	Secale sylvestre Host	0	n	1	0	Poaceae Barnhart
Iz_2_1009	DJI_0527	Sempervivum ruthenicum Schnittsp. & C.B. Lehm.	0	n	1	0	Crassulaceae J.St.-Hil.
Iz_2_1009	DJI_0527	Setaria viridis (L.) P. Beauv.	0	n	1	0	Poaceae Barnhart
Iz_2_1009	DJI_0527	Silene borysthenica (Gruner) Walters	0	n	1	0	Caryophyllaceae Juss.
Iz_2_1009	DJI_0527	Silene latifolia subsp. alba (Mill.) Greuter & Burdet	0	n	1	0	Caryophyllaceae Juss.
Iz_2_1009	DJI_0527	Solidago virgaurea L.	2	n	1	0	Asteraceae Bercht. & J.Presl
Iz_2_1009	DJI_0527	Sorbus aucuparia L.	0	n	1	1	Rosaceae Juss.
Iz_2_1009	DJI_0527	Stachys recta L.	0	n	1	0	Lamiacea Lindl.
Iz_2_1009	DJI_0527	Tanacetum vulgare L.	0	n	1	0	Asteraceae Bercht. & J.Presl
Iz_2_1009	DJI_0527	Thymus pallasianus Heinr.Braun	0	n	1	0	Lamiacea Lindl.
Iz_2_1009	DJI_0527	Tilia cordata Mill.	0	n	1	1	Malvaceae Juss.
Iz_2_1009	DJI_0527	Trifolium alpestre L.	0	n	1	0	Fabaceae Lindl.
Iz_2_1009	DJI_0527	Trifolium arvense L.	0	n	1	0	Fabaceae Lindl.

Iz_2_1009	DJI_0527	Ulmus glabra Huds.	0	n	1	1	Ulmaceae Mirb.
Iz_2_1009	DJI_0527	Verbascum densiflorum Bertol.	0	n	1	0	Scrophulariaceae Juss.
Iz_2_1009	DJI_0527	Veronica austriaca L.	0	n	1	0	Plantaginaceae Juss.
Iz_2_1009	DJI_0527	Veronica incana L.	0	n	1	0	Plantaginaceae Juss.
Iz_2_1009	DJI_0527	Veronica verna L.	0	n	1	0	Plantaginaceae Juss.
Iz_2_1009	DJI_0527	Vincetoxicum hirundinaria Medik	0	n	1	0	Apocynaceae Juss.
Iz_2_1009	DJI_0529	Acer tataricum L.	0	n	1	1	Sapindaceae Juss.
Iz_2_1009	DJI_0529	Achillea millefolium L. s.l.	0	n	1	0	Asteraceae Bercht. & J.Presl
Iz_2_1009	DJI_0529	Allium oleraceum L.	0	n	1	0	Amaryllidaceae J.St.-Hil.
Iz_2_1009	DJI_0529	Ambrosia artemisiifolia L.	0	a	0	0	Asteraceae Bercht. & J.Presl
Iz_2_1009	DJI_0529	Amorpha fruticosa L.	0	a	0	0	Fabaceae Lindl.
Iz_2_1009	DJI_0529	Anthericum ramosum L.	0	n	1	0	Asparagaceae Juss.
Iz_2_1009	DJI_0529	Artemisia marschalliana Spreng.	0	n	1	0	Asteraceae Bercht. & J.Presl
Iz_2_1009	DJI_0529	Asparagus officinalis L.	0	n	1	0	Asparagaceae Juss.
Iz_2_1009	DJI_0529	Astragalus cicer L.	0	n	1	0	Fabaceae Lindl.
Iz_2_1009	DJI_0529	Bassia laniflora (S.G.Gmel.) A.J.Scott	0	n	1	0	Amaranthaceae Juss.
Iz_2_1009	DJI_0529	Berberis vulgaris L.	0	n	1	0	Berberidaceae Juss.
Iz_2_1009	DJI_0529	Betula pendula Roth.	0	n	1	1	Betulaceae Gray
Iz_2_1009	DJI_0529	Betula pendula Roth.	0	n	1	0	Poaceae Barnhart
Iz_2_1009	DJI_0529	Bromus inermis Leyss.	0		NA	NA	
Iz_2_1009	DJI_0529	Bromus squarrosus L.	0	a	0	0	Poaceae Barnhart
Iz_2_1009	DJI_0529	Bromus tectorum L.	0	a	0	0	Poaceae Barnhart
Iz_2_1009	DJI_0529	Calamagrostis epigejos (L.) Roth	15	n	1	0	Poaceae Barnhart
Iz_2_1009	DJI_0529	Carduus acanthoides L.	0	n	1	0	Asteraceae Bercht. & J.Presl
Iz_2_1009	DJI_0529	Carex sp.	0	n	1	0	Cyperaceae Juss.
Iz_2_1009	DJI_0529	Centaurea majorovii Dumbadze	0	n	1	0	Asteraceae Bercht. & J.Presl
Iz_2_1009	DJI_0529	Cf. Solidago virgaurea	0		NA	NA	
Iz_2_1009	DJI_0529	Chamaecytisus ruthenicus (Fisch.ex Wol.) Klaskova	2	n	1	0	Fabaceae Lindl.
Iz_2_1009	DJI_0529	Chelidonium majus L.	0	n	1	0	Papaveraceae Juss.
Iz_2_1009	DJI_0529	Chenopodium betaceum Andrz.	0	n	1	0	Amaranthaceae Juss.
Iz_2_1009	DJI_0529	Chenopodium cf. album	0	n	1	0	Amaranthaceae Juss.
Iz_2_1009	DJI_0529	Chenopodium sp.	0		NA	0	Amaranthaceae Juss.
Iz_2_1009	DJI_0529	Chondrilla juncea L.	0	n	1	0	Asteraceae Bercht. & J.Presl
Iz_2_1009	DJI_0529	Cirsium vulgare (Savi) Ten.	0	n	1	0	Asteraceae Bercht. & J.Presl
Iz_2_1009	DJI_0529	Clinopodium acinos (L.) Kuntze	0	n	1	0	Lamiacea Lindl.
Iz_2_1009	DJI_0529	Convallaria majalis L.	0	n	1	0	Asparagaceae Juss.
Iz_2_1009	DJI_0529	Crataegus rhipidophylla Gand.	0	n	1	1	Rosaceae Juss.
Iz_2_1009	DJI_0529	Crepis foetida L.	0	n	1	0	Asteraceae Bercht. & J.Presl
Iz_2_1009	DJI_0529	Dactylis glomerata L.	0	n	1	0	Poaceae Barnhart
Iz_2_1009	DJI_0529	Dianthus campestris M.Bieb.	0	n	1	0	Caryophyllaceae Juss.
Iz_2_1009	DJI_0529	Digitaria sanguinalis (L.) Scop.	0	a	0	0	Poaceae Barnhart
Iz_2_1009	DJI_0529	Elymus repens (L.) Gould	0	n	1	0	Poaceae Barnhart
Iz_2_1009	DJI_0529	Epilobium tetragonum L.	0	n	1	0	Onagraceae Juss.
Iz_2_1009	DJI_0529	Eragrostis minor Host	0	a	0	0	Poaceae Barnhart
Iz_2_1009	DJI_0529	Erigeron canadensis L.	0	a	0	0	Asteraceae Bercht. & J.Presl
Iz_2_1009	DJI_0529	Euphorbia seguieriana Neck.	0	n	1	0	Euphorbiaceae Juss.
Iz_2_1009	DJI_0529	Fallopia dumetorum (L.) Holub	0	n	1	0	Polygonaceae Juss.
Iz_2_1009	DJI_0529	Festuca beckeri (Hack.) Trautv.	0	n	1	0	Poaceae Barnhart
Iz_2_1009	DJI_0529	Fragaria vesca L.	0	n	1	0	Rosaceae Juss.
Iz_2_1009	DJI_0529	Frangula alnus Mill.	0	n	1	1	Rhamnaceae Juss.
Iz_2_1009	DJI_0529	Fraxinus pennsylvanica Marshall	0	a	0	1	Oleaceae Hoffmanns. & Link
Iz_2_1009	DJI_0529	Galeopsis bifida Boenn.	0	n	1	0	Lamiacea Lindl.
Iz_2_1009	DJI_0529	Galium verum L.	0	n	1	0	Rubiaceae Juss.
Iz_2_1009	DJI_0529	Genista tinctoria L.	0	n	1	0	Fabaceae Lindl.
Iz_2_1009	DJI_0529	Geranium robertianum L.	0	n	1	0	Geraniaceae Juss.
Iz_2_1009	DJI_0529	Geranium sanquineum L.	0	n	1	0	Geraniaceae Juss.
Iz_2_1009	DJI_0529	Gypsophila paniculata L.	0	n	1	0	Caryophyllaceae Juss.
Iz_2_1009	DJI_0529	Helichrysum arenarium (L.) Moench	0	n	1	0	Asteraceae Bercht. & J.Presl
Iz_2_1009	DJI_0529	Herniaria polygama J.Gay	0	n	1	0	Caryophyllaceae Juss.
Iz_2_1009	DJI_0529	Hieracium robustum Fr.	0	n	1	0	Asteraceae Bercht. & J.Presl
Iz_2_1009	DJI_0529	Hieracium umbellatum L.	0	n	1	NA	Asteraceae Bercht. & J.Presl
Iz_2_1009	DJI_0529	Hylotelephium maximum (L.) Holub	0	n	1	0	Crassulaceae J.St.-Hil.
Iz_2_1009	DJI_0529	Jacobaea borysthenica (DC.) B.Nord. & Greuter	0	n	1	0	Asteraceae Bercht. & J.Presl
Iz_2_1009	DJI_0529	Jasione montana L.	0	n	1	0	Campanulaceae Juss.
Iz_2_1009	DJI_0529	Jurinea cyanoides (L.) Rchb.s.l.	0	n	1	0	Asteraceae Bercht. & J.Presl
Iz_2_1009	DJI_0529	Koeleria glauca (Spreng.) DC.	0	n	1	0	Poaceae Barnhart
Iz_2_1009	DJI_0529	Lactuca serriola L.	0	a	0	0	Asteraceae Bercht. & J.Presl
Iz_2_1009	DJI_0529	Linaria genistifolia (L.) Mill.	0	n	1	0	Plantaginaceae Juss.
Iz_2_1009	DJI_0529	Lomelosia argentea (L.) Greuter & Burdet	0	n	1	0	Caprifoliaceae Juss.
Iz_2_1009	DJI_0529	Melampyrum pratense L.	0	n	1	0	Orobanchaceae Vent.
Iz_2_1009	DJI_0529	Oenothera biennis L.	0	a	0	0	Onagraceae Juss.
Iz_2_1009	DJI_0529	Pentanema britannica (L.) D.Gut.Larr., Santos-Vicente, Anderb., E.Rico & M.M.Mart.Ort.	0	n	1	0	Asteraceae Bercht. & J.Presl
Iz_2_1009	DJI_0529	Peucedanum oreoselinum (L.) Moench	1	n	1	0	Apiaceae Lindl.
Iz_2_1009	DJI_0529	Peucedanum ruthenicum M.Bieb.	0	n	1	0	Apiaceae Lindl.
Iz_2_1009	DJI_0529	Pilosella echioides (Lumn.) F.W.Schultz & Sch.Bip.	0	n	1	0	Asteraceae Bercht. & J.Presl
Iz_2_1009	DJI_0529	Pilosella officinarum F. Schultz et Sch. Bip.	0	n	1	0	Asteraceae Bercht. & J.Presl
Iz_2_1009	DJI_0529	Pilosella sp.	0		NA	0	Asteraceae Bercht. & J.Presl
Iz_2_1009	DJI_0529	Pinus sylvestris L.	0	n	1	1	Pinaceae Spreng. ex F.Rudolphi
Iz_2_1009	DJI_0529	Poa annua L.	0	n	1	0	Poaceae Barnhart
Iz_2_1009	DJI_0529	Poa nemoralis L.	0	n	1	0	Poaceae Barnhart
Iz_2_1009	DJI_0529	Poa sp.	0		NA	0	Poaceae Barnhart
Iz_2_1009	DJI_0529	Polygonatum odoratum (Mill.) Druce	0	n	1	0	Asparagaceae Juss.
Iz_2_1009	DJI_0529	Polygonum arenarium Waldst. & Kit.	0	n	1	0	Polygonaceae Juss.
Iz_2_1009	DJI_0529	Populus nigra L.	0	n	1	1	Salicaceae Mirb.
Iz_2_1009	DJI_0529	Populus tremula L.	0	n	1	1	Salicaceae Mirb.
Iz_2_1009	DJI_0529	Populus Ã canescens (Aiton) Sm.	0	n	1	1	Salicaceae Mirb.
Iz_2_1009	DJI_0529	Potentilla argentea L.	0	n	1	0	Rosaceae Juss.
Iz_2_1009	DJI_0529	Psammophiliella muralis (L.) Ikonn.	0	n	1	0	Caryophyllaceae Juss.
Iz_2_1009	DJI_0529	Psephellus sumensis (Kalen.) Greuter	0	n	1	0	Asteraceae Bercht. & J.Presl
Iz_2_1009	DJI_0529	Pulsatilla pratensis (L.) Mill. s.l.	0	n	1	0	Ranunculaceae Juss.
Iz_2_1009	DJI_0529	Pyrus communis L.	0	a	0	1	Rosaceae Juss.
Iz_2_1009	DJI_0529	Quercus robur L.	0	n	1	1	Fagaceae Dumort.
Iz_2_1009	DJI_0529	Robinia pseudoacacia L.	0	a	0	1	Fabaceae Lindl.
Iz_2_1009	DJI_0529	Rosa sp.	0		NA	1	Rosaceae Juss.
Iz_2_1009	DJI_0529	Rubus idaeus L.	0	n	1	0	Rosaceae Juss.
Iz_2_1009	DJI_0529	Rumex acetosella L.	0	n	1	0	Polygonaceae Juss.
Iz_2_1009	DJI_0529	Rumex thyrsiflorus Fingerh.	0	n	1	0	Polygonaceae Juss.
Iz_2_1009	DJI_0529	Salix alba L.	0	n	1	1	Salicaceae Mirb.

Iz_2_1009	DJI_0529	Sambucus nigra L.	0	n	1	1	Viburnaceae Raf.
Iz_2_1009	DJI_0529	Secale sylvestre Host	0	n	1	0	Poaceae Barnhart
Iz_2_1009	DJI_0529	Sempervivum ruthenicum Schnittsp. & C.B. Lehm.	0	n	1	0	Crassulaceae J.St.-Hil.
Iz_2_1009	DJI_0529	Setaria viridis (L.) P. Beauv.	0	n	1	0	Poaceae Barnhart
Iz_2_1009	DJI_0529	Silene borysthenica (Gruner) Walters	0	n	1	0	Caryophyllaceae Juss.
Iz_2_1009	DJI_0529	Silene latifolia subsp. alba (Mill.) Greuter & Burdet	0	n	1	0	Caryophyllaceae Juss.
Iz_2_1009	DJI_0529	Solidago virgaurea L.	2	n	1	0	Asteraceae Bercht. & J.Presl
Iz_2_1009	DJI_0529	Sorbus aucuparia L.	0	n	1	1	Rosaceae Juss.
Iz_2_1009	DJI_0529	Stachys recta L.	0	n	1	0	Lamiacea Lindl.
Iz_2_1009	DJI_0529	Tanacetum vulgare L.	0	n	1	0	Asteraceae Bercht. & J.Presl
Iz_2_1009	DJI_0529	Thymus pallasianus Heinr.Braun	0	n	1	0	Lamiacea Lindl.
Iz_2_1009	DJI_0529	Tilia cordata Mill.	0	n	1	1	Malvaceae Juss.
Iz_2_1009	DJI_0529	Trifolium alpestre L.	0	n	1	0	Fabaceae Lindl.
Iz_2_1009	DJI_0529	Trifolium arvense L.	0	n	1	0	Fabaceae Lindl.
Iz_2_1009	DJI_0529	Ulmus glabra Huds.	0	n	1	1	Ulmaceae Mirb.
Iz_2_1009	DJI_0529	Verbascum densiflorum Bertol.	0	n	1	0	Scrophulariaceae Juss.
Iz_2_1009	DJI_0529	Veronica austriaca L.	0	n	1	0	Plantaginaceae Juss.
Iz_2_1009	DJI_0529	Veronica incana L.	0	n	1	0	Plantaginaceae Juss.
Iz_2_1009	DJI_0529	Veronica verna L.	0	n	1	0	Plantaginaceae Juss.
Iz_2_1009	DJI_0529	Vincetoxicum hirundinaria Medik	0	n	1	0	Apocynaceae Juss.
Iz_2_1009	DJI_0530	Acer tataricum L.	0	n	1	1	Sapindaceae Juss.
Iz_2_1009	DJI_0530	Achillea millefolium L. s.l.	0	n	1	0	Asteraceae Bercht. & J.Presl
Iz_2_1009	DJI_0530	Allium oleraceum L.	0	n	1	0	Amaryllidaceae J.St.-Hil.
Iz_2_1009	DJI_0530	Ambrosia artemisiifolia L.	0	a	0	0	Asteraceae Bercht. & J.Presl
Iz_2_1009	DJI_0530	Amorpha fruticosa L.	0	a	0	0	Fabaceae Lindl.
Iz_2_1009	DJI_0530	Anthericum ramosum L.	0	n	1	0	Asparagaceae Juss.
Iz_2_1009	DJI_0530	Artemisia marschalliana Spreng.	0	n	1	0	Asteraceae Bercht. & J.Presl
Iz_2_1009	DJI_0530	Asparagus officinalis L.	0	n	1	0	Asparagaceae Juss.
Iz_2_1009	DJI_0530	Astragalus cicer L.	0	n	1	0	Fabaceae Lindl.
Iz_2_1009	DJI_0530	Bassia laniflora (S.G.Gmel.) A.J.Scott	0	n	1	0	Amaranthaceae Juss.
Iz_2_1009	DJI_0530	Berberis vulgaris L.	0	n	1	0	Berberidaceae Juss.
Iz_2_1009	DJI_0530	Betula pendula Roth.	0	n	1	1	Betulaceae Gray
Iz_2_1009	DJI_0530	Betula pendula Roth.	0	n	1	0	Poaceae Barnhart
Iz_2_1009	DJI_0530	Bromus inermis Leyss.	0		NA	NA	
Iz_2_1009	DJI_0530	Bromus squarrosus L.	0	a	0	0	Poaceae Barnhart
Iz_2_1009	DJI_0530	Bromus tectorum L.	0	a	0	0	Poaceae Barnhart
Iz_2_1009	DJI_0530	Calamagrostis epigejos (L.) Roth	60	n	1	0	Poaceae Barnhart
Iz_2_1009	DJI_0530	Carduus acanthoides L.	0	n	1	0	Asteraceae Bercht. & J.Presl
Iz_2_1009	DJI_0530	Carex sp.	0	n	1	0	Cyperaceae Juss.
Iz_2_1009	DJI_0530	Centaurea majorovii Dumbadze	0	n	1	0	Asteraceae Bercht. & J.Presl
Iz_2_1009	DJI_0530	Cf. Solidago virgaurea	0		NA	NA	
Iz_2_1009	DJI_0530	Chamaecytisus ruthenicus (Fisch.ex Wol.) Klaskova	0	n	1	0	Fabaceae Lindl.
Iz_2_1009	DJI_0530	Chelidonium majus L.	0	n	1	0	Papaveraceae Juss.
Iz_2_1009	DJI_0530	Chenopodium betaceum Andrz.	0	n	1	0	Amaranthaceae Juss.
Iz_2_1009	DJI_0530	Chenopodium cf. album	0	n	1	0	Amaranthaceae Juss.
Iz_2_1009	DJI_0530	Chenopodium sp.	0		NA	0	Amaranthaceae Juss.
Iz_2_1009	DJI_0530	Chondrilla juncea L.	0	n	1	0	Asteraceae Bercht. & J.Presl
Iz_2_1009	DJI_0530	Cirsium vulgare (Savi) Ten.	0	n	1	0	Asteraceae Bercht. & J.Presl
Iz_2_1009	DJI_0530	Clinopodium acinos (L.) Kuntze	0	n	1	0	Lamiacea Lindl.
Iz_2_1009	DJI_0530	Convallaria majalis L.	0	n	1	0	Asparagaceae Juss.
Iz_2_1009	DJI_0530	Crataegus rhipidophylla Gand.	0	n	1	1	Rosaceae Juss.
Iz_2_1009	DJI_0530	Crepis foetida L.	0	n	1	0	Asteraceae Bercht. & J.Presl
Iz_2_1009	DJI_0530	Dactylis glomerata L.	0	n	1	0	Poaceae Barnhart
Iz_2_1009	DJI_0530	Dianthus campestris M.Bieb.	0	n	1	0	Caryophyllaceae Juss.
Iz_2_1009	DJI_0530	Digitaria sanguinalis (L.) Scop.	0	a	0	0	Poaceae Barnhart
Iz_2_1009	DJI_0530	Elymus repens (L.) Gould	0	n	1	0	Poaceae Barnhart
Iz_2_1009	DJI_0530	Epilobium tetragonum L.	0	n	1	0	Onagraceae Juss.
Iz_2_1009	DJI_0530	Eragrostis minor Host	0	a	0	0	Poaceae Barnhart
Iz_2_1009	DJI_0530	Erigeron canadensis L.	0	a	0	0	Asteraceae Bercht. & J.Presl
Iz_2_1009	DJI_0530	Euphorbia seguieriana Neck.	0	n	1	0	Euphorbiaceae Juss.
Iz_2_1009	DJI_0530	Fallopia dumetorum (L.) Holub	0	n	1	0	Polygonaceae Juss.
Iz_2_1009	DJI_0530	Festuca beckeri (Hack.) Trautv.	0	n	1	0	Poaceae Barnhart
Iz_2_1009	DJI_0530	Fragaria vesca L.	0	n	1	0	Rosaceae Juss.
Iz_2_1009	DJI_0530	Frangula alnus Mill.	0	n	1	1	Rhamnaceae Juss.
Iz_2_1009	DJI_0530	Fraxinus pennsylvanica Marshall	0	a	0	1	Oleaceae Hoffmanns. & Link
Iz_2_1009	DJI_0530	Galeopsis bifida Boenn.	0	n	1	0	Lamiacea Lindl.
Iz_2_1009	DJI_0530	Galium verum L.	0	n	1	0	Rubiaceae Juss.
Iz_2_1009	DJI_0530	Genista tinctoria L.	0	n	1	0	Fabaceae Lindl.
Iz_2_1009	DJI_0530	Geranium robertianum L.	0	n	1	0	Geraniaceae Juss.
Iz_2_1009	DJI_0530	Geranium sanquineum L.	0	n	1	0	Geraniaceae Juss.
Iz_2_1009	DJI_0530	Gypsophila paniculata L.	0	n	1	0	Caryophyllaceae Juss.
Iz_2_1009	DJI_0530	Helichrysum arenarium (L.) Moench	0	n	1	0	Asteraceae Bercht. & J.Presl
Iz_2_1009	DJI_0530	Herniaria polygama J.Gay	0	n	1	0	Caryophyllaceae Juss.
Iz_2_1009	DJI_0530	Hieracium robustum Fr.	0	n	1	0	Asteraceae Bercht. & J.Presl
Iz_2_1009	DJI_0530	Hieracium umbellatum L.	0	n	1	NA	Asteraceae Bercht. & J.Presl
Iz_2_1009	DJI_0530	Hylotelephium maximum (L.) Holub	0	n	1	0	Crassulaceae J.St.-Hil.
Iz_2_1009	DJI_0530	Jacobaea borysthenica (DC.) B.Nord. & Greuter	0	n	1	0	Asteraceae Bercht. & J.Presl
Iz_2_1009	DJI_0530	Jasione montana L.	0	n	1	0	Campanulaceae Juss.
Iz_2_1009	DJI_0530	Jurinea cyanoides (L.) Rchb.s.l.	0	n	1	0	Asteraceae Bercht. & J.Presl
Iz_2_1009	DJI_0530	Koeleria glauca (Spreng.) DC.	0	n	1	0	Poaceae Barnhart
Iz_2_1009	DJI_0530	Lactuca serriola L.	0	a	0	0	Asteraceae Bercht. & J.Presl
Iz_2_1009	DJI_0530	Linaria genistifolia (L.) Mill.	0	n	1	0	Plantaginaceae Juss.
Iz_2_1009	DJI_0530	Lomelosia argentea (L.) Greuter & Burdet	0	n	1	0	Caprifoliaceae Juss.
Iz_2_1009	DJI_0530	Melampyrum pratense L.	0	n	1	0	Orobanchaceae Vent.
Iz_2_1009	DJI_0530	Oenothera biennis L.	0	a	0	0	Onagraceae Juss.
Iz_2_1009	DJI_0530	Pentanema britannica (L.) D.Gut.Larr., Santos-Vicente, Anderb., E.Rico & M.M.Mart.Ort.	0	n	1	0	Asteraceae Bercht. & J.Presl
Iz_2_1009	DJI_0530	Peucedanum oreoselinum (L.) Moench	0.5	n	1	0	Apiaceae Lindl.
Iz_2_1009	DJI_0530	Peucedanum ruthenicum M.Bieb.	0	n	1	0	Apiaceae Lindl.
Iz_2_1009	DJI_0530	Pilosella echioides (Lumn.) F.W.Schultz & Sch.Bip.	0	n	1	0	Asteraceae Bercht. & J.Presl
Iz_2_1009	DJI_0530	Pilosella officinarum F. Schultz et Sch. Bip.	0	n	1	0	Asteraceae Bercht. & J.Presl
Iz_2_1009	DJI_0530	Pilosella sp.	0		NA	0	Asteraceae Bercht. & J.Presl
Iz_2_1009	DJI_0530	Pinus sylvestris L.	0	n	1	1	Pinaceae Spreng. ex F.Rudolphi
Iz_2_1009	DJI_0530	Poa annua L.	0	n	1	0	Poaceae Barnhart
Iz_2_1009	DJI_0530	Poa nemoralis L.	0	n	1	0	Poaceae Barnhart
Iz_2_1009	DJI_0530	Poa sp.	0		NA	0	Poaceae Barnhart
Iz_2_1009	DJI_0530	Polygonatum odoratum (Mill.) Druce	0	n	1	0	Asparagaceae Juss.
Iz_2_1009	DJI_0530	Polygonum arenarium Waldst. & Kit.	0	n	1	0	Polygonaceae Juss.
Iz_2_1009	DJI_0530	Populus nigra L.	0	n	1	1	Salicaceae Mirb.

Iz_2_1009	DJI_0530	Populus tremula L.	0	n	1	1	Salicaceae Mirb.
Iz_2_1009	DJI_0530	Populus Ã canescens (Aiton) Sm.	0	n	1	1	Salicaceae Mirb.
Iz_2_1009	DJI_0530	Potentilla argentea L.	0	n	1	0	Rosaceae Juss.
Iz_2_1009	DJI_0530	Psammophiliella muralis (L.) Ikonn.	0	n	1	0	Caryophyllaceae Juss.
Iz_2_1009	DJI_0530	Psephellus sumensis (Kalen.) Greuter	0	n	1	0	Asteraceae Bercht. & J.Presl
Iz_2_1009	DJI_0530	Pulsatilla pratensis (L.) Mill. s.l.	0	n	1	0	Ranunculaceae Juss.
Iz_2_1009	DJI_0530	Pyrus communis L.	0	a	0	1	Rosaceae Juss.
Iz_2_1009	DJI_0530	Quercus robur L.	0	n	1	1	Fagaceae Dumort.
Iz_2_1009	DJI_0530	Robinia pseudoacacia L.	0	a	0	1	Fabaceae Lindl.
Iz_2_1009	DJI_0530	Rosa sp.	0		NA	1	Rosaceae Juss.
Iz_2_1009	DJI_0530	Rubus idaeus L.	0	n	1	0	Rosaceae Juss.
Iz_2_1009	DJI_0530	Rumex acetosella L.	0	n	1	0	Polygonaceae Juss.
Iz_2_1009	DJI_0530	Rumex thyrsiflorus Fingerh.	0	n	1	0	Polygonaceae Juss.
Iz_2_1009	DJI_0530	Salix alba L.	0	n	1	1	Salicaceae Mirb.
Iz_2_1009	DJI_0530	Sambucus nigra L.	0	n	1	1	Viburnaceae Raf.
Iz_2_1009	DJI_0530	Secale sylvestre Host	0	n	1	0	Poaceae Barnhart
Iz_2_1009	DJI_0530	Sempervivum ruthenicum Schnittsp. & C.B. Lehm.	0	n	1	0	Crassulaceae J.St.-Hil.
Iz_2_1009	DJI_0530	Setaria viridis (L.) P. Beauv.	0	n	1	0	Poaceae Barnhart
Iz_2_1009	DJI_0530	Silene borysthenica (Gruner) Walters	0	n	1	0	Caryophyllaceae Juss.
Iz_2_1009	DJI_0530	Silene latifolia subsp. alba (Mill.) Greuter & Burdet	0	n	1	0	Caryophyllaceae Juss.
Iz_2_1009	DJI_0530	Solidago virgaurea L.	0	n	1	0	Asteraceae Bercht. & J.Presl
Iz_2_1009	DJI_0530	Sorbus aucuparia L.	0	n	1	1	Rosaceae Juss.
Iz_2_1009	DJI_0530	Stachys recta L.	0	n	1	0	Lamiacea Lindl.
Iz_2_1009	DJI_0530	Tanacetum vulgare L.	0	n	1	0	Asteraceae Bercht. & J.Presl
Iz_2_1009	DJI_0530	Thymus pallasianus Heinr.Braun	0	n	1	0	Lamiacea Lindl.
Iz_2_1009	DJI_0530	Tilia cordata Mill.	0	n	1	1	Malvaceae Juss.
Iz_2_1009	DJI_0530	Trifolium alpestre L.	0	n	1	0	Fabaceae Lindl.
Iz_2_1009	DJI_0530	Trifolium arvense L.	0	n	1	0	Fabaceae Lindl.
Iz_2_1009	DJI_0530	Ulmus glabra Huds.	0	n	1	1	Ulmaceae Mirb.
Iz_2_1009	DJI_0530	Verbascum densiflorum Bertol.	0	n	1	0	Scrophulariaceae Juss.
Iz_2_1009	DJI_0530	Veronica austriaca L.	0	n	1	0	Plantaginaceae Juss.
Iz_2_1009	DJI_0530	Veronica incana L.	0	n	1	0	Plantaginaceae Juss.
Iz_2_1009	DJI_0530	Veronica verna L.	0	n	1	0	Plantaginaceae Juss.
Iz_2_1009	DJI_0530	Vincetoxicum hirundinaria Medik	0	n	1	0	Apocynaceae Juss.
Iz_2_1009	DJI_0533	Acer tataricum L.	0	n	1	1	Sapindaceae Juss.
Iz_2_1009	DJI_0533	Achillea millefolium L. s.l.	0	n	1	0	Asteraceae Bercht. & J.Presl
Iz_2_1009	DJI_0533	Allium oleraceum L.	0	n	1	0	Amaryllidaceae J.St.-Hil.
Iz_2_1009	DJI_0533	Ambrosia artemisiifolia L.	0	a	0	0	Asteraceae Bercht. & J.Presl
Iz_2_1009	DJI_0533	Amorpha fruticosa L.	0	a	0	0	Fabaceae Lindl.
Iz_2_1009	DJI_0533	Anthericum ramosum L.	0	n	1	0	Asparagaceae Juss.
Iz_2_1009	DJI_0533	Artemisia marschalliana Spreng.	0	n	1	0	Asteraceae Bercht. & J.Presl
Iz_2_1009	DJI_0533	Asparagus officinalis L.	0	n	1	0	Asparagaceae Juss.
Iz_2_1009	DJI_0533	Astragalus cicer L.	0	n	1	0	Fabaceae Lindl.
Iz_2_1009	DJI_0533	Bassia laniflora (S.G.Gmel.) A.J.Scott	0	n	1	0	Amaranthaceae Juss.
Iz_2_1009	DJI_0533	Berberis vulgaris L.	0	n	1	0	Berberidaceae Juss.
Iz_2_1009	DJI_0533	Betula pendula Roth.	0	n	1	1	Betulaceae Gray
Iz_2_1009	DJI_0533	Betula pendula Roth.	0	n	1	0	Poaceae Barnhart
Iz_2_1009	DJI_0533	Bromus inermis Leyss.	0		NA	NA	
Iz_2_1009	DJI_0533	Bromus squarrosus L.	0	a	0	0	Poaceae Barnhart
Iz_2_1009	DJI_0533	Bromus tectorum L.	0	a	0	0	Poaceae Barnhart
Iz_2_1009	DJI_0533	Calamagrostis epigejos (L.) Roth	50	n	1	0	Poaceae Barnhart
Iz_2_1009	DJI_0533	Carduus acanthoides L.	0	n	1	0	Asteraceae Bercht. & J.Presl
Iz_2_1009	DJI_0533	Carex sp.	0	n	1	0	Cyperaceae Juss.
Iz_2_1009	DJI_0533	Centaurea majorovii Dumbadze	0	n	1	0	Asteraceae Bercht. & J.Presl
Iz_2_1009	DJI_0533	Cf. Solidago virgaurea	0		NA	NA	
Iz_2_1009	DJI_0533	Chamaecytisus ruthenicus (Fisch.ex Wol.) Klaskova	0	n	1	0	Fabaceae Lindl.
Iz_2_1009	DJI_0533	Chelidonium majus L.	0	n	1	0	Papaveraceae Juss.
Iz_2_1009	DJI_0533	Chenopodium betaceum Andrz.	0	n	1	0	Amaranthaceae Juss.
Iz_2_1009	DJI_0533	Chenopodium cf. album	0	n	1	0	Amaranthaceae Juss.
Iz_2_1009	DJI_0533	Chenopodium sp.	0		NA	0	Amaranthaceae Juss.
Iz_2_1009	DJI_0533	Chondrilla juncea L.	0	n	1	0	Asteraceae Bercht. & J.Presl
Iz_2_1009	DJI_0533	Cirsium vulgare (Savi) Ten.	0	n	1	0	Asteraceae Bercht. & J.Presl
Iz_2_1009	DJI_0533	Clinopodium acinos (L.) Kuntze	0	n	1	0	Lamiacea Lindl.
Iz_2_1009	DJI_0533	Convallaria majalis L.	0	n	1	0	Asparagaceae Juss.
Iz_2_1009	DJI_0533	Crataegus rhipidophylla Gand.	0	n	1	1	Rosaceae Juss.
Iz_2_1009	DJI_0533	Crepis foetida L.	0	n	1	0	Asteraceae Bercht. & J.Presl
Iz_2_1009	DJI_0533	Dactylis glomerata L.	0	n	1	0	Poaceae Barnhart
Iz_2_1009	DJI_0533	Dianthus campestris M.Bieb.	0	n	1	0	Caryophyllaceae Juss.
Iz_2_1009	DJI_0533	Digitaria sanguinalis (L.) Scop.	0	a	0	0	Poaceae Barnhart
Iz_2_1009	DJI_0533	Elymus repens (L.) Gould	0	n	1	0	Poaceae Barnhart
Iz_2_1009	DJI_0533	Epilobium tetragonum L.	0	n	1	0	Onagraceae Juss.
Iz_2_1009	DJI_0533	Eragrostis minor Host	0	a	0	0	Poaceae Barnhart
Iz_2_1009	DJI_0533	Erigeron canadensis L.	0	a	0	0	Asteraceae Bercht. & J.Presl
Iz_2_1009	DJI_0533	Euphorbia seguieriana Neck.	0	n	1	0	Euphorbiaceae Juss.
Iz_2_1009	DJI_0533	Fallopia dumetorum (L.) Holub	0	n	1	0	Polygonaceae Juss.
Iz_2_1009	DJI_0533	Festuca beckeri (Hack.) Trautv.	0	n	1	0	Poaceae Barnhart
Iz_2_1009	DJI_0533	Fragaria vesca L.	0	n	1	0	Rosaceae Juss.
Iz_2_1009	DJI_0533	Frangula alnus Mill.	0	n	1	1	Rhamnaceae Juss.
Iz_2_1009	DJI_0533	Fraxinus pennsylvanica Marshall	0	a	0	1	Oleaceae Hoffmanns. & Link
Iz_2_1009	DJI_0533	Galeopsis bifida Boenn.	0	n	1	0	Lamiacea Lindl.
Iz_2_1009	DJI_0533	Galium verum L.	0	n	1	0	Rubiaceae Juss.
Iz_2_1009	DJI_0533	Genista tinctoria L.	0	n	1	0	Fabaceae Lindl.
Iz_2_1009	DJI_0533	Geranium robertianum L.	0	n	1	0	Geraniaceae Juss.
Iz_2_1009	DJI_0533	Geranium sanquineum L.	0	n	1	0	Geraniaceae Juss.
Iz_2_1009	DJI_0533	Gypsophila paniculata L.	0	n	1	0	Caryophyllaceae Juss.
Iz_2_1009	DJI_0533	Helichrysum arenarium (L.) Moench	0	n	1	0	Asteraceae Bercht. & J.Presl
Iz_2_1009	DJI_0533	Herniaria polygama J.Gay	0	n	1	0	Caryophyllaceae Juss.
Iz_2_1009	DJI_0533	Hieracium robustum Fr.	0	n	1	0	Asteraceae Bercht. & J.Presl
Iz_2_1009	DJI_0533	Hieracium umbellatum L.	0	n	1	NA	Asteraceae Bercht. & J.Presl
Iz_2_1009	DJI_0533	Hylotelephium maximum (L.) Holub	0	n	1	0	Crassulaceae J.St.-Hil.
Iz_2_1009	DJI_0533	Jacobaea borysthenica (DC.) B.Nord. & Greuter	0	n	1	0	Asteraceae Bercht. & J.Presl
Iz_2_1009	DJI_0533	Jasione montana L.	0	n	1	0	Campanulaceae Juss.
Iz_2_1009	DJI_0533	Jurinea cyanoides (L.) Rchb.s.l.	0	n	1	0	Asteraceae Bercht. & J.Presl
Iz_2_1009	DJI_0533	Koeleria glauca (Spreng.) DC.	0	n	1	0	Poaceae Barnhart
Iz_2_1009	DJI_0533	Lactuca serriola L.	0	a	0	0	Asteraceae Bercht. & J.Presl
Iz_2_1009	DJI_0533	Linaria genistifolia (L.) Mill.	0	n	1	0	Plantaginaceae Juss.
Iz_2_1009	DJI_0533	Lomelosia argentea (L.) Greuter & Burdet	0	n	1	0	Caprifoliaceae Juss.
Iz_2_1009	DJI_0533	Melampyrum pratense L.	0	n	1	0	Orobanchaceae Vent.

Iz_2_1009	DJI_0533	Oenothera biennis L.	0	a	0	0	Onagraceae Juss.
Iz_2_1009	DJI_0533	Pentanema britannica (L.) D.Gut.Larr., Santos-Vicente, Anderb., E.Rico & M.M.Mart.Ort.	0	n	1	0	Asteraceae Bercht. & J.Presl
Iz_2_1009	DJI_0533	Peucedanum oreoselinum (L.) Moench	0	n	1	0	Apiaceae Lindl.
Iz_2_1009	DJI_0533	Peucedanum ruthenicum M.Bieb.	0	n	1	0	Apiaceae Lindl.
Iz_2_1009	DJI_0533	Pilosella echioides (Lumn.) F.W.Schultz & Sch.Bip.	0	n	1	0	Asteraceae Bercht. & J.Presl
Iz_2_1009	DJI_0533	Pilosella officinarum F. Schultz et Sch. Bip.	0	n	1	0	Asteraceae Bercht. & J.Presl
Iz_2_1009	DJI_0533	Pilosella sp.	0		NA	0	Asteraceae Bercht. & J.Presl
Iz_2_1009	DJI_0533	Pinus sylvestris L.	0	n	1	1	Pinaceae Spreng. ex F.Rudolphi
Iz_2_1009	DJI_0533	Poa annua L.	0	n	1	0	Poaceae Barnhart
Iz_2_1009	DJI_0533	Poa nemoralis L.	0	n	1	0	Poaceae Barnhart
Iz_2_1009	DJI_0533	Poa sp.	0		NA	0	Poaceae Barnhart
Iz_2_1009	DJI_0533	Polygonatum odoratum (Mill.) Druce	0	n	1	0	Asparagaceae Juss.
Iz_2_1009	DJI_0533	Polygonum arenarium Waldst. & Kit.	0	n	1	0	Polygonaceae Juss.
Iz_2_1009	DJI_0533	Populus nigra L.	0	n	1	1	Salicaceae Mirb.
Iz_2_1009	DJI_0533	Populus tremula L.	0	n	1	1	Salicaceae Mirb.
Iz_2_1009	DJI_0533	Populus Ã canescens (Aiton) Sm.	0	n	1	1	Salicaceae Mirb.
Iz_2_1009	DJI_0533	Potentilla argentea L.	0	n	1	0	Rosaceae Juss.
Iz_2_1009	DJI_0533	Psammophiliella muralis (L.) Ikonn.	0	n	1	0	Caryophyllaceae Juss.
Iz_2_1009	DJI_0533	Psephellus sumensis (Kalen.) Greuter	0	n	1	0	Asteraceae Bercht. & J.Presl
Iz_2_1009	DJI_0533	Pulsatilla pratensis (L.) Mill. s.l.	0	n	1	0	Ranunculaceae Juss.
Iz_2_1009	DJI_0533	Pyrus communis L.	0	a	0	1	Rosaceae Juss.
Iz_2_1009	DJI_0533	Quercus robur L.	0	n	1	1	Fagaceae Dumort.
Iz_2_1009	DJI_0533	Robinia pseudoacacia L.	0	a	0	1	Fabaceae Lindl.
Iz_2_1009	DJI_0533	Rosa sp.	0		NA	1	Rosaceae Juss.
Iz_2_1009	DJI_0533	Rubus idaeus L.	0	n	1	0	Rosaceae Juss.
Iz_2_1009	DJI_0533	Rumex acetosella L.	0	n	1	0	Polygonaceae Juss.
Iz_2_1009	DJI_0533	Rumex thyrsiflorus Fingerh.	0	n	1	0	Polygonaceae Juss.
Iz_2_1009	DJI_0533	Salix alba L.	0	n	1	1	Salicaceae Mirb.
Iz_2_1009	DJI_0533	Sambucus nigra L.	0	n	1	1	Viburnaceae Raf.
Iz_2_1009	DJI_0533	Secale sylvestre Host	0	n	1	0	Poaceae Barnhart
Iz_2_1009	DJI_0533	Sempervivum ruthenicum Schnittsp. & C.B. Lehm.	0	n	1	0	Crassulaceae J.St.-Hil.
Iz_2_1009	DJI_0533	Setaria viridis (L.) P. Beauv.	0	n	1	0	Poaceae Barnhart
Iz_2_1009	DJI_0533	Silene borysthenica (Gruner) Walters	0	n	1	0	Caryophyllaceae Juss.
Iz_2_1009	DJI_0533	Silene latifolia subsp. alba (Mill.) Greuter & Burdet	0	n	1	0	Caryophyllaceae Juss.
Iz_2_1009	DJI_0533	Solidago virgaurea L.	2	n	1	0	Asteraceae Bercht. & J.Presl
Iz_2_1009	DJI_0533	Sorbus aucuparia L.	0	n	1	1	Rosaceae Juss.
Iz_2_1009	DJI_0533	Stachys recta L.	0	n	1	0	Lamiacea Lindl.
Iz_2_1009	DJI_0533	Tanacetum vulgare L.	0	n	1	0	Asteraceae Bercht. & J.Presl
Iz_2_1009	DJI_0533	Thymus pallasianus Heinr.Braun	0	n	1	0	Lamiacea Lindl.
Iz_2_1009	DJI_0533	Tilia cordata Mill.	0	n	1	1	Malvaceae Juss.
Iz_2_1009	DJI_0533	Trifolium alpestre L.	0	n	1	0	Fabaceae Lindl.
Iz_2_1009	DJI_0533	Trifolium arvense L.	0	n	1	0	Fabaceae Lindl.
Iz_2_1009	DJI_0533	Ulmus glabra Huds.	0	n	1	1	Ulmaceae Mirb.
Iz_2_1009	DJI_0533	Verbascum densiflorum Bertol.	0	n	1	0	Scrophulariaceae Juss.
Iz_2_1009	DJI_0533	Veronica austriaca L.	0	n	1	0	Plantaginaceae Juss.
Iz_2_1009	DJI_0533	Veronica incana L.	0	n	1	0	Plantaginaceae Juss.
Iz_2_1009	DJI_0533	Veronica verna L.	0	n	1	0	Plantaginaceae Juss.
Iz_2_1009	DJI_0533	Vincetoxicum hirundinaria Medik	0	n	1	0	Apocynaceae Juss.
Iz_2_1009	DJI_0534	Acer tataricum L.	0	n	1	1	Sapindaceae Juss.
Iz_2_1009	DJI_0534	Achillea millefolium L. s.l.	0	n	1	0	Asteraceae Bercht. & J.Presl
Iz_2_1009	DJI_0534	Allium oleraceum L.	0	n	1	0	Amaryllidaceae J.St.-Hil.
Iz_2_1009	DJI_0534	Ambrosia artemisiifolia L.	0	a	0	0	Asteraceae Bercht. & J.Presl
Iz_2_1009	DJI_0534	Amorpha fruticosa L.	0	a	0	0	Fabaceae Lindl.
Iz_2_1009	DJI_0534	Anthericum ramosum L.	0	n	1	0	Asparagaceae Juss.
Iz_2_1009	DJI_0534	Artemisia marschalliana Spreng.	0	n	1	0	Asteraceae Bercht. & J.Presl
Iz_2_1009	DJI_0534	Asparagus officinalis L.	0	n	1	0	Asparagaceae Juss.
Iz_2_1009	DJI_0534	Astragalus cicer L.	0	n	1	0	Fabaceae Lindl.
Iz_2_1009	DJI_0534	Bassia laniflora (S.G.Gmel.) A.J.Scott	0	n	1	0	Amaranthaceae Juss.
Iz_2_1009	DJI_0534	Berberis vulgaris L.	0	n	1	0	Berberidaceae Juss.
Iz_2_1009	DJI_0534	Betula pendula Roth.	0	n	1	1	Betulaceae Gray
Iz_2_1009	DJI_0534	Betula pendula Roth.	0	n	1	0	Poaceae Barnhart
Iz_2_1009	DJI_0534	Bromus inermis Leyss.	0		NA	NA	
Iz_2_1009	DJI_0534	Bromus squarrosus L.	0	a	0	0	Poaceae Barnhart
Iz_2_1009	DJI_0534	Bromus tectorum L.	0	a	0	0	Poaceae Barnhart
Iz_2_1009	DJI_0534	Calamagrostis epigejos (L.) Roth	40	n	1	0	Poaceae Barnhart
Iz_2_1009	DJI_0534	Carduus acanthoides L.	0	n	1	0	Asteraceae Bercht. & J.Presl
Iz_2_1009	DJI_0534	Carex sp.	0	n	1	0	Cyperaceae Juss.
Iz_2_1009	DJI_0534	Centaurea majorovii Dumbadze	0	n	1	0	Asteraceae Bercht. & J.Presl
Iz_2_1009	DJI_0534	Cf. Solidago virgaurea	0		NA	NA	
Iz_2_1009	DJI_0534	Chamaecytisus ruthenicus (Fisch.ex Wol.) Klaskova	0	n	1	0	Fabaceae Lindl.
Iz_2_1009	DJI_0534	Chelidonium majus L.	0	n	1	0	Papaveraceae Juss.
Iz_2_1009	DJI_0534	Chenopodium betaceum Andrz.	0	n	1	0	Amaranthaceae Juss.
Iz_2_1009	DJI_0534	Chenopodium cf. album	1	n	1	0	Amaranthaceae Juss.
Iz_2_1009	DJI_0534	Chenopodium sp.	0		NA	0	Amaranthaceae Juss.
Iz_2_1009	DJI_0534	Chondrilla juncea L.	0	n	1	0	Asteraceae Bercht. & J.Presl
Iz_2_1009	DJI_0534	Cirsium vulgare (Savi) Ten.	0	n	1	0	Asteraceae Bercht. & J.Presl
Iz_2_1009	DJI_0534	Clinopodium acinos (L.) Kuntze	0	n	1	0	Lamiacea Lindl.
Iz_2_1009	DJI_0534	Convallaria majalis L.	0	n	1	0	Asparagaceae Juss.
Iz_2_1009	DJI_0534	Crataegus rhipidophylla Gand.	0	n	1	1	Rosaceae Juss.
Iz_2_1009	DJI_0534	Crepis foetida L.	0	n	1	0	Asteraceae Bercht. & J.Presl
Iz_2_1009	DJI_0534	Dactylis glomerata L.	0	n	1	0	Poaceae Barnhart
Iz_2_1009	DJI_0534	Dianthus campestris M.Bieb.	0	n	1	0	Caryophyllaceae Juss.
Iz_2_1009	DJI_0534	Digitaria sanguinalis (L.) Scop.	0	a	0	0	Poaceae Barnhart
Iz_2_1009	DJI_0534	Elymus repens (L.) Gould	0	n	1	0	Poaceae Barnhart
Iz_2_1009	DJI_0534	Epilobium tetragonum L.	0	n	1	0	Onagraceae Juss.
Iz_2_1009	DJI_0534	Eragrostis minor Host	0	a	0	0	Poaceae Barnhart
Iz_2_1009	DJI_0534	Erigeron canadensis L.	0	a	0	0	Asteraceae Bercht. & J.Presl
Iz_2_1009	DJI_0534	Euphorbia seguieriana Neck.	0	n	1	0	Euphorbiaceae Juss.
Iz_2_1009	DJI_0534	Fallopia dumetorum (L.) Holub	0	n	1	0	Polygonaceae Juss.
Iz_2_1009	DJI_0534	Festuca beckeri (Hack.) Trautv.	0	n	1	0	Poaceae Barnhart
Iz_2_1009	DJI_0534	Fragaria vesca L.	0	n	1	0	Rosaceae Juss.
Iz_2_1009	DJI_0534	Frangula alnus Mill.	0	n	1	1	Rhamnaceae Juss.
Iz_2_1009	DJI_0534	Fraxinus pennsylvanica Marshall	0	a	0	1	Oleaceae Hoffmanns. & Link
Iz_2_1009	DJI_0534	Galeopsis bifida Boenn.	0	n	1	0	Lamiacea Lindl.
Iz_2_1009	DJI_0534	Galium verum L.	0	n	1	0	Rubiaceae Juss.
Iz_2_1009	DJI_0534	Genista tinctoria L.	0	n	1	0	Fabaceae Lindl.
Iz_2_1009	DJI_0534	Geranium robertianum L.	0	n	1	0	Geraniaceae Juss.
Iz_2_1009	DJI_0534	Geranium sanquineum L.	0	n	1	0	Geraniaceae Juss.

Iz_2_1009	DJI_0534	Gypsophila paniculata L.	0	n	1	0	Caryophyllaceae Juss.
Iz_2_1009	DJI_0534	Helichrysum arenarium (L.) Moench	0	n	1	0	Asteraceae Bercht. & J.Presl
Iz_2_1009	DJI_0534	Herniaria polygama J.Gay	0	n	1	0	Caryophyllaceae Juss.
Iz_2_1009	DJI_0534	Hieracium robustum Fr.	0	n	1	0	Asteraceae Bercht. & J.Presl
Iz_2_1009	DJI_0534	Hieracium umbellatum L.	0	n	1	NA	Asteraceae Bercht. & J.Presl
Iz_2_1009	DJI_0534	Hylotelephium maximum (L.) Holub	0	n	1	0	Crassulaceae J.St.-Hil.
Iz_2_1009	DJI_0534	Jacobaea borysthenica (DC.) B.Nord. & Greuter	0	n	1	0	Asteraceae Bercht. & J.Presl
Iz_2_1009	DJI_0534	Jasione montana L.	0	n	1	0	Campanulaceae Juss.
Iz_2_1009	DJI_0534	Jurinea cyanoides (L.) Rchb.s.l.	0	n	1	0	Asteraceae Bercht. & J.Presl
Iz_2_1009	DJI_0534	Koeleria glauca (Spreng.) DC.	0	n	1	0	Poaceae Barnhart
Iz_2_1009	DJI_0534	Lactuca serriola L.	0	a	0	0	Asteraceae Bercht. & J.Presl
Iz_2_1009	DJI_0534	Linaria genistifolia (L.) Mill.	0	n	1	0	Plantaginaceae Juss.
Iz_2_1009	DJI_0534	Lomelosia argentea (L.) Greuter & Burdet	0	n	1	0	Caprifoliaceae Juss.
Iz_2_1009	DJI_0534	Melampyrum pratense L.	0	n	1	0	Orobanchaceae Vent.
Iz_2_1009	DJI_0534	Oenothera biennis L.	0	a	0	0	Onagraceae Juss.
Iz_2_1009	DJI_0534	Pentanema britannica (L.) D.Gut.Larr., Santos-Vicente, Anderb., E.Rico & M.M.Mart.Ort.	0	n	1	0	Asteraceae Bercht. & J.Presl
Iz_2_1009	DJI_0534	Peucedanum oreoselinum (L.) Moench	1	n	1	0	Apiaceae Lindl.
Iz_2_1009	DJI_0534	Peucedanum ruthenicum M.Bieb.	0	n	1	0	Apiaceae Lindl.
Iz_2_1009	DJI_0534	Pilosella echioides (Lumn.) F.W.Schultz & Sch.Bip.	0	n	1	0	Asteraceae Bercht. & J.Presl
Iz_2_1009	DJI_0534	Pilosella officinarum F. Schultz et Sch. Bip.	0	n	1	0	Asteraceae Bercht. & J.Presl
Iz_2_1009	DJI_0534	Pilosella sp.	0		NA	0	Asteraceae Bercht. & J.Presl
Iz_2_1009	DJI_0534	Pinus sylvestris L.	0	n	1	1	Pinaceae Spreng. ex F.Rudolphi
Iz_2_1009	DJI_0534	Poa annua L.	0	n	1	0	Poaceae Barnhart
Iz_2_1009	DJI_0534	Poa nemoralis L.	0	n	1	0	Poaceae Barnhart
Iz_2_1009	DJI_0534	Poa sp.	0		NA	0	Poaceae Barnhart
Iz_2_1009	DJI_0534	Polygonatum odoratum (Mill.) Druce	0	n	1	0	Asparagaceae Juss.
Iz_2_1009	DJI_0534	Polygonum arenarium Waldst. & Kit.	0	n	1	0	Polygonaceae Juss.
Iz_2_1009	DJI_0534	Populus nigra L.	0	n	1	1	Salicaceae Mirb.
Iz_2_1009	DJI_0534	Populus tremula L.	0	n	1	1	Salicaceae Mirb.
Iz_2_1009	DJI_0534	Populus Ã canescens (Aiton) Sm.	0	n	1	1	Salicaceae Mirb.
Iz_2_1009	DJI_0534	Potentilla argentea L.	0	n	1	0	Rosaceae Juss.
Iz_2_1009	DJI_0534	Psammophiliella muralis (L.) Ikonn.	0	n	1	0	Caryophyllaceae Juss.
Iz_2_1009	DJI_0534	Psephellus sumensis (Kalen.) Greuter	0	n	1	0	Asteraceae Bercht. & J.Presl
Iz_2_1009	DJI_0534	Pulsatilla pratensis (L.) Mill. s.l.	0	n	1	0	Ranunculaceae Juss.
Iz_2_1009	DJI_0534	Pyrus communis L.	0	a	0	1	Rosaceae Juss.
Iz_2_1009	DJI_0534	Quercus robur L.	0	n	1	1	Fagaceae Dumort.
Iz_2_1009	DJI_0534	Robinia pseudoacacia L.	0	a	0	1	Fabaceae Lindl.
Iz_2_1009	DJI_0534	Rosa sp.	0		NA	1	Rosaceae Juss.
Iz_2_1009	DJI_0534	Rubus idaeus L.	0	n	1	0	Rosaceae Juss.
Iz_2_1009	DJI_0534	Rumex acetosella L.	0	n	1	0	Polygonaceae Juss.
Iz_2_1009	DJI_0534	Rumex thyrsiflorus Fingerh.	0	n	1	0	Polygonaceae Juss.
Iz_2_1009	DJI_0534	Salix alba L.	0	n	1	1	Salicaceae Mirb.
Iz_2_1009	DJI_0534	Sambucus nigra L.	0	n	1	1	Viburnaceae Raf.
Iz_2_1009	DJI_0534	Secale sylvestre Host	0	n	1	0	Poaceae Barnhart
Iz_2_1009	DJI_0534	Sempervivum ruthenicum Schnittsp. & C.B. Lehm.	0	n	1	0	Crassulaceae J.St.-Hil.
Iz_2_1009	DJI_0534	Setaria viridis (L.) P. Beauv.	0	n	1	0	Poaceae Barnhart
Iz_2_1009	DJI_0534	Silene borysthenica (Gruner) Walters	0	n	1	0	Caryophyllaceae Juss.
Iz_2_1009	DJI_0534	Silene latifolia subsp. alba (Mill.) Greuter & Burdet	0	n	1	0	Caryophyllaceae Juss.
Iz_2_1009	DJI_0534	Solidago virgaurea L.	0.5	n	1	0	Asteraceae Bercht. & J.Presl
Iz_2_1009	DJI_0534	Sorbus aucuparia L.	0	n	1	1	Rosaceae Juss.
Iz_2_1009	DJI_0534	Stachys recta L.	0	n	1	0	Lamiacea Lindl.
Iz_2_1009	DJI_0534	Tanacetum vulgare L.	0	n	1	0	Asteraceae Bercht. & J.Presl
Iz_2_1009	DJI_0534	Thymus pallasianus Heinr.Braun	0	n	1	0	Lamiacea Lindl.
Iz_2_1009	DJI_0534	Tilia cordata Mill.	0	n	1	1	Malvaceae Juss.
Iz_2_1009	DJI_0534	Trifolium alpestre L.	0	n	1	0	Fabaceae Lindl.
Iz_2_1009	DJI_0534	Trifolium arvense L.	0	n	1	0	Fabaceae Lindl.
Iz_2_1009	DJI_0534	Ulmus glabra Huds.	0	n	1	1	Ulmaceae Mirb.
Iz_2_1009	DJI_0534	Verbascum densiflorum Bertol.	0	n	1	0	Scrophulariaceae Juss.
Iz_2_1009	DJI_0534	Veronica austriaca L.	0	n	1	0	Plantaginaceae Juss.
Iz_2_1009	DJI_0534	Veronica incana L.	0	n	1	0	Plantaginaceae Juss.
Iz_2_1009	DJI_0534	Veronica verna L.	0	n	1	0	Plantaginaceae Juss.
Iz_2_1009	DJI_0534	Vincetoxicum hirundinaria Medik	0	n	1	0	Apocynaceae Juss.
Iz_2_1009	DJI_0536	Acer tataricum L.	0	n	1	1	Sapindaceae Juss.
Iz_2_1009	DJI_0536	Achillea millefolium L. s.l.	0	n	1	0	Asteraceae Bercht. & J.Presl
Iz_2_1009	DJI_0536	Allium oleraceum L.	0	n	1	0	Amaryllidaceae J.St.-Hil.
Iz_2_1009	DJI_0536	Ambrosia artemisiifolia L.	0	a	0	0	Asteraceae Bercht. & J.Presl
Iz_2_1009	DJI_0536	Amorpha fruticosa L.	0	a	0	0	Fabaceae Lindl.
Iz_2_1009	DJI_0536	Anthericum ramosum L.	0	n	1	0	Asparagaceae Juss.
Iz_2_1009	DJI_0536	Artemisia marschalliana Spreng.	0	n	1	0	Asteraceae Bercht. & J.Presl
Iz_2_1009	DJI_0536	Asparagus officinalis L.	0	n	1	0	Asparagaceae Juss.
Iz_2_1009	DJI_0536	Astragalus cicer L.	0	n	1	0	Fabaceae Lindl.
Iz_2_1009	DJI_0536	Bassia laniflora (S.G.Gmel.) A.J.Scott	0	n	1	0	Amaranthaceae Juss.
Iz_2_1009	DJI_0536	Berberis vulgaris L.	0	n	1	0	Berberidaceae Juss.
Iz_2_1009	DJI_0536	Betula pendula Roth.	0	n	1	1	Betulaceae Gray
Iz_2_1009	DJI_0536	Betula pendula Roth.	0	n	1	0	Poaceae Barnhart
Iz_2_1009	DJI_0536	Bromus inermis Leyss.	0		NA	NA	
Iz_2_1009	DJI_0536	Bromus squarrosus L.	0	a	0	0	Poaceae Barnhart
Iz_2_1009	DJI_0536	Bromus tectorum L.	0	a	0	0	Poaceae Barnhart
Iz_2_1009	DJI_0536	Calamagrostis epigejos (L.) Roth	45	n	1	0	Poaceae Barnhart
Iz_2_1009	DJI_0536	Carduus acanthoides L.	0	n	1	0	Asteraceae Bercht. & J.Presl
Iz_2_1009	DJI_0536	Carex sp.	0	n	1	0	Cyperaceae Juss.
Iz_2_1009	DJI_0536	Centaurea majorovii Dumbadze	0	n	1	0	Asteraceae Bercht. & J.Presl
Iz_2_1009	DJI_0536	Cf. Solidago virgaurea	0		NA	NA	
Iz_2_1009	DJI_0536	Chamaecytisus ruthenicus (Fisch.ex Wol.) Klaskova	0	n	1	0	Fabaceae Lindl.
Iz_2_1009	DJI_0536	Chelidonium majus L.	0	n	1	0	Papaveraceae Juss.
Iz_2_1009	DJI_0536	Chenopodium betaceum Andrz.	0	n	1	0	Amaranthaceae Juss.
Iz_2_1009	DJI_0536	Chenopodium cf. album	0	n	1	0	Amaranthaceae Juss.
Iz_2_1009	DJI_0536	Chenopodium sp.	0		NA	0	Amaranthaceae Juss.
Iz_2_1009	DJI_0536	Chondrilla juncea L.	0	n	1	0	Asteraceae Bercht. & J.Presl
Iz_2_1009	DJI_0536	Cirsium vulgare (Savi) Ten.	0	n	1	0	Asteraceae Bercht. & J.Presl
Iz_2_1009	DJI_0536	Clinopodium acinos (L.) Kuntze	0	n	1	0	Lamiacea Lindl.
Iz_2_1009	DJI_0536	Convallaria majalis L.	0	n	1	0	Asparagaceae Juss.
Iz_2_1009	DJI_0536	Crataegus rhipidophylla Gand.	0	n	1	1	Rosaceae Juss.
Iz_2_1009	DJI_0536	Crepis foetida L.	0	n	1	0	Asteraceae Bercht. & J.Presl
Iz_2_1009	DJI_0536	Dactylis glomerata L.	0	n	1	0	Poaceae Barnhart
Iz_2_1009	DJI_0536	Dianthus campestris M.Bieb.	0	n	1	0	Caryophyllaceae Juss.
Iz_2_1009	DJI_0536	Digitaria sanguinalis (L.) Scop.	0	a	0	0	Poaceae Barnhart
Iz_2_1009	DJI_0536	Elymus repens (L.) Gould	0	n	1	0	Poaceae Barnhart

Iz_2_1009	DJI_0536	Epilobium tetragonum L.	0	n	1	0	Onagraceae Juss.
Iz_2_1009	DJI_0536	Eragrostis minor Host	0	a	0	0	Poaceae Barnhart
Iz_2_1009	DJI_0536	Erigeron canadensis L.	5	a	0	0	Asteraceae Bercht. & J.Presl
Iz_2_1009	DJI_0536	Euphorbia seguieriana Neck.	0	n	1	0	Euphorbiaceae Juss.
Iz_2_1009	DJI_0536	Fallopia dumetorum (L.) Holub	0	n	1	0	Polygonaceae Juss.
Iz_2_1009	DJI_0536	Festuca beckeri (Hack.) Trautv.	0	n	1	0	Poaceae Barnhart
Iz_2_1009	DJI_0536	Fragaria vesca L.	0	n	1	0	Rosaceae Juss.
Iz_2_1009	DJI_0536	Frangula alnus Mill.	0	n	1	1	Rhamnaceae Juss.
Iz_2_1009	DJI_0536	Fraxinus pennsylvanica Marshall	0	a	0	1	Oleaceae Hoffmanns. & Link
Iz_2_1009	DJI_0536	Galeopsis bifida Boenn.	0	n	1	0	Lamiacea Lindl.
Iz_2_1009	DJI_0536	Galium verum L.	0	n	1	0	Rubiaceae Juss.
Iz_2_1009	DJI_0536	Genista tinctoria L.	0	n	1	0	Fabaceae Lindl.
Iz_2_1009	DJI_0536	Geranium robertianum L.	0	n	1	0	Geraniaceae Juss.
Iz_2_1009	DJI_0536	Geranium sanquineum L.	0	n	1	0	Geraniaceae Juss.
Iz_2_1009	DJI_0536	Gypsophila paniculata L.	0	n	1	0	Caryophyllaceae Juss.
Iz_2_1009	DJI_0536	Helichrysum arenarium (L.) Moench	0	n	1	0	Asteraceae Bercht. & J.Presl
Iz_2_1009	DJI_0536	Herniaria polygama J.Gay	0	n	1	0	Caryophyllaceae Juss.
Iz_2_1009	DJI_0536	Hieracium robustum Fr.	0	n	1	0	Asteraceae Bercht. & J.Presl
Iz_2_1009	DJI_0536	Hieracium umbellatum L.	0	n	1	NA	Asteraceae Bercht. & J.Presl
Iz_2_1009	DJI_0536	Hylotelephium maximum (L.) Holub	0	n	1	0	Crassulaceae J.St.-Hil.
Iz_2_1009	DJI_0536	Jacobaea borysthenica (DC.) B.Nord. & Greuter	0	n	1	0	Asteraceae Bercht. & J.Presl
Iz_2_1009	DJI_0536	Jasione montana L.	0	n	1	0	Campanulaceae Juss.
Iz_2_1009	DJI_0536	Jurinea cyanoides (L.) Rchb.s.l.	0	n	1	0	Asteraceae Bercht. & J.Presl
Iz_2_1009	DJI_0536	Koeleria glauca (Spreng.) DC.	0	n	1	0	Poaceae Barnhart
Iz_2_1009	DJI_0536	Lactuca serriola L.	0	a	0	0	Asteraceae Bercht. & J.Presl
Iz_2_1009	DJI_0536	Linaria genistifolia (L.) Mill.	0	n	1	0	Plantaginaceae Juss.
Iz_2_1009	DJI_0536	Lomelosia argentea (L.) Greuter & Burdet	0	n	1	0	Caprifoliaceae Juss.
Iz_2_1009	DJI_0536	Melampyrum pratense L.	0	n	1	0	Orobanchaceae Vent.
Iz_2_1009	DJI_0536	Oenothera biennis L.	0	a	0	0	Onagraceae Juss.
Iz_2_1009	DJI_0536	Pentanema britannica (L.) D.Gut.Larr., Santos-Vicente, Anderb., E.Rico & M.M.Mart.Ort.	0	n	1	0	Asteraceae Bercht. & J.Presl
Iz_2_1009	DJI_0536	Peucedanum oreoselinum (L.) Moench	0	n	1	0	Apiaceae Lindl.
Iz_2_1009	DJI_0536	Peucedanum ruthenicum M.Bieb.	0	n	1	0	Apiaceae Lindl.
Iz_2_1009	DJI_0536	Pilosella echioides (Lumn.) F.W.Schultz & Sch.Bip.	0	n	1	0	Asteraceae Bercht. & J.Presl
Iz_2_1009	DJI_0536	Pilosella officinarum F. Schultz et Sch. Bip.	0	n	1	0	Asteraceae Bercht. & J.Presl
Iz_2_1009	DJI_0536	Pilosella sp.	0		NA	0	Asteraceae Bercht. & J.Presl
Iz_2_1009	DJI_0536	Pinus sylvestris L.	0	n	1	1	Pinaceae Spreng. ex F.Rudolphi
Iz_2_1009	DJI_0536	Poa annua L.	0	n	1	0	Poaceae Barnhart
Iz_2_1009	DJI_0536	Poa nemoralis L.	0	n	1	0	Poaceae Barnhart
Iz_2_1009	DJI_0536	Poa sp.	0		NA	0	Poaceae Barnhart
Iz_2_1009	DJI_0536	Polygonatum odoratum (Mill.) Druce	0	n	1	0	Asparagaceae Juss.
Iz_2_1009	DJI_0536	Polygonum arenarium Waldst. & Kit.	0	n	1	0	Polygonaceae Juss.
Iz_2_1009	DJI_0536	Populus nigra L.	0	n	1	1	Salicaceae Mirb.
Iz_2_1009	DJI_0536	Populus tremula L.	0	n	1	1	Salicaceae Mirb.
Iz_2_1009	DJI_0536	Populus Ã canescens (Aiton) Sm.	0	n	1	1	Salicaceae Mirb.
Iz_2_1009	DJI_0536	Potentilla argentea L.	0	n	1	0	Rosaceae Juss.
Iz_2_1009	DJI_0536	Psammophiliella muralis (L.) Ikonn.	0	n	1	0	Caryophyllaceae Juss.
Iz_2_1009	DJI_0536	Psephellus sumensis (Kalen.) Greuter	0	n	1	0	Asteraceae Bercht. & J.Presl
Iz_2_1009	DJI_0536	Pulsatilla pratensis (L.) Mill. s.l.	0	n	1	0	Ranunculaceae Juss.
Iz_2_1009	DJI_0536	Pyrus communis L.	0	a	0	1	Rosaceae Juss.
Iz_2_1009	DJI_0536	Quercus robur L.	0	n	1	1	Fagaceae Dumort.
Iz_2_1009	DJI_0536	Robinia pseudoacacia L.	0	a	0	1	Fabaceae Lindl.
Iz_2_1009	DJI_0536	Rosa sp.	0		NA	1	Rosaceae Juss.
Iz_2_1009	DJI_0536	Rubus idaeus L.	0	n	1	0	Rosaceae Juss.
Iz_2_1009	DJI_0536	Rumex acetosella L.	0	n	1	0	Polygonaceae Juss.
Iz_2_1009	DJI_0536	Rumex thyrsiflorus Fingerh.	0	n	1	0	Polygonaceae Juss.
Iz_2_1009	DJI_0536	Salix alba L.	0	n	1	1	Salicaceae Mirb.
Iz_2_1009	DJI_0536	Sambucus nigra L.	0	n	1	1	Viburnaceae Raf.
Iz_2_1009	DJI_0536	Secale sylvestre Host	0	n	1	0	Poaceae Barnhart
Iz_2_1009	DJI_0536	Sempervivum ruthenicum Schnittsp. & C.B. Lehm.	0	n	1	0	Crassulaceae J.St.-Hil.
Iz_2_1009	DJI_0536	Setaria viridis (L.) P. Beauv.	0	n	1	0	Poaceae Barnhart
Iz_2_1009	DJI_0536	Silene borysthenica (Gruner) Walters	0	n	1	0	Caryophyllaceae Juss.
Iz_2_1009	DJI_0536	Silene latifolia subsp. alba (Mill.) Greuter & Burdet	0	n	1	0	Caryophyllaceae Juss.
Iz_2_1009	DJI_0536	Solidago virgaurea L.	3	n	1	0	Asteraceae Bercht. & J.Presl
Iz_2_1009	DJI_0536	Sorbus aucuparia L.	0	n	1	1	Rosaceae Juss.
Iz_2_1009	DJI_0536	Stachys recta L.	0	n	1	0	Lamiacea Lindl.
Iz_2_1009	DJI_0536	Tanacetum vulgare L.	0	n	1	0	Asteraceae Bercht. & J.Presl
Iz_2_1009	DJI_0536	Thymus pallasianus Heinr.Braun	0	n	1	0	Lamiacea Lindl.
Iz_2_1009	DJI_0536	Tilia cordata Mill.	0	n	1	1	Malvaceae Juss.
Iz_2_1009	DJI_0536	Trifolium alpestre L.	0	n	1	0	Fabaceae Lindl.
Iz_2_1009	DJI_0536	Trifolium arvense L.	0	n	1	0	Fabaceae Lindl.
Iz_2_1009	DJI_0536	Ulmus glabra Huds.	0	n	1	1	Ulmaceae Mirb.
Iz_2_1009	DJI_0536	Verbascum densiflorum Bertol.	0	n	1	0	Scrophulariaceae Juss.
Iz_2_1009	DJI_0536	Veronica austriaca L.	0	n	1	0	Plantaginaceae Juss.
Iz_2_1009	DJI_0536	Veronica incana L.	0	n	1	0	Plantaginaceae Juss.
Iz_2_1009	DJI_0536	Veronica verna L.	0	n	1	0	Plantaginaceae Juss.
Iz_2_1009	DJI_0536	Vincetoxicum hirundinaria Medik	0	n	1	0	Apocynaceae Juss.
Iz_2_1009	DJI_0537	Acer tataricum L.	0	n	1	1	Sapindaceae Juss.
Iz_2_1009	DJI_0537	Achillea millefolium L. s.l.	0	n	1	0	Asteraceae Bercht. & J.Presl
Iz_2_1009	DJI_0537	Allium oleraceum L.	0	n	1	0	Amaryllidaceae J.St.-Hil.
Iz_2_1009	DJI_0537	Ambrosia artemisiifolia L.	0	a	0	0	Asteraceae Bercht. & J.Presl
Iz_2_1009	DJI_0537	Amorpha fruticosa L.	0	a	0	0	Fabaceae Lindl.
Iz_2_1009	DJI_0537	Anthericum ramosum L.	0	n	1	0	Asparagaceae Juss.
Iz_2_1009	DJI_0537	Artemisia marschalliana Spreng.	0	n	1	0	Asteraceae Bercht. & J.Presl
Iz_2_1009	DJI_0537	Asparagus officinalis L.	0	n	1	0	Asparagaceae Juss.
Iz_2_1009	DJI_0537	Astragalus cicer L.	0	n	1	0	Fabaceae Lindl.
Iz_2_1009	DJI_0537	Bassia laniflora (S.G.Gmel.) A.J.Scott	0	n	1	0	Amaranthaceae Juss.
Iz_2_1009	DJI_0537	Berberis vulgaris L.	0	n	1	0	Berberidaceae Juss.
Iz_2_1009	DJI_0537	Betula pendula Roth.	0	n	1	1	Betulaceae Gray
Iz_2_1009	DJI_0537	Betula pendula Roth.	0	n	1	0	Poaceae Barnhart
Iz_2_1009	DJI_0537	Bromus inermis Leyss.	0		NA	NA	
Iz_2_1009	DJI_0537	Bromus squarrosus L.	0	a	0	0	Poaceae Barnhart
Iz_2_1009	DJI_0537	Bromus tectorum L.	0	a	0	0	Poaceae Barnhart
Iz_2_1009	DJI_0537	Calamagrostis epigejos (L.) Roth	45	n	1	0	Poaceae Barnhart
Iz_2_1009	DJI_0537	Carduus acanthoides L.	0	n	1	0	Asteraceae Bercht. & J.Presl
Iz_2_1009	DJI_0537	Carex sp.	0	n	1	0	Cyperaceae Juss.
Iz_2_1009	DJI_0537	Centaurea majorovii Dumbadze	0	n	1	0	Asteraceae Bercht. & J.Presl
Iz_2_1009	DJI_0537	Cf. Solidago virgaurea	0		NA	NA	
Iz_2_1009	DJI_0537	Chamaecytisus ruthenicus (Fisch.ex Wol.) Klaskova	0	n	1	0	Fabaceae Lindl.

Iz_2_1009	DJI_0537	Chelidonium majus L.	0	n	1	0	Papaveraceae Juss.
Iz_2_1009	DJI_0537	Chenopodium betaceum Andrz.	0	n	1	0	Amaranthaceae Juss.
Iz_2_1009	DJI_0537	Chenopodium cf. album	0	n	1	0	Amaranthaceae Juss.
Iz_2_1009	DJI_0537	Chenopodium sp.	0		NA	0	Amaranthaceae Juss.
Iz_2_1009	DJI_0537	Chondrilla juncea L.	0	n	1	0	Asteraceae Bercht. & J.Presl
Iz_2_1009	DJI_0537	Cirsium vulgare (Savi) Ten.	0	n	1	0	Asteraceae Bercht. & J.Presl
Iz_2_1009	DJI_0537	Clinopodium acinos (L.) Kuntze	0	n	1	0	Lamiacea Lindl.
Iz_2_1009	DJI_0537	Convallaria majalis L.	0	n	1	0	Asparagaceae Juss.
Iz_2_1009	DJI_0537	Crataegus rhipidophylla Gand.	0	n	1	1	Rosaceae Juss.
Iz_2_1009	DJI_0537	Crepis foetida L.	0	n	1	0	Asteraceae Bercht. & J.Presl
Iz_2_1009	DJI_0537	Dactylis glomerata L.	0	n	1	0	Poaceae Barnhart
Iz_2_1009	DJI_0537	Dianthus campestris M.Bieb.	0	n	1	0	Caryophyllaceae Juss.
Iz_2_1009	DJI_0537	Digitaria sanguinalis (L.) Scop.	0	a	0	0	Poaceae Barnhart
Iz_2_1009	DJI_0537	Elymus repens (L.) Gould	0	n	1	0	Poaceae Barnhart
Iz_2_1009	DJI_0537	Epilobium tetragonum L.	0	n	1	0	Onagraceae Juss.
Iz_2_1009	DJI_0537	Eragrostis minor Host	0	a	0	0	Poaceae Barnhart
Iz_2_1009	DJI_0537	Erigeron canadensis L.	5	a	0	0	Asteraceae Bercht. & J.Presl
Iz_2_1009	DJI_0537	Euphorbia seguieriana Neck.	0	n	1	0	Euphorbiaceae Juss.
Iz_2_1009	DJI_0537	Fallopia dumetorum (L.) Holub	0	n	1	0	Polygonaceae Juss.
Iz_2_1009	DJI_0537	Festuca beckeri (Hack.) Trautv.	0	n	1	0	Poaceae Barnhart
Iz_2_1009	DJI_0537	Fragaria vesca L.	0	n	1	0	Rosaceae Juss.
Iz_2_1009	DJI_0537	Frangula alnus Mill.	0	n	1	1	Rhamnaceae Juss.
Iz_2_1009	DJI_0537	Fraxinus pennsylvanica Marshall	0	a	0	1	Oleaceae Hoffmanns. & Link
Iz_2_1009	DJI_0537	Galeopsis bifida Boenn.	0	n	1	0	Lamiacea Lindl.
Iz_2_1009	DJI_0537	Galium verum L.	0	n	1	0	Rubiaceae Juss.
Iz_2_1009	DJI_0537	Genista tinctoria L.	0	n	1	0	Fabaceae Lindl.
Iz_2_1009	DJI_0537	Geranium robertianum L.	0	n	1	0	Geraniaceae Juss.
Iz_2_1009	DJI_0537	Geranium sanquineum L.	0	n	1	0	Geraniaceae Juss.
Iz_2_1009	DJI_0537	Gypsophila paniculata L.	0	n	1	0	Caryophyllaceae Juss.
Iz_2_1009	DJI_0537	Helichrysum arenarium (L.) Moench	0	n	1	0	Asteraceae Bercht. & J.Presl
Iz_2_1009	DJI_0537	Herniaria polygama J.Gay	0	n	1	0	Caryophyllaceae Juss.
Iz_2_1009	DJI_0537	Hieracium robustum Fr.	0	n	1	0	Asteraceae Bercht. & J.Presl
Iz_2_1009	DJI_0537	Hieracium umbellatum L.	0	n	1	NA	Asteraceae Bercht. & J.Presl
Iz_2_1009	DJI_0537	Hylotelephium maximum (L.) Holub	0	n	1	0	Crassulaceae J.St.-Hil.
Iz_2_1009	DJI_0537	Jacobaea borysthenica (DC.) B.Nord. & Greuter	0	n	1	0	Asteraceae Bercht. & J.Presl
Iz_2_1009	DJI_0537	Jasione montana L.	0	n	1	0	Campanulaceae Juss.
Iz_2_1009	DJI_0537	Jurinea cyanoides (L.) Rchb.s.l.	0	n	1	0	Asteraceae Bercht. & J.Presl
Iz_2_1009	DJI_0537	Koeleria glauca (Spreng.) DC.	0	n	1	0	Poaceae Barnhart
Iz_2_1009	DJI_0537	Lactuca serriola L.	0	a	0	0	Asteraceae Bercht. & J.Presl
Iz_2_1009	DJI_0537	Linaria genistifolia (L.) Mill.	0	n	1	0	Plantaginaceae Juss.
Iz_2_1009	DJI_0537	Lomelosia argentea (L.) Greuter & Burdet	0	n	1	0	Caprifoliaceae Juss.
Iz_2_1009	DJI_0537	Melampyrum pratense L.	0	n	1	0	Orobanchaceae Vent.
Iz_2_1009	DJI_0537	Oenothera biennis L.	0	a	0	0	Onagraceae Juss.
Iz_2_1009	DJI_0537	Pentanema britannica (L.) D.Gut.Larr., Santos-Vicente, Anderb., E.Rico & M.M.Mart.Ort.	0	n	1	0	Asteraceae Bercht. & J.Presl
Iz_2_1009	DJI_0537	Peucedanum oreoselinum (L.) Moench	0	n	1	0	Apiaceae Lindl.
Iz_2_1009	DJI_0537	Peucedanum ruthenicum M.Bieb.	0	n	1	0	Apiaceae Lindl.
Iz_2_1009	DJI_0537	Pilosella echioides (Lumn.) F.W.Schultz & Sch.Bip.	0	n	1	0	Asteraceae Bercht. & J.Presl
Iz_2_1009	DJI_0537	Pilosella officinarum F. Schultz et Sch. Bip.	0	n	1	0	Asteraceae Bercht. & J.Presl
Iz_2_1009	DJI_0537	Pilosella sp.	0		NA	0	Asteraceae Bercht. & J.Presl
Iz_2_1009	DJI_0537	Pinus sylvestris L.	0	n	1	1	Pinaceae Spreng. ex F.Rudolphi
Iz_2_1009	DJI_0537	Poa annua L.	0	n	1	0	Poaceae Barnhart
Iz_2_1009	DJI_0537	Poa nemoralis L.	0	n	1	0	Poaceae Barnhart
Iz_2_1009	DJI_0537	Poa sp.	0		NA	0	Poaceae Barnhart
Iz_2_1009	DJI_0537	Polygonatum odoratum (Mill.) Druce	0	n	1	0	Asparagaceae Juss.
Iz_2_1009	DJI_0537	Polygonum arenarium Waldst. & Kit.	0	n	1	0	Polygonaceae Juss.
Iz_2_1009	DJI_0537	Populus nigra L.	0	n	1	1	Salicaceae Mirb.
Iz_2_1009	DJI_0537	Populus tremula L.	0	n	1	1	Salicaceae Mirb.
Iz_2_1009	DJI_0537	Populus Ã canescens (Aiton) Sm.	0	n	1	1	Salicaceae Mirb.
Iz_2_1009	DJI_0537	Potentilla argentea L.	0	n	1	0	Rosaceae Juss.
Iz_2_1009	DJI_0537	Psammophiliella muralis (L.) Ikonn.	0	n	1	0	Caryophyllaceae Juss.
Iz_2_1009	DJI_0537	Psephellus sumensis (Kalen.) Greuter	0	n	1	0	Asteraceae Bercht. & J.Presl
Iz_2_1009	DJI_0537	Pulsatilla pratensis (L.) Mill. s.l.	0	n	1	0	Ranunculaceae Juss.
Iz_2_1009	DJI_0537	Pyrus communis L.	0	a	0	1	Rosaceae Juss.
Iz_2_1009	DJI_0537	Quercus robur L.	0	n	1	1	Fagaceae Dumort.
Iz_2_1009	DJI_0537	Robinia pseudoacacia L.	0	a	0	1	Fabaceae Lindl.
Iz_2_1009	DJI_0537	Rosa sp.	0		NA	1	Rosaceae Juss.
Iz_2_1009	DJI_0537	Rubus idaeus L.	0	n	1	0	Rosaceae Juss.
Iz_2_1009	DJI_0537	Rumex acetosella L.	0	n	1	0	Polygonaceae Juss.
Iz_2_1009	DJI_0537	Rumex thyrsiflorus Fingerh.	0	n	1	0	Polygonaceae Juss.
Iz_2_1009	DJI_0537	Salix alba L.	0	n	1	1	Salicaceae Mirb.
Iz_2_1009	DJI_0537	Sambucus nigra L.	0	n	1	1	Viburnaceae Raf.
Iz_2_1009	DJI_0537	Secale sylvestre Host	0	n	1	0	Poaceae Barnhart
Iz_2_1009	DJI_0537	Sempervivum ruthenicum Schnittsp. & C.B. Lehm.	0	n	1	0	Crassulaceae J.St.-Hil.
Iz_2_1009	DJI_0537	Setaria viridis (L.) P. Beauv.	0	n	1	0	Poaceae Barnhart
Iz_2_1009	DJI_0537	Silene borysthenica (Gruner) Walters	0	n	1	0	Caryophyllaceae Juss.
Iz_2_1009	DJI_0537	Silene latifolia subsp. alba (Mill.) Greuter & Burdet	0	n	1	0	Caryophyllaceae Juss.
Iz_2_1009	DJI_0537	Solidago virgaurea L.	0.5	n	1	0	Asteraceae Bercht. & J.Presl
Iz_2_1009	DJI_0537	Sorbus aucuparia L.	0	n	1	1	Rosaceae Juss.
Iz_2_1009	DJI_0537	Stachys recta L.	0	n	1	0	Lamiacea Lindl.
Iz_2_1009	DJI_0537	Tanacetum vulgare L.	0	n	1	0	Asteraceae Bercht. & J.Presl
Iz_2_1009	DJI_0537	Thymus pallasianus Heinr.Braun	0	n	1	0	Lamiacea Lindl.
Iz_2_1009	DJI_0537	Tilia cordata Mill.	0	n	1	1	Malvaceae Juss.
Iz_2_1009	DJI_0537	Trifolium alpestre L.	0	n	1	0	Fabaceae Lindl.
Iz_2_1009	DJI_0537	Trifolium arvense L.	0	n	1	0	Fabaceae Lindl.
Iz_2_1009	DJI_0537	Ulmus glabra Huds.	0	n	1	1	Ulmaceae Mirb.
Iz_2_1009	DJI_0537	Verbascum densiflorum Bertol.	0	n	1	0	Scrophulariaceae Juss.
Iz_2_1009	DJI_0537	Veronica austriaca L.	0	n	1	0	Plantaginaceae Juss.
Iz_2_1009	DJI_0537	Veronica incana L.	0	n	1	0	Plantaginaceae Juss.
Iz_2_1009	DJI_0537	Veronica verna L.	0	n	1	0	Plantaginaceae Juss.
Iz_2_1009	DJI_0537	Vincetoxicum hirundinaria Medik	0	n	1	0	Apocynaceae Juss.
Iz_2_1009	DJI_0539	Acer tataricum L.	0	n	1	1	Sapindaceae Juss.
Iz_2_1009	DJI_0539	Achillea millefolium L. s.l.	0	n	1	0	Asteraceae Bercht. & J.Presl
Iz_2_1009	DJI_0539	Allium oleraceum L.	0	n	1	0	Amaryllidaceae J.St.-Hil.
Iz_2_1009	DJI_0539	Ambrosia artemisiifolia L.	0	a	0	0	Asteraceae Bercht. & J.Presl
Iz_2_1009	DJI_0539	Amorpha fruticosa L.	0	a	0	0	Fabaceae Lindl.
Iz_2_1009	DJI_0539	Anthericum ramosum L.	0	n	1	0	Asparagaceae Juss.
Iz_2_1009	DJI_0539	Artemisia marschalliana Spreng.	0	n	1	0	Asteraceae Bercht. & J.Presl
Iz_2_1009	DJI_0539	Asparagus officinalis L.	0	n	1	0	Asparagaceae Juss.

Iz_2_1009	DJI_0539	Astragalus cicer L.	0	n	1	0	Fabaceae Lindl.
Iz_2_1009	DJI_0539	Bassia laniflora (S.G.Gmel.) A.J.Scott	0	n	1	0	Amaranthaceae Juss.
Iz_2_1009	DJI_0539	Berberis vulgaris L.	0	n	1	0	Berberidaceae Juss.
Iz_2_1009	DJI_0539	Betula pendula Roth.	0	n	1	1	Betulaceae Gray
Iz_2_1009	DJI_0539	Betula pendula Roth.	0	n	1	0	Poaceae Barnhart
Iz_2_1009	DJI_0539	Bromus inermis Leyss.	0		NA	NA	
Iz_2_1009	DJI_0539	Bromus squarrosus L.	0	a	0	0	Poaceae Barnhart
Iz_2_1009	DJI_0539	Bromus tectorum L.	0	a	0	0	Poaceae Barnhart
Iz_2_1009	DJI_0539	Calamagrostis epigejos (L.) Roth	40	n	1	0	Poaceae Barnhart
Iz_2_1009	DJI_0539	Carduus acanthoides L.	0	n	1	0	Asteraceae Bercht. & J.Presl
Iz_2_1009	DJI_0539	Carex sp.	0	n	1	0	Cyperaceae Juss.
Iz_2_1009	DJI_0539	Centaurea majorovii Dumbadze	0	n	1	0	Asteraceae Bercht. & J.Presl
Iz_2_1009	DJI_0539	Cf. Solidago virgaurea	0		NA	NA	
Iz_2_1009	DJI_0539	Chamaecytisus ruthenicus (Fisch.ex Wol.) Klaskova	0	n	1	0	Fabaceae Lindl.
Iz_2_1009	DJI_0539	Chelidonium majus L.	0	n	1	0	Papaveraceae Juss.
Iz_2_1009	DJI_0539	Chenopodium betaceum Andrz.	0	n	1	0	Amaranthaceae Juss.
Iz_2_1009	DJI_0539	Chenopodium cf. album	0	n	1	0	Amaranthaceae Juss.
Iz_2_1009	DJI_0539	Chenopodium sp.	0		NA	0	Amaranthaceae Juss.
Iz_2_1009	DJI_0539	Chondrilla juncea L.	0	n	1	0	Asteraceae Bercht. & J.Presl
Iz_2_1009	DJI_0539	Cirsium vulgare (Savi) Ten.	0	n	1	0	Asteraceae Bercht. & J.Presl
Iz_2_1009	DJI_0539	Clinopodium acinos (L.) Kuntze	0	n	1	0	Lamiacea Lindl.
Iz_2_1009	DJI_0539	Convallaria majalis L.	0	n	1	0	Asparagaceae Juss.
Iz_2_1009	DJI_0539	Crataegus rhipidophylla Gand.	0	n	1	1	Rosaceae Juss.
Iz_2_1009	DJI_0539	Crepis foetida L.	0	n	1	0	Asteraceae Bercht. & J.Presl
Iz_2_1009	DJI_0539	Dactylis glomerata L.	0	n	1	0	Poaceae Barnhart
Iz_2_1009	DJI_0539	Dianthus campestris M.Bieb.	0	n	1	0	Caryophyllaceae Juss.
Iz_2_1009	DJI_0539	Digitaria sanguinalis (L.) Scop.	0	a	0	0	Poaceae Barnhart
Iz_2_1009	DJI_0539	Elymus repens (L.) Gould	0	n	1	0	Poaceae Barnhart
Iz_2_1009	DJI_0539	Epilobium tetragonum L.	0	n	1	0	Onagraceae Juss.
Iz_2_1009	DJI_0539	Eragrostis minor Host	0	a	0	0	Poaceae Barnhart
Iz_2_1009	DJI_0539	Erigeron canadensis L.	0	a	0	0	Asteraceae Bercht. & J.Presl
Iz_2_1009	DJI_0539	Euphorbia seguieriana Neck.	0	n	1	0	Euphorbiaceae Juss.
Iz_2_1009	DJI_0539	Fallopia dumetorum (L.) Holub	0	n	1	0	Polygonaceae Juss.
Iz_2_1009	DJI_0539	Festuca beckeri (Hack.) Trautv.	0	n	1	0	Poaceae Barnhart
Iz_2_1009	DJI_0539	Fragaria vesca L.	0	n	1	0	Rosaceae Juss.
Iz_2_1009	DJI_0539	Frangula alnus Mill.	0	n	1	1	Rhamnaceae Juss.
Iz_2_1009	DJI_0539	Fraxinus pennsylvanica Marshall	0	a	0	1	Oleaceae Hoffmanns. & Link
Iz_2_1009	DJI_0539	Galeopsis bifida Boenn.	0	n	1	0	Lamiacea Lindl.
Iz_2_1009	DJI_0539	Galium verum L.	0	n	1	0	Rubiaceae Juss.
Iz_2_1009	DJI_0539	Genista tinctoria L.	0	n	1	0	Fabaceae Lindl.
Iz_2_1009	DJI_0539	Geranium robertianum L.	0	n	1	0	Geraniaceae Juss.
Iz_2_1009	DJI_0539	Geranium sanquineum L.	0	n	1	0	Geraniaceae Juss.
Iz_2_1009	DJI_0539	Gypsophila paniculata L.	0	n	1	0	Caryophyllaceae Juss.
Iz_2_1009	DJI_0539	Helichrysum arenarium (L.) Moench	0	n	1	0	Asteraceae Bercht. & J.Presl
Iz_2_1009	DJI_0539	Herniaria polygama J.Gay	0	n	1	0	Caryophyllaceae Juss.
Iz_2_1009	DJI_0539	Hieracium robustum Fr.	0	n	1	0	Asteraceae Bercht. & J.Presl
Iz_2_1009	DJI_0539	Hieracium umbellatum L.	0	n	1	NA	Asteraceae Bercht. & J.Presl
Iz_2_1009	DJI_0539	Hylotelephium maximum (L.) Holub	0	n	1	0	Crassulaceae J.St.-Hil.
Iz_2_1009	DJI_0539	Jacobaea borysthenica (DC.) B.Nord. & Greuter	0	n	1	0	Asteraceae Bercht. & J.Presl
Iz_2_1009	DJI_0539	Jasione montana L.	0	n	1	0	Campanulaceae Juss.
Iz_2_1009	DJI_0539	Jurinea cyanoides (L.) Rchb.s.l.	0	n	1	0	Asteraceae Bercht. & J.Presl
Iz_2_1009	DJI_0539	Koeleria glauca (Spreng.) DC.	0	n	1	0	Poaceae Barnhart
Iz_2_1009	DJI_0539	Lactuca serriola L.	0	a	0	0	Asteraceae Bercht. & J.Presl
Iz_2_1009	DJI_0539	Linaria genistifolia (L.) Mill.	0	n	1	0	Plantaginaceae Juss.
Iz_2_1009	DJI_0539	Lomelosia argentea (L.) Greuter & Burdet	0	n	1	0	Caprifoliaceae Juss.
Iz_2_1009	DJI_0539	Melampyrum pratense L.	0	n	1	0	Orobanchaceae Vent.
Iz_2_1009	DJI_0539	Oenothera biennis L.	0	a	0	0	Onagraceae Juss.
Iz_2_1009	DJI_0539	Pentanema britannica (L.) D.Gut.Larr., Santos-Vicente, Anderb., E.Rico & M.M.Mart.Ort.	0	n	1	0	Asteraceae Bercht. & J.Presl
Iz_2_1009	DJI_0539	Peucedanum oreoselinum (L.) Moench	0	n	1	0	Apiaceae Lindl.
Iz_2_1009	DJI_0539	Peucedanum ruthenicum M.Bieb.	0	n	1	0	Apiaceae Lindl.
Iz_2_1009	DJI_0539	Pilosella echioides (Lumn.) F.W.Schultz & Sch.Bip.	0	n	1	0	Asteraceae Bercht. & J.Presl
Iz_2_1009	DJI_0539	Pilosella officinarum F. Schultz et Sch. Bip.	0	n	1	0	Asteraceae Bercht. & J.Presl
Iz_2_1009	DJI_0539	Pilosella sp.	0		NA	0	Asteraceae Bercht. & J.Presl
Iz_2_1009	DJI_0539	Pinus sylvestris L.	0	n	1	1	Pinaceae Spreng. ex F.Rudolphi
Iz_2_1009	DJI_0539	Poa annua L.	0	n	1	0	Poaceae Barnhart
Iz_2_1009	DJI_0539	Poa nemoralis L.	0	n	1	0	Poaceae Barnhart
Iz_2_1009	DJI_0539	Poa sp.	0		NA	0	Poaceae Barnhart
Iz_2_1009	DJI_0539	Polygonatum odoratum (Mill.) Druce	0	n	1	0	Asparagaceae Juss.
Iz_2_1009	DJI_0539	Polygonum arenarium Waldst. & Kit.	0	n	1	0	Polygonaceae Juss.
Iz_2_1009	DJI_0539	Populus nigra L.	0	n	1	1	Salicaceae Mirb.
Iz_2_1009	DJI_0539	Populus tremula L.	0	n	1	1	Salicaceae Mirb.
Iz_2_1009	DJI_0539	Populus Ã canescens (Aiton) Sm.	0	n	1	1	Salicaceae Mirb.
Iz_2_1009	DJI_0539	Potentilla argentea L.	0	n	1	0	Rosaceae Juss.
Iz_2_1009	DJI_0539	Psammophiliella muralis (L.) Ikonn.	0	n	1	0	Caryophyllaceae Juss.
Iz_2_1009	DJI_0539	Psephellus sumensis (Kalen.) Greuter	0	n	1	0	Asteraceae Bercht. & J.Presl
Iz_2_1009	DJI_0539	Pulsatilla pratensis (L.) Mill. s.l.	0	n	1	0	Ranunculaceae Juss.
Iz_2_1009	DJI_0539	Pyrus communis L.	0	a	0	1	Rosaceae Juss.
Iz_2_1009	DJI_0539	Quercus robur L.	0	n	1	1	Fagaceae Dumort.
Iz_2_1009	DJI_0539	Robinia pseudoacacia L.	0	a	0	1	Fabaceae Lindl.
Iz_2_1009	DJI_0539	Rosa sp.	0		NA	1	Rosaceae Juss.
Iz_2_1009	DJI_0539	Rubus idaeus L.	0	n	1	0	Rosaceae Juss.
Iz_2_1009	DJI_0539	Rumex acetosella L.	0	n	1	0	Polygonaceae Juss.
Iz_2_1009	DJI_0539	Rumex thyrsiflorus Fingerh.	0	n	1	0	Polygonaceae Juss.
Iz_2_1009	DJI_0539	Salix alba L.	0	n	1	1	Salicaceae Mirb.
Iz_2_1009	DJI_0539	Sambucus nigra L.	0	n	1	1	Viburnaceae Raf.
Iz_2_1009	DJI_0539	Secale sylvestre Host	0	n	1	0	Poaceae Barnhart
Iz_2_1009	DJI_0539	Sempervivum ruthenicum Schnittsp. & C.B. Lehm.	0	n	1	0	Crassulaceae J.St.-Hil.
Iz_2_1009	DJI_0539	Setaria viridis (L.) P. Beauv.	0	n	1	0	Poaceae Barnhart
Iz_2_1009	DJI_0539	Silene borysthenica (Gruner) Walters	0	n	1	0	Caryophyllaceae Juss.
Iz_2_1009	DJI_0539	Silene latifolia subsp. alba (Mill.) Greuter & Burdet	0	n	1	0	Caryophyllaceae Juss.
Iz_2_1009	DJI_0539	Solidago virgaurea L.	3	n	1	0	Asteraceae Bercht. & J.Presl
Iz_2_1009	DJI_0539	Sorbus aucuparia L.	0	n	1	1	Rosaceae Juss.
Iz_2_1009	DJI_0539	Stachys recta L.	0	n	1	0	Lamiacea Lindl.
Iz_2_1009	DJI_0539	Tanacetum vulgare L.	0	n	1	0	Asteraceae Bercht. & J.Presl
Iz_2_1009	DJI_0539	Thymus pallasianus Heinr.Braun	0	n	1	0	Lamiacea Lindl.
Iz_2_1009	DJI_0539	Tilia cordata Mill.	0	n	1	1	Malvaceae Juss.
Iz_2_1009	DJI_0539	Trifolium alpestre L.	0	n	1	0	Fabaceae Lindl.
Iz_2_1009	DJI_0539	Trifolium arvense L.	0	n	1	0	Fabaceae Lindl.

Iz_2_1009	DJI_0539	Ulmus glabra Huds.	0	n	1	1	Ulmaceae Mirb.
Iz_2_1009	DJI_0539	Verbascum densiflorum Bertol.	0	n	1	0	Scrophulariaceae Juss.
Iz_2_1009	DJI_0539	Veronica austriaca L.	0	n	1	0	Plantaginaceae Juss.
Iz_2_1009	DJI_0539	Veronica incana L.	0	n	1	0	Plantaginaceae Juss.
Iz_2_1009	DJI_0539	Veronica verna L.	0	n	1	0	Plantaginaceae Juss.
Iz_2_1009	DJI_0539	Vincetoxicum hirundinaria Medik	0	n	1	0	Apocynaceae Juss.
Iz_2_1009	DJI_0540	Acer tataricum L.	0	n	1	1	Sapindaceae Juss.
Iz_2_1009	DJI_0540	Achillea millefolium L. s.l.	0	n	1	0	Asteraceae Bercht. & J.Presl
Iz_2_1009	DJI_0540	Allium oleraceum L.	0	n	1	0	Amaryllidaceae J.St.-Hil.
Iz_2_1009	DJI_0540	Ambrosia artemisiifolia L.	0	a	0	0	Asteraceae Bercht. & J.Presl
Iz_2_1009	DJI_0540	Amorpha fruticosa L.	0	a	0	0	Fabaceae Lindl.
Iz_2_1009	DJI_0540	Anthericum ramosum L.	0	n	1	0	Asparagaceae Juss.
Iz_2_1009	DJI_0540	Artemisia marschalliana Spreng.	0	n	1	0	Asteraceae Bercht. & J.Presl
Iz_2_1009	DJI_0540	Asparagus officinalis L.	0	n	1	0	Asparagaceae Juss.
Iz_2_1009	DJI_0540	Astragalus cicer L.	0	n	1	0	Fabaceae Lindl.
Iz_2_1009	DJI_0540	Bassia laniflora (S.G.Gmel.) A.J.Scott	0	n	1	0	Amaranthaceae Juss.
Iz_2_1009	DJI_0540	Berberis vulgaris L.	0	n	1	0	Berberidaceae Juss.
Iz_2_1009	DJI_0540	Betula pendula Roth.	0	n	1	1	Betulaceae Gray
Iz_2_1009	DJI_0540	Betula pendula Roth.	0	n	1	0	Poaceae Barnhart
Iz_2_1009	DJI_0540	Bromus inermis Leyss.	0		NA	NA	
Iz_2_1009	DJI_0540	Bromus squarrosus L.	0	a	0	0	Poaceae Barnhart
Iz_2_1009	DJI_0540	Bromus tectorum L.	0	a	0	0	Poaceae Barnhart
Iz_2_1009	DJI_0540	Calamagrostis epigejos (L.) Roth	25	n	1	0	Poaceae Barnhart
Iz_2_1009	DJI_0540	Carduus acanthoides L.	0	n	1	0	Asteraceae Bercht. & J.Presl
Iz_2_1009	DJI_0540	Carex sp.	0	n	1	0	Cyperaceae Juss.
Iz_2_1009	DJI_0540	Centaurea majorovii Dumbadze	0	n	1	0	Asteraceae Bercht. & J.Presl
Iz_2_1009	DJI_0540	Cf. Solidago virgaurea	0		NA	NA	
Iz_2_1009	DJI_0540	Chamaecytisus ruthenicus (Fisch.ex Wol.) Klaskova	0	n	1	0	Fabaceae Lindl.
Iz_2_1009	DJI_0540	Chelidonium majus L.	0	n	1	0	Papaveraceae Juss.
Iz_2_1009	DJI_0540	Chenopodium betaceum Andrz.	0	n	1	0	Amaranthaceae Juss.
Iz_2_1009	DJI_0540	Chenopodium cf. album	0	n	1	0	Amaranthaceae Juss.
Iz_2_1009	DJI_0540	Chenopodium sp.	0		NA	0	Amaranthaceae Juss.
Iz_2_1009	DJI_0540	Chondrilla juncea L.	0	n	1	0	Asteraceae Bercht. & J.Presl
Iz_2_1009	DJI_0540	Cirsium vulgare (Savi) Ten.	0	n	1	0	Asteraceae Bercht. & J.Presl
Iz_2_1009	DJI_0540	Clinopodium acinos (L.) Kuntze	0	n	1	0	Lamiacea Lindl.
Iz_2_1009	DJI_0540	Convallaria majalis L.	0	n	1	0	Asparagaceae Juss.
Iz_2_1009	DJI_0540	Crataegus rhipidophylla Gand.	0	n	1	1	Rosaceae Juss.
Iz_2_1009	DJI_0540	Crepis foetida L.	0	n	1	0	Asteraceae Bercht. & J.Presl
Iz_2_1009	DJI_0540	Dactylis glomerata L.	0	n	1	0	Poaceae Barnhart
Iz_2_1009	DJI_0540	Dianthus campestris M.Bieb.	0	n	1	0	Caryophyllaceae Juss.
Iz_2_1009	DJI_0540	Digitaria sanguinalis (L.) Scop.	0	a	0	0	Poaceae Barnhart
Iz_2_1009	DJI_0540	Elymus repens (L.) Gould	0	n	1	0	Poaceae Barnhart
Iz_2_1009	DJI_0540	Epilobium tetragonum L.	0	n	1	0	Onagraceae Juss.
Iz_2_1009	DJI_0540	Eragrostis minor Host	0	a	0	0	Poaceae Barnhart
Iz_2_1009	DJI_0540	Erigeron canadensis L.	2	a	0	0	Asteraceae Bercht. & J.Presl
Iz_2_1009	DJI_0540	Euphorbia seguieriana Neck.	0	n	1	0	Euphorbiaceae Juss.
Iz_2_1009	DJI_0540	Fallopia dumetorum (L.) Holub	0	n	1	0	Polygonaceae Juss.
Iz_2_1009	DJI_0540	Festuca beckeri (Hack.) Trautv.	0	n	1	0	Poaceae Barnhart
Iz_2_1009	DJI_0540	Fragaria vesca L.	0	n	1	0	Rosaceae Juss.
Iz_2_1009	DJI_0540	Frangula alnus Mill.	0	n	1	1	Rhamnaceae Juss.
Iz_2_1009	DJI_0540	Fraxinus pennsylvanica Marshall	0	a	0	1	Oleaceae Hoffmanns. & Link
Iz_2_1009	DJI_0540	Galeopsis bifida Boenn.	0	n	1	0	Lamiacea Lindl.
Iz_2_1009	DJI_0540	Galium verum L.	0	n	1	0	Rubiaceae Juss.
Iz_2_1009	DJI_0540	Genista tinctoria L.	0	n	1	0	Fabaceae Lindl.
Iz_2_1009	DJI_0540	Geranium robertianum L.	0	n	1	0	Geraniaceae Juss.
Iz_2_1009	DJI_0540	Geranium sanquineum L.	0	n	1	0	Geraniaceae Juss.
Iz_2_1009	DJI_0540	Gypsophila paniculata L.	0	n	1	0	Caryophyllaceae Juss.
Iz_2_1009	DJI_0540	Helichrysum arenarium (L.) Moench	0	n	1	0	Asteraceae Bercht. & J.Presl
Iz_2_1009	DJI_0540	Herniaria polygama J.Gay	0	n	1	0	Caryophyllaceae Juss.
Iz_2_1009	DJI_0540	Hieracium robustum Fr.	0	n	1	0	Asteraceae Bercht. & J.Presl
Iz_2_1009	DJI_0540	Hieracium umbellatum L.	0	n	1	NA	Asteraceae Bercht. & J.Presl
Iz_2_1009	DJI_0540	Hylotelephium maximum (L.) Holub	0	n	1	0	Crassulaceae J.St.-Hil.
Iz_2_1009	DJI_0540	Jacobaea borysthenica (DC.) B.Nord. & Greuter	0	n	1	0	Asteraceae Bercht. & J.Presl
Iz_2_1009	DJI_0540	Jasione montana L.	0	n	1	0	Campanulaceae Juss.
Iz_2_1009	DJI_0540	Jurinea cyanoides (L.) Rchb.s.l.	0	n	1	0	Asteraceae Bercht. & J.Presl
Iz_2_1009	DJI_0540	Koeleria glauca (Spreng.) DC.	0	n	1	0	Poaceae Barnhart
Iz_2_1009	DJI_0540	Lactuca serriola L.	0	a	0	0	Asteraceae Bercht. & J.Presl
Iz_2_1009	DJI_0540	Linaria genistifolia (L.) Mill.	0	n	1	0	Plantaginaceae Juss.
Iz_2_1009	DJI_0540	Lomelosia argentea (L.) Greuter & Burdet	0	n	1	0	Caprifoliaceae Juss.
Iz_2_1009	DJI_0540	Melampyrum pratense L.	0	n	1	0	Orobanchaceae Vent.
Iz_2_1009	DJI_0540	Oenothera biennis L.	0	a	0	0	Onagraceae Juss.
Iz_2_1009	DJI_0540	Pentanema britannica (L.) D.Gut.Larr., Santos-Vicente, Anderb., E.Rico & M.M.Mart.Ort.	0	n	1	0	Asteraceae Bercht. & J.Presl
Iz_2_1009	DJI_0540	Peucedanum oreoselinum (L.) Moench	0	n	1	0	Apiaceae Lindl.
Iz_2_1009	DJI_0540	Peucedanum ruthenicum M.Bieb.	0	n	1	0	Apiaceae Lindl.
Iz_2_1009	DJI_0540	Pilosella echioides (Lumn.) F.W.Schultz & Sch.Bip.	0	n	1	0	Asteraceae Bercht. & J.Presl
Iz_2_1009	DJI_0540	Pilosella officinarum F. Schultz et Sch. Bip.	0	n	1	0	Asteraceae Bercht. & J.Presl
Iz_2_1009	DJI_0540	Pilosella sp.	0		NA	0	Asteraceae Bercht. & J.Presl
Iz_2_1009	DJI_0540	Pinus sylvestris L.	0	n	1	1	Pinaceae Spreng. ex F.Rudolphi
Iz_2_1009	DJI_0540	Poa annua L.	0	n	1	0	Poaceae Barnhart
Iz_2_1009	DJI_0540	Poa nemoralis L.	0	n	1	0	Poaceae Barnhart
Iz_2_1009	DJI_0540	Poa sp.	0		NA	0	Poaceae Barnhart
Iz_2_1009	DJI_0540	Polygonatum odoratum (Mill.) Druce	0	n	1	0	Asparagaceae Juss.
Iz_2_1009	DJI_0540	Polygonum arenarium Waldst. & Kit.	0	n	1	0	Polygonaceae Juss.
Iz_2_1009	DJI_0540	Populus nigra L.	0	n	1	1	Salicaceae Mirb.
Iz_2_1009	DJI_0540	Populus tremula L.	0	n	1	1	Salicaceae Mirb.
Iz_2_1009	DJI_0540	Populus Ã canescens (Aiton) Sm.	0	n	1	1	Salicaceae Mirb.
Iz_2_1009	DJI_0540	Potentilla argentea L.	0	n	1	0	Rosaceae Juss.
Iz_2_1009	DJI_0540	Psammophiliella muralis (L.) Ikonn.	0	n	1	0	Caryophyllaceae Juss.
Iz_2_1009	DJI_0540	Psephellus sumensis (Kalen.) Greuter	0	n	1	0	Asteraceae Bercht. & J.Presl
Iz_2_1009	DJI_0540	Pulsatilla pratensis (L.) Mill. s.l.	0	n	1	0	Ranunculaceae Juss.
Iz_2_1009	DJI_0540	Pyrus communis L.	0	a	0	1	Rosaceae Juss.
Iz_2_1009	DJI_0540	Quercus robur L.	0	n	1	1	Fagaceae Dumort.
Iz_2_1009	DJI_0540	Robinia pseudoacacia L.	0	a	0	1	Fabaceae Lindl.
Iz_2_1009	DJI_0540	Rosa sp.	0		NA	1	Rosaceae Juss.
Iz_2_1009	DJI_0540	Rubus idaeus L.	0	n	1	0	Rosaceae Juss.
Iz_2_1009	DJI_0540	Rumex acetosella L.	0	n	1	0	Polygonaceae Juss.
Iz_2_1009	DJI_0540	Rumex thyrsiflorus Fingerh.	0	n	1	0	Polygonaceae Juss.
Iz_2_1009	DJI_0540	Salix alba L.	0	n	1	1	Salicaceae Mirb.

Iz_2_1009	DJI_0540	Sambucus nigra L.	0	n	1	1	Viburnaceae Raf.
Iz_2_1009	DJI_0540	Secale sylvestre Host	0	n	1	0	Poaceae Barnhart
Iz_2_1009	DJI_0540	Sempervivum ruthenicum Schnittsp. & C.B. Lehm.	0	n	1	0	Crassulaceae J.St.-Hil.
Iz_2_1009	DJI_0540	Setaria viridis (L.) P. Beauv.	0	n	1	0	Poaceae Barnhart
Iz_2_1009	DJI_0540	Silene borysthenica (Gruner) Walters	0	n	1	0	Caryophyllaceae Juss.
Iz_2_1009	DJI_0540	Silene latifolia subsp. alba (Mill.) Greuter & Burdet	0	n	1	0	Caryophyllaceae Juss.
Iz_2_1009	DJI_0540	Solidago virgaurea L.	3	n	1	0	Asteraceae Bercht. & J.Presl
Iz_2_1009	DJI_0540	Sorbus aucuparia L.	0	n	1	1	Rosaceae Juss.
Iz_2_1009	DJI_0540	Stachys recta L.	0	n	1	0	Lamiacea Lindl.
Iz_2_1009	DJI_0540	Tanacetum vulgare L.	0	n	1	0	Asteraceae Bercht. & J.Presl
Iz_2_1009	DJI_0540	Thymus pallasianus Heinr.Braun	0	n	1	0	Lamiacea Lindl.
Iz_2_1009	DJI_0540	Tilia cordata Mill.	0	n	1	1	Malvaceae Juss.
Iz_2_1009	DJI_0540	Trifolium alpestre L.	0	n	1	0	Fabaceae Lindl.
Iz_2_1009	DJI_0540	Trifolium arvense L.	0	n	1	0	Fabaceae Lindl.
Iz_2_1009	DJI_0540	Ulmus glabra Huds.	0	n	1	1	Ulmaceae Mirb.
Iz_2_1009	DJI_0540	Verbascum densiflorum Bertol.	0	n	1	0	Scrophulariaceae Juss.
Iz_2_1009	DJI_0540	Veronica austriaca L.	0	n	1	0	Plantaginaceae Juss.
Iz_2_1009	DJI_0540	Veronica incana L.	0	n	1	0	Plantaginaceae Juss.
Iz_2_1009	DJI_0540	Veronica verna L.	0	n	1	0	Plantaginaceae Juss.
Iz_2_1009	DJI_0540	Vincetoxicum hirundinaria Medik	0	n	1	0	Apocynaceae Juss.
Iz_2_1009	DJI_0542	Acer tataricum L.	0	n	1	1	Sapindaceae Juss.
Iz_2_1009	DJI_0542	Achillea millefolium L. s.l.	0	n	1	0	Asteraceae Bercht. & J.Presl
Iz_2_1009	DJI_0542	Allium oleraceum L.	0	n	1	0	Amaryllidaceae J.St.-Hil.
Iz_2_1009	DJI_0542	Ambrosia artemisiifolia L.	0	a	0	0	Asteraceae Bercht. & J.Presl
Iz_2_1009	DJI_0542	Amorpha fruticosa L.	0	a	0	0	Fabaceae Lindl.
Iz_2_1009	DJI_0542	Anthericum ramosum L.	0	n	1	0	Asparagaceae Juss.
Iz_2_1009	DJI_0542	Artemisia marschalliana Spreng.	0	n	1	0	Asteraceae Bercht. & J.Presl
Iz_2_1009	DJI_0542	Asparagus officinalis L.	0	n	1	0	Asparagaceae Juss.
Iz_2_1009	DJI_0542	Astragalus cicer L.	0	n	1	0	Fabaceae Lindl.
Iz_2_1009	DJI_0542	Bassia laniflora (S.G.Gmel.) A.J.Scott	0	n	1	0	Amaranthaceae Juss.
Iz_2_1009	DJI_0542	Berberis vulgaris L.	0	n	1	0	Berberidaceae Juss.
Iz_2_1009	DJI_0542	Betula pendula Roth.	0	n	1	1	Betulaceae Gray
Iz_2_1009	DJI_0542	Betula pendula Roth.	0	n	1	0	Poaceae Barnhart
Iz_2_1009	DJI_0542	Bromus inermis Leyss.	0		NA	NA	
Iz_2_1009	DJI_0542	Bromus squarrosus L.	0	a	0	0	Poaceae Barnhart
Iz_2_1009	DJI_0542	Bromus tectorum L.	0	a	0	0	Poaceae Barnhart
Iz_2_1009	DJI_0542	Calamagrostis epigejos (L.) Roth	20	n	1	0	Poaceae Barnhart
Iz_2_1009	DJI_0542	Carduus acanthoides L.	0	n	1	0	Asteraceae Bercht. & J.Presl
Iz_2_1009	DJI_0542	Carex sp.	0	n	1	0	Cyperaceae Juss.
Iz_2_1009	DJI_0542	Centaurea majorovii Dumbadze	0	n	1	0	Asteraceae Bercht. & J.Presl
Iz_2_1009	DJI_0542	Cf. Solidago virgaurea	0		NA	NA	
Iz_2_1009	DJI_0542	Chamaecytisus ruthenicus (Fisch.ex Wol.) Klaskova	0	n	1	0	Fabaceae Lindl.
Iz_2_1009	DJI_0542	Chelidonium majus L.	0	n	1	0	Papaveraceae Juss.
Iz_2_1009	DJI_0542	Chenopodium betaceum Andrz.	0	n	1	0	Amaranthaceae Juss.
Iz_2_1009	DJI_0542	Chenopodium cf. album	0	n	1	0	Amaranthaceae Juss.
Iz_2_1009	DJI_0542	Chenopodium sp.	0		NA	0	Amaranthaceae Juss.
Iz_2_1009	DJI_0542	Chondrilla juncea L.	0	n	1	0	Asteraceae Bercht. & J.Presl
Iz_2_1009	DJI_0542	Cirsium vulgare (Savi) Ten.	0	n	1	0	Asteraceae Bercht. & J.Presl
Iz_2_1009	DJI_0542	Clinopodium acinos (L.) Kuntze	0	n	1	0	Lamiacea Lindl.
Iz_2_1009	DJI_0542	Convallaria majalis L.	0	n	1	0	Asparagaceae Juss.
Iz_2_1009	DJI_0542	Crataegus rhipidophylla Gand.	0	n	1	1	Rosaceae Juss.
Iz_2_1009	DJI_0542	Crepis foetida L.	0	n	1	0	Asteraceae Bercht. & J.Presl
Iz_2_1009	DJI_0542	Dactylis glomerata L.	0	n	1	0	Poaceae Barnhart
Iz_2_1009	DJI_0542	Dianthus campestris M.Bieb.	0	n	1	0	Caryophyllaceae Juss.
Iz_2_1009	DJI_0542	Digitaria sanguinalis (L.) Scop.	0	a	0	0	Poaceae Barnhart
Iz_2_1009	DJI_0542	Elymus repens (L.) Gould	0	n	1	0	Poaceae Barnhart
Iz_2_1009	DJI_0542	Epilobium tetragonum L.	0	n	1	0	Onagraceae Juss.
Iz_2_1009	DJI_0542	Eragrostis minor Host	0	a	0	0	Poaceae Barnhart
Iz_2_1009	DJI_0542	Erigeron canadensis L.	8	a	0	0	Asteraceae Bercht. & J.Presl
Iz_2_1009	DJI_0542	Euphorbia seguieriana Neck.	0	n	1	0	Euphorbiaceae Juss.
Iz_2_1009	DJI_0542	Fallopia dumetorum (L.) Holub	0	n	1	0	Polygonaceae Juss.
Iz_2_1009	DJI_0542	Festuca beckeri (Hack.) Trautv.	0	n	1	0	Poaceae Barnhart
Iz_2_1009	DJI_0542	Fragaria vesca L.	0	n	1	0	Rosaceae Juss.
Iz_2_1009	DJI_0542	Frangula alnus Mill.	0	n	1	1	Rhamnaceae Juss.
Iz_2_1009	DJI_0542	Fraxinus pennsylvanica Marshall	0	a	0	1	Oleaceae Hoffmanns. & Link
Iz_2_1009	DJI_0542	Galeopsis bifida Boenn.	0	n	1	0	Lamiacea Lindl.
Iz_2_1009	DJI_0542	Galium verum L.	0	n	1	0	Rubiaceae Juss.
Iz_2_1009	DJI_0542	Genista tinctoria L.	0	n	1	0	Fabaceae Lindl.
Iz_2_1009	DJI_0542	Geranium robertianum L.	0	n	1	0	Geraniaceae Juss.
Iz_2_1009	DJI_0542	Geranium sanquineum L.	0	n	1	0	Geraniaceae Juss.
Iz_2_1009	DJI_0542	Gypsophila paniculata L.	0	n	1	0	Caryophyllaceae Juss.
Iz_2_1009	DJI_0542	Helichrysum arenarium (L.) Moench	0	n	1	0	Asteraceae Bercht. & J.Presl
Iz_2_1009	DJI_0542	Herniaria polygama J.Gay	0	n	1	0	Caryophyllaceae Juss.
Iz_2_1009	DJI_0542	Hieracium robustum Fr.	0	n	1	0	Asteraceae Bercht. & J.Presl
Iz_2_1009	DJI_0542	Hieracium umbellatum L.	0	n	1	NA	Asteraceae Bercht. & J.Presl
Iz_2_1009	DJI_0542	Hylotelephium maximum (L.) Holub	0	n	1	0	Crassulaceae J.St.-Hil.
Iz_2_1009	DJI_0542	Jacobaea borysthenica (DC.) B.Nord. & Greuter	0	n	1	0	Asteraceae Bercht. & J.Presl
Iz_2_1009	DJI_0542	Jasione montana L.	0	n	1	0	Campanulaceae Juss.
Iz_2_1009	DJI_0542	Jurinea cyanoides (L.) Rchb.s.l.	0	n	1	0	Asteraceae Bercht. & J.Presl
Iz_2_1009	DJI_0542	Koeleria glauca (Spreng.) DC.	0	n	1	0	Poaceae Barnhart
Iz_2_1009	DJI_0542	Lactuca serriola L.	0	a	0	0	Asteraceae Bercht. & J.Presl
Iz_2_1009	DJI_0542	Linaria genistifolia (L.) Mill.	0	n	1	0	Plantaginaceae Juss.
Iz_2_1009	DJI_0542	Lomelosia argentea (L.) Greuter & Burdet	0	n	1	0	Caprifoliaceae Juss.
Iz_2_1009	DJI_0542	Melampyrum pratense L.	0	n	1	0	Orobanchaceae Vent.
Iz_2_1009	DJI_0542	Oenothera biennis L.	0	a	0	0	Onagraceae Juss.
Iz_2_1009	DJI_0542	Pentanema britannica (L.) D.Gut.Larr., Santos-Vicente, Anderb., E.Rico & M.M.Mart.Ort.	0	n	1	0	Asteraceae Bercht. & J.Presl
Iz_2_1009	DJI_0542	Peucedanum oreoselinum (L.) Moench	0	n	1	0	Apiaceae Lindl.
Iz_2_1009	DJI_0542	Peucedanum ruthenicum M.Bieb.	0	n	1	0	Apiaceae Lindl.
Iz_2_1009	DJI_0542	Pilosella echioides (Lumn.) F.W.Schultz & Sch.Bip.	0	n	1	0	Asteraceae Bercht. & J.Presl
Iz_2_1009	DJI_0542	Pilosella officinarum F. Schultz et Sch. Bip.	0	n	1	0	Asteraceae Bercht. & J.Presl
Iz_2_1009	DJI_0542	Pilosella sp.	0		NA	0	Asteraceae Bercht. & J.Presl
Iz_2_1009	DJI_0542	Pinus sylvestris L.	0	n	1	1	Pinaceae Spreng. ex F.Rudolphi
Iz_2_1009	DJI_0542	Poa annua L.	0	n	1	0	Poaceae Barnhart
Iz_2_1009	DJI_0542	Poa nemoralis L.	0	n	1	0	Poaceae Barnhart
Iz_2_1009	DJI_0542	Poa sp.	0		NA	0	Poaceae Barnhart
Iz_2_1009	DJI_0542	Polygonatum odoratum (Mill.) Druce	0	n	1	0	Asparagaceae Juss.
Iz_2_1009	DJI_0542	Polygonum arenarium Waldst. & Kit.	0	n	1	0	Polygonaceae Juss.
Iz_2_1009	DJI_0542	Populus nigra L.	0	n	1	1	Salicaceae Mirb.

Iz_2_1009	DJI_0542	Populus tremula L.	0	n	1	1	Salicaceae Mirb.
Iz_2_1009	DJI_0542	Populus Ã canescens (Aiton) Sm.	0	n	1	1	Salicaceae Mirb.
Iz_2_1009	DJI_0542	Potentilla argentea L.	0	n	1	0	Rosaceae Juss.
Iz_2_1009	DJI_0542	Psammophiliella muralis (L.) Ikonn.	0	n	1	0	Caryophyllaceae Juss.
Iz_2_1009	DJI_0542	Psephellus sumensis (Kalen.) Greuter	0	n	1	0	Asteraceae Bercht. & J.Presl
Iz_2_1009	DJI_0542	Pulsatilla pratensis (L.) Mill. s.l.	0	n	1	0	Ranunculaceae Juss.
Iz_2_1009	DJI_0542	Pyrus communis L.	0	a	0	1	Rosaceae Juss.
Iz_2_1009	DJI_0542	Quercus robur L.	0	n	1	1	Fagaceae Dumort.
Iz_2_1009	DJI_0542	Robinia pseudoacacia L.	0	a	0	1	Fabaceae Lindl.
Iz_2_1009	DJI_0542	Rosa sp.	0		NA	1	Rosaceae Juss.
Iz_2_1009	DJI_0542	Rubus idaeus L.	0	n	1	0	Rosaceae Juss.
Iz_2_1009	DJI_0542	Rumex acetosella L.	0	n	1	0	Polygonaceae Juss.
Iz_2_1009	DJI_0542	Rumex thyrsiflorus Fingerh.	0	n	1	0	Polygonaceae Juss.
Iz_2_1009	DJI_0542	Salix alba L.	0	n	1	1	Salicaceae Mirb.
Iz_2_1009	DJI_0542	Sambucus nigra L.	0	n	1	1	Viburnaceae Raf.
Iz_2_1009	DJI_0542	Secale sylvestre Host	0	n	1	0	Poaceae Barnhart
Iz_2_1009	DJI_0542	Sempervivum ruthenicum Schnittsp. & C.B. Lehm.	0	n	1	0	Crassulaceae J.St.-Hil.
Iz_2_1009	DJI_0542	Setaria viridis (L.) P. Beauv.	0	n	1	0	Poaceae Barnhart
Iz_2_1009	DJI_0542	Silene borysthenica (Gruner) Walters	0	n	1	0	Caryophyllaceae Juss.
Iz_2_1009	DJI_0542	Silene latifolia subsp. alba (Mill.) Greuter & Burdet	0	n	1	0	Caryophyllaceae Juss.
Iz_2_1009	DJI_0542	Solidago virgaurea L.	1	n	1	0	Asteraceae Bercht. & J.Presl
Iz_2_1009	DJI_0542	Sorbus aucuparia L.	0	n	1	1	Rosaceae Juss.
Iz_2_1009	DJI_0542	Stachys recta L.	0	n	1	0	Lamiacea Lindl.
Iz_2_1009	DJI_0542	Tanacetum vulgare L.	0	n	1	0	Asteraceae Bercht. & J.Presl
Iz_2_1009	DJI_0542	Thymus pallasianus Heinr.Braun	0	n	1	0	Lamiacea Lindl.
Iz_2_1009	DJI_0542	Tilia cordata Mill.	0	n	1	1	Malvaceae Juss.
Iz_2_1009	DJI_0542	Trifolium alpestre L.	0	n	1	0	Fabaceae Lindl.
Iz_2_1009	DJI_0542	Trifolium arvense L.	0	n	1	0	Fabaceae Lindl.
Iz_2_1009	DJI_0542	Ulmus glabra Huds.	0	n	1	1	Ulmaceae Mirb.
Iz_2_1009	DJI_0542	Verbascum densiflorum Bertol.	0	n	1	0	Scrophulariaceae Juss.
Iz_2_1009	DJI_0542	Veronica austriaca L.	0	n	1	0	Plantaginaceae Juss.
Iz_2_1009	DJI_0542	Veronica incana L.	0	n	1	0	Plantaginaceae Juss.
Iz_2_1009	DJI_0542	Veronica verna L.	0	n	1	0	Plantaginaceae Juss.
Iz_2_1009	DJI_0542	Vincetoxicum hirundinaria Medik	0	n	1	0	Apocynaceae Juss.
Iz_2_1009	DJI_0545	Acer tataricum L.	0	n	1	1	Sapindaceae Juss.
Iz_2_1009	DJI_0545	Achillea millefolium L. s.l.	0	n	1	0	Asteraceae Bercht. & J.Presl
Iz_2_1009	DJI_0545	Allium oleraceum L.	0	n	1	0	Amaryllidaceae J.St.-Hil.
Iz_2_1009	DJI_0545	Ambrosia artemisiifolia L.	0	a	0	0	Asteraceae Bercht. & J.Presl
Iz_2_1009	DJI_0545	Amorpha fruticosa L.	0	a	0	0	Fabaceae Lindl.
Iz_2_1009	DJI_0545	Anthericum ramosum L.	0	n	1	0	Asparagaceae Juss.
Iz_2_1009	DJI_0545	Artemisia marschalliana Spreng.	0	n	1	0	Asteraceae Bercht. & J.Presl
Iz_2_1009	DJI_0545	Asparagus officinalis L.	0	n	1	0	Asparagaceae Juss.
Iz_2_1009	DJI_0545	Astragalus cicer L.	0	n	1	0	Fabaceae Lindl.
Iz_2_1009	DJI_0545	Bassia laniflora (S.G.Gmel.) A.J.Scott	0	n	1	0	Amaranthaceae Juss.
Iz_2_1009	DJI_0545	Berberis vulgaris L.	0	n	1	0	Berberidaceae Juss.
Iz_2_1009	DJI_0545	Betula pendula Roth.	0	n	1	1	Betulaceae Gray
Iz_2_1009	DJI_0545	Betula pendula Roth.	0	n	1	0	Poaceae Barnhart
Iz_2_1009	DJI_0545	Bromus inermis Leyss.	0		NA	NA	
Iz_2_1009	DJI_0545	Bromus squarrosus L.	0	a	0	0	Poaceae Barnhart
Iz_2_1009	DJI_0545	Bromus tectorum L.	0	a	0	0	Poaceae Barnhart
Iz_2_1009	DJI_0545	Calamagrostis epigejos (L.) Roth	30	n	1	0	Poaceae Barnhart
Iz_2_1009	DJI_0545	Carduus acanthoides L.	0	n	1	0	Asteraceae Bercht. & J.Presl
Iz_2_1009	DJI_0545	Carex sp.	0	n	1	0	Cyperaceae Juss.
Iz_2_1009	DJI_0545	Centaurea majorovii Dumbadze	0	n	1	0	Asteraceae Bercht. & J.Presl
Iz_2_1009	DJI_0545	Cf. Solidago virgaurea	0		NA	NA	
Iz_2_1009	DJI_0545	Chamaecytisus ruthenicus (Fisch.ex Wol.) Klaskova	0	n	1	0	Fabaceae Lindl.
Iz_2_1009	DJI_0545	Chelidonium majus L.	0	n	1	0	Papaveraceae Juss.
Iz_2_1009	DJI_0545	Chenopodium betaceum Andrz.	0	n	1	0	Amaranthaceae Juss.
Iz_2_1009	DJI_0545	Chenopodium cf. album	0	n	1	0	Amaranthaceae Juss.
Iz_2_1009	DJI_0545	Chenopodium sp.	0		NA	0	Amaranthaceae Juss.
Iz_2_1009	DJI_0545	Chondrilla juncea L.	0	n	1	0	Asteraceae Bercht. & J.Presl
Iz_2_1009	DJI_0545	Cirsium vulgare (Savi) Ten.	0	n	1	0	Asteraceae Bercht. & J.Presl
Iz_2_1009	DJI_0545	Clinopodium acinos (L.) Kuntze	0	n	1	0	Lamiacea Lindl.
Iz_2_1009	DJI_0545	Convallaria majalis L.	0	n	1	0	Asparagaceae Juss.
Iz_2_1009	DJI_0545	Crataegus rhipidophylla Gand.	0	n	1	1	Rosaceae Juss.
Iz_2_1009	DJI_0545	Crepis foetida L.	0	n	1	0	Asteraceae Bercht. & J.Presl
Iz_2_1009	DJI_0545	Dactylis glomerata L.	0	n	1	0	Poaceae Barnhart
Iz_2_1009	DJI_0545	Dianthus campestris M.Bieb.	0	n	1	0	Caryophyllaceae Juss.
Iz_2_1009	DJI_0545	Digitaria sanguinalis (L.) Scop.	0	a	0	0	Poaceae Barnhart
Iz_2_1009	DJI_0545	Elymus repens (L.) Gould	0	n	1	0	Poaceae Barnhart
Iz_2_1009	DJI_0545	Epilobium tetragonum L.	0	n	1	0	Onagraceae Juss.
Iz_2_1009	DJI_0545	Eragrostis minor Host	0	a	0	0	Poaceae Barnhart
Iz_2_1009	DJI_0545	Erigeron canadensis L.	5	a	0	0	Asteraceae Bercht. & J.Presl
Iz_2_1009	DJI_0545	Euphorbia seguieriana Neck.	0	n	1	0	Euphorbiaceae Juss.
Iz_2_1009	DJI_0545	Fallopia dumetorum (L.) Holub	0	n	1	0	Polygonaceae Juss.
Iz_2_1009	DJI_0545	Festuca beckeri (Hack.) Trautv.	0	n	1	0	Poaceae Barnhart
Iz_2_1009	DJI_0545	Fragaria vesca L.	0	n	1	0	Rosaceae Juss.
Iz_2_1009	DJI_0545	Frangula alnus Mill.	0	n	1	1	Rhamnaceae Juss.
Iz_2_1009	DJI_0545	Fraxinus pennsylvanica Marshall	0	a	0	1	Oleaceae Hoffmanns. & Link
Iz_2_1009	DJI_0545	Galeopsis bifida Boenn.	0	n	1	0	Lamiacea Lindl.
Iz_2_1009	DJI_0545	Galium verum L.	0	n	1	0	Rubiaceae Juss.
Iz_2_1009	DJI_0545	Genista tinctoria L.	0	n	1	0	Fabaceae Lindl.
Iz_2_1009	DJI_0545	Geranium robertianum L.	0	n	1	0	Geraniaceae Juss.
Iz_2_1009	DJI_0545	Geranium sanquineum L.	0	n	1	0	Geraniaceae Juss.
Iz_2_1009	DJI_0545	Gypsophila paniculata L.	0	n	1	0	Caryophyllaceae Juss.
Iz_2_1009	DJI_0545	Helichrysum arenarium (L.) Moench	0	n	1	0	Asteraceae Bercht. & J.Presl
Iz_2_1009	DJI_0545	Herniaria polygama J.Gay	0	n	1	0	Caryophyllaceae Juss.
Iz_2_1009	DJI_0545	Hieracium robustum Fr.	0	n	1	0	Asteraceae Bercht. & J.Presl
Iz_2_1009	DJI_0545	Hieracium umbellatum L.	0	n	1	NA	Asteraceae Bercht. & J.Presl
Iz_2_1009	DJI_0545	Hylotelephium maximum (L.) Holub	0	n	1	0	Crassulaceae J.St.-Hil.
Iz_2_1009	DJI_0545	Jacobaea borysthenica (DC.) B.Nord. & Greuter	0	n	1	0	Asteraceae Bercht. & J.Presl
Iz_2_1009	DJI_0545	Jasione montana L.	0	n	1	0	Campanulaceae Juss.
Iz_2_1009	DJI_0545	Jurinea cyanoides (L.) Rchb.s.l.	0	n	1	0	Asteraceae Bercht. & J.Presl
Iz_2_1009	DJI_0545	Koeleria glauca (Spreng.) DC.	0	n	1	0	Poaceae Barnhart
Iz_2_1009	DJI_0545	Lactuca serriola L.	0	a	0	0	Asteraceae Bercht. & J.Presl
Iz_2_1009	DJI_0545	Linaria genistifolia (L.) Mill.	0	n	1	0	Plantaginaceae Juss.
Iz_2_1009	DJI_0545	Lomelosia argentea (L.) Greuter & Burdet	0	n	1	0	Caprifoliaceae Juss.
Iz_2_1009	DJI_0545	Melampyrum pratense L.	0	n	1	0	Orobanchaceae Vent.

Iz_2_1009	DJI_0545	Oenothera biennis L.	0	a	0	0	Onagraceae Juss.
Iz_2_1009	DJI_0545	Pentanema britannica (L.) D.Gut.Larr., Santos-Vicente, Anderb., E.Rico & M.M.Mart.Ort.	0	n	1	0	Asteraceae Bercht. & J.Presl
Iz_2_1009	DJI_0545	Peucedanum oreoselinum (L.) Moench	0	n	1	0	Apiaceae Lindl.
Iz_2_1009	DJI_0545	Peucedanum ruthenicum M.Bieb.	0	n	1	0	Apiaceae Lindl.
Iz_2_1009	DJI_0545	Pilosella echioides (Lumn.) F.W.Schultz & Sch.Bip.	0	n	1	0	Asteraceae Bercht. & J.Presl
Iz_2_1009	DJI_0545	Pilosella officinarum F. Schultz et Sch. Bip.	0	n	1	0	Asteraceae Bercht. & J.Presl
Iz_2_1009	DJI_0545	Pilosella sp.	0		NA	0	Asteraceae Bercht. & J.Presl
Iz_2_1009	DJI_0545	Pinus sylvestris L.	0	n	1	1	Pinaceae Spreng. ex F.Rudolphi
Iz_2_1009	DJI_0545	Poa annua L.	0	n	1	0	Poaceae Barnhart
Iz_2_1009	DJI_0545	Poa nemoralis L.	0	n	1	0	Poaceae Barnhart
Iz_2_1009	DJI_0545	Poa sp.	0		NA	0	Poaceae Barnhart
Iz_2_1009	DJI_0545	Polygonatum odoratum (Mill.) Druce	0	n	1	0	Asparagaceae Juss.
Iz_2_1009	DJI_0545	Polygonum arenarium Waldst. & Kit.	0	n	1	0	Polygonaceae Juss.
Iz_2_1009	DJI_0545	Populus nigra L.	0	n	1	1	Salicaceae Mirb.
Iz_2_1009	DJI_0545	Populus tremula L.	0	n	1	1	Salicaceae Mirb.
Iz_2_1009	DJI_0545	Populus Ã canescens (Aiton) Sm.	0	n	1	1	Salicaceae Mirb.
Iz_2_1009	DJI_0545	Potentilla argentea L.	0	n	1	0	Rosaceae Juss.
Iz_2_1009	DJI_0545	Psammophiliella muralis (L.) Ikonn.	0	n	1	0	Caryophyllaceae Juss.
Iz_2_1009	DJI_0545	Psephellus sumensis (Kalen.) Greuter	0	n	1	0	Asteraceae Bercht. & J.Presl
Iz_2_1009	DJI_0545	Pulsatilla pratensis (L.) Mill. s.l.	0	n	1	0	Ranunculaceae Juss.
Iz_2_1009	DJI_0545	Pyrus communis L.	0	a	0	1	Rosaceae Juss.
Iz_2_1009	DJI_0545	Quercus robur L.	0	n	1	1	Fagaceae Dumort.
Iz_2_1009	DJI_0545	Robinia pseudoacacia L.	0	a	0	1	Fabaceae Lindl.
Iz_2_1009	DJI_0545	Rosa sp.	0		NA	1	Rosaceae Juss.
Iz_2_1009	DJI_0545	Rubus idaeus L.	0	n	1	0	Rosaceae Juss.
Iz_2_1009	DJI_0545	Rumex acetosella L.	0	n	1	0	Polygonaceae Juss.
Iz_2_1009	DJI_0545	Rumex thyrsiflorus Fingerh.	0	n	1	0	Polygonaceae Juss.
Iz_2_1009	DJI_0545	Salix alba L.	0	n	1	1	Salicaceae Mirb.
Iz_2_1009	DJI_0545	Sambucus nigra L.	0	n	1	1	Viburnaceae Raf.
Iz_2_1009	DJI_0545	Secale sylvestre Host	0	n	1	0	Poaceae Barnhart
Iz_2_1009	DJI_0545	Sempervivum ruthenicum Schnittsp. & C.B. Lehm.	0	n	1	0	Crassulaceae J.St.-Hil.
Iz_2_1009	DJI_0545	Setaria viridis (L.) P. Beauv.	0	n	1	0	Poaceae Barnhart
Iz_2_1009	DJI_0545	Silene borysthenica (Gruner) Walters	0	n	1	0	Caryophyllaceae Juss.
Iz_2_1009	DJI_0545	Silene latifolia subsp. alba (Mill.) Greuter & Burdet	0	n	1	0	Caryophyllaceae Juss.
Iz_2_1009	DJI_0545	Solidago virgaurea L.	2	n	1	0	Asteraceae Bercht. & J.Presl
Iz_2_1009	DJI_0545	Sorbus aucuparia L.	0	n	1	1	Rosaceae Juss.
Iz_2_1009	DJI_0545	Stachys recta L.	0	n	1	0	Lamiacea Lindl.
Iz_2_1009	DJI_0545	Tanacetum vulgare L.	0	n	1	0	Asteraceae Bercht. & J.Presl
Iz_2_1009	DJI_0545	Thymus pallasianus Heinr.Braun	0	n	1	0	Lamiacea Lindl.
Iz_2_1009	DJI_0545	Tilia cordata Mill.	0	n	1	1	Malvaceae Juss.
Iz_2_1009	DJI_0545	Trifolium alpestre L.	0	n	1	0	Fabaceae Lindl.
Iz_2_1009	DJI_0545	Trifolium arvense L.	0	n	1	0	Fabaceae Lindl.
Iz_2_1009	DJI_0545	Ulmus glabra Huds.	0	n	1	1	Ulmaceae Mirb.
Iz_2_1009	DJI_0545	Verbascum densiflorum Bertol.	0	n	1	0	Scrophulariaceae Juss.
Iz_2_1009	DJI_0545	Veronica austriaca L.	0	n	1	0	Plantaginaceae Juss.
Iz_2_1009	DJI_0545	Veronica incana L.	0	n	1	0	Plantaginaceae Juss.
Iz_2_1009	DJI_0545	Veronica verna L.	0	n	1	0	Plantaginaceae Juss.
Iz_2_1009	DJI_0545	Vincetoxicum hirundinaria Medik	0	n	1	0	Apocynaceae Juss.
Iz_2_1009	DJI_0547	Acer tataricum L.	0	n	1	1	Sapindaceae Juss.
Iz_2_1009	DJI_0547	Achillea millefolium L. s.l.	0	n	1	0	Asteraceae Bercht. & J.Presl
Iz_2_1009	DJI_0547	Allium oleraceum L.	0	n	1	0	Amaryllidaceae J.St.-Hil.
Iz_2_1009	DJI_0547	Ambrosia artemisiifolia L.	0	a	0	0	Asteraceae Bercht. & J.Presl
Iz_2_1009	DJI_0547	Amorpha fruticosa L.	0	a	0	0	Fabaceae Lindl.
Iz_2_1009	DJI_0547	Anthericum ramosum L.	0	n	1	0	Asparagaceae Juss.
Iz_2_1009	DJI_0547	Artemisia marschalliana Spreng.	0	n	1	0	Asteraceae Bercht. & J.Presl
Iz_2_1009	DJI_0547	Asparagus officinalis L.	0	n	1	0	Asparagaceae Juss.
Iz_2_1009	DJI_0547	Astragalus cicer L.	0	n	1	0	Fabaceae Lindl.
Iz_2_1009	DJI_0547	Bassia laniflora (S.G.Gmel.) A.J.Scott	0	n	1	0	Amaranthaceae Juss.
Iz_2_1009	DJI_0547	Berberis vulgaris L.	0	n	1	0	Berberidaceae Juss.
Iz_2_1009	DJI_0547	Betula pendula Roth.	0	n	1	1	Betulaceae Gray
Iz_2_1009	DJI_0547	Betula pendula Roth.	0	n	1	0	Poaceae Barnhart
Iz_2_1009	DJI_0547	Bromus inermis Leyss.	0		NA	NA	
Iz_2_1009	DJI_0547	Bromus squarrosus L.	0	a	0	0	Poaceae Barnhart
Iz_2_1009	DJI_0547	Bromus tectorum L.	0	a	0	0	Poaceae Barnhart
Iz_2_1009	DJI_0547	Calamagrostis epigejos (L.) Roth	60	n	1	0	Poaceae Barnhart
Iz_2_1009	DJI_0547	Carduus acanthoides L.	0	n	1	0	Asteraceae Bercht. & J.Presl
Iz_2_1009	DJI_0547	Carex sp.	0	n	1	0	Cyperaceae Juss.
Iz_2_1009	DJI_0547	Centaurea majorovii Dumbadze	0	n	1	0	Asteraceae Bercht. & J.Presl
Iz_2_1009	DJI_0547	Cf. Solidago virgaurea	0		NA	NA	
Iz_2_1009	DJI_0547	Chamaecytisus ruthenicus (Fisch.ex Wol.) Klaskova	0	n	1	0	Fabaceae Lindl.
Iz_2_1009	DJI_0547	Chelidonium majus L.	0	n	1	0	Papaveraceae Juss.
Iz_2_1009	DJI_0547	Chenopodium betaceum Andrz.	0	n	1	0	Amaranthaceae Juss.
Iz_2_1009	DJI_0547	Chenopodium cf. album	0	n	1	0	Amaranthaceae Juss.
Iz_2_1009	DJI_0547	Chenopodium sp.	0		NA	0	Amaranthaceae Juss.
Iz_2_1009	DJI_0547	Chondrilla juncea L.	0	n	1	0	Asteraceae Bercht. & J.Presl
Iz_2_1009	DJI_0547	Cirsium vulgare (Savi) Ten.	0	n	1	0	Asteraceae Bercht. & J.Presl
Iz_2_1009	DJI_0547	Clinopodium acinos (L.) Kuntze	0	n	1	0	Lamiacea Lindl.
Iz_2_1009	DJI_0547	Convallaria majalis L.	0	n	1	0	Asparagaceae Juss.
Iz_2_1009	DJI_0547	Crataegus rhipidophylla Gand.	0	n	1	1	Rosaceae Juss.
Iz_2_1009	DJI_0547	Crepis foetida L.	0	n	1	0	Asteraceae Bercht. & J.Presl
Iz_2_1009	DJI_0547	Dactylis glomerata L.	0	n	1	0	Poaceae Barnhart
Iz_2_1009	DJI_0547	Dianthus campestris M.Bieb.	0	n	1	0	Caryophyllaceae Juss.
Iz_2_1009	DJI_0547	Digitaria sanguinalis (L.) Scop.	0	a	0	0	Poaceae Barnhart
Iz_2_1009	DJI_0547	Elymus repens (L.) Gould	0	n	1	0	Poaceae Barnhart
Iz_2_1009	DJI_0547	Epilobium tetragonum L.	0	n	1	0	Onagraceae Juss.
Iz_2_1009	DJI_0547	Eragrostis minor Host	0	a	0	0	Poaceae Barnhart
Iz_2_1009	DJI_0547	Erigeron canadensis L.	0	a	0	0	Asteraceae Bercht. & J.Presl
Iz_2_1009	DJI_0547	Euphorbia seguieriana Neck.	0	n	1	0	Euphorbiaceae Juss.
Iz_2_1009	DJI_0547	Fallopia dumetorum (L.) Holub	0	n	1	0	Polygonaceae Juss.
Iz_2_1009	DJI_0547	Festuca beckeri (Hack.) Trautv.	0	n	1	0	Poaceae Barnhart
Iz_2_1009	DJI_0547	Fragaria vesca L.	0	n	1	0	Rosaceae Juss.
Iz_2_1009	DJI_0547	Frangula alnus Mill.	0	n	1	1	Rhamnaceae Juss.
Iz_2_1009	DJI_0547	Fraxinus pennsylvanica Marshall	0	a	0	1	Oleaceae Hoffmanns. & Link
Iz_2_1009	DJI_0547	Galeopsis bifida Boenn.	0	n	1	0	Lamiacea Lindl.
Iz_2_1009	DJI_0547	Galium verum L.	0	n	1	0	Rubiaceae Juss.
Iz_2_1009	DJI_0547	Genista tinctoria L.	0	n	1	0	Fabaceae Lindl.
Iz_2_1009	DJI_0547	Geranium robertianum L.	0	n	1	0	Geraniaceae Juss.
Iz_2_1009	DJI_0547	Geranium sanquineum L.	0	n	1	0	Geraniaceae Juss.

Iz_2_1009	DJI_0547	Gypsophila paniculata L.	0	n	1	0	Caryophyllaceae Juss.
Iz_2_1009	DJI_0547	Helichrysum arenarium (L.) Moench	0	n	1	0	Asteraceae Bercht. & J.Presl
Iz_2_1009	DJI_0547	Herniaria polygama J.Gay	0	n	1	0	Caryophyllaceae Juss.
Iz_2_1009	DJI_0547	Hieracium robustum Fr.	0	n	1	0	Asteraceae Bercht. & J.Presl
Iz_2_1009	DJI_0547	Hieracium umbellatum L.	0	n	1	NA	Asteraceae Bercht. & J.Presl
Iz_2_1009	DJI_0547	Hylotelephium maximum (L.) Holub	0	n	1	0	Crassulaceae J.St.-Hil.
Iz_2_1009	DJI_0547	Jacobaea borysthenica (DC.) B.Nord. & Greuter	0	n	1	0	Asteraceae Bercht. & J.Presl
Iz_2_1009	DJI_0547	Jasione montana L.	0	n	1	0	Campanulaceae Juss.
Iz_2_1009	DJI_0547	Jurinea cyanoides (L.) Rchb.s.l.	0	n	1	0	Asteraceae Bercht. & J.Presl
Iz_2_1009	DJI_0547	Koeleria glauca (Spreng.) DC.	0	n	1	0	Poaceae Barnhart
Iz_2_1009	DJI_0547	Lactuca serriola L.	0	a	0	0	Asteraceae Bercht. & J.Presl
Iz_2_1009	DJI_0547	Linaria genistifolia (L.) Mill.	0	n	1	0	Plantaginaceae Juss.
Iz_2_1009	DJI_0547	Lomelosia argentea (L.) Greuter & Burdet	0	n	1	0	Caprifoliaceae Juss.
Iz_2_1009	DJI_0547	Melampyrum pratense L.	0	n	1	0	Orobanchaceae Vent.
Iz_2_1009	DJI_0547	Oenothera biennis L.	0	a	0	0	Onagraceae Juss.
Iz_2_1009	DJI_0547	Pentanema britannica (L.) D.Gut.Larr., Santos-Vicente, Anderb., E.Rico & M.M.Mart.Ort.	0	n	1	0	Asteraceae Bercht. & J.Presl
Iz_2_1009	DJI_0547	Peucedanum oreoselinum (L.) Moench	0	n	1	0	Apiaceae Lindl.
Iz_2_1009	DJI_0547	Peucedanum ruthenicum M.Bieb.	0	n	1	0	Apiaceae Lindl.
Iz_2_1009	DJI_0547	Pilosella echioides (Lumn.) F.W.Schultz & Sch.Bip.	0	n	1	0	Asteraceae Bercht. & J.Presl
Iz_2_1009	DJI_0547	Pilosella officinarum F. Schultz et Sch. Bip.	0	n	1	0	Asteraceae Bercht. & J.Presl
Iz_2_1009	DJI_0547	Pilosella sp.	0		NA	0	Asteraceae Bercht. & J.Presl
Iz_2_1009	DJI_0547	Pinus sylvestris L.	0	n	1	1	Pinaceae Spreng. ex F.Rudolphi
Iz_2_1009	DJI_0547	Poa annua L.	0	n	1	0	Poaceae Barnhart
Iz_2_1009	DJI_0547	Poa nemoralis L.	0	n	1	0	Poaceae Barnhart
Iz_2_1009	DJI_0547	Poa sp.	0		NA	0	Poaceae Barnhart
Iz_2_1009	DJI_0547	Polygonatum odoratum (Mill.) Druce	0	n	1	0	Asparagaceae Juss.
Iz_2_1009	DJI_0547	Polygonum arenarium Waldst. & Kit.	0	n	1	0	Polygonaceae Juss.
Iz_2_1009	DJI_0547	Populus nigra L.	0	n	1	1	Salicaceae Mirb.
Iz_2_1009	DJI_0547	Populus tremula L.	0	n	1	1	Salicaceae Mirb.
Iz_2_1009	DJI_0547	Populus Ã canescens (Aiton) Sm.	0	n	1	1	Salicaceae Mirb.
Iz_2_1009	DJI_0547	Potentilla argentea L.	0	n	1	0	Rosaceae Juss.
Iz_2_1009	DJI_0547	Psammophiliella muralis (L.) Ikonn.	0	n	1	0	Caryophyllaceae Juss.
Iz_2_1009	DJI_0547	Psephellus sumensis (Kalen.) Greuter	0	n	1	0	Asteraceae Bercht. & J.Presl
Iz_2_1009	DJI_0547	Pulsatilla pratensis (L.) Mill. s.l.	0	n	1	0	Ranunculaceae Juss.
Iz_2_1009	DJI_0547	Pyrus communis L.	0	a	0	1	Rosaceae Juss.
Iz_2_1009	DJI_0547	Quercus robur L.	0	n	1	1	Fagaceae Dumort.
Iz_2_1009	DJI_0547	Robinia pseudoacacia L.	0	a	0	1	Fabaceae Lindl.
Iz_2_1009	DJI_0547	Rosa sp.	0		NA	1	Rosaceae Juss.
Iz_2_1009	DJI_0547	Rubus idaeus L.	0	n	1	0	Rosaceae Juss.
Iz_2_1009	DJI_0547	Rumex acetosella L.	0	n	1	0	Polygonaceae Juss.
Iz_2_1009	DJI_0547	Rumex thyrsiflorus Fingerh.	0	n	1	0	Polygonaceae Juss.
Iz_2_1009	DJI_0547	Salix alba L.	0	n	1	1	Salicaceae Mirb.
Iz_2_1009	DJI_0547	Sambucus nigra L.	0	n	1	1	Viburnaceae Raf.
Iz_2_1009	DJI_0547	Secale sylvestre Host	0	n	1	0	Poaceae Barnhart
Iz_2_1009	DJI_0547	Sempervivum ruthenicum Schnittsp. & C.B. Lehm.	0	n	1	0	Crassulaceae J.St.-Hil.
Iz_2_1009	DJI_0547	Setaria viridis (L.) P. Beauv.	0	n	1	0	Poaceae Barnhart
Iz_2_1009	DJI_0547	Silene borysthenica (Gruner) Walters	0	n	1	0	Caryophyllaceae Juss.
Iz_2_1009	DJI_0547	Silene latifolia subsp. alba (Mill.) Greuter & Burdet	0	n	1	0	Caryophyllaceae Juss.
Iz_2_1009	DJI_0547	Solidago virgaurea L.	0	n	1	0	Asteraceae Bercht. & J.Presl
Iz_2_1009	DJI_0547	Sorbus aucuparia L.	0	n	1	1	Rosaceae Juss.
Iz_2_1009	DJI_0547	Stachys recta L.	0	n	1	0	Lamiacea Lindl.
Iz_2_1009	DJI_0547	Tanacetum vulgare L.	0	n	1	0	Asteraceae Bercht. & J.Presl
Iz_2_1009	DJI_0547	Thymus pallasianus Heinr.Braun	0	n	1	0	Lamiacea Lindl.
Iz_2_1009	DJI_0547	Tilia cordata Mill.	0	n	1	1	Malvaceae Juss.
Iz_2_1009	DJI_0547	Trifolium alpestre L.	0	n	1	0	Fabaceae Lindl.
Iz_2_1009	DJI_0547	Trifolium arvense L.	0	n	1	0	Fabaceae Lindl.
Iz_2_1009	DJI_0547	Ulmus glabra Huds.	0	n	1	1	Ulmaceae Mirb.
Iz_2_1009	DJI_0547	Verbascum densiflorum Bertol.	0	n	1	0	Scrophulariaceae Juss.
Iz_2_1009	DJI_0547	Veronica austriaca L.	0	n	1	0	Plantaginaceae Juss.
Iz_2_1009	DJI_0547	Veronica incana L.	0	n	1	0	Plantaginaceae Juss.
Iz_2_1009	DJI_0547	Veronica verna L.	0	n	1	0	Plantaginaceae Juss.
Iz_2_1009	DJI_0547	Vincetoxicum hirundinaria Medik	0	n	1	0	Apocynaceae Juss.
Iz_2_1009	DJI_0549	Acer tataricum L.	0	n	1	1	Sapindaceae Juss.
Iz_2_1009	DJI_0549	Achillea millefolium L. s.l.	0	n	1	0	Asteraceae Bercht. & J.Presl
Iz_2_1009	DJI_0549	Allium oleraceum L.	0	n	1	0	Amaryllidaceae J.St.-Hil.
Iz_2_1009	DJI_0549	Ambrosia artemisiifolia L.	0	a	0	0	Asteraceae Bercht. & J.Presl
Iz_2_1009	DJI_0549	Amorpha fruticosa L.	0	a	0	0	Fabaceae Lindl.
Iz_2_1009	DJI_0549	Anthericum ramosum L.	0	n	1	0	Asparagaceae Juss.
Iz_2_1009	DJI_0549	Artemisia marschalliana Spreng.	0	n	1	0	Asteraceae Bercht. & J.Presl
Iz_2_1009	DJI_0549	Asparagus officinalis L.	0	n	1	0	Asparagaceae Juss.
Iz_2_1009	DJI_0549	Astragalus cicer L.	0	n	1	0	Fabaceae Lindl.
Iz_2_1009	DJI_0549	Bassia laniflora (S.G.Gmel.) A.J.Scott	0	n	1	0	Amaranthaceae Juss.
Iz_2_1009	DJI_0549	Berberis vulgaris L.	0	n	1	0	Berberidaceae Juss.
Iz_2_1009	DJI_0549	Betula pendula Roth.	0	n	1	1	Betulaceae Gray
Iz_2_1009	DJI_0549	Betula pendula Roth.	0	n	1	0	Poaceae Barnhart
Iz_2_1009	DJI_0549	Bromus inermis Leyss.	0		NA	NA	
Iz_2_1009	DJI_0549	Bromus squarrosus L.	0	a	0	0	Poaceae Barnhart
Iz_2_1009	DJI_0549	Bromus tectorum L.	0	a	0	0	Poaceae Barnhart
Iz_2_1009	DJI_0549	Calamagrostis epigejos (L.) Roth	25	n	1	0	Poaceae Barnhart
Iz_2_1009	DJI_0549	Carduus acanthoides L.	0	n	1	0	Asteraceae Bercht. & J.Presl
Iz_2_1009	DJI_0549	Carex sp.	0	n	1	0	Cyperaceae Juss.
Iz_2_1009	DJI_0549	Centaurea majorovii Dumbadze	0	n	1	0	Asteraceae Bercht. & J.Presl
Iz_2_1009	DJI_0549	Cf. Solidago virgaurea	0		NA	NA	
Iz_2_1009	DJI_0549	Chamaecytisus ruthenicus (Fisch.ex Wol.) Klaskova	0	n	1	0	Fabaceae Lindl.
Iz_2_1009	DJI_0549	Chelidonium majus L.	0	n	1	0	Papaveraceae Juss.
Iz_2_1009	DJI_0549	Chenopodium betaceum Andrz.	0	n	1	0	Amaranthaceae Juss.
Iz_2_1009	DJI_0549	Chenopodium cf. album	0	n	1	0	Amaranthaceae Juss.
Iz_2_1009	DJI_0549	Chenopodium sp.	0		NA	0	Amaranthaceae Juss.
Iz_2_1009	DJI_0549	Chondrilla juncea L.	0	n	1	0	Asteraceae Bercht. & J.Presl
Iz_2_1009	DJI_0549	Cirsium vulgare (Savi) Ten.	0	n	1	0	Asteraceae Bercht. & J.Presl
Iz_2_1009	DJI_0549	Clinopodium acinos (L.) Kuntze	0	n	1	0	Lamiacea Lindl.
Iz_2_1009	DJI_0549	Convallaria majalis L.	0	n	1	0	Asparagaceae Juss.
Iz_2_1009	DJI_0549	Crataegus rhipidophylla Gand.	0	n	1	1	Rosaceae Juss.
Iz_2_1009	DJI_0549	Crepis foetida L.	0	n	1	0	Asteraceae Bercht. & J.Presl
Iz_2_1009	DJI_0549	Dactylis glomerata L.	0	n	1	0	Poaceae Barnhart
Iz_2_1009	DJI_0549	Dianthus campestris M.Bieb.	0	n	1	0	Caryophyllaceae Juss.
Iz_2_1009	DJI_0549	Digitaria sanguinalis (L.) Scop.	0	a	0	0	Poaceae Barnhart
Iz_2_1009	DJI_0549	Elymus repens (L.) Gould	0	n	1	0	Poaceae Barnhart

Iz_2_1009	DJI_0549	Epilobium tetragonum L.	0	n	1	0	Onagraceae Juss.
Iz_2_1009	DJI_0549	Eragrostis minor Host	0	a	0	0	Poaceae Barnhart
Iz_2_1009	DJI_0549	Erigeron canadensis L.	0	a	0	0	Asteraceae Bercht. & J.Presl
Iz_2_1009	DJI_0549	Euphorbia seguieriana Neck.	0	n	1	0	Euphorbiaceae Juss.
Iz_2_1009	DJI_0549	Fallopia dumetorum (L.) Holub	0	n	1	0	Polygonaceae Juss.
Iz_2_1009	DJI_0549	Festuca beckeri (Hack.) Trautv.	0	n	1	0	Poaceae Barnhart
Iz_2_1009	DJI_0549	Fragaria vesca L.	0	n	1	0	Rosaceae Juss.
Iz_2_1009	DJI_0549	Frangula alnus Mill.	0	n	1	1	Rhamnaceae Juss.
Iz_2_1009	DJI_0549	Fraxinus pennsylvanica Marshall	0	a	0	1	Oleaceae Hoffmanns. & Link
Iz_2_1009	DJI_0549	Galeopsis bifida Boenn.	0	n	1	0	Lamiacea Lindl.
Iz_2_1009	DJI_0549	Galium verum L.	0	n	1	0	Rubiaceae Juss.
Iz_2_1009	DJI_0549	Genista tinctoria L.	0	n	1	0	Fabaceae Lindl.
Iz_2_1009	DJI_0549	Geranium robertianum L.	0	n	1	0	Geraniaceae Juss.
Iz_2_1009	DJI_0549	Geranium sanquineum L.	0	n	1	0	Geraniaceae Juss.
Iz_2_1009	DJI_0549	Gypsophila paniculata L.	0	n	1	0	Caryophyllaceae Juss.
Iz_2_1009	DJI_0549	Helichrysum arenarium (L.) Moench	0	n	1	0	Asteraceae Bercht. & J.Presl
Iz_2_1009	DJI_0549	Herniaria polygama J.Gay	0	n	1	0	Caryophyllaceae Juss.
Iz_2_1009	DJI_0549	Hieracium robustum Fr.	0	n	1	0	Asteraceae Bercht. & J.Presl
Iz_2_1009	DJI_0549	Hieracium umbellatum L.	0	n	1	NA	Asteraceae Bercht. & J.Presl
Iz_2_1009	DJI_0549	Hylotelephium maximum (L.) Holub	0	n	1	0	Crassulaceae J.St.-Hil.
Iz_2_1009	DJI_0549	Jacobaea borysthenica (DC.) B.Nord. & Greuter	0	n	1	0	Asteraceae Bercht. & J.Presl
Iz_2_1009	DJI_0549	Jasione montana L.	0	n	1	0	Campanulaceae Juss.
Iz_2_1009	DJI_0549	Jurinea cyanoides (L.) Rchb.s.l.	0	n	1	0	Asteraceae Bercht. & J.Presl
Iz_2_1009	DJI_0549	Koeleria glauca (Spreng.) DC.	0	n	1	0	Poaceae Barnhart
Iz_2_1009	DJI_0549	Lactuca serriola L.	0	a	0	0	Asteraceae Bercht. & J.Presl
Iz_2_1009	DJI_0549	Linaria genistifolia (L.) Mill.	0	n	1	0	Plantaginaceae Juss.
Iz_2_1009	DJI_0549	Lomelosia argentea (L.) Greuter & Burdet	0	n	1	0	Caprifoliaceae Juss.
Iz_2_1009	DJI_0549	Melampyrum pratense L.	0	n	1	0	Orobanchaceae Vent.
Iz_2_1009	DJI_0549	Oenothera biennis L.	0	a	0	0	Onagraceae Juss.
Iz_2_1009	DJI_0549	Pentanema britannica (L.) D.Gut.Larr., Santos-Vicente, Anderb., E.Rico & M.M.Mart.Ort.	0	n	1	0	Asteraceae Bercht. & J.Presl
Iz_2_1009	DJI_0549	Peucedanum oreoselinum (L.) Moench	0	n	1	0	Apiaceae Lindl.
Iz_2_1009	DJI_0549	Peucedanum ruthenicum M.Bieb.	0	n	1	0	Apiaceae Lindl.
Iz_2_1009	DJI_0549	Pilosella echioides (Lumn.) F.W.Schultz & Sch.Bip.	0	n	1	0	Asteraceae Bercht. & J.Presl
Iz_2_1009	DJI_0549	Pilosella officinarum F. Schultz et Sch. Bip.	0	n	1	0	Asteraceae Bercht. & J.Presl
Iz_2_1009	DJI_0549	Pilosella sp.	0		NA	0	Asteraceae Bercht. & J.Presl
Iz_2_1009	DJI_0549	Pinus sylvestris L.	0	n	1	1	Pinaceae Spreng. ex F.Rudolphi
Iz_2_1009	DJI_0549	Poa annua L.	0	n	1	0	Poaceae Barnhart
Iz_2_1009	DJI_0549	Poa nemoralis L.	0	n	1	0	Poaceae Barnhart
Iz_2_1009	DJI_0549	Poa sp.	0		NA	0	Poaceae Barnhart
Iz_2_1009	DJI_0549	Polygonatum odoratum (Mill.) Druce	0	n	1	0	Asparagaceae Juss.
Iz_2_1009	DJI_0549	Polygonum arenarium Waldst. & Kit.	0	n	1	0	Polygonaceae Juss.
Iz_2_1009	DJI_0549	Populus nigra L.	0	n	1	1	Salicaceae Mirb.
Iz_2_1009	DJI_0549	Populus tremula L.	0	n	1	1	Salicaceae Mirb.
Iz_2_1009	DJI_0549	Populus Ã canescens (Aiton) Sm.	0	n	1	1	Salicaceae Mirb.
Iz_2_1009	DJI_0549	Potentilla argentea L.	0	n	1	0	Rosaceae Juss.
Iz_2_1009	DJI_0549	Psammophiliella muralis (L.) Ikonn.	0	n	1	0	Caryophyllaceae Juss.
Iz_2_1009	DJI_0549	Psephellus sumensis (Kalen.) Greuter	0	n	1	0	Asteraceae Bercht. & J.Presl
Iz_2_1009	DJI_0549	Pulsatilla pratensis (L.) Mill. s.l.	0	n	1	0	Ranunculaceae Juss.
Iz_2_1009	DJI_0549	Pyrus communis L.	0	a	0	1	Rosaceae Juss.
Iz_2_1009	DJI_0549	Quercus robur L.	0	n	1	1	Fagaceae Dumort.
Iz_2_1009	DJI_0549	Robinia pseudoacacia L.	0	a	0	1	Fabaceae Lindl.
Iz_2_1009	DJI_0549	Rosa sp.	0		NA	1	Rosaceae Juss.
Iz_2_1009	DJI_0549	Rubus idaeus L.	0	n	1	0	Rosaceae Juss.
Iz_2_1009	DJI_0549	Rumex acetosella L.	0	n	1	0	Polygonaceae Juss.
Iz_2_1009	DJI_0549	Rumex thyrsiflorus Fingerh.	0	n	1	0	Polygonaceae Juss.
Iz_2_1009	DJI_0549	Salix alba L.	0	n	1	1	Salicaceae Mirb.
Iz_2_1009	DJI_0549	Sambucus nigra L.	0	n	1	1	Viburnaceae Raf.
Iz_2_1009	DJI_0549	Secale sylvestre Host	0	n	1	0	Poaceae Barnhart
Iz_2_1009	DJI_0549	Sempervivum ruthenicum Schnittsp. & C.B. Lehm.	0	n	1	0	Crassulaceae J.St.-Hil.
Iz_2_1009	DJI_0549	Setaria viridis (L.) P. Beauv.	0	n	1	0	Poaceae Barnhart
Iz_2_1009	DJI_0549	Silene borysthenica (Gruner) Walters	0	n	1	0	Caryophyllaceae Juss.
Iz_2_1009	DJI_0549	Silene latifolia subsp. alba (Mill.) Greuter & Burdet	0	n	1	0	Caryophyllaceae Juss.
Iz_2_1009	DJI_0549	Solidago virgaurea L.	0	n	1	0	Asteraceae Bercht. & J.Presl
Iz_2_1009	DJI_0549	Sorbus aucuparia L.	0	n	1	1	Rosaceae Juss.
Iz_2_1009	DJI_0549	Stachys recta L.	0	n	1	0	Lamiacea Lindl.
Iz_2_1009	DJI_0549	Tanacetum vulgare L.	0	n	1	0	Asteraceae Bercht. & J.Presl
Iz_2_1009	DJI_0549	Thymus pallasianus Heinr.Braun	0	n	1	0	Lamiacea Lindl.
Iz_2_1009	DJI_0549	Tilia cordata Mill.	0	n	1	1	Malvaceae Juss.
Iz_2_1009	DJI_0549	Trifolium alpestre L.	0	n	1	0	Fabaceae Lindl.
Iz_2_1009	DJI_0549	Trifolium arvense L.	0	n	1	0	Fabaceae Lindl.
Iz_2_1009	DJI_0549	Ulmus glabra Huds.	0	n	1	1	Ulmaceae Mirb.
Iz_2_1009	DJI_0549	Verbascum densiflorum Bertol.	0	n	1	0	Scrophulariaceae Juss.
Iz_2_1009	DJI_0549	Veronica austriaca L.	0	n	1	0	Plantaginaceae Juss.
Iz_2_1009	DJI_0549	Veronica incana L.	0	n	1	0	Plantaginaceae Juss.
Iz_2_1009	DJI_0549	Veronica verna L.	0	n	1	0	Plantaginaceae Juss.
Iz_2_1009	DJI_0549	Vincetoxicum hirundinaria Medik	0	n	1	0	Apocynaceae Juss.
Iz_2_1009	DJI_0551	Acer tataricum L.	0	n	1	1	Sapindaceae Juss.
Iz_2_1009	DJI_0551	Achillea millefolium L. s.l.	0	n	1	0	Asteraceae Bercht. & J.Presl
Iz_2_1009	DJI_0551	Allium oleraceum L.	0	n	1	0	Amaryllidaceae J.St.-Hil.
Iz_2_1009	DJI_0551	Ambrosia artemisiifolia L.	0	a	0	0	Asteraceae Bercht. & J.Presl
Iz_2_1009	DJI_0551	Amorpha fruticosa L.	0	a	0	0	Fabaceae Lindl.
Iz_2_1009	DJI_0551	Anthericum ramosum L.	0	n	1	0	Asparagaceae Juss.
Iz_2_1009	DJI_0551	Artemisia marschalliana Spreng.	0	n	1	0	Asteraceae Bercht. & J.Presl
Iz_2_1009	DJI_0551	Asparagus officinalis L.	0	n	1	0	Asparagaceae Juss.
Iz_2_1009	DJI_0551	Astragalus cicer L.	0	n	1	0	Fabaceae Lindl.
Iz_2_1009	DJI_0551	Bassia laniflora (S.G.Gmel.) A.J.Scott	0	n	1	0	Amaranthaceae Juss.
Iz_2_1009	DJI_0551	Berberis vulgaris L.	0	n	1	0	Berberidaceae Juss.
Iz_2_1009	DJI_0551	Betula pendula Roth.	0	n	1	1	Betulaceae Gray
Iz_2_1009	DJI_0551	Betula pendula Roth.	0	n	1	0	Poaceae Barnhart
Iz_2_1009	DJI_0551	Bromus inermis Leyss.	0		NA	NA	
Iz_2_1009	DJI_0551	Bromus squarrosus L.	0	a	0	0	Poaceae Barnhart
Iz_2_1009	DJI_0551	Bromus tectorum L.	0	a	0	0	Poaceae Barnhart
Iz_2_1009	DJI_0551	Calamagrostis epigejos (L.) Roth	50	n	1	0	Poaceae Barnhart
Iz_2_1009	DJI_0551	Carduus acanthoides L.	0	n	1	0	Asteraceae Bercht. & J.Presl
Iz_2_1009	DJI_0551	Carex sp.	0	n	1	0	Cyperaceae Juss.
Iz_2_1009	DJI_0551	Centaurea majorovii Dumbadze	0	n	1	0	Asteraceae Bercht. & J.Presl
Iz_2_1009	DJI_0551	Cf. Solidago virgaurea	0		NA	NA	
Iz_2_1009	DJI_0551	Chamaecytisus ruthenicus (Fisch.ex Wol.) Klaskova	0	n	1	0	Fabaceae Lindl.

Iz_2_1009	DJI_0551	Chelidonium majus L.	0	n	1	0	Papaveraceae Juss.
Iz_2_1009	DJI_0551	Chenopodium betaceum Andrz.	0	n	1	0	Amaranthaceae Juss.
Iz_2_1009	DJI_0551	Chenopodium cf. album	0	n	1	0	Amaranthaceae Juss.
Iz_2_1009	DJI_0551	Chenopodium sp.	0		NA	0	Amaranthaceae Juss.
Iz_2_1009	DJI_0551	Chondrilla juncea L.	0	n	1	0	Asteraceae Bercht. & J.Presl
Iz_2_1009	DJI_0551	Cirsium vulgare (Savi) Ten.	0	n	1	0	Asteraceae Bercht. & J.Presl
Iz_2_1009	DJI_0551	Clinopodium acinos (L.) Kuntze	0	n	1	0	Lamiacea Lindl.
Iz_2_1009	DJI_0551	Convallaria majalis L.	0	n	1	0	Asparagaceae Juss.
Iz_2_1009	DJI_0551	Crataegus rhipidophylla Gand.	0	n	1	1	Rosaceae Juss.
Iz_2_1009	DJI_0551	Crepis foetida L.	0	n	1	0	Asteraceae Bercht. & J.Presl
Iz_2_1009	DJI_0551	Dactylis glomerata L.	0	n	1	0	Poaceae Barnhart
Iz_2_1009	DJI_0551	Dianthus campestris M.Bieb.	0	n	1	0	Caryophyllaceae Juss.
Iz_2_1009	DJI_0551	Digitaria sanguinalis (L.) Scop.	0	a	0	0	Poaceae Barnhart
Iz_2_1009	DJI_0551	Elymus repens (L.) Gould	0	n	1	0	Poaceae Barnhart
Iz_2_1009	DJI_0551	Epilobium tetragonum L.	0	n	1	0	Onagraceae Juss.
Iz_2_1009	DJI_0551	Eragrostis minor Host	0	a	0	0	Poaceae Barnhart
Iz_2_1009	DJI_0551	Erigeron canadensis L.	0	a	0	0	Asteraceae Bercht. & J.Presl
Iz_2_1009	DJI_0551	Euphorbia seguieriana Neck.	0	n	1	0	Euphorbiaceae Juss.
Iz_2_1009	DJI_0551	Fallopia dumetorum (L.) Holub	0	n	1	0	Polygonaceae Juss.
Iz_2_1009	DJI_0551	Festuca beckeri (Hack.) Trautv.	0	n	1	0	Poaceae Barnhart
Iz_2_1009	DJI_0551	Fragaria vesca L.	0	n	1	0	Rosaceae Juss.
Iz_2_1009	DJI_0551	Frangula alnus Mill.	0	n	1	1	Rhamnaceae Juss.
Iz_2_1009	DJI_0551	Fraxinus pennsylvanica Marshall	0	a	0	1	Oleaceae Hoffmanns. & Link
Iz_2_1009	DJI_0551	Galeopsis bifida Boenn.	0	n	1	0	Lamiacea Lindl.
Iz_2_1009	DJI_0551	Galium verum L.	0	n	1	0	Rubiaceae Juss.
Iz_2_1009	DJI_0551	Genista tinctoria L.	0	n	1	0	Fabaceae Lindl.
Iz_2_1009	DJI_0551	Geranium robertianum L.	0	n	1	0	Geraniaceae Juss.
Iz_2_1009	DJI_0551	Geranium sanquineum L.	0	n	1	0	Geraniaceae Juss.
Iz_2_1009	DJI_0551	Gypsophila paniculata L.	0	n	1	0	Caryophyllaceae Juss.
Iz_2_1009	DJI_0551	Helichrysum arenarium (L.) Moench	0	n	1	0	Asteraceae Bercht. & J.Presl
Iz_2_1009	DJI_0551	Herniaria polygama J.Gay	0	n	1	0	Caryophyllaceae Juss.
Iz_2_1009	DJI_0551	Hieracium robustum Fr.	0	n	1	0	Asteraceae Bercht. & J.Presl
Iz_2_1009	DJI_0551	Hieracium umbellatum L.	0	n	1	NA	Asteraceae Bercht. & J.Presl
Iz_2_1009	DJI_0551	Hylotelephium maximum (L.) Holub	0	n	1	0	Crassulaceae J.St.-Hil.
Iz_2_1009	DJI_0551	Jacobaea borysthenica (DC.) B.Nord. & Greuter	0	n	1	0	Asteraceae Bercht. & J.Presl
Iz_2_1009	DJI_0551	Jasione montana L.	0	n	1	0	Campanulaceae Juss.
Iz_2_1009	DJI_0551	Jurinea cyanoides (L.) Rchb.s.l.	0	n	1	0	Asteraceae Bercht. & J.Presl
Iz_2_1009	DJI_0551	Koeleria glauca (Spreng.) DC.	0	n	1	0	Poaceae Barnhart
Iz_2_1009	DJI_0551	Lactuca serriola L.	0	a	0	0	Asteraceae Bercht. & J.Presl
Iz_2_1009	DJI_0551	Linaria genistifolia (L.) Mill.	0	n	1	0	Plantaginaceae Juss.
Iz_2_1009	DJI_0551	Lomelosia argentea (L.) Greuter & Burdet	0	n	1	0	Caprifoliaceae Juss.
Iz_2_1009	DJI_0551	Melampyrum pratense L.	0	n	1	0	Orobanchaceae Vent.
Iz_2_1009	DJI_0551	Oenothera biennis L.	0	a	0	0	Onagraceae Juss.
Iz_2_1009	DJI_0551	Pentanema britannica (L.) D.Gut.Larr., Santos-Vicente, Anderb., E.Rico & M.M.Mart.Ort.	0	n	1	0	Asteraceae Bercht. & J.Presl
Iz_2_1009	DJI_0551	Peucedanum oreoselinum (L.) Moench	0	n	1	0	Apiaceae Lindl.
Iz_2_1009	DJI_0551	Peucedanum ruthenicum M.Bieb.	0	n	1	0	Apiaceae Lindl.
Iz_2_1009	DJI_0551	Pilosella echioides (Lumn.) F.W.Schultz & Sch.Bip.	0	n	1	0	Asteraceae Bercht. & J.Presl
Iz_2_1009	DJI_0551	Pilosella officinarum F. Schultz et Sch. Bip.	0	n	1	0	Asteraceae Bercht. & J.Presl
Iz_2_1009	DJI_0551	Pilosella sp.	0		NA	0	Asteraceae Bercht. & J.Presl
Iz_2_1009	DJI_0551	Pinus sylvestris L.	0	n	1	1	Pinaceae Spreng. ex F.Rudolphi
Iz_2_1009	DJI_0551	Poa annua L.	0	n	1	0	Poaceae Barnhart
Iz_2_1009	DJI_0551	Poa nemoralis L.	0	n	1	0	Poaceae Barnhart
Iz_2_1009	DJI_0551	Poa sp.	0		NA	0	Poaceae Barnhart
Iz_2_1009	DJI_0551	Polygonatum odoratum (Mill.) Druce	0	n	1	0	Asparagaceae Juss.
Iz_2_1009	DJI_0551	Polygonum arenarium Waldst. & Kit.	0	n	1	0	Polygonaceae Juss.
Iz_2_1009	DJI_0551	Populus nigra L.	0	n	1	1	Salicaceae Mirb.
Iz_2_1009	DJI_0551	Populus tremula L.	0	n	1	1	Salicaceae Mirb.
Iz_2_1009	DJI_0551	Populus Ã canescens (Aiton) Sm.	0	n	1	1	Salicaceae Mirb.
Iz_2_1009	DJI_0551	Potentilla argentea L.	0	n	1	0	Rosaceae Juss.
Iz_2_1009	DJI_0551	Psammophiliella muralis (L.) Ikonn.	0	n	1	0	Caryophyllaceae Juss.
Iz_2_1009	DJI_0551	Psephellus sumensis (Kalen.) Greuter	0	n	1	0	Asteraceae Bercht. & J.Presl
Iz_2_1009	DJI_0551	Pulsatilla pratensis (L.) Mill. s.l.	0	n	1	0	Ranunculaceae Juss.
Iz_2_1009	DJI_0551	Pyrus communis L.	0	a	0	1	Rosaceae Juss.
Iz_2_1009	DJI_0551	Quercus robur L.	0	n	1	1	Fagaceae Dumort.
Iz_2_1009	DJI_0551	Robinia pseudoacacia L.	0	a	0	1	Fabaceae Lindl.
Iz_2_1009	DJI_0551	Rosa sp.	0		NA	1	Rosaceae Juss.
Iz_2_1009	DJI_0551	Rubus idaeus L.	0	n	1	0	Rosaceae Juss.
Iz_2_1009	DJI_0551	Rumex acetosella L.	0	n	1	0	Polygonaceae Juss.
Iz_2_1009	DJI_0551	Rumex thyrsiflorus Fingerh.	0	n	1	0	Polygonaceae Juss.
Iz_2_1009	DJI_0551	Salix alba L.	0	n	1	1	Salicaceae Mirb.
Iz_2_1009	DJI_0551	Sambucus nigra L.	0	n	1	1	Viburnaceae Raf.
Iz_2_1009	DJI_0551	Secale sylvestre Host	0	n	1	0	Poaceae Barnhart
Iz_2_1009	DJI_0551	Sempervivum ruthenicum Schnittsp. & C.B. Lehm.	0	n	1	0	Crassulaceae J.St.-Hil.
Iz_2_1009	DJI_0551	Setaria viridis (L.) P. Beauv.	0	n	1	0	Poaceae Barnhart
Iz_2_1009	DJI_0551	Silene borysthenica (Gruner) Walters	0	n	1	0	Caryophyllaceae Juss.
Iz_2_1009	DJI_0551	Silene latifolia subsp. alba (Mill.) Greuter & Burdet	0	n	1	0	Caryophyllaceae Juss.
Iz_2_1009	DJI_0551	Solidago virgaurea L.	0	n	1	0	Asteraceae Bercht. & J.Presl
Iz_2_1009	DJI_0551	Sorbus aucuparia L.	0	n	1	1	Rosaceae Juss.
Iz_2_1009	DJI_0551	Stachys recta L.	0	n	1	0	Lamiacea Lindl.
Iz_2_1009	DJI_0551	Tanacetum vulgare L.	0	n	1	0	Asteraceae Bercht. & J.Presl
Iz_2_1009	DJI_0551	Thymus pallasianus Heinr.Braun	0	n	1	0	Lamiacea Lindl.
Iz_2_1009	DJI_0551	Tilia cordata Mill.	0	n	1	1	Malvaceae Juss.
Iz_2_1009	DJI_0551	Trifolium alpestre L.	0	n	1	0	Fabaceae Lindl.
Iz_2_1009	DJI_0551	Trifolium arvense L.	0	n	1	0	Fabaceae Lindl.
Iz_2_1009	DJI_0551	Ulmus glabra Huds.	0	n	1	1	Ulmaceae Mirb.
Iz_2_1009	DJI_0551	Verbascum densiflorum Bertol.	0	n	1	0	Scrophulariaceae Juss.
Iz_2_1009	DJI_0551	Veronica austriaca L.	0	n	1	0	Plantaginaceae Juss.
Iz_2_1009	DJI_0551	Veronica incana L.	0	n	1	0	Plantaginaceae Juss.
Iz_2_1009	DJI_0551	Veronica verna L.	0	n	1	0	Plantaginaceae Juss.
Iz_2_1009	DJI_0551	Vincetoxicum hirundinaria Medik	0	n	1	0	Apocynaceae Juss.
Iz_2_1009	DJI_0553	Acer tataricum L.	0	n	1	1	Sapindaceae Juss.
Iz_2_1009	DJI_0553	Achillea millefolium L. s.l.	0	n	1	0	Asteraceae Bercht. & J.Presl
Iz_2_1009	DJI_0553	Allium oleraceum L.	0	n	1	0	Amaryllidaceae J.St.-Hil.
Iz_2_1009	DJI_0553	Ambrosia artemisiifolia L.	0	a	0	0	Asteraceae Bercht. & J.Presl
Iz_2_1009	DJI_0553	Amorpha fruticosa L.	0	a	0	0	Fabaceae Lindl.
Iz_2_1009	DJI_0553	Anthericum ramosum L.	0	n	1	0	Asparagaceae Juss.
Iz_2_1009	DJI_0553	Artemisia marschalliana Spreng.	0	n	1	0	Asteraceae Bercht. & J.Presl
Iz_2_1009	DJI_0553	Asparagus officinalis L.	0	n	1	0	Asparagaceae Juss.

Iz_2_1009	DJI_0553	Astragalus cicer L.	0	n	1	0	Fabaceae Lindl.
Iz_2_1009	DJI_0553	Bassia laniflora (S.G.Gmel.) A.J.Scott	0	n	1	0	Amaranthaceae Juss.
Iz_2_1009	DJI_0553	Berberis vulgaris L.	0	n	1	0	Berberidaceae Juss.
Iz_2_1009	DJI_0553	Betula pendula Roth.	0	n	1	1	Betulaceae Gray
Iz_2_1009	DJI_0553	Betula pendula Roth.	0	n	1	0	Poaceae Barnhart
Iz_2_1009	DJI_0553	Bromus inermis Leyss.	0		NA	NA	
Iz_2_1009	DJI_0553	Bromus squarrosus L.	0	a	0	0	Poaceae Barnhart
Iz_2_1009	DJI_0553	Bromus tectorum L.	0	a	0	0	Poaceae Barnhart
Iz_2_1009	DJI_0553	Calamagrostis epigejos (L.) Roth	0.2	n	1	0	Poaceae Barnhart
Iz_2_1009	DJI_0553	Carduus acanthoides L.	0	n	1	0	Asteraceae Bercht. & J.Presl
Iz_2_1009	DJI_0553	Carex sp.	0	n	1	0	Cyperaceae Juss.
Iz_2_1009	DJI_0553	Centaurea majorovii Dumbadze	0	n	1	0	Asteraceae Bercht. & J.Presl
Iz_2_1009	DJI_0553	Cf. Solidago virgaurea	0		NA	NA	
Iz_2_1009	DJI_0553	Chamaecytisus ruthenicus (Fisch.ex Wol.) Klaskova	0	n	1	0	Fabaceae Lindl.
Iz_2_1009	DJI_0553	Chelidonium majus L.	0	n	1	0	Papaveraceae Juss.
Iz_2_1009	DJI_0553	Chenopodium betaceum Andrz.	0	n	1	0	Amaranthaceae Juss.
Iz_2_1009	DJI_0553	Chenopodium cf. album	0	n	1	0	Amaranthaceae Juss.
Iz_2_1009	DJI_0553	Chenopodium sp.	0		NA	0	Amaranthaceae Juss.
Iz_2_1009	DJI_0553	Chondrilla juncea L.	0	n	1	0	Asteraceae Bercht. & J.Presl
Iz_2_1009	DJI_0553	Cirsium vulgare (Savi) Ten.	0	n	1	0	Asteraceae Bercht. & J.Presl
Iz_2_1009	DJI_0553	Clinopodium acinos (L.) Kuntze	0	n	1	0	Lamiacea Lindl.
Iz_2_1009	DJI_0553	Convallaria majalis L.	0	n	1	0	Asparagaceae Juss.
Iz_2_1009	DJI_0553	Crataegus rhipidophylla Gand.	0	n	1	1	Rosaceae Juss.
Iz_2_1009	DJI_0553	Crepis foetida L.	0	n	1	0	Asteraceae Bercht. & J.Presl
Iz_2_1009	DJI_0553	Dactylis glomerata L.	0	n	1	0	Poaceae Barnhart
Iz_2_1009	DJI_0553	Dianthus campestris M.Bieb.	0	n	1	0	Caryophyllaceae Juss.
Iz_2_1009	DJI_0553	Digitaria sanguinalis (L.) Scop.	0	a	0	0	Poaceae Barnhart
Iz_2_1009	DJI_0553	Elymus repens (L.) Gould	0	n	1	0	Poaceae Barnhart
Iz_2_1009	DJI_0553	Epilobium tetragonum L.	0	n	1	0	Onagraceae Juss.
Iz_2_1009	DJI_0553	Eragrostis minor Host	0	a	0	0	Poaceae Barnhart
Iz_2_1009	DJI_0553	Erigeron canadensis L.	0	a	0	0	Asteraceae Bercht. & J.Presl
Iz_2_1009	DJI_0553	Euphorbia seguieriana Neck.	0	n	1	0	Euphorbiaceae Juss.
Iz_2_1009	DJI_0553	Fallopia dumetorum (L.) Holub	0	n	1	0	Polygonaceae Juss.
Iz_2_1009	DJI_0553	Festuca beckeri (Hack.) Trautv.	0	n	1	0	Poaceae Barnhart
Iz_2_1009	DJI_0553	Fragaria vesca L.	0	n	1	0	Rosaceae Juss.
Iz_2_1009	DJI_0553	Frangula alnus Mill.	0	n	1	1	Rhamnaceae Juss.
Iz_2_1009	DJI_0553	Fraxinus pennsylvanica Marshall	0	a	0	1	Oleaceae Hoffmanns. & Link
Iz_2_1009	DJI_0553	Galeopsis bifida Boenn.	0	n	1	0	Lamiacea Lindl.
Iz_2_1009	DJI_0553	Galium verum L.	0	n	1	0	Rubiaceae Juss.
Iz_2_1009	DJI_0553	Genista tinctoria L.	0	n	1	0	Fabaceae Lindl.
Iz_2_1009	DJI_0553	Geranium robertianum L.	0	n	1	0	Geraniaceae Juss.
Iz_2_1009	DJI_0553	Geranium sanquineum L.	0	n	1	0	Geraniaceae Juss.
Iz_2_1009	DJI_0553	Gypsophila paniculata L.	0	n	1	0	Caryophyllaceae Juss.
Iz_2_1009	DJI_0553	Helichrysum arenarium (L.) Moench	0	n	1	0	Asteraceae Bercht. & J.Presl
Iz_2_1009	DJI_0553	Herniaria polygama J.Gay	0	n	1	0	Caryophyllaceae Juss.
Iz_2_1009	DJI_0553	Hieracium robustum Fr.	0	n	1	0	Asteraceae Bercht. & J.Presl
Iz_2_1009	DJI_0553	Hieracium umbellatum L.	0	n	1	NA	Asteraceae Bercht. & J.Presl
Iz_2_1009	DJI_0553	Hylotelephium maximum (L.) Holub	0	n	1	0	Crassulaceae J.St.-Hil.
Iz_2_1009	DJI_0553	Jacobaea borysthenica (DC.) B.Nord. & Greuter	0	n	1	0	Asteraceae Bercht. & J.Presl
Iz_2_1009	DJI_0553	Jasione montana L.	0	n	1	0	Campanulaceae Juss.
Iz_2_1009	DJI_0553	Jurinea cyanoides (L.) Rchb.s.l.	0	n	1	0	Asteraceae Bercht. & J.Presl
Iz_2_1009	DJI_0553	Koeleria glauca (Spreng.) DC.	0	n	1	0	Poaceae Barnhart
Iz_2_1009	DJI_0553	Lactuca serriola L.	0	a	0	0	Asteraceae Bercht. & J.Presl
Iz_2_1009	DJI_0553	Linaria genistifolia (L.) Mill.	0	n	1	0	Plantaginaceae Juss.
Iz_2_1009	DJI_0553	Lomelosia argentea (L.) Greuter & Burdet	0	n	1	0	Caprifoliaceae Juss.
Iz_2_1009	DJI_0553	Melampyrum pratense L.	0	n	1	0	Orobanchaceae Vent.
Iz_2_1009	DJI_0553	Oenothera biennis L.	0	a	0	0	Onagraceae Juss.
Iz_2_1009	DJI_0553	Pentanema britannica (L.) D.Gut.Larr., Santos-Vicente, Anderb., E.Rico & M.M.Mart.Ort.	0	n	1	0	Asteraceae Bercht. & J.Presl
Iz_2_1009	DJI_0553	Peucedanum oreoselinum (L.) Moench	0	n	1	0	Apiaceae Lindl.
Iz_2_1009	DJI_0553	Peucedanum ruthenicum M.Bieb.	0	n	1	0	Apiaceae Lindl.
Iz_2_1009	DJI_0553	Pilosella echioides (Lumn.) F.W.Schultz & Sch.Bip.	0	n	1	0	Asteraceae Bercht. & J.Presl
Iz_2_1009	DJI_0553	Pilosella officinarum F. Schultz et Sch. Bip.	0	n	1	0	Asteraceae Bercht. & J.Presl
Iz_2_1009	DJI_0553	Pilosella sp.	0		NA	0	Asteraceae Bercht. & J.Presl
Iz_2_1009	DJI_0553	Pinus sylvestris L.	0	n	1	1	Pinaceae Spreng. ex F.Rudolphi
Iz_2_1009	DJI_0553	Poa annua L.	0	n	1	0	Poaceae Barnhart
Iz_2_1009	DJI_0553	Poa nemoralis L.	0	n	1	0	Poaceae Barnhart
Iz_2_1009	DJI_0553	Poa sp.	0		NA	0	Poaceae Barnhart
Iz_2_1009	DJI_0553	Polygonatum odoratum (Mill.) Druce	0	n	1	0	Asparagaceae Juss.
Iz_2_1009	DJI_0553	Polygonum arenarium Waldst. & Kit.	0	n	1	0	Polygonaceae Juss.
Iz_2_1009	DJI_0553	Populus nigra L.	0	n	1	1	Salicaceae Mirb.
Iz_2_1009	DJI_0553	Populus tremula L.	0	n	1	1	Salicaceae Mirb.
Iz_2_1009	DJI_0553	Populus Ã canescens (Aiton) Sm.	0	n	1	1	Salicaceae Mirb.
Iz_2_1009	DJI_0553	Potentilla argentea L.	0	n	1	0	Rosaceae Juss.
Iz_2_1009	DJI_0553	Psammophiliella muralis (L.) Ikonn.	0	n	1	0	Caryophyllaceae Juss.
Iz_2_1009	DJI_0553	Psephellus sumensis (Kalen.) Greuter	0	n	1	0	Asteraceae Bercht. & J.Presl
Iz_2_1009	DJI_0553	Pulsatilla pratensis (L.) Mill. s.l.	0	n	1	0	Ranunculaceae Juss.
Iz_2_1009	DJI_0553	Pyrus communis L.	0	a	0	1	Rosaceae Juss.
Iz_2_1009	DJI_0553	Quercus robur L.	0	n	1	1	Fagaceae Dumort.
Iz_2_1009	DJI_0553	Robinia pseudoacacia L.	0	a	0	1	Fabaceae Lindl.
Iz_2_1009	DJI_0553	Rosa sp.	0		NA	1	Rosaceae Juss.
Iz_2_1009	DJI_0553	Rubus idaeus L.	0	n	1	0	Rosaceae Juss.
Iz_2_1009	DJI_0553	Rumex acetosella L.	0	n	1	0	Polygonaceae Juss.
Iz_2_1009	DJI_0553	Rumex thyrsiflorus Fingerh.	0	n	1	0	Polygonaceae Juss.
Iz_2_1009	DJI_0553	Salix alba L.	0	n	1	1	Salicaceae Mirb.
Iz_2_1009	DJI_0553	Sambucus nigra L.	0	n	1	1	Viburnaceae Raf.
Iz_2_1009	DJI_0553	Secale sylvestre Host	0	n	1	0	Poaceae Barnhart
Iz_2_1009	DJI_0553	Sempervivum ruthenicum Schnittsp. & C.B. Lehm.	0	n	1	0	Crassulaceae J.St.-Hil.
Iz_2_1009	DJI_0553	Setaria viridis (L.) P. Beauv.	0	n	1	0	Poaceae Barnhart
Iz_2_1009	DJI_0553	Silene borysthenica (Gruner) Walters	0	n	1	0	Caryophyllaceae Juss.
Iz_2_1009	DJI_0553	Silene latifolia subsp. alba (Mill.) Greuter & Burdet	0	n	1	0	Caryophyllaceae Juss.
Iz_2_1009	DJI_0553	Solidago virgaurea L.	0	n	1	0	Asteraceae Bercht. & J.Presl
Iz_2_1009	DJI_0553	Sorbus aucuparia L.	0	n	1	1	Rosaceae Juss.
Iz_2_1009	DJI_0553	Stachys recta L.	0	n	1	0	Lamiacea Lindl.
Iz_2_1009	DJI_0553	Tanacetum vulgare L.	0	n	1	0	Asteraceae Bercht. & J.Presl
Iz_2_1009	DJI_0553	Thymus pallasianus Heinr.Braun	0	n	1	0	Lamiacea Lindl.
Iz_2_1009	DJI_0553	Tilia cordata Mill.	0	n	1	1	Malvaceae Juss.
Iz_2_1009	DJI_0553	Trifolium alpestre L.	0	n	1	0	Fabaceae Lindl.
Iz_2_1009	DJI_0553	Trifolium arvense L.	0	n	1	0	Fabaceae Lindl.

Iz_2_1009	DJI_0553	Ulmus glabra Huds.	0	n	1	1	Ulmaceae Mirb.
Iz_2_1009	DJI_0553	Verbascum densiflorum Bertol.	0	n	1	0	Scrophulariaceae Juss.
Iz_2_1009	DJI_0553	Veronica austriaca L.	0	n	1	0	Plantaginaceae Juss.
Iz_2_1009	DJI_0553	Veronica incana L.	0	n	1	0	Plantaginaceae Juss.
Iz_2_1009	DJI_0553	Veronica verna L.	0	n	1	0	Plantaginaceae Juss.
Iz_2_1009	DJI_0553	Vincetoxicum hirundinaria Medik	0	n	1	0	Apocynaceae Juss.
Iz_2_1009	DJI_0558	Acer tataricum L.	0	n	1	1	Sapindaceae Juss.
Iz_2_1009	DJI_0558	Achillea millefolium L. s.l.	0	n	1	0	Asteraceae Bercht. & J.Presl
Iz_2_1009	DJI_0558	Allium oleraceum L.	0	n	1	0	Amaryllidaceae J.St.-Hil.
Iz_2_1009	DJI_0558	Ambrosia artemisiifolia L.	0	a	0	0	Asteraceae Bercht. & J.Presl
Iz_2_1009	DJI_0558	Amorpha fruticosa L.	0	a	0	0	Fabaceae Lindl.
Iz_2_1009	DJI_0558	Anthericum ramosum L.	0	n	1	0	Asparagaceae Juss.
Iz_2_1009	DJI_0558	Artemisia marschalliana Spreng.	0	n	1	0	Asteraceae Bercht. & J.Presl
Iz_2_1009	DJI_0558	Asparagus officinalis L.	0	n	1	0	Asparagaceae Juss.
Iz_2_1009	DJI_0558	Astragalus cicer L.	0	n	1	0	Fabaceae Lindl.
Iz_2_1009	DJI_0558	Bassia laniflora (S.G.Gmel.) A.J.Scott	0	n	1	0	Amaranthaceae Juss.
Iz_2_1009	DJI_0558	Berberis vulgaris L.	0	n	1	0	Berberidaceae Juss.
Iz_2_1009	DJI_0558	Betula pendula Roth.	0	n	1	1	Betulaceae Gray
Iz_2_1009	DJI_0558	Betula pendula Roth.	0	n	1	0	Poaceae Barnhart
Iz_2_1009	DJI_0558	Bromus inermis Leyss.	0		NA	NA	
Iz_2_1009	DJI_0558	Bromus squarrosus L.	0	a	0	0	Poaceae Barnhart
Iz_2_1009	DJI_0558	Bromus tectorum L.	0	a	0	0	Poaceae Barnhart
Iz_2_1009	DJI_0558	Calamagrostis epigejos (L.) Roth	0.1	n	1	0	Poaceae Barnhart
Iz_2_1009	DJI_0558	Carduus acanthoides L.	0	n	1	0	Asteraceae Bercht. & J.Presl
Iz_2_1009	DJI_0558	Carex sp.	0	n	1	0	Cyperaceae Juss.
Iz_2_1009	DJI_0558	Centaurea majorovii Dumbadze	0	n	1	0	Asteraceae Bercht. & J.Presl
Iz_2_1009	DJI_0558	Cf. Solidago virgaurea	0		NA	NA	
Iz_2_1009	DJI_0558	Chamaecytisus ruthenicus (Fisch.ex Wol.) Klaskova	0	n	1	0	Fabaceae Lindl.
Iz_2_1009	DJI_0558	Chelidonium majus L.	0	n	1	0	Papaveraceae Juss.
Iz_2_1009	DJI_0558	Chenopodium betaceum Andrz.	0	n	1	0	Amaranthaceae Juss.
Iz_2_1009	DJI_0558	Chenopodium cf. album	0	n	1	0	Amaranthaceae Juss.
Iz_2_1009	DJI_0558	Chenopodium sp.	0		NA	0	Amaranthaceae Juss.
Iz_2_1009	DJI_0558	Chondrilla juncea L.	0	n	1	0	Asteraceae Bercht. & J.Presl
Iz_2_1009	DJI_0558	Cirsium vulgare (Savi) Ten.	0	n	1	0	Asteraceae Bercht. & J.Presl
Iz_2_1009	DJI_0558	Clinopodium acinos (L.) Kuntze	0	n	1	0	Lamiacea Lindl.
Iz_2_1009	DJI_0558	Convallaria majalis L.	0	n	1	0	Asparagaceae Juss.
Iz_2_1009	DJI_0558	Crataegus rhipidophylla Gand.	0	n	1	1	Rosaceae Juss.
Iz_2_1009	DJI_0558	Crepis foetida L.	0	n	1	0	Asteraceae Bercht. & J.Presl
Iz_2_1009	DJI_0558	Dactylis glomerata L.	0	n	1	0	Poaceae Barnhart
Iz_2_1009	DJI_0558	Dianthus campestris M.Bieb.	0	n	1	0	Caryophyllaceae Juss.
Iz_2_1009	DJI_0558	Digitaria sanguinalis (L.) Scop.	0	a	0	0	Poaceae Barnhart
Iz_2_1009	DJI_0558	Elymus repens (L.) Gould	0	n	1	0	Poaceae Barnhart
Iz_2_1009	DJI_0558	Epilobium tetragonum L.	0	n	1	0	Onagraceae Juss.
Iz_2_1009	DJI_0558	Eragrostis minor Host	0	a	0	0	Poaceae Barnhart
Iz_2_1009	DJI_0558	Erigeron canadensis L.	0	a	0	0	Asteraceae Bercht. & J.Presl
Iz_2_1009	DJI_0558	Euphorbia seguieriana Neck.	0	n	1	0	Euphorbiaceae Juss.
Iz_2_1009	DJI_0558	Fallopia dumetorum (L.) Holub	0	n	1	0	Polygonaceae Juss.
Iz_2_1009	DJI_0558	Festuca beckeri (Hack.) Trautv.	0	n	1	0	Poaceae Barnhart
Iz_2_1009	DJI_0558	Fragaria vesca L.	0	n	1	0	Rosaceae Juss.
Iz_2_1009	DJI_0558	Frangula alnus Mill.	0	n	1	1	Rhamnaceae Juss.
Iz_2_1009	DJI_0558	Fraxinus pennsylvanica Marshall	0	a	0	1	Oleaceae Hoffmanns. & Link
Iz_2_1009	DJI_0558	Galeopsis bifida Boenn.	0	n	1	0	Lamiacea Lindl.
Iz_2_1009	DJI_0558	Galium verum L.	0	n	1	0	Rubiaceae Juss.
Iz_2_1009	DJI_0558	Genista tinctoria L.	0	n	1	0	Fabaceae Lindl.
Iz_2_1009	DJI_0558	Geranium robertianum L.	0	n	1	0	Geraniaceae Juss.
Iz_2_1009	DJI_0558	Geranium sanquineum L.	0	n	1	0	Geraniaceae Juss.
Iz_2_1009	DJI_0558	Gypsophila paniculata L.	0	n	1	0	Caryophyllaceae Juss.
Iz_2_1009	DJI_0558	Helichrysum arenarium (L.) Moench	0	n	1	0	Asteraceae Bercht. & J.Presl
Iz_2_1009	DJI_0558	Herniaria polygama J.Gay	0	n	1	0	Caryophyllaceae Juss.
Iz_2_1009	DJI_0558	Hieracium robustum Fr.	0	n	1	0	Asteraceae Bercht. & J.Presl
Iz_2_1009	DJI_0558	Hieracium umbellatum L.	0	n	1	NA	Asteraceae Bercht. & J.Presl
Iz_2_1009	DJI_0558	Hylotelephium maximum (L.) Holub	0	n	1	0	Crassulaceae J.St.-Hil.
Iz_2_1009	DJI_0558	Jacobaea borysthenica (DC.) B.Nord. & Greuter	0	n	1	0	Asteraceae Bercht. & J.Presl
Iz_2_1009	DJI_0558	Jasione montana L.	0	n	1	0	Campanulaceae Juss.
Iz_2_1009	DJI_0558	Jurinea cyanoides (L.) Rchb.s.l.	0	n	1	0	Asteraceae Bercht. & J.Presl
Iz_2_1009	DJI_0558	Koeleria glauca (Spreng.) DC.	0	n	1	0	Poaceae Barnhart
Iz_2_1009	DJI_0558	Lactuca serriola L.	0	a	0	0	Asteraceae Bercht. & J.Presl
Iz_2_1009	DJI_0558	Linaria genistifolia (L.) Mill.	0	n	1	0	Plantaginaceae Juss.
Iz_2_1009	DJI_0558	Lomelosia argentea (L.) Greuter & Burdet	0	n	1	0	Caprifoliaceae Juss.
Iz_2_1009	DJI_0558	Melampyrum pratense L.	0	n	1	0	Orobanchaceae Vent.
Iz_2_1009	DJI_0558	Oenothera biennis L.	0	a	0	0	Onagraceae Juss.
Iz_2_1009	DJI_0558	Pentanema britannica (L.) D.Gut.Larr., Santos-Vicente, Anderb., E.Rico & M.M.Mart.Ort.	0	n	1	0	Asteraceae Bercht. & J.Presl
Iz_2_1009	DJI_0558	Peucedanum oreoselinum (L.) Moench	0	n	1	0	Apiaceae Lindl.
Iz_2_1009	DJI_0558	Peucedanum ruthenicum M.Bieb.	0	n	1	0	Apiaceae Lindl.
Iz_2_1009	DJI_0558	Pilosella echioides (Lumn.) F.W.Schultz & Sch.Bip.	0	n	1	0	Asteraceae Bercht. & J.Presl
Iz_2_1009	DJI_0558	Pilosella officinarum F. Schultz et Sch. Bip.	0	n	1	0	Asteraceae Bercht. & J.Presl
Iz_2_1009	DJI_0558	Pilosella sp.	0		NA	0	Asteraceae Bercht. & J.Presl
Iz_2_1009	DJI_0558	Pinus sylvestris L.	0	n	1	1	Pinaceae Spreng. ex F.Rudolphi
Iz_2_1009	DJI_0558	Poa annua L.	0	n	1	0	Poaceae Barnhart
Iz_2_1009	DJI_0558	Poa nemoralis L.	0	n	1	0	Poaceae Barnhart
Iz_2_1009	DJI_0558	Poa sp.	0		NA	0	Poaceae Barnhart
Iz_2_1009	DJI_0558	Polygonatum odoratum (Mill.) Druce	0	n	1	0	Asparagaceae Juss.
Iz_2_1009	DJI_0558	Polygonum arenarium Waldst. & Kit.	0	n	1	0	Polygonaceae Juss.
Iz_2_1009	DJI_0558	Populus nigra L.	0	n	1	1	Salicaceae Mirb.
Iz_2_1009	DJI_0558	Populus tremula L.	0	n	1	1	Salicaceae Mirb.
Iz_2_1009	DJI_0558	Populus Ã canescens (Aiton) Sm.	0	n	1	1	Salicaceae Mirb.
Iz_2_1009	DJI_0558	Potentilla argentea L.	0	n	1	0	Rosaceae Juss.
Iz_2_1009	DJI_0558	Psammophiliella muralis (L.) Ikonn.	0	n	1	0	Caryophyllaceae Juss.
Iz_2_1009	DJI_0558	Psephellus sumensis (Kalen.) Greuter	0	n	1	0	Asteraceae Bercht. & J.Presl
Iz_2_1009	DJI_0558	Pulsatilla pratensis (L.) Mill. s.l.	0	n	1	0	Ranunculaceae Juss.
Iz_2_1009	DJI_0558	Pyrus communis L.	0	a	0	1	Rosaceae Juss.
Iz_2_1009	DJI_0558	Quercus robur L.	0	n	1	1	Fagaceae Dumort.
Iz_2_1009	DJI_0558	Robinia pseudoacacia L.	0	a	0	1	Fabaceae Lindl.
Iz_2_1009	DJI_0558	Rosa sp.	0		NA	1	Rosaceae Juss.
Iz_2_1009	DJI_0558	Rubus idaeus L.	0	n	1	0	Rosaceae Juss.
Iz_2_1009	DJI_0558	Rumex acetosella L.	0	n	1	0	Polygonaceae Juss.
Iz_2_1009	DJI_0558	Rumex thyrsiflorus Fingerh.	0	n	1	0	Polygonaceae Juss.
Iz_2_1009	DJI_0558	Salix alba L.	0	n	1	1	Salicaceae Mirb.

Iz_2_1009	DJI_0558	Sambucus nigra L.	0	n	1	1	Viburnaceae Raf.
Iz_2_1009	DJI_0558	Secale sylvestre Host	0	n	1	0	Poaceae Barnhart
Iz_2_1009	DJI_0558	Sempervivum ruthenicum Schnittsp. & C.B. Lehm.	0	n	1	0	Crassulaceae J.St.-Hil.
Iz_2_1009	DJI_0558	Setaria viridis (L.) P. Beauv.	0	n	1	0	Poaceae Barnhart
Iz_2_1009	DJI_0558	Silene borysthenica (Gruner) Walters	0	n	1	0	Caryophyllaceae Juss.
Iz_2_1009	DJI_0558	Silene latifolia subsp. alba (Mill.) Greuter & Burdet	0	n	1	0	Caryophyllaceae Juss.
Iz_2_1009	DJI_0558	Solidago virgaurea L.	0	n	1	0	Asteraceae Bercht. & J.Presl
Iz_2_1009	DJI_0558	Sorbus aucuparia L.	0	n	1	1	Rosaceae Juss.
Iz_2_1009	DJI_0558	Stachys recta L.	0	n	1	0	Lamiacea Lindl.
Iz_2_1009	DJI_0558	Tanacetum vulgare L.	0	n	1	0	Asteraceae Bercht. & J.Presl
Iz_2_1009	DJI_0558	Thymus pallasianus Heinr.Braun	0	n	1	0	Lamiacea Lindl.
Iz_2_1009	DJI_0558	Tilia cordata Mill.	0	n	1	1	Malvaceae Juss.
Iz_2_1009	DJI_0558	Trifolium alpestre L.	0	n	1	0	Fabaceae Lindl.
Iz_2_1009	DJI_0558	Trifolium arvense L.	0	n	1	0	Fabaceae Lindl.
Iz_2_1009	DJI_0558	Ulmus glabra Huds.	0	n	1	1	Ulmaceae Mirb.
Iz_2_1009	DJI_0558	Verbascum densiflorum Bertol.	0	n	1	0	Scrophulariaceae Juss.
Iz_2_1009	DJI_0558	Veronica austriaca L.	0	n	1	0	Plantaginaceae Juss.
Iz_2_1009	DJI_0558	Veronica incana L.	0	n	1	0	Plantaginaceae Juss.
Iz_2_1009	DJI_0558	Veronica verna L.	0	n	1	0	Plantaginaceae Juss.
Iz_2_1009	DJI_0558	Vincetoxicum hirundinaria Medik	0	n	1	0	Apocynaceae Juss.
Iz_2_1009	DJI_0560	Acer tataricum L.	0	n	1	1	Sapindaceae Juss.
Iz_2_1009	DJI_0560	Achillea millefolium L. s.l.	0	n	1	0	Asteraceae Bercht. & J.Presl
Iz_2_1009	DJI_0560	Allium oleraceum L.	0	n	1	0	Amaryllidaceae J.St.-Hil.
Iz_2_1009	DJI_0560	Ambrosia artemisiifolia L.	0	a	0	0	Asteraceae Bercht. & J.Presl
Iz_2_1009	DJI_0560	Amorpha fruticosa L.	0	a	0	0	Fabaceae Lindl.
Iz_2_1009	DJI_0560	Anthericum ramosum L.	0	n	1	0	Asparagaceae Juss.
Iz_2_1009	DJI_0560	Artemisia marschalliana Spreng.	0	n	1	0	Asteraceae Bercht. & J.Presl
Iz_2_1009	DJI_0560	Asparagus officinalis L.	0	n	1	0	Asparagaceae Juss.
Iz_2_1009	DJI_0560	Astragalus cicer L.	0	n	1	0	Fabaceae Lindl.
Iz_2_1009	DJI_0560	Bassia laniflora (S.G.Gmel.) A.J.Scott	0	n	1	0	Amaranthaceae Juss.
Iz_2_1009	DJI_0560	Berberis vulgaris L.	0	n	1	0	Berberidaceae Juss.
Iz_2_1009	DJI_0560	Betula pendula Roth.	0	n	1	1	Betulaceae Gray
Iz_2_1009	DJI_0560	Betula pendula Roth.	0	n	1	0	Poaceae Barnhart
Iz_2_1009	DJI_0560	Bromus inermis Leyss.	0		NA	NA	
Iz_2_1009	DJI_0560	Bromus squarrosus L.	0	a	0	0	Poaceae Barnhart
Iz_2_1009	DJI_0560	Bromus tectorum L.	0	a	0	0	Poaceae Barnhart
Iz_2_1009	DJI_0560	Calamagrostis epigejos (L.) Roth	0.1	n	1	0	Poaceae Barnhart
Iz_2_1009	DJI_0560	Carduus acanthoides L.	0	n	1	0	Asteraceae Bercht. & J.Presl
Iz_2_1009	DJI_0560	Carex sp.	0	n	1	0	Cyperaceae Juss.
Iz_2_1009	DJI_0560	Centaurea majorovii Dumbadze	0	n	1	0	Asteraceae Bercht. & J.Presl
Iz_2_1009	DJI_0560	Cf. Solidago virgaurea	0		NA	NA	
Iz_2_1009	DJI_0560	Chamaecytisus ruthenicus (Fisch.ex Wol.) Klaskova	0	n	1	0	Fabaceae Lindl.
Iz_2_1009	DJI_0560	Chelidonium majus L.	0	n	1	0	Papaveraceae Juss.
Iz_2_1009	DJI_0560	Chenopodium betaceum Andrz.	0	n	1	0	Amaranthaceae Juss.
Iz_2_1009	DJI_0560	Chenopodium cf. album	0	n	1	0	Amaranthaceae Juss.
Iz_2_1009	DJI_0560	Chenopodium sp.	0		NA	0	Amaranthaceae Juss.
Iz_2_1009	DJI_0560	Chondrilla juncea L.	0	n	1	0	Asteraceae Bercht. & J.Presl
Iz_2_1009	DJI_0560	Cirsium vulgare (Savi) Ten.	0	n	1	0	Asteraceae Bercht. & J.Presl
Iz_2_1009	DJI_0560	Clinopodium acinos (L.) Kuntze	0	n	1	0	Lamiacea Lindl.
Iz_2_1009	DJI_0560	Convallaria majalis L.	0	n	1	0	Asparagaceae Juss.
Iz_2_1009	DJI_0560	Crataegus rhipidophylla Gand.	0	n	1	1	Rosaceae Juss.
Iz_2_1009	DJI_0560	Crepis foetida L.	0	n	1	0	Asteraceae Bercht. & J.Presl
Iz_2_1009	DJI_0560	Dactylis glomerata L.	0	n	1	0	Poaceae Barnhart
Iz_2_1009	DJI_0560	Dianthus campestris M.Bieb.	0	n	1	0	Caryophyllaceae Juss.
Iz_2_1009	DJI_0560	Digitaria sanguinalis (L.) Scop.	0	a	0	0	Poaceae Barnhart
Iz_2_1009	DJI_0560	Elymus repens (L.) Gould	0	n	1	0	Poaceae Barnhart
Iz_2_1009	DJI_0560	Epilobium tetragonum L.	0	n	1	0	Onagraceae Juss.
Iz_2_1009	DJI_0560	Eragrostis minor Host	0	a	0	0	Poaceae Barnhart
Iz_2_1009	DJI_0560	Erigeron canadensis L.	0	a	0	0	Asteraceae Bercht. & J.Presl
Iz_2_1009	DJI_0560	Euphorbia seguieriana Neck.	0	n	1	0	Euphorbiaceae Juss.
Iz_2_1009	DJI_0560	Fallopia dumetorum (L.) Holub	0	n	1	0	Polygonaceae Juss.
Iz_2_1009	DJI_0560	Festuca beckeri (Hack.) Trautv.	0	n	1	0	Poaceae Barnhart
Iz_2_1009	DJI_0560	Fragaria vesca L.	0	n	1	0	Rosaceae Juss.
Iz_2_1009	DJI_0560	Frangula alnus Mill.	0	n	1	1	Rhamnaceae Juss.
Iz_2_1009	DJI_0560	Fraxinus pennsylvanica Marshall	0	a	0	1	Oleaceae Hoffmanns. & Link
Iz_2_1009	DJI_0560	Galeopsis bifida Boenn.	0	n	1	0	Lamiacea Lindl.
Iz_2_1009	DJI_0560	Galium verum L.	0	n	1	0	Rubiaceae Juss.
Iz_2_1009	DJI_0560	Genista tinctoria L.	0	n	1	0	Fabaceae Lindl.
Iz_2_1009	DJI_0560	Geranium robertianum L.	0	n	1	0	Geraniaceae Juss.
Iz_2_1009	DJI_0560	Geranium sanquineum L.	0	n	1	0	Geraniaceae Juss.
Iz_2_1009	DJI_0560	Gypsophila paniculata L.	0	n	1	0	Caryophyllaceae Juss.
Iz_2_1009	DJI_0560	Helichrysum arenarium (L.) Moench	0	n	1	0	Asteraceae Bercht. & J.Presl
Iz_2_1009	DJI_0560	Herniaria polygama J.Gay	0	n	1	0	Caryophyllaceae Juss.
Iz_2_1009	DJI_0560	Hieracium robustum Fr.	0	n	1	0	Asteraceae Bercht. & J.Presl
Iz_2_1009	DJI_0560	Hieracium umbellatum L.	0	n	1	NA	Asteraceae Bercht. & J.Presl
Iz_2_1009	DJI_0560	Hylotelephium maximum (L.) Holub	0	n	1	0	Crassulaceae J.St.-Hil.
Iz_2_1009	DJI_0560	Jacobaea borysthenica (DC.) B.Nord. & Greuter	0	n	1	0	Asteraceae Bercht. & J.Presl
Iz_2_1009	DJI_0560	Jasione montana L.	0	n	1	0	Campanulaceae Juss.
Iz_2_1009	DJI_0560	Jurinea cyanoides (L.) Rchb.s.l.	0	n	1	0	Asteraceae Bercht. & J.Presl
Iz_2_1009	DJI_0560	Koeleria glauca (Spreng.) DC.	0	n	1	0	Poaceae Barnhart
Iz_2_1009	DJI_0560	Lactuca serriola L.	0	a	0	0	Asteraceae Bercht. & J.Presl
Iz_2_1009	DJI_0560	Linaria genistifolia (L.) Mill.	0	n	1	0	Plantaginaceae Juss.
Iz_2_1009	DJI_0560	Lomelosia argentea (L.) Greuter & Burdet	0	n	1	0	Caprifoliaceae Juss.
Iz_2_1009	DJI_0560	Melampyrum pratense L.	0	n	1	0	Orobanchaceae Vent.
Iz_2_1009	DJI_0560	Oenothera biennis L.	0	a	0	0	Onagraceae Juss.
Iz_2_1009	DJI_0560	Pentanema britannica (L.) D.Gut.Larr., Santos-Vicente, Anderb., E.Rico & M.M.Mart.Ort.	0	n	1	0	Asteraceae Bercht. & J.Presl
Iz_2_1009	DJI_0560	Peucedanum oreoselinum (L.) Moench	0	n	1	0	Apiaceae Lindl.
Iz_2_1009	DJI_0560	Peucedanum ruthenicum M.Bieb.	0	n	1	0	Apiaceae Lindl.
Iz_2_1009	DJI_0560	Pilosella echioides (Lumn.) F.W.Schultz & Sch.Bip.	0	n	1	0	Asteraceae Bercht. & J.Presl
Iz_2_1009	DJI_0560	Pilosella officinarum F. Schultz et Sch. Bip.	0	n	1	0	Asteraceae Bercht. & J.Presl
Iz_2_1009	DJI_0560	Pilosella sp.	0		NA	0	Asteraceae Bercht. & J.Presl
Iz_2_1009	DJI_0560	Pinus sylvestris L.	0	n	1	1	Pinaceae Spreng. ex F.Rudolphi
Iz_2_1009	DJI_0560	Poa annua L.	0	n	1	0	Poaceae Barnhart
Iz_2_1009	DJI_0560	Poa nemoralis L.	0	n	1	0	Poaceae Barnhart
Iz_2_1009	DJI_0560	Poa sp.	0		NA	0	Poaceae Barnhart
Iz_2_1009	DJI_0560	Polygonatum odoratum (Mill.) Druce	0	n	1	0	Asparagaceae Juss.
Iz_2_1009	DJI_0560	Polygonum arenarium Waldst. & Kit.	0	n	1	0	Polygonaceae Juss.
Iz_2_1009	DJI_0560	Populus nigra L.	0	n	1	1	Salicaceae Mirb.

Iz_2_1009	DJI_0560	Populus tremula L.	0	n	1	1	Salicaceae Mirb.
Iz_2_1009	DJI_0560	Populus Ã canescens (Aiton) Sm.	0	n	1	1	Salicaceae Mirb.
Iz_2_1009	DJI_0560	Potentilla argentea L.	0	n	1	0	Rosaceae Juss.
Iz_2_1009	DJI_0560	Psammophiliella muralis (L.) Ikonn.	0	n	1	0	Caryophyllaceae Juss.
Iz_2_1009	DJI_0560	Psephellus sumensis (Kalen.) Greuter	0	n	1	0	Asteraceae Bercht. & J.Presl
Iz_2_1009	DJI_0560	Pulsatilla pratensis (L.) Mill. s.l.	0	n	1	0	Ranunculaceae Juss.
Iz_2_1009	DJI_0560	Pyrus communis L.	0	a	0	1	Rosaceae Juss.
Iz_2_1009	DJI_0560	Quercus robur L.	0	n	1	1	Fagaceae Dumort.
Iz_2_1009	DJI_0560	Robinia pseudoacacia L.	0	a	0	1	Fabaceae Lindl.
Iz_2_1009	DJI_0560	Rosa sp.	0		NA	1	Rosaceae Juss.
Iz_2_1009	DJI_0560	Rubus idaeus L.	0	n	1	0	Rosaceae Juss.
Iz_2_1009	DJI_0560	Rumex acetosella L.	0	n	1	0	Polygonaceae Juss.
Iz_2_1009	DJI_0560	Rumex thyrsiflorus Fingerh.	0	n	1	0	Polygonaceae Juss.
Iz_2_1009	DJI_0560	Salix alba L.	0	n	1	1	Salicaceae Mirb.
Iz_2_1009	DJI_0560	Sambucus nigra L.	0	n	1	1	Viburnaceae Raf.
Iz_2_1009	DJI_0560	Secale sylvestre Host	0	n	1	0	Poaceae Barnhart
Iz_2_1009	DJI_0560	Sempervivum ruthenicum Schnittsp. & C.B. Lehm.	0	n	1	0	Crassulaceae J.St.-Hil.
Iz_2_1009	DJI_0560	Setaria viridis (L.) P. Beauv.	0	n	1	0	Poaceae Barnhart
Iz_2_1009	DJI_0560	Silene borysthenica (Gruner) Walters	0	n	1	0	Caryophyllaceae Juss.
Iz_2_1009	DJI_0560	Silene latifolia subsp. alba (Mill.) Greuter & Burdet	0	n	1	0	Caryophyllaceae Juss.
Iz_2_1009	DJI_0560	Solidago virgaurea L.	0	n	1	0	Asteraceae Bercht. & J.Presl
Iz_2_1009	DJI_0560	Sorbus aucuparia L.	0	n	1	1	Rosaceae Juss.
Iz_2_1009	DJI_0560	Stachys recta L.	0	n	1	0	Lamiacea Lindl.
Iz_2_1009	DJI_0560	Tanacetum vulgare L.	0	n	1	0	Asteraceae Bercht. & J.Presl
Iz_2_1009	DJI_0560	Thymus pallasianus Heinr.Braun	0	n	1	0	Lamiacea Lindl.
Iz_2_1009	DJI_0560	Tilia cordata Mill.	0	n	1	1	Malvaceae Juss.
Iz_2_1009	DJI_0560	Trifolium alpestre L.	0	n	1	0	Fabaceae Lindl.
Iz_2_1009	DJI_0560	Trifolium arvense L.	0	n	1	0	Fabaceae Lindl.
Iz_2_1009	DJI_0560	Ulmus glabra Huds.	0	n	1	1	Ulmaceae Mirb.
Iz_2_1009	DJI_0560	Verbascum densiflorum Bertol.	0	n	1	0	Scrophulariaceae Juss.
Iz_2_1009	DJI_0560	Veronica austriaca L.	0	n	1	0	Plantaginaceae Juss.
Iz_2_1009	DJI_0560	Veronica incana L.	0	n	1	0	Plantaginaceae Juss.
Iz_2_1009	DJI_0560	Veronica verna L.	0	n	1	0	Plantaginaceae Juss.
Iz_2_1009	DJI_0560	Vincetoxicum hirundinaria Medik	0	n	1	0	Apocynaceae Juss.
Iz_2_1009	DJI_0562	Acer tataricum L.	0	n	1	1	Sapindaceae Juss.
Iz_2_1009	DJI_0562	Achillea millefolium L. s.l.	0	n	1	0	Asteraceae Bercht. & J.Presl
Iz_2_1009	DJI_0562	Allium oleraceum L.	0	n	1	0	Amaryllidaceae J.St.-Hil.
Iz_2_1009	DJI_0562	Ambrosia artemisiifolia L.	0	a	0	0	Asteraceae Bercht. & J.Presl
Iz_2_1009	DJI_0562	Amorpha fruticosa L.	0	a	0	0	Fabaceae Lindl.
Iz_2_1009	DJI_0562	Anthericum ramosum L.	0	n	1	0	Asparagaceae Juss.
Iz_2_1009	DJI_0562	Artemisia marschalliana Spreng.	0	n	1	0	Asteraceae Bercht. & J.Presl
Iz_2_1009	DJI_0562	Asparagus officinalis L.	0	n	1	0	Asparagaceae Juss.
Iz_2_1009	DJI_0562	Astragalus cicer L.	0	n	1	0	Fabaceae Lindl.
Iz_2_1009	DJI_0562	Bassia laniflora (S.G.Gmel.) A.J.Scott	0	n	1	0	Amaranthaceae Juss.
Iz_2_1009	DJI_0562	Berberis vulgaris L.	0	n	1	0	Berberidaceae Juss.
Iz_2_1009	DJI_0562	Betula pendula Roth.	0	n	1	1	Betulaceae Gray
Iz_2_1009	DJI_0562	Betula pendula Roth.	0	n	1	0	Poaceae Barnhart
Iz_2_1009	DJI_0562	Bromus inermis Leyss.	0		NA	NA	
Iz_2_1009	DJI_0562	Bromus squarrosus L.	0	a	0	0	Poaceae Barnhart
Iz_2_1009	DJI_0562	Bromus tectorum L.	0	a	0	0	Poaceae Barnhart
Iz_2_1009	DJI_0562	Calamagrostis epigejos (L.) Roth	50	n	1	0	Poaceae Barnhart
Iz_2_1009	DJI_0562	Carduus acanthoides L.	0	n	1	0	Asteraceae Bercht. & J.Presl
Iz_2_1009	DJI_0562	Carex sp.	0	n	1	0	Cyperaceae Juss.
Iz_2_1009	DJI_0562	Centaurea majorovii Dumbadze	0	n	1	0	Asteraceae Bercht. & J.Presl
Iz_2_1009	DJI_0562	Cf. Solidago virgaurea	0		NA	NA	
Iz_2_1009	DJI_0562	Chamaecytisus ruthenicus (Fisch.ex Wol.) Klaskova	0	n	1	0	Fabaceae Lindl.
Iz_2_1009	DJI_0562	Chelidonium majus L.	0	n	1	0	Papaveraceae Juss.
Iz_2_1009	DJI_0562	Chenopodium betaceum Andrz.	0	n	1	0	Amaranthaceae Juss.
Iz_2_1009	DJI_0562	Chenopodium cf. album	0	n	1	0	Amaranthaceae Juss.
Iz_2_1009	DJI_0562	Chenopodium sp.	0		NA	0	Amaranthaceae Juss.
Iz_2_1009	DJI_0562	Chondrilla juncea L.	0	n	1	0	Asteraceae Bercht. & J.Presl
Iz_2_1009	DJI_0562	Cirsium vulgare (Savi) Ten.	0	n	1	0	Asteraceae Bercht. & J.Presl
Iz_2_1009	DJI_0562	Clinopodium acinos (L.) Kuntze	0	n	1	0	Lamiacea Lindl.
Iz_2_1009	DJI_0562	Convallaria majalis L.	0	n	1	0	Asparagaceae Juss.
Iz_2_1009	DJI_0562	Crataegus rhipidophylla Gand.	0	n	1	1	Rosaceae Juss.
Iz_2_1009	DJI_0562	Crepis foetida L.	0	n	1	0	Asteraceae Bercht. & J.Presl
Iz_2_1009	DJI_0562	Dactylis glomerata L.	0	n	1	0	Poaceae Barnhart
Iz_2_1009	DJI_0562	Dianthus campestris M.Bieb.	0	n	1	0	Caryophyllaceae Juss.
Iz_2_1009	DJI_0562	Digitaria sanguinalis (L.) Scop.	0	a	0	0	Poaceae Barnhart
Iz_2_1009	DJI_0562	Elymus repens (L.) Gould	0	n	1	0	Poaceae Barnhart
Iz_2_1009	DJI_0562	Epilobium tetragonum L.	0	n	1	0	Onagraceae Juss.
Iz_2_1009	DJI_0562	Eragrostis minor Host	0	a	0	0	Poaceae Barnhart
Iz_2_1009	DJI_0562	Erigeron canadensis L.	0	a	0	0	Asteraceae Bercht. & J.Presl
Iz_2_1009	DJI_0562	Euphorbia seguieriana Neck.	0	n	1	0	Euphorbiaceae Juss.
Iz_2_1009	DJI_0562	Fallopia dumetorum (L.) Holub	0	n	1	0	Polygonaceae Juss.
Iz_2_1009	DJI_0562	Festuca beckeri (Hack.) Trautv.	0	n	1	0	Poaceae Barnhart
Iz_2_1009	DJI_0562	Fragaria vesca L.	0	n	1	0	Rosaceae Juss.
Iz_2_1009	DJI_0562	Frangula alnus Mill.	0	n	1	1	Rhamnaceae Juss.
Iz_2_1009	DJI_0562	Fraxinus pennsylvanica Marshall	0	a	0	1	Oleaceae Hoffmanns. & Link
Iz_2_1009	DJI_0562	Galeopsis bifida Boenn.	0	n	1	0	Lamiacea Lindl.
Iz_2_1009	DJI_0562	Galium verum L.	0	n	1	0	Rubiaceae Juss.
Iz_2_1009	DJI_0562	Genista tinctoria L.	0	n	1	0	Fabaceae Lindl.
Iz_2_1009	DJI_0562	Geranium robertianum L.	0	n	1	0	Geraniaceae Juss.
Iz_2_1009	DJI_0562	Geranium sanquineum L.	0	n	1	0	Geraniaceae Juss.
Iz_2_1009	DJI_0562	Gypsophila paniculata L.	0	n	1	0	Caryophyllaceae Juss.
Iz_2_1009	DJI_0562	Helichrysum arenarium (L.) Moench	0	n	1	0	Asteraceae Bercht. & J.Presl
Iz_2_1009	DJI_0562	Herniaria polygama J.Gay	0	n	1	0	Caryophyllaceae Juss.
Iz_2_1009	DJI_0562	Hieracium robustum Fr.	0	n	1	0	Asteraceae Bercht. & J.Presl
Iz_2_1009	DJI_0562	Hieracium umbellatum L.	0	n	1	NA	Asteraceae Bercht. & J.Presl
Iz_2_1009	DJI_0562	Hylotelephium maximum (L.) Holub	0	n	1	0	Crassulaceae J.St.-Hil.
Iz_2_1009	DJI_0562	Jacobaea borysthenica (DC.) B.Nord. & Greuter	0	n	1	0	Asteraceae Bercht. & J.Presl
Iz_2_1009	DJI_0562	Jasione montana L.	0	n	1	0	Campanulaceae Juss.
Iz_2_1009	DJI_0562	Jurinea cyanoides (L.) Rchb.s.l.	0	n	1	0	Asteraceae Bercht. & J.Presl
Iz_2_1009	DJI_0562	Koeleria glauca (Spreng.) DC.	0	n	1	0	Poaceae Barnhart
Iz_2_1009	DJI_0562	Lactuca serriola L.	0	a	0	0	Asteraceae Bercht. & J.Presl
Iz_2_1009	DJI_0562	Linaria genistifolia (L.) Mill.	0	n	1	0	Plantaginaceae Juss.
Iz_2_1009	DJI_0562	Lomelosia argentea (L.) Greuter & Burdet	0	n	1	0	Caprifoliaceae Juss.
Iz_2_1009	DJI_0562	Melampyrum pratense L.	0	n	1	0	Orobanchaceae Vent.

Iz_2_1009	DJI_0562	Oenothera biennis L.	0	a	0	0	Onagraceae Juss.
Iz_2_1009	DJI_0562	Pentanema britannica (L.) D.Gut.Larr., Santos-Vicente, Anderb., E.Rico & M.M.Mart.Ort.	0	n	1	0	Asteraceae Bercht. & J.Presl
Iz_2_1009	DJI_0562	Peucedanum oreoselinum (L.) Moench	0	n	1	0	Apiaceae Lindl.
Iz_2_1009	DJI_0562	Peucedanum ruthenicum M.Bieb.	0	n	1	0	Apiaceae Lindl.
Iz_2_1009	DJI_0562	Pilosella echioides (Lumn.) F.W.Schultz & Sch.Bip.	0	n	1	0	Asteraceae Bercht. & J.Presl
Iz_2_1009	DJI_0562	Pilosella officinarum F. Schultz et Sch. Bip.	0	n	1	0	Asteraceae Bercht. & J.Presl
Iz_2_1009	DJI_0562	Pilosella sp.	0		NA	0	Asteraceae Bercht. & J.Presl
Iz_2_1009	DJI_0562	Pinus sylvestris L.	0	n	1	1	Pinaceae Spreng. ex F.Rudolphi
Iz_2_1009	DJI_0562	Poa annua L.	0	n	1	0	Poaceae Barnhart
Iz_2_1009	DJI_0562	Poa nemoralis L.	0	n	1	0	Poaceae Barnhart
Iz_2_1009	DJI_0562	Poa sp.	0		NA	0	Poaceae Barnhart
Iz_2_1009	DJI_0562	Polygonatum odoratum (Mill.) Druce	0	n	1	0	Asparagaceae Juss.
Iz_2_1009	DJI_0562	Polygonum arenarium Waldst. & Kit.	0	n	1	0	Polygonaceae Juss.
Iz_2_1009	DJI_0562	Populus nigra L.	0	n	1	1	Salicaceae Mirb.
Iz_2_1009	DJI_0562	Populus tremula L.	0	n	1	1	Salicaceae Mirb.
Iz_2_1009	DJI_0562	Populus Ã canescens (Aiton) Sm.	0	n	1	1	Salicaceae Mirb.
Iz_2_1009	DJI_0562	Potentilla argentea L.	0	n	1	0	Rosaceae Juss.
Iz_2_1009	DJI_0562	Psammophiliella muralis (L.) Ikonn.	0	n	1	0	Caryophyllaceae Juss.
Iz_2_1009	DJI_0562	Psephellus sumensis (Kalen.) Greuter	0	n	1	0	Asteraceae Bercht. & J.Presl
Iz_2_1009	DJI_0562	Pulsatilla pratensis (L.) Mill. s.l.	0	n	1	0	Ranunculaceae Juss.
Iz_2_1009	DJI_0562	Pyrus communis L.	0	a	0	1	Rosaceae Juss.
Iz_2_1009	DJI_0562	Quercus robur L.	0	n	1	1	Fagaceae Dumort.
Iz_2_1009	DJI_0562	Robinia pseudoacacia L.	0	a	0	1	Fabaceae Lindl.
Iz_2_1009	DJI_0562	Rosa sp.	0		NA	1	Rosaceae Juss.
Iz_2_1009	DJI_0562	Rubus idaeus L.	0	n	1	0	Rosaceae Juss.
Iz_2_1009	DJI_0562	Rumex acetosella L.	0	n	1	0	Polygonaceae Juss.
Iz_2_1009	DJI_0562	Rumex thyrsiflorus Fingerh.	0	n	1	0	Polygonaceae Juss.
Iz_2_1009	DJI_0562	Salix alba L.	0	n	1	1	Salicaceae Mirb.
Iz_2_1009	DJI_0562	Sambucus nigra L.	0	n	1	1	Viburnaceae Raf.
Iz_2_1009	DJI_0562	Secale sylvestre Host	0	n	1	0	Poaceae Barnhart
Iz_2_1009	DJI_0562	Sempervivum ruthenicum Schnittsp. & C.B. Lehm.	0	n	1	0	Crassulaceae J.St.-Hil.
Iz_2_1009	DJI_0562	Setaria viridis (L.) P. Beauv.	0	n	1	0	Poaceae Barnhart
Iz_2_1009	DJI_0562	Silene borysthenica (Gruner) Walters	0	n	1	0	Caryophyllaceae Juss.
Iz_2_1009	DJI_0562	Silene latifolia subsp. alba (Mill.) Greuter & Burdet	0	n	1	0	Caryophyllaceae Juss.
Iz_2_1009	DJI_0562	Solidago virgaurea L.	0	n	1	0	Asteraceae Bercht. & J.Presl
Iz_2_1009	DJI_0562	Sorbus aucuparia L.	0	n	1	1	Rosaceae Juss.
Iz_2_1009	DJI_0562	Stachys recta L.	0	n	1	0	Lamiacea Lindl.
Iz_2_1009	DJI_0562	Tanacetum vulgare L.	0	n	1	0	Asteraceae Bercht. & J.Presl
Iz_2_1009	DJI_0562	Thymus pallasianus Heinr.Braun	0	n	1	0	Lamiacea Lindl.
Iz_2_1009	DJI_0562	Tilia cordata Mill.	0	n	1	1	Malvaceae Juss.
Iz_2_1009	DJI_0562	Trifolium alpestre L.	0	n	1	0	Fabaceae Lindl.
Iz_2_1009	DJI_0562	Trifolium arvense L.	0	n	1	0	Fabaceae Lindl.
Iz_2_1009	DJI_0562	Ulmus glabra Huds.	0	n	1	1	Ulmaceae Mirb.
Iz_2_1009	DJI_0562	Verbascum densiflorum Bertol.	0	n	1	0	Scrophulariaceae Juss.
Iz_2_1009	DJI_0562	Veronica austriaca L.	0	n	1	0	Plantaginaceae Juss.
Iz_2_1009	DJI_0562	Veronica incana L.	0	n	1	0	Plantaginaceae Juss.
Iz_2_1009	DJI_0562	Veronica verna L.	0	n	1	0	Plantaginaceae Juss.
Iz_2_1009	DJI_0562	Vincetoxicum hirundinaria Medik	0	n	1	0	Apocynaceae Juss.
Iz_2_1009	DJI_0569	Acer tataricum L.	0	n	1	1	Sapindaceae Juss.
Iz_2_1009	DJI_0569	Achillea millefolium L. s.l.	0	n	1	0	Asteraceae Bercht. & J.Presl
Iz_2_1009	DJI_0569	Allium oleraceum L.	0	n	1	0	Amaryllidaceae J.St.-Hil.
Iz_2_1009	DJI_0569	Ambrosia artemisiifolia L.	0	a	0	0	Asteraceae Bercht. & J.Presl
Iz_2_1009	DJI_0569	Amorpha fruticosa L.	0	a	0	0	Fabaceae Lindl.
Iz_2_1009	DJI_0569	Anthericum ramosum L.	0	n	1	0	Asparagaceae Juss.
Iz_2_1009	DJI_0569	Artemisia marschalliana Spreng.	0	n	1	0	Asteraceae Bercht. & J.Presl
Iz_2_1009	DJI_0569	Asparagus officinalis L.	0	n	1	0	Asparagaceae Juss.
Iz_2_1009	DJI_0569	Astragalus cicer L.	0	n	1	0	Fabaceae Lindl.
Iz_2_1009	DJI_0569	Bassia laniflora (S.G.Gmel.) A.J.Scott	0	n	1	0	Amaranthaceae Juss.
Iz_2_1009	DJI_0569	Berberis vulgaris L.	0	n	1	0	Berberidaceae Juss.
Iz_2_1009	DJI_0569	Betula pendula Roth.	0	n	1	1	Betulaceae Gray
Iz_2_1009	DJI_0569	Betula pendula Roth.	0	n	1	0	Poaceae Barnhart
Iz_2_1009	DJI_0569	Bromus inermis Leyss.	0		NA	NA	
Iz_2_1009	DJI_0569	Bromus squarrosus L.	0	a	0	0	Poaceae Barnhart
Iz_2_1009	DJI_0569	Bromus tectorum L.	0	a	0	0	Poaceae Barnhart
Iz_2_1009	DJI_0569	Calamagrostis epigejos (L.) Roth	30	n	1	0	Poaceae Barnhart
Iz_2_1009	DJI_0569	Carduus acanthoides L.	0	n	1	0	Asteraceae Bercht. & J.Presl
Iz_2_1009	DJI_0569	Carex sp.	0	n	1	0	Cyperaceae Juss.
Iz_2_1009	DJI_0569	Centaurea majorovii Dumbadze	0	n	1	0	Asteraceae Bercht. & J.Presl
Iz_2_1009	DJI_0569	Cf. Solidago virgaurea	0		NA	NA	
Iz_2_1009	DJI_0569	Chamaecytisus ruthenicus (Fisch.ex Wol.) Klaskova	0	n	1	0	Fabaceae Lindl.
Iz_2_1009	DJI_0569	Chelidonium majus L.	0	n	1	0	Papaveraceae Juss.
Iz_2_1009	DJI_0569	Chenopodium betaceum Andrz.	0	n	1	0	Amaranthaceae Juss.
Iz_2_1009	DJI_0569	Chenopodium cf. album	0	n	1	0	Amaranthaceae Juss.
Iz_2_1009	DJI_0569	Chenopodium sp.	0		NA	0	Amaranthaceae Juss.
Iz_2_1009	DJI_0569	Chondrilla juncea L.	0	n	1	0	Asteraceae Bercht. & J.Presl
Iz_2_1009	DJI_0569	Cirsium vulgare (Savi) Ten.	0	n	1	0	Asteraceae Bercht. & J.Presl
Iz_2_1009	DJI_0569	Clinopodium acinos (L.) Kuntze	0	n	1	0	Lamiacea Lindl.
Iz_2_1009	DJI_0569	Convallaria majalis L.	0	n	1	0	Asparagaceae Juss.
Iz_2_1009	DJI_0569	Crataegus rhipidophylla Gand.	0	n	1	1	Rosaceae Juss.
Iz_2_1009	DJI_0569	Crepis foetida L.	0	n	1	0	Asteraceae Bercht. & J.Presl
Iz_2_1009	DJI_0569	Dactylis glomerata L.	0	n	1	0	Poaceae Barnhart
Iz_2_1009	DJI_0569	Dianthus campestris M.Bieb.	0	n	1	0	Caryophyllaceae Juss.
Iz_2_1009	DJI_0569	Digitaria sanguinalis (L.) Scop.	0	a	0	0	Poaceae Barnhart
Iz_2_1009	DJI_0569	Elymus repens (L.) Gould	0	n	1	0	Poaceae Barnhart
Iz_2_1009	DJI_0569	Epilobium tetragonum L.	0	n	1	0	Onagraceae Juss.
Iz_2_1009	DJI_0569	Eragrostis minor Host	0	a	0	0	Poaceae Barnhart
Iz_2_1009	DJI_0569	Erigeron canadensis L.	0	a	0	0	Asteraceae Bercht. & J.Presl
Iz_2_1009	DJI_0569	Euphorbia seguieriana Neck.	0	n	1	0	Euphorbiaceae Juss.
Iz_2_1009	DJI_0569	Fallopia dumetorum (L.) Holub	0	n	1	0	Polygonaceae Juss.
Iz_2_1009	DJI_0569	Festuca beckeri (Hack.) Trautv.	0	n	1	0	Poaceae Barnhart
Iz_2_1009	DJI_0569	Fragaria vesca L.	0	n	1	0	Rosaceae Juss.
Iz_2_1009	DJI_0569	Frangula alnus Mill.	0	n	1	1	Rhamnaceae Juss.
Iz_2_1009	DJI_0569	Fraxinus pennsylvanica Marshall	0	a	0	1	Oleaceae Hoffmanns. & Link
Iz_2_1009	DJI_0569	Galeopsis bifida Boenn.	0	n	1	0	Lamiacea Lindl.
Iz_2_1009	DJI_0569	Galium verum L.	0	n	1	0	Rubiaceae Juss.
Iz_2_1009	DJI_0569	Genista tinctoria L.	0	n	1	0	Fabaceae Lindl.
Iz_2_1009	DJI_0569	Geranium robertianum L.	1	n	1	0	Geraniaceae Juss.
Iz_2_1009	DJI_0569	Geranium sanquineum L.	0	n	1	0	Geraniaceae Juss.

Iz_2_1009	DJI_0569	Gypsophila paniculata L.	0	n	1	0	Caryophyllaceae Juss.
Iz_2_1009	DJI_0569	Helichrysum arenarium (L.) Moench	0	n	1	0	Asteraceae Bercht. & J.Presl
Iz_2_1009	DJI_0569	Herniaria polygama J.Gay	0	n	1	0	Caryophyllaceae Juss.
Iz_2_1009	DJI_0569	Hieracium robustum Fr.	0	n	1	0	Asteraceae Bercht. & J.Presl
Iz_2_1009	DJI_0569	Hieracium umbellatum L.	0	n	1	NA	Asteraceae Bercht. & J.Presl
Iz_2_1009	DJI_0569	Hylotelephium maximum (L.) Holub	0	n	1	0	Crassulaceae J.St.-Hil.
Iz_2_1009	DJI_0569	Jacobaea borysthenica (DC.) B.Nord. & Greuter	0	n	1	0	Asteraceae Bercht. & J.Presl
Iz_2_1009	DJI_0569	Jasione montana L.	0	n	1	0	Campanulaceae Juss.
Iz_2_1009	DJI_0569	Jurinea cyanoides (L.) Rchb.s.l.	0	n	1	0	Asteraceae Bercht. & J.Presl
Iz_2_1009	DJI_0569	Koeleria glauca (Spreng.) DC.	0	n	1	0	Poaceae Barnhart
Iz_2_1009	DJI_0569	Lactuca serriola L.	0	a	0	0	Asteraceae Bercht. & J.Presl
Iz_2_1009	DJI_0569	Linaria genistifolia (L.) Mill.	0	n	1	0	Plantaginaceae Juss.
Iz_2_1009	DJI_0569	Lomelosia argentea (L.) Greuter & Burdet	0	n	1	0	Caprifoliaceae Juss.
Iz_2_1009	DJI_0569	Melampyrum pratense L.	0	n	1	0	Orobanchaceae Vent.
Iz_2_1009	DJI_0569	Oenothera biennis L.	0	a	0	0	Onagraceae Juss.
Iz_2_1009	DJI_0569	Pentanema britannica (L.) D.Gut.Larr., Santos-Vicente, Anderb., E.Rico & M.M.Mart.Ort.	0	n	1	0	Asteraceae Bercht. & J.Presl
Iz_2_1009	DJI_0569	Peucedanum oreoselinum (L.) Moench	0	n	1	0	Apiaceae Lindl.
Iz_2_1009	DJI_0569	Peucedanum ruthenicum M.Bieb.	0	n	1	0	Apiaceae Lindl.
Iz_2_1009	DJI_0569	Pilosella echioides (Lumn.) F.W.Schultz & Sch.Bip.	0	n	1	0	Asteraceae Bercht. & J.Presl
Iz_2_1009	DJI_0569	Pilosella officinarum F. Schultz et Sch. Bip.	0	n	1	0	Asteraceae Bercht. & J.Presl
Iz_2_1009	DJI_0569	Pilosella sp.	0		NA	0	Asteraceae Bercht. & J.Presl
Iz_2_1009	DJI_0569	Pinus sylvestris L.	0	n	1	1	Pinaceae Spreng. ex F.Rudolphi
Iz_2_1009	DJI_0569	Poa annua L.	0	n	1	0	Poaceae Barnhart
Iz_2_1009	DJI_0569	Poa nemoralis L.	0	n	1	0	Poaceae Barnhart
Iz_2_1009	DJI_0569	Poa sp.	0		NA	0	Poaceae Barnhart
Iz_2_1009	DJI_0569	Polygonatum odoratum (Mill.) Druce	0	n	1	0	Asparagaceae Juss.
Iz_2_1009	DJI_0569	Polygonum arenarium Waldst. & Kit.	0	n	1	0	Polygonaceae Juss.
Iz_2_1009	DJI_0569	Populus nigra L.	0	n	1	1	Salicaceae Mirb.
Iz_2_1009	DJI_0569	Populus tremula L.	0	n	1	1	Salicaceae Mirb.
Iz_2_1009	DJI_0569	Populus Ã canescens (Aiton) Sm.	0	n	1	1	Salicaceae Mirb.
Iz_2_1009	DJI_0569	Potentilla argentea L.	0	n	1	0	Rosaceae Juss.
Iz_2_1009	DJI_0569	Psammophiliella muralis (L.) Ikonn.	0	n	1	0	Caryophyllaceae Juss.
Iz_2_1009	DJI_0569	Psephellus sumensis (Kalen.) Greuter	0	n	1	0	Asteraceae Bercht. & J.Presl
Iz_2_1009	DJI_0569	Pulsatilla pratensis (L.) Mill. s.l.	0	n	1	0	Ranunculaceae Juss.
Iz_2_1009	DJI_0569	Pyrus communis L.	0	a	0	1	Rosaceae Juss.
Iz_2_1009	DJI_0569	Quercus robur L.	0	n	1	1	Fagaceae Dumort.
Iz_2_1009	DJI_0569	Robinia pseudoacacia L.	0	a	0	1	Fabaceae Lindl.
Iz_2_1009	DJI_0569	Rosa sp.	0		NA	1	Rosaceae Juss.
Iz_2_1009	DJI_0569	Rubus idaeus L.	0	n	1	0	Rosaceae Juss.
Iz_2_1009	DJI_0569	Rumex acetosella L.	0	n	1	0	Polygonaceae Juss.
Iz_2_1009	DJI_0569	Rumex thyrsiflorus Fingerh.	0	n	1	0	Polygonaceae Juss.
Iz_2_1009	DJI_0569	Salix alba L.	0	n	1	1	Salicaceae Mirb.
Iz_2_1009	DJI_0569	Sambucus nigra L.	0	n	1	1	Viburnaceae Raf.
Iz_2_1009	DJI_0569	Secale sylvestre Host	0	n	1	0	Poaceae Barnhart
Iz_2_1009	DJI_0569	Sempervivum ruthenicum Schnittsp. & C.B. Lehm.	0	n	1	0	Crassulaceae J.St.-Hil.
Iz_2_1009	DJI_0569	Setaria viridis (L.) P. Beauv.	0	n	1	0	Poaceae Barnhart
Iz_2_1009	DJI_0569	Silene borysthenica (Gruner) Walters	0	n	1	0	Caryophyllaceae Juss.
Iz_2_1009	DJI_0569	Silene latifolia subsp. alba (Mill.) Greuter & Burdet	0	n	1	0	Caryophyllaceae Juss.
Iz_2_1009	DJI_0569	Solidago virgaurea L.	0	n	1	0	Asteraceae Bercht. & J.Presl
Iz_2_1009	DJI_0569	Sorbus aucuparia L.	0	n	1	1	Rosaceae Juss.
Iz_2_1009	DJI_0569	Stachys recta L.	0	n	1	0	Lamiacea Lindl.
Iz_2_1009	DJI_0569	Tanacetum vulgare L.	0	n	1	0	Asteraceae Bercht. & J.Presl
Iz_2_1009	DJI_0569	Thymus pallasianus Heinr.Braun	0	n	1	0	Lamiacea Lindl.
Iz_2_1009	DJI_0569	Tilia cordata Mill.	0	n	1	1	Malvaceae Juss.
Iz_2_1009	DJI_0569	Trifolium alpestre L.	0	n	1	0	Fabaceae Lindl.
Iz_2_1009	DJI_0569	Trifolium arvense L.	0	n	1	0	Fabaceae Lindl.
Iz_2_1009	DJI_0569	Ulmus glabra Huds.	0	n	1	1	Ulmaceae Mirb.
Iz_2_1009	DJI_0569	Verbascum densiflorum Bertol.	0	n	1	0	Scrophulariaceae Juss.
Iz_2_1009	DJI_0569	Veronica austriaca L.	0	n	1	0	Plantaginaceae Juss.
Iz_2_1009	DJI_0569	Veronica incana L.	0	n	1	0	Plantaginaceae Juss.
Iz_2_1009	DJI_0569	Veronica verna L.	0	n	1	0	Plantaginaceae Juss.
Iz_2_1009	DJI_0569	Vincetoxicum hirundinaria Medik	0	n	1	0	Apocynaceae Juss.
Iz_2_1009	DJI_0572	Acer tataricum L.	0	n	1	1	Sapindaceae Juss.
Iz_2_1009	DJI_0572	Achillea millefolium L. s.l.	0	n	1	0	Asteraceae Bercht. & J.Presl
Iz_2_1009	DJI_0572	Allium oleraceum L.	0	n	1	0	Amaryllidaceae J.St.-Hil.
Iz_2_1009	DJI_0572	Ambrosia artemisiifolia L.	0	a	0	0	Asteraceae Bercht. & J.Presl
Iz_2_1009	DJI_0572	Amorpha fruticosa L.	0	a	0	0	Fabaceae Lindl.
Iz_2_1009	DJI_0572	Anthericum ramosum L.	0	n	1	0	Asparagaceae Juss.
Iz_2_1009	DJI_0572	Artemisia marschalliana Spreng.	0	n	1	0	Asteraceae Bercht. & J.Presl
Iz_2_1009	DJI_0572	Asparagus officinalis L.	0	n	1	0	Asparagaceae Juss.
Iz_2_1009	DJI_0572	Astragalus cicer L.	0	n	1	0	Fabaceae Lindl.
Iz_2_1009	DJI_0572	Bassia laniflora (S.G.Gmel.) A.J.Scott	0	n	1	0	Amaranthaceae Juss.
Iz_2_1009	DJI_0572	Berberis vulgaris L.	0	n	1	0	Berberidaceae Juss.
Iz_2_1009	DJI_0572	Betula pendula Roth.	0	n	1	1	Betulaceae Gray
Iz_2_1009	DJI_0572	Betula pendula Roth.	0	n	1	0	Poaceae Barnhart
Iz_2_1009	DJI_0572	Bromus inermis Leyss.	0		NA	NA	
Iz_2_1009	DJI_0572	Bromus squarrosus L.	0	a	0	0	Poaceae Barnhart
Iz_2_1009	DJI_0572	Bromus tectorum L.	0	a	0	0	Poaceae Barnhart
Iz_2_1009	DJI_0572	Calamagrostis epigejos (L.) Roth	25	n	1	0	Poaceae Barnhart
Iz_2_1009	DJI_0572	Carduus acanthoides L.	0	n	1	0	Asteraceae Bercht. & J.Presl
Iz_2_1009	DJI_0572	Carex sp.	0	n	1	0	Cyperaceae Juss.
Iz_2_1009	DJI_0572	Centaurea majorovii Dumbadze	0	n	1	0	Asteraceae Bercht. & J.Presl
Iz_2_1009	DJI_0572	Cf. Solidago virgaurea	0		NA	NA	
Iz_2_1009	DJI_0572	Chamaecytisus ruthenicus (Fisch.ex Wol.) Klaskova	0	n	1	0	Fabaceae Lindl.
Iz_2_1009	DJI_0572	Chelidonium majus L.	0	n	1	0	Papaveraceae Juss.
Iz_2_1009	DJI_0572	Chenopodium betaceum Andrz.	0	n	1	0	Amaranthaceae Juss.
Iz_2_1009	DJI_0572	Chenopodium cf. album	0	n	1	0	Amaranthaceae Juss.
Iz_2_1009	DJI_0572	Chenopodium sp.	0		NA	0	Amaranthaceae Juss.
Iz_2_1009	DJI_0572	Chondrilla juncea L.	0	n	1	0	Asteraceae Bercht. & J.Presl
Iz_2_1009	DJI_0572	Cirsium vulgare (Savi) Ten.	0	n	1	0	Asteraceae Bercht. & J.Presl
Iz_2_1009	DJI_0572	Clinopodium acinos (L.) Kuntze	0	n	1	0	Lamiacea Lindl.
Iz_2_1009	DJI_0572	Convallaria majalis L.	0	n	1	0	Asparagaceae Juss.
Iz_2_1009	DJI_0572	Crataegus rhipidophylla Gand.	0	n	1	1	Rosaceae Juss.
Iz_2_1009	DJI_0572	Crepis foetida L.	0	n	1	0	Asteraceae Bercht. & J.Presl
Iz_2_1009	DJI_0572	Dactylis glomerata L.	0	n	1	0	Poaceae Barnhart
Iz_2_1009	DJI_0572	Dianthus campestris M.Bieb.	0	n	1	0	Caryophyllaceae Juss.
Iz_2_1009	DJI_0572	Digitaria sanguinalis (L.) Scop.	0	a	0	0	Poaceae Barnhart
Iz_2_1009	DJI_0572	Elymus repens (L.) Gould	0	n	1	0	Poaceae Barnhart

Iz_2_1009	DJI_0572	Epilobium tetragonum L.	0	n	1	0	Onagraceae Juss.
Iz_2_1009	DJI_0572	Eragrostis minor Host	0	a	0	0	Poaceae Barnhart
Iz_2_1009	DJI_0572	Erigeron canadensis L.	0	a	0	0	Asteraceae Bercht. & J.Presl
Iz_2_1009	DJI_0572	Euphorbia seguieriana Neck.	0	n	1	0	Euphorbiaceae Juss.
Iz_2_1009	DJI_0572	Fallopia dumetorum (L.) Holub	0	n	1	0	Polygonaceae Juss.
Iz_2_1009	DJI_0572	Festuca beckeri (Hack.) Trautv.	0	n	1	0	Poaceae Barnhart
Iz_2_1009	DJI_0572	Fragaria vesca L.	0	n	1	0	Rosaceae Juss.
Iz_2_1009	DJI_0572	Frangula alnus Mill.	0	n	1	1	Rhamnaceae Juss.
Iz_2_1009	DJI_0572	Fraxinus pennsylvanica Marshall	0	a	0	1	Oleaceae Hoffmanns. & Link
Iz_2_1009	DJI_0572	Galeopsis bifida Boenn.	0	n	1	0	Lamiacea Lindl.
Iz_2_1009	DJI_0572	Galium verum L.	0	n	1	0	Rubiaceae Juss.
Iz_2_1009	DJI_0572	Genista tinctoria L.	0	n	1	0	Fabaceae Lindl.
Iz_2_1009	DJI_0572	Geranium robertianum L.	0	n	1	0	Geraniaceae Juss.
Iz_2_1009	DJI_0572	Geranium sanquineum L.	0	n	1	0	Geraniaceae Juss.
Iz_2_1009	DJI_0572	Gypsophila paniculata L.	0	n	1	0	Caryophyllaceae Juss.
Iz_2_1009	DJI_0572	Helichrysum arenarium (L.) Moench	0	n	1	0	Asteraceae Bercht. & J.Presl
Iz_2_1009	DJI_0572	Herniaria polygama J.Gay	0	n	1	0	Caryophyllaceae Juss.
Iz_2_1009	DJI_0572	Hieracium robustum Fr.	0	n	1	0	Asteraceae Bercht. & J.Presl
Iz_2_1009	DJI_0572	Hieracium umbellatum L.	0	n	1	NA	Asteraceae Bercht. & J.Presl
Iz_2_1009	DJI_0572	Hylotelephium maximum (L.) Holub	0	n	1	0	Crassulaceae J.St.-Hil.
Iz_2_1009	DJI_0572	Jacobaea borysthenica (DC.) B.Nord. & Greuter	0	n	1	0	Asteraceae Bercht. & J.Presl
Iz_2_1009	DJI_0572	Jasione montana L.	0	n	1	0	Campanulaceae Juss.
Iz_2_1009	DJI_0572	Jurinea cyanoides (L.) Rchb.s.l.	0	n	1	0	Asteraceae Bercht. & J.Presl
Iz_2_1009	DJI_0572	Koeleria glauca (Spreng.) DC.	0	n	1	0	Poaceae Barnhart
Iz_2_1009	DJI_0572	Lactuca serriola L.	0	a	0	0	Asteraceae Bercht. & J.Presl
Iz_2_1009	DJI_0572	Linaria genistifolia (L.) Mill.	0	n	1	0	Plantaginaceae Juss.
Iz_2_1009	DJI_0572	Lomelosia argentea (L.) Greuter & Burdet	0	n	1	0	Caprifoliaceae Juss.
Iz_2_1009	DJI_0572	Melampyrum pratense L.	0	n	1	0	Orobanchaceae Vent.
Iz_2_1009	DJI_0572	Oenothera biennis L.	0	a	0	0	Onagraceae Juss.
Iz_2_1009	DJI_0572	Pentanema britannica (L.) D.Gut.Larr., Santos-Vicente, Anderb., E.Rico & M.M.Mart.Ort.	0	n	1	0	Asteraceae Bercht. & J.Presl
Iz_2_1009	DJI_0572	Peucedanum oreoselinum (L.) Moench	0	n	1	0	Apiaceae Lindl.
Iz_2_1009	DJI_0572	Peucedanum ruthenicum M.Bieb.	0	n	1	0	Apiaceae Lindl.
Iz_2_1009	DJI_0572	Pilosella echioides (Lumn.) F.W.Schultz & Sch.Bip.	0	n	1	0	Asteraceae Bercht. & J.Presl
Iz_2_1009	DJI_0572	Pilosella officinarum F. Schultz et Sch. Bip.	0	n	1	0	Asteraceae Bercht. & J.Presl
Iz_2_1009	DJI_0572	Pilosella sp.	0		NA	0	Asteraceae Bercht. & J.Presl
Iz_2_1009	DJI_0572	Pinus sylvestris L.	0	n	1	1	Pinaceae Spreng. ex F.Rudolphi
Iz_2_1009	DJI_0572	Poa annua L.	0	n	1	0	Poaceae Barnhart
Iz_2_1009	DJI_0572	Poa nemoralis L.	0	n	1	0	Poaceae Barnhart
Iz_2_1009	DJI_0572	Poa sp.	0		NA	0	Poaceae Barnhart
Iz_2_1009	DJI_0572	Polygonatum odoratum (Mill.) Druce	0	n	1	0	Asparagaceae Juss.
Iz_2_1009	DJI_0572	Polygonum arenarium Waldst. & Kit.	0	n	1	0	Polygonaceae Juss.
Iz_2_1009	DJI_0572	Populus nigra L.	0	n	1	1	Salicaceae Mirb.
Iz_2_1009	DJI_0572	Populus tremula L.	0	n	1	1	Salicaceae Mirb.
Iz_2_1009	DJI_0572	Populus Ã canescens (Aiton) Sm.	0	n	1	1	Salicaceae Mirb.
Iz_2_1009	DJI_0572	Potentilla argentea L.	0	n	1	0	Rosaceae Juss.
Iz_2_1009	DJI_0572	Psammophiliella muralis (L.) Ikonn.	0	n	1	0	Caryophyllaceae Juss.
Iz_2_1009	DJI_0572	Psephellus sumensis (Kalen.) Greuter	0	n	1	0	Asteraceae Bercht. & J.Presl
Iz_2_1009	DJI_0572	Pulsatilla pratensis (L.) Mill. s.l.	0	n	1	0	Ranunculaceae Juss.
Iz_2_1009	DJI_0572	Pyrus communis L.	0	a	0	1	Rosaceae Juss.
Iz_2_1009	DJI_0572	Quercus robur L.	0	n	1	1	Fagaceae Dumort.
Iz_2_1009	DJI_0572	Robinia pseudoacacia L.	0	a	0	1	Fabaceae Lindl.
Iz_2_1009	DJI_0572	Rosa sp.	0		NA	1	Rosaceae Juss.
Iz_2_1009	DJI_0572	Rubus idaeus L.	0	n	1	0	Rosaceae Juss.
Iz_2_1009	DJI_0572	Rumex acetosella L.	0	n	1	0	Polygonaceae Juss.
Iz_2_1009	DJI_0572	Rumex thyrsiflorus Fingerh.	0	n	1	0	Polygonaceae Juss.
Iz_2_1009	DJI_0572	Salix alba L.	0	n	1	1	Salicaceae Mirb.
Iz_2_1009	DJI_0572	Sambucus nigra L.	0	n	1	1	Viburnaceae Raf.
Iz_2_1009	DJI_0572	Secale sylvestre Host	0	n	1	0	Poaceae Barnhart
Iz_2_1009	DJI_0572	Sempervivum ruthenicum Schnittsp. & C.B. Lehm.	0	n	1	0	Crassulaceae J.St.-Hil.
Iz_2_1009	DJI_0572	Setaria viridis (L.) P. Beauv.	0	n	1	0	Poaceae Barnhart
Iz_2_1009	DJI_0572	Silene borysthenica (Gruner) Walters	0	n	1	0	Caryophyllaceae Juss.
Iz_2_1009	DJI_0572	Silene latifolia subsp. alba (Mill.) Greuter & Burdet	0	n	1	0	Caryophyllaceae Juss.
Iz_2_1009	DJI_0572	Solidago virgaurea L.	0	n	1	0	Asteraceae Bercht. & J.Presl
Iz_2_1009	DJI_0572	Sorbus aucuparia L.	0	n	1	1	Rosaceae Juss.
Iz_2_1009	DJI_0572	Stachys recta L.	0	n	1	0	Lamiacea Lindl.
Iz_2_1009	DJI_0572	Tanacetum vulgare L.	0	n	1	0	Asteraceae Bercht. & J.Presl
Iz_2_1009	DJI_0572	Thymus pallasianus Heinr.Braun	0	n	1	0	Lamiacea Lindl.
Iz_2_1009	DJI_0572	Tilia cordata Mill.	0	n	1	1	Malvaceae Juss.
Iz_2_1009	DJI_0572	Trifolium alpestre L.	0	n	1	0	Fabaceae Lindl.
Iz_2_1009	DJI_0572	Trifolium arvense L.	0	n	1	0	Fabaceae Lindl.
Iz_2_1009	DJI_0572	Ulmus glabra Huds.	0	n	1	1	Ulmaceae Mirb.
Iz_2_1009	DJI_0572	Verbascum densiflorum Bertol.	0	n	1	0	Scrophulariaceae Juss.
Iz_2_1009	DJI_0572	Veronica austriaca L.	0	n	1	0	Plantaginaceae Juss.
Iz_2_1009	DJI_0572	Veronica incana L.	0	n	1	0	Plantaginaceae Juss.
Iz_2_1009	DJI_0572	Veronica verna L.	0	n	1	0	Plantaginaceae Juss.
Iz_2_1009	DJI_0572	Vincetoxicum hirundinaria Medik	0	n	1	0	Apocynaceae Juss.
Iz_2_1109	DJI_0222	Acer tataricum L.	0	n	1	1	Sapindaceae Juss.
Iz_2_1109	DJI_0222	Achillea millefolium L. s.l.	0	n	1	0	Asteraceae Bercht. & J.Presl
Iz_2_1109	DJI_0222	Allium oleraceum L.	0	n	1	0	Amaryllidaceae J.St.-Hil.
Iz_2_1109	DJI_0222	Ambrosia artemisiifolia L.	0	a	0	0	Asteraceae Bercht. & J.Presl
Iz_2_1109	DJI_0222	Amorpha fruticosa L.	0	a	0	0	Fabaceae Lindl.
Iz_2_1109	DJI_0222	Anthericum ramosum L.	0	n	1	0	Asparagaceae Juss.
Iz_2_1109	DJI_0222	Artemisia marschalliana Spreng.	0	n	1	0	Asteraceae Bercht. & J.Presl
Iz_2_1109	DJI_0222	Asparagus officinalis L.	0	n	1	0	Asparagaceae Juss.
Iz_2_1109	DJI_0222	Astragalus cicer L.	0	n	1	0	Fabaceae Lindl.
Iz_2_1109	DJI_0222	Bassia laniflora (S.G.Gmel.) A.J.Scott	0	n	1	0	Amaranthaceae Juss.
Iz_2_1109	DJI_0222	Berberis vulgaris L.	0	n	1	0	Berberidaceae Juss.
Iz_2_1109	DJI_0222	Betula pendula Roth.	0	n	1	1	Betulaceae Gray
Iz_2_1109	DJI_0222	Betula pendula Roth.	0	n	1	0	Poaceae Barnhart
Iz_2_1109	DJI_0222	Bromus inermis Leyss.	0		NA	NA	
Iz_2_1109	DJI_0222	Bromus squarrosus L.	0	a	0	0	Poaceae Barnhart
Iz_2_1109	DJI_0222	Bromus tectorum L.	0	a	0	0	Poaceae Barnhart
Iz_2_1109	DJI_0222	Calamagrostis epigejos (L.) Roth	2	n	1	0	Poaceae Barnhart
Iz_2_1109	DJI_0222	Carduus acanthoides L.	0	n	1	0	Asteraceae Bercht. & J.Presl
Iz_2_1109	DJI_0222	Carex sp.	0	n	1	0	Cyperaceae Juss.
Iz_2_1109	DJI_0222	Centaurea majorovii Dumbadze	0	n	1	0	Asteraceae Bercht. & J.Presl
Iz_2_1109	DJI_0222	Cf. Euporbia/Linaria	0		NA	0	
Iz_2_1109	DJI_0222	Chamaecytisus ruthenicus (Fisch.ex Wol.) Klaskova	0	n	1	0	Fabaceae Lindl.

Iz_2_1109	DJI_0222	Chelidonium majus L.	0	n	1	0	Papaveraceae Juss.
Iz_2_1109	DJI_0222	Chenopodium betaceum Andrz.	0	n	1	0	Amaranthaceae Juss.
Iz_2_1109	DJI_0222	Chenopodium cf. album	0	n	1	0	Amaranthaceae Juss.
Iz_2_1109	DJI_0222	Chenopodium sp.	0		NA	0	Amaranthaceae Juss.
Iz_2_1109	DJI_0222	Chondrilla juncea L.	0	n	1	0	Asteraceae Bercht. & J.Presl
Iz_2_1109	DJI_0222	Cirsium vulgare (Savi) Ten.	0	n	1	0	Asteraceae Bercht. & J.Presl
Iz_2_1109	DJI_0222	Clinopodium acinos (L.) Kuntze	0	n	1	0	Lamiacea Lindl.
Iz_2_1109	DJI_0222	Convallaria majalis L.	0	n	1	0	Asparagaceae Juss.
Iz_2_1109	DJI_0222	Crataegus rhipidophylla Gand.	0	n	1	1	Rosaceae Juss.
Iz_2_1109	DJI_0222	Crepis foetida L.	0	n	1	0	Asteraceae Bercht. & J.Presl
Iz_2_1109	DJI_0222	Dactylis glomerata L.	0	n	1	0	Poaceae Barnhart
Iz_2_1109	DJI_0222	Dianthus campestris M.Bieb.	0	n	1	0	Caryophyllaceae Juss.
Iz_2_1109	DJI_0222	Digitaria sanguinalis (L.) Scop.	0	a	0	0	Poaceae Barnhart
Iz_2_1109	DJI_0222	Elymus repens (L.) Gould	0	n	1	0	Poaceae Barnhart
Iz_2_1109	DJI_0222	Epilobium tetragonum L.	0	n	1	0	Onagraceae Juss.
Iz_2_1109	DJI_0222	Eragrostis minor Host	0	a	0	0	Poaceae Barnhart
Iz_2_1109	DJI_0222	Erigeron annuus (L.) Desf.	0	a	0	0	Asteraceae Bercht. & J.Presl
Iz_2_1109	DJI_0222	Erigeron canadensis L.	6	a	0	0	Asteraceae Bercht. & J.Presl
Iz_2_1109	DJI_0222	Euphorbia seguieriana Neck.	0	n	1	0	Euphorbiaceae Juss.
Iz_2_1109	DJI_0222	Fallopia dumetorum (L.) Holub	1	n	1	0	Polygonaceae Juss.
Iz_2_1109	DJI_0222	Festuca beckeri (Hack.) Trautv.	0	n	1	0	Poaceae Barnhart
Iz_2_1109	DJI_0222	Fragaria vesca L.	0	n	1	0	Rosaceae Juss.
Iz_2_1109	DJI_0222	Frangula alnus Mill.	0	n	1	1	Rhamnaceae Juss.
Iz_2_1109	DJI_0222	Fraxinus pennsylvanica Marshall	0	a	0	1	Oleaceae Hoffmanns. & Link
Iz_2_1109	DJI_0222	Galeopsis bifida Boenn.	0	n	1	0	Lamiacea Lindl.
Iz_2_1109	DJI_0222	Galium verum L.	0	n	1	0	Rubiaceae Juss.
Iz_2_1109	DJI_0222	Genista tinctoria L.	0	n	1	0	Fabaceae Lindl.
Iz_2_1109	DJI_0222	Geranium robertianum L.	0	n	1	0	Geraniaceae Juss.
Iz_2_1109	DJI_0222	Geranium sanquineum L.	0	n	1	0	Geraniaceae Juss.
Iz_2_1109	DJI_0222	Gypsophila paniculata L.	0	n	1	0	Caryophyllaceae Juss.
Iz_2_1109	DJI_0222	Helichrysum arenarium (L.) Moench	0	n	1	0	Asteraceae Bercht. & J.Presl
Iz_2_1109	DJI_0222	Herniaria polygama J.Gay	0	n	1	0	Caryophyllaceae Juss.
Iz_2_1109	DJI_0222	Hieracium robustum Fr.	0	n	1	0	Asteraceae Bercht. & J.Presl
Iz_2_1109	DJI_0222	Hieracium umbellatum L.	0	n	1	NA	Asteraceae Bercht. & J.Presl
Iz_2_1109	DJI_0222	Hylotelephium maximum (L.) Holub	0	n	1	0	Crassulaceae J.St.-Hil.
Iz_2_1109	DJI_0222	Jacobaea borysthenica (DC.) B.Nord. & Greuter	0	n	1	0	Asteraceae Bercht. & J.Presl
Iz_2_1109	DJI_0222	Jasione montana L.	0	n	1	0	Campanulaceae Juss.
Iz_2_1109	DJI_0222	Jurinea cyanoides (L.) Rchb.s.l.	0	n	1	0	Asteraceae Bercht. & J.Presl
Iz_2_1109	DJI_0222	Koeleria glauca (Spreng.) DC.	0	n	1	0	Poaceae Barnhart
Iz_2_1109	DJI_0222	Lactuca serriola L.	0	a	0	0	Asteraceae Bercht. & J.Presl
Iz_2_1109	DJI_0222	Linaria genistifolia (L.) Mill.	0	n	1	0	Plantaginaceae Juss.
Iz_2_1109	DJI_0222	Lomelosia argentea (L.) Greuter & Burdet	0	n	1	0	Caprifoliaceae Juss.
Iz_2_1109	DJI_0222	Melampyrum pratense L.	0	n	1	0	Orobanchaceae Vent.
Iz_2_1109	DJI_0222	Oenothera biennis L.	0	a	0	0	Onagraceae Juss.
Iz_2_1109	DJI_0222	Pentanema britannica (L.) D.Gut.Larr., Santos-Vicente, Anderb., E.Rico & M.M.Mart.Ort.	0	n	1	0	Asteraceae Bercht. & J.Presl
Iz_2_1109	DJI_0222	Peucedanum oreoselinum (L.) Moench	0	n	1	0	Apiaceae Lindl.
Iz_2_1109	DJI_0222	Peucedanum ruthenicum M.Bieb.	0	n	1	0	Apiaceae Lindl.
Iz_2_1109	DJI_0222	Pilosella echioides (Lumn.) F.W.Schultz & Sch.Bip.	0	n	1	0	Asteraceae Bercht. & J.Presl
Iz_2_1109	DJI_0222	Pilosella officinarum F. Schultz et Sch. Bip.	0	n	1	0	Asteraceae Bercht. & J.Presl
Iz_2_1109	DJI_0222	Pilosella sp.	0		NA	0	Asteraceae Bercht. & J.Presl
Iz_2_1109	DJI_0222	Pinus sylvestris L.	0	n	1	1	Pinaceae Spreng. ex F.Rudolphi
Iz_2_1109	DJI_0222	Poa annua L.	0	n	1	0	Poaceae Barnhart
Iz_2_1109	DJI_0222	Poa nemoralis L.	0	n	1	0	Poaceae Barnhart
Iz_2_1109	DJI_0222	Poa sp.	0		NA	0	Poaceae Barnhart
Iz_2_1109	DJI_0222	Polygonatum odoratum (Mill.) Druce	0	n	1	0	Asparagaceae Juss.
Iz_2_1109	DJI_0222	Polygonum arenarium Waldst. & Kit.	0	n	1	0	Polygonaceae Juss.
Iz_2_1109	DJI_0222	Populus nigra L.	0	n	1	1	Salicaceae Mirb.
Iz_2_1109	DJI_0222	Populus tremula L.	0	n	1	1	Salicaceae Mirb.
Iz_2_1109	DJI_0222	Populus Ã canescens (Aiton) Sm.	0	n	1	1	Salicaceae Mirb.
Iz_2_1109	DJI_0222	Potentilla argentea L.	0	n	1	0	Rosaceae Juss.
Iz_2_1109	DJI_0222	Psammophiliella muralis (L.) Ikonn.	0	n	1	0	Caryophyllaceae Juss.
Iz_2_1109	DJI_0222	Psephellus sumensis (Kalen.) Greuter	0	n	1	0	Asteraceae Bercht. & J.Presl
Iz_2_1109	DJI_0222	Pulsatilla pratensis (L.) Mill. s.l.	0	n	1	0	Ranunculaceae Juss.
Iz_2_1109	DJI_0222	Pyrus communis L.	0	a	0	1	Rosaceae Juss.
Iz_2_1109	DJI_0222	Quercus robur L.	0	n	1	1	Fagaceae Dumort.
Iz_2_1109	DJI_0222	Robinia pseudoacacia L.	0	a	0	1	Fabaceae Lindl.
Iz_2_1109	DJI_0222	Rosa sp.	0		NA	1	Rosaceae Juss.
Iz_2_1109	DJI_0222	Rubus idaeus L.	0	n	1	0	Rosaceae Juss.
Iz_2_1109	DJI_0222	Rumex acetosella L.	3	n	1	0	Polygonaceae Juss.
Iz_2_1109	DJI_0222	Rumex thyrsiflorus Fingerh.	0	n	1	0	Polygonaceae Juss.
Iz_2_1109	DJI_0222	Salix alba L.	0	n	1	1	Salicaceae Mirb.
Iz_2_1109	DJI_0222	Sambucus nigra L.	0	n	1	1	Viburnaceae Raf.
Iz_2_1109	DJI_0222	Secale sylvestre Host	0	n	1	0	Poaceae Barnhart
Iz_2_1109	DJI_0222	Sempervivum ruthenicum Schnittsp. & C.B. Lehm.	0	n	1	0	Crassulaceae J.St.-Hil.
Iz_2_1109	DJI_0222	Setaria viridis (L.) P. Beauv.	0	n	1	0	Poaceae Barnhart
Iz_2_1109	DJI_0222	Silene borysthenica (Gruner) Walters	0	n	1	0	Caryophyllaceae Juss.
Iz_2_1109	DJI_0222	Silene latifolia subsp. alba (Mill.) Greuter & Burdet	0	n	1	0	Caryophyllaceae Juss.
Iz_2_1109	DJI_0222	Solidago virgaurea L.	0	n	1	0	Asteraceae Bercht. & J.Presl
Iz_2_1109	DJI_0222	Sorbus aucuparia L.	0	n	1	1	Rosaceae Juss.
Iz_2_1109	DJI_0222	Stachys recta L.	2	n	1	0	Lamiacea Lindl.
Iz_2_1109	DJI_0222	Tanacetum vulgare L.	0	n	1	0	Asteraceae Bercht. & J.Presl
Iz_2_1109	DJI_0222	Thymus pallasianus Heinr.Braun	0	n	1	0	Lamiacea Lindl.
Iz_2_1109	DJI_0222	Tilia cordata Mill.	0	n	1	1	Malvaceae Juss.
Iz_2_1109	DJI_0222	Trifolium alpestre L.	0	n	1	0	Fabaceae Lindl.
Iz_2_1109	DJI_0222	Trifolium arvense L.	0	n	1	0	Fabaceae Lindl.
Iz_2_1109	DJI_0222	Ulmus glabra Huds.	0	n	1	1	Ulmaceae Mirb.
Iz_2_1109	DJI_0222	Verbascum densiflorum Bertol.	0	n	1	0	Scrophulariaceae Juss.
Iz_2_1109	DJI_0222	Verbascum sp.	0	n	1	0	Scrophulariaceae Juss.
Iz_2_1109	DJI_0222	Veronica austriaca L.	0	n	1	0	Plantaginaceae Juss.
Iz_2_1109	DJI_0222	Veronica incana L.	0	n	1	0	Plantaginaceae Juss.
Iz_2_1109	DJI_0222	Veronica verna L.	0	n	1	0	Plantaginaceae Juss.
Iz_2_1109	DJI_0222	Vincetoxicum hirundinaria Medik	0	n	1	0	Apocynaceae Juss.
Iz_2_1109	DJI_0225	Acer tataricum L.	0	n	1	1	Sapindaceae Juss.
Iz_2_1109	DJI_0225	Achillea millefolium L. s.l.	0	n	1	0	Asteraceae Bercht. & J.Presl
Iz_2_1109	DJI_0225	Allium oleraceum L.	0	n	1	0	Amaryllidaceae J.St.-Hil.
Iz_2_1109	DJI_0225	Ambrosia artemisiifolia L.	0	a	0	0	Asteraceae Bercht. & J.Presl
Iz_2_1109	DJI_0225	Amorpha fruticosa L.	0	a	0	0	Fabaceae Lindl.
Iz_2_1109	DJI_0225	Anthericum ramosum L.	0	n	1	0	Asparagaceae Juss.

Iz_2_1109	DJI_0225	Artemisia marschalliana Spreng.	0	n	1	0	Asteraceae Bercht. & J.Presl
Iz_2_1109	DJI_0225	Asparagus officinalis L.	0	n	1	0	Asparagaceae Juss.
Iz_2_1109	DJI_0225	Astragalus cicer L.	0	n	1	0	Fabaceae Lindl.
Iz_2_1109	DJI_0225	Bassia laniflora (S.G.Gmel.) A.J.Scott	0	n	1	0	Amaranthaceae Juss.
Iz_2_1109	DJI_0225	Berberis vulgaris L.	0	n	1	0	Berberidaceae Juss.
Iz_2_1109	DJI_0225	Betula pendula Roth.	0	n	1	1	Betulaceae Gray
Iz_2_1109	DJI_0225	Betula pendula Roth.	0	n	1	0	Poaceae Barnhart
Iz_2_1109	DJI_0225	Bromus inermis Leyss.	0		NA	NA	
Iz_2_1109	DJI_0225	Bromus squarrosus L.	0	a	0	0	Poaceae Barnhart
Iz_2_1109	DJI_0225	Bromus tectorum L.	0	a	0	0	Poaceae Barnhart
Iz_2_1109	DJI_0225	Calamagrostis epigejos (L.) Roth	0.2	n	1	0	Poaceae Barnhart
Iz_2_1109	DJI_0225	Carduus acanthoides L.	0	n	1	0	Asteraceae Bercht. & J.Presl
Iz_2_1109	DJI_0225	Carex sp.	0	n	1	0	Cyperaceae Juss.
Iz_2_1109	DJI_0225	Centaurea majorovii Dumbadze	0	n	1	0	Asteraceae Bercht. & J.Presl
Iz_2_1109	DJI_0225	Cf. Euporbia/Linaria	0		NA	0	
Iz_2_1109	DJI_0225	Chamaecytisus ruthenicus (Fisch.ex Wol.) Klaskova	0	n	1	0	Fabaceae Lindl.
Iz_2_1109	DJI_0225	Chelidonium majus L.	0	n	1	0	Papaveraceae Juss.
Iz_2_1109	DJI_0225	Chenopodium betaceum Andrz.	0	n	1	0	Amaranthaceae Juss.
Iz_2_1109	DJI_0225	Chenopodium cf. album	0	n	1	0	Amaranthaceae Juss.
Iz_2_1109	DJI_0225	Chenopodium sp.	0		NA	0	Amaranthaceae Juss.
Iz_2_1109	DJI_0225	Chondrilla juncea L.	0	n	1	0	Asteraceae Bercht. & J.Presl
Iz_2_1109	DJI_0225	Cirsium vulgare (Savi) Ten.	0	n	1	0	Asteraceae Bercht. & J.Presl
Iz_2_1109	DJI_0225	Clinopodium acinos (L.) Kuntze	0	n	1	0	Lamiacea Lindl.
Iz_2_1109	DJI_0225	Convallaria majalis L.	0	n	1	0	Asparagaceae Juss.
Iz_2_1109	DJI_0225	Crataegus rhipidophylla Gand.	0	n	1	1	Rosaceae Juss.
Iz_2_1109	DJI_0225	Crepis foetida L.	0	n	1	0	Asteraceae Bercht. & J.Presl
Iz_2_1109	DJI_0225	Dactylis glomerata L.	0	n	1	0	Poaceae Barnhart
Iz_2_1109	DJI_0225	Dianthus campestris M.Bieb.	0	n	1	0	Caryophyllaceae Juss.
Iz_2_1109	DJI_0225	Digitaria sanguinalis (L.) Scop.	0	a	0	0	Poaceae Barnhart
Iz_2_1109	DJI_0225	Elymus repens (L.) Gould	0	n	1	0	Poaceae Barnhart
Iz_2_1109	DJI_0225	Epilobium tetragonum L.	0	n	1	0	Onagraceae Juss.
Iz_2_1109	DJI_0225	Eragrostis minor Host	0	a	0	0	Poaceae Barnhart
Iz_2_1109	DJI_0225	Erigeron annuus (L.) Desf.	0	a	0	0	Asteraceae Bercht. & J.Presl
Iz_2_1109	DJI_0225	Erigeron canadensis L.	3	a	0	0	Asteraceae Bercht. & J.Presl
Iz_2_1109	DJI_0225	Euphorbia seguieriana Neck.	0	n	1	0	Euphorbiaceae Juss.
Iz_2_1109	DJI_0225	Fallopia dumetorum (L.) Holub	0	n	1	0	Polygonaceae Juss.
Iz_2_1109	DJI_0225	Festuca beckeri (Hack.) Trautv.	0	n	1	0	Poaceae Barnhart
Iz_2_1109	DJI_0225	Fragaria vesca L.	0	n	1	0	Rosaceae Juss.
Iz_2_1109	DJI_0225	Frangula alnus Mill.	0	n	1	1	Rhamnaceae Juss.
Iz_2_1109	DJI_0225	Fraxinus pennsylvanica Marshall	0	a	0	1	Oleaceae Hoffmanns. & Link
Iz_2_1109	DJI_0225	Galeopsis bifida Boenn.	0	n	1	0	Lamiacea Lindl.
Iz_2_1109	DJI_0225	Galium verum L.	0	n	1	0	Rubiaceae Juss.
Iz_2_1109	DJI_0225	Genista tinctoria L.	0	n	1	0	Fabaceae Lindl.
Iz_2_1109	DJI_0225	Geranium robertianum L.	0	n	1	0	Geraniaceae Juss.
Iz_2_1109	DJI_0225	Geranium sanquineum L.	0	n	1	0	Geraniaceae Juss.
Iz_2_1109	DJI_0225	Gypsophila paniculata L.	0	n	1	0	Caryophyllaceae Juss.
Iz_2_1109	DJI_0225	Helichrysum arenarium (L.) Moench	0	n	1	0	Asteraceae Bercht. & J.Presl
Iz_2_1109	DJI_0225	Herniaria polygama J.Gay	0	n	1	0	Caryophyllaceae Juss.
Iz_2_1109	DJI_0225	Hieracium robustum Fr.	0	n	1	0	Asteraceae Bercht. & J.Presl
Iz_2_1109	DJI_0225	Hieracium umbellatum L.	0	n	1	NA	Asteraceae Bercht. & J.Presl
Iz_2_1109	DJI_0225	Hylotelephium maximum (L.) Holub	0	n	1	0	Crassulaceae J.St.-Hil.
Iz_2_1109	DJI_0225	Jacobaea borysthenica (DC.) B.Nord. & Greuter	0	n	1	0	Asteraceae Bercht. & J.Presl
Iz_2_1109	DJI_0225	Jasione montana L.	0	n	1	0	Campanulaceae Juss.
Iz_2_1109	DJI_0225	Jurinea cyanoides (L.) Rchb.s.l.	0	n	1	0	Asteraceae Bercht. & J.Presl
Iz_2_1109	DJI_0225	Koeleria glauca (Spreng.) DC.	0	n	1	0	Poaceae Barnhart
Iz_2_1109	DJI_0225	Lactuca serriola L.	0	a	0	0	Asteraceae Bercht. & J.Presl
Iz_2_1109	DJI_0225	Linaria genistifolia (L.) Mill.	0	n	1	0	Plantaginaceae Juss.
Iz_2_1109	DJI_0225	Lomelosia argentea (L.) Greuter & Burdet	0	n	1	0	Caprifoliaceae Juss.
Iz_2_1109	DJI_0225	Melampyrum pratense L.	0	n	1	0	Orobanchaceae Vent.
Iz_2_1109	DJI_0225	Oenothera biennis L.	0	a	0	0	Onagraceae Juss.
Iz_2_1109	DJI_0225	Pentanema britannica (L.) D.Gut.Larr., Santos-Vicente, Anderb., E.Rico & M.M.Mart.Ort.	0	n	1	0	Asteraceae Bercht. & J.Presl
Iz_2_1109	DJI_0225	Peucedanum oreoselinum (L.) Moench	0	n	1	0	Apiaceae Lindl.
Iz_2_1109	DJI_0225	Peucedanum ruthenicum M.Bieb.	0	n	1	0	Apiaceae Lindl.
Iz_2_1109	DJI_0225	Pilosella echioides (Lumn.) F.W.Schultz & Sch.Bip.	0	n	1	0	Asteraceae Bercht. & J.Presl
Iz_2_1109	DJI_0225	Pilosella officinarum F. Schultz et Sch. Bip.	0	n	1	0	Asteraceae Bercht. & J.Presl
Iz_2_1109	DJI_0225	Pilosella sp.	0		NA	0	Asteraceae Bercht. & J.Presl
Iz_2_1109	DJI_0225	Pinus sylvestris L.	0	n	1	1	Pinaceae Spreng. ex F.Rudolphi
Iz_2_1109	DJI_0225	Poa annua L.	0	n	1	0	Poaceae Barnhart
Iz_2_1109	DJI_0225	Poa nemoralis L.	0	n	1	0	Poaceae Barnhart
Iz_2_1109	DJI_0225	Poa sp.	0		NA	0	Poaceae Barnhart
Iz_2_1109	DJI_0225	Polygonatum odoratum (Mill.) Druce	0	n	1	0	Asparagaceae Juss.
Iz_2_1109	DJI_0225	Polygonum arenarium Waldst. & Kit.	0	n	1	0	Polygonaceae Juss.
Iz_2_1109	DJI_0225	Populus nigra L.	0	n	1	1	Salicaceae Mirb.
Iz_2_1109	DJI_0225	Populus tremula L.	0	n	1	1	Salicaceae Mirb.
Iz_2_1109	DJI_0225	Populus Ã canescens (Aiton) Sm.	0	n	1	1	Salicaceae Mirb.
Iz_2_1109	DJI_0225	Potentilla argentea L.	0	n	1	0	Rosaceae Juss.
Iz_2_1109	DJI_0225	Psammophiliella muralis (L.) Ikonn.	0	n	1	0	Caryophyllaceae Juss.
Iz_2_1109	DJI_0225	Psephellus sumensis (Kalen.) Greuter	0	n	1	0	Asteraceae Bercht. & J.Presl
Iz_2_1109	DJI_0225	Pulsatilla pratensis (L.) Mill. s.l.	0	n	1	0	Ranunculaceae Juss.
Iz_2_1109	DJI_0225	Pyrus communis L.	0	a	0	1	Rosaceae Juss.
Iz_2_1109	DJI_0225	Quercus robur L.	0	n	1	1	Fagaceae Dumort.
Iz_2_1109	DJI_0225	Robinia pseudoacacia L.	0	a	0	1	Fabaceae Lindl.
Iz_2_1109	DJI_0225	Rosa sp.	0		NA	1	Rosaceae Juss.
Iz_2_1109	DJI_0225	Rubus idaeus L.	0	n	1	0	Rosaceae Juss.
Iz_2_1109	DJI_0225	Rumex acetosella L.	0	n	1	0	Polygonaceae Juss.
Iz_2_1109	DJI_0225	Rumex thyrsiflorus Fingerh.	0	n	1	0	Polygonaceae Juss.
Iz_2_1109	DJI_0225	Salix alba L.	0	n	1	1	Salicaceae Mirb.
Iz_2_1109	DJI_0225	Sambucus nigra L.	0	n	1	1	Viburnaceae Raf.
Iz_2_1109	DJI_0225	Secale sylvestre Host	0	n	1	0	Poaceae Barnhart
Iz_2_1109	DJI_0225	Sempervivum ruthenicum Schnittsp. & C.B. Lehm.	0	n	1	0	Crassulaceae J.St.-Hil.
Iz_2_1109	DJI_0225	Setaria viridis (L.) P. Beauv.	0	n	1	0	Poaceae Barnhart
Iz_2_1109	DJI_0225	Silene borysthenica (Gruner) Walters	0	n	1	0	Caryophyllaceae Juss.
Iz_2_1109	DJI_0225	Silene latifolia subsp. alba (Mill.) Greuter & Burdet	0	n	1	0	Caryophyllaceae Juss.
Iz_2_1109	DJI_0225	Solidago virgaurea L.	0	n	1	0	Asteraceae Bercht. & J.Presl
Iz_2_1109	DJI_0225	Sorbus aucuparia L.	0	n	1	1	Rosaceae Juss.
Iz_2_1109	DJI_0225	Stachys recta L.	0	n	1	0	Lamiacea Lindl.
Iz_2_1109	DJI_0225	Tanacetum vulgare L.	0	n	1	0	Asteraceae Bercht. & J.Presl
Iz_2_1109	DJI_0225	Thymus pallasianus Heinr.Braun	0	n	1	0	Lamiacea Lindl.

Iz_2_1109	DJI_0225	Tilia cordata Mill.	0	n	1	1	Malvaceae Juss.
Iz_2_1109	DJI_0225	Trifolium alpestre L.	0	n	1	0	Fabaceae Lindl.
Iz_2_1109	DJI_0225	Trifolium arvense L.	0	n	1	0	Fabaceae Lindl.
Iz_2_1109	DJI_0225	Ulmus glabra Huds.	0	n	1	1	Ulmaceae Mirb.
Iz_2_1109	DJI_0225	Verbascum densiflorum Bertol.	0	n	1	0	Scrophulariaceae Juss.
Iz_2_1109	DJI_0225	Verbascum sp.	0	n	1	0	Scrophulariaceae Juss.
Iz_2_1109	DJI_0225	Veronica austriaca L.	0	n	1	0	Plantaginaceae Juss.
Iz_2_1109	DJI_0225	Veronica incana L.	0	n	1	0	Plantaginaceae Juss.
Iz_2_1109	DJI_0225	Veronica verna L.	0	n	1	0	Plantaginaceae Juss.
Iz_2_1109	DJI_0225	Vincetoxicum hirundinaria Medik	0	n	1	0	Apocynaceae Juss.
Iz_2_1109	DJI_0229	Acer tataricum L.	0	n	1	1	Sapindaceae Juss.
Iz_2_1109	DJI_0229	Achillea millefolium L. s.l.	0	n	1	0	Asteraceae Bercht. & J.Presl
Iz_2_1109	DJI_0229	Allium oleraceum L.	0	n	1	0	Amaryllidaceae J.St.-Hil.
Iz_2_1109	DJI_0229	Ambrosia artemisiifolia L.	0	a	0	0	Asteraceae Bercht. & J.Presl
Iz_2_1109	DJI_0229	Amorpha fruticosa L.	0	a	0	0	Fabaceae Lindl.
Iz_2_1109	DJI_0229	Anthericum ramosum L.	0	n	1	0	Asparagaceae Juss.
Iz_2_1109	DJI_0229	Artemisia marschalliana Spreng.	0	n	1	0	Asteraceae Bercht. & J.Presl
Iz_2_1109	DJI_0229	Asparagus officinalis L.	0	n	1	0	Asparagaceae Juss.
Iz_2_1109	DJI_0229	Astragalus cicer L.	0	n	1	0	Fabaceae Lindl.
Iz_2_1109	DJI_0229	Bassia laniflora (S.G.Gmel.) A.J.Scott	0	n	1	0	Amaranthaceae Juss.
Iz_2_1109	DJI_0229	Berberis vulgaris L.	0	n	1	0	Berberidaceae Juss.
Iz_2_1109	DJI_0229	Betula pendula Roth.	0	n	1	1	Betulaceae Gray
Iz_2_1109	DJI_0229	Betula pendula Roth.	0	n	1	0	Poaceae Barnhart
Iz_2_1109	DJI_0229	Bromus inermis Leyss.	0		NA	NA	
Iz_2_1109	DJI_0229	Bromus squarrosus L.	0	a	0	0	Poaceae Barnhart
Iz_2_1109	DJI_0229	Bromus tectorum L.	0	a	0	0	Poaceae Barnhart
Iz_2_1109	DJI_0229	Calamagrostis epigejos (L.) Roth	0.1	n	1	0	Poaceae Barnhart
Iz_2_1109	DJI_0229	Carduus acanthoides L.	0	n	1	0	Asteraceae Bercht. & J.Presl
Iz_2_1109	DJI_0229	Carex sp.	0	n	1	0	Cyperaceae Juss.
Iz_2_1109	DJI_0229	Centaurea majorovii Dumbadze	0	n	1	0	Asteraceae Bercht. & J.Presl
Iz_2_1109	DJI_0229	Cf. Euporbia/Linaria	0		NA	0	
Iz_2_1109	DJI_0229	Chamaecytisus ruthenicus (Fisch.ex Wol.) Klaskova	0	n	1	0	Fabaceae Lindl.
Iz_2_1109	DJI_0229	Chelidonium majus L.	0.5	n	1	0	Papaveraceae Juss.
Iz_2_1109	DJI_0229	Chenopodium betaceum Andrz.	0	n	1	0	Amaranthaceae Juss.
Iz_2_1109	DJI_0229	Chenopodium cf. album	0	n	1	0	Amaranthaceae Juss.
Iz_2_1109	DJI_0229	Chenopodium sp.	0		NA	0	Amaranthaceae Juss.
Iz_2_1109	DJI_0229	Chondrilla juncea L.	0	n	1	0	Asteraceae Bercht. & J.Presl
Iz_2_1109	DJI_0229	Cirsium vulgare (Savi) Ten.	0	n	1	0	Asteraceae Bercht. & J.Presl
Iz_2_1109	DJI_0229	Clinopodium acinos (L.) Kuntze	0	n	1	0	Lamiacea Lindl.
Iz_2_1109	DJI_0229	Convallaria majalis L.	0	n	1	0	Asparagaceae Juss.
Iz_2_1109	DJI_0229	Crataegus rhipidophylla Gand.	0	n	1	1	Rosaceae Juss.
Iz_2_1109	DJI_0229	Crepis foetida L.	0	n	1	0	Asteraceae Bercht. & J.Presl
Iz_2_1109	DJI_0229	Dactylis glomerata L.	0	n	1	0	Poaceae Barnhart
Iz_2_1109	DJI_0229	Dianthus campestris M.Bieb.	0	n	1	0	Caryophyllaceae Juss.
Iz_2_1109	DJI_0229	Digitaria sanguinalis (L.) Scop.	0	a	0	0	Poaceae Barnhart
Iz_2_1109	DJI_0229	Elymus repens (L.) Gould	0	n	1	0	Poaceae Barnhart
Iz_2_1109	DJI_0229	Epilobium tetragonum L.	0	n	1	0	Onagraceae Juss.
Iz_2_1109	DJI_0229	Eragrostis minor Host	0	a	0	0	Poaceae Barnhart
Iz_2_1109	DJI_0229	Erigeron annuus (L.) Desf.	0	a	0	0	Asteraceae Bercht. & J.Presl
Iz_2_1109	DJI_0229	Erigeron canadensis L.	10	a	0	0	Asteraceae Bercht. & J.Presl
Iz_2_1109	DJI_0229	Euphorbia seguieriana Neck.	0	n	1	0	Euphorbiaceae Juss.
Iz_2_1109	DJI_0229	Fallopia dumetorum (L.) Holub	0.2	n	1	0	Polygonaceae Juss.
Iz_2_1109	DJI_0229	Festuca beckeri (Hack.) Trautv.	0	n	1	0	Poaceae Barnhart
Iz_2_1109	DJI_0229	Fragaria vesca L.	0	n	1	0	Rosaceae Juss.
Iz_2_1109	DJI_0229	Frangula alnus Mill.	0	n	1	1	Rhamnaceae Juss.
Iz_2_1109	DJI_0229	Fraxinus pennsylvanica Marshall	0	a	0	1	Oleaceae Hoffmanns. & Link
Iz_2_1109	DJI_0229	Galeopsis bifida Boenn.	0	n	1	0	Lamiacea Lindl.
Iz_2_1109	DJI_0229	Galium verum L.	0	n	1	0	Rubiaceae Juss.
Iz_2_1109	DJI_0229	Genista tinctoria L.	0	n	1	0	Fabaceae Lindl.
Iz_2_1109	DJI_0229	Geranium robertianum L.	0	n	1	0	Geraniaceae Juss.
Iz_2_1109	DJI_0229	Geranium sanquineum L.	0	n	1	0	Geraniaceae Juss.
Iz_2_1109	DJI_0229	Gypsophila paniculata L.	0	n	1	0	Caryophyllaceae Juss.
Iz_2_1109	DJI_0229	Helichrysum arenarium (L.) Moench	0	n	1	0	Asteraceae Bercht. & J.Presl
Iz_2_1109	DJI_0229	Herniaria polygama J.Gay	0	n	1	0	Caryophyllaceae Juss.
Iz_2_1109	DJI_0229	Hieracium robustum Fr.	0	n	1	0	Asteraceae Bercht. & J.Presl
Iz_2_1109	DJI_0229	Hieracium umbellatum L.	0	n	1	NA	Asteraceae Bercht. & J.Presl
Iz_2_1109	DJI_0229	Hylotelephium maximum (L.) Holub	0	n	1	0	Crassulaceae J.St.-Hil.
Iz_2_1109	DJI_0229	Jacobaea borysthenica (DC.) B.Nord. & Greuter	0	n	1	0	Asteraceae Bercht. & J.Presl
Iz_2_1109	DJI_0229	Jasione montana L.	0	n	1	0	Campanulaceae Juss.
Iz_2_1109	DJI_0229	Jurinea cyanoides (L.) Rchb.s.l.	0	n	1	0	Asteraceae Bercht. & J.Presl
Iz_2_1109	DJI_0229	Koeleria glauca (Spreng.) DC.	0	n	1	0	Poaceae Barnhart
Iz_2_1109	DJI_0229	Lactuca serriola L.	0	a	0	0	Asteraceae Bercht. & J.Presl
Iz_2_1109	DJI_0229	Linaria genistifolia (L.) Mill.	0	n	1	0	Plantaginaceae Juss.
Iz_2_1109	DJI_0229	Lomelosia argentea (L.) Greuter & Burdet	0	n	1	0	Caprifoliaceae Juss.
Iz_2_1109	DJI_0229	Melampyrum pratense L.	0	n	1	0	Orobanchaceae Vent.
Iz_2_1109	DJI_0229	Oenothera biennis L.	0	a	0	0	Onagraceae Juss.
Iz_2_1109	DJI_0229	Pentanema britannica (L.) D.Gut.Larr., Santos-Vicente, Anderb., E.Rico & M.M.Mart.Ort.	0	n	1	0	Asteraceae Bercht. & J.Presl
Iz_2_1109	DJI_0229	Peucedanum oreoselinum (L.) Moench	0	n	1	0	Apiaceae Lindl.
Iz_2_1109	DJI_0229	Peucedanum ruthenicum M.Bieb.	0	n	1	0	Apiaceae Lindl.
Iz_2_1109	DJI_0229	Pilosella echioides (Lumn.) F.W.Schultz & Sch.Bip.	0	n	1	0	Asteraceae Bercht. & J.Presl
Iz_2_1109	DJI_0229	Pilosella officinarum F. Schultz et Sch. Bip.	0	n	1	0	Asteraceae Bercht. & J.Presl
Iz_2_1109	DJI_0229	Pilosella sp.	0		NA	0	Asteraceae Bercht. & J.Presl
Iz_2_1109	DJI_0229	Pinus sylvestris L.	0	n	1	1	Pinaceae Spreng. ex F.Rudolphi
Iz_2_1109	DJI_0229	Poa annua L.	0	n	1	0	Poaceae Barnhart
Iz_2_1109	DJI_0229	Poa nemoralis L.	0	n	1	0	Poaceae Barnhart
Iz_2_1109	DJI_0229	Poa sp.	0		NA	0	Poaceae Barnhart
Iz_2_1109	DJI_0229	Polygonatum odoratum (Mill.) Druce	0	n	1	0	Asparagaceae Juss.
Iz_2_1109	DJI_0229	Polygonum arenarium Waldst. & Kit.	0	n	1	0	Polygonaceae Juss.
Iz_2_1109	DJI_0229	Populus nigra L.	0	n	1	1	Salicaceae Mirb.
Iz_2_1109	DJI_0229	Populus tremula L.	0	n	1	1	Salicaceae Mirb.
Iz_2_1109	DJI_0229	Populus Ã canescens (Aiton) Sm.	0	n	1	1	Salicaceae Mirb.
Iz_2_1109	DJI_0229	Potentilla argentea L.	0	n	1	0	Rosaceae Juss.
Iz_2_1109	DJI_0229	Psammophiliella muralis (L.) Ikonn.	0	n	1	0	Caryophyllaceae Juss.
Iz_2_1109	DJI_0229	Psephellus sumensis (Kalen.) Greuter	0	n	1	0	Asteraceae Bercht. & J.Presl
Iz_2_1109	DJI_0229	Pulsatilla pratensis (L.) Mill. s.l.	0	n	1	0	Ranunculaceae Juss.
Iz_2_1109	DJI_0229	Pyrus communis L.	0	a	0	1	Rosaceae Juss.
Iz_2_1109	DJI_0229	Quercus robur L.	0	n	1	1	Fagaceae Dumort.
Iz_2_1109	DJI_0229	Robinia pseudoacacia L.	0	a	0	1	Fabaceae Lindl.

Iz_2_1109	DJI_0229	Rosa sp.	0		NA	1	Rosaceae Juss.
Iz_2_1109	DJI_0229	Rubus idaeus L.	0	n	1	0	Rosaceae Juss.
Iz_2_1109	DJI_0229	Rumex acetosella L.	0	n	1	0	Polygonaceae Juss.
Iz_2_1109	DJI_0229	Rumex thyrsiflorus Fingerh.	0	n	1	0	Polygonaceae Juss.
Iz_2_1109	DJI_0229	Salix alba L.	0	n	1	1	Salicaceae Mirb.
Iz_2_1109	DJI_0229	Sambucus nigra L.	0	n	1	1	Viburnaceae Raf.
Iz_2_1109	DJI_0229	Secale sylvestre Host	0	n	1	0	Poaceae Barnhart
Iz_2_1109	DJI_0229	Sempervivum ruthenicum Schnittsp. & C.B. Lehm.	0	n	1	0	Crassulaceae J.St.-Hil.
Iz_2_1109	DJI_0229	Setaria viridis (L.) P. Beauv.	0	n	1	0	Poaceae Barnhart
Iz_2_1109	DJI_0229	Silene borysthenica (Gruner) Walters	0	n	1	0	Caryophyllaceae Juss.
Iz_2_1109	DJI_0229	Silene latifolia subsp. alba (Mill.) Greuter & Burdet	0	n	1	0	Caryophyllaceae Juss.
Iz_2_1109	DJI_0229	Solidago virgaurea L.	0	n	1	0	Asteraceae Bercht. & J.Presl
Iz_2_1109	DJI_0229	Sorbus aucuparia L.	0	n	1	1	Rosaceae Juss.
Iz_2_1109	DJI_0229	Stachys recta L.	0	n	1	0	Lamiacea Lindl.
Iz_2_1109	DJI_0229	Tanacetum vulgare L.	0	n	1	0	Asteraceae Bercht. & J.Presl
Iz_2_1109	DJI_0229	Thymus pallasianus Heinr.Braun	0	n	1	0	Lamiacea Lindl.
Iz_2_1109	DJI_0229	Tilia cordata Mill.	0	n	1	1	Malvaceae Juss.
Iz_2_1109	DJI_0229	Trifolium alpestre L.	0	n	1	0	Fabaceae Lindl.
Iz_2_1109	DJI_0229	Trifolium arvense L.	0	n	1	0	Fabaceae Lindl.
Iz_2_1109	DJI_0229	Ulmus glabra Huds.	0	n	1	1	Ulmaceae Mirb.
Iz_2_1109	DJI_0229	Verbascum densiflorum Bertol.	0	n	1	0	Scrophulariaceae Juss.
Iz_2_1109	DJI_0229	Verbascum sp.	0	n	1	0	Scrophulariaceae Juss.
Iz_2_1109	DJI_0229	Veronica austriaca L.	0	n	1	0	Plantaginaceae Juss.
Iz_2_1109	DJI_0229	Veronica incana L.	0	n	1	0	Plantaginaceae Juss.
Iz_2_1109	DJI_0229	Veronica verna L.	0	n	1	0	Plantaginaceae Juss.
Iz_2_1109	DJI_0229	Vincetoxicum hirundinaria Medik	0	n	1	0	Apocynaceae Juss.
Iz_2_1109	DJI_0231	Acer tataricum L.	0	n	1	1	Sapindaceae Juss.
Iz_2_1109	DJI_0231	Achillea millefolium L. s.l.	0	n	1	0	Asteraceae Bercht. & J.Presl
Iz_2_1109	DJI_0231	Allium oleraceum L.	0	n	1	0	Amaryllidaceae J.St.-Hil.
Iz_2_1109	DJI_0231	Ambrosia artemisiifolia L.	0	a	0	0	Asteraceae Bercht. & J.Presl
Iz_2_1109	DJI_0231	Amorpha fruticosa L.	0	a	0	0	Fabaceae Lindl.
Iz_2_1109	DJI_0231	Anthericum ramosum L.	0	n	1	0	Asparagaceae Juss.
Iz_2_1109	DJI_0231	Artemisia marschalliana Spreng.	0	n	1	0	Asteraceae Bercht. & J.Presl
Iz_2_1109	DJI_0231	Asparagus officinalis L.	0	n	1	0	Asparagaceae Juss.
Iz_2_1109	DJI_0231	Astragalus cicer L.	0	n	1	0	Fabaceae Lindl.
Iz_2_1109	DJI_0231	Bassia laniflora (S.G.Gmel.) A.J.Scott	0	n	1	0	Amaranthaceae Juss.
Iz_2_1109	DJI_0231	Berberis vulgaris L.	0	n	1	0	Berberidaceae Juss.
Iz_2_1109	DJI_0231	Betula pendula Roth.	0	n	1	1	Betulaceae Gray
Iz_2_1109	DJI_0231	Betula pendula Roth.	0	n	1	0	Poaceae Barnhart
Iz_2_1109	DJI_0231	Bromus inermis Leyss.	0		NA	NA	
Iz_2_1109	DJI_0231	Bromus squarrosus L.	0	a	0	0	Poaceae Barnhart
Iz_2_1109	DJI_0231	Bromus tectorum L.	0	a	0	0	Poaceae Barnhart
Iz_2_1109	DJI_0231	Calamagrostis epigejos (L.) Roth	0.2	n	1	0	Poaceae Barnhart
Iz_2_1109	DJI_0231	Carduus acanthoides L.	0	n	1	0	Asteraceae Bercht. & J.Presl
Iz_2_1109	DJI_0231	Carex sp.	0	n	1	0	Cyperaceae Juss.
Iz_2_1109	DJI_0231	Centaurea majorovii Dumbadze	0	n	1	0	Asteraceae Bercht. & J.Presl
Iz_2_1109	DJI_0231	Cf. Euporbia/Linaria	0		NA	0	
Iz_2_1109	DJI_0231	Chamaecytisus ruthenicus (Fisch.ex Wol.) Klaskova	0	n	1	0	Fabaceae Lindl.
Iz_2_1109	DJI_0231	Chelidonium majus L.	1	n	1	0	Papaveraceae Juss.
Iz_2_1109	DJI_0231	Chenopodium betaceum Andrz.	0	n	1	0	Amaranthaceae Juss.
Iz_2_1109	DJI_0231	Chenopodium cf. album	0	n	1	0	Amaranthaceae Juss.
Iz_2_1109	DJI_0231	Chenopodium sp.	0		NA	0	Amaranthaceae Juss.
Iz_2_1109	DJI_0231	Chondrilla juncea L.	0	n	1	0	Asteraceae Bercht. & J.Presl
Iz_2_1109	DJI_0231	Cirsium vulgare (Savi) Ten.	0	n	1	0	Asteraceae Bercht. & J.Presl
Iz_2_1109	DJI_0231	Clinopodium acinos (L.) Kuntze	0	n	1	0	Lamiacea Lindl.
Iz_2_1109	DJI_0231	Convallaria majalis L.	0	n	1	0	Asparagaceae Juss.
Iz_2_1109	DJI_0231	Crataegus rhipidophylla Gand.	0	n	1	1	Rosaceae Juss.
Iz_2_1109	DJI_0231	Crepis foetida L.	0	n	1	0	Asteraceae Bercht. & J.Presl
Iz_2_1109	DJI_0231	Dactylis glomerata L.	0	n	1	0	Poaceae Barnhart
Iz_2_1109	DJI_0231	Dianthus campestris M.Bieb.	0	n	1	0	Caryophyllaceae Juss.
Iz_2_1109	DJI_0231	Digitaria sanguinalis (L.) Scop.	0	a	0	0	Poaceae Barnhart
Iz_2_1109	DJI_0231	Elymus repens (L.) Gould	0	n	1	0	Poaceae Barnhart
Iz_2_1109	DJI_0231	Epilobium tetragonum L.	0	n	1	0	Onagraceae Juss.
Iz_2_1109	DJI_0231	Eragrostis minor Host	0	a	0	0	Poaceae Barnhart
Iz_2_1109	DJI_0231	Erigeron annuus (L.) Desf.	0.2	a	0	0	Asteraceae Bercht. & J.Presl
Iz_2_1109	DJI_0231	Erigeron canadensis L.	2	a	0	0	Asteraceae Bercht. & J.Presl
Iz_2_1109	DJI_0231	Euphorbia seguieriana Neck.	0	n	1	0	Euphorbiaceae Juss.
Iz_2_1109	DJI_0231	Fallopia dumetorum (L.) Holub	15	n	1	0	Polygonaceae Juss.
Iz_2_1109	DJI_0231	Festuca beckeri (Hack.) Trautv.	0	n	1	0	Poaceae Barnhart
Iz_2_1109	DJI_0231	Fragaria vesca L.	0	n	1	0	Rosaceae Juss.
Iz_2_1109	DJI_0231	Frangula alnus Mill.	0	n	1	1	Rhamnaceae Juss.
Iz_2_1109	DJI_0231	Fraxinus pennsylvanica Marshall	0	a	0	1	Oleaceae Hoffmanns. & Link
Iz_2_1109	DJI_0231	Galeopsis bifida Boenn.	0	n	1	0	Lamiacea Lindl.
Iz_2_1109	DJI_0231	Galium verum L.	0	n	1	0	Rubiaceae Juss.
Iz_2_1109	DJI_0231	Genista tinctoria L.	0	n	1	0	Fabaceae Lindl.
Iz_2_1109	DJI_0231	Geranium robertianum L.	0	n	1	0	Geraniaceae Juss.
Iz_2_1109	DJI_0231	Geranium sanquineum L.	0	n	1	0	Geraniaceae Juss.
Iz_2_1109	DJI_0231	Gypsophila paniculata L.	0	n	1	0	Caryophyllaceae Juss.
Iz_2_1109	DJI_0231	Helichrysum arenarium (L.) Moench	0	n	1	0	Asteraceae Bercht. & J.Presl
Iz_2_1109	DJI_0231	Herniaria polygama J.Gay	0	n	1	0	Caryophyllaceae Juss.
Iz_2_1109	DJI_0231	Hieracium robustum Fr.	0	n	1	0	Asteraceae Bercht. & J.Presl
Iz_2_1109	DJI_0231	Hieracium umbellatum L.	1	n	1	NA	Asteraceae Bercht. & J.Presl
Iz_2_1109	DJI_0231	Hylotelephium maximum (L.) Holub	0.2	n	1	0	Crassulaceae J.St.-Hil.
Iz_2_1109	DJI_0231	Jacobaea borysthenica (DC.) B.Nord. & Greuter	0	n	1	0	Asteraceae Bercht. & J.Presl
Iz_2_1109	DJI_0231	Jasione montana L.	0	n	1	0	Campanulaceae Juss.
Iz_2_1109	DJI_0231	Jurinea cyanoides (L.) Rchb.s.l.	0	n	1	0	Asteraceae Bercht. & J.Presl
Iz_2_1109	DJI_0231	Koeleria glauca (Spreng.) DC.	0	n	1	0	Poaceae Barnhart
Iz_2_1109	DJI_0231	Lactuca serriola L.	0	a	0	0	Asteraceae Bercht. & J.Presl
Iz_2_1109	DJI_0231	Linaria genistifolia (L.) Mill.	0	n	1	0	Plantaginaceae Juss.
Iz_2_1109	DJI_0231	Lomelosia argentea (L.) Greuter & Burdet	0	n	1	0	Caprifoliaceae Juss.
Iz_2_1109	DJI_0231	Melampyrum pratense L.	0	n	1	0	Orobanchaceae Vent.
Iz_2_1109	DJI_0231	Oenothera biennis L.	0	a	0	0	Onagraceae Juss.
Iz_2_1109	DJI_0231	Pentanema britannica (L.) D.Gut.Larr., Santos-Vicente, Anderb., E.Rico & M.M.Mart.Ort.	0	n	1	0	Asteraceae Bercht. & J.Presl
Iz_2_1109	DJI_0231	Peucedanum oreoselinum (L.) Moench	0	n	1	0	Apiaceae Lindl.
Iz_2_1109	DJI_0231	Peucedanum ruthenicum M.Bieb.	0	n	1	0	Apiaceae Lindl.
Iz_2_1109	DJI_0231	Pilosella echioides (Lumn.) F.W.Schultz & Sch.Bip.	0	n	1	0	Asteraceae Bercht. & J.Presl
Iz_2_1109	DJI_0231	Pilosella officinarum F. Schultz et Sch. Bip.	0	n	1	0	Asteraceae Bercht. & J.Presl
Iz_2_1109	DJI_0231	Pilosella sp.	0		NA	0	Asteraceae Bercht. & J.Presl

Iz_2_1109	DJI_0231	Pinus sylvestris L.	0	n	1	1	Pinaceae Spreng. ex F.Rudolphi
Iz_2_1109	DJI_0231	Poa annua L.	0	n	1	0	Poaceae Barnhart
Iz_2_1109	DJI_0231	Poa nemoralis L.	0	n	1	0	Poaceae Barnhart
Iz_2_1109	DJI_0231	Poa sp.	0		NA	0	Poaceae Barnhart
Iz_2_1109	DJI_0231	Polygonatum odoratum (Mill.) Druce	0	n	1	0	Asparagaceae Juss.
Iz_2_1109	DJI_0231	Polygonum arenarium Waldst. & Kit.	0	n	1	0	Polygonaceae Juss.
Iz_2_1109	DJI_0231	Populus nigra L.	0	n	1	1	Salicaceae Mirb.
Iz_2_1109	DJI_0231	Populus tremula L.	1	n	1	1	Salicaceae Mirb.
Iz_2_1109	DJI_0231	Populus Ã canescens (Aiton) Sm.	0	n	1	1	Salicaceae Mirb.
Iz_2_1109	DJI_0231	Potentilla argentea L.	0	n	1	0	Rosaceae Juss.
Iz_2_1109	DJI_0231	Psammophiliella muralis (L.) Ikonn.	0	n	1	0	Caryophyllaceae Juss.
Iz_2_1109	DJI_0231	Psephellus sumensis (Kalen.) Greuter	0	n	1	0	Asteraceae Bercht. & J.Presl
Iz_2_1109	DJI_0231	Pulsatilla pratensis (L.) Mill. s.l.	0	n	1	0	Ranunculaceae Juss.
Iz_2_1109	DJI_0231	Pyrus communis L.	0	a	0	1	Rosaceae Juss.
Iz_2_1109	DJI_0231	Quercus robur L.	0	n	1	1	Fagaceae Dumort.
Iz_2_1109	DJI_0231	Robinia pseudoacacia L.	0	a	0	1	Fabaceae Lindl.
Iz_2_1109	DJI_0231	Rosa sp.	0		NA	1	Rosaceae Juss.
Iz_2_1109	DJI_0231	Rubus idaeus L.	0	n	1	0	Rosaceae Juss.
Iz_2_1109	DJI_0231	Rumex acetosella L.	0	n	1	0	Polygonaceae Juss.
Iz_2_1109	DJI_0231	Rumex thyrsiflorus Fingerh.	0	n	1	0	Polygonaceae Juss.
Iz_2_1109	DJI_0231	Salix alba L.	0	n	1	1	Salicaceae Mirb.
Iz_2_1109	DJI_0231	Sambucus nigra L.	0	n	1	1	Viburnaceae Raf.
Iz_2_1109	DJI_0231	Secale sylvestre Host	0	n	1	0	Poaceae Barnhart
Iz_2_1109	DJI_0231	Sempervivum ruthenicum Schnittsp. & C.B. Lehm.	0	n	1	0	Crassulaceae J.St.-Hil.
Iz_2_1109	DJI_0231	Setaria viridis (L.) P. Beauv.	0	n	1	0	Poaceae Barnhart
Iz_2_1109	DJI_0231	Silene borysthenica (Gruner) Walters	0	n	1	0	Caryophyllaceae Juss.
Iz_2_1109	DJI_0231	Silene latifolia subsp. alba (Mill.) Greuter & Burdet	0	n	1	0	Caryophyllaceae Juss.
Iz_2_1109	DJI_0231	Solidago virgaurea L.	0	n	1	0	Asteraceae Bercht. & J.Presl
Iz_2_1109	DJI_0231	Sorbus aucuparia L.	0	n	1	1	Rosaceae Juss.
Iz_2_1109	DJI_0231	Stachys recta L.	0	n	1	0	Lamiacea Lindl.
Iz_2_1109	DJI_0231	Tanacetum vulgare L.	0	n	1	0	Asteraceae Bercht. & J.Presl
Iz_2_1109	DJI_0231	Thymus pallasianus Heinr.Braun	0	n	1	0	Lamiacea Lindl.
Iz_2_1109	DJI_0231	Tilia cordata Mill.	0	n	1	1	Malvaceae Juss.
Iz_2_1109	DJI_0231	Trifolium alpestre L.	0	n	1	0	Fabaceae Lindl.
Iz_2_1109	DJI_0231	Trifolium arvense L.	0	n	1	0	Fabaceae Lindl.
Iz_2_1109	DJI_0231	Ulmus glabra Huds.	0	n	1	1	Ulmaceae Mirb.
Iz_2_1109	DJI_0231	Verbascum densiflorum Bertol.	0	n	1	0	Scrophulariaceae Juss.
Iz_2_1109	DJI_0231	Verbascum sp.	0	n	1	0	Scrophulariaceae Juss.
Iz_2_1109	DJI_0231	Veronica austriaca L.	0	n	1	0	Plantaginaceae Juss.
Iz_2_1109	DJI_0231	Veronica incana L.	0	n	1	0	Plantaginaceae Juss.
Iz_2_1109	DJI_0231	Veronica verna L.	0	n	1	0	Plantaginaceae Juss.
Iz_2_1109	DJI_0231	Vincetoxicum hirundinaria Medik	0	n	1	0	Apocynaceae Juss.
Iz_2_1109	DJI_0237	Acer tataricum L.	0	n	1	1	Sapindaceae Juss.
Iz_2_1109	DJI_0237	Achillea millefolium L. s.l.	0	n	1	0	Asteraceae Bercht. & J.Presl
Iz_2_1109	DJI_0237	Allium oleraceum L.	0	n	1	0	Amaryllidaceae J.St.-Hil.
Iz_2_1109	DJI_0237	Ambrosia artemisiifolia L.	0	a	0	0	Asteraceae Bercht. & J.Presl
Iz_2_1109	DJI_0237	Amorpha fruticosa L.	0	a	0	0	Fabaceae Lindl.
Iz_2_1109	DJI_0237	Anthericum ramosum L.	0	n	1	0	Asparagaceae Juss.
Iz_2_1109	DJI_0237	Artemisia marschalliana Spreng.	0	n	1	0	Asteraceae Bercht. & J.Presl
Iz_2_1109	DJI_0237	Asparagus officinalis L.	0	n	1	0	Asparagaceae Juss.
Iz_2_1109	DJI_0237	Astragalus cicer L.	0	n	1	0	Fabaceae Lindl.
Iz_2_1109	DJI_0237	Bassia laniflora (S.G.Gmel.) A.J.Scott	0	n	1	0	Amaranthaceae Juss.
Iz_2_1109	DJI_0237	Berberis vulgaris L.	0	n	1	0	Berberidaceae Juss.
Iz_2_1109	DJI_0237	Betula pendula Roth.	0	n	1	1	Betulaceae Gray
Iz_2_1109	DJI_0237	Betula pendula Roth.	0	n	1	0	Poaceae Barnhart
Iz_2_1109	DJI_0237	Bromus inermis Leyss.	0		NA	NA	
Iz_2_1109	DJI_0237	Bromus squarrosus L.	0	a	0	0	Poaceae Barnhart
Iz_2_1109	DJI_0237	Bromus tectorum L.	0	a	0	0	Poaceae Barnhart
Iz_2_1109	DJI_0237	Calamagrostis epigejos (L.) Roth	0.5	n	1	0	Poaceae Barnhart
Iz_2_1109	DJI_0237	Carduus acanthoides L.	0	n	1	0	Asteraceae Bercht. & J.Presl
Iz_2_1109	DJI_0237	Carex sp.	0	n	1	0	Cyperaceae Juss.
Iz_2_1109	DJI_0237	Centaurea majorovii Dumbadze	0	n	1	0	Asteraceae Bercht. & J.Presl
Iz_2_1109	DJI_0237	Cf. Euporbia/Linaria	0		NA	0	
Iz_2_1109	DJI_0237	Chamaecytisus ruthenicus (Fisch.ex Wol.) Klaskova	0	n	1	0	Fabaceae Lindl.
Iz_2_1109	DJI_0237	Chelidonium majus L.	0	n	1	0	Papaveraceae Juss.
Iz_2_1109	DJI_0237	Chenopodium betaceum Andrz.	0	n	1	0	Amaranthaceae Juss.
Iz_2_1109	DJI_0237	Chenopodium cf. album	0	n	1	0	Amaranthaceae Juss.
Iz_2_1109	DJI_0237	Chenopodium sp.	0		NA	0	Amaranthaceae Juss.
Iz_2_1109	DJI_0237	Chondrilla juncea L.	0	n	1	0	Asteraceae Bercht. & J.Presl
Iz_2_1109	DJI_0237	Cirsium vulgare (Savi) Ten.	0	n	1	0	Asteraceae Bercht. & J.Presl
Iz_2_1109	DJI_0237	Clinopodium acinos (L.) Kuntze	0	n	1	0	Lamiacea Lindl.
Iz_2_1109	DJI_0237	Convallaria majalis L.	0	n	1	0	Asparagaceae Juss.
Iz_2_1109	DJI_0237	Crataegus rhipidophylla Gand.	0	n	1	1	Rosaceae Juss.
Iz_2_1109	DJI_0237	Crepis foetida L.	0	n	1	0	Asteraceae Bercht. & J.Presl
Iz_2_1109	DJI_0237	Dactylis glomerata L.	0	n	1	0	Poaceae Barnhart
Iz_2_1109	DJI_0237	Dianthus campestris M.Bieb.	0	n	1	0	Caryophyllaceae Juss.
Iz_2_1109	DJI_0237	Digitaria sanguinalis (L.) Scop.	0	a	0	0	Poaceae Barnhart
Iz_2_1109	DJI_0237	Elymus repens (L.) Gould	0	n	1	0	Poaceae Barnhart
Iz_2_1109	DJI_0237	Epilobium tetragonum L.	0	n	1	0	Onagraceae Juss.
Iz_2_1109	DJI_0237	Eragrostis minor Host	0	a	0	0	Poaceae Barnhart
Iz_2_1109	DJI_0237	Erigeron annuus (L.) Desf.	0	a	0	0	Asteraceae Bercht. & J.Presl
Iz_2_1109	DJI_0237	Erigeron canadensis L.	4	a	0	0	Asteraceae Bercht. & J.Presl
Iz_2_1109	DJI_0237	Euphorbia seguieriana Neck.	0	n	1	0	Euphorbiaceae Juss.
Iz_2_1109	DJI_0237	Fallopia dumetorum (L.) Holub	2	n	1	0	Polygonaceae Juss.
Iz_2_1109	DJI_0237	Festuca beckeri (Hack.) Trautv.	0	n	1	0	Poaceae Barnhart
Iz_2_1109	DJI_0237	Fragaria vesca L.	0	n	1	0	Rosaceae Juss.
Iz_2_1109	DJI_0237	Frangula alnus Mill.	0	n	1	1	Rhamnaceae Juss.
Iz_2_1109	DJI_0237	Fraxinus pennsylvanica Marshall	0	a	0	1	Oleaceae Hoffmanns. & Link
Iz_2_1109	DJI_0237	Galeopsis bifida Boenn.	0	n	1	0	Lamiacea Lindl.
Iz_2_1109	DJI_0237	Galium verum L.	0	n	1	0	Rubiaceae Juss.
Iz_2_1109	DJI_0237	Genista tinctoria L.	0	n	1	0	Fabaceae Lindl.
Iz_2_1109	DJI_0237	Geranium robertianum L.	0	n	1	0	Geraniaceae Juss.
Iz_2_1109	DJI_0237	Geranium sanquineum L.	0	n	1	0	Geraniaceae Juss.
Iz_2_1109	DJI_0237	Gypsophila paniculata L.	0	n	1	0	Caryophyllaceae Juss.
Iz_2_1109	DJI_0237	Helichrysum arenarium (L.) Moench	0	n	1	0	Asteraceae Bercht. & J.Presl
Iz_2_1109	DJI_0237	Herniaria polygama J.Gay	0	n	1	0	Caryophyllaceae Juss.
Iz_2_1109	DJI_0237	Hieracium robustum Fr.	0	n	1	0	Asteraceae Bercht. & J.Presl
Iz_2_1109	DJI_0237	Hieracium umbellatum L.	0	n	1	NA	Asteraceae Bercht. & J.Presl

Iz_2_1109	DJI_0237	Hylotelephium maximum (L.) Holub	0	n	1	0	Crassulaceae J.St.-Hil.
Iz_2_1109	DJI_0237	Jacobaea borysthenica (DC.) B.Nord. & Greuter	0	n	1	0	Asteraceae Bercht. & J.Presl
Iz_2_1109	DJI_0237	Jasione montana L.	0	n	1	0	Campanulaceae Juss.
Iz_2_1109	DJI_0237	Jurinea cyanoides (L.) Rchb.s.l.	0	n	1	0	Asteraceae Bercht. & J.Presl
Iz_2_1109	DJI_0237	Koeleria glauca (Spreng.) DC.	0	n	1	0	Poaceae Barnhart
Iz_2_1109	DJI_0237	Lactuca serriola L.	0	a	0	0	Asteraceae Bercht. & J.Presl
Iz_2_1109	DJI_0237	Linaria genistifolia (L.) Mill.	0	n	1	0	Plantaginaceae Juss.
Iz_2_1109	DJI_0237	Lomelosia argentea (L.) Greuter & Burdet	0	n	1	0	Caprifoliaceae Juss.
Iz_2_1109	DJI_0237	Melampyrum pratense L.	0	n	1	0	Orobanchaceae Vent.
Iz_2_1109	DJI_0237	Oenothera biennis L.	0	a	0	0	Onagraceae Juss.
Iz_2_1109	DJI_0237	Pentanema britannica (L.) D.Gut.Larr., Santos-Vicente, Anderb., E.Rico & M.M.Mart.Ort.	0	n	1	0	Asteraceae Bercht. & J.Presl
Iz_2_1109	DJI_0237	Peucedanum oreoselinum (L.) Moench	0	n	1	0	Apiaceae Lindl.
Iz_2_1109	DJI_0237	Peucedanum ruthenicum M.Bieb.	0	n	1	0	Apiaceae Lindl.
Iz_2_1109	DJI_0237	Pilosella echioides (Lumn.) F.W.Schultz & Sch.Bip.	0	n	1	0	Asteraceae Bercht. & J.Presl
Iz_2_1109	DJI_0237	Pilosella officinarum F. Schultz et Sch. Bip.	0	n	1	0	Asteraceae Bercht. & J.Presl
Iz_2_1109	DJI_0237	Pilosella sp.	0		NA	0	Asteraceae Bercht. & J.Presl
Iz_2_1109	DJI_0237	Pinus sylvestris L.	0	n	1	1	Pinaceae Spreng. ex F.Rudolphi
Iz_2_1109	DJI_0237	Poa annua L.	0	n	1	0	Poaceae Barnhart
Iz_2_1109	DJI_0237	Poa nemoralis L.	0	n	1	0	Poaceae Barnhart
Iz_2_1109	DJI_0237	Poa sp.	0		NA	0	Poaceae Barnhart
Iz_2_1109	DJI_0237	Polygonatum odoratum (Mill.) Druce	0	n	1	0	Asparagaceae Juss.
Iz_2_1109	DJI_0237	Polygonum arenarium Waldst. & Kit.	0	n	1	0	Polygonaceae Juss.
Iz_2_1109	DJI_0237	Populus nigra L.	0	n	1	1	Salicaceae Mirb.
Iz_2_1109	DJI_0237	Populus tremula L.	0	n	1	1	Salicaceae Mirb.
Iz_2_1109	DJI_0237	Populus Ã canescens (Aiton) Sm.	0	n	1	1	Salicaceae Mirb.
Iz_2_1109	DJI_0237	Potentilla argentea L.	0	n	1	0	Rosaceae Juss.
Iz_2_1109	DJI_0237	Psammophiliella muralis (L.) Ikonn.	0	n	1	0	Caryophyllaceae Juss.
Iz_2_1109	DJI_0237	Psephellus sumensis (Kalen.) Greuter	0	n	1	0	Asteraceae Bercht. & J.Presl
Iz_2_1109	DJI_0237	Pulsatilla pratensis (L.) Mill. s.l.	0	n	1	0	Ranunculaceae Juss.
Iz_2_1109	DJI_0237	Pyrus communis L.	0	a	0	1	Rosaceae Juss.
Iz_2_1109	DJI_0237	Quercus robur L.	0	n	1	1	Fagaceae Dumort.
Iz_2_1109	DJI_0237	Robinia pseudoacacia L.	0	a	0	1	Fabaceae Lindl.
Iz_2_1109	DJI_0237	Rosa sp.	0		NA	1	Rosaceae Juss.
Iz_2_1109	DJI_0237	Rubus idaeus L.	0	n	1	0	Rosaceae Juss.
Iz_2_1109	DJI_0237	Rumex acetosella L.	0	n	1	0	Polygonaceae Juss.
Iz_2_1109	DJI_0237	Rumex thyrsiflorus Fingerh.	0	n	1	0	Polygonaceae Juss.
Iz_2_1109	DJI_0237	Salix alba L.	0	n	1	1	Salicaceae Mirb.
Iz_2_1109	DJI_0237	Sambucus nigra L.	0	n	1	1	Viburnaceae Raf.
Iz_2_1109	DJI_0237	Secale sylvestre Host	0	n	1	0	Poaceae Barnhart
Iz_2_1109	DJI_0237	Sempervivum ruthenicum Schnittsp. & C.B. Lehm.	0	n	1	0	Crassulaceae J.St.-Hil.
Iz_2_1109	DJI_0237	Setaria viridis (L.) P. Beauv.	0	n	1	0	Poaceae Barnhart
Iz_2_1109	DJI_0237	Silene borysthenica (Gruner) Walters	0	n	1	0	Caryophyllaceae Juss.
Iz_2_1109	DJI_0237	Silene latifolia subsp. alba (Mill.) Greuter & Burdet	0	n	1	0	Caryophyllaceae Juss.
Iz_2_1109	DJI_0237	Solidago virgaurea L.	0	n	1	0	Asteraceae Bercht. & J.Presl
Iz_2_1109	DJI_0237	Sorbus aucuparia L.	0	n	1	1	Rosaceae Juss.
Iz_2_1109	DJI_0237	Stachys recta L.	0	n	1	0	Lamiacea Lindl.
Iz_2_1109	DJI_0237	Tanacetum vulgare L.	0	n	1	0	Asteraceae Bercht. & J.Presl
Iz_2_1109	DJI_0237	Thymus pallasianus Heinr.Braun	0	n	1	0	Lamiacea Lindl.
Iz_2_1109	DJI_0237	Tilia cordata Mill.	0	n	1	1	Malvaceae Juss.
Iz_2_1109	DJI_0237	Trifolium alpestre L.	0	n	1	0	Fabaceae Lindl.
Iz_2_1109	DJI_0237	Trifolium arvense L.	0	n	1	0	Fabaceae Lindl.
Iz_2_1109	DJI_0237	Ulmus glabra Huds.	0	n	1	1	Ulmaceae Mirb.
Iz_2_1109	DJI_0237	Verbascum densiflorum Bertol.	0	n	1	0	Scrophulariaceae Juss.
Iz_2_1109	DJI_0237	Verbascum sp.	1	n	1	0	Scrophulariaceae Juss.
Iz_2_1109	DJI_0237	Veronica austriaca L.	0	n	1	0	Plantaginaceae Juss.
Iz_2_1109	DJI_0237	Veronica incana L.	0	n	1	0	Plantaginaceae Juss.
Iz_2_1109	DJI_0237	Veronica verna L.	0	n	1	0	Plantaginaceae Juss.
Iz_2_1109	DJI_0237	Vincetoxicum hirundinaria Medik	0	n	1	0	Apocynaceae Juss.
Iz_2_1109	DJI_0238	Acer tataricum L.	0	n	1	1	Sapindaceae Juss.
Iz_2_1109	DJI_0238	Achillea millefolium L. s.l.	0	n	1	0	Asteraceae Bercht. & J.Presl
Iz_2_1109	DJI_0238	Allium oleraceum L.	0	n	1	0	Amaryllidaceae J.St.-Hil.
Iz_2_1109	DJI_0238	Ambrosia artemisiifolia L.	0	a	0	0	Asteraceae Bercht. & J.Presl
Iz_2_1109	DJI_0238	Amorpha fruticosa L.	0	a	0	0	Fabaceae Lindl.
Iz_2_1109	DJI_0238	Anthericum ramosum L.	0	n	1	0	Asparagaceae Juss.
Iz_2_1109	DJI_0238	Artemisia marschalliana Spreng.	0	n	1	0	Asteraceae Bercht. & J.Presl
Iz_2_1109	DJI_0238	Asparagus officinalis L.	0	n	1	0	Asparagaceae Juss.
Iz_2_1109	DJI_0238	Astragalus cicer L.	0	n	1	0	Fabaceae Lindl.
Iz_2_1109	DJI_0238	Bassia laniflora (S.G.Gmel.) A.J.Scott	0	n	1	0	Amaranthaceae Juss.
Iz_2_1109	DJI_0238	Berberis vulgaris L.	0	n	1	0	Berberidaceae Juss.
Iz_2_1109	DJI_0238	Betula pendula Roth.	0	n	1	1	Betulaceae Gray
Iz_2_1109	DJI_0238	Betula pendula Roth.	0	n	1	0	Poaceae Barnhart
Iz_2_1109	DJI_0238	Bromus inermis Leyss.	0		NA	NA	
Iz_2_1109	DJI_0238	Bromus squarrosus L.	0	a	0	0	Poaceae Barnhart
Iz_2_1109	DJI_0238	Bromus tectorum L.	0	a	0	0	Poaceae Barnhart
Iz_2_1109	DJI_0238	Calamagrostis epigejos (L.) Roth	0.5	n	1	0	Poaceae Barnhart
Iz_2_1109	DJI_0238	Carduus acanthoides L.	0	n	1	0	Asteraceae Bercht. & J.Presl
Iz_2_1109	DJI_0238	Carex sp.	0	n	1	0	Cyperaceae Juss.
Iz_2_1109	DJI_0238	Centaurea majorovii Dumbadze	0	n	1	0	Asteraceae Bercht. & J.Presl
Iz_2_1109	DJI_0238	Cf. Euporbia/Linaria	0.5		NA	0	
Iz_2_1109	DJI_0238	Chamaecytisus ruthenicus (Fisch.ex Wol.) Klaskova	0	n	1	0	Fabaceae Lindl.
Iz_2_1109	DJI_0238	Chelidonium majus L.	0	n	1	0	Papaveraceae Juss.
Iz_2_1109	DJI_0238	Chenopodium betaceum Andrz.	0	n	1	0	Amaranthaceae Juss.
Iz_2_1109	DJI_0238	Chenopodium cf. album	0	n	1	0	Amaranthaceae Juss.
Iz_2_1109	DJI_0238	Chenopodium sp.	0		NA	0	Amaranthaceae Juss.
Iz_2_1109	DJI_0238	Chondrilla juncea L.	0	n	1	0	Asteraceae Bercht. & J.Presl
Iz_2_1109	DJI_0238	Cirsium vulgare (Savi) Ten.	0	n	1	0	Asteraceae Bercht. & J.Presl
Iz_2_1109	DJI_0238	Clinopodium acinos (L.) Kuntze	0	n	1	0	Lamiacea Lindl.
Iz_2_1109	DJI_0238	Convallaria majalis L.	0	n	1	0	Asparagaceae Juss.
Iz_2_1109	DJI_0238	Crataegus rhipidophylla Gand.	0	n	1	1	Rosaceae Juss.
Iz_2_1109	DJI_0238	Crepis foetida L.	0	n	1	0	Asteraceae Bercht. & J.Presl
Iz_2_1109	DJI_0238	Dactylis glomerata L.	0	n	1	0	Poaceae Barnhart
Iz_2_1109	DJI_0238	Dianthus campestris M.Bieb.	0	n	1	0	Caryophyllaceae Juss.
Iz_2_1109	DJI_0238	Digitaria sanguinalis (L.) Scop.	0	a	0	0	Poaceae Barnhart
Iz_2_1109	DJI_0238	Elymus repens (L.) Gould	0	n	1	0	Poaceae Barnhart
Iz_2_1109	DJI_0238	Epilobium tetragonum L.	0	n	1	0	Onagraceae Juss.
Iz_2_1109	DJI_0238	Eragrostis minor Host	0	a	0	0	Poaceae Barnhart
Iz_2_1109	DJI_0238	Erigeron annuus (L.) Desf.	0	a	0	0	Asteraceae Bercht. & J.Presl
Iz_2_1109	DJI_0238	Erigeron canadensis L.	5	a	0	0	Asteraceae Bercht. & J.Presl

Iz_2_1109	DJI_0238	Euphorbia seguieriana Neck.	0	n	1	0	Euphorbiaceae Juss.
Iz_2_1109	DJI_0238	Fallopia dumetorum (L.) Holub	0	n	1	0	Polygonaceae Juss.
Iz_2_1109	DJI_0238	Festuca beckeri (Hack.) Trautv.	0	n	1	0	Poaceae Barnhart
Iz_2_1109	DJI_0238	Fragaria vesca L.	0	n	1	0	Rosaceae Juss.
Iz_2_1109	DJI_0238	Frangula alnus Mill.	0	n	1	1	Rhamnaceae Juss.
Iz_2_1109	DJI_0238	Fraxinus pennsylvanica Marshall	0	a	0	1	Oleaceae Hoffmanns. & Link
Iz_2_1109	DJI_0238	Galeopsis bifida Boenn.	0	n	1	0	Lamiacea Lindl.
Iz_2_1109	DJI_0238	Galium verum L.	0	n	1	0	Rubiaceae Juss.
Iz_2_1109	DJI_0238	Genista tinctoria L.	0	n	1	0	Fabaceae Lindl.
Iz_2_1109	DJI_0238	Geranium robertianum L.	0	n	1	0	Geraniaceae Juss.
Iz_2_1109	DJI_0238	Geranium sanquineum L.	0	n	1	0	Geraniaceae Juss.
Iz_2_1109	DJI_0238	Gypsophila paniculata L.	0	n	1	0	Caryophyllaceae Juss.
Iz_2_1109	DJI_0238	Helichrysum arenarium (L.) Moench	0	n	1	0	Asteraceae Bercht. & J.Presl
Iz_2_1109	DJI_0238	Herniaria polygama J.Gay	0	n	1	0	Caryophyllaceae Juss.
Iz_2_1109	DJI_0238	Hieracium robustum Fr.	0	n	1	0	Asteraceae Bercht. & J.Presl
Iz_2_1109	DJI_0238	Hieracium umbellatum L.	0	n	1	NA	Asteraceae Bercht. & J.Presl
Iz_2_1109	DJI_0238	Hylotelephium maximum (L.) Holub	0	n	1	0	Crassulaceae J.St.-Hil.
Iz_2_1109	DJI_0238	Jacobaea borysthenica (DC.) B.Nord. & Greuter	0	n	1	0	Asteraceae Bercht. & J.Presl
Iz_2_1109	DJI_0238	Jasione montana L.	0	n	1	0	Campanulaceae Juss.
Iz_2_1109	DJI_0238	Jurinea cyanoides (L.) Rchb.s.l.	0	n	1	0	Asteraceae Bercht. & J.Presl
Iz_2_1109	DJI_0238	Koeleria glauca (Spreng.) DC.	0	n	1	0	Poaceae Barnhart
Iz_2_1109	DJI_0238	Lactuca serriola L.	0	a	0	0	Asteraceae Bercht. & J.Presl
Iz_2_1109	DJI_0238	Linaria genistifolia (L.) Mill.	0	n	1	0	Plantaginaceae Juss.
Iz_2_1109	DJI_0238	Lomelosia argentea (L.) Greuter & Burdet	0	n	1	0	Caprifoliaceae Juss.
Iz_2_1109	DJI_0238	Melampyrum pratense L.	0	n	1	0	Orobanchaceae Vent.
Iz_2_1109	DJI_0238	Oenothera biennis L.	0	a	0	0	Onagraceae Juss.
Iz_2_1109	DJI_0238	Pentanema britannica (L.) D.Gut.Larr., Santos-Vicente, Anderb., E.Rico & M.M.Mart.Ort.	0	n	1	0	Asteraceae Bercht. & J.Presl
Iz_2_1109	DJI_0238	Peucedanum oreoselinum (L.) Moench	0	n	1	0	Apiaceae Lindl.
Iz_2_1109	DJI_0238	Peucedanum ruthenicum M.Bieb.	0	n	1	0	Apiaceae Lindl.
Iz_2_1109	DJI_0238	Pilosella echioides (Lumn.) F.W.Schultz & Sch.Bip.	0	n	1	0	Asteraceae Bercht. & J.Presl
Iz_2_1109	DJI_0238	Pilosella officinarum F. Schultz et Sch. Bip.	0	n	1	0	Asteraceae Bercht. & J.Presl
Iz_2_1109	DJI_0238	Pilosella sp.	0		NA	0	Asteraceae Bercht. & J.Presl
Iz_2_1109	DJI_0238	Pinus sylvestris L.	0	n	1	1	Pinaceae Spreng. ex F.Rudolphi
Iz_2_1109	DJI_0238	Poa annua L.	0	n	1	0	Poaceae Barnhart
Iz_2_1109	DJI_0238	Poa nemoralis L.	5	n	1	0	Poaceae Barnhart
Iz_2_1109	DJI_0238	Poa sp.	0		NA	0	Poaceae Barnhart
Iz_2_1109	DJI_0238	Polygonatum odoratum (Mill.) Druce	0	n	1	0	Asparagaceae Juss.
Iz_2_1109	DJI_0238	Polygonum arenarium Waldst. & Kit.	0	n	1	0	Polygonaceae Juss.
Iz_2_1109	DJI_0238	Populus nigra L.	0	n	1	1	Salicaceae Mirb.
Iz_2_1109	DJI_0238	Populus tremula L.	0	n	1	1	Salicaceae Mirb.
Iz_2_1109	DJI_0238	Populus Ã canescens (Aiton) Sm.	0	n	1	1	Salicaceae Mirb.
Iz_2_1109	DJI_0238	Potentilla argentea L.	0	n	1	0	Rosaceae Juss.
Iz_2_1109	DJI_0238	Psammophiliella muralis (L.) Ikonn.	0	n	1	0	Caryophyllaceae Juss.
Iz_2_1109	DJI_0238	Psephellus sumensis (Kalen.) Greuter	0	n	1	0	Asteraceae Bercht. & J.Presl
Iz_2_1109	DJI_0238	Pulsatilla pratensis (L.) Mill. s.l.	0	n	1	0	Ranunculaceae Juss.
Iz_2_1109	DJI_0238	Pyrus communis L.	0	a	0	1	Rosaceae Juss.
Iz_2_1109	DJI_0238	Quercus robur L.	0	n	1	1	Fagaceae Dumort.
Iz_2_1109	DJI_0238	Robinia pseudoacacia L.	0	a	0	1	Fabaceae Lindl.
Iz_2_1109	DJI_0238	Rosa sp.	0		NA	1	Rosaceae Juss.
Iz_2_1109	DJI_0238	Rubus idaeus L.	0	n	1	0	Rosaceae Juss.
Iz_2_1109	DJI_0238	Rumex acetosella L.	0	n	1	0	Polygonaceae Juss.
Iz_2_1109	DJI_0238	Rumex thyrsiflorus Fingerh.	0	n	1	0	Polygonaceae Juss.
Iz_2_1109	DJI_0238	Salix alba L.	0	n	1	1	Salicaceae Mirb.
Iz_2_1109	DJI_0238	Sambucus nigra L.	0	n	1	1	Viburnaceae Raf.
Iz_2_1109	DJI_0238	Secale sylvestre Host	0	n	1	0	Poaceae Barnhart
Iz_2_1109	DJI_0238	Sempervivum ruthenicum Schnittsp. & C.B. Lehm.	0	n	1	0	Crassulaceae J.St.-Hil.
Iz_2_1109	DJI_0238	Setaria viridis (L.) P. Beauv.	0	n	1	0	Poaceae Barnhart
Iz_2_1109	DJI_0238	Silene borysthenica (Gruner) Walters	0	n	1	0	Caryophyllaceae Juss.
Iz_2_1109	DJI_0238	Silene latifolia subsp. alba (Mill.) Greuter & Burdet	0	n	1	0	Caryophyllaceae Juss.
Iz_2_1109	DJI_0238	Solidago virgaurea L.	0	n	1	0	Asteraceae Bercht. & J.Presl
Iz_2_1109	DJI_0238	Sorbus aucuparia L.	0	n	1	1	Rosaceae Juss.
Iz_2_1109	DJI_0238	Stachys recta L.	0	n	1	0	Lamiacea Lindl.
Iz_2_1109	DJI_0238	Tanacetum vulgare L.	0	n	1	0	Asteraceae Bercht. & J.Presl
Iz_2_1109	DJI_0238	Thymus pallasianus Heinr.Braun	0	n	1	0	Lamiacea Lindl.
Iz_2_1109	DJI_0238	Tilia cordata Mill.	0	n	1	1	Malvaceae Juss.
Iz_2_1109	DJI_0238	Trifolium alpestre L.	0	n	1	0	Fabaceae Lindl.
Iz_2_1109	DJI_0238	Trifolium arvense L.	0	n	1	0	Fabaceae Lindl.
Iz_2_1109	DJI_0238	Ulmus glabra Huds.	0	n	1	1	Ulmaceae Mirb.
Iz_2_1109	DJI_0238	Verbascum densiflorum Bertol.	0	n	1	0	Scrophulariaceae Juss.
Iz_2_1109	DJI_0238	Verbascum sp.	1	n	1	0	Scrophulariaceae Juss.
Iz_2_1109	DJI_0238	Veronica austriaca L.	0	n	1	0	Plantaginaceae Juss.
Iz_2_1109	DJI_0238	Veronica incana L.	0	n	1	0	Plantaginaceae Juss.
Iz_2_1109	DJI_0238	Veronica verna L.	0	n	1	0	Plantaginaceae Juss.
Iz_2_1109	DJI_0238	Vincetoxicum hirundinaria Medik	0.2	n	1	0	Apocynaceae Juss.
Iz_2_1109	DJI_0245	Acer tataricum L.	0	n	1	1	Sapindaceae Juss.
Iz_2_1109	DJI_0245	Achillea millefolium L. s.l.	0	n	1	0	Asteraceae Bercht. & J.Presl
Iz_2_1109	DJI_0245	Allium oleraceum L.	0	n	1	0	Amaryllidaceae J.St.-Hil.
Iz_2_1109	DJI_0245	Ambrosia artemisiifolia L.	0	a	0	0	Asteraceae Bercht. & J.Presl
Iz_2_1109	DJI_0245	Amorpha fruticosa L.	0	a	0	0	Fabaceae Lindl.
Iz_2_1109	DJI_0245	Anthericum ramosum L.	0	n	1	0	Asparagaceae Juss.
Iz_2_1109	DJI_0245	Artemisia marschalliana Spreng.	0	n	1	0	Asteraceae Bercht. & J.Presl
Iz_2_1109	DJI_0245	Asparagus officinalis L.	0	n	1	0	Asparagaceae Juss.
Iz_2_1109	DJI_0245	Astragalus cicer L.	0	n	1	0	Fabaceae Lindl.
Iz_2_1109	DJI_0245	Bassia laniflora (S.G.Gmel.) A.J.Scott	0	n	1	0	Amaranthaceae Juss.
Iz_2_1109	DJI_0245	Berberis vulgaris L.	0	n	1	0	Berberidaceae Juss.
Iz_2_1109	DJI_0245	Betula pendula Roth.	0	n	1	1	Betulaceae Gray
Iz_2_1109	DJI_0245	Betula pendula Roth.	0	n	1	0	Poaceae Barnhart
Iz_2_1109	DJI_0245	Bromus inermis Leyss.	0		NA	NA	
Iz_2_1109	DJI_0245	Bromus squarrosus L.	0	a	0	0	Poaceae Barnhart
Iz_2_1109	DJI_0245	Bromus tectorum L.	0	a	0	0	Poaceae Barnhart
Iz_2_1109	DJI_0245	Calamagrostis epigejos (L.) Roth	3	n	1	0	Poaceae Barnhart
Iz_2_1109	DJI_0245	Carduus acanthoides L.	0	n	1	0	Asteraceae Bercht. & J.Presl
Iz_2_1109	DJI_0245	Carex sp.	0	n	1	0	Cyperaceae Juss.
Iz_2_1109	DJI_0245	Centaurea majorovii Dumbadze	0	n	1	0	Asteraceae Bercht. & J.Presl
Iz_2_1109	DJI_0245	Cf. Euporbia/Linaria	0		NA	0	
Iz_2_1109	DJI_0245	Chamaecytisus ruthenicus (Fisch.ex Wol.) Klaskova	0	n	1	0	Fabaceae Lindl.
Iz_2_1109	DJI_0245	Chelidonium majus L.	0	n	1	0	Papaveraceae Juss.
Iz_2_1109	DJI_0245	Chenopodium betaceum Andrz.	0	n	1	0	Amaranthaceae Juss.

Iz_2_1109	DJI_0245	Chenopodium cf. album	0	n	1	0	Amaranthaceae Juss.
Iz_2_1109	DJI_0245	Chenopodium sp.	0		NA	0	Amaranthaceae Juss.
Iz_2_1109	DJI_0245	Chondrilla juncea L.	0	n	1	0	Asteraceae Bercht. & J.Presl
Iz_2_1109	DJI_0245	Cirsium vulgare (Savi) Ten.	0	n	1	0	Asteraceae Bercht. & J.Presl
Iz_2_1109	DJI_0245	Clinopodium acinos (L.) Kuntze	0	n	1	0	Lamiacea Lindl.
Iz_2_1109	DJI_0245	Convallaria majalis L.	0	n	1	0	Asparagaceae Juss.
Iz_2_1109	DJI_0245	Crataegus rhipidophylla Gand.	0	n	1	1	Rosaceae Juss.
Iz_2_1109	DJI_0245	Crepis foetida L.	0	n	1	0	Asteraceae Bercht. & J.Presl
Iz_2_1109	DJI_0245	Dactylis glomerata L.	0	n	1	0	Poaceae Barnhart
Iz_2_1109	DJI_0245	Dianthus campestris M.Bieb.	0	n	1	0	Caryophyllaceae Juss.
Iz_2_1109	DJI_0245	Digitaria sanguinalis (L.) Scop.	0	a	0	0	Poaceae Barnhart
Iz_2_1109	DJI_0245	Elymus repens (L.) Gould	0	n	1	0	Poaceae Barnhart
Iz_2_1109	DJI_0245	Epilobium tetragonum L.	0	n	1	0	Onagraceae Juss.
Iz_2_1109	DJI_0245	Eragrostis minor Host	0	a	0	0	Poaceae Barnhart
Iz_2_1109	DJI_0245	Erigeron annuus (L.) Desf.	0	a	0	0	Asteraceae Bercht. & J.Presl
Iz_2_1109	DJI_0245	Erigeron canadensis L.	17	a	0	0	Asteraceae Bercht. & J.Presl
Iz_2_1109	DJI_0245	Euphorbia seguieriana Neck.	0	n	1	0	Euphorbiaceae Juss.
Iz_2_1109	DJI_0245	Fallopia dumetorum (L.) Holub	0	n	1	0	Polygonaceae Juss.
Iz_2_1109	DJI_0245	Festuca beckeri (Hack.) Trautv.	0	n	1	0	Poaceae Barnhart
Iz_2_1109	DJI_0245	Fragaria vesca L.	0	n	1	0	Rosaceae Juss.
Iz_2_1109	DJI_0245	Frangula alnus Mill.	0	n	1	1	Rhamnaceae Juss.
Iz_2_1109	DJI_0245	Fraxinus pennsylvanica Marshall	0	a	0	1	Oleaceae Hoffmanns. & Link
Iz_2_1109	DJI_0245	Galeopsis bifida Boenn.	0	n	1	0	Lamiacea Lindl.
Iz_2_1109	DJI_0245	Galium verum L.	0	n	1	0	Rubiaceae Juss.
Iz_2_1109	DJI_0245	Genista tinctoria L.	0	n	1	0	Fabaceae Lindl.
Iz_2_1109	DJI_0245	Geranium robertianum L.	0	n	1	0	Geraniaceae Juss.
Iz_2_1109	DJI_0245	Geranium sanquineum L.	0	n	1	0	Geraniaceae Juss.
Iz_2_1109	DJI_0245	Gypsophila paniculata L.	0	n	1	0	Caryophyllaceae Juss.
Iz_2_1109	DJI_0245	Helichrysum arenarium (L.) Moench	0	n	1	0	Asteraceae Bercht. & J.Presl
Iz_2_1109	DJI_0245	Herniaria polygama J.Gay	0	n	1	0	Caryophyllaceae Juss.
Iz_2_1109	DJI_0245	Hieracium robustum Fr.	0	n	1	0	Asteraceae Bercht. & J.Presl
Iz_2_1109	DJI_0245	Hieracium umbellatum L.	0	n	1	NA	Asteraceae Bercht. & J.Presl
Iz_2_1109	DJI_0245	Hylotelephium maximum (L.) Holub	0	n	1	0	Crassulaceae J.St.-Hil.
Iz_2_1109	DJI_0245	Jacobaea borysthenica (DC.) B.Nord. & Greuter	0	n	1	0	Asteraceae Bercht. & J.Presl
Iz_2_1109	DJI_0245	Jasione montana L.	0	n	1	0	Campanulaceae Juss.
Iz_2_1109	DJI_0245	Jurinea cyanoides (L.) Rchb.s.l.	0	n	1	0	Asteraceae Bercht. & J.Presl
Iz_2_1109	DJI_0245	Koeleria glauca (Spreng.) DC.	0	n	1	0	Poaceae Barnhart
Iz_2_1109	DJI_0245	Lactuca serriola L.	0	a	0	0	Asteraceae Bercht. & J.Presl
Iz_2_1109	DJI_0245	Linaria genistifolia (L.) Mill.	0	n	1	0	Plantaginaceae Juss.
Iz_2_1109	DJI_0245	Lomelosia argentea (L.) Greuter & Burdet	0	n	1	0	Caprifoliaceae Juss.
Iz_2_1109	DJI_0245	Melampyrum pratense L.	0	n	1	0	Orobanchaceae Vent.
Iz_2_1109	DJI_0245	Oenothera biennis L.	0	a	0	0	Onagraceae Juss.
Iz_2_1109	DJI_0245	Pentanema britannica (L.) D.Gut.Larr., Santos-Vicente, Anderb., E.Rico & M.M.Mart.Ort.	0	n	1	0	Asteraceae Bercht. & J.Presl
Iz_2_1109	DJI_0245	Peucedanum oreoselinum (L.) Moench	0	n	1	0	Apiaceae Lindl.
Iz_2_1109	DJI_0245	Peucedanum ruthenicum M.Bieb.	0	n	1	0	Apiaceae Lindl.
Iz_2_1109	DJI_0245	Pilosella echioides (Lumn.) F.W.Schultz & Sch.Bip.	0	n	1	0	Asteraceae Bercht. & J.Presl
Iz_2_1109	DJI_0245	Pilosella officinarum F. Schultz et Sch. Bip.	0	n	1	0	Asteraceae Bercht. & J.Presl
Iz_2_1109	DJI_0245	Pilosella sp.	1		NA	0	Asteraceae Bercht. & J.Presl
Iz_2_1109	DJI_0245	Pinus sylvestris L.	0	n	1	1	Pinaceae Spreng. ex F.Rudolphi
Iz_2_1109	DJI_0245	Poa annua L.	0	n	1	0	Poaceae Barnhart
Iz_2_1109	DJI_0245	Poa nemoralis L.	0	n	1	0	Poaceae Barnhart
Iz_2_1109	DJI_0245	Poa sp.	0		NA	0	Poaceae Barnhart
Iz_2_1109	DJI_0245	Polygonatum odoratum (Mill.) Druce	0	n	1	0	Asparagaceae Juss.
Iz_2_1109	DJI_0245	Polygonum arenarium Waldst. & Kit.	0	n	1	0	Polygonaceae Juss.
Iz_2_1109	DJI_0245	Populus nigra L.	0	n	1	1	Salicaceae Mirb.
Iz_2_1109	DJI_0245	Populus tremula L.	0	n	1	1	Salicaceae Mirb.
Iz_2_1109	DJI_0245	Populus Ã canescens (Aiton) Sm.	0	n	1	1	Salicaceae Mirb.
Iz_2_1109	DJI_0245	Potentilla argentea L.	0	n	1	0	Rosaceae Juss.
Iz_2_1109	DJI_0245	Psammophiliella muralis (L.) Ikonn.	0	n	1	0	Caryophyllaceae Juss.
Iz_2_1109	DJI_0245	Psephellus sumensis (Kalen.) Greuter	0	n	1	0	Asteraceae Bercht. & J.Presl
Iz_2_1109	DJI_0245	Pulsatilla pratensis (L.) Mill. s.l.	0	n	1	0	Ranunculaceae Juss.
Iz_2_1109	DJI_0245	Pyrus communis L.	0	a	0	1	Rosaceae Juss.
Iz_2_1109	DJI_0245	Quercus robur L.	0	n	1	1	Fagaceae Dumort.
Iz_2_1109	DJI_0245	Robinia pseudoacacia L.	0	a	0	1	Fabaceae Lindl.
Iz_2_1109	DJI_0245	Rosa sp.	0		NA	1	Rosaceae Juss.
Iz_2_1109	DJI_0245	Rubus idaeus L.	0	n	1	0	Rosaceae Juss.
Iz_2_1109	DJI_0245	Rumex acetosella L.	0	n	1	0	Polygonaceae Juss.
Iz_2_1109	DJI_0245	Rumex thyrsiflorus Fingerh.	0	n	1	0	Polygonaceae Juss.
Iz_2_1109	DJI_0245	Salix alba L.	0	n	1	1	Salicaceae Mirb.
Iz_2_1109	DJI_0245	Sambucus nigra L.	0	n	1	1	Viburnaceae Raf.
Iz_2_1109	DJI_0245	Secale sylvestre Host	0	n	1	0	Poaceae Barnhart
Iz_2_1109	DJI_0245	Sempervivum ruthenicum Schnittsp. & C.B. Lehm.	0	n	1	0	Crassulaceae J.St.-Hil.
Iz_2_1109	DJI_0245	Setaria viridis (L.) P. Beauv.	0	n	1	0	Poaceae Barnhart
Iz_2_1109	DJI_0245	Silene borysthenica (Gruner) Walters	0	n	1	0	Caryophyllaceae Juss.
Iz_2_1109	DJI_0245	Silene latifolia subsp. alba (Mill.) Greuter & Burdet	0	n	1	0	Caryophyllaceae Juss.
Iz_2_1109	DJI_0245	Solidago virgaurea L.	0	n	1	0	Asteraceae Bercht. & J.Presl
Iz_2_1109	DJI_0245	Sorbus aucuparia L.	0	n	1	1	Rosaceae Juss.
Iz_2_1109	DJI_0245	Stachys recta L.	0	n	1	0	Lamiacea Lindl.
Iz_2_1109	DJI_0245	Tanacetum vulgare L.	0	n	1	0	Asteraceae Bercht. & J.Presl
Iz_2_1109	DJI_0245	Thymus pallasianus Heinr.Braun	0	n	1	0	Lamiacea Lindl.
Iz_2_1109	DJI_0245	Tilia cordata Mill.	0	n	1	1	Malvaceae Juss.
Iz_2_1109	DJI_0245	Trifolium alpestre L.	0	n	1	0	Fabaceae Lindl.
Iz_2_1109	DJI_0245	Trifolium arvense L.	0	n	1	0	Fabaceae Lindl.
Iz_2_1109	DJI_0245	Ulmus glabra Huds.	0	n	1	1	Ulmaceae Mirb.
Iz_2_1109	DJI_0245	Verbascum densiflorum Bertol.	0	n	1	0	Scrophulariaceae Juss.
Iz_2_1109	DJI_0245	Verbascum sp.	0	n	1	0	Scrophulariaceae Juss.
Iz_2_1109	DJI_0245	Veronica austriaca L.	0	n	1	0	Plantaginaceae Juss.
Iz_2_1109	DJI_0245	Veronica incana L.	0	n	1	0	Plantaginaceae Juss.
Iz_2_1109	DJI_0245	Veronica verna L.	0	n	1	0	Plantaginaceae Juss.
Iz_2_1109	DJI_0245	Vincetoxicum hirundinaria Medik	0	n	1	0	Apocynaceae Juss.
Iz_2_1109	DJI_0250	Acer tataricum L.	0	n	1	1	Sapindaceae Juss.
Iz_2_1109	DJI_0250	Achillea millefolium L. s.l.	0	n	1	0	Asteraceae Bercht. & J.Presl
Iz_2_1109	DJI_0250	Allium oleraceum L.	0	n	1	0	Amaryllidaceae J.St.-Hil.
Iz_2_1109	DJI_0250	Ambrosia artemisiifolia L.	0	a	0	0	Asteraceae Bercht. & J.Presl
Iz_2_1109	DJI_0250	Amorpha fruticosa L.	0	a	0	0	Fabaceae Lindl.
Iz_2_1109	DJI_0250	Anthericum ramosum L.	0	n	1	0	Asparagaceae Juss.
Iz_2_1109	DJI_0250	Artemisia marschalliana Spreng.	0	n	1	0	Asteraceae Bercht. & J.Presl
Iz_2_1109	DJI_0250	Asparagus officinalis L.	0	n	1	0	Asparagaceae Juss.

Iz_2_1109	DJI_0250	Astragalus cicer L.	0	n	1	0	Fabaceae Lindl.
Iz_2_1109	DJI_0250	Bassia laniflora (S.G.Gmel.) A.J.Scott	0	n	1	0	Amaranthaceae Juss.
Iz_2_1109	DJI_0250	Berberis vulgaris L.	0	n	1	0	Berberidaceae Juss.
Iz_2_1109	DJI_0250	Betula pendula Roth.	0	n	1	1	Betulaceae Gray
Iz_2_1109	DJI_0250	Betula pendula Roth.	0	n	1	0	Poaceae Barnhart
Iz_2_1109	DJI_0250	Bromus inermis Leyss.	0		NA	NA	
Iz_2_1109	DJI_0250	Bromus squarrosus L.	0	a	0	0	Poaceae Barnhart
Iz_2_1109	DJI_0250	Bromus tectorum L.	0	a	0	0	Poaceae Barnhart
Iz_2_1109	DJI_0250	Calamagrostis epigejos (L.) Roth	10	n	1	0	Poaceae Barnhart
Iz_2_1109	DJI_0250	Carduus acanthoides L.	0	n	1	0	Asteraceae Bercht. & J.Presl
Iz_2_1109	DJI_0250	Carex sp.	0	n	1	0	Cyperaceae Juss.
Iz_2_1109	DJI_0250	Centaurea majorovii Dumbadze	0	n	1	0	Asteraceae Bercht. & J.Presl
Iz_2_1109	DJI_0250	Cf. Euporbia/Linaria	0		NA	0	
Iz_2_1109	DJI_0250	Chamaecytisus ruthenicus (Fisch.ex Wol.) Klaskova	0	n	1	0	Fabaceae Lindl.
Iz_2_1109	DJI_0250	Chelidonium majus L.	0	n	1	0	Papaveraceae Juss.
Iz_2_1109	DJI_0250	Chenopodium betaceum Andrz.	0	n	1	0	Amaranthaceae Juss.
Iz_2_1109	DJI_0250	Chenopodium cf. album	0	n	1	0	Amaranthaceae Juss.
Iz_2_1109	DJI_0250	Chenopodium sp.	0		NA	0	Amaranthaceae Juss.
Iz_2_1109	DJI_0250	Chondrilla juncea L.	0	n	1	0	Asteraceae Bercht. & J.Presl
Iz_2_1109	DJI_0250	Cirsium vulgare (Savi) Ten.	0	n	1	0	Asteraceae Bercht. & J.Presl
Iz_2_1109	DJI_0250	Clinopodium acinos (L.) Kuntze	0	n	1	0	Lamiacea Lindl.
Iz_2_1109	DJI_0250	Convallaria majalis L.	0	n	1	0	Asparagaceae Juss.
Iz_2_1109	DJI_0250	Crataegus rhipidophylla Gand.	0	n	1	1	Rosaceae Juss.
Iz_2_1109	DJI_0250	Crepis foetida L.	0	n	1	0	Asteraceae Bercht. & J.Presl
Iz_2_1109	DJI_0250	Dactylis glomerata L.	0	n	1	0	Poaceae Barnhart
Iz_2_1109	DJI_0250	Dianthus campestris M.Bieb.	0	n	1	0	Caryophyllaceae Juss.
Iz_2_1109	DJI_0250	Digitaria sanguinalis (L.) Scop.	0	a	0	0	Poaceae Barnhart
Iz_2_1109	DJI_0250	Elymus repens (L.) Gould	0	n	1	0	Poaceae Barnhart
Iz_2_1109	DJI_0250	Epilobium tetragonum L.	0	n	1	0	Onagraceae Juss.
Iz_2_1109	DJI_0250	Eragrostis minor Host	0	a	0	0	Poaceae Barnhart
Iz_2_1109	DJI_0250	Erigeron annuus (L.) Desf.	0	a	0	0	Asteraceae Bercht. & J.Presl
Iz_2_1109	DJI_0250	Erigeron canadensis L.	10	a	0	0	Asteraceae Bercht. & J.Presl
Iz_2_1109	DJI_0250	Euphorbia seguieriana Neck.	0	n	1	0	Euphorbiaceae Juss.
Iz_2_1109	DJI_0250	Fallopia dumetorum (L.) Holub	0	n	1	0	Polygonaceae Juss.
Iz_2_1109	DJI_0250	Festuca beckeri (Hack.) Trautv.	0	n	1	0	Poaceae Barnhart
Iz_2_1109	DJI_0250	Fragaria vesca L.	0	n	1	0	Rosaceae Juss.
Iz_2_1109	DJI_0250	Frangula alnus Mill.	0	n	1	1	Rhamnaceae Juss.
Iz_2_1109	DJI_0250	Fraxinus pennsylvanica Marshall	0	a	0	1	Oleaceae Hoffmanns. & Link
Iz_2_1109	DJI_0250	Galeopsis bifida Boenn.	0	n	1	0	Lamiacea Lindl.
Iz_2_1109	DJI_0250	Galium verum L.	0	n	1	0	Rubiaceae Juss.
Iz_2_1109	DJI_0250	Genista tinctoria L.	0	n	1	0	Fabaceae Lindl.
Iz_2_1109	DJI_0250	Geranium robertianum L.	0	n	1	0	Geraniaceae Juss.
Iz_2_1109	DJI_0250	Geranium sanquineum L.	0	n	1	0	Geraniaceae Juss.
Iz_2_1109	DJI_0250	Gypsophila paniculata L.	0	n	1	0	Caryophyllaceae Juss.
Iz_2_1109	DJI_0250	Helichrysum arenarium (L.) Moench	0	n	1	0	Asteraceae Bercht. & J.Presl
Iz_2_1109	DJI_0250	Herniaria polygama J.Gay	0	n	1	0	Caryophyllaceae Juss.
Iz_2_1109	DJI_0250	Hieracium robustum Fr.	0	n	1	0	Asteraceae Bercht. & J.Presl
Iz_2_1109	DJI_0250	Hieracium umbellatum L.	0	n	1	NA	Asteraceae Bercht. & J.Presl
Iz_2_1109	DJI_0250	Hylotelephium maximum (L.) Holub	0	n	1	0	Crassulaceae J.St.-Hil.
Iz_2_1109	DJI_0250	Jacobaea borysthenica (DC.) B.Nord. & Greuter	0	n	1	0	Asteraceae Bercht. & J.Presl
Iz_2_1109	DJI_0250	Jasione montana L.	0	n	1	0	Campanulaceae Juss.
Iz_2_1109	DJI_0250	Jurinea cyanoides (L.) Rchb.s.l.	3	n	1	0	Asteraceae Bercht. & J.Presl
Iz_2_1109	DJI_0250	Koeleria glauca (Spreng.) DC.	0	n	1	0	Poaceae Barnhart
Iz_2_1109	DJI_0250	Lactuca serriola L.	0	a	0	0	Asteraceae Bercht. & J.Presl
Iz_2_1109	DJI_0250	Linaria genistifolia (L.) Mill.	0	n	1	0	Plantaginaceae Juss.
Iz_2_1109	DJI_0250	Lomelosia argentea (L.) Greuter & Burdet	0	n	1	0	Caprifoliaceae Juss.
Iz_2_1109	DJI_0250	Melampyrum pratense L.	0	n	1	0	Orobanchaceae Vent.
Iz_2_1109	DJI_0250	Oenothera biennis L.	0	a	0	0	Onagraceae Juss.
Iz_2_1109	DJI_0250	Pentanema britannica (L.) D.Gut.Larr., Santos-Vicente, Anderb., E.Rico & M.M.Mart.Ort.	0	n	1	0	Asteraceae Bercht. & J.Presl
Iz_2_1109	DJI_0250	Peucedanum oreoselinum (L.) Moench	0	n	1	0	Apiaceae Lindl.
Iz_2_1109	DJI_0250	Peucedanum ruthenicum M.Bieb.	0	n	1	0	Apiaceae Lindl.
Iz_2_1109	DJI_0250	Pilosella echioides (Lumn.) F.W.Schultz & Sch.Bip.	0	n	1	0	Asteraceae Bercht. & J.Presl
Iz_2_1109	DJI_0250	Pilosella officinarum F. Schultz et Sch. Bip.	0	n	1	0	Asteraceae Bercht. & J.Presl
Iz_2_1109	DJI_0250	Pilosella sp.	0		NA	0	Asteraceae Bercht. & J.Presl
Iz_2_1109	DJI_0250	Pinus sylvestris L.	0	n	1	1	Pinaceae Spreng. ex F.Rudolphi
Iz_2_1109	DJI_0250	Poa annua L.	0	n	1	0	Poaceae Barnhart
Iz_2_1109	DJI_0250	Poa nemoralis L.	0	n	1	0	Poaceae Barnhart
Iz_2_1109	DJI_0250	Poa sp.	0		NA	0	Poaceae Barnhart
Iz_2_1109	DJI_0250	Polygonatum odoratum (Mill.) Druce	0	n	1	0	Asparagaceae Juss.
Iz_2_1109	DJI_0250	Polygonum arenarium Waldst. & Kit.	0	n	1	0	Polygonaceae Juss.
Iz_2_1109	DJI_0250	Populus nigra L.	0	n	1	1	Salicaceae Mirb.
Iz_2_1109	DJI_0250	Populus tremula L.	0	n	1	1	Salicaceae Mirb.
Iz_2_1109	DJI_0250	Populus Ã canescens (Aiton) Sm.	0	n	1	1	Salicaceae Mirb.
Iz_2_1109	DJI_0250	Potentilla argentea L.	0	n	1	0	Rosaceae Juss.
Iz_2_1109	DJI_0250	Psammophiliella muralis (L.) Ikonn.	0	n	1	0	Caryophyllaceae Juss.
Iz_2_1109	DJI_0250	Psephellus sumensis (Kalen.) Greuter	0	n	1	0	Asteraceae Bercht. & J.Presl
Iz_2_1109	DJI_0250	Pulsatilla pratensis (L.) Mill. s.l.	0	n	1	0	Ranunculaceae Juss.
Iz_2_1109	DJI_0250	Pyrus communis L.	0	a	0	1	Rosaceae Juss.
Iz_2_1109	DJI_0250	Quercus robur L.	0	n	1	1	Fagaceae Dumort.
Iz_2_1109	DJI_0250	Robinia pseudoacacia L.	0	a	0	1	Fabaceae Lindl.
Iz_2_1109	DJI_0250	Rosa sp.	0		NA	1	Rosaceae Juss.
Iz_2_1109	DJI_0250	Rubus idaeus L.	0	n	1	0	Rosaceae Juss.
Iz_2_1109	DJI_0250	Rumex acetosella L.	0	n	1	0	Polygonaceae Juss.
Iz_2_1109	DJI_0250	Rumex thyrsiflorus Fingerh.	0	n	1	0	Polygonaceae Juss.
Iz_2_1109	DJI_0250	Salix alba L.	0	n	1	1	Salicaceae Mirb.
Iz_2_1109	DJI_0250	Sambucus nigra L.	0	n	1	1	Viburnaceae Raf.
Iz_2_1109	DJI_0250	Secale sylvestre Host	0	n	1	0	Poaceae Barnhart
Iz_2_1109	DJI_0250	Sempervivum ruthenicum Schnittsp. & C.B. Lehm.	0	n	1	0	Crassulaceae J.St.-Hil.
Iz_2_1109	DJI_0250	Setaria viridis (L.) P. Beauv.	0	n	1	0	Poaceae Barnhart
Iz_2_1109	DJI_0250	Silene borysthenica (Gruner) Walters	0	n	1	0	Caryophyllaceae Juss.
Iz_2_1109	DJI_0250	Silene latifolia subsp. alba (Mill.) Greuter & Burdet	0	n	1	0	Caryophyllaceae Juss.
Iz_2_1109	DJI_0250	Solidago virgaurea L.	0	n	1	0	Asteraceae Bercht. & J.Presl
Iz_2_1109	DJI_0250	Sorbus aucuparia L.	0	n	1	1	Rosaceae Juss.
Iz_2_1109	DJI_0250	Stachys recta L.	0	n	1	0	Lamiacea Lindl.
Iz_2_1109	DJI_0250	Tanacetum vulgare L.	0	n	1	0	Asteraceae Bercht. & J.Presl
Iz_2_1109	DJI_0250	Thymus pallasianus Heinr.Braun	0	n	1	0	Lamiacea Lindl.
Iz_2_1109	DJI_0250	Tilia cordata Mill.	0	n	1	1	Malvaceae Juss.
Iz_2_1109	DJI_0250	Trifolium alpestre L.	0	n	1	0	Fabaceae Lindl.

Iz_2_1109	DJI_0250	Trifolium arvense L.	0	n	1	0	Fabaceae Lindl.
Iz_2_1109	DJI_0250	Ulmus glabra Huds.	0	n	1	1	Ulmaceae Mirb.
Iz_2_1109	DJI_0250	Verbascum densiflorum Bertol.	0	n	1	0	Scrophulariaceae Juss.
Iz_2_1109	DJI_0250	Verbascum sp.	0	n	1	0	Scrophulariaceae Juss.
Iz_2_1109	DJI_0250	Veronica austriaca L.	0	n	1	0	Plantaginaceae Juss.
Iz_2_1109	DJI_0250	Veronica incana L.	0	n	1	0	Plantaginaceae Juss.
Iz_2_1109	DJI_0250	Veronica verna L.	0	n	1	0	Plantaginaceae Juss.
Iz_2_1109	DJI_0250	Vincetoxicum hirundinaria Medik	0	n	1	0	Apocynaceae Juss.
Iz_2_1109	DJI_0255	Acer tataricum L.	0	n	1	1	Sapindaceae Juss.
Iz_2_1109	DJI_0255	Achillea millefolium L. s.l.	0	n	1	0	Asteraceae Bercht. & J.Presl
Iz_2_1109	DJI_0255	Allium oleraceum L.	0	n	1	0	Amaryllidaceae J.St.-Hil.
Iz_2_1109	DJI_0255	Ambrosia artemisiifolia L.	0	a	0	0	Asteraceae Bercht. & J.Presl
Iz_2_1109	DJI_0255	Amorpha fruticosa L.	0	a	0	0	Fabaceae Lindl.
Iz_2_1109	DJI_0255	Anthericum ramosum L.	0	n	1	0	Asparagaceae Juss.
Iz_2_1109	DJI_0255	Artemisia marschalliana Spreng.	0	n	1	0	Asteraceae Bercht. & J.Presl
Iz_2_1109	DJI_0255	Asparagus officinalis L.	0	n	1	0	Asparagaceae Juss.
Iz_2_1109	DJI_0255	Astragalus cicer L.	0	n	1	0	Fabaceae Lindl.
Iz_2_1109	DJI_0255	Bassia laniflora (S.G.Gmel.) A.J.Scott	0	n	1	0	Amaranthaceae Juss.
Iz_2_1109	DJI_0255	Berberis vulgaris L.	0	n	1	0	Berberidaceae Juss.
Iz_2_1109	DJI_0255	Betula pendula Roth.	0	n	1	1	Betulaceae Gray
Iz_2_1109	DJI_0255	Betula pendula Roth.	0	n	1	0	Poaceae Barnhart
Iz_2_1109	DJI_0255	Bromus inermis Leyss.	0		NA	NA	
Iz_2_1109	DJI_0255	Bromus squarrosus L.	0	a	0	0	Poaceae Barnhart
Iz_2_1109	DJI_0255	Bromus tectorum L.	0	a	0	0	Poaceae Barnhart
Iz_2_1109	DJI_0255	Calamagrostis epigejos (L.) Roth	0	n	1	0	Poaceae Barnhart
Iz_2_1109	DJI_0255	Carduus acanthoides L.	0	n	1	0	Asteraceae Bercht. & J.Presl
Iz_2_1109	DJI_0255	Carex sp.	0	n	1	0	Cyperaceae Juss.
Iz_2_1109	DJI_0255	Centaurea majorovii Dumbadze	0	n	1	0	Asteraceae Bercht. & J.Presl
Iz_2_1109	DJI_0255	Cf. Euporbia/Linaria	0		NA	0	
Iz_2_1109	DJI_0255	Chamaecytisus ruthenicus (Fisch.ex Wol.) Klaskova	0	n	1	0	Fabaceae Lindl.
Iz_2_1109	DJI_0255	Chelidonium majus L.	0	n	1	0	Papaveraceae Juss.
Iz_2_1109	DJI_0255	Chenopodium betaceum Andrz.	0	n	1	0	Amaranthaceae Juss.
Iz_2_1109	DJI_0255	Chenopodium cf. album	0	n	1	0	Amaranthaceae Juss.
Iz_2_1109	DJI_0255	Chenopodium sp.	0		NA	0	Amaranthaceae Juss.
Iz_2_1109	DJI_0255	Chondrilla juncea L.	0	n	1	0	Asteraceae Bercht. & J.Presl
Iz_2_1109	DJI_0255	Cirsium vulgare (Savi) Ten.	0	n	1	0	Asteraceae Bercht. & J.Presl
Iz_2_1109	DJI_0255	Clinopodium acinos (L.) Kuntze	0	n	1	0	Lamiacea Lindl.
Iz_2_1109	DJI_0255	Convallaria majalis L.	0	n	1	0	Asparagaceae Juss.
Iz_2_1109	DJI_0255	Crataegus rhipidophylla Gand.	0	n	1	1	Rosaceae Juss.
Iz_2_1109	DJI_0255	Crepis foetida L.	0	n	1	0	Asteraceae Bercht. & J.Presl
Iz_2_1109	DJI_0255	Dactylis glomerata L.	0	n	1	0	Poaceae Barnhart
Iz_2_1109	DJI_0255	Dianthus campestris M.Bieb.	0	n	1	0	Caryophyllaceae Juss.
Iz_2_1109	DJI_0255	Digitaria sanguinalis (L.) Scop.	0	a	0	0	Poaceae Barnhart
Iz_2_1109	DJI_0255	Elymus repens (L.) Gould	0	n	1	0	Poaceae Barnhart
Iz_2_1109	DJI_0255	Epilobium tetragonum L.	0	n	1	0	Onagraceae Juss.
Iz_2_1109	DJI_0255	Eragrostis minor Host	0	a	0	0	Poaceae Barnhart
Iz_2_1109	DJI_0255	Erigeron annuus (L.) Desf.	0	a	0	0	Asteraceae Bercht. & J.Presl
Iz_2_1109	DJI_0255	Erigeron canadensis L.	10	a	0	0	Asteraceae Bercht. & J.Presl
Iz_2_1109	DJI_0255	Euphorbia seguieriana Neck.	0	n	1	0	Euphorbiaceae Juss.
Iz_2_1109	DJI_0255	Fallopia dumetorum (L.) Holub	0	n	1	0	Polygonaceae Juss.
Iz_2_1109	DJI_0255	Festuca beckeri (Hack.) Trautv.	0	n	1	0	Poaceae Barnhart
Iz_2_1109	DJI_0255	Fragaria vesca L.	0	n	1	0	Rosaceae Juss.
Iz_2_1109	DJI_0255	Frangula alnus Mill.	0	n	1	1	Rhamnaceae Juss.
Iz_2_1109	DJI_0255	Fraxinus pennsylvanica Marshall	0	a	0	1	Oleaceae Hoffmanns. & Link
Iz_2_1109	DJI_0255	Galeopsis bifida Boenn.	0	n	1	0	Lamiacea Lindl.
Iz_2_1109	DJI_0255	Galium verum L.	0	n	1	0	Rubiaceae Juss.
Iz_2_1109	DJI_0255	Genista tinctoria L.	0	n	1	0	Fabaceae Lindl.
Iz_2_1109	DJI_0255	Geranium robertianum L.	0	n	1	0	Geraniaceae Juss.
Iz_2_1109	DJI_0255	Geranium sanquineum L.	0	n	1	0	Geraniaceae Juss.
Iz_2_1109	DJI_0255	Gypsophila paniculata L.	0	n	1	0	Caryophyllaceae Juss.
Iz_2_1109	DJI_0255	Helichrysum arenarium (L.) Moench	0	n	1	0	Asteraceae Bercht. & J.Presl
Iz_2_1109	DJI_0255	Herniaria polygama J.Gay	0	n	1	0	Caryophyllaceae Juss.
Iz_2_1109	DJI_0255	Hieracium robustum Fr.	0	n	1	0	Asteraceae Bercht. & J.Presl
Iz_2_1109	DJI_0255	Hieracium umbellatum L.	0	n	1	NA	Asteraceae Bercht. & J.Presl
Iz_2_1109	DJI_0255	Hylotelephium maximum (L.) Holub	0	n	1	0	Crassulaceae J.St.-Hil.
Iz_2_1109	DJI_0255	Jacobaea borysthenica (DC.) B.Nord. & Greuter	0	n	1	0	Asteraceae Bercht. & J.Presl
Iz_2_1109	DJI_0255	Jasione montana L.	0	n	1	0	Campanulaceae Juss.
Iz_2_1109	DJI_0255	Jurinea cyanoides (L.) Rchb.s.l.	0	n	1	0	Asteraceae Bercht. & J.Presl
Iz_2_1109	DJI_0255	Koeleria glauca (Spreng.) DC.	0	n	1	0	Poaceae Barnhart
Iz_2_1109	DJI_0255	Lactuca serriola L.	0	a	0	0	Asteraceae Bercht. & J.Presl
Iz_2_1109	DJI_0255	Linaria genistifolia (L.) Mill.	0	n	1	0	Plantaginaceae Juss.
Iz_2_1109	DJI_0255	Lomelosia argentea (L.) Greuter & Burdet	0	n	1	0	Caprifoliaceae Juss.
Iz_2_1109	DJI_0255	Melampyrum pratense L.	0	n	1	0	Orobanchaceae Vent.
Iz_2_1109	DJI_0255	Oenothera biennis L.	0	a	0	0	Onagraceae Juss.
Iz_2_1109	DJI_0255	Pentanema britannica (L.) D.Gut.Larr., Santos-Vicente, Anderb., E.Rico & M.M.Mart.Ort.	0	n	1	0	Asteraceae Bercht. & J.Presl
Iz_2_1109	DJI_0255	Peucedanum oreoselinum (L.) Moench	0	n	1	0	Apiaceae Lindl.
Iz_2_1109	DJI_0255	Peucedanum ruthenicum M.Bieb.	0	n	1	0	Apiaceae Lindl.
Iz_2_1109	DJI_0255	Pilosella echioides (Lumn.) F.W.Schultz & Sch.Bip.	0	n	1	0	Asteraceae Bercht. & J.Presl
Iz_2_1109	DJI_0255	Pilosella officinarum F. Schultz et Sch. Bip.	0	n	1	0	Asteraceae Bercht. & J.Presl
Iz_2_1109	DJI_0255	Pilosella sp.	0		NA	0	Asteraceae Bercht. & J.Presl
Iz_2_1109	DJI_0255	Pinus sylvestris L.	0	n	1	1	Pinaceae Spreng. ex F.Rudolphi
Iz_2_1109	DJI_0255	Poa annua L.	0	n	1	0	Poaceae Barnhart
Iz_2_1109	DJI_0255	Poa nemoralis L.	0	n	1	0	Poaceae Barnhart
Iz_2_1109	DJI_0255	Poa sp.	0		NA	0	Poaceae Barnhart
Iz_2_1109	DJI_0255	Polygonatum odoratum (Mill.) Druce	0	n	1	0	Asparagaceae Juss.
Iz_2_1109	DJI_0255	Polygonum arenarium Waldst. & Kit.	0	n	1	0	Polygonaceae Juss.
Iz_2_1109	DJI_0255	Populus nigra L.	0	n	1	1	Salicaceae Mirb.
Iz_2_1109	DJI_0255	Populus tremula L.	0	n	1	1	Salicaceae Mirb.
Iz_2_1109	DJI_0255	Populus Ã canescens (Aiton) Sm.	0	n	1	1	Salicaceae Mirb.
Iz_2_1109	DJI_0255	Potentilla argentea L.	0	n	1	0	Rosaceae Juss.
Iz_2_1109	DJI_0255	Psammophiliella muralis (L.) Ikonn.	0	n	1	0	Caryophyllaceae Juss.
Iz_2_1109	DJI_0255	Psephellus sumensis (Kalen.) Greuter	0	n	1	0	Asteraceae Bercht. & J.Presl
Iz_2_1109	DJI_0255	Pulsatilla pratensis (L.) Mill. s.l.	0	n	1	0	Ranunculaceae Juss.
Iz_2_1109	DJI_0255	Pyrus communis L.	0	a	0	1	Rosaceae Juss.
Iz_2_1109	DJI_0255	Quercus robur L.	0	n	1	1	Fagaceae Dumort.
Iz_2_1109	DJI_0255	Robinia pseudoacacia L.	0	a	0	1	Fabaceae Lindl.
Iz_2_1109	DJI_0255	Rosa sp.	0		NA	1	Rosaceae Juss.
Iz_2_1109	DJI_0255	Rubus idaeus L.	0	n	1	0	Rosaceae Juss.

Iz_2_1109	DJI_0255	Rumex acetosella L.	1	n	1	0	Polygonaceae Juss.
Iz_2_1109	DJI_0255	Rumex thyrsiflorus Fingerh.	0	n	1	0	Polygonaceae Juss.
Iz_2_1109	DJI_0255	Salix alba L.	0	n	1	1	Salicaceae Mirb.
Iz_2_1109	DJI_0255	Sambucus nigra L.	0	n	1	1	Viburnaceae Raf.
Iz_2_1109	DJI_0255	Secale sylvestre Host	0	n	1	0	Poaceae Barnhart
Iz_2_1109	DJI_0255	Sempervivum ruthenicum Schnittsp. & C.B. Lehm.	0	n	1	0	Crassulaceae J.St.-Hil.
Iz_2_1109	DJI_0255	Setaria viridis (L.) P. Beauv.	0	n	1	0	Poaceae Barnhart
Iz_2_1109	DJI_0255	Silene borysthenica (Gruner) Walters	0	n	1	0	Caryophyllaceae Juss.
Iz_2_1109	DJI_0255	Silene latifolia subsp. alba (Mill.) Greuter & Burdet	0	n	1	0	Caryophyllaceae Juss.
Iz_2_1109	DJI_0255	Solidago virgaurea L.	0	n	1	0	Asteraceae Bercht. & J.Presl
Iz_2_1109	DJI_0255	Sorbus aucuparia L.	0	n	1	1	Rosaceae Juss.
Iz_2_1109	DJI_0255	Stachys recta L.	0	n	1	0	Lamiacea Lindl.
Iz_2_1109	DJI_0255	Tanacetum vulgare L.	0	n	1	0	Asteraceae Bercht. & J.Presl
Iz_2_1109	DJI_0255	Thymus pallasianus Heinr.Braun	0	n	1	0	Lamiacea Lindl.
Iz_2_1109	DJI_0255	Tilia cordata Mill.	0	n	1	1	Malvaceae Juss.
Iz_2_1109	DJI_0255	Trifolium alpestre L.	0	n	1	0	Fabaceae Lindl.
Iz_2_1109	DJI_0255	Trifolium arvense L.	0	n	1	0	Fabaceae Lindl.
Iz_2_1109	DJI_0255	Ulmus glabra Huds.	0	n	1	1	Ulmaceae Mirb.
Iz_2_1109	DJI_0255	Verbascum densiflorum Bertol.	0	n	1	0	Scrophulariaceae Juss.
Iz_2_1109	DJI_0255	Verbascum sp.	0	n	1	0	Scrophulariaceae Juss.
Iz_2_1109	DJI_0255	Veronica austriaca L.	0	n	1	0	Plantaginaceae Juss.
Iz_2_1109	DJI_0255	Veronica incana L.	0	n	1	0	Plantaginaceae Juss.
Iz_2_1109	DJI_0255	Veronica verna L.	0	n	1	0	Plantaginaceae Juss.
Iz_2_1109	DJI_0255	Vincetoxicum hirundinaria Medik	0	n	1	0	Apocynaceae Juss.
Iz_2_1209	DJI_0043	Acer tataricum L.	0	n	1	1	Sapindaceae Juss.
Iz_2_1209	DJI_0043	Achillea millefolium L. s.l.	0	n	1	0	Asteraceae Bercht. & J.Presl
Iz_2_1209	DJI_0043	Allium oleraceum L.	0	n	1	0	Amaryllidaceae J.St.-Hil.
Iz_2_1209	DJI_0043	Ambrosia artemisiifolia L.	0	a	0	0	Asteraceae Bercht. & J.Presl
Iz_2_1209	DJI_0043	Amorpha fruticosa L.	0	a	0	0	Fabaceae Lindl.
Iz_2_1209	DJI_0043	Anthericum ramosum L.	0	n	1	0	Asparagaceae Juss.
Iz_2_1209	DJI_0043	Artemisia marschalliana Spreng.	0	n	1	0	Asteraceae Bercht. & J.Presl
Iz_2_1209	DJI_0043	Asparagus officinalis L.	0	n	1	0	Asparagaceae Juss.
Iz_2_1209	DJI_0043	Astragalus cicer L.	0	n	1	0	Fabaceae Lindl.
Iz_2_1209	DJI_0043	Bassia laniflora (S.G.Gmel.) A.J.Scott	0	n	1	0	Amaranthaceae Juss.
Iz_2_1209	DJI_0043	Berberis vulgaris L.	0	n	1	0	Berberidaceae Juss.
Iz_2_1209	DJI_0043	Betula pendula Roth.	0	n	1	1	Betulaceae Gray
Iz_2_1209	DJI_0043	Betula pendula Roth.	0	n	1	0	Poaceae Barnhart
Iz_2_1209	DJI_0043	Bromus inermis Leyss.	0		NA	NA	
Iz_2_1209	DJI_0043	Bromus squarrosus L.	0	a	0	0	Poaceae Barnhart
Iz_2_1209	DJI_0043	Bromus tectorum L.	0	a	0	0	Poaceae Barnhart
Iz_2_1209	DJI_0043	Calamagrostis epigejos (L.) Roth	30	n	1	0	Poaceae Barnhart
Iz_2_1209	DJI_0043	Carduus acanthoides L.	0	n	1	0	Asteraceae Bercht. & J.Presl
Iz_2_1209	DJI_0043	Carex sp.	0	n	1	0	Cyperaceae Juss.
Iz_2_1209	DJI_0043	Centaurea majorovii Dumbadze	0	n	1	0	Asteraceae Bercht. & J.Presl
Iz_2_1209	DJI_0043	Chamaecytisus ruthenicus (Fisch.ex Wol.) Klaskova	0	n	1	0	Fabaceae Lindl.
Iz_2_1209	DJI_0043	Chelidonium majus L.	0	n	1	0	Papaveraceae Juss.
Iz_2_1209	DJI_0043	Chenopodium betaceum Andrz.	0	n	1	0	Amaranthaceae Juss.
Iz_2_1209	DJI_0043	Chenopodium cf. album	0	n	1	0	Amaranthaceae Juss.
Iz_2_1209	DJI_0043	Chenopodium sp.	0		NA	0	Amaranthaceae Juss.
Iz_2_1209	DJI_0043	Chondrilla juncea L.	0	n	1	0	Asteraceae Bercht. & J.Presl
Iz_2_1209	DJI_0043	Cirsium vulgare (Savi) Ten.	0	n	1	0	Asteraceae Bercht. & J.Presl
Iz_2_1209	DJI_0043	Clinopodium acinos (L.) Kuntze	0	n	1	0	Lamiacea Lindl.
Iz_2_1209	DJI_0043	Convallaria majalis L.	0	n	1	0	Asparagaceae Juss.
Iz_2_1209	DJI_0043	Crataegus rhipidophylla Gand.	0	n	1	1	Rosaceae Juss.
Iz_2_1209	DJI_0043	Crepis foetida L.	0	n	1	0	Asteraceae Bercht. & J.Presl
Iz_2_1209	DJI_0043	Dactylis glomerata L.	0	n	1	0	Poaceae Barnhart
Iz_2_1209	DJI_0043	Dianthus campestris M.Bieb.	1	n	1	0	Caryophyllaceae Juss.
Iz_2_1209	DJI_0043	Digitaria sanguinalis (L.) Scop.	0	a	0	0	Poaceae Barnhart
Iz_2_1209	DJI_0043	Elymus repens (L.) Gould	0	n	1	0	Poaceae Barnhart
Iz_2_1209	DJI_0043	Epilobium tetragonum L.	0	n	1	0	Onagraceae Juss.
Iz_2_1209	DJI_0043	Eragrostis minor Host	0	a	0	0	Poaceae Barnhart
Iz_2_1209	DJI_0043	Erigeron canadensis L.	0	a	0	0	Asteraceae Bercht. & J.Presl
Iz_2_1209	DJI_0043	Euphorbia seguieriana Neck.	0	n	1	0	Euphorbiaceae Juss.
Iz_2_1209	DJI_0043	Fallopia dumetorum (L.) Holub	0	n	1	0	Polygonaceae Juss.
Iz_2_1209	DJI_0043	Festuca beckeri (Hack.) Trautv.	0	n	1	0	Poaceae Barnhart
Iz_2_1209	DJI_0043	Fragaria vesca L.	0	n	1	0	Rosaceae Juss.
Iz_2_1209	DJI_0043	Frangula alnus Mill.	0	n	1	1	Rhamnaceae Juss.
Iz_2_1209	DJI_0043	Fraxinus pennsylvanica Marshall	0	a	0	1	Oleaceae Hoffmanns. & Link
Iz_2_1209	DJI_0043	Galeopsis bifida Boenn.	0	n	1	0	Lamiacea Lindl.
Iz_2_1209	DJI_0043	Galium verum L.	0	n	1	0	Rubiaceae Juss.
Iz_2_1209	DJI_0043	Genista tinctoria L.	0	n	1	0	Fabaceae Lindl.
Iz_2_1209	DJI_0043	Geranium robertianum L.	0	n	1	0	Geraniaceae Juss.
Iz_2_1209	DJI_0043	Geranium sanquineum L.	0	n	1	0	Geraniaceae Juss.
Iz_2_1209	DJI_0043	Gypsophila paniculata L.	0	n	1	0	Caryophyllaceae Juss.
Iz_2_1209	DJI_0043	Helichrysum arenarium (L.) Moench	0	n	1	0	Asteraceae Bercht. & J.Presl
Iz_2_1209	DJI_0043	Herniaria polygama J.Gay	0	n	1	0	Caryophyllaceae Juss.
Iz_2_1209	DJI_0043	Hieracium robustum Fr.	0	n	1	0	Asteraceae Bercht. & J.Presl
Iz_2_1209	DJI_0043	Hieracium umbellatum L.	0	n	1	NA	Asteraceae Bercht. & J.Presl
Iz_2_1209	DJI_0043	Hylotelephium maximum (L.) Holub	1	n	1	0	Crassulaceae J.St.-Hil.
Iz_2_1209	DJI_0043	Jacobaea borysthenica (DC.) B.Nord. & Greuter	0	n	1	0	Asteraceae Bercht. & J.Presl
Iz_2_1209	DJI_0043	Jasione montana L.	0	n	1	0	Campanulaceae Juss.
Iz_2_1209	DJI_0043	Jurinea cyanoides (L.) Rchb.s.l.	0	n	1	0	Asteraceae Bercht. & J.Presl
Iz_2_1209	DJI_0043	Koeleria glauca (Spreng.) DC.	0	n	1	0	Poaceae Barnhart
Iz_2_1209	DJI_0043	Lactuca serriola L.	0	a	0	0	Asteraceae Bercht. & J.Presl
Iz_2_1209	DJI_0043	Linaria genistifolia (L.) Mill.	0	n	1	0	Plantaginaceae Juss.
Iz_2_1209	DJI_0043	Lomelosia argentea (L.) Greuter & Burdet	0	n	1	0	Caprifoliaceae Juss.
Iz_2_1209	DJI_0043	Melampyrum pratense L.	0	n	1	0	Orobanchaceae Vent.
Iz_2_1209	DJI_0043	Oenothera biennis L.	0	a	0	0	Onagraceae Juss.
Iz_2_1209	DJI_0043	Pentanema britannica (L.) D.Gut.Larr., Santos-Vicente, Anderb., E.Rico & M.M.Mart.Ort.	0	n	1	0	Asteraceae Bercht. & J.Presl
Iz_2_1209	DJI_0043	Peucedanum oreoselinum (L.) Moench	2	n	1	0	Apiaceae Lindl.
Iz_2_1209	DJI_0043	Peucedanum ruthenicum M.Bieb.	0	n	1	0	Apiaceae Lindl.
Iz_2_1209	DJI_0043	Pilosella echioides (Lumn.) F.W.Schultz & Sch.Bip.	0	n	1	0	Asteraceae Bercht. & J.Presl
Iz_2_1209	DJI_0043	Pilosella officinarum F. Schultz et Sch. Bip.	0	n	1	0	Asteraceae Bercht. & J.Presl
Iz_2_1209	DJI_0043	Pilosella sp.	0		NA	0	Asteraceae Bercht. & J.Presl
Iz_2_1209	DJI_0043	Pinus sylvestris L.	0	n	1	1	Pinaceae Spreng. ex F.Rudolphi
Iz_2_1209	DJI_0043	Poa annua L.	0	n	1	0	Poaceae Barnhart
Iz_2_1209	DJI_0043	Poa nemoralis L.	0	n	1	0	Poaceae Barnhart
Iz_2_1209	DJI_0043	Poa sp.	0		NA	0	Poaceae Barnhart

Iz_2_1209	DJI_0043	Polygonatum odoratum (Mill.) Druce	0	n	1	0	Asparagaceae Juss.
Iz_2_1209	DJI_0043	Polygonum arenarium Waldst. & Kit.	0	n	1	0	Polygonaceae Juss.
Iz_2_1209	DJI_0043	Populus nigra L.	0	n	1	1	Salicaceae Mirb.
Iz_2_1209	DJI_0043	Populus tremula L.	0	n	1	1	Salicaceae Mirb.
Iz_2_1209	DJI_0043	Populus Ã canescens (Aiton) Sm.	0	n	1	1	Salicaceae Mirb.
Iz_2_1209	DJI_0043	Potentilla argentea L.	0	n	1	0	Rosaceae Juss.
Iz_2_1209	DJI_0043	Psammophiliella muralis (L.) Ikonn.	0	n	1	0	Caryophyllaceae Juss.
Iz_2_1209	DJI_0043	Psephellus sumensis (Kalen.) Greuter	4	n	1	0	Asteraceae Bercht. & J.Presl
Iz_2_1209	DJI_0043	Pulsatilla pratensis (L.) Mill. s.l.	0	n	1	0	Ranunculaceae Juss.
Iz_2_1209	DJI_0043	Pyrus communis L.	0	a	0	1	Rosaceae Juss.
Iz_2_1209	DJI_0043	Quercus robur L.	0	n	1	1	Fagaceae Dumort.
Iz_2_1209	DJI_0043	Robinia pseudoacacia L.	0	a	0	1	Fabaceae Lindl.
Iz_2_1209	DJI_0043	Rosa sp.	0		NA	1	Rosaceae Juss.
Iz_2_1209	DJI_0043	Rubus idaeus L.	0	n	1	0	Rosaceae Juss.
Iz_2_1209	DJI_0043	Rumex acetosella L.	0	n	1	0	Polygonaceae Juss.
Iz_2_1209	DJI_0043	Rumex thyrsiflorus Fingerh.	0	n	1	0	Polygonaceae Juss.
Iz_2_1209	DJI_0043	Salix alba L.	0	n	1	1	Salicaceae Mirb.
Iz_2_1209	DJI_0043	Sambucus nigra L.	0	n	1	1	Viburnaceae Raf.
Iz_2_1209	DJI_0043	Secale sylvestre Host	0	n	1	0	Poaceae Barnhart
Iz_2_1209	DJI_0043	Sempervivum ruthenicum Schnittsp. & C.B. Lehm.	0	n	1	0	Crassulaceae J.St.-Hil.
Iz_2_1209	DJI_0043	Setaria viridis (L.) P. Beauv.	0	n	1	0	Poaceae Barnhart
Iz_2_1209	DJI_0043	Silene borysthenica (Gruner) Walters	0	n	1	0	Caryophyllaceae Juss.
Iz_2_1209	DJI_0043	Silene latifolia subsp. alba (Mill.) Greuter & Burdet	0	n	1	0	Caryophyllaceae Juss.
Iz_2_1209	DJI_0043	Solidago virgaurea L.	1	n	1	0	Asteraceae Bercht. & J.Presl
Iz_2_1209	DJI_0043	Sorbus aucuparia L.	0	n	1	1	Rosaceae Juss.
Iz_2_1209	DJI_0043	Stachys recta L.	0	n	1	0	Lamiacea Lindl.
Iz_2_1209	DJI_0043	Tanacetum vulgare L.	0	n	1	0	Asteraceae Bercht. & J.Presl
Iz_2_1209	DJI_0043	Thymus pallasianus Heinr.Braun	0	n	1	0	Lamiacea Lindl.
Iz_2_1209	DJI_0043	Tilia cordata Mill.	0	n	1	1	Malvaceae Juss.
Iz_2_1209	DJI_0043	Trifolium alpestre L.	0	n	1	0	Fabaceae Lindl.
Iz_2_1209	DJI_0043	Trifolium arvense L.	0	n	1	0	Fabaceae Lindl.
Iz_2_1209	DJI_0043	Ulmus glabra Huds.	0	n	1	1	Ulmaceae Mirb.
Iz_2_1209	DJI_0043	Verbascum densiflorum Bertol.	0	n	1	0	Scrophulariaceae Juss.
Iz_2_1209	DJI_0043	Veronica austriaca L.	0	n	1	0	Plantaginaceae Juss.
Iz_2_1209	DJI_0043	Veronica incana L.	0	n	1	0	Plantaginaceae Juss.
Iz_2_1209	DJI_0043	Veronica verna L.	0	n	1	0	Plantaginaceae Juss.
Iz_2_1209	DJI_0043	Vincetoxicum hirundinaria Medik	0	n	1	0	Apocynaceae Juss.
Iz_2_1209	DJI_0046	Acer tataricum L.	0	n	1	1	Sapindaceae Juss.
Iz_2_1209	DJI_0046	Achillea millefolium L. s.l.	0	n	1	0	Asteraceae Bercht. & J.Presl
Iz_2_1209	DJI_0046	Allium oleraceum L.	0	n	1	0	Amaryllidaceae J.St.-Hil.
Iz_2_1209	DJI_0046	Ambrosia artemisiifolia L.	0	a	0	0	Asteraceae Bercht. & J.Presl
Iz_2_1209	DJI_0046	Amorpha fruticosa L.	0	a	0	0	Fabaceae Lindl.
Iz_2_1209	DJI_0046	Anthericum ramosum L.	0	n	1	0	Asparagaceae Juss.
Iz_2_1209	DJI_0046	Artemisia marschalliana Spreng.	0	n	1	0	Asteraceae Bercht. & J.Presl
Iz_2_1209	DJI_0046	Asparagus officinalis L.	0	n	1	0	Asparagaceae Juss.
Iz_2_1209	DJI_0046	Astragalus cicer L.	0	n	1	0	Fabaceae Lindl.
Iz_2_1209	DJI_0046	Bassia laniflora (S.G.Gmel.) A.J.Scott	0	n	1	0	Amaranthaceae Juss.
Iz_2_1209	DJI_0046	Berberis vulgaris L.	0	n	1	0	Berberidaceae Juss.
Iz_2_1209	DJI_0046	Betula pendula Roth.	0	n	1	1	Betulaceae Gray
Iz_2_1209	DJI_0046	Betula pendula Roth.	0	n	1	0	Poaceae Barnhart
Iz_2_1209	DJI_0046	Bromus inermis Leyss.	0		NA	NA	
Iz_2_1209	DJI_0046	Bromus squarrosus L.	0	a	0	0	Poaceae Barnhart
Iz_2_1209	DJI_0046	Bromus tectorum L.	0	a	0	0	Poaceae Barnhart
Iz_2_1209	DJI_0046	Calamagrostis epigejos (L.) Roth	70	n	1	0	Poaceae Barnhart
Iz_2_1209	DJI_0046	Carduus acanthoides L.	0	n	1	0	Asteraceae Bercht. & J.Presl
Iz_2_1209	DJI_0046	Carex sp.	0	n	1	0	Cyperaceae Juss.
Iz_2_1209	DJI_0046	Centaurea majorovii Dumbadze	0	n	1	0	Asteraceae Bercht. & J.Presl
Iz_2_1209	DJI_0046	Chamaecytisus ruthenicus (Fisch.ex Wol.) Klaskova	15	n	1	0	Fabaceae Lindl.
Iz_2_1209	DJI_0046	Chelidonium majus L.	0	n	1	0	Papaveraceae Juss.
Iz_2_1209	DJI_0046	Chenopodium betaceum Andrz.	0	n	1	0	Amaranthaceae Juss.
Iz_2_1209	DJI_0046	Chenopodium cf. album	0	n	1	0	Amaranthaceae Juss.
Iz_2_1209	DJI_0046	Chenopodium sp.	0		NA	0	Amaranthaceae Juss.
Iz_2_1209	DJI_0046	Chondrilla juncea L.	0	n	1	0	Asteraceae Bercht. & J.Presl
Iz_2_1209	DJI_0046	Cirsium vulgare (Savi) Ten.	0	n	1	0	Asteraceae Bercht. & J.Presl
Iz_2_1209	DJI_0046	Clinopodium acinos (L.) Kuntze	0	n	1	0	Lamiacea Lindl.
Iz_2_1209	DJI_0046	Convallaria majalis L.	0	n	1	0	Asparagaceae Juss.
Iz_2_1209	DJI_0046	Crataegus rhipidophylla Gand.	0	n	1	1	Rosaceae Juss.
Iz_2_1209	DJI_0046	Crepis foetida L.	0	n	1	0	Asteraceae Bercht. & J.Presl
Iz_2_1209	DJI_0046	Dactylis glomerata L.	0	n	1	0	Poaceae Barnhart
Iz_2_1209	DJI_0046	Dianthus campestris M.Bieb.	0	n	1	0	Caryophyllaceae Juss.
Iz_2_1209	DJI_0046	Digitaria sanguinalis (L.) Scop.	0	a	0	0	Poaceae Barnhart
Iz_2_1209	DJI_0046	Elymus repens (L.) Gould	0	n	1	0	Poaceae Barnhart
Iz_2_1209	DJI_0046	Epilobium tetragonum L.	0	n	1	0	Onagraceae Juss.
Iz_2_1209	DJI_0046	Eragrostis minor Host	0	a	0	0	Poaceae Barnhart
Iz_2_1209	DJI_0046	Erigeron canadensis L.	0	a	0	0	Asteraceae Bercht. & J.Presl
Iz_2_1209	DJI_0046	Euphorbia seguieriana Neck.	0	n	1	0	Euphorbiaceae Juss.
Iz_2_1209	DJI_0046	Fallopia dumetorum (L.) Holub	0	n	1	0	Polygonaceae Juss.
Iz_2_1209	DJI_0046	Festuca beckeri (Hack.) Trautv.	0	n	1	0	Poaceae Barnhart
Iz_2_1209	DJI_0046	Fragaria vesca L.	0	n	1	0	Rosaceae Juss.
Iz_2_1209	DJI_0046	Frangula alnus Mill.	0	n	1	1	Rhamnaceae Juss.
Iz_2_1209	DJI_0046	Fraxinus pennsylvanica Marshall	0	a	0	1	Oleaceae Hoffmanns. & Link
Iz_2_1209	DJI_0046	Galeopsis bifida Boenn.	0	n	1	0	Lamiacea Lindl.
Iz_2_1209	DJI_0046	Galium verum L.	0	n	1	0	Rubiaceae Juss.
Iz_2_1209	DJI_0046	Genista tinctoria L.	0	n	1	0	Fabaceae Lindl.
Iz_2_1209	DJI_0046	Geranium robertianum L.	0	n	1	0	Geraniaceae Juss.
Iz_2_1209	DJI_0046	Geranium sanquineum L.	0	n	1	0	Geraniaceae Juss.
Iz_2_1209	DJI_0046	Gypsophila paniculata L.	0	n	1	0	Caryophyllaceae Juss.
Iz_2_1209	DJI_0046	Helichrysum arenarium (L.) Moench	0	n	1	0	Asteraceae Bercht. & J.Presl
Iz_2_1209	DJI_0046	Herniaria polygama J.Gay	0	n	1	0	Caryophyllaceae Juss.
Iz_2_1209	DJI_0046	Hieracium robustum Fr.	0	n	1	0	Asteraceae Bercht. & J.Presl
Iz_2_1209	DJI_0046	Hieracium umbellatum L.	0	n	1	NA	Asteraceae Bercht. & J.Presl
Iz_2_1209	DJI_0046	Hylotelephium maximum (L.) Holub	0	n	1	0	Crassulaceae J.St.-Hil.
Iz_2_1209	DJI_0046	Jacobaea borysthenica (DC.) B.Nord. & Greuter	0	n	1	0	Asteraceae Bercht. & J.Presl
Iz_2_1209	DJI_0046	Jasione montana L.	0	n	1	0	Campanulaceae Juss.
Iz_2_1209	DJI_0046	Jurinea cyanoides (L.) Rchb.s.l.	0	n	1	0	Asteraceae Bercht. & J.Presl
Iz_2_1209	DJI_0046	Koeleria glauca (Spreng.) DC.	0	n	1	0	Poaceae Barnhart
Iz_2_1209	DJI_0046	Lactuca serriola L.	0	a	0	0	Asteraceae Bercht. & J.Presl
Iz_2_1209	DJI_0046	Linaria genistifolia (L.) Mill.	0	n	1	0	Plantaginaceae Juss.

Iz_2_1209	DJI_0046	Lomelosia argentea (L.) Greuter & Burdet	0	n	1	0	Caprifoliaceae Juss.
Iz_2_1209	DJI_0046	Melampyrum pratense L.	0	n	1	0	Orobanchaceae Vent.
Iz_2_1209	DJI_0046	Oenothera biennis L.	0	a	0	0	Onagraceae Juss.
Iz_2_1209	DJI_0046	Pentanema britannica (L.) D.Gut.Larr., Santos-Vicente, Anderb., E.Rico & M.M.Mart.Ort.	0	n	1	0	Asteraceae Bercht. & J.Presl
Iz_2_1209	DJI_0046	Peucedanum oreoselinum (L.) Moench	5	n	1	0	Apiaceae Lindl.
Iz_2_1209	DJI_0046	Peucedanum ruthenicum M.Bieb.	0	n	1	0	Apiaceae Lindl.
Iz_2_1209	DJI_0046	Pilosella echioides (Lumn.) F.W.Schultz & Sch.Bip.	0	n	1	0	Asteraceae Bercht. & J.Presl
Iz_2_1209	DJI_0046	Pilosella officinarum F. Schultz et Sch. Bip.	0	n	1	0	Asteraceae Bercht. & J.Presl
Iz_2_1209	DJI_0046	Pilosella sp.	0		NA	0	Asteraceae Bercht. & J.Presl
Iz_2_1209	DJI_0046	Pinus sylvestris L.	0	n	1	1	Pinaceae Spreng. ex F.Rudolphi
Iz_2_1209	DJI_0046	Poa annua L.	0	n	1	0	Poaceae Barnhart
Iz_2_1209	DJI_0046	Poa nemoralis L.	0	n	1	0	Poaceae Barnhart
Iz_2_1209	DJI_0046	Poa sp.	0		NA	0	Poaceae Barnhart
Iz_2_1209	DJI_0046	Polygonatum odoratum (Mill.) Druce	0	n	1	0	Asparagaceae Juss.
Iz_2_1209	DJI_0046	Polygonum arenarium Waldst. & Kit.	0	n	1	0	Polygonaceae Juss.
Iz_2_1209	DJI_0046	Populus nigra L.	0	n	1	1	Salicaceae Mirb.
Iz_2_1209	DJI_0046	Populus tremula L.	0	n	1	1	Salicaceae Mirb.
Iz_2_1209	DJI_0046	Populus Ã canescens (Aiton) Sm.	0	n	1	1	Salicaceae Mirb.
Iz_2_1209	DJI_0046	Potentilla argentea L.	0	n	1	0	Rosaceae Juss.
Iz_2_1209	DJI_0046	Psammophiliella muralis (L.) Ikonn.	0	n	1	0	Caryophyllaceae Juss.
Iz_2_1209	DJI_0046	Psephellus sumensis (Kalen.) Greuter	2	n	1	0	Asteraceae Bercht. & J.Presl
Iz_2_1209	DJI_0046	Pulsatilla pratensis (L.) Mill. s.l.	0	n	1	0	Ranunculaceae Juss.
Iz_2_1209	DJI_0046	Pyrus communis L.	0	a	0	1	Rosaceae Juss.
Iz_2_1209	DJI_0046	Quercus robur L.	0	n	1	1	Fagaceae Dumort.
Iz_2_1209	DJI_0046	Robinia pseudoacacia L.	0	a	0	1	Fabaceae Lindl.
Iz_2_1209	DJI_0046	Rosa sp.	0		NA	1	Rosaceae Juss.
Iz_2_1209	DJI_0046	Rubus idaeus L.	0	n	1	0	Rosaceae Juss.
Iz_2_1209	DJI_0046	Rumex acetosella L.	0	n	1	0	Polygonaceae Juss.
Iz_2_1209	DJI_0046	Rumex thyrsiflorus Fingerh.	0	n	1	0	Polygonaceae Juss.
Iz_2_1209	DJI_0046	Salix alba L.	0	n	1	1	Salicaceae Mirb.
Iz_2_1209	DJI_0046	Sambucus nigra L.	0	n	1	1	Viburnaceae Raf.
Iz_2_1209	DJI_0046	Secale sylvestre Host	0	n	1	0	Poaceae Barnhart
Iz_2_1209	DJI_0046	Sempervivum ruthenicum Schnittsp. & C.B. Lehm.	0	n	1	0	Crassulaceae J.St.-Hil.
Iz_2_1209	DJI_0046	Setaria viridis (L.) P. Beauv.	0	n	1	0	Poaceae Barnhart
Iz_2_1209	DJI_0046	Silene borysthenica (Gruner) Walters	0	n	1	0	Caryophyllaceae Juss.
Iz_2_1209	DJI_0046	Silene latifolia subsp. alba (Mill.) Greuter & Burdet	0	n	1	0	Caryophyllaceae Juss.
Iz_2_1209	DJI_0046	Solidago virgaurea L.	1	n	1	0	Asteraceae Bercht. & J.Presl
Iz_2_1209	DJI_0046	Sorbus aucuparia L.	0	n	1	1	Rosaceae Juss.
Iz_2_1209	DJI_0046	Stachys recta L.	0	n	1	0	Lamiacea Lindl.
Iz_2_1209	DJI_0046	Tanacetum vulgare L.	0	n	1	0	Asteraceae Bercht. & J.Presl
Iz_2_1209	DJI_0046	Thymus pallasianus Heinr.Braun	0	n	1	0	Lamiacea Lindl.
Iz_2_1209	DJI_0046	Tilia cordata Mill.	0	n	1	1	Malvaceae Juss.
Iz_2_1209	DJI_0046	Trifolium alpestre L.	0	n	1	0	Fabaceae Lindl.
Iz_2_1209	DJI_0046	Trifolium arvense L.	0	n	1	0	Fabaceae Lindl.
Iz_2_1209	DJI_0046	Ulmus glabra Huds.	0	n	1	1	Ulmaceae Mirb.
Iz_2_1209	DJI_0046	Verbascum densiflorum Bertol.	0	n	1	0	Scrophulariaceae Juss.
Iz_2_1209	DJI_0046	Veronica austriaca L.	0	n	1	0	Plantaginaceae Juss.
Iz_2_1209	DJI_0046	Veronica incana L.	0	n	1	0	Plantaginaceae Juss.
Iz_2_1209	DJI_0046	Veronica verna L.	0	n	1	0	Plantaginaceae Juss.
Iz_2_1209	DJI_0046	Vincetoxicum hirundinaria Medik	0	n	1	0	Apocynaceae Juss.
Iz_2_1209	DJI_0050	Acer tataricum L.	0	n	1	1	Sapindaceae Juss.
Iz_2_1209	DJI_0050	Achillea millefolium L. s.l.	0	n	1	0	Asteraceae Bercht. & J.Presl
Iz_2_1209	DJI_0050	Allium oleraceum L.	0	n	1	0	Amaryllidaceae J.St.-Hil.
Iz_2_1209	DJI_0050	Ambrosia artemisiifolia L.	0	a	0	0	Asteraceae Bercht. & J.Presl
Iz_2_1209	DJI_0050	Amorpha fruticosa L.	0	a	0	0	Fabaceae Lindl.
Iz_2_1209	DJI_0050	Anthericum ramosum L.	0	n	1	0	Asparagaceae Juss.
Iz_2_1209	DJI_0050	Artemisia marschalliana Spreng.	0	n	1	0	Asteraceae Bercht. & J.Presl
Iz_2_1209	DJI_0050	Asparagus officinalis L.	0	n	1	0	Asparagaceae Juss.
Iz_2_1209	DJI_0050	Astragalus cicer L.	0	n	1	0	Fabaceae Lindl.
Iz_2_1209	DJI_0050	Bassia laniflora (S.G.Gmel.) A.J.Scott	0	n	1	0	Amaranthaceae Juss.
Iz_2_1209	DJI_0050	Berberis vulgaris L.	0	n	1	0	Berberidaceae Juss.
Iz_2_1209	DJI_0050	Betula pendula Roth.	0	n	1	1	Betulaceae Gray
Iz_2_1209	DJI_0050	Betula pendula Roth.	0	n	1	0	Poaceae Barnhart
Iz_2_1209	DJI_0050	Bromus inermis Leyss.	0		NA	NA	
Iz_2_1209	DJI_0050	Bromus squarrosus L.	0	a	0	0	Poaceae Barnhart
Iz_2_1209	DJI_0050	Bromus tectorum L.	0	a	0	0	Poaceae Barnhart
Iz_2_1209	DJI_0050	Calamagrostis epigejos (L.) Roth	30	n	1	0	Poaceae Barnhart
Iz_2_1209	DJI_0050	Carduus acanthoides L.	0	n	1	0	Asteraceae Bercht. & J.Presl
Iz_2_1209	DJI_0050	Carex sp.	0	n	1	0	Cyperaceae Juss.
Iz_2_1209	DJI_0050	Centaurea majorovii Dumbadze	0	n	1	0	Asteraceae Bercht. & J.Presl
Iz_2_1209	DJI_0050	Chamaecytisus ruthenicus (Fisch.ex Wol.) Klaskova	0	n	1	0	Fabaceae Lindl.
Iz_2_1209	DJI_0050	Chelidonium majus L.	1	n	1	0	Papaveraceae Juss.
Iz_2_1209	DJI_0050	Chenopodium betaceum Andrz.	0	n	1	0	Amaranthaceae Juss.
Iz_2_1209	DJI_0050	Chenopodium cf. album	0	n	1	0	Amaranthaceae Juss.
Iz_2_1209	DJI_0050	Chenopodium sp.	0		NA	0	Amaranthaceae Juss.
Iz_2_1209	DJI_0050	Chondrilla juncea L.	0	n	1	0	Asteraceae Bercht. & J.Presl
Iz_2_1209	DJI_0050	Cirsium vulgare (Savi) Ten.	0	n	1	0	Asteraceae Bercht. & J.Presl
Iz_2_1209	DJI_0050	Clinopodium acinos (L.) Kuntze	0	n	1	0	Lamiacea Lindl.
Iz_2_1209	DJI_0050	Convallaria majalis L.	0	n	1	0	Asparagaceae Juss.
Iz_2_1209	DJI_0050	Crataegus rhipidophylla Gand.	0	n	1	1	Rosaceae Juss.
Iz_2_1209	DJI_0050	Crepis foetida L.	0	n	1	0	Asteraceae Bercht. & J.Presl
Iz_2_1209	DJI_0050	Dactylis glomerata L.	0	n	1	0	Poaceae Barnhart
Iz_2_1209	DJI_0050	Dianthus campestris M.Bieb.	0	n	1	0	Caryophyllaceae Juss.
Iz_2_1209	DJI_0050	Digitaria sanguinalis (L.) Scop.	0	a	0	0	Poaceae Barnhart
Iz_2_1209	DJI_0050	Elymus repens (L.) Gould	0	n	1	0	Poaceae Barnhart
Iz_2_1209	DJI_0050	Epilobium tetragonum L.	0	n	1	0	Onagraceae Juss.
Iz_2_1209	DJI_0050	Eragrostis minor Host	0	a	0	0	Poaceae Barnhart
Iz_2_1209	DJI_0050	Erigeron canadensis L.	0.1	a	0	0	Asteraceae Bercht. & J.Presl
Iz_2_1209	DJI_0050	Euphorbia seguieriana Neck.	0	n	1	0	Euphorbiaceae Juss.
Iz_2_1209	DJI_0050	Fallopia dumetorum (L.) Holub	0	n	1	0	Polygonaceae Juss.
Iz_2_1209	DJI_0050	Festuca beckeri (Hack.) Trautv.	0	n	1	0	Poaceae Barnhart
Iz_2_1209	DJI_0050	Fragaria vesca L.	0	n	1	0	Rosaceae Juss.
Iz_2_1209	DJI_0050	Frangula alnus Mill.	0	n	1	1	Rhamnaceae Juss.
Iz_2_1209	DJI_0050	Fraxinus pennsylvanica Marshall	0	a	0	1	Oleaceae Hoffmanns. & Link
Iz_2_1209	DJI_0050	Galeopsis bifida Boenn.	0	n	1	0	Lamiacea Lindl.
Iz_2_1209	DJI_0050	Galium verum L.	0	n	1	0	Rubiaceae Juss.
Iz_2_1209	DJI_0050	Genista tinctoria L.	0	n	1	0	Fabaceae Lindl.
Iz_2_1209	DJI_0050	Geranium robertianum L.	0	n	1	0	Geraniaceae Juss.

Iz_2_1209	DJI_0050	Geranium sanquineum L.	0	n	1	0	Geraniaceae Juss.
Iz_2_1209	DJI_0050	Gypsophila paniculata L.	0	n	1	0	Caryophyllaceae Juss.
Iz_2_1209	DJI_0050	Helichrysum arenarium (L.) Moench	0	n	1	0	Asteraceae Bercht. & J.Presl
Iz_2_1209	DJI_0050	Herniaria polygama J.Gay	0	n	1	0	Caryophyllaceae Juss.
Iz_2_1209	DJI_0050	Hieracium robustum Fr.	0	n	1	0	Asteraceae Bercht. & J.Presl
Iz_2_1209	DJI_0050	Hieracium umbellatum L.	0	n	1	NA	Asteraceae Bercht. & J.Presl
Iz_2_1209	DJI_0050	Hylotelephium maximum (L.) Holub	0	n	1	0	Crassulaceae J.St.-Hil.
Iz_2_1209	DJI_0050	Jacobaea borysthenica (DC.) B.Nord. & Greuter	0	n	1	0	Asteraceae Bercht. & J.Presl
Iz_2_1209	DJI_0050	Jasione montana L.	0	n	1	0	Campanulaceae Juss.
Iz_2_1209	DJI_0050	Jurinea cyanoides (L.) Rchb.s.l.	0	n	1	0	Asteraceae Bercht. & J.Presl
Iz_2_1209	DJI_0050	Koeleria glauca (Spreng.) DC.	0	n	1	0	Poaceae Barnhart
Iz_2_1209	DJI_0050	Lactuca serriola L.	0.2	a	0	0	Asteraceae Bercht. & J.Presl
Iz_2_1209	DJI_0050	Linaria genistifolia (L.) Mill.	0	n	1	0	Plantaginaceae Juss.
Iz_2_1209	DJI_0050	Lomelosia argentea (L.) Greuter & Burdet	0	n	1	0	Caprifoliaceae Juss.
Iz_2_1209	DJI_0050	Melampyrum pratense L.	0	n	1	0	Orobanchaceae Vent.
Iz_2_1209	DJI_0050	Oenothera biennis L.	0	a	0	0	Onagraceae Juss.
Iz_2_1209	DJI_0050	Pentanema britannica (L.) D.Gut.Larr., Santos-Vicente, Anderb., E.Rico & M.M.Mart.Ort.	0	n	1	0	Asteraceae Bercht. & J.Presl
Iz_2_1209	DJI_0050	Peucedanum oreoselinum (L.) Moench	0	n	1	0	Apiaceae Lindl.
Iz_2_1209	DJI_0050	Peucedanum ruthenicum M.Bieb.	0	n	1	0	Apiaceae Lindl.
Iz_2_1209	DJI_0050	Pilosella echioides (Lumn.) F.W.Schultz & Sch.Bip.	0	n	1	0	Asteraceae Bercht. & J.Presl
Iz_2_1209	DJI_0050	Pilosella officinarum F. Schultz et Sch. Bip.	0	n	1	0	Asteraceae Bercht. & J.Presl
Iz_2_1209	DJI_0050	Pilosella sp.	0		NA	0	Asteraceae Bercht. & J.Presl
Iz_2_1209	DJI_0050	Pinus sylvestris L.	0	n	1	1	Pinaceae Spreng. ex F.Rudolphi
Iz_2_1209	DJI_0050	Poa annua L.	0	n	1	0	Poaceae Barnhart
Iz_2_1209	DJI_0050	Poa nemoralis L.	0	n	1	0	Poaceae Barnhart
Iz_2_1209	DJI_0050	Poa sp.	0		NA	0	Poaceae Barnhart
Iz_2_1209	DJI_0050	Polygonatum odoratum (Mill.) Druce	0	n	1	0	Asparagaceae Juss.
Iz_2_1209	DJI_0050	Polygonum arenarium Waldst. & Kit.	0	n	1	0	Polygonaceae Juss.
Iz_2_1209	DJI_0050	Populus nigra L.	0	n	1	1	Salicaceae Mirb.
Iz_2_1209	DJI_0050	Populus tremula L.	0	n	1	1	Salicaceae Mirb.
Iz_2_1209	DJI_0050	Populus Ã canescens (Aiton) Sm.	0	n	1	1	Salicaceae Mirb.
Iz_2_1209	DJI_0050	Potentilla argentea L.	0	n	1	0	Rosaceae Juss.
Iz_2_1209	DJI_0050	Psammophiliella muralis (L.) Ikonn.	0	n	1	0	Caryophyllaceae Juss.
Iz_2_1209	DJI_0050	Psephellus sumensis (Kalen.) Greuter	0	n	1	0	Asteraceae Bercht. & J.Presl
Iz_2_1209	DJI_0050	Pulsatilla pratensis (L.) Mill. s.l.	0	n	1	0	Ranunculaceae Juss.
Iz_2_1209	DJI_0050	Pyrus communis L.	0	a	0	1	Rosaceae Juss.
Iz_2_1209	DJI_0050	Quercus robur L.	0	n	1	1	Fagaceae Dumort.
Iz_2_1209	DJI_0050	Robinia pseudoacacia L.	0	a	0	1	Fabaceae Lindl.
Iz_2_1209	DJI_0050	Rosa sp.	0		NA	1	Rosaceae Juss.
Iz_2_1209	DJI_0050	Rubus idaeus L.	0	n	1	0	Rosaceae Juss.
Iz_2_1209	DJI_0050	Rumex acetosella L.	0	n	1	0	Polygonaceae Juss.
Iz_2_1209	DJI_0050	Rumex thyrsiflorus Fingerh.	0	n	1	0	Polygonaceae Juss.
Iz_2_1209	DJI_0050	Salix alba L.	0	n	1	1	Salicaceae Mirb.
Iz_2_1209	DJI_0050	Sambucus nigra L.	0	n	1	1	Viburnaceae Raf.
Iz_2_1209	DJI_0050	Secale sylvestre Host	0	n	1	0	Poaceae Barnhart
Iz_2_1209	DJI_0050	Sempervivum ruthenicum Schnittsp. & C.B. Lehm.	0	n	1	0	Crassulaceae J.St.-Hil.
Iz_2_1209	DJI_0050	Setaria viridis (L.) P. Beauv.	0	n	1	0	Poaceae Barnhart
Iz_2_1209	DJI_0050	Silene borysthenica (Gruner) Walters	0	n	1	0	Caryophyllaceae Juss.
Iz_2_1209	DJI_0050	Silene latifolia subsp. alba (Mill.) Greuter & Burdet	0	n	1	0	Caryophyllaceae Juss.
Iz_2_1209	DJI_0050	Solidago virgaurea L.	0	n	1	0	Asteraceae Bercht. & J.Presl
Iz_2_1209	DJI_0050	Sorbus aucuparia L.	0	n	1	1	Rosaceae Juss.
Iz_2_1209	DJI_0050	Stachys recta L.	0	n	1	0	Lamiacea Lindl.
Iz_2_1209	DJI_0050	Tanacetum vulgare L.	0	n	1	0	Asteraceae Bercht. & J.Presl
Iz_2_1209	DJI_0050	Thymus pallasianus Heinr.Braun	0	n	1	0	Lamiacea Lindl.
Iz_2_1209	DJI_0050	Tilia cordata Mill.	0	n	1	1	Malvaceae Juss.
Iz_2_1209	DJI_0050	Trifolium alpestre L.	0	n	1	0	Fabaceae Lindl.
Iz_2_1209	DJI_0050	Trifolium arvense L.	0	n	1	0	Fabaceae Lindl.
Iz_2_1209	DJI_0050	Ulmus glabra Huds.	0	n	1	1	Ulmaceae Mirb.
Iz_2_1209	DJI_0050	Verbascum densiflorum Bertol.	0	n	1	0	Scrophulariaceae Juss.
Iz_2_1209	DJI_0050	Veronica austriaca L.	0	n	1	0	Plantaginaceae Juss.
Iz_2_1209	DJI_0050	Veronica incana L.	0	n	1	0	Plantaginaceae Juss.
Iz_2_1209	DJI_0050	Veronica verna L.	0	n	1	0	Plantaginaceae Juss.
Iz_2_1209	DJI_0050	Vincetoxicum hirundinaria Medik	0	n	1	0	Apocynaceae Juss.
Iz_2_1209	DJI_0053	Acer tataricum L.	0	n	1	1	Sapindaceae Juss.
Iz_2_1209	DJI_0053	Achillea millefolium L. s.l.	0	n	1	0	Asteraceae Bercht. & J.Presl
Iz_2_1209	DJI_0053	Allium oleraceum L.	0	n	1	0	Amaryllidaceae J.St.-Hil.
Iz_2_1209	DJI_0053	Ambrosia artemisiifolia L.	0	a	0	0	Asteraceae Bercht. & J.Presl
Iz_2_1209	DJI_0053	Amorpha fruticosa L.	0	a	0	0	Fabaceae Lindl.
Iz_2_1209	DJI_0053	Anthericum ramosum L.	0	n	1	0	Asparagaceae Juss.
Iz_2_1209	DJI_0053	Artemisia marschalliana Spreng.	0	n	1	0	Asteraceae Bercht. & J.Presl
Iz_2_1209	DJI_0053	Asparagus officinalis L.	0	n	1	0	Asparagaceae Juss.
Iz_2_1209	DJI_0053	Astragalus cicer L.	0	n	1	0	Fabaceae Lindl.
Iz_2_1209	DJI_0053	Bassia laniflora (S.G.Gmel.) A.J.Scott	0	n	1	0	Amaranthaceae Juss.
Iz_2_1209	DJI_0053	Berberis vulgaris L.	0	n	1	0	Berberidaceae Juss.
Iz_2_1209	DJI_0053	Betula pendula Roth.	0	n	1	1	Betulaceae Gray
Iz_2_1209	DJI_0053	Betula pendula Roth.	0	n	1	0	Poaceae Barnhart
Iz_2_1209	DJI_0053	Bromus inermis Leyss.	0		NA	NA	
Iz_2_1209	DJI_0053	Bromus squarrosus L.	0	a	0	0	Poaceae Barnhart
Iz_2_1209	DJI_0053	Bromus tectorum L.	0	a	0	0	Poaceae Barnhart
Iz_2_1209	DJI_0053	Calamagrostis epigejos (L.) Roth	40	n	1	0	Poaceae Barnhart
Iz_2_1209	DJI_0053	Carduus acanthoides L.	0	n	1	0	Asteraceae Bercht. & J.Presl
Iz_2_1209	DJI_0053	Carex sp.	0	n	1	0	Cyperaceae Juss.
Iz_2_1209	DJI_0053	Centaurea majorovii Dumbadze	0	n	1	0	Asteraceae Bercht. & J.Presl
Iz_2_1209	DJI_0053	Chamaecytisus ruthenicus (Fisch.ex Wol.) Klaskova	0	n	1	0	Fabaceae Lindl.
Iz_2_1209	DJI_0053	Chelidonium majus L.	0	n	1	0	Papaveraceae Juss.
Iz_2_1209	DJI_0053	Chenopodium betaceum Andrz.	0	n	1	0	Amaranthaceae Juss.
Iz_2_1209	DJI_0053	Chenopodium cf. album	0	n	1	0	Amaranthaceae Juss.
Iz_2_1209	DJI_0053	Chenopodium sp.	0		NA	0	Amaranthaceae Juss.
Iz_2_1209	DJI_0053	Chondrilla juncea L.	0	n	1	0	Asteraceae Bercht. & J.Presl
Iz_2_1209	DJI_0053	Cirsium vulgare (Savi) Ten.	0	n	1	0	Asteraceae Bercht. & J.Presl
Iz_2_1209	DJI_0053	Clinopodium acinos (L.) Kuntze	0	n	1	0	Lamiacea Lindl.
Iz_2_1209	DJI_0053	Convallaria majalis L.	0	n	1	0	Asparagaceae Juss.
Iz_2_1209	DJI_0053	Crataegus rhipidophylla Gand.	0	n	1	1	Rosaceae Juss.
Iz_2_1209	DJI_0053	Crepis foetida L.	0	n	1	0	Asteraceae Bercht. & J.Presl
Iz_2_1209	DJI_0053	Dactylis glomerata L.	0	n	1	0	Poaceae Barnhart
Iz_2_1209	DJI_0053	Dianthus campestris M.Bieb.	1	n	1	0	Caryophyllaceae Juss.
Iz_2_1209	DJI_0053	Digitaria sanguinalis (L.) Scop.	0	a	0	0	Poaceae Barnhart
Iz_2_1209	DJI_0053	Elymus repens (L.) Gould	0	n	1	0	Poaceae Barnhart

Iz_2_1209	DJI_0053	Epilobium tetragonum L.	0	n	1	0	Onagraceae Juss.
Iz_2_1209	DJI_0053	Eragrostis minor Host	0	a	0	0	Poaceae Barnhart
Iz_2_1209	DJI_0053	Erigeron canadensis L.	0	a	0	0	Asteraceae Bercht. & J.Presl
Iz_2_1209	DJI_0053	Euphorbia seguieriana Neck.	0	n	1	0	Euphorbiaceae Juss.
Iz_2_1209	DJI_0053	Fallopia dumetorum (L.) Holub	0	n	1	0	Polygonaceae Juss.
Iz_2_1209	DJI_0053	Festuca beckeri (Hack.) Trautv.	0	n	1	0	Poaceae Barnhart
Iz_2_1209	DJI_0053	Fragaria vesca L.	0	n	1	0	Rosaceae Juss.
Iz_2_1209	DJI_0053	Frangula alnus Mill.	0	n	1	1	Rhamnaceae Juss.
Iz_2_1209	DJI_0053	Fraxinus pennsylvanica Marshall	0	a	0	1	Oleaceae Hoffmanns. & Link
Iz_2_1209	DJI_0053	Galeopsis bifida Boenn.	0	n	1	0	Lamiacea Lindl.
Iz_2_1209	DJI_0053	Galium verum L.	0	n	1	0	Rubiaceae Juss.
Iz_2_1209	DJI_0053	Genista tinctoria L.	0	n	1	0	Fabaceae Lindl.
Iz_2_1209	DJI_0053	Geranium robertianum L.	0	n	1	0	Geraniaceae Juss.
Iz_2_1209	DJI_0053	Geranium sanquineum L.	0	n	1	0	Geraniaceae Juss.
Iz_2_1209	DJI_0053	Gypsophila paniculata L.	0	n	1	0	Caryophyllaceae Juss.
Iz_2_1209	DJI_0053	Helichrysum arenarium (L.) Moench	0	n	1	0	Asteraceae Bercht. & J.Presl
Iz_2_1209	DJI_0053	Herniaria polygama J.Gay	0	n	1	0	Caryophyllaceae Juss.
Iz_2_1209	DJI_0053	Hieracium robustum Fr.	0	n	1	0	Asteraceae Bercht. & J.Presl
Iz_2_1209	DJI_0053	Hieracium umbellatum L.	1	n	1	NA	Asteraceae Bercht. & J.Presl
Iz_2_1209	DJI_0053	Hylotelephium maximum (L.) Holub	0	n	1	0	Crassulaceae J.St.-Hil.
Iz_2_1209	DJI_0053	Jacobaea borysthenica (DC.) B.Nord. & Greuter	0	n	1	0	Asteraceae Bercht. & J.Presl
Iz_2_1209	DJI_0053	Jasione montana L.	0	n	1	0	Campanulaceae Juss.
Iz_2_1209	DJI_0053	Jurinea cyanoides (L.) Rchb.s.l.	0	n	1	0	Asteraceae Bercht. & J.Presl
Iz_2_1209	DJI_0053	Koeleria glauca (Spreng.) DC.	0	n	1	0	Poaceae Barnhart
Iz_2_1209	DJI_0053	Lactuca serriola L.	0	a	0	0	Asteraceae Bercht. & J.Presl
Iz_2_1209	DJI_0053	Linaria genistifolia (L.) Mill.	0	n	1	0	Plantaginaceae Juss.
Iz_2_1209	DJI_0053	Lomelosia argentea (L.) Greuter & Burdet	0	n	1	0	Caprifoliaceae Juss.
Iz_2_1209	DJI_0053	Melampyrum pratense L.	0	n	1	0	Orobanchaceae Vent.
Iz_2_1209	DJI_0053	Oenothera biennis L.	0	a	0	0	Onagraceae Juss.
Iz_2_1209	DJI_0053	Pentanema britannica (L.) D.Gut.Larr., Santos-Vicente, Anderb., E.Rico & M.M.Mart.Ort.	0	n	1	0	Asteraceae Bercht. & J.Presl
Iz_2_1209	DJI_0053	Peucedanum oreoselinum (L.) Moench	0	n	1	0	Apiaceae Lindl.
Iz_2_1209	DJI_0053	Peucedanum ruthenicum M.Bieb.	0	n	1	0	Apiaceae Lindl.
Iz_2_1209	DJI_0053	Pilosella echioides (Lumn.) F.W.Schultz & Sch.Bip.	0	n	1	0	Asteraceae Bercht. & J.Presl
Iz_2_1209	DJI_0053	Pilosella officinarum F. Schultz et Sch. Bip.	0	n	1	0	Asteraceae Bercht. & J.Presl
Iz_2_1209	DJI_0053	Pilosella sp.	0		NA	0	Asteraceae Bercht. & J.Presl
Iz_2_1209	DJI_0053	Pinus sylvestris L.	0	n	1	1	Pinaceae Spreng. ex F.Rudolphi
Iz_2_1209	DJI_0053	Poa annua L.	0	n	1	0	Poaceae Barnhart
Iz_2_1209	DJI_0053	Poa nemoralis L.	0	n	1	0	Poaceae Barnhart
Iz_2_1209	DJI_0053	Poa sp.	0		NA	0	Poaceae Barnhart
Iz_2_1209	DJI_0053	Polygonatum odoratum (Mill.) Druce	0	n	1	0	Asparagaceae Juss.
Iz_2_1209	DJI_0053	Polygonum arenarium Waldst. & Kit.	0	n	1	0	Polygonaceae Juss.
Iz_2_1209	DJI_0053	Populus nigra L.	0	n	1	1	Salicaceae Mirb.
Iz_2_1209	DJI_0053	Populus tremula L.	0	n	1	1	Salicaceae Mirb.
Iz_2_1209	DJI_0053	Populus Ã canescens (Aiton) Sm.	0	n	1	1	Salicaceae Mirb.
Iz_2_1209	DJI_0053	Potentilla argentea L.	0	n	1	0	Rosaceae Juss.
Iz_2_1209	DJI_0053	Psammophiliella muralis (L.) Ikonn.	0	n	1	0	Caryophyllaceae Juss.
Iz_2_1209	DJI_0053	Psephellus sumensis (Kalen.) Greuter	0	n	1	0	Asteraceae Bercht. & J.Presl
Iz_2_1209	DJI_0053	Pulsatilla pratensis (L.) Mill. s.l.	0	n	1	0	Ranunculaceae Juss.
Iz_2_1209	DJI_0053	Pyrus communis L.	0	a	0	1	Rosaceae Juss.
Iz_2_1209	DJI_0053	Quercus robur L.	0	n	1	1	Fagaceae Dumort.
Iz_2_1209	DJI_0053	Robinia pseudoacacia L.	0	a	0	1	Fabaceae Lindl.
Iz_2_1209	DJI_0053	Rosa sp.	0		NA	1	Rosaceae Juss.
Iz_2_1209	DJI_0053	Rubus idaeus L.	0	n	1	0	Rosaceae Juss.
Iz_2_1209	DJI_0053	Rumex acetosella L.	0	n	1	0	Polygonaceae Juss.
Iz_2_1209	DJI_0053	Rumex thyrsiflorus Fingerh.	0	n	1	0	Polygonaceae Juss.
Iz_2_1209	DJI_0053	Salix alba L.	0	n	1	1	Salicaceae Mirb.
Iz_2_1209	DJI_0053	Sambucus nigra L.	0	n	1	1	Viburnaceae Raf.
Iz_2_1209	DJI_0053	Secale sylvestre Host	0	n	1	0	Poaceae Barnhart
Iz_2_1209	DJI_0053	Sempervivum ruthenicum Schnittsp. & C.B. Lehm.	0	n	1	0	Crassulaceae J.St.-Hil.
Iz_2_1209	DJI_0053	Setaria viridis (L.) P. Beauv.	0	n	1	0	Poaceae Barnhart
Iz_2_1209	DJI_0053	Silene borysthenica (Gruner) Walters	0	n	1	0	Caryophyllaceae Juss.
Iz_2_1209	DJI_0053	Silene latifolia subsp. alba (Mill.) Greuter & Burdet	0	n	1	0	Caryophyllaceae Juss.
Iz_2_1209	DJI_0053	Solidago virgaurea L.	0	n	1	0	Asteraceae Bercht. & J.Presl
Iz_2_1209	DJI_0053	Sorbus aucuparia L.	0	n	1	1	Rosaceae Juss.
Iz_2_1209	DJI_0053	Stachys recta L.	0	n	1	0	Lamiacea Lindl.
Iz_2_1209	DJI_0053	Tanacetum vulgare L.	0	n	1	0	Asteraceae Bercht. & J.Presl
Iz_2_1209	DJI_0053	Thymus pallasianus Heinr.Braun	0	n	1	0	Lamiacea Lindl.
Iz_2_1209	DJI_0053	Tilia cordata Mill.	0	n	1	1	Malvaceae Juss.
Iz_2_1209	DJI_0053	Trifolium alpestre L.	0	n	1	0	Fabaceae Lindl.
Iz_2_1209	DJI_0053	Trifolium arvense L.	0	n	1	0	Fabaceae Lindl.
Iz_2_1209	DJI_0053	Ulmus glabra Huds.	0	n	1	1	Ulmaceae Mirb.
Iz_2_1209	DJI_0053	Verbascum densiflorum Bertol.	0	n	1	0	Scrophulariaceae Juss.
Iz_2_1209	DJI_0053	Veronica austriaca L.	0	n	1	0	Plantaginaceae Juss.
Iz_2_1209	DJI_0053	Veronica incana L.	0	n	1	0	Plantaginaceae Juss.
Iz_2_1209	DJI_0053	Veronica verna L.	0	n	1	0	Plantaginaceae Juss.
Iz_2_1209	DJI_0053	Vincetoxicum hirundinaria Medik	0	n	1	0	Apocynaceae Juss.
Iz_2_1209	DJI_0056	Acer tataricum L.	0	n	1	1	Sapindaceae Juss.
Iz_2_1209	DJI_0056	Achillea millefolium L. s.l.	0	n	1	0	Asteraceae Bercht. & J.Presl
Iz_2_1209	DJI_0056	Allium oleraceum L.	0	n	1	0	Amaryllidaceae J.St.-Hil.
Iz_2_1209	DJI_0056	Ambrosia artemisiifolia L.	0	a	0	0	Asteraceae Bercht. & J.Presl
Iz_2_1209	DJI_0056	Amorpha fruticosa L.	0	a	0	0	Fabaceae Lindl.
Iz_2_1209	DJI_0056	Anthericum ramosum L.	0	n	1	0	Asparagaceae Juss.
Iz_2_1209	DJI_0056	Artemisia marschalliana Spreng.	0	n	1	0	Asteraceae Bercht. & J.Presl
Iz_2_1209	DJI_0056	Asparagus officinalis L.	0	n	1	0	Asparagaceae Juss.
Iz_2_1209	DJI_0056	Astragalus cicer L.	0	n	1	0	Fabaceae Lindl.
Iz_2_1209	DJI_0056	Bassia laniflora (S.G.Gmel.) A.J.Scott	0	n	1	0	Amaranthaceae Juss.
Iz_2_1209	DJI_0056	Berberis vulgaris L.	0	n	1	0	Berberidaceae Juss.
Iz_2_1209	DJI_0056	Betula pendula Roth.	0	n	1	1	Betulaceae Gray
Iz_2_1209	DJI_0056	Betula pendula Roth.	0	n	1	0	Poaceae Barnhart
Iz_2_1209	DJI_0056	Bromus inermis Leyss.	0		NA	NA	
Iz_2_1209	DJI_0056	Bromus squarrosus L.	0	a	0	0	Poaceae Barnhart
Iz_2_1209	DJI_0056	Bromus tectorum L.	0	a	0	0	Poaceae Barnhart
Iz_2_1209	DJI_0056	Calamagrostis epigejos (L.) Roth	30	n	1	0	Poaceae Barnhart
Iz_2_1209	DJI_0056	Carduus acanthoides L.	0	n	1	0	Asteraceae Bercht. & J.Presl
Iz_2_1209	DJI_0056	Carex sp.	0	n	1	0	Cyperaceae Juss.
Iz_2_1209	DJI_0056	Centaurea majorovii Dumbadze	0	n	1	0	Asteraceae Bercht. & J.Presl
Iz_2_1209	DJI_0056	Chamaecytisus ruthenicus (Fisch.ex Wol.) Klaskova	0	n	1	0	Fabaceae Lindl.
Iz_2_1209	DJI_0056	Chelidonium majus L.	0	n	1	0	Papaveraceae Juss.

Iz_2_1209	DJI_0056	Chenopodium betaceum Andrz.	0	n	1	0	Amaranthaceae Juss.
Iz_2_1209	DJI_0056	Chenopodium cf. album	0	n	1	0	Amaranthaceae Juss.
Iz_2_1209	DJI_0056	Chenopodium sp.	0		NA	0	Amaranthaceae Juss.
Iz_2_1209	DJI_0056	Chondrilla juncea L.	0	n	1	0	Asteraceae Bercht. & J.Presl
Iz_2_1209	DJI_0056	Cirsium vulgare (Savi) Ten.	0	n	1	0	Asteraceae Bercht. & J.Presl
Iz_2_1209	DJI_0056	Clinopodium acinos (L.) Kuntze	0	n	1	0	Lamiacea Lindl.
Iz_2_1209	DJI_0056	Convallaria majalis L.	0	n	1	0	Asparagaceae Juss.
Iz_2_1209	DJI_0056	Crataegus rhipidophylla Gand.	0	n	1	1	Rosaceae Juss.
Iz_2_1209	DJI_0056	Crepis foetida L.	0	n	1	0	Asteraceae Bercht. & J.Presl
Iz_2_1209	DJI_0056	Dactylis glomerata L.	0	n	1	0	Poaceae Barnhart
Iz_2_1209	DJI_0056	Dianthus campestris M.Bieb.	0	n	1	0	Caryophyllaceae Juss.
Iz_2_1209	DJI_0056	Digitaria sanguinalis (L.) Scop.	0	a	0	0	Poaceae Barnhart
Iz_2_1209	DJI_0056	Elymus repens (L.) Gould	0	n	1	0	Poaceae Barnhart
Iz_2_1209	DJI_0056	Epilobium tetragonum L.	0	n	1	0	Onagraceae Juss.
Iz_2_1209	DJI_0056	Eragrostis minor Host	0	a	0	0	Poaceae Barnhart
Iz_2_1209	DJI_0056	Erigeron canadensis L.	0	a	0	0	Asteraceae Bercht. & J.Presl
Iz_2_1209	DJI_0056	Euphorbia seguieriana Neck.	0	n	1	0	Euphorbiaceae Juss.
Iz_2_1209	DJI_0056	Fallopia dumetorum (L.) Holub	0	n	1	0	Polygonaceae Juss.
Iz_2_1209	DJI_0056	Festuca beckeri (Hack.) Trautv.	0	n	1	0	Poaceae Barnhart
Iz_2_1209	DJI_0056	Fragaria vesca L.	0	n	1	0	Rosaceae Juss.
Iz_2_1209	DJI_0056	Frangula alnus Mill.	0	n	1	1	Rhamnaceae Juss.
Iz_2_1209	DJI_0056	Fraxinus pennsylvanica Marshall	0	a	0	1	Oleaceae Hoffmanns. & Link
Iz_2_1209	DJI_0056	Galeopsis bifida Boenn.	0	n	1	0	Lamiacea Lindl.
Iz_2_1209	DJI_0056	Galium verum L.	0	n	1	0	Rubiaceae Juss.
Iz_2_1209	DJI_0056	Genista tinctoria L.	0	n	1	0	Fabaceae Lindl.
Iz_2_1209	DJI_0056	Geranium robertianum L.	0	n	1	0	Geraniaceae Juss.
Iz_2_1209	DJI_0056	Geranium sanquineum L.	0	n	1	0	Geraniaceae Juss.
Iz_2_1209	DJI_0056	Gypsophila paniculata L.	0	n	1	0	Caryophyllaceae Juss.
Iz_2_1209	DJI_0056	Helichrysum arenarium (L.) Moench	0	n	1	0	Asteraceae Bercht. & J.Presl
Iz_2_1209	DJI_0056	Herniaria polygama J.Gay	0	n	1	0	Caryophyllaceae Juss.
Iz_2_1209	DJI_0056	Hieracium robustum Fr.	0	n	1	0	Asteraceae Bercht. & J.Presl
Iz_2_1209	DJI_0056	Hieracium umbellatum L.	0	n	1	NA	Asteraceae Bercht. & J.Presl
Iz_2_1209	DJI_0056	Hylotelephium maximum (L.) Holub	0	n	1	0	Crassulaceae J.St.-Hil.
Iz_2_1209	DJI_0056	Jacobaea borysthenica (DC.) B.Nord. & Greuter	0	n	1	0	Asteraceae Bercht. & J.Presl
Iz_2_1209	DJI_0056	Jasione montana L.	0	n	1	0	Campanulaceae Juss.
Iz_2_1209	DJI_0056	Jurinea cyanoides (L.) Rchb.s.l.	0	n	1	0	Asteraceae Bercht. & J.Presl
Iz_2_1209	DJI_0056	Koeleria glauca (Spreng.) DC.	0	n	1	0	Poaceae Barnhart
Iz_2_1209	DJI_0056	Lactuca serriola L.	0	a	0	0	Asteraceae Bercht. & J.Presl
Iz_2_1209	DJI_0056	Linaria genistifolia (L.) Mill.	0	n	1	0	Plantaginaceae Juss.
Iz_2_1209	DJI_0056	Lomelosia argentea (L.) Greuter & Burdet	0	n	1	0	Caprifoliaceae Juss.
Iz_2_1209	DJI_0056	Melampyrum pratense L.	0	n	1	0	Orobanchaceae Vent.
Iz_2_1209	DJI_0056	Oenothera biennis L.	0	a	0	0	Onagraceae Juss.
Iz_2_1209	DJI_0056	Pentanema britannica (L.) D.Gut.Larr., Santos-Vicente, Anderb., E.Rico & M.M.Mart.Ort.	0	n	1	0	Asteraceae Bercht. & J.Presl
Iz_2_1209	DJI_0056	Peucedanum oreoselinum (L.) Moench	10	n	1	0	Apiaceae Lindl.
Iz_2_1209	DJI_0056	Peucedanum ruthenicum M.Bieb.	0	n	1	0	Apiaceae Lindl.
Iz_2_1209	DJI_0056	Pilosella echioides (Lumn.) F.W.Schultz & Sch.Bip.	0	n	1	0	Asteraceae Bercht. & J.Presl
Iz_2_1209	DJI_0056	Pilosella officinarum F. Schultz et Sch. Bip.	0	n	1	0	Asteraceae Bercht. & J.Presl
Iz_2_1209	DJI_0056	Pilosella sp.	0		NA	0	Asteraceae Bercht. & J.Presl
Iz_2_1209	DJI_0056	Pinus sylvestris L.	0	n	1	1	Pinaceae Spreng. ex F.Rudolphi
Iz_2_1209	DJI_0056	Poa annua L.	0	n	1	0	Poaceae Barnhart
Iz_2_1209	DJI_0056	Poa nemoralis L.	0	n	1	0	Poaceae Barnhart
Iz_2_1209	DJI_0056	Poa sp.	0		NA	0	Poaceae Barnhart
Iz_2_1209	DJI_0056	Polygonatum odoratum (Mill.) Druce	0	n	1	0	Asparagaceae Juss.
Iz_2_1209	DJI_0056	Polygonum arenarium Waldst. & Kit.	0	n	1	0	Polygonaceae Juss.
Iz_2_1209	DJI_0056	Populus nigra L.	0	n	1	1	Salicaceae Mirb.
Iz_2_1209	DJI_0056	Populus tremula L.	0	n	1	1	Salicaceae Mirb.
Iz_2_1209	DJI_0056	Populus Ã canescens (Aiton) Sm.	0	n	1	1	Salicaceae Mirb.
Iz_2_1209	DJI_0056	Potentilla argentea L.	0	n	1	0	Rosaceae Juss.
Iz_2_1209	DJI_0056	Psammophiliella muralis (L.) Ikonn.	0	n	1	0	Caryophyllaceae Juss.
Iz_2_1209	DJI_0056	Psephellus sumensis (Kalen.) Greuter	0	n	1	0	Asteraceae Bercht. & J.Presl
Iz_2_1209	DJI_0056	Pulsatilla pratensis (L.) Mill. s.l.	0	n	1	0	Ranunculaceae Juss.
Iz_2_1209	DJI_0056	Pyrus communis L.	0	a	0	1	Rosaceae Juss.
Iz_2_1209	DJI_0056	Quercus robur L.	0	n	1	1	Fagaceae Dumort.
Iz_2_1209	DJI_0056	Robinia pseudoacacia L.	0	a	0	1	Fabaceae Lindl.
Iz_2_1209	DJI_0056	Rosa sp.	0		NA	1	Rosaceae Juss.
Iz_2_1209	DJI_0056	Rubus idaeus L.	0	n	1	0	Rosaceae Juss.
Iz_2_1209	DJI_0056	Rumex acetosella L.	0	n	1	0	Polygonaceae Juss.
Iz_2_1209	DJI_0056	Rumex thyrsiflorus Fingerh.	0	n	1	0	Polygonaceae Juss.
Iz_2_1209	DJI_0056	Salix alba L.	0	n	1	1	Salicaceae Mirb.
Iz_2_1209	DJI_0056	Sambucus nigra L.	0	n	1	1	Viburnaceae Raf.
Iz_2_1209	DJI_0056	Secale sylvestre Host	0	n	1	0	Poaceae Barnhart
Iz_2_1209	DJI_0056	Sempervivum ruthenicum Schnittsp. & C.B. Lehm.	0	n	1	0	Crassulaceae J.St.-Hil.
Iz_2_1209	DJI_0056	Setaria viridis (L.) P. Beauv.	0	n	1	0	Poaceae Barnhart
Iz_2_1209	DJI_0056	Silene borysthenica (Gruner) Walters	0	n	1	0	Caryophyllaceae Juss.
Iz_2_1209	DJI_0056	Silene latifolia subsp. alba (Mill.) Greuter & Burdet	0	n	1	0	Caryophyllaceae Juss.
Iz_2_1209	DJI_0056	Solidago virgaurea L.	0	n	1	0	Asteraceae Bercht. & J.Presl
Iz_2_1209	DJI_0056	Sorbus aucuparia L.	0	n	1	1	Rosaceae Juss.
Iz_2_1209	DJI_0056	Stachys recta L.	0	n	1	0	Lamiacea Lindl.
Iz_2_1209	DJI_0056	Tanacetum vulgare L.	0	n	1	0	Asteraceae Bercht. & J.Presl
Iz_2_1209	DJI_0056	Thymus pallasianus Heinr.Braun	0	n	1	0	Lamiacea Lindl.
Iz_2_1209	DJI_0056	Tilia cordata Mill.	0	n	1	1	Malvaceae Juss.
Iz_2_1209	DJI_0056	Trifolium alpestre L.	0	n	1	0	Fabaceae Lindl.
Iz_2_1209	DJI_0056	Trifolium arvense L.	0	n	1	0	Fabaceae Lindl.
Iz_2_1209	DJI_0056	Ulmus glabra Huds.	0	n	1	1	Ulmaceae Mirb.
Iz_2_1209	DJI_0056	Verbascum densiflorum Bertol.	0	n	1	0	Scrophulariaceae Juss.
Iz_2_1209	DJI_0056	Veronica austriaca L.	0	n	1	0	Plantaginaceae Juss.
Iz_2_1209	DJI_0056	Veronica incana L.	0	n	1	0	Plantaginaceae Juss.
Iz_2_1209	DJI_0056	Veronica verna L.	0	n	1	0	Plantaginaceae Juss.
Iz_2_1209	DJI_0056	Vincetoxicum hirundinaria Medik	0	n	1	0	Apocynaceae Juss.
Iz_2_1209	DJI_0059	Acer tataricum L.	0	n	1	1	Sapindaceae Juss.
Iz_2_1209	DJI_0059	Achillea millefolium L. s.l.	0	n	1	0	Asteraceae Bercht. & J.Presl
Iz_2_1209	DJI_0059	Allium oleraceum L.	0	n	1	0	Amaryllidaceae J.St.-Hil.
Iz_2_1209	DJI_0059	Ambrosia artemisiifolia L.	0	a	0	0	Asteraceae Bercht. & J.Presl
Iz_2_1209	DJI_0059	Amorpha fruticosa L.	0	a	0	0	Fabaceae Lindl.
Iz_2_1209	DJI_0059	Anthericum ramosum L.	0	n	1	0	Asparagaceae Juss.
Iz_2_1209	DJI_0059	Artemisia marschalliana Spreng.	0	n	1	0	Asteraceae Bercht. & J.Presl
Iz_2_1209	DJI_0059	Asparagus officinalis L.	0	n	1	0	Asparagaceae Juss.
Iz_2_1209	DJI_0059	Astragalus cicer L.	0	n	1	0	Fabaceae Lindl.

Iz_2_1209	DJI_0059	Bassia laniflora (S.G.Gmel.) A.J.Scott	0	n	1	0	Amaranthaceae Juss.
Iz_2_1209	DJI_0059	Berberis vulgaris L.	0	n	1	0	Berberidaceae Juss.
Iz_2_1209	DJI_0059	Betula pendula Roth.	0	n	1	1	Betulaceae Gray
Iz_2_1209	DJI_0059	Betula pendula Roth.	0	n	1	0	Poaceae Barnhart
Iz_2_1209	DJI_0059	Bromus inermis Leyss.	0		NA	NA	
Iz_2_1209	DJI_0059	Bromus squarrosus L.	0	a	0	0	Poaceae Barnhart
Iz_2_1209	DJI_0059	Bromus tectorum L.	0	a	0	0	Poaceae Barnhart
Iz_2_1209	DJI_0059	Calamagrostis epigejos (L.) Roth	65	n	1	0	Poaceae Barnhart
Iz_2_1209	DJI_0059	Carduus acanthoides L.	0	n	1	0	Asteraceae Bercht. & J.Presl
Iz_2_1209	DJI_0059	Carex sp.	0	n	1	0	Cyperaceae Juss.
Iz_2_1209	DJI_0059	Centaurea majorovii Dumbadze	0	n	1	0	Asteraceae Bercht. & J.Presl
Iz_2_1209	DJI_0059	Chamaecytisus ruthenicus (Fisch.ex Wol.) Klaskova	0	n	1	0	Fabaceae Lindl.
Iz_2_1209	DJI_0059	Chelidonium majus L.	0	n	1	0	Papaveraceae Juss.
Iz_2_1209	DJI_0059	Chenopodium betaceum Andrz.	0.5	n	1	0	Amaranthaceae Juss.
Iz_2_1209	DJI_0059	Chenopodium cf. album	0	n	1	0	Amaranthaceae Juss.
Iz_2_1209	DJI_0059	Chenopodium sp.	0		NA	0	Amaranthaceae Juss.
Iz_2_1209	DJI_0059	Chondrilla juncea L.	0	n	1	0	Asteraceae Bercht. & J.Presl
Iz_2_1209	DJI_0059	Cirsium vulgare (Savi) Ten.	0	n	1	0	Asteraceae Bercht. & J.Presl
Iz_2_1209	DJI_0059	Clinopodium acinos (L.) Kuntze	0	n	1	0	Lamiacea Lindl.
Iz_2_1209	DJI_0059	Convallaria majalis L.	0	n	1	0	Asparagaceae Juss.
Iz_2_1209	DJI_0059	Crataegus rhipidophylla Gand.	0	n	1	1	Rosaceae Juss.
Iz_2_1209	DJI_0059	Crepis foetida L.	0	n	1	0	Asteraceae Bercht. & J.Presl
Iz_2_1209	DJI_0059	Dactylis glomerata L.	0	n	1	0	Poaceae Barnhart
Iz_2_1209	DJI_0059	Dianthus campestris M.Bieb.	0	n	1	0	Caryophyllaceae Juss.
Iz_2_1209	DJI_0059	Digitaria sanguinalis (L.) Scop.	0	a	0	0	Poaceae Barnhart
Iz_2_1209	DJI_0059	Elymus repens (L.) Gould	0	n	1	0	Poaceae Barnhart
Iz_2_1209	DJI_0059	Epilobium tetragonum L.	0	n	1	0	Onagraceae Juss.
Iz_2_1209	DJI_0059	Eragrostis minor Host	0	a	0	0	Poaceae Barnhart
Iz_2_1209	DJI_0059	Erigeron canadensis L.	0	a	0	0	Asteraceae Bercht. & J.Presl
Iz_2_1209	DJI_0059	Euphorbia seguieriana Neck.	0	n	1	0	Euphorbiaceae Juss.
Iz_2_1209	DJI_0059	Fallopia dumetorum (L.) Holub	0	n	1	0	Polygonaceae Juss.
Iz_2_1209	DJI_0059	Festuca beckeri (Hack.) Trautv.	0	n	1	0	Poaceae Barnhart
Iz_2_1209	DJI_0059	Fragaria vesca L.	0	n	1	0	Rosaceae Juss.
Iz_2_1209	DJI_0059	Frangula alnus Mill.	0	n	1	1	Rhamnaceae Juss.
Iz_2_1209	DJI_0059	Fraxinus pennsylvanica Marshall	0	a	0	1	Oleaceae Hoffmanns. & Link
Iz_2_1209	DJI_0059	Galeopsis bifida Boenn.	0	n	1	0	Lamiacea Lindl.
Iz_2_1209	DJI_0059	Galium verum L.	0	n	1	0	Rubiaceae Juss.
Iz_2_1209	DJI_0059	Genista tinctoria L.	0	n	1	0	Fabaceae Lindl.
Iz_2_1209	DJI_0059	Geranium robertianum L.	0	n	1	0	Geraniaceae Juss.
Iz_2_1209	DJI_0059	Geranium sanquineum L.	0	n	1	0	Geraniaceae Juss.
Iz_2_1209	DJI_0059	Gypsophila paniculata L.	0	n	1	0	Caryophyllaceae Juss.
Iz_2_1209	DJI_0059	Helichrysum arenarium (L.) Moench	0	n	1	0	Asteraceae Bercht. & J.Presl
Iz_2_1209	DJI_0059	Herniaria polygama J.Gay	0	n	1	0	Caryophyllaceae Juss.
Iz_2_1209	DJI_0059	Hieracium robustum Fr.	0	n	1	0	Asteraceae Bercht. & J.Presl
Iz_2_1209	DJI_0059	Hieracium umbellatum L.	0	n	1	NA	Asteraceae Bercht. & J.Presl
Iz_2_1209	DJI_0059	Hylotelephium maximum (L.) Holub	0	n	1	0	Crassulaceae J.St.-Hil.
Iz_2_1209	DJI_0059	Jacobaea borysthenica (DC.) B.Nord. & Greuter	0	n	1	0	Asteraceae Bercht. & J.Presl
Iz_2_1209	DJI_0059	Jasione montana L.	0	n	1	0	Campanulaceae Juss.
Iz_2_1209	DJI_0059	Jurinea cyanoides (L.) Rchb.s.l.	0	n	1	0	Asteraceae Bercht. & J.Presl
Iz_2_1209	DJI_0059	Koeleria glauca (Spreng.) DC.	0	n	1	0	Poaceae Barnhart
Iz_2_1209	DJI_0059	Lactuca serriola L.	0	a	0	0	Asteraceae Bercht. & J.Presl
Iz_2_1209	DJI_0059	Linaria genistifolia (L.) Mill.	0	n	1	0	Plantaginaceae Juss.
Iz_2_1209	DJI_0059	Lomelosia argentea (L.) Greuter & Burdet	0	n	1	0	Caprifoliaceae Juss.
Iz_2_1209	DJI_0059	Melampyrum pratense L.	0	n	1	0	Orobanchaceae Vent.
Iz_2_1209	DJI_0059	Oenothera biennis L.	0	a	0	0	Onagraceae Juss.
Iz_2_1209	DJI_0059	Pentanema britannica (L.) D.Gut.Larr., Santos-Vicente, Anderb., E.Rico & M.M.Mart.Ort.	0	n	1	0	Asteraceae Bercht. & J.Presl
Iz_2_1209	DJI_0059	Peucedanum oreoselinum (L.) Moench	1	n	1	0	Apiaceae Lindl.
Iz_2_1209	DJI_0059	Peucedanum ruthenicum M.Bieb.	0	n	1	0	Apiaceae Lindl.
Iz_2_1209	DJI_0059	Pilosella echioides (Lumn.) F.W.Schultz & Sch.Bip.	0	n	1	0	Asteraceae Bercht. & J.Presl
Iz_2_1209	DJI_0059	Pilosella officinarum F. Schultz et Sch. Bip.	0	n	1	0	Asteraceae Bercht. & J.Presl
Iz_2_1209	DJI_0059	Pilosella sp.	0		NA	0	Asteraceae Bercht. & J.Presl
Iz_2_1209	DJI_0059	Pinus sylvestris L.	0	n	1	1	Pinaceae Spreng. ex F.Rudolphi
Iz_2_1209	DJI_0059	Poa annua L.	0	n	1	0	Poaceae Barnhart
Iz_2_1209	DJI_0059	Poa nemoralis L.	0	n	1	0	Poaceae Barnhart
Iz_2_1209	DJI_0059	Poa sp.	0		NA	0	Poaceae Barnhart
Iz_2_1209	DJI_0059	Polygonatum odoratum (Mill.) Druce	0	n	1	0	Asparagaceae Juss.
Iz_2_1209	DJI_0059	Polygonum arenarium Waldst. & Kit.	0	n	1	0	Polygonaceae Juss.
Iz_2_1209	DJI_0059	Populus nigra L.	0	n	1	1	Salicaceae Mirb.
Iz_2_1209	DJI_0059	Populus tremula L.	0	n	1	1	Salicaceae Mirb.
Iz_2_1209	DJI_0059	Populus Ã canescens (Aiton) Sm.	0	n	1	1	Salicaceae Mirb.
Iz_2_1209	DJI_0059	Potentilla argentea L.	0	n	1	0	Rosaceae Juss.
Iz_2_1209	DJI_0059	Psammophiliella muralis (L.) Ikonn.	0	n	1	0	Caryophyllaceae Juss.
Iz_2_1209	DJI_0059	Psephellus sumensis (Kalen.) Greuter	0	n	1	0	Asteraceae Bercht. & J.Presl
Iz_2_1209	DJI_0059	Pulsatilla pratensis (L.) Mill. s.l.	0	n	1	0	Ranunculaceae Juss.
Iz_2_1209	DJI_0059	Pyrus communis L.	0	a	0	1	Rosaceae Juss.
Iz_2_1209	DJI_0059	Quercus robur L.	0	n	1	1	Fagaceae Dumort.
Iz_2_1209	DJI_0059	Robinia pseudoacacia L.	0	a	0	1	Fabaceae Lindl.
Iz_2_1209	DJI_0059	Rosa sp.	0		NA	1	Rosaceae Juss.
Iz_2_1209	DJI_0059	Rubus idaeus L.	0	n	1	0	Rosaceae Juss.
Iz_2_1209	DJI_0059	Rumex acetosella L.	0	n	1	0	Polygonaceae Juss.
Iz_2_1209	DJI_0059	Rumex thyrsiflorus Fingerh.	0	n	1	0	Polygonaceae Juss.
Iz_2_1209	DJI_0059	Salix alba L.	0	n	1	1	Salicaceae Mirb.
Iz_2_1209	DJI_0059	Sambucus nigra L.	0	n	1	1	Viburnaceae Raf.
Iz_2_1209	DJI_0059	Secale sylvestre Host	0	n	1	0	Poaceae Barnhart
Iz_2_1209	DJI_0059	Sempervivum ruthenicum Schnittsp. & C.B. Lehm.	0	n	1	0	Crassulaceae J.St.-Hil.
Iz_2_1209	DJI_0059	Setaria viridis (L.) P. Beauv.	0	n	1	0	Poaceae Barnhart
Iz_2_1209	DJI_0059	Silene borysthenica (Gruner) Walters	0	n	1	0	Caryophyllaceae Juss.
Iz_2_1209	DJI_0059	Silene latifolia subsp. alba (Mill.) Greuter & Burdet	0	n	1	0	Caryophyllaceae Juss.
Iz_2_1209	DJI_0059	Solidago virgaurea L.	0	n	1	0	Asteraceae Bercht. & J.Presl
Iz_2_1209	DJI_0059	Sorbus aucuparia L.	0	n	1	1	Rosaceae Juss.
Iz_2_1209	DJI_0059	Stachys recta L.	0	n	1	0	Lamiacea Lindl.
Iz_2_1209	DJI_0059	Tanacetum vulgare L.	0	n	1	0	Asteraceae Bercht. & J.Presl
Iz_2_1209	DJI_0059	Thymus pallasianus Heinr.Braun	0	n	1	0	Lamiacea Lindl.
Iz_2_1209	DJI_0059	Tilia cordata Mill.	0	n	1	1	Malvaceae Juss.
Iz_2_1209	DJI_0059	Trifolium alpestre L.	0	n	1	0	Fabaceae Lindl.
Iz_2_1209	DJI_0059	Trifolium arvense L.	0	n	1	0	Fabaceae Lindl.
Iz_2_1209	DJI_0059	Ulmus glabra Huds.	0	n	1	1	Ulmaceae Mirb.
Iz_2_1209	DJI_0059	Verbascum densiflorum Bertol.	0	n	1	0	Scrophulariaceae Juss.

Iz_2_1209	DJI_0059	Veronica austriaca L.	0	n	1	0	Plantaginaceae Juss.
Iz_2_1209	DJI_0059	Veronica incana L.	0	n	1	0	Plantaginaceae Juss.
Iz_2_1209	DJI_0059	Veronica verna L.	0	n	1	0	Plantaginaceae Juss.
Iz_2_1209	DJI_0059	Vincetoxicum hirundinaria Medik	0	n	1	0	Apocynaceae Juss.
Iz_2_1209	DJI_0064	Acer tataricum L.	0	n	1	1	Sapindaceae Juss.
Iz_2_1209	DJI_0064	Achillea millefolium L. s.l.	0	n	1	0	Asteraceae Bercht. & J.Presl
Iz_2_1209	DJI_0064	Allium oleraceum L.	0	n	1	0	Amaryllidaceae J.St.-Hil.
Iz_2_1209	DJI_0064	Ambrosia artemisiifolia L.	0	a	0	0	Asteraceae Bercht. & J.Presl
Iz_2_1209	DJI_0064	Amorpha fruticosa L.	0	a	0	0	Fabaceae Lindl.
Iz_2_1209	DJI_0064	Anthericum ramosum L.	0	n	1	0	Asparagaceae Juss.
Iz_2_1209	DJI_0064	Artemisia marschalliana Spreng.	0	n	1	0	Asteraceae Bercht. & J.Presl
Iz_2_1209	DJI_0064	Asparagus officinalis L.	0	n	1	0	Asparagaceae Juss.
Iz_2_1209	DJI_0064	Astragalus cicer L.	0	n	1	0	Fabaceae Lindl.
Iz_2_1209	DJI_0064	Bassia laniflora (S.G.Gmel.) A.J.Scott	0	n	1	0	Amaranthaceae Juss.
Iz_2_1209	DJI_0064	Berberis vulgaris L.	0	n	1	0	Berberidaceae Juss.
Iz_2_1209	DJI_0064	Betula pendula Roth.	0	n	1	1	Betulaceae Gray
Iz_2_1209	DJI_0064	Betula pendula Roth.	0	n	1	0	Poaceae Barnhart
Iz_2_1209	DJI_0064	Bromus inermis Leyss.	0		NA	NA	
Iz_2_1209	DJI_0064	Bromus squarrosus L.	0	a	0	0	Poaceae Barnhart
Iz_2_1209	DJI_0064	Bromus tectorum L.	0	a	0	0	Poaceae Barnhart
Iz_2_1209	DJI_0064	Calamagrostis epigejos (L.) Roth	50	n	1	0	Poaceae Barnhart
Iz_2_1209	DJI_0064	Carduus acanthoides L.	0	n	1	0	Asteraceae Bercht. & J.Presl
Iz_2_1209	DJI_0064	Carex sp.	0	n	1	0	Cyperaceae Juss.
Iz_2_1209	DJI_0064	Centaurea majorovii Dumbadze	0	n	1	0	Asteraceae Bercht. & J.Presl
Iz_2_1209	DJI_0064	Chamaecytisus ruthenicus (Fisch.ex Wol.) Klaskova	5	n	1	0	Fabaceae Lindl.
Iz_2_1209	DJI_0064	Chelidonium majus L.	0.5	n	1	0	Papaveraceae Juss.
Iz_2_1209	DJI_0064	Chenopodium betaceum Andrz.	0	n	1	0	Amaranthaceae Juss.
Iz_2_1209	DJI_0064	Chenopodium cf. album	0	n	1	0	Amaranthaceae Juss.
Iz_2_1209	DJI_0064	Chenopodium sp.	0		NA	0	Amaranthaceae Juss.
Iz_2_1209	DJI_0064	Chondrilla juncea L.	0	n	1	0	Asteraceae Bercht. & J.Presl
Iz_2_1209	DJI_0064	Cirsium vulgare (Savi) Ten.	0	n	1	0	Asteraceae Bercht. & J.Presl
Iz_2_1209	DJI_0064	Clinopodium acinos (L.) Kuntze	0	n	1	0	Lamiacea Lindl.
Iz_2_1209	DJI_0064	Convallaria majalis L.	0	n	1	0	Asparagaceae Juss.
Iz_2_1209	DJI_0064	Crataegus rhipidophylla Gand.	0	n	1	1	Rosaceae Juss.
Iz_2_1209	DJI_0064	Crepis foetida L.	0	n	1	0	Asteraceae Bercht. & J.Presl
Iz_2_1209	DJI_0064	Dactylis glomerata L.	0	n	1	0	Poaceae Barnhart
Iz_2_1209	DJI_0064	Dianthus campestris M.Bieb.	0	n	1	0	Caryophyllaceae Juss.
Iz_2_1209	DJI_0064	Digitaria sanguinalis (L.) Scop.	0	a	0	0	Poaceae Barnhart
Iz_2_1209	DJI_0064	Elymus repens (L.) Gould	0	n	1	0	Poaceae Barnhart
Iz_2_1209	DJI_0064	Epilobium tetragonum L.	0	n	1	0	Onagraceae Juss.
Iz_2_1209	DJI_0064	Eragrostis minor Host	0	a	0	0	Poaceae Barnhart
Iz_2_1209	DJI_0064	Erigeron canadensis L.	0	a	0	0	Asteraceae Bercht. & J.Presl
Iz_2_1209	DJI_0064	Euphorbia seguieriana Neck.	0	n	1	0	Euphorbiaceae Juss.
Iz_2_1209	DJI_0064	Fallopia dumetorum (L.) Holub	0	n	1	0	Polygonaceae Juss.
Iz_2_1209	DJI_0064	Festuca beckeri (Hack.) Trautv.	0	n	1	0	Poaceae Barnhart
Iz_2_1209	DJI_0064	Fragaria vesca L.	0	n	1	0	Rosaceae Juss.
Iz_2_1209	DJI_0064	Frangula alnus Mill.	0	n	1	1	Rhamnaceae Juss.
Iz_2_1209	DJI_0064	Fraxinus pennsylvanica Marshall	0	a	0	1	Oleaceae Hoffmanns. & Link
Iz_2_1209	DJI_0064	Galeopsis bifida Boenn.	0	n	1	0	Lamiacea Lindl.
Iz_2_1209	DJI_0064	Galium verum L.	0	n	1	0	Rubiaceae Juss.
Iz_2_1209	DJI_0064	Genista tinctoria L.	0	n	1	0	Fabaceae Lindl.
Iz_2_1209	DJI_0064	Geranium robertianum L.	0	n	1	0	Geraniaceae Juss.
Iz_2_1209	DJI_0064	Geranium sanquineum L.	0	n	1	0	Geraniaceae Juss.
Iz_2_1209	DJI_0064	Gypsophila paniculata L.	0	n	1	0	Caryophyllaceae Juss.
Iz_2_1209	DJI_0064	Helichrysum arenarium (L.) Moench	0	n	1	0	Asteraceae Bercht. & J.Presl
Iz_2_1209	DJI_0064	Herniaria polygama J.Gay	0	n	1	0	Caryophyllaceae Juss.
Iz_2_1209	DJI_0064	Hieracium robustum Fr.	0	n	1	0	Asteraceae Bercht. & J.Presl
Iz_2_1209	DJI_0064	Hieracium umbellatum L.	0.5	n	1	NA	Asteraceae Bercht. & J.Presl
Iz_2_1209	DJI_0064	Hylotelephium maximum (L.) Holub	0	n	1	0	Crassulaceae J.St.-Hil.
Iz_2_1209	DJI_0064	Jacobaea borysthenica (DC.) B.Nord. & Greuter	0	n	1	0	Asteraceae Bercht. & J.Presl
Iz_2_1209	DJI_0064	Jasione montana L.	0	n	1	0	Campanulaceae Juss.
Iz_2_1209	DJI_0064	Jurinea cyanoides (L.) Rchb.s.l.	0	n	1	0	Asteraceae Bercht. & J.Presl
Iz_2_1209	DJI_0064	Koeleria glauca (Spreng.) DC.	0	n	1	0	Poaceae Barnhart
Iz_2_1209	DJI_0064	Lactuca serriola L.	0	a	0	0	Asteraceae Bercht. & J.Presl
Iz_2_1209	DJI_0064	Linaria genistifolia (L.) Mill.	0	n	1	0	Plantaginaceae Juss.
Iz_2_1209	DJI_0064	Lomelosia argentea (L.) Greuter & Burdet	0	n	1	0	Caprifoliaceae Juss.
Iz_2_1209	DJI_0064	Melampyrum pratense L.	0	n	1	0	Orobanchaceae Vent.
Iz_2_1209	DJI_0064	Oenothera biennis L.	0	a	0	0	Onagraceae Juss.
Iz_2_1209	DJI_0064	Pentanema britannica (L.) D.Gut.Larr., Santos-Vicente, Anderb., E.Rico & M.M.Mart.Ort.	0	n	1	0	Asteraceae Bercht. & J.Presl
Iz_2_1209	DJI_0064	Peucedanum oreoselinum (L.) Moench	3	n	1	0	Apiaceae Lindl.
Iz_2_1209	DJI_0064	Peucedanum ruthenicum M.Bieb.	0	n	1	0	Apiaceae Lindl.
Iz_2_1209	DJI_0064	Pilosella echioides (Lumn.) F.W.Schultz & Sch.Bip.	0	n	1	0	Asteraceae Bercht. & J.Presl
Iz_2_1209	DJI_0064	Pilosella officinarum F. Schultz et Sch. Bip.	0	n	1	0	Asteraceae Bercht. & J.Presl
Iz_2_1209	DJI_0064	Pilosella sp.	0		NA	0	Asteraceae Bercht. & J.Presl
Iz_2_1209	DJI_0064	Pinus sylvestris L.	0	n	1	1	Pinaceae Spreng. ex F.Rudolphi
Iz_2_1209	DJI_0064	Poa annua L.	0	n	1	0	Poaceae Barnhart
Iz_2_1209	DJI_0064	Poa nemoralis L.	0	n	1	0	Poaceae Barnhart
Iz_2_1209	DJI_0064	Poa sp.	0		NA	0	Poaceae Barnhart
Iz_2_1209	DJI_0064	Polygonatum odoratum (Mill.) Druce	0	n	1	0	Asparagaceae Juss.
Iz_2_1209	DJI_0064	Polygonum arenarium Waldst. & Kit.	0	n	1	0	Polygonaceae Juss.
Iz_2_1209	DJI_0064	Populus nigra L.	0	n	1	1	Salicaceae Mirb.
Iz_2_1209	DJI_0064	Populus tremula L.	0	n	1	1	Salicaceae Mirb.
Iz_2_1209	DJI_0064	Populus Ã canescens (Aiton) Sm.	0	n	1	1	Salicaceae Mirb.
Iz_2_1209	DJI_0064	Potentilla argentea L.	0	n	1	0	Rosaceae Juss.
Iz_2_1209	DJI_0064	Psammophiliella muralis (L.) Ikonn.	0	n	1	0	Caryophyllaceae Juss.
Iz_2_1209	DJI_0064	Psephellus sumensis (Kalen.) Greuter	0	n	1	0	Asteraceae Bercht. & J.Presl
Iz_2_1209	DJI_0064	Pulsatilla pratensis (L.) Mill. s.l.	0	n	1	0	Ranunculaceae Juss.
Iz_2_1209	DJI_0064	Pyrus communis L.	0	a	0	1	Rosaceae Juss.
Iz_2_1209	DJI_0064	Quercus robur L.	0	n	1	1	Fagaceae Dumort.
Iz_2_1209	DJI_0064	Robinia pseudoacacia L.	0	a	0	1	Fabaceae Lindl.
Iz_2_1209	DJI_0064	Rosa sp.	0		NA	1	Rosaceae Juss.
Iz_2_1209	DJI_0064	Rubus idaeus L.	0	n	1	0	Rosaceae Juss.
Iz_2_1209	DJI_0064	Rumex acetosella L.	0	n	1	0	Polygonaceae Juss.
Iz_2_1209	DJI_0064	Rumex thyrsiflorus Fingerh.	0	n	1	0	Polygonaceae Juss.
Iz_2_1209	DJI_0064	Salix alba L.	0	n	1	1	Salicaceae Mirb.
Iz_2_1209	DJI_0064	Sambucus nigra L.	0	n	1	1	Viburnaceae Raf.
Iz_2_1209	DJI_0064	Secale sylvestre Host	0	n	1	0	Poaceae Barnhart
Iz_2_1209	DJI_0064	Sempervivum ruthenicum Schnittsp. & C.B. Lehm.	0	n	1	0	Crassulaceae J.St.-Hil.

Iz_2_1209	DJI_0064	Setaria viridis (L.) P. Beauv.	0	n	1	0	Poaceae Barnhart
Iz_2_1209	DJI_0064	Silene borysthenica (Gruner) Walters	0	n	1	0	Caryophyllaceae Juss.
Iz_2_1209	DJI_0064	Silene latifolia subsp. alba (Mill.) Greuter & Burdet	0	n	1	0	Caryophyllaceae Juss.
Iz_2_1209	DJI_0064	Solidago virgaurea L.	0	n	1	0	Asteraceae Bercht. & J.Presl
Iz_2_1209	DJI_0064	Sorbus aucuparia L.	0	n	1	1	Rosaceae Juss.
Iz_2_1209	DJI_0064	Stachys recta L.	0	n	1	0	Lamiacea Lindl.
Iz_2_1209	DJI_0064	Tanacetum vulgare L.	0	n	1	0	Asteraceae Bercht. & J.Presl
Iz_2_1209	DJI_0064	Thymus pallasianus Heinr.Braun	0	n	1	0	Lamiacea Lindl.
Iz_2_1209	DJI_0064	Tilia cordata Mill.	0	n	1	1	Malvaceae Juss.
Iz_2_1209	DJI_0064	Trifolium alpestre L.	0	n	1	0	Fabaceae Lindl.
Iz_2_1209	DJI_0064	Trifolium arvense L.	0	n	1	0	Fabaceae Lindl.
Iz_2_1209	DJI_0064	Ulmus glabra Huds.	0	n	1	1	Ulmaceae Mirb.
Iz_2_1209	DJI_0064	Verbascum densiflorum Bertol.	0	n	1	0	Scrophulariaceae Juss.
Iz_2_1209	DJI_0064	Veronica austriaca L.	0	n	1	0	Plantaginaceae Juss.
Iz_2_1209	DJI_0064	Veronica incana L.	0	n	1	0	Plantaginaceae Juss.
Iz_2_1209	DJI_0064	Veronica verna L.	0	n	1	0	Plantaginaceae Juss.
Iz_2_1209	DJI_0064	Vincetoxicum hirundinaria Medik	0	n	1	0	Apocynaceae Juss.
Iz_2_1209	DJI_0067	Acer tataricum L.	0	n	1	1	Sapindaceae Juss.
Iz_2_1209	DJI_0067	Achillea millefolium L. s.l.	0	n	1	0	Asteraceae Bercht. & J.Presl
Iz_2_1209	DJI_0067	Allium oleraceum L.	0	n	1	0	Amaryllidaceae J.St.-Hil.
Iz_2_1209	DJI_0067	Ambrosia artemisiifolia L.	0	a	0	0	Asteraceae Bercht. & J.Presl
Iz_2_1209	DJI_0067	Amorpha fruticosa L.	0	a	0	0	Fabaceae Lindl.
Iz_2_1209	DJI_0067	Anthericum ramosum L.	0	n	1	0	Asparagaceae Juss.
Iz_2_1209	DJI_0067	Artemisia marschalliana Spreng.	0	n	1	0	Asteraceae Bercht. & J.Presl
Iz_2_1209	DJI_0067	Asparagus officinalis L.	0	n	1	0	Asparagaceae Juss.
Iz_2_1209	DJI_0067	Astragalus cicer L.	0	n	1	0	Fabaceae Lindl.
Iz_2_1209	DJI_0067	Bassia laniflora (S.G.Gmel.) A.J.Scott	0	n	1	0	Amaranthaceae Juss.
Iz_2_1209	DJI_0067	Berberis vulgaris L.	0	n	1	0	Berberidaceae Juss.
Iz_2_1209	DJI_0067	Betula pendula Roth.	0	n	1	1	Betulaceae Gray
Iz_2_1209	DJI_0067	Betula pendula Roth.	0	n	1	0	Poaceae Barnhart
Iz_2_1209	DJI_0067	Bromus inermis Leyss.	0		NA	NA	
Iz_2_1209	DJI_0067	Bromus squarrosus L.	0	a	0	0	Poaceae Barnhart
Iz_2_1209	DJI_0067	Bromus tectorum L.	0	a	0	0	Poaceae Barnhart
Iz_2_1209	DJI_0067	Calamagrostis epigejos (L.) Roth	40	n	1	0	Poaceae Barnhart
Iz_2_1209	DJI_0067	Carduus acanthoides L.	0	n	1	0	Asteraceae Bercht. & J.Presl
Iz_2_1209	DJI_0067	Carex sp.	0	n	1	0	Cyperaceae Juss.
Iz_2_1209	DJI_0067	Centaurea majorovii Dumbadze	0	n	1	0	Asteraceae Bercht. & J.Presl
Iz_2_1209	DJI_0067	Chamaecytisus ruthenicus (Fisch.ex Wol.) Klaskova	0	n	1	0	Fabaceae Lindl.
Iz_2_1209	DJI_0067	Chelidonium majus L.	0	n	1	0	Papaveraceae Juss.
Iz_2_1209	DJI_0067	Chenopodium betaceum Andrz.	0.5	n	1	0	Amaranthaceae Juss.
Iz_2_1209	DJI_0067	Chenopodium cf. album	0	n	1	0	Amaranthaceae Juss.
Iz_2_1209	DJI_0067	Chenopodium sp.	0		NA	0	Amaranthaceae Juss.
Iz_2_1209	DJI_0067	Chondrilla juncea L.	0	n	1	0	Asteraceae Bercht. & J.Presl
Iz_2_1209	DJI_0067	Cirsium vulgare (Savi) Ten.	0	n	1	0	Asteraceae Bercht. & J.Presl
Iz_2_1209	DJI_0067	Clinopodium acinos (L.) Kuntze	0	n	1	0	Lamiacea Lindl.
Iz_2_1209	DJI_0067	Convallaria majalis L.	0	n	1	0	Asparagaceae Juss.
Iz_2_1209	DJI_0067	Crataegus rhipidophylla Gand.	0	n	1	1	Rosaceae Juss.
Iz_2_1209	DJI_0067	Crepis foetida L.	0	n	1	0	Asteraceae Bercht. & J.Presl
Iz_2_1209	DJI_0067	Dactylis glomerata L.	0	n	1	0	Poaceae Barnhart
Iz_2_1209	DJI_0067	Dianthus campestris M.Bieb.	0	n	1	0	Caryophyllaceae Juss.
Iz_2_1209	DJI_0067	Digitaria sanguinalis (L.) Scop.	0	a	0	0	Poaceae Barnhart
Iz_2_1209	DJI_0067	Elymus repens (L.) Gould	0	n	1	0	Poaceae Barnhart
Iz_2_1209	DJI_0067	Epilobium tetragonum L.	0	n	1	0	Onagraceae Juss.
Iz_2_1209	DJI_0067	Eragrostis minor Host	0	a	0	0	Poaceae Barnhart
Iz_2_1209	DJI_0067	Erigeron canadensis L.	0	a	0	0	Asteraceae Bercht. & J.Presl
Iz_2_1209	DJI_0067	Euphorbia seguieriana Neck.	0	n	1	0	Euphorbiaceae Juss.
Iz_2_1209	DJI_0067	Fallopia dumetorum (L.) Holub	0	n	1	0	Polygonaceae Juss.
Iz_2_1209	DJI_0067	Festuca beckeri (Hack.) Trautv.	0	n	1	0	Poaceae Barnhart
Iz_2_1209	DJI_0067	Fragaria vesca L.	0	n	1	0	Rosaceae Juss.
Iz_2_1209	DJI_0067	Frangula alnus Mill.	0	n	1	1	Rhamnaceae Juss.
Iz_2_1209	DJI_0067	Fraxinus pennsylvanica Marshall	0	a	0	1	Oleaceae Hoffmanns. & Link
Iz_2_1209	DJI_0067	Galeopsis bifida Boenn.	0	n	1	0	Lamiacea Lindl.
Iz_2_1209	DJI_0067	Galium verum L.	0	n	1	0	Rubiaceae Juss.
Iz_2_1209	DJI_0067	Genista tinctoria L.	0	n	1	0	Fabaceae Lindl.
Iz_2_1209	DJI_0067	Geranium robertianum L.	0	n	1	0	Geraniaceae Juss.
Iz_2_1209	DJI_0067	Geranium sanquineum L.	0	n	1	0	Geraniaceae Juss.
Iz_2_1209	DJI_0067	Gypsophila paniculata L.	0	n	1	0	Caryophyllaceae Juss.
Iz_2_1209	DJI_0067	Helichrysum arenarium (L.) Moench	0	n	1	0	Asteraceae Bercht. & J.Presl
Iz_2_1209	DJI_0067	Herniaria polygama J.Gay	0	n	1	0	Caryophyllaceae Juss.
Iz_2_1209	DJI_0067	Hieracium robustum Fr.	0	n	1	0	Asteraceae Bercht. & J.Presl
Iz_2_1209	DJI_0067	Hieracium umbellatum L.	0	n	1	NA	Asteraceae Bercht. & J.Presl
Iz_2_1209	DJI_0067	Hylotelephium maximum (L.) Holub	0	n	1	0	Crassulaceae J.St.-Hil.
Iz_2_1209	DJI_0067	Jacobaea borysthenica (DC.) B.Nord. & Greuter	0	n	1	0	Asteraceae Bercht. & J.Presl
Iz_2_1209	DJI_0067	Jasione montana L.	0	n	1	0	Campanulaceae Juss.
Iz_2_1209	DJI_0067	Jurinea cyanoides (L.) Rchb.s.l.	0	n	1	0	Asteraceae Bercht. & J.Presl
Iz_2_1209	DJI_0067	Koeleria glauca (Spreng.) DC.	0	n	1	0	Poaceae Barnhart
Iz_2_1209	DJI_0067	Lactuca serriola L.	0	a	0	0	Asteraceae Bercht. & J.Presl
Iz_2_1209	DJI_0067	Linaria genistifolia (L.) Mill.	0	n	1	0	Plantaginaceae Juss.
Iz_2_1209	DJI_0067	Lomelosia argentea (L.) Greuter & Burdet	0	n	1	0	Caprifoliaceae Juss.
Iz_2_1209	DJI_0067	Melampyrum pratense L.	0	n	1	0	Orobanchaceae Vent.
Iz_2_1209	DJI_0067	Oenothera biennis L.	0	a	0	0	Onagraceae Juss.
Iz_2_1209	DJI_0067	Pentanema britannica (L.) D.Gut.Larr., Santos-Vicente, Anderb., E.Rico & M.M.Mart.Ort.	0	n	1	0	Asteraceae Bercht. & J.Presl
Iz_2_1209	DJI_0067	Peucedanum oreoselinum (L.) Moench	4	n	1	0	Apiaceae Lindl.
Iz_2_1209	DJI_0067	Peucedanum ruthenicum M.Bieb.	0	n	1	0	Apiaceae Lindl.
Iz_2_1209	DJI_0067	Pilosella echioides (Lumn.) F.W.Schultz & Sch.Bip.	0	n	1	0	Asteraceae Bercht. & J.Presl
Iz_2_1209	DJI_0067	Pilosella officinarum F. Schultz et Sch. Bip.	0	n	1	0	Asteraceae Bercht. & J.Presl
Iz_2_1209	DJI_0067	Pilosella sp.	0		NA	0	Asteraceae Bercht. & J.Presl
Iz_2_1209	DJI_0067	Pinus sylvestris L.	0	n	1	1	Pinaceae Spreng. ex F.Rudolphi
Iz_2_1209	DJI_0067	Poa annua L.	0	n	1	0	Poaceae Barnhart
Iz_2_1209	DJI_0067	Poa nemoralis L.	0	n	1	0	Poaceae Barnhart
Iz_2_1209	DJI_0067	Poa sp.	0		NA	0	Poaceae Barnhart
Iz_2_1209	DJI_0067	Polygonatum odoratum (Mill.) Druce	0	n	1	0	Asparagaceae Juss.
Iz_2_1209	DJI_0067	Polygonum arenarium Waldst. & Kit.	0	n	1	0	Polygonaceae Juss.
Iz_2_1209	DJI_0067	Populus nigra L.	0	n	1	1	Salicaceae Mirb.
Iz_2_1209	DJI_0067	Populus tremula L.	0	n	1	1	Salicaceae Mirb.
Iz_2_1209	DJI_0067	Populus Ã canescens (Aiton) Sm.	0	n	1	1	Salicaceae Mirb.
Iz_2_1209	DJI_0067	Potentilla argentea L.	0	n	1	0	Rosaceae Juss.
Iz_2_1209	DJI_0067	Psammophiliella muralis (L.) Ikonn.	0	n	1	0	Caryophyllaceae Juss.

Iz_2_1209	DJI_0067	Psephellus sumensis (Kalen.) Greuter	0	n	1	0	Asteraceae Bercht. & J.Presl
Iz_2_1209	DJI_0067	Pulsatilla pratensis (L.) Mill. s.l.	0	n	1	0	Ranunculaceae Juss.
Iz_2_1209	DJI_0067	Pyrus communis L.	0	a	0	1	Rosaceae Juss.
Iz_2_1209	DJI_0067	Quercus robur L.	0	n	1	1	Fagaceae Dumort.
Iz_2_1209	DJI_0067	Robinia pseudoacacia L.	0	a	0	1	Fabaceae Lindl.
Iz_2_1209	DJI_0067	Rosa sp.	0		NA	1	Rosaceae Juss.
Iz_2_1209	DJI_0067	Rubus idaeus L.	0	n	1	0	Rosaceae Juss.
Iz_2_1209	DJI_0067	Rumex acetosella L.	0	n	1	0	Polygonaceae Juss.
Iz_2_1209	DJI_0067	Rumex thyrsiflorus Fingerh.	0	n	1	0	Polygonaceae Juss.
Iz_2_1209	DJI_0067	Salix alba L.	0	n	1	1	Salicaceae Mirb.
Iz_2_1209	DJI_0067	Sambucus nigra L.	0	n	1	1	Viburnaceae Raf.
Iz_2_1209	DJI_0067	Secale sylvestre Host	0	n	1	0	Poaceae Barnhart
Iz_2_1209	DJI_0067	Sempervivum ruthenicum Schnittsp. & C.B. Lehm.	0	n	1	0	Crassulaceae J.St.-Hil.
Iz_2_1209	DJI_0067	Setaria viridis (L.) P. Beauv.	0	n	1	0	Poaceae Barnhart
Iz_2_1209	DJI_0067	Silene borysthenica (Gruner) Walters	0	n	1	0	Caryophyllaceae Juss.
Iz_2_1209	DJI_0067	Silene latifolia subsp. alba (Mill.) Greuter & Burdet	0	n	1	0	Caryophyllaceae Juss.
Iz_2_1209	DJI_0067	Solidago virgaurea L.	0	n	1	0	Asteraceae Bercht. & J.Presl
Iz_2_1209	DJI_0067	Sorbus aucuparia L.	0	n	1	1	Rosaceae Juss.
Iz_2_1209	DJI_0067	Stachys recta L.	0	n	1	0	Lamiacea Lindl.
Iz_2_1209	DJI_0067	Tanacetum vulgare L.	0	n	1	0	Asteraceae Bercht. & J.Presl
Iz_2_1209	DJI_0067	Thymus pallasianus Heinr.Braun	0	n	1	0	Lamiacea Lindl.
Iz_2_1209	DJI_0067	Tilia cordata Mill.	0	n	1	1	Malvaceae Juss.
Iz_2_1209	DJI_0067	Trifolium alpestre L.	0	n	1	0	Fabaceae Lindl.
Iz_2_1209	DJI_0067	Trifolium arvense L.	0	n	1	0	Fabaceae Lindl.
Iz_2_1209	DJI_0067	Ulmus glabra Huds.	0	n	1	1	Ulmaceae Mirb.
Iz_2_1209	DJI_0067	Verbascum densiflorum Bertol.	0	n	1	0	Scrophulariaceae Juss.
Iz_2_1209	DJI_0067	Veronica austriaca L.	0	n	1	0	Plantaginaceae Juss.
Iz_2_1209	DJI_0067	Veronica incana L.	0	n	1	0	Plantaginaceae Juss.
Iz_2_1209	DJI_0067	Veronica verna L.	0	n	1	0	Plantaginaceae Juss.
Iz_2_1209	DJI_0067	Vincetoxicum hirundinaria Medik	0	n	1	0	Apocynaceae Juss.
Iz_3_1009	DJI_0001	Acer tataricum L.	0	n	1	1	Sapindaceae Juss.
Iz_3_1009	DJI_0001	Achillea millefolium L. s.l.	0	n	1	0	Asteraceae Bercht. & J.Presl
Iz_3_1009	DJI_0001	Allium oleraceum L.	0	n	1	0	Amaryllidaceae J.St.-Hil.
Iz_3_1009	DJI_0001	Ambrosia artemisiifolia L.	0	a	0	0	Asteraceae Bercht. & J.Presl
Iz_3_1009	DJI_0001	Amorpha fruticosa L.	0	a	0	0	Fabaceae Lindl.
Iz_3_1009	DJI_0001	Anthericum ramosum L.	0	n	1	0	Asparagaceae Juss.
Iz_3_1009	DJI_0001	Artemisia marschalliana Spreng.	0	n	1	0	Asteraceae Bercht. & J.Presl
Iz_3_1009	DJI_0001	Asparagus officinalis L.	0	n	1	0	Asparagaceae Juss.
Iz_3_1009	DJI_0001	Astragalus cicer L.	0	n	1	0	Fabaceae Lindl.
Iz_3_1009	DJI_0001	Bassia laniflora (S.G.Gmel.) A.J.Scott	0	n	1	0	Amaranthaceae Juss.
Iz_3_1009	DJI_0001	Berberis vulgaris L.	0	n	1	0	Berberidaceae Juss.
Iz_3_1009	DJI_0001	Betula pendula Roth.	0	n	1	1	Betulaceae Gray
Iz_3_1009	DJI_0001	Betula pendula Roth.	0	n	1	0	Poaceae Barnhart
Iz_3_1009	DJI_0001	Bromus inermis Leyss.	0		NA	NA	
Iz_3_1009	DJI_0001	Bromus squarrosus L.	0	a	0	0	Poaceae Barnhart
Iz_3_1009	DJI_0001	Bromus tectorum L.	0	a	0	0	Poaceae Barnhart
Iz_3_1009	DJI_0001	Calamagrostis epigejos (L.) Roth	2	n	1	0	Poaceae Barnhart
Iz_3_1009	DJI_0001	Carduus acanthoides L.	0	n	1	0	Asteraceae Bercht. & J.Presl
Iz_3_1009	DJI_0001	Carex sp.	0	n	1	0	Cyperaceae Juss.
Iz_3_1009	DJI_0001	Centaurea majorovii Dumbadze	0	n	1	0	Asteraceae Bercht. & J.Presl
Iz_3_1009	DJI_0001	Chamaecytisus ruthenicus (Fisch.ex Wol.) Klaskova	0	n	1	0	Fabaceae Lindl.
Iz_3_1009	DJI_0001	Chelidonium majus L.	3	n	1	0	Papaveraceae Juss.
Iz_3_1009	DJI_0001	Chenopodium betaceum Andrz.	0	n	1	0	Amaranthaceae Juss.
Iz_3_1009	DJI_0001	Chenopodium cf. album	1	n	1	0	Amaranthaceae Juss.
Iz_3_1009	DJI_0001	Chenopodium sp.	0		NA	0	Amaranthaceae Juss.
Iz_3_1009	DJI_0001	Chondrilla juncea L.	0	n	1	0	Asteraceae Bercht. & J.Presl
Iz_3_1009	DJI_0001	Cirsium vulgare (Savi) Ten.	0	n	1	0	Asteraceae Bercht. & J.Presl
Iz_3_1009	DJI_0001	Clinopodium acinos (L.) Kuntze	0	n	1	0	Lamiacea Lindl.
Iz_3_1009	DJI_0001	Convallaria majalis L.	0	n	1	0	Asparagaceae Juss.
Iz_3_1009	DJI_0001	Crataegus rhipidophylla Gand.	0	n	1	1	Rosaceae Juss.
Iz_3_1009	DJI_0001	Crepis foetida L.	0	n	1	0	Asteraceae Bercht. & J.Presl
Iz_3_1009	DJI_0001	Dactylis glomerata L.	0	n	1	0	Poaceae Barnhart
Iz_3_1009	DJI_0001	Dianthus campestris M.Bieb.	0	n	1	0	Caryophyllaceae Juss.
Iz_3_1009	DJI_0001	Digitaria sanguinalis (L.) Scop.	0	a	0	0	Poaceae Barnhart
Iz_3_1009	DJI_0001	Elymus repens (L.) Gould	0	n	1	0	Poaceae Barnhart
Iz_3_1009	DJI_0001	Epilobium tetragonum L.	0	n	1	0	Onagraceae Juss.
Iz_3_1009	DJI_0001	Eragrostis minor Host	0	a	0	0	Poaceae Barnhart
Iz_3_1009	DJI_0001	Erigeron canadensis L.	0	a	0	0	Asteraceae Bercht. & J.Presl
Iz_3_1009	DJI_0001	Euphorbia seguieriana Neck.	0	n	1	0	Euphorbiaceae Juss.
Iz_3_1009	DJI_0001	Fallopia dumetorum (L.) Holub	1	n	1	0	Polygonaceae Juss.
Iz_3_1009	DJI_0001	Festuca beckeri (Hack.) Trautv.	0	n	1	0	Poaceae Barnhart
Iz_3_1009	DJI_0001	Fragaria vesca L.	0	n	1	0	Rosaceae Juss.
Iz_3_1009	DJI_0001	Frangula alnus Mill.	0	n	1	1	Rhamnaceae Juss.
Iz_3_1009	DJI_0001	Fraxinus pennsylvanica Marshall	0	a	0	1	Oleaceae Hoffmanns. & Link
Iz_3_1009	DJI_0001	Galeopsis bifida Boenn.	0	n	1	0	Lamiacea Lindl.
Iz_3_1009	DJI_0001	Galium verum L.	0	n	1	0	Rubiaceae Juss.
Iz_3_1009	DJI_0001	Genista tinctoria L.	0	n	1	0	Fabaceae Lindl.
Iz_3_1009	DJI_0001	Geranium robertianum L.	1	n	1	0	Geraniaceae Juss.
Iz_3_1009	DJI_0001	Geranium sanquineum L.	0	n	1	0	Geraniaceae Juss.
Iz_3_1009	DJI_0001	Gypsophila paniculata L.	0	n	1	0	Caryophyllaceae Juss.
Iz_3_1009	DJI_0001	Helichrysum arenarium (L.) Moench	0	n	1	0	Asteraceae Bercht. & J.Presl
Iz_3_1009	DJI_0001	Herniaria polygama J.Gay	0	n	1	0	Caryophyllaceae Juss.
Iz_3_1009	DJI_0001	Hieracium robustum Fr.	0	n	1	0	Asteraceae Bercht. & J.Presl
Iz_3_1009	DJI_0001	Hieracium umbellatum L.	0	n	1	NA	Asteraceae Bercht. & J.Presl
Iz_3_1009	DJI_0001	Hylotelephium maximum (L.) Holub	0	n	1	0	Crassulaceae J.St.-Hil.
Iz_3_1009	DJI_0001	Jacobaea borysthenica (DC.) B.Nord. & Greuter	0	n	1	0	Asteraceae Bercht. & J.Presl
Iz_3_1009	DJI_0001	Jasione montana L.	0	n	1	0	Campanulaceae Juss.
Iz_3_1009	DJI_0001	Jurinea cyanoides (L.) Rchb.s.l.	0	n	1	0	Asteraceae Bercht. & J.Presl
Iz_3_1009	DJI_0001	Koeleria glauca (Spreng.) DC.	0	n	1	0	Poaceae Barnhart
Iz_3_1009	DJI_0001	Lactuca serriola L.	0	a	0	0	Asteraceae Bercht. & J.Presl
Iz_3_1009	DJI_0001	Linaria genistifolia (L.) Mill.	0	n	1	0	Plantaginaceae Juss.
Iz_3_1009	DJI_0001	Lomelosia argentea (L.) Greuter & Burdet	0	n	1	0	Caprifoliaceae Juss.
Iz_3_1009	DJI_0001	Melampyrum pratense L.	0	n	1	0	Orobanchaceae Vent.
Iz_3_1009	DJI_0001	Oenothera biennis L.	0	a	0	0	Onagraceae Juss.
Iz_3_1009	DJI_0001	Pentanema britannica (L.) D.Gut.Larr., Santos-Vicente, Anderb., E.Rico & M.M.Mart.Ort.	0	n	1	0	Asteraceae Bercht. & J.Presl
Iz_3_1009	DJI_0001	Peucedanum oreoselinum (L.) Moench	0	n	1	0	Apiaceae Lindl.
Iz_3_1009	DJI_0001	Peucedanum ruthenicum M.Bieb.	0	n	1	0	Apiaceae Lindl.
Iz_3_1009	DJI_0001	Pilosella echioides (Lumn.) F.W.Schultz & Sch.Bip.	0	n	1	0	Asteraceae Bercht. & J.Presl

Iz_3_1009	DJI_0001	Pilosella officinarum F. Schultz et Sch. Bip.	0	n	1	0	Asteraceae Bercht. & J.Presl
Iz_3_1009	DJI_0001	Pilosella sp.	0		NA	0	Asteraceae Bercht. & J.Presl
Iz_3_1009	DJI_0001	Pinus sylvestris L.	0	n	1	1	Pinaceae Spreng. ex F.Rudolphi
Iz_3_1009	DJI_0001	Poa annua L.	0	n	1	0	Poaceae Barnhart
Iz_3_1009	DJI_0001	Poa nemoralis L.	0	n	1	0	Poaceae Barnhart
Iz_3_1009	DJI_0001	Poa sp.	0		NA	0	Poaceae Barnhart
Iz_3_1009	DJI_0001	Polygonatum odoratum (Mill.) Druce	0	n	1	0	Asparagaceae Juss.
Iz_3_1009	DJI_0001	Polygonum arenarium Waldst. & Kit.	0	n	1	0	Polygonaceae Juss.
Iz_3_1009	DJI_0001	Populus nigra L.	0	n	1	1	Salicaceae Mirb.
Iz_3_1009	DJI_0001	Populus tremula L.	0	n	1	1	Salicaceae Mirb.
Iz_3_1009	DJI_0001	Populus Ã canescens (Aiton) Sm.	0	n	1	1	Salicaceae Mirb.
Iz_3_1009	DJI_0001	Potentilla argentea L.	0	n	1	0	Rosaceae Juss.
Iz_3_1009	DJI_0001	Psammophiliella muralis (L.) Ikonn.	0	n	1	0	Caryophyllaceae Juss.
Iz_3_1009	DJI_0001	Psephellus sumensis (Kalen.) Greuter	0	n	1	0	Asteraceae Bercht. & J.Presl
Iz_3_1009	DJI_0001	Pulsatilla pratensis (L.) Mill. s.l.	0	n	1	0	Ranunculaceae Juss.
Iz_3_1009	DJI_0001	Pyrus communis L.	0	a	0	1	Rosaceae Juss.
Iz_3_1009	DJI_0001	Quercus robur L.	0	n	1	1	Fagaceae Dumort.
Iz_3_1009	DJI_0001	Robinia pseudoacacia L.	0	a	0	1	Fabaceae Lindl.
Iz_3_1009	DJI_0001	Rosa sp.	0		NA	1	Rosaceae Juss.
Iz_3_1009	DJI_0001	Rubus idaeus L.	0	n	1	0	Rosaceae Juss.
Iz_3_1009	DJI_0001	Rumex acetosella L.	0	n	1	0	Polygonaceae Juss.
Iz_3_1009	DJI_0001	Rumex thyrsiflorus Fingerh.	0	n	1	0	Polygonaceae Juss.
Iz_3_1009	DJI_0001	Salix alba L.	0	n	1	1	Salicaceae Mirb.
Iz_3_1009	DJI_0001	Sambucus nigra L.	0	n	1	1	Viburnaceae Raf.
Iz_3_1009	DJI_0001	Secale sylvestre Host	0	n	1	0	Poaceae Barnhart
Iz_3_1009	DJI_0001	Sempervivum ruthenicum Schnittsp. & C.B. Lehm.	0	n	1	0	Crassulaceae J.St.-Hil.
Iz_3_1009	DJI_0001	Setaria viridis (L.) P. Beauv.	0	n	1	0	Poaceae Barnhart
Iz_3_1009	DJI_0001	Silene borysthenica (Gruner) Walters	0	n	1	0	Caryophyllaceae Juss.
Iz_3_1009	DJI_0001	Silene latifolia subsp. alba (Mill.) Greuter & Burdet	0	n	1	0	Caryophyllaceae Juss.
Iz_3_1009	DJI_0001	Solidago virgaurea L.	0	n	1	0	Asteraceae Bercht. & J.Presl
Iz_3_1009	DJI_0001	Sonchus oleraceus L.	0	a	0	0	Asteraceae Bercht. & J.Presl
Iz_3_1009	DJI_0001	Sorbus aucuparia L.	0	n	1	1	Rosaceae Juss.
Iz_3_1009	DJI_0001	Stachys recta L.	0	n	1	0	Lamiacea Lindl.
Iz_3_1009	DJI_0001	Tanacetum vulgare L.	0	n	1	0	Asteraceae Bercht. & J.Presl
Iz_3_1009	DJI_0001	Thymus pallasianus Heinr.Braun	0	n	1	0	Lamiacea Lindl.
Iz_3_1009	DJI_0001	Tilia cordata Mill.	0	n	1	1	Malvaceae Juss.
Iz_3_1009	DJI_0001	Trifolium alpestre L.	0	n	1	0	Fabaceae Lindl.
Iz_3_1009	DJI_0001	Trifolium arvense L.	0	n	1	0	Fabaceae Lindl.
Iz_3_1009	DJI_0001	Ulmus glabra Huds.	0	n	1	1	Ulmaceae Mirb.
Iz_3_1009	DJI_0001	Verbascum densiflorum Bertol.	0	n	1	0	Scrophulariaceae Juss.
Iz_3_1009	DJI_0001	Veronica austriaca L.	0	n	1	0	Plantaginaceae Juss.
Iz_3_1009	DJI_0001	Veronica incana L.	0	n	1	0	Plantaginaceae Juss.
Iz_3_1009	DJI_0001	Veronica verna L.	0	n	1	0	Plantaginaceae Juss.
Iz_3_1009	DJI_0001	Vincetoxicum hirundinaria Medik	0	n	1	0	Apocynaceae Juss.
Iz_3_1009	DJI_0002	Acer tataricum L.	0	n	1	1	Sapindaceae Juss.
Iz_3_1009	DJI_0002	Achillea millefolium L. s.l.	0	n	1	0	Asteraceae Bercht. & J.Presl
Iz_3_1009	DJI_0002	Allium oleraceum L.	0	n	1	0	Amaryllidaceae J.St.-Hil.
Iz_3_1009	DJI_0002	Ambrosia artemisiifolia L.	0	a	0	0	Asteraceae Bercht. & J.Presl
Iz_3_1009	DJI_0002	Amorpha fruticosa L.	0	a	0	0	Fabaceae Lindl.
Iz_3_1009	DJI_0002	Anthericum ramosum L.	0	n	1	0	Asparagaceae Juss.
Iz_3_1009	DJI_0002	Artemisia marschalliana Spreng.	0	n	1	0	Asteraceae Bercht. & J.Presl
Iz_3_1009	DJI_0002	Asparagus officinalis L.	0	n	1	0	Asparagaceae Juss.
Iz_3_1009	DJI_0002	Astragalus cicer L.	0	n	1	0	Fabaceae Lindl.
Iz_3_1009	DJI_0002	Bassia laniflora (S.G.Gmel.) A.J.Scott	0	n	1	0	Amaranthaceae Juss.
Iz_3_1009	DJI_0002	Berberis vulgaris L.	0	n	1	0	Berberidaceae Juss.
Iz_3_1009	DJI_0002	Betula pendula Roth.	0	n	1	1	Betulaceae Gray
Iz_3_1009	DJI_0002	Betula pendula Roth.	0	n	1	0	Poaceae Barnhart
Iz_3_1009	DJI_0002	Bromus inermis Leyss.	0		NA	NA	
Iz_3_1009	DJI_0002	Bromus squarrosus L.	0	a	0	0	Poaceae Barnhart
Iz_3_1009	DJI_0002	Bromus tectorum L.	0	a	0	0	Poaceae Barnhart
Iz_3_1009	DJI_0002	Calamagrostis epigejos (L.) Roth	3	n	1	0	Poaceae Barnhart
Iz_3_1009	DJI_0002	Carduus acanthoides L.	0	n	1	0	Asteraceae Bercht. & J.Presl
Iz_3_1009	DJI_0002	Carex sp.	0	n	1	0	Cyperaceae Juss.
Iz_3_1009	DJI_0002	Centaurea majorovii Dumbadze	0	n	1	0	Asteraceae Bercht. & J.Presl
Iz_3_1009	DJI_0002	Chamaecytisus ruthenicus (Fisch.ex Wol.) Klaskova	0	n	1	0	Fabaceae Lindl.
Iz_3_1009	DJI_0002	Chelidonium majus L.	2	n	1	0	Papaveraceae Juss.
Iz_3_1009	DJI_0002	Chenopodium betaceum Andrz.	0	n	1	0	Amaranthaceae Juss.
Iz_3_1009	DJI_0002	Chenopodium cf. album	0	n	1	0	Amaranthaceae Juss.
Iz_3_1009	DJI_0002	Chenopodium sp.	0		NA	0	Amaranthaceae Juss.
Iz_3_1009	DJI_0002	Chondrilla juncea L.	0	n	1	0	Asteraceae Bercht. & J.Presl
Iz_3_1009	DJI_0002	Cirsium vulgare (Savi) Ten.	0	n	1	0	Asteraceae Bercht. & J.Presl
Iz_3_1009	DJI_0002	Clinopodium acinos (L.) Kuntze	0	n	1	0	Lamiacea Lindl.
Iz_3_1009	DJI_0002	Convallaria majalis L.	0	n	1	0	Asparagaceae Juss.
Iz_3_1009	DJI_0002	Crataegus rhipidophylla Gand.	0	n	1	1	Rosaceae Juss.
Iz_3_1009	DJI_0002	Crepis foetida L.	0	n	1	0	Asteraceae Bercht. & J.Presl
Iz_3_1009	DJI_0002	Dactylis glomerata L.	0	n	1	0	Poaceae Barnhart
Iz_3_1009	DJI_0002	Dianthus campestris M.Bieb.	0	n	1	0	Caryophyllaceae Juss.
Iz_3_1009	DJI_0002	Digitaria sanguinalis (L.) Scop.	0	a	0	0	Poaceae Barnhart
Iz_3_1009	DJI_0002	Elymus repens (L.) Gould	0	n	1	0	Poaceae Barnhart
Iz_3_1009	DJI_0002	Epilobium tetragonum L.	0	n	1	0	Onagraceae Juss.
Iz_3_1009	DJI_0002	Eragrostis minor Host	0	a	0	0	Poaceae Barnhart
Iz_3_1009	DJI_0002	Erigeron canadensis L.	0	a	0	0	Asteraceae Bercht. & J.Presl
Iz_3_1009	DJI_0002	Euphorbia seguieriana Neck.	0	n	1	0	Euphorbiaceae Juss.
Iz_3_1009	DJI_0002	Fallopia dumetorum (L.) Holub	1	n	1	0	Polygonaceae Juss.
Iz_3_1009	DJI_0002	Festuca beckeri (Hack.) Trautv.	0	n	1	0	Poaceae Barnhart
Iz_3_1009	DJI_0002	Fragaria vesca L.	0	n	1	0	Rosaceae Juss.
Iz_3_1009	DJI_0002	Frangula alnus Mill.	0	n	1	1	Rhamnaceae Juss.
Iz_3_1009	DJI_0002	Fraxinus pennsylvanica Marshall	0	a	0	1	Oleaceae Hoffmanns. & Link
Iz_3_1009	DJI_0002	Galeopsis bifida Boenn.	0	n	1	0	Lamiacea Lindl.
Iz_3_1009	DJI_0002	Galium verum L.	0	n	1	0	Rubiaceae Juss.
Iz_3_1009	DJI_0002	Genista tinctoria L.	0	n	1	0	Fabaceae Lindl.
Iz_3_1009	DJI_0002	Geranium robertianum L.	0	n	1	0	Geraniaceae Juss.
Iz_3_1009	DJI_0002	Geranium sanquineum L.	0	n	1	0	Geraniaceae Juss.
Iz_3_1009	DJI_0002	Gypsophila paniculata L.	0	n	1	0	Caryophyllaceae Juss.
Iz_3_1009	DJI_0002	Helichrysum arenarium (L.) Moench	0	n	1	0	Asteraceae Bercht. & J.Presl
Iz_3_1009	DJI_0002	Herniaria polygama J.Gay	0	n	1	0	Caryophyllaceae Juss.
Iz_3_1009	DJI_0002	Hieracium robustum Fr.	0	n	1	0	Asteraceae Bercht. & J.Presl
Iz_3_1009	DJI_0002	Hieracium umbellatum L.	0	n	1	NA	Asteraceae Bercht. & J.Presl

Iz_3_1009	DJI_0002	Hylotelephium maximum (L.) Holub	0	n	1	0	Crassulaceae J.St.-Hil.
Iz_3_1009	DJI_0002	Jacobaea borysthenica (DC.) B.Nord. & Greuter	0	n	1	0	Asteraceae Bercht. & J.Presl
Iz_3_1009	DJI_0002	Jasione montana L.	0	n	1	0	Campanulaceae Juss.
Iz_3_1009	DJI_0002	Jurinea cyanoides (L.) Rchb.s.l.	0	n	1	0	Asteraceae Bercht. & J.Presl
Iz_3_1009	DJI_0002	Koeleria glauca (Spreng.) DC.	0	n	1	0	Poaceae Barnhart
Iz_3_1009	DJI_0002	Lactuca serriola L.	0	a	0	0	Asteraceae Bercht. & J.Presl
Iz_3_1009	DJI_0002	Linaria genistifolia (L.) Mill.	0	n	1	0	Plantaginaceae Juss.
Iz_3_1009	DJI_0002	Lomelosia argentea (L.) Greuter & Burdet	0	n	1	0	Caprifoliaceae Juss.
Iz_3_1009	DJI_0002	Melampyrum pratense L.	0	n	1	0	Orobanchaceae Vent.
Iz_3_1009	DJI_0002	Oenothera biennis L.	0	a	0	0	Onagraceae Juss.
Iz_3_1009	DJI_0002	Pentanema britannica (L.) D.Gut.Larr., Santos-Vicente, Anderb., E.Rico & M.M.Mart.Ort.	0	n	1	0	Asteraceae Bercht. & J.Presl
Iz_3_1009	DJI_0002	Peucedanum oreoselinum (L.) Moench	0	n	1	0	Apiaceae Lindl.
Iz_3_1009	DJI_0002	Peucedanum ruthenicum M.Bieb.	0	n	1	0	Apiaceae Lindl.
Iz_3_1009	DJI_0002	Pilosella echioides (Lumn.) F.W.Schultz & Sch.Bip.	0	n	1	0	Asteraceae Bercht. & J.Presl
Iz_3_1009	DJI_0002	Pilosella officinarum F. Schultz et Sch. Bip.	0	n	1	0	Asteraceae Bercht. & J.Presl
Iz_3_1009	DJI_0002	Pilosella sp.	0		NA	0	Asteraceae Bercht. & J.Presl
Iz_3_1009	DJI_0002	Pinus sylvestris L.	0	n	1	1	Pinaceae Spreng. ex F.Rudolphi
Iz_3_1009	DJI_0002	Poa annua L.	0	n	1	0	Poaceae Barnhart
Iz_3_1009	DJI_0002	Poa nemoralis L.	0	n	1	0	Poaceae Barnhart
Iz_3_1009	DJI_0002	Poa sp.	0		NA	0	Poaceae Barnhart
Iz_3_1009	DJI_0002	Polygonatum odoratum (Mill.) Druce	0	n	1	0	Asparagaceae Juss.
Iz_3_1009	DJI_0002	Polygonum arenarium Waldst. & Kit.	0	n	1	0	Polygonaceae Juss.
Iz_3_1009	DJI_0002	Populus nigra L.	0	n	1	1	Salicaceae Mirb.
Iz_3_1009	DJI_0002	Populus tremula L.	0	n	1	1	Salicaceae Mirb.
Iz_3_1009	DJI_0002	Populus Ã canescens (Aiton) Sm.	0	n	1	1	Salicaceae Mirb.
Iz_3_1009	DJI_0002	Potentilla argentea L.	0	n	1	0	Rosaceae Juss.
Iz_3_1009	DJI_0002	Psammophiliella muralis (L.) Ikonn.	0	n	1	0	Caryophyllaceae Juss.
Iz_3_1009	DJI_0002	Psephellus sumensis (Kalen.) Greuter	0	n	1	0	Asteraceae Bercht. & J.Presl
Iz_3_1009	DJI_0002	Pulsatilla pratensis (L.) Mill. s.l.	0	n	1	0	Ranunculaceae Juss.
Iz_3_1009	DJI_0002	Pyrus communis L.	0	a	0	1	Rosaceae Juss.
Iz_3_1009	DJI_0002	Quercus robur L.	0	n	1	1	Fagaceae Dumort.
Iz_3_1009	DJI_0002	Robinia pseudoacacia L.	0	a	0	1	Fabaceae Lindl.
Iz_3_1009	DJI_0002	Rosa sp.	0		NA	1	Rosaceae Juss.
Iz_3_1009	DJI_0002	Rubus idaeus L.	0	n	1	0	Rosaceae Juss.
Iz_3_1009	DJI_0002	Rumex acetosella L.	0	n	1	0	Polygonaceae Juss.
Iz_3_1009	DJI_0002	Rumex thyrsiflorus Fingerh.	0	n	1	0	Polygonaceae Juss.
Iz_3_1009	DJI_0002	Salix alba L.	0	n	1	1	Salicaceae Mirb.
Iz_3_1009	DJI_0002	Sambucus nigra L.	0	n	1	1	Viburnaceae Raf.
Iz_3_1009	DJI_0002	Secale sylvestre Host	0	n	1	0	Poaceae Barnhart
Iz_3_1009	DJI_0002	Sempervivum ruthenicum Schnittsp. & C.B. Lehm.	0	n	1	0	Crassulaceae J.St.-Hil.
Iz_3_1009	DJI_0002	Setaria viridis (L.) P. Beauv.	0	n	1	0	Poaceae Barnhart
Iz_3_1009	DJI_0002	Silene borysthenica (Gruner) Walters	0	n	1	0	Caryophyllaceae Juss.
Iz_3_1009	DJI_0002	Silene latifolia subsp. alba (Mill.) Greuter & Burdet	0	n	1	0	Caryophyllaceae Juss.
Iz_3_1009	DJI_0002	Solidago virgaurea L.	0	n	1	0	Asteraceae Bercht. & J.Presl
Iz_3_1009	DJI_0002	Sonchus oleraceus L.	0	a	0	0	Asteraceae Bercht. & J.Presl
Iz_3_1009	DJI_0002	Sorbus aucuparia L.	0	n	1	1	Rosaceae Juss.
Iz_3_1009	DJI_0002	Stachys recta L.	0	n	1	0	Lamiacea Lindl.
Iz_3_1009	DJI_0002	Tanacetum vulgare L.	0	n	1	0	Asteraceae Bercht. & J.Presl
Iz_3_1009	DJI_0002	Thymus pallasianus Heinr.Braun	0	n	1	0	Lamiacea Lindl.
Iz_3_1009	DJI_0002	Tilia cordata Mill.	0	n	1	1	Malvaceae Juss.
Iz_3_1009	DJI_0002	Trifolium alpestre L.	0	n	1	0	Fabaceae Lindl.
Iz_3_1009	DJI_0002	Trifolium arvense L.	0	n	1	0	Fabaceae Lindl.
Iz_3_1009	DJI_0002	Ulmus glabra Huds.	0	n	1	1	Ulmaceae Mirb.
Iz_3_1009	DJI_0002	Verbascum densiflorum Bertol.	0	n	1	0	Scrophulariaceae Juss.
Iz_3_1009	DJI_0002	Veronica austriaca L.	0	n	1	0	Plantaginaceae Juss.
Iz_3_1009	DJI_0002	Veronica incana L.	0	n	1	0	Plantaginaceae Juss.
Iz_3_1009	DJI_0002	Veronica verna L.	0	n	1	0	Plantaginaceae Juss.
Iz_3_1009	DJI_0002	Vincetoxicum hirundinaria Medik	0	n	1	0	Apocynaceae Juss.
Iz_3_1009	DJI_0004	Acer tataricum L.	0	n	1	1	Sapindaceae Juss.
Iz_3_1009	DJI_0004	Achillea millefolium L. s.l.	0	n	1	0	Asteraceae Bercht. & J.Presl
Iz_3_1009	DJI_0004	Allium oleraceum L.	0	n	1	0	Amaryllidaceae J.St.-Hil.
Iz_3_1009	DJI_0004	Ambrosia artemisiifolia L.	0	a	0	0	Asteraceae Bercht. & J.Presl
Iz_3_1009	DJI_0004	Amorpha fruticosa L.	0	a	0	0	Fabaceae Lindl.
Iz_3_1009	DJI_0004	Anthericum ramosum L.	0	n	1	0	Asparagaceae Juss.
Iz_3_1009	DJI_0004	Artemisia marschalliana Spreng.	0	n	1	0	Asteraceae Bercht. & J.Presl
Iz_3_1009	DJI_0004	Asparagus officinalis L.	0	n	1	0	Asparagaceae Juss.
Iz_3_1009	DJI_0004	Astragalus cicer L.	0	n	1	0	Fabaceae Lindl.
Iz_3_1009	DJI_0004	Bassia laniflora (S.G.Gmel.) A.J.Scott	0	n	1	0	Amaranthaceae Juss.
Iz_3_1009	DJI_0004	Berberis vulgaris L.	0	n	1	0	Berberidaceae Juss.
Iz_3_1009	DJI_0004	Betula pendula Roth.	0	n	1	1	Betulaceae Gray
Iz_3_1009	DJI_0004	Betula pendula Roth.	0	n	1	0	Poaceae Barnhart
Iz_3_1009	DJI_0004	Bromus inermis Leyss.	0		NA	NA	
Iz_3_1009	DJI_0004	Bromus squarrosus L.	0	a	0	0	Poaceae Barnhart
Iz_3_1009	DJI_0004	Bromus tectorum L.	0	a	0	0	Poaceae Barnhart
Iz_3_1009	DJI_0004	Calamagrostis epigejos (L.) Roth	1	n	1	0	Poaceae Barnhart
Iz_3_1009	DJI_0004	Carduus acanthoides L.	0	n	1	0	Asteraceae Bercht. & J.Presl
Iz_3_1009	DJI_0004	Carex sp.	0	n	1	0	Cyperaceae Juss.
Iz_3_1009	DJI_0004	Centaurea majorovii Dumbadze	0	n	1	0	Asteraceae Bercht. & J.Presl
Iz_3_1009	DJI_0004	Chamaecytisus ruthenicus (Fisch.ex Wol.) Klaskova	5	n	1	0	Fabaceae Lindl.
Iz_3_1009	DJI_0004	Chelidonium majus L.	1	n	1	0	Papaveraceae Juss.
Iz_3_1009	DJI_0004	Chenopodium betaceum Andrz.	0	n	1	0	Amaranthaceae Juss.
Iz_3_1009	DJI_0004	Chenopodium cf. album	0	n	1	0	Amaranthaceae Juss.
Iz_3_1009	DJI_0004	Chenopodium sp.	0		NA	0	Amaranthaceae Juss.
Iz_3_1009	DJI_0004	Chondrilla juncea L.	0	n	1	0	Asteraceae Bercht. & J.Presl
Iz_3_1009	DJI_0004	Cirsium vulgare (Savi) Ten.	0	n	1	0	Asteraceae Bercht. & J.Presl
Iz_3_1009	DJI_0004	Clinopodium acinos (L.) Kuntze	0	n	1	0	Lamiacea Lindl.
Iz_3_1009	DJI_0004	Convallaria majalis L.	0	n	1	0	Asparagaceae Juss.
Iz_3_1009	DJI_0004	Crataegus rhipidophylla Gand.	0	n	1	1	Rosaceae Juss.
Iz_3_1009	DJI_0004	Crepis foetida L.	0	n	1	0	Asteraceae Bercht. & J.Presl
Iz_3_1009	DJI_0004	Dactylis glomerata L.	0	n	1	0	Poaceae Barnhart
Iz_3_1009	DJI_0004	Dianthus campestris M.Bieb.	0	n	1	0	Caryophyllaceae Juss.
Iz_3_1009	DJI_0004	Digitaria sanguinalis (L.) Scop.	0	a	0	0	Poaceae Barnhart
Iz_3_1009	DJI_0004	Elymus repens (L.) Gould	0	n	1	0	Poaceae Barnhart
Iz_3_1009	DJI_0004	Epilobium tetragonum L.	0	n	1	0	Onagraceae Juss.
Iz_3_1009	DJI_0004	Eragrostis minor Host	0	a	0	0	Poaceae Barnhart
Iz_3_1009	DJI_0004	Erigeron canadensis L.	0	a	0	0	Asteraceae Bercht. & J.Presl
Iz_3_1009	DJI_0004	Euphorbia seguieriana Neck.	0	n	1	0	Euphorbiaceae Juss.
Iz_3_1009	DJI_0004	Fallopia dumetorum (L.) Holub	0	n	1	0	Polygonaceae Juss.

Iz_3_1009	DJI_0004	Festuca beckeri (Hack.) Trautv.	0	n	1	0	Poaceae Barnhart
Iz_3_1009	DJI_0004	Fragaria vesca L.	0	n	1	0	Rosaceae Juss.
Iz_3_1009	DJI_0004	Frangula alnus Mill.	0	n	1	1	Rhamnaceae Juss.
Iz_3_1009	DJI_0004	Fraxinus pennsylvanica Marshall	0	a	0	1	Oleaceae Hoffmanns. & Link
Iz_3_1009	DJI_0004	Galeopsis bifida Boenn.	0	n	1	0	Lamiacea Lindl.
Iz_3_1009	DJI_0004	Galium verum L.	0	n	1	0	Rubiaceae Juss.
Iz_3_1009	DJI_0004	Genista tinctoria L.	0	n	1	0	Fabaceae Lindl.
Iz_3_1009	DJI_0004	Geranium robertianum L.	0	n	1	0	Geraniaceae Juss.
Iz_3_1009	DJI_0004	Geranium sanquineum L.	0	n	1	0	Geraniaceae Juss.
Iz_3_1009	DJI_0004	Gypsophila paniculata L.	0	n	1	0	Caryophyllaceae Juss.
Iz_3_1009	DJI_0004	Helichrysum arenarium (L.) Moench	0	n	1	0	Asteraceae Bercht. & J.Presl
Iz_3_1009	DJI_0004	Herniaria polygama J.Gay	0	n	1	0	Caryophyllaceae Juss.
Iz_3_1009	DJI_0004	Hieracium robustum Fr.	0	n	1	0	Asteraceae Bercht. & J.Presl
Iz_3_1009	DJI_0004	Hieracium umbellatum L.	0	n	1	NA	Asteraceae Bercht. & J.Presl
Iz_3_1009	DJI_0004	Hylotelephium maximum (L.) Holub	0	n	1	0	Crassulaceae J.St.-Hil.
Iz_3_1009	DJI_0004	Jacobaea borysthenica (DC.) B.Nord. & Greuter	0	n	1	0	Asteraceae Bercht. & J.Presl
Iz_3_1009	DJI_0004	Jasione montana L.	0	n	1	0	Campanulaceae Juss.
Iz_3_1009	DJI_0004	Jurinea cyanoides (L.) Rchb.s.l.	0	n	1	0	Asteraceae Bercht. & J.Presl
Iz_3_1009	DJI_0004	Koeleria glauca (Spreng.) DC.	0	n	1	0	Poaceae Barnhart
Iz_3_1009	DJI_0004	Lactuca serriola L.	0	a	0	0	Asteraceae Bercht. & J.Presl
Iz_3_1009	DJI_0004	Linaria genistifolia (L.) Mill.	0	n	1	0	Plantaginaceae Juss.
Iz_3_1009	DJI_0004	Lomelosia argentea (L.) Greuter & Burdet	0	n	1	0	Caprifoliaceae Juss.
Iz_3_1009	DJI_0004	Melampyrum pratense L.	0	n	1	0	Orobanchaceae Vent.
Iz_3_1009	DJI_0004	Oenothera biennis L.	0	a	0	0	Onagraceae Juss.
Iz_3_1009	DJI_0004	Pentanema britannica (L.) D.Gut.Larr., Santos-Vicente, Anderb., E.Rico & M.M.Mart.Ort.	0	n	1	0	Asteraceae Bercht. & J.Presl
Iz_3_1009	DJI_0004	Peucedanum oreoselinum (L.) Moench	2	n	1	0	Apiaceae Lindl.
Iz_3_1009	DJI_0004	Peucedanum ruthenicum M.Bieb.	0	n	1	0	Apiaceae Lindl.
Iz_3_1009	DJI_0004	Pilosella echioides (Lumn.) F.W.Schultz & Sch.Bip.	0	n	1	0	Asteraceae Bercht. & J.Presl
Iz_3_1009	DJI_0004	Pilosella officinarum F. Schultz et Sch. Bip.	0	n	1	0	Asteraceae Bercht. & J.Presl
Iz_3_1009	DJI_0004	Pilosella sp.	0		NA	0	Asteraceae Bercht. & J.Presl
Iz_3_1009	DJI_0004	Pinus sylvestris L.	0	n	1	1	Pinaceae Spreng. ex F.Rudolphi
Iz_3_1009	DJI_0004	Poa annua L.	0	n	1	0	Poaceae Barnhart
Iz_3_1009	DJI_0004	Poa nemoralis L.	0	n	1	0	Poaceae Barnhart
Iz_3_1009	DJI_0004	Poa sp.	0		NA	0	Poaceae Barnhart
Iz_3_1009	DJI_0004	Polygonatum odoratum (Mill.) Druce	0	n	1	0	Asparagaceae Juss.
Iz_3_1009	DJI_0004	Polygonum arenarium Waldst. & Kit.	0	n	1	0	Polygonaceae Juss.
Iz_3_1009	DJI_0004	Populus nigra L.	0	n	1	1	Salicaceae Mirb.
Iz_3_1009	DJI_0004	Populus tremula L.	0	n	1	1	Salicaceae Mirb.
Iz_3_1009	DJI_0004	Populus Ã canescens (Aiton) Sm.	0	n	1	1	Salicaceae Mirb.
Iz_3_1009	DJI_0004	Potentilla argentea L.	0	n	1	0	Rosaceae Juss.
Iz_3_1009	DJI_0004	Psammophiliella muralis (L.) Ikonn.	0	n	1	0	Caryophyllaceae Juss.
Iz_3_1009	DJI_0004	Psephellus sumensis (Kalen.) Greuter	0	n	1	0	Asteraceae Bercht. & J.Presl
Iz_3_1009	DJI_0004	Pulsatilla pratensis (L.) Mill. s.l.	0	n	1	0	Ranunculaceae Juss.
Iz_3_1009	DJI_0004	Pyrus communis L.	0	a	0	1	Rosaceae Juss.
Iz_3_1009	DJI_0004	Quercus robur L.	0	n	1	1	Fagaceae Dumort.
Iz_3_1009	DJI_0004	Robinia pseudoacacia L.	0	a	0	1	Fabaceae Lindl.
Iz_3_1009	DJI_0004	Rosa sp.	0		NA	1	Rosaceae Juss.
Iz_3_1009	DJI_0004	Rubus idaeus L.	0	n	1	0	Rosaceae Juss.
Iz_3_1009	DJI_0004	Rumex acetosella L.	0	n	1	0	Polygonaceae Juss.
Iz_3_1009	DJI_0004	Rumex thyrsiflorus Fingerh.	0	n	1	0	Polygonaceae Juss.
Iz_3_1009	DJI_0004	Salix alba L.	0	n	1	1	Salicaceae Mirb.
Iz_3_1009	DJI_0004	Sambucus nigra L.	0	n	1	1	Viburnaceae Raf.
Iz_3_1009	DJI_0004	Secale sylvestre Host	0	n	1	0	Poaceae Barnhart
Iz_3_1009	DJI_0004	Sempervivum ruthenicum Schnittsp. & C.B. Lehm.	0	n	1	0	Crassulaceae J.St.-Hil.
Iz_3_1009	DJI_0004	Setaria viridis (L.) P. Beauv.	0	n	1	0	Poaceae Barnhart
Iz_3_1009	DJI_0004	Silene borysthenica (Gruner) Walters	0	n	1	0	Caryophyllaceae Juss.
Iz_3_1009	DJI_0004	Silene latifolia subsp. alba (Mill.) Greuter & Burdet	0	n	1	0	Caryophyllaceae Juss.
Iz_3_1009	DJI_0004	Solidago virgaurea L.	0	n	1	0	Asteraceae Bercht. & J.Presl
Iz_3_1009	DJI_0004	Sonchus oleraceus L.	0	a	0	0	Asteraceae Bercht. & J.Presl
Iz_3_1009	DJI_0004	Sorbus aucuparia L.	0	n	1	1	Rosaceae Juss.
Iz_3_1009	DJI_0004	Stachys recta L.	0	n	1	0	Lamiacea Lindl.
Iz_3_1009	DJI_0004	Tanacetum vulgare L.	0	n	1	0	Asteraceae Bercht. & J.Presl
Iz_3_1009	DJI_0004	Thymus pallasianus Heinr.Braun	0	n	1	0	Lamiacea Lindl.
Iz_3_1009	DJI_0004	Tilia cordata Mill.	0	n	1	1	Malvaceae Juss.
Iz_3_1009	DJI_0004	Trifolium alpestre L.	0	n	1	0	Fabaceae Lindl.
Iz_3_1009	DJI_0004	Trifolium arvense L.	0	n	1	0	Fabaceae Lindl.
Iz_3_1009	DJI_0004	Ulmus glabra Huds.	0	n	1	1	Ulmaceae Mirb.
Iz_3_1009	DJI_0004	Verbascum densiflorum Bertol.	0	n	1	0	Scrophulariaceae Juss.
Iz_3_1009	DJI_0004	Veronica austriaca L.	0	n	1	0	Plantaginaceae Juss.
Iz_3_1009	DJI_0004	Veronica incana L.	0	n	1	0	Plantaginaceae Juss.
Iz_3_1009	DJI_0004	Veronica verna L.	0	n	1	0	Plantaginaceae Juss.
Iz_3_1009	DJI_0004	Vincetoxicum hirundinaria Medik	0	n	1	0	Apocynaceae Juss.
Iz_3_1009	DJI_0831	Acer tataricum L.	0	n	1	1	Sapindaceae Juss.
Iz_3_1009	DJI_0831	Achillea millefolium L. s.l.	0	n	1	0	Asteraceae Bercht. & J.Presl
Iz_3_1009	DJI_0831	Allium oleraceum L.	0	n	1	0	Amaryllidaceae J.St.-Hil.
Iz_3_1009	DJI_0831	Ambrosia artemisiifolia L.	0	a	0	0	Asteraceae Bercht. & J.Presl
Iz_3_1009	DJI_0831	Amorpha fruticosa L.	0	a	0	0	Fabaceae Lindl.
Iz_3_1009	DJI_0831	Anthericum ramosum L.	0	n	1	0	Asparagaceae Juss.
Iz_3_1009	DJI_0831	Artemisia marschalliana Spreng.	0	n	1	0	Asteraceae Bercht. & J.Presl
Iz_3_1009	DJI_0831	Asparagus officinalis L.	0	n	1	0	Asparagaceae Juss.
Iz_3_1009	DJI_0831	Astragalus cicer L.	0	n	1	0	Fabaceae Lindl.
Iz_3_1009	DJI_0831	Bassia laniflora (S.G.Gmel.) A.J.Scott	0	n	1	0	Amaranthaceae Juss.
Iz_3_1009	DJI_0831	Berberis vulgaris L.	0	n	1	0	Berberidaceae Juss.
Iz_3_1009	DJI_0831	Betula pendula Roth.	0	n	1	1	Betulaceae Gray
Iz_3_1009	DJI_0831	Betula pendula Roth.	0	n	1	0	Poaceae Barnhart
Iz_3_1009	DJI_0831	Bromus inermis Leyss.	0		NA	NA	
Iz_3_1009	DJI_0831	Bromus squarrosus L.	0	a	0	0	Poaceae Barnhart
Iz_3_1009	DJI_0831	Bromus tectorum L.	0	a	0	0	Poaceae Barnhart
Iz_3_1009	DJI_0831	Calamagrostis epigejos (L.) Roth	25	n	1	0	Poaceae Barnhart
Iz_3_1009	DJI_0831	Carduus acanthoides L.	0	n	1	0	Asteraceae Bercht. & J.Presl
Iz_3_1009	DJI_0831	Carex sp.	0	n	1	0	Cyperaceae Juss.
Iz_3_1009	DJI_0831	Centaurea majorovii Dumbadze	0	n	1	0	Asteraceae Bercht. & J.Presl
Iz_3_1009	DJI_0831	Chamaecytisus ruthenicus (Fisch.ex Wol.) Klaskova	0	n	1	0	Fabaceae Lindl.
Iz_3_1009	DJI_0831	Chelidonium majus L.	0	n	1	0	Papaveraceae Juss.
Iz_3_1009	DJI_0831	Chenopodium betaceum Andrz.	0	n	1	0	Amaranthaceae Juss.
Iz_3_1009	DJI_0831	Chenopodium cf. album	0	n	1	0	Amaranthaceae Juss.
Iz_3_1009	DJI_0831	Chenopodium sp.	0		NA	0	Amaranthaceae Juss.
Iz_3_1009	DJI_0831	Chondrilla juncea L.	0	n	1	0	Asteraceae Bercht. & J.Presl

Iz_3_1009	DJI_0831	Cirsium vulgare (Savi) Ten.	0	n	1	0	Asteraceae Bercht. & J.Presl
Iz_3_1009	DJI_0831	Clinopodium acinos (L.) Kuntze	0	n	1	0	Lamiacea Lindl.
Iz_3_1009	DJI_0831	Convallaria majalis L.	0	n	1	0	Asparagaceae Juss.
Iz_3_1009	DJI_0831	Crataegus rhipidophylla Gand.	0	n	1	1	Rosaceae Juss.
Iz_3_1009	DJI_0831	Crepis foetida L.	0	n	1	0	Asteraceae Bercht. & J.Presl
Iz_3_1009	DJI_0831	Dactylis glomerata L.	0	n	1	0	Poaceae Barnhart
Iz_3_1009	DJI_0831	Dianthus campestris M.Bieb.	0	n	1	0	Caryophyllaceae Juss.
Iz_3_1009	DJI_0831	Digitaria sanguinalis (L.) Scop.	0	a	0	0	Poaceae Barnhart
Iz_3_1009	DJI_0831	Elymus repens (L.) Gould	0	n	1	0	Poaceae Barnhart
Iz_3_1009	DJI_0831	Epilobium tetragonum L.	0	n	1	0	Onagraceae Juss.
Iz_3_1009	DJI_0831	Eragrostis minor Host	0	a	0	0	Poaceae Barnhart
Iz_3_1009	DJI_0831	Erigeron canadensis L.	1	a	0	0	Asteraceae Bercht. & J.Presl
Iz_3_1009	DJI_0831	Euphorbia seguieriana Neck.	0	n	1	0	Euphorbiaceae Juss.
Iz_3_1009	DJI_0831	Fallopia dumetorum (L.) Holub	0	n	1	0	Polygonaceae Juss.
Iz_3_1009	DJI_0831	Festuca beckeri (Hack.) Trautv.	0	n	1	0	Poaceae Barnhart
Iz_3_1009	DJI_0831	Fragaria vesca L.	0	n	1	0	Rosaceae Juss.
Iz_3_1009	DJI_0831	Frangula alnus Mill.	0	n	1	1	Rhamnaceae Juss.
Iz_3_1009	DJI_0831	Fraxinus pennsylvanica Marshall	0	a	0	1	Oleaceae Hoffmanns. & Link
Iz_3_1009	DJI_0831	Galeopsis bifida Boenn.	0	n	1	0	Lamiacea Lindl.
Iz_3_1009	DJI_0831	Galium verum L.	0	n	1	0	Rubiaceae Juss.
Iz_3_1009	DJI_0831	Genista tinctoria L.	0	n	1	0	Fabaceae Lindl.
Iz_3_1009	DJI_0831	Geranium robertianum L.	0	n	1	0	Geraniaceae Juss.
Iz_3_1009	DJI_0831	Geranium sanquineum L.	0	n	1	0	Geraniaceae Juss.
Iz_3_1009	DJI_0831	Gypsophila paniculata L.	0	n	1	0	Caryophyllaceae Juss.
Iz_3_1009	DJI_0831	Helichrysum arenarium (L.) Moench	0	n	1	0	Asteraceae Bercht. & J.Presl
Iz_3_1009	DJI_0831	Herniaria polygama J.Gay	0	n	1	0	Caryophyllaceae Juss.
Iz_3_1009	DJI_0831	Hieracium robustum Fr.	0	n	1	0	Asteraceae Bercht. & J.Presl
Iz_3_1009	DJI_0831	Hieracium umbellatum L.	0	n	1	NA	Asteraceae Bercht. & J.Presl
Iz_3_1009	DJI_0831	Hylotelephium maximum (L.) Holub	0	n	1	0	Crassulaceae J.St.-Hil.
Iz_3_1009	DJI_0831	Jacobaea borysthenica (DC.) B.Nord. & Greuter	0	n	1	0	Asteraceae Bercht. & J.Presl
Iz_3_1009	DJI_0831	Jasione montana L.	0	n	1	0	Campanulaceae Juss.
Iz_3_1009	DJI_0831	Jurinea cyanoides (L.) Rchb.s.l.	0	n	1	0	Asteraceae Bercht. & J.Presl
Iz_3_1009	DJI_0831	Koeleria glauca (Spreng.) DC.	0	n	1	0	Poaceae Barnhart
Iz_3_1009	DJI_0831	Lactuca serriola L.	0	a	0	0	Asteraceae Bercht. & J.Presl
Iz_3_1009	DJI_0831	Linaria genistifolia (L.) Mill.	0	n	1	0	Plantaginaceae Juss.
Iz_3_1009	DJI_0831	Lomelosia argentea (L.) Greuter & Burdet	0	n	1	0	Caprifoliaceae Juss.
Iz_3_1009	DJI_0831	Melampyrum pratense L.	0	n	1	0	Orobanchaceae Vent.
Iz_3_1009	DJI_0831	Oenothera biennis L.	0	a	0	0	Onagraceae Juss.
Iz_3_1009	DJI_0831	Pentanema britannica (L.) D.Gut.Larr., Santos-Vicente, Anderb., E.Rico & M.M.Mart.Ort.	0	n	1	0	Asteraceae Bercht. & J.Presl
Iz_3_1009	DJI_0831	Peucedanum oreoselinum (L.) Moench	0	n	1	0	Apiaceae Lindl.
Iz_3_1009	DJI_0831	Peucedanum ruthenicum M.Bieb.	0	n	1	0	Apiaceae Lindl.
Iz_3_1009	DJI_0831	Pilosella echioides (Lumn.) F.W.Schultz & Sch.Bip.	0	n	1	0	Asteraceae Bercht. & J.Presl
Iz_3_1009	DJI_0831	Pilosella officinarum F. Schultz et Sch. Bip.	0	n	1	0	Asteraceae Bercht. & J.Presl
Iz_3_1009	DJI_0831	Pilosella sp.	0		NA	0	Asteraceae Bercht. & J.Presl
Iz_3_1009	DJI_0831	Pinus sylvestris L.	0	n	1	1	Pinaceae Spreng. ex F.Rudolphi
Iz_3_1009	DJI_0831	Poa annua L.	0	n	1	0	Poaceae Barnhart
Iz_3_1009	DJI_0831	Poa nemoralis L.	0	n	1	0	Poaceae Barnhart
Iz_3_1009	DJI_0831	Poa sp.	0		NA	0	Poaceae Barnhart
Iz_3_1009	DJI_0831	Polygonatum odoratum (Mill.) Druce	0	n	1	0	Asparagaceae Juss.
Iz_3_1009	DJI_0831	Polygonum arenarium Waldst. & Kit.	0	n	1	0	Polygonaceae Juss.
Iz_3_1009	DJI_0831	Populus nigra L.	0	n	1	1	Salicaceae Mirb.
Iz_3_1009	DJI_0831	Populus tremula L.	0	n	1	1	Salicaceae Mirb.
Iz_3_1009	DJI_0831	Populus Ã canescens (Aiton) Sm.	0	n	1	1	Salicaceae Mirb.
Iz_3_1009	DJI_0831	Potentilla argentea L.	0	n	1	0	Rosaceae Juss.
Iz_3_1009	DJI_0831	Psammophiliella muralis (L.) Ikonn.	0	n	1	0	Caryophyllaceae Juss.
Iz_3_1009	DJI_0831	Psephellus sumensis (Kalen.) Greuter	0	n	1	0	Asteraceae Bercht. & J.Presl
Iz_3_1009	DJI_0831	Pulsatilla pratensis (L.) Mill. s.l.	0	n	1	0	Ranunculaceae Juss.
Iz_3_1009	DJI_0831	Pyrus communis L.	0	a	0	1	Rosaceae Juss.
Iz_3_1009	DJI_0831	Quercus robur L.	7	n	1	1	Fagaceae Dumort.
Iz_3_1009	DJI_0831	Robinia pseudoacacia L.	0	a	0	1	Fabaceae Lindl.
Iz_3_1009	DJI_0831	Rosa sp.	0		NA	1	Rosaceae Juss.
Iz_3_1009	DJI_0831	Rubus idaeus L.	0	n	1	0	Rosaceae Juss.
Iz_3_1009	DJI_0831	Rumex acetosella L.	0	n	1	0	Polygonaceae Juss.
Iz_3_1009	DJI_0831	Rumex thyrsiflorus Fingerh.	0	n	1	0	Polygonaceae Juss.
Iz_3_1009	DJI_0831	Salix alba L.	0	n	1	1	Salicaceae Mirb.
Iz_3_1009	DJI_0831	Sambucus nigra L.	0	n	1	1	Viburnaceae Raf.
Iz_3_1009	DJI_0831	Secale sylvestre Host	0	n	1	0	Poaceae Barnhart
Iz_3_1009	DJI_0831	Sempervivum ruthenicum Schnittsp. & C.B. Lehm.	0	n	1	0	Crassulaceae J.St.-Hil.
Iz_3_1009	DJI_0831	Setaria viridis (L.) P. Beauv.	0	n	1	0	Poaceae Barnhart
Iz_3_1009	DJI_0831	Silene borysthenica (Gruner) Walters	0	n	1	0	Caryophyllaceae Juss.
Iz_3_1009	DJI_0831	Silene latifolia subsp. alba (Mill.) Greuter & Burdet	0	n	1	0	Caryophyllaceae Juss.
Iz_3_1009	DJI_0831	Solidago virgaurea L.	0	n	1	0	Asteraceae Bercht. & J.Presl
Iz_3_1009	DJI_0831	Sonchus oleraceus L.	0	a	0	0	Asteraceae Bercht. & J.Presl
Iz_3_1009	DJI_0831	Sorbus aucuparia L.	0	n	1	1	Rosaceae Juss.
Iz_3_1009	DJI_0831	Stachys recta L.	0	n	1	0	Lamiacea Lindl.
Iz_3_1009	DJI_0831	Tanacetum vulgare L.	0	n	1	0	Asteraceae Bercht. & J.Presl
Iz_3_1009	DJI_0831	Thymus pallasianus Heinr.Braun	0	n	1	0	Lamiacea Lindl.
Iz_3_1009	DJI_0831	Tilia cordata Mill.	0	n	1	1	Malvaceae Juss.
Iz_3_1009	DJI_0831	Trifolium alpestre L.	0	n	1	0	Fabaceae Lindl.
Iz_3_1009	DJI_0831	Trifolium arvense L.	0	n	1	0	Fabaceae Lindl.
Iz_3_1009	DJI_0831	Ulmus glabra Huds.	0	n	1	1	Ulmaceae Mirb.
Iz_3_1009	DJI_0831	Verbascum densiflorum Bertol.	0	n	1	0	Scrophulariaceae Juss.
Iz_3_1009	DJI_0831	Veronica austriaca L.	0	n	1	0	Plantaginaceae Juss.
Iz_3_1009	DJI_0831	Veronica incana L.	0	n	1	0	Plantaginaceae Juss.
Iz_3_1009	DJI_0831	Veronica verna L.	0	n	1	0	Plantaginaceae Juss.
Iz_3_1009	DJI_0831	Vincetoxicum hirundinaria Medik	0	n	1	0	Apocynaceae Juss.
Iz_3_1009	DJI_0832	Acer tataricum L.	0	n	1	1	Sapindaceae Juss.
Iz_3_1009	DJI_0832	Achillea millefolium L. s.l.	0	n	1	0	Asteraceae Bercht. & J.Presl
Iz_3_1009	DJI_0832	Allium oleraceum L.	0	n	1	0	Amaryllidaceae J.St.-Hil.
Iz_3_1009	DJI_0832	Ambrosia artemisiifolia L.	0	a	0	0	Asteraceae Bercht. & J.Presl
Iz_3_1009	DJI_0832	Amorpha fruticosa L.	0	a	0	0	Fabaceae Lindl.
Iz_3_1009	DJI_0832	Anthericum ramosum L.	0	n	1	0	Asparagaceae Juss.
Iz_3_1009	DJI_0832	Artemisia marschalliana Spreng.	0	n	1	0	Asteraceae Bercht. & J.Presl
Iz_3_1009	DJI_0832	Asparagus officinalis L.	0	n	1	0	Asparagaceae Juss.
Iz_3_1009	DJI_0832	Astragalus cicer L.	0	n	1	0	Fabaceae Lindl.
Iz_3_1009	DJI_0832	Bassia laniflora (S.G.Gmel.) A.J.Scott	0	n	1	0	Amaranthaceae Juss.
Iz_3_1009	DJI_0832	Berberis vulgaris L.	0	n	1	0	Berberidaceae Juss.
Iz_3_1009	DJI_0832	Betula pendula Roth.	0	n	1	1	Betulaceae Gray

Iz_3_1009	DJI_0832	Betula pendula Roth.	0	n	1	0	Poaceae Barnhart
Iz_3_1009	DJI_0832	Bromus inermis Leyss.	0		NA	NA	
Iz_3_1009	DJI_0832	Bromus squarrosus L.	0	a	0	0	Poaceae Barnhart
Iz_3_1009	DJI_0832	Bromus tectorum L.	0	a	0	0	Poaceae Barnhart
Iz_3_1009	DJI_0832	Calamagrostis epigejos (L.) Roth	50	n	1	0	Poaceae Barnhart
Iz_3_1009	DJI_0832	Carduus acanthoides L.	0	n	1	0	Asteraceae Bercht. & J.Presl
Iz_3_1009	DJI_0832	Carex sp.	0	n	1	0	Cyperaceae Juss.
Iz_3_1009	DJI_0832	Centaurea majorovii Dumbadze	0	n	1	0	Asteraceae Bercht. & J.Presl
Iz_3_1009	DJI_0832	Chamaecytisus ruthenicus (Fisch.ex Wol.) Klaskova	0	n	1	0	Fabaceae Lindl.
Iz_3_1009	DJI_0832	Chelidonium majus L.	0	n	1	0	Papaveraceae Juss.
Iz_3_1009	DJI_0832	Chenopodium betaceum Andrz.	0	n	1	0	Amaranthaceae Juss.
Iz_3_1009	DJI_0832	Chenopodium cf. album	0	n	1	0	Amaranthaceae Juss.
Iz_3_1009	DJI_0832	Chenopodium sp.	0		NA	0	Amaranthaceae Juss.
Iz_3_1009	DJI_0832	Chondrilla juncea L.	0	n	1	0	Asteraceae Bercht. & J.Presl
Iz_3_1009	DJI_0832	Cirsium vulgare (Savi) Ten.	0	n	1	0	Asteraceae Bercht. & J.Presl
Iz_3_1009	DJI_0832	Clinopodium acinos (L.) Kuntze	0	n	1	0	Lamiacea Lindl.
Iz_3_1009	DJI_0832	Convallaria majalis L.	0	n	1	0	Asparagaceae Juss.
Iz_3_1009	DJI_0832	Crataegus rhipidophylla Gand.	0	n	1	1	Rosaceae Juss.
Iz_3_1009	DJI_0832	Crepis foetida L.	0	n	1	0	Asteraceae Bercht. & J.Presl
Iz_3_1009	DJI_0832	Dactylis glomerata L.	0	n	1	0	Poaceae Barnhart
Iz_3_1009	DJI_0832	Dianthus campestris M.Bieb.	0	n	1	0	Caryophyllaceae Juss.
Iz_3_1009	DJI_0832	Digitaria sanguinalis (L.) Scop.	0	a	0	0	Poaceae Barnhart
Iz_3_1009	DJI_0832	Elymus repens (L.) Gould	0	n	1	0	Poaceae Barnhart
Iz_3_1009	DJI_0832	Epilobium tetragonum L.	0	n	1	0	Onagraceae Juss.
Iz_3_1009	DJI_0832	Eragrostis minor Host	0	a	0	0	Poaceae Barnhart
Iz_3_1009	DJI_0832	Erigeron canadensis L.	0	a	0	0	Asteraceae Bercht. & J.Presl
Iz_3_1009	DJI_0832	Euphorbia seguieriana Neck.	0	n	1	0	Euphorbiaceae Juss.
Iz_3_1009	DJI_0832	Fallopia dumetorum (L.) Holub	0	n	1	0	Polygonaceae Juss.
Iz_3_1009	DJI_0832	Festuca beckeri (Hack.) Trautv.	0	n	1	0	Poaceae Barnhart
Iz_3_1009	DJI_0832	Fragaria vesca L.	0	n	1	0	Rosaceae Juss.
Iz_3_1009	DJI_0832	Frangula alnus Mill.	0	n	1	1	Rhamnaceae Juss.
Iz_3_1009	DJI_0832	Fraxinus pennsylvanica Marshall	0	a	0	1	Oleaceae Hoffmanns. & Link
Iz_3_1009	DJI_0832	Galeopsis bifida Boenn.	0	n	1	0	Lamiacea Lindl.
Iz_3_1009	DJI_0832	Galium verum L.	0	n	1	0	Rubiaceae Juss.
Iz_3_1009	DJI_0832	Genista tinctoria L.	0	n	1	0	Fabaceae Lindl.
Iz_3_1009	DJI_0832	Geranium robertianum L.	0	n	1	0	Geraniaceae Juss.
Iz_3_1009	DJI_0832	Geranium sanquineum L.	0	n	1	0	Geraniaceae Juss.
Iz_3_1009	DJI_0832	Gypsophila paniculata L.	0	n	1	0	Caryophyllaceae Juss.
Iz_3_1009	DJI_0832	Helichrysum arenarium (L.) Moench	0	n	1	0	Asteraceae Bercht. & J.Presl
Iz_3_1009	DJI_0832	Herniaria polygama J.Gay	0	n	1	0	Caryophyllaceae Juss.
Iz_3_1009	DJI_0832	Hieracium robustum Fr.	0	n	1	0	Asteraceae Bercht. & J.Presl
Iz_3_1009	DJI_0832	Hieracium umbellatum L.	0	n	1	NA	Asteraceae Bercht. & J.Presl
Iz_3_1009	DJI_0832	Hylotelephium maximum (L.) Holub	0	n	1	0	Crassulaceae J.St.-Hil.
Iz_3_1009	DJI_0832	Jacobaea borysthenica (DC.) B.Nord. & Greuter	0	n	1	0	Asteraceae Bercht. & J.Presl
Iz_3_1009	DJI_0832	Jasione montana L.	0	n	1	0	Campanulaceae Juss.
Iz_3_1009	DJI_0832	Jurinea cyanoides (L.) Rchb.s.l.	0	n	1	0	Asteraceae Bercht. & J.Presl
Iz_3_1009	DJI_0832	Koeleria glauca (Spreng.) DC.	0	n	1	0	Poaceae Barnhart
Iz_3_1009	DJI_0832	Lactuca serriola L.	0	a	0	0	Asteraceae Bercht. & J.Presl
Iz_3_1009	DJI_0832	Linaria genistifolia (L.) Mill.	0	n	1	0	Plantaginaceae Juss.
Iz_3_1009	DJI_0832	Lomelosia argentea (L.) Greuter & Burdet	0	n	1	0	Caprifoliaceae Juss.
Iz_3_1009	DJI_0832	Melampyrum pratense L.	0	n	1	0	Orobanchaceae Vent.
Iz_3_1009	DJI_0832	Oenothera biennis L.	0	a	0	0	Onagraceae Juss.
Iz_3_1009	DJI_0832	Pentanema britannica (L.) D.Gut.Larr., Santos-Vicente, Anderb., E.Rico & M.M.Mart.Ort.	0	n	1	0	Asteraceae Bercht. & J.Presl
Iz_3_1009	DJI_0832	Peucedanum oreoselinum (L.) Moench	0	n	1	0	Apiaceae Lindl.
Iz_3_1009	DJI_0832	Peucedanum ruthenicum M.Bieb.	0	n	1	0	Apiaceae Lindl.
Iz_3_1009	DJI_0832	Pilosella echioides (Lumn.) F.W.Schultz & Sch.Bip.	0	n	1	0	Asteraceae Bercht. & J.Presl
Iz_3_1009	DJI_0832	Pilosella officinarum F. Schultz et Sch. Bip.	0	n	1	0	Asteraceae Bercht. & J.Presl
Iz_3_1009	DJI_0832	Pilosella sp.	0		NA	0	Asteraceae Bercht. & J.Presl
Iz_3_1009	DJI_0832	Pinus sylvestris L.	0	n	1	1	Pinaceae Spreng. ex F.Rudolphi
Iz_3_1009	DJI_0832	Poa annua L.	0	n	1	0	Poaceae Barnhart
Iz_3_1009	DJI_0832	Poa nemoralis L.	0	n	1	0	Poaceae Barnhart
Iz_3_1009	DJI_0832	Poa sp.	0		NA	0	Poaceae Barnhart
Iz_3_1009	DJI_0832	Polygonatum odoratum (Mill.) Druce	0	n	1	0	Asparagaceae Juss.
Iz_3_1009	DJI_0832	Polygonum arenarium Waldst. & Kit.	0	n	1	0	Polygonaceae Juss.
Iz_3_1009	DJI_0832	Populus nigra L.	0	n	1	1	Salicaceae Mirb.
Iz_3_1009	DJI_0832	Populus tremula L.	0	n	1	1	Salicaceae Mirb.
Iz_3_1009	DJI_0832	Populus Ã canescens (Aiton) Sm.	0	n	1	1	Salicaceae Mirb.
Iz_3_1009	DJI_0832	Potentilla argentea L.	0	n	1	0	Rosaceae Juss.
Iz_3_1009	DJI_0832	Psammophiliella muralis (L.) Ikonn.	0	n	1	0	Caryophyllaceae Juss.
Iz_3_1009	DJI_0832	Psephellus sumensis (Kalen.) Greuter	0	n	1	0	Asteraceae Bercht. & J.Presl
Iz_3_1009	DJI_0832	Pulsatilla pratensis (L.) Mill. s.l.	0	n	1	0	Ranunculaceae Juss.
Iz_3_1009	DJI_0832	Pyrus communis L.	0	a	0	1	Rosaceae Juss.
Iz_3_1009	DJI_0832	Quercus robur L.	0	n	1	1	Fagaceae Dumort.
Iz_3_1009	DJI_0832	Robinia pseudoacacia L.	0	a	0	1	Fabaceae Lindl.
Iz_3_1009	DJI_0832	Rosa sp.	0		NA	1	Rosaceae Juss.
Iz_3_1009	DJI_0832	Rubus idaeus L.	0	n	1	0	Rosaceae Juss.
Iz_3_1009	DJI_0832	Rumex acetosella L.	0	n	1	0	Polygonaceae Juss.
Iz_3_1009	DJI_0832	Rumex thyrsiflorus Fingerh.	0	n	1	0	Polygonaceae Juss.
Iz_3_1009	DJI_0832	Salix alba L.	0	n	1	1	Salicaceae Mirb.
Iz_3_1009	DJI_0832	Sambucus nigra L.	0	n	1	1	Viburnaceae Raf.
Iz_3_1009	DJI_0832	Secale sylvestre Host	0	n	1	0	Poaceae Barnhart
Iz_3_1009	DJI_0832	Sempervivum ruthenicum Schnittsp. & C.B. Lehm.	0	n	1	0	Crassulaceae J.St.-Hil.
Iz_3_1009	DJI_0832	Setaria viridis (L.) P. Beauv.	0	n	1	0	Poaceae Barnhart
Iz_3_1009	DJI_0832	Silene borysthenica (Gruner) Walters	0	n	1	0	Caryophyllaceae Juss.
Iz_3_1009	DJI_0832	Silene latifolia subsp. alba (Mill.) Greuter & Burdet	0	n	1	0	Caryophyllaceae Juss.
Iz_3_1009	DJI_0832	Solidago virgaurea L.	1	n	1	0	Asteraceae Bercht. & J.Presl
Iz_3_1009	DJI_0832	Sonchus oleraceus L.	0	a	0	0	Asteraceae Bercht. & J.Presl
Iz_3_1009	DJI_0832	Sorbus aucuparia L.	0	n	1	1	Rosaceae Juss.
Iz_3_1009	DJI_0832	Stachys recta L.	0	n	1	0	Lamiacea Lindl.
Iz_3_1009	DJI_0832	Tanacetum vulgare L.	0	n	1	0	Asteraceae Bercht. & J.Presl
Iz_3_1009	DJI_0832	Thymus pallasianus Heinr.Braun	0	n	1	0	Lamiacea Lindl.
Iz_3_1009	DJI_0832	Tilia cordata Mill.	0	n	1	1	Malvaceae Juss.
Iz_3_1009	DJI_0832	Trifolium alpestre L.	0	n	1	0	Fabaceae Lindl.
Iz_3_1009	DJI_0832	Trifolium arvense L.	0	n	1	0	Fabaceae Lindl.
Iz_3_1009	DJI_0832	Ulmus glabra Huds.	0	n	1	1	Ulmaceae Mirb.
Iz_3_1009	DJI_0832	Verbascum densiflorum Bertol.	0	n	1	0	Scrophulariaceae Juss.
Iz_3_1009	DJI_0832	Veronica austriaca L.	0	n	1	0	Plantaginaceae Juss.
Iz_3_1009	DJI_0832	Veronica incana L.	0	n	1	0	Plantaginaceae Juss.

Iz_3_1009	DJI_0832	Veronica verna L.	0	n	1	0	Plantaginaceae Juss.
Iz_3_1009	DJI_0832	Vincetoxicum hirundinaria Medik	0	n	1	0	Apocynaceae Juss.
Iz_3_1009	DJI_0833	Acer tataricum L.	0	n	1	1	Sapindaceae Juss.
Iz_3_1009	DJI_0833	Achillea millefolium L. s.l.	0	n	1	0	Asteraceae Bercht. & J.Presl
Iz_3_1009	DJI_0833	Allium oleraceum L.	0	n	1	0	Amaryllidaceae J.St.-Hil.
Iz_3_1009	DJI_0833	Ambrosia artemisiifolia L.	0	a	0	0	Asteraceae Bercht. & J.Presl
Iz_3_1009	DJI_0833	Amorpha fruticosa L.	0	a	0	0	Fabaceae Lindl.
Iz_3_1009	DJI_0833	Anthericum ramosum L.	0	n	1	0	Asparagaceae Juss.
Iz_3_1009	DJI_0833	Artemisia marschalliana Spreng.	0	n	1	0	Asteraceae Bercht. & J.Presl
Iz_3_1009	DJI_0833	Asparagus officinalis L.	0	n	1	0	Asparagaceae Juss.
Iz_3_1009	DJI_0833	Astragalus cicer L.	0	n	1	0	Fabaceae Lindl.
Iz_3_1009	DJI_0833	Bassia laniflora (S.G.Gmel.) A.J.Scott	0	n	1	0	Amaranthaceae Juss.
Iz_3_1009	DJI_0833	Berberis vulgaris L.	0	n	1	0	Berberidaceae Juss.
Iz_3_1009	DJI_0833	Betula pendula Roth.	0	n	1	1	Betulaceae Gray
Iz_3_1009	DJI_0833	Betula pendula Roth.	0	n	1	0	Poaceae Barnhart
Iz_3_1009	DJI_0833	Bromus inermis Leyss.	0		NA	NA	
Iz_3_1009	DJI_0833	Bromus squarrosus L.	0	a	0	0	Poaceae Barnhart
Iz_3_1009	DJI_0833	Bromus tectorum L.	0	a	0	0	Poaceae Barnhart
Iz_3_1009	DJI_0833	Calamagrostis epigejos (L.) Roth	60	n	1	0	Poaceae Barnhart
Iz_3_1009	DJI_0833	Carduus acanthoides L.	0	n	1	0	Asteraceae Bercht. & J.Presl
Iz_3_1009	DJI_0833	Carex sp.	0	n	1	0	Cyperaceae Juss.
Iz_3_1009	DJI_0833	Centaurea majorovii Dumbadze	0	n	1	0	Asteraceae Bercht. & J.Presl
Iz_3_1009	DJI_0833	Chamaecytisus ruthenicus (Fisch.ex Wol.) Klaskova	0	n	1	0	Fabaceae Lindl.
Iz_3_1009	DJI_0833	Chelidonium majus L.	0	n	1	0	Papaveraceae Juss.
Iz_3_1009	DJI_0833	Chenopodium betaceum Andrz.	0	n	1	0	Amaranthaceae Juss.
Iz_3_1009	DJI_0833	Chenopodium cf. album	0	n	1	0	Amaranthaceae Juss.
Iz_3_1009	DJI_0833	Chenopodium sp.	0		NA	0	Amaranthaceae Juss.
Iz_3_1009	DJI_0833	Chondrilla juncea L.	0	n	1	0	Asteraceae Bercht. & J.Presl
Iz_3_1009	DJI_0833	Cirsium vulgare (Savi) Ten.	0	n	1	0	Asteraceae Bercht. & J.Presl
Iz_3_1009	DJI_0833	Clinopodium acinos (L.) Kuntze	0	n	1	0	Lamiacea Lindl.
Iz_3_1009	DJI_0833	Convallaria majalis L.	0	n	1	0	Asparagaceae Juss.
Iz_3_1009	DJI_0833	Crataegus rhipidophylla Gand.	0	n	1	1	Rosaceae Juss.
Iz_3_1009	DJI_0833	Crepis foetida L.	0	n	1	0	Asteraceae Bercht. & J.Presl
Iz_3_1009	DJI_0833	Dactylis glomerata L.	0	n	1	0	Poaceae Barnhart
Iz_3_1009	DJI_0833	Dianthus campestris M.Bieb.	0	n	1	0	Caryophyllaceae Juss.
Iz_3_1009	DJI_0833	Digitaria sanguinalis (L.) Scop.	0	a	0	0	Poaceae Barnhart
Iz_3_1009	DJI_0833	Elymus repens (L.) Gould	0	n	1	0	Poaceae Barnhart
Iz_3_1009	DJI_0833	Epilobium tetragonum L.	0	n	1	0	Onagraceae Juss.
Iz_3_1009	DJI_0833	Eragrostis minor Host	0	a	0	0	Poaceae Barnhart
Iz_3_1009	DJI_0833	Erigeron canadensis L.	2	a	0	0	Asteraceae Bercht. & J.Presl
Iz_3_1009	DJI_0833	Euphorbia seguieriana Neck.	0	n	1	0	Euphorbiaceae Juss.
Iz_3_1009	DJI_0833	Fallopia dumetorum (L.) Holub	0	n	1	0	Polygonaceae Juss.
Iz_3_1009	DJI_0833	Festuca beckeri (Hack.) Trautv.	0	n	1	0	Poaceae Barnhart
Iz_3_1009	DJI_0833	Fragaria vesca L.	0	n	1	0	Rosaceae Juss.
Iz_3_1009	DJI_0833	Frangula alnus Mill.	0	n	1	1	Rhamnaceae Juss.
Iz_3_1009	DJI_0833	Fraxinus pennsylvanica Marshall	0	a	0	1	Oleaceae Hoffmanns. & Link
Iz_3_1009	DJI_0833	Galeopsis bifida Boenn.	0	n	1	0	Lamiacea Lindl.
Iz_3_1009	DJI_0833	Galium verum L.	0	n	1	0	Rubiaceae Juss.
Iz_3_1009	DJI_0833	Genista tinctoria L.	0	n	1	0	Fabaceae Lindl.
Iz_3_1009	DJI_0833	Geranium robertianum L.	0	n	1	0	Geraniaceae Juss.
Iz_3_1009	DJI_0833	Geranium sanquineum L.	0	n	1	0	Geraniaceae Juss.
Iz_3_1009	DJI_0833	Gypsophila paniculata L.	0	n	1	0	Caryophyllaceae Juss.
Iz_3_1009	DJI_0833	Helichrysum arenarium (L.) Moench	0	n	1	0	Asteraceae Bercht. & J.Presl
Iz_3_1009	DJI_0833	Herniaria polygama J.Gay	0	n	1	0	Caryophyllaceae Juss.
Iz_3_1009	DJI_0833	Hieracium robustum Fr.	0	n	1	0	Asteraceae Bercht. & J.Presl
Iz_3_1009	DJI_0833	Hieracium umbellatum L.	0	n	1	NA	Asteraceae Bercht. & J.Presl
Iz_3_1009	DJI_0833	Hylotelephium maximum (L.) Holub	0	n	1	0	Crassulaceae J.St.-Hil.
Iz_3_1009	DJI_0833	Jacobaea borysthenica (DC.) B.Nord. & Greuter	0	n	1	0	Asteraceae Bercht. & J.Presl
Iz_3_1009	DJI_0833	Jasione montana L.	0	n	1	0	Campanulaceae Juss.
Iz_3_1009	DJI_0833	Jurinea cyanoides (L.) Rchb.s.l.	0	n	1	0	Asteraceae Bercht. & J.Presl
Iz_3_1009	DJI_0833	Koeleria glauca (Spreng.) DC.	0	n	1	0	Poaceae Barnhart
Iz_3_1009	DJI_0833	Lactuca serriola L.	0	a	0	0	Asteraceae Bercht. & J.Presl
Iz_3_1009	DJI_0833	Linaria genistifolia (L.) Mill.	0	n	1	0	Plantaginaceae Juss.
Iz_3_1009	DJI_0833	Lomelosia argentea (L.) Greuter & Burdet	0	n	1	0	Caprifoliaceae Juss.
Iz_3_1009	DJI_0833	Melampyrum pratense L.	0	n	1	0	Orobanchaceae Vent.
Iz_3_1009	DJI_0833	Oenothera biennis L.	0	a	0	0	Onagraceae Juss.
Iz_3_1009	DJI_0833	Pentanema britannica (L.) D.Gut.Larr., Santos-Vicente, Anderb., E.Rico & M.M.Mart.Ort.	0	n	1	0	Asteraceae Bercht. & J.Presl
Iz_3_1009	DJI_0833	Peucedanum oreoselinum (L.) Moench	1	n	1	0	Apiaceae Lindl.
Iz_3_1009	DJI_0833	Peucedanum ruthenicum M.Bieb.	0	n	1	0	Apiaceae Lindl.
Iz_3_1009	DJI_0833	Pilosella echioides (Lumn.) F.W.Schultz & Sch.Bip.	0	n	1	0	Asteraceae Bercht. & J.Presl
Iz_3_1009	DJI_0833	Pilosella officinarum F. Schultz et Sch. Bip.	0	n	1	0	Asteraceae Bercht. & J.Presl
Iz_3_1009	DJI_0833	Pilosella sp.	0		NA	0	Asteraceae Bercht. & J.Presl
Iz_3_1009	DJI_0833	Pinus sylvestris L.	0	n	1	1	Pinaceae Spreng. ex F.Rudolphi
Iz_3_1009	DJI_0833	Poa annua L.	0	n	1	0	Poaceae Barnhart
Iz_3_1009	DJI_0833	Poa nemoralis L.	0	n	1	0	Poaceae Barnhart
Iz_3_1009	DJI_0833	Poa sp.	0		NA	0	Poaceae Barnhart
Iz_3_1009	DJI_0833	Polygonatum odoratum (Mill.) Druce	0	n	1	0	Asparagaceae Juss.
Iz_3_1009	DJI_0833	Polygonum arenarium Waldst. & Kit.	0	n	1	0	Polygonaceae Juss.
Iz_3_1009	DJI_0833	Populus nigra L.	0	n	1	1	Salicaceae Mirb.
Iz_3_1009	DJI_0833	Populus tremula L.	0	n	1	1	Salicaceae Mirb.
Iz_3_1009	DJI_0833	Populus Ã canescens (Aiton) Sm.	0	n	1	1	Salicaceae Mirb.
Iz_3_1009	DJI_0833	Potentilla argentea L.	0	n	1	0	Rosaceae Juss.
Iz_3_1009	DJI_0833	Psammophiliella muralis (L.) Ikonn.	0	n	1	0	Caryophyllaceae Juss.
Iz_3_1009	DJI_0833	Psephellus sumensis (Kalen.) Greuter	0	n	1	0	Asteraceae Bercht. & J.Presl
Iz_3_1009	DJI_0833	Pulsatilla pratensis (L.) Mill. s.l.	0	n	1	0	Ranunculaceae Juss.
Iz_3_1009	DJI_0833	Pyrus communis L.	0	a	0	1	Rosaceae Juss.
Iz_3_1009	DJI_0833	Quercus robur L.	0	n	1	1	Fagaceae Dumort.
Iz_3_1009	DJI_0833	Robinia pseudoacacia L.	0	a	0	1	Fabaceae Lindl.
Iz_3_1009	DJI_0833	Rosa sp.	0		NA	1	Rosaceae Juss.
Iz_3_1009	DJI_0833	Rubus idaeus L.	0	n	1	0	Rosaceae Juss.
Iz_3_1009	DJI_0833	Rumex acetosella L.	0	n	1	0	Polygonaceae Juss.
Iz_3_1009	DJI_0833	Rumex thyrsiflorus Fingerh.	0	n	1	0	Polygonaceae Juss.
Iz_3_1009	DJI_0833	Salix alba L.	0	n	1	1	Salicaceae Mirb.
Iz_3_1009	DJI_0833	Sambucus nigra L.	0	n	1	1	Viburnaceae Raf.
Iz_3_1009	DJI_0833	Secale sylvestre Host	0	n	1	0	Poaceae Barnhart
Iz_3_1009	DJI_0833	Sempervivum ruthenicum Schnittsp. & C.B. Lehm.	0	n	1	0	Crassulaceae J.St.-Hil.
Iz_3_1009	DJI_0833	Setaria viridis (L.) P. Beauv.	0	n	1	0	Poaceae Barnhart
Iz_3_1009	DJI_0833	Silene borysthenica (Gruner) Walters	0	n	1	0	Caryophyllaceae Juss.

Iz_3_1009	DJI_0833	Silene latifolia subsp. alba (Mill.) Greuter & Burdet	0	n	1	0	Caryophyllaceae Juss.
Iz_3_1009	DJI_0833	Solidago virgaurea L.	0.5	n	1	0	Asteraceae Bercht. & J.Presl
Iz_3_1009	DJI_0833	Sonchus oleraceus L.	0	a	0	0	Asteraceae Bercht. & J.Presl
Iz_3_1009	DJI_0833	Sorbus aucuparia L.	0	n	1	1	Rosaceae Juss.
Iz_3_1009	DJI_0833	Stachys recta L.	0	n	1	0	Lamiacea Lindl.
Iz_3_1009	DJI_0833	Tanacetum vulgare L.	0	n	1	0	Asteraceae Bercht. & J.Presl
Iz_3_1009	DJI_0833	Thymus pallasianus Heinr.Braun	0	n	1	0	Lamiacea Lindl.
Iz_3_1009	DJI_0833	Tilia cordata Mill.	0	n	1	1	Malvaceae Juss.
Iz_3_1009	DJI_0833	Trifolium alpestre L.	0	n	1	0	Fabaceae Lindl.
Iz_3_1009	DJI_0833	Trifolium arvense L.	0	n	1	0	Fabaceae Lindl.
Iz_3_1009	DJI_0833	Ulmus glabra Huds.	0	n	1	1	Ulmaceae Mirb.
Iz_3_1009	DJI_0833	Verbascum densiflorum Bertol.	0	n	1	0	Scrophulariaceae Juss.
Iz_3_1009	DJI_0833	Veronica austriaca L.	0	n	1	0	Plantaginaceae Juss.
Iz_3_1009	DJI_0833	Veronica incana L.	0	n	1	0	Plantaginaceae Juss.
Iz_3_1009	DJI_0833	Veronica verna L.	0	n	1	0	Plantaginaceae Juss.
Iz_3_1009	DJI_0833	Vincetoxicum hirundinaria Medik	0	n	1	0	Apocynaceae Juss.
Iz_3_1009	DJI_0837	Acer tataricum L.	0	n	1	1	Sapindaceae Juss.
Iz_3_1009	DJI_0837	Achillea millefolium L. s.l.	0	n	1	0	Asteraceae Bercht. & J.Presl
Iz_3_1009	DJI_0837	Allium oleraceum L.	0	n	1	0	Amaryllidaceae J.St.-Hil.
Iz_3_1009	DJI_0837	Ambrosia artemisiifolia L.	0	a	0	0	Asteraceae Bercht. & J.Presl
Iz_3_1009	DJI_0837	Amorpha fruticosa L.	0	a	0	0	Fabaceae Lindl.
Iz_3_1009	DJI_0837	Anthericum ramosum L.	0	n	1	0	Asparagaceae Juss.
Iz_3_1009	DJI_0837	Artemisia marschalliana Spreng.	0	n	1	0	Asteraceae Bercht. & J.Presl
Iz_3_1009	DJI_0837	Asparagus officinalis L.	0	n	1	0	Asparagaceae Juss.
Iz_3_1009	DJI_0837	Astragalus cicer L.	0	n	1	0	Fabaceae Lindl.
Iz_3_1009	DJI_0837	Bassia laniflora (S.G.Gmel.) A.J.Scott	0	n	1	0	Amaranthaceae Juss.
Iz_3_1009	DJI_0837	Berberis vulgaris L.	0	n	1	0	Berberidaceae Juss.
Iz_3_1009	DJI_0837	Betula pendula Roth.	0	n	1	1	Betulaceae Gray
Iz_3_1009	DJI_0837	Betula pendula Roth.	0	n	1	0	Poaceae Barnhart
Iz_3_1009	DJI_0837	Bromus inermis Leyss.	0		NA	NA	
Iz_3_1009	DJI_0837	Bromus squarrosus L.	0	a	0	0	Poaceae Barnhart
Iz_3_1009	DJI_0837	Bromus tectorum L.	0	a	0	0	Poaceae Barnhart
Iz_3_1009	DJI_0837	Calamagrostis epigejos (L.) Roth	40	n	1	0	Poaceae Barnhart
Iz_3_1009	DJI_0837	Carduus acanthoides L.	0	n	1	0	Asteraceae Bercht. & J.Presl
Iz_3_1009	DJI_0837	Carex sp.	0	n	1	0	Cyperaceae Juss.
Iz_3_1009	DJI_0837	Centaurea majorovii Dumbadze	0	n	1	0	Asteraceae Bercht. & J.Presl
Iz_3_1009	DJI_0837	Chamaecytisus ruthenicus (Fisch.ex Wol.) Klaskova	0	n	1	0	Fabaceae Lindl.
Iz_3_1009	DJI_0837	Chelidonium majus L.	0	n	1	0	Papaveraceae Juss.
Iz_3_1009	DJI_0837	Chenopodium betaceum Andrz.	0	n	1	0	Amaranthaceae Juss.
Iz_3_1009	DJI_0837	Chenopodium cf. album	0	n	1	0	Amaranthaceae Juss.
Iz_3_1009	DJI_0837	Chenopodium sp.	0		NA	0	Amaranthaceae Juss.
Iz_3_1009	DJI_0837	Chondrilla juncea L.	0	n	1	0	Asteraceae Bercht. & J.Presl
Iz_3_1009	DJI_0837	Cirsium vulgare (Savi) Ten.	0	n	1	0	Asteraceae Bercht. & J.Presl
Iz_3_1009	DJI_0837	Clinopodium acinos (L.) Kuntze	0	n	1	0	Lamiacea Lindl.
Iz_3_1009	DJI_0837	Convallaria majalis L.	0	n	1	0	Asparagaceae Juss.
Iz_3_1009	DJI_0837	Crataegus rhipidophylla Gand.	0	n	1	1	Rosaceae Juss.
Iz_3_1009	DJI_0837	Crepis foetida L.	0	n	1	0	Asteraceae Bercht. & J.Presl
Iz_3_1009	DJI_0837	Dactylis glomerata L.	0	n	1	0	Poaceae Barnhart
Iz_3_1009	DJI_0837	Dianthus campestris M.Bieb.	0	n	1	0	Caryophyllaceae Juss.
Iz_3_1009	DJI_0837	Digitaria sanguinalis (L.) Scop.	0	a	0	0	Poaceae Barnhart
Iz_3_1009	DJI_0837	Elymus repens (L.) Gould	0	n	1	0	Poaceae Barnhart
Iz_3_1009	DJI_0837	Epilobium tetragonum L.	0	n	1	0	Onagraceae Juss.
Iz_3_1009	DJI_0837	Eragrostis minor Host	0	a	0	0	Poaceae Barnhart
Iz_3_1009	DJI_0837	Erigeron canadensis L.	4	a	0	0	Asteraceae Bercht. & J.Presl
Iz_3_1009	DJI_0837	Euphorbia seguieriana Neck.	0	n	1	0	Euphorbiaceae Juss.
Iz_3_1009	DJI_0837	Fallopia dumetorum (L.) Holub	0	n	1	0	Polygonaceae Juss.
Iz_3_1009	DJI_0837	Festuca beckeri (Hack.) Trautv.	0	n	1	0	Poaceae Barnhart
Iz_3_1009	DJI_0837	Fragaria vesca L.	0	n	1	0	Rosaceae Juss.
Iz_3_1009	DJI_0837	Frangula alnus Mill.	0	n	1	1	Rhamnaceae Juss.
Iz_3_1009	DJI_0837	Fraxinus pennsylvanica Marshall	0	a	0	1	Oleaceae Hoffmanns. & Link
Iz_3_1009	DJI_0837	Galeopsis bifida Boenn.	0	n	1	0	Lamiacea Lindl.
Iz_3_1009	DJI_0837	Galium verum L.	0	n	1	0	Rubiaceae Juss.
Iz_3_1009	DJI_0837	Genista tinctoria L.	0	n	1	0	Fabaceae Lindl.
Iz_3_1009	DJI_0837	Geranium robertianum L.	0	n	1	0	Geraniaceae Juss.
Iz_3_1009	DJI_0837	Geranium sanquineum L.	0	n	1	0	Geraniaceae Juss.
Iz_3_1009	DJI_0837	Gypsophila paniculata L.	0	n	1	0	Caryophyllaceae Juss.
Iz_3_1009	DJI_0837	Helichrysum arenarium (L.) Moench	0	n	1	0	Asteraceae Bercht. & J.Presl
Iz_3_1009	DJI_0837	Herniaria polygama J.Gay	0	n	1	0	Caryophyllaceae Juss.
Iz_3_1009	DJI_0837	Hieracium robustum Fr.	0	n	1	0	Asteraceae Bercht. & J.Presl
Iz_3_1009	DJI_0837	Hieracium umbellatum L.	0	n	1	NA	Asteraceae Bercht. & J.Presl
Iz_3_1009	DJI_0837	Hylotelephium maximum (L.) Holub	0	n	1	0	Crassulaceae J.St.-Hil.
Iz_3_1009	DJI_0837	Jacobaea borysthenica (DC.) B.Nord. & Greuter	0	n	1	0	Asteraceae Bercht. & J.Presl
Iz_3_1009	DJI_0837	Jasione montana L.	0	n	1	0	Campanulaceae Juss.
Iz_3_1009	DJI_0837	Jurinea cyanoides (L.) Rchb.s.l.	0	n	1	0	Asteraceae Bercht. & J.Presl
Iz_3_1009	DJI_0837	Koeleria glauca (Spreng.) DC.	0	n	1	0	Poaceae Barnhart
Iz_3_1009	DJI_0837	Lactuca serriola L.	1	a	0	0	Asteraceae Bercht. & J.Presl
Iz_3_1009	DJI_0837	Linaria genistifolia (L.) Mill.	0	n	1	0	Plantaginaceae Juss.
Iz_3_1009	DJI_0837	Lomelosia argentea (L.) Greuter & Burdet	0	n	1	0	Caprifoliaceae Juss.
Iz_3_1009	DJI_0837	Melampyrum pratense L.	0	n	1	0	Orobanchaceae Vent.
Iz_3_1009	DJI_0837	Oenothera biennis L.	0	a	0	0	Onagraceae Juss.
Iz_3_1009	DJI_0837	Pentanema britannica (L.) D.Gut.Larr., Santos-Vicente, Anderb., E.Rico & M.M.Mart.Ort.	0	n	1	0	Asteraceae Bercht. & J.Presl
Iz_3_1009	DJI_0837	Peucedanum oreoselinum (L.) Moench	3	n	1	0	Apiaceae Lindl.
Iz_3_1009	DJI_0837	Peucedanum ruthenicum M.Bieb.	0	n	1	0	Apiaceae Lindl.
Iz_3_1009	DJI_0837	Pilosella echioides (Lumn.) F.W.Schultz & Sch.Bip.	0	n	1	0	Asteraceae Bercht. & J.Presl
Iz_3_1009	DJI_0837	Pilosella officinarum F. Schultz et Sch. Bip.	0	n	1	0	Asteraceae Bercht. & J.Presl
Iz_3_1009	DJI_0837	Pilosella sp.	0		NA	0	Asteraceae Bercht. & J.Presl
Iz_3_1009	DJI_0837	Pinus sylvestris L.	0	n	1	1	Pinaceae Spreng. ex F.Rudolphi
Iz_3_1009	DJI_0837	Poa annua L.	0	n	1	0	Poaceae Barnhart
Iz_3_1009	DJI_0837	Poa nemoralis L.	0	n	1	0	Poaceae Barnhart
Iz_3_1009	DJI_0837	Poa sp.	0		NA	0	Poaceae Barnhart
Iz_3_1009	DJI_0837	Polygonatum odoratum (Mill.) Druce	0	n	1	0	Asparagaceae Juss.
Iz_3_1009	DJI_0837	Polygonum arenarium Waldst. & Kit.	0	n	1	0	Polygonaceae Juss.
Iz_3_1009	DJI_0837	Populus nigra L.	0	n	1	1	Salicaceae Mirb.
Iz_3_1009	DJI_0837	Populus tremula L.	0	n	1	1	Salicaceae Mirb.
Iz_3_1009	DJI_0837	Populus Ã canescens (Aiton) Sm.	0	n	1	1	Salicaceae Mirb.
Iz_3_1009	DJI_0837	Potentilla argentea L.	0	n	1	0	Rosaceae Juss.
Iz_3_1009	DJI_0837	Psammophiliella muralis (L.) Ikonn.	0	n	1	0	Caryophyllaceae Juss.
Iz_3_1009	DJI_0837	Psephellus sumensis (Kalen.) Greuter	0	n	1	0	Asteraceae Bercht. & J.Presl

Iz_3_1009	DJI_0837	Pulsatilla pratensis (L.) Mill. s.l.	0	n	1	0	Ranunculaceae Juss.
Iz_3_1009	DJI_0837	Pyrus communis L.	0	a	0	1	Rosaceae Juss.
Iz_3_1009	DJI_0837	Quercus robur L.	0	n	1	1	Fagaceae Dumort.
Iz_3_1009	DJI_0837	Robinia pseudoacacia L.	0	a	0	1	Fabaceae Lindl.
Iz_3_1009	DJI_0837	Rosa sp.	0		NA	1	Rosaceae Juss.
Iz_3_1009	DJI_0837	Rubus idaeus L.	0	n	1	0	Rosaceae Juss.
Iz_3_1009	DJI_0837	Rumex acetosella L.	0	n	1	0	Polygonaceae Juss.
Iz_3_1009	DJI_0837	Rumex thyrsiflorus Fingerh.	0	n	1	0	Polygonaceae Juss.
Iz_3_1009	DJI_0837	Salix alba L.	0	n	1	1	Salicaceae Mirb.
Iz_3_1009	DJI_0837	Sambucus nigra L.	0	n	1	1	Viburnaceae Raf.
Iz_3_1009	DJI_0837	Secale sylvestre Host	0	n	1	0	Poaceae Barnhart
Iz_3_1009	DJI_0837	Sempervivum ruthenicum Schnittsp. & C.B. Lehm.	0	n	1	0	Crassulaceae J.St.-Hil.
Iz_3_1009	DJI_0837	Setaria viridis (L.) P. Beauv.	0	n	1	0	Poaceae Barnhart
Iz_3_1009	DJI_0837	Silene borysthenica (Gruner) Walters	0	n	1	0	Caryophyllaceae Juss.
Iz_3_1009	DJI_0837	Silene latifolia subsp. alba (Mill.) Greuter & Burdet	0	n	1	0	Caryophyllaceae Juss.
Iz_3_1009	DJI_0837	Solidago virgaurea L.	0	n	1	0	Asteraceae Bercht. & J.Presl
Iz_3_1009	DJI_0837	Sonchus oleraceus L.	0	a	0	0	Asteraceae Bercht. & J.Presl
Iz_3_1009	DJI_0837	Sorbus aucuparia L.	0	n	1	1	Rosaceae Juss.
Iz_3_1009	DJI_0837	Stachys recta L.	0	n	1	0	Lamiacea Lindl.
Iz_3_1009	DJI_0837	Tanacetum vulgare L.	0	n	1	0	Asteraceae Bercht. & J.Presl
Iz_3_1009	DJI_0837	Thymus pallasianus Heinr.Braun	0	n	1	0	Lamiacea Lindl.
Iz_3_1009	DJI_0837	Tilia cordata Mill.	0	n	1	1	Malvaceae Juss.
Iz_3_1009	DJI_0837	Trifolium alpestre L.	0	n	1	0	Fabaceae Lindl.
Iz_3_1009	DJI_0837	Trifolium arvense L.	0	n	1	0	Fabaceae Lindl.
Iz_3_1009	DJI_0837	Ulmus glabra Huds.	0	n	1	1	Ulmaceae Mirb.
Iz_3_1009	DJI_0837	Verbascum densiflorum Bertol.	0	n	1	0	Scrophulariaceae Juss.
Iz_3_1009	DJI_0837	Veronica austriaca L.	0	n	1	0	Plantaginaceae Juss.
Iz_3_1009	DJI_0837	Veronica incana L.	0	n	1	0	Plantaginaceae Juss.
Iz_3_1009	DJI_0837	Veronica verna L.	0	n	1	0	Plantaginaceae Juss.
Iz_3_1009	DJI_0837	Vincetoxicum hirundinaria Medik	0	n	1	0	Apocynaceae Juss.
Iz_3_1009	DJI_0838	Acer tataricum L.	0	n	1	1	Sapindaceae Juss.
Iz_3_1009	DJI_0838	Achillea millefolium L. s.l.	0	n	1	0	Asteraceae Bercht. & J.Presl
Iz_3_1009	DJI_0838	Allium oleraceum L.	0	n	1	0	Amaryllidaceae J.St.-Hil.
Iz_3_1009	DJI_0838	Ambrosia artemisiifolia L.	0	a	0	0	Asteraceae Bercht. & J.Presl
Iz_3_1009	DJI_0838	Amorpha fruticosa L.	0	a	0	0	Fabaceae Lindl.
Iz_3_1009	DJI_0838	Anthericum ramosum L.	0	n	1	0	Asparagaceae Juss.
Iz_3_1009	DJI_0838	Artemisia marschalliana Spreng.	0	n	1	0	Asteraceae Bercht. & J.Presl
Iz_3_1009	DJI_0838	Asparagus officinalis L.	0	n	1	0	Asparagaceae Juss.
Iz_3_1009	DJI_0838	Astragalus cicer L.	0	n	1	0	Fabaceae Lindl.
Iz_3_1009	DJI_0838	Bassia laniflora (S.G.Gmel.) A.J.Scott	0	n	1	0	Amaranthaceae Juss.
Iz_3_1009	DJI_0838	Berberis vulgaris L.	0	n	1	0	Berberidaceae Juss.
Iz_3_1009	DJI_0838	Betula pendula Roth.	0	n	1	1	Betulaceae Gray
Iz_3_1009	DJI_0838	Betula pendula Roth.	0	n	1	0	Poaceae Barnhart
Iz_3_1009	DJI_0838	Bromus inermis Leyss.	0		NA	NA	
Iz_3_1009	DJI_0838	Bromus squarrosus L.	0	a	0	0	Poaceae Barnhart
Iz_3_1009	DJI_0838	Bromus tectorum L.	0	a	0	0	Poaceae Barnhart
Iz_3_1009	DJI_0838	Calamagrostis epigejos (L.) Roth	50	n	1	0	Poaceae Barnhart
Iz_3_1009	DJI_0838	Carduus acanthoides L.	0	n	1	0	Asteraceae Bercht. & J.Presl
Iz_3_1009	DJI_0838	Carex sp.	0	n	1	0	Cyperaceae Juss.
Iz_3_1009	DJI_0838	Centaurea majorovii Dumbadze	0	n	1	0	Asteraceae Bercht. & J.Presl
Iz_3_1009	DJI_0838	Chamaecytisus ruthenicus (Fisch.ex Wol.) Klaskova	0	n	1	0	Fabaceae Lindl.
Iz_3_1009	DJI_0838	Chelidonium majus L.	0	n	1	0	Papaveraceae Juss.
Iz_3_1009	DJI_0838	Chenopodium betaceum Andrz.	0	n	1	0	Amaranthaceae Juss.
Iz_3_1009	DJI_0838	Chenopodium cf. album	0	n	1	0	Amaranthaceae Juss.
Iz_3_1009	DJI_0838	Chenopodium sp.	0		NA	0	Amaranthaceae Juss.
Iz_3_1009	DJI_0838	Chondrilla juncea L.	0	n	1	0	Asteraceae Bercht. & J.Presl
Iz_3_1009	DJI_0838	Cirsium vulgare (Savi) Ten.	0	n	1	0	Asteraceae Bercht. & J.Presl
Iz_3_1009	DJI_0838	Clinopodium acinos (L.) Kuntze	0	n	1	0	Lamiacea Lindl.
Iz_3_1009	DJI_0838	Convallaria majalis L.	0	n	1	0	Asparagaceae Juss.
Iz_3_1009	DJI_0838	Crataegus rhipidophylla Gand.	0	n	1	1	Rosaceae Juss.
Iz_3_1009	DJI_0838	Crepis foetida L.	0	n	1	0	Asteraceae Bercht. & J.Presl
Iz_3_1009	DJI_0838	Dactylis glomerata L.	0	n	1	0	Poaceae Barnhart
Iz_3_1009	DJI_0838	Dianthus campestris M.Bieb.	0	n	1	0	Caryophyllaceae Juss.
Iz_3_1009	DJI_0838	Digitaria sanguinalis (L.) Scop.	0	a	0	0	Poaceae Barnhart
Iz_3_1009	DJI_0838	Elymus repens (L.) Gould	0	n	1	0	Poaceae Barnhart
Iz_3_1009	DJI_0838	Epilobium tetragonum L.	0	n	1	0	Onagraceae Juss.
Iz_3_1009	DJI_0838	Eragrostis minor Host	0	a	0	0	Poaceae Barnhart
Iz_3_1009	DJI_0838	Erigeron canadensis L.	10	a	0	0	Asteraceae Bercht. & J.Presl
Iz_3_1009	DJI_0838	Euphorbia seguieriana Neck.	0	n	1	0	Euphorbiaceae Juss.
Iz_3_1009	DJI_0838	Fallopia dumetorum (L.) Holub	0	n	1	0	Polygonaceae Juss.
Iz_3_1009	DJI_0838	Festuca beckeri (Hack.) Trautv.	0	n	1	0	Poaceae Barnhart
Iz_3_1009	DJI_0838	Fragaria vesca L.	0	n	1	0	Rosaceae Juss.
Iz_3_1009	DJI_0838	Frangula alnus Mill.	0	n	1	1	Rhamnaceae Juss.
Iz_3_1009	DJI_0838	Fraxinus pennsylvanica Marshall	0	a	0	1	Oleaceae Hoffmanns. & Link
Iz_3_1009	DJI_0838	Galeopsis bifida Boenn.	0	n	1	0	Lamiacea Lindl.
Iz_3_1009	DJI_0838	Galium verum L.	0	n	1	0	Rubiaceae Juss.
Iz_3_1009	DJI_0838	Genista tinctoria L.	0	n	1	0	Fabaceae Lindl.
Iz_3_1009	DJI_0838	Geranium robertianum L.	0	n	1	0	Geraniaceae Juss.
Iz_3_1009	DJI_0838	Geranium sanquineum L.	0	n	1	0	Geraniaceae Juss.
Iz_3_1009	DJI_0838	Gypsophila paniculata L.	0	n	1	0	Caryophyllaceae Juss.
Iz_3_1009	DJI_0838	Helichrysum arenarium (L.) Moench	0	n	1	0	Asteraceae Bercht. & J.Presl
Iz_3_1009	DJI_0838	Herniaria polygama J.Gay	0	n	1	0	Caryophyllaceae Juss.
Iz_3_1009	DJI_0838	Hieracium robustum Fr.	0	n	1	0	Asteraceae Bercht. & J.Presl
Iz_3_1009	DJI_0838	Hieracium umbellatum L.	0	n	1	NA	Asteraceae Bercht. & J.Presl
Iz_3_1009	DJI_0838	Hylotelephium maximum (L.) Holub	0	n	1	0	Crassulaceae J.St.-Hil.
Iz_3_1009	DJI_0838	Jacobaea borysthenica (DC.) B.Nord. & Greuter	0	n	1	0	Asteraceae Bercht. & J.Presl
Iz_3_1009	DJI_0838	Jasione montana L.	0	n	1	0	Campanulaceae Juss.
Iz_3_1009	DJI_0838	Jurinea cyanoides (L.) Rchb.s.l.	0	n	1	0	Asteraceae Bercht. & J.Presl
Iz_3_1009	DJI_0838	Koeleria glauca (Spreng.) DC.	0	n	1	0	Poaceae Barnhart
Iz_3_1009	DJI_0838	Lactuca serriola L.	0.5	a	0	0	Asteraceae Bercht. & J.Presl
Iz_3_1009	DJI_0838	Linaria genistifolia (L.) Mill.	0	n	1	0	Plantaginaceae Juss.
Iz_3_1009	DJI_0838	Lomelosia argentea (L.) Greuter & Burdet	0	n	1	0	Caprifoliaceae Juss.
Iz_3_1009	DJI_0838	Melampyrum pratense L.	0	n	1	0	Orobanchaceae Vent.
Iz_3_1009	DJI_0838	Oenothera biennis L.	0	a	0	0	Onagraceae Juss.
Iz_3_1009	DJI_0838	Pentanema britannica (L.) D.Gut.Larr., Santos-Vicente, Anderb., E.Rico & M.M.Mart.Ort.	0	n	1	0	Asteraceae Bercht. & J.Presl
Iz_3_1009	DJI_0838	Peucedanum oreoselinum (L.) Moench	2	n	1	0	Apiaceae Lindl.
Iz_3_1009	DJI_0838	Peucedanum ruthenicum M.Bieb.	0	n	1	0	Apiaceae Lindl.
Iz_3_1009	DJI_0838	Pilosella echioides (Lumn.) F.W.Schultz & Sch.Bip.	0	n	1	0	Asteraceae Bercht. & J.Presl

Iz_3_1009	DJI_0838	Pilosella officinarum F. Schultz et Sch. Bip.	0	n	1	0	Asteraceae Bercht. & J.Presl
Iz_3_1009	DJI_0838	Pilosella sp.	0		NA	0	Asteraceae Bercht. & J.Presl
Iz_3_1009	DJI_0838	Pinus sylvestris L.	0	n	1	1	Pinaceae Spreng. ex F.Rudolphi
Iz_3_1009	DJI_0838	Poa annua L.	0	n	1	0	Poaceae Barnhart
Iz_3_1009	DJI_0838	Poa nemoralis L.	0	n	1	0	Poaceae Barnhart
Iz_3_1009	DJI_0838	Poa sp.	0		NA	0	Poaceae Barnhart
Iz_3_1009	DJI_0838	Polygonatum odoratum (Mill.) Druce	0	n	1	0	Asparagaceae Juss.
Iz_3_1009	DJI_0838	Polygonum arenarium Waldst. & Kit.	0	n	1	0	Polygonaceae Juss.
Iz_3_1009	DJI_0838	Populus nigra L.	0	n	1	1	Salicaceae Mirb.
Iz_3_1009	DJI_0838	Populus tremula L.	0	n	1	1	Salicaceae Mirb.
Iz_3_1009	DJI_0838	Populus Ã canescens (Aiton) Sm.	0	n	1	1	Salicaceae Mirb.
Iz_3_1009	DJI_0838	Potentilla argentea L.	0	n	1	0	Rosaceae Juss.
Iz_3_1009	DJI_0838	Psammophiliella muralis (L.) Ikonn.	0	n	1	0	Caryophyllaceae Juss.
Iz_3_1009	DJI_0838	Psephellus sumensis (Kalen.) Greuter	0	n	1	0	Asteraceae Bercht. & J.Presl
Iz_3_1009	DJI_0838	Pulsatilla pratensis (L.) Mill. s.l.	0	n	1	0	Ranunculaceae Juss.
Iz_3_1009	DJI_0838	Pyrus communis L.	0	a	0	1	Rosaceae Juss.
Iz_3_1009	DJI_0838	Quercus robur L.	0	n	1	1	Fagaceae Dumort.
Iz_3_1009	DJI_0838	Robinia pseudoacacia L.	0	a	0	1	Fabaceae Lindl.
Iz_3_1009	DJI_0838	Rosa sp.	0		NA	1	Rosaceae Juss.
Iz_3_1009	DJI_0838	Rubus idaeus L.	0	n	1	0	Rosaceae Juss.
Iz_3_1009	DJI_0838	Rumex acetosella L.	0	n	1	0	Polygonaceae Juss.
Iz_3_1009	DJI_0838	Rumex thyrsiflorus Fingerh.	0	n	1	0	Polygonaceae Juss.
Iz_3_1009	DJI_0838	Salix alba L.	0	n	1	1	Salicaceae Mirb.
Iz_3_1009	DJI_0838	Sambucus nigra L.	0	n	1	1	Viburnaceae Raf.
Iz_3_1009	DJI_0838	Secale sylvestre Host	0	n	1	0	Poaceae Barnhart
Iz_3_1009	DJI_0838	Sempervivum ruthenicum Schnittsp. & C.B. Lehm.	0	n	1	0	Crassulaceae J.St.-Hil.
Iz_3_1009	DJI_0838	Setaria viridis (L.) P. Beauv.	0	n	1	0	Poaceae Barnhart
Iz_3_1009	DJI_0838	Silene borysthenica (Gruner) Walters	0	n	1	0	Caryophyllaceae Juss.
Iz_3_1009	DJI_0838	Silene latifolia subsp. alba (Mill.) Greuter & Burdet	0	n	1	0	Caryophyllaceae Juss.
Iz_3_1009	DJI_0838	Solidago virgaurea L.	0	n	1	0	Asteraceae Bercht. & J.Presl
Iz_3_1009	DJI_0838	Sonchus oleraceus L.	0	a	0	0	Asteraceae Bercht. & J.Presl
Iz_3_1009	DJI_0838	Sorbus aucuparia L.	0	n	1	1	Rosaceae Juss.
Iz_3_1009	DJI_0838	Stachys recta L.	0	n	1	0	Lamiacea Lindl.
Iz_3_1009	DJI_0838	Tanacetum vulgare L.	0	n	1	0	Asteraceae Bercht. & J.Presl
Iz_3_1009	DJI_0838	Thymus pallasianus Heinr.Braun	0	n	1	0	Lamiacea Lindl.
Iz_3_1009	DJI_0838	Tilia cordata Mill.	0	n	1	1	Malvaceae Juss.
Iz_3_1009	DJI_0838	Trifolium alpestre L.	0	n	1	0	Fabaceae Lindl.
Iz_3_1009	DJI_0838	Trifolium arvense L.	0	n	1	0	Fabaceae Lindl.
Iz_3_1009	DJI_0838	Ulmus glabra Huds.	0	n	1	1	Ulmaceae Mirb.
Iz_3_1009	DJI_0838	Verbascum densiflorum Bertol.	0	n	1	0	Scrophulariaceae Juss.
Iz_3_1009	DJI_0838	Veronica austriaca L.	0	n	1	0	Plantaginaceae Juss.
Iz_3_1009	DJI_0838	Veronica incana L.	0	n	1	0	Plantaginaceae Juss.
Iz_3_1009	DJI_0838	Veronica verna L.	0	n	1	0	Plantaginaceae Juss.
Iz_3_1009	DJI_0838	Vincetoxicum hirundinaria Medik	0	n	1	0	Apocynaceae Juss.
Iz_3_1009	DJI_0842	Acer tataricum L.	0	n	1	1	Sapindaceae Juss.
Iz_3_1009	DJI_0842	Achillea millefolium L. s.l.	0	n	1	0	Asteraceae Bercht. & J.Presl
Iz_3_1009	DJI_0842	Allium oleraceum L.	0	n	1	0	Amaryllidaceae J.St.-Hil.
Iz_3_1009	DJI_0842	Ambrosia artemisiifolia L.	0	a	0	0	Asteraceae Bercht. & J.Presl
Iz_3_1009	DJI_0842	Amorpha fruticosa L.	0	a	0	0	Fabaceae Lindl.
Iz_3_1009	DJI_0842	Anthericum ramosum L.	0	n	1	0	Asparagaceae Juss.
Iz_3_1009	DJI_0842	Artemisia marschalliana Spreng.	0	n	1	0	Asteraceae Bercht. & J.Presl
Iz_3_1009	DJI_0842	Asparagus officinalis L.	0	n	1	0	Asparagaceae Juss.
Iz_3_1009	DJI_0842	Astragalus cicer L.	0	n	1	0	Fabaceae Lindl.
Iz_3_1009	DJI_0842	Bassia laniflora (S.G.Gmel.) A.J.Scott	0	n	1	0	Amaranthaceae Juss.
Iz_3_1009	DJI_0842	Berberis vulgaris L.	0	n	1	0	Berberidaceae Juss.
Iz_3_1009	DJI_0842	Betula pendula Roth.	0	n	1	1	Betulaceae Gray
Iz_3_1009	DJI_0842	Betula pendula Roth.	0	n	1	0	Poaceae Barnhart
Iz_3_1009	DJI_0842	Bromus inermis Leyss.	0		NA	NA	
Iz_3_1009	DJI_0842	Bromus squarrosus L.	0	a	0	0	Poaceae Barnhart
Iz_3_1009	DJI_0842	Bromus tectorum L.	0	a	0	0	Poaceae Barnhart
Iz_3_1009	DJI_0842	Calamagrostis epigejos (L.) Roth	40	n	1	0	Poaceae Barnhart
Iz_3_1009	DJI_0842	Carduus acanthoides L.	0	n	1	0	Asteraceae Bercht. & J.Presl
Iz_3_1009	DJI_0842	Carex sp.	0	n	1	0	Cyperaceae Juss.
Iz_3_1009	DJI_0842	Centaurea majorovii Dumbadze	0	n	1	0	Asteraceae Bercht. & J.Presl
Iz_3_1009	DJI_0842	Chamaecytisus ruthenicus (Fisch.ex Wol.) Klaskova	0	n	1	0	Fabaceae Lindl.
Iz_3_1009	DJI_0842	Chelidonium majus L.	0	n	1	0	Papaveraceae Juss.
Iz_3_1009	DJI_0842	Chenopodium betaceum Andrz.	0	n	1	0	Amaranthaceae Juss.
Iz_3_1009	DJI_0842	Chenopodium cf. album	0	n	1	0	Amaranthaceae Juss.
Iz_3_1009	DJI_0842	Chenopodium sp.	0		NA	0	Amaranthaceae Juss.
Iz_3_1009	DJI_0842	Chondrilla juncea L.	0	n	1	0	Asteraceae Bercht. & J.Presl
Iz_3_1009	DJI_0842	Cirsium vulgare (Savi) Ten.	0	n	1	0	Asteraceae Bercht. & J.Presl
Iz_3_1009	DJI_0842	Clinopodium acinos (L.) Kuntze	0	n	1	0	Lamiacea Lindl.
Iz_3_1009	DJI_0842	Convallaria majalis L.	0	n	1	0	Asparagaceae Juss.
Iz_3_1009	DJI_0842	Crataegus rhipidophylla Gand.	0	n	1	1	Rosaceae Juss.
Iz_3_1009	DJI_0842	Crepis foetida L.	0	n	1	0	Asteraceae Bercht. & J.Presl
Iz_3_1009	DJI_0842	Dactylis glomerata L.	0	n	1	0	Poaceae Barnhart
Iz_3_1009	DJI_0842	Dianthus campestris M.Bieb.	0	n	1	0	Caryophyllaceae Juss.
Iz_3_1009	DJI_0842	Digitaria sanguinalis (L.) Scop.	0	a	0	0	Poaceae Barnhart
Iz_3_1009	DJI_0842	Elymus repens (L.) Gould	0	n	1	0	Poaceae Barnhart
Iz_3_1009	DJI_0842	Epilobium tetragonum L.	0	n	1	0	Onagraceae Juss.
Iz_3_1009	DJI_0842	Eragrostis minor Host	0	a	0	0	Poaceae Barnhart
Iz_3_1009	DJI_0842	Erigeron canadensis L.	0	a	0	0	Asteraceae Bercht. & J.Presl
Iz_3_1009	DJI_0842	Euphorbia seguieriana Neck.	0	n	1	0	Euphorbiaceae Juss.
Iz_3_1009	DJI_0842	Fallopia dumetorum (L.) Holub	0	n	1	0	Polygonaceae Juss.
Iz_3_1009	DJI_0842	Festuca beckeri (Hack.) Trautv.	0	n	1	0	Poaceae Barnhart
Iz_3_1009	DJI_0842	Fragaria vesca L.	0	n	1	0	Rosaceae Juss.
Iz_3_1009	DJI_0842	Frangula alnus Mill.	0	n	1	1	Rhamnaceae Juss.
Iz_3_1009	DJI_0842	Fraxinus pennsylvanica Marshall	0	a	0	1	Oleaceae Hoffmanns. & Link
Iz_3_1009	DJI_0842	Galeopsis bifida Boenn.	0	n	1	0	Lamiacea Lindl.
Iz_3_1009	DJI_0842	Galium verum L.	0	n	1	0	Rubiaceae Juss.
Iz_3_1009	DJI_0842	Genista tinctoria L.	0	n	1	0	Fabaceae Lindl.
Iz_3_1009	DJI_0842	Geranium robertianum L.	0	n	1	0	Geraniaceae Juss.
Iz_3_1009	DJI_0842	Geranium sanquineum L.	0	n	1	0	Geraniaceae Juss.
Iz_3_1009	DJI_0842	Gypsophila paniculata L.	0	n	1	0	Caryophyllaceae Juss.
Iz_3_1009	DJI_0842	Helichrysum arenarium (L.) Moench	0	n	1	0	Asteraceae Bercht. & J.Presl
Iz_3_1009	DJI_0842	Herniaria polygama J.Gay	0	n	1	0	Caryophyllaceae Juss.
Iz_3_1009	DJI_0842	Hieracium robustum Fr.	0	n	1	0	Asteraceae Bercht. & J.Presl
Iz_3_1009	DJI_0842	Hieracium umbellatum L.	0	n	1	NA	Asteraceae Bercht. & J.Presl

Iz_3_1009	DJI_0842	Hylotelephium maximum (L.) Holub	0	n	1	0	Crassulaceae J.St.-Hil.
Iz_3_1009	DJI_0842	Jacobaea borysthenica (DC.) B.Nord. & Greuter	0	n	1	0	Asteraceae Bercht. & J.Presl
Iz_3_1009	DJI_0842	Jasione montana L.	0	n	1	0	Campanulaceae Juss.
Iz_3_1009	DJI_0842	Jurinea cyanoides (L.) Rchb.s.l.	0	n	1	0	Asteraceae Bercht. & J.Presl
Iz_3_1009	DJI_0842	Koeleria glauca (Spreng.) DC.	0	n	1	0	Poaceae Barnhart
Iz_3_1009	DJI_0842	Lactuca serriola L.	0	a	0	0	Asteraceae Bercht. & J.Presl
Iz_3_1009	DJI_0842	Linaria genistifolia (L.) Mill.	0	n	1	0	Plantaginaceae Juss.
Iz_3_1009	DJI_0842	Lomelosia argentea (L.) Greuter & Burdet	0	n	1	0	Caprifoliaceae Juss.
Iz_3_1009	DJI_0842	Melampyrum pratense L.	0	n	1	0	Orobanchaceae Vent.
Iz_3_1009	DJI_0842	Oenothera biennis L.	0	a	0	0	Onagraceae Juss.
Iz_3_1009	DJI_0842	Pentanema britannica (L.) D.Gut.Larr., Santos-Vicente, Anderb., E.Rico & M.M.Mart.Ort.	0	n	1	0	Asteraceae Bercht. & J.Presl
Iz_3_1009	DJI_0842	Peucedanum oreoselinum (L.) Moench	0	n	1	0	Apiaceae Lindl.
Iz_3_1009	DJI_0842	Peucedanum ruthenicum M.Bieb.	0	n	1	0	Apiaceae Lindl.
Iz_3_1009	DJI_0842	Pilosella echioides (Lumn.) F.W.Schultz & Sch.Bip.	0	n	1	0	Asteraceae Bercht. & J.Presl
Iz_3_1009	DJI_0842	Pilosella officinarum F. Schultz et Sch. Bip.	0	n	1	0	Asteraceae Bercht. & J.Presl
Iz_3_1009	DJI_0842	Pilosella sp.	0		NA	0	Asteraceae Bercht. & J.Presl
Iz_3_1009	DJI_0842	Pinus sylvestris L.	0	n	1	1	Pinaceae Spreng. ex F.Rudolphi
Iz_3_1009	DJI_0842	Poa annua L.	0	n	1	0	Poaceae Barnhart
Iz_3_1009	DJI_0842	Poa nemoralis L.	0	n	1	0	Poaceae Barnhart
Iz_3_1009	DJI_0842	Poa sp.	0		NA	0	Poaceae Barnhart
Iz_3_1009	DJI_0842	Polygonatum odoratum (Mill.) Druce	0	n	1	0	Asparagaceae Juss.
Iz_3_1009	DJI_0842	Polygonum arenarium Waldst. & Kit.	0	n	1	0	Polygonaceae Juss.
Iz_3_1009	DJI_0842	Populus nigra L.	0	n	1	1	Salicaceae Mirb.
Iz_3_1009	DJI_0842	Populus tremula L.	0	n	1	1	Salicaceae Mirb.
Iz_3_1009	DJI_0842	Populus Ã canescens (Aiton) Sm.	0	n	1	1	Salicaceae Mirb.
Iz_3_1009	DJI_0842	Potentilla argentea L.	0	n	1	0	Rosaceae Juss.
Iz_3_1009	DJI_0842	Psammophiliella muralis (L.) Ikonn.	0	n	1	0	Caryophyllaceae Juss.
Iz_3_1009	DJI_0842	Psephellus sumensis (Kalen.) Greuter	0	n	1	0	Asteraceae Bercht. & J.Presl
Iz_3_1009	DJI_0842	Pulsatilla pratensis (L.) Mill. s.l.	0	n	1	0	Ranunculaceae Juss.
Iz_3_1009	DJI_0842	Pyrus communis L.	0	a	0	1	Rosaceae Juss.
Iz_3_1009	DJI_0842	Quercus robur L.	0	n	1	1	Fagaceae Dumort.
Iz_3_1009	DJI_0842	Robinia pseudoacacia L.	0	a	0	1	Fabaceae Lindl.
Iz_3_1009	DJI_0842	Rosa sp.	0		NA	1	Rosaceae Juss.
Iz_3_1009	DJI_0842	Rubus idaeus L.	0	n	1	0	Rosaceae Juss.
Iz_3_1009	DJI_0842	Rumex acetosella L.	0	n	1	0	Polygonaceae Juss.
Iz_3_1009	DJI_0842	Rumex thyrsiflorus Fingerh.	0	n	1	0	Polygonaceae Juss.
Iz_3_1009	DJI_0842	Salix alba L.	0	n	1	1	Salicaceae Mirb.
Iz_3_1009	DJI_0842	Sambucus nigra L.	0	n	1	1	Viburnaceae Raf.
Iz_3_1009	DJI_0842	Secale sylvestre Host	0	n	1	0	Poaceae Barnhart
Iz_3_1009	DJI_0842	Sempervivum ruthenicum Schnittsp. & C.B. Lehm.	0	n	1	0	Crassulaceae J.St.-Hil.
Iz_3_1009	DJI_0842	Setaria viridis (L.) P. Beauv.	0	n	1	0	Poaceae Barnhart
Iz_3_1009	DJI_0842	Silene borysthenica (Gruner) Walters	0	n	1	0	Caryophyllaceae Juss.
Iz_3_1009	DJI_0842	Silene latifolia subsp. alba (Mill.) Greuter & Burdet	0	n	1	0	Caryophyllaceae Juss.
Iz_3_1009	DJI_0842	Solidago virgaurea L.	0	n	1	0	Asteraceae Bercht. & J.Presl
Iz_3_1009	DJI_0842	Sonchus oleraceus L.	1	a	0	0	Asteraceae Bercht. & J.Presl
Iz_3_1009	DJI_0842	Sorbus aucuparia L.	0	n	1	1	Rosaceae Juss.
Iz_3_1009	DJI_0842	Stachys recta L.	0	n	1	0	Lamiacea Lindl.
Iz_3_1009	DJI_0842	Tanacetum vulgare L.	0	n	1	0	Asteraceae Bercht. & J.Presl
Iz_3_1009	DJI_0842	Thymus pallasianus Heinr.Braun	0	n	1	0	Lamiacea Lindl.
Iz_3_1009	DJI_0842	Tilia cordata Mill.	0	n	1	1	Malvaceae Juss.
Iz_3_1009	DJI_0842	Trifolium alpestre L.	0	n	1	0	Fabaceae Lindl.
Iz_3_1009	DJI_0842	Trifolium arvense L.	0	n	1	0	Fabaceae Lindl.
Iz_3_1009	DJI_0842	Ulmus glabra Huds.	0	n	1	1	Ulmaceae Mirb.
Iz_3_1009	DJI_0842	Verbascum densiflorum Bertol.	0	n	1	0	Scrophulariaceae Juss.
Iz_3_1009	DJI_0842	Veronica austriaca L.	0	n	1	0	Plantaginaceae Juss.
Iz_3_1009	DJI_0842	Veronica incana L.	0	n	1	0	Plantaginaceae Juss.
Iz_3_1009	DJI_0842	Veronica verna L.	0	n	1	0	Plantaginaceae Juss.
Iz_3_1009	DJI_0842	Vincetoxicum hirundinaria Medik	0	n	1	0	Apocynaceae Juss.
Iz_3_1009	DJI_0843	Acer tataricum L.	0	n	1	1	Sapindaceae Juss.
Iz_3_1009	DJI_0843	Achillea millefolium L. s.l.	0	n	1	0	Asteraceae Bercht. & J.Presl
Iz_3_1009	DJI_0843	Allium oleraceum L.	0	n	1	0	Amaryllidaceae J.St.-Hil.
Iz_3_1009	DJI_0843	Ambrosia artemisiifolia L.	0	a	0	0	Asteraceae Bercht. & J.Presl
Iz_3_1009	DJI_0843	Amorpha fruticosa L.	0	a	0	0	Fabaceae Lindl.
Iz_3_1009	DJI_0843	Anthericum ramosum L.	0	n	1	0	Asparagaceae Juss.
Iz_3_1009	DJI_0843	Artemisia marschalliana Spreng.	0	n	1	0	Asteraceae Bercht. & J.Presl
Iz_3_1009	DJI_0843	Asparagus officinalis L.	0	n	1	0	Asparagaceae Juss.
Iz_3_1009	DJI_0843	Astragalus cicer L.	0	n	1	0	Fabaceae Lindl.
Iz_3_1009	DJI_0843	Bassia laniflora (S.G.Gmel.) A.J.Scott	0	n	1	0	Amaranthaceae Juss.
Iz_3_1009	DJI_0843	Berberis vulgaris L.	0	n	1	0	Berberidaceae Juss.
Iz_3_1009	DJI_0843	Betula pendula Roth.	0	n	1	1	Betulaceae Gray
Iz_3_1009	DJI_0843	Betula pendula Roth.	0	n	1	0	Poaceae Barnhart
Iz_3_1009	DJI_0843	Bromus inermis Leyss.	0		NA	NA	
Iz_3_1009	DJI_0843	Bromus squarrosus L.	0	a	0	0	Poaceae Barnhart
Iz_3_1009	DJI_0843	Bromus tectorum L.	0	a	0	0	Poaceae Barnhart
Iz_3_1009	DJI_0843	Calamagrostis epigejos (L.) Roth	60	n	1	0	Poaceae Barnhart
Iz_3_1009	DJI_0843	Carduus acanthoides L.	0	n	1	0	Asteraceae Bercht. & J.Presl
Iz_3_1009	DJI_0843	Carex sp.	0	n	1	0	Cyperaceae Juss.
Iz_3_1009	DJI_0843	Centaurea majorovii Dumbadze	0	n	1	0	Asteraceae Bercht. & J.Presl
Iz_3_1009	DJI_0843	Chamaecytisus ruthenicus (Fisch.ex Wol.) Klaskova	0	n	1	0	Fabaceae Lindl.
Iz_3_1009	DJI_0843	Chelidonium majus L.	0	n	1	0	Papaveraceae Juss.
Iz_3_1009	DJI_0843	Chenopodium betaceum Andrz.	0	n	1	0	Amaranthaceae Juss.
Iz_3_1009	DJI_0843	Chenopodium cf. album	0	n	1	0	Amaranthaceae Juss.
Iz_3_1009	DJI_0843	Chenopodium sp.	0		NA	0	Amaranthaceae Juss.
Iz_3_1009	DJI_0843	Chondrilla juncea L.	0	n	1	0	Asteraceae Bercht. & J.Presl
Iz_3_1009	DJI_0843	Cirsium vulgare (Savi) Ten.	0	n	1	0	Asteraceae Bercht. & J.Presl
Iz_3_1009	DJI_0843	Clinopodium acinos (L.) Kuntze	0	n	1	0	Lamiacea Lindl.
Iz_3_1009	DJI_0843	Convallaria majalis L.	0	n	1	0	Asparagaceae Juss.
Iz_3_1009	DJI_0843	Crataegus rhipidophylla Gand.	0	n	1	1	Rosaceae Juss.
Iz_3_1009	DJI_0843	Crepis foetida L.	0	n	1	0	Asteraceae Bercht. & J.Presl
Iz_3_1009	DJI_0843	Dactylis glomerata L.	0	n	1	0	Poaceae Barnhart
Iz_3_1009	DJI_0843	Dianthus campestris M.Bieb.	0	n	1	0	Caryophyllaceae Juss.
Iz_3_1009	DJI_0843	Digitaria sanguinalis (L.) Scop.	0	a	0	0	Poaceae Barnhart
Iz_3_1009	DJI_0843	Elymus repens (L.) Gould	0	n	1	0	Poaceae Barnhart
Iz_3_1009	DJI_0843	Epilobium tetragonum L.	0	n	1	0	Onagraceae Juss.
Iz_3_1009	DJI_0843	Eragrostis minor Host	0	a	0	0	Poaceae Barnhart
Iz_3_1009	DJI_0843	Erigeron canadensis L.	0	a	0	0	Asteraceae Bercht. & J.Presl
Iz_3_1009	DJI_0843	Euphorbia seguieriana Neck.	0	n	1	0	Euphorbiaceae Juss.
Iz_3_1009	DJI_0843	Fallopia dumetorum (L.) Holub	0	n	1	0	Polygonaceae Juss.

Iz_3_1009	DJI_0843	Festuca beckeri (Hack.) Trautv.	0	n	1	0	Poaceae Barnhart
Iz_3_1009	DJI_0843	Fragaria vesca L.	0	n	1	0	Rosaceae Juss.
Iz_3_1009	DJI_0843	Frangula alnus Mill.	0	n	1	1	Rhamnaceae Juss.
Iz_3_1009	DJI_0843	Fraxinus pennsylvanica Marshall	0	a	0	1	Oleaceae Hoffmanns. & Link
Iz_3_1009	DJI_0843	Galeopsis bifida Boenn.	0	n	1	0	Lamiacea Lindl.
Iz_3_1009	DJI_0843	Galium verum L.	0	n	1	0	Rubiaceae Juss.
Iz_3_1009	DJI_0843	Genista tinctoria L.	0	n	1	0	Fabaceae Lindl.
Iz_3_1009	DJI_0843	Geranium robertianum L.	0	n	1	0	Geraniaceae Juss.
Iz_3_1009	DJI_0843	Geranium sanquineum L.	0	n	1	0	Geraniaceae Juss.
Iz_3_1009	DJI_0843	Gypsophila paniculata L.	0	n	1	0	Caryophyllaceae Juss.
Iz_3_1009	DJI_0843	Helichrysum arenarium (L.) Moench	0	n	1	0	Asteraceae Bercht. & J.Presl
Iz_3_1009	DJI_0843	Herniaria polygama J.Gay	0	n	1	0	Caryophyllaceae Juss.
Iz_3_1009	DJI_0843	Hieracium robustum Fr.	0	n	1	0	Asteraceae Bercht. & J.Presl
Iz_3_1009	DJI_0843	Hieracium umbellatum L.	0	n	1	NA	Asteraceae Bercht. & J.Presl
Iz_3_1009	DJI_0843	Hylotelephium maximum (L.) Holub	0	n	1	0	Crassulaceae J.St.-Hil.
Iz_3_1009	DJI_0843	Jacobaea borysthenica (DC.) B.Nord. & Greuter	0	n	1	0	Asteraceae Bercht. & J.Presl
Iz_3_1009	DJI_0843	Jasione montana L.	0	n	1	0	Campanulaceae Juss.
Iz_3_1009	DJI_0843	Jurinea cyanoides (L.) Rchb.s.l.	0	n	1	0	Asteraceae Bercht. & J.Presl
Iz_3_1009	DJI_0843	Koeleria glauca (Spreng.) DC.	0	n	1	0	Poaceae Barnhart
Iz_3_1009	DJI_0843	Lactuca serriola L.	0	a	0	0	Asteraceae Bercht. & J.Presl
Iz_3_1009	DJI_0843	Linaria genistifolia (L.) Mill.	0	n	1	0	Plantaginaceae Juss.
Iz_3_1009	DJI_0843	Lomelosia argentea (L.) Greuter & Burdet	0	n	1	0	Caprifoliaceae Juss.
Iz_3_1009	DJI_0843	Melampyrum pratense L.	0	n	1	0	Orobanchaceae Vent.
Iz_3_1009	DJI_0843	Oenothera biennis L.	0	a	0	0	Onagraceae Juss.
Iz_3_1009	DJI_0843	Pentanema britannica (L.) D.Gut.Larr., Santos-Vicente, Anderb., E.Rico & M.M.Mart.Ort.	0	n	1	0	Asteraceae Bercht. & J.Presl
Iz_3_1009	DJI_0843	Peucedanum oreoselinum (L.) Moench	3	n	1	0	Apiaceae Lindl.
Iz_3_1009	DJI_0843	Peucedanum ruthenicum M.Bieb.	0	n	1	0	Apiaceae Lindl.
Iz_3_1009	DJI_0843	Pilosella echioides (Lumn.) F.W.Schultz & Sch.Bip.	0	n	1	0	Asteraceae Bercht. & J.Presl
Iz_3_1009	DJI_0843	Pilosella officinarum F. Schultz et Sch. Bip.	0	n	1	0	Asteraceae Bercht. & J.Presl
Iz_3_1009	DJI_0843	Pilosella sp.	0		NA	0	Asteraceae Bercht. & J.Presl
Iz_3_1009	DJI_0843	Pinus sylvestris L.	0	n	1	1	Pinaceae Spreng. ex F.Rudolphi
Iz_3_1009	DJI_0843	Poa annua L.	0	n	1	0	Poaceae Barnhart
Iz_3_1009	DJI_0843	Poa nemoralis L.	0	n	1	0	Poaceae Barnhart
Iz_3_1009	DJI_0843	Poa sp.	0		NA	0	Poaceae Barnhart
Iz_3_1009	DJI_0843	Polygonatum odoratum (Mill.) Druce	0.5	n	1	0	Asparagaceae Juss.
Iz_3_1009	DJI_0843	Polygonum arenarium Waldst. & Kit.	0	n	1	0	Polygonaceae Juss.
Iz_3_1009	DJI_0843	Populus nigra L.	0	n	1	1	Salicaceae Mirb.
Iz_3_1009	DJI_0843	Populus tremula L.	0	n	1	1	Salicaceae Mirb.
Iz_3_1009	DJI_0843	Populus Ã canescens (Aiton) Sm.	0	n	1	1	Salicaceae Mirb.
Iz_3_1009	DJI_0843	Potentilla argentea L.	0	n	1	0	Rosaceae Juss.
Iz_3_1009	DJI_0843	Psammophiliella muralis (L.) Ikonn.	0	n	1	0	Caryophyllaceae Juss.
Iz_3_1009	DJI_0843	Psephellus sumensis (Kalen.) Greuter	0	n	1	0	Asteraceae Bercht. & J.Presl
Iz_3_1009	DJI_0843	Pulsatilla pratensis (L.) Mill. s.l.	0	n	1	0	Ranunculaceae Juss.
Iz_3_1009	DJI_0843	Pyrus communis L.	0	a	0	1	Rosaceae Juss.
Iz_3_1009	DJI_0843	Quercus robur L.	0	n	1	1	Fagaceae Dumort.
Iz_3_1009	DJI_0843	Robinia pseudoacacia L.	0	a	0	1	Fabaceae Lindl.
Iz_3_1009	DJI_0843	Rosa sp.	0		NA	1	Rosaceae Juss.
Iz_3_1009	DJI_0843	Rubus idaeus L.	0	n	1	0	Rosaceae Juss.
Iz_3_1009	DJI_0843	Rumex acetosella L.	0	n	1	0	Polygonaceae Juss.
Iz_3_1009	DJI_0843	Rumex thyrsiflorus Fingerh.	0	n	1	0	Polygonaceae Juss.
Iz_3_1009	DJI_0843	Salix alba L.	0	n	1	1	Salicaceae Mirb.
Iz_3_1009	DJI_0843	Sambucus nigra L.	0	n	1	1	Viburnaceae Raf.
Iz_3_1009	DJI_0843	Secale sylvestre Host	0	n	1	0	Poaceae Barnhart
Iz_3_1009	DJI_0843	Sempervivum ruthenicum Schnittsp. & C.B. Lehm.	0	n	1	0	Crassulaceae J.St.-Hil.
Iz_3_1009	DJI_0843	Setaria viridis (L.) P. Beauv.	0	n	1	0	Poaceae Barnhart
Iz_3_1009	DJI_0843	Silene borysthenica (Gruner) Walters	0	n	1	0	Caryophyllaceae Juss.
Iz_3_1009	DJI_0843	Silene latifolia subsp. alba (Mill.) Greuter & Burdet	0	n	1	0	Caryophyllaceae Juss.
Iz_3_1009	DJI_0843	Solidago virgaurea L.	0	n	1	0	Asteraceae Bercht. & J.Presl
Iz_3_1009	DJI_0843	Sonchus oleraceus L.	0	a	0	0	Asteraceae Bercht. & J.Presl
Iz_3_1009	DJI_0843	Sorbus aucuparia L.	0	n	1	1	Rosaceae Juss.
Iz_3_1009	DJI_0843	Stachys recta L.	0	n	1	0	Lamiacea Lindl.
Iz_3_1009	DJI_0843	Tanacetum vulgare L.	0	n	1	0	Asteraceae Bercht. & J.Presl
Iz_3_1009	DJI_0843	Thymus pallasianus Heinr.Braun	0	n	1	0	Lamiacea Lindl.
Iz_3_1009	DJI_0843	Tilia cordata Mill.	0	n	1	1	Malvaceae Juss.
Iz_3_1009	DJI_0843	Trifolium alpestre L.	0	n	1	0	Fabaceae Lindl.
Iz_3_1009	DJI_0843	Trifolium arvense L.	0	n	1	0	Fabaceae Lindl.
Iz_3_1009	DJI_0843	Ulmus glabra Huds.	0	n	1	1	Ulmaceae Mirb.
Iz_3_1009	DJI_0843	Verbascum densiflorum Bertol.	0	n	1	0	Scrophulariaceae Juss.
Iz_3_1009	DJI_0843	Veronica austriaca L.	0	n	1	0	Plantaginaceae Juss.
Iz_3_1009	DJI_0843	Veronica incana L.	0	n	1	0	Plantaginaceae Juss.
Iz_3_1009	DJI_0843	Veronica verna L.	0	n	1	0	Plantaginaceae Juss.
Iz_3_1009	DJI_0843	Vincetoxicum hirundinaria Medik	0	n	1	0	Apocynaceae Juss.
Iz_3_1009	DJI_0845	Acer tataricum L.	0	n	1	1	Sapindaceae Juss.
Iz_3_1009	DJI_0845	Achillea millefolium L. s.l.	0	n	1	0	Asteraceae Bercht. & J.Presl
Iz_3_1009	DJI_0845	Allium oleraceum L.	0	n	1	0	Amaryllidaceae J.St.-Hil.
Iz_3_1009	DJI_0845	Ambrosia artemisiifolia L.	0	a	0	0	Asteraceae Bercht. & J.Presl
Iz_3_1009	DJI_0845	Amorpha fruticosa L.	0	a	0	0	Fabaceae Lindl.
Iz_3_1009	DJI_0845	Anthericum ramosum L.	0	n	1	0	Asparagaceae Juss.
Iz_3_1009	DJI_0845	Artemisia marschalliana Spreng.	0	n	1	0	Asteraceae Bercht. & J.Presl
Iz_3_1009	DJI_0845	Asparagus officinalis L.	0	n	1	0	Asparagaceae Juss.
Iz_3_1009	DJI_0845	Astragalus cicer L.	0	n	1	0	Fabaceae Lindl.
Iz_3_1009	DJI_0845	Bassia laniflora (S.G.Gmel.) A.J.Scott	0	n	1	0	Amaranthaceae Juss.
Iz_3_1009	DJI_0845	Berberis vulgaris L.	0	n	1	0	Berberidaceae Juss.
Iz_3_1009	DJI_0845	Betula pendula Roth.	0	n	1	1	Betulaceae Gray
Iz_3_1009	DJI_0845	Betula pendula Roth.	0	n	1	0	Poaceae Barnhart
Iz_3_1009	DJI_0845	Bromus inermis Leyss.	0		NA	NA	
Iz_3_1009	DJI_0845	Bromus squarrosus L.	0	a	0	0	Poaceae Barnhart
Iz_3_1009	DJI_0845	Bromus tectorum L.	0	a	0	0	Poaceae Barnhart
Iz_3_1009	DJI_0845	Calamagrostis epigejos (L.) Roth	40	n	1	0	Poaceae Barnhart
Iz_3_1009	DJI_0845	Carduus acanthoides L.	0	n	1	0	Asteraceae Bercht. & J.Presl
Iz_3_1009	DJI_0845	Carex sp.	0	n	1	0	Cyperaceae Juss.
Iz_3_1009	DJI_0845	Centaurea majorovii Dumbadze	0	n	1	0	Asteraceae Bercht. & J.Presl
Iz_3_1009	DJI_0845	Chamaecytisus ruthenicus (Fisch.ex Wol.) Klaskova	0	n	1	0	Fabaceae Lindl.
Iz_3_1009	DJI_0845	Chelidonium majus L.	0	n	1	0	Papaveraceae Juss.
Iz_3_1009	DJI_0845	Chenopodium betaceum Andrz.	0	n	1	0	Amaranthaceae Juss.
Iz_3_1009	DJI_0845	Chenopodium cf. album	0	n	1	0	Amaranthaceae Juss.
Iz_3_1009	DJI_0845	Chenopodium sp.	0		NA	0	Amaranthaceae Juss.
Iz_3_1009	DJI_0845	Chondrilla juncea L.	0	n	1	0	Asteraceae Bercht. & J.Presl

Iz_3_1009	DJI_0845	Cirsium vulgare (Savi) Ten.	0	n	1	0	Asteraceae Bercht. & J.Presl
Iz_3_1009	DJI_0845	Clinopodium acinos (L.) Kuntze	0	n	1	0	Lamiacea Lindl.
Iz_3_1009	DJI_0845	Convallaria majalis L.	0	n	1	0	Asparagaceae Juss.
Iz_3_1009	DJI_0845	Crataegus rhipidophylla Gand.	0	n	1	1	Rosaceae Juss.
Iz_3_1009	DJI_0845	Crepis foetida L.	0	n	1	0	Asteraceae Bercht. & J.Presl
Iz_3_1009	DJI_0845	Dactylis glomerata L.	0	n	1	0	Poaceae Barnhart
Iz_3_1009	DJI_0845	Dianthus campestris M.Bieb.	0	n	1	0	Caryophyllaceae Juss.
Iz_3_1009	DJI_0845	Digitaria sanguinalis (L.) Scop.	0	a	0	0	Poaceae Barnhart
Iz_3_1009	DJI_0845	Elymus repens (L.) Gould	0	n	1	0	Poaceae Barnhart
Iz_3_1009	DJI_0845	Epilobium tetragonum L.	0	n	1	0	Onagraceae Juss.
Iz_3_1009	DJI_0845	Eragrostis minor Host	0	a	0	0	Poaceae Barnhart
Iz_3_1009	DJI_0845	Erigeron canadensis L.	0	a	0	0	Asteraceae Bercht. & J.Presl
Iz_3_1009	DJI_0845	Euphorbia seguieriana Neck.	0	n	1	0	Euphorbiaceae Juss.
Iz_3_1009	DJI_0845	Fallopia dumetorum (L.) Holub	0	n	1	0	Polygonaceae Juss.
Iz_3_1009	DJI_0845	Festuca beckeri (Hack.) Trautv.	0	n	1	0	Poaceae Barnhart
Iz_3_1009	DJI_0845	Fragaria vesca L.	0	n	1	0	Rosaceae Juss.
Iz_3_1009	DJI_0845	Frangula alnus Mill.	0	n	1	1	Rhamnaceae Juss.
Iz_3_1009	DJI_0845	Fraxinus pennsylvanica Marshall	0	a	0	1	Oleaceae Hoffmanns. & Link
Iz_3_1009	DJI_0845	Galeopsis bifida Boenn.	0	n	1	0	Lamiacea Lindl.
Iz_3_1009	DJI_0845	Galium verum L.	0	n	1	0	Rubiaceae Juss.
Iz_3_1009	DJI_0845	Genista tinctoria L.	0	n	1	0	Fabaceae Lindl.
Iz_3_1009	DJI_0845	Geranium robertianum L.	0	n	1	0	Geraniaceae Juss.
Iz_3_1009	DJI_0845	Geranium sanquineum L.	0	n	1	0	Geraniaceae Juss.
Iz_3_1009	DJI_0845	Gypsophila paniculata L.	0	n	1	0	Caryophyllaceae Juss.
Iz_3_1009	DJI_0845	Helichrysum arenarium (L.) Moench	0	n	1	0	Asteraceae Bercht. & J.Presl
Iz_3_1009	DJI_0845	Herniaria polygama J.Gay	0	n	1	0	Caryophyllaceae Juss.
Iz_3_1009	DJI_0845	Hieracium robustum Fr.	0	n	1	0	Asteraceae Bercht. & J.Presl
Iz_3_1009	DJI_0845	Hieracium umbellatum L.	0	n	1	NA	Asteraceae Bercht. & J.Presl
Iz_3_1009	DJI_0845	Hylotelephium maximum (L.) Holub	0	n	1	0	Crassulaceae J.St.-Hil.
Iz_3_1009	DJI_0845	Jacobaea borysthenica (DC.) B.Nord. & Greuter	0	n	1	0	Asteraceae Bercht. & J.Presl
Iz_3_1009	DJI_0845	Jasione montana L.	0	n	1	0	Campanulaceae Juss.
Iz_3_1009	DJI_0845	Jurinea cyanoides (L.) Rchb.s.l.	0	n	1	0	Asteraceae Bercht. & J.Presl
Iz_3_1009	DJI_0845	Koeleria glauca (Spreng.) DC.	0	n	1	0	Poaceae Barnhart
Iz_3_1009	DJI_0845	Lactuca serriola L.	0	a	0	0	Asteraceae Bercht. & J.Presl
Iz_3_1009	DJI_0845	Linaria genistifolia (L.) Mill.	0	n	1	0	Plantaginaceae Juss.
Iz_3_1009	DJI_0845	Lomelosia argentea (L.) Greuter & Burdet	0	n	1	0	Caprifoliaceae Juss.
Iz_3_1009	DJI_0845	Melampyrum pratense L.	0	n	1	0	Orobanchaceae Vent.
Iz_3_1009	DJI_0845	Oenothera biennis L.	0	a	0	0	Onagraceae Juss.
Iz_3_1009	DJI_0845	Pentanema britannica (L.) D.Gut.Larr., Santos-Vicente, Anderb., E.Rico & M.M.Mart.Ort.	0	n	1	0	Asteraceae Bercht. & J.Presl
Iz_3_1009	DJI_0845	Peucedanum oreoselinum (L.) Moench	10	n	1	0	Apiaceae Lindl.
Iz_3_1009	DJI_0845	Peucedanum ruthenicum M.Bieb.	0	n	1	0	Apiaceae Lindl.
Iz_3_1009	DJI_0845	Pilosella echioides (Lumn.) F.W.Schultz & Sch.Bip.	0	n	1	0	Asteraceae Bercht. & J.Presl
Iz_3_1009	DJI_0845	Pilosella officinarum F. Schultz et Sch. Bip.	0	n	1	0	Asteraceae Bercht. & J.Presl
Iz_3_1009	DJI_0845	Pilosella sp.	0		NA	0	Asteraceae Bercht. & J.Presl
Iz_3_1009	DJI_0845	Pinus sylvestris L.	0	n	1	1	Pinaceae Spreng. ex F.Rudolphi
Iz_3_1009	DJI_0845	Poa annua L.	0	n	1	0	Poaceae Barnhart
Iz_3_1009	DJI_0845	Poa nemoralis L.	0	n	1	0	Poaceae Barnhart
Iz_3_1009	DJI_0845	Poa sp.	0		NA	0	Poaceae Barnhart
Iz_3_1009	DJI_0845	Polygonatum odoratum (Mill.) Druce	0	n	1	0	Asparagaceae Juss.
Iz_3_1009	DJI_0845	Polygonum arenarium Waldst. & Kit.	0	n	1	0	Polygonaceae Juss.
Iz_3_1009	DJI_0845	Populus nigra L.	0	n	1	1	Salicaceae Mirb.
Iz_3_1009	DJI_0845	Populus tremula L.	0	n	1	1	Salicaceae Mirb.
Iz_3_1009	DJI_0845	Populus Ã canescens (Aiton) Sm.	0	n	1	1	Salicaceae Mirb.
Iz_3_1009	DJI_0845	Potentilla argentea L.	0	n	1	0	Rosaceae Juss.
Iz_3_1009	DJI_0845	Psammophiliella muralis (L.) Ikonn.	0	n	1	0	Caryophyllaceae Juss.
Iz_3_1009	DJI_0845	Psephellus sumensis (Kalen.) Greuter	0	n	1	0	Asteraceae Bercht. & J.Presl
Iz_3_1009	DJI_0845	Pulsatilla pratensis (L.) Mill. s.l.	0	n	1	0	Ranunculaceae Juss.
Iz_3_1009	DJI_0845	Pyrus communis L.	0	a	0	1	Rosaceae Juss.
Iz_3_1009	DJI_0845	Quercus robur L.	0	n	1	1	Fagaceae Dumort.
Iz_3_1009	DJI_0845	Robinia pseudoacacia L.	0	a	0	1	Fabaceae Lindl.
Iz_3_1009	DJI_0845	Rosa sp.	0		NA	1	Rosaceae Juss.
Iz_3_1009	DJI_0845	Rubus idaeus L.	0	n	1	0	Rosaceae Juss.
Iz_3_1009	DJI_0845	Rumex acetosella L.	0	n	1	0	Polygonaceae Juss.
Iz_3_1009	DJI_0845	Rumex thyrsiflorus Fingerh.	0	n	1	0	Polygonaceae Juss.
Iz_3_1009	DJI_0845	Salix alba L.	0	n	1	1	Salicaceae Mirb.
Iz_3_1009	DJI_0845	Sambucus nigra L.	0	n	1	1	Viburnaceae Raf.
Iz_3_1009	DJI_0845	Secale sylvestre Host	0	n	1	0	Poaceae Barnhart
Iz_3_1009	DJI_0845	Sempervivum ruthenicum Schnittsp. & C.B. Lehm.	0	n	1	0	Crassulaceae J.St.-Hil.
Iz_3_1009	DJI_0845	Setaria viridis (L.) P. Beauv.	0	n	1	0	Poaceae Barnhart
Iz_3_1009	DJI_0845	Silene borysthenica (Gruner) Walters	0	n	1	0	Caryophyllaceae Juss.
Iz_3_1009	DJI_0845	Silene latifolia subsp. alba (Mill.) Greuter & Burdet	0	n	1	0	Caryophyllaceae Juss.
Iz_3_1009	DJI_0845	Solidago virgaurea L.	0	n	1	0	Asteraceae Bercht. & J.Presl
Iz_3_1009	DJI_0845	Sonchus oleraceus L.	0	a	0	0	Asteraceae Bercht. & J.Presl
Iz_3_1009	DJI_0845	Sorbus aucuparia L.	0	n	1	1	Rosaceae Juss.
Iz_3_1009	DJI_0845	Stachys recta L.	0	n	1	0	Lamiacea Lindl.
Iz_3_1009	DJI_0845	Tanacetum vulgare L.	0	n	1	0	Asteraceae Bercht. & J.Presl
Iz_3_1009	DJI_0845	Thymus pallasianus Heinr.Braun	0	n	1	0	Lamiacea Lindl.
Iz_3_1009	DJI_0845	Tilia cordata Mill.	0	n	1	1	Malvaceae Juss.
Iz_3_1009	DJI_0845	Trifolium alpestre L.	0	n	1	0	Fabaceae Lindl.
Iz_3_1009	DJI_0845	Trifolium arvense L.	0	n	1	0	Fabaceae Lindl.
Iz_3_1009	DJI_0845	Ulmus glabra Huds.	0	n	1	1	Ulmaceae Mirb.
Iz_3_1009	DJI_0845	Verbascum densiflorum Bertol.	0	n	1	0	Scrophulariaceae Juss.
Iz_3_1009	DJI_0845	Veronica austriaca L.	0	n	1	0	Plantaginaceae Juss.
Iz_3_1009	DJI_0845	Veronica incana L.	0	n	1	0	Plantaginaceae Juss.
Iz_3_1009	DJI_0845	Veronica verna L.	0	n	1	0	Plantaginaceae Juss.
Iz_3_1009	DJI_0845	Vincetoxicum hirundinaria Medik	0	n	1	0	Apocynaceae Juss.
Iz_3_1009	DJI_0850	Acer tataricum L.	0	n	1	1	Sapindaceae Juss.
Iz_3_1009	DJI_0850	Achillea millefolium L. s.l.	0	n	1	0	Asteraceae Bercht. & J.Presl
Iz_3_1009	DJI_0850	Allium oleraceum L.	0	n	1	0	Amaryllidaceae J.St.-Hil.
Iz_3_1009	DJI_0850	Ambrosia artemisiifolia L.	0	a	0	0	Asteraceae Bercht. & J.Presl
Iz_3_1009	DJI_0850	Amorpha fruticosa L.	0	a	0	0	Fabaceae Lindl.
Iz_3_1009	DJI_0850	Anthericum ramosum L.	0	n	1	0	Asparagaceae Juss.
Iz_3_1009	DJI_0850	Artemisia marschalliana Spreng.	0	n	1	0	Asteraceae Bercht. & J.Presl
Iz_3_1009	DJI_0850	Asparagus officinalis L.	0	n	1	0	Asparagaceae Juss.
Iz_3_1009	DJI_0850	Astragalus cicer L.	0	n	1	0	Fabaceae Lindl.
Iz_3_1009	DJI_0850	Bassia laniflora (S.G.Gmel.) A.J.Scott	0	n	1	0	Amaranthaceae Juss.
Iz_3_1009	DJI_0850	Berberis vulgaris L.	0	n	1	0	Berberidaceae Juss.
Iz_3_1009	DJI_0850	Betula pendula Roth.	0	n	1	1	Betulaceae Gray

Iz_3_1009	DJI_0850	Betula pendula Roth.	0	n	1	0	Poaceae Barnhart
Iz_3_1009	DJI_0850	Bromus inermis Leyss.	0		NA	NA	
Iz_3_1009	DJI_0850	Bromus squarrosus L.	0	a	0	0	Poaceae Barnhart
Iz_3_1009	DJI_0850	Bromus tectorum L.	0	a	0	0	Poaceae Barnhart
Iz_3_1009	DJI_0850	Calamagrostis epigejos (L.) Roth	25	n	1	0	Poaceae Barnhart
Iz_3_1009	DJI_0850	Carduus acanthoides L.	0	n	1	0	Asteraceae Bercht. & J.Presl
Iz_3_1009	DJI_0850	Carex sp.	0	n	1	0	Cyperaceae Juss.
Iz_3_1009	DJI_0850	Centaurea majorovii Dumbadze	0	n	1	0	Asteraceae Bercht. & J.Presl
Iz_3_1009	DJI_0850	Chamaecytisus ruthenicus (Fisch.ex Wol.) Klaskova	0	n	1	0	Fabaceae Lindl.
Iz_3_1009	DJI_0850	Chelidonium majus L.	0	n	1	0	Papaveraceae Juss.
Iz_3_1009	DJI_0850	Chenopodium betaceum Andrz.	0	n	1	0	Amaranthaceae Juss.
Iz_3_1009	DJI_0850	Chenopodium cf. album	0	n	1	0	Amaranthaceae Juss.
Iz_3_1009	DJI_0850	Chenopodium sp.	0		NA	0	Amaranthaceae Juss.
Iz_3_1009	DJI_0850	Chondrilla juncea L.	0	n	1	0	Asteraceae Bercht. & J.Presl
Iz_3_1009	DJI_0850	Cirsium vulgare (Savi) Ten.	0	n	1	0	Asteraceae Bercht. & J.Presl
Iz_3_1009	DJI_0850	Clinopodium acinos (L.) Kuntze	0	n	1	0	Lamiacea Lindl.
Iz_3_1009	DJI_0850	Convallaria majalis L.	0	n	1	0	Asparagaceae Juss.
Iz_3_1009	DJI_0850	Crataegus rhipidophylla Gand.	0	n	1	1	Rosaceae Juss.
Iz_3_1009	DJI_0850	Crepis foetida L.	0	n	1	0	Asteraceae Bercht. & J.Presl
Iz_3_1009	DJI_0850	Dactylis glomerata L.	0	n	1	0	Poaceae Barnhart
Iz_3_1009	DJI_0850	Dianthus campestris M.Bieb.	0	n	1	0	Caryophyllaceae Juss.
Iz_3_1009	DJI_0850	Digitaria sanguinalis (L.) Scop.	0	a	0	0	Poaceae Barnhart
Iz_3_1009	DJI_0850	Elymus repens (L.) Gould	0	n	1	0	Poaceae Barnhart
Iz_3_1009	DJI_0850	Epilobium tetragonum L.	0	n	1	0	Onagraceae Juss.
Iz_3_1009	DJI_0850	Eragrostis minor Host	0	a	0	0	Poaceae Barnhart
Iz_3_1009	DJI_0850	Erigeron canadensis L.	0	a	0	0	Asteraceae Bercht. & J.Presl
Iz_3_1009	DJI_0850	Euphorbia seguieriana Neck.	0	n	1	0	Euphorbiaceae Juss.
Iz_3_1009	DJI_0850	Fallopia dumetorum (L.) Holub	0.2	n	1	0	Polygonaceae Juss.
Iz_3_1009	DJI_0850	Festuca beckeri (Hack.) Trautv.	0	n	1	0	Poaceae Barnhart
Iz_3_1009	DJI_0850	Fragaria vesca L.	0	n	1	0	Rosaceae Juss.
Iz_3_1009	DJI_0850	Frangula alnus Mill.	0	n	1	1	Rhamnaceae Juss.
Iz_3_1009	DJI_0850	Fraxinus pennsylvanica Marshall	0	a	0	1	Oleaceae Hoffmanns. & Link
Iz_3_1009	DJI_0850	Galeopsis bifida Boenn.	0	n	1	0	Lamiacea Lindl.
Iz_3_1009	DJI_0850	Galium verum L.	0	n	1	0	Rubiaceae Juss.
Iz_3_1009	DJI_0850	Genista tinctoria L.	0	n	1	0	Fabaceae Lindl.
Iz_3_1009	DJI_0850	Geranium robertianum L.	0	n	1	0	Geraniaceae Juss.
Iz_3_1009	DJI_0850	Geranium sanquineum L.	0	n	1	0	Geraniaceae Juss.
Iz_3_1009	DJI_0850	Gypsophila paniculata L.	0	n	1	0	Caryophyllaceae Juss.
Iz_3_1009	DJI_0850	Helichrysum arenarium (L.) Moench	0	n	1	0	Asteraceae Bercht. & J.Presl
Iz_3_1009	DJI_0850	Herniaria polygama J.Gay	0	n	1	0	Caryophyllaceae Juss.
Iz_3_1009	DJI_0850	Hieracium robustum Fr.	0	n	1	0	Asteraceae Bercht. & J.Presl
Iz_3_1009	DJI_0850	Hieracium umbellatum L.	0	n	1	NA	Asteraceae Bercht. & J.Presl
Iz_3_1009	DJI_0850	Hylotelephium maximum (L.) Holub	0	n	1	0	Crassulaceae J.St.-Hil.
Iz_3_1009	DJI_0850	Jacobaea borysthenica (DC.) B.Nord. & Greuter	0	n	1	0	Asteraceae Bercht. & J.Presl
Iz_3_1009	DJI_0850	Jasione montana L.	0	n	1	0	Campanulaceae Juss.
Iz_3_1009	DJI_0850	Jurinea cyanoides (L.) Rchb.s.l.	0	n	1	0	Asteraceae Bercht. & J.Presl
Iz_3_1009	DJI_0850	Koeleria glauca (Spreng.) DC.	0	n	1	0	Poaceae Barnhart
Iz_3_1009	DJI_0850	Lactuca serriola L.	0	a	0	0	Asteraceae Bercht. & J.Presl
Iz_3_1009	DJI_0850	Linaria genistifolia (L.) Mill.	0	n	1	0	Plantaginaceae Juss.
Iz_3_1009	DJI_0850	Lomelosia argentea (L.) Greuter & Burdet	0	n	1	0	Caprifoliaceae Juss.
Iz_3_1009	DJI_0850	Melampyrum pratense L.	0	n	1	0	Orobanchaceae Vent.
Iz_3_1009	DJI_0850	Oenothera biennis L.	0	a	0	0	Onagraceae Juss.
Iz_3_1009	DJI_0850	Pentanema britannica (L.) D.Gut.Larr., Santos-Vicente, Anderb., E.Rico & M.M.Mart.Ort.	0	n	1	0	Asteraceae Bercht. & J.Presl
Iz_3_1009	DJI_0850	Peucedanum oreoselinum (L.) Moench	3	n	1	0	Apiaceae Lindl.
Iz_3_1009	DJI_0850	Peucedanum ruthenicum M.Bieb.	0	n	1	0	Apiaceae Lindl.
Iz_3_1009	DJI_0850	Pilosella echioides (Lumn.) F.W.Schultz & Sch.Bip.	0	n	1	0	Asteraceae Bercht. & J.Presl
Iz_3_1009	DJI_0850	Pilosella officinarum F. Schultz et Sch. Bip.	0	n	1	0	Asteraceae Bercht. & J.Presl
Iz_3_1009	DJI_0850	Pilosella sp.	0		NA	0	Asteraceae Bercht. & J.Presl
Iz_3_1009	DJI_0850	Pinus sylvestris L.	0	n	1	1	Pinaceae Spreng. ex F.Rudolphi
Iz_3_1009	DJI_0850	Poa annua L.	0	n	1	0	Poaceae Barnhart
Iz_3_1009	DJI_0850	Poa nemoralis L.	0	n	1	0	Poaceae Barnhart
Iz_3_1009	DJI_0850	Poa sp.	0		NA	0	Poaceae Barnhart
Iz_3_1009	DJI_0850	Polygonatum odoratum (Mill.) Druce	0	n	1	0	Asparagaceae Juss.
Iz_3_1009	DJI_0850	Polygonum arenarium Waldst. & Kit.	0	n	1	0	Polygonaceae Juss.
Iz_3_1009	DJI_0850	Populus nigra L.	0	n	1	1	Salicaceae Mirb.
Iz_3_1009	DJI_0850	Populus tremula L.	0	n	1	1	Salicaceae Mirb.
Iz_3_1009	DJI_0850	Populus Ã canescens (Aiton) Sm.	0	n	1	1	Salicaceae Mirb.
Iz_3_1009	DJI_0850	Potentilla argentea L.	0	n	1	0	Rosaceae Juss.
Iz_3_1009	DJI_0850	Psammophiliella muralis (L.) Ikonn.	0	n	1	0	Caryophyllaceae Juss.
Iz_3_1009	DJI_0850	Psephellus sumensis (Kalen.) Greuter	0	n	1	0	Asteraceae Bercht. & J.Presl
Iz_3_1009	DJI_0850	Pulsatilla pratensis (L.) Mill. s.l.	0	n	1	0	Ranunculaceae Juss.
Iz_3_1009	DJI_0850	Pyrus communis L.	0	a	0	1	Rosaceae Juss.
Iz_3_1009	DJI_0850	Quercus robur L.	0	n	1	1	Fagaceae Dumort.
Iz_3_1009	DJI_0850	Robinia pseudoacacia L.	0	a	0	1	Fabaceae Lindl.
Iz_3_1009	DJI_0850	Rosa sp.	0		NA	1	Rosaceae Juss.
Iz_3_1009	DJI_0850	Rubus idaeus L.	0	n	1	0	Rosaceae Juss.
Iz_3_1009	DJI_0850	Rumex acetosella L.	0	n	1	0	Polygonaceae Juss.
Iz_3_1009	DJI_0850	Rumex thyrsiflorus Fingerh.	0	n	1	0	Polygonaceae Juss.
Iz_3_1009	DJI_0850	Salix alba L.	0	n	1	1	Salicaceae Mirb.
Iz_3_1009	DJI_0850	Sambucus nigra L.	0	n	1	1	Viburnaceae Raf.
Iz_3_1009	DJI_0850	Secale sylvestre Host	0	n	1	0	Poaceae Barnhart
Iz_3_1009	DJI_0850	Sempervivum ruthenicum Schnittsp. & C.B. Lehm.	0	n	1	0	Crassulaceae J.St.-Hil.
Iz_3_1009	DJI_0850	Setaria viridis (L.) P. Beauv.	0	n	1	0	Poaceae Barnhart
Iz_3_1009	DJI_0850	Silene borysthenica (Gruner) Walters	0	n	1	0	Caryophyllaceae Juss.
Iz_3_1009	DJI_0850	Silene latifolia subsp. alba (Mill.) Greuter & Burdet	0	n	1	0	Caryophyllaceae Juss.
Iz_3_1009	DJI_0850	Solidago virgaurea L.	0	n	1	0	Asteraceae Bercht. & J.Presl
Iz_3_1009	DJI_0850	Sonchus oleraceus L.	0	a	0	0	Asteraceae Bercht. & J.Presl
Iz_3_1009	DJI_0850	Sorbus aucuparia L.	0	n	1	1	Rosaceae Juss.
Iz_3_1009	DJI_0850	Stachys recta L.	0	n	1	0	Lamiacea Lindl.
Iz_3_1009	DJI_0850	Tanacetum vulgare L.	0	n	1	0	Asteraceae Bercht. & J.Presl
Iz_3_1009	DJI_0850	Thymus pallasianus Heinr.Braun	0	n	1	0	Lamiacea Lindl.
Iz_3_1009	DJI_0850	Tilia cordata Mill.	0	n	1	1	Malvaceae Juss.
Iz_3_1009	DJI_0850	Trifolium alpestre L.	0	n	1	0	Fabaceae Lindl.
Iz_3_1009	DJI_0850	Trifolium arvense L.	0	n	1	0	Fabaceae Lindl.
Iz_3_1009	DJI_0850	Ulmus glabra Huds.	0	n	1	1	Ulmaceae Mirb.
Iz_3_1009	DJI_0850	Verbascum densiflorum Bertol.	0	n	1	0	Scrophulariaceae Juss.
Iz_3_1009	DJI_0850	Veronica austriaca L.	0	n	1	0	Plantaginaceae Juss.
Iz_3_1009	DJI_0850	Veronica incana L.	0	n	1	0	Plantaginaceae Juss.

Iz_3_1009	DJI_0850	Veronica verna L.	0	n	1	0	Plantaginaceae Juss.
Iz_3_1009	DJI_0850	Vincetoxicum hirundinaria Medik	0	n	1	0	Apocynaceae Juss.
Iz_3_1009	DJI_0852	Acer tataricum L.	0	n	1	1	Sapindaceae Juss.
Iz_3_1009	DJI_0852	Achillea millefolium L. s.l.	0	n	1	0	Asteraceae Bercht. & J.Presl
Iz_3_1009	DJI_0852	Allium oleraceum L.	0	n	1	0	Amaryllidaceae J.St.-Hil.
Iz_3_1009	DJI_0852	Ambrosia artemisiifolia L.	0	a	0	0	Asteraceae Bercht. & J.Presl
Iz_3_1009	DJI_0852	Amorpha fruticosa L.	0	a	0	0	Fabaceae Lindl.
Iz_3_1009	DJI_0852	Anthericum ramosum L.	0	n	1	0	Asparagaceae Juss.
Iz_3_1009	DJI_0852	Artemisia marschalliana Spreng.	0	n	1	0	Asteraceae Bercht. & J.Presl
Iz_3_1009	DJI_0852	Asparagus officinalis L.	0	n	1	0	Asparagaceae Juss.
Iz_3_1009	DJI_0852	Astragalus cicer L.	0	n	1	0	Fabaceae Lindl.
Iz_3_1009	DJI_0852	Bassia laniflora (S.G.Gmel.) A.J.Scott	0	n	1	0	Amaranthaceae Juss.
Iz_3_1009	DJI_0852	Berberis vulgaris L.	0	n	1	0	Berberidaceae Juss.
Iz_3_1009	DJI_0852	Betula pendula Roth.	0	n	1	1	Betulaceae Gray
Iz_3_1009	DJI_0852	Betula pendula Roth.	0	n	1	0	Poaceae Barnhart
Iz_3_1009	DJI_0852	Bromus inermis Leyss.	0		NA	NA	
Iz_3_1009	DJI_0852	Bromus squarrosus L.	0	a	0	0	Poaceae Barnhart
Iz_3_1009	DJI_0852	Bromus tectorum L.	0	a	0	0	Poaceae Barnhart
Iz_3_1009	DJI_0852	Calamagrostis epigejos (L.) Roth	40	n	1	0	Poaceae Barnhart
Iz_3_1009	DJI_0852	Carduus acanthoides L.	0	n	1	0	Asteraceae Bercht. & J.Presl
Iz_3_1009	DJI_0852	Carex sp.	0	n	1	0	Cyperaceae Juss.
Iz_3_1009	DJI_0852	Centaurea majorovii Dumbadze	0	n	1	0	Asteraceae Bercht. & J.Presl
Iz_3_1009	DJI_0852	Chamaecytisus ruthenicus (Fisch.ex Wol.) Klaskova	0	n	1	0	Fabaceae Lindl.
Iz_3_1009	DJI_0852	Chelidonium majus L.	0	n	1	0	Papaveraceae Juss.
Iz_3_1009	DJI_0852	Chenopodium betaceum Andrz.	0	n	1	0	Amaranthaceae Juss.
Iz_3_1009	DJI_0852	Chenopodium cf. album	0	n	1	0	Amaranthaceae Juss.
Iz_3_1009	DJI_0852	Chenopodium sp.	0		NA	0	Amaranthaceae Juss.
Iz_3_1009	DJI_0852	Chondrilla juncea L.	0	n	1	0	Asteraceae Bercht. & J.Presl
Iz_3_1009	DJI_0852	Cirsium vulgare (Savi) Ten.	0	n	1	0	Asteraceae Bercht. & J.Presl
Iz_3_1009	DJI_0852	Clinopodium acinos (L.) Kuntze	0	n	1	0	Lamiacea Lindl.
Iz_3_1009	DJI_0852	Convallaria majalis L.	0	n	1	0	Asparagaceae Juss.
Iz_3_1009	DJI_0852	Crataegus rhipidophylla Gand.	0	n	1	1	Rosaceae Juss.
Iz_3_1009	DJI_0852	Crepis foetida L.	0	n	1	0	Asteraceae Bercht. & J.Presl
Iz_3_1009	DJI_0852	Dactylis glomerata L.	0	n	1	0	Poaceae Barnhart
Iz_3_1009	DJI_0852	Dianthus campestris M.Bieb.	0	n	1	0	Caryophyllaceae Juss.
Iz_3_1009	DJI_0852	Digitaria sanguinalis (L.) Scop.	0	a	0	0	Poaceae Barnhart
Iz_3_1009	DJI_0852	Elymus repens (L.) Gould	0	n	1	0	Poaceae Barnhart
Iz_3_1009	DJI_0852	Epilobium tetragonum L.	0	n	1	0	Onagraceae Juss.
Iz_3_1009	DJI_0852	Eragrostis minor Host	0	a	0	0	Poaceae Barnhart
Iz_3_1009	DJI_0852	Erigeron canadensis L.	0	a	0	0	Asteraceae Bercht. & J.Presl
Iz_3_1009	DJI_0852	Euphorbia seguieriana Neck.	0	n	1	0	Euphorbiaceae Juss.
Iz_3_1009	DJI_0852	Fallopia dumetorum (L.) Holub	0	n	1	0	Polygonaceae Juss.
Iz_3_1009	DJI_0852	Festuca beckeri (Hack.) Trautv.	0	n	1	0	Poaceae Barnhart
Iz_3_1009	DJI_0852	Fragaria vesca L.	0	n	1	0	Rosaceae Juss.
Iz_3_1009	DJI_0852	Frangula alnus Mill.	0	n	1	1	Rhamnaceae Juss.
Iz_3_1009	DJI_0852	Fraxinus pennsylvanica Marshall	0	a	0	1	Oleaceae Hoffmanns. & Link
Iz_3_1009	DJI_0852	Galeopsis bifida Boenn.	0	n	1	0	Lamiacea Lindl.
Iz_3_1009	DJI_0852	Galium verum L.	0	n	1	0	Rubiaceae Juss.
Iz_3_1009	DJI_0852	Genista tinctoria L.	0	n	1	0	Fabaceae Lindl.
Iz_3_1009	DJI_0852	Geranium robertianum L.	0	n	1	0	Geraniaceae Juss.
Iz_3_1009	DJI_0852	Geranium sanquineum L.	0	n	1	0	Geraniaceae Juss.
Iz_3_1009	DJI_0852	Gypsophila paniculata L.	0	n	1	0	Caryophyllaceae Juss.
Iz_3_1009	DJI_0852	Helichrysum arenarium (L.) Moench	0	n	1	0	Asteraceae Bercht. & J.Presl
Iz_3_1009	DJI_0852	Herniaria polygama J.Gay	0	n	1	0	Caryophyllaceae Juss.
Iz_3_1009	DJI_0852	Hieracium robustum Fr.	0	n	1	0	Asteraceae Bercht. & J.Presl
Iz_3_1009	DJI_0852	Hieracium umbellatum L.	0	n	1	NA	Asteraceae Bercht. & J.Presl
Iz_3_1009	DJI_0852	Hylotelephium maximum (L.) Holub	0	n	1	0	Crassulaceae J.St.-Hil.
Iz_3_1009	DJI_0852	Jacobaea borysthenica (DC.) B.Nord. & Greuter	0	n	1	0	Asteraceae Bercht. & J.Presl
Iz_3_1009	DJI_0852	Jasione montana L.	0	n	1	0	Campanulaceae Juss.
Iz_3_1009	DJI_0852	Jurinea cyanoides (L.) Rchb.s.l.	0	n	1	0	Asteraceae Bercht. & J.Presl
Iz_3_1009	DJI_0852	Koeleria glauca (Spreng.) DC.	0	n	1	0	Poaceae Barnhart
Iz_3_1009	DJI_0852	Lactuca serriola L.	0	a	0	0	Asteraceae Bercht. & J.Presl
Iz_3_1009	DJI_0852	Linaria genistifolia (L.) Mill.	0	n	1	0	Plantaginaceae Juss.
Iz_3_1009	DJI_0852	Lomelosia argentea (L.) Greuter & Burdet	0	n	1	0	Caprifoliaceae Juss.
Iz_3_1009	DJI_0852	Melampyrum pratense L.	0	n	1	0	Orobanchaceae Vent.
Iz_3_1009	DJI_0852	Oenothera biennis L.	0	a	0	0	Onagraceae Juss.
Iz_3_1009	DJI_0852	Pentanema britannica (L.) D.Gut.Larr., Santos-Vicente, Anderb., E.Rico & M.M.Mart.Ort.	0	n	1	0	Asteraceae Bercht. & J.Presl
Iz_3_1009	DJI_0852	Peucedanum oreoselinum (L.) Moench	5	n	1	0	Apiaceae Lindl.
Iz_3_1009	DJI_0852	Peucedanum ruthenicum M.Bieb.	0	n	1	0	Apiaceae Lindl.
Iz_3_1009	DJI_0852	Pilosella echioides (Lumn.) F.W.Schultz & Sch.Bip.	0	n	1	0	Asteraceae Bercht. & J.Presl
Iz_3_1009	DJI_0852	Pilosella officinarum F. Schultz et Sch. Bip.	0	n	1	0	Asteraceae Bercht. & J.Presl
Iz_3_1009	DJI_0852	Pilosella sp.	0		NA	0	Asteraceae Bercht. & J.Presl
Iz_3_1009	DJI_0852	Pinus sylvestris L.	0	n	1	1	Pinaceae Spreng. ex F.Rudolphi
Iz_3_1009	DJI_0852	Poa annua L.	0	n	1	0	Poaceae Barnhart
Iz_3_1009	DJI_0852	Poa nemoralis L.	0	n	1	0	Poaceae Barnhart
Iz_3_1009	DJI_0852	Poa sp.	0		NA	0	Poaceae Barnhart
Iz_3_1009	DJI_0852	Polygonatum odoratum (Mill.) Druce	0	n	1	0	Asparagaceae Juss.
Iz_3_1009	DJI_0852	Polygonum arenarium Waldst. & Kit.	0	n	1	0	Polygonaceae Juss.
Iz_3_1009	DJI_0852	Populus nigra L.	0	n	1	1	Salicaceae Mirb.
Iz_3_1009	DJI_0852	Populus tremula L.	0	n	1	1	Salicaceae Mirb.
Iz_3_1009	DJI_0852	Populus Ã canescens (Aiton) Sm.	0	n	1	1	Salicaceae Mirb.
Iz_3_1009	DJI_0852	Potentilla argentea L.	0	n	1	0	Rosaceae Juss.
Iz_3_1009	DJI_0852	Psammophiliella muralis (L.) Ikonn.	0	n	1	0	Caryophyllaceae Juss.
Iz_3_1009	DJI_0852	Psephellus sumensis (Kalen.) Greuter	0	n	1	0	Asteraceae Bercht. & J.Presl
Iz_3_1009	DJI_0852	Pulsatilla pratensis (L.) Mill. s.l.	0	n	1	0	Ranunculaceae Juss.
Iz_3_1009	DJI_0852	Pyrus communis L.	0	a	0	1	Rosaceae Juss.
Iz_3_1009	DJI_0852	Quercus robur L.	0	n	1	1	Fagaceae Dumort.
Iz_3_1009	DJI_0852	Robinia pseudoacacia L.	0	a	0	1	Fabaceae Lindl.
Iz_3_1009	DJI_0852	Rosa sp.	0		NA	1	Rosaceae Juss.
Iz_3_1009	DJI_0852	Rubus idaeus L.	0	n	1	0	Rosaceae Juss.
Iz_3_1009	DJI_0852	Rumex acetosella L.	0	n	1	0	Polygonaceae Juss.
Iz_3_1009	DJI_0852	Rumex thyrsiflorus Fingerh.	0	n	1	0	Polygonaceae Juss.
Iz_3_1009	DJI_0852	Salix alba L.	0	n	1	1	Salicaceae Mirb.
Iz_3_1009	DJI_0852	Sambucus nigra L.	0	n	1	1	Viburnaceae Raf.
Iz_3_1009	DJI_0852	Secale sylvestre Host	0	n	1	0	Poaceae Barnhart
Iz_3_1009	DJI_0852	Sempervivum ruthenicum Schnittsp. & C.B. Lehm.	0	n	1	0	Crassulaceae J.St.-Hil.
Iz_3_1009	DJI_0852	Setaria viridis (L.) P. Beauv.	0	n	1	0	Poaceae Barnhart
Iz_3_1009	DJI_0852	Silene borysthenica (Gruner) Walters	0	n	1	0	Caryophyllaceae Juss.

Iz_3_1009	DJI_0852	Silene latifolia subsp. alba (Mill.) Greuter & Burdet	0	n	1	0	Caryophyllaceae Juss.
Iz_3_1009	DJI_0852	Solidago virgaurea L.	0	n	1	0	Asteraceae Bercht. & J.Presl
Iz_3_1009	DJI_0852	Sonchus oleraceus L.	0	a	0	0	Asteraceae Bercht. & J.Presl
Iz_3_1009	DJI_0852	Sorbus aucuparia L.	0	n	1	1	Rosaceae Juss.
Iz_3_1009	DJI_0852	Stachys recta L.	0	n	1	0	Lamiacea Lindl.
Iz_3_1009	DJI_0852	Tanacetum vulgare L.	0	n	1	0	Asteraceae Bercht. & J.Presl
Iz_3_1009	DJI_0852	Thymus pallasianus Heinr.Braun	0	n	1	0	Lamiacea Lindl.
Iz_3_1009	DJI_0852	Tilia cordata Mill.	0	n	1	1	Malvaceae Juss.
Iz_3_1009	DJI_0852	Trifolium alpestre L.	0	n	1	0	Fabaceae Lindl.
Iz_3_1009	DJI_0852	Trifolium arvense L.	0	n	1	0	Fabaceae Lindl.
Iz_3_1009	DJI_0852	Ulmus glabra Huds.	0	n	1	1	Ulmaceae Mirb.
Iz_3_1009	DJI_0852	Verbascum densiflorum Bertol.	0	n	1	0	Scrophulariaceae Juss.
Iz_3_1009	DJI_0852	Veronica austriaca L.	0	n	1	0	Plantaginaceae Juss.
Iz_3_1009	DJI_0852	Veronica incana L.	0	n	1	0	Plantaginaceae Juss.
Iz_3_1009	DJI_0852	Veronica verna L.	0	n	1	0	Plantaginaceae Juss.
Iz_3_1009	DJI_0852	Vincetoxicum hirundinaria Medik	0	n	1	0	Apocynaceae Juss.
Iz_3_1009	DJI_0853	Acer tataricum L.	0	n	1	1	Sapindaceae Juss.
Iz_3_1009	DJI_0853	Achillea millefolium L. s.l.	0	n	1	0	Asteraceae Bercht. & J.Presl
Iz_3_1009	DJI_0853	Allium oleraceum L.	0	n	1	0	Amaryllidaceae J.St.-Hil.
Iz_3_1009	DJI_0853	Ambrosia artemisiifolia L.	0	a	0	0	Asteraceae Bercht. & J.Presl
Iz_3_1009	DJI_0853	Amorpha fruticosa L.	0	a	0	0	Fabaceae Lindl.
Iz_3_1009	DJI_0853	Anthericum ramosum L.	0	n	1	0	Asparagaceae Juss.
Iz_3_1009	DJI_0853	Artemisia marschalliana Spreng.	0	n	1	0	Asteraceae Bercht. & J.Presl
Iz_3_1009	DJI_0853	Asparagus officinalis L.	0	n	1	0	Asparagaceae Juss.
Iz_3_1009	DJI_0853	Astragalus cicer L.	0	n	1	0	Fabaceae Lindl.
Iz_3_1009	DJI_0853	Bassia laniflora (S.G.Gmel.) A.J.Scott	0	n	1	0	Amaranthaceae Juss.
Iz_3_1009	DJI_0853	Berberis vulgaris L.	0	n	1	0	Berberidaceae Juss.
Iz_3_1009	DJI_0853	Betula pendula Roth.	1	n	1	1	Betulaceae Gray
Iz_3_1009	DJI_0853	Betula pendula Roth.	1	n	1	0	Poaceae Barnhart
Iz_3_1009	DJI_0853	Bromus inermis Leyss.	0		NA	NA	
Iz_3_1009	DJI_0853	Bromus squarrosus L.	0	a	0	0	Poaceae Barnhart
Iz_3_1009	DJI_0853	Bromus tectorum L.	0	a	0	0	Poaceae Barnhart
Iz_3_1009	DJI_0853	Calamagrostis epigejos (L.) Roth	40	n	1	0	Poaceae Barnhart
Iz_3_1009	DJI_0853	Carduus acanthoides L.	0	n	1	0	Asteraceae Bercht. & J.Presl
Iz_3_1009	DJI_0853	Carex sp.	0	n	1	0	Cyperaceae Juss.
Iz_3_1009	DJI_0853	Centaurea majorovii Dumbadze	0	n	1	0	Asteraceae Bercht. & J.Presl
Iz_3_1009	DJI_0853	Chamaecytisus ruthenicus (Fisch.ex Wol.) Klaskova	0	n	1	0	Fabaceae Lindl.
Iz_3_1009	DJI_0853	Chelidonium majus L.	0	n	1	0	Papaveraceae Juss.
Iz_3_1009	DJI_0853	Chenopodium betaceum Andrz.	0	n	1	0	Amaranthaceae Juss.
Iz_3_1009	DJI_0853	Chenopodium cf. album	0	n	1	0	Amaranthaceae Juss.
Iz_3_1009	DJI_0853	Chenopodium sp.	0		NA	0	Amaranthaceae Juss.
Iz_3_1009	DJI_0853	Chondrilla juncea L.	0	n	1	0	Asteraceae Bercht. & J.Presl
Iz_3_1009	DJI_0853	Cirsium vulgare (Savi) Ten.	0	n	1	0	Asteraceae Bercht. & J.Presl
Iz_3_1009	DJI_0853	Clinopodium acinos (L.) Kuntze	0	n	1	0	Lamiacea Lindl.
Iz_3_1009	DJI_0853	Convallaria majalis L.	0	n	1	0	Asparagaceae Juss.
Iz_3_1009	DJI_0853	Crataegus rhipidophylla Gand.	0	n	1	1	Rosaceae Juss.
Iz_3_1009	DJI_0853	Crepis foetida L.	0	n	1	0	Asteraceae Bercht. & J.Presl
Iz_3_1009	DJI_0853	Dactylis glomerata L.	0	n	1	0	Poaceae Barnhart
Iz_3_1009	DJI_0853	Dianthus campestris M.Bieb.	0	n	1	0	Caryophyllaceae Juss.
Iz_3_1009	DJI_0853	Digitaria sanguinalis (L.) Scop.	0	a	0	0	Poaceae Barnhart
Iz_3_1009	DJI_0853	Elymus repens (L.) Gould	0	n	1	0	Poaceae Barnhart
Iz_3_1009	DJI_0853	Epilobium tetragonum L.	0	n	1	0	Onagraceae Juss.
Iz_3_1009	DJI_0853	Eragrostis minor Host	0	a	0	0	Poaceae Barnhart
Iz_3_1009	DJI_0853	Erigeron canadensis L.	0	a	0	0	Asteraceae Bercht. & J.Presl
Iz_3_1009	DJI_0853	Euphorbia seguieriana Neck.	0	n	1	0	Euphorbiaceae Juss.
Iz_3_1009	DJI_0853	Fallopia dumetorum (L.) Holub	0	n	1	0	Polygonaceae Juss.
Iz_3_1009	DJI_0853	Festuca beckeri (Hack.) Trautv.	0	n	1	0	Poaceae Barnhart
Iz_3_1009	DJI_0853	Fragaria vesca L.	0	n	1	0	Rosaceae Juss.
Iz_3_1009	DJI_0853	Frangula alnus Mill.	0	n	1	1	Rhamnaceae Juss.
Iz_3_1009	DJI_0853	Fraxinus pennsylvanica Marshall	0	a	0	1	Oleaceae Hoffmanns. & Link
Iz_3_1009	DJI_0853	Galeopsis bifida Boenn.	0	n	1	0	Lamiacea Lindl.
Iz_3_1009	DJI_0853	Galium verum L.	0	n	1	0	Rubiaceae Juss.
Iz_3_1009	DJI_0853	Genista tinctoria L.	0	n	1	0	Fabaceae Lindl.
Iz_3_1009	DJI_0853	Geranium robertianum L.	0	n	1	0	Geraniaceae Juss.
Iz_3_1009	DJI_0853	Geranium sanquineum L.	2	n	1	0	Geraniaceae Juss.
Iz_3_1009	DJI_0853	Gypsophila paniculata L.	0	n	1	0	Caryophyllaceae Juss.
Iz_3_1009	DJI_0853	Helichrysum arenarium (L.) Moench	0	n	1	0	Asteraceae Bercht. & J.Presl
Iz_3_1009	DJI_0853	Herniaria polygama J.Gay	0	n	1	0	Caryophyllaceae Juss.
Iz_3_1009	DJI_0853	Hieracium robustum Fr.	0	n	1	0	Asteraceae Bercht. & J.Presl
Iz_3_1009	DJI_0853	Hieracium umbellatum L.	0	n	1	NA	Asteraceae Bercht. & J.Presl
Iz_3_1009	DJI_0853	Hylotelephium maximum (L.) Holub	0	n	1	0	Crassulaceae J.St.-Hil.
Iz_3_1009	DJI_0853	Jacobaea borysthenica (DC.) B.Nord. & Greuter	0	n	1	0	Asteraceae Bercht. & J.Presl
Iz_3_1009	DJI_0853	Jasione montana L.	0	n	1	0	Campanulaceae Juss.
Iz_3_1009	DJI_0853	Jurinea cyanoides (L.) Rchb.s.l.	0	n	1	0	Asteraceae Bercht. & J.Presl
Iz_3_1009	DJI_0853	Koeleria glauca (Spreng.) DC.	0	n	1	0	Poaceae Barnhart
Iz_3_1009	DJI_0853	Lactuca serriola L.	0	a	0	0	Asteraceae Bercht. & J.Presl
Iz_3_1009	DJI_0853	Linaria genistifolia (L.) Mill.	0	n	1	0	Plantaginaceae Juss.
Iz_3_1009	DJI_0853	Lomelosia argentea (L.) Greuter & Burdet	0	n	1	0	Caprifoliaceae Juss.
Iz_3_1009	DJI_0853	Melampyrum pratense L.	0	n	1	0	Orobanchaceae Vent.
Iz_3_1009	DJI_0853	Oenothera biennis L.	0	a	0	0	Onagraceae Juss.
Iz_3_1009	DJI_0853	Pentanema britannica (L.) D.Gut.Larr., Santos-Vicente, Anderb., E.Rico & M.M.Mart.Ort.	0	n	1	0	Asteraceae Bercht. & J.Presl
Iz_3_1009	DJI_0853	Peucedanum oreoselinum (L.) Moench	2	n	1	0	Apiaceae Lindl.
Iz_3_1009	DJI_0853	Peucedanum ruthenicum M.Bieb.	0	n	1	0	Apiaceae Lindl.
Iz_3_1009	DJI_0853	Pilosella echioides (Lumn.) F.W.Schultz & Sch.Bip.	0	n	1	0	Asteraceae Bercht. & J.Presl
Iz_3_1009	DJI_0853	Pilosella officinarum F. Schultz et Sch. Bip.	0	n	1	0	Asteraceae Bercht. & J.Presl
Iz_3_1009	DJI_0853	Pilosella sp.	0		NA	0	Asteraceae Bercht. & J.Presl
Iz_3_1009	DJI_0853	Pinus sylvestris L.	0	n	1	1	Pinaceae Spreng. ex F.Rudolphi
Iz_3_1009	DJI_0853	Poa annua L.	0	n	1	0	Poaceae Barnhart
Iz_3_1009	DJI_0853	Poa nemoralis L.	0	n	1	0	Poaceae Barnhart
Iz_3_1009	DJI_0853	Poa sp.	0		NA	0	Poaceae Barnhart
Iz_3_1009	DJI_0853	Polygonatum odoratum (Mill.) Druce	0.5	n	1	0	Asparagaceae Juss.
Iz_3_1009	DJI_0853	Polygonum arenarium Waldst. & Kit.	0	n	1	0	Polygonaceae Juss.
Iz_3_1009	DJI_0853	Populus nigra L.	0	n	1	1	Salicaceae Mirb.
Iz_3_1009	DJI_0853	Populus tremula L.	0	n	1	1	Salicaceae Mirb.
Iz_3_1009	DJI_0853	Populus Ã canescens (Aiton) Sm.	0	n	1	1	Salicaceae Mirb.
Iz_3_1009	DJI_0853	Potentilla argentea L.	0	n	1	0	Rosaceae Juss.
Iz_3_1009	DJI_0853	Psammophiliella muralis (L.) Ikonn.	0	n	1	0	Caryophyllaceae Juss.
Iz_3_1009	DJI_0853	Psephellus sumensis (Kalen.) Greuter	0	n	1	0	Asteraceae Bercht. & J.Presl

Iz_3_1009	DJI_0853	Pulsatilla pratensis (L.) Mill. s.l.	0	n	1	0	Ranunculaceae Juss.
Iz_3_1009	DJI_0853	Pyrus communis L.	0	a	0	1	Rosaceae Juss.
Iz_3_1009	DJI_0853	Quercus robur L.	2	n	1	1	Fagaceae Dumort.
Iz_3_1009	DJI_0853	Robinia pseudoacacia L.	0	a	0	1	Fabaceae Lindl.
Iz_3_1009	DJI_0853	Rosa sp.	0		NA	1	Rosaceae Juss.
Iz_3_1009	DJI_0853	Rubus idaeus L.	0	n	1	0	Rosaceae Juss.
Iz_3_1009	DJI_0853	Rumex acetosella L.	0	n	1	0	Polygonaceae Juss.
Iz_3_1009	DJI_0853	Rumex thyrsiflorus Fingerh.	0	n	1	0	Polygonaceae Juss.
Iz_3_1009	DJI_0853	Salix alba L.	0	n	1	1	Salicaceae Mirb.
Iz_3_1009	DJI_0853	Sambucus nigra L.	0	n	1	1	Viburnaceae Raf.
Iz_3_1009	DJI_0853	Secale sylvestre Host	0	n	1	0	Poaceae Barnhart
Iz_3_1009	DJI_0853	Sempervivum ruthenicum Schnittsp. & C.B. Lehm.	0	n	1	0	Crassulaceae J.St.-Hil.
Iz_3_1009	DJI_0853	Setaria viridis (L.) P. Beauv.	0	n	1	0	Poaceae Barnhart
Iz_3_1009	DJI_0853	Silene borysthenica (Gruner) Walters	0	n	1	0	Caryophyllaceae Juss.
Iz_3_1009	DJI_0853	Silene latifolia subsp. alba (Mill.) Greuter & Burdet	0	n	1	0	Caryophyllaceae Juss.
Iz_3_1009	DJI_0853	Solidago virgaurea L.	0	n	1	0	Asteraceae Bercht. & J.Presl
Iz_3_1009	DJI_0853	Sonchus oleraceus L.	0	a	0	0	Asteraceae Bercht. & J.Presl
Iz_3_1009	DJI_0853	Sorbus aucuparia L.	0	n	1	1	Rosaceae Juss.
Iz_3_1009	DJI_0853	Stachys recta L.	0	n	1	0	Lamiacea Lindl.
Iz_3_1009	DJI_0853	Tanacetum vulgare L.	0	n	1	0	Asteraceae Bercht. & J.Presl
Iz_3_1009	DJI_0853	Thymus pallasianus Heinr.Braun	0	n	1	0	Lamiacea Lindl.
Iz_3_1009	DJI_0853	Tilia cordata Mill.	0	n	1	1	Malvaceae Juss.
Iz_3_1009	DJI_0853	Trifolium alpestre L.	0	n	1	0	Fabaceae Lindl.
Iz_3_1009	DJI_0853	Trifolium arvense L.	0	n	1	0	Fabaceae Lindl.
Iz_3_1009	DJI_0853	Ulmus glabra Huds.	0	n	1	1	Ulmaceae Mirb.
Iz_3_1009	DJI_0853	Verbascum densiflorum Bertol.	0	n	1	0	Scrophulariaceae Juss.
Iz_3_1009	DJI_0853	Veronica austriaca L.	0	n	1	0	Plantaginaceae Juss.
Iz_3_1009	DJI_0853	Veronica incana L.	0	n	1	0	Plantaginaceae Juss.
Iz_3_1009	DJI_0853	Veronica verna L.	0	n	1	0	Plantaginaceae Juss.
Iz_3_1009	DJI_0853	Vincetoxicum hirundinaria Medik	0	n	1	0	Apocynaceae Juss.
Iz_3_1009	DJI_0855	Acer tataricum L.	0	n	1	1	Sapindaceae Juss.
Iz_3_1009	DJI_0855	Achillea millefolium L. s.l.	0	n	1	0	Asteraceae Bercht. & J.Presl
Iz_3_1009	DJI_0855	Allium oleraceum L.	0	n	1	0	Amaryllidaceae J.St.-Hil.
Iz_3_1009	DJI_0855	Ambrosia artemisiifolia L.	0	a	0	0	Asteraceae Bercht. & J.Presl
Iz_3_1009	DJI_0855	Amorpha fruticosa L.	0	a	0	0	Fabaceae Lindl.
Iz_3_1009	DJI_0855	Anthericum ramosum L.	0	n	1	0	Asparagaceae Juss.
Iz_3_1009	DJI_0855	Artemisia marschalliana Spreng.	0	n	1	0	Asteraceae Bercht. & J.Presl
Iz_3_1009	DJI_0855	Asparagus officinalis L.	0	n	1	0	Asparagaceae Juss.
Iz_3_1009	DJI_0855	Astragalus cicer L.	0	n	1	0	Fabaceae Lindl.
Iz_3_1009	DJI_0855	Bassia laniflora (S.G.Gmel.) A.J.Scott	0	n	1	0	Amaranthaceae Juss.
Iz_3_1009	DJI_0855	Berberis vulgaris L.	0	n	1	0	Berberidaceae Juss.
Iz_3_1009	DJI_0855	Betula pendula Roth.	0	n	1	1	Betulaceae Gray
Iz_3_1009	DJI_0855	Betula pendula Roth.	0	n	1	0	Poaceae Barnhart
Iz_3_1009	DJI_0855	Bromus inermis Leyss.	0		NA	NA	
Iz_3_1009	DJI_0855	Bromus squarrosus L.	0	a	0	0	Poaceae Barnhart
Iz_3_1009	DJI_0855	Bromus tectorum L.	0	a	0	0	Poaceae Barnhart
Iz_3_1009	DJI_0855	Calamagrostis epigejos (L.) Roth	70	n	1	0	Poaceae Barnhart
Iz_3_1009	DJI_0855	Carduus acanthoides L.	0	n	1	0	Asteraceae Bercht. & J.Presl
Iz_3_1009	DJI_0855	Carex sp.	0	n	1	0	Cyperaceae Juss.
Iz_3_1009	DJI_0855	Centaurea majorovii Dumbadze	0	n	1	0	Asteraceae Bercht. & J.Presl
Iz_3_1009	DJI_0855	Chamaecytisus ruthenicus (Fisch.ex Wol.) Klaskova	0	n	1	0	Fabaceae Lindl.
Iz_3_1009	DJI_0855	Chelidonium majus L.	0	n	1	0	Papaveraceae Juss.
Iz_3_1009	DJI_0855	Chenopodium betaceum Andrz.	0	n	1	0	Amaranthaceae Juss.
Iz_3_1009	DJI_0855	Chenopodium cf. album	0	n	1	0	Amaranthaceae Juss.
Iz_3_1009	DJI_0855	Chenopodium sp.	0		NA	0	Amaranthaceae Juss.
Iz_3_1009	DJI_0855	Chondrilla juncea L.	0	n	1	0	Asteraceae Bercht. & J.Presl
Iz_3_1009	DJI_0855	Cirsium vulgare (Savi) Ten.	0	n	1	0	Asteraceae Bercht. & J.Presl
Iz_3_1009	DJI_0855	Clinopodium acinos (L.) Kuntze	0	n	1	0	Lamiacea Lindl.
Iz_3_1009	DJI_0855	Convallaria majalis L.	0	n	1	0	Asparagaceae Juss.
Iz_3_1009	DJI_0855	Crataegus rhipidophylla Gand.	0	n	1	1	Rosaceae Juss.
Iz_3_1009	DJI_0855	Crepis foetida L.	0	n	1	0	Asteraceae Bercht. & J.Presl
Iz_3_1009	DJI_0855	Dactylis glomerata L.	0	n	1	0	Poaceae Barnhart
Iz_3_1009	DJI_0855	Dianthus campestris M.Bieb.	0	n	1	0	Caryophyllaceae Juss.
Iz_3_1009	DJI_0855	Digitaria sanguinalis (L.) Scop.	0	a	0	0	Poaceae Barnhart
Iz_3_1009	DJI_0855	Elymus repens (L.) Gould	0	n	1	0	Poaceae Barnhart
Iz_3_1009	DJI_0855	Epilobium tetragonum L.	0	n	1	0	Onagraceae Juss.
Iz_3_1009	DJI_0855	Eragrostis minor Host	0	a	0	0	Poaceae Barnhart
Iz_3_1009	DJI_0855	Erigeron canadensis L.	0	a	0	0	Asteraceae Bercht. & J.Presl
Iz_3_1009	DJI_0855	Euphorbia seguieriana Neck.	0	n	1	0	Euphorbiaceae Juss.
Iz_3_1009	DJI_0855	Fallopia dumetorum (L.) Holub	0	n	1	0	Polygonaceae Juss.
Iz_3_1009	DJI_0855	Festuca beckeri (Hack.) Trautv.	0	n	1	0	Poaceae Barnhart
Iz_3_1009	DJI_0855	Fragaria vesca L.	0	n	1	0	Rosaceae Juss.
Iz_3_1009	DJI_0855	Frangula alnus Mill.	0	n	1	1	Rhamnaceae Juss.
Iz_3_1009	DJI_0855	Fraxinus pennsylvanica Marshall	0	a	0	1	Oleaceae Hoffmanns. & Link
Iz_3_1009	DJI_0855	Galeopsis bifida Boenn.	0	n	1	0	Lamiacea Lindl.
Iz_3_1009	DJI_0855	Galium verum L.	0	n	1	0	Rubiaceae Juss.
Iz_3_1009	DJI_0855	Genista tinctoria L.	0	n	1	0	Fabaceae Lindl.
Iz_3_1009	DJI_0855	Geranium robertianum L.	0	n	1	0	Geraniaceae Juss.
Iz_3_1009	DJI_0855	Geranium sanquineum L.	0	n	1	0	Geraniaceae Juss.
Iz_3_1009	DJI_0855	Gypsophila paniculata L.	0	n	1	0	Caryophyllaceae Juss.
Iz_3_1009	DJI_0855	Helichrysum arenarium (L.) Moench	0	n	1	0	Asteraceae Bercht. & J.Presl
Iz_3_1009	DJI_0855	Herniaria polygama J.Gay	0	n	1	0	Caryophyllaceae Juss.
Iz_3_1009	DJI_0855	Hieracium robustum Fr.	0	n	1	0	Asteraceae Bercht. & J.Presl
Iz_3_1009	DJI_0855	Hieracium umbellatum L.	0	n	1	NA	Asteraceae Bercht. & J.Presl
Iz_3_1009	DJI_0855	Hylotelephium maximum (L.) Holub	0	n	1	0	Crassulaceae J.St.-Hil.
Iz_3_1009	DJI_0855	Jacobaea borysthenica (DC.) B.Nord. & Greuter	0	n	1	0	Asteraceae Bercht. & J.Presl
Iz_3_1009	DJI_0855	Jasione montana L.	0	n	1	0	Campanulaceae Juss.
Iz_3_1009	DJI_0855	Jurinea cyanoides (L.) Rchb.s.l.	0	n	1	0	Asteraceae Bercht. & J.Presl
Iz_3_1009	DJI_0855	Koeleria glauca (Spreng.) DC.	0	n	1	0	Poaceae Barnhart
Iz_3_1009	DJI_0855	Lactuca serriola L.	0	a	0	0	Asteraceae Bercht. & J.Presl
Iz_3_1009	DJI_0855	Linaria genistifolia (L.) Mill.	0	n	1	0	Plantaginaceae Juss.
Iz_3_1009	DJI_0855	Lomelosia argentea (L.) Greuter & Burdet	0	n	1	0	Caprifoliaceae Juss.
Iz_3_1009	DJI_0855	Melampyrum pratense L.	0	n	1	0	Orobanchaceae Vent.
Iz_3_1009	DJI_0855	Oenothera biennis L.	0	a	0	0	Onagraceae Juss.
Iz_3_1009	DJI_0855	Pentanema britannica (L.) D.Gut.Larr., Santos-Vicente, Anderb., E.Rico & M.M.Mart.Ort.	0	n	1	0	Asteraceae Bercht. & J.Presl
Iz_3_1009	DJI_0855	Peucedanum oreoselinum (L.) Moench	5	n	1	0	Apiaceae Lindl.
Iz_3_1009	DJI_0855	Peucedanum ruthenicum M.Bieb.	0	n	1	0	Apiaceae Lindl.
Iz_3_1009	DJI_0855	Pilosella echioides (Lumn.) F.W.Schultz & Sch.Bip.	0	n	1	0	Asteraceae Bercht. & J.Presl

Iz_3_1009	DJI_0855	Pilosella officinarum F. Schultz et Sch. Bip.	0	n	1	0	Asteraceae Bercht. & J.Presl
Iz_3_1009	DJI_0855	Pilosella sp.	0		NA	0	Asteraceae Bercht. & J.Presl
Iz_3_1009	DJI_0855	Pinus sylvestris L.	0	n	1	1	Pinaceae Spreng. ex F.Rudolphi
Iz_3_1009	DJI_0855	Poa annua L.	0	n	1	0	Poaceae Barnhart
Iz_3_1009	DJI_0855	Poa nemoralis L.	0	n	1	0	Poaceae Barnhart
Iz_3_1009	DJI_0855	Poa sp.	0		NA	0	Poaceae Barnhart
Iz_3_1009	DJI_0855	Polygonatum odoratum (Mill.) Druce	0.5	n	1	0	Asparagaceae Juss.
Iz_3_1009	DJI_0855	Polygonum arenarium Waldst. & Kit.	0	n	1	0	Polygonaceae Juss.
Iz_3_1009	DJI_0855	Populus nigra L.	0	n	1	1	Salicaceae Mirb.
Iz_3_1009	DJI_0855	Populus tremula L.	0	n	1	1	Salicaceae Mirb.
Iz_3_1009	DJI_0855	Populus Ã canescens (Aiton) Sm.	0	n	1	1	Salicaceae Mirb.
Iz_3_1009	DJI_0855	Potentilla argentea L.	0	n	1	0	Rosaceae Juss.
Iz_3_1009	DJI_0855	Psammophiliella muralis (L.) Ikonn.	0	n	1	0	Caryophyllaceae Juss.
Iz_3_1009	DJI_0855	Psephellus sumensis (Kalen.) Greuter	0	n	1	0	Asteraceae Bercht. & J.Presl
Iz_3_1009	DJI_0855	Pulsatilla pratensis (L.) Mill. s.l.	0	n	1	0	Ranunculaceae Juss.
Iz_3_1009	DJI_0855	Pyrus communis L.	0	a	0	1	Rosaceae Juss.
Iz_3_1009	DJI_0855	Quercus robur L.	0	n	1	1	Fagaceae Dumort.
Iz_3_1009	DJI_0855	Robinia pseudoacacia L.	0	a	0	1	Fabaceae Lindl.
Iz_3_1009	DJI_0855	Rosa sp.	0		NA	1	Rosaceae Juss.
Iz_3_1009	DJI_0855	Rubus idaeus L.	0	n	1	0	Rosaceae Juss.
Iz_3_1009	DJI_0855	Rumex acetosella L.	0	n	1	0	Polygonaceae Juss.
Iz_3_1009	DJI_0855	Rumex thyrsiflorus Fingerh.	0	n	1	0	Polygonaceae Juss.
Iz_3_1009	DJI_0855	Salix alba L.	0	n	1	1	Salicaceae Mirb.
Iz_3_1009	DJI_0855	Sambucus nigra L.	0	n	1	1	Viburnaceae Raf.
Iz_3_1009	DJI_0855	Secale sylvestre Host	0	n	1	0	Poaceae Barnhart
Iz_3_1009	DJI_0855	Sempervivum ruthenicum Schnittsp. & C.B. Lehm.	0	n	1	0	Crassulaceae J.St.-Hil.
Iz_3_1009	DJI_0855	Setaria viridis (L.) P. Beauv.	0	n	1	0	Poaceae Barnhart
Iz_3_1009	DJI_0855	Silene borysthenica (Gruner) Walters	0	n	1	0	Caryophyllaceae Juss.
Iz_3_1009	DJI_0855	Silene latifolia subsp. alba (Mill.) Greuter & Burdet	0	n	1	0	Caryophyllaceae Juss.
Iz_3_1009	DJI_0855	Solidago virgaurea L.	0	n	1	0	Asteraceae Bercht. & J.Presl
Iz_3_1009	DJI_0855	Sonchus oleraceus L.	0	a	0	0	Asteraceae Bercht. & J.Presl
Iz_3_1009	DJI_0855	Sorbus aucuparia L.	0	n	1	1	Rosaceae Juss.
Iz_3_1009	DJI_0855	Stachys recta L.	0	n	1	0	Lamiacea Lindl.
Iz_3_1009	DJI_0855	Tanacetum vulgare L.	0	n	1	0	Asteraceae Bercht. & J.Presl
Iz_3_1009	DJI_0855	Thymus pallasianus Heinr.Braun	0	n	1	0	Lamiacea Lindl.
Iz_3_1009	DJI_0855	Tilia cordata Mill.	0	n	1	1	Malvaceae Juss.
Iz_3_1009	DJI_0855	Trifolium alpestre L.	0	n	1	0	Fabaceae Lindl.
Iz_3_1009	DJI_0855	Trifolium arvense L.	0	n	1	0	Fabaceae Lindl.
Iz_3_1009	DJI_0855	Ulmus glabra Huds.	0	n	1	1	Ulmaceae Mirb.
Iz_3_1009	DJI_0855	Verbascum densiflorum Bertol.	0	n	1	0	Scrophulariaceae Juss.
Iz_3_1009	DJI_0855	Veronica austriaca L.	0	n	1	0	Plantaginaceae Juss.
Iz_3_1009	DJI_0855	Veronica incana L.	0	n	1	0	Plantaginaceae Juss.
Iz_3_1009	DJI_0855	Veronica verna L.	0	n	1	0	Plantaginaceae Juss.
Iz_3_1009	DJI_0855	Vincetoxicum hirundinaria Medik	0	n	1	0	Apocynaceae Juss.
Iz_3_1009	DJI_0857	Acer tataricum L.	0	n	1	1	Sapindaceae Juss.
Iz_3_1009	DJI_0857	Achillea millefolium L. s.l.	0	n	1	0	Asteraceae Bercht. & J.Presl
Iz_3_1009	DJI_0857	Allium oleraceum L.	0	n	1	0	Amaryllidaceae J.St.-Hil.
Iz_3_1009	DJI_0857	Ambrosia artemisiifolia L.	0	a	0	0	Asteraceae Bercht. & J.Presl
Iz_3_1009	DJI_0857	Amorpha fruticosa L.	0	a	0	0	Fabaceae Lindl.
Iz_3_1009	DJI_0857	Anthericum ramosum L.	0	n	1	0	Asparagaceae Juss.
Iz_3_1009	DJI_0857	Artemisia marschalliana Spreng.	0	n	1	0	Asteraceae Bercht. & J.Presl
Iz_3_1009	DJI_0857	Asparagus officinalis L.	0	n	1	0	Asparagaceae Juss.
Iz_3_1009	DJI_0857	Astragalus cicer L.	0	n	1	0	Fabaceae Lindl.
Iz_3_1009	DJI_0857	Bassia laniflora (S.G.Gmel.) A.J.Scott	0	n	1	0	Amaranthaceae Juss.
Iz_3_1009	DJI_0857	Berberis vulgaris L.	0	n	1	0	Berberidaceae Juss.
Iz_3_1009	DJI_0857	Betula pendula Roth.	0	n	1	1	Betulaceae Gray
Iz_3_1009	DJI_0857	Betula pendula Roth.	0	n	1	0	Poaceae Barnhart
Iz_3_1009	DJI_0857	Bromus inermis Leyss.	0		NA	NA	
Iz_3_1009	DJI_0857	Bromus squarrosus L.	0	a	0	0	Poaceae Barnhart
Iz_3_1009	DJI_0857	Bromus tectorum L.	0	a	0	0	Poaceae Barnhart
Iz_3_1009	DJI_0857	Calamagrostis epigejos (L.) Roth	40	n	1	0	Poaceae Barnhart
Iz_3_1009	DJI_0857	Carduus acanthoides L.	0	n	1	0	Asteraceae Bercht. & J.Presl
Iz_3_1009	DJI_0857	Carex sp.	0	n	1	0	Cyperaceae Juss.
Iz_3_1009	DJI_0857	Centaurea majorovii Dumbadze	0	n	1	0	Asteraceae Bercht. & J.Presl
Iz_3_1009	DJI_0857	Chamaecytisus ruthenicus (Fisch.ex Wol.) Klaskova	0	n	1	0	Fabaceae Lindl.
Iz_3_1009	DJI_0857	Chelidonium majus L.	0.2	n	1	0	Papaveraceae Juss.
Iz_3_1009	DJI_0857	Chenopodium betaceum Andrz.	0	n	1	0	Amaranthaceae Juss.
Iz_3_1009	DJI_0857	Chenopodium cf. album	0	n	1	0	Amaranthaceae Juss.
Iz_3_1009	DJI_0857	Chenopodium sp.	0		NA	0	Amaranthaceae Juss.
Iz_3_1009	DJI_0857	Chondrilla juncea L.	0	n	1	0	Asteraceae Bercht. & J.Presl
Iz_3_1009	DJI_0857	Cirsium vulgare (Savi) Ten.	0	n	1	0	Asteraceae Bercht. & J.Presl
Iz_3_1009	DJI_0857	Clinopodium acinos (L.) Kuntze	0	n	1	0	Lamiacea Lindl.
Iz_3_1009	DJI_0857	Convallaria majalis L.	0	n	1	0	Asparagaceae Juss.
Iz_3_1009	DJI_0857	Crataegus rhipidophylla Gand.	0	n	1	1	Rosaceae Juss.
Iz_3_1009	DJI_0857	Crepis foetida L.	0	n	1	0	Asteraceae Bercht. & J.Presl
Iz_3_1009	DJI_0857	Dactylis glomerata L.	0	n	1	0	Poaceae Barnhart
Iz_3_1009	DJI_0857	Dianthus campestris M.Bieb.	0	n	1	0	Caryophyllaceae Juss.
Iz_3_1009	DJI_0857	Digitaria sanguinalis (L.) Scop.	0	a	0	0	Poaceae Barnhart
Iz_3_1009	DJI_0857	Elymus repens (L.) Gould	0	n	1	0	Poaceae Barnhart
Iz_3_1009	DJI_0857	Epilobium tetragonum L.	0	n	1	0	Onagraceae Juss.
Iz_3_1009	DJI_0857	Eragrostis minor Host	0	a	0	0	Poaceae Barnhart
Iz_3_1009	DJI_0857	Erigeron canadensis L.	0	a	0	0	Asteraceae Bercht. & J.Presl
Iz_3_1009	DJI_0857	Euphorbia seguieriana Neck.	0	n	1	0	Euphorbiaceae Juss.
Iz_3_1009	DJI_0857	Fallopia dumetorum (L.) Holub	0	n	1	0	Polygonaceae Juss.
Iz_3_1009	DJI_0857	Festuca beckeri (Hack.) Trautv.	0	n	1	0	Poaceae Barnhart
Iz_3_1009	DJI_0857	Fragaria vesca L.	0	n	1	0	Rosaceae Juss.
Iz_3_1009	DJI_0857	Frangula alnus Mill.	0	n	1	1	Rhamnaceae Juss.
Iz_3_1009	DJI_0857	Fraxinus pennsylvanica Marshall	0	a	0	1	Oleaceae Hoffmanns. & Link
Iz_3_1009	DJI_0857	Galeopsis bifida Boenn.	0	n	1	0	Lamiacea Lindl.
Iz_3_1009	DJI_0857	Galium verum L.	0	n	1	0	Rubiaceae Juss.
Iz_3_1009	DJI_0857	Genista tinctoria L.	0	n	1	0	Fabaceae Lindl.
Iz_3_1009	DJI_0857	Geranium robertianum L.	0	n	1	0	Geraniaceae Juss.
Iz_3_1009	DJI_0857	Geranium sanquineum L.	0	n	1	0	Geraniaceae Juss.
Iz_3_1009	DJI_0857	Gypsophila paniculata L.	0	n	1	0	Caryophyllaceae Juss.
Iz_3_1009	DJI_0857	Helichrysum arenarium (L.) Moench	0	n	1	0	Asteraceae Bercht. & J.Presl
Iz_3_1009	DJI_0857	Herniaria polygama J.Gay	0	n	1	0	Caryophyllaceae Juss.
Iz_3_1009	DJI_0857	Hieracium robustum Fr.	0	n	1	0	Asteraceae Bercht. & J.Presl
Iz_3_1009	DJI_0857	Hieracium umbellatum L.	0	n	1	NA	Asteraceae Bercht. & J.Presl

Iz_3_1009	DJI_0857	Hylotelephium maximum (L.) Holub	0	n	1	0	Crassulaceae J.St.-Hil.
Iz_3_1009	DJI_0857	Jacobaea borysthenica (DC.) B.Nord. & Greuter	0	n	1	0	Asteraceae Bercht. & J.Presl
Iz_3_1009	DJI_0857	Jasione montana L.	0	n	1	0	Campanulaceae Juss.
Iz_3_1009	DJI_0857	Jurinea cyanoides (L.) Rchb.s.l.	0	n	1	0	Asteraceae Bercht. & J.Presl
Iz_3_1009	DJI_0857	Koeleria glauca (Spreng.) DC.	0	n	1	0	Poaceae Barnhart
Iz_3_1009	DJI_0857	Lactuca serriola L.	0	a	0	0	Asteraceae Bercht. & J.Presl
Iz_3_1009	DJI_0857	Linaria genistifolia (L.) Mill.	0	n	1	0	Plantaginaceae Juss.
Iz_3_1009	DJI_0857	Lomelosia argentea (L.) Greuter & Burdet	0	n	1	0	Caprifoliaceae Juss.
Iz_3_1009	DJI_0857	Melampyrum pratense L.	0	n	1	0	Orobanchaceae Vent.
Iz_3_1009	DJI_0857	Oenothera biennis L.	0	a	0	0	Onagraceae Juss.
Iz_3_1009	DJI_0857	Pentanema britannica (L.) D.Gut.Larr., Santos-Vicente, Anderb., E.Rico & M.M.Mart.Ort.	0	n	1	0	Asteraceae Bercht. & J.Presl
Iz_3_1009	DJI_0857	Peucedanum oreoselinum (L.) Moench	2	n	1	0	Apiaceae Lindl.
Iz_3_1009	DJI_0857	Peucedanum ruthenicum M.Bieb.	0	n	1	0	Apiaceae Lindl.
Iz_3_1009	DJI_0857	Pilosella echioides (Lumn.) F.W.Schultz & Sch.Bip.	0	n	1	0	Asteraceae Bercht. & J.Presl
Iz_3_1009	DJI_0857	Pilosella officinarum F. Schultz et Sch. Bip.	0	n	1	0	Asteraceae Bercht. & J.Presl
Iz_3_1009	DJI_0857	Pilosella sp.	0		NA	0	Asteraceae Bercht. & J.Presl
Iz_3_1009	DJI_0857	Pinus sylvestris L.	0	n	1	1	Pinaceae Spreng. ex F.Rudolphi
Iz_3_1009	DJI_0857	Poa annua L.	0	n	1	0	Poaceae Barnhart
Iz_3_1009	DJI_0857	Poa nemoralis L.	0	n	1	0	Poaceae Barnhart
Iz_3_1009	DJI_0857	Poa sp.	0		NA	0	Poaceae Barnhart
Iz_3_1009	DJI_0857	Polygonatum odoratum (Mill.) Druce	0	n	1	0	Asparagaceae Juss.
Iz_3_1009	DJI_0857	Polygonum arenarium Waldst. & Kit.	0	n	1	0	Polygonaceae Juss.
Iz_3_1009	DJI_0857	Populus nigra L.	0	n	1	1	Salicaceae Mirb.
Iz_3_1009	DJI_0857	Populus tremula L.	0	n	1	1	Salicaceae Mirb.
Iz_3_1009	DJI_0857	Populus Ã canescens (Aiton) Sm.	0	n	1	1	Salicaceae Mirb.
Iz_3_1009	DJI_0857	Potentilla argentea L.	0	n	1	0	Rosaceae Juss.
Iz_3_1009	DJI_0857	Psammophiliella muralis (L.) Ikonn.	0	n	1	0	Caryophyllaceae Juss.
Iz_3_1009	DJI_0857	Psephellus sumensis (Kalen.) Greuter	0	n	1	0	Asteraceae Bercht. & J.Presl
Iz_3_1009	DJI_0857	Pulsatilla pratensis (L.) Mill. s.l.	0	n	1	0	Ranunculaceae Juss.
Iz_3_1009	DJI_0857	Pyrus communis L.	0	a	0	1	Rosaceae Juss.
Iz_3_1009	DJI_0857	Quercus robur L.	0	n	1	1	Fagaceae Dumort.
Iz_3_1009	DJI_0857	Robinia pseudoacacia L.	0	a	0	1	Fabaceae Lindl.
Iz_3_1009	DJI_0857	Rosa sp.	0		NA	1	Rosaceae Juss.
Iz_3_1009	DJI_0857	Rubus idaeus L.	0	n	1	0	Rosaceae Juss.
Iz_3_1009	DJI_0857	Rumex acetosella L.	0	n	1	0	Polygonaceae Juss.
Iz_3_1009	DJI_0857	Rumex thyrsiflorus Fingerh.	0	n	1	0	Polygonaceae Juss.
Iz_3_1009	DJI_0857	Salix alba L.	0	n	1	1	Salicaceae Mirb.
Iz_3_1009	DJI_0857	Sambucus nigra L.	0	n	1	1	Viburnaceae Raf.
Iz_3_1009	DJI_0857	Secale sylvestre Host	0	n	1	0	Poaceae Barnhart
Iz_3_1009	DJI_0857	Sempervivum ruthenicum Schnittsp. & C.B. Lehm.	0	n	1	0	Crassulaceae J.St.-Hil.
Iz_3_1009	DJI_0857	Setaria viridis (L.) P. Beauv.	0	n	1	0	Poaceae Barnhart
Iz_3_1009	DJI_0857	Silene borysthenica (Gruner) Walters	0	n	1	0	Caryophyllaceae Juss.
Iz_3_1009	DJI_0857	Silene latifolia subsp. alba (Mill.) Greuter & Burdet	0	n	1	0	Caryophyllaceae Juss.
Iz_3_1009	DJI_0857	Solidago virgaurea L.	0	n	1	0	Asteraceae Bercht. & J.Presl
Iz_3_1009	DJI_0857	Sonchus oleraceus L.	0	a	0	0	Asteraceae Bercht. & J.Presl
Iz_3_1009	DJI_0857	Sorbus aucuparia L.	0	n	1	1	Rosaceae Juss.
Iz_3_1009	DJI_0857	Stachys recta L.	0	n	1	0	Lamiacea Lindl.
Iz_3_1009	DJI_0857	Tanacetum vulgare L.	0	n	1	0	Asteraceae Bercht. & J.Presl
Iz_3_1009	DJI_0857	Thymus pallasianus Heinr.Braun	0	n	1	0	Lamiacea Lindl.
Iz_3_1009	DJI_0857	Tilia cordata Mill.	0	n	1	1	Malvaceae Juss.
Iz_3_1009	DJI_0857	Trifolium alpestre L.	0	n	1	0	Fabaceae Lindl.
Iz_3_1009	DJI_0857	Trifolium arvense L.	0	n	1	0	Fabaceae Lindl.
Iz_3_1009	DJI_0857	Ulmus glabra Huds.	0	n	1	1	Ulmaceae Mirb.
Iz_3_1009	DJI_0857	Verbascum densiflorum Bertol.	0	n	1	0	Scrophulariaceae Juss.
Iz_3_1009	DJI_0857	Veronica austriaca L.	0	n	1	0	Plantaginaceae Juss.
Iz_3_1009	DJI_0857	Veronica incana L.	0	n	1	0	Plantaginaceae Juss.
Iz_3_1009	DJI_0857	Veronica verna L.	0	n	1	0	Plantaginaceae Juss.
Iz_3_1009	DJI_0857	Vincetoxicum hirundinaria Medik	0	n	1	0	Apocynaceae Juss.
Iz_3_1009	DJI_0858	Acer tataricum L.	0	n	1	1	Sapindaceae Juss.
Iz_3_1009	DJI_0858	Achillea millefolium L. s.l.	0	n	1	0	Asteraceae Bercht. & J.Presl
Iz_3_1009	DJI_0858	Allium oleraceum L.	0	n	1	0	Amaryllidaceae J.St.-Hil.
Iz_3_1009	DJI_0858	Ambrosia artemisiifolia L.	0	a	0	0	Asteraceae Bercht. & J.Presl
Iz_3_1009	DJI_0858	Amorpha fruticosa L.	0	a	0	0	Fabaceae Lindl.
Iz_3_1009	DJI_0858	Anthericum ramosum L.	0	n	1	0	Asparagaceae Juss.
Iz_3_1009	DJI_0858	Artemisia marschalliana Spreng.	0	n	1	0	Asteraceae Bercht. & J.Presl
Iz_3_1009	DJI_0858	Asparagus officinalis L.	0	n	1	0	Asparagaceae Juss.
Iz_3_1009	DJI_0858	Astragalus cicer L.	0	n	1	0	Fabaceae Lindl.
Iz_3_1009	DJI_0858	Bassia laniflora (S.G.Gmel.) A.J.Scott	0	n	1	0	Amaranthaceae Juss.
Iz_3_1009	DJI_0858	Berberis vulgaris L.	0	n	1	0	Berberidaceae Juss.
Iz_3_1009	DJI_0858	Betula pendula Roth.	0	n	1	1	Betulaceae Gray
Iz_3_1009	DJI_0858	Betula pendula Roth.	0	n	1	0	Poaceae Barnhart
Iz_3_1009	DJI_0858	Bromus inermis Leyss.	0		NA	NA	
Iz_3_1009	DJI_0858	Bromus squarrosus L.	0	a	0	0	Poaceae Barnhart
Iz_3_1009	DJI_0858	Bromus tectorum L.	0	a	0	0	Poaceae Barnhart
Iz_3_1009	DJI_0858	Calamagrostis epigejos (L.) Roth	10	n	1	0	Poaceae Barnhart
Iz_3_1009	DJI_0858	Carduus acanthoides L.	0	n	1	0	Asteraceae Bercht. & J.Presl
Iz_3_1009	DJI_0858	Carex sp.	0	n	1	0	Cyperaceae Juss.
Iz_3_1009	DJI_0858	Centaurea majorovii Dumbadze	0	n	1	0	Asteraceae Bercht. & J.Presl
Iz_3_1009	DJI_0858	Chamaecytisus ruthenicus (Fisch.ex Wol.) Klaskova	0	n	1	0	Fabaceae Lindl.
Iz_3_1009	DJI_0858	Chelidonium majus L.	2	n	1	0	Papaveraceae Juss.
Iz_3_1009	DJI_0858	Chenopodium betaceum Andrz.	0	n	1	0	Amaranthaceae Juss.
Iz_3_1009	DJI_0858	Chenopodium cf. album	0	n	1	0	Amaranthaceae Juss.
Iz_3_1009	DJI_0858	Chenopodium sp.	0		NA	0	Amaranthaceae Juss.
Iz_3_1009	DJI_0858	Chondrilla juncea L.	0	n	1	0	Asteraceae Bercht. & J.Presl
Iz_3_1009	DJI_0858	Cirsium vulgare (Savi) Ten.	0	n	1	0	Asteraceae Bercht. & J.Presl
Iz_3_1009	DJI_0858	Clinopodium acinos (L.) Kuntze	0	n	1	0	Lamiacea Lindl.
Iz_3_1009	DJI_0858	Convallaria majalis L.	0	n	1	0	Asparagaceae Juss.
Iz_3_1009	DJI_0858	Crataegus rhipidophylla Gand.	0	n	1	1	Rosaceae Juss.
Iz_3_1009	DJI_0858	Crepis foetida L.	0	n	1	0	Asteraceae Bercht. & J.Presl
Iz_3_1009	DJI_0858	Dactylis glomerata L.	0	n	1	0	Poaceae Barnhart
Iz_3_1009	DJI_0858	Dianthus campestris M.Bieb.	0	n	1	0	Caryophyllaceae Juss.
Iz_3_1009	DJI_0858	Digitaria sanguinalis (L.) Scop.	0	a	0	0	Poaceae Barnhart
Iz_3_1009	DJI_0858	Elymus repens (L.) Gould	0	n	1	0	Poaceae Barnhart
Iz_3_1009	DJI_0858	Epilobium tetragonum L.	0	n	1	0	Onagraceae Juss.
Iz_3_1009	DJI_0858	Eragrostis minor Host	0	a	0	0	Poaceae Barnhart
Iz_3_1009	DJI_0858	Erigeron canadensis L.	0	a	0	0	Asteraceae Bercht. & J.Presl
Iz_3_1009	DJI_0858	Euphorbia seguieriana Neck.	0	n	1	0	Euphorbiaceae Juss.
Iz_3_1009	DJI_0858	Fallopia dumetorum (L.) Holub	0	n	1	0	Polygonaceae Juss.

Iz_3_1009	DJI_0858	Festuca beckeri (Hack.) Trautv.	0	n	1	0	Poaceae Barnhart
Iz_3_1009	DJI_0858	Fragaria vesca L.	0	n	1	0	Rosaceae Juss.
Iz_3_1009	DJI_0858	Frangula alnus Mill.	0	n	1	1	Rhamnaceae Juss.
Iz_3_1009	DJI_0858	Fraxinus pennsylvanica Marshall	0	a	0	1	Oleaceae Hoffmanns. & Link
Iz_3_1009	DJI_0858	Galeopsis bifida Boenn.	0	n	1	0	Lamiacea Lindl.
Iz_3_1009	DJI_0858	Galium verum L.	0	n	1	0	Rubiaceae Juss.
Iz_3_1009	DJI_0858	Genista tinctoria L.	0	n	1	0	Fabaceae Lindl.
Iz_3_1009	DJI_0858	Geranium robertianum L.	0	n	1	0	Geraniaceae Juss.
Iz_3_1009	DJI_0858	Geranium sanquineum L.	0	n	1	0	Geraniaceae Juss.
Iz_3_1009	DJI_0858	Gypsophila paniculata L.	0	n	1	0	Caryophyllaceae Juss.
Iz_3_1009	DJI_0858	Helichrysum arenarium (L.) Moench	0	n	1	0	Asteraceae Bercht. & J.Presl
Iz_3_1009	DJI_0858	Herniaria polygama J.Gay	0	n	1	0	Caryophyllaceae Juss.
Iz_3_1009	DJI_0858	Hieracium robustum Fr.	0	n	1	0	Asteraceae Bercht. & J.Presl
Iz_3_1009	DJI_0858	Hieracium umbellatum L.	0	n	1	NA	Asteraceae Bercht. & J.Presl
Iz_3_1009	DJI_0858	Hylotelephium maximum (L.) Holub	0	n	1	0	Crassulaceae J.St.-Hil.
Iz_3_1009	DJI_0858	Jacobaea borysthenica (DC.) B.Nord. & Greuter	0	n	1	0	Asteraceae Bercht. & J.Presl
Iz_3_1009	DJI_0858	Jasione montana L.	0	n	1	0	Campanulaceae Juss.
Iz_3_1009	DJI_0858	Jurinea cyanoides (L.) Rchb.s.l.	0	n	1	0	Asteraceae Bercht. & J.Presl
Iz_3_1009	DJI_0858	Koeleria glauca (Spreng.) DC.	0	n	1	0	Poaceae Barnhart
Iz_3_1009	DJI_0858	Lactuca serriola L.	0	a	0	0	Asteraceae Bercht. & J.Presl
Iz_3_1009	DJI_0858	Linaria genistifolia (L.) Mill.	0	n	1	0	Plantaginaceae Juss.
Iz_3_1009	DJI_0858	Lomelosia argentea (L.) Greuter & Burdet	0	n	1	0	Caprifoliaceae Juss.
Iz_3_1009	DJI_0858	Melampyrum pratense L.	0	n	1	0	Orobanchaceae Vent.
Iz_3_1009	DJI_0858	Oenothera biennis L.	0	a	0	0	Onagraceae Juss.
Iz_3_1009	DJI_0858	Pentanema britannica (L.) D.Gut.Larr., Santos-Vicente, Anderb., E.Rico & M.M.Mart.Ort.	0	n	1	0	Asteraceae Bercht. & J.Presl
Iz_3_1009	DJI_0858	Peucedanum oreoselinum (L.) Moench	2	n	1	0	Apiaceae Lindl.
Iz_3_1009	DJI_0858	Peucedanum ruthenicum M.Bieb.	0	n	1	0	Apiaceae Lindl.
Iz_3_1009	DJI_0858	Pilosella echioides (Lumn.) F.W.Schultz & Sch.Bip.	0	n	1	0	Asteraceae Bercht. & J.Presl
Iz_3_1009	DJI_0858	Pilosella officinarum F. Schultz et Sch. Bip.	0	n	1	0	Asteraceae Bercht. & J.Presl
Iz_3_1009	DJI_0858	Pilosella sp.	0		NA	0	Asteraceae Bercht. & J.Presl
Iz_3_1009	DJI_0858	Pinus sylvestris L.	0	n	1	1	Pinaceae Spreng. ex F.Rudolphi
Iz_3_1009	DJI_0858	Poa annua L.	0	n	1	0	Poaceae Barnhart
Iz_3_1009	DJI_0858	Poa nemoralis L.	0	n	1	0	Poaceae Barnhart
Iz_3_1009	DJI_0858	Poa sp.	0		NA	0	Poaceae Barnhart
Iz_3_1009	DJI_0858	Polygonatum odoratum (Mill.) Druce	0	n	1	0	Asparagaceae Juss.
Iz_3_1009	DJI_0858	Polygonum arenarium Waldst. & Kit.	0	n	1	0	Polygonaceae Juss.
Iz_3_1009	DJI_0858	Populus nigra L.	0	n	1	1	Salicaceae Mirb.
Iz_3_1009	DJI_0858	Populus tremula L.	0	n	1	1	Salicaceae Mirb.
Iz_3_1009	DJI_0858	Populus Ã canescens (Aiton) Sm.	0	n	1	1	Salicaceae Mirb.
Iz_3_1009	DJI_0858	Potentilla argentea L.	0	n	1	0	Rosaceae Juss.
Iz_3_1009	DJI_0858	Psammophiliella muralis (L.) Ikonn.	0	n	1	0	Caryophyllaceae Juss.
Iz_3_1009	DJI_0858	Psephellus sumensis (Kalen.) Greuter	0	n	1	0	Asteraceae Bercht. & J.Presl
Iz_3_1009	DJI_0858	Pulsatilla pratensis (L.) Mill. s.l.	0	n	1	0	Ranunculaceae Juss.
Iz_3_1009	DJI_0858	Pyrus communis L.	0	a	0	1	Rosaceae Juss.
Iz_3_1009	DJI_0858	Quercus robur L.	0	n	1	1	Fagaceae Dumort.
Iz_3_1009	DJI_0858	Robinia pseudoacacia L.	0	a	0	1	Fabaceae Lindl.
Iz_3_1009	DJI_0858	Rosa sp.	0		NA	1	Rosaceae Juss.
Iz_3_1009	DJI_0858	Rubus idaeus L.	0	n	1	0	Rosaceae Juss.
Iz_3_1009	DJI_0858	Rumex acetosella L.	0	n	1	0	Polygonaceae Juss.
Iz_3_1009	DJI_0858	Rumex thyrsiflorus Fingerh.	0	n	1	0	Polygonaceae Juss.
Iz_3_1009	DJI_0858	Salix alba L.	0	n	1	1	Salicaceae Mirb.
Iz_3_1009	DJI_0858	Sambucus nigra L.	0	n	1	1	Viburnaceae Raf.
Iz_3_1009	DJI_0858	Secale sylvestre Host	0	n	1	0	Poaceae Barnhart
Iz_3_1009	DJI_0858	Sempervivum ruthenicum Schnittsp. & C.B. Lehm.	0	n	1	0	Crassulaceae J.St.-Hil.
Iz_3_1009	DJI_0858	Setaria viridis (L.) P. Beauv.	0	n	1	0	Poaceae Barnhart
Iz_3_1009	DJI_0858	Silene borysthenica (Gruner) Walters	0	n	1	0	Caryophyllaceae Juss.
Iz_3_1009	DJI_0858	Silene latifolia subsp. alba (Mill.) Greuter & Burdet	0	n	1	0	Caryophyllaceae Juss.
Iz_3_1009	DJI_0858	Solidago virgaurea L.	0	n	1	0	Asteraceae Bercht. & J.Presl
Iz_3_1009	DJI_0858	Sonchus oleraceus L.	0	a	0	0	Asteraceae Bercht. & J.Presl
Iz_3_1009	DJI_0858	Sorbus aucuparia L.	0	n	1	1	Rosaceae Juss.
Iz_3_1009	DJI_0858	Stachys recta L.	0	n	1	0	Lamiacea Lindl.
Iz_3_1009	DJI_0858	Tanacetum vulgare L.	0	n	1	0	Asteraceae Bercht. & J.Presl
Iz_3_1009	DJI_0858	Thymus pallasianus Heinr.Braun	0	n	1	0	Lamiacea Lindl.
Iz_3_1009	DJI_0858	Tilia cordata Mill.	0	n	1	1	Malvaceae Juss.
Iz_3_1009	DJI_0858	Trifolium alpestre L.	0	n	1	0	Fabaceae Lindl.
Iz_3_1009	DJI_0858	Trifolium arvense L.	0	n	1	0	Fabaceae Lindl.
Iz_3_1009	DJI_0858	Ulmus glabra Huds.	0	n	1	1	Ulmaceae Mirb.
Iz_3_1009	DJI_0858	Verbascum densiflorum Bertol.	0	n	1	0	Scrophulariaceae Juss.
Iz_3_1009	DJI_0858	Veronica austriaca L.	0	n	1	0	Plantaginaceae Juss.
Iz_3_1009	DJI_0858	Veronica incana L.	0	n	1	0	Plantaginaceae Juss.
Iz_3_1009	DJI_0858	Veronica verna L.	0	n	1	0	Plantaginaceae Juss.
Iz_3_1009	DJI_0858	Vincetoxicum hirundinaria Medik	0	n	1	0	Apocynaceae Juss.
Iz_3_1009	DJI_0859	Acer tataricum L.	0	n	1	1	Sapindaceae Juss.
Iz_3_1009	DJI_0859	Achillea millefolium L. s.l.	0	n	1	0	Asteraceae Bercht. & J.Presl
Iz_3_1009	DJI_0859	Allium oleraceum L.	0	n	1	0	Amaryllidaceae J.St.-Hil.
Iz_3_1009	DJI_0859	Ambrosia artemisiifolia L.	0	a	0	0	Asteraceae Bercht. & J.Presl
Iz_3_1009	DJI_0859	Amorpha fruticosa L.	0	a	0	0	Fabaceae Lindl.
Iz_3_1009	DJI_0859	Anthericum ramosum L.	0	n	1	0	Asparagaceae Juss.
Iz_3_1009	DJI_0859	Artemisia marschalliana Spreng.	0	n	1	0	Asteraceae Bercht. & J.Presl
Iz_3_1009	DJI_0859	Asparagus officinalis L.	0	n	1	0	Asparagaceae Juss.
Iz_3_1009	DJI_0859	Astragalus cicer L.	0	n	1	0	Fabaceae Lindl.
Iz_3_1009	DJI_0859	Bassia laniflora (S.G.Gmel.) A.J.Scott	0	n	1	0	Amaranthaceae Juss.
Iz_3_1009	DJI_0859	Berberis vulgaris L.	0	n	1	0	Berberidaceae Juss.
Iz_3_1009	DJI_0859	Betula pendula Roth.	0	n	1	1	Betulaceae Gray
Iz_3_1009	DJI_0859	Betula pendula Roth.	0	n	1	0	Poaceae Barnhart
Iz_3_1009	DJI_0859	Bromus inermis Leyss.	0		NA	NA	
Iz_3_1009	DJI_0859	Bromus squarrosus L.	0	a	0	0	Poaceae Barnhart
Iz_3_1009	DJI_0859	Bromus tectorum L.	0	a	0	0	Poaceae Barnhart
Iz_3_1009	DJI_0859	Calamagrostis epigejos (L.) Roth	30	n	1	0	Poaceae Barnhart
Iz_3_1009	DJI_0859	Carduus acanthoides L.	0	n	1	0	Asteraceae Bercht. & J.Presl
Iz_3_1009	DJI_0859	Carex sp.	0	n	1	0	Cyperaceae Juss.
Iz_3_1009	DJI_0859	Centaurea majorovii Dumbadze	0	n	1	0	Asteraceae Bercht. & J.Presl
Iz_3_1009	DJI_0859	Chamaecytisus ruthenicus (Fisch.ex Wol.) Klaskova	0	n	1	0	Fabaceae Lindl.
Iz_3_1009	DJI_0859	Chelidonium majus L.	0.2	n	1	0	Papaveraceae Juss.
Iz_3_1009	DJI_0859	Chenopodium betaceum Andrz.	0	n	1	0	Amaranthaceae Juss.
Iz_3_1009	DJI_0859	Chenopodium cf. album	0	n	1	0	Amaranthaceae Juss.
Iz_3_1009	DJI_0859	Chenopodium sp.	0		NA	0	Amaranthaceae Juss.
Iz_3_1009	DJI_0859	Chondrilla juncea L.	0	n	1	0	Asteraceae Bercht. & J.Presl

Iz_3_1009	DJI_0859	Cirsium vulgare (Savi) Ten.	0	n	1	0	Asteraceae Bercht. & J.Presl
Iz_3_1009	DJI_0859	Clinopodium acinos (L.) Kuntze	0	n	1	0	Lamiacea Lindl.
Iz_3_1009	DJI_0859	Convallaria majalis L.	0	n	1	0	Asparagaceae Juss.
Iz_3_1009	DJI_0859	Crataegus rhipidophylla Gand.	0	n	1	1	Rosaceae Juss.
Iz_3_1009	DJI_0859	Crepis foetida L.	0	n	1	0	Asteraceae Bercht. & J.Presl
Iz_3_1009	DJI_0859	Dactylis glomerata L.	0	n	1	0	Poaceae Barnhart
Iz_3_1009	DJI_0859	Dianthus campestris M.Bieb.	0	n	1	0	Caryophyllaceae Juss.
Iz_3_1009	DJI_0859	Digitaria sanguinalis (L.) Scop.	0	a	0	0	Poaceae Barnhart
Iz_3_1009	DJI_0859	Elymus repens (L.) Gould	0	n	1	0	Poaceae Barnhart
Iz_3_1009	DJI_0859	Epilobium tetragonum L.	0	n	1	0	Onagraceae Juss.
Iz_3_1009	DJI_0859	Eragrostis minor Host	0	a	0	0	Poaceae Barnhart
Iz_3_1009	DJI_0859	Erigeron canadensis L.	0	a	0	0	Asteraceae Bercht. & J.Presl
Iz_3_1009	DJI_0859	Euphorbia seguieriana Neck.	0	n	1	0	Euphorbiaceae Juss.
Iz_3_1009	DJI_0859	Fallopia dumetorum (L.) Holub	0.2	n	1	0	Polygonaceae Juss.
Iz_3_1009	DJI_0859	Festuca beckeri (Hack.) Trautv.	0	n	1	0	Poaceae Barnhart
Iz_3_1009	DJI_0859	Fragaria vesca L.	0	n	1	0	Rosaceae Juss.
Iz_3_1009	DJI_0859	Frangula alnus Mill.	0	n	1	1	Rhamnaceae Juss.
Iz_3_1009	DJI_0859	Fraxinus pennsylvanica Marshall	0	a	0	1	Oleaceae Hoffmanns. & Link
Iz_3_1009	DJI_0859	Galeopsis bifida Boenn.	0	n	1	0	Lamiacea Lindl.
Iz_3_1009	DJI_0859	Galium verum L.	0	n	1	0	Rubiaceae Juss.
Iz_3_1009	DJI_0859	Genista tinctoria L.	0	n	1	0	Fabaceae Lindl.
Iz_3_1009	DJI_0859	Geranium robertianum L.	0	n	1	0	Geraniaceae Juss.
Iz_3_1009	DJI_0859	Geranium sanquineum L.	0	n	1	0	Geraniaceae Juss.
Iz_3_1009	DJI_0859	Gypsophila paniculata L.	0	n	1	0	Caryophyllaceae Juss.
Iz_3_1009	DJI_0859	Helichrysum arenarium (L.) Moench	0	n	1	0	Asteraceae Bercht. & J.Presl
Iz_3_1009	DJI_0859	Herniaria polygama J.Gay	0	n	1	0	Caryophyllaceae Juss.
Iz_3_1009	DJI_0859	Hieracium robustum Fr.	0	n	1	0	Asteraceae Bercht. & J.Presl
Iz_3_1009	DJI_0859	Hieracium umbellatum L.	0	n	1	NA	Asteraceae Bercht. & J.Presl
Iz_3_1009	DJI_0859	Hylotelephium maximum (L.) Holub	0	n	1	0	Crassulaceae J.St.-Hil.
Iz_3_1009	DJI_0859	Jacobaea borysthenica (DC.) B.Nord. & Greuter	0	n	1	0	Asteraceae Bercht. & J.Presl
Iz_3_1009	DJI_0859	Jasione montana L.	0	n	1	0	Campanulaceae Juss.
Iz_3_1009	DJI_0859	Jurinea cyanoides (L.) Rchb.s.l.	0	n	1	0	Asteraceae Bercht. & J.Presl
Iz_3_1009	DJI_0859	Koeleria glauca (Spreng.) DC.	0	n	1	0	Poaceae Barnhart
Iz_3_1009	DJI_0859	Lactuca serriola L.	0	a	0	0	Asteraceae Bercht. & J.Presl
Iz_3_1009	DJI_0859	Linaria genistifolia (L.) Mill.	0	n	1	0	Plantaginaceae Juss.
Iz_3_1009	DJI_0859	Lomelosia argentea (L.) Greuter & Burdet	0	n	1	0	Caprifoliaceae Juss.
Iz_3_1009	DJI_0859	Melampyrum pratense L.	0	n	1	0	Orobanchaceae Vent.
Iz_3_1009	DJI_0859	Oenothera biennis L.	0	a	0	0	Onagraceae Juss.
Iz_3_1009	DJI_0859	Pentanema britannica (L.) D.Gut.Larr., Santos-Vicente, Anderb., E.Rico & M.M.Mart.Ort.	0	n	1	0	Asteraceae Bercht. & J.Presl
Iz_3_1009	DJI_0859	Peucedanum oreoselinum (L.) Moench	6	n	1	0	Apiaceae Lindl.
Iz_3_1009	DJI_0859	Peucedanum ruthenicum M.Bieb.	0	n	1	0	Apiaceae Lindl.
Iz_3_1009	DJI_0859	Pilosella echioides (Lumn.) F.W.Schultz & Sch.Bip.	0	n	1	0	Asteraceae Bercht. & J.Presl
Iz_3_1009	DJI_0859	Pilosella officinarum F. Schultz et Sch. Bip.	0	n	1	0	Asteraceae Bercht. & J.Presl
Iz_3_1009	DJI_0859	Pilosella sp.	0		NA	0	Asteraceae Bercht. & J.Presl
Iz_3_1009	DJI_0859	Pinus sylvestris L.	0	n	1	1	Pinaceae Spreng. ex F.Rudolphi
Iz_3_1009	DJI_0859	Poa annua L.	0	n	1	0	Poaceae Barnhart
Iz_3_1009	DJI_0859	Poa nemoralis L.	0	n	1	0	Poaceae Barnhart
Iz_3_1009	DJI_0859	Poa sp.	0		NA	0	Poaceae Barnhart
Iz_3_1009	DJI_0859	Polygonatum odoratum (Mill.) Druce	0	n	1	0	Asparagaceae Juss.
Iz_3_1009	DJI_0859	Polygonum arenarium Waldst. & Kit.	0	n	1	0	Polygonaceae Juss.
Iz_3_1009	DJI_0859	Populus nigra L.	0	n	1	1	Salicaceae Mirb.
Iz_3_1009	DJI_0859	Populus tremula L.	0	n	1	1	Salicaceae Mirb.
Iz_3_1009	DJI_0859	Populus Ã canescens (Aiton) Sm.	0	n	1	1	Salicaceae Mirb.
Iz_3_1009	DJI_0859	Potentilla argentea L.	0	n	1	0	Rosaceae Juss.
Iz_3_1009	DJI_0859	Psammophiliella muralis (L.) Ikonn.	0	n	1	0	Caryophyllaceae Juss.
Iz_3_1009	DJI_0859	Psephellus sumensis (Kalen.) Greuter	0	n	1	0	Asteraceae Bercht. & J.Presl
Iz_3_1009	DJI_0859	Pulsatilla pratensis (L.) Mill. s.l.	0	n	1	0	Ranunculaceae Juss.
Iz_3_1009	DJI_0859	Pyrus communis L.	0	a	0	1	Rosaceae Juss.
Iz_3_1009	DJI_0859	Quercus robur L.	0	n	1	1	Fagaceae Dumort.
Iz_3_1009	DJI_0859	Robinia pseudoacacia L.	0	a	0	1	Fabaceae Lindl.
Iz_3_1009	DJI_0859	Rosa sp.	0		NA	1	Rosaceae Juss.
Iz_3_1009	DJI_0859	Rubus idaeus L.	0	n	1	0	Rosaceae Juss.
Iz_3_1009	DJI_0859	Rumex acetosella L.	0	n	1	0	Polygonaceae Juss.
Iz_3_1009	DJI_0859	Rumex thyrsiflorus Fingerh.	0	n	1	0	Polygonaceae Juss.
Iz_3_1009	DJI_0859	Salix alba L.	0	n	1	1	Salicaceae Mirb.
Iz_3_1009	DJI_0859	Sambucus nigra L.	0	n	1	1	Viburnaceae Raf.
Iz_3_1009	DJI_0859	Secale sylvestre Host	0	n	1	0	Poaceae Barnhart
Iz_3_1009	DJI_0859	Sempervivum ruthenicum Schnittsp. & C.B. Lehm.	0	n	1	0	Crassulaceae J.St.-Hil.
Iz_3_1009	DJI_0859	Setaria viridis (L.) P. Beauv.	0	n	1	0	Poaceae Barnhart
Iz_3_1009	DJI_0859	Silene borysthenica (Gruner) Walters	0	n	1	0	Caryophyllaceae Juss.
Iz_3_1009	DJI_0859	Silene latifolia subsp. alba (Mill.) Greuter & Burdet	0	n	1	0	Caryophyllaceae Juss.
Iz_3_1009	DJI_0859	Solidago virgaurea L.	0	n	1	0	Asteraceae Bercht. & J.Presl
Iz_3_1009	DJI_0859	Sonchus oleraceus L.	0	a	0	0	Asteraceae Bercht. & J.Presl
Iz_3_1009	DJI_0859	Sorbus aucuparia L.	0	n	1	1	Rosaceae Juss.
Iz_3_1009	DJI_0859	Stachys recta L.	0	n	1	0	Lamiacea Lindl.
Iz_3_1009	DJI_0859	Tanacetum vulgare L.	0	n	1	0	Asteraceae Bercht. & J.Presl
Iz_3_1009	DJI_0859	Thymus pallasianus Heinr.Braun	0	n	1	0	Lamiacea Lindl.
Iz_3_1009	DJI_0859	Tilia cordata Mill.	0	n	1	1	Malvaceae Juss.
Iz_3_1009	DJI_0859	Trifolium alpestre L.	0	n	1	0	Fabaceae Lindl.
Iz_3_1009	DJI_0859	Trifolium arvense L.	0	n	1	0	Fabaceae Lindl.
Iz_3_1009	DJI_0859	Ulmus glabra Huds.	0	n	1	1	Ulmaceae Mirb.
Iz_3_1009	DJI_0859	Verbascum densiflorum Bertol.	0	n	1	0	Scrophulariaceae Juss.
Iz_3_1009	DJI_0859	Veronica austriaca L.	0	n	1	0	Plantaginaceae Juss.
Iz_3_1009	DJI_0859	Veronica incana L.	0	n	1	0	Plantaginaceae Juss.
Iz_3_1009	DJI_0859	Veronica verna L.	0	n	1	0	Plantaginaceae Juss.
Iz_3_1009	DJI_0859	Vincetoxicum hirundinaria Medik	0	n	1	0	Apocynaceae Juss.
Iz_3_1009	DJI_0860	Acer tataricum L.	0	n	1	1	Sapindaceae Juss.
Iz_3_1009	DJI_0860	Achillea millefolium L. s.l.	0	n	1	0	Asteraceae Bercht. & J.Presl
Iz_3_1009	DJI_0860	Allium oleraceum L.	0	n	1	0	Amaryllidaceae J.St.-Hil.
Iz_3_1009	DJI_0860	Ambrosia artemisiifolia L.	0	a	0	0	Asteraceae Bercht. & J.Presl
Iz_3_1009	DJI_0860	Amorpha fruticosa L.	0	a	0	0	Fabaceae Lindl.
Iz_3_1009	DJI_0860	Anthericum ramosum L.	0	n	1	0	Asparagaceae Juss.
Iz_3_1009	DJI_0860	Artemisia marschalliana Spreng.	0	n	1	0	Asteraceae Bercht. & J.Presl
Iz_3_1009	DJI_0860	Asparagus officinalis L.	0	n	1	0	Asparagaceae Juss.
Iz_3_1009	DJI_0860	Astragalus cicer L.	0	n	1	0	Fabaceae Lindl.
Iz_3_1009	DJI_0860	Bassia laniflora (S.G.Gmel.) A.J.Scott	0	n	1	0	Amaranthaceae Juss.
Iz_3_1009	DJI_0860	Berberis vulgaris L.	0	n	1	0	Berberidaceae Juss.
Iz_3_1009	DJI_0860	Betula pendula Roth.	0	n	1	1	Betulaceae Gray

Iz_3_1009	DJI_0860	Betula pendula Roth.	0	n	1	0	Poaceae Barnhart
Iz_3_1009	DJI_0860	Bromus inermis Leyss.	0		NA	NA	
Iz_3_1009	DJI_0860	Bromus squarrosus L.	0	a	0	0	Poaceae Barnhart
Iz_3_1009	DJI_0860	Bromus tectorum L.	0	a	0	0	Poaceae Barnhart
Iz_3_1009	DJI_0860	Calamagrostis epigejos (L.) Roth	10	n	1	0	Poaceae Barnhart
Iz_3_1009	DJI_0860	Carduus acanthoides L.	0	n	1	0	Asteraceae Bercht. & J.Presl
Iz_3_1009	DJI_0860	Carex sp.	0	n	1	0	Cyperaceae Juss.
Iz_3_1009	DJI_0860	Centaurea majorovii Dumbadze	0	n	1	0	Asteraceae Bercht. & J.Presl
Iz_3_1009	DJI_0860	Chamaecytisus ruthenicus (Fisch.ex Wol.) Klaskova	0	n	1	0	Fabaceae Lindl.
Iz_3_1009	DJI_0860	Chelidonium majus L.	0	n	1	0	Papaveraceae Juss.
Iz_3_1009	DJI_0860	Chenopodium betaceum Andrz.	0	n	1	0	Amaranthaceae Juss.
Iz_3_1009	DJI_0860	Chenopodium cf. album	0	n	1	0	Amaranthaceae Juss.
Iz_3_1009	DJI_0860	Chenopodium sp.	0		NA	0	Amaranthaceae Juss.
Iz_3_1009	DJI_0860	Chondrilla juncea L.	0	n	1	0	Asteraceae Bercht. & J.Presl
Iz_3_1009	DJI_0860	Cirsium vulgare (Savi) Ten.	0	n	1	0	Asteraceae Bercht. & J.Presl
Iz_3_1009	DJI_0860	Clinopodium acinos (L.) Kuntze	0	n	1	0	Lamiacea Lindl.
Iz_3_1009	DJI_0860	Convallaria majalis L.	0	n	1	0	Asparagaceae Juss.
Iz_3_1009	DJI_0860	Crataegus rhipidophylla Gand.	0	n	1	1	Rosaceae Juss.
Iz_3_1009	DJI_0860	Crepis foetida L.	0	n	1	0	Asteraceae Bercht. & J.Presl
Iz_3_1009	DJI_0860	Dactylis glomerata L.	0	n	1	0	Poaceae Barnhart
Iz_3_1009	DJI_0860	Dianthus campestris M.Bieb.	0	n	1	0	Caryophyllaceae Juss.
Iz_3_1009	DJI_0860	Digitaria sanguinalis (L.) Scop.	0	a	0	0	Poaceae Barnhart
Iz_3_1009	DJI_0860	Elymus repens (L.) Gould	0	n	1	0	Poaceae Barnhart
Iz_3_1009	DJI_0860	Epilobium tetragonum L.	0	n	1	0	Onagraceae Juss.
Iz_3_1009	DJI_0860	Eragrostis minor Host	0	a	0	0	Poaceae Barnhart
Iz_3_1009	DJI_0860	Erigeron canadensis L.	0	a	0	0	Asteraceae Bercht. & J.Presl
Iz_3_1009	DJI_0860	Euphorbia seguieriana Neck.	0	n	1	0	Euphorbiaceae Juss.
Iz_3_1009	DJI_0860	Fallopia dumetorum (L.) Holub	0	n	1	0	Polygonaceae Juss.
Iz_3_1009	DJI_0860	Festuca beckeri (Hack.) Trautv.	0	n	1	0	Poaceae Barnhart
Iz_3_1009	DJI_0860	Fragaria vesca L.	0	n	1	0	Rosaceae Juss.
Iz_3_1009	DJI_0860	Frangula alnus Mill.	0	n	1	1	Rhamnaceae Juss.
Iz_3_1009	DJI_0860	Fraxinus pennsylvanica Marshall	0	a	0	1	Oleaceae Hoffmanns. & Link
Iz_3_1009	DJI_0860	Galeopsis bifida Boenn.	0	n	1	0	Lamiacea Lindl.
Iz_3_1009	DJI_0860	Galium verum L.	0	n	1	0	Rubiaceae Juss.
Iz_3_1009	DJI_0860	Genista tinctoria L.	0	n	1	0	Fabaceae Lindl.
Iz_3_1009	DJI_0860	Geranium robertianum L.	0	n	1	0	Geraniaceae Juss.
Iz_3_1009	DJI_0860	Geranium sanquineum L.	0	n	1	0	Geraniaceae Juss.
Iz_3_1009	DJI_0860	Gypsophila paniculata L.	0	n	1	0	Caryophyllaceae Juss.
Iz_3_1009	DJI_0860	Helichrysum arenarium (L.) Moench	0	n	1	0	Asteraceae Bercht. & J.Presl
Iz_3_1009	DJI_0860	Herniaria polygama J.Gay	0	n	1	0	Caryophyllaceae Juss.
Iz_3_1009	DJI_0860	Hieracium robustum Fr.	0	n	1	0	Asteraceae Bercht. & J.Presl
Iz_3_1009	DJI_0860	Hieracium umbellatum L.	0	n	1	NA	Asteraceae Bercht. & J.Presl
Iz_3_1009	DJI_0860	Hylotelephium maximum (L.) Holub	0	n	1	0	Crassulaceae J.St.-Hil.
Iz_3_1009	DJI_0860	Jacobaea borysthenica (DC.) B.Nord. & Greuter	0	n	1	0	Asteraceae Bercht. & J.Presl
Iz_3_1009	DJI_0860	Jasione montana L.	0	n	1	0	Campanulaceae Juss.
Iz_3_1009	DJI_0860	Jurinea cyanoides (L.) Rchb.s.l.	0	n	1	0	Asteraceae Bercht. & J.Presl
Iz_3_1009	DJI_0860	Koeleria glauca (Spreng.) DC.	0	n	1	0	Poaceae Barnhart
Iz_3_1009	DJI_0860	Lactuca serriola L.	0	a	0	0	Asteraceae Bercht. & J.Presl
Iz_3_1009	DJI_0860	Linaria genistifolia (L.) Mill.	0	n	1	0	Plantaginaceae Juss.
Iz_3_1009	DJI_0860	Lomelosia argentea (L.) Greuter & Burdet	0	n	1	0	Caprifoliaceae Juss.
Iz_3_1009	DJI_0860	Melampyrum pratense L.	0	n	1	0	Orobanchaceae Vent.
Iz_3_1009	DJI_0860	Oenothera biennis L.	0	a	0	0	Onagraceae Juss.
Iz_3_1009	DJI_0860	Pentanema britannica (L.) D.Gut.Larr., Santos-Vicente, Anderb., E.Rico & M.M.Mart.Ort.	0	n	1	0	Asteraceae Bercht. & J.Presl
Iz_3_1009	DJI_0860	Peucedanum oreoselinum (L.) Moench	5	n	1	0	Apiaceae Lindl.
Iz_3_1009	DJI_0860	Peucedanum ruthenicum M.Bieb.	0	n	1	0	Apiaceae Lindl.
Iz_3_1009	DJI_0860	Pilosella echioides (Lumn.) F.W.Schultz & Sch.Bip.	0	n	1	0	Asteraceae Bercht. & J.Presl
Iz_3_1009	DJI_0860	Pilosella officinarum F. Schultz et Sch. Bip.	0	n	1	0	Asteraceae Bercht. & J.Presl
Iz_3_1009	DJI_0860	Pilosella sp.	0		NA	0	Asteraceae Bercht. & J.Presl
Iz_3_1009	DJI_0860	Pinus sylvestris L.	0	n	1	1	Pinaceae Spreng. ex F.Rudolphi
Iz_3_1009	DJI_0860	Poa annua L.	0	n	1	0	Poaceae Barnhart
Iz_3_1009	DJI_0860	Poa nemoralis L.	0	n	1	0	Poaceae Barnhart
Iz_3_1009	DJI_0860	Poa sp.	0		NA	0	Poaceae Barnhart
Iz_3_1009	DJI_0860	Polygonatum odoratum (Mill.) Druce	0	n	1	0	Asparagaceae Juss.
Iz_3_1009	DJI_0860	Polygonum arenarium Waldst. & Kit.	0	n	1	0	Polygonaceae Juss.
Iz_3_1009	DJI_0860	Populus nigra L.	0	n	1	1	Salicaceae Mirb.
Iz_3_1009	DJI_0860	Populus tremula L.	0	n	1	1	Salicaceae Mirb.
Iz_3_1009	DJI_0860	Populus Ã canescens (Aiton) Sm.	0	n	1	1	Salicaceae Mirb.
Iz_3_1009	DJI_0860	Potentilla argentea L.	0	n	1	0	Rosaceae Juss.
Iz_3_1009	DJI_0860	Psammophiliella muralis (L.) Ikonn.	0	n	1	0	Caryophyllaceae Juss.
Iz_3_1009	DJI_0860	Psephellus sumensis (Kalen.) Greuter	1	n	1	0	Asteraceae Bercht. & J.Presl
Iz_3_1009	DJI_0860	Pulsatilla pratensis (L.) Mill. s.l.	0	n	1	0	Ranunculaceae Juss.
Iz_3_1009	DJI_0860	Pyrus communis L.	0	a	0	1	Rosaceae Juss.
Iz_3_1009	DJI_0860	Quercus robur L.	0	n	1	1	Fagaceae Dumort.
Iz_3_1009	DJI_0860	Robinia pseudoacacia L.	0	a	0	1	Fabaceae Lindl.
Iz_3_1009	DJI_0860	Rosa sp.	0		NA	1	Rosaceae Juss.
Iz_3_1009	DJI_0860	Rubus idaeus L.	0	n	1	0	Rosaceae Juss.
Iz_3_1009	DJI_0860	Rumex acetosella L.	0	n	1	0	Polygonaceae Juss.
Iz_3_1009	DJI_0860	Rumex thyrsiflorus Fingerh.	0	n	1	0	Polygonaceae Juss.
Iz_3_1009	DJI_0860	Salix alba L.	0	n	1	1	Salicaceae Mirb.
Iz_3_1009	DJI_0860	Sambucus nigra L.	0	n	1	1	Viburnaceae Raf.
Iz_3_1009	DJI_0860	Secale sylvestre Host	0	n	1	0	Poaceae Barnhart
Iz_3_1009	DJI_0860	Sempervivum ruthenicum Schnittsp. & C.B. Lehm.	0	n	1	0	Crassulaceae J.St.-Hil.
Iz_3_1009	DJI_0860	Setaria viridis (L.) P. Beauv.	0	n	1	0	Poaceae Barnhart
Iz_3_1009	DJI_0860	Silene borysthenica (Gruner) Walters	0	n	1	0	Caryophyllaceae Juss.
Iz_3_1009	DJI_0860	Silene latifolia subsp. alba (Mill.) Greuter & Burdet	0	n	1	0	Caryophyllaceae Juss.
Iz_3_1009	DJI_0860	Solidago virgaurea L.	0	n	1	0	Asteraceae Bercht. & J.Presl
Iz_3_1009	DJI_0860	Sonchus oleraceus L.	0	a	0	0	Asteraceae Bercht. & J.Presl
Iz_3_1009	DJI_0860	Sorbus aucuparia L.	0	n	1	1	Rosaceae Juss.
Iz_3_1009	DJI_0860	Stachys recta L.	0	n	1	0	Lamiacea Lindl.
Iz_3_1009	DJI_0860	Tanacetum vulgare L.	0	n	1	0	Asteraceae Bercht. & J.Presl
Iz_3_1009	DJI_0860	Thymus pallasianus Heinr.Braun	0	n	1	0	Lamiacea Lindl.
Iz_3_1009	DJI_0860	Tilia cordata Mill.	0	n	1	1	Malvaceae Juss.
Iz_3_1009	DJI_0860	Trifolium alpestre L.	0	n	1	0	Fabaceae Lindl.
Iz_3_1009	DJI_0860	Trifolium arvense L.	0	n	1	0	Fabaceae Lindl.
Iz_3_1009	DJI_0860	Ulmus glabra Huds.	0	n	1	1	Ulmaceae Mirb.
Iz_3_1009	DJI_0860	Verbascum densiflorum Bertol.	0	n	1	0	Scrophulariaceae Juss.
Iz_3_1009	DJI_0860	Veronica austriaca L.	0	n	1	0	Plantaginaceae Juss.
Iz_3_1009	DJI_0860	Veronica incana L.	0	n	1	0	Plantaginaceae Juss.

Iz_3_1009	DJI_0860	Veronica verna L.	0	n	1	0	Plantaginaceae Juss.
Iz_3_1009	DJI_0860	Vincetoxicum hirundinaria Medik	0	n	1	0	Apocynaceae Juss.
Iz_3_1009	DJI_0861	Acer tataricum L.	0	n	1	1	Sapindaceae Juss.
Iz_3_1009	DJI_0861	Achillea millefolium L. s.l.	0	n	1	0	Asteraceae Bercht. & J.Presl
Iz_3_1009	DJI_0861	Allium oleraceum L.	0	n	1	0	Amaryllidaceae J.St.-Hil.
Iz_3_1009	DJI_0861	Ambrosia artemisiifolia L.	0	a	0	0	Asteraceae Bercht. & J.Presl
Iz_3_1009	DJI_0861	Amorpha fruticosa L.	0	a	0	0	Fabaceae Lindl.
Iz_3_1009	DJI_0861	Anthericum ramosum L.	0	n	1	0	Asparagaceae Juss.
Iz_3_1009	DJI_0861	Artemisia marschalliana Spreng.	0	n	1	0	Asteraceae Bercht. & J.Presl
Iz_3_1009	DJI_0861	Asparagus officinalis L.	0	n	1	0	Asparagaceae Juss.
Iz_3_1009	DJI_0861	Astragalus cicer L.	0	n	1	0	Fabaceae Lindl.
Iz_3_1009	DJI_0861	Bassia laniflora (S.G.Gmel.) A.J.Scott	0	n	1	0	Amaranthaceae Juss.
Iz_3_1009	DJI_0861	Berberis vulgaris L.	0	n	1	0	Berberidaceae Juss.
Iz_3_1009	DJI_0861	Betula pendula Roth.	0	n	1	1	Betulaceae Gray
Iz_3_1009	DJI_0861	Betula pendula Roth.	0	n	1	0	Poaceae Barnhart
Iz_3_1009	DJI_0861	Bromus inermis Leyss.	0		NA	NA	
Iz_3_1009	DJI_0861	Bromus squarrosus L.	0	a	0	0	Poaceae Barnhart
Iz_3_1009	DJI_0861	Bromus tectorum L.	0	a	0	0	Poaceae Barnhart
Iz_3_1009	DJI_0861	Calamagrostis epigejos (L.) Roth	10	n	1	0	Poaceae Barnhart
Iz_3_1009	DJI_0861	Carduus acanthoides L.	0	n	1	0	Asteraceae Bercht. & J.Presl
Iz_3_1009	DJI_0861	Carex sp.	0	n	1	0	Cyperaceae Juss.
Iz_3_1009	DJI_0861	Centaurea majorovii Dumbadze	0	n	1	0	Asteraceae Bercht. & J.Presl
Iz_3_1009	DJI_0861	Chamaecytisus ruthenicus (Fisch.ex Wol.) Klaskova	0	n	1	0	Fabaceae Lindl.
Iz_3_1009	DJI_0861	Chelidonium majus L.	0.2	n	1	0	Papaveraceae Juss.
Iz_3_1009	DJI_0861	Chenopodium betaceum Andrz.	0	n	1	0	Amaranthaceae Juss.
Iz_3_1009	DJI_0861	Chenopodium cf. album	0	n	1	0	Amaranthaceae Juss.
Iz_3_1009	DJI_0861	Chenopodium sp.	0		NA	0	Amaranthaceae Juss.
Iz_3_1009	DJI_0861	Chondrilla juncea L.	0	n	1	0	Asteraceae Bercht. & J.Presl
Iz_3_1009	DJI_0861	Cirsium vulgare (Savi) Ten.	0	n	1	0	Asteraceae Bercht. & J.Presl
Iz_3_1009	DJI_0861	Clinopodium acinos (L.) Kuntze	0	n	1	0	Lamiacea Lindl.
Iz_3_1009	DJI_0861	Convallaria majalis L.	0	n	1	0	Asparagaceae Juss.
Iz_3_1009	DJI_0861	Crataegus rhipidophylla Gand.	0	n	1	1	Rosaceae Juss.
Iz_3_1009	DJI_0861	Crepis foetida L.	0	n	1	0	Asteraceae Bercht. & J.Presl
Iz_3_1009	DJI_0861	Dactylis glomerata L.	0	n	1	0	Poaceae Barnhart
Iz_3_1009	DJI_0861	Dianthus campestris M.Bieb.	0	n	1	0	Caryophyllaceae Juss.
Iz_3_1009	DJI_0861	Digitaria sanguinalis (L.) Scop.	0	a	0	0	Poaceae Barnhart
Iz_3_1009	DJI_0861	Elymus repens (L.) Gould	0	n	1	0	Poaceae Barnhart
Iz_3_1009	DJI_0861	Epilobium tetragonum L.	0	n	1	0	Onagraceae Juss.
Iz_3_1009	DJI_0861	Eragrostis minor Host	0	a	0	0	Poaceae Barnhart
Iz_3_1009	DJI_0861	Erigeron canadensis L.	0	a	0	0	Asteraceae Bercht. & J.Presl
Iz_3_1009	DJI_0861	Euphorbia seguieriana Neck.	0	n	1	0	Euphorbiaceae Juss.
Iz_3_1009	DJI_0861	Fallopia dumetorum (L.) Holub	0	n	1	0	Polygonaceae Juss.
Iz_3_1009	DJI_0861	Festuca beckeri (Hack.) Trautv.	0	n	1	0	Poaceae Barnhart
Iz_3_1009	DJI_0861	Fragaria vesca L.	0	n	1	0	Rosaceae Juss.
Iz_3_1009	DJI_0861	Frangula alnus Mill.	0	n	1	1	Rhamnaceae Juss.
Iz_3_1009	DJI_0861	Fraxinus pennsylvanica Marshall	0	a	0	1	Oleaceae Hoffmanns. & Link
Iz_3_1009	DJI_0861	Galeopsis bifida Boenn.	0	n	1	0	Lamiacea Lindl.
Iz_3_1009	DJI_0861	Galium verum L.	0	n	1	0	Rubiaceae Juss.
Iz_3_1009	DJI_0861	Genista tinctoria L.	0	n	1	0	Fabaceae Lindl.
Iz_3_1009	DJI_0861	Geranium robertianum L.	0	n	1	0	Geraniaceae Juss.
Iz_3_1009	DJI_0861	Geranium sanquineum L.	0	n	1	0	Geraniaceae Juss.
Iz_3_1009	DJI_0861	Gypsophila paniculata L.	0	n	1	0	Caryophyllaceae Juss.
Iz_3_1009	DJI_0861	Helichrysum arenarium (L.) Moench	0	n	1	0	Asteraceae Bercht. & J.Presl
Iz_3_1009	DJI_0861	Herniaria polygama J.Gay	0	n	1	0	Caryophyllaceae Juss.
Iz_3_1009	DJI_0861	Hieracium robustum Fr.	0	n	1	0	Asteraceae Bercht. & J.Presl
Iz_3_1009	DJI_0861	Hieracium umbellatum L.	0	n	1	NA	Asteraceae Bercht. & J.Presl
Iz_3_1009	DJI_0861	Hylotelephium maximum (L.) Holub	0	n	1	0	Crassulaceae J.St.-Hil.
Iz_3_1009	DJI_0861	Jacobaea borysthenica (DC.) B.Nord. & Greuter	0	n	1	0	Asteraceae Bercht. & J.Presl
Iz_3_1009	DJI_0861	Jasione montana L.	0	n	1	0	Campanulaceae Juss.
Iz_3_1009	DJI_0861	Jurinea cyanoides (L.) Rchb.s.l.	0	n	1	0	Asteraceae Bercht. & J.Presl
Iz_3_1009	DJI_0861	Koeleria glauca (Spreng.) DC.	0	n	1	0	Poaceae Barnhart
Iz_3_1009	DJI_0861	Lactuca serriola L.	0	a	0	0	Asteraceae Bercht. & J.Presl
Iz_3_1009	DJI_0861	Linaria genistifolia (L.) Mill.	0	n	1	0	Plantaginaceae Juss.
Iz_3_1009	DJI_0861	Lomelosia argentea (L.) Greuter & Burdet	0	n	1	0	Caprifoliaceae Juss.
Iz_3_1009	DJI_0861	Melampyrum pratense L.	0	n	1	0	Orobanchaceae Vent.
Iz_3_1009	DJI_0861	Oenothera biennis L.	0	a	0	0	Onagraceae Juss.
Iz_3_1009	DJI_0861	Pentanema britannica (L.) D.Gut.Larr., Santos-Vicente, Anderb., E.Rico & M.M.Mart.Ort.	0	n	1	0	Asteraceae Bercht. & J.Presl
Iz_3_1009	DJI_0861	Peucedanum oreoselinum (L.) Moench	5	n	1	0	Apiaceae Lindl.
Iz_3_1009	DJI_0861	Peucedanum ruthenicum M.Bieb.	0	n	1	0	Apiaceae Lindl.
Iz_3_1009	DJI_0861	Pilosella echioides (Lumn.) F.W.Schultz & Sch.Bip.	0	n	1	0	Asteraceae Bercht. & J.Presl
Iz_3_1009	DJI_0861	Pilosella officinarum F. Schultz et Sch. Bip.	0	n	1	0	Asteraceae Bercht. & J.Presl
Iz_3_1009	DJI_0861	Pilosella sp.	0		NA	0	Asteraceae Bercht. & J.Presl
Iz_3_1009	DJI_0861	Pinus sylvestris L.	0	n	1	1	Pinaceae Spreng. ex F.Rudolphi
Iz_3_1009	DJI_0861	Poa annua L.	0	n	1	0	Poaceae Barnhart
Iz_3_1009	DJI_0861	Poa nemoralis L.	0	n	1	0	Poaceae Barnhart
Iz_3_1009	DJI_0861	Poa sp.	0		NA	0	Poaceae Barnhart
Iz_3_1009	DJI_0861	Polygonatum odoratum (Mill.) Druce	0	n	1	0	Asparagaceae Juss.
Iz_3_1009	DJI_0861	Polygonum arenarium Waldst. & Kit.	0	n	1	0	Polygonaceae Juss.
Iz_3_1009	DJI_0861	Populus nigra L.	0	n	1	1	Salicaceae Mirb.
Iz_3_1009	DJI_0861	Populus tremula L.	0	n	1	1	Salicaceae Mirb.
Iz_3_1009	DJI_0861	Populus Ã canescens (Aiton) Sm.	0	n	1	1	Salicaceae Mirb.
Iz_3_1009	DJI_0861	Potentilla argentea L.	0	n	1	0	Rosaceae Juss.
Iz_3_1009	DJI_0861	Psammophiliella muralis (L.) Ikonn.	0	n	1	0	Caryophyllaceae Juss.
Iz_3_1009	DJI_0861	Psephellus sumensis (Kalen.) Greuter	0	n	1	0	Asteraceae Bercht. & J.Presl
Iz_3_1009	DJI_0861	Pulsatilla pratensis (L.) Mill. s.l.	0	n	1	0	Ranunculaceae Juss.
Iz_3_1009	DJI_0861	Pyrus communis L.	0	a	0	1	Rosaceae Juss.
Iz_3_1009	DJI_0861	Quercus robur L.	0	n	1	1	Fagaceae Dumort.
Iz_3_1009	DJI_0861	Robinia pseudoacacia L.	0	a	0	1	Fabaceae Lindl.
Iz_3_1009	DJI_0861	Rosa sp.	0		NA	1	Rosaceae Juss.
Iz_3_1009	DJI_0861	Rubus idaeus L.	0	n	1	0	Rosaceae Juss.
Iz_3_1009	DJI_0861	Rumex acetosella L.	0	n	1	0	Polygonaceae Juss.
Iz_3_1009	DJI_0861	Rumex thyrsiflorus Fingerh.	0	n	1	0	Polygonaceae Juss.
Iz_3_1009	DJI_0861	Salix alba L.	0	n	1	1	Salicaceae Mirb.
Iz_3_1009	DJI_0861	Sambucus nigra L.	0	n	1	1	Viburnaceae Raf.
Iz_3_1009	DJI_0861	Secale sylvestre Host	0	n	1	0	Poaceae Barnhart
Iz_3_1009	DJI_0861	Sempervivum ruthenicum Schnittsp. & C.B. Lehm.	0	n	1	0	Crassulaceae J.St.-Hil.
Iz_3_1009	DJI_0861	Setaria viridis (L.) P. Beauv.	0	n	1	0	Poaceae Barnhart
Iz_3_1009	DJI_0861	Silene borysthenica (Gruner) Walters	0	n	1	0	Caryophyllaceae Juss.

Iz_3_1009	DJI_0861	Silene latifolia subsp. alba (Mill.) Greuter & Burdet	0	n	1	0	Caryophyllaceae Juss.
Iz_3_1009	DJI_0861	Solidago virgaurea L.	0	n	1	0	Asteraceae Bercht. & J.Presl
Iz_3_1009	DJI_0861	Sonchus oleraceus L.	0	a	0	0	Asteraceae Bercht. & J.Presl
Iz_3_1009	DJI_0861	Sorbus aucuparia L.	0	n	1	1	Rosaceae Juss.
Iz_3_1009	DJI_0861	Stachys recta L.	0	n	1	0	Lamiacea Lindl.
Iz_3_1009	DJI_0861	Tanacetum vulgare L.	0	n	1	0	Asteraceae Bercht. & J.Presl
Iz_3_1009	DJI_0861	Thymus pallasianus Heinr.Braun	0	n	1	0	Lamiacea Lindl.
Iz_3_1009	DJI_0861	Tilia cordata Mill.	0	n	1	1	Malvaceae Juss.
Iz_3_1009	DJI_0861	Trifolium alpestre L.	0	n	1	0	Fabaceae Lindl.
Iz_3_1009	DJI_0861	Trifolium arvense L.	0	n	1	0	Fabaceae Lindl.
Iz_3_1009	DJI_0861	Ulmus glabra Huds.	0	n	1	1	Ulmaceae Mirb.
Iz_3_1009	DJI_0861	Verbascum densiflorum Bertol.	0	n	1	0	Scrophulariaceae Juss.
Iz_3_1009	DJI_0861	Veronica austriaca L.	0	n	1	0	Plantaginaceae Juss.
Iz_3_1009	DJI_0861	Veronica incana L.	0	n	1	0	Plantaginaceae Juss.
Iz_3_1009	DJI_0861	Veronica verna L.	0	n	1	0	Plantaginaceae Juss.
Iz_3_1009	DJI_0861	Vincetoxicum hirundinaria Medik	0	n	1	0	Apocynaceae Juss.
Iz_3_1009	DJI_0862	Acer tataricum L.	0	n	1	1	Sapindaceae Juss.
Iz_3_1009	DJI_0862	Achillea millefolium L. s.l.	0	n	1	0	Asteraceae Bercht. & J.Presl
Iz_3_1009	DJI_0862	Allium oleraceum L.	0	n	1	0	Amaryllidaceae J.St.-Hil.
Iz_3_1009	DJI_0862	Ambrosia artemisiifolia L.	0	a	0	0	Asteraceae Bercht. & J.Presl
Iz_3_1009	DJI_0862	Amorpha fruticosa L.	0	a	0	0	Fabaceae Lindl.
Iz_3_1009	DJI_0862	Anthericum ramosum L.	0	n	1	0	Asparagaceae Juss.
Iz_3_1009	DJI_0862	Artemisia marschalliana Spreng.	0	n	1	0	Asteraceae Bercht. & J.Presl
Iz_3_1009	DJI_0862	Asparagus officinalis L.	0	n	1	0	Asparagaceae Juss.
Iz_3_1009	DJI_0862	Astragalus cicer L.	0	n	1	0	Fabaceae Lindl.
Iz_3_1009	DJI_0862	Bassia laniflora (S.G.Gmel.) A.J.Scott	0	n	1	0	Amaranthaceae Juss.
Iz_3_1009	DJI_0862	Berberis vulgaris L.	0	n	1	0	Berberidaceae Juss.
Iz_3_1009	DJI_0862	Betula pendula Roth.	0	n	1	1	Betulaceae Gray
Iz_3_1009	DJI_0862	Betula pendula Roth.	0	n	1	0	Poaceae Barnhart
Iz_3_1009	DJI_0862	Bromus inermis Leyss.	0		NA	NA	
Iz_3_1009	DJI_0862	Bromus squarrosus L.	0	a	0	0	Poaceae Barnhart
Iz_3_1009	DJI_0862	Bromus tectorum L.	0	a	0	0	Poaceae Barnhart
Iz_3_1009	DJI_0862	Calamagrostis epigejos (L.) Roth	10	n	1	0	Poaceae Barnhart
Iz_3_1009	DJI_0862	Carduus acanthoides L.	0	n	1	0	Asteraceae Bercht. & J.Presl
Iz_3_1009	DJI_0862	Carex sp.	0	n	1	0	Cyperaceae Juss.
Iz_3_1009	DJI_0862	Centaurea majorovii Dumbadze	0	n	1	0	Asteraceae Bercht. & J.Presl
Iz_3_1009	DJI_0862	Chamaecytisus ruthenicus (Fisch.ex Wol.) Klaskova	0	n	1	0	Fabaceae Lindl.
Iz_3_1009	DJI_0862	Chelidonium majus L.	1	n	1	0	Papaveraceae Juss.
Iz_3_1009	DJI_0862	Chenopodium betaceum Andrz.	0	n	1	0	Amaranthaceae Juss.
Iz_3_1009	DJI_0862	Chenopodium cf. album	0	n	1	0	Amaranthaceae Juss.
Iz_3_1009	DJI_0862	Chenopodium sp.	0		NA	0	Amaranthaceae Juss.
Iz_3_1009	DJI_0862	Chondrilla juncea L.	0	n	1	0	Asteraceae Bercht. & J.Presl
Iz_3_1009	DJI_0862	Cirsium vulgare (Savi) Ten.	0	n	1	0	Asteraceae Bercht. & J.Presl
Iz_3_1009	DJI_0862	Clinopodium acinos (L.) Kuntze	0	n	1	0	Lamiacea Lindl.
Iz_3_1009	DJI_0862	Convallaria majalis L.	0	n	1	0	Asparagaceae Juss.
Iz_3_1009	DJI_0862	Crataegus rhipidophylla Gand.	0	n	1	1	Rosaceae Juss.
Iz_3_1009	DJI_0862	Crepis foetida L.	0	n	1	0	Asteraceae Bercht. & J.Presl
Iz_3_1009	DJI_0862	Dactylis glomerata L.	0	n	1	0	Poaceae Barnhart
Iz_3_1009	DJI_0862	Dianthus campestris M.Bieb.	0	n	1	0	Caryophyllaceae Juss.
Iz_3_1009	DJI_0862	Digitaria sanguinalis (L.) Scop.	0	a	0	0	Poaceae Barnhart
Iz_3_1009	DJI_0862	Elymus repens (L.) Gould	0	n	1	0	Poaceae Barnhart
Iz_3_1009	DJI_0862	Epilobium tetragonum L.	0	n	1	0	Onagraceae Juss.
Iz_3_1009	DJI_0862	Eragrostis minor Host	0	a	0	0	Poaceae Barnhart
Iz_3_1009	DJI_0862	Erigeron canadensis L.	0	a	0	0	Asteraceae Bercht. & J.Presl
Iz_3_1009	DJI_0862	Euphorbia seguieriana Neck.	0	n	1	0	Euphorbiaceae Juss.
Iz_3_1009	DJI_0862	Fallopia dumetorum (L.) Holub	0	n	1	0	Polygonaceae Juss.
Iz_3_1009	DJI_0862	Festuca beckeri (Hack.) Trautv.	0	n	1	0	Poaceae Barnhart
Iz_3_1009	DJI_0862	Fragaria vesca L.	0	n	1	0	Rosaceae Juss.
Iz_3_1009	DJI_0862	Frangula alnus Mill.	0	n	1	1	Rhamnaceae Juss.
Iz_3_1009	DJI_0862	Fraxinus pennsylvanica Marshall	0	a	0	1	Oleaceae Hoffmanns. & Link
Iz_3_1009	DJI_0862	Galeopsis bifida Boenn.	0	n	1	0	Lamiacea Lindl.
Iz_3_1009	DJI_0862	Galium verum L.	0	n	1	0	Rubiaceae Juss.
Iz_3_1009	DJI_0862	Genista tinctoria L.	0	n	1	0	Fabaceae Lindl.
Iz_3_1009	DJI_0862	Geranium robertianum L.	0	n	1	0	Geraniaceae Juss.
Iz_3_1009	DJI_0862	Geranium sanquineum L.	0	n	1	0	Geraniaceae Juss.
Iz_3_1009	DJI_0862	Gypsophila paniculata L.	0	n	1	0	Caryophyllaceae Juss.
Iz_3_1009	DJI_0862	Helichrysum arenarium (L.) Moench	0	n	1	0	Asteraceae Bercht. & J.Presl
Iz_3_1009	DJI_0862	Herniaria polygama J.Gay	0	n	1	0	Caryophyllaceae Juss.
Iz_3_1009	DJI_0862	Hieracium robustum Fr.	0	n	1	0	Asteraceae Bercht. & J.Presl
Iz_3_1009	DJI_0862	Hieracium umbellatum L.	0	n	1	NA	Asteraceae Bercht. & J.Presl
Iz_3_1009	DJI_0862	Hylotelephium maximum (L.) Holub	0	n	1	0	Crassulaceae J.St.-Hil.
Iz_3_1009	DJI_0862	Jacobaea borysthenica (DC.) B.Nord. & Greuter	0	n	1	0	Asteraceae Bercht. & J.Presl
Iz_3_1009	DJI_0862	Jasione montana L.	0	n	1	0	Campanulaceae Juss.
Iz_3_1009	DJI_0862	Jurinea cyanoides (L.) Rchb.s.l.	0	n	1	0	Asteraceae Bercht. & J.Presl
Iz_3_1009	DJI_0862	Koeleria glauca (Spreng.) DC.	0	n	1	0	Poaceae Barnhart
Iz_3_1009	DJI_0862	Lactuca serriola L.	0	a	0	0	Asteraceae Bercht. & J.Presl
Iz_3_1009	DJI_0862	Linaria genistifolia (L.) Mill.	0	n	1	0	Plantaginaceae Juss.
Iz_3_1009	DJI_0862	Lomelosia argentea (L.) Greuter & Burdet	0	n	1	0	Caprifoliaceae Juss.
Iz_3_1009	DJI_0862	Melampyrum pratense L.	0	n	1	0	Orobanchaceae Vent.
Iz_3_1009	DJI_0862	Oenothera biennis L.	0	a	0	0	Onagraceae Juss.
Iz_3_1009	DJI_0862	Pentanema britannica (L.) D.Gut.Larr., Santos-Vicente, Anderb., E.Rico & M.M.Mart.Ort.	0	n	1	0	Asteraceae Bercht. & J.Presl
Iz_3_1009	DJI_0862	Peucedanum oreoselinum (L.) Moench	5	n	1	0	Apiaceae Lindl.
Iz_3_1009	DJI_0862	Peucedanum ruthenicum M.Bieb.	0	n	1	0	Apiaceae Lindl.
Iz_3_1009	DJI_0862	Pilosella echioides (Lumn.) F.W.Schultz & Sch.Bip.	0	n	1	0	Asteraceae Bercht. & J.Presl
Iz_3_1009	DJI_0862	Pilosella officinarum F. Schultz et Sch. Bip.	0	n	1	0	Asteraceae Bercht. & J.Presl
Iz_3_1009	DJI_0862	Pilosella sp.	0		NA	0	Asteraceae Bercht. & J.Presl
Iz_3_1009	DJI_0862	Pinus sylvestris L.	0	n	1	1	Pinaceae Spreng. ex F.Rudolphi
Iz_3_1009	DJI_0862	Poa annua L.	0	n	1	0	Poaceae Barnhart
Iz_3_1009	DJI_0862	Poa nemoralis L.	0	n	1	0	Poaceae Barnhart
Iz_3_1009	DJI_0862	Poa sp.	0		NA	0	Poaceae Barnhart
Iz_3_1009	DJI_0862	Polygonatum odoratum (Mill.) Druce	0	n	1	0	Asparagaceae Juss.
Iz_3_1009	DJI_0862	Polygonum arenarium Waldst. & Kit.	0	n	1	0	Polygonaceae Juss.
Iz_3_1009	DJI_0862	Populus nigra L.	0	n	1	1	Salicaceae Mirb.
Iz_3_1009	DJI_0862	Populus tremula L.	0	n	1	1	Salicaceae Mirb.
Iz_3_1009	DJI_0862	Populus Ã canescens (Aiton) Sm.	0	n	1	1	Salicaceae Mirb.
Iz_3_1009	DJI_0862	Potentilla argentea L.	0	n	1	0	Rosaceae Juss.
Iz_3_1009	DJI_0862	Psammophiliella muralis (L.) Ikonn.	0	n	1	0	Caryophyllaceae Juss.
Iz_3_1009	DJI_0862	Psephellus sumensis (Kalen.) Greuter	0	n	1	0	Asteraceae Bercht. & J.Presl

Iz_3_1009	DJI_0862	Pulsatilla pratensis (L.) Mill. s.l.	0	n	1	0	Ranunculaceae Juss.
Iz_3_1009	DJI_0862	Pyrus communis L.	0	a	0	1	Rosaceae Juss.
Iz_3_1009	DJI_0862	Quercus robur L.	0	n	1	1	Fagaceae Dumort.
Iz_3_1009	DJI_0862	Robinia pseudoacacia L.	0	a	0	1	Fabaceae Lindl.
Iz_3_1009	DJI_0862	Rosa sp.	0		NA	1	Rosaceae Juss.
Iz_3_1009	DJI_0862	Rubus idaeus L.	0	n	1	0	Rosaceae Juss.
Iz_3_1009	DJI_0862	Rumex acetosella L.	0	n	1	0	Polygonaceae Juss.
Iz_3_1009	DJI_0862	Rumex thyrsiflorus Fingerh.	0	n	1	0	Polygonaceae Juss.
Iz_3_1009	DJI_0862	Salix alba L.	0	n	1	1	Salicaceae Mirb.
Iz_3_1009	DJI_0862	Sambucus nigra L.	0	n	1	1	Viburnaceae Raf.
Iz_3_1009	DJI_0862	Secale sylvestre Host	0	n	1	0	Poaceae Barnhart
Iz_3_1009	DJI_0862	Sempervivum ruthenicum Schnittsp. & C.B. Lehm.	0	n	1	0	Crassulaceae J.St.-Hil.
Iz_3_1009	DJI_0862	Setaria viridis (L.) P. Beauv.	0	n	1	0	Poaceae Barnhart
Iz_3_1009	DJI_0862	Silene borysthenica (Gruner) Walters	0	n	1	0	Caryophyllaceae Juss.
Iz_3_1009	DJI_0862	Silene latifolia subsp. alba (Mill.) Greuter & Burdet	0	n	1	0	Caryophyllaceae Juss.
Iz_3_1009	DJI_0862	Solidago virgaurea L.	0	n	1	0	Asteraceae Bercht. & J.Presl
Iz_3_1009	DJI_0862	Sonchus oleraceus L.	0	a	0	0	Asteraceae Bercht. & J.Presl
Iz_3_1009	DJI_0862	Sorbus aucuparia L.	0	n	1	1	Rosaceae Juss.
Iz_3_1009	DJI_0862	Stachys recta L.	0	n	1	0	Lamiacea Lindl.
Iz_3_1009	DJI_0862	Tanacetum vulgare L.	0	n	1	0	Asteraceae Bercht. & J.Presl
Iz_3_1009	DJI_0862	Thymus pallasianus Heinr.Braun	0	n	1	0	Lamiacea Lindl.
Iz_3_1009	DJI_0862	Tilia cordata Mill.	0	n	1	1	Malvaceae Juss.
Iz_3_1009	DJI_0862	Trifolium alpestre L.	0	n	1	0	Fabaceae Lindl.
Iz_3_1009	DJI_0862	Trifolium arvense L.	0	n	1	0	Fabaceae Lindl.
Iz_3_1009	DJI_0862	Ulmus glabra Huds.	0	n	1	1	Ulmaceae Mirb.
Iz_3_1009	DJI_0862	Verbascum densiflorum Bertol.	0	n	1	0	Scrophulariaceae Juss.
Iz_3_1009	DJI_0862	Veronica austriaca L.	0	n	1	0	Plantaginaceae Juss.
Iz_3_1009	DJI_0862	Veronica incana L.	0	n	1	0	Plantaginaceae Juss.
Iz_3_1009	DJI_0862	Veronica verna L.	0	n	1	0	Plantaginaceae Juss.
Iz_3_1009	DJI_0862	Vincetoxicum hirundinaria Medik	0	n	1	0	Apocynaceae Juss.
Iz_3_1009	DJI_0863	Acer tataricum L.	0	n	1	1	Sapindaceae Juss.
Iz_3_1009	DJI_0863	Achillea millefolium L. s.l.	0	n	1	0	Asteraceae Bercht. & J.Presl
Iz_3_1009	DJI_0863	Allium oleraceum L.	0	n	1	0	Amaryllidaceae J.St.-Hil.
Iz_3_1009	DJI_0863	Ambrosia artemisiifolia L.	0	a	0	0	Asteraceae Bercht. & J.Presl
Iz_3_1009	DJI_0863	Amorpha fruticosa L.	0	a	0	0	Fabaceae Lindl.
Iz_3_1009	DJI_0863	Anthericum ramosum L.	0	n	1	0	Asparagaceae Juss.
Iz_3_1009	DJI_0863	Artemisia marschalliana Spreng.	0	n	1	0	Asteraceae Bercht. & J.Presl
Iz_3_1009	DJI_0863	Asparagus officinalis L.	0	n	1	0	Asparagaceae Juss.
Iz_3_1009	DJI_0863	Astragalus cicer L.	0	n	1	0	Fabaceae Lindl.
Iz_3_1009	DJI_0863	Bassia laniflora (S.G.Gmel.) A.J.Scott	0	n	1	0	Amaranthaceae Juss.
Iz_3_1009	DJI_0863	Berberis vulgaris L.	0	n	1	0	Berberidaceae Juss.
Iz_3_1009	DJI_0863	Betula pendula Roth.	0	n	1	1	Betulaceae Gray
Iz_3_1009	DJI_0863	Betula pendula Roth.	0	n	1	0	Poaceae Barnhart
Iz_3_1009	DJI_0863	Bromus inermis Leyss.	0		NA	NA	
Iz_3_1009	DJI_0863	Bromus squarrosus L.	0	a	0	0	Poaceae Barnhart
Iz_3_1009	DJI_0863	Bromus tectorum L.	0	a	0	0	Poaceae Barnhart
Iz_3_1009	DJI_0863	Calamagrostis epigejos (L.) Roth	10	n	1	0	Poaceae Barnhart
Iz_3_1009	DJI_0863	Carduus acanthoides L.	0	n	1	0	Asteraceae Bercht. & J.Presl
Iz_3_1009	DJI_0863	Carex sp.	0	n	1	0	Cyperaceae Juss.
Iz_3_1009	DJI_0863	Centaurea majorovii Dumbadze	0	n	1	0	Asteraceae Bercht. & J.Presl
Iz_3_1009	DJI_0863	Chamaecytisus ruthenicus (Fisch.ex Wol.) Klaskova	8	n	1	0	Fabaceae Lindl.
Iz_3_1009	DJI_0863	Chelidonium majus L.	0	n	1	0	Papaveraceae Juss.
Iz_3_1009	DJI_0863	Chenopodium betaceum Andrz.	0	n	1	0	Amaranthaceae Juss.
Iz_3_1009	DJI_0863	Chenopodium cf. album	0	n	1	0	Amaranthaceae Juss.
Iz_3_1009	DJI_0863	Chenopodium sp.	0		NA	0	Amaranthaceae Juss.
Iz_3_1009	DJI_0863	Chondrilla juncea L.	0	n	1	0	Asteraceae Bercht. & J.Presl
Iz_3_1009	DJI_0863	Cirsium vulgare (Savi) Ten.	0	n	1	0	Asteraceae Bercht. & J.Presl
Iz_3_1009	DJI_0863	Clinopodium acinos (L.) Kuntze	0	n	1	0	Lamiacea Lindl.
Iz_3_1009	DJI_0863	Convallaria majalis L.	0	n	1	0	Asparagaceae Juss.
Iz_3_1009	DJI_0863	Crataegus rhipidophylla Gand.	0	n	1	1	Rosaceae Juss.
Iz_3_1009	DJI_0863	Crepis foetida L.	0	n	1	0	Asteraceae Bercht. & J.Presl
Iz_3_1009	DJI_0863	Dactylis glomerata L.	0	n	1	0	Poaceae Barnhart
Iz_3_1009	DJI_0863	Dianthus campestris M.Bieb.	0	n	1	0	Caryophyllaceae Juss.
Iz_3_1009	DJI_0863	Digitaria sanguinalis (L.) Scop.	0	a	0	0	Poaceae Barnhart
Iz_3_1009	DJI_0863	Elymus repens (L.) Gould	0	n	1	0	Poaceae Barnhart
Iz_3_1009	DJI_0863	Epilobium tetragonum L.	0	n	1	0	Onagraceae Juss.
Iz_3_1009	DJI_0863	Eragrostis minor Host	0	a	0	0	Poaceae Barnhart
Iz_3_1009	DJI_0863	Erigeron canadensis L.	0	a	0	0	Asteraceae Bercht. & J.Presl
Iz_3_1009	DJI_0863	Euphorbia seguieriana Neck.	0	n	1	0	Euphorbiaceae Juss.
Iz_3_1009	DJI_0863	Fallopia dumetorum (L.) Holub	0	n	1	0	Polygonaceae Juss.
Iz_3_1009	DJI_0863	Festuca beckeri (Hack.) Trautv.	0	n	1	0	Poaceae Barnhart
Iz_3_1009	DJI_0863	Fragaria vesca L.	0	n	1	0	Rosaceae Juss.
Iz_3_1009	DJI_0863	Frangula alnus Mill.	0	n	1	1	Rhamnaceae Juss.
Iz_3_1009	DJI_0863	Fraxinus pennsylvanica Marshall	0	a	0	1	Oleaceae Hoffmanns. & Link
Iz_3_1009	DJI_0863	Galeopsis bifida Boenn.	0	n	1	0	Lamiacea Lindl.
Iz_3_1009	DJI_0863	Galium verum L.	0	n	1	0	Rubiaceae Juss.
Iz_3_1009	DJI_0863	Genista tinctoria L.	0	n	1	0	Fabaceae Lindl.
Iz_3_1009	DJI_0863	Geranium robertianum L.	0	n	1	0	Geraniaceae Juss.
Iz_3_1009	DJI_0863	Geranium sanquineum L.	0	n	1	0	Geraniaceae Juss.
Iz_3_1009	DJI_0863	Gypsophila paniculata L.	0	n	1	0	Caryophyllaceae Juss.
Iz_3_1009	DJI_0863	Helichrysum arenarium (L.) Moench	0	n	1	0	Asteraceae Bercht. & J.Presl
Iz_3_1009	DJI_0863	Herniaria polygama J.Gay	0	n	1	0	Caryophyllaceae Juss.
Iz_3_1009	DJI_0863	Hieracium robustum Fr.	0	n	1	0	Asteraceae Bercht. & J.Presl
Iz_3_1009	DJI_0863	Hieracium umbellatum L.	0	n	1	NA	Asteraceae Bercht. & J.Presl
Iz_3_1009	DJI_0863	Hylotelephium maximum (L.) Holub	0	n	1	0	Crassulaceae J.St.-Hil.
Iz_3_1009	DJI_0863	Jacobaea borysthenica (DC.) B.Nord. & Greuter	0	n	1	0	Asteraceae Bercht. & J.Presl
Iz_3_1009	DJI_0863	Jasione montana L.	0	n	1	0	Campanulaceae Juss.
Iz_3_1009	DJI_0863	Jurinea cyanoides (L.) Rchb.s.l.	0	n	1	0	Asteraceae Bercht. & J.Presl
Iz_3_1009	DJI_0863	Koeleria glauca (Spreng.) DC.	0	n	1	0	Poaceae Barnhart
Iz_3_1009	DJI_0863	Lactuca serriola L.	0	a	0	0	Asteraceae Bercht. & J.Presl
Iz_3_1009	DJI_0863	Linaria genistifolia (L.) Mill.	0	n	1	0	Plantaginaceae Juss.
Iz_3_1009	DJI_0863	Lomelosia argentea (L.) Greuter & Burdet	0	n	1	0	Caprifoliaceae Juss.
Iz_3_1009	DJI_0863	Melampyrum pratense L.	0	n	1	0	Orobanchaceae Vent.
Iz_3_1009	DJI_0863	Oenothera biennis L.	0	a	0	0	Onagraceae Juss.
Iz_3_1009	DJI_0863	Pentanema britannica (L.) D.Gut.Larr., Santos-Vicente, Anderb., E.Rico & M.M.Mart.Ort.	0	n	1	0	Asteraceae Bercht. & J.Presl
Iz_3_1009	DJI_0863	Peucedanum oreoselinum (L.) Moench	3	n	1	0	Apiaceae Lindl.
Iz_3_1009	DJI_0863	Peucedanum ruthenicum M.Bieb.	0	n	1	0	Apiaceae Lindl.
Iz_3_1009	DJI_0863	Pilosella echioides (Lumn.) F.W.Schultz & Sch.Bip.	0	n	1	0	Asteraceae Bercht. & J.Presl

Iz_3_1009	DJI_0863	Pilosella officinarum F. Schultz et Sch. Bip.	0	n	1	0	Asteraceae Bercht. & J.Presl
Iz_3_1009	DJI_0863	Pilosella sp.	0		NA	0	Asteraceae Bercht. & J.Presl
Iz_3_1009	DJI_0863	Pinus sylvestris L.	0	n	1	1	Pinaceae Spreng. ex F.Rudolphi
Iz_3_1009	DJI_0863	Poa annua L.	0	n	1	0	Poaceae Barnhart
Iz_3_1009	DJI_0863	Poa nemoralis L.	0	n	1	0	Poaceae Barnhart
Iz_3_1009	DJI_0863	Poa sp.	0		NA	0	Poaceae Barnhart
Iz_3_1009	DJI_0863	Polygonatum odoratum (Mill.) Druce	1	n	1	0	Asparagaceae Juss.
Iz_3_1009	DJI_0863	Polygonum arenarium Waldst. & Kit.	0	n	1	0	Polygonaceae Juss.
Iz_3_1009	DJI_0863	Populus nigra L.	0	n	1	1	Salicaceae Mirb.
Iz_3_1009	DJI_0863	Populus tremula L.	0	n	1	1	Salicaceae Mirb.
Iz_3_1009	DJI_0863	Populus Ã canescens (Aiton) Sm.	0	n	1	1	Salicaceae Mirb.
Iz_3_1009	DJI_0863	Potentilla argentea L.	0	n	1	0	Rosaceae Juss.
Iz_3_1009	DJI_0863	Psammophiliella muralis (L.) Ikonn.	0	n	1	0	Caryophyllaceae Juss.
Iz_3_1009	DJI_0863	Psephellus sumensis (Kalen.) Greuter	0	n	1	0	Asteraceae Bercht. & J.Presl
Iz_3_1009	DJI_0863	Pulsatilla pratensis (L.) Mill. s.l.	0	n	1	0	Ranunculaceae Juss.
Iz_3_1009	DJI_0863	Pyrus communis L.	0	a	0	1	Rosaceae Juss.
Iz_3_1009	DJI_0863	Quercus robur L.	0	n	1	1	Fagaceae Dumort.
Iz_3_1009	DJI_0863	Robinia pseudoacacia L.	0	a	0	1	Fabaceae Lindl.
Iz_3_1009	DJI_0863	Rosa sp.	0		NA	1	Rosaceae Juss.
Iz_3_1009	DJI_0863	Rubus idaeus L.	0	n	1	0	Rosaceae Juss.
Iz_3_1009	DJI_0863	Rumex acetosella L.	0	n	1	0	Polygonaceae Juss.
Iz_3_1009	DJI_0863	Rumex thyrsiflorus Fingerh.	0	n	1	0	Polygonaceae Juss.
Iz_3_1009	DJI_0863	Salix alba L.	0	n	1	1	Salicaceae Mirb.
Iz_3_1009	DJI_0863	Sambucus nigra L.	0	n	1	1	Viburnaceae Raf.
Iz_3_1009	DJI_0863	Secale sylvestre Host	0	n	1	0	Poaceae Barnhart
Iz_3_1009	DJI_0863	Sempervivum ruthenicum Schnittsp. & C.B. Lehm.	0	n	1	0	Crassulaceae J.St.-Hil.
Iz_3_1009	DJI_0863	Setaria viridis (L.) P. Beauv.	0	n	1	0	Poaceae Barnhart
Iz_3_1009	DJI_0863	Silene borysthenica (Gruner) Walters	0	n	1	0	Caryophyllaceae Juss.
Iz_3_1009	DJI_0863	Silene latifolia subsp. alba (Mill.) Greuter & Burdet	0	n	1	0	Caryophyllaceae Juss.
Iz_3_1009	DJI_0863	Solidago virgaurea L.	0	n	1	0	Asteraceae Bercht. & J.Presl
Iz_3_1009	DJI_0863	Sonchus oleraceus L.	0	a	0	0	Asteraceae Bercht. & J.Presl
Iz_3_1009	DJI_0863	Sorbus aucuparia L.	0	n	1	1	Rosaceae Juss.
Iz_3_1009	DJI_0863	Stachys recta L.	0	n	1	0	Lamiacea Lindl.
Iz_3_1009	DJI_0863	Tanacetum vulgare L.	0	n	1	0	Asteraceae Bercht. & J.Presl
Iz_3_1009	DJI_0863	Thymus pallasianus Heinr.Braun	0	n	1	0	Lamiacea Lindl.
Iz_3_1009	DJI_0863	Tilia cordata Mill.	0	n	1	1	Malvaceae Juss.
Iz_3_1009	DJI_0863	Trifolium alpestre L.	0	n	1	0	Fabaceae Lindl.
Iz_3_1009	DJI_0863	Trifolium arvense L.	0	n	1	0	Fabaceae Lindl.
Iz_3_1009	DJI_0863	Ulmus glabra Huds.	0	n	1	1	Ulmaceae Mirb.
Iz_3_1009	DJI_0863	Verbascum densiflorum Bertol.	0	n	1	0	Scrophulariaceae Juss.
Iz_3_1009	DJI_0863	Veronica austriaca L.	0	n	1	0	Plantaginaceae Juss.
Iz_3_1009	DJI_0863	Veronica incana L.	0	n	1	0	Plantaginaceae Juss.
Iz_3_1009	DJI_0863	Veronica verna L.	0	n	1	0	Plantaginaceae Juss.
Iz_3_1009	DJI_0863	Vincetoxicum hirundinaria Medik	0	n	1	0	Apocynaceae Juss.
Iz_3_1009	DJI_0864	Acer tataricum L.	0	n	1	1	Sapindaceae Juss.
Iz_3_1009	DJI_0864	Achillea millefolium L. s.l.	0	n	1	0	Asteraceae Bercht. & J.Presl
Iz_3_1009	DJI_0864	Allium oleraceum L.	0	n	1	0	Amaryllidaceae J.St.-Hil.
Iz_3_1009	DJI_0864	Ambrosia artemisiifolia L.	0	a	0	0	Asteraceae Bercht. & J.Presl
Iz_3_1009	DJI_0864	Amorpha fruticosa L.	0	a	0	0	Fabaceae Lindl.
Iz_3_1009	DJI_0864	Anthericum ramosum L.	0	n	1	0	Asparagaceae Juss.
Iz_3_1009	DJI_0864	Artemisia marschalliana Spreng.	0	n	1	0	Asteraceae Bercht. & J.Presl
Iz_3_1009	DJI_0864	Asparagus officinalis L.	0	n	1	0	Asparagaceae Juss.
Iz_3_1009	DJI_0864	Astragalus cicer L.	0	n	1	0	Fabaceae Lindl.
Iz_3_1009	DJI_0864	Bassia laniflora (S.G.Gmel.) A.J.Scott	0	n	1	0	Amaranthaceae Juss.
Iz_3_1009	DJI_0864	Berberis vulgaris L.	0	n	1	0	Berberidaceae Juss.
Iz_3_1009	DJI_0864	Betula pendula Roth.	0	n	1	1	Betulaceae Gray
Iz_3_1009	DJI_0864	Betula pendula Roth.	0	n	1	0	Poaceae Barnhart
Iz_3_1009	DJI_0864	Bromus inermis Leyss.	0		NA	NA	
Iz_3_1009	DJI_0864	Bromus squarrosus L.	0	a	0	0	Poaceae Barnhart
Iz_3_1009	DJI_0864	Bromus tectorum L.	0	a	0	0	Poaceae Barnhart
Iz_3_1009	DJI_0864	Calamagrostis epigejos (L.) Roth	10	n	1	0	Poaceae Barnhart
Iz_3_1009	DJI_0864	Carduus acanthoides L.	0	n	1	0	Asteraceae Bercht. & J.Presl
Iz_3_1009	DJI_0864	Carex sp.	0	n	1	0	Cyperaceae Juss.
Iz_3_1009	DJI_0864	Centaurea majorovii Dumbadze	0	n	1	0	Asteraceae Bercht. & J.Presl
Iz_3_1009	DJI_0864	Chamaecytisus ruthenicus (Fisch.ex Wol.) Klaskova	3	n	1	0	Fabaceae Lindl.
Iz_3_1009	DJI_0864	Chelidonium majus L.	0.2	n	1	0	Papaveraceae Juss.
Iz_3_1009	DJI_0864	Chenopodium betaceum Andrz.	0	n	1	0	Amaranthaceae Juss.
Iz_3_1009	DJI_0864	Chenopodium cf. album	0	n	1	0	Amaranthaceae Juss.
Iz_3_1009	DJI_0864	Chenopodium sp.	0		NA	0	Amaranthaceae Juss.
Iz_3_1009	DJI_0864	Chondrilla juncea L.	0	n	1	0	Asteraceae Bercht. & J.Presl
Iz_3_1009	DJI_0864	Cirsium vulgare (Savi) Ten.	0	n	1	0	Asteraceae Bercht. & J.Presl
Iz_3_1009	DJI_0864	Clinopodium acinos (L.) Kuntze	0	n	1	0	Lamiacea Lindl.
Iz_3_1009	DJI_0864	Convallaria majalis L.	0	n	1	0	Asparagaceae Juss.
Iz_3_1009	DJI_0864	Crataegus rhipidophylla Gand.	0	n	1	1	Rosaceae Juss.
Iz_3_1009	DJI_0864	Crepis foetida L.	0	n	1	0	Asteraceae Bercht. & J.Presl
Iz_3_1009	DJI_0864	Dactylis glomerata L.	0	n	1	0	Poaceae Barnhart
Iz_3_1009	DJI_0864	Dianthus campestris M.Bieb.	0	n	1	0	Caryophyllaceae Juss.
Iz_3_1009	DJI_0864	Digitaria sanguinalis (L.) Scop.	0	a	0	0	Poaceae Barnhart
Iz_3_1009	DJI_0864	Elymus repens (L.) Gould	0	n	1	0	Poaceae Barnhart
Iz_3_1009	DJI_0864	Epilobium tetragonum L.	0	n	1	0	Onagraceae Juss.
Iz_3_1009	DJI_0864	Eragrostis minor Host	0	a	0	0	Poaceae Barnhart
Iz_3_1009	DJI_0864	Erigeron canadensis L.	0	a	0	0	Asteraceae Bercht. & J.Presl
Iz_3_1009	DJI_0864	Euphorbia seguieriana Neck.	0	n	1	0	Euphorbiaceae Juss.
Iz_3_1009	DJI_0864	Fallopia dumetorum (L.) Holub	0	n	1	0	Polygonaceae Juss.
Iz_3_1009	DJI_0864	Festuca beckeri (Hack.) Trautv.	0	n	1	0	Poaceae Barnhart
Iz_3_1009	DJI_0864	Fragaria vesca L.	0	n	1	0	Rosaceae Juss.
Iz_3_1009	DJI_0864	Frangula alnus Mill.	0	n	1	1	Rhamnaceae Juss.
Iz_3_1009	DJI_0864	Fraxinus pennsylvanica Marshall	0	a	0	1	Oleaceae Hoffmanns. & Link
Iz_3_1009	DJI_0864	Galeopsis bifida Boenn.	0	n	1	0	Lamiacea Lindl.
Iz_3_1009	DJI_0864	Galium verum L.	0	n	1	0	Rubiaceae Juss.
Iz_3_1009	DJI_0864	Genista tinctoria L.	0	n	1	0	Fabaceae Lindl.
Iz_3_1009	DJI_0864	Geranium robertianum L.	0	n	1	0	Geraniaceae Juss.
Iz_3_1009	DJI_0864	Geranium sanquineum L.	0	n	1	0	Geraniaceae Juss.
Iz_3_1009	DJI_0864	Gypsophila paniculata L.	0	n	1	0	Caryophyllaceae Juss.
Iz_3_1009	DJI_0864	Helichrysum arenarium (L.) Moench	0	n	1	0	Asteraceae Bercht. & J.Presl
Iz_3_1009	DJI_0864	Herniaria polygama J.Gay	0	n	1	0	Caryophyllaceae Juss.
Iz_3_1009	DJI_0864	Hieracium robustum Fr.	0	n	1	0	Asteraceae Bercht. & J.Presl
Iz_3_1009	DJI_0864	Hieracium umbellatum L.	0	n	1	NA	Asteraceae Bercht. & J.Presl

Iz_3_1009	DJI_0864	Hylotelephium maximum (L.) Holub	0	n	1	0	Crassulaceae J.St.-Hil.
Iz_3_1009	DJI_0864	Jacobaea borysthenica (DC.) B.Nord. & Greuter	0	n	1	0	Asteraceae Bercht. & J.Presl
Iz_3_1009	DJI_0864	Jasione montana L.	0	n	1	0	Campanulaceae Juss.
Iz_3_1009	DJI_0864	Jurinea cyanoides (L.) Rchb.s.l.	0	n	1	0	Asteraceae Bercht. & J.Presl
Iz_3_1009	DJI_0864	Koeleria glauca (Spreng.) DC.	0	n	1	0	Poaceae Barnhart
Iz_3_1009	DJI_0864	Lactuca serriola L.	0	a	0	0	Asteraceae Bercht. & J.Presl
Iz_3_1009	DJI_0864	Linaria genistifolia (L.) Mill.	0	n	1	0	Plantaginaceae Juss.
Iz_3_1009	DJI_0864	Lomelosia argentea (L.) Greuter & Burdet	0	n	1	0	Caprifoliaceae Juss.
Iz_3_1009	DJI_0864	Melampyrum pratense L.	0	n	1	0	Orobanchaceae Vent.
Iz_3_1009	DJI_0864	Oenothera biennis L.	0	a	0	0	Onagraceae Juss.
Iz_3_1009	DJI_0864	Pentanema britannica (L.) D.Gut.Larr., Santos-Vicente, Anderb., E.Rico & M.M.Mart.Ort.	0	n	1	0	Asteraceae Bercht. & J.Presl
Iz_3_1009	DJI_0864	Peucedanum oreoselinum (L.) Moench	5	n	1	0	Apiaceae Lindl.
Iz_3_1009	DJI_0864	Peucedanum ruthenicum M.Bieb.	0	n	1	0	Apiaceae Lindl.
Iz_3_1009	DJI_0864	Pilosella echioides (Lumn.) F.W.Schultz & Sch.Bip.	0	n	1	0	Asteraceae Bercht. & J.Presl
Iz_3_1009	DJI_0864	Pilosella officinarum F. Schultz et Sch. Bip.	0	n	1	0	Asteraceae Bercht. & J.Presl
Iz_3_1009	DJI_0864	Pilosella sp.	0		NA	0	Asteraceae Bercht. & J.Presl
Iz_3_1009	DJI_0864	Pinus sylvestris L.	0	n	1	1	Pinaceae Spreng. ex F.Rudolphi
Iz_3_1009	DJI_0864	Poa annua L.	0	n	1	0	Poaceae Barnhart
Iz_3_1009	DJI_0864	Poa nemoralis L.	0	n	1	0	Poaceae Barnhart
Iz_3_1009	DJI_0864	Poa sp.	0		NA	0	Poaceae Barnhart
Iz_3_1009	DJI_0864	Polygonatum odoratum (Mill.) Druce	0	n	1	0	Asparagaceae Juss.
Iz_3_1009	DJI_0864	Polygonum arenarium Waldst. & Kit.	0	n	1	0	Polygonaceae Juss.
Iz_3_1009	DJI_0864	Populus nigra L.	0	n	1	1	Salicaceae Mirb.
Iz_3_1009	DJI_0864	Populus tremula L.	0	n	1	1	Salicaceae Mirb.
Iz_3_1009	DJI_0864	Populus Ã canescens (Aiton) Sm.	0	n	1	1	Salicaceae Mirb.
Iz_3_1009	DJI_0864	Potentilla argentea L.	0	n	1	0	Rosaceae Juss.
Iz_3_1009	DJI_0864	Psammophiliella muralis (L.) Ikonn.	0	n	1	0	Caryophyllaceae Juss.
Iz_3_1009	DJI_0864	Psephellus sumensis (Kalen.) Greuter	0	n	1	0	Asteraceae Bercht. & J.Presl
Iz_3_1009	DJI_0864	Pulsatilla pratensis (L.) Mill. s.l.	0	n	1	0	Ranunculaceae Juss.
Iz_3_1009	DJI_0864	Pyrus communis L.	0	a	0	1	Rosaceae Juss.
Iz_3_1009	DJI_0864	Quercus robur L.	0	n	1	1	Fagaceae Dumort.
Iz_3_1009	DJI_0864	Robinia pseudoacacia L.	0	a	0	1	Fabaceae Lindl.
Iz_3_1009	DJI_0864	Rosa sp.	0		NA	1	Rosaceae Juss.
Iz_3_1009	DJI_0864	Rubus idaeus L.	0	n	1	0	Rosaceae Juss.
Iz_3_1009	DJI_0864	Rumex acetosella L.	0	n	1	0	Polygonaceae Juss.
Iz_3_1009	DJI_0864	Rumex thyrsiflorus Fingerh.	0	n	1	0	Polygonaceae Juss.
Iz_3_1009	DJI_0864	Salix alba L.	0	n	1	1	Salicaceae Mirb.
Iz_3_1009	DJI_0864	Sambucus nigra L.	0	n	1	1	Viburnaceae Raf.
Iz_3_1009	DJI_0864	Secale sylvestre Host	0	n	1	0	Poaceae Barnhart
Iz_3_1009	DJI_0864	Sempervivum ruthenicum Schnittsp. & C.B. Lehm.	0	n	1	0	Crassulaceae J.St.-Hil.
Iz_3_1009	DJI_0864	Setaria viridis (L.) P. Beauv.	0	n	1	0	Poaceae Barnhart
Iz_3_1009	DJI_0864	Silene borysthenica (Gruner) Walters	0	n	1	0	Caryophyllaceae Juss.
Iz_3_1009	DJI_0864	Silene latifolia subsp. alba (Mill.) Greuter & Burdet	0	n	1	0	Caryophyllaceae Juss.
Iz_3_1009	DJI_0864	Solidago virgaurea L.	0	n	1	0	Asteraceae Bercht. & J.Presl
Iz_3_1009	DJI_0864	Sonchus oleraceus L.	0	a	0	0	Asteraceae Bercht. & J.Presl
Iz_3_1009	DJI_0864	Sorbus aucuparia L.	0	n	1	1	Rosaceae Juss.
Iz_3_1009	DJI_0864	Stachys recta L.	0	n	1	0	Lamiacea Lindl.
Iz_3_1009	DJI_0864	Tanacetum vulgare L.	0	n	1	0	Asteraceae Bercht. & J.Presl
Iz_3_1009	DJI_0864	Thymus pallasianus Heinr.Braun	0	n	1	0	Lamiacea Lindl.
Iz_3_1009	DJI_0864	Tilia cordata Mill.	0	n	1	1	Malvaceae Juss.
Iz_3_1009	DJI_0864	Trifolium alpestre L.	0	n	1	0	Fabaceae Lindl.
Iz_3_1009	DJI_0864	Trifolium arvense L.	0	n	1	0	Fabaceae Lindl.
Iz_3_1009	DJI_0864	Ulmus glabra Huds.	0	n	1	1	Ulmaceae Mirb.
Iz_3_1009	DJI_0864	Verbascum densiflorum Bertol.	0	n	1	0	Scrophulariaceae Juss.
Iz_3_1009	DJI_0864	Veronica austriaca L.	0	n	1	0	Plantaginaceae Juss.
Iz_3_1009	DJI_0864	Veronica incana L.	0	n	1	0	Plantaginaceae Juss.
Iz_3_1009	DJI_0864	Veronica verna L.	0	n	1	0	Plantaginaceae Juss.
Iz_3_1009	DJI_0864	Vincetoxicum hirundinaria Medik	0	n	1	0	Apocynaceae Juss.
Iz_3_1009	DJI_0865	Acer tataricum L.	0	n	1	1	Sapindaceae Juss.
Iz_3_1009	DJI_0865	Achillea millefolium L. s.l.	0	n	1	0	Asteraceae Bercht. & J.Presl
Iz_3_1009	DJI_0865	Allium oleraceum L.	0	n	1	0	Amaryllidaceae J.St.-Hil.
Iz_3_1009	DJI_0865	Ambrosia artemisiifolia L.	0	a	0	0	Asteraceae Bercht. & J.Presl
Iz_3_1009	DJI_0865	Amorpha fruticosa L.	0	a	0	0	Fabaceae Lindl.
Iz_3_1009	DJI_0865	Anthericum ramosum L.	0	n	1	0	Asparagaceae Juss.
Iz_3_1009	DJI_0865	Artemisia marschalliana Spreng.	0	n	1	0	Asteraceae Bercht. & J.Presl
Iz_3_1009	DJI_0865	Asparagus officinalis L.	0	n	1	0	Asparagaceae Juss.
Iz_3_1009	DJI_0865	Astragalus cicer L.	0	n	1	0	Fabaceae Lindl.
Iz_3_1009	DJI_0865	Bassia laniflora (S.G.Gmel.) A.J.Scott	0	n	1	0	Amaranthaceae Juss.
Iz_3_1009	DJI_0865	Berberis vulgaris L.	0	n	1	0	Berberidaceae Juss.
Iz_3_1009	DJI_0865	Betula pendula Roth.	0	n	1	1	Betulaceae Gray
Iz_3_1009	DJI_0865	Betula pendula Roth.	0	n	1	0	Poaceae Barnhart
Iz_3_1009	DJI_0865	Bromus inermis Leyss.	0		NA	NA	
Iz_3_1009	DJI_0865	Bromus squarrosus L.	0	a	0	0	Poaceae Barnhart
Iz_3_1009	DJI_0865	Bromus tectorum L.	0	a	0	0	Poaceae Barnhart
Iz_3_1009	DJI_0865	Calamagrostis epigejos (L.) Roth	10	n	1	0	Poaceae Barnhart
Iz_3_1009	DJI_0865	Carduus acanthoides L.	0	n	1	0	Asteraceae Bercht. & J.Presl
Iz_3_1009	DJI_0865	Carex sp.	0	n	1	0	Cyperaceae Juss.
Iz_3_1009	DJI_0865	Centaurea majorovii Dumbadze	0	n	1	0	Asteraceae Bercht. & J.Presl
Iz_3_1009	DJI_0865	Chamaecytisus ruthenicus (Fisch.ex Wol.) Klaskova	3	n	1	0	Fabaceae Lindl.
Iz_3_1009	DJI_0865	Chelidonium majus L.	0.2	n	1	0	Papaveraceae Juss.
Iz_3_1009	DJI_0865	Chenopodium betaceum Andrz.	0	n	1	0	Amaranthaceae Juss.
Iz_3_1009	DJI_0865	Chenopodium cf. album	0	n	1	0	Amaranthaceae Juss.
Iz_3_1009	DJI_0865	Chenopodium sp.	0		NA	0	Amaranthaceae Juss.
Iz_3_1009	DJI_0865	Chondrilla juncea L.	0	n	1	0	Asteraceae Bercht. & J.Presl
Iz_3_1009	DJI_0865	Cirsium vulgare (Savi) Ten.	0	n	1	0	Asteraceae Bercht. & J.Presl
Iz_3_1009	DJI_0865	Clinopodium acinos (L.) Kuntze	0	n	1	0	Lamiacea Lindl.
Iz_3_1009	DJI_0865	Convallaria majalis L.	0	n	1	0	Asparagaceae Juss.
Iz_3_1009	DJI_0865	Crataegus rhipidophylla Gand.	0	n	1	1	Rosaceae Juss.
Iz_3_1009	DJI_0865	Crepis foetida L.	0	n	1	0	Asteraceae Bercht. & J.Presl
Iz_3_1009	DJI_0865	Dactylis glomerata L.	0	n	1	0	Poaceae Barnhart
Iz_3_1009	DJI_0865	Dianthus campestris M.Bieb.	0	n	1	0	Caryophyllaceae Juss.
Iz_3_1009	DJI_0865	Digitaria sanguinalis (L.) Scop.	0	a	0	0	Poaceae Barnhart
Iz_3_1009	DJI_0865	Elymus repens (L.) Gould	0	n	1	0	Poaceae Barnhart
Iz_3_1009	DJI_0865	Epilobium tetragonum L.	0	n	1	0	Onagraceae Juss.
Iz_3_1009	DJI_0865	Eragrostis minor Host	0	a	0	0	Poaceae Barnhart
Iz_3_1009	DJI_0865	Erigeron canadensis L.	0	a	0	0	Asteraceae Bercht. & J.Presl
Iz_3_1009	DJI_0865	Euphorbia seguieriana Neck.	0	n	1	0	Euphorbiaceae Juss.
Iz_3_1009	DJI_0865	Fallopia dumetorum (L.) Holub	0	n	1	0	Polygonaceae Juss.

Iz_3_1009	DJI_0865	Festuca beckeri (Hack.) Trautv.	0	n	1	0	Poaceae Barnhart
Iz_3_1009	DJI_0865	Fragaria vesca L.	0	n	1	0	Rosaceae Juss.
Iz_3_1009	DJI_0865	Frangula alnus Mill.	0	n	1	1	Rhamnaceae Juss.
Iz_3_1009	DJI_0865	Fraxinus pennsylvanica Marshall	0	a	0	1	Oleaceae Hoffmanns. & Link
Iz_3_1009	DJI_0865	Galeopsis bifida Boenn.	0	n	1	0	Lamiacea Lindl.
Iz_3_1009	DJI_0865	Galium verum L.	0	n	1	0	Rubiaceae Juss.
Iz_3_1009	DJI_0865	Genista tinctoria L.	0	n	1	0	Fabaceae Lindl.
Iz_3_1009	DJI_0865	Geranium robertianum L.	0	n	1	0	Geraniaceae Juss.
Iz_3_1009	DJI_0865	Geranium sanquineum L.	0	n	1	0	Geraniaceae Juss.
Iz_3_1009	DJI_0865	Gypsophila paniculata L.	0	n	1	0	Caryophyllaceae Juss.
Iz_3_1009	DJI_0865	Helichrysum arenarium (L.) Moench	0	n	1	0	Asteraceae Bercht. & J.Presl
Iz_3_1009	DJI_0865	Herniaria polygama J.Gay	0	n	1	0	Caryophyllaceae Juss.
Iz_3_1009	DJI_0865	Hieracium robustum Fr.	0	n	1	0	Asteraceae Bercht. & J.Presl
Iz_3_1009	DJI_0865	Hieracium umbellatum L.	0	n	1	NA	Asteraceae Bercht. & J.Presl
Iz_3_1009	DJI_0865	Hylotelephium maximum (L.) Holub	0	n	1	0	Crassulaceae J.St.-Hil.
Iz_3_1009	DJI_0865	Jacobaea borysthenica (DC.) B.Nord. & Greuter	0	n	1	0	Asteraceae Bercht. & J.Presl
Iz_3_1009	DJI_0865	Jasione montana L.	0	n	1	0	Campanulaceae Juss.
Iz_3_1009	DJI_0865	Jurinea cyanoides (L.) Rchb.s.l.	0	n	1	0	Asteraceae Bercht. & J.Presl
Iz_3_1009	DJI_0865	Koeleria glauca (Spreng.) DC.	0	n	1	0	Poaceae Barnhart
Iz_3_1009	DJI_0865	Lactuca serriola L.	0	a	0	0	Asteraceae Bercht. & J.Presl
Iz_3_1009	DJI_0865	Linaria genistifolia (L.) Mill.	0	n	1	0	Plantaginaceae Juss.
Iz_3_1009	DJI_0865	Lomelosia argentea (L.) Greuter & Burdet	0	n	1	0	Caprifoliaceae Juss.
Iz_3_1009	DJI_0865	Melampyrum pratense L.	0	n	1	0	Orobanchaceae Vent.
Iz_3_1009	DJI_0865	Oenothera biennis L.	0	a	0	0	Onagraceae Juss.
Iz_3_1009	DJI_0865	Pentanema britannica (L.) D.Gut.Larr., Santos-Vicente, Anderb., E.Rico & M.M.Mart.Ort.	0	n	1	0	Asteraceae Bercht. & J.Presl
Iz_3_1009	DJI_0865	Peucedanum oreoselinum (L.) Moench	3	n	1	0	Apiaceae Lindl.
Iz_3_1009	DJI_0865	Peucedanum ruthenicum M.Bieb.	0	n	1	0	Apiaceae Lindl.
Iz_3_1009	DJI_0865	Pilosella echioides (Lumn.) F.W.Schultz & Sch.Bip.	0	n	1	0	Asteraceae Bercht. & J.Presl
Iz_3_1009	DJI_0865	Pilosella officinarum F. Schultz et Sch. Bip.	0	n	1	0	Asteraceae Bercht. & J.Presl
Iz_3_1009	DJI_0865	Pilosella sp.	0		NA	0	Asteraceae Bercht. & J.Presl
Iz_3_1009	DJI_0865	Pinus sylvestris L.	0	n	1	1	Pinaceae Spreng. ex F.Rudolphi
Iz_3_1009	DJI_0865	Poa annua L.	0	n	1	0	Poaceae Barnhart
Iz_3_1009	DJI_0865	Poa nemoralis L.	0	n	1	0	Poaceae Barnhart
Iz_3_1009	DJI_0865	Poa sp.	0		NA	0	Poaceae Barnhart
Iz_3_1009	DJI_0865	Polygonatum odoratum (Mill.) Druce	0	n	1	0	Asparagaceae Juss.
Iz_3_1009	DJI_0865	Polygonum arenarium Waldst. & Kit.	0	n	1	0	Polygonaceae Juss.
Iz_3_1009	DJI_0865	Populus nigra L.	0	n	1	1	Salicaceae Mirb.
Iz_3_1009	DJI_0865	Populus tremula L.	0	n	1	1	Salicaceae Mirb.
Iz_3_1009	DJI_0865	Populus Ã canescens (Aiton) Sm.	0	n	1	1	Salicaceae Mirb.
Iz_3_1009	DJI_0865	Potentilla argentea L.	0	n	1	0	Rosaceae Juss.
Iz_3_1009	DJI_0865	Psammophiliella muralis (L.) Ikonn.	0	n	1	0	Caryophyllaceae Juss.
Iz_3_1009	DJI_0865	Psephellus sumensis (Kalen.) Greuter	0	n	1	0	Asteraceae Bercht. & J.Presl
Iz_3_1009	DJI_0865	Pulsatilla pratensis (L.) Mill. s.l.	0	n	1	0	Ranunculaceae Juss.
Iz_3_1009	DJI_0865	Pyrus communis L.	0	a	0	1	Rosaceae Juss.
Iz_3_1009	DJI_0865	Quercus robur L.	0	n	1	1	Fagaceae Dumort.
Iz_3_1009	DJI_0865	Robinia pseudoacacia L.	0	a	0	1	Fabaceae Lindl.
Iz_3_1009	DJI_0865	Rosa sp.	0		NA	1	Rosaceae Juss.
Iz_3_1009	DJI_0865	Rubus idaeus L.	0	n	1	0	Rosaceae Juss.
Iz_3_1009	DJI_0865	Rumex acetosella L.	0	n	1	0	Polygonaceae Juss.
Iz_3_1009	DJI_0865	Rumex thyrsiflorus Fingerh.	0	n	1	0	Polygonaceae Juss.
Iz_3_1009	DJI_0865	Salix alba L.	0	n	1	1	Salicaceae Mirb.
Iz_3_1009	DJI_0865	Sambucus nigra L.	0	n	1	1	Viburnaceae Raf.
Iz_3_1009	DJI_0865	Secale sylvestre Host	0	n	1	0	Poaceae Barnhart
Iz_3_1009	DJI_0865	Sempervivum ruthenicum Schnittsp. & C.B. Lehm.	0	n	1	0	Crassulaceae J.St.-Hil.
Iz_3_1009	DJI_0865	Setaria viridis (L.) P. Beauv.	0	n	1	0	Poaceae Barnhart
Iz_3_1009	DJI_0865	Silene borysthenica (Gruner) Walters	0	n	1	0	Caryophyllaceae Juss.
Iz_3_1009	DJI_0865	Silene latifolia subsp. alba (Mill.) Greuter & Burdet	0	n	1	0	Caryophyllaceae Juss.
Iz_3_1009	DJI_0865	Solidago virgaurea L.	0	n	1	0	Asteraceae Bercht. & J.Presl
Iz_3_1009	DJI_0865	Sonchus oleraceus L.	0	a	0	0	Asteraceae Bercht. & J.Presl
Iz_3_1009	DJI_0865	Sorbus aucuparia L.	0	n	1	1	Rosaceae Juss.
Iz_3_1009	DJI_0865	Stachys recta L.	0	n	1	0	Lamiacea Lindl.
Iz_3_1009	DJI_0865	Tanacetum vulgare L.	0	n	1	0	Asteraceae Bercht. & J.Presl
Iz_3_1009	DJI_0865	Thymus pallasianus Heinr.Braun	0	n	1	0	Lamiacea Lindl.
Iz_3_1009	DJI_0865	Tilia cordata Mill.	0	n	1	1	Malvaceae Juss.
Iz_3_1009	DJI_0865	Trifolium alpestre L.	0	n	1	0	Fabaceae Lindl.
Iz_3_1009	DJI_0865	Trifolium arvense L.	0	n	1	0	Fabaceae Lindl.
Iz_3_1009	DJI_0865	Ulmus glabra Huds.	0	n	1	1	Ulmaceae Mirb.
Iz_3_1009	DJI_0865	Verbascum densiflorum Bertol.	0	n	1	0	Scrophulariaceae Juss.
Iz_3_1009	DJI_0865	Veronica austriaca L.	0	n	1	0	Plantaginaceae Juss.
Iz_3_1009	DJI_0865	Veronica incana L.	0	n	1	0	Plantaginaceae Juss.
Iz_3_1009	DJI_0865	Veronica verna L.	0	n	1	0	Plantaginaceae Juss.
Iz_3_1009	DJI_0865	Vincetoxicum hirundinaria Medik	0	n	1	0	Apocynaceae Juss.
Iz_3_1009	DJI_0866	Acer tataricum L.	0	n	1	1	Sapindaceae Juss.
Iz_3_1009	DJI_0866	Achillea millefolium L. s.l.	0	n	1	0	Asteraceae Bercht. & J.Presl
Iz_3_1009	DJI_0866	Allium oleraceum L.	0	n	1	0	Amaryllidaceae J.St.-Hil.
Iz_3_1009	DJI_0866	Ambrosia artemisiifolia L.	0	a	0	0	Asteraceae Bercht. & J.Presl
Iz_3_1009	DJI_0866	Amorpha fruticosa L.	0	a	0	0	Fabaceae Lindl.
Iz_3_1009	DJI_0866	Anthericum ramosum L.	0	n	1	0	Asparagaceae Juss.
Iz_3_1009	DJI_0866	Artemisia marschalliana Spreng.	0	n	1	0	Asteraceae Bercht. & J.Presl
Iz_3_1009	DJI_0866	Asparagus officinalis L.	0	n	1	0	Asparagaceae Juss.
Iz_3_1009	DJI_0866	Astragalus cicer L.	0	n	1	0	Fabaceae Lindl.
Iz_3_1009	DJI_0866	Bassia laniflora (S.G.Gmel.) A.J.Scott	0	n	1	0	Amaranthaceae Juss.
Iz_3_1009	DJI_0866	Berberis vulgaris L.	0	n	1	0	Berberidaceae Juss.
Iz_3_1009	DJI_0866	Betula pendula Roth.	0	n	1	1	Betulaceae Gray
Iz_3_1009	DJI_0866	Betula pendula Roth.	0	n	1	0	Poaceae Barnhart
Iz_3_1009	DJI_0866	Bromus inermis Leyss.	0		NA	NA	
Iz_3_1009	DJI_0866	Bromus squarrosus L.	0	a	0	0	Poaceae Barnhart
Iz_3_1009	DJI_0866	Bromus tectorum L.	0	a	0	0	Poaceae Barnhart
Iz_3_1009	DJI_0866	Calamagrostis epigejos (L.) Roth	40	n	1	0	Poaceae Barnhart
Iz_3_1009	DJI_0866	Carduus acanthoides L.	0	n	1	0	Asteraceae Bercht. & J.Presl
Iz_3_1009	DJI_0866	Carex sp.	0	n	1	0	Cyperaceae Juss.
Iz_3_1009	DJI_0866	Centaurea majorovii Dumbadze	0	n	1	0	Asteraceae Bercht. & J.Presl
Iz_3_1009	DJI_0866	Chamaecytisus ruthenicus (Fisch.ex Wol.) Klaskova	4	n	1	0	Fabaceae Lindl.
Iz_3_1009	DJI_0866	Chelidonium majus L.	0	n	1	0	Papaveraceae Juss.
Iz_3_1009	DJI_0866	Chenopodium betaceum Andrz.	0	n	1	0	Amaranthaceae Juss.
Iz_3_1009	DJI_0866	Chenopodium cf. album	0	n	1	0	Amaranthaceae Juss.
Iz_3_1009	DJI_0866	Chenopodium sp.	0		NA	0	Amaranthaceae Juss.
Iz_3_1009	DJI_0866	Chondrilla juncea L.	0	n	1	0	Asteraceae Bercht. & J.Presl

Iz_3_1009	DJI_0866	Cirsium vulgare (Savi) Ten.	0	n	1	0	Asteraceae Bercht. & J.Presl
Iz_3_1009	DJI_0866	Clinopodium acinos (L.) Kuntze	0	n	1	0	Lamiacea Lindl.
Iz_3_1009	DJI_0866	Convallaria majalis L.	0	n	1	0	Asparagaceae Juss.
Iz_3_1009	DJI_0866	Crataegus rhipidophylla Gand.	0	n	1	1	Rosaceae Juss.
Iz_3_1009	DJI_0866	Crepis foetida L.	0	n	1	0	Asteraceae Bercht. & J.Presl
Iz_3_1009	DJI_0866	Dactylis glomerata L.	0	n	1	0	Poaceae Barnhart
Iz_3_1009	DJI_0866	Dianthus campestris M.Bieb.	0	n	1	0	Caryophyllaceae Juss.
Iz_3_1009	DJI_0866	Digitaria sanguinalis (L.) Scop.	0	a	0	0	Poaceae Barnhart
Iz_3_1009	DJI_0866	Elymus repens (L.) Gould	0	n	1	0	Poaceae Barnhart
Iz_3_1009	DJI_0866	Epilobium tetragonum L.	0	n	1	0	Onagraceae Juss.
Iz_3_1009	DJI_0866	Eragrostis minor Host	0	a	0	0	Poaceae Barnhart
Iz_3_1009	DJI_0866	Erigeron canadensis L.	0	a	0	0	Asteraceae Bercht. & J.Presl
Iz_3_1009	DJI_0866	Euphorbia seguieriana Neck.	0	n	1	0	Euphorbiaceae Juss.
Iz_3_1009	DJI_0866	Fallopia dumetorum (L.) Holub	0	n	1	0	Polygonaceae Juss.
Iz_3_1009	DJI_0866	Festuca beckeri (Hack.) Trautv.	0	n	1	0	Poaceae Barnhart
Iz_3_1009	DJI_0866	Fragaria vesca L.	0	n	1	0	Rosaceae Juss.
Iz_3_1009	DJI_0866	Frangula alnus Mill.	0	n	1	1	Rhamnaceae Juss.
Iz_3_1009	DJI_0866	Fraxinus pennsylvanica Marshall	0	a	0	1	Oleaceae Hoffmanns. & Link
Iz_3_1009	DJI_0866	Galeopsis bifida Boenn.	0	n	1	0	Lamiacea Lindl.
Iz_3_1009	DJI_0866	Galium verum L.	0	n	1	0	Rubiaceae Juss.
Iz_3_1009	DJI_0866	Genista tinctoria L.	0	n	1	0	Fabaceae Lindl.
Iz_3_1009	DJI_0866	Geranium robertianum L.	0	n	1	0	Geraniaceae Juss.
Iz_3_1009	DJI_0866	Geranium sanquineum L.	0	n	1	0	Geraniaceae Juss.
Iz_3_1009	DJI_0866	Gypsophila paniculata L.	0	n	1	0	Caryophyllaceae Juss.
Iz_3_1009	DJI_0866	Helichrysum arenarium (L.) Moench	0	n	1	0	Asteraceae Bercht. & J.Presl
Iz_3_1009	DJI_0866	Herniaria polygama J.Gay	0	n	1	0	Caryophyllaceae Juss.
Iz_3_1009	DJI_0866	Hieracium robustum Fr.	0	n	1	0	Asteraceae Bercht. & J.Presl
Iz_3_1009	DJI_0866	Hieracium umbellatum L.	0	n	1	NA	Asteraceae Bercht. & J.Presl
Iz_3_1009	DJI_0866	Hylotelephium maximum (L.) Holub	0	n	1	0	Crassulaceae J.St.-Hil.
Iz_3_1009	DJI_0866	Jacobaea borysthenica (DC.) B.Nord. & Greuter	0	n	1	0	Asteraceae Bercht. & J.Presl
Iz_3_1009	DJI_0866	Jasione montana L.	0	n	1	0	Campanulaceae Juss.
Iz_3_1009	DJI_0866	Jurinea cyanoides (L.) Rchb.s.l.	0	n	1	0	Asteraceae Bercht. & J.Presl
Iz_3_1009	DJI_0866	Koeleria glauca (Spreng.) DC.	0	n	1	0	Poaceae Barnhart
Iz_3_1009	DJI_0866	Lactuca serriola L.	0	a	0	0	Asteraceae Bercht. & J.Presl
Iz_3_1009	DJI_0866	Linaria genistifolia (L.) Mill.	0	n	1	0	Plantaginaceae Juss.
Iz_3_1009	DJI_0866	Lomelosia argentea (L.) Greuter & Burdet	0	n	1	0	Caprifoliaceae Juss.
Iz_3_1009	DJI_0866	Melampyrum pratense L.	0	n	1	0	Orobanchaceae Vent.
Iz_3_1009	DJI_0866	Oenothera biennis L.	0	a	0	0	Onagraceae Juss.
Iz_3_1009	DJI_0866	Pentanema britannica (L.) D.Gut.Larr., Santos-Vicente, Anderb., E.Rico & M.M.Mart.Ort.	0	n	1	0	Asteraceae Bercht. & J.Presl
Iz_3_1009	DJI_0866	Peucedanum oreoselinum (L.) Moench	2	n	1	0	Apiaceae Lindl.
Iz_3_1009	DJI_0866	Peucedanum ruthenicum M.Bieb.	0	n	1	0	Apiaceae Lindl.
Iz_3_1009	DJI_0866	Pilosella echioides (Lumn.) F.W.Schultz & Sch.Bip.	0	n	1	0	Asteraceae Bercht. & J.Presl
Iz_3_1009	DJI_0866	Pilosella officinarum F. Schultz et Sch. Bip.	0	n	1	0	Asteraceae Bercht. & J.Presl
Iz_3_1009	DJI_0866	Pilosella sp.	0		NA	0	Asteraceae Bercht. & J.Presl
Iz_3_1009	DJI_0866	Pinus sylvestris L.	0	n	1	1	Pinaceae Spreng. ex F.Rudolphi
Iz_3_1009	DJI_0866	Poa annua L.	0	n	1	0	Poaceae Barnhart
Iz_3_1009	DJI_0866	Poa nemoralis L.	0	n	1	0	Poaceae Barnhart
Iz_3_1009	DJI_0866	Poa sp.	0		NA	0	Poaceae Barnhart
Iz_3_1009	DJI_0866	Polygonatum odoratum (Mill.) Druce	0	n	1	0	Asparagaceae Juss.
Iz_3_1009	DJI_0866	Polygonum arenarium Waldst. & Kit.	0	n	1	0	Polygonaceae Juss.
Iz_3_1009	DJI_0866	Populus nigra L.	0	n	1	1	Salicaceae Mirb.
Iz_3_1009	DJI_0866	Populus tremula L.	0	n	1	1	Salicaceae Mirb.
Iz_3_1009	DJI_0866	Populus Ã canescens (Aiton) Sm.	0	n	1	1	Salicaceae Mirb.
Iz_3_1009	DJI_0866	Potentilla argentea L.	0	n	1	0	Rosaceae Juss.
Iz_3_1009	DJI_0866	Psammophiliella muralis (L.) Ikonn.	0	n	1	0	Caryophyllaceae Juss.
Iz_3_1009	DJI_0866	Psephellus sumensis (Kalen.) Greuter	0	n	1	0	Asteraceae Bercht. & J.Presl
Iz_3_1009	DJI_0866	Pulsatilla pratensis (L.) Mill. s.l.	0	n	1	0	Ranunculaceae Juss.
Iz_3_1009	DJI_0866	Pyrus communis L.	0	a	0	1	Rosaceae Juss.
Iz_3_1009	DJI_0866	Quercus robur L.	0	n	1	1	Fagaceae Dumort.
Iz_3_1009	DJI_0866	Robinia pseudoacacia L.	0	a	0	1	Fabaceae Lindl.
Iz_3_1009	DJI_0866	Rosa sp.	0		NA	1	Rosaceae Juss.
Iz_3_1009	DJI_0866	Rubus idaeus L.	0	n	1	0	Rosaceae Juss.
Iz_3_1009	DJI_0866	Rumex acetosella L.	0	n	1	0	Polygonaceae Juss.
Iz_3_1009	DJI_0866	Rumex thyrsiflorus Fingerh.	0	n	1	0	Polygonaceae Juss.
Iz_3_1009	DJI_0866	Salix alba L.	0	n	1	1	Salicaceae Mirb.
Iz_3_1009	DJI_0866	Sambucus nigra L.	0	n	1	1	Viburnaceae Raf.
Iz_3_1009	DJI_0866	Secale sylvestre Host	0	n	1	0	Poaceae Barnhart
Iz_3_1009	DJI_0866	Sempervivum ruthenicum Schnittsp. & C.B. Lehm.	0	n	1	0	Crassulaceae J.St.-Hil.
Iz_3_1009	DJI_0866	Setaria viridis (L.) P. Beauv.	0	n	1	0	Poaceae Barnhart
Iz_3_1009	DJI_0866	Silene borysthenica (Gruner) Walters	0	n	1	0	Caryophyllaceae Juss.
Iz_3_1009	DJI_0866	Silene latifolia subsp. alba (Mill.) Greuter & Burdet	0	n	1	0	Caryophyllaceae Juss.
Iz_3_1009	DJI_0866	Solidago virgaurea L.	0	n	1	0	Asteraceae Bercht. & J.Presl
Iz_3_1009	DJI_0866	Sonchus oleraceus L.	0	a	0	0	Asteraceae Bercht. & J.Presl
Iz_3_1009	DJI_0866	Sorbus aucuparia L.	0	n	1	1	Rosaceae Juss.
Iz_3_1009	DJI_0866	Stachys recta L.	0	n	1	0	Lamiacea Lindl.
Iz_3_1009	DJI_0866	Tanacetum vulgare L.	0	n	1	0	Asteraceae Bercht. & J.Presl
Iz_3_1009	DJI_0866	Thymus pallasianus Heinr.Braun	0	n	1	0	Lamiacea Lindl.
Iz_3_1009	DJI_0866	Tilia cordata Mill.	0	n	1	1	Malvaceae Juss.
Iz_3_1009	DJI_0866	Trifolium alpestre L.	0	n	1	0	Fabaceae Lindl.
Iz_3_1009	DJI_0866	Trifolium arvense L.	0	n	1	0	Fabaceae Lindl.
Iz_3_1009	DJI_0866	Ulmus glabra Huds.	0	n	1	1	Ulmaceae Mirb.
Iz_3_1009	DJI_0866	Verbascum densiflorum Bertol.	0	n	1	0	Scrophulariaceae Juss.
Iz_3_1009	DJI_0866	Veronica austriaca L.	0	n	1	0	Plantaginaceae Juss.
Iz_3_1009	DJI_0866	Veronica incana L.	0	n	1	0	Plantaginaceae Juss.
Iz_3_1009	DJI_0866	Veronica verna L.	0	n	1	0	Plantaginaceae Juss.
Iz_3_1009	DJI_0866	Vincetoxicum hirundinaria Medik	0	n	1	0	Apocynaceae Juss.
Iz_3_1009	DJI_0867	Acer tataricum L.	0	n	1	1	Sapindaceae Juss.
Iz_3_1009	DJI_0867	Achillea millefolium L. s.l.	0	n	1	0	Asteraceae Bercht. & J.Presl
Iz_3_1009	DJI_0867	Allium oleraceum L.	0	n	1	0	Amaryllidaceae J.St.-Hil.
Iz_3_1009	DJI_0867	Ambrosia artemisiifolia L.	0	a	0	0	Asteraceae Bercht. & J.Presl
Iz_3_1009	DJI_0867	Amorpha fruticosa L.	0	a	0	0	Fabaceae Lindl.
Iz_3_1009	DJI_0867	Anthericum ramosum L.	0	n	1	0	Asparagaceae Juss.
Iz_3_1009	DJI_0867	Artemisia marschalliana Spreng.	0	n	1	0	Asteraceae Bercht. & J.Presl
Iz_3_1009	DJI_0867	Asparagus officinalis L.	0	n	1	0	Asparagaceae Juss.
Iz_3_1009	DJI_0867	Astragalus cicer L.	0	n	1	0	Fabaceae Lindl.
Iz_3_1009	DJI_0867	Bassia laniflora (S.G.Gmel.) A.J.Scott	0	n	1	0	Amaranthaceae Juss.
Iz_3_1009	DJI_0867	Berberis vulgaris L.	0	n	1	0	Berberidaceae Juss.
Iz_3_1009	DJI_0867	Betula pendula Roth.	0	n	1	1	Betulaceae Gray

Iz_3_1009	DJI_0867	Betula pendula Roth.	0	n	1	0	Poaceae Barnhart
Iz_3_1009	DJI_0867	Bromus inermis Leyss.	0		NA	NA	
Iz_3_1009	DJI_0867	Bromus squarrosus L.	0	a	0	0	Poaceae Barnhart
Iz_3_1009	DJI_0867	Bromus tectorum L.	0	a	0	0	Poaceae Barnhart
Iz_3_1009	DJI_0867	Calamagrostis epigejos (L.) Roth	50	n	1	0	Poaceae Barnhart
Iz_3_1009	DJI_0867	Carduus acanthoides L.	0	n	1	0	Asteraceae Bercht. & J.Presl
Iz_3_1009	DJI_0867	Carex sp.	0	n	1	0	Cyperaceae Juss.
Iz_3_1009	DJI_0867	Centaurea majorovii Dumbadze	0	n	1	0	Asteraceae Bercht. & J.Presl
Iz_3_1009	DJI_0867	Chamaecytisus ruthenicus (Fisch.ex Wol.) Klaskova	3	n	1	0	Fabaceae Lindl.
Iz_3_1009	DJI_0867	Chelidonium majus L.	0	n	1	0	Papaveraceae Juss.
Iz_3_1009	DJI_0867	Chenopodium betaceum Andrz.	0	n	1	0	Amaranthaceae Juss.
Iz_3_1009	DJI_0867	Chenopodium cf. album	0	n	1	0	Amaranthaceae Juss.
Iz_3_1009	DJI_0867	Chenopodium sp.	0		NA	0	Amaranthaceae Juss.
Iz_3_1009	DJI_0867	Chondrilla juncea L.	0	n	1	0	Asteraceae Bercht. & J.Presl
Iz_3_1009	DJI_0867	Cirsium vulgare (Savi) Ten.	0	n	1	0	Asteraceae Bercht. & J.Presl
Iz_3_1009	DJI_0867	Clinopodium acinos (L.) Kuntze	0	n	1	0	Lamiacea Lindl.
Iz_3_1009	DJI_0867	Convallaria majalis L.	0	n	1	0	Asparagaceae Juss.
Iz_3_1009	DJI_0867	Crataegus rhipidophylla Gand.	0	n	1	1	Rosaceae Juss.
Iz_3_1009	DJI_0867	Crepis foetida L.	0	n	1	0	Asteraceae Bercht. & J.Presl
Iz_3_1009	DJI_0867	Dactylis glomerata L.	0	n	1	0	Poaceae Barnhart
Iz_3_1009	DJI_0867	Dianthus campestris M.Bieb.	0	n	1	0	Caryophyllaceae Juss.
Iz_3_1009	DJI_0867	Digitaria sanguinalis (L.) Scop.	0	a	0	0	Poaceae Barnhart
Iz_3_1009	DJI_0867	Elymus repens (L.) Gould	0	n	1	0	Poaceae Barnhart
Iz_3_1009	DJI_0867	Epilobium tetragonum L.	0	n	1	0	Onagraceae Juss.
Iz_3_1009	DJI_0867	Eragrostis minor Host	0	a	0	0	Poaceae Barnhart
Iz_3_1009	DJI_0867	Erigeron canadensis L.	0	a	0	0	Asteraceae Bercht. & J.Presl
Iz_3_1009	DJI_0867	Euphorbia seguieriana Neck.	0	n	1	0	Euphorbiaceae Juss.
Iz_3_1009	DJI_0867	Fallopia dumetorum (L.) Holub	0	n	1	0	Polygonaceae Juss.
Iz_3_1009	DJI_0867	Festuca beckeri (Hack.) Trautv.	0	n	1	0	Poaceae Barnhart
Iz_3_1009	DJI_0867	Fragaria vesca L.	0	n	1	0	Rosaceae Juss.
Iz_3_1009	DJI_0867	Frangula alnus Mill.	0	n	1	1	Rhamnaceae Juss.
Iz_3_1009	DJI_0867	Fraxinus pennsylvanica Marshall	0	a	0	1	Oleaceae Hoffmanns. & Link
Iz_3_1009	DJI_0867	Galeopsis bifida Boenn.	0	n	1	0	Lamiacea Lindl.
Iz_3_1009	DJI_0867	Galium verum L.	0	n	1	0	Rubiaceae Juss.
Iz_3_1009	DJI_0867	Genista tinctoria L.	0	n	1	0	Fabaceae Lindl.
Iz_3_1009	DJI_0867	Geranium robertianum L.	0	n	1	0	Geraniaceae Juss.
Iz_3_1009	DJI_0867	Geranium sanquineum L.	0	n	1	0	Geraniaceae Juss.
Iz_3_1009	DJI_0867	Gypsophila paniculata L.	0	n	1	0	Caryophyllaceae Juss.
Iz_3_1009	DJI_0867	Helichrysum arenarium (L.) Moench	0	n	1	0	Asteraceae Bercht. & J.Presl
Iz_3_1009	DJI_0867	Herniaria polygama J.Gay	0	n	1	0	Caryophyllaceae Juss.
Iz_3_1009	DJI_0867	Hieracium robustum Fr.	0	n	1	0	Asteraceae Bercht. & J.Presl
Iz_3_1009	DJI_0867	Hieracium umbellatum L.	0	n	1	NA	Asteraceae Bercht. & J.Presl
Iz_3_1009	DJI_0867	Hylotelephium maximum (L.) Holub	0	n	1	0	Crassulaceae J.St.-Hil.
Iz_3_1009	DJI_0867	Jacobaea borysthenica (DC.) B.Nord. & Greuter	0	n	1	0	Asteraceae Bercht. & J.Presl
Iz_3_1009	DJI_0867	Jasione montana L.	0	n	1	0	Campanulaceae Juss.
Iz_3_1009	DJI_0867	Jurinea cyanoides (L.) Rchb.s.l.	0	n	1	0	Asteraceae Bercht. & J.Presl
Iz_3_1009	DJI_0867	Koeleria glauca (Spreng.) DC.	0	n	1	0	Poaceae Barnhart
Iz_3_1009	DJI_0867	Lactuca serriola L.	0	a	0	0	Asteraceae Bercht. & J.Presl
Iz_3_1009	DJI_0867	Linaria genistifolia (L.) Mill.	0	n	1	0	Plantaginaceae Juss.
Iz_3_1009	DJI_0867	Lomelosia argentea (L.) Greuter & Burdet	0	n	1	0	Caprifoliaceae Juss.
Iz_3_1009	DJI_0867	Melampyrum pratense L.	0	n	1	0	Orobanchaceae Vent.
Iz_3_1009	DJI_0867	Oenothera biennis L.	0	a	0	0	Onagraceae Juss.
Iz_3_1009	DJI_0867	Pentanema britannica (L.) D.Gut.Larr., Santos-Vicente, Anderb., E.Rico & M.M.Mart.Ort.	0	n	1	0	Asteraceae Bercht. & J.Presl
Iz_3_1009	DJI_0867	Peucedanum oreoselinum (L.) Moench	5	n	1	0	Apiaceae Lindl.
Iz_3_1009	DJI_0867	Peucedanum ruthenicum M.Bieb.	0	n	1	0	Apiaceae Lindl.
Iz_3_1009	DJI_0867	Pilosella echioides (Lumn.) F.W.Schultz & Sch.Bip.	0	n	1	0	Asteraceae Bercht. & J.Presl
Iz_3_1009	DJI_0867	Pilosella officinarum F. Schultz et Sch. Bip.	0	n	1	0	Asteraceae Bercht. & J.Presl
Iz_3_1009	DJI_0867	Pilosella sp.	0		NA	0	Asteraceae Bercht. & J.Presl
Iz_3_1009	DJI_0867	Pinus sylvestris L.	0	n	1	1	Pinaceae Spreng. ex F.Rudolphi
Iz_3_1009	DJI_0867	Poa annua L.	0	n	1	0	Poaceae Barnhart
Iz_3_1009	DJI_0867	Poa nemoralis L.	0	n	1	0	Poaceae Barnhart
Iz_3_1009	DJI_0867	Poa sp.	0		NA	0	Poaceae Barnhart
Iz_3_1009	DJI_0867	Polygonatum odoratum (Mill.) Druce	0	n	1	0	Asparagaceae Juss.
Iz_3_1009	DJI_0867	Polygonum arenarium Waldst. & Kit.	0	n	1	0	Polygonaceae Juss.
Iz_3_1009	DJI_0867	Populus nigra L.	0	n	1	1	Salicaceae Mirb.
Iz_3_1009	DJI_0867	Populus tremula L.	0	n	1	1	Salicaceae Mirb.
Iz_3_1009	DJI_0867	Populus Ã canescens (Aiton) Sm.	0	n	1	1	Salicaceae Mirb.
Iz_3_1009	DJI_0867	Potentilla argentea L.	0	n	1	0	Rosaceae Juss.
Iz_3_1009	DJI_0867	Psammophiliella muralis (L.) Ikonn.	0	n	1	0	Caryophyllaceae Juss.
Iz_3_1009	DJI_0867	Psephellus sumensis (Kalen.) Greuter	0	n	1	0	Asteraceae Bercht. & J.Presl
Iz_3_1009	DJI_0867	Pulsatilla pratensis (L.) Mill. s.l.	0	n	1	0	Ranunculaceae Juss.
Iz_3_1009	DJI_0867	Pyrus communis L.	0	a	0	1	Rosaceae Juss.
Iz_3_1009	DJI_0867	Quercus robur L.	0	n	1	1	Fagaceae Dumort.
Iz_3_1009	DJI_0867	Robinia pseudoacacia L.	0	a	0	1	Fabaceae Lindl.
Iz_3_1009	DJI_0867	Rosa sp.	0		NA	1	Rosaceae Juss.
Iz_3_1009	DJI_0867	Rubus idaeus L.	0	n	1	0	Rosaceae Juss.
Iz_3_1009	DJI_0867	Rumex acetosella L.	0	n	1	0	Polygonaceae Juss.
Iz_3_1009	DJI_0867	Rumex thyrsiflorus Fingerh.	0	n	1	0	Polygonaceae Juss.
Iz_3_1009	DJI_0867	Salix alba L.	0	n	1	1	Salicaceae Mirb.
Iz_3_1009	DJI_0867	Sambucus nigra L.	0	n	1	1	Viburnaceae Raf.
Iz_3_1009	DJI_0867	Secale sylvestre Host	0	n	1	0	Poaceae Barnhart
Iz_3_1009	DJI_0867	Sempervivum ruthenicum Schnittsp. & C.B. Lehm.	0	n	1	0	Crassulaceae J.St.-Hil.
Iz_3_1009	DJI_0867	Setaria viridis (L.) P. Beauv.	0	n	1	0	Poaceae Barnhart
Iz_3_1009	DJI_0867	Silene borysthenica (Gruner) Walters	0	n	1	0	Caryophyllaceae Juss.
Iz_3_1009	DJI_0867	Silene latifolia subsp. alba (Mill.) Greuter & Burdet	0	n	1	0	Caryophyllaceae Juss.
Iz_3_1009	DJI_0867	Solidago virgaurea L.	0	n	1	0	Asteraceae Bercht. & J.Presl
Iz_3_1009	DJI_0867	Sonchus oleraceus L.	0	a	0	0	Asteraceae Bercht. & J.Presl
Iz_3_1009	DJI_0867	Sorbus aucuparia L.	0	n	1	1	Rosaceae Juss.
Iz_3_1009	DJI_0867	Stachys recta L.	0	n	1	0	Lamiacea Lindl.
Iz_3_1009	DJI_0867	Tanacetum vulgare L.	0	n	1	0	Asteraceae Bercht. & J.Presl
Iz_3_1009	DJI_0867	Thymus pallasianus Heinr.Braun	0	n	1	0	Lamiacea Lindl.
Iz_3_1009	DJI_0867	Tilia cordata Mill.	0	n	1	1	Malvaceae Juss.
Iz_3_1009	DJI_0867	Trifolium alpestre L.	0	n	1	0	Fabaceae Lindl.
Iz_3_1009	DJI_0867	Trifolium arvense L.	0	n	1	0	Fabaceae Lindl.
Iz_3_1009	DJI_0867	Ulmus glabra Huds.	0	n	1	1	Ulmaceae Mirb.
Iz_3_1009	DJI_0867	Verbascum densiflorum Bertol.	0	n	1	0	Scrophulariaceae Juss.
Iz_3_1009	DJI_0867	Veronica austriaca L.	0	n	1	0	Plantaginaceae Juss.
Iz_3_1009	DJI_0867	Veronica incana L.	0	n	1	0	Plantaginaceae Juss.

Iz_3_1009	DJI_0867	Veronica verna L.	0	n	1	0	Plantaginaceae Juss.
Iz_3_1009	DJI_0867	Vincetoxicum hirundinaria Medik	0	n	1	0	Apocynaceae Juss.
Iz_3_1009	DJI_0868	Acer tataricum L.	0	n	1	1	Sapindaceae Juss.
Iz_3_1009	DJI_0868	Achillea millefolium L. s.l.	0	n	1	0	Asteraceae Bercht. & J.Presl
Iz_3_1009	DJI_0868	Allium oleraceum L.	0	n	1	0	Amaryllidaceae J.St.-Hil.
Iz_3_1009	DJI_0868	Ambrosia artemisiifolia L.	0	a	0	0	Asteraceae Bercht. & J.Presl
Iz_3_1009	DJI_0868	Amorpha fruticosa L.	0	a	0	0	Fabaceae Lindl.
Iz_3_1009	DJI_0868	Anthericum ramosum L.	0	n	1	0	Asparagaceae Juss.
Iz_3_1009	DJI_0868	Artemisia marschalliana Spreng.	0	n	1	0	Asteraceae Bercht. & J.Presl
Iz_3_1009	DJI_0868	Asparagus officinalis L.	0	n	1	0	Asparagaceae Juss.
Iz_3_1009	DJI_0868	Astragalus cicer L.	0	n	1	0	Fabaceae Lindl.
Iz_3_1009	DJI_0868	Bassia laniflora (S.G.Gmel.) A.J.Scott	0	n	1	0	Amaranthaceae Juss.
Iz_3_1009	DJI_0868	Berberis vulgaris L.	0	n	1	0	Berberidaceae Juss.
Iz_3_1009	DJI_0868	Betula pendula Roth.	0	n	1	1	Betulaceae Gray
Iz_3_1009	DJI_0868	Betula pendula Roth.	0	n	1	0	Poaceae Barnhart
Iz_3_1009	DJI_0868	Bromus inermis Leyss.	0		NA	NA	
Iz_3_1009	DJI_0868	Bromus squarrosus L.	0	a	0	0	Poaceae Barnhart
Iz_3_1009	DJI_0868	Bromus tectorum L.	0	a	0	0	Poaceae Barnhart
Iz_3_1009	DJI_0868	Calamagrostis epigejos (L.) Roth	60	n	1	0	Poaceae Barnhart
Iz_3_1009	DJI_0868	Carduus acanthoides L.	0	n	1	0	Asteraceae Bercht. & J.Presl
Iz_3_1009	DJI_0868	Carex sp.	1	n	1	0	Cyperaceae Juss.
Iz_3_1009	DJI_0868	Centaurea majorovii Dumbadze	0	n	1	0	Asteraceae Bercht. & J.Presl
Iz_3_1009	DJI_0868	Chamaecytisus ruthenicus (Fisch.ex Wol.) Klaskova	0	n	1	0	Fabaceae Lindl.
Iz_3_1009	DJI_0868	Chelidonium majus L.	0	n	1	0	Papaveraceae Juss.
Iz_3_1009	DJI_0868	Chenopodium betaceum Andrz.	0	n	1	0	Amaranthaceae Juss.
Iz_3_1009	DJI_0868	Chenopodium cf. album	0	n	1	0	Amaranthaceae Juss.
Iz_3_1009	DJI_0868	Chenopodium sp.	0		NA	0	Amaranthaceae Juss.
Iz_3_1009	DJI_0868	Chondrilla juncea L.	0	n	1	0	Asteraceae Bercht. & J.Presl
Iz_3_1009	DJI_0868	Cirsium vulgare (Savi) Ten.	0	n	1	0	Asteraceae Bercht. & J.Presl
Iz_3_1009	DJI_0868	Clinopodium acinos (L.) Kuntze	0	n	1	0	Lamiacea Lindl.
Iz_3_1009	DJI_0868	Convallaria majalis L.	0	n	1	0	Asparagaceae Juss.
Iz_3_1009	DJI_0868	Crataegus rhipidophylla Gand.	0	n	1	1	Rosaceae Juss.
Iz_3_1009	DJI_0868	Crepis foetida L.	0	n	1	0	Asteraceae Bercht. & J.Presl
Iz_3_1009	DJI_0868	Dactylis glomerata L.	0	n	1	0	Poaceae Barnhart
Iz_3_1009	DJI_0868	Dianthus campestris M.Bieb.	0	n	1	0	Caryophyllaceae Juss.
Iz_3_1009	DJI_0868	Digitaria sanguinalis (L.) Scop.	0	a	0	0	Poaceae Barnhart
Iz_3_1009	DJI_0868	Elymus repens (L.) Gould	0	n	1	0	Poaceae Barnhart
Iz_3_1009	DJI_0868	Epilobium tetragonum L.	0	n	1	0	Onagraceae Juss.
Iz_3_1009	DJI_0868	Eragrostis minor Host	0	a	0	0	Poaceae Barnhart
Iz_3_1009	DJI_0868	Erigeron canadensis L.	0.2	a	0	0	Asteraceae Bercht. & J.Presl
Iz_3_1009	DJI_0868	Euphorbia seguieriana Neck.	0	n	1	0	Euphorbiaceae Juss.
Iz_3_1009	DJI_0868	Fallopia dumetorum (L.) Holub	0	n	1	0	Polygonaceae Juss.
Iz_3_1009	DJI_0868	Festuca beckeri (Hack.) Trautv.	0	n	1	0	Poaceae Barnhart
Iz_3_1009	DJI_0868	Fragaria vesca L.	0	n	1	0	Rosaceae Juss.
Iz_3_1009	DJI_0868	Frangula alnus Mill.	0	n	1	1	Rhamnaceae Juss.
Iz_3_1009	DJI_0868	Fraxinus pennsylvanica Marshall	0	a	0	1	Oleaceae Hoffmanns. & Link
Iz_3_1009	DJI_0868	Galeopsis bifida Boenn.	0	n	1	0	Lamiacea Lindl.
Iz_3_1009	DJI_0868	Galium verum L.	0	n	1	0	Rubiaceae Juss.
Iz_3_1009	DJI_0868	Genista tinctoria L.	0	n	1	0	Fabaceae Lindl.
Iz_3_1009	DJI_0868	Geranium robertianum L.	0	n	1	0	Geraniaceae Juss.
Iz_3_1009	DJI_0868	Geranium sanquineum L.	0	n	1	0	Geraniaceae Juss.
Iz_3_1009	DJI_0868	Gypsophila paniculata L.	0	n	1	0	Caryophyllaceae Juss.
Iz_3_1009	DJI_0868	Helichrysum arenarium (L.) Moench	0	n	1	0	Asteraceae Bercht. & J.Presl
Iz_3_1009	DJI_0868	Herniaria polygama J.Gay	0	n	1	0	Caryophyllaceae Juss.
Iz_3_1009	DJI_0868	Hieracium robustum Fr.	0	n	1	0	Asteraceae Bercht. & J.Presl
Iz_3_1009	DJI_0868	Hieracium umbellatum L.	0	n	1	NA	Asteraceae Bercht. & J.Presl
Iz_3_1009	DJI_0868	Hylotelephium maximum (L.) Holub	0	n	1	0	Crassulaceae J.St.-Hil.
Iz_3_1009	DJI_0868	Jacobaea borysthenica (DC.) B.Nord. & Greuter	0	n	1	0	Asteraceae Bercht. & J.Presl
Iz_3_1009	DJI_0868	Jasione montana L.	0	n	1	0	Campanulaceae Juss.
Iz_3_1009	DJI_0868	Jurinea cyanoides (L.) Rchb.s.l.	0	n	1	0	Asteraceae Bercht. & J.Presl
Iz_3_1009	DJI_0868	Koeleria glauca (Spreng.) DC.	0	n	1	0	Poaceae Barnhart
Iz_3_1009	DJI_0868	Lactuca serriola L.	0	a	0	0	Asteraceae Bercht. & J.Presl
Iz_3_1009	DJI_0868	Linaria genistifolia (L.) Mill.	0	n	1	0	Plantaginaceae Juss.
Iz_3_1009	DJI_0868	Lomelosia argentea (L.) Greuter & Burdet	0	n	1	0	Caprifoliaceae Juss.
Iz_3_1009	DJI_0868	Melampyrum pratense L.	0	n	1	0	Orobanchaceae Vent.
Iz_3_1009	DJI_0868	Oenothera biennis L.	0	a	0	0	Onagraceae Juss.
Iz_3_1009	DJI_0868	Pentanema britannica (L.) D.Gut.Larr., Santos-Vicente, Anderb., E.Rico & M.M.Mart.Ort.	0	n	1	0	Asteraceae Bercht. & J.Presl
Iz_3_1009	DJI_0868	Peucedanum oreoselinum (L.) Moench	2	n	1	0	Apiaceae Lindl.
Iz_3_1009	DJI_0868	Peucedanum ruthenicum M.Bieb.	0	n	1	0	Apiaceae Lindl.
Iz_3_1009	DJI_0868	Pilosella echioides (Lumn.) F.W.Schultz & Sch.Bip.	0	n	1	0	Asteraceae Bercht. & J.Presl
Iz_3_1009	DJI_0868	Pilosella officinarum F. Schultz et Sch. Bip.	0	n	1	0	Asteraceae Bercht. & J.Presl
Iz_3_1009	DJI_0868	Pilosella sp.	0		NA	0	Asteraceae Bercht. & J.Presl
Iz_3_1009	DJI_0868	Pinus sylvestris L.	0	n	1	1	Pinaceae Spreng. ex F.Rudolphi
Iz_3_1009	DJI_0868	Poa annua L.	0	n	1	0	Poaceae Barnhart
Iz_3_1009	DJI_0868	Poa nemoralis L.	0	n	1	0	Poaceae Barnhart
Iz_3_1009	DJI_0868	Poa sp.	0		NA	0	Poaceae Barnhart
Iz_3_1009	DJI_0868	Polygonatum odoratum (Mill.) Druce	0	n	1	0	Asparagaceae Juss.
Iz_3_1009	DJI_0868	Polygonum arenarium Waldst. & Kit.	0	n	1	0	Polygonaceae Juss.
Iz_3_1009	DJI_0868	Populus nigra L.	0	n	1	1	Salicaceae Mirb.
Iz_3_1009	DJI_0868	Populus tremula L.	0	n	1	1	Salicaceae Mirb.
Iz_3_1009	DJI_0868	Populus Ã canescens (Aiton) Sm.	0	n	1	1	Salicaceae Mirb.
Iz_3_1009	DJI_0868	Potentilla argentea L.	0	n	1	0	Rosaceae Juss.
Iz_3_1009	DJI_0868	Psammophiliella muralis (L.) Ikonn.	0	n	1	0	Caryophyllaceae Juss.
Iz_3_1009	DJI_0868	Psephellus sumensis (Kalen.) Greuter	0	n	1	0	Asteraceae Bercht. & J.Presl
Iz_3_1009	DJI_0868	Pulsatilla pratensis (L.) Mill. s.l.	0	n	1	0	Ranunculaceae Juss.
Iz_3_1009	DJI_0868	Pyrus communis L.	0	a	0	1	Rosaceae Juss.
Iz_3_1009	DJI_0868	Quercus robur L.	1	n	1	1	Fagaceae Dumort.
Iz_3_1009	DJI_0868	Robinia pseudoacacia L.	0	a	0	1	Fabaceae Lindl.
Iz_3_1009	DJI_0868	Rosa sp.	0		NA	1	Rosaceae Juss.
Iz_3_1009	DJI_0868	Rubus idaeus L.	0	n	1	0	Rosaceae Juss.
Iz_3_1009	DJI_0868	Rumex acetosella L.	0	n	1	0	Polygonaceae Juss.
Iz_3_1009	DJI_0868	Rumex thyrsiflorus Fingerh.	0	n	1	0	Polygonaceae Juss.
Iz_3_1009	DJI_0868	Salix alba L.	0	n	1	1	Salicaceae Mirb.
Iz_3_1009	DJI_0868	Sambucus nigra L.	0	n	1	1	Viburnaceae Raf.
Iz_3_1009	DJI_0868	Secale sylvestre Host	0	n	1	0	Poaceae Barnhart
Iz_3_1009	DJI_0868	Sempervivum ruthenicum Schnittsp. & C.B. Lehm.	0	n	1	0	Crassulaceae J.St.-Hil.
Iz_3_1009	DJI_0868	Setaria viridis (L.) P. Beauv.	0	n	1	0	Poaceae Barnhart
Iz_3_1009	DJI_0868	Silene borysthenica (Gruner) Walters	0	n	1	0	Caryophyllaceae Juss.

Iz_3_1009	DJI_0868	Silene latifolia subsp. alba (Mill.) Greuter & Burdet	0	n	1	0	Caryophyllaceae Juss.
Iz_3_1009	DJI_0868	Solidago virgaurea L.	0	n	1	0	Asteraceae Bercht. & J.Presl
Iz_3_1009	DJI_0868	Sonchus oleraceus L.	0	a	0	0	Asteraceae Bercht. & J.Presl
Iz_3_1009	DJI_0868	Sorbus aucuparia L.	0	n	1	1	Rosaceae Juss.
Iz_3_1009	DJI_0868	Stachys recta L.	0	n	1	0	Lamiacea Lindl.
Iz_3_1009	DJI_0868	Tanacetum vulgare L.	0	n	1	0	Asteraceae Bercht. & J.Presl
Iz_3_1009	DJI_0868	Thymus pallasianus Heinr.Braun	0	n	1	0	Lamiacea Lindl.
Iz_3_1009	DJI_0868	Tilia cordata Mill.	0	n	1	1	Malvaceae Juss.
Iz_3_1009	DJI_0868	Trifolium alpestre L.	0	n	1	0	Fabaceae Lindl.
Iz_3_1009	DJI_0868	Trifolium arvense L.	0	n	1	0	Fabaceae Lindl.
Iz_3_1009	DJI_0868	Ulmus glabra Huds.	0	n	1	1	Ulmaceae Mirb.
Iz_3_1009	DJI_0868	Verbascum densiflorum Bertol.	0	n	1	0	Scrophulariaceae Juss.
Iz_3_1009	DJI_0868	Veronica austriaca L.	0	n	1	0	Plantaginaceae Juss.
Iz_3_1009	DJI_0868	Veronica incana L.	0	n	1	0	Plantaginaceae Juss.
Iz_3_1009	DJI_0868	Veronica verna L.	0	n	1	0	Plantaginaceae Juss.
Iz_3_1009	DJI_0868	Vincetoxicum hirundinaria Medik	0	n	1	0	Apocynaceae Juss.
Iz_3_1009	DJI_0869	Acer tataricum L.	0	n	1	1	Sapindaceae Juss.
Iz_3_1009	DJI_0869	Achillea millefolium L. s.l.	0	n	1	0	Asteraceae Bercht. & J.Presl
Iz_3_1009	DJI_0869	Allium oleraceum L.	0	n	1	0	Amaryllidaceae J.St.-Hil.
Iz_3_1009	DJI_0869	Ambrosia artemisiifolia L.	0	a	0	0	Asteraceae Bercht. & J.Presl
Iz_3_1009	DJI_0869	Amorpha fruticosa L.	0	a	0	0	Fabaceae Lindl.
Iz_3_1009	DJI_0869	Anthericum ramosum L.	0	n	1	0	Asparagaceae Juss.
Iz_3_1009	DJI_0869	Artemisia marschalliana Spreng.	0	n	1	0	Asteraceae Bercht. & J.Presl
Iz_3_1009	DJI_0869	Asparagus officinalis L.	0	n	1	0	Asparagaceae Juss.
Iz_3_1009	DJI_0869	Astragalus cicer L.	0	n	1	0	Fabaceae Lindl.
Iz_3_1009	DJI_0869	Bassia laniflora (S.G.Gmel.) A.J.Scott	0	n	1	0	Amaranthaceae Juss.
Iz_3_1009	DJI_0869	Berberis vulgaris L.	0	n	1	0	Berberidaceae Juss.
Iz_3_1009	DJI_0869	Betula pendula Roth.	0	n	1	1	Betulaceae Gray
Iz_3_1009	DJI_0869	Betula pendula Roth.	0	n	1	0	Poaceae Barnhart
Iz_3_1009	DJI_0869	Bromus inermis Leyss.	0		NA	NA	
Iz_3_1009	DJI_0869	Bromus squarrosus L.	0	a	0	0	Poaceae Barnhart
Iz_3_1009	DJI_0869	Bromus tectorum L.	0	a	0	0	Poaceae Barnhart
Iz_3_1009	DJI_0869	Calamagrostis epigejos (L.) Roth	15	n	1	0	Poaceae Barnhart
Iz_3_1009	DJI_0869	Carduus acanthoides L.	0	n	1	0	Asteraceae Bercht. & J.Presl
Iz_3_1009	DJI_0869	Carex sp.	0	n	1	0	Cyperaceae Juss.
Iz_3_1009	DJI_0869	Centaurea majorovii Dumbadze	0	n	1	0	Asteraceae Bercht. & J.Presl
Iz_3_1009	DJI_0869	Chamaecytisus ruthenicus (Fisch.ex Wol.) Klaskova	1	n	1	0	Fabaceae Lindl.
Iz_3_1009	DJI_0869	Chelidonium majus L.	1	n	1	0	Papaveraceae Juss.
Iz_3_1009	DJI_0869	Chenopodium betaceum Andrz.	0	n	1	0	Amaranthaceae Juss.
Iz_3_1009	DJI_0869	Chenopodium cf. album	0	n	1	0	Amaranthaceae Juss.
Iz_3_1009	DJI_0869	Chenopodium sp.	0		NA	0	Amaranthaceae Juss.
Iz_3_1009	DJI_0869	Chondrilla juncea L.	2	n	1	0	Asteraceae Bercht. & J.Presl
Iz_3_1009	DJI_0869	Cirsium vulgare (Savi) Ten.	0	n	1	0	Asteraceae Bercht. & J.Presl
Iz_3_1009	DJI_0869	Clinopodium acinos (L.) Kuntze	0	n	1	0	Lamiacea Lindl.
Iz_3_1009	DJI_0869	Convallaria majalis L.	0	n	1	0	Asparagaceae Juss.
Iz_3_1009	DJI_0869	Crataegus rhipidophylla Gand.	0	n	1	1	Rosaceae Juss.
Iz_3_1009	DJI_0869	Crepis foetida L.	0	n	1	0	Asteraceae Bercht. & J.Presl
Iz_3_1009	DJI_0869	Dactylis glomerata L.	0	n	1	0	Poaceae Barnhart
Iz_3_1009	DJI_0869	Dianthus campestris M.Bieb.	0	n	1	0	Caryophyllaceae Juss.
Iz_3_1009	DJI_0869	Digitaria sanguinalis (L.) Scop.	0	a	0	0	Poaceae Barnhart
Iz_3_1009	DJI_0869	Elymus repens (L.) Gould	0	n	1	0	Poaceae Barnhart
Iz_3_1009	DJI_0869	Epilobium tetragonum L.	0	n	1	0	Onagraceae Juss.
Iz_3_1009	DJI_0869	Eragrostis minor Host	0	a	0	0	Poaceae Barnhart
Iz_3_1009	DJI_0869	Erigeron canadensis L.	2	a	0	0	Asteraceae Bercht. & J.Presl
Iz_3_1009	DJI_0869	Euphorbia seguieriana Neck.	0	n	1	0	Euphorbiaceae Juss.
Iz_3_1009	DJI_0869	Fallopia dumetorum (L.) Holub	0	n	1	0	Polygonaceae Juss.
Iz_3_1009	DJI_0869	Festuca beckeri (Hack.) Trautv.	0	n	1	0	Poaceae Barnhart
Iz_3_1009	DJI_0869	Fragaria vesca L.	0	n	1	0	Rosaceae Juss.
Iz_3_1009	DJI_0869	Frangula alnus Mill.	0	n	1	1	Rhamnaceae Juss.
Iz_3_1009	DJI_0869	Fraxinus pennsylvanica Marshall	0	a	0	1	Oleaceae Hoffmanns. & Link
Iz_3_1009	DJI_0869	Galeopsis bifida Boenn.	0	n	1	0	Lamiacea Lindl.
Iz_3_1009	DJI_0869	Galium verum L.	0	n	1	0	Rubiaceae Juss.
Iz_3_1009	DJI_0869	Genista tinctoria L.	0	n	1	0	Fabaceae Lindl.
Iz_3_1009	DJI_0869	Geranium robertianum L.	0	n	1	0	Geraniaceae Juss.
Iz_3_1009	DJI_0869	Geranium sanquineum L.	0	n	1	0	Geraniaceae Juss.
Iz_3_1009	DJI_0869	Gypsophila paniculata L.	0	n	1	0	Caryophyllaceae Juss.
Iz_3_1009	DJI_0869	Helichrysum arenarium (L.) Moench	0	n	1	0	Asteraceae Bercht. & J.Presl
Iz_3_1009	DJI_0869	Herniaria polygama J.Gay	0	n	1	0	Caryophyllaceae Juss.
Iz_3_1009	DJI_0869	Hieracium robustum Fr.	0	n	1	0	Asteraceae Bercht. & J.Presl
Iz_3_1009	DJI_0869	Hieracium umbellatum L.	0	n	1	NA	Asteraceae Bercht. & J.Presl
Iz_3_1009	DJI_0869	Hylotelephium maximum (L.) Holub	0	n	1	0	Crassulaceae J.St.-Hil.
Iz_3_1009	DJI_0869	Jacobaea borysthenica (DC.) B.Nord. & Greuter	0	n	1	0	Asteraceae Bercht. & J.Presl
Iz_3_1009	DJI_0869	Jasione montana L.	0	n	1	0	Campanulaceae Juss.
Iz_3_1009	DJI_0869	Jurinea cyanoides (L.) Rchb.s.l.	0	n	1	0	Asteraceae Bercht. & J.Presl
Iz_3_1009	DJI_0869	Koeleria glauca (Spreng.) DC.	0	n	1	0	Poaceae Barnhart
Iz_3_1009	DJI_0869	Lactuca serriola L.	0	a	0	0	Asteraceae Bercht. & J.Presl
Iz_3_1009	DJI_0869	Linaria genistifolia (L.) Mill.	0	n	1	0	Plantaginaceae Juss.
Iz_3_1009	DJI_0869	Lomelosia argentea (L.) Greuter & Burdet	0	n	1	0	Caprifoliaceae Juss.
Iz_3_1009	DJI_0869	Melampyrum pratense L.	0	n	1	0	Orobanchaceae Vent.
Iz_3_1009	DJI_0869	Oenothera biennis L.	0	a	0	0	Onagraceae Juss.
Iz_3_1009	DJI_0869	Pentanema britannica (L.) D.Gut.Larr., Santos-Vicente, Anderb., E.Rico & M.M.Mart.Ort.	0	n	1	0	Asteraceae Bercht. & J.Presl
Iz_3_1009	DJI_0869	Peucedanum oreoselinum (L.) Moench	4	n	1	0	Apiaceae Lindl.
Iz_3_1009	DJI_0869	Peucedanum ruthenicum M.Bieb.	0	n	1	0	Apiaceae Lindl.
Iz_3_1009	DJI_0869	Pilosella echioides (Lumn.) F.W.Schultz & Sch.Bip.	0	n	1	0	Asteraceae Bercht. & J.Presl
Iz_3_1009	DJI_0869	Pilosella officinarum F. Schultz et Sch. Bip.	0	n	1	0	Asteraceae Bercht. & J.Presl
Iz_3_1009	DJI_0869	Pilosella sp.	0		NA	0	Asteraceae Bercht. & J.Presl
Iz_3_1009	DJI_0869	Pinus sylvestris L.	0	n	1	1	Pinaceae Spreng. ex F.Rudolphi
Iz_3_1009	DJI_0869	Poa annua L.	0	n	1	0	Poaceae Barnhart
Iz_3_1009	DJI_0869	Poa nemoralis L.	0	n	1	0	Poaceae Barnhart
Iz_3_1009	DJI_0869	Poa sp.	0		NA	0	Poaceae Barnhart
Iz_3_1009	DJI_0869	Polygonatum odoratum (Mill.) Druce	0	n	1	0	Asparagaceae Juss.
Iz_3_1009	DJI_0869	Polygonum arenarium Waldst. & Kit.	0	n	1	0	Polygonaceae Juss.
Iz_3_1009	DJI_0869	Populus nigra L.	0	n	1	1	Salicaceae Mirb.
Iz_3_1009	DJI_0869	Populus tremula L.	0	n	1	1	Salicaceae Mirb.
Iz_3_1009	DJI_0869	Populus Ã canescens (Aiton) Sm.	0	n	1	1	Salicaceae Mirb.
Iz_3_1009	DJI_0869	Potentilla argentea L.	0	n	1	0	Rosaceae Juss.
Iz_3_1009	DJI_0869	Psammophiliella muralis (L.) Ikonn.	0	n	1	0	Caryophyllaceae Juss.
Iz_3_1009	DJI_0869	Psephellus sumensis (Kalen.) Greuter	2	n	1	0	Asteraceae Bercht. & J.Presl

Iz_3_1009	DJI_0869	Pulsatilla pratensis (L.) Mill. s.l.	0	n	1	0	Ranunculaceae Juss.
Iz_3_1009	DJI_0869	Pyrus communis L.	0	a	0	1	Rosaceae Juss.
Iz_3_1009	DJI_0869	Quercus robur L.	0	n	1	1	Fagaceae Dumort.
Iz_3_1009	DJI_0869	Robinia pseudoacacia L.	0	a	0	1	Fabaceae Lindl.
Iz_3_1009	DJI_0869	Rosa sp.	0		NA	1	Rosaceae Juss.
Iz_3_1009	DJI_0869	Rubus idaeus L.	0	n	1	0	Rosaceae Juss.
Iz_3_1009	DJI_0869	Rumex acetosella L.	0	n	1	0	Polygonaceae Juss.
Iz_3_1009	DJI_0869	Rumex thyrsiflorus Fingerh.	0	n	1	0	Polygonaceae Juss.
Iz_3_1009	DJI_0869	Salix alba L.	0	n	1	1	Salicaceae Mirb.
Iz_3_1009	DJI_0869	Sambucus nigra L.	0	n	1	1	Viburnaceae Raf.
Iz_3_1009	DJI_0869	Secale sylvestre Host	0	n	1	0	Poaceae Barnhart
Iz_3_1009	DJI_0869	Sempervivum ruthenicum Schnittsp. & C.B. Lehm.	0	n	1	0	Crassulaceae J.St.-Hil.
Iz_3_1009	DJI_0869	Setaria viridis (L.) P. Beauv.	0	n	1	0	Poaceae Barnhart
Iz_3_1009	DJI_0869	Silene borysthenica (Gruner) Walters	0	n	1	0	Caryophyllaceae Juss.
Iz_3_1009	DJI_0869	Silene latifolia subsp. alba (Mill.) Greuter & Burdet	0	n	1	0	Caryophyllaceae Juss.
Iz_3_1009	DJI_0869	Solidago virgaurea L.	0	n	1	0	Asteraceae Bercht. & J.Presl
Iz_3_1009	DJI_0869	Sonchus oleraceus L.	0	a	0	0	Asteraceae Bercht. & J.Presl
Iz_3_1009	DJI_0869	Sorbus aucuparia L.	0	n	1	1	Rosaceae Juss.
Iz_3_1009	DJI_0869	Stachys recta L.	0	n	1	0	Lamiacea Lindl.
Iz_3_1009	DJI_0869	Tanacetum vulgare L.	0	n	1	0	Asteraceae Bercht. & J.Presl
Iz_3_1009	DJI_0869	Thymus pallasianus Heinr.Braun	0	n	1	0	Lamiacea Lindl.
Iz_3_1009	DJI_0869	Tilia cordata Mill.	0	n	1	1	Malvaceae Juss.
Iz_3_1009	DJI_0869	Trifolium alpestre L.	0	n	1	0	Fabaceae Lindl.
Iz_3_1009	DJI_0869	Trifolium arvense L.	0	n	1	0	Fabaceae Lindl.
Iz_3_1009	DJI_0869	Ulmus glabra Huds.	0	n	1	1	Ulmaceae Mirb.
Iz_3_1009	DJI_0869	Verbascum densiflorum Bertol.	0	n	1	0	Scrophulariaceae Juss.
Iz_3_1009	DJI_0869	Veronica austriaca L.	0	n	1	0	Plantaginaceae Juss.
Iz_3_1009	DJI_0869	Veronica incana L.	0	n	1	0	Plantaginaceae Juss.
Iz_3_1009	DJI_0869	Veronica verna L.	0	n	1	0	Plantaginaceae Juss.
Iz_3_1009	DJI_0869	Vincetoxicum hirundinaria Medik	0	n	1	0	Apocynaceae Juss.
Iz_3_1009	DJI_0870	Acer tataricum L.	0	n	1	1	Sapindaceae Juss.
Iz_3_1009	DJI_0870	Achillea millefolium L. s.l.	0	n	1	0	Asteraceae Bercht. & J.Presl
Iz_3_1009	DJI_0870	Allium oleraceum L.	0	n	1	0	Amaryllidaceae J.St.-Hil.
Iz_3_1009	DJI_0870	Ambrosia artemisiifolia L.	0	a	0	0	Asteraceae Bercht. & J.Presl
Iz_3_1009	DJI_0870	Amorpha fruticosa L.	0	a	0	0	Fabaceae Lindl.
Iz_3_1009	DJI_0870	Anthericum ramosum L.	0	n	1	0	Asparagaceae Juss.
Iz_3_1009	DJI_0870	Artemisia marschalliana Spreng.	0	n	1	0	Asteraceae Bercht. & J.Presl
Iz_3_1009	DJI_0870	Asparagus officinalis L.	0	n	1	0	Asparagaceae Juss.
Iz_3_1009	DJI_0870	Astragalus cicer L.	0	n	1	0	Fabaceae Lindl.
Iz_3_1009	DJI_0870	Bassia laniflora (S.G.Gmel.) A.J.Scott	0	n	1	0	Amaranthaceae Juss.
Iz_3_1009	DJI_0870	Berberis vulgaris L.	0	n	1	0	Berberidaceae Juss.
Iz_3_1009	DJI_0870	Betula pendula Roth.	0	n	1	1	Betulaceae Gray
Iz_3_1009	DJI_0870	Betula pendula Roth.	0	n	1	0	Poaceae Barnhart
Iz_3_1009	DJI_0870	Bromus inermis Leyss.	0		NA	NA	
Iz_3_1009	DJI_0870	Bromus squarrosus L.	0	a	0	0	Poaceae Barnhart
Iz_3_1009	DJI_0870	Bromus tectorum L.	0	a	0	0	Poaceae Barnhart
Iz_3_1009	DJI_0870	Calamagrostis epigejos (L.) Roth	25	n	1	0	Poaceae Barnhart
Iz_3_1009	DJI_0870	Carduus acanthoides L.	0	n	1	0	Asteraceae Bercht. & J.Presl
Iz_3_1009	DJI_0870	Carex sp.	0	n	1	0	Cyperaceae Juss.
Iz_3_1009	DJI_0870	Centaurea majorovii Dumbadze	0	n	1	0	Asteraceae Bercht. & J.Presl
Iz_3_1009	DJI_0870	Chamaecytisus ruthenicus (Fisch.ex Wol.) Klaskova	0	n	1	0	Fabaceae Lindl.
Iz_3_1009	DJI_0870	Chelidonium majus L.	0	n	1	0	Papaveraceae Juss.
Iz_3_1009	DJI_0870	Chenopodium betaceum Andrz.	0	n	1	0	Amaranthaceae Juss.
Iz_3_1009	DJI_0870	Chenopodium cf. album	0	n	1	0	Amaranthaceae Juss.
Iz_3_1009	DJI_0870	Chenopodium sp.	0		NA	0	Amaranthaceae Juss.
Iz_3_1009	DJI_0870	Chondrilla juncea L.	0	n	1	0	Asteraceae Bercht. & J.Presl
Iz_3_1009	DJI_0870	Cirsium vulgare (Savi) Ten.	0	n	1	0	Asteraceae Bercht. & J.Presl
Iz_3_1009	DJI_0870	Clinopodium acinos (L.) Kuntze	0	n	1	0	Lamiacea Lindl.
Iz_3_1009	DJI_0870	Convallaria majalis L.	0	n	1	0	Asparagaceae Juss.
Iz_3_1009	DJI_0870	Crataegus rhipidophylla Gand.	0	n	1	1	Rosaceae Juss.
Iz_3_1009	DJI_0870	Crepis foetida L.	0	n	1	0	Asteraceae Bercht. & J.Presl
Iz_3_1009	DJI_0870	Dactylis glomerata L.	0	n	1	0	Poaceae Barnhart
Iz_3_1009	DJI_0870	Dianthus campestris M.Bieb.	0	n	1	0	Caryophyllaceae Juss.
Iz_3_1009	DJI_0870	Digitaria sanguinalis (L.) Scop.	0	a	0	0	Poaceae Barnhart
Iz_3_1009	DJI_0870	Elymus repens (L.) Gould	0	n	1	0	Poaceae Barnhart
Iz_3_1009	DJI_0870	Epilobium tetragonum L.	0	n	1	0	Onagraceae Juss.
Iz_3_1009	DJI_0870	Eragrostis minor Host	0	a	0	0	Poaceae Barnhart
Iz_3_1009	DJI_0870	Erigeron canadensis L.	2	a	0	0	Asteraceae Bercht. & J.Presl
Iz_3_1009	DJI_0870	Euphorbia seguieriana Neck.	0	n	1	0	Euphorbiaceae Juss.
Iz_3_1009	DJI_0870	Fallopia dumetorum (L.) Holub	0	n	1	0	Polygonaceae Juss.
Iz_3_1009	DJI_0870	Festuca beckeri (Hack.) Trautv.	0	n	1	0	Poaceae Barnhart
Iz_3_1009	DJI_0870	Fragaria vesca L.	0	n	1	0	Rosaceae Juss.
Iz_3_1009	DJI_0870	Frangula alnus Mill.	0	n	1	1	Rhamnaceae Juss.
Iz_3_1009	DJI_0870	Fraxinus pennsylvanica Marshall	0	a	0	1	Oleaceae Hoffmanns. & Link
Iz_3_1009	DJI_0870	Galeopsis bifida Boenn.	0	n	1	0	Lamiacea Lindl.
Iz_3_1009	DJI_0870	Galium verum L.	0	n	1	0	Rubiaceae Juss.
Iz_3_1009	DJI_0870	Genista tinctoria L.	0	n	1	0	Fabaceae Lindl.
Iz_3_1009	DJI_0870	Geranium robertianum L.	0	n	1	0	Geraniaceae Juss.
Iz_3_1009	DJI_0870	Geranium sanquineum L.	0	n	1	0	Geraniaceae Juss.
Iz_3_1009	DJI_0870	Gypsophila paniculata L.	0	n	1	0	Caryophyllaceae Juss.
Iz_3_1009	DJI_0870	Helichrysum arenarium (L.) Moench	0	n	1	0	Asteraceae Bercht. & J.Presl
Iz_3_1009	DJI_0870	Herniaria polygama J.Gay	0	n	1	0	Caryophyllaceae Juss.
Iz_3_1009	DJI_0870	Hieracium robustum Fr.	0	n	1	0	Asteraceae Bercht. & J.Presl
Iz_3_1009	DJI_0870	Hieracium umbellatum L.	0	n	1	NA	Asteraceae Bercht. & J.Presl
Iz_3_1009	DJI_0870	Hylotelephium maximum (L.) Holub	0	n	1	0	Crassulaceae J.St.-Hil.
Iz_3_1009	DJI_0870	Jacobaea borysthenica (DC.) B.Nord. & Greuter	0	n	1	0	Asteraceae Bercht. & J.Presl
Iz_3_1009	DJI_0870	Jasione montana L.	0	n	1	0	Campanulaceae Juss.
Iz_3_1009	DJI_0870	Jurinea cyanoides (L.) Rchb.s.l.	0	n	1	0	Asteraceae Bercht. & J.Presl
Iz_3_1009	DJI_0870	Koeleria glauca (Spreng.) DC.	0	n	1	0	Poaceae Barnhart
Iz_3_1009	DJI_0870	Lactuca serriola L.	0	a	0	0	Asteraceae Bercht. & J.Presl
Iz_3_1009	DJI_0870	Linaria genistifolia (L.) Mill.	0	n	1	0	Plantaginaceae Juss.
Iz_3_1009	DJI_0870	Lomelosia argentea (L.) Greuter & Burdet	0	n	1	0	Caprifoliaceae Juss.
Iz_3_1009	DJI_0870	Melampyrum pratense L.	0	n	1	0	Orobanchaceae Vent.
Iz_3_1009	DJI_0870	Oenothera biennis L.	0	a	0	0	Onagraceae Juss.
Iz_3_1009	DJI_0870	Pentanema britannica (L.) D.Gut.Larr., Santos-Vicente, Anderb., E.Rico & M.M.Mart.Ort.	0	n	1	0	Asteraceae Bercht. & J.Presl
Iz_3_1009	DJI_0870	Peucedanum oreoselinum (L.) Moench	3	n	1	0	Apiaceae Lindl.
Iz_3_1009	DJI_0870	Peucedanum ruthenicum M.Bieb.	0	n	1	0	Apiaceae Lindl.
Iz_3_1009	DJI_0870	Pilosella echioides (Lumn.) F.W.Schultz & Sch.Bip.	0	n	1	0	Asteraceae Bercht. & J.Presl

Iz_3_1009	DJI_0870	Pilosella officinarum F. Schultz et Sch. Bip.	0	n	1	0	Asteraceae Bercht. & J.Presl
Iz_3_1009	DJI_0870	Pilosella sp.	0		NA	0	Asteraceae Bercht. & J.Presl
Iz_3_1009	DJI_0870	Pinus sylvestris L.	0	n	1	1	Pinaceae Spreng. ex F.Rudolphi
Iz_3_1009	DJI_0870	Poa annua L.	0	n	1	0	Poaceae Barnhart
Iz_3_1009	DJI_0870	Poa nemoralis L.	0	n	1	0	Poaceae Barnhart
Iz_3_1009	DJI_0870	Poa sp.	0		NA	0	Poaceae Barnhart
Iz_3_1009	DJI_0870	Polygonatum odoratum (Mill.) Druce	0	n	1	0	Asparagaceae Juss.
Iz_3_1009	DJI_0870	Polygonum arenarium Waldst. & Kit.	0	n	1	0	Polygonaceae Juss.
Iz_3_1009	DJI_0870	Populus nigra L.	0	n	1	1	Salicaceae Mirb.
Iz_3_1009	DJI_0870	Populus tremula L.	0	n	1	1	Salicaceae Mirb.
Iz_3_1009	DJI_0870	Populus Ã canescens (Aiton) Sm.	0	n	1	1	Salicaceae Mirb.
Iz_3_1009	DJI_0870	Potentilla argentea L.	0	n	1	0	Rosaceae Juss.
Iz_3_1009	DJI_0870	Psammophiliella muralis (L.) Ikonn.	0	n	1	0	Caryophyllaceae Juss.
Iz_3_1009	DJI_0870	Psephellus sumensis (Kalen.) Greuter	0	n	1	0	Asteraceae Bercht. & J.Presl
Iz_3_1009	DJI_0870	Pulsatilla pratensis (L.) Mill. s.l.	0	n	1	0	Ranunculaceae Juss.
Iz_3_1009	DJI_0870	Pyrus communis L.	0	a	0	1	Rosaceae Juss.
Iz_3_1009	DJI_0870	Quercus robur L.	20	n	1	1	Fagaceae Dumort.
Iz_3_1009	DJI_0870	Robinia pseudoacacia L.	0	a	0	1	Fabaceae Lindl.
Iz_3_1009	DJI_0870	Rosa sp.	0		NA	1	Rosaceae Juss.
Iz_3_1009	DJI_0870	Rubus idaeus L.	0	n	1	0	Rosaceae Juss.
Iz_3_1009	DJI_0870	Rumex acetosella L.	0	n	1	0	Polygonaceae Juss.
Iz_3_1009	DJI_0870	Rumex thyrsiflorus Fingerh.	0	n	1	0	Polygonaceae Juss.
Iz_3_1009	DJI_0870	Salix alba L.	0	n	1	1	Salicaceae Mirb.
Iz_3_1009	DJI_0870	Sambucus nigra L.	0	n	1	1	Viburnaceae Raf.
Iz_3_1009	DJI_0870	Secale sylvestre Host	0	n	1	0	Poaceae Barnhart
Iz_3_1009	DJI_0870	Sempervivum ruthenicum Schnittsp. & C.B. Lehm.	0	n	1	0	Crassulaceae J.St.-Hil.
Iz_3_1009	DJI_0870	Setaria viridis (L.) P. Beauv.	0	n	1	0	Poaceae Barnhart
Iz_3_1009	DJI_0870	Silene borysthenica (Gruner) Walters	0	n	1	0	Caryophyllaceae Juss.
Iz_3_1009	DJI_0870	Silene latifolia subsp. alba (Mill.) Greuter & Burdet	0	n	1	0	Caryophyllaceae Juss.
Iz_3_1009	DJI_0870	Solidago virgaurea L.	0	n	1	0	Asteraceae Bercht. & J.Presl
Iz_3_1009	DJI_0870	Sonchus oleraceus L.	0	a	0	0	Asteraceae Bercht. & J.Presl
Iz_3_1009	DJI_0870	Sorbus aucuparia L.	0	n	1	1	Rosaceae Juss.
Iz_3_1009	DJI_0870	Stachys recta L.	0	n	1	0	Lamiacea Lindl.
Iz_3_1009	DJI_0870	Tanacetum vulgare L.	0	n	1	0	Asteraceae Bercht. & J.Presl
Iz_3_1009	DJI_0870	Thymus pallasianus Heinr.Braun	0	n	1	0	Lamiacea Lindl.
Iz_3_1009	DJI_0870	Tilia cordata Mill.	0	n	1	1	Malvaceae Juss.
Iz_3_1009	DJI_0870	Trifolium alpestre L.	0	n	1	0	Fabaceae Lindl.
Iz_3_1009	DJI_0870	Trifolium arvense L.	0	n	1	0	Fabaceae Lindl.
Iz_3_1009	DJI_0870	Ulmus glabra Huds.	0	n	1	1	Ulmaceae Mirb.
Iz_3_1009	DJI_0870	Verbascum densiflorum Bertol.	0	n	1	0	Scrophulariaceae Juss.
Iz_3_1009	DJI_0870	Veronica austriaca L.	0	n	1	0	Plantaginaceae Juss.
Iz_3_1009	DJI_0870	Veronica incana L.	0	n	1	0	Plantaginaceae Juss.
Iz_3_1009	DJI_0870	Veronica verna L.	0	n	1	0	Plantaginaceae Juss.
Iz_3_1009	DJI_0870	Vincetoxicum hirundinaria Medik	0	n	1	0	Apocynaceae Juss.
Iz_3_1009	DJI_0871	Acer tataricum L.	0	n	1	1	Sapindaceae Juss.
Iz_3_1009	DJI_0871	Achillea millefolium L. s.l.	0	n	1	0	Asteraceae Bercht. & J.Presl
Iz_3_1009	DJI_0871	Allium oleraceum L.	0	n	1	0	Amaryllidaceae J.St.-Hil.
Iz_3_1009	DJI_0871	Ambrosia artemisiifolia L.	0	a	0	0	Asteraceae Bercht. & J.Presl
Iz_3_1009	DJI_0871	Amorpha fruticosa L.	0	a	0	0	Fabaceae Lindl.
Iz_3_1009	DJI_0871	Anthericum ramosum L.	0	n	1	0	Asparagaceae Juss.
Iz_3_1009	DJI_0871	Artemisia marschalliana Spreng.	0	n	1	0	Asteraceae Bercht. & J.Presl
Iz_3_1009	DJI_0871	Asparagus officinalis L.	0	n	1	0	Asparagaceae Juss.
Iz_3_1009	DJI_0871	Astragalus cicer L.	0	n	1	0	Fabaceae Lindl.
Iz_3_1009	DJI_0871	Bassia laniflora (S.G.Gmel.) A.J.Scott	0	n	1	0	Amaranthaceae Juss.
Iz_3_1009	DJI_0871	Berberis vulgaris L.	0	n	1	0	Berberidaceae Juss.
Iz_3_1009	DJI_0871	Betula pendula Roth.	0	n	1	1	Betulaceae Gray
Iz_3_1009	DJI_0871	Betula pendula Roth.	0	n	1	0	Poaceae Barnhart
Iz_3_1009	DJI_0871	Bromus inermis Leyss.	0		NA	NA	
Iz_3_1009	DJI_0871	Bromus squarrosus L.	0	a	0	0	Poaceae Barnhart
Iz_3_1009	DJI_0871	Bromus tectorum L.	0	a	0	0	Poaceae Barnhart
Iz_3_1009	DJI_0871	Calamagrostis epigejos (L.) Roth	10	n	1	0	Poaceae Barnhart
Iz_3_1009	DJI_0871	Carduus acanthoides L.	0	n	1	0	Asteraceae Bercht. & J.Presl
Iz_3_1009	DJI_0871	Carex sp.	0	n	1	0	Cyperaceae Juss.
Iz_3_1009	DJI_0871	Centaurea majorovii Dumbadze	0	n	1	0	Asteraceae Bercht. & J.Presl
Iz_3_1009	DJI_0871	Chamaecytisus ruthenicus (Fisch.ex Wol.) Klaskova	20	n	1	0	Fabaceae Lindl.
Iz_3_1009	DJI_0871	Chelidonium majus L.	2	n	1	0	Papaveraceae Juss.
Iz_3_1009	DJI_0871	Chenopodium betaceum Andrz.	0	n	1	0	Amaranthaceae Juss.
Iz_3_1009	DJI_0871	Chenopodium cf. album	0	n	1	0	Amaranthaceae Juss.
Iz_3_1009	DJI_0871	Chenopodium sp.	0		NA	0	Amaranthaceae Juss.
Iz_3_1009	DJI_0871	Chondrilla juncea L.	0	n	1	0	Asteraceae Bercht. & J.Presl
Iz_3_1009	DJI_0871	Cirsium vulgare (Savi) Ten.	0	n	1	0	Asteraceae Bercht. & J.Presl
Iz_3_1009	DJI_0871	Clinopodium acinos (L.) Kuntze	0	n	1	0	Lamiacea Lindl.
Iz_3_1009	DJI_0871	Convallaria majalis L.	0	n	1	0	Asparagaceae Juss.
Iz_3_1009	DJI_0871	Crataegus rhipidophylla Gand.	0	n	1	1	Rosaceae Juss.
Iz_3_1009	DJI_0871	Crepis foetida L.	0	n	1	0	Asteraceae Bercht. & J.Presl
Iz_3_1009	DJI_0871	Dactylis glomerata L.	0	n	1	0	Poaceae Barnhart
Iz_3_1009	DJI_0871	Dianthus campestris M.Bieb.	0	n	1	0	Caryophyllaceae Juss.
Iz_3_1009	DJI_0871	Digitaria sanguinalis (L.) Scop.	0	a	0	0	Poaceae Barnhart
Iz_3_1009	DJI_0871	Elymus repens (L.) Gould	0	n	1	0	Poaceae Barnhart
Iz_3_1009	DJI_0871	Epilobium tetragonum L.	0	n	1	0	Onagraceae Juss.
Iz_3_1009	DJI_0871	Eragrostis minor Host	0	a	0	0	Poaceae Barnhart
Iz_3_1009	DJI_0871	Erigeron canadensis L.	1	a	0	0	Asteraceae Bercht. & J.Presl
Iz_3_1009	DJI_0871	Euphorbia seguieriana Neck.	0	n	1	0	Euphorbiaceae Juss.
Iz_3_1009	DJI_0871	Fallopia dumetorum (L.) Holub	0	n	1	0	Polygonaceae Juss.
Iz_3_1009	DJI_0871	Festuca beckeri (Hack.) Trautv.	0	n	1	0	Poaceae Barnhart
Iz_3_1009	DJI_0871	Fragaria vesca L.	0	n	1	0	Rosaceae Juss.
Iz_3_1009	DJI_0871	Frangula alnus Mill.	0	n	1	1	Rhamnaceae Juss.
Iz_3_1009	DJI_0871	Fraxinus pennsylvanica Marshall	0	a	0	1	Oleaceae Hoffmanns. & Link
Iz_3_1009	DJI_0871	Galeopsis bifida Boenn.	0	n	1	0	Lamiacea Lindl.
Iz_3_1009	DJI_0871	Galium verum L.	0	n	1	0	Rubiaceae Juss.
Iz_3_1009	DJI_0871	Genista tinctoria L.	0	n	1	0	Fabaceae Lindl.
Iz_3_1009	DJI_0871	Geranium robertianum L.	0	n	1	0	Geraniaceae Juss.
Iz_3_1009	DJI_0871	Geranium sanquineum L.	0	n	1	0	Geraniaceae Juss.
Iz_3_1009	DJI_0871	Gypsophila paniculata L.	0	n	1	0	Caryophyllaceae Juss.
Iz_3_1009	DJI_0871	Helichrysum arenarium (L.) Moench	0	n	1	0	Asteraceae Bercht. & J.Presl
Iz_3_1009	DJI_0871	Herniaria polygama J.Gay	0	n	1	0	Caryophyllaceae Juss.
Iz_3_1009	DJI_0871	Hieracium robustum Fr.	0	n	1	0	Asteraceae Bercht. & J.Presl
Iz_3_1009	DJI_0871	Hieracium umbellatum L.	0	n	1	NA	Asteraceae Bercht. & J.Presl

Iz_3_1009	DJI_0871	Hylotelephium maximum (L.) Holub	0	n	1	0	Crassulaceae J.St.-Hil.
Iz_3_1009	DJI_0871	Jacobaea borysthenica (DC.) B.Nord. & Greuter	0	n	1	0	Asteraceae Bercht. & J.Presl
Iz_3_1009	DJI_0871	Jasione montana L.	0	n	1	0	Campanulaceae Juss.
Iz_3_1009	DJI_0871	Jurinea cyanoides (L.) Rchb.s.l.	0	n	1	0	Asteraceae Bercht. & J.Presl
Iz_3_1009	DJI_0871	Koeleria glauca (Spreng.) DC.	0	n	1	0	Poaceae Barnhart
Iz_3_1009	DJI_0871	Lactuca serriola L.	0	a	0	0	Asteraceae Bercht. & J.Presl
Iz_3_1009	DJI_0871	Linaria genistifolia (L.) Mill.	0	n	1	0	Plantaginaceae Juss.
Iz_3_1009	DJI_0871	Lomelosia argentea (L.) Greuter & Burdet	0	n	1	0	Caprifoliaceae Juss.
Iz_3_1009	DJI_0871	Melampyrum pratense L.	0	n	1	0	Orobanchaceae Vent.
Iz_3_1009	DJI_0871	Oenothera biennis L.	0	a	0	0	Onagraceae Juss.
Iz_3_1009	DJI_0871	Pentanema britannica (L.) D.Gut.Larr., Santos-Vicente, Anderb., E.Rico & M.M.Mart.Ort.	0	n	1	0	Asteraceae Bercht. & J.Presl
Iz_3_1009	DJI_0871	Peucedanum oreoselinum (L.) Moench	2	n	1	0	Apiaceae Lindl.
Iz_3_1009	DJI_0871	Peucedanum ruthenicum M.Bieb.	0	n	1	0	Apiaceae Lindl.
Iz_3_1009	DJI_0871	Pilosella echioides (Lumn.) F.W.Schultz & Sch.Bip.	0	n	1	0	Asteraceae Bercht. & J.Presl
Iz_3_1009	DJI_0871	Pilosella officinarum F. Schultz et Sch. Bip.	0	n	1	0	Asteraceae Bercht. & J.Presl
Iz_3_1009	DJI_0871	Pilosella sp.	0		NA	0	Asteraceae Bercht. & J.Presl
Iz_3_1009	DJI_0871	Pinus sylvestris L.	0	n	1	1	Pinaceae Spreng. ex F.Rudolphi
Iz_3_1009	DJI_0871	Poa annua L.	0	n	1	0	Poaceae Barnhart
Iz_3_1009	DJI_0871	Poa nemoralis L.	0	n	1	0	Poaceae Barnhart
Iz_3_1009	DJI_0871	Poa sp.	0		NA	0	Poaceae Barnhart
Iz_3_1009	DJI_0871	Polygonatum odoratum (Mill.) Druce	0.5	n	1	0	Asparagaceae Juss.
Iz_3_1009	DJI_0871	Polygonum arenarium Waldst. & Kit.	0	n	1	0	Polygonaceae Juss.
Iz_3_1009	DJI_0871	Populus nigra L.	0	n	1	1	Salicaceae Mirb.
Iz_3_1009	DJI_0871	Populus tremula L.	0	n	1	1	Salicaceae Mirb.
Iz_3_1009	DJI_0871	Populus Ã canescens (Aiton) Sm.	0	n	1	1	Salicaceae Mirb.
Iz_3_1009	DJI_0871	Potentilla argentea L.	0	n	1	0	Rosaceae Juss.
Iz_3_1009	DJI_0871	Psammophiliella muralis (L.) Ikonn.	0	n	1	0	Caryophyllaceae Juss.
Iz_3_1009	DJI_0871	Psephellus sumensis (Kalen.) Greuter	0	n	1	0	Asteraceae Bercht. & J.Presl
Iz_3_1009	DJI_0871	Pulsatilla pratensis (L.) Mill. s.l.	0	n	1	0	Ranunculaceae Juss.
Iz_3_1009	DJI_0871	Pyrus communis L.	0	a	0	1	Rosaceae Juss.
Iz_3_1009	DJI_0871	Quercus robur L.	2	n	1	1	Fagaceae Dumort.
Iz_3_1009	DJI_0871	Robinia pseudoacacia L.	0	a	0	1	Fabaceae Lindl.
Iz_3_1009	DJI_0871	Rosa sp.	0		NA	1	Rosaceae Juss.
Iz_3_1009	DJI_0871	Rubus idaeus L.	0	n	1	0	Rosaceae Juss.
Iz_3_1009	DJI_0871	Rumex acetosella L.	0	n	1	0	Polygonaceae Juss.
Iz_3_1009	DJI_0871	Rumex thyrsiflorus Fingerh.	0	n	1	0	Polygonaceae Juss.
Iz_3_1009	DJI_0871	Salix alba L.	0	n	1	1	Salicaceae Mirb.
Iz_3_1009	DJI_0871	Sambucus nigra L.	0	n	1	1	Viburnaceae Raf.
Iz_3_1009	DJI_0871	Secale sylvestre Host	0	n	1	0	Poaceae Barnhart
Iz_3_1009	DJI_0871	Sempervivum ruthenicum Schnittsp. & C.B. Lehm.	0	n	1	0	Crassulaceae J.St.-Hil.
Iz_3_1009	DJI_0871	Setaria viridis (L.) P. Beauv.	0	n	1	0	Poaceae Barnhart
Iz_3_1009	DJI_0871	Silene borysthenica (Gruner) Walters	0	n	1	0	Caryophyllaceae Juss.
Iz_3_1009	DJI_0871	Silene latifolia subsp. alba (Mill.) Greuter & Burdet	0	n	1	0	Caryophyllaceae Juss.
Iz_3_1009	DJI_0871	Solidago virgaurea L.	0	n	1	0	Asteraceae Bercht. & J.Presl
Iz_3_1009	DJI_0871	Sonchus oleraceus L.	0	a	0	0	Asteraceae Bercht. & J.Presl
Iz_3_1009	DJI_0871	Sorbus aucuparia L.	0	n	1	1	Rosaceae Juss.
Iz_3_1009	DJI_0871	Stachys recta L.	0	n	1	0	Lamiacea Lindl.
Iz_3_1009	DJI_0871	Tanacetum vulgare L.	0	n	1	0	Asteraceae Bercht. & J.Presl
Iz_3_1009	DJI_0871	Thymus pallasianus Heinr.Braun	0	n	1	0	Lamiacea Lindl.
Iz_3_1009	DJI_0871	Tilia cordata Mill.	0	n	1	1	Malvaceae Juss.
Iz_3_1009	DJI_0871	Trifolium alpestre L.	0	n	1	0	Fabaceae Lindl.
Iz_3_1009	DJI_0871	Trifolium arvense L.	0	n	1	0	Fabaceae Lindl.
Iz_3_1009	DJI_0871	Ulmus glabra Huds.	0	n	1	1	Ulmaceae Mirb.
Iz_3_1009	DJI_0871	Verbascum densiflorum Bertol.	0	n	1	0	Scrophulariaceae Juss.
Iz_3_1009	DJI_0871	Veronica austriaca L.	0	n	1	0	Plantaginaceae Juss.
Iz_3_1009	DJI_0871	Veronica incana L.	0	n	1	0	Plantaginaceae Juss.
Iz_3_1009	DJI_0871	Veronica verna L.	0	n	1	0	Plantaginaceae Juss.
Iz_3_1009	DJI_0871	Vincetoxicum hirundinaria Medik	0	n	1	0	Apocynaceae Juss.
Iz_3_1009	DJI_0874	Acer tataricum L.	0	n	1	1	Sapindaceae Juss.
Iz_3_1009	DJI_0874	Achillea millefolium L. s.l.	0	n	1	0	Asteraceae Bercht. & J.Presl
Iz_3_1009	DJI_0874	Allium oleraceum L.	0	n	1	0	Amaryllidaceae J.St.-Hil.
Iz_3_1009	DJI_0874	Ambrosia artemisiifolia L.	0	a	0	0	Asteraceae Bercht. & J.Presl
Iz_3_1009	DJI_0874	Amorpha fruticosa L.	0	a	0	0	Fabaceae Lindl.
Iz_3_1009	DJI_0874	Anthericum ramosum L.	0	n	1	0	Asparagaceae Juss.
Iz_3_1009	DJI_0874	Artemisia marschalliana Spreng.	0	n	1	0	Asteraceae Bercht. & J.Presl
Iz_3_1009	DJI_0874	Asparagus officinalis L.	0	n	1	0	Asparagaceae Juss.
Iz_3_1009	DJI_0874	Astragalus cicer L.	0	n	1	0	Fabaceae Lindl.
Iz_3_1009	DJI_0874	Bassia laniflora (S.G.Gmel.) A.J.Scott	0	n	1	0	Amaranthaceae Juss.
Iz_3_1009	DJI_0874	Berberis vulgaris L.	0	n	1	0	Berberidaceae Juss.
Iz_3_1009	DJI_0874	Betula pendula Roth.	0	n	1	1	Betulaceae Gray
Iz_3_1009	DJI_0874	Betula pendula Roth.	0	n	1	0	Poaceae Barnhart
Iz_3_1009	DJI_0874	Bromus inermis Leyss.	0		NA	NA	
Iz_3_1009	DJI_0874	Bromus squarrosus L.	0	a	0	0	Poaceae Barnhart
Iz_3_1009	DJI_0874	Bromus tectorum L.	0	a	0	0	Poaceae Barnhart
Iz_3_1009	DJI_0874	Calamagrostis epigejos (L.) Roth	5	n	1	0	Poaceae Barnhart
Iz_3_1009	DJI_0874	Carduus acanthoides L.	0	n	1	0	Asteraceae Bercht. & J.Presl
Iz_3_1009	DJI_0874	Carex sp.	0	n	1	0	Cyperaceae Juss.
Iz_3_1009	DJI_0874	Centaurea majorovii Dumbadze	0	n	1	0	Asteraceae Bercht. & J.Presl
Iz_3_1009	DJI_0874	Chamaecytisus ruthenicus (Fisch.ex Wol.) Klaskova	3	n	1	0	Fabaceae Lindl.
Iz_3_1009	DJI_0874	Chelidonium majus L.	5	n	1	0	Papaveraceae Juss.
Iz_3_1009	DJI_0874	Chenopodium betaceum Andrz.	0	n	1	0	Amaranthaceae Juss.
Iz_3_1009	DJI_0874	Chenopodium cf. album	0	n	1	0	Amaranthaceae Juss.
Iz_3_1009	DJI_0874	Chenopodium sp.	0		NA	0	Amaranthaceae Juss.
Iz_3_1009	DJI_0874	Chondrilla juncea L.	0	n	1	0	Asteraceae Bercht. & J.Presl
Iz_3_1009	DJI_0874	Cirsium vulgare (Savi) Ten.	0	n	1	0	Asteraceae Bercht. & J.Presl
Iz_3_1009	DJI_0874	Clinopodium acinos (L.) Kuntze	0	n	1	0	Lamiacea Lindl.
Iz_3_1009	DJI_0874	Convallaria majalis L.	0	n	1	0	Asparagaceae Juss.
Iz_3_1009	DJI_0874	Crataegus rhipidophylla Gand.	0	n	1	1	Rosaceae Juss.
Iz_3_1009	DJI_0874	Crepis foetida L.	0	n	1	0	Asteraceae Bercht. & J.Presl
Iz_3_1009	DJI_0874	Dactylis glomerata L.	0	n	1	0	Poaceae Barnhart
Iz_3_1009	DJI_0874	Dianthus campestris M.Bieb.	0	n	1	0	Caryophyllaceae Juss.
Iz_3_1009	DJI_0874	Digitaria sanguinalis (L.) Scop.	0	a	0	0	Poaceae Barnhart
Iz_3_1009	DJI_0874	Elymus repens (L.) Gould	0	n	1	0	Poaceae Barnhart
Iz_3_1009	DJI_0874	Epilobium tetragonum L.	0	n	1	0	Onagraceae Juss.
Iz_3_1009	DJI_0874	Eragrostis minor Host	0	a	0	0	Poaceae Barnhart
Iz_3_1009	DJI_0874	Erigeron canadensis L.	0	a	0	0	Asteraceae Bercht. & J.Presl
Iz_3_1009	DJI_0874	Euphorbia seguieriana Neck.	0	n	1	0	Euphorbiaceae Juss.
Iz_3_1009	DJI_0874	Fallopia dumetorum (L.) Holub	5	n	1	0	Polygonaceae Juss.

Iz_3_1009	DJI_0874	Festuca beckeri (Hack.) Trautv.	0	n	1	0	Poaceae Barnhart
Iz_3_1009	DJI_0874	Fragaria vesca L.	0	n	1	0	Rosaceae Juss.
Iz_3_1009	DJI_0874	Frangula alnus Mill.	0	n	1	1	Rhamnaceae Juss.
Iz_3_1009	DJI_0874	Fraxinus pennsylvanica Marshall	0	a	0	1	Oleaceae Hoffmanns. & Link
Iz_3_1009	DJI_0874	Galeopsis bifida Boenn.	0	n	1	0	Lamiacea Lindl.
Iz_3_1009	DJI_0874	Galium verum L.	0	n	1	0	Rubiaceae Juss.
Iz_3_1009	DJI_0874	Genista tinctoria L.	0	n	1	0	Fabaceae Lindl.
Iz_3_1009	DJI_0874	Geranium robertianum L.	0	n	1	0	Geraniaceae Juss.
Iz_3_1009	DJI_0874	Geranium sanquineum L.	0	n	1	0	Geraniaceae Juss.
Iz_3_1009	DJI_0874	Gypsophila paniculata L.	0	n	1	0	Caryophyllaceae Juss.
Iz_3_1009	DJI_0874	Helichrysum arenarium (L.) Moench	0	n	1	0	Asteraceae Bercht. & J.Presl
Iz_3_1009	DJI_0874	Herniaria polygama J.Gay	0	n	1	0	Caryophyllaceae Juss.
Iz_3_1009	DJI_0874	Hieracium robustum Fr.	0	n	1	0	Asteraceae Bercht. & J.Presl
Iz_3_1009	DJI_0874	Hieracium umbellatum L.	0	n	1	NA	Asteraceae Bercht. & J.Presl
Iz_3_1009	DJI_0874	Hylotelephium maximum (L.) Holub	0	n	1	0	Crassulaceae J.St.-Hil.
Iz_3_1009	DJI_0874	Jacobaea borysthenica (DC.) B.Nord. & Greuter	0	n	1	0	Asteraceae Bercht. & J.Presl
Iz_3_1009	DJI_0874	Jasione montana L.	0	n	1	0	Campanulaceae Juss.
Iz_3_1009	DJI_0874	Jurinea cyanoides (L.) Rchb.s.l.	0	n	1	0	Asteraceae Bercht. & J.Presl
Iz_3_1009	DJI_0874	Koeleria glauca (Spreng.) DC.	0	n	1	0	Poaceae Barnhart
Iz_3_1009	DJI_0874	Lactuca serriola L.	0	a	0	0	Asteraceae Bercht. & J.Presl
Iz_3_1009	DJI_0874	Linaria genistifolia (L.) Mill.	0	n	1	0	Plantaginaceae Juss.
Iz_3_1009	DJI_0874	Lomelosia argentea (L.) Greuter & Burdet	0	n	1	0	Caprifoliaceae Juss.
Iz_3_1009	DJI_0874	Melampyrum pratense L.	0	n	1	0	Orobanchaceae Vent.
Iz_3_1009	DJI_0874	Oenothera biennis L.	0	a	0	0	Onagraceae Juss.
Iz_3_1009	DJI_0874	Pentanema britannica (L.) D.Gut.Larr., Santos-Vicente, Anderb., E.Rico & M.M.Mart.Ort.	0	n	1	0	Asteraceae Bercht. & J.Presl
Iz_3_1009	DJI_0874	Peucedanum oreoselinum (L.) Moench	2	n	1	0	Apiaceae Lindl.
Iz_3_1009	DJI_0874	Peucedanum ruthenicum M.Bieb.	0	n	1	0	Apiaceae Lindl.
Iz_3_1009	DJI_0874	Pilosella echioides (Lumn.) F.W.Schultz & Sch.Bip.	0	n	1	0	Asteraceae Bercht. & J.Presl
Iz_3_1009	DJI_0874	Pilosella officinarum F. Schultz et Sch. Bip.	0	n	1	0	Asteraceae Bercht. & J.Presl
Iz_3_1009	DJI_0874	Pilosella sp.	0		NA	0	Asteraceae Bercht. & J.Presl
Iz_3_1009	DJI_0874	Pinus sylvestris L.	0	n	1	1	Pinaceae Spreng. ex F.Rudolphi
Iz_3_1009	DJI_0874	Poa annua L.	0	n	1	0	Poaceae Barnhart
Iz_3_1009	DJI_0874	Poa nemoralis L.	0	n	1	0	Poaceae Barnhart
Iz_3_1009	DJI_0874	Poa sp.	0		NA	0	Poaceae Barnhart
Iz_3_1009	DJI_0874	Polygonatum odoratum (Mill.) Druce	0	n	1	0	Asparagaceae Juss.
Iz_3_1009	DJI_0874	Polygonum arenarium Waldst. & Kit.	0	n	1	0	Polygonaceae Juss.
Iz_3_1009	DJI_0874	Populus nigra L.	0	n	1	1	Salicaceae Mirb.
Iz_3_1009	DJI_0874	Populus tremula L.	0	n	1	1	Salicaceae Mirb.
Iz_3_1009	DJI_0874	Populus Ã canescens (Aiton) Sm.	0	n	1	1	Salicaceae Mirb.
Iz_3_1009	DJI_0874	Potentilla argentea L.	0	n	1	0	Rosaceae Juss.
Iz_3_1009	DJI_0874	Psammophiliella muralis (L.) Ikonn.	0	n	1	0	Caryophyllaceae Juss.
Iz_3_1009	DJI_0874	Psephellus sumensis (Kalen.) Greuter	0	n	1	0	Asteraceae Bercht. & J.Presl
Iz_3_1009	DJI_0874	Pulsatilla pratensis (L.) Mill. s.l.	0	n	1	0	Ranunculaceae Juss.
Iz_3_1009	DJI_0874	Pyrus communis L.	0	a	0	1	Rosaceae Juss.
Iz_3_1009	DJI_0874	Quercus robur L.	0	n	1	1	Fagaceae Dumort.
Iz_3_1009	DJI_0874	Robinia pseudoacacia L.	0	a	0	1	Fabaceae Lindl.
Iz_3_1009	DJI_0874	Rosa sp.	0		NA	1	Rosaceae Juss.
Iz_3_1009	DJI_0874	Rubus idaeus L.	0	n	1	0	Rosaceae Juss.
Iz_3_1009	DJI_0874	Rumex acetosella L.	0	n	1	0	Polygonaceae Juss.
Iz_3_1009	DJI_0874	Rumex thyrsiflorus Fingerh.	0	n	1	0	Polygonaceae Juss.
Iz_3_1009	DJI_0874	Salix alba L.	0	n	1	1	Salicaceae Mirb.
Iz_3_1009	DJI_0874	Sambucus nigra L.	0	n	1	1	Viburnaceae Raf.
Iz_3_1009	DJI_0874	Secale sylvestre Host	0	n	1	0	Poaceae Barnhart
Iz_3_1009	DJI_0874	Sempervivum ruthenicum Schnittsp. & C.B. Lehm.	0	n	1	0	Crassulaceae J.St.-Hil.
Iz_3_1009	DJI_0874	Setaria viridis (L.) P. Beauv.	0	n	1	0	Poaceae Barnhart
Iz_3_1009	DJI_0874	Silene borysthenica (Gruner) Walters	0	n	1	0	Caryophyllaceae Juss.
Iz_3_1009	DJI_0874	Silene latifolia subsp. alba (Mill.) Greuter & Burdet	0	n	1	0	Caryophyllaceae Juss.
Iz_3_1009	DJI_0874	Solidago virgaurea L.	0	n	1	0	Asteraceae Bercht. & J.Presl
Iz_3_1009	DJI_0874	Sonchus oleraceus L.	0	a	0	0	Asteraceae Bercht. & J.Presl
Iz_3_1009	DJI_0874	Sorbus aucuparia L.	0	n	1	1	Rosaceae Juss.
Iz_3_1009	DJI_0874	Stachys recta L.	0	n	1	0	Lamiacea Lindl.
Iz_3_1009	DJI_0874	Tanacetum vulgare L.	0	n	1	0	Asteraceae Bercht. & J.Presl
Iz_3_1009	DJI_0874	Thymus pallasianus Heinr.Braun	0	n	1	0	Lamiacea Lindl.
Iz_3_1009	DJI_0874	Tilia cordata Mill.	0	n	1	1	Malvaceae Juss.
Iz_3_1009	DJI_0874	Trifolium alpestre L.	0	n	1	0	Fabaceae Lindl.
Iz_3_1009	DJI_0874	Trifolium arvense L.	0	n	1	0	Fabaceae Lindl.
Iz_3_1009	DJI_0874	Ulmus glabra Huds.	0	n	1	1	Ulmaceae Mirb.
Iz_3_1009	DJI_0874	Verbascum densiflorum Bertol.	0	n	1	0	Scrophulariaceae Juss.
Iz_3_1009	DJI_0874	Veronica austriaca L.	0	n	1	0	Plantaginaceae Juss.
Iz_3_1009	DJI_0874	Veronica incana L.	0	n	1	0	Plantaginaceae Juss.
Iz_3_1009	DJI_0874	Veronica verna L.	0	n	1	0	Plantaginaceae Juss.
Iz_3_1009	DJI_0874	Vincetoxicum hirundinaria Medik	0	n	1	0	Apocynaceae Juss.
Iz_3_1009	DJI_0875	Acer tataricum L.	0	n	1	1	Sapindaceae Juss.
Iz_3_1009	DJI_0875	Achillea millefolium L. s.l.	0	n	1	0	Asteraceae Bercht. & J.Presl
Iz_3_1009	DJI_0875	Allium oleraceum L.	0	n	1	0	Amaryllidaceae J.St.-Hil.
Iz_3_1009	DJI_0875	Ambrosia artemisiifolia L.	0	a	0	0	Asteraceae Bercht. & J.Presl
Iz_3_1009	DJI_0875	Amorpha fruticosa L.	0	a	0	0	Fabaceae Lindl.
Iz_3_1009	DJI_0875	Anthericum ramosum L.	0	n	1	0	Asparagaceae Juss.
Iz_3_1009	DJI_0875	Artemisia marschalliana Spreng.	0	n	1	0	Asteraceae Bercht. & J.Presl
Iz_3_1009	DJI_0875	Asparagus officinalis L.	0	n	1	0	Asparagaceae Juss.
Iz_3_1009	DJI_0875	Astragalus cicer L.	0	n	1	0	Fabaceae Lindl.
Iz_3_1009	DJI_0875	Bassia laniflora (S.G.Gmel.) A.J.Scott	0	n	1	0	Amaranthaceae Juss.
Iz_3_1009	DJI_0875	Berberis vulgaris L.	0	n	1	0	Berberidaceae Juss.
Iz_3_1009	DJI_0875	Betula pendula Roth.	2	n	1	1	Betulaceae Gray
Iz_3_1009	DJI_0875	Betula pendula Roth.	2	n	1	0	Poaceae Barnhart
Iz_3_1009	DJI_0875	Bromus inermis Leyss.	0		NA	NA	
Iz_3_1009	DJI_0875	Bromus squarrosus L.	0	a	0	0	Poaceae Barnhart
Iz_3_1009	DJI_0875	Bromus tectorum L.	0	a	0	0	Poaceae Barnhart
Iz_3_1009	DJI_0875	Calamagrostis epigejos (L.) Roth	15	n	1	0	Poaceae Barnhart
Iz_3_1009	DJI_0875	Carduus acanthoides L.	0	n	1	0	Asteraceae Bercht. & J.Presl
Iz_3_1009	DJI_0875	Carex sp.	0	n	1	0	Cyperaceae Juss.
Iz_3_1009	DJI_0875	Centaurea majorovii Dumbadze	0	n	1	0	Asteraceae Bercht. & J.Presl
Iz_3_1009	DJI_0875	Chamaecytisus ruthenicus (Fisch.ex Wol.) Klaskova	4	n	1	0	Fabaceae Lindl.
Iz_3_1009	DJI_0875	Chelidonium majus L.	1	n	1	0	Papaveraceae Juss.
Iz_3_1009	DJI_0875	Chenopodium betaceum Andrz.	0	n	1	0	Amaranthaceae Juss.
Iz_3_1009	DJI_0875	Chenopodium cf. album	0	n	1	0	Amaranthaceae Juss.
Iz_3_1009	DJI_0875	Chenopodium sp.	0		NA	0	Amaranthaceae Juss.
Iz_3_1009	DJI_0875	Chondrilla juncea L.	0	n	1	0	Asteraceae Bercht. & J.Presl

Iz_3_1009	DJI_0875	Cirsium vulgare (Savi) Ten.	0	n	1	0	Asteraceae Bercht. & J.Presl
Iz_3_1009	DJI_0875	Clinopodium acinos (L.) Kuntze	0	n	1	0	Lamiacea Lindl.
Iz_3_1009	DJI_0875	Convallaria majalis L.	0	n	1	0	Asparagaceae Juss.
Iz_3_1009	DJI_0875	Crataegus rhipidophylla Gand.	0	n	1	1	Rosaceae Juss.
Iz_3_1009	DJI_0875	Crepis foetida L.	0	n	1	0	Asteraceae Bercht. & J.Presl
Iz_3_1009	DJI_0875	Dactylis glomerata L.	0	n	1	0	Poaceae Barnhart
Iz_3_1009	DJI_0875	Dianthus campestris M.Bieb.	0	n	1	0	Caryophyllaceae Juss.
Iz_3_1009	DJI_0875	Digitaria sanguinalis (L.) Scop.	0	a	0	0	Poaceae Barnhart
Iz_3_1009	DJI_0875	Elymus repens (L.) Gould	0	n	1	0	Poaceae Barnhart
Iz_3_1009	DJI_0875	Epilobium tetragonum L.	0	n	1	0	Onagraceae Juss.
Iz_3_1009	DJI_0875	Eragrostis minor Host	0	a	0	0	Poaceae Barnhart
Iz_3_1009	DJI_0875	Erigeron canadensis L.	0	a	0	0	Asteraceae Bercht. & J.Presl
Iz_3_1009	DJI_0875	Euphorbia seguieriana Neck.	0	n	1	0	Euphorbiaceae Juss.
Iz_3_1009	DJI_0875	Fallopia dumetorum (L.) Holub	0	n	1	0	Polygonaceae Juss.
Iz_3_1009	DJI_0875	Festuca beckeri (Hack.) Trautv.	0	n	1	0	Poaceae Barnhart
Iz_3_1009	DJI_0875	Fragaria vesca L.	0	n	1	0	Rosaceae Juss.
Iz_3_1009	DJI_0875	Frangula alnus Mill.	0	n	1	1	Rhamnaceae Juss.
Iz_3_1009	DJI_0875	Fraxinus pennsylvanica Marshall	0	a	0	1	Oleaceae Hoffmanns. & Link
Iz_3_1009	DJI_0875	Galeopsis bifida Boenn.	0	n	1	0	Lamiacea Lindl.
Iz_3_1009	DJI_0875	Galium verum L.	0	n	1	0	Rubiaceae Juss.
Iz_3_1009	DJI_0875	Genista tinctoria L.	0	n	1	0	Fabaceae Lindl.
Iz_3_1009	DJI_0875	Geranium robertianum L.	0	n	1	0	Geraniaceae Juss.
Iz_3_1009	DJI_0875	Geranium sanquineum L.	0	n	1	0	Geraniaceae Juss.
Iz_3_1009	DJI_0875	Gypsophila paniculata L.	0	n	1	0	Caryophyllaceae Juss.
Iz_3_1009	DJI_0875	Helichrysum arenarium (L.) Moench	0	n	1	0	Asteraceae Bercht. & J.Presl
Iz_3_1009	DJI_0875	Herniaria polygama J.Gay	0	n	1	0	Caryophyllaceae Juss.
Iz_3_1009	DJI_0875	Hieracium robustum Fr.	0	n	1	0	Asteraceae Bercht. & J.Presl
Iz_3_1009	DJI_0875	Hieracium umbellatum L.	0	n	1	NA	Asteraceae Bercht. & J.Presl
Iz_3_1009	DJI_0875	Hylotelephium maximum (L.) Holub	0	n	1	0	Crassulaceae J.St.-Hil.
Iz_3_1009	DJI_0875	Jacobaea borysthenica (DC.) B.Nord. & Greuter	0	n	1	0	Asteraceae Bercht. & J.Presl
Iz_3_1009	DJI_0875	Jasione montana L.	0	n	1	0	Campanulaceae Juss.
Iz_3_1009	DJI_0875	Jurinea cyanoides (L.) Rchb.s.l.	0	n	1	0	Asteraceae Bercht. & J.Presl
Iz_3_1009	DJI_0875	Koeleria glauca (Spreng.) DC.	0	n	1	0	Poaceae Barnhart
Iz_3_1009	DJI_0875	Lactuca serriola L.	0	a	0	0	Asteraceae Bercht. & J.Presl
Iz_3_1009	DJI_0875	Linaria genistifolia (L.) Mill.	0	n	1	0	Plantaginaceae Juss.
Iz_3_1009	DJI_0875	Lomelosia argentea (L.) Greuter & Burdet	0	n	1	0	Caprifoliaceae Juss.
Iz_3_1009	DJI_0875	Melampyrum pratense L.	0	n	1	0	Orobanchaceae Vent.
Iz_3_1009	DJI_0875	Oenothera biennis L.	0	a	0	0	Onagraceae Juss.
Iz_3_1009	DJI_0875	Pentanema britannica (L.) D.Gut.Larr., Santos-Vicente, Anderb., E.Rico & M.M.Mart.Ort.	0	n	1	0	Asteraceae Bercht. & J.Presl
Iz_3_1009	DJI_0875	Peucedanum oreoselinum (L.) Moench	5	n	1	0	Apiaceae Lindl.
Iz_3_1009	DJI_0875	Peucedanum ruthenicum M.Bieb.	0	n	1	0	Apiaceae Lindl.
Iz_3_1009	DJI_0875	Pilosella echioides (Lumn.) F.W.Schultz & Sch.Bip.	0	n	1	0	Asteraceae Bercht. & J.Presl
Iz_3_1009	DJI_0875	Pilosella officinarum F. Schultz et Sch. Bip.	0	n	1	0	Asteraceae Bercht. & J.Presl
Iz_3_1009	DJI_0875	Pilosella sp.	0		NA	0	Asteraceae Bercht. & J.Presl
Iz_3_1009	DJI_0875	Pinus sylvestris L.	0	n	1	1	Pinaceae Spreng. ex F.Rudolphi
Iz_3_1009	DJI_0875	Poa annua L.	0	n	1	0	Poaceae Barnhart
Iz_3_1009	DJI_0875	Poa nemoralis L.	0	n	1	0	Poaceae Barnhart
Iz_3_1009	DJI_0875	Poa sp.	0		NA	0	Poaceae Barnhart
Iz_3_1009	DJI_0875	Polygonatum odoratum (Mill.) Druce	0	n	1	0	Asparagaceae Juss.
Iz_3_1009	DJI_0875	Polygonum arenarium Waldst. & Kit.	0	n	1	0	Polygonaceae Juss.
Iz_3_1009	DJI_0875	Populus nigra L.	0	n	1	1	Salicaceae Mirb.
Iz_3_1009	DJI_0875	Populus tremula L.	0	n	1	1	Salicaceae Mirb.
Iz_3_1009	DJI_0875	Populus Ã canescens (Aiton) Sm.	0	n	1	1	Salicaceae Mirb.
Iz_3_1009	DJI_0875	Potentilla argentea L.	0	n	1	0	Rosaceae Juss.
Iz_3_1009	DJI_0875	Psammophiliella muralis (L.) Ikonn.	0	n	1	0	Caryophyllaceae Juss.
Iz_3_1009	DJI_0875	Psephellus sumensis (Kalen.) Greuter	0.5	n	1	0	Asteraceae Bercht. & J.Presl
Iz_3_1009	DJI_0875	Pulsatilla pratensis (L.) Mill. s.l.	0	n	1	0	Ranunculaceae Juss.
Iz_3_1009	DJI_0875	Pyrus communis L.	0	a	0	1	Rosaceae Juss.
Iz_3_1009	DJI_0875	Quercus robur L.	0	n	1	1	Fagaceae Dumort.
Iz_3_1009	DJI_0875	Robinia pseudoacacia L.	0	a	0	1	Fabaceae Lindl.
Iz_3_1009	DJI_0875	Rosa sp.	0		NA	1	Rosaceae Juss.
Iz_3_1009	DJI_0875	Rubus idaeus L.	0	n	1	0	Rosaceae Juss.
Iz_3_1009	DJI_0875	Rumex acetosella L.	0	n	1	0	Polygonaceae Juss.
Iz_3_1009	DJI_0875	Rumex thyrsiflorus Fingerh.	0	n	1	0	Polygonaceae Juss.
Iz_3_1009	DJI_0875	Salix alba L.	0	n	1	1	Salicaceae Mirb.
Iz_3_1009	DJI_0875	Sambucus nigra L.	0	n	1	1	Viburnaceae Raf.
Iz_3_1009	DJI_0875	Secale sylvestre Host	0	n	1	0	Poaceae Barnhart
Iz_3_1009	DJI_0875	Sempervivum ruthenicum Schnittsp. & C.B. Lehm.	0	n	1	0	Crassulaceae J.St.-Hil.
Iz_3_1009	DJI_0875	Setaria viridis (L.) P. Beauv.	0	n	1	0	Poaceae Barnhart
Iz_3_1009	DJI_0875	Silene borysthenica (Gruner) Walters	0	n	1	0	Caryophyllaceae Juss.
Iz_3_1009	DJI_0875	Silene latifolia subsp. alba (Mill.) Greuter & Burdet	0	n	1	0	Caryophyllaceae Juss.
Iz_3_1009	DJI_0875	Solidago virgaurea L.	0	n	1	0	Asteraceae Bercht. & J.Presl
Iz_3_1009	DJI_0875	Sonchus oleraceus L.	0	a	0	0	Asteraceae Bercht. & J.Presl
Iz_3_1009	DJI_0875	Sorbus aucuparia L.	0	n	1	1	Rosaceae Juss.
Iz_3_1009	DJI_0875	Stachys recta L.	0	n	1	0	Lamiacea Lindl.
Iz_3_1009	DJI_0875	Tanacetum vulgare L.	0	n	1	0	Asteraceae Bercht. & J.Presl
Iz_3_1009	DJI_0875	Thymus pallasianus Heinr.Braun	0	n	1	0	Lamiacea Lindl.
Iz_3_1009	DJI_0875	Tilia cordata Mill.	0	n	1	1	Malvaceae Juss.
Iz_3_1009	DJI_0875	Trifolium alpestre L.	0	n	1	0	Fabaceae Lindl.
Iz_3_1009	DJI_0875	Trifolium arvense L.	0	n	1	0	Fabaceae Lindl.
Iz_3_1009	DJI_0875	Ulmus glabra Huds.	0	n	1	1	Ulmaceae Mirb.
Iz_3_1009	DJI_0875	Verbascum densiflorum Bertol.	0	n	1	0	Scrophulariaceae Juss.
Iz_3_1009	DJI_0875	Veronica austriaca L.	0	n	1	0	Plantaginaceae Juss.
Iz_3_1009	DJI_0875	Veronica incana L.	0	n	1	0	Plantaginaceae Juss.
Iz_3_1009	DJI_0875	Veronica verna L.	0	n	1	0	Plantaginaceae Juss.
Iz_3_1009	DJI_0875	Vincetoxicum hirundinaria Medik	0	n	1	0	Apocynaceae Juss.
Iz_3_1009	DJI_0876	Acer tataricum L.	2	n	1	1	Sapindaceae Juss.
Iz_3_1009	DJI_0876	Achillea millefolium L. s.l.	0	n	1	0	Asteraceae Bercht. & J.Presl
Iz_3_1009	DJI_0876	Allium oleraceum L.	0	n	1	0	Amaryllidaceae J.St.-Hil.
Iz_3_1009	DJI_0876	Ambrosia artemisiifolia L.	0	a	0	0	Asteraceae Bercht. & J.Presl
Iz_3_1009	DJI_0876	Amorpha fruticosa L.	0	a	0	0	Fabaceae Lindl.
Iz_3_1009	DJI_0876	Anthericum ramosum L.	0	n	1	0	Asparagaceae Juss.
Iz_3_1009	DJI_0876	Artemisia marschalliana Spreng.	0	n	1	0	Asteraceae Bercht. & J.Presl
Iz_3_1009	DJI_0876	Asparagus officinalis L.	0	n	1	0	Asparagaceae Juss.
Iz_3_1009	DJI_0876	Astragalus cicer L.	0	n	1	0	Fabaceae Lindl.
Iz_3_1009	DJI_0876	Bassia laniflora (S.G.Gmel.) A.J.Scott	0	n	1	0	Amaranthaceae Juss.
Iz_3_1009	DJI_0876	Berberis vulgaris L.	0	n	1	0	Berberidaceae Juss.
Iz_3_1009	DJI_0876	Betula pendula Roth.	0	n	1	1	Betulaceae Gray

Iz_3_1009	DJI_0876	Betula pendula Roth.	0	n	1	0	Poaceae Barnhart
Iz_3_1009	DJI_0876	Bromus inermis Leyss.	0		NA	NA	
Iz_3_1009	DJI_0876	Bromus squarrosus L.	0	a	0	0	Poaceae Barnhart
Iz_3_1009	DJI_0876	Bromus tectorum L.	0	a	0	0	Poaceae Barnhart
Iz_3_1009	DJI_0876	Calamagrostis epigejos (L.) Roth	10	n	1	0	Poaceae Barnhart
Iz_3_1009	DJI_0876	Carduus acanthoides L.	0	n	1	0	Asteraceae Bercht. & J.Presl
Iz_3_1009	DJI_0876	Carex sp.	0	n	1	0	Cyperaceae Juss.
Iz_3_1009	DJI_0876	Centaurea majorovii Dumbadze	0	n	1	0	Asteraceae Bercht. & J.Presl
Iz_3_1009	DJI_0876	Chamaecytisus ruthenicus (Fisch.ex Wol.) Klaskova	5	n	1	0	Fabaceae Lindl.
Iz_3_1009	DJI_0876	Chelidonium majus L.	0	n	1	0	Papaveraceae Juss.
Iz_3_1009	DJI_0876	Chenopodium betaceum Andrz.	0	n	1	0	Amaranthaceae Juss.
Iz_3_1009	DJI_0876	Chenopodium cf. album	0	n	1	0	Amaranthaceae Juss.
Iz_3_1009	DJI_0876	Chenopodium sp.	0		NA	0	Amaranthaceae Juss.
Iz_3_1009	DJI_0876	Chondrilla juncea L.	0	n	1	0	Asteraceae Bercht. & J.Presl
Iz_3_1009	DJI_0876	Cirsium vulgare (Savi) Ten.	0	n	1	0	Asteraceae Bercht. & J.Presl
Iz_3_1009	DJI_0876	Clinopodium acinos (L.) Kuntze	0	n	1	0	Lamiacea Lindl.
Iz_3_1009	DJI_0876	Convallaria majalis L.	0	n	1	0	Asparagaceae Juss.
Iz_3_1009	DJI_0876	Crataegus rhipidophylla Gand.	0	n	1	1	Rosaceae Juss.
Iz_3_1009	DJI_0876	Crepis foetida L.	0	n	1	0	Asteraceae Bercht. & J.Presl
Iz_3_1009	DJI_0876	Dactylis glomerata L.	0	n	1	0	Poaceae Barnhart
Iz_3_1009	DJI_0876	Dianthus campestris M.Bieb.	1	n	1	0	Caryophyllaceae Juss.
Iz_3_1009	DJI_0876	Digitaria sanguinalis (L.) Scop.	0	a	0	0	Poaceae Barnhart
Iz_3_1009	DJI_0876	Elymus repens (L.) Gould	0	n	1	0	Poaceae Barnhart
Iz_3_1009	DJI_0876	Epilobium tetragonum L.	0.2	n	1	0	Onagraceae Juss.
Iz_3_1009	DJI_0876	Eragrostis minor Host	0	a	0	0	Poaceae Barnhart
Iz_3_1009	DJI_0876	Erigeron canadensis L.	0	a	0	0	Asteraceae Bercht. & J.Presl
Iz_3_1009	DJI_0876	Euphorbia seguieriana Neck.	0	n	1	0	Euphorbiaceae Juss.
Iz_3_1009	DJI_0876	Fallopia dumetorum (L.) Holub	0	n	1	0	Polygonaceae Juss.
Iz_3_1009	DJI_0876	Festuca beckeri (Hack.) Trautv.	0	n	1	0	Poaceae Barnhart
Iz_3_1009	DJI_0876	Fragaria vesca L.	0	n	1	0	Rosaceae Juss.
Iz_3_1009	DJI_0876	Frangula alnus Mill.	0	n	1	1	Rhamnaceae Juss.
Iz_3_1009	DJI_0876	Fraxinus pennsylvanica Marshall	0	a	0	1	Oleaceae Hoffmanns. & Link
Iz_3_1009	DJI_0876	Galeopsis bifida Boenn.	0	n	1	0	Lamiacea Lindl.
Iz_3_1009	DJI_0876	Galium verum L.	0	n	1	0	Rubiaceae Juss.
Iz_3_1009	DJI_0876	Genista tinctoria L.	0	n	1	0	Fabaceae Lindl.
Iz_3_1009	DJI_0876	Geranium robertianum L.	0	n	1	0	Geraniaceae Juss.
Iz_3_1009	DJI_0876	Geranium sanquineum L.	0	n	1	0	Geraniaceae Juss.
Iz_3_1009	DJI_0876	Gypsophila paniculata L.	0	n	1	0	Caryophyllaceae Juss.
Iz_3_1009	DJI_0876	Helichrysum arenarium (L.) Moench	0	n	1	0	Asteraceae Bercht. & J.Presl
Iz_3_1009	DJI_0876	Herniaria polygama J.Gay	0	n	1	0	Caryophyllaceae Juss.
Iz_3_1009	DJI_0876	Hieracium robustum Fr.	0	n	1	0	Asteraceae Bercht. & J.Presl
Iz_3_1009	DJI_0876	Hieracium umbellatum L.	0	n	1	NA	Asteraceae Bercht. & J.Presl
Iz_3_1009	DJI_0876	Hylotelephium maximum (L.) Holub	0	n	1	0	Crassulaceae J.St.-Hil.
Iz_3_1009	DJI_0876	Jacobaea borysthenica (DC.) B.Nord. & Greuter	0	n	1	0	Asteraceae Bercht. & J.Presl
Iz_3_1009	DJI_0876	Jasione montana L.	0	n	1	0	Campanulaceae Juss.
Iz_3_1009	DJI_0876	Jurinea cyanoides (L.) Rchb.s.l.	0	n	1	0	Asteraceae Bercht. & J.Presl
Iz_3_1009	DJI_0876	Koeleria glauca (Spreng.) DC.	0	n	1	0	Poaceae Barnhart
Iz_3_1009	DJI_0876	Lactuca serriola L.	0	a	0	0	Asteraceae Bercht. & J.Presl
Iz_3_1009	DJI_0876	Linaria genistifolia (L.) Mill.	0	n	1	0	Plantaginaceae Juss.
Iz_3_1009	DJI_0876	Lomelosia argentea (L.) Greuter & Burdet	0	n	1	0	Caprifoliaceae Juss.
Iz_3_1009	DJI_0876	Melampyrum pratense L.	0	n	1	0	Orobanchaceae Vent.
Iz_3_1009	DJI_0876	Oenothera biennis L.	0	a	0	0	Onagraceae Juss.
Iz_3_1009	DJI_0876	Pentanema britannica (L.) D.Gut.Larr., Santos-Vicente, Anderb., E.Rico & M.M.Mart.Ort.	0	n	1	0	Asteraceae Bercht. & J.Presl
Iz_3_1009	DJI_0876	Peucedanum oreoselinum (L.) Moench	10	n	1	0	Apiaceae Lindl.
Iz_3_1009	DJI_0876	Peucedanum ruthenicum M.Bieb.	0	n	1	0	Apiaceae Lindl.
Iz_3_1009	DJI_0876	Pilosella echioides (Lumn.) F.W.Schultz & Sch.Bip.	0	n	1	0	Asteraceae Bercht. & J.Presl
Iz_3_1009	DJI_0876	Pilosella officinarum F. Schultz et Sch. Bip.	0	n	1	0	Asteraceae Bercht. & J.Presl
Iz_3_1009	DJI_0876	Pilosella sp.	0		NA	0	Asteraceae Bercht. & J.Presl
Iz_3_1009	DJI_0876	Pinus sylvestris L.	0	n	1	1	Pinaceae Spreng. ex F.Rudolphi
Iz_3_1009	DJI_0876	Poa annua L.	0	n	1	0	Poaceae Barnhart
Iz_3_1009	DJI_0876	Poa nemoralis L.	0	n	1	0	Poaceae Barnhart
Iz_3_1009	DJI_0876	Poa sp.	0		NA	0	Poaceae Barnhart
Iz_3_1009	DJI_0876	Polygonatum odoratum (Mill.) Druce	0	n	1	0	Asparagaceae Juss.
Iz_3_1009	DJI_0876	Polygonum arenarium Waldst. & Kit.	0	n	1	0	Polygonaceae Juss.
Iz_3_1009	DJI_0876	Populus nigra L.	0	n	1	1	Salicaceae Mirb.
Iz_3_1009	DJI_0876	Populus tremula L.	0	n	1	1	Salicaceae Mirb.
Iz_3_1009	DJI_0876	Populus Ã canescens (Aiton) Sm.	0	n	1	1	Salicaceae Mirb.
Iz_3_1009	DJI_0876	Potentilla argentea L.	0	n	1	0	Rosaceae Juss.
Iz_3_1009	DJI_0876	Psammophiliella muralis (L.) Ikonn.	0	n	1	0	Caryophyllaceae Juss.
Iz_3_1009	DJI_0876	Psephellus sumensis (Kalen.) Greuter	0	n	1	0	Asteraceae Bercht. & J.Presl
Iz_3_1009	DJI_0876	Pulsatilla pratensis (L.) Mill. s.l.	0	n	1	0	Ranunculaceae Juss.
Iz_3_1009	DJI_0876	Pyrus communis L.	0	a	0	1	Rosaceae Juss.
Iz_3_1009	DJI_0876	Quercus robur L.	0	n	1	1	Fagaceae Dumort.
Iz_3_1009	DJI_0876	Robinia pseudoacacia L.	0	a	0	1	Fabaceae Lindl.
Iz_3_1009	DJI_0876	Rosa sp.	0		NA	1	Rosaceae Juss.
Iz_3_1009	DJI_0876	Rubus idaeus L.	0	n	1	0	Rosaceae Juss.
Iz_3_1009	DJI_0876	Rumex acetosella L.	0	n	1	0	Polygonaceae Juss.
Iz_3_1009	DJI_0876	Rumex thyrsiflorus Fingerh.	0	n	1	0	Polygonaceae Juss.
Iz_3_1009	DJI_0876	Salix alba L.	0	n	1	1	Salicaceae Mirb.
Iz_3_1009	DJI_0876	Sambucus nigra L.	0	n	1	1	Viburnaceae Raf.
Iz_3_1009	DJI_0876	Secale sylvestre Host	0	n	1	0	Poaceae Barnhart
Iz_3_1009	DJI_0876	Sempervivum ruthenicum Schnittsp. & C.B. Lehm.	0	n	1	0	Crassulaceae J.St.-Hil.
Iz_3_1009	DJI_0876	Setaria viridis (L.) P. Beauv.	0	n	1	0	Poaceae Barnhart
Iz_3_1009	DJI_0876	Silene borysthenica (Gruner) Walters	0	n	1	0	Caryophyllaceae Juss.
Iz_3_1009	DJI_0876	Silene latifolia subsp. alba (Mill.) Greuter & Burdet	0	n	1	0	Caryophyllaceae Juss.
Iz_3_1009	DJI_0876	Solidago virgaurea L.	1	n	1	0	Asteraceae Bercht. & J.Presl
Iz_3_1009	DJI_0876	Sonchus oleraceus L.	0	a	0	0	Asteraceae Bercht. & J.Presl
Iz_3_1009	DJI_0876	Sorbus aucuparia L.	0	n	1	1	Rosaceae Juss.
Iz_3_1009	DJI_0876	Stachys recta L.	0	n	1	0	Lamiacea Lindl.
Iz_3_1009	DJI_0876	Tanacetum vulgare L.	0	n	1	0	Asteraceae Bercht. & J.Presl
Iz_3_1009	DJI_0876	Thymus pallasianus Heinr.Braun	0	n	1	0	Lamiacea Lindl.
Iz_3_1009	DJI_0876	Tilia cordata Mill.	0	n	1	1	Malvaceae Juss.
Iz_3_1009	DJI_0876	Trifolium alpestre L.	0	n	1	0	Fabaceae Lindl.
Iz_3_1009	DJI_0876	Trifolium arvense L.	0	n	1	0	Fabaceae Lindl.
Iz_3_1009	DJI_0876	Ulmus glabra Huds.	0	n	1	1	Ulmaceae Mirb.
Iz_3_1009	DJI_0876	Verbascum densiflorum Bertol.	0	n	1	0	Scrophulariaceae Juss.
Iz_3_1009	DJI_0876	Veronica austriaca L.	0	n	1	0	Plantaginaceae Juss.
Iz_3_1009	DJI_0876	Veronica incana L.	0	n	1	0	Plantaginaceae Juss.

Iz_3_1009	DJI_0876	Veronica verna L.	0	n	1	0	Plantaginaceae Juss.
Iz_3_1009	DJI_0876	Vincetoxicum hirundinaria Medik	0	n	1	0	Apocynaceae Juss.
Iz_3_1009	DJI_0880	Acer tataricum L.	3	n	1	1	Sapindaceae Juss.
Iz_3_1009	DJI_0880	Achillea millefolium L. s.l.	0	n	1	0	Asteraceae Bercht. & J.Presl
Iz_3_1009	DJI_0880	Allium oleraceum L.	0	n	1	0	Amaryllidaceae J.St.-Hil.
Iz_3_1009	DJI_0880	Ambrosia artemisiifolia L.	0	a	0	0	Asteraceae Bercht. & J.Presl
Iz_3_1009	DJI_0880	Amorpha fruticosa L.	0	a	0	0	Fabaceae Lindl.
Iz_3_1009	DJI_0880	Anthericum ramosum L.	0	n	1	0	Asparagaceae Juss.
Iz_3_1009	DJI_0880	Artemisia marschalliana Spreng.	0	n	1	0	Asteraceae Bercht. & J.Presl
Iz_3_1009	DJI_0880	Asparagus officinalis L.	0	n	1	0	Asparagaceae Juss.
Iz_3_1009	DJI_0880	Astragalus cicer L.	0	n	1	0	Fabaceae Lindl.
Iz_3_1009	DJI_0880	Bassia laniflora (S.G.Gmel.) A.J.Scott	0	n	1	0	Amaranthaceae Juss.
Iz_3_1009	DJI_0880	Berberis vulgaris L.	0	n	1	0	Berberidaceae Juss.
Iz_3_1009	DJI_0880	Betula pendula Roth.	0	n	1	1	Betulaceae Gray
Iz_3_1009	DJI_0880	Betula pendula Roth.	0	n	1	0	Poaceae Barnhart
Iz_3_1009	DJI_0880	Bromus inermis Leyss.	0		NA	NA	
Iz_3_1009	DJI_0880	Bromus squarrosus L.	0	a	0	0	Poaceae Barnhart
Iz_3_1009	DJI_0880	Bromus tectorum L.	0	a	0	0	Poaceae Barnhart
Iz_3_1009	DJI_0880	Calamagrostis epigejos (L.) Roth	8	n	1	0	Poaceae Barnhart
Iz_3_1009	DJI_0880	Carduus acanthoides L.	0	n	1	0	Asteraceae Bercht. & J.Presl
Iz_3_1009	DJI_0880	Carex sp.	0	n	1	0	Cyperaceae Juss.
Iz_3_1009	DJI_0880	Centaurea majorovii Dumbadze	0	n	1	0	Asteraceae Bercht. & J.Presl
Iz_3_1009	DJI_0880	Chamaecytisus ruthenicus (Fisch.ex Wol.) Klaskova	0	n	1	0	Fabaceae Lindl.
Iz_3_1009	DJI_0880	Chelidonium majus L.	0	n	1	0	Papaveraceae Juss.
Iz_3_1009	DJI_0880	Chenopodium betaceum Andrz.	0	n	1	0	Amaranthaceae Juss.
Iz_3_1009	DJI_0880	Chenopodium cf. album	0	n	1	0	Amaranthaceae Juss.
Iz_3_1009	DJI_0880	Chenopodium sp.	0		NA	0	Amaranthaceae Juss.
Iz_3_1009	DJI_0880	Chondrilla juncea L.	0	n	1	0	Asteraceae Bercht. & J.Presl
Iz_3_1009	DJI_0880	Cirsium vulgare (Savi) Ten.	0	n	1	0	Asteraceae Bercht. & J.Presl
Iz_3_1009	DJI_0880	Clinopodium acinos (L.) Kuntze	0	n	1	0	Lamiacea Lindl.
Iz_3_1009	DJI_0880	Convallaria majalis L.	0	n	1	0	Asparagaceae Juss.
Iz_3_1009	DJI_0880	Crataegus rhipidophylla Gand.	0	n	1	1	Rosaceae Juss.
Iz_3_1009	DJI_0880	Crepis foetida L.	0	n	1	0	Asteraceae Bercht. & J.Presl
Iz_3_1009	DJI_0880	Dactylis glomerata L.	0	n	1	0	Poaceae Barnhart
Iz_3_1009	DJI_0880	Dianthus campestris M.Bieb.	0	n	1	0	Caryophyllaceae Juss.
Iz_3_1009	DJI_0880	Digitaria sanguinalis (L.) Scop.	0	a	0	0	Poaceae Barnhart
Iz_3_1009	DJI_0880	Elymus repens (L.) Gould	0	n	1	0	Poaceae Barnhart
Iz_3_1009	DJI_0880	Epilobium tetragonum L.	0	n	1	0	Onagraceae Juss.
Iz_3_1009	DJI_0880	Eragrostis minor Host	0	a	0	0	Poaceae Barnhart
Iz_3_1009	DJI_0880	Erigeron canadensis L.	0	a	0	0	Asteraceae Bercht. & J.Presl
Iz_3_1009	DJI_0880	Euphorbia seguieriana Neck.	0	n	1	0	Euphorbiaceae Juss.
Iz_3_1009	DJI_0880	Fallopia dumetorum (L.) Holub	0	n	1	0	Polygonaceae Juss.
Iz_3_1009	DJI_0880	Festuca beckeri (Hack.) Trautv.	0	n	1	0	Poaceae Barnhart
Iz_3_1009	DJI_0880	Fragaria vesca L.	0	n	1	0	Rosaceae Juss.
Iz_3_1009	DJI_0880	Frangula alnus Mill.	0	n	1	1	Rhamnaceae Juss.
Iz_3_1009	DJI_0880	Fraxinus pennsylvanica Marshall	0	a	0	1	Oleaceae Hoffmanns. & Link
Iz_3_1009	DJI_0880	Galeopsis bifida Boenn.	0	n	1	0	Lamiacea Lindl.
Iz_3_1009	DJI_0880	Galium verum L.	0	n	1	0	Rubiaceae Juss.
Iz_3_1009	DJI_0880	Genista tinctoria L.	0	n	1	0	Fabaceae Lindl.
Iz_3_1009	DJI_0880	Geranium robertianum L.	0	n	1	0	Geraniaceae Juss.
Iz_3_1009	DJI_0880	Geranium sanquineum L.	0	n	1	0	Geraniaceae Juss.
Iz_3_1009	DJI_0880	Gypsophila paniculata L.	0	n	1	0	Caryophyllaceae Juss.
Iz_3_1009	DJI_0880	Helichrysum arenarium (L.) Moench	0	n	1	0	Asteraceae Bercht. & J.Presl
Iz_3_1009	DJI_0880	Herniaria polygama J.Gay	0	n	1	0	Caryophyllaceae Juss.
Iz_3_1009	DJI_0880	Hieracium robustum Fr.	0	n	1	0	Asteraceae Bercht. & J.Presl
Iz_3_1009	DJI_0880	Hieracium umbellatum L.	0	n	1	NA	Asteraceae Bercht. & J.Presl
Iz_3_1009	DJI_0880	Hylotelephium maximum (L.) Holub	0	n	1	0	Crassulaceae J.St.-Hil.
Iz_3_1009	DJI_0880	Jacobaea borysthenica (DC.) B.Nord. & Greuter	0	n	1	0	Asteraceae Bercht. & J.Presl
Iz_3_1009	DJI_0880	Jasione montana L.	0	n	1	0	Campanulaceae Juss.
Iz_3_1009	DJI_0880	Jurinea cyanoides (L.) Rchb.s.l.	0	n	1	0	Asteraceae Bercht. & J.Presl
Iz_3_1009	DJI_0880	Koeleria glauca (Spreng.) DC.	0	n	1	0	Poaceae Barnhart
Iz_3_1009	DJI_0880	Lactuca serriola L.	0	a	0	0	Asteraceae Bercht. & J.Presl
Iz_3_1009	DJI_0880	Linaria genistifolia (L.) Mill.	0	n	1	0	Plantaginaceae Juss.
Iz_3_1009	DJI_0880	Lomelosia argentea (L.) Greuter & Burdet	0	n	1	0	Caprifoliaceae Juss.
Iz_3_1009	DJI_0880	Melampyrum pratense L.	0	n	1	0	Orobanchaceae Vent.
Iz_3_1009	DJI_0880	Oenothera biennis L.	0	a	0	0	Onagraceae Juss.
Iz_3_1009	DJI_0880	Pentanema britannica (L.) D.Gut.Larr., Santos-Vicente, Anderb., E.Rico & M.M.Mart.Ort.	0	n	1	0	Asteraceae Bercht. & J.Presl
Iz_3_1009	DJI_0880	Peucedanum oreoselinum (L.) Moench	10	n	1	0	Apiaceae Lindl.
Iz_3_1009	DJI_0880	Peucedanum ruthenicum M.Bieb.	0	n	1	0	Apiaceae Lindl.
Iz_3_1009	DJI_0880	Pilosella echioides (Lumn.) F.W.Schultz & Sch.Bip.	0	n	1	0	Asteraceae Bercht. & J.Presl
Iz_3_1009	DJI_0880	Pilosella officinarum F. Schultz et Sch. Bip.	0	n	1	0	Asteraceae Bercht. & J.Presl
Iz_3_1009	DJI_0880	Pilosella sp.	0		NA	0	Asteraceae Bercht. & J.Presl
Iz_3_1009	DJI_0880	Pinus sylvestris L.	0	n	1	1	Pinaceae Spreng. ex F.Rudolphi
Iz_3_1009	DJI_0880	Poa annua L.	0	n	1	0	Poaceae Barnhart
Iz_3_1009	DJI_0880	Poa nemoralis L.	0	n	1	0	Poaceae Barnhart
Iz_3_1009	DJI_0880	Poa sp.	0		NA	0	Poaceae Barnhart
Iz_3_1009	DJI_0880	Polygonatum odoratum (Mill.) Druce	0	n	1	0	Asparagaceae Juss.
Iz_3_1009	DJI_0880	Polygonum arenarium Waldst. & Kit.	0	n	1	0	Polygonaceae Juss.
Iz_3_1009	DJI_0880	Populus nigra L.	0	n	1	1	Salicaceae Mirb.
Iz_3_1009	DJI_0880	Populus tremula L.	0	n	1	1	Salicaceae Mirb.
Iz_3_1009	DJI_0880	Populus Ã canescens (Aiton) Sm.	0	n	1	1	Salicaceae Mirb.
Iz_3_1009	DJI_0880	Potentilla argentea L.	0	n	1	0	Rosaceae Juss.
Iz_3_1009	DJI_0880	Psammophiliella muralis (L.) Ikonn.	0	n	1	0	Caryophyllaceae Juss.
Iz_3_1009	DJI_0880	Psephellus sumensis (Kalen.) Greuter	0	n	1	0	Asteraceae Bercht. & J.Presl
Iz_3_1009	DJI_0880	Pulsatilla pratensis (L.) Mill. s.l.	0.5	n	1	0	Ranunculaceae Juss.
Iz_3_1009	DJI_0880	Pyrus communis L.	0	a	0	1	Rosaceae Juss.
Iz_3_1009	DJI_0880	Quercus robur L.	0	n	1	1	Fagaceae Dumort.
Iz_3_1009	DJI_0880	Robinia pseudoacacia L.	0	a	0	1	Fabaceae Lindl.
Iz_3_1009	DJI_0880	Rosa sp.	0		NA	1	Rosaceae Juss.
Iz_3_1009	DJI_0880	Rubus idaeus L.	0	n	1	0	Rosaceae Juss.
Iz_3_1009	DJI_0880	Rumex acetosella L.	0	n	1	0	Polygonaceae Juss.
Iz_3_1009	DJI_0880	Rumex thyrsiflorus Fingerh.	0	n	1	0	Polygonaceae Juss.
Iz_3_1009	DJI_0880	Salix alba L.	0	n	1	1	Salicaceae Mirb.
Iz_3_1009	DJI_0880	Sambucus nigra L.	0	n	1	1	Viburnaceae Raf.
Iz_3_1009	DJI_0880	Secale sylvestre Host	0	n	1	0	Poaceae Barnhart
Iz_3_1009	DJI_0880	Sempervivum ruthenicum Schnittsp. & C.B. Lehm.	0	n	1	0	Crassulaceae J.St.-Hil.
Iz_3_1009	DJI_0880	Setaria viridis (L.) P. Beauv.	0	n	1	0	Poaceae Barnhart
Iz_3_1009	DJI_0880	Silene borysthenica (Gruner) Walters	0	n	1	0	Caryophyllaceae Juss.

Iz_3_1009	DJI_0880	Silene latifolia subsp. alba (Mill.) Greuter & Burdet	0	n	1	0	Caryophyllaceae Juss.
Iz_3_1009	DJI_0880	Solidago virgaurea L.	0	n	1	0	Asteraceae Bercht. & J.Presl
Iz_3_1009	DJI_0880	Sonchus oleraceus L.	0	a	0	0	Asteraceae Bercht. & J.Presl
Iz_3_1009	DJI_0880	Sorbus aucuparia L.	0	n	1	1	Rosaceae Juss.
Iz_3_1009	DJI_0880	Stachys recta L.	0	n	1	0	Lamiacea Lindl.
Iz_3_1009	DJI_0880	Tanacetum vulgare L.	0	n	1	0	Asteraceae Bercht. & J.Presl
Iz_3_1009	DJI_0880	Thymus pallasianus Heinr.Braun	0	n	1	0	Lamiacea Lindl.
Iz_3_1009	DJI_0880	Tilia cordata Mill.	0	n	1	1	Malvaceae Juss.
Iz_3_1009	DJI_0880	Trifolium alpestre L.	0	n	1	0	Fabaceae Lindl.
Iz_3_1009	DJI_0880	Trifolium arvense L.	0	n	1	0	Fabaceae Lindl.
Iz_3_1009	DJI_0880	Ulmus glabra Huds.	0	n	1	1	Ulmaceae Mirb.
Iz_3_1009	DJI_0880	Verbascum densiflorum Bertol.	0	n	1	0	Scrophulariaceae Juss.
Iz_3_1009	DJI_0880	Veronica austriaca L.	0	n	1	0	Plantaginaceae Juss.
Iz_3_1009	DJI_0880	Veronica incana L.	0	n	1	0	Plantaginaceae Juss.
Iz_3_1009	DJI_0880	Veronica verna L.	0	n	1	0	Plantaginaceae Juss.
Iz_3_1009	DJI_0880	Vincetoxicum hirundinaria Medik	0	n	1	0	Apocynaceae Juss.
Iz_3_1009	DJI_0887	Acer tataricum L.	0	n	1	1	Sapindaceae Juss.
Iz_3_1009	DJI_0887	Achillea millefolium L. s.l.	0	n	1	0	Asteraceae Bercht. & J.Presl
Iz_3_1009	DJI_0887	Allium oleraceum L.	0	n	1	0	Amaryllidaceae J.St.-Hil.
Iz_3_1009	DJI_0887	Ambrosia artemisiifolia L.	0	a	0	0	Asteraceae Bercht. & J.Presl
Iz_3_1009	DJI_0887	Amorpha fruticosa L.	0	a	0	0	Fabaceae Lindl.
Iz_3_1009	DJI_0887	Anthericum ramosum L.	0	n	1	0	Asparagaceae Juss.
Iz_3_1009	DJI_0887	Artemisia marschalliana Spreng.	0	n	1	0	Asteraceae Bercht. & J.Presl
Iz_3_1009	DJI_0887	Asparagus officinalis L.	0	n	1	0	Asparagaceae Juss.
Iz_3_1009	DJI_0887	Astragalus cicer L.	0	n	1	0	Fabaceae Lindl.
Iz_3_1009	DJI_0887	Bassia laniflora (S.G.Gmel.) A.J.Scott	0	n	1	0	Amaranthaceae Juss.
Iz_3_1009	DJI_0887	Berberis vulgaris L.	0	n	1	0	Berberidaceae Juss.
Iz_3_1009	DJI_0887	Betula pendula Roth.	0	n	1	1	Betulaceae Gray
Iz_3_1009	DJI_0887	Betula pendula Roth.	0	n	1	0	Poaceae Barnhart
Iz_3_1009	DJI_0887	Bromus inermis Leyss.	0		NA	NA	
Iz_3_1009	DJI_0887	Bromus squarrosus L.	0	a	0	0	Poaceae Barnhart
Iz_3_1009	DJI_0887	Bromus tectorum L.	0	a	0	0	Poaceae Barnhart
Iz_3_1009	DJI_0887	Calamagrostis epigejos (L.) Roth	15	n	1	0	Poaceae Barnhart
Iz_3_1009	DJI_0887	Carduus acanthoides L.	0	n	1	0	Asteraceae Bercht. & J.Presl
Iz_3_1009	DJI_0887	Carex sp.	0	n	1	0	Cyperaceae Juss.
Iz_3_1009	DJI_0887	Centaurea majorovii Dumbadze	0	n	1	0	Asteraceae Bercht. & J.Presl
Iz_3_1009	DJI_0887	Chamaecytisus ruthenicus (Fisch.ex Wol.) Klaskova	0	n	1	0	Fabaceae Lindl.
Iz_3_1009	DJI_0887	Chelidonium majus L.	0	n	1	0	Papaveraceae Juss.
Iz_3_1009	DJI_0887	Chenopodium betaceum Andrz.	0	n	1	0	Amaranthaceae Juss.
Iz_3_1009	DJI_0887	Chenopodium cf. album	0	n	1	0	Amaranthaceae Juss.
Iz_3_1009	DJI_0887	Chenopodium sp.	0		NA	0	Amaranthaceae Juss.
Iz_3_1009	DJI_0887	Chondrilla juncea L.	0	n	1	0	Asteraceae Bercht. & J.Presl
Iz_3_1009	DJI_0887	Cirsium vulgare (Savi) Ten.	0	n	1	0	Asteraceae Bercht. & J.Presl
Iz_3_1009	DJI_0887	Clinopodium acinos (L.) Kuntze	0	n	1	0	Lamiacea Lindl.
Iz_3_1009	DJI_0887	Convallaria majalis L.	0	n	1	0	Asparagaceae Juss.
Iz_3_1009	DJI_0887	Crataegus rhipidophylla Gand.	0	n	1	1	Rosaceae Juss.
Iz_3_1009	DJI_0887	Crepis foetida L.	0	n	1	0	Asteraceae Bercht. & J.Presl
Iz_3_1009	DJI_0887	Dactylis glomerata L.	0	n	1	0	Poaceae Barnhart
Iz_3_1009	DJI_0887	Dianthus campestris M.Bieb.	0	n	1	0	Caryophyllaceae Juss.
Iz_3_1009	DJI_0887	Digitaria sanguinalis (L.) Scop.	0	a	0	0	Poaceae Barnhart
Iz_3_1009	DJI_0887	Elymus repens (L.) Gould	0	n	1	0	Poaceae Barnhart
Iz_3_1009	DJI_0887	Epilobium tetragonum L.	0	n	1	0	Onagraceae Juss.
Iz_3_1009	DJI_0887	Eragrostis minor Host	0	a	0	0	Poaceae Barnhart
Iz_3_1009	DJI_0887	Erigeron canadensis L.	5	a	0	0	Asteraceae Bercht. & J.Presl
Iz_3_1009	DJI_0887	Euphorbia seguieriana Neck.	0	n	1	0	Euphorbiaceae Juss.
Iz_3_1009	DJI_0887	Fallopia dumetorum (L.) Holub	0.5	n	1	0	Polygonaceae Juss.
Iz_3_1009	DJI_0887	Festuca beckeri (Hack.) Trautv.	0	n	1	0	Poaceae Barnhart
Iz_3_1009	DJI_0887	Fragaria vesca L.	0	n	1	0	Rosaceae Juss.
Iz_3_1009	DJI_0887	Frangula alnus Mill.	0	n	1	1	Rhamnaceae Juss.
Iz_3_1009	DJI_0887	Fraxinus pennsylvanica Marshall	0	a	0	1	Oleaceae Hoffmanns. & Link
Iz_3_1009	DJI_0887	Galeopsis bifida Boenn.	0	n	1	0	Lamiacea Lindl.
Iz_3_1009	DJI_0887	Galium verum L.	0	n	1	0	Rubiaceae Juss.
Iz_3_1009	DJI_0887	Genista tinctoria L.	0	n	1	0	Fabaceae Lindl.
Iz_3_1009	DJI_0887	Geranium robertianum L.	0	n	1	0	Geraniaceae Juss.
Iz_3_1009	DJI_0887	Geranium sanquineum L.	0	n	1	0	Geraniaceae Juss.
Iz_3_1009	DJI_0887	Gypsophila paniculata L.	0	n	1	0	Caryophyllaceae Juss.
Iz_3_1009	DJI_0887	Helichrysum arenarium (L.) Moench	0	n	1	0	Asteraceae Bercht. & J.Presl
Iz_3_1009	DJI_0887	Herniaria polygama J.Gay	0	n	1	0	Caryophyllaceae Juss.
Iz_3_1009	DJI_0887	Hieracium robustum Fr.	0	n	1	0	Asteraceae Bercht. & J.Presl
Iz_3_1009	DJI_0887	Hieracium umbellatum L.	0	n	1	NA	Asteraceae Bercht. & J.Presl
Iz_3_1009	DJI_0887	Hylotelephium maximum (L.) Holub	0	n	1	0	Crassulaceae J.St.-Hil.
Iz_3_1009	DJI_0887	Jacobaea borysthenica (DC.) B.Nord. & Greuter	0	n	1	0	Asteraceae Bercht. & J.Presl
Iz_3_1009	DJI_0887	Jasione montana L.	0	n	1	0	Campanulaceae Juss.
Iz_3_1009	DJI_0887	Jurinea cyanoides (L.) Rchb.s.l.	0	n	1	0	Asteraceae Bercht. & J.Presl
Iz_3_1009	DJI_0887	Koeleria glauca (Spreng.) DC.	0	n	1	0	Poaceae Barnhart
Iz_3_1009	DJI_0887	Lactuca serriola L.	0	a	0	0	Asteraceae Bercht. & J.Presl
Iz_3_1009	DJI_0887	Linaria genistifolia (L.) Mill.	0	n	1	0	Plantaginaceae Juss.
Iz_3_1009	DJI_0887	Lomelosia argentea (L.) Greuter & Burdet	0	n	1	0	Caprifoliaceae Juss.
Iz_3_1009	DJI_0887	Melampyrum pratense L.	0	n	1	0	Orobanchaceae Vent.
Iz_3_1009	DJI_0887	Oenothera biennis L.	0	a	0	0	Onagraceae Juss.
Iz_3_1009	DJI_0887	Pentanema britannica (L.) D.Gut.Larr., Santos-Vicente, Anderb., E.Rico & M.M.Mart.Ort.	0	n	1	0	Asteraceae Bercht. & J.Presl
Iz_3_1009	DJI_0887	Peucedanum oreoselinum (L.) Moench	8	n	1	0	Apiaceae Lindl.
Iz_3_1009	DJI_0887	Peucedanum ruthenicum M.Bieb.	0	n	1	0	Apiaceae Lindl.
Iz_3_1009	DJI_0887	Pilosella echioides (Lumn.) F.W.Schultz & Sch.Bip.	0	n	1	0	Asteraceae Bercht. & J.Presl
Iz_3_1009	DJI_0887	Pilosella officinarum F. Schultz et Sch. Bip.	0	n	1	0	Asteraceae Bercht. & J.Presl
Iz_3_1009	DJI_0887	Pilosella sp.	0		NA	0	Asteraceae Bercht. & J.Presl
Iz_3_1009	DJI_0887	Pinus sylvestris L.	0	n	1	1	Pinaceae Spreng. ex F.Rudolphi
Iz_3_1009	DJI_0887	Poa annua L.	0	n	1	0	Poaceae Barnhart
Iz_3_1009	DJI_0887	Poa nemoralis L.	0	n	1	0	Poaceae Barnhart
Iz_3_1009	DJI_0887	Poa sp.	2		NA	0	Poaceae Barnhart
Iz_3_1009	DJI_0887	Polygonatum odoratum (Mill.) Druce	2	n	1	0	Asparagaceae Juss.
Iz_3_1009	DJI_0887	Polygonum arenarium Waldst. & Kit.	0	n	1	0	Polygonaceae Juss.
Iz_3_1009	DJI_0887	Populus nigra L.	0	n	1	1	Salicaceae Mirb.
Iz_3_1009	DJI_0887	Populus tremula L.	0	n	1	1	Salicaceae Mirb.
Iz_3_1009	DJI_0887	Populus Ã canescens (Aiton) Sm.	0	n	1	1	Salicaceae Mirb.
Iz_3_1009	DJI_0887	Potentilla argentea L.	0	n	1	0	Rosaceae Juss.
Iz_3_1009	DJI_0887	Psammophiliella muralis (L.) Ikonn.	0	n	1	0	Caryophyllaceae Juss.
Iz_3_1009	DJI_0887	Psephellus sumensis (Kalen.) Greuter	0	n	1	0	Asteraceae Bercht. & J.Presl

Iz_3_1009	DJI_0887	Pulsatilla pratensis (L.) Mill. s.l.	0	n	1	0	Ranunculaceae Juss.
Iz_3_1009	DJI_0887	Pyrus communis L.	0	a	0	1	Rosaceae Juss.
Iz_3_1009	DJI_0887	Quercus robur L.	0	n	1	1	Fagaceae Dumort.
Iz_3_1009	DJI_0887	Robinia pseudoacacia L.	0	a	0	1	Fabaceae Lindl.
Iz_3_1009	DJI_0887	Rosa sp.	0		NA	1	Rosaceae Juss.
Iz_3_1009	DJI_0887	Rubus idaeus L.	0	n	1	0	Rosaceae Juss.
Iz_3_1009	DJI_0887	Rumex acetosella L.	0	n	1	0	Polygonaceae Juss.
Iz_3_1009	DJI_0887	Rumex thyrsiflorus Fingerh.	0	n	1	0	Polygonaceae Juss.
Iz_3_1009	DJI_0887	Salix alba L.	0	n	1	1	Salicaceae Mirb.
Iz_3_1009	DJI_0887	Sambucus nigra L.	0	n	1	1	Viburnaceae Raf.
Iz_3_1009	DJI_0887	Secale sylvestre Host	0	n	1	0	Poaceae Barnhart
Iz_3_1009	DJI_0887	Sempervivum ruthenicum Schnittsp. & C.B. Lehm.	0	n	1	0	Crassulaceae J.St.-Hil.
Iz_3_1009	DJI_0887	Setaria viridis (L.) P. Beauv.	0	n	1	0	Poaceae Barnhart
Iz_3_1009	DJI_0887	Silene borysthenica (Gruner) Walters	0	n	1	0	Caryophyllaceae Juss.
Iz_3_1009	DJI_0887	Silene latifolia subsp. alba (Mill.) Greuter & Burdet	0	n	1	0	Caryophyllaceae Juss.
Iz_3_1009	DJI_0887	Solidago virgaurea L.	0	n	1	0	Asteraceae Bercht. & J.Presl
Iz_3_1009	DJI_0887	Sonchus oleraceus L.	0	a	0	0	Asteraceae Bercht. & J.Presl
Iz_3_1009	DJI_0887	Sorbus aucuparia L.	0	n	1	1	Rosaceae Juss.
Iz_3_1009	DJI_0887	Stachys recta L.	0	n	1	0	Lamiacea Lindl.
Iz_3_1009	DJI_0887	Tanacetum vulgare L.	0	n	1	0	Asteraceae Bercht. & J.Presl
Iz_3_1009	DJI_0887	Thymus pallasianus Heinr.Braun	0	n	1	0	Lamiacea Lindl.
Iz_3_1009	DJI_0887	Tilia cordata Mill.	0	n	1	1	Malvaceae Juss.
Iz_3_1009	DJI_0887	Trifolium alpestre L.	0	n	1	0	Fabaceae Lindl.
Iz_3_1009	DJI_0887	Trifolium arvense L.	0	n	1	0	Fabaceae Lindl.
Iz_3_1009	DJI_0887	Ulmus glabra Huds.	0	n	1	1	Ulmaceae Mirb.
Iz_3_1009	DJI_0887	Verbascum densiflorum Bertol.	0	n	1	0	Scrophulariaceae Juss.
Iz_3_1009	DJI_0887	Veronica austriaca L.	0	n	1	0	Plantaginaceae Juss.
Iz_3_1009	DJI_0887	Veronica incana L.	0	n	1	0	Plantaginaceae Juss.
Iz_3_1009	DJI_0887	Veronica verna L.	0	n	1	0	Plantaginaceae Juss.
Iz_3_1009	DJI_0887	Vincetoxicum hirundinaria Medik	0	n	1	0	Apocynaceae Juss.
Iz_3_1009	DJI_0888	Acer tataricum L.	0	n	1	1	Sapindaceae Juss.
Iz_3_1009	DJI_0888	Achillea millefolium L. s.l.	0	n	1	0	Asteraceae Bercht. & J.Presl
Iz_3_1009	DJI_0888	Allium oleraceum L.	0	n	1	0	Amaryllidaceae J.St.-Hil.
Iz_3_1009	DJI_0888	Ambrosia artemisiifolia L.	0	a	0	0	Asteraceae Bercht. & J.Presl
Iz_3_1009	DJI_0888	Amorpha fruticosa L.	0	a	0	0	Fabaceae Lindl.
Iz_3_1009	DJI_0888	Anthericum ramosum L.	0	n	1	0	Asparagaceae Juss.
Iz_3_1009	DJI_0888	Artemisia marschalliana Spreng.	0	n	1	0	Asteraceae Bercht. & J.Presl
Iz_3_1009	DJI_0888	Asparagus officinalis L.	0	n	1	0	Asparagaceae Juss.
Iz_3_1009	DJI_0888	Astragalus cicer L.	0	n	1	0	Fabaceae Lindl.
Iz_3_1009	DJI_0888	Bassia laniflora (S.G.Gmel.) A.J.Scott	0	n	1	0	Amaranthaceae Juss.
Iz_3_1009	DJI_0888	Berberis vulgaris L.	0	n	1	0	Berberidaceae Juss.
Iz_3_1009	DJI_0888	Betula pendula Roth.	0	n	1	1	Betulaceae Gray
Iz_3_1009	DJI_0888	Betula pendula Roth.	0	n	1	0	Poaceae Barnhart
Iz_3_1009	DJI_0888	Bromus inermis Leyss.	0		NA	NA	
Iz_3_1009	DJI_0888	Bromus squarrosus L.	0	a	0	0	Poaceae Barnhart
Iz_3_1009	DJI_0888	Bromus tectorum L.	0	a	0	0	Poaceae Barnhart
Iz_3_1009	DJI_0888	Calamagrostis epigejos (L.) Roth	10	n	1	0	Poaceae Barnhart
Iz_3_1009	DJI_0888	Carduus acanthoides L.	0	n	1	0	Asteraceae Bercht. & J.Presl
Iz_3_1009	DJI_0888	Carex sp.	0	n	1	0	Cyperaceae Juss.
Iz_3_1009	DJI_0888	Centaurea majorovii Dumbadze	0	n	1	0	Asteraceae Bercht. & J.Presl
Iz_3_1009	DJI_0888	Chamaecytisus ruthenicus (Fisch.ex Wol.) Klaskova	3	n	1	0	Fabaceae Lindl.
Iz_3_1009	DJI_0888	Chelidonium majus L.	0	n	1	0	Papaveraceae Juss.
Iz_3_1009	DJI_0888	Chenopodium betaceum Andrz.	0	n	1	0	Amaranthaceae Juss.
Iz_3_1009	DJI_0888	Chenopodium cf. album	0	n	1	0	Amaranthaceae Juss.
Iz_3_1009	DJI_0888	Chenopodium sp.	0		NA	0	Amaranthaceae Juss.
Iz_3_1009	DJI_0888	Chondrilla juncea L.	0	n	1	0	Asteraceae Bercht. & J.Presl
Iz_3_1009	DJI_0888	Cirsium vulgare (Savi) Ten.	0	n	1	0	Asteraceae Bercht. & J.Presl
Iz_3_1009	DJI_0888	Clinopodium acinos (L.) Kuntze	0	n	1	0	Lamiacea Lindl.
Iz_3_1009	DJI_0888	Convallaria majalis L.	0	n	1	0	Asparagaceae Juss.
Iz_3_1009	DJI_0888	Crataegus rhipidophylla Gand.	0	n	1	1	Rosaceae Juss.
Iz_3_1009	DJI_0888	Crepis foetida L.	0	n	1	0	Asteraceae Bercht. & J.Presl
Iz_3_1009	DJI_0888	Dactylis glomerata L.	0	n	1	0	Poaceae Barnhart
Iz_3_1009	DJI_0888	Dianthus campestris M.Bieb.	0	n	1	0	Caryophyllaceae Juss.
Iz_3_1009	DJI_0888	Digitaria sanguinalis (L.) Scop.	0	a	0	0	Poaceae Barnhart
Iz_3_1009	DJI_0888	Elymus repens (L.) Gould	0	n	1	0	Poaceae Barnhart
Iz_3_1009	DJI_0888	Epilobium tetragonum L.	0	n	1	0	Onagraceae Juss.
Iz_3_1009	DJI_0888	Eragrostis minor Host	0	a	0	0	Poaceae Barnhart
Iz_3_1009	DJI_0888	Erigeron canadensis L.	1	a	0	0	Asteraceae Bercht. & J.Presl
Iz_3_1009	DJI_0888	Euphorbia seguieriana Neck.	0	n	1	0	Euphorbiaceae Juss.
Iz_3_1009	DJI_0888	Fallopia dumetorum (L.) Holub	0	n	1	0	Polygonaceae Juss.
Iz_3_1009	DJI_0888	Festuca beckeri (Hack.) Trautv.	0	n	1	0	Poaceae Barnhart
Iz_3_1009	DJI_0888	Fragaria vesca L.	0	n	1	0	Rosaceae Juss.
Iz_3_1009	DJI_0888	Frangula alnus Mill.	0	n	1	1	Rhamnaceae Juss.
Iz_3_1009	DJI_0888	Fraxinus pennsylvanica Marshall	6	a	0	1	Oleaceae Hoffmanns. & Link
Iz_3_1009	DJI_0888	Galeopsis bifida Boenn.	0	n	1	0	Lamiacea Lindl.
Iz_3_1009	DJI_0888	Galium verum L.	0	n	1	0	Rubiaceae Juss.
Iz_3_1009	DJI_0888	Genista tinctoria L.	0	n	1	0	Fabaceae Lindl.
Iz_3_1009	DJI_0888	Geranium robertianum L.	0	n	1	0	Geraniaceae Juss.
Iz_3_1009	DJI_0888	Geranium sanquineum L.	0	n	1	0	Geraniaceae Juss.
Iz_3_1009	DJI_0888	Gypsophila paniculata L.	0	n	1	0	Caryophyllaceae Juss.
Iz_3_1009	DJI_0888	Helichrysum arenarium (L.) Moench	0	n	1	0	Asteraceae Bercht. & J.Presl
Iz_3_1009	DJI_0888	Herniaria polygama J.Gay	0	n	1	0	Caryophyllaceae Juss.
Iz_3_1009	DJI_0888	Hieracium robustum Fr.	0	n	1	0	Asteraceae Bercht. & J.Presl
Iz_3_1009	DJI_0888	Hieracium umbellatum L.	0	n	1	NA	Asteraceae Bercht. & J.Presl
Iz_3_1009	DJI_0888	Hylotelephium maximum (L.) Holub	0	n	1	0	Crassulaceae J.St.-Hil.
Iz_3_1009	DJI_0888	Jacobaea borysthenica (DC.) B.Nord. & Greuter	0	n	1	0	Asteraceae Bercht. & J.Presl
Iz_3_1009	DJI_0888	Jasione montana L.	0	n	1	0	Campanulaceae Juss.
Iz_3_1009	DJI_0888	Jurinea cyanoides (L.) Rchb.s.l.	0	n	1	0	Asteraceae Bercht. & J.Presl
Iz_3_1009	DJI_0888	Koeleria glauca (Spreng.) DC.	0	n	1	0	Poaceae Barnhart
Iz_3_1009	DJI_0888	Lactuca serriola L.	0	a	0	0	Asteraceae Bercht. & J.Presl
Iz_3_1009	DJI_0888	Linaria genistifolia (L.) Mill.	0	n	1	0	Plantaginaceae Juss.
Iz_3_1009	DJI_0888	Lomelosia argentea (L.) Greuter & Burdet	0	n	1	0	Caprifoliaceae Juss.
Iz_3_1009	DJI_0888	Melampyrum pratense L.	0	n	1	0	Orobanchaceae Vent.
Iz_3_1009	DJI_0888	Oenothera biennis L.	0	a	0	0	Onagraceae Juss.
Iz_3_1009	DJI_0888	Pentanema britannica (L.) D.Gut.Larr., Santos-Vicente, Anderb., E.Rico & M.M.Mart.Ort.	0	n	1	0	Asteraceae Bercht. & J.Presl
Iz_3_1009	DJI_0888	Peucedanum oreoselinum (L.) Moench	10	n	1	0	Apiaceae Lindl.
Iz_3_1009	DJI_0888	Peucedanum ruthenicum M.Bieb.	0	n	1	0	Apiaceae Lindl.
Iz_3_1009	DJI_0888	Pilosella echioides (Lumn.) F.W.Schultz & Sch.Bip.	0	n	1	0	Asteraceae Bercht. & J.Presl

Iz_3_1009	DJI_0888	Pilosella officinarum F. Schultz et Sch. Bip.	0	n	1	0	Asteraceae Bercht. & J.Presl
Iz_3_1009	DJI_0888	Pilosella sp.	0		NA	0	Asteraceae Bercht. & J.Presl
Iz_3_1009	DJI_0888	Pinus sylvestris L.	0	n	1	1	Pinaceae Spreng. ex F.Rudolphi
Iz_3_1009	DJI_0888	Poa annua L.	0	n	1	0	Poaceae Barnhart
Iz_3_1009	DJI_0888	Poa nemoralis L.	0	n	1	0	Poaceae Barnhart
Iz_3_1009	DJI_0888	Poa sp.	0		NA	0	Poaceae Barnhart
Iz_3_1009	DJI_0888	Polygonatum odoratum (Mill.) Druce	0	n	1	0	Asparagaceae Juss.
Iz_3_1009	DJI_0888	Polygonum arenarium Waldst. & Kit.	0	n	1	0	Polygonaceae Juss.
Iz_3_1009	DJI_0888	Populus nigra L.	0	n	1	1	Salicaceae Mirb.
Iz_3_1009	DJI_0888	Populus tremula L.	0	n	1	1	Salicaceae Mirb.
Iz_3_1009	DJI_0888	Populus Ã canescens (Aiton) Sm.	0	n	1	1	Salicaceae Mirb.
Iz_3_1009	DJI_0888	Potentilla argentea L.	0	n	1	0	Rosaceae Juss.
Iz_3_1009	DJI_0888	Psammophiliella muralis (L.) Ikonn.	0	n	1	0	Caryophyllaceae Juss.
Iz_3_1009	DJI_0888	Psephellus sumensis (Kalen.) Greuter	0	n	1	0	Asteraceae Bercht. & J.Presl
Iz_3_1009	DJI_0888	Pulsatilla pratensis (L.) Mill. s.l.	0	n	1	0	Ranunculaceae Juss.
Iz_3_1009	DJI_0888	Pyrus communis L.	0	a	0	1	Rosaceae Juss.
Iz_3_1009	DJI_0888	Quercus robur L.	0	n	1	1	Fagaceae Dumort.
Iz_3_1009	DJI_0888	Robinia pseudoacacia L.	0	a	0	1	Fabaceae Lindl.
Iz_3_1009	DJI_0888	Rosa sp.	0		NA	1	Rosaceae Juss.
Iz_3_1009	DJI_0888	Rubus idaeus L.	0	n	1	0	Rosaceae Juss.
Iz_3_1009	DJI_0888	Rumex acetosella L.	0	n	1	0	Polygonaceae Juss.
Iz_3_1009	DJI_0888	Rumex thyrsiflorus Fingerh.	0	n	1	0	Polygonaceae Juss.
Iz_3_1009	DJI_0888	Salix alba L.	0	n	1	1	Salicaceae Mirb.
Iz_3_1009	DJI_0888	Sambucus nigra L.	0	n	1	1	Viburnaceae Raf.
Iz_3_1009	DJI_0888	Secale sylvestre Host	0	n	1	0	Poaceae Barnhart
Iz_3_1009	DJI_0888	Sempervivum ruthenicum Schnittsp. & C.B. Lehm.	0	n	1	0	Crassulaceae J.St.-Hil.
Iz_3_1009	DJI_0888	Setaria viridis (L.) P. Beauv.	0	n	1	0	Poaceae Barnhart
Iz_3_1009	DJI_0888	Silene borysthenica (Gruner) Walters	0	n	1	0	Caryophyllaceae Juss.
Iz_3_1009	DJI_0888	Silene latifolia subsp. alba (Mill.) Greuter & Burdet	0	n	1	0	Caryophyllaceae Juss.
Iz_3_1009	DJI_0888	Solidago virgaurea L.	0	n	1	0	Asteraceae Bercht. & J.Presl
Iz_3_1009	DJI_0888	Sonchus oleraceus L.	0	a	0	0	Asteraceae Bercht. & J.Presl
Iz_3_1009	DJI_0888	Sorbus aucuparia L.	0	n	1	1	Rosaceae Juss.
Iz_3_1009	DJI_0888	Stachys recta L.	0	n	1	0	Lamiacea Lindl.
Iz_3_1009	DJI_0888	Tanacetum vulgare L.	0	n	1	0	Asteraceae Bercht. & J.Presl
Iz_3_1009	DJI_0888	Thymus pallasianus Heinr.Braun	0	n	1	0	Lamiacea Lindl.
Iz_3_1009	DJI_0888	Tilia cordata Mill.	0	n	1	1	Malvaceae Juss.
Iz_3_1009	DJI_0888	Trifolium alpestre L.	0	n	1	0	Fabaceae Lindl.
Iz_3_1009	DJI_0888	Trifolium arvense L.	0	n	1	0	Fabaceae Lindl.
Iz_3_1009	DJI_0888	Ulmus glabra Huds.	0	n	1	1	Ulmaceae Mirb.
Iz_3_1009	DJI_0888	Verbascum densiflorum Bertol.	0	n	1	0	Scrophulariaceae Juss.
Iz_3_1009	DJI_0888	Veronica austriaca L.	0	n	1	0	Plantaginaceae Juss.
Iz_3_1009	DJI_0888	Veronica incana L.	0	n	1	0	Plantaginaceae Juss.
Iz_3_1009	DJI_0888	Veronica verna L.	0	n	1	0	Plantaginaceae Juss.
Iz_3_1009	DJI_0888	Vincetoxicum hirundinaria Medik	0	n	1	0	Apocynaceae Juss.
Iz_3_1009	DJI_0893	Acer tataricum L.	0	n	1	1	Sapindaceae Juss.
Iz_3_1009	DJI_0893	Achillea millefolium L. s.l.	0	n	1	0	Asteraceae Bercht. & J.Presl
Iz_3_1009	DJI_0893	Allium oleraceum L.	0	n	1	0	Amaryllidaceae J.St.-Hil.
Iz_3_1009	DJI_0893	Ambrosia artemisiifolia L.	0	a	0	0	Asteraceae Bercht. & J.Presl
Iz_3_1009	DJI_0893	Amorpha fruticosa L.	0	a	0	0	Fabaceae Lindl.
Iz_3_1009	DJI_0893	Anthericum ramosum L.	0	n	1	0	Asparagaceae Juss.
Iz_3_1009	DJI_0893	Artemisia marschalliana Spreng.	0	n	1	0	Asteraceae Bercht. & J.Presl
Iz_3_1009	DJI_0893	Asparagus officinalis L.	0	n	1	0	Asparagaceae Juss.
Iz_3_1009	DJI_0893	Astragalus cicer L.	0	n	1	0	Fabaceae Lindl.
Iz_3_1009	DJI_0893	Bassia laniflora (S.G.Gmel.) A.J.Scott	0	n	1	0	Amaranthaceae Juss.
Iz_3_1009	DJI_0893	Berberis vulgaris L.	0	n	1	0	Berberidaceae Juss.
Iz_3_1009	DJI_0893	Betula pendula Roth.	0	n	1	1	Betulaceae Gray
Iz_3_1009	DJI_0893	Betula pendula Roth.	0	n	1	0	Poaceae Barnhart
Iz_3_1009	DJI_0893	Bromus inermis Leyss.	0		NA	NA	
Iz_3_1009	DJI_0893	Bromus squarrosus L.	0	a	0	0	Poaceae Barnhart
Iz_3_1009	DJI_0893	Bromus tectorum L.	0	a	0	0	Poaceae Barnhart
Iz_3_1009	DJI_0893	Calamagrostis epigejos (L.) Roth	0.5	n	1	0	Poaceae Barnhart
Iz_3_1009	DJI_0893	Carduus acanthoides L.	0	n	1	0	Asteraceae Bercht. & J.Presl
Iz_3_1009	DJI_0893	Carex sp.	0	n	1	0	Cyperaceae Juss.
Iz_3_1009	DJI_0893	Centaurea majorovii Dumbadze	0	n	1	0	Asteraceae Bercht. & J.Presl
Iz_3_1009	DJI_0893	Chamaecytisus ruthenicus (Fisch.ex Wol.) Klaskova	0	n	1	0	Fabaceae Lindl.
Iz_3_1009	DJI_0893	Chelidonium majus L.	0	n	1	0	Papaveraceae Juss.
Iz_3_1009	DJI_0893	Chenopodium betaceum Andrz.	0	n	1	0	Amaranthaceae Juss.
Iz_3_1009	DJI_0893	Chenopodium cf. album	0	n	1	0	Amaranthaceae Juss.
Iz_3_1009	DJI_0893	Chenopodium sp.	0		NA	0	Amaranthaceae Juss.
Iz_3_1009	DJI_0893	Chondrilla juncea L.	0	n	1	0	Asteraceae Bercht. & J.Presl
Iz_3_1009	DJI_0893	Cirsium vulgare (Savi) Ten.	0	n	1	0	Asteraceae Bercht. & J.Presl
Iz_3_1009	DJI_0893	Clinopodium acinos (L.) Kuntze	0	n	1	0	Lamiacea Lindl.
Iz_3_1009	DJI_0893	Convallaria majalis L.	0	n	1	0	Asparagaceae Juss.
Iz_3_1009	DJI_0893	Crataegus rhipidophylla Gand.	0	n	1	1	Rosaceae Juss.
Iz_3_1009	DJI_0893	Crepis foetida L.	0	n	1	0	Asteraceae Bercht. & J.Presl
Iz_3_1009	DJI_0893	Dactylis glomerata L.	0	n	1	0	Poaceae Barnhart
Iz_3_1009	DJI_0893	Dianthus campestris M.Bieb.	0	n	1	0	Caryophyllaceae Juss.
Iz_3_1009	DJI_0893	Digitaria sanguinalis (L.) Scop.	0	a	0	0	Poaceae Barnhart
Iz_3_1009	DJI_0893	Elymus repens (L.) Gould	0	n	1	0	Poaceae Barnhart
Iz_3_1009	DJI_0893	Epilobium tetragonum L.	0	n	1	0	Onagraceae Juss.
Iz_3_1009	DJI_0893	Eragrostis minor Host	0	a	0	0	Poaceae Barnhart
Iz_3_1009	DJI_0893	Erigeron canadensis L.	0	a	0	0	Asteraceae Bercht. & J.Presl
Iz_3_1009	DJI_0893	Euphorbia seguieriana Neck.	0	n	1	0	Euphorbiaceae Juss.
Iz_3_1009	DJI_0893	Fallopia dumetorum (L.) Holub	5	n	1	0	Polygonaceae Juss.
Iz_3_1009	DJI_0893	Festuca beckeri (Hack.) Trautv.	0	n	1	0	Poaceae Barnhart
Iz_3_1009	DJI_0893	Fragaria vesca L.	0	n	1	0	Rosaceae Juss.
Iz_3_1009	DJI_0893	Frangula alnus Mill.	0	n	1	1	Rhamnaceae Juss.
Iz_3_1009	DJI_0893	Fraxinus pennsylvanica Marshall	0	a	0	1	Oleaceae Hoffmanns. & Link
Iz_3_1009	DJI_0893	Galeopsis bifida Boenn.	0	n	1	0	Lamiacea Lindl.
Iz_3_1009	DJI_0893	Galium verum L.	0	n	1	0	Rubiaceae Juss.
Iz_3_1009	DJI_0893	Genista tinctoria L.	0	n	1	0	Fabaceae Lindl.
Iz_3_1009	DJI_0893	Geranium robertianum L.	0	n	1	0	Geraniaceae Juss.
Iz_3_1009	DJI_0893	Geranium sanquineum L.	0	n	1	0	Geraniaceae Juss.
Iz_3_1009	DJI_0893	Gypsophila paniculata L.	0	n	1	0	Caryophyllaceae Juss.
Iz_3_1009	DJI_0893	Helichrysum arenarium (L.) Moench	0	n	1	0	Asteraceae Bercht. & J.Presl
Iz_3_1009	DJI_0893	Herniaria polygama J.Gay	0	n	1	0	Caryophyllaceae Juss.
Iz_3_1009	DJI_0893	Hieracium robustum Fr.	0	n	1	0	Asteraceae Bercht. & J.Presl
Iz_3_1009	DJI_0893	Hieracium umbellatum L.	0	n	1	NA	Asteraceae Bercht. & J.Presl

Iz_3_1009	DJI_0893	Hylotelephium maximum (L.) Holub	0	n	1	0	Crassulaceae J.St.-Hil.
Iz_3_1009	DJI_0893	Jacobaea borysthenica (DC.) B.Nord. & Greuter	0	n	1	0	Asteraceae Bercht. & J.Presl
Iz_3_1009	DJI_0893	Jasione montana L.	0	n	1	0	Campanulaceae Juss.
Iz_3_1009	DJI_0893	Jurinea cyanoides (L.) Rchb.s.l.	0	n	1	0	Asteraceae Bercht. & J.Presl
Iz_3_1009	DJI_0893	Koeleria glauca (Spreng.) DC.	0	n	1	0	Poaceae Barnhart
Iz_3_1009	DJI_0893	Lactuca serriola L.	0	a	0	0	Asteraceae Bercht. & J.Presl
Iz_3_1009	DJI_0893	Linaria genistifolia (L.) Mill.	0	n	1	0	Plantaginaceae Juss.
Iz_3_1009	DJI_0893	Lomelosia argentea (L.) Greuter & Burdet	0	n	1	0	Caprifoliaceae Juss.
Iz_3_1009	DJI_0893	Melampyrum pratense L.	0	n	1	0	Orobanchaceae Vent.
Iz_3_1009	DJI_0893	Oenothera biennis L.	0	a	0	0	Onagraceae Juss.
Iz_3_1009	DJI_0893	Pentanema britannica (L.) D.Gut.Larr., Santos-Vicente, Anderb., E.Rico & M.M.Mart.Ort.	0	n	1	0	Asteraceae Bercht. & J.Presl
Iz_3_1009	DJI_0893	Peucedanum oreoselinum (L.) Moench	0	n	1	0	Apiaceae Lindl.
Iz_3_1009	DJI_0893	Peucedanum ruthenicum M.Bieb.	0	n	1	0	Apiaceae Lindl.
Iz_3_1009	DJI_0893	Pilosella echioides (Lumn.) F.W.Schultz & Sch.Bip.	0	n	1	0	Asteraceae Bercht. & J.Presl
Iz_3_1009	DJI_0893	Pilosella officinarum F. Schultz et Sch. Bip.	0	n	1	0	Asteraceae Bercht. & J.Presl
Iz_3_1009	DJI_0893	Pilosella sp.	0		NA	0	Asteraceae Bercht. & J.Presl
Iz_3_1009	DJI_0893	Pinus sylvestris L.	0	n	1	1	Pinaceae Spreng. ex F.Rudolphi
Iz_3_1009	DJI_0893	Poa annua L.	0	n	1	0	Poaceae Barnhart
Iz_3_1009	DJI_0893	Poa nemoralis L.	0	n	1	0	Poaceae Barnhart
Iz_3_1009	DJI_0893	Poa sp.	0		NA	0	Poaceae Barnhart
Iz_3_1009	DJI_0893	Polygonatum odoratum (Mill.) Druce	0	n	1	0	Asparagaceae Juss.
Iz_3_1009	DJI_0893	Polygonum arenarium Waldst. & Kit.	0	n	1	0	Polygonaceae Juss.
Iz_3_1009	DJI_0893	Populus nigra L.	0	n	1	1	Salicaceae Mirb.
Iz_3_1009	DJI_0893	Populus tremula L.	0	n	1	1	Salicaceae Mirb.
Iz_3_1009	DJI_0893	Populus Ã canescens (Aiton) Sm.	0	n	1	1	Salicaceae Mirb.
Iz_3_1009	DJI_0893	Potentilla argentea L.	0	n	1	0	Rosaceae Juss.
Iz_3_1009	DJI_0893	Psammophiliella muralis (L.) Ikonn.	0	n	1	0	Caryophyllaceae Juss.
Iz_3_1009	DJI_0893	Psephellus sumensis (Kalen.) Greuter	0	n	1	0	Asteraceae Bercht. & J.Presl
Iz_3_1009	DJI_0893	Pulsatilla pratensis (L.) Mill. s.l.	0	n	1	0	Ranunculaceae Juss.
Iz_3_1009	DJI_0893	Pyrus communis L.	0	a	0	1	Rosaceae Juss.
Iz_3_1009	DJI_0893	Quercus robur L.	0	n	1	1	Fagaceae Dumort.
Iz_3_1009	DJI_0893	Robinia pseudoacacia L.	0	a	0	1	Fabaceae Lindl.
Iz_3_1009	DJI_0893	Rosa sp.	0		NA	1	Rosaceae Juss.
Iz_3_1009	DJI_0893	Rubus idaeus L.	0	n	1	0	Rosaceae Juss.
Iz_3_1009	DJI_0893	Rumex acetosella L.	0	n	1	0	Polygonaceae Juss.
Iz_3_1009	DJI_0893	Rumex thyrsiflorus Fingerh.	0	n	1	0	Polygonaceae Juss.
Iz_3_1009	DJI_0893	Salix alba L.	0	n	1	1	Salicaceae Mirb.
Iz_3_1009	DJI_0893	Sambucus nigra L.	0	n	1	1	Viburnaceae Raf.
Iz_3_1009	DJI_0893	Secale sylvestre Host	0	n	1	0	Poaceae Barnhart
Iz_3_1009	DJI_0893	Sempervivum ruthenicum Schnittsp. & C.B. Lehm.	0	n	1	0	Crassulaceae J.St.-Hil.
Iz_3_1009	DJI_0893	Setaria viridis (L.) P. Beauv.	0	n	1	0	Poaceae Barnhart
Iz_3_1009	DJI_0893	Silene borysthenica (Gruner) Walters	0	n	1	0	Caryophyllaceae Juss.
Iz_3_1009	DJI_0893	Silene latifolia subsp. alba (Mill.) Greuter & Burdet	0	n	1	0	Caryophyllaceae Juss.
Iz_3_1009	DJI_0893	Solidago virgaurea L.	0	n	1	0	Asteraceae Bercht. & J.Presl
Iz_3_1009	DJI_0893	Sonchus oleraceus L.	0	a	0	0	Asteraceae Bercht. & J.Presl
Iz_3_1009	DJI_0893	Sorbus aucuparia L.	0	n	1	1	Rosaceae Juss.
Iz_3_1009	DJI_0893	Stachys recta L.	0	n	1	0	Lamiacea Lindl.
Iz_3_1009	DJI_0893	Tanacetum vulgare L.	0	n	1	0	Asteraceae Bercht. & J.Presl
Iz_3_1009	DJI_0893	Thymus pallasianus Heinr.Braun	0	n	1	0	Lamiacea Lindl.
Iz_3_1009	DJI_0893	Tilia cordata Mill.	0	n	1	1	Malvaceae Juss.
Iz_3_1009	DJI_0893	Trifolium alpestre L.	0	n	1	0	Fabaceae Lindl.
Iz_3_1009	DJI_0893	Trifolium arvense L.	0	n	1	0	Fabaceae Lindl.
Iz_3_1009	DJI_0893	Ulmus glabra Huds.	0	n	1	1	Ulmaceae Mirb.
Iz_3_1009	DJI_0893	Verbascum densiflorum Bertol.	0	n	1	0	Scrophulariaceae Juss.
Iz_3_1009	DJI_0893	Veronica austriaca L.	0	n	1	0	Plantaginaceae Juss.
Iz_3_1009	DJI_0893	Veronica incana L.	0	n	1	0	Plantaginaceae Juss.
Iz_3_1009	DJI_0893	Veronica verna L.	0	n	1	0	Plantaginaceae Juss.
Iz_3_1009	DJI_0893	Vincetoxicum hirundinaria Medik	0	n	1	0	Apocynaceae Juss.
Iz_3_1009	DJI_0895	Acer tataricum L.	0	n	1	1	Sapindaceae Juss.
Iz_3_1009	DJI_0895	Achillea millefolium L. s.l.	0	n	1	0	Asteraceae Bercht. & J.Presl
Iz_3_1009	DJI_0895	Allium oleraceum L.	0	n	1	0	Amaryllidaceae J.St.-Hil.
Iz_3_1009	DJI_0895	Ambrosia artemisiifolia L.	0	a	0	0	Asteraceae Bercht. & J.Presl
Iz_3_1009	DJI_0895	Amorpha fruticosa L.	0	a	0	0	Fabaceae Lindl.
Iz_3_1009	DJI_0895	Anthericum ramosum L.	0	n	1	0	Asparagaceae Juss.
Iz_3_1009	DJI_0895	Artemisia marschalliana Spreng.	0	n	1	0	Asteraceae Bercht. & J.Presl
Iz_3_1009	DJI_0895	Asparagus officinalis L.	0	n	1	0	Asparagaceae Juss.
Iz_3_1009	DJI_0895	Astragalus cicer L.	0	n	1	0	Fabaceae Lindl.
Iz_3_1009	DJI_0895	Bassia laniflora (S.G.Gmel.) A.J.Scott	0	n	1	0	Amaranthaceae Juss.
Iz_3_1009	DJI_0895	Berberis vulgaris L.	0	n	1	0	Berberidaceae Juss.
Iz_3_1009	DJI_0895	Betula pendula Roth.	0	n	1	1	Betulaceae Gray
Iz_3_1009	DJI_0895	Betula pendula Roth.	0	n	1	0	Poaceae Barnhart
Iz_3_1009	DJI_0895	Bromus inermis Leyss.	0		NA	NA	
Iz_3_1009	DJI_0895	Bromus squarrosus L.	0	a	0	0	Poaceae Barnhart
Iz_3_1009	DJI_0895	Bromus tectorum L.	0	a	0	0	Poaceae Barnhart
Iz_3_1009	DJI_0895	Calamagrostis epigejos (L.) Roth	3	n	1	0	Poaceae Barnhart
Iz_3_1009	DJI_0895	Carduus acanthoides L.	0	n	1	0	Asteraceae Bercht. & J.Presl
Iz_3_1009	DJI_0895	Carex sp.	0	n	1	0	Cyperaceae Juss.
Iz_3_1009	DJI_0895	Centaurea majorovii Dumbadze	0	n	1	0	Asteraceae Bercht. & J.Presl
Iz_3_1009	DJI_0895	Chamaecytisus ruthenicus (Fisch.ex Wol.) Klaskova	0	n	1	0	Fabaceae Lindl.
Iz_3_1009	DJI_0895	Chelidonium majus L.	0	n	1	0	Papaveraceae Juss.
Iz_3_1009	DJI_0895	Chenopodium betaceum Andrz.	0	n	1	0	Amaranthaceae Juss.
Iz_3_1009	DJI_0895	Chenopodium cf. album	0.5	n	1	0	Amaranthaceae Juss.
Iz_3_1009	DJI_0895	Chenopodium sp.	0		NA	0	Amaranthaceae Juss.
Iz_3_1009	DJI_0895	Chondrilla juncea L.	0	n	1	0	Asteraceae Bercht. & J.Presl
Iz_3_1009	DJI_0895	Cirsium vulgare (Savi) Ten.	0	n	1	0	Asteraceae Bercht. & J.Presl
Iz_3_1009	DJI_0895	Clinopodium acinos (L.) Kuntze	0	n	1	0	Lamiacea Lindl.
Iz_3_1009	DJI_0895	Convallaria majalis L.	0	n	1	0	Asparagaceae Juss.
Iz_3_1009	DJI_0895	Crataegus rhipidophylla Gand.	0	n	1	1	Rosaceae Juss.
Iz_3_1009	DJI_0895	Crepis foetida L.	0	n	1	0	Asteraceae Bercht. & J.Presl
Iz_3_1009	DJI_0895	Dactylis glomerata L.	0	n	1	0	Poaceae Barnhart
Iz_3_1009	DJI_0895	Dianthus campestris M.Bieb.	0	n	1	0	Caryophyllaceae Juss.
Iz_3_1009	DJI_0895	Digitaria sanguinalis (L.) Scop.	0	a	0	0	Poaceae Barnhart
Iz_3_1009	DJI_0895	Elymus repens (L.) Gould	0	n	1	0	Poaceae Barnhart
Iz_3_1009	DJI_0895	Epilobium tetragonum L.	0	n	1	0	Onagraceae Juss.
Iz_3_1009	DJI_0895	Eragrostis minor Host	0	a	0	0	Poaceae Barnhart
Iz_3_1009	DJI_0895	Erigeron canadensis L.	0	a	0	0	Asteraceae Bercht. & J.Presl
Iz_3_1009	DJI_0895	Euphorbia seguieriana Neck.	0	n	1	0	Euphorbiaceae Juss.
Iz_3_1009	DJI_0895	Fallopia dumetorum (L.) Holub	3	n	1	0	Polygonaceae Juss.

Iz_3_1009	DJI_0895	Festuca beckeri (Hack.) Trautv.	0	n	1	0	Poaceae Barnhart
Iz_3_1009	DJI_0895	Fragaria vesca L.	0	n	1	0	Rosaceae Juss.
Iz_3_1009	DJI_0895	Frangula alnus Mill.	0	n	1	1	Rhamnaceae Juss.
Iz_3_1009	DJI_0895	Fraxinus pennsylvanica Marshall	0	a	0	1	Oleaceae Hoffmanns. & Link
Iz_3_1009	DJI_0895	Galeopsis bifida Boenn.	0	n	1	0	Lamiacea Lindl.
Iz_3_1009	DJI_0895	Galium verum L.	0	n	1	0	Rubiaceae Juss.
Iz_3_1009	DJI_0895	Genista tinctoria L.	0	n	1	0	Fabaceae Lindl.
Iz_3_1009	DJI_0895	Geranium robertianum L.	0	n	1	0	Geraniaceae Juss.
Iz_3_1009	DJI_0895	Geranium sanquineum L.	0	n	1	0	Geraniaceae Juss.
Iz_3_1009	DJI_0895	Gypsophila paniculata L.	0	n	1	0	Caryophyllaceae Juss.
Iz_3_1009	DJI_0895	Helichrysum arenarium (L.) Moench	0	n	1	0	Asteraceae Bercht. & J.Presl
Iz_3_1009	DJI_0895	Herniaria polygama J.Gay	0	n	1	0	Caryophyllaceae Juss.
Iz_3_1009	DJI_0895	Hieracium robustum Fr.	0	n	1	0	Asteraceae Bercht. & J.Presl
Iz_3_1009	DJI_0895	Hieracium umbellatum L.	0	n	1	NA	Asteraceae Bercht. & J.Presl
Iz_3_1009	DJI_0895	Hylotelephium maximum (L.) Holub	0	n	1	0	Crassulaceae J.St.-Hil.
Iz_3_1009	DJI_0895	Jacobaea borysthenica (DC.) B.Nord. & Greuter	0	n	1	0	Asteraceae Bercht. & J.Presl
Iz_3_1009	DJI_0895	Jasione montana L.	0	n	1	0	Campanulaceae Juss.
Iz_3_1009	DJI_0895	Jurinea cyanoides (L.) Rchb.s.l.	0	n	1	0	Asteraceae Bercht. & J.Presl
Iz_3_1009	DJI_0895	Koeleria glauca (Spreng.) DC.	0	n	1	0	Poaceae Barnhart
Iz_3_1009	DJI_0895	Lactuca serriola L.	0	a	0	0	Asteraceae Bercht. & J.Presl
Iz_3_1009	DJI_0895	Linaria genistifolia (L.) Mill.	0	n	1	0	Plantaginaceae Juss.
Iz_3_1009	DJI_0895	Lomelosia argentea (L.) Greuter & Burdet	0	n	1	0	Caprifoliaceae Juss.
Iz_3_1009	DJI_0895	Melampyrum pratense L.	0	n	1	0	Orobanchaceae Vent.
Iz_3_1009	DJI_0895	Oenothera biennis L.	0	a	0	0	Onagraceae Juss.
Iz_3_1009	DJI_0895	Pentanema britannica (L.) D.Gut.Larr., Santos-Vicente, Anderb., E.Rico & M.M.Mart.Ort.	0	n	1	0	Asteraceae Bercht. & J.Presl
Iz_3_1009	DJI_0895	Peucedanum oreoselinum (L.) Moench	0	n	1	0	Apiaceae Lindl.
Iz_3_1009	DJI_0895	Peucedanum ruthenicum M.Bieb.	0	n	1	0	Apiaceae Lindl.
Iz_3_1009	DJI_0895	Pilosella echioides (Lumn.) F.W.Schultz & Sch.Bip.	0	n	1	0	Asteraceae Bercht. & J.Presl
Iz_3_1009	DJI_0895	Pilosella officinarum F. Schultz et Sch. Bip.	0	n	1	0	Asteraceae Bercht. & J.Presl
Iz_3_1009	DJI_0895	Pilosella sp.	0		NA	0	Asteraceae Bercht. & J.Presl
Iz_3_1009	DJI_0895	Pinus sylvestris L.	0	n	1	1	Pinaceae Spreng. ex F.Rudolphi
Iz_3_1009	DJI_0895	Poa annua L.	0	n	1	0	Poaceae Barnhart
Iz_3_1009	DJI_0895	Poa nemoralis L.	0	n	1	0	Poaceae Barnhart
Iz_3_1009	DJI_0895	Poa sp.	0		NA	0	Poaceae Barnhart
Iz_3_1009	DJI_0895	Polygonatum odoratum (Mill.) Druce	0	n	1	0	Asparagaceae Juss.
Iz_3_1009	DJI_0895	Polygonum arenarium Waldst. & Kit.	0	n	1	0	Polygonaceae Juss.
Iz_3_1009	DJI_0895	Populus nigra L.	0	n	1	1	Salicaceae Mirb.
Iz_3_1009	DJI_0895	Populus tremula L.	0	n	1	1	Salicaceae Mirb.
Iz_3_1009	DJI_0895	Populus Ã canescens (Aiton) Sm.	0	n	1	1	Salicaceae Mirb.
Iz_3_1009	DJI_0895	Potentilla argentea L.	0	n	1	0	Rosaceae Juss.
Iz_3_1009	DJI_0895	Psammophiliella muralis (L.) Ikonn.	0	n	1	0	Caryophyllaceae Juss.
Iz_3_1009	DJI_0895	Psephellus sumensis (Kalen.) Greuter	0	n	1	0	Asteraceae Bercht. & J.Presl
Iz_3_1009	DJI_0895	Pulsatilla pratensis (L.) Mill. s.l.	0	n	1	0	Ranunculaceae Juss.
Iz_3_1009	DJI_0895	Pyrus communis L.	0	a	0	1	Rosaceae Juss.
Iz_3_1009	DJI_0895	Quercus robur L.	0	n	1	1	Fagaceae Dumort.
Iz_3_1009	DJI_0895	Robinia pseudoacacia L.	0	a	0	1	Fabaceae Lindl.
Iz_3_1009	DJI_0895	Rosa sp.	0		NA	1	Rosaceae Juss.
Iz_3_1009	DJI_0895	Rubus idaeus L.	0	n	1	0	Rosaceae Juss.
Iz_3_1009	DJI_0895	Rumex acetosella L.	0	n	1	0	Polygonaceae Juss.
Iz_3_1009	DJI_0895	Rumex thyrsiflorus Fingerh.	0	n	1	0	Polygonaceae Juss.
Iz_3_1009	DJI_0895	Salix alba L.	0	n	1	1	Salicaceae Mirb.
Iz_3_1009	DJI_0895	Sambucus nigra L.	0	n	1	1	Viburnaceae Raf.
Iz_3_1009	DJI_0895	Secale sylvestre Host	0	n	1	0	Poaceae Barnhart
Iz_3_1009	DJI_0895	Sempervivum ruthenicum Schnittsp. & C.B. Lehm.	0	n	1	0	Crassulaceae J.St.-Hil.
Iz_3_1009	DJI_0895	Setaria viridis (L.) P. Beauv.	0	n	1	0	Poaceae Barnhart
Iz_3_1009	DJI_0895	Silene borysthenica (Gruner) Walters	0	n	1	0	Caryophyllaceae Juss.
Iz_3_1009	DJI_0895	Silene latifolia subsp. alba (Mill.) Greuter & Burdet	0	n	1	0	Caryophyllaceae Juss.
Iz_3_1009	DJI_0895	Solidago virgaurea L.	0	n	1	0	Asteraceae Bercht. & J.Presl
Iz_3_1009	DJI_0895	Sonchus oleraceus L.	0	a	0	0	Asteraceae Bercht. & J.Presl
Iz_3_1009	DJI_0895	Sorbus aucuparia L.	0	n	1	1	Rosaceae Juss.
Iz_3_1009	DJI_0895	Stachys recta L.	0	n	1	0	Lamiacea Lindl.
Iz_3_1009	DJI_0895	Tanacetum vulgare L.	0	n	1	0	Asteraceae Bercht. & J.Presl
Iz_3_1009	DJI_0895	Thymus pallasianus Heinr.Braun	0	n	1	0	Lamiacea Lindl.
Iz_3_1009	DJI_0895	Tilia cordata Mill.	0	n	1	1	Malvaceae Juss.
Iz_3_1009	DJI_0895	Trifolium alpestre L.	0	n	1	0	Fabaceae Lindl.
Iz_3_1009	DJI_0895	Trifolium arvense L.	0	n	1	0	Fabaceae Lindl.
Iz_3_1009	DJI_0895	Ulmus glabra Huds.	0	n	1	1	Ulmaceae Mirb.
Iz_3_1009	DJI_0895	Verbascum densiflorum Bertol.	0	n	1	0	Scrophulariaceae Juss.
Iz_3_1009	DJI_0895	Veronica austriaca L.	0	n	1	0	Plantaginaceae Juss.
Iz_3_1009	DJI_0895	Veronica incana L.	0	n	1	0	Plantaginaceae Juss.
Iz_3_1009	DJI_0895	Veronica verna L.	0	n	1	0	Plantaginaceae Juss.
Iz_3_1009	DJI_0895	Vincetoxicum hirundinaria Medik	0	n	1	0	Apocynaceae Juss.
Iz_3_1009	DJI_0896	Acer tataricum L.	0	n	1	1	Sapindaceae Juss.
Iz_3_1009	DJI_0896	Achillea millefolium L. s.l.	0	n	1	0	Asteraceae Bercht. & J.Presl
Iz_3_1009	DJI_0896	Allium oleraceum L.	0	n	1	0	Amaryllidaceae J.St.-Hil.
Iz_3_1009	DJI_0896	Ambrosia artemisiifolia L.	0	a	0	0	Asteraceae Bercht. & J.Presl
Iz_3_1009	DJI_0896	Amorpha fruticosa L.	0	a	0	0	Fabaceae Lindl.
Iz_3_1009	DJI_0896	Anthericum ramosum L.	0	n	1	0	Asparagaceae Juss.
Iz_3_1009	DJI_0896	Artemisia marschalliana Spreng.	0	n	1	0	Asteraceae Bercht. & J.Presl
Iz_3_1009	DJI_0896	Asparagus officinalis L.	0	n	1	0	Asparagaceae Juss.
Iz_3_1009	DJI_0896	Astragalus cicer L.	0	n	1	0	Fabaceae Lindl.
Iz_3_1009	DJI_0896	Bassia laniflora (S.G.Gmel.) A.J.Scott	0	n	1	0	Amaranthaceae Juss.
Iz_3_1009	DJI_0896	Berberis vulgaris L.	0	n	1	0	Berberidaceae Juss.
Iz_3_1009	DJI_0896	Betula pendula Roth.	0	n	1	1	Betulaceae Gray
Iz_3_1009	DJI_0896	Betula pendula Roth.	0	n	1	0	Poaceae Barnhart
Iz_3_1009	DJI_0896	Bromus inermis Leyss.	0		NA	NA	
Iz_3_1009	DJI_0896	Bromus squarrosus L.	0	a	0	0	Poaceae Barnhart
Iz_3_1009	DJI_0896	Bromus tectorum L.	0	a	0	0	Poaceae Barnhart
Iz_3_1009	DJI_0896	Calamagrostis epigejos (L.) Roth	20	n	1	0	Poaceae Barnhart
Iz_3_1009	DJI_0896	Carduus acanthoides L.	0	n	1	0	Asteraceae Bercht. & J.Presl
Iz_3_1009	DJI_0896	Carex sp.	0	n	1	0	Cyperaceae Juss.
Iz_3_1009	DJI_0896	Centaurea majorovii Dumbadze	0	n	1	0	Asteraceae Bercht. & J.Presl
Iz_3_1009	DJI_0896	Chamaecytisus ruthenicus (Fisch.ex Wol.) Klaskova	0	n	1	0	Fabaceae Lindl.
Iz_3_1009	DJI_0896	Chelidonium majus L.	0	n	1	0	Papaveraceae Juss.
Iz_3_1009	DJI_0896	Chenopodium betaceum Andrz.	0	n	1	0	Amaranthaceae Juss.
Iz_3_1009	DJI_0896	Chenopodium cf. album	0	n	1	0	Amaranthaceae Juss.
Iz_3_1009	DJI_0896	Chenopodium sp.	0		NA	0	Amaranthaceae Juss.
Iz_3_1009	DJI_0896	Chondrilla juncea L.	0	n	1	0	Asteraceae Bercht. & J.Presl

Iz_3_1009	DJI_0896	Cirsium vulgare (Savi) Ten.	0	n	1	0	Asteraceae Bercht. & J.Presl
Iz_3_1009	DJI_0896	Clinopodium acinos (L.) Kuntze	0	n	1	0	Lamiacea Lindl.
Iz_3_1009	DJI_0896	Convallaria majalis L.	0	n	1	0	Asparagaceae Juss.
Iz_3_1009	DJI_0896	Crataegus rhipidophylla Gand.	0	n	1	1	Rosaceae Juss.
Iz_3_1009	DJI_0896	Crepis foetida L.	0	n	1	0	Asteraceae Bercht. & J.Presl
Iz_3_1009	DJI_0896	Dactylis glomerata L.	0	n	1	0	Poaceae Barnhart
Iz_3_1009	DJI_0896	Dianthus campestris M.Bieb.	0	n	1	0	Caryophyllaceae Juss.
Iz_3_1009	DJI_0896	Digitaria sanguinalis (L.) Scop.	0	a	0	0	Poaceae Barnhart
Iz_3_1009	DJI_0896	Elymus repens (L.) Gould	0	n	1	0	Poaceae Barnhart
Iz_3_1009	DJI_0896	Epilobium tetragonum L.	0	n	1	0	Onagraceae Juss.
Iz_3_1009	DJI_0896	Eragrostis minor Host	0	a	0	0	Poaceae Barnhart
Iz_3_1009	DJI_0896	Erigeron canadensis L.	0	a	0	0	Asteraceae Bercht. & J.Presl
Iz_3_1009	DJI_0896	Euphorbia seguieriana Neck.	0	n	1	0	Euphorbiaceae Juss.
Iz_3_1009	DJI_0896	Fallopia dumetorum (L.) Holub	1	n	1	0	Polygonaceae Juss.
Iz_3_1009	DJI_0896	Festuca beckeri (Hack.) Trautv.	0	n	1	0	Poaceae Barnhart
Iz_3_1009	DJI_0896	Fragaria vesca L.	0	n	1	0	Rosaceae Juss.
Iz_3_1009	DJI_0896	Frangula alnus Mill.	0	n	1	1	Rhamnaceae Juss.
Iz_3_1009	DJI_0896	Fraxinus pennsylvanica Marshall	0	a	0	1	Oleaceae Hoffmanns. & Link
Iz_3_1009	DJI_0896	Galeopsis bifida Boenn.	0	n	1	0	Lamiacea Lindl.
Iz_3_1009	DJI_0896	Galium verum L.	0	n	1	0	Rubiaceae Juss.
Iz_3_1009	DJI_0896	Genista tinctoria L.	0	n	1	0	Fabaceae Lindl.
Iz_3_1009	DJI_0896	Geranium robertianum L.	0	n	1	0	Geraniaceae Juss.
Iz_3_1009	DJI_0896	Geranium sanquineum L.	0	n	1	0	Geraniaceae Juss.
Iz_3_1009	DJI_0896	Gypsophila paniculata L.	0	n	1	0	Caryophyllaceae Juss.
Iz_3_1009	DJI_0896	Helichrysum arenarium (L.) Moench	0	n	1	0	Asteraceae Bercht. & J.Presl
Iz_3_1009	DJI_0896	Herniaria polygama J.Gay	0	n	1	0	Caryophyllaceae Juss.
Iz_3_1009	DJI_0896	Hieracium robustum Fr.	0	n	1	0	Asteraceae Bercht. & J.Presl
Iz_3_1009	DJI_0896	Hieracium umbellatum L.	0	n	1	NA	Asteraceae Bercht. & J.Presl
Iz_3_1009	DJI_0896	Hylotelephium maximum (L.) Holub	0	n	1	0	Crassulaceae J.St.-Hil.
Iz_3_1009	DJI_0896	Jacobaea borysthenica (DC.) B.Nord. & Greuter	0	n	1	0	Asteraceae Bercht. & J.Presl
Iz_3_1009	DJI_0896	Jasione montana L.	0	n	1	0	Campanulaceae Juss.
Iz_3_1009	DJI_0896	Jurinea cyanoides (L.) Rchb.s.l.	0	n	1	0	Asteraceae Bercht. & J.Presl
Iz_3_1009	DJI_0896	Koeleria glauca (Spreng.) DC.	0	n	1	0	Poaceae Barnhart
Iz_3_1009	DJI_0896	Lactuca serriola L.	0	a	0	0	Asteraceae Bercht. & J.Presl
Iz_3_1009	DJI_0896	Linaria genistifolia (L.) Mill.	0	n	1	0	Plantaginaceae Juss.
Iz_3_1009	DJI_0896	Lomelosia argentea (L.) Greuter & Burdet	0	n	1	0	Caprifoliaceae Juss.
Iz_3_1009	DJI_0896	Melampyrum pratense L.	0	n	1	0	Orobanchaceae Vent.
Iz_3_1009	DJI_0896	Oenothera biennis L.	0	a	0	0	Onagraceae Juss.
Iz_3_1009	DJI_0896	Pentanema britannica (L.) D.Gut.Larr., Santos-Vicente, Anderb., E.Rico & M.M.Mart.Ort.	0	n	1	0	Asteraceae Bercht. & J.Presl
Iz_3_1009	DJI_0896	Peucedanum oreoselinum (L.) Moench	0	n	1	0	Apiaceae Lindl.
Iz_3_1009	DJI_0896	Peucedanum ruthenicum M.Bieb.	0	n	1	0	Apiaceae Lindl.
Iz_3_1009	DJI_0896	Pilosella echioides (Lumn.) F.W.Schultz & Sch.Bip.	0	n	1	0	Asteraceae Bercht. & J.Presl
Iz_3_1009	DJI_0896	Pilosella officinarum F. Schultz et Sch. Bip.	0	n	1	0	Asteraceae Bercht. & J.Presl
Iz_3_1009	DJI_0896	Pilosella sp.	0		NA	0	Asteraceae Bercht. & J.Presl
Iz_3_1009	DJI_0896	Pinus sylvestris L.	0	n	1	1	Pinaceae Spreng. ex F.Rudolphi
Iz_3_1009	DJI_0896	Poa annua L.	0	n	1	0	Poaceae Barnhart
Iz_3_1009	DJI_0896	Poa nemoralis L.	0	n	1	0	Poaceae Barnhart
Iz_3_1009	DJI_0896	Poa sp.	0		NA	0	Poaceae Barnhart
Iz_3_1009	DJI_0896	Polygonatum odoratum (Mill.) Druce	0	n	1	0	Asparagaceae Juss.
Iz_3_1009	DJI_0896	Polygonum arenarium Waldst. & Kit.	0	n	1	0	Polygonaceae Juss.
Iz_3_1009	DJI_0896	Populus nigra L.	0	n	1	1	Salicaceae Mirb.
Iz_3_1009	DJI_0896	Populus tremula L.	0	n	1	1	Salicaceae Mirb.
Iz_3_1009	DJI_0896	Populus Ã canescens (Aiton) Sm.	0	n	1	1	Salicaceae Mirb.
Iz_3_1009	DJI_0896	Potentilla argentea L.	0	n	1	0	Rosaceae Juss.
Iz_3_1009	DJI_0896	Psammophiliella muralis (L.) Ikonn.	0	n	1	0	Caryophyllaceae Juss.
Iz_3_1009	DJI_0896	Psephellus sumensis (Kalen.) Greuter	0	n	1	0	Asteraceae Bercht. & J.Presl
Iz_3_1009	DJI_0896	Pulsatilla pratensis (L.) Mill. s.l.	0	n	1	0	Ranunculaceae Juss.
Iz_3_1009	DJI_0896	Pyrus communis L.	0	a	0	1	Rosaceae Juss.
Iz_3_1009	DJI_0896	Quercus robur L.	2	n	1	1	Fagaceae Dumort.
Iz_3_1009	DJI_0896	Robinia pseudoacacia L.	0	a	0	1	Fabaceae Lindl.
Iz_3_1009	DJI_0896	Rosa sp.	0		NA	1	Rosaceae Juss.
Iz_3_1009	DJI_0896	Rubus idaeus L.	0	n	1	0	Rosaceae Juss.
Iz_3_1009	DJI_0896	Rumex acetosella L.	0	n	1	0	Polygonaceae Juss.
Iz_3_1009	DJI_0896	Rumex thyrsiflorus Fingerh.	0	n	1	0	Polygonaceae Juss.
Iz_3_1009	DJI_0896	Salix alba L.	0	n	1	1	Salicaceae Mirb.
Iz_3_1009	DJI_0896	Sambucus nigra L.	0	n	1	1	Viburnaceae Raf.
Iz_3_1009	DJI_0896	Secale sylvestre Host	0	n	1	0	Poaceae Barnhart
Iz_3_1009	DJI_0896	Sempervivum ruthenicum Schnittsp. & C.B. Lehm.	0	n	1	0	Crassulaceae J.St.-Hil.
Iz_3_1009	DJI_0896	Setaria viridis (L.) P. Beauv.	0	n	1	0	Poaceae Barnhart
Iz_3_1009	DJI_0896	Silene borysthenica (Gruner) Walters	0	n	1	0	Caryophyllaceae Juss.
Iz_3_1009	DJI_0896	Silene latifolia subsp. alba (Mill.) Greuter & Burdet	0	n	1	0	Caryophyllaceae Juss.
Iz_3_1009	DJI_0896	Solidago virgaurea L.	0	n	1	0	Asteraceae Bercht. & J.Presl
Iz_3_1009	DJI_0896	Sonchus oleraceus L.	0	a	0	0	Asteraceae Bercht. & J.Presl
Iz_3_1009	DJI_0896	Sorbus aucuparia L.	0	n	1	1	Rosaceae Juss.
Iz_3_1009	DJI_0896	Stachys recta L.	0	n	1	0	Lamiacea Lindl.
Iz_3_1009	DJI_0896	Tanacetum vulgare L.	0	n	1	0	Asteraceae Bercht. & J.Presl
Iz_3_1009	DJI_0896	Thymus pallasianus Heinr.Braun	0	n	1	0	Lamiacea Lindl.
Iz_3_1009	DJI_0896	Tilia cordata Mill.	0	n	1	1	Malvaceae Juss.
Iz_3_1009	DJI_0896	Trifolium alpestre L.	0	n	1	0	Fabaceae Lindl.
Iz_3_1009	DJI_0896	Trifolium arvense L.	0	n	1	0	Fabaceae Lindl.
Iz_3_1009	DJI_0896	Ulmus glabra Huds.	0	n	1	1	Ulmaceae Mirb.
Iz_3_1009	DJI_0896	Verbascum densiflorum Bertol.	0	n	1	0	Scrophulariaceae Juss.
Iz_3_1009	DJI_0896	Veronica austriaca L.	0	n	1	0	Plantaginaceae Juss.
Iz_3_1009	DJI_0896	Veronica incana L.	0	n	1	0	Plantaginaceae Juss.
Iz_3_1009	DJI_0896	Veronica verna L.	0	n	1	0	Plantaginaceae Juss.
Iz_3_1009	DJI_0896	Vincetoxicum hirundinaria Medik	0	n	1	0	Apocynaceae Juss.
Iz_3_1009	DJI_0897	Acer tataricum L.	0	n	1	1	Sapindaceae Juss.
Iz_3_1009	DJI_0897	Achillea millefolium L. s.l.	0	n	1	0	Asteraceae Bercht. & J.Presl
Iz_3_1009	DJI_0897	Allium oleraceum L.	0	n	1	0	Amaryllidaceae J.St.-Hil.
Iz_3_1009	DJI_0897	Ambrosia artemisiifolia L.	0	a	0	0	Asteraceae Bercht. & J.Presl
Iz_3_1009	DJI_0897	Amorpha fruticosa L.	0	a	0	0	Fabaceae Lindl.
Iz_3_1009	DJI_0897	Anthericum ramosum L.	0	n	1	0	Asparagaceae Juss.
Iz_3_1009	DJI_0897	Artemisia marschalliana Spreng.	0	n	1	0	Asteraceae Bercht. & J.Presl
Iz_3_1009	DJI_0897	Asparagus officinalis L.	0	n	1	0	Asparagaceae Juss.
Iz_3_1009	DJI_0897	Astragalus cicer L.	0	n	1	0	Fabaceae Lindl.
Iz_3_1009	DJI_0897	Bassia laniflora (S.G.Gmel.) A.J.Scott	0	n	1	0	Amaranthaceae Juss.
Iz_3_1009	DJI_0897	Berberis vulgaris L.	0	n	1	0	Berberidaceae Juss.
Iz_3_1009	DJI_0897	Betula pendula Roth.	0	n	1	1	Betulaceae Gray

Iz_3_1009	DJI_0897	Betula pendula Roth.	0	n	1	0	Poaceae Barnhart
Iz_3_1009	DJI_0897	Bromus inermis Leyss.	0		NA	NA	
Iz_3_1009	DJI_0897	Bromus squarrosus L.	0	a	0	0	Poaceae Barnhart
Iz_3_1009	DJI_0897	Bromus tectorum L.	0	a	0	0	Poaceae Barnhart
Iz_3_1009	DJI_0897	Calamagrostis epigejos (L.) Roth	10	n	1	0	Poaceae Barnhart
Iz_3_1009	DJI_0897	Carduus acanthoides L.	0	n	1	0	Asteraceae Bercht. & J.Presl
Iz_3_1009	DJI_0897	Carex sp.	0	n	1	0	Cyperaceae Juss.
Iz_3_1009	DJI_0897	Centaurea majorovii Dumbadze	0	n	1	0	Asteraceae Bercht. & J.Presl
Iz_3_1009	DJI_0897	Chamaecytisus ruthenicus (Fisch.ex Wol.) Klaskova	3	n	1	0	Fabaceae Lindl.
Iz_3_1009	DJI_0897	Chelidonium majus L.	0	n	1	0	Papaveraceae Juss.
Iz_3_1009	DJI_0897	Chenopodium betaceum Andrz.	0	n	1	0	Amaranthaceae Juss.
Iz_3_1009	DJI_0897	Chenopodium cf. album	0	n	1	0	Amaranthaceae Juss.
Iz_3_1009	DJI_0897	Chenopodium sp.	0		NA	0	Amaranthaceae Juss.
Iz_3_1009	DJI_0897	Chondrilla juncea L.	0	n	1	0	Asteraceae Bercht. & J.Presl
Iz_3_1009	DJI_0897	Cirsium vulgare (Savi) Ten.	0	n	1	0	Asteraceae Bercht. & J.Presl
Iz_3_1009	DJI_0897	Clinopodium acinos (L.) Kuntze	0	n	1	0	Lamiacea Lindl.
Iz_3_1009	DJI_0897	Convallaria majalis L.	0	n	1	0	Asparagaceae Juss.
Iz_3_1009	DJI_0897	Crataegus rhipidophylla Gand.	0	n	1	1	Rosaceae Juss.
Iz_3_1009	DJI_0897	Crepis foetida L.	0	n	1	0	Asteraceae Bercht. & J.Presl
Iz_3_1009	DJI_0897	Dactylis glomerata L.	0	n	1	0	Poaceae Barnhart
Iz_3_1009	DJI_0897	Dianthus campestris M.Bieb.	0	n	1	0	Caryophyllaceae Juss.
Iz_3_1009	DJI_0897	Digitaria sanguinalis (L.) Scop.	0	a	0	0	Poaceae Barnhart
Iz_3_1009	DJI_0897	Elymus repens (L.) Gould	0	n	1	0	Poaceae Barnhart
Iz_3_1009	DJI_0897	Epilobium tetragonum L.	0	n	1	0	Onagraceae Juss.
Iz_3_1009	DJI_0897	Eragrostis minor Host	0	a	0	0	Poaceae Barnhart
Iz_3_1009	DJI_0897	Erigeron canadensis L.	0	a	0	0	Asteraceae Bercht. & J.Presl
Iz_3_1009	DJI_0897	Euphorbia seguieriana Neck.	0	n	1	0	Euphorbiaceae Juss.
Iz_3_1009	DJI_0897	Fallopia dumetorum (L.) Holub	1	n	1	0	Polygonaceae Juss.
Iz_3_1009	DJI_0897	Festuca beckeri (Hack.) Trautv.	0	n	1	0	Poaceae Barnhart
Iz_3_1009	DJI_0897	Fragaria vesca L.	0	n	1	0	Rosaceae Juss.
Iz_3_1009	DJI_0897	Frangula alnus Mill.	0	n	1	1	Rhamnaceae Juss.
Iz_3_1009	DJI_0897	Fraxinus pennsylvanica Marshall	0	a	0	1	Oleaceae Hoffmanns. & Link
Iz_3_1009	DJI_0897	Galeopsis bifida Boenn.	0	n	1	0	Lamiacea Lindl.
Iz_3_1009	DJI_0897	Galium verum L.	0	n	1	0	Rubiaceae Juss.
Iz_3_1009	DJI_0897	Genista tinctoria L.	0	n	1	0	Fabaceae Lindl.
Iz_3_1009	DJI_0897	Geranium robertianum L.	0	n	1	0	Geraniaceae Juss.
Iz_3_1009	DJI_0897	Geranium sanquineum L.	0	n	1	0	Geraniaceae Juss.
Iz_3_1009	DJI_0897	Gypsophila paniculata L.	0	n	1	0	Caryophyllaceae Juss.
Iz_3_1009	DJI_0897	Helichrysum arenarium (L.) Moench	0	n	1	0	Asteraceae Bercht. & J.Presl
Iz_3_1009	DJI_0897	Herniaria polygama J.Gay	0	n	1	0	Caryophyllaceae Juss.
Iz_3_1009	DJI_0897	Hieracium robustum Fr.	0	n	1	0	Asteraceae Bercht. & J.Presl
Iz_3_1009	DJI_0897	Hieracium umbellatum L.	0	n	1	NA	Asteraceae Bercht. & J.Presl
Iz_3_1009	DJI_0897	Hylotelephium maximum (L.) Holub	0	n	1	0	Crassulaceae J.St.-Hil.
Iz_3_1009	DJI_0897	Jacobaea borysthenica (DC.) B.Nord. & Greuter	0	n	1	0	Asteraceae Bercht. & J.Presl
Iz_3_1009	DJI_0897	Jasione montana L.	0	n	1	0	Campanulaceae Juss.
Iz_3_1009	DJI_0897	Jurinea cyanoides (L.) Rchb.s.l.	0	n	1	0	Asteraceae Bercht. & J.Presl
Iz_3_1009	DJI_0897	Koeleria glauca (Spreng.) DC.	0	n	1	0	Poaceae Barnhart
Iz_3_1009	DJI_0897	Lactuca serriola L.	0	a	0	0	Asteraceae Bercht. & J.Presl
Iz_3_1009	DJI_0897	Linaria genistifolia (L.) Mill.	0	n	1	0	Plantaginaceae Juss.
Iz_3_1009	DJI_0897	Lomelosia argentea (L.) Greuter & Burdet	0	n	1	0	Caprifoliaceae Juss.
Iz_3_1009	DJI_0897	Melampyrum pratense L.	0	n	1	0	Orobanchaceae Vent.
Iz_3_1009	DJI_0897	Oenothera biennis L.	0	a	0	0	Onagraceae Juss.
Iz_3_1009	DJI_0897	Pentanema britannica (L.) D.Gut.Larr., Santos-Vicente, Anderb., E.Rico & M.M.Mart.Ort.	0	n	1	0	Asteraceae Bercht. & J.Presl
Iz_3_1009	DJI_0897	Peucedanum oreoselinum (L.) Moench	7	n	1	0	Apiaceae Lindl.
Iz_3_1009	DJI_0897	Peucedanum ruthenicum M.Bieb.	0	n	1	0	Apiaceae Lindl.
Iz_3_1009	DJI_0897	Pilosella echioides (Lumn.) F.W.Schultz & Sch.Bip.	0	n	1	0	Asteraceae Bercht. & J.Presl
Iz_3_1009	DJI_0897	Pilosella officinarum F. Schultz et Sch. Bip.	0	n	1	0	Asteraceae Bercht. & J.Presl
Iz_3_1009	DJI_0897	Pilosella sp.	0		NA	0	Asteraceae Bercht. & J.Presl
Iz_3_1009	DJI_0897	Pinus sylvestris L.	0	n	1	1	Pinaceae Spreng. ex F.Rudolphi
Iz_3_1009	DJI_0897	Poa annua L.	0	n	1	0	Poaceae Barnhart
Iz_3_1009	DJI_0897	Poa nemoralis L.	0	n	1	0	Poaceae Barnhart
Iz_3_1009	DJI_0897	Poa sp.	0		NA	0	Poaceae Barnhart
Iz_3_1009	DJI_0897	Polygonatum odoratum (Mill.) Druce	0.2	n	1	0	Asparagaceae Juss.
Iz_3_1009	DJI_0897	Polygonum arenarium Waldst. & Kit.	0	n	1	0	Polygonaceae Juss.
Iz_3_1009	DJI_0897	Populus nigra L.	0	n	1	1	Salicaceae Mirb.
Iz_3_1009	DJI_0897	Populus tremula L.	0	n	1	1	Salicaceae Mirb.
Iz_3_1009	DJI_0897	Populus Ã canescens (Aiton) Sm.	0	n	1	1	Salicaceae Mirb.
Iz_3_1009	DJI_0897	Potentilla argentea L.	0	n	1	0	Rosaceae Juss.
Iz_3_1009	DJI_0897	Psammophiliella muralis (L.) Ikonn.	0	n	1	0	Caryophyllaceae Juss.
Iz_3_1009	DJI_0897	Psephellus sumensis (Kalen.) Greuter	0	n	1	0	Asteraceae Bercht. & J.Presl
Iz_3_1009	DJI_0897	Pulsatilla pratensis (L.) Mill. s.l.	0	n	1	0	Ranunculaceae Juss.
Iz_3_1009	DJI_0897	Pyrus communis L.	0	a	0	1	Rosaceae Juss.
Iz_3_1009	DJI_0897	Quercus robur L.	0	n	1	1	Fagaceae Dumort.
Iz_3_1009	DJI_0897	Robinia pseudoacacia L.	0	a	0	1	Fabaceae Lindl.
Iz_3_1009	DJI_0897	Rosa sp.	0		NA	1	Rosaceae Juss.
Iz_3_1009	DJI_0897	Rubus idaeus L.	0	n	1	0	Rosaceae Juss.
Iz_3_1009	DJI_0897	Rumex acetosella L.	0	n	1	0	Polygonaceae Juss.
Iz_3_1009	DJI_0897	Rumex thyrsiflorus Fingerh.	0	n	1	0	Polygonaceae Juss.
Iz_3_1009	DJI_0897	Salix alba L.	0	n	1	1	Salicaceae Mirb.
Iz_3_1009	DJI_0897	Sambucus nigra L.	0	n	1	1	Viburnaceae Raf.
Iz_3_1009	DJI_0897	Secale sylvestre Host	0	n	1	0	Poaceae Barnhart
Iz_3_1009	DJI_0897	Sempervivum ruthenicum Schnittsp. & C.B. Lehm.	0	n	1	0	Crassulaceae J.St.-Hil.
Iz_3_1009	DJI_0897	Setaria viridis (L.) P. Beauv.	0	n	1	0	Poaceae Barnhart
Iz_3_1009	DJI_0897	Silene borysthenica (Gruner) Walters	0	n	1	0	Caryophyllaceae Juss.
Iz_3_1009	DJI_0897	Silene latifolia subsp. alba (Mill.) Greuter & Burdet	0	n	1	0	Caryophyllaceae Juss.
Iz_3_1009	DJI_0897	Solidago virgaurea L.	0	n	1	0	Asteraceae Bercht. & J.Presl
Iz_3_1009	DJI_0897	Sonchus oleraceus L.	0	a	0	0	Asteraceae Bercht. & J.Presl
Iz_3_1009	DJI_0897	Sorbus aucuparia L.	0	n	1	1	Rosaceae Juss.
Iz_3_1009	DJI_0897	Stachys recta L.	0	n	1	0	Lamiacea Lindl.
Iz_3_1009	DJI_0897	Tanacetum vulgare L.	0	n	1	0	Asteraceae Bercht. & J.Presl
Iz_3_1009	DJI_0897	Thymus pallasianus Heinr.Braun	0	n	1	0	Lamiacea Lindl.
Iz_3_1009	DJI_0897	Tilia cordata Mill.	0	n	1	1	Malvaceae Juss.
Iz_3_1009	DJI_0897	Trifolium alpestre L.	0	n	1	0	Fabaceae Lindl.
Iz_3_1009	DJI_0897	Trifolium arvense L.	0	n	1	0	Fabaceae Lindl.
Iz_3_1009	DJI_0897	Ulmus glabra Huds.	0	n	1	1	Ulmaceae Mirb.
Iz_3_1009	DJI_0897	Verbascum densiflorum Bertol.	0	n	1	0	Scrophulariaceae Juss.
Iz_3_1009	DJI_0897	Veronica austriaca L.	0	n	1	0	Plantaginaceae Juss.
Iz_3_1009	DJI_0897	Veronica incana L.	0	n	1	0	Plantaginaceae Juss.

Iz_3_1009	DJI_0897	Veronica verna L.	0	n	1	0	Plantaginaceae Juss.
Iz_3_1009	DJI_0897	Vincetoxicum hirundinaria Medik	0	n	1	0	Apocynaceae Juss.
Iz_3_1009	DJI_0900	Acer tataricum L.	0	n	1	1	Sapindaceae Juss.
Iz_3_1009	DJI_0900	Achillea millefolium L. s.l.	0	n	1	0	Asteraceae Bercht. & J.Presl
Iz_3_1009	DJI_0900	Allium oleraceum L.	0	n	1	0	Amaryllidaceae J.St.-Hil.
Iz_3_1009	DJI_0900	Ambrosia artemisiifolia L.	0	a	0	0	Asteraceae Bercht. & J.Presl
Iz_3_1009	DJI_0900	Amorpha fruticosa L.	0	a	0	0	Fabaceae Lindl.
Iz_3_1009	DJI_0900	Anthericum ramosum L.	0	n	1	0	Asparagaceae Juss.
Iz_3_1009	DJI_0900	Artemisia marschalliana Spreng.	0	n	1	0	Asteraceae Bercht. & J.Presl
Iz_3_1009	DJI_0900	Asparagus officinalis L.	0	n	1	0	Asparagaceae Juss.
Iz_3_1009	DJI_0900	Astragalus cicer L.	0	n	1	0	Fabaceae Lindl.
Iz_3_1009	DJI_0900	Bassia laniflora (S.G.Gmel.) A.J.Scott	0	n	1	0	Amaranthaceae Juss.
Iz_3_1009	DJI_0900	Berberis vulgaris L.	0	n	1	0	Berberidaceae Juss.
Iz_3_1009	DJI_0900	Betula pendula Roth.	0	n	1	1	Betulaceae Gray
Iz_3_1009	DJI_0900	Betula pendula Roth.	0	n	1	0	Poaceae Barnhart
Iz_3_1009	DJI_0900	Bromus inermis Leyss.	0		NA	NA	
Iz_3_1009	DJI_0900	Bromus squarrosus L.	0	a	0	0	Poaceae Barnhart
Iz_3_1009	DJI_0900	Bromus tectorum L.	0	a	0	0	Poaceae Barnhart
Iz_3_1009	DJI_0900	Calamagrostis epigejos (L.) Roth	35	n	1	0	Poaceae Barnhart
Iz_3_1009	DJI_0900	Carduus acanthoides L.	0	n	1	0	Asteraceae Bercht. & J.Presl
Iz_3_1009	DJI_0900	Carex sp.	0	n	1	0	Cyperaceae Juss.
Iz_3_1009	DJI_0900	Centaurea majorovii Dumbadze	0	n	1	0	Asteraceae Bercht. & J.Presl
Iz_3_1009	DJI_0900	Chamaecytisus ruthenicus (Fisch.ex Wol.) Klaskova	0	n	1	0	Fabaceae Lindl.
Iz_3_1009	DJI_0900	Chelidonium majus L.	0	n	1	0	Papaveraceae Juss.
Iz_3_1009	DJI_0900	Chenopodium betaceum Andrz.	0	n	1	0	Amaranthaceae Juss.
Iz_3_1009	DJI_0900	Chenopodium cf. album	0	n	1	0	Amaranthaceae Juss.
Iz_3_1009	DJI_0900	Chenopodium sp.	0		NA	0	Amaranthaceae Juss.
Iz_3_1009	DJI_0900	Chondrilla juncea L.	0	n	1	0	Asteraceae Bercht. & J.Presl
Iz_3_1009	DJI_0900	Cirsium vulgare (Savi) Ten.	0	n	1	0	Asteraceae Bercht. & J.Presl
Iz_3_1009	DJI_0900	Clinopodium acinos (L.) Kuntze	0	n	1	0	Lamiacea Lindl.
Iz_3_1009	DJI_0900	Convallaria majalis L.	0	n	1	0	Asparagaceae Juss.
Iz_3_1009	DJI_0900	Crataegus rhipidophylla Gand.	2	n	1	1	Rosaceae Juss.
Iz_3_1009	DJI_0900	Crepis foetida L.	0	n	1	0	Asteraceae Bercht. & J.Presl
Iz_3_1009	DJI_0900	Dactylis glomerata L.	0	n	1	0	Poaceae Barnhart
Iz_3_1009	DJI_0900	Dianthus campestris M.Bieb.	0	n	1	0	Caryophyllaceae Juss.
Iz_3_1009	DJI_0900	Digitaria sanguinalis (L.) Scop.	0	a	0	0	Poaceae Barnhart
Iz_3_1009	DJI_0900	Elymus repens (L.) Gould	0	n	1	0	Poaceae Barnhart
Iz_3_1009	DJI_0900	Epilobium tetragonum L.	0	n	1	0	Onagraceae Juss.
Iz_3_1009	DJI_0900	Eragrostis minor Host	0	a	0	0	Poaceae Barnhart
Iz_3_1009	DJI_0900	Erigeron canadensis L.	0	a	0	0	Asteraceae Bercht. & J.Presl
Iz_3_1009	DJI_0900	Euphorbia seguieriana Neck.	0	n	1	0	Euphorbiaceae Juss.
Iz_3_1009	DJI_0900	Fallopia dumetorum (L.) Holub	1	n	1	0	Polygonaceae Juss.
Iz_3_1009	DJI_0900	Festuca beckeri (Hack.) Trautv.	0	n	1	0	Poaceae Barnhart
Iz_3_1009	DJI_0900	Fragaria vesca L.	0	n	1	0	Rosaceae Juss.
Iz_3_1009	DJI_0900	Frangula alnus Mill.	0	n	1	1	Rhamnaceae Juss.
Iz_3_1009	DJI_0900	Fraxinus pennsylvanica Marshall	0	a	0	1	Oleaceae Hoffmanns. & Link
Iz_3_1009	DJI_0900	Galeopsis bifida Boenn.	0	n	1	0	Lamiacea Lindl.
Iz_3_1009	DJI_0900	Galium verum L.	0	n	1	0	Rubiaceae Juss.
Iz_3_1009	DJI_0900	Genista tinctoria L.	0	n	1	0	Fabaceae Lindl.
Iz_3_1009	DJI_0900	Geranium robertianum L.	0	n	1	0	Geraniaceae Juss.
Iz_3_1009	DJI_0900	Geranium sanquineum L.	0	n	1	0	Geraniaceae Juss.
Iz_3_1009	DJI_0900	Gypsophila paniculata L.	0	n	1	0	Caryophyllaceae Juss.
Iz_3_1009	DJI_0900	Helichrysum arenarium (L.) Moench	0	n	1	0	Asteraceae Bercht. & J.Presl
Iz_3_1009	DJI_0900	Herniaria polygama J.Gay	0	n	1	0	Caryophyllaceae Juss.
Iz_3_1009	DJI_0900	Hieracium robustum Fr.	0	n	1	0	Asteraceae Bercht. & J.Presl
Iz_3_1009	DJI_0900	Hieracium umbellatum L.	0	n	1	NA	Asteraceae Bercht. & J.Presl
Iz_3_1009	DJI_0900	Hylotelephium maximum (L.) Holub	0	n	1	0	Crassulaceae J.St.-Hil.
Iz_3_1009	DJI_0900	Jacobaea borysthenica (DC.) B.Nord. & Greuter	0	n	1	0	Asteraceae Bercht. & J.Presl
Iz_3_1009	DJI_0900	Jasione montana L.	0	n	1	0	Campanulaceae Juss.
Iz_3_1009	DJI_0900	Jurinea cyanoides (L.) Rchb.s.l.	0	n	1	0	Asteraceae Bercht. & J.Presl
Iz_3_1009	DJI_0900	Koeleria glauca (Spreng.) DC.	0	n	1	0	Poaceae Barnhart
Iz_3_1009	DJI_0900	Lactuca serriola L.	0	a	0	0	Asteraceae Bercht. & J.Presl
Iz_3_1009	DJI_0900	Linaria genistifolia (L.) Mill.	0	n	1	0	Plantaginaceae Juss.
Iz_3_1009	DJI_0900	Lomelosia argentea (L.) Greuter & Burdet	0	n	1	0	Caprifoliaceae Juss.
Iz_3_1009	DJI_0900	Melampyrum pratense L.	0	n	1	0	Orobanchaceae Vent.
Iz_3_1009	DJI_0900	Oenothera biennis L.	0	a	0	0	Onagraceae Juss.
Iz_3_1009	DJI_0900	Pentanema britannica (L.) D.Gut.Larr., Santos-Vicente, Anderb., E.Rico & M.M.Mart.Ort.	0	n	1	0	Asteraceae Bercht. & J.Presl
Iz_3_1009	DJI_0900	Peucedanum oreoselinum (L.) Moench	3	n	1	0	Apiaceae Lindl.
Iz_3_1009	DJI_0900	Peucedanum ruthenicum M.Bieb.	0	n	1	0	Apiaceae Lindl.
Iz_3_1009	DJI_0900	Pilosella echioides (Lumn.) F.W.Schultz & Sch.Bip.	0	n	1	0	Asteraceae Bercht. & J.Presl
Iz_3_1009	DJI_0900	Pilosella officinarum F. Schultz et Sch. Bip.	0	n	1	0	Asteraceae Bercht. & J.Presl
Iz_3_1009	DJI_0900	Pilosella sp.	0		NA	0	Asteraceae Bercht. & J.Presl
Iz_3_1009	DJI_0900	Pinus sylvestris L.	0	n	1	1	Pinaceae Spreng. ex F.Rudolphi
Iz_3_1009	DJI_0900	Poa annua L.	0	n	1	0	Poaceae Barnhart
Iz_3_1009	DJI_0900	Poa nemoralis L.	0	n	1	0	Poaceae Barnhart
Iz_3_1009	DJI_0900	Poa sp.	0		NA	0	Poaceae Barnhart
Iz_3_1009	DJI_0900	Polygonatum odoratum (Mill.) Druce	0	n	1	0	Asparagaceae Juss.
Iz_3_1009	DJI_0900	Polygonum arenarium Waldst. & Kit.	0	n	1	0	Polygonaceae Juss.
Iz_3_1009	DJI_0900	Populus nigra L.	0	n	1	1	Salicaceae Mirb.
Iz_3_1009	DJI_0900	Populus tremula L.	0	n	1	1	Salicaceae Mirb.
Iz_3_1009	DJI_0900	Populus Ã canescens (Aiton) Sm.	0	n	1	1	Salicaceae Mirb.
Iz_3_1009	DJI_0900	Potentilla argentea L.	0	n	1	0	Rosaceae Juss.
Iz_3_1009	DJI_0900	Psammophiliella muralis (L.) Ikonn.	0	n	1	0	Caryophyllaceae Juss.
Iz_3_1009	DJI_0900	Psephellus sumensis (Kalen.) Greuter	0	n	1	0	Asteraceae Bercht. & J.Presl
Iz_3_1009	DJI_0900	Pulsatilla pratensis (L.) Mill. s.l.	0	n	1	0	Ranunculaceae Juss.
Iz_3_1009	DJI_0900	Pyrus communis L.	0	a	0	1	Rosaceae Juss.
Iz_3_1009	DJI_0900	Quercus robur L.	0	n	1	1	Fagaceae Dumort.
Iz_3_1009	DJI_0900	Robinia pseudoacacia L.	0	a	0	1	Fabaceae Lindl.
Iz_3_1009	DJI_0900	Rosa sp.	0		NA	1	Rosaceae Juss.
Iz_3_1009	DJI_0900	Rubus idaeus L.	0	n	1	0	Rosaceae Juss.
Iz_3_1009	DJI_0900	Rumex acetosella L.	0	n	1	0	Polygonaceae Juss.
Iz_3_1009	DJI_0900	Rumex thyrsiflorus Fingerh.	0	n	1	0	Polygonaceae Juss.
Iz_3_1009	DJI_0900	Salix alba L.	0	n	1	1	Salicaceae Mirb.
Iz_3_1009	DJI_0900	Sambucus nigra L.	0	n	1	1	Viburnaceae Raf.
Iz_3_1009	DJI_0900	Secale sylvestre Host	0	n	1	0	Poaceae Barnhart
Iz_3_1009	DJI_0900	Sempervivum ruthenicum Schnittsp. & C.B. Lehm.	0	n	1	0	Crassulaceae J.St.-Hil.
Iz_3_1009	DJI_0900	Setaria viridis (L.) P. Beauv.	0	n	1	0	Poaceae Barnhart
Iz_3_1009	DJI_0900	Silene borysthenica (Gruner) Walters	0	n	1	0	Caryophyllaceae Juss.

Iz_3_1009	DJI_0900	Silene latifolia subsp. alba (Mill.) Greuter & Burdet	0	n	1	0	Caryophyllaceae Juss.
Iz_3_1009	DJI_0900	Solidago virgaurea L.	0	n	1	0	Asteraceae Bercht. & J.Presl
Iz_3_1009	DJI_0900	Sonchus oleraceus L.	0	a	0	0	Asteraceae Bercht. & J.Presl
Iz_3_1009	DJI_0900	Sorbus aucuparia L.	0	n	1	1	Rosaceae Juss.
Iz_3_1009	DJI_0900	Stachys recta L.	0	n	1	0	Lamiacea Lindl.
Iz_3_1009	DJI_0900	Tanacetum vulgare L.	0	n	1	0	Asteraceae Bercht. & J.Presl
Iz_3_1009	DJI_0900	Thymus pallasianus Heinr.Braun	0	n	1	0	Lamiacea Lindl.
Iz_3_1009	DJI_0900	Tilia cordata Mill.	0	n	1	1	Malvaceae Juss.
Iz_3_1009	DJI_0900	Trifolium alpestre L.	0	n	1	0	Fabaceae Lindl.
Iz_3_1009	DJI_0900	Trifolium arvense L.	0	n	1	0	Fabaceae Lindl.
Iz_3_1009	DJI_0900	Ulmus glabra Huds.	0	n	1	1	Ulmaceae Mirb.
Iz_3_1009	DJI_0900	Verbascum densiflorum Bertol.	0	n	1	0	Scrophulariaceae Juss.
Iz_3_1009	DJI_0900	Veronica austriaca L.	0	n	1	0	Plantaginaceae Juss.
Iz_3_1009	DJI_0900	Veronica incana L.	0	n	1	0	Plantaginaceae Juss.
Iz_3_1009	DJI_0900	Veronica verna L.	0	n	1	0	Plantaginaceae Juss.
Iz_3_1009	DJI_0900	Vincetoxicum hirundinaria Medik	0	n	1	0	Apocynaceae Juss.
Iz_3_1009	DJI_0901	Acer tataricum L.	0	n	1	1	Sapindaceae Juss.
Iz_3_1009	DJI_0901	Achillea millefolium L. s.l.	0	n	1	0	Asteraceae Bercht. & J.Presl
Iz_3_1009	DJI_0901	Allium oleraceum L.	0	n	1	0	Amaryllidaceae J.St.-Hil.
Iz_3_1009	DJI_0901	Ambrosia artemisiifolia L.	0	a	0	0	Asteraceae Bercht. & J.Presl
Iz_3_1009	DJI_0901	Amorpha fruticosa L.	0	a	0	0	Fabaceae Lindl.
Iz_3_1009	DJI_0901	Anthericum ramosum L.	0	n	1	0	Asparagaceae Juss.
Iz_3_1009	DJI_0901	Artemisia marschalliana Spreng.	0	n	1	0	Asteraceae Bercht. & J.Presl
Iz_3_1009	DJI_0901	Asparagus officinalis L.	0	n	1	0	Asparagaceae Juss.
Iz_3_1009	DJI_0901	Astragalus cicer L.	0	n	1	0	Fabaceae Lindl.
Iz_3_1009	DJI_0901	Bassia laniflora (S.G.Gmel.) A.J.Scott	0	n	1	0	Amaranthaceae Juss.
Iz_3_1009	DJI_0901	Berberis vulgaris L.	0	n	1	0	Berberidaceae Juss.
Iz_3_1009	DJI_0901	Betula pendula Roth.	0	n	1	1	Betulaceae Gray
Iz_3_1009	DJI_0901	Betula pendula Roth.	0	n	1	0	Poaceae Barnhart
Iz_3_1009	DJI_0901	Bromus inermis Leyss.	0		NA	NA	
Iz_3_1009	DJI_0901	Bromus squarrosus L.	0	a	0	0	Poaceae Barnhart
Iz_3_1009	DJI_0901	Bromus tectorum L.	0	a	0	0	Poaceae Barnhart
Iz_3_1009	DJI_0901	Calamagrostis epigejos (L.) Roth	25	n	1	0	Poaceae Barnhart
Iz_3_1009	DJI_0901	Carduus acanthoides L.	0	n	1	0	Asteraceae Bercht. & J.Presl
Iz_3_1009	DJI_0901	Carex sp.	0	n	1	0	Cyperaceae Juss.
Iz_3_1009	DJI_0901	Centaurea majorovii Dumbadze	0	n	1	0	Asteraceae Bercht. & J.Presl
Iz_3_1009	DJI_0901	Chamaecytisus ruthenicus (Fisch.ex Wol.) Klaskova	0	n	1	0	Fabaceae Lindl.
Iz_3_1009	DJI_0901	Chelidonium majus L.	0	n	1	0	Papaveraceae Juss.
Iz_3_1009	DJI_0901	Chenopodium betaceum Andrz.	0	n	1	0	Amaranthaceae Juss.
Iz_3_1009	DJI_0901	Chenopodium cf. album	0	n	1	0	Amaranthaceae Juss.
Iz_3_1009	DJI_0901	Chenopodium sp.	0		NA	0	Amaranthaceae Juss.
Iz_3_1009	DJI_0901	Chondrilla juncea L.	0	n	1	0	Asteraceae Bercht. & J.Presl
Iz_3_1009	DJI_0901	Cirsium vulgare (Savi) Ten.	0	n	1	0	Asteraceae Bercht. & J.Presl
Iz_3_1009	DJI_0901	Clinopodium acinos (L.) Kuntze	0	n	1	0	Lamiacea Lindl.
Iz_3_1009	DJI_0901	Convallaria majalis L.	0	n	1	0	Asparagaceae Juss.
Iz_3_1009	DJI_0901	Crataegus rhipidophylla Gand.	0	n	1	1	Rosaceae Juss.
Iz_3_1009	DJI_0901	Crepis foetida L.	0	n	1	0	Asteraceae Bercht. & J.Presl
Iz_3_1009	DJI_0901	Dactylis glomerata L.	0	n	1	0	Poaceae Barnhart
Iz_3_1009	DJI_0901	Dianthus campestris M.Bieb.	0	n	1	0	Caryophyllaceae Juss.
Iz_3_1009	DJI_0901	Digitaria sanguinalis (L.) Scop.	0	a	0	0	Poaceae Barnhart
Iz_3_1009	DJI_0901	Elymus repens (L.) Gould	0	n	1	0	Poaceae Barnhart
Iz_3_1009	DJI_0901	Epilobium tetragonum L.	0	n	1	0	Onagraceae Juss.
Iz_3_1009	DJI_0901	Eragrostis minor Host	0	a	0	0	Poaceae Barnhart
Iz_3_1009	DJI_0901	Erigeron canadensis L.	0	a	0	0	Asteraceae Bercht. & J.Presl
Iz_3_1009	DJI_0901	Euphorbia seguieriana Neck.	0	n	1	0	Euphorbiaceae Juss.
Iz_3_1009	DJI_0901	Fallopia dumetorum (L.) Holub	2	n	1	0	Polygonaceae Juss.
Iz_3_1009	DJI_0901	Festuca beckeri (Hack.) Trautv.	0	n	1	0	Poaceae Barnhart
Iz_3_1009	DJI_0901	Fragaria vesca L.	0	n	1	0	Rosaceae Juss.
Iz_3_1009	DJI_0901	Frangula alnus Mill.	0	n	1	1	Rhamnaceae Juss.
Iz_3_1009	DJI_0901	Fraxinus pennsylvanica Marshall	0	a	0	1	Oleaceae Hoffmanns. & Link
Iz_3_1009	DJI_0901	Galeopsis bifida Boenn.	0	n	1	0	Lamiacea Lindl.
Iz_3_1009	DJI_0901	Galium verum L.	0	n	1	0	Rubiaceae Juss.
Iz_3_1009	DJI_0901	Genista tinctoria L.	0	n	1	0	Fabaceae Lindl.
Iz_3_1009	DJI_0901	Geranium robertianum L.	0	n	1	0	Geraniaceae Juss.
Iz_3_1009	DJI_0901	Geranium sanquineum L.	0	n	1	0	Geraniaceae Juss.
Iz_3_1009	DJI_0901	Gypsophila paniculata L.	0	n	1	0	Caryophyllaceae Juss.
Iz_3_1009	DJI_0901	Helichrysum arenarium (L.) Moench	0	n	1	0	Asteraceae Bercht. & J.Presl
Iz_3_1009	DJI_0901	Herniaria polygama J.Gay	0	n	1	0	Caryophyllaceae Juss.
Iz_3_1009	DJI_0901	Hieracium robustum Fr.	0	n	1	0	Asteraceae Bercht. & J.Presl
Iz_3_1009	DJI_0901	Hieracium umbellatum L.	0	n	1	NA	Asteraceae Bercht. & J.Presl
Iz_3_1009	DJI_0901	Hylotelephium maximum (L.) Holub	0	n	1	0	Crassulaceae J.St.-Hil.
Iz_3_1009	DJI_0901	Jacobaea borysthenica (DC.) B.Nord. & Greuter	0	n	1	0	Asteraceae Bercht. & J.Presl
Iz_3_1009	DJI_0901	Jasione montana L.	0	n	1	0	Campanulaceae Juss.
Iz_3_1009	DJI_0901	Jurinea cyanoides (L.) Rchb.s.l.	0	n	1	0	Asteraceae Bercht. & J.Presl
Iz_3_1009	DJI_0901	Koeleria glauca (Spreng.) DC.	0	n	1	0	Poaceae Barnhart
Iz_3_1009	DJI_0901	Lactuca serriola L.	0	a	0	0	Asteraceae Bercht. & J.Presl
Iz_3_1009	DJI_0901	Linaria genistifolia (L.) Mill.	0	n	1	0	Plantaginaceae Juss.
Iz_3_1009	DJI_0901	Lomelosia argentea (L.) Greuter & Burdet	0	n	1	0	Caprifoliaceae Juss.
Iz_3_1009	DJI_0901	Melampyrum pratense L.	0	n	1	0	Orobanchaceae Vent.
Iz_3_1009	DJI_0901	Oenothera biennis L.	0	a	0	0	Onagraceae Juss.
Iz_3_1009	DJI_0901	Pentanema britannica (L.) D.Gut.Larr., Santos-Vicente, Anderb., E.Rico & M.M.Mart.Ort.	0	n	1	0	Asteraceae Bercht. & J.Presl
Iz_3_1009	DJI_0901	Peucedanum oreoselinum (L.) Moench	0	n	1	0	Apiaceae Lindl.
Iz_3_1009	DJI_0901	Peucedanum ruthenicum M.Bieb.	0	n	1	0	Apiaceae Lindl.
Iz_3_1009	DJI_0901	Pilosella echioides (Lumn.) F.W.Schultz & Sch.Bip.	0	n	1	0	Asteraceae Bercht. & J.Presl
Iz_3_1009	DJI_0901	Pilosella officinarum F. Schultz et Sch. Bip.	0	n	1	0	Asteraceae Bercht. & J.Presl
Iz_3_1009	DJI_0901	Pilosella sp.	0		NA	0	Asteraceae Bercht. & J.Presl
Iz_3_1009	DJI_0901	Pinus sylvestris L.	0	n	1	1	Pinaceae Spreng. ex F.Rudolphi
Iz_3_1009	DJI_0901	Poa annua L.	0	n	1	0	Poaceae Barnhart
Iz_3_1009	DJI_0901	Poa nemoralis L.	0	n	1	0	Poaceae Barnhart
Iz_3_1009	DJI_0901	Poa sp.	0		NA	0	Poaceae Barnhart
Iz_3_1009	DJI_0901	Polygonatum odoratum (Mill.) Druce	0	n	1	0	Asparagaceae Juss.
Iz_3_1009	DJI_0901	Polygonum arenarium Waldst. & Kit.	0	n	1	0	Polygonaceae Juss.
Iz_3_1009	DJI_0901	Populus nigra L.	0	n	1	1	Salicaceae Mirb.
Iz_3_1009	DJI_0901	Populus tremula L.	0	n	1	1	Salicaceae Mirb.
Iz_3_1009	DJI_0901	Populus Ã canescens (Aiton) Sm.	0	n	1	1	Salicaceae Mirb.
Iz_3_1009	DJI_0901	Potentilla argentea L.	0	n	1	0	Rosaceae Juss.
Iz_3_1009	DJI_0901	Psammophiliella muralis (L.) Ikonn.	0	n	1	0	Caryophyllaceae Juss.
Iz_3_1009	DJI_0901	Psephellus sumensis (Kalen.) Greuter	0	n	1	0	Asteraceae Bercht. & J.Presl

Iz_3_1009	DJI_0901	Pulsatilla pratensis (L.) Mill. s.l.	0	n	1	0	Ranunculaceae Juss.
Iz_3_1009	DJI_0901	Pyrus communis L.	0	a	0	1	Rosaceae Juss.
Iz_3_1009	DJI_0901	Quercus robur L.	0	n	1	1	Fagaceae Dumort.
Iz_3_1009	DJI_0901	Robinia pseudoacacia L.	0	a	0	1	Fabaceae Lindl.
Iz_3_1009	DJI_0901	Rosa sp.	0		NA	1	Rosaceae Juss.
Iz_3_1009	DJI_0901	Rubus idaeus L.	0	n	1	0	Rosaceae Juss.
Iz_3_1009	DJI_0901	Rumex acetosella L.	0	n	1	0	Polygonaceae Juss.
Iz_3_1009	DJI_0901	Rumex thyrsiflorus Fingerh.	0	n	1	0	Polygonaceae Juss.
Iz_3_1009	DJI_0901	Salix alba L.	0	n	1	1	Salicaceae Mirb.
Iz_3_1009	DJI_0901	Sambucus nigra L.	0	n	1	1	Viburnaceae Raf.
Iz_3_1009	DJI_0901	Secale sylvestre Host	0	n	1	0	Poaceae Barnhart
Iz_3_1009	DJI_0901	Sempervivum ruthenicum Schnittsp. & C.B. Lehm.	0	n	1	0	Crassulaceae J.St.-Hil.
Iz_3_1009	DJI_0901	Setaria viridis (L.) P. Beauv.	0	n	1	0	Poaceae Barnhart
Iz_3_1009	DJI_0901	Silene borysthenica (Gruner) Walters	0	n	1	0	Caryophyllaceae Juss.
Iz_3_1009	DJI_0901	Silene latifolia subsp. alba (Mill.) Greuter & Burdet	0	n	1	0	Caryophyllaceae Juss.
Iz_3_1009	DJI_0901	Solidago virgaurea L.	0.2	n	1	0	Asteraceae Bercht. & J.Presl
Iz_3_1009	DJI_0901	Sonchus oleraceus L.	0	a	0	0	Asteraceae Bercht. & J.Presl
Iz_3_1009	DJI_0901	Sorbus aucuparia L.	0	n	1	1	Rosaceae Juss.
Iz_3_1009	DJI_0901	Stachys recta L.	0	n	1	0	Lamiacea Lindl.
Iz_3_1009	DJI_0901	Tanacetum vulgare L.	0	n	1	0	Asteraceae Bercht. & J.Presl
Iz_3_1009	DJI_0901	Thymus pallasianus Heinr.Braun	0	n	1	0	Lamiacea Lindl.
Iz_3_1009	DJI_0901	Tilia cordata Mill.	0	n	1	1	Malvaceae Juss.
Iz_3_1009	DJI_0901	Trifolium alpestre L.	0	n	1	0	Fabaceae Lindl.
Iz_3_1009	DJI_0901	Trifolium arvense L.	0	n	1	0	Fabaceae Lindl.
Iz_3_1009	DJI_0901	Ulmus glabra Huds.	0	n	1	1	Ulmaceae Mirb.
Iz_3_1009	DJI_0901	Verbascum densiflorum Bertol.	0	n	1	0	Scrophulariaceae Juss.
Iz_3_1009	DJI_0901	Veronica austriaca L.	0	n	1	0	Plantaginaceae Juss.
Iz_3_1009	DJI_0901	Veronica incana L.	0	n	1	0	Plantaginaceae Juss.
Iz_3_1009	DJI_0901	Veronica verna L.	0	n	1	0	Plantaginaceae Juss.
Iz_3_1009	DJI_0901	Vincetoxicum hirundinaria Medik	0	n	1	0	Apocynaceae Juss.
Iz_3_1009	DJI_0902	Acer tataricum L.	0	n	1	1	Sapindaceae Juss.
Iz_3_1009	DJI_0902	Achillea millefolium L. s.l.	0	n	1	0	Asteraceae Bercht. & J.Presl
Iz_3_1009	DJI_0902	Allium oleraceum L.	0	n	1	0	Amaryllidaceae J.St.-Hil.
Iz_3_1009	DJI_0902	Ambrosia artemisiifolia L.	0	a	0	0	Asteraceae Bercht. & J.Presl
Iz_3_1009	DJI_0902	Amorpha fruticosa L.	0	a	0	0	Fabaceae Lindl.
Iz_3_1009	DJI_0902	Anthericum ramosum L.	0	n	1	0	Asparagaceae Juss.
Iz_3_1009	DJI_0902	Artemisia marschalliana Spreng.	0	n	1	0	Asteraceae Bercht. & J.Presl
Iz_3_1009	DJI_0902	Asparagus officinalis L.	0	n	1	0	Asparagaceae Juss.
Iz_3_1009	DJI_0902	Astragalus cicer L.	0	n	1	0	Fabaceae Lindl.
Iz_3_1009	DJI_0902	Bassia laniflora (S.G.Gmel.) A.J.Scott	0	n	1	0	Amaranthaceae Juss.
Iz_3_1009	DJI_0902	Berberis vulgaris L.	0	n	1	0	Berberidaceae Juss.
Iz_3_1009	DJI_0902	Betula pendula Roth.	0	n	1	1	Betulaceae Gray
Iz_3_1009	DJI_0902	Betula pendula Roth.	0	n	1	0	Poaceae Barnhart
Iz_3_1009	DJI_0902	Bromus inermis Leyss.	0		NA	NA	
Iz_3_1009	DJI_0902	Bromus squarrosus L.	0	a	0	0	Poaceae Barnhart
Iz_3_1009	DJI_0902	Bromus tectorum L.	0	a	0	0	Poaceae Barnhart
Iz_3_1009	DJI_0902	Calamagrostis epigejos (L.) Roth	50	n	1	0	Poaceae Barnhart
Iz_3_1009	DJI_0902	Carduus acanthoides L.	0	n	1	0	Asteraceae Bercht. & J.Presl
Iz_3_1009	DJI_0902	Carex sp.	0	n	1	0	Cyperaceae Juss.
Iz_3_1009	DJI_0902	Centaurea majorovii Dumbadze	0	n	1	0	Asteraceae Bercht. & J.Presl
Iz_3_1009	DJI_0902	Chamaecytisus ruthenicus (Fisch.ex Wol.) Klaskova	0	n	1	0	Fabaceae Lindl.
Iz_3_1009	DJI_0902	Chelidonium majus L.	0	n	1	0	Papaveraceae Juss.
Iz_3_1009	DJI_0902	Chenopodium betaceum Andrz.	0	n	1	0	Amaranthaceae Juss.
Iz_3_1009	DJI_0902	Chenopodium cf. album	0	n	1	0	Amaranthaceae Juss.
Iz_3_1009	DJI_0902	Chenopodium sp.	0		NA	0	Amaranthaceae Juss.
Iz_3_1009	DJI_0902	Chondrilla juncea L.	0	n	1	0	Asteraceae Bercht. & J.Presl
Iz_3_1009	DJI_0902	Cirsium vulgare (Savi) Ten.	0	n	1	0	Asteraceae Bercht. & J.Presl
Iz_3_1009	DJI_0902	Clinopodium acinos (L.) Kuntze	0	n	1	0	Lamiacea Lindl.
Iz_3_1009	DJI_0902	Convallaria majalis L.	0	n	1	0	Asparagaceae Juss.
Iz_3_1009	DJI_0902	Crataegus rhipidophylla Gand.	0	n	1	1	Rosaceae Juss.
Iz_3_1009	DJI_0902	Crepis foetida L.	0	n	1	0	Asteraceae Bercht. & J.Presl
Iz_3_1009	DJI_0902	Dactylis glomerata L.	0	n	1	0	Poaceae Barnhart
Iz_3_1009	DJI_0902	Dianthus campestris M.Bieb.	0	n	1	0	Caryophyllaceae Juss.
Iz_3_1009	DJI_0902	Digitaria sanguinalis (L.) Scop.	0	a	0	0	Poaceae Barnhart
Iz_3_1009	DJI_0902	Elymus repens (L.) Gould	0	n	1	0	Poaceae Barnhart
Iz_3_1009	DJI_0902	Epilobium tetragonum L.	0	n	1	0	Onagraceae Juss.
Iz_3_1009	DJI_0902	Eragrostis minor Host	0	a	0	0	Poaceae Barnhart
Iz_3_1009	DJI_0902	Erigeron canadensis L.	0	a	0	0	Asteraceae Bercht. & J.Presl
Iz_3_1009	DJI_0902	Euphorbia seguieriana Neck.	0	n	1	0	Euphorbiaceae Juss.
Iz_3_1009	DJI_0902	Fallopia dumetorum (L.) Holub	2	n	1	0	Polygonaceae Juss.
Iz_3_1009	DJI_0902	Festuca beckeri (Hack.) Trautv.	0	n	1	0	Poaceae Barnhart
Iz_3_1009	DJI_0902	Fragaria vesca L.	0	n	1	0	Rosaceae Juss.
Iz_3_1009	DJI_0902	Frangula alnus Mill.	0	n	1	1	Rhamnaceae Juss.
Iz_3_1009	DJI_0902	Fraxinus pennsylvanica Marshall	0	a	0	1	Oleaceae Hoffmanns. & Link
Iz_3_1009	DJI_0902	Galeopsis bifida Boenn.	0	n	1	0	Lamiacea Lindl.
Iz_3_1009	DJI_0902	Galium verum L.	0	n	1	0	Rubiaceae Juss.
Iz_3_1009	DJI_0902	Genista tinctoria L.	0	n	1	0	Fabaceae Lindl.
Iz_3_1009	DJI_0902	Geranium robertianum L.	0	n	1	0	Geraniaceae Juss.
Iz_3_1009	DJI_0902	Geranium sanquineum L.	0	n	1	0	Geraniaceae Juss.
Iz_3_1009	DJI_0902	Gypsophila paniculata L.	0	n	1	0	Caryophyllaceae Juss.
Iz_3_1009	DJI_0902	Helichrysum arenarium (L.) Moench	0	n	1	0	Asteraceae Bercht. & J.Presl
Iz_3_1009	DJI_0902	Herniaria polygama J.Gay	0	n	1	0	Caryophyllaceae Juss.
Iz_3_1009	DJI_0902	Hieracium robustum Fr.	0	n	1	0	Asteraceae Bercht. & J.Presl
Iz_3_1009	DJI_0902	Hieracium umbellatum L.	0	n	1	NA	Asteraceae Bercht. & J.Presl
Iz_3_1009	DJI_0902	Hylotelephium maximum (L.) Holub	0.2	n	1	0	Crassulaceae J.St.-Hil.
Iz_3_1009	DJI_0902	Jacobaea borysthenica (DC.) B.Nord. & Greuter	0	n	1	0	Asteraceae Bercht. & J.Presl
Iz_3_1009	DJI_0902	Jasione montana L.	0	n	1	0	Campanulaceae Juss.
Iz_3_1009	DJI_0902	Jurinea cyanoides (L.) Rchb.s.l.	0	n	1	0	Asteraceae Bercht. & J.Presl
Iz_3_1009	DJI_0902	Koeleria glauca (Spreng.) DC.	0	n	1	0	Poaceae Barnhart
Iz_3_1009	DJI_0902	Lactuca serriola L.	0	a	0	0	Asteraceae Bercht. & J.Presl
Iz_3_1009	DJI_0902	Linaria genistifolia (L.) Mill.	0	n	1	0	Plantaginaceae Juss.
Iz_3_1009	DJI_0902	Lomelosia argentea (L.) Greuter & Burdet	0	n	1	0	Caprifoliaceae Juss.
Iz_3_1009	DJI_0902	Melampyrum pratense L.	0	n	1	0	Orobanchaceae Vent.
Iz_3_1009	DJI_0902	Oenothera biennis L.	0	a	0	0	Onagraceae Juss.
Iz_3_1009	DJI_0902	Pentanema britannica (L.) D.Gut.Larr., Santos-Vicente, Anderb., E.Rico & M.M.Mart.Ort.	0	n	1	0	Asteraceae Bercht. & J.Presl
Iz_3_1009	DJI_0902	Peucedanum oreoselinum (L.) Moench	0	n	1	0	Apiaceae Lindl.
Iz_3_1009	DJI_0902	Peucedanum ruthenicum M.Bieb.	0	n	1	0	Apiaceae Lindl.
Iz_3_1009	DJI_0902	Pilosella echioides (Lumn.) F.W.Schultz & Sch.Bip.	0	n	1	0	Asteraceae Bercht. & J.Presl

Iz_3_1009	DJI_0902	Pilosella officinarum F. Schultz et Sch. Bip.	0	n	1	0	Asteraceae Bercht. & J.Presl
Iz_3_1009	DJI_0902	Pilosella sp.	0		NA	0	Asteraceae Bercht. & J.Presl
Iz_3_1009	DJI_0902	Pinus sylvestris L.	0	n	1	1	Pinaceae Spreng. ex F.Rudolphi
Iz_3_1009	DJI_0902	Poa annua L.	0	n	1	0	Poaceae Barnhart
Iz_3_1009	DJI_0902	Poa nemoralis L.	0	n	1	0	Poaceae Barnhart
Iz_3_1009	DJI_0902	Poa sp.	0		NA	0	Poaceae Barnhart
Iz_3_1009	DJI_0902	Polygonatum odoratum (Mill.) Druce	0	n	1	0	Asparagaceae Juss.
Iz_3_1009	DJI_0902	Polygonum arenarium Waldst. & Kit.	0	n	1	0	Polygonaceae Juss.
Iz_3_1009	DJI_0902	Populus nigra L.	0	n	1	1	Salicaceae Mirb.
Iz_3_1009	DJI_0902	Populus tremula L.	0	n	1	1	Salicaceae Mirb.
Iz_3_1009	DJI_0902	Populus Ã canescens (Aiton) Sm.	0	n	1	1	Salicaceae Mirb.
Iz_3_1009	DJI_0902	Potentilla argentea L.	0	n	1	0	Rosaceae Juss.
Iz_3_1009	DJI_0902	Psammophiliella muralis (L.) Ikonn.	0	n	1	0	Caryophyllaceae Juss.
Iz_3_1009	DJI_0902	Psephellus sumensis (Kalen.) Greuter	0	n	1	0	Asteraceae Bercht. & J.Presl
Iz_3_1009	DJI_0902	Pulsatilla pratensis (L.) Mill. s.l.	0	n	1	0	Ranunculaceae Juss.
Iz_3_1009	DJI_0902	Pyrus communis L.	0	a	0	1	Rosaceae Juss.
Iz_3_1009	DJI_0902	Quercus robur L.	0	n	1	1	Fagaceae Dumort.
Iz_3_1009	DJI_0902	Robinia pseudoacacia L.	0	a	0	1	Fabaceae Lindl.
Iz_3_1009	DJI_0902	Rosa sp.	0		NA	1	Rosaceae Juss.
Iz_3_1009	DJI_0902	Rubus idaeus L.	0	n	1	0	Rosaceae Juss.
Iz_3_1009	DJI_0902	Rumex acetosella L.	0	n	1	0	Polygonaceae Juss.
Iz_3_1009	DJI_0902	Rumex thyrsiflorus Fingerh.	0	n	1	0	Polygonaceae Juss.
Iz_3_1009	DJI_0902	Salix alba L.	0	n	1	1	Salicaceae Mirb.
Iz_3_1009	DJI_0902	Sambucus nigra L.	0	n	1	1	Viburnaceae Raf.
Iz_3_1009	DJI_0902	Secale sylvestre Host	0	n	1	0	Poaceae Barnhart
Iz_3_1009	DJI_0902	Sempervivum ruthenicum Schnittsp. & C.B. Lehm.	0	n	1	0	Crassulaceae J.St.-Hil.
Iz_3_1009	DJI_0902	Setaria viridis (L.) P. Beauv.	0	n	1	0	Poaceae Barnhart
Iz_3_1009	DJI_0902	Silene borysthenica (Gruner) Walters	0	n	1	0	Caryophyllaceae Juss.
Iz_3_1009	DJI_0902	Silene latifolia subsp. alba (Mill.) Greuter & Burdet	0	n	1	0	Caryophyllaceae Juss.
Iz_3_1009	DJI_0902	Solidago virgaurea L.	0	n	1	0	Asteraceae Bercht. & J.Presl
Iz_3_1009	DJI_0902	Sonchus oleraceus L.	0	a	0	0	Asteraceae Bercht. & J.Presl
Iz_3_1009	DJI_0902	Sorbus aucuparia L.	0	n	1	1	Rosaceae Juss.
Iz_3_1009	DJI_0902	Stachys recta L.	0	n	1	0	Lamiacea Lindl.
Iz_3_1009	DJI_0902	Tanacetum vulgare L.	0	n	1	0	Asteraceae Bercht. & J.Presl
Iz_3_1009	DJI_0902	Thymus pallasianus Heinr.Braun	0	n	1	0	Lamiacea Lindl.
Iz_3_1009	DJI_0902	Tilia cordata Mill.	0	n	1	1	Malvaceae Juss.
Iz_3_1009	DJI_0902	Trifolium alpestre L.	0	n	1	0	Fabaceae Lindl.
Iz_3_1009	DJI_0902	Trifolium arvense L.	0	n	1	0	Fabaceae Lindl.
Iz_3_1009	DJI_0902	Ulmus glabra Huds.	0	n	1	1	Ulmaceae Mirb.
Iz_3_1009	DJI_0902	Verbascum densiflorum Bertol.	0	n	1	0	Scrophulariaceae Juss.
Iz_3_1009	DJI_0902	Veronica austriaca L.	0	n	1	0	Plantaginaceae Juss.
Iz_3_1009	DJI_0902	Veronica incana L.	0	n	1	0	Plantaginaceae Juss.
Iz_3_1009	DJI_0902	Veronica verna L.	0	n	1	0	Plantaginaceae Juss.
Iz_3_1009	DJI_0902	Vincetoxicum hirundinaria Medik	0	n	1	0	Apocynaceae Juss.
Iz_3_1009	DJI_0903	Acer tataricum L.	0	n	1	1	Sapindaceae Juss.
Iz_3_1009	DJI_0903	Achillea millefolium L. s.l.	0	n	1	0	Asteraceae Bercht. & J.Presl
Iz_3_1009	DJI_0903	Allium oleraceum L.	0	n	1	0	Amaryllidaceae J.St.-Hil.
Iz_3_1009	DJI_0903	Ambrosia artemisiifolia L.	0	a	0	0	Asteraceae Bercht. & J.Presl
Iz_3_1009	DJI_0903	Amorpha fruticosa L.	0	a	0	0	Fabaceae Lindl.
Iz_3_1009	DJI_0903	Anthericum ramosum L.	0	n	1	0	Asparagaceae Juss.
Iz_3_1009	DJI_0903	Artemisia marschalliana Spreng.	0	n	1	0	Asteraceae Bercht. & J.Presl
Iz_3_1009	DJI_0903	Asparagus officinalis L.	0	n	1	0	Asparagaceae Juss.
Iz_3_1009	DJI_0903	Astragalus cicer L.	0	n	1	0	Fabaceae Lindl.
Iz_3_1009	DJI_0903	Bassia laniflora (S.G.Gmel.) A.J.Scott	0	n	1	0	Amaranthaceae Juss.
Iz_3_1009	DJI_0903	Berberis vulgaris L.	0	n	1	0	Berberidaceae Juss.
Iz_3_1009	DJI_0903	Betula pendula Roth.	0	n	1	1	Betulaceae Gray
Iz_3_1009	DJI_0903	Betula pendula Roth.	0	n	1	0	Poaceae Barnhart
Iz_3_1009	DJI_0903	Bromus inermis Leyss.	0		NA	NA	
Iz_3_1009	DJI_0903	Bromus squarrosus L.	0	a	0	0	Poaceae Barnhart
Iz_3_1009	DJI_0903	Bromus tectorum L.	0	a	0	0	Poaceae Barnhart
Iz_3_1009	DJI_0903	Calamagrostis epigejos (L.) Roth	25	n	1	0	Poaceae Barnhart
Iz_3_1009	DJI_0903	Carduus acanthoides L.	0	n	1	0	Asteraceae Bercht. & J.Presl
Iz_3_1009	DJI_0903	Carex sp.	0	n	1	0	Cyperaceae Juss.
Iz_3_1009	DJI_0903	Centaurea majorovii Dumbadze	0	n	1	0	Asteraceae Bercht. & J.Presl
Iz_3_1009	DJI_0903	Chamaecytisus ruthenicus (Fisch.ex Wol.) Klaskova	0	n	1	0	Fabaceae Lindl.
Iz_3_1009	DJI_0903	Chelidonium majus L.	0.2	n	1	0	Papaveraceae Juss.
Iz_3_1009	DJI_0903	Chenopodium betaceum Andrz.	0	n	1	0	Amaranthaceae Juss.
Iz_3_1009	DJI_0903	Chenopodium cf. album	0	n	1	0	Amaranthaceae Juss.
Iz_3_1009	DJI_0903	Chenopodium sp.	0		NA	0	Amaranthaceae Juss.
Iz_3_1009	DJI_0903	Chondrilla juncea L.	0	n	1	0	Asteraceae Bercht. & J.Presl
Iz_3_1009	DJI_0903	Cirsium vulgare (Savi) Ten.	0	n	1	0	Asteraceae Bercht. & J.Presl
Iz_3_1009	DJI_0903	Clinopodium acinos (L.) Kuntze	0	n	1	0	Lamiacea Lindl.
Iz_3_1009	DJI_0903	Convallaria majalis L.	0	n	1	0	Asparagaceae Juss.
Iz_3_1009	DJI_0903	Crataegus rhipidophylla Gand.	0	n	1	1	Rosaceae Juss.
Iz_3_1009	DJI_0903	Crepis foetida L.	0	n	1	0	Asteraceae Bercht. & J.Presl
Iz_3_1009	DJI_0903	Dactylis glomerata L.	0	n	1	0	Poaceae Barnhart
Iz_3_1009	DJI_0903	Dianthus campestris M.Bieb.	0	n	1	0	Caryophyllaceae Juss.
Iz_3_1009	DJI_0903	Digitaria sanguinalis (L.) Scop.	0	a	0	0	Poaceae Barnhart
Iz_3_1009	DJI_0903	Elymus repens (L.) Gould	0	n	1	0	Poaceae Barnhart
Iz_3_1009	DJI_0903	Epilobium tetragonum L.	0	n	1	0	Onagraceae Juss.
Iz_3_1009	DJI_0903	Eragrostis minor Host	0	a	0	0	Poaceae Barnhart
Iz_3_1009	DJI_0903	Erigeron canadensis L.	0	a	0	0	Asteraceae Bercht. & J.Presl
Iz_3_1009	DJI_0903	Euphorbia seguieriana Neck.	0	n	1	0	Euphorbiaceae Juss.
Iz_3_1009	DJI_0903	Fallopia dumetorum (L.) Holub	3	n	1	0	Polygonaceae Juss.
Iz_3_1009	DJI_0903	Festuca beckeri (Hack.) Trautv.	0	n	1	0	Poaceae Barnhart
Iz_3_1009	DJI_0903	Fragaria vesca L.	0	n	1	0	Rosaceae Juss.
Iz_3_1009	DJI_0903	Frangula alnus Mill.	0	n	1	1	Rhamnaceae Juss.
Iz_3_1009	DJI_0903	Fraxinus pennsylvanica Marshall	0	a	0	1	Oleaceae Hoffmanns. & Link
Iz_3_1009	DJI_0903	Galeopsis bifida Boenn.	0	n	1	0	Lamiacea Lindl.
Iz_3_1009	DJI_0903	Galium verum L.	0	n	1	0	Rubiaceae Juss.
Iz_3_1009	DJI_0903	Genista tinctoria L.	0	n	1	0	Fabaceae Lindl.
Iz_3_1009	DJI_0903	Geranium robertianum L.	0	n	1	0	Geraniaceae Juss.
Iz_3_1009	DJI_0903	Geranium sanquineum L.	0	n	1	0	Geraniaceae Juss.
Iz_3_1009	DJI_0903	Gypsophila paniculata L.	0	n	1	0	Caryophyllaceae Juss.
Iz_3_1009	DJI_0903	Helichrysum arenarium (L.) Moench	0	n	1	0	Asteraceae Bercht. & J.Presl
Iz_3_1009	DJI_0903	Herniaria polygama J.Gay	0	n	1	0	Caryophyllaceae Juss.
Iz_3_1009	DJI_0903	Hieracium robustum Fr.	0	n	1	0	Asteraceae Bercht. & J.Presl
Iz_3_1009	DJI_0903	Hieracium umbellatum L.	0	n	1	NA	Asteraceae Bercht. & J.Presl

Iz_3_1009	DJI_0903	Hylotelephium maximum (L.) Holub	0	n	1	0	Crassulaceae J.St.-Hil.
Iz_3_1009	DJI_0903	Jacobaea borysthenica (DC.) B.Nord. & Greuter	0	n	1	0	Asteraceae Bercht. & J.Presl
Iz_3_1009	DJI_0903	Jasione montana L.	0	n	1	0	Campanulaceae Juss.
Iz_3_1009	DJI_0903	Jurinea cyanoides (L.) Rchb.s.l.	0	n	1	0	Asteraceae Bercht. & J.Presl
Iz_3_1009	DJI_0903	Koeleria glauca (Spreng.) DC.	0	n	1	0	Poaceae Barnhart
Iz_3_1009	DJI_0903	Lactuca serriola L.	0	a	0	0	Asteraceae Bercht. & J.Presl
Iz_3_1009	DJI_0903	Linaria genistifolia (L.) Mill.	0	n	1	0	Plantaginaceae Juss.
Iz_3_1009	DJI_0903	Lomelosia argentea (L.) Greuter & Burdet	0	n	1	0	Caprifoliaceae Juss.
Iz_3_1009	DJI_0903	Melampyrum pratense L.	0	n	1	0	Orobanchaceae Vent.
Iz_3_1009	DJI_0903	Oenothera biennis L.	0	a	0	0	Onagraceae Juss.
Iz_3_1009	DJI_0903	Pentanema britannica (L.) D.Gut.Larr., Santos-Vicente, Anderb., E.Rico & M.M.Mart.Ort.	0	n	1	0	Asteraceae Bercht. & J.Presl
Iz_3_1009	DJI_0903	Peucedanum oreoselinum (L.) Moench	1	n	1	0	Apiaceae Lindl.
Iz_3_1009	DJI_0903	Peucedanum ruthenicum M.Bieb.	0	n	1	0	Apiaceae Lindl.
Iz_3_1009	DJI_0903	Pilosella echioides (Lumn.) F.W.Schultz & Sch.Bip.	0	n	1	0	Asteraceae Bercht. & J.Presl
Iz_3_1009	DJI_0903	Pilosella officinarum F. Schultz et Sch. Bip.	0	n	1	0	Asteraceae Bercht. & J.Presl
Iz_3_1009	DJI_0903	Pilosella sp.	0		NA	0	Asteraceae Bercht. & J.Presl
Iz_3_1009	DJI_0903	Pinus sylvestris L.	0	n	1	1	Pinaceae Spreng. ex F.Rudolphi
Iz_3_1009	DJI_0903	Poa annua L.	0	n	1	0	Poaceae Barnhart
Iz_3_1009	DJI_0903	Poa nemoralis L.	0	n	1	0	Poaceae Barnhart
Iz_3_1009	DJI_0903	Poa sp.	0		NA	0	Poaceae Barnhart
Iz_3_1009	DJI_0903	Polygonatum odoratum (Mill.) Druce	0.3	n	1	0	Asparagaceae Juss.
Iz_3_1009	DJI_0903	Polygonum arenarium Waldst. & Kit.	0	n	1	0	Polygonaceae Juss.
Iz_3_1009	DJI_0903	Populus nigra L.	0	n	1	1	Salicaceae Mirb.
Iz_3_1009	DJI_0903	Populus tremula L.	0	n	1	1	Salicaceae Mirb.
Iz_3_1009	DJI_0903	Populus Ã canescens (Aiton) Sm.	0	n	1	1	Salicaceae Mirb.
Iz_3_1009	DJI_0903	Potentilla argentea L.	0	n	1	0	Rosaceae Juss.
Iz_3_1009	DJI_0903	Psammophiliella muralis (L.) Ikonn.	0	n	1	0	Caryophyllaceae Juss.
Iz_3_1009	DJI_0903	Psephellus sumensis (Kalen.) Greuter	0	n	1	0	Asteraceae Bercht. & J.Presl
Iz_3_1009	DJI_0903	Pulsatilla pratensis (L.) Mill. s.l.	0	n	1	0	Ranunculaceae Juss.
Iz_3_1009	DJI_0903	Pyrus communis L.	0	a	0	1	Rosaceae Juss.
Iz_3_1009	DJI_0903	Quercus robur L.	0	n	1	1	Fagaceae Dumort.
Iz_3_1009	DJI_0903	Robinia pseudoacacia L.	0	a	0	1	Fabaceae Lindl.
Iz_3_1009	DJI_0903	Rosa sp.	0		NA	1	Rosaceae Juss.
Iz_3_1009	DJI_0903	Rubus idaeus L.	0	n	1	0	Rosaceae Juss.
Iz_3_1009	DJI_0903	Rumex acetosella L.	0	n	1	0	Polygonaceae Juss.
Iz_3_1009	DJI_0903	Rumex thyrsiflorus Fingerh.	0	n	1	0	Polygonaceae Juss.
Iz_3_1009	DJI_0903	Salix alba L.	0	n	1	1	Salicaceae Mirb.
Iz_3_1009	DJI_0903	Sambucus nigra L.	0	n	1	1	Viburnaceae Raf.
Iz_3_1009	DJI_0903	Secale sylvestre Host	0	n	1	0	Poaceae Barnhart
Iz_3_1009	DJI_0903	Sempervivum ruthenicum Schnittsp. & C.B. Lehm.	0	n	1	0	Crassulaceae J.St.-Hil.
Iz_3_1009	DJI_0903	Setaria viridis (L.) P. Beauv.	0	n	1	0	Poaceae Barnhart
Iz_3_1009	DJI_0903	Silene borysthenica (Gruner) Walters	0	n	1	0	Caryophyllaceae Juss.
Iz_3_1009	DJI_0903	Silene latifolia subsp. alba (Mill.) Greuter & Burdet	0	n	1	0	Caryophyllaceae Juss.
Iz_3_1009	DJI_0903	Solidago virgaurea L.	0	n	1	0	Asteraceae Bercht. & J.Presl
Iz_3_1009	DJI_0903	Sonchus oleraceus L.	0	a	0	0	Asteraceae Bercht. & J.Presl
Iz_3_1009	DJI_0903	Sorbus aucuparia L.	0	n	1	1	Rosaceae Juss.
Iz_3_1009	DJI_0903	Stachys recta L.	0	n	1	0	Lamiacea Lindl.
Iz_3_1009	DJI_0903	Tanacetum vulgare L.	0	n	1	0	Asteraceae Bercht. & J.Presl
Iz_3_1009	DJI_0903	Thymus pallasianus Heinr.Braun	0	n	1	0	Lamiacea Lindl.
Iz_3_1009	DJI_0903	Tilia cordata Mill.	0	n	1	1	Malvaceae Juss.
Iz_3_1009	DJI_0903	Trifolium alpestre L.	0	n	1	0	Fabaceae Lindl.
Iz_3_1009	DJI_0903	Trifolium arvense L.	0	n	1	0	Fabaceae Lindl.
Iz_3_1009	DJI_0903	Ulmus glabra Huds.	0	n	1	1	Ulmaceae Mirb.
Iz_3_1009	DJI_0903	Verbascum densiflorum Bertol.	0	n	1	0	Scrophulariaceae Juss.
Iz_3_1009	DJI_0903	Veronica austriaca L.	0	n	1	0	Plantaginaceae Juss.
Iz_3_1009	DJI_0903	Veronica incana L.	0	n	1	0	Plantaginaceae Juss.
Iz_3_1009	DJI_0903	Veronica verna L.	0	n	1	0	Plantaginaceae Juss.
Iz_3_1009	DJI_0903	Vincetoxicum hirundinaria Medik	0	n	1	0	Apocynaceae Juss.
Iz_3_1009	DJI_0904	Acer tataricum L.	0	n	1	1	Sapindaceae Juss.
Iz_3_1009	DJI_0904	Achillea millefolium L. s.l.	0	n	1	0	Asteraceae Bercht. & J.Presl
Iz_3_1009	DJI_0904	Allium oleraceum L.	0	n	1	0	Amaryllidaceae J.St.-Hil.
Iz_3_1009	DJI_0904	Ambrosia artemisiifolia L.	0	a	0	0	Asteraceae Bercht. & J.Presl
Iz_3_1009	DJI_0904	Amorpha fruticosa L.	0	a	0	0	Fabaceae Lindl.
Iz_3_1009	DJI_0904	Anthericum ramosum L.	0	n	1	0	Asparagaceae Juss.
Iz_3_1009	DJI_0904	Artemisia marschalliana Spreng.	0	n	1	0	Asteraceae Bercht. & J.Presl
Iz_3_1009	DJI_0904	Asparagus officinalis L.	0	n	1	0	Asparagaceae Juss.
Iz_3_1009	DJI_0904	Astragalus cicer L.	0	n	1	0	Fabaceae Lindl.
Iz_3_1009	DJI_0904	Bassia laniflora (S.G.Gmel.) A.J.Scott	0	n	1	0	Amaranthaceae Juss.
Iz_3_1009	DJI_0904	Berberis vulgaris L.	0	n	1	0	Berberidaceae Juss.
Iz_3_1009	DJI_0904	Betula pendula Roth.	0	n	1	1	Betulaceae Gray
Iz_3_1009	DJI_0904	Betula pendula Roth.	0	n	1	0	Poaceae Barnhart
Iz_3_1009	DJI_0904	Bromus inermis Leyss.	0		NA	NA	
Iz_3_1009	DJI_0904	Bromus squarrosus L.	0	a	0	0	Poaceae Barnhart
Iz_3_1009	DJI_0904	Bromus tectorum L.	0	a	0	0	Poaceae Barnhart
Iz_3_1009	DJI_0904	Calamagrostis epigejos (L.) Roth	50	n	1	0	Poaceae Barnhart
Iz_3_1009	DJI_0904	Carduus acanthoides L.	0	n	1	0	Asteraceae Bercht. & J.Presl
Iz_3_1009	DJI_0904	Carex sp.	0	n	1	0	Cyperaceae Juss.
Iz_3_1009	DJI_0904	Centaurea majorovii Dumbadze	0	n	1	0	Asteraceae Bercht. & J.Presl
Iz_3_1009	DJI_0904	Chamaecytisus ruthenicus (Fisch.ex Wol.) Klaskova	0	n	1	0	Fabaceae Lindl.
Iz_3_1009	DJI_0904	Chelidonium majus L.	1	n	1	0	Papaveraceae Juss.
Iz_3_1009	DJI_0904	Chenopodium betaceum Andrz.	0	n	1	0	Amaranthaceae Juss.
Iz_3_1009	DJI_0904	Chenopodium cf. album	0.2	n	1	0	Amaranthaceae Juss.
Iz_3_1009	DJI_0904	Chenopodium sp.	0		NA	0	Amaranthaceae Juss.
Iz_3_1009	DJI_0904	Chondrilla juncea L.	0	n	1	0	Asteraceae Bercht. & J.Presl
Iz_3_1009	DJI_0904	Cirsium vulgare (Savi) Ten.	0	n	1	0	Asteraceae Bercht. & J.Presl
Iz_3_1009	DJI_0904	Clinopodium acinos (L.) Kuntze	0	n	1	0	Lamiacea Lindl.
Iz_3_1009	DJI_0904	Convallaria majalis L.	0	n	1	0	Asparagaceae Juss.
Iz_3_1009	DJI_0904	Crataegus rhipidophylla Gand.	0	n	1	1	Rosaceae Juss.
Iz_3_1009	DJI_0904	Crepis foetida L.	0	n	1	0	Asteraceae Bercht. & J.Presl
Iz_3_1009	DJI_0904	Dactylis glomerata L.	0	n	1	0	Poaceae Barnhart
Iz_3_1009	DJI_0904	Dianthus campestris M.Bieb.	0	n	1	0	Caryophyllaceae Juss.
Iz_3_1009	DJI_0904	Digitaria sanguinalis (L.) Scop.	0	a	0	0	Poaceae Barnhart
Iz_3_1009	DJI_0904	Elymus repens (L.) Gould	0	n	1	0	Poaceae Barnhart
Iz_3_1009	DJI_0904	Epilobium tetragonum L.	0	n	1	0	Onagraceae Juss.
Iz_3_1009	DJI_0904	Eragrostis minor Host	0	a	0	0	Poaceae Barnhart
Iz_3_1009	DJI_0904	Erigeron canadensis L.	0	a	0	0	Asteraceae Bercht. & J.Presl
Iz_3_1009	DJI_0904	Euphorbia seguieriana Neck.	0	n	1	0	Euphorbiaceae Juss.
Iz_3_1009	DJI_0904	Fallopia dumetorum (L.) Holub	1	n	1	0	Polygonaceae Juss.

Iz_3_1009	DJI_0904	Festuca beckeri (Hack.) Trautv.	0	n	1	0	Poaceae Barnhart
Iz_3_1009	DJI_0904	Fragaria vesca L.	0	n	1	0	Rosaceae Juss.
Iz_3_1009	DJI_0904	Frangula alnus Mill.	0	n	1	1	Rhamnaceae Juss.
Iz_3_1009	DJI_0904	Fraxinus pennsylvanica Marshall	0	a	0	1	Oleaceae Hoffmanns. & Link
Iz_3_1009	DJI_0904	Galeopsis bifida Boenn.	0	n	1	0	Lamiacea Lindl.
Iz_3_1009	DJI_0904	Galium verum L.	0	n	1	0	Rubiaceae Juss.
Iz_3_1009	DJI_0904	Genista tinctoria L.	0	n	1	0	Fabaceae Lindl.
Iz_3_1009	DJI_0904	Geranium robertianum L.	0	n	1	0	Geraniaceae Juss.
Iz_3_1009	DJI_0904	Geranium sanquineum L.	0	n	1	0	Geraniaceae Juss.
Iz_3_1009	DJI_0904	Gypsophila paniculata L.	0	n	1	0	Caryophyllaceae Juss.
Iz_3_1009	DJI_0904	Helichrysum arenarium (L.) Moench	0	n	1	0	Asteraceae Bercht. & J.Presl
Iz_3_1009	DJI_0904	Herniaria polygama J.Gay	0	n	1	0	Caryophyllaceae Juss.
Iz_3_1009	DJI_0904	Hieracium robustum Fr.	0	n	1	0	Asteraceae Bercht. & J.Presl
Iz_3_1009	DJI_0904	Hieracium umbellatum L.	0	n	1	NA	Asteraceae Bercht. & J.Presl
Iz_3_1009	DJI_0904	Hylotelephium maximum (L.) Holub	0	n	1	0	Crassulaceae J.St.-Hil.
Iz_3_1009	DJI_0904	Jacobaea borysthenica (DC.) B.Nord. & Greuter	0	n	1	0	Asteraceae Bercht. & J.Presl
Iz_3_1009	DJI_0904	Jasione montana L.	0	n	1	0	Campanulaceae Juss.
Iz_3_1009	DJI_0904	Jurinea cyanoides (L.) Rchb.s.l.	0	n	1	0	Asteraceae Bercht. & J.Presl
Iz_3_1009	DJI_0904	Koeleria glauca (Spreng.) DC.	0	n	1	0	Poaceae Barnhart
Iz_3_1009	DJI_0904	Lactuca serriola L.	0	a	0	0	Asteraceae Bercht. & J.Presl
Iz_3_1009	DJI_0904	Linaria genistifolia (L.) Mill.	0	n	1	0	Plantaginaceae Juss.
Iz_3_1009	DJI_0904	Lomelosia argentea (L.) Greuter & Burdet	0	n	1	0	Caprifoliaceae Juss.
Iz_3_1009	DJI_0904	Melampyrum pratense L.	0	n	1	0	Orobanchaceae Vent.
Iz_3_1009	DJI_0904	Oenothera biennis L.	0	a	0	0	Onagraceae Juss.
Iz_3_1009	DJI_0904	Pentanema britannica (L.) D.Gut.Larr., Santos-Vicente, Anderb., E.Rico & M.M.Mart.Ort.	0	n	1	0	Asteraceae Bercht. & J.Presl
Iz_3_1009	DJI_0904	Peucedanum oreoselinum (L.) Moench	3	n	1	0	Apiaceae Lindl.
Iz_3_1009	DJI_0904	Peucedanum ruthenicum M.Bieb.	0	n	1	0	Apiaceae Lindl.
Iz_3_1009	DJI_0904	Pilosella echioides (Lumn.) F.W.Schultz & Sch.Bip.	0	n	1	0	Asteraceae Bercht. & J.Presl
Iz_3_1009	DJI_0904	Pilosella officinarum F. Schultz et Sch. Bip.	0	n	1	0	Asteraceae Bercht. & J.Presl
Iz_3_1009	DJI_0904	Pilosella sp.	0		NA	0	Asteraceae Bercht. & J.Presl
Iz_3_1009	DJI_0904	Pinus sylvestris L.	0	n	1	1	Pinaceae Spreng. ex F.Rudolphi
Iz_3_1009	DJI_0904	Poa annua L.	0	n	1	0	Poaceae Barnhart
Iz_3_1009	DJI_0904	Poa nemoralis L.	0	n	1	0	Poaceae Barnhart
Iz_3_1009	DJI_0904	Poa sp.	0		NA	0	Poaceae Barnhart
Iz_3_1009	DJI_0904	Polygonatum odoratum (Mill.) Druce	0	n	1	0	Asparagaceae Juss.
Iz_3_1009	DJI_0904	Polygonum arenarium Waldst. & Kit.	0	n	1	0	Polygonaceae Juss.
Iz_3_1009	DJI_0904	Populus nigra L.	0	n	1	1	Salicaceae Mirb.
Iz_3_1009	DJI_0904	Populus tremula L.	0	n	1	1	Salicaceae Mirb.
Iz_3_1009	DJI_0904	Populus Ã canescens (Aiton) Sm.	0	n	1	1	Salicaceae Mirb.
Iz_3_1009	DJI_0904	Potentilla argentea L.	0	n	1	0	Rosaceae Juss.
Iz_3_1009	DJI_0904	Psammophiliella muralis (L.) Ikonn.	0	n	1	0	Caryophyllaceae Juss.
Iz_3_1009	DJI_0904	Psephellus sumensis (Kalen.) Greuter	0	n	1	0	Asteraceae Bercht. & J.Presl
Iz_3_1009	DJI_0904	Pulsatilla pratensis (L.) Mill. s.l.	0	n	1	0	Ranunculaceae Juss.
Iz_3_1009	DJI_0904	Pyrus communis L.	0	a	0	1	Rosaceae Juss.
Iz_3_1009	DJI_0904	Quercus robur L.	0	n	1	1	Fagaceae Dumort.
Iz_3_1009	DJI_0904	Robinia pseudoacacia L.	0	a	0	1	Fabaceae Lindl.
Iz_3_1009	DJI_0904	Rosa sp.	0		NA	1	Rosaceae Juss.
Iz_3_1009	DJI_0904	Rubus idaeus L.	0	n	1	0	Rosaceae Juss.
Iz_3_1009	DJI_0904	Rumex acetosella L.	0	n	1	0	Polygonaceae Juss.
Iz_3_1009	DJI_0904	Rumex thyrsiflorus Fingerh.	0	n	1	0	Polygonaceae Juss.
Iz_3_1009	DJI_0904	Salix alba L.	0	n	1	1	Salicaceae Mirb.
Iz_3_1009	DJI_0904	Sambucus nigra L.	0	n	1	1	Viburnaceae Raf.
Iz_3_1009	DJI_0904	Secale sylvestre Host	0	n	1	0	Poaceae Barnhart
Iz_3_1009	DJI_0904	Sempervivum ruthenicum Schnittsp. & C.B. Lehm.	0	n	1	0	Crassulaceae J.St.-Hil.
Iz_3_1009	DJI_0904	Setaria viridis (L.) P. Beauv.	0	n	1	0	Poaceae Barnhart
Iz_3_1009	DJI_0904	Silene borysthenica (Gruner) Walters	0	n	1	0	Caryophyllaceae Juss.
Iz_3_1009	DJI_0904	Silene latifolia subsp. alba (Mill.) Greuter & Burdet	0	n	1	0	Caryophyllaceae Juss.
Iz_3_1009	DJI_0904	Solidago virgaurea L.	0	n	1	0	Asteraceae Bercht. & J.Presl
Iz_3_1009	DJI_0904	Sonchus oleraceus L.	0	a	0	0	Asteraceae Bercht. & J.Presl
Iz_3_1009	DJI_0904	Sorbus aucuparia L.	0	n	1	1	Rosaceae Juss.
Iz_3_1009	DJI_0904	Stachys recta L.	0	n	1	0	Lamiacea Lindl.
Iz_3_1009	DJI_0904	Tanacetum vulgare L.	0	n	1	0	Asteraceae Bercht. & J.Presl
Iz_3_1009	DJI_0904	Thymus pallasianus Heinr.Braun	0	n	1	0	Lamiacea Lindl.
Iz_3_1009	DJI_0904	Tilia cordata Mill.	0	n	1	1	Malvaceae Juss.
Iz_3_1009	DJI_0904	Trifolium alpestre L.	0	n	1	0	Fabaceae Lindl.
Iz_3_1009	DJI_0904	Trifolium arvense L.	0	n	1	0	Fabaceae Lindl.
Iz_3_1009	DJI_0904	Ulmus glabra Huds.	0	n	1	1	Ulmaceae Mirb.
Iz_3_1009	DJI_0904	Verbascum densiflorum Bertol.	0	n	1	0	Scrophulariaceae Juss.
Iz_3_1009	DJI_0904	Veronica austriaca L.	0	n	1	0	Plantaginaceae Juss.
Iz_3_1009	DJI_0904	Veronica incana L.	0	n	1	0	Plantaginaceae Juss.
Iz_3_1009	DJI_0904	Veronica verna L.	0	n	1	0	Plantaginaceae Juss.
Iz_3_1009	DJI_0904	Vincetoxicum hirundinaria Medik	0	n	1	0	Apocynaceae Juss.
Iz_3_1009	DJI_0908	Acer tataricum L.	0	n	1	1	Sapindaceae Juss.
Iz_3_1009	DJI_0908	Achillea millefolium L. s.l.	0	n	1	0	Asteraceae Bercht. & J.Presl
Iz_3_1009	DJI_0908	Allium oleraceum L.	0	n	1	0	Amaryllidaceae J.St.-Hil.
Iz_3_1009	DJI_0908	Ambrosia artemisiifolia L.	0	a	0	0	Asteraceae Bercht. & J.Presl
Iz_3_1009	DJI_0908	Amorpha fruticosa L.	0	a	0	0	Fabaceae Lindl.
Iz_3_1009	DJI_0908	Anthericum ramosum L.	0	n	1	0	Asparagaceae Juss.
Iz_3_1009	DJI_0908	Artemisia marschalliana Spreng.	0	n	1	0	Asteraceae Bercht. & J.Presl
Iz_3_1009	DJI_0908	Asparagus officinalis L.	0	n	1	0	Asparagaceae Juss.
Iz_3_1009	DJI_0908	Astragalus cicer L.	0	n	1	0	Fabaceae Lindl.
Iz_3_1009	DJI_0908	Bassia laniflora (S.G.Gmel.) A.J.Scott	0	n	1	0	Amaranthaceae Juss.
Iz_3_1009	DJI_0908	Berberis vulgaris L.	0	n	1	0	Berberidaceae Juss.
Iz_3_1009	DJI_0908	Betula pendula Roth.	0	n	1	1	Betulaceae Gray
Iz_3_1009	DJI_0908	Betula pendula Roth.	0	n	1	0	Poaceae Barnhart
Iz_3_1009	DJI_0908	Bromus inermis Leyss.	0		NA	NA	
Iz_3_1009	DJI_0908	Bromus squarrosus L.	0	a	0	0	Poaceae Barnhart
Iz_3_1009	DJI_0908	Bromus tectorum L.	0	a	0	0	Poaceae Barnhart
Iz_3_1009	DJI_0908	Calamagrostis epigejos (L.) Roth	35	n	1	0	Poaceae Barnhart
Iz_3_1009	DJI_0908	Carduus acanthoides L.	0	n	1	0	Asteraceae Bercht. & J.Presl
Iz_3_1009	DJI_0908	Carex sp.	0	n	1	0	Cyperaceae Juss.
Iz_3_1009	DJI_0908	Centaurea majorovii Dumbadze	0	n	1	0	Asteraceae Bercht. & J.Presl
Iz_3_1009	DJI_0908	Chamaecytisus ruthenicus (Fisch.ex Wol.) Klaskova	0	n	1	0	Fabaceae Lindl.
Iz_3_1009	DJI_0908	Chelidonium majus L.	0	n	1	0	Papaveraceae Juss.
Iz_3_1009	DJI_0908	Chenopodium betaceum Andrz.	0	n	1	0	Amaranthaceae Juss.
Iz_3_1009	DJI_0908	Chenopodium cf. album	0	n	1	0	Amaranthaceae Juss.
Iz_3_1009	DJI_0908	Chenopodium sp.	0		NA	0	Amaranthaceae Juss.
Iz_3_1009	DJI_0908	Chondrilla juncea L.	0	n	1	0	Asteraceae Bercht. & J.Presl

Iz_3_1009	DJI_0908	Cirsium vulgare (Savi) Ten.	0	n	1	0	Asteraceae Bercht. & J.Presl
Iz_3_1009	DJI_0908	Clinopodium acinos (L.) Kuntze	0	n	1	0	Lamiacea Lindl.
Iz_3_1009	DJI_0908	Convallaria majalis L.	0	n	1	0	Asparagaceae Juss.
Iz_3_1009	DJI_0908	Crataegus rhipidophylla Gand.	0	n	1	1	Rosaceae Juss.
Iz_3_1009	DJI_0908	Crepis foetida L.	0	n	1	0	Asteraceae Bercht. & J.Presl
Iz_3_1009	DJI_0908	Dactylis glomerata L.	0	n	1	0	Poaceae Barnhart
Iz_3_1009	DJI_0908	Dianthus campestris M.Bieb.	0	n	1	0	Caryophyllaceae Juss.
Iz_3_1009	DJI_0908	Digitaria sanguinalis (L.) Scop.	0	a	0	0	Poaceae Barnhart
Iz_3_1009	DJI_0908	Elymus repens (L.) Gould	0	n	1	0	Poaceae Barnhart
Iz_3_1009	DJI_0908	Epilobium tetragonum L.	0	n	1	0	Onagraceae Juss.
Iz_3_1009	DJI_0908	Eragrostis minor Host	0	a	0	0	Poaceae Barnhart
Iz_3_1009	DJI_0908	Erigeron canadensis L.	0	a	0	0	Asteraceae Bercht. & J.Presl
Iz_3_1009	DJI_0908	Euphorbia seguieriana Neck.	0	n	1	0	Euphorbiaceae Juss.
Iz_3_1009	DJI_0908	Fallopia dumetorum (L.) Holub	0.5	n	1	0	Polygonaceae Juss.
Iz_3_1009	DJI_0908	Festuca beckeri (Hack.) Trautv.	0	n	1	0	Poaceae Barnhart
Iz_3_1009	DJI_0908	Fragaria vesca L.	0	n	1	0	Rosaceae Juss.
Iz_3_1009	DJI_0908	Frangula alnus Mill.	0	n	1	1	Rhamnaceae Juss.
Iz_3_1009	DJI_0908	Fraxinus pennsylvanica Marshall	0	a	0	1	Oleaceae Hoffmanns. & Link
Iz_3_1009	DJI_0908	Galeopsis bifida Boenn.	0	n	1	0	Lamiacea Lindl.
Iz_3_1009	DJI_0908	Galium verum L.	0	n	1	0	Rubiaceae Juss.
Iz_3_1009	DJI_0908	Genista tinctoria L.	0	n	1	0	Fabaceae Lindl.
Iz_3_1009	DJI_0908	Geranium robertianum L.	0	n	1	0	Geraniaceae Juss.
Iz_3_1009	DJI_0908	Geranium sanquineum L.	0	n	1	0	Geraniaceae Juss.
Iz_3_1009	DJI_0908	Gypsophila paniculata L.	0	n	1	0	Caryophyllaceae Juss.
Iz_3_1009	DJI_0908	Helichrysum arenarium (L.) Moench	0	n	1	0	Asteraceae Bercht. & J.Presl
Iz_3_1009	DJI_0908	Herniaria polygama J.Gay	0	n	1	0	Caryophyllaceae Juss.
Iz_3_1009	DJI_0908	Hieracium robustum Fr.	0	n	1	0	Asteraceae Bercht. & J.Presl
Iz_3_1009	DJI_0908	Hieracium umbellatum L.	0	n	1	NA	Asteraceae Bercht. & J.Presl
Iz_3_1009	DJI_0908	Hylotelephium maximum (L.) Holub	0	n	1	0	Crassulaceae J.St.-Hil.
Iz_3_1009	DJI_0908	Jacobaea borysthenica (DC.) B.Nord. & Greuter	0	n	1	0	Asteraceae Bercht. & J.Presl
Iz_3_1009	DJI_0908	Jasione montana L.	0	n	1	0	Campanulaceae Juss.
Iz_3_1009	DJI_0908	Jurinea cyanoides (L.) Rchb.s.l.	0	n	1	0	Asteraceae Bercht. & J.Presl
Iz_3_1009	DJI_0908	Koeleria glauca (Spreng.) DC.	0	n	1	0	Poaceae Barnhart
Iz_3_1009	DJI_0908	Lactuca serriola L.	0	a	0	0	Asteraceae Bercht. & J.Presl
Iz_3_1009	DJI_0908	Linaria genistifolia (L.) Mill.	0	n	1	0	Plantaginaceae Juss.
Iz_3_1009	DJI_0908	Lomelosia argentea (L.) Greuter & Burdet	0	n	1	0	Caprifoliaceae Juss.
Iz_3_1009	DJI_0908	Melampyrum pratense L.	0	n	1	0	Orobanchaceae Vent.
Iz_3_1009	DJI_0908	Oenothera biennis L.	0	a	0	0	Onagraceae Juss.
Iz_3_1009	DJI_0908	Pentanema britannica (L.) D.Gut.Larr., Santos-Vicente, Anderb., E.Rico & M.M.Mart.Ort.	0	n	1	0	Asteraceae Bercht. & J.Presl
Iz_3_1009	DJI_0908	Peucedanum oreoselinum (L.) Moench	4	n	1	0	Apiaceae Lindl.
Iz_3_1009	DJI_0908	Peucedanum ruthenicum M.Bieb.	0	n	1	0	Apiaceae Lindl.
Iz_3_1009	DJI_0908	Pilosella echioides (Lumn.) F.W.Schultz & Sch.Bip.	0	n	1	0	Asteraceae Bercht. & J.Presl
Iz_3_1009	DJI_0908	Pilosella officinarum F. Schultz et Sch. Bip.	0	n	1	0	Asteraceae Bercht. & J.Presl
Iz_3_1009	DJI_0908	Pilosella sp.	0		NA	0	Asteraceae Bercht. & J.Presl
Iz_3_1009	DJI_0908	Pinus sylvestris L.	0	n	1	1	Pinaceae Spreng. ex F.Rudolphi
Iz_3_1009	DJI_0908	Poa annua L.	0	n	1	0	Poaceae Barnhart
Iz_3_1009	DJI_0908	Poa nemoralis L.	0	n	1	0	Poaceae Barnhart
Iz_3_1009	DJI_0908	Poa sp.	0		NA	0	Poaceae Barnhart
Iz_3_1009	DJI_0908	Polygonatum odoratum (Mill.) Druce	0.5	n	1	0	Asparagaceae Juss.
Iz_3_1009	DJI_0908	Polygonum arenarium Waldst. & Kit.	0	n	1	0	Polygonaceae Juss.
Iz_3_1009	DJI_0908	Populus nigra L.	0	n	1	1	Salicaceae Mirb.
Iz_3_1009	DJI_0908	Populus tremula L.	0	n	1	1	Salicaceae Mirb.
Iz_3_1009	DJI_0908	Populus Ã canescens (Aiton) Sm.	0	n	1	1	Salicaceae Mirb.
Iz_3_1009	DJI_0908	Potentilla argentea L.	0	n	1	0	Rosaceae Juss.
Iz_3_1009	DJI_0908	Psammophiliella muralis (L.) Ikonn.	0	n	1	0	Caryophyllaceae Juss.
Iz_3_1009	DJI_0908	Psephellus sumensis (Kalen.) Greuter	0	n	1	0	Asteraceae Bercht. & J.Presl
Iz_3_1009	DJI_0908	Pulsatilla pratensis (L.) Mill. s.l.	0	n	1	0	Ranunculaceae Juss.
Iz_3_1009	DJI_0908	Pyrus communis L.	0	a	0	1	Rosaceae Juss.
Iz_3_1009	DJI_0908	Quercus robur L.	0	n	1	1	Fagaceae Dumort.
Iz_3_1009	DJI_0908	Robinia pseudoacacia L.	0	a	0	1	Fabaceae Lindl.
Iz_3_1009	DJI_0908	Rosa sp.	0		NA	1	Rosaceae Juss.
Iz_3_1009	DJI_0908	Rubus idaeus L.	0	n	1	0	Rosaceae Juss.
Iz_3_1009	DJI_0908	Rumex acetosella L.	0	n	1	0	Polygonaceae Juss.
Iz_3_1009	DJI_0908	Rumex thyrsiflorus Fingerh.	0	n	1	0	Polygonaceae Juss.
Iz_3_1009	DJI_0908	Salix alba L.	0	n	1	1	Salicaceae Mirb.
Iz_3_1009	DJI_0908	Sambucus nigra L.	0	n	1	1	Viburnaceae Raf.
Iz_3_1009	DJI_0908	Secale sylvestre Host	0	n	1	0	Poaceae Barnhart
Iz_3_1009	DJI_0908	Sempervivum ruthenicum Schnittsp. & C.B. Lehm.	0	n	1	0	Crassulaceae J.St.-Hil.
Iz_3_1009	DJI_0908	Setaria viridis (L.) P. Beauv.	0	n	1	0	Poaceae Barnhart
Iz_3_1009	DJI_0908	Silene borysthenica (Gruner) Walters	0	n	1	0	Caryophyllaceae Juss.
Iz_3_1009	DJI_0908	Silene latifolia subsp. alba (Mill.) Greuter & Burdet	0	n	1	0	Caryophyllaceae Juss.
Iz_3_1009	DJI_0908	Solidago virgaurea L.	0	n	1	0	Asteraceae Bercht. & J.Presl
Iz_3_1009	DJI_0908	Sonchus oleraceus L.	0	a	0	0	Asteraceae Bercht. & J.Presl
Iz_3_1009	DJI_0908	Sorbus aucuparia L.	0	n	1	1	Rosaceae Juss.
Iz_3_1009	DJI_0908	Stachys recta L.	0	n	1	0	Lamiacea Lindl.
Iz_3_1009	DJI_0908	Tanacetum vulgare L.	0	n	1	0	Asteraceae Bercht. & J.Presl
Iz_3_1009	DJI_0908	Thymus pallasianus Heinr.Braun	0	n	1	0	Lamiacea Lindl.
Iz_3_1009	DJI_0908	Tilia cordata Mill.	0	n	1	1	Malvaceae Juss.
Iz_3_1009	DJI_0908	Trifolium alpestre L.	0	n	1	0	Fabaceae Lindl.
Iz_3_1009	DJI_0908	Trifolium arvense L.	0	n	1	0	Fabaceae Lindl.
Iz_3_1009	DJI_0908	Ulmus glabra Huds.	0	n	1	1	Ulmaceae Mirb.
Iz_3_1009	DJI_0908	Verbascum densiflorum Bertol.	0	n	1	0	Scrophulariaceae Juss.
Iz_3_1009	DJI_0908	Veronica austriaca L.	0	n	1	0	Plantaginaceae Juss.
Iz_3_1009	DJI_0908	Veronica incana L.	0	n	1	0	Plantaginaceae Juss.
Iz_3_1009	DJI_0908	Veronica verna L.	0	n	1	0	Plantaginaceae Juss.
Iz_3_1009	DJI_0908	Vincetoxicum hirundinaria Medik	0	n	1	0	Apocynaceae Juss.
Iz_3_1009	DJI_0911	Acer tataricum L.	0	n	1	1	Sapindaceae Juss.
Iz_3_1009	DJI_0911	Achillea millefolium L. s.l.	0	n	1	0	Asteraceae Bercht. & J.Presl
Iz_3_1009	DJI_0911	Allium oleraceum L.	0	n	1	0	Amaryllidaceae J.St.-Hil.
Iz_3_1009	DJI_0911	Ambrosia artemisiifolia L.	0	a	0	0	Asteraceae Bercht. & J.Presl
Iz_3_1009	DJI_0911	Amorpha fruticosa L.	0	a	0	0	Fabaceae Lindl.
Iz_3_1009	DJI_0911	Anthericum ramosum L.	0	n	1	0	Asparagaceae Juss.
Iz_3_1009	DJI_0911	Artemisia marschalliana Spreng.	0	n	1	0	Asteraceae Bercht. & J.Presl
Iz_3_1009	DJI_0911	Asparagus officinalis L.	0	n	1	0	Asparagaceae Juss.
Iz_3_1009	DJI_0911	Astragalus cicer L.	0	n	1	0	Fabaceae Lindl.
Iz_3_1009	DJI_0911	Bassia laniflora (S.G.Gmel.) A.J.Scott	0	n	1	0	Amaranthaceae Juss.
Iz_3_1009	DJI_0911	Berberis vulgaris L.	0	n	1	0	Berberidaceae Juss.
Iz_3_1009	DJI_0911	Betula pendula Roth.	0	n	1	1	Betulaceae Gray

Iz_3_1009	DJI_0911	Betula pendula Roth.	0	n	1	0	Poaceae Barnhart
Iz_3_1009	DJI_0911	Bromus inermis Leyss.	0		NA	NA	
Iz_3_1009	DJI_0911	Bromus squarrosus L.	0	a	0	0	Poaceae Barnhart
Iz_3_1009	DJI_0911	Bromus tectorum L.	0	a	0	0	Poaceae Barnhart
Iz_3_1009	DJI_0911	Calamagrostis epigejos (L.) Roth	80	n	1	0	Poaceae Barnhart
Iz_3_1009	DJI_0911	Carduus acanthoides L.	0	n	1	0	Asteraceae Bercht. & J.Presl
Iz_3_1009	DJI_0911	Carex sp.	0	n	1	0	Cyperaceae Juss.
Iz_3_1009	DJI_0911	Centaurea majorovii Dumbadze	0	n	1	0	Asteraceae Bercht. & J.Presl
Iz_3_1009	DJI_0911	Chamaecytisus ruthenicus (Fisch.ex Wol.) Klaskova	0	n	1	0	Fabaceae Lindl.
Iz_3_1009	DJI_0911	Chelidonium majus L.	0	n	1	0	Papaveraceae Juss.
Iz_3_1009	DJI_0911	Chenopodium betaceum Andrz.	0	n	1	0	Amaranthaceae Juss.
Iz_3_1009	DJI_0911	Chenopodium cf. album	0	n	1	0	Amaranthaceae Juss.
Iz_3_1009	DJI_0911	Chenopodium sp.	0		NA	0	Amaranthaceae Juss.
Iz_3_1009	DJI_0911	Chondrilla juncea L.	0	n	1	0	Asteraceae Bercht. & J.Presl
Iz_3_1009	DJI_0911	Cirsium vulgare (Savi) Ten.	0	n	1	0	Asteraceae Bercht. & J.Presl
Iz_3_1009	DJI_0911	Clinopodium acinos (L.) Kuntze	0	n	1	0	Lamiacea Lindl.
Iz_3_1009	DJI_0911	Convallaria majalis L.	0	n	1	0	Asparagaceae Juss.
Iz_3_1009	DJI_0911	Crataegus rhipidophylla Gand.	0	n	1	1	Rosaceae Juss.
Iz_3_1009	DJI_0911	Crepis foetida L.	0	n	1	0	Asteraceae Bercht. & J.Presl
Iz_3_1009	DJI_0911	Dactylis glomerata L.	0	n	1	0	Poaceae Barnhart
Iz_3_1009	DJI_0911	Dianthus campestris M.Bieb.	0	n	1	0	Caryophyllaceae Juss.
Iz_3_1009	DJI_0911	Digitaria sanguinalis (L.) Scop.	0	a	0	0	Poaceae Barnhart
Iz_3_1009	DJI_0911	Elymus repens (L.) Gould	0	n	1	0	Poaceae Barnhart
Iz_3_1009	DJI_0911	Epilobium tetragonum L.	0	n	1	0	Onagraceae Juss.
Iz_3_1009	DJI_0911	Eragrostis minor Host	0	a	0	0	Poaceae Barnhart
Iz_3_1009	DJI_0911	Erigeron canadensis L.	0	a	0	0	Asteraceae Bercht. & J.Presl
Iz_3_1009	DJI_0911	Euphorbia seguieriana Neck.	0	n	1	0	Euphorbiaceae Juss.
Iz_3_1009	DJI_0911	Fallopia dumetorum (L.) Holub	0	n	1	0	Polygonaceae Juss.
Iz_3_1009	DJI_0911	Festuca beckeri (Hack.) Trautv.	0	n	1	0	Poaceae Barnhart
Iz_3_1009	DJI_0911	Fragaria vesca L.	0	n	1	0	Rosaceae Juss.
Iz_3_1009	DJI_0911	Frangula alnus Mill.	0	n	1	1	Rhamnaceae Juss.
Iz_3_1009	DJI_0911	Fraxinus pennsylvanica Marshall	0	a	0	1	Oleaceae Hoffmanns. & Link
Iz_3_1009	DJI_0911	Galeopsis bifida Boenn.	0	n	1	0	Lamiacea Lindl.
Iz_3_1009	DJI_0911	Galium verum L.	0	n	1	0	Rubiaceae Juss.
Iz_3_1009	DJI_0911	Genista tinctoria L.	0	n	1	0	Fabaceae Lindl.
Iz_3_1009	DJI_0911	Geranium robertianum L.	0	n	1	0	Geraniaceae Juss.
Iz_3_1009	DJI_0911	Geranium sanquineum L.	0	n	1	0	Geraniaceae Juss.
Iz_3_1009	DJI_0911	Gypsophila paniculata L.	0	n	1	0	Caryophyllaceae Juss.
Iz_3_1009	DJI_0911	Helichrysum arenarium (L.) Moench	0	n	1	0	Asteraceae Bercht. & J.Presl
Iz_3_1009	DJI_0911	Herniaria polygama J.Gay	0	n	1	0	Caryophyllaceae Juss.
Iz_3_1009	DJI_0911	Hieracium robustum Fr.	0	n	1	0	Asteraceae Bercht. & J.Presl
Iz_3_1009	DJI_0911	Hieracium umbellatum L.	0	n	1	NA	Asteraceae Bercht. & J.Presl
Iz_3_1009	DJI_0911	Hylotelephium maximum (L.) Holub	0	n	1	0	Crassulaceae J.St.-Hil.
Iz_3_1009	DJI_0911	Jacobaea borysthenica (DC.) B.Nord. & Greuter	0	n	1	0	Asteraceae Bercht. & J.Presl
Iz_3_1009	DJI_0911	Jasione montana L.	0	n	1	0	Campanulaceae Juss.
Iz_3_1009	DJI_0911	Jurinea cyanoides (L.) Rchb.s.l.	0	n	1	0	Asteraceae Bercht. & J.Presl
Iz_3_1009	DJI_0911	Koeleria glauca (Spreng.) DC.	0	n	1	0	Poaceae Barnhart
Iz_3_1009	DJI_0911	Lactuca serriola L.	0.5	a	0	0	Asteraceae Bercht. & J.Presl
Iz_3_1009	DJI_0911	Linaria genistifolia (L.) Mill.	0	n	1	0	Plantaginaceae Juss.
Iz_3_1009	DJI_0911	Lomelosia argentea (L.) Greuter & Burdet	0	n	1	0	Caprifoliaceae Juss.
Iz_3_1009	DJI_0911	Melampyrum pratense L.	0	n	1	0	Orobanchaceae Vent.
Iz_3_1009	DJI_0911	Oenothera biennis L.	0	a	0	0	Onagraceae Juss.
Iz_3_1009	DJI_0911	Pentanema britannica (L.) D.Gut.Larr., Santos-Vicente, Anderb., E.Rico & M.M.Mart.Ort.	0	n	1	0	Asteraceae Bercht. & J.Presl
Iz_3_1009	DJI_0911	Peucedanum oreoselinum (L.) Moench	8	n	1	0	Apiaceae Lindl.
Iz_3_1009	DJI_0911	Peucedanum ruthenicum M.Bieb.	0	n	1	0	Apiaceae Lindl.
Iz_3_1009	DJI_0911	Pilosella echioides (Lumn.) F.W.Schultz & Sch.Bip.	0	n	1	0	Asteraceae Bercht. & J.Presl
Iz_3_1009	DJI_0911	Pilosella officinarum F. Schultz et Sch. Bip.	0	n	1	0	Asteraceae Bercht. & J.Presl
Iz_3_1009	DJI_0911	Pilosella sp.	0		NA	0	Asteraceae Bercht. & J.Presl
Iz_3_1009	DJI_0911	Pinus sylvestris L.	0	n	1	1	Pinaceae Spreng. ex F.Rudolphi
Iz_3_1009	DJI_0911	Poa annua L.	0	n	1	0	Poaceae Barnhart
Iz_3_1009	DJI_0911	Poa nemoralis L.	0	n	1	0	Poaceae Barnhart
Iz_3_1009	DJI_0911	Poa sp.	0		NA	0	Poaceae Barnhart
Iz_3_1009	DJI_0911	Polygonatum odoratum (Mill.) Druce	0.5	n	1	0	Asparagaceae Juss.
Iz_3_1009	DJI_0911	Polygonum arenarium Waldst. & Kit.	0	n	1	0	Polygonaceae Juss.
Iz_3_1009	DJI_0911	Populus nigra L.	0	n	1	1	Salicaceae Mirb.
Iz_3_1009	DJI_0911	Populus tremula L.	0	n	1	1	Salicaceae Mirb.
Iz_3_1009	DJI_0911	Populus Ã canescens (Aiton) Sm.	0	n	1	1	Salicaceae Mirb.
Iz_3_1009	DJI_0911	Potentilla argentea L.	0	n	1	0	Rosaceae Juss.
Iz_3_1009	DJI_0911	Psammophiliella muralis (L.) Ikonn.	0	n	1	0	Caryophyllaceae Juss.
Iz_3_1009	DJI_0911	Psephellus sumensis (Kalen.) Greuter	0	n	1	0	Asteraceae Bercht. & J.Presl
Iz_3_1009	DJI_0911	Pulsatilla pratensis (L.) Mill. s.l.	0	n	1	0	Ranunculaceae Juss.
Iz_3_1009	DJI_0911	Pyrus communis L.	0	a	0	1	Rosaceae Juss.
Iz_3_1009	DJI_0911	Quercus robur L.	0	n	1	1	Fagaceae Dumort.
Iz_3_1009	DJI_0911	Robinia pseudoacacia L.	0	a	0	1	Fabaceae Lindl.
Iz_3_1009	DJI_0911	Rosa sp.	0		NA	1	Rosaceae Juss.
Iz_3_1009	DJI_0911	Rubus idaeus L.	0	n	1	0	Rosaceae Juss.
Iz_3_1009	DJI_0911	Rumex acetosella L.	0	n	1	0	Polygonaceae Juss.
Iz_3_1009	DJI_0911	Rumex thyrsiflorus Fingerh.	0	n	1	0	Polygonaceae Juss.
Iz_3_1009	DJI_0911	Salix alba L.	0	n	1	1	Salicaceae Mirb.
Iz_3_1009	DJI_0911	Sambucus nigra L.	0	n	1	1	Viburnaceae Raf.
Iz_3_1009	DJI_0911	Secale sylvestre Host	0	n	1	0	Poaceae Barnhart
Iz_3_1009	DJI_0911	Sempervivum ruthenicum Schnittsp. & C.B. Lehm.	0	n	1	0	Crassulaceae J.St.-Hil.
Iz_3_1009	DJI_0911	Setaria viridis (L.) P. Beauv.	0	n	1	0	Poaceae Barnhart
Iz_3_1009	DJI_0911	Silene borysthenica (Gruner) Walters	0	n	1	0	Caryophyllaceae Juss.
Iz_3_1009	DJI_0911	Silene latifolia subsp. alba (Mill.) Greuter & Burdet	0	n	1	0	Caryophyllaceae Juss.
Iz_3_1009	DJI_0911	Solidago virgaurea L.	0	n	1	0	Asteraceae Bercht. & J.Presl
Iz_3_1009	DJI_0911	Sonchus oleraceus L.	0	a	0	0	Asteraceae Bercht. & J.Presl
Iz_3_1009	DJI_0911	Sorbus aucuparia L.	0	n	1	1	Rosaceae Juss.
Iz_3_1009	DJI_0911	Stachys recta L.	0	n	1	0	Lamiacea Lindl.
Iz_3_1009	DJI_0911	Tanacetum vulgare L.	0	n	1	0	Asteraceae Bercht. & J.Presl
Iz_3_1009	DJI_0911	Thymus pallasianus Heinr.Braun	0	n	1	0	Lamiacea Lindl.
Iz_3_1009	DJI_0911	Tilia cordata Mill.	0	n	1	1	Malvaceae Juss.
Iz_3_1009	DJI_0911	Trifolium alpestre L.	0	n	1	0	Fabaceae Lindl.
Iz_3_1009	DJI_0911	Trifolium arvense L.	0	n	1	0	Fabaceae Lindl.
Iz_3_1009	DJI_0911	Ulmus glabra Huds.	0	n	1	1	Ulmaceae Mirb.
Iz_3_1009	DJI_0911	Verbascum densiflorum Bertol.	0	n	1	0	Scrophulariaceae Juss.
Iz_3_1009	DJI_0911	Veronica austriaca L.	0	n	1	0	Plantaginaceae Juss.
Iz_3_1009	DJI_0911	Veronica incana L.	0	n	1	0	Plantaginaceae Juss.

Iz_3_1009	DJI_0911	Veronica verna L.	0	n	1	0	Plantaginaceae Juss.
Iz_3_1009	DJI_0911	Vincetoxicum hirundinaria Medik	0	n	1	0	Apocynaceae Juss.
Iz_3_1009	DJI_0926	Acer tataricum L.	0	n	1	1	Sapindaceae Juss.
Iz_3_1009	DJI_0926	Achillea millefolium L. s.l.	0	n	1	0	Asteraceae Bercht. & J.Presl
Iz_3_1009	DJI_0926	Allium oleraceum L.	0	n	1	0	Amaryllidaceae J.St.-Hil.
Iz_3_1009	DJI_0926	Ambrosia artemisiifolia L.	0	a	0	0	Asteraceae Bercht. & J.Presl
Iz_3_1009	DJI_0926	Amorpha fruticosa L.	0	a	0	0	Fabaceae Lindl.
Iz_3_1009	DJI_0926	Anthericum ramosum L.	0	n	1	0	Asparagaceae Juss.
Iz_3_1009	DJI_0926	Artemisia marschalliana Spreng.	0	n	1	0	Asteraceae Bercht. & J.Presl
Iz_3_1009	DJI_0926	Asparagus officinalis L.	0	n	1	0	Asparagaceae Juss.
Iz_3_1009	DJI_0926	Astragalus cicer L.	0	n	1	0	Fabaceae Lindl.
Iz_3_1009	DJI_0926	Bassia laniflora (S.G.Gmel.) A.J.Scott	0	n	1	0	Amaranthaceae Juss.
Iz_3_1009	DJI_0926	Berberis vulgaris L.	0	n	1	0	Berberidaceae Juss.
Iz_3_1009	DJI_0926	Betula pendula Roth.	0	n	1	1	Betulaceae Gray
Iz_3_1009	DJI_0926	Betula pendula Roth.	0	n	1	0	Poaceae Barnhart
Iz_3_1009	DJI_0926	Bromus inermis Leyss.	0		NA	NA	
Iz_3_1009	DJI_0926	Bromus squarrosus L.	0	a	0	0	Poaceae Barnhart
Iz_3_1009	DJI_0926	Bromus tectorum L.	0	a	0	0	Poaceae Barnhart
Iz_3_1009	DJI_0926	Calamagrostis epigejos (L.) Roth	40	n	1	0	Poaceae Barnhart
Iz_3_1009	DJI_0926	Carduus acanthoides L.	0	n	1	0	Asteraceae Bercht. & J.Presl
Iz_3_1009	DJI_0926	Carex sp.	0	n	1	0	Cyperaceae Juss.
Iz_3_1009	DJI_0926	Centaurea majorovii Dumbadze	0	n	1	0	Asteraceae Bercht. & J.Presl
Iz_3_1009	DJI_0926	Chamaecytisus ruthenicus (Fisch.ex Wol.) Klaskova	0	n	1	0	Fabaceae Lindl.
Iz_3_1009	DJI_0926	Chelidonium majus L.	0	n	1	0	Papaveraceae Juss.
Iz_3_1009	DJI_0926	Chenopodium betaceum Andrz.	0	n	1	0	Amaranthaceae Juss.
Iz_3_1009	DJI_0926	Chenopodium cf. album	0	n	1	0	Amaranthaceae Juss.
Iz_3_1009	DJI_0926	Chenopodium sp.	0		NA	0	Amaranthaceae Juss.
Iz_3_1009	DJI_0926	Chondrilla juncea L.	0	n	1	0	Asteraceae Bercht. & J.Presl
Iz_3_1009	DJI_0926	Cirsium vulgare (Savi) Ten.	0	n	1	0	Asteraceae Bercht. & J.Presl
Iz_3_1009	DJI_0926	Clinopodium acinos (L.) Kuntze	0	n	1	0	Lamiacea Lindl.
Iz_3_1009	DJI_0926	Convallaria majalis L.	0	n	1	0	Asparagaceae Juss.
Iz_3_1009	DJI_0926	Crataegus rhipidophylla Gand.	0	n	1	1	Rosaceae Juss.
Iz_3_1009	DJI_0926	Crepis foetida L.	0	n	1	0	Asteraceae Bercht. & J.Presl
Iz_3_1009	DJI_0926	Dactylis glomerata L.	0	n	1	0	Poaceae Barnhart
Iz_3_1009	DJI_0926	Dianthus campestris M.Bieb.	0	n	1	0	Caryophyllaceae Juss.
Iz_3_1009	DJI_0926	Digitaria sanguinalis (L.) Scop.	0	a	0	0	Poaceae Barnhart
Iz_3_1009	DJI_0926	Elymus repens (L.) Gould	0	n	1	0	Poaceae Barnhart
Iz_3_1009	DJI_0926	Epilobium tetragonum L.	0	n	1	0	Onagraceae Juss.
Iz_3_1009	DJI_0926	Eragrostis minor Host	0	a	0	0	Poaceae Barnhart
Iz_3_1009	DJI_0926	Erigeron canadensis L.	4	a	0	0	Asteraceae Bercht. & J.Presl
Iz_3_1009	DJI_0926	Euphorbia seguieriana Neck.	0	n	1	0	Euphorbiaceae Juss.
Iz_3_1009	DJI_0926	Fallopia dumetorum (L.) Holub	0	n	1	0	Polygonaceae Juss.
Iz_3_1009	DJI_0926	Festuca beckeri (Hack.) Trautv.	0	n	1	0	Poaceae Barnhart
Iz_3_1009	DJI_0926	Fragaria vesca L.	0	n	1	0	Rosaceae Juss.
Iz_3_1009	DJI_0926	Frangula alnus Mill.	0	n	1	1	Rhamnaceae Juss.
Iz_3_1009	DJI_0926	Fraxinus pennsylvanica Marshall	0	a	0	1	Oleaceae Hoffmanns. & Link
Iz_3_1009	DJI_0926	Galeopsis bifida Boenn.	0	n	1	0	Lamiacea Lindl.
Iz_3_1009	DJI_0926	Galium verum L.	0	n	1	0	Rubiaceae Juss.
Iz_3_1009	DJI_0926	Genista tinctoria L.	0	n	1	0	Fabaceae Lindl.
Iz_3_1009	DJI_0926	Geranium robertianum L.	0	n	1	0	Geraniaceae Juss.
Iz_3_1009	DJI_0926	Geranium sanquineum L.	0	n	1	0	Geraniaceae Juss.
Iz_3_1009	DJI_0926	Gypsophila paniculata L.	0	n	1	0	Caryophyllaceae Juss.
Iz_3_1009	DJI_0926	Helichrysum arenarium (L.) Moench	0	n	1	0	Asteraceae Bercht. & J.Presl
Iz_3_1009	DJI_0926	Herniaria polygama J.Gay	0	n	1	0	Caryophyllaceae Juss.
Iz_3_1009	DJI_0926	Hieracium robustum Fr.	0	n	1	0	Asteraceae Bercht. & J.Presl
Iz_3_1009	DJI_0926	Hieracium umbellatum L.	0	n	1	NA	Asteraceae Bercht. & J.Presl
Iz_3_1009	DJI_0926	Hylotelephium maximum (L.) Holub	0	n	1	0	Crassulaceae J.St.-Hil.
Iz_3_1009	DJI_0926	Jacobaea borysthenica (DC.) B.Nord. & Greuter	0	n	1	0	Asteraceae Bercht. & J.Presl
Iz_3_1009	DJI_0926	Jasione montana L.	0	n	1	0	Campanulaceae Juss.
Iz_3_1009	DJI_0926	Jurinea cyanoides (L.) Rchb.s.l.	0	n	1	0	Asteraceae Bercht. & J.Presl
Iz_3_1009	DJI_0926	Koeleria glauca (Spreng.) DC.	0	n	1	0	Poaceae Barnhart
Iz_3_1009	DJI_0926	Lactuca serriola L.	0	a	0	0	Asteraceae Bercht. & J.Presl
Iz_3_1009	DJI_0926	Linaria genistifolia (L.) Mill.	0	n	1	0	Plantaginaceae Juss.
Iz_3_1009	DJI_0926	Lomelosia argentea (L.) Greuter & Burdet	0	n	1	0	Caprifoliaceae Juss.
Iz_3_1009	DJI_0926	Melampyrum pratense L.	0	n	1	0	Orobanchaceae Vent.
Iz_3_1009	DJI_0926	Oenothera biennis L.	0	a	0	0	Onagraceae Juss.
Iz_3_1009	DJI_0926	Pentanema britannica (L.) D.Gut.Larr., Santos-Vicente, Anderb., E.Rico & M.M.Mart.Ort.	0	n	1	0	Asteraceae Bercht. & J.Presl
Iz_3_1009	DJI_0926	Peucedanum oreoselinum (L.) Moench	2	n	1	0	Apiaceae Lindl.
Iz_3_1009	DJI_0926	Peucedanum ruthenicum M.Bieb.	0	n	1	0	Apiaceae Lindl.
Iz_3_1009	DJI_0926	Pilosella echioides (Lumn.) F.W.Schultz & Sch.Bip.	0	n	1	0	Asteraceae Bercht. & J.Presl
Iz_3_1009	DJI_0926	Pilosella officinarum F. Schultz et Sch. Bip.	0	n	1	0	Asteraceae Bercht. & J.Presl
Iz_3_1009	DJI_0926	Pilosella sp.	0		NA	0	Asteraceae Bercht. & J.Presl
Iz_3_1009	DJI_0926	Pinus sylvestris L.	0	n	1	1	Pinaceae Spreng. ex F.Rudolphi
Iz_3_1009	DJI_0926	Poa annua L.	0	n	1	0	Poaceae Barnhart
Iz_3_1009	DJI_0926	Poa nemoralis L.	0	n	1	0	Poaceae Barnhart
Iz_3_1009	DJI_0926	Poa sp.	0		NA	0	Poaceae Barnhart
Iz_3_1009	DJI_0926	Polygonatum odoratum (Mill.) Druce	0	n	1	0	Asparagaceae Juss.
Iz_3_1009	DJI_0926	Polygonum arenarium Waldst. & Kit.	0	n	1	0	Polygonaceae Juss.
Iz_3_1009	DJI_0926	Populus nigra L.	0	n	1	1	Salicaceae Mirb.
Iz_3_1009	DJI_0926	Populus tremula L.	0	n	1	1	Salicaceae Mirb.
Iz_3_1009	DJI_0926	Populus Ã canescens (Aiton) Sm.	0	n	1	1	Salicaceae Mirb.
Iz_3_1009	DJI_0926	Potentilla argentea L.	0	n	1	0	Rosaceae Juss.
Iz_3_1009	DJI_0926	Psammophiliella muralis (L.) Ikonn.	0	n	1	0	Caryophyllaceae Juss.
Iz_3_1009	DJI_0926	Psephellus sumensis (Kalen.) Greuter	0	n	1	0	Asteraceae Bercht. & J.Presl
Iz_3_1009	DJI_0926	Pulsatilla pratensis (L.) Mill. s.l.	0	n	1	0	Ranunculaceae Juss.
Iz_3_1009	DJI_0926	Pyrus communis L.	0	a	0	1	Rosaceae Juss.
Iz_3_1009	DJI_0926	Quercus robur L.	0	n	1	1	Fagaceae Dumort.
Iz_3_1009	DJI_0926	Robinia pseudoacacia L.	0	a	0	1	Fabaceae Lindl.
Iz_3_1009	DJI_0926	Rosa sp.	0		NA	1	Rosaceae Juss.
Iz_3_1009	DJI_0926	Rubus idaeus L.	0	n	1	0	Rosaceae Juss.
Iz_3_1009	DJI_0926	Rumex acetosella L.	0	n	1	0	Polygonaceae Juss.
Iz_3_1009	DJI_0926	Rumex thyrsiflorus Fingerh.	0	n	1	0	Polygonaceae Juss.
Iz_3_1009	DJI_0926	Salix alba L.	0	n	1	1	Salicaceae Mirb.
Iz_3_1009	DJI_0926	Sambucus nigra L.	0	n	1	1	Viburnaceae Raf.
Iz_3_1009	DJI_0926	Secale sylvestre Host	0	n	1	0	Poaceae Barnhart
Iz_3_1009	DJI_0926	Sempervivum ruthenicum Schnittsp. & C.B. Lehm.	0	n	1	0	Crassulaceae J.St.-Hil.
Iz_3_1009	DJI_0926	Setaria viridis (L.) P. Beauv.	0	n	1	0	Poaceae Barnhart
Iz_3_1009	DJI_0926	Silene borysthenica (Gruner) Walters	0	n	1	0	Caryophyllaceae Juss.

Iz_3_1009	DJI_0926	Silene latifolia subsp. alba (Mill.) Greuter & Burdet	0	n	1	0	Caryophyllaceae Juss.
Iz_3_1009	DJI_0926	Solidago virgaurea L.	0	n	1	0	Asteraceae Bercht. & J.Presl
Iz_3_1009	DJI_0926	Sonchus oleraceus L.	0	a	0	0	Asteraceae Bercht. & J.Presl
Iz_3_1009	DJI_0926	Sorbus aucuparia L.	0	n	1	1	Rosaceae Juss.
Iz_3_1009	DJI_0926	Stachys recta L.	0	n	1	0	Lamiacea Lindl.
Iz_3_1009	DJI_0926	Tanacetum vulgare L.	0	n	1	0	Asteraceae Bercht. & J.Presl
Iz_3_1009	DJI_0926	Thymus pallasianus Heinr.Braun	0	n	1	0	Lamiacea Lindl.
Iz_3_1009	DJI_0926	Tilia cordata Mill.	0	n	1	1	Malvaceae Juss.
Iz_3_1009	DJI_0926	Trifolium alpestre L.	0	n	1	0	Fabaceae Lindl.
Iz_3_1009	DJI_0926	Trifolium arvense L.	0	n	1	0	Fabaceae Lindl.
Iz_3_1009	DJI_0926	Ulmus glabra Huds.	0	n	1	1	Ulmaceae Mirb.
Iz_3_1009	DJI_0926	Verbascum densiflorum Bertol.	0	n	1	0	Scrophulariaceae Juss.
Iz_3_1009	DJI_0926	Veronica austriaca L.	0	n	1	0	Plantaginaceae Juss.
Iz_3_1009	DJI_0926	Veronica incana L.	0	n	1	0	Plantaginaceae Juss.
Iz_3_1009	DJI_0926	Veronica verna L.	0	n	1	0	Plantaginaceae Juss.
Iz_3_1009	DJI_0926	Vincetoxicum hirundinaria Medik	0	n	1	0	Apocynaceae Juss.
Iz_3_1009	DJI_0929	Acer tataricum L.	0	n	1	1	Sapindaceae Juss.
Iz_3_1009	DJI_0929	Achillea millefolium L. s.l.	0	n	1	0	Asteraceae Bercht. & J.Presl
Iz_3_1009	DJI_0929	Allium oleraceum L.	0	n	1	0	Amaryllidaceae J.St.-Hil.
Iz_3_1009	DJI_0929	Ambrosia artemisiifolia L.	0	a	0	0	Asteraceae Bercht. & J.Presl
Iz_3_1009	DJI_0929	Amorpha fruticosa L.	0	a	0	0	Fabaceae Lindl.
Iz_3_1009	DJI_0929	Anthericum ramosum L.	0	n	1	0	Asparagaceae Juss.
Iz_3_1009	DJI_0929	Artemisia marschalliana Spreng.	0	n	1	0	Asteraceae Bercht. & J.Presl
Iz_3_1009	DJI_0929	Asparagus officinalis L.	0	n	1	0	Asparagaceae Juss.
Iz_3_1009	DJI_0929	Astragalus cicer L.	0	n	1	0	Fabaceae Lindl.
Iz_3_1009	DJI_0929	Bassia laniflora (S.G.Gmel.) A.J.Scott	0	n	1	0	Amaranthaceae Juss.
Iz_3_1009	DJI_0929	Berberis vulgaris L.	0	n	1	0	Berberidaceae Juss.
Iz_3_1009	DJI_0929	Betula pendula Roth.	0	n	1	1	Betulaceae Gray
Iz_3_1009	DJI_0929	Betula pendula Roth.	0	n	1	0	Poaceae Barnhart
Iz_3_1009	DJI_0929	Bromus inermis Leyss.	0		NA	NA	
Iz_3_1009	DJI_0929	Bromus squarrosus L.	0	a	0	0	Poaceae Barnhart
Iz_3_1009	DJI_0929	Bromus tectorum L.	0	a	0	0	Poaceae Barnhart
Iz_3_1009	DJI_0929	Calamagrostis epigejos (L.) Roth	25	n	1	0	Poaceae Barnhart
Iz_3_1009	DJI_0929	Carduus acanthoides L.	0	n	1	0	Asteraceae Bercht. & J.Presl
Iz_3_1009	DJI_0929	Carex sp.	0	n	1	0	Cyperaceae Juss.
Iz_3_1009	DJI_0929	Centaurea majorovii Dumbadze	0	n	1	0	Asteraceae Bercht. & J.Presl
Iz_3_1009	DJI_0929	Chamaecytisus ruthenicus (Fisch.ex Wol.) Klaskova	0	n	1	0	Fabaceae Lindl.
Iz_3_1009	DJI_0929	Chelidonium majus L.	0	n	1	0	Papaveraceae Juss.
Iz_3_1009	DJI_0929	Chenopodium betaceum Andrz.	0	n	1	0	Amaranthaceae Juss.
Iz_3_1009	DJI_0929	Chenopodium cf. album	0	n	1	0	Amaranthaceae Juss.
Iz_3_1009	DJI_0929	Chenopodium sp.	0		NA	0	Amaranthaceae Juss.
Iz_3_1009	DJI_0929	Chondrilla juncea L.	0	n	1	0	Asteraceae Bercht. & J.Presl
Iz_3_1009	DJI_0929	Cirsium vulgare (Savi) Ten.	0	n	1	0	Asteraceae Bercht. & J.Presl
Iz_3_1009	DJI_0929	Clinopodium acinos (L.) Kuntze	0	n	1	0	Lamiacea Lindl.
Iz_3_1009	DJI_0929	Convallaria majalis L.	0	n	1	0	Asparagaceae Juss.
Iz_3_1009	DJI_0929	Crataegus rhipidophylla Gand.	0	n	1	1	Rosaceae Juss.
Iz_3_1009	DJI_0929	Crepis foetida L.	0	n	1	0	Asteraceae Bercht. & J.Presl
Iz_3_1009	DJI_0929	Dactylis glomerata L.	0	n	1	0	Poaceae Barnhart
Iz_3_1009	DJI_0929	Dianthus campestris M.Bieb.	0	n	1	0	Caryophyllaceae Juss.
Iz_3_1009	DJI_0929	Digitaria sanguinalis (L.) Scop.	0	a	0	0	Poaceae Barnhart
Iz_3_1009	DJI_0929	Elymus repens (L.) Gould	0	n	1	0	Poaceae Barnhart
Iz_3_1009	DJI_0929	Epilobium tetragonum L.	0	n	1	0	Onagraceae Juss.
Iz_3_1009	DJI_0929	Eragrostis minor Host	0	a	0	0	Poaceae Barnhart
Iz_3_1009	DJI_0929	Erigeron canadensis L.	0	a	0	0	Asteraceae Bercht. & J.Presl
Iz_3_1009	DJI_0929	Euphorbia seguieriana Neck.	0	n	1	0	Euphorbiaceae Juss.
Iz_3_1009	DJI_0929	Fallopia dumetorum (L.) Holub	0	n	1	0	Polygonaceae Juss.
Iz_3_1009	DJI_0929	Festuca beckeri (Hack.) Trautv.	0	n	1	0	Poaceae Barnhart
Iz_3_1009	DJI_0929	Fragaria vesca L.	0	n	1	0	Rosaceae Juss.
Iz_3_1009	DJI_0929	Frangula alnus Mill.	0	n	1	1	Rhamnaceae Juss.
Iz_3_1009	DJI_0929	Fraxinus pennsylvanica Marshall	0	a	0	1	Oleaceae Hoffmanns. & Link
Iz_3_1009	DJI_0929	Galeopsis bifida Boenn.	0	n	1	0	Lamiacea Lindl.
Iz_3_1009	DJI_0929	Galium verum L.	0	n	1	0	Rubiaceae Juss.
Iz_3_1009	DJI_0929	Genista tinctoria L.	0	n	1	0	Fabaceae Lindl.
Iz_3_1009	DJI_0929	Geranium robertianum L.	0	n	1	0	Geraniaceae Juss.
Iz_3_1009	DJI_0929	Geranium sanquineum L.	0	n	1	0	Geraniaceae Juss.
Iz_3_1009	DJI_0929	Gypsophila paniculata L.	0	n	1	0	Caryophyllaceae Juss.
Iz_3_1009	DJI_0929	Helichrysum arenarium (L.) Moench	0	n	1	0	Asteraceae Bercht. & J.Presl
Iz_3_1009	DJI_0929	Herniaria polygama J.Gay	0	n	1	0	Caryophyllaceae Juss.
Iz_3_1009	DJI_0929	Hieracium robustum Fr.	0	n	1	0	Asteraceae Bercht. & J.Presl
Iz_3_1009	DJI_0929	Hieracium umbellatum L.	0	n	1	NA	Asteraceae Bercht. & J.Presl
Iz_3_1009	DJI_0929	Hylotelephium maximum (L.) Holub	0	n	1	0	Crassulaceae J.St.-Hil.
Iz_3_1009	DJI_0929	Jacobaea borysthenica (DC.) B.Nord. & Greuter	0	n	1	0	Asteraceae Bercht. & J.Presl
Iz_3_1009	DJI_0929	Jasione montana L.	0	n	1	0	Campanulaceae Juss.
Iz_3_1009	DJI_0929	Jurinea cyanoides (L.) Rchb.s.l.	0	n	1	0	Asteraceae Bercht. & J.Presl
Iz_3_1009	DJI_0929	Koeleria glauca (Spreng.) DC.	0	n	1	0	Poaceae Barnhart
Iz_3_1009	DJI_0929	Lactuca serriola L.	0	a	0	0	Asteraceae Bercht. & J.Presl
Iz_3_1009	DJI_0929	Linaria genistifolia (L.) Mill.	0	n	1	0	Plantaginaceae Juss.
Iz_3_1009	DJI_0929	Lomelosia argentea (L.) Greuter & Burdet	0	n	1	0	Caprifoliaceae Juss.
Iz_3_1009	DJI_0929	Melampyrum pratense L.	0	n	1	0	Orobanchaceae Vent.
Iz_3_1009	DJI_0929	Oenothera biennis L.	0	a	0	0	Onagraceae Juss.
Iz_3_1009	DJI_0929	Pentanema britannica (L.) D.Gut.Larr., Santos-Vicente, Anderb., E.Rico & M.M.Mart.Ort.	0	n	1	0	Asteraceae Bercht. & J.Presl
Iz_3_1009	DJI_0929	Peucedanum oreoselinum (L.) Moench	2	n	1	0	Apiaceae Lindl.
Iz_3_1009	DJI_0929	Peucedanum ruthenicum M.Bieb.	0	n	1	0	Apiaceae Lindl.
Iz_3_1009	DJI_0929	Pilosella echioides (Lumn.) F.W.Schultz & Sch.Bip.	0	n	1	0	Asteraceae Bercht. & J.Presl
Iz_3_1009	DJI_0929	Pilosella officinarum F. Schultz et Sch. Bip.	0	n	1	0	Asteraceae Bercht. & J.Presl
Iz_3_1009	DJI_0929	Pilosella sp.	0		NA	0	Asteraceae Bercht. & J.Presl
Iz_3_1009	DJI_0929	Pinus sylvestris L.	0	n	1	1	Pinaceae Spreng. ex F.Rudolphi
Iz_3_1009	DJI_0929	Poa annua L.	0	n	1	0	Poaceae Barnhart
Iz_3_1009	DJI_0929	Poa nemoralis L.	0	n	1	0	Poaceae Barnhart
Iz_3_1009	DJI_0929	Poa sp.	0		NA	0	Poaceae Barnhart
Iz_3_1009	DJI_0929	Polygonatum odoratum (Mill.) Druce	0.5	n	1	0	Asparagaceae Juss.
Iz_3_1009	DJI_0929	Polygonum arenarium Waldst. & Kit.	0	n	1	0	Polygonaceae Juss.
Iz_3_1009	DJI_0929	Populus nigra L.	0	n	1	1	Salicaceae Mirb.
Iz_3_1009	DJI_0929	Populus tremula L.	0	n	1	1	Salicaceae Mirb.
Iz_3_1009	DJI_0929	Populus Ã canescens (Aiton) Sm.	0	n	1	1	Salicaceae Mirb.
Iz_3_1009	DJI_0929	Potentilla argentea L.	0	n	1	0	Rosaceae Juss.
Iz_3_1009	DJI_0929	Psammophiliella muralis (L.) Ikonn.	0	n	1	0	Caryophyllaceae Juss.
Iz_3_1009	DJI_0929	Psephellus sumensis (Kalen.) Greuter	0	n	1	0	Asteraceae Bercht. & J.Presl

Iz_3_1009	DJI_0929	Pulsatilla pratensis (L.) Mill. s.l.	0	n	1	0	Ranunculaceae Juss.
Iz_3_1009	DJI_0929	Pyrus communis L.	0	a	0	1	Rosaceae Juss.
Iz_3_1009	DJI_0929	Quercus robur L.	1.5	n	1	1	Fagaceae Dumort.
Iz_3_1009	DJI_0929	Robinia pseudoacacia L.	0	a	0	1	Fabaceae Lindl.
Iz_3_1009	DJI_0929	Rosa sp.	0		NA	1	Rosaceae Juss.
Iz_3_1009	DJI_0929	Rubus idaeus L.	0	n	1	0	Rosaceae Juss.
Iz_3_1009	DJI_0929	Rumex acetosella L.	0	n	1	0	Polygonaceae Juss.
Iz_3_1009	DJI_0929	Rumex thyrsiflorus Fingerh.	0	n	1	0	Polygonaceae Juss.
Iz_3_1009	DJI_0929	Salix alba L.	0	n	1	1	Salicaceae Mirb.
Iz_3_1009	DJI_0929	Sambucus nigra L.	0	n	1	1	Viburnaceae Raf.
Iz_3_1009	DJI_0929	Secale sylvestre Host	0	n	1	0	Poaceae Barnhart
Iz_3_1009	DJI_0929	Sempervivum ruthenicum Schnittsp. & C.B. Lehm.	0	n	1	0	Crassulaceae J.St.-Hil.
Iz_3_1009	DJI_0929	Setaria viridis (L.) P. Beauv.	0	n	1	0	Poaceae Barnhart
Iz_3_1009	DJI_0929	Silene borysthenica (Gruner) Walters	0	n	1	0	Caryophyllaceae Juss.
Iz_3_1009	DJI_0929	Silene latifolia subsp. alba (Mill.) Greuter & Burdet	0	n	1	0	Caryophyllaceae Juss.
Iz_3_1009	DJI_0929	Solidago virgaurea L.	2	n	1	0	Asteraceae Bercht. & J.Presl
Iz_3_1009	DJI_0929	Sonchus oleraceus L.	0	a	0	0	Asteraceae Bercht. & J.Presl
Iz_3_1009	DJI_0929	Sorbus aucuparia L.	0	n	1	1	Rosaceae Juss.
Iz_3_1009	DJI_0929	Stachys recta L.	0	n	1	0	Lamiacea Lindl.
Iz_3_1009	DJI_0929	Tanacetum vulgare L.	0	n	1	0	Asteraceae Bercht. & J.Presl
Iz_3_1009	DJI_0929	Thymus pallasianus Heinr.Braun	0	n	1	0	Lamiacea Lindl.
Iz_3_1009	DJI_0929	Tilia cordata Mill.	0	n	1	1	Malvaceae Juss.
Iz_3_1009	DJI_0929	Trifolium alpestre L.	0	n	1	0	Fabaceae Lindl.
Iz_3_1009	DJI_0929	Trifolium arvense L.	0	n	1	0	Fabaceae Lindl.
Iz_3_1009	DJI_0929	Ulmus glabra Huds.	0	n	1	1	Ulmaceae Mirb.
Iz_3_1009	DJI_0929	Verbascum densiflorum Bertol.	0	n	1	0	Scrophulariaceae Juss.
Iz_3_1009	DJI_0929	Veronica austriaca L.	0	n	1	0	Plantaginaceae Juss.
Iz_3_1009	DJI_0929	Veronica incana L.	0	n	1	0	Plantaginaceae Juss.
Iz_3_1009	DJI_0929	Veronica verna L.	0	n	1	0	Plantaginaceae Juss.
Iz_3_1009	DJI_0929	Vincetoxicum hirundinaria Medik	0	n	1	0	Apocynaceae Juss.
Iz_3_1009	DJI_0931	Acer tataricum L.	0	n	1	1	Sapindaceae Juss.
Iz_3_1009	DJI_0931	Achillea millefolium L. s.l.	0	n	1	0	Asteraceae Bercht. & J.Presl
Iz_3_1009	DJI_0931	Allium oleraceum L.	0	n	1	0	Amaryllidaceae J.St.-Hil.
Iz_3_1009	DJI_0931	Ambrosia artemisiifolia L.	0	a	0	0	Asteraceae Bercht. & J.Presl
Iz_3_1009	DJI_0931	Amorpha fruticosa L.	0	a	0	0	Fabaceae Lindl.
Iz_3_1009	DJI_0931	Anthericum ramosum L.	0	n	1	0	Asparagaceae Juss.
Iz_3_1009	DJI_0931	Artemisia marschalliana Spreng.	0	n	1	0	Asteraceae Bercht. & J.Presl
Iz_3_1009	DJI_0931	Asparagus officinalis L.	0	n	1	0	Asparagaceae Juss.
Iz_3_1009	DJI_0931	Astragalus cicer L.	0	n	1	0	Fabaceae Lindl.
Iz_3_1009	DJI_0931	Bassia laniflora (S.G.Gmel.) A.J.Scott	0	n	1	0	Amaranthaceae Juss.
Iz_3_1009	DJI_0931	Berberis vulgaris L.	0	n	1	0	Berberidaceae Juss.
Iz_3_1009	DJI_0931	Betula pendula Roth.	0	n	1	1	Betulaceae Gray
Iz_3_1009	DJI_0931	Betula pendula Roth.	0	n	1	0	Poaceae Barnhart
Iz_3_1009	DJI_0931	Bromus inermis Leyss.	0		NA	NA	
Iz_3_1009	DJI_0931	Bromus squarrosus L.	0	a	0	0	Poaceae Barnhart
Iz_3_1009	DJI_0931	Bromus tectorum L.	0	a	0	0	Poaceae Barnhart
Iz_3_1009	DJI_0931	Calamagrostis epigejos (L.) Roth	25	n	1	0	Poaceae Barnhart
Iz_3_1009	DJI_0931	Carduus acanthoides L.	0	n	1	0	Asteraceae Bercht. & J.Presl
Iz_3_1009	DJI_0931	Carex sp.	0	n	1	0	Cyperaceae Juss.
Iz_3_1009	DJI_0931	Centaurea majorovii Dumbadze	0	n	1	0	Asteraceae Bercht. & J.Presl
Iz_3_1009	DJI_0931	Chamaecytisus ruthenicus (Fisch.ex Wol.) Klaskova	0	n	1	0	Fabaceae Lindl.
Iz_3_1009	DJI_0931	Chelidonium majus L.	0	n	1	0	Papaveraceae Juss.
Iz_3_1009	DJI_0931	Chenopodium betaceum Andrz.	0	n	1	0	Amaranthaceae Juss.
Iz_3_1009	DJI_0931	Chenopodium cf. album	0	n	1	0	Amaranthaceae Juss.
Iz_3_1009	DJI_0931	Chenopodium sp.	0		NA	0	Amaranthaceae Juss.
Iz_3_1009	DJI_0931	Chondrilla juncea L.	0	n	1	0	Asteraceae Bercht. & J.Presl
Iz_3_1009	DJI_0931	Cirsium vulgare (Savi) Ten.	0	n	1	0	Asteraceae Bercht. & J.Presl
Iz_3_1009	DJI_0931	Clinopodium acinos (L.) Kuntze	0	n	1	0	Lamiacea Lindl.
Iz_3_1009	DJI_0931	Convallaria majalis L.	0	n	1	0	Asparagaceae Juss.
Iz_3_1009	DJI_0931	Crataegus rhipidophylla Gand.	0	n	1	1	Rosaceae Juss.
Iz_3_1009	DJI_0931	Crepis foetida L.	0	n	1	0	Asteraceae Bercht. & J.Presl
Iz_3_1009	DJI_0931	Dactylis glomerata L.	0	n	1	0	Poaceae Barnhart
Iz_3_1009	DJI_0931	Dianthus campestris M.Bieb.	0	n	1	0	Caryophyllaceae Juss.
Iz_3_1009	DJI_0931	Digitaria sanguinalis (L.) Scop.	0	a	0	0	Poaceae Barnhart
Iz_3_1009	DJI_0931	Elymus repens (L.) Gould	0	n	1	0	Poaceae Barnhart
Iz_3_1009	DJI_0931	Epilobium tetragonum L.	0	n	1	0	Onagraceae Juss.
Iz_3_1009	DJI_0931	Eragrostis minor Host	0	a	0	0	Poaceae Barnhart
Iz_3_1009	DJI_0931	Erigeron canadensis L.	0	a	0	0	Asteraceae Bercht. & J.Presl
Iz_3_1009	DJI_0931	Euphorbia seguieriana Neck.	0	n	1	0	Euphorbiaceae Juss.
Iz_3_1009	DJI_0931	Fallopia dumetorum (L.) Holub	0	n	1	0	Polygonaceae Juss.
Iz_3_1009	DJI_0931	Festuca beckeri (Hack.) Trautv.	0	n	1	0	Poaceae Barnhart
Iz_3_1009	DJI_0931	Fragaria vesca L.	0	n	1	0	Rosaceae Juss.
Iz_3_1009	DJI_0931	Frangula alnus Mill.	0	n	1	1	Rhamnaceae Juss.
Iz_3_1009	DJI_0931	Fraxinus pennsylvanica Marshall	0	a	0	1	Oleaceae Hoffmanns. & Link
Iz_3_1009	DJI_0931	Galeopsis bifida Boenn.	0	n	1	0	Lamiacea Lindl.
Iz_3_1009	DJI_0931	Galium verum L.	0	n	1	0	Rubiaceae Juss.
Iz_3_1009	DJI_0931	Genista tinctoria L.	0	n	1	0	Fabaceae Lindl.
Iz_3_1009	DJI_0931	Geranium robertianum L.	0	n	1	0	Geraniaceae Juss.
Iz_3_1009	DJI_0931	Geranium sanquineum L.	0	n	1	0	Geraniaceae Juss.
Iz_3_1009	DJI_0931	Gypsophila paniculata L.	0	n	1	0	Caryophyllaceae Juss.
Iz_3_1009	DJI_0931	Helichrysum arenarium (L.) Moench	0	n	1	0	Asteraceae Bercht. & J.Presl
Iz_3_1009	DJI_0931	Herniaria polygama J.Gay	0	n	1	0	Caryophyllaceae Juss.
Iz_3_1009	DJI_0931	Hieracium robustum Fr.	0	n	1	0	Asteraceae Bercht. & J.Presl
Iz_3_1009	DJI_0931	Hieracium umbellatum L.	0	n	1	NA	Asteraceae Bercht. & J.Presl
Iz_3_1009	DJI_0931	Hylotelephium maximum (L.) Holub	0	n	1	0	Crassulaceae J.St.-Hil.
Iz_3_1009	DJI_0931	Jacobaea borysthenica (DC.) B.Nord. & Greuter	0	n	1	0	Asteraceae Bercht. & J.Presl
Iz_3_1009	DJI_0931	Jasione montana L.	0	n	1	0	Campanulaceae Juss.
Iz_3_1009	DJI_0931	Jurinea cyanoides (L.) Rchb.s.l.	0	n	1	0	Asteraceae Bercht. & J.Presl
Iz_3_1009	DJI_0931	Koeleria glauca (Spreng.) DC.	0	n	1	0	Poaceae Barnhart
Iz_3_1009	DJI_0931	Lactuca serriola L.	0	a	0	0	Asteraceae Bercht. & J.Presl
Iz_3_1009	DJI_0931	Linaria genistifolia (L.) Mill.	0	n	1	0	Plantaginaceae Juss.
Iz_3_1009	DJI_0931	Lomelosia argentea (L.) Greuter & Burdet	0	n	1	0	Caprifoliaceae Juss.
Iz_3_1009	DJI_0931	Melampyrum pratense L.	0	n	1	0	Orobanchaceae Vent.
Iz_3_1009	DJI_0931	Oenothera biennis L.	0	a	0	0	Onagraceae Juss.
Iz_3_1009	DJI_0931	Pentanema britannica (L.) D.Gut.Larr., Santos-Vicente, Anderb., E.Rico & M.M.Mart.Ort.	0	n	1	0	Asteraceae Bercht. & J.Presl
Iz_3_1009	DJI_0931	Peucedanum oreoselinum (L.) Moench	4	n	1	0	Apiaceae Lindl.
Iz_3_1009	DJI_0931	Peucedanum ruthenicum M.Bieb.	0	n	1	0	Apiaceae Lindl.
Iz_3_1009	DJI_0931	Pilosella echioides (Lumn.) F.W.Schultz & Sch.Bip.	0	n	1	0	Asteraceae Bercht. & J.Presl

Iz_3_1009	DJI_0931	Pilosella officinarum F. Schultz et Sch. Bip.	0	n	1	0	Asteraceae Bercht. & J.Presl
Iz_3_1009	DJI_0931	Pilosella sp.	0		NA	0	Asteraceae Bercht. & J.Presl
Iz_3_1009	DJI_0931	Pinus sylvestris L.	0	n	1	1	Pinaceae Spreng. ex F.Rudolphi
Iz_3_1009	DJI_0931	Poa annua L.	0	n	1	0	Poaceae Barnhart
Iz_3_1009	DJI_0931	Poa nemoralis L.	0	n	1	0	Poaceae Barnhart
Iz_3_1009	DJI_0931	Poa sp.	5		NA	0	Poaceae Barnhart
Iz_3_1009	DJI_0931	Polygonatum odoratum (Mill.) Druce	0.5	n	1	0	Asparagaceae Juss.
Iz_3_1009	DJI_0931	Polygonum arenarium Waldst. & Kit.	0	n	1	0	Polygonaceae Juss.
Iz_3_1009	DJI_0931	Populus nigra L.	0	n	1	1	Salicaceae Mirb.
Iz_3_1009	DJI_0931	Populus tremula L.	0	n	1	1	Salicaceae Mirb.
Iz_3_1009	DJI_0931	Populus Ã canescens (Aiton) Sm.	0	n	1	1	Salicaceae Mirb.
Iz_3_1009	DJI_0931	Potentilla argentea L.	0	n	1	0	Rosaceae Juss.
Iz_3_1009	DJI_0931	Psammophiliella muralis (L.) Ikonn.	0	n	1	0	Caryophyllaceae Juss.
Iz_3_1009	DJI_0931	Psephellus sumensis (Kalen.) Greuter	0	n	1	0	Asteraceae Bercht. & J.Presl
Iz_3_1009	DJI_0931	Pulsatilla pratensis (L.) Mill. s.l.	0	n	1	0	Ranunculaceae Juss.
Iz_3_1009	DJI_0931	Pyrus communis L.	0	a	0	1	Rosaceae Juss.
Iz_3_1009	DJI_0931	Quercus robur L.	1.5	n	1	1	Fagaceae Dumort.
Iz_3_1009	DJI_0931	Robinia pseudoacacia L.	0	a	0	1	Fabaceae Lindl.
Iz_3_1009	DJI_0931	Rosa sp.	0		NA	1	Rosaceae Juss.
Iz_3_1009	DJI_0931	Rubus idaeus L.	0	n	1	0	Rosaceae Juss.
Iz_3_1009	DJI_0931	Rumex acetosella L.	0	n	1	0	Polygonaceae Juss.
Iz_3_1009	DJI_0931	Rumex thyrsiflorus Fingerh.	0	n	1	0	Polygonaceae Juss.
Iz_3_1009	DJI_0931	Salix alba L.	0	n	1	1	Salicaceae Mirb.
Iz_3_1009	DJI_0931	Sambucus nigra L.	0	n	1	1	Viburnaceae Raf.
Iz_3_1009	DJI_0931	Secale sylvestre Host	0	n	1	0	Poaceae Barnhart
Iz_3_1009	DJI_0931	Sempervivum ruthenicum Schnittsp. & C.B. Lehm.	0	n	1	0	Crassulaceae J.St.-Hil.
Iz_3_1009	DJI_0931	Setaria viridis (L.) P. Beauv.	0	n	1	0	Poaceae Barnhart
Iz_3_1009	DJI_0931	Silene borysthenica (Gruner) Walters	0	n	1	0	Caryophyllaceae Juss.
Iz_3_1009	DJI_0931	Silene latifolia subsp. alba (Mill.) Greuter & Burdet	0	n	1	0	Caryophyllaceae Juss.
Iz_3_1009	DJI_0931	Solidago virgaurea L.	0	n	1	0	Asteraceae Bercht. & J.Presl
Iz_3_1009	DJI_0931	Sonchus oleraceus L.	0	a	0	0	Asteraceae Bercht. & J.Presl
Iz_3_1009	DJI_0931	Sorbus aucuparia L.	0	n	1	1	Rosaceae Juss.
Iz_3_1009	DJI_0931	Stachys recta L.	0	n	1	0	Lamiacea Lindl.
Iz_3_1009	DJI_0931	Tanacetum vulgare L.	0	n	1	0	Asteraceae Bercht. & J.Presl
Iz_3_1009	DJI_0931	Thymus pallasianus Heinr.Braun	0	n	1	0	Lamiacea Lindl.
Iz_3_1009	DJI_0931	Tilia cordata Mill.	0	n	1	1	Malvaceae Juss.
Iz_3_1009	DJI_0931	Trifolium alpestre L.	0	n	1	0	Fabaceae Lindl.
Iz_3_1009	DJI_0931	Trifolium arvense L.	0	n	1	0	Fabaceae Lindl.
Iz_3_1009	DJI_0931	Ulmus glabra Huds.	0	n	1	1	Ulmaceae Mirb.
Iz_3_1009	DJI_0931	Verbascum densiflorum Bertol.	0	n	1	0	Scrophulariaceae Juss.
Iz_3_1009	DJI_0931	Veronica austriaca L.	0	n	1	0	Plantaginaceae Juss.
Iz_3_1009	DJI_0931	Veronica incana L.	0	n	1	0	Plantaginaceae Juss.
Iz_3_1009	DJI_0931	Veronica verna L.	0	n	1	0	Plantaginaceae Juss.
Iz_3_1009	DJI_0931	Vincetoxicum hirundinaria Medik	0	n	1	0	Apocynaceae Juss.
Iz_3_1009	DJI_0933	Acer tataricum L.	0	n	1	1	Sapindaceae Juss.
Iz_3_1009	DJI_0933	Achillea millefolium L. s.l.	0	n	1	0	Asteraceae Bercht. & J.Presl
Iz_3_1009	DJI_0933	Allium oleraceum L.	0	n	1	0	Amaryllidaceae J.St.-Hil.
Iz_3_1009	DJI_0933	Ambrosia artemisiifolia L.	0	a	0	0	Asteraceae Bercht. & J.Presl
Iz_3_1009	DJI_0933	Amorpha fruticosa L.	0	a	0	0	Fabaceae Lindl.
Iz_3_1009	DJI_0933	Anthericum ramosum L.	0	n	1	0	Asparagaceae Juss.
Iz_3_1009	DJI_0933	Artemisia marschalliana Spreng.	0	n	1	0	Asteraceae Bercht. & J.Presl
Iz_3_1009	DJI_0933	Asparagus officinalis L.	0	n	1	0	Asparagaceae Juss.
Iz_3_1009	DJI_0933	Astragalus cicer L.	0	n	1	0	Fabaceae Lindl.
Iz_3_1009	DJI_0933	Bassia laniflora (S.G.Gmel.) A.J.Scott	0	n	1	0	Amaranthaceae Juss.
Iz_3_1009	DJI_0933	Berberis vulgaris L.	0	n	1	0	Berberidaceae Juss.
Iz_3_1009	DJI_0933	Betula pendula Roth.	0	n	1	1	Betulaceae Gray
Iz_3_1009	DJI_0933	Betula pendula Roth.	0	n	1	0	Poaceae Barnhart
Iz_3_1009	DJI_0933	Bromus inermis Leyss.	0		NA	NA	
Iz_3_1009	DJI_0933	Bromus squarrosus L.	0	a	0	0	Poaceae Barnhart
Iz_3_1009	DJI_0933	Bromus tectorum L.	0	a	0	0	Poaceae Barnhart
Iz_3_1009	DJI_0933	Calamagrostis epigejos (L.) Roth	15	n	1	0	Poaceae Barnhart
Iz_3_1009	DJI_0933	Carduus acanthoides L.	0	n	1	0	Asteraceae Bercht. & J.Presl
Iz_3_1009	DJI_0933	Carex sp.	0	n	1	0	Cyperaceae Juss.
Iz_3_1009	DJI_0933	Centaurea majorovii Dumbadze	0	n	1	0	Asteraceae Bercht. & J.Presl
Iz_3_1009	DJI_0933	Chamaecytisus ruthenicus (Fisch.ex Wol.) Klaskova	0	n	1	0	Fabaceae Lindl.
Iz_3_1009	DJI_0933	Chelidonium majus L.	0	n	1	0	Papaveraceae Juss.
Iz_3_1009	DJI_0933	Chenopodium betaceum Andrz.	0	n	1	0	Amaranthaceae Juss.
Iz_3_1009	DJI_0933	Chenopodium cf. album	0	n	1	0	Amaranthaceae Juss.
Iz_3_1009	DJI_0933	Chenopodium sp.	0		NA	0	Amaranthaceae Juss.
Iz_3_1009	DJI_0933	Chondrilla juncea L.	0	n	1	0	Asteraceae Bercht. & J.Presl
Iz_3_1009	DJI_0933	Cirsium vulgare (Savi) Ten.	0	n	1	0	Asteraceae Bercht. & J.Presl
Iz_3_1009	DJI_0933	Clinopodium acinos (L.) Kuntze	0	n	1	0	Lamiacea Lindl.
Iz_3_1009	DJI_0933	Convallaria majalis L.	0	n	1	0	Asparagaceae Juss.
Iz_3_1009	DJI_0933	Crataegus rhipidophylla Gand.	0	n	1	1	Rosaceae Juss.
Iz_3_1009	DJI_0933	Crepis foetida L.	0	n	1	0	Asteraceae Bercht. & J.Presl
Iz_3_1009	DJI_0933	Dactylis glomerata L.	0	n	1	0	Poaceae Barnhart
Iz_3_1009	DJI_0933	Dianthus campestris M.Bieb.	0	n	1	0	Caryophyllaceae Juss.
Iz_3_1009	DJI_0933	Digitaria sanguinalis (L.) Scop.	0	a	0	0	Poaceae Barnhart
Iz_3_1009	DJI_0933	Elymus repens (L.) Gould	0	n	1	0	Poaceae Barnhart
Iz_3_1009	DJI_0933	Epilobium tetragonum L.	0	n	1	0	Onagraceae Juss.
Iz_3_1009	DJI_0933	Eragrostis minor Host	0	a	0	0	Poaceae Barnhart
Iz_3_1009	DJI_0933	Erigeron canadensis L.	0	a	0	0	Asteraceae Bercht. & J.Presl
Iz_3_1009	DJI_0933	Euphorbia seguieriana Neck.	0	n	1	0	Euphorbiaceae Juss.
Iz_3_1009	DJI_0933	Fallopia dumetorum (L.) Holub	0	n	1	0	Polygonaceae Juss.
Iz_3_1009	DJI_0933	Festuca beckeri (Hack.) Trautv.	0	n	1	0	Poaceae Barnhart
Iz_3_1009	DJI_0933	Fragaria vesca L.	0	n	1	0	Rosaceae Juss.
Iz_3_1009	DJI_0933	Frangula alnus Mill.	0	n	1	1	Rhamnaceae Juss.
Iz_3_1009	DJI_0933	Fraxinus pennsylvanica Marshall	0	a	0	1	Oleaceae Hoffmanns. & Link
Iz_3_1009	DJI_0933	Galeopsis bifida Boenn.	0	n	1	0	Lamiacea Lindl.
Iz_3_1009	DJI_0933	Galium verum L.	0	n	1	0	Rubiaceae Juss.
Iz_3_1009	DJI_0933	Genista tinctoria L.	0	n	1	0	Fabaceae Lindl.
Iz_3_1009	DJI_0933	Geranium robertianum L.	0	n	1	0	Geraniaceae Juss.
Iz_3_1009	DJI_0933	Geranium sanquineum L.	0	n	1	0	Geraniaceae Juss.
Iz_3_1009	DJI_0933	Gypsophila paniculata L.	0	n	1	0	Caryophyllaceae Juss.
Iz_3_1009	DJI_0933	Helichrysum arenarium (L.) Moench	0	n	1	0	Asteraceae Bercht. & J.Presl
Iz_3_1009	DJI_0933	Herniaria polygama J.Gay	0	n	1	0	Caryophyllaceae Juss.
Iz_3_1009	DJI_0933	Hieracium robustum Fr.	0	n	1	0	Asteraceae Bercht. & J.Presl
Iz_3_1009	DJI_0933	Hieracium umbellatum L.	0	n	1	NA	Asteraceae Bercht. & J.Presl

Iz_3_1009	DJI_0933	Hylotelephium maximum (L.) Holub	0	n	1	0	Crassulaceae J.St.-Hil.
Iz_3_1009	DJI_0933	Jacobaea borysthenica (DC.) B.Nord. & Greuter	0	n	1	0	Asteraceae Bercht. & J.Presl
Iz_3_1009	DJI_0933	Jasione montana L.	0	n	1	0	Campanulaceae Juss.
Iz_3_1009	DJI_0933	Jurinea cyanoides (L.) Rchb.s.l.	0	n	1	0	Asteraceae Bercht. & J.Presl
Iz_3_1009	DJI_0933	Koeleria glauca (Spreng.) DC.	0	n	1	0	Poaceae Barnhart
Iz_3_1009	DJI_0933	Lactuca serriola L.	0	a	0	0	Asteraceae Bercht. & J.Presl
Iz_3_1009	DJI_0933	Linaria genistifolia (L.) Mill.	0	n	1	0	Plantaginaceae Juss.
Iz_3_1009	DJI_0933	Lomelosia argentea (L.) Greuter & Burdet	0	n	1	0	Caprifoliaceae Juss.
Iz_3_1009	DJI_0933	Melampyrum pratense L.	0	n	1	0	Orobanchaceae Vent.
Iz_3_1009	DJI_0933	Oenothera biennis L.	0	a	0	0	Onagraceae Juss.
Iz_3_1009	DJI_0933	Pentanema britannica (L.) D.Gut.Larr., Santos-Vicente, Anderb., E.Rico & M.M.Mart.Ort.	0	n	1	0	Asteraceae Bercht. & J.Presl
Iz_3_1009	DJI_0933	Peucedanum oreoselinum (L.) Moench	4	n	1	0	Apiaceae Lindl.
Iz_3_1009	DJI_0933	Peucedanum ruthenicum M.Bieb.	0	n	1	0	Apiaceae Lindl.
Iz_3_1009	DJI_0933	Pilosella echioides (Lumn.) F.W.Schultz & Sch.Bip.	0	n	1	0	Asteraceae Bercht. & J.Presl
Iz_3_1009	DJI_0933	Pilosella officinarum F. Schultz et Sch. Bip.	0	n	1	0	Asteraceae Bercht. & J.Presl
Iz_3_1009	DJI_0933	Pilosella sp.	0		NA	0	Asteraceae Bercht. & J.Presl
Iz_3_1009	DJI_0933	Pinus sylvestris L.	0	n	1	1	Pinaceae Spreng. ex F.Rudolphi
Iz_3_1009	DJI_0933	Poa annua L.	0	n	1	0	Poaceae Barnhart
Iz_3_1009	DJI_0933	Poa nemoralis L.	0	n	1	0	Poaceae Barnhart
Iz_3_1009	DJI_0933	Poa sp.	0		NA	0	Poaceae Barnhart
Iz_3_1009	DJI_0933	Polygonatum odoratum (Mill.) Druce	0.5	n	1	0	Asparagaceae Juss.
Iz_3_1009	DJI_0933	Polygonum arenarium Waldst. & Kit.	0	n	1	0	Polygonaceae Juss.
Iz_3_1009	DJI_0933	Populus nigra L.	0	n	1	1	Salicaceae Mirb.
Iz_3_1009	DJI_0933	Populus tremula L.	0	n	1	1	Salicaceae Mirb.
Iz_3_1009	DJI_0933	Populus Ã canescens (Aiton) Sm.	0	n	1	1	Salicaceae Mirb.
Iz_3_1009	DJI_0933	Potentilla argentea L.	0	n	1	0	Rosaceae Juss.
Iz_3_1009	DJI_0933	Psammophiliella muralis (L.) Ikonn.	0	n	1	0	Caryophyllaceae Juss.
Iz_3_1009	DJI_0933	Psephellus sumensis (Kalen.) Greuter	0	n	1	0	Asteraceae Bercht. & J.Presl
Iz_3_1009	DJI_0933	Pulsatilla pratensis (L.) Mill. s.l.	0	n	1	0	Ranunculaceae Juss.
Iz_3_1009	DJI_0933	Pyrus communis L.	0	a	0	1	Rosaceae Juss.
Iz_3_1009	DJI_0933	Quercus robur L.	0	n	1	1	Fagaceae Dumort.
Iz_3_1009	DJI_0933	Robinia pseudoacacia L.	0	a	0	1	Fabaceae Lindl.
Iz_3_1009	DJI_0933	Rosa sp.	0		NA	1	Rosaceae Juss.
Iz_3_1009	DJI_0933	Rubus idaeus L.	0	n	1	0	Rosaceae Juss.
Iz_3_1009	DJI_0933	Rumex acetosella L.	0	n	1	0	Polygonaceae Juss.
Iz_3_1009	DJI_0933	Rumex thyrsiflorus Fingerh.	0	n	1	0	Polygonaceae Juss.
Iz_3_1009	DJI_0933	Salix alba L.	0	n	1	1	Salicaceae Mirb.
Iz_3_1009	DJI_0933	Sambucus nigra L.	0	n	1	1	Viburnaceae Raf.
Iz_3_1009	DJI_0933	Secale sylvestre Host	0	n	1	0	Poaceae Barnhart
Iz_3_1009	DJI_0933	Sempervivum ruthenicum Schnittsp. & C.B. Lehm.	0	n	1	0	Crassulaceae J.St.-Hil.
Iz_3_1009	DJI_0933	Setaria viridis (L.) P. Beauv.	0	n	1	0	Poaceae Barnhart
Iz_3_1009	DJI_0933	Silene borysthenica (Gruner) Walters	0	n	1	0	Caryophyllaceae Juss.
Iz_3_1009	DJI_0933	Silene latifolia subsp. alba (Mill.) Greuter & Burdet	0	n	1	0	Caryophyllaceae Juss.
Iz_3_1009	DJI_0933	Solidago virgaurea L.	0	n	1	0	Asteraceae Bercht. & J.Presl
Iz_3_1009	DJI_0933	Sonchus oleraceus L.	0	a	0	0	Asteraceae Bercht. & J.Presl
Iz_3_1009	DJI_0933	Sorbus aucuparia L.	0	n	1	1	Rosaceae Juss.
Iz_3_1009	DJI_0933	Stachys recta L.	0	n	1	0	Lamiacea Lindl.
Iz_3_1009	DJI_0933	Tanacetum vulgare L.	0	n	1	0	Asteraceae Bercht. & J.Presl
Iz_3_1009	DJI_0933	Thymus pallasianus Heinr.Braun	0	n	1	0	Lamiacea Lindl.
Iz_3_1009	DJI_0933	Tilia cordata Mill.	0	n	1	1	Malvaceae Juss.
Iz_3_1009	DJI_0933	Trifolium alpestre L.	0	n	1	0	Fabaceae Lindl.
Iz_3_1009	DJI_0933	Trifolium arvense L.	0	n	1	0	Fabaceae Lindl.
Iz_3_1009	DJI_0933	Ulmus glabra Huds.	0	n	1	1	Ulmaceae Mirb.
Iz_3_1009	DJI_0933	Verbascum densiflorum Bertol.	0	n	1	0	Scrophulariaceae Juss.
Iz_3_1009	DJI_0933	Veronica austriaca L.	0	n	1	0	Plantaginaceae Juss.
Iz_3_1009	DJI_0933	Veronica incana L.	0	n	1	0	Plantaginaceae Juss.
Iz_3_1009	DJI_0933	Veronica verna L.	0	n	1	0	Plantaginaceae Juss.
Iz_3_1009	DJI_0933	Vincetoxicum hirundinaria Medik	0	n	1	0	Apocynaceae Juss.
Iz_3_1009	DJI_0935	Acer tataricum L.	0	n	1	1	Sapindaceae Juss.
Iz_3_1009	DJI_0935	Achillea millefolium L. s.l.	0	n	1	0	Asteraceae Bercht. & J.Presl
Iz_3_1009	DJI_0935	Allium oleraceum L.	0	n	1	0	Amaryllidaceae J.St.-Hil.
Iz_3_1009	DJI_0935	Ambrosia artemisiifolia L.	0	a	0	0	Asteraceae Bercht. & J.Presl
Iz_3_1009	DJI_0935	Amorpha fruticosa L.	0	a	0	0	Fabaceae Lindl.
Iz_3_1009	DJI_0935	Anthericum ramosum L.	0	n	1	0	Asparagaceae Juss.
Iz_3_1009	DJI_0935	Artemisia marschalliana Spreng.	0	n	1	0	Asteraceae Bercht. & J.Presl
Iz_3_1009	DJI_0935	Asparagus officinalis L.	0	n	1	0	Asparagaceae Juss.
Iz_3_1009	DJI_0935	Astragalus cicer L.	0	n	1	0	Fabaceae Lindl.
Iz_3_1009	DJI_0935	Bassia laniflora (S.G.Gmel.) A.J.Scott	0	n	1	0	Amaranthaceae Juss.
Iz_3_1009	DJI_0935	Berberis vulgaris L.	0	n	1	0	Berberidaceae Juss.
Iz_3_1009	DJI_0935	Betula pendula Roth.	0	n	1	1	Betulaceae Gray
Iz_3_1009	DJI_0935	Betula pendula Roth.	0	n	1	0	Poaceae Barnhart
Iz_3_1009	DJI_0935	Bromus inermis Leyss.	0		NA	NA	
Iz_3_1009	DJI_0935	Bromus squarrosus L.	0	a	0	0	Poaceae Barnhart
Iz_3_1009	DJI_0935	Bromus tectorum L.	0	a	0	0	Poaceae Barnhart
Iz_3_1009	DJI_0935	Calamagrostis epigejos (L.) Roth	40	n	1	0	Poaceae Barnhart
Iz_3_1009	DJI_0935	Carduus acanthoides L.	0	n	1	0	Asteraceae Bercht. & J.Presl
Iz_3_1009	DJI_0935	Carex sp.	0	n	1	0	Cyperaceae Juss.
Iz_3_1009	DJI_0935	Centaurea majorovii Dumbadze	0	n	1	0	Asteraceae Bercht. & J.Presl
Iz_3_1009	DJI_0935	Chamaecytisus ruthenicus (Fisch.ex Wol.) Klaskova	0	n	1	0	Fabaceae Lindl.
Iz_3_1009	DJI_0935	Chelidonium majus L.	2	n	1	0	Papaveraceae Juss.
Iz_3_1009	DJI_0935	Chenopodium betaceum Andrz.	0	n	1	0	Amaranthaceae Juss.
Iz_3_1009	DJI_0935	Chenopodium cf. album	0	n	1	0	Amaranthaceae Juss.
Iz_3_1009	DJI_0935	Chenopodium sp.	0		NA	0	Amaranthaceae Juss.
Iz_3_1009	DJI_0935	Chondrilla juncea L.	0	n	1	0	Asteraceae Bercht. & J.Presl
Iz_3_1009	DJI_0935	Cirsium vulgare (Savi) Ten.	0	n	1	0	Asteraceae Bercht. & J.Presl
Iz_3_1009	DJI_0935	Clinopodium acinos (L.) Kuntze	0	n	1	0	Lamiacea Lindl.
Iz_3_1009	DJI_0935	Convallaria majalis L.	0	n	1	0	Asparagaceae Juss.
Iz_3_1009	DJI_0935	Crataegus rhipidophylla Gand.	0	n	1	1	Rosaceae Juss.
Iz_3_1009	DJI_0935	Crepis foetida L.	0	n	1	0	Asteraceae Bercht. & J.Presl
Iz_3_1009	DJI_0935	Dactylis glomerata L.	0	n	1	0	Poaceae Barnhart
Iz_3_1009	DJI_0935	Dianthus campestris M.Bieb.	0	n	1	0	Caryophyllaceae Juss.
Iz_3_1009	DJI_0935	Digitaria sanguinalis (L.) Scop.	0	a	0	0	Poaceae Barnhart
Iz_3_1009	DJI_0935	Elymus repens (L.) Gould	0	n	1	0	Poaceae Barnhart
Iz_3_1009	DJI_0935	Epilobium tetragonum L.	0	n	1	0	Onagraceae Juss.
Iz_3_1009	DJI_0935	Eragrostis minor Host	0	a	0	0	Poaceae Barnhart
Iz_3_1009	DJI_0935	Erigeron canadensis L.	0	a	0	0	Asteraceae Bercht. & J.Presl
Iz_3_1009	DJI_0935	Euphorbia seguieriana Neck.	0	n	1	0	Euphorbiaceae Juss.
Iz_3_1009	DJI_0935	Fallopia dumetorum (L.) Holub	3	n	1	0	Polygonaceae Juss.

Iz_3_1009	DJI_0935	Festuca beckeri (Hack.) Trautv.	0	n	1	0	Poaceae Barnhart
Iz_3_1009	DJI_0935	Fragaria vesca L.	0	n	1	0	Rosaceae Juss.
Iz_3_1009	DJI_0935	Frangula alnus Mill.	0	n	1	1	Rhamnaceae Juss.
Iz_3_1009	DJI_0935	Fraxinus pennsylvanica Marshall	0	a	0	1	Oleaceae Hoffmanns. & Link
Iz_3_1009	DJI_0935	Galeopsis bifida Boenn.	0	n	1	0	Lamiacea Lindl.
Iz_3_1009	DJI_0935	Galium verum L.	0	n	1	0	Rubiaceae Juss.
Iz_3_1009	DJI_0935	Genista tinctoria L.	0	n	1	0	Fabaceae Lindl.
Iz_3_1009	DJI_0935	Geranium robertianum L.	0	n	1	0	Geraniaceae Juss.
Iz_3_1009	DJI_0935	Geranium sanquineum L.	0	n	1	0	Geraniaceae Juss.
Iz_3_1009	DJI_0935	Gypsophila paniculata L.	0	n	1	0	Caryophyllaceae Juss.
Iz_3_1009	DJI_0935	Helichrysum arenarium (L.) Moench	0	n	1	0	Asteraceae Bercht. & J.Presl
Iz_3_1009	DJI_0935	Herniaria polygama J.Gay	0	n	1	0	Caryophyllaceae Juss.
Iz_3_1009	DJI_0935	Hieracium robustum Fr.	0	n	1	0	Asteraceae Bercht. & J.Presl
Iz_3_1009	DJI_0935	Hieracium umbellatum L.	0	n	1	NA	Asteraceae Bercht. & J.Presl
Iz_3_1009	DJI_0935	Hylotelephium maximum (L.) Holub	0	n	1	0	Crassulaceae J.St.-Hil.
Iz_3_1009	DJI_0935	Jacobaea borysthenica (DC.) B.Nord. & Greuter	0	n	1	0	Asteraceae Bercht. & J.Presl
Iz_3_1009	DJI_0935	Jasione montana L.	0	n	1	0	Campanulaceae Juss.
Iz_3_1009	DJI_0935	Jurinea cyanoides (L.) Rchb.s.l.	0	n	1	0	Asteraceae Bercht. & J.Presl
Iz_3_1009	DJI_0935	Koeleria glauca (Spreng.) DC.	0	n	1	0	Poaceae Barnhart
Iz_3_1009	DJI_0935	Lactuca serriola L.	0	a	0	0	Asteraceae Bercht. & J.Presl
Iz_3_1009	DJI_0935	Linaria genistifolia (L.) Mill.	0	n	1	0	Plantaginaceae Juss.
Iz_3_1009	DJI_0935	Lomelosia argentea (L.) Greuter & Burdet	0	n	1	0	Caprifoliaceae Juss.
Iz_3_1009	DJI_0935	Melampyrum pratense L.	0	n	1	0	Orobanchaceae Vent.
Iz_3_1009	DJI_0935	Oenothera biennis L.	0	a	0	0	Onagraceae Juss.
Iz_3_1009	DJI_0935	Pentanema britannica (L.) D.Gut.Larr., Santos-Vicente, Anderb., E.Rico & M.M.Mart.Ort.	0	n	1	0	Asteraceae Bercht. & J.Presl
Iz_3_1009	DJI_0935	Peucedanum oreoselinum (L.) Moench	2	n	1	0	Apiaceae Lindl.
Iz_3_1009	DJI_0935	Peucedanum ruthenicum M.Bieb.	0	n	1	0	Apiaceae Lindl.
Iz_3_1009	DJI_0935	Pilosella echioides (Lumn.) F.W.Schultz & Sch.Bip.	0	n	1	0	Asteraceae Bercht. & J.Presl
Iz_3_1009	DJI_0935	Pilosella officinarum F. Schultz et Sch. Bip.	0	n	1	0	Asteraceae Bercht. & J.Presl
Iz_3_1009	DJI_0935	Pilosella sp.	0		NA	0	Asteraceae Bercht. & J.Presl
Iz_3_1009	DJI_0935	Pinus sylvestris L.	0	n	1	1	Pinaceae Spreng. ex F.Rudolphi
Iz_3_1009	DJI_0935	Poa annua L.	0	n	1	0	Poaceae Barnhart
Iz_3_1009	DJI_0935	Poa nemoralis L.	0	n	1	0	Poaceae Barnhart
Iz_3_1009	DJI_0935	Poa sp.	0		NA	0	Poaceae Barnhart
Iz_3_1009	DJI_0935	Polygonatum odoratum (Mill.) Druce	1	n	1	0	Asparagaceae Juss.
Iz_3_1009	DJI_0935	Polygonum arenarium Waldst. & Kit.	0	n	1	0	Polygonaceae Juss.
Iz_3_1009	DJI_0935	Populus nigra L.	0	n	1	1	Salicaceae Mirb.
Iz_3_1009	DJI_0935	Populus tremula L.	0	n	1	1	Salicaceae Mirb.
Iz_3_1009	DJI_0935	Populus Ã canescens (Aiton) Sm.	0	n	1	1	Salicaceae Mirb.
Iz_3_1009	DJI_0935	Potentilla argentea L.	0	n	1	0	Rosaceae Juss.
Iz_3_1009	DJI_0935	Psammophiliella muralis (L.) Ikonn.	0	n	1	0	Caryophyllaceae Juss.
Iz_3_1009	DJI_0935	Psephellus sumensis (Kalen.) Greuter	0	n	1	0	Asteraceae Bercht. & J.Presl
Iz_3_1009	DJI_0935	Pulsatilla pratensis (L.) Mill. s.l.	0	n	1	0	Ranunculaceae Juss.
Iz_3_1009	DJI_0935	Pyrus communis L.	0	a	0	1	Rosaceae Juss.
Iz_3_1009	DJI_0935	Quercus robur L.	10	n	1	1	Fagaceae Dumort.
Iz_3_1009	DJI_0935	Robinia pseudoacacia L.	0	a	0	1	Fabaceae Lindl.
Iz_3_1009	DJI_0935	Rosa sp.	0		NA	1	Rosaceae Juss.
Iz_3_1009	DJI_0935	Rubus idaeus L.	0	n	1	0	Rosaceae Juss.
Iz_3_1009	DJI_0935	Rumex acetosella L.	0	n	1	0	Polygonaceae Juss.
Iz_3_1009	DJI_0935	Rumex thyrsiflorus Fingerh.	0	n	1	0	Polygonaceae Juss.
Iz_3_1009	DJI_0935	Salix alba L.	0	n	1	1	Salicaceae Mirb.
Iz_3_1009	DJI_0935	Sambucus nigra L.	0	n	1	1	Viburnaceae Raf.
Iz_3_1009	DJI_0935	Secale sylvestre Host	0	n	1	0	Poaceae Barnhart
Iz_3_1009	DJI_0935	Sempervivum ruthenicum Schnittsp. & C.B. Lehm.	0	n	1	0	Crassulaceae J.St.-Hil.
Iz_3_1009	DJI_0935	Setaria viridis (L.) P. Beauv.	0	n	1	0	Poaceae Barnhart
Iz_3_1009	DJI_0935	Silene borysthenica (Gruner) Walters	0	n	1	0	Caryophyllaceae Juss.
Iz_3_1009	DJI_0935	Silene latifolia subsp. alba (Mill.) Greuter & Burdet	0	n	1	0	Caryophyllaceae Juss.
Iz_3_1009	DJI_0935	Solidago virgaurea L.	0	n	1	0	Asteraceae Bercht. & J.Presl
Iz_3_1009	DJI_0935	Sonchus oleraceus L.	0	a	0	0	Asteraceae Bercht. & J.Presl
Iz_3_1009	DJI_0935	Sorbus aucuparia L.	0	n	1	1	Rosaceae Juss.
Iz_3_1009	DJI_0935	Stachys recta L.	0	n	1	0	Lamiacea Lindl.
Iz_3_1009	DJI_0935	Tanacetum vulgare L.	0	n	1	0	Asteraceae Bercht. & J.Presl
Iz_3_1009	DJI_0935	Thymus pallasianus Heinr.Braun	0	n	1	0	Lamiacea Lindl.
Iz_3_1009	DJI_0935	Tilia cordata Mill.	0	n	1	1	Malvaceae Juss.
Iz_3_1009	DJI_0935	Trifolium alpestre L.	0	n	1	0	Fabaceae Lindl.
Iz_3_1009	DJI_0935	Trifolium arvense L.	0	n	1	0	Fabaceae Lindl.
Iz_3_1009	DJI_0935	Ulmus glabra Huds.	0	n	1	1	Ulmaceae Mirb.
Iz_3_1009	DJI_0935	Verbascum densiflorum Bertol.	0	n	1	0	Scrophulariaceae Juss.
Iz_3_1009	DJI_0935	Veronica austriaca L.	0	n	1	0	Plantaginaceae Juss.
Iz_3_1009	DJI_0935	Veronica incana L.	0	n	1	0	Plantaginaceae Juss.
Iz_3_1009	DJI_0935	Veronica verna L.	0	n	1	0	Plantaginaceae Juss.
Iz_3_1009	DJI_0935	Vincetoxicum hirundinaria Medik	0	n	1	0	Apocynaceae Juss.
Iz_3_1009	DJI_0938	Acer tataricum L.	0	n	1	1	Sapindaceae Juss.
Iz_3_1009	DJI_0938	Achillea millefolium L. s.l.	0	n	1	0	Asteraceae Bercht. & J.Presl
Iz_3_1009	DJI_0938	Allium oleraceum L.	0	n	1	0	Amaryllidaceae J.St.-Hil.
Iz_3_1009	DJI_0938	Ambrosia artemisiifolia L.	0	a	0	0	Asteraceae Bercht. & J.Presl
Iz_3_1009	DJI_0938	Amorpha fruticosa L.	0	a	0	0	Fabaceae Lindl.
Iz_3_1009	DJI_0938	Anthericum ramosum L.	0	n	1	0	Asparagaceae Juss.
Iz_3_1009	DJI_0938	Artemisia marschalliana Spreng.	0	n	1	0	Asteraceae Bercht. & J.Presl
Iz_3_1009	DJI_0938	Asparagus officinalis L.	0	n	1	0	Asparagaceae Juss.
Iz_3_1009	DJI_0938	Astragalus cicer L.	0	n	1	0	Fabaceae Lindl.
Iz_3_1009	DJI_0938	Bassia laniflora (S.G.Gmel.) A.J.Scott	0	n	1	0	Amaranthaceae Juss.
Iz_3_1009	DJI_0938	Berberis vulgaris L.	0	n	1	0	Berberidaceae Juss.
Iz_3_1009	DJI_0938	Betula pendula Roth.	0.2	n	1	1	Betulaceae Gray
Iz_3_1009	DJI_0938	Betula pendula Roth.	0.2	n	1	0	Poaceae Barnhart
Iz_3_1009	DJI_0938	Bromus inermis Leyss.	0		NA	NA	
Iz_3_1009	DJI_0938	Bromus squarrosus L.	0	a	0	0	Poaceae Barnhart
Iz_3_1009	DJI_0938	Bromus tectorum L.	0	a	0	0	Poaceae Barnhart
Iz_3_1009	DJI_0938	Calamagrostis epigejos (L.) Roth	15	n	1	0	Poaceae Barnhart
Iz_3_1009	DJI_0938	Carduus acanthoides L.	0	n	1	0	Asteraceae Bercht. & J.Presl
Iz_3_1009	DJI_0938	Carex sp.	0	n	1	0	Cyperaceae Juss.
Iz_3_1009	DJI_0938	Centaurea majorovii Dumbadze	0	n	1	0	Asteraceae Bercht. & J.Presl
Iz_3_1009	DJI_0938	Chamaecytisus ruthenicus (Fisch.ex Wol.) Klaskova	5	n	1	0	Fabaceae Lindl.
Iz_3_1009	DJI_0938	Chelidonium majus L.	0.2	n	1	0	Papaveraceae Juss.
Iz_3_1009	DJI_0938	Chenopodium betaceum Andrz.	0	n	1	0	Amaranthaceae Juss.
Iz_3_1009	DJI_0938	Chenopodium cf. album	0	n	1	0	Amaranthaceae Juss.
Iz_3_1009	DJI_0938	Chenopodium sp.	0		NA	0	Amaranthaceae Juss.
Iz_3_1009	DJI_0938	Chondrilla juncea L.	0	n	1	0	Asteraceae Bercht. & J.Presl

Iz_3_1009	DJI_0938	Cirsium vulgare (Savi) Ten.	0	n	1	0	Asteraceae Bercht. & J.Presl
Iz_3_1009	DJI_0938	Clinopodium acinos (L.) Kuntze	0	n	1	0	Lamiacea Lindl.
Iz_3_1009	DJI_0938	Convallaria majalis L.	0	n	1	0	Asparagaceae Juss.
Iz_3_1009	DJI_0938	Crataegus rhipidophylla Gand.	0	n	1	1	Rosaceae Juss.
Iz_3_1009	DJI_0938	Crepis foetida L.	0	n	1	0	Asteraceae Bercht. & J.Presl
Iz_3_1009	DJI_0938	Dactylis glomerata L.	0	n	1	0	Poaceae Barnhart
Iz_3_1009	DJI_0938	Dianthus campestris M.Bieb.	0	n	1	0	Caryophyllaceae Juss.
Iz_3_1009	DJI_0938	Digitaria sanguinalis (L.) Scop.	0	a	0	0	Poaceae Barnhart
Iz_3_1009	DJI_0938	Elymus repens (L.) Gould	0	n	1	0	Poaceae Barnhart
Iz_3_1009	DJI_0938	Epilobium tetragonum L.	0	n	1	0	Onagraceae Juss.
Iz_3_1009	DJI_0938	Eragrostis minor Host	0	a	0	0	Poaceae Barnhart
Iz_3_1009	DJI_0938	Erigeron canadensis L.	0	a	0	0	Asteraceae Bercht. & J.Presl
Iz_3_1009	DJI_0938	Euphorbia seguieriana Neck.	0	n	1	0	Euphorbiaceae Juss.
Iz_3_1009	DJI_0938	Fallopia dumetorum (L.) Holub	0	n	1	0	Polygonaceae Juss.
Iz_3_1009	DJI_0938	Festuca beckeri (Hack.) Trautv.	0	n	1	0	Poaceae Barnhart
Iz_3_1009	DJI_0938	Fragaria vesca L.	0	n	1	0	Rosaceae Juss.
Iz_3_1009	DJI_0938	Frangula alnus Mill.	0	n	1	1	Rhamnaceae Juss.
Iz_3_1009	DJI_0938	Fraxinus pennsylvanica Marshall	0	a	0	1	Oleaceae Hoffmanns. & Link
Iz_3_1009	DJI_0938	Galeopsis bifida Boenn.	0	n	1	0	Lamiacea Lindl.
Iz_3_1009	DJI_0938	Galium verum L.	0	n	1	0	Rubiaceae Juss.
Iz_3_1009	DJI_0938	Genista tinctoria L.	0	n	1	0	Fabaceae Lindl.
Iz_3_1009	DJI_0938	Geranium robertianum L.	0	n	1	0	Geraniaceae Juss.
Iz_3_1009	DJI_0938	Geranium sanquineum L.	0	n	1	0	Geraniaceae Juss.
Iz_3_1009	DJI_0938	Gypsophila paniculata L.	0	n	1	0	Caryophyllaceae Juss.
Iz_3_1009	DJI_0938	Helichrysum arenarium (L.) Moench	0	n	1	0	Asteraceae Bercht. & J.Presl
Iz_3_1009	DJI_0938	Herniaria polygama J.Gay	0	n	1	0	Caryophyllaceae Juss.
Iz_3_1009	DJI_0938	Hieracium robustum Fr.	0	n	1	0	Asteraceae Bercht. & J.Presl
Iz_3_1009	DJI_0938	Hieracium umbellatum L.	0	n	1	NA	Asteraceae Bercht. & J.Presl
Iz_3_1009	DJI_0938	Hylotelephium maximum (L.) Holub	0	n	1	0	Crassulaceae J.St.-Hil.
Iz_3_1009	DJI_0938	Jacobaea borysthenica (DC.) B.Nord. & Greuter	0	n	1	0	Asteraceae Bercht. & J.Presl
Iz_3_1009	DJI_0938	Jasione montana L.	0	n	1	0	Campanulaceae Juss.
Iz_3_1009	DJI_0938	Jurinea cyanoides (L.) Rchb.s.l.	0	n	1	0	Asteraceae Bercht. & J.Presl
Iz_3_1009	DJI_0938	Koeleria glauca (Spreng.) DC.	0	n	1	0	Poaceae Barnhart
Iz_3_1009	DJI_0938	Lactuca serriola L.	0	a	0	0	Asteraceae Bercht. & J.Presl
Iz_3_1009	DJI_0938	Linaria genistifolia (L.) Mill.	0	n	1	0	Plantaginaceae Juss.
Iz_3_1009	DJI_0938	Lomelosia argentea (L.) Greuter & Burdet	0	n	1	0	Caprifoliaceae Juss.
Iz_3_1009	DJI_0938	Melampyrum pratense L.	0	n	1	0	Orobanchaceae Vent.
Iz_3_1009	DJI_0938	Oenothera biennis L.	0	a	0	0	Onagraceae Juss.
Iz_3_1009	DJI_0938	Pentanema britannica (L.) D.Gut.Larr., Santos-Vicente, Anderb., E.Rico & M.M.Mart.Ort.	0	n	1	0	Asteraceae Bercht. & J.Presl
Iz_3_1009	DJI_0938	Peucedanum oreoselinum (L.) Moench	10	n	1	0	Apiaceae Lindl.
Iz_3_1009	DJI_0938	Peucedanum ruthenicum M.Bieb.	0	n	1	0	Apiaceae Lindl.
Iz_3_1009	DJI_0938	Pilosella echioides (Lumn.) F.W.Schultz & Sch.Bip.	0	n	1	0	Asteraceae Bercht. & J.Presl
Iz_3_1009	DJI_0938	Pilosella officinarum F. Schultz et Sch. Bip.	0	n	1	0	Asteraceae Bercht. & J.Presl
Iz_3_1009	DJI_0938	Pilosella sp.	0		NA	0	Asteraceae Bercht. & J.Presl
Iz_3_1009	DJI_0938	Pinus sylvestris L.	0	n	1	1	Pinaceae Spreng. ex F.Rudolphi
Iz_3_1009	DJI_0938	Poa annua L.	0	n	1	0	Poaceae Barnhart
Iz_3_1009	DJI_0938	Poa nemoralis L.	0	n	1	0	Poaceae Barnhart
Iz_3_1009	DJI_0938	Poa sp.	0		NA	0	Poaceae Barnhart
Iz_3_1009	DJI_0938	Polygonatum odoratum (Mill.) Druce	0.5	n	1	0	Asparagaceae Juss.
Iz_3_1009	DJI_0938	Polygonum arenarium Waldst. & Kit.	0	n	1	0	Polygonaceae Juss.
Iz_3_1009	DJI_0938	Populus nigra L.	0	n	1	1	Salicaceae Mirb.
Iz_3_1009	DJI_0938	Populus tremula L.	0	n	1	1	Salicaceae Mirb.
Iz_3_1009	DJI_0938	Populus Ã canescens (Aiton) Sm.	0	n	1	1	Salicaceae Mirb.
Iz_3_1009	DJI_0938	Potentilla argentea L.	0	n	1	0	Rosaceae Juss.
Iz_3_1009	DJI_0938	Psammophiliella muralis (L.) Ikonn.	0	n	1	0	Caryophyllaceae Juss.
Iz_3_1009	DJI_0938	Psephellus sumensis (Kalen.) Greuter	0	n	1	0	Asteraceae Bercht. & J.Presl
Iz_3_1009	DJI_0938	Pulsatilla pratensis (L.) Mill. s.l.	0	n	1	0	Ranunculaceae Juss.
Iz_3_1009	DJI_0938	Pyrus communis L.	0	a	0	1	Rosaceae Juss.
Iz_3_1009	DJI_0938	Quercus robur L.	0	n	1	1	Fagaceae Dumort.
Iz_3_1009	DJI_0938	Robinia pseudoacacia L.	0	a	0	1	Fabaceae Lindl.
Iz_3_1009	DJI_0938	Rosa sp.	0		NA	1	Rosaceae Juss.
Iz_3_1009	DJI_0938	Rubus idaeus L.	0	n	1	0	Rosaceae Juss.
Iz_3_1009	DJI_0938	Rumex acetosella L.	0	n	1	0	Polygonaceae Juss.
Iz_3_1009	DJI_0938	Rumex thyrsiflorus Fingerh.	0	n	1	0	Polygonaceae Juss.
Iz_3_1009	DJI_0938	Salix alba L.	0	n	1	1	Salicaceae Mirb.
Iz_3_1009	DJI_0938	Sambucus nigra L.	0	n	1	1	Viburnaceae Raf.
Iz_3_1009	DJI_0938	Secale sylvestre Host	0	n	1	0	Poaceae Barnhart
Iz_3_1009	DJI_0938	Sempervivum ruthenicum Schnittsp. & C.B. Lehm.	0	n	1	0	Crassulaceae J.St.-Hil.
Iz_3_1009	DJI_0938	Setaria viridis (L.) P. Beauv.	0	n	1	0	Poaceae Barnhart
Iz_3_1009	DJI_0938	Silene borysthenica (Gruner) Walters	0	n	1	0	Caryophyllaceae Juss.
Iz_3_1009	DJI_0938	Silene latifolia subsp. alba (Mill.) Greuter & Burdet	0	n	1	0	Caryophyllaceae Juss.
Iz_3_1009	DJI_0938	Solidago virgaurea L.	0	n	1	0	Asteraceae Bercht. & J.Presl
Iz_3_1009	DJI_0938	Sonchus oleraceus L.	0	a	0	0	Asteraceae Bercht. & J.Presl
Iz_3_1009	DJI_0938	Sorbus aucuparia L.	0	n	1	1	Rosaceae Juss.
Iz_3_1009	DJI_0938	Stachys recta L.	0	n	1	0	Lamiacea Lindl.
Iz_3_1009	DJI_0938	Tanacetum vulgare L.	0	n	1	0	Asteraceae Bercht. & J.Presl
Iz_3_1009	DJI_0938	Thymus pallasianus Heinr.Braun	0	n	1	0	Lamiacea Lindl.
Iz_3_1009	DJI_0938	Tilia cordata Mill.	0	n	1	1	Malvaceae Juss.
Iz_3_1009	DJI_0938	Trifolium alpestre L.	0	n	1	0	Fabaceae Lindl.
Iz_3_1009	DJI_0938	Trifolium arvense L.	0	n	1	0	Fabaceae Lindl.
Iz_3_1009	DJI_0938	Ulmus glabra Huds.	0	n	1	1	Ulmaceae Mirb.
Iz_3_1009	DJI_0938	Verbascum densiflorum Bertol.	0	n	1	0	Scrophulariaceae Juss.
Iz_3_1009	DJI_0938	Veronica austriaca L.	0	n	1	0	Plantaginaceae Juss.
Iz_3_1009	DJI_0938	Veronica incana L.	0	n	1	0	Plantaginaceae Juss.
Iz_3_1009	DJI_0938	Veronica verna L.	0	n	1	0	Plantaginaceae Juss.
Iz_3_1009	DJI_0938	Vincetoxicum hirundinaria Medik	0	n	1	0	Apocynaceae Juss.
Iz_3_1009	DJI_0945	Acer tataricum L.	0	n	1	1	Sapindaceae Juss.
Iz_3_1009	DJI_0945	Achillea millefolium L. s.l.	0	n	1	0	Asteraceae Bercht. & J.Presl
Iz_3_1009	DJI_0945	Allium oleraceum L.	0	n	1	0	Amaryllidaceae J.St.-Hil.
Iz_3_1009	DJI_0945	Ambrosia artemisiifolia L.	0	a	0	0	Asteraceae Bercht. & J.Presl
Iz_3_1009	DJI_0945	Amorpha fruticosa L.	0	a	0	0	Fabaceae Lindl.
Iz_3_1009	DJI_0945	Anthericum ramosum L.	0	n	1	0	Asparagaceae Juss.
Iz_3_1009	DJI_0945	Artemisia marschalliana Spreng.	0	n	1	0	Asteraceae Bercht. & J.Presl
Iz_3_1009	DJI_0945	Asparagus officinalis L.	0	n	1	0	Asparagaceae Juss.
Iz_3_1009	DJI_0945	Astragalus cicer L.	0	n	1	0	Fabaceae Lindl.
Iz_3_1009	DJI_0945	Bassia laniflora (S.G.Gmel.) A.J.Scott	0	n	1	0	Amaranthaceae Juss.
Iz_3_1009	DJI_0945	Berberis vulgaris L.	0	n	1	0	Berberidaceae Juss.
Iz_3_1009	DJI_0945	Betula pendula Roth.	0	n	1	1	Betulaceae Gray

Iz_3_1009	DJI_0945	Betula pendula Roth.	0	n	1	0	Poaceae Barnhart
Iz_3_1009	DJI_0945	Bromus inermis Leyss.	0		NA	NA	
Iz_3_1009	DJI_0945	Bromus squarrosus L.	0	a	0	0	Poaceae Barnhart
Iz_3_1009	DJI_0945	Bromus tectorum L.	0	a	0	0	Poaceae Barnhart
Iz_3_1009	DJI_0945	Calamagrostis epigejos (L.) Roth	15	n	1	0	Poaceae Barnhart
Iz_3_1009	DJI_0945	Carduus acanthoides L.	0	n	1	0	Asteraceae Bercht. & J.Presl
Iz_3_1009	DJI_0945	Carex sp.	0	n	1	0	Cyperaceae Juss.
Iz_3_1009	DJI_0945	Centaurea majorovii Dumbadze	0	n	1	0	Asteraceae Bercht. & J.Presl
Iz_3_1009	DJI_0945	Chamaecytisus ruthenicus (Fisch.ex Wol.) Klaskova	0	n	1	0	Fabaceae Lindl.
Iz_3_1009	DJI_0945	Chelidonium majus L.	0	n	1	0	Papaveraceae Juss.
Iz_3_1009	DJI_0945	Chenopodium betaceum Andrz.	0	n	1	0	Amaranthaceae Juss.
Iz_3_1009	DJI_0945	Chenopodium cf. album	0	n	1	0	Amaranthaceae Juss.
Iz_3_1009	DJI_0945	Chenopodium sp.	0		NA	0	Amaranthaceae Juss.
Iz_3_1009	DJI_0945	Chondrilla juncea L.	0	n	1	0	Asteraceae Bercht. & J.Presl
Iz_3_1009	DJI_0945	Cirsium vulgare (Savi) Ten.	0	n	1	0	Asteraceae Bercht. & J.Presl
Iz_3_1009	DJI_0945	Clinopodium acinos (L.) Kuntze	0	n	1	0	Lamiacea Lindl.
Iz_3_1009	DJI_0945	Convallaria majalis L.	0	n	1	0	Asparagaceae Juss.
Iz_3_1009	DJI_0945	Crataegus rhipidophylla Gand.	0	n	1	1	Rosaceae Juss.
Iz_3_1009	DJI_0945	Crepis foetida L.	0	n	1	0	Asteraceae Bercht. & J.Presl
Iz_3_1009	DJI_0945	Dactylis glomerata L.	0	n	1	0	Poaceae Barnhart
Iz_3_1009	DJI_0945	Dianthus campestris M.Bieb.	0	n	1	0	Caryophyllaceae Juss.
Iz_3_1009	DJI_0945	Digitaria sanguinalis (L.) Scop.	0	a	0	0	Poaceae Barnhart
Iz_3_1009	DJI_0945	Elymus repens (L.) Gould	0	n	1	0	Poaceae Barnhart
Iz_3_1009	DJI_0945	Epilobium tetragonum L.	0	n	1	0	Onagraceae Juss.
Iz_3_1009	DJI_0945	Eragrostis minor Host	0	a	0	0	Poaceae Barnhart
Iz_3_1009	DJI_0945	Erigeron canadensis L.	0	a	0	0	Asteraceae Bercht. & J.Presl
Iz_3_1009	DJI_0945	Euphorbia seguieriana Neck.	0	n	1	0	Euphorbiaceae Juss.
Iz_3_1009	DJI_0945	Fallopia dumetorum (L.) Holub	0	n	1	0	Polygonaceae Juss.
Iz_3_1009	DJI_0945	Festuca beckeri (Hack.) Trautv.	0	n	1	0	Poaceae Barnhart
Iz_3_1009	DJI_0945	Fragaria vesca L.	0	n	1	0	Rosaceae Juss.
Iz_3_1009	DJI_0945	Frangula alnus Mill.	0	n	1	1	Rhamnaceae Juss.
Iz_3_1009	DJI_0945	Fraxinus pennsylvanica Marshall	0	a	0	1	Oleaceae Hoffmanns. & Link
Iz_3_1009	DJI_0945	Galeopsis bifida Boenn.	0	n	1	0	Lamiacea Lindl.
Iz_3_1009	DJI_0945	Galium verum L.	0	n	1	0	Rubiaceae Juss.
Iz_3_1009	DJI_0945	Genista tinctoria L.	0	n	1	0	Fabaceae Lindl.
Iz_3_1009	DJI_0945	Geranium robertianum L.	0	n	1	0	Geraniaceae Juss.
Iz_3_1009	DJI_0945	Geranium sanquineum L.	0	n	1	0	Geraniaceae Juss.
Iz_3_1009	DJI_0945	Gypsophila paniculata L.	0	n	1	0	Caryophyllaceae Juss.
Iz_3_1009	DJI_0945	Helichrysum arenarium (L.) Moench	0	n	1	0	Asteraceae Bercht. & J.Presl
Iz_3_1009	DJI_0945	Herniaria polygama J.Gay	0	n	1	0	Caryophyllaceae Juss.
Iz_3_1009	DJI_0945	Hieracium robustum Fr.	0	n	1	0	Asteraceae Bercht. & J.Presl
Iz_3_1009	DJI_0945	Hieracium umbellatum L.	0	n	1	NA	Asteraceae Bercht. & J.Presl
Iz_3_1009	DJI_0945	Hylotelephium maximum (L.) Holub	0	n	1	0	Crassulaceae J.St.-Hil.
Iz_3_1009	DJI_0945	Jacobaea borysthenica (DC.) B.Nord. & Greuter	0	n	1	0	Asteraceae Bercht. & J.Presl
Iz_3_1009	DJI_0945	Jasione montana L.	0	n	1	0	Campanulaceae Juss.
Iz_3_1009	DJI_0945	Jurinea cyanoides (L.) Rchb.s.l.	0	n	1	0	Asteraceae Bercht. & J.Presl
Iz_3_1009	DJI_0945	Koeleria glauca (Spreng.) DC.	0	n	1	0	Poaceae Barnhart
Iz_3_1009	DJI_0945	Lactuca serriola L.	0	a	0	0	Asteraceae Bercht. & J.Presl
Iz_3_1009	DJI_0945	Linaria genistifolia (L.) Mill.	0	n	1	0	Plantaginaceae Juss.
Iz_3_1009	DJI_0945	Lomelosia argentea (L.) Greuter & Burdet	0	n	1	0	Caprifoliaceae Juss.
Iz_3_1009	DJI_0945	Melampyrum pratense L.	0	n	1	0	Orobanchaceae Vent.
Iz_3_1009	DJI_0945	Oenothera biennis L.	0	a	0	0	Onagraceae Juss.
Iz_3_1009	DJI_0945	Pentanema britannica (L.) D.Gut.Larr., Santos-Vicente, Anderb., E.Rico & M.M.Mart.Ort.	0	n	1	0	Asteraceae Bercht. & J.Presl
Iz_3_1009	DJI_0945	Peucedanum oreoselinum (L.) Moench	8	n	1	0	Apiaceae Lindl.
Iz_3_1009	DJI_0945	Peucedanum ruthenicum M.Bieb.	0	n	1	0	Apiaceae Lindl.
Iz_3_1009	DJI_0945	Pilosella echioides (Lumn.) F.W.Schultz & Sch.Bip.	0	n	1	0	Asteraceae Bercht. & J.Presl
Iz_3_1009	DJI_0945	Pilosella officinarum F. Schultz et Sch. Bip.	0	n	1	0	Asteraceae Bercht. & J.Presl
Iz_3_1009	DJI_0945	Pilosella sp.	0		NA	0	Asteraceae Bercht. & J.Presl
Iz_3_1009	DJI_0945	Pinus sylvestris L.	0	n	1	1	Pinaceae Spreng. ex F.Rudolphi
Iz_3_1009	DJI_0945	Poa annua L.	0	n	1	0	Poaceae Barnhart
Iz_3_1009	DJI_0945	Poa nemoralis L.	0	n	1	0	Poaceae Barnhart
Iz_3_1009	DJI_0945	Poa sp.	0		NA	0	Poaceae Barnhart
Iz_3_1009	DJI_0945	Polygonatum odoratum (Mill.) Druce	0	n	1	0	Asparagaceae Juss.
Iz_3_1009	DJI_0945	Polygonum arenarium Waldst. & Kit.	0	n	1	0	Polygonaceae Juss.
Iz_3_1009	DJI_0945	Populus nigra L.	0	n	1	1	Salicaceae Mirb.
Iz_3_1009	DJI_0945	Populus tremula L.	0	n	1	1	Salicaceae Mirb.
Iz_3_1009	DJI_0945	Populus Ã canescens (Aiton) Sm.	0	n	1	1	Salicaceae Mirb.
Iz_3_1009	DJI_0945	Potentilla argentea L.	0	n	1	0	Rosaceae Juss.
Iz_3_1009	DJI_0945	Psammophiliella muralis (L.) Ikonn.	0	n	1	0	Caryophyllaceae Juss.
Iz_3_1009	DJI_0945	Psephellus sumensis (Kalen.) Greuter	0	n	1	0	Asteraceae Bercht. & J.Presl
Iz_3_1009	DJI_0945	Pulsatilla pratensis (L.) Mill. s.l.	0	n	1	0	Ranunculaceae Juss.
Iz_3_1009	DJI_0945	Pyrus communis L.	0	a	0	1	Rosaceae Juss.
Iz_3_1009	DJI_0945	Quercus robur L.	0	n	1	1	Fagaceae Dumort.
Iz_3_1009	DJI_0945	Robinia pseudoacacia L.	0	a	0	1	Fabaceae Lindl.
Iz_3_1009	DJI_0945	Rosa sp.	0		NA	1	Rosaceae Juss.
Iz_3_1009	DJI_0945	Rubus idaeus L.	0	n	1	0	Rosaceae Juss.
Iz_3_1009	DJI_0945	Rumex acetosella L.	0	n	1	0	Polygonaceae Juss.
Iz_3_1009	DJI_0945	Rumex thyrsiflorus Fingerh.	0	n	1	0	Polygonaceae Juss.
Iz_3_1009	DJI_0945	Salix alba L.	0	n	1	1	Salicaceae Mirb.
Iz_3_1009	DJI_0945	Sambucus nigra L.	0	n	1	1	Viburnaceae Raf.
Iz_3_1009	DJI_0945	Secale sylvestre Host	0	n	1	0	Poaceae Barnhart
Iz_3_1009	DJI_0945	Sempervivum ruthenicum Schnittsp. & C.B. Lehm.	0	n	1	0	Crassulaceae J.St.-Hil.
Iz_3_1009	DJI_0945	Setaria viridis (L.) P. Beauv.	0	n	1	0	Poaceae Barnhart
Iz_3_1009	DJI_0945	Silene borysthenica (Gruner) Walters	0	n	1	0	Caryophyllaceae Juss.
Iz_3_1009	DJI_0945	Silene latifolia subsp. alba (Mill.) Greuter & Burdet	0	n	1	0	Caryophyllaceae Juss.
Iz_3_1009	DJI_0945	Solidago virgaurea L.	0	n	1	0	Asteraceae Bercht. & J.Presl
Iz_3_1009	DJI_0945	Sonchus oleraceus L.	0	a	0	0	Asteraceae Bercht. & J.Presl
Iz_3_1009	DJI_0945	Sorbus aucuparia L.	0	n	1	1	Rosaceae Juss.
Iz_3_1009	DJI_0945	Stachys recta L.	0	n	1	0	Lamiacea Lindl.
Iz_3_1009	DJI_0945	Tanacetum vulgare L.	0	n	1	0	Asteraceae Bercht. & J.Presl
Iz_3_1009	DJI_0945	Thymus pallasianus Heinr.Braun	0	n	1	0	Lamiacea Lindl.
Iz_3_1009	DJI_0945	Tilia cordata Mill.	0	n	1	1	Malvaceae Juss.
Iz_3_1009	DJI_0945	Trifolium alpestre L.	0	n	1	0	Fabaceae Lindl.
Iz_3_1009	DJI_0945	Trifolium arvense L.	0	n	1	0	Fabaceae Lindl.
Iz_3_1009	DJI_0945	Ulmus glabra Huds.	0	n	1	1	Ulmaceae Mirb.
Iz_3_1009	DJI_0945	Verbascum densiflorum Bertol.	0	n	1	0	Scrophulariaceae Juss.
Iz_3_1009	DJI_0945	Veronica austriaca L.	0	n	1	0	Plantaginaceae Juss.
Iz_3_1009	DJI_0945	Veronica incana L.	0	n	1	0	Plantaginaceae Juss.

Iz_3_1009	DJI_0945	Veronica verna L.	0	n	1	0	Plantaginaceae Juss.
Iz_3_1009	DJI_0945	Vincetoxicum hirundinaria Medik	0	n	1	0	Apocynaceae Juss.
Iz_3_1009	DJI_0951	Acer tataricum L.	0	n	1	1	Sapindaceae Juss.
Iz_3_1009	DJI_0951	Achillea millefolium L. s.l.	0	n	1	0	Asteraceae Bercht. & J.Presl
Iz_3_1009	DJI_0951	Allium oleraceum L.	0	n	1	0	Amaryllidaceae J.St.-Hil.
Iz_3_1009	DJI_0951	Ambrosia artemisiifolia L.	0	a	0	0	Asteraceae Bercht. & J.Presl
Iz_3_1009	DJI_0951	Amorpha fruticosa L.	0	a	0	0	Fabaceae Lindl.
Iz_3_1009	DJI_0951	Anthericum ramosum L.	0	n	1	0	Asparagaceae Juss.
Iz_3_1009	DJI_0951	Artemisia marschalliana Spreng.	0	n	1	0	Asteraceae Bercht. & J.Presl
Iz_3_1009	DJI_0951	Asparagus officinalis L.	0	n	1	0	Asparagaceae Juss.
Iz_3_1009	DJI_0951	Astragalus cicer L.	0	n	1	0	Fabaceae Lindl.
Iz_3_1009	DJI_0951	Bassia laniflora (S.G.Gmel.) A.J.Scott	0	n	1	0	Amaranthaceae Juss.
Iz_3_1009	DJI_0951	Berberis vulgaris L.	0	n	1	0	Berberidaceae Juss.
Iz_3_1009	DJI_0951	Betula pendula Roth.	0	n	1	1	Betulaceae Gray
Iz_3_1009	DJI_0951	Betula pendula Roth.	0	n	1	0	Poaceae Barnhart
Iz_3_1009	DJI_0951	Bromus inermis Leyss.	0		NA	NA	
Iz_3_1009	DJI_0951	Bromus squarrosus L.	0	a	0	0	Poaceae Barnhart
Iz_3_1009	DJI_0951	Bromus tectorum L.	0	a	0	0	Poaceae Barnhart
Iz_3_1009	DJI_0951	Calamagrostis epigejos (L.) Roth	5	n	1	0	Poaceae Barnhart
Iz_3_1009	DJI_0951	Carduus acanthoides L.	0	n	1	0	Asteraceae Bercht. & J.Presl
Iz_3_1009	DJI_0951	Carex sp.	0	n	1	0	Cyperaceae Juss.
Iz_3_1009	DJI_0951	Centaurea majorovii Dumbadze	0	n	1	0	Asteraceae Bercht. & J.Presl
Iz_3_1009	DJI_0951	Chamaecytisus ruthenicus (Fisch.ex Wol.) Klaskova	0	n	1	0	Fabaceae Lindl.
Iz_3_1009	DJI_0951	Chelidonium majus L.	0	n	1	0	Papaveraceae Juss.
Iz_3_1009	DJI_0951	Chenopodium betaceum Andrz.	0	n	1	0	Amaranthaceae Juss.
Iz_3_1009	DJI_0951	Chenopodium cf. album	0	n	1	0	Amaranthaceae Juss.
Iz_3_1009	DJI_0951	Chenopodium sp.	0		NA	0	Amaranthaceae Juss.
Iz_3_1009	DJI_0951	Chondrilla juncea L.	0	n	1	0	Asteraceae Bercht. & J.Presl
Iz_3_1009	DJI_0951	Cirsium vulgare (Savi) Ten.	0	n	1	0	Asteraceae Bercht. & J.Presl
Iz_3_1009	DJI_0951	Clinopodium acinos (L.) Kuntze	0	n	1	0	Lamiacea Lindl.
Iz_3_1009	DJI_0951	Convallaria majalis L.	0	n	1	0	Asparagaceae Juss.
Iz_3_1009	DJI_0951	Crataegus rhipidophylla Gand.	0	n	1	1	Rosaceae Juss.
Iz_3_1009	DJI_0951	Crepis foetida L.	0	n	1	0	Asteraceae Bercht. & J.Presl
Iz_3_1009	DJI_0951	Dactylis glomerata L.	0	n	1	0	Poaceae Barnhart
Iz_3_1009	DJI_0951	Dianthus campestris M.Bieb.	0	n	1	0	Caryophyllaceae Juss.
Iz_3_1009	DJI_0951	Digitaria sanguinalis (L.) Scop.	0	a	0	0	Poaceae Barnhart
Iz_3_1009	DJI_0951	Elymus repens (L.) Gould	0	n	1	0	Poaceae Barnhart
Iz_3_1009	DJI_0951	Epilobium tetragonum L.	0	n	1	0	Onagraceae Juss.
Iz_3_1009	DJI_0951	Eragrostis minor Host	0	a	0	0	Poaceae Barnhart
Iz_3_1009	DJI_0951	Erigeron canadensis L.	0	a	0	0	Asteraceae Bercht. & J.Presl
Iz_3_1009	DJI_0951	Euphorbia seguieriana Neck.	0	n	1	0	Euphorbiaceae Juss.
Iz_3_1009	DJI_0951	Fallopia dumetorum (L.) Holub	0.5	n	1	0	Polygonaceae Juss.
Iz_3_1009	DJI_0951	Festuca beckeri (Hack.) Trautv.	0	n	1	0	Poaceae Barnhart
Iz_3_1009	DJI_0951	Fragaria vesca L.	0	n	1	0	Rosaceae Juss.
Iz_3_1009	DJI_0951	Frangula alnus Mill.	0	n	1	1	Rhamnaceae Juss.
Iz_3_1009	DJI_0951	Fraxinus pennsylvanica Marshall	0	a	0	1	Oleaceae Hoffmanns. & Link
Iz_3_1009	DJI_0951	Galeopsis bifida Boenn.	0	n	1	0	Lamiacea Lindl.
Iz_3_1009	DJI_0951	Galium verum L.	0	n	1	0	Rubiaceae Juss.
Iz_3_1009	DJI_0951	Genista tinctoria L.	0	n	1	0	Fabaceae Lindl.
Iz_3_1009	DJI_0951	Geranium robertianum L.	0	n	1	0	Geraniaceae Juss.
Iz_3_1009	DJI_0951	Geranium sanquineum L.	0	n	1	0	Geraniaceae Juss.
Iz_3_1009	DJI_0951	Gypsophila paniculata L.	0	n	1	0	Caryophyllaceae Juss.
Iz_3_1009	DJI_0951	Helichrysum arenarium (L.) Moench	0	n	1	0	Asteraceae Bercht. & J.Presl
Iz_3_1009	DJI_0951	Herniaria polygama J.Gay	0	n	1	0	Caryophyllaceae Juss.
Iz_3_1009	DJI_0951	Hieracium robustum Fr.	0	n	1	0	Asteraceae Bercht. & J.Presl
Iz_3_1009	DJI_0951	Hieracium umbellatum L.	0	n	1	NA	Asteraceae Bercht. & J.Presl
Iz_3_1009	DJI_0951	Hylotelephium maximum (L.) Holub	0	n	1	0	Crassulaceae J.St.-Hil.
Iz_3_1009	DJI_0951	Jacobaea borysthenica (DC.) B.Nord. & Greuter	0	n	1	0	Asteraceae Bercht. & J.Presl
Iz_3_1009	DJI_0951	Jasione montana L.	0	n	1	0	Campanulaceae Juss.
Iz_3_1009	DJI_0951	Jurinea cyanoides (L.) Rchb.s.l.	0	n	1	0	Asteraceae Bercht. & J.Presl
Iz_3_1009	DJI_0951	Koeleria glauca (Spreng.) DC.	0	n	1	0	Poaceae Barnhart
Iz_3_1009	DJI_0951	Lactuca serriola L.	0	a	0	0	Asteraceae Bercht. & J.Presl
Iz_3_1009	DJI_0951	Linaria genistifolia (L.) Mill.	0	n	1	0	Plantaginaceae Juss.
Iz_3_1009	DJI_0951	Lomelosia argentea (L.) Greuter & Burdet	0	n	1	0	Caprifoliaceae Juss.
Iz_3_1009	DJI_0951	Melampyrum pratense L.	0	n	1	0	Orobanchaceae Vent.
Iz_3_1009	DJI_0951	Oenothera biennis L.	0	a	0	0	Onagraceae Juss.
Iz_3_1009	DJI_0951	Pentanema britannica (L.) D.Gut.Larr., Santos-Vicente, Anderb., E.Rico & M.M.Mart.Ort.	0	n	1	0	Asteraceae Bercht. & J.Presl
Iz_3_1009	DJI_0951	Peucedanum oreoselinum (L.) Moench	0	n	1	0	Apiaceae Lindl.
Iz_3_1009	DJI_0951	Peucedanum ruthenicum M.Bieb.	0	n	1	0	Apiaceae Lindl.
Iz_3_1009	DJI_0951	Pilosella echioides (Lumn.) F.W.Schultz & Sch.Bip.	0	n	1	0	Asteraceae Bercht. & J.Presl
Iz_3_1009	DJI_0951	Pilosella officinarum F. Schultz et Sch. Bip.	0	n	1	0	Asteraceae Bercht. & J.Presl
Iz_3_1009	DJI_0951	Pilosella sp.	0		NA	0	Asteraceae Bercht. & J.Presl
Iz_3_1009	DJI_0951	Pinus sylvestris L.	0	n	1	1	Pinaceae Spreng. ex F.Rudolphi
Iz_3_1009	DJI_0951	Poa annua L.	0	n	1	0	Poaceae Barnhart
Iz_3_1009	DJI_0951	Poa nemoralis L.	0	n	1	0	Poaceae Barnhart
Iz_3_1009	DJI_0951	Poa sp.	0		NA	0	Poaceae Barnhart
Iz_3_1009	DJI_0951	Polygonatum odoratum (Mill.) Druce	0	n	1	0	Asparagaceae Juss.
Iz_3_1009	DJI_0951	Polygonum arenarium Waldst. & Kit.	0	n	1	0	Polygonaceae Juss.
Iz_3_1009	DJI_0951	Populus nigra L.	0	n	1	1	Salicaceae Mirb.
Iz_3_1009	DJI_0951	Populus tremula L.	0	n	1	1	Salicaceae Mirb.
Iz_3_1009	DJI_0951	Populus Ã canescens (Aiton) Sm.	0	n	1	1	Salicaceae Mirb.
Iz_3_1009	DJI_0951	Potentilla argentea L.	0	n	1	0	Rosaceae Juss.
Iz_3_1009	DJI_0951	Psammophiliella muralis (L.) Ikonn.	0	n	1	0	Caryophyllaceae Juss.
Iz_3_1009	DJI_0951	Psephellus sumensis (Kalen.) Greuter	0	n	1	0	Asteraceae Bercht. & J.Presl
Iz_3_1009	DJI_0951	Pulsatilla pratensis (L.) Mill. s.l.	0	n	1	0	Ranunculaceae Juss.
Iz_3_1009	DJI_0951	Pyrus communis L.	0	a	0	1	Rosaceae Juss.
Iz_3_1009	DJI_0951	Quercus robur L.	0	n	1	1	Fagaceae Dumort.
Iz_3_1009	DJI_0951	Robinia pseudoacacia L.	0	a	0	1	Fabaceae Lindl.
Iz_3_1009	DJI_0951	Rosa sp.	0		NA	1	Rosaceae Juss.
Iz_3_1009	DJI_0951	Rubus idaeus L.	0	n	1	0	Rosaceae Juss.
Iz_3_1009	DJI_0951	Rumex acetosella L.	0	n	1	0	Polygonaceae Juss.
Iz_3_1009	DJI_0951	Rumex thyrsiflorus Fingerh.	0	n	1	0	Polygonaceae Juss.
Iz_3_1009	DJI_0951	Salix alba L.	0	n	1	1	Salicaceae Mirb.
Iz_3_1009	DJI_0951	Sambucus nigra L.	0	n	1	1	Viburnaceae Raf.
Iz_3_1009	DJI_0951	Secale sylvestre Host	0	n	1	0	Poaceae Barnhart
Iz_3_1009	DJI_0951	Sempervivum ruthenicum Schnittsp. & C.B. Lehm.	0	n	1	0	Crassulaceae J.St.-Hil.
Iz_3_1009	DJI_0951	Setaria viridis (L.) P. Beauv.	0	n	1	0	Poaceae Barnhart
Iz_3_1009	DJI_0951	Silene borysthenica (Gruner) Walters	0	n	1	0	Caryophyllaceae Juss.

Iz_3_1009	DJI_0951	Silene latifolia subsp. alba (Mill.) Greuter & Burdet	0	n	1	0	Caryophyllaceae Juss.
Iz_3_1009	DJI_0951	Solidago virgaurea L.	0	n	1	0	Asteraceae Bercht. & J.Presl
Iz_3_1009	DJI_0951	Sonchus oleraceus L.	0	a	0	0	Asteraceae Bercht. & J.Presl
Iz_3_1009	DJI_0951	Sorbus aucuparia L.	0	n	1	1	Rosaceae Juss.
Iz_3_1009	DJI_0951	Stachys recta L.	0	n	1	0	Lamiacea Lindl.
Iz_3_1009	DJI_0951	Tanacetum vulgare L.	0	n	1	0	Asteraceae Bercht. & J.Presl
Iz_3_1009	DJI_0951	Thymus pallasianus Heinr.Braun	0	n	1	0	Lamiacea Lindl.
Iz_3_1009	DJI_0951	Tilia cordata Mill.	0	n	1	1	Malvaceae Juss.
Iz_3_1009	DJI_0951	Trifolium alpestre L.	0	n	1	0	Fabaceae Lindl.
Iz_3_1009	DJI_0951	Trifolium arvense L.	0	n	1	0	Fabaceae Lindl.
Iz_3_1009	DJI_0951	Ulmus glabra Huds.	0	n	1	1	Ulmaceae Mirb.
Iz_3_1009	DJI_0951	Verbascum densiflorum Bertol.	0	n	1	0	Scrophulariaceae Juss.
Iz_3_1009	DJI_0951	Veronica austriaca L.	0	n	1	0	Plantaginaceae Juss.
Iz_3_1009	DJI_0951	Veronica incana L.	0	n	1	0	Plantaginaceae Juss.
Iz_3_1009	DJI_0951	Veronica verna L.	0	n	1	0	Plantaginaceae Juss.
Iz_3_1009	DJI_0951	Vincetoxicum hirundinaria Medik	0	n	1	0	Apocynaceae Juss.
Iz_3_1009	DJI_0954	Acer tataricum L.	0	n	1	1	Sapindaceae Juss.
Iz_3_1009	DJI_0954	Achillea millefolium L. s.l.	0	n	1	0	Asteraceae Bercht. & J.Presl
Iz_3_1009	DJI_0954	Allium oleraceum L.	0	n	1	0	Amaryllidaceae J.St.-Hil.
Iz_3_1009	DJI_0954	Ambrosia artemisiifolia L.	0	a	0	0	Asteraceae Bercht. & J.Presl
Iz_3_1009	DJI_0954	Amorpha fruticosa L.	0	a	0	0	Fabaceae Lindl.
Iz_3_1009	DJI_0954	Anthericum ramosum L.	0	n	1	0	Asparagaceae Juss.
Iz_3_1009	DJI_0954	Artemisia marschalliana Spreng.	0	n	1	0	Asteraceae Bercht. & J.Presl
Iz_3_1009	DJI_0954	Asparagus officinalis L.	0	n	1	0	Asparagaceae Juss.
Iz_3_1009	DJI_0954	Astragalus cicer L.	0	n	1	0	Fabaceae Lindl.
Iz_3_1009	DJI_0954	Bassia laniflora (S.G.Gmel.) A.J.Scott	0	n	1	0	Amaranthaceae Juss.
Iz_3_1009	DJI_0954	Berberis vulgaris L.	0	n	1	0	Berberidaceae Juss.
Iz_3_1009	DJI_0954	Betula pendula Roth.	0	n	1	1	Betulaceae Gray
Iz_3_1009	DJI_0954	Betula pendula Roth.	0	n	1	0	Poaceae Barnhart
Iz_3_1009	DJI_0954	Bromus inermis Leyss.	0		NA	NA	
Iz_3_1009	DJI_0954	Bromus squarrosus L.	0	a	0	0	Poaceae Barnhart
Iz_3_1009	DJI_0954	Bromus tectorum L.	0	a	0	0	Poaceae Barnhart
Iz_3_1009	DJI_0954	Calamagrostis epigejos (L.) Roth	15	n	1	0	Poaceae Barnhart
Iz_3_1009	DJI_0954	Carduus acanthoides L.	0	n	1	0	Asteraceae Bercht. & J.Presl
Iz_3_1009	DJI_0954	Carex sp.	0	n	1	0	Cyperaceae Juss.
Iz_3_1009	DJI_0954	Centaurea majorovii Dumbadze	0	n	1	0	Asteraceae Bercht. & J.Presl
Iz_3_1009	DJI_0954	Chamaecytisus ruthenicus (Fisch.ex Wol.) Klaskova	10	n	1	0	Fabaceae Lindl.
Iz_3_1009	DJI_0954	Chelidonium majus L.	0.5	n	1	0	Papaveraceae Juss.
Iz_3_1009	DJI_0954	Chenopodium betaceum Andrz.	0	n	1	0	Amaranthaceae Juss.
Iz_3_1009	DJI_0954	Chenopodium cf. album	0	n	1	0	Amaranthaceae Juss.
Iz_3_1009	DJI_0954	Chenopodium sp.	0		NA	0	Amaranthaceae Juss.
Iz_3_1009	DJI_0954	Chondrilla juncea L.	0	n	1	0	Asteraceae Bercht. & J.Presl
Iz_3_1009	DJI_0954	Cirsium vulgare (Savi) Ten.	0	n	1	0	Asteraceae Bercht. & J.Presl
Iz_3_1009	DJI_0954	Clinopodium acinos (L.) Kuntze	0	n	1	0	Lamiacea Lindl.
Iz_3_1009	DJI_0954	Convallaria majalis L.	0	n	1	0	Asparagaceae Juss.
Iz_3_1009	DJI_0954	Crataegus rhipidophylla Gand.	0	n	1	1	Rosaceae Juss.
Iz_3_1009	DJI_0954	Crepis foetida L.	0	n	1	0	Asteraceae Bercht. & J.Presl
Iz_3_1009	DJI_0954	Dactylis glomerata L.	0	n	1	0	Poaceae Barnhart
Iz_3_1009	DJI_0954	Dianthus campestris M.Bieb.	0	n	1	0	Caryophyllaceae Juss.
Iz_3_1009	DJI_0954	Digitaria sanguinalis (L.) Scop.	0	a	0	0	Poaceae Barnhart
Iz_3_1009	DJI_0954	Elymus repens (L.) Gould	0	n	1	0	Poaceae Barnhart
Iz_3_1009	DJI_0954	Epilobium tetragonum L.	0	n	1	0	Onagraceae Juss.
Iz_3_1009	DJI_0954	Eragrostis minor Host	0	a	0	0	Poaceae Barnhart
Iz_3_1009	DJI_0954	Erigeron canadensis L.	0	a	0	0	Asteraceae Bercht. & J.Presl
Iz_3_1009	DJI_0954	Euphorbia seguieriana Neck.	0	n	1	0	Euphorbiaceae Juss.
Iz_3_1009	DJI_0954	Fallopia dumetorum (L.) Holub	0.5	n	1	0	Polygonaceae Juss.
Iz_3_1009	DJI_0954	Festuca beckeri (Hack.) Trautv.	0	n	1	0	Poaceae Barnhart
Iz_3_1009	DJI_0954	Fragaria vesca L.	0	n	1	0	Rosaceae Juss.
Iz_3_1009	DJI_0954	Frangula alnus Mill.	0	n	1	1	Rhamnaceae Juss.
Iz_3_1009	DJI_0954	Fraxinus pennsylvanica Marshall	0	a	0	1	Oleaceae Hoffmanns. & Link
Iz_3_1009	DJI_0954	Galeopsis bifida Boenn.	0	n	1	0	Lamiacea Lindl.
Iz_3_1009	DJI_0954	Galium verum L.	0	n	1	0	Rubiaceae Juss.
Iz_3_1009	DJI_0954	Genista tinctoria L.	0	n	1	0	Fabaceae Lindl.
Iz_3_1009	DJI_0954	Geranium robertianum L.	0	n	1	0	Geraniaceae Juss.
Iz_3_1009	DJI_0954	Geranium sanquineum L.	0	n	1	0	Geraniaceae Juss.
Iz_3_1009	DJI_0954	Gypsophila paniculata L.	0	n	1	0	Caryophyllaceae Juss.
Iz_3_1009	DJI_0954	Helichrysum arenarium (L.) Moench	0	n	1	0	Asteraceae Bercht. & J.Presl
Iz_3_1009	DJI_0954	Herniaria polygama J.Gay	0	n	1	0	Caryophyllaceae Juss.
Iz_3_1009	DJI_0954	Hieracium robustum Fr.	0	n	1	0	Asteraceae Bercht. & J.Presl
Iz_3_1009	DJI_0954	Hieracium umbellatum L.	0	n	1	NA	Asteraceae Bercht. & J.Presl
Iz_3_1009	DJI_0954	Hylotelephium maximum (L.) Holub	0	n	1	0	Crassulaceae J.St.-Hil.
Iz_3_1009	DJI_0954	Jacobaea borysthenica (DC.) B.Nord. & Greuter	0	n	1	0	Asteraceae Bercht. & J.Presl
Iz_3_1009	DJI_0954	Jasione montana L.	0	n	1	0	Campanulaceae Juss.
Iz_3_1009	DJI_0954	Jurinea cyanoides (L.) Rchb.s.l.	0	n	1	0	Asteraceae Bercht. & J.Presl
Iz_3_1009	DJI_0954	Koeleria glauca (Spreng.) DC.	0	n	1	0	Poaceae Barnhart
Iz_3_1009	DJI_0954	Lactuca serriola L.	0	a	0	0	Asteraceae Bercht. & J.Presl
Iz_3_1009	DJI_0954	Linaria genistifolia (L.) Mill.	0	n	1	0	Plantaginaceae Juss.
Iz_3_1009	DJI_0954	Lomelosia argentea (L.) Greuter & Burdet	0	n	1	0	Caprifoliaceae Juss.
Iz_3_1009	DJI_0954	Melampyrum pratense L.	0	n	1	0	Orobanchaceae Vent.
Iz_3_1009	DJI_0954	Oenothera biennis L.	0	a	0	0	Onagraceae Juss.
Iz_3_1009	DJI_0954	Pentanema britannica (L.) D.Gut.Larr., Santos-Vicente, Anderb., E.Rico & M.M.Mart.Ort.	0	n	1	0	Asteraceae Bercht. & J.Presl
Iz_3_1009	DJI_0954	Peucedanum oreoselinum (L.) Moench	0	n	1	0	Apiaceae Lindl.
Iz_3_1009	DJI_0954	Peucedanum ruthenicum M.Bieb.	0	n	1	0	Apiaceae Lindl.
Iz_3_1009	DJI_0954	Pilosella echioides (Lumn.) F.W.Schultz & Sch.Bip.	0	n	1	0	Asteraceae Bercht. & J.Presl
Iz_3_1009	DJI_0954	Pilosella officinarum F. Schultz et Sch. Bip.	0	n	1	0	Asteraceae Bercht. & J.Presl
Iz_3_1009	DJI_0954	Pilosella sp.	0		NA	0	Asteraceae Bercht. & J.Presl
Iz_3_1009	DJI_0954	Pinus sylvestris L.	0	n	1	1	Pinaceae Spreng. ex F.Rudolphi
Iz_3_1009	DJI_0954	Poa annua L.	0	n	1	0	Poaceae Barnhart
Iz_3_1009	DJI_0954	Poa nemoralis L.	0	n	1	0	Poaceae Barnhart
Iz_3_1009	DJI_0954	Poa sp.	0		NA	0	Poaceae Barnhart
Iz_3_1009	DJI_0954	Polygonatum odoratum (Mill.) Druce	0	n	1	0	Asparagaceae Juss.
Iz_3_1009	DJI_0954	Polygonum arenarium Waldst. & Kit.	0	n	1	0	Polygonaceae Juss.
Iz_3_1009	DJI_0954	Populus nigra L.	0	n	1	1	Salicaceae Mirb.
Iz_3_1009	DJI_0954	Populus tremula L.	0	n	1	1	Salicaceae Mirb.
Iz_3_1009	DJI_0954	Populus Ã canescens (Aiton) Sm.	0	n	1	1	Salicaceae Mirb.
Iz_3_1009	DJI_0954	Potentilla argentea L.	0	n	1	0	Rosaceae Juss.
Iz_3_1009	DJI_0954	Psammophiliella muralis (L.) Ikonn.	0	n	1	0	Caryophyllaceae Juss.
Iz_3_1009	DJI_0954	Psephellus sumensis (Kalen.) Greuter	0	n	1	0	Asteraceae Bercht. & J.Presl

Iz_3_1009	DJI_0954	Pulsatilla pratensis (L.) Mill. s.l.	0	n	1	0	Ranunculaceae Juss.
Iz_3_1009	DJI_0954	Pyrus communis L.	0	a	0	1	Rosaceae Juss.
Iz_3_1009	DJI_0954	Quercus robur L.	0	n	1	1	Fagaceae Dumort.
Iz_3_1009	DJI_0954	Robinia pseudoacacia L.	0	a	0	1	Fabaceae Lindl.
Iz_3_1009	DJI_0954	Rosa sp.	0		NA	1	Rosaceae Juss.
Iz_3_1009	DJI_0954	Rubus idaeus L.	0	n	1	0	Rosaceae Juss.
Iz_3_1009	DJI_0954	Rumex acetosella L.	0	n	1	0	Polygonaceae Juss.
Iz_3_1009	DJI_0954	Rumex thyrsiflorus Fingerh.	0	n	1	0	Polygonaceae Juss.
Iz_3_1009	DJI_0954	Salix alba L.	0	n	1	1	Salicaceae Mirb.
Iz_3_1009	DJI_0954	Sambucus nigra L.	0	n	1	1	Viburnaceae Raf.
Iz_3_1009	DJI_0954	Secale sylvestre Host	0	n	1	0	Poaceae Barnhart
Iz_3_1009	DJI_0954	Sempervivum ruthenicum Schnittsp. & C.B. Lehm.	0	n	1	0	Crassulaceae J.St.-Hil.
Iz_3_1009	DJI_0954	Setaria viridis (L.) P. Beauv.	0	n	1	0	Poaceae Barnhart
Iz_3_1009	DJI_0954	Silene borysthenica (Gruner) Walters	0	n	1	0	Caryophyllaceae Juss.
Iz_3_1009	DJI_0954	Silene latifolia subsp. alba (Mill.) Greuter & Burdet	0	n	1	0	Caryophyllaceae Juss.
Iz_3_1009	DJI_0954	Solidago virgaurea L.	0	n	1	0	Asteraceae Bercht. & J.Presl
Iz_3_1009	DJI_0954	Sonchus oleraceus L.	0	a	0	0	Asteraceae Bercht. & J.Presl
Iz_3_1009	DJI_0954	Sorbus aucuparia L.	0	n	1	1	Rosaceae Juss.
Iz_3_1009	DJI_0954	Stachys recta L.	0	n	1	0	Lamiacea Lindl.
Iz_3_1009	DJI_0954	Tanacetum vulgare L.	0	n	1	0	Asteraceae Bercht. & J.Presl
Iz_3_1009	DJI_0954	Thymus pallasianus Heinr.Braun	0	n	1	0	Lamiacea Lindl.
Iz_3_1009	DJI_0954	Tilia cordata Mill.	0	n	1	1	Malvaceae Juss.
Iz_3_1009	DJI_0954	Trifolium alpestre L.	0	n	1	0	Fabaceae Lindl.
Iz_3_1009	DJI_0954	Trifolium arvense L.	0	n	1	0	Fabaceae Lindl.
Iz_3_1009	DJI_0954	Ulmus glabra Huds.	0	n	1	1	Ulmaceae Mirb.
Iz_3_1009	DJI_0954	Verbascum densiflorum Bertol.	0	n	1	0	Scrophulariaceae Juss.
Iz_3_1009	DJI_0954	Veronica austriaca L.	0	n	1	0	Plantaginaceae Juss.
Iz_3_1009	DJI_0954	Veronica incana L.	0	n	1	0	Plantaginaceae Juss.
Iz_3_1009	DJI_0954	Veronica verna L.	0	n	1	0	Plantaginaceae Juss.
Iz_3_1009	DJI_0954	Vincetoxicum hirundinaria Medik	0	n	1	0	Apocynaceae Juss.
Iz_3_1009	DJI_0956	Acer tataricum L.	0	n	1	1	Sapindaceae Juss.
Iz_3_1009	DJI_0956	Achillea millefolium L. s.l.	0	n	1	0	Asteraceae Bercht. & J.Presl
Iz_3_1009	DJI_0956	Allium oleraceum L.	0	n	1	0	Amaryllidaceae J.St.-Hil.
Iz_3_1009	DJI_0956	Ambrosia artemisiifolia L.	0	a	0	0	Asteraceae Bercht. & J.Presl
Iz_3_1009	DJI_0956	Amorpha fruticosa L.	0	a	0	0	Fabaceae Lindl.
Iz_3_1009	DJI_0956	Anthericum ramosum L.	0	n	1	0	Asparagaceae Juss.
Iz_3_1009	DJI_0956	Artemisia marschalliana Spreng.	0	n	1	0	Asteraceae Bercht. & J.Presl
Iz_3_1009	DJI_0956	Asparagus officinalis L.	0	n	1	0	Asparagaceae Juss.
Iz_3_1009	DJI_0956	Astragalus cicer L.	0	n	1	0	Fabaceae Lindl.
Iz_3_1009	DJI_0956	Bassia laniflora (S.G.Gmel.) A.J.Scott	0	n	1	0	Amaranthaceae Juss.
Iz_3_1009	DJI_0956	Berberis vulgaris L.	0	n	1	0	Berberidaceae Juss.
Iz_3_1009	DJI_0956	Betula pendula Roth.	0	n	1	1	Betulaceae Gray
Iz_3_1009	DJI_0956	Betula pendula Roth.	0	n	1	0	Poaceae Barnhart
Iz_3_1009	DJI_0956	Bromus inermis Leyss.	0		NA	NA	
Iz_3_1009	DJI_0956	Bromus squarrosus L.	0	a	0	0	Poaceae Barnhart
Iz_3_1009	DJI_0956	Bromus tectorum L.	0	a	0	0	Poaceae Barnhart
Iz_3_1009	DJI_0956	Calamagrostis epigejos (L.) Roth	20	n	1	0	Poaceae Barnhart
Iz_3_1009	DJI_0956	Carduus acanthoides L.	0	n	1	0	Asteraceae Bercht. & J.Presl
Iz_3_1009	DJI_0956	Carex sp.	0	n	1	0	Cyperaceae Juss.
Iz_3_1009	DJI_0956	Centaurea majorovii Dumbadze	0	n	1	0	Asteraceae Bercht. & J.Presl
Iz_3_1009	DJI_0956	Chamaecytisus ruthenicus (Fisch.ex Wol.) Klaskova	0	n	1	0	Fabaceae Lindl.
Iz_3_1009	DJI_0956	Chelidonium majus L.	0.5	n	1	0	Papaveraceae Juss.
Iz_3_1009	DJI_0956	Chenopodium betaceum Andrz.	0	n	1	0	Amaranthaceae Juss.
Iz_3_1009	DJI_0956	Chenopodium cf. album	0	n	1	0	Amaranthaceae Juss.
Iz_3_1009	DJI_0956	Chenopodium sp.	0		NA	0	Amaranthaceae Juss.
Iz_3_1009	DJI_0956	Chondrilla juncea L.	0	n	1	0	Asteraceae Bercht. & J.Presl
Iz_3_1009	DJI_0956	Cirsium vulgare (Savi) Ten.	0	n	1	0	Asteraceae Bercht. & J.Presl
Iz_3_1009	DJI_0956	Clinopodium acinos (L.) Kuntze	0	n	1	0	Lamiacea Lindl.
Iz_3_1009	DJI_0956	Convallaria majalis L.	0	n	1	0	Asparagaceae Juss.
Iz_3_1009	DJI_0956	Crataegus rhipidophylla Gand.	0	n	1	1	Rosaceae Juss.
Iz_3_1009	DJI_0956	Crepis foetida L.	0	n	1	0	Asteraceae Bercht. & J.Presl
Iz_3_1009	DJI_0956	Dactylis glomerata L.	0	n	1	0	Poaceae Barnhart
Iz_3_1009	DJI_0956	Dianthus campestris M.Bieb.	0	n	1	0	Caryophyllaceae Juss.
Iz_3_1009	DJI_0956	Digitaria sanguinalis (L.) Scop.	0	a	0	0	Poaceae Barnhart
Iz_3_1009	DJI_0956	Elymus repens (L.) Gould	0	n	1	0	Poaceae Barnhart
Iz_3_1009	DJI_0956	Epilobium tetragonum L.	0	n	1	0	Onagraceae Juss.
Iz_3_1009	DJI_0956	Eragrostis minor Host	0	a	0	0	Poaceae Barnhart
Iz_3_1009	DJI_0956	Erigeron canadensis L.	0	a	0	0	Asteraceae Bercht. & J.Presl
Iz_3_1009	DJI_0956	Euphorbia seguieriana Neck.	0	n	1	0	Euphorbiaceae Juss.
Iz_3_1009	DJI_0956	Fallopia dumetorum (L.) Holub	1	n	1	0	Polygonaceae Juss.
Iz_3_1009	DJI_0956	Festuca beckeri (Hack.) Trautv.	0	n	1	0	Poaceae Barnhart
Iz_3_1009	DJI_0956	Fragaria vesca L.	0	n	1	0	Rosaceae Juss.
Iz_3_1009	DJI_0956	Frangula alnus Mill.	0	n	1	1	Rhamnaceae Juss.
Iz_3_1009	DJI_0956	Fraxinus pennsylvanica Marshall	0	a	0	1	Oleaceae Hoffmanns. & Link
Iz_3_1009	DJI_0956	Galeopsis bifida Boenn.	0	n	1	0	Lamiacea Lindl.
Iz_3_1009	DJI_0956	Galium verum L.	0	n	1	0	Rubiaceae Juss.
Iz_3_1009	DJI_0956	Genista tinctoria L.	0	n	1	0	Fabaceae Lindl.
Iz_3_1009	DJI_0956	Geranium robertianum L.	0	n	1	0	Geraniaceae Juss.
Iz_3_1009	DJI_0956	Geranium sanquineum L.	0	n	1	0	Geraniaceae Juss.
Iz_3_1009	DJI_0956	Gypsophila paniculata L.	0	n	1	0	Caryophyllaceae Juss.
Iz_3_1009	DJI_0956	Helichrysum arenarium (L.) Moench	0	n	1	0	Asteraceae Bercht. & J.Presl
Iz_3_1009	DJI_0956	Herniaria polygama J.Gay	0	n	1	0	Caryophyllaceae Juss.
Iz_3_1009	DJI_0956	Hieracium robustum Fr.	0	n	1	0	Asteraceae Bercht. & J.Presl
Iz_3_1009	DJI_0956	Hieracium umbellatum L.	0	n	1	NA	Asteraceae Bercht. & J.Presl
Iz_3_1009	DJI_0956	Hylotelephium maximum (L.) Holub	0	n	1	0	Crassulaceae J.St.-Hil.
Iz_3_1009	DJI_0956	Jacobaea borysthenica (DC.) B.Nord. & Greuter	0	n	1	0	Asteraceae Bercht. & J.Presl
Iz_3_1009	DJI_0956	Jasione montana L.	0	n	1	0	Campanulaceae Juss.
Iz_3_1009	DJI_0956	Jurinea cyanoides (L.) Rchb.s.l.	0	n	1	0	Asteraceae Bercht. & J.Presl
Iz_3_1009	DJI_0956	Koeleria glauca (Spreng.) DC.	0	n	1	0	Poaceae Barnhart
Iz_3_1009	DJI_0956	Lactuca serriola L.	0	a	0	0	Asteraceae Bercht. & J.Presl
Iz_3_1009	DJI_0956	Linaria genistifolia (L.) Mill.	0	n	1	0	Plantaginaceae Juss.
Iz_3_1009	DJI_0956	Lomelosia argentea (L.) Greuter & Burdet	0	n	1	0	Caprifoliaceae Juss.
Iz_3_1009	DJI_0956	Melampyrum pratense L.	0	n	1	0	Orobanchaceae Vent.
Iz_3_1009	DJI_0956	Oenothera biennis L.	0	a	0	0	Onagraceae Juss.
Iz_3_1009	DJI_0956	Pentanema britannica (L.) D.Gut.Larr., Santos-Vicente, Anderb., E.Rico & M.M.Mart.Ort.	0	n	1	0	Asteraceae Bercht. & J.Presl
Iz_3_1009	DJI_0956	Peucedanum oreoselinum (L.) Moench	0	n	1	0	Apiaceae Lindl.
Iz_3_1009	DJI_0956	Peucedanum ruthenicum M.Bieb.	0	n	1	0	Apiaceae Lindl.
Iz_3_1009	DJI_0956	Pilosella echioides (Lumn.) F.W.Schultz & Sch.Bip.	0	n	1	0	Asteraceae Bercht. & J.Presl

Iz_3_1009	DJI_0956	Pilosella officinarum F. Schultz et Sch. Bip.	0	n	1	0	Asteraceae Bercht. & J.Presl
Iz_3_1009	DJI_0956	Pilosella sp.	0		NA	0	Asteraceae Bercht. & J.Presl
Iz_3_1009	DJI_0956	Pinus sylvestris L.	0	n	1	1	Pinaceae Spreng. ex F.Rudolphi
Iz_3_1009	DJI_0956	Poa annua L.	0	n	1	0	Poaceae Barnhart
Iz_3_1009	DJI_0956	Poa nemoralis L.	0	n	1	0	Poaceae Barnhart
Iz_3_1009	DJI_0956	Poa sp.	0		NA	0	Poaceae Barnhart
Iz_3_1009	DJI_0956	Polygonatum odoratum (Mill.) Druce	0	n	1	0	Asparagaceae Juss.
Iz_3_1009	DJI_0956	Polygonum arenarium Waldst. & Kit.	0	n	1	0	Polygonaceae Juss.
Iz_3_1009	DJI_0956	Populus nigra L.	0	n	1	1	Salicaceae Mirb.
Iz_3_1009	DJI_0956	Populus tremula L.	0	n	1	1	Salicaceae Mirb.
Iz_3_1009	DJI_0956	Populus Ã canescens (Aiton) Sm.	0	n	1	1	Salicaceae Mirb.
Iz_3_1009	DJI_0956	Potentilla argentea L.	0	n	1	0	Rosaceae Juss.
Iz_3_1009	DJI_0956	Psammophiliella muralis (L.) Ikonn.	0	n	1	0	Caryophyllaceae Juss.
Iz_3_1009	DJI_0956	Psephellus sumensis (Kalen.) Greuter	0	n	1	0	Asteraceae Bercht. & J.Presl
Iz_3_1009	DJI_0956	Pulsatilla pratensis (L.) Mill. s.l.	0	n	1	0	Ranunculaceae Juss.
Iz_3_1009	DJI_0956	Pyrus communis L.	0	a	0	1	Rosaceae Juss.
Iz_3_1009	DJI_0956	Quercus robur L.	0	n	1	1	Fagaceae Dumort.
Iz_3_1009	DJI_0956	Robinia pseudoacacia L.	0	a	0	1	Fabaceae Lindl.
Iz_3_1009	DJI_0956	Rosa sp.	0		NA	1	Rosaceae Juss.
Iz_3_1009	DJI_0956	Rubus idaeus L.	0	n	1	0	Rosaceae Juss.
Iz_3_1009	DJI_0956	Rumex acetosella L.	0	n	1	0	Polygonaceae Juss.
Iz_3_1009	DJI_0956	Rumex thyrsiflorus Fingerh.	0	n	1	0	Polygonaceae Juss.
Iz_3_1009	DJI_0956	Salix alba L.	0	n	1	1	Salicaceae Mirb.
Iz_3_1009	DJI_0956	Sambucus nigra L.	0	n	1	1	Viburnaceae Raf.
Iz_3_1009	DJI_0956	Secale sylvestre Host	0	n	1	0	Poaceae Barnhart
Iz_3_1009	DJI_0956	Sempervivum ruthenicum Schnittsp. & C.B. Lehm.	0	n	1	0	Crassulaceae J.St.-Hil.
Iz_3_1009	DJI_0956	Setaria viridis (L.) P. Beauv.	0	n	1	0	Poaceae Barnhart
Iz_3_1009	DJI_0956	Silene borysthenica (Gruner) Walters	0	n	1	0	Caryophyllaceae Juss.
Iz_3_1009	DJI_0956	Silene latifolia subsp. alba (Mill.) Greuter & Burdet	0	n	1	0	Caryophyllaceae Juss.
Iz_3_1009	DJI_0956	Solidago virgaurea L.	0	n	1	0	Asteraceae Bercht. & J.Presl
Iz_3_1009	DJI_0956	Sonchus oleraceus L.	0	a	0	0	Asteraceae Bercht. & J.Presl
Iz_3_1009	DJI_0956	Sorbus aucuparia L.	0	n	1	1	Rosaceae Juss.
Iz_3_1009	DJI_0956	Stachys recta L.	0	n	1	0	Lamiacea Lindl.
Iz_3_1009	DJI_0956	Tanacetum vulgare L.	0	n	1	0	Asteraceae Bercht. & J.Presl
Iz_3_1009	DJI_0956	Thymus pallasianus Heinr.Braun	0	n	1	0	Lamiacea Lindl.
Iz_3_1009	DJI_0956	Tilia cordata Mill.	0	n	1	1	Malvaceae Juss.
Iz_3_1009	DJI_0956	Trifolium alpestre L.	0	n	1	0	Fabaceae Lindl.
Iz_3_1009	DJI_0956	Trifolium arvense L.	0	n	1	0	Fabaceae Lindl.
Iz_3_1009	DJI_0956	Ulmus glabra Huds.	0	n	1	1	Ulmaceae Mirb.
Iz_3_1009	DJI_0956	Verbascum densiflorum Bertol.	0	n	1	0	Scrophulariaceae Juss.
Iz_3_1009	DJI_0956	Veronica austriaca L.	0	n	1	0	Plantaginaceae Juss.
Iz_3_1009	DJI_0956	Veronica incana L.	0	n	1	0	Plantaginaceae Juss.
Iz_3_1009	DJI_0956	Veronica verna L.	0	n	1	0	Plantaginaceae Juss.
Iz_3_1009	DJI_0956	Vincetoxicum hirundinaria Medik	0	n	1	0	Apocynaceae Juss.
Iz_3_1009	DJI_0966	Acer tataricum L.	0	n	1	1	Sapindaceae Juss.
Iz_3_1009	DJI_0966	Achillea millefolium L. s.l.	0	n	1	0	Asteraceae Bercht. & J.Presl
Iz_3_1009	DJI_0966	Allium oleraceum L.	0	n	1	0	Amaryllidaceae J.St.-Hil.
Iz_3_1009	DJI_0966	Ambrosia artemisiifolia L.	0	a	0	0	Asteraceae Bercht. & J.Presl
Iz_3_1009	DJI_0966	Amorpha fruticosa L.	0	a	0	0	Fabaceae Lindl.
Iz_3_1009	DJI_0966	Anthericum ramosum L.	0	n	1	0	Asparagaceae Juss.
Iz_3_1009	DJI_0966	Artemisia marschalliana Spreng.	0	n	1	0	Asteraceae Bercht. & J.Presl
Iz_3_1009	DJI_0966	Asparagus officinalis L.	0	n	1	0	Asparagaceae Juss.
Iz_3_1009	DJI_0966	Astragalus cicer L.	0	n	1	0	Fabaceae Lindl.
Iz_3_1009	DJI_0966	Bassia laniflora (S.G.Gmel.) A.J.Scott	0	n	1	0	Amaranthaceae Juss.
Iz_3_1009	DJI_0966	Berberis vulgaris L.	0	n	1	0	Berberidaceae Juss.
Iz_3_1009	DJI_0966	Betula pendula Roth.	0	n	1	1	Betulaceae Gray
Iz_3_1009	DJI_0966	Betula pendula Roth.	0	n	1	0	Poaceae Barnhart
Iz_3_1009	DJI_0966	Bromus inermis Leyss.	0		NA	NA	
Iz_3_1009	DJI_0966	Bromus squarrosus L.	0	a	0	0	Poaceae Barnhart
Iz_3_1009	DJI_0966	Bromus tectorum L.	0	a	0	0	Poaceae Barnhart
Iz_3_1009	DJI_0966	Calamagrostis epigejos (L.) Roth	10	n	1	0	Poaceae Barnhart
Iz_3_1009	DJI_0966	Carduus acanthoides L.	0	n	1	0	Asteraceae Bercht. & J.Presl
Iz_3_1009	DJI_0966	Carex sp.	0	n	1	0	Cyperaceae Juss.
Iz_3_1009	DJI_0966	Centaurea majorovii Dumbadze	0	n	1	0	Asteraceae Bercht. & J.Presl
Iz_3_1009	DJI_0966	Chamaecytisus ruthenicus (Fisch.ex Wol.) Klaskova	0	n	1	0	Fabaceae Lindl.
Iz_3_1009	DJI_0966	Chelidonium majus L.	0.5	n	1	0	Papaveraceae Juss.
Iz_3_1009	DJI_0966	Chenopodium betaceum Andrz.	0	n	1	0	Amaranthaceae Juss.
Iz_3_1009	DJI_0966	Chenopodium cf. album	0	n	1	0	Amaranthaceae Juss.
Iz_3_1009	DJI_0966	Chenopodium sp.	0		NA	0	Amaranthaceae Juss.
Iz_3_1009	DJI_0966	Chondrilla juncea L.	0	n	1	0	Asteraceae Bercht. & J.Presl
Iz_3_1009	DJI_0966	Cirsium vulgare (Savi) Ten.	0	n	1	0	Asteraceae Bercht. & J.Presl
Iz_3_1009	DJI_0966	Clinopodium acinos (L.) Kuntze	0	n	1	0	Lamiacea Lindl.
Iz_3_1009	DJI_0966	Convallaria majalis L.	0	n	1	0	Asparagaceae Juss.
Iz_3_1009	DJI_0966	Crataegus rhipidophylla Gand.	0	n	1	1	Rosaceae Juss.
Iz_3_1009	DJI_0966	Crepis foetida L.	0	n	1	0	Asteraceae Bercht. & J.Presl
Iz_3_1009	DJI_0966	Dactylis glomerata L.	0	n	1	0	Poaceae Barnhart
Iz_3_1009	DJI_0966	Dianthus campestris M.Bieb.	0	n	1	0	Caryophyllaceae Juss.
Iz_3_1009	DJI_0966	Digitaria sanguinalis (L.) Scop.	0	a	0	0	Poaceae Barnhart
Iz_3_1009	DJI_0966	Elymus repens (L.) Gould	0	n	1	0	Poaceae Barnhart
Iz_3_1009	DJI_0966	Epilobium tetragonum L.	0	n	1	0	Onagraceae Juss.
Iz_3_1009	DJI_0966	Eragrostis minor Host	0	a	0	0	Poaceae Barnhart
Iz_3_1009	DJI_0966	Erigeron canadensis L.	0	a	0	0	Asteraceae Bercht. & J.Presl
Iz_3_1009	DJI_0966	Euphorbia seguieriana Neck.	0	n	1	0	Euphorbiaceae Juss.
Iz_3_1009	DJI_0966	Fallopia dumetorum (L.) Holub	0.5	n	1	0	Polygonaceae Juss.
Iz_3_1009	DJI_0966	Festuca beckeri (Hack.) Trautv.	0	n	1	0	Poaceae Barnhart
Iz_3_1009	DJI_0966	Fragaria vesca L.	0	n	1	0	Rosaceae Juss.
Iz_3_1009	DJI_0966	Frangula alnus Mill.	0	n	1	1	Rhamnaceae Juss.
Iz_3_1009	DJI_0966	Fraxinus pennsylvanica Marshall	0	a	0	1	Oleaceae Hoffmanns. & Link
Iz_3_1009	DJI_0966	Galeopsis bifida Boenn.	0	n	1	0	Lamiacea Lindl.
Iz_3_1009	DJI_0966	Galium verum L.	0	n	1	0	Rubiaceae Juss.
Iz_3_1009	DJI_0966	Genista tinctoria L.	0	n	1	0	Fabaceae Lindl.
Iz_3_1009	DJI_0966	Geranium robertianum L.	0	n	1	0	Geraniaceae Juss.
Iz_3_1009	DJI_0966	Geranium sanquineum L.	0	n	1	0	Geraniaceae Juss.
Iz_3_1009	DJI_0966	Gypsophila paniculata L.	0	n	1	0	Caryophyllaceae Juss.
Iz_3_1009	DJI_0966	Helichrysum arenarium (L.) Moench	0	n	1	0	Asteraceae Bercht. & J.Presl
Iz_3_1009	DJI_0966	Herniaria polygama J.Gay	0	n	1	0	Caryophyllaceae Juss.
Iz_3_1009	DJI_0966	Hieracium robustum Fr.	0	n	1	0	Asteraceae Bercht. & J.Presl
Iz_3_1009	DJI_0966	Hieracium umbellatum L.	0	n	1	NA	Asteraceae Bercht. & J.Presl

Iz_3_1009	DJI_0966	Hylotelephium maximum (L.) Holub	0	n	1	0	Crassulaceae J.St.-Hil.
Iz_3_1009	DJI_0966	Jacobaea borysthenica (DC.) B.Nord. & Greuter	0	n	1	0	Asteraceae Bercht. & J.Presl
Iz_3_1009	DJI_0966	Jasione montana L.	0	n	1	0	Campanulaceae Juss.
Iz_3_1009	DJI_0966	Jurinea cyanoides (L.) Rchb.s.l.	0	n	1	0	Asteraceae Bercht. & J.Presl
Iz_3_1009	DJI_0966	Koeleria glauca (Spreng.) DC.	0	n	1	0	Poaceae Barnhart
Iz_3_1009	DJI_0966	Lactuca serriola L.	0	a	0	0	Asteraceae Bercht. & J.Presl
Iz_3_1009	DJI_0966	Linaria genistifolia (L.) Mill.	0	n	1	0	Plantaginaceae Juss.
Iz_3_1009	DJI_0966	Lomelosia argentea (L.) Greuter & Burdet	0	n	1	0	Caprifoliaceae Juss.
Iz_3_1009	DJI_0966	Melampyrum pratense L.	0	n	1	0	Orobanchaceae Vent.
Iz_3_1009	DJI_0966	Oenothera biennis L.	0	a	0	0	Onagraceae Juss.
Iz_3_1009	DJI_0966	Pentanema britannica (L.) D.Gut.Larr., Santos-Vicente, Anderb., E.Rico & M.M.Mart.Ort.	0	n	1	0	Asteraceae Bercht. & J.Presl
Iz_3_1009	DJI_0966	Peucedanum oreoselinum (L.) Moench	0	n	1	0	Apiaceae Lindl.
Iz_3_1009	DJI_0966	Peucedanum ruthenicum M.Bieb.	0	n	1	0	Apiaceae Lindl.
Iz_3_1009	DJI_0966	Pilosella echioides (Lumn.) F.W.Schultz & Sch.Bip.	0	n	1	0	Asteraceae Bercht. & J.Presl
Iz_3_1009	DJI_0966	Pilosella officinarum F. Schultz et Sch. Bip.	0	n	1	0	Asteraceae Bercht. & J.Presl
Iz_3_1009	DJI_0966	Pilosella sp.	0		NA	0	Asteraceae Bercht. & J.Presl
Iz_3_1009	DJI_0966	Pinus sylvestris L.	0	n	1	1	Pinaceae Spreng. ex F.Rudolphi
Iz_3_1009	DJI_0966	Poa annua L.	0	n	1	0	Poaceae Barnhart
Iz_3_1009	DJI_0966	Poa nemoralis L.	0	n	1	0	Poaceae Barnhart
Iz_3_1009	DJI_0966	Poa sp.	0		NA	0	Poaceae Barnhart
Iz_3_1009	DJI_0966	Polygonatum odoratum (Mill.) Druce	0	n	1	0	Asparagaceae Juss.
Iz_3_1009	DJI_0966	Polygonum arenarium Waldst. & Kit.	0	n	1	0	Polygonaceae Juss.
Iz_3_1009	DJI_0966	Populus nigra L.	0	n	1	1	Salicaceae Mirb.
Iz_3_1009	DJI_0966	Populus tremula L.	0	n	1	1	Salicaceae Mirb.
Iz_3_1009	DJI_0966	Populus Ã canescens (Aiton) Sm.	0	n	1	1	Salicaceae Mirb.
Iz_3_1009	DJI_0966	Potentilla argentea L.	0	n	1	0	Rosaceae Juss.
Iz_3_1009	DJI_0966	Psammophiliella muralis (L.) Ikonn.	0	n	1	0	Caryophyllaceae Juss.
Iz_3_1009	DJI_0966	Psephellus sumensis (Kalen.) Greuter	0	n	1	0	Asteraceae Bercht. & J.Presl
Iz_3_1009	DJI_0966	Pulsatilla pratensis (L.) Mill. s.l.	0	n	1	0	Ranunculaceae Juss.
Iz_3_1009	DJI_0966	Pyrus communis L.	0	a	0	1	Rosaceae Juss.
Iz_3_1009	DJI_0966	Quercus robur L.	0	n	1	1	Fagaceae Dumort.
Iz_3_1009	DJI_0966	Robinia pseudoacacia L.	0	a	0	1	Fabaceae Lindl.
Iz_3_1009	DJI_0966	Rosa sp.	0		NA	1	Rosaceae Juss.
Iz_3_1009	DJI_0966	Rubus idaeus L.	0	n	1	0	Rosaceae Juss.
Iz_3_1009	DJI_0966	Rumex acetosella L.	0	n	1	0	Polygonaceae Juss.
Iz_3_1009	DJI_0966	Rumex thyrsiflorus Fingerh.	0	n	1	0	Polygonaceae Juss.
Iz_3_1009	DJI_0966	Salix alba L.	0	n	1	1	Salicaceae Mirb.
Iz_3_1009	DJI_0966	Sambucus nigra L.	0	n	1	1	Viburnaceae Raf.
Iz_3_1009	DJI_0966	Secale sylvestre Host	0	n	1	0	Poaceae Barnhart
Iz_3_1009	DJI_0966	Sempervivum ruthenicum Schnittsp. & C.B. Lehm.	0	n	1	0	Crassulaceae J.St.-Hil.
Iz_3_1009	DJI_0966	Setaria viridis (L.) P. Beauv.	0	n	1	0	Poaceae Barnhart
Iz_3_1009	DJI_0966	Silene borysthenica (Gruner) Walters	0	n	1	0	Caryophyllaceae Juss.
Iz_3_1009	DJI_0966	Silene latifolia subsp. alba (Mill.) Greuter & Burdet	0	n	1	0	Caryophyllaceae Juss.
Iz_3_1009	DJI_0966	Solidago virgaurea L.	0	n	1	0	Asteraceae Bercht. & J.Presl
Iz_3_1009	DJI_0966	Sonchus oleraceus L.	0	a	0	0	Asteraceae Bercht. & J.Presl
Iz_3_1009	DJI_0966	Sorbus aucuparia L.	0	n	1	1	Rosaceae Juss.
Iz_3_1009	DJI_0966	Stachys recta L.	0	n	1	0	Lamiacea Lindl.
Iz_3_1009	DJI_0966	Tanacetum vulgare L.	0	n	1	0	Asteraceae Bercht. & J.Presl
Iz_3_1009	DJI_0966	Thymus pallasianus Heinr.Braun	0	n	1	0	Lamiacea Lindl.
Iz_3_1009	DJI_0966	Tilia cordata Mill.	0	n	1	1	Malvaceae Juss.
Iz_3_1009	DJI_0966	Trifolium alpestre L.	0	n	1	0	Fabaceae Lindl.
Iz_3_1009	DJI_0966	Trifolium arvense L.	0	n	1	0	Fabaceae Lindl.
Iz_3_1009	DJI_0966	Ulmus glabra Huds.	0	n	1	1	Ulmaceae Mirb.
Iz_3_1009	DJI_0966	Verbascum densiflorum Bertol.	0	n	1	0	Scrophulariaceae Juss.
Iz_3_1009	DJI_0966	Veronica austriaca L.	0	n	1	0	Plantaginaceae Juss.
Iz_3_1009	DJI_0966	Veronica incana L.	0	n	1	0	Plantaginaceae Juss.
Iz_3_1009	DJI_0966	Veronica verna L.	0	n	1	0	Plantaginaceae Juss.
Iz_3_1009	DJI_0966	Vincetoxicum hirundinaria Medik	0	n	1	0	Apocynaceae Juss.
Iz_3_1009	DJI_0969	Acer tataricum L.	0	n	1	1	Sapindaceae Juss.
Iz_3_1009	DJI_0969	Achillea millefolium L. s.l.	0	n	1	0	Asteraceae Bercht. & J.Presl
Iz_3_1009	DJI_0969	Allium oleraceum L.	0	n	1	0	Amaryllidaceae J.St.-Hil.
Iz_3_1009	DJI_0969	Ambrosia artemisiifolia L.	0	a	0	0	Asteraceae Bercht. & J.Presl
Iz_3_1009	DJI_0969	Amorpha fruticosa L.	0	a	0	0	Fabaceae Lindl.
Iz_3_1009	DJI_0969	Anthericum ramosum L.	0	n	1	0	Asparagaceae Juss.
Iz_3_1009	DJI_0969	Artemisia marschalliana Spreng.	0	n	1	0	Asteraceae Bercht. & J.Presl
Iz_3_1009	DJI_0969	Asparagus officinalis L.	0	n	1	0	Asparagaceae Juss.
Iz_3_1009	DJI_0969	Astragalus cicer L.	0	n	1	0	Fabaceae Lindl.
Iz_3_1009	DJI_0969	Bassia laniflora (S.G.Gmel.) A.J.Scott	0	n	1	0	Amaranthaceae Juss.
Iz_3_1009	DJI_0969	Berberis vulgaris L.	0	n	1	0	Berberidaceae Juss.
Iz_3_1009	DJI_0969	Betula pendula Roth.	0	n	1	1	Betulaceae Gray
Iz_3_1009	DJI_0969	Betula pendula Roth.	0	n	1	0	Poaceae Barnhart
Iz_3_1009	DJI_0969	Bromus inermis Leyss.	0		NA	NA	
Iz_3_1009	DJI_0969	Bromus squarrosus L.	0	a	0	0	Poaceae Barnhart
Iz_3_1009	DJI_0969	Bromus tectorum L.	0	a	0	0	Poaceae Barnhart
Iz_3_1009	DJI_0969	Calamagrostis epigejos (L.) Roth	20	n	1	0	Poaceae Barnhart
Iz_3_1009	DJI_0969	Carduus acanthoides L.	0	n	1	0	Asteraceae Bercht. & J.Presl
Iz_3_1009	DJI_0969	Carex sp.	0	n	1	0	Cyperaceae Juss.
Iz_3_1009	DJI_0969	Centaurea majorovii Dumbadze	0	n	1	0	Asteraceae Bercht. & J.Presl
Iz_3_1009	DJI_0969	Chamaecytisus ruthenicus (Fisch.ex Wol.) Klaskova	0	n	1	0	Fabaceae Lindl.
Iz_3_1009	DJI_0969	Chelidonium majus L.	0.5	n	1	0	Papaveraceae Juss.
Iz_3_1009	DJI_0969	Chenopodium betaceum Andrz.	0	n	1	0	Amaranthaceae Juss.
Iz_3_1009	DJI_0969	Chenopodium cf. album	0	n	1	0	Amaranthaceae Juss.
Iz_3_1009	DJI_0969	Chenopodium sp.	0		NA	0	Amaranthaceae Juss.
Iz_3_1009	DJI_0969	Chondrilla juncea L.	0	n	1	0	Asteraceae Bercht. & J.Presl
Iz_3_1009	DJI_0969	Cirsium vulgare (Savi) Ten.	0	n	1	0	Asteraceae Bercht. & J.Presl
Iz_3_1009	DJI_0969	Clinopodium acinos (L.) Kuntze	0	n	1	0	Lamiacea Lindl.
Iz_3_1009	DJI_0969	Convallaria majalis L.	0	n	1	0	Asparagaceae Juss.
Iz_3_1009	DJI_0969	Crataegus rhipidophylla Gand.	0	n	1	1	Rosaceae Juss.
Iz_3_1009	DJI_0969	Crepis foetida L.	0	n	1	0	Asteraceae Bercht. & J.Presl
Iz_3_1009	DJI_0969	Dactylis glomerata L.	0	n	1	0	Poaceae Barnhart
Iz_3_1009	DJI_0969	Dianthus campestris M.Bieb.	0	n	1	0	Caryophyllaceae Juss.
Iz_3_1009	DJI_0969	Digitaria sanguinalis (L.) Scop.	0	a	0	0	Poaceae Barnhart
Iz_3_1009	DJI_0969	Elymus repens (L.) Gould	0	n	1	0	Poaceae Barnhart
Iz_3_1009	DJI_0969	Epilobium tetragonum L.	0	n	1	0	Onagraceae Juss.
Iz_3_1009	DJI_0969	Eragrostis minor Host	0	a	0	0	Poaceae Barnhart
Iz_3_1009	DJI_0969	Erigeron canadensis L.	0.5	a	0	0	Asteraceae Bercht. & J.Presl
Iz_3_1009	DJI_0969	Euphorbia seguieriana Neck.	0	n	1	0	Euphorbiaceae Juss.
Iz_3_1009	DJI_0969	Fallopia dumetorum (L.) Holub	1	n	1	0	Polygonaceae Juss.

Iz_3_1009	DJI_0969	Festuca beckeri (Hack.) Trautv.	0	n	1	0	Poaceae Barnhart
Iz_3_1009	DJI_0969	Fragaria vesca L.	0	n	1	0	Rosaceae Juss.
Iz_3_1009	DJI_0969	Frangula alnus Mill.	0	n	1	1	Rhamnaceae Juss.
Iz_3_1009	DJI_0969	Fraxinus pennsylvanica Marshall	0	a	0	1	Oleaceae Hoffmanns. & Link
Iz_3_1009	DJI_0969	Galeopsis bifida Boenn.	0	n	1	0	Lamiacea Lindl.
Iz_3_1009	DJI_0969	Galium verum L.	0	n	1	0	Rubiaceae Juss.
Iz_3_1009	DJI_0969	Genista tinctoria L.	0	n	1	0	Fabaceae Lindl.
Iz_3_1009	DJI_0969	Geranium robertianum L.	0	n	1	0	Geraniaceae Juss.
Iz_3_1009	DJI_0969	Geranium sanquineum L.	0	n	1	0	Geraniaceae Juss.
Iz_3_1009	DJI_0969	Gypsophila paniculata L.	0	n	1	0	Caryophyllaceae Juss.
Iz_3_1009	DJI_0969	Helichrysum arenarium (L.) Moench	0	n	1	0	Asteraceae Bercht. & J.Presl
Iz_3_1009	DJI_0969	Herniaria polygama J.Gay	0	n	1	0	Caryophyllaceae Juss.
Iz_3_1009	DJI_0969	Hieracium robustum Fr.	0	n	1	0	Asteraceae Bercht. & J.Presl
Iz_3_1009	DJI_0969	Hieracium umbellatum L.	0	n	1	NA	Asteraceae Bercht. & J.Presl
Iz_3_1009	DJI_0969	Hylotelephium maximum (L.) Holub	0	n	1	0	Crassulaceae J.St.-Hil.
Iz_3_1009	DJI_0969	Jacobaea borysthenica (DC.) B.Nord. & Greuter	0	n	1	0	Asteraceae Bercht. & J.Presl
Iz_3_1009	DJI_0969	Jasione montana L.	0	n	1	0	Campanulaceae Juss.
Iz_3_1009	DJI_0969	Jurinea cyanoides (L.) Rchb.s.l.	0	n	1	0	Asteraceae Bercht. & J.Presl
Iz_3_1009	DJI_0969	Koeleria glauca (Spreng.) DC.	0	n	1	0	Poaceae Barnhart
Iz_3_1009	DJI_0969	Lactuca serriola L.	0	a	0	0	Asteraceae Bercht. & J.Presl
Iz_3_1009	DJI_0969	Linaria genistifolia (L.) Mill.	0	n	1	0	Plantaginaceae Juss.
Iz_3_1009	DJI_0969	Lomelosia argentea (L.) Greuter & Burdet	0	n	1	0	Caprifoliaceae Juss.
Iz_3_1009	DJI_0969	Melampyrum pratense L.	0	n	1	0	Orobanchaceae Vent.
Iz_3_1009	DJI_0969	Oenothera biennis L.	0	a	0	0	Onagraceae Juss.
Iz_3_1009	DJI_0969	Pentanema britannica (L.) D.Gut.Larr., Santos-Vicente, Anderb., E.Rico & M.M.Mart.Ort.	0	n	1	0	Asteraceae Bercht. & J.Presl
Iz_3_1009	DJI_0969	Peucedanum oreoselinum (L.) Moench	0	n	1	0	Apiaceae Lindl.
Iz_3_1009	DJI_0969	Peucedanum ruthenicum M.Bieb.	0	n	1	0	Apiaceae Lindl.
Iz_3_1009	DJI_0969	Pilosella echioides (Lumn.) F.W.Schultz & Sch.Bip.	0	n	1	0	Asteraceae Bercht. & J.Presl
Iz_3_1009	DJI_0969	Pilosella officinarum F. Schultz et Sch. Bip.	0	n	1	0	Asteraceae Bercht. & J.Presl
Iz_3_1009	DJI_0969	Pilosella sp.	0		NA	0	Asteraceae Bercht. & J.Presl
Iz_3_1009	DJI_0969	Pinus sylvestris L.	0	n	1	1	Pinaceae Spreng. ex F.Rudolphi
Iz_3_1009	DJI_0969	Poa annua L.	0	n	1	0	Poaceae Barnhart
Iz_3_1009	DJI_0969	Poa nemoralis L.	0	n	1	0	Poaceae Barnhart
Iz_3_1009	DJI_0969	Poa sp.	0		NA	0	Poaceae Barnhart
Iz_3_1009	DJI_0969	Polygonatum odoratum (Mill.) Druce	0	n	1	0	Asparagaceae Juss.
Iz_3_1009	DJI_0969	Polygonum arenarium Waldst. & Kit.	0	n	1	0	Polygonaceae Juss.
Iz_3_1009	DJI_0969	Populus nigra L.	0	n	1	1	Salicaceae Mirb.
Iz_3_1009	DJI_0969	Populus tremula L.	0	n	1	1	Salicaceae Mirb.
Iz_3_1009	DJI_0969	Populus Ã canescens (Aiton) Sm.	0	n	1	1	Salicaceae Mirb.
Iz_3_1009	DJI_0969	Potentilla argentea L.	0	n	1	0	Rosaceae Juss.
Iz_3_1009	DJI_0969	Psammophiliella muralis (L.) Ikonn.	0	n	1	0	Caryophyllaceae Juss.
Iz_3_1009	DJI_0969	Psephellus sumensis (Kalen.) Greuter	0	n	1	0	Asteraceae Bercht. & J.Presl
Iz_3_1009	DJI_0969	Pulsatilla pratensis (L.) Mill. s.l.	0	n	1	0	Ranunculaceae Juss.
Iz_3_1009	DJI_0969	Pyrus communis L.	0	a	0	1	Rosaceae Juss.
Iz_3_1009	DJI_0969	Quercus robur L.	10	n	1	1	Fagaceae Dumort.
Iz_3_1009	DJI_0969	Robinia pseudoacacia L.	0	a	0	1	Fabaceae Lindl.
Iz_3_1009	DJI_0969	Rosa sp.	0		NA	1	Rosaceae Juss.
Iz_3_1009	DJI_0969	Rubus idaeus L.	0	n	1	0	Rosaceae Juss.
Iz_3_1009	DJI_0969	Rumex acetosella L.	0	n	1	0	Polygonaceae Juss.
Iz_3_1009	DJI_0969	Rumex thyrsiflorus Fingerh.	0	n	1	0	Polygonaceae Juss.
Iz_3_1009	DJI_0969	Salix alba L.	0	n	1	1	Salicaceae Mirb.
Iz_3_1009	DJI_0969	Sambucus nigra L.	0	n	1	1	Viburnaceae Raf.
Iz_3_1009	DJI_0969	Secale sylvestre Host	0	n	1	0	Poaceae Barnhart
Iz_3_1009	DJI_0969	Sempervivum ruthenicum Schnittsp. & C.B. Lehm.	0	n	1	0	Crassulaceae J.St.-Hil.
Iz_3_1009	DJI_0969	Setaria viridis (L.) P. Beauv.	0	n	1	0	Poaceae Barnhart
Iz_3_1009	DJI_0969	Silene borysthenica (Gruner) Walters	0	n	1	0	Caryophyllaceae Juss.
Iz_3_1009	DJI_0969	Silene latifolia subsp. alba (Mill.) Greuter & Burdet	0	n	1	0	Caryophyllaceae Juss.
Iz_3_1009	DJI_0969	Solidago virgaurea L.	0	n	1	0	Asteraceae Bercht. & J.Presl
Iz_3_1009	DJI_0969	Sonchus oleraceus L.	0	a	0	0	Asteraceae Bercht. & J.Presl
Iz_3_1009	DJI_0969	Sorbus aucuparia L.	0	n	1	1	Rosaceae Juss.
Iz_3_1009	DJI_0969	Stachys recta L.	0	n	1	0	Lamiacea Lindl.
Iz_3_1009	DJI_0969	Tanacetum vulgare L.	0	n	1	0	Asteraceae Bercht. & J.Presl
Iz_3_1009	DJI_0969	Thymus pallasianus Heinr.Braun	0	n	1	0	Lamiacea Lindl.
Iz_3_1009	DJI_0969	Tilia cordata Mill.	0	n	1	1	Malvaceae Juss.
Iz_3_1009	DJI_0969	Trifolium alpestre L.	0	n	1	0	Fabaceae Lindl.
Iz_3_1009	DJI_0969	Trifolium arvense L.	0	n	1	0	Fabaceae Lindl.
Iz_3_1009	DJI_0969	Ulmus glabra Huds.	0	n	1	1	Ulmaceae Mirb.
Iz_3_1009	DJI_0969	Verbascum densiflorum Bertol.	0	n	1	0	Scrophulariaceae Juss.
Iz_3_1009	DJI_0969	Veronica austriaca L.	0	n	1	0	Plantaginaceae Juss.
Iz_3_1009	DJI_0969	Veronica incana L.	0	n	1	0	Plantaginaceae Juss.
Iz_3_1009	DJI_0969	Veronica verna L.	0	n	1	0	Plantaginaceae Juss.
Iz_3_1009	DJI_0969	Vincetoxicum hirundinaria Medik	0	n	1	0	Apocynaceae Juss.
Iz_3_1009	DJI_0977	Acer tataricum L.	0	n	1	1	Sapindaceae Juss.
Iz_3_1009	DJI_0977	Achillea millefolium L. s.l.	0	n	1	0	Asteraceae Bercht. & J.Presl
Iz_3_1009	DJI_0977	Allium oleraceum L.	0	n	1	0	Amaryllidaceae J.St.-Hil.
Iz_3_1009	DJI_0977	Ambrosia artemisiifolia L.	0	a	0	0	Asteraceae Bercht. & J.Presl
Iz_3_1009	DJI_0977	Amorpha fruticosa L.	0	a	0	0	Fabaceae Lindl.
Iz_3_1009	DJI_0977	Anthericum ramosum L.	0	n	1	0	Asparagaceae Juss.
Iz_3_1009	DJI_0977	Artemisia marschalliana Spreng.	0	n	1	0	Asteraceae Bercht. & J.Presl
Iz_3_1009	DJI_0977	Asparagus officinalis L.	0	n	1	0	Asparagaceae Juss.
Iz_3_1009	DJI_0977	Astragalus cicer L.	0	n	1	0	Fabaceae Lindl.
Iz_3_1009	DJI_0977	Bassia laniflora (S.G.Gmel.) A.J.Scott	0	n	1	0	Amaranthaceae Juss.
Iz_3_1009	DJI_0977	Berberis vulgaris L.	0	n	1	0	Berberidaceae Juss.
Iz_3_1009	DJI_0977	Betula pendula Roth.	0	n	1	1	Betulaceae Gray
Iz_3_1009	DJI_0977	Betula pendula Roth.	0	n	1	0	Poaceae Barnhart
Iz_3_1009	DJI_0977	Bromus inermis Leyss.	0		NA	NA	
Iz_3_1009	DJI_0977	Bromus squarrosus L.	0	a	0	0	Poaceae Barnhart
Iz_3_1009	DJI_0977	Bromus tectorum L.	0	a	0	0	Poaceae Barnhart
Iz_3_1009	DJI_0977	Calamagrostis epigejos (L.) Roth	80	n	1	0	Poaceae Barnhart
Iz_3_1009	DJI_0977	Carduus acanthoides L.	0	n	1	0	Asteraceae Bercht. & J.Presl
Iz_3_1009	DJI_0977	Carex sp.	0	n	1	0	Cyperaceae Juss.
Iz_3_1009	DJI_0977	Centaurea majorovii Dumbadze	0	n	1	0	Asteraceae Bercht. & J.Presl
Iz_3_1009	DJI_0977	Chamaecytisus ruthenicus (Fisch.ex Wol.) Klaskova	0	n	1	0	Fabaceae Lindl.
Iz_3_1009	DJI_0977	Chelidonium majus L.	0	n	1	0	Papaveraceae Juss.
Iz_3_1009	DJI_0977	Chenopodium betaceum Andrz.	0	n	1	0	Amaranthaceae Juss.
Iz_3_1009	DJI_0977	Chenopodium cf. album	0	n	1	0	Amaranthaceae Juss.
Iz_3_1009	DJI_0977	Chenopodium sp.	0		NA	0	Amaranthaceae Juss.
Iz_3_1009	DJI_0977	Chondrilla juncea L.	0	n	1	0	Asteraceae Bercht. & J.Presl

Iz_3_1009	DJI_0977	Cirsium vulgare (Savi) Ten.	0	n	1	0	Asteraceae Bercht. & J.Presl
Iz_3_1009	DJI_0977	Clinopodium acinos (L.) Kuntze	0	n	1	0	Lamiacea Lindl.
Iz_3_1009	DJI_0977	Convallaria majalis L.	0	n	1	0	Asparagaceae Juss.
Iz_3_1009	DJI_0977	Crataegus rhipidophylla Gand.	0	n	1	1	Rosaceae Juss.
Iz_3_1009	DJI_0977	Crepis foetida L.	0	n	1	0	Asteraceae Bercht. & J.Presl
Iz_3_1009	DJI_0977	Dactylis glomerata L.	0	n	1	0	Poaceae Barnhart
Iz_3_1009	DJI_0977	Dianthus campestris M.Bieb.	0	n	1	0	Caryophyllaceae Juss.
Iz_3_1009	DJI_0977	Digitaria sanguinalis (L.) Scop.	0	a	0	0	Poaceae Barnhart
Iz_3_1009	DJI_0977	Elymus repens (L.) Gould	0	n	1	0	Poaceae Barnhart
Iz_3_1009	DJI_0977	Epilobium tetragonum L.	0	n	1	0	Onagraceae Juss.
Iz_3_1009	DJI_0977	Eragrostis minor Host	0	a	0	0	Poaceae Barnhart
Iz_3_1009	DJI_0977	Erigeron canadensis L.	0	a	0	0	Asteraceae Bercht. & J.Presl
Iz_3_1009	DJI_0977	Euphorbia seguieriana Neck.	0	n	1	0	Euphorbiaceae Juss.
Iz_3_1009	DJI_0977	Fallopia dumetorum (L.) Holub	0	n	1	0	Polygonaceae Juss.
Iz_3_1009	DJI_0977	Festuca beckeri (Hack.) Trautv.	0	n	1	0	Poaceae Barnhart
Iz_3_1009	DJI_0977	Fragaria vesca L.	0	n	1	0	Rosaceae Juss.
Iz_3_1009	DJI_0977	Frangula alnus Mill.	0	n	1	1	Rhamnaceae Juss.
Iz_3_1009	DJI_0977	Fraxinus pennsylvanica Marshall	0	a	0	1	Oleaceae Hoffmanns. & Link
Iz_3_1009	DJI_0977	Galeopsis bifida Boenn.	0	n	1	0	Lamiacea Lindl.
Iz_3_1009	DJI_0977	Galium verum L.	0	n	1	0	Rubiaceae Juss.
Iz_3_1009	DJI_0977	Genista tinctoria L.	0	n	1	0	Fabaceae Lindl.
Iz_3_1009	DJI_0977	Geranium robertianum L.	0	n	1	0	Geraniaceae Juss.
Iz_3_1009	DJI_0977	Geranium sanquineum L.	0	n	1	0	Geraniaceae Juss.
Iz_3_1009	DJI_0977	Gypsophila paniculata L.	0	n	1	0	Caryophyllaceae Juss.
Iz_3_1009	DJI_0977	Helichrysum arenarium (L.) Moench	0	n	1	0	Asteraceae Bercht. & J.Presl
Iz_3_1009	DJI_0977	Herniaria polygama J.Gay	0	n	1	0	Caryophyllaceae Juss.
Iz_3_1009	DJI_0977	Hieracium robustum Fr.	0	n	1	0	Asteraceae Bercht. & J.Presl
Iz_3_1009	DJI_0977	Hieracium umbellatum L.	0	n	1	NA	Asteraceae Bercht. & J.Presl
Iz_3_1009	DJI_0977	Hylotelephium maximum (L.) Holub	0	n	1	0	Crassulaceae J.St.-Hil.
Iz_3_1009	DJI_0977	Jacobaea borysthenica (DC.) B.Nord. & Greuter	0	n	1	0	Asteraceae Bercht. & J.Presl
Iz_3_1009	DJI_0977	Jasione montana L.	0	n	1	0	Campanulaceae Juss.
Iz_3_1009	DJI_0977	Jurinea cyanoides (L.) Rchb.s.l.	0	n	1	0	Asteraceae Bercht. & J.Presl
Iz_3_1009	DJI_0977	Koeleria glauca (Spreng.) DC.	0	n	1	0	Poaceae Barnhart
Iz_3_1009	DJI_0977	Lactuca serriola L.	0	a	0	0	Asteraceae Bercht. & J.Presl
Iz_3_1009	DJI_0977	Linaria genistifolia (L.) Mill.	0	n	1	0	Plantaginaceae Juss.
Iz_3_1009	DJI_0977	Lomelosia argentea (L.) Greuter & Burdet	0	n	1	0	Caprifoliaceae Juss.
Iz_3_1009	DJI_0977	Melampyrum pratense L.	0	n	1	0	Orobanchaceae Vent.
Iz_3_1009	DJI_0977	Oenothera biennis L.	0	a	0	0	Onagraceae Juss.
Iz_3_1009	DJI_0977	Pentanema britannica (L.) D.Gut.Larr., Santos-Vicente, Anderb., E.Rico & M.M.Mart.Ort.	0	n	1	0	Asteraceae Bercht. & J.Presl
Iz_3_1009	DJI_0977	Peucedanum oreoselinum (L.) Moench	10	n	1	0	Apiaceae Lindl.
Iz_3_1009	DJI_0977	Peucedanum ruthenicum M.Bieb.	0	n	1	0	Apiaceae Lindl.
Iz_3_1009	DJI_0977	Pilosella echioides (Lumn.) F.W.Schultz & Sch.Bip.	0	n	1	0	Asteraceae Bercht. & J.Presl
Iz_3_1009	DJI_0977	Pilosella officinarum F. Schultz et Sch. Bip.	0	n	1	0	Asteraceae Bercht. & J.Presl
Iz_3_1009	DJI_0977	Pilosella sp.	0		NA	0	Asteraceae Bercht. & J.Presl
Iz_3_1009	DJI_0977	Pinus sylvestris L.	0	n	1	1	Pinaceae Spreng. ex F.Rudolphi
Iz_3_1009	DJI_0977	Poa annua L.	0	n	1	0	Poaceae Barnhart
Iz_3_1009	DJI_0977	Poa nemoralis L.	0	n	1	0	Poaceae Barnhart
Iz_3_1009	DJI_0977	Poa sp.	0		NA	0	Poaceae Barnhart
Iz_3_1009	DJI_0977	Polygonatum odoratum (Mill.) Druce	0.5	n	1	0	Asparagaceae Juss.
Iz_3_1009	DJI_0977	Polygonum arenarium Waldst. & Kit.	0	n	1	0	Polygonaceae Juss.
Iz_3_1009	DJI_0977	Populus nigra L.	0	n	1	1	Salicaceae Mirb.
Iz_3_1009	DJI_0977	Populus tremula L.	0	n	1	1	Salicaceae Mirb.
Iz_3_1009	DJI_0977	Populus Ã canescens (Aiton) Sm.	0	n	1	1	Salicaceae Mirb.
Iz_3_1009	DJI_0977	Potentilla argentea L.	0	n	1	0	Rosaceae Juss.
Iz_3_1009	DJI_0977	Psammophiliella muralis (L.) Ikonn.	0	n	1	0	Caryophyllaceae Juss.
Iz_3_1009	DJI_0977	Psephellus sumensis (Kalen.) Greuter	0	n	1	0	Asteraceae Bercht. & J.Presl
Iz_3_1009	DJI_0977	Pulsatilla pratensis (L.) Mill. s.l.	0	n	1	0	Ranunculaceae Juss.
Iz_3_1009	DJI_0977	Pyrus communis L.	0	a	0	1	Rosaceae Juss.
Iz_3_1009	DJI_0977	Quercus robur L.	0	n	1	1	Fagaceae Dumort.
Iz_3_1009	DJI_0977	Robinia pseudoacacia L.	0	a	0	1	Fabaceae Lindl.
Iz_3_1009	DJI_0977	Rosa sp.	0		NA	1	Rosaceae Juss.
Iz_3_1009	DJI_0977	Rubus idaeus L.	0	n	1	0	Rosaceae Juss.
Iz_3_1009	DJI_0977	Rumex acetosella L.	0	n	1	0	Polygonaceae Juss.
Iz_3_1009	DJI_0977	Rumex thyrsiflorus Fingerh.	0	n	1	0	Polygonaceae Juss.
Iz_3_1009	DJI_0977	Salix alba L.	0	n	1	1	Salicaceae Mirb.
Iz_3_1009	DJI_0977	Sambucus nigra L.	0	n	1	1	Viburnaceae Raf.
Iz_3_1009	DJI_0977	Secale sylvestre Host	0	n	1	0	Poaceae Barnhart
Iz_3_1009	DJI_0977	Sempervivum ruthenicum Schnittsp. & C.B. Lehm.	0	n	1	0	Crassulaceae J.St.-Hil.
Iz_3_1009	DJI_0977	Setaria viridis (L.) P. Beauv.	0	n	1	0	Poaceae Barnhart
Iz_3_1009	DJI_0977	Silene borysthenica (Gruner) Walters	0	n	1	0	Caryophyllaceae Juss.
Iz_3_1009	DJI_0977	Silene latifolia subsp. alba (Mill.) Greuter & Burdet	0	n	1	0	Caryophyllaceae Juss.
Iz_3_1009	DJI_0977	Solidago virgaurea L.	0	n	1	0	Asteraceae Bercht. & J.Presl
Iz_3_1009	DJI_0977	Sonchus oleraceus L.	0	a	0	0	Asteraceae Bercht. & J.Presl
Iz_3_1009	DJI_0977	Sorbus aucuparia L.	0	n	1	1	Rosaceae Juss.
Iz_3_1009	DJI_0977	Stachys recta L.	0	n	1	0	Lamiacea Lindl.
Iz_3_1009	DJI_0977	Tanacetum vulgare L.	0	n	1	0	Asteraceae Bercht. & J.Presl
Iz_3_1009	DJI_0977	Thymus pallasianus Heinr.Braun	0	n	1	0	Lamiacea Lindl.
Iz_3_1009	DJI_0977	Tilia cordata Mill.	0	n	1	1	Malvaceae Juss.
Iz_3_1009	DJI_0977	Trifolium alpestre L.	0	n	1	0	Fabaceae Lindl.
Iz_3_1009	DJI_0977	Trifolium arvense L.	0	n	1	0	Fabaceae Lindl.
Iz_3_1009	DJI_0977	Ulmus glabra Huds.	0	n	1	1	Ulmaceae Mirb.
Iz_3_1009	DJI_0977	Verbascum densiflorum Bertol.	0	n	1	0	Scrophulariaceae Juss.
Iz_3_1009	DJI_0977	Veronica austriaca L.	0	n	1	0	Plantaginaceae Juss.
Iz_3_1009	DJI_0977	Veronica incana L.	0	n	1	0	Plantaginaceae Juss.
Iz_3_1009	DJI_0977	Veronica verna L.	0	n	1	0	Plantaginaceae Juss.
Iz_3_1009	DJI_0977	Vincetoxicum hirundinaria Medik	0	n	1	0	Apocynaceae Juss.
Iz_3_1009	DJI_0978	Acer tataricum L.	0	n	1	1	Sapindaceae Juss.
Iz_3_1009	DJI_0978	Achillea millefolium L. s.l.	0	n	1	0	Asteraceae Bercht. & J.Presl
Iz_3_1009	DJI_0978	Allium oleraceum L.	0	n	1	0	Amaryllidaceae J.St.-Hil.
Iz_3_1009	DJI_0978	Ambrosia artemisiifolia L.	0	a	0	0	Asteraceae Bercht. & J.Presl
Iz_3_1009	DJI_0978	Amorpha fruticosa L.	0	a	0	0	Fabaceae Lindl.
Iz_3_1009	DJI_0978	Anthericum ramosum L.	0	n	1	0	Asparagaceae Juss.
Iz_3_1009	DJI_0978	Artemisia marschalliana Spreng.	0	n	1	0	Asteraceae Bercht. & J.Presl
Iz_3_1009	DJI_0978	Asparagus officinalis L.	0	n	1	0	Asparagaceae Juss.
Iz_3_1009	DJI_0978	Astragalus cicer L.	0	n	1	0	Fabaceae Lindl.
Iz_3_1009	DJI_0978	Bassia laniflora (S.G.Gmel.) A.J.Scott	0	n	1	0	Amaranthaceae Juss.
Iz_3_1009	DJI_0978	Berberis vulgaris L.	0	n	1	0	Berberidaceae Juss.
Iz_3_1009	DJI_0978	Betula pendula Roth.	0	n	1	1	Betulaceae Gray

Iz_3_1009	DJI_0978	Betula pendula Roth.	0	n	1	0	Poaceae Barnhart
Iz_3_1009	DJI_0978	Bromus inermis Leyss.	0		NA	NA	
Iz_3_1009	DJI_0978	Bromus squarrosus L.	0	a	0	0	Poaceae Barnhart
Iz_3_1009	DJI_0978	Bromus tectorum L.	0	a	0	0	Poaceae Barnhart
Iz_3_1009	DJI_0978	Calamagrostis epigejos (L.) Roth	80	n	1	0	Poaceae Barnhart
Iz_3_1009	DJI_0978	Carduus acanthoides L.	0	n	1	0	Asteraceae Bercht. & J.Presl
Iz_3_1009	DJI_0978	Carex sp.	0	n	1	0	Cyperaceae Juss.
Iz_3_1009	DJI_0978	Centaurea majorovii Dumbadze	0	n	1	0	Asteraceae Bercht. & J.Presl
Iz_3_1009	DJI_0978	Chamaecytisus ruthenicus (Fisch.ex Wol.) Klaskova	0	n	1	0	Fabaceae Lindl.
Iz_3_1009	DJI_0978	Chelidonium majus L.	0	n	1	0	Papaveraceae Juss.
Iz_3_1009	DJI_0978	Chenopodium betaceum Andrz.	0	n	1	0	Amaranthaceae Juss.
Iz_3_1009	DJI_0978	Chenopodium cf. album	0	n	1	0	Amaranthaceae Juss.
Iz_3_1009	DJI_0978	Chenopodium sp.	0		NA	0	Amaranthaceae Juss.
Iz_3_1009	DJI_0978	Chondrilla juncea L.	0	n	1	0	Asteraceae Bercht. & J.Presl
Iz_3_1009	DJI_0978	Cirsium vulgare (Savi) Ten.	0	n	1	0	Asteraceae Bercht. & J.Presl
Iz_3_1009	DJI_0978	Clinopodium acinos (L.) Kuntze	0	n	1	0	Lamiacea Lindl.
Iz_3_1009	DJI_0978	Convallaria majalis L.	0	n	1	0	Asparagaceae Juss.
Iz_3_1009	DJI_0978	Crataegus rhipidophylla Gand.	0	n	1	1	Rosaceae Juss.
Iz_3_1009	DJI_0978	Crepis foetida L.	0	n	1	0	Asteraceae Bercht. & J.Presl
Iz_3_1009	DJI_0978	Dactylis glomerata L.	0	n	1	0	Poaceae Barnhart
Iz_3_1009	DJI_0978	Dianthus campestris M.Bieb.	0	n	1	0	Caryophyllaceae Juss.
Iz_3_1009	DJI_0978	Digitaria sanguinalis (L.) Scop.	0	a	0	0	Poaceae Barnhart
Iz_3_1009	DJI_0978	Elymus repens (L.) Gould	0	n	1	0	Poaceae Barnhart
Iz_3_1009	DJI_0978	Epilobium tetragonum L.	0	n	1	0	Onagraceae Juss.
Iz_3_1009	DJI_0978	Eragrostis minor Host	0	a	0	0	Poaceae Barnhart
Iz_3_1009	DJI_0978	Erigeron canadensis L.	0	a	0	0	Asteraceae Bercht. & J.Presl
Iz_3_1009	DJI_0978	Euphorbia seguieriana Neck.	0	n	1	0	Euphorbiaceae Juss.
Iz_3_1009	DJI_0978	Fallopia dumetorum (L.) Holub	0	n	1	0	Polygonaceae Juss.
Iz_3_1009	DJI_0978	Festuca beckeri (Hack.) Trautv.	0	n	1	0	Poaceae Barnhart
Iz_3_1009	DJI_0978	Fragaria vesca L.	0	n	1	0	Rosaceae Juss.
Iz_3_1009	DJI_0978	Frangula alnus Mill.	0	n	1	1	Rhamnaceae Juss.
Iz_3_1009	DJI_0978	Fraxinus pennsylvanica Marshall	0	a	0	1	Oleaceae Hoffmanns. & Link
Iz_3_1009	DJI_0978	Galeopsis bifida Boenn.	0	n	1	0	Lamiacea Lindl.
Iz_3_1009	DJI_0978	Galium verum L.	0	n	1	0	Rubiaceae Juss.
Iz_3_1009	DJI_0978	Genista tinctoria L.	0	n	1	0	Fabaceae Lindl.
Iz_3_1009	DJI_0978	Geranium robertianum L.	0	n	1	0	Geraniaceae Juss.
Iz_3_1009	DJI_0978	Geranium sanquineum L.	0	n	1	0	Geraniaceae Juss.
Iz_3_1009	DJI_0978	Gypsophila paniculata L.	0	n	1	0	Caryophyllaceae Juss.
Iz_3_1009	DJI_0978	Helichrysum arenarium (L.) Moench	0	n	1	0	Asteraceae Bercht. & J.Presl
Iz_3_1009	DJI_0978	Herniaria polygama J.Gay	0	n	1	0	Caryophyllaceae Juss.
Iz_3_1009	DJI_0978	Hieracium robustum Fr.	0	n	1	0	Asteraceae Bercht. & J.Presl
Iz_3_1009	DJI_0978	Hieracium umbellatum L.	0	n	1	NA	Asteraceae Bercht. & J.Presl
Iz_3_1009	DJI_0978	Hylotelephium maximum (L.) Holub	0	n	1	0	Crassulaceae J.St.-Hil.
Iz_3_1009	DJI_0978	Jacobaea borysthenica (DC.) B.Nord. & Greuter	0	n	1	0	Asteraceae Bercht. & J.Presl
Iz_3_1009	DJI_0978	Jasione montana L.	0	n	1	0	Campanulaceae Juss.
Iz_3_1009	DJI_0978	Jurinea cyanoides (L.) Rchb.s.l.	0	n	1	0	Asteraceae Bercht. & J.Presl
Iz_3_1009	DJI_0978	Koeleria glauca (Spreng.) DC.	0	n	1	0	Poaceae Barnhart
Iz_3_1009	DJI_0978	Lactuca serriola L.	0	a	0	0	Asteraceae Bercht. & J.Presl
Iz_3_1009	DJI_0978	Linaria genistifolia (L.) Mill.	0	n	1	0	Plantaginaceae Juss.
Iz_3_1009	DJI_0978	Lomelosia argentea (L.) Greuter & Burdet	0	n	1	0	Caprifoliaceae Juss.
Iz_3_1009	DJI_0978	Melampyrum pratense L.	0	n	1	0	Orobanchaceae Vent.
Iz_3_1009	DJI_0978	Oenothera biennis L.	0	a	0	0	Onagraceae Juss.
Iz_3_1009	DJI_0978	Pentanema britannica (L.) D.Gut.Larr., Santos-Vicente, Anderb., E.Rico & M.M.Mart.Ort.	0	n	1	0	Asteraceae Bercht. & J.Presl
Iz_3_1009	DJI_0978	Peucedanum oreoselinum (L.) Moench	2	n	1	0	Apiaceae Lindl.
Iz_3_1009	DJI_0978	Peucedanum ruthenicum M.Bieb.	0	n	1	0	Apiaceae Lindl.
Iz_3_1009	DJI_0978	Pilosella echioides (Lumn.) F.W.Schultz & Sch.Bip.	0	n	1	0	Asteraceae Bercht. & J.Presl
Iz_3_1009	DJI_0978	Pilosella officinarum F. Schultz et Sch. Bip.	0	n	1	0	Asteraceae Bercht. & J.Presl
Iz_3_1009	DJI_0978	Pilosella sp.	0		NA	0	Asteraceae Bercht. & J.Presl
Iz_3_1009	DJI_0978	Pinus sylvestris L.	0	n	1	1	Pinaceae Spreng. ex F.Rudolphi
Iz_3_1009	DJI_0978	Poa annua L.	0	n	1	0	Poaceae Barnhart
Iz_3_1009	DJI_0978	Poa nemoralis L.	0	n	1	0	Poaceae Barnhart
Iz_3_1009	DJI_0978	Poa sp.	0		NA	0	Poaceae Barnhart
Iz_3_1009	DJI_0978	Polygonatum odoratum (Mill.) Druce	1	n	1	0	Asparagaceae Juss.
Iz_3_1009	DJI_0978	Polygonum arenarium Waldst. & Kit.	0	n	1	0	Polygonaceae Juss.
Iz_3_1009	DJI_0978	Populus nigra L.	0	n	1	1	Salicaceae Mirb.
Iz_3_1009	DJI_0978	Populus tremula L.	0	n	1	1	Salicaceae Mirb.
Iz_3_1009	DJI_0978	Populus Ã canescens (Aiton) Sm.	0	n	1	1	Salicaceae Mirb.
Iz_3_1009	DJI_0978	Potentilla argentea L.	0	n	1	0	Rosaceae Juss.
Iz_3_1009	DJI_0978	Psammophiliella muralis (L.) Ikonn.	0	n	1	0	Caryophyllaceae Juss.
Iz_3_1009	DJI_0978	Psephellus sumensis (Kalen.) Greuter	0	n	1	0	Asteraceae Bercht. & J.Presl
Iz_3_1009	DJI_0978	Pulsatilla pratensis (L.) Mill. s.l.	0	n	1	0	Ranunculaceae Juss.
Iz_3_1009	DJI_0978	Pyrus communis L.	0	a	0	1	Rosaceae Juss.
Iz_3_1009	DJI_0978	Quercus robur L.	5	n	1	1	Fagaceae Dumort.
Iz_3_1009	DJI_0978	Robinia pseudoacacia L.	0	a	0	1	Fabaceae Lindl.
Iz_3_1009	DJI_0978	Rosa sp.	0		NA	1	Rosaceae Juss.
Iz_3_1009	DJI_0978	Rubus idaeus L.	0	n	1	0	Rosaceae Juss.
Iz_3_1009	DJI_0978	Rumex acetosella L.	0	n	1	0	Polygonaceae Juss.
Iz_3_1009	DJI_0978	Rumex thyrsiflorus Fingerh.	0	n	1	0	Polygonaceae Juss.
Iz_3_1009	DJI_0978	Salix alba L.	0	n	1	1	Salicaceae Mirb.
Iz_3_1009	DJI_0978	Sambucus nigra L.	0	n	1	1	Viburnaceae Raf.
Iz_3_1009	DJI_0978	Secale sylvestre Host	0	n	1	0	Poaceae Barnhart
Iz_3_1009	DJI_0978	Sempervivum ruthenicum Schnittsp. & C.B. Lehm.	0	n	1	0	Crassulaceae J.St.-Hil.
Iz_3_1009	DJI_0978	Setaria viridis (L.) P. Beauv.	0	n	1	0	Poaceae Barnhart
Iz_3_1009	DJI_0978	Silene borysthenica (Gruner) Walters	0	n	1	0	Caryophyllaceae Juss.
Iz_3_1009	DJI_0978	Silene latifolia subsp. alba (Mill.) Greuter & Burdet	0	n	1	0	Caryophyllaceae Juss.
Iz_3_1009	DJI_0978	Solidago virgaurea L.	0	n	1	0	Asteraceae Bercht. & J.Presl
Iz_3_1009	DJI_0978	Sonchus oleraceus L.	0	a	0	0	Asteraceae Bercht. & J.Presl
Iz_3_1009	DJI_0978	Sorbus aucuparia L.	0	n	1	1	Rosaceae Juss.
Iz_3_1009	DJI_0978	Stachys recta L.	0	n	1	0	Lamiacea Lindl.
Iz_3_1009	DJI_0978	Tanacetum vulgare L.	0	n	1	0	Asteraceae Bercht. & J.Presl
Iz_3_1009	DJI_0978	Thymus pallasianus Heinr.Braun	0	n	1	0	Lamiacea Lindl.
Iz_3_1009	DJI_0978	Tilia cordata Mill.	0	n	1	1	Malvaceae Juss.
Iz_3_1009	DJI_0978	Trifolium alpestre L.	0	n	1	0	Fabaceae Lindl.
Iz_3_1009	DJI_0978	Trifolium arvense L.	0	n	1	0	Fabaceae Lindl.
Iz_3_1009	DJI_0978	Ulmus glabra Huds.	0	n	1	1	Ulmaceae Mirb.
Iz_3_1009	DJI_0978	Verbascum densiflorum Bertol.	0	n	1	0	Scrophulariaceae Juss.
Iz_3_1009	DJI_0978	Veronica austriaca L.	0	n	1	0	Plantaginaceae Juss.
Iz_3_1009	DJI_0978	Veronica incana L.	0	n	1	0	Plantaginaceae Juss.

Iz_3_1009	DJI_0978	Veronica verna L.	0	n	1	0	Plantaginaceae Juss.
Iz_3_1009	DJI_0978	Vincetoxicum hirundinaria Medik	0	n	1	0	Apocynaceae Juss.
Iz_3_1009	DJI_0979	Acer tataricum L.	0	n	1	1	Sapindaceae Juss.
Iz_3_1009	DJI_0979	Achillea millefolium L. s.l.	0	n	1	0	Asteraceae Bercht. & J.Presl
Iz_3_1009	DJI_0979	Allium oleraceum L.	0	n	1	0	Amaryllidaceae J.St.-Hil.
Iz_3_1009	DJI_0979	Ambrosia artemisiifolia L.	0	a	0	0	Asteraceae Bercht. & J.Presl
Iz_3_1009	DJI_0979	Amorpha fruticosa L.	0	a	0	0	Fabaceae Lindl.
Iz_3_1009	DJI_0979	Anthericum ramosum L.	0	n	1	0	Asparagaceae Juss.
Iz_3_1009	DJI_0979	Artemisia marschalliana Spreng.	0	n	1	0	Asteraceae Bercht. & J.Presl
Iz_3_1009	DJI_0979	Asparagus officinalis L.	0	n	1	0	Asparagaceae Juss.
Iz_3_1009	DJI_0979	Astragalus cicer L.	0	n	1	0	Fabaceae Lindl.
Iz_3_1009	DJI_0979	Bassia laniflora (S.G.Gmel.) A.J.Scott	0	n	1	0	Amaranthaceae Juss.
Iz_3_1009	DJI_0979	Berberis vulgaris L.	0	n	1	0	Berberidaceae Juss.
Iz_3_1009	DJI_0979	Betula pendula Roth.	0	n	1	1	Betulaceae Gray
Iz_3_1009	DJI_0979	Betula pendula Roth.	0	n	1	0	Poaceae Barnhart
Iz_3_1009	DJI_0979	Bromus inermis Leyss.	0		NA	NA	
Iz_3_1009	DJI_0979	Bromus squarrosus L.	0	a	0	0	Poaceae Barnhart
Iz_3_1009	DJI_0979	Bromus tectorum L.	0	a	0	0	Poaceae Barnhart
Iz_3_1009	DJI_0979	Calamagrostis epigejos (L.) Roth	25	n	1	0	Poaceae Barnhart
Iz_3_1009	DJI_0979	Carduus acanthoides L.	0	n	1	0	Asteraceae Bercht. & J.Presl
Iz_3_1009	DJI_0979	Carex sp.	0	n	1	0	Cyperaceae Juss.
Iz_3_1009	DJI_0979	Centaurea majorovii Dumbadze	0	n	1	0	Asteraceae Bercht. & J.Presl
Iz_3_1009	DJI_0979	Chamaecytisus ruthenicus (Fisch.ex Wol.) Klaskova	0	n	1	0	Fabaceae Lindl.
Iz_3_1009	DJI_0979	Chelidonium majus L.	0	n	1	0	Papaveraceae Juss.
Iz_3_1009	DJI_0979	Chenopodium betaceum Andrz.	0	n	1	0	Amaranthaceae Juss.
Iz_3_1009	DJI_0979	Chenopodium cf. album	0	n	1	0	Amaranthaceae Juss.
Iz_3_1009	DJI_0979	Chenopodium sp.	0		NA	0	Amaranthaceae Juss.
Iz_3_1009	DJI_0979	Chondrilla juncea L.	0	n	1	0	Asteraceae Bercht. & J.Presl
Iz_3_1009	DJI_0979	Cirsium vulgare (Savi) Ten.	0	n	1	0	Asteraceae Bercht. & J.Presl
Iz_3_1009	DJI_0979	Clinopodium acinos (L.) Kuntze	0	n	1	0	Lamiacea Lindl.
Iz_3_1009	DJI_0979	Convallaria majalis L.	0	n	1	0	Asparagaceae Juss.
Iz_3_1009	DJI_0979	Crataegus rhipidophylla Gand.	0	n	1	1	Rosaceae Juss.
Iz_3_1009	DJI_0979	Crepis foetida L.	0	n	1	0	Asteraceae Bercht. & J.Presl
Iz_3_1009	DJI_0979	Dactylis glomerata L.	0	n	1	0	Poaceae Barnhart
Iz_3_1009	DJI_0979	Dianthus campestris M.Bieb.	0	n	1	0	Caryophyllaceae Juss.
Iz_3_1009	DJI_0979	Digitaria sanguinalis (L.) Scop.	0	a	0	0	Poaceae Barnhart
Iz_3_1009	DJI_0979	Elymus repens (L.) Gould	0	n	1	0	Poaceae Barnhart
Iz_3_1009	DJI_0979	Epilobium tetragonum L.	0	n	1	0	Onagraceae Juss.
Iz_3_1009	DJI_0979	Eragrostis minor Host	0	a	0	0	Poaceae Barnhart
Iz_3_1009	DJI_0979	Erigeron canadensis L.	0	a	0	0	Asteraceae Bercht. & J.Presl
Iz_3_1009	DJI_0979	Euphorbia seguieriana Neck.	0	n	1	0	Euphorbiaceae Juss.
Iz_3_1009	DJI_0979	Fallopia dumetorum (L.) Holub	0	n	1	0	Polygonaceae Juss.
Iz_3_1009	DJI_0979	Festuca beckeri (Hack.) Trautv.	0	n	1	0	Poaceae Barnhart
Iz_3_1009	DJI_0979	Fragaria vesca L.	0	n	1	0	Rosaceae Juss.
Iz_3_1009	DJI_0979	Frangula alnus Mill.	0	n	1	1	Rhamnaceae Juss.
Iz_3_1009	DJI_0979	Fraxinus pennsylvanica Marshall	0	a	0	1	Oleaceae Hoffmanns. & Link
Iz_3_1009	DJI_0979	Galeopsis bifida Boenn.	0	n	1	0	Lamiacea Lindl.
Iz_3_1009	DJI_0979	Galium verum L.	0	n	1	0	Rubiaceae Juss.
Iz_3_1009	DJI_0979	Genista tinctoria L.	0	n	1	0	Fabaceae Lindl.
Iz_3_1009	DJI_0979	Geranium robertianum L.	0	n	1	0	Geraniaceae Juss.
Iz_3_1009	DJI_0979	Geranium sanquineum L.	0	n	1	0	Geraniaceae Juss.
Iz_3_1009	DJI_0979	Gypsophila paniculata L.	0	n	1	0	Caryophyllaceae Juss.
Iz_3_1009	DJI_0979	Helichrysum arenarium (L.) Moench	0	n	1	0	Asteraceae Bercht. & J.Presl
Iz_3_1009	DJI_0979	Herniaria polygama J.Gay	0	n	1	0	Caryophyllaceae Juss.
Iz_3_1009	DJI_0979	Hieracium robustum Fr.	0	n	1	0	Asteraceae Bercht. & J.Presl
Iz_3_1009	DJI_0979	Hieracium umbellatum L.	0	n	1	NA	Asteraceae Bercht. & J.Presl
Iz_3_1009	DJI_0979	Hylotelephium maximum (L.) Holub	0	n	1	0	Crassulaceae J.St.-Hil.
Iz_3_1009	DJI_0979	Jacobaea borysthenica (DC.) B.Nord. & Greuter	0	n	1	0	Asteraceae Bercht. & J.Presl
Iz_3_1009	DJI_0979	Jasione montana L.	0	n	1	0	Campanulaceae Juss.
Iz_3_1009	DJI_0979	Jurinea cyanoides (L.) Rchb.s.l.	0	n	1	0	Asteraceae Bercht. & J.Presl
Iz_3_1009	DJI_0979	Koeleria glauca (Spreng.) DC.	0	n	1	0	Poaceae Barnhart
Iz_3_1009	DJI_0979	Lactuca serriola L.	0	a	0	0	Asteraceae Bercht. & J.Presl
Iz_3_1009	DJI_0979	Linaria genistifolia (L.) Mill.	0	n	1	0	Plantaginaceae Juss.
Iz_3_1009	DJI_0979	Lomelosia argentea (L.) Greuter & Burdet	0	n	1	0	Caprifoliaceae Juss.
Iz_3_1009	DJI_0979	Melampyrum pratense L.	0	n	1	0	Orobanchaceae Vent.
Iz_3_1009	DJI_0979	Oenothera biennis L.	0	a	0	0	Onagraceae Juss.
Iz_3_1009	DJI_0979	Pentanema britannica (L.) D.Gut.Larr., Santos-Vicente, Anderb., E.Rico & M.M.Mart.Ort.	0	n	1	0	Asteraceae Bercht. & J.Presl
Iz_3_1009	DJI_0979	Peucedanum oreoselinum (L.) Moench	3	n	1	0	Apiaceae Lindl.
Iz_3_1009	DJI_0979	Peucedanum ruthenicum M.Bieb.	0	n	1	0	Apiaceae Lindl.
Iz_3_1009	DJI_0979	Pilosella echioides (Lumn.) F.W.Schultz & Sch.Bip.	0	n	1	0	Asteraceae Bercht. & J.Presl
Iz_3_1009	DJI_0979	Pilosella officinarum F. Schultz et Sch. Bip.	0	n	1	0	Asteraceae Bercht. & J.Presl
Iz_3_1009	DJI_0979	Pilosella sp.	0		NA	0	Asteraceae Bercht. & J.Presl
Iz_3_1009	DJI_0979	Pinus sylvestris L.	0	n	1	1	Pinaceae Spreng. ex F.Rudolphi
Iz_3_1009	DJI_0979	Poa annua L.	0	n	1	0	Poaceae Barnhart
Iz_3_1009	DJI_0979	Poa nemoralis L.	0	n	1	0	Poaceae Barnhart
Iz_3_1009	DJI_0979	Poa sp.	0		NA	0	Poaceae Barnhart
Iz_3_1009	DJI_0979	Polygonatum odoratum (Mill.) Druce	2	n	1	0	Asparagaceae Juss.
Iz_3_1009	DJI_0979	Polygonum arenarium Waldst. & Kit.	0	n	1	0	Polygonaceae Juss.
Iz_3_1009	DJI_0979	Populus nigra L.	0	n	1	1	Salicaceae Mirb.
Iz_3_1009	DJI_0979	Populus tremula L.	0	n	1	1	Salicaceae Mirb.
Iz_3_1009	DJI_0979	Populus Ã canescens (Aiton) Sm.	0	n	1	1	Salicaceae Mirb.
Iz_3_1009	DJI_0979	Potentilla argentea L.	0	n	1	0	Rosaceae Juss.
Iz_3_1009	DJI_0979	Psammophiliella muralis (L.) Ikonn.	0	n	1	0	Caryophyllaceae Juss.
Iz_3_1009	DJI_0979	Psephellus sumensis (Kalen.) Greuter	0	n	1	0	Asteraceae Bercht. & J.Presl
Iz_3_1009	DJI_0979	Pulsatilla pratensis (L.) Mill. s.l.	0	n	1	0	Ranunculaceae Juss.
Iz_3_1009	DJI_0979	Pyrus communis L.	0	a	0	1	Rosaceae Juss.
Iz_3_1009	DJI_0979	Quercus robur L.	0	n	1	1	Fagaceae Dumort.
Iz_3_1009	DJI_0979	Robinia pseudoacacia L.	0	a	0	1	Fabaceae Lindl.
Iz_3_1009	DJI_0979	Rosa sp.	0		NA	1	Rosaceae Juss.
Iz_3_1009	DJI_0979	Rubus idaeus L.	0	n	1	0	Rosaceae Juss.
Iz_3_1009	DJI_0979	Rumex acetosella L.	0	n	1	0	Polygonaceae Juss.
Iz_3_1009	DJI_0979	Rumex thyrsiflorus Fingerh.	0	n	1	0	Polygonaceae Juss.
Iz_3_1009	DJI_0979	Salix alba L.	0	n	1	1	Salicaceae Mirb.
Iz_3_1009	DJI_0979	Sambucus nigra L.	0	n	1	1	Viburnaceae Raf.
Iz_3_1009	DJI_0979	Secale sylvestre Host	0	n	1	0	Poaceae Barnhart
Iz_3_1009	DJI_0979	Sempervivum ruthenicum Schnittsp. & C.B. Lehm.	0	n	1	0	Crassulaceae J.St.-Hil.
Iz_3_1009	DJI_0979	Setaria viridis (L.) P. Beauv.	0	n	1	0	Poaceae Barnhart
Iz_3_1009	DJI_0979	Silene borysthenica (Gruner) Walters	0	n	1	0	Caryophyllaceae Juss.

Iz_3_1009	DJI_0979	Silene latifolia subsp. alba (Mill.) Greuter & Burdet	0	n	1	0	Caryophyllaceae Juss.
Iz_3_1009	DJI_0979	Solidago virgaurea L.	0	n	1	0	Asteraceae Bercht. & J.Presl
Iz_3_1009	DJI_0979	Sonchus oleraceus L.	0	a	0	0	Asteraceae Bercht. & J.Presl
Iz_3_1009	DJI_0979	Sorbus aucuparia L.	0	n	1	1	Rosaceae Juss.
Iz_3_1009	DJI_0979	Stachys recta L.	0	n	1	0	Lamiacea Lindl.
Iz_3_1009	DJI_0979	Tanacetum vulgare L.	0	n	1	0	Asteraceae Bercht. & J.Presl
Iz_3_1009	DJI_0979	Thymus pallasianus Heinr.Braun	0	n	1	0	Lamiacea Lindl.
Iz_3_1009	DJI_0979	Tilia cordata Mill.	0	n	1	1	Malvaceae Juss.
Iz_3_1009	DJI_0979	Trifolium alpestre L.	0	n	1	0	Fabaceae Lindl.
Iz_3_1009	DJI_0979	Trifolium arvense L.	0	n	1	0	Fabaceae Lindl.
Iz_3_1009	DJI_0979	Ulmus glabra Huds.	0	n	1	1	Ulmaceae Mirb.
Iz_3_1009	DJI_0979	Verbascum densiflorum Bertol.	0	n	1	0	Scrophulariaceae Juss.
Iz_3_1009	DJI_0979	Veronica austriaca L.	0	n	1	0	Plantaginaceae Juss.
Iz_3_1009	DJI_0979	Veronica incana L.	0	n	1	0	Plantaginaceae Juss.
Iz_3_1009	DJI_0979	Veronica verna L.	0	n	1	0	Plantaginaceae Juss.
Iz_3_1009	DJI_0979	Vincetoxicum hirundinaria Medik	0	n	1	0	Apocynaceae Juss.
Iz_3_1009	DJI_0991	Acer tataricum L.	0	n	1	1	Sapindaceae Juss.
Iz_3_1009	DJI_0991	Achillea millefolium L. s.l.	0	n	1	0	Asteraceae Bercht. & J.Presl
Iz_3_1009	DJI_0991	Allium oleraceum L.	0	n	1	0	Amaryllidaceae J.St.-Hil.
Iz_3_1009	DJI_0991	Ambrosia artemisiifolia L.	0	a	0	0	Asteraceae Bercht. & J.Presl
Iz_3_1009	DJI_0991	Amorpha fruticosa L.	0	a	0	0	Fabaceae Lindl.
Iz_3_1009	DJI_0991	Anthericum ramosum L.	0	n	1	0	Asparagaceae Juss.
Iz_3_1009	DJI_0991	Artemisia marschalliana Spreng.	0	n	1	0	Asteraceae Bercht. & J.Presl
Iz_3_1009	DJI_0991	Asparagus officinalis L.	0	n	1	0	Asparagaceae Juss.
Iz_3_1009	DJI_0991	Astragalus cicer L.	0	n	1	0	Fabaceae Lindl.
Iz_3_1009	DJI_0991	Bassia laniflora (S.G.Gmel.) A.J.Scott	0	n	1	0	Amaranthaceae Juss.
Iz_3_1009	DJI_0991	Berberis vulgaris L.	0	n	1	0	Berberidaceae Juss.
Iz_3_1009	DJI_0991	Betula pendula Roth.	0	n	1	1	Betulaceae Gray
Iz_3_1009	DJI_0991	Betula pendula Roth.	0	n	1	0	Poaceae Barnhart
Iz_3_1009	DJI_0991	Bromus inermis Leyss.	0		NA	NA	
Iz_3_1009	DJI_0991	Bromus squarrosus L.	0	a	0	0	Poaceae Barnhart
Iz_3_1009	DJI_0991	Bromus tectorum L.	0	a	0	0	Poaceae Barnhart
Iz_3_1009	DJI_0991	Calamagrostis epigejos (L.) Roth	3	n	1	0	Poaceae Barnhart
Iz_3_1009	DJI_0991	Carduus acanthoides L.	0	n	1	0	Asteraceae Bercht. & J.Presl
Iz_3_1009	DJI_0991	Carex sp.	0	n	1	0	Cyperaceae Juss.
Iz_3_1009	DJI_0991	Centaurea majorovii Dumbadze	0	n	1	0	Asteraceae Bercht. & J.Presl
Iz_3_1009	DJI_0991	Chamaecytisus ruthenicus (Fisch.ex Wol.) Klaskova	2	n	1	0	Fabaceae Lindl.
Iz_3_1009	DJI_0991	Chelidonium majus L.	2	n	1	0	Papaveraceae Juss.
Iz_3_1009	DJI_0991	Chenopodium betaceum Andrz.	0	n	1	0	Amaranthaceae Juss.
Iz_3_1009	DJI_0991	Chenopodium cf. album	0	n	1	0	Amaranthaceae Juss.
Iz_3_1009	DJI_0991	Chenopodium sp.	0		NA	0	Amaranthaceae Juss.
Iz_3_1009	DJI_0991	Chondrilla juncea L.	0	n	1	0	Asteraceae Bercht. & J.Presl
Iz_3_1009	DJI_0991	Cirsium vulgare (Savi) Ten.	0	n	1	0	Asteraceae Bercht. & J.Presl
Iz_3_1009	DJI_0991	Clinopodium acinos (L.) Kuntze	0	n	1	0	Lamiacea Lindl.
Iz_3_1009	DJI_0991	Convallaria majalis L.	0	n	1	0	Asparagaceae Juss.
Iz_3_1009	DJI_0991	Crataegus rhipidophylla Gand.	0	n	1	1	Rosaceae Juss.
Iz_3_1009	DJI_0991	Crepis foetida L.	0	n	1	0	Asteraceae Bercht. & J.Presl
Iz_3_1009	DJI_0991	Dactylis glomerata L.	0	n	1	0	Poaceae Barnhart
Iz_3_1009	DJI_0991	Dianthus campestris M.Bieb.	0	n	1	0	Caryophyllaceae Juss.
Iz_3_1009	DJI_0991	Digitaria sanguinalis (L.) Scop.	0	a	0	0	Poaceae Barnhart
Iz_3_1009	DJI_0991	Elymus repens (L.) Gould	0	n	1	0	Poaceae Barnhart
Iz_3_1009	DJI_0991	Epilobium tetragonum L.	0	n	1	0	Onagraceae Juss.
Iz_3_1009	DJI_0991	Eragrostis minor Host	0	a	0	0	Poaceae Barnhart
Iz_3_1009	DJI_0991	Erigeron canadensis L.	0	a	0	0	Asteraceae Bercht. & J.Presl
Iz_3_1009	DJI_0991	Euphorbia seguieriana Neck.	0	n	1	0	Euphorbiaceae Juss.
Iz_3_1009	DJI_0991	Fallopia dumetorum (L.) Holub	1	n	1	0	Polygonaceae Juss.
Iz_3_1009	DJI_0991	Festuca beckeri (Hack.) Trautv.	0	n	1	0	Poaceae Barnhart
Iz_3_1009	DJI_0991	Fragaria vesca L.	0	n	1	0	Rosaceae Juss.
Iz_3_1009	DJI_0991	Frangula alnus Mill.	0	n	1	1	Rhamnaceae Juss.
Iz_3_1009	DJI_0991	Fraxinus pennsylvanica Marshall	0	a	0	1	Oleaceae Hoffmanns. & Link
Iz_3_1009	DJI_0991	Galeopsis bifida Boenn.	0	n	1	0	Lamiacea Lindl.
Iz_3_1009	DJI_0991	Galium verum L.	0	n	1	0	Rubiaceae Juss.
Iz_3_1009	DJI_0991	Genista tinctoria L.	0	n	1	0	Fabaceae Lindl.
Iz_3_1009	DJI_0991	Geranium robertianum L.	0	n	1	0	Geraniaceae Juss.
Iz_3_1009	DJI_0991	Geranium sanquineum L.	0	n	1	0	Geraniaceae Juss.
Iz_3_1009	DJI_0991	Gypsophila paniculata L.	0	n	1	0	Caryophyllaceae Juss.
Iz_3_1009	DJI_0991	Helichrysum arenarium (L.) Moench	0	n	1	0	Asteraceae Bercht. & J.Presl
Iz_3_1009	DJI_0991	Herniaria polygama J.Gay	0	n	1	0	Caryophyllaceae Juss.
Iz_3_1009	DJI_0991	Hieracium robustum Fr.	0	n	1	0	Asteraceae Bercht. & J.Presl
Iz_3_1009	DJI_0991	Hieracium umbellatum L.	0	n	1	NA	Asteraceae Bercht. & J.Presl
Iz_3_1009	DJI_0991	Hylotelephium maximum (L.) Holub	0	n	1	0	Crassulaceae J.St.-Hil.
Iz_3_1009	DJI_0991	Jacobaea borysthenica (DC.) B.Nord. & Greuter	0	n	1	0	Asteraceae Bercht. & J.Presl
Iz_3_1009	DJI_0991	Jasione montana L.	0	n	1	0	Campanulaceae Juss.
Iz_3_1009	DJI_0991	Jurinea cyanoides (L.) Rchb.s.l.	0	n	1	0	Asteraceae Bercht. & J.Presl
Iz_3_1009	DJI_0991	Koeleria glauca (Spreng.) DC.	0	n	1	0	Poaceae Barnhart
Iz_3_1009	DJI_0991	Lactuca serriola L.	0	a	0	0	Asteraceae Bercht. & J.Presl
Iz_3_1009	DJI_0991	Linaria genistifolia (L.) Mill.	0	n	1	0	Plantaginaceae Juss.
Iz_3_1009	DJI_0991	Lomelosia argentea (L.) Greuter & Burdet	0	n	1	0	Caprifoliaceae Juss.
Iz_3_1009	DJI_0991	Melampyrum pratense L.	0	n	1	0	Orobanchaceae Vent.
Iz_3_1009	DJI_0991	Oenothera biennis L.	0	a	0	0	Onagraceae Juss.
Iz_3_1009	DJI_0991	Pentanema britannica (L.) D.Gut.Larr., Santos-Vicente, Anderb., E.Rico & M.M.Mart.Ort.	0	n	1	0	Asteraceae Bercht. & J.Presl
Iz_3_1009	DJI_0991	Peucedanum oreoselinum (L.) Moench	0	n	1	0	Apiaceae Lindl.
Iz_3_1009	DJI_0991	Peucedanum ruthenicum M.Bieb.	0	n	1	0	Apiaceae Lindl.
Iz_3_1009	DJI_0991	Pilosella echioides (Lumn.) F.W.Schultz & Sch.Bip.	0	n	1	0	Asteraceae Bercht. & J.Presl
Iz_3_1009	DJI_0991	Pilosella officinarum F. Schultz et Sch. Bip.	0	n	1	0	Asteraceae Bercht. & J.Presl
Iz_3_1009	DJI_0991	Pilosella sp.	0		NA	0	Asteraceae Bercht. & J.Presl
Iz_3_1009	DJI_0991	Pinus sylvestris L.	0	n	1	1	Pinaceae Spreng. ex F.Rudolphi
Iz_3_1009	DJI_0991	Poa annua L.	0	n	1	0	Poaceae Barnhart
Iz_3_1009	DJI_0991	Poa nemoralis L.	0	n	1	0	Poaceae Barnhart
Iz_3_1009	DJI_0991	Poa sp.	0		NA	0	Poaceae Barnhart
Iz_3_1009	DJI_0991	Polygonatum odoratum (Mill.) Druce	0	n	1	0	Asparagaceae Juss.
Iz_3_1009	DJI_0991	Polygonum arenarium Waldst. & Kit.	0	n	1	0	Polygonaceae Juss.
Iz_3_1009	DJI_0991	Populus nigra L.	0	n	1	1	Salicaceae Mirb.
Iz_3_1009	DJI_0991	Populus tremula L.	0	n	1	1	Salicaceae Mirb.
Iz_3_1009	DJI_0991	Populus Ã canescens (Aiton) Sm.	0	n	1	1	Salicaceae Mirb.
Iz_3_1009	DJI_0991	Potentilla argentea L.	0	n	1	0	Rosaceae Juss.
Iz_3_1009	DJI_0991	Psammophiliella muralis (L.) Ikonn.	0	n	1	0	Caryophyllaceae Juss.
Iz_3_1009	DJI_0991	Psephellus sumensis (Kalen.) Greuter	0	n	1	0	Asteraceae Bercht. & J.Presl

Iz_3_1009	DJI_0991	Pulsatilla pratensis (L.) Mill. s.l.	0	n	1	0	Ranunculaceae Juss.
Iz_3_1009	DJI_0991	Pyrus communis L.	0	a	0	1	Rosaceae Juss.
Iz_3_1009	DJI_0991	Quercus robur L.	2	n	1	1	Fagaceae Dumort.
Iz_3_1009	DJI_0991	Robinia pseudoacacia L.	0	a	0	1	Fabaceae Lindl.
Iz_3_1009	DJI_0991	Rosa sp.	0		NA	1	Rosaceae Juss.
Iz_3_1009	DJI_0991	Rubus idaeus L.	0	n	1	0	Rosaceae Juss.
Iz_3_1009	DJI_0991	Rumex acetosella L.	0	n	1	0	Polygonaceae Juss.
Iz_3_1009	DJI_0991	Rumex thyrsiflorus Fingerh.	0	n	1	0	Polygonaceae Juss.
Iz_3_1009	DJI_0991	Salix alba L.	0	n	1	1	Salicaceae Mirb.
Iz_3_1009	DJI_0991	Sambucus nigra L.	0	n	1	1	Viburnaceae Raf.
Iz_3_1009	DJI_0991	Secale sylvestre Host	0	n	1	0	Poaceae Barnhart
Iz_3_1009	DJI_0991	Sempervivum ruthenicum Schnittsp. & C.B. Lehm.	0	n	1	0	Crassulaceae J.St.-Hil.
Iz_3_1009	DJI_0991	Setaria viridis (L.) P. Beauv.	0	n	1	0	Poaceae Barnhart
Iz_3_1009	DJI_0991	Silene borysthenica (Gruner) Walters	0	n	1	0	Caryophyllaceae Juss.
Iz_3_1009	DJI_0991	Silene latifolia subsp. alba (Mill.) Greuter & Burdet	0	n	1	0	Caryophyllaceae Juss.
Iz_3_1009	DJI_0991	Solidago virgaurea L.	0	n	1	0	Asteraceae Bercht. & J.Presl
Iz_3_1009	DJI_0991	Sonchus oleraceus L.	0	a	0	0	Asteraceae Bercht. & J.Presl
Iz_3_1009	DJI_0991	Sorbus aucuparia L.	0	n	1	1	Rosaceae Juss.
Iz_3_1009	DJI_0991	Stachys recta L.	0	n	1	0	Lamiacea Lindl.
Iz_3_1009	DJI_0991	Tanacetum vulgare L.	0	n	1	0	Asteraceae Bercht. & J.Presl
Iz_3_1009	DJI_0991	Thymus pallasianus Heinr.Braun	0	n	1	0	Lamiacea Lindl.
Iz_3_1009	DJI_0991	Tilia cordata Mill.	0	n	1	1	Malvaceae Juss.
Iz_3_1009	DJI_0991	Trifolium alpestre L.	0	n	1	0	Fabaceae Lindl.
Iz_3_1009	DJI_0991	Trifolium arvense L.	0	n	1	0	Fabaceae Lindl.
Iz_3_1009	DJI_0991	Ulmus glabra Huds.	0	n	1	1	Ulmaceae Mirb.
Iz_3_1009	DJI_0991	Verbascum densiflorum Bertol.	0	n	1	0	Scrophulariaceae Juss.
Iz_3_1009	DJI_0991	Veronica austriaca L.	0	n	1	0	Plantaginaceae Juss.
Iz_3_1009	DJI_0991	Veronica incana L.	0	n	1	0	Plantaginaceae Juss.
Iz_3_1009	DJI_0991	Veronica verna L.	0	n	1	0	Plantaginaceae Juss.
Iz_3_1009	DJI_0991	Vincetoxicum hirundinaria Medik	0	n	1	0	Apocynaceae Juss.
Iz_3_1109	DJI_0009	Acer tataricum L.	0	n	1	1	Sapindaceae Juss.
Iz_3_1109	DJI_0009	Achillea millefolium L. s.l.	0	n	1	0	Asteraceae Bercht. & J.Presl
Iz_3_1109	DJI_0009	Allium oleraceum L.	0	n	1	0	Amaryllidaceae J.St.-Hil.
Iz_3_1109	DJI_0009	Ambrosia artemisiifolia L.	0	a	0	0	Asteraceae Bercht. & J.Presl
Iz_3_1109	DJI_0009	Amorpha fruticosa L.	0	a	0	0	Fabaceae Lindl.
Iz_3_1109	DJI_0009	Anthericum ramosum L.	0	n	1	0	Asparagaceae Juss.
Iz_3_1109	DJI_0009	Artemisia marschalliana Spreng.	0	n	1	0	Asteraceae Bercht. & J.Presl
Iz_3_1109	DJI_0009	Asparagus officinalis L.	0	n	1	0	Asparagaceae Juss.
Iz_3_1109	DJI_0009	Astragalus cicer L.	0	n	1	0	Fabaceae Lindl.
Iz_3_1109	DJI_0009	Bassia laniflora (S.G.Gmel.) A.J.Scott	0	n	1	0	Amaranthaceae Juss.
Iz_3_1109	DJI_0009	Berberis vulgaris L.	0	n	1	0	Berberidaceae Juss.
Iz_3_1109	DJI_0009	Betula pendula Roth.	0	n	1	1	Betulaceae Gray
Iz_3_1109	DJI_0009	Betula pendula Roth.	0	n	1	0	Poaceae Barnhart
Iz_3_1109	DJI_0009	Bromus inermis Leyss.	0		NA	NA	
Iz_3_1109	DJI_0009	Bromus squarrosus L.	0	a	0	0	Poaceae Barnhart
Iz_3_1109	DJI_0009	Bromus tectorum L.	0	a	0	0	Poaceae Barnhart
Iz_3_1109	DJI_0009	Calamagrostis epigejos (L.) Roth	5	n	1	0	Poaceae Barnhart
Iz_3_1109	DJI_0009	Carduus acanthoides L.	0	n	1	0	Asteraceae Bercht. & J.Presl
Iz_3_1109	DJI_0009	Carex sp.	0	n	1	0	Cyperaceae Juss.
Iz_3_1109	DJI_0009	Centaurea majorovii Dumbadze	0	n	1	0	Asteraceae Bercht. & J.Presl
Iz_3_1109	DJI_0009	Chamaecytisus ruthenicus (Fisch.ex Wol.) Klaskova	0	n	1	0	Fabaceae Lindl.
Iz_3_1109	DJI_0009	Chelidonium majus L.	0	n	1	0	Papaveraceae Juss.
Iz_3_1109	DJI_0009	Chenopodium betaceum Andrz.	0	n	1	0	Amaranthaceae Juss.
Iz_3_1109	DJI_0009	Chenopodium cf. album	0	n	1	0	Amaranthaceae Juss.
Iz_3_1109	DJI_0009	Chenopodium sp.	0		NA	0	Amaranthaceae Juss.
Iz_3_1109	DJI_0009	Chondrilla juncea L.	0	n	1	0	Asteraceae Bercht. & J.Presl
Iz_3_1109	DJI_0009	Cirsium vulgare (Savi) Ten.	0	n	1	0	Asteraceae Bercht. & J.Presl
Iz_3_1109	DJI_0009	Clinopodium acinos (L.) Kuntze	0	n	1	0	Lamiacea Lindl.
Iz_3_1109	DJI_0009	Convallaria majalis L.	0	n	1	0	Asparagaceae Juss.
Iz_3_1109	DJI_0009	Crataegus rhipidophylla Gand.	0	n	1	1	Rosaceae Juss.
Iz_3_1109	DJI_0009	Crepis foetida L.	0	n	1	0	Asteraceae Bercht. & J.Presl
Iz_3_1109	DJI_0009	Dactylis glomerata L.	0	n	1	0	Poaceae Barnhart
Iz_3_1109	DJI_0009	Dianthus campestris M.Bieb.	0	n	1	0	Caryophyllaceae Juss.
Iz_3_1109	DJI_0009	Digitaria sanguinalis (L.) Scop.	0	a	0	0	Poaceae Barnhart
Iz_3_1109	DJI_0009	Elymus repens (L.) Gould	0	n	1	0	Poaceae Barnhart
Iz_3_1109	DJI_0009	Epilobium tetragonum L.	0	n	1	0	Onagraceae Juss.
Iz_3_1109	DJI_0009	Eragrostis minor Host	0	a	0	0	Poaceae Barnhart
Iz_3_1109	DJI_0009	Erigeron canadensis L.	1	a	0	0	Asteraceae Bercht. & J.Presl
Iz_3_1109	DJI_0009	Euphorbia seguieriana Neck.	0	n	1	0	Euphorbiaceae Juss.
Iz_3_1109	DJI_0009	Fallopia dumetorum (L.) Holub	0	n	1	0	Polygonaceae Juss.
Iz_3_1109	DJI_0009	Festuca beckeri (Hack.) Trautv.	0	n	1	0	Poaceae Barnhart
Iz_3_1109	DJI_0009	Fragaria vesca L.	0	n	1	0	Rosaceae Juss.
Iz_3_1109	DJI_0009	Frangula alnus Mill.	0	n	1	1	Rhamnaceae Juss.
Iz_3_1109	DJI_0009	Fraxinus pennsylvanica Marshall	0	a	0	1	Oleaceae Hoffmanns. & Link
Iz_3_1109	DJI_0009	Galeopsis bifida Boenn.	0	n	1	0	Lamiacea Lindl.
Iz_3_1109	DJI_0009	Galium verum L.	0	n	1	0	Rubiaceae Juss.
Iz_3_1109	DJI_0009	Genista tinctoria L.	0	n	1	0	Fabaceae Lindl.
Iz_3_1109	DJI_0009	Geranium robertianum L.	0	n	1	0	Geraniaceae Juss.
Iz_3_1109	DJI_0009	Geranium sanquineum L.	0	n	1	0	Geraniaceae Juss.
Iz_3_1109	DJI_0009	Gypsophila paniculata L.	0	n	1	0	Caryophyllaceae Juss.
Iz_3_1109	DJI_0009	Helichrysum arenarium (L.) Moench	0	n	1	0	Asteraceae Bercht. & J.Presl
Iz_3_1109	DJI_0009	Herniaria polygama J.Gay	0	n	1	0	Caryophyllaceae Juss.
Iz_3_1109	DJI_0009	Hieracium robustum Fr.	0	n	1	0	Asteraceae Bercht. & J.Presl
Iz_3_1109	DJI_0009	Hieracium umbellatum L.	0	n	1	NA	Asteraceae Bercht. & J.Presl
Iz_3_1109	DJI_0009	Hylotelephium maximum (L.) Holub	0	n	1	0	Crassulaceae J.St.-Hil.
Iz_3_1109	DJI_0009	Jacobaea borysthenica (DC.) B.Nord. & Greuter	0	n	1	0	Asteraceae Bercht. & J.Presl
Iz_3_1109	DJI_0009	Jasione montana L.	0	n	1	0	Campanulaceae Juss.
Iz_3_1109	DJI_0009	Jurinea cyanoides (L.) Rchb.s.l.	0	n	1	0	Asteraceae Bercht. & J.Presl
Iz_3_1109	DJI_0009	Koeleria glauca (Spreng.) DC.	0	n	1	0	Poaceae Barnhart
Iz_3_1109	DJI_0009	Lactuca serriola L.	0	a	0	0	Asteraceae Bercht. & J.Presl
Iz_3_1109	DJI_0009	Linaria genistifolia (L.) Mill.	0	n	1	0	Plantaginaceae Juss.
Iz_3_1109	DJI_0009	Lomelosia argentea (L.) Greuter & Burdet	0	n	1	0	Caprifoliaceae Juss.
Iz_3_1109	DJI_0009	Melampyrum pratense L.	0	n	1	0	Orobanchaceae Vent.
Iz_3_1109	DJI_0009	Oenothera biennis L.	0	a	0	0	Onagraceae Juss.
Iz_3_1109	DJI_0009	Pentanema britannica (L.) D.Gut.Larr., Santos-Vicente, Anderb., E.Rico & M.M.Mart.Ort.	0	n	1	0	Asteraceae Bercht. & J.Presl
Iz_3_1109	DJI_0009	Peucedanum oreoselinum (L.) Moench	0	n	1	0	Apiaceae Lindl.
Iz_3_1109	DJI_0009	Peucedanum ruthenicum M.Bieb.	0	n	1	0	Apiaceae Lindl.
Iz_3_1109	DJI_0009	Pilosella echioides (Lumn.) F.W.Schultz & Sch.Bip.	0	n	1	0	Asteraceae Bercht. & J.Presl

Iz_3_1109	DJI_0009	Pilosella officinarum F. Schultz et Sch. Bip.	0	n	1	0	Asteraceae Bercht. & J.Presl
Iz_3_1109	DJI_0009	Pilosella sp.	0		NA	0	Asteraceae Bercht. & J.Presl
Iz_3_1109	DJI_0009	Pinus sylvestris L.	0	n	1	1	Pinaceae Spreng. ex F.Rudolphi
Iz_3_1109	DJI_0009	Poa annua L.	0	n	1	0	Poaceae Barnhart
Iz_3_1109	DJI_0009	Poa nemoralis L.	0	n	1	0	Poaceae Barnhart
Iz_3_1109	DJI_0009	Poa sp.	0		NA	0	Poaceae Barnhart
Iz_3_1109	DJI_0009	Polygonatum odoratum (Mill.) Druce	0	n	1	0	Asparagaceae Juss.
Iz_3_1109	DJI_0009	Polygonum arenarium Waldst. & Kit.	0	n	1	0	Polygonaceae Juss.
Iz_3_1109	DJI_0009	Populus nigra L.	0	n	1	1	Salicaceae Mirb.
Iz_3_1109	DJI_0009	Populus tremula L.	0	n	1	1	Salicaceae Mirb.
Iz_3_1109	DJI_0009	Populus Ã canescens (Aiton) Sm.	0	n	1	1	Salicaceae Mirb.
Iz_3_1109	DJI_0009	Potentilla argentea L.	0	n	1	0	Rosaceae Juss.
Iz_3_1109	DJI_0009	Psammophiliella muralis (L.) Ikonn.	0	n	1	0	Caryophyllaceae Juss.
Iz_3_1109	DJI_0009	Psephellus sumensis (Kalen.) Greuter	0	n	1	0	Asteraceae Bercht. & J.Presl
Iz_3_1109	DJI_0009	Pulsatilla pratensis (L.) Mill. s.l.	0	n	1	0	Ranunculaceae Juss.
Iz_3_1109	DJI_0009	Pyrus communis L.	0	a	0	1	Rosaceae Juss.
Iz_3_1109	DJI_0009	Quercus robur L.	0	n	1	1	Fagaceae Dumort.
Iz_3_1109	DJI_0009	Robinia pseudoacacia L.	0	a	0	1	Fabaceae Lindl.
Iz_3_1109	DJI_0009	Rosa sp.	0		NA	1	Rosaceae Juss.
Iz_3_1109	DJI_0009	Rubus idaeus L.	0	n	1	0	Rosaceae Juss.
Iz_3_1109	DJI_0009	Rumex acetosella L.	0	n	1	0	Polygonaceae Juss.
Iz_3_1109	DJI_0009	Rumex thyrsiflorus Fingerh.	0	n	1	0	Polygonaceae Juss.
Iz_3_1109	DJI_0009	Salix alba L.	0	n	1	1	Salicaceae Mirb.
Iz_3_1109	DJI_0009	Sambucus nigra L.	0	n	1	1	Viburnaceae Raf.
Iz_3_1109	DJI_0009	Secale sylvestre Host	0	n	1	0	Poaceae Barnhart
Iz_3_1109	DJI_0009	Sempervivum ruthenicum Schnittsp. & C.B. Lehm.	0	n	1	0	Crassulaceae J.St.-Hil.
Iz_3_1109	DJI_0009	Setaria viridis (L.) P. Beauv.	0	n	1	0	Poaceae Barnhart
Iz_3_1109	DJI_0009	Silene borysthenica (Gruner) Walters	0	n	1	0	Caryophyllaceae Juss.
Iz_3_1109	DJI_0009	Silene latifolia subsp. alba (Mill.) Greuter & Burdet	0	n	1	0	Caryophyllaceae Juss.
Iz_3_1109	DJI_0009	Solidago virgaurea L.	0	n	1	0	Asteraceae Bercht. & J.Presl
Iz_3_1109	DJI_0009	Sorbus aucuparia L.	0	n	1	1	Rosaceae Juss.
Iz_3_1109	DJI_0009	Species 11.09_3_0045	0		NA	0	
Iz_3_1109	DJI_0009	Species 11.09_3_0058	0		NA	0	
Iz_3_1109	DJI_0009	Stachys recta L.	0	n	1	0	Lamiacea Lindl.
Iz_3_1109	DJI_0009	Tanacetum vulgare L.	0	n	1	0	Asteraceae Bercht. & J.Presl
Iz_3_1109	DJI_0009	Thymus pallasianus Heinr.Braun	0	n	1	0	Lamiacea Lindl.
Iz_3_1109	DJI_0009	Tilia cordata Mill.	0	n	1	1	Malvaceae Juss.
Iz_3_1109	DJI_0009	Trifolium alpestre L.	0	n	1	0	Fabaceae Lindl.
Iz_3_1109	DJI_0009	Trifolium arvense L.	0	n	1	0	Fabaceae Lindl.
Iz_3_1109	DJI_0009	Ulmus glabra Huds.	0	n	1	1	Ulmaceae Mirb.
Iz_3_1109	DJI_0009	Verbascum densiflorum Bertol.	0	n	1	0	Scrophulariaceae Juss.
Iz_3_1109	DJI_0009	Veronica austriaca L.	0	n	1	0	Plantaginaceae Juss.
Iz_3_1109	DJI_0009	Veronica incana L.	0	n	1	0	Plantaginaceae Juss.
Iz_3_1109	DJI_0009	Veronica verna L.	0	n	1	0	Plantaginaceae Juss.
Iz_3_1109	DJI_0009	Vincetoxicum hirundinaria Medik	0	n	1	0	Apocynaceae Juss.
Iz_3_1109	DJI_0014	Acer tataricum L.	0	n	1	1	Sapindaceae Juss.
Iz_3_1109	DJI_0014	Achillea millefolium L. s.l.	0	n	1	0	Asteraceae Bercht. & J.Presl
Iz_3_1109	DJI_0014	Allium oleraceum L.	0	n	1	0	Amaryllidaceae J.St.-Hil.
Iz_3_1109	DJI_0014	Ambrosia artemisiifolia L.	0	a	0	0	Asteraceae Bercht. & J.Presl
Iz_3_1109	DJI_0014	Amorpha fruticosa L.	0	a	0	0	Fabaceae Lindl.
Iz_3_1109	DJI_0014	Anthericum ramosum L.	0	n	1	0	Asparagaceae Juss.
Iz_3_1109	DJI_0014	Artemisia marschalliana Spreng.	0	n	1	0	Asteraceae Bercht. & J.Presl
Iz_3_1109	DJI_0014	Asparagus officinalis L.	0	n	1	0	Asparagaceae Juss.
Iz_3_1109	DJI_0014	Astragalus cicer L.	0	n	1	0	Fabaceae Lindl.
Iz_3_1109	DJI_0014	Bassia laniflora (S.G.Gmel.) A.J.Scott	0	n	1	0	Amaranthaceae Juss.
Iz_3_1109	DJI_0014	Berberis vulgaris L.	0	n	1	0	Berberidaceae Juss.
Iz_3_1109	DJI_0014	Betula pendula Roth.	0	n	1	1	Betulaceae Gray
Iz_3_1109	DJI_0014	Betula pendula Roth.	0	n	1	0	Poaceae Barnhart
Iz_3_1109	DJI_0014	Bromus inermis Leyss.	0		NA	NA	
Iz_3_1109	DJI_0014	Bromus squarrosus L.	0	a	0	0	Poaceae Barnhart
Iz_3_1109	DJI_0014	Bromus tectorum L.	0	a	0	0	Poaceae Barnhart
Iz_3_1109	DJI_0014	Calamagrostis epigejos (L.) Roth	7	n	1	0	Poaceae Barnhart
Iz_3_1109	DJI_0014	Carduus acanthoides L.	0	n	1	0	Asteraceae Bercht. & J.Presl
Iz_3_1109	DJI_0014	Carex sp.	0	n	1	0	Cyperaceae Juss.
Iz_3_1109	DJI_0014	Centaurea majorovii Dumbadze	0	n	1	0	Asteraceae Bercht. & J.Presl
Iz_3_1109	DJI_0014	Chamaecytisus ruthenicus (Fisch.ex Wol.) Klaskova	0	n	1	0	Fabaceae Lindl.
Iz_3_1109	DJI_0014	Chelidonium majus L.	0	n	1	0	Papaveraceae Juss.
Iz_3_1109	DJI_0014	Chenopodium betaceum Andrz.	0	n	1	0	Amaranthaceae Juss.
Iz_3_1109	DJI_0014	Chenopodium cf. album	0	n	1	0	Amaranthaceae Juss.
Iz_3_1109	DJI_0014	Chenopodium sp.	0		NA	0	Amaranthaceae Juss.
Iz_3_1109	DJI_0014	Chondrilla juncea L.	0	n	1	0	Asteraceae Bercht. & J.Presl
Iz_3_1109	DJI_0014	Cirsium vulgare (Savi) Ten.	0	n	1	0	Asteraceae Bercht. & J.Presl
Iz_3_1109	DJI_0014	Clinopodium acinos (L.) Kuntze	0	n	1	0	Lamiacea Lindl.
Iz_3_1109	DJI_0014	Convallaria majalis L.	0	n	1	0	Asparagaceae Juss.
Iz_3_1109	DJI_0014	Crataegus rhipidophylla Gand.	0	n	1	1	Rosaceae Juss.
Iz_3_1109	DJI_0014	Crepis foetida L.	0	n	1	0	Asteraceae Bercht. & J.Presl
Iz_3_1109	DJI_0014	Dactylis glomerata L.	0	n	1	0	Poaceae Barnhart
Iz_3_1109	DJI_0014	Dianthus campestris M.Bieb.	0	n	1	0	Caryophyllaceae Juss.
Iz_3_1109	DJI_0014	Digitaria sanguinalis (L.) Scop.	0	a	0	0	Poaceae Barnhart
Iz_3_1109	DJI_0014	Elymus repens (L.) Gould	0	n	1	0	Poaceae Barnhart
Iz_3_1109	DJI_0014	Epilobium tetragonum L.	0	n	1	0	Onagraceae Juss.
Iz_3_1109	DJI_0014	Eragrostis minor Host	0	a	0	0	Poaceae Barnhart
Iz_3_1109	DJI_0014	Erigeron canadensis L.	8	a	0	0	Asteraceae Bercht. & J.Presl
Iz_3_1109	DJI_0014	Euphorbia seguieriana Neck.	0	n	1	0	Euphorbiaceae Juss.
Iz_3_1109	DJI_0014	Fallopia dumetorum (L.) Holub	0	n	1	0	Polygonaceae Juss.
Iz_3_1109	DJI_0014	Festuca beckeri (Hack.) Trautv.	0	n	1	0	Poaceae Barnhart
Iz_3_1109	DJI_0014	Fragaria vesca L.	0	n	1	0	Rosaceae Juss.
Iz_3_1109	DJI_0014	Frangula alnus Mill.	0	n	1	1	Rhamnaceae Juss.
Iz_3_1109	DJI_0014	Fraxinus pennsylvanica Marshall	0	a	0	1	Oleaceae Hoffmanns. & Link
Iz_3_1109	DJI_0014	Galeopsis bifida Boenn.	0	n	1	0	Lamiacea Lindl.
Iz_3_1109	DJI_0014	Galium verum L.	0	n	1	0	Rubiaceae Juss.
Iz_3_1109	DJI_0014	Genista tinctoria L.	0	n	1	0	Fabaceae Lindl.
Iz_3_1109	DJI_0014	Geranium robertianum L.	0	n	1	0	Geraniaceae Juss.
Iz_3_1109	DJI_0014	Geranium sanquineum L.	0	n	1	0	Geraniaceae Juss.
Iz_3_1109	DJI_0014	Gypsophila paniculata L.	0	n	1	0	Caryophyllaceae Juss.
Iz_3_1109	DJI_0014	Helichrysum arenarium (L.) Moench	0	n	1	0	Asteraceae Bercht. & J.Presl
Iz_3_1109	DJI_0014	Herniaria polygama J.Gay	0	n	1	0	Caryophyllaceae Juss.
Iz_3_1109	DJI_0014	Hieracium robustum Fr.	0	n	1	0	Asteraceae Bercht. & J.Presl

Iz_3_1109	DJI_0014	Hieracium umbellatum L.	0	n	1	NA	Asteraceae Bercht. & J.Presl
Iz_3_1109	DJI_0014	Hylotelephium maximum (L.) Holub	0	n	1	0	Crassulaceae J.St.-Hil.
Iz_3_1109	DJI_0014	Jacobaea borysthenica (DC.) B.Nord. & Greuter	0	n	1	0	Asteraceae Bercht. & J.Presl
Iz_3_1109	DJI_0014	Jasione montana L.	0	n	1	0	Campanulaceae Juss.
Iz_3_1109	DJI_0014	Jurinea cyanoides (L.) Rchb.s.l.	0	n	1	0	Asteraceae Bercht. & J.Presl
Iz_3_1109	DJI_0014	Koeleria glauca (Spreng.) DC.	0	n	1	0	Poaceae Barnhart
Iz_3_1109	DJI_0014	Lactuca serriola L.	0	a	0	0	Asteraceae Bercht. & J.Presl
Iz_3_1109	DJI_0014	Linaria genistifolia (L.) Mill.	0	n	1	0	Plantaginaceae Juss.
Iz_3_1109	DJI_0014	Lomelosia argentea (L.) Greuter & Burdet	0	n	1	0	Caprifoliaceae Juss.
Iz_3_1109	DJI_0014	Melampyrum pratense L.	0	n	1	0	Orobanchaceae Vent.
Iz_3_1109	DJI_0014	Oenothera biennis L.	0	a	0	0	Onagraceae Juss.
Iz_3_1109	DJI_0014	Pentanema britannica (L.) D.Gut.Larr., Santos-Vicente, Anderb., E.Rico & M.M.Mart.Ort.	0	n	1	0	Asteraceae Bercht. & J.Presl
Iz_3_1109	DJI_0014	Peucedanum oreoselinum (L.) Moench	0	n	1	0	Apiaceae Lindl.
Iz_3_1109	DJI_0014	Peucedanum ruthenicum M.Bieb.	0	n	1	0	Apiaceae Lindl.
Iz_3_1109	DJI_0014	Pilosella echioides (Lumn.) F.W.Schultz & Sch.Bip.	0	n	1	0	Asteraceae Bercht. & J.Presl
Iz_3_1109	DJI_0014	Pilosella officinarum F. Schultz et Sch. Bip.	0	n	1	0	Asteraceae Bercht. & J.Presl
Iz_3_1109	DJI_0014	Pilosella sp.	0		NA	0	Asteraceae Bercht. & J.Presl
Iz_3_1109	DJI_0014	Pinus sylvestris L.	0	n	1	1	Pinaceae Spreng. ex F.Rudolphi
Iz_3_1109	DJI_0014	Poa annua L.	0	n	1	0	Poaceae Barnhart
Iz_3_1109	DJI_0014	Poa nemoralis L.	0	n	1	0	Poaceae Barnhart
Iz_3_1109	DJI_0014	Poa sp.	0		NA	0	Poaceae Barnhart
Iz_3_1109	DJI_0014	Polygonatum odoratum (Mill.) Druce	0	n	1	0	Asparagaceae Juss.
Iz_3_1109	DJI_0014	Polygonum arenarium Waldst. & Kit.	0	n	1	0	Polygonaceae Juss.
Iz_3_1109	DJI_0014	Populus nigra L.	0	n	1	1	Salicaceae Mirb.
Iz_3_1109	DJI_0014	Populus tremula L.	0	n	1	1	Salicaceae Mirb.
Iz_3_1109	DJI_0014	Populus Ã canescens (Aiton) Sm.	0	n	1	1	Salicaceae Mirb.
Iz_3_1109	DJI_0014	Potentilla argentea L.	0	n	1	0	Rosaceae Juss.
Iz_3_1109	DJI_0014	Psammophiliella muralis (L.) Ikonn.	0	n	1	0	Caryophyllaceae Juss.
Iz_3_1109	DJI_0014	Psephellus sumensis (Kalen.) Greuter	0	n	1	0	Asteraceae Bercht. & J.Presl
Iz_3_1109	DJI_0014	Pulsatilla pratensis (L.) Mill. s.l.	0	n	1	0	Ranunculaceae Juss.
Iz_3_1109	DJI_0014	Pyrus communis L.	0	a	0	1	Rosaceae Juss.
Iz_3_1109	DJI_0014	Quercus robur L.	0	n	1	1	Fagaceae Dumort.
Iz_3_1109	DJI_0014	Robinia pseudoacacia L.	0	a	0	1	Fabaceae Lindl.
Iz_3_1109	DJI_0014	Rosa sp.	0		NA	1	Rosaceae Juss.
Iz_3_1109	DJI_0014	Rubus idaeus L.	0	n	1	0	Rosaceae Juss.
Iz_3_1109	DJI_0014	Rumex acetosella L.	0	n	1	0	Polygonaceae Juss.
Iz_3_1109	DJI_0014	Rumex thyrsiflorus Fingerh.	0	n	1	0	Polygonaceae Juss.
Iz_3_1109	DJI_0014	Salix alba L.	0	n	1	1	Salicaceae Mirb.
Iz_3_1109	DJI_0014	Sambucus nigra L.	0	n	1	1	Viburnaceae Raf.
Iz_3_1109	DJI_0014	Secale sylvestre Host	0	n	1	0	Poaceae Barnhart
Iz_3_1109	DJI_0014	Sempervivum ruthenicum Schnittsp. & C.B. Lehm.	0	n	1	0	Crassulaceae J.St.-Hil.
Iz_3_1109	DJI_0014	Setaria viridis (L.) P. Beauv.	0	n	1	0	Poaceae Barnhart
Iz_3_1109	DJI_0014	Silene borysthenica (Gruner) Walters	0	n	1	0	Caryophyllaceae Juss.
Iz_3_1109	DJI_0014	Silene latifolia subsp. alba (Mill.) Greuter & Burdet	0	n	1	0	Caryophyllaceae Juss.
Iz_3_1109	DJI_0014	Solidago virgaurea L.	0	n	1	0	Asteraceae Bercht. & J.Presl
Iz_3_1109	DJI_0014	Sorbus aucuparia L.	0	n	1	1	Rosaceae Juss.
Iz_3_1109	DJI_0014	Species 11.09_3_0045	0		NA	0	
Iz_3_1109	DJI_0014	Species 11.09_3_0058	0		NA	0	
Iz_3_1109	DJI_0014	Stachys recta L.	0	n	1	0	Lamiacea Lindl.
Iz_3_1109	DJI_0014	Tanacetum vulgare L.	0	n	1	0	Asteraceae Bercht. & J.Presl
Iz_3_1109	DJI_0014	Thymus pallasianus Heinr.Braun	0	n	1	0	Lamiacea Lindl.
Iz_3_1109	DJI_0014	Tilia cordata Mill.	0	n	1	1	Malvaceae Juss.
Iz_3_1109	DJI_0014	Trifolium alpestre L.	0	n	1	0	Fabaceae Lindl.
Iz_3_1109	DJI_0014	Trifolium arvense L.	0	n	1	0	Fabaceae Lindl.
Iz_3_1109	DJI_0014	Ulmus glabra Huds.	0	n	1	1	Ulmaceae Mirb.
Iz_3_1109	DJI_0014	Verbascum densiflorum Bertol.	0	n	1	0	Scrophulariaceae Juss.
Iz_3_1109	DJI_0014	Veronica austriaca L.	0	n	1	0	Plantaginaceae Juss.
Iz_3_1109	DJI_0014	Veronica incana L.	0	n	1	0	Plantaginaceae Juss.
Iz_3_1109	DJI_0014	Veronica verna L.	0	n	1	0	Plantaginaceae Juss.
Iz_3_1109	DJI_0014	Vincetoxicum hirundinaria Medik	0	n	1	0	Apocynaceae Juss.
Iz_3_1109	DJI_0016	Acer tataricum L.	0	n	1	1	Sapindaceae Juss.
Iz_3_1109	DJI_0016	Achillea millefolium L. s.l.	0	n	1	0	Asteraceae Bercht. & J.Presl
Iz_3_1109	DJI_0016	Allium oleraceum L.	0	n	1	0	Amaryllidaceae J.St.-Hil.
Iz_3_1109	DJI_0016	Ambrosia artemisiifolia L.	0	a	0	0	Asteraceae Bercht. & J.Presl
Iz_3_1109	DJI_0016	Amorpha fruticosa L.	0	a	0	0	Fabaceae Lindl.
Iz_3_1109	DJI_0016	Anthericum ramosum L.	0	n	1	0	Asparagaceae Juss.
Iz_3_1109	DJI_0016	Artemisia marschalliana Spreng.	0	n	1	0	Asteraceae Bercht. & J.Presl
Iz_3_1109	DJI_0016	Asparagus officinalis L.	0	n	1	0	Asparagaceae Juss.
Iz_3_1109	DJI_0016	Astragalus cicer L.	0	n	1	0	Fabaceae Lindl.
Iz_3_1109	DJI_0016	Bassia laniflora (S.G.Gmel.) A.J.Scott	0	n	1	0	Amaranthaceae Juss.
Iz_3_1109	DJI_0016	Berberis vulgaris L.	0	n	1	0	Berberidaceae Juss.
Iz_3_1109	DJI_0016	Betula pendula Roth.	0	n	1	1	Betulaceae Gray
Iz_3_1109	DJI_0016	Betula pendula Roth.	0	n	1	0	Poaceae Barnhart
Iz_3_1109	DJI_0016	Bromus inermis Leyss.	0		NA	NA	
Iz_3_1109	DJI_0016	Bromus squarrosus L.	0	a	0	0	Poaceae Barnhart
Iz_3_1109	DJI_0016	Bromus tectorum L.	0	a	0	0	Poaceae Barnhart
Iz_3_1109	DJI_0016	Calamagrostis epigejos (L.) Roth	0	n	1	0	Poaceae Barnhart
Iz_3_1109	DJI_0016	Carduus acanthoides L.	0	n	1	0	Asteraceae Bercht. & J.Presl
Iz_3_1109	DJI_0016	Carex sp.	0	n	1	0	Cyperaceae Juss.
Iz_3_1109	DJI_0016	Centaurea majorovii Dumbadze	0	n	1	0	Asteraceae Bercht. & J.Presl
Iz_3_1109	DJI_0016	Chamaecytisus ruthenicus (Fisch.ex Wol.) Klaskova	0	n	1	0	Fabaceae Lindl.
Iz_3_1109	DJI_0016	Chelidonium majus L.	0	n	1	0	Papaveraceae Juss.
Iz_3_1109	DJI_0016	Chenopodium betaceum Andrz.	0	n	1	0	Amaranthaceae Juss.
Iz_3_1109	DJI_0016	Chenopodium cf. album	0	n	1	0	Amaranthaceae Juss.
Iz_3_1109	DJI_0016	Chenopodium sp.	0		NA	0	Amaranthaceae Juss.
Iz_3_1109	DJI_0016	Chondrilla juncea L.	0	n	1	0	Asteraceae Bercht. & J.Presl
Iz_3_1109	DJI_0016	Cirsium vulgare (Savi) Ten.	0	n	1	0	Asteraceae Bercht. & J.Presl
Iz_3_1109	DJI_0016	Clinopodium acinos (L.) Kuntze	0	n	1	0	Lamiacea Lindl.
Iz_3_1109	DJI_0016	Convallaria majalis L.	0	n	1	0	Asparagaceae Juss.
Iz_3_1109	DJI_0016	Crataegus rhipidophylla Gand.	0	n	1	1	Rosaceae Juss.
Iz_3_1109	DJI_0016	Crepis foetida L.	0	n	1	0	Asteraceae Bercht. & J.Presl
Iz_3_1109	DJI_0016	Dactylis glomerata L.	0	n	1	0	Poaceae Barnhart
Iz_3_1109	DJI_0016	Dianthus campestris M.Bieb.	0	n	1	0	Caryophyllaceae Juss.
Iz_3_1109	DJI_0016	Digitaria sanguinalis (L.) Scop.	0	a	0	0	Poaceae Barnhart
Iz_3_1109	DJI_0016	Elymus repens (L.) Gould	0	n	1	0	Poaceae Barnhart
Iz_3_1109	DJI_0016	Epilobium tetragonum L.	0	n	1	0	Onagraceae Juss.
Iz_3_1109	DJI_0016	Eragrostis minor Host	0	a	0	0	Poaceae Barnhart
Iz_3_1109	DJI_0016	Erigeron canadensis L.	2	a	0	0	Asteraceae Bercht. & J.Presl

Iz_3_1109	DJI_0016	Euphorbia seguieriana Neck.	0	n	1	0	Euphorbiaceae Juss.
Iz_3_1109	DJI_0016	Fallopia dumetorum (L.) Holub	0	n	1	0	Polygonaceae Juss.
Iz_3_1109	DJI_0016	Festuca beckeri (Hack.) Trautv.	0	n	1	0	Poaceae Barnhart
Iz_3_1109	DJI_0016	Fragaria vesca L.	0	n	1	0	Rosaceae Juss.
Iz_3_1109	DJI_0016	Frangula alnus Mill.	0	n	1	1	Rhamnaceae Juss.
Iz_3_1109	DJI_0016	Fraxinus pennsylvanica Marshall	0	a	0	1	Oleaceae Hoffmanns. & Link
Iz_3_1109	DJI_0016	Galeopsis bifida Boenn.	0	n	1	0	Lamiacea Lindl.
Iz_3_1109	DJI_0016	Galium verum L.	0	n	1	0	Rubiaceae Juss.
Iz_3_1109	DJI_0016	Genista tinctoria L.	0	n	1	0	Fabaceae Lindl.
Iz_3_1109	DJI_0016	Geranium robertianum L.	0	n	1	0	Geraniaceae Juss.
Iz_3_1109	DJI_0016	Geranium sanquineum L.	0	n	1	0	Geraniaceae Juss.
Iz_3_1109	DJI_0016	Gypsophila paniculata L.	0	n	1	0	Caryophyllaceae Juss.
Iz_3_1109	DJI_0016	Helichrysum arenarium (L.) Moench	0	n	1	0	Asteraceae Bercht. & J.Presl
Iz_3_1109	DJI_0016	Herniaria polygama J.Gay	0	n	1	0	Caryophyllaceae Juss.
Iz_3_1109	DJI_0016	Hieracium robustum Fr.	0	n	1	0	Asteraceae Bercht. & J.Presl
Iz_3_1109	DJI_0016	Hieracium umbellatum L.	0	n	1	NA	Asteraceae Bercht. & J.Presl
Iz_3_1109	DJI_0016	Hylotelephium maximum (L.) Holub	0	n	1	0	Crassulaceae J.St.-Hil.
Iz_3_1109	DJI_0016	Jacobaea borysthenica (DC.) B.Nord. & Greuter	0	n	1	0	Asteraceae Bercht. & J.Presl
Iz_3_1109	DJI_0016	Jasione montana L.	0	n	1	0	Campanulaceae Juss.
Iz_3_1109	DJI_0016	Jurinea cyanoides (L.) Rchb.s.l.	0	n	1	0	Asteraceae Bercht. & J.Presl
Iz_3_1109	DJI_0016	Koeleria glauca (Spreng.) DC.	0	n	1	0	Poaceae Barnhart
Iz_3_1109	DJI_0016	Lactuca serriola L.	0	a	0	0	Asteraceae Bercht. & J.Presl
Iz_3_1109	DJI_0016	Linaria genistifolia (L.) Mill.	0	n	1	0	Plantaginaceae Juss.
Iz_3_1109	DJI_0016	Lomelosia argentea (L.) Greuter & Burdet	0	n	1	0	Caprifoliaceae Juss.
Iz_3_1109	DJI_0016	Melampyrum pratense L.	0	n	1	0	Orobanchaceae Vent.
Iz_3_1109	DJI_0016	Oenothera biennis L.	0	a	0	0	Onagraceae Juss.
Iz_3_1109	DJI_0016	Pentanema britannica (L.) D.Gut.Larr., Santos-Vicente, Anderb., E.Rico & M.M.Mart.Ort.	0	n	1	0	Asteraceae Bercht. & J.Presl
Iz_3_1109	DJI_0016	Peucedanum oreoselinum (L.) Moench	0	n	1	0	Apiaceae Lindl.
Iz_3_1109	DJI_0016	Peucedanum ruthenicum M.Bieb.	0	n	1	0	Apiaceae Lindl.
Iz_3_1109	DJI_0016	Pilosella echioides (Lumn.) F.W.Schultz & Sch.Bip.	0	n	1	0	Asteraceae Bercht. & J.Presl
Iz_3_1109	DJI_0016	Pilosella officinarum F. Schultz et Sch. Bip.	0	n	1	0	Asteraceae Bercht. & J.Presl
Iz_3_1109	DJI_0016	Pilosella sp.	0.2		NA	0	Asteraceae Bercht. & J.Presl
Iz_3_1109	DJI_0016	Pinus sylvestris L.	0	n	1	1	Pinaceae Spreng. ex F.Rudolphi
Iz_3_1109	DJI_0016	Poa annua L.	0	n	1	0	Poaceae Barnhart
Iz_3_1109	DJI_0016	Poa nemoralis L.	0	n	1	0	Poaceae Barnhart
Iz_3_1109	DJI_0016	Poa sp.	0		NA	0	Poaceae Barnhart
Iz_3_1109	DJI_0016	Polygonatum odoratum (Mill.) Druce	0	n	1	0	Asparagaceae Juss.
Iz_3_1109	DJI_0016	Polygonum arenarium Waldst. & Kit.	0	n	1	0	Polygonaceae Juss.
Iz_3_1109	DJI_0016	Populus nigra L.	0	n	1	1	Salicaceae Mirb.
Iz_3_1109	DJI_0016	Populus tremula L.	0	n	1	1	Salicaceae Mirb.
Iz_3_1109	DJI_0016	Populus Ã canescens (Aiton) Sm.	0	n	1	1	Salicaceae Mirb.
Iz_3_1109	DJI_0016	Potentilla argentea L.	0	n	1	0	Rosaceae Juss.
Iz_3_1109	DJI_0016	Psammophiliella muralis (L.) Ikonn.	0	n	1	0	Caryophyllaceae Juss.
Iz_3_1109	DJI_0016	Psephellus sumensis (Kalen.) Greuter	0	n	1	0	Asteraceae Bercht. & J.Presl
Iz_3_1109	DJI_0016	Pulsatilla pratensis (L.) Mill. s.l.	0	n	1	0	Ranunculaceae Juss.
Iz_3_1109	DJI_0016	Pyrus communis L.	0	a	0	1	Rosaceae Juss.
Iz_3_1109	DJI_0016	Quercus robur L.	0	n	1	1	Fagaceae Dumort.
Iz_3_1109	DJI_0016	Robinia pseudoacacia L.	0	a	0	1	Fabaceae Lindl.
Iz_3_1109	DJI_0016	Rosa sp.	0		NA	1	Rosaceae Juss.
Iz_3_1109	DJI_0016	Rubus idaeus L.	0	n	1	0	Rosaceae Juss.
Iz_3_1109	DJI_0016	Rumex acetosella L.	0	n	1	0	Polygonaceae Juss.
Iz_3_1109	DJI_0016	Rumex thyrsiflorus Fingerh.	0	n	1	0	Polygonaceae Juss.
Iz_3_1109	DJI_0016	Salix alba L.	0	n	1	1	Salicaceae Mirb.
Iz_3_1109	DJI_0016	Sambucus nigra L.	0	n	1	1	Viburnaceae Raf.
Iz_3_1109	DJI_0016	Secale sylvestre Host	0	n	1	0	Poaceae Barnhart
Iz_3_1109	DJI_0016	Sempervivum ruthenicum Schnittsp. & C.B. Lehm.	0	n	1	0	Crassulaceae J.St.-Hil.
Iz_3_1109	DJI_0016	Setaria viridis (L.) P. Beauv.	0	n	1	0	Poaceae Barnhart
Iz_3_1109	DJI_0016	Silene borysthenica (Gruner) Walters	0	n	1	0	Caryophyllaceae Juss.
Iz_3_1109	DJI_0016	Silene latifolia subsp. alba (Mill.) Greuter & Burdet	0	n	1	0	Caryophyllaceae Juss.
Iz_3_1109	DJI_0016	Solidago virgaurea L.	0	n	1	0	Asteraceae Bercht. & J.Presl
Iz_3_1109	DJI_0016	Sorbus aucuparia L.	0	n	1	1	Rosaceae Juss.
Iz_3_1109	DJI_0016	Species 11.09_3_0045	0		NA	0	
Iz_3_1109	DJI_0016	Species 11.09_3_0058	0		NA	0	
Iz_3_1109	DJI_0016	Stachys recta L.	0	n	1	0	Lamiacea Lindl.
Iz_3_1109	DJI_0016	Tanacetum vulgare L.	0	n	1	0	Asteraceae Bercht. & J.Presl
Iz_3_1109	DJI_0016	Thymus pallasianus Heinr.Braun	0	n	1	0	Lamiacea Lindl.
Iz_3_1109	DJI_0016	Tilia cordata Mill.	0	n	1	1	Malvaceae Juss.
Iz_3_1109	DJI_0016	Trifolium alpestre L.	0	n	1	0	Fabaceae Lindl.
Iz_3_1109	DJI_0016	Trifolium arvense L.	0	n	1	0	Fabaceae Lindl.
Iz_3_1109	DJI_0016	Ulmus glabra Huds.	0	n	1	1	Ulmaceae Mirb.
Iz_3_1109	DJI_0016	Verbascum densiflorum Bertol.	0	n	1	0	Scrophulariaceae Juss.
Iz_3_1109	DJI_0016	Veronica austriaca L.	0	n	1	0	Plantaginaceae Juss.
Iz_3_1109	DJI_0016	Veronica incana L.	0	n	1	0	Plantaginaceae Juss.
Iz_3_1109	DJI_0016	Veronica verna L.	0	n	1	0	Plantaginaceae Juss.
Iz_3_1109	DJI_0016	Vincetoxicum hirundinaria Medik	0	n	1	0	Apocynaceae Juss.
Iz_3_1109	DJI_0020	Acer tataricum L.	0	n	1	1	Sapindaceae Juss.
Iz_3_1109	DJI_0020	Achillea millefolium L. s.l.	0	n	1	0	Asteraceae Bercht. & J.Presl
Iz_3_1109	DJI_0020	Allium oleraceum L.	0	n	1	0	Amaryllidaceae J.St.-Hil.
Iz_3_1109	DJI_0020	Ambrosia artemisiifolia L.	0	a	0	0	Asteraceae Bercht. & J.Presl
Iz_3_1109	DJI_0020	Amorpha fruticosa L.	0	a	0	0	Fabaceae Lindl.
Iz_3_1109	DJI_0020	Anthericum ramosum L.	0	n	1	0	Asparagaceae Juss.
Iz_3_1109	DJI_0020	Artemisia marschalliana Spreng.	0	n	1	0	Asteraceae Bercht. & J.Presl
Iz_3_1109	DJI_0020	Asparagus officinalis L.	0	n	1	0	Asparagaceae Juss.
Iz_3_1109	DJI_0020	Astragalus cicer L.	0	n	1	0	Fabaceae Lindl.
Iz_3_1109	DJI_0020	Bassia laniflora (S.G.Gmel.) A.J.Scott	0	n	1	0	Amaranthaceae Juss.
Iz_3_1109	DJI_0020	Berberis vulgaris L.	0	n	1	0	Berberidaceae Juss.
Iz_3_1109	DJI_0020	Betula pendula Roth.	0	n	1	1	Betulaceae Gray
Iz_3_1109	DJI_0020	Betula pendula Roth.	0	n	1	0	Poaceae Barnhart
Iz_3_1109	DJI_0020	Bromus inermis Leyss.	0		NA	NA	
Iz_3_1109	DJI_0020	Bromus squarrosus L.	0	a	0	0	Poaceae Barnhart
Iz_3_1109	DJI_0020	Bromus tectorum L.	0	a	0	0	Poaceae Barnhart
Iz_3_1109	DJI_0020	Calamagrostis epigejos (L.) Roth	15	n	1	0	Poaceae Barnhart
Iz_3_1109	DJI_0020	Carduus acanthoides L.	0	n	1	0	Asteraceae Bercht. & J.Presl
Iz_3_1109	DJI_0020	Carex sp.	0	n	1	0	Cyperaceae Juss.
Iz_3_1109	DJI_0020	Centaurea majorovii Dumbadze	0	n	1	0	Asteraceae Bercht. & J.Presl
Iz_3_1109	DJI_0020	Chamaecytisus ruthenicus (Fisch.ex Wol.) Klaskova	0	n	1	0	Fabaceae Lindl.
Iz_3_1109	DJI_0020	Chelidonium majus L.	0	n	1	0	Papaveraceae Juss.
Iz_3_1109	DJI_0020	Chenopodium betaceum Andrz.	0	n	1	0	Amaranthaceae Juss.

Iz_3_1109	DJI_0020	Chenopodium cf. album	0	n	1	0	Amaranthaceae Juss.
Iz_3_1109	DJI_0020	Chenopodium sp.	0		NA	0	Amaranthaceae Juss.
Iz_3_1109	DJI_0020	Chondrilla juncea L.	0	n	1	0	Asteraceae Bercht. & J.Presl
Iz_3_1109	DJI_0020	Cirsium vulgare (Savi) Ten.	0	n	1	0	Asteraceae Bercht. & J.Presl
Iz_3_1109	DJI_0020	Clinopodium acinos (L.) Kuntze	0	n	1	0	Lamiacea Lindl.
Iz_3_1109	DJI_0020	Convallaria majalis L.	0	n	1	0	Asparagaceae Juss.
Iz_3_1109	DJI_0020	Crataegus rhipidophylla Gand.	0	n	1	1	Rosaceae Juss.
Iz_3_1109	DJI_0020	Crepis foetida L.	0	n	1	0	Asteraceae Bercht. & J.Presl
Iz_3_1109	DJI_0020	Dactylis glomerata L.	0	n	1	0	Poaceae Barnhart
Iz_3_1109	DJI_0020	Dianthus campestris M.Bieb.	0	n	1	0	Caryophyllaceae Juss.
Iz_3_1109	DJI_0020	Digitaria sanguinalis (L.) Scop.	0	a	0	0	Poaceae Barnhart
Iz_3_1109	DJI_0020	Elymus repens (L.) Gould	0	n	1	0	Poaceae Barnhart
Iz_3_1109	DJI_0020	Epilobium tetragonum L.	0	n	1	0	Onagraceae Juss.
Iz_3_1109	DJI_0020	Eragrostis minor Host	0	a	0	0	Poaceae Barnhart
Iz_3_1109	DJI_0020	Erigeron canadensis L.	0	a	0	0	Asteraceae Bercht. & J.Presl
Iz_3_1109	DJI_0020	Euphorbia seguieriana Neck.	0	n	1	0	Euphorbiaceae Juss.
Iz_3_1109	DJI_0020	Fallopia dumetorum (L.) Holub	0	n	1	0	Polygonaceae Juss.
Iz_3_1109	DJI_0020	Festuca beckeri (Hack.) Trautv.	0	n	1	0	Poaceae Barnhart
Iz_3_1109	DJI_0020	Fragaria vesca L.	0	n	1	0	Rosaceae Juss.
Iz_3_1109	DJI_0020	Frangula alnus Mill.	0	n	1	1	Rhamnaceae Juss.
Iz_3_1109	DJI_0020	Fraxinus pennsylvanica Marshall	0	a	0	1	Oleaceae Hoffmanns. & Link
Iz_3_1109	DJI_0020	Galeopsis bifida Boenn.	0	n	1	0	Lamiacea Lindl.
Iz_3_1109	DJI_0020	Galium verum L.	0	n	1	0	Rubiaceae Juss.
Iz_3_1109	DJI_0020	Genista tinctoria L.	0	n	1	0	Fabaceae Lindl.
Iz_3_1109	DJI_0020	Geranium robertianum L.	0	n	1	0	Geraniaceae Juss.
Iz_3_1109	DJI_0020	Geranium sanquineum L.	0	n	1	0	Geraniaceae Juss.
Iz_3_1109	DJI_0020	Gypsophila paniculata L.	0	n	1	0	Caryophyllaceae Juss.
Iz_3_1109	DJI_0020	Helichrysum arenarium (L.) Moench	0	n	1	0	Asteraceae Bercht. & J.Presl
Iz_3_1109	DJI_0020	Herniaria polygama J.Gay	0	n	1	0	Caryophyllaceae Juss.
Iz_3_1109	DJI_0020	Hieracium robustum Fr.	0	n	1	0	Asteraceae Bercht. & J.Presl
Iz_3_1109	DJI_0020	Hieracium umbellatum L.	0	n	1	NA	Asteraceae Bercht. & J.Presl
Iz_3_1109	DJI_0020	Hylotelephium maximum (L.) Holub	0	n	1	0	Crassulaceae J.St.-Hil.
Iz_3_1109	DJI_0020	Jacobaea borysthenica (DC.) B.Nord. & Greuter	0	n	1	0	Asteraceae Bercht. & J.Presl
Iz_3_1109	DJI_0020	Jasione montana L.	0	n	1	0	Campanulaceae Juss.
Iz_3_1109	DJI_0020	Jurinea cyanoides (L.) Rchb.s.l.	0	n	1	0	Asteraceae Bercht. & J.Presl
Iz_3_1109	DJI_0020	Koeleria glauca (Spreng.) DC.	0	n	1	0	Poaceae Barnhart
Iz_3_1109	DJI_0020	Lactuca serriola L.	0	a	0	0	Asteraceae Bercht. & J.Presl
Iz_3_1109	DJI_0020	Linaria genistifolia (L.) Mill.	0	n	1	0	Plantaginaceae Juss.
Iz_3_1109	DJI_0020	Lomelosia argentea (L.) Greuter & Burdet	0	n	1	0	Caprifoliaceae Juss.
Iz_3_1109	DJI_0020	Melampyrum pratense L.	0	n	1	0	Orobanchaceae Vent.
Iz_3_1109	DJI_0020	Oenothera biennis L.	0	a	0	0	Onagraceae Juss.
Iz_3_1109	DJI_0020	Pentanema britannica (L.) D.Gut.Larr., Santos-Vicente, Anderb., E.Rico & M.M.Mart.Ort.	0	n	1	0	Asteraceae Bercht. & J.Presl
Iz_3_1109	DJI_0020	Peucedanum oreoselinum (L.) Moench	0	n	1	0	Apiaceae Lindl.
Iz_3_1109	DJI_0020	Peucedanum ruthenicum M.Bieb.	0	n	1	0	Apiaceae Lindl.
Iz_3_1109	DJI_0020	Pilosella echioides (Lumn.) F.W.Schultz & Sch.Bip.	0	n	1	0	Asteraceae Bercht. & J.Presl
Iz_3_1109	DJI_0020	Pilosella officinarum F. Schultz et Sch. Bip.	0	n	1	0	Asteraceae Bercht. & J.Presl
Iz_3_1109	DJI_0020	Pilosella sp.	0		NA	0	Asteraceae Bercht. & J.Presl
Iz_3_1109	DJI_0020	Pinus sylvestris L.	0	n	1	1	Pinaceae Spreng. ex F.Rudolphi
Iz_3_1109	DJI_0020	Poa annua L.	0	n	1	0	Poaceae Barnhart
Iz_3_1109	DJI_0020	Poa nemoralis L.	0	n	1	0	Poaceae Barnhart
Iz_3_1109	DJI_0020	Poa sp.	0		NA	0	Poaceae Barnhart
Iz_3_1109	DJI_0020	Polygonatum odoratum (Mill.) Druce	0	n	1	0	Asparagaceae Juss.
Iz_3_1109	DJI_0020	Polygonum arenarium Waldst. & Kit.	0	n	1	0	Polygonaceae Juss.
Iz_3_1109	DJI_0020	Populus nigra L.	0	n	1	1	Salicaceae Mirb.
Iz_3_1109	DJI_0020	Populus tremula L.	0	n	1	1	Salicaceae Mirb.
Iz_3_1109	DJI_0020	Populus Ã canescens (Aiton) Sm.	0	n	1	1	Salicaceae Mirb.
Iz_3_1109	DJI_0020	Potentilla argentea L.	0	n	1	0	Rosaceae Juss.
Iz_3_1109	DJI_0020	Psammophiliella muralis (L.) Ikonn.	0	n	1	0	Caryophyllaceae Juss.
Iz_3_1109	DJI_0020	Psephellus sumensis (Kalen.) Greuter	0	n	1	0	Asteraceae Bercht. & J.Presl
Iz_3_1109	DJI_0020	Pulsatilla pratensis (L.) Mill. s.l.	0	n	1	0	Ranunculaceae Juss.
Iz_3_1109	DJI_0020	Pyrus communis L.	0	a	0	1	Rosaceae Juss.
Iz_3_1109	DJI_0020	Quercus robur L.	0	n	1	1	Fagaceae Dumort.
Iz_3_1109	DJI_0020	Robinia pseudoacacia L.	0	a	0	1	Fabaceae Lindl.
Iz_3_1109	DJI_0020	Rosa sp.	0		NA	1	Rosaceae Juss.
Iz_3_1109	DJI_0020	Rubus idaeus L.	0	n	1	0	Rosaceae Juss.
Iz_3_1109	DJI_0020	Rumex acetosella L.	3	n	1	0	Polygonaceae Juss.
Iz_3_1109	DJI_0020	Rumex thyrsiflorus Fingerh.	0	n	1	0	Polygonaceae Juss.
Iz_3_1109	DJI_0020	Salix alba L.	0	n	1	1	Salicaceae Mirb.
Iz_3_1109	DJI_0020	Sambucus nigra L.	0	n	1	1	Viburnaceae Raf.
Iz_3_1109	DJI_0020	Secale sylvestre Host	0	n	1	0	Poaceae Barnhart
Iz_3_1109	DJI_0020	Sempervivum ruthenicum Schnittsp. & C.B. Lehm.	0	n	1	0	Crassulaceae J.St.-Hil.
Iz_3_1109	DJI_0020	Setaria viridis (L.) P. Beauv.	0	n	1	0	Poaceae Barnhart
Iz_3_1109	DJI_0020	Silene borysthenica (Gruner) Walters	0	n	1	0	Caryophyllaceae Juss.
Iz_3_1109	DJI_0020	Silene latifolia subsp. alba (Mill.) Greuter & Burdet	0	n	1	0	Caryophyllaceae Juss.
Iz_3_1109	DJI_0020	Solidago virgaurea L.	0	n	1	0	Asteraceae Bercht. & J.Presl
Iz_3_1109	DJI_0020	Sorbus aucuparia L.	0	n	1	1	Rosaceae Juss.
Iz_3_1109	DJI_0020	Species 11.09_3_0045	0		NA	0	
Iz_3_1109	DJI_0020	Species 11.09_3_0058	0		NA	0	
Iz_3_1109	DJI_0020	Stachys recta L.	0	n	1	0	Lamiacea Lindl.
Iz_3_1109	DJI_0020	Tanacetum vulgare L.	0	n	1	0	Asteraceae Bercht. & J.Presl
Iz_3_1109	DJI_0020	Thymus pallasianus Heinr.Braun	0	n	1	0	Lamiacea Lindl.
Iz_3_1109	DJI_0020	Tilia cordata Mill.	0	n	1	1	Malvaceae Juss.
Iz_3_1109	DJI_0020	Trifolium alpestre L.	0	n	1	0	Fabaceae Lindl.
Iz_3_1109	DJI_0020	Trifolium arvense L.	0	n	1	0	Fabaceae Lindl.
Iz_3_1109	DJI_0020	Ulmus glabra Huds.	0	n	1	1	Ulmaceae Mirb.
Iz_3_1109	DJI_0020	Verbascum densiflorum Bertol.	0	n	1	0	Scrophulariaceae Juss.
Iz_3_1109	DJI_0020	Veronica austriaca L.	0	n	1	0	Plantaginaceae Juss.
Iz_3_1109	DJI_0020	Veronica incana L.	0	n	1	0	Plantaginaceae Juss.
Iz_3_1109	DJI_0020	Veronica verna L.	0	n	1	0	Plantaginaceae Juss.
Iz_3_1109	DJI_0020	Vincetoxicum hirundinaria Medik	0	n	1	0	Apocynaceae Juss.
Iz_3_1109	DJI_0023	Acer tataricum L.	0	n	1	1	Sapindaceae Juss.
Iz_3_1109	DJI_0023	Achillea millefolium L. s.l.	0	n	1	0	Asteraceae Bercht. & J.Presl
Iz_3_1109	DJI_0023	Allium oleraceum L.	0	n	1	0	Amaryllidaceae J.St.-Hil.
Iz_3_1109	DJI_0023	Ambrosia artemisiifolia L.	0	a	0	0	Asteraceae Bercht. & J.Presl
Iz_3_1109	DJI_0023	Amorpha fruticosa L.	0	a	0	0	Fabaceae Lindl.
Iz_3_1109	DJI_0023	Anthericum ramosum L.	0	n	1	0	Asparagaceae Juss.
Iz_3_1109	DJI_0023	Artemisia marschalliana Spreng.	0	n	1	0	Asteraceae Bercht. & J.Presl
Iz_3_1109	DJI_0023	Asparagus officinalis L.	0	n	1	0	Asparagaceae Juss.

Iz_3_1109	DJI_0023	Astragalus cicer L.	0	n	1	0	Fabaceae Lindl.
Iz_3_1109	DJI_0023	Bassia laniflora (S.G.Gmel.) A.J.Scott	0	n	1	0	Amaranthaceae Juss.
Iz_3_1109	DJI_0023	Berberis vulgaris L.	0	n	1	0	Berberidaceae Juss.
Iz_3_1109	DJI_0023	Betula pendula Roth.	0	n	1	1	Betulaceae Gray
Iz_3_1109	DJI_0023	Betula pendula Roth.	0	n	1	0	Poaceae Barnhart
Iz_3_1109	DJI_0023	Bromus inermis Leyss.	0		NA	NA	
Iz_3_1109	DJI_0023	Bromus squarrosus L.	0	a	0	0	Poaceae Barnhart
Iz_3_1109	DJI_0023	Bromus tectorum L.	0	a	0	0	Poaceae Barnhart
Iz_3_1109	DJI_0023	Calamagrostis epigejos (L.) Roth	0	n	1	0	Poaceae Barnhart
Iz_3_1109	DJI_0023	Carduus acanthoides L.	0	n	1	0	Asteraceae Bercht. & J.Presl
Iz_3_1109	DJI_0023	Carex sp.	0	n	1	0	Cyperaceae Juss.
Iz_3_1109	DJI_0023	Centaurea majorovii Dumbadze	0	n	1	0	Asteraceae Bercht. & J.Presl
Iz_3_1109	DJI_0023	Chamaecytisus ruthenicus (Fisch.ex Wol.) Klaskova	0	n	1	0	Fabaceae Lindl.
Iz_3_1109	DJI_0023	Chelidonium majus L.	0	n	1	0	Papaveraceae Juss.
Iz_3_1109	DJI_0023	Chenopodium betaceum Andrz.	0	n	1	0	Amaranthaceae Juss.
Iz_3_1109	DJI_0023	Chenopodium cf. album	0	n	1	0	Amaranthaceae Juss.
Iz_3_1109	DJI_0023	Chenopodium sp.	0		NA	0	Amaranthaceae Juss.
Iz_3_1109	DJI_0023	Chondrilla juncea L.	0	n	1	0	Asteraceae Bercht. & J.Presl
Iz_3_1109	DJI_0023	Cirsium vulgare (Savi) Ten.	0	n	1	0	Asteraceae Bercht. & J.Presl
Iz_3_1109	DJI_0023	Clinopodium acinos (L.) Kuntze	0	n	1	0	Lamiacea Lindl.
Iz_3_1109	DJI_0023	Convallaria majalis L.	0	n	1	0	Asparagaceae Juss.
Iz_3_1109	DJI_0023	Crataegus rhipidophylla Gand.	0	n	1	1	Rosaceae Juss.
Iz_3_1109	DJI_0023	Crepis foetida L.	0	n	1	0	Asteraceae Bercht. & J.Presl
Iz_3_1109	DJI_0023	Dactylis glomerata L.	0	n	1	0	Poaceae Barnhart
Iz_3_1109	DJI_0023	Dianthus campestris M.Bieb.	0	n	1	0	Caryophyllaceae Juss.
Iz_3_1109	DJI_0023	Digitaria sanguinalis (L.) Scop.	0	a	0	0	Poaceae Barnhart
Iz_3_1109	DJI_0023	Elymus repens (L.) Gould	0	n	1	0	Poaceae Barnhart
Iz_3_1109	DJI_0023	Epilobium tetragonum L.	0	n	1	0	Onagraceae Juss.
Iz_3_1109	DJI_0023	Eragrostis minor Host	0	a	0	0	Poaceae Barnhart
Iz_3_1109	DJI_0023	Erigeron canadensis L.	0.2	a	0	0	Asteraceae Bercht. & J.Presl
Iz_3_1109	DJI_0023	Euphorbia seguieriana Neck.	0	n	1	0	Euphorbiaceae Juss.
Iz_3_1109	DJI_0023	Fallopia dumetorum (L.) Holub	0	n	1	0	Polygonaceae Juss.
Iz_3_1109	DJI_0023	Festuca beckeri (Hack.) Trautv.	0	n	1	0	Poaceae Barnhart
Iz_3_1109	DJI_0023	Fragaria vesca L.	0	n	1	0	Rosaceae Juss.
Iz_3_1109	DJI_0023	Frangula alnus Mill.	0	n	1	1	Rhamnaceae Juss.
Iz_3_1109	DJI_0023	Fraxinus pennsylvanica Marshall	0	a	0	1	Oleaceae Hoffmanns. & Link
Iz_3_1109	DJI_0023	Galeopsis bifida Boenn.	0	n	1	0	Lamiacea Lindl.
Iz_3_1109	DJI_0023	Galium verum L.	0	n	1	0	Rubiaceae Juss.
Iz_3_1109	DJI_0023	Genista tinctoria L.	0	n	1	0	Fabaceae Lindl.
Iz_3_1109	DJI_0023	Geranium robertianum L.	0	n	1	0	Geraniaceae Juss.
Iz_3_1109	DJI_0023	Geranium sanquineum L.	0	n	1	0	Geraniaceae Juss.
Iz_3_1109	DJI_0023	Gypsophila paniculata L.	0	n	1	0	Caryophyllaceae Juss.
Iz_3_1109	DJI_0023	Helichrysum arenarium (L.) Moench	0	n	1	0	Asteraceae Bercht. & J.Presl
Iz_3_1109	DJI_0023	Herniaria polygama J.Gay	0	n	1	0	Caryophyllaceae Juss.
Iz_3_1109	DJI_0023	Hieracium robustum Fr.	0	n	1	0	Asteraceae Bercht. & J.Presl
Iz_3_1109	DJI_0023	Hieracium umbellatum L.	0	n	1	NA	Asteraceae Bercht. & J.Presl
Iz_3_1109	DJI_0023	Hylotelephium maximum (L.) Holub	0	n	1	0	Crassulaceae J.St.-Hil.
Iz_3_1109	DJI_0023	Jacobaea borysthenica (DC.) B.Nord. & Greuter	0	n	1	0	Asteraceae Bercht. & J.Presl
Iz_3_1109	DJI_0023	Jasione montana L.	0	n	1	0	Campanulaceae Juss.
Iz_3_1109	DJI_0023	Jurinea cyanoides (L.) Rchb.s.l.	0	n	1	0	Asteraceae Bercht. & J.Presl
Iz_3_1109	DJI_0023	Koeleria glauca (Spreng.) DC.	0	n	1	0	Poaceae Barnhart
Iz_3_1109	DJI_0023	Lactuca serriola L.	0	a	0	0	Asteraceae Bercht. & J.Presl
Iz_3_1109	DJI_0023	Linaria genistifolia (L.) Mill.	0	n	1	0	Plantaginaceae Juss.
Iz_3_1109	DJI_0023	Lomelosia argentea (L.) Greuter & Burdet	0	n	1	0	Caprifoliaceae Juss.
Iz_3_1109	DJI_0023	Melampyrum pratense L.	0	n	1	0	Orobanchaceae Vent.
Iz_3_1109	DJI_0023	Oenothera biennis L.	0	a	0	0	Onagraceae Juss.
Iz_3_1109	DJI_0023	Pentanema britannica (L.) D.Gut.Larr., Santos-Vicente, Anderb., E.Rico & M.M.Mart.Ort.	0	n	1	0	Asteraceae Bercht. & J.Presl
Iz_3_1109	DJI_0023	Peucedanum oreoselinum (L.) Moench	0	n	1	0	Apiaceae Lindl.
Iz_3_1109	DJI_0023	Peucedanum ruthenicum M.Bieb.	0	n	1	0	Apiaceae Lindl.
Iz_3_1109	DJI_0023	Pilosella echioides (Lumn.) F.W.Schultz & Sch.Bip.	0	n	1	0	Asteraceae Bercht. & J.Presl
Iz_3_1109	DJI_0023	Pilosella officinarum F. Schultz et Sch. Bip.	0	n	1	0	Asteraceae Bercht. & J.Presl
Iz_3_1109	DJI_0023	Pilosella sp.	0.2		NA	0	Asteraceae Bercht. & J.Presl
Iz_3_1109	DJI_0023	Pinus sylvestris L.	0	n	1	1	Pinaceae Spreng. ex F.Rudolphi
Iz_3_1109	DJI_0023	Poa annua L.	0	n	1	0	Poaceae Barnhart
Iz_3_1109	DJI_0023	Poa nemoralis L.	0	n	1	0	Poaceae Barnhart
Iz_3_1109	DJI_0023	Poa sp.	0		NA	0	Poaceae Barnhart
Iz_3_1109	DJI_0023	Polygonatum odoratum (Mill.) Druce	0	n	1	0	Asparagaceae Juss.
Iz_3_1109	DJI_0023	Polygonum arenarium Waldst. & Kit.	0	n	1	0	Polygonaceae Juss.
Iz_3_1109	DJI_0023	Populus nigra L.	0	n	1	1	Salicaceae Mirb.
Iz_3_1109	DJI_0023	Populus tremula L.	0	n	1	1	Salicaceae Mirb.
Iz_3_1109	DJI_0023	Populus Ã canescens (Aiton) Sm.	0	n	1	1	Salicaceae Mirb.
Iz_3_1109	DJI_0023	Potentilla argentea L.	0	n	1	0	Rosaceae Juss.
Iz_3_1109	DJI_0023	Psammophiliella muralis (L.) Ikonn.	0	n	1	0	Caryophyllaceae Juss.
Iz_3_1109	DJI_0023	Psephellus sumensis (Kalen.) Greuter	0	n	1	0	Asteraceae Bercht. & J.Presl
Iz_3_1109	DJI_0023	Pulsatilla pratensis (L.) Mill. s.l.	0	n	1	0	Ranunculaceae Juss.
Iz_3_1109	DJI_0023	Pyrus communis L.	0	a	0	1	Rosaceae Juss.
Iz_3_1109	DJI_0023	Quercus robur L.	0	n	1	1	Fagaceae Dumort.
Iz_3_1109	DJI_0023	Robinia pseudoacacia L.	0	a	0	1	Fabaceae Lindl.
Iz_3_1109	DJI_0023	Rosa sp.	0		NA	1	Rosaceae Juss.
Iz_3_1109	DJI_0023	Rubus idaeus L.	0	n	1	0	Rosaceae Juss.
Iz_3_1109	DJI_0023	Rumex acetosella L.	0	n	1	0	Polygonaceae Juss.
Iz_3_1109	DJI_0023	Rumex thyrsiflorus Fingerh.	0	n	1	0	Polygonaceae Juss.
Iz_3_1109	DJI_0023	Salix alba L.	0	n	1	1	Salicaceae Mirb.
Iz_3_1109	DJI_0023	Sambucus nigra L.	0	n	1	1	Viburnaceae Raf.
Iz_3_1109	DJI_0023	Secale sylvestre Host	0	n	1	0	Poaceae Barnhart
Iz_3_1109	DJI_0023	Sempervivum ruthenicum Schnittsp. & C.B. Lehm.	0	n	1	0	Crassulaceae J.St.-Hil.
Iz_3_1109	DJI_0023	Setaria viridis (L.) P. Beauv.	0	n	1	0	Poaceae Barnhart
Iz_3_1109	DJI_0023	Silene borysthenica (Gruner) Walters	0	n	1	0	Caryophyllaceae Juss.
Iz_3_1109	DJI_0023	Silene latifolia subsp. alba (Mill.) Greuter & Burdet	0	n	1	0	Caryophyllaceae Juss.
Iz_3_1109	DJI_0023	Solidago virgaurea L.	0	n	1	0	Asteraceae Bercht. & J.Presl
Iz_3_1109	DJI_0023	Sorbus aucuparia L.	0	n	1	1	Rosaceae Juss.
Iz_3_1109	DJI_0023	Species 11.09_3_0045	0		NA	0	
Iz_3_1109	DJI_0023	Species 11.09_3_0058	0		NA	0	
Iz_3_1109	DJI_0023	Stachys recta L.	0	n	1	0	Lamiacea Lindl.
Iz_3_1109	DJI_0023	Tanacetum vulgare L.	0	n	1	0	Asteraceae Bercht. & J.Presl
Iz_3_1109	DJI_0023	Thymus pallasianus Heinr.Braun	0	n	1	0	Lamiacea Lindl.
Iz_3_1109	DJI_0023	Tilia cordata Mill.	0	n	1	1	Malvaceae Juss.
Iz_3_1109	DJI_0023	Trifolium alpestre L.	0	n	1	0	Fabaceae Lindl.

Iz_3_1109	DJI_0023	Trifolium arvense L.	0	n	1	0	Fabaceae Lindl.
Iz_3_1109	DJI_0023	Ulmus glabra Huds.	0	n	1	1	Ulmaceae Mirb.
Iz_3_1109	DJI_0023	Verbascum densiflorum Bertol.	0	n	1	0	Scrophulariaceae Juss.
Iz_3_1109	DJI_0023	Veronica austriaca L.	0	n	1	0	Plantaginaceae Juss.
Iz_3_1109	DJI_0023	Veronica incana L.	0	n	1	0	Plantaginaceae Juss.
Iz_3_1109	DJI_0023	Veronica verna L.	0	n	1	0	Plantaginaceae Juss.
Iz_3_1109	DJI_0023	Vincetoxicum hirundinaria Medik	0	n	1	0	Apocynaceae Juss.
Iz_3_1109	DJI_0027	Acer tataricum L.	0	n	1	1	Sapindaceae Juss.
Iz_3_1109	DJI_0027	Achillea millefolium L. s.l.	0	n	1	0	Asteraceae Bercht. & J.Presl
Iz_3_1109	DJI_0027	Allium oleraceum L.	0	n	1	0	Amaryllidaceae J.St.-Hil.
Iz_3_1109	DJI_0027	Ambrosia artemisiifolia L.	0	a	0	0	Asteraceae Bercht. & J.Presl
Iz_3_1109	DJI_0027	Amorpha fruticosa L.	0	a	0	0	Fabaceae Lindl.
Iz_3_1109	DJI_0027	Anthericum ramosum L.	0	n	1	0	Asparagaceae Juss.
Iz_3_1109	DJI_0027	Artemisia marschalliana Spreng.	0	n	1	0	Asteraceae Bercht. & J.Presl
Iz_3_1109	DJI_0027	Asparagus officinalis L.	0	n	1	0	Asparagaceae Juss.
Iz_3_1109	DJI_0027	Astragalus cicer L.	0	n	1	0	Fabaceae Lindl.
Iz_3_1109	DJI_0027	Bassia laniflora (S.G.Gmel.) A.J.Scott	0	n	1	0	Amaranthaceae Juss.
Iz_3_1109	DJI_0027	Berberis vulgaris L.	0	n	1	0	Berberidaceae Juss.
Iz_3_1109	DJI_0027	Betula pendula Roth.	0	n	1	1	Betulaceae Gray
Iz_3_1109	DJI_0027	Betula pendula Roth.	0	n	1	0	Poaceae Barnhart
Iz_3_1109	DJI_0027	Bromus inermis Leyss.	0		NA	NA	
Iz_3_1109	DJI_0027	Bromus squarrosus L.	0	a	0	0	Poaceae Barnhart
Iz_3_1109	DJI_0027	Bromus tectorum L.	0	a	0	0	Poaceae Barnhart
Iz_3_1109	DJI_0027	Calamagrostis epigejos (L.) Roth	0	n	1	0	Poaceae Barnhart
Iz_3_1109	DJI_0027	Carduus acanthoides L.	0	n	1	0	Asteraceae Bercht. & J.Presl
Iz_3_1109	DJI_0027	Carex sp.	0	n	1	0	Cyperaceae Juss.
Iz_3_1109	DJI_0027	Centaurea majorovii Dumbadze	0	n	1	0	Asteraceae Bercht. & J.Presl
Iz_3_1109	DJI_0027	Chamaecytisus ruthenicus (Fisch.ex Wol.) Klaskova	0	n	1	0	Fabaceae Lindl.
Iz_3_1109	DJI_0027	Chelidonium majus L.	0	n	1	0	Papaveraceae Juss.
Iz_3_1109	DJI_0027	Chenopodium betaceum Andrz.	0	n	1	0	Amaranthaceae Juss.
Iz_3_1109	DJI_0027	Chenopodium cf. album	0	n	1	0	Amaranthaceae Juss.
Iz_3_1109	DJI_0027	Chenopodium sp.	0		NA	0	Amaranthaceae Juss.
Iz_3_1109	DJI_0027	Chondrilla juncea L.	0	n	1	0	Asteraceae Bercht. & J.Presl
Iz_3_1109	DJI_0027	Cirsium vulgare (Savi) Ten.	0	n	1	0	Asteraceae Bercht. & J.Presl
Iz_3_1109	DJI_0027	Clinopodium acinos (L.) Kuntze	0	n	1	0	Lamiacea Lindl.
Iz_3_1109	DJI_0027	Convallaria majalis L.	0	n	1	0	Asparagaceae Juss.
Iz_3_1109	DJI_0027	Crataegus rhipidophylla Gand.	0	n	1	1	Rosaceae Juss.
Iz_3_1109	DJI_0027	Crepis foetida L.	0	n	1	0	Asteraceae Bercht. & J.Presl
Iz_3_1109	DJI_0027	Dactylis glomerata L.	0	n	1	0	Poaceae Barnhart
Iz_3_1109	DJI_0027	Dianthus campestris M.Bieb.	0	n	1	0	Caryophyllaceae Juss.
Iz_3_1109	DJI_0027	Digitaria sanguinalis (L.) Scop.	0	a	0	0	Poaceae Barnhart
Iz_3_1109	DJI_0027	Elymus repens (L.) Gould	0	n	1	0	Poaceae Barnhart
Iz_3_1109	DJI_0027	Epilobium tetragonum L.	0	n	1	0	Onagraceae Juss.
Iz_3_1109	DJI_0027	Eragrostis minor Host	0	a	0	0	Poaceae Barnhart
Iz_3_1109	DJI_0027	Erigeron canadensis L.	0.1	a	0	0	Asteraceae Bercht. & J.Presl
Iz_3_1109	DJI_0027	Euphorbia seguieriana Neck.	0	n	1	0	Euphorbiaceae Juss.
Iz_3_1109	DJI_0027	Fallopia dumetorum (L.) Holub	0	n	1	0	Polygonaceae Juss.
Iz_3_1109	DJI_0027	Festuca beckeri (Hack.) Trautv.	0	n	1	0	Poaceae Barnhart
Iz_3_1109	DJI_0027	Fragaria vesca L.	0	n	1	0	Rosaceae Juss.
Iz_3_1109	DJI_0027	Frangula alnus Mill.	0	n	1	1	Rhamnaceae Juss.
Iz_3_1109	DJI_0027	Fraxinus pennsylvanica Marshall	0	a	0	1	Oleaceae Hoffmanns. & Link
Iz_3_1109	DJI_0027	Galeopsis bifida Boenn.	0	n	1	0	Lamiacea Lindl.
Iz_3_1109	DJI_0027	Galium verum L.	0	n	1	0	Rubiaceae Juss.
Iz_3_1109	DJI_0027	Genista tinctoria L.	0	n	1	0	Fabaceae Lindl.
Iz_3_1109	DJI_0027	Geranium robertianum L.	0	n	1	0	Geraniaceae Juss.
Iz_3_1109	DJI_0027	Geranium sanquineum L.	0	n	1	0	Geraniaceae Juss.
Iz_3_1109	DJI_0027	Gypsophila paniculata L.	0	n	1	0	Caryophyllaceae Juss.
Iz_3_1109	DJI_0027	Helichrysum arenarium (L.) Moench	0	n	1	0	Asteraceae Bercht. & J.Presl
Iz_3_1109	DJI_0027	Herniaria polygama J.Gay	0	n	1	0	Caryophyllaceae Juss.
Iz_3_1109	DJI_0027	Hieracium robustum Fr.	0	n	1	0	Asteraceae Bercht. & J.Presl
Iz_3_1109	DJI_0027	Hieracium umbellatum L.	0	n	1	NA	Asteraceae Bercht. & J.Presl
Iz_3_1109	DJI_0027	Hylotelephium maximum (L.) Holub	0	n	1	0	Crassulaceae J.St.-Hil.
Iz_3_1109	DJI_0027	Jacobaea borysthenica (DC.) B.Nord. & Greuter	0	n	1	0	Asteraceae Bercht. & J.Presl
Iz_3_1109	DJI_0027	Jasione montana L.	0	n	1	0	Campanulaceae Juss.
Iz_3_1109	DJI_0027	Jurinea cyanoides (L.) Rchb.s.l.	0	n	1	0	Asteraceae Bercht. & J.Presl
Iz_3_1109	DJI_0027	Koeleria glauca (Spreng.) DC.	0	n	1	0	Poaceae Barnhart
Iz_3_1109	DJI_0027	Lactuca serriola L.	0	a	0	0	Asteraceae Bercht. & J.Presl
Iz_3_1109	DJI_0027	Linaria genistifolia (L.) Mill.	0	n	1	0	Plantaginaceae Juss.
Iz_3_1109	DJI_0027	Lomelosia argentea (L.) Greuter & Burdet	0	n	1	0	Caprifoliaceae Juss.
Iz_3_1109	DJI_0027	Melampyrum pratense L.	0	n	1	0	Orobanchaceae Vent.
Iz_3_1109	DJI_0027	Oenothera biennis L.	0	a	0	0	Onagraceae Juss.
Iz_3_1109	DJI_0027	Pentanema britannica (L.) D.Gut.Larr., Santos-Vicente, Anderb., E.Rico & M.M.Mart.Ort.	0	n	1	0	Asteraceae Bercht. & J.Presl
Iz_3_1109	DJI_0027	Peucedanum oreoselinum (L.) Moench	0	n	1	0	Apiaceae Lindl.
Iz_3_1109	DJI_0027	Peucedanum ruthenicum M.Bieb.	0	n	1	0	Apiaceae Lindl.
Iz_3_1109	DJI_0027	Pilosella echioides (Lumn.) F.W.Schultz & Sch.Bip.	0	n	1	0	Asteraceae Bercht. & J.Presl
Iz_3_1109	DJI_0027	Pilosella officinarum F. Schultz et Sch. Bip.	0	n	1	0	Asteraceae Bercht. & J.Presl
Iz_3_1109	DJI_0027	Pilosella sp.	0		NA	0	Asteraceae Bercht. & J.Presl
Iz_3_1109	DJI_0027	Pinus sylvestris L.	0	n	1	1	Pinaceae Spreng. ex F.Rudolphi
Iz_3_1109	DJI_0027	Poa annua L.	0	n	1	0	Poaceae Barnhart
Iz_3_1109	DJI_0027	Poa nemoralis L.	0	n	1	0	Poaceae Barnhart
Iz_3_1109	DJI_0027	Poa sp.	0		NA	0	Poaceae Barnhart
Iz_3_1109	DJI_0027	Polygonatum odoratum (Mill.) Druce	0	n	1	0	Asparagaceae Juss.
Iz_3_1109	DJI_0027	Polygonum arenarium Waldst. & Kit.	0	n	1	0	Polygonaceae Juss.
Iz_3_1109	DJI_0027	Populus nigra L.	0	n	1	1	Salicaceae Mirb.
Iz_3_1109	DJI_0027	Populus tremula L.	0	n	1	1	Salicaceae Mirb.
Iz_3_1109	DJI_0027	Populus Ã canescens (Aiton) Sm.	0	n	1	1	Salicaceae Mirb.
Iz_3_1109	DJI_0027	Potentilla argentea L.	0	n	1	0	Rosaceae Juss.
Iz_3_1109	DJI_0027	Psammophiliella muralis (L.) Ikonn.	0	n	1	0	Caryophyllaceae Juss.
Iz_3_1109	DJI_0027	Psephellus sumensis (Kalen.) Greuter	0	n	1	0	Asteraceae Bercht. & J.Presl
Iz_3_1109	DJI_0027	Pulsatilla pratensis (L.) Mill. s.l.	0	n	1	0	Ranunculaceae Juss.
Iz_3_1109	DJI_0027	Pyrus communis L.	0	a	0	1	Rosaceae Juss.
Iz_3_1109	DJI_0027	Quercus robur L.	0	n	1	1	Fagaceae Dumort.
Iz_3_1109	DJI_0027	Robinia pseudoacacia L.	0	a	0	1	Fabaceae Lindl.
Iz_3_1109	DJI_0027	Rosa sp.	0		NA	1	Rosaceae Juss.
Iz_3_1109	DJI_0027	Rubus idaeus L.	0	n	1	0	Rosaceae Juss.
Iz_3_1109	DJI_0027	Rumex acetosella L.	0	n	1	0	Polygonaceae Juss.
Iz_3_1109	DJI_0027	Rumex thyrsiflorus Fingerh.	0	n	1	0	Polygonaceae Juss.
Iz_3_1109	DJI_0027	Salix alba L.	0	n	1	1	Salicaceae Mirb.

Iz_3_1109	DJI_0027	Sambucus nigra L.	0	n	1	1	Viburnaceae Raf.
Iz_3_1109	DJI_0027	Secale sylvestre Host	0	n	1	0	Poaceae Barnhart
Iz_3_1109	DJI_0027	Sempervivum ruthenicum Schnittsp. & C.B. Lehm.	0	n	1	0	Crassulaceae J.St.-Hil.
Iz_3_1109	DJI_0027	Setaria viridis (L.) P. Beauv.	0	n	1	0	Poaceae Barnhart
Iz_3_1109	DJI_0027	Silene borysthenica (Gruner) Walters	0	n	1	0	Caryophyllaceae Juss.
Iz_3_1109	DJI_0027	Silene latifolia subsp. alba (Mill.) Greuter & Burdet	0	n	1	0	Caryophyllaceae Juss.
Iz_3_1109	DJI_0027	Solidago virgaurea L.	0	n	1	0	Asteraceae Bercht. & J.Presl
Iz_3_1109	DJI_0027	Sorbus aucuparia L.	0	n	1	1	Rosaceae Juss.
Iz_3_1109	DJI_0027	Species 11.09_3_0045	0		NA	0	
Iz_3_1109	DJI_0027	Species 11.09_3_0058	0		NA	0	
Iz_3_1109	DJI_0027	Stachys recta L.	0	n	1	0	Lamiacea Lindl.
Iz_3_1109	DJI_0027	Tanacetum vulgare L.	0	n	1	0	Asteraceae Bercht. & J.Presl
Iz_3_1109	DJI_0027	Thymus pallasianus Heinr.Braun	0	n	1	0	Lamiacea Lindl.
Iz_3_1109	DJI_0027	Tilia cordata Mill.	0	n	1	1	Malvaceae Juss.
Iz_3_1109	DJI_0027	Trifolium alpestre L.	0	n	1	0	Fabaceae Lindl.
Iz_3_1109	DJI_0027	Trifolium arvense L.	0	n	1	0	Fabaceae Lindl.
Iz_3_1109	DJI_0027	Ulmus glabra Huds.	0	n	1	1	Ulmaceae Mirb.
Iz_3_1109	DJI_0027	Verbascum densiflorum Bertol.	0	n	1	0	Scrophulariaceae Juss.
Iz_3_1109	DJI_0027	Veronica austriaca L.	0	n	1	0	Plantaginaceae Juss.
Iz_3_1109	DJI_0027	Veronica incana L.	0	n	1	0	Plantaginaceae Juss.
Iz_3_1109	DJI_0027	Veronica verna L.	0	n	1	0	Plantaginaceae Juss.
Iz_3_1109	DJI_0027	Vincetoxicum hirundinaria Medik	0	n	1	0	Apocynaceae Juss.
Iz_3_1109	DJI_0031	Acer tataricum L.	0	n	1	1	Sapindaceae Juss.
Iz_3_1109	DJI_0031	Achillea millefolium L. s.l.	0	n	1	0	Asteraceae Bercht. & J.Presl
Iz_3_1109	DJI_0031	Allium oleraceum L.	0	n	1	0	Amaryllidaceae J.St.-Hil.
Iz_3_1109	DJI_0031	Ambrosia artemisiifolia L.	0	a	0	0	Asteraceae Bercht. & J.Presl
Iz_3_1109	DJI_0031	Amorpha fruticosa L.	0	a	0	0	Fabaceae Lindl.
Iz_3_1109	DJI_0031	Anthericum ramosum L.	0	n	1	0	Asparagaceae Juss.
Iz_3_1109	DJI_0031	Artemisia marschalliana Spreng.	0	n	1	0	Asteraceae Bercht. & J.Presl
Iz_3_1109	DJI_0031	Asparagus officinalis L.	0	n	1	0	Asparagaceae Juss.
Iz_3_1109	DJI_0031	Astragalus cicer L.	0	n	1	0	Fabaceae Lindl.
Iz_3_1109	DJI_0031	Bassia laniflora (S.G.Gmel.) A.J.Scott	0	n	1	0	Amaranthaceae Juss.
Iz_3_1109	DJI_0031	Berberis vulgaris L.	0	n	1	0	Berberidaceae Juss.
Iz_3_1109	DJI_0031	Betula pendula Roth.	0	n	1	1	Betulaceae Gray
Iz_3_1109	DJI_0031	Betula pendula Roth.	0	n	1	0	Poaceae Barnhart
Iz_3_1109	DJI_0031	Bromus inermis Leyss.	0		NA	NA	
Iz_3_1109	DJI_0031	Bromus squarrosus L.	0	a	0	0	Poaceae Barnhart
Iz_3_1109	DJI_0031	Bromus tectorum L.	0	a	0	0	Poaceae Barnhart
Iz_3_1109	DJI_0031	Calamagrostis epigejos (L.) Roth	0	n	1	0	Poaceae Barnhart
Iz_3_1109	DJI_0031	Carduus acanthoides L.	0	n	1	0	Asteraceae Bercht. & J.Presl
Iz_3_1109	DJI_0031	Carex sp.	0	n	1	0	Cyperaceae Juss.
Iz_3_1109	DJI_0031	Centaurea majorovii Dumbadze	0	n	1	0	Asteraceae Bercht. & J.Presl
Iz_3_1109	DJI_0031	Chamaecytisus ruthenicus (Fisch.ex Wol.) Klaskova	0	n	1	0	Fabaceae Lindl.
Iz_3_1109	DJI_0031	Chelidonium majus L.	0	n	1	0	Papaveraceae Juss.
Iz_3_1109	DJI_0031	Chenopodium betaceum Andrz.	0	n	1	0	Amaranthaceae Juss.
Iz_3_1109	DJI_0031	Chenopodium cf. album	0	n	1	0	Amaranthaceae Juss.
Iz_3_1109	DJI_0031	Chenopodium sp.	0		NA	0	Amaranthaceae Juss.
Iz_3_1109	DJI_0031	Chondrilla juncea L.	0	n	1	0	Asteraceae Bercht. & J.Presl
Iz_3_1109	DJI_0031	Cirsium vulgare (Savi) Ten.	0	n	1	0	Asteraceae Bercht. & J.Presl
Iz_3_1109	DJI_0031	Clinopodium acinos (L.) Kuntze	0	n	1	0	Lamiacea Lindl.
Iz_3_1109	DJI_0031	Convallaria majalis L.	0	n	1	0	Asparagaceae Juss.
Iz_3_1109	DJI_0031	Crataegus rhipidophylla Gand.	0	n	1	1	Rosaceae Juss.
Iz_3_1109	DJI_0031	Crepis foetida L.	0	n	1	0	Asteraceae Bercht. & J.Presl
Iz_3_1109	DJI_0031	Dactylis glomerata L.	0	n	1	0	Poaceae Barnhart
Iz_3_1109	DJI_0031	Dianthus campestris M.Bieb.	0	n	1	0	Caryophyllaceae Juss.
Iz_3_1109	DJI_0031	Digitaria sanguinalis (L.) Scop.	0	a	0	0	Poaceae Barnhart
Iz_3_1109	DJI_0031	Elymus repens (L.) Gould	0	n	1	0	Poaceae Barnhart
Iz_3_1109	DJI_0031	Epilobium tetragonum L.	0	n	1	0	Onagraceae Juss.
Iz_3_1109	DJI_0031	Eragrostis minor Host	0	a	0	0	Poaceae Barnhart
Iz_3_1109	DJI_0031	Erigeron canadensis L.	0.1	a	0	0	Asteraceae Bercht. & J.Presl
Iz_3_1109	DJI_0031	Euphorbia seguieriana Neck.	0	n	1	0	Euphorbiaceae Juss.
Iz_3_1109	DJI_0031	Fallopia dumetorum (L.) Holub	0	n	1	0	Polygonaceae Juss.
Iz_3_1109	DJI_0031	Festuca beckeri (Hack.) Trautv.	0	n	1	0	Poaceae Barnhart
Iz_3_1109	DJI_0031	Fragaria vesca L.	0	n	1	0	Rosaceae Juss.
Iz_3_1109	DJI_0031	Frangula alnus Mill.	0	n	1	1	Rhamnaceae Juss.
Iz_3_1109	DJI_0031	Fraxinus pennsylvanica Marshall	0	a	0	1	Oleaceae Hoffmanns. & Link
Iz_3_1109	DJI_0031	Galeopsis bifida Boenn.	0	n	1	0	Lamiacea Lindl.
Iz_3_1109	DJI_0031	Galium verum L.	0	n	1	0	Rubiaceae Juss.
Iz_3_1109	DJI_0031	Genista tinctoria L.	0	n	1	0	Fabaceae Lindl.
Iz_3_1109	DJI_0031	Geranium robertianum L.	0	n	1	0	Geraniaceae Juss.
Iz_3_1109	DJI_0031	Geranium sanquineum L.	0	n	1	0	Geraniaceae Juss.
Iz_3_1109	DJI_0031	Gypsophila paniculata L.	0	n	1	0	Caryophyllaceae Juss.
Iz_3_1109	DJI_0031	Helichrysum arenarium (L.) Moench	0	n	1	0	Asteraceae Bercht. & J.Presl
Iz_3_1109	DJI_0031	Herniaria polygama J.Gay	0	n	1	0	Caryophyllaceae Juss.
Iz_3_1109	DJI_0031	Hieracium robustum Fr.	0	n	1	0	Asteraceae Bercht. & J.Presl
Iz_3_1109	DJI_0031	Hieracium umbellatum L.	0	n	1	NA	Asteraceae Bercht. & J.Presl
Iz_3_1109	DJI_0031	Hylotelephium maximum (L.) Holub	0	n	1	0	Crassulaceae J.St.-Hil.
Iz_3_1109	DJI_0031	Jacobaea borysthenica (DC.) B.Nord. & Greuter	0	n	1	0	Asteraceae Bercht. & J.Presl
Iz_3_1109	DJI_0031	Jasione montana L.	0	n	1	0	Campanulaceae Juss.
Iz_3_1109	DJI_0031	Jurinea cyanoides (L.) Rchb.s.l.	0	n	1	0	Asteraceae Bercht. & J.Presl
Iz_3_1109	DJI_0031	Koeleria glauca (Spreng.) DC.	0	n	1	0	Poaceae Barnhart
Iz_3_1109	DJI_0031	Lactuca serriola L.	0	a	0	0	Asteraceae Bercht. & J.Presl
Iz_3_1109	DJI_0031	Linaria genistifolia (L.) Mill.	0	n	1	0	Plantaginaceae Juss.
Iz_3_1109	DJI_0031	Lomelosia argentea (L.) Greuter & Burdet	0	n	1	0	Caprifoliaceae Juss.
Iz_3_1109	DJI_0031	Melampyrum pratense L.	0	n	1	0	Orobanchaceae Vent.
Iz_3_1109	DJI_0031	Oenothera biennis L.	0	a	0	0	Onagraceae Juss.
Iz_3_1109	DJI_0031	Pentanema britannica (L.) D.Gut.Larr., Santos-Vicente, Anderb., E.Rico & M.M.Mart.Ort.	0	n	1	0	Asteraceae Bercht. & J.Presl
Iz_3_1109	DJI_0031	Peucedanum oreoselinum (L.) Moench	0	n	1	0	Apiaceae Lindl.
Iz_3_1109	DJI_0031	Peucedanum ruthenicum M.Bieb.	0	n	1	0	Apiaceae Lindl.
Iz_3_1109	DJI_0031	Pilosella echioides (Lumn.) F.W.Schultz & Sch.Bip.	0.1	n	1	0	Asteraceae Bercht. & J.Presl
Iz_3_1109	DJI_0031	Pilosella officinarum F. Schultz et Sch. Bip.	0	n	1	0	Asteraceae Bercht. & J.Presl
Iz_3_1109	DJI_0031	Pilosella sp.	0		NA	0	Asteraceae Bercht. & J.Presl
Iz_3_1109	DJI_0031	Pinus sylvestris L.	0	n	1	1	Pinaceae Spreng. ex F.Rudolphi
Iz_3_1109	DJI_0031	Poa annua L.	0	n	1	0	Poaceae Barnhart
Iz_3_1109	DJI_0031	Poa nemoralis L.	0	n	1	0	Poaceae Barnhart
Iz_3_1109	DJI_0031	Poa sp.	0		NA	0	Poaceae Barnhart
Iz_3_1109	DJI_0031	Polygonatum odoratum (Mill.) Druce	0	n	1	0	Asparagaceae Juss.
Iz_3_1109	DJI_0031	Polygonum arenarium Waldst. & Kit.	0	n	1	0	Polygonaceae Juss.

Iz_3_1109	DJI_0031	Populus nigra L.	0	n	1	1	Salicaceae Mirb.
Iz_3_1109	DJI_0031	Populus tremula L.	0	n	1	1	Salicaceae Mirb.
Iz_3_1109	DJI_0031	Populus Ã canescens (Aiton) Sm.	0	n	1	1	Salicaceae Mirb.
Iz_3_1109	DJI_0031	Potentilla argentea L.	0	n	1	0	Rosaceae Juss.
Iz_3_1109	DJI_0031	Psammophiliella muralis (L.) Ikonn.	0	n	1	0	Caryophyllaceae Juss.
Iz_3_1109	DJI_0031	Psephellus sumensis (Kalen.) Greuter	0	n	1	0	Asteraceae Bercht. & J.Presl
Iz_3_1109	DJI_0031	Pulsatilla pratensis (L.) Mill. s.l.	0	n	1	0	Ranunculaceae Juss.
Iz_3_1109	DJI_0031	Pyrus communis L.	0	a	0	1	Rosaceae Juss.
Iz_3_1109	DJI_0031	Quercus robur L.	0	n	1	1	Fagaceae Dumort.
Iz_3_1109	DJI_0031	Robinia pseudoacacia L.	0	a	0	1	Fabaceae Lindl.
Iz_3_1109	DJI_0031	Rosa sp.	0		NA	1	Rosaceae Juss.
Iz_3_1109	DJI_0031	Rubus idaeus L.	0	n	1	0	Rosaceae Juss.
Iz_3_1109	DJI_0031	Rumex acetosella L.	0	n	1	0	Polygonaceae Juss.
Iz_3_1109	DJI_0031	Rumex thyrsiflorus Fingerh.	0	n	1	0	Polygonaceae Juss.
Iz_3_1109	DJI_0031	Salix alba L.	0	n	1	1	Salicaceae Mirb.
Iz_3_1109	DJI_0031	Sambucus nigra L.	0	n	1	1	Viburnaceae Raf.
Iz_3_1109	DJI_0031	Secale sylvestre Host	0	n	1	0	Poaceae Barnhart
Iz_3_1109	DJI_0031	Sempervivum ruthenicum Schnittsp. & C.B. Lehm.	0	n	1	0	Crassulaceae J.St.-Hil.
Iz_3_1109	DJI_0031	Setaria viridis (L.) P. Beauv.	0	n	1	0	Poaceae Barnhart
Iz_3_1109	DJI_0031	Silene borysthenica (Gruner) Walters	0	n	1	0	Caryophyllaceae Juss.
Iz_3_1109	DJI_0031	Silene latifolia subsp. alba (Mill.) Greuter & Burdet	0	n	1	0	Caryophyllaceae Juss.
Iz_3_1109	DJI_0031	Solidago virgaurea L.	0	n	1	0	Asteraceae Bercht. & J.Presl
Iz_3_1109	DJI_0031	Sorbus aucuparia L.	0	n	1	1	Rosaceae Juss.
Iz_3_1109	DJI_0031	Species 11.09_3_0045	0		NA	0	
Iz_3_1109	DJI_0031	Species 11.09_3_0058	0		NA	0	
Iz_3_1109	DJI_0031	Stachys recta L.	0	n	1	0	Lamiacea Lindl.
Iz_3_1109	DJI_0031	Tanacetum vulgare L.	0	n	1	0	Asteraceae Bercht. & J.Presl
Iz_3_1109	DJI_0031	Thymus pallasianus Heinr.Braun	0	n	1	0	Lamiacea Lindl.
Iz_3_1109	DJI_0031	Tilia cordata Mill.	0	n	1	1	Malvaceae Juss.
Iz_3_1109	DJI_0031	Trifolium alpestre L.	0	n	1	0	Fabaceae Lindl.
Iz_3_1109	DJI_0031	Trifolium arvense L.	0	n	1	0	Fabaceae Lindl.
Iz_3_1109	DJI_0031	Ulmus glabra Huds.	0	n	1	1	Ulmaceae Mirb.
Iz_3_1109	DJI_0031	Verbascum densiflorum Bertol.	0	n	1	0	Scrophulariaceae Juss.
Iz_3_1109	DJI_0031	Veronica austriaca L.	0	n	1	0	Plantaginaceae Juss.
Iz_3_1109	DJI_0031	Veronica incana L.	0	n	1	0	Plantaginaceae Juss.
Iz_3_1109	DJI_0031	Veronica verna L.	0	n	1	0	Plantaginaceae Juss.
Iz_3_1109	DJI_0031	Vincetoxicum hirundinaria Medik	0	n	1	0	Apocynaceae Juss.
Iz_3_1109	DJI_0033	Acer tataricum L.	0	n	1	1	Sapindaceae Juss.
Iz_3_1109	DJI_0033	Achillea millefolium L. s.l.	0	n	1	0	Asteraceae Bercht. & J.Presl
Iz_3_1109	DJI_0033	Allium oleraceum L.	0	n	1	0	Amaryllidaceae J.St.-Hil.
Iz_3_1109	DJI_0033	Ambrosia artemisiifolia L.	0	a	0	0	Asteraceae Bercht. & J.Presl
Iz_3_1109	DJI_0033	Amorpha fruticosa L.	0	a	0	0	Fabaceae Lindl.
Iz_3_1109	DJI_0033	Anthericum ramosum L.	0	n	1	0	Asparagaceae Juss.
Iz_3_1109	DJI_0033	Artemisia marschalliana Spreng.	0	n	1	0	Asteraceae Bercht. & J.Presl
Iz_3_1109	DJI_0033	Asparagus officinalis L.	0	n	1	0	Asparagaceae Juss.
Iz_3_1109	DJI_0033	Astragalus cicer L.	0	n	1	0	Fabaceae Lindl.
Iz_3_1109	DJI_0033	Bassia laniflora (S.G.Gmel.) A.J.Scott	0	n	1	0	Amaranthaceae Juss.
Iz_3_1109	DJI_0033	Berberis vulgaris L.	0	n	1	0	Berberidaceae Juss.
Iz_3_1109	DJI_0033	Betula pendula Roth.	0	n	1	1	Betulaceae Gray
Iz_3_1109	DJI_0033	Betula pendula Roth.	0	n	1	0	Poaceae Barnhart
Iz_3_1109	DJI_0033	Bromus inermis Leyss.	0		NA	NA	
Iz_3_1109	DJI_0033	Bromus squarrosus L.	0	a	0	0	Poaceae Barnhart
Iz_3_1109	DJI_0033	Bromus tectorum L.	0	a	0	0	Poaceae Barnhart
Iz_3_1109	DJI_0033	Calamagrostis epigejos (L.) Roth	0.2	n	1	0	Poaceae Barnhart
Iz_3_1109	DJI_0033	Carduus acanthoides L.	0	n	1	0	Asteraceae Bercht. & J.Presl
Iz_3_1109	DJI_0033	Carex sp.	0	n	1	0	Cyperaceae Juss.
Iz_3_1109	DJI_0033	Centaurea majorovii Dumbadze	0	n	1	0	Asteraceae Bercht. & J.Presl
Iz_3_1109	DJI_0033	Chamaecytisus ruthenicus (Fisch.ex Wol.) Klaskova	0	n	1	0	Fabaceae Lindl.
Iz_3_1109	DJI_0033	Chelidonium majus L.	0	n	1	0	Papaveraceae Juss.
Iz_3_1109	DJI_0033	Chenopodium betaceum Andrz.	0	n	1	0	Amaranthaceae Juss.
Iz_3_1109	DJI_0033	Chenopodium cf. album	0	n	1	0	Amaranthaceae Juss.
Iz_3_1109	DJI_0033	Chenopodium sp.	0		NA	0	Amaranthaceae Juss.
Iz_3_1109	DJI_0033	Chondrilla juncea L.	0	n	1	0	Asteraceae Bercht. & J.Presl
Iz_3_1109	DJI_0033	Cirsium vulgare (Savi) Ten.	0	n	1	0	Asteraceae Bercht. & J.Presl
Iz_3_1109	DJI_0033	Clinopodium acinos (L.) Kuntze	0	n	1	0	Lamiacea Lindl.
Iz_3_1109	DJI_0033	Convallaria majalis L.	0	n	1	0	Asparagaceae Juss.
Iz_3_1109	DJI_0033	Crataegus rhipidophylla Gand.	0	n	1	1	Rosaceae Juss.
Iz_3_1109	DJI_0033	Crepis foetida L.	0	n	1	0	Asteraceae Bercht. & J.Presl
Iz_3_1109	DJI_0033	Dactylis glomerata L.	0	n	1	0	Poaceae Barnhart
Iz_3_1109	DJI_0033	Dianthus campestris M.Bieb.	0	n	1	0	Caryophyllaceae Juss.
Iz_3_1109	DJI_0033	Digitaria sanguinalis (L.) Scop.	0	a	0	0	Poaceae Barnhart
Iz_3_1109	DJI_0033	Elymus repens (L.) Gould	0	n	1	0	Poaceae Barnhart
Iz_3_1109	DJI_0033	Epilobium tetragonum L.	0	n	1	0	Onagraceae Juss.
Iz_3_1109	DJI_0033	Eragrostis minor Host	0	a	0	0	Poaceae Barnhart
Iz_3_1109	DJI_0033	Erigeron canadensis L.	0.2	a	0	0	Asteraceae Bercht. & J.Presl
Iz_3_1109	DJI_0033	Euphorbia seguieriana Neck.	0	n	1	0	Euphorbiaceae Juss.
Iz_3_1109	DJI_0033	Fallopia dumetorum (L.) Holub	0	n	1	0	Polygonaceae Juss.
Iz_3_1109	DJI_0033	Festuca beckeri (Hack.) Trautv.	0	n	1	0	Poaceae Barnhart
Iz_3_1109	DJI_0033	Fragaria vesca L.	0	n	1	0	Rosaceae Juss.
Iz_3_1109	DJI_0033	Frangula alnus Mill.	0	n	1	1	Rhamnaceae Juss.
Iz_3_1109	DJI_0033	Fraxinus pennsylvanica Marshall	0	a	0	1	Oleaceae Hoffmanns. & Link
Iz_3_1109	DJI_0033	Galeopsis bifida Boenn.	0	n	1	0	Lamiacea Lindl.
Iz_3_1109	DJI_0033	Galium verum L.	0	n	1	0	Rubiaceae Juss.
Iz_3_1109	DJI_0033	Genista tinctoria L.	0	n	1	0	Fabaceae Lindl.
Iz_3_1109	DJI_0033	Geranium robertianum L.	0	n	1	0	Geraniaceae Juss.
Iz_3_1109	DJI_0033	Geranium sanquineum L.	0	n	1	0	Geraniaceae Juss.
Iz_3_1109	DJI_0033	Gypsophila paniculata L.	0	n	1	0	Caryophyllaceae Juss.
Iz_3_1109	DJI_0033	Helichrysum arenarium (L.) Moench	0	n	1	0	Asteraceae Bercht. & J.Presl
Iz_3_1109	DJI_0033	Herniaria polygama J.Gay	0	n	1	0	Caryophyllaceae Juss.
Iz_3_1109	DJI_0033	Hieracium robustum Fr.	0	n	1	0	Asteraceae Bercht. & J.Presl
Iz_3_1109	DJI_0033	Hieracium umbellatum L.	0	n	1	NA	Asteraceae Bercht. & J.Presl
Iz_3_1109	DJI_0033	Hylotelephium maximum (L.) Holub	0	n	1	0	Crassulaceae J.St.-Hil.
Iz_3_1109	DJI_0033	Jacobaea borysthenica (DC.) B.Nord. & Greuter	0	n	1	0	Asteraceae Bercht. & J.Presl
Iz_3_1109	DJI_0033	Jasione montana L.	0	n	1	0	Campanulaceae Juss.
Iz_3_1109	DJI_0033	Jurinea cyanoides (L.) Rchb.s.l.	0	n	1	0	Asteraceae Bercht. & J.Presl
Iz_3_1109	DJI_0033	Koeleria glauca (Spreng.) DC.	0	n	1	0	Poaceae Barnhart
Iz_3_1109	DJI_0033	Lactuca serriola L.	0	a	0	0	Asteraceae Bercht. & J.Presl
Iz_3_1109	DJI_0033	Linaria genistifolia (L.) Mill.	0	n	1	0	Plantaginaceae Juss.

Iz_3_1109	DJI_0033	Lomelosia argentea (L.) Greuter & Burdet	0	n	1	0	Caprifoliaceae Juss.
Iz_3_1109	DJI_0033	Melampyrum pratense L.	0	n	1	0	Orobanchaceae Vent.
Iz_3_1109	DJI_0033	Oenothera biennis L.	0	a	0	0	Onagraceae Juss.
Iz_3_1109	DJI_0033	Pentanema britannica (L.) D.Gut.Larr., Santos-Vicente, Anderb., E.Rico & M.M.Mart.Ort.	0	n	1	0	Asteraceae Bercht. & J.Presl
Iz_3_1109	DJI_0033	Peucedanum oreoselinum (L.) Moench	0	n	1	0	Apiaceae Lindl.
Iz_3_1109	DJI_0033	Peucedanum ruthenicum M.Bieb.	0	n	1	0	Apiaceae Lindl.
Iz_3_1109	DJI_0033	Pilosella echioides (Lumn.) F.W.Schultz & Sch.Bip.	0	n	1	0	Asteraceae Bercht. & J.Presl
Iz_3_1109	DJI_0033	Pilosella officinarum F. Schultz et Sch. Bip.	0	n	1	0	Asteraceae Bercht. & J.Presl
Iz_3_1109	DJI_0033	Pilosella sp.	0		NA	0	Asteraceae Bercht. & J.Presl
Iz_3_1109	DJI_0033	Pinus sylvestris L.	0	n	1	1	Pinaceae Spreng. ex F.Rudolphi
Iz_3_1109	DJI_0033	Poa annua L.	0	n	1	0	Poaceae Barnhart
Iz_3_1109	DJI_0033	Poa nemoralis L.	0	n	1	0	Poaceae Barnhart
Iz_3_1109	DJI_0033	Poa sp.	0		NA	0	Poaceae Barnhart
Iz_3_1109	DJI_0033	Polygonatum odoratum (Mill.) Druce	0	n	1	0	Asparagaceae Juss.
Iz_3_1109	DJI_0033	Polygonum arenarium Waldst. & Kit.	0	n	1	0	Polygonaceae Juss.
Iz_3_1109	DJI_0033	Populus nigra L.	0	n	1	1	Salicaceae Mirb.
Iz_3_1109	DJI_0033	Populus tremula L.	0	n	1	1	Salicaceae Mirb.
Iz_3_1109	DJI_0033	Populus Ã canescens (Aiton) Sm.	0	n	1	1	Salicaceae Mirb.
Iz_3_1109	DJI_0033	Potentilla argentea L.	0	n	1	0	Rosaceae Juss.
Iz_3_1109	DJI_0033	Psammophiliella muralis (L.) Ikonn.	0	n	1	0	Caryophyllaceae Juss.
Iz_3_1109	DJI_0033	Psephellus sumensis (Kalen.) Greuter	0	n	1	0	Asteraceae Bercht. & J.Presl
Iz_3_1109	DJI_0033	Pulsatilla pratensis (L.) Mill. s.l.	0	n	1	0	Ranunculaceae Juss.
Iz_3_1109	DJI_0033	Pyrus communis L.	0	a	0	1	Rosaceae Juss.
Iz_3_1109	DJI_0033	Quercus robur L.	0	n	1	1	Fagaceae Dumort.
Iz_3_1109	DJI_0033	Robinia pseudoacacia L.	0	a	0	1	Fabaceae Lindl.
Iz_3_1109	DJI_0033	Rosa sp.	0		NA	1	Rosaceae Juss.
Iz_3_1109	DJI_0033	Rubus idaeus L.	0	n	1	0	Rosaceae Juss.
Iz_3_1109	DJI_0033	Rumex acetosella L.	0	n	1	0	Polygonaceae Juss.
Iz_3_1109	DJI_0033	Rumex thyrsiflorus Fingerh.	0	n	1	0	Polygonaceae Juss.
Iz_3_1109	DJI_0033	Salix alba L.	0	n	1	1	Salicaceae Mirb.
Iz_3_1109	DJI_0033	Sambucus nigra L.	0	n	1	1	Viburnaceae Raf.
Iz_3_1109	DJI_0033	Secale sylvestre Host	0	n	1	0	Poaceae Barnhart
Iz_3_1109	DJI_0033	Sempervivum ruthenicum Schnittsp. & C.B. Lehm.	0	n	1	0	Crassulaceae J.St.-Hil.
Iz_3_1109	DJI_0033	Setaria viridis (L.) P. Beauv.	0	n	1	0	Poaceae Barnhart
Iz_3_1109	DJI_0033	Silene borysthenica (Gruner) Walters	0	n	1	0	Caryophyllaceae Juss.
Iz_3_1109	DJI_0033	Silene latifolia subsp. alba (Mill.) Greuter & Burdet	0	n	1	0	Caryophyllaceae Juss.
Iz_3_1109	DJI_0033	Solidago virgaurea L.	0	n	1	0	Asteraceae Bercht. & J.Presl
Iz_3_1109	DJI_0033	Sorbus aucuparia L.	0	n	1	1	Rosaceae Juss.
Iz_3_1109	DJI_0033	Species 11.09_3_0045	0		NA	0	
Iz_3_1109	DJI_0033	Species 11.09_3_0058	0		NA	0	
Iz_3_1109	DJI_0033	Stachys recta L.	0	n	1	0	Lamiacea Lindl.
Iz_3_1109	DJI_0033	Tanacetum vulgare L.	0	n	1	0	Asteraceae Bercht. & J.Presl
Iz_3_1109	DJI_0033	Thymus pallasianus Heinr.Braun	0	n	1	0	Lamiacea Lindl.
Iz_3_1109	DJI_0033	Tilia cordata Mill.	0	n	1	1	Malvaceae Juss.
Iz_3_1109	DJI_0033	Trifolium alpestre L.	0	n	1	0	Fabaceae Lindl.
Iz_3_1109	DJI_0033	Trifolium arvense L.	0	n	1	0	Fabaceae Lindl.
Iz_3_1109	DJI_0033	Ulmus glabra Huds.	0	n	1	1	Ulmaceae Mirb.
Iz_3_1109	DJI_0033	Verbascum densiflorum Bertol.	0	n	1	0	Scrophulariaceae Juss.
Iz_3_1109	DJI_0033	Veronica austriaca L.	0	n	1	0	Plantaginaceae Juss.
Iz_3_1109	DJI_0033	Veronica incana L.	0	n	1	0	Plantaginaceae Juss.
Iz_3_1109	DJI_0033	Veronica verna L.	0	n	1	0	Plantaginaceae Juss.
Iz_3_1109	DJI_0033	Vincetoxicum hirundinaria Medik	0	n	1	0	Apocynaceae Juss.
Iz_3_1109	DJI_0035	Acer tataricum L.	0	n	1	1	Sapindaceae Juss.
Iz_3_1109	DJI_0035	Achillea millefolium L. s.l.	0	n	1	0	Asteraceae Bercht. & J.Presl
Iz_3_1109	DJI_0035	Allium oleraceum L.	0	n	1	0	Amaryllidaceae J.St.-Hil.
Iz_3_1109	DJI_0035	Ambrosia artemisiifolia L.	0	a	0	0	Asteraceae Bercht. & J.Presl
Iz_3_1109	DJI_0035	Amorpha fruticosa L.	0	a	0	0	Fabaceae Lindl.
Iz_3_1109	DJI_0035	Anthericum ramosum L.	0	n	1	0	Asparagaceae Juss.
Iz_3_1109	DJI_0035	Artemisia marschalliana Spreng.	0	n	1	0	Asteraceae Bercht. & J.Presl
Iz_3_1109	DJI_0035	Asparagus officinalis L.	0	n	1	0	Asparagaceae Juss.
Iz_3_1109	DJI_0035	Astragalus cicer L.	0	n	1	0	Fabaceae Lindl.
Iz_3_1109	DJI_0035	Bassia laniflora (S.G.Gmel.) A.J.Scott	0	n	1	0	Amaranthaceae Juss.
Iz_3_1109	DJI_0035	Berberis vulgaris L.	0	n	1	0	Berberidaceae Juss.
Iz_3_1109	DJI_0035	Betula pendula Roth.	0	n	1	1	Betulaceae Gray
Iz_3_1109	DJI_0035	Betula pendula Roth.	0	n	1	0	Poaceae Barnhart
Iz_3_1109	DJI_0035	Bromus inermis Leyss.	0		NA	NA	
Iz_3_1109	DJI_0035	Bromus squarrosus L.	0	a	0	0	Poaceae Barnhart
Iz_3_1109	DJI_0035	Bromus tectorum L.	0	a	0	0	Poaceae Barnhart
Iz_3_1109	DJI_0035	Calamagrostis epigejos (L.) Roth	0	n	1	0	Poaceae Barnhart
Iz_3_1109	DJI_0035	Carduus acanthoides L.	0	n	1	0	Asteraceae Bercht. & J.Presl
Iz_3_1109	DJI_0035	Carex sp.	0	n	1	0	Cyperaceae Juss.
Iz_3_1109	DJI_0035	Centaurea majorovii Dumbadze	0	n	1	0	Asteraceae Bercht. & J.Presl
Iz_3_1109	DJI_0035	Chamaecytisus ruthenicus (Fisch.ex Wol.) Klaskova	0	n	1	0	Fabaceae Lindl.
Iz_3_1109	DJI_0035	Chelidonium majus L.	0	n	1	0	Papaveraceae Juss.
Iz_3_1109	DJI_0035	Chenopodium betaceum Andrz.	0	n	1	0	Amaranthaceae Juss.
Iz_3_1109	DJI_0035	Chenopodium cf. album	0	n	1	0	Amaranthaceae Juss.
Iz_3_1109	DJI_0035	Chenopodium sp.	0		NA	0	Amaranthaceae Juss.
Iz_3_1109	DJI_0035	Chondrilla juncea L.	0	n	1	0	Asteraceae Bercht. & J.Presl
Iz_3_1109	DJI_0035	Cirsium vulgare (Savi) Ten.	0	n	1	0	Asteraceae Bercht. & J.Presl
Iz_3_1109	DJI_0035	Clinopodium acinos (L.) Kuntze	0	n	1	0	Lamiacea Lindl.
Iz_3_1109	DJI_0035	Convallaria majalis L.	0	n	1	0	Asparagaceae Juss.
Iz_3_1109	DJI_0035	Crataegus rhipidophylla Gand.	0	n	1	1	Rosaceae Juss.
Iz_3_1109	DJI_0035	Crepis foetida L.	0	n	1	0	Asteraceae Bercht. & J.Presl
Iz_3_1109	DJI_0035	Dactylis glomerata L.	0	n	1	0	Poaceae Barnhart
Iz_3_1109	DJI_0035	Dianthus campestris M.Bieb.	0	n	1	0	Caryophyllaceae Juss.
Iz_3_1109	DJI_0035	Digitaria sanguinalis (L.) Scop.	0	a	0	0	Poaceae Barnhart
Iz_3_1109	DJI_0035	Elymus repens (L.) Gould	0	n	1	0	Poaceae Barnhart
Iz_3_1109	DJI_0035	Epilobium tetragonum L.	0	n	1	0	Onagraceae Juss.
Iz_3_1109	DJI_0035	Eragrostis minor Host	0	a	0	0	Poaceae Barnhart
Iz_3_1109	DJI_0035	Erigeron canadensis L.	1	a	0	0	Asteraceae Bercht. & J.Presl
Iz_3_1109	DJI_0035	Euphorbia seguieriana Neck.	0	n	1	0	Euphorbiaceae Juss.
Iz_3_1109	DJI_0035	Fallopia dumetorum (L.) Holub	0	n	1	0	Polygonaceae Juss.
Iz_3_1109	DJI_0035	Festuca beckeri (Hack.) Trautv.	0	n	1	0	Poaceae Barnhart
Iz_3_1109	DJI_0035	Fragaria vesca L.	0	n	1	0	Rosaceae Juss.
Iz_3_1109	DJI_0035	Frangula alnus Mill.	0	n	1	1	Rhamnaceae Juss.
Iz_3_1109	DJI_0035	Fraxinus pennsylvanica Marshall	0	a	0	1	Oleaceae Hoffmanns. & Link
Iz_3_1109	DJI_0035	Galeopsis bifida Boenn.	0	n	1	0	Lamiacea Lindl.
Iz_3_1109	DJI_0035	Galium verum L.	0	n	1	0	Rubiaceae Juss.

Iz_3_1109	DJI_0035	Genista tinctoria L.	0	n	1	0	Fabaceae Lindl.
Iz_3_1109	DJI_0035	Geranium robertianum L.	0	n	1	0	Geraniaceae Juss.
Iz_3_1109	DJI_0035	Geranium sanquineum L.	0	n	1	0	Geraniaceae Juss.
Iz_3_1109	DJI_0035	Gypsophila paniculata L.	0	n	1	0	Caryophyllaceae Juss.
Iz_3_1109	DJI_0035	Helichrysum arenarium (L.) Moench	0	n	1	0	Asteraceae Bercht. & J.Presl
Iz_3_1109	DJI_0035	Herniaria polygama J.Gay	0	n	1	0	Caryophyllaceae Juss.
Iz_3_1109	DJI_0035	Hieracium robustum Fr.	0	n	1	0	Asteraceae Bercht. & J.Presl
Iz_3_1109	DJI_0035	Hieracium umbellatum L.	0	n	1	NA	Asteraceae Bercht. & J.Presl
Iz_3_1109	DJI_0035	Hylotelephium maximum (L.) Holub	0	n	1	0	Crassulaceae J.St.-Hil.
Iz_3_1109	DJI_0035	Jacobaea borysthenica (DC.) B.Nord. & Greuter	0	n	1	0	Asteraceae Bercht. & J.Presl
Iz_3_1109	DJI_0035	Jasione montana L.	0	n	1	0	Campanulaceae Juss.
Iz_3_1109	DJI_0035	Jurinea cyanoides (L.) Rchb.s.l.	0	n	1	0	Asteraceae Bercht. & J.Presl
Iz_3_1109	DJI_0035	Koeleria glauca (Spreng.) DC.	0	n	1	0	Poaceae Barnhart
Iz_3_1109	DJI_0035	Lactuca serriola L.	0	a	0	0	Asteraceae Bercht. & J.Presl
Iz_3_1109	DJI_0035	Linaria genistifolia (L.) Mill.	0	n	1	0	Plantaginaceae Juss.
Iz_3_1109	DJI_0035	Lomelosia argentea (L.) Greuter & Burdet	0	n	1	0	Caprifoliaceae Juss.
Iz_3_1109	DJI_0035	Melampyrum pratense L.	0	n	1	0	Orobanchaceae Vent.
Iz_3_1109	DJI_0035	Oenothera biennis L.	0	a	0	0	Onagraceae Juss.
Iz_3_1109	DJI_0035	Pentanema britannica (L.) D.Gut.Larr., Santos-Vicente, Anderb., E.Rico & M.M.Mart.Ort.	0	n	1	0	Asteraceae Bercht. & J.Presl
Iz_3_1109	DJI_0035	Peucedanum oreoselinum (L.) Moench	0	n	1	0	Apiaceae Lindl.
Iz_3_1109	DJI_0035	Peucedanum ruthenicum M.Bieb.	0	n	1	0	Apiaceae Lindl.
Iz_3_1109	DJI_0035	Pilosella echioides (Lumn.) F.W.Schultz & Sch.Bip.	0	n	1	0	Asteraceae Bercht. & J.Presl
Iz_3_1109	DJI_0035	Pilosella officinarum F. Schultz et Sch. Bip.	0	n	1	0	Asteraceae Bercht. & J.Presl
Iz_3_1109	DJI_0035	Pilosella sp.	0		NA	0	Asteraceae Bercht. & J.Presl
Iz_3_1109	DJI_0035	Pinus sylvestris L.	0	n	1	1	Pinaceae Spreng. ex F.Rudolphi
Iz_3_1109	DJI_0035	Poa annua L.	0	n	1	0	Poaceae Barnhart
Iz_3_1109	DJI_0035	Poa nemoralis L.	0	n	1	0	Poaceae Barnhart
Iz_3_1109	DJI_0035	Poa sp.	0		NA	0	Poaceae Barnhart
Iz_3_1109	DJI_0035	Polygonatum odoratum (Mill.) Druce	0	n	1	0	Asparagaceae Juss.
Iz_3_1109	DJI_0035	Polygonum arenarium Waldst. & Kit.	0	n	1	0	Polygonaceae Juss.
Iz_3_1109	DJI_0035	Populus nigra L.	0	n	1	1	Salicaceae Mirb.
Iz_3_1109	DJI_0035	Populus tremula L.	0	n	1	1	Salicaceae Mirb.
Iz_3_1109	DJI_0035	Populus Ã canescens (Aiton) Sm.	0	n	1	1	Salicaceae Mirb.
Iz_3_1109	DJI_0035	Potentilla argentea L.	0	n	1	0	Rosaceae Juss.
Iz_3_1109	DJI_0035	Psammophiliella muralis (L.) Ikonn.	0	n	1	0	Caryophyllaceae Juss.
Iz_3_1109	DJI_0035	Psephellus sumensis (Kalen.) Greuter	0	n	1	0	Asteraceae Bercht. & J.Presl
Iz_3_1109	DJI_0035	Pulsatilla pratensis (L.) Mill. s.l.	0	n	1	0	Ranunculaceae Juss.
Iz_3_1109	DJI_0035	Pyrus communis L.	0	a	0	1	Rosaceae Juss.
Iz_3_1109	DJI_0035	Quercus robur L.	0	n	1	1	Fagaceae Dumort.
Iz_3_1109	DJI_0035	Robinia pseudoacacia L.	0	a	0	1	Fabaceae Lindl.
Iz_3_1109	DJI_0035	Rosa sp.	0		NA	1	Rosaceae Juss.
Iz_3_1109	DJI_0035	Rubus idaeus L.	0	n	1	0	Rosaceae Juss.
Iz_3_1109	DJI_0035	Rumex acetosella L.	0	n	1	0	Polygonaceae Juss.
Iz_3_1109	DJI_0035	Rumex thyrsiflorus Fingerh.	0	n	1	0	Polygonaceae Juss.
Iz_3_1109	DJI_0035	Salix alba L.	0	n	1	1	Salicaceae Mirb.
Iz_3_1109	DJI_0035	Sambucus nigra L.	0	n	1	1	Viburnaceae Raf.
Iz_3_1109	DJI_0035	Secale sylvestre Host	0	n	1	0	Poaceae Barnhart
Iz_3_1109	DJI_0035	Sempervivum ruthenicum Schnittsp. & C.B. Lehm.	0	n	1	0	Crassulaceae J.St.-Hil.
Iz_3_1109	DJI_0035	Setaria viridis (L.) P. Beauv.	0	n	1	0	Poaceae Barnhart
Iz_3_1109	DJI_0035	Silene borysthenica (Gruner) Walters	0	n	1	0	Caryophyllaceae Juss.
Iz_3_1109	DJI_0035	Silene latifolia subsp. alba (Mill.) Greuter & Burdet	0	n	1	0	Caryophyllaceae Juss.
Iz_3_1109	DJI_0035	Solidago virgaurea L.	0	n	1	0	Asteraceae Bercht. & J.Presl
Iz_3_1109	DJI_0035	Sorbus aucuparia L.	0	n	1	1	Rosaceae Juss.
Iz_3_1109	DJI_0035	Species 11.09_3_0045	0		NA	0	
Iz_3_1109	DJI_0035	Species 11.09_3_0058	0		NA	0	
Iz_3_1109	DJI_0035	Stachys recta L.	0	n	1	0	Lamiacea Lindl.
Iz_3_1109	DJI_0035	Tanacetum vulgare L.	0	n	1	0	Asteraceae Bercht. & J.Presl
Iz_3_1109	DJI_0035	Thymus pallasianus Heinr.Braun	0	n	1	0	Lamiacea Lindl.
Iz_3_1109	DJI_0035	Tilia cordata Mill.	0	n	1	1	Malvaceae Juss.
Iz_3_1109	DJI_0035	Trifolium alpestre L.	0	n	1	0	Fabaceae Lindl.
Iz_3_1109	DJI_0035	Trifolium arvense L.	0	n	1	0	Fabaceae Lindl.
Iz_3_1109	DJI_0035	Ulmus glabra Huds.	0	n	1	1	Ulmaceae Mirb.
Iz_3_1109	DJI_0035	Verbascum densiflorum Bertol.	0	n	1	0	Scrophulariaceae Juss.
Iz_3_1109	DJI_0035	Veronica austriaca L.	0	n	1	0	Plantaginaceae Juss.
Iz_3_1109	DJI_0035	Veronica incana L.	0	n	1	0	Plantaginaceae Juss.
Iz_3_1109	DJI_0035	Veronica verna L.	0	n	1	0	Plantaginaceae Juss.
Iz_3_1109	DJI_0035	Vincetoxicum hirundinaria Medik	0	n	1	0	Apocynaceae Juss.
Iz_3_1109	DJI_0038	Acer tataricum L.	0	n	1	1	Sapindaceae Juss.
Iz_3_1109	DJI_0038	Achillea millefolium L. s.l.	0	n	1	0	Asteraceae Bercht. & J.Presl
Iz_3_1109	DJI_0038	Allium oleraceum L.	0	n	1	0	Amaryllidaceae J.St.-Hil.
Iz_3_1109	DJI_0038	Ambrosia artemisiifolia L.	0	a	0	0	Asteraceae Bercht. & J.Presl
Iz_3_1109	DJI_0038	Amorpha fruticosa L.	0	a	0	0	Fabaceae Lindl.
Iz_3_1109	DJI_0038	Anthericum ramosum L.	0	n	1	0	Asparagaceae Juss.
Iz_3_1109	DJI_0038	Artemisia marschalliana Spreng.	0	n	1	0	Asteraceae Bercht. & J.Presl
Iz_3_1109	DJI_0038	Asparagus officinalis L.	0	n	1	0	Asparagaceae Juss.
Iz_3_1109	DJI_0038	Astragalus cicer L.	0	n	1	0	Fabaceae Lindl.
Iz_3_1109	DJI_0038	Bassia laniflora (S.G.Gmel.) A.J.Scott	0	n	1	0	Amaranthaceae Juss.
Iz_3_1109	DJI_0038	Berberis vulgaris L.	0	n	1	0	Berberidaceae Juss.
Iz_3_1109	DJI_0038	Betula pendula Roth.	0	n	1	1	Betulaceae Gray
Iz_3_1109	DJI_0038	Betula pendula Roth.	0	n	1	0	Poaceae Barnhart
Iz_3_1109	DJI_0038	Bromus inermis Leyss.	0		NA	NA	
Iz_3_1109	DJI_0038	Bromus squarrosus L.	0	a	0	0	Poaceae Barnhart
Iz_3_1109	DJI_0038	Bromus tectorum L.	0	a	0	0	Poaceae Barnhart
Iz_3_1109	DJI_0038	Calamagrostis epigejos (L.) Roth	0.2	n	1	0	Poaceae Barnhart
Iz_3_1109	DJI_0038	Carduus acanthoides L.	0	n	1	0	Asteraceae Bercht. & J.Presl
Iz_3_1109	DJI_0038	Carex sp.	0	n	1	0	Cyperaceae Juss.
Iz_3_1109	DJI_0038	Centaurea majorovii Dumbadze	0	n	1	0	Asteraceae Bercht. & J.Presl
Iz_3_1109	DJI_0038	Chamaecytisus ruthenicus (Fisch.ex Wol.) Klaskova	0	n	1	0	Fabaceae Lindl.
Iz_3_1109	DJI_0038	Chelidonium majus L.	0	n	1	0	Papaveraceae Juss.
Iz_3_1109	DJI_0038	Chenopodium betaceum Andrz.	0	n	1	0	Amaranthaceae Juss.
Iz_3_1109	DJI_0038	Chenopodium cf. album	0	n	1	0	Amaranthaceae Juss.
Iz_3_1109	DJI_0038	Chenopodium sp.	0		NA	0	Amaranthaceae Juss.
Iz_3_1109	DJI_0038	Chondrilla juncea L.	0	n	1	0	Asteraceae Bercht. & J.Presl
Iz_3_1109	DJI_0038	Cirsium vulgare (Savi) Ten.	0	n	1	0	Asteraceae Bercht. & J.Presl
Iz_3_1109	DJI_0038	Clinopodium acinos (L.) Kuntze	0	n	1	0	Lamiacea Lindl.
Iz_3_1109	DJI_0038	Convallaria majalis L.	0	n	1	0	Asparagaceae Juss.
Iz_3_1109	DJI_0038	Crataegus rhipidophylla Gand.	0	n	1	1	Rosaceae Juss.
Iz_3_1109	DJI_0038	Crepis foetida L.	0	n	1	0	Asteraceae Bercht. & J.Presl

Iz_3_1109	DJI_0038	Dactylis glomerata L.	0	n	1	0	Poaceae Barnhart
Iz_3_1109	DJI_0038	Dianthus campestris M.Bieb.	0	n	1	0	Caryophyllaceae Juss.
Iz_3_1109	DJI_0038	Digitaria sanguinalis (L.) Scop.	0	a	0	0	Poaceae Barnhart
Iz_3_1109	DJI_0038	Elymus repens (L.) Gould	0	n	1	0	Poaceae Barnhart
Iz_3_1109	DJI_0038	Epilobium tetragonum L.	0	n	1	0	Onagraceae Juss.
Iz_3_1109	DJI_0038	Eragrostis minor Host	0	a	0	0	Poaceae Barnhart
Iz_3_1109	DJI_0038	Erigeron canadensis L.	0.3	a	0	0	Asteraceae Bercht. & J.Presl
Iz_3_1109	DJI_0038	Euphorbia seguieriana Neck.	0	n	1	0	Euphorbiaceae Juss.
Iz_3_1109	DJI_0038	Fallopia dumetorum (L.) Holub	0	n	1	0	Polygonaceae Juss.
Iz_3_1109	DJI_0038	Festuca beckeri (Hack.) Trautv.	0	n	1	0	Poaceae Barnhart
Iz_3_1109	DJI_0038	Fragaria vesca L.	0	n	1	0	Rosaceae Juss.
Iz_3_1109	DJI_0038	Frangula alnus Mill.	0	n	1	1	Rhamnaceae Juss.
Iz_3_1109	DJI_0038	Fraxinus pennsylvanica Marshall	0	a	0	1	Oleaceae Hoffmanns. & Link
Iz_3_1109	DJI_0038	Galeopsis bifida Boenn.	0	n	1	0	Lamiacea Lindl.
Iz_3_1109	DJI_0038	Galium verum L.	0	n	1	0	Rubiaceae Juss.
Iz_3_1109	DJI_0038	Genista tinctoria L.	0	n	1	0	Fabaceae Lindl.
Iz_3_1109	DJI_0038	Geranium robertianum L.	0	n	1	0	Geraniaceae Juss.
Iz_3_1109	DJI_0038	Geranium sanquineum L.	0	n	1	0	Geraniaceae Juss.
Iz_3_1109	DJI_0038	Gypsophila paniculata L.	0	n	1	0	Caryophyllaceae Juss.
Iz_3_1109	DJI_0038	Helichrysum arenarium (L.) Moench	0	n	1	0	Asteraceae Bercht. & J.Presl
Iz_3_1109	DJI_0038	Herniaria polygama J.Gay	0	n	1	0	Caryophyllaceae Juss.
Iz_3_1109	DJI_0038	Hieracium robustum Fr.	0	n	1	0	Asteraceae Bercht. & J.Presl
Iz_3_1109	DJI_0038	Hieracium umbellatum L.	0	n	1	NA	Asteraceae Bercht. & J.Presl
Iz_3_1109	DJI_0038	Hylotelephium maximum (L.) Holub	0	n	1	0	Crassulaceae J.St.-Hil.
Iz_3_1109	DJI_0038	Jacobaea borysthenica (DC.) B.Nord. & Greuter	0	n	1	0	Asteraceae Bercht. & J.Presl
Iz_3_1109	DJI_0038	Jasione montana L.	0	n	1	0	Campanulaceae Juss.
Iz_3_1109	DJI_0038	Jurinea cyanoides (L.) Rchb.s.l.	0	n	1	0	Asteraceae Bercht. & J.Presl
Iz_3_1109	DJI_0038	Koeleria glauca (Spreng.) DC.	0	n	1	0	Poaceae Barnhart
Iz_3_1109	DJI_0038	Lactuca serriola L.	0	a	0	0	Asteraceae Bercht. & J.Presl
Iz_3_1109	DJI_0038	Linaria genistifolia (L.) Mill.	0	n	1	0	Plantaginaceae Juss.
Iz_3_1109	DJI_0038	Lomelosia argentea (L.) Greuter & Burdet	0	n	1	0	Caprifoliaceae Juss.
Iz_3_1109	DJI_0038	Melampyrum pratense L.	0	n	1	0	Orobanchaceae Vent.
Iz_3_1109	DJI_0038	Oenothera biennis L.	0	a	0	0	Onagraceae Juss.
Iz_3_1109	DJI_0038	Pentanema britannica (L.) D.Gut.Larr., Santos-Vicente, Anderb., E.Rico & M.M.Mart.Ort.	0	n	1	0	Asteraceae Bercht. & J.Presl
Iz_3_1109	DJI_0038	Peucedanum oreoselinum (L.) Moench	0	n	1	0	Apiaceae Lindl.
Iz_3_1109	DJI_0038	Peucedanum ruthenicum M.Bieb.	0	n	1	0	Apiaceae Lindl.
Iz_3_1109	DJI_0038	Pilosella echioides (Lumn.) F.W.Schultz & Sch.Bip.	0	n	1	0	Asteraceae Bercht. & J.Presl
Iz_3_1109	DJI_0038	Pilosella officinarum F. Schultz et Sch. Bip.	0	n	1	0	Asteraceae Bercht. & J.Presl
Iz_3_1109	DJI_0038	Pilosella sp.	0		NA	0	Asteraceae Bercht. & J.Presl
Iz_3_1109	DJI_0038	Pinus sylvestris L.	0	n	1	1	Pinaceae Spreng. ex F.Rudolphi
Iz_3_1109	DJI_0038	Poa annua L.	0	n	1	0	Poaceae Barnhart
Iz_3_1109	DJI_0038	Poa nemoralis L.	0	n	1	0	Poaceae Barnhart
Iz_3_1109	DJI_0038	Poa sp.	0		NA	0	Poaceae Barnhart
Iz_3_1109	DJI_0038	Polygonatum odoratum (Mill.) Druce	0	n	1	0	Asparagaceae Juss.
Iz_3_1109	DJI_0038	Polygonum arenarium Waldst. & Kit.	0	n	1	0	Polygonaceae Juss.
Iz_3_1109	DJI_0038	Populus nigra L.	0	n	1	1	Salicaceae Mirb.
Iz_3_1109	DJI_0038	Populus tremula L.	0	n	1	1	Salicaceae Mirb.
Iz_3_1109	DJI_0038	Populus Ã canescens (Aiton) Sm.	0	n	1	1	Salicaceae Mirb.
Iz_3_1109	DJI_0038	Potentilla argentea L.	0	n	1	0	Rosaceae Juss.
Iz_3_1109	DJI_0038	Psammophiliella muralis (L.) Ikonn.	0	n	1	0	Caryophyllaceae Juss.
Iz_3_1109	DJI_0038	Psephellus sumensis (Kalen.) Greuter	0	n	1	0	Asteraceae Bercht. & J.Presl
Iz_3_1109	DJI_0038	Pulsatilla pratensis (L.) Mill. s.l.	0	n	1	0	Ranunculaceae Juss.
Iz_3_1109	DJI_0038	Pyrus communis L.	0	a	0	1	Rosaceae Juss.
Iz_3_1109	DJI_0038	Quercus robur L.	0	n	1	1	Fagaceae Dumort.
Iz_3_1109	DJI_0038	Robinia pseudoacacia L.	0	a	0	1	Fabaceae Lindl.
Iz_3_1109	DJI_0038	Rosa sp.	0		NA	1	Rosaceae Juss.
Iz_3_1109	DJI_0038	Rubus idaeus L.	0	n	1	0	Rosaceae Juss.
Iz_3_1109	DJI_0038	Rumex acetosella L.	0	n	1	0	Polygonaceae Juss.
Iz_3_1109	DJI_0038	Rumex thyrsiflorus Fingerh.	0	n	1	0	Polygonaceae Juss.
Iz_3_1109	DJI_0038	Salix alba L.	0	n	1	1	Salicaceae Mirb.
Iz_3_1109	DJI_0038	Sambucus nigra L.	0	n	1	1	Viburnaceae Raf.
Iz_3_1109	DJI_0038	Secale sylvestre Host	0	n	1	0	Poaceae Barnhart
Iz_3_1109	DJI_0038	Sempervivum ruthenicum Schnittsp. & C.B. Lehm.	0	n	1	0	Crassulaceae J.St.-Hil.
Iz_3_1109	DJI_0038	Setaria viridis (L.) P. Beauv.	0	n	1	0	Poaceae Barnhart
Iz_3_1109	DJI_0038	Silene borysthenica (Gruner) Walters	0	n	1	0	Caryophyllaceae Juss.
Iz_3_1109	DJI_0038	Silene latifolia subsp. alba (Mill.) Greuter & Burdet	0	n	1	0	Caryophyllaceae Juss.
Iz_3_1109	DJI_0038	Solidago virgaurea L.	0	n	1	0	Asteraceae Bercht. & J.Presl
Iz_3_1109	DJI_0038	Sorbus aucuparia L.	0	n	1	1	Rosaceae Juss.
Iz_3_1109	DJI_0038	Species 11.09_3_0045	0		NA	0	
Iz_3_1109	DJI_0038	Species 11.09_3_0058	0		NA	0	
Iz_3_1109	DJI_0038	Stachys recta L.	0	n	1	0	Lamiacea Lindl.
Iz_3_1109	DJI_0038	Tanacetum vulgare L.	0	n	1	0	Asteraceae Bercht. & J.Presl
Iz_3_1109	DJI_0038	Thymus pallasianus Heinr.Braun	0	n	1	0	Lamiacea Lindl.
Iz_3_1109	DJI_0038	Tilia cordata Mill.	0	n	1	1	Malvaceae Juss.
Iz_3_1109	DJI_0038	Trifolium alpestre L.	0	n	1	0	Fabaceae Lindl.
Iz_3_1109	DJI_0038	Trifolium arvense L.	0	n	1	0	Fabaceae Lindl.
Iz_3_1109	DJI_0038	Ulmus glabra Huds.	0	n	1	1	Ulmaceae Mirb.
Iz_3_1109	DJI_0038	Verbascum densiflorum Bertol.	0	n	1	0	Scrophulariaceae Juss.
Iz_3_1109	DJI_0038	Veronica austriaca L.	0	n	1	0	Plantaginaceae Juss.
Iz_3_1109	DJI_0038	Veronica incana L.	0	n	1	0	Plantaginaceae Juss.
Iz_3_1109	DJI_0038	Veronica verna L.	0	n	1	0	Plantaginaceae Juss.
Iz_3_1109	DJI_0038	Vincetoxicum hirundinaria Medik	0	n	1	0	Apocynaceae Juss.
Iz_3_1109	DJI_0042	Acer tataricum L.	0	n	1	1	Sapindaceae Juss.
Iz_3_1109	DJI_0042	Achillea millefolium L. s.l.	0	n	1	0	Asteraceae Bercht. & J.Presl
Iz_3_1109	DJI_0042	Allium oleraceum L.	0	n	1	0	Amaryllidaceae J.St.-Hil.
Iz_3_1109	DJI_0042	Ambrosia artemisiifolia L.	0	a	0	0	Asteraceae Bercht. & J.Presl
Iz_3_1109	DJI_0042	Amorpha fruticosa L.	0	a	0	0	Fabaceae Lindl.
Iz_3_1109	DJI_0042	Anthericum ramosum L.	0	n	1	0	Asparagaceae Juss.
Iz_3_1109	DJI_0042	Artemisia marschalliana Spreng.	0	n	1	0	Asteraceae Bercht. & J.Presl
Iz_3_1109	DJI_0042	Asparagus officinalis L.	0	n	1	0	Asparagaceae Juss.
Iz_3_1109	DJI_0042	Astragalus cicer L.	0	n	1	0	Fabaceae Lindl.
Iz_3_1109	DJI_0042	Bassia laniflora (S.G.Gmel.) A.J.Scott	0	n	1	0	Amaranthaceae Juss.
Iz_3_1109	DJI_0042	Berberis vulgaris L.	0	n	1	0	Berberidaceae Juss.
Iz_3_1109	DJI_0042	Betula pendula Roth.	0	n	1	1	Betulaceae Gray
Iz_3_1109	DJI_0042	Betula pendula Roth.	0	n	1	0	Poaceae Barnhart
Iz_3_1109	DJI_0042	Bromus inermis Leyss.	0		NA	NA	
Iz_3_1109	DJI_0042	Bromus squarrosus L.	0	a	0	0	Poaceae Barnhart
Iz_3_1109	DJI_0042	Bromus tectorum L.	0	a	0	0	Poaceae Barnhart

Iz_3_1109	DJI_0042	Calamagrostis epigejos (L.) Roth	0	n	1	0	Poaceae Barnhart
Iz_3_1109	DJI_0042	Carduus acanthoides L.	0	n	1	0	Asteraceae Bercht. & J.Presl
Iz_3_1109	DJI_0042	Carex sp.	0	n	1	0	Cyperaceae Juss.
Iz_3_1109	DJI_0042	Centaurea majorovii Dumbadze	0	n	1	0	Asteraceae Bercht. & J.Presl
Iz_3_1109	DJI_0042	Chamaecytisus ruthenicus (Fisch.ex Wol.) Klaskova	0	n	1	0	Fabaceae Lindl.
Iz_3_1109	DJI_0042	Chelidonium majus L.	0	n	1	0	Papaveraceae Juss.
Iz_3_1109	DJI_0042	Chenopodium betaceum Andrz.	0	n	1	0	Amaranthaceae Juss.
Iz_3_1109	DJI_0042	Chenopodium cf. album	0	n	1	0	Amaranthaceae Juss.
Iz_3_1109	DJI_0042	Chenopodium sp.	0		NA	0	Amaranthaceae Juss.
Iz_3_1109	DJI_0042	Chondrilla juncea L.	0	n	1	0	Asteraceae Bercht. & J.Presl
Iz_3_1109	DJI_0042	Cirsium vulgare (Savi) Ten.	0	n	1	0	Asteraceae Bercht. & J.Presl
Iz_3_1109	DJI_0042	Clinopodium acinos (L.) Kuntze	0	n	1	0	Lamiacea Lindl.
Iz_3_1109	DJI_0042	Convallaria majalis L.	0	n	1	0	Asparagaceae Juss.
Iz_3_1109	DJI_0042	Crataegus rhipidophylla Gand.	0	n	1	1	Rosaceae Juss.
Iz_3_1109	DJI_0042	Crepis foetida L.	0	n	1	0	Asteraceae Bercht. & J.Presl
Iz_3_1109	DJI_0042	Dactylis glomerata L.	0	n	1	0	Poaceae Barnhart
Iz_3_1109	DJI_0042	Dianthus campestris M.Bieb.	0	n	1	0	Caryophyllaceae Juss.
Iz_3_1109	DJI_0042	Digitaria sanguinalis (L.) Scop.	0	a	0	0	Poaceae Barnhart
Iz_3_1109	DJI_0042	Elymus repens (L.) Gould	0	n	1	0	Poaceae Barnhart
Iz_3_1109	DJI_0042	Epilobium tetragonum L.	0	n	1	0	Onagraceae Juss.
Iz_3_1109	DJI_0042	Eragrostis minor Host	0	a	0	0	Poaceae Barnhart
Iz_3_1109	DJI_0042	Erigeron canadensis L.	1	a	0	0	Asteraceae Bercht. & J.Presl
Iz_3_1109	DJI_0042	Euphorbia seguieriana Neck.	0	n	1	0	Euphorbiaceae Juss.
Iz_3_1109	DJI_0042	Fallopia dumetorum (L.) Holub	0	n	1	0	Polygonaceae Juss.
Iz_3_1109	DJI_0042	Festuca beckeri (Hack.) Trautv.	0	n	1	0	Poaceae Barnhart
Iz_3_1109	DJI_0042	Fragaria vesca L.	0	n	1	0	Rosaceae Juss.
Iz_3_1109	DJI_0042	Frangula alnus Mill.	0	n	1	1	Rhamnaceae Juss.
Iz_3_1109	DJI_0042	Fraxinus pennsylvanica Marshall	0	a	0	1	Oleaceae Hoffmanns. & Link
Iz_3_1109	DJI_0042	Galeopsis bifida Boenn.	0	n	1	0	Lamiacea Lindl.
Iz_3_1109	DJI_0042	Galium verum L.	0	n	1	0	Rubiaceae Juss.
Iz_3_1109	DJI_0042	Genista tinctoria L.	0	n	1	0	Fabaceae Lindl.
Iz_3_1109	DJI_0042	Geranium robertianum L.	0	n	1	0	Geraniaceae Juss.
Iz_3_1109	DJI_0042	Geranium sanquineum L.	0	n	1	0	Geraniaceae Juss.
Iz_3_1109	DJI_0042	Gypsophila paniculata L.	0	n	1	0	Caryophyllaceae Juss.
Iz_3_1109	DJI_0042	Helichrysum arenarium (L.) Moench	0	n	1	0	Asteraceae Bercht. & J.Presl
Iz_3_1109	DJI_0042	Herniaria polygama J.Gay	0	n	1	0	Caryophyllaceae Juss.
Iz_3_1109	DJI_0042	Hieracium robustum Fr.	0	n	1	0	Asteraceae Bercht. & J.Presl
Iz_3_1109	DJI_0042	Hieracium umbellatum L.	0	n	1	NA	Asteraceae Bercht. & J.Presl
Iz_3_1109	DJI_0042	Hylotelephium maximum (L.) Holub	0	n	1	0	Crassulaceae J.St.-Hil.
Iz_3_1109	DJI_0042	Jacobaea borysthenica (DC.) B.Nord. & Greuter	0	n	1	0	Asteraceae Bercht. & J.Presl
Iz_3_1109	DJI_0042	Jasione montana L.	0	n	1	0	Campanulaceae Juss.
Iz_3_1109	DJI_0042	Jurinea cyanoides (L.) Rchb.s.l.	0	n	1	0	Asteraceae Bercht. & J.Presl
Iz_3_1109	DJI_0042	Koeleria glauca (Spreng.) DC.	0	n	1	0	Poaceae Barnhart
Iz_3_1109	DJI_0042	Lactuca serriola L.	0	a	0	0	Asteraceae Bercht. & J.Presl
Iz_3_1109	DJI_0042	Linaria genistifolia (L.) Mill.	0	n	1	0	Plantaginaceae Juss.
Iz_3_1109	DJI_0042	Lomelosia argentea (L.) Greuter & Burdet	0	n	1	0	Caprifoliaceae Juss.
Iz_3_1109	DJI_0042	Melampyrum pratense L.	0	n	1	0	Orobanchaceae Vent.
Iz_3_1109	DJI_0042	Oenothera biennis L.	0	a	0	0	Onagraceae Juss.
Iz_3_1109	DJI_0042	Pentanema britannica (L.) D.Gut.Larr., Santos-Vicente, Anderb., E.Rico & M.M.Mart.Ort.	0	n	1	0	Asteraceae Bercht. & J.Presl
Iz_3_1109	DJI_0042	Peucedanum oreoselinum (L.) Moench	0.2	n	1	0	Apiaceae Lindl.
Iz_3_1109	DJI_0042	Peucedanum ruthenicum M.Bieb.	1	n	1	0	Apiaceae Lindl.
Iz_3_1109	DJI_0042	Pilosella echioides (Lumn.) F.W.Schultz & Sch.Bip.	0	n	1	0	Asteraceae Bercht. & J.Presl
Iz_3_1109	DJI_0042	Pilosella officinarum F. Schultz et Sch. Bip.	0	n	1	0	Asteraceae Bercht. & J.Presl
Iz_3_1109	DJI_0042	Pilosella sp.	0		NA	0	Asteraceae Bercht. & J.Presl
Iz_3_1109	DJI_0042	Pinus sylvestris L.	0	n	1	1	Pinaceae Spreng. ex F.Rudolphi
Iz_3_1109	DJI_0042	Poa annua L.	0	n	1	0	Poaceae Barnhart
Iz_3_1109	DJI_0042	Poa nemoralis L.	0	n	1	0	Poaceae Barnhart
Iz_3_1109	DJI_0042	Poa sp.	0		NA	0	Poaceae Barnhart
Iz_3_1109	DJI_0042	Polygonatum odoratum (Mill.) Druce	0	n	1	0	Asparagaceae Juss.
Iz_3_1109	DJI_0042	Polygonum arenarium Waldst. & Kit.	0	n	1	0	Polygonaceae Juss.
Iz_3_1109	DJI_0042	Populus nigra L.	0	n	1	1	Salicaceae Mirb.
Iz_3_1109	DJI_0042	Populus tremula L.	0	n	1	1	Salicaceae Mirb.
Iz_3_1109	DJI_0042	Populus Ã canescens (Aiton) Sm.	0	n	1	1	Salicaceae Mirb.
Iz_3_1109	DJI_0042	Potentilla argentea L.	0	n	1	0	Rosaceae Juss.
Iz_3_1109	DJI_0042	Psammophiliella muralis (L.) Ikonn.	0	n	1	0	Caryophyllaceae Juss.
Iz_3_1109	DJI_0042	Psephellus sumensis (Kalen.) Greuter	0	n	1	0	Asteraceae Bercht. & J.Presl
Iz_3_1109	DJI_0042	Pulsatilla pratensis (L.) Mill. s.l.	0	n	1	0	Ranunculaceae Juss.
Iz_3_1109	DJI_0042	Pyrus communis L.	0	a	0	1	Rosaceae Juss.
Iz_3_1109	DJI_0042	Quercus robur L.	0	n	1	1	Fagaceae Dumort.
Iz_3_1109	DJI_0042	Robinia pseudoacacia L.	0	a	0	1	Fabaceae Lindl.
Iz_3_1109	DJI_0042	Rosa sp.	0		NA	1	Rosaceae Juss.
Iz_3_1109	DJI_0042	Rubus idaeus L.	0	n	1	0	Rosaceae Juss.
Iz_3_1109	DJI_0042	Rumex acetosella L.	0	n	1	0	Polygonaceae Juss.
Iz_3_1109	DJI_0042	Rumex thyrsiflorus Fingerh.	0	n	1	0	Polygonaceae Juss.
Iz_3_1109	DJI_0042	Salix alba L.	0	n	1	1	Salicaceae Mirb.
Iz_3_1109	DJI_0042	Sambucus nigra L.	0	n	1	1	Viburnaceae Raf.
Iz_3_1109	DJI_0042	Secale sylvestre Host	0	n	1	0	Poaceae Barnhart
Iz_3_1109	DJI_0042	Sempervivum ruthenicum Schnittsp. & C.B. Lehm.	0	n	1	0	Crassulaceae J.St.-Hil.
Iz_3_1109	DJI_0042	Setaria viridis (L.) P. Beauv.	0	n	1	0	Poaceae Barnhart
Iz_3_1109	DJI_0042	Silene borysthenica (Gruner) Walters	0	n	1	0	Caryophyllaceae Juss.
Iz_3_1109	DJI_0042	Silene latifolia subsp. alba (Mill.) Greuter & Burdet	0	n	1	0	Caryophyllaceae Juss.
Iz_3_1109	DJI_0042	Solidago virgaurea L.	0	n	1	0	Asteraceae Bercht. & J.Presl
Iz_3_1109	DJI_0042	Sorbus aucuparia L.	0	n	1	1	Rosaceae Juss.
Iz_3_1109	DJI_0042	Species 11.09_3_0045	0		NA	0	
Iz_3_1109	DJI_0042	Species 11.09_3_0058	0		NA	0	
Iz_3_1109	DJI_0042	Stachys recta L.	0	n	1	0	Lamiacea Lindl.
Iz_3_1109	DJI_0042	Tanacetum vulgare L.	0	n	1	0	Asteraceae Bercht. & J.Presl
Iz_3_1109	DJI_0042	Thymus pallasianus Heinr.Braun	0	n	1	0	Lamiacea Lindl.
Iz_3_1109	DJI_0042	Tilia cordata Mill.	0	n	1	1	Malvaceae Juss.
Iz_3_1109	DJI_0042	Trifolium alpestre L.	0	n	1	0	Fabaceae Lindl.
Iz_3_1109	DJI_0042	Trifolium arvense L.	0	n	1	0	Fabaceae Lindl.
Iz_3_1109	DJI_0042	Ulmus glabra Huds.	0	n	1	1	Ulmaceae Mirb.
Iz_3_1109	DJI_0042	Verbascum densiflorum Bertol.	0	n	1	0	Scrophulariaceae Juss.
Iz_3_1109	DJI_0042	Veronica austriaca L.	0	n	1	0	Plantaginaceae Juss.
Iz_3_1109	DJI_0042	Veronica incana L.	0	n	1	0	Plantaginaceae Juss.
Iz_3_1109	DJI_0042	Veronica verna L.	0	n	1	0	Plantaginaceae Juss.
Iz_3_1109	DJI_0042	Vincetoxicum hirundinaria Medik	0	n	1	0	Apocynaceae Juss.
Iz_3_1109	DJI_0045	Acer tataricum L.	0	n	1	1	Sapindaceae Juss.

Iz_3_1109	DJI_0045	Achillea millefolium L. s.l.	0	n	1	0	Asteraceae Bercht. & J.Presl
Iz_3_1109	DJI_0045	Allium oleraceum L.	0	n	1	0	Amaryllidaceae J.St.-Hil.
Iz_3_1109	DJI_0045	Ambrosia artemisiifolia L.	0	a	0	0	Asteraceae Bercht. & J.Presl
Iz_3_1109	DJI_0045	Amorpha fruticosa L.	0	a	0	0	Fabaceae Lindl.
Iz_3_1109	DJI_0045	Anthericum ramosum L.	0	n	1	0	Asparagaceae Juss.
Iz_3_1109	DJI_0045	Artemisia marschalliana Spreng.	0	n	1	0	Asteraceae Bercht. & J.Presl
Iz_3_1109	DJI_0045	Asparagus officinalis L.	0	n	1	0	Asparagaceae Juss.
Iz_3_1109	DJI_0045	Astragalus cicer L.	0	n	1	0	Fabaceae Lindl.
Iz_3_1109	DJI_0045	Bassia laniflora (S.G.Gmel.) A.J.Scott	0	n	1	0	Amaranthaceae Juss.
Iz_3_1109	DJI_0045	Berberis vulgaris L.	0	n	1	0	Berberidaceae Juss.
Iz_3_1109	DJI_0045	Betula pendula Roth.	0	n	1	1	Betulaceae Gray
Iz_3_1109	DJI_0045	Betula pendula Roth.	0	n	1	0	Poaceae Barnhart
Iz_3_1109	DJI_0045	Bromus inermis Leyss.	0		NA	NA	
Iz_3_1109	DJI_0045	Bromus squarrosus L.	0	a	0	0	Poaceae Barnhart
Iz_3_1109	DJI_0045	Bromus tectorum L.	0	a	0	0	Poaceae Barnhart
Iz_3_1109	DJI_0045	Calamagrostis epigejos (L.) Roth	0	n	1	0	Poaceae Barnhart
Iz_3_1109	DJI_0045	Carduus acanthoides L.	0	n	1	0	Asteraceae Bercht. & J.Presl
Iz_3_1109	DJI_0045	Carex sp.	0	n	1	0	Cyperaceae Juss.
Iz_3_1109	DJI_0045	Centaurea majorovii Dumbadze	0	n	1	0	Asteraceae Bercht. & J.Presl
Iz_3_1109	DJI_0045	Chamaecytisus ruthenicus (Fisch.ex Wol.) Klaskova	0	n	1	0	Fabaceae Lindl.
Iz_3_1109	DJI_0045	Chelidonium majus L.	0	n	1	0	Papaveraceae Juss.
Iz_3_1109	DJI_0045	Chenopodium betaceum Andrz.	0	n	1	0	Amaranthaceae Juss.
Iz_3_1109	DJI_0045	Chenopodium cf. album	0	n	1	0	Amaranthaceae Juss.
Iz_3_1109	DJI_0045	Chenopodium sp.	0		NA	0	Amaranthaceae Juss.
Iz_3_1109	DJI_0045	Chondrilla juncea L.	0	n	1	0	Asteraceae Bercht. & J.Presl
Iz_3_1109	DJI_0045	Cirsium vulgare (Savi) Ten.	0	n	1	0	Asteraceae Bercht. & J.Presl
Iz_3_1109	DJI_0045	Clinopodium acinos (L.) Kuntze	0	n	1	0	Lamiacea Lindl.
Iz_3_1109	DJI_0045	Convallaria majalis L.	0	n	1	0	Asparagaceae Juss.
Iz_3_1109	DJI_0045	Crataegus rhipidophylla Gand.	0	n	1	1	Rosaceae Juss.
Iz_3_1109	DJI_0045	Crepis foetida L.	0	n	1	0	Asteraceae Bercht. & J.Presl
Iz_3_1109	DJI_0045	Dactylis glomerata L.	0	n	1	0	Poaceae Barnhart
Iz_3_1109	DJI_0045	Dianthus campestris M.Bieb.	0	n	1	0	Caryophyllaceae Juss.
Iz_3_1109	DJI_0045	Digitaria sanguinalis (L.) Scop.	0	a	0	0	Poaceae Barnhart
Iz_3_1109	DJI_0045	Elymus repens (L.) Gould	0	n	1	0	Poaceae Barnhart
Iz_3_1109	DJI_0045	Epilobium tetragonum L.	0	n	1	0	Onagraceae Juss.
Iz_3_1109	DJI_0045	Eragrostis minor Host	0	a	0	0	Poaceae Barnhart
Iz_3_1109	DJI_0045	Erigeron canadensis L.	0.2	a	0	0	Asteraceae Bercht. & J.Presl
Iz_3_1109	DJI_0045	Euphorbia seguieriana Neck.	0	n	1	0	Euphorbiaceae Juss.
Iz_3_1109	DJI_0045	Fallopia dumetorum (L.) Holub	0	n	1	0	Polygonaceae Juss.
Iz_3_1109	DJI_0045	Festuca beckeri (Hack.) Trautv.	0	n	1	0	Poaceae Barnhart
Iz_3_1109	DJI_0045	Fragaria vesca L.	0	n	1	0	Rosaceae Juss.
Iz_3_1109	DJI_0045	Frangula alnus Mill.	0	n	1	1	Rhamnaceae Juss.
Iz_3_1109	DJI_0045	Fraxinus pennsylvanica Marshall	0	a	0	1	Oleaceae Hoffmanns. & Link
Iz_3_1109	DJI_0045	Galeopsis bifida Boenn.	0	n	1	0	Lamiacea Lindl.
Iz_3_1109	DJI_0045	Galium verum L.	0	n	1	0	Rubiaceae Juss.
Iz_3_1109	DJI_0045	Genista tinctoria L.	0	n	1	0	Fabaceae Lindl.
Iz_3_1109	DJI_0045	Geranium robertianum L.	0	n	1	0	Geraniaceae Juss.
Iz_3_1109	DJI_0045	Geranium sanquineum L.	0	n	1	0	Geraniaceae Juss.
Iz_3_1109	DJI_0045	Gypsophila paniculata L.	0	n	1	0	Caryophyllaceae Juss.
Iz_3_1109	DJI_0045	Helichrysum arenarium (L.) Moench	0	n	1	0	Asteraceae Bercht. & J.Presl
Iz_3_1109	DJI_0045	Herniaria polygama J.Gay	0	n	1	0	Caryophyllaceae Juss.
Iz_3_1109	DJI_0045	Hieracium robustum Fr.	0	n	1	0	Asteraceae Bercht. & J.Presl
Iz_3_1109	DJI_0045	Hieracium umbellatum L.	0	n	1	NA	Asteraceae Bercht. & J.Presl
Iz_3_1109	DJI_0045	Hylotelephium maximum (L.) Holub	0	n	1	0	Crassulaceae J.St.-Hil.
Iz_3_1109	DJI_0045	Jacobaea borysthenica (DC.) B.Nord. & Greuter	0	n	1	0	Asteraceae Bercht. & J.Presl
Iz_3_1109	DJI_0045	Jasione montana L.	0	n	1	0	Campanulaceae Juss.
Iz_3_1109	DJI_0045	Jurinea cyanoides (L.) Rchb.s.l.	0	n	1	0	Asteraceae Bercht. & J.Presl
Iz_3_1109	DJI_0045	Koeleria glauca (Spreng.) DC.	0	n	1	0	Poaceae Barnhart
Iz_3_1109	DJI_0045	Lactuca serriola L.	0	a	0	0	Asteraceae Bercht. & J.Presl
Iz_3_1109	DJI_0045	Linaria genistifolia (L.) Mill.	0	n	1	0	Plantaginaceae Juss.
Iz_3_1109	DJI_0045	Lomelosia argentea (L.) Greuter & Burdet	0	n	1	0	Caprifoliaceae Juss.
Iz_3_1109	DJI_0045	Melampyrum pratense L.	0	n	1	0	Orobanchaceae Vent.
Iz_3_1109	DJI_0045	Oenothera biennis L.	0	a	0	0	Onagraceae Juss.
Iz_3_1109	DJI_0045	Pentanema britannica (L.) D.Gut.Larr., Santos-Vicente, Anderb., E.Rico & M.M.Mart.Ort.	0	n	1	0	Asteraceae Bercht. & J.Presl
Iz_3_1109	DJI_0045	Peucedanum oreoselinum (L.) Moench	0	n	1	0	Apiaceae Lindl.
Iz_3_1109	DJI_0045	Peucedanum ruthenicum M.Bieb.	0	n	1	0	Apiaceae Lindl.
Iz_3_1109	DJI_0045	Pilosella echioides (Lumn.) F.W.Schultz & Sch.Bip.	0	n	1	0	Asteraceae Bercht. & J.Presl
Iz_3_1109	DJI_0045	Pilosella officinarum F. Schultz et Sch. Bip.	0	n	1	0	Asteraceae Bercht. & J.Presl
Iz_3_1109	DJI_0045	Pilosella sp.	0		NA	0	Asteraceae Bercht. & J.Presl
Iz_3_1109	DJI_0045	Pinus sylvestris L.	0	n	1	1	Pinaceae Spreng. ex F.Rudolphi
Iz_3_1109	DJI_0045	Poa annua L.	0	n	1	0	Poaceae Barnhart
Iz_3_1109	DJI_0045	Poa nemoralis L.	0	n	1	0	Poaceae Barnhart
Iz_3_1109	DJI_0045	Poa sp.	0		NA	0	Poaceae Barnhart
Iz_3_1109	DJI_0045	Polygonatum odoratum (Mill.) Druce	0	n	1	0	Asparagaceae Juss.
Iz_3_1109	DJI_0045	Polygonum arenarium Waldst. & Kit.	0	n	1	0	Polygonaceae Juss.
Iz_3_1109	DJI_0045	Populus nigra L.	0	n	1	1	Salicaceae Mirb.
Iz_3_1109	DJI_0045	Populus tremula L.	0.2	n	1	1	Salicaceae Mirb.
Iz_3_1109	DJI_0045	Populus Ã canescens (Aiton) Sm.	0	n	1	1	Salicaceae Mirb.
Iz_3_1109	DJI_0045	Potentilla argentea L.	0	n	1	0	Rosaceae Juss.
Iz_3_1109	DJI_0045	Psammophiliella muralis (L.) Ikonn.	0	n	1	0	Caryophyllaceae Juss.
Iz_3_1109	DJI_0045	Psephellus sumensis (Kalen.) Greuter	0	n	1	0	Asteraceae Bercht. & J.Presl
Iz_3_1109	DJI_0045	Pulsatilla pratensis (L.) Mill. s.l.	0	n	1	0	Ranunculaceae Juss.
Iz_3_1109	DJI_0045	Pyrus communis L.	0	a	0	1	Rosaceae Juss.
Iz_3_1109	DJI_0045	Quercus robur L.	0	n	1	1	Fagaceae Dumort.
Iz_3_1109	DJI_0045	Robinia pseudoacacia L.	0	a	0	1	Fabaceae Lindl.
Iz_3_1109	DJI_0045	Rosa sp.	0		NA	1	Rosaceae Juss.
Iz_3_1109	DJI_0045	Rubus idaeus L.	0	n	1	0	Rosaceae Juss.
Iz_3_1109	DJI_0045	Rumex acetosella L.	0	n	1	0	Polygonaceae Juss.
Iz_3_1109	DJI_0045	Rumex thyrsiflorus Fingerh.	0	n	1	0	Polygonaceae Juss.
Iz_3_1109	DJI_0045	Salix alba L.	0	n	1	1	Salicaceae Mirb.
Iz_3_1109	DJI_0045	Sambucus nigra L.	0	n	1	1	Viburnaceae Raf.
Iz_3_1109	DJI_0045	Secale sylvestre Host	0	n	1	0	Poaceae Barnhart
Iz_3_1109	DJI_0045	Sempervivum ruthenicum Schnittsp. & C.B. Lehm.	0	n	1	0	Crassulaceae J.St.-Hil.
Iz_3_1109	DJI_0045	Setaria viridis (L.) P. Beauv.	0	n	1	0	Poaceae Barnhart
Iz_3_1109	DJI_0045	Silene borysthenica (Gruner) Walters	0	n	1	0	Caryophyllaceae Juss.
Iz_3_1109	DJI_0045	Silene latifolia subsp. alba (Mill.) Greuter & Burdet	0	n	1	0	Caryophyllaceae Juss.
Iz_3_1109	DJI_0045	Solidago virgaurea L.	0	n	1	0	Asteraceae Bercht. & J.Presl
Iz_3_1109	DJI_0045	Sorbus aucuparia L.	0	n	1	1	Rosaceae Juss.

Iz_3_1109	DJI_0045	Species 11.09_3_0045	0.2		NA	0	
Iz_3_1109	DJI_0045	Species 11.09_3_0058	0		NA	0	
Iz_3_1109	DJI_0045	Stachys recta L.	0	n	1	0	Lamiacea Lindl.
Iz_3_1109	DJI_0045	Tanacetum vulgare L.	0	n	1	0	Asteraceae Bercht. & J.Presl
Iz_3_1109	DJI_0045	Thymus pallasianus Heinr.Braun	0	n	1	0	Lamiacea Lindl.
Iz_3_1109	DJI_0045	Tilia cordata Mill.	0	n	1	1	Malvaceae Juss.
Iz_3_1109	DJI_0045	Trifolium alpestre L.	0	n	1	0	Fabaceae Lindl.
Iz_3_1109	DJI_0045	Trifolium arvense L.	0	n	1	0	Fabaceae Lindl.
Iz_3_1109	DJI_0045	Ulmus glabra Huds.	0	n	1	1	Ulmaceae Mirb.
Iz_3_1109	DJI_0045	Verbascum densiflorum Bertol.	0	n	1	0	Scrophulariaceae Juss.
Iz_3_1109	DJI_0045	Veronica austriaca L.	0	n	1	0	Plantaginaceae Juss.
Iz_3_1109	DJI_0045	Veronica incana L.	0	n	1	0	Plantaginaceae Juss.
Iz_3_1109	DJI_0045	Veronica verna L.	0	n	1	0	Plantaginaceae Juss.
Iz_3_1109	DJI_0045	Vincetoxicum hirundinaria Medik	0	n	1	0	Apocynaceae Juss.
Iz_3_1109	DJI_0048	Acer tataricum L.	0	n	1	1	Sapindaceae Juss.
Iz_3_1109	DJI_0048	Achillea millefolium L. s.l.	0	n	1	0	Asteraceae Bercht. & J.Presl
Iz_3_1109	DJI_0048	Allium oleraceum L.	0	n	1	0	Amaryllidaceae J.St.-Hil.
Iz_3_1109	DJI_0048	Ambrosia artemisiifolia L.	0	a	0	0	Asteraceae Bercht. & J.Presl
Iz_3_1109	DJI_0048	Amorpha fruticosa L.	0	a	0	0	Fabaceae Lindl.
Iz_3_1109	DJI_0048	Anthericum ramosum L.	0	n	1	0	Asparagaceae Juss.
Iz_3_1109	DJI_0048	Artemisia marschalliana Spreng.	0	n	1	0	Asteraceae Bercht. & J.Presl
Iz_3_1109	DJI_0048	Asparagus officinalis L.	0	n	1	0	Asparagaceae Juss.
Iz_3_1109	DJI_0048	Astragalus cicer L.	0	n	1	0	Fabaceae Lindl.
Iz_3_1109	DJI_0048	Bassia laniflora (S.G.Gmel.) A.J.Scott	0	n	1	0	Amaranthaceae Juss.
Iz_3_1109	DJI_0048	Berberis vulgaris L.	0	n	1	0	Berberidaceae Juss.
Iz_3_1109	DJI_0048	Betula pendula Roth.	0	n	1	1	Betulaceae Gray
Iz_3_1109	DJI_0048	Betula pendula Roth.	0	n	1	0	Poaceae Barnhart
Iz_3_1109	DJI_0048	Bromus inermis Leyss.	0		NA	NA	
Iz_3_1109	DJI_0048	Bromus squarrosus L.	0	a	0	0	Poaceae Barnhart
Iz_3_1109	DJI_0048	Bromus tectorum L.	0	a	0	0	Poaceae Barnhart
Iz_3_1109	DJI_0048	Calamagrostis epigejos (L.) Roth	2	n	1	0	Poaceae Barnhart
Iz_3_1109	DJI_0048	Carduus acanthoides L.	0	n	1	0	Asteraceae Bercht. & J.Presl
Iz_3_1109	DJI_0048	Carex sp.	0	n	1	0	Cyperaceae Juss.
Iz_3_1109	DJI_0048	Centaurea majorovii Dumbadze	0	n	1	0	Asteraceae Bercht. & J.Presl
Iz_3_1109	DJI_0048	Chamaecytisus ruthenicus (Fisch.ex Wol.) Klaskova	0	n	1	0	Fabaceae Lindl.
Iz_3_1109	DJI_0048	Chelidonium majus L.	0	n	1	0	Papaveraceae Juss.
Iz_3_1109	DJI_0048	Chenopodium betaceum Andrz.	0	n	1	0	Amaranthaceae Juss.
Iz_3_1109	DJI_0048	Chenopodium cf. album	0	n	1	0	Amaranthaceae Juss.
Iz_3_1109	DJI_0048	Chenopodium sp.	0		NA	0	Amaranthaceae Juss.
Iz_3_1109	DJI_0048	Chondrilla juncea L.	0	n	1	0	Asteraceae Bercht. & J.Presl
Iz_3_1109	DJI_0048	Cirsium vulgare (Savi) Ten.	0	n	1	0	Asteraceae Bercht. & J.Presl
Iz_3_1109	DJI_0048	Clinopodium acinos (L.) Kuntze	0	n	1	0	Lamiacea Lindl.
Iz_3_1109	DJI_0048	Convallaria majalis L.	0	n	1	0	Asparagaceae Juss.
Iz_3_1109	DJI_0048	Crataegus rhipidophylla Gand.	0	n	1	1	Rosaceae Juss.
Iz_3_1109	DJI_0048	Crepis foetida L.	0	n	1	0	Asteraceae Bercht. & J.Presl
Iz_3_1109	DJI_0048	Dactylis glomerata L.	0	n	1	0	Poaceae Barnhart
Iz_3_1109	DJI_0048	Dianthus campestris M.Bieb.	0	n	1	0	Caryophyllaceae Juss.
Iz_3_1109	DJI_0048	Digitaria sanguinalis (L.) Scop.	0	a	0	0	Poaceae Barnhart
Iz_3_1109	DJI_0048	Elymus repens (L.) Gould	0	n	1	0	Poaceae Barnhart
Iz_3_1109	DJI_0048	Epilobium tetragonum L.	0	n	1	0	Onagraceae Juss.
Iz_3_1109	DJI_0048	Eragrostis minor Host	0	a	0	0	Poaceae Barnhart
Iz_3_1109	DJI_0048	Erigeron canadensis L.	1.5	a	0	0	Asteraceae Bercht. & J.Presl
Iz_3_1109	DJI_0048	Euphorbia seguieriana Neck.	0	n	1	0	Euphorbiaceae Juss.
Iz_3_1109	DJI_0048	Fallopia dumetorum (L.) Holub	0	n	1	0	Polygonaceae Juss.
Iz_3_1109	DJI_0048	Festuca beckeri (Hack.) Trautv.	0	n	1	0	Poaceae Barnhart
Iz_3_1109	DJI_0048	Fragaria vesca L.	0	n	1	0	Rosaceae Juss.
Iz_3_1109	DJI_0048	Frangula alnus Mill.	0	n	1	1	Rhamnaceae Juss.
Iz_3_1109	DJI_0048	Fraxinus pennsylvanica Marshall	0	a	0	1	Oleaceae Hoffmanns. & Link
Iz_3_1109	DJI_0048	Galeopsis bifida Boenn.	0	n	1	0	Lamiacea Lindl.
Iz_3_1109	DJI_0048	Galium verum L.	0	n	1	0	Rubiaceae Juss.
Iz_3_1109	DJI_0048	Genista tinctoria L.	0	n	1	0	Fabaceae Lindl.
Iz_3_1109	DJI_0048	Geranium robertianum L.	0	n	1	0	Geraniaceae Juss.
Iz_3_1109	DJI_0048	Geranium sanquineum L.	0	n	1	0	Geraniaceae Juss.
Iz_3_1109	DJI_0048	Gypsophila paniculata L.	0	n	1	0	Caryophyllaceae Juss.
Iz_3_1109	DJI_0048	Helichrysum arenarium (L.) Moench	0	n	1	0	Asteraceae Bercht. & J.Presl
Iz_3_1109	DJI_0048	Herniaria polygama J.Gay	0	n	1	0	Caryophyllaceae Juss.
Iz_3_1109	DJI_0048	Hieracium robustum Fr.	0	n	1	0	Asteraceae Bercht. & J.Presl
Iz_3_1109	DJI_0048	Hieracium umbellatum L.	0	n	1	NA	Asteraceae Bercht. & J.Presl
Iz_3_1109	DJI_0048	Hylotelephium maximum (L.) Holub	0	n	1	0	Crassulaceae J.St.-Hil.
Iz_3_1109	DJI_0048	Jacobaea borysthenica (DC.) B.Nord. & Greuter	0	n	1	0	Asteraceae Bercht. & J.Presl
Iz_3_1109	DJI_0048	Jasione montana L.	0	n	1	0	Campanulaceae Juss.
Iz_3_1109	DJI_0048	Jurinea cyanoides (L.) Rchb.s.l.	0	n	1	0	Asteraceae Bercht. & J.Presl
Iz_3_1109	DJI_0048	Koeleria glauca (Spreng.) DC.	0	n	1	0	Poaceae Barnhart
Iz_3_1109	DJI_0048	Lactuca serriola L.	0	a	0	0	Asteraceae Bercht. & J.Presl
Iz_3_1109	DJI_0048	Linaria genistifolia (L.) Mill.	0	n	1	0	Plantaginaceae Juss.
Iz_3_1109	DJI_0048	Lomelosia argentea (L.) Greuter & Burdet	0	n	1	0	Caprifoliaceae Juss.
Iz_3_1109	DJI_0048	Melampyrum pratense L.	0	n	1	0	Orobanchaceae Vent.
Iz_3_1109	DJI_0048	Oenothera biennis L.	0	a	0	0	Onagraceae Juss.
Iz_3_1109	DJI_0048	Pentanema britannica (L.) D.Gut.Larr., Santos-Vicente, Anderb., E.Rico & M.M.Mart.Ort.	0	n	1	0	Asteraceae Bercht. & J.Presl
Iz_3_1109	DJI_0048	Peucedanum oreoselinum (L.) Moench	0	n	1	0	Apiaceae Lindl.
Iz_3_1109	DJI_0048	Peucedanum ruthenicum M.Bieb.	0	n	1	0	Apiaceae Lindl.
Iz_3_1109	DJI_0048	Pilosella echioides (Lumn.) F.W.Schultz & Sch.Bip.	0	n	1	0	Asteraceae Bercht. & J.Presl
Iz_3_1109	DJI_0048	Pilosella officinarum F. Schultz et Sch. Bip.	0	n	1	0	Asteraceae Bercht. & J.Presl
Iz_3_1109	DJI_0048	Pilosella sp.	0		NA	0	Asteraceae Bercht. & J.Presl
Iz_3_1109	DJI_0048	Pinus sylvestris L.	0	n	1	1	Pinaceae Spreng. ex F.Rudolphi
Iz_3_1109	DJI_0048	Poa annua L.	0	n	1	0	Poaceae Barnhart
Iz_3_1109	DJI_0048	Poa nemoralis L.	0	n	1	0	Poaceae Barnhart
Iz_3_1109	DJI_0048	Poa sp.	0		NA	0	Poaceae Barnhart
Iz_3_1109	DJI_0048	Polygonatum odoratum (Mill.) Druce	0	n	1	0	Asparagaceae Juss.
Iz_3_1109	DJI_0048	Polygonum arenarium Waldst. & Kit.	0	n	1	0	Polygonaceae Juss.
Iz_3_1109	DJI_0048	Populus nigra L.	0	n	1	1	Salicaceae Mirb.
Iz_3_1109	DJI_0048	Populus tremula L.	0	n	1	1	Salicaceae Mirb.
Iz_3_1109	DJI_0048	Populus Ã canescens (Aiton) Sm.	0	n	1	1	Salicaceae Mirb.
Iz_3_1109	DJI_0048	Potentilla argentea L.	0	n	1	0	Rosaceae Juss.
Iz_3_1109	DJI_0048	Psammophiliella muralis (L.) Ikonn.	0	n	1	0	Caryophyllaceae Juss.
Iz_3_1109	DJI_0048	Psephellus sumensis (Kalen.) Greuter	0	n	1	0	Asteraceae Bercht. & J.Presl
Iz_3_1109	DJI_0048	Pulsatilla pratensis (L.) Mill. s.l.	0	n	1	0	Ranunculaceae Juss.
Iz_3_1109	DJI_0048	Pyrus communis L.	0	a	0	1	Rosaceae Juss.

Iz_3_1109	DJI_0048	Quercus robur L.	0	n	1	1	Fagaceae Dumort.
Iz_3_1109	DJI_0048	Robinia pseudoacacia L.	0	a	0	1	Fabaceae Lindl.
Iz_3_1109	DJI_0048	Rosa sp.	0		NA	1	Rosaceae Juss.
Iz_3_1109	DJI_0048	Rubus idaeus L.	0	n	1	0	Rosaceae Juss.
Iz_3_1109	DJI_0048	Rumex acetosella L.	0	n	1	0	Polygonaceae Juss.
Iz_3_1109	DJI_0048	Rumex thyrsiflorus Fingerh.	0	n	1	0	Polygonaceae Juss.
Iz_3_1109	DJI_0048	Salix alba L.	0	n	1	1	Salicaceae Mirb.
Iz_3_1109	DJI_0048	Sambucus nigra L.	0	n	1	1	Viburnaceae Raf.
Iz_3_1109	DJI_0048	Secale sylvestre Host	0	n	1	0	Poaceae Barnhart
Iz_3_1109	DJI_0048	Sempervivum ruthenicum Schnittsp. & C.B. Lehm.	0	n	1	0	Crassulaceae J.St.-Hil.
Iz_3_1109	DJI_0048	Setaria viridis (L.) P. Beauv.	0	n	1	0	Poaceae Barnhart
Iz_3_1109	DJI_0048	Silene borysthenica (Gruner) Walters	0	n	1	0	Caryophyllaceae Juss.
Iz_3_1109	DJI_0048	Silene latifolia subsp. alba (Mill.) Greuter & Burdet	0	n	1	0	Caryophyllaceae Juss.
Iz_3_1109	DJI_0048	Solidago virgaurea L.	0	n	1	0	Asteraceae Bercht. & J.Presl
Iz_3_1109	DJI_0048	Sorbus aucuparia L.	0	n	1	1	Rosaceae Juss.
Iz_3_1109	DJI_0048	Species 11.09_3_0045	0		NA	0	
Iz_3_1109	DJI_0048	Species 11.09_3_0058	0		NA	0	
Iz_3_1109	DJI_0048	Stachys recta L.	0	n	1	0	Lamiacea Lindl.
Iz_3_1109	DJI_0048	Tanacetum vulgare L.	0	n	1	0	Asteraceae Bercht. & J.Presl
Iz_3_1109	DJI_0048	Thymus pallasianus Heinr.Braun	0	n	1	0	Lamiacea Lindl.
Iz_3_1109	DJI_0048	Tilia cordata Mill.	0	n	1	1	Malvaceae Juss.
Iz_3_1109	DJI_0048	Trifolium alpestre L.	0	n	1	0	Fabaceae Lindl.
Iz_3_1109	DJI_0048	Trifolium arvense L.	0	n	1	0	Fabaceae Lindl.
Iz_3_1109	DJI_0048	Ulmus glabra Huds.	0	n	1	1	Ulmaceae Mirb.
Iz_3_1109	DJI_0048	Verbascum densiflorum Bertol.	0	n	1	0	Scrophulariaceae Juss.
Iz_3_1109	DJI_0048	Veronica austriaca L.	0	n	1	0	Plantaginaceae Juss.
Iz_3_1109	DJI_0048	Veronica incana L.	0	n	1	0	Plantaginaceae Juss.
Iz_3_1109	DJI_0048	Veronica verna L.	0	n	1	0	Plantaginaceae Juss.
Iz_3_1109	DJI_0048	Vincetoxicum hirundinaria Medik	0	n	1	0	Apocynaceae Juss.
Iz_3_1109	DJI_0051	Acer tataricum L.	0	n	1	1	Sapindaceae Juss.
Iz_3_1109	DJI_0051	Achillea millefolium L. s.l.	0	n	1	0	Asteraceae Bercht. & J.Presl
Iz_3_1109	DJI_0051	Allium oleraceum L.	0	n	1	0	Amaryllidaceae J.St.-Hil.
Iz_3_1109	DJI_0051	Ambrosia artemisiifolia L.	0	a	0	0	Asteraceae Bercht. & J.Presl
Iz_3_1109	DJI_0051	Amorpha fruticosa L.	0	a	0	0	Fabaceae Lindl.
Iz_3_1109	DJI_0051	Anthericum ramosum L.	0	n	1	0	Asparagaceae Juss.
Iz_3_1109	DJI_0051	Artemisia marschalliana Spreng.	0	n	1	0	Asteraceae Bercht. & J.Presl
Iz_3_1109	DJI_0051	Asparagus officinalis L.	0	n	1	0	Asparagaceae Juss.
Iz_3_1109	DJI_0051	Astragalus cicer L.	0	n	1	0	Fabaceae Lindl.
Iz_3_1109	DJI_0051	Bassia laniflora (S.G.Gmel.) A.J.Scott	0	n	1	0	Amaranthaceae Juss.
Iz_3_1109	DJI_0051	Berberis vulgaris L.	0	n	1	0	Berberidaceae Juss.
Iz_3_1109	DJI_0051	Betula pendula Roth.	0	n	1	1	Betulaceae Gray
Iz_3_1109	DJI_0051	Betula pendula Roth.	0	n	1	0	Poaceae Barnhart
Iz_3_1109	DJI_0051	Bromus inermis Leyss.	0		NA	NA	
Iz_3_1109	DJI_0051	Bromus squarrosus L.	0	a	0	0	Poaceae Barnhart
Iz_3_1109	DJI_0051	Bromus tectorum L.	0	a	0	0	Poaceae Barnhart
Iz_3_1109	DJI_0051	Calamagrostis epigejos (L.) Roth	3	n	1	0	Poaceae Barnhart
Iz_3_1109	DJI_0051	Carduus acanthoides L.	0	n	1	0	Asteraceae Bercht. & J.Presl
Iz_3_1109	DJI_0051	Carex sp.	0	n	1	0	Cyperaceae Juss.
Iz_3_1109	DJI_0051	Centaurea majorovii Dumbadze	0	n	1	0	Asteraceae Bercht. & J.Presl
Iz_3_1109	DJI_0051	Chamaecytisus ruthenicus (Fisch.ex Wol.) Klaskova	0	n	1	0	Fabaceae Lindl.
Iz_3_1109	DJI_0051	Chelidonium majus L.	0	n	1	0	Papaveraceae Juss.
Iz_3_1109	DJI_0051	Chenopodium betaceum Andrz.	0	n	1	0	Amaranthaceae Juss.
Iz_3_1109	DJI_0051	Chenopodium cf. album	0	n	1	0	Amaranthaceae Juss.
Iz_3_1109	DJI_0051	Chenopodium sp.	0		NA	0	Amaranthaceae Juss.
Iz_3_1109	DJI_0051	Chondrilla juncea L.	0	n	1	0	Asteraceae Bercht. & J.Presl
Iz_3_1109	DJI_0051	Cirsium vulgare (Savi) Ten.	0	n	1	0	Asteraceae Bercht. & J.Presl
Iz_3_1109	DJI_0051	Clinopodium acinos (L.) Kuntze	0	n	1	0	Lamiacea Lindl.
Iz_3_1109	DJI_0051	Convallaria majalis L.	0	n	1	0	Asparagaceae Juss.
Iz_3_1109	DJI_0051	Crataegus rhipidophylla Gand.	0	n	1	1	Rosaceae Juss.
Iz_3_1109	DJI_0051	Crepis foetida L.	0	n	1	0	Asteraceae Bercht. & J.Presl
Iz_3_1109	DJI_0051	Dactylis glomerata L.	0	n	1	0	Poaceae Barnhart
Iz_3_1109	DJI_0051	Dianthus campestris M.Bieb.	0	n	1	0	Caryophyllaceae Juss.
Iz_3_1109	DJI_0051	Digitaria sanguinalis (L.) Scop.	0	a	0	0	Poaceae Barnhart
Iz_3_1109	DJI_0051	Elymus repens (L.) Gould	0	n	1	0	Poaceae Barnhart
Iz_3_1109	DJI_0051	Epilobium tetragonum L.	0	n	1	0	Onagraceae Juss.
Iz_3_1109	DJI_0051	Eragrostis minor Host	0	a	0	0	Poaceae Barnhart
Iz_3_1109	DJI_0051	Erigeron canadensis L.	3	a	0	0	Asteraceae Bercht. & J.Presl
Iz_3_1109	DJI_0051	Euphorbia seguieriana Neck.	0	n	1	0	Euphorbiaceae Juss.
Iz_3_1109	DJI_0051	Fallopia dumetorum (L.) Holub	0.5	n	1	0	Polygonaceae Juss.
Iz_3_1109	DJI_0051	Festuca beckeri (Hack.) Trautv.	0	n	1	0	Poaceae Barnhart
Iz_3_1109	DJI_0051	Fragaria vesca L.	0	n	1	0	Rosaceae Juss.
Iz_3_1109	DJI_0051	Frangula alnus Mill.	0	n	1	1	Rhamnaceae Juss.
Iz_3_1109	DJI_0051	Fraxinus pennsylvanica Marshall	0	a	0	1	Oleaceae Hoffmanns. & Link
Iz_3_1109	DJI_0051	Galeopsis bifida Boenn.	0	n	1	0	Lamiacea Lindl.
Iz_3_1109	DJI_0051	Galium verum L.	0	n	1	0	Rubiaceae Juss.
Iz_3_1109	DJI_0051	Genista tinctoria L.	0	n	1	0	Fabaceae Lindl.
Iz_3_1109	DJI_0051	Geranium robertianum L.	0	n	1	0	Geraniaceae Juss.
Iz_3_1109	DJI_0051	Geranium sanquineum L.	0	n	1	0	Geraniaceae Juss.
Iz_3_1109	DJI_0051	Gypsophila paniculata L.	0	n	1	0	Caryophyllaceae Juss.
Iz_3_1109	DJI_0051	Helichrysum arenarium (L.) Moench	0	n	1	0	Asteraceae Bercht. & J.Presl
Iz_3_1109	DJI_0051	Herniaria polygama J.Gay	0	n	1	0	Caryophyllaceae Juss.
Iz_3_1109	DJI_0051	Hieracium robustum Fr.	0	n	1	0	Asteraceae Bercht. & J.Presl
Iz_3_1109	DJI_0051	Hieracium umbellatum L.	0	n	1	NA	Asteraceae Bercht. & J.Presl
Iz_3_1109	DJI_0051	Hylotelephium maximum (L.) Holub	0	n	1	0	Crassulaceae J.St.-Hil.
Iz_3_1109	DJI_0051	Jacobaea borysthenica (DC.) B.Nord. & Greuter	0	n	1	0	Asteraceae Bercht. & J.Presl
Iz_3_1109	DJI_0051	Jasione montana L.	0	n	1	0	Campanulaceae Juss.
Iz_3_1109	DJI_0051	Jurinea cyanoides (L.) Rchb.s.l.	0	n	1	0	Asteraceae Bercht. & J.Presl
Iz_3_1109	DJI_0051	Koeleria glauca (Spreng.) DC.	0	n	1	0	Poaceae Barnhart
Iz_3_1109	DJI_0051	Lactuca serriola L.	0	a	0	0	Asteraceae Bercht. & J.Presl
Iz_3_1109	DJI_0051	Linaria genistifolia (L.) Mill.	0	n	1	0	Plantaginaceae Juss.
Iz_3_1109	DJI_0051	Lomelosia argentea (L.) Greuter & Burdet	0	n	1	0	Caprifoliaceae Juss.
Iz_3_1109	DJI_0051	Melampyrum pratense L.	0	n	1	0	Orobanchaceae Vent.
Iz_3_1109	DJI_0051	Oenothera biennis L.	0	a	0	0	Onagraceae Juss.
Iz_3_1109	DJI_0051	Pentanema britannica (L.) D.Gut.Larr., Santos-Vicente, Anderb., E.Rico & M.M.Mart.Ort.	0	n	1	0	Asteraceae Bercht. & J.Presl
Iz_3_1109	DJI_0051	Peucedanum oreoselinum (L.) Moench	0	n	1	0	Apiaceae Lindl.
Iz_3_1109	DJI_0051	Peucedanum ruthenicum M.Bieb.	0	n	1	0	Apiaceae Lindl.
Iz_3_1109	DJI_0051	Pilosella echioides (Lumn.) F.W.Schultz & Sch.Bip.	0	n	1	0	Asteraceae Bercht. & J.Presl
Iz_3_1109	DJI_0051	Pilosella officinarum F. Schultz et Sch. Bip.	0	n	1	0	Asteraceae Bercht. & J.Presl

Iz_3_1109	DJI_0051	Pilosella sp.	0		NA	0	Asteraceae Bercht. & J.Presl
Iz_3_1109	DJI_0051	Pinus sylvestris L.	0	n	1	1	Pinaceae Spreng. ex F.Rudolphi
Iz_3_1109	DJI_0051	Poa annua L.	0	n	1	0	Poaceae Barnhart
Iz_3_1109	DJI_0051	Poa nemoralis L.	0	n	1	0	Poaceae Barnhart
Iz_3_1109	DJI_0051	Poa sp.	0		NA	0	Poaceae Barnhart
Iz_3_1109	DJI_0051	Polygonatum odoratum (Mill.) Druce	0	n	1	0	Asparagaceae Juss.
Iz_3_1109	DJI_0051	Polygonum arenarium Waldst. & Kit.	0	n	1	0	Polygonaceae Juss.
Iz_3_1109	DJI_0051	Populus nigra L.	0	n	1	1	Salicaceae Mirb.
Iz_3_1109	DJI_0051	Populus tremula L.	0	n	1	1	Salicaceae Mirb.
Iz_3_1109	DJI_0051	Populus Ã canescens (Aiton) Sm.	0	n	1	1	Salicaceae Mirb.
Iz_3_1109	DJI_0051	Potentilla argentea L.	0	n	1	0	Rosaceae Juss.
Iz_3_1109	DJI_0051	Psammophiliella muralis (L.) Ikonn.	0	n	1	0	Caryophyllaceae Juss.
Iz_3_1109	DJI_0051	Psephellus sumensis (Kalen.) Greuter	0	n	1	0	Asteraceae Bercht. & J.Presl
Iz_3_1109	DJI_0051	Pulsatilla pratensis (L.) Mill. s.l.	0	n	1	0	Ranunculaceae Juss.
Iz_3_1109	DJI_0051	Pyrus communis L.	0	a	0	1	Rosaceae Juss.
Iz_3_1109	DJI_0051	Quercus robur L.	0	n	1	1	Fagaceae Dumort.
Iz_3_1109	DJI_0051	Robinia pseudoacacia L.	0	a	0	1	Fabaceae Lindl.
Iz_3_1109	DJI_0051	Rosa sp.	0		NA	1	Rosaceae Juss.
Iz_3_1109	DJI_0051	Rubus idaeus L.	0	n	1	0	Rosaceae Juss.
Iz_3_1109	DJI_0051	Rumex acetosella L.	0	n	1	0	Polygonaceae Juss.
Iz_3_1109	DJI_0051	Rumex thyrsiflorus Fingerh.	0	n	1	0	Polygonaceae Juss.
Iz_3_1109	DJI_0051	Salix alba L.	0	n	1	1	Salicaceae Mirb.
Iz_3_1109	DJI_0051	Sambucus nigra L.	0	n	1	1	Viburnaceae Raf.
Iz_3_1109	DJI_0051	Secale sylvestre Host	0	n	1	0	Poaceae Barnhart
Iz_3_1109	DJI_0051	Sempervivum ruthenicum Schnittsp. & C.B. Lehm.	0	n	1	0	Crassulaceae J.St.-Hil.
Iz_3_1109	DJI_0051	Setaria viridis (L.) P. Beauv.	0	n	1	0	Poaceae Barnhart
Iz_3_1109	DJI_0051	Silene borysthenica (Gruner) Walters	0	n	1	0	Caryophyllaceae Juss.
Iz_3_1109	DJI_0051	Silene latifolia subsp. alba (Mill.) Greuter & Burdet	0	n	1	0	Caryophyllaceae Juss.
Iz_3_1109	DJI_0051	Solidago virgaurea L.	0	n	1	0	Asteraceae Bercht. & J.Presl
Iz_3_1109	DJI_0051	Sorbus aucuparia L.	0	n	1	1	Rosaceae Juss.
Iz_3_1109	DJI_0051	Species 11.09_3_0045	0		NA	0	
Iz_3_1109	DJI_0051	Species 11.09_3_0058	0		NA	0	
Iz_3_1109	DJI_0051	Stachys recta L.	0	n	1	0	Lamiacea Lindl.
Iz_3_1109	DJI_0051	Tanacetum vulgare L.	0	n	1	0	Asteraceae Bercht. & J.Presl
Iz_3_1109	DJI_0051	Thymus pallasianus Heinr.Braun	0	n	1	0	Lamiacea Lindl.
Iz_3_1109	DJI_0051	Tilia cordata Mill.	0	n	1	1	Malvaceae Juss.
Iz_3_1109	DJI_0051	Trifolium alpestre L.	0	n	1	0	Fabaceae Lindl.
Iz_3_1109	DJI_0051	Trifolium arvense L.	0	n	1	0	Fabaceae Lindl.
Iz_3_1109	DJI_0051	Ulmus glabra Huds.	0	n	1	1	Ulmaceae Mirb.
Iz_3_1109	DJI_0051	Verbascum densiflorum Bertol.	0	n	1	0	Scrophulariaceae Juss.
Iz_3_1109	DJI_0051	Veronica austriaca L.	0	n	1	0	Plantaginaceae Juss.
Iz_3_1109	DJI_0051	Veronica incana L.	0	n	1	0	Plantaginaceae Juss.
Iz_3_1109	DJI_0051	Veronica verna L.	0	n	1	0	Plantaginaceae Juss.
Iz_3_1109	DJI_0051	Vincetoxicum hirundinaria Medik	0	n	1	0	Apocynaceae Juss.
Iz_3_1109	DJI_0055	Acer tataricum L.	0	n	1	1	Sapindaceae Juss.
Iz_3_1109	DJI_0055	Achillea millefolium L. s.l.	0	n	1	0	Asteraceae Bercht. & J.Presl
Iz_3_1109	DJI_0055	Allium oleraceum L.	0	n	1	0	Amaryllidaceae J.St.-Hil.
Iz_3_1109	DJI_0055	Ambrosia artemisiifolia L.	0	a	0	0	Asteraceae Bercht. & J.Presl
Iz_3_1109	DJI_0055	Amorpha fruticosa L.	0	a	0	0	Fabaceae Lindl.
Iz_3_1109	DJI_0055	Anthericum ramosum L.	0	n	1	0	Asparagaceae Juss.
Iz_3_1109	DJI_0055	Artemisia marschalliana Spreng.	0	n	1	0	Asteraceae Bercht. & J.Presl
Iz_3_1109	DJI_0055	Asparagus officinalis L.	0	n	1	0	Asparagaceae Juss.
Iz_3_1109	DJI_0055	Astragalus cicer L.	0	n	1	0	Fabaceae Lindl.
Iz_3_1109	DJI_0055	Bassia laniflora (S.G.Gmel.) A.J.Scott	0	n	1	0	Amaranthaceae Juss.
Iz_3_1109	DJI_0055	Berberis vulgaris L.	0	n	1	0	Berberidaceae Juss.
Iz_3_1109	DJI_0055	Betula pendula Roth.	0	n	1	1	Betulaceae Gray
Iz_3_1109	DJI_0055	Betula pendula Roth.	0	n	1	0	Poaceae Barnhart
Iz_3_1109	DJI_0055	Bromus inermis Leyss.	0		NA	NA	
Iz_3_1109	DJI_0055	Bromus squarrosus L.	0	a	0	0	Poaceae Barnhart
Iz_3_1109	DJI_0055	Bromus tectorum L.	0	a	0	0	Poaceae Barnhart
Iz_3_1109	DJI_0055	Calamagrostis epigejos (L.) Roth	20	n	1	0	Poaceae Barnhart
Iz_3_1109	DJI_0055	Carduus acanthoides L.	0	n	1	0	Asteraceae Bercht. & J.Presl
Iz_3_1109	DJI_0055	Carex sp.	1	n	1	0	Cyperaceae Juss.
Iz_3_1109	DJI_0055	Centaurea majorovii Dumbadze	0	n	1	0	Asteraceae Bercht. & J.Presl
Iz_3_1109	DJI_0055	Chamaecytisus ruthenicus (Fisch.ex Wol.) Klaskova	0	n	1	0	Fabaceae Lindl.
Iz_3_1109	DJI_0055	Chelidonium majus L.	1	n	1	0	Papaveraceae Juss.
Iz_3_1109	DJI_0055	Chenopodium betaceum Andrz.	0	n	1	0	Amaranthaceae Juss.
Iz_3_1109	DJI_0055	Chenopodium cf. album	0	n	1	0	Amaranthaceae Juss.
Iz_3_1109	DJI_0055	Chenopodium sp.	0		NA	0	Amaranthaceae Juss.
Iz_3_1109	DJI_0055	Chondrilla juncea L.	0	n	1	0	Asteraceae Bercht. & J.Presl
Iz_3_1109	DJI_0055	Cirsium vulgare (Savi) Ten.	0	n	1	0	Asteraceae Bercht. & J.Presl
Iz_3_1109	DJI_0055	Clinopodium acinos (L.) Kuntze	0	n	1	0	Lamiacea Lindl.
Iz_3_1109	DJI_0055	Convallaria majalis L.	0	n	1	0	Asparagaceae Juss.
Iz_3_1109	DJI_0055	Crataegus rhipidophylla Gand.	0	n	1	1	Rosaceae Juss.
Iz_3_1109	DJI_0055	Crepis foetida L.	0	n	1	0	Asteraceae Bercht. & J.Presl
Iz_3_1109	DJI_0055	Dactylis glomerata L.	0	n	1	0	Poaceae Barnhart
Iz_3_1109	DJI_0055	Dianthus campestris M.Bieb.	0	n	1	0	Caryophyllaceae Juss.
Iz_3_1109	DJI_0055	Digitaria sanguinalis (L.) Scop.	0	a	0	0	Poaceae Barnhart
Iz_3_1109	DJI_0055	Elymus repens (L.) Gould	0	n	1	0	Poaceae Barnhart
Iz_3_1109	DJI_0055	Epilobium tetragonum L.	0	n	1	0	Onagraceae Juss.
Iz_3_1109	DJI_0055	Eragrostis minor Host	0	a	0	0	Poaceae Barnhart
Iz_3_1109	DJI_0055	Erigeron canadensis L.	4	a	0	0	Asteraceae Bercht. & J.Presl
Iz_3_1109	DJI_0055	Euphorbia seguieriana Neck.	0	n	1	0	Euphorbiaceae Juss.
Iz_3_1109	DJI_0055	Fallopia dumetorum (L.) Holub	0	n	1	0	Polygonaceae Juss.
Iz_3_1109	DJI_0055	Festuca beckeri (Hack.) Trautv.	0	n	1	0	Poaceae Barnhart
Iz_3_1109	DJI_0055	Fragaria vesca L.	0	n	1	0	Rosaceae Juss.
Iz_3_1109	DJI_0055	Frangula alnus Mill.	0	n	1	1	Rhamnaceae Juss.
Iz_3_1109	DJI_0055	Fraxinus pennsylvanica Marshall	0	a	0	1	Oleaceae Hoffmanns. & Link
Iz_3_1109	DJI_0055	Galeopsis bifida Boenn.	0	n	1	0	Lamiacea Lindl.
Iz_3_1109	DJI_0055	Galium verum L.	0	n	1	0	Rubiaceae Juss.
Iz_3_1109	DJI_0055	Genista tinctoria L.	0	n	1	0	Fabaceae Lindl.
Iz_3_1109	DJI_0055	Geranium robertianum L.	0	n	1	0	Geraniaceae Juss.
Iz_3_1109	DJI_0055	Geranium sanquineum L.	0	n	1	0	Geraniaceae Juss.
Iz_3_1109	DJI_0055	Gypsophila paniculata L.	0	n	1	0	Caryophyllaceae Juss.
Iz_3_1109	DJI_0055	Helichrysum arenarium (L.) Moench	0	n	1	0	Asteraceae Bercht. & J.Presl
Iz_3_1109	DJI_0055	Herniaria polygama J.Gay	0	n	1	0	Caryophyllaceae Juss.
Iz_3_1109	DJI_0055	Hieracium robustum Fr.	0	n	1	0	Asteraceae Bercht. & J.Presl
Iz_3_1109	DJI_0055	Hieracium umbellatum L.	0	n	1	NA	Asteraceae Bercht. & J.Presl

Iz_3_1109	DJI_0055	Hylotelephium maximum (L.) Holub	0	n	1	0	Crassulaceae J.St.-Hil.
Iz_3_1109	DJI_0055	Jacobaea borysthenica (DC.) B.Nord. & Greuter	0	n	1	0	Asteraceae Bercht. & J.Presl
Iz_3_1109	DJI_0055	Jasione montana L.	0	n	1	0	Campanulaceae Juss.
Iz_3_1109	DJI_0055	Jurinea cyanoides (L.) Rchb.s.l.	0	n	1	0	Asteraceae Bercht. & J.Presl
Iz_3_1109	DJI_0055	Koeleria glauca (Spreng.) DC.	0	n	1	0	Poaceae Barnhart
Iz_3_1109	DJI_0055	Lactuca serriola L.	0	a	0	0	Asteraceae Bercht. & J.Presl
Iz_3_1109	DJI_0055	Linaria genistifolia (L.) Mill.	0	n	1	0	Plantaginaceae Juss.
Iz_3_1109	DJI_0055	Lomelosia argentea (L.) Greuter & Burdet	0	n	1	0	Caprifoliaceae Juss.
Iz_3_1109	DJI_0055	Melampyrum pratense L.	0	n	1	0	Orobanchaceae Vent.
Iz_3_1109	DJI_0055	Oenothera biennis L.	0	a	0	0	Onagraceae Juss.
Iz_3_1109	DJI_0055	Pentanema britannica (L.) D.Gut.Larr., Santos-Vicente, Anderb., E.Rico & M.M.Mart.Ort.	0	n	1	0	Asteraceae Bercht. & J.Presl
Iz_3_1109	DJI_0055	Peucedanum oreoselinum (L.) Moench	0	n	1	0	Apiaceae Lindl.
Iz_3_1109	DJI_0055	Peucedanum ruthenicum M.Bieb.	0	n	1	0	Apiaceae Lindl.
Iz_3_1109	DJI_0055	Pilosella echioides (Lumn.) F.W.Schultz & Sch.Bip.	0	n	1	0	Asteraceae Bercht. & J.Presl
Iz_3_1109	DJI_0055	Pilosella officinarum F. Schultz et Sch. Bip.	0	n	1	0	Asteraceae Bercht. & J.Presl
Iz_3_1109	DJI_0055	Pilosella sp.	0		NA	0	Asteraceae Bercht. & J.Presl
Iz_3_1109	DJI_0055	Pinus sylvestris L.	0	n	1	1	Pinaceae Spreng. ex F.Rudolphi
Iz_3_1109	DJI_0055	Poa annua L.	0	n	1	0	Poaceae Barnhart
Iz_3_1109	DJI_0055	Poa nemoralis L.	0	n	1	0	Poaceae Barnhart
Iz_3_1109	DJI_0055	Poa sp.	0		NA	0	Poaceae Barnhart
Iz_3_1109	DJI_0055	Polygonatum odoratum (Mill.) Druce	0	n	1	0	Asparagaceae Juss.
Iz_3_1109	DJI_0055	Polygonum arenarium Waldst. & Kit.	0	n	1	0	Polygonaceae Juss.
Iz_3_1109	DJI_0055	Populus nigra L.	0	n	1	1	Salicaceae Mirb.
Iz_3_1109	DJI_0055	Populus tremula L.	0	n	1	1	Salicaceae Mirb.
Iz_3_1109	DJI_0055	Populus Ã canescens (Aiton) Sm.	0	n	1	1	Salicaceae Mirb.
Iz_3_1109	DJI_0055	Potentilla argentea L.	0	n	1	0	Rosaceae Juss.
Iz_3_1109	DJI_0055	Psammophiliella muralis (L.) Ikonn.	0	n	1	0	Caryophyllaceae Juss.
Iz_3_1109	DJI_0055	Psephellus sumensis (Kalen.) Greuter	0	n	1	0	Asteraceae Bercht. & J.Presl
Iz_3_1109	DJI_0055	Pulsatilla pratensis (L.) Mill. s.l.	0	n	1	0	Ranunculaceae Juss.
Iz_3_1109	DJI_0055	Pyrus communis L.	0	a	0	1	Rosaceae Juss.
Iz_3_1109	DJI_0055	Quercus robur L.	0	n	1	1	Fagaceae Dumort.
Iz_3_1109	DJI_0055	Robinia pseudoacacia L.	0	a	0	1	Fabaceae Lindl.
Iz_3_1109	DJI_0055	Rosa sp.	0		NA	1	Rosaceae Juss.
Iz_3_1109	DJI_0055	Rubus idaeus L.	0	n	1	0	Rosaceae Juss.
Iz_3_1109	DJI_0055	Rumex acetosella L.	0	n	1	0	Polygonaceae Juss.
Iz_3_1109	DJI_0055	Rumex thyrsiflorus Fingerh.	0	n	1	0	Polygonaceae Juss.
Iz_3_1109	DJI_0055	Salix alba L.	0	n	1	1	Salicaceae Mirb.
Iz_3_1109	DJI_0055	Sambucus nigra L.	0	n	1	1	Viburnaceae Raf.
Iz_3_1109	DJI_0055	Secale sylvestre Host	0	n	1	0	Poaceae Barnhart
Iz_3_1109	DJI_0055	Sempervivum ruthenicum Schnittsp. & C.B. Lehm.	0	n	1	0	Crassulaceae J.St.-Hil.
Iz_3_1109	DJI_0055	Setaria viridis (L.) P. Beauv.	0	n	1	0	Poaceae Barnhart
Iz_3_1109	DJI_0055	Silene borysthenica (Gruner) Walters	0	n	1	0	Caryophyllaceae Juss.
Iz_3_1109	DJI_0055	Silene latifolia subsp. alba (Mill.) Greuter & Burdet	0	n	1	0	Caryophyllaceae Juss.
Iz_3_1109	DJI_0055	Solidago virgaurea L.	0	n	1	0	Asteraceae Bercht. & J.Presl
Iz_3_1109	DJI_0055	Sorbus aucuparia L.	0	n	1	1	Rosaceae Juss.
Iz_3_1109	DJI_0055	Species 11.09_3_0045	0		NA	0	
Iz_3_1109	DJI_0055	Species 11.09_3_0058	0		NA	0	
Iz_3_1109	DJI_0055	Stachys recta L.	0	n	1	0	Lamiacea Lindl.
Iz_3_1109	DJI_0055	Tanacetum vulgare L.	0	n	1	0	Asteraceae Bercht. & J.Presl
Iz_3_1109	DJI_0055	Thymus pallasianus Heinr.Braun	0	n	1	0	Lamiacea Lindl.
Iz_3_1109	DJI_0055	Tilia cordata Mill.	0	n	1	1	Malvaceae Juss.
Iz_3_1109	DJI_0055	Trifolium alpestre L.	0	n	1	0	Fabaceae Lindl.
Iz_3_1109	DJI_0055	Trifolium arvense L.	0	n	1	0	Fabaceae Lindl.
Iz_3_1109	DJI_0055	Ulmus glabra Huds.	0	n	1	1	Ulmaceae Mirb.
Iz_3_1109	DJI_0055	Verbascum densiflorum Bertol.	0	n	1	0	Scrophulariaceae Juss.
Iz_3_1109	DJI_0055	Veronica austriaca L.	0	n	1	0	Plantaginaceae Juss.
Iz_3_1109	DJI_0055	Veronica incana L.	0	n	1	0	Plantaginaceae Juss.
Iz_3_1109	DJI_0055	Veronica verna L.	0	n	1	0	Plantaginaceae Juss.
Iz_3_1109	DJI_0055	Vincetoxicum hirundinaria Medik	0	n	1	0	Apocynaceae Juss.
Iz_3_1109	DJI_0058	Acer tataricum L.	0	n	1	1	Sapindaceae Juss.
Iz_3_1109	DJI_0058	Achillea millefolium L. s.l.	0	n	1	0	Asteraceae Bercht. & J.Presl
Iz_3_1109	DJI_0058	Allium oleraceum L.	0	n	1	0	Amaryllidaceae J.St.-Hil.
Iz_3_1109	DJI_0058	Ambrosia artemisiifolia L.	0	a	0	0	Asteraceae Bercht. & J.Presl
Iz_3_1109	DJI_0058	Amorpha fruticosa L.	0	a	0	0	Fabaceae Lindl.
Iz_3_1109	DJI_0058	Anthericum ramosum L.	0	n	1	0	Asparagaceae Juss.
Iz_3_1109	DJI_0058	Artemisia marschalliana Spreng.	0	n	1	0	Asteraceae Bercht. & J.Presl
Iz_3_1109	DJI_0058	Asparagus officinalis L.	0	n	1	0	Asparagaceae Juss.
Iz_3_1109	DJI_0058	Astragalus cicer L.	0	n	1	0	Fabaceae Lindl.
Iz_3_1109	DJI_0058	Bassia laniflora (S.G.Gmel.) A.J.Scott	0	n	1	0	Amaranthaceae Juss.
Iz_3_1109	DJI_0058	Berberis vulgaris L.	0	n	1	0	Berberidaceae Juss.
Iz_3_1109	DJI_0058	Betula pendula Roth.	0	n	1	1	Betulaceae Gray
Iz_3_1109	DJI_0058	Betula pendula Roth.	0	n	1	0	Poaceae Barnhart
Iz_3_1109	DJI_0058	Bromus inermis Leyss.	0		NA	NA	
Iz_3_1109	DJI_0058	Bromus squarrosus L.	0	a	0	0	Poaceae Barnhart
Iz_3_1109	DJI_0058	Bromus tectorum L.	0	a	0	0	Poaceae Barnhart
Iz_3_1109	DJI_0058	Calamagrostis epigejos (L.) Roth	7	n	1	0	Poaceae Barnhart
Iz_3_1109	DJI_0058	Carduus acanthoides L.	0	n	1	0	Asteraceae Bercht. & J.Presl
Iz_3_1109	DJI_0058	Carex sp.	0	n	1	0	Cyperaceae Juss.
Iz_3_1109	DJI_0058	Centaurea majorovii Dumbadze	0	n	1	0	Asteraceae Bercht. & J.Presl
Iz_3_1109	DJI_0058	Chamaecytisus ruthenicus (Fisch.ex Wol.) Klaskova	0	n	1	0	Fabaceae Lindl.
Iz_3_1109	DJI_0058	Chelidonium majus L.	0	n	1	0	Papaveraceae Juss.
Iz_3_1109	DJI_0058	Chenopodium betaceum Andrz.	0	n	1	0	Amaranthaceae Juss.
Iz_3_1109	DJI_0058	Chenopodium cf. album	0	n	1	0	Amaranthaceae Juss.
Iz_3_1109	DJI_0058	Chenopodium sp.	0		NA	0	Amaranthaceae Juss.
Iz_3_1109	DJI_0058	Chondrilla juncea L.	0	n	1	0	Asteraceae Bercht. & J.Presl
Iz_3_1109	DJI_0058	Cirsium vulgare (Savi) Ten.	0	n	1	0	Asteraceae Bercht. & J.Presl
Iz_3_1109	DJI_0058	Clinopodium acinos (L.) Kuntze	0	n	1	0	Lamiacea Lindl.
Iz_3_1109	DJI_0058	Convallaria majalis L.	0	n	1	0	Asparagaceae Juss.
Iz_3_1109	DJI_0058	Crataegus rhipidophylla Gand.	0	n	1	1	Rosaceae Juss.
Iz_3_1109	DJI_0058	Crepis foetida L.	0	n	1	0	Asteraceae Bercht. & J.Presl
Iz_3_1109	DJI_0058	Dactylis glomerata L.	0	n	1	0	Poaceae Barnhart
Iz_3_1109	DJI_0058	Dianthus campestris M.Bieb.	0	n	1	0	Caryophyllaceae Juss.
Iz_3_1109	DJI_0058	Digitaria sanguinalis (L.) Scop.	0	a	0	0	Poaceae Barnhart
Iz_3_1109	DJI_0058	Elymus repens (L.) Gould	0	n	1	0	Poaceae Barnhart
Iz_3_1109	DJI_0058	Epilobium tetragonum L.	0	n	1	0	Onagraceae Juss.
Iz_3_1109	DJI_0058	Eragrostis minor Host	0	a	0	0	Poaceae Barnhart
Iz_3_1109	DJI_0058	Erigeron canadensis L.	0.2	a	0	0	Asteraceae Bercht. & J.Presl
Iz_3_1109	DJI_0058	Euphorbia seguieriana Neck.	0	n	1	0	Euphorbiaceae Juss.

Iz_3_1109	DJI_0058	Fallopia dumetorum (L.) Holub	0.2	n	1	0	Polygonaceae Juss.
Iz_3_1109	DJI_0058	Festuca beckeri (Hack.) Trautv.	0	n	1	0	Poaceae Barnhart
Iz_3_1109	DJI_0058	Fragaria vesca L.	0	n	1	0	Rosaceae Juss.
Iz_3_1109	DJI_0058	Frangula alnus Mill.	0	n	1	1	Rhamnaceae Juss.
Iz_3_1109	DJI_0058	Fraxinus pennsylvanica Marshall	0	a	0	1	Oleaceae Hoffmanns. & Link
Iz_3_1109	DJI_0058	Galeopsis bifida Boenn.	0	n	1	0	Lamiacea Lindl.
Iz_3_1109	DJI_0058	Galium verum L.	0	n	1	0	Rubiaceae Juss.
Iz_3_1109	DJI_0058	Genista tinctoria L.	0	n	1	0	Fabaceae Lindl.
Iz_3_1109	DJI_0058	Geranium robertianum L.	0	n	1	0	Geraniaceae Juss.
Iz_3_1109	DJI_0058	Geranium sanquineum L.	0	n	1	0	Geraniaceae Juss.
Iz_3_1109	DJI_0058	Gypsophila paniculata L.	0	n	1	0	Caryophyllaceae Juss.
Iz_3_1109	DJI_0058	Helichrysum arenarium (L.) Moench	0	n	1	0	Asteraceae Bercht. & J.Presl
Iz_3_1109	DJI_0058	Herniaria polygama J.Gay	0	n	1	0	Caryophyllaceae Juss.
Iz_3_1109	DJI_0058	Hieracium robustum Fr.	0	n	1	0	Asteraceae Bercht. & J.Presl
Iz_3_1109	DJI_0058	Hieracium umbellatum L.	0	n	1	NA	Asteraceae Bercht. & J.Presl
Iz_3_1109	DJI_0058	Hylotelephium maximum (L.) Holub	0	n	1	0	Crassulaceae J.St.-Hil.
Iz_3_1109	DJI_0058	Jacobaea borysthenica (DC.) B.Nord. & Greuter	0	n	1	0	Asteraceae Bercht. & J.Presl
Iz_3_1109	DJI_0058	Jasione montana L.	0	n	1	0	Campanulaceae Juss.
Iz_3_1109	DJI_0058	Jurinea cyanoides (L.) Rchb.s.l.	0	n	1	0	Asteraceae Bercht. & J.Presl
Iz_3_1109	DJI_0058	Koeleria glauca (Spreng.) DC.	0	n	1	0	Poaceae Barnhart
Iz_3_1109	DJI_0058	Lactuca serriola L.	0	a	0	0	Asteraceae Bercht. & J.Presl
Iz_3_1109	DJI_0058	Linaria genistifolia (L.) Mill.	0	n	1	0	Plantaginaceae Juss.
Iz_3_1109	DJI_0058	Lomelosia argentea (L.) Greuter & Burdet	0	n	1	0	Caprifoliaceae Juss.
Iz_3_1109	DJI_0058	Melampyrum pratense L.	0	n	1	0	Orobanchaceae Vent.
Iz_3_1109	DJI_0058	Oenothera biennis L.	0	a	0	0	Onagraceae Juss.
Iz_3_1109	DJI_0058	Pentanema britannica (L.) D.Gut.Larr., Santos-Vicente, Anderb., E.Rico & M.M.Mart.Ort.	0	n	1	0	Asteraceae Bercht. & J.Presl
Iz_3_1109	DJI_0058	Peucedanum oreoselinum (L.) Moench	0	n	1	0	Apiaceae Lindl.
Iz_3_1109	DJI_0058	Peucedanum ruthenicum M.Bieb.	0	n	1	0	Apiaceae Lindl.
Iz_3_1109	DJI_0058	Pilosella echioides (Lumn.) F.W.Schultz & Sch.Bip.	0	n	1	0	Asteraceae Bercht. & J.Presl
Iz_3_1109	DJI_0058	Pilosella officinarum F. Schultz et Sch. Bip.	0	n	1	0	Asteraceae Bercht. & J.Presl
Iz_3_1109	DJI_0058	Pilosella sp.	0		NA	0	Asteraceae Bercht. & J.Presl
Iz_3_1109	DJI_0058	Pinus sylvestris L.	0	n	1	1	Pinaceae Spreng. ex F.Rudolphi
Iz_3_1109	DJI_0058	Poa annua L.	0	n	1	0	Poaceae Barnhart
Iz_3_1109	DJI_0058	Poa nemoralis L.	0	n	1	0	Poaceae Barnhart
Iz_3_1109	DJI_0058	Poa sp.	0		NA	0	Poaceae Barnhart
Iz_3_1109	DJI_0058	Polygonatum odoratum (Mill.) Druce	0	n	1	0	Asparagaceae Juss.
Iz_3_1109	DJI_0058	Polygonum arenarium Waldst. & Kit.	0	n	1	0	Polygonaceae Juss.
Iz_3_1109	DJI_0058	Populus nigra L.	0	n	1	1	Salicaceae Mirb.
Iz_3_1109	DJI_0058	Populus tremula L.	0	n	1	1	Salicaceae Mirb.
Iz_3_1109	DJI_0058	Populus Ã canescens (Aiton) Sm.	0	n	1	1	Salicaceae Mirb.
Iz_3_1109	DJI_0058	Potentilla argentea L.	0	n	1	0	Rosaceae Juss.
Iz_3_1109	DJI_0058	Psammophiliella muralis (L.) Ikonn.	0	n	1	0	Caryophyllaceae Juss.
Iz_3_1109	DJI_0058	Psephellus sumensis (Kalen.) Greuter	0	n	1	0	Asteraceae Bercht. & J.Presl
Iz_3_1109	DJI_0058	Pulsatilla pratensis (L.) Mill. s.l.	0	n	1	0	Ranunculaceae Juss.
Iz_3_1109	DJI_0058	Pyrus communis L.	0	a	0	1	Rosaceae Juss.
Iz_3_1109	DJI_0058	Quercus robur L.	0	n	1	1	Fagaceae Dumort.
Iz_3_1109	DJI_0058	Robinia pseudoacacia L.	0	a	0	1	Fabaceae Lindl.
Iz_3_1109	DJI_0058	Rosa sp.	0		NA	1	Rosaceae Juss.
Iz_3_1109	DJI_0058	Rubus idaeus L.	0	n	1	0	Rosaceae Juss.
Iz_3_1109	DJI_0058	Rumex acetosella L.	1	n	1	0	Polygonaceae Juss.
Iz_3_1109	DJI_0058	Rumex thyrsiflorus Fingerh.	0	n	1	0	Polygonaceae Juss.
Iz_3_1109	DJI_0058	Salix alba L.	0	n	1	1	Salicaceae Mirb.
Iz_3_1109	DJI_0058	Sambucus nigra L.	0	n	1	1	Viburnaceae Raf.
Iz_3_1109	DJI_0058	Secale sylvestre Host	0	n	1	0	Poaceae Barnhart
Iz_3_1109	DJI_0058	Sempervivum ruthenicum Schnittsp. & C.B. Lehm.	0	n	1	0	Crassulaceae J.St.-Hil.
Iz_3_1109	DJI_0058	Setaria viridis (L.) P. Beauv.	0	n	1	0	Poaceae Barnhart
Iz_3_1109	DJI_0058	Silene borysthenica (Gruner) Walters	0	n	1	0	Caryophyllaceae Juss.
Iz_3_1109	DJI_0058	Silene latifolia subsp. alba (Mill.) Greuter & Burdet	0	n	1	0	Caryophyllaceae Juss.
Iz_3_1109	DJI_0058	Solidago virgaurea L.	0	n	1	0	Asteraceae Bercht. & J.Presl
Iz_3_1109	DJI_0058	Sorbus aucuparia L.	0	n	1	1	Rosaceae Juss.
Iz_3_1109	DJI_0058	Species 11.09_3_0045	0		NA	0	
Iz_3_1109	DJI_0058	Species 11.09_3_0058	0.2		NA	0	
Iz_3_1109	DJI_0058	Stachys recta L.	0	n	1	0	Lamiacea Lindl.
Iz_3_1109	DJI_0058	Tanacetum vulgare L.	0	n	1	0	Asteraceae Bercht. & J.Presl
Iz_3_1109	DJI_0058	Thymus pallasianus Heinr.Braun	0	n	1	0	Lamiacea Lindl.
Iz_3_1109	DJI_0058	Tilia cordata Mill.	0	n	1	1	Malvaceae Juss.
Iz_3_1109	DJI_0058	Trifolium alpestre L.	0	n	1	0	Fabaceae Lindl.
Iz_3_1109	DJI_0058	Trifolium arvense L.	0	n	1	0	Fabaceae Lindl.
Iz_3_1109	DJI_0058	Ulmus glabra Huds.	0	n	1	1	Ulmaceae Mirb.
Iz_3_1109	DJI_0058	Verbascum densiflorum Bertol.	0	n	1	0	Scrophulariaceae Juss.
Iz_3_1109	DJI_0058	Veronica austriaca L.	0	n	1	0	Plantaginaceae Juss.
Iz_3_1109	DJI_0058	Veronica incana L.	0	n	1	0	Plantaginaceae Juss.
Iz_3_1109	DJI_0058	Veronica verna L.	0	n	1	0	Plantaginaceae Juss.
Iz_3_1109	DJI_0058	Vincetoxicum hirundinaria Medik	0	n	1	0	Apocynaceae Juss.
Iz_3_1109	DJI_0063	Acer tataricum L.	0	n	1	1	Sapindaceae Juss.
Iz_3_1109	DJI_0063	Achillea millefolium L. s.l.	0	n	1	0	Asteraceae Bercht. & J.Presl
Iz_3_1109	DJI_0063	Allium oleraceum L.	0	n	1	0	Amaryllidaceae J.St.-Hil.
Iz_3_1109	DJI_0063	Ambrosia artemisiifolia L.	0	a	0	0	Asteraceae Bercht. & J.Presl
Iz_3_1109	DJI_0063	Amorpha fruticosa L.	0	a	0	0	Fabaceae Lindl.
Iz_3_1109	DJI_0063	Anthericum ramosum L.	0	n	1	0	Asparagaceae Juss.
Iz_3_1109	DJI_0063	Artemisia marschalliana Spreng.	0	n	1	0	Asteraceae Bercht. & J.Presl
Iz_3_1109	DJI_0063	Asparagus officinalis L.	0	n	1	0	Asparagaceae Juss.
Iz_3_1109	DJI_0063	Astragalus cicer L.	0	n	1	0	Fabaceae Lindl.
Iz_3_1109	DJI_0063	Bassia laniflora (S.G.Gmel.) A.J.Scott	0	n	1	0	Amaranthaceae Juss.
Iz_3_1109	DJI_0063	Berberis vulgaris L.	0	n	1	0	Berberidaceae Juss.
Iz_3_1109	DJI_0063	Betula pendula Roth.	0	n	1	1	Betulaceae Gray
Iz_3_1109	DJI_0063	Betula pendula Roth.	0	n	1	0	Poaceae Barnhart
Iz_3_1109	DJI_0063	Bromus inermis Leyss.	0		NA	NA	
Iz_3_1109	DJI_0063	Bromus squarrosus L.	0	a	0	0	Poaceae Barnhart
Iz_3_1109	DJI_0063	Bromus tectorum L.	0	a	0	0	Poaceae Barnhart
Iz_3_1109	DJI_0063	Calamagrostis epigejos (L.) Roth	0.5	n	1	0	Poaceae Barnhart
Iz_3_1109	DJI_0063	Carduus acanthoides L.	0	n	1	0	Asteraceae Bercht. & J.Presl
Iz_3_1109	DJI_0063	Carex sp.	0	n	1	0	Cyperaceae Juss.
Iz_3_1109	DJI_0063	Centaurea majorovii Dumbadze	0	n	1	0	Asteraceae Bercht. & J.Presl
Iz_3_1109	DJI_0063	Chamaecytisus ruthenicus (Fisch.ex Wol.) Klaskova	0	n	1	0	Fabaceae Lindl.
Iz_3_1109	DJI_0063	Chelidonium majus L.	0.5	n	1	0	Papaveraceae Juss.
Iz_3_1109	DJI_0063	Chenopodium betaceum Andrz.	0	n	1	0	Amaranthaceae Juss.
Iz_3_1109	DJI_0063	Chenopodium cf. album	0	n	1	0	Amaranthaceae Juss.

Iz_3_1109	DJI_0063	Chenopodium sp.	0		NA	0	Amaranthaceae Juss.
Iz_3_1109	DJI_0063	Chondrilla juncea L.	0	n	1	0	Asteraceae Bercht. & J.Presl
Iz_3_1109	DJI_0063	Cirsium vulgare (Savi) Ten.	0	n	1	0	Asteraceae Bercht. & J.Presl
Iz_3_1109	DJI_0063	Clinopodium acinos (L.) Kuntze	0	n	1	0	Lamiacea Lindl.
Iz_3_1109	DJI_0063	Convallaria majalis L.	0	n	1	0	Asparagaceae Juss.
Iz_3_1109	DJI_0063	Crataegus rhipidophylla Gand.	0	n	1	1	Rosaceae Juss.
Iz_3_1109	DJI_0063	Crepis foetida L.	0	n	1	0	Asteraceae Bercht. & J.Presl
Iz_3_1109	DJI_0063	Dactylis glomerata L.	0	n	1	0	Poaceae Barnhart
Iz_3_1109	DJI_0063	Dianthus campestris M.Bieb.	0	n	1	0	Caryophyllaceae Juss.
Iz_3_1109	DJI_0063	Digitaria sanguinalis (L.) Scop.	0	a	0	0	Poaceae Barnhart
Iz_3_1109	DJI_0063	Elymus repens (L.) Gould	0	n	1	0	Poaceae Barnhart
Iz_3_1109	DJI_0063	Epilobium tetragonum L.	0	n	1	0	Onagraceae Juss.
Iz_3_1109	DJI_0063	Eragrostis minor Host	0	a	0	0	Poaceae Barnhart
Iz_3_1109	DJI_0063	Erigeron canadensis L.	0	a	0	0	Asteraceae Bercht. & J.Presl
Iz_3_1109	DJI_0063	Euphorbia seguieriana Neck.	0	n	1	0	Euphorbiaceae Juss.
Iz_3_1109	DJI_0063	Fallopia dumetorum (L.) Holub	0.2	n	1	0	Polygonaceae Juss.
Iz_3_1109	DJI_0063	Festuca beckeri (Hack.) Trautv.	0	n	1	0	Poaceae Barnhart
Iz_3_1109	DJI_0063	Fragaria vesca L.	0	n	1	0	Rosaceae Juss.
Iz_3_1109	DJI_0063	Frangula alnus Mill.	0	n	1	1	Rhamnaceae Juss.
Iz_3_1109	DJI_0063	Fraxinus pennsylvanica Marshall	0	a	0	1	Oleaceae Hoffmanns. & Link
Iz_3_1109	DJI_0063	Galeopsis bifida Boenn.	0	n	1	0	Lamiacea Lindl.
Iz_3_1109	DJI_0063	Galium verum L.	0	n	1	0	Rubiaceae Juss.
Iz_3_1109	DJI_0063	Genista tinctoria L.	0	n	1	0	Fabaceae Lindl.
Iz_3_1109	DJI_0063	Geranium robertianum L.	0	n	1	0	Geraniaceae Juss.
Iz_3_1109	DJI_0063	Geranium sanquineum L.	0	n	1	0	Geraniaceae Juss.
Iz_3_1109	DJI_0063	Gypsophila paniculata L.	0	n	1	0	Caryophyllaceae Juss.
Iz_3_1109	DJI_0063	Helichrysum arenarium (L.) Moench	0	n	1	0	Asteraceae Bercht. & J.Presl
Iz_3_1109	DJI_0063	Herniaria polygama J.Gay	0	n	1	0	Caryophyllaceae Juss.
Iz_3_1109	DJI_0063	Hieracium robustum Fr.	0	n	1	0	Asteraceae Bercht. & J.Presl
Iz_3_1109	DJI_0063	Hieracium umbellatum L.	0	n	1	NA	Asteraceae Bercht. & J.Presl
Iz_3_1109	DJI_0063	Hylotelephium maximum (L.) Holub	0	n	1	0	Crassulaceae J.St.-Hil.
Iz_3_1109	DJI_0063	Jacobaea borysthenica (DC.) B.Nord. & Greuter	0	n	1	0	Asteraceae Bercht. & J.Presl
Iz_3_1109	DJI_0063	Jasione montana L.	0	n	1	0	Campanulaceae Juss.
Iz_3_1109	DJI_0063	Jurinea cyanoides (L.) Rchb.s.l.	0	n	1	0	Asteraceae Bercht. & J.Presl
Iz_3_1109	DJI_0063	Koeleria glauca (Spreng.) DC.	0	n	1	0	Poaceae Barnhart
Iz_3_1109	DJI_0063	Lactuca serriola L.	0	a	0	0	Asteraceae Bercht. & J.Presl
Iz_3_1109	DJI_0063	Linaria genistifolia (L.) Mill.	0	n	1	0	Plantaginaceae Juss.
Iz_3_1109	DJI_0063	Lomelosia argentea (L.) Greuter & Burdet	0	n	1	0	Caprifoliaceae Juss.
Iz_3_1109	DJI_0063	Melampyrum pratense L.	0	n	1	0	Orobanchaceae Vent.
Iz_3_1109	DJI_0063	Oenothera biennis L.	0	a	0	0	Onagraceae Juss.
Iz_3_1109	DJI_0063	Pentanema britannica (L.) D.Gut.Larr., Santos-Vicente, Anderb., E.Rico & M.M.Mart.Ort.	0	n	1	0	Asteraceae Bercht. & J.Presl
Iz_3_1109	DJI_0063	Peucedanum oreoselinum (L.) Moench	0	n	1	0	Apiaceae Lindl.
Iz_3_1109	DJI_0063	Peucedanum ruthenicum M.Bieb.	0	n	1	0	Apiaceae Lindl.
Iz_3_1109	DJI_0063	Pilosella echioides (Lumn.) F.W.Schultz & Sch.Bip.	0	n	1	0	Asteraceae Bercht. & J.Presl
Iz_3_1109	DJI_0063	Pilosella officinarum F. Schultz et Sch. Bip.	0	n	1	0	Asteraceae Bercht. & J.Presl
Iz_3_1109	DJI_0063	Pilosella sp.	0		NA	0	Asteraceae Bercht. & J.Presl
Iz_3_1109	DJI_0063	Pinus sylvestris L.	0	n	1	1	Pinaceae Spreng. ex F.Rudolphi
Iz_3_1109	DJI_0063	Poa annua L.	0	n	1	0	Poaceae Barnhart
Iz_3_1109	DJI_0063	Poa nemoralis L.	0	n	1	0	Poaceae Barnhart
Iz_3_1109	DJI_0063	Poa sp.	0		NA	0	Poaceae Barnhart
Iz_3_1109	DJI_0063	Polygonatum odoratum (Mill.) Druce	0	n	1	0	Asparagaceae Juss.
Iz_3_1109	DJI_0063	Polygonum arenarium Waldst. & Kit.	0	n	1	0	Polygonaceae Juss.
Iz_3_1109	DJI_0063	Populus nigra L.	0	n	1	1	Salicaceae Mirb.
Iz_3_1109	DJI_0063	Populus tremula L.	0	n	1	1	Salicaceae Mirb.
Iz_3_1109	DJI_0063	Populus Ã canescens (Aiton) Sm.	0	n	1	1	Salicaceae Mirb.
Iz_3_1109	DJI_0063	Potentilla argentea L.	0	n	1	0	Rosaceae Juss.
Iz_3_1109	DJI_0063	Psammophiliella muralis (L.) Ikonn.	0	n	1	0	Caryophyllaceae Juss.
Iz_3_1109	DJI_0063	Psephellus sumensis (Kalen.) Greuter	0	n	1	0	Asteraceae Bercht. & J.Presl
Iz_3_1109	DJI_0063	Pulsatilla pratensis (L.) Mill. s.l.	0	n	1	0	Ranunculaceae Juss.
Iz_3_1109	DJI_0063	Pyrus communis L.	0	a	0	1	Rosaceae Juss.
Iz_3_1109	DJI_0063	Quercus robur L.	0	n	1	1	Fagaceae Dumort.
Iz_3_1109	DJI_0063	Robinia pseudoacacia L.	0	a	0	1	Fabaceae Lindl.
Iz_3_1109	DJI_0063	Rosa sp.	0		NA	1	Rosaceae Juss.
Iz_3_1109	DJI_0063	Rubus idaeus L.	0	n	1	0	Rosaceae Juss.
Iz_3_1109	DJI_0063	Rumex acetosella L.	0	n	1	0	Polygonaceae Juss.
Iz_3_1109	DJI_0063	Rumex thyrsiflorus Fingerh.	0	n	1	0	Polygonaceae Juss.
Iz_3_1109	DJI_0063	Salix alba L.	0	n	1	1	Salicaceae Mirb.
Iz_3_1109	DJI_0063	Sambucus nigra L.	0	n	1	1	Viburnaceae Raf.
Iz_3_1109	DJI_0063	Secale sylvestre Host	0	n	1	0	Poaceae Barnhart
Iz_3_1109	DJI_0063	Sempervivum ruthenicum Schnittsp. & C.B. Lehm.	0	n	1	0	Crassulaceae J.St.-Hil.
Iz_3_1109	DJI_0063	Setaria viridis (L.) P. Beauv.	0	n	1	0	Poaceae Barnhart
Iz_3_1109	DJI_0063	Silene borysthenica (Gruner) Walters	0	n	1	0	Caryophyllaceae Juss.
Iz_3_1109	DJI_0063	Silene latifolia subsp. alba (Mill.) Greuter & Burdet	0	n	1	0	Caryophyllaceae Juss.
Iz_3_1109	DJI_0063	Solidago virgaurea L.	0	n	1	0	Asteraceae Bercht. & J.Presl
Iz_3_1109	DJI_0063	Sorbus aucuparia L.	0	n	1	1	Rosaceae Juss.
Iz_3_1109	DJI_0063	Species 11.09_3_0045	0		NA	0	
Iz_3_1109	DJI_0063	Species 11.09_3_0058	0		NA	0	
Iz_3_1109	DJI_0063	Stachys recta L.	0	n	1	0	Lamiacea Lindl.
Iz_3_1109	DJI_0063	Tanacetum vulgare L.	0	n	1	0	Asteraceae Bercht. & J.Presl
Iz_3_1109	DJI_0063	Thymus pallasianus Heinr.Braun	0	n	1	0	Lamiacea Lindl.
Iz_3_1109	DJI_0063	Tilia cordata Mill.	0	n	1	1	Malvaceae Juss.
Iz_3_1109	DJI_0063	Trifolium alpestre L.	0	n	1	0	Fabaceae Lindl.
Iz_3_1109	DJI_0063	Trifolium arvense L.	0	n	1	0	Fabaceae Lindl.
Iz_3_1109	DJI_0063	Ulmus glabra Huds.	0	n	1	1	Ulmaceae Mirb.
Iz_3_1109	DJI_0063	Verbascum densiflorum Bertol.	0	n	1	0	Scrophulariaceae Juss.
Iz_3_1109	DJI_0063	Veronica austriaca L.	0	n	1	0	Plantaginaceae Juss.
Iz_3_1109	DJI_0063	Veronica incana L.	0	n	1	0	Plantaginaceae Juss.
Iz_3_1109	DJI_0063	Veronica verna L.	0	n	1	0	Plantaginaceae Juss.
Iz_3_1109	DJI_0063	Vincetoxicum hirundinaria Medik	0	n	1	0	Apocynaceae Juss.
Iz_3_1109	DJI_0070	Acer tataricum L.	0	n	1	1	Sapindaceae Juss.
Iz_3_1109	DJI_0070	Achillea millefolium L. s.l.	0	n	1	0	Asteraceae Bercht. & J.Presl
Iz_3_1109	DJI_0070	Allium oleraceum L.	0	n	1	0	Amaryllidaceae J.St.-Hil.
Iz_3_1109	DJI_0070	Ambrosia artemisiifolia L.	0	a	0	0	Asteraceae Bercht. & J.Presl
Iz_3_1109	DJI_0070	Amorpha fruticosa L.	0	a	0	0	Fabaceae Lindl.
Iz_3_1109	DJI_0070	Anthericum ramosum L.	0	n	1	0	Asparagaceae Juss.
Iz_3_1109	DJI_0070	Artemisia marschalliana Spreng.	0	n	1	0	Asteraceae Bercht. & J.Presl
Iz_3_1109	DJI_0070	Asparagus officinalis L.	0	n	1	0	Asparagaceae Juss.
Iz_3_1109	DJI_0070	Astragalus cicer L.	0	n	1	0	Fabaceae Lindl.

Iz_3_1109	DJI_0070	Bassia laniflora (S.G.Gmel.) A.J.Scott	0	n	1	0	Amaranthaceae Juss.
Iz_3_1109	DJI_0070	Berberis vulgaris L.	0	n	1	0	Berberidaceae Juss.
Iz_3_1109	DJI_0070	Betula pendula Roth.	0	n	1	1	Betulaceae Gray
Iz_3_1109	DJI_0070	Betula pendula Roth.	0	n	1	0	Poaceae Barnhart
Iz_3_1109	DJI_0070	Bromus inermis Leyss.	0		NA	NA	
Iz_3_1109	DJI_0070	Bromus squarrosus L.	0	a	0	0	Poaceae Barnhart
Iz_3_1109	DJI_0070	Bromus tectorum L.	0	a	0	0	Poaceae Barnhart
Iz_3_1109	DJI_0070	Calamagrostis epigejos (L.) Roth	0	n	1	0	Poaceae Barnhart
Iz_3_1109	DJI_0070	Carduus acanthoides L.	0	n	1	0	Asteraceae Bercht. & J.Presl
Iz_3_1109	DJI_0070	Carex sp.	0	n	1	0	Cyperaceae Juss.
Iz_3_1109	DJI_0070	Centaurea majorovii Dumbadze	0	n	1	0	Asteraceae Bercht. & J.Presl
Iz_3_1109	DJI_0070	Chamaecytisus ruthenicus (Fisch.ex Wol.) Klaskova	0	n	1	0	Fabaceae Lindl.
Iz_3_1109	DJI_0070	Chelidonium majus L.	0	n	1	0	Papaveraceae Juss.
Iz_3_1109	DJI_0070	Chenopodium betaceum Andrz.	0	n	1	0	Amaranthaceae Juss.
Iz_3_1109	DJI_0070	Chenopodium cf. album	0	n	1	0	Amaranthaceae Juss.
Iz_3_1109	DJI_0070	Chenopodium sp.	0		NA	0	Amaranthaceae Juss.
Iz_3_1109	DJI_0070	Chondrilla juncea L.	0	n	1	0	Asteraceae Bercht. & J.Presl
Iz_3_1109	DJI_0070	Cirsium vulgare (Savi) Ten.	0	n	1	0	Asteraceae Bercht. & J.Presl
Iz_3_1109	DJI_0070	Clinopodium acinos (L.) Kuntze	0	n	1	0	Lamiacea Lindl.
Iz_3_1109	DJI_0070	Convallaria majalis L.	0	n	1	0	Asparagaceae Juss.
Iz_3_1109	DJI_0070	Crataegus rhipidophylla Gand.	0	n	1	1	Rosaceae Juss.
Iz_3_1109	DJI_0070	Crepis foetida L.	0	n	1	0	Asteraceae Bercht. & J.Presl
Iz_3_1109	DJI_0070	Dactylis glomerata L.	0	n	1	0	Poaceae Barnhart
Iz_3_1109	DJI_0070	Dianthus campestris M.Bieb.	0	n	1	0	Caryophyllaceae Juss.
Iz_3_1109	DJI_0070	Digitaria sanguinalis (L.) Scop.	0	a	0	0	Poaceae Barnhart
Iz_3_1109	DJI_0070	Elymus repens (L.) Gould	0	n	1	0	Poaceae Barnhart
Iz_3_1109	DJI_0070	Epilobium tetragonum L.	0	n	1	0	Onagraceae Juss.
Iz_3_1109	DJI_0070	Eragrostis minor Host	0	a	0	0	Poaceae Barnhart
Iz_3_1109	DJI_0070	Erigeron canadensis L.	6	a	0	0	Asteraceae Bercht. & J.Presl
Iz_3_1109	DJI_0070	Euphorbia seguieriana Neck.	0	n	1	0	Euphorbiaceae Juss.
Iz_3_1109	DJI_0070	Fallopia dumetorum (L.) Holub	0	n	1	0	Polygonaceae Juss.
Iz_3_1109	DJI_0070	Festuca beckeri (Hack.) Trautv.	0	n	1	0	Poaceae Barnhart
Iz_3_1109	DJI_0070	Fragaria vesca L.	0	n	1	0	Rosaceae Juss.
Iz_3_1109	DJI_0070	Frangula alnus Mill.	0	n	1	1	Rhamnaceae Juss.
Iz_3_1109	DJI_0070	Fraxinus pennsylvanica Marshall	0	a	0	1	Oleaceae Hoffmanns. & Link
Iz_3_1109	DJI_0070	Galeopsis bifida Boenn.	0	n	1	0	Lamiacea Lindl.
Iz_3_1109	DJI_0070	Galium verum L.	0	n	1	0	Rubiaceae Juss.
Iz_3_1109	DJI_0070	Genista tinctoria L.	0	n	1	0	Fabaceae Lindl.
Iz_3_1109	DJI_0070	Geranium robertianum L.	0	n	1	0	Geraniaceae Juss.
Iz_3_1109	DJI_0070	Geranium sanquineum L.	0	n	1	0	Geraniaceae Juss.
Iz_3_1109	DJI_0070	Gypsophila paniculata L.	0	n	1	0	Caryophyllaceae Juss.
Iz_3_1109	DJI_0070	Helichrysum arenarium (L.) Moench	0	n	1	0	Asteraceae Bercht. & J.Presl
Iz_3_1109	DJI_0070	Herniaria polygama J.Gay	0	n	1	0	Caryophyllaceae Juss.
Iz_3_1109	DJI_0070	Hieracium robustum Fr.	0	n	1	0	Asteraceae Bercht. & J.Presl
Iz_3_1109	DJI_0070	Hieracium umbellatum L.	0	n	1	NA	Asteraceae Bercht. & J.Presl
Iz_3_1109	DJI_0070	Hylotelephium maximum (L.) Holub	0	n	1	0	Crassulaceae J.St.-Hil.
Iz_3_1109	DJI_0070	Jacobaea borysthenica (DC.) B.Nord. & Greuter	0	n	1	0	Asteraceae Bercht. & J.Presl
Iz_3_1109	DJI_0070	Jasione montana L.	0	n	1	0	Campanulaceae Juss.
Iz_3_1109	DJI_0070	Jurinea cyanoides (L.) Rchb.s.l.	0	n	1	0	Asteraceae Bercht. & J.Presl
Iz_3_1109	DJI_0070	Koeleria glauca (Spreng.) DC.	0	n	1	0	Poaceae Barnhart
Iz_3_1109	DJI_0070	Lactuca serriola L.	0	a	0	0	Asteraceae Bercht. & J.Presl
Iz_3_1109	DJI_0070	Linaria genistifolia (L.) Mill.	0	n	1	0	Plantaginaceae Juss.
Iz_3_1109	DJI_0070	Lomelosia argentea (L.) Greuter & Burdet	0	n	1	0	Caprifoliaceae Juss.
Iz_3_1109	DJI_0070	Melampyrum pratense L.	0	n	1	0	Orobanchaceae Vent.
Iz_3_1109	DJI_0070	Oenothera biennis L.	0	a	0	0	Onagraceae Juss.
Iz_3_1109	DJI_0070	Pentanema britannica (L.) D.Gut.Larr., Santos-Vicente, Anderb., E.Rico & M.M.Mart.Ort.	0	n	1	0	Asteraceae Bercht. & J.Presl
Iz_3_1109	DJI_0070	Peucedanum oreoselinum (L.) Moench	0	n	1	0	Apiaceae Lindl.
Iz_3_1109	DJI_0070	Peucedanum ruthenicum M.Bieb.	0	n	1	0	Apiaceae Lindl.
Iz_3_1109	DJI_0070	Pilosella echioides (Lumn.) F.W.Schultz & Sch.Bip.	0	n	1	0	Asteraceae Bercht. & J.Presl
Iz_3_1109	DJI_0070	Pilosella officinarum F. Schultz et Sch. Bip.	0	n	1	0	Asteraceae Bercht. & J.Presl
Iz_3_1109	DJI_0070	Pilosella sp.	0		NA	0	Asteraceae Bercht. & J.Presl
Iz_3_1109	DJI_0070	Pinus sylvestris L.	0	n	1	1	Pinaceae Spreng. ex F.Rudolphi
Iz_3_1109	DJI_0070	Poa annua L.	0	n	1	0	Poaceae Barnhart
Iz_3_1109	DJI_0070	Poa nemoralis L.	0	n	1	0	Poaceae Barnhart
Iz_3_1109	DJI_0070	Poa sp.	0		NA	0	Poaceae Barnhart
Iz_3_1109	DJI_0070	Polygonatum odoratum (Mill.) Druce	0	n	1	0	Asparagaceae Juss.
Iz_3_1109	DJI_0070	Polygonum arenarium Waldst. & Kit.	0	n	1	0	Polygonaceae Juss.
Iz_3_1109	DJI_0070	Populus nigra L.	0	n	1	1	Salicaceae Mirb.
Iz_3_1109	DJI_0070	Populus tremula L.	0	n	1	1	Salicaceae Mirb.
Iz_3_1109	DJI_0070	Populus Ã canescens (Aiton) Sm.	0	n	1	1	Salicaceae Mirb.
Iz_3_1109	DJI_0070	Potentilla argentea L.	0	n	1	0	Rosaceae Juss.
Iz_3_1109	DJI_0070	Psammophiliella muralis (L.) Ikonn.	0	n	1	0	Caryophyllaceae Juss.
Iz_3_1109	DJI_0070	Psephellus sumensis (Kalen.) Greuter	0	n	1	0	Asteraceae Bercht. & J.Presl
Iz_3_1109	DJI_0070	Pulsatilla pratensis (L.) Mill. s.l.	0	n	1	0	Ranunculaceae Juss.
Iz_3_1109	DJI_0070	Pyrus communis L.	0	a	0	1	Rosaceae Juss.
Iz_3_1109	DJI_0070	Quercus robur L.	0	n	1	1	Fagaceae Dumort.
Iz_3_1109	DJI_0070	Robinia pseudoacacia L.	0	a	0	1	Fabaceae Lindl.
Iz_3_1109	DJI_0070	Rosa sp.	0		NA	1	Rosaceae Juss.
Iz_3_1109	DJI_0070	Rubus idaeus L.	0	n	1	0	Rosaceae Juss.
Iz_3_1109	DJI_0070	Rumex acetosella L.	0	n	1	0	Polygonaceae Juss.
Iz_3_1109	DJI_0070	Rumex thyrsiflorus Fingerh.	0	n	1	0	Polygonaceae Juss.
Iz_3_1109	DJI_0070	Salix alba L.	0	n	1	1	Salicaceae Mirb.
Iz_3_1109	DJI_0070	Sambucus nigra L.	0	n	1	1	Viburnaceae Raf.
Iz_3_1109	DJI_0070	Secale sylvestre Host	0	n	1	0	Poaceae Barnhart
Iz_3_1109	DJI_0070	Sempervivum ruthenicum Schnittsp. & C.B. Lehm.	0	n	1	0	Crassulaceae J.St.-Hil.
Iz_3_1109	DJI_0070	Setaria viridis (L.) P. Beauv.	0	n	1	0	Poaceae Barnhart
Iz_3_1109	DJI_0070	Silene borysthenica (Gruner) Walters	0	n	1	0	Caryophyllaceae Juss.
Iz_3_1109	DJI_0070	Silene latifolia subsp. alba (Mill.) Greuter & Burdet	0	n	1	0	Caryophyllaceae Juss.
Iz_3_1109	DJI_0070	Solidago virgaurea L.	0	n	1	0	Asteraceae Bercht. & J.Presl
Iz_3_1109	DJI_0070	Sorbus aucuparia L.	0	n	1	1	Rosaceae Juss.
Iz_3_1109	DJI_0070	Species 11.09_3_0045	0		NA	0	
Iz_3_1109	DJI_0070	Species 11.09_3_0058	0		NA	0	
Iz_3_1109	DJI_0070	Stachys recta L.	0	n	1	0	Lamiacea Lindl.
Iz_3_1109	DJI_0070	Tanacetum vulgare L.	0	n	1	0	Asteraceae Bercht. & J.Presl
Iz_3_1109	DJI_0070	Thymus pallasianus Heinr.Braun	0	n	1	0	Lamiacea Lindl.
Iz_3_1109	DJI_0070	Tilia cordata Mill.	0	n	1	1	Malvaceae Juss.
Iz_3_1109	DJI_0070	Trifolium alpestre L.	0	n	1	0	Fabaceae Lindl.
Iz_3_1109	DJI_0070	Trifolium arvense L.	0	n	1	0	Fabaceae Lindl.

Iz_3_1109	DJI_0070	Ulmus glabra Huds.	0	n	1	1	Ulmaceae Mirb.
Iz_3_1109	DJI_0070	Verbascum densiflorum Bertol.	0	n	1	0	Scrophulariaceae Juss.
Iz_3_1109	DJI_0070	Veronica austriaca L.	0	n	1	0	Plantaginaceae Juss.
Iz_3_1109	DJI_0070	Veronica incana L.	0	n	1	0	Plantaginaceae Juss.
Iz_3_1109	DJI_0070	Veronica verna L.	0	n	1	0	Plantaginaceae Juss.
Iz_3_1109	DJI_0070	Vincetoxicum hirundinaria Medik	0	n	1	0	Apocynaceae Juss.
Iz_3_1109	DJI_0073	Acer tataricum L.	0	n	1	1	Sapindaceae Juss.
Iz_3_1109	DJI_0073	Achillea millefolium L. s.l.	0	n	1	0	Asteraceae Bercht. & J.Presl
Iz_3_1109	DJI_0073	Allium oleraceum L.	0	n	1	0	Amaryllidaceae J.St.-Hil.
Iz_3_1109	DJI_0073	Ambrosia artemisiifolia L.	0	a	0	0	Asteraceae Bercht. & J.Presl
Iz_3_1109	DJI_0073	Amorpha fruticosa L.	0	a	0	0	Fabaceae Lindl.
Iz_3_1109	DJI_0073	Anthericum ramosum L.	0	n	1	0	Asparagaceae Juss.
Iz_3_1109	DJI_0073	Artemisia marschalliana Spreng.	0	n	1	0	Asteraceae Bercht. & J.Presl
Iz_3_1109	DJI_0073	Asparagus officinalis L.	0	n	1	0	Asparagaceae Juss.
Iz_3_1109	DJI_0073	Astragalus cicer L.	0	n	1	0	Fabaceae Lindl.
Iz_3_1109	DJI_0073	Bassia laniflora (S.G.Gmel.) A.J.Scott	0	n	1	0	Amaranthaceae Juss.
Iz_3_1109	DJI_0073	Berberis vulgaris L.	0	n	1	0	Berberidaceae Juss.
Iz_3_1109	DJI_0073	Betula pendula Roth.	0	n	1	1	Betulaceae Gray
Iz_3_1109	DJI_0073	Betula pendula Roth.	0	n	1	0	Poaceae Barnhart
Iz_3_1109	DJI_0073	Bromus inermis Leyss.	0		NA	NA	
Iz_3_1109	DJI_0073	Bromus squarrosus L.	0	a	0	0	Poaceae Barnhart
Iz_3_1109	DJI_0073	Bromus tectorum L.	0	a	0	0	Poaceae Barnhart
Iz_3_1109	DJI_0073	Calamagrostis epigejos (L.) Roth	15	n	1	0	Poaceae Barnhart
Iz_3_1109	DJI_0073	Carduus acanthoides L.	0	n	1	0	Asteraceae Bercht. & J.Presl
Iz_3_1109	DJI_0073	Carex sp.	0	n	1	0	Cyperaceae Juss.
Iz_3_1109	DJI_0073	Centaurea majorovii Dumbadze	0	n	1	0	Asteraceae Bercht. & J.Presl
Iz_3_1109	DJI_0073	Chamaecytisus ruthenicus (Fisch.ex Wol.) Klaskova	0	n	1	0	Fabaceae Lindl.
Iz_3_1109	DJI_0073	Chelidonium majus L.	0	n	1	0	Papaveraceae Juss.
Iz_3_1109	DJI_0073	Chenopodium betaceum Andrz.	0	n	1	0	Amaranthaceae Juss.
Iz_3_1109	DJI_0073	Chenopodium cf. album	0	n	1	0	Amaranthaceae Juss.
Iz_3_1109	DJI_0073	Chenopodium sp.	0		NA	0	Amaranthaceae Juss.
Iz_3_1109	DJI_0073	Chondrilla juncea L.	0	n	1	0	Asteraceae Bercht. & J.Presl
Iz_3_1109	DJI_0073	Cirsium vulgare (Savi) Ten.	0	n	1	0	Asteraceae Bercht. & J.Presl
Iz_3_1109	DJI_0073	Clinopodium acinos (L.) Kuntze	0	n	1	0	Lamiacea Lindl.
Iz_3_1109	DJI_0073	Convallaria majalis L.	0	n	1	0	Asparagaceae Juss.
Iz_3_1109	DJI_0073	Crataegus rhipidophylla Gand.	0	n	1	1	Rosaceae Juss.
Iz_3_1109	DJI_0073	Crepis foetida L.	0	n	1	0	Asteraceae Bercht. & J.Presl
Iz_3_1109	DJI_0073	Dactylis glomerata L.	0	n	1	0	Poaceae Barnhart
Iz_3_1109	DJI_0073	Dianthus campestris M.Bieb.	0	n	1	0	Caryophyllaceae Juss.
Iz_3_1109	DJI_0073	Digitaria sanguinalis (L.) Scop.	0	a	0	0	Poaceae Barnhart
Iz_3_1109	DJI_0073	Elymus repens (L.) Gould	0	n	1	0	Poaceae Barnhart
Iz_3_1109	DJI_0073	Epilobium tetragonum L.	0	n	1	0	Onagraceae Juss.
Iz_3_1109	DJI_0073	Eragrostis minor Host	0	a	0	0	Poaceae Barnhart
Iz_3_1109	DJI_0073	Erigeron canadensis L.	5	a	0	0	Asteraceae Bercht. & J.Presl
Iz_3_1109	DJI_0073	Euphorbia seguieriana Neck.	0	n	1	0	Euphorbiaceae Juss.
Iz_3_1109	DJI_0073	Fallopia dumetorum (L.) Holub	1	n	1	0	Polygonaceae Juss.
Iz_3_1109	DJI_0073	Festuca beckeri (Hack.) Trautv.	0	n	1	0	Poaceae Barnhart
Iz_3_1109	DJI_0073	Fragaria vesca L.	0	n	1	0	Rosaceae Juss.
Iz_3_1109	DJI_0073	Frangula alnus Mill.	0	n	1	1	Rhamnaceae Juss.
Iz_3_1109	DJI_0073	Fraxinus pennsylvanica Marshall	0	a	0	1	Oleaceae Hoffmanns. & Link
Iz_3_1109	DJI_0073	Galeopsis bifida Boenn.	0	n	1	0	Lamiacea Lindl.
Iz_3_1109	DJI_0073	Galium verum L.	0	n	1	0	Rubiaceae Juss.
Iz_3_1109	DJI_0073	Genista tinctoria L.	0	n	1	0	Fabaceae Lindl.
Iz_3_1109	DJI_0073	Geranium robertianum L.	0	n	1	0	Geraniaceae Juss.
Iz_3_1109	DJI_0073	Geranium sanquineum L.	0	n	1	0	Geraniaceae Juss.
Iz_3_1109	DJI_0073	Gypsophila paniculata L.	0	n	1	0	Caryophyllaceae Juss.
Iz_3_1109	DJI_0073	Helichrysum arenarium (L.) Moench	0	n	1	0	Asteraceae Bercht. & J.Presl
Iz_3_1109	DJI_0073	Herniaria polygama J.Gay	0	n	1	0	Caryophyllaceae Juss.
Iz_3_1109	DJI_0073	Hieracium robustum Fr.	0	n	1	0	Asteraceae Bercht. & J.Presl
Iz_3_1109	DJI_0073	Hieracium umbellatum L.	0	n	1	NA	Asteraceae Bercht. & J.Presl
Iz_3_1109	DJI_0073	Hylotelephium maximum (L.) Holub	0	n	1	0	Crassulaceae J.St.-Hil.
Iz_3_1109	DJI_0073	Jacobaea borysthenica (DC.) B.Nord. & Greuter	0	n	1	0	Asteraceae Bercht. & J.Presl
Iz_3_1109	DJI_0073	Jasione montana L.	0	n	1	0	Campanulaceae Juss.
Iz_3_1109	DJI_0073	Jurinea cyanoides (L.) Rchb.s.l.	0	n	1	0	Asteraceae Bercht. & J.Presl
Iz_3_1109	DJI_0073	Koeleria glauca (Spreng.) DC.	0	n	1	0	Poaceae Barnhart
Iz_3_1109	DJI_0073	Lactuca serriola L.	0	a	0	0	Asteraceae Bercht. & J.Presl
Iz_3_1109	DJI_0073	Linaria genistifolia (L.) Mill.	0	n	1	0	Plantaginaceae Juss.
Iz_3_1109	DJI_0073	Lomelosia argentea (L.) Greuter & Burdet	0	n	1	0	Caprifoliaceae Juss.
Iz_3_1109	DJI_0073	Melampyrum pratense L.	0	n	1	0	Orobanchaceae Vent.
Iz_3_1109	DJI_0073	Oenothera biennis L.	0	a	0	0	Onagraceae Juss.
Iz_3_1109	DJI_0073	Pentanema britannica (L.) D.Gut.Larr., Santos-Vicente, Anderb., E.Rico & M.M.Mart.Ort.	0	n	1	0	Asteraceae Bercht. & J.Presl
Iz_3_1109	DJI_0073	Peucedanum oreoselinum (L.) Moench	0	n	1	0	Apiaceae Lindl.
Iz_3_1109	DJI_0073	Peucedanum ruthenicum M.Bieb.	0	n	1	0	Apiaceae Lindl.
Iz_3_1109	DJI_0073	Pilosella echioides (Lumn.) F.W.Schultz & Sch.Bip.	0	n	1	0	Asteraceae Bercht. & J.Presl
Iz_3_1109	DJI_0073	Pilosella officinarum F. Schultz et Sch. Bip.	0	n	1	0	Asteraceae Bercht. & J.Presl
Iz_3_1109	DJI_0073	Pilosella sp.	0		NA	0	Asteraceae Bercht. & J.Presl
Iz_3_1109	DJI_0073	Pinus sylvestris L.	0	n	1	1	Pinaceae Spreng. ex F.Rudolphi
Iz_3_1109	DJI_0073	Poa annua L.	0	n	1	0	Poaceae Barnhart
Iz_3_1109	DJI_0073	Poa nemoralis L.	0	n	1	0	Poaceae Barnhart
Iz_3_1109	DJI_0073	Poa sp.	0		NA	0	Poaceae Barnhart
Iz_3_1109	DJI_0073	Polygonatum odoratum (Mill.) Druce	0	n	1	0	Asparagaceae Juss.
Iz_3_1109	DJI_0073	Polygonum arenarium Waldst. & Kit.	0	n	1	0	Polygonaceae Juss.
Iz_3_1109	DJI_0073	Populus nigra L.	0	n	1	1	Salicaceae Mirb.
Iz_3_1109	DJI_0073	Populus tremula L.	0	n	1	1	Salicaceae Mirb.
Iz_3_1109	DJI_0073	Populus Ã canescens (Aiton) Sm.	0	n	1	1	Salicaceae Mirb.
Iz_3_1109	DJI_0073	Potentilla argentea L.	0	n	1	0	Rosaceae Juss.
Iz_3_1109	DJI_0073	Psammophiliella muralis (L.) Ikonn.	0	n	1	0	Caryophyllaceae Juss.
Iz_3_1109	DJI_0073	Psephellus sumensis (Kalen.) Greuter	0	n	1	0	Asteraceae Bercht. & J.Presl
Iz_3_1109	DJI_0073	Pulsatilla pratensis (L.) Mill. s.l.	0	n	1	0	Ranunculaceae Juss.
Iz_3_1109	DJI_0073	Pyrus communis L.	0	a	0	1	Rosaceae Juss.
Iz_3_1109	DJI_0073	Quercus robur L.	0	n	1	1	Fagaceae Dumort.
Iz_3_1109	DJI_0073	Robinia pseudoacacia L.	0	a	0	1	Fabaceae Lindl.
Iz_3_1109	DJI_0073	Rosa sp.	0		NA	1	Rosaceae Juss.
Iz_3_1109	DJI_0073	Rubus idaeus L.	0	n	1	0	Rosaceae Juss.
Iz_3_1109	DJI_0073	Rumex acetosella L.	0	n	1	0	Polygonaceae Juss.
Iz_3_1109	DJI_0073	Rumex thyrsiflorus Fingerh.	0	n	1	0	Polygonaceae Juss.
Iz_3_1109	DJI_0073	Salix alba L.	0	n	1	1	Salicaceae Mirb.
Iz_3_1109	DJI_0073	Sambucus nigra L.	0	n	1	1	Viburnaceae Raf.

Iz_3_1109	DJI_0073	Secale sylvestre Host	0	n	1	0	Poaceae Barnhart
Iz_3_1109	DJI_0073	Sempervivum ruthenicum Schnittsp. & C.B. Lehm.	0	n	1	0	Crassulaceae J.St.-Hil.
Iz_3_1109	DJI_0073	Setaria viridis (L.) P. Beauv.	0	n	1	0	Poaceae Barnhart
Iz_3_1109	DJI_0073	Silene borysthenica (Gruner) Walters	0	n	1	0	Caryophyllaceae Juss.
Iz_3_1109	DJI_0073	Silene latifolia subsp. alba (Mill.) Greuter & Burdet	0	n	1	0	Caryophyllaceae Juss.
Iz_3_1109	DJI_0073	Solidago virgaurea L.	0	n	1	0	Asteraceae Bercht. & J.Presl
Iz_3_1109	DJI_0073	Sorbus aucuparia L.	0	n	1	1	Rosaceae Juss.
Iz_3_1109	DJI_0073	Species 11.09_3_0045	0		NA	0	
Iz_3_1109	DJI_0073	Species 11.09_3_0058	0		NA	0	
Iz_3_1109	DJI_0073	Stachys recta L.	0	n	1	0	Lamiacea Lindl.
Iz_3_1109	DJI_0073	Tanacetum vulgare L.	0	n	1	0	Asteraceae Bercht. & J.Presl
Iz_3_1109	DJI_0073	Thymus pallasianus Heinr.Braun	0	n	1	0	Lamiacea Lindl.
Iz_3_1109	DJI_0073	Tilia cordata Mill.	0	n	1	1	Malvaceae Juss.
Iz_3_1109	DJI_0073	Trifolium alpestre L.	0	n	1	0	Fabaceae Lindl.
Iz_3_1109	DJI_0073	Trifolium arvense L.	0	n	1	0	Fabaceae Lindl.
Iz_3_1109	DJI_0073	Ulmus glabra Huds.	0	n	1	1	Ulmaceae Mirb.
Iz_3_1109	DJI_0073	Verbascum densiflorum Bertol.	0	n	1	0	Scrophulariaceae Juss.
Iz_3_1109	DJI_0073	Veronica austriaca L.	0	n	1	0	Plantaginaceae Juss.
Iz_3_1109	DJI_0073	Veronica incana L.	0	n	1	0	Plantaginaceae Juss.
Iz_3_1109	DJI_0073	Veronica verna L.	0	n	1	0	Plantaginaceae Juss.
Iz_3_1109	DJI_0073	Vincetoxicum hirundinaria Medik	0	n	1	0	Apocynaceae Juss.
Iz_3_1109	DJI_0075	Acer tataricum L.	0	n	1	1	Sapindaceae Juss.
Iz_3_1109	DJI_0075	Achillea millefolium L. s.l.	0	n	1	0	Asteraceae Bercht. & J.Presl
Iz_3_1109	DJI_0075	Allium oleraceum L.	0	n	1	0	Amaryllidaceae J.St.-Hil.
Iz_3_1109	DJI_0075	Ambrosia artemisiifolia L.	0	a	0	0	Asteraceae Bercht. & J.Presl
Iz_3_1109	DJI_0075	Amorpha fruticosa L.	0	a	0	0	Fabaceae Lindl.
Iz_3_1109	DJI_0075	Anthericum ramosum L.	0	n	1	0	Asparagaceae Juss.
Iz_3_1109	DJI_0075	Artemisia marschalliana Spreng.	0	n	1	0	Asteraceae Bercht. & J.Presl
Iz_3_1109	DJI_0075	Asparagus officinalis L.	0	n	1	0	Asparagaceae Juss.
Iz_3_1109	DJI_0075	Astragalus cicer L.	0	n	1	0	Fabaceae Lindl.
Iz_3_1109	DJI_0075	Bassia laniflora (S.G.Gmel.) A.J.Scott	0	n	1	0	Amaranthaceae Juss.
Iz_3_1109	DJI_0075	Berberis vulgaris L.	0	n	1	0	Berberidaceae Juss.
Iz_3_1109	DJI_0075	Betula pendula Roth.	0	n	1	1	Betulaceae Gray
Iz_3_1109	DJI_0075	Betula pendula Roth.	0	n	1	0	Poaceae Barnhart
Iz_3_1109	DJI_0075	Bromus inermis Leyss.	0		NA	NA	
Iz_3_1109	DJI_0075	Bromus squarrosus L.	0	a	0	0	Poaceae Barnhart
Iz_3_1109	DJI_0075	Bromus tectorum L.	0	a	0	0	Poaceae Barnhart
Iz_3_1109	DJI_0075	Calamagrostis epigejos (L.) Roth	3	n	1	0	Poaceae Barnhart
Iz_3_1109	DJI_0075	Carduus acanthoides L.	0	n	1	0	Asteraceae Bercht. & J.Presl
Iz_3_1109	DJI_0075	Carex sp.	0	n	1	0	Cyperaceae Juss.
Iz_3_1109	DJI_0075	Centaurea majorovii Dumbadze	0	n	1	0	Asteraceae Bercht. & J.Presl
Iz_3_1109	DJI_0075	Chamaecytisus ruthenicus (Fisch.ex Wol.) Klaskova	0	n	1	0	Fabaceae Lindl.
Iz_3_1109	DJI_0075	Chelidonium majus L.	0	n	1	0	Papaveraceae Juss.
Iz_3_1109	DJI_0075	Chenopodium betaceum Andrz.	0	n	1	0	Amaranthaceae Juss.
Iz_3_1109	DJI_0075	Chenopodium cf. album	0	n	1	0	Amaranthaceae Juss.
Iz_3_1109	DJI_0075	Chenopodium sp.	0		NA	0	Amaranthaceae Juss.
Iz_3_1109	DJI_0075	Chondrilla juncea L.	0	n	1	0	Asteraceae Bercht. & J.Presl
Iz_3_1109	DJI_0075	Cirsium vulgare (Savi) Ten.	0	n	1	0	Asteraceae Bercht. & J.Presl
Iz_3_1109	DJI_0075	Clinopodium acinos (L.) Kuntze	0	n	1	0	Lamiacea Lindl.
Iz_3_1109	DJI_0075	Convallaria majalis L.	0	n	1	0	Asparagaceae Juss.
Iz_3_1109	DJI_0075	Crataegus rhipidophylla Gand.	0	n	1	1	Rosaceae Juss.
Iz_3_1109	DJI_0075	Crepis foetida L.	0	n	1	0	Asteraceae Bercht. & J.Presl
Iz_3_1109	DJI_0075	Dactylis glomerata L.	0	n	1	0	Poaceae Barnhart
Iz_3_1109	DJI_0075	Dianthus campestris M.Bieb.	0	n	1	0	Caryophyllaceae Juss.
Iz_3_1109	DJI_0075	Digitaria sanguinalis (L.) Scop.	0	a	0	0	Poaceae Barnhart
Iz_3_1109	DJI_0075	Elymus repens (L.) Gould	0	n	1	0	Poaceae Barnhart
Iz_3_1109	DJI_0075	Epilobium tetragonum L.	0	n	1	0	Onagraceae Juss.
Iz_3_1109	DJI_0075	Eragrostis minor Host	0	a	0	0	Poaceae Barnhart
Iz_3_1109	DJI_0075	Erigeron canadensis L.	7	a	0	0	Asteraceae Bercht. & J.Presl
Iz_3_1109	DJI_0075	Euphorbia seguieriana Neck.	0	n	1	0	Euphorbiaceae Juss.
Iz_3_1109	DJI_0075	Fallopia dumetorum (L.) Holub	0	n	1	0	Polygonaceae Juss.
Iz_3_1109	DJI_0075	Festuca beckeri (Hack.) Trautv.	0	n	1	0	Poaceae Barnhart
Iz_3_1109	DJI_0075	Fragaria vesca L.	0	n	1	0	Rosaceae Juss.
Iz_3_1109	DJI_0075	Frangula alnus Mill.	0	n	1	1	Rhamnaceae Juss.
Iz_3_1109	DJI_0075	Fraxinus pennsylvanica Marshall	0	a	0	1	Oleaceae Hoffmanns. & Link
Iz_3_1109	DJI_0075	Galeopsis bifida Boenn.	0	n	1	0	Lamiacea Lindl.
Iz_3_1109	DJI_0075	Galium verum L.	0	n	1	0	Rubiaceae Juss.
Iz_3_1109	DJI_0075	Genista tinctoria L.	0	n	1	0	Fabaceae Lindl.
Iz_3_1109	DJI_0075	Geranium robertianum L.	0	n	1	0	Geraniaceae Juss.
Iz_3_1109	DJI_0075	Geranium sanquineum L.	0	n	1	0	Geraniaceae Juss.
Iz_3_1109	DJI_0075	Gypsophila paniculata L.	0	n	1	0	Caryophyllaceae Juss.
Iz_3_1109	DJI_0075	Helichrysum arenarium (L.) Moench	0	n	1	0	Asteraceae Bercht. & J.Presl
Iz_3_1109	DJI_0075	Herniaria polygama J.Gay	0	n	1	0	Caryophyllaceae Juss.
Iz_3_1109	DJI_0075	Hieracium robustum Fr.	0	n	1	0	Asteraceae Bercht. & J.Presl
Iz_3_1109	DJI_0075	Hieracium umbellatum L.	0	n	1	NA	Asteraceae Bercht. & J.Presl
Iz_3_1109	DJI_0075	Hylotelephium maximum (L.) Holub	0	n	1	0	Crassulaceae J.St.-Hil.
Iz_3_1109	DJI_0075	Jacobaea borysthenica (DC.) B.Nord. & Greuter	0	n	1	0	Asteraceae Bercht. & J.Presl
Iz_3_1109	DJI_0075	Jasione montana L.	0	n	1	0	Campanulaceae Juss.
Iz_3_1109	DJI_0075	Jurinea cyanoides (L.) Rchb.s.l.	0	n	1	0	Asteraceae Bercht. & J.Presl
Iz_3_1109	DJI_0075	Koeleria glauca (Spreng.) DC.	0	n	1	0	Poaceae Barnhart
Iz_3_1109	DJI_0075	Lactuca serriola L.	0	a	0	0	Asteraceae Bercht. & J.Presl
Iz_3_1109	DJI_0075	Linaria genistifolia (L.) Mill.	0	n	1	0	Plantaginaceae Juss.
Iz_3_1109	DJI_0075	Lomelosia argentea (L.) Greuter & Burdet	0	n	1	0	Caprifoliaceae Juss.
Iz_3_1109	DJI_0075	Melampyrum pratense L.	0	n	1	0	Orobanchaceae Vent.
Iz_3_1109	DJI_0075	Oenothera biennis L.	0	a	0	0	Onagraceae Juss.
Iz_3_1109	DJI_0075	Pentanema britannica (L.) D.Gut.Larr., Santos-Vicente, Anderb., E.Rico & M.M.Mart.Ort.	0	n	1	0	Asteraceae Bercht. & J.Presl
Iz_3_1109	DJI_0075	Peucedanum oreoselinum (L.) Moench	0	n	1	0	Apiaceae Lindl.
Iz_3_1109	DJI_0075	Peucedanum ruthenicum M.Bieb.	0	n	1	0	Apiaceae Lindl.
Iz_3_1109	DJI_0075	Pilosella echioides (Lumn.) F.W.Schultz & Sch.Bip.	0	n	1	0	Asteraceae Bercht. & J.Presl
Iz_3_1109	DJI_0075	Pilosella officinarum F. Schultz et Sch. Bip.	0	n	1	0	Asteraceae Bercht. & J.Presl
Iz_3_1109	DJI_0075	Pilosella sp.	0		NA	0	Asteraceae Bercht. & J.Presl
Iz_3_1109	DJI_0075	Pinus sylvestris L.	0	n	1	1	Pinaceae Spreng. ex F.Rudolphi
Iz_3_1109	DJI_0075	Poa annua L.	0	n	1	0	Poaceae Barnhart
Iz_3_1109	DJI_0075	Poa nemoralis L.	0	n	1	0	Poaceae Barnhart
Iz_3_1109	DJI_0075	Poa sp.	0		NA	0	Poaceae Barnhart
Iz_3_1109	DJI_0075	Polygonatum odoratum (Mill.) Druce	0	n	1	0	Asparagaceae Juss.
Iz_3_1109	DJI_0075	Polygonum arenarium Waldst. & Kit.	0	n	1	0	Polygonaceae Juss.
Iz_3_1109	DJI_0075	Populus nigra L.	0	n	1	1	Salicaceae Mirb.

Iz_3_1109	DJI_0075	Populus tremula L.	0	n	1	1	Salicaceae Mirb.
Iz_3_1109	DJI_0075	Populus Ã canescens (Aiton) Sm.	0	n	1	1	Salicaceae Mirb.
Iz_3_1109	DJI_0075	Potentilla argentea L.	0	n	1	0	Rosaceae Juss.
Iz_3_1109	DJI_0075	Psammophiliella muralis (L.) Ikonn.	0	n	1	0	Caryophyllaceae Juss.
Iz_3_1109	DJI_0075	Psephellus sumensis (Kalen.) Greuter	0	n	1	0	Asteraceae Bercht. & J.Presl
Iz_3_1109	DJI_0075	Pulsatilla pratensis (L.) Mill. s.l.	0	n	1	0	Ranunculaceae Juss.
Iz_3_1109	DJI_0075	Pyrus communis L.	0	a	0	1	Rosaceae Juss.
Iz_3_1109	DJI_0075	Quercus robur L.	0	n	1	1	Fagaceae Dumort.
Iz_3_1109	DJI_0075	Robinia pseudoacacia L.	0	a	0	1	Fabaceae Lindl.
Iz_3_1109	DJI_0075	Rosa sp.	0		NA	1	Rosaceae Juss.
Iz_3_1109	DJI_0075	Rubus idaeus L.	0	n	1	0	Rosaceae Juss.
Iz_3_1109	DJI_0075	Rumex acetosella L.	0	n	1	0	Polygonaceae Juss.
Iz_3_1109	DJI_0075	Rumex thyrsiflorus Fingerh.	0	n	1	0	Polygonaceae Juss.
Iz_3_1109	DJI_0075	Salix alba L.	0	n	1	1	Salicaceae Mirb.
Iz_3_1109	DJI_0075	Sambucus nigra L.	0	n	1	1	Viburnaceae Raf.
Iz_3_1109	DJI_0075	Secale sylvestre Host	0	n	1	0	Poaceae Barnhart
Iz_3_1109	DJI_0075	Sempervivum ruthenicum Schnittsp. & C.B. Lehm.	0	n	1	0	Crassulaceae J.St.-Hil.
Iz_3_1109	DJI_0075	Setaria viridis (L.) P. Beauv.	0	n	1	0	Poaceae Barnhart
Iz_3_1109	DJI_0075	Silene borysthenica (Gruner) Walters	0	n	1	0	Caryophyllaceae Juss.
Iz_3_1109	DJI_0075	Silene latifolia subsp. alba (Mill.) Greuter & Burdet	0	n	1	0	Caryophyllaceae Juss.
Iz_3_1109	DJI_0075	Solidago virgaurea L.	0	n	1	0	Asteraceae Bercht. & J.Presl
Iz_3_1109	DJI_0075	Sorbus aucuparia L.	0	n	1	1	Rosaceae Juss.
Iz_3_1109	DJI_0075	Species 11.09_3_0045	0		NA	0	
Iz_3_1109	DJI_0075	Species 11.09_3_0058	0		NA	0	
Iz_3_1109	DJI_0075	Stachys recta L.	0	n	1	0	Lamiacea Lindl.
Iz_3_1109	DJI_0075	Tanacetum vulgare L.	0	n	1	0	Asteraceae Bercht. & J.Presl
Iz_3_1109	DJI_0075	Thymus pallasianus Heinr.Braun	0	n	1	0	Lamiacea Lindl.
Iz_3_1109	DJI_0075	Tilia cordata Mill.	0	n	1	1	Malvaceae Juss.
Iz_3_1109	DJI_0075	Trifolium alpestre L.	0	n	1	0	Fabaceae Lindl.
Iz_3_1109	DJI_0075	Trifolium arvense L.	0	n	1	0	Fabaceae Lindl.
Iz_3_1109	DJI_0075	Ulmus glabra Huds.	0	n	1	1	Ulmaceae Mirb.
Iz_3_1109	DJI_0075	Verbascum densiflorum Bertol.	0	n	1	0	Scrophulariaceae Juss.
Iz_3_1109	DJI_0075	Veronica austriaca L.	0	n	1	0	Plantaginaceae Juss.
Iz_3_1109	DJI_0075	Veronica incana L.	0	n	1	0	Plantaginaceae Juss.
Iz_3_1109	DJI_0075	Veronica verna L.	0	n	1	0	Plantaginaceae Juss.
Iz_3_1109	DJI_0075	Vincetoxicum hirundinaria Medik	0	n	1	0	Apocynaceae Juss.
Iz_3_1109	DJI_0095	Acer tataricum L.	0	n	1	1	Sapindaceae Juss.
Iz_3_1109	DJI_0095	Achillea millefolium L. s.l.	0	n	1	0	Asteraceae Bercht. & J.Presl
Iz_3_1109	DJI_0095	Allium oleraceum L.	0	n	1	0	Amaryllidaceae J.St.-Hil.
Iz_3_1109	DJI_0095	Ambrosia artemisiifolia L.	0	a	0	0	Asteraceae Bercht. & J.Presl
Iz_3_1109	DJI_0095	Amorpha fruticosa L.	0	a	0	0	Fabaceae Lindl.
Iz_3_1109	DJI_0095	Anthericum ramosum L.	0	n	1	0	Asparagaceae Juss.
Iz_3_1109	DJI_0095	Artemisia marschalliana Spreng.	0	n	1	0	Asteraceae Bercht. & J.Presl
Iz_3_1109	DJI_0095	Asparagus officinalis L.	0	n	1	0	Asparagaceae Juss.
Iz_3_1109	DJI_0095	Astragalus cicer L.	0	n	1	0	Fabaceae Lindl.
Iz_3_1109	DJI_0095	Bassia laniflora (S.G.Gmel.) A.J.Scott	0	n	1	0	Amaranthaceae Juss.
Iz_3_1109	DJI_0095	Berberis vulgaris L.	0	n	1	0	Berberidaceae Juss.
Iz_3_1109	DJI_0095	Betula pendula Roth.	0	n	1	1	Betulaceae Gray
Iz_3_1109	DJI_0095	Betula pendula Roth.	0	n	1	0	Poaceae Barnhart
Iz_3_1109	DJI_0095	Bromus inermis Leyss.	0		NA	NA	
Iz_3_1109	DJI_0095	Bromus squarrosus L.	0	a	0	0	Poaceae Barnhart
Iz_3_1109	DJI_0095	Bromus tectorum L.	0	a	0	0	Poaceae Barnhart
Iz_3_1109	DJI_0095	Calamagrostis epigejos (L.) Roth	0.1	n	1	0	Poaceae Barnhart
Iz_3_1109	DJI_0095	Carduus acanthoides L.	0	n	1	0	Asteraceae Bercht. & J.Presl
Iz_3_1109	DJI_0095	Carex sp.	0	n	1	0	Cyperaceae Juss.
Iz_3_1109	DJI_0095	Centaurea majorovii Dumbadze	0	n	1	0	Asteraceae Bercht. & J.Presl
Iz_3_1109	DJI_0095	Chamaecytisus ruthenicus (Fisch.ex Wol.) Klaskova	0	n	1	0	Fabaceae Lindl.
Iz_3_1109	DJI_0095	Chelidonium majus L.	0	n	1	0	Papaveraceae Juss.
Iz_3_1109	DJI_0095	Chenopodium betaceum Andrz.	0	n	1	0	Amaranthaceae Juss.
Iz_3_1109	DJI_0095	Chenopodium cf. album	0	n	1	0	Amaranthaceae Juss.
Iz_3_1109	DJI_0095	Chenopodium sp.	0		NA	0	Amaranthaceae Juss.
Iz_3_1109	DJI_0095	Chondrilla juncea L.	0	n	1	0	Asteraceae Bercht. & J.Presl
Iz_3_1109	DJI_0095	Cirsium vulgare (Savi) Ten.	0	n	1	0	Asteraceae Bercht. & J.Presl
Iz_3_1109	DJI_0095	Clinopodium acinos (L.) Kuntze	0	n	1	0	Lamiacea Lindl.
Iz_3_1109	DJI_0095	Convallaria majalis L.	0	n	1	0	Asparagaceae Juss.
Iz_3_1109	DJI_0095	Crataegus rhipidophylla Gand.	0	n	1	1	Rosaceae Juss.
Iz_3_1109	DJI_0095	Crepis foetida L.	0	n	1	0	Asteraceae Bercht. & J.Presl
Iz_3_1109	DJI_0095	Dactylis glomerata L.	0	n	1	0	Poaceae Barnhart
Iz_3_1109	DJI_0095	Dianthus campestris M.Bieb.	0	n	1	0	Caryophyllaceae Juss.
Iz_3_1109	DJI_0095	Digitaria sanguinalis (L.) Scop.	0	a	0	0	Poaceae Barnhart
Iz_3_1109	DJI_0095	Elymus repens (L.) Gould	0	n	1	0	Poaceae Barnhart
Iz_3_1109	DJI_0095	Epilobium tetragonum L.	0	n	1	0	Onagraceae Juss.
Iz_3_1109	DJI_0095	Eragrostis minor Host	0	a	0	0	Poaceae Barnhart
Iz_3_1109	DJI_0095	Erigeron canadensis L.	0.5	a	0	0	Asteraceae Bercht. & J.Presl
Iz_3_1109	DJI_0095	Euphorbia seguieriana Neck.	0	n	1	0	Euphorbiaceae Juss.
Iz_3_1109	DJI_0095	Fallopia dumetorum (L.) Holub	0	n	1	0	Polygonaceae Juss.
Iz_3_1109	DJI_0095	Festuca beckeri (Hack.) Trautv.	0	n	1	0	Poaceae Barnhart
Iz_3_1109	DJI_0095	Fragaria vesca L.	0	n	1	0	Rosaceae Juss.
Iz_3_1109	DJI_0095	Frangula alnus Mill.	0	n	1	1	Rhamnaceae Juss.
Iz_3_1109	DJI_0095	Fraxinus pennsylvanica Marshall	0	a	0	1	Oleaceae Hoffmanns. & Link
Iz_3_1109	DJI_0095	Galeopsis bifida Boenn.	0	n	1	0	Lamiacea Lindl.
Iz_3_1109	DJI_0095	Galium verum L.	0	n	1	0	Rubiaceae Juss.
Iz_3_1109	DJI_0095	Genista tinctoria L.	0	n	1	0	Fabaceae Lindl.
Iz_3_1109	DJI_0095	Geranium robertianum L.	0	n	1	0	Geraniaceae Juss.
Iz_3_1109	DJI_0095	Geranium sanquineum L.	0	n	1	0	Geraniaceae Juss.
Iz_3_1109	DJI_0095	Gypsophila paniculata L.	0	n	1	0	Caryophyllaceae Juss.
Iz_3_1109	DJI_0095	Helichrysum arenarium (L.) Moench	0	n	1	0	Asteraceae Bercht. & J.Presl
Iz_3_1109	DJI_0095	Herniaria polygama J.Gay	0	n	1	0	Caryophyllaceae Juss.
Iz_3_1109	DJI_0095	Hieracium robustum Fr.	0	n	1	0	Asteraceae Bercht. & J.Presl
Iz_3_1109	DJI_0095	Hieracium umbellatum L.	0	n	1	NA	Asteraceae Bercht. & J.Presl
Iz_3_1109	DJI_0095	Hylotelephium maximum (L.) Holub	0	n	1	0	Crassulaceae J.St.-Hil.
Iz_3_1109	DJI_0095	Jacobaea borysthenica (DC.) B.Nord. & Greuter	0	n	1	0	Asteraceae Bercht. & J.Presl
Iz_3_1109	DJI_0095	Jasione montana L.	0	n	1	0	Campanulaceae Juss.
Iz_3_1109	DJI_0095	Jurinea cyanoides (L.) Rchb.s.l.	0	n	1	0	Asteraceae Bercht. & J.Presl
Iz_3_1109	DJI_0095	Koeleria glauca (Spreng.) DC.	0	n	1	0	Poaceae Barnhart
Iz_3_1109	DJI_0095	Lactuca serriola L.	0	a	0	0	Asteraceae Bercht. & J.Presl
Iz_3_1109	DJI_0095	Linaria genistifolia (L.) Mill.	0	n	1	0	Plantaginaceae Juss.
Iz_3_1109	DJI_0095	Lomelosia argentea (L.) Greuter & Burdet	0	n	1	0	Caprifoliaceae Juss.

Iz_3_1109	DJI_0095	Melampyrum pratense L.	0	n	1	0	Orobanchaceae Vent.
Iz_3_1109	DJI_0095	Oenothera biennis L.	0	a	0	0	Onagraceae Juss.
Iz_3_1109	DJI_0095	Pentanema britannica (L.) D.Gut.Larr., Santos-Vicente, Anderb., E.Rico & M.M.Mart.Ort.	0	n	1	0	Asteraceae Bercht. & J.Presl
Iz_3_1109	DJI_0095	Peucedanum oreoselinum (L.) Moench	0	n	1	0	Apiaceae Lindl.
Iz_3_1109	DJI_0095	Peucedanum ruthenicum M.Bieb.	0	n	1	0	Apiaceae Lindl.
Iz_3_1109	DJI_0095	Pilosella echioides (Lumn.) F.W.Schultz & Sch.Bip.	0	n	1	0	Asteraceae Bercht. & J.Presl
Iz_3_1109	DJI_0095	Pilosella officinarum F. Schultz et Sch. Bip.	0	n	1	0	Asteraceae Bercht. & J.Presl
Iz_3_1109	DJI_0095	Pilosella sp.	0		NA	0	Asteraceae Bercht. & J.Presl
Iz_3_1109	DJI_0095	Pinus sylvestris L.	0	n	1	1	Pinaceae Spreng. ex F.Rudolphi
Iz_3_1109	DJI_0095	Poa annua L.	0	n	1	0	Poaceae Barnhart
Iz_3_1109	DJI_0095	Poa nemoralis L.	0	n	1	0	Poaceae Barnhart
Iz_3_1109	DJI_0095	Poa sp.	0		NA	0	Poaceae Barnhart
Iz_3_1109	DJI_0095	Polygonatum odoratum (Mill.) Druce	0	n	1	0	Asparagaceae Juss.
Iz_3_1109	DJI_0095	Polygonum arenarium Waldst. & Kit.	0	n	1	0	Polygonaceae Juss.
Iz_3_1109	DJI_0095	Populus nigra L.	0	n	1	1	Salicaceae Mirb.
Iz_3_1109	DJI_0095	Populus tremula L.	0	n	1	1	Salicaceae Mirb.
Iz_3_1109	DJI_0095	Populus Ã canescens (Aiton) Sm.	0	n	1	1	Salicaceae Mirb.
Iz_3_1109	DJI_0095	Potentilla argentea L.	0	n	1	0	Rosaceae Juss.
Iz_3_1109	DJI_0095	Psammophiliella muralis (L.) Ikonn.	0	n	1	0	Caryophyllaceae Juss.
Iz_3_1109	DJI_0095	Psephellus sumensis (Kalen.) Greuter	0	n	1	0	Asteraceae Bercht. & J.Presl
Iz_3_1109	DJI_0095	Pulsatilla pratensis (L.) Mill. s.l.	0	n	1	0	Ranunculaceae Juss.
Iz_3_1109	DJI_0095	Pyrus communis L.	0	a	0	1	Rosaceae Juss.
Iz_3_1109	DJI_0095	Quercus robur L.	0	n	1	1	Fagaceae Dumort.
Iz_3_1109	DJI_0095	Robinia pseudoacacia L.	0	a	0	1	Fabaceae Lindl.
Iz_3_1109	DJI_0095	Rosa sp.	0		NA	1	Rosaceae Juss.
Iz_3_1109	DJI_0095	Rubus idaeus L.	0	n	1	0	Rosaceae Juss.
Iz_3_1109	DJI_0095	Rumex acetosella L.	0	n	1	0	Polygonaceae Juss.
Iz_3_1109	DJI_0095	Rumex thyrsiflorus Fingerh.	0	n	1	0	Polygonaceae Juss.
Iz_3_1109	DJI_0095	Salix alba L.	0	n	1	1	Salicaceae Mirb.
Iz_3_1109	DJI_0095	Sambucus nigra L.	0	n	1	1	Viburnaceae Raf.
Iz_3_1109	DJI_0095	Secale sylvestre Host	0	n	1	0	Poaceae Barnhart
Iz_3_1109	DJI_0095	Sempervivum ruthenicum Schnittsp. & C.B. Lehm.	0	n	1	0	Crassulaceae J.St.-Hil.
Iz_3_1109	DJI_0095	Setaria viridis (L.) P. Beauv.	0	n	1	0	Poaceae Barnhart
Iz_3_1109	DJI_0095	Silene borysthenica (Gruner) Walters	0	n	1	0	Caryophyllaceae Juss.
Iz_3_1109	DJI_0095	Silene latifolia subsp. alba (Mill.) Greuter & Burdet	0	n	1	0	Caryophyllaceae Juss.
Iz_3_1109	DJI_0095	Solidago virgaurea L.	0	n	1	0	Asteraceae Bercht. & J.Presl
Iz_3_1109	DJI_0095	Sorbus aucuparia L.	0	n	1	1	Rosaceae Juss.
Iz_3_1109	DJI_0095	Species 11.09_3_0045	0		NA	0	
Iz_3_1109	DJI_0095	Species 11.09_3_0058	0		NA	0	
Iz_3_1109	DJI_0095	Stachys recta L.	0	n	1	0	Lamiacea Lindl.
Iz_3_1109	DJI_0095	Tanacetum vulgare L.	0	n	1	0	Asteraceae Bercht. & J.Presl
Iz_3_1109	DJI_0095	Thymus pallasianus Heinr.Braun	0	n	1	0	Lamiacea Lindl.
Iz_3_1109	DJI_0095	Tilia cordata Mill.	0	n	1	1	Malvaceae Juss.
Iz_3_1109	DJI_0095	Trifolium alpestre L.	0	n	1	0	Fabaceae Lindl.
Iz_3_1109	DJI_0095	Trifolium arvense L.	0	n	1	0	Fabaceae Lindl.
Iz_3_1109	DJI_0095	Ulmus glabra Huds.	0	n	1	1	Ulmaceae Mirb.
Iz_3_1109	DJI_0095	Verbascum densiflorum Bertol.	0	n	1	0	Scrophulariaceae Juss.
Iz_3_1109	DJI_0095	Veronica austriaca L.	0	n	1	0	Plantaginaceae Juss.
Iz_3_1109	DJI_0095	Veronica incana L.	0	n	1	0	Plantaginaceae Juss.
Iz_3_1109	DJI_0095	Veronica verna L.	0	n	1	0	Plantaginaceae Juss.
Iz_3_1109	DJI_0095	Vincetoxicum hirundinaria Medik	0	n	1	0	Apocynaceae Juss.
Iz_3_1109	DJI_0097	Acer tataricum L.	0	n	1	1	Sapindaceae Juss.
Iz_3_1109	DJI_0097	Achillea millefolium L. s.l.	0	n	1	0	Asteraceae Bercht. & J.Presl
Iz_3_1109	DJI_0097	Allium oleraceum L.	0	n	1	0	Amaryllidaceae J.St.-Hil.
Iz_3_1109	DJI_0097	Ambrosia artemisiifolia L.	0	a	0	0	Asteraceae Bercht. & J.Presl
Iz_3_1109	DJI_0097	Amorpha fruticosa L.	0	a	0	0	Fabaceae Lindl.
Iz_3_1109	DJI_0097	Anthericum ramosum L.	0	n	1	0	Asparagaceae Juss.
Iz_3_1109	DJI_0097	Artemisia marschalliana Spreng.	0	n	1	0	Asteraceae Bercht. & J.Presl
Iz_3_1109	DJI_0097	Asparagus officinalis L.	0	n	1	0	Asparagaceae Juss.
Iz_3_1109	DJI_0097	Astragalus cicer L.	0	n	1	0	Fabaceae Lindl.
Iz_3_1109	DJI_0097	Bassia laniflora (S.G.Gmel.) A.J.Scott	0	n	1	0	Amaranthaceae Juss.
Iz_3_1109	DJI_0097	Berberis vulgaris L.	0	n	1	0	Berberidaceae Juss.
Iz_3_1109	DJI_0097	Betula pendula Roth.	0	n	1	1	Betulaceae Gray
Iz_3_1109	DJI_0097	Betula pendula Roth.	0	n	1	0	Poaceae Barnhart
Iz_3_1109	DJI_0097	Bromus inermis Leyss.	0		NA	NA	
Iz_3_1109	DJI_0097	Bromus squarrosus L.	0	a	0	0	Poaceae Barnhart
Iz_3_1109	DJI_0097	Bromus tectorum L.	0	a	0	0	Poaceae Barnhart
Iz_3_1109	DJI_0097	Calamagrostis epigejos (L.) Roth	0.2	n	1	0	Poaceae Barnhart
Iz_3_1109	DJI_0097	Carduus acanthoides L.	0	n	1	0	Asteraceae Bercht. & J.Presl
Iz_3_1109	DJI_0097	Carex sp.	0	n	1	0	Cyperaceae Juss.
Iz_3_1109	DJI_0097	Centaurea majorovii Dumbadze	0	n	1	0	Asteraceae Bercht. & J.Presl
Iz_3_1109	DJI_0097	Chamaecytisus ruthenicus (Fisch.ex Wol.) Klaskova	0	n	1	0	Fabaceae Lindl.
Iz_3_1109	DJI_0097	Chelidonium majus L.	0	n	1	0	Papaveraceae Juss.
Iz_3_1109	DJI_0097	Chenopodium betaceum Andrz.	0	n	1	0	Amaranthaceae Juss.
Iz_3_1109	DJI_0097	Chenopodium cf. album	0	n	1	0	Amaranthaceae Juss.
Iz_3_1109	DJI_0097	Chenopodium sp.	0		NA	0	Amaranthaceae Juss.
Iz_3_1109	DJI_0097	Chondrilla juncea L.	0	n	1	0	Asteraceae Bercht. & J.Presl
Iz_3_1109	DJI_0097	Cirsium vulgare (Savi) Ten.	0	n	1	0	Asteraceae Bercht. & J.Presl
Iz_3_1109	DJI_0097	Clinopodium acinos (L.) Kuntze	0	n	1	0	Lamiacea Lindl.
Iz_3_1109	DJI_0097	Convallaria majalis L.	0	n	1	0	Asparagaceae Juss.
Iz_3_1109	DJI_0097	Crataegus rhipidophylla Gand.	0	n	1	1	Rosaceae Juss.
Iz_3_1109	DJI_0097	Crepis foetida L.	0	n	1	0	Asteraceae Bercht. & J.Presl
Iz_3_1109	DJI_0097	Dactylis glomerata L.	0	n	1	0	Poaceae Barnhart
Iz_3_1109	DJI_0097	Dianthus campestris M.Bieb.	0	n	1	0	Caryophyllaceae Juss.
Iz_3_1109	DJI_0097	Digitaria sanguinalis (L.) Scop.	0	a	0	0	Poaceae Barnhart
Iz_3_1109	DJI_0097	Elymus repens (L.) Gould	0	n	1	0	Poaceae Barnhart
Iz_3_1109	DJI_0097	Epilobium tetragonum L.	0	n	1	0	Onagraceae Juss.
Iz_3_1109	DJI_0097	Eragrostis minor Host	0	a	0	0	Poaceae Barnhart
Iz_3_1109	DJI_0097	Erigeron canadensis L.	4	a	0	0	Asteraceae Bercht. & J.Presl
Iz_3_1109	DJI_0097	Euphorbia seguieriana Neck.	0	n	1	0	Euphorbiaceae Juss.
Iz_3_1109	DJI_0097	Fallopia dumetorum (L.) Holub	0	n	1	0	Polygonaceae Juss.
Iz_3_1109	DJI_0097	Festuca beckeri (Hack.) Trautv.	0	n	1	0	Poaceae Barnhart
Iz_3_1109	DJI_0097	Fragaria vesca L.	0	n	1	0	Rosaceae Juss.
Iz_3_1109	DJI_0097	Frangula alnus Mill.	0	n	1	1	Rhamnaceae Juss.
Iz_3_1109	DJI_0097	Fraxinus pennsylvanica Marshall	0	a	0	1	Oleaceae Hoffmanns. & Link
Iz_3_1109	DJI_0097	Galeopsis bifida Boenn.	0	n	1	0	Lamiacea Lindl.
Iz_3_1109	DJI_0097	Galium verum L.	0	n	1	0	Rubiaceae Juss.
Iz_3_1109	DJI_0097	Genista tinctoria L.	0	n	1	0	Fabaceae Lindl.

Iz_3_1109	DJI_0097	Geranium robertianum L.	0	n	1	0	Geraniaceae Juss.
Iz_3_1109	DJI_0097	Geranium sanquineum L.	0	n	1	0	Geraniaceae Juss.
Iz_3_1109	DJI_0097	Gypsophila paniculata L.	0	n	1	0	Caryophyllaceae Juss.
Iz_3_1109	DJI_0097	Helichrysum arenarium (L.) Moench	0	n	1	0	Asteraceae Bercht. & J.Presl
Iz_3_1109	DJI_0097	Herniaria polygama J.Gay	0	n	1	0	Caryophyllaceae Juss.
Iz_3_1109	DJI_0097	Hieracium robustum Fr.	0	n	1	0	Asteraceae Bercht. & J.Presl
Iz_3_1109	DJI_0097	Hieracium umbellatum L.	0	n	1	NA	Asteraceae Bercht. & J.Presl
Iz_3_1109	DJI_0097	Hylotelephium maximum (L.) Holub	0	n	1	0	Crassulaceae J.St.-Hil.
Iz_3_1109	DJI_0097	Jacobaea borysthenica (DC.) B.Nord. & Greuter	0	n	1	0	Asteraceae Bercht. & J.Presl
Iz_3_1109	DJI_0097	Jasione montana L.	0	n	1	0	Campanulaceae Juss.
Iz_3_1109	DJI_0097	Jurinea cyanoides (L.) Rchb.s.l.	0	n	1	0	Asteraceae Bercht. & J.Presl
Iz_3_1109	DJI_0097	Koeleria glauca (Spreng.) DC.	0	n	1	0	Poaceae Barnhart
Iz_3_1109	DJI_0097	Lactuca serriola L.	0	a	0	0	Asteraceae Bercht. & J.Presl
Iz_3_1109	DJI_0097	Linaria genistifolia (L.) Mill.	0	n	1	0	Plantaginaceae Juss.
Iz_3_1109	DJI_0097	Lomelosia argentea (L.) Greuter & Burdet	0	n	1	0	Caprifoliaceae Juss.
Iz_3_1109	DJI_0097	Melampyrum pratense L.	0	n	1	0	Orobanchaceae Vent.
Iz_3_1109	DJI_0097	Oenothera biennis L.	0	a	0	0	Onagraceae Juss.
Iz_3_1109	DJI_0097	Pentanema britannica (L.) D.Gut.Larr., Santos-Vicente, Anderb., E.Rico & M.M.Mart.Ort.	0	n	1	0	Asteraceae Bercht. & J.Presl
Iz_3_1109	DJI_0097	Peucedanum oreoselinum (L.) Moench	0.2	n	1	0	Apiaceae Lindl.
Iz_3_1109	DJI_0097	Peucedanum ruthenicum M.Bieb.	0	n	1	0	Apiaceae Lindl.
Iz_3_1109	DJI_0097	Pilosella echioides (Lumn.) F.W.Schultz & Sch.Bip.	0	n	1	0	Asteraceae Bercht. & J.Presl
Iz_3_1109	DJI_0097	Pilosella officinarum F. Schultz et Sch. Bip.	0	n	1	0	Asteraceae Bercht. & J.Presl
Iz_3_1109	DJI_0097	Pilosella sp.	0		NA	0	Asteraceae Bercht. & J.Presl
Iz_3_1109	DJI_0097	Pinus sylvestris L.	0	n	1	1	Pinaceae Spreng. ex F.Rudolphi
Iz_3_1109	DJI_0097	Poa annua L.	0	n	1	0	Poaceae Barnhart
Iz_3_1109	DJI_0097	Poa nemoralis L.	0	n	1	0	Poaceae Barnhart
Iz_3_1109	DJI_0097	Poa sp.	0		NA	0	Poaceae Barnhart
Iz_3_1109	DJI_0097	Polygonatum odoratum (Mill.) Druce	0	n	1	0	Asparagaceae Juss.
Iz_3_1109	DJI_0097	Polygonum arenarium Waldst. & Kit.	0	n	1	0	Polygonaceae Juss.
Iz_3_1109	DJI_0097	Populus nigra L.	0	n	1	1	Salicaceae Mirb.
Iz_3_1109	DJI_0097	Populus tremula L.	0	n	1	1	Salicaceae Mirb.
Iz_3_1109	DJI_0097	Populus Ã canescens (Aiton) Sm.	0	n	1	1	Salicaceae Mirb.
Iz_3_1109	DJI_0097	Potentilla argentea L.	0	n	1	0	Rosaceae Juss.
Iz_3_1109	DJI_0097	Psammophiliella muralis (L.) Ikonn.	0	n	1	0	Caryophyllaceae Juss.
Iz_3_1109	DJI_0097	Psephellus sumensis (Kalen.) Greuter	0	n	1	0	Asteraceae Bercht. & J.Presl
Iz_3_1109	DJI_0097	Pulsatilla pratensis (L.) Mill. s.l.	0	n	1	0	Ranunculaceae Juss.
Iz_3_1109	DJI_0097	Pyrus communis L.	0	a	0	1	Rosaceae Juss.
Iz_3_1109	DJI_0097	Quercus robur L.	0	n	1	1	Fagaceae Dumort.
Iz_3_1109	DJI_0097	Robinia pseudoacacia L.	0	a	0	1	Fabaceae Lindl.
Iz_3_1109	DJI_0097	Rosa sp.	0		NA	1	Rosaceae Juss.
Iz_3_1109	DJI_0097	Rubus idaeus L.	0	n	1	0	Rosaceae Juss.
Iz_3_1109	DJI_0097	Rumex acetosella L.	0	n	1	0	Polygonaceae Juss.
Iz_3_1109	DJI_0097	Rumex thyrsiflorus Fingerh.	0	n	1	0	Polygonaceae Juss.
Iz_3_1109	DJI_0097	Salix alba L.	0	n	1	1	Salicaceae Mirb.
Iz_3_1109	DJI_0097	Sambucus nigra L.	0	n	1	1	Viburnaceae Raf.
Iz_3_1109	DJI_0097	Secale sylvestre Host	0	n	1	0	Poaceae Barnhart
Iz_3_1109	DJI_0097	Sempervivum ruthenicum Schnittsp. & C.B. Lehm.	0	n	1	0	Crassulaceae J.St.-Hil.
Iz_3_1109	DJI_0097	Setaria viridis (L.) P. Beauv.	0	n	1	0	Poaceae Barnhart
Iz_3_1109	DJI_0097	Silene borysthenica (Gruner) Walters	0	n	1	0	Caryophyllaceae Juss.
Iz_3_1109	DJI_0097	Silene latifolia subsp. alba (Mill.) Greuter & Burdet	0	n	1	0	Caryophyllaceae Juss.
Iz_3_1109	DJI_0097	Solidago virgaurea L.	0	n	1	0	Asteraceae Bercht. & J.Presl
Iz_3_1109	DJI_0097	Sorbus aucuparia L.	0	n	1	1	Rosaceae Juss.
Iz_3_1109	DJI_0097	Species 11.09_3_0045	0		NA	0	
Iz_3_1109	DJI_0097	Species 11.09_3_0058	0		NA	0	
Iz_3_1109	DJI_0097	Stachys recta L.	0	n	1	0	Lamiacea Lindl.
Iz_3_1109	DJI_0097	Tanacetum vulgare L.	0	n	1	0	Asteraceae Bercht. & J.Presl
Iz_3_1109	DJI_0097	Thymus pallasianus Heinr.Braun	0	n	1	0	Lamiacea Lindl.
Iz_3_1109	DJI_0097	Tilia cordata Mill.	0	n	1	1	Malvaceae Juss.
Iz_3_1109	DJI_0097	Trifolium alpestre L.	0	n	1	0	Fabaceae Lindl.
Iz_3_1109	DJI_0097	Trifolium arvense L.	0	n	1	0	Fabaceae Lindl.
Iz_3_1109	DJI_0097	Ulmus glabra Huds.	0	n	1	1	Ulmaceae Mirb.
Iz_3_1109	DJI_0097	Verbascum densiflorum Bertol.	0	n	1	0	Scrophulariaceae Juss.
Iz_3_1109	DJI_0097	Veronica austriaca L.	0	n	1	0	Plantaginaceae Juss.
Iz_3_1109	DJI_0097	Veronica incana L.	0	n	1	0	Plantaginaceae Juss.
Iz_3_1109	DJI_0097	Veronica verna L.	0	n	1	0	Plantaginaceae Juss.
Iz_3_1109	DJI_0097	Vincetoxicum hirundinaria Medik	0	n	1	0	Apocynaceae Juss.
Iz_3_1109	DJI_0099	Acer tataricum L.	0	n	1	1	Sapindaceae Juss.
Iz_3_1109	DJI_0099	Achillea millefolium L. s.l.	0	n	1	0	Asteraceae Bercht. & J.Presl
Iz_3_1109	DJI_0099	Allium oleraceum L.	0	n	1	0	Amaryllidaceae J.St.-Hil.
Iz_3_1109	DJI_0099	Ambrosia artemisiifolia L.	0	a	0	0	Asteraceae Bercht. & J.Presl
Iz_3_1109	DJI_0099	Amorpha fruticosa L.	0	a	0	0	Fabaceae Lindl.
Iz_3_1109	DJI_0099	Anthericum ramosum L.	0	n	1	0	Asparagaceae Juss.
Iz_3_1109	DJI_0099	Artemisia marschalliana Spreng.	0	n	1	0	Asteraceae Bercht. & J.Presl
Iz_3_1109	DJI_0099	Asparagus officinalis L.	0	n	1	0	Asparagaceae Juss.
Iz_3_1109	DJI_0099	Astragalus cicer L.	0	n	1	0	Fabaceae Lindl.
Iz_3_1109	DJI_0099	Bassia laniflora (S.G.Gmel.) A.J.Scott	0	n	1	0	Amaranthaceae Juss.
Iz_3_1109	DJI_0099	Berberis vulgaris L.	0	n	1	0	Berberidaceae Juss.
Iz_3_1109	DJI_0099	Betula pendula Roth.	0	n	1	1	Betulaceae Gray
Iz_3_1109	DJI_0099	Betula pendula Roth.	0	n	1	0	Poaceae Barnhart
Iz_3_1109	DJI_0099	Bromus inermis Leyss.	0		NA	NA	
Iz_3_1109	DJI_0099	Bromus squarrosus L.	0	a	0	0	Poaceae Barnhart
Iz_3_1109	DJI_0099	Bromus tectorum L.	0	a	0	0	Poaceae Barnhart
Iz_3_1109	DJI_0099	Calamagrostis epigejos (L.) Roth	3	n	1	0	Poaceae Barnhart
Iz_3_1109	DJI_0099	Carduus acanthoides L.	0	n	1	0	Asteraceae Bercht. & J.Presl
Iz_3_1109	DJI_0099	Carex sp.	0	n	1	0	Cyperaceae Juss.
Iz_3_1109	DJI_0099	Centaurea majorovii Dumbadze	0	n	1	0	Asteraceae Bercht. & J.Presl
Iz_3_1109	DJI_0099	Chamaecytisus ruthenicus (Fisch.ex Wol.) Klaskova	0	n	1	0	Fabaceae Lindl.
Iz_3_1109	DJI_0099	Chelidonium majus L.	0	n	1	0	Papaveraceae Juss.
Iz_3_1109	DJI_0099	Chenopodium betaceum Andrz.	0	n	1	0	Amaranthaceae Juss.
Iz_3_1109	DJI_0099	Chenopodium cf. album	0	n	1	0	Amaranthaceae Juss.
Iz_3_1109	DJI_0099	Chenopodium sp.	0		NA	0	Amaranthaceae Juss.
Iz_3_1109	DJI_0099	Chondrilla juncea L.	0	n	1	0	Asteraceae Bercht. & J.Presl
Iz_3_1109	DJI_0099	Cirsium vulgare (Savi) Ten.	0	n	1	0	Asteraceae Bercht. & J.Presl
Iz_3_1109	DJI_0099	Clinopodium acinos (L.) Kuntze	0	n	1	0	Lamiacea Lindl.
Iz_3_1109	DJI_0099	Convallaria majalis L.	0	n	1	0	Asparagaceae Juss.
Iz_3_1109	DJI_0099	Crataegus rhipidophylla Gand.	0	n	1	1	Rosaceae Juss.
Iz_3_1109	DJI_0099	Crepis foetida L.	0	n	1	0	Asteraceae Bercht. & J.Presl
Iz_3_1109	DJI_0099	Dactylis glomerata L.	0	n	1	0	Poaceae Barnhart

Iz_3_1109	DJI_0099	Dianthus campestris M.Bieb.	0	n	1	0	Caryophyllaceae Juss.
Iz_3_1109	DJI_0099	Digitaria sanguinalis (L.) Scop.	0	a	0	0	Poaceae Barnhart
Iz_3_1109	DJI_0099	Elymus repens (L.) Gould	0	n	1	0	Poaceae Barnhart
Iz_3_1109	DJI_0099	Epilobium tetragonum L.	0	n	1	0	Onagraceae Juss.
Iz_3_1109	DJI_0099	Eragrostis minor Host	0	a	0	0	Poaceae Barnhart
Iz_3_1109	DJI_0099	Erigeron canadensis L.	3	a	0	0	Asteraceae Bercht. & J.Presl
Iz_3_1109	DJI_0099	Euphorbia seguieriana Neck.	0	n	1	0	Euphorbiaceae Juss.
Iz_3_1109	DJI_0099	Fallopia dumetorum (L.) Holub	0	n	1	0	Polygonaceae Juss.
Iz_3_1109	DJI_0099	Festuca beckeri (Hack.) Trautv.	0	n	1	0	Poaceae Barnhart
Iz_3_1109	DJI_0099	Fragaria vesca L.	0	n	1	0	Rosaceae Juss.
Iz_3_1109	DJI_0099	Frangula alnus Mill.	0	n	1	1	Rhamnaceae Juss.
Iz_3_1109	DJI_0099	Fraxinus pennsylvanica Marshall	0	a	0	1	Oleaceae Hoffmanns. & Link
Iz_3_1109	DJI_0099	Galeopsis bifida Boenn.	0	n	1	0	Lamiacea Lindl.
Iz_3_1109	DJI_0099	Galium verum L.	0	n	1	0	Rubiaceae Juss.
Iz_3_1109	DJI_0099	Genista tinctoria L.	0	n	1	0	Fabaceae Lindl.
Iz_3_1109	DJI_0099	Geranium robertianum L.	0	n	1	0	Geraniaceae Juss.
Iz_3_1109	DJI_0099	Geranium sanquineum L.	0	n	1	0	Geraniaceae Juss.
Iz_3_1109	DJI_0099	Gypsophila paniculata L.	0	n	1	0	Caryophyllaceae Juss.
Iz_3_1109	DJI_0099	Helichrysum arenarium (L.) Moench	0	n	1	0	Asteraceae Bercht. & J.Presl
Iz_3_1109	DJI_0099	Herniaria polygama J.Gay	0	n	1	0	Caryophyllaceae Juss.
Iz_3_1109	DJI_0099	Hieracium robustum Fr.	0	n	1	0	Asteraceae Bercht. & J.Presl
Iz_3_1109	DJI_0099	Hieracium umbellatum L.	0	n	1	NA	Asteraceae Bercht. & J.Presl
Iz_3_1109	DJI_0099	Hylotelephium maximum (L.) Holub	0	n	1	0	Crassulaceae J.St.-Hil.
Iz_3_1109	DJI_0099	Jacobaea borysthenica (DC.) B.Nord. & Greuter	0	n	1	0	Asteraceae Bercht. & J.Presl
Iz_3_1109	DJI_0099	Jasione montana L.	0	n	1	0	Campanulaceae Juss.
Iz_3_1109	DJI_0099	Jurinea cyanoides (L.) Rchb.s.l.	0	n	1	0	Asteraceae Bercht. & J.Presl
Iz_3_1109	DJI_0099	Koeleria glauca (Spreng.) DC.	0	n	1	0	Poaceae Barnhart
Iz_3_1109	DJI_0099	Lactuca serriola L.	0	a	0	0	Asteraceae Bercht. & J.Presl
Iz_3_1109	DJI_0099	Linaria genistifolia (L.) Mill.	0	n	1	0	Plantaginaceae Juss.
Iz_3_1109	DJI_0099	Lomelosia argentea (L.) Greuter & Burdet	0	n	1	0	Caprifoliaceae Juss.
Iz_3_1109	DJI_0099	Melampyrum pratense L.	0	n	1	0	Orobanchaceae Vent.
Iz_3_1109	DJI_0099	Oenothera biennis L.	0	a	0	0	Onagraceae Juss.
Iz_3_1109	DJI_0099	Pentanema britannica (L.) D.Gut.Larr., Santos-Vicente, Anderb., E.Rico & M.M.Mart.Ort.	0	n	1	0	Asteraceae Bercht. & J.Presl
Iz_3_1109	DJI_0099	Peucedanum oreoselinum (L.) Moench	0	n	1	0	Apiaceae Lindl.
Iz_3_1109	DJI_0099	Peucedanum ruthenicum M.Bieb.	0	n	1	0	Apiaceae Lindl.
Iz_3_1109	DJI_0099	Pilosella echioides (Lumn.) F.W.Schultz & Sch.Bip.	0	n	1	0	Asteraceae Bercht. & J.Presl
Iz_3_1109	DJI_0099	Pilosella officinarum F. Schultz et Sch. Bip.	0	n	1	0	Asteraceae Bercht. & J.Presl
Iz_3_1109	DJI_0099	Pilosella sp.	0		NA	0	Asteraceae Bercht. & J.Presl
Iz_3_1109	DJI_0099	Pinus sylvestris L.	0	n	1	1	Pinaceae Spreng. ex F.Rudolphi
Iz_3_1109	DJI_0099	Poa annua L.	0	n	1	0	Poaceae Barnhart
Iz_3_1109	DJI_0099	Poa nemoralis L.	0	n	1	0	Poaceae Barnhart
Iz_3_1109	DJI_0099	Poa sp.	0		NA	0	Poaceae Barnhart
Iz_3_1109	DJI_0099	Polygonatum odoratum (Mill.) Druce	0	n	1	0	Asparagaceae Juss.
Iz_3_1109	DJI_0099	Polygonum arenarium Waldst. & Kit.	0	n	1	0	Polygonaceae Juss.
Iz_3_1109	DJI_0099	Populus nigra L.	0	n	1	1	Salicaceae Mirb.
Iz_3_1109	DJI_0099	Populus tremula L.	0	n	1	1	Salicaceae Mirb.
Iz_3_1109	DJI_0099	Populus Ã canescens (Aiton) Sm.	0	n	1	1	Salicaceae Mirb.
Iz_3_1109	DJI_0099	Potentilla argentea L.	0	n	1	0	Rosaceae Juss.
Iz_3_1109	DJI_0099	Psammophiliella muralis (L.) Ikonn.	0	n	1	0	Caryophyllaceae Juss.
Iz_3_1109	DJI_0099	Psephellus sumensis (Kalen.) Greuter	0	n	1	0	Asteraceae Bercht. & J.Presl
Iz_3_1109	DJI_0099	Pulsatilla pratensis (L.) Mill. s.l.	0	n	1	0	Ranunculaceae Juss.
Iz_3_1109	DJI_0099	Pyrus communis L.	0	a	0	1	Rosaceae Juss.
Iz_3_1109	DJI_0099	Quercus robur L.	0	n	1	1	Fagaceae Dumort.
Iz_3_1109	DJI_0099	Robinia pseudoacacia L.	0	a	0	1	Fabaceae Lindl.
Iz_3_1109	DJI_0099	Rosa sp.	0		NA	1	Rosaceae Juss.
Iz_3_1109	DJI_0099	Rubus idaeus L.	0	n	1	0	Rosaceae Juss.
Iz_3_1109	DJI_0099	Rumex acetosella L.	0	n	1	0	Polygonaceae Juss.
Iz_3_1109	DJI_0099	Rumex thyrsiflorus Fingerh.	0	n	1	0	Polygonaceae Juss.
Iz_3_1109	DJI_0099	Salix alba L.	0	n	1	1	Salicaceae Mirb.
Iz_3_1109	DJI_0099	Sambucus nigra L.	0	n	1	1	Viburnaceae Raf.
Iz_3_1109	DJI_0099	Secale sylvestre Host	0	n	1	0	Poaceae Barnhart
Iz_3_1109	DJI_0099	Sempervivum ruthenicum Schnittsp. & C.B. Lehm.	0	n	1	0	Crassulaceae J.St.-Hil.
Iz_3_1109	DJI_0099	Setaria viridis (L.) P. Beauv.	0	n	1	0	Poaceae Barnhart
Iz_3_1109	DJI_0099	Silene borysthenica (Gruner) Walters	0	n	1	0	Caryophyllaceae Juss.
Iz_3_1109	DJI_0099	Silene latifolia subsp. alba (Mill.) Greuter & Burdet	0	n	1	0	Caryophyllaceae Juss.
Iz_3_1109	DJI_0099	Solidago virgaurea L.	0	n	1	0	Asteraceae Bercht. & J.Presl
Iz_3_1109	DJI_0099	Sorbus aucuparia L.	0	n	1	1	Rosaceae Juss.
Iz_3_1109	DJI_0099	Species 11.09_3_0045	0		NA	0	
Iz_3_1109	DJI_0099	Species 11.09_3_0058	0		NA	0	
Iz_3_1109	DJI_0099	Stachys recta L.	0	n	1	0	Lamiacea Lindl.
Iz_3_1109	DJI_0099	Tanacetum vulgare L.	0	n	1	0	Asteraceae Bercht. & J.Presl
Iz_3_1109	DJI_0099	Thymus pallasianus Heinr.Braun	0	n	1	0	Lamiacea Lindl.
Iz_3_1109	DJI_0099	Tilia cordata Mill.	0	n	1	1	Malvaceae Juss.
Iz_3_1109	DJI_0099	Trifolium alpestre L.	0	n	1	0	Fabaceae Lindl.
Iz_3_1109	DJI_0099	Trifolium arvense L.	0	n	1	0	Fabaceae Lindl.
Iz_3_1109	DJI_0099	Ulmus glabra Huds.	0	n	1	1	Ulmaceae Mirb.
Iz_3_1109	DJI_0099	Verbascum densiflorum Bertol.	0	n	1	0	Scrophulariaceae Juss.
Iz_3_1109	DJI_0099	Veronica austriaca L.	0	n	1	0	Plantaginaceae Juss.
Iz_3_1109	DJI_0099	Veronica incana L.	0	n	1	0	Plantaginaceae Juss.
Iz_3_1109	DJI_0099	Veronica verna L.	0	n	1	0	Plantaginaceae Juss.
Iz_3_1109	DJI_0099	Vincetoxicum hirundinaria Medik	0	n	1	0	Apocynaceae Juss.
Iz_3_1109	DJI_0101	Acer tataricum L.	0	n	1	1	Sapindaceae Juss.
Iz_3_1109	DJI_0101	Achillea millefolium L. s.l.	0	n	1	0	Asteraceae Bercht. & J.Presl
Iz_3_1109	DJI_0101	Allium oleraceum L.	0	n	1	0	Amaryllidaceae J.St.-Hil.
Iz_3_1109	DJI_0101	Ambrosia artemisiifolia L.	0	a	0	0	Asteraceae Bercht. & J.Presl
Iz_3_1109	DJI_0101	Amorpha fruticosa L.	0	a	0	0	Fabaceae Lindl.
Iz_3_1109	DJI_0101	Anthericum ramosum L.	0	n	1	0	Asparagaceae Juss.
Iz_3_1109	DJI_0101	Artemisia marschalliana Spreng.	0	n	1	0	Asteraceae Bercht. & J.Presl
Iz_3_1109	DJI_0101	Asparagus officinalis L.	0	n	1	0	Asparagaceae Juss.
Iz_3_1109	DJI_0101	Astragalus cicer L.	0	n	1	0	Fabaceae Lindl.
Iz_3_1109	DJI_0101	Bassia laniflora (S.G.Gmel.) A.J.Scott	0	n	1	0	Amaranthaceae Juss.
Iz_3_1109	DJI_0101	Berberis vulgaris L.	0	n	1	0	Berberidaceae Juss.
Iz_3_1109	DJI_0101	Betula pendula Roth.	0	n	1	1	Betulaceae Gray
Iz_3_1109	DJI_0101	Betula pendula Roth.	0	n	1	0	Poaceae Barnhart
Iz_3_1109	DJI_0101	Bromus inermis Leyss.	0		NA	NA	
Iz_3_1109	DJI_0101	Bromus squarrosus L.	0	a	0	0	Poaceae Barnhart
Iz_3_1109	DJI_0101	Bromus tectorum L.	0	a	0	0	Poaceae Barnhart
Iz_3_1109	DJI_0101	Calamagrostis epigejos (L.) Roth	0.5	n	1	0	Poaceae Barnhart

Iz_3_1109	DJI_0101	Carduus acanthoides L.	0	n	1	0	Asteraceae Bercht. & J.Presl
Iz_3_1109	DJI_0101	Carex sp.	0	n	1	0	Cyperaceae Juss.
Iz_3_1109	DJI_0101	Centaurea majorovii Dumbadze	0	n	1	0	Asteraceae Bercht. & J.Presl
Iz_3_1109	DJI_0101	Chamaecytisus ruthenicus (Fisch.ex Wol.) Klaskova	0	n	1	0	Fabaceae Lindl.
Iz_3_1109	DJI_0101	Chelidonium majus L.	0	n	1	0	Papaveraceae Juss.
Iz_3_1109	DJI_0101	Chenopodium betaceum Andrz.	0	n	1	0	Amaranthaceae Juss.
Iz_3_1109	DJI_0101	Chenopodium cf. album	0	n	1	0	Amaranthaceae Juss.
Iz_3_1109	DJI_0101	Chenopodium sp.	0		NA	0	Amaranthaceae Juss.
Iz_3_1109	DJI_0101	Chondrilla juncea L.	0	n	1	0	Asteraceae Bercht. & J.Presl
Iz_3_1109	DJI_0101	Cirsium vulgare (Savi) Ten.	0	n	1	0	Asteraceae Bercht. & J.Presl
Iz_3_1109	DJI_0101	Clinopodium acinos (L.) Kuntze	0	n	1	0	Lamiacea Lindl.
Iz_3_1109	DJI_0101	Convallaria majalis L.	0	n	1	0	Asparagaceae Juss.
Iz_3_1109	DJI_0101	Crataegus rhipidophylla Gand.	0	n	1	1	Rosaceae Juss.
Iz_3_1109	DJI_0101	Crepis foetida L.	0	n	1	0	Asteraceae Bercht. & J.Presl
Iz_3_1109	DJI_0101	Dactylis glomerata L.	0	n	1	0	Poaceae Barnhart
Iz_3_1109	DJI_0101	Dianthus campestris M.Bieb.	0	n	1	0	Caryophyllaceae Juss.
Iz_3_1109	DJI_0101	Digitaria sanguinalis (L.) Scop.	0	a	0	0	Poaceae Barnhart
Iz_3_1109	DJI_0101	Elymus repens (L.) Gould	0	n	1	0	Poaceae Barnhart
Iz_3_1109	DJI_0101	Epilobium tetragonum L.	0	n	1	0	Onagraceae Juss.
Iz_3_1109	DJI_0101	Eragrostis minor Host	0	a	0	0	Poaceae Barnhart
Iz_3_1109	DJI_0101	Erigeron canadensis L.	3	a	0	0	Asteraceae Bercht. & J.Presl
Iz_3_1109	DJI_0101	Euphorbia seguieriana Neck.	0	n	1	0	Euphorbiaceae Juss.
Iz_3_1109	DJI_0101	Fallopia dumetorum (L.) Holub	0	n	1	0	Polygonaceae Juss.
Iz_3_1109	DJI_0101	Festuca beckeri (Hack.) Trautv.	0	n	1	0	Poaceae Barnhart
Iz_3_1109	DJI_0101	Fragaria vesca L.	0	n	1	0	Rosaceae Juss.
Iz_3_1109	DJI_0101	Frangula alnus Mill.	0	n	1	1	Rhamnaceae Juss.
Iz_3_1109	DJI_0101	Fraxinus pennsylvanica Marshall	0	a	0	1	Oleaceae Hoffmanns. & Link
Iz_3_1109	DJI_0101	Galeopsis bifida Boenn.	0	n	1	0	Lamiacea Lindl.
Iz_3_1109	DJI_0101	Galium verum L.	0	n	1	0	Rubiaceae Juss.
Iz_3_1109	DJI_0101	Genista tinctoria L.	0	n	1	0	Fabaceae Lindl.
Iz_3_1109	DJI_0101	Geranium robertianum L.	0	n	1	0	Geraniaceae Juss.
Iz_3_1109	DJI_0101	Geranium sanquineum L.	0	n	1	0	Geraniaceae Juss.
Iz_3_1109	DJI_0101	Gypsophila paniculata L.	0	n	1	0	Caryophyllaceae Juss.
Iz_3_1109	DJI_0101	Helichrysum arenarium (L.) Moench	0	n	1	0	Asteraceae Bercht. & J.Presl
Iz_3_1109	DJI_0101	Herniaria polygama J.Gay	0	n	1	0	Caryophyllaceae Juss.
Iz_3_1109	DJI_0101	Hieracium robustum Fr.	0	n	1	0	Asteraceae Bercht. & J.Presl
Iz_3_1109	DJI_0101	Hieracium umbellatum L.	0	n	1	NA	Asteraceae Bercht. & J.Presl
Iz_3_1109	DJI_0101	Hylotelephium maximum (L.) Holub	0	n	1	0	Crassulaceae J.St.-Hil.
Iz_3_1109	DJI_0101	Jacobaea borysthenica (DC.) B.Nord. & Greuter	0	n	1	0	Asteraceae Bercht. & J.Presl
Iz_3_1109	DJI_0101	Jasione montana L.	0	n	1	0	Campanulaceae Juss.
Iz_3_1109	DJI_0101	Jurinea cyanoides (L.) Rchb.s.l.	0	n	1	0	Asteraceae Bercht. & J.Presl
Iz_3_1109	DJI_0101	Koeleria glauca (Spreng.) DC.	0	n	1	0	Poaceae Barnhart
Iz_3_1109	DJI_0101	Lactuca serriola L.	0	a	0	0	Asteraceae Bercht. & J.Presl
Iz_3_1109	DJI_0101	Linaria genistifolia (L.) Mill.	0	n	1	0	Plantaginaceae Juss.
Iz_3_1109	DJI_0101	Lomelosia argentea (L.) Greuter & Burdet	0	n	1	0	Caprifoliaceae Juss.
Iz_3_1109	DJI_0101	Melampyrum pratense L.	0	n	1	0	Orobanchaceae Vent.
Iz_3_1109	DJI_0101	Oenothera biennis L.	0	a	0	0	Onagraceae Juss.
Iz_3_1109	DJI_0101	Pentanema britannica (L.) D.Gut.Larr., Santos-Vicente, Anderb., E.Rico & M.M.Mart.Ort.	0	n	1	0	Asteraceae Bercht. & J.Presl
Iz_3_1109	DJI_0101	Peucedanum oreoselinum (L.) Moench	0	n	1	0	Apiaceae Lindl.
Iz_3_1109	DJI_0101	Peucedanum ruthenicum M.Bieb.	0	n	1	0	Apiaceae Lindl.
Iz_3_1109	DJI_0101	Pilosella echioides (Lumn.) F.W.Schultz & Sch.Bip.	0	n	1	0	Asteraceae Bercht. & J.Presl
Iz_3_1109	DJI_0101	Pilosella officinarum F. Schultz et Sch. Bip.	0	n	1	0	Asteraceae Bercht. & J.Presl
Iz_3_1109	DJI_0101	Pilosella sp.	0		NA	0	Asteraceae Bercht. & J.Presl
Iz_3_1109	DJI_0101	Pinus sylvestris L.	0	n	1	1	Pinaceae Spreng. ex F.Rudolphi
Iz_3_1109	DJI_0101	Poa annua L.	0	n	1	0	Poaceae Barnhart
Iz_3_1109	DJI_0101	Poa nemoralis L.	0	n	1	0	Poaceae Barnhart
Iz_3_1109	DJI_0101	Poa sp.	0		NA	0	Poaceae Barnhart
Iz_3_1109	DJI_0101	Polygonatum odoratum (Mill.) Druce	0	n	1	0	Asparagaceae Juss.
Iz_3_1109	DJI_0101	Polygonum arenarium Waldst. & Kit.	0	n	1	0	Polygonaceae Juss.
Iz_3_1109	DJI_0101	Populus nigra L.	0	n	1	1	Salicaceae Mirb.
Iz_3_1109	DJI_0101	Populus tremula L.	0	n	1	1	Salicaceae Mirb.
Iz_3_1109	DJI_0101	Populus Ã canescens (Aiton) Sm.	0	n	1	1	Salicaceae Mirb.
Iz_3_1109	DJI_0101	Potentilla argentea L.	0	n	1	0	Rosaceae Juss.
Iz_3_1109	DJI_0101	Psammophiliella muralis (L.) Ikonn.	0	n	1	0	Caryophyllaceae Juss.
Iz_3_1109	DJI_0101	Psephellus sumensis (Kalen.) Greuter	0	n	1	0	Asteraceae Bercht. & J.Presl
Iz_3_1109	DJI_0101	Pulsatilla pratensis (L.) Mill. s.l.	0	n	1	0	Ranunculaceae Juss.
Iz_3_1109	DJI_0101	Pyrus communis L.	0	a	0	1	Rosaceae Juss.
Iz_3_1109	DJI_0101	Quercus robur L.	0	n	1	1	Fagaceae Dumort.
Iz_3_1109	DJI_0101	Robinia pseudoacacia L.	0	a	0	1	Fabaceae Lindl.
Iz_3_1109	DJI_0101	Rosa sp.	0		NA	1	Rosaceae Juss.
Iz_3_1109	DJI_0101	Rubus idaeus L.	0	n	1	0	Rosaceae Juss.
Iz_3_1109	DJI_0101	Rumex acetosella L.	0	n	1	0	Polygonaceae Juss.
Iz_3_1109	DJI_0101	Rumex thyrsiflorus Fingerh.	0	n	1	0	Polygonaceae Juss.
Iz_3_1109	DJI_0101	Salix alba L.	0	n	1	1	Salicaceae Mirb.
Iz_3_1109	DJI_0101	Sambucus nigra L.	0	n	1	1	Viburnaceae Raf.
Iz_3_1109	DJI_0101	Secale sylvestre Host	0	n	1	0	Poaceae Barnhart
Iz_3_1109	DJI_0101	Sempervivum ruthenicum Schnittsp. & C.B. Lehm.	0	n	1	0	Crassulaceae J.St.-Hil.
Iz_3_1109	DJI_0101	Setaria viridis (L.) P. Beauv.	0	n	1	0	Poaceae Barnhart
Iz_3_1109	DJI_0101	Silene borysthenica (Gruner) Walters	0	n	1	0	Caryophyllaceae Juss.
Iz_3_1109	DJI_0101	Silene latifolia subsp. alba (Mill.) Greuter & Burdet	0	n	1	0	Caryophyllaceae Juss.
Iz_3_1109	DJI_0101	Solidago virgaurea L.	0	n	1	0	Asteraceae Bercht. & J.Presl
Iz_3_1109	DJI_0101	Sorbus aucuparia L.	0	n	1	1	Rosaceae Juss.
Iz_3_1109	DJI_0101	Species 11.09_3_0045	0		NA	0	
Iz_3_1109	DJI_0101	Species 11.09_3_0058	0		NA	0	
Iz_3_1109	DJI_0101	Stachys recta L.	0	n	1	0	Lamiacea Lindl.
Iz_3_1109	DJI_0101	Tanacetum vulgare L.	0	n	1	0	Asteraceae Bercht. & J.Presl
Iz_3_1109	DJI_0101	Thymus pallasianus Heinr.Braun	0	n	1	0	Lamiacea Lindl.
Iz_3_1109	DJI_0101	Tilia cordata Mill.	0	n	1	1	Malvaceae Juss.
Iz_3_1109	DJI_0101	Trifolium alpestre L.	0	n	1	0	Fabaceae Lindl.
Iz_3_1109	DJI_0101	Trifolium arvense L.	0	n	1	0	Fabaceae Lindl.
Iz_3_1109	DJI_0101	Ulmus glabra Huds.	0	n	1	1	Ulmaceae Mirb.
Iz_3_1109	DJI_0101	Verbascum densiflorum Bertol.	0	n	1	0	Scrophulariaceae Juss.
Iz_3_1109	DJI_0101	Veronica austriaca L.	0	n	1	0	Plantaginaceae Juss.
Iz_3_1109	DJI_0101	Veronica incana L.	0	n	1	0	Plantaginaceae Juss.
Iz_3_1109	DJI_0101	Veronica verna L.	0	n	1	0	Plantaginaceae Juss.
Iz_3_1109	DJI_0101	Vincetoxicum hirundinaria Medik	0	n	1	0	Apocynaceae Juss.
Iz_3_1109	DJI_0104	Acer tataricum L.	0	n	1	1	Sapindaceae Juss.
Iz_3_1109	DJI_0104	Achillea millefolium L. s.l.	0	n	1	0	Asteraceae Bercht. & J.Presl

Iz_3_1109	DJI_0104	Allium oleraceum L.	0	n	1	0	Amaryllidaceae J.St.-Hil.
Iz_3_1109	DJI_0104	Ambrosia artemisiifolia L.	0	a	0	0	Asteraceae Bercht. & J.Presl
Iz_3_1109	DJI_0104	Amorpha fruticosa L.	0	a	0	0	Fabaceae Lindl.
Iz_3_1109	DJI_0104	Anthericum ramosum L.	0	n	1	0	Asparagaceae Juss.
Iz_3_1109	DJI_0104	Artemisia marschalliana Spreng.	0	n	1	0	Asteraceae Bercht. & J.Presl
Iz_3_1109	DJI_0104	Asparagus officinalis L.	0	n	1	0	Asparagaceae Juss.
Iz_3_1109	DJI_0104	Astragalus cicer L.	0	n	1	0	Fabaceae Lindl.
Iz_3_1109	DJI_0104	Bassia laniflora (S.G.Gmel.) A.J.Scott	0	n	1	0	Amaranthaceae Juss.
Iz_3_1109	DJI_0104	Berberis vulgaris L.	0	n	1	0	Berberidaceae Juss.
Iz_3_1109	DJI_0104	Betula pendula Roth.	0	n	1	1	Betulaceae Gray
Iz_3_1109	DJI_0104	Betula pendula Roth.	0	n	1	0	Poaceae Barnhart
Iz_3_1109	DJI_0104	Bromus inermis Leyss.	0		NA	NA	
Iz_3_1109	DJI_0104	Bromus squarrosus L.	0	a	0	0	Poaceae Barnhart
Iz_3_1109	DJI_0104	Bromus tectorum L.	0	a	0	0	Poaceae Barnhart
Iz_3_1109	DJI_0104	Calamagrostis epigejos (L.) Roth	0.2	n	1	0	Poaceae Barnhart
Iz_3_1109	DJI_0104	Carduus acanthoides L.	0	n	1	0	Asteraceae Bercht. & J.Presl
Iz_3_1109	DJI_0104	Carex sp.	0	n	1	0	Cyperaceae Juss.
Iz_3_1109	DJI_0104	Centaurea majorovii Dumbadze	0	n	1	0	Asteraceae Bercht. & J.Presl
Iz_3_1109	DJI_0104	Chamaecytisus ruthenicus (Fisch.ex Wol.) Klaskova	0	n	1	0	Fabaceae Lindl.
Iz_3_1109	DJI_0104	Chelidonium majus L.	0	n	1	0	Papaveraceae Juss.
Iz_3_1109	DJI_0104	Chenopodium betaceum Andrz.	0	n	1	0	Amaranthaceae Juss.
Iz_3_1109	DJI_0104	Chenopodium cf. album	0	n	1	0	Amaranthaceae Juss.
Iz_3_1109	DJI_0104	Chenopodium sp.	0		NA	0	Amaranthaceae Juss.
Iz_3_1109	DJI_0104	Chondrilla juncea L.	0	n	1	0	Asteraceae Bercht. & J.Presl
Iz_3_1109	DJI_0104	Cirsium vulgare (Savi) Ten.	0	n	1	0	Asteraceae Bercht. & J.Presl
Iz_3_1109	DJI_0104	Clinopodium acinos (L.) Kuntze	0	n	1	0	Lamiacea Lindl.
Iz_3_1109	DJI_0104	Convallaria majalis L.	0	n	1	0	Asparagaceae Juss.
Iz_3_1109	DJI_0104	Crataegus rhipidophylla Gand.	0	n	1	1	Rosaceae Juss.
Iz_3_1109	DJI_0104	Crepis foetida L.	0	n	1	0	Asteraceae Bercht. & J.Presl
Iz_3_1109	DJI_0104	Dactylis glomerata L.	0	n	1	0	Poaceae Barnhart
Iz_3_1109	DJI_0104	Dianthus campestris M.Bieb.	0	n	1	0	Caryophyllaceae Juss.
Iz_3_1109	DJI_0104	Digitaria sanguinalis (L.) Scop.	0	a	0	0	Poaceae Barnhart
Iz_3_1109	DJI_0104	Elymus repens (L.) Gould	0	n	1	0	Poaceae Barnhart
Iz_3_1109	DJI_0104	Epilobium tetragonum L.	0	n	1	0	Onagraceae Juss.
Iz_3_1109	DJI_0104	Eragrostis minor Host	0	a	0	0	Poaceae Barnhart
Iz_3_1109	DJI_0104	Erigeron canadensis L.	7	a	0	0	Asteraceae Bercht. & J.Presl
Iz_3_1109	DJI_0104	Euphorbia seguieriana Neck.	0	n	1	0	Euphorbiaceae Juss.
Iz_3_1109	DJI_0104	Fallopia dumetorum (L.) Holub	2	n	1	0	Polygonaceae Juss.
Iz_3_1109	DJI_0104	Festuca beckeri (Hack.) Trautv.	0	n	1	0	Poaceae Barnhart
Iz_3_1109	DJI_0104	Fragaria vesca L.	0	n	1	0	Rosaceae Juss.
Iz_3_1109	DJI_0104	Frangula alnus Mill.	0	n	1	1	Rhamnaceae Juss.
Iz_3_1109	DJI_0104	Fraxinus pennsylvanica Marshall	0	a	0	1	Oleaceae Hoffmanns. & Link
Iz_3_1109	DJI_0104	Galeopsis bifida Boenn.	0	n	1	0	Lamiacea Lindl.
Iz_3_1109	DJI_0104	Galium verum L.	0	n	1	0	Rubiaceae Juss.
Iz_3_1109	DJI_0104	Genista tinctoria L.	0	n	1	0	Fabaceae Lindl.
Iz_3_1109	DJI_0104	Geranium robertianum L.	0	n	1	0	Geraniaceae Juss.
Iz_3_1109	DJI_0104	Geranium sanquineum L.	0	n	1	0	Geraniaceae Juss.
Iz_3_1109	DJI_0104	Gypsophila paniculata L.	0	n	1	0	Caryophyllaceae Juss.
Iz_3_1109	DJI_0104	Helichrysum arenarium (L.) Moench	0	n	1	0	Asteraceae Bercht. & J.Presl
Iz_3_1109	DJI_0104	Herniaria polygama J.Gay	0	n	1	0	Caryophyllaceae Juss.
Iz_3_1109	DJI_0104	Hieracium robustum Fr.	0	n	1	0	Asteraceae Bercht. & J.Presl
Iz_3_1109	DJI_0104	Hieracium umbellatum L.	0	n	1	NA	Asteraceae Bercht. & J.Presl
Iz_3_1109	DJI_0104	Hylotelephium maximum (L.) Holub	0	n	1	0	Crassulaceae J.St.-Hil.
Iz_3_1109	DJI_0104	Jacobaea borysthenica (DC.) B.Nord. & Greuter	0	n	1	0	Asteraceae Bercht. & J.Presl
Iz_3_1109	DJI_0104	Jasione montana L.	0	n	1	0	Campanulaceae Juss.
Iz_3_1109	DJI_0104	Jurinea cyanoides (L.) Rchb.s.l.	0	n	1	0	Asteraceae Bercht. & J.Presl
Iz_3_1109	DJI_0104	Koeleria glauca (Spreng.) DC.	0	n	1	0	Poaceae Barnhart
Iz_3_1109	DJI_0104	Lactuca serriola L.	0	a	0	0	Asteraceae Bercht. & J.Presl
Iz_3_1109	DJI_0104	Linaria genistifolia (L.) Mill.	0	n	1	0	Plantaginaceae Juss.
Iz_3_1109	DJI_0104	Lomelosia argentea (L.) Greuter & Burdet	0	n	1	0	Caprifoliaceae Juss.
Iz_3_1109	DJI_0104	Melampyrum pratense L.	0	n	1	0	Orobanchaceae Vent.
Iz_3_1109	DJI_0104	Oenothera biennis L.	0	a	0	0	Onagraceae Juss.
Iz_3_1109	DJI_0104	Pentanema britannica (L.) D.Gut.Larr., Santos-Vicente, Anderb., E.Rico & M.M.Mart.Ort.	0	n	1	0	Asteraceae Bercht. & J.Presl
Iz_3_1109	DJI_0104	Peucedanum oreoselinum (L.) Moench	0	n	1	0	Apiaceae Lindl.
Iz_3_1109	DJI_0104	Peucedanum ruthenicum M.Bieb.	0	n	1	0	Apiaceae Lindl.
Iz_3_1109	DJI_0104	Pilosella echioides (Lumn.) F.W.Schultz & Sch.Bip.	0	n	1	0	Asteraceae Bercht. & J.Presl
Iz_3_1109	DJI_0104	Pilosella officinarum F. Schultz et Sch. Bip.	0	n	1	0	Asteraceae Bercht. & J.Presl
Iz_3_1109	DJI_0104	Pilosella sp.	0		NA	0	Asteraceae Bercht. & J.Presl
Iz_3_1109	DJI_0104	Pinus sylvestris L.	0	n	1	1	Pinaceae Spreng. ex F.Rudolphi
Iz_3_1109	DJI_0104	Poa annua L.	0	n	1	0	Poaceae Barnhart
Iz_3_1109	DJI_0104	Poa nemoralis L.	0	n	1	0	Poaceae Barnhart
Iz_3_1109	DJI_0104	Poa sp.	0		NA	0	Poaceae Barnhart
Iz_3_1109	DJI_0104	Polygonatum odoratum (Mill.) Druce	0	n	1	0	Asparagaceae Juss.
Iz_3_1109	DJI_0104	Polygonum arenarium Waldst. & Kit.	0	n	1	0	Polygonaceae Juss.
Iz_3_1109	DJI_0104	Populus nigra L.	0	n	1	1	Salicaceae Mirb.
Iz_3_1109	DJI_0104	Populus tremula L.	0	n	1	1	Salicaceae Mirb.
Iz_3_1109	DJI_0104	Populus Ã canescens (Aiton) Sm.	0	n	1	1	Salicaceae Mirb.
Iz_3_1109	DJI_0104	Potentilla argentea L.	0	n	1	0	Rosaceae Juss.
Iz_3_1109	DJI_0104	Psammophiliella muralis (L.) Ikonn.	0	n	1	0	Caryophyllaceae Juss.
Iz_3_1109	DJI_0104	Psephellus sumensis (Kalen.) Greuter	0	n	1	0	Asteraceae Bercht. & J.Presl
Iz_3_1109	DJI_0104	Pulsatilla pratensis (L.) Mill. s.l.	0	n	1	0	Ranunculaceae Juss.
Iz_3_1109	DJI_0104	Pyrus communis L.	0	a	0	1	Rosaceae Juss.
Iz_3_1109	DJI_0104	Quercus robur L.	0	n	1	1	Fagaceae Dumort.
Iz_3_1109	DJI_0104	Robinia pseudoacacia L.	0	a	0	1	Fabaceae Lindl.
Iz_3_1109	DJI_0104	Rosa sp.	0		NA	1	Rosaceae Juss.
Iz_3_1109	DJI_0104	Rubus idaeus L.	0	n	1	0	Rosaceae Juss.
Iz_3_1109	DJI_0104	Rumex acetosella L.	0	n	1	0	Polygonaceae Juss.
Iz_3_1109	DJI_0104	Rumex thyrsiflorus Fingerh.	0	n	1	0	Polygonaceae Juss.
Iz_3_1109	DJI_0104	Salix alba L.	0	n	1	1	Salicaceae Mirb.
Iz_3_1109	DJI_0104	Sambucus nigra L.	0	n	1	1	Viburnaceae Raf.
Iz_3_1109	DJI_0104	Secale sylvestre Host	0	n	1	0	Poaceae Barnhart
Iz_3_1109	DJI_0104	Sempervivum ruthenicum Schnittsp. & C.B. Lehm.	0	n	1	0	Crassulaceae J.St.-Hil.
Iz_3_1109	DJI_0104	Setaria viridis (L.) P. Beauv.	0	n	1	0	Poaceae Barnhart
Iz_3_1109	DJI_0104	Silene borysthenica (Gruner) Walters	0	n	1	0	Caryophyllaceae Juss.
Iz_3_1109	DJI_0104	Silene latifolia subsp. alba (Mill.) Greuter & Burdet	0	n	1	0	Caryophyllaceae Juss.
Iz_3_1109	DJI_0104	Solidago virgaurea L.	0	n	1	0	Asteraceae Bercht. & J.Presl
Iz_3_1109	DJI_0104	Sorbus aucuparia L.	0	n	1	1	Rosaceae Juss.
Iz_3_1109	DJI_0104	Species 11.09_3_0045	0		NA	0	

Iz_3_1109	DJI_0104	Species 11.09_3_0058	0		NA	0	
Iz_3_1109	DJI_0104	Stachys recta L.	0	n	1	0	Lamiacea Lindl.
Iz_3_1109	DJI_0104	Tanacetum vulgare L.	0	n	1	0	Asteraceae Bercht. & J.Presl
Iz_3_1109	DJI_0104	Thymus pallasianus Heinr.Braun	0	n	1	0	Lamiacea Lindl.
Iz_3_1109	DJI_0104	Tilia cordata Mill.	0	n	1	1	Malvaceae Juss.
Iz_3_1109	DJI_0104	Trifolium alpestre L.	0	n	1	0	Fabaceae Lindl.
Iz_3_1109	DJI_0104	Trifolium arvense L.	0	n	1	0	Fabaceae Lindl.
Iz_3_1109	DJI_0104	Ulmus glabra Huds.	0	n	1	1	Ulmaceae Mirb.
Iz_3_1109	DJI_0104	Verbascum densiflorum Bertol.	0	n	1	0	Scrophulariaceae Juss.
Iz_3_1109	DJI_0104	Veronica austriaca L.	0	n	1	0	Plantaginaceae Juss.
Iz_3_1109	DJI_0104	Veronica incana L.	0	n	1	0	Plantaginaceae Juss.
Iz_3_1109	DJI_0104	Veronica verna L.	0	n	1	0	Plantaginaceae Juss.
Iz_3_1109	DJI_0104	Vincetoxicum hirundinaria Medik	0	n	1	0	Apocynaceae Juss.
Iz_3_1109	DJI_0107	Acer tataricum L.	0	n	1	1	Sapindaceae Juss.
Iz_3_1109	DJI_0107	Achillea millefolium L. s.l.	0	n	1	0	Asteraceae Bercht. & J.Presl
Iz_3_1109	DJI_0107	Allium oleraceum L.	0	n	1	0	Amaryllidaceae J.St.-Hil.
Iz_3_1109	DJI_0107	Ambrosia artemisiifolia L.	0	a	0	0	Asteraceae Bercht. & J.Presl
Iz_3_1109	DJI_0107	Amorpha fruticosa L.	0	a	0	0	Fabaceae Lindl.
Iz_3_1109	DJI_0107	Anthericum ramosum L.	0	n	1	0	Asparagaceae Juss.
Iz_3_1109	DJI_0107	Artemisia marschalliana Spreng.	0	n	1	0	Asteraceae Bercht. & J.Presl
Iz_3_1109	DJI_0107	Asparagus officinalis L.	0	n	1	0	Asparagaceae Juss.
Iz_3_1109	DJI_0107	Astragalus cicer L.	0	n	1	0	Fabaceae Lindl.
Iz_3_1109	DJI_0107	Bassia laniflora (S.G.Gmel.) A.J.Scott	0	n	1	0	Amaranthaceae Juss.
Iz_3_1109	DJI_0107	Berberis vulgaris L.	0	n	1	0	Berberidaceae Juss.
Iz_3_1109	DJI_0107	Betula pendula Roth.	0	n	1	1	Betulaceae Gray
Iz_3_1109	DJI_0107	Betula pendula Roth.	0	n	1	0	Poaceae Barnhart
Iz_3_1109	DJI_0107	Bromus inermis Leyss.	0		NA	NA	
Iz_3_1109	DJI_0107	Bromus squarrosus L.	0	a	0	0	Poaceae Barnhart
Iz_3_1109	DJI_0107	Bromus tectorum L.	0	a	0	0	Poaceae Barnhart
Iz_3_1109	DJI_0107	Calamagrostis epigejos (L.) Roth	0.1	n	1	0	Poaceae Barnhart
Iz_3_1109	DJI_0107	Carduus acanthoides L.	0	n	1	0	Asteraceae Bercht. & J.Presl
Iz_3_1109	DJI_0107	Carex sp.	0.5	n	1	0	Cyperaceae Juss.
Iz_3_1109	DJI_0107	Centaurea majorovii Dumbadze	0	n	1	0	Asteraceae Bercht. & J.Presl
Iz_3_1109	DJI_0107	Chamaecytisus ruthenicus (Fisch.ex Wol.) Klaskova	0	n	1	0	Fabaceae Lindl.
Iz_3_1109	DJI_0107	Chelidonium majus L.	0	n	1	0	Papaveraceae Juss.
Iz_3_1109	DJI_0107	Chenopodium betaceum Andrz.	0	n	1	0	Amaranthaceae Juss.
Iz_3_1109	DJI_0107	Chenopodium cf. album	0	n	1	0	Amaranthaceae Juss.
Iz_3_1109	DJI_0107	Chenopodium sp.	0		NA	0	Amaranthaceae Juss.
Iz_3_1109	DJI_0107	Chondrilla juncea L.	0	n	1	0	Asteraceae Bercht. & J.Presl
Iz_3_1109	DJI_0107	Cirsium vulgare (Savi) Ten.	0	n	1	0	Asteraceae Bercht. & J.Presl
Iz_3_1109	DJI_0107	Clinopodium acinos (L.) Kuntze	0	n	1	0	Lamiacea Lindl.
Iz_3_1109	DJI_0107	Convallaria majalis L.	0	n	1	0	Asparagaceae Juss.
Iz_3_1109	DJI_0107	Crataegus rhipidophylla Gand.	0	n	1	1	Rosaceae Juss.
Iz_3_1109	DJI_0107	Crepis foetida L.	0	n	1	0	Asteraceae Bercht. & J.Presl
Iz_3_1109	DJI_0107	Dactylis glomerata L.	0	n	1	0	Poaceae Barnhart
Iz_3_1109	DJI_0107	Dianthus campestris M.Bieb.	0	n	1	0	Caryophyllaceae Juss.
Iz_3_1109	DJI_0107	Digitaria sanguinalis (L.) Scop.	0	a	0	0	Poaceae Barnhart
Iz_3_1109	DJI_0107	Elymus repens (L.) Gould	0	n	1	0	Poaceae Barnhart
Iz_3_1109	DJI_0107	Epilobium tetragonum L.	0	n	1	0	Onagraceae Juss.
Iz_3_1109	DJI_0107	Eragrostis minor Host	0	a	0	0	Poaceae Barnhart
Iz_3_1109	DJI_0107	Erigeron canadensis L.	0.5	a	0	0	Asteraceae Bercht. & J.Presl
Iz_3_1109	DJI_0107	Euphorbia seguieriana Neck.	0	n	1	0	Euphorbiaceae Juss.
Iz_3_1109	DJI_0107	Fallopia dumetorum (L.) Holub	0	n	1	0	Polygonaceae Juss.
Iz_3_1109	DJI_0107	Festuca beckeri (Hack.) Trautv.	0	n	1	0	Poaceae Barnhart
Iz_3_1109	DJI_0107	Fragaria vesca L.	0	n	1	0	Rosaceae Juss.
Iz_3_1109	DJI_0107	Frangula alnus Mill.	0	n	1	1	Rhamnaceae Juss.
Iz_3_1109	DJI_0107	Fraxinus pennsylvanica Marshall	0	a	0	1	Oleaceae Hoffmanns. & Link
Iz_3_1109	DJI_0107	Galeopsis bifida Boenn.	0	n	1	0	Lamiacea Lindl.
Iz_3_1109	DJI_0107	Galium verum L.	0	n	1	0	Rubiaceae Juss.
Iz_3_1109	DJI_0107	Genista tinctoria L.	0	n	1	0	Fabaceae Lindl.
Iz_3_1109	DJI_0107	Geranium robertianum L.	0	n	1	0	Geraniaceae Juss.
Iz_3_1109	DJI_0107	Geranium sanquineum L.	0	n	1	0	Geraniaceae Juss.
Iz_3_1109	DJI_0107	Gypsophila paniculata L.	0	n	1	0	Caryophyllaceae Juss.
Iz_3_1109	DJI_0107	Helichrysum arenarium (L.) Moench	0	n	1	0	Asteraceae Bercht. & J.Presl
Iz_3_1109	DJI_0107	Herniaria polygama J.Gay	0	n	1	0	Caryophyllaceae Juss.
Iz_3_1109	DJI_0107	Hieracium robustum Fr.	0	n	1	0	Asteraceae Bercht. & J.Presl
Iz_3_1109	DJI_0107	Hieracium umbellatum L.	0	n	1	NA	Asteraceae Bercht. & J.Presl
Iz_3_1109	DJI_0107	Hylotelephium maximum (L.) Holub	0	n	1	0	Crassulaceae J.St.-Hil.
Iz_3_1109	DJI_0107	Jacobaea borysthenica (DC.) B.Nord. & Greuter	0	n	1	0	Asteraceae Bercht. & J.Presl
Iz_3_1109	DJI_0107	Jasione montana L.	0	n	1	0	Campanulaceae Juss.
Iz_3_1109	DJI_0107	Jurinea cyanoides (L.) Rchb.s.l.	0	n	1	0	Asteraceae Bercht. & J.Presl
Iz_3_1109	DJI_0107	Koeleria glauca (Spreng.) DC.	0	n	1	0	Poaceae Barnhart
Iz_3_1109	DJI_0107	Lactuca serriola L.	0	a	0	0	Asteraceae Bercht. & J.Presl
Iz_3_1109	DJI_0107	Linaria genistifolia (L.) Mill.	0	n	1	0	Plantaginaceae Juss.
Iz_3_1109	DJI_0107	Lomelosia argentea (L.) Greuter & Burdet	0	n	1	0	Caprifoliaceae Juss.
Iz_3_1109	DJI_0107	Melampyrum pratense L.	0	n	1	0	Orobanchaceae Vent.
Iz_3_1109	DJI_0107	Oenothera biennis L.	0	a	0	0	Onagraceae Juss.
Iz_3_1109	DJI_0107	Pentanema britannica (L.) D.Gut.Larr., Santos-Vicente, Anderb., E.Rico & M.M.Mart.Ort.	0	n	1	0	Asteraceae Bercht. & J.Presl
Iz_3_1109	DJI_0107	Peucedanum oreoselinum (L.) Moench	0	n	1	0	Apiaceae Lindl.
Iz_3_1109	DJI_0107	Peucedanum ruthenicum M.Bieb.	0	n	1	0	Apiaceae Lindl.
Iz_3_1109	DJI_0107	Pilosella echioides (Lumn.) F.W.Schultz & Sch.Bip.	0	n	1	0	Asteraceae Bercht. & J.Presl
Iz_3_1109	DJI_0107	Pilosella officinarum F. Schultz et Sch. Bip.	0	n	1	0	Asteraceae Bercht. & J.Presl
Iz_3_1109	DJI_0107	Pilosella sp.	0		NA	0	Asteraceae Bercht. & J.Presl
Iz_3_1109	DJI_0107	Pinus sylvestris L.	0	n	1	1	Pinaceae Spreng. ex F.Rudolphi
Iz_3_1109	DJI_0107	Poa annua L.	0	n	1	0	Poaceae Barnhart
Iz_3_1109	DJI_0107	Poa nemoralis L.	0	n	1	0	Poaceae Barnhart
Iz_3_1109	DJI_0107	Poa sp.	0		NA	0	Poaceae Barnhart
Iz_3_1109	DJI_0107	Polygonatum odoratum (Mill.) Druce	0	n	1	0	Asparagaceae Juss.
Iz_3_1109	DJI_0107	Polygonum arenarium Waldst. & Kit.	0	n	1	0	Polygonaceae Juss.
Iz_3_1109	DJI_0107	Populus nigra L.	0	n	1	1	Salicaceae Mirb.
Iz_3_1109	DJI_0107	Populus tremula L.	0	n	1	1	Salicaceae Mirb.
Iz_3_1109	DJI_0107	Populus Ã canescens (Aiton) Sm.	0	n	1	1	Salicaceae Mirb.
Iz_3_1109	DJI_0107	Potentilla argentea L.	0	n	1	0	Rosaceae Juss.
Iz_3_1109	DJI_0107	Psammophiliella muralis (L.) Ikonn.	0	n	1	0	Caryophyllaceae Juss.
Iz_3_1109	DJI_0107	Psephellus sumensis (Kalen.) Greuter	0	n	1	0	Asteraceae Bercht. & J.Presl
Iz_3_1109	DJI_0107	Pulsatilla pratensis (L.) Mill. s.l.	0	n	1	0	Ranunculaceae Juss.
Iz_3_1109	DJI_0107	Pyrus communis L.	0	a	0	1	Rosaceae Juss.
Iz_3_1109	DJI_0107	Quercus robur L.	0	n	1	1	Fagaceae Dumort.

Iz_3_1109	DJI_0107	Robinia pseudoacacia L.	0	a	0	1	Fabaceae Lindl.
Iz_3_1109	DJI_0107	Rosa sp.	0		NA	1	Rosaceae Juss.
Iz_3_1109	DJI_0107	Rubus idaeus L.	0	n	1	0	Rosaceae Juss.
Iz_3_1109	DJI_0107	Rumex acetosella L.	0	n	1	0	Polygonaceae Juss.
Iz_3_1109	DJI_0107	Rumex thyrsiflorus Fingerh.	0	n	1	0	Polygonaceae Juss.
Iz_3_1109	DJI_0107	Salix alba L.	0	n	1	1	Salicaceae Mirb.
Iz_3_1109	DJI_0107	Sambucus nigra L.	0	n	1	1	Viburnaceae Raf.
Iz_3_1109	DJI_0107	Secale sylvestre Host	0	n	1	0	Poaceae Barnhart
Iz_3_1109	DJI_0107	Sempervivum ruthenicum Schnittsp. & C.B. Lehm.	0	n	1	0	Crassulaceae J.St.-Hil.
Iz_3_1109	DJI_0107	Setaria viridis (L.) P. Beauv.	0	n	1	0	Poaceae Barnhart
Iz_3_1109	DJI_0107	Silene borysthenica (Gruner) Walters	0	n	1	0	Caryophyllaceae Juss.
Iz_3_1109	DJI_0107	Silene latifolia subsp. alba (Mill.) Greuter & Burdet	0	n	1	0	Caryophyllaceae Juss.
Iz_3_1109	DJI_0107	Solidago virgaurea L.	0	n	1	0	Asteraceae Bercht. & J.Presl
Iz_3_1109	DJI_0107	Sorbus aucuparia L.	0	n	1	1	Rosaceae Juss.
Iz_3_1109	DJI_0107	Species 11.09_3_0045	0		NA	0	
Iz_3_1109	DJI_0107	Species 11.09_3_0058	0		NA	0	
Iz_3_1109	DJI_0107	Stachys recta L.	0	n	1	0	Lamiacea Lindl.
Iz_3_1109	DJI_0107	Tanacetum vulgare L.	0	n	1	0	Asteraceae Bercht. & J.Presl
Iz_3_1109	DJI_0107	Thymus pallasianus Heinr.Braun	0	n	1	0	Lamiacea Lindl.
Iz_3_1109	DJI_0107	Tilia cordata Mill.	0	n	1	1	Malvaceae Juss.
Iz_3_1109	DJI_0107	Trifolium alpestre L.	0	n	1	0	Fabaceae Lindl.
Iz_3_1109	DJI_0107	Trifolium arvense L.	0	n	1	0	Fabaceae Lindl.
Iz_3_1109	DJI_0107	Ulmus glabra Huds.	0	n	1	1	Ulmaceae Mirb.
Iz_3_1109	DJI_0107	Verbascum densiflorum Bertol.	0	n	1	0	Scrophulariaceae Juss.
Iz_3_1109	DJI_0107	Veronica austriaca L.	0	n	1	0	Plantaginaceae Juss.
Iz_3_1109	DJI_0107	Veronica incana L.	0	n	1	0	Plantaginaceae Juss.
Iz_3_1109	DJI_0107	Veronica verna L.	0	n	1	0	Plantaginaceae Juss.
Iz_3_1109	DJI_0107	Vincetoxicum hirundinaria Medik	0	n	1	0	Apocynaceae Juss.
Iz_3_1109	DJI_0110	Acer tataricum L.	0	n	1	1	Sapindaceae Juss.
Iz_3_1109	DJI_0110	Achillea millefolium L. s.l.	0	n	1	0	Asteraceae Bercht. & J.Presl
Iz_3_1109	DJI_0110	Allium oleraceum L.	0	n	1	0	Amaryllidaceae J.St.-Hil.
Iz_3_1109	DJI_0110	Ambrosia artemisiifolia L.	0	a	0	0	Asteraceae Bercht. & J.Presl
Iz_3_1109	DJI_0110	Amorpha fruticosa L.	0	a	0	0	Fabaceae Lindl.
Iz_3_1109	DJI_0110	Anthericum ramosum L.	0	n	1	0	Asparagaceae Juss.
Iz_3_1109	DJI_0110	Artemisia marschalliana Spreng.	0	n	1	0	Asteraceae Bercht. & J.Presl
Iz_3_1109	DJI_0110	Asparagus officinalis L.	0	n	1	0	Asparagaceae Juss.
Iz_3_1109	DJI_0110	Astragalus cicer L.	0	n	1	0	Fabaceae Lindl.
Iz_3_1109	DJI_0110	Bassia laniflora (S.G.Gmel.) A.J.Scott	0	n	1	0	Amaranthaceae Juss.
Iz_3_1109	DJI_0110	Berberis vulgaris L.	0	n	1	0	Berberidaceae Juss.
Iz_3_1109	DJI_0110	Betula pendula Roth.	0	n	1	1	Betulaceae Gray
Iz_3_1109	DJI_0110	Betula pendula Roth.	0	n	1	0	Poaceae Barnhart
Iz_3_1109	DJI_0110	Bromus inermis Leyss.	0		NA	NA	
Iz_3_1109	DJI_0110	Bromus squarrosus L.	0	a	0	0	Poaceae Barnhart
Iz_3_1109	DJI_0110	Bromus tectorum L.	0	a	0	0	Poaceae Barnhart
Iz_3_1109	DJI_0110	Calamagrostis epigejos (L.) Roth	0	n	1	0	Poaceae Barnhart
Iz_3_1109	DJI_0110	Carduus acanthoides L.	0	n	1	0	Asteraceae Bercht. & J.Presl
Iz_3_1109	DJI_0110	Carex sp.	0	n	1	0	Cyperaceae Juss.
Iz_3_1109	DJI_0110	Centaurea majorovii Dumbadze	0	n	1	0	Asteraceae Bercht. & J.Presl
Iz_3_1109	DJI_0110	Chamaecytisus ruthenicus (Fisch.ex Wol.) Klaskova	0	n	1	0	Fabaceae Lindl.
Iz_3_1109	DJI_0110	Chelidonium majus L.	0	n	1	0	Papaveraceae Juss.
Iz_3_1109	DJI_0110	Chenopodium betaceum Andrz.	0	n	1	0	Amaranthaceae Juss.
Iz_3_1109	DJI_0110	Chenopodium cf. album	0	n	1	0	Amaranthaceae Juss.
Iz_3_1109	DJI_0110	Chenopodium sp.	0		NA	0	Amaranthaceae Juss.
Iz_3_1109	DJI_0110	Chondrilla juncea L.	0	n	1	0	Asteraceae Bercht. & J.Presl
Iz_3_1109	DJI_0110	Cirsium vulgare (Savi) Ten.	0	n	1	0	Asteraceae Bercht. & J.Presl
Iz_3_1109	DJI_0110	Clinopodium acinos (L.) Kuntze	0	n	1	0	Lamiacea Lindl.
Iz_3_1109	DJI_0110	Convallaria majalis L.	0	n	1	0	Asparagaceae Juss.
Iz_3_1109	DJI_0110	Crataegus rhipidophylla Gand.	0	n	1	1	Rosaceae Juss.
Iz_3_1109	DJI_0110	Crepis foetida L.	0	n	1	0	Asteraceae Bercht. & J.Presl
Iz_3_1109	DJI_0110	Dactylis glomerata L.	0	n	1	0	Poaceae Barnhart
Iz_3_1109	DJI_0110	Dianthus campestris M.Bieb.	0	n	1	0	Caryophyllaceae Juss.
Iz_3_1109	DJI_0110	Digitaria sanguinalis (L.) Scop.	0	a	0	0	Poaceae Barnhart
Iz_3_1109	DJI_0110	Elymus repens (L.) Gould	0	n	1	0	Poaceae Barnhart
Iz_3_1109	DJI_0110	Epilobium tetragonum L.	0	n	1	0	Onagraceae Juss.
Iz_3_1109	DJI_0110	Eragrostis minor Host	0	a	0	0	Poaceae Barnhart
Iz_3_1109	DJI_0110	Erigeron canadensis L.	0	a	0	0	Asteraceae Bercht. & J.Presl
Iz_3_1109	DJI_0110	Euphorbia seguieriana Neck.	0	n	1	0	Euphorbiaceae Juss.
Iz_3_1109	DJI_0110	Fallopia dumetorum (L.) Holub	0	n	1	0	Polygonaceae Juss.
Iz_3_1109	DJI_0110	Festuca beckeri (Hack.) Trautv.	0	n	1	0	Poaceae Barnhart
Iz_3_1109	DJI_0110	Fragaria vesca L.	0	n	1	0	Rosaceae Juss.
Iz_3_1109	DJI_0110	Frangula alnus Mill.	0	n	1	1	Rhamnaceae Juss.
Iz_3_1109	DJI_0110	Fraxinus pennsylvanica Marshall	0	a	0	1	Oleaceae Hoffmanns. & Link
Iz_3_1109	DJI_0110	Galeopsis bifida Boenn.	0	n	1	0	Lamiacea Lindl.
Iz_3_1109	DJI_0110	Galium verum L.	0	n	1	0	Rubiaceae Juss.
Iz_3_1109	DJI_0110	Genista tinctoria L.	0	n	1	0	Fabaceae Lindl.
Iz_3_1109	DJI_0110	Geranium robertianum L.	0	n	1	0	Geraniaceae Juss.
Iz_3_1109	DJI_0110	Geranium sanquineum L.	0	n	1	0	Geraniaceae Juss.
Iz_3_1109	DJI_0110	Gypsophila paniculata L.	0	n	1	0	Caryophyllaceae Juss.
Iz_3_1109	DJI_0110	Helichrysum arenarium (L.) Moench	0	n	1	0	Asteraceae Bercht. & J.Presl
Iz_3_1109	DJI_0110	Herniaria polygama J.Gay	0	n	1	0	Caryophyllaceae Juss.
Iz_3_1109	DJI_0110	Hieracium robustum Fr.	0	n	1	0	Asteraceae Bercht. & J.Presl
Iz_3_1109	DJI_0110	Hieracium umbellatum L.	0	n	1	NA	Asteraceae Bercht. & J.Presl
Iz_3_1109	DJI_0110	Hylotelephium maximum (L.) Holub	0	n	1	0	Crassulaceae J.St.-Hil.
Iz_3_1109	DJI_0110	Jacobaea borysthenica (DC.) B.Nord. & Greuter	0	n	1	0	Asteraceae Bercht. & J.Presl
Iz_3_1109	DJI_0110	Jasione montana L.	0	n	1	0	Campanulaceae Juss.
Iz_3_1109	DJI_0110	Jurinea cyanoides (L.) Rchb.s.l.	0	n	1	0	Asteraceae Bercht. & J.Presl
Iz_3_1109	DJI_0110	Koeleria glauca (Spreng.) DC.	0	n	1	0	Poaceae Barnhart
Iz_3_1109	DJI_0110	Lactuca serriola L.	0	a	0	0	Asteraceae Bercht. & J.Presl
Iz_3_1109	DJI_0110	Linaria genistifolia (L.) Mill.	0	n	1	0	Plantaginaceae Juss.
Iz_3_1109	DJI_0110	Lomelosia argentea (L.) Greuter & Burdet	0	n	1	0	Caprifoliaceae Juss.
Iz_3_1109	DJI_0110	Melampyrum pratense L.	0	n	1	0	Orobanchaceae Vent.
Iz_3_1109	DJI_0110	Oenothera biennis L.	0	a	0	0	Onagraceae Juss.
Iz_3_1109	DJI_0110	Pentanema britannica (L.) D.Gut.Larr., Santos-Vicente, Anderb., E.Rico & M.M.Mart.Ort.	0	n	1	0	Asteraceae Bercht. & J.Presl
Iz_3_1109	DJI_0110	Peucedanum oreoselinum (L.) Moench	0	n	1	0	Apiaceae Lindl.
Iz_3_1109	DJI_0110	Peucedanum ruthenicum M.Bieb.	0	n	1	0	Apiaceae Lindl.
Iz_3_1109	DJI_0110	Pilosella echioides (Lumn.) F.W.Schultz & Sch.Bip.	0	n	1	0	Asteraceae Bercht. & J.Presl
Iz_3_1109	DJI_0110	Pilosella officinarum F. Schultz et Sch. Bip.	0	n	1	0	Asteraceae Bercht. & J.Presl
Iz_3_1109	DJI_0110	Pilosella sp.	0		NA	0	Asteraceae Bercht. & J.Presl

Iz_3_1109	DJI_0110	Pinus sylvestris L.	0	n	1	1	Pinaceae Spreng. ex F.Rudolphi
Iz_3_1109	DJI_0110	Poa annua L.	0	n	1	0	Poaceae Barnhart
Iz_3_1109	DJI_0110	Poa nemoralis L.	0	n	1	0	Poaceae Barnhart
Iz_3_1109	DJI_0110	Poa sp.	0		NA	0	Poaceae Barnhart
Iz_3_1109	DJI_0110	Polygonatum odoratum (Mill.) Druce	0	n	1	0	Asparagaceae Juss.
Iz_3_1109	DJI_0110	Polygonum arenarium Waldst. & Kit.	0	n	1	0	Polygonaceae Juss.
Iz_3_1109	DJI_0110	Populus nigra L.	0	n	1	1	Salicaceae Mirb.
Iz_3_1109	DJI_0110	Populus tremula L.	0	n	1	1	Salicaceae Mirb.
Iz_3_1109	DJI_0110	Populus Ã canescens (Aiton) Sm.	0	n	1	1	Salicaceae Mirb.
Iz_3_1109	DJI_0110	Potentilla argentea L.	0	n	1	0	Rosaceae Juss.
Iz_3_1109	DJI_0110	Psammophiliella muralis (L.) Ikonn.	0	n	1	0	Caryophyllaceae Juss.
Iz_3_1109	DJI_0110	Psephellus sumensis (Kalen.) Greuter	0	n	1	0	Asteraceae Bercht. & J.Presl
Iz_3_1109	DJI_0110	Pulsatilla pratensis (L.) Mill. s.l.	0	n	1	0	Ranunculaceae Juss.
Iz_3_1109	DJI_0110	Pyrus communis L.	0	a	0	1	Rosaceae Juss.
Iz_3_1109	DJI_0110	Quercus robur L.	0	n	1	1	Fagaceae Dumort.
Iz_3_1109	DJI_0110	Robinia pseudoacacia L.	0	a	0	1	Fabaceae Lindl.
Iz_3_1109	DJI_0110	Rosa sp.	0		NA	1	Rosaceae Juss.
Iz_3_1109	DJI_0110	Rubus idaeus L.	0	n	1	0	Rosaceae Juss.
Iz_3_1109	DJI_0110	Rumex acetosella L.	0	n	1	0	Polygonaceae Juss.
Iz_3_1109	DJI_0110	Rumex thyrsiflorus Fingerh.	0	n	1	0	Polygonaceae Juss.
Iz_3_1109	DJI_0110	Salix alba L.	0	n	1	1	Salicaceae Mirb.
Iz_3_1109	DJI_0110	Sambucus nigra L.	0	n	1	1	Viburnaceae Raf.
Iz_3_1109	DJI_0110	Secale sylvestre Host	0	n	1	0	Poaceae Barnhart
Iz_3_1109	DJI_0110	Sempervivum ruthenicum Schnittsp. & C.B. Lehm.	0	n	1	0	Crassulaceae J.St.-Hil.
Iz_3_1109	DJI_0110	Setaria viridis (L.) P. Beauv.	0	n	1	0	Poaceae Barnhart
Iz_3_1109	DJI_0110	Silene borysthenica (Gruner) Walters	0	n	1	0	Caryophyllaceae Juss.
Iz_3_1109	DJI_0110	Silene latifolia subsp. alba (Mill.) Greuter & Burdet	0	n	1	0	Caryophyllaceae Juss.
Iz_3_1109	DJI_0110	Solidago virgaurea L.	0	n	1	0	Asteraceae Bercht. & J.Presl
Iz_3_1109	DJI_0110	Sorbus aucuparia L.	0	n	1	1	Rosaceae Juss.
Iz_3_1109	DJI_0110	Species 11.09_3_0045	0		NA	0	
Iz_3_1109	DJI_0110	Species 11.09_3_0058	0		NA	0	
Iz_3_1109	DJI_0110	Stachys recta L.	0	n	1	0	Lamiacea Lindl.
Iz_3_1109	DJI_0110	Tanacetum vulgare L.	0	n	1	0	Asteraceae Bercht. & J.Presl
Iz_3_1109	DJI_0110	Thymus pallasianus Heinr.Braun	0	n	1	0	Lamiacea Lindl.
Iz_3_1109	DJI_0110	Tilia cordata Mill.	0	n	1	1	Malvaceae Juss.
Iz_3_1109	DJI_0110	Trifolium alpestre L.	0	n	1	0	Fabaceae Lindl.
Iz_3_1109	DJI_0110	Trifolium arvense L.	0	n	1	0	Fabaceae Lindl.
Iz_3_1109	DJI_0110	Ulmus glabra Huds.	0	n	1	1	Ulmaceae Mirb.
Iz_3_1109	DJI_0110	Verbascum densiflorum Bertol.	0	n	1	0	Scrophulariaceae Juss.
Iz_3_1109	DJI_0110	Veronica austriaca L.	0	n	1	0	Plantaginaceae Juss.
Iz_3_1109	DJI_0110	Veronica incana L.	0	n	1	0	Plantaginaceae Juss.
Iz_3_1109	DJI_0110	Veronica verna L.	0	n	1	0	Plantaginaceae Juss.
Iz_3_1109	DJI_0110	Vincetoxicum hirundinaria Medik	0	n	1	0	Apocynaceae Juss.
Iz_3_1109	DJI_0114	Acer tataricum L.	0	n	1	1	Sapindaceae Juss.
Iz_3_1109	DJI_0114	Achillea millefolium L. s.l.	0	n	1	0	Asteraceae Bercht. & J.Presl
Iz_3_1109	DJI_0114	Allium oleraceum L.	0	n	1	0	Amaryllidaceae J.St.-Hil.
Iz_3_1109	DJI_0114	Ambrosia artemisiifolia L.	0	a	0	0	Asteraceae Bercht. & J.Presl
Iz_3_1109	DJI_0114	Amorpha fruticosa L.	0	a	0	0	Fabaceae Lindl.
Iz_3_1109	DJI_0114	Anthericum ramosum L.	0	n	1	0	Asparagaceae Juss.
Iz_3_1109	DJI_0114	Artemisia marschalliana Spreng.	0	n	1	0	Asteraceae Bercht. & J.Presl
Iz_3_1109	DJI_0114	Asparagus officinalis L.	0	n	1	0	Asparagaceae Juss.
Iz_3_1109	DJI_0114	Astragalus cicer L.	0	n	1	0	Fabaceae Lindl.
Iz_3_1109	DJI_0114	Bassia laniflora (S.G.Gmel.) A.J.Scott	0	n	1	0	Amaranthaceae Juss.
Iz_3_1109	DJI_0114	Berberis vulgaris L.	0	n	1	0	Berberidaceae Juss.
Iz_3_1109	DJI_0114	Betula pendula Roth.	0	n	1	1	Betulaceae Gray
Iz_3_1109	DJI_0114	Betula pendula Roth.	0	n	1	0	Poaceae Barnhart
Iz_3_1109	DJI_0114	Bromus inermis Leyss.	0		NA	NA	
Iz_3_1109	DJI_0114	Bromus squarrosus L.	0	a	0	0	Poaceae Barnhart
Iz_3_1109	DJI_0114	Bromus tectorum L.	0	a	0	0	Poaceae Barnhart
Iz_3_1109	DJI_0114	Calamagrostis epigejos (L.) Roth	0.1	n	1	0	Poaceae Barnhart
Iz_3_1109	DJI_0114	Carduus acanthoides L.	0	n	1	0	Asteraceae Bercht. & J.Presl
Iz_3_1109	DJI_0114	Carex sp.	0	n	1	0	Cyperaceae Juss.
Iz_3_1109	DJI_0114	Centaurea majorovii Dumbadze	0	n	1	0	Asteraceae Bercht. & J.Presl
Iz_3_1109	DJI_0114	Chamaecytisus ruthenicus (Fisch.ex Wol.) Klaskova	0	n	1	0	Fabaceae Lindl.
Iz_3_1109	DJI_0114	Chelidonium majus L.	0.1	n	1	0	Papaveraceae Juss.
Iz_3_1109	DJI_0114	Chenopodium betaceum Andrz.	0	n	1	0	Amaranthaceae Juss.
Iz_3_1109	DJI_0114	Chenopodium cf. album	0	n	1	0	Amaranthaceae Juss.
Iz_3_1109	DJI_0114	Chenopodium sp.	0		NA	0	Amaranthaceae Juss.
Iz_3_1109	DJI_0114	Chondrilla juncea L.	0	n	1	0	Asteraceae Bercht. & J.Presl
Iz_3_1109	DJI_0114	Cirsium vulgare (Savi) Ten.	0	n	1	0	Asteraceae Bercht. & J.Presl
Iz_3_1109	DJI_0114	Clinopodium acinos (L.) Kuntze	0	n	1	0	Lamiacea Lindl.
Iz_3_1109	DJI_0114	Convallaria majalis L.	0	n	1	0	Asparagaceae Juss.
Iz_3_1109	DJI_0114	Crataegus rhipidophylla Gand.	0	n	1	1	Rosaceae Juss.
Iz_3_1109	DJI_0114	Crepis foetida L.	0	n	1	0	Asteraceae Bercht. & J.Presl
Iz_3_1109	DJI_0114	Dactylis glomerata L.	0	n	1	0	Poaceae Barnhart
Iz_3_1109	DJI_0114	Dianthus campestris M.Bieb.	0	n	1	0	Caryophyllaceae Juss.
Iz_3_1109	DJI_0114	Digitaria sanguinalis (L.) Scop.	0	a	0	0	Poaceae Barnhart
Iz_3_1109	DJI_0114	Elymus repens (L.) Gould	0	n	1	0	Poaceae Barnhart
Iz_3_1109	DJI_0114	Epilobium tetragonum L.	0	n	1	0	Onagraceae Juss.
Iz_3_1109	DJI_0114	Eragrostis minor Host	0	a	0	0	Poaceae Barnhart
Iz_3_1109	DJI_0114	Erigeron canadensis L.	0.1	a	0	0	Asteraceae Bercht. & J.Presl
Iz_3_1109	DJI_0114	Euphorbia seguieriana Neck.	0	n	1	0	Euphorbiaceae Juss.
Iz_3_1109	DJI_0114	Fallopia dumetorum (L.) Holub	0.2	n	1	0	Polygonaceae Juss.
Iz_3_1109	DJI_0114	Festuca beckeri (Hack.) Trautv.	0	n	1	0	Poaceae Barnhart
Iz_3_1109	DJI_0114	Fragaria vesca L.	0	n	1	0	Rosaceae Juss.
Iz_3_1109	DJI_0114	Frangula alnus Mill.	0	n	1	1	Rhamnaceae Juss.
Iz_3_1109	DJI_0114	Fraxinus pennsylvanica Marshall	0	a	0	1	Oleaceae Hoffmanns. & Link
Iz_3_1109	DJI_0114	Galeopsis bifida Boenn.	0	n	1	0	Lamiacea Lindl.
Iz_3_1109	DJI_0114	Galium verum L.	0	n	1	0	Rubiaceae Juss.
Iz_3_1109	DJI_0114	Genista tinctoria L.	0	n	1	0	Fabaceae Lindl.
Iz_3_1109	DJI_0114	Geranium robertianum L.	0	n	1	0	Geraniaceae Juss.
Iz_3_1109	DJI_0114	Geranium sanquineum L.	0	n	1	0	Geraniaceae Juss.
Iz_3_1109	DJI_0114	Gypsophila paniculata L.	0	n	1	0	Caryophyllaceae Juss.
Iz_3_1109	DJI_0114	Helichrysum arenarium (L.) Moench	0	n	1	0	Asteraceae Bercht. & J.Presl
Iz_3_1109	DJI_0114	Herniaria polygama J.Gay	0	n	1	0	Caryophyllaceae Juss.
Iz_3_1109	DJI_0114	Hieracium robustum Fr.	0	n	1	0	Asteraceae Bercht. & J.Presl
Iz_3_1109	DJI_0114	Hieracium umbellatum L.	0	n	1	NA	Asteraceae Bercht. & J.Presl
Iz_3_1109	DJI_0114	Hylotelephium maximum (L.) Holub	0	n	1	0	Crassulaceae J.St.-Hil.

Iz_3_1109	DJI_0114	Jacobaea borysthenica (DC.) B.Nord. & Greuter	0	n	1	0	Asteraceae Bercht. & J.Presl
Iz_3_1109	DJI_0114	Jasione montana L.	0	n	1	0	Campanulaceae Juss.
Iz_3_1109	DJI_0114	Jurinea cyanoides (L.) Rchb.s.l.	0	n	1	0	Asteraceae Bercht. & J.Presl
Iz_3_1109	DJI_0114	Koeleria glauca (Spreng.) DC.	0	n	1	0	Poaceae Barnhart
Iz_3_1109	DJI_0114	Lactuca serriola L.	0	a	0	0	Asteraceae Bercht. & J.Presl
Iz_3_1109	DJI_0114	Linaria genistifolia (L.) Mill.	0	n	1	0	Plantaginaceae Juss.
Iz_3_1109	DJI_0114	Lomelosia argentea (L.) Greuter & Burdet	0	n	1	0	Caprifoliaceae Juss.
Iz_3_1109	DJI_0114	Melampyrum pratense L.	0	n	1	0	Orobanchaceae Vent.
Iz_3_1109	DJI_0114	Oenothera biennis L.	0	a	0	0	Onagraceae Juss.
Iz_3_1109	DJI_0114	Pentanema britannica (L.) D.Gut.Larr., Santos-Vicente, Anderb., E.Rico & M.M.Mart.Ort.	0	n	1	0	Asteraceae Bercht. & J.Presl
Iz_3_1109	DJI_0114	Peucedanum oreoselinum (L.) Moench	0	n	1	0	Apiaceae Lindl.
Iz_3_1109	DJI_0114	Peucedanum ruthenicum M.Bieb.	0	n	1	0	Apiaceae Lindl.
Iz_3_1109	DJI_0114	Pilosella echioides (Lumn.) F.W.Schultz & Sch.Bip.	0	n	1	0	Asteraceae Bercht. & J.Presl
Iz_3_1109	DJI_0114	Pilosella officinarum F. Schultz et Sch. Bip.	0	n	1	0	Asteraceae Bercht. & J.Presl
Iz_3_1109	DJI_0114	Pilosella sp.	0		NA	0	Asteraceae Bercht. & J.Presl
Iz_3_1109	DJI_0114	Pinus sylvestris L.	0	n	1	1	Pinaceae Spreng. ex F.Rudolphi
Iz_3_1109	DJI_0114	Poa annua L.	0	n	1	0	Poaceae Barnhart
Iz_3_1109	DJI_0114	Poa nemoralis L.	0	n	1	0	Poaceae Barnhart
Iz_3_1109	DJI_0114	Poa sp.	0		NA	0	Poaceae Barnhart
Iz_3_1109	DJI_0114	Polygonatum odoratum (Mill.) Druce	0	n	1	0	Asparagaceae Juss.
Iz_3_1109	DJI_0114	Polygonum arenarium Waldst. & Kit.	0	n	1	0	Polygonaceae Juss.
Iz_3_1109	DJI_0114	Populus nigra L.	0	n	1	1	Salicaceae Mirb.
Iz_3_1109	DJI_0114	Populus tremula L.	0	n	1	1	Salicaceae Mirb.
Iz_3_1109	DJI_0114	Populus Ã canescens (Aiton) Sm.	0	n	1	1	Salicaceae Mirb.
Iz_3_1109	DJI_0114	Potentilla argentea L.	0	n	1	0	Rosaceae Juss.
Iz_3_1109	DJI_0114	Psammophiliella muralis (L.) Ikonn.	0	n	1	0	Caryophyllaceae Juss.
Iz_3_1109	DJI_0114	Psephellus sumensis (Kalen.) Greuter	0	n	1	0	Asteraceae Bercht. & J.Presl
Iz_3_1109	DJI_0114	Pulsatilla pratensis (L.) Mill. s.l.	0	n	1	0	Ranunculaceae Juss.
Iz_3_1109	DJI_0114	Pyrus communis L.	0	a	0	1	Rosaceae Juss.
Iz_3_1109	DJI_0114	Quercus robur L.	0	n	1	1	Fagaceae Dumort.
Iz_3_1109	DJI_0114	Robinia pseudoacacia L.	0	a	0	1	Fabaceae Lindl.
Iz_3_1109	DJI_0114	Rosa sp.	0		NA	1	Rosaceae Juss.
Iz_3_1109	DJI_0114	Rubus idaeus L.	0	n	1	0	Rosaceae Juss.
Iz_3_1109	DJI_0114	Rumex acetosella L.	0	n	1	0	Polygonaceae Juss.
Iz_3_1109	DJI_0114	Rumex thyrsiflorus Fingerh.	0	n	1	0	Polygonaceae Juss.
Iz_3_1109	DJI_0114	Salix alba L.	0	n	1	1	Salicaceae Mirb.
Iz_3_1109	DJI_0114	Sambucus nigra L.	0	n	1	1	Viburnaceae Raf.
Iz_3_1109	DJI_0114	Secale sylvestre Host	0	n	1	0	Poaceae Barnhart
Iz_3_1109	DJI_0114	Sempervivum ruthenicum Schnittsp. & C.B. Lehm.	0	n	1	0	Crassulaceae J.St.-Hil.
Iz_3_1109	DJI_0114	Setaria viridis (L.) P. Beauv.	0	n	1	0	Poaceae Barnhart
Iz_3_1109	DJI_0114	Silene borysthenica (Gruner) Walters	0	n	1	0	Caryophyllaceae Juss.
Iz_3_1109	DJI_0114	Silene latifolia subsp. alba (Mill.) Greuter & Burdet	0	n	1	0	Caryophyllaceae Juss.
Iz_3_1109	DJI_0114	Solidago virgaurea L.	0	n	1	0	Asteraceae Bercht. & J.Presl
Iz_3_1109	DJI_0114	Sorbus aucuparia L.	0	n	1	1	Rosaceae Juss.
Iz_3_1109	DJI_0114	Species 11.09_3_0045	0		NA	0	
Iz_3_1109	DJI_0114	Species 11.09_3_0058	0		NA	0	
Iz_3_1109	DJI_0114	Stachys recta L.	0	n	1	0	Lamiacea Lindl.
Iz_3_1109	DJI_0114	Tanacetum vulgare L.	0	n	1	0	Asteraceae Bercht. & J.Presl
Iz_3_1109	DJI_0114	Thymus pallasianus Heinr.Braun	0	n	1	0	Lamiacea Lindl.
Iz_3_1109	DJI_0114	Tilia cordata Mill.	0	n	1	1	Malvaceae Juss.
Iz_3_1109	DJI_0114	Trifolium alpestre L.	0	n	1	0	Fabaceae Lindl.
Iz_3_1109	DJI_0114	Trifolium arvense L.	0	n	1	0	Fabaceae Lindl.
Iz_3_1109	DJI_0114	Ulmus glabra Huds.	0	n	1	1	Ulmaceae Mirb.
Iz_3_1109	DJI_0114	Verbascum densiflorum Bertol.	0	n	1	0	Scrophulariaceae Juss.
Iz_3_1109	DJI_0114	Veronica austriaca L.	0	n	1	0	Plantaginaceae Juss.
Iz_3_1109	DJI_0114	Veronica incana L.	0	n	1	0	Plantaginaceae Juss.
Iz_3_1109	DJI_0114	Veronica verna L.	0	n	1	0	Plantaginaceae Juss.
Iz_3_1109	DJI_0114	Vincetoxicum hirundinaria Medik	0	n	1	0	Apocynaceae Juss.
Iz_3_1109	DJI_0116	Acer tataricum L.	0	n	1	1	Sapindaceae Juss.
Iz_3_1109	DJI_0116	Achillea millefolium L. s.l.	0	n	1	0	Asteraceae Bercht. & J.Presl
Iz_3_1109	DJI_0116	Allium oleraceum L.	0	n	1	0	Amaryllidaceae J.St.-Hil.
Iz_3_1109	DJI_0116	Ambrosia artemisiifolia L.	0	a	0	0	Asteraceae Bercht. & J.Presl
Iz_3_1109	DJI_0116	Amorpha fruticosa L.	0	a	0	0	Fabaceae Lindl.
Iz_3_1109	DJI_0116	Anthericum ramosum L.	0	n	1	0	Asparagaceae Juss.
Iz_3_1109	DJI_0116	Artemisia marschalliana Spreng.	0	n	1	0	Asteraceae Bercht. & J.Presl
Iz_3_1109	DJI_0116	Asparagus officinalis L.	0	n	1	0	Asparagaceae Juss.
Iz_3_1109	DJI_0116	Astragalus cicer L.	0	n	1	0	Fabaceae Lindl.
Iz_3_1109	DJI_0116	Bassia laniflora (S.G.Gmel.) A.J.Scott	0	n	1	0	Amaranthaceae Juss.
Iz_3_1109	DJI_0116	Berberis vulgaris L.	0	n	1	0	Berberidaceae Juss.
Iz_3_1109	DJI_0116	Betula pendula Roth.	0	n	1	1	Betulaceae Gray
Iz_3_1109	DJI_0116	Betula pendula Roth.	0	n	1	0	Poaceae Barnhart
Iz_3_1109	DJI_0116	Bromus inermis Leyss.	0		NA	NA	
Iz_3_1109	DJI_0116	Bromus squarrosus L.	0	a	0	0	Poaceae Barnhart
Iz_3_1109	DJI_0116	Bromus tectorum L.	0	a	0	0	Poaceae Barnhart
Iz_3_1109	DJI_0116	Calamagrostis epigejos (L.) Roth	0.1	n	1	0	Poaceae Barnhart
Iz_3_1109	DJI_0116	Carduus acanthoides L.	0	n	1	0	Asteraceae Bercht. & J.Presl
Iz_3_1109	DJI_0116	Carex sp.	0	n	1	0	Cyperaceae Juss.
Iz_3_1109	DJI_0116	Centaurea majorovii Dumbadze	0	n	1	0	Asteraceae Bercht. & J.Presl
Iz_3_1109	DJI_0116	Chamaecytisus ruthenicus (Fisch.ex Wol.) Klaskova	0	n	1	0	Fabaceae Lindl.
Iz_3_1109	DJI_0116	Chelidonium majus L.	0	n	1	0	Papaveraceae Juss.
Iz_3_1109	DJI_0116	Chenopodium betaceum Andrz.	0	n	1	0	Amaranthaceae Juss.
Iz_3_1109	DJI_0116	Chenopodium cf. album	0	n	1	0	Amaranthaceae Juss.
Iz_3_1109	DJI_0116	Chenopodium sp.	0		NA	0	Amaranthaceae Juss.
Iz_3_1109	DJI_0116	Chondrilla juncea L.	0	n	1	0	Asteraceae Bercht. & J.Presl
Iz_3_1109	DJI_0116	Cirsium vulgare (Savi) Ten.	0	n	1	0	Asteraceae Bercht. & J.Presl
Iz_3_1109	DJI_0116	Clinopodium acinos (L.) Kuntze	0	n	1	0	Lamiacea Lindl.
Iz_3_1109	DJI_0116	Convallaria majalis L.	0	n	1	0	Asparagaceae Juss.
Iz_3_1109	DJI_0116	Crataegus rhipidophylla Gand.	0	n	1	1	Rosaceae Juss.
Iz_3_1109	DJI_0116	Crepis foetida L.	0	n	1	0	Asteraceae Bercht. & J.Presl
Iz_3_1109	DJI_0116	Dactylis glomerata L.	0	n	1	0	Poaceae Barnhart
Iz_3_1109	DJI_0116	Dianthus campestris M.Bieb.	0	n	1	0	Caryophyllaceae Juss.
Iz_3_1109	DJI_0116	Digitaria sanguinalis (L.) Scop.	0	a	0	0	Poaceae Barnhart
Iz_3_1109	DJI_0116	Elymus repens (L.) Gould	0	n	1	0	Poaceae Barnhart
Iz_3_1109	DJI_0116	Epilobium tetragonum L.	0	n	1	0	Onagraceae Juss.
Iz_3_1109	DJI_0116	Eragrostis minor Host	0	a	0	0	Poaceae Barnhart
Iz_3_1109	DJI_0116	Erigeron canadensis L.	0	a	0	0	Asteraceae Bercht. & J.Presl
Iz_3_1109	DJI_0116	Euphorbia seguieriana Neck.	0	n	1	0	Euphorbiaceae Juss.
Iz_3_1109	DJI_0116	Fallopia dumetorum (L.) Holub	0.2	n	1	0	Polygonaceae Juss.

Iz_3_1109	DJI_0116	Festuca beckeri (Hack.) Trautv.	0	n	1	0	Poaceae Barnhart
Iz_3_1109	DJI_0116	Fragaria vesca L.	0	n	1	0	Rosaceae Juss.
Iz_3_1109	DJI_0116	Frangula alnus Mill.	0	n	1	1	Rhamnaceae Juss.
Iz_3_1109	DJI_0116	Fraxinus pennsylvanica Marshall	0	a	0	1	Oleaceae Hoffmanns. & Link
Iz_3_1109	DJI_0116	Galeopsis bifida Boenn.	0	n	1	0	Lamiacea Lindl.
Iz_3_1109	DJI_0116	Galium verum L.	0	n	1	0	Rubiaceae Juss.
Iz_3_1109	DJI_0116	Genista tinctoria L.	0	n	1	0	Fabaceae Lindl.
Iz_3_1109	DJI_0116	Geranium robertianum L.	0	n	1	0	Geraniaceae Juss.
Iz_3_1109	DJI_0116	Geranium sanquineum L.	0	n	1	0	Geraniaceae Juss.
Iz_3_1109	DJI_0116	Gypsophila paniculata L.	0	n	1	0	Caryophyllaceae Juss.
Iz_3_1109	DJI_0116	Helichrysum arenarium (L.) Moench	0	n	1	0	Asteraceae Bercht. & J.Presl
Iz_3_1109	DJI_0116	Herniaria polygama J.Gay	0	n	1	0	Caryophyllaceae Juss.
Iz_3_1109	DJI_0116	Hieracium robustum Fr.	0	n	1	0	Asteraceae Bercht. & J.Presl
Iz_3_1109	DJI_0116	Hieracium umbellatum L.	0	n	1	NA	Asteraceae Bercht. & J.Presl
Iz_3_1109	DJI_0116	Hylotelephium maximum (L.) Holub	0	n	1	0	Crassulaceae J.St.-Hil.
Iz_3_1109	DJI_0116	Jacobaea borysthenica (DC.) B.Nord. & Greuter	0	n	1	0	Asteraceae Bercht. & J.Presl
Iz_3_1109	DJI_0116	Jasione montana L.	0	n	1	0	Campanulaceae Juss.
Iz_3_1109	DJI_0116	Jurinea cyanoides (L.) Rchb.s.l.	0	n	1	0	Asteraceae Bercht. & J.Presl
Iz_3_1109	DJI_0116	Koeleria glauca (Spreng.) DC.	0	n	1	0	Poaceae Barnhart
Iz_3_1109	DJI_0116	Lactuca serriola L.	0	a	0	0	Asteraceae Bercht. & J.Presl
Iz_3_1109	DJI_0116	Linaria genistifolia (L.) Mill.	0	n	1	0	Plantaginaceae Juss.
Iz_3_1109	DJI_0116	Lomelosia argentea (L.) Greuter & Burdet	0	n	1	0	Caprifoliaceae Juss.
Iz_3_1109	DJI_0116	Melampyrum pratense L.	0	n	1	0	Orobanchaceae Vent.
Iz_3_1109	DJI_0116	Oenothera biennis L.	0	a	0	0	Onagraceae Juss.
Iz_3_1109	DJI_0116	Pentanema britannica (L.) D.Gut.Larr., Santos-Vicente, Anderb., E.Rico & M.M.Mart.Ort.	0	n	1	0	Asteraceae Bercht. & J.Presl
Iz_3_1109	DJI_0116	Peucedanum oreoselinum (L.) Moench	0	n	1	0	Apiaceae Lindl.
Iz_3_1109	DJI_0116	Peucedanum ruthenicum M.Bieb.	0	n	1	0	Apiaceae Lindl.
Iz_3_1109	DJI_0116	Pilosella echioides (Lumn.) F.W.Schultz & Sch.Bip.	0	n	1	0	Asteraceae Bercht. & J.Presl
Iz_3_1109	DJI_0116	Pilosella officinarum F. Schultz et Sch. Bip.	0	n	1	0	Asteraceae Bercht. & J.Presl
Iz_3_1109	DJI_0116	Pilosella sp.	0		NA	0	Asteraceae Bercht. & J.Presl
Iz_3_1109	DJI_0116	Pinus sylvestris L.	0	n	1	1	Pinaceae Spreng. ex F.Rudolphi
Iz_3_1109	DJI_0116	Poa annua L.	0	n	1	0	Poaceae Barnhart
Iz_3_1109	DJI_0116	Poa nemoralis L.	0	n	1	0	Poaceae Barnhart
Iz_3_1109	DJI_0116	Poa sp.	0		NA	0	Poaceae Barnhart
Iz_3_1109	DJI_0116	Polygonatum odoratum (Mill.) Druce	0	n	1	0	Asparagaceae Juss.
Iz_3_1109	DJI_0116	Polygonum arenarium Waldst. & Kit.	0	n	1	0	Polygonaceae Juss.
Iz_3_1109	DJI_0116	Populus nigra L.	0	n	1	1	Salicaceae Mirb.
Iz_3_1109	DJI_0116	Populus tremula L.	0	n	1	1	Salicaceae Mirb.
Iz_3_1109	DJI_0116	Populus Ã canescens (Aiton) Sm.	0	n	1	1	Salicaceae Mirb.
Iz_3_1109	DJI_0116	Potentilla argentea L.	0	n	1	0	Rosaceae Juss.
Iz_3_1109	DJI_0116	Psammophiliella muralis (L.) Ikonn.	0	n	1	0	Caryophyllaceae Juss.
Iz_3_1109	DJI_0116	Psephellus sumensis (Kalen.) Greuter	0	n	1	0	Asteraceae Bercht. & J.Presl
Iz_3_1109	DJI_0116	Pulsatilla pratensis (L.) Mill. s.l.	0	n	1	0	Ranunculaceae Juss.
Iz_3_1109	DJI_0116	Pyrus communis L.	0	a	0	1	Rosaceae Juss.
Iz_3_1109	DJI_0116	Quercus robur L.	0	n	1	1	Fagaceae Dumort.
Iz_3_1109	DJI_0116	Robinia pseudoacacia L.	0	a	0	1	Fabaceae Lindl.
Iz_3_1109	DJI_0116	Rosa sp.	0		NA	1	Rosaceae Juss.
Iz_3_1109	DJI_0116	Rubus idaeus L.	0	n	1	0	Rosaceae Juss.
Iz_3_1109	DJI_0116	Rumex acetosella L.	0	n	1	0	Polygonaceae Juss.
Iz_3_1109	DJI_0116	Rumex thyrsiflorus Fingerh.	0	n	1	0	Polygonaceae Juss.
Iz_3_1109	DJI_0116	Salix alba L.	0	n	1	1	Salicaceae Mirb.
Iz_3_1109	DJI_0116	Sambucus nigra L.	0	n	1	1	Viburnaceae Raf.
Iz_3_1109	DJI_0116	Secale sylvestre Host	0	n	1	0	Poaceae Barnhart
Iz_3_1109	DJI_0116	Sempervivum ruthenicum Schnittsp. & C.B. Lehm.	0	n	1	0	Crassulaceae J.St.-Hil.
Iz_3_1109	DJI_0116	Setaria viridis (L.) P. Beauv.	0	n	1	0	Poaceae Barnhart
Iz_3_1109	DJI_0116	Silene borysthenica (Gruner) Walters	0	n	1	0	Caryophyllaceae Juss.
Iz_3_1109	DJI_0116	Silene latifolia subsp. alba (Mill.) Greuter & Burdet	0	n	1	0	Caryophyllaceae Juss.
Iz_3_1109	DJI_0116	Solidago virgaurea L.	0	n	1	0	Asteraceae Bercht. & J.Presl
Iz_3_1109	DJI_0116	Sorbus aucuparia L.	0	n	1	1	Rosaceae Juss.
Iz_3_1109	DJI_0116	Species 11.09_3_0045	0		NA	0	
Iz_3_1109	DJI_0116	Species 11.09_3_0058	0		NA	0	
Iz_3_1109	DJI_0116	Stachys recta L.	0	n	1	0	Lamiacea Lindl.
Iz_3_1109	DJI_0116	Tanacetum vulgare L.	0	n	1	0	Asteraceae Bercht. & J.Presl
Iz_3_1109	DJI_0116	Thymus pallasianus Heinr.Braun	0	n	1	0	Lamiacea Lindl.
Iz_3_1109	DJI_0116	Tilia cordata Mill.	0	n	1	1	Malvaceae Juss.
Iz_3_1109	DJI_0116	Trifolium alpestre L.	0	n	1	0	Fabaceae Lindl.
Iz_3_1109	DJI_0116	Trifolium arvense L.	0	n	1	0	Fabaceae Lindl.
Iz_3_1109	DJI_0116	Ulmus glabra Huds.	0	n	1	1	Ulmaceae Mirb.
Iz_3_1109	DJI_0116	Verbascum densiflorum Bertol.	0	n	1	0	Scrophulariaceae Juss.
Iz_3_1109	DJI_0116	Veronica austriaca L.	0	n	1	0	Plantaginaceae Juss.
Iz_3_1109	DJI_0116	Veronica incana L.	0	n	1	0	Plantaginaceae Juss.
Iz_3_1109	DJI_0116	Veronica verna L.	0	n	1	0	Plantaginaceae Juss.
Iz_3_1109	DJI_0116	Vincetoxicum hirundinaria Medik	0	n	1	0	Apocynaceae Juss.
Iz_3_1109	DJI_0118	Acer tataricum L.	0	n	1	1	Sapindaceae Juss.
Iz_3_1109	DJI_0118	Achillea millefolium L. s.l.	0	n	1	0	Asteraceae Bercht. & J.Presl
Iz_3_1109	DJI_0118	Allium oleraceum L.	0	n	1	0	Amaryllidaceae J.St.-Hil.
Iz_3_1109	DJI_0118	Ambrosia artemisiifolia L.	0	a	0	0	Asteraceae Bercht. & J.Presl
Iz_3_1109	DJI_0118	Amorpha fruticosa L.	0	a	0	0	Fabaceae Lindl.
Iz_3_1109	DJI_0118	Anthericum ramosum L.	0	n	1	0	Asparagaceae Juss.
Iz_3_1109	DJI_0118	Artemisia marschalliana Spreng.	0	n	1	0	Asteraceae Bercht. & J.Presl
Iz_3_1109	DJI_0118	Asparagus officinalis L.	0	n	1	0	Asparagaceae Juss.
Iz_3_1109	DJI_0118	Astragalus cicer L.	0	n	1	0	Fabaceae Lindl.
Iz_3_1109	DJI_0118	Bassia laniflora (S.G.Gmel.) A.J.Scott	0	n	1	0	Amaranthaceae Juss.
Iz_3_1109	DJI_0118	Berberis vulgaris L.	0	n	1	0	Berberidaceae Juss.
Iz_3_1109	DJI_0118	Betula pendula Roth.	0	n	1	1	Betulaceae Gray
Iz_3_1109	DJI_0118	Betula pendula Roth.	0	n	1	0	Poaceae Barnhart
Iz_3_1109	DJI_0118	Bromus inermis Leyss.	0		NA	NA	
Iz_3_1109	DJI_0118	Bromus squarrosus L.	0	a	0	0	Poaceae Barnhart
Iz_3_1109	DJI_0118	Bromus tectorum L.	0	a	0	0	Poaceae Barnhart
Iz_3_1109	DJI_0118	Calamagrostis epigejos (L.) Roth	0	n	1	0	Poaceae Barnhart
Iz_3_1109	DJI_0118	Carduus acanthoides L.	0	n	1	0	Asteraceae Bercht. & J.Presl
Iz_3_1109	DJI_0118	Carex sp.	0	n	1	0	Cyperaceae Juss.
Iz_3_1109	DJI_0118	Centaurea majorovii Dumbadze	0	n	1	0	Asteraceae Bercht. & J.Presl
Iz_3_1109	DJI_0118	Chamaecytisus ruthenicus (Fisch.ex Wol.) Klaskova	0	n	1	0	Fabaceae Lindl.
Iz_3_1109	DJI_0118	Chelidonium majus L.	0.2	n	1	0	Papaveraceae Juss.
Iz_3_1109	DJI_0118	Chenopodium betaceum Andrz.	0	n	1	0	Amaranthaceae Juss.
Iz_3_1109	DJI_0118	Chenopodium cf. album	0	n	1	0	Amaranthaceae Juss.
Iz_3_1109	DJI_0118	Chenopodium sp.	0		NA	0	Amaranthaceae Juss.

Iz_3_1109	DJI_0118	Chondrilla juncea L.	0	n	1	0	Asteraceae Bercht. & J.Presl
Iz_3_1109	DJI_0118	Cirsium vulgare (Savi) Ten.	0	n	1	0	Asteraceae Bercht. & J.Presl
Iz_3_1109	DJI_0118	Clinopodium acinos (L.) Kuntze	0	n	1	0	Lamiacea Lindl.
Iz_3_1109	DJI_0118	Convallaria majalis L.	0	n	1	0	Asparagaceae Juss.
Iz_3_1109	DJI_0118	Crataegus rhipidophylla Gand.	0	n	1	1	Rosaceae Juss.
Iz_3_1109	DJI_0118	Crepis foetida L.	0	n	1	0	Asteraceae Bercht. & J.Presl
Iz_3_1109	DJI_0118	Dactylis glomerata L.	0	n	1	0	Poaceae Barnhart
Iz_3_1109	DJI_0118	Dianthus campestris M.Bieb.	0	n	1	0	Caryophyllaceae Juss.
Iz_3_1109	DJI_0118	Digitaria sanguinalis (L.) Scop.	0	a	0	0	Poaceae Barnhart
Iz_3_1109	DJI_0118	Elymus repens (L.) Gould	0	n	1	0	Poaceae Barnhart
Iz_3_1109	DJI_0118	Epilobium tetragonum L.	0	n	1	0	Onagraceae Juss.
Iz_3_1109	DJI_0118	Eragrostis minor Host	0	a	0	0	Poaceae Barnhart
Iz_3_1109	DJI_0118	Erigeron canadensis L.	1	a	0	0	Asteraceae Bercht. & J.Presl
Iz_3_1109	DJI_0118	Euphorbia seguieriana Neck.	0	n	1	0	Euphorbiaceae Juss.
Iz_3_1109	DJI_0118	Fallopia dumetorum (L.) Holub	2	n	1	0	Polygonaceae Juss.
Iz_3_1109	DJI_0118	Festuca beckeri (Hack.) Trautv.	0	n	1	0	Poaceae Barnhart
Iz_3_1109	DJI_0118	Fragaria vesca L.	0	n	1	0	Rosaceae Juss.
Iz_3_1109	DJI_0118	Frangula alnus Mill.	0	n	1	1	Rhamnaceae Juss.
Iz_3_1109	DJI_0118	Fraxinus pennsylvanica Marshall	0	a	0	1	Oleaceae Hoffmanns. & Link
Iz_3_1109	DJI_0118	Galeopsis bifida Boenn.	0	n	1	0	Lamiacea Lindl.
Iz_3_1109	DJI_0118	Galium verum L.	0	n	1	0	Rubiaceae Juss.
Iz_3_1109	DJI_0118	Genista tinctoria L.	0	n	1	0	Fabaceae Lindl.
Iz_3_1109	DJI_0118	Geranium robertianum L.	0	n	1	0	Geraniaceae Juss.
Iz_3_1109	DJI_0118	Geranium sanquineum L.	0	n	1	0	Geraniaceae Juss.
Iz_3_1109	DJI_0118	Gypsophila paniculata L.	0	n	1	0	Caryophyllaceae Juss.
Iz_3_1109	DJI_0118	Helichrysum arenarium (L.) Moench	0	n	1	0	Asteraceae Bercht. & J.Presl
Iz_3_1109	DJI_0118	Herniaria polygama J.Gay	0	n	1	0	Caryophyllaceae Juss.
Iz_3_1109	DJI_0118	Hieracium robustum Fr.	0	n	1	0	Asteraceae Bercht. & J.Presl
Iz_3_1109	DJI_0118	Hieracium umbellatum L.	0	n	1	NA	Asteraceae Bercht. & J.Presl
Iz_3_1109	DJI_0118	Hylotelephium maximum (L.) Holub	0	n	1	0	Crassulaceae J.St.-Hil.
Iz_3_1109	DJI_0118	Jacobaea borysthenica (DC.) B.Nord. & Greuter	0	n	1	0	Asteraceae Bercht. & J.Presl
Iz_3_1109	DJI_0118	Jasione montana L.	0	n	1	0	Campanulaceae Juss.
Iz_3_1109	DJI_0118	Jurinea cyanoides (L.) Rchb.s.l.	0	n	1	0	Asteraceae Bercht. & J.Presl
Iz_3_1109	DJI_0118	Koeleria glauca (Spreng.) DC.	0	n	1	0	Poaceae Barnhart
Iz_3_1109	DJI_0118	Lactuca serriola L.	0	a	0	0	Asteraceae Bercht. & J.Presl
Iz_3_1109	DJI_0118	Linaria genistifolia (L.) Mill.	0	n	1	0	Plantaginaceae Juss.
Iz_3_1109	DJI_0118	Lomelosia argentea (L.) Greuter & Burdet	0	n	1	0	Caprifoliaceae Juss.
Iz_3_1109	DJI_0118	Melampyrum pratense L.	0	n	1	0	Orobanchaceae Vent.
Iz_3_1109	DJI_0118	Oenothera biennis L.	0	a	0	0	Onagraceae Juss.
Iz_3_1109	DJI_0118	Pentanema britannica (L.) D.Gut.Larr., Santos-Vicente, Anderb., E.Rico & M.M.Mart.Ort.	0	n	1	0	Asteraceae Bercht. & J.Presl
Iz_3_1109	DJI_0118	Peucedanum oreoselinum (L.) Moench	0	n	1	0	Apiaceae Lindl.
Iz_3_1109	DJI_0118	Peucedanum ruthenicum M.Bieb.	0	n	1	0	Apiaceae Lindl.
Iz_3_1109	DJI_0118	Pilosella echioides (Lumn.) F.W.Schultz & Sch.Bip.	0	n	1	0	Asteraceae Bercht. & J.Presl
Iz_3_1109	DJI_0118	Pilosella officinarum F. Schultz et Sch. Bip.	0	n	1	0	Asteraceae Bercht. & J.Presl
Iz_3_1109	DJI_0118	Pilosella sp.	0		NA	0	Asteraceae Bercht. & J.Presl
Iz_3_1109	DJI_0118	Pinus sylvestris L.	0	n	1	1	Pinaceae Spreng. ex F.Rudolphi
Iz_3_1109	DJI_0118	Poa annua L.	0	n	1	0	Poaceae Barnhart
Iz_3_1109	DJI_0118	Poa nemoralis L.	0	n	1	0	Poaceae Barnhart
Iz_3_1109	DJI_0118	Poa sp.	0		NA	0	Poaceae Barnhart
Iz_3_1109	DJI_0118	Polygonatum odoratum (Mill.) Druce	0	n	1	0	Asparagaceae Juss.
Iz_3_1109	DJI_0118	Polygonum arenarium Waldst. & Kit.	0	n	1	0	Polygonaceae Juss.
Iz_3_1109	DJI_0118	Populus nigra L.	0	n	1	1	Salicaceae Mirb.
Iz_3_1109	DJI_0118	Populus tremula L.	0	n	1	1	Salicaceae Mirb.
Iz_3_1109	DJI_0118	Populus Ã canescens (Aiton) Sm.	0	n	1	1	Salicaceae Mirb.
Iz_3_1109	DJI_0118	Potentilla argentea L.	0	n	1	0	Rosaceae Juss.
Iz_3_1109	DJI_0118	Psammophiliella muralis (L.) Ikonn.	0	n	1	0	Caryophyllaceae Juss.
Iz_3_1109	DJI_0118	Psephellus sumensis (Kalen.) Greuter	0	n	1	0	Asteraceae Bercht. & J.Presl
Iz_3_1109	DJI_0118	Pulsatilla pratensis (L.) Mill. s.l.	0	n	1	0	Ranunculaceae Juss.
Iz_3_1109	DJI_0118	Pyrus communis L.	0	a	0	1	Rosaceae Juss.
Iz_3_1109	DJI_0118	Quercus robur L.	0	n	1	1	Fagaceae Dumort.
Iz_3_1109	DJI_0118	Robinia pseudoacacia L.	0	a	0	1	Fabaceae Lindl.
Iz_3_1109	DJI_0118	Rosa sp.	0		NA	1	Rosaceae Juss.
Iz_3_1109	DJI_0118	Rubus idaeus L.	0	n	1	0	Rosaceae Juss.
Iz_3_1109	DJI_0118	Rumex acetosella L.	0	n	1	0	Polygonaceae Juss.
Iz_3_1109	DJI_0118	Rumex thyrsiflorus Fingerh.	0	n	1	0	Polygonaceae Juss.
Iz_3_1109	DJI_0118	Salix alba L.	0	n	1	1	Salicaceae Mirb.
Iz_3_1109	DJI_0118	Sambucus nigra L.	0	n	1	1	Viburnaceae Raf.
Iz_3_1109	DJI_0118	Secale sylvestre Host	0	n	1	0	Poaceae Barnhart
Iz_3_1109	DJI_0118	Sempervivum ruthenicum Schnittsp. & C.B. Lehm.	0	n	1	0	Crassulaceae J.St.-Hil.
Iz_3_1109	DJI_0118	Setaria viridis (L.) P. Beauv.	0	n	1	0	Poaceae Barnhart
Iz_3_1109	DJI_0118	Silene borysthenica (Gruner) Walters	0	n	1	0	Caryophyllaceae Juss.
Iz_3_1109	DJI_0118	Silene latifolia subsp. alba (Mill.) Greuter & Burdet	0	n	1	0	Caryophyllaceae Juss.
Iz_3_1109	DJI_0118	Solidago virgaurea L.	0	n	1	0	Asteraceae Bercht. & J.Presl
Iz_3_1109	DJI_0118	Sorbus aucuparia L.	0	n	1	1	Rosaceae Juss.
Iz_3_1109	DJI_0118	Species 11.09_3_0045	0		NA	0	
Iz_3_1109	DJI_0118	Species 11.09_3_0058	0		NA	0	
Iz_3_1109	DJI_0118	Stachys recta L.	0	n	1	0	Lamiacea Lindl.
Iz_3_1109	DJI_0118	Tanacetum vulgare L.	0	n	1	0	Asteraceae Bercht. & J.Presl
Iz_3_1109	DJI_0118	Thymus pallasianus Heinr.Braun	0	n	1	0	Lamiacea Lindl.
Iz_3_1109	DJI_0118	Tilia cordata Mill.	0	n	1	1	Malvaceae Juss.
Iz_3_1109	DJI_0118	Trifolium alpestre L.	0	n	1	0	Fabaceae Lindl.
Iz_3_1109	DJI_0118	Trifolium arvense L.	0	n	1	0	Fabaceae Lindl.
Iz_3_1109	DJI_0118	Ulmus glabra Huds.	0	n	1	1	Ulmaceae Mirb.
Iz_3_1109	DJI_0118	Verbascum densiflorum Bertol.	0	n	1	0	Scrophulariaceae Juss.
Iz_3_1109	DJI_0118	Veronica austriaca L.	0	n	1	0	Plantaginaceae Juss.
Iz_3_1109	DJI_0118	Veronica incana L.	0	n	1	0	Plantaginaceae Juss.
Iz_3_1109	DJI_0118	Veronica verna L.	0	n	1	0	Plantaginaceae Juss.
Iz_3_1109	DJI_0118	Vincetoxicum hirundinaria Medik	0	n	1	0	Apocynaceae Juss.
Iz_3_1109	DJI_0120	Acer tataricum L.	0	n	1	1	Sapindaceae Juss.
Iz_3_1109	DJI_0120	Achillea millefolium L. s.l.	0	n	1	0	Asteraceae Bercht. & J.Presl
Iz_3_1109	DJI_0120	Allium oleraceum L.	0	n	1	0	Amaryllidaceae J.St.-Hil.
Iz_3_1109	DJI_0120	Ambrosia artemisiifolia L.	0	a	0	0	Asteraceae Bercht. & J.Presl
Iz_3_1109	DJI_0120	Amorpha fruticosa L.	0	a	0	0	Fabaceae Lindl.
Iz_3_1109	DJI_0120	Anthericum ramosum L.	0	n	1	0	Asparagaceae Juss.
Iz_3_1109	DJI_0120	Artemisia marschalliana Spreng.	0	n	1	0	Asteraceae Bercht. & J.Presl
Iz_3_1109	DJI_0120	Asparagus officinalis L.	0	n	1	0	Asparagaceae Juss.
Iz_3_1109	DJI_0120	Astragalus cicer L.	0	n	1	0	Fabaceae Lindl.
Iz_3_1109	DJI_0120	Bassia laniflora (S.G.Gmel.) A.J.Scott	0	n	1	0	Amaranthaceae Juss.

Iz_3_1109	DJI_0120	Berberis vulgaris L.	0	n	1	0	Berberidaceae Juss.
Iz_3_1109	DJI_0120	Betula pendula Roth.	0	n	1	1	Betulaceae Gray
Iz_3_1109	DJI_0120	Betula pendula Roth.	0	n	1	0	Poaceae Barnhart
Iz_3_1109	DJI_0120	Bromus inermis Leyss.	0		NA	NA	
Iz_3_1109	DJI_0120	Bromus squarrosus L.	0	a	0	0	Poaceae Barnhart
Iz_3_1109	DJI_0120	Bromus tectorum L.	0	a	0	0	Poaceae Barnhart
Iz_3_1109	DJI_0120	Calamagrostis epigejos (L.) Roth	3	n	1	0	Poaceae Barnhart
Iz_3_1109	DJI_0120	Carduus acanthoides L.	0	n	1	0	Asteraceae Bercht. & J.Presl
Iz_3_1109	DJI_0120	Carex sp.	0	n	1	0	Cyperaceae Juss.
Iz_3_1109	DJI_0120	Centaurea majorovii Dumbadze	0	n	1	0	Asteraceae Bercht. & J.Presl
Iz_3_1109	DJI_0120	Chamaecytisus ruthenicus (Fisch.ex Wol.) Klaskova	0	n	1	0	Fabaceae Lindl.
Iz_3_1109	DJI_0120	Chelidonium majus L.	0	n	1	0	Papaveraceae Juss.
Iz_3_1109	DJI_0120	Chenopodium betaceum Andrz.	0	n	1	0	Amaranthaceae Juss.
Iz_3_1109	DJI_0120	Chenopodium cf. album	0	n	1	0	Amaranthaceae Juss.
Iz_3_1109	DJI_0120	Chenopodium sp.	0		NA	0	Amaranthaceae Juss.
Iz_3_1109	DJI_0120	Chondrilla juncea L.	0	n	1	0	Asteraceae Bercht. & J.Presl
Iz_3_1109	DJI_0120	Cirsium vulgare (Savi) Ten.	0	n	1	0	Asteraceae Bercht. & J.Presl
Iz_3_1109	DJI_0120	Clinopodium acinos (L.) Kuntze	0	n	1	0	Lamiacea Lindl.
Iz_3_1109	DJI_0120	Convallaria majalis L.	0	n	1	0	Asparagaceae Juss.
Iz_3_1109	DJI_0120	Crataegus rhipidophylla Gand.	0	n	1	1	Rosaceae Juss.
Iz_3_1109	DJI_0120	Crepis foetida L.	0	n	1	0	Asteraceae Bercht. & J.Presl
Iz_3_1109	DJI_0120	Dactylis glomerata L.	0	n	1	0	Poaceae Barnhart
Iz_3_1109	DJI_0120	Dianthus campestris M.Bieb.	0	n	1	0	Caryophyllaceae Juss.
Iz_3_1109	DJI_0120	Digitaria sanguinalis (L.) Scop.	0	a	0	0	Poaceae Barnhart
Iz_3_1109	DJI_0120	Elymus repens (L.) Gould	0	n	1	0	Poaceae Barnhart
Iz_3_1109	DJI_0120	Epilobium tetragonum L.	0	n	1	0	Onagraceae Juss.
Iz_3_1109	DJI_0120	Eragrostis minor Host	0	a	0	0	Poaceae Barnhart
Iz_3_1109	DJI_0120	Erigeron canadensis L.	0.2	a	0	0	Asteraceae Bercht. & J.Presl
Iz_3_1109	DJI_0120	Euphorbia seguieriana Neck.	0	n	1	0	Euphorbiaceae Juss.
Iz_3_1109	DJI_0120	Fallopia dumetorum (L.) Holub	0.2	n	1	0	Polygonaceae Juss.
Iz_3_1109	DJI_0120	Festuca beckeri (Hack.) Trautv.	0	n	1	0	Poaceae Barnhart
Iz_3_1109	DJI_0120	Fragaria vesca L.	0	n	1	0	Rosaceae Juss.
Iz_3_1109	DJI_0120	Frangula alnus Mill.	0	n	1	1	Rhamnaceae Juss.
Iz_3_1109	DJI_0120	Fraxinus pennsylvanica Marshall	0	a	0	1	Oleaceae Hoffmanns. & Link
Iz_3_1109	DJI_0120	Galeopsis bifida Boenn.	0	n	1	0	Lamiacea Lindl.
Iz_3_1109	DJI_0120	Galium verum L.	0	n	1	0	Rubiaceae Juss.
Iz_3_1109	DJI_0120	Genista tinctoria L.	0	n	1	0	Fabaceae Lindl.
Iz_3_1109	DJI_0120	Geranium robertianum L.	0	n	1	0	Geraniaceae Juss.
Iz_3_1109	DJI_0120	Geranium sanquineum L.	0	n	1	0	Geraniaceae Juss.
Iz_3_1109	DJI_0120	Gypsophila paniculata L.	0	n	1	0	Caryophyllaceae Juss.
Iz_3_1109	DJI_0120	Helichrysum arenarium (L.) Moench	0	n	1	0	Asteraceae Bercht. & J.Presl
Iz_3_1109	DJI_0120	Herniaria polygama J.Gay	0	n	1	0	Caryophyllaceae Juss.
Iz_3_1109	DJI_0120	Hieracium robustum Fr.	0	n	1	0	Asteraceae Bercht. & J.Presl
Iz_3_1109	DJI_0120	Hieracium umbellatum L.	0	n	1	NA	Asteraceae Bercht. & J.Presl
Iz_3_1109	DJI_0120	Hylotelephium maximum (L.) Holub	0	n	1	0	Crassulaceae J.St.-Hil.
Iz_3_1109	DJI_0120	Jacobaea borysthenica (DC.) B.Nord. & Greuter	0	n	1	0	Asteraceae Bercht. & J.Presl
Iz_3_1109	DJI_0120	Jasione montana L.	0	n	1	0	Campanulaceae Juss.
Iz_3_1109	DJI_0120	Jurinea cyanoides (L.) Rchb.s.l.	0	n	1	0	Asteraceae Bercht. & J.Presl
Iz_3_1109	DJI_0120	Koeleria glauca (Spreng.) DC.	0	n	1	0	Poaceae Barnhart
Iz_3_1109	DJI_0120	Lactuca serriola L.	0	a	0	0	Asteraceae Bercht. & J.Presl
Iz_3_1109	DJI_0120	Linaria genistifolia (L.) Mill.	0	n	1	0	Plantaginaceae Juss.
Iz_3_1109	DJI_0120	Lomelosia argentea (L.) Greuter & Burdet	0	n	1	0	Caprifoliaceae Juss.
Iz_3_1109	DJI_0120	Melampyrum pratense L.	0	n	1	0	Orobanchaceae Vent.
Iz_3_1109	DJI_0120	Oenothera biennis L.	0	a	0	0	Onagraceae Juss.
Iz_3_1109	DJI_0120	Pentanema britannica (L.) D.Gut.Larr., Santos-Vicente, Anderb., E.Rico & M.M.Mart.Ort.	0	n	1	0	Asteraceae Bercht. & J.Presl
Iz_3_1109	DJI_0120	Peucedanum oreoselinum (L.) Moench	0	n	1	0	Apiaceae Lindl.
Iz_3_1109	DJI_0120	Peucedanum ruthenicum M.Bieb.	0	n	1	0	Apiaceae Lindl.
Iz_3_1109	DJI_0120	Pilosella echioides (Lumn.) F.W.Schultz & Sch.Bip.	0	n	1	0	Asteraceae Bercht. & J.Presl
Iz_3_1109	DJI_0120	Pilosella officinarum F. Schultz et Sch. Bip.	0	n	1	0	Asteraceae Bercht. & J.Presl
Iz_3_1109	DJI_0120	Pilosella sp.	0		NA	0	Asteraceae Bercht. & J.Presl
Iz_3_1109	DJI_0120	Pinus sylvestris L.	0	n	1	1	Pinaceae Spreng. ex F.Rudolphi
Iz_3_1109	DJI_0120	Poa annua L.	0	n	1	0	Poaceae Barnhart
Iz_3_1109	DJI_0120	Poa nemoralis L.	0	n	1	0	Poaceae Barnhart
Iz_3_1109	DJI_0120	Poa sp.	0		NA	0	Poaceae Barnhart
Iz_3_1109	DJI_0120	Polygonatum odoratum (Mill.) Druce	0	n	1	0	Asparagaceae Juss.
Iz_3_1109	DJI_0120	Polygonum arenarium Waldst. & Kit.	0	n	1	0	Polygonaceae Juss.
Iz_3_1109	DJI_0120	Populus nigra L.	0	n	1	1	Salicaceae Mirb.
Iz_3_1109	DJI_0120	Populus tremula L.	0	n	1	1	Salicaceae Mirb.
Iz_3_1109	DJI_0120	Populus Ã canescens (Aiton) Sm.	0	n	1	1	Salicaceae Mirb.
Iz_3_1109	DJI_0120	Potentilla argentea L.	0	n	1	0	Rosaceae Juss.
Iz_3_1109	DJI_0120	Psammophiliella muralis (L.) Ikonn.	0	n	1	0	Caryophyllaceae Juss.
Iz_3_1109	DJI_0120	Psephellus sumensis (Kalen.) Greuter	0	n	1	0	Asteraceae Bercht. & J.Presl
Iz_3_1109	DJI_0120	Pulsatilla pratensis (L.) Mill. s.l.	0	n	1	0	Ranunculaceae Juss.
Iz_3_1109	DJI_0120	Pyrus communis L.	0	a	0	1	Rosaceae Juss.
Iz_3_1109	DJI_0120	Quercus robur L.	0	n	1	1	Fagaceae Dumort.
Iz_3_1109	DJI_0120	Robinia pseudoacacia L.	0	a	0	1	Fabaceae Lindl.
Iz_3_1109	DJI_0120	Rosa sp.	0		NA	1	Rosaceae Juss.
Iz_3_1109	DJI_0120	Rubus idaeus L.	0	n	1	0	Rosaceae Juss.
Iz_3_1109	DJI_0120	Rumex acetosella L.	0	n	1	0	Polygonaceae Juss.
Iz_3_1109	DJI_0120	Rumex thyrsiflorus Fingerh.	0	n	1	0	Polygonaceae Juss.
Iz_3_1109	DJI_0120	Salix alba L.	0	n	1	1	Salicaceae Mirb.
Iz_3_1109	DJI_0120	Sambucus nigra L.	0	n	1	1	Viburnaceae Raf.
Iz_3_1109	DJI_0120	Secale sylvestre Host	0	n	1	0	Poaceae Barnhart
Iz_3_1109	DJI_0120	Sempervivum ruthenicum Schnittsp. & C.B. Lehm.	0	n	1	0	Crassulaceae J.St.-Hil.
Iz_3_1109	DJI_0120	Setaria viridis (L.) P. Beauv.	0	n	1	0	Poaceae Barnhart
Iz_3_1109	DJI_0120	Silene borysthenica (Gruner) Walters	0	n	1	0	Caryophyllaceae Juss.
Iz_3_1109	DJI_0120	Silene latifolia subsp. alba (Mill.) Greuter & Burdet	0	n	1	0	Caryophyllaceae Juss.
Iz_3_1109	DJI_0120	Solidago virgaurea L.	0	n	1	0	Asteraceae Bercht. & J.Presl
Iz_3_1109	DJI_0120	Sorbus aucuparia L.	0	n	1	1	Rosaceae Juss.
Iz_3_1109	DJI_0120	Species 11.09_3_0045	0		NA	0	
Iz_3_1109	DJI_0120	Species 11.09_3_0058	0		NA	0	
Iz_3_1109	DJI_0120	Stachys recta L.	0	n	1	0	Lamiacea Lindl.
Iz_3_1109	DJI_0120	Tanacetum vulgare L.	0	n	1	0	Asteraceae Bercht. & J.Presl
Iz_3_1109	DJI_0120	Thymus pallasianus Heinr.Braun	0	n	1	0	Lamiacea Lindl.
Iz_3_1109	DJI_0120	Tilia cordata Mill.	0	n	1	1	Malvaceae Juss.
Iz_3_1109	DJI_0120	Trifolium alpestre L.	0	n	1	0	Fabaceae Lindl.
Iz_3_1109	DJI_0120	Trifolium arvense L.	0	n	1	0	Fabaceae Lindl.
Iz_3_1109	DJI_0120	Ulmus glabra Huds.	0	n	1	1	Ulmaceae Mirb.

Iz_3_1109	DJI_0120	Verbascum densiflorum Bertol.	0	n	1	0	Scrophulariaceae Juss.
Iz_3_1109	DJI_0120	Veronica austriaca L.	0	n	1	0	Plantaginaceae Juss.
Iz_3_1109	DJI_0120	Veronica incana L.	0	n	1	0	Plantaginaceae Juss.
Iz_3_1109	DJI_0120	Veronica verna L.	0	n	1	0	Plantaginaceae Juss.
Iz_3_1109	DJI_0120	Vincetoxicum hirundinaria Medik	0	n	1	0	Apocynaceae Juss.
Iz_3_1109	DJI_0123	Acer tataricum L.	0	n	1	1	Sapindaceae Juss.
Iz_3_1109	DJI_0123	Achillea millefolium L. s.l.	0	n	1	0	Asteraceae Bercht. & J.Presl
Iz_3_1109	DJI_0123	Allium oleraceum L.	0	n	1	0	Amaryllidaceae J.St.-Hil.
Iz_3_1109	DJI_0123	Ambrosia artemisiifolia L.	0	a	0	0	Asteraceae Bercht. & J.Presl
Iz_3_1109	DJI_0123	Amorpha fruticosa L.	0	a	0	0	Fabaceae Lindl.
Iz_3_1109	DJI_0123	Anthericum ramosum L.	0	n	1	0	Asparagaceae Juss.
Iz_3_1109	DJI_0123	Artemisia marschalliana Spreng.	0	n	1	0	Asteraceae Bercht. & J.Presl
Iz_3_1109	DJI_0123	Asparagus officinalis L.	0	n	1	0	Asparagaceae Juss.
Iz_3_1109	DJI_0123	Astragalus cicer L.	0	n	1	0	Fabaceae Lindl.
Iz_3_1109	DJI_0123	Bassia laniflora (S.G.Gmel.) A.J.Scott	0	n	1	0	Amaranthaceae Juss.
Iz_3_1109	DJI_0123	Berberis vulgaris L.	0	n	1	0	Berberidaceae Juss.
Iz_3_1109	DJI_0123	Betula pendula Roth.	0	n	1	1	Betulaceae Gray
Iz_3_1109	DJI_0123	Betula pendula Roth.	0	n	1	0	Poaceae Barnhart
Iz_3_1109	DJI_0123	Bromus inermis Leyss.	0		NA	NA	
Iz_3_1109	DJI_0123	Bromus squarrosus L.	0	a	0	0	Poaceae Barnhart
Iz_3_1109	DJI_0123	Bromus tectorum L.	0	a	0	0	Poaceae Barnhart
Iz_3_1109	DJI_0123	Calamagrostis epigejos (L.) Roth	0	n	1	0	Poaceae Barnhart
Iz_3_1109	DJI_0123	Carduus acanthoides L.	0	n	1	0	Asteraceae Bercht. & J.Presl
Iz_3_1109	DJI_0123	Carex sp.	0	n	1	0	Cyperaceae Juss.
Iz_3_1109	DJI_0123	Centaurea majorovii Dumbadze	0	n	1	0	Asteraceae Bercht. & J.Presl
Iz_3_1109	DJI_0123	Chamaecytisus ruthenicus (Fisch.ex Wol.) Klaskova	0	n	1	0	Fabaceae Lindl.
Iz_3_1109	DJI_0123	Chelidonium majus L.	0	n	1	0	Papaveraceae Juss.
Iz_3_1109	DJI_0123	Chenopodium betaceum Andrz.	0	n	1	0	Amaranthaceae Juss.
Iz_3_1109	DJI_0123	Chenopodium cf. album	0	n	1	0	Amaranthaceae Juss.
Iz_3_1109	DJI_0123	Chenopodium sp.	0		NA	0	Amaranthaceae Juss.
Iz_3_1109	DJI_0123	Chondrilla juncea L.	0	n	1	0	Asteraceae Bercht. & J.Presl
Iz_3_1109	DJI_0123	Cirsium vulgare (Savi) Ten.	0	n	1	0	Asteraceae Bercht. & J.Presl
Iz_3_1109	DJI_0123	Clinopodium acinos (L.) Kuntze	0	n	1	0	Lamiacea Lindl.
Iz_3_1109	DJI_0123	Convallaria majalis L.	0	n	1	0	Asparagaceae Juss.
Iz_3_1109	DJI_0123	Crataegus rhipidophylla Gand.	0	n	1	1	Rosaceae Juss.
Iz_3_1109	DJI_0123	Crepis foetida L.	0	n	1	0	Asteraceae Bercht. & J.Presl
Iz_3_1109	DJI_0123	Dactylis glomerata L.	0	n	1	0	Poaceae Barnhart
Iz_3_1109	DJI_0123	Dianthus campestris M.Bieb.	0	n	1	0	Caryophyllaceae Juss.
Iz_3_1109	DJI_0123	Digitaria sanguinalis (L.) Scop.	0	a	0	0	Poaceae Barnhart
Iz_3_1109	DJI_0123	Elymus repens (L.) Gould	0	n	1	0	Poaceae Barnhart
Iz_3_1109	DJI_0123	Epilobium tetragonum L.	0	n	1	0	Onagraceae Juss.
Iz_3_1109	DJI_0123	Eragrostis minor Host	0	a	0	0	Poaceae Barnhart
Iz_3_1109	DJI_0123	Erigeron canadensis L.	0.1	a	0	0	Asteraceae Bercht. & J.Presl
Iz_3_1109	DJI_0123	Euphorbia seguieriana Neck.	0	n	1	0	Euphorbiaceae Juss.
Iz_3_1109	DJI_0123	Fallopia dumetorum (L.) Holub	0	n	1	0	Polygonaceae Juss.
Iz_3_1109	DJI_0123	Festuca beckeri (Hack.) Trautv.	0	n	1	0	Poaceae Barnhart
Iz_3_1109	DJI_0123	Fragaria vesca L.	0	n	1	0	Rosaceae Juss.
Iz_3_1109	DJI_0123	Frangula alnus Mill.	0	n	1	1	Rhamnaceae Juss.
Iz_3_1109	DJI_0123	Fraxinus pennsylvanica Marshall	0	a	0	1	Oleaceae Hoffmanns. & Link
Iz_3_1109	DJI_0123	Galeopsis bifida Boenn.	0	n	1	0	Lamiacea Lindl.
Iz_3_1109	DJI_0123	Galium verum L.	0	n	1	0	Rubiaceae Juss.
Iz_3_1109	DJI_0123	Genista tinctoria L.	0	n	1	0	Fabaceae Lindl.
Iz_3_1109	DJI_0123	Geranium robertianum L.	0	n	1	0	Geraniaceae Juss.
Iz_3_1109	DJI_0123	Geranium sanquineum L.	0	n	1	0	Geraniaceae Juss.
Iz_3_1109	DJI_0123	Gypsophila paniculata L.	0	n	1	0	Caryophyllaceae Juss.
Iz_3_1109	DJI_0123	Helichrysum arenarium (L.) Moench	0	n	1	0	Asteraceae Bercht. & J.Presl
Iz_3_1109	DJI_0123	Herniaria polygama J.Gay	0	n	1	0	Caryophyllaceae Juss.
Iz_3_1109	DJI_0123	Hieracium robustum Fr.	0	n	1	0	Asteraceae Bercht. & J.Presl
Iz_3_1109	DJI_0123	Hieracium umbellatum L.	0	n	1	NA	Asteraceae Bercht. & J.Presl
Iz_3_1109	DJI_0123	Hylotelephium maximum (L.) Holub	0	n	1	0	Crassulaceae J.St.-Hil.
Iz_3_1109	DJI_0123	Jacobaea borysthenica (DC.) B.Nord. & Greuter	0	n	1	0	Asteraceae Bercht. & J.Presl
Iz_3_1109	DJI_0123	Jasione montana L.	0	n	1	0	Campanulaceae Juss.
Iz_3_1109	DJI_0123	Jurinea cyanoides (L.) Rchb.s.l.	0	n	1	0	Asteraceae Bercht. & J.Presl
Iz_3_1109	DJI_0123	Koeleria glauca (Spreng.) DC.	0	n	1	0	Poaceae Barnhart
Iz_3_1109	DJI_0123	Lactuca serriola L.	0	a	0	0	Asteraceae Bercht. & J.Presl
Iz_3_1109	DJI_0123	Linaria genistifolia (L.) Mill.	0	n	1	0	Plantaginaceae Juss.
Iz_3_1109	DJI_0123	Lomelosia argentea (L.) Greuter & Burdet	0	n	1	0	Caprifoliaceae Juss.
Iz_3_1109	DJI_0123	Melampyrum pratense L.	0	n	1	0	Orobanchaceae Vent.
Iz_3_1109	DJI_0123	Oenothera biennis L.	0	a	0	0	Onagraceae Juss.
Iz_3_1109	DJI_0123	Pentanema britannica (L.) D.Gut.Larr., Santos-Vicente, Anderb., E.Rico & M.M.Mart.Ort.	0	n	1	0	Asteraceae Bercht. & J.Presl
Iz_3_1109	DJI_0123	Peucedanum oreoselinum (L.) Moench	0	n	1	0	Apiaceae Lindl.
Iz_3_1109	DJI_0123	Peucedanum ruthenicum M.Bieb.	0	n	1	0	Apiaceae Lindl.
Iz_3_1109	DJI_0123	Pilosella echioides (Lumn.) F.W.Schultz & Sch.Bip.	0	n	1	0	Asteraceae Bercht. & J.Presl
Iz_3_1109	DJI_0123	Pilosella officinarum F. Schultz et Sch. Bip.	0	n	1	0	Asteraceae Bercht. & J.Presl
Iz_3_1109	DJI_0123	Pilosella sp.	0		NA	0	Asteraceae Bercht. & J.Presl
Iz_3_1109	DJI_0123	Pinus sylvestris L.	0	n	1	1	Pinaceae Spreng. ex F.Rudolphi
Iz_3_1109	DJI_0123	Poa annua L.	0	n	1	0	Poaceae Barnhart
Iz_3_1109	DJI_0123	Poa nemoralis L.	0	n	1	0	Poaceae Barnhart
Iz_3_1109	DJI_0123	Poa sp.	0		NA	0	Poaceae Barnhart
Iz_3_1109	DJI_0123	Polygonatum odoratum (Mill.) Druce	0	n	1	0	Asparagaceae Juss.
Iz_3_1109	DJI_0123	Polygonum arenarium Waldst. & Kit.	0	n	1	0	Polygonaceae Juss.
Iz_3_1109	DJI_0123	Populus nigra L.	0	n	1	1	Salicaceae Mirb.
Iz_3_1109	DJI_0123	Populus tremula L.	0	n	1	1	Salicaceae Mirb.
Iz_3_1109	DJI_0123	Populus Ã canescens (Aiton) Sm.	0	n	1	1	Salicaceae Mirb.
Iz_3_1109	DJI_0123	Potentilla argentea L.	0	n	1	0	Rosaceae Juss.
Iz_3_1109	DJI_0123	Psammophiliella muralis (L.) Ikonn.	0	n	1	0	Caryophyllaceae Juss.
Iz_3_1109	DJI_0123	Psephellus sumensis (Kalen.) Greuter	0	n	1	0	Asteraceae Bercht. & J.Presl
Iz_3_1109	DJI_0123	Pulsatilla pratensis (L.) Mill. s.l.	0	n	1	0	Ranunculaceae Juss.
Iz_3_1109	DJI_0123	Pyrus communis L.	0	a	0	1	Rosaceae Juss.
Iz_3_1109	DJI_0123	Quercus robur L.	0	n	1	1	Fagaceae Dumort.
Iz_3_1109	DJI_0123	Robinia pseudoacacia L.	0	a	0	1	Fabaceae Lindl.
Iz_3_1109	DJI_0123	Rosa sp.	0		NA	1	Rosaceae Juss.
Iz_3_1109	DJI_0123	Rubus idaeus L.	0	n	1	0	Rosaceae Juss.
Iz_3_1109	DJI_0123	Rumex acetosella L.	0	n	1	0	Polygonaceae Juss.
Iz_3_1109	DJI_0123	Rumex thyrsiflorus Fingerh.	0	n	1	0	Polygonaceae Juss.
Iz_3_1109	DJI_0123	Salix alba L.	0	n	1	1	Salicaceae Mirb.
Iz_3_1109	DJI_0123	Sambucus nigra L.	0	n	1	1	Viburnaceae Raf.
Iz_3_1109	DJI_0123	Secale sylvestre Host	0	n	1	0	Poaceae Barnhart

Iz_3_1109	DJI_0123	Sempervivum ruthenicum Schnittsp. & C.B. Lehm.	0	n	1	0	Crassulaceae J.St.-Hil.
Iz_3_1109	DJI_0123	Setaria viridis (L.) P. Beauv.	0	n	1	0	Poaceae Barnhart
Iz_3_1109	DJI_0123	Silene borysthenica (Gruner) Walters	0	n	1	0	Caryophyllaceae Juss.
Iz_3_1109	DJI_0123	Silene latifolia subsp. alba (Mill.) Greuter & Burdet	0	n	1	0	Caryophyllaceae Juss.
Iz_3_1109	DJI_0123	Solidago virgaurea L.	0	n	1	0	Asteraceae Bercht. & J.Presl
Iz_3_1109	DJI_0123	Sorbus aucuparia L.	0	n	1	1	Rosaceae Juss.
Iz_3_1109	DJI_0123	Species 11.09_3_0045	0		NA	0	
Iz_3_1109	DJI_0123	Species 11.09_3_0058	0		NA	0	
Iz_3_1109	DJI_0123	Stachys recta L.	0	n	1	0	Lamiacea Lindl.
Iz_3_1109	DJI_0123	Tanacetum vulgare L.	0	n	1	0	Asteraceae Bercht. & J.Presl
Iz_3_1109	DJI_0123	Thymus pallasianus Heinr.Braun	0	n	1	0	Lamiacea Lindl.
Iz_3_1109	DJI_0123	Tilia cordata Mill.	0	n	1	1	Malvaceae Juss.
Iz_3_1109	DJI_0123	Trifolium alpestre L.	0	n	1	0	Fabaceae Lindl.
Iz_3_1109	DJI_0123	Trifolium arvense L.	0	n	1	0	Fabaceae Lindl.
Iz_3_1109	DJI_0123	Ulmus glabra Huds.	0	n	1	1	Ulmaceae Mirb.
Iz_3_1109	DJI_0123	Verbascum densiflorum Bertol.	0	n	1	0	Scrophulariaceae Juss.
Iz_3_1109	DJI_0123	Veronica austriaca L.	0	n	1	0	Plantaginaceae Juss.
Iz_3_1109	DJI_0123	Veronica incana L.	0	n	1	0	Plantaginaceae Juss.
Iz_3_1109	DJI_0123	Veronica verna L.	0	n	1	0	Plantaginaceae Juss.
Iz_3_1109	DJI_0123	Vincetoxicum hirundinaria Medik	0	n	1	0	Apocynaceae Juss.
Iz_3_1109	DJI_0126	Acer tataricum L.	0	n	1	1	Sapindaceae Juss.
Iz_3_1109	DJI_0126	Achillea millefolium L. s.l.	0	n	1	0	Asteraceae Bercht. & J.Presl
Iz_3_1109	DJI_0126	Allium oleraceum L.	0	n	1	0	Amaryllidaceae J.St.-Hil.
Iz_3_1109	DJI_0126	Ambrosia artemisiifolia L.	0	a	0	0	Asteraceae Bercht. & J.Presl
Iz_3_1109	DJI_0126	Amorpha fruticosa L.	0	a	0	0	Fabaceae Lindl.
Iz_3_1109	DJI_0126	Anthericum ramosum L.	0	n	1	0	Asparagaceae Juss.
Iz_3_1109	DJI_0126	Artemisia marschalliana Spreng.	0	n	1	0	Asteraceae Bercht. & J.Presl
Iz_3_1109	DJI_0126	Asparagus officinalis L.	0	n	1	0	Asparagaceae Juss.
Iz_3_1109	DJI_0126	Astragalus cicer L.	0	n	1	0	Fabaceae Lindl.
Iz_3_1109	DJI_0126	Bassia laniflora (S.G.Gmel.) A.J.Scott	0	n	1	0	Amaranthaceae Juss.
Iz_3_1109	DJI_0126	Berberis vulgaris L.	0	n	1	0	Berberidaceae Juss.
Iz_3_1109	DJI_0126	Betula pendula Roth.	0	n	1	1	Betulaceae Gray
Iz_3_1109	DJI_0126	Betula pendula Roth.	0	n	1	0	Poaceae Barnhart
Iz_3_1109	DJI_0126	Bromus inermis Leyss.	0		NA	NA	
Iz_3_1109	DJI_0126	Bromus squarrosus L.	0	a	0	0	Poaceae Barnhart
Iz_3_1109	DJI_0126	Bromus tectorum L.	0	a	0	0	Poaceae Barnhart
Iz_3_1109	DJI_0126	Calamagrostis epigejos (L.) Roth	0.2	n	1	0	Poaceae Barnhart
Iz_3_1109	DJI_0126	Carduus acanthoides L.	0	n	1	0	Asteraceae Bercht. & J.Presl
Iz_3_1109	DJI_0126	Carex sp.	0	n	1	0	Cyperaceae Juss.
Iz_3_1109	DJI_0126	Centaurea majorovii Dumbadze	0	n	1	0	Asteraceae Bercht. & J.Presl
Iz_3_1109	DJI_0126	Chamaecytisus ruthenicus (Fisch.ex Wol.) Klaskova	0	n	1	0	Fabaceae Lindl.
Iz_3_1109	DJI_0126	Chelidonium majus L.	0	n	1	0	Papaveraceae Juss.
Iz_3_1109	DJI_0126	Chenopodium betaceum Andrz.	0	n	1	0	Amaranthaceae Juss.
Iz_3_1109	DJI_0126	Chenopodium cf. album	0	n	1	0	Amaranthaceae Juss.
Iz_3_1109	DJI_0126	Chenopodium sp.	0		NA	0	Amaranthaceae Juss.
Iz_3_1109	DJI_0126	Chondrilla juncea L.	0	n	1	0	Asteraceae Bercht. & J.Presl
Iz_3_1109	DJI_0126	Cirsium vulgare (Savi) Ten.	0	n	1	0	Asteraceae Bercht. & J.Presl
Iz_3_1109	DJI_0126	Clinopodium acinos (L.) Kuntze	0	n	1	0	Lamiacea Lindl.
Iz_3_1109	DJI_0126	Convallaria majalis L.	0	n	1	0	Asparagaceae Juss.
Iz_3_1109	DJI_0126	Crataegus rhipidophylla Gand.	0	n	1	1	Rosaceae Juss.
Iz_3_1109	DJI_0126	Crepis foetida L.	0	n	1	0	Asteraceae Bercht. & J.Presl
Iz_3_1109	DJI_0126	Dactylis glomerata L.	0	n	1	0	Poaceae Barnhart
Iz_3_1109	DJI_0126	Dianthus campestris M.Bieb.	0	n	1	0	Caryophyllaceae Juss.
Iz_3_1109	DJI_0126	Digitaria sanguinalis (L.) Scop.	0	a	0	0	Poaceae Barnhart
Iz_3_1109	DJI_0126	Elymus repens (L.) Gould	0	n	1	0	Poaceae Barnhart
Iz_3_1109	DJI_0126	Epilobium tetragonum L.	0	n	1	0	Onagraceae Juss.
Iz_3_1109	DJI_0126	Eragrostis minor Host	0	a	0	0	Poaceae Barnhart
Iz_3_1109	DJI_0126	Erigeron canadensis L.	2	a	0	0	Asteraceae Bercht. & J.Presl
Iz_3_1109	DJI_0126	Euphorbia seguieriana Neck.	0	n	1	0	Euphorbiaceae Juss.
Iz_3_1109	DJI_0126	Fallopia dumetorum (L.) Holub	0.2	n	1	0	Polygonaceae Juss.
Iz_3_1109	DJI_0126	Festuca beckeri (Hack.) Trautv.	0	n	1	0	Poaceae Barnhart
Iz_3_1109	DJI_0126	Fragaria vesca L.	0	n	1	0	Rosaceae Juss.
Iz_3_1109	DJI_0126	Frangula alnus Mill.	0	n	1	1	Rhamnaceae Juss.
Iz_3_1109	DJI_0126	Fraxinus pennsylvanica Marshall	0	a	0	1	Oleaceae Hoffmanns. & Link
Iz_3_1109	DJI_0126	Galeopsis bifida Boenn.	0	n	1	0	Lamiacea Lindl.
Iz_3_1109	DJI_0126	Galium verum L.	0	n	1	0	Rubiaceae Juss.
Iz_3_1109	DJI_0126	Genista tinctoria L.	0	n	1	0	Fabaceae Lindl.
Iz_3_1109	DJI_0126	Geranium robertianum L.	0	n	1	0	Geraniaceae Juss.
Iz_3_1109	DJI_0126	Geranium sanquineum L.	0	n	1	0	Geraniaceae Juss.
Iz_3_1109	DJI_0126	Gypsophila paniculata L.	0	n	1	0	Caryophyllaceae Juss.
Iz_3_1109	DJI_0126	Helichrysum arenarium (L.) Moench	0	n	1	0	Asteraceae Bercht. & J.Presl
Iz_3_1109	DJI_0126	Herniaria polygama J.Gay	0	n	1	0	Caryophyllaceae Juss.
Iz_3_1109	DJI_0126	Hieracium robustum Fr.	0	n	1	0	Asteraceae Bercht. & J.Presl
Iz_3_1109	DJI_0126	Hieracium umbellatum L.	0	n	1	NA	Asteraceae Bercht. & J.Presl
Iz_3_1109	DJI_0126	Hylotelephium maximum (L.) Holub	0	n	1	0	Crassulaceae J.St.-Hil.
Iz_3_1109	DJI_0126	Jacobaea borysthenica (DC.) B.Nord. & Greuter	0	n	1	0	Asteraceae Bercht. & J.Presl
Iz_3_1109	DJI_0126	Jasione montana L.	0	n	1	0	Campanulaceae Juss.
Iz_3_1109	DJI_0126	Jurinea cyanoides (L.) Rchb.s.l.	0	n	1	0	Asteraceae Bercht. & J.Presl
Iz_3_1109	DJI_0126	Koeleria glauca (Spreng.) DC.	0	n	1	0	Poaceae Barnhart
Iz_3_1109	DJI_0126	Lactuca serriola L.	0	a	0	0	Asteraceae Bercht. & J.Presl
Iz_3_1109	DJI_0126	Linaria genistifolia (L.) Mill.	0	n	1	0	Plantaginaceae Juss.
Iz_3_1109	DJI_0126	Lomelosia argentea (L.) Greuter & Burdet	0	n	1	0	Caprifoliaceae Juss.
Iz_3_1109	DJI_0126	Melampyrum pratense L.	0	n	1	0	Orobanchaceae Vent.
Iz_3_1109	DJI_0126	Oenothera biennis L.	0	a	0	0	Onagraceae Juss.
Iz_3_1109	DJI_0126	Pentanema britannica (L.) D.Gut.Larr., Santos-Vicente, Anderb., E.Rico & M.M.Mart.Ort.	0	n	1	0	Asteraceae Bercht. & J.Presl
Iz_3_1109	DJI_0126	Peucedanum oreoselinum (L.) Moench	0	n	1	0	Apiaceae Lindl.
Iz_3_1109	DJI_0126	Peucedanum ruthenicum M.Bieb.	0	n	1	0	Apiaceae Lindl.
Iz_3_1109	DJI_0126	Pilosella echioides (Lumn.) F.W.Schultz & Sch.Bip.	0	n	1	0	Asteraceae Bercht. & J.Presl
Iz_3_1109	DJI_0126	Pilosella officinarum F. Schultz et Sch. Bip.	0	n	1	0	Asteraceae Bercht. & J.Presl
Iz_3_1109	DJI_0126	Pilosella sp.	0		NA	0	Asteraceae Bercht. & J.Presl
Iz_3_1109	DJI_0126	Pinus sylvestris L.	0	n	1	1	Pinaceae Spreng. ex F.Rudolphi
Iz_3_1109	DJI_0126	Poa annua L.	0	n	1	0	Poaceae Barnhart
Iz_3_1109	DJI_0126	Poa nemoralis L.	0	n	1	0	Poaceae Barnhart
Iz_3_1109	DJI_0126	Poa sp.	0		NA	0	Poaceae Barnhart
Iz_3_1109	DJI_0126	Polygonatum odoratum (Mill.) Druce	0	n	1	0	Asparagaceae Juss.
Iz_3_1109	DJI_0126	Polygonum arenarium Waldst. & Kit.	0	n	1	0	Polygonaceae Juss.
Iz_3_1109	DJI_0126	Populus nigra L.	0	n	1	1	Salicaceae Mirb.
Iz_3_1109	DJI_0126	Populus tremula L.	0	n	1	1	Salicaceae Mirb.

Iz_3_1109	DJI_0126	Populus Ã canescens (Aiton) Sm.	0	n	1	1	Salicaceae Mirb.
Iz_3_1109	DJI_0126	Potentilla argentea L.	0	n	1	0	Rosaceae Juss.
Iz_3_1109	DJI_0126	Psammophiliella muralis (L.) Ikonn.	0	n	1	0	Caryophyllaceae Juss.
Iz_3_1109	DJI_0126	Psephellus sumensis (Kalen.) Greuter	0	n	1	0	Asteraceae Bercht. & J.Presl
Iz_3_1109	DJI_0126	Pulsatilla pratensis (L.) Mill. s.l.	0	n	1	0	Ranunculaceae Juss.
Iz_3_1109	DJI_0126	Pyrus communis L.	0	a	0	1	Rosaceae Juss.
Iz_3_1109	DJI_0126	Quercus robur L.	0	n	1	1	Fagaceae Dumort.
Iz_3_1109	DJI_0126	Robinia pseudoacacia L.	0	a	0	1	Fabaceae Lindl.
Iz_3_1109	DJI_0126	Rosa sp.	0		NA	1	Rosaceae Juss.
Iz_3_1109	DJI_0126	Rubus idaeus L.	0	n	1	0	Rosaceae Juss.
Iz_3_1109	DJI_0126	Rumex acetosella L.	0	n	1	0	Polygonaceae Juss.
Iz_3_1109	DJI_0126	Rumex thyrsiflorus Fingerh.	0	n	1	0	Polygonaceae Juss.
Iz_3_1109	DJI_0126	Salix alba L.	0	n	1	1	Salicaceae Mirb.
Iz_3_1109	DJI_0126	Sambucus nigra L.	0	n	1	1	Viburnaceae Raf.
Iz_3_1109	DJI_0126	Secale sylvestre Host	0	n	1	0	Poaceae Barnhart
Iz_3_1109	DJI_0126	Sempervivum ruthenicum Schnittsp. & C.B. Lehm.	0	n	1	0	Crassulaceae J.St.-Hil.
Iz_3_1109	DJI_0126	Setaria viridis (L.) P. Beauv.	0	n	1	0	Poaceae Barnhart
Iz_3_1109	DJI_0126	Silene borysthenica (Gruner) Walters	0	n	1	0	Caryophyllaceae Juss.
Iz_3_1109	DJI_0126	Silene latifolia subsp. alba (Mill.) Greuter & Burdet	0	n	1	0	Caryophyllaceae Juss.
Iz_3_1109	DJI_0126	Solidago virgaurea L.	0	n	1	0	Asteraceae Bercht. & J.Presl
Iz_3_1109	DJI_0126	Sorbus aucuparia L.	0	n	1	1	Rosaceae Juss.
Iz_3_1109	DJI_0126	Species 11.09_3_0045	0		NA	0	
Iz_3_1109	DJI_0126	Species 11.09_3_0058	0		NA	0	
Iz_3_1109	DJI_0126	Stachys recta L.	0	n	1	0	Lamiacea Lindl.
Iz_3_1109	DJI_0126	Tanacetum vulgare L.	0	n	1	0	Asteraceae Bercht. & J.Presl
Iz_3_1109	DJI_0126	Thymus pallasianus Heinr.Braun	0	n	1	0	Lamiacea Lindl.
Iz_3_1109	DJI_0126	Tilia cordata Mill.	0	n	1	1	Malvaceae Juss.
Iz_3_1109	DJI_0126	Trifolium alpestre L.	0	n	1	0	Fabaceae Lindl.
Iz_3_1109	DJI_0126	Trifolium arvense L.	0	n	1	0	Fabaceae Lindl.
Iz_3_1109	DJI_0126	Ulmus glabra Huds.	0	n	1	1	Ulmaceae Mirb.
Iz_3_1109	DJI_0126	Verbascum densiflorum Bertol.	0	n	1	0	Scrophulariaceae Juss.
Iz_3_1109	DJI_0126	Veronica austriaca L.	0	n	1	0	Plantaginaceae Juss.
Iz_3_1109	DJI_0126	Veronica incana L.	0	n	1	0	Plantaginaceae Juss.
Iz_3_1109	DJI_0126	Veronica verna L.	0	n	1	0	Plantaginaceae Juss.
Iz_3_1109	DJI_0126	Vincetoxicum hirundinaria Medik	0	n	1	0	Apocynaceae Juss.
Iz_3_1109	DJI_0128	Acer tataricum L.	0	n	1	1	Sapindaceae Juss.
Iz_3_1109	DJI_0128	Achillea millefolium L. s.l.	0	n	1	0	Asteraceae Bercht. & J.Presl
Iz_3_1109	DJI_0128	Allium oleraceum L.	0	n	1	0	Amaryllidaceae J.St.-Hil.
Iz_3_1109	DJI_0128	Ambrosia artemisiifolia L.	0	a	0	0	Asteraceae Bercht. & J.Presl
Iz_3_1109	DJI_0128	Amorpha fruticosa L.	0	a	0	0	Fabaceae Lindl.
Iz_3_1109	DJI_0128	Anthericum ramosum L.	0	n	1	0	Asparagaceae Juss.
Iz_3_1109	DJI_0128	Artemisia marschalliana Spreng.	0	n	1	0	Asteraceae Bercht. & J.Presl
Iz_3_1109	DJI_0128	Asparagus officinalis L.	0	n	1	0	Asparagaceae Juss.
Iz_3_1109	DJI_0128	Astragalus cicer L.	0	n	1	0	Fabaceae Lindl.
Iz_3_1109	DJI_0128	Bassia laniflora (S.G.Gmel.) A.J.Scott	0	n	1	0	Amaranthaceae Juss.
Iz_3_1109	DJI_0128	Berberis vulgaris L.	0	n	1	0	Berberidaceae Juss.
Iz_3_1109	DJI_0128	Betula pendula Roth.	0	n	1	1	Betulaceae Gray
Iz_3_1109	DJI_0128	Betula pendula Roth.	0	n	1	0	Poaceae Barnhart
Iz_3_1109	DJI_0128	Bromus inermis Leyss.	0		NA	NA	
Iz_3_1109	DJI_0128	Bromus squarrosus L.	0	a	0	0	Poaceae Barnhart
Iz_3_1109	DJI_0128	Bromus tectorum L.	0	a	0	0	Poaceae Barnhart
Iz_3_1109	DJI_0128	Calamagrostis epigejos (L.) Roth	0	n	1	0	Poaceae Barnhart
Iz_3_1109	DJI_0128	Carduus acanthoides L.	0	n	1	0	Asteraceae Bercht. & J.Presl
Iz_3_1109	DJI_0128	Carex sp.	0	n	1	0	Cyperaceae Juss.
Iz_3_1109	DJI_0128	Centaurea majorovii Dumbadze	0	n	1	0	Asteraceae Bercht. & J.Presl
Iz_3_1109	DJI_0128	Chamaecytisus ruthenicus (Fisch.ex Wol.) Klaskova	0	n	1	0	Fabaceae Lindl.
Iz_3_1109	DJI_0128	Chelidonium majus L.	0	n	1	0	Papaveraceae Juss.
Iz_3_1109	DJI_0128	Chenopodium betaceum Andrz.	0	n	1	0	Amaranthaceae Juss.
Iz_3_1109	DJI_0128	Chenopodium cf. album	0	n	1	0	Amaranthaceae Juss.
Iz_3_1109	DJI_0128	Chenopodium sp.	0		NA	0	Amaranthaceae Juss.
Iz_3_1109	DJI_0128	Chondrilla juncea L.	0	n	1	0	Asteraceae Bercht. & J.Presl
Iz_3_1109	DJI_0128	Cirsium vulgare (Savi) Ten.	0	n	1	0	Asteraceae Bercht. & J.Presl
Iz_3_1109	DJI_0128	Clinopodium acinos (L.) Kuntze	0	n	1	0	Lamiacea Lindl.
Iz_3_1109	DJI_0128	Convallaria majalis L.	0	n	1	0	Asparagaceae Juss.
Iz_3_1109	DJI_0128	Crataegus rhipidophylla Gand.	0	n	1	1	Rosaceae Juss.
Iz_3_1109	DJI_0128	Crepis foetida L.	0	n	1	0	Asteraceae Bercht. & J.Presl
Iz_3_1109	DJI_0128	Dactylis glomerata L.	0	n	1	0	Poaceae Barnhart
Iz_3_1109	DJI_0128	Dianthus campestris M.Bieb.	0	n	1	0	Caryophyllaceae Juss.
Iz_3_1109	DJI_0128	Digitaria sanguinalis (L.) Scop.	0	a	0	0	Poaceae Barnhart
Iz_3_1109	DJI_0128	Elymus repens (L.) Gould	0	n	1	0	Poaceae Barnhart
Iz_3_1109	DJI_0128	Epilobium tetragonum L.	0	n	1	0	Onagraceae Juss.
Iz_3_1109	DJI_0128	Eragrostis minor Host	0	a	0	0	Poaceae Barnhart
Iz_3_1109	DJI_0128	Erigeron canadensis L.	0.2	a	0	0	Asteraceae Bercht. & J.Presl
Iz_3_1109	DJI_0128	Euphorbia seguieriana Neck.	0	n	1	0	Euphorbiaceae Juss.
Iz_3_1109	DJI_0128	Fallopia dumetorum (L.) Holub	0.5	n	1	0	Polygonaceae Juss.
Iz_3_1109	DJI_0128	Festuca beckeri (Hack.) Trautv.	0	n	1	0	Poaceae Barnhart
Iz_3_1109	DJI_0128	Fragaria vesca L.	0	n	1	0	Rosaceae Juss.
Iz_3_1109	DJI_0128	Frangula alnus Mill.	0	n	1	1	Rhamnaceae Juss.
Iz_3_1109	DJI_0128	Fraxinus pennsylvanica Marshall	0	a	0	1	Oleaceae Hoffmanns. & Link
Iz_3_1109	DJI_0128	Galeopsis bifida Boenn.	0	n	1	0	Lamiacea Lindl.
Iz_3_1109	DJI_0128	Galium verum L.	0	n	1	0	Rubiaceae Juss.
Iz_3_1109	DJI_0128	Genista tinctoria L.	0	n	1	0	Fabaceae Lindl.
Iz_3_1109	DJI_0128	Geranium robertianum L.	0	n	1	0	Geraniaceae Juss.
Iz_3_1109	DJI_0128	Geranium sanquineum L.	0	n	1	0	Geraniaceae Juss.
Iz_3_1109	DJI_0128	Gypsophila paniculata L.	0	n	1	0	Caryophyllaceae Juss.
Iz_3_1109	DJI_0128	Helichrysum arenarium (L.) Moench	0	n	1	0	Asteraceae Bercht. & J.Presl
Iz_3_1109	DJI_0128	Herniaria polygama J.Gay	0	n	1	0	Caryophyllaceae Juss.
Iz_3_1109	DJI_0128	Hieracium robustum Fr.	0	n	1	0	Asteraceae Bercht. & J.Presl
Iz_3_1109	DJI_0128	Hieracium umbellatum L.	0	n	1	NA	Asteraceae Bercht. & J.Presl
Iz_3_1109	DJI_0128	Hylotelephium maximum (L.) Holub	0	n	1	0	Crassulaceae J.St.-Hil.
Iz_3_1109	DJI_0128	Jacobaea borysthenica (DC.) B.Nord. & Greuter	0	n	1	0	Asteraceae Bercht. & J.Presl
Iz_3_1109	DJI_0128	Jasione montana L.	0	n	1	0	Campanulaceae Juss.
Iz_3_1109	DJI_0128	Jurinea cyanoides (L.) Rchb.s.l.	0	n	1	0	Asteraceae Bercht. & J.Presl
Iz_3_1109	DJI_0128	Koeleria glauca (Spreng.) DC.	0	n	1	0	Poaceae Barnhart
Iz_3_1109	DJI_0128	Lactuca serriola L.	0	a	0	0	Asteraceae Bercht. & J.Presl
Iz_3_1109	DJI_0128	Linaria genistifolia (L.) Mill.	0	n	1	0	Plantaginaceae Juss.
Iz_3_1109	DJI_0128	Lomelosia argentea (L.) Greuter & Burdet	0	n	1	0	Caprifoliaceae Juss.
Iz_3_1109	DJI_0128	Melampyrum pratense L.	0	n	1	0	Orobanchaceae Vent.

Iz_3_1109	DJI_0128	Oenothera biennis L.	0	a	0	0	Onagraceae Juss.
Iz_3_1109	DJI_0128	Pentanema britannica (L.) D.Gut.Larr., Santos-Vicente, Anderb., E.Rico & M.M.Mart.Ort.	0	n	1	0	Asteraceae Bercht. & J.Presl
Iz_3_1109	DJI_0128	Peucedanum oreoselinum (L.) Moench	0	n	1	0	Apiaceae Lindl.
Iz_3_1109	DJI_0128	Peucedanum ruthenicum M.Bieb.	0	n	1	0	Apiaceae Lindl.
Iz_3_1109	DJI_0128	Pilosella echioides (Lumn.) F.W.Schultz & Sch.Bip.	0	n	1	0	Asteraceae Bercht. & J.Presl
Iz_3_1109	DJI_0128	Pilosella officinarum F. Schultz et Sch. Bip.	0	n	1	0	Asteraceae Bercht. & J.Presl
Iz_3_1109	DJI_0128	Pilosella sp.	0		NA	0	Asteraceae Bercht. & J.Presl
Iz_3_1109	DJI_0128	Pinus sylvestris L.	0	n	1	1	Pinaceae Spreng. ex F.Rudolphi
Iz_3_1109	DJI_0128	Poa annua L.	0	n	1	0	Poaceae Barnhart
Iz_3_1109	DJI_0128	Poa nemoralis L.	0	n	1	0	Poaceae Barnhart
Iz_3_1109	DJI_0128	Poa sp.	0		NA	0	Poaceae Barnhart
Iz_3_1109	DJI_0128	Polygonatum odoratum (Mill.) Druce	0	n	1	0	Asparagaceae Juss.
Iz_3_1109	DJI_0128	Polygonum arenarium Waldst. & Kit.	0	n	1	0	Polygonaceae Juss.
Iz_3_1109	DJI_0128	Populus nigra L.	0	n	1	1	Salicaceae Mirb.
Iz_3_1109	DJI_0128	Populus tremula L.	0	n	1	1	Salicaceae Mirb.
Iz_3_1109	DJI_0128	Populus Ã canescens (Aiton) Sm.	0	n	1	1	Salicaceae Mirb.
Iz_3_1109	DJI_0128	Potentilla argentea L.	0	n	1	0	Rosaceae Juss.
Iz_3_1109	DJI_0128	Psammophiliella muralis (L.) Ikonn.	0	n	1	0	Caryophyllaceae Juss.
Iz_3_1109	DJI_0128	Psephellus sumensis (Kalen.) Greuter	0	n	1	0	Asteraceae Bercht. & J.Presl
Iz_3_1109	DJI_0128	Pulsatilla pratensis (L.) Mill. s.l.	0	n	1	0	Ranunculaceae Juss.
Iz_3_1109	DJI_0128	Pyrus communis L.	0	a	0	1	Rosaceae Juss.
Iz_3_1109	DJI_0128	Quercus robur L.	0	n	1	1	Fagaceae Dumort.
Iz_3_1109	DJI_0128	Robinia pseudoacacia L.	0	a	0	1	Fabaceae Lindl.
Iz_3_1109	DJI_0128	Rosa sp.	0		NA	1	Rosaceae Juss.
Iz_3_1109	DJI_0128	Rubus idaeus L.	0	n	1	0	Rosaceae Juss.
Iz_3_1109	DJI_0128	Rumex acetosella L.	0	n	1	0	Polygonaceae Juss.
Iz_3_1109	DJI_0128	Rumex thyrsiflorus Fingerh.	0	n	1	0	Polygonaceae Juss.
Iz_3_1109	DJI_0128	Salix alba L.	0	n	1	1	Salicaceae Mirb.
Iz_3_1109	DJI_0128	Sambucus nigra L.	0	n	1	1	Viburnaceae Raf.
Iz_3_1109	DJI_0128	Secale sylvestre Host	0	n	1	0	Poaceae Barnhart
Iz_3_1109	DJI_0128	Sempervivum ruthenicum Schnittsp. & C.B. Lehm.	0	n	1	0	Crassulaceae J.St.-Hil.
Iz_3_1109	DJI_0128	Setaria viridis (L.) P. Beauv.	0	n	1	0	Poaceae Barnhart
Iz_3_1109	DJI_0128	Silene borysthenica (Gruner) Walters	0	n	1	0	Caryophyllaceae Juss.
Iz_3_1109	DJI_0128	Silene latifolia subsp. alba (Mill.) Greuter & Burdet	0	n	1	0	Caryophyllaceae Juss.
Iz_3_1109	DJI_0128	Solidago virgaurea L.	0	n	1	0	Asteraceae Bercht. & J.Presl
Iz_3_1109	DJI_0128	Sorbus aucuparia L.	0	n	1	1	Rosaceae Juss.
Iz_3_1109	DJI_0128	Species 11.09_3_0045	0		NA	0	
Iz_3_1109	DJI_0128	Species 11.09_3_0058	0		NA	0	
Iz_3_1109	DJI_0128	Stachys recta L.	0	n	1	0	Lamiacea Lindl.
Iz_3_1109	DJI_0128	Tanacetum vulgare L.	0	n	1	0	Asteraceae Bercht. & J.Presl
Iz_3_1109	DJI_0128	Thymus pallasianus Heinr.Braun	0	n	1	0	Lamiacea Lindl.
Iz_3_1109	DJI_0128	Tilia cordata Mill.	0	n	1	1	Malvaceae Juss.
Iz_3_1109	DJI_0128	Trifolium alpestre L.	0	n	1	0	Fabaceae Lindl.
Iz_3_1109	DJI_0128	Trifolium arvense L.	0	n	1	0	Fabaceae Lindl.
Iz_3_1109	DJI_0128	Ulmus glabra Huds.	0	n	1	1	Ulmaceae Mirb.
Iz_3_1109	DJI_0128	Verbascum densiflorum Bertol.	0.1	n	1	0	Scrophulariaceae Juss.
Iz_3_1109	DJI_0128	Veronica austriaca L.	0	n	1	0	Plantaginaceae Juss.
Iz_3_1109	DJI_0128	Veronica incana L.	0	n	1	0	Plantaginaceae Juss.
Iz_3_1109	DJI_0128	Veronica verna L.	0	n	1	0	Plantaginaceae Juss.
Iz_3_1109	DJI_0128	Vincetoxicum hirundinaria Medik	0	n	1	0	Apocynaceae Juss.
Iz_3_1109	DJI_0132	Acer tataricum L.	0	n	1	1	Sapindaceae Juss.
Iz_3_1109	DJI_0132	Achillea millefolium L. s.l.	0	n	1	0	Asteraceae Bercht. & J.Presl
Iz_3_1109	DJI_0132	Allium oleraceum L.	0	n	1	0	Amaryllidaceae J.St.-Hil.
Iz_3_1109	DJI_0132	Ambrosia artemisiifolia L.	0	a	0	0	Asteraceae Bercht. & J.Presl
Iz_3_1109	DJI_0132	Amorpha fruticosa L.	0	a	0	0	Fabaceae Lindl.
Iz_3_1109	DJI_0132	Anthericum ramosum L.	0	n	1	0	Asparagaceae Juss.
Iz_3_1109	DJI_0132	Artemisia marschalliana Spreng.	0	n	1	0	Asteraceae Bercht. & J.Presl
Iz_3_1109	DJI_0132	Asparagus officinalis L.	0	n	1	0	Asparagaceae Juss.
Iz_3_1109	DJI_0132	Astragalus cicer L.	0	n	1	0	Fabaceae Lindl.
Iz_3_1109	DJI_0132	Bassia laniflora (S.G.Gmel.) A.J.Scott	0	n	1	0	Amaranthaceae Juss.
Iz_3_1109	DJI_0132	Berberis vulgaris L.	0	n	1	0	Berberidaceae Juss.
Iz_3_1109	DJI_0132	Betula pendula Roth.	0	n	1	1	Betulaceae Gray
Iz_3_1109	DJI_0132	Betula pendula Roth.	0	n	1	0	Poaceae Barnhart
Iz_3_1109	DJI_0132	Bromus inermis Leyss.	0		NA	NA	
Iz_3_1109	DJI_0132	Bromus squarrosus L.	0	a	0	0	Poaceae Barnhart
Iz_3_1109	DJI_0132	Bromus tectorum L.	0	a	0	0	Poaceae Barnhart
Iz_3_1109	DJI_0132	Calamagrostis epigejos (L.) Roth	0	n	1	0	Poaceae Barnhart
Iz_3_1109	DJI_0132	Carduus acanthoides L.	0	n	1	0	Asteraceae Bercht. & J.Presl
Iz_3_1109	DJI_0132	Carex sp.	0	n	1	0	Cyperaceae Juss.
Iz_3_1109	DJI_0132	Centaurea majorovii Dumbadze	0	n	1	0	Asteraceae Bercht. & J.Presl
Iz_3_1109	DJI_0132	Chamaecytisus ruthenicus (Fisch.ex Wol.) Klaskova	0	n	1	0	Fabaceae Lindl.
Iz_3_1109	DJI_0132	Chelidonium majus L.	0	n	1	0	Papaveraceae Juss.
Iz_3_1109	DJI_0132	Chenopodium betaceum Andrz.	0	n	1	0	Amaranthaceae Juss.
Iz_3_1109	DJI_0132	Chenopodium cf. album	0	n	1	0	Amaranthaceae Juss.
Iz_3_1109	DJI_0132	Chenopodium sp.	0		NA	0	Amaranthaceae Juss.
Iz_3_1109	DJI_0132	Chondrilla juncea L.	0	n	1	0	Asteraceae Bercht. & J.Presl
Iz_3_1109	DJI_0132	Cirsium vulgare (Savi) Ten.	0	n	1	0	Asteraceae Bercht. & J.Presl
Iz_3_1109	DJI_0132	Clinopodium acinos (L.) Kuntze	0	n	1	0	Lamiacea Lindl.
Iz_3_1109	DJI_0132	Convallaria majalis L.	0	n	1	0	Asparagaceae Juss.
Iz_3_1109	DJI_0132	Crataegus rhipidophylla Gand.	0	n	1	1	Rosaceae Juss.
Iz_3_1109	DJI_0132	Crepis foetida L.	0	n	1	0	Asteraceae Bercht. & J.Presl
Iz_3_1109	DJI_0132	Dactylis glomerata L.	0	n	1	0	Poaceae Barnhart
Iz_3_1109	DJI_0132	Dianthus campestris M.Bieb.	0	n	1	0	Caryophyllaceae Juss.
Iz_3_1109	DJI_0132	Digitaria sanguinalis (L.) Scop.	0	a	0	0	Poaceae Barnhart
Iz_3_1109	DJI_0132	Elymus repens (L.) Gould	0	n	1	0	Poaceae Barnhart
Iz_3_1109	DJI_0132	Epilobium tetragonum L.	0	n	1	0	Onagraceae Juss.
Iz_3_1109	DJI_0132	Eragrostis minor Host	0	a	0	0	Poaceae Barnhart
Iz_3_1109	DJI_0132	Erigeron canadensis L.	0.2	a	0	0	Asteraceae Bercht. & J.Presl
Iz_3_1109	DJI_0132	Euphorbia seguieriana Neck.	0	n	1	0	Euphorbiaceae Juss.
Iz_3_1109	DJI_0132	Fallopia dumetorum (L.) Holub	0.5	n	1	0	Polygonaceae Juss.
Iz_3_1109	DJI_0132	Festuca beckeri (Hack.) Trautv.	0	n	1	0	Poaceae Barnhart
Iz_3_1109	DJI_0132	Fragaria vesca L.	0	n	1	0	Rosaceae Juss.
Iz_3_1109	DJI_0132	Frangula alnus Mill.	0	n	1	1	Rhamnaceae Juss.
Iz_3_1109	DJI_0132	Fraxinus pennsylvanica Marshall	0	a	0	1	Oleaceae Hoffmanns. & Link
Iz_3_1109	DJI_0132	Galeopsis bifida Boenn.	0	n	1	0	Lamiacea Lindl.
Iz_3_1109	DJI_0132	Galium verum L.	0	n	1	0	Rubiaceae Juss.
Iz_3_1109	DJI_0132	Genista tinctoria L.	0	n	1	0	Fabaceae Lindl.
Iz_3_1109	DJI_0132	Geranium robertianum L.	0	n	1	0	Geraniaceae Juss.

Iz_3_1109	DJI_0132	Geranium sanquineum L.	0	n	1	0	Geraniaceae Juss.
Iz_3_1109	DJI_0132	Gypsophila paniculata L.	0	n	1	0	Caryophyllaceae Juss.
Iz_3_1109	DJI_0132	Helichrysum arenarium (L.) Moench	0	n	1	0	Asteraceae Bercht. & J.Presl
Iz_3_1109	DJI_0132	Herniaria polygama J.Gay	0	n	1	0	Caryophyllaceae Juss.
Iz_3_1109	DJI_0132	Hieracium robustum Fr.	0	n	1	0	Asteraceae Bercht. & J.Presl
Iz_3_1109	DJI_0132	Hieracium umbellatum L.	0	n	1	NA	Asteraceae Bercht. & J.Presl
Iz_3_1109	DJI_0132	Hylotelephium maximum (L.) Holub	0	n	1	0	Crassulaceae J.St.-Hil.
Iz_3_1109	DJI_0132	Jacobaea borysthenica (DC.) B.Nord. & Greuter	0	n	1	0	Asteraceae Bercht. & J.Presl
Iz_3_1109	DJI_0132	Jasione montana L.	0	n	1	0	Campanulaceae Juss.
Iz_3_1109	DJI_0132	Jurinea cyanoides (L.) Rchb.s.l.	0	n	1	0	Asteraceae Bercht. & J.Presl
Iz_3_1109	DJI_0132	Koeleria glauca (Spreng.) DC.	0	n	1	0	Poaceae Barnhart
Iz_3_1109	DJI_0132	Lactuca serriola L.	0	a	0	0	Asteraceae Bercht. & J.Presl
Iz_3_1109	DJI_0132	Linaria genistifolia (L.) Mill.	0	n	1	0	Plantaginaceae Juss.
Iz_3_1109	DJI_0132	Lomelosia argentea (L.) Greuter & Burdet	0	n	1	0	Caprifoliaceae Juss.
Iz_3_1109	DJI_0132	Melampyrum pratense L.	0	n	1	0	Orobanchaceae Vent.
Iz_3_1109	DJI_0132	Oenothera biennis L.	0	a	0	0	Onagraceae Juss.
Iz_3_1109	DJI_0132	Pentanema britannica (L.) D.Gut.Larr., Santos-Vicente, Anderb., E.Rico & M.M.Mart.Ort.	0	n	1	0	Asteraceae Bercht. & J.Presl
Iz_3_1109	DJI_0132	Peucedanum oreoselinum (L.) Moench	0	n	1	0	Apiaceae Lindl.
Iz_3_1109	DJI_0132	Peucedanum ruthenicum M.Bieb.	0	n	1	0	Apiaceae Lindl.
Iz_3_1109	DJI_0132	Pilosella echioides (Lumn.) F.W.Schultz & Sch.Bip.	0	n	1	0	Asteraceae Bercht. & J.Presl
Iz_3_1109	DJI_0132	Pilosella officinarum F. Schultz et Sch. Bip.	0	n	1	0	Asteraceae Bercht. & J.Presl
Iz_3_1109	DJI_0132	Pilosella sp.	0		NA	0	Asteraceae Bercht. & J.Presl
Iz_3_1109	DJI_0132	Pinus sylvestris L.	0	n	1	1	Pinaceae Spreng. ex F.Rudolphi
Iz_3_1109	DJI_0132	Poa annua L.	0	n	1	0	Poaceae Barnhart
Iz_3_1109	DJI_0132	Poa nemoralis L.	0	n	1	0	Poaceae Barnhart
Iz_3_1109	DJI_0132	Poa sp.	0		NA	0	Poaceae Barnhart
Iz_3_1109	DJI_0132	Polygonatum odoratum (Mill.) Druce	0	n	1	0	Asparagaceae Juss.
Iz_3_1109	DJI_0132	Polygonum arenarium Waldst. & Kit.	0	n	1	0	Polygonaceae Juss.
Iz_3_1109	DJI_0132	Populus nigra L.	0	n	1	1	Salicaceae Mirb.
Iz_3_1109	DJI_0132	Populus tremula L.	0	n	1	1	Salicaceae Mirb.
Iz_3_1109	DJI_0132	Populus Ã canescens (Aiton) Sm.	0	n	1	1	Salicaceae Mirb.
Iz_3_1109	DJI_0132	Potentilla argentea L.	0	n	1	0	Rosaceae Juss.
Iz_3_1109	DJI_0132	Psammophiliella muralis (L.) Ikonn.	0	n	1	0	Caryophyllaceae Juss.
Iz_3_1109	DJI_0132	Psephellus sumensis (Kalen.) Greuter	0	n	1	0	Asteraceae Bercht. & J.Presl
Iz_3_1109	DJI_0132	Pulsatilla pratensis (L.) Mill. s.l.	0	n	1	0	Ranunculaceae Juss.
Iz_3_1109	DJI_0132	Pyrus communis L.	0	a	0	1	Rosaceae Juss.
Iz_3_1109	DJI_0132	Quercus robur L.	0	n	1	1	Fagaceae Dumort.
Iz_3_1109	DJI_0132	Robinia pseudoacacia L.	0	a	0	1	Fabaceae Lindl.
Iz_3_1109	DJI_0132	Rosa sp.	0		NA	1	Rosaceae Juss.
Iz_3_1109	DJI_0132	Rubus idaeus L.	0	n	1	0	Rosaceae Juss.
Iz_3_1109	DJI_0132	Rumex acetosella L.	0	n	1	0	Polygonaceae Juss.
Iz_3_1109	DJI_0132	Rumex thyrsiflorus Fingerh.	0	n	1	0	Polygonaceae Juss.
Iz_3_1109	DJI_0132	Salix alba L.	0	n	1	1	Salicaceae Mirb.
Iz_3_1109	DJI_0132	Sambucus nigra L.	0	n	1	1	Viburnaceae Raf.
Iz_3_1109	DJI_0132	Secale sylvestre Host	0	n	1	0	Poaceae Barnhart
Iz_3_1109	DJI_0132	Sempervivum ruthenicum Schnittsp. & C.B. Lehm.	0	n	1	0	Crassulaceae J.St.-Hil.
Iz_3_1109	DJI_0132	Setaria viridis (L.) P. Beauv.	0	n	1	0	Poaceae Barnhart
Iz_3_1109	DJI_0132	Silene borysthenica (Gruner) Walters	0	n	1	0	Caryophyllaceae Juss.
Iz_3_1109	DJI_0132	Silene latifolia subsp. alba (Mill.) Greuter & Burdet	0	n	1	0	Caryophyllaceae Juss.
Iz_3_1109	DJI_0132	Solidago virgaurea L.	0	n	1	0	Asteraceae Bercht. & J.Presl
Iz_3_1109	DJI_0132	Sorbus aucuparia L.	0	n	1	1	Rosaceae Juss.
Iz_3_1109	DJI_0132	Species 11.09_3_0045	0		NA	0	
Iz_3_1109	DJI_0132	Species 11.09_3_0058	0		NA	0	
Iz_3_1109	DJI_0132	Stachys recta L.	0	n	1	0	Lamiacea Lindl.
Iz_3_1109	DJI_0132	Tanacetum vulgare L.	0	n	1	0	Asteraceae Bercht. & J.Presl
Iz_3_1109	DJI_0132	Thymus pallasianus Heinr.Braun	0	n	1	0	Lamiacea Lindl.
Iz_3_1109	DJI_0132	Tilia cordata Mill.	0	n	1	1	Malvaceae Juss.
Iz_3_1109	DJI_0132	Trifolium alpestre L.	0	n	1	0	Fabaceae Lindl.
Iz_3_1109	DJI_0132	Trifolium arvense L.	0	n	1	0	Fabaceae Lindl.
Iz_3_1109	DJI_0132	Ulmus glabra Huds.	0	n	1	1	Ulmaceae Mirb.
Iz_3_1109	DJI_0132	Verbascum densiflorum Bertol.	0	n	1	0	Scrophulariaceae Juss.
Iz_3_1109	DJI_0132	Veronica austriaca L.	0	n	1	0	Plantaginaceae Juss.
Iz_3_1109	DJI_0132	Veronica incana L.	0	n	1	0	Plantaginaceae Juss.
Iz_3_1109	DJI_0132	Veronica verna L.	0	n	1	0	Plantaginaceae Juss.
Iz_3_1109	DJI_0132	Vincetoxicum hirundinaria Medik	0	n	1	0	Apocynaceae Juss.
Iz_3_1109	DJI_0135	Acer tataricum L.	0	n	1	1	Sapindaceae Juss.
Iz_3_1109	DJI_0135	Achillea millefolium L. s.l.	0	n	1	0	Asteraceae Bercht. & J.Presl
Iz_3_1109	DJI_0135	Allium oleraceum L.	0	n	1	0	Amaryllidaceae J.St.-Hil.
Iz_3_1109	DJI_0135	Ambrosia artemisiifolia L.	0	a	0	0	Asteraceae Bercht. & J.Presl
Iz_3_1109	DJI_0135	Amorpha fruticosa L.	0	a	0	0	Fabaceae Lindl.
Iz_3_1109	DJI_0135	Anthericum ramosum L.	0	n	1	0	Asparagaceae Juss.
Iz_3_1109	DJI_0135	Artemisia marschalliana Spreng.	0	n	1	0	Asteraceae Bercht. & J.Presl
Iz_3_1109	DJI_0135	Asparagus officinalis L.	0	n	1	0	Asparagaceae Juss.
Iz_3_1109	DJI_0135	Astragalus cicer L.	0	n	1	0	Fabaceae Lindl.
Iz_3_1109	DJI_0135	Bassia laniflora (S.G.Gmel.) A.J.Scott	0	n	1	0	Amaranthaceae Juss.
Iz_3_1109	DJI_0135	Berberis vulgaris L.	0	n	1	0	Berberidaceae Juss.
Iz_3_1109	DJI_0135	Betula pendula Roth.	0	n	1	1	Betulaceae Gray
Iz_3_1109	DJI_0135	Betula pendula Roth.	0	n	1	0	Poaceae Barnhart
Iz_3_1109	DJI_0135	Bromus inermis Leyss.	0		NA	NA	
Iz_3_1109	DJI_0135	Bromus squarrosus L.	0	a	0	0	Poaceae Barnhart
Iz_3_1109	DJI_0135	Bromus tectorum L.	0	a	0	0	Poaceae Barnhart
Iz_3_1109	DJI_0135	Calamagrostis epigejos (L.) Roth	0	n	1	0	Poaceae Barnhart
Iz_3_1109	DJI_0135	Carduus acanthoides L.	0	n	1	0	Asteraceae Bercht. & J.Presl
Iz_3_1109	DJI_0135	Carex sp.	0	n	1	0	Cyperaceae Juss.
Iz_3_1109	DJI_0135	Centaurea majorovii Dumbadze	0	n	1	0	Asteraceae Bercht. & J.Presl
Iz_3_1109	DJI_0135	Chamaecytisus ruthenicus (Fisch.ex Wol.) Klaskova	0	n	1	0	Fabaceae Lindl.
Iz_3_1109	DJI_0135	Chelidonium majus L.	0	n	1	0	Papaveraceae Juss.
Iz_3_1109	DJI_0135	Chenopodium betaceum Andrz.	0	n	1	0	Amaranthaceae Juss.
Iz_3_1109	DJI_0135	Chenopodium cf. album	0	n	1	0	Amaranthaceae Juss.
Iz_3_1109	DJI_0135	Chenopodium sp.	0		NA	0	Amaranthaceae Juss.
Iz_3_1109	DJI_0135	Chondrilla juncea L.	0	n	1	0	Asteraceae Bercht. & J.Presl
Iz_3_1109	DJI_0135	Cirsium vulgare (Savi) Ten.	0	n	1	0	Asteraceae Bercht. & J.Presl
Iz_3_1109	DJI_0135	Clinopodium acinos (L.) Kuntze	0	n	1	0	Lamiacea Lindl.
Iz_3_1109	DJI_0135	Convallaria majalis L.	0	n	1	0	Asparagaceae Juss.
Iz_3_1109	DJI_0135	Crataegus rhipidophylla Gand.	0	n	1	1	Rosaceae Juss.
Iz_3_1109	DJI_0135	Crepis foetida L.	0	n	1	0	Asteraceae Bercht. & J.Presl
Iz_3_1109	DJI_0135	Dactylis glomerata L.	0	n	1	0	Poaceae Barnhart
Iz_3_1109	DJI_0135	Dianthus campestris M.Bieb.	0	n	1	0	Caryophyllaceae Juss.

Iz_3_1109	DJI_0135	Digitaria sanguinalis (L.) Scop.	0	a	0	0	Poaceae Barnhart
Iz_3_1109	DJI_0135	Elymus repens (L.) Gould	0	n	1	0	Poaceae Barnhart
Iz_3_1109	DJI_0135	Epilobium tetragonum L.	0	n	1	0	Onagraceae Juss.
Iz_3_1109	DJI_0135	Eragrostis minor Host	0	a	0	0	Poaceae Barnhart
Iz_3_1109	DJI_0135	Erigeron canadensis L.	0	a	0	0	Asteraceae Bercht. & J.Presl
Iz_3_1109	DJI_0135	Euphorbia seguieriana Neck.	0	n	1	0	Euphorbiaceae Juss.
Iz_3_1109	DJI_0135	Fallopia dumetorum (L.) Holub	0.5	n	1	0	Polygonaceae Juss.
Iz_3_1109	DJI_0135	Festuca beckeri (Hack.) Trautv.	0	n	1	0	Poaceae Barnhart
Iz_3_1109	DJI_0135	Fragaria vesca L.	0	n	1	0	Rosaceae Juss.
Iz_3_1109	DJI_0135	Frangula alnus Mill.	0	n	1	1	Rhamnaceae Juss.
Iz_3_1109	DJI_0135	Fraxinus pennsylvanica Marshall	0	a	0	1	Oleaceae Hoffmanns. & Link
Iz_3_1109	DJI_0135	Galeopsis bifida Boenn.	0	n	1	0	Lamiacea Lindl.
Iz_3_1109	DJI_0135	Galium verum L.	0	n	1	0	Rubiaceae Juss.
Iz_3_1109	DJI_0135	Genista tinctoria L.	0	n	1	0	Fabaceae Lindl.
Iz_3_1109	DJI_0135	Geranium robertianum L.	0	n	1	0	Geraniaceae Juss.
Iz_3_1109	DJI_0135	Geranium sanquineum L.	0	n	1	0	Geraniaceae Juss.
Iz_3_1109	DJI_0135	Gypsophila paniculata L.	0	n	1	0	Caryophyllaceae Juss.
Iz_3_1109	DJI_0135	Helichrysum arenarium (L.) Moench	0	n	1	0	Asteraceae Bercht. & J.Presl
Iz_3_1109	DJI_0135	Herniaria polygama J.Gay	0	n	1	0	Caryophyllaceae Juss.
Iz_3_1109	DJI_0135	Hieracium robustum Fr.	0	n	1	0	Asteraceae Bercht. & J.Presl
Iz_3_1109	DJI_0135	Hieracium umbellatum L.	0	n	1	NA	Asteraceae Bercht. & J.Presl
Iz_3_1109	DJI_0135	Hylotelephium maximum (L.) Holub	0	n	1	0	Crassulaceae J.St.-Hil.
Iz_3_1109	DJI_0135	Jacobaea borysthenica (DC.) B.Nord. & Greuter	0	n	1	0	Asteraceae Bercht. & J.Presl
Iz_3_1109	DJI_0135	Jasione montana L.	0	n	1	0	Campanulaceae Juss.
Iz_3_1109	DJI_0135	Jurinea cyanoides (L.) Rchb.s.l.	0	n	1	0	Asteraceae Bercht. & J.Presl
Iz_3_1109	DJI_0135	Koeleria glauca (Spreng.) DC.	0	n	1	0	Poaceae Barnhart
Iz_3_1109	DJI_0135	Lactuca serriola L.	0	a	0	0	Asteraceae Bercht. & J.Presl
Iz_3_1109	DJI_0135	Linaria genistifolia (L.) Mill.	0	n	1	0	Plantaginaceae Juss.
Iz_3_1109	DJI_0135	Lomelosia argentea (L.) Greuter & Burdet	0	n	1	0	Caprifoliaceae Juss.
Iz_3_1109	DJI_0135	Melampyrum pratense L.	0	n	1	0	Orobanchaceae Vent.
Iz_3_1109	DJI_0135	Oenothera biennis L.	0	a	0	0	Onagraceae Juss.
Iz_3_1109	DJI_0135	Pentanema britannica (L.) D.Gut.Larr., Santos-Vicente, Anderb., E.Rico & M.M.Mart.Ort.	0	n	1	0	Asteraceae Bercht. & J.Presl
Iz_3_1109	DJI_0135	Peucedanum oreoselinum (L.) Moench	0	n	1	0	Apiaceae Lindl.
Iz_3_1109	DJI_0135	Peucedanum ruthenicum M.Bieb.	0	n	1	0	Apiaceae Lindl.
Iz_3_1109	DJI_0135	Pilosella echioides (Lumn.) F.W.Schultz & Sch.Bip.	0	n	1	0	Asteraceae Bercht. & J.Presl
Iz_3_1109	DJI_0135	Pilosella officinarum F. Schultz et Sch. Bip.	0	n	1	0	Asteraceae Bercht. & J.Presl
Iz_3_1109	DJI_0135	Pilosella sp.	0		NA	0	Asteraceae Bercht. & J.Presl
Iz_3_1109	DJI_0135	Pinus sylvestris L.	0	n	1	1	Pinaceae Spreng. ex F.Rudolphi
Iz_3_1109	DJI_0135	Poa annua L.	0	n	1	0	Poaceae Barnhart
Iz_3_1109	DJI_0135	Poa nemoralis L.	0	n	1	0	Poaceae Barnhart
Iz_3_1109	DJI_0135	Poa sp.	0		NA	0	Poaceae Barnhart
Iz_3_1109	DJI_0135	Polygonatum odoratum (Mill.) Druce	0	n	1	0	Asparagaceae Juss.
Iz_3_1109	DJI_0135	Polygonum arenarium Waldst. & Kit.	0	n	1	0	Polygonaceae Juss.
Iz_3_1109	DJI_0135	Populus nigra L.	0	n	1	1	Salicaceae Mirb.
Iz_3_1109	DJI_0135	Populus tremula L.	0	n	1	1	Salicaceae Mirb.
Iz_3_1109	DJI_0135	Populus Ã canescens (Aiton) Sm.	0	n	1	1	Salicaceae Mirb.
Iz_3_1109	DJI_0135	Potentilla argentea L.	0	n	1	0	Rosaceae Juss.
Iz_3_1109	DJI_0135	Psammophiliella muralis (L.) Ikonn.	0	n	1	0	Caryophyllaceae Juss.
Iz_3_1109	DJI_0135	Psephellus sumensis (Kalen.) Greuter	0	n	1	0	Asteraceae Bercht. & J.Presl
Iz_3_1109	DJI_0135	Pulsatilla pratensis (L.) Mill. s.l.	0	n	1	0	Ranunculaceae Juss.
Iz_3_1109	DJI_0135	Pyrus communis L.	0	a	0	1	Rosaceae Juss.
Iz_3_1109	DJI_0135	Quercus robur L.	0	n	1	1	Fagaceae Dumort.
Iz_3_1109	DJI_0135	Robinia pseudoacacia L.	0	a	0	1	Fabaceae Lindl.
Iz_3_1109	DJI_0135	Rosa sp.	0		NA	1	Rosaceae Juss.
Iz_3_1109	DJI_0135	Rubus idaeus L.	0	n	1	0	Rosaceae Juss.
Iz_3_1109	DJI_0135	Rumex acetosella L.	0	n	1	0	Polygonaceae Juss.
Iz_3_1109	DJI_0135	Rumex thyrsiflorus Fingerh.	0	n	1	0	Polygonaceae Juss.
Iz_3_1109	DJI_0135	Salix alba L.	0	n	1	1	Salicaceae Mirb.
Iz_3_1109	DJI_0135	Sambucus nigra L.	0	n	1	1	Viburnaceae Raf.
Iz_3_1109	DJI_0135	Secale sylvestre Host	0	n	1	0	Poaceae Barnhart
Iz_3_1109	DJI_0135	Sempervivum ruthenicum Schnittsp. & C.B. Lehm.	0	n	1	0	Crassulaceae J.St.-Hil.
Iz_3_1109	DJI_0135	Setaria viridis (L.) P. Beauv.	0	n	1	0	Poaceae Barnhart
Iz_3_1109	DJI_0135	Silene borysthenica (Gruner) Walters	0	n	1	0	Caryophyllaceae Juss.
Iz_3_1109	DJI_0135	Silene latifolia subsp. alba (Mill.) Greuter & Burdet	0	n	1	0	Caryophyllaceae Juss.
Iz_3_1109	DJI_0135	Solidago virgaurea L.	0	n	1	0	Asteraceae Bercht. & J.Presl
Iz_3_1109	DJI_0135	Sorbus aucuparia L.	0	n	1	1	Rosaceae Juss.
Iz_3_1109	DJI_0135	Species 11.09_3_0045	0		NA	0	
Iz_3_1109	DJI_0135	Species 11.09_3_0058	0		NA	0	
Iz_3_1109	DJI_0135	Stachys recta L.	0	n	1	0	Lamiacea Lindl.
Iz_3_1109	DJI_0135	Tanacetum vulgare L.	0	n	1	0	Asteraceae Bercht. & J.Presl
Iz_3_1109	DJI_0135	Thymus pallasianus Heinr.Braun	0	n	1	0	Lamiacea Lindl.
Iz_3_1109	DJI_0135	Tilia cordata Mill.	0	n	1	1	Malvaceae Juss.
Iz_3_1109	DJI_0135	Trifolium alpestre L.	0	n	1	0	Fabaceae Lindl.
Iz_3_1109	DJI_0135	Trifolium arvense L.	0	n	1	0	Fabaceae Lindl.
Iz_3_1109	DJI_0135	Ulmus glabra Huds.	0	n	1	1	Ulmaceae Mirb.
Iz_3_1109	DJI_0135	Verbascum densiflorum Bertol.	0	n	1	0	Scrophulariaceae Juss.
Iz_3_1109	DJI_0135	Veronica austriaca L.	0	n	1	0	Plantaginaceae Juss.
Iz_3_1109	DJI_0135	Veronica incana L.	0	n	1	0	Plantaginaceae Juss.
Iz_3_1109	DJI_0135	Veronica verna L.	0	n	1	0	Plantaginaceae Juss.
Iz_3_1109	DJI_0135	Vincetoxicum hirundinaria Medik	0	n	1	0	Apocynaceae Juss.
Iz_3_1109	DJI_0137	Acer tataricum L.	0	n	1	1	Sapindaceae Juss.
Iz_3_1109	DJI_0137	Achillea millefolium L. s.l.	0	n	1	0	Asteraceae Bercht. & J.Presl
Iz_3_1109	DJI_0137	Allium oleraceum L.	0	n	1	0	Amaryllidaceae J.St.-Hil.
Iz_3_1109	DJI_0137	Ambrosia artemisiifolia L.	0	a	0	0	Asteraceae Bercht. & J.Presl
Iz_3_1109	DJI_0137	Amorpha fruticosa L.	0	a	0	0	Fabaceae Lindl.
Iz_3_1109	DJI_0137	Anthericum ramosum L.	0	n	1	0	Asparagaceae Juss.
Iz_3_1109	DJI_0137	Artemisia marschalliana Spreng.	0	n	1	0	Asteraceae Bercht. & J.Presl
Iz_3_1109	DJI_0137	Asparagus officinalis L.	0	n	1	0	Asparagaceae Juss.
Iz_3_1109	DJI_0137	Astragalus cicer L.	0	n	1	0	Fabaceae Lindl.
Iz_3_1109	DJI_0137	Bassia laniflora (S.G.Gmel.) A.J.Scott	0	n	1	0	Amaranthaceae Juss.
Iz_3_1109	DJI_0137	Berberis vulgaris L.	0	n	1	0	Berberidaceae Juss.
Iz_3_1109	DJI_0137	Betula pendula Roth.	0	n	1	1	Betulaceae Gray
Iz_3_1109	DJI_0137	Betula pendula Roth.	0	n	1	0	Poaceae Barnhart
Iz_3_1109	DJI_0137	Bromus inermis Leyss.	0		NA	NA	
Iz_3_1109	DJI_0137	Bromus squarrosus L.	0	a	0	0	Poaceae Barnhart
Iz_3_1109	DJI_0137	Bromus tectorum L.	0	a	0	0	Poaceae Barnhart
Iz_3_1109	DJI_0137	Calamagrostis epigejos (L.) Roth	0	n	1	0	Poaceae Barnhart
Iz_3_1109	DJI_0137	Carduus acanthoides L.	0	n	1	0	Asteraceae Bercht. & J.Presl

Iz_3_1109	DJI_0137	Carex sp.	0	n	1	0	Cyperaceae Juss.
Iz_3_1109	DJI_0137	Centaurea majorovii Dumbadze	0	n	1	0	Asteraceae Bercht. & J.Presl
Iz_3_1109	DJI_0137	Chamaecytisus ruthenicus (Fisch.ex Wol.) Klaskova	0	n	1	0	Fabaceae Lindl.
Iz_3_1109	DJI_0137	Chelidonium majus L.	0.1	n	1	0	Papaveraceae Juss.
Iz_3_1109	DJI_0137	Chenopodium betaceum Andrz.	0	n	1	0	Amaranthaceae Juss.
Iz_3_1109	DJI_0137	Chenopodium cf. album	0	n	1	0	Amaranthaceae Juss.
Iz_3_1109	DJI_0137	Chenopodium sp.	0		NA	0	Amaranthaceae Juss.
Iz_3_1109	DJI_0137	Chondrilla juncea L.	0	n	1	0	Asteraceae Bercht. & J.Presl
Iz_3_1109	DJI_0137	Cirsium vulgare (Savi) Ten.	0	n	1	0	Asteraceae Bercht. & J.Presl
Iz_3_1109	DJI_0137	Clinopodium acinos (L.) Kuntze	0	n	1	0	Lamiacea Lindl.
Iz_3_1109	DJI_0137	Convallaria majalis L.	0	n	1	0	Asparagaceae Juss.
Iz_3_1109	DJI_0137	Crataegus rhipidophylla Gand.	0	n	1	1	Rosaceae Juss.
Iz_3_1109	DJI_0137	Crepis foetida L.	0	n	1	0	Asteraceae Bercht. & J.Presl
Iz_3_1109	DJI_0137	Dactylis glomerata L.	0	n	1	0	Poaceae Barnhart
Iz_3_1109	DJI_0137	Dianthus campestris M.Bieb.	0	n	1	0	Caryophyllaceae Juss.
Iz_3_1109	DJI_0137	Digitaria sanguinalis (L.) Scop.	0	a	0	0	Poaceae Barnhart
Iz_3_1109	DJI_0137	Elymus repens (L.) Gould	0	n	1	0	Poaceae Barnhart
Iz_3_1109	DJI_0137	Epilobium tetragonum L.	0	n	1	0	Onagraceae Juss.
Iz_3_1109	DJI_0137	Eragrostis minor Host	0	a	0	0	Poaceae Barnhart
Iz_3_1109	DJI_0137	Erigeron canadensis L.	0	a	0	0	Asteraceae Bercht. & J.Presl
Iz_3_1109	DJI_0137	Euphorbia seguieriana Neck.	0	n	1	0	Euphorbiaceae Juss.
Iz_3_1109	DJI_0137	Fallopia dumetorum (L.) Holub	3	n	1	0	Polygonaceae Juss.
Iz_3_1109	DJI_0137	Festuca beckeri (Hack.) Trautv.	0	n	1	0	Poaceae Barnhart
Iz_3_1109	DJI_0137	Fragaria vesca L.	0	n	1	0	Rosaceae Juss.
Iz_3_1109	DJI_0137	Frangula alnus Mill.	0	n	1	1	Rhamnaceae Juss.
Iz_3_1109	DJI_0137	Fraxinus pennsylvanica Marshall	0	a	0	1	Oleaceae Hoffmanns. & Link
Iz_3_1109	DJI_0137	Galeopsis bifida Boenn.	0	n	1	0	Lamiacea Lindl.
Iz_3_1109	DJI_0137	Galium verum L.	0	n	1	0	Rubiaceae Juss.
Iz_3_1109	DJI_0137	Genista tinctoria L.	0	n	1	0	Fabaceae Lindl.
Iz_3_1109	DJI_0137	Geranium robertianum L.	0	n	1	0	Geraniaceae Juss.
Iz_3_1109	DJI_0137	Geranium sanquineum L.	0	n	1	0	Geraniaceae Juss.
Iz_3_1109	DJI_0137	Gypsophila paniculata L.	0	n	1	0	Caryophyllaceae Juss.
Iz_3_1109	DJI_0137	Helichrysum arenarium (L.) Moench	0	n	1	0	Asteraceae Bercht. & J.Presl
Iz_3_1109	DJI_0137	Herniaria polygama J.Gay	0	n	1	0	Caryophyllaceae Juss.
Iz_3_1109	DJI_0137	Hieracium robustum Fr.	0	n	1	0	Asteraceae Bercht. & J.Presl
Iz_3_1109	DJI_0137	Hieracium umbellatum L.	0	n	1	NA	Asteraceae Bercht. & J.Presl
Iz_3_1109	DJI_0137	Hylotelephium maximum (L.) Holub	0	n	1	0	Crassulaceae J.St.-Hil.
Iz_3_1109	DJI_0137	Jacobaea borysthenica (DC.) B.Nord. & Greuter	0	n	1	0	Asteraceae Bercht. & J.Presl
Iz_3_1109	DJI_0137	Jasione montana L.	0	n	1	0	Campanulaceae Juss.
Iz_3_1109	DJI_0137	Jurinea cyanoides (L.) Rchb.s.l.	0	n	1	0	Asteraceae Bercht. & J.Presl
Iz_3_1109	DJI_0137	Koeleria glauca (Spreng.) DC.	0	n	1	0	Poaceae Barnhart
Iz_3_1109	DJI_0137	Lactuca serriola L.	0	a	0	0	Asteraceae Bercht. & J.Presl
Iz_3_1109	DJI_0137	Linaria genistifolia (L.) Mill.	0	n	1	0	Plantaginaceae Juss.
Iz_3_1109	DJI_0137	Lomelosia argentea (L.) Greuter & Burdet	0	n	1	0	Caprifoliaceae Juss.
Iz_3_1109	DJI_0137	Melampyrum pratense L.	0	n	1	0	Orobanchaceae Vent.
Iz_3_1109	DJI_0137	Oenothera biennis L.	0	a	0	0	Onagraceae Juss.
Iz_3_1109	DJI_0137	Pentanema britannica (L.) D.Gut.Larr., Santos-Vicente, Anderb., E.Rico & M.M.Mart.Ort.	0	n	1	0	Asteraceae Bercht. & J.Presl
Iz_3_1109	DJI_0137	Peucedanum oreoselinum (L.) Moench	0	n	1	0	Apiaceae Lindl.
Iz_3_1109	DJI_0137	Peucedanum ruthenicum M.Bieb.	0	n	1	0	Apiaceae Lindl.
Iz_3_1109	DJI_0137	Pilosella echioides (Lumn.) F.W.Schultz & Sch.Bip.	0	n	1	0	Asteraceae Bercht. & J.Presl
Iz_3_1109	DJI_0137	Pilosella officinarum F. Schultz et Sch. Bip.	0	n	1	0	Asteraceae Bercht. & J.Presl
Iz_3_1109	DJI_0137	Pilosella sp.	0		NA	0	Asteraceae Bercht. & J.Presl
Iz_3_1109	DJI_0137	Pinus sylvestris L.	0	n	1	1	Pinaceae Spreng. ex F.Rudolphi
Iz_3_1109	DJI_0137	Poa annua L.	0	n	1	0	Poaceae Barnhart
Iz_3_1109	DJI_0137	Poa nemoralis L.	0	n	1	0	Poaceae Barnhart
Iz_3_1109	DJI_0137	Poa sp.	0		NA	0	Poaceae Barnhart
Iz_3_1109	DJI_0137	Polygonatum odoratum (Mill.) Druce	0	n	1	0	Asparagaceae Juss.
Iz_3_1109	DJI_0137	Polygonum arenarium Waldst. & Kit.	0	n	1	0	Polygonaceae Juss.
Iz_3_1109	DJI_0137	Populus nigra L.	0	n	1	1	Salicaceae Mirb.
Iz_3_1109	DJI_0137	Populus tremula L.	0	n	1	1	Salicaceae Mirb.
Iz_3_1109	DJI_0137	Populus Ã canescens (Aiton) Sm.	0	n	1	1	Salicaceae Mirb.
Iz_3_1109	DJI_0137	Potentilla argentea L.	0	n	1	0	Rosaceae Juss.
Iz_3_1109	DJI_0137	Psammophiliella muralis (L.) Ikonn.	0	n	1	0	Caryophyllaceae Juss.
Iz_3_1109	DJI_0137	Psephellus sumensis (Kalen.) Greuter	0	n	1	0	Asteraceae Bercht. & J.Presl
Iz_3_1109	DJI_0137	Pulsatilla pratensis (L.) Mill. s.l.	0	n	1	0	Ranunculaceae Juss.
Iz_3_1109	DJI_0137	Pyrus communis L.	0	a	0	1	Rosaceae Juss.
Iz_3_1109	DJI_0137	Quercus robur L.	0	n	1	1	Fagaceae Dumort.
Iz_3_1109	DJI_0137	Robinia pseudoacacia L.	0	a	0	1	Fabaceae Lindl.
Iz_3_1109	DJI_0137	Rosa sp.	0		NA	1	Rosaceae Juss.
Iz_3_1109	DJI_0137	Rubus idaeus L.	0	n	1	0	Rosaceae Juss.
Iz_3_1109	DJI_0137	Rumex acetosella L.	0	n	1	0	Polygonaceae Juss.
Iz_3_1109	DJI_0137	Rumex thyrsiflorus Fingerh.	0	n	1	0	Polygonaceae Juss.
Iz_3_1109	DJI_0137	Salix alba L.	0	n	1	1	Salicaceae Mirb.
Iz_3_1109	DJI_0137	Sambucus nigra L.	0	n	1	1	Viburnaceae Raf.
Iz_3_1109	DJI_0137	Secale sylvestre Host	0	n	1	0	Poaceae Barnhart
Iz_3_1109	DJI_0137	Sempervivum ruthenicum Schnittsp. & C.B. Lehm.	0	n	1	0	Crassulaceae J.St.-Hil.
Iz_3_1109	DJI_0137	Setaria viridis (L.) P. Beauv.	0	n	1	0	Poaceae Barnhart
Iz_3_1109	DJI_0137	Silene borysthenica (Gruner) Walters	0	n	1	0	Caryophyllaceae Juss.
Iz_3_1109	DJI_0137	Silene latifolia subsp. alba (Mill.) Greuter & Burdet	0	n	1	0	Caryophyllaceae Juss.
Iz_3_1109	DJI_0137	Solidago virgaurea L.	0	n	1	0	Asteraceae Bercht. & J.Presl
Iz_3_1109	DJI_0137	Sorbus aucuparia L.	0	n	1	1	Rosaceae Juss.
Iz_3_1109	DJI_0137	Species 11.09_3_0045	0		NA	0	
Iz_3_1109	DJI_0137	Species 11.09_3_0058	0		NA	0	
Iz_3_1109	DJI_0137	Stachys recta L.	0	n	1	0	Lamiacea Lindl.
Iz_3_1109	DJI_0137	Tanacetum vulgare L.	0	n	1	0	Asteraceae Bercht. & J.Presl
Iz_3_1109	DJI_0137	Thymus pallasianus Heinr.Braun	0	n	1	0	Lamiacea Lindl.
Iz_3_1109	DJI_0137	Tilia cordata Mill.	0	n	1	1	Malvaceae Juss.
Iz_3_1109	DJI_0137	Trifolium alpestre L.	0	n	1	0	Fabaceae Lindl.
Iz_3_1109	DJI_0137	Trifolium arvense L.	0	n	1	0	Fabaceae Lindl.
Iz_3_1109	DJI_0137	Ulmus glabra Huds.	0	n	1	1	Ulmaceae Mirb.
Iz_3_1109	DJI_0137	Verbascum densiflorum Bertol.	0	n	1	0	Scrophulariaceae Juss.
Iz_3_1109	DJI_0137	Veronica austriaca L.	0	n	1	0	Plantaginaceae Juss.
Iz_3_1109	DJI_0137	Veronica incana L.	0	n	1	0	Plantaginaceae Juss.
Iz_3_1109	DJI_0137	Veronica verna L.	0	n	1	0	Plantaginaceae Juss.
Iz_3_1109	DJI_0137	Vincetoxicum hirundinaria Medik	0	n	1	0	Apocynaceae Juss.
Iz_3_1109	DJI_0139	Acer tataricum L.	0	n	1	1	Sapindaceae Juss.
Iz_3_1109	DJI_0139	Achillea millefolium L. s.l.	0	n	1	0	Asteraceae Bercht. & J.Presl
Iz_3_1109	DJI_0139	Allium oleraceum L.	0	n	1	0	Amaryllidaceae J.St.-Hil.

Iz_3_1109	DJI_0139	Ambrosia artemisiifolia L.	0	a	0	0	Asteraceae Bercht. & J.Presl
Iz_3_1109	DJI_0139	Amorpha fruticosa L.	0	a	0	0	Fabaceae Lindl.
Iz_3_1109	DJI_0139	Anthericum ramosum L.	0	n	1	0	Asparagaceae Juss.
Iz_3_1109	DJI_0139	Artemisia marschalliana Spreng.	0	n	1	0	Asteraceae Bercht. & J.Presl
Iz_3_1109	DJI_0139	Asparagus officinalis L.	0	n	1	0	Asparagaceae Juss.
Iz_3_1109	DJI_0139	Astragalus cicer L.	0	n	1	0	Fabaceae Lindl.
Iz_3_1109	DJI_0139	Bassia laniflora (S.G.Gmel.) A.J.Scott	0	n	1	0	Amaranthaceae Juss.
Iz_3_1109	DJI_0139	Berberis vulgaris L.	0	n	1	0	Berberidaceae Juss.
Iz_3_1109	DJI_0139	Betula pendula Roth.	0	n	1	1	Betulaceae Gray
Iz_3_1109	DJI_0139	Betula pendula Roth.	0	n	1	0	Poaceae Barnhart
Iz_3_1109	DJI_0139	Bromus inermis Leyss.	0		NA	NA	
Iz_3_1109	DJI_0139	Bromus squarrosus L.	0	a	0	0	Poaceae Barnhart
Iz_3_1109	DJI_0139	Bromus tectorum L.	0	a	0	0	Poaceae Barnhart
Iz_3_1109	DJI_0139	Calamagrostis epigejos (L.) Roth	0	n	1	0	Poaceae Barnhart
Iz_3_1109	DJI_0139	Carduus acanthoides L.	0	n	1	0	Asteraceae Bercht. & J.Presl
Iz_3_1109	DJI_0139	Carex sp.	0	n	1	0	Cyperaceae Juss.
Iz_3_1109	DJI_0139	Centaurea majorovii Dumbadze	0	n	1	0	Asteraceae Bercht. & J.Presl
Iz_3_1109	DJI_0139	Chamaecytisus ruthenicus (Fisch.ex Wol.) Klaskova	0	n	1	0	Fabaceae Lindl.
Iz_3_1109	DJI_0139	Chelidonium majus L.	0.5	n	1	0	Papaveraceae Juss.
Iz_3_1109	DJI_0139	Chenopodium betaceum Andrz.	0	n	1	0	Amaranthaceae Juss.
Iz_3_1109	DJI_0139	Chenopodium cf. album	0	n	1	0	Amaranthaceae Juss.
Iz_3_1109	DJI_0139	Chenopodium sp.	0		NA	0	Amaranthaceae Juss.
Iz_3_1109	DJI_0139	Chondrilla juncea L.	0	n	1	0	Asteraceae Bercht. & J.Presl
Iz_3_1109	DJI_0139	Cirsium vulgare (Savi) Ten.	0	n	1	0	Asteraceae Bercht. & J.Presl
Iz_3_1109	DJI_0139	Clinopodium acinos (L.) Kuntze	0	n	1	0	Lamiacea Lindl.
Iz_3_1109	DJI_0139	Convallaria majalis L.	0	n	1	0	Asparagaceae Juss.
Iz_3_1109	DJI_0139	Crataegus rhipidophylla Gand.	0	n	1	1	Rosaceae Juss.
Iz_3_1109	DJI_0139	Crepis foetida L.	0	n	1	0	Asteraceae Bercht. & J.Presl
Iz_3_1109	DJI_0139	Dactylis glomerata L.	0	n	1	0	Poaceae Barnhart
Iz_3_1109	DJI_0139	Dianthus campestris M.Bieb.	0	n	1	0	Caryophyllaceae Juss.
Iz_3_1109	DJI_0139	Digitaria sanguinalis (L.) Scop.	0	a	0	0	Poaceae Barnhart
Iz_3_1109	DJI_0139	Elymus repens (L.) Gould	0	n	1	0	Poaceae Barnhart
Iz_3_1109	DJI_0139	Epilobium tetragonum L.	0	n	1	0	Onagraceae Juss.
Iz_3_1109	DJI_0139	Eragrostis minor Host	0	a	0	0	Poaceae Barnhart
Iz_3_1109	DJI_0139	Erigeron canadensis L.	0	a	0	0	Asteraceae Bercht. & J.Presl
Iz_3_1109	DJI_0139	Euphorbia seguieriana Neck.	0	n	1	0	Euphorbiaceae Juss.
Iz_3_1109	DJI_0139	Fallopia dumetorum (L.) Holub	1	n	1	0	Polygonaceae Juss.
Iz_3_1109	DJI_0139	Festuca beckeri (Hack.) Trautv.	0	n	1	0	Poaceae Barnhart
Iz_3_1109	DJI_0139	Fragaria vesca L.	0	n	1	0	Rosaceae Juss.
Iz_3_1109	DJI_0139	Frangula alnus Mill.	0	n	1	1	Rhamnaceae Juss.
Iz_3_1109	DJI_0139	Fraxinus pennsylvanica Marshall	0	a	0	1	Oleaceae Hoffmanns. & Link
Iz_3_1109	DJI_0139	Galeopsis bifida Boenn.	0	n	1	0	Lamiacea Lindl.
Iz_3_1109	DJI_0139	Galium verum L.	0	n	1	0	Rubiaceae Juss.
Iz_3_1109	DJI_0139	Genista tinctoria L.	0	n	1	0	Fabaceae Lindl.
Iz_3_1109	DJI_0139	Geranium robertianum L.	0	n	1	0	Geraniaceae Juss.
Iz_3_1109	DJI_0139	Geranium sanquineum L.	0	n	1	0	Geraniaceae Juss.
Iz_3_1109	DJI_0139	Gypsophila paniculata L.	0	n	1	0	Caryophyllaceae Juss.
Iz_3_1109	DJI_0139	Helichrysum arenarium (L.) Moench	0	n	1	0	Asteraceae Bercht. & J.Presl
Iz_3_1109	DJI_0139	Herniaria polygama J.Gay	0	n	1	0	Caryophyllaceae Juss.
Iz_3_1109	DJI_0139	Hieracium robustum Fr.	0	n	1	0	Asteraceae Bercht. & J.Presl
Iz_3_1109	DJI_0139	Hieracium umbellatum L.	0	n	1	NA	Asteraceae Bercht. & J.Presl
Iz_3_1109	DJI_0139	Hylotelephium maximum (L.) Holub	0	n	1	0	Crassulaceae J.St.-Hil.
Iz_3_1109	DJI_0139	Jacobaea borysthenica (DC.) B.Nord. & Greuter	0	n	1	0	Asteraceae Bercht. & J.Presl
Iz_3_1109	DJI_0139	Jasione montana L.	0	n	1	0	Campanulaceae Juss.
Iz_3_1109	DJI_0139	Jurinea cyanoides (L.) Rchb.s.l.	0	n	1	0	Asteraceae Bercht. & J.Presl
Iz_3_1109	DJI_0139	Koeleria glauca (Spreng.) DC.	0	n	1	0	Poaceae Barnhart
Iz_3_1109	DJI_0139	Lactuca serriola L.	0	a	0	0	Asteraceae Bercht. & J.Presl
Iz_3_1109	DJI_0139	Linaria genistifolia (L.) Mill.	0	n	1	0	Plantaginaceae Juss.
Iz_3_1109	DJI_0139	Lomelosia argentea (L.) Greuter & Burdet	0	n	1	0	Caprifoliaceae Juss.
Iz_3_1109	DJI_0139	Melampyrum pratense L.	0	n	1	0	Orobanchaceae Vent.
Iz_3_1109	DJI_0139	Oenothera biennis L.	0	a	0	0	Onagraceae Juss.
Iz_3_1109	DJI_0139	Pentanema britannica (L.) D.Gut.Larr., Santos-Vicente, Anderb., E.Rico & M.M.Mart.Ort.	0	n	1	0	Asteraceae Bercht. & J.Presl
Iz_3_1109	DJI_0139	Peucedanum oreoselinum (L.) Moench	0	n	1	0	Apiaceae Lindl.
Iz_3_1109	DJI_0139	Peucedanum ruthenicum M.Bieb.	0	n	1	0	Apiaceae Lindl.
Iz_3_1109	DJI_0139	Pilosella echioides (Lumn.) F.W.Schultz & Sch.Bip.	0	n	1	0	Asteraceae Bercht. & J.Presl
Iz_3_1109	DJI_0139	Pilosella officinarum F. Schultz et Sch. Bip.	0	n	1	0	Asteraceae Bercht. & J.Presl
Iz_3_1109	DJI_0139	Pilosella sp.	0		NA	0	Asteraceae Bercht. & J.Presl
Iz_3_1109	DJI_0139	Pinus sylvestris L.	0	n	1	1	Pinaceae Spreng. ex F.Rudolphi
Iz_3_1109	DJI_0139	Poa annua L.	0	n	1	0	Poaceae Barnhart
Iz_3_1109	DJI_0139	Poa nemoralis L.	0	n	1	0	Poaceae Barnhart
Iz_3_1109	DJI_0139	Poa sp.	0		NA	0	Poaceae Barnhart
Iz_3_1109	DJI_0139	Polygonatum odoratum (Mill.) Druce	0	n	1	0	Asparagaceae Juss.
Iz_3_1109	DJI_0139	Polygonum arenarium Waldst. & Kit.	0	n	1	0	Polygonaceae Juss.
Iz_3_1109	DJI_0139	Populus nigra L.	0	n	1	1	Salicaceae Mirb.
Iz_3_1109	DJI_0139	Populus tremula L.	0	n	1	1	Salicaceae Mirb.
Iz_3_1109	DJI_0139	Populus Ã canescens (Aiton) Sm.	0	n	1	1	Salicaceae Mirb.
Iz_3_1109	DJI_0139	Potentilla argentea L.	0	n	1	0	Rosaceae Juss.
Iz_3_1109	DJI_0139	Psammophiliella muralis (L.) Ikonn.	0	n	1	0	Caryophyllaceae Juss.
Iz_3_1109	DJI_0139	Psephellus sumensis (Kalen.) Greuter	0	n	1	0	Asteraceae Bercht. & J.Presl
Iz_3_1109	DJI_0139	Pulsatilla pratensis (L.) Mill. s.l.	0	n	1	0	Ranunculaceae Juss.
Iz_3_1109	DJI_0139	Pyrus communis L.	0	a	0	1	Rosaceae Juss.
Iz_3_1109	DJI_0139	Quercus robur L.	0	n	1	1	Fagaceae Dumort.
Iz_3_1109	DJI_0139	Robinia pseudoacacia L.	0	a	0	1	Fabaceae Lindl.
Iz_3_1109	DJI_0139	Rosa sp.	0		NA	1	Rosaceae Juss.
Iz_3_1109	DJI_0139	Rubus idaeus L.	0	n	1	0	Rosaceae Juss.
Iz_3_1109	DJI_0139	Rumex acetosella L.	0	n	1	0	Polygonaceae Juss.
Iz_3_1109	DJI_0139	Rumex thyrsiflorus Fingerh.	0	n	1	0	Polygonaceae Juss.
Iz_3_1109	DJI_0139	Salix alba L.	0	n	1	1	Salicaceae Mirb.
Iz_3_1109	DJI_0139	Sambucus nigra L.	0	n	1	1	Viburnaceae Raf.
Iz_3_1109	DJI_0139	Secale sylvestre Host	0	n	1	0	Poaceae Barnhart
Iz_3_1109	DJI_0139	Sempervivum ruthenicum Schnittsp. & C.B. Lehm.	0	n	1	0	Crassulaceae J.St.-Hil.
Iz_3_1109	DJI_0139	Setaria viridis (L.) P. Beauv.	0	n	1	0	Poaceae Barnhart
Iz_3_1109	DJI_0139	Silene borysthenica (Gruner) Walters	0	n	1	0	Caryophyllaceae Juss.
Iz_3_1109	DJI_0139	Silene latifolia subsp. alba (Mill.) Greuter & Burdet	0	n	1	0	Caryophyllaceae Juss.
Iz_3_1109	DJI_0139	Solidago virgaurea L.	0	n	1	0	Asteraceae Bercht. & J.Presl
Iz_3_1109	DJI_0139	Sorbus aucuparia L.	0	n	1	1	Rosaceae Juss.
Iz_3_1109	DJI_0139	Species 11.09_3_0045	0		NA	0	
Iz_3_1109	DJI_0139	Species 11.09_3_0058	0		NA	0	

Iz_3_1109	DJI_0139	Stachys recta L.	0	n	1	0	Lamiacea Lindl.
Iz_3_1109	DJI_0139	Tanacetum vulgare L.	0	n	1	0	Asteraceae Bercht. & J.Presl
Iz_3_1109	DJI_0139	Thymus pallasianus Heinr.Braun	0	n	1	0	Lamiacea Lindl.
Iz_3_1109	DJI_0139	Tilia cordata Mill.	0	n	1	1	Malvaceae Juss.
Iz_3_1109	DJI_0139	Trifolium alpestre L.	0	n	1	0	Fabaceae Lindl.
Iz_3_1109	DJI_0139	Trifolium arvense L.	0	n	1	0	Fabaceae Lindl.
Iz_3_1109	DJI_0139	Ulmus glabra Huds.	0	n	1	1	Ulmaceae Mirb.
Iz_3_1109	DJI_0139	Verbascum densiflorum Bertol.	0	n	1	0	Scrophulariaceae Juss.
Iz_3_1109	DJI_0139	Veronica austriaca L.	0	n	1	0	Plantaginaceae Juss.
Iz_3_1109	DJI_0139	Veronica incana L.	0	n	1	0	Plantaginaceae Juss.
Iz_3_1109	DJI_0139	Veronica verna L.	0	n	1	0	Plantaginaceae Juss.
Iz_3_1109	DJI_0139	Vincetoxicum hirundinaria Medik	0	n	1	0	Apocynaceae Juss.
Iz_3_1109	DJI_0140	Acer tataricum L.	0	n	1	1	Sapindaceae Juss.
Iz_3_1109	DJI_0140	Achillea millefolium L. s.l.	0	n	1	0	Asteraceae Bercht. & J.Presl
Iz_3_1109	DJI_0140	Allium oleraceum L.	0	n	1	0	Amaryllidaceae J.St.-Hil.
Iz_3_1109	DJI_0140	Ambrosia artemisiifolia L.	0	a	0	0	Asteraceae Bercht. & J.Presl
Iz_3_1109	DJI_0140	Amorpha fruticosa L.	0	a	0	0	Fabaceae Lindl.
Iz_3_1109	DJI_0140	Anthericum ramosum L.	0	n	1	0	Asparagaceae Juss.
Iz_3_1109	DJI_0140	Artemisia marschalliana Spreng.	0	n	1	0	Asteraceae Bercht. & J.Presl
Iz_3_1109	DJI_0140	Asparagus officinalis L.	0	n	1	0	Asparagaceae Juss.
Iz_3_1109	DJI_0140	Astragalus cicer L.	0	n	1	0	Fabaceae Lindl.
Iz_3_1109	DJI_0140	Bassia laniflora (S.G.Gmel.) A.J.Scott	0	n	1	0	Amaranthaceae Juss.
Iz_3_1109	DJI_0140	Berberis vulgaris L.	0	n	1	0	Berberidaceae Juss.
Iz_3_1109	DJI_0140	Betula pendula Roth.	0	n	1	1	Betulaceae Gray
Iz_3_1109	DJI_0140	Betula pendula Roth.	0	n	1	0	Poaceae Barnhart
Iz_3_1109	DJI_0140	Bromus inermis Leyss.	0		NA	NA	
Iz_3_1109	DJI_0140	Bromus squarrosus L.	0	a	0	0	Poaceae Barnhart
Iz_3_1109	DJI_0140	Bromus tectorum L.	0	a	0	0	Poaceae Barnhart
Iz_3_1109	DJI_0140	Calamagrostis epigejos (L.) Roth	0.2	n	1	0	Poaceae Barnhart
Iz_3_1109	DJI_0140	Carduus acanthoides L.	0	n	1	0	Asteraceae Bercht. & J.Presl
Iz_3_1109	DJI_0140	Carex sp.	0	n	1	0	Cyperaceae Juss.
Iz_3_1109	DJI_0140	Centaurea majorovii Dumbadze	0	n	1	0	Asteraceae Bercht. & J.Presl
Iz_3_1109	DJI_0140	Chamaecytisus ruthenicus (Fisch.ex Wol.) Klaskova	0	n	1	0	Fabaceae Lindl.
Iz_3_1109	DJI_0140	Chelidonium majus L.	0.2	n	1	0	Papaveraceae Juss.
Iz_3_1109	DJI_0140	Chenopodium betaceum Andrz.	0	n	1	0	Amaranthaceae Juss.
Iz_3_1109	DJI_0140	Chenopodium cf. album	0	n	1	0	Amaranthaceae Juss.
Iz_3_1109	DJI_0140	Chenopodium sp.	0		NA	0	Amaranthaceae Juss.
Iz_3_1109	DJI_0140	Chondrilla juncea L.	0	n	1	0	Asteraceae Bercht. & J.Presl
Iz_3_1109	DJI_0140	Cirsium vulgare (Savi) Ten.	0	n	1	0	Asteraceae Bercht. & J.Presl
Iz_3_1109	DJI_0140	Clinopodium acinos (L.) Kuntze	0	n	1	0	Lamiacea Lindl.
Iz_3_1109	DJI_0140	Convallaria majalis L.	0	n	1	0	Asparagaceae Juss.
Iz_3_1109	DJI_0140	Crataegus rhipidophylla Gand.	0	n	1	1	Rosaceae Juss.
Iz_3_1109	DJI_0140	Crepis foetida L.	0	n	1	0	Asteraceae Bercht. & J.Presl
Iz_3_1109	DJI_0140	Dactylis glomerata L.	0	n	1	0	Poaceae Barnhart
Iz_3_1109	DJI_0140	Dianthus campestris M.Bieb.	0	n	1	0	Caryophyllaceae Juss.
Iz_3_1109	DJI_0140	Digitaria sanguinalis (L.) Scop.	0	a	0	0	Poaceae Barnhart
Iz_3_1109	DJI_0140	Elymus repens (L.) Gould	0	n	1	0	Poaceae Barnhart
Iz_3_1109	DJI_0140	Epilobium tetragonum L.	0	n	1	0	Onagraceae Juss.
Iz_3_1109	DJI_0140	Eragrostis minor Host	0	a	0	0	Poaceae Barnhart
Iz_3_1109	DJI_0140	Erigeron canadensis L.	0	a	0	0	Asteraceae Bercht. & J.Presl
Iz_3_1109	DJI_0140	Euphorbia seguieriana Neck.	0	n	1	0	Euphorbiaceae Juss.
Iz_3_1109	DJI_0140	Fallopia dumetorum (L.) Holub	3	n	1	0	Polygonaceae Juss.
Iz_3_1109	DJI_0140	Festuca beckeri (Hack.) Trautv.	0	n	1	0	Poaceae Barnhart
Iz_3_1109	DJI_0140	Fragaria vesca L.	0	n	1	0	Rosaceae Juss.
Iz_3_1109	DJI_0140	Frangula alnus Mill.	0	n	1	1	Rhamnaceae Juss.
Iz_3_1109	DJI_0140	Fraxinus pennsylvanica Marshall	0	a	0	1	Oleaceae Hoffmanns. & Link
Iz_3_1109	DJI_0140	Galeopsis bifida Boenn.	0	n	1	0	Lamiacea Lindl.
Iz_3_1109	DJI_0140	Galium verum L.	0	n	1	0	Rubiaceae Juss.
Iz_3_1109	DJI_0140	Genista tinctoria L.	0	n	1	0	Fabaceae Lindl.
Iz_3_1109	DJI_0140	Geranium robertianum L.	0	n	1	0	Geraniaceae Juss.
Iz_3_1109	DJI_0140	Geranium sanquineum L.	0	n	1	0	Geraniaceae Juss.
Iz_3_1109	DJI_0140	Gypsophila paniculata L.	0	n	1	0	Caryophyllaceae Juss.
Iz_3_1109	DJI_0140	Helichrysum arenarium (L.) Moench	0	n	1	0	Asteraceae Bercht. & J.Presl
Iz_3_1109	DJI_0140	Herniaria polygama J.Gay	0	n	1	0	Caryophyllaceae Juss.
Iz_3_1109	DJI_0140	Hieracium robustum Fr.	0	n	1	0	Asteraceae Bercht. & J.Presl
Iz_3_1109	DJI_0140	Hieracium umbellatum L.	0	n	1	NA	Asteraceae Bercht. & J.Presl
Iz_3_1109	DJI_0140	Hylotelephium maximum (L.) Holub	0	n	1	0	Crassulaceae J.St.-Hil.
Iz_3_1109	DJI_0140	Jacobaea borysthenica (DC.) B.Nord. & Greuter	0	n	1	0	Asteraceae Bercht. & J.Presl
Iz_3_1109	DJI_0140	Jasione montana L.	0	n	1	0	Campanulaceae Juss.
Iz_3_1109	DJI_0140	Jurinea cyanoides (L.) Rchb.s.l.	0	n	1	0	Asteraceae Bercht. & J.Presl
Iz_3_1109	DJI_0140	Koeleria glauca (Spreng.) DC.	0	n	1	0	Poaceae Barnhart
Iz_3_1109	DJI_0140	Lactuca serriola L.	0	a	0	0	Asteraceae Bercht. & J.Presl
Iz_3_1109	DJI_0140	Linaria genistifolia (L.) Mill.	0	n	1	0	Plantaginaceae Juss.
Iz_3_1109	DJI_0140	Lomelosia argentea (L.) Greuter & Burdet	0	n	1	0	Caprifoliaceae Juss.
Iz_3_1109	DJI_0140	Melampyrum pratense L.	0	n	1	0	Orobanchaceae Vent.
Iz_3_1109	DJI_0140	Oenothera biennis L.	0	a	0	0	Onagraceae Juss.
Iz_3_1109	DJI_0140	Pentanema britannica (L.) D.Gut.Larr., Santos-Vicente, Anderb., E.Rico & M.M.Mart.Ort.	0	n	1	0	Asteraceae Bercht. & J.Presl
Iz_3_1109	DJI_0140	Peucedanum oreoselinum (L.) Moench	0	n	1	0	Apiaceae Lindl.
Iz_3_1109	DJI_0140	Peucedanum ruthenicum M.Bieb.	0	n	1	0	Apiaceae Lindl.
Iz_3_1109	DJI_0140	Pilosella echioides (Lumn.) F.W.Schultz & Sch.Bip.	0	n	1	0	Asteraceae Bercht. & J.Presl
Iz_3_1109	DJI_0140	Pilosella officinarum F. Schultz et Sch. Bip.	0	n	1	0	Asteraceae Bercht. & J.Presl
Iz_3_1109	DJI_0140	Pilosella sp.	0		NA	0	Asteraceae Bercht. & J.Presl
Iz_3_1109	DJI_0140	Pinus sylvestris L.	0	n	1	1	Pinaceae Spreng. ex F.Rudolphi
Iz_3_1109	DJI_0140	Poa annua L.	0	n	1	0	Poaceae Barnhart
Iz_3_1109	DJI_0140	Poa nemoralis L.	0	n	1	0	Poaceae Barnhart
Iz_3_1109	DJI_0140	Poa sp.	0		NA	0	Poaceae Barnhart
Iz_3_1109	DJI_0140	Polygonatum odoratum (Mill.) Druce	0	n	1	0	Asparagaceae Juss.
Iz_3_1109	DJI_0140	Polygonum arenarium Waldst. & Kit.	0	n	1	0	Polygonaceae Juss.
Iz_3_1109	DJI_0140	Populus nigra L.	0	n	1	1	Salicaceae Mirb.
Iz_3_1109	DJI_0140	Populus tremula L.	0	n	1	1	Salicaceae Mirb.
Iz_3_1109	DJI_0140	Populus Ã canescens (Aiton) Sm.	0	n	1	1	Salicaceae Mirb.
Iz_3_1109	DJI_0140	Potentilla argentea L.	0	n	1	0	Rosaceae Juss.
Iz_3_1109	DJI_0140	Psammophiliella muralis (L.) Ikonn.	0	n	1	0	Caryophyllaceae Juss.
Iz_3_1109	DJI_0140	Psephellus sumensis (Kalen.) Greuter	0	n	1	0	Asteraceae Bercht. & J.Presl
Iz_3_1109	DJI_0140	Pulsatilla pratensis (L.) Mill. s.l.	0	n	1	0	Ranunculaceae Juss.
Iz_3_1109	DJI_0140	Pyrus communis L.	0	a	0	1	Rosaceae Juss.
Iz_3_1109	DJI_0140	Quercus robur L.	0	n	1	1	Fagaceae Dumort.
Iz_3_1109	DJI_0140	Robinia pseudoacacia L.	0	a	0	1	Fabaceae Lindl.

Iz_3_1109	DJI_0140	Rosa sp.	0		NA	1	Rosaceae Juss.
Iz_3_1109	DJI_0140	Rubus idaeus L.	0	n	1	0	Rosaceae Juss.
Iz_3_1109	DJI_0140	Rumex acetosella L.	3	n	1	0	Polygonaceae Juss.
Iz_3_1109	DJI_0140	Rumex thyrsiflorus Fingerh.	0	n	1	0	Polygonaceae Juss.
Iz_3_1109	DJI_0140	Salix alba L.	0	n	1	1	Salicaceae Mirb.
Iz_3_1109	DJI_0140	Sambucus nigra L.	0	n	1	1	Viburnaceae Raf.
Iz_3_1109	DJI_0140	Secale sylvestre Host	0	n	1	0	Poaceae Barnhart
Iz_3_1109	DJI_0140	Sempervivum ruthenicum Schnittsp. & C.B. Lehm.	0	n	1	0	Crassulaceae J.St.-Hil.
Iz_3_1109	DJI_0140	Setaria viridis (L.) P. Beauv.	0	n	1	0	Poaceae Barnhart
Iz_3_1109	DJI_0140	Silene borysthenica (Gruner) Walters	0	n	1	0	Caryophyllaceae Juss.
Iz_3_1109	DJI_0140	Silene latifolia subsp. alba (Mill.) Greuter & Burdet	0	n	1	0	Caryophyllaceae Juss.
Iz_3_1109	DJI_0140	Solidago virgaurea L.	0	n	1	0	Asteraceae Bercht. & J.Presl
Iz_3_1109	DJI_0140	Sorbus aucuparia L.	0	n	1	1	Rosaceae Juss.
Iz_3_1109	DJI_0140	Species 11.09_3_0045	0		NA	0	
Iz_3_1109	DJI_0140	Species 11.09_3_0058	0		NA	0	
Iz_3_1109	DJI_0140	Stachys recta L.	0	n	1	0	Lamiacea Lindl.
Iz_3_1109	DJI_0140	Tanacetum vulgare L.	0	n	1	0	Asteraceae Bercht. & J.Presl
Iz_3_1109	DJI_0140	Thymus pallasianus Heinr.Braun	0	n	1	0	Lamiacea Lindl.
Iz_3_1109	DJI_0140	Tilia cordata Mill.	0	n	1	1	Malvaceae Juss.
Iz_3_1109	DJI_0140	Trifolium alpestre L.	0	n	1	0	Fabaceae Lindl.
Iz_3_1109	DJI_0140	Trifolium arvense L.	0	n	1	0	Fabaceae Lindl.
Iz_3_1109	DJI_0140	Ulmus glabra Huds.	0	n	1	1	Ulmaceae Mirb.
Iz_3_1109	DJI_0140	Verbascum densiflorum Bertol.	0	n	1	0	Scrophulariaceae Juss.
Iz_3_1109	DJI_0140	Veronica austriaca L.	0	n	1	0	Plantaginaceae Juss.
Iz_3_1109	DJI_0140	Veronica incana L.	0	n	1	0	Plantaginaceae Juss.
Iz_3_1109	DJI_0140	Veronica verna L.	0	n	1	0	Plantaginaceae Juss.
Iz_3_1109	DJI_0140	Vincetoxicum hirundinaria Medik	0	n	1	0	Apocynaceae Juss.
Iz_3_1209	DJI_0097	Acer tataricum L.	0	n	1	1	Sapindaceae Juss.
Iz_3_1209	DJI_0097	Achillea millefolium L. s.l.	0	n	1	0	Asteraceae Bercht. & J.Presl
Iz_3_1209	DJI_0097	Allium oleraceum L.	0	n	1	0	Amaryllidaceae J.St.-Hil.
Iz_3_1209	DJI_0097	Ambrosia artemisiifolia L.	0	a	0	0	Asteraceae Bercht. & J.Presl
Iz_3_1209	DJI_0097	Amorpha fruticosa L.	0	a	0	0	Fabaceae Lindl.
Iz_3_1209	DJI_0097	Anthericum ramosum L.	0	n	1	0	Asparagaceae Juss.
Iz_3_1209	DJI_0097	Artemisia marschalliana Spreng.	0	n	1	0	Asteraceae Bercht. & J.Presl
Iz_3_1209	DJI_0097	Asparagus officinalis L.	0	n	1	0	Asparagaceae Juss.
Iz_3_1209	DJI_0097	Astragalus cicer L.	0	n	1	0	Fabaceae Lindl.
Iz_3_1209	DJI_0097	Bassia laniflora (S.G.Gmel.) A.J.Scott	0	n	1	0	Amaranthaceae Juss.
Iz_3_1209	DJI_0097	Berberis vulgaris L.	0	n	1	0	Berberidaceae Juss.
Iz_3_1209	DJI_0097	Betula pendula Roth.	0	n	1	1	Betulaceae Gray
Iz_3_1209	DJI_0097	Betula pendula Roth.	0	n	1	0	Poaceae Barnhart
Iz_3_1209	DJI_0097	Bromus inermis Leyss.	0		NA	NA	
Iz_3_1209	DJI_0097	Bromus squarrosus L.	0	a	0	0	Poaceae Barnhart
Iz_3_1209	DJI_0097	Bromus tectorum L.	0	a	0	0	Poaceae Barnhart
Iz_3_1209	DJI_0097	Calamagrostis epigejos (L.) Roth	3	n	1	0	Poaceae Barnhart
Iz_3_1209	DJI_0097	Carduus acanthoides L.	0	n	1	0	Asteraceae Bercht. & J.Presl
Iz_3_1209	DJI_0097	Carex sp.	0	n	1	0	Cyperaceae Juss.
Iz_3_1209	DJI_0097	Centaurea majorovii Dumbadze	0	n	1	0	Asteraceae Bercht. & J.Presl
Iz_3_1209	DJI_0097	Chamaecytisus ruthenicus (Fisch.ex Wol.) Klaskova	0	n	1	0	Fabaceae Lindl.
Iz_3_1209	DJI_0097	Chelidonium majus L.	5	n	1	0	Papaveraceae Juss.
Iz_3_1209	DJI_0097	Chenopodium betaceum Andrz.	0	n	1	0	Amaranthaceae Juss.
Iz_3_1209	DJI_0097	Chenopodium cf. album	0	n	1	0	Amaranthaceae Juss.
Iz_3_1209	DJI_0097	Chenopodium sp.	0		NA	0	Amaranthaceae Juss.
Iz_3_1209	DJI_0097	Chondrilla juncea L.	0	n	1	0	Asteraceae Bercht. & J.Presl
Iz_3_1209	DJI_0097	Cirsium vulgare (Savi) Ten.	0	n	1	0	Asteraceae Bercht. & J.Presl
Iz_3_1209	DJI_0097	Clinopodium acinos (L.) Kuntze	0	n	1	0	Lamiacea Lindl.
Iz_3_1209	DJI_0097	Convallaria majalis L.	0	n	1	0	Asparagaceae Juss.
Iz_3_1209	DJI_0097	Crataegus rhipidophylla Gand.	0	n	1	1	Rosaceae Juss.
Iz_3_1209	DJI_0097	Crepis foetida L.	0	n	1	0	Asteraceae Bercht. & J.Presl
Iz_3_1209	DJI_0097	Dactylis glomerata L.	0	n	1	0	Poaceae Barnhart
Iz_3_1209	DJI_0097	Dianthus campestris M.Bieb.	0	n	1	0	Caryophyllaceae Juss.
Iz_3_1209	DJI_0097	Digitaria sanguinalis (L.) Scop.	0	a	0	0	Poaceae Barnhart
Iz_3_1209	DJI_0097	Elymus repens (L.) Gould	0	n	1	0	Poaceae Barnhart
Iz_3_1209	DJI_0097	Epilobium tetragonum L.	0	n	1	0	Onagraceae Juss.
Iz_3_1209	DJI_0097	Eragrostis minor Host	0	a	0	0	Poaceae Barnhart
Iz_3_1209	DJI_0097	Erigeron canadensis L.	13	a	0	0	Asteraceae Bercht. & J.Presl
Iz_3_1209	DJI_0097	Euphorbia seguieriana Neck.	0	n	1	0	Euphorbiaceae Juss.
Iz_3_1209	DJI_0097	Fallopia dumetorum (L.) Holub	0	n	1	0	Polygonaceae Juss.
Iz_3_1209	DJI_0097	Festuca beckeri (Hack.) Trautv.	0	n	1	0	Poaceae Barnhart
Iz_3_1209	DJI_0097	Fragaria vesca L.	0	n	1	0	Rosaceae Juss.
Iz_3_1209	DJI_0097	Frangula alnus Mill.	0	n	1	1	Rhamnaceae Juss.
Iz_3_1209	DJI_0097	Fraxinus pennsylvanica Marshall	0	a	0	1	Oleaceae Hoffmanns. & Link
Iz_3_1209	DJI_0097	Galeopsis bifida Boenn.	0	n	1	0	Lamiacea Lindl.
Iz_3_1209	DJI_0097	Galium aparine L.	0		NA	NA	
Iz_3_1209	DJI_0097	Galium verum L.	0	n	1	0	Rubiaceae Juss.
Iz_3_1209	DJI_0097	Genista tinctoria L.	0	n	1	0	Fabaceae Lindl.
Iz_3_1209	DJI_0097	Geranium robertianum L.	0	n	1	0	Geraniaceae Juss.
Iz_3_1209	DJI_0097	Geranium sanquineum L.	0	n	1	0	Geraniaceae Juss.
Iz_3_1209	DJI_0097	Gypsophila paniculata L.	0	n	1	0	Caryophyllaceae Juss.
Iz_3_1209	DJI_0097	Helichrysum arenarium (L.) Moench	0	n	1	0	Asteraceae Bercht. & J.Presl
Iz_3_1209	DJI_0097	Herniaria polygama J.Gay	0	n	1	0	Caryophyllaceae Juss.
Iz_3_1209	DJI_0097	Hieracium robustum Fr.	0	n	1	0	Asteraceae Bercht. & J.Presl
Iz_3_1209	DJI_0097	Hieracium umbellatum L.	0	n	1	NA	Asteraceae Bercht. & J.Presl
Iz_3_1209	DJI_0097	Hylotelephium maximum (L.) Holub	0	n	1	0	Crassulaceae J.St.-Hil.
Iz_3_1209	DJI_0097	Jacobaea borysthenica (DC.) B.Nord. & Greuter	0	n	1	0	Asteraceae Bercht. & J.Presl
Iz_3_1209	DJI_0097	Jasione montana L.	0	n	1	0	Campanulaceae Juss.
Iz_3_1209	DJI_0097	Jurinea cyanoides (L.) Rchb.s.l.	0	n	1	0	Asteraceae Bercht. & J.Presl
Iz_3_1209	DJI_0097	Koeleria glauca (Spreng.) DC.	0	n	1	0	Poaceae Barnhart
Iz_3_1209	DJI_0097	Lactuca serriola L.	0	a	0	0	Asteraceae Bercht. & J.Presl
Iz_3_1209	DJI_0097	Linaria genistifolia (L.) Mill.	0	n	1	0	Plantaginaceae Juss.
Iz_3_1209	DJI_0097	Lomelosia argentea (L.) Greuter & Burdet	0	n	1	0	Caprifoliaceae Juss.
Iz_3_1209	DJI_0097	Melampyrum pratense L.	0	n	1	0	Orobanchaceae Vent.
Iz_3_1209	DJI_0097	Oenothera biennis L.	0	a	0	0	Onagraceae Juss.
Iz_3_1209	DJI_0097	Pentanema britannica (L.) D.Gut.Larr., Santos-Vicente, Anderb., E.Rico & M.M.Mart.Ort.	0	n	1	0	Asteraceae Bercht. & J.Presl
Iz_3_1209	DJI_0097	Peucedanum oreoselinum (L.) Moench	0	n	1	0	Apiaceae Lindl.
Iz_3_1209	DJI_0097	Peucedanum ruthenicum M.Bieb.	0	n	1	0	Apiaceae Lindl.
Iz_3_1209	DJI_0097	Pilosella echioides (Lumn.) F.W.Schultz & Sch.Bip.	0	n	1	0	Asteraceae Bercht. & J.Presl
Iz_3_1209	DJI_0097	Pilosella officinarum F. Schultz et Sch. Bip.	0	n	1	0	Asteraceae Bercht. & J.Presl
Iz_3_1209	DJI_0097	Pilosella sp.	0		NA	0	Asteraceae Bercht. & J.Presl

Iz_3_1209	DJI_0097	Pinus sylvestris L.	0	n	1	1	Pinaceae Spreng. ex F.Rudolphi
Iz_3_1209	DJI_0097	Poa annua L.	0	n	1	0	Poaceae Barnhart
Iz_3_1209	DJI_0097	Poa nemoralis L.	0	n	1	0	Poaceae Barnhart
Iz_3_1209	DJI_0097	Poa sp.	0		NA	0	Poaceae Barnhart
Iz_3_1209	DJI_0097	Polygonatum odoratum (Mill.) Druce	0	n	1	0	Asparagaceae Juss.
Iz_3_1209	DJI_0097	Polygonum arenarium Waldst. & Kit.	0	n	1	0	Polygonaceae Juss.
Iz_3_1209	DJI_0097	Populus nigra L.	0	n	1	1	Salicaceae Mirb.
Iz_3_1209	DJI_0097	Populus tremula L.	0	n	1	1	Salicaceae Mirb.
Iz_3_1209	DJI_0097	Populus Ã canescens (Aiton) Sm.	0	n	1	1	Salicaceae Mirb.
Iz_3_1209	DJI_0097	Potentilla argentea L.	0	n	1	0	Rosaceae Juss.
Iz_3_1209	DJI_0097	Psammophiliella muralis (L.) Ikonn.	0	n	1	0	Caryophyllaceae Juss.
Iz_3_1209	DJI_0097	Psephellus sumensis (Kalen.) Greuter	0	n	1	0	Asteraceae Bercht. & J.Presl
Iz_3_1209	DJI_0097	Pulsatilla pratensis (L.) Mill. s.l.	0	n	1	0	Ranunculaceae Juss.
Iz_3_1209	DJI_0097	Pyrus communis L.	0	a	0	1	Rosaceae Juss.
Iz_3_1209	DJI_0097	Quercus robur L.	0	n	1	1	Fagaceae Dumort.
Iz_3_1209	DJI_0097	Robinia pseudoacacia L.	0	a	0	1	Fabaceae Lindl.
Iz_3_1209	DJI_0097	Rosa sp.	0		NA	1	Rosaceae Juss.
Iz_3_1209	DJI_0097	Rubus idaeus L.	0	n	1	0	Rosaceae Juss.
Iz_3_1209	DJI_0097	Rumex acetosella L.	0	n	1	0	Polygonaceae Juss.
Iz_3_1209	DJI_0097	Rumex thyrsiflorus Fingerh.	0	n	1	0	Polygonaceae Juss.
Iz_3_1209	DJI_0097	Salix alba L.	0	n	1	1	Salicaceae Mirb.
Iz_3_1209	DJI_0097	Sambucus nigra L.	0	n	1	1	Viburnaceae Raf.
Iz_3_1209	DJI_0097	Secale sylvestre Host	0	n	1	0	Poaceae Barnhart
Iz_3_1209	DJI_0097	Sempervivum ruthenicum Schnittsp. & C.B. Lehm.	0	n	1	0	Crassulaceae J.St.-Hil.
Iz_3_1209	DJI_0097	Setaria viridis (L.) P. Beauv.	0	n	1	0	Poaceae Barnhart
Iz_3_1209	DJI_0097	Silene borysthenica (Gruner) Walters	0	n	1	0	Caryophyllaceae Juss.
Iz_3_1209	DJI_0097	Silene latifolia subsp. alba (Mill.) Greuter & Burdet	0	n	1	0	Caryophyllaceae Juss.
Iz_3_1209	DJI_0097	Solidago virgaurea L.	0	n	1	0	Asteraceae Bercht. & J.Presl
Iz_3_1209	DJI_0097	Sorbus aucuparia L.	0	n	1	1	Rosaceae Juss.
Iz_3_1209	DJI_0097	Stachys recta L.	0	n	1	0	Lamiacea Lindl.
Iz_3_1209	DJI_0097	Tanacetum vulgare L.	0	n	1	0	Asteraceae Bercht. & J.Presl
Iz_3_1209	DJI_0097	Thymus pallasianus Heinr.Braun	0	n	1	0	Lamiacea Lindl.
Iz_3_1209	DJI_0097	Tilia cordata Mill.	0	n	1	1	Malvaceae Juss.
Iz_3_1209	DJI_0097	Trifolium alpestre L.	0	n	1	0	Fabaceae Lindl.
Iz_3_1209	DJI_0097	Trifolium arvense L.	0	n	1	0	Fabaceae Lindl.
Iz_3_1209	DJI_0097	Ulmus glabra Huds.	0	n	1	1	Ulmaceae Mirb.
Iz_3_1209	DJI_0097	Verbascum densiflorum Bertol.	0	n	1	0	Scrophulariaceae Juss.
Iz_3_1209	DJI_0097	Veronica austriaca L.	0	n	1	0	Plantaginaceae Juss.
Iz_3_1209	DJI_0097	Veronica incana L.	0	n	1	0	Plantaginaceae Juss.
Iz_3_1209	DJI_0097	Veronica verna L.	0	n	1	0	Plantaginaceae Juss.
Iz_3_1209	DJI_0097	Vincetoxicum hirundinaria Medik	0	n	1	0	Apocynaceae Juss.
Iz_3_1209	DJI_0100	Acer tataricum L.	0	n	1	1	Sapindaceae Juss.
Iz_3_1209	DJI_0100	Achillea millefolium L. s.l.	0	n	1	0	Asteraceae Bercht. & J.Presl
Iz_3_1209	DJI_0100	Allium oleraceum L.	0	n	1	0	Amaryllidaceae J.St.-Hil.
Iz_3_1209	DJI_0100	Ambrosia artemisiifolia L.	0	a	0	0	Asteraceae Bercht. & J.Presl
Iz_3_1209	DJI_0100	Amorpha fruticosa L.	0	a	0	0	Fabaceae Lindl.
Iz_3_1209	DJI_0100	Anthericum ramosum L.	0	n	1	0	Asparagaceae Juss.
Iz_3_1209	DJI_0100	Artemisia marschalliana Spreng.	0	n	1	0	Asteraceae Bercht. & J.Presl
Iz_3_1209	DJI_0100	Asparagus officinalis L.	0	n	1	0	Asparagaceae Juss.
Iz_3_1209	DJI_0100	Astragalus cicer L.	0	n	1	0	Fabaceae Lindl.
Iz_3_1209	DJI_0100	Bassia laniflora (S.G.Gmel.) A.J.Scott	0	n	1	0	Amaranthaceae Juss.
Iz_3_1209	DJI_0100	Berberis vulgaris L.	0	n	1	0	Berberidaceae Juss.
Iz_3_1209	DJI_0100	Betula pendula Roth.	0	n	1	1	Betulaceae Gray
Iz_3_1209	DJI_0100	Betula pendula Roth.	0	n	1	0	Poaceae Barnhart
Iz_3_1209	DJI_0100	Bromus inermis Leyss.	0		NA	NA	
Iz_3_1209	DJI_0100	Bromus squarrosus L.	0	a	0	0	Poaceae Barnhart
Iz_3_1209	DJI_0100	Bromus tectorum L.	0	a	0	0	Poaceae Barnhart
Iz_3_1209	DJI_0100	Calamagrostis epigejos (L.) Roth	0.2	n	1	0	Poaceae Barnhart
Iz_3_1209	DJI_0100	Carduus acanthoides L.	0	n	1	0	Asteraceae Bercht. & J.Presl
Iz_3_1209	DJI_0100	Carex sp.	0	n	1	0	Cyperaceae Juss.
Iz_3_1209	DJI_0100	Centaurea majorovii Dumbadze	0	n	1	0	Asteraceae Bercht. & J.Presl
Iz_3_1209	DJI_0100	Chamaecytisus ruthenicus (Fisch.ex Wol.) Klaskova	0	n	1	0	Fabaceae Lindl.
Iz_3_1209	DJI_0100	Chelidonium majus L.	8	n	1	0	Papaveraceae Juss.
Iz_3_1209	DJI_0100	Chenopodium betaceum Andrz.	0	n	1	0	Amaranthaceae Juss.
Iz_3_1209	DJI_0100	Chenopodium cf. album	0	n	1	0	Amaranthaceae Juss.
Iz_3_1209	DJI_0100	Chenopodium sp.	0		NA	0	Amaranthaceae Juss.
Iz_3_1209	DJI_0100	Chondrilla juncea L.	0	n	1	0	Asteraceae Bercht. & J.Presl
Iz_3_1209	DJI_0100	Cirsium vulgare (Savi) Ten.	0	n	1	0	Asteraceae Bercht. & J.Presl
Iz_3_1209	DJI_0100	Clinopodium acinos (L.) Kuntze	0	n	1	0	Lamiacea Lindl.
Iz_3_1209	DJI_0100	Convallaria majalis L.	0	n	1	0	Asparagaceae Juss.
Iz_3_1209	DJI_0100	Crataegus rhipidophylla Gand.	0	n	1	1	Rosaceae Juss.
Iz_3_1209	DJI_0100	Crepis foetida L.	0	n	1	0	Asteraceae Bercht. & J.Presl
Iz_3_1209	DJI_0100	Dactylis glomerata L.	0	n	1	0	Poaceae Barnhart
Iz_3_1209	DJI_0100	Dianthus campestris M.Bieb.	0	n	1	0	Caryophyllaceae Juss.
Iz_3_1209	DJI_0100	Digitaria sanguinalis (L.) Scop.	0	a	0	0	Poaceae Barnhart
Iz_3_1209	DJI_0100	Elymus repens (L.) Gould	0	n	1	0	Poaceae Barnhart
Iz_3_1209	DJI_0100	Epilobium tetragonum L.	0	n	1	0	Onagraceae Juss.
Iz_3_1209	DJI_0100	Eragrostis minor Host	0	a	0	0	Poaceae Barnhart
Iz_3_1209	DJI_0100	Erigeron canadensis L.	5	a	0	0	Asteraceae Bercht. & J.Presl
Iz_3_1209	DJI_0100	Euphorbia seguieriana Neck.	0	n	1	0	Euphorbiaceae Juss.
Iz_3_1209	DJI_0100	Fallopia dumetorum (L.) Holub	0.2	n	1	0	Polygonaceae Juss.
Iz_3_1209	DJI_0100	Festuca beckeri (Hack.) Trautv.	0	n	1	0	Poaceae Barnhart
Iz_3_1209	DJI_0100	Fragaria vesca L.	0	n	1	0	Rosaceae Juss.
Iz_3_1209	DJI_0100	Frangula alnus Mill.	0	n	1	1	Rhamnaceae Juss.
Iz_3_1209	DJI_0100	Fraxinus pennsylvanica Marshall	0	a	0	1	Oleaceae Hoffmanns. & Link
Iz_3_1209	DJI_0100	Galeopsis bifida Boenn.	0	n	1	0	Lamiacea Lindl.
Iz_3_1209	DJI_0100	Galium aparine L.	0		NA	NA	
Iz_3_1209	DJI_0100	Galium verum L.	0	n	1	0	Rubiaceae Juss.
Iz_3_1209	DJI_0100	Genista tinctoria L.	0	n	1	0	Fabaceae Lindl.
Iz_3_1209	DJI_0100	Geranium robertianum L.	3	n	1	0	Geraniaceae Juss.
Iz_3_1209	DJI_0100	Geranium sanquineum L.	0	n	1	0	Geraniaceae Juss.
Iz_3_1209	DJI_0100	Gypsophila paniculata L.	0	n	1	0	Caryophyllaceae Juss.
Iz_3_1209	DJI_0100	Helichrysum arenarium (L.) Moench	0	n	1	0	Asteraceae Bercht. & J.Presl
Iz_3_1209	DJI_0100	Herniaria polygama J.Gay	0	n	1	0	Caryophyllaceae Juss.
Iz_3_1209	DJI_0100	Hieracium robustum Fr.	0	n	1	0	Asteraceae Bercht. & J.Presl
Iz_3_1209	DJI_0100	Hieracium umbellatum L.	0	n	1	NA	Asteraceae Bercht. & J.Presl
Iz_3_1209	DJI_0100	Hylotelephium maximum (L.) Holub	0	n	1	0	Crassulaceae J.St.-Hil.
Iz_3_1209	DJI_0100	Jacobaea borysthenica (DC.) B.Nord. & Greuter	0	n	1	0	Asteraceae Bercht. & J.Presl

Iz_3_1209	DJI_0100	Jasione montana L.	0	n	1	0	Campanulaceae Juss.
Iz_3_1209	DJI_0100	Jurinea cyanoides (L.) Rchb.s.l.	0	n	1	0	Asteraceae Bercht. & J.Presl
Iz_3_1209	DJI_0100	Koeleria glauca (Spreng.) DC.	0	n	1	0	Poaceae Barnhart
Iz_3_1209	DJI_0100	Lactuca serriola L.	0	a	0	0	Asteraceae Bercht. & J.Presl
Iz_3_1209	DJI_0100	Linaria genistifolia (L.) Mill.	0	n	1	0	Plantaginaceae Juss.
Iz_3_1209	DJI_0100	Lomelosia argentea (L.) Greuter & Burdet	0	n	1	0	Caprifoliaceae Juss.
Iz_3_1209	DJI_0100	Melampyrum pratense L.	0	n	1	0	Orobanchaceae Vent.
Iz_3_1209	DJI_0100	Oenothera biennis L.	0	a	0	0	Onagraceae Juss.
Iz_3_1209	DJI_0100	Pentanema britannica (L.) D.Gut.Larr., Santos-Vicente, Anderb., E.Rico & M.M.Mart.Ort.	0	n	1	0	Asteraceae Bercht. & J.Presl
Iz_3_1209	DJI_0100	Peucedanum oreoselinum (L.) Moench	0	n	1	0	Apiaceae Lindl.
Iz_3_1209	DJI_0100	Peucedanum ruthenicum M.Bieb.	0	n	1	0	Apiaceae Lindl.
Iz_3_1209	DJI_0100	Pilosella echioides (Lumn.) F.W.Schultz & Sch.Bip.	0	n	1	0	Asteraceae Bercht. & J.Presl
Iz_3_1209	DJI_0100	Pilosella officinarum F. Schultz et Sch. Bip.	0	n	1	0	Asteraceae Bercht. & J.Presl
Iz_3_1209	DJI_0100	Pilosella sp.	0		NA	0	Asteraceae Bercht. & J.Presl
Iz_3_1209	DJI_0100	Pinus sylvestris L.	0	n	1	1	Pinaceae Spreng. ex F.Rudolphi
Iz_3_1209	DJI_0100	Poa annua L.	0	n	1	0	Poaceae Barnhart
Iz_3_1209	DJI_0100	Poa nemoralis L.	0	n	1	0	Poaceae Barnhart
Iz_3_1209	DJI_0100	Poa sp.	0		NA	0	Poaceae Barnhart
Iz_3_1209	DJI_0100	Polygonatum odoratum (Mill.) Druce	0	n	1	0	Asparagaceae Juss.
Iz_3_1209	DJI_0100	Polygonum arenarium Waldst. & Kit.	0	n	1	0	Polygonaceae Juss.
Iz_3_1209	DJI_0100	Populus nigra L.	0	n	1	1	Salicaceae Mirb.
Iz_3_1209	DJI_0100	Populus tremula L.	0	n	1	1	Salicaceae Mirb.
Iz_3_1209	DJI_0100	Populus Ã canescens (Aiton) Sm.	0	n	1	1	Salicaceae Mirb.
Iz_3_1209	DJI_0100	Potentilla argentea L.	0	n	1	0	Rosaceae Juss.
Iz_3_1209	DJI_0100	Psammophiliella muralis (L.) Ikonn.	0	n	1	0	Caryophyllaceae Juss.
Iz_3_1209	DJI_0100	Psephellus sumensis (Kalen.) Greuter	0	n	1	0	Asteraceae Bercht. & J.Presl
Iz_3_1209	DJI_0100	Pulsatilla pratensis (L.) Mill. s.l.	0	n	1	0	Ranunculaceae Juss.
Iz_3_1209	DJI_0100	Pyrus communis L.	0	a	0	1	Rosaceae Juss.
Iz_3_1209	DJI_0100	Quercus robur L.	0	n	1	1	Fagaceae Dumort.
Iz_3_1209	DJI_0100	Robinia pseudoacacia L.	0	a	0	1	Fabaceae Lindl.
Iz_3_1209	DJI_0100	Rosa sp.	0		NA	1	Rosaceae Juss.
Iz_3_1209	DJI_0100	Rubus idaeus L.	0	n	1	0	Rosaceae Juss.
Iz_3_1209	DJI_0100	Rumex acetosella L.	0	n	1	0	Polygonaceae Juss.
Iz_3_1209	DJI_0100	Rumex thyrsiflorus Fingerh.	0	n	1	0	Polygonaceae Juss.
Iz_3_1209	DJI_0100	Salix alba L.	0	n	1	1	Salicaceae Mirb.
Iz_3_1209	DJI_0100	Sambucus nigra L.	0	n	1	1	Viburnaceae Raf.
Iz_3_1209	DJI_0100	Secale sylvestre Host	0	n	1	0	Poaceae Barnhart
Iz_3_1209	DJI_0100	Sempervivum ruthenicum Schnittsp. & C.B. Lehm.	0	n	1	0	Crassulaceae J.St.-Hil.
Iz_3_1209	DJI_0100	Setaria viridis (L.) P. Beauv.	0	n	1	0	Poaceae Barnhart
Iz_3_1209	DJI_0100	Silene borysthenica (Gruner) Walters	0	n	1	0	Caryophyllaceae Juss.
Iz_3_1209	DJI_0100	Silene latifolia subsp. alba (Mill.) Greuter & Burdet	0	n	1	0	Caryophyllaceae Juss.
Iz_3_1209	DJI_0100	Solidago virgaurea L.	0	n	1	0	Asteraceae Bercht. & J.Presl
Iz_3_1209	DJI_0100	Sorbus aucuparia L.	0	n	1	1	Rosaceae Juss.
Iz_3_1209	DJI_0100	Stachys recta L.	0	n	1	0	Lamiacea Lindl.
Iz_3_1209	DJI_0100	Tanacetum vulgare L.	0	n	1	0	Asteraceae Bercht. & J.Presl
Iz_3_1209	DJI_0100	Thymus pallasianus Heinr.Braun	0	n	1	0	Lamiacea Lindl.
Iz_3_1209	DJI_0100	Tilia cordata Mill.	0	n	1	1	Malvaceae Juss.
Iz_3_1209	DJI_0100	Trifolium alpestre L.	0	n	1	0	Fabaceae Lindl.
Iz_3_1209	DJI_0100	Trifolium arvense L.	0	n	1	0	Fabaceae Lindl.
Iz_3_1209	DJI_0100	Ulmus glabra Huds.	0	n	1	1	Ulmaceae Mirb.
Iz_3_1209	DJI_0100	Verbascum densiflorum Bertol.	0	n	1	0	Scrophulariaceae Juss.
Iz_3_1209	DJI_0100	Veronica austriaca L.	0	n	1	0	Plantaginaceae Juss.
Iz_3_1209	DJI_0100	Veronica incana L.	0	n	1	0	Plantaginaceae Juss.
Iz_3_1209	DJI_0100	Veronica verna L.	0	n	1	0	Plantaginaceae Juss.
Iz_3_1209	DJI_0100	Vincetoxicum hirundinaria Medik	0	n	1	0	Apocynaceae Juss.
Iz_3_1209	DJI_0103	Acer tataricum L.	0	n	1	1	Sapindaceae Juss.
Iz_3_1209	DJI_0103	Achillea millefolium L. s.l.	0	n	1	0	Asteraceae Bercht. & J.Presl
Iz_3_1209	DJI_0103	Allium oleraceum L.	0	n	1	0	Amaryllidaceae J.St.-Hil.
Iz_3_1209	DJI_0103	Ambrosia artemisiifolia L.	0	a	0	0	Asteraceae Bercht. & J.Presl
Iz_3_1209	DJI_0103	Amorpha fruticosa L.	0	a	0	0	Fabaceae Lindl.
Iz_3_1209	DJI_0103	Anthericum ramosum L.	0	n	1	0	Asparagaceae Juss.
Iz_3_1209	DJI_0103	Artemisia marschalliana Spreng.	0	n	1	0	Asteraceae Bercht. & J.Presl
Iz_3_1209	DJI_0103	Asparagus officinalis L.	0	n	1	0	Asparagaceae Juss.
Iz_3_1209	DJI_0103	Astragalus cicer L.	0	n	1	0	Fabaceae Lindl.
Iz_3_1209	DJI_0103	Bassia laniflora (S.G.Gmel.) A.J.Scott	0	n	1	0	Amaranthaceae Juss.
Iz_3_1209	DJI_0103	Berberis vulgaris L.	0	n	1	0	Berberidaceae Juss.
Iz_3_1209	DJI_0103	Betula pendula Roth.	0	n	1	1	Betulaceae Gray
Iz_3_1209	DJI_0103	Betula pendula Roth.	0	n	1	0	Poaceae Barnhart
Iz_3_1209	DJI_0103	Bromus inermis Leyss.	0		NA	NA	
Iz_3_1209	DJI_0103	Bromus squarrosus L.	0	a	0	0	Poaceae Barnhart
Iz_3_1209	DJI_0103	Bromus tectorum L.	0	a	0	0	Poaceae Barnhart
Iz_3_1209	DJI_0103	Calamagrostis epigejos (L.) Roth	70	n	1	0	Poaceae Barnhart
Iz_3_1209	DJI_0103	Carduus acanthoides L.	0	n	1	0	Asteraceae Bercht. & J.Presl
Iz_3_1209	DJI_0103	Carex sp.	0	n	1	0	Cyperaceae Juss.
Iz_3_1209	DJI_0103	Centaurea majorovii Dumbadze	0	n	1	0	Asteraceae Bercht. & J.Presl
Iz_3_1209	DJI_0103	Chamaecytisus ruthenicus (Fisch.ex Wol.) Klaskova	0	n	1	0	Fabaceae Lindl.
Iz_3_1209	DJI_0103	Chelidonium majus L.	0	n	1	0	Papaveraceae Juss.
Iz_3_1209	DJI_0103	Chenopodium betaceum Andrz.	0	n	1	0	Amaranthaceae Juss.
Iz_3_1209	DJI_0103	Chenopodium cf. album	0	n	1	0	Amaranthaceae Juss.
Iz_3_1209	DJI_0103	Chenopodium sp.	0		NA	0	Amaranthaceae Juss.
Iz_3_1209	DJI_0103	Chondrilla juncea L.	0	n	1	0	Asteraceae Bercht. & J.Presl
Iz_3_1209	DJI_0103	Cirsium vulgare (Savi) Ten.	0	n	1	0	Asteraceae Bercht. & J.Presl
Iz_3_1209	DJI_0103	Clinopodium acinos (L.) Kuntze	0	n	1	0	Lamiacea Lindl.
Iz_3_1209	DJI_0103	Convallaria majalis L.	0	n	1	0	Asparagaceae Juss.
Iz_3_1209	DJI_0103	Crataegus rhipidophylla Gand.	0	n	1	1	Rosaceae Juss.
Iz_3_1209	DJI_0103	Crepis foetida L.	0	n	1	0	Asteraceae Bercht. & J.Presl
Iz_3_1209	DJI_0103	Dactylis glomerata L.	0	n	1	0	Poaceae Barnhart
Iz_3_1209	DJI_0103	Dianthus campestris M.Bieb.	0	n	1	0	Caryophyllaceae Juss.
Iz_3_1209	DJI_0103	Digitaria sanguinalis (L.) Scop.	0	a	0	0	Poaceae Barnhart
Iz_3_1209	DJI_0103	Elymus repens (L.) Gould	0	n	1	0	Poaceae Barnhart
Iz_3_1209	DJI_0103	Epilobium tetragonum L.	0	n	1	0	Onagraceae Juss.
Iz_3_1209	DJI_0103	Eragrostis minor Host	0	a	0	0	Poaceae Barnhart
Iz_3_1209	DJI_0103	Erigeron canadensis L.	0	a	0	0	Asteraceae Bercht. & J.Presl
Iz_3_1209	DJI_0103	Euphorbia seguieriana Neck.	0	n	1	0	Euphorbiaceae Juss.
Iz_3_1209	DJI_0103	Fallopia dumetorum (L.) Holub	0	n	1	0	Polygonaceae Juss.
Iz_3_1209	DJI_0103	Festuca beckeri (Hack.) Trautv.	0	n	1	0	Poaceae Barnhart
Iz_3_1209	DJI_0103	Fragaria vesca L.	0	n	1	0	Rosaceae Juss.
Iz_3_1209	DJI_0103	Frangula alnus Mill.	0	n	1	1	Rhamnaceae Juss.

Iz_3_1209	DJI_0103	Fraxinus pennsylvanica Marshall	0	a	0	1	Oleaceae Hoffmanns. & Link
Iz_3_1209	DJI_0103	Galeopsis bifida Boenn.	0	n	1	0	Lamiacea Lindl.
Iz_3_1209	DJI_0103	Galium aparine L.	0		NA	NA	
Iz_3_1209	DJI_0103	Galium verum L.	0	n	1	0	Rubiaceae Juss.
Iz_3_1209	DJI_0103	Genista tinctoria L.	0	n	1	0	Fabaceae Lindl.
Iz_3_1209	DJI_0103	Geranium robertianum L.	0	n	1	0	Geraniaceae Juss.
Iz_3_1209	DJI_0103	Geranium sanquineum L.	0	n	1	0	Geraniaceae Juss.
Iz_3_1209	DJI_0103	Gypsophila paniculata L.	0	n	1	0	Caryophyllaceae Juss.
Iz_3_1209	DJI_0103	Helichrysum arenarium (L.) Moench	0	n	1	0	Asteraceae Bercht. & J.Presl
Iz_3_1209	DJI_0103	Herniaria polygama J.Gay	0	n	1	0	Caryophyllaceae Juss.
Iz_3_1209	DJI_0103	Hieracium robustum Fr.	0	n	1	0	Asteraceae Bercht. & J.Presl
Iz_3_1209	DJI_0103	Hieracium umbellatum L.	0	n	1	NA	Asteraceae Bercht. & J.Presl
Iz_3_1209	DJI_0103	Hylotelephium maximum (L.) Holub	0	n	1	0	Crassulaceae J.St.-Hil.
Iz_3_1209	DJI_0103	Jacobaea borysthenica (DC.) B.Nord. & Greuter	0	n	1	0	Asteraceae Bercht. & J.Presl
Iz_3_1209	DJI_0103	Jasione montana L.	0	n	1	0	Campanulaceae Juss.
Iz_3_1209	DJI_0103	Jurinea cyanoides (L.) Rchb.s.l.	0	n	1	0	Asteraceae Bercht. & J.Presl
Iz_3_1209	DJI_0103	Koeleria glauca (Spreng.) DC.	0	n	1	0	Poaceae Barnhart
Iz_3_1209	DJI_0103	Lactuca serriola L.	0	a	0	0	Asteraceae Bercht. & J.Presl
Iz_3_1209	DJI_0103	Linaria genistifolia (L.) Mill.	0	n	1	0	Plantaginaceae Juss.
Iz_3_1209	DJI_0103	Lomelosia argentea (L.) Greuter & Burdet	0	n	1	0	Caprifoliaceae Juss.
Iz_3_1209	DJI_0103	Melampyrum pratense L.	0	n	1	0	Orobanchaceae Vent.
Iz_3_1209	DJI_0103	Oenothera biennis L.	0	a	0	0	Onagraceae Juss.
Iz_3_1209	DJI_0103	Pentanema britannica (L.) D.Gut.Larr., Santos-Vicente, Anderb., E.Rico & M.M.Mart.Ort.	0	n	1	0	Asteraceae Bercht. & J.Presl
Iz_3_1209	DJI_0103	Peucedanum oreoselinum (L.) Moench	0	n	1	0	Apiaceae Lindl.
Iz_3_1209	DJI_0103	Peucedanum ruthenicum M.Bieb.	0	n	1	0	Apiaceae Lindl.
Iz_3_1209	DJI_0103	Pilosella echioides (Lumn.) F.W.Schultz & Sch.Bip.	0	n	1	0	Asteraceae Bercht. & J.Presl
Iz_3_1209	DJI_0103	Pilosella officinarum F. Schultz et Sch. Bip.	0	n	1	0	Asteraceae Bercht. & J.Presl
Iz_3_1209	DJI_0103	Pilosella sp.	0		NA	0	Asteraceae Bercht. & J.Presl
Iz_3_1209	DJI_0103	Pinus sylvestris L.	0	n	1	1	Pinaceae Spreng. ex F.Rudolphi
Iz_3_1209	DJI_0103	Poa annua L.	0	n	1	0	Poaceae Barnhart
Iz_3_1209	DJI_0103	Poa nemoralis L.	0	n	1	0	Poaceae Barnhart
Iz_3_1209	DJI_0103	Poa sp.	0		NA	0	Poaceae Barnhart
Iz_3_1209	DJI_0103	Polygonatum odoratum (Mill.) Druce	0	n	1	0	Asparagaceae Juss.
Iz_3_1209	DJI_0103	Polygonum arenarium Waldst. & Kit.	0	n	1	0	Polygonaceae Juss.
Iz_3_1209	DJI_0103	Populus nigra L.	0	n	1	1	Salicaceae Mirb.
Iz_3_1209	DJI_0103	Populus tremula L.	0	n	1	1	Salicaceae Mirb.
Iz_3_1209	DJI_0103	Populus Ã canescens (Aiton) Sm.	0	n	1	1	Salicaceae Mirb.
Iz_3_1209	DJI_0103	Potentilla argentea L.	0	n	1	0	Rosaceae Juss.
Iz_3_1209	DJI_0103	Psammophiliella muralis (L.) Ikonn.	0	n	1	0	Caryophyllaceae Juss.
Iz_3_1209	DJI_0103	Psephellus sumensis (Kalen.) Greuter	0	n	1	0	Asteraceae Bercht. & J.Presl
Iz_3_1209	DJI_0103	Pulsatilla pratensis (L.) Mill. s.l.	0	n	1	0	Ranunculaceae Juss.
Iz_3_1209	DJI_0103	Pyrus communis L.	0	a	0	1	Rosaceae Juss.
Iz_3_1209	DJI_0103	Quercus robur L.	0	n	1	1	Fagaceae Dumort.
Iz_3_1209	DJI_0103	Robinia pseudoacacia L.	0	a	0	1	Fabaceae Lindl.
Iz_3_1209	DJI_0103	Rosa sp.	0		NA	1	Rosaceae Juss.
Iz_3_1209	DJI_0103	Rubus idaeus L.	0	n	1	0	Rosaceae Juss.
Iz_3_1209	DJI_0103	Rumex acetosella L.	0	n	1	0	Polygonaceae Juss.
Iz_3_1209	DJI_0103	Rumex thyrsiflorus Fingerh.	0	n	1	0	Polygonaceae Juss.
Iz_3_1209	DJI_0103	Salix alba L.	0	n	1	1	Salicaceae Mirb.
Iz_3_1209	DJI_0103	Sambucus nigra L.	0	n	1	1	Viburnaceae Raf.
Iz_3_1209	DJI_0103	Secale sylvestre Host	0	n	1	0	Poaceae Barnhart
Iz_3_1209	DJI_0103	Sempervivum ruthenicum Schnittsp. & C.B. Lehm.	0	n	1	0	Crassulaceae J.St.-Hil.
Iz_3_1209	DJI_0103	Setaria viridis (L.) P. Beauv.	0	n	1	0	Poaceae Barnhart
Iz_3_1209	DJI_0103	Silene borysthenica (Gruner) Walters	0	n	1	0	Caryophyllaceae Juss.
Iz_3_1209	DJI_0103	Silene latifolia subsp. alba (Mill.) Greuter & Burdet	0	n	1	0	Caryophyllaceae Juss.
Iz_3_1209	DJI_0103	Solidago virgaurea L.	0	n	1	0	Asteraceae Bercht. & J.Presl
Iz_3_1209	DJI_0103	Sorbus aucuparia L.	0	n	1	1	Rosaceae Juss.
Iz_3_1209	DJI_0103	Stachys recta L.	0	n	1	0	Lamiacea Lindl.
Iz_3_1209	DJI_0103	Tanacetum vulgare L.	0	n	1	0	Asteraceae Bercht. & J.Presl
Iz_3_1209	DJI_0103	Thymus pallasianus Heinr.Braun	0	n	1	0	Lamiacea Lindl.
Iz_3_1209	DJI_0103	Tilia cordata Mill.	0	n	1	1	Malvaceae Juss.
Iz_3_1209	DJI_0103	Trifolium alpestre L.	0	n	1	0	Fabaceae Lindl.
Iz_3_1209	DJI_0103	Trifolium arvense L.	0	n	1	0	Fabaceae Lindl.
Iz_3_1209	DJI_0103	Ulmus glabra Huds.	0	n	1	1	Ulmaceae Mirb.
Iz_3_1209	DJI_0103	Verbascum densiflorum Bertol.	0	n	1	0	Scrophulariaceae Juss.
Iz_3_1209	DJI_0103	Veronica austriaca L.	0	n	1	0	Plantaginaceae Juss.
Iz_3_1209	DJI_0103	Veronica incana L.	0	n	1	0	Plantaginaceae Juss.
Iz_3_1209	DJI_0103	Veronica verna L.	0	n	1	0	Plantaginaceae Juss.
Iz_3_1209	DJI_0103	Vincetoxicum hirundinaria Medik	0	n	1	0	Apocynaceae Juss.
Iz_3_1209	DJI_0106	Acer tataricum L.	0	n	1	1	Sapindaceae Juss.
Iz_3_1209	DJI_0106	Achillea millefolium L. s.l.	0	n	1	0	Asteraceae Bercht. & J.Presl
Iz_3_1209	DJI_0106	Allium oleraceum L.	0	n	1	0	Amaryllidaceae J.St.-Hil.
Iz_3_1209	DJI_0106	Ambrosia artemisiifolia L.	0	a	0	0	Asteraceae Bercht. & J.Presl
Iz_3_1209	DJI_0106	Amorpha fruticosa L.	0	a	0	0	Fabaceae Lindl.
Iz_3_1209	DJI_0106	Anthericum ramosum L.	0	n	1	0	Asparagaceae Juss.
Iz_3_1209	DJI_0106	Artemisia marschalliana Spreng.	0	n	1	0	Asteraceae Bercht. & J.Presl
Iz_3_1209	DJI_0106	Asparagus officinalis L.	0	n	1	0	Asparagaceae Juss.
Iz_3_1209	DJI_0106	Astragalus cicer L.	0	n	1	0	Fabaceae Lindl.
Iz_3_1209	DJI_0106	Bassia laniflora (S.G.Gmel.) A.J.Scott	0	n	1	0	Amaranthaceae Juss.
Iz_3_1209	DJI_0106	Berberis vulgaris L.	0	n	1	0	Berberidaceae Juss.
Iz_3_1209	DJI_0106	Betula pendula Roth.	0	n	1	1	Betulaceae Gray
Iz_3_1209	DJI_0106	Betula pendula Roth.	0	n	1	0	Poaceae Barnhart
Iz_3_1209	DJI_0106	Bromus inermis Leyss.	0		NA	NA	
Iz_3_1209	DJI_0106	Bromus squarrosus L.	0	a	0	0	Poaceae Barnhart
Iz_3_1209	DJI_0106	Bromus tectorum L.	0	a	0	0	Poaceae Barnhart
Iz_3_1209	DJI_0106	Calamagrostis epigejos (L.) Roth	10	n	1	0	Poaceae Barnhart
Iz_3_1209	DJI_0106	Carduus acanthoides L.	0	n	1	0	Asteraceae Bercht. & J.Presl
Iz_3_1209	DJI_0106	Carex sp.	0	n	1	0	Cyperaceae Juss.
Iz_3_1209	DJI_0106	Centaurea majorovii Dumbadze	0	n	1	0	Asteraceae Bercht. & J.Presl
Iz_3_1209	DJI_0106	Chamaecytisus ruthenicus (Fisch.ex Wol.) Klaskova	0	n	1	0	Fabaceae Lindl.
Iz_3_1209	DJI_0106	Chelidonium majus L.	0	n	1	0	Papaveraceae Juss.
Iz_3_1209	DJI_0106	Chenopodium betaceum Andrz.	0	n	1	0	Amaranthaceae Juss.
Iz_3_1209	DJI_0106	Chenopodium cf. album	0	n	1	0	Amaranthaceae Juss.
Iz_3_1209	DJI_0106	Chenopodium sp.	0		NA	0	Amaranthaceae Juss.
Iz_3_1209	DJI_0106	Chondrilla juncea L.	0	n	1	0	Asteraceae Bercht. & J.Presl
Iz_3_1209	DJI_0106	Cirsium vulgare (Savi) Ten.	0	n	1	0	Asteraceae Bercht. & J.Presl
Iz_3_1209	DJI_0106	Clinopodium acinos (L.) Kuntze	0	n	1	0	Lamiacea Lindl.
Iz_3_1209	DJI_0106	Convallaria majalis L.	0	n	1	0	Asparagaceae Juss.

Iz_3_1209	DJI_0106	Crataegus rhipidophylla Gand.	0	n	1	1	Rosaceae Juss.
Iz_3_1209	DJI_0106	Crepis foetida L.	0	n	1	0	Asteraceae Bercht. & J.Presl
Iz_3_1209	DJI_0106	Dactylis glomerata L.	0	n	1	0	Poaceae Barnhart
Iz_3_1209	DJI_0106	Dianthus campestris M.Bieb.	0	n	1	0	Caryophyllaceae Juss.
Iz_3_1209	DJI_0106	Digitaria sanguinalis (L.) Scop.	0	a	0	0	Poaceae Barnhart
Iz_3_1209	DJI_0106	Elymus repens (L.) Gould	0	n	1	0	Poaceae Barnhart
Iz_3_1209	DJI_0106	Epilobium tetragonum L.	0	n	1	0	Onagraceae Juss.
Iz_3_1209	DJI_0106	Eragrostis minor Host	0	a	0	0	Poaceae Barnhart
Iz_3_1209	DJI_0106	Erigeron canadensis L.	0	a	0	0	Asteraceae Bercht. & J.Presl
Iz_3_1209	DJI_0106	Euphorbia seguieriana Neck.	0	n	1	0	Euphorbiaceae Juss.
Iz_3_1209	DJI_0106	Fallopia dumetorum (L.) Holub	0.5	n	1	0	Polygonaceae Juss.
Iz_3_1209	DJI_0106	Festuca beckeri (Hack.) Trautv.	0	n	1	0	Poaceae Barnhart
Iz_3_1209	DJI_0106	Fragaria vesca L.	0	n	1	0	Rosaceae Juss.
Iz_3_1209	DJI_0106	Frangula alnus Mill.	0	n	1	1	Rhamnaceae Juss.
Iz_3_1209	DJI_0106	Fraxinus pennsylvanica Marshall	0	a	0	1	Oleaceae Hoffmanns. & Link
Iz_3_1209	DJI_0106	Galeopsis bifida Boenn.	0	n	1	0	Lamiacea Lindl.
Iz_3_1209	DJI_0106	Galium aparine L.	0		NA	NA	
Iz_3_1209	DJI_0106	Galium verum L.	0	n	1	0	Rubiaceae Juss.
Iz_3_1209	DJI_0106	Genista tinctoria L.	0	n	1	0	Fabaceae Lindl.
Iz_3_1209	DJI_0106	Geranium robertianum L.	0	n	1	0	Geraniaceae Juss.
Iz_3_1209	DJI_0106	Geranium sanquineum L.	0	n	1	0	Geraniaceae Juss.
Iz_3_1209	DJI_0106	Gypsophila paniculata L.	0	n	1	0	Caryophyllaceae Juss.
Iz_3_1209	DJI_0106	Helichrysum arenarium (L.) Moench	0	n	1	0	Asteraceae Bercht. & J.Presl
Iz_3_1209	DJI_0106	Herniaria polygama J.Gay	0	n	1	0	Caryophyllaceae Juss.
Iz_3_1209	DJI_0106	Hieracium robustum Fr.	0	n	1	0	Asteraceae Bercht. & J.Presl
Iz_3_1209	DJI_0106	Hieracium umbellatum L.	0	n	1	NA	Asteraceae Bercht. & J.Presl
Iz_3_1209	DJI_0106	Hylotelephium maximum (L.) Holub	0	n	1	0	Crassulaceae J.St.-Hil.
Iz_3_1209	DJI_0106	Jacobaea borysthenica (DC.) B.Nord. & Greuter	0	n	1	0	Asteraceae Bercht. & J.Presl
Iz_3_1209	DJI_0106	Jasione montana L.	0	n	1	0	Campanulaceae Juss.
Iz_3_1209	DJI_0106	Jurinea cyanoides (L.) Rchb.s.l.	0	n	1	0	Asteraceae Bercht. & J.Presl
Iz_3_1209	DJI_0106	Koeleria glauca (Spreng.) DC.	0	n	1	0	Poaceae Barnhart
Iz_3_1209	DJI_0106	Lactuca serriola L.	0	a	0	0	Asteraceae Bercht. & J.Presl
Iz_3_1209	DJI_0106	Linaria genistifolia (L.) Mill.	0	n	1	0	Plantaginaceae Juss.
Iz_3_1209	DJI_0106	Lomelosia argentea (L.) Greuter & Burdet	0	n	1	0	Caprifoliaceae Juss.
Iz_3_1209	DJI_0106	Melampyrum pratense L.	0	n	1	0	Orobanchaceae Vent.
Iz_3_1209	DJI_0106	Oenothera biennis L.	0	a	0	0	Onagraceae Juss.
Iz_3_1209	DJI_0106	Pentanema britannica (L.) D.Gut.Larr., Santos-Vicente, Anderb., E.Rico & M.M.Mart.Ort.	0	n	1	0	Asteraceae Bercht. & J.Presl
Iz_3_1209	DJI_0106	Peucedanum oreoselinum (L.) Moench	5	n	1	0	Apiaceae Lindl.
Iz_3_1209	DJI_0106	Peucedanum ruthenicum M.Bieb.	0	n	1	0	Apiaceae Lindl.
Iz_3_1209	DJI_0106	Pilosella echioides (Lumn.) F.W.Schultz & Sch.Bip.	0	n	1	0	Asteraceae Bercht. & J.Presl
Iz_3_1209	DJI_0106	Pilosella officinarum F. Schultz et Sch. Bip.	0	n	1	0	Asteraceae Bercht. & J.Presl
Iz_3_1209	DJI_0106	Pilosella sp.	0		NA	0	Asteraceae Bercht. & J.Presl
Iz_3_1209	DJI_0106	Pinus sylvestris L.	0	n	1	1	Pinaceae Spreng. ex F.Rudolphi
Iz_3_1209	DJI_0106	Poa annua L.	0	n	1	0	Poaceae Barnhart
Iz_3_1209	DJI_0106	Poa nemoralis L.	0	n	1	0	Poaceae Barnhart
Iz_3_1209	DJI_0106	Poa sp.	0		NA	0	Poaceae Barnhart
Iz_3_1209	DJI_0106	Polygonatum odoratum (Mill.) Druce	0	n	1	0	Asparagaceae Juss.
Iz_3_1209	DJI_0106	Polygonum arenarium Waldst. & Kit.	0	n	1	0	Polygonaceae Juss.
Iz_3_1209	DJI_0106	Populus nigra L.	0	n	1	1	Salicaceae Mirb.
Iz_3_1209	DJI_0106	Populus tremula L.	0	n	1	1	Salicaceae Mirb.
Iz_3_1209	DJI_0106	Populus Ã canescens (Aiton) Sm.	0	n	1	1	Salicaceae Mirb.
Iz_3_1209	DJI_0106	Potentilla argentea L.	0	n	1	0	Rosaceae Juss.
Iz_3_1209	DJI_0106	Psammophiliella muralis (L.) Ikonn.	0	n	1	0	Caryophyllaceae Juss.
Iz_3_1209	DJI_0106	Psephellus sumensis (Kalen.) Greuter	0	n	1	0	Asteraceae Bercht. & J.Presl
Iz_3_1209	DJI_0106	Pulsatilla pratensis (L.) Mill. s.l.	0	n	1	0	Ranunculaceae Juss.
Iz_3_1209	DJI_0106	Pyrus communis L.	0	a	0	1	Rosaceae Juss.
Iz_3_1209	DJI_0106	Quercus robur L.	0	n	1	1	Fagaceae Dumort.
Iz_3_1209	DJI_0106	Robinia pseudoacacia L.	0	a	0	1	Fabaceae Lindl.
Iz_3_1209	DJI_0106	Rosa sp.	0		NA	1	Rosaceae Juss.
Iz_3_1209	DJI_0106	Rubus idaeus L.	0	n	1	0	Rosaceae Juss.
Iz_3_1209	DJI_0106	Rumex acetosella L.	0	n	1	0	Polygonaceae Juss.
Iz_3_1209	DJI_0106	Rumex thyrsiflorus Fingerh.	0	n	1	0	Polygonaceae Juss.
Iz_3_1209	DJI_0106	Salix alba L.	0	n	1	1	Salicaceae Mirb.
Iz_3_1209	DJI_0106	Sambucus nigra L.	0	n	1	1	Viburnaceae Raf.
Iz_3_1209	DJI_0106	Secale sylvestre Host	0	n	1	0	Poaceae Barnhart
Iz_3_1209	DJI_0106	Sempervivum ruthenicum Schnittsp. & C.B. Lehm.	0	n	1	0	Crassulaceae J.St.-Hil.
Iz_3_1209	DJI_0106	Setaria viridis (L.) P. Beauv.	0	n	1	0	Poaceae Barnhart
Iz_3_1209	DJI_0106	Silene borysthenica (Gruner) Walters	0	n	1	0	Caryophyllaceae Juss.
Iz_3_1209	DJI_0106	Silene latifolia subsp. alba (Mill.) Greuter & Burdet	0	n	1	0	Caryophyllaceae Juss.
Iz_3_1209	DJI_0106	Solidago virgaurea L.	0	n	1	0	Asteraceae Bercht. & J.Presl
Iz_3_1209	DJI_0106	Sorbus aucuparia L.	0	n	1	1	Rosaceae Juss.
Iz_3_1209	DJI_0106	Stachys recta L.	0	n	1	0	Lamiacea Lindl.
Iz_3_1209	DJI_0106	Tanacetum vulgare L.	0	n	1	0	Asteraceae Bercht. & J.Presl
Iz_3_1209	DJI_0106	Thymus pallasianus Heinr.Braun	0	n	1	0	Lamiacea Lindl.
Iz_3_1209	DJI_0106	Tilia cordata Mill.	0	n	1	1	Malvaceae Juss.
Iz_3_1209	DJI_0106	Trifolium alpestre L.	0	n	1	0	Fabaceae Lindl.
Iz_3_1209	DJI_0106	Trifolium arvense L.	0	n	1	0	Fabaceae Lindl.
Iz_3_1209	DJI_0106	Ulmus glabra Huds.	0	n	1	1	Ulmaceae Mirb.
Iz_3_1209	DJI_0106	Verbascum densiflorum Bertol.	0	n	1	0	Scrophulariaceae Juss.
Iz_3_1209	DJI_0106	Veronica austriaca L.	0	n	1	0	Plantaginaceae Juss.
Iz_3_1209	DJI_0106	Veronica incana L.	0	n	1	0	Plantaginaceae Juss.
Iz_3_1209	DJI_0106	Veronica verna L.	0	n	1	0	Plantaginaceae Juss.
Iz_3_1209	DJI_0106	Vincetoxicum hirundinaria Medik	0	n	1	0	Apocynaceae Juss.
Iz_3_1209	DJI_0109	Acer tataricum L.	0	n	1	1	Sapindaceae Juss.
Iz_3_1209	DJI_0109	Achillea millefolium L. s.l.	0	n	1	0	Asteraceae Bercht. & J.Presl
Iz_3_1209	DJI_0109	Allium oleraceum L.	0	n	1	0	Amaryllidaceae J.St.-Hil.
Iz_3_1209	DJI_0109	Ambrosia artemisiifolia L.	0	a	0	0	Asteraceae Bercht. & J.Presl
Iz_3_1209	DJI_0109	Amorpha fruticosa L.	0	a	0	0	Fabaceae Lindl.
Iz_3_1209	DJI_0109	Anthericum ramosum L.	0	n	1	0	Asparagaceae Juss.
Iz_3_1209	DJI_0109	Artemisia marschalliana Spreng.	0	n	1	0	Asteraceae Bercht. & J.Presl
Iz_3_1209	DJI_0109	Asparagus officinalis L.	0	n	1	0	Asparagaceae Juss.
Iz_3_1209	DJI_0109	Astragalus cicer L.	0	n	1	0	Fabaceae Lindl.
Iz_3_1209	DJI_0109	Bassia laniflora (S.G.Gmel.) A.J.Scott	0	n	1	0	Amaranthaceae Juss.
Iz_3_1209	DJI_0109	Berberis vulgaris L.	0	n	1	0	Berberidaceae Juss.
Iz_3_1209	DJI_0109	Betula pendula Roth.	0	n	1	1	Betulaceae Gray
Iz_3_1209	DJI_0109	Betula pendula Roth.	0	n	1	0	Poaceae Barnhart
Iz_3_1209	DJI_0109	Bromus inermis Leyss.	0		NA	NA	
Iz_3_1209	DJI_0109	Bromus squarrosus L.	0	a	0	0	Poaceae Barnhart

Iz_3_1209	DJI_0109	Bromus tectorum L.	0	a	0	0	Poaceae Barnhart
Iz_3_1209	DJI_0109	Calamagrostis epigejos (L.) Roth	4	n	1	0	Poaceae Barnhart
Iz_3_1209	DJI_0109	Carduus acanthoides L.	0	n	1	0	Asteraceae Bercht. & J.Presl
Iz_3_1209	DJI_0109	Carex sp.	0	n	1	0	Cyperaceae Juss.
Iz_3_1209	DJI_0109	Centaurea majorovii Dumbadze	0	n	1	0	Asteraceae Bercht. & J.Presl
Iz_3_1209	DJI_0109	Chamaecytisus ruthenicus (Fisch.ex Wol.) Klaskova	0	n	1	0	Fabaceae Lindl.
Iz_3_1209	DJI_0109	Chelidonium majus L.	0	n	1	0	Papaveraceae Juss.
Iz_3_1209	DJI_0109	Chenopodium betaceum Andrz.	0	n	1	0	Amaranthaceae Juss.
Iz_3_1209	DJI_0109	Chenopodium cf. album	0	n	1	0	Amaranthaceae Juss.
Iz_3_1209	DJI_0109	Chenopodium sp.	0		NA	0	Amaranthaceae Juss.
Iz_3_1209	DJI_0109	Chondrilla juncea L.	0	n	1	0	Asteraceae Bercht. & J.Presl
Iz_3_1209	DJI_0109	Cirsium vulgare (Savi) Ten.	0	n	1	0	Asteraceae Bercht. & J.Presl
Iz_3_1209	DJI_0109	Clinopodium acinos (L.) Kuntze	0	n	1	0	Lamiacea Lindl.
Iz_3_1209	DJI_0109	Convallaria majalis L.	0	n	1	0	Asparagaceae Juss.
Iz_3_1209	DJI_0109	Crataegus rhipidophylla Gand.	0	n	1	1	Rosaceae Juss.
Iz_3_1209	DJI_0109	Crepis foetida L.	0	n	1	0	Asteraceae Bercht. & J.Presl
Iz_3_1209	DJI_0109	Dactylis glomerata L.	0	n	1	0	Poaceae Barnhart
Iz_3_1209	DJI_0109	Dianthus campestris M.Bieb.	0	n	1	0	Caryophyllaceae Juss.
Iz_3_1209	DJI_0109	Digitaria sanguinalis (L.) Scop.	0	a	0	0	Poaceae Barnhart
Iz_3_1209	DJI_0109	Elymus repens (L.) Gould	0	n	1	0	Poaceae Barnhart
Iz_3_1209	DJI_0109	Epilobium tetragonum L.	0	n	1	0	Onagraceae Juss.
Iz_3_1209	DJI_0109	Eragrostis minor Host	0	a	0	0	Poaceae Barnhart
Iz_3_1209	DJI_0109	Erigeron canadensis L.	0	a	0	0	Asteraceae Bercht. & J.Presl
Iz_3_1209	DJI_0109	Euphorbia seguieriana Neck.	0	n	1	0	Euphorbiaceae Juss.
Iz_3_1209	DJI_0109	Fallopia dumetorum (L.) Holub	0	n	1	0	Polygonaceae Juss.
Iz_3_1209	DJI_0109	Festuca beckeri (Hack.) Trautv.	0	n	1	0	Poaceae Barnhart
Iz_3_1209	DJI_0109	Fragaria vesca L.	0	n	1	0	Rosaceae Juss.
Iz_3_1209	DJI_0109	Frangula alnus Mill.	0	n	1	1	Rhamnaceae Juss.
Iz_3_1209	DJI_0109	Fraxinus pennsylvanica Marshall	0	a	0	1	Oleaceae Hoffmanns. & Link
Iz_3_1209	DJI_0109	Galeopsis bifida Boenn.	0	n	1	0	Lamiacea Lindl.
Iz_3_1209	DJI_0109	Galium aparine L.	0.2		NA	NA	
Iz_3_1209	DJI_0109	Galium verum L.	0	n	1	0	Rubiaceae Juss.
Iz_3_1209	DJI_0109	Genista tinctoria L.	0	n	1	0	Fabaceae Lindl.
Iz_3_1209	DJI_0109	Geranium robertianum L.	0	n	1	0	Geraniaceae Juss.
Iz_3_1209	DJI_0109	Geranium sanquineum L.	0	n	1	0	Geraniaceae Juss.
Iz_3_1209	DJI_0109	Gypsophila paniculata L.	0	n	1	0	Caryophyllaceae Juss.
Iz_3_1209	DJI_0109	Helichrysum arenarium (L.) Moench	0	n	1	0	Asteraceae Bercht. & J.Presl
Iz_3_1209	DJI_0109	Herniaria polygama J.Gay	0	n	1	0	Caryophyllaceae Juss.
Iz_3_1209	DJI_0109	Hieracium robustum Fr.	0	n	1	0	Asteraceae Bercht. & J.Presl
Iz_3_1209	DJI_0109	Hieracium umbellatum L.	0	n	1	NA	Asteraceae Bercht. & J.Presl
Iz_3_1209	DJI_0109	Hylotelephium maximum (L.) Holub	0	n	1	0	Crassulaceae J.St.-Hil.
Iz_3_1209	DJI_0109	Jacobaea borysthenica (DC.) B.Nord. & Greuter	0	n	1	0	Asteraceae Bercht. & J.Presl
Iz_3_1209	DJI_0109	Jasione montana L.	0	n	1	0	Campanulaceae Juss.
Iz_3_1209	DJI_0109	Jurinea cyanoides (L.) Rchb.s.l.	0	n	1	0	Asteraceae Bercht. & J.Presl
Iz_3_1209	DJI_0109	Koeleria glauca (Spreng.) DC.	0	n	1	0	Poaceae Barnhart
Iz_3_1209	DJI_0109	Lactuca serriola L.	0	a	0	0	Asteraceae Bercht. & J.Presl
Iz_3_1209	DJI_0109	Linaria genistifolia (L.) Mill.	0	n	1	0	Plantaginaceae Juss.
Iz_3_1209	DJI_0109	Lomelosia argentea (L.) Greuter & Burdet	0	n	1	0	Caprifoliaceae Juss.
Iz_3_1209	DJI_0109	Melampyrum pratense L.	0	n	1	0	Orobanchaceae Vent.
Iz_3_1209	DJI_0109	Oenothera biennis L.	0	a	0	0	Onagraceae Juss.
Iz_3_1209	DJI_0109	Pentanema britannica (L.) D.Gut.Larr., Santos-Vicente, Anderb., E.Rico & M.M.Mart.Ort.	0	n	1	0	Asteraceae Bercht. & J.Presl
Iz_3_1209	DJI_0109	Peucedanum oreoselinum (L.) Moench	8	n	1	0	Apiaceae Lindl.
Iz_3_1209	DJI_0109	Peucedanum ruthenicum M.Bieb.	0	n	1	0	Apiaceae Lindl.
Iz_3_1209	DJI_0109	Pilosella echioides (Lumn.) F.W.Schultz & Sch.Bip.	0	n	1	0	Asteraceae Bercht. & J.Presl
Iz_3_1209	DJI_0109	Pilosella officinarum F. Schultz et Sch. Bip.	0	n	1	0	Asteraceae Bercht. & J.Presl
Iz_3_1209	DJI_0109	Pilosella sp.	0		NA	0	Asteraceae Bercht. & J.Presl
Iz_3_1209	DJI_0109	Pinus sylvestris L.	0	n	1	1	Pinaceae Spreng. ex F.Rudolphi
Iz_3_1209	DJI_0109	Poa annua L.	0	n	1	0	Poaceae Barnhart
Iz_3_1209	DJI_0109	Poa nemoralis L.	0	n	1	0	Poaceae Barnhart
Iz_3_1209	DJI_0109	Poa sp.	0		NA	0	Poaceae Barnhart
Iz_3_1209	DJI_0109	Polygonatum odoratum (Mill.) Druce	0	n	1	0	Asparagaceae Juss.
Iz_3_1209	DJI_0109	Polygonum arenarium Waldst. & Kit.	0	n	1	0	Polygonaceae Juss.
Iz_3_1209	DJI_0109	Populus nigra L.	0	n	1	1	Salicaceae Mirb.
Iz_3_1209	DJI_0109	Populus tremula L.	0	n	1	1	Salicaceae Mirb.
Iz_3_1209	DJI_0109	Populus Ã canescens (Aiton) Sm.	0	n	1	1	Salicaceae Mirb.
Iz_3_1209	DJI_0109	Potentilla argentea L.	0	n	1	0	Rosaceae Juss.
Iz_3_1209	DJI_0109	Psammophiliella muralis (L.) Ikonn.	0	n	1	0	Caryophyllaceae Juss.
Iz_3_1209	DJI_0109	Psephellus sumensis (Kalen.) Greuter	0	n	1	0	Asteraceae Bercht. & J.Presl
Iz_3_1209	DJI_0109	Pulsatilla pratensis (L.) Mill. s.l.	0	n	1	0	Ranunculaceae Juss.
Iz_3_1209	DJI_0109	Pyrus communis L.	0	a	0	1	Rosaceae Juss.
Iz_3_1209	DJI_0109	Quercus robur L.	0	n	1	1	Fagaceae Dumort.
Iz_3_1209	DJI_0109	Robinia pseudoacacia L.	0	a	0	1	Fabaceae Lindl.
Iz_3_1209	DJI_0109	Rosa sp.	0		NA	1	Rosaceae Juss.
Iz_3_1209	DJI_0109	Rubus idaeus L.	0	n	1	0	Rosaceae Juss.
Iz_3_1209	DJI_0109	Rumex acetosella L.	0	n	1	0	Polygonaceae Juss.
Iz_3_1209	DJI_0109	Rumex thyrsiflorus Fingerh.	0	n	1	0	Polygonaceae Juss.
Iz_3_1209	DJI_0109	Salix alba L.	0	n	1	1	Salicaceae Mirb.
Iz_3_1209	DJI_0109	Sambucus nigra L.	0	n	1	1	Viburnaceae Raf.
Iz_3_1209	DJI_0109	Secale sylvestre Host	0	n	1	0	Poaceae Barnhart
Iz_3_1209	DJI_0109	Sempervivum ruthenicum Schnittsp. & C.B. Lehm.	0	n	1	0	Crassulaceae J.St.-Hil.
Iz_3_1209	DJI_0109	Setaria viridis (L.) P. Beauv.	0	n	1	0	Poaceae Barnhart
Iz_3_1209	DJI_0109	Silene borysthenica (Gruner) Walters	0	n	1	0	Caryophyllaceae Juss.
Iz_3_1209	DJI_0109	Silene latifolia subsp. alba (Mill.) Greuter & Burdet	0	n	1	0	Caryophyllaceae Juss.
Iz_3_1209	DJI_0109	Solidago virgaurea L.	0	n	1	0	Asteraceae Bercht. & J.Presl
Iz_3_1209	DJI_0109	Sorbus aucuparia L.	0	n	1	1	Rosaceae Juss.
Iz_3_1209	DJI_0109	Stachys recta L.	0	n	1	0	Lamiacea Lindl.
Iz_3_1209	DJI_0109	Tanacetum vulgare L.	0	n	1	0	Asteraceae Bercht. & J.Presl
Iz_3_1209	DJI_0109	Thymus pallasianus Heinr.Braun	0	n	1	0	Lamiacea Lindl.
Iz_3_1209	DJI_0109	Tilia cordata Mill.	0	n	1	1	Malvaceae Juss.
Iz_3_1209	DJI_0109	Trifolium alpestre L.	0	n	1	0	Fabaceae Lindl.
Iz_3_1209	DJI_0109	Trifolium arvense L.	0	n	1	0	Fabaceae Lindl.
Iz_3_1209	DJI_0109	Ulmus glabra Huds.	0	n	1	1	Ulmaceae Mirb.
Iz_3_1209	DJI_0109	Verbascum densiflorum Bertol.	0	n	1	0	Scrophulariaceae Juss.
Iz_3_1209	DJI_0109	Veronica austriaca L.	0	n	1	0	Plantaginaceae Juss.
Iz_3_1209	DJI_0109	Veronica incana L.	0	n	1	0	Plantaginaceae Juss.
Iz_3_1209	DJI_0109	Veronica verna L.	0	n	1	0	Plantaginaceae Juss.
Iz_3_1209	DJI_0109	Vincetoxicum hirundinaria Medik	0	n	1	0	Apocynaceae Juss.
Iz_3_1209	DJI_0113	Acer tataricum L.	0	n	1	1	Sapindaceae Juss.

Iz_3_1209	DJI_0113	Achillea millefolium L. s.l.	0	n	1	0	Asteraceae Bercht. & J.Presl
Iz_3_1209	DJI_0113	Allium oleraceum L.	0	n	1	0	Amaryllidaceae J.St.-Hil.
Iz_3_1209	DJI_0113	Ambrosia artemisiifolia L.	0	a	0	0	Asteraceae Bercht. & J.Presl
Iz_3_1209	DJI_0113	Amorpha fruticosa L.	0	a	0	0	Fabaceae Lindl.
Iz_3_1209	DJI_0113	Anthericum ramosum L.	0	n	1	0	Asparagaceae Juss.
Iz_3_1209	DJI_0113	Artemisia marschalliana Spreng.	0	n	1	0	Asteraceae Bercht. & J.Presl
Iz_3_1209	DJI_0113	Asparagus officinalis L.	0	n	1	0	Asparagaceae Juss.
Iz_3_1209	DJI_0113	Astragalus cicer L.	0	n	1	0	Fabaceae Lindl.
Iz_3_1209	DJI_0113	Bassia laniflora (S.G.Gmel.) A.J.Scott	0	n	1	0	Amaranthaceae Juss.
Iz_3_1209	DJI_0113	Berberis vulgaris L.	0	n	1	0	Berberidaceae Juss.
Iz_3_1209	DJI_0113	Betula pendula Roth.	0	n	1	1	Betulaceae Gray
Iz_3_1209	DJI_0113	Betula pendula Roth.	0	n	1	0	Poaceae Barnhart
Iz_3_1209	DJI_0113	Bromus inermis Leyss.	0		NA	NA	
Iz_3_1209	DJI_0113	Bromus squarrosus L.	0	a	0	0	Poaceae Barnhart
Iz_3_1209	DJI_0113	Bromus tectorum L.	0	a	0	0	Poaceae Barnhart
Iz_3_1209	DJI_0113	Calamagrostis epigejos (L.) Roth	3	n	1	0	Poaceae Barnhart
Iz_3_1209	DJI_0113	Carduus acanthoides L.	0	n	1	0	Asteraceae Bercht. & J.Presl
Iz_3_1209	DJI_0113	Carex sp.	0	n	1	0	Cyperaceae Juss.
Iz_3_1209	DJI_0113	Centaurea majorovii Dumbadze	0	n	1	0	Asteraceae Bercht. & J.Presl
Iz_3_1209	DJI_0113	Chamaecytisus ruthenicus (Fisch.ex Wol.) Klaskova	0	n	1	0	Fabaceae Lindl.
Iz_3_1209	DJI_0113	Chelidonium majus L.	0	n	1	0	Papaveraceae Juss.
Iz_3_1209	DJI_0113	Chenopodium betaceum Andrz.	0	n	1	0	Amaranthaceae Juss.
Iz_3_1209	DJI_0113	Chenopodium cf. album	0.5	n	1	0	Amaranthaceae Juss.
Iz_3_1209	DJI_0113	Chenopodium sp.	0		NA	0	Amaranthaceae Juss.
Iz_3_1209	DJI_0113	Chondrilla juncea L.	0	n	1	0	Asteraceae Bercht. & J.Presl
Iz_3_1209	DJI_0113	Cirsium vulgare (Savi) Ten.	0	n	1	0	Asteraceae Bercht. & J.Presl
Iz_3_1209	DJI_0113	Clinopodium acinos (L.) Kuntze	0	n	1	0	Lamiacea Lindl.
Iz_3_1209	DJI_0113	Convallaria majalis L.	0	n	1	0	Asparagaceae Juss.
Iz_3_1209	DJI_0113	Crataegus rhipidophylla Gand.	2	n	1	1	Rosaceae Juss.
Iz_3_1209	DJI_0113	Crepis foetida L.	0	n	1	0	Asteraceae Bercht. & J.Presl
Iz_3_1209	DJI_0113	Dactylis glomerata L.	0	n	1	0	Poaceae Barnhart
Iz_3_1209	DJI_0113	Dianthus campestris M.Bieb.	0	n	1	0	Caryophyllaceae Juss.
Iz_3_1209	DJI_0113	Digitaria sanguinalis (L.) Scop.	0	a	0	0	Poaceae Barnhart
Iz_3_1209	DJI_0113	Elymus repens (L.) Gould	0	n	1	0	Poaceae Barnhart
Iz_3_1209	DJI_0113	Epilobium tetragonum L.	0	n	1	0	Onagraceae Juss.
Iz_3_1209	DJI_0113	Eragrostis minor Host	0	a	0	0	Poaceae Barnhart
Iz_3_1209	DJI_0113	Erigeron canadensis L.	0	a	0	0	Asteraceae Bercht. & J.Presl
Iz_3_1209	DJI_0113	Euphorbia seguieriana Neck.	0	n	1	0	Euphorbiaceae Juss.
Iz_3_1209	DJI_0113	Fallopia dumetorum (L.) Holub	1	n	1	0	Polygonaceae Juss.
Iz_3_1209	DJI_0113	Festuca beckeri (Hack.) Trautv.	0	n	1	0	Poaceae Barnhart
Iz_3_1209	DJI_0113	Fragaria vesca L.	0	n	1	0	Rosaceae Juss.
Iz_3_1209	DJI_0113	Frangula alnus Mill.	0	n	1	1	Rhamnaceae Juss.
Iz_3_1209	DJI_0113	Fraxinus pennsylvanica Marshall	0	a	0	1	Oleaceae Hoffmanns. & Link
Iz_3_1209	DJI_0113	Galeopsis bifida Boenn.	0	n	1	0	Lamiacea Lindl.
Iz_3_1209	DJI_0113	Galium aparine L.	0		NA	NA	
Iz_3_1209	DJI_0113	Galium verum L.	0	n	1	0	Rubiaceae Juss.
Iz_3_1209	DJI_0113	Genista tinctoria L.	0	n	1	0	Fabaceae Lindl.
Iz_3_1209	DJI_0113	Geranium robertianum L.	0	n	1	0	Geraniaceae Juss.
Iz_3_1209	DJI_0113	Geranium sanquineum L.	0	n	1	0	Geraniaceae Juss.
Iz_3_1209	DJI_0113	Gypsophila paniculata L.	0	n	1	0	Caryophyllaceae Juss.
Iz_3_1209	DJI_0113	Helichrysum arenarium (L.) Moench	0	n	1	0	Asteraceae Bercht. & J.Presl
Iz_3_1209	DJI_0113	Herniaria polygama J.Gay	0	n	1	0	Caryophyllaceae Juss.
Iz_3_1209	DJI_0113	Hieracium robustum Fr.	0	n	1	0	Asteraceae Bercht. & J.Presl
Iz_3_1209	DJI_0113	Hieracium umbellatum L.	0	n	1	NA	Asteraceae Bercht. & J.Presl
Iz_3_1209	DJI_0113	Hylotelephium maximum (L.) Holub	0	n	1	0	Crassulaceae J.St.-Hil.
Iz_3_1209	DJI_0113	Jacobaea borysthenica (DC.) B.Nord. & Greuter	0	n	1	0	Asteraceae Bercht. & J.Presl
Iz_3_1209	DJI_0113	Jasione montana L.	0	n	1	0	Campanulaceae Juss.
Iz_3_1209	DJI_0113	Jurinea cyanoides (L.) Rchb.s.l.	0	n	1	0	Asteraceae Bercht. & J.Presl
Iz_3_1209	DJI_0113	Koeleria glauca (Spreng.) DC.	0	n	1	0	Poaceae Barnhart
Iz_3_1209	DJI_0113	Lactuca serriola L.	0	a	0	0	Asteraceae Bercht. & J.Presl
Iz_3_1209	DJI_0113	Linaria genistifolia (L.) Mill.	0	n	1	0	Plantaginaceae Juss.
Iz_3_1209	DJI_0113	Lomelosia argentea (L.) Greuter & Burdet	0	n	1	0	Caprifoliaceae Juss.
Iz_3_1209	DJI_0113	Melampyrum pratense L.	0	n	1	0	Orobanchaceae Vent.
Iz_3_1209	DJI_0113	Oenothera biennis L.	0	a	0	0	Onagraceae Juss.
Iz_3_1209	DJI_0113	Pentanema britannica (L.) D.Gut.Larr., Santos-Vicente, Anderb., E.Rico & M.M.Mart.Ort.	0	n	1	0	Asteraceae Bercht. & J.Presl
Iz_3_1209	DJI_0113	Peucedanum oreoselinum (L.) Moench	0.5	n	1	0	Apiaceae Lindl.
Iz_3_1209	DJI_0113	Peucedanum ruthenicum M.Bieb.	0	n	1	0	Apiaceae Lindl.
Iz_3_1209	DJI_0113	Pilosella echioides (Lumn.) F.W.Schultz & Sch.Bip.	0	n	1	0	Asteraceae Bercht. & J.Presl
Iz_3_1209	DJI_0113	Pilosella officinarum F. Schultz et Sch. Bip.	0	n	1	0	Asteraceae Bercht. & J.Presl
Iz_3_1209	DJI_0113	Pilosella sp.	0		NA	0	Asteraceae Bercht. & J.Presl
Iz_3_1209	DJI_0113	Pinus sylvestris L.	0	n	1	1	Pinaceae Spreng. ex F.Rudolphi
Iz_3_1209	DJI_0113	Poa annua L.	0	n	1	0	Poaceae Barnhart
Iz_3_1209	DJI_0113	Poa nemoralis L.	0	n	1	0	Poaceae Barnhart
Iz_3_1209	DJI_0113	Poa sp.	0		NA	0	Poaceae Barnhart
Iz_3_1209	DJI_0113	Polygonatum odoratum (Mill.) Druce	0	n	1	0	Asparagaceae Juss.
Iz_3_1209	DJI_0113	Polygonum arenarium Waldst. & Kit.	0	n	1	0	Polygonaceae Juss.
Iz_3_1209	DJI_0113	Populus nigra L.	0	n	1	1	Salicaceae Mirb.
Iz_3_1209	DJI_0113	Populus tremula L.	0	n	1	1	Salicaceae Mirb.
Iz_3_1209	DJI_0113	Populus Ã canescens (Aiton) Sm.	0	n	1	1	Salicaceae Mirb.
Iz_3_1209	DJI_0113	Potentilla argentea L.	0	n	1	0	Rosaceae Juss.
Iz_3_1209	DJI_0113	Psammophiliella muralis (L.) Ikonn.	0	n	1	0	Caryophyllaceae Juss.
Iz_3_1209	DJI_0113	Psephellus sumensis (Kalen.) Greuter	0	n	1	0	Asteraceae Bercht. & J.Presl
Iz_3_1209	DJI_0113	Pulsatilla pratensis (L.) Mill. s.l.	0	n	1	0	Ranunculaceae Juss.
Iz_3_1209	DJI_0113	Pyrus communis L.	0	a	0	1	Rosaceae Juss.
Iz_3_1209	DJI_0113	Quercus robur L.	0	n	1	1	Fagaceae Dumort.
Iz_3_1209	DJI_0113	Robinia pseudoacacia L.	0	a	0	1	Fabaceae Lindl.
Iz_3_1209	DJI_0113	Rosa sp.	0		NA	1	Rosaceae Juss.
Iz_3_1209	DJI_0113	Rubus idaeus L.	0	n	1	0	Rosaceae Juss.
Iz_3_1209	DJI_0113	Rumex acetosella L.	0	n	1	0	Polygonaceae Juss.
Iz_3_1209	DJI_0113	Rumex thyrsiflorus Fingerh.	0	n	1	0	Polygonaceae Juss.
Iz_3_1209	DJI_0113	Salix alba L.	0	n	1	1	Salicaceae Mirb.
Iz_3_1209	DJI_0113	Sambucus nigra L.	0	n	1	1	Viburnaceae Raf.
Iz_3_1209	DJI_0113	Secale sylvestre Host	0	n	1	0	Poaceae Barnhart
Iz_3_1209	DJI_0113	Sempervivum ruthenicum Schnittsp. & C.B. Lehm.	0	n	1	0	Crassulaceae J.St.-Hil.
Iz_3_1209	DJI_0113	Setaria viridis (L.) P. Beauv.	0	n	1	0	Poaceae Barnhart
Iz_3_1209	DJI_0113	Silene borysthenica (Gruner) Walters	0	n	1	0	Caryophyllaceae Juss.
Iz_3_1209	DJI_0113	Silene latifolia subsp. alba (Mill.) Greuter & Burdet	0	n	1	0	Caryophyllaceae Juss.
Iz_3_1209	DJI_0113	Solidago virgaurea L.	0	n	1	0	Asteraceae Bercht. & J.Presl

Iz_3_1209	DJI_0113	Sorbus aucuparia L.	0	n	1	1	Rosaceae Juss.
Iz_3_1209	DJI_0113	Stachys recta L.	0	n	1	0	Lamiacea Lindl.
Iz_3_1209	DJI_0113	Tanacetum vulgare L.	0	n	1	0	Asteraceae Bercht. & J.Presl
Iz_3_1209	DJI_0113	Thymus pallasianus Heinr.Braun	0	n	1	0	Lamiacea Lindl.
Iz_3_1209	DJI_0113	Tilia cordata Mill.	0	n	1	1	Malvaceae Juss.
Iz_3_1209	DJI_0113	Trifolium alpestre L.	0	n	1	0	Fabaceae Lindl.
Iz_3_1209	DJI_0113	Trifolium arvense L.	0	n	1	0	Fabaceae Lindl.
Iz_3_1209	DJI_0113	Ulmus glabra Huds.	0	n	1	1	Ulmaceae Mirb.
Iz_3_1209	DJI_0113	Verbascum densiflorum Bertol.	0	n	1	0	Scrophulariaceae Juss.
Iz_3_1209	DJI_0113	Veronica austriaca L.	0	n	1	0	Plantaginaceae Juss.
Iz_3_1209	DJI_0113	Veronica incana L.	0	n	1	0	Plantaginaceae Juss.
Iz_3_1209	DJI_0113	Veronica verna L.	0	n	1	0	Plantaginaceae Juss.
Iz_3_1209	DJI_0113	Vincetoxicum hirundinaria Medik	0	n	1	0	Apocynaceae Juss.
Iz_3_1209	DJI_0116	Acer tataricum L.	0	n	1	1	Sapindaceae Juss.
Iz_3_1209	DJI_0116	Achillea millefolium L. s.l.	0	n	1	0	Asteraceae Bercht. & J.Presl
Iz_3_1209	DJI_0116	Allium oleraceum L.	0	n	1	0	Amaryllidaceae J.St.-Hil.
Iz_3_1209	DJI_0116	Ambrosia artemisiifolia L.	0	a	0	0	Asteraceae Bercht. & J.Presl
Iz_3_1209	DJI_0116	Amorpha fruticosa L.	0	a	0	0	Fabaceae Lindl.
Iz_3_1209	DJI_0116	Anthericum ramosum L.	0	n	1	0	Asparagaceae Juss.
Iz_3_1209	DJI_0116	Artemisia marschalliana Spreng.	0	n	1	0	Asteraceae Bercht. & J.Presl
Iz_3_1209	DJI_0116	Asparagus officinalis L.	0	n	1	0	Asparagaceae Juss.
Iz_3_1209	DJI_0116	Astragalus cicer L.	0	n	1	0	Fabaceae Lindl.
Iz_3_1209	DJI_0116	Bassia laniflora (S.G.Gmel.) A.J.Scott	0	n	1	0	Amaranthaceae Juss.
Iz_3_1209	DJI_0116	Berberis vulgaris L.	0	n	1	0	Berberidaceae Juss.
Iz_3_1209	DJI_0116	Betula pendula Roth.	0	n	1	1	Betulaceae Gray
Iz_3_1209	DJI_0116	Betula pendula Roth.	0	n	1	0	Poaceae Barnhart
Iz_3_1209	DJI_0116	Bromus inermis Leyss.	0		NA	NA	
Iz_3_1209	DJI_0116	Bromus squarrosus L.	0	a	0	0	Poaceae Barnhart
Iz_3_1209	DJI_0116	Bromus tectorum L.	0	a	0	0	Poaceae Barnhart
Iz_3_1209	DJI_0116	Calamagrostis epigejos (L.) Roth	15	n	1	0	Poaceae Barnhart
Iz_3_1209	DJI_0116	Carduus acanthoides L.	0	n	1	0	Asteraceae Bercht. & J.Presl
Iz_3_1209	DJI_0116	Carex sp.	0	n	1	0	Cyperaceae Juss.
Iz_3_1209	DJI_0116	Centaurea majorovii Dumbadze	0	n	1	0	Asteraceae Bercht. & J.Presl
Iz_3_1209	DJI_0116	Chamaecytisus ruthenicus (Fisch.ex Wol.) Klaskova	0	n	1	0	Fabaceae Lindl.
Iz_3_1209	DJI_0116	Chelidonium majus L.	0	n	1	0	Papaveraceae Juss.
Iz_3_1209	DJI_0116	Chenopodium betaceum Andrz.	0	n	1	0	Amaranthaceae Juss.
Iz_3_1209	DJI_0116	Chenopodium cf. album	0	n	1	0	Amaranthaceae Juss.
Iz_3_1209	DJI_0116	Chenopodium sp.	0		NA	0	Amaranthaceae Juss.
Iz_3_1209	DJI_0116	Chondrilla juncea L.	0	n	1	0	Asteraceae Bercht. & J.Presl
Iz_3_1209	DJI_0116	Cirsium vulgare (Savi) Ten.	0	n	1	0	Asteraceae Bercht. & J.Presl
Iz_3_1209	DJI_0116	Clinopodium acinos (L.) Kuntze	0	n	1	0	Lamiacea Lindl.
Iz_3_1209	DJI_0116	Convallaria majalis L.	0	n	1	0	Asparagaceae Juss.
Iz_3_1209	DJI_0116	Crataegus rhipidophylla Gand.	0	n	1	1	Rosaceae Juss.
Iz_3_1209	DJI_0116	Crepis foetida L.	0	n	1	0	Asteraceae Bercht. & J.Presl
Iz_3_1209	DJI_0116	Dactylis glomerata L.	0	n	1	0	Poaceae Barnhart
Iz_3_1209	DJI_0116	Dianthus campestris M.Bieb.	0	n	1	0	Caryophyllaceae Juss.
Iz_3_1209	DJI_0116	Digitaria sanguinalis (L.) Scop.	0	a	0	0	Poaceae Barnhart
Iz_3_1209	DJI_0116	Elymus repens (L.) Gould	0	n	1	0	Poaceae Barnhart
Iz_3_1209	DJI_0116	Epilobium tetragonum L.	0	n	1	0	Onagraceae Juss.
Iz_3_1209	DJI_0116	Eragrostis minor Host	0	a	0	0	Poaceae Barnhart
Iz_3_1209	DJI_0116	Erigeron canadensis L.	0.1	a	0	0	Asteraceae Bercht. & J.Presl
Iz_3_1209	DJI_0116	Euphorbia seguieriana Neck.	0	n	1	0	Euphorbiaceae Juss.
Iz_3_1209	DJI_0116	Fallopia dumetorum (L.) Holub	0	n	1	0	Polygonaceae Juss.
Iz_3_1209	DJI_0116	Festuca beckeri (Hack.) Trautv.	0	n	1	0	Poaceae Barnhart
Iz_3_1209	DJI_0116	Fragaria vesca L.	0	n	1	0	Rosaceae Juss.
Iz_3_1209	DJI_0116	Frangula alnus Mill.	0	n	1	1	Rhamnaceae Juss.
Iz_3_1209	DJI_0116	Fraxinus pennsylvanica Marshall	0	a	0	1	Oleaceae Hoffmanns. & Link
Iz_3_1209	DJI_0116	Galeopsis bifida Boenn.	0	n	1	0	Lamiacea Lindl.
Iz_3_1209	DJI_0116	Galium aparine L.	0		NA	NA	
Iz_3_1209	DJI_0116	Galium verum L.	0	n	1	0	Rubiaceae Juss.
Iz_3_1209	DJI_0116	Genista tinctoria L.	0	n	1	0	Fabaceae Lindl.
Iz_3_1209	DJI_0116	Geranium robertianum L.	0	n	1	0	Geraniaceae Juss.
Iz_3_1209	DJI_0116	Geranium sanquineum L.	0	n	1	0	Geraniaceae Juss.
Iz_3_1209	DJI_0116	Gypsophila paniculata L.	0	n	1	0	Caryophyllaceae Juss.
Iz_3_1209	DJI_0116	Helichrysum arenarium (L.) Moench	0	n	1	0	Asteraceae Bercht. & J.Presl
Iz_3_1209	DJI_0116	Herniaria polygama J.Gay	0	n	1	0	Caryophyllaceae Juss.
Iz_3_1209	DJI_0116	Hieracium robustum Fr.	0	n	1	0	Asteraceae Bercht. & J.Presl
Iz_3_1209	DJI_0116	Hieracium umbellatum L.	0	n	1	NA	Asteraceae Bercht. & J.Presl
Iz_3_1209	DJI_0116	Hylotelephium maximum (L.) Holub	0	n	1	0	Crassulaceae J.St.-Hil.
Iz_3_1209	DJI_0116	Jacobaea borysthenica (DC.) B.Nord. & Greuter	0	n	1	0	Asteraceae Bercht. & J.Presl
Iz_3_1209	DJI_0116	Jasione montana L.	0	n	1	0	Campanulaceae Juss.
Iz_3_1209	DJI_0116	Jurinea cyanoides (L.) Rchb.s.l.	0	n	1	0	Asteraceae Bercht. & J.Presl
Iz_3_1209	DJI_0116	Koeleria glauca (Spreng.) DC.	0	n	1	0	Poaceae Barnhart
Iz_3_1209	DJI_0116	Lactuca serriola L.	0.5	a	0	0	Asteraceae Bercht. & J.Presl
Iz_3_1209	DJI_0116	Linaria genistifolia (L.) Mill.	0	n	1	0	Plantaginaceae Juss.
Iz_3_1209	DJI_0116	Lomelosia argentea (L.) Greuter & Burdet	0	n	1	0	Caprifoliaceae Juss.
Iz_3_1209	DJI_0116	Melampyrum pratense L.	0	n	1	0	Orobanchaceae Vent.
Iz_3_1209	DJI_0116	Oenothera biennis L.	0	a	0	0	Onagraceae Juss.
Iz_3_1209	DJI_0116	Pentanema britannica (L.) D.Gut.Larr., Santos-Vicente, Anderb., E.Rico & M.M.Mart.Ort.	0	n	1	0	Asteraceae Bercht. & J.Presl
Iz_3_1209	DJI_0116	Peucedanum oreoselinum (L.) Moench	0	n	1	0	Apiaceae Lindl.
Iz_3_1209	DJI_0116	Peucedanum ruthenicum M.Bieb.	0	n	1	0	Apiaceae Lindl.
Iz_3_1209	DJI_0116	Pilosella echioides (Lumn.) F.W.Schultz & Sch.Bip.	0	n	1	0	Asteraceae Bercht. & J.Presl
Iz_3_1209	DJI_0116	Pilosella officinarum F. Schultz et Sch. Bip.	0	n	1	0	Asteraceae Bercht. & J.Presl
Iz_3_1209	DJI_0116	Pilosella sp.	0		NA	0	Asteraceae Bercht. & J.Presl
Iz_3_1209	DJI_0116	Pinus sylvestris L.	0	n	1	1	Pinaceae Spreng. ex F.Rudolphi
Iz_3_1209	DJI_0116	Poa annua L.	0	n	1	0	Poaceae Barnhart
Iz_3_1209	DJI_0116	Poa nemoralis L.	0	n	1	0	Poaceae Barnhart
Iz_3_1209	DJI_0116	Poa sp.	0		NA	0	Poaceae Barnhart
Iz_3_1209	DJI_0116	Polygonatum odoratum (Mill.) Druce	0	n	1	0	Asparagaceae Juss.
Iz_3_1209	DJI_0116	Polygonum arenarium Waldst. & Kit.	0	n	1	0	Polygonaceae Juss.
Iz_3_1209	DJI_0116	Populus nigra L.	0	n	1	1	Salicaceae Mirb.
Iz_3_1209	DJI_0116	Populus tremula L.	0	n	1	1	Salicaceae Mirb.
Iz_3_1209	DJI_0116	Populus Ã canescens (Aiton) Sm.	0	n	1	1	Salicaceae Mirb.
Iz_3_1209	DJI_0116	Potentilla argentea L.	0	n	1	0	Rosaceae Juss.
Iz_3_1209	DJI_0116	Psammophiliella muralis (L.) Ikonn.	0	n	1	0	Caryophyllaceae Juss.
Iz_3_1209	DJI_0116	Psephellus sumensis (Kalen.) Greuter	0	n	1	0	Asteraceae Bercht. & J.Presl
Iz_3_1209	DJI_0116	Pulsatilla pratensis (L.) Mill. s.l.	0	n	1	0	Ranunculaceae Juss.
Iz_3_1209	DJI_0116	Pyrus communis L.	0	a	0	1	Rosaceae Juss.

Iz_3_1209	DJI_0116	Quercus robur L.	0	n	1	1	Fagaceae Dumort.
Iz_3_1209	DJI_0116	Robinia pseudoacacia L.	0	a	0	1	Fabaceae Lindl.
Iz_3_1209	DJI_0116	Rosa sp.	0		NA	1	Rosaceae Juss.
Iz_3_1209	DJI_0116	Rubus idaeus L.	0	n	1	0	Rosaceae Juss.
Iz_3_1209	DJI_0116	Rumex acetosella L.	0	n	1	0	Polygonaceae Juss.
Iz_3_1209	DJI_0116	Rumex thyrsiflorus Fingerh.	0	n	1	0	Polygonaceae Juss.
Iz_3_1209	DJI_0116	Salix alba L.	0	n	1	1	Salicaceae Mirb.
Iz_3_1209	DJI_0116	Sambucus nigra L.	0	n	1	1	Viburnaceae Raf.
Iz_3_1209	DJI_0116	Secale sylvestre Host	0	n	1	0	Poaceae Barnhart
Iz_3_1209	DJI_0116	Sempervivum ruthenicum Schnittsp. & C.B. Lehm.	0	n	1	0	Crassulaceae J.St.-Hil.
Iz_3_1209	DJI_0116	Setaria viridis (L.) P. Beauv.	0	n	1	0	Poaceae Barnhart
Iz_3_1209	DJI_0116	Silene borysthenica (Gruner) Walters	0	n	1	0	Caryophyllaceae Juss.
Iz_3_1209	DJI_0116	Silene latifolia subsp. alba (Mill.) Greuter & Burdet	0	n	1	0	Caryophyllaceae Juss.
Iz_3_1209	DJI_0116	Solidago virgaurea L.	0	n	1	0	Asteraceae Bercht. & J.Presl
Iz_3_1209	DJI_0116	Sorbus aucuparia L.	0	n	1	1	Rosaceae Juss.
Iz_3_1209	DJI_0116	Stachys recta L.	0	n	1	0	Lamiacea Lindl.
Iz_3_1209	DJI_0116	Tanacetum vulgare L.	0	n	1	0	Asteraceae Bercht. & J.Presl
Iz_3_1209	DJI_0116	Thymus pallasianus Heinr.Braun	0	n	1	0	Lamiacea Lindl.
Iz_3_1209	DJI_0116	Tilia cordata Mill.	0	n	1	1	Malvaceae Juss.
Iz_3_1209	DJI_0116	Trifolium alpestre L.	0	n	1	0	Fabaceae Lindl.
Iz_3_1209	DJI_0116	Trifolium arvense L.	0	n	1	0	Fabaceae Lindl.
Iz_3_1209	DJI_0116	Ulmus glabra Huds.	0	n	1	1	Ulmaceae Mirb.
Iz_3_1209	DJI_0116	Verbascum densiflorum Bertol.	0	n	1	0	Scrophulariaceae Juss.
Iz_3_1209	DJI_0116	Veronica austriaca L.	0	n	1	0	Plantaginaceae Juss.
Iz_3_1209	DJI_0116	Veronica incana L.	0	n	1	0	Plantaginaceae Juss.
Iz_3_1209	DJI_0116	Veronica verna L.	0	n	1	0	Plantaginaceae Juss.
Iz_3_1209	DJI_0116	Vincetoxicum hirundinaria Medik	0	n	1	0	Apocynaceae Juss.
Iz_3_1209	DJI_0119	Acer tataricum L.	0	n	1	1	Sapindaceae Juss.
Iz_3_1209	DJI_0119	Achillea millefolium L. s.l.	0	n	1	0	Asteraceae Bercht. & J.Presl
Iz_3_1209	DJI_0119	Allium oleraceum L.	0	n	1	0	Amaryllidaceae J.St.-Hil.
Iz_3_1209	DJI_0119	Ambrosia artemisiifolia L.	0	a	0	0	Asteraceae Bercht. & J.Presl
Iz_3_1209	DJI_0119	Amorpha fruticosa L.	0	a	0	0	Fabaceae Lindl.
Iz_3_1209	DJI_0119	Anthericum ramosum L.	0	n	1	0	Asparagaceae Juss.
Iz_3_1209	DJI_0119	Artemisia marschalliana Spreng.	0	n	1	0	Asteraceae Bercht. & J.Presl
Iz_3_1209	DJI_0119	Asparagus officinalis L.	0	n	1	0	Asparagaceae Juss.
Iz_3_1209	DJI_0119	Astragalus cicer L.	0	n	1	0	Fabaceae Lindl.
Iz_3_1209	DJI_0119	Bassia laniflora (S.G.Gmel.) A.J.Scott	0	n	1	0	Amaranthaceae Juss.
Iz_3_1209	DJI_0119	Berberis vulgaris L.	0	n	1	0	Berberidaceae Juss.
Iz_3_1209	DJI_0119	Betula pendula Roth.	0	n	1	1	Betulaceae Gray
Iz_3_1209	DJI_0119	Betula pendula Roth.	0	n	1	0	Poaceae Barnhart
Iz_3_1209	DJI_0119	Bromus inermis Leyss.	0		NA	NA	
Iz_3_1209	DJI_0119	Bromus squarrosus L.	0	a	0	0	Poaceae Barnhart
Iz_3_1209	DJI_0119	Bromus tectorum L.	0	a	0	0	Poaceae Barnhart
Iz_3_1209	DJI_0119	Calamagrostis epigejos (L.) Roth	70	n	1	0	Poaceae Barnhart
Iz_3_1209	DJI_0119	Carduus acanthoides L.	0	n	1	0	Asteraceae Bercht. & J.Presl
Iz_3_1209	DJI_0119	Carex sp.	0	n	1	0	Cyperaceae Juss.
Iz_3_1209	DJI_0119	Centaurea majorovii Dumbadze	0	n	1	0	Asteraceae Bercht. & J.Presl
Iz_3_1209	DJI_0119	Chamaecytisus ruthenicus (Fisch.ex Wol.) Klaskova	0	n	1	0	Fabaceae Lindl.
Iz_3_1209	DJI_0119	Chelidonium majus L.	0	n	1	0	Papaveraceae Juss.
Iz_3_1209	DJI_0119	Chenopodium betaceum Andrz.	0	n	1	0	Amaranthaceae Juss.
Iz_3_1209	DJI_0119	Chenopodium cf. album	0	n	1	0	Amaranthaceae Juss.
Iz_3_1209	DJI_0119	Chenopodium sp.	0		NA	0	Amaranthaceae Juss.
Iz_3_1209	DJI_0119	Chondrilla juncea L.	0	n	1	0	Asteraceae Bercht. & J.Presl
Iz_3_1209	DJI_0119	Cirsium vulgare (Savi) Ten.	0	n	1	0	Asteraceae Bercht. & J.Presl
Iz_3_1209	DJI_0119	Clinopodium acinos (L.) Kuntze	0	n	1	0	Lamiacea Lindl.
Iz_3_1209	DJI_0119	Convallaria majalis L.	0	n	1	0	Asparagaceae Juss.
Iz_3_1209	DJI_0119	Crataegus rhipidophylla Gand.	0	n	1	1	Rosaceae Juss.
Iz_3_1209	DJI_0119	Crepis foetida L.	0	n	1	0	Asteraceae Bercht. & J.Presl
Iz_3_1209	DJI_0119	Dactylis glomerata L.	0	n	1	0	Poaceae Barnhart
Iz_3_1209	DJI_0119	Dianthus campestris M.Bieb.	0	n	1	0	Caryophyllaceae Juss.
Iz_3_1209	DJI_0119	Digitaria sanguinalis (L.) Scop.	0	a	0	0	Poaceae Barnhart
Iz_3_1209	DJI_0119	Elymus repens (L.) Gould	0	n	1	0	Poaceae Barnhart
Iz_3_1209	DJI_0119	Epilobium tetragonum L.	0	n	1	0	Onagraceae Juss.
Iz_3_1209	DJI_0119	Eragrostis minor Host	0	a	0	0	Poaceae Barnhart
Iz_3_1209	DJI_0119	Erigeron canadensis L.	0	a	0	0	Asteraceae Bercht. & J.Presl
Iz_3_1209	DJI_0119	Euphorbia seguieriana Neck.	0	n	1	0	Euphorbiaceae Juss.
Iz_3_1209	DJI_0119	Fallopia dumetorum (L.) Holub	1	n	1	0	Polygonaceae Juss.
Iz_3_1209	DJI_0119	Festuca beckeri (Hack.) Trautv.	0	n	1	0	Poaceae Barnhart
Iz_3_1209	DJI_0119	Fragaria vesca L.	0	n	1	0	Rosaceae Juss.
Iz_3_1209	DJI_0119	Frangula alnus Mill.	0	n	1	1	Rhamnaceae Juss.
Iz_3_1209	DJI_0119	Fraxinus pennsylvanica Marshall	0	a	0	1	Oleaceae Hoffmanns. & Link
Iz_3_1209	DJI_0119	Galeopsis bifida Boenn.	0	n	1	0	Lamiacea Lindl.
Iz_3_1209	DJI_0119	Galium aparine L.	0		NA	NA	
Iz_3_1209	DJI_0119	Galium verum L.	0	n	1	0	Rubiaceae Juss.
Iz_3_1209	DJI_0119	Genista tinctoria L.	0	n	1	0	Fabaceae Lindl.
Iz_3_1209	DJI_0119	Geranium robertianum L.	0	n	1	0	Geraniaceae Juss.
Iz_3_1209	DJI_0119	Geranium sanquineum L.	0	n	1	0	Geraniaceae Juss.
Iz_3_1209	DJI_0119	Gypsophila paniculata L.	0	n	1	0	Caryophyllaceae Juss.
Iz_3_1209	DJI_0119	Helichrysum arenarium (L.) Moench	0	n	1	0	Asteraceae Bercht. & J.Presl
Iz_3_1209	DJI_0119	Herniaria polygama J.Gay	0	n	1	0	Caryophyllaceae Juss.
Iz_3_1209	DJI_0119	Hieracium robustum Fr.	0	n	1	0	Asteraceae Bercht. & J.Presl
Iz_3_1209	DJI_0119	Hieracium umbellatum L.	0	n	1	NA	Asteraceae Bercht. & J.Presl
Iz_3_1209	DJI_0119	Hylotelephium maximum (L.) Holub	0	n	1	0	Crassulaceae J.St.-Hil.
Iz_3_1209	DJI_0119	Jacobaea borysthenica (DC.) B.Nord. & Greuter	0	n	1	0	Asteraceae Bercht. & J.Presl
Iz_3_1209	DJI_0119	Jasione montana L.	0	n	1	0	Campanulaceae Juss.
Iz_3_1209	DJI_0119	Jurinea cyanoides (L.) Rchb.s.l.	0	n	1	0	Asteraceae Bercht. & J.Presl
Iz_3_1209	DJI_0119	Koeleria glauca (Spreng.) DC.	0	n	1	0	Poaceae Barnhart
Iz_3_1209	DJI_0119	Lactuca serriola L.	0	a	0	0	Asteraceae Bercht. & J.Presl
Iz_3_1209	DJI_0119	Linaria genistifolia (L.) Mill.	0	n	1	0	Plantaginaceae Juss.
Iz_3_1209	DJI_0119	Lomelosia argentea (L.) Greuter & Burdet	0	n	1	0	Caprifoliaceae Juss.
Iz_3_1209	DJI_0119	Melampyrum pratense L.	0	n	1	0	Orobanchaceae Vent.
Iz_3_1209	DJI_0119	Oenothera biennis L.	0	a	0	0	Onagraceae Juss.
Iz_3_1209	DJI_0119	Pentanema britannica (L.) D.Gut.Larr., Santos-Vicente, Anderb., E.Rico & M.M.Mart.Ort.	0	n	1	0	Asteraceae Bercht. & J.Presl
Iz_3_1209	DJI_0119	Peucedanum oreoselinum (L.) Moench	0	n	1	0	Apiaceae Lindl.
Iz_3_1209	DJI_0119	Peucedanum ruthenicum M.Bieb.	0	n	1	0	Apiaceae Lindl.
Iz_3_1209	DJI_0119	Pilosella echioides (Lumn.) F.W.Schultz & Sch.Bip.	0	n	1	0	Asteraceae Bercht. & J.Presl
Iz_3_1209	DJI_0119	Pilosella officinarum F. Schultz et Sch. Bip.	0	n	1	0	Asteraceae Bercht. & J.Presl
Iz_3_1209	DJI_0119	Pilosella sp.	0		NA	0	Asteraceae Bercht. & J.Presl

Iz_3_1209	DJI_0119	Pinus sylvestris L.	0	n	1	1	Pinaceae Spreng. ex F.Rudolphi
Iz_3_1209	DJI_0119	Poa annua L.	0	n	1	0	Poaceae Barnhart
Iz_3_1209	DJI_0119	Poa nemoralis L.	0	n	1	0	Poaceae Barnhart
Iz_3_1209	DJI_0119	Poa sp.	0		NA	0	Poaceae Barnhart
Iz_3_1209	DJI_0119	Polygonatum odoratum (Mill.) Druce	0	n	1	0	Asparagaceae Juss.
Iz_3_1209	DJI_0119	Polygonum arenarium Waldst. & Kit.	0	n	1	0	Polygonaceae Juss.
Iz_3_1209	DJI_0119	Populus nigra L.	0	n	1	1	Salicaceae Mirb.
Iz_3_1209	DJI_0119	Populus tremula L.	0	n	1	1	Salicaceae Mirb.
Iz_3_1209	DJI_0119	Populus Ã canescens (Aiton) Sm.	0	n	1	1	Salicaceae Mirb.
Iz_3_1209	DJI_0119	Potentilla argentea L.	0	n	1	0	Rosaceae Juss.
Iz_3_1209	DJI_0119	Psammophiliella muralis (L.) Ikonn.	0	n	1	0	Caryophyllaceae Juss.
Iz_3_1209	DJI_0119	Psephellus sumensis (Kalen.) Greuter	0	n	1	0	Asteraceae Bercht. & J.Presl
Iz_3_1209	DJI_0119	Pulsatilla pratensis (L.) Mill. s.l.	0	n	1	0	Ranunculaceae Juss.
Iz_3_1209	DJI_0119	Pyrus communis L.	0	a	0	1	Rosaceae Juss.
Iz_3_1209	DJI_0119	Quercus robur L.	0	n	1	1	Fagaceae Dumort.
Iz_3_1209	DJI_0119	Robinia pseudoacacia L.	0	a	0	1	Fabaceae Lindl.
Iz_3_1209	DJI_0119	Rosa sp.	0		NA	1	Rosaceae Juss.
Iz_3_1209	DJI_0119	Rubus idaeus L.	0	n	1	0	Rosaceae Juss.
Iz_3_1209	DJI_0119	Rumex acetosella L.	0	n	1	0	Polygonaceae Juss.
Iz_3_1209	DJI_0119	Rumex thyrsiflorus Fingerh.	0	n	1	0	Polygonaceae Juss.
Iz_3_1209	DJI_0119	Salix alba L.	0	n	1	1	Salicaceae Mirb.
Iz_3_1209	DJI_0119	Sambucus nigra L.	0	n	1	1	Viburnaceae Raf.
Iz_3_1209	DJI_0119	Secale sylvestre Host	0	n	1	0	Poaceae Barnhart
Iz_3_1209	DJI_0119	Sempervivum ruthenicum Schnittsp. & C.B. Lehm.	0	n	1	0	Crassulaceae J.St.-Hil.
Iz_3_1209	DJI_0119	Setaria viridis (L.) P. Beauv.	0	n	1	0	Poaceae Barnhart
Iz_3_1209	DJI_0119	Silene borysthenica (Gruner) Walters	0	n	1	0	Caryophyllaceae Juss.
Iz_3_1209	DJI_0119	Silene latifolia subsp. alba (Mill.) Greuter & Burdet	0	n	1	0	Caryophyllaceae Juss.
Iz_3_1209	DJI_0119	Solidago virgaurea L.	2	n	1	0	Asteraceae Bercht. & J.Presl
Iz_3_1209	DJI_0119	Sorbus aucuparia L.	0	n	1	1	Rosaceae Juss.
Iz_3_1209	DJI_0119	Stachys recta L.	0	n	1	0	Lamiacea Lindl.
Iz_3_1209	DJI_0119	Tanacetum vulgare L.	0	n	1	0	Asteraceae Bercht. & J.Presl
Iz_3_1209	DJI_0119	Thymus pallasianus Heinr.Braun	0	n	1	0	Lamiacea Lindl.
Iz_3_1209	DJI_0119	Tilia cordata Mill.	0	n	1	1	Malvaceae Juss.
Iz_3_1209	DJI_0119	Trifolium alpestre L.	0	n	1	0	Fabaceae Lindl.
Iz_3_1209	DJI_0119	Trifolium arvense L.	0	n	1	0	Fabaceae Lindl.
Iz_3_1209	DJI_0119	Ulmus glabra Huds.	0	n	1	1	Ulmaceae Mirb.
Iz_3_1209	DJI_0119	Verbascum densiflorum Bertol.	0	n	1	0	Scrophulariaceae Juss.
Iz_3_1209	DJI_0119	Veronica austriaca L.	0	n	1	0	Plantaginaceae Juss.
Iz_3_1209	DJI_0119	Veronica incana L.	0	n	1	0	Plantaginaceae Juss.
Iz_3_1209	DJI_0119	Veronica verna L.	0	n	1	0	Plantaginaceae Juss.
Iz_3_1209	DJI_0119	Vincetoxicum hirundinaria Medik	0	n	1	0	Apocynaceae Juss.
Iz_3_1209	DJI_0122	Acer tataricum L.	0	n	1	1	Sapindaceae Juss.
Iz_3_1209	DJI_0122	Achillea millefolium L. s.l.	0	n	1	0	Asteraceae Bercht. & J.Presl
Iz_3_1209	DJI_0122	Allium oleraceum L.	0	n	1	0	Amaryllidaceae J.St.-Hil.
Iz_3_1209	DJI_0122	Ambrosia artemisiifolia L.	0	a	0	0	Asteraceae Bercht. & J.Presl
Iz_3_1209	DJI_0122	Amorpha fruticosa L.	0	a	0	0	Fabaceae Lindl.
Iz_3_1209	DJI_0122	Anthericum ramosum L.	0	n	1	0	Asparagaceae Juss.
Iz_3_1209	DJI_0122	Artemisia marschalliana Spreng.	0	n	1	0	Asteraceae Bercht. & J.Presl
Iz_3_1209	DJI_0122	Asparagus officinalis L.	0	n	1	0	Asparagaceae Juss.
Iz_3_1209	DJI_0122	Astragalus cicer L.	0	n	1	0	Fabaceae Lindl.
Iz_3_1209	DJI_0122	Bassia laniflora (S.G.Gmel.) A.J.Scott	0	n	1	0	Amaranthaceae Juss.
Iz_3_1209	DJI_0122	Berberis vulgaris L.	0	n	1	0	Berberidaceae Juss.
Iz_3_1209	DJI_0122	Betula pendula Roth.	0	n	1	1	Betulaceae Gray
Iz_3_1209	DJI_0122	Betula pendula Roth.	0	n	1	0	Poaceae Barnhart
Iz_3_1209	DJI_0122	Bromus inermis Leyss.	0		NA	NA	
Iz_3_1209	DJI_0122	Bromus squarrosus L.	0	a	0	0	Poaceae Barnhart
Iz_3_1209	DJI_0122	Bromus tectorum L.	0	a	0	0	Poaceae Barnhart
Iz_3_1209	DJI_0122	Calamagrostis epigejos (L.) Roth	25	n	1	0	Poaceae Barnhart
Iz_3_1209	DJI_0122	Carduus acanthoides L.	0	n	1	0	Asteraceae Bercht. & J.Presl
Iz_3_1209	DJI_0122	Carex sp.	0	n	1	0	Cyperaceae Juss.
Iz_3_1209	DJI_0122	Centaurea majorovii Dumbadze	0	n	1	0	Asteraceae Bercht. & J.Presl
Iz_3_1209	DJI_0122	Chamaecytisus ruthenicus (Fisch.ex Wol.) Klaskova	0	n	1	0	Fabaceae Lindl.
Iz_3_1209	DJI_0122	Chelidonium majus L.	0.5	n	1	0	Papaveraceae Juss.
Iz_3_1209	DJI_0122	Chenopodium betaceum Andrz.	0	n	1	0	Amaranthaceae Juss.
Iz_3_1209	DJI_0122	Chenopodium cf. album	0	n	1	0	Amaranthaceae Juss.
Iz_3_1209	DJI_0122	Chenopodium sp.	0		NA	0	Amaranthaceae Juss.
Iz_3_1209	DJI_0122	Chondrilla juncea L.	0	n	1	0	Asteraceae Bercht. & J.Presl
Iz_3_1209	DJI_0122	Cirsium vulgare (Savi) Ten.	0	n	1	0	Asteraceae Bercht. & J.Presl
Iz_3_1209	DJI_0122	Clinopodium acinos (L.) Kuntze	0	n	1	0	Lamiacea Lindl.
Iz_3_1209	DJI_0122	Convallaria majalis L.	0	n	1	0	Asparagaceae Juss.
Iz_3_1209	DJI_0122	Crataegus rhipidophylla Gand.	0	n	1	1	Rosaceae Juss.
Iz_3_1209	DJI_0122	Crepis foetida L.	0	n	1	0	Asteraceae Bercht. & J.Presl
Iz_3_1209	DJI_0122	Dactylis glomerata L.	0	n	1	0	Poaceae Barnhart
Iz_3_1209	DJI_0122	Dianthus campestris M.Bieb.	0	n	1	0	Caryophyllaceae Juss.
Iz_3_1209	DJI_0122	Digitaria sanguinalis (L.) Scop.	0	a	0	0	Poaceae Barnhart
Iz_3_1209	DJI_0122	Elymus repens (L.) Gould	0	n	1	0	Poaceae Barnhart
Iz_3_1209	DJI_0122	Epilobium tetragonum L.	0	n	1	0	Onagraceae Juss.
Iz_3_1209	DJI_0122	Eragrostis minor Host	0	a	0	0	Poaceae Barnhart
Iz_3_1209	DJI_0122	Erigeron canadensis L.	0	a	0	0	Asteraceae Bercht. & J.Presl
Iz_3_1209	DJI_0122	Euphorbia seguieriana Neck.	0	n	1	0	Euphorbiaceae Juss.
Iz_3_1209	DJI_0122	Fallopia dumetorum (L.) Holub	0	n	1	0	Polygonaceae Juss.
Iz_3_1209	DJI_0122	Festuca beckeri (Hack.) Trautv.	0	n	1	0	Poaceae Barnhart
Iz_3_1209	DJI_0122	Fragaria vesca L.	0	n	1	0	Rosaceae Juss.
Iz_3_1209	DJI_0122	Frangula alnus Mill.	0	n	1	1	Rhamnaceae Juss.
Iz_3_1209	DJI_0122	Fraxinus pennsylvanica Marshall	0	a	0	1	Oleaceae Hoffmanns. & Link
Iz_3_1209	DJI_0122	Galeopsis bifida Boenn.	0	n	1	0	Lamiacea Lindl.
Iz_3_1209	DJI_0122	Galium aparine L.	0		NA	NA	
Iz_3_1209	DJI_0122	Galium verum L.	0	n	1	0	Rubiaceae Juss.
Iz_3_1209	DJI_0122	Genista tinctoria L.	0	n	1	0	Fabaceae Lindl.
Iz_3_1209	DJI_0122	Geranium robertianum L.	0	n	1	0	Geraniaceae Juss.
Iz_3_1209	DJI_0122	Geranium sanquineum L.	0	n	1	0	Geraniaceae Juss.
Iz_3_1209	DJI_0122	Gypsophila paniculata L.	0	n	1	0	Caryophyllaceae Juss.
Iz_3_1209	DJI_0122	Helichrysum arenarium (L.) Moench	0	n	1	0	Asteraceae Bercht. & J.Presl
Iz_3_1209	DJI_0122	Herniaria polygama J.Gay	0	n	1	0	Caryophyllaceae Juss.
Iz_3_1209	DJI_0122	Hieracium robustum Fr.	0	n	1	0	Asteraceae Bercht. & J.Presl
Iz_3_1209	DJI_0122	Hieracium umbellatum L.	0.5	n	1	NA	Asteraceae Bercht. & J.Presl
Iz_3_1209	DJI_0122	Hylotelephium maximum (L.) Holub	0	n	1	0	Crassulaceae J.St.-Hil.
Iz_3_1209	DJI_0122	Jacobaea borysthenica (DC.) B.Nord. & Greuter	0	n	1	0	Asteraceae Bercht. & J.Presl

Iz_3_1209	DJI_0122	Jasione montana L.	0	n	1	0	Campanulaceae Juss.
Iz_3_1209	DJI_0122	Jurinea cyanoides (L.) Rchb.s.l.	0	n	1	0	Asteraceae Bercht. & J.Presl
Iz_3_1209	DJI_0122	Koeleria glauca (Spreng.) DC.	0	n	1	0	Poaceae Barnhart
Iz_3_1209	DJI_0122	Lactuca serriola L.	0	a	0	0	Asteraceae Bercht. & J.Presl
Iz_3_1209	DJI_0122	Linaria genistifolia (L.) Mill.	0	n	1	0	Plantaginaceae Juss.
Iz_3_1209	DJI_0122	Lomelosia argentea (L.) Greuter & Burdet	0	n	1	0	Caprifoliaceae Juss.
Iz_3_1209	DJI_0122	Melampyrum pratense L.	0	n	1	0	Orobanchaceae Vent.
Iz_3_1209	DJI_0122	Oenothera biennis L.	0	a	0	0	Onagraceae Juss.
Iz_3_1209	DJI_0122	Pentanema britannica (L.) D.Gut.Larr., Santos-Vicente, Anderb., E.Rico & M.M.Mart.Ort.	0	n	1	0	Asteraceae Bercht. & J.Presl
Iz_3_1209	DJI_0122	Peucedanum oreoselinum (L.) Moench	0	n	1	0	Apiaceae Lindl.
Iz_3_1209	DJI_0122	Peucedanum ruthenicum M.Bieb.	0	n	1	0	Apiaceae Lindl.
Iz_3_1209	DJI_0122	Pilosella echioides (Lumn.) F.W.Schultz & Sch.Bip.	0	n	1	0	Asteraceae Bercht. & J.Presl
Iz_3_1209	DJI_0122	Pilosella officinarum F. Schultz et Sch. Bip.	0	n	1	0	Asteraceae Bercht. & J.Presl
Iz_3_1209	DJI_0122	Pilosella sp.	0		NA	0	Asteraceae Bercht. & J.Presl
Iz_3_1209	DJI_0122	Pinus sylvestris L.	0	n	1	1	Pinaceae Spreng. ex F.Rudolphi
Iz_3_1209	DJI_0122	Poa annua L.	0	n	1	0	Poaceae Barnhart
Iz_3_1209	DJI_0122	Poa nemoralis L.	0	n	1	0	Poaceae Barnhart
Iz_3_1209	DJI_0122	Poa sp.	0		NA	0	Poaceae Barnhart
Iz_3_1209	DJI_0122	Polygonatum odoratum (Mill.) Druce	0	n	1	0	Asparagaceae Juss.
Iz_3_1209	DJI_0122	Polygonum arenarium Waldst. & Kit.	0	n	1	0	Polygonaceae Juss.
Iz_3_1209	DJI_0122	Populus nigra L.	0	n	1	1	Salicaceae Mirb.
Iz_3_1209	DJI_0122	Populus tremula L.	0	n	1	1	Salicaceae Mirb.
Iz_3_1209	DJI_0122	Populus Ã canescens (Aiton) Sm.	0	n	1	1	Salicaceae Mirb.
Iz_3_1209	DJI_0122	Potentilla argentea L.	0	n	1	0	Rosaceae Juss.
Iz_3_1209	DJI_0122	Psammophiliella muralis (L.) Ikonn.	0	n	1	0	Caryophyllaceae Juss.
Iz_3_1209	DJI_0122	Psephellus sumensis (Kalen.) Greuter	0	n	1	0	Asteraceae Bercht. & J.Presl
Iz_3_1209	DJI_0122	Pulsatilla pratensis (L.) Mill. s.l.	0	n	1	0	Ranunculaceae Juss.
Iz_3_1209	DJI_0122	Pyrus communis L.	0	a	0	1	Rosaceae Juss.
Iz_3_1209	DJI_0122	Quercus robur L.	0	n	1	1	Fagaceae Dumort.
Iz_3_1209	DJI_0122	Robinia pseudoacacia L.	0	a	0	1	Fabaceae Lindl.
Iz_3_1209	DJI_0122	Rosa sp.	0		NA	1	Rosaceae Juss.
Iz_3_1209	DJI_0122	Rubus idaeus L.	0	n	1	0	Rosaceae Juss.
Iz_3_1209	DJI_0122	Rumex acetosella L.	0	n	1	0	Polygonaceae Juss.
Iz_3_1209	DJI_0122	Rumex thyrsiflorus Fingerh.	0	n	1	0	Polygonaceae Juss.
Iz_3_1209	DJI_0122	Salix alba L.	0	n	1	1	Salicaceae Mirb.
Iz_3_1209	DJI_0122	Sambucus nigra L.	0	n	1	1	Viburnaceae Raf.
Iz_3_1209	DJI_0122	Secale sylvestre Host	0	n	1	0	Poaceae Barnhart
Iz_3_1209	DJI_0122	Sempervivum ruthenicum Schnittsp. & C.B. Lehm.	0	n	1	0	Crassulaceae J.St.-Hil.
Iz_3_1209	DJI_0122	Setaria viridis (L.) P. Beauv.	0	n	1	0	Poaceae Barnhart
Iz_3_1209	DJI_0122	Silene borysthenica (Gruner) Walters	0	n	1	0	Caryophyllaceae Juss.
Iz_3_1209	DJI_0122	Silene latifolia subsp. alba (Mill.) Greuter & Burdet	0	n	1	0	Caryophyllaceae Juss.
Iz_3_1209	DJI_0122	Solidago virgaurea L.	0	n	1	0	Asteraceae Bercht. & J.Presl
Iz_3_1209	DJI_0122	Sorbus aucuparia L.	0	n	1	1	Rosaceae Juss.
Iz_3_1209	DJI_0122	Stachys recta L.	0	n	1	0	Lamiacea Lindl.
Iz_3_1209	DJI_0122	Tanacetum vulgare L.	0	n	1	0	Asteraceae Bercht. & J.Presl
Iz_3_1209	DJI_0122	Thymus pallasianus Heinr.Braun	0	n	1	0	Lamiacea Lindl.
Iz_3_1209	DJI_0122	Tilia cordata Mill.	0	n	1	1	Malvaceae Juss.
Iz_3_1209	DJI_0122	Trifolium alpestre L.	0	n	1	0	Fabaceae Lindl.
Iz_3_1209	DJI_0122	Trifolium arvense L.	0	n	1	0	Fabaceae Lindl.
Iz_3_1209	DJI_0122	Ulmus glabra Huds.	0	n	1	1	Ulmaceae Mirb.
Iz_3_1209	DJI_0122	Verbascum densiflorum Bertol.	0	n	1	0	Scrophulariaceae Juss.
Iz_3_1209	DJI_0122	Veronica austriaca L.	0	n	1	0	Plantaginaceae Juss.
Iz_3_1209	DJI_0122	Veronica incana L.	0	n	1	0	Plantaginaceae Juss.
Iz_3_1209	DJI_0122	Veronica verna L.	0	n	1	0	Plantaginaceae Juss.
Iz_3_1209	DJI_0122	Vincetoxicum hirundinaria Medik	0	n	1	0	Apocynaceae Juss.
Iz_3_1209	DJI_0125	Acer tataricum L.	0	n	1	1	Sapindaceae Juss.
Iz_3_1209	DJI_0125	Achillea millefolium L. s.l.	0	n	1	0	Asteraceae Bercht. & J.Presl
Iz_3_1209	DJI_0125	Allium oleraceum L.	0	n	1	0	Amaryllidaceae J.St.-Hil.
Iz_3_1209	DJI_0125	Ambrosia artemisiifolia L.	0	a	0	0	Asteraceae Bercht. & J.Presl
Iz_3_1209	DJI_0125	Amorpha fruticosa L.	0	a	0	0	Fabaceae Lindl.
Iz_3_1209	DJI_0125	Anthericum ramosum L.	0	n	1	0	Asparagaceae Juss.
Iz_3_1209	DJI_0125	Artemisia marschalliana Spreng.	0	n	1	0	Asteraceae Bercht. & J.Presl
Iz_3_1209	DJI_0125	Asparagus officinalis L.	0	n	1	0	Asparagaceae Juss.
Iz_3_1209	DJI_0125	Astragalus cicer L.	0	n	1	0	Fabaceae Lindl.
Iz_3_1209	DJI_0125	Bassia laniflora (S.G.Gmel.) A.J.Scott	0	n	1	0	Amaranthaceae Juss.
Iz_3_1209	DJI_0125	Berberis vulgaris L.	0	n	1	0	Berberidaceae Juss.
Iz_3_1209	DJI_0125	Betula pendula Roth.	0	n	1	1	Betulaceae Gray
Iz_3_1209	DJI_0125	Betula pendula Roth.	0	n	1	0	Poaceae Barnhart
Iz_3_1209	DJI_0125	Bromus inermis Leyss.	0		NA	NA	
Iz_3_1209	DJI_0125	Bromus squarrosus L.	0	a	0	0	Poaceae Barnhart
Iz_3_1209	DJI_0125	Bromus tectorum L.	0	a	0	0	Poaceae Barnhart
Iz_3_1209	DJI_0125	Calamagrostis epigejos (L.) Roth	15	n	1	0	Poaceae Barnhart
Iz_3_1209	DJI_0125	Carduus acanthoides L.	0	n	1	0	Asteraceae Bercht. & J.Presl
Iz_3_1209	DJI_0125	Carex sp.	0.5	n	1	0	Cyperaceae Juss.
Iz_3_1209	DJI_0125	Centaurea majorovii Dumbadze	0	n	1	0	Asteraceae Bercht. & J.Presl
Iz_3_1209	DJI_0125	Chamaecytisus ruthenicus (Fisch.ex Wol.) Klaskova	0	n	1	0	Fabaceae Lindl.
Iz_3_1209	DJI_0125	Chelidonium majus L.	0	n	1	0	Papaveraceae Juss.
Iz_3_1209	DJI_0125	Chenopodium betaceum Andrz.	0	n	1	0	Amaranthaceae Juss.
Iz_3_1209	DJI_0125	Chenopodium cf. album	0	n	1	0	Amaranthaceae Juss.
Iz_3_1209	DJI_0125	Chenopodium sp.	0		NA	0	Amaranthaceae Juss.
Iz_3_1209	DJI_0125	Chondrilla juncea L.	0	n	1	0	Asteraceae Bercht. & J.Presl
Iz_3_1209	DJI_0125	Cirsium vulgare (Savi) Ten.	0	n	1	0	Asteraceae Bercht. & J.Presl
Iz_3_1209	DJI_0125	Clinopodium acinos (L.) Kuntze	0	n	1	0	Lamiacea Lindl.
Iz_3_1209	DJI_0125	Convallaria majalis L.	0	n	1	0	Asparagaceae Juss.
Iz_3_1209	DJI_0125	Crataegus rhipidophylla Gand.	0	n	1	1	Rosaceae Juss.
Iz_3_1209	DJI_0125	Crepis foetida L.	0	n	1	0	Asteraceae Bercht. & J.Presl
Iz_3_1209	DJI_0125	Dactylis glomerata L.	0	n	1	0	Poaceae Barnhart
Iz_3_1209	DJI_0125	Dianthus campestris M.Bieb.	0	n	1	0	Caryophyllaceae Juss.
Iz_3_1209	DJI_0125	Digitaria sanguinalis (L.) Scop.	0	a	0	0	Poaceae Barnhart
Iz_3_1209	DJI_0125	Elymus repens (L.) Gould	0	n	1	0	Poaceae Barnhart
Iz_3_1209	DJI_0125	Epilobium tetragonum L.	0	n	1	0	Onagraceae Juss.
Iz_3_1209	DJI_0125	Eragrostis minor Host	0	a	0	0	Poaceae Barnhart
Iz_3_1209	DJI_0125	Erigeron canadensis L.	0	a	0	0	Asteraceae Bercht. & J.Presl
Iz_3_1209	DJI_0125	Euphorbia seguieriana Neck.	0	n	1	0	Euphorbiaceae Juss.
Iz_3_1209	DJI_0125	Fallopia dumetorum (L.) Holub	0	n	1	0	Polygonaceae Juss.
Iz_3_1209	DJI_0125	Festuca beckeri (Hack.) Trautv.	0	n	1	0	Poaceae Barnhart
Iz_3_1209	DJI_0125	Fragaria vesca L.	0	n	1	0	Rosaceae Juss.
Iz_3_1209	DJI_0125	Frangula alnus Mill.	0	n	1	1	Rhamnaceae Juss.

Iz_3_1209	DJI_0125	Fraxinus pennsylvanica Marshall	0	a	0	1	Oleaceae Hoffmanns. & Link
Iz_3_1209	DJI_0125	Galeopsis bifida Boenn.	0	n	1	0	Lamiacea Lindl.
Iz_3_1209	DJI_0125	Galium aparine L.	0		NA	NA	
Iz_3_1209	DJI_0125	Galium verum L.	0	n	1	0	Rubiaceae Juss.
Iz_3_1209	DJI_0125	Genista tinctoria L.	0	n	1	0	Fabaceae Lindl.
Iz_3_1209	DJI_0125	Geranium robertianum L.	0	n	1	0	Geraniaceae Juss.
Iz_3_1209	DJI_0125	Geranium sanquineum L.	0	n	1	0	Geraniaceae Juss.
Iz_3_1209	DJI_0125	Gypsophila paniculata L.	0	n	1	0	Caryophyllaceae Juss.
Iz_3_1209	DJI_0125	Helichrysum arenarium (L.) Moench	0	n	1	0	Asteraceae Bercht. & J.Presl
Iz_3_1209	DJI_0125	Herniaria polygama J.Gay	0	n	1	0	Caryophyllaceae Juss.
Iz_3_1209	DJI_0125	Hieracium robustum Fr.	0	n	1	0	Asteraceae Bercht. & J.Presl
Iz_3_1209	DJI_0125	Hieracium umbellatum L.	0	n	1	NA	Asteraceae Bercht. & J.Presl
Iz_3_1209	DJI_0125	Hylotelephium maximum (L.) Holub	0	n	1	0	Crassulaceae J.St.-Hil.
Iz_3_1209	DJI_0125	Jacobaea borysthenica (DC.) B.Nord. & Greuter	0	n	1	0	Asteraceae Bercht. & J.Presl
Iz_3_1209	DJI_0125	Jasione montana L.	0	n	1	0	Campanulaceae Juss.
Iz_3_1209	DJI_0125	Jurinea cyanoides (L.) Rchb.s.l.	0	n	1	0	Asteraceae Bercht. & J.Presl
Iz_3_1209	DJI_0125	Koeleria glauca (Spreng.) DC.	0	n	1	0	Poaceae Barnhart
Iz_3_1209	DJI_0125	Lactuca serriola L.	0	a	0	0	Asteraceae Bercht. & J.Presl
Iz_3_1209	DJI_0125	Linaria genistifolia (L.) Mill.	0	n	1	0	Plantaginaceae Juss.
Iz_3_1209	DJI_0125	Lomelosia argentea (L.) Greuter & Burdet	0	n	1	0	Caprifoliaceae Juss.
Iz_3_1209	DJI_0125	Melampyrum pratense L.	0	n	1	0	Orobanchaceae Vent.
Iz_3_1209	DJI_0125	Oenothera biennis L.	0	a	0	0	Onagraceae Juss.
Iz_3_1209	DJI_0125	Pentanema britannica (L.) D.Gut.Larr., Santos-Vicente, Anderb., E.Rico & M.M.Mart.Ort.	0	n	1	0	Asteraceae Bercht. & J.Presl
Iz_3_1209	DJI_0125	Peucedanum oreoselinum (L.) Moench	5	n	1	0	Apiaceae Lindl.
Iz_3_1209	DJI_0125	Peucedanum ruthenicum M.Bieb.	0	n	1	0	Apiaceae Lindl.
Iz_3_1209	DJI_0125	Pilosella echioides (Lumn.) F.W.Schultz & Sch.Bip.	0	n	1	0	Asteraceae Bercht. & J.Presl
Iz_3_1209	DJI_0125	Pilosella officinarum F. Schultz et Sch. Bip.	0	n	1	0	Asteraceae Bercht. & J.Presl
Iz_3_1209	DJI_0125	Pilosella sp.	0		NA	0	Asteraceae Bercht. & J.Presl
Iz_3_1209	DJI_0125	Pinus sylvestris L.	0	n	1	1	Pinaceae Spreng. ex F.Rudolphi
Iz_3_1209	DJI_0125	Poa annua L.	0	n	1	0	Poaceae Barnhart
Iz_3_1209	DJI_0125	Poa nemoralis L.	0	n	1	0	Poaceae Barnhart
Iz_3_1209	DJI_0125	Poa sp.	0		NA	0	Poaceae Barnhart
Iz_3_1209	DJI_0125	Polygonatum odoratum (Mill.) Druce	0	n	1	0	Asparagaceae Juss.
Iz_3_1209	DJI_0125	Polygonum arenarium Waldst. & Kit.	0	n	1	0	Polygonaceae Juss.
Iz_3_1209	DJI_0125	Populus nigra L.	0	n	1	1	Salicaceae Mirb.
Iz_3_1209	DJI_0125	Populus tremula L.	0	n	1	1	Salicaceae Mirb.
Iz_3_1209	DJI_0125	Populus Ã canescens (Aiton) Sm.	0	n	1	1	Salicaceae Mirb.
Iz_3_1209	DJI_0125	Potentilla argentea L.	0	n	1	0	Rosaceae Juss.
Iz_3_1209	DJI_0125	Psammophiliella muralis (L.) Ikonn.	0	n	1	0	Caryophyllaceae Juss.
Iz_3_1209	DJI_0125	Psephellus sumensis (Kalen.) Greuter	0	n	1	0	Asteraceae Bercht. & J.Presl
Iz_3_1209	DJI_0125	Pulsatilla pratensis (L.) Mill. s.l.	0	n	1	0	Ranunculaceae Juss.
Iz_3_1209	DJI_0125	Pyrus communis L.	0	a	0	1	Rosaceae Juss.
Iz_3_1209	DJI_0125	Quercus robur L.	0	n	1	1	Fagaceae Dumort.
Iz_3_1209	DJI_0125	Robinia pseudoacacia L.	0	a	0	1	Fabaceae Lindl.
Iz_3_1209	DJI_0125	Rosa sp.	0		NA	1	Rosaceae Juss.
Iz_3_1209	DJI_0125	Rubus idaeus L.	0	n	1	0	Rosaceae Juss.
Iz_3_1209	DJI_0125	Rumex acetosella L.	0	n	1	0	Polygonaceae Juss.
Iz_3_1209	DJI_0125	Rumex thyrsiflorus Fingerh.	0	n	1	0	Polygonaceae Juss.
Iz_3_1209	DJI_0125	Salix alba L.	0	n	1	1	Salicaceae Mirb.
Iz_3_1209	DJI_0125	Sambucus nigra L.	0	n	1	1	Viburnaceae Raf.
Iz_3_1209	DJI_0125	Secale sylvestre Host	0	n	1	0	Poaceae Barnhart
Iz_3_1209	DJI_0125	Sempervivum ruthenicum Schnittsp. & C.B. Lehm.	0	n	1	0	Crassulaceae J.St.-Hil.
Iz_3_1209	DJI_0125	Setaria viridis (L.) P. Beauv.	0	n	1	0	Poaceae Barnhart
Iz_3_1209	DJI_0125	Silene borysthenica (Gruner) Walters	0	n	1	0	Caryophyllaceae Juss.
Iz_3_1209	DJI_0125	Silene latifolia subsp. alba (Mill.) Greuter & Burdet	0	n	1	0	Caryophyllaceae Juss.
Iz_3_1209	DJI_0125	Solidago virgaurea L.	5	n	1	0	Asteraceae Bercht. & J.Presl
Iz_3_1209	DJI_0125	Sorbus aucuparia L.	0	n	1	1	Rosaceae Juss.
Iz_3_1209	DJI_0125	Stachys recta L.	0	n	1	0	Lamiacea Lindl.
Iz_3_1209	DJI_0125	Tanacetum vulgare L.	0	n	1	0	Asteraceae Bercht. & J.Presl
Iz_3_1209	DJI_0125	Thymus pallasianus Heinr.Braun	0	n	1	0	Lamiacea Lindl.
Iz_3_1209	DJI_0125	Tilia cordata Mill.	0	n	1	1	Malvaceae Juss.
Iz_3_1209	DJI_0125	Trifolium alpestre L.	0	n	1	0	Fabaceae Lindl.
Iz_3_1209	DJI_0125	Trifolium arvense L.	0	n	1	0	Fabaceae Lindl.
Iz_3_1209	DJI_0125	Ulmus glabra Huds.	0	n	1	1	Ulmaceae Mirb.
Iz_3_1209	DJI_0125	Verbascum densiflorum Bertol.	0	n	1	0	Scrophulariaceae Juss.
Iz_3_1209	DJI_0125	Veronica austriaca L.	0	n	1	0	Plantaginaceae Juss.
Iz_3_1209	DJI_0125	Veronica incana L.	0	n	1	0	Plantaginaceae Juss.
Iz_3_1209	DJI_0125	Veronica verna L.	0	n	1	0	Plantaginaceae Juss.
Iz_3_1209	DJI_0125	Vincetoxicum hirundinaria Medik	0	n	1	0	Apocynaceae Juss.
Iz_3_1209	DJI_0128	Acer tataricum L.	0	n	1	1	Sapindaceae Juss.
Iz_3_1209	DJI_0128	Achillea millefolium L. s.l.	0	n	1	0	Asteraceae Bercht. & J.Presl
Iz_3_1209	DJI_0128	Allium oleraceum L.	0	n	1	0	Amaryllidaceae J.St.-Hil.
Iz_3_1209	DJI_0128	Ambrosia artemisiifolia L.	0	a	0	0	Asteraceae Bercht. & J.Presl
Iz_3_1209	DJI_0128	Amorpha fruticosa L.	0	a	0	0	Fabaceae Lindl.
Iz_3_1209	DJI_0128	Anthericum ramosum L.	0	n	1	0	Asparagaceae Juss.
Iz_3_1209	DJI_0128	Artemisia marschalliana Spreng.	0	n	1	0	Asteraceae Bercht. & J.Presl
Iz_3_1209	DJI_0128	Asparagus officinalis L.	0	n	1	0	Asparagaceae Juss.
Iz_3_1209	DJI_0128	Astragalus cicer L.	0	n	1	0	Fabaceae Lindl.
Iz_3_1209	DJI_0128	Bassia laniflora (S.G.Gmel.) A.J.Scott	0	n	1	0	Amaranthaceae Juss.
Iz_3_1209	DJI_0128	Berberis vulgaris L.	0	n	1	0	Berberidaceae Juss.
Iz_3_1209	DJI_0128	Betula pendula Roth.	0	n	1	1	Betulaceae Gray
Iz_3_1209	DJI_0128	Betula pendula Roth.	0	n	1	0	Poaceae Barnhart
Iz_3_1209	DJI_0128	Bromus inermis Leyss.	0		NA	NA	
Iz_3_1209	DJI_0128	Bromus squarrosus L.	0	a	0	0	Poaceae Barnhart
Iz_3_1209	DJI_0128	Bromus tectorum L.	0	a	0	0	Poaceae Barnhart
Iz_3_1209	DJI_0128	Calamagrostis epigejos (L.) Roth	10	n	1	0	Poaceae Barnhart
Iz_3_1209	DJI_0128	Carduus acanthoides L.	0	n	1	0	Asteraceae Bercht. & J.Presl
Iz_3_1209	DJI_0128	Carex sp.	0	n	1	0	Cyperaceae Juss.
Iz_3_1209	DJI_0128	Centaurea majorovii Dumbadze	0	n	1	0	Asteraceae Bercht. & J.Presl
Iz_3_1209	DJI_0128	Chamaecytisus ruthenicus (Fisch.ex Wol.) Klaskova	0	n	1	0	Fabaceae Lindl.
Iz_3_1209	DJI_0128	Chelidonium majus L.	0.1	n	1	0	Papaveraceae Juss.
Iz_3_1209	DJI_0128	Chenopodium betaceum Andrz.	0	n	1	0	Amaranthaceae Juss.
Iz_3_1209	DJI_0128	Chenopodium cf. album	0	n	1	0	Amaranthaceae Juss.
Iz_3_1209	DJI_0128	Chenopodium sp.	0		NA	0	Amaranthaceae Juss.
Iz_3_1209	DJI_0128	Chondrilla juncea L.	0	n	1	0	Asteraceae Bercht. & J.Presl
Iz_3_1209	DJI_0128	Cirsium vulgare (Savi) Ten.	0	n	1	0	Asteraceae Bercht. & J.Presl
Iz_3_1209	DJI_0128	Clinopodium acinos (L.) Kuntze	0	n	1	0	Lamiacea Lindl.
Iz_3_1209	DJI_0128	Convallaria majalis L.	0	n	1	0	Asparagaceae Juss.

Iz_3_1209	DJI_0128	Crataegus rhipidophylla Gand.	0	n	1	1	Rosaceae Juss.
Iz_3_1209	DJI_0128	Crepis foetida L.	0	n	1	0	Asteraceae Bercht. & J.Presl
Iz_3_1209	DJI_0128	Dactylis glomerata L.	0	n	1	0	Poaceae Barnhart
Iz_3_1209	DJI_0128	Dianthus campestris M.Bieb.	0	n	1	0	Caryophyllaceae Juss.
Iz_3_1209	DJI_0128	Digitaria sanguinalis (L.) Scop.	0	a	0	0	Poaceae Barnhart
Iz_3_1209	DJI_0128	Elymus repens (L.) Gould	0	n	1	0	Poaceae Barnhart
Iz_3_1209	DJI_0128	Epilobium tetragonum L.	0	n	1	0	Onagraceae Juss.
Iz_3_1209	DJI_0128	Eragrostis minor Host	0	a	0	0	Poaceae Barnhart
Iz_3_1209	DJI_0128	Erigeron canadensis L.	0	a	0	0	Asteraceae Bercht. & J.Presl
Iz_3_1209	DJI_0128	Euphorbia seguieriana Neck.	0	n	1	0	Euphorbiaceae Juss.
Iz_3_1209	DJI_0128	Fallopia dumetorum (L.) Holub	0.2	n	1	0	Polygonaceae Juss.
Iz_3_1209	DJI_0128	Festuca beckeri (Hack.) Trautv.	0	n	1	0	Poaceae Barnhart
Iz_3_1209	DJI_0128	Fragaria vesca L.	0	n	1	0	Rosaceae Juss.
Iz_3_1209	DJI_0128	Frangula alnus Mill.	0	n	1	1	Rhamnaceae Juss.
Iz_3_1209	DJI_0128	Fraxinus pennsylvanica Marshall	0	a	0	1	Oleaceae Hoffmanns. & Link
Iz_3_1209	DJI_0128	Galeopsis bifida Boenn.	0	n	1	0	Lamiacea Lindl.
Iz_3_1209	DJI_0128	Galium aparine L.	0		NA	NA	
Iz_3_1209	DJI_0128	Galium verum L.	0	n	1	0	Rubiaceae Juss.
Iz_3_1209	DJI_0128	Genista tinctoria L.	0	n	1	0	Fabaceae Lindl.
Iz_3_1209	DJI_0128	Geranium robertianum L.	0	n	1	0	Geraniaceae Juss.
Iz_3_1209	DJI_0128	Geranium sanquineum L.	0	n	1	0	Geraniaceae Juss.
Iz_3_1209	DJI_0128	Gypsophila paniculata L.	0	n	1	0	Caryophyllaceae Juss.
Iz_3_1209	DJI_0128	Helichrysum arenarium (L.) Moench	0	n	1	0	Asteraceae Bercht. & J.Presl
Iz_3_1209	DJI_0128	Herniaria polygama J.Gay	0	n	1	0	Caryophyllaceae Juss.
Iz_3_1209	DJI_0128	Hieracium robustum Fr.	0	n	1	0	Asteraceae Bercht. & J.Presl
Iz_3_1209	DJI_0128	Hieracium umbellatum L.	0	n	1	NA	Asteraceae Bercht. & J.Presl
Iz_3_1209	DJI_0128	Hylotelephium maximum (L.) Holub	0	n	1	0	Crassulaceae J.St.-Hil.
Iz_3_1209	DJI_0128	Jacobaea borysthenica (DC.) B.Nord. & Greuter	0	n	1	0	Asteraceae Bercht. & J.Presl
Iz_3_1209	DJI_0128	Jasione montana L.	0	n	1	0	Campanulaceae Juss.
Iz_3_1209	DJI_0128	Jurinea cyanoides (L.) Rchb.s.l.	0	n	1	0	Asteraceae Bercht. & J.Presl
Iz_3_1209	DJI_0128	Koeleria glauca (Spreng.) DC.	0	n	1	0	Poaceae Barnhart
Iz_3_1209	DJI_0128	Lactuca serriola L.	0	a	0	0	Asteraceae Bercht. & J.Presl
Iz_3_1209	DJI_0128	Linaria genistifolia (L.) Mill.	0	n	1	0	Plantaginaceae Juss.
Iz_3_1209	DJI_0128	Lomelosia argentea (L.) Greuter & Burdet	0	n	1	0	Caprifoliaceae Juss.
Iz_3_1209	DJI_0128	Melampyrum pratense L.	0	n	1	0	Orobanchaceae Vent.
Iz_3_1209	DJI_0128	Oenothera biennis L.	0	a	0	0	Onagraceae Juss.
Iz_3_1209	DJI_0128	Pentanema britannica (L.) D.Gut.Larr., Santos-Vicente, Anderb., E.Rico & M.M.Mart.Ort.	0	n	1	0	Asteraceae Bercht. & J.Presl
Iz_3_1209	DJI_0128	Peucedanum oreoselinum (L.) Moench	5	n	1	0	Apiaceae Lindl.
Iz_3_1209	DJI_0128	Peucedanum ruthenicum M.Bieb.	0	n	1	0	Apiaceae Lindl.
Iz_3_1209	DJI_0128	Pilosella echioides (Lumn.) F.W.Schultz & Sch.Bip.	0	n	1	0	Asteraceae Bercht. & J.Presl
Iz_3_1209	DJI_0128	Pilosella officinarum F. Schultz et Sch. Bip.	0	n	1	0	Asteraceae Bercht. & J.Presl
Iz_3_1209	DJI_0128	Pilosella sp.	0		NA	0	Asteraceae Bercht. & J.Presl
Iz_3_1209	DJI_0128	Pinus sylvestris L.	0	n	1	1	Pinaceae Spreng. ex F.Rudolphi
Iz_3_1209	DJI_0128	Poa annua L.	0	n	1	0	Poaceae Barnhart
Iz_3_1209	DJI_0128	Poa nemoralis L.	0	n	1	0	Poaceae Barnhart
Iz_3_1209	DJI_0128	Poa sp.	0		NA	0	Poaceae Barnhart
Iz_3_1209	DJI_0128	Polygonatum odoratum (Mill.) Druce	0	n	1	0	Asparagaceae Juss.
Iz_3_1209	DJI_0128	Polygonum arenarium Waldst. & Kit.	0	n	1	0	Polygonaceae Juss.
Iz_3_1209	DJI_0128	Populus nigra L.	0	n	1	1	Salicaceae Mirb.
Iz_3_1209	DJI_0128	Populus tremula L.	0	n	1	1	Salicaceae Mirb.
Iz_3_1209	DJI_0128	Populus Ã canescens (Aiton) Sm.	0	n	1	1	Salicaceae Mirb.
Iz_3_1209	DJI_0128	Potentilla argentea L.	0	n	1	0	Rosaceae Juss.
Iz_3_1209	DJI_0128	Psammophiliella muralis (L.) Ikonn.	0	n	1	0	Caryophyllaceae Juss.
Iz_3_1209	DJI_0128	Psephellus sumensis (Kalen.) Greuter	0	n	1	0	Asteraceae Bercht. & J.Presl
Iz_3_1209	DJI_0128	Pulsatilla pratensis (L.) Mill. s.l.	0	n	1	0	Ranunculaceae Juss.
Iz_3_1209	DJI_0128	Pyrus communis L.	0	a	0	1	Rosaceae Juss.
Iz_3_1209	DJI_0128	Quercus robur L.	7	n	1	1	Fagaceae Dumort.
Iz_3_1209	DJI_0128	Robinia pseudoacacia L.	0	a	0	1	Fabaceae Lindl.
Iz_3_1209	DJI_0128	Rosa sp.	0		NA	1	Rosaceae Juss.
Iz_3_1209	DJI_0128	Rubus idaeus L.	0	n	1	0	Rosaceae Juss.
Iz_3_1209	DJI_0128	Rumex acetosella L.	0	n	1	0	Polygonaceae Juss.
Iz_3_1209	DJI_0128	Rumex thyrsiflorus Fingerh.	0	n	1	0	Polygonaceae Juss.
Iz_3_1209	DJI_0128	Salix alba L.	0	n	1	1	Salicaceae Mirb.
Iz_3_1209	DJI_0128	Sambucus nigra L.	0	n	1	1	Viburnaceae Raf.
Iz_3_1209	DJI_0128	Secale sylvestre Host	0	n	1	0	Poaceae Barnhart
Iz_3_1209	DJI_0128	Sempervivum ruthenicum Schnittsp. & C.B. Lehm.	0	n	1	0	Crassulaceae J.St.-Hil.
Iz_3_1209	DJI_0128	Setaria viridis (L.) P. Beauv.	0	n	1	0	Poaceae Barnhart
Iz_3_1209	DJI_0128	Silene borysthenica (Gruner) Walters	0	n	1	0	Caryophyllaceae Juss.
Iz_3_1209	DJI_0128	Silene latifolia subsp. alba (Mill.) Greuter & Burdet	0	n	1	0	Caryophyllaceae Juss.
Iz_3_1209	DJI_0128	Solidago virgaurea L.	4	n	1	0	Asteraceae Bercht. & J.Presl
Iz_3_1209	DJI_0128	Sorbus aucuparia L.	0	n	1	1	Rosaceae Juss.
Iz_3_1209	DJI_0128	Stachys recta L.	0	n	1	0	Lamiacea Lindl.
Iz_3_1209	DJI_0128	Tanacetum vulgare L.	0	n	1	0	Asteraceae Bercht. & J.Presl
Iz_3_1209	DJI_0128	Thymus pallasianus Heinr.Braun	0	n	1	0	Lamiacea Lindl.
Iz_3_1209	DJI_0128	Tilia cordata Mill.	0	n	1	1	Malvaceae Juss.
Iz_3_1209	DJI_0128	Trifolium alpestre L.	0	n	1	0	Fabaceae Lindl.
Iz_3_1209	DJI_0128	Trifolium arvense L.	0	n	1	0	Fabaceae Lindl.
Iz_3_1209	DJI_0128	Ulmus glabra Huds.	0	n	1	1	Ulmaceae Mirb.
Iz_3_1209	DJI_0128	Verbascum densiflorum Bertol.	0	n	1	0	Scrophulariaceae Juss.
Iz_3_1209	DJI_0128	Veronica austriaca L.	0	n	1	0	Plantaginaceae Juss.
Iz_3_1209	DJI_0128	Veronica incana L.	0	n	1	0	Plantaginaceae Juss.
Iz_3_1209	DJI_0128	Veronica verna L.	0	n	1	0	Plantaginaceae Juss.
Iz_3_1209	DJI_0128	Vincetoxicum hirundinaria Medik	0	n	1	0	Apocynaceae Juss.
Iz_3_1209	DJI_0132	Acer tataricum L.	0	n	1	1	Sapindaceae Juss.
Iz_3_1209	DJI_0132	Achillea millefolium L. s.l.	0	n	1	0	Asteraceae Bercht. & J.Presl
Iz_3_1209	DJI_0132	Allium oleraceum L.	0	n	1	0	Amaryllidaceae J.St.-Hil.
Iz_3_1209	DJI_0132	Ambrosia artemisiifolia L.	0	a	0	0	Asteraceae Bercht. & J.Presl
Iz_3_1209	DJI_0132	Amorpha fruticosa L.	0	a	0	0	Fabaceae Lindl.
Iz_3_1209	DJI_0132	Anthericum ramosum L.	0	n	1	0	Asparagaceae Juss.
Iz_3_1209	DJI_0132	Artemisia marschalliana Spreng.	0	n	1	0	Asteraceae Bercht. & J.Presl
Iz_3_1209	DJI_0132	Asparagus officinalis L.	0	n	1	0	Asparagaceae Juss.
Iz_3_1209	DJI_0132	Astragalus cicer L.	0	n	1	0	Fabaceae Lindl.
Iz_3_1209	DJI_0132	Bassia laniflora (S.G.Gmel.) A.J.Scott	0	n	1	0	Amaranthaceae Juss.
Iz_3_1209	DJI_0132	Berberis vulgaris L.	0	n	1	0	Berberidaceae Juss.
Iz_3_1209	DJI_0132	Betula pendula Roth.	0	n	1	1	Betulaceae Gray
Iz_3_1209	DJI_0132	Betula pendula Roth.	0	n	1	0	Poaceae Barnhart
Iz_3_1209	DJI_0132	Bromus inermis Leyss.	0		NA	NA	
Iz_3_1209	DJI_0132	Bromus squarrosus L.	0	a	0	0	Poaceae Barnhart

Iz_3_1209	DJI_0132	Bromus tectorum L.	0	a	0	0	Poaceae Barnhart
Iz_3_1209	DJI_0132	Calamagrostis epigejos (L.) Roth	20	n	1	0	Poaceae Barnhart
Iz_3_1209	DJI_0132	Carduus acanthoides L.	0	n	1	0	Asteraceae Bercht. & J.Presl
Iz_3_1209	DJI_0132	Carex sp.	0	n	1	0	Cyperaceae Juss.
Iz_3_1209	DJI_0132	Centaurea majorovii Dumbadze	0	n	1	0	Asteraceae Bercht. & J.Presl
Iz_3_1209	DJI_0132	Chamaecytisus ruthenicus (Fisch.ex Wol.) Klaskova	0	n	1	0	Fabaceae Lindl.
Iz_3_1209	DJI_0132	Chelidonium majus L.	0	n	1	0	Papaveraceae Juss.
Iz_3_1209	DJI_0132	Chenopodium betaceum Andrz.	0	n	1	0	Amaranthaceae Juss.
Iz_3_1209	DJI_0132	Chenopodium cf. album	0	n	1	0	Amaranthaceae Juss.
Iz_3_1209	DJI_0132	Chenopodium sp.	0		NA	0	Amaranthaceae Juss.
Iz_3_1209	DJI_0132	Chondrilla juncea L.	0	n	1	0	Asteraceae Bercht. & J.Presl
Iz_3_1209	DJI_0132	Cirsium vulgare (Savi) Ten.	0	n	1	0	Asteraceae Bercht. & J.Presl
Iz_3_1209	DJI_0132	Clinopodium acinos (L.) Kuntze	0	n	1	0	Lamiacea Lindl.
Iz_3_1209	DJI_0132	Convallaria majalis L.	0	n	1	0	Asparagaceae Juss.
Iz_3_1209	DJI_0132	Crataegus rhipidophylla Gand.	0	n	1	1	Rosaceae Juss.
Iz_3_1209	DJI_0132	Crepis foetida L.	0	n	1	0	Asteraceae Bercht. & J.Presl
Iz_3_1209	DJI_0132	Dactylis glomerata L.	0	n	1	0	Poaceae Barnhart
Iz_3_1209	DJI_0132	Dianthus campestris M.Bieb.	0	n	1	0	Caryophyllaceae Juss.
Iz_3_1209	DJI_0132	Digitaria sanguinalis (L.) Scop.	0	a	0	0	Poaceae Barnhart
Iz_3_1209	DJI_0132	Elymus repens (L.) Gould	0	n	1	0	Poaceae Barnhart
Iz_3_1209	DJI_0132	Epilobium tetragonum L.	0	n	1	0	Onagraceae Juss.
Iz_3_1209	DJI_0132	Eragrostis minor Host	0	a	0	0	Poaceae Barnhart
Iz_3_1209	DJI_0132	Erigeron canadensis L.	0	a	0	0	Asteraceae Bercht. & J.Presl
Iz_3_1209	DJI_0132	Euphorbia seguieriana Neck.	0	n	1	0	Euphorbiaceae Juss.
Iz_3_1209	DJI_0132	Fallopia dumetorum (L.) Holub	0	n	1	0	Polygonaceae Juss.
Iz_3_1209	DJI_0132	Festuca beckeri (Hack.) Trautv.	0	n	1	0	Poaceae Barnhart
Iz_3_1209	DJI_0132	Fragaria vesca L.	0	n	1	0	Rosaceae Juss.
Iz_3_1209	DJI_0132	Frangula alnus Mill.	0	n	1	1	Rhamnaceae Juss.
Iz_3_1209	DJI_0132	Fraxinus pennsylvanica Marshall	0	a	0	1	Oleaceae Hoffmanns. & Link
Iz_3_1209	DJI_0132	Galeopsis bifida Boenn.	0	n	1	0	Lamiacea Lindl.
Iz_3_1209	DJI_0132	Galium aparine L.	0		NA	NA	
Iz_3_1209	DJI_0132	Galium verum L.	0	n	1	0	Rubiaceae Juss.
Iz_3_1209	DJI_0132	Genista tinctoria L.	0	n	1	0	Fabaceae Lindl.
Iz_3_1209	DJI_0132	Geranium robertianum L.	0	n	1	0	Geraniaceae Juss.
Iz_3_1209	DJI_0132	Geranium sanquineum L.	0	n	1	0	Geraniaceae Juss.
Iz_3_1209	DJI_0132	Gypsophila paniculata L.	0	n	1	0	Caryophyllaceae Juss.
Iz_3_1209	DJI_0132	Helichrysum arenarium (L.) Moench	0	n	1	0	Asteraceae Bercht. & J.Presl
Iz_3_1209	DJI_0132	Herniaria polygama J.Gay	0	n	1	0	Caryophyllaceae Juss.
Iz_3_1209	DJI_0132	Hieracium robustum Fr.	0	n	1	0	Asteraceae Bercht. & J.Presl
Iz_3_1209	DJI_0132	Hieracium umbellatum L.	0	n	1	NA	Asteraceae Bercht. & J.Presl
Iz_3_1209	DJI_0132	Hylotelephium maximum (L.) Holub	0	n	1	0	Crassulaceae J.St.-Hil.
Iz_3_1209	DJI_0132	Jacobaea borysthenica (DC.) B.Nord. & Greuter	0	n	1	0	Asteraceae Bercht. & J.Presl
Iz_3_1209	DJI_0132	Jasione montana L.	0	n	1	0	Campanulaceae Juss.
Iz_3_1209	DJI_0132	Jurinea cyanoides (L.) Rchb.s.l.	0	n	1	0	Asteraceae Bercht. & J.Presl
Iz_3_1209	DJI_0132	Koeleria glauca (Spreng.) DC.	0	n	1	0	Poaceae Barnhart
Iz_3_1209	DJI_0132	Lactuca serriola L.	0	a	0	0	Asteraceae Bercht. & J.Presl
Iz_3_1209	DJI_0132	Linaria genistifolia (L.) Mill.	0	n	1	0	Plantaginaceae Juss.
Iz_3_1209	DJI_0132	Lomelosia argentea (L.) Greuter & Burdet	0	n	1	0	Caprifoliaceae Juss.
Iz_3_1209	DJI_0132	Melampyrum pratense L.	0	n	1	0	Orobanchaceae Vent.
Iz_3_1209	DJI_0132	Oenothera biennis L.	0	a	0	0	Onagraceae Juss.
Iz_3_1209	DJI_0132	Pentanema britannica (L.) D.Gut.Larr., Santos-Vicente, Anderb., E.Rico & M.M.Mart.Ort.	0	n	1	0	Asteraceae Bercht. & J.Presl
Iz_3_1209	DJI_0132	Peucedanum oreoselinum (L.) Moench	0	n	1	0	Apiaceae Lindl.
Iz_3_1209	DJI_0132	Peucedanum ruthenicum M.Bieb.	0	n	1	0	Apiaceae Lindl.
Iz_3_1209	DJI_0132	Pilosella echioides (Lumn.) F.W.Schultz & Sch.Bip.	0	n	1	0	Asteraceae Bercht. & J.Presl
Iz_3_1209	DJI_0132	Pilosella officinarum F. Schultz et Sch. Bip.	0	n	1	0	Asteraceae Bercht. & J.Presl
Iz_3_1209	DJI_0132	Pilosella sp.	0		NA	0	Asteraceae Bercht. & J.Presl
Iz_3_1209	DJI_0132	Pinus sylvestris L.	0	n	1	1	Pinaceae Spreng. ex F.Rudolphi
Iz_3_1209	DJI_0132	Poa annua L.	0	n	1	0	Poaceae Barnhart
Iz_3_1209	DJI_0132	Poa nemoralis L.	0	n	1	0	Poaceae Barnhart
Iz_3_1209	DJI_0132	Poa sp.	0		NA	0	Poaceae Barnhart
Iz_3_1209	DJI_0132	Polygonatum odoratum (Mill.) Druce	0	n	1	0	Asparagaceae Juss.
Iz_3_1209	DJI_0132	Polygonum arenarium Waldst. & Kit.	0	n	1	0	Polygonaceae Juss.
Iz_3_1209	DJI_0132	Populus nigra L.	0	n	1	1	Salicaceae Mirb.
Iz_3_1209	DJI_0132	Populus tremula L.	0	n	1	1	Salicaceae Mirb.
Iz_3_1209	DJI_0132	Populus Ã canescens (Aiton) Sm.	0	n	1	1	Salicaceae Mirb.
Iz_3_1209	DJI_0132	Potentilla argentea L.	0	n	1	0	Rosaceae Juss.
Iz_3_1209	DJI_0132	Psammophiliella muralis (L.) Ikonn.	0	n	1	0	Caryophyllaceae Juss.
Iz_3_1209	DJI_0132	Psephellus sumensis (Kalen.) Greuter	0	n	1	0	Asteraceae Bercht. & J.Presl
Iz_3_1209	DJI_0132	Pulsatilla pratensis (L.) Mill. s.l.	0	n	1	0	Ranunculaceae Juss.
Iz_3_1209	DJI_0132	Pyrus communis L.	0	a	0	1	Rosaceae Juss.
Iz_3_1209	DJI_0132	Quercus robur L.	0	n	1	1	Fagaceae Dumort.
Iz_3_1209	DJI_0132	Robinia pseudoacacia L.	0	a	0	1	Fabaceae Lindl.
Iz_3_1209	DJI_0132	Rosa sp.	0		NA	1	Rosaceae Juss.
Iz_3_1209	DJI_0132	Rubus idaeus L.	0	n	1	0	Rosaceae Juss.
Iz_3_1209	DJI_0132	Rumex acetosella L.	0	n	1	0	Polygonaceae Juss.
Iz_3_1209	DJI_0132	Rumex thyrsiflorus Fingerh.	0	n	1	0	Polygonaceae Juss.
Iz_3_1209	DJI_0132	Salix alba L.	0	n	1	1	Salicaceae Mirb.
Iz_3_1209	DJI_0132	Sambucus nigra L.	0	n	1	1	Viburnaceae Raf.
Iz_3_1209	DJI_0132	Secale sylvestre Host	0	n	1	0	Poaceae Barnhart
Iz_3_1209	DJI_0132	Sempervivum ruthenicum Schnittsp. & C.B. Lehm.	0	n	1	0	Crassulaceae J.St.-Hil.
Iz_3_1209	DJI_0132	Setaria viridis (L.) P. Beauv.	0	n	1	0	Poaceae Barnhart
Iz_3_1209	DJI_0132	Silene borysthenica (Gruner) Walters	0	n	1	0	Caryophyllaceae Juss.
Iz_3_1209	DJI_0132	Silene latifolia subsp. alba (Mill.) Greuter & Burdet	0	n	1	0	Caryophyllaceae Juss.
Iz_3_1209	DJI_0132	Solidago virgaurea L.	0	n	1	0	Asteraceae Bercht. & J.Presl
Iz_3_1209	DJI_0132	Sorbus aucuparia L.	0	n	1	1	Rosaceae Juss.
Iz_3_1209	DJI_0132	Stachys recta L.	0	n	1	0	Lamiacea Lindl.
Iz_3_1209	DJI_0132	Tanacetum vulgare L.	0	n	1	0	Asteraceae Bercht. & J.Presl
Iz_3_1209	DJI_0132	Thymus pallasianus Heinr.Braun	0	n	1	0	Lamiacea Lindl.
Iz_3_1209	DJI_0132	Tilia cordata Mill.	0	n	1	1	Malvaceae Juss.
Iz_3_1209	DJI_0132	Trifolium alpestre L.	0	n	1	0	Fabaceae Lindl.
Iz_3_1209	DJI_0132	Trifolium arvense L.	0	n	1	0	Fabaceae Lindl.
Iz_3_1209	DJI_0132	Ulmus glabra Huds.	0	n	1	1	Ulmaceae Mirb.
Iz_3_1209	DJI_0132	Verbascum densiflorum Bertol.	0	n	1	0	Scrophulariaceae Juss.
Iz_3_1209	DJI_0132	Veronica austriaca L.	0	n	1	0	Plantaginaceae Juss.
Iz_3_1209	DJI_0132	Veronica incana L.	0	n	1	0	Plantaginaceae Juss.
Iz_3_1209	DJI_0132	Veronica verna L.	0	n	1	0	Plantaginaceae Juss.
Iz_3_1209	DJI_0132	Vincetoxicum hirundinaria Medik	0	n	1	0	Apocynaceae Juss.
Iz_4_1009	DJI_0287	Acer tataricum L.	0	n	1	1	Sapindaceae Juss.

Iz_4_1009	DJI_0287	Achillea millefolium L. s.l.	0	n	1	0	Asteraceae Bercht. & J.Presl
Iz_4_1009	DJI_0287	Allium oleraceum L.	0	n	1	0	Amaryllidaceae J.St.-Hil.
Iz_4_1009	DJI_0287	Ambrosia artemisiifolia L.	0	a	0	0	Asteraceae Bercht. & J.Presl
Iz_4_1009	DJI_0287	Amorpha fruticosa L.	0	a	0	0	Fabaceae Lindl.
Iz_4_1009	DJI_0287	Anthericum ramosum L.	0	n	1	0	Asparagaceae Juss.
Iz_4_1009	DJI_0287	Artemisia marschalliana Spreng.	0	n	1	0	Asteraceae Bercht. & J.Presl
Iz_4_1009	DJI_0287	Asparagus officinalis L.	0	n	1	0	Asparagaceae Juss.
Iz_4_1009	DJI_0287	Astragalus cicer L.	0	n	1	0	Fabaceae Lindl.
Iz_4_1009	DJI_0287	Bassia laniflora (S.G.Gmel.) A.J.Scott	0	n	1	0	Amaranthaceae Juss.
Iz_4_1009	DJI_0287	Berberis vulgaris L.	0	n	1	0	Berberidaceae Juss.
Iz_4_1009	DJI_0287	Betula pendula Roth.	0	n	1	1	Betulaceae Gray
Iz_4_1009	DJI_0287	Betula pendula Roth.	0	n	1	0	Poaceae Barnhart
Iz_4_1009	DJI_0287	Bromus inermis Leyss.	0		NA	NA	
Iz_4_1009	DJI_0287	Bromus squarrosus L.	0	a	0	0	Poaceae Barnhart
Iz_4_1009	DJI_0287	Bromus tectorum L.	0	a	0	0	Poaceae Barnhart
Iz_4_1009	DJI_0287	Calamagrostis epigejos (L.) Roth	40	n	1	0	Poaceae Barnhart
Iz_4_1009	DJI_0287	Carduus acanthoides L.	0	n	1	0	Asteraceae Bercht. & J.Presl
Iz_4_1009	DJI_0287	Carex sp.	0	n	1	0	Cyperaceae Juss.
Iz_4_1009	DJI_0287	Centaurea majorovii Dumbadze	0	n	1	0	Asteraceae Bercht. & J.Presl
Iz_4_1009	DJI_0287	Chamaecytisus ruthenicus (Fisch.ex Wol.) Klaskova	0	n	1	0	Fabaceae Lindl.
Iz_4_1009	DJI_0287	Chelidonium majus L.	0	n	1	0	Papaveraceae Juss.
Iz_4_1009	DJI_0287	Chenopodium betaceum Andrz.	0	n	1	0	Amaranthaceae Juss.
Iz_4_1009	DJI_0287	Chenopodium cf. album	0	n	1	0	Amaranthaceae Juss.
Iz_4_1009	DJI_0287	Chenopodium sp.	0		NA	0	Amaranthaceae Juss.
Iz_4_1009	DJI_0287	Chondrilla juncea L.	0	n	1	0	Asteraceae Bercht. & J.Presl
Iz_4_1009	DJI_0287	Cirsium vulgare (Savi) Ten.	0	n	1	0	Asteraceae Bercht. & J.Presl
Iz_4_1009	DJI_0287	Clinopodium acinos (L.) Kuntze	0	n	1	0	Lamiacea Lindl.
Iz_4_1009	DJI_0287	Convallaria majalis L.	0	n	1	0	Asparagaceae Juss.
Iz_4_1009	DJI_0287	Crataegus rhipidophylla Gand.	0	n	1	1	Rosaceae Juss.
Iz_4_1009	DJI_0287	Crepis foetida L.	0	n	1	0	Asteraceae Bercht. & J.Presl
Iz_4_1009	DJI_0287	Dactylis glomerata L.	0	n	1	0	Poaceae Barnhart
Iz_4_1009	DJI_0287	Dianthus campestris M.Bieb.	0	n	1	0	Caryophyllaceae Juss.
Iz_4_1009	DJI_0287	Digitaria sanguinalis (L.) Scop.	0	a	0	0	Poaceae Barnhart
Iz_4_1009	DJI_0287	Elymus repens (L.) Gould	0	n	1	0	Poaceae Barnhart
Iz_4_1009	DJI_0287	Epilobium tetragonum L.	0	n	1	0	Onagraceae Juss.
Iz_4_1009	DJI_0287	Eragrostis minor Host	0	a	0	0	Poaceae Barnhart
Iz_4_1009	DJI_0287	Erigeron canadensis L.	0	a	0	0	Asteraceae Bercht. & J.Presl
Iz_4_1009	DJI_0287	Euphorbia seguieriana Neck.	0	n	1	0	Euphorbiaceae Juss.
Iz_4_1009	DJI_0287	Fallopia dumetorum (L.) Holub	0	n	1	0	Polygonaceae Juss.
Iz_4_1009	DJI_0287	Festuca beckeri (Hack.) Trautv.	0	n	1	0	Poaceae Barnhart
Iz_4_1009	DJI_0287	Fragaria vesca L.	0	n	1	0	Rosaceae Juss.
Iz_4_1009	DJI_0287	Frangula alnus Mill.	0	n	1	1	Rhamnaceae Juss.
Iz_4_1009	DJI_0287	Fraxinus pennsylvanica Marshall	0	a	0	1	Oleaceae Hoffmanns. & Link
Iz_4_1009	DJI_0287	Galeopsis bifida Boenn.	0	n	1	0	Lamiacea Lindl.
Iz_4_1009	DJI_0287	Galium verum L.	0	n	1	0	Rubiaceae Juss.
Iz_4_1009	DJI_0287	Genista tinctoria L.	0	n	1	0	Fabaceae Lindl.
Iz_4_1009	DJI_0287	Geranium robertianum L.	0	n	1	0	Geraniaceae Juss.
Iz_4_1009	DJI_0287	Geranium sanquineum L.	0	n	1	0	Geraniaceae Juss.
Iz_4_1009	DJI_0287	Gypsophila paniculata L.	0	n	1	0	Caryophyllaceae Juss.
Iz_4_1009	DJI_0287	Helichrysum arenarium (L.) Moench	0	n	1	0	Asteraceae Bercht. & J.Presl
Iz_4_1009	DJI_0287	Herniaria polygama J.Gay	0	n	1	0	Caryophyllaceae Juss.
Iz_4_1009	DJI_0287	Hieracium robustum Fr.	0	n	1	0	Asteraceae Bercht. & J.Presl
Iz_4_1009	DJI_0287	Hieracium umbellatum L.	0	n	1	NA	Asteraceae Bercht. & J.Presl
Iz_4_1009	DJI_0287	Hylotelephium maximum (L.) Holub	0	n	1	0	Crassulaceae J.St.-Hil.
Iz_4_1009	DJI_0287	Jacobaea borysthenica (DC.) B.Nord. & Greuter	0	n	1	0	Asteraceae Bercht. & J.Presl
Iz_4_1009	DJI_0287	Jasione montana L.	0	n	1	0	Campanulaceae Juss.
Iz_4_1009	DJI_0287	Jurinea cyanoides (L.) Rchb.s.l.	0	n	1	0	Asteraceae Bercht. & J.Presl
Iz_4_1009	DJI_0287	Koeleria glauca (Spreng.) DC.	0	n	1	0	Poaceae Barnhart
Iz_4_1009	DJI_0287	Lactuca serriola L.	0	a	0	0	Asteraceae Bercht. & J.Presl
Iz_4_1009	DJI_0287	Linaria genistifolia (L.) Mill.	0	n	1	0	Plantaginaceae Juss.
Iz_4_1009	DJI_0287	Lomelosia argentea (L.) Greuter & Burdet	0	n	1	0	Caprifoliaceae Juss.
Iz_4_1009	DJI_0287	Melampyrum pratense L.	0	n	1	0	Orobanchaceae Vent.
Iz_4_1009	DJI_0287	Oenothera biennis L.	0	a	0	0	Onagraceae Juss.
Iz_4_1009	DJI_0287	Pentanema britannica (L.) D.Gut.Larr., Santos-Vicente, Anderb., E.Rico & M.M.Mart.Ort.	0	n	1	0	Asteraceae Bercht. & J.Presl
Iz_4_1009	DJI_0287	Peucedanum oreoselinum (L.) Moench	0	n	1	0	Apiaceae Lindl.
Iz_4_1009	DJI_0287	Peucedanum ruthenicum M.Bieb.	0	n	1	0	Apiaceae Lindl.
Iz_4_1009	DJI_0287	Pilosella echioides (Lumn.) F.W.Schultz & Sch.Bip.	0	n	1	0	Asteraceae Bercht. & J.Presl
Iz_4_1009	DJI_0287	Pilosella officinarum F. Schultz et Sch. Bip.	0	n	1	0	Asteraceae Bercht. & J.Presl
Iz_4_1009	DJI_0287	Pilosella sp.	0		NA	0	Asteraceae Bercht. & J.Presl
Iz_4_1009	DJI_0287	Pinus sylvestris L.	0	n	1	1	Pinaceae Spreng. ex F.Rudolphi
Iz_4_1009	DJI_0287	Poa annua L.	0	n	1	0	Poaceae Barnhart
Iz_4_1009	DJI_0287	Poa nemoralis L.	0	n	1	0	Poaceae Barnhart
Iz_4_1009	DJI_0287	Poa sp.	0		NA	0	Poaceae Barnhart
Iz_4_1009	DJI_0287	Polygonatum odoratum (Mill.) Druce	0	n	1	0	Asparagaceae Juss.
Iz_4_1009	DJI_0287	Polygonum arenarium Waldst. & Kit.	0	n	1	0	Polygonaceae Juss.
Iz_4_1009	DJI_0287	Populus nigra L.	0	n	1	1	Salicaceae Mirb.
Iz_4_1009	DJI_0287	Populus tremula L.	0	n	1	1	Salicaceae Mirb.
Iz_4_1009	DJI_0287	Populus Ã canescens (Aiton) Sm.	0	n	1	1	Salicaceae Mirb.
Iz_4_1009	DJI_0287	Potentilla argentea L.	0	n	1	0	Rosaceae Juss.
Iz_4_1009	DJI_0287	Psammophiliella muralis (L.) Ikonn.	0	n	1	0	Caryophyllaceae Juss.
Iz_4_1009	DJI_0287	Psephellus sumensis (Kalen.) Greuter	0	n	1	0	Asteraceae Bercht. & J.Presl
Iz_4_1009	DJI_0287	Pulsatilla pratensis (L.) Mill. s.l.	0	n	1	0	Ranunculaceae Juss.
Iz_4_1009	DJI_0287	Pyrus communis L.	0	a	0	1	Rosaceae Juss.
Iz_4_1009	DJI_0287	Quercus robur L.	0	n	1	1	Fagaceae Dumort.
Iz_4_1009	DJI_0287	Robinia pseudoacacia L.	0	a	0	1	Fabaceae Lindl.
Iz_4_1009	DJI_0287	Rosa sp.	0		NA	1	Rosaceae Juss.
Iz_4_1009	DJI_0287	Rubus idaeus L.	0	n	1	0	Rosaceae Juss.
Iz_4_1009	DJI_0287	Rumex acetosella L.	2	n	1	0	Polygonaceae Juss.
Iz_4_1009	DJI_0287	Rumex thyrsiflorus Fingerh.	0	n	1	0	Polygonaceae Juss.
Iz_4_1009	DJI_0287	Salix alba L.	0	n	1	1	Salicaceae Mirb.
Iz_4_1009	DJI_0287	Sambucus nigra L.	0	n	1	1	Viburnaceae Raf.
Iz_4_1009	DJI_0287	Secale sylvestre Host	0	n	1	0	Poaceae Barnhart
Iz_4_1009	DJI_0287	Sempervivum ruthenicum Schnittsp. & C.B. Lehm.	0	n	1	0	Crassulaceae J.St.-Hil.
Iz_4_1009	DJI_0287	Setaria viridis (L.) P. Beauv.	0	n	1	0	Poaceae Barnhart
Iz_4_1009	DJI_0287	Silene borysthenica (Gruner) Walters	0	n	1	0	Caryophyllaceae Juss.
Iz_4_1009	DJI_0287	Silene latifolia subsp. alba (Mill.) Greuter & Burdet	0	n	1	0	Caryophyllaceae Juss.
Iz_4_1009	DJI_0287	Solidago virgaurea L.	0	n	1	0	Asteraceae Bercht. & J.Presl
Iz_4_1009	DJI_0287	Sorbus aucuparia L.	0	n	1	1	Rosaceae Juss.

Iz_4_1009	DJI_0287	Stachys recta L.	0	n	1	0	Lamiacea Lindl.
Iz_4_1009	DJI_0287	Tanacetum vulgare L.	0	n	1	0	Asteraceae Bercht. & J.Presl
Iz_4_1009	DJI_0287	Thymus pallasianus Heinr.Braun	0	n	1	0	Lamiacea Lindl.
Iz_4_1009	DJI_0287	Tilia cordata Mill.	0	n	1	1	Malvaceae Juss.
Iz_4_1009	DJI_0287	Trifolium alpestre L.	0	n	1	0	Fabaceae Lindl.
Iz_4_1009	DJI_0287	Trifolium arvense L.	0	n	1	0	Fabaceae Lindl.
Iz_4_1009	DJI_0287	Ulmus glabra Huds.	0	n	1	1	Ulmaceae Mirb.
Iz_4_1009	DJI_0287	Verbascum densiflorum Bertol.	0	n	1	0	Scrophulariaceae Juss.
Iz_4_1009	DJI_0287	Verbascum nigrum L.	0	n	1	0	Scrophulariaceae Juss.
Iz_4_1009	DJI_0287	Veronica austriaca L.	0	n	1	0	Plantaginaceae Juss.
Iz_4_1009	DJI_0287	Veronica incana L.	0	n	1	0	Plantaginaceae Juss.
Iz_4_1009	DJI_0287	Veronica verna L.	0	n	1	0	Plantaginaceae Juss.
Iz_4_1009	DJI_0287	Vincetoxicum hirundinaria Medik	0	n	1	0	Apocynaceae Juss.
Iz_4_1009	DJI_0288	Acer tataricum L.	0	n	1	1	Sapindaceae Juss.
Iz_4_1009	DJI_0288	Achillea millefolium L. s.l.	0	n	1	0	Asteraceae Bercht. & J.Presl
Iz_4_1009	DJI_0288	Allium oleraceum L.	0	n	1	0	Amaryllidaceae J.St.-Hil.
Iz_4_1009	DJI_0288	Ambrosia artemisiifolia L.	0	a	0	0	Asteraceae Bercht. & J.Presl
Iz_4_1009	DJI_0288	Amorpha fruticosa L.	0	a	0	0	Fabaceae Lindl.
Iz_4_1009	DJI_0288	Anthericum ramosum L.	0	n	1	0	Asparagaceae Juss.
Iz_4_1009	DJI_0288	Artemisia marschalliana Spreng.	0	n	1	0	Asteraceae Bercht. & J.Presl
Iz_4_1009	DJI_0288	Asparagus officinalis L.	0	n	1	0	Asparagaceae Juss.
Iz_4_1009	DJI_0288	Astragalus cicer L.	0	n	1	0	Fabaceae Lindl.
Iz_4_1009	DJI_0288	Bassia laniflora (S.G.Gmel.) A.J.Scott	0	n	1	0	Amaranthaceae Juss.
Iz_4_1009	DJI_0288	Berberis vulgaris L.	0	n	1	0	Berberidaceae Juss.
Iz_4_1009	DJI_0288	Betula pendula Roth.	0	n	1	1	Betulaceae Gray
Iz_4_1009	DJI_0288	Betula pendula Roth.	0	n	1	0	Poaceae Barnhart
Iz_4_1009	DJI_0288	Bromus inermis Leyss.	0		NA	NA	
Iz_4_1009	DJI_0288	Bromus squarrosus L.	0	a	0	0	Poaceae Barnhart
Iz_4_1009	DJI_0288	Bromus tectorum L.	0	a	0	0	Poaceae Barnhart
Iz_4_1009	DJI_0288	Calamagrostis epigejos (L.) Roth	40	n	1	0	Poaceae Barnhart
Iz_4_1009	DJI_0288	Carduus acanthoides L.	0	n	1	0	Asteraceae Bercht. & J.Presl
Iz_4_1009	DJI_0288	Carex sp.	0	n	1	0	Cyperaceae Juss.
Iz_4_1009	DJI_0288	Centaurea majorovii Dumbadze	0	n	1	0	Asteraceae Bercht. & J.Presl
Iz_4_1009	DJI_0288	Chamaecytisus ruthenicus (Fisch.ex Wol.) Klaskova	0	n	1	0	Fabaceae Lindl.
Iz_4_1009	DJI_0288	Chelidonium majus L.	0	n	1	0	Papaveraceae Juss.
Iz_4_1009	DJI_0288	Chenopodium betaceum Andrz.	0	n	1	0	Amaranthaceae Juss.
Iz_4_1009	DJI_0288	Chenopodium cf. album	0	n	1	0	Amaranthaceae Juss.
Iz_4_1009	DJI_0288	Chenopodium sp.	0		NA	0	Amaranthaceae Juss.
Iz_4_1009	DJI_0288	Chondrilla juncea L.	0	n	1	0	Asteraceae Bercht. & J.Presl
Iz_4_1009	DJI_0288	Cirsium vulgare (Savi) Ten.	0	n	1	0	Asteraceae Bercht. & J.Presl
Iz_4_1009	DJI_0288	Clinopodium acinos (L.) Kuntze	0	n	1	0	Lamiacea Lindl.
Iz_4_1009	DJI_0288	Convallaria majalis L.	0	n	1	0	Asparagaceae Juss.
Iz_4_1009	DJI_0288	Crataegus rhipidophylla Gand.	0	n	1	1	Rosaceae Juss.
Iz_4_1009	DJI_0288	Crepis foetida L.	0	n	1	0	Asteraceae Bercht. & J.Presl
Iz_4_1009	DJI_0288	Dactylis glomerata L.	0	n	1	0	Poaceae Barnhart
Iz_4_1009	DJI_0288	Dianthus campestris M.Bieb.	0	n	1	0	Caryophyllaceae Juss.
Iz_4_1009	DJI_0288	Digitaria sanguinalis (L.) Scop.	0	a	0	0	Poaceae Barnhart
Iz_4_1009	DJI_0288	Elymus repens (L.) Gould	0	n	1	0	Poaceae Barnhart
Iz_4_1009	DJI_0288	Epilobium tetragonum L.	0	n	1	0	Onagraceae Juss.
Iz_4_1009	DJI_0288	Eragrostis minor Host	0	a	0	0	Poaceae Barnhart
Iz_4_1009	DJI_0288	Erigeron canadensis L.	0	a	0	0	Asteraceae Bercht. & J.Presl
Iz_4_1009	DJI_0288	Euphorbia seguieriana Neck.	0	n	1	0	Euphorbiaceae Juss.
Iz_4_1009	DJI_0288	Fallopia dumetorum (L.) Holub	0	n	1	0	Polygonaceae Juss.
Iz_4_1009	DJI_0288	Festuca beckeri (Hack.) Trautv.	2	n	1	0	Poaceae Barnhart
Iz_4_1009	DJI_0288	Fragaria vesca L.	0	n	1	0	Rosaceae Juss.
Iz_4_1009	DJI_0288	Frangula alnus Mill.	0	n	1	1	Rhamnaceae Juss.
Iz_4_1009	DJI_0288	Fraxinus pennsylvanica Marshall	0	a	0	1	Oleaceae Hoffmanns. & Link
Iz_4_1009	DJI_0288	Galeopsis bifida Boenn.	0	n	1	0	Lamiacea Lindl.
Iz_4_1009	DJI_0288	Galium verum L.	0	n	1	0	Rubiaceae Juss.
Iz_4_1009	DJI_0288	Genista tinctoria L.	0	n	1	0	Fabaceae Lindl.
Iz_4_1009	DJI_0288	Geranium robertianum L.	0	n	1	0	Geraniaceae Juss.
Iz_4_1009	DJI_0288	Geranium sanquineum L.	0	n	1	0	Geraniaceae Juss.
Iz_4_1009	DJI_0288	Gypsophila paniculata L.	0	n	1	0	Caryophyllaceae Juss.
Iz_4_1009	DJI_0288	Helichrysum arenarium (L.) Moench	0	n	1	0	Asteraceae Bercht. & J.Presl
Iz_4_1009	DJI_0288	Herniaria polygama J.Gay	0	n	1	0	Caryophyllaceae Juss.
Iz_4_1009	DJI_0288	Hieracium robustum Fr.	0	n	1	0	Asteraceae Bercht. & J.Presl
Iz_4_1009	DJI_0288	Hieracium umbellatum L.	0	n	1	NA	Asteraceae Bercht. & J.Presl
Iz_4_1009	DJI_0288	Hylotelephium maximum (L.) Holub	0	n	1	0	Crassulaceae J.St.-Hil.
Iz_4_1009	DJI_0288	Jacobaea borysthenica (DC.) B.Nord. & Greuter	0	n	1	0	Asteraceae Bercht. & J.Presl
Iz_4_1009	DJI_0288	Jasione montana L.	0	n	1	0	Campanulaceae Juss.
Iz_4_1009	DJI_0288	Jurinea cyanoides (L.) Rchb.s.l.	0	n	1	0	Asteraceae Bercht. & J.Presl
Iz_4_1009	DJI_0288	Koeleria glauca (Spreng.) DC.	0	n	1	0	Poaceae Barnhart
Iz_4_1009	DJI_0288	Lactuca serriola L.	0	a	0	0	Asteraceae Bercht. & J.Presl
Iz_4_1009	DJI_0288	Linaria genistifolia (L.) Mill.	0	n	1	0	Plantaginaceae Juss.
Iz_4_1009	DJI_0288	Lomelosia argentea (L.) Greuter & Burdet	0	n	1	0	Caprifoliaceae Juss.
Iz_4_1009	DJI_0288	Melampyrum pratense L.	0	n	1	0	Orobanchaceae Vent.
Iz_4_1009	DJI_0288	Oenothera biennis L.	0	a	0	0	Onagraceae Juss.
Iz_4_1009	DJI_0288	Pentanema britannica (L.) D.Gut.Larr., Santos-Vicente, Anderb., E.Rico & M.M.Mart.Ort.	0	n	1	0	Asteraceae Bercht. & J.Presl
Iz_4_1009	DJI_0288	Peucedanum oreoselinum (L.) Moench	0	n	1	0	Apiaceae Lindl.
Iz_4_1009	DJI_0288	Peucedanum ruthenicum M.Bieb.	0	n	1	0	Apiaceae Lindl.
Iz_4_1009	DJI_0288	Pilosella echioides (Lumn.) F.W.Schultz & Sch.Bip.	0	n	1	0	Asteraceae Bercht. & J.Presl
Iz_4_1009	DJI_0288	Pilosella officinarum F. Schultz et Sch. Bip.	0	n	1	0	Asteraceae Bercht. & J.Presl
Iz_4_1009	DJI_0288	Pilosella sp.	0		NA	0	Asteraceae Bercht. & J.Presl
Iz_4_1009	DJI_0288	Pinus sylvestris L.	0	n	1	1	Pinaceae Spreng. ex F.Rudolphi
Iz_4_1009	DJI_0288	Poa annua L.	0	n	1	0	Poaceae Barnhart
Iz_4_1009	DJI_0288	Poa nemoralis L.	0	n	1	0	Poaceae Barnhart
Iz_4_1009	DJI_0288	Poa sp.	0		NA	0	Poaceae Barnhart
Iz_4_1009	DJI_0288	Polygonatum odoratum (Mill.) Druce	0	n	1	0	Asparagaceae Juss.
Iz_4_1009	DJI_0288	Polygonum arenarium Waldst. & Kit.	0	n	1	0	Polygonaceae Juss.
Iz_4_1009	DJI_0288	Populus nigra L.	0	n	1	1	Salicaceae Mirb.
Iz_4_1009	DJI_0288	Populus tremula L.	0	n	1	1	Salicaceae Mirb.
Iz_4_1009	DJI_0288	Populus Ã canescens (Aiton) Sm.	0	n	1	1	Salicaceae Mirb.
Iz_4_1009	DJI_0288	Potentilla argentea L.	0	n	1	0	Rosaceae Juss.
Iz_4_1009	DJI_0288	Psammophiliella muralis (L.) Ikonn.	0	n	1	0	Caryophyllaceae Juss.
Iz_4_1009	DJI_0288	Psephellus sumensis (Kalen.) Greuter	0	n	1	0	Asteraceae Bercht. & J.Presl
Iz_4_1009	DJI_0288	Pulsatilla pratensis (L.) Mill. s.l.	0	n	1	0	Ranunculaceae Juss.
Iz_4_1009	DJI_0288	Pyrus communis L.	0	a	0	1	Rosaceae Juss.
Iz_4_1009	DJI_0288	Quercus robur L.	0	n	1	1	Fagaceae Dumort.

Iz_4_1009	DJI_0288	Robinia pseudoacacia L.	0	a	0	1	Fabaceae Lindl.
Iz_4_1009	DJI_0288	Rosa sp.	0		NA	1	Rosaceae Juss.
Iz_4_1009	DJI_0288	Rubus idaeus L.	0	n	1	0	Rosaceae Juss.
Iz_4_1009	DJI_0288	Rumex acetosella L.	0	n	1	0	Polygonaceae Juss.
Iz_4_1009	DJI_0288	Rumex thyrsiflorus Fingerh.	0	n	1	0	Polygonaceae Juss.
Iz_4_1009	DJI_0288	Salix alba L.	0	n	1	1	Salicaceae Mirb.
Iz_4_1009	DJI_0288	Sambucus nigra L.	0	n	1	1	Viburnaceae Raf.
Iz_4_1009	DJI_0288	Secale sylvestre Host	0	n	1	0	Poaceae Barnhart
Iz_4_1009	DJI_0288	Sempervivum ruthenicum Schnittsp. & C.B. Lehm.	0	n	1	0	Crassulaceae J.St.-Hil.
Iz_4_1009	DJI_0288	Setaria viridis (L.) P. Beauv.	0	n	1	0	Poaceae Barnhart
Iz_4_1009	DJI_0288	Silene borysthenica (Gruner) Walters	0	n	1	0	Caryophyllaceae Juss.
Iz_4_1009	DJI_0288	Silene latifolia subsp. alba (Mill.) Greuter & Burdet	0	n	1	0	Caryophyllaceae Juss.
Iz_4_1009	DJI_0288	Solidago virgaurea L.	0	n	1	0	Asteraceae Bercht. & J.Presl
Iz_4_1009	DJI_0288	Sorbus aucuparia L.	0	n	1	1	Rosaceae Juss.
Iz_4_1009	DJI_0288	Stachys recta L.	0	n	1	0	Lamiacea Lindl.
Iz_4_1009	DJI_0288	Tanacetum vulgare L.	0	n	1	0	Asteraceae Bercht. & J.Presl
Iz_4_1009	DJI_0288	Thymus pallasianus Heinr.Braun	0	n	1	0	Lamiacea Lindl.
Iz_4_1009	DJI_0288	Tilia cordata Mill.	0	n	1	1	Malvaceae Juss.
Iz_4_1009	DJI_0288	Trifolium alpestre L.	0	n	1	0	Fabaceae Lindl.
Iz_4_1009	DJI_0288	Trifolium arvense L.	0	n	1	0	Fabaceae Lindl.
Iz_4_1009	DJI_0288	Ulmus glabra Huds.	0	n	1	1	Ulmaceae Mirb.
Iz_4_1009	DJI_0288	Verbascum densiflorum Bertol.	0	n	1	0	Scrophulariaceae Juss.
Iz_4_1009	DJI_0288	Verbascum nigrum L.	0	n	1	0	Scrophulariaceae Juss.
Iz_4_1009	DJI_0288	Veronica austriaca L.	0	n	1	0	Plantaginaceae Juss.
Iz_4_1009	DJI_0288	Veronica incana L.	0	n	1	0	Plantaginaceae Juss.
Iz_4_1009	DJI_0288	Veronica verna L.	0	n	1	0	Plantaginaceae Juss.
Iz_4_1009	DJI_0288	Vincetoxicum hirundinaria Medik	0	n	1	0	Apocynaceae Juss.
Iz_4_1009	DJI_0292	Acer tataricum L.	0	n	1	1	Sapindaceae Juss.
Iz_4_1009	DJI_0292	Achillea millefolium L. s.l.	0	n	1	0	Asteraceae Bercht. & J.Presl
Iz_4_1009	DJI_0292	Allium oleraceum L.	0	n	1	0	Amaryllidaceae J.St.-Hil.
Iz_4_1009	DJI_0292	Ambrosia artemisiifolia L.	0	a	0	0	Asteraceae Bercht. & J.Presl
Iz_4_1009	DJI_0292	Amorpha fruticosa L.	0	a	0	0	Fabaceae Lindl.
Iz_4_1009	DJI_0292	Anthericum ramosum L.	0	n	1	0	Asparagaceae Juss.
Iz_4_1009	DJI_0292	Artemisia marschalliana Spreng.	0	n	1	0	Asteraceae Bercht. & J.Presl
Iz_4_1009	DJI_0292	Asparagus officinalis L.	0	n	1	0	Asparagaceae Juss.
Iz_4_1009	DJI_0292	Astragalus cicer L.	0	n	1	0	Fabaceae Lindl.
Iz_4_1009	DJI_0292	Bassia laniflora (S.G.Gmel.) A.J.Scott	0	n	1	0	Amaranthaceae Juss.
Iz_4_1009	DJI_0292	Berberis vulgaris L.	0	n	1	0	Berberidaceae Juss.
Iz_4_1009	DJI_0292	Betula pendula Roth.	0	n	1	1	Betulaceae Gray
Iz_4_1009	DJI_0292	Betula pendula Roth.	0	n	1	0	Poaceae Barnhart
Iz_4_1009	DJI_0292	Bromus inermis Leyss.	0		NA	NA	
Iz_4_1009	DJI_0292	Bromus squarrosus L.	0	a	0	0	Poaceae Barnhart
Iz_4_1009	DJI_0292	Bromus tectorum L.	0	a	0	0	Poaceae Barnhart
Iz_4_1009	DJI_0292	Calamagrostis epigejos (L.) Roth	40	n	1	0	Poaceae Barnhart
Iz_4_1009	DJI_0292	Carduus acanthoides L.	0	n	1	0	Asteraceae Bercht. & J.Presl
Iz_4_1009	DJI_0292	Carex sp.	0	n	1	0	Cyperaceae Juss.
Iz_4_1009	DJI_0292	Centaurea majorovii Dumbadze	0	n	1	0	Asteraceae Bercht. & J.Presl
Iz_4_1009	DJI_0292	Chamaecytisus ruthenicus (Fisch.ex Wol.) Klaskova	0	n	1	0	Fabaceae Lindl.
Iz_4_1009	DJI_0292	Chelidonium majus L.	0	n	1	0	Papaveraceae Juss.
Iz_4_1009	DJI_0292	Chenopodium betaceum Andrz.	0	n	1	0	Amaranthaceae Juss.
Iz_4_1009	DJI_0292	Chenopodium cf. album	0	n	1	0	Amaranthaceae Juss.
Iz_4_1009	DJI_0292	Chenopodium sp.	0		NA	0	Amaranthaceae Juss.
Iz_4_1009	DJI_0292	Chondrilla juncea L.	0	n	1	0	Asteraceae Bercht. & J.Presl
Iz_4_1009	DJI_0292	Cirsium vulgare (Savi) Ten.	0	n	1	0	Asteraceae Bercht. & J.Presl
Iz_4_1009	DJI_0292	Clinopodium acinos (L.) Kuntze	0	n	1	0	Lamiacea Lindl.
Iz_4_1009	DJI_0292	Convallaria majalis L.	0	n	1	0	Asparagaceae Juss.
Iz_4_1009	DJI_0292	Crataegus rhipidophylla Gand.	0	n	1	1	Rosaceae Juss.
Iz_4_1009	DJI_0292	Crepis foetida L.	0	n	1	0	Asteraceae Bercht. & J.Presl
Iz_4_1009	DJI_0292	Dactylis glomerata L.	0	n	1	0	Poaceae Barnhart
Iz_4_1009	DJI_0292	Dianthus campestris M.Bieb.	0	n	1	0	Caryophyllaceae Juss.
Iz_4_1009	DJI_0292	Digitaria sanguinalis (L.) Scop.	0	a	0	0	Poaceae Barnhart
Iz_4_1009	DJI_0292	Elymus repens (L.) Gould	0	n	1	0	Poaceae Barnhart
Iz_4_1009	DJI_0292	Epilobium tetragonum L.	0	n	1	0	Onagraceae Juss.
Iz_4_1009	DJI_0292	Eragrostis minor Host	0	a	0	0	Poaceae Barnhart
Iz_4_1009	DJI_0292	Erigeron canadensis L.	0	a	0	0	Asteraceae Bercht. & J.Presl
Iz_4_1009	DJI_0292	Euphorbia seguieriana Neck.	0	n	1	0	Euphorbiaceae Juss.
Iz_4_1009	DJI_0292	Fallopia dumetorum (L.) Holub	0	n	1	0	Polygonaceae Juss.
Iz_4_1009	DJI_0292	Festuca beckeri (Hack.) Trautv.	0	n	1	0	Poaceae Barnhart
Iz_4_1009	DJI_0292	Fragaria vesca L.	0	n	1	0	Rosaceae Juss.
Iz_4_1009	DJI_0292	Frangula alnus Mill.	0	n	1	1	Rhamnaceae Juss.
Iz_4_1009	DJI_0292	Fraxinus pennsylvanica Marshall	0	a	0	1	Oleaceae Hoffmanns. & Link
Iz_4_1009	DJI_0292	Galeopsis bifida Boenn.	0	n	1	0	Lamiacea Lindl.
Iz_4_1009	DJI_0292	Galium verum L.	0	n	1	0	Rubiaceae Juss.
Iz_4_1009	DJI_0292	Genista tinctoria L.	0	n	1	0	Fabaceae Lindl.
Iz_4_1009	DJI_0292	Geranium robertianum L.	0	n	1	0	Geraniaceae Juss.
Iz_4_1009	DJI_0292	Geranium sanquineum L.	0	n	1	0	Geraniaceae Juss.
Iz_4_1009	DJI_0292	Gypsophila paniculata L.	0	n	1	0	Caryophyllaceae Juss.
Iz_4_1009	DJI_0292	Helichrysum arenarium (L.) Moench	0	n	1	0	Asteraceae Bercht. & J.Presl
Iz_4_1009	DJI_0292	Herniaria polygama J.Gay	0	n	1	0	Caryophyllaceae Juss.
Iz_4_1009	DJI_0292	Hieracium robustum Fr.	0	n	1	0	Asteraceae Bercht. & J.Presl
Iz_4_1009	DJI_0292	Hieracium umbellatum L.	0	n	1	NA	Asteraceae Bercht. & J.Presl
Iz_4_1009	DJI_0292	Hylotelephium maximum (L.) Holub	0	n	1	0	Crassulaceae J.St.-Hil.
Iz_4_1009	DJI_0292	Jacobaea borysthenica (DC.) B.Nord. & Greuter	0	n	1	0	Asteraceae Bercht. & J.Presl
Iz_4_1009	DJI_0292	Jasione montana L.	0	n	1	0	Campanulaceae Juss.
Iz_4_1009	DJI_0292	Jurinea cyanoides (L.) Rchb.s.l.	0	n	1	0	Asteraceae Bercht. & J.Presl
Iz_4_1009	DJI_0292	Koeleria glauca (Spreng.) DC.	0	n	1	0	Poaceae Barnhart
Iz_4_1009	DJI_0292	Lactuca serriola L.	0	a	0	0	Asteraceae Bercht. & J.Presl
Iz_4_1009	DJI_0292	Linaria genistifolia (L.) Mill.	0	n	1	0	Plantaginaceae Juss.
Iz_4_1009	DJI_0292	Lomelosia argentea (L.) Greuter & Burdet	0	n	1	0	Caprifoliaceae Juss.
Iz_4_1009	DJI_0292	Melampyrum pratense L.	0	n	1	0	Orobanchaceae Vent.
Iz_4_1009	DJI_0292	Oenothera biennis L.	0	a	0	0	Onagraceae Juss.
Iz_4_1009	DJI_0292	Pentanema britannica (L.) D.Gut.Larr., Santos-Vicente, Anderb., E.Rico & M.M.Mart.Ort.	0	n	1	0	Asteraceae Bercht. & J.Presl
Iz_4_1009	DJI_0292	Peucedanum oreoselinum (L.) Moench	0	n	1	0	Apiaceae Lindl.
Iz_4_1009	DJI_0292	Peucedanum ruthenicum M.Bieb.	0	n	1	0	Apiaceae Lindl.
Iz_4_1009	DJI_0292	Pilosella echioides (Lumn.) F.W.Schultz & Sch.Bip.	0	n	1	0	Asteraceae Bercht. & J.Presl
Iz_4_1009	DJI_0292	Pilosella officinarum F. Schultz et Sch. Bip.	0	n	1	0	Asteraceae Bercht. & J.Presl
Iz_4_1009	DJI_0292	Pilosella sp.	0		NA	0	Asteraceae Bercht. & J.Presl
Iz_4_1009	DJI_0292	Pinus sylvestris L.	0	n	1	1	Pinaceae Spreng. ex F.Rudolphi

Iz_4_1009	DJI_0292	Poa annua L.	0	n	1	0	Poaceae Barnhart
Iz_4_1009	DJI_0292	Poa nemoralis L.	0	n	1	0	Poaceae Barnhart
Iz_4_1009	DJI_0292	Poa sp.	0		NA	0	Poaceae Barnhart
Iz_4_1009	DJI_0292	Polygonatum odoratum (Mill.) Druce	0	n	1	0	Asparagaceae Juss.
Iz_4_1009	DJI_0292	Polygonum arenarium Waldst. & Kit.	0	n	1	0	Polygonaceae Juss.
Iz_4_1009	DJI_0292	Populus nigra L.	0	n	1	1	Salicaceae Mirb.
Iz_4_1009	DJI_0292	Populus tremula L.	0	n	1	1	Salicaceae Mirb.
Iz_4_1009	DJI_0292	Populus Ã canescens (Aiton) Sm.	0	n	1	1	Salicaceae Mirb.
Iz_4_1009	DJI_0292	Potentilla argentea L.	0	n	1	0	Rosaceae Juss.
Iz_4_1009	DJI_0292	Psammophiliella muralis (L.) Ikonn.	0	n	1	0	Caryophyllaceae Juss.
Iz_4_1009	DJI_0292	Psephellus sumensis (Kalen.) Greuter	0	n	1	0	Asteraceae Bercht. & J.Presl
Iz_4_1009	DJI_0292	Pulsatilla pratensis (L.) Mill. s.l.	0	n	1	0	Ranunculaceae Juss.
Iz_4_1009	DJI_0292	Pyrus communis L.	0	a	0	1	Rosaceae Juss.
Iz_4_1009	DJI_0292	Quercus robur L.	0	n	1	1	Fagaceae Dumort.
Iz_4_1009	DJI_0292	Robinia pseudoacacia L.	0	a	0	1	Fabaceae Lindl.
Iz_4_1009	DJI_0292	Rosa sp.	0		NA	1	Rosaceae Juss.
Iz_4_1009	DJI_0292	Rubus idaeus L.	0	n	1	0	Rosaceae Juss.
Iz_4_1009	DJI_0292	Rumex acetosella L.	0	n	1	0	Polygonaceae Juss.
Iz_4_1009	DJI_0292	Rumex thyrsiflorus Fingerh.	0	n	1	0	Polygonaceae Juss.
Iz_4_1009	DJI_0292	Salix alba L.	0	n	1	1	Salicaceae Mirb.
Iz_4_1009	DJI_0292	Sambucus nigra L.	0	n	1	1	Viburnaceae Raf.
Iz_4_1009	DJI_0292	Secale sylvestre Host	0	n	1	0	Poaceae Barnhart
Iz_4_1009	DJI_0292	Sempervivum ruthenicum Schnittsp. & C.B. Lehm.	0	n	1	0	Crassulaceae J.St.-Hil.
Iz_4_1009	DJI_0292	Setaria viridis (L.) P. Beauv.	0	n	1	0	Poaceae Barnhart
Iz_4_1009	DJI_0292	Silene borysthenica (Gruner) Walters	0	n	1	0	Caryophyllaceae Juss.
Iz_4_1009	DJI_0292	Silene latifolia subsp. alba (Mill.) Greuter & Burdet	0	n	1	0	Caryophyllaceae Juss.
Iz_4_1009	DJI_0292	Solidago virgaurea L.	0.5	n	1	0	Asteraceae Bercht. & J.Presl
Iz_4_1009	DJI_0292	Sorbus aucuparia L.	0	n	1	1	Rosaceae Juss.
Iz_4_1009	DJI_0292	Stachys recta L.	0	n	1	0	Lamiacea Lindl.
Iz_4_1009	DJI_0292	Tanacetum vulgare L.	0	n	1	0	Asteraceae Bercht. & J.Presl
Iz_4_1009	DJI_0292	Thymus pallasianus Heinr.Braun	0	n	1	0	Lamiacea Lindl.
Iz_4_1009	DJI_0292	Tilia cordata Mill.	0	n	1	1	Malvaceae Juss.
Iz_4_1009	DJI_0292	Trifolium alpestre L.	0	n	1	0	Fabaceae Lindl.
Iz_4_1009	DJI_0292	Trifolium arvense L.	0	n	1	0	Fabaceae Lindl.
Iz_4_1009	DJI_0292	Ulmus glabra Huds.	0	n	1	1	Ulmaceae Mirb.
Iz_4_1009	DJI_0292	Verbascum densiflorum Bertol.	0	n	1	0	Scrophulariaceae Juss.
Iz_4_1009	DJI_0292	Verbascum nigrum L.	0	n	1	0	Scrophulariaceae Juss.
Iz_4_1009	DJI_0292	Veronica austriaca L.	0	n	1	0	Plantaginaceae Juss.
Iz_4_1009	DJI_0292	Veronica incana L.	0	n	1	0	Plantaginaceae Juss.
Iz_4_1009	DJI_0292	Veronica verna L.	0	n	1	0	Plantaginaceae Juss.
Iz_4_1009	DJI_0292	Vincetoxicum hirundinaria Medik	0	n	1	0	Apocynaceae Juss.
Iz_4_1009	DJI_0297	Acer tataricum L.	0	n	1	1	Sapindaceae Juss.
Iz_4_1009	DJI_0297	Achillea millefolium L. s.l.	0	n	1	0	Asteraceae Bercht. & J.Presl
Iz_4_1009	DJI_0297	Allium oleraceum L.	0	n	1	0	Amaryllidaceae J.St.-Hil.
Iz_4_1009	DJI_0297	Ambrosia artemisiifolia L.	0	a	0	0	Asteraceae Bercht. & J.Presl
Iz_4_1009	DJI_0297	Amorpha fruticosa L.	0	a	0	0	Fabaceae Lindl.
Iz_4_1009	DJI_0297	Anthericum ramosum L.	0	n	1	0	Asparagaceae Juss.
Iz_4_1009	DJI_0297	Artemisia marschalliana Spreng.	0	n	1	0	Asteraceae Bercht. & J.Presl
Iz_4_1009	DJI_0297	Asparagus officinalis L.	0	n	1	0	Asparagaceae Juss.
Iz_4_1009	DJI_0297	Astragalus cicer L.	0	n	1	0	Fabaceae Lindl.
Iz_4_1009	DJI_0297	Bassia laniflora (S.G.Gmel.) A.J.Scott	0	n	1	0	Amaranthaceae Juss.
Iz_4_1009	DJI_0297	Berberis vulgaris L.	0	n	1	0	Berberidaceae Juss.
Iz_4_1009	DJI_0297	Betula pendula Roth.	0	n	1	1	Betulaceae Gray
Iz_4_1009	DJI_0297	Betula pendula Roth.	0	n	1	0	Poaceae Barnhart
Iz_4_1009	DJI_0297	Bromus inermis Leyss.	0		NA	NA	
Iz_4_1009	DJI_0297	Bromus squarrosus L.	0	a	0	0	Poaceae Barnhart
Iz_4_1009	DJI_0297	Bromus tectorum L.	0	a	0	0	Poaceae Barnhart
Iz_4_1009	DJI_0297	Calamagrostis epigejos (L.) Roth	60	n	1	0	Poaceae Barnhart
Iz_4_1009	DJI_0297	Carduus acanthoides L.	0	n	1	0	Asteraceae Bercht. & J.Presl
Iz_4_1009	DJI_0297	Carex sp.	0	n	1	0	Cyperaceae Juss.
Iz_4_1009	DJI_0297	Centaurea majorovii Dumbadze	0	n	1	0	Asteraceae Bercht. & J.Presl
Iz_4_1009	DJI_0297	Chamaecytisus ruthenicus (Fisch.ex Wol.) Klaskova	0	n	1	0	Fabaceae Lindl.
Iz_4_1009	DJI_0297	Chelidonium majus L.	0	n	1	0	Papaveraceae Juss.
Iz_4_1009	DJI_0297	Chenopodium betaceum Andrz.	0	n	1	0	Amaranthaceae Juss.
Iz_4_1009	DJI_0297	Chenopodium cf. album	0	n	1	0	Amaranthaceae Juss.
Iz_4_1009	DJI_0297	Chenopodium sp.	0		NA	0	Amaranthaceae Juss.
Iz_4_1009	DJI_0297	Chondrilla juncea L.	0	n	1	0	Asteraceae Bercht. & J.Presl
Iz_4_1009	DJI_0297	Cirsium vulgare (Savi) Ten.	0	n	1	0	Asteraceae Bercht. & J.Presl
Iz_4_1009	DJI_0297	Clinopodium acinos (L.) Kuntze	0	n	1	0	Lamiacea Lindl.
Iz_4_1009	DJI_0297	Convallaria majalis L.	0	n	1	0	Asparagaceae Juss.
Iz_4_1009	DJI_0297	Crataegus rhipidophylla Gand.	0	n	1	1	Rosaceae Juss.
Iz_4_1009	DJI_0297	Crepis foetida L.	0	n	1	0	Asteraceae Bercht. & J.Presl
Iz_4_1009	DJI_0297	Dactylis glomerata L.	0	n	1	0	Poaceae Barnhart
Iz_4_1009	DJI_0297	Dianthus campestris M.Bieb.	0	n	1	0	Caryophyllaceae Juss.
Iz_4_1009	DJI_0297	Digitaria sanguinalis (L.) Scop.	0	a	0	0	Poaceae Barnhart
Iz_4_1009	DJI_0297	Elymus repens (L.) Gould	0	n	1	0	Poaceae Barnhart
Iz_4_1009	DJI_0297	Epilobium tetragonum L.	0	n	1	0	Onagraceae Juss.
Iz_4_1009	DJI_0297	Eragrostis minor Host	0	a	0	0	Poaceae Barnhart
Iz_4_1009	DJI_0297	Erigeron canadensis L.	0	a	0	0	Asteraceae Bercht. & J.Presl
Iz_4_1009	DJI_0297	Euphorbia seguieriana Neck.	0	n	1	0	Euphorbiaceae Juss.
Iz_4_1009	DJI_0297	Fallopia dumetorum (L.) Holub	0	n	1	0	Polygonaceae Juss.
Iz_4_1009	DJI_0297	Festuca beckeri (Hack.) Trautv.	0	n	1	0	Poaceae Barnhart
Iz_4_1009	DJI_0297	Fragaria vesca L.	0	n	1	0	Rosaceae Juss.
Iz_4_1009	DJI_0297	Frangula alnus Mill.	0	n	1	1	Rhamnaceae Juss.
Iz_4_1009	DJI_0297	Fraxinus pennsylvanica Marshall	0	a	0	1	Oleaceae Hoffmanns. & Link
Iz_4_1009	DJI_0297	Galeopsis bifida Boenn.	0	n	1	0	Lamiacea Lindl.
Iz_4_1009	DJI_0297	Galium verum L.	0	n	1	0	Rubiaceae Juss.
Iz_4_1009	DJI_0297	Genista tinctoria L.	0	n	1	0	Fabaceae Lindl.
Iz_4_1009	DJI_0297	Geranium robertianum L.	0	n	1	0	Geraniaceae Juss.
Iz_4_1009	DJI_0297	Geranium sanquineum L.	0	n	1	0	Geraniaceae Juss.
Iz_4_1009	DJI_0297	Gypsophila paniculata L.	0	n	1	0	Caryophyllaceae Juss.
Iz_4_1009	DJI_0297	Helichrysum arenarium (L.) Moench	0	n	1	0	Asteraceae Bercht. & J.Presl
Iz_4_1009	DJI_0297	Herniaria polygama J.Gay	0	n	1	0	Caryophyllaceae Juss.
Iz_4_1009	DJI_0297	Hieracium robustum Fr.	0	n	1	0	Asteraceae Bercht. & J.Presl
Iz_4_1009	DJI_0297	Hieracium umbellatum L.	0	n	1	NA	Asteraceae Bercht. & J.Presl
Iz_4_1009	DJI_0297	Hylotelephium maximum (L.) Holub	0	n	1	0	Crassulaceae J.St.-Hil.
Iz_4_1009	DJI_0297	Jacobaea borysthenica (DC.) B.Nord. & Greuter	0	n	1	0	Asteraceae Bercht. & J.Presl
Iz_4_1009	DJI_0297	Jasione montana L.	0	n	1	0	Campanulaceae Juss.

Iz_4_1009	DJI_0297	Jurinea cyanoides (L.) Rchb.s.l.	0	n	1	0	Asteraceae Bercht. & J.Presl
Iz_4_1009	DJI_0297	Koeleria glauca (Spreng.) DC.	0	n	1	0	Poaceae Barnhart
Iz_4_1009	DJI_0297	Lactuca serriola L.	0	a	0	0	Asteraceae Bercht. & J.Presl
Iz_4_1009	DJI_0297	Linaria genistifolia (L.) Mill.	0	n	1	0	Plantaginaceae Juss.
Iz_4_1009	DJI_0297	Lomelosia argentea (L.) Greuter & Burdet	0	n	1	0	Caprifoliaceae Juss.
Iz_4_1009	DJI_0297	Melampyrum pratense L.	0	n	1	0	Orobanchaceae Vent.
Iz_4_1009	DJI_0297	Oenothera biennis L.	0	a	0	0	Onagraceae Juss.
Iz_4_1009	DJI_0297	Pentanema britannica (L.) D.Gut.Larr., Santos-Vicente, Anderb., E.Rico & M.M.Mart.Ort.	0	n	1	0	Asteraceae Bercht. & J.Presl
Iz_4_1009	DJI_0297	Peucedanum oreoselinum (L.) Moench	0	n	1	0	Apiaceae Lindl.
Iz_4_1009	DJI_0297	Peucedanum ruthenicum M.Bieb.	0	n	1	0	Apiaceae Lindl.
Iz_4_1009	DJI_0297	Pilosella echioides (Lumn.) F.W.Schultz & Sch.Bip.	0	n	1	0	Asteraceae Bercht. & J.Presl
Iz_4_1009	DJI_0297	Pilosella officinarum F. Schultz et Sch. Bip.	0	n	1	0	Asteraceae Bercht. & J.Presl
Iz_4_1009	DJI_0297	Pilosella sp.	0		NA	0	Asteraceae Bercht. & J.Presl
Iz_4_1009	DJI_0297	Pinus sylvestris L.	0	n	1	1	Pinaceae Spreng. ex F.Rudolphi
Iz_4_1009	DJI_0297	Poa annua L.	0	n	1	0	Poaceae Barnhart
Iz_4_1009	DJI_0297	Poa nemoralis L.	0	n	1	0	Poaceae Barnhart
Iz_4_1009	DJI_0297	Poa sp.	0		NA	0	Poaceae Barnhart
Iz_4_1009	DJI_0297	Polygonatum odoratum (Mill.) Druce	0	n	1	0	Asparagaceae Juss.
Iz_4_1009	DJI_0297	Polygonum arenarium Waldst. & Kit.	0	n	1	0	Polygonaceae Juss.
Iz_4_1009	DJI_0297	Populus nigra L.	0	n	1	1	Salicaceae Mirb.
Iz_4_1009	DJI_0297	Populus tremula L.	0	n	1	1	Salicaceae Mirb.
Iz_4_1009	DJI_0297	Populus Ã canescens (Aiton) Sm.	0	n	1	1	Salicaceae Mirb.
Iz_4_1009	DJI_0297	Potentilla argentea L.	0	n	1	0	Rosaceae Juss.
Iz_4_1009	DJI_0297	Psammophiliella muralis (L.) Ikonn.	0	n	1	0	Caryophyllaceae Juss.
Iz_4_1009	DJI_0297	Psephellus sumensis (Kalen.) Greuter	0	n	1	0	Asteraceae Bercht. & J.Presl
Iz_4_1009	DJI_0297	Pulsatilla pratensis (L.) Mill. s.l.	0	n	1	0	Ranunculaceae Juss.
Iz_4_1009	DJI_0297	Pyrus communis L.	0	a	0	1	Rosaceae Juss.
Iz_4_1009	DJI_0297	Quercus robur L.	0	n	1	1	Fagaceae Dumort.
Iz_4_1009	DJI_0297	Robinia pseudoacacia L.	0	a	0	1	Fabaceae Lindl.
Iz_4_1009	DJI_0297	Rosa sp.	0		NA	1	Rosaceae Juss.
Iz_4_1009	DJI_0297	Rubus idaeus L.	0	n	1	0	Rosaceae Juss.
Iz_4_1009	DJI_0297	Rumex acetosella L.	0	n	1	0	Polygonaceae Juss.
Iz_4_1009	DJI_0297	Rumex thyrsiflorus Fingerh.	0	n	1	0	Polygonaceae Juss.
Iz_4_1009	DJI_0297	Salix alba L.	0	n	1	1	Salicaceae Mirb.
Iz_4_1009	DJI_0297	Sambucus nigra L.	0	n	1	1	Viburnaceae Raf.
Iz_4_1009	DJI_0297	Secale sylvestre Host	0	n	1	0	Poaceae Barnhart
Iz_4_1009	DJI_0297	Sempervivum ruthenicum Schnittsp. & C.B. Lehm.	0	n	1	0	Crassulaceae J.St.-Hil.
Iz_4_1009	DJI_0297	Setaria viridis (L.) P. Beauv.	0	n	1	0	Poaceae Barnhart
Iz_4_1009	DJI_0297	Silene borysthenica (Gruner) Walters	0	n	1	0	Caryophyllaceae Juss.
Iz_4_1009	DJI_0297	Silene latifolia subsp. alba (Mill.) Greuter & Burdet	0	n	1	0	Caryophyllaceae Juss.
Iz_4_1009	DJI_0297	Solidago virgaurea L.	0	n	1	0	Asteraceae Bercht. & J.Presl
Iz_4_1009	DJI_0297	Sorbus aucuparia L.	0	n	1	1	Rosaceae Juss.
Iz_4_1009	DJI_0297	Stachys recta L.	0	n	1	0	Lamiacea Lindl.
Iz_4_1009	DJI_0297	Tanacetum vulgare L.	0	n	1	0	Asteraceae Bercht. & J.Presl
Iz_4_1009	DJI_0297	Thymus pallasianus Heinr.Braun	0	n	1	0	Lamiacea Lindl.
Iz_4_1009	DJI_0297	Tilia cordata Mill.	0	n	1	1	Malvaceae Juss.
Iz_4_1009	DJI_0297	Trifolium alpestre L.	0	n	1	0	Fabaceae Lindl.
Iz_4_1009	DJI_0297	Trifolium arvense L.	0	n	1	0	Fabaceae Lindl.
Iz_4_1009	DJI_0297	Ulmus glabra Huds.	0	n	1	1	Ulmaceae Mirb.
Iz_4_1009	DJI_0297	Verbascum densiflorum Bertol.	0	n	1	0	Scrophulariaceae Juss.
Iz_4_1009	DJI_0297	Verbascum nigrum L.	0	n	1	0	Scrophulariaceae Juss.
Iz_4_1009	DJI_0297	Veronica austriaca L.	0	n	1	0	Plantaginaceae Juss.
Iz_4_1009	DJI_0297	Veronica incana L.	0	n	1	0	Plantaginaceae Juss.
Iz_4_1009	DJI_0297	Veronica verna L.	0	n	1	0	Plantaginaceae Juss.
Iz_4_1009	DJI_0297	Vincetoxicum hirundinaria Medik	0	n	1	0	Apocynaceae Juss.
Iz_4_1009	DJI_0299	Acer tataricum L.	0	n	1	1	Sapindaceae Juss.
Iz_4_1009	DJI_0299	Achillea millefolium L. s.l.	0	n	1	0	Asteraceae Bercht. & J.Presl
Iz_4_1009	DJI_0299	Allium oleraceum L.	0	n	1	0	Amaryllidaceae J.St.-Hil.
Iz_4_1009	DJI_0299	Ambrosia artemisiifolia L.	0	a	0	0	Asteraceae Bercht. & J.Presl
Iz_4_1009	DJI_0299	Amorpha fruticosa L.	0	a	0	0	Fabaceae Lindl.
Iz_4_1009	DJI_0299	Anthericum ramosum L.	0	n	1	0	Asparagaceae Juss.
Iz_4_1009	DJI_0299	Artemisia marschalliana Spreng.	0	n	1	0	Asteraceae Bercht. & J.Presl
Iz_4_1009	DJI_0299	Asparagus officinalis L.	0	n	1	0	Asparagaceae Juss.
Iz_4_1009	DJI_0299	Astragalus cicer L.	0	n	1	0	Fabaceae Lindl.
Iz_4_1009	DJI_0299	Bassia laniflora (S.G.Gmel.) A.J.Scott	0	n	1	0	Amaranthaceae Juss.
Iz_4_1009	DJI_0299	Berberis vulgaris L.	0	n	1	0	Berberidaceae Juss.
Iz_4_1009	DJI_0299	Betula pendula Roth.	0	n	1	1	Betulaceae Gray
Iz_4_1009	DJI_0299	Betula pendula Roth.	0	n	1	0	Poaceae Barnhart
Iz_4_1009	DJI_0299	Bromus inermis Leyss.	0		NA	NA	
Iz_4_1009	DJI_0299	Bromus squarrosus L.	0	a	0	0	Poaceae Barnhart
Iz_4_1009	DJI_0299	Bromus tectorum L.	0	a	0	0	Poaceae Barnhart
Iz_4_1009	DJI_0299	Calamagrostis epigejos (L.) Roth	60	n	1	0	Poaceae Barnhart
Iz_4_1009	DJI_0299	Carduus acanthoides L.	0	n	1	0	Asteraceae Bercht. & J.Presl
Iz_4_1009	DJI_0299	Carex sp.	0	n	1	0	Cyperaceae Juss.
Iz_4_1009	DJI_0299	Centaurea majorovii Dumbadze	0	n	1	0	Asteraceae Bercht. & J.Presl
Iz_4_1009	DJI_0299	Chamaecytisus ruthenicus (Fisch.ex Wol.) Klaskova	8	n	1	0	Fabaceae Lindl.
Iz_4_1009	DJI_0299	Chelidonium majus L.	0	n	1	0	Papaveraceae Juss.
Iz_4_1009	DJI_0299	Chenopodium betaceum Andrz.	0	n	1	0	Amaranthaceae Juss.
Iz_4_1009	DJI_0299	Chenopodium cf. album	0	n	1	0	Amaranthaceae Juss.
Iz_4_1009	DJI_0299	Chenopodium sp.	0		NA	0	Amaranthaceae Juss.
Iz_4_1009	DJI_0299	Chondrilla juncea L.	0	n	1	0	Asteraceae Bercht. & J.Presl
Iz_4_1009	DJI_0299	Cirsium vulgare (Savi) Ten.	0	n	1	0	Asteraceae Bercht. & J.Presl
Iz_4_1009	DJI_0299	Clinopodium acinos (L.) Kuntze	0	n	1	0	Lamiacea Lindl.
Iz_4_1009	DJI_0299	Convallaria majalis L.	0	n	1	0	Asparagaceae Juss.
Iz_4_1009	DJI_0299	Crataegus rhipidophylla Gand.	0	n	1	1	Rosaceae Juss.
Iz_4_1009	DJI_0299	Crepis foetida L.	0	n	1	0	Asteraceae Bercht. & J.Presl
Iz_4_1009	DJI_0299	Dactylis glomerata L.	0	n	1	0	Poaceae Barnhart
Iz_4_1009	DJI_0299	Dianthus campestris M.Bieb.	0	n	1	0	Caryophyllaceae Juss.
Iz_4_1009	DJI_0299	Digitaria sanguinalis (L.) Scop.	0	a	0	0	Poaceae Barnhart
Iz_4_1009	DJI_0299	Elymus repens (L.) Gould	0	n	1	0	Poaceae Barnhart
Iz_4_1009	DJI_0299	Epilobium tetragonum L.	0	n	1	0	Onagraceae Juss.
Iz_4_1009	DJI_0299	Eragrostis minor Host	0	a	0	0	Poaceae Barnhart
Iz_4_1009	DJI_0299	Erigeron canadensis L.	0	a	0	0	Asteraceae Bercht. & J.Presl
Iz_4_1009	DJI_0299	Euphorbia seguieriana Neck.	0	n	1	0	Euphorbiaceae Juss.
Iz_4_1009	DJI_0299	Fallopia dumetorum (L.) Holub	0	n	1	0	Polygonaceae Juss.
Iz_4_1009	DJI_0299	Festuca beckeri (Hack.) Trautv.	0	n	1	0	Poaceae Barnhart
Iz_4_1009	DJI_0299	Fragaria vesca L.	0	n	1	0	Rosaceae Juss.
Iz_4_1009	DJI_0299	Frangula alnus Mill.	0	n	1	1	Rhamnaceae Juss.

Iz_4_1009	DJI_0299	Fraxinus pennsylvanica Marshall	0	a	0	1	Oleaceae Hoffmanns. & Link
Iz_4_1009	DJI_0299	Galeopsis bifida Boenn.	0	n	1	0	Lamiacea Lindl.
Iz_4_1009	DJI_0299	Galium verum L.	0	n	1	0	Rubiaceae Juss.
Iz_4_1009	DJI_0299	Genista tinctoria L.	0	n	1	0	Fabaceae Lindl.
Iz_4_1009	DJI_0299	Geranium robertianum L.	0	n	1	0	Geraniaceae Juss.
Iz_4_1009	DJI_0299	Geranium sanquineum L.	0	n	1	0	Geraniaceae Juss.
Iz_4_1009	DJI_0299	Gypsophila paniculata L.	0	n	1	0	Caryophyllaceae Juss.
Iz_4_1009	DJI_0299	Helichrysum arenarium (L.) Moench	0	n	1	0	Asteraceae Bercht. & J.Presl
Iz_4_1009	DJI_0299	Herniaria polygama J.Gay	0	n	1	0	Caryophyllaceae Juss.
Iz_4_1009	DJI_0299	Hieracium robustum Fr.	0	n	1	0	Asteraceae Bercht. & J.Presl
Iz_4_1009	DJI_0299	Hieracium umbellatum L.	0	n	1	NA	Asteraceae Bercht. & J.Presl
Iz_4_1009	DJI_0299	Hylotelephium maximum (L.) Holub	0	n	1	0	Crassulaceae J.St.-Hil.
Iz_4_1009	DJI_0299	Jacobaea borysthenica (DC.) B.Nord. & Greuter	0	n	1	0	Asteraceae Bercht. & J.Presl
Iz_4_1009	DJI_0299	Jasione montana L.	0	n	1	0	Campanulaceae Juss.
Iz_4_1009	DJI_0299	Jurinea cyanoides (L.) Rchb.s.l.	0	n	1	0	Asteraceae Bercht. & J.Presl
Iz_4_1009	DJI_0299	Koeleria glauca (Spreng.) DC.	0	n	1	0	Poaceae Barnhart
Iz_4_1009	DJI_0299	Lactuca serriola L.	0	a	0	0	Asteraceae Bercht. & J.Presl
Iz_4_1009	DJI_0299	Linaria genistifolia (L.) Mill.	0	n	1	0	Plantaginaceae Juss.
Iz_4_1009	DJI_0299	Lomelosia argentea (L.) Greuter & Burdet	0	n	1	0	Caprifoliaceae Juss.
Iz_4_1009	DJI_0299	Melampyrum pratense L.	0	n	1	0	Orobanchaceae Vent.
Iz_4_1009	DJI_0299	Oenothera biennis L.	0	a	0	0	Onagraceae Juss.
Iz_4_1009	DJI_0299	Pentanema britannica (L.) D.Gut.Larr., Santos-Vicente, Anderb., E.Rico & M.M.Mart.Ort.	0	n	1	0	Asteraceae Bercht. & J.Presl
Iz_4_1009	DJI_0299	Peucedanum oreoselinum (L.) Moench	0	n	1	0	Apiaceae Lindl.
Iz_4_1009	DJI_0299	Peucedanum ruthenicum M.Bieb.	0	n	1	0	Apiaceae Lindl.
Iz_4_1009	DJI_0299	Pilosella echioides (Lumn.) F.W.Schultz & Sch.Bip.	0	n	1	0	Asteraceae Bercht. & J.Presl
Iz_4_1009	DJI_0299	Pilosella officinarum F. Schultz et Sch. Bip.	0	n	1	0	Asteraceae Bercht. & J.Presl
Iz_4_1009	DJI_0299	Pilosella sp.	0		NA	0	Asteraceae Bercht. & J.Presl
Iz_4_1009	DJI_0299	Pinus sylvestris L.	0	n	1	1	Pinaceae Spreng. ex F.Rudolphi
Iz_4_1009	DJI_0299	Poa annua L.	0	n	1	0	Poaceae Barnhart
Iz_4_1009	DJI_0299	Poa nemoralis L.	0	n	1	0	Poaceae Barnhart
Iz_4_1009	DJI_0299	Poa sp.	0		NA	0	Poaceae Barnhart
Iz_4_1009	DJI_0299	Polygonatum odoratum (Mill.) Druce	0	n	1	0	Asparagaceae Juss.
Iz_4_1009	DJI_0299	Polygonum arenarium Waldst. & Kit.	0	n	1	0	Polygonaceae Juss.
Iz_4_1009	DJI_0299	Populus nigra L.	0	n	1	1	Salicaceae Mirb.
Iz_4_1009	DJI_0299	Populus tremula L.	0	n	1	1	Salicaceae Mirb.
Iz_4_1009	DJI_0299	Populus Ã canescens (Aiton) Sm.	0	n	1	1	Salicaceae Mirb.
Iz_4_1009	DJI_0299	Potentilla argentea L.	0	n	1	0	Rosaceae Juss.
Iz_4_1009	DJI_0299	Psammophiliella muralis (L.) Ikonn.	0	n	1	0	Caryophyllaceae Juss.
Iz_4_1009	DJI_0299	Psephellus sumensis (Kalen.) Greuter	0	n	1	0	Asteraceae Bercht. & J.Presl
Iz_4_1009	DJI_0299	Pulsatilla pratensis (L.) Mill. s.l.	0	n	1	0	Ranunculaceae Juss.
Iz_4_1009	DJI_0299	Pyrus communis L.	0	a	0	1	Rosaceae Juss.
Iz_4_1009	DJI_0299	Quercus robur L.	0	n	1	1	Fagaceae Dumort.
Iz_4_1009	DJI_0299	Robinia pseudoacacia L.	0	a	0	1	Fabaceae Lindl.
Iz_4_1009	DJI_0299	Rosa sp.	0		NA	1	Rosaceae Juss.
Iz_4_1009	DJI_0299	Rubus idaeus L.	0	n	1	0	Rosaceae Juss.
Iz_4_1009	DJI_0299	Rumex acetosella L.	0	n	1	0	Polygonaceae Juss.
Iz_4_1009	DJI_0299	Rumex thyrsiflorus Fingerh.	0	n	1	0	Polygonaceae Juss.
Iz_4_1009	DJI_0299	Salix alba L.	0	n	1	1	Salicaceae Mirb.
Iz_4_1009	DJI_0299	Sambucus nigra L.	0	n	1	1	Viburnaceae Raf.
Iz_4_1009	DJI_0299	Secale sylvestre Host	0	n	1	0	Poaceae Barnhart
Iz_4_1009	DJI_0299	Sempervivum ruthenicum Schnittsp. & C.B. Lehm.	0	n	1	0	Crassulaceae J.St.-Hil.
Iz_4_1009	DJI_0299	Setaria viridis (L.) P. Beauv.	0	n	1	0	Poaceae Barnhart
Iz_4_1009	DJI_0299	Silene borysthenica (Gruner) Walters	0	n	1	0	Caryophyllaceae Juss.
Iz_4_1009	DJI_0299	Silene latifolia subsp. alba (Mill.) Greuter & Burdet	0	n	1	0	Caryophyllaceae Juss.
Iz_4_1009	DJI_0299	Solidago virgaurea L.	0	n	1	0	Asteraceae Bercht. & J.Presl
Iz_4_1009	DJI_0299	Sorbus aucuparia L.	0	n	1	1	Rosaceae Juss.
Iz_4_1009	DJI_0299	Stachys recta L.	0	n	1	0	Lamiacea Lindl.
Iz_4_1009	DJI_0299	Tanacetum vulgare L.	0	n	1	0	Asteraceae Bercht. & J.Presl
Iz_4_1009	DJI_0299	Thymus pallasianus Heinr.Braun	0	n	1	0	Lamiacea Lindl.
Iz_4_1009	DJI_0299	Tilia cordata Mill.	0	n	1	1	Malvaceae Juss.
Iz_4_1009	DJI_0299	Trifolium alpestre L.	0	n	1	0	Fabaceae Lindl.
Iz_4_1009	DJI_0299	Trifolium arvense L.	0	n	1	0	Fabaceae Lindl.
Iz_4_1009	DJI_0299	Ulmus glabra Huds.	0	n	1	1	Ulmaceae Mirb.
Iz_4_1009	DJI_0299	Verbascum densiflorum Bertol.	0	n	1	0	Scrophulariaceae Juss.
Iz_4_1009	DJI_0299	Verbascum nigrum L.	0	n	1	0	Scrophulariaceae Juss.
Iz_4_1009	DJI_0299	Veronica austriaca L.	0	n	1	0	Plantaginaceae Juss.
Iz_4_1009	DJI_0299	Veronica incana L.	0	n	1	0	Plantaginaceae Juss.
Iz_4_1009	DJI_0299	Veronica verna L.	0	n	1	0	Plantaginaceae Juss.
Iz_4_1009	DJI_0299	Vincetoxicum hirundinaria Medik	0	n	1	0	Apocynaceae Juss.
Iz_4_1009	DJI_0300	Acer tataricum L.	0	n	1	1	Sapindaceae Juss.
Iz_4_1009	DJI_0300	Achillea millefolium L. s.l.	0	n	1	0	Asteraceae Bercht. & J.Presl
Iz_4_1009	DJI_0300	Allium oleraceum L.	0	n	1	0	Amaryllidaceae J.St.-Hil.
Iz_4_1009	DJI_0300	Ambrosia artemisiifolia L.	0	a	0	0	Asteraceae Bercht. & J.Presl
Iz_4_1009	DJI_0300	Amorpha fruticosa L.	0	a	0	0	Fabaceae Lindl.
Iz_4_1009	DJI_0300	Anthericum ramosum L.	0	n	1	0	Asparagaceae Juss.
Iz_4_1009	DJI_0300	Artemisia marschalliana Spreng.	0	n	1	0	Asteraceae Bercht. & J.Presl
Iz_4_1009	DJI_0300	Asparagus officinalis L.	0	n	1	0	Asparagaceae Juss.
Iz_4_1009	DJI_0300	Astragalus cicer L.	0	n	1	0	Fabaceae Lindl.
Iz_4_1009	DJI_0300	Bassia laniflora (S.G.Gmel.) A.J.Scott	0	n	1	0	Amaranthaceae Juss.
Iz_4_1009	DJI_0300	Berberis vulgaris L.	0	n	1	0	Berberidaceae Juss.
Iz_4_1009	DJI_0300	Betula pendula Roth.	0	n	1	1	Betulaceae Gray
Iz_4_1009	DJI_0300	Betula pendula Roth.	0	n	1	0	Poaceae Barnhart
Iz_4_1009	DJI_0300	Bromus inermis Leyss.	0		NA	NA	
Iz_4_1009	DJI_0300	Bromus squarrosus L.	0	a	0	0	Poaceae Barnhart
Iz_4_1009	DJI_0300	Bromus tectorum L.	0	a	0	0	Poaceae Barnhart
Iz_4_1009	DJI_0300	Calamagrostis epigejos (L.) Roth	1	n	1	0	Poaceae Barnhart
Iz_4_1009	DJI_0300	Carduus acanthoides L.	0	n	1	0	Asteraceae Bercht. & J.Presl
Iz_4_1009	DJI_0300	Carex sp.	5	n	1	0	Cyperaceae Juss.
Iz_4_1009	DJI_0300	Centaurea majorovii Dumbadze	0	n	1	0	Asteraceae Bercht. & J.Presl
Iz_4_1009	DJI_0300	Chamaecytisus ruthenicus (Fisch.ex Wol.) Klaskova	0	n	1	0	Fabaceae Lindl.
Iz_4_1009	DJI_0300	Chelidonium majus L.	0	n	1	0	Papaveraceae Juss.
Iz_4_1009	DJI_0300	Chenopodium betaceum Andrz.	0	n	1	0	Amaranthaceae Juss.
Iz_4_1009	DJI_0300	Chenopodium cf. album	0	n	1	0	Amaranthaceae Juss.
Iz_4_1009	DJI_0300	Chenopodium sp.	0		NA	0	Amaranthaceae Juss.
Iz_4_1009	DJI_0300	Chondrilla juncea L.	0	n	1	0	Asteraceae Bercht. & J.Presl
Iz_4_1009	DJI_0300	Cirsium vulgare (Savi) Ten.	0	n	1	0	Asteraceae Bercht. & J.Presl
Iz_4_1009	DJI_0300	Clinopodium acinos (L.) Kuntze	0	n	1	0	Lamiacea Lindl.
Iz_4_1009	DJI_0300	Convallaria majalis L.	0	n	1	0	Asparagaceae Juss.

Iz_4_1009	DJI_0300	Crataegus rhipidophylla Gand.	0	n	1	1	Rosaceae Juss.
Iz_4_1009	DJI_0300	Crepis foetida L.	0	n	1	0	Asteraceae Bercht. & J.Presl
Iz_4_1009	DJI_0300	Dactylis glomerata L.	0	n	1	0	Poaceae Barnhart
Iz_4_1009	DJI_0300	Dianthus campestris M.Bieb.	0	n	1	0	Caryophyllaceae Juss.
Iz_4_1009	DJI_0300	Digitaria sanguinalis (L.) Scop.	0	a	0	0	Poaceae Barnhart
Iz_4_1009	DJI_0300	Elymus repens (L.) Gould	0	n	1	0	Poaceae Barnhart
Iz_4_1009	DJI_0300	Epilobium tetragonum L.	0	n	1	0	Onagraceae Juss.
Iz_4_1009	DJI_0300	Eragrostis minor Host	0	a	0	0	Poaceae Barnhart
Iz_4_1009	DJI_0300	Erigeron canadensis L.	0	a	0	0	Asteraceae Bercht. & J.Presl
Iz_4_1009	DJI_0300	Euphorbia seguieriana Neck.	0	n	1	0	Euphorbiaceae Juss.
Iz_4_1009	DJI_0300	Fallopia dumetorum (L.) Holub	0	n	1	0	Polygonaceae Juss.
Iz_4_1009	DJI_0300	Festuca beckeri (Hack.) Trautv.	0	n	1	0	Poaceae Barnhart
Iz_4_1009	DJI_0300	Fragaria vesca L.	0	n	1	0	Rosaceae Juss.
Iz_4_1009	DJI_0300	Frangula alnus Mill.	0	n	1	1	Rhamnaceae Juss.
Iz_4_1009	DJI_0300	Fraxinus pennsylvanica Marshall	0	a	0	1	Oleaceae Hoffmanns. & Link
Iz_4_1009	DJI_0300	Galeopsis bifida Boenn.	0	n	1	0	Lamiacea Lindl.
Iz_4_1009	DJI_0300	Galium verum L.	0	n	1	0	Rubiaceae Juss.
Iz_4_1009	DJI_0300	Genista tinctoria L.	0	n	1	0	Fabaceae Lindl.
Iz_4_1009	DJI_0300	Geranium robertianum L.	0	n	1	0	Geraniaceae Juss.
Iz_4_1009	DJI_0300	Geranium sanquineum L.	0	n	1	0	Geraniaceae Juss.
Iz_4_1009	DJI_0300	Gypsophila paniculata L.	0	n	1	0	Caryophyllaceae Juss.
Iz_4_1009	DJI_0300	Helichrysum arenarium (L.) Moench	0	n	1	0	Asteraceae Bercht. & J.Presl
Iz_4_1009	DJI_0300	Herniaria polygama J.Gay	0	n	1	0	Caryophyllaceae Juss.
Iz_4_1009	DJI_0300	Hieracium robustum Fr.	0	n	1	0	Asteraceae Bercht. & J.Presl
Iz_4_1009	DJI_0300	Hieracium umbellatum L.	0	n	1	NA	Asteraceae Bercht. & J.Presl
Iz_4_1009	DJI_0300	Hylotelephium maximum (L.) Holub	0	n	1	0	Crassulaceae J.St.-Hil.
Iz_4_1009	DJI_0300	Jacobaea borysthenica (DC.) B.Nord. & Greuter	0	n	1	0	Asteraceae Bercht. & J.Presl
Iz_4_1009	DJI_0300	Jasione montana L.	0	n	1	0	Campanulaceae Juss.
Iz_4_1009	DJI_0300	Jurinea cyanoides (L.) Rchb.s.l.	0	n	1	0	Asteraceae Bercht. & J.Presl
Iz_4_1009	DJI_0300	Koeleria glauca (Spreng.) DC.	0	n	1	0	Poaceae Barnhart
Iz_4_1009	DJI_0300	Lactuca serriola L.	0	a	0	0	Asteraceae Bercht. & J.Presl
Iz_4_1009	DJI_0300	Linaria genistifolia (L.) Mill.	0	n	1	0	Plantaginaceae Juss.
Iz_4_1009	DJI_0300	Lomelosia argentea (L.) Greuter & Burdet	0	n	1	0	Caprifoliaceae Juss.
Iz_4_1009	DJI_0300	Melampyrum pratense L.	0	n	1	0	Orobanchaceae Vent.
Iz_4_1009	DJI_0300	Oenothera biennis L.	0	a	0	0	Onagraceae Juss.
Iz_4_1009	DJI_0300	Pentanema britannica (L.) D.Gut.Larr., Santos-Vicente, Anderb., E.Rico & M.M.Mart.Ort.	0	n	1	0	Asteraceae Bercht. & J.Presl
Iz_4_1009	DJI_0300	Peucedanum oreoselinum (L.) Moench	0	n	1	0	Apiaceae Lindl.
Iz_4_1009	DJI_0300	Peucedanum ruthenicum M.Bieb.	0	n	1	0	Apiaceae Lindl.
Iz_4_1009	DJI_0300	Pilosella echioides (Lumn.) F.W.Schultz & Sch.Bip.	0	n	1	0	Asteraceae Bercht. & J.Presl
Iz_4_1009	DJI_0300	Pilosella officinarum F. Schultz et Sch. Bip.	0	n	1	0	Asteraceae Bercht. & J.Presl
Iz_4_1009	DJI_0300	Pilosella sp.	0		NA	0	Asteraceae Bercht. & J.Presl
Iz_4_1009	DJI_0300	Pinus sylvestris L.	0	n	1	1	Pinaceae Spreng. ex F.Rudolphi
Iz_4_1009	DJI_0300	Poa annua L.	0	n	1	0	Poaceae Barnhart
Iz_4_1009	DJI_0300	Poa nemoralis L.	0	n	1	0	Poaceae Barnhart
Iz_4_1009	DJI_0300	Poa sp.	0		NA	0	Poaceae Barnhart
Iz_4_1009	DJI_0300	Polygonatum odoratum (Mill.) Druce	0	n	1	0	Asparagaceae Juss.
Iz_4_1009	DJI_0300	Polygonum arenarium Waldst. & Kit.	0	n	1	0	Polygonaceae Juss.
Iz_4_1009	DJI_0300	Populus nigra L.	0	n	1	1	Salicaceae Mirb.
Iz_4_1009	DJI_0300	Populus tremula L.	0	n	1	1	Salicaceae Mirb.
Iz_4_1009	DJI_0300	Populus Ã canescens (Aiton) Sm.	0	n	1	1	Salicaceae Mirb.
Iz_4_1009	DJI_0300	Potentilla argentea L.	0	n	1	0	Rosaceae Juss.
Iz_4_1009	DJI_0300	Psammophiliella muralis (L.) Ikonn.	0	n	1	0	Caryophyllaceae Juss.
Iz_4_1009	DJI_0300	Psephellus sumensis (Kalen.) Greuter	0	n	1	0	Asteraceae Bercht. & J.Presl
Iz_4_1009	DJI_0300	Pulsatilla pratensis (L.) Mill. s.l.	0	n	1	0	Ranunculaceae Juss.
Iz_4_1009	DJI_0300	Pyrus communis L.	0	a	0	1	Rosaceae Juss.
Iz_4_1009	DJI_0300	Quercus robur L.	0	n	1	1	Fagaceae Dumort.
Iz_4_1009	DJI_0300	Robinia pseudoacacia L.	0	a	0	1	Fabaceae Lindl.
Iz_4_1009	DJI_0300	Rosa sp.	0		NA	1	Rosaceae Juss.
Iz_4_1009	DJI_0300	Rubus idaeus L.	0	n	1	0	Rosaceae Juss.
Iz_4_1009	DJI_0300	Rumex acetosella L.	0	n	1	0	Polygonaceae Juss.
Iz_4_1009	DJI_0300	Rumex thyrsiflorus Fingerh.	0	n	1	0	Polygonaceae Juss.
Iz_4_1009	DJI_0300	Salix alba L.	0	n	1	1	Salicaceae Mirb.
Iz_4_1009	DJI_0300	Sambucus nigra L.	0	n	1	1	Viburnaceae Raf.
Iz_4_1009	DJI_0300	Secale sylvestre Host	0	n	1	0	Poaceae Barnhart
Iz_4_1009	DJI_0300	Sempervivum ruthenicum Schnittsp. & C.B. Lehm.	0	n	1	0	Crassulaceae J.St.-Hil.
Iz_4_1009	DJI_0300	Setaria viridis (L.) P. Beauv.	0	n	1	0	Poaceae Barnhart
Iz_4_1009	DJI_0300	Silene borysthenica (Gruner) Walters	0	n	1	0	Caryophyllaceae Juss.
Iz_4_1009	DJI_0300	Silene latifolia subsp. alba (Mill.) Greuter & Burdet	0	n	1	0	Caryophyllaceae Juss.
Iz_4_1009	DJI_0300	Solidago virgaurea L.	0	n	1	0	Asteraceae Bercht. & J.Presl
Iz_4_1009	DJI_0300	Sorbus aucuparia L.	0	n	1	1	Rosaceae Juss.
Iz_4_1009	DJI_0300	Stachys recta L.	0	n	1	0	Lamiacea Lindl.
Iz_4_1009	DJI_0300	Tanacetum vulgare L.	0	n	1	0	Asteraceae Bercht. & J.Presl
Iz_4_1009	DJI_0300	Thymus pallasianus Heinr.Braun	0	n	1	0	Lamiacea Lindl.
Iz_4_1009	DJI_0300	Tilia cordata Mill.	0	n	1	1	Malvaceae Juss.
Iz_4_1009	DJI_0300	Trifolium alpestre L.	0	n	1	0	Fabaceae Lindl.
Iz_4_1009	DJI_0300	Trifolium arvense L.	0	n	1	0	Fabaceae Lindl.
Iz_4_1009	DJI_0300	Ulmus glabra Huds.	0	n	1	1	Ulmaceae Mirb.
Iz_4_1009	DJI_0300	Verbascum densiflorum Bertol.	0	n	1	0	Scrophulariaceae Juss.
Iz_4_1009	DJI_0300	Verbascum nigrum L.	0	n	1	0	Scrophulariaceae Juss.
Iz_4_1009	DJI_0300	Veronica austriaca L.	0	n	1	0	Plantaginaceae Juss.
Iz_4_1009	DJI_0300	Veronica incana L.	0	n	1	0	Plantaginaceae Juss.
Iz_4_1009	DJI_0300	Veronica verna L.	0	n	1	0	Plantaginaceae Juss.
Iz_4_1009	DJI_0300	Vincetoxicum hirundinaria Medik	0	n	1	0	Apocynaceae Juss.
Iz_4_1009	DJI_0303	Acer tataricum L.	0	n	1	1	Sapindaceae Juss.
Iz_4_1009	DJI_0303	Achillea millefolium L. s.l.	0	n	1	0	Asteraceae Bercht. & J.Presl
Iz_4_1009	DJI_0303	Allium oleraceum L.	0	n	1	0	Amaryllidaceae J.St.-Hil.
Iz_4_1009	DJI_0303	Ambrosia artemisiifolia L.	0	a	0	0	Asteraceae Bercht. & J.Presl
Iz_4_1009	DJI_0303	Amorpha fruticosa L.	0	a	0	0	Fabaceae Lindl.
Iz_4_1009	DJI_0303	Anthericum ramosum L.	0	n	1	0	Asparagaceae Juss.
Iz_4_1009	DJI_0303	Artemisia marschalliana Spreng.	0	n	1	0	Asteraceae Bercht. & J.Presl
Iz_4_1009	DJI_0303	Asparagus officinalis L.	0	n	1	0	Asparagaceae Juss.
Iz_4_1009	DJI_0303	Astragalus cicer L.	0	n	1	0	Fabaceae Lindl.
Iz_4_1009	DJI_0303	Bassia laniflora (S.G.Gmel.) A.J.Scott	0	n	1	0	Amaranthaceae Juss.
Iz_4_1009	DJI_0303	Berberis vulgaris L.	0	n	1	0	Berberidaceae Juss.
Iz_4_1009	DJI_0303	Betula pendula Roth.	0	n	1	1	Betulaceae Gray
Iz_4_1009	DJI_0303	Betula pendula Roth.	0	n	1	0	Poaceae Barnhart
Iz_4_1009	DJI_0303	Bromus inermis Leyss.	0		NA	NA	
Iz_4_1009	DJI_0303	Bromus squarrosus L.	0	a	0	0	Poaceae Barnhart

Iz_4_1009	DJI_0303	Bromus tectorum L.	0	a	0	0	Poaceae Barnhart
Iz_4_1009	DJI_0303	Calamagrostis epigejos (L.) Roth	40	n	1	0	Poaceae Barnhart
Iz_4_1009	DJI_0303	Carduus acanthoides L.	0	n	1	0	Asteraceae Bercht. & J.Presl
Iz_4_1009	DJI_0303	Carex sp.	0	n	1	0	Cyperaceae Juss.
Iz_4_1009	DJI_0303	Centaurea majorovii Dumbadze	0	n	1	0	Asteraceae Bercht. & J.Presl
Iz_4_1009	DJI_0303	Chamaecytisus ruthenicus (Fisch.ex Wol.) Klaskova	0	n	1	0	Fabaceae Lindl.
Iz_4_1009	DJI_0303	Chelidonium majus L.	0	n	1	0	Papaveraceae Juss.
Iz_4_1009	DJI_0303	Chenopodium betaceum Andrz.	0	n	1	0	Amaranthaceae Juss.
Iz_4_1009	DJI_0303	Chenopodium cf. album	0	n	1	0	Amaranthaceae Juss.
Iz_4_1009	DJI_0303	Chenopodium sp.	0		NA	0	Amaranthaceae Juss.
Iz_4_1009	DJI_0303	Chondrilla juncea L.	0	n	1	0	Asteraceae Bercht. & J.Presl
Iz_4_1009	DJI_0303	Cirsium vulgare (Savi) Ten.	0	n	1	0	Asteraceae Bercht. & J.Presl
Iz_4_1009	DJI_0303	Clinopodium acinos (L.) Kuntze	0	n	1	0	Lamiacea Lindl.
Iz_4_1009	DJI_0303	Convallaria majalis L.	0	n	1	0	Asparagaceae Juss.
Iz_4_1009	DJI_0303	Crataegus rhipidophylla Gand.	0	n	1	1	Rosaceae Juss.
Iz_4_1009	DJI_0303	Crepis foetida L.	0	n	1	0	Asteraceae Bercht. & J.Presl
Iz_4_1009	DJI_0303	Dactylis glomerata L.	0	n	1	0	Poaceae Barnhart
Iz_4_1009	DJI_0303	Dianthus campestris M.Bieb.	0	n	1	0	Caryophyllaceae Juss.
Iz_4_1009	DJI_0303	Digitaria sanguinalis (L.) Scop.	0	a	0	0	Poaceae Barnhart
Iz_4_1009	DJI_0303	Elymus repens (L.) Gould	0	n	1	0	Poaceae Barnhart
Iz_4_1009	DJI_0303	Epilobium tetragonum L.	0	n	1	0	Onagraceae Juss.
Iz_4_1009	DJI_0303	Eragrostis minor Host	0	a	0	0	Poaceae Barnhart
Iz_4_1009	DJI_0303	Erigeron canadensis L.	0	a	0	0	Asteraceae Bercht. & J.Presl
Iz_4_1009	DJI_0303	Euphorbia seguieriana Neck.	0	n	1	0	Euphorbiaceae Juss.
Iz_4_1009	DJI_0303	Fallopia dumetorum (L.) Holub	0	n	1	0	Polygonaceae Juss.
Iz_4_1009	DJI_0303	Festuca beckeri (Hack.) Trautv.	0	n	1	0	Poaceae Barnhart
Iz_4_1009	DJI_0303	Fragaria vesca L.	0	n	1	0	Rosaceae Juss.
Iz_4_1009	DJI_0303	Frangula alnus Mill.	0	n	1	1	Rhamnaceae Juss.
Iz_4_1009	DJI_0303	Fraxinus pennsylvanica Marshall	0	a	0	1	Oleaceae Hoffmanns. & Link
Iz_4_1009	DJI_0303	Galeopsis bifida Boenn.	0	n	1	0	Lamiacea Lindl.
Iz_4_1009	DJI_0303	Galium verum L.	0	n	1	0	Rubiaceae Juss.
Iz_4_1009	DJI_0303	Genista tinctoria L.	0	n	1	0	Fabaceae Lindl.
Iz_4_1009	DJI_0303	Geranium robertianum L.	0	n	1	0	Geraniaceae Juss.
Iz_4_1009	DJI_0303	Geranium sanquineum L.	0	n	1	0	Geraniaceae Juss.
Iz_4_1009	DJI_0303	Gypsophila paniculata L.	0	n	1	0	Caryophyllaceae Juss.
Iz_4_1009	DJI_0303	Helichrysum arenarium (L.) Moench	0	n	1	0	Asteraceae Bercht. & J.Presl
Iz_4_1009	DJI_0303	Herniaria polygama J.Gay	0	n	1	0	Caryophyllaceae Juss.
Iz_4_1009	DJI_0303	Hieracium robustum Fr.	0	n	1	0	Asteraceae Bercht. & J.Presl
Iz_4_1009	DJI_0303	Hieracium umbellatum L.	2	n	1	NA	Asteraceae Bercht. & J.Presl
Iz_4_1009	DJI_0303	Hylotelephium maximum (L.) Holub	0	n	1	0	Crassulaceae J.St.-Hil.
Iz_4_1009	DJI_0303	Jacobaea borysthenica (DC.) B.Nord. & Greuter	0	n	1	0	Asteraceae Bercht. & J.Presl
Iz_4_1009	DJI_0303	Jasione montana L.	0	n	1	0	Campanulaceae Juss.
Iz_4_1009	DJI_0303	Jurinea cyanoides (L.) Rchb.s.l.	0	n	1	0	Asteraceae Bercht. & J.Presl
Iz_4_1009	DJI_0303	Koeleria glauca (Spreng.) DC.	0	n	1	0	Poaceae Barnhart
Iz_4_1009	DJI_0303	Lactuca serriola L.	0	a	0	0	Asteraceae Bercht. & J.Presl
Iz_4_1009	DJI_0303	Linaria genistifolia (L.) Mill.	0	n	1	0	Plantaginaceae Juss.
Iz_4_1009	DJI_0303	Lomelosia argentea (L.) Greuter & Burdet	0	n	1	0	Caprifoliaceae Juss.
Iz_4_1009	DJI_0303	Melampyrum pratense L.	0	n	1	0	Orobanchaceae Vent.
Iz_4_1009	DJI_0303	Oenothera biennis L.	0	a	0	0	Onagraceae Juss.
Iz_4_1009	DJI_0303	Pentanema britannica (L.) D.Gut.Larr., Santos-Vicente, Anderb., E.Rico & M.M.Mart.Ort.	0	n	1	0	Asteraceae Bercht. & J.Presl
Iz_4_1009	DJI_0303	Peucedanum oreoselinum (L.) Moench	0	n	1	0	Apiaceae Lindl.
Iz_4_1009	DJI_0303	Peucedanum ruthenicum M.Bieb.	0	n	1	0	Apiaceae Lindl.
Iz_4_1009	DJI_0303	Pilosella echioides (Lumn.) F.W.Schultz & Sch.Bip.	0	n	1	0	Asteraceae Bercht. & J.Presl
Iz_4_1009	DJI_0303	Pilosella officinarum F. Schultz et Sch. Bip.	2	n	1	0	Asteraceae Bercht. & J.Presl
Iz_4_1009	DJI_0303	Pilosella sp.	0		NA	0	Asteraceae Bercht. & J.Presl
Iz_4_1009	DJI_0303	Pinus sylvestris L.	0	n	1	1	Pinaceae Spreng. ex F.Rudolphi
Iz_4_1009	DJI_0303	Poa annua L.	0	n	1	0	Poaceae Barnhart
Iz_4_1009	DJI_0303	Poa nemoralis L.	0	n	1	0	Poaceae Barnhart
Iz_4_1009	DJI_0303	Poa sp.	0		NA	0	Poaceae Barnhart
Iz_4_1009	DJI_0303	Polygonatum odoratum (Mill.) Druce	0	n	1	0	Asparagaceae Juss.
Iz_4_1009	DJI_0303	Polygonum arenarium Waldst. & Kit.	0	n	1	0	Polygonaceae Juss.
Iz_4_1009	DJI_0303	Populus nigra L.	0	n	1	1	Salicaceae Mirb.
Iz_4_1009	DJI_0303	Populus tremula L.	0	n	1	1	Salicaceae Mirb.
Iz_4_1009	DJI_0303	Populus Ã canescens (Aiton) Sm.	0	n	1	1	Salicaceae Mirb.
Iz_4_1009	DJI_0303	Potentilla argentea L.	0	n	1	0	Rosaceae Juss.
Iz_4_1009	DJI_0303	Psammophiliella muralis (L.) Ikonn.	0	n	1	0	Caryophyllaceae Juss.
Iz_4_1009	DJI_0303	Psephellus sumensis (Kalen.) Greuter	0	n	1	0	Asteraceae Bercht. & J.Presl
Iz_4_1009	DJI_0303	Pulsatilla pratensis (L.) Mill. s.l.	0	n	1	0	Ranunculaceae Juss.
Iz_4_1009	DJI_0303	Pyrus communis L.	0	a	0	1	Rosaceae Juss.
Iz_4_1009	DJI_0303	Quercus robur L.	0	n	1	1	Fagaceae Dumort.
Iz_4_1009	DJI_0303	Robinia pseudoacacia L.	0	a	0	1	Fabaceae Lindl.
Iz_4_1009	DJI_0303	Rosa sp.	0		NA	1	Rosaceae Juss.
Iz_4_1009	DJI_0303	Rubus idaeus L.	0	n	1	0	Rosaceae Juss.
Iz_4_1009	DJI_0303	Rumex acetosella L.	1	n	1	0	Polygonaceae Juss.
Iz_4_1009	DJI_0303	Rumex thyrsiflorus Fingerh.	0	n	1	0	Polygonaceae Juss.
Iz_4_1009	DJI_0303	Salix alba L.	0	n	1	1	Salicaceae Mirb.
Iz_4_1009	DJI_0303	Sambucus nigra L.	0	n	1	1	Viburnaceae Raf.
Iz_4_1009	DJI_0303	Secale sylvestre Host	0	n	1	0	Poaceae Barnhart
Iz_4_1009	DJI_0303	Sempervivum ruthenicum Schnittsp. & C.B. Lehm.	0	n	1	0	Crassulaceae J.St.-Hil.
Iz_4_1009	DJI_0303	Setaria viridis (L.) P. Beauv.	0	n	1	0	Poaceae Barnhart
Iz_4_1009	DJI_0303	Silene borysthenica (Gruner) Walters	0	n	1	0	Caryophyllaceae Juss.
Iz_4_1009	DJI_0303	Silene latifolia subsp. alba (Mill.) Greuter & Burdet	0	n	1	0	Caryophyllaceae Juss.
Iz_4_1009	DJI_0303	Solidago virgaurea L.	0	n	1	0	Asteraceae Bercht. & J.Presl
Iz_4_1009	DJI_0303	Sorbus aucuparia L.	0	n	1	1	Rosaceae Juss.
Iz_4_1009	DJI_0303	Stachys recta L.	0	n	1	0	Lamiacea Lindl.
Iz_4_1009	DJI_0303	Tanacetum vulgare L.	0	n	1	0	Asteraceae Bercht. & J.Presl
Iz_4_1009	DJI_0303	Thymus pallasianus Heinr.Braun	0	n	1	0	Lamiacea Lindl.
Iz_4_1009	DJI_0303	Tilia cordata Mill.	0	n	1	1	Malvaceae Juss.
Iz_4_1009	DJI_0303	Trifolium alpestre L.	0	n	1	0	Fabaceae Lindl.
Iz_4_1009	DJI_0303	Trifolium arvense L.	0	n	1	0	Fabaceae Lindl.
Iz_4_1009	DJI_0303	Ulmus glabra Huds.	0	n	1	1	Ulmaceae Mirb.
Iz_4_1009	DJI_0303	Verbascum densiflorum Bertol.	0	n	1	0	Scrophulariaceae Juss.
Iz_4_1009	DJI_0303	Verbascum nigrum L.	0	n	1	0	Scrophulariaceae Juss.
Iz_4_1009	DJI_0303	Veronica austriaca L.	0	n	1	0	Plantaginaceae Juss.
Iz_4_1009	DJI_0303	Veronica incana L.	0	n	1	0	Plantaginaceae Juss.
Iz_4_1009	DJI_0303	Veronica verna L.	0	n	1	0	Plantaginaceae Juss.
Iz_4_1009	DJI_0303	Vincetoxicum hirundinaria Medik	0	n	1	0	Apocynaceae Juss.
Iz_4_1009	DJI_0309	Acer tataricum L.	0	n	1	1	Sapindaceae Juss.

Iz_4_1009	DJI_0309	Achillea millefolium L. s.l.	0	n	1	0	Asteraceae Bercht. & J.Presl
Iz_4_1009	DJI_0309	Allium oleraceum L.	0	n	1	0	Amaryllidaceae J.St.-Hil.
Iz_4_1009	DJI_0309	Ambrosia artemisiifolia L.	0	a	0	0	Asteraceae Bercht. & J.Presl
Iz_4_1009	DJI_0309	Amorpha fruticosa L.	0	a	0	0	Fabaceae Lindl.
Iz_4_1009	DJI_0309	Anthericum ramosum L.	0	n	1	0	Asparagaceae Juss.
Iz_4_1009	DJI_0309	Artemisia marschalliana Spreng.	0	n	1	0	Asteraceae Bercht. & J.Presl
Iz_4_1009	DJI_0309	Asparagus officinalis L.	0	n	1	0	Asparagaceae Juss.
Iz_4_1009	DJI_0309	Astragalus cicer L.	0	n	1	0	Fabaceae Lindl.
Iz_4_1009	DJI_0309	Bassia laniflora (S.G.Gmel.) A.J.Scott	0	n	1	0	Amaranthaceae Juss.
Iz_4_1009	DJI_0309	Berberis vulgaris L.	0	n	1	0	Berberidaceae Juss.
Iz_4_1009	DJI_0309	Betula pendula Roth.	0	n	1	1	Betulaceae Gray
Iz_4_1009	DJI_0309	Betula pendula Roth.	0	n	1	0	Poaceae Barnhart
Iz_4_1009	DJI_0309	Bromus inermis Leyss.	0		NA	NA	
Iz_4_1009	DJI_0309	Bromus squarrosus L.	0	a	0	0	Poaceae Barnhart
Iz_4_1009	DJI_0309	Bromus tectorum L.	0	a	0	0	Poaceae Barnhart
Iz_4_1009	DJI_0309	Calamagrostis epigejos (L.) Roth	2	n	1	0	Poaceae Barnhart
Iz_4_1009	DJI_0309	Carduus acanthoides L.	0	n	1	0	Asteraceae Bercht. & J.Presl
Iz_4_1009	DJI_0309	Carex sp.	0	n	1	0	Cyperaceae Juss.
Iz_4_1009	DJI_0309	Centaurea majorovii Dumbadze	0	n	1	0	Asteraceae Bercht. & J.Presl
Iz_4_1009	DJI_0309	Chamaecytisus ruthenicus (Fisch.ex Wol.) Klaskova	0	n	1	0	Fabaceae Lindl.
Iz_4_1009	DJI_0309	Chelidonium majus L.	0	n	1	0	Papaveraceae Juss.
Iz_4_1009	DJI_0309	Chenopodium betaceum Andrz.	0	n	1	0	Amaranthaceae Juss.
Iz_4_1009	DJI_0309	Chenopodium cf. album	0	n	1	0	Amaranthaceae Juss.
Iz_4_1009	DJI_0309	Chenopodium sp.	0		NA	0	Amaranthaceae Juss.
Iz_4_1009	DJI_0309	Chondrilla juncea L.	0	n	1	0	Asteraceae Bercht. & J.Presl
Iz_4_1009	DJI_0309	Cirsium vulgare (Savi) Ten.	0	n	1	0	Asteraceae Bercht. & J.Presl
Iz_4_1009	DJI_0309	Clinopodium acinos (L.) Kuntze	0	n	1	0	Lamiacea Lindl.
Iz_4_1009	DJI_0309	Convallaria majalis L.	0	n	1	0	Asparagaceae Juss.
Iz_4_1009	DJI_0309	Crataegus rhipidophylla Gand.	0	n	1	1	Rosaceae Juss.
Iz_4_1009	DJI_0309	Crepis foetida L.	0	n	1	0	Asteraceae Bercht. & J.Presl
Iz_4_1009	DJI_0309	Dactylis glomerata L.	0	n	1	0	Poaceae Barnhart
Iz_4_1009	DJI_0309	Dianthus campestris M.Bieb.	0	n	1	0	Caryophyllaceae Juss.
Iz_4_1009	DJI_0309	Digitaria sanguinalis (L.) Scop.	0	a	0	0	Poaceae Barnhart
Iz_4_1009	DJI_0309	Elymus repens (L.) Gould	0	n	1	0	Poaceae Barnhart
Iz_4_1009	DJI_0309	Epilobium tetragonum L.	0	n	1	0	Onagraceae Juss.
Iz_4_1009	DJI_0309	Eragrostis minor Host	0	a	0	0	Poaceae Barnhart
Iz_4_1009	DJI_0309	Erigeron canadensis L.	10	a	0	0	Asteraceae Bercht. & J.Presl
Iz_4_1009	DJI_0309	Euphorbia seguieriana Neck.	0	n	1	0	Euphorbiaceae Juss.
Iz_4_1009	DJI_0309	Fallopia dumetorum (L.) Holub	0	n	1	0	Polygonaceae Juss.
Iz_4_1009	DJI_0309	Festuca beckeri (Hack.) Trautv.	0	n	1	0	Poaceae Barnhart
Iz_4_1009	DJI_0309	Fragaria vesca L.	0	n	1	0	Rosaceae Juss.
Iz_4_1009	DJI_0309	Frangula alnus Mill.	0	n	1	1	Rhamnaceae Juss.
Iz_4_1009	DJI_0309	Fraxinus pennsylvanica Marshall	0	a	0	1	Oleaceae Hoffmanns. & Link
Iz_4_1009	DJI_0309	Galeopsis bifida Boenn.	0	n	1	0	Lamiacea Lindl.
Iz_4_1009	DJI_0309	Galium verum L.	0	n	1	0	Rubiaceae Juss.
Iz_4_1009	DJI_0309	Genista tinctoria L.	0	n	1	0	Fabaceae Lindl.
Iz_4_1009	DJI_0309	Geranium robertianum L.	0	n	1	0	Geraniaceae Juss.
Iz_4_1009	DJI_0309	Geranium sanquineum L.	0	n	1	0	Geraniaceae Juss.
Iz_4_1009	DJI_0309	Gypsophila paniculata L.	0	n	1	0	Caryophyllaceae Juss.
Iz_4_1009	DJI_0309	Helichrysum arenarium (L.) Moench	0	n	1	0	Asteraceae Bercht. & J.Presl
Iz_4_1009	DJI_0309	Herniaria polygama J.Gay	0	n	1	0	Caryophyllaceae Juss.
Iz_4_1009	DJI_0309	Hieracium robustum Fr.	0	n	1	0	Asteraceae Bercht. & J.Presl
Iz_4_1009	DJI_0309	Hieracium umbellatum L.	0	n	1	NA	Asteraceae Bercht. & J.Presl
Iz_4_1009	DJI_0309	Hylotelephium maximum (L.) Holub	0	n	1	0	Crassulaceae J.St.-Hil.
Iz_4_1009	DJI_0309	Jacobaea borysthenica (DC.) B.Nord. & Greuter	0	n	1	0	Asteraceae Bercht. & J.Presl
Iz_4_1009	DJI_0309	Jasione montana L.	0	n	1	0	Campanulaceae Juss.
Iz_4_1009	DJI_0309	Jurinea cyanoides (L.) Rchb.s.l.	0	n	1	0	Asteraceae Bercht. & J.Presl
Iz_4_1009	DJI_0309	Koeleria glauca (Spreng.) DC.	0	n	1	0	Poaceae Barnhart
Iz_4_1009	DJI_0309	Lactuca serriola L.	0	a	0	0	Asteraceae Bercht. & J.Presl
Iz_4_1009	DJI_0309	Linaria genistifolia (L.) Mill.	0	n	1	0	Plantaginaceae Juss.
Iz_4_1009	DJI_0309	Lomelosia argentea (L.) Greuter & Burdet	0	n	1	0	Caprifoliaceae Juss.
Iz_4_1009	DJI_0309	Melampyrum pratense L.	0	n	1	0	Orobanchaceae Vent.
Iz_4_1009	DJI_0309	Oenothera biennis L.	0	a	0	0	Onagraceae Juss.
Iz_4_1009	DJI_0309	Pentanema britannica (L.) D.Gut.Larr., Santos-Vicente, Anderb., E.Rico & M.M.Mart.Ort.	0	n	1	0	Asteraceae Bercht. & J.Presl
Iz_4_1009	DJI_0309	Peucedanum oreoselinum (L.) Moench	0	n	1	0	Apiaceae Lindl.
Iz_4_1009	DJI_0309	Peucedanum ruthenicum M.Bieb.	0	n	1	0	Apiaceae Lindl.
Iz_4_1009	DJI_0309	Pilosella echioides (Lumn.) F.W.Schultz & Sch.Bip.	0	n	1	0	Asteraceae Bercht. & J.Presl
Iz_4_1009	DJI_0309	Pilosella officinarum F. Schultz et Sch. Bip.	6	n	1	0	Asteraceae Bercht. & J.Presl
Iz_4_1009	DJI_0309	Pilosella sp.	0		NA	0	Asteraceae Bercht. & J.Presl
Iz_4_1009	DJI_0309	Pinus sylvestris L.	0	n	1	1	Pinaceae Spreng. ex F.Rudolphi
Iz_4_1009	DJI_0309	Poa annua L.	0	n	1	0	Poaceae Barnhart
Iz_4_1009	DJI_0309	Poa nemoralis L.	0	n	1	0	Poaceae Barnhart
Iz_4_1009	DJI_0309	Poa sp.	0		NA	0	Poaceae Barnhart
Iz_4_1009	DJI_0309	Polygonatum odoratum (Mill.) Druce	0	n	1	0	Asparagaceae Juss.
Iz_4_1009	DJI_0309	Polygonum arenarium Waldst. & Kit.	0	n	1	0	Polygonaceae Juss.
Iz_4_1009	DJI_0309	Populus nigra L.	0	n	1	1	Salicaceae Mirb.
Iz_4_1009	DJI_0309	Populus tremula L.	0	n	1	1	Salicaceae Mirb.
Iz_4_1009	DJI_0309	Populus Ã canescens (Aiton) Sm.	0	n	1	1	Salicaceae Mirb.
Iz_4_1009	DJI_0309	Potentilla argentea L.	0	n	1	0	Rosaceae Juss.
Iz_4_1009	DJI_0309	Psammophiliella muralis (L.) Ikonn.	0	n	1	0	Caryophyllaceae Juss.
Iz_4_1009	DJI_0309	Psephellus sumensis (Kalen.) Greuter	0	n	1	0	Asteraceae Bercht. & J.Presl
Iz_4_1009	DJI_0309	Pulsatilla pratensis (L.) Mill. s.l.	0	n	1	0	Ranunculaceae Juss.
Iz_4_1009	DJI_0309	Pyrus communis L.	0	a	0	1	Rosaceae Juss.
Iz_4_1009	DJI_0309	Quercus robur L.	0	n	1	1	Fagaceae Dumort.
Iz_4_1009	DJI_0309	Robinia pseudoacacia L.	0	a	0	1	Fabaceae Lindl.
Iz_4_1009	DJI_0309	Rosa sp.	0		NA	1	Rosaceae Juss.
Iz_4_1009	DJI_0309	Rubus idaeus L.	0	n	1	0	Rosaceae Juss.
Iz_4_1009	DJI_0309	Rumex acetosella L.	0	n	1	0	Polygonaceae Juss.
Iz_4_1009	DJI_0309	Rumex thyrsiflorus Fingerh.	0	n	1	0	Polygonaceae Juss.
Iz_4_1009	DJI_0309	Salix alba L.	0	n	1	1	Salicaceae Mirb.
Iz_4_1009	DJI_0309	Sambucus nigra L.	0	n	1	1	Viburnaceae Raf.
Iz_4_1009	DJI_0309	Secale sylvestre Host	0	n	1	0	Poaceae Barnhart
Iz_4_1009	DJI_0309	Sempervivum ruthenicum Schnittsp. & C.B. Lehm.	0	n	1	0	Crassulaceae J.St.-Hil.
Iz_4_1009	DJI_0309	Setaria viridis (L.) P. Beauv.	0	n	1	0	Poaceae Barnhart
Iz_4_1009	DJI_0309	Silene borysthenica (Gruner) Walters	0	n	1	0	Caryophyllaceae Juss.
Iz_4_1009	DJI_0309	Silene latifolia subsp. alba (Mill.) Greuter & Burdet	0	n	1	0	Caryophyllaceae Juss.
Iz_4_1009	DJI_0309	Solidago virgaurea L.	0	n	1	0	Asteraceae Bercht. & J.Presl
Iz_4_1009	DJI_0309	Sorbus aucuparia L.	0	n	1	1	Rosaceae Juss.

Iz_4_1009	DJI_0309	Stachys recta L.	0	n	1	0	Lamiacea Lindl.
Iz_4_1009	DJI_0309	Tanacetum vulgare L.	0	n	1	0	Asteraceae Bercht. & J.Presl
Iz_4_1009	DJI_0309	Thymus pallasianus Heinr.Braun	0	n	1	0	Lamiacea Lindl.
Iz_4_1009	DJI_0309	Tilia cordata Mill.	0	n	1	1	Malvaceae Juss.
Iz_4_1009	DJI_0309	Trifolium alpestre L.	0	n	1	0	Fabaceae Lindl.
Iz_4_1009	DJI_0309	Trifolium arvense L.	0	n	1	0	Fabaceae Lindl.
Iz_4_1009	DJI_0309	Ulmus glabra Huds.	0	n	1	1	Ulmaceae Mirb.
Iz_4_1009	DJI_0309	Verbascum densiflorum Bertol.	0	n	1	0	Scrophulariaceae Juss.
Iz_4_1009	DJI_0309	Verbascum nigrum L.	0.2	n	1	0	Scrophulariaceae Juss.
Iz_4_1009	DJI_0309	Veronica austriaca L.	0	n	1	0	Plantaginaceae Juss.
Iz_4_1009	DJI_0309	Veronica incana L.	0	n	1	0	Plantaginaceae Juss.
Iz_4_1009	DJI_0309	Veronica verna L.	0	n	1	0	Plantaginaceae Juss.
Iz_4_1009	DJI_0309	Vincetoxicum hirundinaria Medik	0	n	1	0	Apocynaceae Juss.
Iz_4_1009	DJI_0314	Acer tataricum L.	0	n	1	1	Sapindaceae Juss.
Iz_4_1009	DJI_0314	Achillea millefolium L. s.l.	0	n	1	0	Asteraceae Bercht. & J.Presl
Iz_4_1009	DJI_0314	Allium oleraceum L.	0	n	1	0	Amaryllidaceae J.St.-Hil.
Iz_4_1009	DJI_0314	Ambrosia artemisiifolia L.	0	a	0	0	Asteraceae Bercht. & J.Presl
Iz_4_1009	DJI_0314	Amorpha fruticosa L.	0	a	0	0	Fabaceae Lindl.
Iz_4_1009	DJI_0314	Anthericum ramosum L.	0	n	1	0	Asparagaceae Juss.
Iz_4_1009	DJI_0314	Artemisia marschalliana Spreng.	0	n	1	0	Asteraceae Bercht. & J.Presl
Iz_4_1009	DJI_0314	Asparagus officinalis L.	0	n	1	0	Asparagaceae Juss.
Iz_4_1009	DJI_0314	Astragalus cicer L.	0	n	1	0	Fabaceae Lindl.
Iz_4_1009	DJI_0314	Bassia laniflora (S.G.Gmel.) A.J.Scott	0	n	1	0	Amaranthaceae Juss.
Iz_4_1009	DJI_0314	Berberis vulgaris L.	0	n	1	0	Berberidaceae Juss.
Iz_4_1009	DJI_0314	Betula pendula Roth.	0	n	1	1	Betulaceae Gray
Iz_4_1009	DJI_0314	Betula pendula Roth.	0	n	1	0	Poaceae Barnhart
Iz_4_1009	DJI_0314	Bromus inermis Leyss.	0		NA	NA	
Iz_4_1009	DJI_0314	Bromus squarrosus L.	0	a	0	0	Poaceae Barnhart
Iz_4_1009	DJI_0314	Bromus tectorum L.	0	a	0	0	Poaceae Barnhart
Iz_4_1009	DJI_0314	Calamagrostis epigejos (L.) Roth	25	n	1	0	Poaceae Barnhart
Iz_4_1009	DJI_0314	Carduus acanthoides L.	0	n	1	0	Asteraceae Bercht. & J.Presl
Iz_4_1009	DJI_0314	Carex sp.	0	n	1	0	Cyperaceae Juss.
Iz_4_1009	DJI_0314	Centaurea majorovii Dumbadze	0	n	1	0	Asteraceae Bercht. & J.Presl
Iz_4_1009	DJI_0314	Chamaecytisus ruthenicus (Fisch.ex Wol.) Klaskova	0	n	1	0	Fabaceae Lindl.
Iz_4_1009	DJI_0314	Chelidonium majus L.	0	n	1	0	Papaveraceae Juss.
Iz_4_1009	DJI_0314	Chenopodium betaceum Andrz.	0	n	1	0	Amaranthaceae Juss.
Iz_4_1009	DJI_0314	Chenopodium cf. album	0	n	1	0	Amaranthaceae Juss.
Iz_4_1009	DJI_0314	Chenopodium sp.	0		NA	0	Amaranthaceae Juss.
Iz_4_1009	DJI_0314	Chondrilla juncea L.	0	n	1	0	Asteraceae Bercht. & J.Presl
Iz_4_1009	DJI_0314	Cirsium vulgare (Savi) Ten.	0	n	1	0	Asteraceae Bercht. & J.Presl
Iz_4_1009	DJI_0314	Clinopodium acinos (L.) Kuntze	0	n	1	0	Lamiacea Lindl.
Iz_4_1009	DJI_0314	Convallaria majalis L.	0	n	1	0	Asparagaceae Juss.
Iz_4_1009	DJI_0314	Crataegus rhipidophylla Gand.	0	n	1	1	Rosaceae Juss.
Iz_4_1009	DJI_0314	Crepis foetida L.	0	n	1	0	Asteraceae Bercht. & J.Presl
Iz_4_1009	DJI_0314	Dactylis glomerata L.	0	n	1	0	Poaceae Barnhart
Iz_4_1009	DJI_0314	Dianthus campestris M.Bieb.	0	n	1	0	Caryophyllaceae Juss.
Iz_4_1009	DJI_0314	Digitaria sanguinalis (L.) Scop.	0	a	0	0	Poaceae Barnhart
Iz_4_1009	DJI_0314	Elymus repens (L.) Gould	0	n	1	0	Poaceae Barnhart
Iz_4_1009	DJI_0314	Epilobium tetragonum L.	0	n	1	0	Onagraceae Juss.
Iz_4_1009	DJI_0314	Eragrostis minor Host	0	a	0	0	Poaceae Barnhart
Iz_4_1009	DJI_0314	Erigeron canadensis L.	1	a	0	0	Asteraceae Bercht. & J.Presl
Iz_4_1009	DJI_0314	Euphorbia seguieriana Neck.	0	n	1	0	Euphorbiaceae Juss.
Iz_4_1009	DJI_0314	Fallopia dumetorum (L.) Holub	0	n	1	0	Polygonaceae Juss.
Iz_4_1009	DJI_0314	Festuca beckeri (Hack.) Trautv.	0	n	1	0	Poaceae Barnhart
Iz_4_1009	DJI_0314	Fragaria vesca L.	0	n	1	0	Rosaceae Juss.
Iz_4_1009	DJI_0314	Frangula alnus Mill.	0	n	1	1	Rhamnaceae Juss.
Iz_4_1009	DJI_0314	Fraxinus pennsylvanica Marshall	0	a	0	1	Oleaceae Hoffmanns. & Link
Iz_4_1009	DJI_0314	Galeopsis bifida Boenn.	0	n	1	0	Lamiacea Lindl.
Iz_4_1009	DJI_0314	Galium verum L.	0	n	1	0	Rubiaceae Juss.
Iz_4_1009	DJI_0314	Genista tinctoria L.	0	n	1	0	Fabaceae Lindl.
Iz_4_1009	DJI_0314	Geranium robertianum L.	0	n	1	0	Geraniaceae Juss.
Iz_4_1009	DJI_0314	Geranium sanquineum L.	0	n	1	0	Geraniaceae Juss.
Iz_4_1009	DJI_0314	Gypsophila paniculata L.	0	n	1	0	Caryophyllaceae Juss.
Iz_4_1009	DJI_0314	Helichrysum arenarium (L.) Moench	0	n	1	0	Asteraceae Bercht. & J.Presl
Iz_4_1009	DJI_0314	Herniaria polygama J.Gay	0	n	1	0	Caryophyllaceae Juss.
Iz_4_1009	DJI_0314	Hieracium robustum Fr.	0	n	1	0	Asteraceae Bercht. & J.Presl
Iz_4_1009	DJI_0314	Hieracium umbellatum L.	0	n	1	NA	Asteraceae Bercht. & J.Presl
Iz_4_1009	DJI_0314	Hylotelephium maximum (L.) Holub	0	n	1	0	Crassulaceae J.St.-Hil.
Iz_4_1009	DJI_0314	Jacobaea borysthenica (DC.) B.Nord. & Greuter	0	n	1	0	Asteraceae Bercht. & J.Presl
Iz_4_1009	DJI_0314	Jasione montana L.	0	n	1	0	Campanulaceae Juss.
Iz_4_1009	DJI_0314	Jurinea cyanoides (L.) Rchb.s.l.	0	n	1	0	Asteraceae Bercht. & J.Presl
Iz_4_1009	DJI_0314	Koeleria glauca (Spreng.) DC.	0	n	1	0	Poaceae Barnhart
Iz_4_1009	DJI_0314	Lactuca serriola L.	0	a	0	0	Asteraceae Bercht. & J.Presl
Iz_4_1009	DJI_0314	Linaria genistifolia (L.) Mill.	0	n	1	0	Plantaginaceae Juss.
Iz_4_1009	DJI_0314	Lomelosia argentea (L.) Greuter & Burdet	0	n	1	0	Caprifoliaceae Juss.
Iz_4_1009	DJI_0314	Melampyrum pratense L.	0	n	1	0	Orobanchaceae Vent.
Iz_4_1009	DJI_0314	Oenothera biennis L.	0	a	0	0	Onagraceae Juss.
Iz_4_1009	DJI_0314	Pentanema britannica (L.) D.Gut.Larr., Santos-Vicente, Anderb., E.Rico & M.M.Mart.Ort.	0	n	1	0	Asteraceae Bercht. & J.Presl
Iz_4_1009	DJI_0314	Peucedanum oreoselinum (L.) Moench	0	n	1	0	Apiaceae Lindl.
Iz_4_1009	DJI_0314	Peucedanum ruthenicum M.Bieb.	0	n	1	0	Apiaceae Lindl.
Iz_4_1009	DJI_0314	Pilosella echioides (Lumn.) F.W.Schultz & Sch.Bip.	0	n	1	0	Asteraceae Bercht. & J.Presl
Iz_4_1009	DJI_0314	Pilosella officinarum F. Schultz et Sch. Bip.	0	n	1	0	Asteraceae Bercht. & J.Presl
Iz_4_1009	DJI_0314	Pilosella sp.	0		NA	0	Asteraceae Bercht. & J.Presl
Iz_4_1009	DJI_0314	Pinus sylvestris L.	0	n	1	1	Pinaceae Spreng. ex F.Rudolphi
Iz_4_1009	DJI_0314	Poa annua L.	0	n	1	0	Poaceae Barnhart
Iz_4_1009	DJI_0314	Poa nemoralis L.	0	n	1	0	Poaceae Barnhart
Iz_4_1009	DJI_0314	Poa sp.	0		NA	0	Poaceae Barnhart
Iz_4_1009	DJI_0314	Polygonatum odoratum (Mill.) Druce	0	n	1	0	Asparagaceae Juss.
Iz_4_1009	DJI_0314	Polygonum arenarium Waldst. & Kit.	0	n	1	0	Polygonaceae Juss.
Iz_4_1009	DJI_0314	Populus nigra L.	0	n	1	1	Salicaceae Mirb.
Iz_4_1009	DJI_0314	Populus tremula L.	0	n	1	1	Salicaceae Mirb.
Iz_4_1009	DJI_0314	Populus Ã canescens (Aiton) Sm.	0	n	1	1	Salicaceae Mirb.
Iz_4_1009	DJI_0314	Potentilla argentea L.	0	n	1	0	Rosaceae Juss.
Iz_4_1009	DJI_0314	Psammophiliella muralis (L.) Ikonn.	0	n	1	0	Caryophyllaceae Juss.
Iz_4_1009	DJI_0314	Psephellus sumensis (Kalen.) Greuter	0	n	1	0	Asteraceae Bercht. & J.Presl
Iz_4_1009	DJI_0314	Pulsatilla pratensis (L.) Mill. s.l.	0	n	1	0	Ranunculaceae Juss.
Iz_4_1009	DJI_0314	Pyrus communis L.	0	a	0	1	Rosaceae Juss.
Iz_4_1009	DJI_0314	Quercus robur L.	0	n	1	1	Fagaceae Dumort.

Iz_4_1009	DJI_0314	Robinia pseudoacacia L.	0	a	0	1	Fabaceae Lindl.
Iz_4_1009	DJI_0314	Rosa sp.	0		NA	1	Rosaceae Juss.
Iz_4_1009	DJI_0314	Rubus idaeus L.	0	n	1	0	Rosaceae Juss.
Iz_4_1009	DJI_0314	Rumex acetosella L.	0	n	1	0	Polygonaceae Juss.
Iz_4_1009	DJI_0314	Rumex thyrsiflorus Fingerh.	0	n	1	0	Polygonaceae Juss.
Iz_4_1009	DJI_0314	Salix alba L.	0	n	1	1	Salicaceae Mirb.
Iz_4_1009	DJI_0314	Sambucus nigra L.	0	n	1	1	Viburnaceae Raf.
Iz_4_1009	DJI_0314	Secale sylvestre Host	0	n	1	0	Poaceae Barnhart
Iz_4_1009	DJI_0314	Sempervivum ruthenicum Schnittsp. & C.B. Lehm.	0	n	1	0	Crassulaceae J.St.-Hil.
Iz_4_1009	DJI_0314	Setaria viridis (L.) P. Beauv.	0	n	1	0	Poaceae Barnhart
Iz_4_1009	DJI_0314	Silene borysthenica (Gruner) Walters	0	n	1	0	Caryophyllaceae Juss.
Iz_4_1009	DJI_0314	Silene latifolia subsp. alba (Mill.) Greuter & Burdet	0	n	1	0	Caryophyllaceae Juss.
Iz_4_1009	DJI_0314	Solidago virgaurea L.	0	n	1	0	Asteraceae Bercht. & J.Presl
Iz_4_1009	DJI_0314	Sorbus aucuparia L.	0	n	1	1	Rosaceae Juss.
Iz_4_1009	DJI_0314	Stachys recta L.	0	n	1	0	Lamiacea Lindl.
Iz_4_1009	DJI_0314	Tanacetum vulgare L.	0	n	1	0	Asteraceae Bercht. & J.Presl
Iz_4_1009	DJI_0314	Thymus pallasianus Heinr.Braun	0	n	1	0	Lamiacea Lindl.
Iz_4_1009	DJI_0314	Tilia cordata Mill.	0	n	1	1	Malvaceae Juss.
Iz_4_1009	DJI_0314	Trifolium alpestre L.	0	n	1	0	Fabaceae Lindl.
Iz_4_1009	DJI_0314	Trifolium arvense L.	0	n	1	0	Fabaceae Lindl.
Iz_4_1009	DJI_0314	Ulmus glabra Huds.	0	n	1	1	Ulmaceae Mirb.
Iz_4_1009	DJI_0314	Verbascum densiflorum Bertol.	0	n	1	0	Scrophulariaceae Juss.
Iz_4_1009	DJI_0314	Verbascum nigrum L.	0	n	1	0	Scrophulariaceae Juss.
Iz_4_1009	DJI_0314	Veronica austriaca L.	0	n	1	0	Plantaginaceae Juss.
Iz_4_1009	DJI_0314	Veronica incana L.	0	n	1	0	Plantaginaceae Juss.
Iz_4_1009	DJI_0314	Veronica verna L.	0	n	1	0	Plantaginaceae Juss.
Iz_4_1009	DJI_0314	Vincetoxicum hirundinaria Medik	0	n	1	0	Apocynaceae Juss.
Iz_4_1009	DJI_0316	Acer tataricum L.	0	n	1	1	Sapindaceae Juss.
Iz_4_1009	DJI_0316	Achillea millefolium L. s.l.	0	n	1	0	Asteraceae Bercht. & J.Presl
Iz_4_1009	DJI_0316	Allium oleraceum L.	0	n	1	0	Amaryllidaceae J.St.-Hil.
Iz_4_1009	DJI_0316	Ambrosia artemisiifolia L.	0	a	0	0	Asteraceae Bercht. & J.Presl
Iz_4_1009	DJI_0316	Amorpha fruticosa L.	0	a	0	0	Fabaceae Lindl.
Iz_4_1009	DJI_0316	Anthericum ramosum L.	0	n	1	0	Asparagaceae Juss.
Iz_4_1009	DJI_0316	Artemisia marschalliana Spreng.	0	n	1	0	Asteraceae Bercht. & J.Presl
Iz_4_1009	DJI_0316	Asparagus officinalis L.	0	n	1	0	Asparagaceae Juss.
Iz_4_1009	DJI_0316	Astragalus cicer L.	0	n	1	0	Fabaceae Lindl.
Iz_4_1009	DJI_0316	Bassia laniflora (S.G.Gmel.) A.J.Scott	0	n	1	0	Amaranthaceae Juss.
Iz_4_1009	DJI_0316	Berberis vulgaris L.	0	n	1	0	Berberidaceae Juss.
Iz_4_1009	DJI_0316	Betula pendula Roth.	0	n	1	1	Betulaceae Gray
Iz_4_1009	DJI_0316	Betula pendula Roth.	0	n	1	0	Poaceae Barnhart
Iz_4_1009	DJI_0316	Bromus inermis Leyss.	0		NA	NA	
Iz_4_1009	DJI_0316	Bromus squarrosus L.	0	a	0	0	Poaceae Barnhart
Iz_4_1009	DJI_0316	Bromus tectorum L.	0	a	0	0	Poaceae Barnhart
Iz_4_1009	DJI_0316	Calamagrostis epigejos (L.) Roth	10	n	1	0	Poaceae Barnhart
Iz_4_1009	DJI_0316	Carduus acanthoides L.	0	n	1	0	Asteraceae Bercht. & J.Presl
Iz_4_1009	DJI_0316	Carex sp.	0	n	1	0	Cyperaceae Juss.
Iz_4_1009	DJI_0316	Centaurea majorovii Dumbadze	0	n	1	0	Asteraceae Bercht. & J.Presl
Iz_4_1009	DJI_0316	Chamaecytisus ruthenicus (Fisch.ex Wol.) Klaskova	0	n	1	0	Fabaceae Lindl.
Iz_4_1009	DJI_0316	Chelidonium majus L.	0	n	1	0	Papaveraceae Juss.
Iz_4_1009	DJI_0316	Chenopodium betaceum Andrz.	0	n	1	0	Amaranthaceae Juss.
Iz_4_1009	DJI_0316	Chenopodium cf. album	0	n	1	0	Amaranthaceae Juss.
Iz_4_1009	DJI_0316	Chenopodium sp.	0		NA	0	Amaranthaceae Juss.
Iz_4_1009	DJI_0316	Chondrilla juncea L.	0	n	1	0	Asteraceae Bercht. & J.Presl
Iz_4_1009	DJI_0316	Cirsium vulgare (Savi) Ten.	0	n	1	0	Asteraceae Bercht. & J.Presl
Iz_4_1009	DJI_0316	Clinopodium acinos (L.) Kuntze	0	n	1	0	Lamiacea Lindl.
Iz_4_1009	DJI_0316	Convallaria majalis L.	0	n	1	0	Asparagaceae Juss.
Iz_4_1009	DJI_0316	Crataegus rhipidophylla Gand.	0	n	1	1	Rosaceae Juss.
Iz_4_1009	DJI_0316	Crepis foetida L.	0	n	1	0	Asteraceae Bercht. & J.Presl
Iz_4_1009	DJI_0316	Dactylis glomerata L.	0	n	1	0	Poaceae Barnhart
Iz_4_1009	DJI_0316	Dianthus campestris M.Bieb.	0	n	1	0	Caryophyllaceae Juss.
Iz_4_1009	DJI_0316	Digitaria sanguinalis (L.) Scop.	0	a	0	0	Poaceae Barnhart
Iz_4_1009	DJI_0316	Elymus repens (L.) Gould	0	n	1	0	Poaceae Barnhart
Iz_4_1009	DJI_0316	Epilobium tetragonum L.	0	n	1	0	Onagraceae Juss.
Iz_4_1009	DJI_0316	Eragrostis minor Host	0	a	0	0	Poaceae Barnhart
Iz_4_1009	DJI_0316	Erigeron canadensis L.	2	a	0	0	Asteraceae Bercht. & J.Presl
Iz_4_1009	DJI_0316	Euphorbia seguieriana Neck.	0	n	1	0	Euphorbiaceae Juss.
Iz_4_1009	DJI_0316	Fallopia dumetorum (L.) Holub	0	n	1	0	Polygonaceae Juss.
Iz_4_1009	DJI_0316	Festuca beckeri (Hack.) Trautv.	0	n	1	0	Poaceae Barnhart
Iz_4_1009	DJI_0316	Fragaria vesca L.	0	n	1	0	Rosaceae Juss.
Iz_4_1009	DJI_0316	Frangula alnus Mill.	0	n	1	1	Rhamnaceae Juss.
Iz_4_1009	DJI_0316	Fraxinus pennsylvanica Marshall	0	a	0	1	Oleaceae Hoffmanns. & Link
Iz_4_1009	DJI_0316	Galeopsis bifida Boenn.	0	n	1	0	Lamiacea Lindl.
Iz_4_1009	DJI_0316	Galium verum L.	0	n	1	0	Rubiaceae Juss.
Iz_4_1009	DJI_0316	Genista tinctoria L.	0	n	1	0	Fabaceae Lindl.
Iz_4_1009	DJI_0316	Geranium robertianum L.	0	n	1	0	Geraniaceae Juss.
Iz_4_1009	DJI_0316	Geranium sanquineum L.	0	n	1	0	Geraniaceae Juss.
Iz_4_1009	DJI_0316	Gypsophila paniculata L.	0	n	1	0	Caryophyllaceae Juss.
Iz_4_1009	DJI_0316	Helichrysum arenarium (L.) Moench	0	n	1	0	Asteraceae Bercht. & J.Presl
Iz_4_1009	DJI_0316	Herniaria polygama J.Gay	0	n	1	0	Caryophyllaceae Juss.
Iz_4_1009	DJI_0316	Hieracium robustum Fr.	0	n	1	0	Asteraceae Bercht. & J.Presl
Iz_4_1009	DJI_0316	Hieracium umbellatum L.	0	n	1	NA	Asteraceae Bercht. & J.Presl
Iz_4_1009	DJI_0316	Hylotelephium maximum (L.) Holub	0	n	1	0	Crassulaceae J.St.-Hil.
Iz_4_1009	DJI_0316	Jacobaea borysthenica (DC.) B.Nord. & Greuter	0	n	1	0	Asteraceae Bercht. & J.Presl
Iz_4_1009	DJI_0316	Jasione montana L.	0	n	1	0	Campanulaceae Juss.
Iz_4_1009	DJI_0316	Jurinea cyanoides (L.) Rchb.s.l.	0	n	1	0	Asteraceae Bercht. & J.Presl
Iz_4_1009	DJI_0316	Koeleria glauca (Spreng.) DC.	0	n	1	0	Poaceae Barnhart
Iz_4_1009	DJI_0316	Lactuca serriola L.	0	a	0	0	Asteraceae Bercht. & J.Presl
Iz_4_1009	DJI_0316	Linaria genistifolia (L.) Mill.	0	n	1	0	Plantaginaceae Juss.
Iz_4_1009	DJI_0316	Lomelosia argentea (L.) Greuter & Burdet	0	n	1	0	Caprifoliaceae Juss.
Iz_4_1009	DJI_0316	Melampyrum pratense L.	0	n	1	0	Orobanchaceae Vent.
Iz_4_1009	DJI_0316	Oenothera biennis L.	0	a	0	0	Onagraceae Juss.
Iz_4_1009	DJI_0316	Pentanema britannica (L.) D.Gut.Larr., Santos-Vicente, Anderb., E.Rico & M.M.Mart.Ort.	0	n	1	0	Asteraceae Bercht. & J.Presl
Iz_4_1009	DJI_0316	Peucedanum oreoselinum (L.) Moench	0	n	1	0	Apiaceae Lindl.
Iz_4_1009	DJI_0316	Peucedanum ruthenicum M.Bieb.	0	n	1	0	Apiaceae Lindl.
Iz_4_1009	DJI_0316	Pilosella echioides (Lumn.) F.W.Schultz & Sch.Bip.	0	n	1	0	Asteraceae Bercht. & J.Presl
Iz_4_1009	DJI_0316	Pilosella officinarum F. Schultz et Sch. Bip.	0	n	1	0	Asteraceae Bercht. & J.Presl
Iz_4_1009	DJI_0316	Pilosella sp.	0		NA	0	Asteraceae Bercht. & J.Presl
Iz_4_1009	DJI_0316	Pinus sylvestris L.	0	n	1	1	Pinaceae Spreng. ex F.Rudolphi

Iz_4_1009	DJI_0316	Poa annua L.	0	n	1	0	Poaceae Barnhart
Iz_4_1009	DJI_0316	Poa nemoralis L.	0	n	1	0	Poaceae Barnhart
Iz_4_1009	DJI_0316	Poa sp.	0		NA	0	Poaceae Barnhart
Iz_4_1009	DJI_0316	Polygonatum odoratum (Mill.) Druce	0	n	1	0	Asparagaceae Juss.
Iz_4_1009	DJI_0316	Polygonum arenarium Waldst. & Kit.	0	n	1	0	Polygonaceae Juss.
Iz_4_1009	DJI_0316	Populus nigra L.	0	n	1	1	Salicaceae Mirb.
Iz_4_1009	DJI_0316	Populus tremula L.	0	n	1	1	Salicaceae Mirb.
Iz_4_1009	DJI_0316	Populus Ã canescens (Aiton) Sm.	0	n	1	1	Salicaceae Mirb.
Iz_4_1009	DJI_0316	Potentilla argentea L.	0	n	1	0	Rosaceae Juss.
Iz_4_1009	DJI_0316	Psammophiliella muralis (L.) Ikonn.	0	n	1	0	Caryophyllaceae Juss.
Iz_4_1009	DJI_0316	Psephellus sumensis (Kalen.) Greuter	0	n	1	0	Asteraceae Bercht. & J.Presl
Iz_4_1009	DJI_0316	Pulsatilla pratensis (L.) Mill. s.l.	0	n	1	0	Ranunculaceae Juss.
Iz_4_1009	DJI_0316	Pyrus communis L.	0	a	0	1	Rosaceae Juss.
Iz_4_1009	DJI_0316	Quercus robur L.	0	n	1	1	Fagaceae Dumort.
Iz_4_1009	DJI_0316	Robinia pseudoacacia L.	0	a	0	1	Fabaceae Lindl.
Iz_4_1009	DJI_0316	Rosa sp.	0		NA	1	Rosaceae Juss.
Iz_4_1009	DJI_0316	Rubus idaeus L.	0	n	1	0	Rosaceae Juss.
Iz_4_1009	DJI_0316	Rumex acetosella L.	0	n	1	0	Polygonaceae Juss.
Iz_4_1009	DJI_0316	Rumex thyrsiflorus Fingerh.	0	n	1	0	Polygonaceae Juss.
Iz_4_1009	DJI_0316	Salix alba L.	0	n	1	1	Salicaceae Mirb.
Iz_4_1009	DJI_0316	Sambucus nigra L.	0	n	1	1	Viburnaceae Raf.
Iz_4_1009	DJI_0316	Secale sylvestre Host	0	n	1	0	Poaceae Barnhart
Iz_4_1009	DJI_0316	Sempervivum ruthenicum Schnittsp. & C.B. Lehm.	0	n	1	0	Crassulaceae J.St.-Hil.
Iz_4_1009	DJI_0316	Setaria viridis (L.) P. Beauv.	0	n	1	0	Poaceae Barnhart
Iz_4_1009	DJI_0316	Silene borysthenica (Gruner) Walters	0	n	1	0	Caryophyllaceae Juss.
Iz_4_1009	DJI_0316	Silene latifolia subsp. alba (Mill.) Greuter & Burdet	0	n	1	0	Caryophyllaceae Juss.
Iz_4_1009	DJI_0316	Solidago virgaurea L.	1	n	1	0	Asteraceae Bercht. & J.Presl
Iz_4_1009	DJI_0316	Sorbus aucuparia L.	0	n	1	1	Rosaceae Juss.
Iz_4_1009	DJI_0316	Stachys recta L.	0	n	1	0	Lamiacea Lindl.
Iz_4_1009	DJI_0316	Tanacetum vulgare L.	0	n	1	0	Asteraceae Bercht. & J.Presl
Iz_4_1009	DJI_0316	Thymus pallasianus Heinr.Braun	0	n	1	0	Lamiacea Lindl.
Iz_4_1009	DJI_0316	Tilia cordata Mill.	0	n	1	1	Malvaceae Juss.
Iz_4_1009	DJI_0316	Trifolium alpestre L.	0	n	1	0	Fabaceae Lindl.
Iz_4_1009	DJI_0316	Trifolium arvense L.	0	n	1	0	Fabaceae Lindl.
Iz_4_1009	DJI_0316	Ulmus glabra Huds.	0	n	1	1	Ulmaceae Mirb.
Iz_4_1009	DJI_0316	Verbascum densiflorum Bertol.	0	n	1	0	Scrophulariaceae Juss.
Iz_4_1009	DJI_0316	Verbascum nigrum L.	0	n	1	0	Scrophulariaceae Juss.
Iz_4_1009	DJI_0316	Veronica austriaca L.	0	n	1	0	Plantaginaceae Juss.
Iz_4_1009	DJI_0316	Veronica incana L.	0	n	1	0	Plantaginaceae Juss.
Iz_4_1009	DJI_0316	Veronica verna L.	0	n	1	0	Plantaginaceae Juss.
Iz_4_1009	DJI_0316	Vincetoxicum hirundinaria Medik	0	n	1	0	Apocynaceae Juss.
Iz_4_1009	DJI_0319	Acer tataricum L.	0	n	1	1	Sapindaceae Juss.
Iz_4_1009	DJI_0319	Achillea millefolium L. s.l.	0	n	1	0	Asteraceae Bercht. & J.Presl
Iz_4_1009	DJI_0319	Allium oleraceum L.	0	n	1	0	Amaryllidaceae J.St.-Hil.
Iz_4_1009	DJI_0319	Ambrosia artemisiifolia L.	0	a	0	0	Asteraceae Bercht. & J.Presl
Iz_4_1009	DJI_0319	Amorpha fruticosa L.	0	a	0	0	Fabaceae Lindl.
Iz_4_1009	DJI_0319	Anthericum ramosum L.	0	n	1	0	Asparagaceae Juss.
Iz_4_1009	DJI_0319	Artemisia marschalliana Spreng.	0	n	1	0	Asteraceae Bercht. & J.Presl
Iz_4_1009	DJI_0319	Asparagus officinalis L.	0	n	1	0	Asparagaceae Juss.
Iz_4_1009	DJI_0319	Astragalus cicer L.	0	n	1	0	Fabaceae Lindl.
Iz_4_1009	DJI_0319	Bassia laniflora (S.G.Gmel.) A.J.Scott	0	n	1	0	Amaranthaceae Juss.
Iz_4_1009	DJI_0319	Berberis vulgaris L.	0	n	1	0	Berberidaceae Juss.
Iz_4_1009	DJI_0319	Betula pendula Roth.	0	n	1	1	Betulaceae Gray
Iz_4_1009	DJI_0319	Betula pendula Roth.	0	n	1	0	Poaceae Barnhart
Iz_4_1009	DJI_0319	Bromus inermis Leyss.	0		NA	NA	
Iz_4_1009	DJI_0319	Bromus squarrosus L.	0	a	0	0	Poaceae Barnhart
Iz_4_1009	DJI_0319	Bromus tectorum L.	0	a	0	0	Poaceae Barnhart
Iz_4_1009	DJI_0319	Calamagrostis epigejos (L.) Roth	3	n	1	0	Poaceae Barnhart
Iz_4_1009	DJI_0319	Carduus acanthoides L.	0	n	1	0	Asteraceae Bercht. & J.Presl
Iz_4_1009	DJI_0319	Carex sp.	0	n	1	0	Cyperaceae Juss.
Iz_4_1009	DJI_0319	Centaurea majorovii Dumbadze	0	n	1	0	Asteraceae Bercht. & J.Presl
Iz_4_1009	DJI_0319	Chamaecytisus ruthenicus (Fisch.ex Wol.) Klaskova	0	n	1	0	Fabaceae Lindl.
Iz_4_1009	DJI_0319	Chelidonium majus L.	0	n	1	0	Papaveraceae Juss.
Iz_4_1009	DJI_0319	Chenopodium betaceum Andrz.	0	n	1	0	Amaranthaceae Juss.
Iz_4_1009	DJI_0319	Chenopodium cf. album	0	n	1	0	Amaranthaceae Juss.
Iz_4_1009	DJI_0319	Chenopodium sp.	0		NA	0	Amaranthaceae Juss.
Iz_4_1009	DJI_0319	Chondrilla juncea L.	0	n	1	0	Asteraceae Bercht. & J.Presl
Iz_4_1009	DJI_0319	Cirsium vulgare (Savi) Ten.	0	n	1	0	Asteraceae Bercht. & J.Presl
Iz_4_1009	DJI_0319	Clinopodium acinos (L.) Kuntze	0	n	1	0	Lamiacea Lindl.
Iz_4_1009	DJI_0319	Convallaria majalis L.	0	n	1	0	Asparagaceae Juss.
Iz_4_1009	DJI_0319	Crataegus rhipidophylla Gand.	0	n	1	1	Rosaceae Juss.
Iz_4_1009	DJI_0319	Crepis foetida L.	0	n	1	0	Asteraceae Bercht. & J.Presl
Iz_4_1009	DJI_0319	Dactylis glomerata L.	0	n	1	0	Poaceae Barnhart
Iz_4_1009	DJI_0319	Dianthus campestris M.Bieb.	0	n	1	0	Caryophyllaceae Juss.
Iz_4_1009	DJI_0319	Digitaria sanguinalis (L.) Scop.	0	a	0	0	Poaceae Barnhart
Iz_4_1009	DJI_0319	Elymus repens (L.) Gould	0	n	1	0	Poaceae Barnhart
Iz_4_1009	DJI_0319	Epilobium tetragonum L.	0	n	1	0	Onagraceae Juss.
Iz_4_1009	DJI_0319	Eragrostis minor Host	0	a	0	0	Poaceae Barnhart
Iz_4_1009	DJI_0319	Erigeron canadensis L.	0.2	a	0	0	Asteraceae Bercht. & J.Presl
Iz_4_1009	DJI_0319	Euphorbia seguieriana Neck.	0	n	1	0	Euphorbiaceae Juss.
Iz_4_1009	DJI_0319	Fallopia dumetorum (L.) Holub	0	n	1	0	Polygonaceae Juss.
Iz_4_1009	DJI_0319	Festuca beckeri (Hack.) Trautv.	0	n	1	0	Poaceae Barnhart
Iz_4_1009	DJI_0319	Fragaria vesca L.	0	n	1	0	Rosaceae Juss.
Iz_4_1009	DJI_0319	Frangula alnus Mill.	0	n	1	1	Rhamnaceae Juss.
Iz_4_1009	DJI_0319	Fraxinus pennsylvanica Marshall	0	a	0	1	Oleaceae Hoffmanns. & Link
Iz_4_1009	DJI_0319	Galeopsis bifida Boenn.	0	n	1	0	Lamiacea Lindl.
Iz_4_1009	DJI_0319	Galium verum L.	0	n	1	0	Rubiaceae Juss.
Iz_4_1009	DJI_0319	Genista tinctoria L.	0	n	1	0	Fabaceae Lindl.
Iz_4_1009	DJI_0319	Geranium robertianum L.	0	n	1	0	Geraniaceae Juss.
Iz_4_1009	DJI_0319	Geranium sanquineum L.	0	n	1	0	Geraniaceae Juss.
Iz_4_1009	DJI_0319	Gypsophila paniculata L.	0	n	1	0	Caryophyllaceae Juss.
Iz_4_1009	DJI_0319	Helichrysum arenarium (L.) Moench	0	n	1	0	Asteraceae Bercht. & J.Presl
Iz_4_1009	DJI_0319	Herniaria polygama J.Gay	0	n	1	0	Caryophyllaceae Juss.
Iz_4_1009	DJI_0319	Hieracium robustum Fr.	0	n	1	0	Asteraceae Bercht. & J.Presl
Iz_4_1009	DJI_0319	Hieracium umbellatum L.	0	n	1	NA	Asteraceae Bercht. & J.Presl
Iz_4_1009	DJI_0319	Hylotelephium maximum (L.) Holub	0	n	1	0	Crassulaceae J.St.-Hil.
Iz_4_1009	DJI_0319	Jacobaea borysthenica (DC.) B.Nord. & Greuter	0	n	1	0	Asteraceae Bercht. & J.Presl
Iz_4_1009	DJI_0319	Jasione montana L.	0	n	1	0	Campanulaceae Juss.

Iz_4_1009	DJI_0319	Jurinea cyanoides (L.) Rchb.s.l.	0	n	1	0	Asteraceae Bercht. & J.Presl
Iz_4_1009	DJI_0319	Koeleria glauca (Spreng.) DC.	0	n	1	0	Poaceae Barnhart
Iz_4_1009	DJI_0319	Lactuca serriola L.	0	a	0	0	Asteraceae Bercht. & J.Presl
Iz_4_1009	DJI_0319	Linaria genistifolia (L.) Mill.	0	n	1	0	Plantaginaceae Juss.
Iz_4_1009	DJI_0319	Lomelosia argentea (L.) Greuter & Burdet	0	n	1	0	Caprifoliaceae Juss.
Iz_4_1009	DJI_0319	Melampyrum pratense L.	0	n	1	0	Orobanchaceae Vent.
Iz_4_1009	DJI_0319	Oenothera biennis L.	0	a	0	0	Onagraceae Juss.
Iz_4_1009	DJI_0319	Pentanema britannica (L.) D.Gut.Larr., Santos-Vicente, Anderb., E.Rico & M.M.Mart.Ort.	0	n	1	0	Asteraceae Bercht. & J.Presl
Iz_4_1009	DJI_0319	Peucedanum oreoselinum (L.) Moench	0	n	1	0	Apiaceae Lindl.
Iz_4_1009	DJI_0319	Peucedanum ruthenicum M.Bieb.	0	n	1	0	Apiaceae Lindl.
Iz_4_1009	DJI_0319	Pilosella echioides (Lumn.) F.W.Schultz & Sch.Bip.	0	n	1	0	Asteraceae Bercht. & J.Presl
Iz_4_1009	DJI_0319	Pilosella officinarum F. Schultz et Sch. Bip.	0	n	1	0	Asteraceae Bercht. & J.Presl
Iz_4_1009	DJI_0319	Pilosella sp.	0		NA	0	Asteraceae Bercht. & J.Presl
Iz_4_1009	DJI_0319	Pinus sylvestris L.	0	n	1	1	Pinaceae Spreng. ex F.Rudolphi
Iz_4_1009	DJI_0319	Poa annua L.	0	n	1	0	Poaceae Barnhart
Iz_4_1009	DJI_0319	Poa nemoralis L.	0	n	1	0	Poaceae Barnhart
Iz_4_1009	DJI_0319	Poa sp.	0		NA	0	Poaceae Barnhart
Iz_4_1009	DJI_0319	Polygonatum odoratum (Mill.) Druce	0	n	1	0	Asparagaceae Juss.
Iz_4_1009	DJI_0319	Polygonum arenarium Waldst. & Kit.	0	n	1	0	Polygonaceae Juss.
Iz_4_1009	DJI_0319	Populus nigra L.	0	n	1	1	Salicaceae Mirb.
Iz_4_1009	DJI_0319	Populus tremula L.	0	n	1	1	Salicaceae Mirb.
Iz_4_1009	DJI_0319	Populus Ã canescens (Aiton) Sm.	0	n	1	1	Salicaceae Mirb.
Iz_4_1009	DJI_0319	Potentilla argentea L.	0	n	1	0	Rosaceae Juss.
Iz_4_1009	DJI_0319	Psammophiliella muralis (L.) Ikonn.	0	n	1	0	Caryophyllaceae Juss.
Iz_4_1009	DJI_0319	Psephellus sumensis (Kalen.) Greuter	0	n	1	0	Asteraceae Bercht. & J.Presl
Iz_4_1009	DJI_0319	Pulsatilla pratensis (L.) Mill. s.l.	0	n	1	0	Ranunculaceae Juss.
Iz_4_1009	DJI_0319	Pyrus communis L.	0	a	0	1	Rosaceae Juss.
Iz_4_1009	DJI_0319	Quercus robur L.	0	n	1	1	Fagaceae Dumort.
Iz_4_1009	DJI_0319	Robinia pseudoacacia L.	0	a	0	1	Fabaceae Lindl.
Iz_4_1009	DJI_0319	Rosa sp.	0		NA	1	Rosaceae Juss.
Iz_4_1009	DJI_0319	Rubus idaeus L.	0	n	1	0	Rosaceae Juss.
Iz_4_1009	DJI_0319	Rumex acetosella L.	0	n	1	0	Polygonaceae Juss.
Iz_4_1009	DJI_0319	Rumex thyrsiflorus Fingerh.	0	n	1	0	Polygonaceae Juss.
Iz_4_1009	DJI_0319	Salix alba L.	0	n	1	1	Salicaceae Mirb.
Iz_4_1009	DJI_0319	Sambucus nigra L.	0	n	1	1	Viburnaceae Raf.
Iz_4_1009	DJI_0319	Secale sylvestre Host	0	n	1	0	Poaceae Barnhart
Iz_4_1009	DJI_0319	Sempervivum ruthenicum Schnittsp. & C.B. Lehm.	0	n	1	0	Crassulaceae J.St.-Hil.
Iz_4_1009	DJI_0319	Setaria viridis (L.) P. Beauv.	0	n	1	0	Poaceae Barnhart
Iz_4_1009	DJI_0319	Silene borysthenica (Gruner) Walters	0	n	1	0	Caryophyllaceae Juss.
Iz_4_1009	DJI_0319	Silene latifolia subsp. alba (Mill.) Greuter & Burdet	0	n	1	0	Caryophyllaceae Juss.
Iz_4_1009	DJI_0319	Solidago virgaurea L.	0	n	1	0	Asteraceae Bercht. & J.Presl
Iz_4_1009	DJI_0319	Sorbus aucuparia L.	0	n	1	1	Rosaceae Juss.
Iz_4_1009	DJI_0319	Stachys recta L.	0	n	1	0	Lamiacea Lindl.
Iz_4_1009	DJI_0319	Tanacetum vulgare L.	0	n	1	0	Asteraceae Bercht. & J.Presl
Iz_4_1009	DJI_0319	Thymus pallasianus Heinr.Braun	0	n	1	0	Lamiacea Lindl.
Iz_4_1009	DJI_0319	Tilia cordata Mill.	0	n	1	1	Malvaceae Juss.
Iz_4_1009	DJI_0319	Trifolium alpestre L.	0	n	1	0	Fabaceae Lindl.
Iz_4_1009	DJI_0319	Trifolium arvense L.	0	n	1	0	Fabaceae Lindl.
Iz_4_1009	DJI_0319	Ulmus glabra Huds.	0	n	1	1	Ulmaceae Mirb.
Iz_4_1009	DJI_0319	Verbascum densiflorum Bertol.	0	n	1	0	Scrophulariaceae Juss.
Iz_4_1009	DJI_0319	Verbascum nigrum L.	0	n	1	0	Scrophulariaceae Juss.
Iz_4_1009	DJI_0319	Veronica austriaca L.	0	n	1	0	Plantaginaceae Juss.
Iz_4_1009	DJI_0319	Veronica incana L.	0	n	1	0	Plantaginaceae Juss.
Iz_4_1009	DJI_0319	Veronica verna L.	0	n	1	0	Plantaginaceae Juss.
Iz_4_1009	DJI_0319	Vincetoxicum hirundinaria Medik	0	n	1	0	Apocynaceae Juss.
Iz_4_1009	DJI_0323	Acer tataricum L.	0	n	1	1	Sapindaceae Juss.
Iz_4_1009	DJI_0323	Achillea millefolium L. s.l.	0	n	1	0	Asteraceae Bercht. & J.Presl
Iz_4_1009	DJI_0323	Allium oleraceum L.	0	n	1	0	Amaryllidaceae J.St.-Hil.
Iz_4_1009	DJI_0323	Ambrosia artemisiifolia L.	0	a	0	0	Asteraceae Bercht. & J.Presl
Iz_4_1009	DJI_0323	Amorpha fruticosa L.	0	a	0	0	Fabaceae Lindl.
Iz_4_1009	DJI_0323	Anthericum ramosum L.	0	n	1	0	Asparagaceae Juss.
Iz_4_1009	DJI_0323	Artemisia marschalliana Spreng.	0	n	1	0	Asteraceae Bercht. & J.Presl
Iz_4_1009	DJI_0323	Asparagus officinalis L.	0	n	1	0	Asparagaceae Juss.
Iz_4_1009	DJI_0323	Astragalus cicer L.	0	n	1	0	Fabaceae Lindl.
Iz_4_1009	DJI_0323	Bassia laniflora (S.G.Gmel.) A.J.Scott	0	n	1	0	Amaranthaceae Juss.
Iz_4_1009	DJI_0323	Berberis vulgaris L.	0	n	1	0	Berberidaceae Juss.
Iz_4_1009	DJI_0323	Betula pendula Roth.	0	n	1	1	Betulaceae Gray
Iz_4_1009	DJI_0323	Betula pendula Roth.	0	n	1	0	Poaceae Barnhart
Iz_4_1009	DJI_0323	Bromus inermis Leyss.	0		NA	NA	
Iz_4_1009	DJI_0323	Bromus squarrosus L.	0	a	0	0	Poaceae Barnhart
Iz_4_1009	DJI_0323	Bromus tectorum L.	0	a	0	0	Poaceae Barnhart
Iz_4_1009	DJI_0323	Calamagrostis epigejos (L.) Roth	10	n	1	0	Poaceae Barnhart
Iz_4_1009	DJI_0323	Carduus acanthoides L.	0	n	1	0	Asteraceae Bercht. & J.Presl
Iz_4_1009	DJI_0323	Carex sp.	0	n	1	0	Cyperaceae Juss.
Iz_4_1009	DJI_0323	Centaurea majorovii Dumbadze	0	n	1	0	Asteraceae Bercht. & J.Presl
Iz_4_1009	DJI_0323	Chamaecytisus ruthenicus (Fisch.ex Wol.) Klaskova	0	n	1	0	Fabaceae Lindl.
Iz_4_1009	DJI_0323	Chelidonium majus L.	0	n	1	0	Papaveraceae Juss.
Iz_4_1009	DJI_0323	Chenopodium betaceum Andrz.	0	n	1	0	Amaranthaceae Juss.
Iz_4_1009	DJI_0323	Chenopodium cf. album	0	n	1	0	Amaranthaceae Juss.
Iz_4_1009	DJI_0323	Chenopodium sp.	0		NA	0	Amaranthaceae Juss.
Iz_4_1009	DJI_0323	Chondrilla juncea L.	0	n	1	0	Asteraceae Bercht. & J.Presl
Iz_4_1009	DJI_0323	Cirsium vulgare (Savi) Ten.	0	n	1	0	Asteraceae Bercht. & J.Presl
Iz_4_1009	DJI_0323	Clinopodium acinos (L.) Kuntze	0	n	1	0	Lamiacea Lindl.
Iz_4_1009	DJI_0323	Convallaria majalis L.	0	n	1	0	Asparagaceae Juss.
Iz_4_1009	DJI_0323	Crataegus rhipidophylla Gand.	0	n	1	1	Rosaceae Juss.
Iz_4_1009	DJI_0323	Crepis foetida L.	0	n	1	0	Asteraceae Bercht. & J.Presl
Iz_4_1009	DJI_0323	Dactylis glomerata L.	0	n	1	0	Poaceae Barnhart
Iz_4_1009	DJI_0323	Dianthus campestris M.Bieb.	0	n	1	0	Caryophyllaceae Juss.
Iz_4_1009	DJI_0323	Digitaria sanguinalis (L.) Scop.	0	a	0	0	Poaceae Barnhart
Iz_4_1009	DJI_0323	Elymus repens (L.) Gould	0	n	1	0	Poaceae Barnhart
Iz_4_1009	DJI_0323	Epilobium tetragonum L.	0	n	1	0	Onagraceae Juss.
Iz_4_1009	DJI_0323	Eragrostis minor Host	0	a	0	0	Poaceae Barnhart
Iz_4_1009	DJI_0323	Erigeron canadensis L.	0	a	0	0	Asteraceae Bercht. & J.Presl
Iz_4_1009	DJI_0323	Euphorbia seguieriana Neck.	0	n	1	0	Euphorbiaceae Juss.
Iz_4_1009	DJI_0323	Fallopia dumetorum (L.) Holub	0	n	1	0	Polygonaceae Juss.
Iz_4_1009	DJI_0323	Festuca beckeri (Hack.) Trautv.	0	n	1	0	Poaceae Barnhart
Iz_4_1009	DJI_0323	Fragaria vesca L.	0	n	1	0	Rosaceae Juss.
Iz_4_1009	DJI_0323	Frangula alnus Mill.	0	n	1	1	Rhamnaceae Juss.

Iz_4_1009	DJI_0323	Fraxinus pennsylvanica Marshall	0	a	0	1	Oleaceae Hoffmanns. & Link
Iz_4_1009	DJI_0323	Galeopsis bifida Boenn.	0	n	1	0	Lamiacea Lindl.
Iz_4_1009	DJI_0323	Galium verum L.	0	n	1	0	Rubiaceae Juss.
Iz_4_1009	DJI_0323	Genista tinctoria L.	0	n	1	0	Fabaceae Lindl.
Iz_4_1009	DJI_0323	Geranium robertianum L.	0	n	1	0	Geraniaceae Juss.
Iz_4_1009	DJI_0323	Geranium sanquineum L.	0	n	1	0	Geraniaceae Juss.
Iz_4_1009	DJI_0323	Gypsophila paniculata L.	0	n	1	0	Caryophyllaceae Juss.
Iz_4_1009	DJI_0323	Helichrysum arenarium (L.) Moench	0	n	1	0	Asteraceae Bercht. & J.Presl
Iz_4_1009	DJI_0323	Herniaria polygama J.Gay	0	n	1	0	Caryophyllaceae Juss.
Iz_4_1009	DJI_0323	Hieracium robustum Fr.	0	n	1	0	Asteraceae Bercht. & J.Presl
Iz_4_1009	DJI_0323	Hieracium umbellatum L.	0	n	1	NA	Asteraceae Bercht. & J.Presl
Iz_4_1009	DJI_0323	Hylotelephium maximum (L.) Holub	0	n	1	0	Crassulaceae J.St.-Hil.
Iz_4_1009	DJI_0323	Jacobaea borysthenica (DC.) B.Nord. & Greuter	0	n	1	0	Asteraceae Bercht. & J.Presl
Iz_4_1009	DJI_0323	Jasione montana L.	0	n	1	0	Campanulaceae Juss.
Iz_4_1009	DJI_0323	Jurinea cyanoides (L.) Rchb.s.l.	0	n	1	0	Asteraceae Bercht. & J.Presl
Iz_4_1009	DJI_0323	Koeleria glauca (Spreng.) DC.	0	n	1	0	Poaceae Barnhart
Iz_4_1009	DJI_0323	Lactuca serriola L.	0	a	0	0	Asteraceae Bercht. & J.Presl
Iz_4_1009	DJI_0323	Linaria genistifolia (L.) Mill.	0	n	1	0	Plantaginaceae Juss.
Iz_4_1009	DJI_0323	Lomelosia argentea (L.) Greuter & Burdet	0	n	1	0	Caprifoliaceae Juss.
Iz_4_1009	DJI_0323	Melampyrum pratense L.	0	n	1	0	Orobanchaceae Vent.
Iz_4_1009	DJI_0323	Oenothera biennis L.	0	a	0	0	Onagraceae Juss.
Iz_4_1009	DJI_0323	Pentanema britannica (L.) D.Gut.Larr., Santos-Vicente, Anderb., E.Rico & M.M.Mart.Ort.	0	n	1	0	Asteraceae Bercht. & J.Presl
Iz_4_1009	DJI_0323	Peucedanum oreoselinum (L.) Moench	0	n	1	0	Apiaceae Lindl.
Iz_4_1009	DJI_0323	Peucedanum ruthenicum M.Bieb.	0	n	1	0	Apiaceae Lindl.
Iz_4_1009	DJI_0323	Pilosella echioides (Lumn.) F.W.Schultz & Sch.Bip.	0	n	1	0	Asteraceae Bercht. & J.Presl
Iz_4_1009	DJI_0323	Pilosella officinarum F. Schultz et Sch. Bip.	0	n	1	0	Asteraceae Bercht. & J.Presl
Iz_4_1009	DJI_0323	Pilosella sp.	0		NA	0	Asteraceae Bercht. & J.Presl
Iz_4_1009	DJI_0323	Pinus sylvestris L.	0	n	1	1	Pinaceae Spreng. ex F.Rudolphi
Iz_4_1009	DJI_0323	Poa annua L.	0	n	1	0	Poaceae Barnhart
Iz_4_1009	DJI_0323	Poa nemoralis L.	0	n	1	0	Poaceae Barnhart
Iz_4_1009	DJI_0323	Poa sp.	0		NA	0	Poaceae Barnhart
Iz_4_1009	DJI_0323	Polygonatum odoratum (Mill.) Druce	0	n	1	0	Asparagaceae Juss.
Iz_4_1009	DJI_0323	Polygonum arenarium Waldst. & Kit.	0	n	1	0	Polygonaceae Juss.
Iz_4_1009	DJI_0323	Populus nigra L.	0	n	1	1	Salicaceae Mirb.
Iz_4_1009	DJI_0323	Populus tremula L.	0	n	1	1	Salicaceae Mirb.
Iz_4_1009	DJI_0323	Populus Ã canescens (Aiton) Sm.	0	n	1	1	Salicaceae Mirb.
Iz_4_1009	DJI_0323	Potentilla argentea L.	0	n	1	0	Rosaceae Juss.
Iz_4_1009	DJI_0323	Psammophiliella muralis (L.) Ikonn.	0	n	1	0	Caryophyllaceae Juss.
Iz_4_1009	DJI_0323	Psephellus sumensis (Kalen.) Greuter	0	n	1	0	Asteraceae Bercht. & J.Presl
Iz_4_1009	DJI_0323	Pulsatilla pratensis (L.) Mill. s.l.	0	n	1	0	Ranunculaceae Juss.
Iz_4_1009	DJI_0323	Pyrus communis L.	0	a	0	1	Rosaceae Juss.
Iz_4_1009	DJI_0323	Quercus robur L.	0	n	1	1	Fagaceae Dumort.
Iz_4_1009	DJI_0323	Robinia pseudoacacia L.	0	a	0	1	Fabaceae Lindl.
Iz_4_1009	DJI_0323	Rosa sp.	0		NA	1	Rosaceae Juss.
Iz_4_1009	DJI_0323	Rubus idaeus L.	0	n	1	0	Rosaceae Juss.
Iz_4_1009	DJI_0323	Rumex acetosella L.	0	n	1	0	Polygonaceae Juss.
Iz_4_1009	DJI_0323	Rumex thyrsiflorus Fingerh.	0	n	1	0	Polygonaceae Juss.
Iz_4_1009	DJI_0323	Salix alba L.	0	n	1	1	Salicaceae Mirb.
Iz_4_1009	DJI_0323	Sambucus nigra L.	0	n	1	1	Viburnaceae Raf.
Iz_4_1009	DJI_0323	Secale sylvestre Host	0	n	1	0	Poaceae Barnhart
Iz_4_1009	DJI_0323	Sempervivum ruthenicum Schnittsp. & C.B. Lehm.	0	n	1	0	Crassulaceae J.St.-Hil.
Iz_4_1009	DJI_0323	Setaria viridis (L.) P. Beauv.	0	n	1	0	Poaceae Barnhart
Iz_4_1009	DJI_0323	Silene borysthenica (Gruner) Walters	0	n	1	0	Caryophyllaceae Juss.
Iz_4_1009	DJI_0323	Silene latifolia subsp. alba (Mill.) Greuter & Burdet	0	n	1	0	Caryophyllaceae Juss.
Iz_4_1009	DJI_0323	Solidago virgaurea L.	0	n	1	0	Asteraceae Bercht. & J.Presl
Iz_4_1009	DJI_0323	Sorbus aucuparia L.	0	n	1	1	Rosaceae Juss.
Iz_4_1009	DJI_0323	Stachys recta L.	0	n	1	0	Lamiacea Lindl.
Iz_4_1009	DJI_0323	Tanacetum vulgare L.	0	n	1	0	Asteraceae Bercht. & J.Presl
Iz_4_1009	DJI_0323	Thymus pallasianus Heinr.Braun	0	n	1	0	Lamiacea Lindl.
Iz_4_1009	DJI_0323	Tilia cordata Mill.	0	n	1	1	Malvaceae Juss.
Iz_4_1009	DJI_0323	Trifolium alpestre L.	0	n	1	0	Fabaceae Lindl.
Iz_4_1009	DJI_0323	Trifolium arvense L.	0	n	1	0	Fabaceae Lindl.
Iz_4_1009	DJI_0323	Ulmus glabra Huds.	0	n	1	1	Ulmaceae Mirb.
Iz_4_1009	DJI_0323	Verbascum densiflorum Bertol.	0	n	1	0	Scrophulariaceae Juss.
Iz_4_1009	DJI_0323	Verbascum nigrum L.	0	n	1	0	Scrophulariaceae Juss.
Iz_4_1009	DJI_0323	Veronica austriaca L.	0	n	1	0	Plantaginaceae Juss.
Iz_4_1009	DJI_0323	Veronica incana L.	0	n	1	0	Plantaginaceae Juss.
Iz_4_1009	DJI_0323	Veronica verna L.	0	n	1	0	Plantaginaceae Juss.
Iz_4_1009	DJI_0323	Vincetoxicum hirundinaria Medik	0	n	1	0	Apocynaceae Juss.
Iz_4_1009	DJI_0325	Acer tataricum L.	0	n	1	1	Sapindaceae Juss.
Iz_4_1009	DJI_0325	Achillea millefolium L. s.l.	0	n	1	0	Asteraceae Bercht. & J.Presl
Iz_4_1009	DJI_0325	Allium oleraceum L.	0	n	1	0	Amaryllidaceae J.St.-Hil.
Iz_4_1009	DJI_0325	Ambrosia artemisiifolia L.	0	a	0	0	Asteraceae Bercht. & J.Presl
Iz_4_1009	DJI_0325	Amorpha fruticosa L.	0	a	0	0	Fabaceae Lindl.
Iz_4_1009	DJI_0325	Anthericum ramosum L.	0	n	1	0	Asparagaceae Juss.
Iz_4_1009	DJI_0325	Artemisia marschalliana Spreng.	0	n	1	0	Asteraceae Bercht. & J.Presl
Iz_4_1009	DJI_0325	Asparagus officinalis L.	0	n	1	0	Asparagaceae Juss.
Iz_4_1009	DJI_0325	Astragalus cicer L.	0	n	1	0	Fabaceae Lindl.
Iz_4_1009	DJI_0325	Bassia laniflora (S.G.Gmel.) A.J.Scott	0	n	1	0	Amaranthaceae Juss.
Iz_4_1009	DJI_0325	Berberis vulgaris L.	0	n	1	0	Berberidaceae Juss.
Iz_4_1009	DJI_0325	Betula pendula Roth.	0	n	1	1	Betulaceae Gray
Iz_4_1009	DJI_0325	Betula pendula Roth.	0	n	1	0	Poaceae Barnhart
Iz_4_1009	DJI_0325	Bromus inermis Leyss.	0		NA	NA	
Iz_4_1009	DJI_0325	Bromus squarrosus L.	0	a	0	0	Poaceae Barnhart
Iz_4_1009	DJI_0325	Bromus tectorum L.	0	a	0	0	Poaceae Barnhart
Iz_4_1009	DJI_0325	Calamagrostis epigejos (L.) Roth	40	n	1	0	Poaceae Barnhart
Iz_4_1009	DJI_0325	Carduus acanthoides L.	0	n	1	0	Asteraceae Bercht. & J.Presl
Iz_4_1009	DJI_0325	Carex sp.	0	n	1	0	Cyperaceae Juss.
Iz_4_1009	DJI_0325	Centaurea majorovii Dumbadze	0	n	1	0	Asteraceae Bercht. & J.Presl
Iz_4_1009	DJI_0325	Chamaecytisus ruthenicus (Fisch.ex Wol.) Klaskova	0	n	1	0	Fabaceae Lindl.
Iz_4_1009	DJI_0325	Chelidonium majus L.	0	n	1	0	Papaveraceae Juss.
Iz_4_1009	DJI_0325	Chenopodium betaceum Andrz.	0	n	1	0	Amaranthaceae Juss.
Iz_4_1009	DJI_0325	Chenopodium cf. album	0	n	1	0	Amaranthaceae Juss.
Iz_4_1009	DJI_0325	Chenopodium sp.	0		NA	0	Amaranthaceae Juss.
Iz_4_1009	DJI_0325	Chondrilla juncea L.	0	n	1	0	Asteraceae Bercht. & J.Presl
Iz_4_1009	DJI_0325	Cirsium vulgare (Savi) Ten.	0	n	1	0	Asteraceae Bercht. & J.Presl
Iz_4_1009	DJI_0325	Clinopodium acinos (L.) Kuntze	0	n	1	0	Lamiacea Lindl.
Iz_4_1009	DJI_0325	Convallaria majalis L.	0	n	1	0	Asparagaceae Juss.

Iz_4_1009	DJI_0325	Crataegus rhipidophylla Gand.	0	n	1	1	Rosaceae Juss.
Iz_4_1009	DJI_0325	Crepis foetida L.	0	n	1	0	Asteraceae Bercht. & J.Presl
Iz_4_1009	DJI_0325	Dactylis glomerata L.	0	n	1	0	Poaceae Barnhart
Iz_4_1009	DJI_0325	Dianthus campestris M.Bieb.	0	n	1	0	Caryophyllaceae Juss.
Iz_4_1009	DJI_0325	Digitaria sanguinalis (L.) Scop.	0	a	0	0	Poaceae Barnhart
Iz_4_1009	DJI_0325	Elymus repens (L.) Gould	0	n	1	0	Poaceae Barnhart
Iz_4_1009	DJI_0325	Epilobium tetragonum L.	0	n	1	0	Onagraceae Juss.
Iz_4_1009	DJI_0325	Eragrostis minor Host	0	a	0	0	Poaceae Barnhart
Iz_4_1009	DJI_0325	Erigeron canadensis L.	3	a	0	0	Asteraceae Bercht. & J.Presl
Iz_4_1009	DJI_0325	Euphorbia seguieriana Neck.	0	n	1	0	Euphorbiaceae Juss.
Iz_4_1009	DJI_0325	Fallopia dumetorum (L.) Holub	0	n	1	0	Polygonaceae Juss.
Iz_4_1009	DJI_0325	Festuca beckeri (Hack.) Trautv.	0	n	1	0	Poaceae Barnhart
Iz_4_1009	DJI_0325	Fragaria vesca L.	0	n	1	0	Rosaceae Juss.
Iz_4_1009	DJI_0325	Frangula alnus Mill.	0	n	1	1	Rhamnaceae Juss.
Iz_4_1009	DJI_0325	Fraxinus pennsylvanica Marshall	0	a	0	1	Oleaceae Hoffmanns. & Link
Iz_4_1009	DJI_0325	Galeopsis bifida Boenn.	0	n	1	0	Lamiacea Lindl.
Iz_4_1009	DJI_0325	Galium verum L.	0	n	1	0	Rubiaceae Juss.
Iz_4_1009	DJI_0325	Genista tinctoria L.	0	n	1	0	Fabaceae Lindl.
Iz_4_1009	DJI_0325	Geranium robertianum L.	0	n	1	0	Geraniaceae Juss.
Iz_4_1009	DJI_0325	Geranium sanquineum L.	0	n	1	0	Geraniaceae Juss.
Iz_4_1009	DJI_0325	Gypsophila paniculata L.	0	n	1	0	Caryophyllaceae Juss.
Iz_4_1009	DJI_0325	Helichrysum arenarium (L.) Moench	0	n	1	0	Asteraceae Bercht. & J.Presl
Iz_4_1009	DJI_0325	Herniaria polygama J.Gay	0	n	1	0	Caryophyllaceae Juss.
Iz_4_1009	DJI_0325	Hieracium robustum Fr.	1	n	1	0	Asteraceae Bercht. & J.Presl
Iz_4_1009	DJI_0325	Hieracium umbellatum L.	0	n	1	NA	Asteraceae Bercht. & J.Presl
Iz_4_1009	DJI_0325	Hylotelephium maximum (L.) Holub	0	n	1	0	Crassulaceae J.St.-Hil.
Iz_4_1009	DJI_0325	Jacobaea borysthenica (DC.) B.Nord. & Greuter	0	n	1	0	Asteraceae Bercht. & J.Presl
Iz_4_1009	DJI_0325	Jasione montana L.	0	n	1	0	Campanulaceae Juss.
Iz_4_1009	DJI_0325	Jurinea cyanoides (L.) Rchb.s.l.	0	n	1	0	Asteraceae Bercht. & J.Presl
Iz_4_1009	DJI_0325	Koeleria glauca (Spreng.) DC.	0	n	1	0	Poaceae Barnhart
Iz_4_1009	DJI_0325	Lactuca serriola L.	0	a	0	0	Asteraceae Bercht. & J.Presl
Iz_4_1009	DJI_0325	Linaria genistifolia (L.) Mill.	0	n	1	0	Plantaginaceae Juss.
Iz_4_1009	DJI_0325	Lomelosia argentea (L.) Greuter & Burdet	0	n	1	0	Caprifoliaceae Juss.
Iz_4_1009	DJI_0325	Melampyrum pratense L.	0	n	1	0	Orobanchaceae Vent.
Iz_4_1009	DJI_0325	Oenothera biennis L.	0	a	0	0	Onagraceae Juss.
Iz_4_1009	DJI_0325	Pentanema britannica (L.) D.Gut.Larr., Santos-Vicente, Anderb., E.Rico & M.M.Mart.Ort.	0	n	1	0	Asteraceae Bercht. & J.Presl
Iz_4_1009	DJI_0325	Peucedanum oreoselinum (L.) Moench	0	n	1	0	Apiaceae Lindl.
Iz_4_1009	DJI_0325	Peucedanum ruthenicum M.Bieb.	0	n	1	0	Apiaceae Lindl.
Iz_4_1009	DJI_0325	Pilosella echioides (Lumn.) F.W.Schultz & Sch.Bip.	0	n	1	0	Asteraceae Bercht. & J.Presl
Iz_4_1009	DJI_0325	Pilosella officinarum F. Schultz et Sch. Bip.	0	n	1	0	Asteraceae Bercht. & J.Presl
Iz_4_1009	DJI_0325	Pilosella sp.	0		NA	0	Asteraceae Bercht. & J.Presl
Iz_4_1009	DJI_0325	Pinus sylvestris L.	0	n	1	1	Pinaceae Spreng. ex F.Rudolphi
Iz_4_1009	DJI_0325	Poa annua L.	0	n	1	0	Poaceae Barnhart
Iz_4_1009	DJI_0325	Poa nemoralis L.	0	n	1	0	Poaceae Barnhart
Iz_4_1009	DJI_0325	Poa sp.	0		NA	0	Poaceae Barnhart
Iz_4_1009	DJI_0325	Polygonatum odoratum (Mill.) Druce	0	n	1	0	Asparagaceae Juss.
Iz_4_1009	DJI_0325	Polygonum arenarium Waldst. & Kit.	0	n	1	0	Polygonaceae Juss.
Iz_4_1009	DJI_0325	Populus nigra L.	0	n	1	1	Salicaceae Mirb.
Iz_4_1009	DJI_0325	Populus tremula L.	0	n	1	1	Salicaceae Mirb.
Iz_4_1009	DJI_0325	Populus Ã canescens (Aiton) Sm.	0	n	1	1	Salicaceae Mirb.
Iz_4_1009	DJI_0325	Potentilla argentea L.	0	n	1	0	Rosaceae Juss.
Iz_4_1009	DJI_0325	Psammophiliella muralis (L.) Ikonn.	0	n	1	0	Caryophyllaceae Juss.
Iz_4_1009	DJI_0325	Psephellus sumensis (Kalen.) Greuter	0	n	1	0	Asteraceae Bercht. & J.Presl
Iz_4_1009	DJI_0325	Pulsatilla pratensis (L.) Mill. s.l.	0	n	1	0	Ranunculaceae Juss.
Iz_4_1009	DJI_0325	Pyrus communis L.	0	a	0	1	Rosaceae Juss.
Iz_4_1009	DJI_0325	Quercus robur L.	0	n	1	1	Fagaceae Dumort.
Iz_4_1009	DJI_0325	Robinia pseudoacacia L.	0	a	0	1	Fabaceae Lindl.
Iz_4_1009	DJI_0325	Rosa sp.	0		NA	1	Rosaceae Juss.
Iz_4_1009	DJI_0325	Rubus idaeus L.	0	n	1	0	Rosaceae Juss.
Iz_4_1009	DJI_0325	Rumex acetosella L.	0	n	1	0	Polygonaceae Juss.
Iz_4_1009	DJI_0325	Rumex thyrsiflorus Fingerh.	0	n	1	0	Polygonaceae Juss.
Iz_4_1009	DJI_0325	Salix alba L.	0	n	1	1	Salicaceae Mirb.
Iz_4_1009	DJI_0325	Sambucus nigra L.	0	n	1	1	Viburnaceae Raf.
Iz_4_1009	DJI_0325	Secale sylvestre Host	0	n	1	0	Poaceae Barnhart
Iz_4_1009	DJI_0325	Sempervivum ruthenicum Schnittsp. & C.B. Lehm.	0	n	1	0	Crassulaceae J.St.-Hil.
Iz_4_1009	DJI_0325	Setaria viridis (L.) P. Beauv.	0	n	1	0	Poaceae Barnhart
Iz_4_1009	DJI_0325	Silene borysthenica (Gruner) Walters	0	n	1	0	Caryophyllaceae Juss.
Iz_4_1009	DJI_0325	Silene latifolia subsp. alba (Mill.) Greuter & Burdet	0	n	1	0	Caryophyllaceae Juss.
Iz_4_1009	DJI_0325	Solidago virgaurea L.	0	n	1	0	Asteraceae Bercht. & J.Presl
Iz_4_1009	DJI_0325	Sorbus aucuparia L.	0	n	1	1	Rosaceae Juss.
Iz_4_1009	DJI_0325	Stachys recta L.	0	n	1	0	Lamiacea Lindl.
Iz_4_1009	DJI_0325	Tanacetum vulgare L.	0	n	1	0	Asteraceae Bercht. & J.Presl
Iz_4_1009	DJI_0325	Thymus pallasianus Heinr.Braun	0	n	1	0	Lamiacea Lindl.
Iz_4_1009	DJI_0325	Tilia cordata Mill.	0	n	1	1	Malvaceae Juss.
Iz_4_1009	DJI_0325	Trifolium alpestre L.	0	n	1	0	Fabaceae Lindl.
Iz_4_1009	DJI_0325	Trifolium arvense L.	0	n	1	0	Fabaceae Lindl.
Iz_4_1009	DJI_0325	Ulmus glabra Huds.	0	n	1	1	Ulmaceae Mirb.
Iz_4_1009	DJI_0325	Verbascum densiflorum Bertol.	0	n	1	0	Scrophulariaceae Juss.
Iz_4_1009	DJI_0325	Verbascum nigrum L.	0	n	1	0	Scrophulariaceae Juss.
Iz_4_1009	DJI_0325	Veronica austriaca L.	0	n	1	0	Plantaginaceae Juss.
Iz_4_1009	DJI_0325	Veronica incana L.	0	n	1	0	Plantaginaceae Juss.
Iz_4_1009	DJI_0325	Veronica verna L.	0	n	1	0	Plantaginaceae Juss.
Iz_4_1009	DJI_0325	Vincetoxicum hirundinaria Medik	0	n	1	0	Apocynaceae Juss.
Iz_4_1009	DJI_0327	Acer tataricum L.	0	n	1	1	Sapindaceae Juss.
Iz_4_1009	DJI_0327	Achillea millefolium L. s.l.	0	n	1	0	Asteraceae Bercht. & J.Presl
Iz_4_1009	DJI_0327	Allium oleraceum L.	0	n	1	0	Amaryllidaceae J.St.-Hil.
Iz_4_1009	DJI_0327	Ambrosia artemisiifolia L.	0	a	0	0	Asteraceae Bercht. & J.Presl
Iz_4_1009	DJI_0327	Amorpha fruticosa L.	0	a	0	0	Fabaceae Lindl.
Iz_4_1009	DJI_0327	Anthericum ramosum L.	0	n	1	0	Asparagaceae Juss.
Iz_4_1009	DJI_0327	Artemisia marschalliana Spreng.	0	n	1	0	Asteraceae Bercht. & J.Presl
Iz_4_1009	DJI_0327	Asparagus officinalis L.	0	n	1	0	Asparagaceae Juss.
Iz_4_1009	DJI_0327	Astragalus cicer L.	0	n	1	0	Fabaceae Lindl.
Iz_4_1009	DJI_0327	Bassia laniflora (S.G.Gmel.) A.J.Scott	0	n	1	0	Amaranthaceae Juss.
Iz_4_1009	DJI_0327	Berberis vulgaris L.	0	n	1	0	Berberidaceae Juss.
Iz_4_1009	DJI_0327	Betula pendula Roth.	0	n	1	1	Betulaceae Gray
Iz_4_1009	DJI_0327	Betula pendula Roth.	0	n	1	0	Poaceae Barnhart
Iz_4_1009	DJI_0327	Bromus inermis Leyss.	0		NA	NA	
Iz_4_1009	DJI_0327	Bromus squarrosus L.	0	a	0	0	Poaceae Barnhart

Iz_4_1009	DJI_0327	Bromus tectorum L.	0	a	0	0	Poaceae Barnhart
Iz_4_1009	DJI_0327	Calamagrostis epigejos (L.) Roth	40	n	1	0	Poaceae Barnhart
Iz_4_1009	DJI_0327	Carduus acanthoides L.	0	n	1	0	Asteraceae Bercht. & J.Presl
Iz_4_1009	DJI_0327	Carex sp.	0	n	1	0	Cyperaceae Juss.
Iz_4_1009	DJI_0327	Centaurea majorovii Dumbadze	0	n	1	0	Asteraceae Bercht. & J.Presl
Iz_4_1009	DJI_0327	Chamaecytisus ruthenicus (Fisch.ex Wol.) Klaskova	0	n	1	0	Fabaceae Lindl.
Iz_4_1009	DJI_0327	Chelidonium majus L.	0	n	1	0	Papaveraceae Juss.
Iz_4_1009	DJI_0327	Chenopodium betaceum Andrz.	0	n	1	0	Amaranthaceae Juss.
Iz_4_1009	DJI_0327	Chenopodium cf. album	0	n	1	0	Amaranthaceae Juss.
Iz_4_1009	DJI_0327	Chenopodium sp.	0		NA	0	Amaranthaceae Juss.
Iz_4_1009	DJI_0327	Chondrilla juncea L.	0	n	1	0	Asteraceae Bercht. & J.Presl
Iz_4_1009	DJI_0327	Cirsium vulgare (Savi) Ten.	0	n	1	0	Asteraceae Bercht. & J.Presl
Iz_4_1009	DJI_0327	Clinopodium acinos (L.) Kuntze	0	n	1	0	Lamiacea Lindl.
Iz_4_1009	DJI_0327	Convallaria majalis L.	0	n	1	0	Asparagaceae Juss.
Iz_4_1009	DJI_0327	Crataegus rhipidophylla Gand.	0	n	1	1	Rosaceae Juss.
Iz_4_1009	DJI_0327	Crepis foetida L.	0	n	1	0	Asteraceae Bercht. & J.Presl
Iz_4_1009	DJI_0327	Dactylis glomerata L.	0	n	1	0	Poaceae Barnhart
Iz_4_1009	DJI_0327	Dianthus campestris M.Bieb.	0	n	1	0	Caryophyllaceae Juss.
Iz_4_1009	DJI_0327	Digitaria sanguinalis (L.) Scop.	0	a	0	0	Poaceae Barnhart
Iz_4_1009	DJI_0327	Elymus repens (L.) Gould	0	n	1	0	Poaceae Barnhart
Iz_4_1009	DJI_0327	Epilobium tetragonum L.	0	n	1	0	Onagraceae Juss.
Iz_4_1009	DJI_0327	Eragrostis minor Host	0	a	0	0	Poaceae Barnhart
Iz_4_1009	DJI_0327	Erigeron canadensis L.	0	a	0	0	Asteraceae Bercht. & J.Presl
Iz_4_1009	DJI_0327	Euphorbia seguieriana Neck.	0	n	1	0	Euphorbiaceae Juss.
Iz_4_1009	DJI_0327	Fallopia dumetorum (L.) Holub	0	n	1	0	Polygonaceae Juss.
Iz_4_1009	DJI_0327	Festuca beckeri (Hack.) Trautv.	0	n	1	0	Poaceae Barnhart
Iz_4_1009	DJI_0327	Fragaria vesca L.	0	n	1	0	Rosaceae Juss.
Iz_4_1009	DJI_0327	Frangula alnus Mill.	0	n	1	1	Rhamnaceae Juss.
Iz_4_1009	DJI_0327	Fraxinus pennsylvanica Marshall	0	a	0	1	Oleaceae Hoffmanns. & Link
Iz_4_1009	DJI_0327	Galeopsis bifida Boenn.	0	n	1	0	Lamiacea Lindl.
Iz_4_1009	DJI_0327	Galium verum L.	0	n	1	0	Rubiaceae Juss.
Iz_4_1009	DJI_0327	Genista tinctoria L.	0	n	1	0	Fabaceae Lindl.
Iz_4_1009	DJI_0327	Geranium robertianum L.	0	n	1	0	Geraniaceae Juss.
Iz_4_1009	DJI_0327	Geranium sanquineum L.	0	n	1	0	Geraniaceae Juss.
Iz_4_1009	DJI_0327	Gypsophila paniculata L.	0	n	1	0	Caryophyllaceae Juss.
Iz_4_1009	DJI_0327	Helichrysum arenarium (L.) Moench	0	n	1	0	Asteraceae Bercht. & J.Presl
Iz_4_1009	DJI_0327	Herniaria polygama J.Gay	0	n	1	0	Caryophyllaceae Juss.
Iz_4_1009	DJI_0327	Hieracium robustum Fr.	0.5	n	1	0	Asteraceae Bercht. & J.Presl
Iz_4_1009	DJI_0327	Hieracium umbellatum L.	0	n	1	NA	Asteraceae Bercht. & J.Presl
Iz_4_1009	DJI_0327	Hylotelephium maximum (L.) Holub	0	n	1	0	Crassulaceae J.St.-Hil.
Iz_4_1009	DJI_0327	Jacobaea borysthenica (DC.) B.Nord. & Greuter	0	n	1	0	Asteraceae Bercht. & J.Presl
Iz_4_1009	DJI_0327	Jasione montana L.	0	n	1	0	Campanulaceae Juss.
Iz_4_1009	DJI_0327	Jurinea cyanoides (L.) Rchb.s.l.	0	n	1	0	Asteraceae Bercht. & J.Presl
Iz_4_1009	DJI_0327	Koeleria glauca (Spreng.) DC.	0	n	1	0	Poaceae Barnhart
Iz_4_1009	DJI_0327	Lactuca serriola L.	0	a	0	0	Asteraceae Bercht. & J.Presl
Iz_4_1009	DJI_0327	Linaria genistifolia (L.) Mill.	0	n	1	0	Plantaginaceae Juss.
Iz_4_1009	DJI_0327	Lomelosia argentea (L.) Greuter & Burdet	0	n	1	0	Caprifoliaceae Juss.
Iz_4_1009	DJI_0327	Melampyrum pratense L.	0	n	1	0	Orobanchaceae Vent.
Iz_4_1009	DJI_0327	Oenothera biennis L.	0	a	0	0	Onagraceae Juss.
Iz_4_1009	DJI_0327	Pentanema britannica (L.) D.Gut.Larr., Santos-Vicente, Anderb., E.Rico & M.M.Mart.Ort.	0	n	1	0	Asteraceae Bercht. & J.Presl
Iz_4_1009	DJI_0327	Peucedanum oreoselinum (L.) Moench	0	n	1	0	Apiaceae Lindl.
Iz_4_1009	DJI_0327	Peucedanum ruthenicum M.Bieb.	0	n	1	0	Apiaceae Lindl.
Iz_4_1009	DJI_0327	Pilosella echioides (Lumn.) F.W.Schultz & Sch.Bip.	0	n	1	0	Asteraceae Bercht. & J.Presl
Iz_4_1009	DJI_0327	Pilosella officinarum F. Schultz et Sch. Bip.	0	n	1	0	Asteraceae Bercht. & J.Presl
Iz_4_1009	DJI_0327	Pilosella sp.	0		NA	0	Asteraceae Bercht. & J.Presl
Iz_4_1009	DJI_0327	Pinus sylvestris L.	0	n	1	1	Pinaceae Spreng. ex F.Rudolphi
Iz_4_1009	DJI_0327	Poa annua L.	0	n	1	0	Poaceae Barnhart
Iz_4_1009	DJI_0327	Poa nemoralis L.	0	n	1	0	Poaceae Barnhart
Iz_4_1009	DJI_0327	Poa sp.	0		NA	0	Poaceae Barnhart
Iz_4_1009	DJI_0327	Polygonatum odoratum (Mill.) Druce	0	n	1	0	Asparagaceae Juss.
Iz_4_1009	DJI_0327	Polygonum arenarium Waldst. & Kit.	0	n	1	0	Polygonaceae Juss.
Iz_4_1009	DJI_0327	Populus nigra L.	0	n	1	1	Salicaceae Mirb.
Iz_4_1009	DJI_0327	Populus tremula L.	0	n	1	1	Salicaceae Mirb.
Iz_4_1009	DJI_0327	Populus Ã canescens (Aiton) Sm.	0	n	1	1	Salicaceae Mirb.
Iz_4_1009	DJI_0327	Potentilla argentea L.	0	n	1	0	Rosaceae Juss.
Iz_4_1009	DJI_0327	Psammophiliella muralis (L.) Ikonn.	0	n	1	0	Caryophyllaceae Juss.
Iz_4_1009	DJI_0327	Psephellus sumensis (Kalen.) Greuter	0	n	1	0	Asteraceae Bercht. & J.Presl
Iz_4_1009	DJI_0327	Pulsatilla pratensis (L.) Mill. s.l.	0	n	1	0	Ranunculaceae Juss.
Iz_4_1009	DJI_0327	Pyrus communis L.	0	a	0	1	Rosaceae Juss.
Iz_4_1009	DJI_0327	Quercus robur L.	0	n	1	1	Fagaceae Dumort.
Iz_4_1009	DJI_0327	Robinia pseudoacacia L.	0	a	0	1	Fabaceae Lindl.
Iz_4_1009	DJI_0327	Rosa sp.	0		NA	1	Rosaceae Juss.
Iz_4_1009	DJI_0327	Rubus idaeus L.	0	n	1	0	Rosaceae Juss.
Iz_4_1009	DJI_0327	Rumex acetosella L.	0	n	1	0	Polygonaceae Juss.
Iz_4_1009	DJI_0327	Rumex thyrsiflorus Fingerh.	0	n	1	0	Polygonaceae Juss.
Iz_4_1009	DJI_0327	Salix alba L.	0	n	1	1	Salicaceae Mirb.
Iz_4_1009	DJI_0327	Sambucus nigra L.	0	n	1	1	Viburnaceae Raf.
Iz_4_1009	DJI_0327	Secale sylvestre Host	0	n	1	0	Poaceae Barnhart
Iz_4_1009	DJI_0327	Sempervivum ruthenicum Schnittsp. & C.B. Lehm.	0	n	1	0	Crassulaceae J.St.-Hil.
Iz_4_1009	DJI_0327	Setaria viridis (L.) P. Beauv.	0	n	1	0	Poaceae Barnhart
Iz_4_1009	DJI_0327	Silene borysthenica (Gruner) Walters	0	n	1	0	Caryophyllaceae Juss.
Iz_4_1009	DJI_0327	Silene latifolia subsp. alba (Mill.) Greuter & Burdet	0	n	1	0	Caryophyllaceae Juss.
Iz_4_1009	DJI_0327	Solidago virgaurea L.	0	n	1	0	Asteraceae Bercht. & J.Presl
Iz_4_1009	DJI_0327	Sorbus aucuparia L.	0	n	1	1	Rosaceae Juss.
Iz_4_1009	DJI_0327	Stachys recta L.	0	n	1	0	Lamiacea Lindl.
Iz_4_1009	DJI_0327	Tanacetum vulgare L.	0	n	1	0	Asteraceae Bercht. & J.Presl
Iz_4_1009	DJI_0327	Thymus pallasianus Heinr.Braun	0	n	1	0	Lamiacea Lindl.
Iz_4_1009	DJI_0327	Tilia cordata Mill.	0	n	1	1	Malvaceae Juss.
Iz_4_1009	DJI_0327	Trifolium alpestre L.	0	n	1	0	Fabaceae Lindl.
Iz_4_1009	DJI_0327	Trifolium arvense L.	0	n	1	0	Fabaceae Lindl.
Iz_4_1009	DJI_0327	Ulmus glabra Huds.	0	n	1	1	Ulmaceae Mirb.
Iz_4_1009	DJI_0327	Verbascum densiflorum Bertol.	0	n	1	0	Scrophulariaceae Juss.
Iz_4_1009	DJI_0327	Verbascum nigrum L.	0	n	1	0	Scrophulariaceae Juss.
Iz_4_1009	DJI_0327	Veronica austriaca L.	0	n	1	0	Plantaginaceae Juss.
Iz_4_1009	DJI_0327	Veronica incana L.	0	n	1	0	Plantaginaceae Juss.
Iz_4_1009	DJI_0327	Veronica verna L.	0	n	1	0	Plantaginaceae Juss.
Iz_4_1009	DJI_0327	Vincetoxicum hirundinaria Medik	0	n	1	0	Apocynaceae Juss.
Iz_4_1009	DJI_0330	Acer tataricum L.	0	n	1	1	Sapindaceae Juss.

Iz_4_1009	DJI_0330	Achillea millefolium L. s.l.	0	n	1	0	Asteraceae Bercht. & J.Presl
Iz_4_1009	DJI_0330	Allium oleraceum L.	0	n	1	0	Amaryllidaceae J.St.-Hil.
Iz_4_1009	DJI_0330	Ambrosia artemisiifolia L.	0	a	0	0	Asteraceae Bercht. & J.Presl
Iz_4_1009	DJI_0330	Amorpha fruticosa L.	0	a	0	0	Fabaceae Lindl.
Iz_4_1009	DJI_0330	Anthericum ramosum L.	0	n	1	0	Asparagaceae Juss.
Iz_4_1009	DJI_0330	Artemisia marschalliana Spreng.	0	n	1	0	Asteraceae Bercht. & J.Presl
Iz_4_1009	DJI_0330	Asparagus officinalis L.	0	n	1	0	Asparagaceae Juss.
Iz_4_1009	DJI_0330	Astragalus cicer L.	0	n	1	0	Fabaceae Lindl.
Iz_4_1009	DJI_0330	Bassia laniflora (S.G.Gmel.) A.J.Scott	0	n	1	0	Amaranthaceae Juss.
Iz_4_1009	DJI_0330	Berberis vulgaris L.	0	n	1	0	Berberidaceae Juss.
Iz_4_1009	DJI_0330	Betula pendula Roth.	0	n	1	1	Betulaceae Gray
Iz_4_1009	DJI_0330	Betula pendula Roth.	0	n	1	0	Poaceae Barnhart
Iz_4_1009	DJI_0330	Bromus inermis Leyss.	0		NA	NA	
Iz_4_1009	DJI_0330	Bromus squarrosus L.	0	a	0	0	Poaceae Barnhart
Iz_4_1009	DJI_0330	Bromus tectorum L.	0	a	0	0	Poaceae Barnhart
Iz_4_1009	DJI_0330	Calamagrostis epigejos (L.) Roth	5	n	1	0	Poaceae Barnhart
Iz_4_1009	DJI_0330	Carduus acanthoides L.	0	n	1	0	Asteraceae Bercht. & J.Presl
Iz_4_1009	DJI_0330	Carex sp.	0	n	1	0	Cyperaceae Juss.
Iz_4_1009	DJI_0330	Centaurea majorovii Dumbadze	0	n	1	0	Asteraceae Bercht. & J.Presl
Iz_4_1009	DJI_0330	Chamaecytisus ruthenicus (Fisch.ex Wol.) Klaskova	0	n	1	0	Fabaceae Lindl.
Iz_4_1009	DJI_0330	Chelidonium majus L.	0	n	1	0	Papaveraceae Juss.
Iz_4_1009	DJI_0330	Chenopodium betaceum Andrz.	0	n	1	0	Amaranthaceae Juss.
Iz_4_1009	DJI_0330	Chenopodium cf. album	0	n	1	0	Amaranthaceae Juss.
Iz_4_1009	DJI_0330	Chenopodium sp.	0		NA	0	Amaranthaceae Juss.
Iz_4_1009	DJI_0330	Chondrilla juncea L.	0	n	1	0	Asteraceae Bercht. & J.Presl
Iz_4_1009	DJI_0330	Cirsium vulgare (Savi) Ten.	0	n	1	0	Asteraceae Bercht. & J.Presl
Iz_4_1009	DJI_0330	Clinopodium acinos (L.) Kuntze	0	n	1	0	Lamiacea Lindl.
Iz_4_1009	DJI_0330	Convallaria majalis L.	0	n	1	0	Asparagaceae Juss.
Iz_4_1009	DJI_0330	Crataegus rhipidophylla Gand.	0	n	1	1	Rosaceae Juss.
Iz_4_1009	DJI_0330	Crepis foetida L.	0	n	1	0	Asteraceae Bercht. & J.Presl
Iz_4_1009	DJI_0330	Dactylis glomerata L.	0	n	1	0	Poaceae Barnhart
Iz_4_1009	DJI_0330	Dianthus campestris M.Bieb.	0	n	1	0	Caryophyllaceae Juss.
Iz_4_1009	DJI_0330	Digitaria sanguinalis (L.) Scop.	0	a	0	0	Poaceae Barnhart
Iz_4_1009	DJI_0330	Elymus repens (L.) Gould	0	n	1	0	Poaceae Barnhart
Iz_4_1009	DJI_0330	Epilobium tetragonum L.	0	n	1	0	Onagraceae Juss.
Iz_4_1009	DJI_0330	Eragrostis minor Host	0	a	0	0	Poaceae Barnhart
Iz_4_1009	DJI_0330	Erigeron canadensis L.	1	a	0	0	Asteraceae Bercht. & J.Presl
Iz_4_1009	DJI_0330	Euphorbia seguieriana Neck.	0	n	1	0	Euphorbiaceae Juss.
Iz_4_1009	DJI_0330	Fallopia dumetorum (L.) Holub	0	n	1	0	Polygonaceae Juss.
Iz_4_1009	DJI_0330	Festuca beckeri (Hack.) Trautv.	0	n	1	0	Poaceae Barnhart
Iz_4_1009	DJI_0330	Fragaria vesca L.	0	n	1	0	Rosaceae Juss.
Iz_4_1009	DJI_0330	Frangula alnus Mill.	0	n	1	1	Rhamnaceae Juss.
Iz_4_1009	DJI_0330	Fraxinus pennsylvanica Marshall	0	a	0	1	Oleaceae Hoffmanns. & Link
Iz_4_1009	DJI_0330	Galeopsis bifida Boenn.	0	n	1	0	Lamiacea Lindl.
Iz_4_1009	DJI_0330	Galium verum L.	0	n	1	0	Rubiaceae Juss.
Iz_4_1009	DJI_0330	Genista tinctoria L.	0	n	1	0	Fabaceae Lindl.
Iz_4_1009	DJI_0330	Geranium robertianum L.	0	n	1	0	Geraniaceae Juss.
Iz_4_1009	DJI_0330	Geranium sanquineum L.	0	n	1	0	Geraniaceae Juss.
Iz_4_1009	DJI_0330	Gypsophila paniculata L.	0	n	1	0	Caryophyllaceae Juss.
Iz_4_1009	DJI_0330	Helichrysum arenarium (L.) Moench	0	n	1	0	Asteraceae Bercht. & J.Presl
Iz_4_1009	DJI_0330	Herniaria polygama J.Gay	0	n	1	0	Caryophyllaceae Juss.
Iz_4_1009	DJI_0330	Hieracium robustum Fr.	0	n	1	0	Asteraceae Bercht. & J.Presl
Iz_4_1009	DJI_0330	Hieracium umbellatum L.	0	n	1	NA	Asteraceae Bercht. & J.Presl
Iz_4_1009	DJI_0330	Hylotelephium maximum (L.) Holub	0	n	1	0	Crassulaceae J.St.-Hil.
Iz_4_1009	DJI_0330	Jacobaea borysthenica (DC.) B.Nord. & Greuter	0	n	1	0	Asteraceae Bercht. & J.Presl
Iz_4_1009	DJI_0330	Jasione montana L.	0	n	1	0	Campanulaceae Juss.
Iz_4_1009	DJI_0330	Jurinea cyanoides (L.) Rchb.s.l.	0	n	1	0	Asteraceae Bercht. & J.Presl
Iz_4_1009	DJI_0330	Koeleria glauca (Spreng.) DC.	0	n	1	0	Poaceae Barnhart
Iz_4_1009	DJI_0330	Lactuca serriola L.	0	a	0	0	Asteraceae Bercht. & J.Presl
Iz_4_1009	DJI_0330	Linaria genistifolia (L.) Mill.	0	n	1	0	Plantaginaceae Juss.
Iz_4_1009	DJI_0330	Lomelosia argentea (L.) Greuter & Burdet	0	n	1	0	Caprifoliaceae Juss.
Iz_4_1009	DJI_0330	Melampyrum pratense L.	0	n	1	0	Orobanchaceae Vent.
Iz_4_1009	DJI_0330	Oenothera biennis L.	0	a	0	0	Onagraceae Juss.
Iz_4_1009	DJI_0330	Pentanema britannica (L.) D.Gut.Larr., Santos-Vicente, Anderb., E.Rico & M.M.Mart.Ort.	0	n	1	0	Asteraceae Bercht. & J.Presl
Iz_4_1009	DJI_0330	Peucedanum oreoselinum (L.) Moench	0	n	1	0	Apiaceae Lindl.
Iz_4_1009	DJI_0330	Peucedanum ruthenicum M.Bieb.	0	n	1	0	Apiaceae Lindl.
Iz_4_1009	DJI_0330	Pilosella echioides (Lumn.) F.W.Schultz & Sch.Bip.	0	n	1	0	Asteraceae Bercht. & J.Presl
Iz_4_1009	DJI_0330	Pilosella officinarum F. Schultz et Sch. Bip.	0	n	1	0	Asteraceae Bercht. & J.Presl
Iz_4_1009	DJI_0330	Pilosella sp.	0		NA	0	Asteraceae Bercht. & J.Presl
Iz_4_1009	DJI_0330	Pinus sylvestris L.	0	n	1	1	Pinaceae Spreng. ex F.Rudolphi
Iz_4_1009	DJI_0330	Poa annua L.	0	n	1	0	Poaceae Barnhart
Iz_4_1009	DJI_0330	Poa nemoralis L.	0	n	1	0	Poaceae Barnhart
Iz_4_1009	DJI_0330	Poa sp.	0		NA	0	Poaceae Barnhart
Iz_4_1009	DJI_0330	Polygonatum odoratum (Mill.) Druce	0	n	1	0	Asparagaceae Juss.
Iz_4_1009	DJI_0330	Polygonum arenarium Waldst. & Kit.	0	n	1	0	Polygonaceae Juss.
Iz_4_1009	DJI_0330	Populus nigra L.	0	n	1	1	Salicaceae Mirb.
Iz_4_1009	DJI_0330	Populus tremula L.	0	n	1	1	Salicaceae Mirb.
Iz_4_1009	DJI_0330	Populus Ã canescens (Aiton) Sm.	0	n	1	1	Salicaceae Mirb.
Iz_4_1009	DJI_0330	Potentilla argentea L.	0	n	1	0	Rosaceae Juss.
Iz_4_1009	DJI_0330	Psammophiliella muralis (L.) Ikonn.	0	n	1	0	Caryophyllaceae Juss.
Iz_4_1009	DJI_0330	Psephellus sumensis (Kalen.) Greuter	0	n	1	0	Asteraceae Bercht. & J.Presl
Iz_4_1009	DJI_0330	Pulsatilla pratensis (L.) Mill. s.l.	0	n	1	0	Ranunculaceae Juss.
Iz_4_1009	DJI_0330	Pyrus communis L.	0	a	0	1	Rosaceae Juss.
Iz_4_1009	DJI_0330	Quercus robur L.	0	n	1	1	Fagaceae Dumort.
Iz_4_1009	DJI_0330	Robinia pseudoacacia L.	0	a	0	1	Fabaceae Lindl.
Iz_4_1009	DJI_0330	Rosa sp.	0		NA	1	Rosaceae Juss.
Iz_4_1009	DJI_0330	Rubus idaeus L.	0	n	1	0	Rosaceae Juss.
Iz_4_1009	DJI_0330	Rumex acetosella L.	1	n	1	0	Polygonaceae Juss.
Iz_4_1009	DJI_0330	Rumex thyrsiflorus Fingerh.	0	n	1	0	Polygonaceae Juss.
Iz_4_1009	DJI_0330	Salix alba L.	0	n	1	1	Salicaceae Mirb.
Iz_4_1009	DJI_0330	Sambucus nigra L.	0	n	1	1	Viburnaceae Raf.
Iz_4_1009	DJI_0330	Secale sylvestre Host	0	n	1	0	Poaceae Barnhart
Iz_4_1009	DJI_0330	Sempervivum ruthenicum Schnittsp. & C.B. Lehm.	0	n	1	0	Crassulaceae J.St.-Hil.
Iz_4_1009	DJI_0330	Setaria viridis (L.) P. Beauv.	0	n	1	0	Poaceae Barnhart
Iz_4_1009	DJI_0330	Silene borysthenica (Gruner) Walters	0	n	1	0	Caryophyllaceae Juss.
Iz_4_1009	DJI_0330	Silene latifolia subsp. alba (Mill.) Greuter & Burdet	0	n	1	0	Caryophyllaceae Juss.
Iz_4_1009	DJI_0330	Solidago virgaurea L.	0	n	1	0	Asteraceae Bercht. & J.Presl
Iz_4_1009	DJI_0330	Sorbus aucuparia L.	0	n	1	1	Rosaceae Juss.

Iz_4_1009	DJI_0330	Stachys recta L.	0	n	1	0	Lamiacea Lindl.
Iz_4_1009	DJI_0330	Tanacetum vulgare L.	0	n	1	0	Asteraceae Bercht. & J.Presl
Iz_4_1009	DJI_0330	Thymus pallasianus Heinr.Braun	0	n	1	0	Lamiacea Lindl.
Iz_4_1009	DJI_0330	Tilia cordata Mill.	0	n	1	1	Malvaceae Juss.
Iz_4_1009	DJI_0330	Trifolium alpestre L.	0	n	1	0	Fabaceae Lindl.
Iz_4_1009	DJI_0330	Trifolium arvense L.	0	n	1	0	Fabaceae Lindl.
Iz_4_1009	DJI_0330	Ulmus glabra Huds.	0	n	1	1	Ulmaceae Mirb.
Iz_4_1009	DJI_0330	Verbascum densiflorum Bertol.	0	n	1	0	Scrophulariaceae Juss.
Iz_4_1009	DJI_0330	Verbascum nigrum L.	0	n	1	0	Scrophulariaceae Juss.
Iz_4_1009	DJI_0330	Veronica austriaca L.	0	n	1	0	Plantaginaceae Juss.
Iz_4_1009	DJI_0330	Veronica incana L.	0	n	1	0	Plantaginaceae Juss.
Iz_4_1009	DJI_0330	Veronica verna L.	0	n	1	0	Plantaginaceae Juss.
Iz_4_1009	DJI_0330	Vincetoxicum hirundinaria Medik	0	n	1	0	Apocynaceae Juss.
Iz_4_1009	DJI_0333	Acer tataricum L.	0	n	1	1	Sapindaceae Juss.
Iz_4_1009	DJI_0333	Achillea millefolium L. s.l.	0	n	1	0	Asteraceae Bercht. & J.Presl
Iz_4_1009	DJI_0333	Allium oleraceum L.	0	n	1	0	Amaryllidaceae J.St.-Hil.
Iz_4_1009	DJI_0333	Ambrosia artemisiifolia L.	0	a	0	0	Asteraceae Bercht. & J.Presl
Iz_4_1009	DJI_0333	Amorpha fruticosa L.	0	a	0	0	Fabaceae Lindl.
Iz_4_1009	DJI_0333	Anthericum ramosum L.	0	n	1	0	Asparagaceae Juss.
Iz_4_1009	DJI_0333	Artemisia marschalliana Spreng.	0	n	1	0	Asteraceae Bercht. & J.Presl
Iz_4_1009	DJI_0333	Asparagus officinalis L.	0	n	1	0	Asparagaceae Juss.
Iz_4_1009	DJI_0333	Astragalus cicer L.	0	n	1	0	Fabaceae Lindl.
Iz_4_1009	DJI_0333	Bassia laniflora (S.G.Gmel.) A.J.Scott	0	n	1	0	Amaranthaceae Juss.
Iz_4_1009	DJI_0333	Berberis vulgaris L.	0	n	1	0	Berberidaceae Juss.
Iz_4_1009	DJI_0333	Betula pendula Roth.	0	n	1	1	Betulaceae Gray
Iz_4_1009	DJI_0333	Betula pendula Roth.	0	n	1	0	Poaceae Barnhart
Iz_4_1009	DJI_0333	Bromus inermis Leyss.	0		NA	NA	
Iz_4_1009	DJI_0333	Bromus squarrosus L.	0	a	0	0	Poaceae Barnhart
Iz_4_1009	DJI_0333	Bromus tectorum L.	0	a	0	0	Poaceae Barnhart
Iz_4_1009	DJI_0333	Calamagrostis epigejos (L.) Roth	2	n	1	0	Poaceae Barnhart
Iz_4_1009	DJI_0333	Carduus acanthoides L.	0	n	1	0	Asteraceae Bercht. & J.Presl
Iz_4_1009	DJI_0333	Carex sp.	0	n	1	0	Cyperaceae Juss.
Iz_4_1009	DJI_0333	Centaurea majorovii Dumbadze	0	n	1	0	Asteraceae Bercht. & J.Presl
Iz_4_1009	DJI_0333	Chamaecytisus ruthenicus (Fisch.ex Wol.) Klaskova	0	n	1	0	Fabaceae Lindl.
Iz_4_1009	DJI_0333	Chelidonium majus L.	0	n	1	0	Papaveraceae Juss.
Iz_4_1009	DJI_0333	Chenopodium betaceum Andrz.	0	n	1	0	Amaranthaceae Juss.
Iz_4_1009	DJI_0333	Chenopodium cf. album	0	n	1	0	Amaranthaceae Juss.
Iz_4_1009	DJI_0333	Chenopodium sp.	0		NA	0	Amaranthaceae Juss.
Iz_4_1009	DJI_0333	Chondrilla juncea L.	0	n	1	0	Asteraceae Bercht. & J.Presl
Iz_4_1009	DJI_0333	Cirsium vulgare (Savi) Ten.	0	n	1	0	Asteraceae Bercht. & J.Presl
Iz_4_1009	DJI_0333	Clinopodium acinos (L.) Kuntze	0	n	1	0	Lamiacea Lindl.
Iz_4_1009	DJI_0333	Convallaria majalis L.	0	n	1	0	Asparagaceae Juss.
Iz_4_1009	DJI_0333	Crataegus rhipidophylla Gand.	0	n	1	1	Rosaceae Juss.
Iz_4_1009	DJI_0333	Crepis foetida L.	0	n	1	0	Asteraceae Bercht. & J.Presl
Iz_4_1009	DJI_0333	Dactylis glomerata L.	0	n	1	0	Poaceae Barnhart
Iz_4_1009	DJI_0333	Dianthus campestris M.Bieb.	0	n	1	0	Caryophyllaceae Juss.
Iz_4_1009	DJI_0333	Digitaria sanguinalis (L.) Scop.	0	a	0	0	Poaceae Barnhart
Iz_4_1009	DJI_0333	Elymus repens (L.) Gould	0	n	1	0	Poaceae Barnhart
Iz_4_1009	DJI_0333	Epilobium tetragonum L.	0	n	1	0	Onagraceae Juss.
Iz_4_1009	DJI_0333	Eragrostis minor Host	0	a	0	0	Poaceae Barnhart
Iz_4_1009	DJI_0333	Erigeron canadensis L.	0	a	0	0	Asteraceae Bercht. & J.Presl
Iz_4_1009	DJI_0333	Euphorbia seguieriana Neck.	0	n	1	0	Euphorbiaceae Juss.
Iz_4_1009	DJI_0333	Fallopia dumetorum (L.) Holub	0	n	1	0	Polygonaceae Juss.
Iz_4_1009	DJI_0333	Festuca beckeri (Hack.) Trautv.	0	n	1	0	Poaceae Barnhart
Iz_4_1009	DJI_0333	Fragaria vesca L.	0	n	1	0	Rosaceae Juss.
Iz_4_1009	DJI_0333	Frangula alnus Mill.	0	n	1	1	Rhamnaceae Juss.
Iz_4_1009	DJI_0333	Fraxinus pennsylvanica Marshall	0	a	0	1	Oleaceae Hoffmanns. & Link
Iz_4_1009	DJI_0333	Galeopsis bifida Boenn.	0	n	1	0	Lamiacea Lindl.
Iz_4_1009	DJI_0333	Galium verum L.	0	n	1	0	Rubiaceae Juss.
Iz_4_1009	DJI_0333	Genista tinctoria L.	0	n	1	0	Fabaceae Lindl.
Iz_4_1009	DJI_0333	Geranium robertianum L.	0	n	1	0	Geraniaceae Juss.
Iz_4_1009	DJI_0333	Geranium sanquineum L.	0	n	1	0	Geraniaceae Juss.
Iz_4_1009	DJI_0333	Gypsophila paniculata L.	0	n	1	0	Caryophyllaceae Juss.
Iz_4_1009	DJI_0333	Helichrysum arenarium (L.) Moench	0	n	1	0	Asteraceae Bercht. & J.Presl
Iz_4_1009	DJI_0333	Herniaria polygama J.Gay	0	n	1	0	Caryophyllaceae Juss.
Iz_4_1009	DJI_0333	Hieracium robustum Fr.	0	n	1	0	Asteraceae Bercht. & J.Presl
Iz_4_1009	DJI_0333	Hieracium umbellatum L.	0	n	1	NA	Asteraceae Bercht. & J.Presl
Iz_4_1009	DJI_0333	Hylotelephium maximum (L.) Holub	0	n	1	0	Crassulaceae J.St.-Hil.
Iz_4_1009	DJI_0333	Jacobaea borysthenica (DC.) B.Nord. & Greuter	0	n	1	0	Asteraceae Bercht. & J.Presl
Iz_4_1009	DJI_0333	Jasione montana L.	0	n	1	0	Campanulaceae Juss.
Iz_4_1009	DJI_0333	Jurinea cyanoides (L.) Rchb.s.l.	0	n	1	0	Asteraceae Bercht. & J.Presl
Iz_4_1009	DJI_0333	Koeleria glauca (Spreng.) DC.	0	n	1	0	Poaceae Barnhart
Iz_4_1009	DJI_0333	Lactuca serriola L.	0	a	0	0	Asteraceae Bercht. & J.Presl
Iz_4_1009	DJI_0333	Linaria genistifolia (L.) Mill.	0	n	1	0	Plantaginaceae Juss.
Iz_4_1009	DJI_0333	Lomelosia argentea (L.) Greuter & Burdet	0	n	1	0	Caprifoliaceae Juss.
Iz_4_1009	DJI_0333	Melampyrum pratense L.	0	n	1	0	Orobanchaceae Vent.
Iz_4_1009	DJI_0333	Oenothera biennis L.	0	a	0	0	Onagraceae Juss.
Iz_4_1009	DJI_0333	Pentanema britannica (L.) D.Gut.Larr., Santos-Vicente, Anderb., E.Rico & M.M.Mart.Ort.	0	n	1	0	Asteraceae Bercht. & J.Presl
Iz_4_1009	DJI_0333	Peucedanum oreoselinum (L.) Moench	0	n	1	0	Apiaceae Lindl.
Iz_4_1009	DJI_0333	Peucedanum ruthenicum M.Bieb.	0	n	1	0	Apiaceae Lindl.
Iz_4_1009	DJI_0333	Pilosella echioides (Lumn.) F.W.Schultz & Sch.Bip.	0	n	1	0	Asteraceae Bercht. & J.Presl
Iz_4_1009	DJI_0333	Pilosella officinarum F. Schultz et Sch. Bip.	0	n	1	0	Asteraceae Bercht. & J.Presl
Iz_4_1009	DJI_0333	Pilosella sp.	0		NA	0	Asteraceae Bercht. & J.Presl
Iz_4_1009	DJI_0333	Pinus sylvestris L.	0	n	1	1	Pinaceae Spreng. ex F.Rudolphi
Iz_4_1009	DJI_0333	Poa annua L.	0	n	1	0	Poaceae Barnhart
Iz_4_1009	DJI_0333	Poa nemoralis L.	0	n	1	0	Poaceae Barnhart
Iz_4_1009	DJI_0333	Poa sp.	0		NA	0	Poaceae Barnhart
Iz_4_1009	DJI_0333	Polygonatum odoratum (Mill.) Druce	0.5	n	1	0	Asparagaceae Juss.
Iz_4_1009	DJI_0333	Polygonum arenarium Waldst. & Kit.	0	n	1	0	Polygonaceae Juss.
Iz_4_1009	DJI_0333	Populus nigra L.	0	n	1	1	Salicaceae Mirb.
Iz_4_1009	DJI_0333	Populus tremula L.	0	n	1	1	Salicaceae Mirb.
Iz_4_1009	DJI_0333	Populus Ã canescens (Aiton) Sm.	0	n	1	1	Salicaceae Mirb.
Iz_4_1009	DJI_0333	Potentilla argentea L.	0	n	1	0	Rosaceae Juss.
Iz_4_1009	DJI_0333	Psammophiliella muralis (L.) Ikonn.	0	n	1	0	Caryophyllaceae Juss.
Iz_4_1009	DJI_0333	Psephellus sumensis (Kalen.) Greuter	0	n	1	0	Asteraceae Bercht. & J.Presl
Iz_4_1009	DJI_0333	Pulsatilla pratensis (L.) Mill. s.l.	0	n	1	0	Ranunculaceae Juss.
Iz_4_1009	DJI_0333	Pyrus communis L.	0	a	0	1	Rosaceae Juss.
Iz_4_1009	DJI_0333	Quercus robur L.	0	n	1	1	Fagaceae Dumort.

Iz_4_1009	DJI_0333	Robinia pseudoacacia L.	0	a	0	1	Fabaceae Lindl.
Iz_4_1009	DJI_0333	Rosa sp.	0		NA	1	Rosaceae Juss.
Iz_4_1009	DJI_0333	Rubus idaeus L.	0	n	1	0	Rosaceae Juss.
Iz_4_1009	DJI_0333	Rumex acetosella L.	0	n	1	0	Polygonaceae Juss.
Iz_4_1009	DJI_0333	Rumex thyrsiflorus Fingerh.	0	n	1	0	Polygonaceae Juss.
Iz_4_1009	DJI_0333	Salix alba L.	0	n	1	1	Salicaceae Mirb.
Iz_4_1009	DJI_0333	Sambucus nigra L.	0	n	1	1	Viburnaceae Raf.
Iz_4_1009	DJI_0333	Secale sylvestre Host	0	n	1	0	Poaceae Barnhart
Iz_4_1009	DJI_0333	Sempervivum ruthenicum Schnittsp. & C.B. Lehm.	0	n	1	0	Crassulaceae J.St.-Hil.
Iz_4_1009	DJI_0333	Setaria viridis (L.) P. Beauv.	0	n	1	0	Poaceae Barnhart
Iz_4_1009	DJI_0333	Silene borysthenica (Gruner) Walters	0	n	1	0	Caryophyllaceae Juss.
Iz_4_1009	DJI_0333	Silene latifolia subsp. alba (Mill.) Greuter & Burdet	0	n	1	0	Caryophyllaceae Juss.
Iz_4_1009	DJI_0333	Solidago virgaurea L.	0	n	1	0	Asteraceae Bercht. & J.Presl
Iz_4_1009	DJI_0333	Sorbus aucuparia L.	0	n	1	1	Rosaceae Juss.
Iz_4_1009	DJI_0333	Stachys recta L.	0	n	1	0	Lamiacea Lindl.
Iz_4_1009	DJI_0333	Tanacetum vulgare L.	0	n	1	0	Asteraceae Bercht. & J.Presl
Iz_4_1009	DJI_0333	Thymus pallasianus Heinr.Braun	0	n	1	0	Lamiacea Lindl.
Iz_4_1009	DJI_0333	Tilia cordata Mill.	0	n	1	1	Malvaceae Juss.
Iz_4_1009	DJI_0333	Trifolium alpestre L.	0	n	1	0	Fabaceae Lindl.
Iz_4_1009	DJI_0333	Trifolium arvense L.	0	n	1	0	Fabaceae Lindl.
Iz_4_1009	DJI_0333	Ulmus glabra Huds.	0	n	1	1	Ulmaceae Mirb.
Iz_4_1009	DJI_0333	Verbascum densiflorum Bertol.	0	n	1	0	Scrophulariaceae Juss.
Iz_4_1009	DJI_0333	Verbascum nigrum L.	0	n	1	0	Scrophulariaceae Juss.
Iz_4_1009	DJI_0333	Veronica austriaca L.	0	n	1	0	Plantaginaceae Juss.
Iz_4_1009	DJI_0333	Veronica incana L.	0	n	1	0	Plantaginaceae Juss.
Iz_4_1009	DJI_0333	Veronica verna L.	0	n	1	0	Plantaginaceae Juss.
Iz_4_1009	DJI_0333	Vincetoxicum hirundinaria Medik	0	n	1	0	Apocynaceae Juss.
Iz_4_1009	DJI_0335	Acer tataricum L.	0	n	1	1	Sapindaceae Juss.
Iz_4_1009	DJI_0335	Achillea millefolium L. s.l.	0	n	1	0	Asteraceae Bercht. & J.Presl
Iz_4_1009	DJI_0335	Allium oleraceum L.	0	n	1	0	Amaryllidaceae J.St.-Hil.
Iz_4_1009	DJI_0335	Ambrosia artemisiifolia L.	0	a	0	0	Asteraceae Bercht. & J.Presl
Iz_4_1009	DJI_0335	Amorpha fruticosa L.	0	a	0	0	Fabaceae Lindl.
Iz_4_1009	DJI_0335	Anthericum ramosum L.	0	n	1	0	Asparagaceae Juss.
Iz_4_1009	DJI_0335	Artemisia marschalliana Spreng.	0	n	1	0	Asteraceae Bercht. & J.Presl
Iz_4_1009	DJI_0335	Asparagus officinalis L.	0	n	1	0	Asparagaceae Juss.
Iz_4_1009	DJI_0335	Astragalus cicer L.	0	n	1	0	Fabaceae Lindl.
Iz_4_1009	DJI_0335	Bassia laniflora (S.G.Gmel.) A.J.Scott	0	n	1	0	Amaranthaceae Juss.
Iz_4_1009	DJI_0335	Berberis vulgaris L.	0	n	1	0	Berberidaceae Juss.
Iz_4_1009	DJI_0335	Betula pendula Roth.	0	n	1	1	Betulaceae Gray
Iz_4_1009	DJI_0335	Betula pendula Roth.	0	n	1	0	Poaceae Barnhart
Iz_4_1009	DJI_0335	Bromus inermis Leyss.	0		NA	NA	
Iz_4_1009	DJI_0335	Bromus squarrosus L.	0	a	0	0	Poaceae Barnhart
Iz_4_1009	DJI_0335	Bromus tectorum L.	0	a	0	0	Poaceae Barnhart
Iz_4_1009	DJI_0335	Calamagrostis epigejos (L.) Roth	5	n	1	0	Poaceae Barnhart
Iz_4_1009	DJI_0335	Carduus acanthoides L.	0	n	1	0	Asteraceae Bercht. & J.Presl
Iz_4_1009	DJI_0335	Carex sp.	0	n	1	0	Cyperaceae Juss.
Iz_4_1009	DJI_0335	Centaurea majorovii Dumbadze	0	n	1	0	Asteraceae Bercht. & J.Presl
Iz_4_1009	DJI_0335	Chamaecytisus ruthenicus (Fisch.ex Wol.) Klaskova	0	n	1	0	Fabaceae Lindl.
Iz_4_1009	DJI_0335	Chelidonium majus L.	0	n	1	0	Papaveraceae Juss.
Iz_4_1009	DJI_0335	Chenopodium betaceum Andrz.	0	n	1	0	Amaranthaceae Juss.
Iz_4_1009	DJI_0335	Chenopodium cf. album	0	n	1	0	Amaranthaceae Juss.
Iz_4_1009	DJI_0335	Chenopodium sp.	0		NA	0	Amaranthaceae Juss.
Iz_4_1009	DJI_0335	Chondrilla juncea L.	0	n	1	0	Asteraceae Bercht. & J.Presl
Iz_4_1009	DJI_0335	Cirsium vulgare (Savi) Ten.	0	n	1	0	Asteraceae Bercht. & J.Presl
Iz_4_1009	DJI_0335	Clinopodium acinos (L.) Kuntze	0	n	1	0	Lamiacea Lindl.
Iz_4_1009	DJI_0335	Convallaria majalis L.	0	n	1	0	Asparagaceae Juss.
Iz_4_1009	DJI_0335	Crataegus rhipidophylla Gand.	0	n	1	1	Rosaceae Juss.
Iz_4_1009	DJI_0335	Crepis foetida L.	0	n	1	0	Asteraceae Bercht. & J.Presl
Iz_4_1009	DJI_0335	Dactylis glomerata L.	0	n	1	0	Poaceae Barnhart
Iz_4_1009	DJI_0335	Dianthus campestris M.Bieb.	0	n	1	0	Caryophyllaceae Juss.
Iz_4_1009	DJI_0335	Digitaria sanguinalis (L.) Scop.	0	a	0	0	Poaceae Barnhart
Iz_4_1009	DJI_0335	Elymus repens (L.) Gould	0	n	1	0	Poaceae Barnhart
Iz_4_1009	DJI_0335	Epilobium tetragonum L.	0	n	1	0	Onagraceae Juss.
Iz_4_1009	DJI_0335	Eragrostis minor Host	0	a	0	0	Poaceae Barnhart
Iz_4_1009	DJI_0335	Erigeron canadensis L.	0	a	0	0	Asteraceae Bercht. & J.Presl
Iz_4_1009	DJI_0335	Euphorbia seguieriana Neck.	0	n	1	0	Euphorbiaceae Juss.
Iz_4_1009	DJI_0335	Fallopia dumetorum (L.) Holub	0	n	1	0	Polygonaceae Juss.
Iz_4_1009	DJI_0335	Festuca beckeri (Hack.) Trautv.	0	n	1	0	Poaceae Barnhart
Iz_4_1009	DJI_0335	Fragaria vesca L.	0	n	1	0	Rosaceae Juss.
Iz_4_1009	DJI_0335	Frangula alnus Mill.	0	n	1	1	Rhamnaceae Juss.
Iz_4_1009	DJI_0335	Fraxinus pennsylvanica Marshall	0	a	0	1	Oleaceae Hoffmanns. & Link
Iz_4_1009	DJI_0335	Galeopsis bifida Boenn.	0	n	1	0	Lamiacea Lindl.
Iz_4_1009	DJI_0335	Galium verum L.	0	n	1	0	Rubiaceae Juss.
Iz_4_1009	DJI_0335	Genista tinctoria L.	0	n	1	0	Fabaceae Lindl.
Iz_4_1009	DJI_0335	Geranium robertianum L.	0	n	1	0	Geraniaceae Juss.
Iz_4_1009	DJI_0335	Geranium sanquineum L.	0	n	1	0	Geraniaceae Juss.
Iz_4_1009	DJI_0335	Gypsophila paniculata L.	0	n	1	0	Caryophyllaceae Juss.
Iz_4_1009	DJI_0335	Helichrysum arenarium (L.) Moench	0	n	1	0	Asteraceae Bercht. & J.Presl
Iz_4_1009	DJI_0335	Herniaria polygama J.Gay	0	n	1	0	Caryophyllaceae Juss.
Iz_4_1009	DJI_0335	Hieracium robustum Fr.	0	n	1	0	Asteraceae Bercht. & J.Presl
Iz_4_1009	DJI_0335	Hieracium umbellatum L.	0	n	1	NA	Asteraceae Bercht. & J.Presl
Iz_4_1009	DJI_0335	Hylotelephium maximum (L.) Holub	0	n	1	0	Crassulaceae J.St.-Hil.
Iz_4_1009	DJI_0335	Jacobaea borysthenica (DC.) B.Nord. & Greuter	0	n	1	0	Asteraceae Bercht. & J.Presl
Iz_4_1009	DJI_0335	Jasione montana L.	0	n	1	0	Campanulaceae Juss.
Iz_4_1009	DJI_0335	Jurinea cyanoides (L.) Rchb.s.l.	0	n	1	0	Asteraceae Bercht. & J.Presl
Iz_4_1009	DJI_0335	Koeleria glauca (Spreng.) DC.	0	n	1	0	Poaceae Barnhart
Iz_4_1009	DJI_0335	Lactuca serriola L.	0	a	0	0	Asteraceae Bercht. & J.Presl
Iz_4_1009	DJI_0335	Linaria genistifolia (L.) Mill.	0	n	1	0	Plantaginaceae Juss.
Iz_4_1009	DJI_0335	Lomelosia argentea (L.) Greuter & Burdet	0	n	1	0	Caprifoliaceae Juss.
Iz_4_1009	DJI_0335	Melampyrum pratense L.	0	n	1	0	Orobanchaceae Vent.
Iz_4_1009	DJI_0335	Oenothera biennis L.	0	a	0	0	Onagraceae Juss.
Iz_4_1009	DJI_0335	Pentanema britannica (L.) D.Gut.Larr., Santos-Vicente, Anderb., E.Rico & M.M.Mart.Ort.	0	n	1	0	Asteraceae Bercht. & J.Presl
Iz_4_1009	DJI_0335	Peucedanum oreoselinum (L.) Moench	0	n	1	0	Apiaceae Lindl.
Iz_4_1009	DJI_0335	Peucedanum ruthenicum M.Bieb.	0	n	1	0	Apiaceae Lindl.
Iz_4_1009	DJI_0335	Pilosella echioides (Lumn.) F.W.Schultz & Sch.Bip.	0	n	1	0	Asteraceae Bercht. & J.Presl
Iz_4_1009	DJI_0335	Pilosella officinarum F. Schultz et Sch. Bip.	0	n	1	0	Asteraceae Bercht. & J.Presl
Iz_4_1009	DJI_0335	Pilosella sp.	0		NA	0	Asteraceae Bercht. & J.Presl
Iz_4_1009	DJI_0335	Pinus sylvestris L.	0	n	1	1	Pinaceae Spreng. ex F.Rudolphi

Iz_4_1009	DJI_0335	Poa annua L.	0	n	1	0	Poaceae Barnhart
Iz_4_1009	DJI_0335	Poa nemoralis L.	0	n	1	0	Poaceae Barnhart
Iz_4_1009	DJI_0335	Poa sp.	0		NA	0	Poaceae Barnhart
Iz_4_1009	DJI_0335	Polygonatum odoratum (Mill.) Druce	0	n	1	0	Asparagaceae Juss.
Iz_4_1009	DJI_0335	Polygonum arenarium Waldst. & Kit.	0	n	1	0	Polygonaceae Juss.
Iz_4_1009	DJI_0335	Populus nigra L.	0	n	1	1	Salicaceae Mirb.
Iz_4_1009	DJI_0335	Populus tremula L.	0	n	1	1	Salicaceae Mirb.
Iz_4_1009	DJI_0335	Populus Ã canescens (Aiton) Sm.	0	n	1	1	Salicaceae Mirb.
Iz_4_1009	DJI_0335	Potentilla argentea L.	0	n	1	0	Rosaceae Juss.
Iz_4_1009	DJI_0335	Psammophiliella muralis (L.) Ikonn.	0	n	1	0	Caryophyllaceae Juss.
Iz_4_1009	DJI_0335	Psephellus sumensis (Kalen.) Greuter	0	n	1	0	Asteraceae Bercht. & J.Presl
Iz_4_1009	DJI_0335	Pulsatilla pratensis (L.) Mill. s.l.	0	n	1	0	Ranunculaceae Juss.
Iz_4_1009	DJI_0335	Pyrus communis L.	0	a	0	1	Rosaceae Juss.
Iz_4_1009	DJI_0335	Quercus robur L.	0	n	1	1	Fagaceae Dumort.
Iz_4_1009	DJI_0335	Robinia pseudoacacia L.	0	a	0	1	Fabaceae Lindl.
Iz_4_1009	DJI_0335	Rosa sp.	0		NA	1	Rosaceae Juss.
Iz_4_1009	DJI_0335	Rubus idaeus L.	0	n	1	0	Rosaceae Juss.
Iz_4_1009	DJI_0335	Rumex acetosella L.	0	n	1	0	Polygonaceae Juss.
Iz_4_1009	DJI_0335	Rumex thyrsiflorus Fingerh.	0	n	1	0	Polygonaceae Juss.
Iz_4_1009	DJI_0335	Salix alba L.	0	n	1	1	Salicaceae Mirb.
Iz_4_1009	DJI_0335	Sambucus nigra L.	0	n	1	1	Viburnaceae Raf.
Iz_4_1009	DJI_0335	Secale sylvestre Host	0	n	1	0	Poaceae Barnhart
Iz_4_1009	DJI_0335	Sempervivum ruthenicum Schnittsp. & C.B. Lehm.	0	n	1	0	Crassulaceae J.St.-Hil.
Iz_4_1009	DJI_0335	Setaria viridis (L.) P. Beauv.	0	n	1	0	Poaceae Barnhart
Iz_4_1009	DJI_0335	Silene borysthenica (Gruner) Walters	0	n	1	0	Caryophyllaceae Juss.
Iz_4_1009	DJI_0335	Silene latifolia subsp. alba (Mill.) Greuter & Burdet	0	n	1	0	Caryophyllaceae Juss.
Iz_4_1009	DJI_0335	Solidago virgaurea L.	0	n	1	0	Asteraceae Bercht. & J.Presl
Iz_4_1009	DJI_0335	Sorbus aucuparia L.	0	n	1	1	Rosaceae Juss.
Iz_4_1009	DJI_0335	Stachys recta L.	0	n	1	0	Lamiacea Lindl.
Iz_4_1009	DJI_0335	Tanacetum vulgare L.	0	n	1	0	Asteraceae Bercht. & J.Presl
Iz_4_1009	DJI_0335	Thymus pallasianus Heinr.Braun	0	n	1	0	Lamiacea Lindl.
Iz_4_1009	DJI_0335	Tilia cordata Mill.	0	n	1	1	Malvaceae Juss.
Iz_4_1009	DJI_0335	Trifolium alpestre L.	0	n	1	0	Fabaceae Lindl.
Iz_4_1009	DJI_0335	Trifolium arvense L.	0	n	1	0	Fabaceae Lindl.
Iz_4_1009	DJI_0335	Ulmus glabra Huds.	0	n	1	1	Ulmaceae Mirb.
Iz_4_1009	DJI_0335	Verbascum densiflorum Bertol.	0	n	1	0	Scrophulariaceae Juss.
Iz_4_1009	DJI_0335	Verbascum nigrum L.	0	n	1	0	Scrophulariaceae Juss.
Iz_4_1009	DJI_0335	Veronica austriaca L.	0	n	1	0	Plantaginaceae Juss.
Iz_4_1009	DJI_0335	Veronica incana L.	0	n	1	0	Plantaginaceae Juss.
Iz_4_1009	DJI_0335	Veronica verna L.	0	n	1	0	Plantaginaceae Juss.
Iz_4_1009	DJI_0335	Vincetoxicum hirundinaria Medik	0	n	1	0	Apocynaceae Juss.
Iz_4_1009	DJI_0336	Acer tataricum L.	0	n	1	1	Sapindaceae Juss.
Iz_4_1009	DJI_0336	Achillea millefolium L. s.l.	0	n	1	0	Asteraceae Bercht. & J.Presl
Iz_4_1009	DJI_0336	Allium oleraceum L.	0	n	1	0	Amaryllidaceae J.St.-Hil.
Iz_4_1009	DJI_0336	Ambrosia artemisiifolia L.	0	a	0	0	Asteraceae Bercht. & J.Presl
Iz_4_1009	DJI_0336	Amorpha fruticosa L.	0	a	0	0	Fabaceae Lindl.
Iz_4_1009	DJI_0336	Anthericum ramosum L.	0	n	1	0	Asparagaceae Juss.
Iz_4_1009	DJI_0336	Artemisia marschalliana Spreng.	0	n	1	0	Asteraceae Bercht. & J.Presl
Iz_4_1009	DJI_0336	Asparagus officinalis L.	0	n	1	0	Asparagaceae Juss.
Iz_4_1009	DJI_0336	Astragalus cicer L.	0	n	1	0	Fabaceae Lindl.
Iz_4_1009	DJI_0336	Bassia laniflora (S.G.Gmel.) A.J.Scott	0	n	1	0	Amaranthaceae Juss.
Iz_4_1009	DJI_0336	Berberis vulgaris L.	0	n	1	0	Berberidaceae Juss.
Iz_4_1009	DJI_0336	Betula pendula Roth.	0	n	1	1	Betulaceae Gray
Iz_4_1009	DJI_0336	Betula pendula Roth.	0	n	1	0	Poaceae Barnhart
Iz_4_1009	DJI_0336	Bromus inermis Leyss.	0		NA	NA	
Iz_4_1009	DJI_0336	Bromus squarrosus L.	0	a	0	0	Poaceae Barnhart
Iz_4_1009	DJI_0336	Bromus tectorum L.	0	a	0	0	Poaceae Barnhart
Iz_4_1009	DJI_0336	Calamagrostis epigejos (L.) Roth	5	n	1	0	Poaceae Barnhart
Iz_4_1009	DJI_0336	Carduus acanthoides L.	0	n	1	0	Asteraceae Bercht. & J.Presl
Iz_4_1009	DJI_0336	Carex sp.	0	n	1	0	Cyperaceae Juss.
Iz_4_1009	DJI_0336	Centaurea majorovii Dumbadze	0	n	1	0	Asteraceae Bercht. & J.Presl
Iz_4_1009	DJI_0336	Chamaecytisus ruthenicus (Fisch.ex Wol.) Klaskova	0	n	1	0	Fabaceae Lindl.
Iz_4_1009	DJI_0336	Chelidonium majus L.	0	n	1	0	Papaveraceae Juss.
Iz_4_1009	DJI_0336	Chenopodium betaceum Andrz.	0	n	1	0	Amaranthaceae Juss.
Iz_4_1009	DJI_0336	Chenopodium cf. album	0	n	1	0	Amaranthaceae Juss.
Iz_4_1009	DJI_0336	Chenopodium sp.	0		NA	0	Amaranthaceae Juss.
Iz_4_1009	DJI_0336	Chondrilla juncea L.	0	n	1	0	Asteraceae Bercht. & J.Presl
Iz_4_1009	DJI_0336	Cirsium vulgare (Savi) Ten.	0	n	1	0	Asteraceae Bercht. & J.Presl
Iz_4_1009	DJI_0336	Clinopodium acinos (L.) Kuntze	0	n	1	0	Lamiacea Lindl.
Iz_4_1009	DJI_0336	Convallaria majalis L.	0	n	1	0	Asparagaceae Juss.
Iz_4_1009	DJI_0336	Crataegus rhipidophylla Gand.	0	n	1	1	Rosaceae Juss.
Iz_4_1009	DJI_0336	Crepis foetida L.	0	n	1	0	Asteraceae Bercht. & J.Presl
Iz_4_1009	DJI_0336	Dactylis glomerata L.	0	n	1	0	Poaceae Barnhart
Iz_4_1009	DJI_0336	Dianthus campestris M.Bieb.	0	n	1	0	Caryophyllaceae Juss.
Iz_4_1009	DJI_0336	Digitaria sanguinalis (L.) Scop.	0	a	0	0	Poaceae Barnhart
Iz_4_1009	DJI_0336	Elymus repens (L.) Gould	0	n	1	0	Poaceae Barnhart
Iz_4_1009	DJI_0336	Epilobium tetragonum L.	0	n	1	0	Onagraceae Juss.
Iz_4_1009	DJI_0336	Eragrostis minor Host	0	a	0	0	Poaceae Barnhart
Iz_4_1009	DJI_0336	Erigeron canadensis L.	0	a	0	0	Asteraceae Bercht. & J.Presl
Iz_4_1009	DJI_0336	Euphorbia seguieriana Neck.	0	n	1	0	Euphorbiaceae Juss.
Iz_4_1009	DJI_0336	Fallopia dumetorum (L.) Holub	0	n	1	0	Polygonaceae Juss.
Iz_4_1009	DJI_0336	Festuca beckeri (Hack.) Trautv.	0	n	1	0	Poaceae Barnhart
Iz_4_1009	DJI_0336	Fragaria vesca L.	0	n	1	0	Rosaceae Juss.
Iz_4_1009	DJI_0336	Frangula alnus Mill.	0	n	1	1	Rhamnaceae Juss.
Iz_4_1009	DJI_0336	Fraxinus pennsylvanica Marshall	0	a	0	1	Oleaceae Hoffmanns. & Link
Iz_4_1009	DJI_0336	Galeopsis bifida Boenn.	0	n	1	0	Lamiacea Lindl.
Iz_4_1009	DJI_0336	Galium verum L.	0	n	1	0	Rubiaceae Juss.
Iz_4_1009	DJI_0336	Genista tinctoria L.	0	n	1	0	Fabaceae Lindl.
Iz_4_1009	DJI_0336	Geranium robertianum L.	0	n	1	0	Geraniaceae Juss.
Iz_4_1009	DJI_0336	Geranium sanquineum L.	0	n	1	0	Geraniaceae Juss.
Iz_4_1009	DJI_0336	Gypsophila paniculata L.	0	n	1	0	Caryophyllaceae Juss.
Iz_4_1009	DJI_0336	Helichrysum arenarium (L.) Moench	0	n	1	0	Asteraceae Bercht. & J.Presl
Iz_4_1009	DJI_0336	Herniaria polygama J.Gay	0	n	1	0	Caryophyllaceae Juss.
Iz_4_1009	DJI_0336	Hieracium robustum Fr.	0	n	1	0	Asteraceae Bercht. & J.Presl
Iz_4_1009	DJI_0336	Hieracium umbellatum L.	0	n	1	NA	Asteraceae Bercht. & J.Presl
Iz_4_1009	DJI_0336	Hylotelephium maximum (L.) Holub	0	n	1	0	Crassulaceae J.St.-Hil.
Iz_4_1009	DJI_0336	Jacobaea borysthenica (DC.) B.Nord. & Greuter	0	n	1	0	Asteraceae Bercht. & J.Presl
Iz_4_1009	DJI_0336	Jasione montana L.	0	n	1	0	Campanulaceae Juss.

Iz_4_1009	DJI_0336	Jurinea cyanoides (L.) Rchb.s.l.	0	n	1	0	Asteraceae Bercht. & J.Presl
Iz_4_1009	DJI_0336	Koeleria glauca (Spreng.) DC.	0	n	1	0	Poaceae Barnhart
Iz_4_1009	DJI_0336	Lactuca serriola L.	0	a	0	0	Asteraceae Bercht. & J.Presl
Iz_4_1009	DJI_0336	Linaria genistifolia (L.) Mill.	0	n	1	0	Plantaginaceae Juss.
Iz_4_1009	DJI_0336	Lomelosia argentea (L.) Greuter & Burdet	0	n	1	0	Caprifoliaceae Juss.
Iz_4_1009	DJI_0336	Melampyrum pratense L.	0	n	1	0	Orobanchaceae Vent.
Iz_4_1009	DJI_0336	Oenothera biennis L.	0	a	0	0	Onagraceae Juss.
Iz_4_1009	DJI_0336	Pentanema britannica (L.) D.Gut.Larr., Santos-Vicente, Anderb., E.Rico & M.M.Mart.Ort.	0	n	1	0	Asteraceae Bercht. & J.Presl
Iz_4_1009	DJI_0336	Peucedanum oreoselinum (L.) Moench	0	n	1	0	Apiaceae Lindl.
Iz_4_1009	DJI_0336	Peucedanum ruthenicum M.Bieb.	0	n	1	0	Apiaceae Lindl.
Iz_4_1009	DJI_0336	Pilosella echioides (Lumn.) F.W.Schultz & Sch.Bip.	0	n	1	0	Asteraceae Bercht. & J.Presl
Iz_4_1009	DJI_0336	Pilosella officinarum F. Schultz et Sch. Bip.	0	n	1	0	Asteraceae Bercht. & J.Presl
Iz_4_1009	DJI_0336	Pilosella sp.	0		NA	0	Asteraceae Bercht. & J.Presl
Iz_4_1009	DJI_0336	Pinus sylvestris L.	0	n	1	1	Pinaceae Spreng. ex F.Rudolphi
Iz_4_1009	DJI_0336	Poa annua L.	0	n	1	0	Poaceae Barnhart
Iz_4_1009	DJI_0336	Poa nemoralis L.	0	n	1	0	Poaceae Barnhart
Iz_4_1009	DJI_0336	Poa sp.	0		NA	0	Poaceae Barnhart
Iz_4_1009	DJI_0336	Polygonatum odoratum (Mill.) Druce	0	n	1	0	Asparagaceae Juss.
Iz_4_1009	DJI_0336	Polygonum arenarium Waldst. & Kit.	0	n	1	0	Polygonaceae Juss.
Iz_4_1009	DJI_0336	Populus nigra L.	0	n	1	1	Salicaceae Mirb.
Iz_4_1009	DJI_0336	Populus tremula L.	0	n	1	1	Salicaceae Mirb.
Iz_4_1009	DJI_0336	Populus Ã canescens (Aiton) Sm.	0	n	1	1	Salicaceae Mirb.
Iz_4_1009	DJI_0336	Potentilla argentea L.	0	n	1	0	Rosaceae Juss.
Iz_4_1009	DJI_0336	Psammophiliella muralis (L.) Ikonn.	0	n	1	0	Caryophyllaceae Juss.
Iz_4_1009	DJI_0336	Psephellus sumensis (Kalen.) Greuter	0	n	1	0	Asteraceae Bercht. & J.Presl
Iz_4_1009	DJI_0336	Pulsatilla pratensis (L.) Mill. s.l.	0	n	1	0	Ranunculaceae Juss.
Iz_4_1009	DJI_0336	Pyrus communis L.	0	a	0	1	Rosaceae Juss.
Iz_4_1009	DJI_0336	Quercus robur L.	0	n	1	1	Fagaceae Dumort.
Iz_4_1009	DJI_0336	Robinia pseudoacacia L.	0	a	0	1	Fabaceae Lindl.
Iz_4_1009	DJI_0336	Rosa sp.	0		NA	1	Rosaceae Juss.
Iz_4_1009	DJI_0336	Rubus idaeus L.	0	n	1	0	Rosaceae Juss.
Iz_4_1009	DJI_0336	Rumex acetosella L.	0	n	1	0	Polygonaceae Juss.
Iz_4_1009	DJI_0336	Rumex thyrsiflorus Fingerh.	0	n	1	0	Polygonaceae Juss.
Iz_4_1009	DJI_0336	Salix alba L.	0	n	1	1	Salicaceae Mirb.
Iz_4_1009	DJI_0336	Sambucus nigra L.	0	n	1	1	Viburnaceae Raf.
Iz_4_1009	DJI_0336	Secale sylvestre Host	0	n	1	0	Poaceae Barnhart
Iz_4_1009	DJI_0336	Sempervivum ruthenicum Schnittsp. & C.B. Lehm.	0	n	1	0	Crassulaceae J.St.-Hil.
Iz_4_1009	DJI_0336	Setaria viridis (L.) P. Beauv.	0	n	1	0	Poaceae Barnhart
Iz_4_1009	DJI_0336	Silene borysthenica (Gruner) Walters	0	n	1	0	Caryophyllaceae Juss.
Iz_4_1009	DJI_0336	Silene latifolia subsp. alba (Mill.) Greuter & Burdet	0	n	1	0	Caryophyllaceae Juss.
Iz_4_1009	DJI_0336	Solidago virgaurea L.	0	n	1	0	Asteraceae Bercht. & J.Presl
Iz_4_1009	DJI_0336	Sorbus aucuparia L.	0	n	1	1	Rosaceae Juss.
Iz_4_1009	DJI_0336	Stachys recta L.	0	n	1	0	Lamiacea Lindl.
Iz_4_1009	DJI_0336	Tanacetum vulgare L.	0	n	1	0	Asteraceae Bercht. & J.Presl
Iz_4_1009	DJI_0336	Thymus pallasianus Heinr.Braun	0	n	1	0	Lamiacea Lindl.
Iz_4_1009	DJI_0336	Tilia cordata Mill.	0	n	1	1	Malvaceae Juss.
Iz_4_1009	DJI_0336	Trifolium alpestre L.	0	n	1	0	Fabaceae Lindl.
Iz_4_1009	DJI_0336	Trifolium arvense L.	0	n	1	0	Fabaceae Lindl.
Iz_4_1009	DJI_0336	Ulmus glabra Huds.	0	n	1	1	Ulmaceae Mirb.
Iz_4_1009	DJI_0336	Verbascum densiflorum Bertol.	0	n	1	0	Scrophulariaceae Juss.
Iz_4_1009	DJI_0336	Verbascum nigrum L.	0	n	1	0	Scrophulariaceae Juss.
Iz_4_1009	DJI_0336	Veronica austriaca L.	0	n	1	0	Plantaginaceae Juss.
Iz_4_1009	DJI_0336	Veronica incana L.	0	n	1	0	Plantaginaceae Juss.
Iz_4_1009	DJI_0336	Veronica verna L.	0	n	1	0	Plantaginaceae Juss.
Iz_4_1009	DJI_0336	Vincetoxicum hirundinaria Medik	0	n	1	0	Apocynaceae Juss.
Iz_4_1009	DJI_0339	Acer tataricum L.	0	n	1	1	Sapindaceae Juss.
Iz_4_1009	DJI_0339	Achillea millefolium L. s.l.	0	n	1	0	Asteraceae Bercht. & J.Presl
Iz_4_1009	DJI_0339	Allium oleraceum L.	0	n	1	0	Amaryllidaceae J.St.-Hil.
Iz_4_1009	DJI_0339	Ambrosia artemisiifolia L.	0	a	0	0	Asteraceae Bercht. & J.Presl
Iz_4_1009	DJI_0339	Amorpha fruticosa L.	0	a	0	0	Fabaceae Lindl.
Iz_4_1009	DJI_0339	Anthericum ramosum L.	0	n	1	0	Asparagaceae Juss.
Iz_4_1009	DJI_0339	Artemisia marschalliana Spreng.	0	n	1	0	Asteraceae Bercht. & J.Presl
Iz_4_1009	DJI_0339	Asparagus officinalis L.	0	n	1	0	Asparagaceae Juss.
Iz_4_1009	DJI_0339	Astragalus cicer L.	0	n	1	0	Fabaceae Lindl.
Iz_4_1009	DJI_0339	Bassia laniflora (S.G.Gmel.) A.J.Scott	0	n	1	0	Amaranthaceae Juss.
Iz_4_1009	DJI_0339	Berberis vulgaris L.	0	n	1	0	Berberidaceae Juss.
Iz_4_1009	DJI_0339	Betula pendula Roth.	0	n	1	1	Betulaceae Gray
Iz_4_1009	DJI_0339	Betula pendula Roth.	0	n	1	0	Poaceae Barnhart
Iz_4_1009	DJI_0339	Bromus inermis Leyss.	0		NA	NA	
Iz_4_1009	DJI_0339	Bromus squarrosus L.	0	a	0	0	Poaceae Barnhart
Iz_4_1009	DJI_0339	Bromus tectorum L.	0	a	0	0	Poaceae Barnhart
Iz_4_1009	DJI_0339	Calamagrostis epigejos (L.) Roth	3	n	1	0	Poaceae Barnhart
Iz_4_1009	DJI_0339	Carduus acanthoides L.	0	n	1	0	Asteraceae Bercht. & J.Presl
Iz_4_1009	DJI_0339	Carex sp.	0	n	1	0	Cyperaceae Juss.
Iz_4_1009	DJI_0339	Centaurea majorovii Dumbadze	0	n	1	0	Asteraceae Bercht. & J.Presl
Iz_4_1009	DJI_0339	Chamaecytisus ruthenicus (Fisch.ex Wol.) Klaskova	0	n	1	0	Fabaceae Lindl.
Iz_4_1009	DJI_0339	Chelidonium majus L.	0	n	1	0	Papaveraceae Juss.
Iz_4_1009	DJI_0339	Chenopodium betaceum Andrz.	0	n	1	0	Amaranthaceae Juss.
Iz_4_1009	DJI_0339	Chenopodium cf. album	0	n	1	0	Amaranthaceae Juss.
Iz_4_1009	DJI_0339	Chenopodium sp.	0		NA	0	Amaranthaceae Juss.
Iz_4_1009	DJI_0339	Chondrilla juncea L.	0	n	1	0	Asteraceae Bercht. & J.Presl
Iz_4_1009	DJI_0339	Cirsium vulgare (Savi) Ten.	0	n	1	0	Asteraceae Bercht. & J.Presl
Iz_4_1009	DJI_0339	Clinopodium acinos (L.) Kuntze	0	n	1	0	Lamiacea Lindl.
Iz_4_1009	DJI_0339	Convallaria majalis L.	0	n	1	0	Asparagaceae Juss.
Iz_4_1009	DJI_0339	Crataegus rhipidophylla Gand.	0	n	1	1	Rosaceae Juss.
Iz_4_1009	DJI_0339	Crepis foetida L.	0	n	1	0	Asteraceae Bercht. & J.Presl
Iz_4_1009	DJI_0339	Dactylis glomerata L.	0	n	1	0	Poaceae Barnhart
Iz_4_1009	DJI_0339	Dianthus campestris M.Bieb.	0	n	1	0	Caryophyllaceae Juss.
Iz_4_1009	DJI_0339	Digitaria sanguinalis (L.) Scop.	0	a	0	0	Poaceae Barnhart
Iz_4_1009	DJI_0339	Elymus repens (L.) Gould	0	n	1	0	Poaceae Barnhart
Iz_4_1009	DJI_0339	Epilobium tetragonum L.	0	n	1	0	Onagraceae Juss.
Iz_4_1009	DJI_0339	Eragrostis minor Host	0	a	0	0	Poaceae Barnhart
Iz_4_1009	DJI_0339	Erigeron canadensis L.	0	a	0	0	Asteraceae Bercht. & J.Presl
Iz_4_1009	DJI_0339	Euphorbia seguieriana Neck.	0	n	1	0	Euphorbiaceae Juss.
Iz_4_1009	DJI_0339	Fallopia dumetorum (L.) Holub	0	n	1	0	Polygonaceae Juss.
Iz_4_1009	DJI_0339	Festuca beckeri (Hack.) Trautv.	0	n	1	0	Poaceae Barnhart
Iz_4_1009	DJI_0339	Fragaria vesca L.	0	n	1	0	Rosaceae Juss.
Iz_4_1009	DJI_0339	Frangula alnus Mill.	0	n	1	1	Rhamnaceae Juss.

Iz_4_1009	DJI_0339	Fraxinus pennsylvanica Marshall	0	a	0	1	Oleaceae Hoffmanns. & Link
Iz_4_1009	DJI_0339	Galeopsis bifida Boenn.	0	n	1	0	Lamiacea Lindl.
Iz_4_1009	DJI_0339	Galium verum L.	0	n	1	0	Rubiaceae Juss.
Iz_4_1009	DJI_0339	Genista tinctoria L.	0	n	1	0	Fabaceae Lindl.
Iz_4_1009	DJI_0339	Geranium robertianum L.	0	n	1	0	Geraniaceae Juss.
Iz_4_1009	DJI_0339	Geranium sanquineum L.	0	n	1	0	Geraniaceae Juss.
Iz_4_1009	DJI_0339	Gypsophila paniculata L.	0	n	1	0	Caryophyllaceae Juss.
Iz_4_1009	DJI_0339	Helichrysum arenarium (L.) Moench	0	n	1	0	Asteraceae Bercht. & J.Presl
Iz_4_1009	DJI_0339	Herniaria polygama J.Gay	0	n	1	0	Caryophyllaceae Juss.
Iz_4_1009	DJI_0339	Hieracium robustum Fr.	0	n	1	0	Asteraceae Bercht. & J.Presl
Iz_4_1009	DJI_0339	Hieracium umbellatum L.	0	n	1	NA	Asteraceae Bercht. & J.Presl
Iz_4_1009	DJI_0339	Hylotelephium maximum (L.) Holub	0	n	1	0	Crassulaceae J.St.-Hil.
Iz_4_1009	DJI_0339	Jacobaea borysthenica (DC.) B.Nord. & Greuter	0	n	1	0	Asteraceae Bercht. & J.Presl
Iz_4_1009	DJI_0339	Jasione montana L.	0	n	1	0	Campanulaceae Juss.
Iz_4_1009	DJI_0339	Jurinea cyanoides (L.) Rchb.s.l.	0	n	1	0	Asteraceae Bercht. & J.Presl
Iz_4_1009	DJI_0339	Koeleria glauca (Spreng.) DC.	0	n	1	0	Poaceae Barnhart
Iz_4_1009	DJI_0339	Lactuca serriola L.	0	a	0	0	Asteraceae Bercht. & J.Presl
Iz_4_1009	DJI_0339	Linaria genistifolia (L.) Mill.	0	n	1	0	Plantaginaceae Juss.
Iz_4_1009	DJI_0339	Lomelosia argentea (L.) Greuter & Burdet	0	n	1	0	Caprifoliaceae Juss.
Iz_4_1009	DJI_0339	Melampyrum pratense L.	0	n	1	0	Orobanchaceae Vent.
Iz_4_1009	DJI_0339	Oenothera biennis L.	0	a	0	0	Onagraceae Juss.
Iz_4_1009	DJI_0339	Pentanema britannica (L.) D.Gut.Larr., Santos-Vicente, Anderb., E.Rico & M.M.Mart.Ort.	0	n	1	0	Asteraceae Bercht. & J.Presl
Iz_4_1009	DJI_0339	Peucedanum oreoselinum (L.) Moench	0	n	1	0	Apiaceae Lindl.
Iz_4_1009	DJI_0339	Peucedanum ruthenicum M.Bieb.	0	n	1	0	Apiaceae Lindl.
Iz_4_1009	DJI_0339	Pilosella echioides (Lumn.) F.W.Schultz & Sch.Bip.	0	n	1	0	Asteraceae Bercht. & J.Presl
Iz_4_1009	DJI_0339	Pilosella officinarum F. Schultz et Sch. Bip.	0	n	1	0	Asteraceae Bercht. & J.Presl
Iz_4_1009	DJI_0339	Pilosella sp.	0		NA	0	Asteraceae Bercht. & J.Presl
Iz_4_1009	DJI_0339	Pinus sylvestris L.	0	n	1	1	Pinaceae Spreng. ex F.Rudolphi
Iz_4_1009	DJI_0339	Poa annua L.	0	n	1	0	Poaceae Barnhart
Iz_4_1009	DJI_0339	Poa nemoralis L.	0	n	1	0	Poaceae Barnhart
Iz_4_1009	DJI_0339	Poa sp.	0		NA	0	Poaceae Barnhart
Iz_4_1009	DJI_0339	Polygonatum odoratum (Mill.) Druce	0	n	1	0	Asparagaceae Juss.
Iz_4_1009	DJI_0339	Polygonum arenarium Waldst. & Kit.	0	n	1	0	Polygonaceae Juss.
Iz_4_1009	DJI_0339	Populus nigra L.	0	n	1	1	Salicaceae Mirb.
Iz_4_1009	DJI_0339	Populus tremula L.	0	n	1	1	Salicaceae Mirb.
Iz_4_1009	DJI_0339	Populus Ã canescens (Aiton) Sm.	0	n	1	1	Salicaceae Mirb.
Iz_4_1009	DJI_0339	Potentilla argentea L.	0	n	1	0	Rosaceae Juss.
Iz_4_1009	DJI_0339	Psammophiliella muralis (L.) Ikonn.	0	n	1	0	Caryophyllaceae Juss.
Iz_4_1009	DJI_0339	Psephellus sumensis (Kalen.) Greuter	0	n	1	0	Asteraceae Bercht. & J.Presl
Iz_4_1009	DJI_0339	Pulsatilla pratensis (L.) Mill. s.l.	0	n	1	0	Ranunculaceae Juss.
Iz_4_1009	DJI_0339	Pyrus communis L.	0	a	0	1	Rosaceae Juss.
Iz_4_1009	DJI_0339	Quercus robur L.	0	n	1	1	Fagaceae Dumort.
Iz_4_1009	DJI_0339	Robinia pseudoacacia L.	0	a	0	1	Fabaceae Lindl.
Iz_4_1009	DJI_0339	Rosa sp.	0		NA	1	Rosaceae Juss.
Iz_4_1009	DJI_0339	Rubus idaeus L.	0	n	1	0	Rosaceae Juss.
Iz_4_1009	DJI_0339	Rumex acetosella L.	0	n	1	0	Polygonaceae Juss.
Iz_4_1009	DJI_0339	Rumex thyrsiflorus Fingerh.	0	n	1	0	Polygonaceae Juss.
Iz_4_1009	DJI_0339	Salix alba L.	0	n	1	1	Salicaceae Mirb.
Iz_4_1009	DJI_0339	Sambucus nigra L.	0	n	1	1	Viburnaceae Raf.
Iz_4_1009	DJI_0339	Secale sylvestre Host	0	n	1	0	Poaceae Barnhart
Iz_4_1009	DJI_0339	Sempervivum ruthenicum Schnittsp. & C.B. Lehm.	0	n	1	0	Crassulaceae J.St.-Hil.
Iz_4_1009	DJI_0339	Setaria viridis (L.) P. Beauv.	0	n	1	0	Poaceae Barnhart
Iz_4_1009	DJI_0339	Silene borysthenica (Gruner) Walters	0	n	1	0	Caryophyllaceae Juss.
Iz_4_1009	DJI_0339	Silene latifolia subsp. alba (Mill.) Greuter & Burdet	0	n	1	0	Caryophyllaceae Juss.
Iz_4_1009	DJI_0339	Solidago virgaurea L.	0	n	1	0	Asteraceae Bercht. & J.Presl
Iz_4_1009	DJI_0339	Sorbus aucuparia L.	0	n	1	1	Rosaceae Juss.
Iz_4_1009	DJI_0339	Stachys recta L.	0	n	1	0	Lamiacea Lindl.
Iz_4_1009	DJI_0339	Tanacetum vulgare L.	0	n	1	0	Asteraceae Bercht. & J.Presl
Iz_4_1009	DJI_0339	Thymus pallasianus Heinr.Braun	0	n	1	0	Lamiacea Lindl.
Iz_4_1009	DJI_0339	Tilia cordata Mill.	0	n	1	1	Malvaceae Juss.
Iz_4_1009	DJI_0339	Trifolium alpestre L.	0	n	1	0	Fabaceae Lindl.
Iz_4_1009	DJI_0339	Trifolium arvense L.	0	n	1	0	Fabaceae Lindl.
Iz_4_1009	DJI_0339	Ulmus glabra Huds.	0	n	1	1	Ulmaceae Mirb.
Iz_4_1009	DJI_0339	Verbascum densiflorum Bertol.	0	n	1	0	Scrophulariaceae Juss.
Iz_4_1009	DJI_0339	Verbascum nigrum L.	0	n	1	0	Scrophulariaceae Juss.
Iz_4_1009	DJI_0339	Veronica austriaca L.	0	n	1	0	Plantaginaceae Juss.
Iz_4_1009	DJI_0339	Veronica incana L.	0	n	1	0	Plantaginaceae Juss.
Iz_4_1009	DJI_0339	Veronica verna L.	0	n	1	0	Plantaginaceae Juss.
Iz_4_1009	DJI_0339	Vincetoxicum hirundinaria Medik	0	n	1	0	Apocynaceae Juss.
Iz_4_1009	DJI_0342	Acer tataricum L.	0	n	1	1	Sapindaceae Juss.
Iz_4_1009	DJI_0342	Achillea millefolium L. s.l.	0	n	1	0	Asteraceae Bercht. & J.Presl
Iz_4_1009	DJI_0342	Allium oleraceum L.	0	n	1	0	Amaryllidaceae J.St.-Hil.
Iz_4_1009	DJI_0342	Ambrosia artemisiifolia L.	0	a	0	0	Asteraceae Bercht. & J.Presl
Iz_4_1009	DJI_0342	Amorpha fruticosa L.	0	a	0	0	Fabaceae Lindl.
Iz_4_1009	DJI_0342	Anthericum ramosum L.	0	n	1	0	Asparagaceae Juss.
Iz_4_1009	DJI_0342	Artemisia marschalliana Spreng.	0	n	1	0	Asteraceae Bercht. & J.Presl
Iz_4_1009	DJI_0342	Asparagus officinalis L.	0	n	1	0	Asparagaceae Juss.
Iz_4_1009	DJI_0342	Astragalus cicer L.	0	n	1	0	Fabaceae Lindl.
Iz_4_1009	DJI_0342	Bassia laniflora (S.G.Gmel.) A.J.Scott	0	n	1	0	Amaranthaceae Juss.
Iz_4_1009	DJI_0342	Berberis vulgaris L.	0	n	1	0	Berberidaceae Juss.
Iz_4_1009	DJI_0342	Betula pendula Roth.	0	n	1	1	Betulaceae Gray
Iz_4_1009	DJI_0342	Betula pendula Roth.	0	n	1	0	Poaceae Barnhart
Iz_4_1009	DJI_0342	Bromus inermis Leyss.	0		NA	NA	
Iz_4_1009	DJI_0342	Bromus squarrosus L.	0	a	0	0	Poaceae Barnhart
Iz_4_1009	DJI_0342	Bromus tectorum L.	0	a	0	0	Poaceae Barnhart
Iz_4_1009	DJI_0342	Calamagrostis epigejos (L.) Roth	8	n	1	0	Poaceae Barnhart
Iz_4_1009	DJI_0342	Carduus acanthoides L.	0	n	1	0	Asteraceae Bercht. & J.Presl
Iz_4_1009	DJI_0342	Carex sp.	0	n	1	0	Cyperaceae Juss.
Iz_4_1009	DJI_0342	Centaurea majorovii Dumbadze	0	n	1	0	Asteraceae Bercht. & J.Presl
Iz_4_1009	DJI_0342	Chamaecytisus ruthenicus (Fisch.ex Wol.) Klaskova	0	n	1	0	Fabaceae Lindl.
Iz_4_1009	DJI_0342	Chelidonium majus L.	0	n	1	0	Papaveraceae Juss.
Iz_4_1009	DJI_0342	Chenopodium betaceum Andrz.	0	n	1	0	Amaranthaceae Juss.
Iz_4_1009	DJI_0342	Chenopodium cf. album	0	n	1	0	Amaranthaceae Juss.
Iz_4_1009	DJI_0342	Chenopodium sp.	0		NA	0	Amaranthaceae Juss.
Iz_4_1009	DJI_0342	Chondrilla juncea L.	0	n	1	0	Asteraceae Bercht. & J.Presl
Iz_4_1009	DJI_0342	Cirsium vulgare (Savi) Ten.	0	n	1	0	Asteraceae Bercht. & J.Presl
Iz_4_1009	DJI_0342	Clinopodium acinos (L.) Kuntze	0	n	1	0	Lamiacea Lindl.
Iz_4_1009	DJI_0342	Convallaria majalis L.	0	n	1	0	Asparagaceae Juss.

Iz_4_1009	DJI_0342	Crataegus rhipidophylla Gand.	0	n	1	1	Rosaceae Juss.
Iz_4_1009	DJI_0342	Crepis foetida L.	0	n	1	0	Asteraceae Bercht. & J.Presl
Iz_4_1009	DJI_0342	Dactylis glomerata L.	0	n	1	0	Poaceae Barnhart
Iz_4_1009	DJI_0342	Dianthus campestris M.Bieb.	0	n	1	0	Caryophyllaceae Juss.
Iz_4_1009	DJI_0342	Digitaria sanguinalis (L.) Scop.	0	a	0	0	Poaceae Barnhart
Iz_4_1009	DJI_0342	Elymus repens (L.) Gould	0	n	1	0	Poaceae Barnhart
Iz_4_1009	DJI_0342	Epilobium tetragonum L.	0	n	1	0	Onagraceae Juss.
Iz_4_1009	DJI_0342	Eragrostis minor Host	0	a	0	0	Poaceae Barnhart
Iz_4_1009	DJI_0342	Erigeron canadensis L.	1	a	0	0	Asteraceae Bercht. & J.Presl
Iz_4_1009	DJI_0342	Euphorbia seguieriana Neck.	0	n	1	0	Euphorbiaceae Juss.
Iz_4_1009	DJI_0342	Fallopia dumetorum (L.) Holub	0	n	1	0	Polygonaceae Juss.
Iz_4_1009	DJI_0342	Festuca beckeri (Hack.) Trautv.	0	n	1	0	Poaceae Barnhart
Iz_4_1009	DJI_0342	Fragaria vesca L.	0	n	1	0	Rosaceae Juss.
Iz_4_1009	DJI_0342	Frangula alnus Mill.	0	n	1	1	Rhamnaceae Juss.
Iz_4_1009	DJI_0342	Fraxinus pennsylvanica Marshall	0	a	0	1	Oleaceae Hoffmanns. & Link
Iz_4_1009	DJI_0342	Galeopsis bifida Boenn.	0	n	1	0	Lamiacea Lindl.
Iz_4_1009	DJI_0342	Galium verum L.	0	n	1	0	Rubiaceae Juss.
Iz_4_1009	DJI_0342	Genista tinctoria L.	0	n	1	0	Fabaceae Lindl.
Iz_4_1009	DJI_0342	Geranium robertianum L.	0	n	1	0	Geraniaceae Juss.
Iz_4_1009	DJI_0342	Geranium sanquineum L.	0	n	1	0	Geraniaceae Juss.
Iz_4_1009	DJI_0342	Gypsophila paniculata L.	0	n	1	0	Caryophyllaceae Juss.
Iz_4_1009	DJI_0342	Helichrysum arenarium (L.) Moench	0	n	1	0	Asteraceae Bercht. & J.Presl
Iz_4_1009	DJI_0342	Herniaria polygama J.Gay	0	n	1	0	Caryophyllaceae Juss.
Iz_4_1009	DJI_0342	Hieracium robustum Fr.	0	n	1	0	Asteraceae Bercht. & J.Presl
Iz_4_1009	DJI_0342	Hieracium umbellatum L.	0	n	1	NA	Asteraceae Bercht. & J.Presl
Iz_4_1009	DJI_0342	Hylotelephium maximum (L.) Holub	0	n	1	0	Crassulaceae J.St.-Hil.
Iz_4_1009	DJI_0342	Jacobaea borysthenica (DC.) B.Nord. & Greuter	0	n	1	0	Asteraceae Bercht. & J.Presl
Iz_4_1009	DJI_0342	Jasione montana L.	0	n	1	0	Campanulaceae Juss.
Iz_4_1009	DJI_0342	Jurinea cyanoides (L.) Rchb.s.l.	0	n	1	0	Asteraceae Bercht. & J.Presl
Iz_4_1009	DJI_0342	Koeleria glauca (Spreng.) DC.	0	n	1	0	Poaceae Barnhart
Iz_4_1009	DJI_0342	Lactuca serriola L.	0	a	0	0	Asteraceae Bercht. & J.Presl
Iz_4_1009	DJI_0342	Linaria genistifolia (L.) Mill.	0	n	1	0	Plantaginaceae Juss.
Iz_4_1009	DJI_0342	Lomelosia argentea (L.) Greuter & Burdet	0	n	1	0	Caprifoliaceae Juss.
Iz_4_1009	DJI_0342	Melampyrum pratense L.	0	n	1	0	Orobanchaceae Vent.
Iz_4_1009	DJI_0342	Oenothera biennis L.	0	a	0	0	Onagraceae Juss.
Iz_4_1009	DJI_0342	Pentanema britannica (L.) D.Gut.Larr., Santos-Vicente, Anderb., E.Rico & M.M.Mart.Ort.	0	n	1	0	Asteraceae Bercht. & J.Presl
Iz_4_1009	DJI_0342	Peucedanum oreoselinum (L.) Moench	0	n	1	0	Apiaceae Lindl.
Iz_4_1009	DJI_0342	Peucedanum ruthenicum M.Bieb.	0	n	1	0	Apiaceae Lindl.
Iz_4_1009	DJI_0342	Pilosella echioides (Lumn.) F.W.Schultz & Sch.Bip.	0	n	1	0	Asteraceae Bercht. & J.Presl
Iz_4_1009	DJI_0342	Pilosella officinarum F. Schultz et Sch. Bip.	0	n	1	0	Asteraceae Bercht. & J.Presl
Iz_4_1009	DJI_0342	Pilosella sp.	0		NA	0	Asteraceae Bercht. & J.Presl
Iz_4_1009	DJI_0342	Pinus sylvestris L.	0	n	1	1	Pinaceae Spreng. ex F.Rudolphi
Iz_4_1009	DJI_0342	Poa annua L.	0	n	1	0	Poaceae Barnhart
Iz_4_1009	DJI_0342	Poa nemoralis L.	0	n	1	0	Poaceae Barnhart
Iz_4_1009	DJI_0342	Poa sp.	0		NA	0	Poaceae Barnhart
Iz_4_1009	DJI_0342	Polygonatum odoratum (Mill.) Druce	0	n	1	0	Asparagaceae Juss.
Iz_4_1009	DJI_0342	Polygonum arenarium Waldst. & Kit.	0	n	1	0	Polygonaceae Juss.
Iz_4_1009	DJI_0342	Populus nigra L.	0	n	1	1	Salicaceae Mirb.
Iz_4_1009	DJI_0342	Populus tremula L.	0	n	1	1	Salicaceae Mirb.
Iz_4_1009	DJI_0342	Populus Ã canescens (Aiton) Sm.	0	n	1	1	Salicaceae Mirb.
Iz_4_1009	DJI_0342	Potentilla argentea L.	0	n	1	0	Rosaceae Juss.
Iz_4_1009	DJI_0342	Psammophiliella muralis (L.) Ikonn.	0	n	1	0	Caryophyllaceae Juss.
Iz_4_1009	DJI_0342	Psephellus sumensis (Kalen.) Greuter	0	n	1	0	Asteraceae Bercht. & J.Presl
Iz_4_1009	DJI_0342	Pulsatilla pratensis (L.) Mill. s.l.	0	n	1	0	Ranunculaceae Juss.
Iz_4_1009	DJI_0342	Pyrus communis L.	0	a	0	1	Rosaceae Juss.
Iz_4_1009	DJI_0342	Quercus robur L.	0	n	1	1	Fagaceae Dumort.
Iz_4_1009	DJI_0342	Robinia pseudoacacia L.	0	a	0	1	Fabaceae Lindl.
Iz_4_1009	DJI_0342	Rosa sp.	0		NA	1	Rosaceae Juss.
Iz_4_1009	DJI_0342	Rubus idaeus L.	0	n	1	0	Rosaceae Juss.
Iz_4_1009	DJI_0342	Rumex acetosella L.	1	n	1	0	Polygonaceae Juss.
Iz_4_1009	DJI_0342	Rumex thyrsiflorus Fingerh.	0	n	1	0	Polygonaceae Juss.
Iz_4_1009	DJI_0342	Salix alba L.	0	n	1	1	Salicaceae Mirb.
Iz_4_1009	DJI_0342	Sambucus nigra L.	0	n	1	1	Viburnaceae Raf.
Iz_4_1009	DJI_0342	Secale sylvestre Host	0	n	1	0	Poaceae Barnhart
Iz_4_1009	DJI_0342	Sempervivum ruthenicum Schnittsp. & C.B. Lehm.	0	n	1	0	Crassulaceae J.St.-Hil.
Iz_4_1009	DJI_0342	Setaria viridis (L.) P. Beauv.	0	n	1	0	Poaceae Barnhart
Iz_4_1009	DJI_0342	Silene borysthenica (Gruner) Walters	0	n	1	0	Caryophyllaceae Juss.
Iz_4_1009	DJI_0342	Silene latifolia subsp. alba (Mill.) Greuter & Burdet	0	n	1	0	Caryophyllaceae Juss.
Iz_4_1009	DJI_0342	Solidago virgaurea L.	0	n	1	0	Asteraceae Bercht. & J.Presl
Iz_4_1009	DJI_0342	Sorbus aucuparia L.	0	n	1	1	Rosaceae Juss.
Iz_4_1009	DJI_0342	Stachys recta L.	0	n	1	0	Lamiacea Lindl.
Iz_4_1009	DJI_0342	Tanacetum vulgare L.	0	n	1	0	Asteraceae Bercht. & J.Presl
Iz_4_1009	DJI_0342	Thymus pallasianus Heinr.Braun	0	n	1	0	Lamiacea Lindl.
Iz_4_1009	DJI_0342	Tilia cordata Mill.	0	n	1	1	Malvaceae Juss.
Iz_4_1009	DJI_0342	Trifolium alpestre L.	0	n	1	0	Fabaceae Lindl.
Iz_4_1009	DJI_0342	Trifolium arvense L.	0	n	1	0	Fabaceae Lindl.
Iz_4_1009	DJI_0342	Ulmus glabra Huds.	0	n	1	1	Ulmaceae Mirb.
Iz_4_1009	DJI_0342	Verbascum densiflorum Bertol.	0	n	1	0	Scrophulariaceae Juss.
Iz_4_1009	DJI_0342	Verbascum nigrum L.	0	n	1	0	Scrophulariaceae Juss.
Iz_4_1009	DJI_0342	Veronica austriaca L.	0	n	1	0	Plantaginaceae Juss.
Iz_4_1009	DJI_0342	Veronica incana L.	0	n	1	0	Plantaginaceae Juss.
Iz_4_1009	DJI_0342	Veronica verna L.	0	n	1	0	Plantaginaceae Juss.
Iz_4_1009	DJI_0342	Vincetoxicum hirundinaria Medik	0	n	1	0	Apocynaceae Juss.
Iz_4_1009	DJI_0343	Acer tataricum L.	0	n	1	1	Sapindaceae Juss.
Iz_4_1009	DJI_0343	Achillea millefolium L. s.l.	0	n	1	0	Asteraceae Bercht. & J.Presl
Iz_4_1009	DJI_0343	Allium oleraceum L.	0	n	1	0	Amaryllidaceae J.St.-Hil.
Iz_4_1009	DJI_0343	Ambrosia artemisiifolia L.	0	a	0	0	Asteraceae Bercht. & J.Presl
Iz_4_1009	DJI_0343	Amorpha fruticosa L.	0	a	0	0	Fabaceae Lindl.
Iz_4_1009	DJI_0343	Anthericum ramosum L.	0	n	1	0	Asparagaceae Juss.
Iz_4_1009	DJI_0343	Artemisia marschalliana Spreng.	0	n	1	0	Asteraceae Bercht. & J.Presl
Iz_4_1009	DJI_0343	Asparagus officinalis L.	0	n	1	0	Asparagaceae Juss.
Iz_4_1009	DJI_0343	Astragalus cicer L.	0	n	1	0	Fabaceae Lindl.
Iz_4_1009	DJI_0343	Bassia laniflora (S.G.Gmel.) A.J.Scott	0	n	1	0	Amaranthaceae Juss.
Iz_4_1009	DJI_0343	Berberis vulgaris L.	0	n	1	0	Berberidaceae Juss.
Iz_4_1009	DJI_0343	Betula pendula Roth.	0	n	1	1	Betulaceae Gray
Iz_4_1009	DJI_0343	Betula pendula Roth.	0	n	1	0	Poaceae Barnhart
Iz_4_1009	DJI_0343	Bromus inermis Leyss.	0		NA	NA	
Iz_4_1009	DJI_0343	Bromus squarrosus L.	0	a	0	0	Poaceae Barnhart

Iz_4_1009	DJI_0343	Bromus tectorum L.	0	a	0	0	Poaceae Barnhart
Iz_4_1009	DJI_0343	Calamagrostis epigejos (L.) Roth	30	n	1	0	Poaceae Barnhart
Iz_4_1009	DJI_0343	Carduus acanthoides L.	0	n	1	0	Asteraceae Bercht. & J.Presl
Iz_4_1009	DJI_0343	Carex sp.	0	n	1	0	Cyperaceae Juss.
Iz_4_1009	DJI_0343	Centaurea majorovii Dumbadze	0	n	1	0	Asteraceae Bercht. & J.Presl
Iz_4_1009	DJI_0343	Chamaecytisus ruthenicus (Fisch.ex Wol.) Klaskova	0	n	1	0	Fabaceae Lindl.
Iz_4_1009	DJI_0343	Chelidonium majus L.	0	n	1	0	Papaveraceae Juss.
Iz_4_1009	DJI_0343	Chenopodium betaceum Andrz.	0	n	1	0	Amaranthaceae Juss.
Iz_4_1009	DJI_0343	Chenopodium cf. album	0	n	1	0	Amaranthaceae Juss.
Iz_4_1009	DJI_0343	Chenopodium sp.	0		NA	0	Amaranthaceae Juss.
Iz_4_1009	DJI_0343	Chondrilla juncea L.	0	n	1	0	Asteraceae Bercht. & J.Presl
Iz_4_1009	DJI_0343	Cirsium vulgare (Savi) Ten.	0	n	1	0	Asteraceae Bercht. & J.Presl
Iz_4_1009	DJI_0343	Clinopodium acinos (L.) Kuntze	0	n	1	0	Lamiacea Lindl.
Iz_4_1009	DJI_0343	Convallaria majalis L.	0	n	1	0	Asparagaceae Juss.
Iz_4_1009	DJI_0343	Crataegus rhipidophylla Gand.	0	n	1	1	Rosaceae Juss.
Iz_4_1009	DJI_0343	Crepis foetida L.	0	n	1	0	Asteraceae Bercht. & J.Presl
Iz_4_1009	DJI_0343	Dactylis glomerata L.	0	n	1	0	Poaceae Barnhart
Iz_4_1009	DJI_0343	Dianthus campestris M.Bieb.	0	n	1	0	Caryophyllaceae Juss.
Iz_4_1009	DJI_0343	Digitaria sanguinalis (L.) Scop.	0	a	0	0	Poaceae Barnhart
Iz_4_1009	DJI_0343	Elymus repens (L.) Gould	0	n	1	0	Poaceae Barnhart
Iz_4_1009	DJI_0343	Epilobium tetragonum L.	0	n	1	0	Onagraceae Juss.
Iz_4_1009	DJI_0343	Eragrostis minor Host	0	a	0	0	Poaceae Barnhart
Iz_4_1009	DJI_0343	Erigeron canadensis L.	2	a	0	0	Asteraceae Bercht. & J.Presl
Iz_4_1009	DJI_0343	Euphorbia seguieriana Neck.	0	n	1	0	Euphorbiaceae Juss.
Iz_4_1009	DJI_0343	Fallopia dumetorum (L.) Holub	0	n	1	0	Polygonaceae Juss.
Iz_4_1009	DJI_0343	Festuca beckeri (Hack.) Trautv.	0	n	1	0	Poaceae Barnhart
Iz_4_1009	DJI_0343	Fragaria vesca L.	0	n	1	0	Rosaceae Juss.
Iz_4_1009	DJI_0343	Frangula alnus Mill.	0	n	1	1	Rhamnaceae Juss.
Iz_4_1009	DJI_0343	Fraxinus pennsylvanica Marshall	0	a	0	1	Oleaceae Hoffmanns. & Link
Iz_4_1009	DJI_0343	Galeopsis bifida Boenn.	0	n	1	0	Lamiacea Lindl.
Iz_4_1009	DJI_0343	Galium verum L.	0	n	1	0	Rubiaceae Juss.
Iz_4_1009	DJI_0343	Genista tinctoria L.	0	n	1	0	Fabaceae Lindl.
Iz_4_1009	DJI_0343	Geranium robertianum L.	0	n	1	0	Geraniaceae Juss.
Iz_4_1009	DJI_0343	Geranium sanquineum L.	0	n	1	0	Geraniaceae Juss.
Iz_4_1009	DJI_0343	Gypsophila paniculata L.	0	n	1	0	Caryophyllaceae Juss.
Iz_4_1009	DJI_0343	Helichrysum arenarium (L.) Moench	0	n	1	0	Asteraceae Bercht. & J.Presl
Iz_4_1009	DJI_0343	Herniaria polygama J.Gay	0	n	1	0	Caryophyllaceae Juss.
Iz_4_1009	DJI_0343	Hieracium robustum Fr.	0	n	1	0	Asteraceae Bercht. & J.Presl
Iz_4_1009	DJI_0343	Hieracium umbellatum L.	0	n	1	NA	Asteraceae Bercht. & J.Presl
Iz_4_1009	DJI_0343	Hylotelephium maximum (L.) Holub	0	n	1	0	Crassulaceae J.St.-Hil.
Iz_4_1009	DJI_0343	Jacobaea borysthenica (DC.) B.Nord. & Greuter	0	n	1	0	Asteraceae Bercht. & J.Presl
Iz_4_1009	DJI_0343	Jasione montana L.	0	n	1	0	Campanulaceae Juss.
Iz_4_1009	DJI_0343	Jurinea cyanoides (L.) Rchb.s.l.	0	n	1	0	Asteraceae Bercht. & J.Presl
Iz_4_1009	DJI_0343	Koeleria glauca (Spreng.) DC.	0	n	1	0	Poaceae Barnhart
Iz_4_1009	DJI_0343	Lactuca serriola L.	0	a	0	0	Asteraceae Bercht. & J.Presl
Iz_4_1009	DJI_0343	Linaria genistifolia (L.) Mill.	0	n	1	0	Plantaginaceae Juss.
Iz_4_1009	DJI_0343	Lomelosia argentea (L.) Greuter & Burdet	0	n	1	0	Caprifoliaceae Juss.
Iz_4_1009	DJI_0343	Melampyrum pratense L.	0	n	1	0	Orobanchaceae Vent.
Iz_4_1009	DJI_0343	Oenothera biennis L.	0	a	0	0	Onagraceae Juss.
Iz_4_1009	DJI_0343	Pentanema britannica (L.) D.Gut.Larr., Santos-Vicente, Anderb., E.Rico & M.M.Mart.Ort.	0	n	1	0	Asteraceae Bercht. & J.Presl
Iz_4_1009	DJI_0343	Peucedanum oreoselinum (L.) Moench	0	n	1	0	Apiaceae Lindl.
Iz_4_1009	DJI_0343	Peucedanum ruthenicum M.Bieb.	0	n	1	0	Apiaceae Lindl.
Iz_4_1009	DJI_0343	Pilosella echioides (Lumn.) F.W.Schultz & Sch.Bip.	0	n	1	0	Asteraceae Bercht. & J.Presl
Iz_4_1009	DJI_0343	Pilosella officinarum F. Schultz et Sch. Bip.	0	n	1	0	Asteraceae Bercht. & J.Presl
Iz_4_1009	DJI_0343	Pilosella sp.	0		NA	0	Asteraceae Bercht. & J.Presl
Iz_4_1009	DJI_0343	Pinus sylvestris L.	0	n	1	1	Pinaceae Spreng. ex F.Rudolphi
Iz_4_1009	DJI_0343	Poa annua L.	0	n	1	0	Poaceae Barnhart
Iz_4_1009	DJI_0343	Poa nemoralis L.	0	n	1	0	Poaceae Barnhart
Iz_4_1009	DJI_0343	Poa sp.	0		NA	0	Poaceae Barnhart
Iz_4_1009	DJI_0343	Polygonatum odoratum (Mill.) Druce	0	n	1	0	Asparagaceae Juss.
Iz_4_1009	DJI_0343	Polygonum arenarium Waldst. & Kit.	0	n	1	0	Polygonaceae Juss.
Iz_4_1009	DJI_0343	Populus nigra L.	0	n	1	1	Salicaceae Mirb.
Iz_4_1009	DJI_0343	Populus tremula L.	0	n	1	1	Salicaceae Mirb.
Iz_4_1009	DJI_0343	Populus Ã canescens (Aiton) Sm.	0	n	1	1	Salicaceae Mirb.
Iz_4_1009	DJI_0343	Potentilla argentea L.	0	n	1	0	Rosaceae Juss.
Iz_4_1009	DJI_0343	Psammophiliella muralis (L.) Ikonn.	0	n	1	0	Caryophyllaceae Juss.
Iz_4_1009	DJI_0343	Psephellus sumensis (Kalen.) Greuter	0	n	1	0	Asteraceae Bercht. & J.Presl
Iz_4_1009	DJI_0343	Pulsatilla pratensis (L.) Mill. s.l.	0	n	1	0	Ranunculaceae Juss.
Iz_4_1009	DJI_0343	Pyrus communis L.	0	a	0	1	Rosaceae Juss.
Iz_4_1009	DJI_0343	Quercus robur L.	0	n	1	1	Fagaceae Dumort.
Iz_4_1009	DJI_0343	Robinia pseudoacacia L.	0	a	0	1	Fabaceae Lindl.
Iz_4_1009	DJI_0343	Rosa sp.	0		NA	1	Rosaceae Juss.
Iz_4_1009	DJI_0343	Rubus idaeus L.	0	n	1	0	Rosaceae Juss.
Iz_4_1009	DJI_0343	Rumex acetosella L.	0	n	1	0	Polygonaceae Juss.
Iz_4_1009	DJI_0343	Rumex thyrsiflorus Fingerh.	0	n	1	0	Polygonaceae Juss.
Iz_4_1009	DJI_0343	Salix alba L.	0	n	1	1	Salicaceae Mirb.
Iz_4_1009	DJI_0343	Sambucus nigra L.	0	n	1	1	Viburnaceae Raf.
Iz_4_1009	DJI_0343	Secale sylvestre Host	0	n	1	0	Poaceae Barnhart
Iz_4_1009	DJI_0343	Sempervivum ruthenicum Schnittsp. & C.B. Lehm.	0	n	1	0	Crassulaceae J.St.-Hil.
Iz_4_1009	DJI_0343	Setaria viridis (L.) P. Beauv.	0	n	1	0	Poaceae Barnhart
Iz_4_1009	DJI_0343	Silene borysthenica (Gruner) Walters	0	n	1	0	Caryophyllaceae Juss.
Iz_4_1009	DJI_0343	Silene latifolia subsp. alba (Mill.) Greuter & Burdet	0	n	1	0	Caryophyllaceae Juss.
Iz_4_1009	DJI_0343	Solidago virgaurea L.	0	n	1	0	Asteraceae Bercht. & J.Presl
Iz_4_1009	DJI_0343	Sorbus aucuparia L.	0	n	1	1	Rosaceae Juss.
Iz_4_1009	DJI_0343	Stachys recta L.	0	n	1	0	Lamiacea Lindl.
Iz_4_1009	DJI_0343	Tanacetum vulgare L.	0	n	1	0	Asteraceae Bercht. & J.Presl
Iz_4_1009	DJI_0343	Thymus pallasianus Heinr.Braun	0	n	1	0	Lamiacea Lindl.
Iz_4_1009	DJI_0343	Tilia cordata Mill.	0	n	1	1	Malvaceae Juss.
Iz_4_1009	DJI_0343	Trifolium alpestre L.	0	n	1	0	Fabaceae Lindl.
Iz_4_1009	DJI_0343	Trifolium arvense L.	0	n	1	0	Fabaceae Lindl.
Iz_4_1009	DJI_0343	Ulmus glabra Huds.	0	n	1	1	Ulmaceae Mirb.
Iz_4_1009	DJI_0343	Verbascum densiflorum Bertol.	0	n	1	0	Scrophulariaceae Juss.
Iz_4_1009	DJI_0343	Verbascum nigrum L.	0	n	1	0	Scrophulariaceae Juss.
Iz_4_1009	DJI_0343	Veronica austriaca L.	0	n	1	0	Plantaginaceae Juss.
Iz_4_1009	DJI_0343	Veronica incana L.	0	n	1	0	Plantaginaceae Juss.
Iz_4_1009	DJI_0343	Veronica verna L.	0	n	1	0	Plantaginaceae Juss.
Iz_4_1009	DJI_0343	Vincetoxicum hirundinaria Medik	0	n	1	0	Apocynaceae Juss.
Iz_4_1009	DJI_0344	Acer tataricum L.	0	n	1	1	Sapindaceae Juss.

Iz_4_1009	DJI_0344	Achillea millefolium L. s.l.	0	n	1	0	Asteraceae Bercht. & J.Presl
Iz_4_1009	DJI_0344	Allium oleraceum L.	0	n	1	0	Amaryllidaceae J.St.-Hil.
Iz_4_1009	DJI_0344	Ambrosia artemisiifolia L.	0	a	0	0	Asteraceae Bercht. & J.Presl
Iz_4_1009	DJI_0344	Amorpha fruticosa L.	0	a	0	0	Fabaceae Lindl.
Iz_4_1009	DJI_0344	Anthericum ramosum L.	0	n	1	0	Asparagaceae Juss.
Iz_4_1009	DJI_0344	Artemisia marschalliana Spreng.	0	n	1	0	Asteraceae Bercht. & J.Presl
Iz_4_1009	DJI_0344	Asparagus officinalis L.	0	n	1	0	Asparagaceae Juss.
Iz_4_1009	DJI_0344	Astragalus cicer L.	0	n	1	0	Fabaceae Lindl.
Iz_4_1009	DJI_0344	Bassia laniflora (S.G.Gmel.) A.J.Scott	0	n	1	0	Amaranthaceae Juss.
Iz_4_1009	DJI_0344	Berberis vulgaris L.	0	n	1	0	Berberidaceae Juss.
Iz_4_1009	DJI_0344	Betula pendula Roth.	0	n	1	1	Betulaceae Gray
Iz_4_1009	DJI_0344	Betula pendula Roth.	0	n	1	0	Poaceae Barnhart
Iz_4_1009	DJI_0344	Bromus inermis Leyss.	0		NA	NA	
Iz_4_1009	DJI_0344	Bromus squarrosus L.	0	a	0	0	Poaceae Barnhart
Iz_4_1009	DJI_0344	Bromus tectorum L.	0	a	0	0	Poaceae Barnhart
Iz_4_1009	DJI_0344	Calamagrostis epigejos (L.) Roth	20	n	1	0	Poaceae Barnhart
Iz_4_1009	DJI_0344	Carduus acanthoides L.	0	n	1	0	Asteraceae Bercht. & J.Presl
Iz_4_1009	DJI_0344	Carex sp.	0	n	1	0	Cyperaceae Juss.
Iz_4_1009	DJI_0344	Centaurea majorovii Dumbadze	0	n	1	0	Asteraceae Bercht. & J.Presl
Iz_4_1009	DJI_0344	Chamaecytisus ruthenicus (Fisch.ex Wol.) Klaskova	0	n	1	0	Fabaceae Lindl.
Iz_4_1009	DJI_0344	Chelidonium majus L.	0	n	1	0	Papaveraceae Juss.
Iz_4_1009	DJI_0344	Chenopodium betaceum Andrz.	0	n	1	0	Amaranthaceae Juss.
Iz_4_1009	DJI_0344	Chenopodium cf. album	0	n	1	0	Amaranthaceae Juss.
Iz_4_1009	DJI_0344	Chenopodium sp.	0		NA	0	Amaranthaceae Juss.
Iz_4_1009	DJI_0344	Chondrilla juncea L.	0	n	1	0	Asteraceae Bercht. & J.Presl
Iz_4_1009	DJI_0344	Cirsium vulgare (Savi) Ten.	0	n	1	0	Asteraceae Bercht. & J.Presl
Iz_4_1009	DJI_0344	Clinopodium acinos (L.) Kuntze	0	n	1	0	Lamiacea Lindl.
Iz_4_1009	DJI_0344	Convallaria majalis L.	0	n	1	0	Asparagaceae Juss.
Iz_4_1009	DJI_0344	Crataegus rhipidophylla Gand.	0	n	1	1	Rosaceae Juss.
Iz_4_1009	DJI_0344	Crepis foetida L.	0	n	1	0	Asteraceae Bercht. & J.Presl
Iz_4_1009	DJI_0344	Dactylis glomerata L.	0	n	1	0	Poaceae Barnhart
Iz_4_1009	DJI_0344	Dianthus campestris M.Bieb.	0	n	1	0	Caryophyllaceae Juss.
Iz_4_1009	DJI_0344	Digitaria sanguinalis (L.) Scop.	0	a	0	0	Poaceae Barnhart
Iz_4_1009	DJI_0344	Elymus repens (L.) Gould	0	n	1	0	Poaceae Barnhart
Iz_4_1009	DJI_0344	Epilobium tetragonum L.	0	n	1	0	Onagraceae Juss.
Iz_4_1009	DJI_0344	Eragrostis minor Host	0	a	0	0	Poaceae Barnhart
Iz_4_1009	DJI_0344	Erigeron canadensis L.	5	a	0	0	Asteraceae Bercht. & J.Presl
Iz_4_1009	DJI_0344	Euphorbia seguieriana Neck.	0	n	1	0	Euphorbiaceae Juss.
Iz_4_1009	DJI_0344	Fallopia dumetorum (L.) Holub	0	n	1	0	Polygonaceae Juss.
Iz_4_1009	DJI_0344	Festuca beckeri (Hack.) Trautv.	0	n	1	0	Poaceae Barnhart
Iz_4_1009	DJI_0344	Fragaria vesca L.	0	n	1	0	Rosaceae Juss.
Iz_4_1009	DJI_0344	Frangula alnus Mill.	0	n	1	1	Rhamnaceae Juss.
Iz_4_1009	DJI_0344	Fraxinus pennsylvanica Marshall	0	a	0	1	Oleaceae Hoffmanns. & Link
Iz_4_1009	DJI_0344	Galeopsis bifida Boenn.	0	n	1	0	Lamiacea Lindl.
Iz_4_1009	DJI_0344	Galium verum L.	0	n	1	0	Rubiaceae Juss.
Iz_4_1009	DJI_0344	Genista tinctoria L.	0	n	1	0	Fabaceae Lindl.
Iz_4_1009	DJI_0344	Geranium robertianum L.	0	n	1	0	Geraniaceae Juss.
Iz_4_1009	DJI_0344	Geranium sanquineum L.	0	n	1	0	Geraniaceae Juss.
Iz_4_1009	DJI_0344	Gypsophila paniculata L.	0	n	1	0	Caryophyllaceae Juss.
Iz_4_1009	DJI_0344	Helichrysum arenarium (L.) Moench	0	n	1	0	Asteraceae Bercht. & J.Presl
Iz_4_1009	DJI_0344	Herniaria polygama J.Gay	0	n	1	0	Caryophyllaceae Juss.
Iz_4_1009	DJI_0344	Hieracium robustum Fr.	0	n	1	0	Asteraceae Bercht. & J.Presl
Iz_4_1009	DJI_0344	Hieracium umbellatum L.	0	n	1	NA	Asteraceae Bercht. & J.Presl
Iz_4_1009	DJI_0344	Hylotelephium maximum (L.) Holub	0	n	1	0	Crassulaceae J.St.-Hil.
Iz_4_1009	DJI_0344	Jacobaea borysthenica (DC.) B.Nord. & Greuter	0	n	1	0	Asteraceae Bercht. & J.Presl
Iz_4_1009	DJI_0344	Jasione montana L.	0	n	1	0	Campanulaceae Juss.
Iz_4_1009	DJI_0344	Jurinea cyanoides (L.) Rchb.s.l.	0	n	1	0	Asteraceae Bercht. & J.Presl
Iz_4_1009	DJI_0344	Koeleria glauca (Spreng.) DC.	0	n	1	0	Poaceae Barnhart
Iz_4_1009	DJI_0344	Lactuca serriola L.	0	a	0	0	Asteraceae Bercht. & J.Presl
Iz_4_1009	DJI_0344	Linaria genistifolia (L.) Mill.	0	n	1	0	Plantaginaceae Juss.
Iz_4_1009	DJI_0344	Lomelosia argentea (L.) Greuter & Burdet	0	n	1	0	Caprifoliaceae Juss.
Iz_4_1009	DJI_0344	Melampyrum pratense L.	0	n	1	0	Orobanchaceae Vent.
Iz_4_1009	DJI_0344	Oenothera biennis L.	0	a	0	0	Onagraceae Juss.
Iz_4_1009	DJI_0344	Pentanema britannica (L.) D.Gut.Larr., Santos-Vicente, Anderb., E.Rico & M.M.Mart.Ort.	0	n	1	0	Asteraceae Bercht. & J.Presl
Iz_4_1009	DJI_0344	Peucedanum oreoselinum (L.) Moench	0	n	1	0	Apiaceae Lindl.
Iz_4_1009	DJI_0344	Peucedanum ruthenicum M.Bieb.	0	n	1	0	Apiaceae Lindl.
Iz_4_1009	DJI_0344	Pilosella echioides (Lumn.) F.W.Schultz & Sch.Bip.	0	n	1	0	Asteraceae Bercht. & J.Presl
Iz_4_1009	DJI_0344	Pilosella officinarum F. Schultz et Sch. Bip.	0	n	1	0	Asteraceae Bercht. & J.Presl
Iz_4_1009	DJI_0344	Pilosella sp.	0		NA	0	Asteraceae Bercht. & J.Presl
Iz_4_1009	DJI_0344	Pinus sylvestris L.	0	n	1	1	Pinaceae Spreng. ex F.Rudolphi
Iz_4_1009	DJI_0344	Poa annua L.	0	n	1	0	Poaceae Barnhart
Iz_4_1009	DJI_0344	Poa nemoralis L.	0	n	1	0	Poaceae Barnhart
Iz_4_1009	DJI_0344	Poa sp.	0		NA	0	Poaceae Barnhart
Iz_4_1009	DJI_0344	Polygonatum odoratum (Mill.) Druce	0	n	1	0	Asparagaceae Juss.
Iz_4_1009	DJI_0344	Polygonum arenarium Waldst. & Kit.	0	n	1	0	Polygonaceae Juss.
Iz_4_1009	DJI_0344	Populus nigra L.	0	n	1	1	Salicaceae Mirb.
Iz_4_1009	DJI_0344	Populus tremula L.	0	n	1	1	Salicaceae Mirb.
Iz_4_1009	DJI_0344	Populus Ã canescens (Aiton) Sm.	0	n	1	1	Salicaceae Mirb.
Iz_4_1009	DJI_0344	Potentilla argentea L.	0	n	1	0	Rosaceae Juss.
Iz_4_1009	DJI_0344	Psammophiliella muralis (L.) Ikonn.	0	n	1	0	Caryophyllaceae Juss.
Iz_4_1009	DJI_0344	Psephellus sumensis (Kalen.) Greuter	0	n	1	0	Asteraceae Bercht. & J.Presl
Iz_4_1009	DJI_0344	Pulsatilla pratensis (L.) Mill. s.l.	0	n	1	0	Ranunculaceae Juss.
Iz_4_1009	DJI_0344	Pyrus communis L.	0	a	0	1	Rosaceae Juss.
Iz_4_1009	DJI_0344	Quercus robur L.	0	n	1	1	Fagaceae Dumort.
Iz_4_1009	DJI_0344	Robinia pseudoacacia L.	0	a	0	1	Fabaceae Lindl.
Iz_4_1009	DJI_0344	Rosa sp.	0		NA	1	Rosaceae Juss.
Iz_4_1009	DJI_0344	Rubus idaeus L.	0	n	1	0	Rosaceae Juss.
Iz_4_1009	DJI_0344	Rumex acetosella L.	0	n	1	0	Polygonaceae Juss.
Iz_4_1009	DJI_0344	Rumex thyrsiflorus Fingerh.	0	n	1	0	Polygonaceae Juss.
Iz_4_1009	DJI_0344	Salix alba L.	0	n	1	1	Salicaceae Mirb.
Iz_4_1009	DJI_0344	Sambucus nigra L.	0	n	1	1	Viburnaceae Raf.
Iz_4_1009	DJI_0344	Secale sylvestre Host	0	n	1	0	Poaceae Barnhart
Iz_4_1009	DJI_0344	Sempervivum ruthenicum Schnittsp. & C.B. Lehm.	0	n	1	0	Crassulaceae J.St.-Hil.
Iz_4_1009	DJI_0344	Setaria viridis (L.) P. Beauv.	0	n	1	0	Poaceae Barnhart
Iz_4_1009	DJI_0344	Silene borysthenica (Gruner) Walters	0	n	1	0	Caryophyllaceae Juss.
Iz_4_1009	DJI_0344	Silene latifolia subsp. alba (Mill.) Greuter & Burdet	0	n	1	0	Caryophyllaceae Juss.
Iz_4_1009	DJI_0344	Solidago virgaurea L.	0	n	1	0	Asteraceae Bercht. & J.Presl
Iz_4_1009	DJI_0344	Sorbus aucuparia L.	0	n	1	1	Rosaceae Juss.

Iz_4_1009	DJI_0344	Stachys recta L.	0	n	1	0	Lamiacea Lindl.
Iz_4_1009	DJI_0344	Tanacetum vulgare L.	0	n	1	0	Asteraceae Bercht. & J.Presl
Iz_4_1009	DJI_0344	Thymus pallasianus Heinr.Braun	0	n	1	0	Lamiacea Lindl.
Iz_4_1009	DJI_0344	Tilia cordata Mill.	0	n	1	1	Malvaceae Juss.
Iz_4_1009	DJI_0344	Trifolium alpestre L.	0	n	1	0	Fabaceae Lindl.
Iz_4_1009	DJI_0344	Trifolium arvense L.	0	n	1	0	Fabaceae Lindl.
Iz_4_1009	DJI_0344	Ulmus glabra Huds.	0	n	1	1	Ulmaceae Mirb.
Iz_4_1009	DJI_0344	Verbascum densiflorum Bertol.	0	n	1	0	Scrophulariaceae Juss.
Iz_4_1009	DJI_0344	Verbascum nigrum L.	0	n	1	0	Scrophulariaceae Juss.
Iz_4_1009	DJI_0344	Veronica austriaca L.	0	n	1	0	Plantaginaceae Juss.
Iz_4_1009	DJI_0344	Veronica incana L.	0	n	1	0	Plantaginaceae Juss.
Iz_4_1009	DJI_0344	Veronica verna L.	0	n	1	0	Plantaginaceae Juss.
Iz_4_1009	DJI_0344	Vincetoxicum hirundinaria Medik	0	n	1	0	Apocynaceae Juss.
Iz_4_1009	DJI_0346	Acer tataricum L.	0	n	1	1	Sapindaceae Juss.
Iz_4_1009	DJI_0346	Achillea millefolium L. s.l.	0	n	1	0	Asteraceae Bercht. & J.Presl
Iz_4_1009	DJI_0346	Allium oleraceum L.	0	n	1	0	Amaryllidaceae J.St.-Hil.
Iz_4_1009	DJI_0346	Ambrosia artemisiifolia L.	0	a	0	0	Asteraceae Bercht. & J.Presl
Iz_4_1009	DJI_0346	Amorpha fruticosa L.	0	a	0	0	Fabaceae Lindl.
Iz_4_1009	DJI_0346	Anthericum ramosum L.	0	n	1	0	Asparagaceae Juss.
Iz_4_1009	DJI_0346	Artemisia marschalliana Spreng.	0	n	1	0	Asteraceae Bercht. & J.Presl
Iz_4_1009	DJI_0346	Asparagus officinalis L.	0	n	1	0	Asparagaceae Juss.
Iz_4_1009	DJI_0346	Astragalus cicer L.	0	n	1	0	Fabaceae Lindl.
Iz_4_1009	DJI_0346	Bassia laniflora (S.G.Gmel.) A.J.Scott	0	n	1	0	Amaranthaceae Juss.
Iz_4_1009	DJI_0346	Berberis vulgaris L.	0	n	1	0	Berberidaceae Juss.
Iz_4_1009	DJI_0346	Betula pendula Roth.	0	n	1	1	Betulaceae Gray
Iz_4_1009	DJI_0346	Betula pendula Roth.	0	n	1	0	Poaceae Barnhart
Iz_4_1009	DJI_0346	Bromus inermis Leyss.	0		NA	NA	
Iz_4_1009	DJI_0346	Bromus squarrosus L.	0	a	0	0	Poaceae Barnhart
Iz_4_1009	DJI_0346	Bromus tectorum L.	0	a	0	0	Poaceae Barnhart
Iz_4_1009	DJI_0346	Calamagrostis epigejos (L.) Roth	10	n	1	0	Poaceae Barnhart
Iz_4_1009	DJI_0346	Carduus acanthoides L.	0	n	1	0	Asteraceae Bercht. & J.Presl
Iz_4_1009	DJI_0346	Carex sp.	0	n	1	0	Cyperaceae Juss.
Iz_4_1009	DJI_0346	Centaurea majorovii Dumbadze	0	n	1	0	Asteraceae Bercht. & J.Presl
Iz_4_1009	DJI_0346	Chamaecytisus ruthenicus (Fisch.ex Wol.) Klaskova	0	n	1	0	Fabaceae Lindl.
Iz_4_1009	DJI_0346	Chelidonium majus L.	0	n	1	0	Papaveraceae Juss.
Iz_4_1009	DJI_0346	Chenopodium betaceum Andrz.	0	n	1	0	Amaranthaceae Juss.
Iz_4_1009	DJI_0346	Chenopodium cf. album	0	n	1	0	Amaranthaceae Juss.
Iz_4_1009	DJI_0346	Chenopodium sp.	0		NA	0	Amaranthaceae Juss.
Iz_4_1009	DJI_0346	Chondrilla juncea L.	0	n	1	0	Asteraceae Bercht. & J.Presl
Iz_4_1009	DJI_0346	Cirsium vulgare (Savi) Ten.	0	n	1	0	Asteraceae Bercht. & J.Presl
Iz_4_1009	DJI_0346	Clinopodium acinos (L.) Kuntze	0	n	1	0	Lamiacea Lindl.
Iz_4_1009	DJI_0346	Convallaria majalis L.	0	n	1	0	Asparagaceae Juss.
Iz_4_1009	DJI_0346	Crataegus rhipidophylla Gand.	0	n	1	1	Rosaceae Juss.
Iz_4_1009	DJI_0346	Crepis foetida L.	0	n	1	0	Asteraceae Bercht. & J.Presl
Iz_4_1009	DJI_0346	Dactylis glomerata L.	0	n	1	0	Poaceae Barnhart
Iz_4_1009	DJI_0346	Dianthus campestris M.Bieb.	0	n	1	0	Caryophyllaceae Juss.
Iz_4_1009	DJI_0346	Digitaria sanguinalis (L.) Scop.	0	a	0	0	Poaceae Barnhart
Iz_4_1009	DJI_0346	Elymus repens (L.) Gould	0	n	1	0	Poaceae Barnhart
Iz_4_1009	DJI_0346	Epilobium tetragonum L.	0	n	1	0	Onagraceae Juss.
Iz_4_1009	DJI_0346	Eragrostis minor Host	0	a	0	0	Poaceae Barnhart
Iz_4_1009	DJI_0346	Erigeron canadensis L.	5	a	0	0	Asteraceae Bercht. & J.Presl
Iz_4_1009	DJI_0346	Euphorbia seguieriana Neck.	0	n	1	0	Euphorbiaceae Juss.
Iz_4_1009	DJI_0346	Fallopia dumetorum (L.) Holub	0	n	1	0	Polygonaceae Juss.
Iz_4_1009	DJI_0346	Festuca beckeri (Hack.) Trautv.	0	n	1	0	Poaceae Barnhart
Iz_4_1009	DJI_0346	Fragaria vesca L.	0	n	1	0	Rosaceae Juss.
Iz_4_1009	DJI_0346	Frangula alnus Mill.	0	n	1	1	Rhamnaceae Juss.
Iz_4_1009	DJI_0346	Fraxinus pennsylvanica Marshall	0	a	0	1	Oleaceae Hoffmanns. & Link
Iz_4_1009	DJI_0346	Galeopsis bifida Boenn.	0	n	1	0	Lamiacea Lindl.
Iz_4_1009	DJI_0346	Galium verum L.	0	n	1	0	Rubiaceae Juss.
Iz_4_1009	DJI_0346	Genista tinctoria L.	0	n	1	0	Fabaceae Lindl.
Iz_4_1009	DJI_0346	Geranium robertianum L.	0	n	1	0	Geraniaceae Juss.
Iz_4_1009	DJI_0346	Geranium sanquineum L.	0	n	1	0	Geraniaceae Juss.
Iz_4_1009	DJI_0346	Gypsophila paniculata L.	0	n	1	0	Caryophyllaceae Juss.
Iz_4_1009	DJI_0346	Helichrysum arenarium (L.) Moench	0	n	1	0	Asteraceae Bercht. & J.Presl
Iz_4_1009	DJI_0346	Herniaria polygama J.Gay	0	n	1	0	Caryophyllaceae Juss.
Iz_4_1009	DJI_0346	Hieracium robustum Fr.	0	n	1	0	Asteraceae Bercht. & J.Presl
Iz_4_1009	DJI_0346	Hieracium umbellatum L.	0	n	1	NA	Asteraceae Bercht. & J.Presl
Iz_4_1009	DJI_0346	Hylotelephium maximum (L.) Holub	0	n	1	0	Crassulaceae J.St.-Hil.
Iz_4_1009	DJI_0346	Jacobaea borysthenica (DC.) B.Nord. & Greuter	0	n	1	0	Asteraceae Bercht. & J.Presl
Iz_4_1009	DJI_0346	Jasione montana L.	0	n	1	0	Campanulaceae Juss.
Iz_4_1009	DJI_0346	Jurinea cyanoides (L.) Rchb.s.l.	0	n	1	0	Asteraceae Bercht. & J.Presl
Iz_4_1009	DJI_0346	Koeleria glauca (Spreng.) DC.	0	n	1	0	Poaceae Barnhart
Iz_4_1009	DJI_0346	Lactuca serriola L.	0	a	0	0	Asteraceae Bercht. & J.Presl
Iz_4_1009	DJI_0346	Linaria genistifolia (L.) Mill.	0	n	1	0	Plantaginaceae Juss.
Iz_4_1009	DJI_0346	Lomelosia argentea (L.) Greuter & Burdet	0	n	1	0	Caprifoliaceae Juss.
Iz_4_1009	DJI_0346	Melampyrum pratense L.	0	n	1	0	Orobanchaceae Vent.
Iz_4_1009	DJI_0346	Oenothera biennis L.	0	a	0	0	Onagraceae Juss.
Iz_4_1009	DJI_0346	Pentanema britannica (L.) D.Gut.Larr., Santos-Vicente, Anderb., E.Rico & M.M.Mart.Ort.	0	n	1	0	Asteraceae Bercht. & J.Presl
Iz_4_1009	DJI_0346	Peucedanum oreoselinum (L.) Moench	0	n	1	0	Apiaceae Lindl.
Iz_4_1009	DJI_0346	Peucedanum ruthenicum M.Bieb.	0	n	1	0	Apiaceae Lindl.
Iz_4_1009	DJI_0346	Pilosella echioides (Lumn.) F.W.Schultz & Sch.Bip.	0	n	1	0	Asteraceae Bercht. & J.Presl
Iz_4_1009	DJI_0346	Pilosella officinarum F. Schultz et Sch. Bip.	0	n	1	0	Asteraceae Bercht. & J.Presl
Iz_4_1009	DJI_0346	Pilosella sp.	0		NA	0	Asteraceae Bercht. & J.Presl
Iz_4_1009	DJI_0346	Pinus sylvestris L.	0	n	1	1	Pinaceae Spreng. ex F.Rudolphi
Iz_4_1009	DJI_0346	Poa annua L.	0	n	1	0	Poaceae Barnhart
Iz_4_1009	DJI_0346	Poa nemoralis L.	0	n	1	0	Poaceae Barnhart
Iz_4_1009	DJI_0346	Poa sp.	0		NA	0	Poaceae Barnhart
Iz_4_1009	DJI_0346	Polygonatum odoratum (Mill.) Druce	0	n	1	0	Asparagaceae Juss.
Iz_4_1009	DJI_0346	Polygonum arenarium Waldst. & Kit.	0	n	1	0	Polygonaceae Juss.
Iz_4_1009	DJI_0346	Populus nigra L.	0	n	1	1	Salicaceae Mirb.
Iz_4_1009	DJI_0346	Populus tremula L.	0	n	1	1	Salicaceae Mirb.
Iz_4_1009	DJI_0346	Populus Ã canescens (Aiton) Sm.	0	n	1	1	Salicaceae Mirb.
Iz_4_1009	DJI_0346	Potentilla argentea L.	0	n	1	0	Rosaceae Juss.
Iz_4_1009	DJI_0346	Psammophiliella muralis (L.) Ikonn.	0	n	1	0	Caryophyllaceae Juss.
Iz_4_1009	DJI_0346	Psephellus sumensis (Kalen.) Greuter	0	n	1	0	Asteraceae Bercht. & J.Presl
Iz_4_1009	DJI_0346	Pulsatilla pratensis (L.) Mill. s.l.	0	n	1	0	Ranunculaceae Juss.
Iz_4_1009	DJI_0346	Pyrus communis L.	0	a	0	1	Rosaceae Juss.
Iz_4_1009	DJI_0346	Quercus robur L.	0	n	1	1	Fagaceae Dumort.

Iz_4_1009	DJI_0346	Robinia pseudoacacia L.	0	a	0	1	Fabaceae Lindl.
Iz_4_1009	DJI_0346	Rosa sp.	0		NA	1	Rosaceae Juss.
Iz_4_1009	DJI_0346	Rubus idaeus L.	0	n	1	0	Rosaceae Juss.
Iz_4_1009	DJI_0346	Rumex acetosella L.	0	n	1	0	Polygonaceae Juss.
Iz_4_1009	DJI_0346	Rumex thyrsiflorus Fingerh.	0	n	1	0	Polygonaceae Juss.
Iz_4_1009	DJI_0346	Salix alba L.	0	n	1	1	Salicaceae Mirb.
Iz_4_1009	DJI_0346	Sambucus nigra L.	0	n	1	1	Viburnaceae Raf.
Iz_4_1009	DJI_0346	Secale sylvestre Host	0	n	1	0	Poaceae Barnhart
Iz_4_1009	DJI_0346	Sempervivum ruthenicum Schnittsp. & C.B. Lehm.	0	n	1	0	Crassulaceae J.St.-Hil.
Iz_4_1009	DJI_0346	Setaria viridis (L.) P. Beauv.	0	n	1	0	Poaceae Barnhart
Iz_4_1009	DJI_0346	Silene borysthenica (Gruner) Walters	0	n	1	0	Caryophyllaceae Juss.
Iz_4_1009	DJI_0346	Silene latifolia subsp. alba (Mill.) Greuter & Burdet	0	n	1	0	Caryophyllaceae Juss.
Iz_4_1009	DJI_0346	Solidago virgaurea L.	0	n	1	0	Asteraceae Bercht. & J.Presl
Iz_4_1009	DJI_0346	Sorbus aucuparia L.	0	n	1	1	Rosaceae Juss.
Iz_4_1009	DJI_0346	Stachys recta L.	0	n	1	0	Lamiacea Lindl.
Iz_4_1009	DJI_0346	Tanacetum vulgare L.	0	n	1	0	Asteraceae Bercht. & J.Presl
Iz_4_1009	DJI_0346	Thymus pallasianus Heinr.Braun	0	n	1	0	Lamiacea Lindl.
Iz_4_1009	DJI_0346	Tilia cordata Mill.	0	n	1	1	Malvaceae Juss.
Iz_4_1009	DJI_0346	Trifolium alpestre L.	0	n	1	0	Fabaceae Lindl.
Iz_4_1009	DJI_0346	Trifolium arvense L.	0	n	1	0	Fabaceae Lindl.
Iz_4_1009	DJI_0346	Ulmus glabra Huds.	0	n	1	1	Ulmaceae Mirb.
Iz_4_1009	DJI_0346	Verbascum densiflorum Bertol.	0	n	1	0	Scrophulariaceae Juss.
Iz_4_1009	DJI_0346	Verbascum nigrum L.	0	n	1	0	Scrophulariaceae Juss.
Iz_4_1009	DJI_0346	Veronica austriaca L.	0	n	1	0	Plantaginaceae Juss.
Iz_4_1009	DJI_0346	Veronica incana L.	0	n	1	0	Plantaginaceae Juss.
Iz_4_1009	DJI_0346	Veronica verna L.	0	n	1	0	Plantaginaceae Juss.
Iz_4_1009	DJI_0346	Vincetoxicum hirundinaria Medik	0	n	1	0	Apocynaceae Juss.
Iz_4_1009	DJI_0348	Acer tataricum L.	0	n	1	1	Sapindaceae Juss.
Iz_4_1009	DJI_0348	Achillea millefolium L. s.l.	0	n	1	0	Asteraceae Bercht. & J.Presl
Iz_4_1009	DJI_0348	Allium oleraceum L.	0	n	1	0	Amaryllidaceae J.St.-Hil.
Iz_4_1009	DJI_0348	Ambrosia artemisiifolia L.	0	a	0	0	Asteraceae Bercht. & J.Presl
Iz_4_1009	DJI_0348	Amorpha fruticosa L.	0	a	0	0	Fabaceae Lindl.
Iz_4_1009	DJI_0348	Anthericum ramosum L.	0	n	1	0	Asparagaceae Juss.
Iz_4_1009	DJI_0348	Artemisia marschalliana Spreng.	0	n	1	0	Asteraceae Bercht. & J.Presl
Iz_4_1009	DJI_0348	Asparagus officinalis L.	0	n	1	0	Asparagaceae Juss.
Iz_4_1009	DJI_0348	Astragalus cicer L.	0	n	1	0	Fabaceae Lindl.
Iz_4_1009	DJI_0348	Bassia laniflora (S.G.Gmel.) A.J.Scott	0	n	1	0	Amaranthaceae Juss.
Iz_4_1009	DJI_0348	Berberis vulgaris L.	0	n	1	0	Berberidaceae Juss.
Iz_4_1009	DJI_0348	Betula pendula Roth.	0	n	1	1	Betulaceae Gray
Iz_4_1009	DJI_0348	Betula pendula Roth.	0	n	1	0	Poaceae Barnhart
Iz_4_1009	DJI_0348	Bromus inermis Leyss.	0		NA	NA	
Iz_4_1009	DJI_0348	Bromus squarrosus L.	0	a	0	0	Poaceae Barnhart
Iz_4_1009	DJI_0348	Bromus tectorum L.	0	a	0	0	Poaceae Barnhart
Iz_4_1009	DJI_0348	Calamagrostis epigejos (L.) Roth	35	n	1	0	Poaceae Barnhart
Iz_4_1009	DJI_0348	Carduus acanthoides L.	0	n	1	0	Asteraceae Bercht. & J.Presl
Iz_4_1009	DJI_0348	Carex sp.	0	n	1	0	Cyperaceae Juss.
Iz_4_1009	DJI_0348	Centaurea majorovii Dumbadze	0	n	1	0	Asteraceae Bercht. & J.Presl
Iz_4_1009	DJI_0348	Chamaecytisus ruthenicus (Fisch.ex Wol.) Klaskova	0	n	1	0	Fabaceae Lindl.
Iz_4_1009	DJI_0348	Chelidonium majus L.	0	n	1	0	Papaveraceae Juss.
Iz_4_1009	DJI_0348	Chenopodium betaceum Andrz.	0	n	1	0	Amaranthaceae Juss.
Iz_4_1009	DJI_0348	Chenopodium cf. album	0	n	1	0	Amaranthaceae Juss.
Iz_4_1009	DJI_0348	Chenopodium sp.	0		NA	0	Amaranthaceae Juss.
Iz_4_1009	DJI_0348	Chondrilla juncea L.	0	n	1	0	Asteraceae Bercht. & J.Presl
Iz_4_1009	DJI_0348	Cirsium vulgare (Savi) Ten.	0	n	1	0	Asteraceae Bercht. & J.Presl
Iz_4_1009	DJI_0348	Clinopodium acinos (L.) Kuntze	0	n	1	0	Lamiacea Lindl.
Iz_4_1009	DJI_0348	Convallaria majalis L.	0	n	1	0	Asparagaceae Juss.
Iz_4_1009	DJI_0348	Crataegus rhipidophylla Gand.	0	n	1	1	Rosaceae Juss.
Iz_4_1009	DJI_0348	Crepis foetida L.	0	n	1	0	Asteraceae Bercht. & J.Presl
Iz_4_1009	DJI_0348	Dactylis glomerata L.	0	n	1	0	Poaceae Barnhart
Iz_4_1009	DJI_0348	Dianthus campestris M.Bieb.	0	n	1	0	Caryophyllaceae Juss.
Iz_4_1009	DJI_0348	Digitaria sanguinalis (L.) Scop.	0	a	0	0	Poaceae Barnhart
Iz_4_1009	DJI_0348	Elymus repens (L.) Gould	0	n	1	0	Poaceae Barnhart
Iz_4_1009	DJI_0348	Epilobium tetragonum L.	0	n	1	0	Onagraceae Juss.
Iz_4_1009	DJI_0348	Eragrostis minor Host	0	a	0	0	Poaceae Barnhart
Iz_4_1009	DJI_0348	Erigeron canadensis L.	4	a	0	0	Asteraceae Bercht. & J.Presl
Iz_4_1009	DJI_0348	Euphorbia seguieriana Neck.	0	n	1	0	Euphorbiaceae Juss.
Iz_4_1009	DJI_0348	Fallopia dumetorum (L.) Holub	0	n	1	0	Polygonaceae Juss.
Iz_4_1009	DJI_0348	Festuca beckeri (Hack.) Trautv.	0	n	1	0	Poaceae Barnhart
Iz_4_1009	DJI_0348	Fragaria vesca L.	0	n	1	0	Rosaceae Juss.
Iz_4_1009	DJI_0348	Frangula alnus Mill.	0	n	1	1	Rhamnaceae Juss.
Iz_4_1009	DJI_0348	Fraxinus pennsylvanica Marshall	0	a	0	1	Oleaceae Hoffmanns. & Link
Iz_4_1009	DJI_0348	Galeopsis bifida Boenn.	0	n	1	0	Lamiacea Lindl.
Iz_4_1009	DJI_0348	Galium verum L.	0	n	1	0	Rubiaceae Juss.
Iz_4_1009	DJI_0348	Genista tinctoria L.	0	n	1	0	Fabaceae Lindl.
Iz_4_1009	DJI_0348	Geranium robertianum L.	0	n	1	0	Geraniaceae Juss.
Iz_4_1009	DJI_0348	Geranium sanquineum L.	0	n	1	0	Geraniaceae Juss.
Iz_4_1009	DJI_0348	Gypsophila paniculata L.	0	n	1	0	Caryophyllaceae Juss.
Iz_4_1009	DJI_0348	Helichrysum arenarium (L.) Moench	0	n	1	0	Asteraceae Bercht. & J.Presl
Iz_4_1009	DJI_0348	Herniaria polygama J.Gay	0	n	1	0	Caryophyllaceae Juss.
Iz_4_1009	DJI_0348	Hieracium robustum Fr.	0	n	1	0	Asteraceae Bercht. & J.Presl
Iz_4_1009	DJI_0348	Hieracium umbellatum L.	0	n	1	NA	Asteraceae Bercht. & J.Presl
Iz_4_1009	DJI_0348	Hylotelephium maximum (L.) Holub	0	n	1	0	Crassulaceae J.St.-Hil.
Iz_4_1009	DJI_0348	Jacobaea borysthenica (DC.) B.Nord. & Greuter	0	n	1	0	Asteraceae Bercht. & J.Presl
Iz_4_1009	DJI_0348	Jasione montana L.	0	n	1	0	Campanulaceae Juss.
Iz_4_1009	DJI_0348	Jurinea cyanoides (L.) Rchb.s.l.	0	n	1	0	Asteraceae Bercht. & J.Presl
Iz_4_1009	DJI_0348	Koeleria glauca (Spreng.) DC.	0	n	1	0	Poaceae Barnhart
Iz_4_1009	DJI_0348	Lactuca serriola L.	0	a	0	0	Asteraceae Bercht. & J.Presl
Iz_4_1009	DJI_0348	Linaria genistifolia (L.) Mill.	0	n	1	0	Plantaginaceae Juss.
Iz_4_1009	DJI_0348	Lomelosia argentea (L.) Greuter & Burdet	0	n	1	0	Caprifoliaceae Juss.
Iz_4_1009	DJI_0348	Melampyrum pratense L.	0	n	1	0	Orobanchaceae Vent.
Iz_4_1009	DJI_0348	Oenothera biennis L.	0	a	0	0	Onagraceae Juss.
Iz_4_1009	DJI_0348	Pentanema britannica (L.) D.Gut.Larr., Santos-Vicente, Anderb., E.Rico & M.M.Mart.Ort.	0	n	1	0	Asteraceae Bercht. & J.Presl
Iz_4_1009	DJI_0348	Peucedanum oreoselinum (L.) Moench	0	n	1	0	Apiaceae Lindl.
Iz_4_1009	DJI_0348	Peucedanum ruthenicum M.Bieb.	0	n	1	0	Apiaceae Lindl.
Iz_4_1009	DJI_0348	Pilosella echioides (Lumn.) F.W.Schultz & Sch.Bip.	0	n	1	0	Asteraceae Bercht. & J.Presl
Iz_4_1009	DJI_0348	Pilosella officinarum F. Schultz et Sch. Bip.	0	n	1	0	Asteraceae Bercht. & J.Presl
Iz_4_1009	DJI_0348	Pilosella sp.	0		NA	0	Asteraceae Bercht. & J.Presl
Iz_4_1009	DJI_0348	Pinus sylvestris L.	0	n	1	1	Pinaceae Spreng. ex F.Rudolphi

Iz_4_1009	DJI_0348	Poa annua L.	0	n	1	0	Poaceae Barnhart
Iz_4_1009	DJI_0348	Poa nemoralis L.	0	n	1	0	Poaceae Barnhart
Iz_4_1009	DJI_0348	Poa sp.	0		NA	0	Poaceae Barnhart
Iz_4_1009	DJI_0348	Polygonatum odoratum (Mill.) Druce	0	n	1	0	Asparagaceae Juss.
Iz_4_1009	DJI_0348	Polygonum arenarium Waldst. & Kit.	0	n	1	0	Polygonaceae Juss.
Iz_4_1009	DJI_0348	Populus nigra L.	0	n	1	1	Salicaceae Mirb.
Iz_4_1009	DJI_0348	Populus tremula L.	0	n	1	1	Salicaceae Mirb.
Iz_4_1009	DJI_0348	Populus Ã canescens (Aiton) Sm.	0	n	1	1	Salicaceae Mirb.
Iz_4_1009	DJI_0348	Potentilla argentea L.	0	n	1	0	Rosaceae Juss.
Iz_4_1009	DJI_0348	Psammophiliella muralis (L.) Ikonn.	0	n	1	0	Caryophyllaceae Juss.
Iz_4_1009	DJI_0348	Psephellus sumensis (Kalen.) Greuter	0	n	1	0	Asteraceae Bercht. & J.Presl
Iz_4_1009	DJI_0348	Pulsatilla pratensis (L.) Mill. s.l.	0	n	1	0	Ranunculaceae Juss.
Iz_4_1009	DJI_0348	Pyrus communis L.	0	a	0	1	Rosaceae Juss.
Iz_4_1009	DJI_0348	Quercus robur L.	0	n	1	1	Fagaceae Dumort.
Iz_4_1009	DJI_0348	Robinia pseudoacacia L.	0	a	0	1	Fabaceae Lindl.
Iz_4_1009	DJI_0348	Rosa sp.	0		NA	1	Rosaceae Juss.
Iz_4_1009	DJI_0348	Rubus idaeus L.	0	n	1	0	Rosaceae Juss.
Iz_4_1009	DJI_0348	Rumex acetosella L.	0	n	1	0	Polygonaceae Juss.
Iz_4_1009	DJI_0348	Rumex thyrsiflorus Fingerh.	0	n	1	0	Polygonaceae Juss.
Iz_4_1009	DJI_0348	Salix alba L.	0	n	1	1	Salicaceae Mirb.
Iz_4_1009	DJI_0348	Sambucus nigra L.	0	n	1	1	Viburnaceae Raf.
Iz_4_1009	DJI_0348	Secale sylvestre Host	0	n	1	0	Poaceae Barnhart
Iz_4_1009	DJI_0348	Sempervivum ruthenicum Schnittsp. & C.B. Lehm.	0	n	1	0	Crassulaceae J.St.-Hil.
Iz_4_1009	DJI_0348	Setaria viridis (L.) P. Beauv.	0	n	1	0	Poaceae Barnhart
Iz_4_1009	DJI_0348	Silene borysthenica (Gruner) Walters	0	n	1	0	Caryophyllaceae Juss.
Iz_4_1009	DJI_0348	Silene latifolia subsp. alba (Mill.) Greuter & Burdet	0	n	1	0	Caryophyllaceae Juss.
Iz_4_1009	DJI_0348	Solidago virgaurea L.	0	n	1	0	Asteraceae Bercht. & J.Presl
Iz_4_1009	DJI_0348	Sorbus aucuparia L.	0	n	1	1	Rosaceae Juss.
Iz_4_1009	DJI_0348	Stachys recta L.	0	n	1	0	Lamiacea Lindl.
Iz_4_1009	DJI_0348	Tanacetum vulgare L.	0	n	1	0	Asteraceae Bercht. & J.Presl
Iz_4_1009	DJI_0348	Thymus pallasianus Heinr.Braun	0	n	1	0	Lamiacea Lindl.
Iz_4_1009	DJI_0348	Tilia cordata Mill.	0	n	1	1	Malvaceae Juss.
Iz_4_1009	DJI_0348	Trifolium alpestre L.	0	n	1	0	Fabaceae Lindl.
Iz_4_1009	DJI_0348	Trifolium arvense L.	0	n	1	0	Fabaceae Lindl.
Iz_4_1009	DJI_0348	Ulmus glabra Huds.	0	n	1	1	Ulmaceae Mirb.
Iz_4_1009	DJI_0348	Verbascum densiflorum Bertol.	0	n	1	0	Scrophulariaceae Juss.
Iz_4_1009	DJI_0348	Verbascum nigrum L.	0	n	1	0	Scrophulariaceae Juss.
Iz_4_1009	DJI_0348	Veronica austriaca L.	0	n	1	0	Plantaginaceae Juss.
Iz_4_1009	DJI_0348	Veronica incana L.	0	n	1	0	Plantaginaceae Juss.
Iz_4_1009	DJI_0348	Veronica verna L.	0	n	1	0	Plantaginaceae Juss.
Iz_4_1009	DJI_0348	Vincetoxicum hirundinaria Medik	0	n	1	0	Apocynaceae Juss.
Iz_4_1009	DJI_0351	Acer tataricum L.	0	n	1	1	Sapindaceae Juss.
Iz_4_1009	DJI_0351	Achillea millefolium L. s.l.	0	n	1	0	Asteraceae Bercht. & J.Presl
Iz_4_1009	DJI_0351	Allium oleraceum L.	0	n	1	0	Amaryllidaceae J.St.-Hil.
Iz_4_1009	DJI_0351	Ambrosia artemisiifolia L.	0	a	0	0	Asteraceae Bercht. & J.Presl
Iz_4_1009	DJI_0351	Amorpha fruticosa L.	0	a	0	0	Fabaceae Lindl.
Iz_4_1009	DJI_0351	Anthericum ramosum L.	0	n	1	0	Asparagaceae Juss.
Iz_4_1009	DJI_0351	Artemisia marschalliana Spreng.	0	n	1	0	Asteraceae Bercht. & J.Presl
Iz_4_1009	DJI_0351	Asparagus officinalis L.	0	n	1	0	Asparagaceae Juss.
Iz_4_1009	DJI_0351	Astragalus cicer L.	0	n	1	0	Fabaceae Lindl.
Iz_4_1009	DJI_0351	Bassia laniflora (S.G.Gmel.) A.J.Scott	0	n	1	0	Amaranthaceae Juss.
Iz_4_1009	DJI_0351	Berberis vulgaris L.	0	n	1	0	Berberidaceae Juss.
Iz_4_1009	DJI_0351	Betula pendula Roth.	0	n	1	1	Betulaceae Gray
Iz_4_1009	DJI_0351	Betula pendula Roth.	0	n	1	0	Poaceae Barnhart
Iz_4_1009	DJI_0351	Bromus inermis Leyss.	0		NA	NA	
Iz_4_1009	DJI_0351	Bromus squarrosus L.	0	a	0	0	Poaceae Barnhart
Iz_4_1009	DJI_0351	Bromus tectorum L.	0	a	0	0	Poaceae Barnhart
Iz_4_1009	DJI_0351	Calamagrostis epigejos (L.) Roth	30	n	1	0	Poaceae Barnhart
Iz_4_1009	DJI_0351	Carduus acanthoides L.	0	n	1	0	Asteraceae Bercht. & J.Presl
Iz_4_1009	DJI_0351	Carex sp.	0	n	1	0	Cyperaceae Juss.
Iz_4_1009	DJI_0351	Centaurea majorovii Dumbadze	0	n	1	0	Asteraceae Bercht. & J.Presl
Iz_4_1009	DJI_0351	Chamaecytisus ruthenicus (Fisch.ex Wol.) Klaskova	0	n	1	0	Fabaceae Lindl.
Iz_4_1009	DJI_0351	Chelidonium majus L.	0	n	1	0	Papaveraceae Juss.
Iz_4_1009	DJI_0351	Chenopodium betaceum Andrz.	0	n	1	0	Amaranthaceae Juss.
Iz_4_1009	DJI_0351	Chenopodium cf. album	0	n	1	0	Amaranthaceae Juss.
Iz_4_1009	DJI_0351	Chenopodium sp.	0		NA	0	Amaranthaceae Juss.
Iz_4_1009	DJI_0351	Chondrilla juncea L.	0	n	1	0	Asteraceae Bercht. & J.Presl
Iz_4_1009	DJI_0351	Cirsium vulgare (Savi) Ten.	0	n	1	0	Asteraceae Bercht. & J.Presl
Iz_4_1009	DJI_0351	Clinopodium acinos (L.) Kuntze	0	n	1	0	Lamiacea Lindl.
Iz_4_1009	DJI_0351	Convallaria majalis L.	0	n	1	0	Asparagaceae Juss.
Iz_4_1009	DJI_0351	Crataegus rhipidophylla Gand.	0	n	1	1	Rosaceae Juss.
Iz_4_1009	DJI_0351	Crepis foetida L.	0	n	1	0	Asteraceae Bercht. & J.Presl
Iz_4_1009	DJI_0351	Dactylis glomerata L.	0	n	1	0	Poaceae Barnhart
Iz_4_1009	DJI_0351	Dianthus campestris M.Bieb.	0	n	1	0	Caryophyllaceae Juss.
Iz_4_1009	DJI_0351	Digitaria sanguinalis (L.) Scop.	0	a	0	0	Poaceae Barnhart
Iz_4_1009	DJI_0351	Elymus repens (L.) Gould	0	n	1	0	Poaceae Barnhart
Iz_4_1009	DJI_0351	Epilobium tetragonum L.	0	n	1	0	Onagraceae Juss.
Iz_4_1009	DJI_0351	Eragrostis minor Host	0	a	0	0	Poaceae Barnhart
Iz_4_1009	DJI_0351	Erigeron canadensis L.	1	a	0	0	Asteraceae Bercht. & J.Presl
Iz_4_1009	DJI_0351	Euphorbia seguieriana Neck.	0	n	1	0	Euphorbiaceae Juss.
Iz_4_1009	DJI_0351	Fallopia dumetorum (L.) Holub	0	n	1	0	Polygonaceae Juss.
Iz_4_1009	DJI_0351	Festuca beckeri (Hack.) Trautv.	0	n	1	0	Poaceae Barnhart
Iz_4_1009	DJI_0351	Fragaria vesca L.	0	n	1	0	Rosaceae Juss.
Iz_4_1009	DJI_0351	Frangula alnus Mill.	0	n	1	1	Rhamnaceae Juss.
Iz_4_1009	DJI_0351	Fraxinus pennsylvanica Marshall	0	a	0	1	Oleaceae Hoffmanns. & Link
Iz_4_1009	DJI_0351	Galeopsis bifida Boenn.	0	n	1	0	Lamiacea Lindl.
Iz_4_1009	DJI_0351	Galium verum L.	0	n	1	0	Rubiaceae Juss.
Iz_4_1009	DJI_0351	Genista tinctoria L.	0	n	1	0	Fabaceae Lindl.
Iz_4_1009	DJI_0351	Geranium robertianum L.	0	n	1	0	Geraniaceae Juss.
Iz_4_1009	DJI_0351	Geranium sanquineum L.	0	n	1	0	Geraniaceae Juss.
Iz_4_1009	DJI_0351	Gypsophila paniculata L.	0	n	1	0	Caryophyllaceae Juss.
Iz_4_1009	DJI_0351	Helichrysum arenarium (L.) Moench	0	n	1	0	Asteraceae Bercht. & J.Presl
Iz_4_1009	DJI_0351	Herniaria polygama J.Gay	0	n	1	0	Caryophyllaceae Juss.
Iz_4_1009	DJI_0351	Hieracium robustum Fr.	0	n	1	0	Asteraceae Bercht. & J.Presl
Iz_4_1009	DJI_0351	Hieracium umbellatum L.	0	n	1	NA	Asteraceae Bercht. & J.Presl
Iz_4_1009	DJI_0351	Hylotelephium maximum (L.) Holub	0	n	1	0	Crassulaceae J.St.-Hil.
Iz_4_1009	DJI_0351	Jacobaea borysthenica (DC.) B.Nord. & Greuter	0	n	1	0	Asteraceae Bercht. & J.Presl
Iz_4_1009	DJI_0351	Jasione montana L.	0	n	1	0	Campanulaceae Juss.

Iz_4_1009	DJI_0351	Jurinea cyanoides (L.) Rchb.s.l.	0	n	1	0	Asteraceae Bercht. & J.Presl
Iz_4_1009	DJI_0351	Koeleria glauca (Spreng.) DC.	0	n	1	0	Poaceae Barnhart
Iz_4_1009	DJI_0351	Lactuca serriola L.	0	a	0	0	Asteraceae Bercht. & J.Presl
Iz_4_1009	DJI_0351	Linaria genistifolia (L.) Mill.	0	n	1	0	Plantaginaceae Juss.
Iz_4_1009	DJI_0351	Lomelosia argentea (L.) Greuter & Burdet	0	n	1	0	Caprifoliaceae Juss.
Iz_4_1009	DJI_0351	Melampyrum pratense L.	0	n	1	0	Orobanchaceae Vent.
Iz_4_1009	DJI_0351	Oenothera biennis L.	0	a	0	0	Onagraceae Juss.
Iz_4_1009	DJI_0351	Pentanema britannica (L.) D.Gut.Larr., Santos-Vicente, Anderb., E.Rico & M.M.Mart.Ort.	0	n	1	0	Asteraceae Bercht. & J.Presl
Iz_4_1009	DJI_0351	Peucedanum oreoselinum (L.) Moench	0	n	1	0	Apiaceae Lindl.
Iz_4_1009	DJI_0351	Peucedanum ruthenicum M.Bieb.	0	n	1	0	Apiaceae Lindl.
Iz_4_1009	DJI_0351	Pilosella echioides (Lumn.) F.W.Schultz & Sch.Bip.	0	n	1	0	Asteraceae Bercht. & J.Presl
Iz_4_1009	DJI_0351	Pilosella officinarum F. Schultz et Sch. Bip.	0	n	1	0	Asteraceae Bercht. & J.Presl
Iz_4_1009	DJI_0351	Pilosella sp.	0		NA	0	Asteraceae Bercht. & J.Presl
Iz_4_1009	DJI_0351	Pinus sylvestris L.	0	n	1	1	Pinaceae Spreng. ex F.Rudolphi
Iz_4_1009	DJI_0351	Poa annua L.	0	n	1	0	Poaceae Barnhart
Iz_4_1009	DJI_0351	Poa nemoralis L.	0	n	1	0	Poaceae Barnhart
Iz_4_1009	DJI_0351	Poa sp.	0		NA	0	Poaceae Barnhart
Iz_4_1009	DJI_0351	Polygonatum odoratum (Mill.) Druce	0	n	1	0	Asparagaceae Juss.
Iz_4_1009	DJI_0351	Polygonum arenarium Waldst. & Kit.	0	n	1	0	Polygonaceae Juss.
Iz_4_1009	DJI_0351	Populus nigra L.	0	n	1	1	Salicaceae Mirb.
Iz_4_1009	DJI_0351	Populus tremula L.	0	n	1	1	Salicaceae Mirb.
Iz_4_1009	DJI_0351	Populus Ã canescens (Aiton) Sm.	0	n	1	1	Salicaceae Mirb.
Iz_4_1009	DJI_0351	Potentilla argentea L.	0	n	1	0	Rosaceae Juss.
Iz_4_1009	DJI_0351	Psammophiliella muralis (L.) Ikonn.	0	n	1	0	Caryophyllaceae Juss.
Iz_4_1009	DJI_0351	Psephellus sumensis (Kalen.) Greuter	0	n	1	0	Asteraceae Bercht. & J.Presl
Iz_4_1009	DJI_0351	Pulsatilla pratensis (L.) Mill. s.l.	0	n	1	0	Ranunculaceae Juss.
Iz_4_1009	DJI_0351	Pyrus communis L.	0	a	0	1	Rosaceae Juss.
Iz_4_1009	DJI_0351	Quercus robur L.	0	n	1	1	Fagaceae Dumort.
Iz_4_1009	DJI_0351	Robinia pseudoacacia L.	0	a	0	1	Fabaceae Lindl.
Iz_4_1009	DJI_0351	Rosa sp.	0		NA	1	Rosaceae Juss.
Iz_4_1009	DJI_0351	Rubus idaeus L.	0	n	1	0	Rosaceae Juss.
Iz_4_1009	DJI_0351	Rumex acetosella L.	0	n	1	0	Polygonaceae Juss.
Iz_4_1009	DJI_0351	Rumex thyrsiflorus Fingerh.	0	n	1	0	Polygonaceae Juss.
Iz_4_1009	DJI_0351	Salix alba L.	0	n	1	1	Salicaceae Mirb.
Iz_4_1009	DJI_0351	Sambucus nigra L.	0	n	1	1	Viburnaceae Raf.
Iz_4_1009	DJI_0351	Secale sylvestre Host	0	n	1	0	Poaceae Barnhart
Iz_4_1009	DJI_0351	Sempervivum ruthenicum Schnittsp. & C.B. Lehm.	0	n	1	0	Crassulaceae J.St.-Hil.
Iz_4_1009	DJI_0351	Setaria viridis (L.) P. Beauv.	0	n	1	0	Poaceae Barnhart
Iz_4_1009	DJI_0351	Silene borysthenica (Gruner) Walters	0	n	1	0	Caryophyllaceae Juss.
Iz_4_1009	DJI_0351	Silene latifolia subsp. alba (Mill.) Greuter & Burdet	0	n	1	0	Caryophyllaceae Juss.
Iz_4_1009	DJI_0351	Solidago virgaurea L.	0	n	1	0	Asteraceae Bercht. & J.Presl
Iz_4_1009	DJI_0351	Sorbus aucuparia L.	0	n	1	1	Rosaceae Juss.
Iz_4_1009	DJI_0351	Stachys recta L.	0	n	1	0	Lamiacea Lindl.
Iz_4_1009	DJI_0351	Tanacetum vulgare L.	0	n	1	0	Asteraceae Bercht. & J.Presl
Iz_4_1009	DJI_0351	Thymus pallasianus Heinr.Braun	0	n	1	0	Lamiacea Lindl.
Iz_4_1009	DJI_0351	Tilia cordata Mill.	0	n	1	1	Malvaceae Juss.
Iz_4_1009	DJI_0351	Trifolium alpestre L.	0	n	1	0	Fabaceae Lindl.
Iz_4_1009	DJI_0351	Trifolium arvense L.	0	n	1	0	Fabaceae Lindl.
Iz_4_1009	DJI_0351	Ulmus glabra Huds.	0	n	1	1	Ulmaceae Mirb.
Iz_4_1009	DJI_0351	Verbascum densiflorum Bertol.	0	n	1	0	Scrophulariaceae Juss.
Iz_4_1009	DJI_0351	Verbascum nigrum L.	0	n	1	0	Scrophulariaceae Juss.
Iz_4_1009	DJI_0351	Veronica austriaca L.	0	n	1	0	Plantaginaceae Juss.
Iz_4_1009	DJI_0351	Veronica incana L.	0	n	1	0	Plantaginaceae Juss.
Iz_4_1009	DJI_0351	Veronica verna L.	0	n	1	0	Plantaginaceae Juss.
Iz_4_1009	DJI_0351	Vincetoxicum hirundinaria Medik	0	n	1	0	Apocynaceae Juss.
Iz_4_1009	DJI_0352	Acer tataricum L.	0	n	1	1	Sapindaceae Juss.
Iz_4_1009	DJI_0352	Achillea millefolium L. s.l.	0	n	1	0	Asteraceae Bercht. & J.Presl
Iz_4_1009	DJI_0352	Allium oleraceum L.	0	n	1	0	Amaryllidaceae J.St.-Hil.
Iz_4_1009	DJI_0352	Ambrosia artemisiifolia L.	0	a	0	0	Asteraceae Bercht. & J.Presl
Iz_4_1009	DJI_0352	Amorpha fruticosa L.	0	a	0	0	Fabaceae Lindl.
Iz_4_1009	DJI_0352	Anthericum ramosum L.	0	n	1	0	Asparagaceae Juss.
Iz_4_1009	DJI_0352	Artemisia marschalliana Spreng.	0	n	1	0	Asteraceae Bercht. & J.Presl
Iz_4_1009	DJI_0352	Asparagus officinalis L.	0	n	1	0	Asparagaceae Juss.
Iz_4_1009	DJI_0352	Astragalus cicer L.	0	n	1	0	Fabaceae Lindl.
Iz_4_1009	DJI_0352	Bassia laniflora (S.G.Gmel.) A.J.Scott	0	n	1	0	Amaranthaceae Juss.
Iz_4_1009	DJI_0352	Berberis vulgaris L.	0	n	1	0	Berberidaceae Juss.
Iz_4_1009	DJI_0352	Betula pendula Roth.	0	n	1	1	Betulaceae Gray
Iz_4_1009	DJI_0352	Betula pendula Roth.	0	n	1	0	Poaceae Barnhart
Iz_4_1009	DJI_0352	Bromus inermis Leyss.	0		NA	NA	
Iz_4_1009	DJI_0352	Bromus squarrosus L.	0	a	0	0	Poaceae Barnhart
Iz_4_1009	DJI_0352	Bromus tectorum L.	0	a	0	0	Poaceae Barnhart
Iz_4_1009	DJI_0352	Calamagrostis epigejos (L.) Roth	30	n	1	0	Poaceae Barnhart
Iz_4_1009	DJI_0352	Carduus acanthoides L.	0	n	1	0	Asteraceae Bercht. & J.Presl
Iz_4_1009	DJI_0352	Carex sp.	0	n	1	0	Cyperaceae Juss.
Iz_4_1009	DJI_0352	Centaurea majorovii Dumbadze	0	n	1	0	Asteraceae Bercht. & J.Presl
Iz_4_1009	DJI_0352	Chamaecytisus ruthenicus (Fisch.ex Wol.) Klaskova	0	n	1	0	Fabaceae Lindl.
Iz_4_1009	DJI_0352	Chelidonium majus L.	0	n	1	0	Papaveraceae Juss.
Iz_4_1009	DJI_0352	Chenopodium betaceum Andrz.	0	n	1	0	Amaranthaceae Juss.
Iz_4_1009	DJI_0352	Chenopodium cf. album	0	n	1	0	Amaranthaceae Juss.
Iz_4_1009	DJI_0352	Chenopodium sp.	0		NA	0	Amaranthaceae Juss.
Iz_4_1009	DJI_0352	Chondrilla juncea L.	0	n	1	0	Asteraceae Bercht. & J.Presl
Iz_4_1009	DJI_0352	Cirsium vulgare (Savi) Ten.	0	n	1	0	Asteraceae Bercht. & J.Presl
Iz_4_1009	DJI_0352	Clinopodium acinos (L.) Kuntze	0	n	1	0	Lamiacea Lindl.
Iz_4_1009	DJI_0352	Convallaria majalis L.	0	n	1	0	Asparagaceae Juss.
Iz_4_1009	DJI_0352	Crataegus rhipidophylla Gand.	0	n	1	1	Rosaceae Juss.
Iz_4_1009	DJI_0352	Crepis foetida L.	0	n	1	0	Asteraceae Bercht. & J.Presl
Iz_4_1009	DJI_0352	Dactylis glomerata L.	0	n	1	0	Poaceae Barnhart
Iz_4_1009	DJI_0352	Dianthus campestris M.Bieb.	0	n	1	0	Caryophyllaceae Juss.
Iz_4_1009	DJI_0352	Digitaria sanguinalis (L.) Scop.	0	a	0	0	Poaceae Barnhart
Iz_4_1009	DJI_0352	Elymus repens (L.) Gould	0	n	1	0	Poaceae Barnhart
Iz_4_1009	DJI_0352	Epilobium tetragonum L.	0	n	1	0	Onagraceae Juss.
Iz_4_1009	DJI_0352	Eragrostis minor Host	0	a	0	0	Poaceae Barnhart
Iz_4_1009	DJI_0352	Erigeron canadensis L.	5	a	0	0	Asteraceae Bercht. & J.Presl
Iz_4_1009	DJI_0352	Euphorbia seguieriana Neck.	0	n	1	0	Euphorbiaceae Juss.
Iz_4_1009	DJI_0352	Fallopia dumetorum (L.) Holub	0	n	1	0	Polygonaceae Juss.
Iz_4_1009	DJI_0352	Festuca beckeri (Hack.) Trautv.	0	n	1	0	Poaceae Barnhart
Iz_4_1009	DJI_0352	Fragaria vesca L.	0	n	1	0	Rosaceae Juss.
Iz_4_1009	DJI_0352	Frangula alnus Mill.	0	n	1	1	Rhamnaceae Juss.

Iz_4_1009	DJI_0352	Fraxinus pennsylvanica Marshall	0	a	0	1	Oleaceae Hoffmanns. & Link
Iz_4_1009	DJI_0352	Galeopsis bifida Boenn.	0	n	1	0	Lamiacea Lindl.
Iz_4_1009	DJI_0352	Galium verum L.	0	n	1	0	Rubiaceae Juss.
Iz_4_1009	DJI_0352	Genista tinctoria L.	0	n	1	0	Fabaceae Lindl.
Iz_4_1009	DJI_0352	Geranium robertianum L.	0	n	1	0	Geraniaceae Juss.
Iz_4_1009	DJI_0352	Geranium sanquineum L.	0	n	1	0	Geraniaceae Juss.
Iz_4_1009	DJI_0352	Gypsophila paniculata L.	0	n	1	0	Caryophyllaceae Juss.
Iz_4_1009	DJI_0352	Helichrysum arenarium (L.) Moench	0	n	1	0	Asteraceae Bercht. & J.Presl
Iz_4_1009	DJI_0352	Herniaria polygama J.Gay	0	n	1	0	Caryophyllaceae Juss.
Iz_4_1009	DJI_0352	Hieracium robustum Fr.	0	n	1	0	Asteraceae Bercht. & J.Presl
Iz_4_1009	DJI_0352	Hieracium umbellatum L.	0	n	1	NA	Asteraceae Bercht. & J.Presl
Iz_4_1009	DJI_0352	Hylotelephium maximum (L.) Holub	0	n	1	0	Crassulaceae J.St.-Hil.
Iz_4_1009	DJI_0352	Jacobaea borysthenica (DC.) B.Nord. & Greuter	0	n	1	0	Asteraceae Bercht. & J.Presl
Iz_4_1009	DJI_0352	Jasione montana L.	0	n	1	0	Campanulaceae Juss.
Iz_4_1009	DJI_0352	Jurinea cyanoides (L.) Rchb.s.l.	0	n	1	0	Asteraceae Bercht. & J.Presl
Iz_4_1009	DJI_0352	Koeleria glauca (Spreng.) DC.	0	n	1	0	Poaceae Barnhart
Iz_4_1009	DJI_0352	Lactuca serriola L.	0	a	0	0	Asteraceae Bercht. & J.Presl
Iz_4_1009	DJI_0352	Linaria genistifolia (L.) Mill.	0	n	1	0	Plantaginaceae Juss.
Iz_4_1009	DJI_0352	Lomelosia argentea (L.) Greuter & Burdet	0	n	1	0	Caprifoliaceae Juss.
Iz_4_1009	DJI_0352	Melampyrum pratense L.	0	n	1	0	Orobanchaceae Vent.
Iz_4_1009	DJI_0352	Oenothera biennis L.	0	a	0	0	Onagraceae Juss.
Iz_4_1009	DJI_0352	Pentanema britannica (L.) D.Gut.Larr., Santos-Vicente, Anderb., E.Rico & M.M.Mart.Ort.	0	n	1	0	Asteraceae Bercht. & J.Presl
Iz_4_1009	DJI_0352	Peucedanum oreoselinum (L.) Moench	0	n	1	0	Apiaceae Lindl.
Iz_4_1009	DJI_0352	Peucedanum ruthenicum M.Bieb.	0	n	1	0	Apiaceae Lindl.
Iz_4_1009	DJI_0352	Pilosella echioides (Lumn.) F.W.Schultz & Sch.Bip.	0	n	1	0	Asteraceae Bercht. & J.Presl
Iz_4_1009	DJI_0352	Pilosella officinarum F. Schultz et Sch. Bip.	0	n	1	0	Asteraceae Bercht. & J.Presl
Iz_4_1009	DJI_0352	Pilosella sp.	0		NA	0	Asteraceae Bercht. & J.Presl
Iz_4_1009	DJI_0352	Pinus sylvestris L.	0	n	1	1	Pinaceae Spreng. ex F.Rudolphi
Iz_4_1009	DJI_0352	Poa annua L.	0	n	1	0	Poaceae Barnhart
Iz_4_1009	DJI_0352	Poa nemoralis L.	0	n	1	0	Poaceae Barnhart
Iz_4_1009	DJI_0352	Poa sp.	0		NA	0	Poaceae Barnhart
Iz_4_1009	DJI_0352	Polygonatum odoratum (Mill.) Druce	0	n	1	0	Asparagaceae Juss.
Iz_4_1009	DJI_0352	Polygonum arenarium Waldst. & Kit.	0	n	1	0	Polygonaceae Juss.
Iz_4_1009	DJI_0352	Populus nigra L.	0	n	1	1	Salicaceae Mirb.
Iz_4_1009	DJI_0352	Populus tremula L.	0	n	1	1	Salicaceae Mirb.
Iz_4_1009	DJI_0352	Populus Ã canescens (Aiton) Sm.	0	n	1	1	Salicaceae Mirb.
Iz_4_1009	DJI_0352	Potentilla argentea L.	0	n	1	0	Rosaceae Juss.
Iz_4_1009	DJI_0352	Psammophiliella muralis (L.) Ikonn.	0	n	1	0	Caryophyllaceae Juss.
Iz_4_1009	DJI_0352	Psephellus sumensis (Kalen.) Greuter	0	n	1	0	Asteraceae Bercht. & J.Presl
Iz_4_1009	DJI_0352	Pulsatilla pratensis (L.) Mill. s.l.	0	n	1	0	Ranunculaceae Juss.
Iz_4_1009	DJI_0352	Pyrus communis L.	0	a	0	1	Rosaceae Juss.
Iz_4_1009	DJI_0352	Quercus robur L.	0	n	1	1	Fagaceae Dumort.
Iz_4_1009	DJI_0352	Robinia pseudoacacia L.	0	a	0	1	Fabaceae Lindl.
Iz_4_1009	DJI_0352	Rosa sp.	0		NA	1	Rosaceae Juss.
Iz_4_1009	DJI_0352	Rubus idaeus L.	0	n	1	0	Rosaceae Juss.
Iz_4_1009	DJI_0352	Rumex acetosella L.	0	n	1	0	Polygonaceae Juss.
Iz_4_1009	DJI_0352	Rumex thyrsiflorus Fingerh.	0	n	1	0	Polygonaceae Juss.
Iz_4_1009	DJI_0352	Salix alba L.	0	n	1	1	Salicaceae Mirb.
Iz_4_1009	DJI_0352	Sambucus nigra L.	0	n	1	1	Viburnaceae Raf.
Iz_4_1009	DJI_0352	Secale sylvestre Host	0	n	1	0	Poaceae Barnhart
Iz_4_1009	DJI_0352	Sempervivum ruthenicum Schnittsp. & C.B. Lehm.	0	n	1	0	Crassulaceae J.St.-Hil.
Iz_4_1009	DJI_0352	Setaria viridis (L.) P. Beauv.	0	n	1	0	Poaceae Barnhart
Iz_4_1009	DJI_0352	Silene borysthenica (Gruner) Walters	0	n	1	0	Caryophyllaceae Juss.
Iz_4_1009	DJI_0352	Silene latifolia subsp. alba (Mill.) Greuter & Burdet	0	n	1	0	Caryophyllaceae Juss.
Iz_4_1009	DJI_0352	Solidago virgaurea L.	0	n	1	0	Asteraceae Bercht. & J.Presl
Iz_4_1009	DJI_0352	Sorbus aucuparia L.	0	n	1	1	Rosaceae Juss.
Iz_4_1009	DJI_0352	Stachys recta L.	0	n	1	0	Lamiacea Lindl.
Iz_4_1009	DJI_0352	Tanacetum vulgare L.	0	n	1	0	Asteraceae Bercht. & J.Presl
Iz_4_1009	DJI_0352	Thymus pallasianus Heinr.Braun	0	n	1	0	Lamiacea Lindl.
Iz_4_1009	DJI_0352	Tilia cordata Mill.	0	n	1	1	Malvaceae Juss.
Iz_4_1009	DJI_0352	Trifolium alpestre L.	0	n	1	0	Fabaceae Lindl.
Iz_4_1009	DJI_0352	Trifolium arvense L.	0	n	1	0	Fabaceae Lindl.
Iz_4_1009	DJI_0352	Ulmus glabra Huds.	0	n	1	1	Ulmaceae Mirb.
Iz_4_1009	DJI_0352	Verbascum densiflorum Bertol.	0	n	1	0	Scrophulariaceae Juss.
Iz_4_1009	DJI_0352	Verbascum nigrum L.	0	n	1	0	Scrophulariaceae Juss.
Iz_4_1009	DJI_0352	Veronica austriaca L.	0	n	1	0	Plantaginaceae Juss.
Iz_4_1009	DJI_0352	Veronica incana L.	0	n	1	0	Plantaginaceae Juss.
Iz_4_1009	DJI_0352	Veronica verna L.	0	n	1	0	Plantaginaceae Juss.
Iz_4_1009	DJI_0352	Vincetoxicum hirundinaria Medik	0	n	1	0	Apocynaceae Juss.
Iz_4_1009	DJI_0355	Acer tataricum L.	0	n	1	1	Sapindaceae Juss.
Iz_4_1009	DJI_0355	Achillea millefolium L. s.l.	0	n	1	0	Asteraceae Bercht. & J.Presl
Iz_4_1009	DJI_0355	Allium oleraceum L.	0	n	1	0	Amaryllidaceae J.St.-Hil.
Iz_4_1009	DJI_0355	Ambrosia artemisiifolia L.	0	a	0	0	Asteraceae Bercht. & J.Presl
Iz_4_1009	DJI_0355	Amorpha fruticosa L.	0	a	0	0	Fabaceae Lindl.
Iz_4_1009	DJI_0355	Anthericum ramosum L.	0	n	1	0	Asparagaceae Juss.
Iz_4_1009	DJI_0355	Artemisia marschalliana Spreng.	0	n	1	0	Asteraceae Bercht. & J.Presl
Iz_4_1009	DJI_0355	Asparagus officinalis L.	0	n	1	0	Asparagaceae Juss.
Iz_4_1009	DJI_0355	Astragalus cicer L.	0	n	1	0	Fabaceae Lindl.
Iz_4_1009	DJI_0355	Bassia laniflora (S.G.Gmel.) A.J.Scott	0	n	1	0	Amaranthaceae Juss.
Iz_4_1009	DJI_0355	Berberis vulgaris L.	0	n	1	0	Berberidaceae Juss.
Iz_4_1009	DJI_0355	Betula pendula Roth.	0	n	1	1	Betulaceae Gray
Iz_4_1009	DJI_0355	Betula pendula Roth.	0	n	1	0	Poaceae Barnhart
Iz_4_1009	DJI_0355	Bromus inermis Leyss.	0		NA	NA	
Iz_4_1009	DJI_0355	Bromus squarrosus L.	0	a	0	0	Poaceae Barnhart
Iz_4_1009	DJI_0355	Bromus tectorum L.	0	a	0	0	Poaceae Barnhart
Iz_4_1009	DJI_0355	Calamagrostis epigejos (L.) Roth	45	n	1	0	Poaceae Barnhart
Iz_4_1009	DJI_0355	Carduus acanthoides L.	0	n	1	0	Asteraceae Bercht. & J.Presl
Iz_4_1009	DJI_0355	Carex sp.	0	n	1	0	Cyperaceae Juss.
Iz_4_1009	DJI_0355	Centaurea majorovii Dumbadze	0	n	1	0	Asteraceae Bercht. & J.Presl
Iz_4_1009	DJI_0355	Chamaecytisus ruthenicus (Fisch.ex Wol.) Klaskova	0	n	1	0	Fabaceae Lindl.
Iz_4_1009	DJI_0355	Chelidonium majus L.	0	n	1	0	Papaveraceae Juss.
Iz_4_1009	DJI_0355	Chenopodium betaceum Andrz.	0	n	1	0	Amaranthaceae Juss.
Iz_4_1009	DJI_0355	Chenopodium cf. album	0	n	1	0	Amaranthaceae Juss.
Iz_4_1009	DJI_0355	Chenopodium sp.	2		NA	0	Amaranthaceae Juss.
Iz_4_1009	DJI_0355	Chondrilla juncea L.	0	n	1	0	Asteraceae Bercht. & J.Presl
Iz_4_1009	DJI_0355	Cirsium vulgare (Savi) Ten.	0	n	1	0	Asteraceae Bercht. & J.Presl
Iz_4_1009	DJI_0355	Clinopodium acinos (L.) Kuntze	0	n	1	0	Lamiacea Lindl.
Iz_4_1009	DJI_0355	Convallaria majalis L.	0	n	1	0	Asparagaceae Juss.

Iz_4_1009	DJI_0355	Crataegus rhipidophylla Gand.	0	n	1	1	Rosaceae Juss.
Iz_4_1009	DJI_0355	Crepis foetida L.	0	n	1	0	Asteraceae Bercht. & J.Presl
Iz_4_1009	DJI_0355	Dactylis glomerata L.	0	n	1	0	Poaceae Barnhart
Iz_4_1009	DJI_0355	Dianthus campestris M.Bieb.	0	n	1	0	Caryophyllaceae Juss.
Iz_4_1009	DJI_0355	Digitaria sanguinalis (L.) Scop.	0	a	0	0	Poaceae Barnhart
Iz_4_1009	DJI_0355	Elymus repens (L.) Gould	0	n	1	0	Poaceae Barnhart
Iz_4_1009	DJI_0355	Epilobium tetragonum L.	0	n	1	0	Onagraceae Juss.
Iz_4_1009	DJI_0355	Eragrostis minor Host	0	a	0	0	Poaceae Barnhart
Iz_4_1009	DJI_0355	Erigeron canadensis L.	10	a	0	0	Asteraceae Bercht. & J.Presl
Iz_4_1009	DJI_0355	Euphorbia seguieriana Neck.	0	n	1	0	Euphorbiaceae Juss.
Iz_4_1009	DJI_0355	Fallopia dumetorum (L.) Holub	0	n	1	0	Polygonaceae Juss.
Iz_4_1009	DJI_0355	Festuca beckeri (Hack.) Trautv.	0	n	1	0	Poaceae Barnhart
Iz_4_1009	DJI_0355	Fragaria vesca L.	0	n	1	0	Rosaceae Juss.
Iz_4_1009	DJI_0355	Frangula alnus Mill.	0	n	1	1	Rhamnaceae Juss.
Iz_4_1009	DJI_0355	Fraxinus pennsylvanica Marshall	0	a	0	1	Oleaceae Hoffmanns. & Link
Iz_4_1009	DJI_0355	Galeopsis bifida Boenn.	0	n	1	0	Lamiacea Lindl.
Iz_4_1009	DJI_0355	Galium verum L.	0	n	1	0	Rubiaceae Juss.
Iz_4_1009	DJI_0355	Genista tinctoria L.	0	n	1	0	Fabaceae Lindl.
Iz_4_1009	DJI_0355	Geranium robertianum L.	0	n	1	0	Geraniaceae Juss.
Iz_4_1009	DJI_0355	Geranium sanquineum L.	0	n	1	0	Geraniaceae Juss.
Iz_4_1009	DJI_0355	Gypsophila paniculata L.	0	n	1	0	Caryophyllaceae Juss.
Iz_4_1009	DJI_0355	Helichrysum arenarium (L.) Moench	0	n	1	0	Asteraceae Bercht. & J.Presl
Iz_4_1009	DJI_0355	Herniaria polygama J.Gay	0	n	1	0	Caryophyllaceae Juss.
Iz_4_1009	DJI_0355	Hieracium robustum Fr.	0	n	1	0	Asteraceae Bercht. & J.Presl
Iz_4_1009	DJI_0355	Hieracium umbellatum L.	0	n	1	NA	Asteraceae Bercht. & J.Presl
Iz_4_1009	DJI_0355	Hylotelephium maximum (L.) Holub	0	n	1	0	Crassulaceae J.St.-Hil.
Iz_4_1009	DJI_0355	Jacobaea borysthenica (DC.) B.Nord. & Greuter	0	n	1	0	Asteraceae Bercht. & J.Presl
Iz_4_1009	DJI_0355	Jasione montana L.	0	n	1	0	Campanulaceae Juss.
Iz_4_1009	DJI_0355	Jurinea cyanoides (L.) Rchb.s.l.	0	n	1	0	Asteraceae Bercht. & J.Presl
Iz_4_1009	DJI_0355	Koeleria glauca (Spreng.) DC.	0	n	1	0	Poaceae Barnhart
Iz_4_1009	DJI_0355	Lactuca serriola L.	0	a	0	0	Asteraceae Bercht. & J.Presl
Iz_4_1009	DJI_0355	Linaria genistifolia (L.) Mill.	0	n	1	0	Plantaginaceae Juss.
Iz_4_1009	DJI_0355	Lomelosia argentea (L.) Greuter & Burdet	0	n	1	0	Caprifoliaceae Juss.
Iz_4_1009	DJI_0355	Melampyrum pratense L.	0	n	1	0	Orobanchaceae Vent.
Iz_4_1009	DJI_0355	Oenothera biennis L.	0	a	0	0	Onagraceae Juss.
Iz_4_1009	DJI_0355	Pentanema britannica (L.) D.Gut.Larr., Santos-Vicente, Anderb., E.Rico & M.M.Mart.Ort.	0	n	1	0	Asteraceae Bercht. & J.Presl
Iz_4_1009	DJI_0355	Peucedanum oreoselinum (L.) Moench	0	n	1	0	Apiaceae Lindl.
Iz_4_1009	DJI_0355	Peucedanum ruthenicum M.Bieb.	0	n	1	0	Apiaceae Lindl.
Iz_4_1009	DJI_0355	Pilosella echioides (Lumn.) F.W.Schultz & Sch.Bip.	0	n	1	0	Asteraceae Bercht. & J.Presl
Iz_4_1009	DJI_0355	Pilosella officinarum F. Schultz et Sch. Bip.	0	n	1	0	Asteraceae Bercht. & J.Presl
Iz_4_1009	DJI_0355	Pilosella sp.	0		NA	0	Asteraceae Bercht. & J.Presl
Iz_4_1009	DJI_0355	Pinus sylvestris L.	0	n	1	1	Pinaceae Spreng. ex F.Rudolphi
Iz_4_1009	DJI_0355	Poa annua L.	0	n	1	0	Poaceae Barnhart
Iz_4_1009	DJI_0355	Poa nemoralis L.	0	n	1	0	Poaceae Barnhart
Iz_4_1009	DJI_0355	Poa sp.	0		NA	0	Poaceae Barnhart
Iz_4_1009	DJI_0355	Polygonatum odoratum (Mill.) Druce	0	n	1	0	Asparagaceae Juss.
Iz_4_1009	DJI_0355	Polygonum arenarium Waldst. & Kit.	0	n	1	0	Polygonaceae Juss.
Iz_4_1009	DJI_0355	Populus nigra L.	0	n	1	1	Salicaceae Mirb.
Iz_4_1009	DJI_0355	Populus tremula L.	0	n	1	1	Salicaceae Mirb.
Iz_4_1009	DJI_0355	Populus Ã canescens (Aiton) Sm.	0	n	1	1	Salicaceae Mirb.
Iz_4_1009	DJI_0355	Potentilla argentea L.	0	n	1	0	Rosaceae Juss.
Iz_4_1009	DJI_0355	Psammophiliella muralis (L.) Ikonn.	0	n	1	0	Caryophyllaceae Juss.
Iz_4_1009	DJI_0355	Psephellus sumensis (Kalen.) Greuter	0	n	1	0	Asteraceae Bercht. & J.Presl
Iz_4_1009	DJI_0355	Pulsatilla pratensis (L.) Mill. s.l.	0	n	1	0	Ranunculaceae Juss.
Iz_4_1009	DJI_0355	Pyrus communis L.	0	a	0	1	Rosaceae Juss.
Iz_4_1009	DJI_0355	Quercus robur L.	0	n	1	1	Fagaceae Dumort.
Iz_4_1009	DJI_0355	Robinia pseudoacacia L.	0	a	0	1	Fabaceae Lindl.
Iz_4_1009	DJI_0355	Rosa sp.	0		NA	1	Rosaceae Juss.
Iz_4_1009	DJI_0355	Rubus idaeus L.	0	n	1	0	Rosaceae Juss.
Iz_4_1009	DJI_0355	Rumex acetosella L.	0	n	1	0	Polygonaceae Juss.
Iz_4_1009	DJI_0355	Rumex thyrsiflorus Fingerh.	0	n	1	0	Polygonaceae Juss.
Iz_4_1009	DJI_0355	Salix alba L.	0	n	1	1	Salicaceae Mirb.
Iz_4_1009	DJI_0355	Sambucus nigra L.	0	n	1	1	Viburnaceae Raf.
Iz_4_1009	DJI_0355	Secale sylvestre Host	0	n	1	0	Poaceae Barnhart
Iz_4_1009	DJI_0355	Sempervivum ruthenicum Schnittsp. & C.B. Lehm.	0	n	1	0	Crassulaceae J.St.-Hil.
Iz_4_1009	DJI_0355	Setaria viridis (L.) P. Beauv.	0	n	1	0	Poaceae Barnhart
Iz_4_1009	DJI_0355	Silene borysthenica (Gruner) Walters	0	n	1	0	Caryophyllaceae Juss.
Iz_4_1009	DJI_0355	Silene latifolia subsp. alba (Mill.) Greuter & Burdet	0	n	1	0	Caryophyllaceae Juss.
Iz_4_1009	DJI_0355	Solidago virgaurea L.	0	n	1	0	Asteraceae Bercht. & J.Presl
Iz_4_1009	DJI_0355	Sorbus aucuparia L.	0	n	1	1	Rosaceae Juss.
Iz_4_1009	DJI_0355	Stachys recta L.	0	n	1	0	Lamiacea Lindl.
Iz_4_1009	DJI_0355	Tanacetum vulgare L.	0	n	1	0	Asteraceae Bercht. & J.Presl
Iz_4_1009	DJI_0355	Thymus pallasianus Heinr.Braun	0	n	1	0	Lamiacea Lindl.
Iz_4_1009	DJI_0355	Tilia cordata Mill.	0	n	1	1	Malvaceae Juss.
Iz_4_1009	DJI_0355	Trifolium alpestre L.	0	n	1	0	Fabaceae Lindl.
Iz_4_1009	DJI_0355	Trifolium arvense L.	0	n	1	0	Fabaceae Lindl.
Iz_4_1009	DJI_0355	Ulmus glabra Huds.	0	n	1	1	Ulmaceae Mirb.
Iz_4_1009	DJI_0355	Verbascum densiflorum Bertol.	0	n	1	0	Scrophulariaceae Juss.
Iz_4_1009	DJI_0355	Verbascum nigrum L.	0	n	1	0	Scrophulariaceae Juss.
Iz_4_1009	DJI_0355	Veronica austriaca L.	0	n	1	0	Plantaginaceae Juss.
Iz_4_1009	DJI_0355	Veronica incana L.	0	n	1	0	Plantaginaceae Juss.
Iz_4_1009	DJI_0355	Veronica verna L.	0	n	1	0	Plantaginaceae Juss.
Iz_4_1009	DJI_0355	Vincetoxicum hirundinaria Medik	0	n	1	0	Apocynaceae Juss.
Iz_4_1009	DJI_0356	Acer tataricum L.	0	n	1	1	Sapindaceae Juss.
Iz_4_1009	DJI_0356	Achillea millefolium L. s.l.	0	n	1	0	Asteraceae Bercht. & J.Presl
Iz_4_1009	DJI_0356	Allium oleraceum L.	0	n	1	0	Amaryllidaceae J.St.-Hil.
Iz_4_1009	DJI_0356	Ambrosia artemisiifolia L.	0	a	0	0	Asteraceae Bercht. & J.Presl
Iz_4_1009	DJI_0356	Amorpha fruticosa L.	0	a	0	0	Fabaceae Lindl.
Iz_4_1009	DJI_0356	Anthericum ramosum L.	0	n	1	0	Asparagaceae Juss.
Iz_4_1009	DJI_0356	Artemisia marschalliana Spreng.	0	n	1	0	Asteraceae Bercht. & J.Presl
Iz_4_1009	DJI_0356	Asparagus officinalis L.	0	n	1	0	Asparagaceae Juss.
Iz_4_1009	DJI_0356	Astragalus cicer L.	0	n	1	0	Fabaceae Lindl.
Iz_4_1009	DJI_0356	Bassia laniflora (S.G.Gmel.) A.J.Scott	0	n	1	0	Amaranthaceae Juss.
Iz_4_1009	DJI_0356	Berberis vulgaris L.	0	n	1	0	Berberidaceae Juss.
Iz_4_1009	DJI_0356	Betula pendula Roth.	0	n	1	1	Betulaceae Gray
Iz_4_1009	DJI_0356	Betula pendula Roth.	0	n	1	0	Poaceae Barnhart
Iz_4_1009	DJI_0356	Bromus inermis Leyss.	0		NA	NA	
Iz_4_1009	DJI_0356	Bromus squarrosus L.	0	a	0	0	Poaceae Barnhart

Iz_4_1009	DJI_0356	Bromus tectorum L.	0	a	0	0	Poaceae Barnhart
Iz_4_1009	DJI_0356	Calamagrostis epigejos (L.) Roth	45	n	1	0	Poaceae Barnhart
Iz_4_1009	DJI_0356	Carduus acanthoides L.	0	n	1	0	Asteraceae Bercht. & J.Presl
Iz_4_1009	DJI_0356	Carex sp.	0	n	1	0	Cyperaceae Juss.
Iz_4_1009	DJI_0356	Centaurea majorovii Dumbadze	0	n	1	0	Asteraceae Bercht. & J.Presl
Iz_4_1009	DJI_0356	Chamaecytisus ruthenicus (Fisch.ex Wol.) Klaskova	0	n	1	0	Fabaceae Lindl.
Iz_4_1009	DJI_0356	Chelidonium majus L.	0	n	1	0	Papaveraceae Juss.
Iz_4_1009	DJI_0356	Chenopodium betaceum Andrz.	0	n	1	0	Amaranthaceae Juss.
Iz_4_1009	DJI_0356	Chenopodium cf. album	0	n	1	0	Amaranthaceae Juss.
Iz_4_1009	DJI_0356	Chenopodium sp.	0		NA	0	Amaranthaceae Juss.
Iz_4_1009	DJI_0356	Chondrilla juncea L.	0	n	1	0	Asteraceae Bercht. & J.Presl
Iz_4_1009	DJI_0356	Cirsium vulgare (Savi) Ten.	0	n	1	0	Asteraceae Bercht. & J.Presl
Iz_4_1009	DJI_0356	Clinopodium acinos (L.) Kuntze	0	n	1	0	Lamiacea Lindl.
Iz_4_1009	DJI_0356	Convallaria majalis L.	0	n	1	0	Asparagaceae Juss.
Iz_4_1009	DJI_0356	Crataegus rhipidophylla Gand.	0	n	1	1	Rosaceae Juss.
Iz_4_1009	DJI_0356	Crepis foetida L.	0	n	1	0	Asteraceae Bercht. & J.Presl
Iz_4_1009	DJI_0356	Dactylis glomerata L.	0	n	1	0	Poaceae Barnhart
Iz_4_1009	DJI_0356	Dianthus campestris M.Bieb.	0	n	1	0	Caryophyllaceae Juss.
Iz_4_1009	DJI_0356	Digitaria sanguinalis (L.) Scop.	0	a	0	0	Poaceae Barnhart
Iz_4_1009	DJI_0356	Elymus repens (L.) Gould	0	n	1	0	Poaceae Barnhart
Iz_4_1009	DJI_0356	Epilobium tetragonum L.	0	n	1	0	Onagraceae Juss.
Iz_4_1009	DJI_0356	Eragrostis minor Host	0	a	0	0	Poaceae Barnhart
Iz_4_1009	DJI_0356	Erigeron canadensis L.	5	a	0	0	Asteraceae Bercht. & J.Presl
Iz_4_1009	DJI_0356	Euphorbia seguieriana Neck.	0	n	1	0	Euphorbiaceae Juss.
Iz_4_1009	DJI_0356	Fallopia dumetorum (L.) Holub	0	n	1	0	Polygonaceae Juss.
Iz_4_1009	DJI_0356	Festuca beckeri (Hack.) Trautv.	0	n	1	0	Poaceae Barnhart
Iz_4_1009	DJI_0356	Fragaria vesca L.	0	n	1	0	Rosaceae Juss.
Iz_4_1009	DJI_0356	Frangula alnus Mill.	0	n	1	1	Rhamnaceae Juss.
Iz_4_1009	DJI_0356	Fraxinus pennsylvanica Marshall	0	a	0	1	Oleaceae Hoffmanns. & Link
Iz_4_1009	DJI_0356	Galeopsis bifida Boenn.	0	n	1	0	Lamiacea Lindl.
Iz_4_1009	DJI_0356	Galium verum L.	0	n	1	0	Rubiaceae Juss.
Iz_4_1009	DJI_0356	Genista tinctoria L.	0	n	1	0	Fabaceae Lindl.
Iz_4_1009	DJI_0356	Geranium robertianum L.	0	n	1	0	Geraniaceae Juss.
Iz_4_1009	DJI_0356	Geranium sanquineum L.	0	n	1	0	Geraniaceae Juss.
Iz_4_1009	DJI_0356	Gypsophila paniculata L.	0	n	1	0	Caryophyllaceae Juss.
Iz_4_1009	DJI_0356	Helichrysum arenarium (L.) Moench	0	n	1	0	Asteraceae Bercht. & J.Presl
Iz_4_1009	DJI_0356	Herniaria polygama J.Gay	0	n	1	0	Caryophyllaceae Juss.
Iz_4_1009	DJI_0356	Hieracium robustum Fr.	0	n	1	0	Asteraceae Bercht. & J.Presl
Iz_4_1009	DJI_0356	Hieracium umbellatum L.	0	n	1	NA	Asteraceae Bercht. & J.Presl
Iz_4_1009	DJI_0356	Hylotelephium maximum (L.) Holub	0	n	1	0	Crassulaceae J.St.-Hil.
Iz_4_1009	DJI_0356	Jacobaea borysthenica (DC.) B.Nord. & Greuter	0	n	1	0	Asteraceae Bercht. & J.Presl
Iz_4_1009	DJI_0356	Jasione montana L.	0	n	1	0	Campanulaceae Juss.
Iz_4_1009	DJI_0356	Jurinea cyanoides (L.) Rchb.s.l.	0	n	1	0	Asteraceae Bercht. & J.Presl
Iz_4_1009	DJI_0356	Koeleria glauca (Spreng.) DC.	0	n	1	0	Poaceae Barnhart
Iz_4_1009	DJI_0356	Lactuca serriola L.	0	a	0	0	Asteraceae Bercht. & J.Presl
Iz_4_1009	DJI_0356	Linaria genistifolia (L.) Mill.	0	n	1	0	Plantaginaceae Juss.
Iz_4_1009	DJI_0356	Lomelosia argentea (L.) Greuter & Burdet	0	n	1	0	Caprifoliaceae Juss.
Iz_4_1009	DJI_0356	Melampyrum pratense L.	0	n	1	0	Orobanchaceae Vent.
Iz_4_1009	DJI_0356	Oenothera biennis L.	0	a	0	0	Onagraceae Juss.
Iz_4_1009	DJI_0356	Pentanema britannica (L.) D.Gut.Larr., Santos-Vicente, Anderb., E.Rico & M.M.Mart.Ort.	0	n	1	0	Asteraceae Bercht. & J.Presl
Iz_4_1009	DJI_0356	Peucedanum oreoselinum (L.) Moench	0	n	1	0	Apiaceae Lindl.
Iz_4_1009	DJI_0356	Peucedanum ruthenicum M.Bieb.	0	n	1	0	Apiaceae Lindl.
Iz_4_1009	DJI_0356	Pilosella echioides (Lumn.) F.W.Schultz & Sch.Bip.	0	n	1	0	Asteraceae Bercht. & J.Presl
Iz_4_1009	DJI_0356	Pilosella officinarum F. Schultz et Sch. Bip.	0	n	1	0	Asteraceae Bercht. & J.Presl
Iz_4_1009	DJI_0356	Pilosella sp.	0		NA	0	Asteraceae Bercht. & J.Presl
Iz_4_1009	DJI_0356	Pinus sylvestris L.	0	n	1	1	Pinaceae Spreng. ex F.Rudolphi
Iz_4_1009	DJI_0356	Poa annua L.	0	n	1	0	Poaceae Barnhart
Iz_4_1009	DJI_0356	Poa nemoralis L.	0	n	1	0	Poaceae Barnhart
Iz_4_1009	DJI_0356	Poa sp.	0		NA	0	Poaceae Barnhart
Iz_4_1009	DJI_0356	Polygonatum odoratum (Mill.) Druce	0	n	1	0	Asparagaceae Juss.
Iz_4_1009	DJI_0356	Polygonum arenarium Waldst. & Kit.	0	n	1	0	Polygonaceae Juss.
Iz_4_1009	DJI_0356	Populus nigra L.	0	n	1	1	Salicaceae Mirb.
Iz_4_1009	DJI_0356	Populus tremula L.	0	n	1	1	Salicaceae Mirb.
Iz_4_1009	DJI_0356	Populus Ã canescens (Aiton) Sm.	0	n	1	1	Salicaceae Mirb.
Iz_4_1009	DJI_0356	Potentilla argentea L.	0	n	1	0	Rosaceae Juss.
Iz_4_1009	DJI_0356	Psammophiliella muralis (L.) Ikonn.	0	n	1	0	Caryophyllaceae Juss.
Iz_4_1009	DJI_0356	Psephellus sumensis (Kalen.) Greuter	0	n	1	0	Asteraceae Bercht. & J.Presl
Iz_4_1009	DJI_0356	Pulsatilla pratensis (L.) Mill. s.l.	0	n	1	0	Ranunculaceae Juss.
Iz_4_1009	DJI_0356	Pyrus communis L.	0	a	0	1	Rosaceae Juss.
Iz_4_1009	DJI_0356	Quercus robur L.	0	n	1	1	Fagaceae Dumort.
Iz_4_1009	DJI_0356	Robinia pseudoacacia L.	0	a	0	1	Fabaceae Lindl.
Iz_4_1009	DJI_0356	Rosa sp.	0		NA	1	Rosaceae Juss.
Iz_4_1009	DJI_0356	Rubus idaeus L.	0	n	1	0	Rosaceae Juss.
Iz_4_1009	DJI_0356	Rumex acetosella L.	0	n	1	0	Polygonaceae Juss.
Iz_4_1009	DJI_0356	Rumex thyrsiflorus Fingerh.	0	n	1	0	Polygonaceae Juss.
Iz_4_1009	DJI_0356	Salix alba L.	0	n	1	1	Salicaceae Mirb.
Iz_4_1009	DJI_0356	Sambucus nigra L.	0	n	1	1	Viburnaceae Raf.
Iz_4_1009	DJI_0356	Secale sylvestre Host	0	n	1	0	Poaceae Barnhart
Iz_4_1009	DJI_0356	Sempervivum ruthenicum Schnittsp. & C.B. Lehm.	0	n	1	0	Crassulaceae J.St.-Hil.
Iz_4_1009	DJI_0356	Setaria viridis (L.) P. Beauv.	0	n	1	0	Poaceae Barnhart
Iz_4_1009	DJI_0356	Silene borysthenica (Gruner) Walters	0	n	1	0	Caryophyllaceae Juss.
Iz_4_1009	DJI_0356	Silene latifolia subsp. alba (Mill.) Greuter & Burdet	0	n	1	0	Caryophyllaceae Juss.
Iz_4_1009	DJI_0356	Solidago virgaurea L.	0	n	1	0	Asteraceae Bercht. & J.Presl
Iz_4_1009	DJI_0356	Sorbus aucuparia L.	0	n	1	1	Rosaceae Juss.
Iz_4_1009	DJI_0356	Stachys recta L.	0	n	1	0	Lamiacea Lindl.
Iz_4_1009	DJI_0356	Tanacetum vulgare L.	0	n	1	0	Asteraceae Bercht. & J.Presl
Iz_4_1009	DJI_0356	Thymus pallasianus Heinr.Braun	0	n	1	0	Lamiacea Lindl.
Iz_4_1009	DJI_0356	Tilia cordata Mill.	0	n	1	1	Malvaceae Juss.
Iz_4_1009	DJI_0356	Trifolium alpestre L.	0	n	1	0	Fabaceae Lindl.
Iz_4_1009	DJI_0356	Trifolium arvense L.	0	n	1	0	Fabaceae Lindl.
Iz_4_1009	DJI_0356	Ulmus glabra Huds.	0	n	1	1	Ulmaceae Mirb.
Iz_4_1009	DJI_0356	Verbascum densiflorum Bertol.	0	n	1	0	Scrophulariaceae Juss.
Iz_4_1009	DJI_0356	Verbascum nigrum L.	0	n	1	0	Scrophulariaceae Juss.
Iz_4_1009	DJI_0356	Veronica austriaca L.	0	n	1	0	Plantaginaceae Juss.
Iz_4_1009	DJI_0356	Veronica incana L.	0	n	1	0	Plantaginaceae Juss.
Iz_4_1009	DJI_0356	Veronica verna L.	0	n	1	0	Plantaginaceae Juss.
Iz_4_1009	DJI_0356	Vincetoxicum hirundinaria Medik	0	n	1	0	Apocynaceae Juss.
Iz_4_1009	DJI_0357	Acer tataricum L.	0	n	1	1	Sapindaceae Juss.

Iz_4_1009	DJI_0357	Achillea millefolium L. s.l.	0	n	1	0	Asteraceae Bercht. & J.Presl
Iz_4_1009	DJI_0357	Allium oleraceum L.	0	n	1	0	Amaryllidaceae J.St.-Hil.
Iz_4_1009	DJI_0357	Ambrosia artemisiifolia L.	0	a	0	0	Asteraceae Bercht. & J.Presl
Iz_4_1009	DJI_0357	Amorpha fruticosa L.	0	a	0	0	Fabaceae Lindl.
Iz_4_1009	DJI_0357	Anthericum ramosum L.	0	n	1	0	Asparagaceae Juss.
Iz_4_1009	DJI_0357	Artemisia marschalliana Spreng.	0	n	1	0	Asteraceae Bercht. & J.Presl
Iz_4_1009	DJI_0357	Asparagus officinalis L.	0	n	1	0	Asparagaceae Juss.
Iz_4_1009	DJI_0357	Astragalus cicer L.	0	n	1	0	Fabaceae Lindl.
Iz_4_1009	DJI_0357	Bassia laniflora (S.G.Gmel.) A.J.Scott	0	n	1	0	Amaranthaceae Juss.
Iz_4_1009	DJI_0357	Berberis vulgaris L.	0	n	1	0	Berberidaceae Juss.
Iz_4_1009	DJI_0357	Betula pendula Roth.	0	n	1	1	Betulaceae Gray
Iz_4_1009	DJI_0357	Betula pendula Roth.	0	n	1	0	Poaceae Barnhart
Iz_4_1009	DJI_0357	Bromus inermis Leyss.	0		NA	NA	
Iz_4_1009	DJI_0357	Bromus squarrosus L.	0	a	0	0	Poaceae Barnhart
Iz_4_1009	DJI_0357	Bromus tectorum L.	0	a	0	0	Poaceae Barnhart
Iz_4_1009	DJI_0357	Calamagrostis epigejos (L.) Roth	40	n	1	0	Poaceae Barnhart
Iz_4_1009	DJI_0357	Carduus acanthoides L.	0	n	1	0	Asteraceae Bercht. & J.Presl
Iz_4_1009	DJI_0357	Carex sp.	0	n	1	0	Cyperaceae Juss.
Iz_4_1009	DJI_0357	Centaurea majorovii Dumbadze	0	n	1	0	Asteraceae Bercht. & J.Presl
Iz_4_1009	DJI_0357	Chamaecytisus ruthenicus (Fisch.ex Wol.) Klaskova	0	n	1	0	Fabaceae Lindl.
Iz_4_1009	DJI_0357	Chelidonium majus L.	0	n	1	0	Papaveraceae Juss.
Iz_4_1009	DJI_0357	Chenopodium betaceum Andrz.	0	n	1	0	Amaranthaceae Juss.
Iz_4_1009	DJI_0357	Chenopodium cf. album	0	n	1	0	Amaranthaceae Juss.
Iz_4_1009	DJI_0357	Chenopodium sp.	0		NA	0	Amaranthaceae Juss.
Iz_4_1009	DJI_0357	Chondrilla juncea L.	0	n	1	0	Asteraceae Bercht. & J.Presl
Iz_4_1009	DJI_0357	Cirsium vulgare (Savi) Ten.	0	n	1	0	Asteraceae Bercht. & J.Presl
Iz_4_1009	DJI_0357	Clinopodium acinos (L.) Kuntze	0	n	1	0	Lamiacea Lindl.
Iz_4_1009	DJI_0357	Convallaria majalis L.	0	n	1	0	Asparagaceae Juss.
Iz_4_1009	DJI_0357	Crataegus rhipidophylla Gand.	0	n	1	1	Rosaceae Juss.
Iz_4_1009	DJI_0357	Crepis foetida L.	0	n	1	0	Asteraceae Bercht. & J.Presl
Iz_4_1009	DJI_0357	Dactylis glomerata L.	0	n	1	0	Poaceae Barnhart
Iz_4_1009	DJI_0357	Dianthus campestris M.Bieb.	0	n	1	0	Caryophyllaceae Juss.
Iz_4_1009	DJI_0357	Digitaria sanguinalis (L.) Scop.	0	a	0	0	Poaceae Barnhart
Iz_4_1009	DJI_0357	Elymus repens (L.) Gould	0	n	1	0	Poaceae Barnhart
Iz_4_1009	DJI_0357	Epilobium tetragonum L.	0	n	1	0	Onagraceae Juss.
Iz_4_1009	DJI_0357	Eragrostis minor Host	0	a	0	0	Poaceae Barnhart
Iz_4_1009	DJI_0357	Erigeron canadensis L.	4	a	0	0	Asteraceae Bercht. & J.Presl
Iz_4_1009	DJI_0357	Euphorbia seguieriana Neck.	0	n	1	0	Euphorbiaceae Juss.
Iz_4_1009	DJI_0357	Fallopia dumetorum (L.) Holub	0	n	1	0	Polygonaceae Juss.
Iz_4_1009	DJI_0357	Festuca beckeri (Hack.) Trautv.	0	n	1	0	Poaceae Barnhart
Iz_4_1009	DJI_0357	Fragaria vesca L.	0	n	1	0	Rosaceae Juss.
Iz_4_1009	DJI_0357	Frangula alnus Mill.	0	n	1	1	Rhamnaceae Juss.
Iz_4_1009	DJI_0357	Fraxinus pennsylvanica Marshall	0	a	0	1	Oleaceae Hoffmanns. & Link
Iz_4_1009	DJI_0357	Galeopsis bifida Boenn.	0	n	1	0	Lamiacea Lindl.
Iz_4_1009	DJI_0357	Galium verum L.	0	n	1	0	Rubiaceae Juss.
Iz_4_1009	DJI_0357	Genista tinctoria L.	0	n	1	0	Fabaceae Lindl.
Iz_4_1009	DJI_0357	Geranium robertianum L.	0	n	1	0	Geraniaceae Juss.
Iz_4_1009	DJI_0357	Geranium sanquineum L.	0	n	1	0	Geraniaceae Juss.
Iz_4_1009	DJI_0357	Gypsophila paniculata L.	0	n	1	0	Caryophyllaceae Juss.
Iz_4_1009	DJI_0357	Helichrysum arenarium (L.) Moench	0	n	1	0	Asteraceae Bercht. & J.Presl
Iz_4_1009	DJI_0357	Herniaria polygama J.Gay	0	n	1	0	Caryophyllaceae Juss.
Iz_4_1009	DJI_0357	Hieracium robustum Fr.	0	n	1	0	Asteraceae Bercht. & J.Presl
Iz_4_1009	DJI_0357	Hieracium umbellatum L.	0	n	1	NA	Asteraceae Bercht. & J.Presl
Iz_4_1009	DJI_0357	Hylotelephium maximum (L.) Holub	0	n	1	0	Crassulaceae J.St.-Hil.
Iz_4_1009	DJI_0357	Jacobaea borysthenica (DC.) B.Nord. & Greuter	0	n	1	0	Asteraceae Bercht. & J.Presl
Iz_4_1009	DJI_0357	Jasione montana L.	0	n	1	0	Campanulaceae Juss.
Iz_4_1009	DJI_0357	Jurinea cyanoides (L.) Rchb.s.l.	0	n	1	0	Asteraceae Bercht. & J.Presl
Iz_4_1009	DJI_0357	Koeleria glauca (Spreng.) DC.	0	n	1	0	Poaceae Barnhart
Iz_4_1009	DJI_0357	Lactuca serriola L.	0	a	0	0	Asteraceae Bercht. & J.Presl
Iz_4_1009	DJI_0357	Linaria genistifolia (L.) Mill.	0	n	1	0	Plantaginaceae Juss.
Iz_4_1009	DJI_0357	Lomelosia argentea (L.) Greuter & Burdet	0	n	1	0	Caprifoliaceae Juss.
Iz_4_1009	DJI_0357	Melampyrum pratense L.	0	n	1	0	Orobanchaceae Vent.
Iz_4_1009	DJI_0357	Oenothera biennis L.	0	a	0	0	Onagraceae Juss.
Iz_4_1009	DJI_0357	Pentanema britannica (L.) D.Gut.Larr., Santos-Vicente, Anderb., E.Rico & M.M.Mart.Ort.	0	n	1	0	Asteraceae Bercht. & J.Presl
Iz_4_1009	DJI_0357	Peucedanum oreoselinum (L.) Moench	0	n	1	0	Apiaceae Lindl.
Iz_4_1009	DJI_0357	Peucedanum ruthenicum M.Bieb.	0	n	1	0	Apiaceae Lindl.
Iz_4_1009	DJI_0357	Pilosella echioides (Lumn.) F.W.Schultz & Sch.Bip.	0	n	1	0	Asteraceae Bercht. & J.Presl
Iz_4_1009	DJI_0357	Pilosella officinarum F. Schultz et Sch. Bip.	0	n	1	0	Asteraceae Bercht. & J.Presl
Iz_4_1009	DJI_0357	Pilosella sp.	0		NA	0	Asteraceae Bercht. & J.Presl
Iz_4_1009	DJI_0357	Pinus sylvestris L.	0	n	1	1	Pinaceae Spreng. ex F.Rudolphi
Iz_4_1009	DJI_0357	Poa annua L.	0	n	1	0	Poaceae Barnhart
Iz_4_1009	DJI_0357	Poa nemoralis L.	0	n	1	0	Poaceae Barnhart
Iz_4_1009	DJI_0357	Poa sp.	0		NA	0	Poaceae Barnhart
Iz_4_1009	DJI_0357	Polygonatum odoratum (Mill.) Druce	0	n	1	0	Asparagaceae Juss.
Iz_4_1009	DJI_0357	Polygonum arenarium Waldst. & Kit.	0	n	1	0	Polygonaceae Juss.
Iz_4_1009	DJI_0357	Populus nigra L.	0	n	1	1	Salicaceae Mirb.
Iz_4_1009	DJI_0357	Populus tremula L.	0	n	1	1	Salicaceae Mirb.
Iz_4_1009	DJI_0357	Populus Ã canescens (Aiton) Sm.	0	n	1	1	Salicaceae Mirb.
Iz_4_1009	DJI_0357	Potentilla argentea L.	0	n	1	0	Rosaceae Juss.
Iz_4_1009	DJI_0357	Psammophiliella muralis (L.) Ikonn.	0	n	1	0	Caryophyllaceae Juss.
Iz_4_1009	DJI_0357	Psephellus sumensis (Kalen.) Greuter	0	n	1	0	Asteraceae Bercht. & J.Presl
Iz_4_1009	DJI_0357	Pulsatilla pratensis (L.) Mill. s.l.	0	n	1	0	Ranunculaceae Juss.
Iz_4_1009	DJI_0357	Pyrus communis L.	0	a	0	1	Rosaceae Juss.
Iz_4_1009	DJI_0357	Quercus robur L.	0	n	1	1	Fagaceae Dumort.
Iz_4_1009	DJI_0357	Robinia pseudoacacia L.	0	a	0	1	Fabaceae Lindl.
Iz_4_1009	DJI_0357	Rosa sp.	0		NA	1	Rosaceae Juss.
Iz_4_1009	DJI_0357	Rubus idaeus L.	0	n	1	0	Rosaceae Juss.
Iz_4_1009	DJI_0357	Rumex acetosella L.	0	n	1	0	Polygonaceae Juss.
Iz_4_1009	DJI_0357	Rumex thyrsiflorus Fingerh.	0	n	1	0	Polygonaceae Juss.
Iz_4_1009	DJI_0357	Salix alba L.	0	n	1	1	Salicaceae Mirb.
Iz_4_1009	DJI_0357	Sambucus nigra L.	0	n	1	1	Viburnaceae Raf.
Iz_4_1009	DJI_0357	Secale sylvestre Host	0	n	1	0	Poaceae Barnhart
Iz_4_1009	DJI_0357	Sempervivum ruthenicum Schnittsp. & C.B. Lehm.	0	n	1	0	Crassulaceae J.St.-Hil.
Iz_4_1009	DJI_0357	Setaria viridis (L.) P. Beauv.	0	n	1	0	Poaceae Barnhart
Iz_4_1009	DJI_0357	Silene borysthenica (Gruner) Walters	0	n	1	0	Caryophyllaceae Juss.
Iz_4_1009	DJI_0357	Silene latifolia subsp. alba (Mill.) Greuter & Burdet	0	n	1	0	Caryophyllaceae Juss.
Iz_4_1009	DJI_0357	Solidago virgaurea L.	0	n	1	0	Asteraceae Bercht. & J.Presl
Iz_4_1009	DJI_0357	Sorbus aucuparia L.	0	n	1	1	Rosaceae Juss.

Iz_4_1009	DJI_0357	Stachys recta L.	0	n	1	0	Lamiacea Lindl.
Iz_4_1009	DJI_0357	Tanacetum vulgare L.	0	n	1	0	Asteraceae Bercht. & J.Presl
Iz_4_1009	DJI_0357	Thymus pallasianus Heinr.Braun	0	n	1	0	Lamiacea Lindl.
Iz_4_1009	DJI_0357	Tilia cordata Mill.	0	n	1	1	Malvaceae Juss.
Iz_4_1009	DJI_0357	Trifolium alpestre L.	0	n	1	0	Fabaceae Lindl.
Iz_4_1009	DJI_0357	Trifolium arvense L.	0	n	1	0	Fabaceae Lindl.
Iz_4_1009	DJI_0357	Ulmus glabra Huds.	0	n	1	1	Ulmaceae Mirb.
Iz_4_1009	DJI_0357	Verbascum densiflorum Bertol.	0	n	1	0	Scrophulariaceae Juss.
Iz_4_1009	DJI_0357	Verbascum nigrum L.	0	n	1	0	Scrophulariaceae Juss.
Iz_4_1009	DJI_0357	Veronica austriaca L.	0	n	1	0	Plantaginaceae Juss.
Iz_4_1009	DJI_0357	Veronica incana L.	0	n	1	0	Plantaginaceae Juss.
Iz_4_1009	DJI_0357	Veronica verna L.	0	n	1	0	Plantaginaceae Juss.
Iz_4_1009	DJI_0357	Vincetoxicum hirundinaria Medik	0	n	1	0	Apocynaceae Juss.
Iz_4_1009	DJI_0358	Acer tataricum L.	0	n	1	1	Sapindaceae Juss.
Iz_4_1009	DJI_0358	Achillea millefolium L. s.l.	0	n	1	0	Asteraceae Bercht. & J.Presl
Iz_4_1009	DJI_0358	Allium oleraceum L.	0	n	1	0	Amaryllidaceae J.St.-Hil.
Iz_4_1009	DJI_0358	Ambrosia artemisiifolia L.	0	a	0	0	Asteraceae Bercht. & J.Presl
Iz_4_1009	DJI_0358	Amorpha fruticosa L.	0	a	0	0	Fabaceae Lindl.
Iz_4_1009	DJI_0358	Anthericum ramosum L.	0	n	1	0	Asparagaceae Juss.
Iz_4_1009	DJI_0358	Artemisia marschalliana Spreng.	0	n	1	0	Asteraceae Bercht. & J.Presl
Iz_4_1009	DJI_0358	Asparagus officinalis L.	0	n	1	0	Asparagaceae Juss.
Iz_4_1009	DJI_0358	Astragalus cicer L.	0	n	1	0	Fabaceae Lindl.
Iz_4_1009	DJI_0358	Bassia laniflora (S.G.Gmel.) A.J.Scott	0	n	1	0	Amaranthaceae Juss.
Iz_4_1009	DJI_0358	Berberis vulgaris L.	0	n	1	0	Berberidaceae Juss.
Iz_4_1009	DJI_0358	Betula pendula Roth.	0	n	1	1	Betulaceae Gray
Iz_4_1009	DJI_0358	Betula pendula Roth.	0	n	1	0	Poaceae Barnhart
Iz_4_1009	DJI_0358	Bromus inermis Leyss.	0		NA	NA	
Iz_4_1009	DJI_0358	Bromus squarrosus L.	0	a	0	0	Poaceae Barnhart
Iz_4_1009	DJI_0358	Bromus tectorum L.	0	a	0	0	Poaceae Barnhart
Iz_4_1009	DJI_0358	Calamagrostis epigejos (L.) Roth	1	n	1	0	Poaceae Barnhart
Iz_4_1009	DJI_0358	Carduus acanthoides L.	0	n	1	0	Asteraceae Bercht. & J.Presl
Iz_4_1009	DJI_0358	Carex sp.	0	n	1	0	Cyperaceae Juss.
Iz_4_1009	DJI_0358	Centaurea majorovii Dumbadze	0	n	1	0	Asteraceae Bercht. & J.Presl
Iz_4_1009	DJI_0358	Chamaecytisus ruthenicus (Fisch.ex Wol.) Klaskova	0	n	1	0	Fabaceae Lindl.
Iz_4_1009	DJI_0358	Chelidonium majus L.	0	n	1	0	Papaveraceae Juss.
Iz_4_1009	DJI_0358	Chenopodium betaceum Andrz.	0	n	1	0	Amaranthaceae Juss.
Iz_4_1009	DJI_0358	Chenopodium cf. album	0	n	1	0	Amaranthaceae Juss.
Iz_4_1009	DJI_0358	Chenopodium sp.	0		NA	0	Amaranthaceae Juss.
Iz_4_1009	DJI_0358	Chondrilla juncea L.	0	n	1	0	Asteraceae Bercht. & J.Presl
Iz_4_1009	DJI_0358	Cirsium vulgare (Savi) Ten.	0	n	1	0	Asteraceae Bercht. & J.Presl
Iz_4_1009	DJI_0358	Clinopodium acinos (L.) Kuntze	0	n	1	0	Lamiacea Lindl.
Iz_4_1009	DJI_0358	Convallaria majalis L.	0	n	1	0	Asparagaceae Juss.
Iz_4_1009	DJI_0358	Crataegus rhipidophylla Gand.	0	n	1	1	Rosaceae Juss.
Iz_4_1009	DJI_0358	Crepis foetida L.	0	n	1	0	Asteraceae Bercht. & J.Presl
Iz_4_1009	DJI_0358	Dactylis glomerata L.	0	n	1	0	Poaceae Barnhart
Iz_4_1009	DJI_0358	Dianthus campestris M.Bieb.	0	n	1	0	Caryophyllaceae Juss.
Iz_4_1009	DJI_0358	Digitaria sanguinalis (L.) Scop.	0	a	0	0	Poaceae Barnhart
Iz_4_1009	DJI_0358	Elymus repens (L.) Gould	0	n	1	0	Poaceae Barnhart
Iz_4_1009	DJI_0358	Epilobium tetragonum L.	0	n	1	0	Onagraceae Juss.
Iz_4_1009	DJI_0358	Eragrostis minor Host	0	a	0	0	Poaceae Barnhart
Iz_4_1009	DJI_0358	Erigeron canadensis L.	0	a	0	0	Asteraceae Bercht. & J.Presl
Iz_4_1009	DJI_0358	Euphorbia seguieriana Neck.	0	n	1	0	Euphorbiaceae Juss.
Iz_4_1009	DJI_0358	Fallopia dumetorum (L.) Holub	0	n	1	0	Polygonaceae Juss.
Iz_4_1009	DJI_0358	Festuca beckeri (Hack.) Trautv.	0	n	1	0	Poaceae Barnhart
Iz_4_1009	DJI_0358	Fragaria vesca L.	0	n	1	0	Rosaceae Juss.
Iz_4_1009	DJI_0358	Frangula alnus Mill.	0	n	1	1	Rhamnaceae Juss.
Iz_4_1009	DJI_0358	Fraxinus pennsylvanica Marshall	0	a	0	1	Oleaceae Hoffmanns. & Link
Iz_4_1009	DJI_0358	Galeopsis bifida Boenn.	0	n	1	0	Lamiacea Lindl.
Iz_4_1009	DJI_0358	Galium verum L.	0	n	1	0	Rubiaceae Juss.
Iz_4_1009	DJI_0358	Genista tinctoria L.	0	n	1	0	Fabaceae Lindl.
Iz_4_1009	DJI_0358	Geranium robertianum L.	0	n	1	0	Geraniaceae Juss.
Iz_4_1009	DJI_0358	Geranium sanquineum L.	0	n	1	0	Geraniaceae Juss.
Iz_4_1009	DJI_0358	Gypsophila paniculata L.	0	n	1	0	Caryophyllaceae Juss.
Iz_4_1009	DJI_0358	Helichrysum arenarium (L.) Moench	0	n	1	0	Asteraceae Bercht. & J.Presl
Iz_4_1009	DJI_0358	Herniaria polygama J.Gay	0	n	1	0	Caryophyllaceae Juss.
Iz_4_1009	DJI_0358	Hieracium robustum Fr.	0	n	1	0	Asteraceae Bercht. & J.Presl
Iz_4_1009	DJI_0358	Hieracium umbellatum L.	0	n	1	NA	Asteraceae Bercht. & J.Presl
Iz_4_1009	DJI_0358	Hylotelephium maximum (L.) Holub	0	n	1	0	Crassulaceae J.St.-Hil.
Iz_4_1009	DJI_0358	Jacobaea borysthenica (DC.) B.Nord. & Greuter	0	n	1	0	Asteraceae Bercht. & J.Presl
Iz_4_1009	DJI_0358	Jasione montana L.	0	n	1	0	Campanulaceae Juss.
Iz_4_1009	DJI_0358	Jurinea cyanoides (L.) Rchb.s.l.	0	n	1	0	Asteraceae Bercht. & J.Presl
Iz_4_1009	DJI_0358	Koeleria glauca (Spreng.) DC.	0	n	1	0	Poaceae Barnhart
Iz_4_1009	DJI_0358	Lactuca serriola L.	0	a	0	0	Asteraceae Bercht. & J.Presl
Iz_4_1009	DJI_0358	Linaria genistifolia (L.) Mill.	0	n	1	0	Plantaginaceae Juss.
Iz_4_1009	DJI_0358	Lomelosia argentea (L.) Greuter & Burdet	0	n	1	0	Caprifoliaceae Juss.
Iz_4_1009	DJI_0358	Melampyrum pratense L.	0	n	1	0	Orobanchaceae Vent.
Iz_4_1009	DJI_0358	Oenothera biennis L.	0	a	0	0	Onagraceae Juss.
Iz_4_1009	DJI_0358	Pentanema britannica (L.) D.Gut.Larr., Santos-Vicente, Anderb., E.Rico & M.M.Mart.Ort.	0	n	1	0	Asteraceae Bercht. & J.Presl
Iz_4_1009	DJI_0358	Peucedanum oreoselinum (L.) Moench	0.5	n	1	0	Apiaceae Lindl.
Iz_4_1009	DJI_0358	Peucedanum ruthenicum M.Bieb.	0	n	1	0	Apiaceae Lindl.
Iz_4_1009	DJI_0358	Pilosella echioides (Lumn.) F.W.Schultz & Sch.Bip.	0	n	1	0	Asteraceae Bercht. & J.Presl
Iz_4_1009	DJI_0358	Pilosella officinarum F. Schultz et Sch. Bip.	0	n	1	0	Asteraceae Bercht. & J.Presl
Iz_4_1009	DJI_0358	Pilosella sp.	0		NA	0	Asteraceae Bercht. & J.Presl
Iz_4_1009	DJI_0358	Pinus sylvestris L.	0	n	1	1	Pinaceae Spreng. ex F.Rudolphi
Iz_4_1009	DJI_0358	Poa annua L.	0	n	1	0	Poaceae Barnhart
Iz_4_1009	DJI_0358	Poa nemoralis L.	0	n	1	0	Poaceae Barnhart
Iz_4_1009	DJI_0358	Poa sp.	0		NA	0	Poaceae Barnhart
Iz_4_1009	DJI_0358	Polygonatum odoratum (Mill.) Druce	0	n	1	0	Asparagaceae Juss.
Iz_4_1009	DJI_0358	Polygonum arenarium Waldst. & Kit.	0	n	1	0	Polygonaceae Juss.
Iz_4_1009	DJI_0358	Populus nigra L.	0	n	1	1	Salicaceae Mirb.
Iz_4_1009	DJI_0358	Populus tremula L.	0	n	1	1	Salicaceae Mirb.
Iz_4_1009	DJI_0358	Populus Ã canescens (Aiton) Sm.	0	n	1	1	Salicaceae Mirb.
Iz_4_1009	DJI_0358	Potentilla argentea L.	0	n	1	0	Rosaceae Juss.
Iz_4_1009	DJI_0358	Psammophiliella muralis (L.) Ikonn.	0	n	1	0	Caryophyllaceae Juss.
Iz_4_1009	DJI_0358	Psephellus sumensis (Kalen.) Greuter	0	n	1	0	Asteraceae Bercht. & J.Presl
Iz_4_1009	DJI_0358	Pulsatilla pratensis (L.) Mill. s.l.	0	n	1	0	Ranunculaceae Juss.
Iz_4_1009	DJI_0358	Pyrus communis L.	0	a	0	1	Rosaceae Juss.
Iz_4_1009	DJI_0358	Quercus robur L.	0	n	1	1	Fagaceae Dumort.

Iz_4_1009	DJI_0358	Robinia pseudoacacia L.	0	a	0	1	Fabaceae Lindl.
Iz_4_1009	DJI_0358	Rosa sp.	0		NA	1	Rosaceae Juss.
Iz_4_1009	DJI_0358	Rubus idaeus L.	0	n	1	0	Rosaceae Juss.
Iz_4_1009	DJI_0358	Rumex acetosella L.	0	n	1	0	Polygonaceae Juss.
Iz_4_1009	DJI_0358	Rumex thyrsiflorus Fingerh.	0	n	1	0	Polygonaceae Juss.
Iz_4_1009	DJI_0358	Salix alba L.	0	n	1	1	Salicaceae Mirb.
Iz_4_1009	DJI_0358	Sambucus nigra L.	0	n	1	1	Viburnaceae Raf.
Iz_4_1009	DJI_0358	Secale sylvestre Host	0	n	1	0	Poaceae Barnhart
Iz_4_1009	DJI_0358	Sempervivum ruthenicum Schnittsp. & C.B. Lehm.	0	n	1	0	Crassulaceae J.St.-Hil.
Iz_4_1009	DJI_0358	Setaria viridis (L.) P. Beauv.	0	n	1	0	Poaceae Barnhart
Iz_4_1009	DJI_0358	Silene borysthenica (Gruner) Walters	0	n	1	0	Caryophyllaceae Juss.
Iz_4_1009	DJI_0358	Silene latifolia subsp. alba (Mill.) Greuter & Burdet	0	n	1	0	Caryophyllaceae Juss.
Iz_4_1009	DJI_0358	Solidago virgaurea L.	0	n	1	0	Asteraceae Bercht. & J.Presl
Iz_4_1009	DJI_0358	Sorbus aucuparia L.	0	n	1	1	Rosaceae Juss.
Iz_4_1009	DJI_0358	Stachys recta L.	0	n	1	0	Lamiacea Lindl.
Iz_4_1009	DJI_0358	Tanacetum vulgare L.	0	n	1	0	Asteraceae Bercht. & J.Presl
Iz_4_1009	DJI_0358	Thymus pallasianus Heinr.Braun	0	n	1	0	Lamiacea Lindl.
Iz_4_1009	DJI_0358	Tilia cordata Mill.	0	n	1	1	Malvaceae Juss.
Iz_4_1009	DJI_0358	Trifolium alpestre L.	0	n	1	0	Fabaceae Lindl.
Iz_4_1009	DJI_0358	Trifolium arvense L.	0	n	1	0	Fabaceae Lindl.
Iz_4_1009	DJI_0358	Ulmus glabra Huds.	0	n	1	1	Ulmaceae Mirb.
Iz_4_1009	DJI_0358	Verbascum densiflorum Bertol.	0	n	1	0	Scrophulariaceae Juss.
Iz_4_1009	DJI_0358	Verbascum nigrum L.	0	n	1	0	Scrophulariaceae Juss.
Iz_4_1009	DJI_0358	Veronica austriaca L.	0	n	1	0	Plantaginaceae Juss.
Iz_4_1009	DJI_0358	Veronica incana L.	0	n	1	0	Plantaginaceae Juss.
Iz_4_1009	DJI_0358	Veronica verna L.	0	n	1	0	Plantaginaceae Juss.
Iz_4_1009	DJI_0358	Vincetoxicum hirundinaria Medik	0	n	1	0	Apocynaceae Juss.
Iz_4_1009	DJI_0359	Acer tataricum L.	0	n	1	1	Sapindaceae Juss.
Iz_4_1009	DJI_0359	Achillea millefolium L. s.l.	0	n	1	0	Asteraceae Bercht. & J.Presl
Iz_4_1009	DJI_0359	Allium oleraceum L.	0	n	1	0	Amaryllidaceae J.St.-Hil.
Iz_4_1009	DJI_0359	Ambrosia artemisiifolia L.	0	a	0	0	Asteraceae Bercht. & J.Presl
Iz_4_1009	DJI_0359	Amorpha fruticosa L.	0	a	0	0	Fabaceae Lindl.
Iz_4_1009	DJI_0359	Anthericum ramosum L.	0	n	1	0	Asparagaceae Juss.
Iz_4_1009	DJI_0359	Artemisia marschalliana Spreng.	0	n	1	0	Asteraceae Bercht. & J.Presl
Iz_4_1009	DJI_0359	Asparagus officinalis L.	0	n	1	0	Asparagaceae Juss.
Iz_4_1009	DJI_0359	Astragalus cicer L.	0	n	1	0	Fabaceae Lindl.
Iz_4_1009	DJI_0359	Bassia laniflora (S.G.Gmel.) A.J.Scott	0	n	1	0	Amaranthaceae Juss.
Iz_4_1009	DJI_0359	Berberis vulgaris L.	0	n	1	0	Berberidaceae Juss.
Iz_4_1009	DJI_0359	Betula pendula Roth.	0	n	1	1	Betulaceae Gray
Iz_4_1009	DJI_0359	Betula pendula Roth.	0	n	1	0	Poaceae Barnhart
Iz_4_1009	DJI_0359	Bromus inermis Leyss.	0		NA	NA	
Iz_4_1009	DJI_0359	Bromus squarrosus L.	0	a	0	0	Poaceae Barnhart
Iz_4_1009	DJI_0359	Bromus tectorum L.	0	a	0	0	Poaceae Barnhart
Iz_4_1009	DJI_0359	Calamagrostis epigejos (L.) Roth	2	n	1	0	Poaceae Barnhart
Iz_4_1009	DJI_0359	Carduus acanthoides L.	0	n	1	0	Asteraceae Bercht. & J.Presl
Iz_4_1009	DJI_0359	Carex sp.	0	n	1	0	Cyperaceae Juss.
Iz_4_1009	DJI_0359	Centaurea majorovii Dumbadze	0	n	1	0	Asteraceae Bercht. & J.Presl
Iz_4_1009	DJI_0359	Chamaecytisus ruthenicus (Fisch.ex Wol.) Klaskova	0	n	1	0	Fabaceae Lindl.
Iz_4_1009	DJI_0359	Chelidonium majus L.	0	n	1	0	Papaveraceae Juss.
Iz_4_1009	DJI_0359	Chenopodium betaceum Andrz.	0	n	1	0	Amaranthaceae Juss.
Iz_4_1009	DJI_0359	Chenopodium cf. album	0	n	1	0	Amaranthaceae Juss.
Iz_4_1009	DJI_0359	Chenopodium sp.	0		NA	0	Amaranthaceae Juss.
Iz_4_1009	DJI_0359	Chondrilla juncea L.	0	n	1	0	Asteraceae Bercht. & J.Presl
Iz_4_1009	DJI_0359	Cirsium vulgare (Savi) Ten.	0	n	1	0	Asteraceae Bercht. & J.Presl
Iz_4_1009	DJI_0359	Clinopodium acinos (L.) Kuntze	0	n	1	0	Lamiacea Lindl.
Iz_4_1009	DJI_0359	Convallaria majalis L.	0	n	1	0	Asparagaceae Juss.
Iz_4_1009	DJI_0359	Crataegus rhipidophylla Gand.	0	n	1	1	Rosaceae Juss.
Iz_4_1009	DJI_0359	Crepis foetida L.	0	n	1	0	Asteraceae Bercht. & J.Presl
Iz_4_1009	DJI_0359	Dactylis glomerata L.	0	n	1	0	Poaceae Barnhart
Iz_4_1009	DJI_0359	Dianthus campestris M.Bieb.	0	n	1	0	Caryophyllaceae Juss.
Iz_4_1009	DJI_0359	Digitaria sanguinalis (L.) Scop.	0	a	0	0	Poaceae Barnhart
Iz_4_1009	DJI_0359	Elymus repens (L.) Gould	0	n	1	0	Poaceae Barnhart
Iz_4_1009	DJI_0359	Epilobium tetragonum L.	0	n	1	0	Onagraceae Juss.
Iz_4_1009	DJI_0359	Eragrostis minor Host	0	a	0	0	Poaceae Barnhart
Iz_4_1009	DJI_0359	Erigeron canadensis L.	0	a	0	0	Asteraceae Bercht. & J.Presl
Iz_4_1009	DJI_0359	Euphorbia seguieriana Neck.	0	n	1	0	Euphorbiaceae Juss.
Iz_4_1009	DJI_0359	Fallopia dumetorum (L.) Holub	0	n	1	0	Polygonaceae Juss.
Iz_4_1009	DJI_0359	Festuca beckeri (Hack.) Trautv.	0	n	1	0	Poaceae Barnhart
Iz_4_1009	DJI_0359	Fragaria vesca L.	0	n	1	0	Rosaceae Juss.
Iz_4_1009	DJI_0359	Frangula alnus Mill.	0	n	1	1	Rhamnaceae Juss.
Iz_4_1009	DJI_0359	Fraxinus pennsylvanica Marshall	0	a	0	1	Oleaceae Hoffmanns. & Link
Iz_4_1009	DJI_0359	Galeopsis bifida Boenn.	0	n	1	0	Lamiacea Lindl.
Iz_4_1009	DJI_0359	Galium verum L.	0	n	1	0	Rubiaceae Juss.
Iz_4_1009	DJI_0359	Genista tinctoria L.	0	n	1	0	Fabaceae Lindl.
Iz_4_1009	DJI_0359	Geranium robertianum L.	0	n	1	0	Geraniaceae Juss.
Iz_4_1009	DJI_0359	Geranium sanquineum L.	0	n	1	0	Geraniaceae Juss.
Iz_4_1009	DJI_0359	Gypsophila paniculata L.	0	n	1	0	Caryophyllaceae Juss.
Iz_4_1009	DJI_0359	Helichrysum arenarium (L.) Moench	0	n	1	0	Asteraceae Bercht. & J.Presl
Iz_4_1009	DJI_0359	Herniaria polygama J.Gay	0	n	1	0	Caryophyllaceae Juss.
Iz_4_1009	DJI_0359	Hieracium robustum Fr.	0	n	1	0	Asteraceae Bercht. & J.Presl
Iz_4_1009	DJI_0359	Hieracium umbellatum L.	0	n	1	NA	Asteraceae Bercht. & J.Presl
Iz_4_1009	DJI_0359	Hylotelephium maximum (L.) Holub	0	n	1	0	Crassulaceae J.St.-Hil.
Iz_4_1009	DJI_0359	Jacobaea borysthenica (DC.) B.Nord. & Greuter	0	n	1	0	Asteraceae Bercht. & J.Presl
Iz_4_1009	DJI_0359	Jasione montana L.	0	n	1	0	Campanulaceae Juss.
Iz_4_1009	DJI_0359	Jurinea cyanoides (L.) Rchb.s.l.	0	n	1	0	Asteraceae Bercht. & J.Presl
Iz_4_1009	DJI_0359	Koeleria glauca (Spreng.) DC.	0	n	1	0	Poaceae Barnhart
Iz_4_1009	DJI_0359	Lactuca serriola L.	0	a	0	0	Asteraceae Bercht. & J.Presl
Iz_4_1009	DJI_0359	Linaria genistifolia (L.) Mill.	0	n	1	0	Plantaginaceae Juss.
Iz_4_1009	DJI_0359	Lomelosia argentea (L.) Greuter & Burdet	0	n	1	0	Caprifoliaceae Juss.
Iz_4_1009	DJI_0359	Melampyrum pratense L.	0	n	1	0	Orobanchaceae Vent.
Iz_4_1009	DJI_0359	Oenothera biennis L.	0	a	0	0	Onagraceae Juss.
Iz_4_1009	DJI_0359	Pentanema britannica (L.) D.Gut.Larr., Santos-Vicente, Anderb., E.Rico & M.M.Mart.Ort.	0	n	1	0	Asteraceae Bercht. & J.Presl
Iz_4_1009	DJI_0359	Peucedanum oreoselinum (L.) Moench	1	n	1	0	Apiaceae Lindl.
Iz_4_1009	DJI_0359	Peucedanum ruthenicum M.Bieb.	0	n	1	0	Apiaceae Lindl.
Iz_4_1009	DJI_0359	Pilosella echioides (Lumn.) F.W.Schultz & Sch.Bip.	0	n	1	0	Asteraceae Bercht. & J.Presl
Iz_4_1009	DJI_0359	Pilosella officinarum F. Schultz et Sch. Bip.	0	n	1	0	Asteraceae Bercht. & J.Presl
Iz_4_1009	DJI_0359	Pilosella sp.	0		NA	0	Asteraceae Bercht. & J.Presl
Iz_4_1009	DJI_0359	Pinus sylvestris L.	0	n	1	1	Pinaceae Spreng. ex F.Rudolphi

Iz_4_1009	DJI_0359	Poa annua L.	0	n	1	0	Poaceae Barnhart
Iz_4_1009	DJI_0359	Poa nemoralis L.	0	n	1	0	Poaceae Barnhart
Iz_4_1009	DJI_0359	Poa sp.	0		NA	0	Poaceae Barnhart
Iz_4_1009	DJI_0359	Polygonatum odoratum (Mill.) Druce	0	n	1	0	Asparagaceae Juss.
Iz_4_1009	DJI_0359	Polygonum arenarium Waldst. & Kit.	0	n	1	0	Polygonaceae Juss.
Iz_4_1009	DJI_0359	Populus nigra L.	0	n	1	1	Salicaceae Mirb.
Iz_4_1009	DJI_0359	Populus tremula L.	0	n	1	1	Salicaceae Mirb.
Iz_4_1009	DJI_0359	Populus Ã canescens (Aiton) Sm.	0	n	1	1	Salicaceae Mirb.
Iz_4_1009	DJI_0359	Potentilla argentea L.	0	n	1	0	Rosaceae Juss.
Iz_4_1009	DJI_0359	Psammophiliella muralis (L.) Ikonn.	0	n	1	0	Caryophyllaceae Juss.
Iz_4_1009	DJI_0359	Psephellus sumensis (Kalen.) Greuter	0	n	1	0	Asteraceae Bercht. & J.Presl
Iz_4_1009	DJI_0359	Pulsatilla pratensis (L.) Mill. s.l.	0	n	1	0	Ranunculaceae Juss.
Iz_4_1009	DJI_0359	Pyrus communis L.	0	a	0	1	Rosaceae Juss.
Iz_4_1009	DJI_0359	Quercus robur L.	0	n	1	1	Fagaceae Dumort.
Iz_4_1009	DJI_0359	Robinia pseudoacacia L.	0	a	0	1	Fabaceae Lindl.
Iz_4_1009	DJI_0359	Rosa sp.	0		NA	1	Rosaceae Juss.
Iz_4_1009	DJI_0359	Rubus idaeus L.	0	n	1	0	Rosaceae Juss.
Iz_4_1009	DJI_0359	Rumex acetosella L.	0	n	1	0	Polygonaceae Juss.
Iz_4_1009	DJI_0359	Rumex thyrsiflorus Fingerh.	0	n	1	0	Polygonaceae Juss.
Iz_4_1009	DJI_0359	Salix alba L.	0	n	1	1	Salicaceae Mirb.
Iz_4_1009	DJI_0359	Sambucus nigra L.	0	n	1	1	Viburnaceae Raf.
Iz_4_1009	DJI_0359	Secale sylvestre Host	0	n	1	0	Poaceae Barnhart
Iz_4_1009	DJI_0359	Sempervivum ruthenicum Schnittsp. & C.B. Lehm.	0	n	1	0	Crassulaceae J.St.-Hil.
Iz_4_1009	DJI_0359	Setaria viridis (L.) P. Beauv.	0	n	1	0	Poaceae Barnhart
Iz_4_1009	DJI_0359	Silene borysthenica (Gruner) Walters	0	n	1	0	Caryophyllaceae Juss.
Iz_4_1009	DJI_0359	Silene latifolia subsp. alba (Mill.) Greuter & Burdet	0	n	1	0	Caryophyllaceae Juss.
Iz_4_1009	DJI_0359	Solidago virgaurea L.	0	n	1	0	Asteraceae Bercht. & J.Presl
Iz_4_1009	DJI_0359	Sorbus aucuparia L.	0	n	1	1	Rosaceae Juss.
Iz_4_1009	DJI_0359	Stachys recta L.	0	n	1	0	Lamiacea Lindl.
Iz_4_1009	DJI_0359	Tanacetum vulgare L.	0	n	1	0	Asteraceae Bercht. & J.Presl
Iz_4_1009	DJI_0359	Thymus pallasianus Heinr.Braun	0	n	1	0	Lamiacea Lindl.
Iz_4_1009	DJI_0359	Tilia cordata Mill.	0	n	1	1	Malvaceae Juss.
Iz_4_1009	DJI_0359	Trifolium alpestre L.	0	n	1	0	Fabaceae Lindl.
Iz_4_1009	DJI_0359	Trifolium arvense L.	0	n	1	0	Fabaceae Lindl.
Iz_4_1009	DJI_0359	Ulmus glabra Huds.	0	n	1	1	Ulmaceae Mirb.
Iz_4_1009	DJI_0359	Verbascum densiflorum Bertol.	0	n	1	0	Scrophulariaceae Juss.
Iz_4_1009	DJI_0359	Verbascum nigrum L.	0	n	1	0	Scrophulariaceae Juss.
Iz_4_1009	DJI_0359	Veronica austriaca L.	0	n	1	0	Plantaginaceae Juss.
Iz_4_1009	DJI_0359	Veronica incana L.	0	n	1	0	Plantaginaceae Juss.
Iz_4_1009	DJI_0359	Veronica verna L.	0	n	1	0	Plantaginaceae Juss.
Iz_4_1009	DJI_0359	Vincetoxicum hirundinaria Medik	0	n	1	0	Apocynaceae Juss.
Iz_4_1009	DJI_0360	Acer tataricum L.	0	n	1	1	Sapindaceae Juss.
Iz_4_1009	DJI_0360	Achillea millefolium L. s.l.	0	n	1	0	Asteraceae Bercht. & J.Presl
Iz_4_1009	DJI_0360	Allium oleraceum L.	0	n	1	0	Amaryllidaceae J.St.-Hil.
Iz_4_1009	DJI_0360	Ambrosia artemisiifolia L.	0	a	0	0	Asteraceae Bercht. & J.Presl
Iz_4_1009	DJI_0360	Amorpha fruticosa L.	0	a	0	0	Fabaceae Lindl.
Iz_4_1009	DJI_0360	Anthericum ramosum L.	0	n	1	0	Asparagaceae Juss.
Iz_4_1009	DJI_0360	Artemisia marschalliana Spreng.	0	n	1	0	Asteraceae Bercht. & J.Presl
Iz_4_1009	DJI_0360	Asparagus officinalis L.	0	n	1	0	Asparagaceae Juss.
Iz_4_1009	DJI_0360	Astragalus cicer L.	0	n	1	0	Fabaceae Lindl.
Iz_4_1009	DJI_0360	Bassia laniflora (S.G.Gmel.) A.J.Scott	0	n	1	0	Amaranthaceae Juss.
Iz_4_1009	DJI_0360	Berberis vulgaris L.	0	n	1	0	Berberidaceae Juss.
Iz_4_1009	DJI_0360	Betula pendula Roth.	0	n	1	1	Betulaceae Gray
Iz_4_1009	DJI_0360	Betula pendula Roth.	0	n	1	0	Poaceae Barnhart
Iz_4_1009	DJI_0360	Bromus inermis Leyss.	0		NA	NA	
Iz_4_1009	DJI_0360	Bromus squarrosus L.	0	a	0	0	Poaceae Barnhart
Iz_4_1009	DJI_0360	Bromus tectorum L.	0	a	0	0	Poaceae Barnhart
Iz_4_1009	DJI_0360	Calamagrostis epigejos (L.) Roth	2	n	1	0	Poaceae Barnhart
Iz_4_1009	DJI_0360	Carduus acanthoides L.	0	n	1	0	Asteraceae Bercht. & J.Presl
Iz_4_1009	DJI_0360	Carex sp.	0	n	1	0	Cyperaceae Juss.
Iz_4_1009	DJI_0360	Centaurea majorovii Dumbadze	0	n	1	0	Asteraceae Bercht. & J.Presl
Iz_4_1009	DJI_0360	Chamaecytisus ruthenicus (Fisch.ex Wol.) Klaskova	0	n	1	0	Fabaceae Lindl.
Iz_4_1009	DJI_0360	Chelidonium majus L.	0	n	1	0	Papaveraceae Juss.
Iz_4_1009	DJI_0360	Chenopodium betaceum Andrz.	0	n	1	0	Amaranthaceae Juss.
Iz_4_1009	DJI_0360	Chenopodium cf. album	0	n	1	0	Amaranthaceae Juss.
Iz_4_1009	DJI_0360	Chenopodium sp.	0		NA	0	Amaranthaceae Juss.
Iz_4_1009	DJI_0360	Chondrilla juncea L.	0	n	1	0	Asteraceae Bercht. & J.Presl
Iz_4_1009	DJI_0360	Cirsium vulgare (Savi) Ten.	0	n	1	0	Asteraceae Bercht. & J.Presl
Iz_4_1009	DJI_0360	Clinopodium acinos (L.) Kuntze	0	n	1	0	Lamiacea Lindl.
Iz_4_1009	DJI_0360	Convallaria majalis L.	0	n	1	0	Asparagaceae Juss.
Iz_4_1009	DJI_0360	Crataegus rhipidophylla Gand.	0	n	1	1	Rosaceae Juss.
Iz_4_1009	DJI_0360	Crepis foetida L.	0	n	1	0	Asteraceae Bercht. & J.Presl
Iz_4_1009	DJI_0360	Dactylis glomerata L.	0	n	1	0	Poaceae Barnhart
Iz_4_1009	DJI_0360	Dianthus campestris M.Bieb.	0	n	1	0	Caryophyllaceae Juss.
Iz_4_1009	DJI_0360	Digitaria sanguinalis (L.) Scop.	0	a	0	0	Poaceae Barnhart
Iz_4_1009	DJI_0360	Elymus repens (L.) Gould	0	n	1	0	Poaceae Barnhart
Iz_4_1009	DJI_0360	Epilobium tetragonum L.	0	n	1	0	Onagraceae Juss.
Iz_4_1009	DJI_0360	Eragrostis minor Host	0	a	0	0	Poaceae Barnhart
Iz_4_1009	DJI_0360	Erigeron canadensis L.	0	a	0	0	Asteraceae Bercht. & J.Presl
Iz_4_1009	DJI_0360	Euphorbia seguieriana Neck.	0	n	1	0	Euphorbiaceae Juss.
Iz_4_1009	DJI_0360	Fallopia dumetorum (L.) Holub	0	n	1	0	Polygonaceae Juss.
Iz_4_1009	DJI_0360	Festuca beckeri (Hack.) Trautv.	0	n	1	0	Poaceae Barnhart
Iz_4_1009	DJI_0360	Fragaria vesca L.	0	n	1	0	Rosaceae Juss.
Iz_4_1009	DJI_0360	Frangula alnus Mill.	0	n	1	1	Rhamnaceae Juss.
Iz_4_1009	DJI_0360	Fraxinus pennsylvanica Marshall	0	a	0	1	Oleaceae Hoffmanns. & Link
Iz_4_1009	DJI_0360	Galeopsis bifida Boenn.	0	n	1	0	Lamiacea Lindl.
Iz_4_1009	DJI_0360	Galium verum L.	0	n	1	0	Rubiaceae Juss.
Iz_4_1009	DJI_0360	Genista tinctoria L.	0	n	1	0	Fabaceae Lindl.
Iz_4_1009	DJI_0360	Geranium robertianum L.	0	n	1	0	Geraniaceae Juss.
Iz_4_1009	DJI_0360	Geranium sanquineum L.	0	n	1	0	Geraniaceae Juss.
Iz_4_1009	DJI_0360	Gypsophila paniculata L.	0	n	1	0	Caryophyllaceae Juss.
Iz_4_1009	DJI_0360	Helichrysum arenarium (L.) Moench	0	n	1	0	Asteraceae Bercht. & J.Presl
Iz_4_1009	DJI_0360	Herniaria polygama J.Gay	0	n	1	0	Caryophyllaceae Juss.
Iz_4_1009	DJI_0360	Hieracium robustum Fr.	0	n	1	0	Asteraceae Bercht. & J.Presl
Iz_4_1009	DJI_0360	Hieracium umbellatum L.	0	n	1	NA	Asteraceae Bercht. & J.Presl
Iz_4_1009	DJI_0360	Hylotelephium maximum (L.) Holub	0	n	1	0	Crassulaceae J.St.-Hil.
Iz_4_1009	DJI_0360	Jacobaea borysthenica (DC.) B.Nord. & Greuter	0	n	1	0	Asteraceae Bercht. & J.Presl
Iz_4_1009	DJI_0360	Jasione montana L.	0	n	1	0	Campanulaceae Juss.

Iz_4_1009	DJI_0360	Jurinea cyanoides (L.) Rchb.s.l.	0	n	1	0	Asteraceae Bercht. & J.Presl
Iz_4_1009	DJI_0360	Koeleria glauca (Spreng.) DC.	0	n	1	0	Poaceae Barnhart
Iz_4_1009	DJI_0360	Lactuca serriola L.	0	a	0	0	Asteraceae Bercht. & J.Presl
Iz_4_1009	DJI_0360	Linaria genistifolia (L.) Mill.	0	n	1	0	Plantaginaceae Juss.
Iz_4_1009	DJI_0360	Lomelosia argentea (L.) Greuter & Burdet	0	n	1	0	Caprifoliaceae Juss.
Iz_4_1009	DJI_0360	Melampyrum pratense L.	0	n	1	0	Orobanchaceae Vent.
Iz_4_1009	DJI_0360	Oenothera biennis L.	0	a	0	0	Onagraceae Juss.
Iz_4_1009	DJI_0360	Pentanema britannica (L.) D.Gut.Larr., Santos-Vicente, Anderb., E.Rico & M.M.Mart.Ort.	0	n	1	0	Asteraceae Bercht. & J.Presl
Iz_4_1009	DJI_0360	Peucedanum oreoselinum (L.) Moench	2	n	1	0	Apiaceae Lindl.
Iz_4_1009	DJI_0360	Peucedanum ruthenicum M.Bieb.	0	n	1	0	Apiaceae Lindl.
Iz_4_1009	DJI_0360	Pilosella echioides (Lumn.) F.W.Schultz & Sch.Bip.	0	n	1	0	Asteraceae Bercht. & J.Presl
Iz_4_1009	DJI_0360	Pilosella officinarum F. Schultz et Sch. Bip.	0	n	1	0	Asteraceae Bercht. & J.Presl
Iz_4_1009	DJI_0360	Pilosella sp.	0		NA	0	Asteraceae Bercht. & J.Presl
Iz_4_1009	DJI_0360	Pinus sylvestris L.	0	n	1	1	Pinaceae Spreng. ex F.Rudolphi
Iz_4_1009	DJI_0360	Poa annua L.	0	n	1	0	Poaceae Barnhart
Iz_4_1009	DJI_0360	Poa nemoralis L.	0	n	1	0	Poaceae Barnhart
Iz_4_1009	DJI_0360	Poa sp.	0		NA	0	Poaceae Barnhart
Iz_4_1009	DJI_0360	Polygonatum odoratum (Mill.) Druce	0	n	1	0	Asparagaceae Juss.
Iz_4_1009	DJI_0360	Polygonum arenarium Waldst. & Kit.	0	n	1	0	Polygonaceae Juss.
Iz_4_1009	DJI_0360	Populus nigra L.	0	n	1	1	Salicaceae Mirb.
Iz_4_1009	DJI_0360	Populus tremula L.	0	n	1	1	Salicaceae Mirb.
Iz_4_1009	DJI_0360	Populus Ã canescens (Aiton) Sm.	0	n	1	1	Salicaceae Mirb.
Iz_4_1009	DJI_0360	Potentilla argentea L.	0	n	1	0	Rosaceae Juss.
Iz_4_1009	DJI_0360	Psammophiliella muralis (L.) Ikonn.	0	n	1	0	Caryophyllaceae Juss.
Iz_4_1009	DJI_0360	Psephellus sumensis (Kalen.) Greuter	0	n	1	0	Asteraceae Bercht. & J.Presl
Iz_4_1009	DJI_0360	Pulsatilla pratensis (L.) Mill. s.l.	0	n	1	0	Ranunculaceae Juss.
Iz_4_1009	DJI_0360	Pyrus communis L.	0	a	0	1	Rosaceae Juss.
Iz_4_1009	DJI_0360	Quercus robur L.	0	n	1	1	Fagaceae Dumort.
Iz_4_1009	DJI_0360	Robinia pseudoacacia L.	0	a	0	1	Fabaceae Lindl.
Iz_4_1009	DJI_0360	Rosa sp.	0		NA	1	Rosaceae Juss.
Iz_4_1009	DJI_0360	Rubus idaeus L.	0	n	1	0	Rosaceae Juss.
Iz_4_1009	DJI_0360	Rumex acetosella L.	0	n	1	0	Polygonaceae Juss.
Iz_4_1009	DJI_0360	Rumex thyrsiflorus Fingerh.	0	n	1	0	Polygonaceae Juss.
Iz_4_1009	DJI_0360	Salix alba L.	0	n	1	1	Salicaceae Mirb.
Iz_4_1009	DJI_0360	Sambucus nigra L.	0	n	1	1	Viburnaceae Raf.
Iz_4_1009	DJI_0360	Secale sylvestre Host	0	n	1	0	Poaceae Barnhart
Iz_4_1009	DJI_0360	Sempervivum ruthenicum Schnittsp. & C.B. Lehm.	0	n	1	0	Crassulaceae J.St.-Hil.
Iz_4_1009	DJI_0360	Setaria viridis (L.) P. Beauv.	0	n	1	0	Poaceae Barnhart
Iz_4_1009	DJI_0360	Silene borysthenica (Gruner) Walters	0	n	1	0	Caryophyllaceae Juss.
Iz_4_1009	DJI_0360	Silene latifolia subsp. alba (Mill.) Greuter & Burdet	0	n	1	0	Caryophyllaceae Juss.
Iz_4_1009	DJI_0360	Solidago virgaurea L.	0	n	1	0	Asteraceae Bercht. & J.Presl
Iz_4_1009	DJI_0360	Sorbus aucuparia L.	0	n	1	1	Rosaceae Juss.
Iz_4_1009	DJI_0360	Stachys recta L.	0	n	1	0	Lamiacea Lindl.
Iz_4_1009	DJI_0360	Tanacetum vulgare L.	0	n	1	0	Asteraceae Bercht. & J.Presl
Iz_4_1009	DJI_0360	Thymus pallasianus Heinr.Braun	0	n	1	0	Lamiacea Lindl.
Iz_4_1009	DJI_0360	Tilia cordata Mill.	0	n	1	1	Malvaceae Juss.
Iz_4_1009	DJI_0360	Trifolium alpestre L.	0	n	1	0	Fabaceae Lindl.
Iz_4_1009	DJI_0360	Trifolium arvense L.	0	n	1	0	Fabaceae Lindl.
Iz_4_1009	DJI_0360	Ulmus glabra Huds.	0	n	1	1	Ulmaceae Mirb.
Iz_4_1009	DJI_0360	Verbascum densiflorum Bertol.	0	n	1	0	Scrophulariaceae Juss.
Iz_4_1009	DJI_0360	Verbascum nigrum L.	0	n	1	0	Scrophulariaceae Juss.
Iz_4_1009	DJI_0360	Veronica austriaca L.	0	n	1	0	Plantaginaceae Juss.
Iz_4_1009	DJI_0360	Veronica incana L.	0	n	1	0	Plantaginaceae Juss.
Iz_4_1009	DJI_0360	Veronica verna L.	0	n	1	0	Plantaginaceae Juss.
Iz_4_1009	DJI_0360	Vincetoxicum hirundinaria Medik	0	n	1	0	Apocynaceae Juss.
Iz_4_1009	DJI_0361	Acer tataricum L.	0	n	1	1	Sapindaceae Juss.
Iz_4_1009	DJI_0361	Achillea millefolium L. s.l.	0	n	1	0	Asteraceae Bercht. & J.Presl
Iz_4_1009	DJI_0361	Allium oleraceum L.	0	n	1	0	Amaryllidaceae J.St.-Hil.
Iz_4_1009	DJI_0361	Ambrosia artemisiifolia L.	0	a	0	0	Asteraceae Bercht. & J.Presl
Iz_4_1009	DJI_0361	Amorpha fruticosa L.	0	a	0	0	Fabaceae Lindl.
Iz_4_1009	DJI_0361	Anthericum ramosum L.	0	n	1	0	Asparagaceae Juss.
Iz_4_1009	DJI_0361	Artemisia marschalliana Spreng.	0	n	1	0	Asteraceae Bercht. & J.Presl
Iz_4_1009	DJI_0361	Asparagus officinalis L.	0	n	1	0	Asparagaceae Juss.
Iz_4_1009	DJI_0361	Astragalus cicer L.	0	n	1	0	Fabaceae Lindl.
Iz_4_1009	DJI_0361	Bassia laniflora (S.G.Gmel.) A.J.Scott	0	n	1	0	Amaranthaceae Juss.
Iz_4_1009	DJI_0361	Berberis vulgaris L.	0	n	1	0	Berberidaceae Juss.
Iz_4_1009	DJI_0361	Betula pendula Roth.	0	n	1	1	Betulaceae Gray
Iz_4_1009	DJI_0361	Betula pendula Roth.	0	n	1	0	Poaceae Barnhart
Iz_4_1009	DJI_0361	Bromus inermis Leyss.	0		NA	NA	
Iz_4_1009	DJI_0361	Bromus squarrosus L.	0	a	0	0	Poaceae Barnhart
Iz_4_1009	DJI_0361	Bromus tectorum L.	0	a	0	0	Poaceae Barnhart
Iz_4_1009	DJI_0361	Calamagrostis epigejos (L.) Roth	2	n	1	0	Poaceae Barnhart
Iz_4_1009	DJI_0361	Carduus acanthoides L.	0	n	1	0	Asteraceae Bercht. & J.Presl
Iz_4_1009	DJI_0361	Carex sp.	0	n	1	0	Cyperaceae Juss.
Iz_4_1009	DJI_0361	Centaurea majorovii Dumbadze	0	n	1	0	Asteraceae Bercht. & J.Presl
Iz_4_1009	DJI_0361	Chamaecytisus ruthenicus (Fisch.ex Wol.) Klaskova	0	n	1	0	Fabaceae Lindl.
Iz_4_1009	DJI_0361	Chelidonium majus L.	0	n	1	0	Papaveraceae Juss.
Iz_4_1009	DJI_0361	Chenopodium betaceum Andrz.	0	n	1	0	Amaranthaceae Juss.
Iz_4_1009	DJI_0361	Chenopodium cf. album	0	n	1	0	Amaranthaceae Juss.
Iz_4_1009	DJI_0361	Chenopodium sp.	0		NA	0	Amaranthaceae Juss.
Iz_4_1009	DJI_0361	Chondrilla juncea L.	0	n	1	0	Asteraceae Bercht. & J.Presl
Iz_4_1009	DJI_0361	Cirsium vulgare (Savi) Ten.	0	n	1	0	Asteraceae Bercht. & J.Presl
Iz_4_1009	DJI_0361	Clinopodium acinos (L.) Kuntze	0	n	1	0	Lamiacea Lindl.
Iz_4_1009	DJI_0361	Convallaria majalis L.	0	n	1	0	Asparagaceae Juss.
Iz_4_1009	DJI_0361	Crataegus rhipidophylla Gand.	0	n	1	1	Rosaceae Juss.
Iz_4_1009	DJI_0361	Crepis foetida L.	0	n	1	0	Asteraceae Bercht. & J.Presl
Iz_4_1009	DJI_0361	Dactylis glomerata L.	0	n	1	0	Poaceae Barnhart
Iz_4_1009	DJI_0361	Dianthus campestris M.Bieb.	0	n	1	0	Caryophyllaceae Juss.
Iz_4_1009	DJI_0361	Digitaria sanguinalis (L.) Scop.	0	a	0	0	Poaceae Barnhart
Iz_4_1009	DJI_0361	Elymus repens (L.) Gould	0	n	1	0	Poaceae Barnhart
Iz_4_1009	DJI_0361	Epilobium tetragonum L.	0	n	1	0	Onagraceae Juss.
Iz_4_1009	DJI_0361	Eragrostis minor Host	0	a	0	0	Poaceae Barnhart
Iz_4_1009	DJI_0361	Erigeron canadensis L.	0.5	a	0	0	Asteraceae Bercht. & J.Presl
Iz_4_1009	DJI_0361	Euphorbia seguieriana Neck.	0	n	1	0	Euphorbiaceae Juss.
Iz_4_1009	DJI_0361	Fallopia dumetorum (L.) Holub	0	n	1	0	Polygonaceae Juss.
Iz_4_1009	DJI_0361	Festuca beckeri (Hack.) Trautv.	0	n	1	0	Poaceae Barnhart
Iz_4_1009	DJI_0361	Fragaria vesca L.	0	n	1	0	Rosaceae Juss.
Iz_4_1009	DJI_0361	Frangula alnus Mill.	0	n	1	1	Rhamnaceae Juss.

Iz_4_1009	DJI_0361	Fraxinus pennsylvanica Marshall	0	a	0	1	Oleaceae Hoffmanns. & Link
Iz_4_1009	DJI_0361	Galeopsis bifida Boenn.	0	n	1	0	Lamiacea Lindl.
Iz_4_1009	DJI_0361	Galium verum L.	0	n	1	0	Rubiaceae Juss.
Iz_4_1009	DJI_0361	Genista tinctoria L.	0	n	1	0	Fabaceae Lindl.
Iz_4_1009	DJI_0361	Geranium robertianum L.	0	n	1	0	Geraniaceae Juss.
Iz_4_1009	DJI_0361	Geranium sanquineum L.	0	n	1	0	Geraniaceae Juss.
Iz_4_1009	DJI_0361	Gypsophila paniculata L.	0	n	1	0	Caryophyllaceae Juss.
Iz_4_1009	DJI_0361	Helichrysum arenarium (L.) Moench	0	n	1	0	Asteraceae Bercht. & J.Presl
Iz_4_1009	DJI_0361	Herniaria polygama J.Gay	0	n	1	0	Caryophyllaceae Juss.
Iz_4_1009	DJI_0361	Hieracium robustum Fr.	0	n	1	0	Asteraceae Bercht. & J.Presl
Iz_4_1009	DJI_0361	Hieracium umbellatum L.	0	n	1	NA	Asteraceae Bercht. & J.Presl
Iz_4_1009	DJI_0361	Hylotelephium maximum (L.) Holub	0	n	1	0	Crassulaceae J.St.-Hil.
Iz_4_1009	DJI_0361	Jacobaea borysthenica (DC.) B.Nord. & Greuter	0	n	1	0	Asteraceae Bercht. & J.Presl
Iz_4_1009	DJI_0361	Jasione montana L.	0	n	1	0	Campanulaceae Juss.
Iz_4_1009	DJI_0361	Jurinea cyanoides (L.) Rchb.s.l.	0	n	1	0	Asteraceae Bercht. & J.Presl
Iz_4_1009	DJI_0361	Koeleria glauca (Spreng.) DC.	0	n	1	0	Poaceae Barnhart
Iz_4_1009	DJI_0361	Lactuca serriola L.	0	a	0	0	Asteraceae Bercht. & J.Presl
Iz_4_1009	DJI_0361	Linaria genistifolia (L.) Mill.	0	n	1	0	Plantaginaceae Juss.
Iz_4_1009	DJI_0361	Lomelosia argentea (L.) Greuter & Burdet	0	n	1	0	Caprifoliaceae Juss.
Iz_4_1009	DJI_0361	Melampyrum pratense L.	0	n	1	0	Orobanchaceae Vent.
Iz_4_1009	DJI_0361	Oenothera biennis L.	0	a	0	0	Onagraceae Juss.
Iz_4_1009	DJI_0361	Pentanema britannica (L.) D.Gut.Larr., Santos-Vicente, Anderb., E.Rico & M.M.Mart.Ort.	0	n	1	0	Asteraceae Bercht. & J.Presl
Iz_4_1009	DJI_0361	Peucedanum oreoselinum (L.) Moench	0	n	1	0	Apiaceae Lindl.
Iz_4_1009	DJI_0361	Peucedanum ruthenicum M.Bieb.	0	n	1	0	Apiaceae Lindl.
Iz_4_1009	DJI_0361	Pilosella echioides (Lumn.) F.W.Schultz & Sch.Bip.	0	n	1	0	Asteraceae Bercht. & J.Presl
Iz_4_1009	DJI_0361	Pilosella officinarum F. Schultz et Sch. Bip.	0	n	1	0	Asteraceae Bercht. & J.Presl
Iz_4_1009	DJI_0361	Pilosella sp.	0		NA	0	Asteraceae Bercht. & J.Presl
Iz_4_1009	DJI_0361	Pinus sylvestris L.	0	n	1	1	Pinaceae Spreng. ex F.Rudolphi
Iz_4_1009	DJI_0361	Poa annua L.	0	n	1	0	Poaceae Barnhart
Iz_4_1009	DJI_0361	Poa nemoralis L.	0	n	1	0	Poaceae Barnhart
Iz_4_1009	DJI_0361	Poa sp.	0		NA	0	Poaceae Barnhart
Iz_4_1009	DJI_0361	Polygonatum odoratum (Mill.) Druce	0	n	1	0	Asparagaceae Juss.
Iz_4_1009	DJI_0361	Polygonum arenarium Waldst. & Kit.	0	n	1	0	Polygonaceae Juss.
Iz_4_1009	DJI_0361	Populus nigra L.	0	n	1	1	Salicaceae Mirb.
Iz_4_1009	DJI_0361	Populus tremula L.	0	n	1	1	Salicaceae Mirb.
Iz_4_1009	DJI_0361	Populus Ã canescens (Aiton) Sm.	0	n	1	1	Salicaceae Mirb.
Iz_4_1009	DJI_0361	Potentilla argentea L.	0	n	1	0	Rosaceae Juss.
Iz_4_1009	DJI_0361	Psammophiliella muralis (L.) Ikonn.	0	n	1	0	Caryophyllaceae Juss.
Iz_4_1009	DJI_0361	Psephellus sumensis (Kalen.) Greuter	0	n	1	0	Asteraceae Bercht. & J.Presl
Iz_4_1009	DJI_0361	Pulsatilla pratensis (L.) Mill. s.l.	0	n	1	0	Ranunculaceae Juss.
Iz_4_1009	DJI_0361	Pyrus communis L.	0	a	0	1	Rosaceae Juss.
Iz_4_1009	DJI_0361	Quercus robur L.	0	n	1	1	Fagaceae Dumort.
Iz_4_1009	DJI_0361	Robinia pseudoacacia L.	0	a	0	1	Fabaceae Lindl.
Iz_4_1009	DJI_0361	Rosa sp.	0		NA	1	Rosaceae Juss.
Iz_4_1009	DJI_0361	Rubus idaeus L.	0	n	1	0	Rosaceae Juss.
Iz_4_1009	DJI_0361	Rumex acetosella L.	0	n	1	0	Polygonaceae Juss.
Iz_4_1009	DJI_0361	Rumex thyrsiflorus Fingerh.	0	n	1	0	Polygonaceae Juss.
Iz_4_1009	DJI_0361	Salix alba L.	0	n	1	1	Salicaceae Mirb.
Iz_4_1009	DJI_0361	Sambucus nigra L.	0	n	1	1	Viburnaceae Raf.
Iz_4_1009	DJI_0361	Secale sylvestre Host	0	n	1	0	Poaceae Barnhart
Iz_4_1009	DJI_0361	Sempervivum ruthenicum Schnittsp. & C.B. Lehm.	0	n	1	0	Crassulaceae J.St.-Hil.
Iz_4_1009	DJI_0361	Setaria viridis (L.) P. Beauv.	0	n	1	0	Poaceae Barnhart
Iz_4_1009	DJI_0361	Silene borysthenica (Gruner) Walters	0	n	1	0	Caryophyllaceae Juss.
Iz_4_1009	DJI_0361	Silene latifolia subsp. alba (Mill.) Greuter & Burdet	0	n	1	0	Caryophyllaceae Juss.
Iz_4_1009	DJI_0361	Solidago virgaurea L.	0	n	1	0	Asteraceae Bercht. & J.Presl
Iz_4_1009	DJI_0361	Sorbus aucuparia L.	0	n	1	1	Rosaceae Juss.
Iz_4_1009	DJI_0361	Stachys recta L.	0	n	1	0	Lamiacea Lindl.
Iz_4_1009	DJI_0361	Tanacetum vulgare L.	0	n	1	0	Asteraceae Bercht. & J.Presl
Iz_4_1009	DJI_0361	Thymus pallasianus Heinr.Braun	0	n	1	0	Lamiacea Lindl.
Iz_4_1009	DJI_0361	Tilia cordata Mill.	0	n	1	1	Malvaceae Juss.
Iz_4_1009	DJI_0361	Trifolium alpestre L.	0	n	1	0	Fabaceae Lindl.
Iz_4_1009	DJI_0361	Trifolium arvense L.	0	n	1	0	Fabaceae Lindl.
Iz_4_1009	DJI_0361	Ulmus glabra Huds.	0	n	1	1	Ulmaceae Mirb.
Iz_4_1009	DJI_0361	Verbascum densiflorum Bertol.	0	n	1	0	Scrophulariaceae Juss.
Iz_4_1009	DJI_0361	Verbascum nigrum L.	0	n	1	0	Scrophulariaceae Juss.
Iz_4_1009	DJI_0361	Veronica austriaca L.	0	n	1	0	Plantaginaceae Juss.
Iz_4_1009	DJI_0361	Veronica incana L.	0	n	1	0	Plantaginaceae Juss.
Iz_4_1009	DJI_0361	Veronica verna L.	0	n	1	0	Plantaginaceae Juss.
Iz_4_1009	DJI_0361	Vincetoxicum hirundinaria Medik	0	n	1	0	Apocynaceae Juss.
Iz_4_1009	DJI_0362	Acer tataricum L.	0	n	1	1	Sapindaceae Juss.
Iz_4_1009	DJI_0362	Achillea millefolium L. s.l.	0	n	1	0	Asteraceae Bercht. & J.Presl
Iz_4_1009	DJI_0362	Allium oleraceum L.	0	n	1	0	Amaryllidaceae J.St.-Hil.
Iz_4_1009	DJI_0362	Ambrosia artemisiifolia L.	0	a	0	0	Asteraceae Bercht. & J.Presl
Iz_4_1009	DJI_0362	Amorpha fruticosa L.	0	a	0	0	Fabaceae Lindl.
Iz_4_1009	DJI_0362	Anthericum ramosum L.	0	n	1	0	Asparagaceae Juss.
Iz_4_1009	DJI_0362	Artemisia marschalliana Spreng.	0	n	1	0	Asteraceae Bercht. & J.Presl
Iz_4_1009	DJI_0362	Asparagus officinalis L.	0	n	1	0	Asparagaceae Juss.
Iz_4_1009	DJI_0362	Astragalus cicer L.	0	n	1	0	Fabaceae Lindl.
Iz_4_1009	DJI_0362	Bassia laniflora (S.G.Gmel.) A.J.Scott	0	n	1	0	Amaranthaceae Juss.
Iz_4_1009	DJI_0362	Berberis vulgaris L.	0	n	1	0	Berberidaceae Juss.
Iz_4_1009	DJI_0362	Betula pendula Roth.	0	n	1	1	Betulaceae Gray
Iz_4_1009	DJI_0362	Betula pendula Roth.	0	n	1	0	Poaceae Barnhart
Iz_4_1009	DJI_0362	Bromus inermis Leyss.	0		NA	NA	
Iz_4_1009	DJI_0362	Bromus squarrosus L.	0	a	0	0	Poaceae Barnhart
Iz_4_1009	DJI_0362	Bromus tectorum L.	0	a	0	0	Poaceae Barnhart
Iz_4_1009	DJI_0362	Calamagrostis epigejos (L.) Roth	2	n	1	0	Poaceae Barnhart
Iz_4_1009	DJI_0362	Carduus acanthoides L.	0	n	1	0	Asteraceae Bercht. & J.Presl
Iz_4_1009	DJI_0362	Carex sp.	0	n	1	0	Cyperaceae Juss.
Iz_4_1009	DJI_0362	Centaurea majorovii Dumbadze	0	n	1	0	Asteraceae Bercht. & J.Presl
Iz_4_1009	DJI_0362	Chamaecytisus ruthenicus (Fisch.ex Wol.) Klaskova	0	n	1	0	Fabaceae Lindl.
Iz_4_1009	DJI_0362	Chelidonium majus L.	0	n	1	0	Papaveraceae Juss.
Iz_4_1009	DJI_0362	Chenopodium betaceum Andrz.	0	n	1	0	Amaranthaceae Juss.
Iz_4_1009	DJI_0362	Chenopodium cf. album	0	n	1	0	Amaranthaceae Juss.
Iz_4_1009	DJI_0362	Chenopodium sp.	0		NA	0	Amaranthaceae Juss.
Iz_4_1009	DJI_0362	Chondrilla juncea L.	0	n	1	0	Asteraceae Bercht. & J.Presl
Iz_4_1009	DJI_0362	Cirsium vulgare (Savi) Ten.	0	n	1	0	Asteraceae Bercht. & J.Presl
Iz_4_1009	DJI_0362	Clinopodium acinos (L.) Kuntze	0	n	1	0	Lamiacea Lindl.
Iz_4_1009	DJI_0362	Convallaria majalis L.	0	n	1	0	Asparagaceae Juss.

Iz_4_1009	DJI_0362	Crataegus rhipidophylla Gand.	0	n	1	1	Rosaceae Juss.
Iz_4_1009	DJI_0362	Crepis foetida L.	0	n	1	0	Asteraceae Bercht. & J.Presl
Iz_4_1009	DJI_0362	Dactylis glomerata L.	0	n	1	0	Poaceae Barnhart
Iz_4_1009	DJI_0362	Dianthus campestris M.Bieb.	0	n	1	0	Caryophyllaceae Juss.
Iz_4_1009	DJI_0362	Digitaria sanguinalis (L.) Scop.	0	a	0	0	Poaceae Barnhart
Iz_4_1009	DJI_0362	Elymus repens (L.) Gould	0	n	1	0	Poaceae Barnhart
Iz_4_1009	DJI_0362	Epilobium tetragonum L.	0	n	1	0	Onagraceae Juss.
Iz_4_1009	DJI_0362	Eragrostis minor Host	0	a	0	0	Poaceae Barnhart
Iz_4_1009	DJI_0362	Erigeron canadensis L.	0	a	0	0	Asteraceae Bercht. & J.Presl
Iz_4_1009	DJI_0362	Euphorbia seguieriana Neck.	0	n	1	0	Euphorbiaceae Juss.
Iz_4_1009	DJI_0362	Fallopia dumetorum (L.) Holub	0	n	1	0	Polygonaceae Juss.
Iz_4_1009	DJI_0362	Festuca beckeri (Hack.) Trautv.	0	n	1	0	Poaceae Barnhart
Iz_4_1009	DJI_0362	Fragaria vesca L.	0	n	1	0	Rosaceae Juss.
Iz_4_1009	DJI_0362	Frangula alnus Mill.	0	n	1	1	Rhamnaceae Juss.
Iz_4_1009	DJI_0362	Fraxinus pennsylvanica Marshall	0	a	0	1	Oleaceae Hoffmanns. & Link
Iz_4_1009	DJI_0362	Galeopsis bifida Boenn.	0	n	1	0	Lamiacea Lindl.
Iz_4_1009	DJI_0362	Galium verum L.	0	n	1	0	Rubiaceae Juss.
Iz_4_1009	DJI_0362	Genista tinctoria L.	0	n	1	0	Fabaceae Lindl.
Iz_4_1009	DJI_0362	Geranium robertianum L.	0	n	1	0	Geraniaceae Juss.
Iz_4_1009	DJI_0362	Geranium sanquineum L.	0	n	1	0	Geraniaceae Juss.
Iz_4_1009	DJI_0362	Gypsophila paniculata L.	0	n	1	0	Caryophyllaceae Juss.
Iz_4_1009	DJI_0362	Helichrysum arenarium (L.) Moench	0	n	1	0	Asteraceae Bercht. & J.Presl
Iz_4_1009	DJI_0362	Herniaria polygama J.Gay	0	n	1	0	Caryophyllaceae Juss.
Iz_4_1009	DJI_0362	Hieracium robustum Fr.	0	n	1	0	Asteraceae Bercht. & J.Presl
Iz_4_1009	DJI_0362	Hieracium umbellatum L.	0	n	1	NA	Asteraceae Bercht. & J.Presl
Iz_4_1009	DJI_0362	Hylotelephium maximum (L.) Holub	0	n	1	0	Crassulaceae J.St.-Hil.
Iz_4_1009	DJI_0362	Jacobaea borysthenica (DC.) B.Nord. & Greuter	0	n	1	0	Asteraceae Bercht. & J.Presl
Iz_4_1009	DJI_0362	Jasione montana L.	0	n	1	0	Campanulaceae Juss.
Iz_4_1009	DJI_0362	Jurinea cyanoides (L.) Rchb.s.l.	0	n	1	0	Asteraceae Bercht. & J.Presl
Iz_4_1009	DJI_0362	Koeleria glauca (Spreng.) DC.	0	n	1	0	Poaceae Barnhart
Iz_4_1009	DJI_0362	Lactuca serriola L.	0	a	0	0	Asteraceae Bercht. & J.Presl
Iz_4_1009	DJI_0362	Linaria genistifolia (L.) Mill.	0	n	1	0	Plantaginaceae Juss.
Iz_4_1009	DJI_0362	Lomelosia argentea (L.) Greuter & Burdet	0	n	1	0	Caprifoliaceae Juss.
Iz_4_1009	DJI_0362	Melampyrum pratense L.	0	n	1	0	Orobanchaceae Vent.
Iz_4_1009	DJI_0362	Oenothera biennis L.	0	a	0	0	Onagraceae Juss.
Iz_4_1009	DJI_0362	Pentanema britannica (L.) D.Gut.Larr., Santos-Vicente, Anderb., E.Rico & M.M.Mart.Ort.	0	n	1	0	Asteraceae Bercht. & J.Presl
Iz_4_1009	DJI_0362	Peucedanum oreoselinum (L.) Moench	0	n	1	0	Apiaceae Lindl.
Iz_4_1009	DJI_0362	Peucedanum ruthenicum M.Bieb.	0	n	1	0	Apiaceae Lindl.
Iz_4_1009	DJI_0362	Pilosella echioides (Lumn.) F.W.Schultz & Sch.Bip.	0	n	1	0	Asteraceae Bercht. & J.Presl
Iz_4_1009	DJI_0362	Pilosella officinarum F. Schultz et Sch. Bip.	0	n	1	0	Asteraceae Bercht. & J.Presl
Iz_4_1009	DJI_0362	Pilosella sp.	0		NA	0	Asteraceae Bercht. & J.Presl
Iz_4_1009	DJI_0362	Pinus sylvestris L.	0	n	1	1	Pinaceae Spreng. ex F.Rudolphi
Iz_4_1009	DJI_0362	Poa annua L.	0	n	1	0	Poaceae Barnhart
Iz_4_1009	DJI_0362	Poa nemoralis L.	0	n	1	0	Poaceae Barnhart
Iz_4_1009	DJI_0362	Poa sp.	0		NA	0	Poaceae Barnhart
Iz_4_1009	DJI_0362	Polygonatum odoratum (Mill.) Druce	0	n	1	0	Asparagaceae Juss.
Iz_4_1009	DJI_0362	Polygonum arenarium Waldst. & Kit.	0	n	1	0	Polygonaceae Juss.
Iz_4_1009	DJI_0362	Populus nigra L.	0	n	1	1	Salicaceae Mirb.
Iz_4_1009	DJI_0362	Populus tremula L.	0	n	1	1	Salicaceae Mirb.
Iz_4_1009	DJI_0362	Populus Ã canescens (Aiton) Sm.	0	n	1	1	Salicaceae Mirb.
Iz_4_1009	DJI_0362	Potentilla argentea L.	0	n	1	0	Rosaceae Juss.
Iz_4_1009	DJI_0362	Psammophiliella muralis (L.) Ikonn.	0	n	1	0	Caryophyllaceae Juss.
Iz_4_1009	DJI_0362	Psephellus sumensis (Kalen.) Greuter	0	n	1	0	Asteraceae Bercht. & J.Presl
Iz_4_1009	DJI_0362	Pulsatilla pratensis (L.) Mill. s.l.	0	n	1	0	Ranunculaceae Juss.
Iz_4_1009	DJI_0362	Pyrus communis L.	0	a	0	1	Rosaceae Juss.
Iz_4_1009	DJI_0362	Quercus robur L.	0	n	1	1	Fagaceae Dumort.
Iz_4_1009	DJI_0362	Robinia pseudoacacia L.	0	a	0	1	Fabaceae Lindl.
Iz_4_1009	DJI_0362	Rosa sp.	0		NA	1	Rosaceae Juss.
Iz_4_1009	DJI_0362	Rubus idaeus L.	0	n	1	0	Rosaceae Juss.
Iz_4_1009	DJI_0362	Rumex acetosella L.	0	n	1	0	Polygonaceae Juss.
Iz_4_1009	DJI_0362	Rumex thyrsiflorus Fingerh.	0	n	1	0	Polygonaceae Juss.
Iz_4_1009	DJI_0362	Salix alba L.	0	n	1	1	Salicaceae Mirb.
Iz_4_1009	DJI_0362	Sambucus nigra L.	0	n	1	1	Viburnaceae Raf.
Iz_4_1009	DJI_0362	Secale sylvestre Host	0	n	1	0	Poaceae Barnhart
Iz_4_1009	DJI_0362	Sempervivum ruthenicum Schnittsp. & C.B. Lehm.	0	n	1	0	Crassulaceae J.St.-Hil.
Iz_4_1009	DJI_0362	Setaria viridis (L.) P. Beauv.	0	n	1	0	Poaceae Barnhart
Iz_4_1009	DJI_0362	Silene borysthenica (Gruner) Walters	0	n	1	0	Caryophyllaceae Juss.
Iz_4_1009	DJI_0362	Silene latifolia subsp. alba (Mill.) Greuter & Burdet	0	n	1	0	Caryophyllaceae Juss.
Iz_4_1009	DJI_0362	Solidago virgaurea L.	0	n	1	0	Asteraceae Bercht. & J.Presl
Iz_4_1009	DJI_0362	Sorbus aucuparia L.	0	n	1	1	Rosaceae Juss.
Iz_4_1009	DJI_0362	Stachys recta L.	0	n	1	0	Lamiacea Lindl.
Iz_4_1009	DJI_0362	Tanacetum vulgare L.	0	n	1	0	Asteraceae Bercht. & J.Presl
Iz_4_1009	DJI_0362	Thymus pallasianus Heinr.Braun	0	n	1	0	Lamiacea Lindl.
Iz_4_1009	DJI_0362	Tilia cordata Mill.	0	n	1	1	Malvaceae Juss.
Iz_4_1009	DJI_0362	Trifolium alpestre L.	0	n	1	0	Fabaceae Lindl.
Iz_4_1009	DJI_0362	Trifolium arvense L.	0	n	1	0	Fabaceae Lindl.
Iz_4_1009	DJI_0362	Ulmus glabra Huds.	0	n	1	1	Ulmaceae Mirb.
Iz_4_1009	DJI_0362	Verbascum densiflorum Bertol.	0	n	1	0	Scrophulariaceae Juss.
Iz_4_1009	DJI_0362	Verbascum nigrum L.	0	n	1	0	Scrophulariaceae Juss.
Iz_4_1009	DJI_0362	Veronica austriaca L.	0	n	1	0	Plantaginaceae Juss.
Iz_4_1009	DJI_0362	Veronica incana L.	0	n	1	0	Plantaginaceae Juss.
Iz_4_1009	DJI_0362	Veronica verna L.	0	n	1	0	Plantaginaceae Juss.
Iz_4_1009	DJI_0362	Vincetoxicum hirundinaria Medik	0	n	1	0	Apocynaceae Juss.
Iz_4_1009	DJI_0363	Acer tataricum L.	0	n	1	1	Sapindaceae Juss.
Iz_4_1009	DJI_0363	Achillea millefolium L. s.l.	0	n	1	0	Asteraceae Bercht. & J.Presl
Iz_4_1009	DJI_0363	Allium oleraceum L.	0	n	1	0	Amaryllidaceae J.St.-Hil.
Iz_4_1009	DJI_0363	Ambrosia artemisiifolia L.	0	a	0	0	Asteraceae Bercht. & J.Presl
Iz_4_1009	DJI_0363	Amorpha fruticosa L.	0	a	0	0	Fabaceae Lindl.
Iz_4_1009	DJI_0363	Anthericum ramosum L.	0	n	1	0	Asparagaceae Juss.
Iz_4_1009	DJI_0363	Artemisia marschalliana Spreng.	0	n	1	0	Asteraceae Bercht. & J.Presl
Iz_4_1009	DJI_0363	Asparagus officinalis L.	0	n	1	0	Asparagaceae Juss.
Iz_4_1009	DJI_0363	Astragalus cicer L.	0	n	1	0	Fabaceae Lindl.
Iz_4_1009	DJI_0363	Bassia laniflora (S.G.Gmel.) A.J.Scott	0	n	1	0	Amaranthaceae Juss.
Iz_4_1009	DJI_0363	Berberis vulgaris L.	0	n	1	0	Berberidaceae Juss.
Iz_4_1009	DJI_0363	Betula pendula Roth.	0	n	1	1	Betulaceae Gray
Iz_4_1009	DJI_0363	Betula pendula Roth.	0	n	1	0	Poaceae Barnhart
Iz_4_1009	DJI_0363	Bromus inermis Leyss.	0		NA	NA	
Iz_4_1009	DJI_0363	Bromus squarrosus L.	0	a	0	0	Poaceae Barnhart

Iz_4_1009	DJI_0363	Bromus tectorum L.	0	a	0	0	Poaceae Barnhart
Iz_4_1009	DJI_0363	Calamagrostis epigejos (L.) Roth	25	n	1	0	Poaceae Barnhart
Iz_4_1009	DJI_0363	Carduus acanthoides L.	0	n	1	0	Asteraceae Bercht. & J.Presl
Iz_4_1009	DJI_0363	Carex sp.	0	n	1	0	Cyperaceae Juss.
Iz_4_1009	DJI_0363	Centaurea majorovii Dumbadze	0	n	1	0	Asteraceae Bercht. & J.Presl
Iz_4_1009	DJI_0363	Chamaecytisus ruthenicus (Fisch.ex Wol.) Klaskova	0	n	1	0	Fabaceae Lindl.
Iz_4_1009	DJI_0363	Chelidonium majus L.	0	n	1	0	Papaveraceae Juss.
Iz_4_1009	DJI_0363	Chenopodium betaceum Andrz.	0	n	1	0	Amaranthaceae Juss.
Iz_4_1009	DJI_0363	Chenopodium cf. album	0	n	1	0	Amaranthaceae Juss.
Iz_4_1009	DJI_0363	Chenopodium sp.	0		NA	0	Amaranthaceae Juss.
Iz_4_1009	DJI_0363	Chondrilla juncea L.	0	n	1	0	Asteraceae Bercht. & J.Presl
Iz_4_1009	DJI_0363	Cirsium vulgare (Savi) Ten.	0	n	1	0	Asteraceae Bercht. & J.Presl
Iz_4_1009	DJI_0363	Clinopodium acinos (L.) Kuntze	0	n	1	0	Lamiacea Lindl.
Iz_4_1009	DJI_0363	Convallaria majalis L.	0	n	1	0	Asparagaceae Juss.
Iz_4_1009	DJI_0363	Crataegus rhipidophylla Gand.	0	n	1	1	Rosaceae Juss.
Iz_4_1009	DJI_0363	Crepis foetida L.	0	n	1	0	Asteraceae Bercht. & J.Presl
Iz_4_1009	DJI_0363	Dactylis glomerata L.	0	n	1	0	Poaceae Barnhart
Iz_4_1009	DJI_0363	Dianthus campestris M.Bieb.	0	n	1	0	Caryophyllaceae Juss.
Iz_4_1009	DJI_0363	Digitaria sanguinalis (L.) Scop.	0	a	0	0	Poaceae Barnhart
Iz_4_1009	DJI_0363	Elymus repens (L.) Gould	0	n	1	0	Poaceae Barnhart
Iz_4_1009	DJI_0363	Epilobium tetragonum L.	0	n	1	0	Onagraceae Juss.
Iz_4_1009	DJI_0363	Eragrostis minor Host	0	a	0	0	Poaceae Barnhart
Iz_4_1009	DJI_0363	Erigeron canadensis L.	3	a	0	0	Asteraceae Bercht. & J.Presl
Iz_4_1009	DJI_0363	Euphorbia seguieriana Neck.	0	n	1	0	Euphorbiaceae Juss.
Iz_4_1009	DJI_0363	Fallopia dumetorum (L.) Holub	0	n	1	0	Polygonaceae Juss.
Iz_4_1009	DJI_0363	Festuca beckeri (Hack.) Trautv.	0	n	1	0	Poaceae Barnhart
Iz_4_1009	DJI_0363	Fragaria vesca L.	0	n	1	0	Rosaceae Juss.
Iz_4_1009	DJI_0363	Frangula alnus Mill.	0	n	1	1	Rhamnaceae Juss.
Iz_4_1009	DJI_0363	Fraxinus pennsylvanica Marshall	0	a	0	1	Oleaceae Hoffmanns. & Link
Iz_4_1009	DJI_0363	Galeopsis bifida Boenn.	0	n	1	0	Lamiacea Lindl.
Iz_4_1009	DJI_0363	Galium verum L.	0	n	1	0	Rubiaceae Juss.
Iz_4_1009	DJI_0363	Genista tinctoria L.	0	n	1	0	Fabaceae Lindl.
Iz_4_1009	DJI_0363	Geranium robertianum L.	0	n	1	0	Geraniaceae Juss.
Iz_4_1009	DJI_0363	Geranium sanquineum L.	0	n	1	0	Geraniaceae Juss.
Iz_4_1009	DJI_0363	Gypsophila paniculata L.	0	n	1	0	Caryophyllaceae Juss.
Iz_4_1009	DJI_0363	Helichrysum arenarium (L.) Moench	0	n	1	0	Asteraceae Bercht. & J.Presl
Iz_4_1009	DJI_0363	Herniaria polygama J.Gay	0	n	1	0	Caryophyllaceae Juss.
Iz_4_1009	DJI_0363	Hieracium robustum Fr.	0	n	1	0	Asteraceae Bercht. & J.Presl
Iz_4_1009	DJI_0363	Hieracium umbellatum L.	0	n	1	NA	Asteraceae Bercht. & J.Presl
Iz_4_1009	DJI_0363	Hylotelephium maximum (L.) Holub	0	n	1	0	Crassulaceae J.St.-Hil.
Iz_4_1009	DJI_0363	Jacobaea borysthenica (DC.) B.Nord. & Greuter	0	n	1	0	Asteraceae Bercht. & J.Presl
Iz_4_1009	DJI_0363	Jasione montana L.	0	n	1	0	Campanulaceae Juss.
Iz_4_1009	DJI_0363	Jurinea cyanoides (L.) Rchb.s.l.	0	n	1	0	Asteraceae Bercht. & J.Presl
Iz_4_1009	DJI_0363	Koeleria glauca (Spreng.) DC.	0	n	1	0	Poaceae Barnhart
Iz_4_1009	DJI_0363	Lactuca serriola L.	0	a	0	0	Asteraceae Bercht. & J.Presl
Iz_4_1009	DJI_0363	Linaria genistifolia (L.) Mill.	0	n	1	0	Plantaginaceae Juss.
Iz_4_1009	DJI_0363	Lomelosia argentea (L.) Greuter & Burdet	0	n	1	0	Caprifoliaceae Juss.
Iz_4_1009	DJI_0363	Melampyrum pratense L.	0	n	1	0	Orobanchaceae Vent.
Iz_4_1009	DJI_0363	Oenothera biennis L.	0	a	0	0	Onagraceae Juss.
Iz_4_1009	DJI_0363	Pentanema britannica (L.) D.Gut.Larr., Santos-Vicente, Anderb., E.Rico & M.M.Mart.Ort.	0	n	1	0	Asteraceae Bercht. & J.Presl
Iz_4_1009	DJI_0363	Peucedanum oreoselinum (L.) Moench	0	n	1	0	Apiaceae Lindl.
Iz_4_1009	DJI_0363	Peucedanum ruthenicum M.Bieb.	0	n	1	0	Apiaceae Lindl.
Iz_4_1009	DJI_0363	Pilosella echioides (Lumn.) F.W.Schultz & Sch.Bip.	0	n	1	0	Asteraceae Bercht. & J.Presl
Iz_4_1009	DJI_0363	Pilosella officinarum F. Schultz et Sch. Bip.	0	n	1	0	Asteraceae Bercht. & J.Presl
Iz_4_1009	DJI_0363	Pilosella sp.	0		NA	0	Asteraceae Bercht. & J.Presl
Iz_4_1009	DJI_0363	Pinus sylvestris L.	0	n	1	1	Pinaceae Spreng. ex F.Rudolphi
Iz_4_1009	DJI_0363	Poa annua L.	0	n	1	0	Poaceae Barnhart
Iz_4_1009	DJI_0363	Poa nemoralis L.	0	n	1	0	Poaceae Barnhart
Iz_4_1009	DJI_0363	Poa sp.	0		NA	0	Poaceae Barnhart
Iz_4_1009	DJI_0363	Polygonatum odoratum (Mill.) Druce	0	n	1	0	Asparagaceae Juss.
Iz_4_1009	DJI_0363	Polygonum arenarium Waldst. & Kit.	0	n	1	0	Polygonaceae Juss.
Iz_4_1009	DJI_0363	Populus nigra L.	0	n	1	1	Salicaceae Mirb.
Iz_4_1009	DJI_0363	Populus tremula L.	0	n	1	1	Salicaceae Mirb.
Iz_4_1009	DJI_0363	Populus Ã canescens (Aiton) Sm.	0	n	1	1	Salicaceae Mirb.
Iz_4_1009	DJI_0363	Potentilla argentea L.	0	n	1	0	Rosaceae Juss.
Iz_4_1009	DJI_0363	Psammophiliella muralis (L.) Ikonn.	0	n	1	0	Caryophyllaceae Juss.
Iz_4_1009	DJI_0363	Psephellus sumensis (Kalen.) Greuter	0	n	1	0	Asteraceae Bercht. & J.Presl
Iz_4_1009	DJI_0363	Pulsatilla pratensis (L.) Mill. s.l.	0	n	1	0	Ranunculaceae Juss.
Iz_4_1009	DJI_0363	Pyrus communis L.	0	a	0	1	Rosaceae Juss.
Iz_4_1009	DJI_0363	Quercus robur L.	0	n	1	1	Fagaceae Dumort.
Iz_4_1009	DJI_0363	Robinia pseudoacacia L.	0	a	0	1	Fabaceae Lindl.
Iz_4_1009	DJI_0363	Rosa sp.	0		NA	1	Rosaceae Juss.
Iz_4_1009	DJI_0363	Rubus idaeus L.	0	n	1	0	Rosaceae Juss.
Iz_4_1009	DJI_0363	Rumex acetosella L.	0	n	1	0	Polygonaceae Juss.
Iz_4_1009	DJI_0363	Rumex thyrsiflorus Fingerh.	0	n	1	0	Polygonaceae Juss.
Iz_4_1009	DJI_0363	Salix alba L.	0	n	1	1	Salicaceae Mirb.
Iz_4_1009	DJI_0363	Sambucus nigra L.	0	n	1	1	Viburnaceae Raf.
Iz_4_1009	DJI_0363	Secale sylvestre Host	0	n	1	0	Poaceae Barnhart
Iz_4_1009	DJI_0363	Sempervivum ruthenicum Schnittsp. & C.B. Lehm.	0	n	1	0	Crassulaceae J.St.-Hil.
Iz_4_1009	DJI_0363	Setaria viridis (L.) P. Beauv.	0	n	1	0	Poaceae Barnhart
Iz_4_1009	DJI_0363	Silene borysthenica (Gruner) Walters	0	n	1	0	Caryophyllaceae Juss.
Iz_4_1009	DJI_0363	Silene latifolia subsp. alba (Mill.) Greuter & Burdet	0	n	1	0	Caryophyllaceae Juss.
Iz_4_1009	DJI_0363	Solidago virgaurea L.	0	n	1	0	Asteraceae Bercht. & J.Presl
Iz_4_1009	DJI_0363	Sorbus aucuparia L.	0	n	1	1	Rosaceae Juss.
Iz_4_1009	DJI_0363	Stachys recta L.	0	n	1	0	Lamiacea Lindl.
Iz_4_1009	DJI_0363	Tanacetum vulgare L.	0	n	1	0	Asteraceae Bercht. & J.Presl
Iz_4_1009	DJI_0363	Thymus pallasianus Heinr.Braun	0	n	1	0	Lamiacea Lindl.
Iz_4_1009	DJI_0363	Tilia cordata Mill.	0	n	1	1	Malvaceae Juss.
Iz_4_1009	DJI_0363	Trifolium alpestre L.	0	n	1	0	Fabaceae Lindl.
Iz_4_1009	DJI_0363	Trifolium arvense L.	0	n	1	0	Fabaceae Lindl.
Iz_4_1009	DJI_0363	Ulmus glabra Huds.	0	n	1	1	Ulmaceae Mirb.
Iz_4_1009	DJI_0363	Verbascum densiflorum Bertol.	0	n	1	0	Scrophulariaceae Juss.
Iz_4_1009	DJI_0363	Verbascum nigrum L.	0	n	1	0	Scrophulariaceae Juss.
Iz_4_1009	DJI_0363	Veronica austriaca L.	0	n	1	0	Plantaginaceae Juss.
Iz_4_1009	DJI_0363	Veronica incana L.	0	n	1	0	Plantaginaceae Juss.
Iz_4_1009	DJI_0363	Veronica verna L.	0	n	1	0	Plantaginaceae Juss.
Iz_4_1009	DJI_0363	Vincetoxicum hirundinaria Medik	0	n	1	0	Apocynaceae Juss.
Iz_4_1009	DJI_0364	Acer tataricum L.	0	n	1	1	Sapindaceae Juss.

Iz_4_1009	DJI_0364	Achillea millefolium L. s.l.	0	n	1	0	Asteraceae Bercht. & J.Presl
Iz_4_1009	DJI_0364	Allium oleraceum L.	0	n	1	0	Amaryllidaceae J.St.-Hil.
Iz_4_1009	DJI_0364	Ambrosia artemisiifolia L.	0	a	0	0	Asteraceae Bercht. & J.Presl
Iz_4_1009	DJI_0364	Amorpha fruticosa L.	0	a	0	0	Fabaceae Lindl.
Iz_4_1009	DJI_0364	Anthericum ramosum L.	0	n	1	0	Asparagaceae Juss.
Iz_4_1009	DJI_0364	Artemisia marschalliana Spreng.	0	n	1	0	Asteraceae Bercht. & J.Presl
Iz_4_1009	DJI_0364	Asparagus officinalis L.	0	n	1	0	Asparagaceae Juss.
Iz_4_1009	DJI_0364	Astragalus cicer L.	0	n	1	0	Fabaceae Lindl.
Iz_4_1009	DJI_0364	Bassia laniflora (S.G.Gmel.) A.J.Scott	0	n	1	0	Amaranthaceae Juss.
Iz_4_1009	DJI_0364	Berberis vulgaris L.	0	n	1	0	Berberidaceae Juss.
Iz_4_1009	DJI_0364	Betula pendula Roth.	0	n	1	1	Betulaceae Gray
Iz_4_1009	DJI_0364	Betula pendula Roth.	0	n	1	0	Poaceae Barnhart
Iz_4_1009	DJI_0364	Bromus inermis Leyss.	0		NA	NA	
Iz_4_1009	DJI_0364	Bromus squarrosus L.	0	a	0	0	Poaceae Barnhart
Iz_4_1009	DJI_0364	Bromus tectorum L.	0	a	0	0	Poaceae Barnhart
Iz_4_1009	DJI_0364	Calamagrostis epigejos (L.) Roth	2	n	1	0	Poaceae Barnhart
Iz_4_1009	DJI_0364	Carduus acanthoides L.	0	n	1	0	Asteraceae Bercht. & J.Presl
Iz_4_1009	DJI_0364	Carex sp.	0	n	1	0	Cyperaceae Juss.
Iz_4_1009	DJI_0364	Centaurea majorovii Dumbadze	0	n	1	0	Asteraceae Bercht. & J.Presl
Iz_4_1009	DJI_0364	Chamaecytisus ruthenicus (Fisch.ex Wol.) Klaskova	0.5	n	1	0	Fabaceae Lindl.
Iz_4_1009	DJI_0364	Chelidonium majus L.	0	n	1	0	Papaveraceae Juss.
Iz_4_1009	DJI_0364	Chenopodium betaceum Andrz.	0	n	1	0	Amaranthaceae Juss.
Iz_4_1009	DJI_0364	Chenopodium cf. album	0	n	1	0	Amaranthaceae Juss.
Iz_4_1009	DJI_0364	Chenopodium sp.	0		NA	0	Amaranthaceae Juss.
Iz_4_1009	DJI_0364	Chondrilla juncea L.	0	n	1	0	Asteraceae Bercht. & J.Presl
Iz_4_1009	DJI_0364	Cirsium vulgare (Savi) Ten.	0	n	1	0	Asteraceae Bercht. & J.Presl
Iz_4_1009	DJI_0364	Clinopodium acinos (L.) Kuntze	0	n	1	0	Lamiacea Lindl.
Iz_4_1009	DJI_0364	Convallaria majalis L.	0	n	1	0	Asparagaceae Juss.
Iz_4_1009	DJI_0364	Crataegus rhipidophylla Gand.	0	n	1	1	Rosaceae Juss.
Iz_4_1009	DJI_0364	Crepis foetida L.	0	n	1	0	Asteraceae Bercht. & J.Presl
Iz_4_1009	DJI_0364	Dactylis glomerata L.	0	n	1	0	Poaceae Barnhart
Iz_4_1009	DJI_0364	Dianthus campestris M.Bieb.	0	n	1	0	Caryophyllaceae Juss.
Iz_4_1009	DJI_0364	Digitaria sanguinalis (L.) Scop.	0	a	0	0	Poaceae Barnhart
Iz_4_1009	DJI_0364	Elymus repens (L.) Gould	0	n	1	0	Poaceae Barnhart
Iz_4_1009	DJI_0364	Epilobium tetragonum L.	0	n	1	0	Onagraceae Juss.
Iz_4_1009	DJI_0364	Eragrostis minor Host	0	a	0	0	Poaceae Barnhart
Iz_4_1009	DJI_0364	Erigeron canadensis L.	0	a	0	0	Asteraceae Bercht. & J.Presl
Iz_4_1009	DJI_0364	Euphorbia seguieriana Neck.	0	n	1	0	Euphorbiaceae Juss.
Iz_4_1009	DJI_0364	Fallopia dumetorum (L.) Holub	0	n	1	0	Polygonaceae Juss.
Iz_4_1009	DJI_0364	Festuca beckeri (Hack.) Trautv.	0	n	1	0	Poaceae Barnhart
Iz_4_1009	DJI_0364	Fragaria vesca L.	0	n	1	0	Rosaceae Juss.
Iz_4_1009	DJI_0364	Frangula alnus Mill.	0	n	1	1	Rhamnaceae Juss.
Iz_4_1009	DJI_0364	Fraxinus pennsylvanica Marshall	0	a	0	1	Oleaceae Hoffmanns. & Link
Iz_4_1009	DJI_0364	Galeopsis bifida Boenn.	0	n	1	0	Lamiacea Lindl.
Iz_4_1009	DJI_0364	Galium verum L.	0	n	1	0	Rubiaceae Juss.
Iz_4_1009	DJI_0364	Genista tinctoria L.	0	n	1	0	Fabaceae Lindl.
Iz_4_1009	DJI_0364	Geranium robertianum L.	0	n	1	0	Geraniaceae Juss.
Iz_4_1009	DJI_0364	Geranium sanquineum L.	0	n	1	0	Geraniaceae Juss.
Iz_4_1009	DJI_0364	Gypsophila paniculata L.	0	n	1	0	Caryophyllaceae Juss.
Iz_4_1009	DJI_0364	Helichrysum arenarium (L.) Moench	0	n	1	0	Asteraceae Bercht. & J.Presl
Iz_4_1009	DJI_0364	Herniaria polygama J.Gay	0	n	1	0	Caryophyllaceae Juss.
Iz_4_1009	DJI_0364	Hieracium robustum Fr.	0	n	1	0	Asteraceae Bercht. & J.Presl
Iz_4_1009	DJI_0364	Hieracium umbellatum L.	0	n	1	NA	Asteraceae Bercht. & J.Presl
Iz_4_1009	DJI_0364	Hylotelephium maximum (L.) Holub	0	n	1	0	Crassulaceae J.St.-Hil.
Iz_4_1009	DJI_0364	Jacobaea borysthenica (DC.) B.Nord. & Greuter	0	n	1	0	Asteraceae Bercht. & J.Presl
Iz_4_1009	DJI_0364	Jasione montana L.	0	n	1	0	Campanulaceae Juss.
Iz_4_1009	DJI_0364	Jurinea cyanoides (L.) Rchb.s.l.	0	n	1	0	Asteraceae Bercht. & J.Presl
Iz_4_1009	DJI_0364	Koeleria glauca (Spreng.) DC.	0	n	1	0	Poaceae Barnhart
Iz_4_1009	DJI_0364	Lactuca serriola L.	0	a	0	0	Asteraceae Bercht. & J.Presl
Iz_4_1009	DJI_0364	Linaria genistifolia (L.) Mill.	0	n	1	0	Plantaginaceae Juss.
Iz_4_1009	DJI_0364	Lomelosia argentea (L.) Greuter & Burdet	0	n	1	0	Caprifoliaceae Juss.
Iz_4_1009	DJI_0364	Melampyrum pratense L.	0	n	1	0	Orobanchaceae Vent.
Iz_4_1009	DJI_0364	Oenothera biennis L.	0	a	0	0	Onagraceae Juss.
Iz_4_1009	DJI_0364	Pentanema britannica (L.) D.Gut.Larr., Santos-Vicente, Anderb., E.Rico & M.M.Mart.Ort.	0	n	1	0	Asteraceae Bercht. & J.Presl
Iz_4_1009	DJI_0364	Peucedanum oreoselinum (L.) Moench	0	n	1	0	Apiaceae Lindl.
Iz_4_1009	DJI_0364	Peucedanum ruthenicum M.Bieb.	0	n	1	0	Apiaceae Lindl.
Iz_4_1009	DJI_0364	Pilosella echioides (Lumn.) F.W.Schultz & Sch.Bip.	0	n	1	0	Asteraceae Bercht. & J.Presl
Iz_4_1009	DJI_0364	Pilosella officinarum F. Schultz et Sch. Bip.	0	n	1	0	Asteraceae Bercht. & J.Presl
Iz_4_1009	DJI_0364	Pilosella sp.	0		NA	0	Asteraceae Bercht. & J.Presl
Iz_4_1009	DJI_0364	Pinus sylvestris L.	0	n	1	1	Pinaceae Spreng. ex F.Rudolphi
Iz_4_1009	DJI_0364	Poa annua L.	0	n	1	0	Poaceae Barnhart
Iz_4_1009	DJI_0364	Poa nemoralis L.	0	n	1	0	Poaceae Barnhart
Iz_4_1009	DJI_0364	Poa sp.	0		NA	0	Poaceae Barnhart
Iz_4_1009	DJI_0364	Polygonatum odoratum (Mill.) Druce	0	n	1	0	Asparagaceae Juss.
Iz_4_1009	DJI_0364	Polygonum arenarium Waldst. & Kit.	0	n	1	0	Polygonaceae Juss.
Iz_4_1009	DJI_0364	Populus nigra L.	0	n	1	1	Salicaceae Mirb.
Iz_4_1009	DJI_0364	Populus tremula L.	0	n	1	1	Salicaceae Mirb.
Iz_4_1009	DJI_0364	Populus Ã canescens (Aiton) Sm.	0	n	1	1	Salicaceae Mirb.
Iz_4_1009	DJI_0364	Potentilla argentea L.	0	n	1	0	Rosaceae Juss.
Iz_4_1009	DJI_0364	Psammophiliella muralis (L.) Ikonn.	0	n	1	0	Caryophyllaceae Juss.
Iz_4_1009	DJI_0364	Psephellus sumensis (Kalen.) Greuter	0	n	1	0	Asteraceae Bercht. & J.Presl
Iz_4_1009	DJI_0364	Pulsatilla pratensis (L.) Mill. s.l.	0	n	1	0	Ranunculaceae Juss.
Iz_4_1009	DJI_0364	Pyrus communis L.	0	a	0	1	Rosaceae Juss.
Iz_4_1009	DJI_0364	Quercus robur L.	0	n	1	1	Fagaceae Dumort.
Iz_4_1009	DJI_0364	Robinia pseudoacacia L.	0	a	0	1	Fabaceae Lindl.
Iz_4_1009	DJI_0364	Rosa sp.	0		NA	1	Rosaceae Juss.
Iz_4_1009	DJI_0364	Rubus idaeus L.	0	n	1	0	Rosaceae Juss.
Iz_4_1009	DJI_0364	Rumex acetosella L.	0	n	1	0	Polygonaceae Juss.
Iz_4_1009	DJI_0364	Rumex thyrsiflorus Fingerh.	0	n	1	0	Polygonaceae Juss.
Iz_4_1009	DJI_0364	Salix alba L.	0	n	1	1	Salicaceae Mirb.
Iz_4_1009	DJI_0364	Sambucus nigra L.	0	n	1	1	Viburnaceae Raf.
Iz_4_1009	DJI_0364	Secale sylvestre Host	0	n	1	0	Poaceae Barnhart
Iz_4_1009	DJI_0364	Sempervivum ruthenicum Schnittsp. & C.B. Lehm.	0	n	1	0	Crassulaceae J.St.-Hil.
Iz_4_1009	DJI_0364	Setaria viridis (L.) P. Beauv.	0	n	1	0	Poaceae Barnhart
Iz_4_1009	DJI_0364	Silene borysthenica (Gruner) Walters	0	n	1	0	Caryophyllaceae Juss.
Iz_4_1009	DJI_0364	Silene latifolia subsp. alba (Mill.) Greuter & Burdet	0	n	1	0	Caryophyllaceae Juss.
Iz_4_1009	DJI_0364	Solidago virgaurea L.	0	n	1	0	Asteraceae Bercht. & J.Presl
Iz_4_1009	DJI_0364	Sorbus aucuparia L.	0	n	1	1	Rosaceae Juss.

Iz_4_1009	DJI_0364	Stachys recta L.	0	n	1	0	Lamiacea Lindl.
Iz_4_1009	DJI_0364	Tanacetum vulgare L.	0	n	1	0	Asteraceae Bercht. & J.Presl
Iz_4_1009	DJI_0364	Thymus pallasianus Heinr.Braun	0	n	1	0	Lamiacea Lindl.
Iz_4_1009	DJI_0364	Tilia cordata Mill.	0	n	1	1	Malvaceae Juss.
Iz_4_1009	DJI_0364	Trifolium alpestre L.	0	n	1	0	Fabaceae Lindl.
Iz_4_1009	DJI_0364	Trifolium arvense L.	0	n	1	0	Fabaceae Lindl.
Iz_4_1009	DJI_0364	Ulmus glabra Huds.	0	n	1	1	Ulmaceae Mirb.
Iz_4_1009	DJI_0364	Verbascum densiflorum Bertol.	0	n	1	0	Scrophulariaceae Juss.
Iz_4_1009	DJI_0364	Verbascum nigrum L.	0	n	1	0	Scrophulariaceae Juss.
Iz_4_1009	DJI_0364	Veronica austriaca L.	0	n	1	0	Plantaginaceae Juss.
Iz_4_1009	DJI_0364	Veronica incana L.	0	n	1	0	Plantaginaceae Juss.
Iz_4_1009	DJI_0364	Veronica verna L.	0	n	1	0	Plantaginaceae Juss.
Iz_4_1009	DJI_0364	Vincetoxicum hirundinaria Medik	0	n	1	0	Apocynaceae Juss.
Iz_4_1009	DJI_0365	Acer tataricum L.	0	n	1	1	Sapindaceae Juss.
Iz_4_1009	DJI_0365	Achillea millefolium L. s.l.	0	n	1	0	Asteraceae Bercht. & J.Presl
Iz_4_1009	DJI_0365	Allium oleraceum L.	0	n	1	0	Amaryllidaceae J.St.-Hil.
Iz_4_1009	DJI_0365	Ambrosia artemisiifolia L.	0	a	0	0	Asteraceae Bercht. & J.Presl
Iz_4_1009	DJI_0365	Amorpha fruticosa L.	0	a	0	0	Fabaceae Lindl.
Iz_4_1009	DJI_0365	Anthericum ramosum L.	0	n	1	0	Asparagaceae Juss.
Iz_4_1009	DJI_0365	Artemisia marschalliana Spreng.	0	n	1	0	Asteraceae Bercht. & J.Presl
Iz_4_1009	DJI_0365	Asparagus officinalis L.	0	n	1	0	Asparagaceae Juss.
Iz_4_1009	DJI_0365	Astragalus cicer L.	0	n	1	0	Fabaceae Lindl.
Iz_4_1009	DJI_0365	Bassia laniflora (S.G.Gmel.) A.J.Scott	0	n	1	0	Amaranthaceae Juss.
Iz_4_1009	DJI_0365	Berberis vulgaris L.	0	n	1	0	Berberidaceae Juss.
Iz_4_1009	DJI_0365	Betula pendula Roth.	0	n	1	1	Betulaceae Gray
Iz_4_1009	DJI_0365	Betula pendula Roth.	0	n	1	0	Poaceae Barnhart
Iz_4_1009	DJI_0365	Bromus inermis Leyss.	0		NA	NA	
Iz_4_1009	DJI_0365	Bromus squarrosus L.	0	a	0	0	Poaceae Barnhart
Iz_4_1009	DJI_0365	Bromus tectorum L.	0	a	0	0	Poaceae Barnhart
Iz_4_1009	DJI_0365	Calamagrostis epigejos (L.) Roth	1	n	1	0	Poaceae Barnhart
Iz_4_1009	DJI_0365	Carduus acanthoides L.	0	n	1	0	Asteraceae Bercht. & J.Presl
Iz_4_1009	DJI_0365	Carex sp.	0	n	1	0	Cyperaceae Juss.
Iz_4_1009	DJI_0365	Centaurea majorovii Dumbadze	0	n	1	0	Asteraceae Bercht. & J.Presl
Iz_4_1009	DJI_0365	Chamaecytisus ruthenicus (Fisch.ex Wol.) Klaskova	1	n	1	0	Fabaceae Lindl.
Iz_4_1009	DJI_0365	Chelidonium majus L.	0	n	1	0	Papaveraceae Juss.
Iz_4_1009	DJI_0365	Chenopodium betaceum Andrz.	0	n	1	0	Amaranthaceae Juss.
Iz_4_1009	DJI_0365	Chenopodium cf. album	0	n	1	0	Amaranthaceae Juss.
Iz_4_1009	DJI_0365	Chenopodium sp.	0		NA	0	Amaranthaceae Juss.
Iz_4_1009	DJI_0365	Chondrilla juncea L.	0	n	1	0	Asteraceae Bercht. & J.Presl
Iz_4_1009	DJI_0365	Cirsium vulgare (Savi) Ten.	0	n	1	0	Asteraceae Bercht. & J.Presl
Iz_4_1009	DJI_0365	Clinopodium acinos (L.) Kuntze	0	n	1	0	Lamiacea Lindl.
Iz_4_1009	DJI_0365	Convallaria majalis L.	0	n	1	0	Asparagaceae Juss.
Iz_4_1009	DJI_0365	Crataegus rhipidophylla Gand.	0	n	1	1	Rosaceae Juss.
Iz_4_1009	DJI_0365	Crepis foetida L.	0	n	1	0	Asteraceae Bercht. & J.Presl
Iz_4_1009	DJI_0365	Dactylis glomerata L.	0	n	1	0	Poaceae Barnhart
Iz_4_1009	DJI_0365	Dianthus campestris M.Bieb.	0	n	1	0	Caryophyllaceae Juss.
Iz_4_1009	DJI_0365	Digitaria sanguinalis (L.) Scop.	0	a	0	0	Poaceae Barnhart
Iz_4_1009	DJI_0365	Elymus repens (L.) Gould	0	n	1	0	Poaceae Barnhart
Iz_4_1009	DJI_0365	Epilobium tetragonum L.	0	n	1	0	Onagraceae Juss.
Iz_4_1009	DJI_0365	Eragrostis minor Host	0	a	0	0	Poaceae Barnhart
Iz_4_1009	DJI_0365	Erigeron canadensis L.	0	a	0	0	Asteraceae Bercht. & J.Presl
Iz_4_1009	DJI_0365	Euphorbia seguieriana Neck.	0	n	1	0	Euphorbiaceae Juss.
Iz_4_1009	DJI_0365	Fallopia dumetorum (L.) Holub	0	n	1	0	Polygonaceae Juss.
Iz_4_1009	DJI_0365	Festuca beckeri (Hack.) Trautv.	0	n	1	0	Poaceae Barnhart
Iz_4_1009	DJI_0365	Fragaria vesca L.	0	n	1	0	Rosaceae Juss.
Iz_4_1009	DJI_0365	Frangula alnus Mill.	0	n	1	1	Rhamnaceae Juss.
Iz_4_1009	DJI_0365	Fraxinus pennsylvanica Marshall	0	a	0	1	Oleaceae Hoffmanns. & Link
Iz_4_1009	DJI_0365	Galeopsis bifida Boenn.	0	n	1	0	Lamiacea Lindl.
Iz_4_1009	DJI_0365	Galium verum L.	0	n	1	0	Rubiaceae Juss.
Iz_4_1009	DJI_0365	Genista tinctoria L.	0	n	1	0	Fabaceae Lindl.
Iz_4_1009	DJI_0365	Geranium robertianum L.	0	n	1	0	Geraniaceae Juss.
Iz_4_1009	DJI_0365	Geranium sanquineum L.	0	n	1	0	Geraniaceae Juss.
Iz_4_1009	DJI_0365	Gypsophila paniculata L.	0	n	1	0	Caryophyllaceae Juss.
Iz_4_1009	DJI_0365	Helichrysum arenarium (L.) Moench	0	n	1	0	Asteraceae Bercht. & J.Presl
Iz_4_1009	DJI_0365	Herniaria polygama J.Gay	0	n	1	0	Caryophyllaceae Juss.
Iz_4_1009	DJI_0365	Hieracium robustum Fr.	0	n	1	0	Asteraceae Bercht. & J.Presl
Iz_4_1009	DJI_0365	Hieracium umbellatum L.	0	n	1	NA	Asteraceae Bercht. & J.Presl
Iz_4_1009	DJI_0365	Hylotelephium maximum (L.) Holub	0	n	1	0	Crassulaceae J.St.-Hil.
Iz_4_1009	DJI_0365	Jacobaea borysthenica (DC.) B.Nord. & Greuter	0	n	1	0	Asteraceae Bercht. & J.Presl
Iz_4_1009	DJI_0365	Jasione montana L.	0	n	1	0	Campanulaceae Juss.
Iz_4_1009	DJI_0365	Jurinea cyanoides (L.) Rchb.s.l.	0	n	1	0	Asteraceae Bercht. & J.Presl
Iz_4_1009	DJI_0365	Koeleria glauca (Spreng.) DC.	0	n	1	0	Poaceae Barnhart
Iz_4_1009	DJI_0365	Lactuca serriola L.	0	a	0	0	Asteraceae Bercht. & J.Presl
Iz_4_1009	DJI_0365	Linaria genistifolia (L.) Mill.	0	n	1	0	Plantaginaceae Juss.
Iz_4_1009	DJI_0365	Lomelosia argentea (L.) Greuter & Burdet	0	n	1	0	Caprifoliaceae Juss.
Iz_4_1009	DJI_0365	Melampyrum pratense L.	0	n	1	0	Orobanchaceae Vent.
Iz_4_1009	DJI_0365	Oenothera biennis L.	0	a	0	0	Onagraceae Juss.
Iz_4_1009	DJI_0365	Pentanema britannica (L.) D.Gut.Larr., Santos-Vicente, Anderb., E.Rico & M.M.Mart.Ort.	0	n	1	0	Asteraceae Bercht. & J.Presl
Iz_4_1009	DJI_0365	Peucedanum oreoselinum (L.) Moench	0	n	1	0	Apiaceae Lindl.
Iz_4_1009	DJI_0365	Peucedanum ruthenicum M.Bieb.	0	n	1	0	Apiaceae Lindl.
Iz_4_1009	DJI_0365	Pilosella echioides (Lumn.) F.W.Schultz & Sch.Bip.	0	n	1	0	Asteraceae Bercht. & J.Presl
Iz_4_1009	DJI_0365	Pilosella officinarum F. Schultz et Sch. Bip.	0	n	1	0	Asteraceae Bercht. & J.Presl
Iz_4_1009	DJI_0365	Pilosella sp.	0		NA	0	Asteraceae Bercht. & J.Presl
Iz_4_1009	DJI_0365	Pinus sylvestris L.	0	n	1	1	Pinaceae Spreng. ex F.Rudolphi
Iz_4_1009	DJI_0365	Poa annua L.	0	n	1	0	Poaceae Barnhart
Iz_4_1009	DJI_0365	Poa nemoralis L.	0	n	1	0	Poaceae Barnhart
Iz_4_1009	DJI_0365	Poa sp.	0		NA	0	Poaceae Barnhart
Iz_4_1009	DJI_0365	Polygonatum odoratum (Mill.) Druce	0	n	1	0	Asparagaceae Juss.
Iz_4_1009	DJI_0365	Polygonum arenarium Waldst. & Kit.	0	n	1	0	Polygonaceae Juss.
Iz_4_1009	DJI_0365	Populus nigra L.	0	n	1	1	Salicaceae Mirb.
Iz_4_1009	DJI_0365	Populus tremula L.	0	n	1	1	Salicaceae Mirb.
Iz_4_1009	DJI_0365	Populus Ã canescens (Aiton) Sm.	0	n	1	1	Salicaceae Mirb.
Iz_4_1009	DJI_0365	Potentilla argentea L.	0	n	1	0	Rosaceae Juss.
Iz_4_1009	DJI_0365	Psammophiliella muralis (L.) Ikonn.	0	n	1	0	Caryophyllaceae Juss.
Iz_4_1009	DJI_0365	Psephellus sumensis (Kalen.) Greuter	0	n	1	0	Asteraceae Bercht. & J.Presl
Iz_4_1009	DJI_0365	Pulsatilla pratensis (L.) Mill. s.l.	0	n	1	0	Ranunculaceae Juss.
Iz_4_1009	DJI_0365	Pyrus communis L.	0	a	0	1	Rosaceae Juss.
Iz_4_1009	DJI_0365	Quercus robur L.	0	n	1	1	Fagaceae Dumort.

Iz_4_1009	DJI_0365	Robinia pseudoacacia L.	0	a	0	1	Fabaceae Lindl.
Iz_4_1009	DJI_0365	Rosa sp.	0		NA	1	Rosaceae Juss.
Iz_4_1009	DJI_0365	Rubus idaeus L.	0	n	1	0	Rosaceae Juss.
Iz_4_1009	DJI_0365	Rumex acetosella L.	0.2	n	1	0	Polygonaceae Juss.
Iz_4_1009	DJI_0365	Rumex thyrsiflorus Fingerh.	0	n	1	0	Polygonaceae Juss.
Iz_4_1009	DJI_0365	Salix alba L.	0	n	1	1	Salicaceae Mirb.
Iz_4_1009	DJI_0365	Sambucus nigra L.	0	n	1	1	Viburnaceae Raf.
Iz_4_1009	DJI_0365	Secale sylvestre Host	0	n	1	0	Poaceae Barnhart
Iz_4_1009	DJI_0365	Sempervivum ruthenicum Schnittsp. & C.B. Lehm.	0	n	1	0	Crassulaceae J.St.-Hil.
Iz_4_1009	DJI_0365	Setaria viridis (L.) P. Beauv.	0	n	1	0	Poaceae Barnhart
Iz_4_1009	DJI_0365	Silene borysthenica (Gruner) Walters	0	n	1	0	Caryophyllaceae Juss.
Iz_4_1009	DJI_0365	Silene latifolia subsp. alba (Mill.) Greuter & Burdet	0	n	1	0	Caryophyllaceae Juss.
Iz_4_1009	DJI_0365	Solidago virgaurea L.	0	n	1	0	Asteraceae Bercht. & J.Presl
Iz_4_1009	DJI_0365	Sorbus aucuparia L.	0	n	1	1	Rosaceae Juss.
Iz_4_1009	DJI_0365	Stachys recta L.	0	n	1	0	Lamiacea Lindl.
Iz_4_1009	DJI_0365	Tanacetum vulgare L.	0	n	1	0	Asteraceae Bercht. & J.Presl
Iz_4_1009	DJI_0365	Thymus pallasianus Heinr.Braun	0	n	1	0	Lamiacea Lindl.
Iz_4_1009	DJI_0365	Tilia cordata Mill.	0	n	1	1	Malvaceae Juss.
Iz_4_1009	DJI_0365	Trifolium alpestre L.	0	n	1	0	Fabaceae Lindl.
Iz_4_1009	DJI_0365	Trifolium arvense L.	0	n	1	0	Fabaceae Lindl.
Iz_4_1009	DJI_0365	Ulmus glabra Huds.	0	n	1	1	Ulmaceae Mirb.
Iz_4_1009	DJI_0365	Verbascum densiflorum Bertol.	0	n	1	0	Scrophulariaceae Juss.
Iz_4_1009	DJI_0365	Verbascum nigrum L.	0	n	1	0	Scrophulariaceae Juss.
Iz_4_1009	DJI_0365	Veronica austriaca L.	0	n	1	0	Plantaginaceae Juss.
Iz_4_1009	DJI_0365	Veronica incana L.	0	n	1	0	Plantaginaceae Juss.
Iz_4_1009	DJI_0365	Veronica verna L.	0	n	1	0	Plantaginaceae Juss.
Iz_4_1009	DJI_0365	Vincetoxicum hirundinaria Medik	0	n	1	0	Apocynaceae Juss.
Iz_4_1009	DJI_0366	Acer tataricum L.	0	n	1	1	Sapindaceae Juss.
Iz_4_1009	DJI_0366	Achillea millefolium L. s.l.	0	n	1	0	Asteraceae Bercht. & J.Presl
Iz_4_1009	DJI_0366	Allium oleraceum L.	0	n	1	0	Amaryllidaceae J.St.-Hil.
Iz_4_1009	DJI_0366	Ambrosia artemisiifolia L.	0	a	0	0	Asteraceae Bercht. & J.Presl
Iz_4_1009	DJI_0366	Amorpha fruticosa L.	0	a	0	0	Fabaceae Lindl.
Iz_4_1009	DJI_0366	Anthericum ramosum L.	0	n	1	0	Asparagaceae Juss.
Iz_4_1009	DJI_0366	Artemisia marschalliana Spreng.	0	n	1	0	Asteraceae Bercht. & J.Presl
Iz_4_1009	DJI_0366	Asparagus officinalis L.	0	n	1	0	Asparagaceae Juss.
Iz_4_1009	DJI_0366	Astragalus cicer L.	0	n	1	0	Fabaceae Lindl.
Iz_4_1009	DJI_0366	Bassia laniflora (S.G.Gmel.) A.J.Scott	0	n	1	0	Amaranthaceae Juss.
Iz_4_1009	DJI_0366	Berberis vulgaris L.	0	n	1	0	Berberidaceae Juss.
Iz_4_1009	DJI_0366	Betula pendula Roth.	0	n	1	1	Betulaceae Gray
Iz_4_1009	DJI_0366	Betula pendula Roth.	0	n	1	0	Poaceae Barnhart
Iz_4_1009	DJI_0366	Bromus inermis Leyss.	0		NA	NA	
Iz_4_1009	DJI_0366	Bromus squarrosus L.	0	a	0	0	Poaceae Barnhart
Iz_4_1009	DJI_0366	Bromus tectorum L.	0	a	0	0	Poaceae Barnhart
Iz_4_1009	DJI_0366	Calamagrostis epigejos (L.) Roth	1	n	1	0	Poaceae Barnhart
Iz_4_1009	DJI_0366	Carduus acanthoides L.	0	n	1	0	Asteraceae Bercht. & J.Presl
Iz_4_1009	DJI_0366	Carex sp.	0	n	1	0	Cyperaceae Juss.
Iz_4_1009	DJI_0366	Centaurea majorovii Dumbadze	0	n	1	0	Asteraceae Bercht. & J.Presl
Iz_4_1009	DJI_0366	Chamaecytisus ruthenicus (Fisch.ex Wol.) Klaskova	1	n	1	0	Fabaceae Lindl.
Iz_4_1009	DJI_0366	Chelidonium majus L.	0	n	1	0	Papaveraceae Juss.
Iz_4_1009	DJI_0366	Chenopodium betaceum Andrz.	0	n	1	0	Amaranthaceae Juss.
Iz_4_1009	DJI_0366	Chenopodium cf. album	0	n	1	0	Amaranthaceae Juss.
Iz_4_1009	DJI_0366	Chenopodium sp.	0		NA	0	Amaranthaceae Juss.
Iz_4_1009	DJI_0366	Chondrilla juncea L.	0	n	1	0	Asteraceae Bercht. & J.Presl
Iz_4_1009	DJI_0366	Cirsium vulgare (Savi) Ten.	0	n	1	0	Asteraceae Bercht. & J.Presl
Iz_4_1009	DJI_0366	Clinopodium acinos (L.) Kuntze	0	n	1	0	Lamiacea Lindl.
Iz_4_1009	DJI_0366	Convallaria majalis L.	0	n	1	0	Asparagaceae Juss.
Iz_4_1009	DJI_0366	Crataegus rhipidophylla Gand.	0	n	1	1	Rosaceae Juss.
Iz_4_1009	DJI_0366	Crepis foetida L.	0	n	1	0	Asteraceae Bercht. & J.Presl
Iz_4_1009	DJI_0366	Dactylis glomerata L.	0	n	1	0	Poaceae Barnhart
Iz_4_1009	DJI_0366	Dianthus campestris M.Bieb.	0	n	1	0	Caryophyllaceae Juss.
Iz_4_1009	DJI_0366	Digitaria sanguinalis (L.) Scop.	0	a	0	0	Poaceae Barnhart
Iz_4_1009	DJI_0366	Elymus repens (L.) Gould	0	n	1	0	Poaceae Barnhart
Iz_4_1009	DJI_0366	Epilobium tetragonum L.	0	n	1	0	Onagraceae Juss.
Iz_4_1009	DJI_0366	Eragrostis minor Host	0	a	0	0	Poaceae Barnhart
Iz_4_1009	DJI_0366	Erigeron canadensis L.	0	a	0	0	Asteraceae Bercht. & J.Presl
Iz_4_1009	DJI_0366	Euphorbia seguieriana Neck.	0	n	1	0	Euphorbiaceae Juss.
Iz_4_1009	DJI_0366	Fallopia dumetorum (L.) Holub	0	n	1	0	Polygonaceae Juss.
Iz_4_1009	DJI_0366	Festuca beckeri (Hack.) Trautv.	0	n	1	0	Poaceae Barnhart
Iz_4_1009	DJI_0366	Fragaria vesca L.	0	n	1	0	Rosaceae Juss.
Iz_4_1009	DJI_0366	Frangula alnus Mill.	0	n	1	1	Rhamnaceae Juss.
Iz_4_1009	DJI_0366	Fraxinus pennsylvanica Marshall	0	a	0	1	Oleaceae Hoffmanns. & Link
Iz_4_1009	DJI_0366	Galeopsis bifida Boenn.	0	n	1	0	Lamiacea Lindl.
Iz_4_1009	DJI_0366	Galium verum L.	0	n	1	0	Rubiaceae Juss.
Iz_4_1009	DJI_0366	Genista tinctoria L.	0	n	1	0	Fabaceae Lindl.
Iz_4_1009	DJI_0366	Geranium robertianum L.	0	n	1	0	Geraniaceae Juss.
Iz_4_1009	DJI_0366	Geranium sanquineum L.	0	n	1	0	Geraniaceae Juss.
Iz_4_1009	DJI_0366	Gypsophila paniculata L.	0	n	1	0	Caryophyllaceae Juss.
Iz_4_1009	DJI_0366	Helichrysum arenarium (L.) Moench	0	n	1	0	Asteraceae Bercht. & J.Presl
Iz_4_1009	DJI_0366	Herniaria polygama J.Gay	0	n	1	0	Caryophyllaceae Juss.
Iz_4_1009	DJI_0366	Hieracium robustum Fr.	0	n	1	0	Asteraceae Bercht. & J.Presl
Iz_4_1009	DJI_0366	Hieracium umbellatum L.	0	n	1	NA	Asteraceae Bercht. & J.Presl
Iz_4_1009	DJI_0366	Hylotelephium maximum (L.) Holub	0	n	1	0	Crassulaceae J.St.-Hil.
Iz_4_1009	DJI_0366	Jacobaea borysthenica (DC.) B.Nord. & Greuter	0	n	1	0	Asteraceae Bercht. & J.Presl
Iz_4_1009	DJI_0366	Jasione montana L.	0	n	1	0	Campanulaceae Juss.
Iz_4_1009	DJI_0366	Jurinea cyanoides (L.) Rchb.s.l.	0	n	1	0	Asteraceae Bercht. & J.Presl
Iz_4_1009	DJI_0366	Koeleria glauca (Spreng.) DC.	0	n	1	0	Poaceae Barnhart
Iz_4_1009	DJI_0366	Lactuca serriola L.	0	a	0	0	Asteraceae Bercht. & J.Presl
Iz_4_1009	DJI_0366	Linaria genistifolia (L.) Mill.	0	n	1	0	Plantaginaceae Juss.
Iz_4_1009	DJI_0366	Lomelosia argentea (L.) Greuter & Burdet	0	n	1	0	Caprifoliaceae Juss.
Iz_4_1009	DJI_0366	Melampyrum pratense L.	0	n	1	0	Orobanchaceae Vent.
Iz_4_1009	DJI_0366	Oenothera biennis L.	0	a	0	0	Onagraceae Juss.
Iz_4_1009	DJI_0366	Pentanema britannica (L.) D.Gut.Larr., Santos-Vicente, Anderb., E.Rico & M.M.Mart.Ort.	0	n	1	0	Asteraceae Bercht. & J.Presl
Iz_4_1009	DJI_0366	Peucedanum oreoselinum (L.) Moench	0	n	1	0	Apiaceae Lindl.
Iz_4_1009	DJI_0366	Peucedanum ruthenicum M.Bieb.	0	n	1	0	Apiaceae Lindl.
Iz_4_1009	DJI_0366	Pilosella echioides (Lumn.) F.W.Schultz & Sch.Bip.	0	n	1	0	Asteraceae Bercht. & J.Presl
Iz_4_1009	DJI_0366	Pilosella officinarum F. Schultz et Sch. Bip.	0	n	1	0	Asteraceae Bercht. & J.Presl
Iz_4_1009	DJI_0366	Pilosella sp.	0		NA	0	Asteraceae Bercht. & J.Presl
Iz_4_1009	DJI_0366	Pinus sylvestris L.	0	n	1	1	Pinaceae Spreng. ex F.Rudolphi

Iz_4_1009	DJI_0366	Poa annua L.	0	n	1	0	Poaceae Barnhart
Iz_4_1009	DJI_0366	Poa nemoralis L.	0	n	1	0	Poaceae Barnhart
Iz_4_1009	DJI_0366	Poa sp.	0		NA	0	Poaceae Barnhart
Iz_4_1009	DJI_0366	Polygonatum odoratum (Mill.) Druce	0	n	1	0	Asparagaceae Juss.
Iz_4_1009	DJI_0366	Polygonum arenarium Waldst. & Kit.	0	n	1	0	Polygonaceae Juss.
Iz_4_1009	DJI_0366	Populus nigra L.	0	n	1	1	Salicaceae Mirb.
Iz_4_1009	DJI_0366	Populus tremula L.	0	n	1	1	Salicaceae Mirb.
Iz_4_1009	DJI_0366	Populus Ã canescens (Aiton) Sm.	0	n	1	1	Salicaceae Mirb.
Iz_4_1009	DJI_0366	Potentilla argentea L.	0	n	1	0	Rosaceae Juss.
Iz_4_1009	DJI_0366	Psammophiliella muralis (L.) Ikonn.	0	n	1	0	Caryophyllaceae Juss.
Iz_4_1009	DJI_0366	Psephellus sumensis (Kalen.) Greuter	0	n	1	0	Asteraceae Bercht. & J.Presl
Iz_4_1009	DJI_0366	Pulsatilla pratensis (L.) Mill. s.l.	0	n	1	0	Ranunculaceae Juss.
Iz_4_1009	DJI_0366	Pyrus communis L.	0	a	0	1	Rosaceae Juss.
Iz_4_1009	DJI_0366	Quercus robur L.	0	n	1	1	Fagaceae Dumort.
Iz_4_1009	DJI_0366	Robinia pseudoacacia L.	0	a	0	1	Fabaceae Lindl.
Iz_4_1009	DJI_0366	Rosa sp.	0		NA	1	Rosaceae Juss.
Iz_4_1009	DJI_0366	Rubus idaeus L.	0	n	1	0	Rosaceae Juss.
Iz_4_1009	DJI_0366	Rumex acetosella L.	0	n	1	0	Polygonaceae Juss.
Iz_4_1009	DJI_0366	Rumex thyrsiflorus Fingerh.	0	n	1	0	Polygonaceae Juss.
Iz_4_1009	DJI_0366	Salix alba L.	0	n	1	1	Salicaceae Mirb.
Iz_4_1009	DJI_0366	Sambucus nigra L.	0	n	1	1	Viburnaceae Raf.
Iz_4_1009	DJI_0366	Secale sylvestre Host	0	n	1	0	Poaceae Barnhart
Iz_4_1009	DJI_0366	Sempervivum ruthenicum Schnittsp. & C.B. Lehm.	0	n	1	0	Crassulaceae J.St.-Hil.
Iz_4_1009	DJI_0366	Setaria viridis (L.) P. Beauv.	0	n	1	0	Poaceae Barnhart
Iz_4_1009	DJI_0366	Silene borysthenica (Gruner) Walters	0	n	1	0	Caryophyllaceae Juss.
Iz_4_1009	DJI_0366	Silene latifolia subsp. alba (Mill.) Greuter & Burdet	0	n	1	0	Caryophyllaceae Juss.
Iz_4_1009	DJI_0366	Solidago virgaurea L.	1	n	1	0	Asteraceae Bercht. & J.Presl
Iz_4_1009	DJI_0366	Sorbus aucuparia L.	0	n	1	1	Rosaceae Juss.
Iz_4_1009	DJI_0366	Stachys recta L.	0	n	1	0	Lamiacea Lindl.
Iz_4_1009	DJI_0366	Tanacetum vulgare L.	0	n	1	0	Asteraceae Bercht. & J.Presl
Iz_4_1009	DJI_0366	Thymus pallasianus Heinr.Braun	0	n	1	0	Lamiacea Lindl.
Iz_4_1009	DJI_0366	Tilia cordata Mill.	0	n	1	1	Malvaceae Juss.
Iz_4_1009	DJI_0366	Trifolium alpestre L.	0	n	1	0	Fabaceae Lindl.
Iz_4_1009	DJI_0366	Trifolium arvense L.	0	n	1	0	Fabaceae Lindl.
Iz_4_1009	DJI_0366	Ulmus glabra Huds.	0	n	1	1	Ulmaceae Mirb.
Iz_4_1009	DJI_0366	Verbascum densiflorum Bertol.	0	n	1	0	Scrophulariaceae Juss.
Iz_4_1009	DJI_0366	Verbascum nigrum L.	0	n	1	0	Scrophulariaceae Juss.
Iz_4_1009	DJI_0366	Veronica austriaca L.	0	n	1	0	Plantaginaceae Juss.
Iz_4_1009	DJI_0366	Veronica incana L.	0	n	1	0	Plantaginaceae Juss.
Iz_4_1009	DJI_0366	Veronica verna L.	0	n	1	0	Plantaginaceae Juss.
Iz_4_1009	DJI_0366	Vincetoxicum hirundinaria Medik	0	n	1	0	Apocynaceae Juss.
Iz_4_1009	DJI_0367	Acer tataricum L.	0	n	1	1	Sapindaceae Juss.
Iz_4_1009	DJI_0367	Achillea millefolium L. s.l.	0	n	1	0	Asteraceae Bercht. & J.Presl
Iz_4_1009	DJI_0367	Allium oleraceum L.	0	n	1	0	Amaryllidaceae J.St.-Hil.
Iz_4_1009	DJI_0367	Ambrosia artemisiifolia L.	0	a	0	0	Asteraceae Bercht. & J.Presl
Iz_4_1009	DJI_0367	Amorpha fruticosa L.	0	a	0	0	Fabaceae Lindl.
Iz_4_1009	DJI_0367	Anthericum ramosum L.	0	n	1	0	Asparagaceae Juss.
Iz_4_1009	DJI_0367	Artemisia marschalliana Spreng.	0	n	1	0	Asteraceae Bercht. & J.Presl
Iz_4_1009	DJI_0367	Asparagus officinalis L.	0	n	1	0	Asparagaceae Juss.
Iz_4_1009	DJI_0367	Astragalus cicer L.	0	n	1	0	Fabaceae Lindl.
Iz_4_1009	DJI_0367	Bassia laniflora (S.G.Gmel.) A.J.Scott	0	n	1	0	Amaranthaceae Juss.
Iz_4_1009	DJI_0367	Berberis vulgaris L.	0	n	1	0	Berberidaceae Juss.
Iz_4_1009	DJI_0367	Betula pendula Roth.	0	n	1	1	Betulaceae Gray
Iz_4_1009	DJI_0367	Betula pendula Roth.	0	n	1	0	Poaceae Barnhart
Iz_4_1009	DJI_0367	Bromus inermis Leyss.	0		NA	NA	
Iz_4_1009	DJI_0367	Bromus squarrosus L.	0	a	0	0	Poaceae Barnhart
Iz_4_1009	DJI_0367	Bromus tectorum L.	0	a	0	0	Poaceae Barnhart
Iz_4_1009	DJI_0367	Calamagrostis epigejos (L.) Roth	3	n	1	0	Poaceae Barnhart
Iz_4_1009	DJI_0367	Carduus acanthoides L.	0	n	1	0	Asteraceae Bercht. & J.Presl
Iz_4_1009	DJI_0367	Carex sp.	0	n	1	0	Cyperaceae Juss.
Iz_4_1009	DJI_0367	Centaurea majorovii Dumbadze	0	n	1	0	Asteraceae Bercht. & J.Presl
Iz_4_1009	DJI_0367	Chamaecytisus ruthenicus (Fisch.ex Wol.) Klaskova	0	n	1	0	Fabaceae Lindl.
Iz_4_1009	DJI_0367	Chelidonium majus L.	0	n	1	0	Papaveraceae Juss.
Iz_4_1009	DJI_0367	Chenopodium betaceum Andrz.	0	n	1	0	Amaranthaceae Juss.
Iz_4_1009	DJI_0367	Chenopodium cf. album	0	n	1	0	Amaranthaceae Juss.
Iz_4_1009	DJI_0367	Chenopodium sp.	0		NA	0	Amaranthaceae Juss.
Iz_4_1009	DJI_0367	Chondrilla juncea L.	0	n	1	0	Asteraceae Bercht. & J.Presl
Iz_4_1009	DJI_0367	Cirsium vulgare (Savi) Ten.	0	n	1	0	Asteraceae Bercht. & J.Presl
Iz_4_1009	DJI_0367	Clinopodium acinos (L.) Kuntze	0	n	1	0	Lamiacea Lindl.
Iz_4_1009	DJI_0367	Convallaria majalis L.	0	n	1	0	Asparagaceae Juss.
Iz_4_1009	DJI_0367	Crataegus rhipidophylla Gand.	0	n	1	1	Rosaceae Juss.
Iz_4_1009	DJI_0367	Crepis foetida L.	0	n	1	0	Asteraceae Bercht. & J.Presl
Iz_4_1009	DJI_0367	Dactylis glomerata L.	0	n	1	0	Poaceae Barnhart
Iz_4_1009	DJI_0367	Dianthus campestris M.Bieb.	0	n	1	0	Caryophyllaceae Juss.
Iz_4_1009	DJI_0367	Digitaria sanguinalis (L.) Scop.	0	a	0	0	Poaceae Barnhart
Iz_4_1009	DJI_0367	Elymus repens (L.) Gould	0	n	1	0	Poaceae Barnhart
Iz_4_1009	DJI_0367	Epilobium tetragonum L.	0	n	1	0	Onagraceae Juss.
Iz_4_1009	DJI_0367	Eragrostis minor Host	0	a	0	0	Poaceae Barnhart
Iz_4_1009	DJI_0367	Erigeron canadensis L.	2	a	0	0	Asteraceae Bercht. & J.Presl
Iz_4_1009	DJI_0367	Euphorbia seguieriana Neck.	0	n	1	0	Euphorbiaceae Juss.
Iz_4_1009	DJI_0367	Fallopia dumetorum (L.) Holub	0	n	1	0	Polygonaceae Juss.
Iz_4_1009	DJI_0367	Festuca beckeri (Hack.) Trautv.	0	n	1	0	Poaceae Barnhart
Iz_4_1009	DJI_0367	Fragaria vesca L.	0	n	1	0	Rosaceae Juss.
Iz_4_1009	DJI_0367	Frangula alnus Mill.	0	n	1	1	Rhamnaceae Juss.
Iz_4_1009	DJI_0367	Fraxinus pennsylvanica Marshall	0	a	0	1	Oleaceae Hoffmanns. & Link
Iz_4_1009	DJI_0367	Galeopsis bifida Boenn.	0	n	1	0	Lamiacea Lindl.
Iz_4_1009	DJI_0367	Galium verum L.	0	n	1	0	Rubiaceae Juss.
Iz_4_1009	DJI_0367	Genista tinctoria L.	0	n	1	0	Fabaceae Lindl.
Iz_4_1009	DJI_0367	Geranium robertianum L.	0	n	1	0	Geraniaceae Juss.
Iz_4_1009	DJI_0367	Geranium sanquineum L.	0	n	1	0	Geraniaceae Juss.
Iz_4_1009	DJI_0367	Gypsophila paniculata L.	0	n	1	0	Caryophyllaceae Juss.
Iz_4_1009	DJI_0367	Helichrysum arenarium (L.) Moench	0	n	1	0	Asteraceae Bercht. & J.Presl
Iz_4_1009	DJI_0367	Herniaria polygama J.Gay	0	n	1	0	Caryophyllaceae Juss.
Iz_4_1009	DJI_0367	Hieracium robustum Fr.	0	n	1	0	Asteraceae Bercht. & J.Presl
Iz_4_1009	DJI_0367	Hieracium umbellatum L.	0	n	1	NA	Asteraceae Bercht. & J.Presl
Iz_4_1009	DJI_0367	Hylotelephium maximum (L.) Holub	0	n	1	0	Crassulaceae J.St.-Hil.
Iz_4_1009	DJI_0367	Jacobaea borysthenica (DC.) B.Nord. & Greuter	0	n	1	0	Asteraceae Bercht. & J.Presl
Iz_4_1009	DJI_0367	Jasione montana L.	0	n	1	0	Campanulaceae Juss.

Iz_4_1009	DJI_0367	Jurinea cyanoides (L.) Rchb.s.l.	0	n	1	0	Asteraceae Bercht. & J.Presl
Iz_4_1009	DJI_0367	Koeleria glauca (Spreng.) DC.	0	n	1	0	Poaceae Barnhart
Iz_4_1009	DJI_0367	Lactuca serriola L.	0	a	0	0	Asteraceae Bercht. & J.Presl
Iz_4_1009	DJI_0367	Linaria genistifolia (L.) Mill.	0	n	1	0	Plantaginaceae Juss.
Iz_4_1009	DJI_0367	Lomelosia argentea (L.) Greuter & Burdet	0	n	1	0	Caprifoliaceae Juss.
Iz_4_1009	DJI_0367	Melampyrum pratense L.	0	n	1	0	Orobanchaceae Vent.
Iz_4_1009	DJI_0367	Oenothera biennis L.	0	a	0	0	Onagraceae Juss.
Iz_4_1009	DJI_0367	Pentanema britannica (L.) D.Gut.Larr., Santos-Vicente, Anderb., E.Rico & M.M.Mart.Ort.	0	n	1	0	Asteraceae Bercht. & J.Presl
Iz_4_1009	DJI_0367	Peucedanum oreoselinum (L.) Moench	0.5	n	1	0	Apiaceae Lindl.
Iz_4_1009	DJI_0367	Peucedanum ruthenicum M.Bieb.	0	n	1	0	Apiaceae Lindl.
Iz_4_1009	DJI_0367	Pilosella echioides (Lumn.) F.W.Schultz & Sch.Bip.	0	n	1	0	Asteraceae Bercht. & J.Presl
Iz_4_1009	DJI_0367	Pilosella officinarum F. Schultz et Sch. Bip.	0	n	1	0	Asteraceae Bercht. & J.Presl
Iz_4_1009	DJI_0367	Pilosella sp.	0		NA	0	Asteraceae Bercht. & J.Presl
Iz_4_1009	DJI_0367	Pinus sylvestris L.	0	n	1	1	Pinaceae Spreng. ex F.Rudolphi
Iz_4_1009	DJI_0367	Poa annua L.	0	n	1	0	Poaceae Barnhart
Iz_4_1009	DJI_0367	Poa nemoralis L.	0	n	1	0	Poaceae Barnhart
Iz_4_1009	DJI_0367	Poa sp.	0		NA	0	Poaceae Barnhart
Iz_4_1009	DJI_0367	Polygonatum odoratum (Mill.) Druce	0	n	1	0	Asparagaceae Juss.
Iz_4_1009	DJI_0367	Polygonum arenarium Waldst. & Kit.	0	n	1	0	Polygonaceae Juss.
Iz_4_1009	DJI_0367	Populus nigra L.	0	n	1	1	Salicaceae Mirb.
Iz_4_1009	DJI_0367	Populus tremula L.	0	n	1	1	Salicaceae Mirb.
Iz_4_1009	DJI_0367	Populus Ã canescens (Aiton) Sm.	0	n	1	1	Salicaceae Mirb.
Iz_4_1009	DJI_0367	Potentilla argentea L.	0	n	1	0	Rosaceae Juss.
Iz_4_1009	DJI_0367	Psammophiliella muralis (L.) Ikonn.	0	n	1	0	Caryophyllaceae Juss.
Iz_4_1009	DJI_0367	Psephellus sumensis (Kalen.) Greuter	0	n	1	0	Asteraceae Bercht. & J.Presl
Iz_4_1009	DJI_0367	Pulsatilla pratensis (L.) Mill. s.l.	0	n	1	0	Ranunculaceae Juss.
Iz_4_1009	DJI_0367	Pyrus communis L.	0	a	0	1	Rosaceae Juss.
Iz_4_1009	DJI_0367	Quercus robur L.	0	n	1	1	Fagaceae Dumort.
Iz_4_1009	DJI_0367	Robinia pseudoacacia L.	0	a	0	1	Fabaceae Lindl.
Iz_4_1009	DJI_0367	Rosa sp.	0		NA	1	Rosaceae Juss.
Iz_4_1009	DJI_0367	Rubus idaeus L.	0	n	1	0	Rosaceae Juss.
Iz_4_1009	DJI_0367	Rumex acetosella L.	0	n	1	0	Polygonaceae Juss.
Iz_4_1009	DJI_0367	Rumex thyrsiflorus Fingerh.	0	n	1	0	Polygonaceae Juss.
Iz_4_1009	DJI_0367	Salix alba L.	0	n	1	1	Salicaceae Mirb.
Iz_4_1009	DJI_0367	Sambucus nigra L.	0	n	1	1	Viburnaceae Raf.
Iz_4_1009	DJI_0367	Secale sylvestre Host	0	n	1	0	Poaceae Barnhart
Iz_4_1009	DJI_0367	Sempervivum ruthenicum Schnittsp. & C.B. Lehm.	0	n	1	0	Crassulaceae J.St.-Hil.
Iz_4_1009	DJI_0367	Setaria viridis (L.) P. Beauv.	0	n	1	0	Poaceae Barnhart
Iz_4_1009	DJI_0367	Silene borysthenica (Gruner) Walters	0	n	1	0	Caryophyllaceae Juss.
Iz_4_1009	DJI_0367	Silene latifolia subsp. alba (Mill.) Greuter & Burdet	0	n	1	0	Caryophyllaceae Juss.
Iz_4_1009	DJI_0367	Solidago virgaurea L.	0	n	1	0	Asteraceae Bercht. & J.Presl
Iz_4_1009	DJI_0367	Sorbus aucuparia L.	0	n	1	1	Rosaceae Juss.
Iz_4_1009	DJI_0367	Stachys recta L.	0	n	1	0	Lamiacea Lindl.
Iz_4_1009	DJI_0367	Tanacetum vulgare L.	0	n	1	0	Asteraceae Bercht. & J.Presl
Iz_4_1009	DJI_0367	Thymus pallasianus Heinr.Braun	0	n	1	0	Lamiacea Lindl.
Iz_4_1009	DJI_0367	Tilia cordata Mill.	0	n	1	1	Malvaceae Juss.
Iz_4_1009	DJI_0367	Trifolium alpestre L.	0	n	1	0	Fabaceae Lindl.
Iz_4_1009	DJI_0367	Trifolium arvense L.	0	n	1	0	Fabaceae Lindl.
Iz_4_1009	DJI_0367	Ulmus glabra Huds.	0	n	1	1	Ulmaceae Mirb.
Iz_4_1009	DJI_0367	Verbascum densiflorum Bertol.	0	n	1	0	Scrophulariaceae Juss.
Iz_4_1009	DJI_0367	Verbascum nigrum L.	0	n	1	0	Scrophulariaceae Juss.
Iz_4_1009	DJI_0367	Veronica austriaca L.	0	n	1	0	Plantaginaceae Juss.
Iz_4_1009	DJI_0367	Veronica incana L.	0	n	1	0	Plantaginaceae Juss.
Iz_4_1009	DJI_0367	Veronica verna L.	0	n	1	0	Plantaginaceae Juss.
Iz_4_1009	DJI_0367	Vincetoxicum hirundinaria Medik	0	n	1	0	Apocynaceae Juss.
Iz_4_1009	DJI_0372	Acer tataricum L.	0	n	1	1	Sapindaceae Juss.
Iz_4_1009	DJI_0372	Achillea millefolium L. s.l.	0	n	1	0	Asteraceae Bercht. & J.Presl
Iz_4_1009	DJI_0372	Allium oleraceum L.	0	n	1	0	Amaryllidaceae J.St.-Hil.
Iz_4_1009	DJI_0372	Ambrosia artemisiifolia L.	0	a	0	0	Asteraceae Bercht. & J.Presl
Iz_4_1009	DJI_0372	Amorpha fruticosa L.	0	a	0	0	Fabaceae Lindl.
Iz_4_1009	DJI_0372	Anthericum ramosum L.	0	n	1	0	Asparagaceae Juss.
Iz_4_1009	DJI_0372	Artemisia marschalliana Spreng.	0	n	1	0	Asteraceae Bercht. & J.Presl
Iz_4_1009	DJI_0372	Asparagus officinalis L.	0	n	1	0	Asparagaceae Juss.
Iz_4_1009	DJI_0372	Astragalus cicer L.	0	n	1	0	Fabaceae Lindl.
Iz_4_1009	DJI_0372	Bassia laniflora (S.G.Gmel.) A.J.Scott	0	n	1	0	Amaranthaceae Juss.
Iz_4_1009	DJI_0372	Berberis vulgaris L.	0	n	1	0	Berberidaceae Juss.
Iz_4_1009	DJI_0372	Betula pendula Roth.	0	n	1	1	Betulaceae Gray
Iz_4_1009	DJI_0372	Betula pendula Roth.	0	n	1	0	Poaceae Barnhart
Iz_4_1009	DJI_0372	Bromus inermis Leyss.	0		NA	NA	
Iz_4_1009	DJI_0372	Bromus squarrosus L.	0	a	0	0	Poaceae Barnhart
Iz_4_1009	DJI_0372	Bromus tectorum L.	0	a	0	0	Poaceae Barnhart
Iz_4_1009	DJI_0372	Calamagrostis epigejos (L.) Roth	40	n	1	0	Poaceae Barnhart
Iz_4_1009	DJI_0372	Carduus acanthoides L.	0	n	1	0	Asteraceae Bercht. & J.Presl
Iz_4_1009	DJI_0372	Carex sp.	0	n	1	0	Cyperaceae Juss.
Iz_4_1009	DJI_0372	Centaurea majorovii Dumbadze	0	n	1	0	Asteraceae Bercht. & J.Presl
Iz_4_1009	DJI_0372	Chamaecytisus ruthenicus (Fisch.ex Wol.) Klaskova	0	n	1	0	Fabaceae Lindl.
Iz_4_1009	DJI_0372	Chelidonium majus L.	0	n	1	0	Papaveraceae Juss.
Iz_4_1009	DJI_0372	Chenopodium betaceum Andrz.	0	n	1	0	Amaranthaceae Juss.
Iz_4_1009	DJI_0372	Chenopodium cf. album	0	n	1	0	Amaranthaceae Juss.
Iz_4_1009	DJI_0372	Chenopodium sp.	0		NA	0	Amaranthaceae Juss.
Iz_4_1009	DJI_0372	Chondrilla juncea L.	0	n	1	0	Asteraceae Bercht. & J.Presl
Iz_4_1009	DJI_0372	Cirsium vulgare (Savi) Ten.	0	n	1	0	Asteraceae Bercht. & J.Presl
Iz_4_1009	DJI_0372	Clinopodium acinos (L.) Kuntze	0	n	1	0	Lamiacea Lindl.
Iz_4_1009	DJI_0372	Convallaria majalis L.	0	n	1	0	Asparagaceae Juss.
Iz_4_1009	DJI_0372	Crataegus rhipidophylla Gand.	0	n	1	1	Rosaceae Juss.
Iz_4_1009	DJI_0372	Crepis foetida L.	0	n	1	0	Asteraceae Bercht. & J.Presl
Iz_4_1009	DJI_0372	Dactylis glomerata L.	0	n	1	0	Poaceae Barnhart
Iz_4_1009	DJI_0372	Dianthus campestris M.Bieb.	0	n	1	0	Caryophyllaceae Juss.
Iz_4_1009	DJI_0372	Digitaria sanguinalis (L.) Scop.	0	a	0	0	Poaceae Barnhart
Iz_4_1009	DJI_0372	Elymus repens (L.) Gould	0	n	1	0	Poaceae Barnhart
Iz_4_1009	DJI_0372	Epilobium tetragonum L.	0	n	1	0	Onagraceae Juss.
Iz_4_1009	DJI_0372	Eragrostis minor Host	0	a	0	0	Poaceae Barnhart
Iz_4_1009	DJI_0372	Erigeron canadensis L.	0	a	0	0	Asteraceae Bercht. & J.Presl
Iz_4_1009	DJI_0372	Euphorbia seguieriana Neck.	0	n	1	0	Euphorbiaceae Juss.
Iz_4_1009	DJI_0372	Fallopia dumetorum (L.) Holub	0	n	1	0	Polygonaceae Juss.
Iz_4_1009	DJI_0372	Festuca beckeri (Hack.) Trautv.	0	n	1	0	Poaceae Barnhart
Iz_4_1009	DJI_0372	Fragaria vesca L.	0	n	1	0	Rosaceae Juss.
Iz_4_1009	DJI_0372	Frangula alnus Mill.	0	n	1	1	Rhamnaceae Juss.

Iz_4_1009	DJI_0372	Fraxinus pennsylvanica Marshall	0	a	0	1	Oleaceae Hoffmanns. & Link
Iz_4_1009	DJI_0372	Galeopsis bifida Boenn.	0	n	1	0	Lamiacea Lindl.
Iz_4_1009	DJI_0372	Galium verum L.	0	n	1	0	Rubiaceae Juss.
Iz_4_1009	DJI_0372	Genista tinctoria L.	0	n	1	0	Fabaceae Lindl.
Iz_4_1009	DJI_0372	Geranium robertianum L.	0	n	1	0	Geraniaceae Juss.
Iz_4_1009	DJI_0372	Geranium sanquineum L.	0	n	1	0	Geraniaceae Juss.
Iz_4_1009	DJI_0372	Gypsophila paniculata L.	0	n	1	0	Caryophyllaceae Juss.
Iz_4_1009	DJI_0372	Helichrysum arenarium (L.) Moench	0	n	1	0	Asteraceae Bercht. & J.Presl
Iz_4_1009	DJI_0372	Herniaria polygama J.Gay	0	n	1	0	Caryophyllaceae Juss.
Iz_4_1009	DJI_0372	Hieracium robustum Fr.	0	n	1	0	Asteraceae Bercht. & J.Presl
Iz_4_1009	DJI_0372	Hieracium umbellatum L.	0	n	1	NA	Asteraceae Bercht. & J.Presl
Iz_4_1009	DJI_0372	Hylotelephium maximum (L.) Holub	0	n	1	0	Crassulaceae J.St.-Hil.
Iz_4_1009	DJI_0372	Jacobaea borysthenica (DC.) B.Nord. & Greuter	0	n	1	0	Asteraceae Bercht. & J.Presl
Iz_4_1009	DJI_0372	Jasione montana L.	0	n	1	0	Campanulaceae Juss.
Iz_4_1009	DJI_0372	Jurinea cyanoides (L.) Rchb.s.l.	0	n	1	0	Asteraceae Bercht. & J.Presl
Iz_4_1009	DJI_0372	Koeleria glauca (Spreng.) DC.	0	n	1	0	Poaceae Barnhart
Iz_4_1009	DJI_0372	Lactuca serriola L.	0	a	0	0	Asteraceae Bercht. & J.Presl
Iz_4_1009	DJI_0372	Linaria genistifolia (L.) Mill.	0	n	1	0	Plantaginaceae Juss.
Iz_4_1009	DJI_0372	Lomelosia argentea (L.) Greuter & Burdet	0	n	1	0	Caprifoliaceae Juss.
Iz_4_1009	DJI_0372	Melampyrum pratense L.	0	n	1	0	Orobanchaceae Vent.
Iz_4_1009	DJI_0372	Oenothera biennis L.	0	a	0	0	Onagraceae Juss.
Iz_4_1009	DJI_0372	Pentanema britannica (L.) D.Gut.Larr., Santos-Vicente, Anderb., E.Rico & M.M.Mart.Ort.	0	n	1	0	Asteraceae Bercht. & J.Presl
Iz_4_1009	DJI_0372	Peucedanum oreoselinum (L.) Moench	0	n	1	0	Apiaceae Lindl.
Iz_4_1009	DJI_0372	Peucedanum ruthenicum M.Bieb.	0	n	1	0	Apiaceae Lindl.
Iz_4_1009	DJI_0372	Pilosella echioides (Lumn.) F.W.Schultz & Sch.Bip.	0	n	1	0	Asteraceae Bercht. & J.Presl
Iz_4_1009	DJI_0372	Pilosella officinarum F. Schultz et Sch. Bip.	0	n	1	0	Asteraceae Bercht. & J.Presl
Iz_4_1009	DJI_0372	Pilosella sp.	0		NA	0	Asteraceae Bercht. & J.Presl
Iz_4_1009	DJI_0372	Pinus sylvestris L.	0	n	1	1	Pinaceae Spreng. ex F.Rudolphi
Iz_4_1009	DJI_0372	Poa annua L.	0	n	1	0	Poaceae Barnhart
Iz_4_1009	DJI_0372	Poa nemoralis L.	0	n	1	0	Poaceae Barnhart
Iz_4_1009	DJI_0372	Poa sp.	0		NA	0	Poaceae Barnhart
Iz_4_1009	DJI_0372	Polygonatum odoratum (Mill.) Druce	0	n	1	0	Asparagaceae Juss.
Iz_4_1009	DJI_0372	Polygonum arenarium Waldst. & Kit.	0	n	1	0	Polygonaceae Juss.
Iz_4_1009	DJI_0372	Populus nigra L.	0	n	1	1	Salicaceae Mirb.
Iz_4_1009	DJI_0372	Populus tremula L.	0	n	1	1	Salicaceae Mirb.
Iz_4_1009	DJI_0372	Populus Ã canescens (Aiton) Sm.	0	n	1	1	Salicaceae Mirb.
Iz_4_1009	DJI_0372	Potentilla argentea L.	0	n	1	0	Rosaceae Juss.
Iz_4_1009	DJI_0372	Psammophiliella muralis (L.) Ikonn.	0	n	1	0	Caryophyllaceae Juss.
Iz_4_1009	DJI_0372	Psephellus sumensis (Kalen.) Greuter	0	n	1	0	Asteraceae Bercht. & J.Presl
Iz_4_1009	DJI_0372	Pulsatilla pratensis (L.) Mill. s.l.	0	n	1	0	Ranunculaceae Juss.
Iz_4_1009	DJI_0372	Pyrus communis L.	0	a	0	1	Rosaceae Juss.
Iz_4_1009	DJI_0372	Quercus robur L.	0	n	1	1	Fagaceae Dumort.
Iz_4_1009	DJI_0372	Robinia pseudoacacia L.	0	a	0	1	Fabaceae Lindl.
Iz_4_1009	DJI_0372	Rosa sp.	0		NA	1	Rosaceae Juss.
Iz_4_1009	DJI_0372	Rubus idaeus L.	0	n	1	0	Rosaceae Juss.
Iz_4_1009	DJI_0372	Rumex acetosella L.	0	n	1	0	Polygonaceae Juss.
Iz_4_1009	DJI_0372	Rumex thyrsiflorus Fingerh.	0	n	1	0	Polygonaceae Juss.
Iz_4_1009	DJI_0372	Salix alba L.	0	n	1	1	Salicaceae Mirb.
Iz_4_1009	DJI_0372	Sambucus nigra L.	0	n	1	1	Viburnaceae Raf.
Iz_4_1009	DJI_0372	Secale sylvestre Host	0	n	1	0	Poaceae Barnhart
Iz_4_1009	DJI_0372	Sempervivum ruthenicum Schnittsp. & C.B. Lehm.	0	n	1	0	Crassulaceae J.St.-Hil.
Iz_4_1009	DJI_0372	Setaria viridis (L.) P. Beauv.	0	n	1	0	Poaceae Barnhart
Iz_4_1009	DJI_0372	Silene borysthenica (Gruner) Walters	0	n	1	0	Caryophyllaceae Juss.
Iz_4_1009	DJI_0372	Silene latifolia subsp. alba (Mill.) Greuter & Burdet	0	n	1	0	Caryophyllaceae Juss.
Iz_4_1009	DJI_0372	Solidago virgaurea L.	0	n	1	0	Asteraceae Bercht. & J.Presl
Iz_4_1009	DJI_0372	Sorbus aucuparia L.	0	n	1	1	Rosaceae Juss.
Iz_4_1009	DJI_0372	Stachys recta L.	0	n	1	0	Lamiacea Lindl.
Iz_4_1009	DJI_0372	Tanacetum vulgare L.	0	n	1	0	Asteraceae Bercht. & J.Presl
Iz_4_1009	DJI_0372	Thymus pallasianus Heinr.Braun	0	n	1	0	Lamiacea Lindl.
Iz_4_1009	DJI_0372	Tilia cordata Mill.	0	n	1	1	Malvaceae Juss.
Iz_4_1009	DJI_0372	Trifolium alpestre L.	0	n	1	0	Fabaceae Lindl.
Iz_4_1009	DJI_0372	Trifolium arvense L.	0	n	1	0	Fabaceae Lindl.
Iz_4_1009	DJI_0372	Ulmus glabra Huds.	0	n	1	1	Ulmaceae Mirb.
Iz_4_1009	DJI_0372	Verbascum densiflorum Bertol.	0	n	1	0	Scrophulariaceae Juss.
Iz_4_1009	DJI_0372	Verbascum nigrum L.	0	n	1	0	Scrophulariaceae Juss.
Iz_4_1009	DJI_0372	Veronica austriaca L.	0	n	1	0	Plantaginaceae Juss.
Iz_4_1009	DJI_0372	Veronica incana L.	0	n	1	0	Plantaginaceae Juss.
Iz_4_1009	DJI_0372	Veronica verna L.	0	n	1	0	Plantaginaceae Juss.
Iz_4_1009	DJI_0372	Vincetoxicum hirundinaria Medik	0	n	1	0	Apocynaceae Juss.
Iz_4_1009	DJI_0373	Acer tataricum L.	0	n	1	1	Sapindaceae Juss.
Iz_4_1009	DJI_0373	Achillea millefolium L. s.l.	0	n	1	0	Asteraceae Bercht. & J.Presl
Iz_4_1009	DJI_0373	Allium oleraceum L.	0	n	1	0	Amaryllidaceae J.St.-Hil.
Iz_4_1009	DJI_0373	Ambrosia artemisiifolia L.	0	a	0	0	Asteraceae Bercht. & J.Presl
Iz_4_1009	DJI_0373	Amorpha fruticosa L.	0	a	0	0	Fabaceae Lindl.
Iz_4_1009	DJI_0373	Anthericum ramosum L.	0	n	1	0	Asparagaceae Juss.
Iz_4_1009	DJI_0373	Artemisia marschalliana Spreng.	0	n	1	0	Asteraceae Bercht. & J.Presl
Iz_4_1009	DJI_0373	Asparagus officinalis L.	0	n	1	0	Asparagaceae Juss.
Iz_4_1009	DJI_0373	Astragalus cicer L.	0	n	1	0	Fabaceae Lindl.
Iz_4_1009	DJI_0373	Bassia laniflora (S.G.Gmel.) A.J.Scott	0	n	1	0	Amaranthaceae Juss.
Iz_4_1009	DJI_0373	Berberis vulgaris L.	0	n	1	0	Berberidaceae Juss.
Iz_4_1009	DJI_0373	Betula pendula Roth.	0	n	1	1	Betulaceae Gray
Iz_4_1009	DJI_0373	Betula pendula Roth.	0	n	1	0	Poaceae Barnhart
Iz_4_1009	DJI_0373	Bromus inermis Leyss.	0		NA	NA	
Iz_4_1009	DJI_0373	Bromus squarrosus L.	0	a	0	0	Poaceae Barnhart
Iz_4_1009	DJI_0373	Bromus tectorum L.	0	a	0	0	Poaceae Barnhart
Iz_4_1009	DJI_0373	Calamagrostis epigejos (L.) Roth	5	n	1	0	Poaceae Barnhart
Iz_4_1009	DJI_0373	Carduus acanthoides L.	0	n	1	0	Asteraceae Bercht. & J.Presl
Iz_4_1009	DJI_0373	Carex sp.	0	n	1	0	Cyperaceae Juss.
Iz_4_1009	DJI_0373	Centaurea majorovii Dumbadze	0	n	1	0	Asteraceae Bercht. & J.Presl
Iz_4_1009	DJI_0373	Chamaecytisus ruthenicus (Fisch.ex Wol.) Klaskova	0	n	1	0	Fabaceae Lindl.
Iz_4_1009	DJI_0373	Chelidonium majus L.	0	n	1	0	Papaveraceae Juss.
Iz_4_1009	DJI_0373	Chenopodium betaceum Andrz.	0	n	1	0	Amaranthaceae Juss.
Iz_4_1009	DJI_0373	Chenopodium cf. album	0	n	1	0	Amaranthaceae Juss.
Iz_4_1009	DJI_0373	Chenopodium sp.	0		NA	0	Amaranthaceae Juss.
Iz_4_1009	DJI_0373	Chondrilla juncea L.	0	n	1	0	Asteraceae Bercht. & J.Presl
Iz_4_1009	DJI_0373	Cirsium vulgare (Savi) Ten.	0	n	1	0	Asteraceae Bercht. & J.Presl
Iz_4_1009	DJI_0373	Clinopodium acinos (L.) Kuntze	0	n	1	0	Lamiacea Lindl.
Iz_4_1009	DJI_0373	Convallaria majalis L.	0	n	1	0	Asparagaceae Juss.

Iz_4_1009	DJI_0373	Crataegus rhipidophylla Gand.	0	n	1	1	Rosaceae Juss.
Iz_4_1009	DJI_0373	Crepis foetida L.	0	n	1	0	Asteraceae Bercht. & J.Presl
Iz_4_1009	DJI_0373	Dactylis glomerata L.	0	n	1	0	Poaceae Barnhart
Iz_4_1009	DJI_0373	Dianthus campestris M.Bieb.	0	n	1	0	Caryophyllaceae Juss.
Iz_4_1009	DJI_0373	Digitaria sanguinalis (L.) Scop.	0	a	0	0	Poaceae Barnhart
Iz_4_1009	DJI_0373	Elymus repens (L.) Gould	0	n	1	0	Poaceae Barnhart
Iz_4_1009	DJI_0373	Epilobium tetragonum L.	0	n	1	0	Onagraceae Juss.
Iz_4_1009	DJI_0373	Eragrostis minor Host	0	a	0	0	Poaceae Barnhart
Iz_4_1009	DJI_0373	Erigeron canadensis L.	0	a	0	0	Asteraceae Bercht. & J.Presl
Iz_4_1009	DJI_0373	Euphorbia seguieriana Neck.	0	n	1	0	Euphorbiaceae Juss.
Iz_4_1009	DJI_0373	Fallopia dumetorum (L.) Holub	0	n	1	0	Polygonaceae Juss.
Iz_4_1009	DJI_0373	Festuca beckeri (Hack.) Trautv.	0	n	1	0	Poaceae Barnhart
Iz_4_1009	DJI_0373	Fragaria vesca L.	0	n	1	0	Rosaceae Juss.
Iz_4_1009	DJI_0373	Frangula alnus Mill.	0	n	1	1	Rhamnaceae Juss.
Iz_4_1009	DJI_0373	Fraxinus pennsylvanica Marshall	0	a	0	1	Oleaceae Hoffmanns. & Link
Iz_4_1009	DJI_0373	Galeopsis bifida Boenn.	0	n	1	0	Lamiacea Lindl.
Iz_4_1009	DJI_0373	Galium verum L.	0	n	1	0	Rubiaceae Juss.
Iz_4_1009	DJI_0373	Genista tinctoria L.	0	n	1	0	Fabaceae Lindl.
Iz_4_1009	DJI_0373	Geranium robertianum L.	0	n	1	0	Geraniaceae Juss.
Iz_4_1009	DJI_0373	Geranium sanquineum L.	0	n	1	0	Geraniaceae Juss.
Iz_4_1009	DJI_0373	Gypsophila paniculata L.	0	n	1	0	Caryophyllaceae Juss.
Iz_4_1009	DJI_0373	Helichrysum arenarium (L.) Moench	0	n	1	0	Asteraceae Bercht. & J.Presl
Iz_4_1009	DJI_0373	Herniaria polygama J.Gay	0	n	1	0	Caryophyllaceae Juss.
Iz_4_1009	DJI_0373	Hieracium robustum Fr.	0	n	1	0	Asteraceae Bercht. & J.Presl
Iz_4_1009	DJI_0373	Hieracium umbellatum L.	0	n	1	NA	Asteraceae Bercht. & J.Presl
Iz_4_1009	DJI_0373	Hylotelephium maximum (L.) Holub	0	n	1	0	Crassulaceae J.St.-Hil.
Iz_4_1009	DJI_0373	Jacobaea borysthenica (DC.) B.Nord. & Greuter	0	n	1	0	Asteraceae Bercht. & J.Presl
Iz_4_1009	DJI_0373	Jasione montana L.	0	n	1	0	Campanulaceae Juss.
Iz_4_1009	DJI_0373	Jurinea cyanoides (L.) Rchb.s.l.	0	n	1	0	Asteraceae Bercht. & J.Presl
Iz_4_1009	DJI_0373	Koeleria glauca (Spreng.) DC.	0	n	1	0	Poaceae Barnhart
Iz_4_1009	DJI_0373	Lactuca serriola L.	0	a	0	0	Asteraceae Bercht. & J.Presl
Iz_4_1009	DJI_0373	Linaria genistifolia (L.) Mill.	0	n	1	0	Plantaginaceae Juss.
Iz_4_1009	DJI_0373	Lomelosia argentea (L.) Greuter & Burdet	0	n	1	0	Caprifoliaceae Juss.
Iz_4_1009	DJI_0373	Melampyrum pratense L.	0	n	1	0	Orobanchaceae Vent.
Iz_4_1009	DJI_0373	Oenothera biennis L.	0	a	0	0	Onagraceae Juss.
Iz_4_1009	DJI_0373	Pentanema britannica (L.) D.Gut.Larr., Santos-Vicente, Anderb., E.Rico & M.M.Mart.Ort.	0	n	1	0	Asteraceae Bercht. & J.Presl
Iz_4_1009	DJI_0373	Peucedanum oreoselinum (L.) Moench	0	n	1	0	Apiaceae Lindl.
Iz_4_1009	DJI_0373	Peucedanum ruthenicum M.Bieb.	0	n	1	0	Apiaceae Lindl.
Iz_4_1009	DJI_0373	Pilosella echioides (Lumn.) F.W.Schultz & Sch.Bip.	0	n	1	0	Asteraceae Bercht. & J.Presl
Iz_4_1009	DJI_0373	Pilosella officinarum F. Schultz et Sch. Bip.	0	n	1	0	Asteraceae Bercht. & J.Presl
Iz_4_1009	DJI_0373	Pilosella sp.	0		NA	0	Asteraceae Bercht. & J.Presl
Iz_4_1009	DJI_0373	Pinus sylvestris L.	0	n	1	1	Pinaceae Spreng. ex F.Rudolphi
Iz_4_1009	DJI_0373	Poa annua L.	0	n	1	0	Poaceae Barnhart
Iz_4_1009	DJI_0373	Poa nemoralis L.	0	n	1	0	Poaceae Barnhart
Iz_4_1009	DJI_0373	Poa sp.	0		NA	0	Poaceae Barnhart
Iz_4_1009	DJI_0373	Polygonatum odoratum (Mill.) Druce	0	n	1	0	Asparagaceae Juss.
Iz_4_1009	DJI_0373	Polygonum arenarium Waldst. & Kit.	0	n	1	0	Polygonaceae Juss.
Iz_4_1009	DJI_0373	Populus nigra L.	0	n	1	1	Salicaceae Mirb.
Iz_4_1009	DJI_0373	Populus tremula L.	0	n	1	1	Salicaceae Mirb.
Iz_4_1009	DJI_0373	Populus Ã canescens (Aiton) Sm.	0	n	1	1	Salicaceae Mirb.
Iz_4_1009	DJI_0373	Potentilla argentea L.	0	n	1	0	Rosaceae Juss.
Iz_4_1009	DJI_0373	Psammophiliella muralis (L.) Ikonn.	0	n	1	0	Caryophyllaceae Juss.
Iz_4_1009	DJI_0373	Psephellus sumensis (Kalen.) Greuter	0	n	1	0	Asteraceae Bercht. & J.Presl
Iz_4_1009	DJI_0373	Pulsatilla pratensis (L.) Mill. s.l.	0	n	1	0	Ranunculaceae Juss.
Iz_4_1009	DJI_0373	Pyrus communis L.	0	a	0	1	Rosaceae Juss.
Iz_4_1009	DJI_0373	Quercus robur L.	0	n	1	1	Fagaceae Dumort.
Iz_4_1009	DJI_0373	Robinia pseudoacacia L.	0	a	0	1	Fabaceae Lindl.
Iz_4_1009	DJI_0373	Rosa sp.	0		NA	1	Rosaceae Juss.
Iz_4_1009	DJI_0373	Rubus idaeus L.	0	n	1	0	Rosaceae Juss.
Iz_4_1009	DJI_0373	Rumex acetosella L.	0.5	n	1	0	Polygonaceae Juss.
Iz_4_1009	DJI_0373	Rumex thyrsiflorus Fingerh.	0	n	1	0	Polygonaceae Juss.
Iz_4_1009	DJI_0373	Salix alba L.	0	n	1	1	Salicaceae Mirb.
Iz_4_1009	DJI_0373	Sambucus nigra L.	0	n	1	1	Viburnaceae Raf.
Iz_4_1009	DJI_0373	Secale sylvestre Host	0	n	1	0	Poaceae Barnhart
Iz_4_1009	DJI_0373	Sempervivum ruthenicum Schnittsp. & C.B. Lehm.	0	n	1	0	Crassulaceae J.St.-Hil.
Iz_4_1009	DJI_0373	Setaria viridis (L.) P. Beauv.	0	n	1	0	Poaceae Barnhart
Iz_4_1009	DJI_0373	Silene borysthenica (Gruner) Walters	0	n	1	0	Caryophyllaceae Juss.
Iz_4_1009	DJI_0373	Silene latifolia subsp. alba (Mill.) Greuter & Burdet	0	n	1	0	Caryophyllaceae Juss.
Iz_4_1009	DJI_0373	Solidago virgaurea L.	0	n	1	0	Asteraceae Bercht. & J.Presl
Iz_4_1009	DJI_0373	Sorbus aucuparia L.	0	n	1	1	Rosaceae Juss.
Iz_4_1009	DJI_0373	Stachys recta L.	0	n	1	0	Lamiacea Lindl.
Iz_4_1009	DJI_0373	Tanacetum vulgare L.	0	n	1	0	Asteraceae Bercht. & J.Presl
Iz_4_1009	DJI_0373	Thymus pallasianus Heinr.Braun	0	n	1	0	Lamiacea Lindl.
Iz_4_1009	DJI_0373	Tilia cordata Mill.	0	n	1	1	Malvaceae Juss.
Iz_4_1009	DJI_0373	Trifolium alpestre L.	0	n	1	0	Fabaceae Lindl.
Iz_4_1009	DJI_0373	Trifolium arvense L.	0	n	1	0	Fabaceae Lindl.
Iz_4_1009	DJI_0373	Ulmus glabra Huds.	0	n	1	1	Ulmaceae Mirb.
Iz_4_1009	DJI_0373	Verbascum densiflorum Bertol.	0	n	1	0	Scrophulariaceae Juss.
Iz_4_1009	DJI_0373	Verbascum nigrum L.	0	n	1	0	Scrophulariaceae Juss.
Iz_4_1009	DJI_0373	Veronica austriaca L.	0	n	1	0	Plantaginaceae Juss.
Iz_4_1009	DJI_0373	Veronica incana L.	0	n	1	0	Plantaginaceae Juss.
Iz_4_1009	DJI_0373	Veronica verna L.	0	n	1	0	Plantaginaceae Juss.
Iz_4_1009	DJI_0373	Vincetoxicum hirundinaria Medik	0	n	1	0	Apocynaceae Juss.
Iz_4_1009	DJI_0375	Acer tataricum L.	0	n	1	1	Sapindaceae Juss.
Iz_4_1009	DJI_0375	Achillea millefolium L. s.l.	0	n	1	0	Asteraceae Bercht. & J.Presl
Iz_4_1009	DJI_0375	Allium oleraceum L.	0	n	1	0	Amaryllidaceae J.St.-Hil.
Iz_4_1009	DJI_0375	Ambrosia artemisiifolia L.	0	a	0	0	Asteraceae Bercht. & J.Presl
Iz_4_1009	DJI_0375	Amorpha fruticosa L.	0	a	0	0	Fabaceae Lindl.
Iz_4_1009	DJI_0375	Anthericum ramosum L.	0	n	1	0	Asparagaceae Juss.
Iz_4_1009	DJI_0375	Artemisia marschalliana Spreng.	0	n	1	0	Asteraceae Bercht. & J.Presl
Iz_4_1009	DJI_0375	Asparagus officinalis L.	0	n	1	0	Asparagaceae Juss.
Iz_4_1009	DJI_0375	Astragalus cicer L.	0	n	1	0	Fabaceae Lindl.
Iz_4_1009	DJI_0375	Bassia laniflora (S.G.Gmel.) A.J.Scott	0	n	1	0	Amaranthaceae Juss.
Iz_4_1009	DJI_0375	Berberis vulgaris L.	0	n	1	0	Berberidaceae Juss.
Iz_4_1009	DJI_0375	Betula pendula Roth.	0	n	1	1	Betulaceae Gray
Iz_4_1009	DJI_0375	Betula pendula Roth.	0	n	1	0	Poaceae Barnhart
Iz_4_1009	DJI_0375	Bromus inermis Leyss.	0		NA	NA	
Iz_4_1009	DJI_0375	Bromus squarrosus L.	0	a	0	0	Poaceae Barnhart

Iz_4_1009	DJI_0375	Bromus tectorum L.	0	a	0	0	Poaceae Barnhart
Iz_4_1009	DJI_0375	Calamagrostis epigejos (L.) Roth	2	n	1	0	Poaceae Barnhart
Iz_4_1009	DJI_0375	Carduus acanthoides L.	0	n	1	0	Asteraceae Bercht. & J.Presl
Iz_4_1009	DJI_0375	Carex sp.	0	n	1	0	Cyperaceae Juss.
Iz_4_1009	DJI_0375	Centaurea majorovii Dumbadze	0	n	1	0	Asteraceae Bercht. & J.Presl
Iz_4_1009	DJI_0375	Chamaecytisus ruthenicus (Fisch.ex Wol.) Klaskova	0	n	1	0	Fabaceae Lindl.
Iz_4_1009	DJI_0375	Chelidonium majus L.	0	n	1	0	Papaveraceae Juss.
Iz_4_1009	DJI_0375	Chenopodium betaceum Andrz.	0	n	1	0	Amaranthaceae Juss.
Iz_4_1009	DJI_0375	Chenopodium cf. album	0	n	1	0	Amaranthaceae Juss.
Iz_4_1009	DJI_0375	Chenopodium sp.	0		NA	0	Amaranthaceae Juss.
Iz_4_1009	DJI_0375	Chondrilla juncea L.	0	n	1	0	Asteraceae Bercht. & J.Presl
Iz_4_1009	DJI_0375	Cirsium vulgare (Savi) Ten.	0	n	1	0	Asteraceae Bercht. & J.Presl
Iz_4_1009	DJI_0375	Clinopodium acinos (L.) Kuntze	0	n	1	0	Lamiacea Lindl.
Iz_4_1009	DJI_0375	Convallaria majalis L.	0	n	1	0	Asparagaceae Juss.
Iz_4_1009	DJI_0375	Crataegus rhipidophylla Gand.	0	n	1	1	Rosaceae Juss.
Iz_4_1009	DJI_0375	Crepis foetida L.	0	n	1	0	Asteraceae Bercht. & J.Presl
Iz_4_1009	DJI_0375	Dactylis glomerata L.	0	n	1	0	Poaceae Barnhart
Iz_4_1009	DJI_0375	Dianthus campestris M.Bieb.	0	n	1	0	Caryophyllaceae Juss.
Iz_4_1009	DJI_0375	Digitaria sanguinalis (L.) Scop.	0	a	0	0	Poaceae Barnhart
Iz_4_1009	DJI_0375	Elymus repens (L.) Gould	0	n	1	0	Poaceae Barnhart
Iz_4_1009	DJI_0375	Epilobium tetragonum L.	0	n	1	0	Onagraceae Juss.
Iz_4_1009	DJI_0375	Eragrostis minor Host	0	a	0	0	Poaceae Barnhart
Iz_4_1009	DJI_0375	Erigeron canadensis L.	0	a	0	0	Asteraceae Bercht. & J.Presl
Iz_4_1009	DJI_0375	Euphorbia seguieriana Neck.	0	n	1	0	Euphorbiaceae Juss.
Iz_4_1009	DJI_0375	Fallopia dumetorum (L.) Holub	0	n	1	0	Polygonaceae Juss.
Iz_4_1009	DJI_0375	Festuca beckeri (Hack.) Trautv.	0	n	1	0	Poaceae Barnhart
Iz_4_1009	DJI_0375	Fragaria vesca L.	0	n	1	0	Rosaceae Juss.
Iz_4_1009	DJI_0375	Frangula alnus Mill.	0	n	1	1	Rhamnaceae Juss.
Iz_4_1009	DJI_0375	Fraxinus pennsylvanica Marshall	0	a	0	1	Oleaceae Hoffmanns. & Link
Iz_4_1009	DJI_0375	Galeopsis bifida Boenn.	0	n	1	0	Lamiacea Lindl.
Iz_4_1009	DJI_0375	Galium verum L.	0	n	1	0	Rubiaceae Juss.
Iz_4_1009	DJI_0375	Genista tinctoria L.	0	n	1	0	Fabaceae Lindl.
Iz_4_1009	DJI_0375	Geranium robertianum L.	0	n	1	0	Geraniaceae Juss.
Iz_4_1009	DJI_0375	Geranium sanquineum L.	0	n	1	0	Geraniaceae Juss.
Iz_4_1009	DJI_0375	Gypsophila paniculata L.	0	n	1	0	Caryophyllaceae Juss.
Iz_4_1009	DJI_0375	Helichrysum arenarium (L.) Moench	0	n	1	0	Asteraceae Bercht. & J.Presl
Iz_4_1009	DJI_0375	Herniaria polygama J.Gay	0	n	1	0	Caryophyllaceae Juss.
Iz_4_1009	DJI_0375	Hieracium robustum Fr.	0	n	1	0	Asteraceae Bercht. & J.Presl
Iz_4_1009	DJI_0375	Hieracium umbellatum L.	0	n	1	NA	Asteraceae Bercht. & J.Presl
Iz_4_1009	DJI_0375	Hylotelephium maximum (L.) Holub	0	n	1	0	Crassulaceae J.St.-Hil.
Iz_4_1009	DJI_0375	Jacobaea borysthenica (DC.) B.Nord. & Greuter	0	n	1	0	Asteraceae Bercht. & J.Presl
Iz_4_1009	DJI_0375	Jasione montana L.	0	n	1	0	Campanulaceae Juss.
Iz_4_1009	DJI_0375	Jurinea cyanoides (L.) Rchb.s.l.	0	n	1	0	Asteraceae Bercht. & J.Presl
Iz_4_1009	DJI_0375	Koeleria glauca (Spreng.) DC.	0	n	1	0	Poaceae Barnhart
Iz_4_1009	DJI_0375	Lactuca serriola L.	0	a	0	0	Asteraceae Bercht. & J.Presl
Iz_4_1009	DJI_0375	Linaria genistifolia (L.) Mill.	0	n	1	0	Plantaginaceae Juss.
Iz_4_1009	DJI_0375	Lomelosia argentea (L.) Greuter & Burdet	0	n	1	0	Caprifoliaceae Juss.
Iz_4_1009	DJI_0375	Melampyrum pratense L.	0	n	1	0	Orobanchaceae Vent.
Iz_4_1009	DJI_0375	Oenothera biennis L.	0	a	0	0	Onagraceae Juss.
Iz_4_1009	DJI_0375	Pentanema britannica (L.) D.Gut.Larr., Santos-Vicente, Anderb., E.Rico & M.M.Mart.Ort.	0	n	1	0	Asteraceae Bercht. & J.Presl
Iz_4_1009	DJI_0375	Peucedanum oreoselinum (L.) Moench	0	n	1	0	Apiaceae Lindl.
Iz_4_1009	DJI_0375	Peucedanum ruthenicum M.Bieb.	0	n	1	0	Apiaceae Lindl.
Iz_4_1009	DJI_0375	Pilosella echioides (Lumn.) F.W.Schultz & Sch.Bip.	0	n	1	0	Asteraceae Bercht. & J.Presl
Iz_4_1009	DJI_0375	Pilosella officinarum F. Schultz et Sch. Bip.	0	n	1	0	Asteraceae Bercht. & J.Presl
Iz_4_1009	DJI_0375	Pilosella sp.	0		NA	0	Asteraceae Bercht. & J.Presl
Iz_4_1009	DJI_0375	Pinus sylvestris L.	0	n	1	1	Pinaceae Spreng. ex F.Rudolphi
Iz_4_1009	DJI_0375	Poa annua L.	0	n	1	0	Poaceae Barnhart
Iz_4_1009	DJI_0375	Poa nemoralis L.	0	n	1	0	Poaceae Barnhart
Iz_4_1009	DJI_0375	Poa sp.	0		NA	0	Poaceae Barnhart
Iz_4_1009	DJI_0375	Polygonatum odoratum (Mill.) Druce	0	n	1	0	Asparagaceae Juss.
Iz_4_1009	DJI_0375	Polygonum arenarium Waldst. & Kit.	0	n	1	0	Polygonaceae Juss.
Iz_4_1009	DJI_0375	Populus nigra L.	0	n	1	1	Salicaceae Mirb.
Iz_4_1009	DJI_0375	Populus tremula L.	0	n	1	1	Salicaceae Mirb.
Iz_4_1009	DJI_0375	Populus Ã canescens (Aiton) Sm.	0	n	1	1	Salicaceae Mirb.
Iz_4_1009	DJI_0375	Potentilla argentea L.	0	n	1	0	Rosaceae Juss.
Iz_4_1009	DJI_0375	Psammophiliella muralis (L.) Ikonn.	0	n	1	0	Caryophyllaceae Juss.
Iz_4_1009	DJI_0375	Psephellus sumensis (Kalen.) Greuter	0	n	1	0	Asteraceae Bercht. & J.Presl
Iz_4_1009	DJI_0375	Pulsatilla pratensis (L.) Mill. s.l.	0	n	1	0	Ranunculaceae Juss.
Iz_4_1009	DJI_0375	Pyrus communis L.	0	a	0	1	Rosaceae Juss.
Iz_4_1009	DJI_0375	Quercus robur L.	0	n	1	1	Fagaceae Dumort.
Iz_4_1009	DJI_0375	Robinia pseudoacacia L.	0	a	0	1	Fabaceae Lindl.
Iz_4_1009	DJI_0375	Rosa sp.	0		NA	1	Rosaceae Juss.
Iz_4_1009	DJI_0375	Rubus idaeus L.	0	n	1	0	Rosaceae Juss.
Iz_4_1009	DJI_0375	Rumex acetosella L.	0	n	1	0	Polygonaceae Juss.
Iz_4_1009	DJI_0375	Rumex thyrsiflorus Fingerh.	0	n	1	0	Polygonaceae Juss.
Iz_4_1009	DJI_0375	Salix alba L.	0	n	1	1	Salicaceae Mirb.
Iz_4_1009	DJI_0375	Sambucus nigra L.	0	n	1	1	Viburnaceae Raf.
Iz_4_1009	DJI_0375	Secale sylvestre Host	0	n	1	0	Poaceae Barnhart
Iz_4_1009	DJI_0375	Sempervivum ruthenicum Schnittsp. & C.B. Lehm.	0	n	1	0	Crassulaceae J.St.-Hil.
Iz_4_1009	DJI_0375	Setaria viridis (L.) P. Beauv.	0	n	1	0	Poaceae Barnhart
Iz_4_1009	DJI_0375	Silene borysthenica (Gruner) Walters	0	n	1	0	Caryophyllaceae Juss.
Iz_4_1009	DJI_0375	Silene latifolia subsp. alba (Mill.) Greuter & Burdet	0	n	1	0	Caryophyllaceae Juss.
Iz_4_1009	DJI_0375	Solidago virgaurea L.	0	n	1	0	Asteraceae Bercht. & J.Presl
Iz_4_1009	DJI_0375	Sorbus aucuparia L.	0	n	1	1	Rosaceae Juss.
Iz_4_1009	DJI_0375	Stachys recta L.	0	n	1	0	Lamiacea Lindl.
Iz_4_1009	DJI_0375	Tanacetum vulgare L.	0	n	1	0	Asteraceae Bercht. & J.Presl
Iz_4_1009	DJI_0375	Thymus pallasianus Heinr.Braun	0	n	1	0	Lamiacea Lindl.
Iz_4_1009	DJI_0375	Tilia cordata Mill.	0	n	1	1	Malvaceae Juss.
Iz_4_1009	DJI_0375	Trifolium alpestre L.	0	n	1	0	Fabaceae Lindl.
Iz_4_1009	DJI_0375	Trifolium arvense L.	0	n	1	0	Fabaceae Lindl.
Iz_4_1009	DJI_0375	Ulmus glabra Huds.	0	n	1	1	Ulmaceae Mirb.
Iz_4_1009	DJI_0375	Verbascum densiflorum Bertol.	0	n	1	0	Scrophulariaceae Juss.
Iz_4_1009	DJI_0375	Verbascum nigrum L.	0	n	1	0	Scrophulariaceae Juss.
Iz_4_1009	DJI_0375	Veronica austriaca L.	0	n	1	0	Plantaginaceae Juss.
Iz_4_1009	DJI_0375	Veronica incana L.	0	n	1	0	Plantaginaceae Juss.
Iz_4_1009	DJI_0375	Veronica verna L.	0	n	1	0	Plantaginaceae Juss.
Iz_4_1009	DJI_0375	Vincetoxicum hirundinaria Medik	0	n	1	0	Apocynaceae Juss.
Iz_4_1009	DJI_0377	Acer tataricum L.	0	n	1	1	Sapindaceae Juss.

Iz_4_1009	DJI_0377	Achillea millefolium L. s.l.	0	n	1	0	Asteraceae Bercht. & J.Presl
Iz_4_1009	DJI_0377	Allium oleraceum L.	0	n	1	0	Amaryllidaceae J.St.-Hil.
Iz_4_1009	DJI_0377	Ambrosia artemisiifolia L.	0	a	0	0	Asteraceae Bercht. & J.Presl
Iz_4_1009	DJI_0377	Amorpha fruticosa L.	0	a	0	0	Fabaceae Lindl.
Iz_4_1009	DJI_0377	Anthericum ramosum L.	0	n	1	0	Asparagaceae Juss.
Iz_4_1009	DJI_0377	Artemisia marschalliana Spreng.	0	n	1	0	Asteraceae Bercht. & J.Presl
Iz_4_1009	DJI_0377	Asparagus officinalis L.	0	n	1	0	Asparagaceae Juss.
Iz_4_1009	DJI_0377	Astragalus cicer L.	0	n	1	0	Fabaceae Lindl.
Iz_4_1009	DJI_0377	Bassia laniflora (S.G.Gmel.) A.J.Scott	0	n	1	0	Amaranthaceae Juss.
Iz_4_1009	DJI_0377	Berberis vulgaris L.	0	n	1	0	Berberidaceae Juss.
Iz_4_1009	DJI_0377	Betula pendula Roth.	0	n	1	1	Betulaceae Gray
Iz_4_1009	DJI_0377	Betula pendula Roth.	0	n	1	0	Poaceae Barnhart
Iz_4_1009	DJI_0377	Bromus inermis Leyss.	0		NA	NA	
Iz_4_1009	DJI_0377	Bromus squarrosus L.	0	a	0	0	Poaceae Barnhart
Iz_4_1009	DJI_0377	Bromus tectorum L.	0	a	0	0	Poaceae Barnhart
Iz_4_1009	DJI_0377	Calamagrostis epigejos (L.) Roth	1	n	1	0	Poaceae Barnhart
Iz_4_1009	DJI_0377	Carduus acanthoides L.	0	n	1	0	Asteraceae Bercht. & J.Presl
Iz_4_1009	DJI_0377	Carex sp.	0	n	1	0	Cyperaceae Juss.
Iz_4_1009	DJI_0377	Centaurea majorovii Dumbadze	0	n	1	0	Asteraceae Bercht. & J.Presl
Iz_4_1009	DJI_0377	Chamaecytisus ruthenicus (Fisch.ex Wol.) Klaskova	0	n	1	0	Fabaceae Lindl.
Iz_4_1009	DJI_0377	Chelidonium majus L.	0	n	1	0	Papaveraceae Juss.
Iz_4_1009	DJI_0377	Chenopodium betaceum Andrz.	0	n	1	0	Amaranthaceae Juss.
Iz_4_1009	DJI_0377	Chenopodium cf. album	0	n	1	0	Amaranthaceae Juss.
Iz_4_1009	DJI_0377	Chenopodium sp.	0		NA	0	Amaranthaceae Juss.
Iz_4_1009	DJI_0377	Chondrilla juncea L.	0	n	1	0	Asteraceae Bercht. & J.Presl
Iz_4_1009	DJI_0377	Cirsium vulgare (Savi) Ten.	0	n	1	0	Asteraceae Bercht. & J.Presl
Iz_4_1009	DJI_0377	Clinopodium acinos (L.) Kuntze	0	n	1	0	Lamiacea Lindl.
Iz_4_1009	DJI_0377	Convallaria majalis L.	0	n	1	0	Asparagaceae Juss.
Iz_4_1009	DJI_0377	Crataegus rhipidophylla Gand.	0	n	1	1	Rosaceae Juss.
Iz_4_1009	DJI_0377	Crepis foetida L.	0	n	1	0	Asteraceae Bercht. & J.Presl
Iz_4_1009	DJI_0377	Dactylis glomerata L.	0	n	1	0	Poaceae Barnhart
Iz_4_1009	DJI_0377	Dianthus campestris M.Bieb.	0	n	1	0	Caryophyllaceae Juss.
Iz_4_1009	DJI_0377	Digitaria sanguinalis (L.) Scop.	0	a	0	0	Poaceae Barnhart
Iz_4_1009	DJI_0377	Elymus repens (L.) Gould	0	n	1	0	Poaceae Barnhart
Iz_4_1009	DJI_0377	Epilobium tetragonum L.	0	n	1	0	Onagraceae Juss.
Iz_4_1009	DJI_0377	Eragrostis minor Host	0	a	0	0	Poaceae Barnhart
Iz_4_1009	DJI_0377	Erigeron canadensis L.	0	a	0	0	Asteraceae Bercht. & J.Presl
Iz_4_1009	DJI_0377	Euphorbia seguieriana Neck.	0	n	1	0	Euphorbiaceae Juss.
Iz_4_1009	DJI_0377	Fallopia dumetorum (L.) Holub	0	n	1	0	Polygonaceae Juss.
Iz_4_1009	DJI_0377	Festuca beckeri (Hack.) Trautv.	0	n	1	0	Poaceae Barnhart
Iz_4_1009	DJI_0377	Fragaria vesca L.	0	n	1	0	Rosaceae Juss.
Iz_4_1009	DJI_0377	Frangula alnus Mill.	0	n	1	1	Rhamnaceae Juss.
Iz_4_1009	DJI_0377	Fraxinus pennsylvanica Marshall	0	a	0	1	Oleaceae Hoffmanns. & Link
Iz_4_1009	DJI_0377	Galeopsis bifida Boenn.	0	n	1	0	Lamiacea Lindl.
Iz_4_1009	DJI_0377	Galium verum L.	0	n	1	0	Rubiaceae Juss.
Iz_4_1009	DJI_0377	Genista tinctoria L.	0	n	1	0	Fabaceae Lindl.
Iz_4_1009	DJI_0377	Geranium robertianum L.	0	n	1	0	Geraniaceae Juss.
Iz_4_1009	DJI_0377	Geranium sanquineum L.	0	n	1	0	Geraniaceae Juss.
Iz_4_1009	DJI_0377	Gypsophila paniculata L.	0	n	1	0	Caryophyllaceae Juss.
Iz_4_1009	DJI_0377	Helichrysum arenarium (L.) Moench	0	n	1	0	Asteraceae Bercht. & J.Presl
Iz_4_1009	DJI_0377	Herniaria polygama J.Gay	0	n	1	0	Caryophyllaceae Juss.
Iz_4_1009	DJI_0377	Hieracium robustum Fr.	0	n	1	0	Asteraceae Bercht. & J.Presl
Iz_4_1009	DJI_0377	Hieracium umbellatum L.	0	n	1	NA	Asteraceae Bercht. & J.Presl
Iz_4_1009	DJI_0377	Hylotelephium maximum (L.) Holub	0	n	1	0	Crassulaceae J.St.-Hil.
Iz_4_1009	DJI_0377	Jacobaea borysthenica (DC.) B.Nord. & Greuter	0	n	1	0	Asteraceae Bercht. & J.Presl
Iz_4_1009	DJI_0377	Jasione montana L.	0	n	1	0	Campanulaceae Juss.
Iz_4_1009	DJI_0377	Jurinea cyanoides (L.) Rchb.s.l.	0	n	1	0	Asteraceae Bercht. & J.Presl
Iz_4_1009	DJI_0377	Koeleria glauca (Spreng.) DC.	0	n	1	0	Poaceae Barnhart
Iz_4_1009	DJI_0377	Lactuca serriola L.	0	a	0	0	Asteraceae Bercht. & J.Presl
Iz_4_1009	DJI_0377	Linaria genistifolia (L.) Mill.	0	n	1	0	Plantaginaceae Juss.
Iz_4_1009	DJI_0377	Lomelosia argentea (L.) Greuter & Burdet	0	n	1	0	Caprifoliaceae Juss.
Iz_4_1009	DJI_0377	Melampyrum pratense L.	0	n	1	0	Orobanchaceae Vent.
Iz_4_1009	DJI_0377	Oenothera biennis L.	0	a	0	0	Onagraceae Juss.
Iz_4_1009	DJI_0377	Pentanema britannica (L.) D.Gut.Larr., Santos-Vicente, Anderb., E.Rico & M.M.Mart.Ort.	0	n	1	0	Asteraceae Bercht. & J.Presl
Iz_4_1009	DJI_0377	Peucedanum oreoselinum (L.) Moench	0	n	1	0	Apiaceae Lindl.
Iz_4_1009	DJI_0377	Peucedanum ruthenicum M.Bieb.	0	n	1	0	Apiaceae Lindl.
Iz_4_1009	DJI_0377	Pilosella echioides (Lumn.) F.W.Schultz & Sch.Bip.	0	n	1	0	Asteraceae Bercht. & J.Presl
Iz_4_1009	DJI_0377	Pilosella officinarum F. Schultz et Sch. Bip.	0	n	1	0	Asteraceae Bercht. & J.Presl
Iz_4_1009	DJI_0377	Pilosella sp.	0		NA	0	Asteraceae Bercht. & J.Presl
Iz_4_1009	DJI_0377	Pinus sylvestris L.	0	n	1	1	Pinaceae Spreng. ex F.Rudolphi
Iz_4_1009	DJI_0377	Poa annua L.	0	n	1	0	Poaceae Barnhart
Iz_4_1009	DJI_0377	Poa nemoralis L.	0	n	1	0	Poaceae Barnhart
Iz_4_1009	DJI_0377	Poa sp.	0		NA	0	Poaceae Barnhart
Iz_4_1009	DJI_0377	Polygonatum odoratum (Mill.) Druce	0	n	1	0	Asparagaceae Juss.
Iz_4_1009	DJI_0377	Polygonum arenarium Waldst. & Kit.	0	n	1	0	Polygonaceae Juss.
Iz_4_1009	DJI_0377	Populus nigra L.	0	n	1	1	Salicaceae Mirb.
Iz_4_1009	DJI_0377	Populus tremula L.	0	n	1	1	Salicaceae Mirb.
Iz_4_1009	DJI_0377	Populus Ã canescens (Aiton) Sm.	0	n	1	1	Salicaceae Mirb.
Iz_4_1009	DJI_0377	Potentilla argentea L.	0	n	1	0	Rosaceae Juss.
Iz_4_1009	DJI_0377	Psammophiliella muralis (L.) Ikonn.	0	n	1	0	Caryophyllaceae Juss.
Iz_4_1009	DJI_0377	Psephellus sumensis (Kalen.) Greuter	0	n	1	0	Asteraceae Bercht. & J.Presl
Iz_4_1009	DJI_0377	Pulsatilla pratensis (L.) Mill. s.l.	0	n	1	0	Ranunculaceae Juss.
Iz_4_1009	DJI_0377	Pyrus communis L.	0	a	0	1	Rosaceae Juss.
Iz_4_1009	DJI_0377	Quercus robur L.	0	n	1	1	Fagaceae Dumort.
Iz_4_1009	DJI_0377	Robinia pseudoacacia L.	0	a	0	1	Fabaceae Lindl.
Iz_4_1009	DJI_0377	Rosa sp.	0		NA	1	Rosaceae Juss.
Iz_4_1009	DJI_0377	Rubus idaeus L.	0	n	1	0	Rosaceae Juss.
Iz_4_1009	DJI_0377	Rumex acetosella L.	0	n	1	0	Polygonaceae Juss.
Iz_4_1009	DJI_0377	Rumex thyrsiflorus Fingerh.	0	n	1	0	Polygonaceae Juss.
Iz_4_1009	DJI_0377	Salix alba L.	0	n	1	1	Salicaceae Mirb.
Iz_4_1009	DJI_0377	Sambucus nigra L.	0	n	1	1	Viburnaceae Raf.
Iz_4_1009	DJI_0377	Secale sylvestre Host	0	n	1	0	Poaceae Barnhart
Iz_4_1009	DJI_0377	Sempervivum ruthenicum Schnittsp. & C.B. Lehm.	0	n	1	0	Crassulaceae J.St.-Hil.
Iz_4_1009	DJI_0377	Setaria viridis (L.) P. Beauv.	0	n	1	0	Poaceae Barnhart
Iz_4_1009	DJI_0377	Silene borysthenica (Gruner) Walters	0	n	1	0	Caryophyllaceae Juss.
Iz_4_1009	DJI_0377	Silene latifolia subsp. alba (Mill.) Greuter & Burdet	0	n	1	0	Caryophyllaceae Juss.
Iz_4_1009	DJI_0377	Solidago virgaurea L.	1	n	1	0	Asteraceae Bercht. & J.Presl
Iz_4_1009	DJI_0377	Sorbus aucuparia L.	0	n	1	1	Rosaceae Juss.

Iz_4_1009	DJI_0377	Stachys recta L.	0	n	1	0	Lamiacea Lindl.
Iz_4_1009	DJI_0377	Tanacetum vulgare L.	0	n	1	0	Asteraceae Bercht. & J.Presl
Iz_4_1009	DJI_0377	Thymus pallasianus Heinr.Braun	0	n	1	0	Lamiacea Lindl.
Iz_4_1009	DJI_0377	Tilia cordata Mill.	0	n	1	1	Malvaceae Juss.
Iz_4_1009	DJI_0377	Trifolium alpestre L.	0	n	1	0	Fabaceae Lindl.
Iz_4_1009	DJI_0377	Trifolium arvense L.	0	n	1	0	Fabaceae Lindl.
Iz_4_1009	DJI_0377	Ulmus glabra Huds.	0	n	1	1	Ulmaceae Mirb.
Iz_4_1009	DJI_0377	Verbascum densiflorum Bertol.	0	n	1	0	Scrophulariaceae Juss.
Iz_4_1009	DJI_0377	Verbascum nigrum L.	0	n	1	0	Scrophulariaceae Juss.
Iz_4_1009	DJI_0377	Veronica austriaca L.	0	n	1	0	Plantaginaceae Juss.
Iz_4_1009	DJI_0377	Veronica incana L.	0	n	1	0	Plantaginaceae Juss.
Iz_4_1009	DJI_0377	Veronica verna L.	0	n	1	0	Plantaginaceae Juss.
Iz_4_1009	DJI_0377	Vincetoxicum hirundinaria Medik	0	n	1	0	Apocynaceae Juss.
Iz_4_1009	DJI_0378	Acer tataricum L.	0	n	1	1	Sapindaceae Juss.
Iz_4_1009	DJI_0378	Achillea millefolium L. s.l.	0	n	1	0	Asteraceae Bercht. & J.Presl
Iz_4_1009	DJI_0378	Allium oleraceum L.	0	n	1	0	Amaryllidaceae J.St.-Hil.
Iz_4_1009	DJI_0378	Ambrosia artemisiifolia L.	0	a	0	0	Asteraceae Bercht. & J.Presl
Iz_4_1009	DJI_0378	Amorpha fruticosa L.	0	a	0	0	Fabaceae Lindl.
Iz_4_1009	DJI_0378	Anthericum ramosum L.	0	n	1	0	Asparagaceae Juss.
Iz_4_1009	DJI_0378	Artemisia marschalliana Spreng.	0	n	1	0	Asteraceae Bercht. & J.Presl
Iz_4_1009	DJI_0378	Asparagus officinalis L.	0	n	1	0	Asparagaceae Juss.
Iz_4_1009	DJI_0378	Astragalus cicer L.	0	n	1	0	Fabaceae Lindl.
Iz_4_1009	DJI_0378	Bassia laniflora (S.G.Gmel.) A.J.Scott	0	n	1	0	Amaranthaceae Juss.
Iz_4_1009	DJI_0378	Berberis vulgaris L.	0	n	1	0	Berberidaceae Juss.
Iz_4_1009	DJI_0378	Betula pendula Roth.	0	n	1	1	Betulaceae Gray
Iz_4_1009	DJI_0378	Betula pendula Roth.	0	n	1	0	Poaceae Barnhart
Iz_4_1009	DJI_0378	Bromus inermis Leyss.	0		NA	NA	
Iz_4_1009	DJI_0378	Bromus squarrosus L.	0	a	0	0	Poaceae Barnhart
Iz_4_1009	DJI_0378	Bromus tectorum L.	0	a	0	0	Poaceae Barnhart
Iz_4_1009	DJI_0378	Calamagrostis epigejos (L.) Roth	0.5	n	1	0	Poaceae Barnhart
Iz_4_1009	DJI_0378	Carduus acanthoides L.	0	n	1	0	Asteraceae Bercht. & J.Presl
Iz_4_1009	DJI_0378	Carex sp.	0	n	1	0	Cyperaceae Juss.
Iz_4_1009	DJI_0378	Centaurea majorovii Dumbadze	0	n	1	0	Asteraceae Bercht. & J.Presl
Iz_4_1009	DJI_0378	Chamaecytisus ruthenicus (Fisch.ex Wol.) Klaskova	0	n	1	0	Fabaceae Lindl.
Iz_4_1009	DJI_0378	Chelidonium majus L.	0	n	1	0	Papaveraceae Juss.
Iz_4_1009	DJI_0378	Chenopodium betaceum Andrz.	0	n	1	0	Amaranthaceae Juss.
Iz_4_1009	DJI_0378	Chenopodium cf. album	0	n	1	0	Amaranthaceae Juss.
Iz_4_1009	DJI_0378	Chenopodium sp.	0		NA	0	Amaranthaceae Juss.
Iz_4_1009	DJI_0378	Chondrilla juncea L.	0	n	1	0	Asteraceae Bercht. & J.Presl
Iz_4_1009	DJI_0378	Cirsium vulgare (Savi) Ten.	0	n	1	0	Asteraceae Bercht. & J.Presl
Iz_4_1009	DJI_0378	Clinopodium acinos (L.) Kuntze	0	n	1	0	Lamiacea Lindl.
Iz_4_1009	DJI_0378	Convallaria majalis L.	0	n	1	0	Asparagaceae Juss.
Iz_4_1009	DJI_0378	Crataegus rhipidophylla Gand.	0	n	1	1	Rosaceae Juss.
Iz_4_1009	DJI_0378	Crepis foetida L.	0	n	1	0	Asteraceae Bercht. & J.Presl
Iz_4_1009	DJI_0378	Dactylis glomerata L.	0	n	1	0	Poaceae Barnhart
Iz_4_1009	DJI_0378	Dianthus campestris M.Bieb.	0	n	1	0	Caryophyllaceae Juss.
Iz_4_1009	DJI_0378	Digitaria sanguinalis (L.) Scop.	0	a	0	0	Poaceae Barnhart
Iz_4_1009	DJI_0378	Elymus repens (L.) Gould	0	n	1	0	Poaceae Barnhart
Iz_4_1009	DJI_0378	Epilobium tetragonum L.	0	n	1	0	Onagraceae Juss.
Iz_4_1009	DJI_0378	Eragrostis minor Host	0	a	0	0	Poaceae Barnhart
Iz_4_1009	DJI_0378	Erigeron canadensis L.	0	a	0	0	Asteraceae Bercht. & J.Presl
Iz_4_1009	DJI_0378	Euphorbia seguieriana Neck.	0	n	1	0	Euphorbiaceae Juss.
Iz_4_1009	DJI_0378	Fallopia dumetorum (L.) Holub	0	n	1	0	Polygonaceae Juss.
Iz_4_1009	DJI_0378	Festuca beckeri (Hack.) Trautv.	0	n	1	0	Poaceae Barnhart
Iz_4_1009	DJI_0378	Fragaria vesca L.	0	n	1	0	Rosaceae Juss.
Iz_4_1009	DJI_0378	Frangula alnus Mill.	0	n	1	1	Rhamnaceae Juss.
Iz_4_1009	DJI_0378	Fraxinus pennsylvanica Marshall	0	a	0	1	Oleaceae Hoffmanns. & Link
Iz_4_1009	DJI_0378	Galeopsis bifida Boenn.	0	n	1	0	Lamiacea Lindl.
Iz_4_1009	DJI_0378	Galium verum L.	0	n	1	0	Rubiaceae Juss.
Iz_4_1009	DJI_0378	Genista tinctoria L.	0	n	1	0	Fabaceae Lindl.
Iz_4_1009	DJI_0378	Geranium robertianum L.	0	n	1	0	Geraniaceae Juss.
Iz_4_1009	DJI_0378	Geranium sanquineum L.	0	n	1	0	Geraniaceae Juss.
Iz_4_1009	DJI_0378	Gypsophila paniculata L.	0	n	1	0	Caryophyllaceae Juss.
Iz_4_1009	DJI_0378	Helichrysum arenarium (L.) Moench	0	n	1	0	Asteraceae Bercht. & J.Presl
Iz_4_1009	DJI_0378	Herniaria polygama J.Gay	0	n	1	0	Caryophyllaceae Juss.
Iz_4_1009	DJI_0378	Hieracium robustum Fr.	0	n	1	0	Asteraceae Bercht. & J.Presl
Iz_4_1009	DJI_0378	Hieracium umbellatum L.	0	n	1	NA	Asteraceae Bercht. & J.Presl
Iz_4_1009	DJI_0378	Hylotelephium maximum (L.) Holub	0	n	1	0	Crassulaceae J.St.-Hil.
Iz_4_1009	DJI_0378	Jacobaea borysthenica (DC.) B.Nord. & Greuter	0	n	1	0	Asteraceae Bercht. & J.Presl
Iz_4_1009	DJI_0378	Jasione montana L.	0	n	1	0	Campanulaceae Juss.
Iz_4_1009	DJI_0378	Jurinea cyanoides (L.) Rchb.s.l.	0	n	1	0	Asteraceae Bercht. & J.Presl
Iz_4_1009	DJI_0378	Koeleria glauca (Spreng.) DC.	0	n	1	0	Poaceae Barnhart
Iz_4_1009	DJI_0378	Lactuca serriola L.	0	a	0	0	Asteraceae Bercht. & J.Presl
Iz_4_1009	DJI_0378	Linaria genistifolia (L.) Mill.	0	n	1	0	Plantaginaceae Juss.
Iz_4_1009	DJI_0378	Lomelosia argentea (L.) Greuter & Burdet	0	n	1	0	Caprifoliaceae Juss.
Iz_4_1009	DJI_0378	Melampyrum pratense L.	0	n	1	0	Orobanchaceae Vent.
Iz_4_1009	DJI_0378	Oenothera biennis L.	0	a	0	0	Onagraceae Juss.
Iz_4_1009	DJI_0378	Pentanema britannica (L.) D.Gut.Larr., Santos-Vicente, Anderb., E.Rico & M.M.Mart.Ort.	0	n	1	0	Asteraceae Bercht. & J.Presl
Iz_4_1009	DJI_0378	Peucedanum oreoselinum (L.) Moench	0	n	1	0	Apiaceae Lindl.
Iz_4_1009	DJI_0378	Peucedanum ruthenicum M.Bieb.	0	n	1	0	Apiaceae Lindl.
Iz_4_1009	DJI_0378	Pilosella echioides (Lumn.) F.W.Schultz & Sch.Bip.	0	n	1	0	Asteraceae Bercht. & J.Presl
Iz_4_1009	DJI_0378	Pilosella officinarum F. Schultz et Sch. Bip.	0	n	1	0	Asteraceae Bercht. & J.Presl
Iz_4_1009	DJI_0378	Pilosella sp.	0		NA	0	Asteraceae Bercht. & J.Presl
Iz_4_1009	DJI_0378	Pinus sylvestris L.	0	n	1	1	Pinaceae Spreng. ex F.Rudolphi
Iz_4_1009	DJI_0378	Poa annua L.	0	n	1	0	Poaceae Barnhart
Iz_4_1009	DJI_0378	Poa nemoralis L.	0	n	1	0	Poaceae Barnhart
Iz_4_1009	DJI_0378	Poa sp.	0		NA	0	Poaceae Barnhart
Iz_4_1009	DJI_0378	Polygonatum odoratum (Mill.) Druce	0	n	1	0	Asparagaceae Juss.
Iz_4_1009	DJI_0378	Polygonum arenarium Waldst. & Kit.	0	n	1	0	Polygonaceae Juss.
Iz_4_1009	DJI_0378	Populus nigra L.	0	n	1	1	Salicaceae Mirb.
Iz_4_1009	DJI_0378	Populus tremula L.	0	n	1	1	Salicaceae Mirb.
Iz_4_1009	DJI_0378	Populus Ã canescens (Aiton) Sm.	0	n	1	1	Salicaceae Mirb.
Iz_4_1009	DJI_0378	Potentilla argentea L.	0	n	1	0	Rosaceae Juss.
Iz_4_1009	DJI_0378	Psammophiliella muralis (L.) Ikonn.	0	n	1	0	Caryophyllaceae Juss.
Iz_4_1009	DJI_0378	Psephellus sumensis (Kalen.) Greuter	0	n	1	0	Asteraceae Bercht. & J.Presl
Iz_4_1009	DJI_0378	Pulsatilla pratensis (L.) Mill. s.l.	0	n	1	0	Ranunculaceae Juss.
Iz_4_1009	DJI_0378	Pyrus communis L.	0	a	0	1	Rosaceae Juss.
Iz_4_1009	DJI_0378	Quercus robur L.	0	n	1	1	Fagaceae Dumort.

Iz_4_1009	DJI_0378	Robinia pseudoacacia L.	0	a	0	1	Fabaceae Lindl.
Iz_4_1009	DJI_0378	Rosa sp.	0		NA	1	Rosaceae Juss.
Iz_4_1009	DJI_0378	Rubus idaeus L.	0	n	1	0	Rosaceae Juss.
Iz_4_1009	DJI_0378	Rumex acetosella L.	0	n	1	0	Polygonaceae Juss.
Iz_4_1009	DJI_0378	Rumex thyrsiflorus Fingerh.	0	n	1	0	Polygonaceae Juss.
Iz_4_1009	DJI_0378	Salix alba L.	0	n	1	1	Salicaceae Mirb.
Iz_4_1009	DJI_0378	Sambucus nigra L.	0	n	1	1	Viburnaceae Raf.
Iz_4_1009	DJI_0378	Secale sylvestre Host	0	n	1	0	Poaceae Barnhart
Iz_4_1009	DJI_0378	Sempervivum ruthenicum Schnittsp. & C.B. Lehm.	0	n	1	0	Crassulaceae J.St.-Hil.
Iz_4_1009	DJI_0378	Setaria viridis (L.) P. Beauv.	0	n	1	0	Poaceae Barnhart
Iz_4_1009	DJI_0378	Silene borysthenica (Gruner) Walters	0	n	1	0	Caryophyllaceae Juss.
Iz_4_1009	DJI_0378	Silene latifolia subsp. alba (Mill.) Greuter & Burdet	0	n	1	0	Caryophyllaceae Juss.
Iz_4_1009	DJI_0378	Solidago virgaurea L.	0.5	n	1	0	Asteraceae Bercht. & J.Presl
Iz_4_1009	DJI_0378	Sorbus aucuparia L.	0	n	1	1	Rosaceae Juss.
Iz_4_1009	DJI_0378	Stachys recta L.	0	n	1	0	Lamiacea Lindl.
Iz_4_1009	DJI_0378	Tanacetum vulgare L.	0	n	1	0	Asteraceae Bercht. & J.Presl
Iz_4_1009	DJI_0378	Thymus pallasianus Heinr.Braun	0	n	1	0	Lamiacea Lindl.
Iz_4_1009	DJI_0378	Tilia cordata Mill.	0	n	1	1	Malvaceae Juss.
Iz_4_1009	DJI_0378	Trifolium alpestre L.	0	n	1	0	Fabaceae Lindl.
Iz_4_1009	DJI_0378	Trifolium arvense L.	0	n	1	0	Fabaceae Lindl.
Iz_4_1009	DJI_0378	Ulmus glabra Huds.	0	n	1	1	Ulmaceae Mirb.
Iz_4_1009	DJI_0378	Verbascum densiflorum Bertol.	0	n	1	0	Scrophulariaceae Juss.
Iz_4_1009	DJI_0378	Verbascum nigrum L.	0	n	1	0	Scrophulariaceae Juss.
Iz_4_1009	DJI_0378	Veronica austriaca L.	0	n	1	0	Plantaginaceae Juss.
Iz_4_1009	DJI_0378	Veronica incana L.	0	n	1	0	Plantaginaceae Juss.
Iz_4_1009	DJI_0378	Veronica verna L.	0	n	1	0	Plantaginaceae Juss.
Iz_4_1009	DJI_0378	Vincetoxicum hirundinaria Medik	0	n	1	0	Apocynaceae Juss.
Iz_4_1009	DJI_0381	Acer tataricum L.	0	n	1	1	Sapindaceae Juss.
Iz_4_1009	DJI_0381	Achillea millefolium L. s.l.	0	n	1	0	Asteraceae Bercht. & J.Presl
Iz_4_1009	DJI_0381	Allium oleraceum L.	0	n	1	0	Amaryllidaceae J.St.-Hil.
Iz_4_1009	DJI_0381	Ambrosia artemisiifolia L.	0	a	0	0	Asteraceae Bercht. & J.Presl
Iz_4_1009	DJI_0381	Amorpha fruticosa L.	0	a	0	0	Fabaceae Lindl.
Iz_4_1009	DJI_0381	Anthericum ramosum L.	0	n	1	0	Asparagaceae Juss.
Iz_4_1009	DJI_0381	Artemisia marschalliana Spreng.	0	n	1	0	Asteraceae Bercht. & J.Presl
Iz_4_1009	DJI_0381	Asparagus officinalis L.	0	n	1	0	Asparagaceae Juss.
Iz_4_1009	DJI_0381	Astragalus cicer L.	0	n	1	0	Fabaceae Lindl.
Iz_4_1009	DJI_0381	Bassia laniflora (S.G.Gmel.) A.J.Scott	0	n	1	0	Amaranthaceae Juss.
Iz_4_1009	DJI_0381	Berberis vulgaris L.	0	n	1	0	Berberidaceae Juss.
Iz_4_1009	DJI_0381	Betula pendula Roth.	0	n	1	1	Betulaceae Gray
Iz_4_1009	DJI_0381	Betula pendula Roth.	0	n	1	0	Poaceae Barnhart
Iz_4_1009	DJI_0381	Bromus inermis Leyss.	0		NA	NA	
Iz_4_1009	DJI_0381	Bromus squarrosus L.	0	a	0	0	Poaceae Barnhart
Iz_4_1009	DJI_0381	Bromus tectorum L.	0	a	0	0	Poaceae Barnhart
Iz_4_1009	DJI_0381	Calamagrostis epigejos (L.) Roth	2	n	1	0	Poaceae Barnhart
Iz_4_1009	DJI_0381	Carduus acanthoides L.	0	n	1	0	Asteraceae Bercht. & J.Presl
Iz_4_1009	DJI_0381	Carex sp.	0	n	1	0	Cyperaceae Juss.
Iz_4_1009	DJI_0381	Centaurea majorovii Dumbadze	0	n	1	0	Asteraceae Bercht. & J.Presl
Iz_4_1009	DJI_0381	Chamaecytisus ruthenicus (Fisch.ex Wol.) Klaskova	0	n	1	0	Fabaceae Lindl.
Iz_4_1009	DJI_0381	Chelidonium majus L.	0	n	1	0	Papaveraceae Juss.
Iz_4_1009	DJI_0381	Chenopodium betaceum Andrz.	0	n	1	0	Amaranthaceae Juss.
Iz_4_1009	DJI_0381	Chenopodium cf. album	0	n	1	0	Amaranthaceae Juss.
Iz_4_1009	DJI_0381	Chenopodium sp.	0		NA	0	Amaranthaceae Juss.
Iz_4_1009	DJI_0381	Chondrilla juncea L.	1	n	1	0	Asteraceae Bercht. & J.Presl
Iz_4_1009	DJI_0381	Cirsium vulgare (Savi) Ten.	0	n	1	0	Asteraceae Bercht. & J.Presl
Iz_4_1009	DJI_0381	Clinopodium acinos (L.) Kuntze	0	n	1	0	Lamiacea Lindl.
Iz_4_1009	DJI_0381	Convallaria majalis L.	0	n	1	0	Asparagaceae Juss.
Iz_4_1009	DJI_0381	Crataegus rhipidophylla Gand.	0	n	1	1	Rosaceae Juss.
Iz_4_1009	DJI_0381	Crepis foetida L.	0	n	1	0	Asteraceae Bercht. & J.Presl
Iz_4_1009	DJI_0381	Dactylis glomerata L.	0	n	1	0	Poaceae Barnhart
Iz_4_1009	DJI_0381	Dianthus campestris M.Bieb.	0	n	1	0	Caryophyllaceae Juss.
Iz_4_1009	DJI_0381	Digitaria sanguinalis (L.) Scop.	0	a	0	0	Poaceae Barnhart
Iz_4_1009	DJI_0381	Elymus repens (L.) Gould	0	n	1	0	Poaceae Barnhart
Iz_4_1009	DJI_0381	Epilobium tetragonum L.	0	n	1	0	Onagraceae Juss.
Iz_4_1009	DJI_0381	Eragrostis minor Host	0	a	0	0	Poaceae Barnhart
Iz_4_1009	DJI_0381	Erigeron canadensis L.	5	a	0	0	Asteraceae Bercht. & J.Presl
Iz_4_1009	DJI_0381	Euphorbia seguieriana Neck.	0	n	1	0	Euphorbiaceae Juss.
Iz_4_1009	DJI_0381	Fallopia dumetorum (L.) Holub	0	n	1	0	Polygonaceae Juss.
Iz_4_1009	DJI_0381	Festuca beckeri (Hack.) Trautv.	0	n	1	0	Poaceae Barnhart
Iz_4_1009	DJI_0381	Fragaria vesca L.	0	n	1	0	Rosaceae Juss.
Iz_4_1009	DJI_0381	Frangula alnus Mill.	0	n	1	1	Rhamnaceae Juss.
Iz_4_1009	DJI_0381	Fraxinus pennsylvanica Marshall	0	a	0	1	Oleaceae Hoffmanns. & Link
Iz_4_1009	DJI_0381	Galeopsis bifida Boenn.	0	n	1	0	Lamiacea Lindl.
Iz_4_1009	DJI_0381	Galium verum L.	0	n	1	0	Rubiaceae Juss.
Iz_4_1009	DJI_0381	Genista tinctoria L.	0	n	1	0	Fabaceae Lindl.
Iz_4_1009	DJI_0381	Geranium robertianum L.	0	n	1	0	Geraniaceae Juss.
Iz_4_1009	DJI_0381	Geranium sanquineum L.	0	n	1	0	Geraniaceae Juss.
Iz_4_1009	DJI_0381	Gypsophila paniculata L.	0	n	1	0	Caryophyllaceae Juss.
Iz_4_1009	DJI_0381	Helichrysum arenarium (L.) Moench	0	n	1	0	Asteraceae Bercht. & J.Presl
Iz_4_1009	DJI_0381	Herniaria polygama J.Gay	0	n	1	0	Caryophyllaceae Juss.
Iz_4_1009	DJI_0381	Hieracium robustum Fr.	0	n	1	0	Asteraceae Bercht. & J.Presl
Iz_4_1009	DJI_0381	Hieracium umbellatum L.	0	n	1	NA	Asteraceae Bercht. & J.Presl
Iz_4_1009	DJI_0381	Hylotelephium maximum (L.) Holub	0	n	1	0	Crassulaceae J.St.-Hil.
Iz_4_1009	DJI_0381	Jacobaea borysthenica (DC.) B.Nord. & Greuter	0	n	1	0	Asteraceae Bercht. & J.Presl
Iz_4_1009	DJI_0381	Jasione montana L.	0	n	1	0	Campanulaceae Juss.
Iz_4_1009	DJI_0381	Jurinea cyanoides (L.) Rchb.s.l.	0	n	1	0	Asteraceae Bercht. & J.Presl
Iz_4_1009	DJI_0381	Koeleria glauca (Spreng.) DC.	0	n	1	0	Poaceae Barnhart
Iz_4_1009	DJI_0381	Lactuca serriola L.	0	a	0	0	Asteraceae Bercht. & J.Presl
Iz_4_1009	DJI_0381	Linaria genistifolia (L.) Mill.	0	n	1	0	Plantaginaceae Juss.
Iz_4_1009	DJI_0381	Lomelosia argentea (L.) Greuter & Burdet	0	n	1	0	Caprifoliaceae Juss.
Iz_4_1009	DJI_0381	Melampyrum pratense L.	0	n	1	0	Orobanchaceae Vent.
Iz_4_1009	DJI_0381	Oenothera biennis L.	0	a	0	0	Onagraceae Juss.
Iz_4_1009	DJI_0381	Pentanema britannica (L.) D.Gut.Larr., Santos-Vicente, Anderb., E.Rico & M.M.Mart.Ort.	0	n	1	0	Asteraceae Bercht. & J.Presl
Iz_4_1009	DJI_0381	Peucedanum oreoselinum (L.) Moench	0	n	1	0	Apiaceae Lindl.
Iz_4_1009	DJI_0381	Peucedanum ruthenicum M.Bieb.	0	n	1	0	Apiaceae Lindl.
Iz_4_1009	DJI_0381	Pilosella echioides (Lumn.) F.W.Schultz & Sch.Bip.	0	n	1	0	Asteraceae Bercht. & J.Presl
Iz_4_1009	DJI_0381	Pilosella officinarum F. Schultz et Sch. Bip.	0	n	1	0	Asteraceae Bercht. & J.Presl
Iz_4_1009	DJI_0381	Pilosella sp.	0		NA	0	Asteraceae Bercht. & J.Presl
Iz_4_1009	DJI_0381	Pinus sylvestris L.	0	n	1	1	Pinaceae Spreng. ex F.Rudolphi

Iz_4_1009	DJI_0381	Poa annua L.	0	n	1	0	Poaceae Barnhart
Iz_4_1009	DJI_0381	Poa nemoralis L.	0	n	1	0	Poaceae Barnhart
Iz_4_1009	DJI_0381	Poa sp.	0		NA	0	Poaceae Barnhart
Iz_4_1009	DJI_0381	Polygonatum odoratum (Mill.) Druce	0	n	1	0	Asparagaceae Juss.
Iz_4_1009	DJI_0381	Polygonum arenarium Waldst. & Kit.	0	n	1	0	Polygonaceae Juss.
Iz_4_1009	DJI_0381	Populus nigra L.	0	n	1	1	Salicaceae Mirb.
Iz_4_1009	DJI_0381	Populus tremula L.	0	n	1	1	Salicaceae Mirb.
Iz_4_1009	DJI_0381	Populus Ã canescens (Aiton) Sm.	0	n	1	1	Salicaceae Mirb.
Iz_4_1009	DJI_0381	Potentilla argentea L.	0	n	1	0	Rosaceae Juss.
Iz_4_1009	DJI_0381	Psammophiliella muralis (L.) Ikonn.	0	n	1	0	Caryophyllaceae Juss.
Iz_4_1009	DJI_0381	Psephellus sumensis (Kalen.) Greuter	0	n	1	0	Asteraceae Bercht. & J.Presl
Iz_4_1009	DJI_0381	Pulsatilla pratensis (L.) Mill. s.l.	0	n	1	0	Ranunculaceae Juss.
Iz_4_1009	DJI_0381	Pyrus communis L.	0	a	0	1	Rosaceae Juss.
Iz_4_1009	DJI_0381	Quercus robur L.	0	n	1	1	Fagaceae Dumort.
Iz_4_1009	DJI_0381	Robinia pseudoacacia L.	0	a	0	1	Fabaceae Lindl.
Iz_4_1009	DJI_0381	Rosa sp.	0		NA	1	Rosaceae Juss.
Iz_4_1009	DJI_0381	Rubus idaeus L.	0	n	1	0	Rosaceae Juss.
Iz_4_1009	DJI_0381	Rumex acetosella L.	0	n	1	0	Polygonaceae Juss.
Iz_4_1009	DJI_0381	Rumex thyrsiflorus Fingerh.	0	n	1	0	Polygonaceae Juss.
Iz_4_1009	DJI_0381	Salix alba L.	0	n	1	1	Salicaceae Mirb.
Iz_4_1009	DJI_0381	Sambucus nigra L.	0	n	1	1	Viburnaceae Raf.
Iz_4_1009	DJI_0381	Secale sylvestre Host	0	n	1	0	Poaceae Barnhart
Iz_4_1009	DJI_0381	Sempervivum ruthenicum Schnittsp. & C.B. Lehm.	0	n	1	0	Crassulaceae J.St.-Hil.
Iz_4_1009	DJI_0381	Setaria viridis (L.) P. Beauv.	0	n	1	0	Poaceae Barnhart
Iz_4_1009	DJI_0381	Silene borysthenica (Gruner) Walters	0	n	1	0	Caryophyllaceae Juss.
Iz_4_1009	DJI_0381	Silene latifolia subsp. alba (Mill.) Greuter & Burdet	0	n	1	0	Caryophyllaceae Juss.
Iz_4_1009	DJI_0381	Solidago virgaurea L.	0	n	1	0	Asteraceae Bercht. & J.Presl
Iz_4_1009	DJI_0381	Sorbus aucuparia L.	0	n	1	1	Rosaceae Juss.
Iz_4_1009	DJI_0381	Stachys recta L.	0	n	1	0	Lamiacea Lindl.
Iz_4_1009	DJI_0381	Tanacetum vulgare L.	0	n	1	0	Asteraceae Bercht. & J.Presl
Iz_4_1009	DJI_0381	Thymus pallasianus Heinr.Braun	0	n	1	0	Lamiacea Lindl.
Iz_4_1009	DJI_0381	Tilia cordata Mill.	0	n	1	1	Malvaceae Juss.
Iz_4_1009	DJI_0381	Trifolium alpestre L.	0	n	1	0	Fabaceae Lindl.
Iz_4_1009	DJI_0381	Trifolium arvense L.	0	n	1	0	Fabaceae Lindl.
Iz_4_1009	DJI_0381	Ulmus glabra Huds.	0	n	1	1	Ulmaceae Mirb.
Iz_4_1009	DJI_0381	Verbascum densiflorum Bertol.	0	n	1	0	Scrophulariaceae Juss.
Iz_4_1009	DJI_0381	Verbascum nigrum L.	0	n	1	0	Scrophulariaceae Juss.
Iz_4_1009	DJI_0381	Veronica austriaca L.	0	n	1	0	Plantaginaceae Juss.
Iz_4_1009	DJI_0381	Veronica incana L.	0	n	1	0	Plantaginaceae Juss.
Iz_4_1009	DJI_0381	Veronica verna L.	0	n	1	0	Plantaginaceae Juss.
Iz_4_1009	DJI_0381	Vincetoxicum hirundinaria Medik	0	n	1	0	Apocynaceae Juss.
Iz_4_1009	DJI_0383	Acer tataricum L.	0	n	1	1	Sapindaceae Juss.
Iz_4_1009	DJI_0383	Achillea millefolium L. s.l.	0	n	1	0	Asteraceae Bercht. & J.Presl
Iz_4_1009	DJI_0383	Allium oleraceum L.	0	n	1	0	Amaryllidaceae J.St.-Hil.
Iz_4_1009	DJI_0383	Ambrosia artemisiifolia L.	0	a	0	0	Asteraceae Bercht. & J.Presl
Iz_4_1009	DJI_0383	Amorpha fruticosa L.	0	a	0	0	Fabaceae Lindl.
Iz_4_1009	DJI_0383	Anthericum ramosum L.	0	n	1	0	Asparagaceae Juss.
Iz_4_1009	DJI_0383	Artemisia marschalliana Spreng.	0	n	1	0	Asteraceae Bercht. & J.Presl
Iz_4_1009	DJI_0383	Asparagus officinalis L.	0	n	1	0	Asparagaceae Juss.
Iz_4_1009	DJI_0383	Astragalus cicer L.	0	n	1	0	Fabaceae Lindl.
Iz_4_1009	DJI_0383	Bassia laniflora (S.G.Gmel.) A.J.Scott	0	n	1	0	Amaranthaceae Juss.
Iz_4_1009	DJI_0383	Berberis vulgaris L.	0	n	1	0	Berberidaceae Juss.
Iz_4_1009	DJI_0383	Betula pendula Roth.	0	n	1	1	Betulaceae Gray
Iz_4_1009	DJI_0383	Betula pendula Roth.	0	n	1	0	Poaceae Barnhart
Iz_4_1009	DJI_0383	Bromus inermis Leyss.	0		NA	NA	
Iz_4_1009	DJI_0383	Bromus squarrosus L.	0	a	0	0	Poaceae Barnhart
Iz_4_1009	DJI_0383	Bromus tectorum L.	0	a	0	0	Poaceae Barnhart
Iz_4_1009	DJI_0383	Calamagrostis epigejos (L.) Roth	0	n	1	0	Poaceae Barnhart
Iz_4_1009	DJI_0383	Carduus acanthoides L.	0	n	1	0	Asteraceae Bercht. & J.Presl
Iz_4_1009	DJI_0383	Carex sp.	0	n	1	0	Cyperaceae Juss.
Iz_4_1009	DJI_0383	Centaurea majorovii Dumbadze	0	n	1	0	Asteraceae Bercht. & J.Presl
Iz_4_1009	DJI_0383	Chamaecytisus ruthenicus (Fisch.ex Wol.) Klaskova	0	n	1	0	Fabaceae Lindl.
Iz_4_1009	DJI_0383	Chelidonium majus L.	0	n	1	0	Papaveraceae Juss.
Iz_4_1009	DJI_0383	Chenopodium betaceum Andrz.	0	n	1	0	Amaranthaceae Juss.
Iz_4_1009	DJI_0383	Chenopodium cf. album	0	n	1	0	Amaranthaceae Juss.
Iz_4_1009	DJI_0383	Chenopodium sp.	0		NA	0	Amaranthaceae Juss.
Iz_4_1009	DJI_0383	Chondrilla juncea L.	0	n	1	0	Asteraceae Bercht. & J.Presl
Iz_4_1009	DJI_0383	Cirsium vulgare (Savi) Ten.	0	n	1	0	Asteraceae Bercht. & J.Presl
Iz_4_1009	DJI_0383	Clinopodium acinos (L.) Kuntze	0	n	1	0	Lamiacea Lindl.
Iz_4_1009	DJI_0383	Convallaria majalis L.	0	n	1	0	Asparagaceae Juss.
Iz_4_1009	DJI_0383	Crataegus rhipidophylla Gand.	0	n	1	1	Rosaceae Juss.
Iz_4_1009	DJI_0383	Crepis foetida L.	0	n	1	0	Asteraceae Bercht. & J.Presl
Iz_4_1009	DJI_0383	Dactylis glomerata L.	0	n	1	0	Poaceae Barnhart
Iz_4_1009	DJI_0383	Dianthus campestris M.Bieb.	0	n	1	0	Caryophyllaceae Juss.
Iz_4_1009	DJI_0383	Digitaria sanguinalis (L.) Scop.	0	a	0	0	Poaceae Barnhart
Iz_4_1009	DJI_0383	Elymus repens (L.) Gould	0	n	1	0	Poaceae Barnhart
Iz_4_1009	DJI_0383	Epilobium tetragonum L.	0	n	1	0	Onagraceae Juss.
Iz_4_1009	DJI_0383	Eragrostis minor Host	0	a	0	0	Poaceae Barnhart
Iz_4_1009	DJI_0383	Erigeron canadensis L.	3	a	0	0	Asteraceae Bercht. & J.Presl
Iz_4_1009	DJI_0383	Euphorbia seguieriana Neck.	0	n	1	0	Euphorbiaceae Juss.
Iz_4_1009	DJI_0383	Fallopia dumetorum (L.) Holub	0	n	1	0	Polygonaceae Juss.
Iz_4_1009	DJI_0383	Festuca beckeri (Hack.) Trautv.	0	n	1	0	Poaceae Barnhart
Iz_4_1009	DJI_0383	Fragaria vesca L.	0	n	1	0	Rosaceae Juss.
Iz_4_1009	DJI_0383	Frangula alnus Mill.	0	n	1	1	Rhamnaceae Juss.
Iz_4_1009	DJI_0383	Fraxinus pennsylvanica Marshall	0	a	0	1	Oleaceae Hoffmanns. & Link
Iz_4_1009	DJI_0383	Galeopsis bifida Boenn.	0	n	1	0	Lamiacea Lindl.
Iz_4_1009	DJI_0383	Galium verum L.	0	n	1	0	Rubiaceae Juss.
Iz_4_1009	DJI_0383	Genista tinctoria L.	0	n	1	0	Fabaceae Lindl.
Iz_4_1009	DJI_0383	Geranium robertianum L.	0	n	1	0	Geraniaceae Juss.
Iz_4_1009	DJI_0383	Geranium sanquineum L.	0	n	1	0	Geraniaceae Juss.
Iz_4_1009	DJI_0383	Gypsophila paniculata L.	0	n	1	0	Caryophyllaceae Juss.
Iz_4_1009	DJI_0383	Helichrysum arenarium (L.) Moench	0	n	1	0	Asteraceae Bercht. & J.Presl
Iz_4_1009	DJI_0383	Herniaria polygama J.Gay	0	n	1	0	Caryophyllaceae Juss.
Iz_4_1009	DJI_0383	Hieracium robustum Fr.	0	n	1	0	Asteraceae Bercht. & J.Presl
Iz_4_1009	DJI_0383	Hieracium umbellatum L.	0	n	1	NA	Asteraceae Bercht. & J.Presl
Iz_4_1009	DJI_0383	Hylotelephium maximum (L.) Holub	0	n	1	0	Crassulaceae J.St.-Hil.
Iz_4_1009	DJI_0383	Jacobaea borysthenica (DC.) B.Nord. & Greuter	0	n	1	0	Asteraceae Bercht. & J.Presl
Iz_4_1009	DJI_0383	Jasione montana L.	0	n	1	0	Campanulaceae Juss.

Iz_4_1009	DJI_0383	Jurinea cyanoides (L.) Rchb.s.l.	0	n	1	0	Asteraceae Bercht. & J.Presl
Iz_4_1009	DJI_0383	Koeleria glauca (Spreng.) DC.	0	n	1	0	Poaceae Barnhart
Iz_4_1009	DJI_0383	Lactuca serriola L.	0	a	0	0	Asteraceae Bercht. & J.Presl
Iz_4_1009	DJI_0383	Linaria genistifolia (L.) Mill.	0	n	1	0	Plantaginaceae Juss.
Iz_4_1009	DJI_0383	Lomelosia argentea (L.) Greuter & Burdet	0	n	1	0	Caprifoliaceae Juss.
Iz_4_1009	DJI_0383	Melampyrum pratense L.	0	n	1	0	Orobanchaceae Vent.
Iz_4_1009	DJI_0383	Oenothera biennis L.	0	a	0	0	Onagraceae Juss.
Iz_4_1009	DJI_0383	Pentanema britannica (L.) D.Gut.Larr., Santos-Vicente, Anderb., E.Rico & M.M.Mart.Ort.	0	n	1	0	Asteraceae Bercht. & J.Presl
Iz_4_1009	DJI_0383	Peucedanum oreoselinum (L.) Moench	0	n	1	0	Apiaceae Lindl.
Iz_4_1009	DJI_0383	Peucedanum ruthenicum M.Bieb.	0	n	1	0	Apiaceae Lindl.
Iz_4_1009	DJI_0383	Pilosella echioides (Lumn.) F.W.Schultz & Sch.Bip.	0	n	1	0	Asteraceae Bercht. & J.Presl
Iz_4_1009	DJI_0383	Pilosella officinarum F. Schultz et Sch. Bip.	0	n	1	0	Asteraceae Bercht. & J.Presl
Iz_4_1009	DJI_0383	Pilosella sp.	0		NA	0	Asteraceae Bercht. & J.Presl
Iz_4_1009	DJI_0383	Pinus sylvestris L.	0	n	1	1	Pinaceae Spreng. ex F.Rudolphi
Iz_4_1009	DJI_0383	Poa annua L.	0	n	1	0	Poaceae Barnhart
Iz_4_1009	DJI_0383	Poa nemoralis L.	0	n	1	0	Poaceae Barnhart
Iz_4_1009	DJI_0383	Poa sp.	0		NA	0	Poaceae Barnhart
Iz_4_1009	DJI_0383	Polygonatum odoratum (Mill.) Druce	0	n	1	0	Asparagaceae Juss.
Iz_4_1009	DJI_0383	Polygonum arenarium Waldst. & Kit.	0	n	1	0	Polygonaceae Juss.
Iz_4_1009	DJI_0383	Populus nigra L.	0	n	1	1	Salicaceae Mirb.
Iz_4_1009	DJI_0383	Populus tremula L.	0	n	1	1	Salicaceae Mirb.
Iz_4_1009	DJI_0383	Populus Ã canescens (Aiton) Sm.	0	n	1	1	Salicaceae Mirb.
Iz_4_1009	DJI_0383	Potentilla argentea L.	0	n	1	0	Rosaceae Juss.
Iz_4_1009	DJI_0383	Psammophiliella muralis (L.) Ikonn.	0	n	1	0	Caryophyllaceae Juss.
Iz_4_1009	DJI_0383	Psephellus sumensis (Kalen.) Greuter	0	n	1	0	Asteraceae Bercht. & J.Presl
Iz_4_1009	DJI_0383	Pulsatilla pratensis (L.) Mill. s.l.	0	n	1	0	Ranunculaceae Juss.
Iz_4_1009	DJI_0383	Pyrus communis L.	0	a	0	1	Rosaceae Juss.
Iz_4_1009	DJI_0383	Quercus robur L.	0	n	1	1	Fagaceae Dumort.
Iz_4_1009	DJI_0383	Robinia pseudoacacia L.	0	a	0	1	Fabaceae Lindl.
Iz_4_1009	DJI_0383	Rosa sp.	0		NA	1	Rosaceae Juss.
Iz_4_1009	DJI_0383	Rubus idaeus L.	0	n	1	0	Rosaceae Juss.
Iz_4_1009	DJI_0383	Rumex acetosella L.	0	n	1	0	Polygonaceae Juss.
Iz_4_1009	DJI_0383	Rumex thyrsiflorus Fingerh.	0	n	1	0	Polygonaceae Juss.
Iz_4_1009	DJI_0383	Salix alba L.	0	n	1	1	Salicaceae Mirb.
Iz_4_1009	DJI_0383	Sambucus nigra L.	0	n	1	1	Viburnaceae Raf.
Iz_4_1009	DJI_0383	Secale sylvestre Host	0	n	1	0	Poaceae Barnhart
Iz_4_1009	DJI_0383	Sempervivum ruthenicum Schnittsp. & C.B. Lehm.	0	n	1	0	Crassulaceae J.St.-Hil.
Iz_4_1009	DJI_0383	Setaria viridis (L.) P. Beauv.	0	n	1	0	Poaceae Barnhart
Iz_4_1009	DJI_0383	Silene borysthenica (Gruner) Walters	0	n	1	0	Caryophyllaceae Juss.
Iz_4_1009	DJI_0383	Silene latifolia subsp. alba (Mill.) Greuter & Burdet	0	n	1	0	Caryophyllaceae Juss.
Iz_4_1009	DJI_0383	Solidago virgaurea L.	0	n	1	0	Asteraceae Bercht. & J.Presl
Iz_4_1009	DJI_0383	Sorbus aucuparia L.	0	n	1	1	Rosaceae Juss.
Iz_4_1009	DJI_0383	Stachys recta L.	0	n	1	0	Lamiacea Lindl.
Iz_4_1009	DJI_0383	Tanacetum vulgare L.	0	n	1	0	Asteraceae Bercht. & J.Presl
Iz_4_1009	DJI_0383	Thymus pallasianus Heinr.Braun	0	n	1	0	Lamiacea Lindl.
Iz_4_1009	DJI_0383	Tilia cordata Mill.	0	n	1	1	Malvaceae Juss.
Iz_4_1009	DJI_0383	Trifolium alpestre L.	0	n	1	0	Fabaceae Lindl.
Iz_4_1009	DJI_0383	Trifolium arvense L.	0	n	1	0	Fabaceae Lindl.
Iz_4_1009	DJI_0383	Ulmus glabra Huds.	0	n	1	1	Ulmaceae Mirb.
Iz_4_1009	DJI_0383	Verbascum densiflorum Bertol.	0	n	1	0	Scrophulariaceae Juss.
Iz_4_1009	DJI_0383	Verbascum nigrum L.	0	n	1	0	Scrophulariaceae Juss.
Iz_4_1009	DJI_0383	Veronica austriaca L.	0	n	1	0	Plantaginaceae Juss.
Iz_4_1009	DJI_0383	Veronica incana L.	0	n	1	0	Plantaginaceae Juss.
Iz_4_1009	DJI_0383	Veronica verna L.	0	n	1	0	Plantaginaceae Juss.
Iz_4_1009	DJI_0383	Vincetoxicum hirundinaria Medik	0	n	1	0	Apocynaceae Juss.
Iz_4_1009	DJI_0385	Acer tataricum L.	0	n	1	1	Sapindaceae Juss.
Iz_4_1009	DJI_0385	Achillea millefolium L. s.l.	0	n	1	0	Asteraceae Bercht. & J.Presl
Iz_4_1009	DJI_0385	Allium oleraceum L.	0	n	1	0	Amaryllidaceae J.St.-Hil.
Iz_4_1009	DJI_0385	Ambrosia artemisiifolia L.	0	a	0	0	Asteraceae Bercht. & J.Presl
Iz_4_1009	DJI_0385	Amorpha fruticosa L.	0	a	0	0	Fabaceae Lindl.
Iz_4_1009	DJI_0385	Anthericum ramosum L.	0	n	1	0	Asparagaceae Juss.
Iz_4_1009	DJI_0385	Artemisia marschalliana Spreng.	0	n	1	0	Asteraceae Bercht. & J.Presl
Iz_4_1009	DJI_0385	Asparagus officinalis L.	0	n	1	0	Asparagaceae Juss.
Iz_4_1009	DJI_0385	Astragalus cicer L.	0	n	1	0	Fabaceae Lindl.
Iz_4_1009	DJI_0385	Bassia laniflora (S.G.Gmel.) A.J.Scott	0	n	1	0	Amaranthaceae Juss.
Iz_4_1009	DJI_0385	Berberis vulgaris L.	0	n	1	0	Berberidaceae Juss.
Iz_4_1009	DJI_0385	Betula pendula Roth.	0	n	1	1	Betulaceae Gray
Iz_4_1009	DJI_0385	Betula pendula Roth.	0	n	1	0	Poaceae Barnhart
Iz_4_1009	DJI_0385	Bromus inermis Leyss.	0		NA	NA	
Iz_4_1009	DJI_0385	Bromus squarrosus L.	0	a	0	0	Poaceae Barnhart
Iz_4_1009	DJI_0385	Bromus tectorum L.	0	a	0	0	Poaceae Barnhart
Iz_4_1009	DJI_0385	Calamagrostis epigejos (L.) Roth	0.2	n	1	0	Poaceae Barnhart
Iz_4_1009	DJI_0385	Carduus acanthoides L.	0	n	1	0	Asteraceae Bercht. & J.Presl
Iz_4_1009	DJI_0385	Carex sp.	0	n	1	0	Cyperaceae Juss.
Iz_4_1009	DJI_0385	Centaurea majorovii Dumbadze	0	n	1	0	Asteraceae Bercht. & J.Presl
Iz_4_1009	DJI_0385	Chamaecytisus ruthenicus (Fisch.ex Wol.) Klaskova	0	n	1	0	Fabaceae Lindl.
Iz_4_1009	DJI_0385	Chelidonium majus L.	0	n	1	0	Papaveraceae Juss.
Iz_4_1009	DJI_0385	Chenopodium betaceum Andrz.	0	n	1	0	Amaranthaceae Juss.
Iz_4_1009	DJI_0385	Chenopodium cf. album	0	n	1	0	Amaranthaceae Juss.
Iz_4_1009	DJI_0385	Chenopodium sp.	0		NA	0	Amaranthaceae Juss.
Iz_4_1009	DJI_0385	Chondrilla juncea L.	0	n	1	0	Asteraceae Bercht. & J.Presl
Iz_4_1009	DJI_0385	Cirsium vulgare (Savi) Ten.	0	n	1	0	Asteraceae Bercht. & J.Presl
Iz_4_1009	DJI_0385	Clinopodium acinos (L.) Kuntze	0	n	1	0	Lamiacea Lindl.
Iz_4_1009	DJI_0385	Convallaria majalis L.	0	n	1	0	Asparagaceae Juss.
Iz_4_1009	DJI_0385	Crataegus rhipidophylla Gand.	0	n	1	1	Rosaceae Juss.
Iz_4_1009	DJI_0385	Crepis foetida L.	0	n	1	0	Asteraceae Bercht. & J.Presl
Iz_4_1009	DJI_0385	Dactylis glomerata L.	0	n	1	0	Poaceae Barnhart
Iz_4_1009	DJI_0385	Dianthus campestris M.Bieb.	0	n	1	0	Caryophyllaceae Juss.
Iz_4_1009	DJI_0385	Digitaria sanguinalis (L.) Scop.	0	a	0	0	Poaceae Barnhart
Iz_4_1009	DJI_0385	Elymus repens (L.) Gould	0	n	1	0	Poaceae Barnhart
Iz_4_1009	DJI_0385	Epilobium tetragonum L.	0	n	1	0	Onagraceae Juss.
Iz_4_1009	DJI_0385	Eragrostis minor Host	0	a	0	0	Poaceae Barnhart
Iz_4_1009	DJI_0385	Erigeron canadensis L.	0.2	a	0	0	Asteraceae Bercht. & J.Presl
Iz_4_1009	DJI_0385	Euphorbia seguieriana Neck.	0	n	1	0	Euphorbiaceae Juss.
Iz_4_1009	DJI_0385	Fallopia dumetorum (L.) Holub	0	n	1	0	Polygonaceae Juss.
Iz_4_1009	DJI_0385	Festuca beckeri (Hack.) Trautv.	0	n	1	0	Poaceae Barnhart
Iz_4_1009	DJI_0385	Fragaria vesca L.	0	n	1	0	Rosaceae Juss.
Iz_4_1009	DJI_0385	Frangula alnus Mill.	0	n	1	1	Rhamnaceae Juss.

Iz_4_1009	DJI_0385	Fraxinus pennsylvanica Marshall	0	a	0	1	Oleaceae Hoffmanns. & Link
Iz_4_1009	DJI_0385	Galeopsis bifida Boenn.	0	n	1	0	Lamiacea Lindl.
Iz_4_1009	DJI_0385	Galium verum L.	0	n	1	0	Rubiaceae Juss.
Iz_4_1009	DJI_0385	Genista tinctoria L.	0	n	1	0	Fabaceae Lindl.
Iz_4_1009	DJI_0385	Geranium robertianum L.	0	n	1	0	Geraniaceae Juss.
Iz_4_1009	DJI_0385	Geranium sanquineum L.	0	n	1	0	Geraniaceae Juss.
Iz_4_1009	DJI_0385	Gypsophila paniculata L.	0	n	1	0	Caryophyllaceae Juss.
Iz_4_1009	DJI_0385	Helichrysum arenarium (L.) Moench	0	n	1	0	Asteraceae Bercht. & J.Presl
Iz_4_1009	DJI_0385	Herniaria polygama J.Gay	0	n	1	0	Caryophyllaceae Juss.
Iz_4_1009	DJI_0385	Hieracium robustum Fr.	0	n	1	0	Asteraceae Bercht. & J.Presl
Iz_4_1009	DJI_0385	Hieracium umbellatum L.	0	n	1	NA	Asteraceae Bercht. & J.Presl
Iz_4_1009	DJI_0385	Hylotelephium maximum (L.) Holub	0	n	1	0	Crassulaceae J.St.-Hil.
Iz_4_1009	DJI_0385	Jacobaea borysthenica (DC.) B.Nord. & Greuter	0	n	1	0	Asteraceae Bercht. & J.Presl
Iz_4_1009	DJI_0385	Jasione montana L.	0	n	1	0	Campanulaceae Juss.
Iz_4_1009	DJI_0385	Jurinea cyanoides (L.) Rchb.s.l.	0	n	1	0	Asteraceae Bercht. & J.Presl
Iz_4_1009	DJI_0385	Koeleria glauca (Spreng.) DC.	0	n	1	0	Poaceae Barnhart
Iz_4_1009	DJI_0385	Lactuca serriola L.	0	a	0	0	Asteraceae Bercht. & J.Presl
Iz_4_1009	DJI_0385	Linaria genistifolia (L.) Mill.	0	n	1	0	Plantaginaceae Juss.
Iz_4_1009	DJI_0385	Lomelosia argentea (L.) Greuter & Burdet	0	n	1	0	Caprifoliaceae Juss.
Iz_4_1009	DJI_0385	Melampyrum pratense L.	0	n	1	0	Orobanchaceae Vent.
Iz_4_1009	DJI_0385	Oenothera biennis L.	0	a	0	0	Onagraceae Juss.
Iz_4_1009	DJI_0385	Pentanema britannica (L.) D.Gut.Larr., Santos-Vicente, Anderb., E.Rico & M.M.Mart.Ort.	0	n	1	0	Asteraceae Bercht. & J.Presl
Iz_4_1009	DJI_0385	Peucedanum oreoselinum (L.) Moench	0	n	1	0	Apiaceae Lindl.
Iz_4_1009	DJI_0385	Peucedanum ruthenicum M.Bieb.	0	n	1	0	Apiaceae Lindl.
Iz_4_1009	DJI_0385	Pilosella echioides (Lumn.) F.W.Schultz & Sch.Bip.	0	n	1	0	Asteraceae Bercht. & J.Presl
Iz_4_1009	DJI_0385	Pilosella officinarum F. Schultz et Sch. Bip.	0	n	1	0	Asteraceae Bercht. & J.Presl
Iz_4_1009	DJI_0385	Pilosella sp.	0		NA	0	Asteraceae Bercht. & J.Presl
Iz_4_1009	DJI_0385	Pinus sylvestris L.	0	n	1	1	Pinaceae Spreng. ex F.Rudolphi
Iz_4_1009	DJI_0385	Poa annua L.	0	n	1	0	Poaceae Barnhart
Iz_4_1009	DJI_0385	Poa nemoralis L.	0	n	1	0	Poaceae Barnhart
Iz_4_1009	DJI_0385	Poa sp.	0		NA	0	Poaceae Barnhart
Iz_4_1009	DJI_0385	Polygonatum odoratum (Mill.) Druce	0	n	1	0	Asparagaceae Juss.
Iz_4_1009	DJI_0385	Polygonum arenarium Waldst. & Kit.	0	n	1	0	Polygonaceae Juss.
Iz_4_1009	DJI_0385	Populus nigra L.	0	n	1	1	Salicaceae Mirb.
Iz_4_1009	DJI_0385	Populus tremula L.	0	n	1	1	Salicaceae Mirb.
Iz_4_1009	DJI_0385	Populus Ã canescens (Aiton) Sm.	0	n	1	1	Salicaceae Mirb.
Iz_4_1009	DJI_0385	Potentilla argentea L.	0	n	1	0	Rosaceae Juss.
Iz_4_1009	DJI_0385	Psammophiliella muralis (L.) Ikonn.	0	n	1	0	Caryophyllaceae Juss.
Iz_4_1009	DJI_0385	Psephellus sumensis (Kalen.) Greuter	0	n	1	0	Asteraceae Bercht. & J.Presl
Iz_4_1009	DJI_0385	Pulsatilla pratensis (L.) Mill. s.l.	0	n	1	0	Ranunculaceae Juss.
Iz_4_1009	DJI_0385	Pyrus communis L.	0	a	0	1	Rosaceae Juss.
Iz_4_1009	DJI_0385	Quercus robur L.	0	n	1	1	Fagaceae Dumort.
Iz_4_1009	DJI_0385	Robinia pseudoacacia L.	0	a	0	1	Fabaceae Lindl.
Iz_4_1009	DJI_0385	Rosa sp.	0		NA	1	Rosaceae Juss.
Iz_4_1009	DJI_0385	Rubus idaeus L.	0	n	1	0	Rosaceae Juss.
Iz_4_1009	DJI_0385	Rumex acetosella L.	0	n	1	0	Polygonaceae Juss.
Iz_4_1009	DJI_0385	Rumex thyrsiflorus Fingerh.	0	n	1	0	Polygonaceae Juss.
Iz_4_1009	DJI_0385	Salix alba L.	0	n	1	1	Salicaceae Mirb.
Iz_4_1009	DJI_0385	Sambucus nigra L.	0	n	1	1	Viburnaceae Raf.
Iz_4_1009	DJI_0385	Secale sylvestre Host	0	n	1	0	Poaceae Barnhart
Iz_4_1009	DJI_0385	Sempervivum ruthenicum Schnittsp. & C.B. Lehm.	0	n	1	0	Crassulaceae J.St.-Hil.
Iz_4_1009	DJI_0385	Setaria viridis (L.) P. Beauv.	0	n	1	0	Poaceae Barnhart
Iz_4_1009	DJI_0385	Silene borysthenica (Gruner) Walters	0	n	1	0	Caryophyllaceae Juss.
Iz_4_1009	DJI_0385	Silene latifolia subsp. alba (Mill.) Greuter & Burdet	0	n	1	0	Caryophyllaceae Juss.
Iz_4_1009	DJI_0385	Solidago virgaurea L.	0	n	1	0	Asteraceae Bercht. & J.Presl
Iz_4_1009	DJI_0385	Sorbus aucuparia L.	0	n	1	1	Rosaceae Juss.
Iz_4_1009	DJI_0385	Stachys recta L.	0	n	1	0	Lamiacea Lindl.
Iz_4_1009	DJI_0385	Tanacetum vulgare L.	0	n	1	0	Asteraceae Bercht. & J.Presl
Iz_4_1009	DJI_0385	Thymus pallasianus Heinr.Braun	0	n	1	0	Lamiacea Lindl.
Iz_4_1009	DJI_0385	Tilia cordata Mill.	0	n	1	1	Malvaceae Juss.
Iz_4_1009	DJI_0385	Trifolium alpestre L.	0	n	1	0	Fabaceae Lindl.
Iz_4_1009	DJI_0385	Trifolium arvense L.	0	n	1	0	Fabaceae Lindl.
Iz_4_1009	DJI_0385	Ulmus glabra Huds.	0	n	1	1	Ulmaceae Mirb.
Iz_4_1009	DJI_0385	Verbascum densiflorum Bertol.	0	n	1	0	Scrophulariaceae Juss.
Iz_4_1009	DJI_0385	Verbascum nigrum L.	0	n	1	0	Scrophulariaceae Juss.
Iz_4_1009	DJI_0385	Veronica austriaca L.	0	n	1	0	Plantaginaceae Juss.
Iz_4_1009	DJI_0385	Veronica incana L.	0	n	1	0	Plantaginaceae Juss.
Iz_4_1009	DJI_0385	Veronica verna L.	0	n	1	0	Plantaginaceae Juss.
Iz_4_1009	DJI_0385	Vincetoxicum hirundinaria Medik	0	n	1	0	Apocynaceae Juss.
Iz_4_1009	DJI_0386	Acer tataricum L.	0	n	1	1	Sapindaceae Juss.
Iz_4_1009	DJI_0386	Achillea millefolium L. s.l.	0	n	1	0	Asteraceae Bercht. & J.Presl
Iz_4_1009	DJI_0386	Allium oleraceum L.	0	n	1	0	Amaryllidaceae J.St.-Hil.
Iz_4_1009	DJI_0386	Ambrosia artemisiifolia L.	0	a	0	0	Asteraceae Bercht. & J.Presl
Iz_4_1009	DJI_0386	Amorpha fruticosa L.	0	a	0	0	Fabaceae Lindl.
Iz_4_1009	DJI_0386	Anthericum ramosum L.	0	n	1	0	Asparagaceae Juss.
Iz_4_1009	DJI_0386	Artemisia marschalliana Spreng.	0	n	1	0	Asteraceae Bercht. & J.Presl
Iz_4_1009	DJI_0386	Asparagus officinalis L.	0	n	1	0	Asparagaceae Juss.
Iz_4_1009	DJI_0386	Astragalus cicer L.	0	n	1	0	Fabaceae Lindl.
Iz_4_1009	DJI_0386	Bassia laniflora (S.G.Gmel.) A.J.Scott	0	n	1	0	Amaranthaceae Juss.
Iz_4_1009	DJI_0386	Berberis vulgaris L.	0	n	1	0	Berberidaceae Juss.
Iz_4_1009	DJI_0386	Betula pendula Roth.	0	n	1	1	Betulaceae Gray
Iz_4_1009	DJI_0386	Betula pendula Roth.	0	n	1	0	Poaceae Barnhart
Iz_4_1009	DJI_0386	Bromus inermis Leyss.	0		NA	NA	
Iz_4_1009	DJI_0386	Bromus squarrosus L.	0	a	0	0	Poaceae Barnhart
Iz_4_1009	DJI_0386	Bromus tectorum L.	0	a	0	0	Poaceae Barnhart
Iz_4_1009	DJI_0386	Calamagrostis epigejos (L.) Roth	0	n	1	0	Poaceae Barnhart
Iz_4_1009	DJI_0386	Carduus acanthoides L.	0	n	1	0	Asteraceae Bercht. & J.Presl
Iz_4_1009	DJI_0386	Carex sp.	0	n	1	0	Cyperaceae Juss.
Iz_4_1009	DJI_0386	Centaurea majorovii Dumbadze	0	n	1	0	Asteraceae Bercht. & J.Presl
Iz_4_1009	DJI_0386	Chamaecytisus ruthenicus (Fisch.ex Wol.) Klaskova	0	n	1	0	Fabaceae Lindl.
Iz_4_1009	DJI_0386	Chelidonium majus L.	0	n	1	0	Papaveraceae Juss.
Iz_4_1009	DJI_0386	Chenopodium betaceum Andrz.	0	n	1	0	Amaranthaceae Juss.
Iz_4_1009	DJI_0386	Chenopodium cf. album	0	n	1	0	Amaranthaceae Juss.
Iz_4_1009	DJI_0386	Chenopodium sp.	0		NA	0	Amaranthaceae Juss.
Iz_4_1009	DJI_0386	Chondrilla juncea L.	0	n	1	0	Asteraceae Bercht. & J.Presl
Iz_4_1009	DJI_0386	Cirsium vulgare (Savi) Ten.	0	n	1	0	Asteraceae Bercht. & J.Presl
Iz_4_1009	DJI_0386	Clinopodium acinos (L.) Kuntze	0	n	1	0	Lamiacea Lindl.
Iz_4_1009	DJI_0386	Convallaria majalis L.	0	n	1	0	Asparagaceae Juss.

Iz_4_1009	DJI_0386	Crataegus rhipidophylla Gand.	0	n	1	1	Rosaceae Juss.
Iz_4_1009	DJI_0386	Crepis foetida L.	0	n	1	0	Asteraceae Bercht. & J.Presl
Iz_4_1009	DJI_0386	Dactylis glomerata L.	0	n	1	0	Poaceae Barnhart
Iz_4_1009	DJI_0386	Dianthus campestris M.Bieb.	0	n	1	0	Caryophyllaceae Juss.
Iz_4_1009	DJI_0386	Digitaria sanguinalis (L.) Scop.	0	a	0	0	Poaceae Barnhart
Iz_4_1009	DJI_0386	Elymus repens (L.) Gould	0	n	1	0	Poaceae Barnhart
Iz_4_1009	DJI_0386	Epilobium tetragonum L.	0	n	1	0	Onagraceae Juss.
Iz_4_1009	DJI_0386	Eragrostis minor Host	0	a	0	0	Poaceae Barnhart
Iz_4_1009	DJI_0386	Erigeron canadensis L.	0	a	0	0	Asteraceae Bercht. & J.Presl
Iz_4_1009	DJI_0386	Euphorbia seguieriana Neck.	0	n	1	0	Euphorbiaceae Juss.
Iz_4_1009	DJI_0386	Fallopia dumetorum (L.) Holub	0	n	1	0	Polygonaceae Juss.
Iz_4_1009	DJI_0386	Festuca beckeri (Hack.) Trautv.	0	n	1	0	Poaceae Barnhart
Iz_4_1009	DJI_0386	Fragaria vesca L.	0	n	1	0	Rosaceae Juss.
Iz_4_1009	DJI_0386	Frangula alnus Mill.	0	n	1	1	Rhamnaceae Juss.
Iz_4_1009	DJI_0386	Fraxinus pennsylvanica Marshall	0	a	0	1	Oleaceae Hoffmanns. & Link
Iz_4_1009	DJI_0386	Galeopsis bifida Boenn.	0	n	1	0	Lamiacea Lindl.
Iz_4_1009	DJI_0386	Galium verum L.	0	n	1	0	Rubiaceae Juss.
Iz_4_1009	DJI_0386	Genista tinctoria L.	0	n	1	0	Fabaceae Lindl.
Iz_4_1009	DJI_0386	Geranium robertianum L.	0	n	1	0	Geraniaceae Juss.
Iz_4_1009	DJI_0386	Geranium sanquineum L.	0	n	1	0	Geraniaceae Juss.
Iz_4_1009	DJI_0386	Gypsophila paniculata L.	0	n	1	0	Caryophyllaceae Juss.
Iz_4_1009	DJI_0386	Helichrysum arenarium (L.) Moench	0	n	1	0	Asteraceae Bercht. & J.Presl
Iz_4_1009	DJI_0386	Herniaria polygama J.Gay	0	n	1	0	Caryophyllaceae Juss.
Iz_4_1009	DJI_0386	Hieracium robustum Fr.	0	n	1	0	Asteraceae Bercht. & J.Presl
Iz_4_1009	DJI_0386	Hieracium umbellatum L.	0	n	1	NA	Asteraceae Bercht. & J.Presl
Iz_4_1009	DJI_0386	Hylotelephium maximum (L.) Holub	0	n	1	0	Crassulaceae J.St.-Hil.
Iz_4_1009	DJI_0386	Jacobaea borysthenica (DC.) B.Nord. & Greuter	0	n	1	0	Asteraceae Bercht. & J.Presl
Iz_4_1009	DJI_0386	Jasione montana L.	0	n	1	0	Campanulaceae Juss.
Iz_4_1009	DJI_0386	Jurinea cyanoides (L.) Rchb.s.l.	0	n	1	0	Asteraceae Bercht. & J.Presl
Iz_4_1009	DJI_0386	Koeleria glauca (Spreng.) DC.	0	n	1	0	Poaceae Barnhart
Iz_4_1009	DJI_0386	Lactuca serriola L.	0	a	0	0	Asteraceae Bercht. & J.Presl
Iz_4_1009	DJI_0386	Linaria genistifolia (L.) Mill.	0	n	1	0	Plantaginaceae Juss.
Iz_4_1009	DJI_0386	Lomelosia argentea (L.) Greuter & Burdet	0	n	1	0	Caprifoliaceae Juss.
Iz_4_1009	DJI_0386	Melampyrum pratense L.	0	n	1	0	Orobanchaceae Vent.
Iz_4_1009	DJI_0386	Oenothera biennis L.	0	a	0	0	Onagraceae Juss.
Iz_4_1009	DJI_0386	Pentanema britannica (L.) D.Gut.Larr., Santos-Vicente, Anderb., E.Rico & M.M.Mart.Ort.	0	n	1	0	Asteraceae Bercht. & J.Presl
Iz_4_1009	DJI_0386	Peucedanum oreoselinum (L.) Moench	0	n	1	0	Apiaceae Lindl.
Iz_4_1009	DJI_0386	Peucedanum ruthenicum M.Bieb.	0	n	1	0	Apiaceae Lindl.
Iz_4_1009	DJI_0386	Pilosella echioides (Lumn.) F.W.Schultz & Sch.Bip.	0	n	1	0	Asteraceae Bercht. & J.Presl
Iz_4_1009	DJI_0386	Pilosella officinarum F. Schultz et Sch. Bip.	0	n	1	0	Asteraceae Bercht. & J.Presl
Iz_4_1009	DJI_0386	Pilosella sp.	0		NA	0	Asteraceae Bercht. & J.Presl
Iz_4_1009	DJI_0386	Pinus sylvestris L.	0	n	1	1	Pinaceae Spreng. ex F.Rudolphi
Iz_4_1009	DJI_0386	Poa annua L.	0	n	1	0	Poaceae Barnhart
Iz_4_1009	DJI_0386	Poa nemoralis L.	0	n	1	0	Poaceae Barnhart
Iz_4_1009	DJI_0386	Poa sp.	0		NA	0	Poaceae Barnhart
Iz_4_1009	DJI_0386	Polygonatum odoratum (Mill.) Druce	0	n	1	0	Asparagaceae Juss.
Iz_4_1009	DJI_0386	Polygonum arenarium Waldst. & Kit.	0	n	1	0	Polygonaceae Juss.
Iz_4_1009	DJI_0386	Populus nigra L.	0	n	1	1	Salicaceae Mirb.
Iz_4_1009	DJI_0386	Populus tremula L.	0	n	1	1	Salicaceae Mirb.
Iz_4_1009	DJI_0386	Populus Ã canescens (Aiton) Sm.	0	n	1	1	Salicaceae Mirb.
Iz_4_1009	DJI_0386	Potentilla argentea L.	0	n	1	0	Rosaceae Juss.
Iz_4_1009	DJI_0386	Psammophiliella muralis (L.) Ikonn.	0	n	1	0	Caryophyllaceae Juss.
Iz_4_1009	DJI_0386	Psephellus sumensis (Kalen.) Greuter	0	n	1	0	Asteraceae Bercht. & J.Presl
Iz_4_1009	DJI_0386	Pulsatilla pratensis (L.) Mill. s.l.	0	n	1	0	Ranunculaceae Juss.
Iz_4_1009	DJI_0386	Pyrus communis L.	0	a	0	1	Rosaceae Juss.
Iz_4_1009	DJI_0386	Quercus robur L.	0	n	1	1	Fagaceae Dumort.
Iz_4_1009	DJI_0386	Robinia pseudoacacia L.	0	a	0	1	Fabaceae Lindl.
Iz_4_1009	DJI_0386	Rosa sp.	0		NA	1	Rosaceae Juss.
Iz_4_1009	DJI_0386	Rubus idaeus L.	0	n	1	0	Rosaceae Juss.
Iz_4_1009	DJI_0386	Rumex acetosella L.	0.5	n	1	0	Polygonaceae Juss.
Iz_4_1009	DJI_0386	Rumex thyrsiflorus Fingerh.	0	n	1	0	Polygonaceae Juss.
Iz_4_1009	DJI_0386	Salix alba L.	0	n	1	1	Salicaceae Mirb.
Iz_4_1009	DJI_0386	Sambucus nigra L.	0	n	1	1	Viburnaceae Raf.
Iz_4_1009	DJI_0386	Secale sylvestre Host	0	n	1	0	Poaceae Barnhart
Iz_4_1009	DJI_0386	Sempervivum ruthenicum Schnittsp. & C.B. Lehm.	0	n	1	0	Crassulaceae J.St.-Hil.
Iz_4_1009	DJI_0386	Setaria viridis (L.) P. Beauv.	0	n	1	0	Poaceae Barnhart
Iz_4_1009	DJI_0386	Silene borysthenica (Gruner) Walters	0	n	1	0	Caryophyllaceae Juss.
Iz_4_1009	DJI_0386	Silene latifolia subsp. alba (Mill.) Greuter & Burdet	0	n	1	0	Caryophyllaceae Juss.
Iz_4_1009	DJI_0386	Solidago virgaurea L.	0	n	1	0	Asteraceae Bercht. & J.Presl
Iz_4_1009	DJI_0386	Sorbus aucuparia L.	0	n	1	1	Rosaceae Juss.
Iz_4_1009	DJI_0386	Stachys recta L.	0	n	1	0	Lamiacea Lindl.
Iz_4_1009	DJI_0386	Tanacetum vulgare L.	0	n	1	0	Asteraceae Bercht. & J.Presl
Iz_4_1009	DJI_0386	Thymus pallasianus Heinr.Braun	0	n	1	0	Lamiacea Lindl.
Iz_4_1009	DJI_0386	Tilia cordata Mill.	0	n	1	1	Malvaceae Juss.
Iz_4_1009	DJI_0386	Trifolium alpestre L.	0	n	1	0	Fabaceae Lindl.
Iz_4_1009	DJI_0386	Trifolium arvense L.	0	n	1	0	Fabaceae Lindl.
Iz_4_1009	DJI_0386	Ulmus glabra Huds.	0	n	1	1	Ulmaceae Mirb.
Iz_4_1009	DJI_0386	Verbascum densiflorum Bertol.	0	n	1	0	Scrophulariaceae Juss.
Iz_4_1009	DJI_0386	Verbascum nigrum L.	0	n	1	0	Scrophulariaceae Juss.
Iz_4_1009	DJI_0386	Veronica austriaca L.	0	n	1	0	Plantaginaceae Juss.
Iz_4_1009	DJI_0386	Veronica incana L.	0	n	1	0	Plantaginaceae Juss.
Iz_4_1009	DJI_0386	Veronica verna L.	0	n	1	0	Plantaginaceae Juss.
Iz_4_1009	DJI_0386	Vincetoxicum hirundinaria Medik	0	n	1	0	Apocynaceae Juss.
Iz_4_1009	DJI_0387	Acer tataricum L.	0	n	1	1	Sapindaceae Juss.
Iz_4_1009	DJI_0387	Achillea millefolium L. s.l.	0	n	1	0	Asteraceae Bercht. & J.Presl
Iz_4_1009	DJI_0387	Allium oleraceum L.	0	n	1	0	Amaryllidaceae J.St.-Hil.
Iz_4_1009	DJI_0387	Ambrosia artemisiifolia L.	0	a	0	0	Asteraceae Bercht. & J.Presl
Iz_4_1009	DJI_0387	Amorpha fruticosa L.	0	a	0	0	Fabaceae Lindl.
Iz_4_1009	DJI_0387	Anthericum ramosum L.	0	n	1	0	Asparagaceae Juss.
Iz_4_1009	DJI_0387	Artemisia marschalliana Spreng.	0	n	1	0	Asteraceae Bercht. & J.Presl
Iz_4_1009	DJI_0387	Asparagus officinalis L.	0	n	1	0	Asparagaceae Juss.
Iz_4_1009	DJI_0387	Astragalus cicer L.	0	n	1	0	Fabaceae Lindl.
Iz_4_1009	DJI_0387	Bassia laniflora (S.G.Gmel.) A.J.Scott	0	n	1	0	Amaranthaceae Juss.
Iz_4_1009	DJI_0387	Berberis vulgaris L.	0	n	1	0	Berberidaceae Juss.
Iz_4_1009	DJI_0387	Betula pendula Roth.	0	n	1	1	Betulaceae Gray
Iz_4_1009	DJI_0387	Betula pendula Roth.	0	n	1	0	Poaceae Barnhart
Iz_4_1009	DJI_0387	Bromus inermis Leyss.	0		NA	NA	
Iz_4_1009	DJI_0387	Bromus squarrosus L.	0	a	0	0	Poaceae Barnhart

Iz_4_1009	DJI_0387	Bromus tectorum L.	0	a	0	0	Poaceae Barnhart
Iz_4_1009	DJI_0387	Calamagrostis epigejos (L.) Roth	0.2	n	1	0	Poaceae Barnhart
Iz_4_1009	DJI_0387	Carduus acanthoides L.	0	n	1	0	Asteraceae Bercht. & J.Presl
Iz_4_1009	DJI_0387	Carex sp.	0	n	1	0	Cyperaceae Juss.
Iz_4_1009	DJI_0387	Centaurea majorovii Dumbadze	0	n	1	0	Asteraceae Bercht. & J.Presl
Iz_4_1009	DJI_0387	Chamaecytisus ruthenicus (Fisch.ex Wol.) Klaskova	0	n	1	0	Fabaceae Lindl.
Iz_4_1009	DJI_0387	Chelidonium majus L.	0	n	1	0	Papaveraceae Juss.
Iz_4_1009	DJI_0387	Chenopodium betaceum Andrz.	0	n	1	0	Amaranthaceae Juss.
Iz_4_1009	DJI_0387	Chenopodium cf. album	0	n	1	0	Amaranthaceae Juss.
Iz_4_1009	DJI_0387	Chenopodium sp.	0		NA	0	Amaranthaceae Juss.
Iz_4_1009	DJI_0387	Chondrilla juncea L.	0	n	1	0	Asteraceae Bercht. & J.Presl
Iz_4_1009	DJI_0387	Cirsium vulgare (Savi) Ten.	0	n	1	0	Asteraceae Bercht. & J.Presl
Iz_4_1009	DJI_0387	Clinopodium acinos (L.) Kuntze	0	n	1	0	Lamiacea Lindl.
Iz_4_1009	DJI_0387	Convallaria majalis L.	0	n	1	0	Asparagaceae Juss.
Iz_4_1009	DJI_0387	Crataegus rhipidophylla Gand.	0	n	1	1	Rosaceae Juss.
Iz_4_1009	DJI_0387	Crepis foetida L.	0	n	1	0	Asteraceae Bercht. & J.Presl
Iz_4_1009	DJI_0387	Dactylis glomerata L.	0	n	1	0	Poaceae Barnhart
Iz_4_1009	DJI_0387	Dianthus campestris M.Bieb.	0	n	1	0	Caryophyllaceae Juss.
Iz_4_1009	DJI_0387	Digitaria sanguinalis (L.) Scop.	0	a	0	0	Poaceae Barnhart
Iz_4_1009	DJI_0387	Elymus repens (L.) Gould	0	n	1	0	Poaceae Barnhart
Iz_4_1009	DJI_0387	Epilobium tetragonum L.	0	n	1	0	Onagraceae Juss.
Iz_4_1009	DJI_0387	Eragrostis minor Host	0	a	0	0	Poaceae Barnhart
Iz_4_1009	DJI_0387	Erigeron canadensis L.	0	a	0	0	Asteraceae Bercht. & J.Presl
Iz_4_1009	DJI_0387	Euphorbia seguieriana Neck.	0	n	1	0	Euphorbiaceae Juss.
Iz_4_1009	DJI_0387	Fallopia dumetorum (L.) Holub	0	n	1	0	Polygonaceae Juss.
Iz_4_1009	DJI_0387	Festuca beckeri (Hack.) Trautv.	0	n	1	0	Poaceae Barnhart
Iz_4_1009	DJI_0387	Fragaria vesca L.	0	n	1	0	Rosaceae Juss.
Iz_4_1009	DJI_0387	Frangula alnus Mill.	0	n	1	1	Rhamnaceae Juss.
Iz_4_1009	DJI_0387	Fraxinus pennsylvanica Marshall	0	a	0	1	Oleaceae Hoffmanns. & Link
Iz_4_1009	DJI_0387	Galeopsis bifida Boenn.	0	n	1	0	Lamiacea Lindl.
Iz_4_1009	DJI_0387	Galium verum L.	0	n	1	0	Rubiaceae Juss.
Iz_4_1009	DJI_0387	Genista tinctoria L.	0	n	1	0	Fabaceae Lindl.
Iz_4_1009	DJI_0387	Geranium robertianum L.	0	n	1	0	Geraniaceae Juss.
Iz_4_1009	DJI_0387	Geranium sanquineum L.	0	n	1	0	Geraniaceae Juss.
Iz_4_1009	DJI_0387	Gypsophila paniculata L.	0	n	1	0	Caryophyllaceae Juss.
Iz_4_1009	DJI_0387	Helichrysum arenarium (L.) Moench	0	n	1	0	Asteraceae Bercht. & J.Presl
Iz_4_1009	DJI_0387	Herniaria polygama J.Gay	0	n	1	0	Caryophyllaceae Juss.
Iz_4_1009	DJI_0387	Hieracium robustum Fr.	0	n	1	0	Asteraceae Bercht. & J.Presl
Iz_4_1009	DJI_0387	Hieracium umbellatum L.	0	n	1	NA	Asteraceae Bercht. & J.Presl
Iz_4_1009	DJI_0387	Hylotelephium maximum (L.) Holub	0	n	1	0	Crassulaceae J.St.-Hil.
Iz_4_1009	DJI_0387	Jacobaea borysthenica (DC.) B.Nord. & Greuter	0	n	1	0	Asteraceae Bercht. & J.Presl
Iz_4_1009	DJI_0387	Jasione montana L.	0	n	1	0	Campanulaceae Juss.
Iz_4_1009	DJI_0387	Jurinea cyanoides (L.) Rchb.s.l.	0	n	1	0	Asteraceae Bercht. & J.Presl
Iz_4_1009	DJI_0387	Koeleria glauca (Spreng.) DC.	0	n	1	0	Poaceae Barnhart
Iz_4_1009	DJI_0387	Lactuca serriola L.	0	a	0	0	Asteraceae Bercht. & J.Presl
Iz_4_1009	DJI_0387	Linaria genistifolia (L.) Mill.	0	n	1	0	Plantaginaceae Juss.
Iz_4_1009	DJI_0387	Lomelosia argentea (L.) Greuter & Burdet	0	n	1	0	Caprifoliaceae Juss.
Iz_4_1009	DJI_0387	Melampyrum pratense L.	0	n	1	0	Orobanchaceae Vent.
Iz_4_1009	DJI_0387	Oenothera biennis L.	0	a	0	0	Onagraceae Juss.
Iz_4_1009	DJI_0387	Pentanema britannica (L.) D.Gut.Larr., Santos-Vicente, Anderb., E.Rico & M.M.Mart.Ort.	0	n	1	0	Asteraceae Bercht. & J.Presl
Iz_4_1009	DJI_0387	Peucedanum oreoselinum (L.) Moench	0	n	1	0	Apiaceae Lindl.
Iz_4_1009	DJI_0387	Peucedanum ruthenicum M.Bieb.	0	n	1	0	Apiaceae Lindl.
Iz_4_1009	DJI_0387	Pilosella echioides (Lumn.) F.W.Schultz & Sch.Bip.	0	n	1	0	Asteraceae Bercht. & J.Presl
Iz_4_1009	DJI_0387	Pilosella officinarum F. Schultz et Sch. Bip.	0	n	1	0	Asteraceae Bercht. & J.Presl
Iz_4_1009	DJI_0387	Pilosella sp.	0		NA	0	Asteraceae Bercht. & J.Presl
Iz_4_1009	DJI_0387	Pinus sylvestris L.	0	n	1	1	Pinaceae Spreng. ex F.Rudolphi
Iz_4_1009	DJI_0387	Poa annua L.	0	n	1	0	Poaceae Barnhart
Iz_4_1009	DJI_0387	Poa nemoralis L.	0	n	1	0	Poaceae Barnhart
Iz_4_1009	DJI_0387	Poa sp.	0		NA	0	Poaceae Barnhart
Iz_4_1009	DJI_0387	Polygonatum odoratum (Mill.) Druce	0	n	1	0	Asparagaceae Juss.
Iz_4_1009	DJI_0387	Polygonum arenarium Waldst. & Kit.	0	n	1	0	Polygonaceae Juss.
Iz_4_1009	DJI_0387	Populus nigra L.	0	n	1	1	Salicaceae Mirb.
Iz_4_1009	DJI_0387	Populus tremula L.	0	n	1	1	Salicaceae Mirb.
Iz_4_1009	DJI_0387	Populus Ã canescens (Aiton) Sm.	0	n	1	1	Salicaceae Mirb.
Iz_4_1009	DJI_0387	Potentilla argentea L.	0	n	1	0	Rosaceae Juss.
Iz_4_1009	DJI_0387	Psammophiliella muralis (L.) Ikonn.	0	n	1	0	Caryophyllaceae Juss.
Iz_4_1009	DJI_0387	Psephellus sumensis (Kalen.) Greuter	0	n	1	0	Asteraceae Bercht. & J.Presl
Iz_4_1009	DJI_0387	Pulsatilla pratensis (L.) Mill. s.l.	0	n	1	0	Ranunculaceae Juss.
Iz_4_1009	DJI_0387	Pyrus communis L.	0	a	0	1	Rosaceae Juss.
Iz_4_1009	DJI_0387	Quercus robur L.	0	n	1	1	Fagaceae Dumort.
Iz_4_1009	DJI_0387	Robinia pseudoacacia L.	0	a	0	1	Fabaceae Lindl.
Iz_4_1009	DJI_0387	Rosa sp.	0		NA	1	Rosaceae Juss.
Iz_4_1009	DJI_0387	Rubus idaeus L.	0	n	1	0	Rosaceae Juss.
Iz_4_1009	DJI_0387	Rumex acetosella L.	0	n	1	0	Polygonaceae Juss.
Iz_4_1009	DJI_0387	Rumex thyrsiflorus Fingerh.	0	n	1	0	Polygonaceae Juss.
Iz_4_1009	DJI_0387	Salix alba L.	0	n	1	1	Salicaceae Mirb.
Iz_4_1009	DJI_0387	Sambucus nigra L.	0	n	1	1	Viburnaceae Raf.
Iz_4_1009	DJI_0387	Secale sylvestre Host	0	n	1	0	Poaceae Barnhart
Iz_4_1009	DJI_0387	Sempervivum ruthenicum Schnittsp. & C.B. Lehm.	0	n	1	0	Crassulaceae J.St.-Hil.
Iz_4_1009	DJI_0387	Setaria viridis (L.) P. Beauv.	0	n	1	0	Poaceae Barnhart
Iz_4_1009	DJI_0387	Silene borysthenica (Gruner) Walters	0	n	1	0	Caryophyllaceae Juss.
Iz_4_1009	DJI_0387	Silene latifolia subsp. alba (Mill.) Greuter & Burdet	0	n	1	0	Caryophyllaceae Juss.
Iz_4_1009	DJI_0387	Solidago virgaurea L.	0	n	1	0	Asteraceae Bercht. & J.Presl
Iz_4_1009	DJI_0387	Sorbus aucuparia L.	0	n	1	1	Rosaceae Juss.
Iz_4_1009	DJI_0387	Stachys recta L.	0	n	1	0	Lamiacea Lindl.
Iz_4_1009	DJI_0387	Tanacetum vulgare L.	0	n	1	0	Asteraceae Bercht. & J.Presl
Iz_4_1009	DJI_0387	Thymus pallasianus Heinr.Braun	0	n	1	0	Lamiacea Lindl.
Iz_4_1009	DJI_0387	Tilia cordata Mill.	0	n	1	1	Malvaceae Juss.
Iz_4_1009	DJI_0387	Trifolium alpestre L.	0	n	1	0	Fabaceae Lindl.
Iz_4_1009	DJI_0387	Trifolium arvense L.	0	n	1	0	Fabaceae Lindl.
Iz_4_1009	DJI_0387	Ulmus glabra Huds.	0	n	1	1	Ulmaceae Mirb.
Iz_4_1009	DJI_0387	Verbascum densiflorum Bertol.	0	n	1	0	Scrophulariaceae Juss.
Iz_4_1009	DJI_0387	Verbascum nigrum L.	0	n	1	0	Scrophulariaceae Juss.
Iz_4_1009	DJI_0387	Veronica austriaca L.	0	n	1	0	Plantaginaceae Juss.
Iz_4_1009	DJI_0387	Veronica incana L.	0	n	1	0	Plantaginaceae Juss.
Iz_4_1009	DJI_0387	Veronica verna L.	0	n	1	0	Plantaginaceae Juss.
Iz_4_1009	DJI_0387	Vincetoxicum hirundinaria Medik	0	n	1	0	Apocynaceae Juss.
Iz_4_1009	DJI_0388	Acer tataricum L.	0	n	1	1	Sapindaceae Juss.

Iz_4_1009	DJI_0388	Achillea millefolium L. s.l.	0	n	1	0	Asteraceae Bercht. & J.Presl
Iz_4_1009	DJI_0388	Allium oleraceum L.	0	n	1	0	Amaryllidaceae J.St.-Hil.
Iz_4_1009	DJI_0388	Ambrosia artemisiifolia L.	0	a	0	0	Asteraceae Bercht. & J.Presl
Iz_4_1009	DJI_0388	Amorpha fruticosa L.	0	a	0	0	Fabaceae Lindl.
Iz_4_1009	DJI_0388	Anthericum ramosum L.	0	n	1	0	Asparagaceae Juss.
Iz_4_1009	DJI_0388	Artemisia marschalliana Spreng.	0	n	1	0	Asteraceae Bercht. & J.Presl
Iz_4_1009	DJI_0388	Asparagus officinalis L.	0	n	1	0	Asparagaceae Juss.
Iz_4_1009	DJI_0388	Astragalus cicer L.	0	n	1	0	Fabaceae Lindl.
Iz_4_1009	DJI_0388	Bassia laniflora (S.G.Gmel.) A.J.Scott	0	n	1	0	Amaranthaceae Juss.
Iz_4_1009	DJI_0388	Berberis vulgaris L.	0	n	1	0	Berberidaceae Juss.
Iz_4_1009	DJI_0388	Betula pendula Roth.	0	n	1	1	Betulaceae Gray
Iz_4_1009	DJI_0388	Betula pendula Roth.	0	n	1	0	Poaceae Barnhart
Iz_4_1009	DJI_0388	Bromus inermis Leyss.	0		NA	NA	
Iz_4_1009	DJI_0388	Bromus squarrosus L.	0	a	0	0	Poaceae Barnhart
Iz_4_1009	DJI_0388	Bromus tectorum L.	0	a	0	0	Poaceae Barnhart
Iz_4_1009	DJI_0388	Calamagrostis epigejos (L.) Roth	0.5	n	1	0	Poaceae Barnhart
Iz_4_1009	DJI_0388	Carduus acanthoides L.	0	n	1	0	Asteraceae Bercht. & J.Presl
Iz_4_1009	DJI_0388	Carex sp.	0	n	1	0	Cyperaceae Juss.
Iz_4_1009	DJI_0388	Centaurea majorovii Dumbadze	0	n	1	0	Asteraceae Bercht. & J.Presl
Iz_4_1009	DJI_0388	Chamaecytisus ruthenicus (Fisch.ex Wol.) Klaskova	0	n	1	0	Fabaceae Lindl.
Iz_4_1009	DJI_0388	Chelidonium majus L.	0	n	1	0	Papaveraceae Juss.
Iz_4_1009	DJI_0388	Chenopodium betaceum Andrz.	0	n	1	0	Amaranthaceae Juss.
Iz_4_1009	DJI_0388	Chenopodium cf. album	0	n	1	0	Amaranthaceae Juss.
Iz_4_1009	DJI_0388	Chenopodium sp.	0		NA	0	Amaranthaceae Juss.
Iz_4_1009	DJI_0388	Chondrilla juncea L.	0	n	1	0	Asteraceae Bercht. & J.Presl
Iz_4_1009	DJI_0388	Cirsium vulgare (Savi) Ten.	0	n	1	0	Asteraceae Bercht. & J.Presl
Iz_4_1009	DJI_0388	Clinopodium acinos (L.) Kuntze	0	n	1	0	Lamiacea Lindl.
Iz_4_1009	DJI_0388	Convallaria majalis L.	0	n	1	0	Asparagaceae Juss.
Iz_4_1009	DJI_0388	Crataegus rhipidophylla Gand.	0	n	1	1	Rosaceae Juss.
Iz_4_1009	DJI_0388	Crepis foetida L.	0	n	1	0	Asteraceae Bercht. & J.Presl
Iz_4_1009	DJI_0388	Dactylis glomerata L.	0	n	1	0	Poaceae Barnhart
Iz_4_1009	DJI_0388	Dianthus campestris M.Bieb.	0	n	1	0	Caryophyllaceae Juss.
Iz_4_1009	DJI_0388	Digitaria sanguinalis (L.) Scop.	0	a	0	0	Poaceae Barnhart
Iz_4_1009	DJI_0388	Elymus repens (L.) Gould	0	n	1	0	Poaceae Barnhart
Iz_4_1009	DJI_0388	Epilobium tetragonum L.	0	n	1	0	Onagraceae Juss.
Iz_4_1009	DJI_0388	Eragrostis minor Host	0	a	0	0	Poaceae Barnhart
Iz_4_1009	DJI_0388	Erigeron canadensis L.	0	a	0	0	Asteraceae Bercht. & J.Presl
Iz_4_1009	DJI_0388	Euphorbia seguieriana Neck.	0	n	1	0	Euphorbiaceae Juss.
Iz_4_1009	DJI_0388	Fallopia dumetorum (L.) Holub	0	n	1	0	Polygonaceae Juss.
Iz_4_1009	DJI_0388	Festuca beckeri (Hack.) Trautv.	0	n	1	0	Poaceae Barnhart
Iz_4_1009	DJI_0388	Fragaria vesca L.	0	n	1	0	Rosaceae Juss.
Iz_4_1009	DJI_0388	Frangula alnus Mill.	0	n	1	1	Rhamnaceae Juss.
Iz_4_1009	DJI_0388	Fraxinus pennsylvanica Marshall	0	a	0	1	Oleaceae Hoffmanns. & Link
Iz_4_1009	DJI_0388	Galeopsis bifida Boenn.	0	n	1	0	Lamiacea Lindl.
Iz_4_1009	DJI_0388	Galium verum L.	0	n	1	0	Rubiaceae Juss.
Iz_4_1009	DJI_0388	Genista tinctoria L.	0	n	1	0	Fabaceae Lindl.
Iz_4_1009	DJI_0388	Geranium robertianum L.	0	n	1	0	Geraniaceae Juss.
Iz_4_1009	DJI_0388	Geranium sanquineum L.	0	n	1	0	Geraniaceae Juss.
Iz_4_1009	DJI_0388	Gypsophila paniculata L.	0	n	1	0	Caryophyllaceae Juss.
Iz_4_1009	DJI_0388	Helichrysum arenarium (L.) Moench	0	n	1	0	Asteraceae Bercht. & J.Presl
Iz_4_1009	DJI_0388	Herniaria polygama J.Gay	0	n	1	0	Caryophyllaceae Juss.
Iz_4_1009	DJI_0388	Hieracium robustum Fr.	0	n	1	0	Asteraceae Bercht. & J.Presl
Iz_4_1009	DJI_0388	Hieracium umbellatum L.	0	n	1	NA	Asteraceae Bercht. & J.Presl
Iz_4_1009	DJI_0388	Hylotelephium maximum (L.) Holub	0	n	1	0	Crassulaceae J.St.-Hil.
Iz_4_1009	DJI_0388	Jacobaea borysthenica (DC.) B.Nord. & Greuter	0	n	1	0	Asteraceae Bercht. & J.Presl
Iz_4_1009	DJI_0388	Jasione montana L.	0	n	1	0	Campanulaceae Juss.
Iz_4_1009	DJI_0388	Jurinea cyanoides (L.) Rchb.s.l.	0	n	1	0	Asteraceae Bercht. & J.Presl
Iz_4_1009	DJI_0388	Koeleria glauca (Spreng.) DC.	0	n	1	0	Poaceae Barnhart
Iz_4_1009	DJI_0388	Lactuca serriola L.	0	a	0	0	Asteraceae Bercht. & J.Presl
Iz_4_1009	DJI_0388	Linaria genistifolia (L.) Mill.	0	n	1	0	Plantaginaceae Juss.
Iz_4_1009	DJI_0388	Lomelosia argentea (L.) Greuter & Burdet	0	n	1	0	Caprifoliaceae Juss.
Iz_4_1009	DJI_0388	Melampyrum pratense L.	0	n	1	0	Orobanchaceae Vent.
Iz_4_1009	DJI_0388	Oenothera biennis L.	0	a	0	0	Onagraceae Juss.
Iz_4_1009	DJI_0388	Pentanema britannica (L.) D.Gut.Larr., Santos-Vicente, Anderb., E.Rico & M.M.Mart.Ort.	0	n	1	0	Asteraceae Bercht. & J.Presl
Iz_4_1009	DJI_0388	Peucedanum oreoselinum (L.) Moench	0	n	1	0	Apiaceae Lindl.
Iz_4_1009	DJI_0388	Peucedanum ruthenicum M.Bieb.	0	n	1	0	Apiaceae Lindl.
Iz_4_1009	DJI_0388	Pilosella echioides (Lumn.) F.W.Schultz & Sch.Bip.	0	n	1	0	Asteraceae Bercht. & J.Presl
Iz_4_1009	DJI_0388	Pilosella officinarum F. Schultz et Sch. Bip.	0	n	1	0	Asteraceae Bercht. & J.Presl
Iz_4_1009	DJI_0388	Pilosella sp.	0		NA	0	Asteraceae Bercht. & J.Presl
Iz_4_1009	DJI_0388	Pinus sylvestris L.	0	n	1	1	Pinaceae Spreng. ex F.Rudolphi
Iz_4_1009	DJI_0388	Poa annua L.	0	n	1	0	Poaceae Barnhart
Iz_4_1009	DJI_0388	Poa nemoralis L.	0	n	1	0	Poaceae Barnhart
Iz_4_1009	DJI_0388	Poa sp.	0		NA	0	Poaceae Barnhart
Iz_4_1009	DJI_0388	Polygonatum odoratum (Mill.) Druce	0	n	1	0	Asparagaceae Juss.
Iz_4_1009	DJI_0388	Polygonum arenarium Waldst. & Kit.	0	n	1	0	Polygonaceae Juss.
Iz_4_1009	DJI_0388	Populus nigra L.	0	n	1	1	Salicaceae Mirb.
Iz_4_1009	DJI_0388	Populus tremula L.	0	n	1	1	Salicaceae Mirb.
Iz_4_1009	DJI_0388	Populus Ã canescens (Aiton) Sm.	0	n	1	1	Salicaceae Mirb.
Iz_4_1009	DJI_0388	Potentilla argentea L.	0	n	1	0	Rosaceae Juss.
Iz_4_1009	DJI_0388	Psammophiliella muralis (L.) Ikonn.	0	n	1	0	Caryophyllaceae Juss.
Iz_4_1009	DJI_0388	Psephellus sumensis (Kalen.) Greuter	0	n	1	0	Asteraceae Bercht. & J.Presl
Iz_4_1009	DJI_0388	Pulsatilla pratensis (L.) Mill. s.l.	0	n	1	0	Ranunculaceae Juss.
Iz_4_1009	DJI_0388	Pyrus communis L.	0	a	0	1	Rosaceae Juss.
Iz_4_1009	DJI_0388	Quercus robur L.	0	n	1	1	Fagaceae Dumort.
Iz_4_1009	DJI_0388	Robinia pseudoacacia L.	0	a	0	1	Fabaceae Lindl.
Iz_4_1009	DJI_0388	Rosa sp.	0		NA	1	Rosaceae Juss.
Iz_4_1009	DJI_0388	Rubus idaeus L.	0	n	1	0	Rosaceae Juss.
Iz_4_1009	DJI_0388	Rumex acetosella L.	0	n	1	0	Polygonaceae Juss.
Iz_4_1009	DJI_0388	Rumex thyrsiflorus Fingerh.	0	n	1	0	Polygonaceae Juss.
Iz_4_1009	DJI_0388	Salix alba L.	0	n	1	1	Salicaceae Mirb.
Iz_4_1009	DJI_0388	Sambucus nigra L.	0	n	1	1	Viburnaceae Raf.
Iz_4_1009	DJI_0388	Secale sylvestre Host	0	n	1	0	Poaceae Barnhart
Iz_4_1009	DJI_0388	Sempervivum ruthenicum Schnittsp. & C.B. Lehm.	0	n	1	0	Crassulaceae J.St.-Hil.
Iz_4_1009	DJI_0388	Setaria viridis (L.) P. Beauv.	0	n	1	0	Poaceae Barnhart
Iz_4_1009	DJI_0388	Silene borysthenica (Gruner) Walters	0	n	1	0	Caryophyllaceae Juss.
Iz_4_1009	DJI_0388	Silene latifolia subsp. alba (Mill.) Greuter & Burdet	0	n	1	0	Caryophyllaceae Juss.
Iz_4_1009	DJI_0388	Solidago virgaurea L.	0	n	1	0	Asteraceae Bercht. & J.Presl
Iz_4_1009	DJI_0388	Sorbus aucuparia L.	0	n	1	1	Rosaceae Juss.

Iz_4_1009	DJI_0388	Stachys recta L.	0	n	1	0	Lamiacea Lindl.
Iz_4_1009	DJI_0388	Tanacetum vulgare L.	0	n	1	0	Asteraceae Bercht. & J.Presl
Iz_4_1009	DJI_0388	Thymus pallasianus Heinr.Braun	0	n	1	0	Lamiacea Lindl.
Iz_4_1009	DJI_0388	Tilia cordata Mill.	0	n	1	1	Malvaceae Juss.
Iz_4_1009	DJI_0388	Trifolium alpestre L.	0	n	1	0	Fabaceae Lindl.
Iz_4_1009	DJI_0388	Trifolium arvense L.	0	n	1	0	Fabaceae Lindl.
Iz_4_1009	DJI_0388	Ulmus glabra Huds.	0	n	1	1	Ulmaceae Mirb.
Iz_4_1009	DJI_0388	Verbascum densiflorum Bertol.	0	n	1	0	Scrophulariaceae Juss.
Iz_4_1009	DJI_0388	Verbascum nigrum L.	0	n	1	0	Scrophulariaceae Juss.
Iz_4_1009	DJI_0388	Veronica austriaca L.	0	n	1	0	Plantaginaceae Juss.
Iz_4_1009	DJI_0388	Veronica incana L.	0	n	1	0	Plantaginaceae Juss.
Iz_4_1009	DJI_0388	Veronica verna L.	0	n	1	0	Plantaginaceae Juss.
Iz_4_1009	DJI_0388	Vincetoxicum hirundinaria Medik	0	n	1	0	Apocynaceae Juss.
Iz_4_1009	DJI_0389	Acer tataricum L.	0	n	1	1	Sapindaceae Juss.
Iz_4_1009	DJI_0389	Achillea millefolium L. s.l.	0	n	1	0	Asteraceae Bercht. & J.Presl
Iz_4_1009	DJI_0389	Allium oleraceum L.	0	n	1	0	Amaryllidaceae J.St.-Hil.
Iz_4_1009	DJI_0389	Ambrosia artemisiifolia L.	0	a	0	0	Asteraceae Bercht. & J.Presl
Iz_4_1009	DJI_0389	Amorpha fruticosa L.	0	a	0	0	Fabaceae Lindl.
Iz_4_1009	DJI_0389	Anthericum ramosum L.	0	n	1	0	Asparagaceae Juss.
Iz_4_1009	DJI_0389	Artemisia marschalliana Spreng.	0	n	1	0	Asteraceae Bercht. & J.Presl
Iz_4_1009	DJI_0389	Asparagus officinalis L.	0	n	1	0	Asparagaceae Juss.
Iz_4_1009	DJI_0389	Astragalus cicer L.	0	n	1	0	Fabaceae Lindl.
Iz_4_1009	DJI_0389	Bassia laniflora (S.G.Gmel.) A.J.Scott	0	n	1	0	Amaranthaceae Juss.
Iz_4_1009	DJI_0389	Berberis vulgaris L.	0	n	1	0	Berberidaceae Juss.
Iz_4_1009	DJI_0389	Betula pendula Roth.	0	n	1	1	Betulaceae Gray
Iz_4_1009	DJI_0389	Betula pendula Roth.	0	n	1	0	Poaceae Barnhart
Iz_4_1009	DJI_0389	Bromus inermis Leyss.	0		NA	NA	
Iz_4_1009	DJI_0389	Bromus squarrosus L.	0	a	0	0	Poaceae Barnhart
Iz_4_1009	DJI_0389	Bromus tectorum L.	0	a	0	0	Poaceae Barnhart
Iz_4_1009	DJI_0389	Calamagrostis epigejos (L.) Roth	2	n	1	0	Poaceae Barnhart
Iz_4_1009	DJI_0389	Carduus acanthoides L.	0	n	1	0	Asteraceae Bercht. & J.Presl
Iz_4_1009	DJI_0389	Carex sp.	0	n	1	0	Cyperaceae Juss.
Iz_4_1009	DJI_0389	Centaurea majorovii Dumbadze	0	n	1	0	Asteraceae Bercht. & J.Presl
Iz_4_1009	DJI_0389	Chamaecytisus ruthenicus (Fisch.ex Wol.) Klaskova	0	n	1	0	Fabaceae Lindl.
Iz_4_1009	DJI_0389	Chelidonium majus L.	0	n	1	0	Papaveraceae Juss.
Iz_4_1009	DJI_0389	Chenopodium betaceum Andrz.	0	n	1	0	Amaranthaceae Juss.
Iz_4_1009	DJI_0389	Chenopodium cf. album	0	n	1	0	Amaranthaceae Juss.
Iz_4_1009	DJI_0389	Chenopodium sp.	0		NA	0	Amaranthaceae Juss.
Iz_4_1009	DJI_0389	Chondrilla juncea L.	0	n	1	0	Asteraceae Bercht. & J.Presl
Iz_4_1009	DJI_0389	Cirsium vulgare (Savi) Ten.	0	n	1	0	Asteraceae Bercht. & J.Presl
Iz_4_1009	DJI_0389	Clinopodium acinos (L.) Kuntze	0	n	1	0	Lamiacea Lindl.
Iz_4_1009	DJI_0389	Convallaria majalis L.	0	n	1	0	Asparagaceae Juss.
Iz_4_1009	DJI_0389	Crataegus rhipidophylla Gand.	0	n	1	1	Rosaceae Juss.
Iz_4_1009	DJI_0389	Crepis foetida L.	0	n	1	0	Asteraceae Bercht. & J.Presl
Iz_4_1009	DJI_0389	Dactylis glomerata L.	0	n	1	0	Poaceae Barnhart
Iz_4_1009	DJI_0389	Dianthus campestris M.Bieb.	0	n	1	0	Caryophyllaceae Juss.
Iz_4_1009	DJI_0389	Digitaria sanguinalis (L.) Scop.	0	a	0	0	Poaceae Barnhart
Iz_4_1009	DJI_0389	Elymus repens (L.) Gould	0	n	1	0	Poaceae Barnhart
Iz_4_1009	DJI_0389	Epilobium tetragonum L.	0	n	1	0	Onagraceae Juss.
Iz_4_1009	DJI_0389	Eragrostis minor Host	0	a	0	0	Poaceae Barnhart
Iz_4_1009	DJI_0389	Erigeron canadensis L.	0	a	0	0	Asteraceae Bercht. & J.Presl
Iz_4_1009	DJI_0389	Euphorbia seguieriana Neck.	0	n	1	0	Euphorbiaceae Juss.
Iz_4_1009	DJI_0389	Fallopia dumetorum (L.) Holub	0	n	1	0	Polygonaceae Juss.
Iz_4_1009	DJI_0389	Festuca beckeri (Hack.) Trautv.	0	n	1	0	Poaceae Barnhart
Iz_4_1009	DJI_0389	Fragaria vesca L.	0	n	1	0	Rosaceae Juss.
Iz_4_1009	DJI_0389	Frangula alnus Mill.	0	n	1	1	Rhamnaceae Juss.
Iz_4_1009	DJI_0389	Fraxinus pennsylvanica Marshall	0	a	0	1	Oleaceae Hoffmanns. & Link
Iz_4_1009	DJI_0389	Galeopsis bifida Boenn.	0	n	1	0	Lamiacea Lindl.
Iz_4_1009	DJI_0389	Galium verum L.	0	n	1	0	Rubiaceae Juss.
Iz_4_1009	DJI_0389	Genista tinctoria L.	0	n	1	0	Fabaceae Lindl.
Iz_4_1009	DJI_0389	Geranium robertianum L.	0	n	1	0	Geraniaceae Juss.
Iz_4_1009	DJI_0389	Geranium sanquineum L.	0	n	1	0	Geraniaceae Juss.
Iz_4_1009	DJI_0389	Gypsophila paniculata L.	0	n	1	0	Caryophyllaceae Juss.
Iz_4_1009	DJI_0389	Helichrysum arenarium (L.) Moench	0	n	1	0	Asteraceae Bercht. & J.Presl
Iz_4_1009	DJI_0389	Herniaria polygama J.Gay	0	n	1	0	Caryophyllaceae Juss.
Iz_4_1009	DJI_0389	Hieracium robustum Fr.	0	n	1	0	Asteraceae Bercht. & J.Presl
Iz_4_1009	DJI_0389	Hieracium umbellatum L.	0	n	1	NA	Asteraceae Bercht. & J.Presl
Iz_4_1009	DJI_0389	Hylotelephium maximum (L.) Holub	0	n	1	0	Crassulaceae J.St.-Hil.
Iz_4_1009	DJI_0389	Jacobaea borysthenica (DC.) B.Nord. & Greuter	0	n	1	0	Asteraceae Bercht. & J.Presl
Iz_4_1009	DJI_0389	Jasione montana L.	0	n	1	0	Campanulaceae Juss.
Iz_4_1009	DJI_0389	Jurinea cyanoides (L.) Rchb.s.l.	0	n	1	0	Asteraceae Bercht. & J.Presl
Iz_4_1009	DJI_0389	Koeleria glauca (Spreng.) DC.	0	n	1	0	Poaceae Barnhart
Iz_4_1009	DJI_0389	Lactuca serriola L.	0	a	0	0	Asteraceae Bercht. & J.Presl
Iz_4_1009	DJI_0389	Linaria genistifolia (L.) Mill.	0	n	1	0	Plantaginaceae Juss.
Iz_4_1009	DJI_0389	Lomelosia argentea (L.) Greuter & Burdet	0	n	1	0	Caprifoliaceae Juss.
Iz_4_1009	DJI_0389	Melampyrum pratense L.	0	n	1	0	Orobanchaceae Vent.
Iz_4_1009	DJI_0389	Oenothera biennis L.	0	a	0	0	Onagraceae Juss.
Iz_4_1009	DJI_0389	Pentanema britannica (L.) D.Gut.Larr., Santos-Vicente, Anderb., E.Rico & M.M.Mart.Ort.	0	n	1	0	Asteraceae Bercht. & J.Presl
Iz_4_1009	DJI_0389	Peucedanum oreoselinum (L.) Moench	0	n	1	0	Apiaceae Lindl.
Iz_4_1009	DJI_0389	Peucedanum ruthenicum M.Bieb.	0	n	1	0	Apiaceae Lindl.
Iz_4_1009	DJI_0389	Pilosella echioides (Lumn.) F.W.Schultz & Sch.Bip.	0	n	1	0	Asteraceae Bercht. & J.Presl
Iz_4_1009	DJI_0389	Pilosella officinarum F. Schultz et Sch. Bip.	0	n	1	0	Asteraceae Bercht. & J.Presl
Iz_4_1009	DJI_0389	Pilosella sp.	0		NA	0	Asteraceae Bercht. & J.Presl
Iz_4_1009	DJI_0389	Pinus sylvestris L.	0	n	1	1	Pinaceae Spreng. ex F.Rudolphi
Iz_4_1009	DJI_0389	Poa annua L.	0	n	1	0	Poaceae Barnhart
Iz_4_1009	DJI_0389	Poa nemoralis L.	0	n	1	0	Poaceae Barnhart
Iz_4_1009	DJI_0389	Poa sp.	0		NA	0	Poaceae Barnhart
Iz_4_1009	DJI_0389	Polygonatum odoratum (Mill.) Druce	0	n	1	0	Asparagaceae Juss.
Iz_4_1009	DJI_0389	Polygonum arenarium Waldst. & Kit.	0	n	1	0	Polygonaceae Juss.
Iz_4_1009	DJI_0389	Populus nigra L.	0	n	1	1	Salicaceae Mirb.
Iz_4_1009	DJI_0389	Populus tremula L.	0	n	1	1	Salicaceae Mirb.
Iz_4_1009	DJI_0389	Populus Ã canescens (Aiton) Sm.	0	n	1	1	Salicaceae Mirb.
Iz_4_1009	DJI_0389	Potentilla argentea L.	0	n	1	0	Rosaceae Juss.
Iz_4_1009	DJI_0389	Psammophiliella muralis (L.) Ikonn.	0	n	1	0	Caryophyllaceae Juss.
Iz_4_1009	DJI_0389	Psephellus sumensis (Kalen.) Greuter	0	n	1	0	Asteraceae Bercht. & J.Presl
Iz_4_1009	DJI_0389	Pulsatilla pratensis (L.) Mill. s.l.	0	n	1	0	Ranunculaceae Juss.
Iz_4_1009	DJI_0389	Pyrus communis L.	0	a	0	1	Rosaceae Juss.
Iz_4_1009	DJI_0389	Quercus robur L.	0	n	1	1	Fagaceae Dumort.

Iz_4_1009	DJI_0389	Robinia pseudoacacia L.	0	a	0	1	Fabaceae Lindl.
Iz_4_1009	DJI_0389	Rosa sp.	0		NA	1	Rosaceae Juss.
Iz_4_1009	DJI_0389	Rubus idaeus L.	0	n	1	0	Rosaceae Juss.
Iz_4_1009	DJI_0389	Rumex acetosella L.	0	n	1	0	Polygonaceae Juss.
Iz_4_1009	DJI_0389	Rumex thyrsiflorus Fingerh.	0	n	1	0	Polygonaceae Juss.
Iz_4_1009	DJI_0389	Salix alba L.	0	n	1	1	Salicaceae Mirb.
Iz_4_1009	DJI_0389	Sambucus nigra L.	0	n	1	1	Viburnaceae Raf.
Iz_4_1009	DJI_0389	Secale sylvestre Host	0	n	1	0	Poaceae Barnhart
Iz_4_1009	DJI_0389	Sempervivum ruthenicum Schnittsp. & C.B. Lehm.	0	n	1	0	Crassulaceae J.St.-Hil.
Iz_4_1009	DJI_0389	Setaria viridis (L.) P. Beauv.	0	n	1	0	Poaceae Barnhart
Iz_4_1009	DJI_0389	Silene borysthenica (Gruner) Walters	0	n	1	0	Caryophyllaceae Juss.
Iz_4_1009	DJI_0389	Silene latifolia subsp. alba (Mill.) Greuter & Burdet	0	n	1	0	Caryophyllaceae Juss.
Iz_4_1009	DJI_0389	Solidago virgaurea L.	0	n	1	0	Asteraceae Bercht. & J.Presl
Iz_4_1009	DJI_0389	Sorbus aucuparia L.	0	n	1	1	Rosaceae Juss.
Iz_4_1009	DJI_0389	Stachys recta L.	0	n	1	0	Lamiacea Lindl.
Iz_4_1009	DJI_0389	Tanacetum vulgare L.	0	n	1	0	Asteraceae Bercht. & J.Presl
Iz_4_1009	DJI_0389	Thymus pallasianus Heinr.Braun	0	n	1	0	Lamiacea Lindl.
Iz_4_1009	DJI_0389	Tilia cordata Mill.	0	n	1	1	Malvaceae Juss.
Iz_4_1009	DJI_0389	Trifolium alpestre L.	0	n	1	0	Fabaceae Lindl.
Iz_4_1009	DJI_0389	Trifolium arvense L.	0	n	1	0	Fabaceae Lindl.
Iz_4_1009	DJI_0389	Ulmus glabra Huds.	0	n	1	1	Ulmaceae Mirb.
Iz_4_1009	DJI_0389	Verbascum densiflorum Bertol.	0	n	1	0	Scrophulariaceae Juss.
Iz_4_1009	DJI_0389	Verbascum nigrum L.	0	n	1	0	Scrophulariaceae Juss.
Iz_4_1009	DJI_0389	Veronica austriaca L.	0	n	1	0	Plantaginaceae Juss.
Iz_4_1009	DJI_0389	Veronica incana L.	0	n	1	0	Plantaginaceae Juss.
Iz_4_1009	DJI_0389	Veronica verna L.	0	n	1	0	Plantaginaceae Juss.
Iz_4_1009	DJI_0389	Vincetoxicum hirundinaria Medik	0	n	1	0	Apocynaceae Juss.
Iz_4_1009	DJI_0392	Acer tataricum L.	0	n	1	1	Sapindaceae Juss.
Iz_4_1009	DJI_0392	Achillea millefolium L. s.l.	0	n	1	0	Asteraceae Bercht. & J.Presl
Iz_4_1009	DJI_0392	Allium oleraceum L.	0	n	1	0	Amaryllidaceae J.St.-Hil.
Iz_4_1009	DJI_0392	Ambrosia artemisiifolia L.	0	a	0	0	Asteraceae Bercht. & J.Presl
Iz_4_1009	DJI_0392	Amorpha fruticosa L.	0	a	0	0	Fabaceae Lindl.
Iz_4_1009	DJI_0392	Anthericum ramosum L.	0	n	1	0	Asparagaceae Juss.
Iz_4_1009	DJI_0392	Artemisia marschalliana Spreng.	0	n	1	0	Asteraceae Bercht. & J.Presl
Iz_4_1009	DJI_0392	Asparagus officinalis L.	0	n	1	0	Asparagaceae Juss.
Iz_4_1009	DJI_0392	Astragalus cicer L.	0	n	1	0	Fabaceae Lindl.
Iz_4_1009	DJI_0392	Bassia laniflora (S.G.Gmel.) A.J.Scott	0	n	1	0	Amaranthaceae Juss.
Iz_4_1009	DJI_0392	Berberis vulgaris L.	0	n	1	0	Berberidaceae Juss.
Iz_4_1009	DJI_0392	Betula pendula Roth.	0	n	1	1	Betulaceae Gray
Iz_4_1009	DJI_0392	Betula pendula Roth.	0	n	1	0	Poaceae Barnhart
Iz_4_1009	DJI_0392	Bromus inermis Leyss.	0		NA	NA	
Iz_4_1009	DJI_0392	Bromus squarrosus L.	0	a	0	0	Poaceae Barnhart
Iz_4_1009	DJI_0392	Bromus tectorum L.	0	a	0	0	Poaceae Barnhart
Iz_4_1009	DJI_0392	Calamagrostis epigejos (L.) Roth	25	n	1	0	Poaceae Barnhart
Iz_4_1009	DJI_0392	Carduus acanthoides L.	0	n	1	0	Asteraceae Bercht. & J.Presl
Iz_4_1009	DJI_0392	Carex sp.	0	n	1	0	Cyperaceae Juss.
Iz_4_1009	DJI_0392	Centaurea majorovii Dumbadze	0	n	1	0	Asteraceae Bercht. & J.Presl
Iz_4_1009	DJI_0392	Chamaecytisus ruthenicus (Fisch.ex Wol.) Klaskova	0	n	1	0	Fabaceae Lindl.
Iz_4_1009	DJI_0392	Chelidonium majus L.	0	n	1	0	Papaveraceae Juss.
Iz_4_1009	DJI_0392	Chenopodium betaceum Andrz.	0	n	1	0	Amaranthaceae Juss.
Iz_4_1009	DJI_0392	Chenopodium cf. album	0	n	1	0	Amaranthaceae Juss.
Iz_4_1009	DJI_0392	Chenopodium sp.	0		NA	0	Amaranthaceae Juss.
Iz_4_1009	DJI_0392	Chondrilla juncea L.	0	n	1	0	Asteraceae Bercht. & J.Presl
Iz_4_1009	DJI_0392	Cirsium vulgare (Savi) Ten.	0	n	1	0	Asteraceae Bercht. & J.Presl
Iz_4_1009	DJI_0392	Clinopodium acinos (L.) Kuntze	0	n	1	0	Lamiacea Lindl.
Iz_4_1009	DJI_0392	Convallaria majalis L.	0	n	1	0	Asparagaceae Juss.
Iz_4_1009	DJI_0392	Crataegus rhipidophylla Gand.	0	n	1	1	Rosaceae Juss.
Iz_4_1009	DJI_0392	Crepis foetida L.	0	n	1	0	Asteraceae Bercht. & J.Presl
Iz_4_1009	DJI_0392	Dactylis glomerata L.	0	n	1	0	Poaceae Barnhart
Iz_4_1009	DJI_0392	Dianthus campestris M.Bieb.	0	n	1	0	Caryophyllaceae Juss.
Iz_4_1009	DJI_0392	Digitaria sanguinalis (L.) Scop.	0	a	0	0	Poaceae Barnhart
Iz_4_1009	DJI_0392	Elymus repens (L.) Gould	0	n	1	0	Poaceae Barnhart
Iz_4_1009	DJI_0392	Epilobium tetragonum L.	0	n	1	0	Onagraceae Juss.
Iz_4_1009	DJI_0392	Eragrostis minor Host	0	a	0	0	Poaceae Barnhart
Iz_4_1009	DJI_0392	Erigeron canadensis L.	0	a	0	0	Asteraceae Bercht. & J.Presl
Iz_4_1009	DJI_0392	Euphorbia seguieriana Neck.	0	n	1	0	Euphorbiaceae Juss.
Iz_4_1009	DJI_0392	Fallopia dumetorum (L.) Holub	0	n	1	0	Polygonaceae Juss.
Iz_4_1009	DJI_0392	Festuca beckeri (Hack.) Trautv.	0	n	1	0	Poaceae Barnhart
Iz_4_1009	DJI_0392	Fragaria vesca L.	0	n	1	0	Rosaceae Juss.
Iz_4_1009	DJI_0392	Frangula alnus Mill.	0	n	1	1	Rhamnaceae Juss.
Iz_4_1009	DJI_0392	Fraxinus pennsylvanica Marshall	0	a	0	1	Oleaceae Hoffmanns. & Link
Iz_4_1009	DJI_0392	Galeopsis bifida Boenn.	0	n	1	0	Lamiacea Lindl.
Iz_4_1009	DJI_0392	Galium verum L.	0	n	1	0	Rubiaceae Juss.
Iz_4_1009	DJI_0392	Genista tinctoria L.	0	n	1	0	Fabaceae Lindl.
Iz_4_1009	DJI_0392	Geranium robertianum L.	0	n	1	0	Geraniaceae Juss.
Iz_4_1009	DJI_0392	Geranium sanquineum L.	0	n	1	0	Geraniaceae Juss.
Iz_4_1009	DJI_0392	Gypsophila paniculata L.	0	n	1	0	Caryophyllaceae Juss.
Iz_4_1009	DJI_0392	Helichrysum arenarium (L.) Moench	0	n	1	0	Asteraceae Bercht. & J.Presl
Iz_4_1009	DJI_0392	Herniaria polygama J.Gay	0	n	1	0	Caryophyllaceae Juss.
Iz_4_1009	DJI_0392	Hieracium robustum Fr.	0	n	1	0	Asteraceae Bercht. & J.Presl
Iz_4_1009	DJI_0392	Hieracium umbellatum L.	0	n	1	NA	Asteraceae Bercht. & J.Presl
Iz_4_1009	DJI_0392	Hylotelephium maximum (L.) Holub	0	n	1	0	Crassulaceae J.St.-Hil.
Iz_4_1009	DJI_0392	Jacobaea borysthenica (DC.) B.Nord. & Greuter	0	n	1	0	Asteraceae Bercht. & J.Presl
Iz_4_1009	DJI_0392	Jasione montana L.	0	n	1	0	Campanulaceae Juss.
Iz_4_1009	DJI_0392	Jurinea cyanoides (L.) Rchb.s.l.	0	n	1	0	Asteraceae Bercht. & J.Presl
Iz_4_1009	DJI_0392	Koeleria glauca (Spreng.) DC.	0	n	1	0	Poaceae Barnhart
Iz_4_1009	DJI_0392	Lactuca serriola L.	0	a	0	0	Asteraceae Bercht. & J.Presl
Iz_4_1009	DJI_0392	Linaria genistifolia (L.) Mill.	0	n	1	0	Plantaginaceae Juss.
Iz_4_1009	DJI_0392	Lomelosia argentea (L.) Greuter & Burdet	0	n	1	0	Caprifoliaceae Juss.
Iz_4_1009	DJI_0392	Melampyrum pratense L.	0	n	1	0	Orobanchaceae Vent.
Iz_4_1009	DJI_0392	Oenothera biennis L.	0	a	0	0	Onagraceae Juss.
Iz_4_1009	DJI_0392	Pentanema britannica (L.) D.Gut.Larr., Santos-Vicente, Anderb., E.Rico & M.M.Mart.Ort.	0	n	1	0	Asteraceae Bercht. & J.Presl
Iz_4_1009	DJI_0392	Peucedanum oreoselinum (L.) Moench	0	n	1	0	Apiaceae Lindl.
Iz_4_1009	DJI_0392	Peucedanum ruthenicum M.Bieb.	0	n	1	0	Apiaceae Lindl.
Iz_4_1009	DJI_0392	Pilosella echioides (Lumn.) F.W.Schultz & Sch.Bip.	0	n	1	0	Asteraceae Bercht. & J.Presl
Iz_4_1009	DJI_0392	Pilosella officinarum F. Schultz et Sch. Bip.	0	n	1	0	Asteraceae Bercht. & J.Presl
Iz_4_1009	DJI_0392	Pilosella sp.	0		NA	0	Asteraceae Bercht. & J.Presl
Iz_4_1009	DJI_0392	Pinus sylvestris L.	0	n	1	1	Pinaceae Spreng. ex F.Rudolphi

Iz_4_1009	DJI_0392	Poa annua L.	0	n	1	0	Poaceae Barnhart
Iz_4_1009	DJI_0392	Poa nemoralis L.	0	n	1	0	Poaceae Barnhart
Iz_4_1009	DJI_0392	Poa sp.	0		NA	0	Poaceae Barnhart
Iz_4_1009	DJI_0392	Polygonatum odoratum (Mill.) Druce	0	n	1	0	Asparagaceae Juss.
Iz_4_1009	DJI_0392	Polygonum arenarium Waldst. & Kit.	0	n	1	0	Polygonaceae Juss.
Iz_4_1009	DJI_0392	Populus nigra L.	0	n	1	1	Salicaceae Mirb.
Iz_4_1009	DJI_0392	Populus tremula L.	0	n	1	1	Salicaceae Mirb.
Iz_4_1009	DJI_0392	Populus Ã canescens (Aiton) Sm.	0	n	1	1	Salicaceae Mirb.
Iz_4_1009	DJI_0392	Potentilla argentea L.	0	n	1	0	Rosaceae Juss.
Iz_4_1009	DJI_0392	Psammophiliella muralis (L.) Ikonn.	0	n	1	0	Caryophyllaceae Juss.
Iz_4_1009	DJI_0392	Psephellus sumensis (Kalen.) Greuter	0	n	1	0	Asteraceae Bercht. & J.Presl
Iz_4_1009	DJI_0392	Pulsatilla pratensis (L.) Mill. s.l.	0	n	1	0	Ranunculaceae Juss.
Iz_4_1009	DJI_0392	Pyrus communis L.	0	a	0	1	Rosaceae Juss.
Iz_4_1009	DJI_0392	Quercus robur L.	0	n	1	1	Fagaceae Dumort.
Iz_4_1009	DJI_0392	Robinia pseudoacacia L.	0	a	0	1	Fabaceae Lindl.
Iz_4_1009	DJI_0392	Rosa sp.	0		NA	1	Rosaceae Juss.
Iz_4_1009	DJI_0392	Rubus idaeus L.	0	n	1	0	Rosaceae Juss.
Iz_4_1009	DJI_0392	Rumex acetosella L.	0	n	1	0	Polygonaceae Juss.
Iz_4_1009	DJI_0392	Rumex thyrsiflorus Fingerh.	0	n	1	0	Polygonaceae Juss.
Iz_4_1009	DJI_0392	Salix alba L.	0	n	1	1	Salicaceae Mirb.
Iz_4_1009	DJI_0392	Sambucus nigra L.	0	n	1	1	Viburnaceae Raf.
Iz_4_1009	DJI_0392	Secale sylvestre Host	0	n	1	0	Poaceae Barnhart
Iz_4_1009	DJI_0392	Sempervivum ruthenicum Schnittsp. & C.B. Lehm.	0	n	1	0	Crassulaceae J.St.-Hil.
Iz_4_1009	DJI_0392	Setaria viridis (L.) P. Beauv.	0	n	1	0	Poaceae Barnhart
Iz_4_1009	DJI_0392	Silene borysthenica (Gruner) Walters	0	n	1	0	Caryophyllaceae Juss.
Iz_4_1009	DJI_0392	Silene latifolia subsp. alba (Mill.) Greuter & Burdet	0	n	1	0	Caryophyllaceae Juss.
Iz_4_1009	DJI_0392	Solidago virgaurea L.	0	n	1	0	Asteraceae Bercht. & J.Presl
Iz_4_1009	DJI_0392	Sorbus aucuparia L.	0	n	1	1	Rosaceae Juss.
Iz_4_1009	DJI_0392	Stachys recta L.	0	n	1	0	Lamiacea Lindl.
Iz_4_1009	DJI_0392	Tanacetum vulgare L.	0	n	1	0	Asteraceae Bercht. & J.Presl
Iz_4_1009	DJI_0392	Thymus pallasianus Heinr.Braun	0	n	1	0	Lamiacea Lindl.
Iz_4_1009	DJI_0392	Tilia cordata Mill.	0	n	1	1	Malvaceae Juss.
Iz_4_1009	DJI_0392	Trifolium alpestre L.	0	n	1	0	Fabaceae Lindl.
Iz_4_1009	DJI_0392	Trifolium arvense L.	0	n	1	0	Fabaceae Lindl.
Iz_4_1009	DJI_0392	Ulmus glabra Huds.	0	n	1	1	Ulmaceae Mirb.
Iz_4_1009	DJI_0392	Verbascum densiflorum Bertol.	0	n	1	0	Scrophulariaceae Juss.
Iz_4_1009	DJI_0392	Verbascum nigrum L.	0	n	1	0	Scrophulariaceae Juss.
Iz_4_1009	DJI_0392	Veronica austriaca L.	0	n	1	0	Plantaginaceae Juss.
Iz_4_1009	DJI_0392	Veronica incana L.	0	n	1	0	Plantaginaceae Juss.
Iz_4_1009	DJI_0392	Veronica verna L.	0	n	1	0	Plantaginaceae Juss.
Iz_4_1009	DJI_0392	Vincetoxicum hirundinaria Medik	0	n	1	0	Apocynaceae Juss.
Iz_4_1009	DJI_0395	Acer tataricum L.	0	n	1	1	Sapindaceae Juss.
Iz_4_1009	DJI_0395	Achillea millefolium L. s.l.	0	n	1	0	Asteraceae Bercht. & J.Presl
Iz_4_1009	DJI_0395	Allium oleraceum L.	0	n	1	0	Amaryllidaceae J.St.-Hil.
Iz_4_1009	DJI_0395	Ambrosia artemisiifolia L.	0	a	0	0	Asteraceae Bercht. & J.Presl
Iz_4_1009	DJI_0395	Amorpha fruticosa L.	0	a	0	0	Fabaceae Lindl.
Iz_4_1009	DJI_0395	Anthericum ramosum L.	0	n	1	0	Asparagaceae Juss.
Iz_4_1009	DJI_0395	Artemisia marschalliana Spreng.	0	n	1	0	Asteraceae Bercht. & J.Presl
Iz_4_1009	DJI_0395	Asparagus officinalis L.	0	n	1	0	Asparagaceae Juss.
Iz_4_1009	DJI_0395	Astragalus cicer L.	0	n	1	0	Fabaceae Lindl.
Iz_4_1009	DJI_0395	Bassia laniflora (S.G.Gmel.) A.J.Scott	0	n	1	0	Amaranthaceae Juss.
Iz_4_1009	DJI_0395	Berberis vulgaris L.	0	n	1	0	Berberidaceae Juss.
Iz_4_1009	DJI_0395	Betula pendula Roth.	0	n	1	1	Betulaceae Gray
Iz_4_1009	DJI_0395	Betula pendula Roth.	0	n	1	0	Poaceae Barnhart
Iz_4_1009	DJI_0395	Bromus inermis Leyss.	0		NA	NA	
Iz_4_1009	DJI_0395	Bromus squarrosus L.	0	a	0	0	Poaceae Barnhart
Iz_4_1009	DJI_0395	Bromus tectorum L.	0	a	0	0	Poaceae Barnhart
Iz_4_1009	DJI_0395	Calamagrostis epigejos (L.) Roth	25	n	1	0	Poaceae Barnhart
Iz_4_1009	DJI_0395	Carduus acanthoides L.	0	n	1	0	Asteraceae Bercht. & J.Presl
Iz_4_1009	DJI_0395	Carex sp.	0	n	1	0	Cyperaceae Juss.
Iz_4_1009	DJI_0395	Centaurea majorovii Dumbadze	0	n	1	0	Asteraceae Bercht. & J.Presl
Iz_4_1009	DJI_0395	Chamaecytisus ruthenicus (Fisch.ex Wol.) Klaskova	0	n	1	0	Fabaceae Lindl.
Iz_4_1009	DJI_0395	Chelidonium majus L.	0	n	1	0	Papaveraceae Juss.
Iz_4_1009	DJI_0395	Chenopodium betaceum Andrz.	0	n	1	0	Amaranthaceae Juss.
Iz_4_1009	DJI_0395	Chenopodium cf. album	0	n	1	0	Amaranthaceae Juss.
Iz_4_1009	DJI_0395	Chenopodium sp.	0		NA	0	Amaranthaceae Juss.
Iz_4_1009	DJI_0395	Chondrilla juncea L.	0	n	1	0	Asteraceae Bercht. & J.Presl
Iz_4_1009	DJI_0395	Cirsium vulgare (Savi) Ten.	0	n	1	0	Asteraceae Bercht. & J.Presl
Iz_4_1009	DJI_0395	Clinopodium acinos (L.) Kuntze	0	n	1	0	Lamiacea Lindl.
Iz_4_1009	DJI_0395	Convallaria majalis L.	0	n	1	0	Asparagaceae Juss.
Iz_4_1009	DJI_0395	Crataegus rhipidophylla Gand.	0	n	1	1	Rosaceae Juss.
Iz_4_1009	DJI_0395	Crepis foetida L.	0	n	1	0	Asteraceae Bercht. & J.Presl
Iz_4_1009	DJI_0395	Dactylis glomerata L.	0	n	1	0	Poaceae Barnhart
Iz_4_1009	DJI_0395	Dianthus campestris M.Bieb.	0	n	1	0	Caryophyllaceae Juss.
Iz_4_1009	DJI_0395	Digitaria sanguinalis (L.) Scop.	0	a	0	0	Poaceae Barnhart
Iz_4_1009	DJI_0395	Elymus repens (L.) Gould	0	n	1	0	Poaceae Barnhart
Iz_4_1009	DJI_0395	Epilobium tetragonum L.	0	n	1	0	Onagraceae Juss.
Iz_4_1009	DJI_0395	Eragrostis minor Host	0	a	0	0	Poaceae Barnhart
Iz_4_1009	DJI_0395	Erigeron canadensis L.	0	a	0	0	Asteraceae Bercht. & J.Presl
Iz_4_1009	DJI_0395	Euphorbia seguieriana Neck.	0	n	1	0	Euphorbiaceae Juss.
Iz_4_1009	DJI_0395	Fallopia dumetorum (L.) Holub	0	n	1	0	Polygonaceae Juss.
Iz_4_1009	DJI_0395	Festuca beckeri (Hack.) Trautv.	0	n	1	0	Poaceae Barnhart
Iz_4_1009	DJI_0395	Fragaria vesca L.	0	n	1	0	Rosaceae Juss.
Iz_4_1009	DJI_0395	Frangula alnus Mill.	0	n	1	1	Rhamnaceae Juss.
Iz_4_1009	DJI_0395	Fraxinus pennsylvanica Marshall	0	a	0	1	Oleaceae Hoffmanns. & Link
Iz_4_1009	DJI_0395	Galeopsis bifida Boenn.	0	n	1	0	Lamiacea Lindl.
Iz_4_1009	DJI_0395	Galium verum L.	0	n	1	0	Rubiaceae Juss.
Iz_4_1009	DJI_0395	Genista tinctoria L.	0	n	1	0	Fabaceae Lindl.
Iz_4_1009	DJI_0395	Geranium robertianum L.	0	n	1	0	Geraniaceae Juss.
Iz_4_1009	DJI_0395	Geranium sanquineum L.	0	n	1	0	Geraniaceae Juss.
Iz_4_1009	DJI_0395	Gypsophila paniculata L.	0	n	1	0	Caryophyllaceae Juss.
Iz_4_1009	DJI_0395	Helichrysum arenarium (L.) Moench	0	n	1	0	Asteraceae Bercht. & J.Presl
Iz_4_1009	DJI_0395	Herniaria polygama J.Gay	0	n	1	0	Caryophyllaceae Juss.
Iz_4_1009	DJI_0395	Hieracium robustum Fr.	0	n	1	0	Asteraceae Bercht. & J.Presl
Iz_4_1009	DJI_0395	Hieracium umbellatum L.	0	n	1	NA	Asteraceae Bercht. & J.Presl
Iz_4_1009	DJI_0395	Hylotelephium maximum (L.) Holub	0	n	1	0	Crassulaceae J.St.-Hil.
Iz_4_1009	DJI_0395	Jacobaea borysthenica (DC.) B.Nord. & Greuter	0	n	1	0	Asteraceae Bercht. & J.Presl
Iz_4_1009	DJI_0395	Jasione montana L.	0	n	1	0	Campanulaceae Juss.

Iz_4_1009	DJI_0395	Jurinea cyanoides (L.) Rchb.s.l.	0	n	1	0	Asteraceae Bercht. & J.Presl
Iz_4_1009	DJI_0395	Koeleria glauca (Spreng.) DC.	0	n	1	0	Poaceae Barnhart
Iz_4_1009	DJI_0395	Lactuca serriola L.	0	a	0	0	Asteraceae Bercht. & J.Presl
Iz_4_1009	DJI_0395	Linaria genistifolia (L.) Mill.	0	n	1	0	Plantaginaceae Juss.
Iz_4_1009	DJI_0395	Lomelosia argentea (L.) Greuter & Burdet	0	n	1	0	Caprifoliaceae Juss.
Iz_4_1009	DJI_0395	Melampyrum pratense L.	0	n	1	0	Orobanchaceae Vent.
Iz_4_1009	DJI_0395	Oenothera biennis L.	0	a	0	0	Onagraceae Juss.
Iz_4_1009	DJI_0395	Pentanema britannica (L.) D.Gut.Larr., Santos-Vicente, Anderb., E.Rico & M.M.Mart.Ort.	0	n	1	0	Asteraceae Bercht. & J.Presl
Iz_4_1009	DJI_0395	Peucedanum oreoselinum (L.) Moench	0	n	1	0	Apiaceae Lindl.
Iz_4_1009	DJI_0395	Peucedanum ruthenicum M.Bieb.	0	n	1	0	Apiaceae Lindl.
Iz_4_1009	DJI_0395	Pilosella echioides (Lumn.) F.W.Schultz & Sch.Bip.	0	n	1	0	Asteraceae Bercht. & J.Presl
Iz_4_1009	DJI_0395	Pilosella officinarum F. Schultz et Sch. Bip.	0	n	1	0	Asteraceae Bercht. & J.Presl
Iz_4_1009	DJI_0395	Pilosella sp.	0		NA	0	Asteraceae Bercht. & J.Presl
Iz_4_1009	DJI_0395	Pinus sylvestris L.	0	n	1	1	Pinaceae Spreng. ex F.Rudolphi
Iz_4_1009	DJI_0395	Poa annua L.	0	n	1	0	Poaceae Barnhart
Iz_4_1009	DJI_0395	Poa nemoralis L.	0	n	1	0	Poaceae Barnhart
Iz_4_1009	DJI_0395	Poa sp.	0		NA	0	Poaceae Barnhart
Iz_4_1009	DJI_0395	Polygonatum odoratum (Mill.) Druce	0	n	1	0	Asparagaceae Juss.
Iz_4_1009	DJI_0395	Polygonum arenarium Waldst. & Kit.	0	n	1	0	Polygonaceae Juss.
Iz_4_1009	DJI_0395	Populus nigra L.	0	n	1	1	Salicaceae Mirb.
Iz_4_1009	DJI_0395	Populus tremula L.	0	n	1	1	Salicaceae Mirb.
Iz_4_1009	DJI_0395	Populus Ã canescens (Aiton) Sm.	0	n	1	1	Salicaceae Mirb.
Iz_4_1009	DJI_0395	Potentilla argentea L.	0	n	1	0	Rosaceae Juss.
Iz_4_1009	DJI_0395	Psammophiliella muralis (L.) Ikonn.	0	n	1	0	Caryophyllaceae Juss.
Iz_4_1009	DJI_0395	Psephellus sumensis (Kalen.) Greuter	0	n	1	0	Asteraceae Bercht. & J.Presl
Iz_4_1009	DJI_0395	Pulsatilla pratensis (L.) Mill. s.l.	0	n	1	0	Ranunculaceae Juss.
Iz_4_1009	DJI_0395	Pyrus communis L.	0	a	0	1	Rosaceae Juss.
Iz_4_1009	DJI_0395	Quercus robur L.	0	n	1	1	Fagaceae Dumort.
Iz_4_1009	DJI_0395	Robinia pseudoacacia L.	0	a	0	1	Fabaceae Lindl.
Iz_4_1009	DJI_0395	Rosa sp.	0		NA	1	Rosaceae Juss.
Iz_4_1009	DJI_0395	Rubus idaeus L.	0	n	1	0	Rosaceae Juss.
Iz_4_1009	DJI_0395	Rumex acetosella L.	0	n	1	0	Polygonaceae Juss.
Iz_4_1009	DJI_0395	Rumex thyrsiflorus Fingerh.	0	n	1	0	Polygonaceae Juss.
Iz_4_1009	DJI_0395	Salix alba L.	0	n	1	1	Salicaceae Mirb.
Iz_4_1009	DJI_0395	Sambucus nigra L.	0	n	1	1	Viburnaceae Raf.
Iz_4_1009	DJI_0395	Secale sylvestre Host	0	n	1	0	Poaceae Barnhart
Iz_4_1009	DJI_0395	Sempervivum ruthenicum Schnittsp. & C.B. Lehm.	0	n	1	0	Crassulaceae J.St.-Hil.
Iz_4_1009	DJI_0395	Setaria viridis (L.) P. Beauv.	0	n	1	0	Poaceae Barnhart
Iz_4_1009	DJI_0395	Silene borysthenica (Gruner) Walters	0	n	1	0	Caryophyllaceae Juss.
Iz_4_1009	DJI_0395	Silene latifolia subsp. alba (Mill.) Greuter & Burdet	0	n	1	0	Caryophyllaceae Juss.
Iz_4_1009	DJI_0395	Solidago virgaurea L.	0	n	1	0	Asteraceae Bercht. & J.Presl
Iz_4_1009	DJI_0395	Sorbus aucuparia L.	0	n	1	1	Rosaceae Juss.
Iz_4_1009	DJI_0395	Stachys recta L.	0	n	1	0	Lamiacea Lindl.
Iz_4_1009	DJI_0395	Tanacetum vulgare L.	0	n	1	0	Asteraceae Bercht. & J.Presl
Iz_4_1009	DJI_0395	Thymus pallasianus Heinr.Braun	0	n	1	0	Lamiacea Lindl.
Iz_4_1009	DJI_0395	Tilia cordata Mill.	0	n	1	1	Malvaceae Juss.
Iz_4_1009	DJI_0395	Trifolium alpestre L.	0	n	1	0	Fabaceae Lindl.
Iz_4_1009	DJI_0395	Trifolium arvense L.	0	n	1	0	Fabaceae Lindl.
Iz_4_1009	DJI_0395	Ulmus glabra Huds.	0	n	1	1	Ulmaceae Mirb.
Iz_4_1009	DJI_0395	Verbascum densiflorum Bertol.	0	n	1	0	Scrophulariaceae Juss.
Iz_4_1009	DJI_0395	Verbascum nigrum L.	0	n	1	0	Scrophulariaceae Juss.
Iz_4_1009	DJI_0395	Veronica austriaca L.	0	n	1	0	Plantaginaceae Juss.
Iz_4_1009	DJI_0395	Veronica incana L.	0	n	1	0	Plantaginaceae Juss.
Iz_4_1009	DJI_0395	Veronica verna L.	0	n	1	0	Plantaginaceae Juss.
Iz_4_1009	DJI_0395	Vincetoxicum hirundinaria Medik	0	n	1	0	Apocynaceae Juss.
Iz_4_1009	DJI_0397	Acer tataricum L.	0	n	1	1	Sapindaceae Juss.
Iz_4_1009	DJI_0397	Achillea millefolium L. s.l.	0	n	1	0	Asteraceae Bercht. & J.Presl
Iz_4_1009	DJI_0397	Allium oleraceum L.	0	n	1	0	Amaryllidaceae J.St.-Hil.
Iz_4_1009	DJI_0397	Ambrosia artemisiifolia L.	0	a	0	0	Asteraceae Bercht. & J.Presl
Iz_4_1009	DJI_0397	Amorpha fruticosa L.	0	a	0	0	Fabaceae Lindl.
Iz_4_1009	DJI_0397	Anthericum ramosum L.	0	n	1	0	Asparagaceae Juss.
Iz_4_1009	DJI_0397	Artemisia marschalliana Spreng.	0	n	1	0	Asteraceae Bercht. & J.Presl
Iz_4_1009	DJI_0397	Asparagus officinalis L.	0	n	1	0	Asparagaceae Juss.
Iz_4_1009	DJI_0397	Astragalus cicer L.	0	n	1	0	Fabaceae Lindl.
Iz_4_1009	DJI_0397	Bassia laniflora (S.G.Gmel.) A.J.Scott	0	n	1	0	Amaranthaceae Juss.
Iz_4_1009	DJI_0397	Berberis vulgaris L.	0	n	1	0	Berberidaceae Juss.
Iz_4_1009	DJI_0397	Betula pendula Roth.	0	n	1	1	Betulaceae Gray
Iz_4_1009	DJI_0397	Betula pendula Roth.	0	n	1	0	Poaceae Barnhart
Iz_4_1009	DJI_0397	Bromus inermis Leyss.	0		NA	NA	
Iz_4_1009	DJI_0397	Bromus squarrosus L.	0	a	0	0	Poaceae Barnhart
Iz_4_1009	DJI_0397	Bromus tectorum L.	0	a	0	0	Poaceae Barnhart
Iz_4_1009	DJI_0397	Calamagrostis epigejos (L.) Roth	60	n	1	0	Poaceae Barnhart
Iz_4_1009	DJI_0397	Carduus acanthoides L.	0	n	1	0	Asteraceae Bercht. & J.Presl
Iz_4_1009	DJI_0397	Carex sp.	0	n	1	0	Cyperaceae Juss.
Iz_4_1009	DJI_0397	Centaurea majorovii Dumbadze	0	n	1	0	Asteraceae Bercht. & J.Presl
Iz_4_1009	DJI_0397	Chamaecytisus ruthenicus (Fisch.ex Wol.) Klaskova	0	n	1	0	Fabaceae Lindl.
Iz_4_1009	DJI_0397	Chelidonium majus L.	0	n	1	0	Papaveraceae Juss.
Iz_4_1009	DJI_0397	Chenopodium betaceum Andrz.	0	n	1	0	Amaranthaceae Juss.
Iz_4_1009	DJI_0397	Chenopodium cf. album	0	n	1	0	Amaranthaceae Juss.
Iz_4_1009	DJI_0397	Chenopodium sp.	0		NA	0	Amaranthaceae Juss.
Iz_4_1009	DJI_0397	Chondrilla juncea L.	0	n	1	0	Asteraceae Bercht. & J.Presl
Iz_4_1009	DJI_0397	Cirsium vulgare (Savi) Ten.	0	n	1	0	Asteraceae Bercht. & J.Presl
Iz_4_1009	DJI_0397	Clinopodium acinos (L.) Kuntze	0	n	1	0	Lamiacea Lindl.
Iz_4_1009	DJI_0397	Convallaria majalis L.	0	n	1	0	Asparagaceae Juss.
Iz_4_1009	DJI_0397	Crataegus rhipidophylla Gand.	0	n	1	1	Rosaceae Juss.
Iz_4_1009	DJI_0397	Crepis foetida L.	0	n	1	0	Asteraceae Bercht. & J.Presl
Iz_4_1009	DJI_0397	Dactylis glomerata L.	0	n	1	0	Poaceae Barnhart
Iz_4_1009	DJI_0397	Dianthus campestris M.Bieb.	0	n	1	0	Caryophyllaceae Juss.
Iz_4_1009	DJI_0397	Digitaria sanguinalis (L.) Scop.	0	a	0	0	Poaceae Barnhart
Iz_4_1009	DJI_0397	Elymus repens (L.) Gould	0	n	1	0	Poaceae Barnhart
Iz_4_1009	DJI_0397	Epilobium tetragonum L.	0	n	1	0	Onagraceae Juss.
Iz_4_1009	DJI_0397	Eragrostis minor Host	0	a	0	0	Poaceae Barnhart
Iz_4_1009	DJI_0397	Erigeron canadensis L.	0	a	0	0	Asteraceae Bercht. & J.Presl
Iz_4_1009	DJI_0397	Euphorbia seguieriana Neck.	0	n	1	0	Euphorbiaceae Juss.
Iz_4_1009	DJI_0397	Fallopia dumetorum (L.) Holub	0	n	1	0	Polygonaceae Juss.
Iz_4_1009	DJI_0397	Festuca beckeri (Hack.) Trautv.	0	n	1	0	Poaceae Barnhart
Iz_4_1009	DJI_0397	Fragaria vesca L.	0	n	1	0	Rosaceae Juss.
Iz_4_1009	DJI_0397	Frangula alnus Mill.	0	n	1	1	Rhamnaceae Juss.

Iz_4_1009	DJI_0397	Fraxinus pennsylvanica Marshall	0	a	0	1	Oleaceae Hoffmanns. & Link
Iz_4_1009	DJI_0397	Galeopsis bifida Boenn.	0	n	1	0	Lamiacea Lindl.
Iz_4_1009	DJI_0397	Galium verum L.	0	n	1	0	Rubiaceae Juss.
Iz_4_1009	DJI_0397	Genista tinctoria L.	0	n	1	0	Fabaceae Lindl.
Iz_4_1009	DJI_0397	Geranium robertianum L.	0	n	1	0	Geraniaceae Juss.
Iz_4_1009	DJI_0397	Geranium sanquineum L.	0	n	1	0	Geraniaceae Juss.
Iz_4_1009	DJI_0397	Gypsophila paniculata L.	0	n	1	0	Caryophyllaceae Juss.
Iz_4_1009	DJI_0397	Helichrysum arenarium (L.) Moench	0	n	1	0	Asteraceae Bercht. & J.Presl
Iz_4_1009	DJI_0397	Herniaria polygama J.Gay	0	n	1	0	Caryophyllaceae Juss.
Iz_4_1009	DJI_0397	Hieracium robustum Fr.	0	n	1	0	Asteraceae Bercht. & J.Presl
Iz_4_1009	DJI_0397	Hieracium umbellatum L.	0	n	1	NA	Asteraceae Bercht. & J.Presl
Iz_4_1009	DJI_0397	Hylotelephium maximum (L.) Holub	0	n	1	0	Crassulaceae J.St.-Hil.
Iz_4_1009	DJI_0397	Jacobaea borysthenica (DC.) B.Nord. & Greuter	0	n	1	0	Asteraceae Bercht. & J.Presl
Iz_4_1009	DJI_0397	Jasione montana L.	0	n	1	0	Campanulaceae Juss.
Iz_4_1009	DJI_0397	Jurinea cyanoides (L.) Rchb.s.l.	0	n	1	0	Asteraceae Bercht. & J.Presl
Iz_4_1009	DJI_0397	Koeleria glauca (Spreng.) DC.	0	n	1	0	Poaceae Barnhart
Iz_4_1009	DJI_0397	Lactuca serriola L.	0	a	0	0	Asteraceae Bercht. & J.Presl
Iz_4_1009	DJI_0397	Linaria genistifolia (L.) Mill.	0	n	1	0	Plantaginaceae Juss.
Iz_4_1009	DJI_0397	Lomelosia argentea (L.) Greuter & Burdet	0	n	1	0	Caprifoliaceae Juss.
Iz_4_1009	DJI_0397	Melampyrum pratense L.	0	n	1	0	Orobanchaceae Vent.
Iz_4_1009	DJI_0397	Oenothera biennis L.	0	a	0	0	Onagraceae Juss.
Iz_4_1009	DJI_0397	Pentanema britannica (L.) D.Gut.Larr., Santos-Vicente, Anderb., E.Rico & M.M.Mart.Ort.	0	n	1	0	Asteraceae Bercht. & J.Presl
Iz_4_1009	DJI_0397	Peucedanum oreoselinum (L.) Moench	0	n	1	0	Apiaceae Lindl.
Iz_4_1009	DJI_0397	Peucedanum ruthenicum M.Bieb.	0	n	1	0	Apiaceae Lindl.
Iz_4_1009	DJI_0397	Pilosella echioides (Lumn.) F.W.Schultz & Sch.Bip.	0	n	1	0	Asteraceae Bercht. & J.Presl
Iz_4_1009	DJI_0397	Pilosella officinarum F. Schultz et Sch. Bip.	0	n	1	0	Asteraceae Bercht. & J.Presl
Iz_4_1009	DJI_0397	Pilosella sp.	0		NA	0	Asteraceae Bercht. & J.Presl
Iz_4_1009	DJI_0397	Pinus sylvestris L.	0	n	1	1	Pinaceae Spreng. ex F.Rudolphi
Iz_4_1009	DJI_0397	Poa annua L.	0	n	1	0	Poaceae Barnhart
Iz_4_1009	DJI_0397	Poa nemoralis L.	0	n	1	0	Poaceae Barnhart
Iz_4_1009	DJI_0397	Poa sp.	0		NA	0	Poaceae Barnhart
Iz_4_1009	DJI_0397	Polygonatum odoratum (Mill.) Druce	0	n	1	0	Asparagaceae Juss.
Iz_4_1009	DJI_0397	Polygonum arenarium Waldst. & Kit.	0	n	1	0	Polygonaceae Juss.
Iz_4_1009	DJI_0397	Populus nigra L.	0	n	1	1	Salicaceae Mirb.
Iz_4_1009	DJI_0397	Populus tremula L.	0	n	1	1	Salicaceae Mirb.
Iz_4_1009	DJI_0397	Populus Ã canescens (Aiton) Sm.	0	n	1	1	Salicaceae Mirb.
Iz_4_1009	DJI_0397	Potentilla argentea L.	0	n	1	0	Rosaceae Juss.
Iz_4_1009	DJI_0397	Psammophiliella muralis (L.) Ikonn.	0	n	1	0	Caryophyllaceae Juss.
Iz_4_1009	DJI_0397	Psephellus sumensis (Kalen.) Greuter	0	n	1	0	Asteraceae Bercht. & J.Presl
Iz_4_1009	DJI_0397	Pulsatilla pratensis (L.) Mill. s.l.	0	n	1	0	Ranunculaceae Juss.
Iz_4_1009	DJI_0397	Pyrus communis L.	0	a	0	1	Rosaceae Juss.
Iz_4_1009	DJI_0397	Quercus robur L.	0	n	1	1	Fagaceae Dumort.
Iz_4_1009	DJI_0397	Robinia pseudoacacia L.	0	a	0	1	Fabaceae Lindl.
Iz_4_1009	DJI_0397	Rosa sp.	0		NA	1	Rosaceae Juss.
Iz_4_1009	DJI_0397	Rubus idaeus L.	0	n	1	0	Rosaceae Juss.
Iz_4_1009	DJI_0397	Rumex acetosella L.	0	n	1	0	Polygonaceae Juss.
Iz_4_1009	DJI_0397	Rumex thyrsiflorus Fingerh.	0	n	1	0	Polygonaceae Juss.
Iz_4_1009	DJI_0397	Salix alba L.	0	n	1	1	Salicaceae Mirb.
Iz_4_1009	DJI_0397	Sambucus nigra L.	0	n	1	1	Viburnaceae Raf.
Iz_4_1009	DJI_0397	Secale sylvestre Host	0	n	1	0	Poaceae Barnhart
Iz_4_1009	DJI_0397	Sempervivum ruthenicum Schnittsp. & C.B. Lehm.	0	n	1	0	Crassulaceae J.St.-Hil.
Iz_4_1009	DJI_0397	Setaria viridis (L.) P. Beauv.	0	n	1	0	Poaceae Barnhart
Iz_4_1009	DJI_0397	Silene borysthenica (Gruner) Walters	0	n	1	0	Caryophyllaceae Juss.
Iz_4_1009	DJI_0397	Silene latifolia subsp. alba (Mill.) Greuter & Burdet	0	n	1	0	Caryophyllaceae Juss.
Iz_4_1009	DJI_0397	Solidago virgaurea L.	0	n	1	0	Asteraceae Bercht. & J.Presl
Iz_4_1009	DJI_0397	Sorbus aucuparia L.	0	n	1	1	Rosaceae Juss.
Iz_4_1009	DJI_0397	Stachys recta L.	0	n	1	0	Lamiacea Lindl.
Iz_4_1009	DJI_0397	Tanacetum vulgare L.	0	n	1	0	Asteraceae Bercht. & J.Presl
Iz_4_1009	DJI_0397	Thymus pallasianus Heinr.Braun	0	n	1	0	Lamiacea Lindl.
Iz_4_1009	DJI_0397	Tilia cordata Mill.	0	n	1	1	Malvaceae Juss.
Iz_4_1009	DJI_0397	Trifolium alpestre L.	0	n	1	0	Fabaceae Lindl.
Iz_4_1009	DJI_0397	Trifolium arvense L.	0	n	1	0	Fabaceae Lindl.
Iz_4_1009	DJI_0397	Ulmus glabra Huds.	0	n	1	1	Ulmaceae Mirb.
Iz_4_1009	DJI_0397	Verbascum densiflorum Bertol.	0	n	1	0	Scrophulariaceae Juss.
Iz_4_1009	DJI_0397	Verbascum nigrum L.	0	n	1	0	Scrophulariaceae Juss.
Iz_4_1009	DJI_0397	Veronica austriaca L.	0	n	1	0	Plantaginaceae Juss.
Iz_4_1009	DJI_0397	Veronica incana L.	0	n	1	0	Plantaginaceae Juss.
Iz_4_1009	DJI_0397	Veronica verna L.	0	n	1	0	Plantaginaceae Juss.
Iz_4_1009	DJI_0397	Vincetoxicum hirundinaria Medik	0	n	1	0	Apocynaceae Juss.
Iz_4_1009	DJI_0399	Acer tataricum L.	0	n	1	1	Sapindaceae Juss.
Iz_4_1009	DJI_0399	Achillea millefolium L. s.l.	0	n	1	0	Asteraceae Bercht. & J.Presl
Iz_4_1009	DJI_0399	Allium oleraceum L.	0	n	1	0	Amaryllidaceae J.St.-Hil.
Iz_4_1009	DJI_0399	Ambrosia artemisiifolia L.	0	a	0	0	Asteraceae Bercht. & J.Presl
Iz_4_1009	DJI_0399	Amorpha fruticosa L.	0	a	0	0	Fabaceae Lindl.
Iz_4_1009	DJI_0399	Anthericum ramosum L.	0	n	1	0	Asparagaceae Juss.
Iz_4_1009	DJI_0399	Artemisia marschalliana Spreng.	0	n	1	0	Asteraceae Bercht. & J.Presl
Iz_4_1009	DJI_0399	Asparagus officinalis L.	0	n	1	0	Asparagaceae Juss.
Iz_4_1009	DJI_0399	Astragalus cicer L.	0	n	1	0	Fabaceae Lindl.
Iz_4_1009	DJI_0399	Bassia laniflora (S.G.Gmel.) A.J.Scott	0	n	1	0	Amaranthaceae Juss.
Iz_4_1009	DJI_0399	Berberis vulgaris L.	0	n	1	0	Berberidaceae Juss.
Iz_4_1009	DJI_0399	Betula pendula Roth.	0	n	1	1	Betulaceae Gray
Iz_4_1009	DJI_0399	Betula pendula Roth.	0	n	1	0	Poaceae Barnhart
Iz_4_1009	DJI_0399	Bromus inermis Leyss.	0		NA	NA	
Iz_4_1009	DJI_0399	Bromus squarrosus L.	0	a	0	0	Poaceae Barnhart
Iz_4_1009	DJI_0399	Bromus tectorum L.	0	a	0	0	Poaceae Barnhart
Iz_4_1009	DJI_0399	Calamagrostis epigejos (L.) Roth	15	n	1	0	Poaceae Barnhart
Iz_4_1009	DJI_0399	Carduus acanthoides L.	0	n	1	0	Asteraceae Bercht. & J.Presl
Iz_4_1009	DJI_0399	Carex sp.	0	n	1	0	Cyperaceae Juss.
Iz_4_1009	DJI_0399	Centaurea majorovii Dumbadze	0	n	1	0	Asteraceae Bercht. & J.Presl
Iz_4_1009	DJI_0399	Chamaecytisus ruthenicus (Fisch.ex Wol.) Klaskova	0	n	1	0	Fabaceae Lindl.
Iz_4_1009	DJI_0399	Chelidonium majus L.	0	n	1	0	Papaveraceae Juss.
Iz_4_1009	DJI_0399	Chenopodium betaceum Andrz.	0	n	1	0	Amaranthaceae Juss.
Iz_4_1009	DJI_0399	Chenopodium cf. album	0	n	1	0	Amaranthaceae Juss.
Iz_4_1009	DJI_0399	Chenopodium sp.	0		NA	0	Amaranthaceae Juss.
Iz_4_1009	DJI_0399	Chondrilla juncea L.	0	n	1	0	Asteraceae Bercht. & J.Presl
Iz_4_1009	DJI_0399	Cirsium vulgare (Savi) Ten.	0	n	1	0	Asteraceae Bercht. & J.Presl
Iz_4_1009	DJI_0399	Clinopodium acinos (L.) Kuntze	0	n	1	0	Lamiacea Lindl.
Iz_4_1009	DJI_0399	Convallaria majalis L.	0	n	1	0	Asparagaceae Juss.

Iz_4_1009	DJI_0399	Crataegus rhipidophylla Gand.	0	n	1	1	Rosaceae Juss.
Iz_4_1009	DJI_0399	Crepis foetida L.	0	n	1	0	Asteraceae Bercht. & J.Presl
Iz_4_1009	DJI_0399	Dactylis glomerata L.	0	n	1	0	Poaceae Barnhart
Iz_4_1009	DJI_0399	Dianthus campestris M.Bieb.	0	n	1	0	Caryophyllaceae Juss.
Iz_4_1009	DJI_0399	Digitaria sanguinalis (L.) Scop.	0	a	0	0	Poaceae Barnhart
Iz_4_1009	DJI_0399	Elymus repens (L.) Gould	0	n	1	0	Poaceae Barnhart
Iz_4_1009	DJI_0399	Epilobium tetragonum L.	0	n	1	0	Onagraceae Juss.
Iz_4_1009	DJI_0399	Eragrostis minor Host	0	a	0	0	Poaceae Barnhart
Iz_4_1009	DJI_0399	Erigeron canadensis L.	0	a	0	0	Asteraceae Bercht. & J.Presl
Iz_4_1009	DJI_0399	Euphorbia seguieriana Neck.	0	n	1	0	Euphorbiaceae Juss.
Iz_4_1009	DJI_0399	Fallopia dumetorum (L.) Holub	0	n	1	0	Polygonaceae Juss.
Iz_4_1009	DJI_0399	Festuca beckeri (Hack.) Trautv.	0	n	1	0	Poaceae Barnhart
Iz_4_1009	DJI_0399	Fragaria vesca L.	0	n	1	0	Rosaceae Juss.
Iz_4_1009	DJI_0399	Frangula alnus Mill.	0	n	1	1	Rhamnaceae Juss.
Iz_4_1009	DJI_0399	Fraxinus pennsylvanica Marshall	0	a	0	1	Oleaceae Hoffmanns. & Link
Iz_4_1009	DJI_0399	Galeopsis bifida Boenn.	0	n	1	0	Lamiacea Lindl.
Iz_4_1009	DJI_0399	Galium verum L.	0	n	1	0	Rubiaceae Juss.
Iz_4_1009	DJI_0399	Genista tinctoria L.	0	n	1	0	Fabaceae Lindl.
Iz_4_1009	DJI_0399	Geranium robertianum L.	0	n	1	0	Geraniaceae Juss.
Iz_4_1009	DJI_0399	Geranium sanquineum L.	0	n	1	0	Geraniaceae Juss.
Iz_4_1009	DJI_0399	Gypsophila paniculata L.	0	n	1	0	Caryophyllaceae Juss.
Iz_4_1009	DJI_0399	Helichrysum arenarium (L.) Moench	0	n	1	0	Asteraceae Bercht. & J.Presl
Iz_4_1009	DJI_0399	Herniaria polygama J.Gay	0	n	1	0	Caryophyllaceae Juss.
Iz_4_1009	DJI_0399	Hieracium robustum Fr.	0	n	1	0	Asteraceae Bercht. & J.Presl
Iz_4_1009	DJI_0399	Hieracium umbellatum L.	0	n	1	NA	Asteraceae Bercht. & J.Presl
Iz_4_1009	DJI_0399	Hylotelephium maximum (L.) Holub	0	n	1	0	Crassulaceae J.St.-Hil.
Iz_4_1009	DJI_0399	Jacobaea borysthenica (DC.) B.Nord. & Greuter	0	n	1	0	Asteraceae Bercht. & J.Presl
Iz_4_1009	DJI_0399	Jasione montana L.	0	n	1	0	Campanulaceae Juss.
Iz_4_1009	DJI_0399	Jurinea cyanoides (L.) Rchb.s.l.	0	n	1	0	Asteraceae Bercht. & J.Presl
Iz_4_1009	DJI_0399	Koeleria glauca (Spreng.) DC.	0	n	1	0	Poaceae Barnhart
Iz_4_1009	DJI_0399	Lactuca serriola L.	0	a	0	0	Asteraceae Bercht. & J.Presl
Iz_4_1009	DJI_0399	Linaria genistifolia (L.) Mill.	0	n	1	0	Plantaginaceae Juss.
Iz_4_1009	DJI_0399	Lomelosia argentea (L.) Greuter & Burdet	0	n	1	0	Caprifoliaceae Juss.
Iz_4_1009	DJI_0399	Melampyrum pratense L.	0	n	1	0	Orobanchaceae Vent.
Iz_4_1009	DJI_0399	Oenothera biennis L.	0	a	0	0	Onagraceae Juss.
Iz_4_1009	DJI_0399	Pentanema britannica (L.) D.Gut.Larr., Santos-Vicente, Anderb., E.Rico & M.M.Mart.Ort.	0	n	1	0	Asteraceae Bercht. & J.Presl
Iz_4_1009	DJI_0399	Peucedanum oreoselinum (L.) Moench	0	n	1	0	Apiaceae Lindl.
Iz_4_1009	DJI_0399	Peucedanum ruthenicum M.Bieb.	0	n	1	0	Apiaceae Lindl.
Iz_4_1009	DJI_0399	Pilosella echioides (Lumn.) F.W.Schultz & Sch.Bip.	0	n	1	0	Asteraceae Bercht. & J.Presl
Iz_4_1009	DJI_0399	Pilosella officinarum F. Schultz et Sch. Bip.	0	n	1	0	Asteraceae Bercht. & J.Presl
Iz_4_1009	DJI_0399	Pilosella sp.	0		NA	0	Asteraceae Bercht. & J.Presl
Iz_4_1009	DJI_0399	Pinus sylvestris L.	0	n	1	1	Pinaceae Spreng. ex F.Rudolphi
Iz_4_1009	DJI_0399	Poa annua L.	0	n	1	0	Poaceae Barnhart
Iz_4_1009	DJI_0399	Poa nemoralis L.	0	n	1	0	Poaceae Barnhart
Iz_4_1009	DJI_0399	Poa sp.	0		NA	0	Poaceae Barnhart
Iz_4_1009	DJI_0399	Polygonatum odoratum (Mill.) Druce	0.5	n	1	0	Asparagaceae Juss.
Iz_4_1009	DJI_0399	Polygonum arenarium Waldst. & Kit.	0	n	1	0	Polygonaceae Juss.
Iz_4_1009	DJI_0399	Populus nigra L.	0	n	1	1	Salicaceae Mirb.
Iz_4_1009	DJI_0399	Populus tremula L.	0	n	1	1	Salicaceae Mirb.
Iz_4_1009	DJI_0399	Populus Ã canescens (Aiton) Sm.	0	n	1	1	Salicaceae Mirb.
Iz_4_1009	DJI_0399	Potentilla argentea L.	0	n	1	0	Rosaceae Juss.
Iz_4_1009	DJI_0399	Psammophiliella muralis (L.) Ikonn.	0	n	1	0	Caryophyllaceae Juss.
Iz_4_1009	DJI_0399	Psephellus sumensis (Kalen.) Greuter	0	n	1	0	Asteraceae Bercht. & J.Presl
Iz_4_1009	DJI_0399	Pulsatilla pratensis (L.) Mill. s.l.	0	n	1	0	Ranunculaceae Juss.
Iz_4_1009	DJI_0399	Pyrus communis L.	0	a	0	1	Rosaceae Juss.
Iz_4_1009	DJI_0399	Quercus robur L.	0	n	1	1	Fagaceae Dumort.
Iz_4_1009	DJI_0399	Robinia pseudoacacia L.	0	a	0	1	Fabaceae Lindl.
Iz_4_1009	DJI_0399	Rosa sp.	0		NA	1	Rosaceae Juss.
Iz_4_1009	DJI_0399	Rubus idaeus L.	0	n	1	0	Rosaceae Juss.
Iz_4_1009	DJI_0399	Rumex acetosella L.	0	n	1	0	Polygonaceae Juss.
Iz_4_1009	DJI_0399	Rumex thyrsiflorus Fingerh.	0	n	1	0	Polygonaceae Juss.
Iz_4_1009	DJI_0399	Salix alba L.	0	n	1	1	Salicaceae Mirb.
Iz_4_1009	DJI_0399	Sambucus nigra L.	0	n	1	1	Viburnaceae Raf.
Iz_4_1009	DJI_0399	Secale sylvestre Host	0	n	1	0	Poaceae Barnhart
Iz_4_1009	DJI_0399	Sempervivum ruthenicum Schnittsp. & C.B. Lehm.	0	n	1	0	Crassulaceae J.St.-Hil.
Iz_4_1009	DJI_0399	Setaria viridis (L.) P. Beauv.	0	n	1	0	Poaceae Barnhart
Iz_4_1009	DJI_0399	Silene borysthenica (Gruner) Walters	0	n	1	0	Caryophyllaceae Juss.
Iz_4_1009	DJI_0399	Silene latifolia subsp. alba (Mill.) Greuter & Burdet	0	n	1	0	Caryophyllaceae Juss.
Iz_4_1009	DJI_0399	Solidago virgaurea L.	0	n	1	0	Asteraceae Bercht. & J.Presl
Iz_4_1009	DJI_0399	Sorbus aucuparia L.	0	n	1	1	Rosaceae Juss.
Iz_4_1009	DJI_0399	Stachys recta L.	0	n	1	0	Lamiacea Lindl.
Iz_4_1009	DJI_0399	Tanacetum vulgare L.	0	n	1	0	Asteraceae Bercht. & J.Presl
Iz_4_1009	DJI_0399	Thymus pallasianus Heinr.Braun	0	n	1	0	Lamiacea Lindl.
Iz_4_1009	DJI_0399	Tilia cordata Mill.	0	n	1	1	Malvaceae Juss.
Iz_4_1009	DJI_0399	Trifolium alpestre L.	0	n	1	0	Fabaceae Lindl.
Iz_4_1009	DJI_0399	Trifolium arvense L.	0	n	1	0	Fabaceae Lindl.
Iz_4_1009	DJI_0399	Ulmus glabra Huds.	0	n	1	1	Ulmaceae Mirb.
Iz_4_1009	DJI_0399	Verbascum densiflorum Bertol.	0	n	1	0	Scrophulariaceae Juss.
Iz_4_1009	DJI_0399	Verbascum nigrum L.	0	n	1	0	Scrophulariaceae Juss.
Iz_4_1009	DJI_0399	Veronica austriaca L.	0	n	1	0	Plantaginaceae Juss.
Iz_4_1009	DJI_0399	Veronica incana L.	0	n	1	0	Plantaginaceae Juss.
Iz_4_1009	DJI_0399	Veronica verna L.	0	n	1	0	Plantaginaceae Juss.
Iz_4_1009	DJI_0399	Vincetoxicum hirundinaria Medik	0	n	1	0	Apocynaceae Juss.
Iz_4_1009	DJI_0405	Acer tataricum L.	0	n	1	1	Sapindaceae Juss.
Iz_4_1009	DJI_0405	Achillea millefolium L. s.l.	0	n	1	0	Asteraceae Bercht. & J.Presl
Iz_4_1009	DJI_0405	Allium oleraceum L.	0	n	1	0	Amaryllidaceae J.St.-Hil.
Iz_4_1009	DJI_0405	Ambrosia artemisiifolia L.	0	a	0	0	Asteraceae Bercht. & J.Presl
Iz_4_1009	DJI_0405	Amorpha fruticosa L.	0	a	0	0	Fabaceae Lindl.
Iz_4_1009	DJI_0405	Anthericum ramosum L.	0	n	1	0	Asparagaceae Juss.
Iz_4_1009	DJI_0405	Artemisia marschalliana Spreng.	0	n	1	0	Asteraceae Bercht. & J.Presl
Iz_4_1009	DJI_0405	Asparagus officinalis L.	0	n	1	0	Asparagaceae Juss.
Iz_4_1009	DJI_0405	Astragalus cicer L.	0	n	1	0	Fabaceae Lindl.
Iz_4_1009	DJI_0405	Bassia laniflora (S.G.Gmel.) A.J.Scott	0	n	1	0	Amaranthaceae Juss.
Iz_4_1009	DJI_0405	Berberis vulgaris L.	0	n	1	0	Berberidaceae Juss.
Iz_4_1009	DJI_0405	Betula pendula Roth.	0	n	1	1	Betulaceae Gray
Iz_4_1009	DJI_0405	Betula pendula Roth.	0	n	1	0	Poaceae Barnhart
Iz_4_1009	DJI_0405	Bromus inermis Leyss.	0		NA	NA	
Iz_4_1009	DJI_0405	Bromus squarrosus L.	0	a	0	0	Poaceae Barnhart

Iz_4_1009	DJI_0405	Bromus tectorum L.	0	a	0	0	Poaceae Barnhart
Iz_4_1009	DJI_0405	Calamagrostis epigejos (L.) Roth	20	n	1	0	Poaceae Barnhart
Iz_4_1009	DJI_0405	Carduus acanthoides L.	0	n	1	0	Asteraceae Bercht. & J.Presl
Iz_4_1009	DJI_0405	Carex sp.	0	n	1	0	Cyperaceae Juss.
Iz_4_1009	DJI_0405	Centaurea majorovii Dumbadze	0	n	1	0	Asteraceae Bercht. & J.Presl
Iz_4_1009	DJI_0405	Chamaecytisus ruthenicus (Fisch.ex Wol.) Klaskova	0	n	1	0	Fabaceae Lindl.
Iz_4_1009	DJI_0405	Chelidonium majus L.	0	n	1	0	Papaveraceae Juss.
Iz_4_1009	DJI_0405	Chenopodium betaceum Andrz.	0	n	1	0	Amaranthaceae Juss.
Iz_4_1009	DJI_0405	Chenopodium cf. album	0	n	1	0	Amaranthaceae Juss.
Iz_4_1009	DJI_0405	Chenopodium sp.	0		NA	0	Amaranthaceae Juss.
Iz_4_1009	DJI_0405	Chondrilla juncea L.	0	n	1	0	Asteraceae Bercht. & J.Presl
Iz_4_1009	DJI_0405	Cirsium vulgare (Savi) Ten.	0	n	1	0	Asteraceae Bercht. & J.Presl
Iz_4_1009	DJI_0405	Clinopodium acinos (L.) Kuntze	0	n	1	0	Lamiacea Lindl.
Iz_4_1009	DJI_0405	Convallaria majalis L.	0	n	1	0	Asparagaceae Juss.
Iz_4_1009	DJI_0405	Crataegus rhipidophylla Gand.	0	n	1	1	Rosaceae Juss.
Iz_4_1009	DJI_0405	Crepis foetida L.	0	n	1	0	Asteraceae Bercht. & J.Presl
Iz_4_1009	DJI_0405	Dactylis glomerata L.	0	n	1	0	Poaceae Barnhart
Iz_4_1009	DJI_0405	Dianthus campestris M.Bieb.	0	n	1	0	Caryophyllaceae Juss.
Iz_4_1009	DJI_0405	Digitaria sanguinalis (L.) Scop.	0	a	0	0	Poaceae Barnhart
Iz_4_1009	DJI_0405	Elymus repens (L.) Gould	0	n	1	0	Poaceae Barnhart
Iz_4_1009	DJI_0405	Epilobium tetragonum L.	0	n	1	0	Onagraceae Juss.
Iz_4_1009	DJI_0405	Eragrostis minor Host	0	a	0	0	Poaceae Barnhart
Iz_4_1009	DJI_0405	Erigeron canadensis L.	0	a	0	0	Asteraceae Bercht. & J.Presl
Iz_4_1009	DJI_0405	Euphorbia seguieriana Neck.	0	n	1	0	Euphorbiaceae Juss.
Iz_4_1009	DJI_0405	Fallopia dumetorum (L.) Holub	0	n	1	0	Polygonaceae Juss.
Iz_4_1009	DJI_0405	Festuca beckeri (Hack.) Trautv.	0	n	1	0	Poaceae Barnhart
Iz_4_1009	DJI_0405	Fragaria vesca L.	0	n	1	0	Rosaceae Juss.
Iz_4_1009	DJI_0405	Frangula alnus Mill.	0	n	1	1	Rhamnaceae Juss.
Iz_4_1009	DJI_0405	Fraxinus pennsylvanica Marshall	0	a	0	1	Oleaceae Hoffmanns. & Link
Iz_4_1009	DJI_0405	Galeopsis bifida Boenn.	0	n	1	0	Lamiacea Lindl.
Iz_4_1009	DJI_0405	Galium verum L.	0	n	1	0	Rubiaceae Juss.
Iz_4_1009	DJI_0405	Genista tinctoria L.	0	n	1	0	Fabaceae Lindl.
Iz_4_1009	DJI_0405	Geranium robertianum L.	0	n	1	0	Geraniaceae Juss.
Iz_4_1009	DJI_0405	Geranium sanquineum L.	0	n	1	0	Geraniaceae Juss.
Iz_4_1009	DJI_0405	Gypsophila paniculata L.	0	n	1	0	Caryophyllaceae Juss.
Iz_4_1009	DJI_0405	Helichrysum arenarium (L.) Moench	0	n	1	0	Asteraceae Bercht. & J.Presl
Iz_4_1009	DJI_0405	Herniaria polygama J.Gay	0	n	1	0	Caryophyllaceae Juss.
Iz_4_1009	DJI_0405	Hieracium robustum Fr.	0	n	1	0	Asteraceae Bercht. & J.Presl
Iz_4_1009	DJI_0405	Hieracium umbellatum L.	0	n	1	NA	Asteraceae Bercht. & J.Presl
Iz_4_1009	DJI_0405	Hylotelephium maximum (L.) Holub	0	n	1	0	Crassulaceae J.St.-Hil.
Iz_4_1009	DJI_0405	Jacobaea borysthenica (DC.) B.Nord. & Greuter	0	n	1	0	Asteraceae Bercht. & J.Presl
Iz_4_1009	DJI_0405	Jasione montana L.	0	n	1	0	Campanulaceae Juss.
Iz_4_1009	DJI_0405	Jurinea cyanoides (L.) Rchb.s.l.	0	n	1	0	Asteraceae Bercht. & J.Presl
Iz_4_1009	DJI_0405	Koeleria glauca (Spreng.) DC.	0	n	1	0	Poaceae Barnhart
Iz_4_1009	DJI_0405	Lactuca serriola L.	0	a	0	0	Asteraceae Bercht. & J.Presl
Iz_4_1009	DJI_0405	Linaria genistifolia (L.) Mill.	0	n	1	0	Plantaginaceae Juss.
Iz_4_1009	DJI_0405	Lomelosia argentea (L.) Greuter & Burdet	0	n	1	0	Caprifoliaceae Juss.
Iz_4_1009	DJI_0405	Melampyrum pratense L.	0	n	1	0	Orobanchaceae Vent.
Iz_4_1009	DJI_0405	Oenothera biennis L.	0	a	0	0	Onagraceae Juss.
Iz_4_1009	DJI_0405	Pentanema britannica (L.) D.Gut.Larr., Santos-Vicente, Anderb., E.Rico & M.M.Mart.Ort.	0	n	1	0	Asteraceae Bercht. & J.Presl
Iz_4_1009	DJI_0405	Peucedanum oreoselinum (L.) Moench	0	n	1	0	Apiaceae Lindl.
Iz_4_1009	DJI_0405	Peucedanum ruthenicum M.Bieb.	0	n	1	0	Apiaceae Lindl.
Iz_4_1009	DJI_0405	Pilosella echioides (Lumn.) F.W.Schultz & Sch.Bip.	0	n	1	0	Asteraceae Bercht. & J.Presl
Iz_4_1009	DJI_0405	Pilosella officinarum F. Schultz et Sch. Bip.	0	n	1	0	Asteraceae Bercht. & J.Presl
Iz_4_1009	DJI_0405	Pilosella sp.	0		NA	0	Asteraceae Bercht. & J.Presl
Iz_4_1009	DJI_0405	Pinus sylvestris L.	0	n	1	1	Pinaceae Spreng. ex F.Rudolphi
Iz_4_1009	DJI_0405	Poa annua L.	0	n	1	0	Poaceae Barnhart
Iz_4_1009	DJI_0405	Poa nemoralis L.	0	n	1	0	Poaceae Barnhart
Iz_4_1009	DJI_0405	Poa sp.	0		NA	0	Poaceae Barnhart
Iz_4_1009	DJI_0405	Polygonatum odoratum (Mill.) Druce	0	n	1	0	Asparagaceae Juss.
Iz_4_1009	DJI_0405	Polygonum arenarium Waldst. & Kit.	0	n	1	0	Polygonaceae Juss.
Iz_4_1009	DJI_0405	Populus nigra L.	0	n	1	1	Salicaceae Mirb.
Iz_4_1009	DJI_0405	Populus tremula L.	0	n	1	1	Salicaceae Mirb.
Iz_4_1009	DJI_0405	Populus Ã canescens (Aiton) Sm.	0	n	1	1	Salicaceae Mirb.
Iz_4_1009	DJI_0405	Potentilla argentea L.	0	n	1	0	Rosaceae Juss.
Iz_4_1009	DJI_0405	Psammophiliella muralis (L.) Ikonn.	0	n	1	0	Caryophyllaceae Juss.
Iz_4_1009	DJI_0405	Psephellus sumensis (Kalen.) Greuter	0	n	1	0	Asteraceae Bercht. & J.Presl
Iz_4_1009	DJI_0405	Pulsatilla pratensis (L.) Mill. s.l.	0	n	1	0	Ranunculaceae Juss.
Iz_4_1009	DJI_0405	Pyrus communis L.	0	a	0	1	Rosaceae Juss.
Iz_4_1009	DJI_0405	Quercus robur L.	0	n	1	1	Fagaceae Dumort.
Iz_4_1009	DJI_0405	Robinia pseudoacacia L.	0	a	0	1	Fabaceae Lindl.
Iz_4_1009	DJI_0405	Rosa sp.	0		NA	1	Rosaceae Juss.
Iz_4_1009	DJI_0405	Rubus idaeus L.	0	n	1	0	Rosaceae Juss.
Iz_4_1009	DJI_0405	Rumex acetosella L.	0	n	1	0	Polygonaceae Juss.
Iz_4_1009	DJI_0405	Rumex thyrsiflorus Fingerh.	0	n	1	0	Polygonaceae Juss.
Iz_4_1009	DJI_0405	Salix alba L.	0	n	1	1	Salicaceae Mirb.
Iz_4_1009	DJI_0405	Sambucus nigra L.	0	n	1	1	Viburnaceae Raf.
Iz_4_1009	DJI_0405	Secale sylvestre Host	0	n	1	0	Poaceae Barnhart
Iz_4_1009	DJI_0405	Sempervivum ruthenicum Schnittsp. & C.B. Lehm.	0	n	1	0	Crassulaceae J.St.-Hil.
Iz_4_1009	DJI_0405	Setaria viridis (L.) P. Beauv.	0	n	1	0	Poaceae Barnhart
Iz_4_1009	DJI_0405	Silene borysthenica (Gruner) Walters	0	n	1	0	Caryophyllaceae Juss.
Iz_4_1009	DJI_0405	Silene latifolia subsp. alba (Mill.) Greuter & Burdet	0	n	1	0	Caryophyllaceae Juss.
Iz_4_1009	DJI_0405	Solidago virgaurea L.	0	n	1	0	Asteraceae Bercht. & J.Presl
Iz_4_1009	DJI_0405	Sorbus aucuparia L.	0	n	1	1	Rosaceae Juss.
Iz_4_1009	DJI_0405	Stachys recta L.	0	n	1	0	Lamiacea Lindl.
Iz_4_1009	DJI_0405	Tanacetum vulgare L.	0	n	1	0	Asteraceae Bercht. & J.Presl
Iz_4_1009	DJI_0405	Thymus pallasianus Heinr.Braun	0	n	1	0	Lamiacea Lindl.
Iz_4_1009	DJI_0405	Tilia cordata Mill.	0	n	1	1	Malvaceae Juss.
Iz_4_1009	DJI_0405	Trifolium alpestre L.	0	n	1	0	Fabaceae Lindl.
Iz_4_1009	DJI_0405	Trifolium arvense L.	0	n	1	0	Fabaceae Lindl.
Iz_4_1009	DJI_0405	Ulmus glabra Huds.	0	n	1	1	Ulmaceae Mirb.
Iz_4_1009	DJI_0405	Verbascum densiflorum Bertol.	0	n	1	0	Scrophulariaceae Juss.
Iz_4_1009	DJI_0405	Verbascum nigrum L.	0	n	1	0	Scrophulariaceae Juss.
Iz_4_1009	DJI_0405	Veronica austriaca L.	0	n	1	0	Plantaginaceae Juss.
Iz_4_1009	DJI_0405	Veronica incana L.	0	n	1	0	Plantaginaceae Juss.
Iz_4_1009	DJI_0405	Veronica verna L.	0	n	1	0	Plantaginaceae Juss.
Iz_4_1009	DJI_0405	Vincetoxicum hirundinaria Medik	0	n	1	0	Apocynaceae Juss.
Iz_4_1009	DJI_0406	Acer tataricum L.	0	n	1	1	Sapindaceae Juss.

Iz_4_1009	DJI_0406	Achillea millefolium L. s.l.	0	n	1	0	Asteraceae Bercht. & J.Presl
Iz_4_1009	DJI_0406	Allium oleraceum L.	0	n	1	0	Amaryllidaceae J.St.-Hil.
Iz_4_1009	DJI_0406	Ambrosia artemisiifolia L.	0	a	0	0	Asteraceae Bercht. & J.Presl
Iz_4_1009	DJI_0406	Amorpha fruticosa L.	0	a	0	0	Fabaceae Lindl.
Iz_4_1009	DJI_0406	Anthericum ramosum L.	0	n	1	0	Asparagaceae Juss.
Iz_4_1009	DJI_0406	Artemisia marschalliana Spreng.	0	n	1	0	Asteraceae Bercht. & J.Presl
Iz_4_1009	DJI_0406	Asparagus officinalis L.	0	n	1	0	Asparagaceae Juss.
Iz_4_1009	DJI_0406	Astragalus cicer L.	0	n	1	0	Fabaceae Lindl.
Iz_4_1009	DJI_0406	Bassia laniflora (S.G.Gmel.) A.J.Scott	0	n	1	0	Amaranthaceae Juss.
Iz_4_1009	DJI_0406	Berberis vulgaris L.	0	n	1	0	Berberidaceae Juss.
Iz_4_1009	DJI_0406	Betula pendula Roth.	0	n	1	1	Betulaceae Gray
Iz_4_1009	DJI_0406	Betula pendula Roth.	0	n	1	0	Poaceae Barnhart
Iz_4_1009	DJI_0406	Bromus inermis Leyss.	0		NA	NA	
Iz_4_1009	DJI_0406	Bromus squarrosus L.	0	a	0	0	Poaceae Barnhart
Iz_4_1009	DJI_0406	Bromus tectorum L.	0	a	0	0	Poaceae Barnhart
Iz_4_1009	DJI_0406	Calamagrostis epigejos (L.) Roth	10	n	1	0	Poaceae Barnhart
Iz_4_1009	DJI_0406	Carduus acanthoides L.	0	n	1	0	Asteraceae Bercht. & J.Presl
Iz_4_1009	DJI_0406	Carex sp.	0	n	1	0	Cyperaceae Juss.
Iz_4_1009	DJI_0406	Centaurea majorovii Dumbadze	0	n	1	0	Asteraceae Bercht. & J.Presl
Iz_4_1009	DJI_0406	Chamaecytisus ruthenicus (Fisch.ex Wol.) Klaskova	0	n	1	0	Fabaceae Lindl.
Iz_4_1009	DJI_0406	Chelidonium majus L.	0	n	1	0	Papaveraceae Juss.
Iz_4_1009	DJI_0406	Chenopodium betaceum Andrz.	0	n	1	0	Amaranthaceae Juss.
Iz_4_1009	DJI_0406	Chenopodium cf. album	0	n	1	0	Amaranthaceae Juss.
Iz_4_1009	DJI_0406	Chenopodium sp.	0		NA	0	Amaranthaceae Juss.
Iz_4_1009	DJI_0406	Chondrilla juncea L.	0	n	1	0	Asteraceae Bercht. & J.Presl
Iz_4_1009	DJI_0406	Cirsium vulgare (Savi) Ten.	0	n	1	0	Asteraceae Bercht. & J.Presl
Iz_4_1009	DJI_0406	Clinopodium acinos (L.) Kuntze	0	n	1	0	Lamiacea Lindl.
Iz_4_1009	DJI_0406	Convallaria majalis L.	0	n	1	0	Asparagaceae Juss.
Iz_4_1009	DJI_0406	Crataegus rhipidophylla Gand.	0	n	1	1	Rosaceae Juss.
Iz_4_1009	DJI_0406	Crepis foetida L.	0	n	1	0	Asteraceae Bercht. & J.Presl
Iz_4_1009	DJI_0406	Dactylis glomerata L.	0	n	1	0	Poaceae Barnhart
Iz_4_1009	DJI_0406	Dianthus campestris M.Bieb.	0	n	1	0	Caryophyllaceae Juss.
Iz_4_1009	DJI_0406	Digitaria sanguinalis (L.) Scop.	0	a	0	0	Poaceae Barnhart
Iz_4_1009	DJI_0406	Elymus repens (L.) Gould	0	n	1	0	Poaceae Barnhart
Iz_4_1009	DJI_0406	Epilobium tetragonum L.	0	n	1	0	Onagraceae Juss.
Iz_4_1009	DJI_0406	Eragrostis minor Host	0	a	0	0	Poaceae Barnhart
Iz_4_1009	DJI_0406	Erigeron canadensis L.	3	a	0	0	Asteraceae Bercht. & J.Presl
Iz_4_1009	DJI_0406	Euphorbia seguieriana Neck.	0	n	1	0	Euphorbiaceae Juss.
Iz_4_1009	DJI_0406	Fallopia dumetorum (L.) Holub	0	n	1	0	Polygonaceae Juss.
Iz_4_1009	DJI_0406	Festuca beckeri (Hack.) Trautv.	0	n	1	0	Poaceae Barnhart
Iz_4_1009	DJI_0406	Fragaria vesca L.	0	n	1	0	Rosaceae Juss.
Iz_4_1009	DJI_0406	Frangula alnus Mill.	0	n	1	1	Rhamnaceae Juss.
Iz_4_1009	DJI_0406	Fraxinus pennsylvanica Marshall	0	a	0	1	Oleaceae Hoffmanns. & Link
Iz_4_1009	DJI_0406	Galeopsis bifida Boenn.	0	n	1	0	Lamiacea Lindl.
Iz_4_1009	DJI_0406	Galium verum L.	0	n	1	0	Rubiaceae Juss.
Iz_4_1009	DJI_0406	Genista tinctoria L.	0	n	1	0	Fabaceae Lindl.
Iz_4_1009	DJI_0406	Geranium robertianum L.	0	n	1	0	Geraniaceae Juss.
Iz_4_1009	DJI_0406	Geranium sanquineum L.	0	n	1	0	Geraniaceae Juss.
Iz_4_1009	DJI_0406	Gypsophila paniculata L.	0	n	1	0	Caryophyllaceae Juss.
Iz_4_1009	DJI_0406	Helichrysum arenarium (L.) Moench	0	n	1	0	Asteraceae Bercht. & J.Presl
Iz_4_1009	DJI_0406	Herniaria polygama J.Gay	0	n	1	0	Caryophyllaceae Juss.
Iz_4_1009	DJI_0406	Hieracium robustum Fr.	0	n	1	0	Asteraceae Bercht. & J.Presl
Iz_4_1009	DJI_0406	Hieracium umbellatum L.	0	n	1	NA	Asteraceae Bercht. & J.Presl
Iz_4_1009	DJI_0406	Hylotelephium maximum (L.) Holub	0	n	1	0	Crassulaceae J.St.-Hil.
Iz_4_1009	DJI_0406	Jacobaea borysthenica (DC.) B.Nord. & Greuter	0	n	1	0	Asteraceae Bercht. & J.Presl
Iz_4_1009	DJI_0406	Jasione montana L.	0	n	1	0	Campanulaceae Juss.
Iz_4_1009	DJI_0406	Jurinea cyanoides (L.) Rchb.s.l.	0	n	1	0	Asteraceae Bercht. & J.Presl
Iz_4_1009	DJI_0406	Koeleria glauca (Spreng.) DC.	0	n	1	0	Poaceae Barnhart
Iz_4_1009	DJI_0406	Lactuca serriola L.	0	a	0	0	Asteraceae Bercht. & J.Presl
Iz_4_1009	DJI_0406	Linaria genistifolia (L.) Mill.	0	n	1	0	Plantaginaceae Juss.
Iz_4_1009	DJI_0406	Lomelosia argentea (L.) Greuter & Burdet	0	n	1	0	Caprifoliaceae Juss.
Iz_4_1009	DJI_0406	Melampyrum pratense L.	0	n	1	0	Orobanchaceae Vent.
Iz_4_1009	DJI_0406	Oenothera biennis L.	0	a	0	0	Onagraceae Juss.
Iz_4_1009	DJI_0406	Pentanema britannica (L.) D.Gut.Larr., Santos-Vicente, Anderb., E.Rico & M.M.Mart.Ort.	0	n	1	0	Asteraceae Bercht. & J.Presl
Iz_4_1009	DJI_0406	Peucedanum oreoselinum (L.) Moench	0	n	1	0	Apiaceae Lindl.
Iz_4_1009	DJI_0406	Peucedanum ruthenicum M.Bieb.	0	n	1	0	Apiaceae Lindl.
Iz_4_1009	DJI_0406	Pilosella echioides (Lumn.) F.W.Schultz & Sch.Bip.	0	n	1	0	Asteraceae Bercht. & J.Presl
Iz_4_1009	DJI_0406	Pilosella officinarum F. Schultz et Sch. Bip.	0	n	1	0	Asteraceae Bercht. & J.Presl
Iz_4_1009	DJI_0406	Pilosella sp.	0		NA	0	Asteraceae Bercht. & J.Presl
Iz_4_1009	DJI_0406	Pinus sylvestris L.	0	n	1	1	Pinaceae Spreng. ex F.Rudolphi
Iz_4_1009	DJI_0406	Poa annua L.	0	n	1	0	Poaceae Barnhart
Iz_4_1009	DJI_0406	Poa nemoralis L.	0	n	1	0	Poaceae Barnhart
Iz_4_1009	DJI_0406	Poa sp.	0		NA	0	Poaceae Barnhart
Iz_4_1009	DJI_0406	Polygonatum odoratum (Mill.) Druce	0	n	1	0	Asparagaceae Juss.
Iz_4_1009	DJI_0406	Polygonum arenarium Waldst. & Kit.	0	n	1	0	Polygonaceae Juss.
Iz_4_1009	DJI_0406	Populus nigra L.	0	n	1	1	Salicaceae Mirb.
Iz_4_1009	DJI_0406	Populus tremula L.	0	n	1	1	Salicaceae Mirb.
Iz_4_1009	DJI_0406	Populus Ã canescens (Aiton) Sm.	0	n	1	1	Salicaceae Mirb.
Iz_4_1009	DJI_0406	Potentilla argentea L.	0	n	1	0	Rosaceae Juss.
Iz_4_1009	DJI_0406	Psammophiliella muralis (L.) Ikonn.	0	n	1	0	Caryophyllaceae Juss.
Iz_4_1009	DJI_0406	Psephellus sumensis (Kalen.) Greuter	0	n	1	0	Asteraceae Bercht. & J.Presl
Iz_4_1009	DJI_0406	Pulsatilla pratensis (L.) Mill. s.l.	0	n	1	0	Ranunculaceae Juss.
Iz_4_1009	DJI_0406	Pyrus communis L.	0	a	0	1	Rosaceae Juss.
Iz_4_1009	DJI_0406	Quercus robur L.	0	n	1	1	Fagaceae Dumort.
Iz_4_1009	DJI_0406	Robinia pseudoacacia L.	0	a	0	1	Fabaceae Lindl.
Iz_4_1009	DJI_0406	Rosa sp.	0		NA	1	Rosaceae Juss.
Iz_4_1009	DJI_0406	Rubus idaeus L.	0	n	1	0	Rosaceae Juss.
Iz_4_1009	DJI_0406	Rumex acetosella L.	0	n	1	0	Polygonaceae Juss.
Iz_4_1009	DJI_0406	Rumex thyrsiflorus Fingerh.	0	n	1	0	Polygonaceae Juss.
Iz_4_1009	DJI_0406	Salix alba L.	0	n	1	1	Salicaceae Mirb.
Iz_4_1009	DJI_0406	Sambucus nigra L.	0	n	1	1	Viburnaceae Raf.
Iz_4_1009	DJI_0406	Secale sylvestre Host	0	n	1	0	Poaceae Barnhart
Iz_4_1009	DJI_0406	Sempervivum ruthenicum Schnittsp. & C.B. Lehm.	0	n	1	0	Crassulaceae J.St.-Hil.
Iz_4_1009	DJI_0406	Setaria viridis (L.) P. Beauv.	0	n	1	0	Poaceae Barnhart
Iz_4_1009	DJI_0406	Silene borysthenica (Gruner) Walters	0	n	1	0	Caryophyllaceae Juss.
Iz_4_1009	DJI_0406	Silene latifolia subsp. alba (Mill.) Greuter & Burdet	0	n	1	0	Caryophyllaceae Juss.
Iz_4_1009	DJI_0406	Solidago virgaurea L.	3	n	1	0	Asteraceae Bercht. & J.Presl
Iz_4_1009	DJI_0406	Sorbus aucuparia L.	0	n	1	1	Rosaceae Juss.

Iz_4_1009	DJI_0406	Stachys recta L.	0	n	1	0	Lamiacea Lindl.
Iz_4_1009	DJI_0406	Tanacetum vulgare L.	0	n	1	0	Asteraceae Bercht. & J.Presl
Iz_4_1009	DJI_0406	Thymus pallasianus Heinr.Braun	0	n	1	0	Lamiacea Lindl.
Iz_4_1009	DJI_0406	Tilia cordata Mill.	0	n	1	1	Malvaceae Juss.
Iz_4_1009	DJI_0406	Trifolium alpestre L.	0	n	1	0	Fabaceae Lindl.
Iz_4_1009	DJI_0406	Trifolium arvense L.	0	n	1	0	Fabaceae Lindl.
Iz_4_1009	DJI_0406	Ulmus glabra Huds.	0	n	1	1	Ulmaceae Mirb.
Iz_4_1009	DJI_0406	Verbascum densiflorum Bertol.	0	n	1	0	Scrophulariaceae Juss.
Iz_4_1009	DJI_0406	Verbascum nigrum L.	0	n	1	0	Scrophulariaceae Juss.
Iz_4_1009	DJI_0406	Veronica austriaca L.	0	n	1	0	Plantaginaceae Juss.
Iz_4_1009	DJI_0406	Veronica incana L.	0	n	1	0	Plantaginaceae Juss.
Iz_4_1009	DJI_0406	Veronica verna L.	0	n	1	0	Plantaginaceae Juss.
Iz_4_1009	DJI_0406	Vincetoxicum hirundinaria Medik	0	n	1	0	Apocynaceae Juss.
Iz_4_1009	DJI_0407	Acer tataricum L.	0	n	1	1	Sapindaceae Juss.
Iz_4_1009	DJI_0407	Achillea millefolium L. s.l.	0	n	1	0	Asteraceae Bercht. & J.Presl
Iz_4_1009	DJI_0407	Allium oleraceum L.	0	n	1	0	Amaryllidaceae J.St.-Hil.
Iz_4_1009	DJI_0407	Ambrosia artemisiifolia L.	0	a	0	0	Asteraceae Bercht. & J.Presl
Iz_4_1009	DJI_0407	Amorpha fruticosa L.	0	a	0	0	Fabaceae Lindl.
Iz_4_1009	DJI_0407	Anthericum ramosum L.	0	n	1	0	Asparagaceae Juss.
Iz_4_1009	DJI_0407	Artemisia marschalliana Spreng.	0	n	1	0	Asteraceae Bercht. & J.Presl
Iz_4_1009	DJI_0407	Asparagus officinalis L.	0	n	1	0	Asparagaceae Juss.
Iz_4_1009	DJI_0407	Astragalus cicer L.	0	n	1	0	Fabaceae Lindl.
Iz_4_1009	DJI_0407	Bassia laniflora (S.G.Gmel.) A.J.Scott	0	n	1	0	Amaranthaceae Juss.
Iz_4_1009	DJI_0407	Berberis vulgaris L.	0	n	1	0	Berberidaceae Juss.
Iz_4_1009	DJI_0407	Betula pendula Roth.	0	n	1	1	Betulaceae Gray
Iz_4_1009	DJI_0407	Betula pendula Roth.	0	n	1	0	Poaceae Barnhart
Iz_4_1009	DJI_0407	Bromus inermis Leyss.	0		NA	NA	
Iz_4_1009	DJI_0407	Bromus squarrosus L.	0	a	0	0	Poaceae Barnhart
Iz_4_1009	DJI_0407	Bromus tectorum L.	0	a	0	0	Poaceae Barnhart
Iz_4_1009	DJI_0407	Calamagrostis epigejos (L.) Roth	5	n	1	0	Poaceae Barnhart
Iz_4_1009	DJI_0407	Carduus acanthoides L.	0	n	1	0	Asteraceae Bercht. & J.Presl
Iz_4_1009	DJI_0407	Carex sp.	0	n	1	0	Cyperaceae Juss.
Iz_4_1009	DJI_0407	Centaurea majorovii Dumbadze	0	n	1	0	Asteraceae Bercht. & J.Presl
Iz_4_1009	DJI_0407	Chamaecytisus ruthenicus (Fisch.ex Wol.) Klaskova	3	n	1	0	Fabaceae Lindl.
Iz_4_1009	DJI_0407	Chelidonium majus L.	0	n	1	0	Papaveraceae Juss.
Iz_4_1009	DJI_0407	Chenopodium betaceum Andrz.	0	n	1	0	Amaranthaceae Juss.
Iz_4_1009	DJI_0407	Chenopodium cf. album	0	n	1	0	Amaranthaceae Juss.
Iz_4_1009	DJI_0407	Chenopodium sp.	0		NA	0	Amaranthaceae Juss.
Iz_4_1009	DJI_0407	Chondrilla juncea L.	0	n	1	0	Asteraceae Bercht. & J.Presl
Iz_4_1009	DJI_0407	Cirsium vulgare (Savi) Ten.	0	n	1	0	Asteraceae Bercht. & J.Presl
Iz_4_1009	DJI_0407	Clinopodium acinos (L.) Kuntze	0	n	1	0	Lamiacea Lindl.
Iz_4_1009	DJI_0407	Convallaria majalis L.	0	n	1	0	Asparagaceae Juss.
Iz_4_1009	DJI_0407	Crataegus rhipidophylla Gand.	0	n	1	1	Rosaceae Juss.
Iz_4_1009	DJI_0407	Crepis foetida L.	0	n	1	0	Asteraceae Bercht. & J.Presl
Iz_4_1009	DJI_0407	Dactylis glomerata L.	0	n	1	0	Poaceae Barnhart
Iz_4_1009	DJI_0407	Dianthus campestris M.Bieb.	0	n	1	0	Caryophyllaceae Juss.
Iz_4_1009	DJI_0407	Digitaria sanguinalis (L.) Scop.	0	a	0	0	Poaceae Barnhart
Iz_4_1009	DJI_0407	Elymus repens (L.) Gould	0	n	1	0	Poaceae Barnhart
Iz_4_1009	DJI_0407	Epilobium tetragonum L.	0	n	1	0	Onagraceae Juss.
Iz_4_1009	DJI_0407	Eragrostis minor Host	0	a	0	0	Poaceae Barnhart
Iz_4_1009	DJI_0407	Erigeron canadensis L.	3	a	0	0	Asteraceae Bercht. & J.Presl
Iz_4_1009	DJI_0407	Euphorbia seguieriana Neck.	0	n	1	0	Euphorbiaceae Juss.
Iz_4_1009	DJI_0407	Fallopia dumetorum (L.) Holub	0	n	1	0	Polygonaceae Juss.
Iz_4_1009	DJI_0407	Festuca beckeri (Hack.) Trautv.	0	n	1	0	Poaceae Barnhart
Iz_4_1009	DJI_0407	Fragaria vesca L.	0	n	1	0	Rosaceae Juss.
Iz_4_1009	DJI_0407	Frangula alnus Mill.	0	n	1	1	Rhamnaceae Juss.
Iz_4_1009	DJI_0407	Fraxinus pennsylvanica Marshall	0	a	0	1	Oleaceae Hoffmanns. & Link
Iz_4_1009	DJI_0407	Galeopsis bifida Boenn.	0	n	1	0	Lamiacea Lindl.
Iz_4_1009	DJI_0407	Galium verum L.	0	n	1	0	Rubiaceae Juss.
Iz_4_1009	DJI_0407	Genista tinctoria L.	0	n	1	0	Fabaceae Lindl.
Iz_4_1009	DJI_0407	Geranium robertianum L.	0	n	1	0	Geraniaceae Juss.
Iz_4_1009	DJI_0407	Geranium sanquineum L.	0	n	1	0	Geraniaceae Juss.
Iz_4_1009	DJI_0407	Gypsophila paniculata L.	0	n	1	0	Caryophyllaceae Juss.
Iz_4_1009	DJI_0407	Helichrysum arenarium (L.) Moench	0	n	1	0	Asteraceae Bercht. & J.Presl
Iz_4_1009	DJI_0407	Herniaria polygama J.Gay	0	n	1	0	Caryophyllaceae Juss.
Iz_4_1009	DJI_0407	Hieracium robustum Fr.	0	n	1	0	Asteraceae Bercht. & J.Presl
Iz_4_1009	DJI_0407	Hieracium umbellatum L.	0	n	1	NA	Asteraceae Bercht. & J.Presl
Iz_4_1009	DJI_0407	Hylotelephium maximum (L.) Holub	0	n	1	0	Crassulaceae J.St.-Hil.
Iz_4_1009	DJI_0407	Jacobaea borysthenica (DC.) B.Nord. & Greuter	0	n	1	0	Asteraceae Bercht. & J.Presl
Iz_4_1009	DJI_0407	Jasione montana L.	0	n	1	0	Campanulaceae Juss.
Iz_4_1009	DJI_0407	Jurinea cyanoides (L.) Rchb.s.l.	0	n	1	0	Asteraceae Bercht. & J.Presl
Iz_4_1009	DJI_0407	Koeleria glauca (Spreng.) DC.	0	n	1	0	Poaceae Barnhart
Iz_4_1009	DJI_0407	Lactuca serriola L.	0	a	0	0	Asteraceae Bercht. & J.Presl
Iz_4_1009	DJI_0407	Linaria genistifolia (L.) Mill.	0	n	1	0	Plantaginaceae Juss.
Iz_4_1009	DJI_0407	Lomelosia argentea (L.) Greuter & Burdet	0	n	1	0	Caprifoliaceae Juss.
Iz_4_1009	DJI_0407	Melampyrum pratense L.	0	n	1	0	Orobanchaceae Vent.
Iz_4_1009	DJI_0407	Oenothera biennis L.	0	a	0	0	Onagraceae Juss.
Iz_4_1009	DJI_0407	Pentanema britannica (L.) D.Gut.Larr., Santos-Vicente, Anderb., E.Rico & M.M.Mart.Ort.	0	n	1	0	Asteraceae Bercht. & J.Presl
Iz_4_1009	DJI_0407	Peucedanum oreoselinum (L.) Moench	0	n	1	0	Apiaceae Lindl.
Iz_4_1009	DJI_0407	Peucedanum ruthenicum M.Bieb.	0	n	1	0	Apiaceae Lindl.
Iz_4_1009	DJI_0407	Pilosella echioides (Lumn.) F.W.Schultz & Sch.Bip.	0	n	1	0	Asteraceae Bercht. & J.Presl
Iz_4_1009	DJI_0407	Pilosella officinarum F. Schultz et Sch. Bip.	0	n	1	0	Asteraceae Bercht. & J.Presl
Iz_4_1009	DJI_0407	Pilosella sp.	0		NA	0	Asteraceae Bercht. & J.Presl
Iz_4_1009	DJI_0407	Pinus sylvestris L.	0	n	1	1	Pinaceae Spreng. ex F.Rudolphi
Iz_4_1009	DJI_0407	Poa annua L.	0	n	1	0	Poaceae Barnhart
Iz_4_1009	DJI_0407	Poa nemoralis L.	0	n	1	0	Poaceae Barnhart
Iz_4_1009	DJI_0407	Poa sp.	0		NA	0	Poaceae Barnhart
Iz_4_1009	DJI_0407	Polygonatum odoratum (Mill.) Druce	0	n	1	0	Asparagaceae Juss.
Iz_4_1009	DJI_0407	Polygonum arenarium Waldst. & Kit.	0	n	1	0	Polygonaceae Juss.
Iz_4_1009	DJI_0407	Populus nigra L.	0	n	1	1	Salicaceae Mirb.
Iz_4_1009	DJI_0407	Populus tremula L.	0	n	1	1	Salicaceae Mirb.
Iz_4_1009	DJI_0407	Populus Ã canescens (Aiton) Sm.	0	n	1	1	Salicaceae Mirb.
Iz_4_1009	DJI_0407	Potentilla argentea L.	0	n	1	0	Rosaceae Juss.
Iz_4_1009	DJI_0407	Psammophiliella muralis (L.) Ikonn.	0	n	1	0	Caryophyllaceae Juss.
Iz_4_1009	DJI_0407	Psephellus sumensis (Kalen.) Greuter	0	n	1	0	Asteraceae Bercht. & J.Presl
Iz_4_1009	DJI_0407	Pulsatilla pratensis (L.) Mill. s.l.	0	n	1	0	Ranunculaceae Juss.
Iz_4_1009	DJI_0407	Pyrus communis L.	0	a	0	1	Rosaceae Juss.
Iz_4_1009	DJI_0407	Quercus robur L.	0	n	1	1	Fagaceae Dumort.

Iz_4_1009	DJI_0407	Robinia pseudoacacia L.	0	a	0	1	Fabaceae Lindl.
Iz_4_1009	DJI_0407	Rosa sp.	0		NA	1	Rosaceae Juss.
Iz_4_1009	DJI_0407	Rubus idaeus L.	0	n	1	0	Rosaceae Juss.
Iz_4_1009	DJI_0407	Rumex acetosella L.	0	n	1	0	Polygonaceae Juss.
Iz_4_1009	DJI_0407	Rumex thyrsiflorus Fingerh.	0	n	1	0	Polygonaceae Juss.
Iz_4_1009	DJI_0407	Salix alba L.	0	n	1	1	Salicaceae Mirb.
Iz_4_1009	DJI_0407	Sambucus nigra L.	0	n	1	1	Viburnaceae Raf.
Iz_4_1009	DJI_0407	Secale sylvestre Host	0	n	1	0	Poaceae Barnhart
Iz_4_1009	DJI_0407	Sempervivum ruthenicum Schnittsp. & C.B. Lehm.	0	n	1	0	Crassulaceae J.St.-Hil.
Iz_4_1009	DJI_0407	Setaria viridis (L.) P. Beauv.	0	n	1	0	Poaceae Barnhart
Iz_4_1009	DJI_0407	Silene borysthenica (Gruner) Walters	0	n	1	0	Caryophyllaceae Juss.
Iz_4_1009	DJI_0407	Silene latifolia subsp. alba (Mill.) Greuter & Burdet	0	n	1	0	Caryophyllaceae Juss.
Iz_4_1009	DJI_0407	Solidago virgaurea L.	1	n	1	0	Asteraceae Bercht. & J.Presl
Iz_4_1009	DJI_0407	Sorbus aucuparia L.	0	n	1	1	Rosaceae Juss.
Iz_4_1009	DJI_0407	Stachys recta L.	0	n	1	0	Lamiacea Lindl.
Iz_4_1009	DJI_0407	Tanacetum vulgare L.	0	n	1	0	Asteraceae Bercht. & J.Presl
Iz_4_1009	DJI_0407	Thymus pallasianus Heinr.Braun	0	n	1	0	Lamiacea Lindl.
Iz_4_1009	DJI_0407	Tilia cordata Mill.	0	n	1	1	Malvaceae Juss.
Iz_4_1009	DJI_0407	Trifolium alpestre L.	0	n	1	0	Fabaceae Lindl.
Iz_4_1009	DJI_0407	Trifolium arvense L.	0	n	1	0	Fabaceae Lindl.
Iz_4_1009	DJI_0407	Ulmus glabra Huds.	0	n	1	1	Ulmaceae Mirb.
Iz_4_1009	DJI_0407	Verbascum densiflorum Bertol.	0	n	1	0	Scrophulariaceae Juss.
Iz_4_1009	DJI_0407	Verbascum nigrum L.	0	n	1	0	Scrophulariaceae Juss.
Iz_4_1009	DJI_0407	Veronica austriaca L.	0	n	1	0	Plantaginaceae Juss.
Iz_4_1009	DJI_0407	Veronica incana L.	0	n	1	0	Plantaginaceae Juss.
Iz_4_1009	DJI_0407	Veronica verna L.	0	n	1	0	Plantaginaceae Juss.
Iz_4_1009	DJI_0407	Vincetoxicum hirundinaria Medik	0	n	1	0	Apocynaceae Juss.
Iz_4_1009	DJI_0408	Acer tataricum L.	0	n	1	1	Sapindaceae Juss.
Iz_4_1009	DJI_0408	Achillea millefolium L. s.l.	0	n	1	0	Asteraceae Bercht. & J.Presl
Iz_4_1009	DJI_0408	Allium oleraceum L.	0	n	1	0	Amaryllidaceae J.St.-Hil.
Iz_4_1009	DJI_0408	Ambrosia artemisiifolia L.	0	a	0	0	Asteraceae Bercht. & J.Presl
Iz_4_1009	DJI_0408	Amorpha fruticosa L.	0	a	0	0	Fabaceae Lindl.
Iz_4_1009	DJI_0408	Anthericum ramosum L.	0	n	1	0	Asparagaceae Juss.
Iz_4_1009	DJI_0408	Artemisia marschalliana Spreng.	0	n	1	0	Asteraceae Bercht. & J.Presl
Iz_4_1009	DJI_0408	Asparagus officinalis L.	0	n	1	0	Asparagaceae Juss.
Iz_4_1009	DJI_0408	Astragalus cicer L.	0	n	1	0	Fabaceae Lindl.
Iz_4_1009	DJI_0408	Bassia laniflora (S.G.Gmel.) A.J.Scott	0	n	1	0	Amaranthaceae Juss.
Iz_4_1009	DJI_0408	Berberis vulgaris L.	0	n	1	0	Berberidaceae Juss.
Iz_4_1009	DJI_0408	Betula pendula Roth.	0	n	1	1	Betulaceae Gray
Iz_4_1009	DJI_0408	Betula pendula Roth.	0	n	1	0	Poaceae Barnhart
Iz_4_1009	DJI_0408	Bromus inermis Leyss.	0		NA	NA	
Iz_4_1009	DJI_0408	Bromus squarrosus L.	0	a	0	0	Poaceae Barnhart
Iz_4_1009	DJI_0408	Bromus tectorum L.	0	a	0	0	Poaceae Barnhart
Iz_4_1009	DJI_0408	Calamagrostis epigejos (L.) Roth	10	n	1	0	Poaceae Barnhart
Iz_4_1009	DJI_0408	Carduus acanthoides L.	0	n	1	0	Asteraceae Bercht. & J.Presl
Iz_4_1009	DJI_0408	Carex sp.	0	n	1	0	Cyperaceae Juss.
Iz_4_1009	DJI_0408	Centaurea majorovii Dumbadze	0	n	1	0	Asteraceae Bercht. & J.Presl
Iz_4_1009	DJI_0408	Chamaecytisus ruthenicus (Fisch.ex Wol.) Klaskova	3	n	1	0	Fabaceae Lindl.
Iz_4_1009	DJI_0408	Chelidonium majus L.	0	n	1	0	Papaveraceae Juss.
Iz_4_1009	DJI_0408	Chenopodium betaceum Andrz.	0	n	1	0	Amaranthaceae Juss.
Iz_4_1009	DJI_0408	Chenopodium cf. album	0	n	1	0	Amaranthaceae Juss.
Iz_4_1009	DJI_0408	Chenopodium sp.	0		NA	0	Amaranthaceae Juss.
Iz_4_1009	DJI_0408	Chondrilla juncea L.	0	n	1	0	Asteraceae Bercht. & J.Presl
Iz_4_1009	DJI_0408	Cirsium vulgare (Savi) Ten.	0	n	1	0	Asteraceae Bercht. & J.Presl
Iz_4_1009	DJI_0408	Clinopodium acinos (L.) Kuntze	0	n	1	0	Lamiacea Lindl.
Iz_4_1009	DJI_0408	Convallaria majalis L.	0	n	1	0	Asparagaceae Juss.
Iz_4_1009	DJI_0408	Crataegus rhipidophylla Gand.	0	n	1	1	Rosaceae Juss.
Iz_4_1009	DJI_0408	Crepis foetida L.	0	n	1	0	Asteraceae Bercht. & J.Presl
Iz_4_1009	DJI_0408	Dactylis glomerata L.	0	n	1	0	Poaceae Barnhart
Iz_4_1009	DJI_0408	Dianthus campestris M.Bieb.	0	n	1	0	Caryophyllaceae Juss.
Iz_4_1009	DJI_0408	Digitaria sanguinalis (L.) Scop.	0	a	0	0	Poaceae Barnhart
Iz_4_1009	DJI_0408	Elymus repens (L.) Gould	0	n	1	0	Poaceae Barnhart
Iz_4_1009	DJI_0408	Epilobium tetragonum L.	0	n	1	0	Onagraceae Juss.
Iz_4_1009	DJI_0408	Eragrostis minor Host	0	a	0	0	Poaceae Barnhart
Iz_4_1009	DJI_0408	Erigeron canadensis L.	5	a	0	0	Asteraceae Bercht. & J.Presl
Iz_4_1009	DJI_0408	Euphorbia seguieriana Neck.	0	n	1	0	Euphorbiaceae Juss.
Iz_4_1009	DJI_0408	Fallopia dumetorum (L.) Holub	0	n	1	0	Polygonaceae Juss.
Iz_4_1009	DJI_0408	Festuca beckeri (Hack.) Trautv.	0	n	1	0	Poaceae Barnhart
Iz_4_1009	DJI_0408	Fragaria vesca L.	0	n	1	0	Rosaceae Juss.
Iz_4_1009	DJI_0408	Frangula alnus Mill.	0	n	1	1	Rhamnaceae Juss.
Iz_4_1009	DJI_0408	Fraxinus pennsylvanica Marshall	0	a	0	1	Oleaceae Hoffmanns. & Link
Iz_4_1009	DJI_0408	Galeopsis bifida Boenn.	0	n	1	0	Lamiacea Lindl.
Iz_4_1009	DJI_0408	Galium verum L.	0	n	1	0	Rubiaceae Juss.
Iz_4_1009	DJI_0408	Genista tinctoria L.	0	n	1	0	Fabaceae Lindl.
Iz_4_1009	DJI_0408	Geranium robertianum L.	0	n	1	0	Geraniaceae Juss.
Iz_4_1009	DJI_0408	Geranium sanquineum L.	0	n	1	0	Geraniaceae Juss.
Iz_4_1009	DJI_0408	Gypsophila paniculata L.	0	n	1	0	Caryophyllaceae Juss.
Iz_4_1009	DJI_0408	Helichrysum arenarium (L.) Moench	0	n	1	0	Asteraceae Bercht. & J.Presl
Iz_4_1009	DJI_0408	Herniaria polygama J.Gay	0	n	1	0	Caryophyllaceae Juss.
Iz_4_1009	DJI_0408	Hieracium robustum Fr.	2	n	1	0	Asteraceae Bercht. & J.Presl
Iz_4_1009	DJI_0408	Hieracium umbellatum L.	0	n	1	NA	Asteraceae Bercht. & J.Presl
Iz_4_1009	DJI_0408	Hylotelephium maximum (L.) Holub	0	n	1	0	Crassulaceae J.St.-Hil.
Iz_4_1009	DJI_0408	Jacobaea borysthenica (DC.) B.Nord. & Greuter	0	n	1	0	Asteraceae Bercht. & J.Presl
Iz_4_1009	DJI_0408	Jasione montana L.	0	n	1	0	Campanulaceae Juss.
Iz_4_1009	DJI_0408	Jurinea cyanoides (L.) Rchb.s.l.	0	n	1	0	Asteraceae Bercht. & J.Presl
Iz_4_1009	DJI_0408	Koeleria glauca (Spreng.) DC.	0	n	1	0	Poaceae Barnhart
Iz_4_1009	DJI_0408	Lactuca serriola L.	0	a	0	0	Asteraceae Bercht. & J.Presl
Iz_4_1009	DJI_0408	Linaria genistifolia (L.) Mill.	0	n	1	0	Plantaginaceae Juss.
Iz_4_1009	DJI_0408	Lomelosia argentea (L.) Greuter & Burdet	0	n	1	0	Caprifoliaceae Juss.
Iz_4_1009	DJI_0408	Melampyrum pratense L.	0	n	1	0	Orobanchaceae Vent.
Iz_4_1009	DJI_0408	Oenothera biennis L.	0	a	0	0	Onagraceae Juss.
Iz_4_1009	DJI_0408	Pentanema britannica (L.) D.Gut.Larr., Santos-Vicente, Anderb., E.Rico & M.M.Mart.Ort.	0	n	1	0	Asteraceae Bercht. & J.Presl
Iz_4_1009	DJI_0408	Peucedanum oreoselinum (L.) Moench	0	n	1	0	Apiaceae Lindl.
Iz_4_1009	DJI_0408	Peucedanum ruthenicum M.Bieb.	0	n	1	0	Apiaceae Lindl.
Iz_4_1009	DJI_0408	Pilosella echioides (Lumn.) F.W.Schultz & Sch.Bip.	0	n	1	0	Asteraceae Bercht. & J.Presl
Iz_4_1009	DJI_0408	Pilosella officinarum F. Schultz et Sch. Bip.	0	n	1	0	Asteraceae Bercht. & J.Presl
Iz_4_1009	DJI_0408	Pilosella sp.	0		NA	0	Asteraceae Bercht. & J.Presl
Iz_4_1009	DJI_0408	Pinus sylvestris L.	0	n	1	1	Pinaceae Spreng. ex F.Rudolphi

Iz_4_1009	DJI_0408	Poa annua L.	0	n	1	0	Poaceae Barnhart
Iz_4_1009	DJI_0408	Poa nemoralis L.	0	n	1	0	Poaceae Barnhart
Iz_4_1009	DJI_0408	Poa sp.	0		NA	0	Poaceae Barnhart
Iz_4_1009	DJI_0408	Polygonatum odoratum (Mill.) Druce	0	n	1	0	Asparagaceae Juss.
Iz_4_1009	DJI_0408	Polygonum arenarium Waldst. & Kit.	0	n	1	0	Polygonaceae Juss.
Iz_4_1009	DJI_0408	Populus nigra L.	0	n	1	1	Salicaceae Mirb.
Iz_4_1009	DJI_0408	Populus tremula L.	0	n	1	1	Salicaceae Mirb.
Iz_4_1009	DJI_0408	Populus Ã canescens (Aiton) Sm.	0	n	1	1	Salicaceae Mirb.
Iz_4_1009	DJI_0408	Potentilla argentea L.	0	n	1	0	Rosaceae Juss.
Iz_4_1009	DJI_0408	Psammophiliella muralis (L.) Ikonn.	0	n	1	0	Caryophyllaceae Juss.
Iz_4_1009	DJI_0408	Psephellus sumensis (Kalen.) Greuter	0	n	1	0	Asteraceae Bercht. & J.Presl
Iz_4_1009	DJI_0408	Pulsatilla pratensis (L.) Mill. s.l.	0	n	1	0	Ranunculaceae Juss.
Iz_4_1009	DJI_0408	Pyrus communis L.	0	a	0	1	Rosaceae Juss.
Iz_4_1009	DJI_0408	Quercus robur L.	0	n	1	1	Fagaceae Dumort.
Iz_4_1009	DJI_0408	Robinia pseudoacacia L.	0	a	0	1	Fabaceae Lindl.
Iz_4_1009	DJI_0408	Rosa sp.	0		NA	1	Rosaceae Juss.
Iz_4_1009	DJI_0408	Rubus idaeus L.	0	n	1	0	Rosaceae Juss.
Iz_4_1009	DJI_0408	Rumex acetosella L.	0	n	1	0	Polygonaceae Juss.
Iz_4_1009	DJI_0408	Rumex thyrsiflorus Fingerh.	0	n	1	0	Polygonaceae Juss.
Iz_4_1009	DJI_0408	Salix alba L.	0	n	1	1	Salicaceae Mirb.
Iz_4_1009	DJI_0408	Sambucus nigra L.	0	n	1	1	Viburnaceae Raf.
Iz_4_1009	DJI_0408	Secale sylvestre Host	0	n	1	0	Poaceae Barnhart
Iz_4_1009	DJI_0408	Sempervivum ruthenicum Schnittsp. & C.B. Lehm.	0	n	1	0	Crassulaceae J.St.-Hil.
Iz_4_1009	DJI_0408	Setaria viridis (L.) P. Beauv.	0	n	1	0	Poaceae Barnhart
Iz_4_1009	DJI_0408	Silene borysthenica (Gruner) Walters	0	n	1	0	Caryophyllaceae Juss.
Iz_4_1009	DJI_0408	Silene latifolia subsp. alba (Mill.) Greuter & Burdet	0	n	1	0	Caryophyllaceae Juss.
Iz_4_1009	DJI_0408	Solidago virgaurea L.	2	n	1	0	Asteraceae Bercht. & J.Presl
Iz_4_1009	DJI_0408	Sorbus aucuparia L.	0	n	1	1	Rosaceae Juss.
Iz_4_1009	DJI_0408	Stachys recta L.	0	n	1	0	Lamiacea Lindl.
Iz_4_1009	DJI_0408	Tanacetum vulgare L.	0	n	1	0	Asteraceae Bercht. & J.Presl
Iz_4_1009	DJI_0408	Thymus pallasianus Heinr.Braun	0	n	1	0	Lamiacea Lindl.
Iz_4_1009	DJI_0408	Tilia cordata Mill.	0	n	1	1	Malvaceae Juss.
Iz_4_1009	DJI_0408	Trifolium alpestre L.	0	n	1	0	Fabaceae Lindl.
Iz_4_1009	DJI_0408	Trifolium arvense L.	0	n	1	0	Fabaceae Lindl.
Iz_4_1009	DJI_0408	Ulmus glabra Huds.	0	n	1	1	Ulmaceae Mirb.
Iz_4_1009	DJI_0408	Verbascum densiflorum Bertol.	0	n	1	0	Scrophulariaceae Juss.
Iz_4_1009	DJI_0408	Verbascum nigrum L.	0	n	1	0	Scrophulariaceae Juss.
Iz_4_1009	DJI_0408	Veronica austriaca L.	0	n	1	0	Plantaginaceae Juss.
Iz_4_1009	DJI_0408	Veronica incana L.	0	n	1	0	Plantaginaceae Juss.
Iz_4_1009	DJI_0408	Veronica verna L.	0	n	1	0	Plantaginaceae Juss.
Iz_4_1009	DJI_0408	Vincetoxicum hirundinaria Medik	0	n	1	0	Apocynaceae Juss.
Iz_4_1009	DJI_0411	Acer tataricum L.	0	n	1	1	Sapindaceae Juss.
Iz_4_1009	DJI_0411	Achillea millefolium L. s.l.	0	n	1	0	Asteraceae Bercht. & J.Presl
Iz_4_1009	DJI_0411	Allium oleraceum L.	0	n	1	0	Amaryllidaceae J.St.-Hil.
Iz_4_1009	DJI_0411	Ambrosia artemisiifolia L.	0	a	0	0	Asteraceae Bercht. & J.Presl
Iz_4_1009	DJI_0411	Amorpha fruticosa L.	0	a	0	0	Fabaceae Lindl.
Iz_4_1009	DJI_0411	Anthericum ramosum L.	0	n	1	0	Asparagaceae Juss.
Iz_4_1009	DJI_0411	Artemisia marschalliana Spreng.	0	n	1	0	Asteraceae Bercht. & J.Presl
Iz_4_1009	DJI_0411	Asparagus officinalis L.	0	n	1	0	Asparagaceae Juss.
Iz_4_1009	DJI_0411	Astragalus cicer L.	0	n	1	0	Fabaceae Lindl.
Iz_4_1009	DJI_0411	Bassia laniflora (S.G.Gmel.) A.J.Scott	0	n	1	0	Amaranthaceae Juss.
Iz_4_1009	DJI_0411	Berberis vulgaris L.	0	n	1	0	Berberidaceae Juss.
Iz_4_1009	DJI_0411	Betula pendula Roth.	0	n	1	1	Betulaceae Gray
Iz_4_1009	DJI_0411	Betula pendula Roth.	0	n	1	0	Poaceae Barnhart
Iz_4_1009	DJI_0411	Bromus inermis Leyss.	0		NA	NA	
Iz_4_1009	DJI_0411	Bromus squarrosus L.	0	a	0	0	Poaceae Barnhart
Iz_4_1009	DJI_0411	Bromus tectorum L.	0	a	0	0	Poaceae Barnhart
Iz_4_1009	DJI_0411	Calamagrostis epigejos (L.) Roth	40	n	1	0	Poaceae Barnhart
Iz_4_1009	DJI_0411	Carduus acanthoides L.	2	n	1	0	Asteraceae Bercht. & J.Presl
Iz_4_1009	DJI_0411	Carex sp.	0	n	1	0	Cyperaceae Juss.
Iz_4_1009	DJI_0411	Centaurea majorovii Dumbadze	0	n	1	0	Asteraceae Bercht. & J.Presl
Iz_4_1009	DJI_0411	Chamaecytisus ruthenicus (Fisch.ex Wol.) Klaskova	0	n	1	0	Fabaceae Lindl.
Iz_4_1009	DJI_0411	Chelidonium majus L.	0	n	1	0	Papaveraceae Juss.
Iz_4_1009	DJI_0411	Chenopodium betaceum Andrz.	0	n	1	0	Amaranthaceae Juss.
Iz_4_1009	DJI_0411	Chenopodium cf. album	0	n	1	0	Amaranthaceae Juss.
Iz_4_1009	DJI_0411	Chenopodium sp.	0		NA	0	Amaranthaceae Juss.
Iz_4_1009	DJI_0411	Chondrilla juncea L.	0.5	n	1	0	Asteraceae Bercht. & J.Presl
Iz_4_1009	DJI_0411	Cirsium vulgare (Savi) Ten.	0	n	1	0	Asteraceae Bercht. & J.Presl
Iz_4_1009	DJI_0411	Clinopodium acinos (L.) Kuntze	0	n	1	0	Lamiacea Lindl.
Iz_4_1009	DJI_0411	Convallaria majalis L.	0	n	1	0	Asparagaceae Juss.
Iz_4_1009	DJI_0411	Crataegus rhipidophylla Gand.	0	n	1	1	Rosaceae Juss.
Iz_4_1009	DJI_0411	Crepis foetida L.	0	n	1	0	Asteraceae Bercht. & J.Presl
Iz_4_1009	DJI_0411	Dactylis glomerata L.	0	n	1	0	Poaceae Barnhart
Iz_4_1009	DJI_0411	Dianthus campestris M.Bieb.	0	n	1	0	Caryophyllaceae Juss.
Iz_4_1009	DJI_0411	Digitaria sanguinalis (L.) Scop.	0	a	0	0	Poaceae Barnhart
Iz_4_1009	DJI_0411	Elymus repens (L.) Gould	0	n	1	0	Poaceae Barnhart
Iz_4_1009	DJI_0411	Epilobium tetragonum L.	0	n	1	0	Onagraceae Juss.
Iz_4_1009	DJI_0411	Eragrostis minor Host	0	a	0	0	Poaceae Barnhart
Iz_4_1009	DJI_0411	Erigeron canadensis L.	5	a	0	0	Asteraceae Bercht. & J.Presl
Iz_4_1009	DJI_0411	Euphorbia seguieriana Neck.	0	n	1	0	Euphorbiaceae Juss.
Iz_4_1009	DJI_0411	Fallopia dumetorum (L.) Holub	0	n	1	0	Polygonaceae Juss.
Iz_4_1009	DJI_0411	Festuca beckeri (Hack.) Trautv.	0	n	1	0	Poaceae Barnhart
Iz_4_1009	DJI_0411	Fragaria vesca L.	0	n	1	0	Rosaceae Juss.
Iz_4_1009	DJI_0411	Frangula alnus Mill.	0	n	1	1	Rhamnaceae Juss.
Iz_4_1009	DJI_0411	Fraxinus pennsylvanica Marshall	0	a	0	1	Oleaceae Hoffmanns. & Link
Iz_4_1009	DJI_0411	Galeopsis bifida Boenn.	0	n	1	0	Lamiacea Lindl.
Iz_4_1009	DJI_0411	Galium verum L.	0	n	1	0	Rubiaceae Juss.
Iz_4_1009	DJI_0411	Genista tinctoria L.	0	n	1	0	Fabaceae Lindl.
Iz_4_1009	DJI_0411	Geranium robertianum L.	0	n	1	0	Geraniaceae Juss.
Iz_4_1009	DJI_0411	Geranium sanquineum L.	0	n	1	0	Geraniaceae Juss.
Iz_4_1009	DJI_0411	Gypsophila paniculata L.	0	n	1	0	Caryophyllaceae Juss.
Iz_4_1009	DJI_0411	Helichrysum arenarium (L.) Moench	0	n	1	0	Asteraceae Bercht. & J.Presl
Iz_4_1009	DJI_0411	Herniaria polygama J.Gay	0	n	1	0	Caryophyllaceae Juss.
Iz_4_1009	DJI_0411	Hieracium robustum Fr.	0	n	1	0	Asteraceae Bercht. & J.Presl
Iz_4_1009	DJI_0411	Hieracium umbellatum L.	0	n	1	NA	Asteraceae Bercht. & J.Presl
Iz_4_1009	DJI_0411	Hylotelephium maximum (L.) Holub	0	n	1	0	Crassulaceae J.St.-Hil.
Iz_4_1009	DJI_0411	Jacobaea borysthenica (DC.) B.Nord. & Greuter	0	n	1	0	Asteraceae Bercht. & J.Presl
Iz_4_1009	DJI_0411	Jasione montana L.	0	n	1	0	Campanulaceae Juss.

Iz_4_1009	DJI_0411	Jurinea cyanoides (L.) Rchb.s.l.	0	n	1	0	Asteraceae Bercht. & J.Presl
Iz_4_1009	DJI_0411	Koeleria glauca (Spreng.) DC.	0	n	1	0	Poaceae Barnhart
Iz_4_1009	DJI_0411	Lactuca serriola L.	0	a	0	0	Asteraceae Bercht. & J.Presl
Iz_4_1009	DJI_0411	Linaria genistifolia (L.) Mill.	0	n	1	0	Plantaginaceae Juss.
Iz_4_1009	DJI_0411	Lomelosia argentea (L.) Greuter & Burdet	0	n	1	0	Caprifoliaceae Juss.
Iz_4_1009	DJI_0411	Melampyrum pratense L.	0	n	1	0	Orobanchaceae Vent.
Iz_4_1009	DJI_0411	Oenothera biennis L.	0	a	0	0	Onagraceae Juss.
Iz_4_1009	DJI_0411	Pentanema britannica (L.) D.Gut.Larr., Santos-Vicente, Anderb., E.Rico & M.M.Mart.Ort.	0	n	1	0	Asteraceae Bercht. & J.Presl
Iz_4_1009	DJI_0411	Peucedanum oreoselinum (L.) Moench	0	n	1	0	Apiaceae Lindl.
Iz_4_1009	DJI_0411	Peucedanum ruthenicum M.Bieb.	0	n	1	0	Apiaceae Lindl.
Iz_4_1009	DJI_0411	Pilosella echioides (Lumn.) F.W.Schultz & Sch.Bip.	0	n	1	0	Asteraceae Bercht. & J.Presl
Iz_4_1009	DJI_0411	Pilosella officinarum F. Schultz et Sch. Bip.	0	n	1	0	Asteraceae Bercht. & J.Presl
Iz_4_1009	DJI_0411	Pilosella sp.	0		NA	0	Asteraceae Bercht. & J.Presl
Iz_4_1009	DJI_0411	Pinus sylvestris L.	0	n	1	1	Pinaceae Spreng. ex F.Rudolphi
Iz_4_1009	DJI_0411	Poa annua L.	0	n	1	0	Poaceae Barnhart
Iz_4_1009	DJI_0411	Poa nemoralis L.	0	n	1	0	Poaceae Barnhart
Iz_4_1009	DJI_0411	Poa sp.	0		NA	0	Poaceae Barnhart
Iz_4_1009	DJI_0411	Polygonatum odoratum (Mill.) Druce	0	n	1	0	Asparagaceae Juss.
Iz_4_1009	DJI_0411	Polygonum arenarium Waldst. & Kit.	0	n	1	0	Polygonaceae Juss.
Iz_4_1009	DJI_0411	Populus nigra L.	0	n	1	1	Salicaceae Mirb.
Iz_4_1009	DJI_0411	Populus tremula L.	0	n	1	1	Salicaceae Mirb.
Iz_4_1009	DJI_0411	Populus Ã canescens (Aiton) Sm.	0	n	1	1	Salicaceae Mirb.
Iz_4_1009	DJI_0411	Potentilla argentea L.	0	n	1	0	Rosaceae Juss.
Iz_4_1009	DJI_0411	Psammophiliella muralis (L.) Ikonn.	0	n	1	0	Caryophyllaceae Juss.
Iz_4_1009	DJI_0411	Psephellus sumensis (Kalen.) Greuter	0	n	1	0	Asteraceae Bercht. & J.Presl
Iz_4_1009	DJI_0411	Pulsatilla pratensis (L.) Mill. s.l.	0	n	1	0	Ranunculaceae Juss.
Iz_4_1009	DJI_0411	Pyrus communis L.	0	a	0	1	Rosaceae Juss.
Iz_4_1009	DJI_0411	Quercus robur L.	0	n	1	1	Fagaceae Dumort.
Iz_4_1009	DJI_0411	Robinia pseudoacacia L.	0	a	0	1	Fabaceae Lindl.
Iz_4_1009	DJI_0411	Rosa sp.	0		NA	1	Rosaceae Juss.
Iz_4_1009	DJI_0411	Rubus idaeus L.	0	n	1	0	Rosaceae Juss.
Iz_4_1009	DJI_0411	Rumex acetosella L.	0	n	1	0	Polygonaceae Juss.
Iz_4_1009	DJI_0411	Rumex thyrsiflorus Fingerh.	0	n	1	0	Polygonaceae Juss.
Iz_4_1009	DJI_0411	Salix alba L.	0	n	1	1	Salicaceae Mirb.
Iz_4_1009	DJI_0411	Sambucus nigra L.	0	n	1	1	Viburnaceae Raf.
Iz_4_1009	DJI_0411	Secale sylvestre Host	0	n	1	0	Poaceae Barnhart
Iz_4_1009	DJI_0411	Sempervivum ruthenicum Schnittsp. & C.B. Lehm.	0	n	1	0	Crassulaceae J.St.-Hil.
Iz_4_1009	DJI_0411	Setaria viridis (L.) P. Beauv.	0	n	1	0	Poaceae Barnhart
Iz_4_1009	DJI_0411	Silene borysthenica (Gruner) Walters	0	n	1	0	Caryophyllaceae Juss.
Iz_4_1009	DJI_0411	Silene latifolia subsp. alba (Mill.) Greuter & Burdet	0	n	1	0	Caryophyllaceae Juss.
Iz_4_1009	DJI_0411	Solidago virgaurea L.	0	n	1	0	Asteraceae Bercht. & J.Presl
Iz_4_1009	DJI_0411	Sorbus aucuparia L.	0	n	1	1	Rosaceae Juss.
Iz_4_1009	DJI_0411	Stachys recta L.	0	n	1	0	Lamiacea Lindl.
Iz_4_1009	DJI_0411	Tanacetum vulgare L.	0	n	1	0	Asteraceae Bercht. & J.Presl
Iz_4_1009	DJI_0411	Thymus pallasianus Heinr.Braun	0	n	1	0	Lamiacea Lindl.
Iz_4_1009	DJI_0411	Tilia cordata Mill.	0	n	1	1	Malvaceae Juss.
Iz_4_1009	DJI_0411	Trifolium alpestre L.	0	n	1	0	Fabaceae Lindl.
Iz_4_1009	DJI_0411	Trifolium arvense L.	0	n	1	0	Fabaceae Lindl.
Iz_4_1009	DJI_0411	Ulmus glabra Huds.	0	n	1	1	Ulmaceae Mirb.
Iz_4_1009	DJI_0411	Verbascum densiflorum Bertol.	0	n	1	0	Scrophulariaceae Juss.
Iz_4_1009	DJI_0411	Verbascum nigrum L.	0	n	1	0	Scrophulariaceae Juss.
Iz_4_1009	DJI_0411	Veronica austriaca L.	0	n	1	0	Plantaginaceae Juss.
Iz_4_1009	DJI_0411	Veronica incana L.	0	n	1	0	Plantaginaceae Juss.
Iz_4_1009	DJI_0411	Veronica verna L.	0	n	1	0	Plantaginaceae Juss.
Iz_4_1009	DJI_0411	Vincetoxicum hirundinaria Medik	0	n	1	0	Apocynaceae Juss.
Iz_4_1109	DJI_0011	Acer tataricum L.	0	n	1	1	Sapindaceae Juss.
Iz_4_1109	DJI_0011	Achillea millefolium L. s.l.	0	n	1	0	Asteraceae Bercht. & J.Presl
Iz_4_1109	DJI_0011	Allium oleraceum L.	0	n	1	0	Amaryllidaceae J.St.-Hil.
Iz_4_1109	DJI_0011	Ambrosia artemisiifolia L.	0	a	0	0	Asteraceae Bercht. & J.Presl
Iz_4_1109	DJI_0011	Amorpha fruticosa L.	0	a	0	0	Fabaceae Lindl.
Iz_4_1109	DJI_0011	Anthericum ramosum L.	0	n	1	0	Asparagaceae Juss.
Iz_4_1109	DJI_0011	Artemisia marschalliana Spreng.	0	n	1	0	Asteraceae Bercht. & J.Presl
Iz_4_1109	DJI_0011	Asparagus officinalis L.	0	n	1	0	Asparagaceae Juss.
Iz_4_1109	DJI_0011	Asteraceae sp.	0	n	1	0	Asteraceae Bercht. & J.Presl
Iz_4_1109	DJI_0011	Astragalus cicer L.	0	n	1	0	Fabaceae Lindl.
Iz_4_1109	DJI_0011	Atriplex sp.	0		NA	NA	
Iz_4_1109	DJI_0011	Bassia laniflora (S.G.Gmel.) A.J.Scott	0	n	1	0	Amaranthaceae Juss.
Iz_4_1109	DJI_0011	Berberis vulgaris L.	0	n	1	0	Berberidaceae Juss.
Iz_4_1109	DJI_0011	Betula pendula Roth.	0	n	1	1	Betulaceae Gray
Iz_4_1109	DJI_0011	Betula pendula Roth.	0	n	1	0	Poaceae Barnhart
Iz_4_1109	DJI_0011	Bromus inermis Leyss.	0		NA	NA	
Iz_4_1109	DJI_0011	Bromus squarrosus L.	0	a	0	0	Poaceae Barnhart
Iz_4_1109	DJI_0011	Bromus tectorum L.	0	a	0	0	Poaceae Barnhart
Iz_4_1109	DJI_0011	Calamagrostis epigejos (L.) Roth	15	n	1	0	Poaceae Barnhart
Iz_4_1109	DJI_0011	Carduus acanthoides L.	0	n	1	0	Asteraceae Bercht. & J.Presl
Iz_4_1109	DJI_0011	Carex sp.	0	n	1	0	Cyperaceae Juss.
Iz_4_1109	DJI_0011	Centaurea majorovii Dumbadze	0	n	1	0	Asteraceae Bercht. & J.Presl
Iz_4_1109	DJI_0011	Chamaecytisus ruthenicus (Fisch.ex Wol.) Klaskova	5	n	1	0	Fabaceae Lindl.
Iz_4_1109	DJI_0011	Chelidonium majus L.	0	n	1	0	Papaveraceae Juss.
Iz_4_1109	DJI_0011	Chenopodium betaceum Andrz.	0	n	1	0	Amaranthaceae Juss.
Iz_4_1109	DJI_0011	Chenopodium cf. album	0	n	1	0	Amaranthaceae Juss.
Iz_4_1109	DJI_0011	Chenopodium sp.	0		NA	0	Amaranthaceae Juss.
Iz_4_1109	DJI_0011	Chondrilla juncea L.	0	n	1	0	Asteraceae Bercht. & J.Presl
Iz_4_1109	DJI_0011	Cirsium vulgare (Savi) Ten.	0	n	1	0	Asteraceae Bercht. & J.Presl
Iz_4_1109	DJI_0011	Clinopodium acinos (L.) Kuntze	0	n	1	0	Lamiacea Lindl.
Iz_4_1109	DJI_0011	Convallaria majalis L.	0	n	1	0	Asparagaceae Juss.
Iz_4_1109	DJI_0011	Crataegus rhipidophylla Gand.	0	n	1	1	Rosaceae Juss.
Iz_4_1109	DJI_0011	Crepis foetida L.	0	n	1	0	Asteraceae Bercht. & J.Presl
Iz_4_1109	DJI_0011	Dactylis glomerata L.	0	n	1	0	Poaceae Barnhart
Iz_4_1109	DJI_0011	Dianthus campestris M.Bieb.	0	n	1	0	Caryophyllaceae Juss.
Iz_4_1109	DJI_0011	Digitaria sanguinalis (L.) Scop.	0	a	0	0	Poaceae Barnhart
Iz_4_1109	DJI_0011	Elymus repens (L.) Gould	0	n	1	0	Poaceae Barnhart
Iz_4_1109	DJI_0011	Epilobium tetragonum L.	0	n	1	0	Onagraceae Juss.
Iz_4_1109	DJI_0011	Eragrostis minor Host	0	a	0	0	Poaceae Barnhart
Iz_4_1109	DJI_0011	Erigeron annuus (L.) Desf.	0	a	0	0	Asteraceae Bercht. & J.Presl
Iz_4_1109	DJI_0011	Erigeron canadensis L.	3	a	0	0	Asteraceae Bercht. & J.Presl
Iz_4_1109	DJI_0011	Euphorbia seguieriana Neck.	0	n	1	0	Euphorbiaceae Juss.
Iz_4_1109	DJI_0011	Fallopia dumetorum (L.) Holub	0	n	1	0	Polygonaceae Juss.

Iz_4_1109	DJI_0011	Festuca beckeri (Hack.) Trautv.	0	n	1	0	Poaceae Barnhart
Iz_4_1109	DJI_0011	Fragaria vesca L.	0	n	1	0	Rosaceae Juss.
Iz_4_1109	DJI_0011	Frangula alnus Mill.	0	n	1	1	Rhamnaceae Juss.
Iz_4_1109	DJI_0011	Fraxinus pennsylvanica Marshall	0	a	0	1	Oleaceae Hoffmanns. & Link
Iz_4_1109	DJI_0011	Galeopsis bifida Boenn.	0	n	1	0	Lamiacea Lindl.
Iz_4_1109	DJI_0011	Galium verum L.	0	n	1	0	Rubiaceae Juss.
Iz_4_1109	DJI_0011	Genista tinctoria L.	0	n	1	0	Fabaceae Lindl.
Iz_4_1109	DJI_0011	Geranium robertianum L.	0	n	1	0	Geraniaceae Juss.
Iz_4_1109	DJI_0011	Geranium sanquineum L.	0	n	1	0	Geraniaceae Juss.
Iz_4_1109	DJI_0011	Gypsophila paniculata L.	0	n	1	0	Caryophyllaceae Juss.
Iz_4_1109	DJI_0011	Helichrysum arenarium (L.) Moench	0	n	1	0	Asteraceae Bercht. & J.Presl
Iz_4_1109	DJI_0011	Herniaria polygama J.Gay	0	n	1	0	Caryophyllaceae Juss.
Iz_4_1109	DJI_0011	Hieracium robustum Fr.	0	n	1	0	Asteraceae Bercht. & J.Presl
Iz_4_1109	DJI_0011	Hieracium umbellatum L.	0	n	1	NA	Asteraceae Bercht. & J.Presl
Iz_4_1109	DJI_0011	Hylotelephium maximum (L.) Holub	0	n	1	0	Crassulaceae J.St.-Hil.
Iz_4_1109	DJI_0011	Jacobaea borysthenica (DC.) B.Nord. & Greuter	0	n	1	0	Asteraceae Bercht. & J.Presl
Iz_4_1109	DJI_0011	Jasione montana L.	0	n	1	0	Campanulaceae Juss.
Iz_4_1109	DJI_0011	Jurinea cyanoides (L.) Rchb.s.l.	0	n	1	0	Asteraceae Bercht. & J.Presl
Iz_4_1109	DJI_0011	Koeleria glauca (Spreng.) DC.	0	n	1	0	Poaceae Barnhart
Iz_4_1109	DJI_0011	Lactuca serriola L.	0	a	0	0	Asteraceae Bercht. & J.Presl
Iz_4_1109	DJI_0011	Linaria genistifolia (L.) Mill.	0	n	1	0	Plantaginaceae Juss.
Iz_4_1109	DJI_0011	Lomelosia argentea (L.) Greuter & Burdet	0	n	1	0	Caprifoliaceae Juss.
Iz_4_1109	DJI_0011	Melampyrum pratense L.	0	n	1	0	Orobanchaceae Vent.
Iz_4_1109	DJI_0011	Oenothera biennis L.	0	a	0	0	Onagraceae Juss.
Iz_4_1109	DJI_0011	Pentanema britannica (L.) D.Gut.Larr., Santos-Vicente, Anderb., E.Rico & M.M.Mart.Ort.	0	n	1	0	Asteraceae Bercht. & J.Presl
Iz_4_1109	DJI_0011	Peucedanum oreoselinum (L.) Moench	0	n	1	0	Apiaceae Lindl.
Iz_4_1109	DJI_0011	Peucedanum ruthenicum M.Bieb.	0	n	1	0	Apiaceae Lindl.
Iz_4_1109	DJI_0011	Pilosella echioides (Lumn.) F.W.Schultz & Sch.Bip.	0	n	1	0	Asteraceae Bercht. & J.Presl
Iz_4_1109	DJI_0011	Pilosella officinarum F. Schultz et Sch. Bip.	0	n	1	0	Asteraceae Bercht. & J.Presl
Iz_4_1109	DJI_0011	Pilosella sp.	0		NA	0	Asteraceae Bercht. & J.Presl
Iz_4_1109	DJI_0011	Pinus sylvestris L.	0	n	1	1	Pinaceae Spreng. ex F.Rudolphi
Iz_4_1109	DJI_0011	Poa annua L.	0	n	1	0	Poaceae Barnhart
Iz_4_1109	DJI_0011	Poa nemoralis L.	0	n	1	0	Poaceae Barnhart
Iz_4_1109	DJI_0011	Poa sp.	0		NA	0	Poaceae Barnhart
Iz_4_1109	DJI_0011	Polygonatum odoratum (Mill.) Druce	0	n	1	0	Asparagaceae Juss.
Iz_4_1109	DJI_0011	Polygonum arenarium Waldst. & Kit.	0	n	1	0	Polygonaceae Juss.
Iz_4_1109	DJI_0011	Populus nigra L.	0	n	1	1	Salicaceae Mirb.
Iz_4_1109	DJI_0011	Populus tremula L.	0	n	1	1	Salicaceae Mirb.
Iz_4_1109	DJI_0011	Populus Ã canescens (Aiton) Sm.	0	n	1	1	Salicaceae Mirb.
Iz_4_1109	DJI_0011	Potentilla argentea L.	0	n	1	0	Rosaceae Juss.
Iz_4_1109	DJI_0011	Psammophiliella muralis (L.) Ikonn.	0	n	1	0	Caryophyllaceae Juss.
Iz_4_1109	DJI_0011	Psephellus sumensis (Kalen.) Greuter	0	n	1	0	Asteraceae Bercht. & J.Presl
Iz_4_1109	DJI_0011	Pulsatilla pratensis (L.) Mill. s.l.	0	n	1	0	Ranunculaceae Juss.
Iz_4_1109	DJI_0011	Pyrus communis L.	0	a	0	1	Rosaceae Juss.
Iz_4_1109	DJI_0011	Quercus robur L.	0	n	1	1	Fagaceae Dumort.
Iz_4_1109	DJI_0011	Robinia pseudoacacia L.	0	a	0	1	Fabaceae Lindl.
Iz_4_1109	DJI_0011	Rosa sp.	0		NA	1	Rosaceae Juss.
Iz_4_1109	DJI_0011	Rubus idaeus L.	0	n	1	0	Rosaceae Juss.
Iz_4_1109	DJI_0011	Rumex acetosella L.	0	n	1	0	Polygonaceae Juss.
Iz_4_1109	DJI_0011	Rumex thyrsiflorus Fingerh.	0	n	1	0	Polygonaceae Juss.
Iz_4_1109	DJI_0011	Salix alba L.	0	n	1	1	Salicaceae Mirb.
Iz_4_1109	DJI_0011	Sambucus nigra L.	0	n	1	1	Viburnaceae Raf.
Iz_4_1109	DJI_0011	Secale sylvestre Host	0	n	1	0	Poaceae Barnhart
Iz_4_1109	DJI_0011	Sempervivum ruthenicum Schnittsp. & C.B. Lehm.	0	n	1	0	Crassulaceae J.St.-Hil.
Iz_4_1109	DJI_0011	Setaria viridis (L.) P. Beauv.	0	n	1	0	Poaceae Barnhart
Iz_4_1109	DJI_0011	Silene borysthenica (Gruner) Walters	0	n	1	0	Caryophyllaceae Juss.
Iz_4_1109	DJI_0011	Silene latifolia subsp. alba (Mill.) Greuter & Burdet	0	n	1	0	Caryophyllaceae Juss.
Iz_4_1109	DJI_0011	Solidago virgaurea L.	0	n	1	0	Asteraceae Bercht. & J.Presl
Iz_4_1109	DJI_0011	Sorbus aucuparia L.	0	n	1	1	Rosaceae Juss.
Iz_4_1109	DJI_0011	Stachys recta L.	0	n	1	0	Lamiacea Lindl.
Iz_4_1109	DJI_0011	Tanacetum vulgare L.	0	n	1	0	Asteraceae Bercht. & J.Presl
Iz_4_1109	DJI_0011	Thymus pallasianus Heinr.Braun	0	n	1	0	Lamiacea Lindl.
Iz_4_1109	DJI_0011	Tilia cordata Mill.	0	n	1	1	Malvaceae Juss.
Iz_4_1109	DJI_0011	Trifolium alpestre L.	0	n	1	0	Fabaceae Lindl.
Iz_4_1109	DJI_0011	Trifolium arvense L.	0	n	1	0	Fabaceae Lindl.
Iz_4_1109	DJI_0011	Ulmus glabra Huds.	0	n	1	1	Ulmaceae Mirb.
Iz_4_1109	DJI_0011	Verbascum densiflorum Bertol.	0	n	1	0	Scrophulariaceae Juss.
Iz_4_1109	DJI_0011	Veronica austriaca L.	0	n	1	0	Plantaginaceae Juss.
Iz_4_1109	DJI_0011	Veronica incana L.	0	n	1	0	Plantaginaceae Juss.
Iz_4_1109	DJI_0011	Veronica verna L.	0	n	1	0	Plantaginaceae Juss.
Iz_4_1109	DJI_0011	Vincetoxicum hirundinaria Medik	0	n	1	0	Apocynaceae Juss.
Iz_4_1109	DJI_0012	Acer tataricum L.	0	n	1	1	Sapindaceae Juss.
Iz_4_1109	DJI_0012	Achillea millefolium L. s.l.	0	n	1	0	Asteraceae Bercht. & J.Presl
Iz_4_1109	DJI_0012	Allium oleraceum L.	0	n	1	0	Amaryllidaceae J.St.-Hil.
Iz_4_1109	DJI_0012	Ambrosia artemisiifolia L.	0	a	0	0	Asteraceae Bercht. & J.Presl
Iz_4_1109	DJI_0012	Amorpha fruticosa L.	0	a	0	0	Fabaceae Lindl.
Iz_4_1109	DJI_0012	Anthericum ramosum L.	0	n	1	0	Asparagaceae Juss.
Iz_4_1109	DJI_0012	Artemisia marschalliana Spreng.	0	n	1	0	Asteraceae Bercht. & J.Presl
Iz_4_1109	DJI_0012	Asparagus officinalis L.	0	n	1	0	Asparagaceae Juss.
Iz_4_1109	DJI_0012	Asteraceae sp.	0	n	1	0	Asteraceae Bercht. & J.Presl
Iz_4_1109	DJI_0012	Astragalus cicer L.	0	n	1	0	Fabaceae Lindl.
Iz_4_1109	DJI_0012	Atriplex sp.	0		NA	NA	
Iz_4_1109	DJI_0012	Bassia laniflora (S.G.Gmel.) A.J.Scott	0	n	1	0	Amaranthaceae Juss.
Iz_4_1109	DJI_0012	Berberis vulgaris L.	0	n	1	0	Berberidaceae Juss.
Iz_4_1109	DJI_0012	Betula pendula Roth.	0	n	1	1	Betulaceae Gray
Iz_4_1109	DJI_0012	Betula pendula Roth.	0	n	1	0	Poaceae Barnhart
Iz_4_1109	DJI_0012	Bromus inermis Leyss.	0		NA	NA	
Iz_4_1109	DJI_0012	Bromus squarrosus L.	0	a	0	0	Poaceae Barnhart
Iz_4_1109	DJI_0012	Bromus tectorum L.	0	a	0	0	Poaceae Barnhart
Iz_4_1109	DJI_0012	Calamagrostis epigejos (L.) Roth	2	n	1	0	Poaceae Barnhart
Iz_4_1109	DJI_0012	Carduus acanthoides L.	0	n	1	0	Asteraceae Bercht. & J.Presl
Iz_4_1109	DJI_0012	Carex sp.	0	n	1	0	Cyperaceae Juss.
Iz_4_1109	DJI_0012	Centaurea majorovii Dumbadze	0	n	1	0	Asteraceae Bercht. & J.Presl
Iz_4_1109	DJI_0012	Chamaecytisus ruthenicus (Fisch.ex Wol.) Klaskova	0	n	1	0	Fabaceae Lindl.
Iz_4_1109	DJI_0012	Chelidonium majus L.	0	n	1	0	Papaveraceae Juss.
Iz_4_1109	DJI_0012	Chenopodium betaceum Andrz.	0	n	1	0	Amaranthaceae Juss.
Iz_4_1109	DJI_0012	Chenopodium cf. album	0	n	1	0	Amaranthaceae Juss.
Iz_4_1109	DJI_0012	Chenopodium sp.	0		NA	0	Amaranthaceae Juss.

Iz_4_1109	DJI_0012	Chondrilla juncea L.	0	n	1	0	Asteraceae Bercht. & J.Presl
Iz_4_1109	DJI_0012	Cirsium vulgare (Savi) Ten.	0	n	1	0	Asteraceae Bercht. & J.Presl
Iz_4_1109	DJI_0012	Clinopodium acinos (L.) Kuntze	0	n	1	0	Lamiacea Lindl.
Iz_4_1109	DJI_0012	Convallaria majalis L.	0	n	1	0	Asparagaceae Juss.
Iz_4_1109	DJI_0012	Crataegus rhipidophylla Gand.	1	n	1	1	Rosaceae Juss.
Iz_4_1109	DJI_0012	Crepis foetida L.	0	n	1	0	Asteraceae Bercht. & J.Presl
Iz_4_1109	DJI_0012	Dactylis glomerata L.	0	n	1	0	Poaceae Barnhart
Iz_4_1109	DJI_0012	Dianthus campestris M.Bieb.	0	n	1	0	Caryophyllaceae Juss.
Iz_4_1109	DJI_0012	Digitaria sanguinalis (L.) Scop.	0	a	0	0	Poaceae Barnhart
Iz_4_1109	DJI_0012	Elymus repens (L.) Gould	0	n	1	0	Poaceae Barnhart
Iz_4_1109	DJI_0012	Epilobium tetragonum L.	0	n	1	0	Onagraceae Juss.
Iz_4_1109	DJI_0012	Eragrostis minor Host	0	a	0	0	Poaceae Barnhart
Iz_4_1109	DJI_0012	Erigeron annuus (L.) Desf.	0	a	0	0	Asteraceae Bercht. & J.Presl
Iz_4_1109	DJI_0012	Erigeron canadensis L.	7	a	0	0	Asteraceae Bercht. & J.Presl
Iz_4_1109	DJI_0012	Euphorbia seguieriana Neck.	0	n	1	0	Euphorbiaceae Juss.
Iz_4_1109	DJI_0012	Fallopia dumetorum (L.) Holub	0	n	1	0	Polygonaceae Juss.
Iz_4_1109	DJI_0012	Festuca beckeri (Hack.) Trautv.	0	n	1	0	Poaceae Barnhart
Iz_4_1109	DJI_0012	Fragaria vesca L.	0	n	1	0	Rosaceae Juss.
Iz_4_1109	DJI_0012	Frangula alnus Mill.	0	n	1	1	Rhamnaceae Juss.
Iz_4_1109	DJI_0012	Fraxinus pennsylvanica Marshall	0	a	0	1	Oleaceae Hoffmanns. & Link
Iz_4_1109	DJI_0012	Galeopsis bifida Boenn.	0	n	1	0	Lamiacea Lindl.
Iz_4_1109	DJI_0012	Galium verum L.	0	n	1	0	Rubiaceae Juss.
Iz_4_1109	DJI_0012	Genista tinctoria L.	0	n	1	0	Fabaceae Lindl.
Iz_4_1109	DJI_0012	Geranium robertianum L.	0	n	1	0	Geraniaceae Juss.
Iz_4_1109	DJI_0012	Geranium sanquineum L.	0	n	1	0	Geraniaceae Juss.
Iz_4_1109	DJI_0012	Gypsophila paniculata L.	0	n	1	0	Caryophyllaceae Juss.
Iz_4_1109	DJI_0012	Helichrysum arenarium (L.) Moench	0	n	1	0	Asteraceae Bercht. & J.Presl
Iz_4_1109	DJI_0012	Herniaria polygama J.Gay	0	n	1	0	Caryophyllaceae Juss.
Iz_4_1109	DJI_0012	Hieracium robustum Fr.	0	n	1	0	Asteraceae Bercht. & J.Presl
Iz_4_1109	DJI_0012	Hieracium umbellatum L.	0	n	1	NA	Asteraceae Bercht. & J.Presl
Iz_4_1109	DJI_0012	Hylotelephium maximum (L.) Holub	0	n	1	0	Crassulaceae J.St.-Hil.
Iz_4_1109	DJI_0012	Jacobaea borysthenica (DC.) B.Nord. & Greuter	0	n	1	0	Asteraceae Bercht. & J.Presl
Iz_4_1109	DJI_0012	Jasione montana L.	0	n	1	0	Campanulaceae Juss.
Iz_4_1109	DJI_0012	Jurinea cyanoides (L.) Rchb.s.l.	0	n	1	0	Asteraceae Bercht. & J.Presl
Iz_4_1109	DJI_0012	Koeleria glauca (Spreng.) DC.	0	n	1	0	Poaceae Barnhart
Iz_4_1109	DJI_0012	Lactuca serriola L.	0	a	0	0	Asteraceae Bercht. & J.Presl
Iz_4_1109	DJI_0012	Linaria genistifolia (L.) Mill.	0	n	1	0	Plantaginaceae Juss.
Iz_4_1109	DJI_0012	Lomelosia argentea (L.) Greuter & Burdet	0	n	1	0	Caprifoliaceae Juss.
Iz_4_1109	DJI_0012	Melampyrum pratense L.	0	n	1	0	Orobanchaceae Vent.
Iz_4_1109	DJI_0012	Oenothera biennis L.	0	a	0	0	Onagraceae Juss.
Iz_4_1109	DJI_0012	Pentanema britannica (L.) D.Gut.Larr., Santos-Vicente, Anderb., E.Rico & M.M.Mart.Ort.	0	n	1	0	Asteraceae Bercht. & J.Presl
Iz_4_1109	DJI_0012	Peucedanum oreoselinum (L.) Moench	0	n	1	0	Apiaceae Lindl.
Iz_4_1109	DJI_0012	Peucedanum ruthenicum M.Bieb.	0	n	1	0	Apiaceae Lindl.
Iz_4_1109	DJI_0012	Pilosella echioides (Lumn.) F.W.Schultz & Sch.Bip.	0	n	1	0	Asteraceae Bercht. & J.Presl
Iz_4_1109	DJI_0012	Pilosella officinarum F. Schultz et Sch. Bip.	0	n	1	0	Asteraceae Bercht. & J.Presl
Iz_4_1109	DJI_0012	Pilosella sp.	0		NA	0	Asteraceae Bercht. & J.Presl
Iz_4_1109	DJI_0012	Pinus sylvestris L.	0	n	1	1	Pinaceae Spreng. ex F.Rudolphi
Iz_4_1109	DJI_0012	Poa annua L.	0	n	1	0	Poaceae Barnhart
Iz_4_1109	DJI_0012	Poa nemoralis L.	0	n	1	0	Poaceae Barnhart
Iz_4_1109	DJI_0012	Poa sp.	0		NA	0	Poaceae Barnhart
Iz_4_1109	DJI_0012	Polygonatum odoratum (Mill.) Druce	0	n	1	0	Asparagaceae Juss.
Iz_4_1109	DJI_0012	Polygonum arenarium Waldst. & Kit.	0	n	1	0	Polygonaceae Juss.
Iz_4_1109	DJI_0012	Populus nigra L.	0	n	1	1	Salicaceae Mirb.
Iz_4_1109	DJI_0012	Populus tremula L.	0	n	1	1	Salicaceae Mirb.
Iz_4_1109	DJI_0012	Populus Ã canescens (Aiton) Sm.	0	n	1	1	Salicaceae Mirb.
Iz_4_1109	DJI_0012	Potentilla argentea L.	0	n	1	0	Rosaceae Juss.
Iz_4_1109	DJI_0012	Psammophiliella muralis (L.) Ikonn.	0	n	1	0	Caryophyllaceae Juss.
Iz_4_1109	DJI_0012	Psephellus sumensis (Kalen.) Greuter	0	n	1	0	Asteraceae Bercht. & J.Presl
Iz_4_1109	DJI_0012	Pulsatilla pratensis (L.) Mill. s.l.	0	n	1	0	Ranunculaceae Juss.
Iz_4_1109	DJI_0012	Pyrus communis L.	0	a	0	1	Rosaceae Juss.
Iz_4_1109	DJI_0012	Quercus robur L.	0	n	1	1	Fagaceae Dumort.
Iz_4_1109	DJI_0012	Robinia pseudoacacia L.	0	a	0	1	Fabaceae Lindl.
Iz_4_1109	DJI_0012	Rosa sp.	0		NA	1	Rosaceae Juss.
Iz_4_1109	DJI_0012	Rubus idaeus L.	0	n	1	0	Rosaceae Juss.
Iz_4_1109	DJI_0012	Rumex acetosella L.	0	n	1	0	Polygonaceae Juss.
Iz_4_1109	DJI_0012	Rumex thyrsiflorus Fingerh.	0	n	1	0	Polygonaceae Juss.
Iz_4_1109	DJI_0012	Salix alba L.	0	n	1	1	Salicaceae Mirb.
Iz_4_1109	DJI_0012	Sambucus nigra L.	0	n	1	1	Viburnaceae Raf.
Iz_4_1109	DJI_0012	Secale sylvestre Host	0	n	1	0	Poaceae Barnhart
Iz_4_1109	DJI_0012	Sempervivum ruthenicum Schnittsp. & C.B. Lehm.	0	n	1	0	Crassulaceae J.St.-Hil.
Iz_4_1109	DJI_0012	Setaria viridis (L.) P. Beauv.	0	n	1	0	Poaceae Barnhart
Iz_4_1109	DJI_0012	Silene borysthenica (Gruner) Walters	0	n	1	0	Caryophyllaceae Juss.
Iz_4_1109	DJI_0012	Silene latifolia subsp. alba (Mill.) Greuter & Burdet	0	n	1	0	Caryophyllaceae Juss.
Iz_4_1109	DJI_0012	Solidago virgaurea L.	0	n	1	0	Asteraceae Bercht. & J.Presl
Iz_4_1109	DJI_0012	Sorbus aucuparia L.	0	n	1	1	Rosaceae Juss.
Iz_4_1109	DJI_0012	Stachys recta L.	0	n	1	0	Lamiacea Lindl.
Iz_4_1109	DJI_0012	Tanacetum vulgare L.	0	n	1	0	Asteraceae Bercht. & J.Presl
Iz_4_1109	DJI_0012	Thymus pallasianus Heinr.Braun	0	n	1	0	Lamiacea Lindl.
Iz_4_1109	DJI_0012	Tilia cordata Mill.	0	n	1	1	Malvaceae Juss.
Iz_4_1109	DJI_0012	Trifolium alpestre L.	0	n	1	0	Fabaceae Lindl.
Iz_4_1109	DJI_0012	Trifolium arvense L.	0	n	1	0	Fabaceae Lindl.
Iz_4_1109	DJI_0012	Ulmus glabra Huds.	0	n	1	1	Ulmaceae Mirb.
Iz_4_1109	DJI_0012	Verbascum densiflorum Bertol.	0	n	1	0	Scrophulariaceae Juss.
Iz_4_1109	DJI_0012	Veronica austriaca L.	0	n	1	0	Plantaginaceae Juss.
Iz_4_1109	DJI_0012	Veronica incana L.	0	n	1	0	Plantaginaceae Juss.
Iz_4_1109	DJI_0012	Veronica verna L.	0	n	1	0	Plantaginaceae Juss.
Iz_4_1109	DJI_0012	Vincetoxicum hirundinaria Medik	0	n	1	0	Apocynaceae Juss.
Iz_4_1109	DJI_0016	Acer tataricum L.	0	n	1	1	Sapindaceae Juss.
Iz_4_1109	DJI_0016	Achillea millefolium L. s.l.	0	n	1	0	Asteraceae Bercht. & J.Presl
Iz_4_1109	DJI_0016	Allium oleraceum L.	0	n	1	0	Amaryllidaceae J.St.-Hil.
Iz_4_1109	DJI_0016	Ambrosia artemisiifolia L.	0	a	0	0	Asteraceae Bercht. & J.Presl
Iz_4_1109	DJI_0016	Amorpha fruticosa L.	0	a	0	0	Fabaceae Lindl.
Iz_4_1109	DJI_0016	Anthericum ramosum L.	0	n	1	0	Asparagaceae Juss.
Iz_4_1109	DJI_0016	Artemisia marschalliana Spreng.	0	n	1	0	Asteraceae Bercht. & J.Presl
Iz_4_1109	DJI_0016	Asparagus officinalis L.	0	n	1	0	Asparagaceae Juss.
Iz_4_1109	DJI_0016	Asteraceae sp.	0	n	1	0	Asteraceae Bercht. & J.Presl
Iz_4_1109	DJI_0016	Astragalus cicer L.	0	n	1	0	Fabaceae Lindl.
Iz_4_1109	DJI_0016	Atriplex sp.	0.2		NA	NA	

Iz_4_1109	DJI_0016	Bassia laniflora (S.G.Gmel.) A.J.Scott	0	n	1	0	Amaranthaceae Juss.
Iz_4_1109	DJI_0016	Berberis vulgaris L.	0	n	1	0	Berberidaceae Juss.
Iz_4_1109	DJI_0016	Betula pendula Roth.	0	n	1	1	Betulaceae Gray
Iz_4_1109	DJI_0016	Betula pendula Roth.	0	n	1	0	Poaceae Barnhart
Iz_4_1109	DJI_0016	Bromus inermis Leyss.	0		NA	NA	
Iz_4_1109	DJI_0016	Bromus squarrosus L.	0	a	0	0	Poaceae Barnhart
Iz_4_1109	DJI_0016	Bromus tectorum L.	0	a	0	0	Poaceae Barnhart
Iz_4_1109	DJI_0016	Calamagrostis epigejos (L.) Roth	0	n	1	0	Poaceae Barnhart
Iz_4_1109	DJI_0016	Carduus acanthoides L.	0	n	1	0	Asteraceae Bercht. & J.Presl
Iz_4_1109	DJI_0016	Carex sp.	0.2	n	1	0	Cyperaceae Juss.
Iz_4_1109	DJI_0016	Centaurea majorovii Dumbadze	0	n	1	0	Asteraceae Bercht. & J.Presl
Iz_4_1109	DJI_0016	Chamaecytisus ruthenicus (Fisch.ex Wol.) Klaskova	0	n	1	0	Fabaceae Lindl.
Iz_4_1109	DJI_0016	Chelidonium majus L.	0	n	1	0	Papaveraceae Juss.
Iz_4_1109	DJI_0016	Chenopodium betaceum Andrz.	0	n	1	0	Amaranthaceae Juss.
Iz_4_1109	DJI_0016	Chenopodium cf. album	0	n	1	0	Amaranthaceae Juss.
Iz_4_1109	DJI_0016	Chenopodium sp.	0		NA	0	Amaranthaceae Juss.
Iz_4_1109	DJI_0016	Chondrilla juncea L.	0	n	1	0	Asteraceae Bercht. & J.Presl
Iz_4_1109	DJI_0016	Cirsium vulgare (Savi) Ten.	0	n	1	0	Asteraceae Bercht. & J.Presl
Iz_4_1109	DJI_0016	Clinopodium acinos (L.) Kuntze	0	n	1	0	Lamiacea Lindl.
Iz_4_1109	DJI_0016	Convallaria majalis L.	0	n	1	0	Asparagaceae Juss.
Iz_4_1109	DJI_0016	Crataegus rhipidophylla Gand.	0	n	1	1	Rosaceae Juss.
Iz_4_1109	DJI_0016	Crepis foetida L.	0	n	1	0	Asteraceae Bercht. & J.Presl
Iz_4_1109	DJI_0016	Dactylis glomerata L.	0	n	1	0	Poaceae Barnhart
Iz_4_1109	DJI_0016	Dianthus campestris M.Bieb.	0	n	1	0	Caryophyllaceae Juss.
Iz_4_1109	DJI_0016	Digitaria sanguinalis (L.) Scop.	0	a	0	0	Poaceae Barnhart
Iz_4_1109	DJI_0016	Elymus repens (L.) Gould	0	n	1	0	Poaceae Barnhart
Iz_4_1109	DJI_0016	Epilobium tetragonum L.	0	n	1	0	Onagraceae Juss.
Iz_4_1109	DJI_0016	Eragrostis minor Host	0	a	0	0	Poaceae Barnhart
Iz_4_1109	DJI_0016	Erigeron annuus (L.) Desf.	0	a	0	0	Asteraceae Bercht. & J.Presl
Iz_4_1109	DJI_0016	Erigeron canadensis L.	0.2	a	0	0	Asteraceae Bercht. & J.Presl
Iz_4_1109	DJI_0016	Euphorbia seguieriana Neck.	0	n	1	0	Euphorbiaceae Juss.
Iz_4_1109	DJI_0016	Fallopia dumetorum (L.) Holub	0	n	1	0	Polygonaceae Juss.
Iz_4_1109	DJI_0016	Festuca beckeri (Hack.) Trautv.	0	n	1	0	Poaceae Barnhart
Iz_4_1109	DJI_0016	Fragaria vesca L.	0	n	1	0	Rosaceae Juss.
Iz_4_1109	DJI_0016	Frangula alnus Mill.	0	n	1	1	Rhamnaceae Juss.
Iz_4_1109	DJI_0016	Fraxinus pennsylvanica Marshall	0	a	0	1	Oleaceae Hoffmanns. & Link
Iz_4_1109	DJI_0016	Galeopsis bifida Boenn.	0	n	1	0	Lamiacea Lindl.
Iz_4_1109	DJI_0016	Galium verum L.	0	n	1	0	Rubiaceae Juss.
Iz_4_1109	DJI_0016	Genista tinctoria L.	0	n	1	0	Fabaceae Lindl.
Iz_4_1109	DJI_0016	Geranium robertianum L.	0	n	1	0	Geraniaceae Juss.
Iz_4_1109	DJI_0016	Geranium sanquineum L.	0	n	1	0	Geraniaceae Juss.
Iz_4_1109	DJI_0016	Gypsophila paniculata L.	0	n	1	0	Caryophyllaceae Juss.
Iz_4_1109	DJI_0016	Helichrysum arenarium (L.) Moench	0	n	1	0	Asteraceae Bercht. & J.Presl
Iz_4_1109	DJI_0016	Herniaria polygama J.Gay	0	n	1	0	Caryophyllaceae Juss.
Iz_4_1109	DJI_0016	Hieracium robustum Fr.	0	n	1	0	Asteraceae Bercht. & J.Presl
Iz_4_1109	DJI_0016	Hieracium umbellatum L.	0	n	1	NA	Asteraceae Bercht. & J.Presl
Iz_4_1109	DJI_0016	Hylotelephium maximum (L.) Holub	0	n	1	0	Crassulaceae J.St.-Hil.
Iz_4_1109	DJI_0016	Jacobaea borysthenica (DC.) B.Nord. & Greuter	0	n	1	0	Asteraceae Bercht. & J.Presl
Iz_4_1109	DJI_0016	Jasione montana L.	0	n	1	0	Campanulaceae Juss.
Iz_4_1109	DJI_0016	Jurinea cyanoides (L.) Rchb.s.l.	0	n	1	0	Asteraceae Bercht. & J.Presl
Iz_4_1109	DJI_0016	Koeleria glauca (Spreng.) DC.	0	n	1	0	Poaceae Barnhart
Iz_4_1109	DJI_0016	Lactuca serriola L.	0	a	0	0	Asteraceae Bercht. & J.Presl
Iz_4_1109	DJI_0016	Linaria genistifolia (L.) Mill.	0	n	1	0	Plantaginaceae Juss.
Iz_4_1109	DJI_0016	Lomelosia argentea (L.) Greuter & Burdet	0	n	1	0	Caprifoliaceae Juss.
Iz_4_1109	DJI_0016	Melampyrum pratense L.	0	n	1	0	Orobanchaceae Vent.
Iz_4_1109	DJI_0016	Oenothera biennis L.	0	a	0	0	Onagraceae Juss.
Iz_4_1109	DJI_0016	Pentanema britannica (L.) D.Gut.Larr., Santos-Vicente, Anderb., E.Rico & M.M.Mart.Ort.	0	n	1	0	Asteraceae Bercht. & J.Presl
Iz_4_1109	DJI_0016	Peucedanum oreoselinum (L.) Moench	0	n	1	0	Apiaceae Lindl.
Iz_4_1109	DJI_0016	Peucedanum ruthenicum M.Bieb.	0	n	1	0	Apiaceae Lindl.
Iz_4_1109	DJI_0016	Pilosella echioides (Lumn.) F.W.Schultz & Sch.Bip.	0	n	1	0	Asteraceae Bercht. & J.Presl
Iz_4_1109	DJI_0016	Pilosella officinarum F. Schultz et Sch. Bip.	0	n	1	0	Asteraceae Bercht. & J.Presl
Iz_4_1109	DJI_0016	Pilosella sp.	0		NA	0	Asteraceae Bercht. & J.Presl
Iz_4_1109	DJI_0016	Pinus sylvestris L.	0	n	1	1	Pinaceae Spreng. ex F.Rudolphi
Iz_4_1109	DJI_0016	Poa annua L.	0	n	1	0	Poaceae Barnhart
Iz_4_1109	DJI_0016	Poa nemoralis L.	0	n	1	0	Poaceae Barnhart
Iz_4_1109	DJI_0016	Poa sp.	0		NA	0	Poaceae Barnhart
Iz_4_1109	DJI_0016	Polygonatum odoratum (Mill.) Druce	0	n	1	0	Asparagaceae Juss.
Iz_4_1109	DJI_0016	Polygonum arenarium Waldst. & Kit.	0	n	1	0	Polygonaceae Juss.
Iz_4_1109	DJI_0016	Populus nigra L.	0	n	1	1	Salicaceae Mirb.
Iz_4_1109	DJI_0016	Populus tremula L.	0	n	1	1	Salicaceae Mirb.
Iz_4_1109	DJI_0016	Populus Ã canescens (Aiton) Sm.	0	n	1	1	Salicaceae Mirb.
Iz_4_1109	DJI_0016	Potentilla argentea L.	0	n	1	0	Rosaceae Juss.
Iz_4_1109	DJI_0016	Psammophiliella muralis (L.) Ikonn.	0	n	1	0	Caryophyllaceae Juss.
Iz_4_1109	DJI_0016	Psephellus sumensis (Kalen.) Greuter	0	n	1	0	Asteraceae Bercht. & J.Presl
Iz_4_1109	DJI_0016	Pulsatilla pratensis (L.) Mill. s.l.	0	n	1	0	Ranunculaceae Juss.
Iz_4_1109	DJI_0016	Pyrus communis L.	0	a	0	1	Rosaceae Juss.
Iz_4_1109	DJI_0016	Quercus robur L.	0	n	1	1	Fagaceae Dumort.
Iz_4_1109	DJI_0016	Robinia pseudoacacia L.	0	a	0	1	Fabaceae Lindl.
Iz_4_1109	DJI_0016	Rosa sp.	0		NA	1	Rosaceae Juss.
Iz_4_1109	DJI_0016	Rubus idaeus L.	0	n	1	0	Rosaceae Juss.
Iz_4_1109	DJI_0016	Rumex acetosella L.	3	n	1	0	Polygonaceae Juss.
Iz_4_1109	DJI_0016	Rumex thyrsiflorus Fingerh.	0	n	1	0	Polygonaceae Juss.
Iz_4_1109	DJI_0016	Salix alba L.	0	n	1	1	Salicaceae Mirb.
Iz_4_1109	DJI_0016	Sambucus nigra L.	0	n	1	1	Viburnaceae Raf.
Iz_4_1109	DJI_0016	Secale sylvestre Host	0	n	1	0	Poaceae Barnhart
Iz_4_1109	DJI_0016	Sempervivum ruthenicum Schnittsp. & C.B. Lehm.	0	n	1	0	Crassulaceae J.St.-Hil.
Iz_4_1109	DJI_0016	Setaria viridis (L.) P. Beauv.	0	n	1	0	Poaceae Barnhart
Iz_4_1109	DJI_0016	Silene borysthenica (Gruner) Walters	0	n	1	0	Caryophyllaceae Juss.
Iz_4_1109	DJI_0016	Silene latifolia subsp. alba (Mill.) Greuter & Burdet	0	n	1	0	Caryophyllaceae Juss.
Iz_4_1109	DJI_0016	Solidago virgaurea L.	0	n	1	0	Asteraceae Bercht. & J.Presl
Iz_4_1109	DJI_0016	Sorbus aucuparia L.	0	n	1	1	Rosaceae Juss.
Iz_4_1109	DJI_0016	Stachys recta L.	0	n	1	0	Lamiacea Lindl.
Iz_4_1109	DJI_0016	Tanacetum vulgare L.	0	n	1	0	Asteraceae Bercht. & J.Presl
Iz_4_1109	DJI_0016	Thymus pallasianus Heinr.Braun	0	n	1	0	Lamiacea Lindl.
Iz_4_1109	DJI_0016	Tilia cordata Mill.	0	n	1	1	Malvaceae Juss.
Iz_4_1109	DJI_0016	Trifolium alpestre L.	0	n	1	0	Fabaceae Lindl.
Iz_4_1109	DJI_0016	Trifolium arvense L.	0	n	1	0	Fabaceae Lindl.
Iz_4_1109	DJI_0016	Ulmus glabra Huds.	0	n	1	1	Ulmaceae Mirb.

Iz_4_1109	DJI_0016	Verbascum densiflorum Bertol.	0	n	1	0	Scrophulariaceae Juss.
Iz_4_1109	DJI_0016	Veronica austriaca L.	0	n	1	0	Plantaginaceae Juss.
Iz_4_1109	DJI_0016	Veronica incana L.	0	n	1	0	Plantaginaceae Juss.
Iz_4_1109	DJI_0016	Veronica verna L.	0	n	1	0	Plantaginaceae Juss.
Iz_4_1109	DJI_0016	Vincetoxicum hirundinaria Medik	0	n	1	0	Apocynaceae Juss.
Iz_4_1109	DJI_0025	Acer tataricum L.	0	n	1	1	Sapindaceae Juss.
Iz_4_1109	DJI_0025	Achillea millefolium L. s.l.	0	n	1	0	Asteraceae Bercht. & J.Presl
Iz_4_1109	DJI_0025	Allium oleraceum L.	0	n	1	0	Amaryllidaceae J.St.-Hil.
Iz_4_1109	DJI_0025	Ambrosia artemisiifolia L.	0	a	0	0	Asteraceae Bercht. & J.Presl
Iz_4_1109	DJI_0025	Amorpha fruticosa L.	0	a	0	0	Fabaceae Lindl.
Iz_4_1109	DJI_0025	Anthericum ramosum L.	0	n	1	0	Asparagaceae Juss.
Iz_4_1109	DJI_0025	Artemisia marschalliana Spreng.	0	n	1	0	Asteraceae Bercht. & J.Presl
Iz_4_1109	DJI_0025	Asparagus officinalis L.	0	n	1	0	Asparagaceae Juss.
Iz_4_1109	DJI_0025	Asteraceae sp.	0	n	1	0	Asteraceae Bercht. & J.Presl
Iz_4_1109	DJI_0025	Astragalus cicer L.	0	n	1	0	Fabaceae Lindl.
Iz_4_1109	DJI_0025	Atriplex sp.	0		NA	NA	
Iz_4_1109	DJI_0025	Bassia laniflora (S.G.Gmel.) A.J.Scott	0	n	1	0	Amaranthaceae Juss.
Iz_4_1109	DJI_0025	Berberis vulgaris L.	0	n	1	0	Berberidaceae Juss.
Iz_4_1109	DJI_0025	Betula pendula Roth.	0	n	1	1	Betulaceae Gray
Iz_4_1109	DJI_0025	Betula pendula Roth.	0	n	1	0	Poaceae Barnhart
Iz_4_1109	DJI_0025	Bromus inermis Leyss.	0.2		NA	NA	
Iz_4_1109	DJI_0025	Bromus squarrosus L.	0	a	0	0	Poaceae Barnhart
Iz_4_1109	DJI_0025	Bromus tectorum L.	0	a	0	0	Poaceae Barnhart
Iz_4_1109	DJI_0025	Calamagrostis epigejos (L.) Roth	5	n	1	0	Poaceae Barnhart
Iz_4_1109	DJI_0025	Carduus acanthoides L.	0	n	1	0	Asteraceae Bercht. & J.Presl
Iz_4_1109	DJI_0025	Carex sp.	0	n	1	0	Cyperaceae Juss.
Iz_4_1109	DJI_0025	Centaurea majorovii Dumbadze	0	n	1	0	Asteraceae Bercht. & J.Presl
Iz_4_1109	DJI_0025	Chamaecytisus ruthenicus (Fisch.ex Wol.) Klaskova	0	n	1	0	Fabaceae Lindl.
Iz_4_1109	DJI_0025	Chelidonium majus L.	0	n	1	0	Papaveraceae Juss.
Iz_4_1109	DJI_0025	Chenopodium betaceum Andrz.	0	n	1	0	Amaranthaceae Juss.
Iz_4_1109	DJI_0025	Chenopodium cf. album	0	n	1	0	Amaranthaceae Juss.
Iz_4_1109	DJI_0025	Chenopodium sp.	0.2		NA	0	Amaranthaceae Juss.
Iz_4_1109	DJI_0025	Chondrilla juncea L.	0	n	1	0	Asteraceae Bercht. & J.Presl
Iz_4_1109	DJI_0025	Cirsium vulgare (Savi) Ten.	0	n	1	0	Asteraceae Bercht. & J.Presl
Iz_4_1109	DJI_0025	Clinopodium acinos (L.) Kuntze	0	n	1	0	Lamiacea Lindl.
Iz_4_1109	DJI_0025	Convallaria majalis L.	0	n	1	0	Asparagaceae Juss.
Iz_4_1109	DJI_0025	Crataegus rhipidophylla Gand.	0	n	1	1	Rosaceae Juss.
Iz_4_1109	DJI_0025	Crepis foetida L.	0	n	1	0	Asteraceae Bercht. & J.Presl
Iz_4_1109	DJI_0025	Dactylis glomerata L.	0	n	1	0	Poaceae Barnhart
Iz_4_1109	DJI_0025	Dianthus campestris M.Bieb.	0	n	1	0	Caryophyllaceae Juss.
Iz_4_1109	DJI_0025	Digitaria sanguinalis (L.) Scop.	0	a	0	0	Poaceae Barnhart
Iz_4_1109	DJI_0025	Elymus repens (L.) Gould	0	n	1	0	Poaceae Barnhart
Iz_4_1109	DJI_0025	Epilobium tetragonum L.	0	n	1	0	Onagraceae Juss.
Iz_4_1109	DJI_0025	Eragrostis minor Host	0	a	0	0	Poaceae Barnhart
Iz_4_1109	DJI_0025	Erigeron annuus (L.) Desf.	0	a	0	0	Asteraceae Bercht. & J.Presl
Iz_4_1109	DJI_0025	Erigeron canadensis L.	15	a	0	0	Asteraceae Bercht. & J.Presl
Iz_4_1109	DJI_0025	Euphorbia seguieriana Neck.	0	n	1	0	Euphorbiaceae Juss.
Iz_4_1109	DJI_0025	Fallopia dumetorum (L.) Holub	0	n	1	0	Polygonaceae Juss.
Iz_4_1109	DJI_0025	Festuca beckeri (Hack.) Trautv.	0	n	1	0	Poaceae Barnhart
Iz_4_1109	DJI_0025	Fragaria vesca L.	0	n	1	0	Rosaceae Juss.
Iz_4_1109	DJI_0025	Frangula alnus Mill.	0	n	1	1	Rhamnaceae Juss.
Iz_4_1109	DJI_0025	Fraxinus pennsylvanica Marshall	0	a	0	1	Oleaceae Hoffmanns. & Link
Iz_4_1109	DJI_0025	Galeopsis bifida Boenn.	0	n	1	0	Lamiacea Lindl.
Iz_4_1109	DJI_0025	Galium verum L.	0	n	1	0	Rubiaceae Juss.
Iz_4_1109	DJI_0025	Genista tinctoria L.	0	n	1	0	Fabaceae Lindl.
Iz_4_1109	DJI_0025	Geranium robertianum L.	0	n	1	0	Geraniaceae Juss.
Iz_4_1109	DJI_0025	Geranium sanquineum L.	0	n	1	0	Geraniaceae Juss.
Iz_4_1109	DJI_0025	Gypsophila paniculata L.	0	n	1	0	Caryophyllaceae Juss.
Iz_4_1109	DJI_0025	Helichrysum arenarium (L.) Moench	0	n	1	0	Asteraceae Bercht. & J.Presl
Iz_4_1109	DJI_0025	Herniaria polygama J.Gay	0	n	1	0	Caryophyllaceae Juss.
Iz_4_1109	DJI_0025	Hieracium robustum Fr.	0	n	1	0	Asteraceae Bercht. & J.Presl
Iz_4_1109	DJI_0025	Hieracium umbellatum L.	0	n	1	NA	Asteraceae Bercht. & J.Presl
Iz_4_1109	DJI_0025	Hylotelephium maximum (L.) Holub	0	n	1	0	Crassulaceae J.St.-Hil.
Iz_4_1109	DJI_0025	Jacobaea borysthenica (DC.) B.Nord. & Greuter	0	n	1	0	Asteraceae Bercht. & J.Presl
Iz_4_1109	DJI_0025	Jasione montana L.	0	n	1	0	Campanulaceae Juss.
Iz_4_1109	DJI_0025	Jurinea cyanoides (L.) Rchb.s.l.	0	n	1	0	Asteraceae Bercht. & J.Presl
Iz_4_1109	DJI_0025	Koeleria glauca (Spreng.) DC.	0	n	1	0	Poaceae Barnhart
Iz_4_1109	DJI_0025	Lactuca serriola L.	0	a	0	0	Asteraceae Bercht. & J.Presl
Iz_4_1109	DJI_0025	Linaria genistifolia (L.) Mill.	0	n	1	0	Plantaginaceae Juss.
Iz_4_1109	DJI_0025	Lomelosia argentea (L.) Greuter & Burdet	0	n	1	0	Caprifoliaceae Juss.
Iz_4_1109	DJI_0025	Melampyrum pratense L.	0	n	1	0	Orobanchaceae Vent.
Iz_4_1109	DJI_0025	Oenothera biennis L.	0	a	0	0	Onagraceae Juss.
Iz_4_1109	DJI_0025	Pentanema britannica (L.) D.Gut.Larr., Santos-Vicente, Anderb., E.Rico & M.M.Mart.Ort.	0	n	1	0	Asteraceae Bercht. & J.Presl
Iz_4_1109	DJI_0025	Peucedanum oreoselinum (L.) Moench	0	n	1	0	Apiaceae Lindl.
Iz_4_1109	DJI_0025	Peucedanum ruthenicum M.Bieb.	0	n	1	0	Apiaceae Lindl.
Iz_4_1109	DJI_0025	Pilosella echioides (Lumn.) F.W.Schultz & Sch.Bip.	0	n	1	0	Asteraceae Bercht. & J.Presl
Iz_4_1109	DJI_0025	Pilosella officinarum F. Schultz et Sch. Bip.	0	n	1	0	Asteraceae Bercht. & J.Presl
Iz_4_1109	DJI_0025	Pilosella sp.	0		NA	0	Asteraceae Bercht. & J.Presl
Iz_4_1109	DJI_0025	Pinus sylvestris L.	0	n	1	1	Pinaceae Spreng. ex F.Rudolphi
Iz_4_1109	DJI_0025	Poa annua L.	0	n	1	0	Poaceae Barnhart
Iz_4_1109	DJI_0025	Poa nemoralis L.	0	n	1	0	Poaceae Barnhart
Iz_4_1109	DJI_0025	Poa sp.	0		NA	0	Poaceae Barnhart
Iz_4_1109	DJI_0025	Polygonatum odoratum (Mill.) Druce	0	n	1	0	Asparagaceae Juss.
Iz_4_1109	DJI_0025	Polygonum arenarium Waldst. & Kit.	0	n	1	0	Polygonaceae Juss.
Iz_4_1109	DJI_0025	Populus nigra L.	0	n	1	1	Salicaceae Mirb.
Iz_4_1109	DJI_0025	Populus tremula L.	0	n	1	1	Salicaceae Mirb.
Iz_4_1109	DJI_0025	Populus Ã canescens (Aiton) Sm.	0	n	1	1	Salicaceae Mirb.
Iz_4_1109	DJI_0025	Potentilla argentea L.	0	n	1	0	Rosaceae Juss.
Iz_4_1109	DJI_0025	Psammophiliella muralis (L.) Ikonn.	0	n	1	0	Caryophyllaceae Juss.
Iz_4_1109	DJI_0025	Psephellus sumensis (Kalen.) Greuter	0	n	1	0	Asteraceae Bercht. & J.Presl
Iz_4_1109	DJI_0025	Pulsatilla pratensis (L.) Mill. s.l.	0	n	1	0	Ranunculaceae Juss.
Iz_4_1109	DJI_0025	Pyrus communis L.	0	a	0	1	Rosaceae Juss.
Iz_4_1109	DJI_0025	Quercus robur L.	0	n	1	1	Fagaceae Dumort.
Iz_4_1109	DJI_0025	Robinia pseudoacacia L.	0	a	0	1	Fabaceae Lindl.
Iz_4_1109	DJI_0025	Rosa sp.	0		NA	1	Rosaceae Juss.
Iz_4_1109	DJI_0025	Rubus idaeus L.	0	n	1	0	Rosaceae Juss.
Iz_4_1109	DJI_0025	Rumex acetosella L.	2	n	1	0	Polygonaceae Juss.
Iz_4_1109	DJI_0025	Rumex thyrsiflorus Fingerh.	0	n	1	0	Polygonaceae Juss.

Iz_4_1109	DJI_0025	Salix alba L.	0	n	1	1	Salicaceae Mirb.
Iz_4_1109	DJI_0025	Sambucus nigra L.	0	n	1	1	Viburnaceae Raf.
Iz_4_1109	DJI_0025	Secale sylvestre Host	0	n	1	0	Poaceae Barnhart
Iz_4_1109	DJI_0025	Sempervivum ruthenicum Schnittsp. & C.B. Lehm.	0	n	1	0	Crassulaceae J.St.-Hil.
Iz_4_1109	DJI_0025	Setaria viridis (L.) P. Beauv.	0	n	1	0	Poaceae Barnhart
Iz_4_1109	DJI_0025	Silene borysthenica (Gruner) Walters	0	n	1	0	Caryophyllaceae Juss.
Iz_4_1109	DJI_0025	Silene latifolia subsp. alba (Mill.) Greuter & Burdet	0	n	1	0	Caryophyllaceae Juss.
Iz_4_1109	DJI_0025	Solidago virgaurea L.	0	n	1	0	Asteraceae Bercht. & J.Presl
Iz_4_1109	DJI_0025	Sorbus aucuparia L.	0	n	1	1	Rosaceae Juss.
Iz_4_1109	DJI_0025	Stachys recta L.	0	n	1	0	Lamiacea Lindl.
Iz_4_1109	DJI_0025	Tanacetum vulgare L.	0	n	1	0	Asteraceae Bercht. & J.Presl
Iz_4_1109	DJI_0025	Thymus pallasianus Heinr.Braun	0	n	1	0	Lamiacea Lindl.
Iz_4_1109	DJI_0025	Tilia cordata Mill.	0	n	1	1	Malvaceae Juss.
Iz_4_1109	DJI_0025	Trifolium alpestre L.	0	n	1	0	Fabaceae Lindl.
Iz_4_1109	DJI_0025	Trifolium arvense L.	0	n	1	0	Fabaceae Lindl.
Iz_4_1109	DJI_0025	Ulmus glabra Huds.	0	n	1	1	Ulmaceae Mirb.
Iz_4_1109	DJI_0025	Verbascum densiflorum Bertol.	0	n	1	0	Scrophulariaceae Juss.
Iz_4_1109	DJI_0025	Veronica austriaca L.	0	n	1	0	Plantaginaceae Juss.
Iz_4_1109	DJI_0025	Veronica incana L.	0	n	1	0	Plantaginaceae Juss.
Iz_4_1109	DJI_0025	Veronica verna L.	0	n	1	0	Plantaginaceae Juss.
Iz_4_1109	DJI_0025	Vincetoxicum hirundinaria Medik	0	n	1	0	Apocynaceae Juss.
Iz_4_1109	DJI_0030	Acer tataricum L.	0	n	1	1	Sapindaceae Juss.
Iz_4_1109	DJI_0030	Achillea millefolium L. s.l.	0	n	1	0	Asteraceae Bercht. & J.Presl
Iz_4_1109	DJI_0030	Allium oleraceum L.	0	n	1	0	Amaryllidaceae J.St.-Hil.
Iz_4_1109	DJI_0030	Ambrosia artemisiifolia L.	0	a	0	0	Asteraceae Bercht. & J.Presl
Iz_4_1109	DJI_0030	Amorpha fruticosa L.	0	a	0	0	Fabaceae Lindl.
Iz_4_1109	DJI_0030	Anthericum ramosum L.	0	n	1	0	Asparagaceae Juss.
Iz_4_1109	DJI_0030	Artemisia marschalliana Spreng.	0	n	1	0	Asteraceae Bercht. & J.Presl
Iz_4_1109	DJI_0030	Asparagus officinalis L.	0	n	1	0	Asparagaceae Juss.
Iz_4_1109	DJI_0030	Asteraceae sp.	0	n	1	0	Asteraceae Bercht. & J.Presl
Iz_4_1109	DJI_0030	Astragalus cicer L.	0	n	1	0	Fabaceae Lindl.
Iz_4_1109	DJI_0030	Atriplex sp.	0		NA	NA	
Iz_4_1109	DJI_0030	Bassia laniflora (S.G.Gmel.) A.J.Scott	0	n	1	0	Amaranthaceae Juss.
Iz_4_1109	DJI_0030	Berberis vulgaris L.	0	n	1	0	Berberidaceae Juss.
Iz_4_1109	DJI_0030	Betula pendula Roth.	0	n	1	1	Betulaceae Gray
Iz_4_1109	DJI_0030	Betula pendula Roth.	0	n	1	0	Poaceae Barnhart
Iz_4_1109	DJI_0030	Bromus inermis Leyss.	0		NA	NA	
Iz_4_1109	DJI_0030	Bromus squarrosus L.	0	a	0	0	Poaceae Barnhart
Iz_4_1109	DJI_0030	Bromus tectorum L.	0	a	0	0	Poaceae Barnhart
Iz_4_1109	DJI_0030	Calamagrostis epigejos (L.) Roth	5	n	1	0	Poaceae Barnhart
Iz_4_1109	DJI_0030	Carduus acanthoides L.	0	n	1	0	Asteraceae Bercht. & J.Presl
Iz_4_1109	DJI_0030	Carex sp.	0	n	1	0	Cyperaceae Juss.
Iz_4_1109	DJI_0030	Centaurea majorovii Dumbadze	0	n	1	0	Asteraceae Bercht. & J.Presl
Iz_4_1109	DJI_0030	Chamaecytisus ruthenicus (Fisch.ex Wol.) Klaskova	0	n	1	0	Fabaceae Lindl.
Iz_4_1109	DJI_0030	Chelidonium majus L.	0	n	1	0	Papaveraceae Juss.
Iz_4_1109	DJI_0030	Chenopodium betaceum Andrz.	0	n	1	0	Amaranthaceae Juss.
Iz_4_1109	DJI_0030	Chenopodium cf. album	0	n	1	0	Amaranthaceae Juss.
Iz_4_1109	DJI_0030	Chenopodium sp.	0		NA	0	Amaranthaceae Juss.
Iz_4_1109	DJI_0030	Chondrilla juncea L.	0	n	1	0	Asteraceae Bercht. & J.Presl
Iz_4_1109	DJI_0030	Cirsium vulgare (Savi) Ten.	0	n	1	0	Asteraceae Bercht. & J.Presl
Iz_4_1109	DJI_0030	Clinopodium acinos (L.) Kuntze	0	n	1	0	Lamiacea Lindl.
Iz_4_1109	DJI_0030	Convallaria majalis L.	0	n	1	0	Asparagaceae Juss.
Iz_4_1109	DJI_0030	Crataegus rhipidophylla Gand.	0	n	1	1	Rosaceae Juss.
Iz_4_1109	DJI_0030	Crepis foetida L.	0	n	1	0	Asteraceae Bercht. & J.Presl
Iz_4_1109	DJI_0030	Dactylis glomerata L.	0	n	1	0	Poaceae Barnhart
Iz_4_1109	DJI_0030	Dianthus campestris M.Bieb.	0	n	1	0	Caryophyllaceae Juss.
Iz_4_1109	DJI_0030	Digitaria sanguinalis (L.) Scop.	0	a	0	0	Poaceae Barnhart
Iz_4_1109	DJI_0030	Elymus repens (L.) Gould	0	n	1	0	Poaceae Barnhart
Iz_4_1109	DJI_0030	Epilobium tetragonum L.	0	n	1	0	Onagraceae Juss.
Iz_4_1109	DJI_0030	Eragrostis minor Host	0	a	0	0	Poaceae Barnhart
Iz_4_1109	DJI_0030	Erigeron annuus (L.) Desf.	0	a	0	0	Asteraceae Bercht. & J.Presl
Iz_4_1109	DJI_0030	Erigeron canadensis L.	0	a	0	0	Asteraceae Bercht. & J.Presl
Iz_4_1109	DJI_0030	Euphorbia seguieriana Neck.	0	n	1	0	Euphorbiaceae Juss.
Iz_4_1109	DJI_0030	Fallopia dumetorum (L.) Holub	0	n	1	0	Polygonaceae Juss.
Iz_4_1109	DJI_0030	Festuca beckeri (Hack.) Trautv.	0	n	1	0	Poaceae Barnhart
Iz_4_1109	DJI_0030	Fragaria vesca L.	0	n	1	0	Rosaceae Juss.
Iz_4_1109	DJI_0030	Frangula alnus Mill.	0	n	1	1	Rhamnaceae Juss.
Iz_4_1109	DJI_0030	Fraxinus pennsylvanica Marshall	0	a	0	1	Oleaceae Hoffmanns. & Link
Iz_4_1109	DJI_0030	Galeopsis bifida Boenn.	0	n	1	0	Lamiacea Lindl.
Iz_4_1109	DJI_0030	Galium verum L.	0	n	1	0	Rubiaceae Juss.
Iz_4_1109	DJI_0030	Genista tinctoria L.	0	n	1	0	Fabaceae Lindl.
Iz_4_1109	DJI_0030	Geranium robertianum L.	0	n	1	0	Geraniaceae Juss.
Iz_4_1109	DJI_0030	Geranium sanquineum L.	0	n	1	0	Geraniaceae Juss.
Iz_4_1109	DJI_0030	Gypsophila paniculata L.	0	n	1	0	Caryophyllaceae Juss.
Iz_4_1109	DJI_0030	Helichrysum arenarium (L.) Moench	0	n	1	0	Asteraceae Bercht. & J.Presl
Iz_4_1109	DJI_0030	Herniaria polygama J.Gay	0	n	1	0	Caryophyllaceae Juss.
Iz_4_1109	DJI_0030	Hieracium robustum Fr.	0	n	1	0	Asteraceae Bercht. & J.Presl
Iz_4_1109	DJI_0030	Hieracium umbellatum L.	0	n	1	NA	Asteraceae Bercht. & J.Presl
Iz_4_1109	DJI_0030	Hylotelephium maximum (L.) Holub	0	n	1	0	Crassulaceae J.St.-Hil.
Iz_4_1109	DJI_0030	Jacobaea borysthenica (DC.) B.Nord. & Greuter	0	n	1	0	Asteraceae Bercht. & J.Presl
Iz_4_1109	DJI_0030	Jasione montana L.	0	n	1	0	Campanulaceae Juss.
Iz_4_1109	DJI_0030	Jurinea cyanoides (L.) Rchb.s.l.	0	n	1	0	Asteraceae Bercht. & J.Presl
Iz_4_1109	DJI_0030	Koeleria glauca (Spreng.) DC.	0	n	1	0	Poaceae Barnhart
Iz_4_1109	DJI_0030	Lactuca serriola L.	0	a	0	0	Asteraceae Bercht. & J.Presl
Iz_4_1109	DJI_0030	Linaria genistifolia (L.) Mill.	0	n	1	0	Plantaginaceae Juss.
Iz_4_1109	DJI_0030	Lomelosia argentea (L.) Greuter & Burdet	0	n	1	0	Caprifoliaceae Juss.
Iz_4_1109	DJI_0030	Melampyrum pratense L.	0	n	1	0	Orobanchaceae Vent.
Iz_4_1109	DJI_0030	Oenothera biennis L.	0	a	0	0	Onagraceae Juss.
Iz_4_1109	DJI_0030	Pentanema britannica (L.) D.Gut.Larr., Santos-Vicente, Anderb., E.Rico & M.M.Mart.Ort.	0	n	1	0	Asteraceae Bercht. & J.Presl
Iz_4_1109	DJI_0030	Peucedanum oreoselinum (L.) Moench	0	n	1	0	Apiaceae Lindl.
Iz_4_1109	DJI_0030	Peucedanum ruthenicum M.Bieb.	0	n	1	0	Apiaceae Lindl.
Iz_4_1109	DJI_0030	Pilosella echioides (Lumn.) F.W.Schultz & Sch.Bip.	0	n	1	0	Asteraceae Bercht. & J.Presl
Iz_4_1109	DJI_0030	Pilosella officinarum F. Schultz et Sch. Bip.	0	n	1	0	Asteraceae Bercht. & J.Presl
Iz_4_1109	DJI_0030	Pilosella sp.	0		NA	0	Asteraceae Bercht. & J.Presl
Iz_4_1109	DJI_0030	Pinus sylvestris L.	0	n	1	1	Pinaceae Spreng. ex F.Rudolphi
Iz_4_1109	DJI_0030	Poa annua L.	0	n	1	0	Poaceae Barnhart
Iz_4_1109	DJI_0030	Poa nemoralis L.	0	n	1	0	Poaceae Barnhart
Iz_4_1109	DJI_0030	Poa sp.	0		NA	0	Poaceae Barnhart

Iz_4_1109	DJI_0030	Polygonatum odoratum (Mill.) Druce	0	n	1	0	Asparagaceae Juss.
Iz_4_1109	DJI_0030	Polygonum arenarium Waldst. & Kit.	0	n	1	0	Polygonaceae Juss.
Iz_4_1109	DJI_0030	Populus nigra L.	0	n	1	1	Salicaceae Mirb.
Iz_4_1109	DJI_0030	Populus tremula L.	0	n	1	1	Salicaceae Mirb.
Iz_4_1109	DJI_0030	Populus Ã canescens (Aiton) Sm.	0	n	1	1	Salicaceae Mirb.
Iz_4_1109	DJI_0030	Potentilla argentea L.	0	n	1	0	Rosaceae Juss.
Iz_4_1109	DJI_0030	Psammophiliella muralis (L.) Ikonn.	0	n	1	0	Caryophyllaceae Juss.
Iz_4_1109	DJI_0030	Psephellus sumensis (Kalen.) Greuter	0	n	1	0	Asteraceae Bercht. & J.Presl
Iz_4_1109	DJI_0030	Pulsatilla pratensis (L.) Mill. s.l.	0	n	1	0	Ranunculaceae Juss.
Iz_4_1109	DJI_0030	Pyrus communis L.	0	a	0	1	Rosaceae Juss.
Iz_4_1109	DJI_0030	Quercus robur L.	0	n	1	1	Fagaceae Dumort.
Iz_4_1109	DJI_0030	Robinia pseudoacacia L.	0	a	0	1	Fabaceae Lindl.
Iz_4_1109	DJI_0030	Rosa sp.	0		NA	1	Rosaceae Juss.
Iz_4_1109	DJI_0030	Rubus idaeus L.	0	n	1	0	Rosaceae Juss.
Iz_4_1109	DJI_0030	Rumex acetosella L.	0	n	1	0	Polygonaceae Juss.
Iz_4_1109	DJI_0030	Rumex thyrsiflorus Fingerh.	0	n	1	0	Polygonaceae Juss.
Iz_4_1109	DJI_0030	Salix alba L.	0	n	1	1	Salicaceae Mirb.
Iz_4_1109	DJI_0030	Sambucus nigra L.	0	n	1	1	Viburnaceae Raf.
Iz_4_1109	DJI_0030	Secale sylvestre Host	0	n	1	0	Poaceae Barnhart
Iz_4_1109	DJI_0030	Sempervivum ruthenicum Schnittsp. & C.B. Lehm.	0	n	1	0	Crassulaceae J.St.-Hil.
Iz_4_1109	DJI_0030	Setaria viridis (L.) P. Beauv.	0	n	1	0	Poaceae Barnhart
Iz_4_1109	DJI_0030	Silene borysthenica (Gruner) Walters	0	n	1	0	Caryophyllaceae Juss.
Iz_4_1109	DJI_0030	Silene latifolia subsp. alba (Mill.) Greuter & Burdet	0	n	1	0	Caryophyllaceae Juss.
Iz_4_1109	DJI_0030	Solidago virgaurea L.	0	n	1	0	Asteraceae Bercht. & J.Presl
Iz_4_1109	DJI_0030	Sorbus aucuparia L.	0	n	1	1	Rosaceae Juss.
Iz_4_1109	DJI_0030	Stachys recta L.	0	n	1	0	Lamiacea Lindl.
Iz_4_1109	DJI_0030	Tanacetum vulgare L.	0	n	1	0	Asteraceae Bercht. & J.Presl
Iz_4_1109	DJI_0030	Thymus pallasianus Heinr.Braun	0	n	1	0	Lamiacea Lindl.
Iz_4_1109	DJI_0030	Tilia cordata Mill.	0	n	1	1	Malvaceae Juss.
Iz_4_1109	DJI_0030	Trifolium alpestre L.	0	n	1	0	Fabaceae Lindl.
Iz_4_1109	DJI_0030	Trifolium arvense L.	0	n	1	0	Fabaceae Lindl.
Iz_4_1109	DJI_0030	Ulmus glabra Huds.	0	n	1	1	Ulmaceae Mirb.
Iz_4_1109	DJI_0030	Verbascum densiflorum Bertol.	0	n	1	0	Scrophulariaceae Juss.
Iz_4_1109	DJI_0030	Veronica austriaca L.	0	n	1	0	Plantaginaceae Juss.
Iz_4_1109	DJI_0030	Veronica incana L.	0	n	1	0	Plantaginaceae Juss.
Iz_4_1109	DJI_0030	Veronica verna L.	0	n	1	0	Plantaginaceae Juss.
Iz_4_1109	DJI_0030	Vincetoxicum hirundinaria Medik	0	n	1	0	Apocynaceae Juss.
Iz_4_1109	DJI_0043	Acer tataricum L.	0	n	1	1	Sapindaceae Juss.
Iz_4_1109	DJI_0043	Achillea millefolium L. s.l.	0	n	1	0	Asteraceae Bercht. & J.Presl
Iz_4_1109	DJI_0043	Allium oleraceum L.	0	n	1	0	Amaryllidaceae J.St.-Hil.
Iz_4_1109	DJI_0043	Ambrosia artemisiifolia L.	0	a	0	0	Asteraceae Bercht. & J.Presl
Iz_4_1109	DJI_0043	Amorpha fruticosa L.	0	a	0	0	Fabaceae Lindl.
Iz_4_1109	DJI_0043	Anthericum ramosum L.	0	n	1	0	Asparagaceae Juss.
Iz_4_1109	DJI_0043	Artemisia marschalliana Spreng.	0	n	1	0	Asteraceae Bercht. & J.Presl
Iz_4_1109	DJI_0043	Asparagus officinalis L.	0	n	1	0	Asparagaceae Juss.
Iz_4_1109	DJI_0043	Asteraceae sp.	0	n	1	0	Asteraceae Bercht. & J.Presl
Iz_4_1109	DJI_0043	Astragalus cicer L.	0	n	1	0	Fabaceae Lindl.
Iz_4_1109	DJI_0043	Atriplex sp.	0		NA	NA	
Iz_4_1109	DJI_0043	Bassia laniflora (S.G.Gmel.) A.J.Scott	0	n	1	0	Amaranthaceae Juss.
Iz_4_1109	DJI_0043	Berberis vulgaris L.	0	n	1	0	Berberidaceae Juss.
Iz_4_1109	DJI_0043	Betula pendula Roth.	0	n	1	1	Betulaceae Gray
Iz_4_1109	DJI_0043	Betula pendula Roth.	0	n	1	0	Poaceae Barnhart
Iz_4_1109	DJI_0043	Bromus inermis Leyss.	0		NA	NA	
Iz_4_1109	DJI_0043	Bromus squarrosus L.	0	a	0	0	Poaceae Barnhart
Iz_4_1109	DJI_0043	Bromus tectorum L.	0	a	0	0	Poaceae Barnhart
Iz_4_1109	DJI_0043	Calamagrostis epigejos (L.) Roth	3	n	1	0	Poaceae Barnhart
Iz_4_1109	DJI_0043	Carduus acanthoides L.	0	n	1	0	Asteraceae Bercht. & J.Presl
Iz_4_1109	DJI_0043	Carex sp.	0	n	1	0	Cyperaceae Juss.
Iz_4_1109	DJI_0043	Centaurea majorovii Dumbadze	0	n	1	0	Asteraceae Bercht. & J.Presl
Iz_4_1109	DJI_0043	Chamaecytisus ruthenicus (Fisch.ex Wol.) Klaskova	0	n	1	0	Fabaceae Lindl.
Iz_4_1109	DJI_0043	Chelidonium majus L.	0	n	1	0	Papaveraceae Juss.
Iz_4_1109	DJI_0043	Chenopodium betaceum Andrz.	0	n	1	0	Amaranthaceae Juss.
Iz_4_1109	DJI_0043	Chenopodium cf. album	0	n	1	0	Amaranthaceae Juss.
Iz_4_1109	DJI_0043	Chenopodium sp.	0		NA	0	Amaranthaceae Juss.
Iz_4_1109	DJI_0043	Chondrilla juncea L.	0	n	1	0	Asteraceae Bercht. & J.Presl
Iz_4_1109	DJI_0043	Cirsium vulgare (Savi) Ten.	0	n	1	0	Asteraceae Bercht. & J.Presl
Iz_4_1109	DJI_0043	Clinopodium acinos (L.) Kuntze	0	n	1	0	Lamiacea Lindl.
Iz_4_1109	DJI_0043	Convallaria majalis L.	0	n	1	0	Asparagaceae Juss.
Iz_4_1109	DJI_0043	Crataegus rhipidophylla Gand.	0	n	1	1	Rosaceae Juss.
Iz_4_1109	DJI_0043	Crepis foetida L.	0	n	1	0	Asteraceae Bercht. & J.Presl
Iz_4_1109	DJI_0043	Dactylis glomerata L.	0	n	1	0	Poaceae Barnhart
Iz_4_1109	DJI_0043	Dianthus campestris M.Bieb.	0	n	1	0	Caryophyllaceae Juss.
Iz_4_1109	DJI_0043	Digitaria sanguinalis (L.) Scop.	0	a	0	0	Poaceae Barnhart
Iz_4_1109	DJI_0043	Elymus repens (L.) Gould	0	n	1	0	Poaceae Barnhart
Iz_4_1109	DJI_0043	Epilobium tetragonum L.	0	n	1	0	Onagraceae Juss.
Iz_4_1109	DJI_0043	Eragrostis minor Host	0	a	0	0	Poaceae Barnhart
Iz_4_1109	DJI_0043	Erigeron annuus (L.) Desf.	0	a	0	0	Asteraceae Bercht. & J.Presl
Iz_4_1109	DJI_0043	Erigeron canadensis L.	2	a	0	0	Asteraceae Bercht. & J.Presl
Iz_4_1109	DJI_0043	Euphorbia seguieriana Neck.	0	n	1	0	Euphorbiaceae Juss.
Iz_4_1109	DJI_0043	Fallopia dumetorum (L.) Holub	3	n	1	0	Polygonaceae Juss.
Iz_4_1109	DJI_0043	Festuca beckeri (Hack.) Trautv.	0	n	1	0	Poaceae Barnhart
Iz_4_1109	DJI_0043	Fragaria vesca L.	0	n	1	0	Rosaceae Juss.
Iz_4_1109	DJI_0043	Frangula alnus Mill.	5	n	1	1	Rhamnaceae Juss.
Iz_4_1109	DJI_0043	Fraxinus pennsylvanica Marshall	0	a	0	1	Oleaceae Hoffmanns. & Link
Iz_4_1109	DJI_0043	Galeopsis bifida Boenn.	0	n	1	0	Lamiacea Lindl.
Iz_4_1109	DJI_0043	Galium verum L.	0	n	1	0	Rubiaceae Juss.
Iz_4_1109	DJI_0043	Genista tinctoria L.	0	n	1	0	Fabaceae Lindl.
Iz_4_1109	DJI_0043	Geranium robertianum L.	0	n	1	0	Geraniaceae Juss.
Iz_4_1109	DJI_0043	Geranium sanquineum L.	0	n	1	0	Geraniaceae Juss.
Iz_4_1109	DJI_0043	Gypsophila paniculata L.	0	n	1	0	Caryophyllaceae Juss.
Iz_4_1109	DJI_0043	Helichrysum arenarium (L.) Moench	0	n	1	0	Asteraceae Bercht. & J.Presl
Iz_4_1109	DJI_0043	Herniaria polygama J.Gay	0	n	1	0	Caryophyllaceae Juss.
Iz_4_1109	DJI_0043	Hieracium robustum Fr.	0	n	1	0	Asteraceae Bercht. & J.Presl
Iz_4_1109	DJI_0043	Hieracium umbellatum L.	0	n	1	NA	Asteraceae Bercht. & J.Presl
Iz_4_1109	DJI_0043	Hylotelephium maximum (L.) Holub	0	n	1	0	Crassulaceae J.St.-Hil.
Iz_4_1109	DJI_0043	Jacobaea borysthenica (DC.) B.Nord. & Greuter	0	n	1	0	Asteraceae Bercht. & J.Presl
Iz_4_1109	DJI_0043	Jasione montana L.	0	n	1	0	Campanulaceae Juss.
Iz_4_1109	DJI_0043	Jurinea cyanoides (L.) Rchb.s.l.	0	n	1	0	Asteraceae Bercht. & J.Presl

Iz_4_1109	DJI_0043	Koeleria glauca (Spreng.) DC.	0	n	1	0	Poaceae Barnhart
Iz_4_1109	DJI_0043	Lactuca serriola L.	0	a	0	0	Asteraceae Bercht. & J.Presl
Iz_4_1109	DJI_0043	Linaria genistifolia (L.) Mill.	0	n	1	0	Plantaginaceae Juss.
Iz_4_1109	DJI_0043	Lomelosia argentea (L.) Greuter & Burdet	0	n	1	0	Caprifoliaceae Juss.
Iz_4_1109	DJI_0043	Melampyrum pratense L.	0	n	1	0	Orobanchaceae Vent.
Iz_4_1109	DJI_0043	Oenothera biennis L.	0	a	0	0	Onagraceae Juss.
Iz_4_1109	DJI_0043	Pentanema britannica (L.) D.Gut.Larr., Santos-Vicente, Anderb., E.Rico & M.M.Mart.Ort.	0	n	1	0	Asteraceae Bercht. & J.Presl
Iz_4_1109	DJI_0043	Peucedanum oreoselinum (L.) Moench	0	n	1	0	Apiaceae Lindl.
Iz_4_1109	DJI_0043	Peucedanum ruthenicum M.Bieb.	0	n	1	0	Apiaceae Lindl.
Iz_4_1109	DJI_0043	Pilosella echioides (Lumn.) F.W.Schultz & Sch.Bip.	0	n	1	0	Asteraceae Bercht. & J.Presl
Iz_4_1109	DJI_0043	Pilosella officinarum F. Schultz et Sch. Bip.	0	n	1	0	Asteraceae Bercht. & J.Presl
Iz_4_1109	DJI_0043	Pilosella sp.	0		NA	0	Asteraceae Bercht. & J.Presl
Iz_4_1109	DJI_0043	Pinus sylvestris L.	0	n	1	1	Pinaceae Spreng. ex F.Rudolphi
Iz_4_1109	DJI_0043	Poa annua L.	0	n	1	0	Poaceae Barnhart
Iz_4_1109	DJI_0043	Poa nemoralis L.	0	n	1	0	Poaceae Barnhart
Iz_4_1109	DJI_0043	Poa sp.	0		NA	0	Poaceae Barnhart
Iz_4_1109	DJI_0043	Polygonatum odoratum (Mill.) Druce	0	n	1	0	Asparagaceae Juss.
Iz_4_1109	DJI_0043	Polygonum arenarium Waldst. & Kit.	0	n	1	0	Polygonaceae Juss.
Iz_4_1109	DJI_0043	Populus nigra L.	0	n	1	1	Salicaceae Mirb.
Iz_4_1109	DJI_0043	Populus tremula L.	0	n	1	1	Salicaceae Mirb.
Iz_4_1109	DJI_0043	Populus Ã canescens (Aiton) Sm.	0	n	1	1	Salicaceae Mirb.
Iz_4_1109	DJI_0043	Potentilla argentea L.	0	n	1	0	Rosaceae Juss.
Iz_4_1109	DJI_0043	Psammophiliella muralis (L.) Ikonn.	0	n	1	0	Caryophyllaceae Juss.
Iz_4_1109	DJI_0043	Psephellus sumensis (Kalen.) Greuter	0	n	1	0	Asteraceae Bercht. & J.Presl
Iz_4_1109	DJI_0043	Pulsatilla pratensis (L.) Mill. s.l.	0	n	1	0	Ranunculaceae Juss.
Iz_4_1109	DJI_0043	Pyrus communis L.	0	a	0	1	Rosaceae Juss.
Iz_4_1109	DJI_0043	Quercus robur L.	0	n	1	1	Fagaceae Dumort.
Iz_4_1109	DJI_0043	Robinia pseudoacacia L.	0	a	0	1	Fabaceae Lindl.
Iz_4_1109	DJI_0043	Rosa sp.	0		NA	1	Rosaceae Juss.
Iz_4_1109	DJI_0043	Rubus idaeus L.	0	n	1	0	Rosaceae Juss.
Iz_4_1109	DJI_0043	Rumex acetosella L.	0	n	1	0	Polygonaceae Juss.
Iz_4_1109	DJI_0043	Rumex thyrsiflorus Fingerh.	0	n	1	0	Polygonaceae Juss.
Iz_4_1109	DJI_0043	Salix alba L.	0	n	1	1	Salicaceae Mirb.
Iz_4_1109	DJI_0043	Sambucus nigra L.	0	n	1	1	Viburnaceae Raf.
Iz_4_1109	DJI_0043	Secale sylvestre Host	0	n	1	0	Poaceae Barnhart
Iz_4_1109	DJI_0043	Sempervivum ruthenicum Schnittsp. & C.B. Lehm.	0	n	1	0	Crassulaceae J.St.-Hil.
Iz_4_1109	DJI_0043	Setaria viridis (L.) P. Beauv.	0	n	1	0	Poaceae Barnhart
Iz_4_1109	DJI_0043	Silene borysthenica (Gruner) Walters	0	n	1	0	Caryophyllaceae Juss.
Iz_4_1109	DJI_0043	Silene latifolia subsp. alba (Mill.) Greuter & Burdet	0	n	1	0	Caryophyllaceae Juss.
Iz_4_1109	DJI_0043	Solidago virgaurea L.	0	n	1	0	Asteraceae Bercht. & J.Presl
Iz_4_1109	DJI_0043	Sorbus aucuparia L.	0	n	1	1	Rosaceae Juss.
Iz_4_1109	DJI_0043	Stachys recta L.	0	n	1	0	Lamiacea Lindl.
Iz_4_1109	DJI_0043	Tanacetum vulgare L.	0	n	1	0	Asteraceae Bercht. & J.Presl
Iz_4_1109	DJI_0043	Thymus pallasianus Heinr.Braun	0	n	1	0	Lamiacea Lindl.
Iz_4_1109	DJI_0043	Tilia cordata Mill.	0	n	1	1	Malvaceae Juss.
Iz_4_1109	DJI_0043	Trifolium alpestre L.	1	n	1	0	Fabaceae Lindl.
Iz_4_1109	DJI_0043	Trifolium arvense L.	0	n	1	0	Fabaceae Lindl.
Iz_4_1109	DJI_0043	Ulmus glabra Huds.	0	n	1	1	Ulmaceae Mirb.
Iz_4_1109	DJI_0043	Verbascum densiflorum Bertol.	0	n	1	0	Scrophulariaceae Juss.
Iz_4_1109	DJI_0043	Veronica austriaca L.	0	n	1	0	Plantaginaceae Juss.
Iz_4_1109	DJI_0043	Veronica incana L.	0	n	1	0	Plantaginaceae Juss.
Iz_4_1109	DJI_0043	Veronica verna L.	0	n	1	0	Plantaginaceae Juss.
Iz_4_1109	DJI_0043	Vincetoxicum hirundinaria Medik	0	n	1	0	Apocynaceae Juss.
Iz_4_1109	DJI_0050	Acer tataricum L.	0	n	1	1	Sapindaceae Juss.
Iz_4_1109	DJI_0050	Achillea millefolium L. s.l.	0	n	1	0	Asteraceae Bercht. & J.Presl
Iz_4_1109	DJI_0050	Allium oleraceum L.	0	n	1	0	Amaryllidaceae J.St.-Hil.
Iz_4_1109	DJI_0050	Ambrosia artemisiifolia L.	0	a	0	0	Asteraceae Bercht. & J.Presl
Iz_4_1109	DJI_0050	Amorpha fruticosa L.	0	a	0	0	Fabaceae Lindl.
Iz_4_1109	DJI_0050	Anthericum ramosum L.	0	n	1	0	Asparagaceae Juss.
Iz_4_1109	DJI_0050	Artemisia marschalliana Spreng.	0	n	1	0	Asteraceae Bercht. & J.Presl
Iz_4_1109	DJI_0050	Asparagus officinalis L.	0	n	1	0	Asparagaceae Juss.
Iz_4_1109	DJI_0050	Asteraceae sp.	0	n	1	0	Asteraceae Bercht. & J.Presl
Iz_4_1109	DJI_0050	Astragalus cicer L.	0	n	1	0	Fabaceae Lindl.
Iz_4_1109	DJI_0050	Atriplex sp.	0		NA	NA	
Iz_4_1109	DJI_0050	Bassia laniflora (S.G.Gmel.) A.J.Scott	0	n	1	0	Amaranthaceae Juss.
Iz_4_1109	DJI_0050	Berberis vulgaris L.	0	n	1	0	Berberidaceae Juss.
Iz_4_1109	DJI_0050	Betula pendula Roth.	3	n	1	1	Betulaceae Gray
Iz_4_1109	DJI_0050	Betula pendula Roth.	3	n	1	0	Poaceae Barnhart
Iz_4_1109	DJI_0050	Bromus inermis Leyss.	0		NA	NA	
Iz_4_1109	DJI_0050	Bromus squarrosus L.	0	a	0	0	Poaceae Barnhart
Iz_4_1109	DJI_0050	Bromus tectorum L.	0	a	0	0	Poaceae Barnhart
Iz_4_1109	DJI_0050	Calamagrostis epigejos (L.) Roth	5	n	1	0	Poaceae Barnhart
Iz_4_1109	DJI_0050	Carduus acanthoides L.	0	n	1	0	Asteraceae Bercht. & J.Presl
Iz_4_1109	DJI_0050	Carex sp.	0	n	1	0	Cyperaceae Juss.
Iz_4_1109	DJI_0050	Centaurea majorovii Dumbadze	0	n	1	0	Asteraceae Bercht. & J.Presl
Iz_4_1109	DJI_0050	Chamaecytisus ruthenicus (Fisch.ex Wol.) Klaskova	0	n	1	0	Fabaceae Lindl.
Iz_4_1109	DJI_0050	Chelidonium majus L.	0	n	1	0	Papaveraceae Juss.
Iz_4_1109	DJI_0050	Chenopodium betaceum Andrz.	0	n	1	0	Amaranthaceae Juss.
Iz_4_1109	DJI_0050	Chenopodium cf. album	0	n	1	0	Amaranthaceae Juss.
Iz_4_1109	DJI_0050	Chenopodium sp.	0		NA	0	Amaranthaceae Juss.
Iz_4_1109	DJI_0050	Chondrilla juncea L.	0	n	1	0	Asteraceae Bercht. & J.Presl
Iz_4_1109	DJI_0050	Cirsium vulgare (Savi) Ten.	0	n	1	0	Asteraceae Bercht. & J.Presl
Iz_4_1109	DJI_0050	Clinopodium acinos (L.) Kuntze	0	n	1	0	Lamiacea Lindl.
Iz_4_1109	DJI_0050	Convallaria majalis L.	0	n	1	0	Asparagaceae Juss.
Iz_4_1109	DJI_0050	Crataegus rhipidophylla Gand.	0	n	1	1	Rosaceae Juss.
Iz_4_1109	DJI_0050	Crepis foetida L.	0	n	1	0	Asteraceae Bercht. & J.Presl
Iz_4_1109	DJI_0050	Dactylis glomerata L.	0	n	1	0	Poaceae Barnhart
Iz_4_1109	DJI_0050	Dianthus campestris M.Bieb.	0	n	1	0	Caryophyllaceae Juss.
Iz_4_1109	DJI_0050	Digitaria sanguinalis (L.) Scop.	0	a	0	0	Poaceae Barnhart
Iz_4_1109	DJI_0050	Elymus repens (L.) Gould	0	n	1	0	Poaceae Barnhart
Iz_4_1109	DJI_0050	Epilobium tetragonum L.	0	n	1	0	Onagraceae Juss.
Iz_4_1109	DJI_0050	Eragrostis minor Host	0	a	0	0	Poaceae Barnhart
Iz_4_1109	DJI_0050	Erigeron annuus (L.) Desf.	0	a	0	0	Asteraceae Bercht. & J.Presl
Iz_4_1109	DJI_0050	Erigeron canadensis L.	12	a	0	0	Asteraceae Bercht. & J.Presl
Iz_4_1109	DJI_0050	Euphorbia seguieriana Neck.	0	n	1	0	Euphorbiaceae Juss.
Iz_4_1109	DJI_0050	Fallopia dumetorum (L.) Holub	3	n	1	0	Polygonaceae Juss.
Iz_4_1109	DJI_0050	Festuca beckeri (Hack.) Trautv.	0	n	1	0	Poaceae Barnhart
Iz_4_1109	DJI_0050	Fragaria vesca L.	0	n	1	0	Rosaceae Juss.

Iz_4_1109	DJI_0050	Frangula alnus Mill.	0	n	1	1	Rhamnaceae Juss.
Iz_4_1109	DJI_0050	Fraxinus pennsylvanica Marshall	0	a	0	1	Oleaceae Hoffmanns. & Link
Iz_4_1109	DJI_0050	Galeopsis bifida Boenn.	0	n	1	0	Lamiacea Lindl.
Iz_4_1109	DJI_0050	Galium verum L.	0	n	1	0	Rubiaceae Juss.
Iz_4_1109	DJI_0050	Genista tinctoria L.	0	n	1	0	Fabaceae Lindl.
Iz_4_1109	DJI_0050	Geranium robertianum L.	0	n	1	0	Geraniaceae Juss.
Iz_4_1109	DJI_0050	Geranium sanquineum L.	0	n	1	0	Geraniaceae Juss.
Iz_4_1109	DJI_0050	Gypsophila paniculata L.	0	n	1	0	Caryophyllaceae Juss.
Iz_4_1109	DJI_0050	Helichrysum arenarium (L.) Moench	0	n	1	0	Asteraceae Bercht. & J.Presl
Iz_4_1109	DJI_0050	Herniaria polygama J.Gay	0	n	1	0	Caryophyllaceae Juss.
Iz_4_1109	DJI_0050	Hieracium robustum Fr.	0	n	1	0	Asteraceae Bercht. & J.Presl
Iz_4_1109	DJI_0050	Hieracium umbellatum L.	0	n	1	NA	Asteraceae Bercht. & J.Presl
Iz_4_1109	DJI_0050	Hylotelephium maximum (L.) Holub	0	n	1	0	Crassulaceae J.St.-Hil.
Iz_4_1109	DJI_0050	Jacobaea borysthenica (DC.) B.Nord. & Greuter	0	n	1	0	Asteraceae Bercht. & J.Presl
Iz_4_1109	DJI_0050	Jasione montana L.	0	n	1	0	Campanulaceae Juss.
Iz_4_1109	DJI_0050	Jurinea cyanoides (L.) Rchb.s.l.	0	n	1	0	Asteraceae Bercht. & J.Presl
Iz_4_1109	DJI_0050	Koeleria glauca (Spreng.) DC.	0	n	1	0	Poaceae Barnhart
Iz_4_1109	DJI_0050	Lactuca serriola L.	2	a	0	0	Asteraceae Bercht. & J.Presl
Iz_4_1109	DJI_0050	Linaria genistifolia (L.) Mill.	0	n	1	0	Plantaginaceae Juss.
Iz_4_1109	DJI_0050	Lomelosia argentea (L.) Greuter & Burdet	0	n	1	0	Caprifoliaceae Juss.
Iz_4_1109	DJI_0050	Melampyrum pratense L.	0	n	1	0	Orobanchaceae Vent.
Iz_4_1109	DJI_0050	Oenothera biennis L.	0	a	0	0	Onagraceae Juss.
Iz_4_1109	DJI_0050	Pentanema britannica (L.) D.Gut.Larr., Santos-Vicente, Anderb., E.Rico & M.M.Mart.Ort.	0	n	1	0	Asteraceae Bercht. & J.Presl
Iz_4_1109	DJI_0050	Peucedanum oreoselinum (L.) Moench	0	n	1	0	Apiaceae Lindl.
Iz_4_1109	DJI_0050	Peucedanum ruthenicum M.Bieb.	0	n	1	0	Apiaceae Lindl.
Iz_4_1109	DJI_0050	Pilosella echioides (Lumn.) F.W.Schultz & Sch.Bip.	0	n	1	0	Asteraceae Bercht. & J.Presl
Iz_4_1109	DJI_0050	Pilosella officinarum F. Schultz et Sch. Bip.	0	n	1	0	Asteraceae Bercht. & J.Presl
Iz_4_1109	DJI_0050	Pilosella sp.	0		NA	0	Asteraceae Bercht. & J.Presl
Iz_4_1109	DJI_0050	Pinus sylvestris L.	0	n	1	1	Pinaceae Spreng. ex F.Rudolphi
Iz_4_1109	DJI_0050	Poa annua L.	0	n	1	0	Poaceae Barnhart
Iz_4_1109	DJI_0050	Poa nemoralis L.	0	n	1	0	Poaceae Barnhart
Iz_4_1109	DJI_0050	Poa sp.	0		NA	0	Poaceae Barnhart
Iz_4_1109	DJI_0050	Polygonatum odoratum (Mill.) Druce	0	n	1	0	Asparagaceae Juss.
Iz_4_1109	DJI_0050	Polygonum arenarium Waldst. & Kit.	0	n	1	0	Polygonaceae Juss.
Iz_4_1109	DJI_0050	Populus nigra L.	0	n	1	1	Salicaceae Mirb.
Iz_4_1109	DJI_0050	Populus tremula L.	0	n	1	1	Salicaceae Mirb.
Iz_4_1109	DJI_0050	Populus Ã canescens (Aiton) Sm.	0	n	1	1	Salicaceae Mirb.
Iz_4_1109	DJI_0050	Potentilla argentea L.	0	n	1	0	Rosaceae Juss.
Iz_4_1109	DJI_0050	Psammophiliella muralis (L.) Ikonn.	0	n	1	0	Caryophyllaceae Juss.
Iz_4_1109	DJI_0050	Psephellus sumensis (Kalen.) Greuter	0	n	1	0	Asteraceae Bercht. & J.Presl
Iz_4_1109	DJI_0050	Pulsatilla pratensis (L.) Mill. s.l.	0	n	1	0	Ranunculaceae Juss.
Iz_4_1109	DJI_0050	Pyrus communis L.	2	a	0	1	Rosaceae Juss.
Iz_4_1109	DJI_0050	Quercus robur L.	0	n	1	1	Fagaceae Dumort.
Iz_4_1109	DJI_0050	Robinia pseudoacacia L.	0	a	0	1	Fabaceae Lindl.
Iz_4_1109	DJI_0050	Rosa sp.	0		NA	1	Rosaceae Juss.
Iz_4_1109	DJI_0050	Rubus idaeus L.	0	n	1	0	Rosaceae Juss.
Iz_4_1109	DJI_0050	Rumex acetosella L.	0	n	1	0	Polygonaceae Juss.
Iz_4_1109	DJI_0050	Rumex thyrsiflorus Fingerh.	0	n	1	0	Polygonaceae Juss.
Iz_4_1109	DJI_0050	Salix alba L.	0	n	1	1	Salicaceae Mirb.
Iz_4_1109	DJI_0050	Sambucus nigra L.	0	n	1	1	Viburnaceae Raf.
Iz_4_1109	DJI_0050	Secale sylvestre Host	0	n	1	0	Poaceae Barnhart
Iz_4_1109	DJI_0050	Sempervivum ruthenicum Schnittsp. & C.B. Lehm.	0	n	1	0	Crassulaceae J.St.-Hil.
Iz_4_1109	DJI_0050	Setaria viridis (L.) P. Beauv.	0	n	1	0	Poaceae Barnhart
Iz_4_1109	DJI_0050	Silene borysthenica (Gruner) Walters	0	n	1	0	Caryophyllaceae Juss.
Iz_4_1109	DJI_0050	Silene latifolia subsp. alba (Mill.) Greuter & Burdet	0	n	1	0	Caryophyllaceae Juss.
Iz_4_1109	DJI_0050	Solidago virgaurea L.	0	n	1	0	Asteraceae Bercht. & J.Presl
Iz_4_1109	DJI_0050	Sorbus aucuparia L.	0	n	1	1	Rosaceae Juss.
Iz_4_1109	DJI_0050	Stachys recta L.	0	n	1	0	Lamiacea Lindl.
Iz_4_1109	DJI_0050	Tanacetum vulgare L.	0	n	1	0	Asteraceae Bercht. & J.Presl
Iz_4_1109	DJI_0050	Thymus pallasianus Heinr.Braun	0	n	1	0	Lamiacea Lindl.
Iz_4_1109	DJI_0050	Tilia cordata Mill.	0	n	1	1	Malvaceae Juss.
Iz_4_1109	DJI_0050	Trifolium alpestre L.	0	n	1	0	Fabaceae Lindl.
Iz_4_1109	DJI_0050	Trifolium arvense L.	0	n	1	0	Fabaceae Lindl.
Iz_4_1109	DJI_0050	Ulmus glabra Huds.	0	n	1	1	Ulmaceae Mirb.
Iz_4_1109	DJI_0050	Verbascum densiflorum Bertol.	0	n	1	0	Scrophulariaceae Juss.
Iz_4_1109	DJI_0050	Veronica austriaca L.	0	n	1	0	Plantaginaceae Juss.
Iz_4_1109	DJI_0050	Veronica incana L.	0	n	1	0	Plantaginaceae Juss.
Iz_4_1109	DJI_0050	Veronica verna L.	0	n	1	0	Plantaginaceae Juss.
Iz_4_1109	DJI_0050	Vincetoxicum hirundinaria Medik	0	n	1	0	Apocynaceae Juss.
Iz_4_1109	DJI_0053	Acer tataricum L.	0	n	1	1	Sapindaceae Juss.
Iz_4_1109	DJI_0053	Achillea millefolium L. s.l.	0	n	1	0	Asteraceae Bercht. & J.Presl
Iz_4_1109	DJI_0053	Allium oleraceum L.	0	n	1	0	Amaryllidaceae J.St.-Hil.
Iz_4_1109	DJI_0053	Ambrosia artemisiifolia L.	0	a	0	0	Asteraceae Bercht. & J.Presl
Iz_4_1109	DJI_0053	Amorpha fruticosa L.	0	a	0	0	Fabaceae Lindl.
Iz_4_1109	DJI_0053	Anthericum ramosum L.	0	n	1	0	Asparagaceae Juss.
Iz_4_1109	DJI_0053	Artemisia marschalliana Spreng.	0	n	1	0	Asteraceae Bercht. & J.Presl
Iz_4_1109	DJI_0053	Asparagus officinalis L.	0	n	1	0	Asparagaceae Juss.
Iz_4_1109	DJI_0053	Asteraceae sp.	0	n	1	0	Asteraceae Bercht. & J.Presl
Iz_4_1109	DJI_0053	Astragalus cicer L.	0	n	1	0	Fabaceae Lindl.
Iz_4_1109	DJI_0053	Atriplex sp.	0		NA	NA	
Iz_4_1109	DJI_0053	Bassia laniflora (S.G.Gmel.) A.J.Scott	0	n	1	0	Amaranthaceae Juss.
Iz_4_1109	DJI_0053	Berberis vulgaris L.	0	n	1	0	Berberidaceae Juss.
Iz_4_1109	DJI_0053	Betula pendula Roth.	0	n	1	1	Betulaceae Gray
Iz_4_1109	DJI_0053	Betula pendula Roth.	0	n	1	0	Poaceae Barnhart
Iz_4_1109	DJI_0053	Bromus inermis Leyss.	0		NA	NA	
Iz_4_1109	DJI_0053	Bromus squarrosus L.	0	a	0	0	Poaceae Barnhart
Iz_4_1109	DJI_0053	Bromus tectorum L.	0	a	0	0	Poaceae Barnhart
Iz_4_1109	DJI_0053	Calamagrostis epigejos (L.) Roth	85	n	1	0	Poaceae Barnhart
Iz_4_1109	DJI_0053	Carduus acanthoides L.	0	n	1	0	Asteraceae Bercht. & J.Presl
Iz_4_1109	DJI_0053	Carex sp.	0	n	1	0	Cyperaceae Juss.
Iz_4_1109	DJI_0053	Centaurea majorovii Dumbadze	0	n	1	0	Asteraceae Bercht. & J.Presl
Iz_4_1109	DJI_0053	Chamaecytisus ruthenicus (Fisch.ex Wol.) Klaskova	0	n	1	0	Fabaceae Lindl.
Iz_4_1109	DJI_0053	Chelidonium majus L.	0	n	1	0	Papaveraceae Juss.
Iz_4_1109	DJI_0053	Chenopodium betaceum Andrz.	0	n	1	0	Amaranthaceae Juss.
Iz_4_1109	DJI_0053	Chenopodium cf. album	0	n	1	0	Amaranthaceae Juss.
Iz_4_1109	DJI_0053	Chenopodium sp.	0		NA	0	Amaranthaceae Juss.
Iz_4_1109	DJI_0053	Chondrilla juncea L.	0	n	1	0	Asteraceae Bercht. & J.Presl
Iz_4_1109	DJI_0053	Cirsium vulgare (Savi) Ten.	0	n	1	0	Asteraceae Bercht. & J.Presl

Iz_4_1109	DJI_0053	Clinopodium acinos (L.) Kuntze	0	n	1	0	Lamiacea Lindl.
Iz_4_1109	DJI_0053	Convallaria majalis L.	0	n	1	0	Asparagaceae Juss.
Iz_4_1109	DJI_0053	Crataegus rhipidophylla Gand.	1	n	1	1	Rosaceae Juss.
Iz_4_1109	DJI_0053	Crepis foetida L.	0	n	1	0	Asteraceae Bercht. & J.Presl
Iz_4_1109	DJI_0053	Dactylis glomerata L.	0	n	1	0	Poaceae Barnhart
Iz_4_1109	DJI_0053	Dianthus campestris M.Bieb.	0	n	1	0	Caryophyllaceae Juss.
Iz_4_1109	DJI_0053	Digitaria sanguinalis (L.) Scop.	0	a	0	0	Poaceae Barnhart
Iz_4_1109	DJI_0053	Elymus repens (L.) Gould	0	n	1	0	Poaceae Barnhart
Iz_4_1109	DJI_0053	Epilobium tetragonum L.	0	n	1	0	Onagraceae Juss.
Iz_4_1109	DJI_0053	Eragrostis minor Host	0	a	0	0	Poaceae Barnhart
Iz_4_1109	DJI_0053	Erigeron annuus (L.) Desf.	0	a	0	0	Asteraceae Bercht. & J.Presl
Iz_4_1109	DJI_0053	Erigeron canadensis L.	4	a	0	0	Asteraceae Bercht. & J.Presl
Iz_4_1109	DJI_0053	Euphorbia seguieriana Neck.	0	n	1	0	Euphorbiaceae Juss.
Iz_4_1109	DJI_0053	Fallopia dumetorum (L.) Holub	6	n	1	0	Polygonaceae Juss.
Iz_4_1109	DJI_0053	Festuca beckeri (Hack.) Trautv.	0	n	1	0	Poaceae Barnhart
Iz_4_1109	DJI_0053	Fragaria vesca L.	0	n	1	0	Rosaceae Juss.
Iz_4_1109	DJI_0053	Frangula alnus Mill.	0	n	1	1	Rhamnaceae Juss.
Iz_4_1109	DJI_0053	Fraxinus pennsylvanica Marshall	0	a	0	1	Oleaceae Hoffmanns. & Link
Iz_4_1109	DJI_0053	Galeopsis bifida Boenn.	0	n	1	0	Lamiacea Lindl.
Iz_4_1109	DJI_0053	Galium verum L.	0	n	1	0	Rubiaceae Juss.
Iz_4_1109	DJI_0053	Genista tinctoria L.	0	n	1	0	Fabaceae Lindl.
Iz_4_1109	DJI_0053	Geranium robertianum L.	0	n	1	0	Geraniaceae Juss.
Iz_4_1109	DJI_0053	Geranium sanquineum L.	0	n	1	0	Geraniaceae Juss.
Iz_4_1109	DJI_0053	Gypsophila paniculata L.	0	n	1	0	Caryophyllaceae Juss.
Iz_4_1109	DJI_0053	Helichrysum arenarium (L.) Moench	0	n	1	0	Asteraceae Bercht. & J.Presl
Iz_4_1109	DJI_0053	Herniaria polygama J.Gay	0	n	1	0	Caryophyllaceae Juss.
Iz_4_1109	DJI_0053	Hieracium robustum Fr.	0	n	1	0	Asteraceae Bercht. & J.Presl
Iz_4_1109	DJI_0053	Hieracium umbellatum L.	0	n	1	NA	Asteraceae Bercht. & J.Presl
Iz_4_1109	DJI_0053	Hylotelephium maximum (L.) Holub	0	n	1	0	Crassulaceae J.St.-Hil.
Iz_4_1109	DJI_0053	Jacobaea borysthenica (DC.) B.Nord. & Greuter	0	n	1	0	Asteraceae Bercht. & J.Presl
Iz_4_1109	DJI_0053	Jasione montana L.	0	n	1	0	Campanulaceae Juss.
Iz_4_1109	DJI_0053	Jurinea cyanoides (L.) Rchb.s.l.	0	n	1	0	Asteraceae Bercht. & J.Presl
Iz_4_1109	DJI_0053	Koeleria glauca (Spreng.) DC.	0	n	1	0	Poaceae Barnhart
Iz_4_1109	DJI_0053	Lactuca serriola L.	0	a	0	0	Asteraceae Bercht. & J.Presl
Iz_4_1109	DJI_0053	Linaria genistifolia (L.) Mill.	0	n	1	0	Plantaginaceae Juss.
Iz_4_1109	DJI_0053	Lomelosia argentea (L.) Greuter & Burdet	0	n	1	0	Caprifoliaceae Juss.
Iz_4_1109	DJI_0053	Melampyrum pratense L.	0	n	1	0	Orobanchaceae Vent.
Iz_4_1109	DJI_0053	Oenothera biennis L.	0	a	0	0	Onagraceae Juss.
Iz_4_1109	DJI_0053	Pentanema britannica (L.) D.Gut.Larr., Santos-Vicente, Anderb., E.Rico & M.M.Mart.Ort.	0	n	1	0	Asteraceae Bercht. & J.Presl
Iz_4_1109	DJI_0053	Peucedanum oreoselinum (L.) Moench	0	n	1	0	Apiaceae Lindl.
Iz_4_1109	DJI_0053	Peucedanum ruthenicum M.Bieb.	0	n	1	0	Apiaceae Lindl.
Iz_4_1109	DJI_0053	Pilosella echioides (Lumn.) F.W.Schultz & Sch.Bip.	0	n	1	0	Asteraceae Bercht. & J.Presl
Iz_4_1109	DJI_0053	Pilosella officinarum F. Schultz et Sch. Bip.	0	n	1	0	Asteraceae Bercht. & J.Presl
Iz_4_1109	DJI_0053	Pilosella sp.	0		NA	0	Asteraceae Bercht. & J.Presl
Iz_4_1109	DJI_0053	Pinus sylvestris L.	0	n	1	1	Pinaceae Spreng. ex F.Rudolphi
Iz_4_1109	DJI_0053	Poa annua L.	0	n	1	0	Poaceae Barnhart
Iz_4_1109	DJI_0053	Poa nemoralis L.	0	n	1	0	Poaceae Barnhart
Iz_4_1109	DJI_0053	Poa sp.	0		NA	0	Poaceae Barnhart
Iz_4_1109	DJI_0053	Polygonatum odoratum (Mill.) Druce	0	n	1	0	Asparagaceae Juss.
Iz_4_1109	DJI_0053	Polygonum arenarium Waldst. & Kit.	0	n	1	0	Polygonaceae Juss.
Iz_4_1109	DJI_0053	Populus nigra L.	0	n	1	1	Salicaceae Mirb.
Iz_4_1109	DJI_0053	Populus tremula L.	0	n	1	1	Salicaceae Mirb.
Iz_4_1109	DJI_0053	Populus Ã canescens (Aiton) Sm.	0	n	1	1	Salicaceae Mirb.
Iz_4_1109	DJI_0053	Potentilla argentea L.	0	n	1	0	Rosaceae Juss.
Iz_4_1109	DJI_0053	Psammophiliella muralis (L.) Ikonn.	0	n	1	0	Caryophyllaceae Juss.
Iz_4_1109	DJI_0053	Psephellus sumensis (Kalen.) Greuter	0	n	1	0	Asteraceae Bercht. & J.Presl
Iz_4_1109	DJI_0053	Pulsatilla pratensis (L.) Mill. s.l.	0	n	1	0	Ranunculaceae Juss.
Iz_4_1109	DJI_0053	Pyrus communis L.	0	a	0	1	Rosaceae Juss.
Iz_4_1109	DJI_0053	Quercus robur L.	0	n	1	1	Fagaceae Dumort.
Iz_4_1109	DJI_0053	Robinia pseudoacacia L.	0	a	0	1	Fabaceae Lindl.
Iz_4_1109	DJI_0053	Rosa sp.	0		NA	1	Rosaceae Juss.
Iz_4_1109	DJI_0053	Rubus idaeus L.	0	n	1	0	Rosaceae Juss.
Iz_4_1109	DJI_0053	Rumex acetosella L.	0	n	1	0	Polygonaceae Juss.
Iz_4_1109	DJI_0053	Rumex thyrsiflorus Fingerh.	0	n	1	0	Polygonaceae Juss.
Iz_4_1109	DJI_0053	Salix alba L.	0	n	1	1	Salicaceae Mirb.
Iz_4_1109	DJI_0053	Sambucus nigra L.	0	n	1	1	Viburnaceae Raf.
Iz_4_1109	DJI_0053	Secale sylvestre Host	0	n	1	0	Poaceae Barnhart
Iz_4_1109	DJI_0053	Sempervivum ruthenicum Schnittsp. & C.B. Lehm.	0	n	1	0	Crassulaceae J.St.-Hil.
Iz_4_1109	DJI_0053	Setaria viridis (L.) P. Beauv.	0	n	1	0	Poaceae Barnhart
Iz_4_1109	DJI_0053	Silene borysthenica (Gruner) Walters	0	n	1	0	Caryophyllaceae Juss.
Iz_4_1109	DJI_0053	Silene latifolia subsp. alba (Mill.) Greuter & Burdet	0	n	1	0	Caryophyllaceae Juss.
Iz_4_1109	DJI_0053	Solidago virgaurea L.	1	n	1	0	Asteraceae Bercht. & J.Presl
Iz_4_1109	DJI_0053	Sorbus aucuparia L.	0	n	1	1	Rosaceae Juss.
Iz_4_1109	DJI_0053	Stachys recta L.	0	n	1	0	Lamiacea Lindl.
Iz_4_1109	DJI_0053	Tanacetum vulgare L.	0	n	1	0	Asteraceae Bercht. & J.Presl
Iz_4_1109	DJI_0053	Thymus pallasianus Heinr.Braun	0	n	1	0	Lamiacea Lindl.
Iz_4_1109	DJI_0053	Tilia cordata Mill.	0	n	1	1	Malvaceae Juss.
Iz_4_1109	DJI_0053	Trifolium alpestre L.	0	n	1	0	Fabaceae Lindl.
Iz_4_1109	DJI_0053	Trifolium arvense L.	0	n	1	0	Fabaceae Lindl.
Iz_4_1109	DJI_0053	Ulmus glabra Huds.	1	n	1	1	Ulmaceae Mirb.
Iz_4_1109	DJI_0053	Verbascum densiflorum Bertol.	0	n	1	0	Scrophulariaceae Juss.
Iz_4_1109	DJI_0053	Veronica austriaca L.	0	n	1	0	Plantaginaceae Juss.
Iz_4_1109	DJI_0053	Veronica incana L.	0	n	1	0	Plantaginaceae Juss.
Iz_4_1109	DJI_0053	Veronica verna L.	0	n	1	0	Plantaginaceae Juss.
Iz_4_1109	DJI_0053	Vincetoxicum hirundinaria Medik	0	n	1	0	Apocynaceae Juss.
Iz_4_1109	DJI_0058	Acer tataricum L.	0	n	1	1	Sapindaceae Juss.
Iz_4_1109	DJI_0058	Achillea millefolium L. s.l.	0	n	1	0	Asteraceae Bercht. & J.Presl
Iz_4_1109	DJI_0058	Allium oleraceum L.	0	n	1	0	Amaryllidaceae J.St.-Hil.
Iz_4_1109	DJI_0058	Ambrosia artemisiifolia L.	0	a	0	0	Asteraceae Bercht. & J.Presl
Iz_4_1109	DJI_0058	Amorpha fruticosa L.	0	a	0	0	Fabaceae Lindl.
Iz_4_1109	DJI_0058	Anthericum ramosum L.	0	n	1	0	Asparagaceae Juss.
Iz_4_1109	DJI_0058	Artemisia marschalliana Spreng.	0	n	1	0	Asteraceae Bercht. & J.Presl
Iz_4_1109	DJI_0058	Asparagus officinalis L.	0	n	1	0	Asparagaceae Juss.
Iz_4_1109	DJI_0058	Asteraceae sp.	0	n	1	0	Asteraceae Bercht. & J.Presl
Iz_4_1109	DJI_0058	Astragalus cicer L.	0	n	1	0	Fabaceae Lindl.
Iz_4_1109	DJI_0058	Atriplex sp.	0		NA	NA	
Iz_4_1109	DJI_0058	Bassia laniflora (S.G.Gmel.) A.J.Scott	0	n	1	0	Amaranthaceae Juss.
Iz_4_1109	DJI_0058	Berberis vulgaris L.	0	n	1	0	Berberidaceae Juss.

Iz_4_1109	DJI_0058	Betula pendula Roth.	0	n	1	1	Betulaceae Gray
Iz_4_1109	DJI_0058	Betula pendula Roth.	0	n	1	0	Poaceae Barnhart
Iz_4_1109	DJI_0058	Bromus inermis Leyss.	0		NA	NA	
Iz_4_1109	DJI_0058	Bromus squarrosus L.	0	a	0	0	Poaceae Barnhart
Iz_4_1109	DJI_0058	Bromus tectorum L.	0	a	0	0	Poaceae Barnhart
Iz_4_1109	DJI_0058	Calamagrostis epigejos (L.) Roth	3	n	1	0	Poaceae Barnhart
Iz_4_1109	DJI_0058	Carduus acanthoides L.	0	n	1	0	Asteraceae Bercht. & J.Presl
Iz_4_1109	DJI_0058	Carex sp.	5	n	1	0	Cyperaceae Juss.
Iz_4_1109	DJI_0058	Centaurea majorovii Dumbadze	0	n	1	0	Asteraceae Bercht. & J.Presl
Iz_4_1109	DJI_0058	Chamaecytisus ruthenicus (Fisch.ex Wol.) Klaskova	0	n	1	0	Fabaceae Lindl.
Iz_4_1109	DJI_0058	Chelidonium majus L.	0	n	1	0	Papaveraceae Juss.
Iz_4_1109	DJI_0058	Chenopodium betaceum Andrz.	0	n	1	0	Amaranthaceae Juss.
Iz_4_1109	DJI_0058	Chenopodium cf. album	0	n	1	0	Amaranthaceae Juss.
Iz_4_1109	DJI_0058	Chenopodium sp.	0		NA	0	Amaranthaceae Juss.
Iz_4_1109	DJI_0058	Chondrilla juncea L.	0	n	1	0	Asteraceae Bercht. & J.Presl
Iz_4_1109	DJI_0058	Cirsium vulgare (Savi) Ten.	0	n	1	0	Asteraceae Bercht. & J.Presl
Iz_4_1109	DJI_0058	Clinopodium acinos (L.) Kuntze	0	n	1	0	Lamiacea Lindl.
Iz_4_1109	DJI_0058	Convallaria majalis L.	0	n	1	0	Asparagaceae Juss.
Iz_4_1109	DJI_0058	Crataegus rhipidophylla Gand.	0	n	1	1	Rosaceae Juss.
Iz_4_1109	DJI_0058	Crepis foetida L.	0	n	1	0	Asteraceae Bercht. & J.Presl
Iz_4_1109	DJI_0058	Dactylis glomerata L.	0	n	1	0	Poaceae Barnhart
Iz_4_1109	DJI_0058	Dianthus campestris M.Bieb.	0	n	1	0	Caryophyllaceae Juss.
Iz_4_1109	DJI_0058	Digitaria sanguinalis (L.) Scop.	0	a	0	0	Poaceae Barnhart
Iz_4_1109	DJI_0058	Elymus repens (L.) Gould	0	n	1	0	Poaceae Barnhart
Iz_4_1109	DJI_0058	Epilobium tetragonum L.	0	n	1	0	Onagraceae Juss.
Iz_4_1109	DJI_0058	Eragrostis minor Host	0	a	0	0	Poaceae Barnhart
Iz_4_1109	DJI_0058	Erigeron annuus (L.) Desf.	0	a	0	0	Asteraceae Bercht. & J.Presl
Iz_4_1109	DJI_0058	Erigeron canadensis L.	15	a	0	0	Asteraceae Bercht. & J.Presl
Iz_4_1109	DJI_0058	Euphorbia seguieriana Neck.	0	n	1	0	Euphorbiaceae Juss.
Iz_4_1109	DJI_0058	Fallopia dumetorum (L.) Holub	4	n	1	0	Polygonaceae Juss.
Iz_4_1109	DJI_0058	Festuca beckeri (Hack.) Trautv.	0	n	1	0	Poaceae Barnhart
Iz_4_1109	DJI_0058	Fragaria vesca L.	0	n	1	0	Rosaceae Juss.
Iz_4_1109	DJI_0058	Frangula alnus Mill.	0	n	1	1	Rhamnaceae Juss.
Iz_4_1109	DJI_0058	Fraxinus pennsylvanica Marshall	0	a	0	1	Oleaceae Hoffmanns. & Link
Iz_4_1109	DJI_0058	Galeopsis bifida Boenn.	0	n	1	0	Lamiacea Lindl.
Iz_4_1109	DJI_0058	Galium verum L.	0	n	1	0	Rubiaceae Juss.
Iz_4_1109	DJI_0058	Genista tinctoria L.	0	n	1	0	Fabaceae Lindl.
Iz_4_1109	DJI_0058	Geranium robertianum L.	0	n	1	0	Geraniaceae Juss.
Iz_4_1109	DJI_0058	Geranium sanquineum L.	0	n	1	0	Geraniaceae Juss.
Iz_4_1109	DJI_0058	Gypsophila paniculata L.	0	n	1	0	Caryophyllaceae Juss.
Iz_4_1109	DJI_0058	Helichrysum arenarium (L.) Moench	0	n	1	0	Asteraceae Bercht. & J.Presl
Iz_4_1109	DJI_0058	Herniaria polygama J.Gay	0	n	1	0	Caryophyllaceae Juss.
Iz_4_1109	DJI_0058	Hieracium robustum Fr.	0	n	1	0	Asteraceae Bercht. & J.Presl
Iz_4_1109	DJI_0058	Hieracium umbellatum L.	0	n	1	NA	Asteraceae Bercht. & J.Presl
Iz_4_1109	DJI_0058	Hylotelephium maximum (L.) Holub	0	n	1	0	Crassulaceae J.St.-Hil.
Iz_4_1109	DJI_0058	Jacobaea borysthenica (DC.) B.Nord. & Greuter	0	n	1	0	Asteraceae Bercht. & J.Presl
Iz_4_1109	DJI_0058	Jasione montana L.	0	n	1	0	Campanulaceae Juss.
Iz_4_1109	DJI_0058	Jurinea cyanoides (L.) Rchb.s.l.	0	n	1	0	Asteraceae Bercht. & J.Presl
Iz_4_1109	DJI_0058	Koeleria glauca (Spreng.) DC.	0	n	1	0	Poaceae Barnhart
Iz_4_1109	DJI_0058	Lactuca serriola L.	0	a	0	0	Asteraceae Bercht. & J.Presl
Iz_4_1109	DJI_0058	Linaria genistifolia (L.) Mill.	0	n	1	0	Plantaginaceae Juss.
Iz_4_1109	DJI_0058	Lomelosia argentea (L.) Greuter & Burdet	0	n	1	0	Caprifoliaceae Juss.
Iz_4_1109	DJI_0058	Melampyrum pratense L.	0	n	1	0	Orobanchaceae Vent.
Iz_4_1109	DJI_0058	Oenothera biennis L.	0	a	0	0	Onagraceae Juss.
Iz_4_1109	DJI_0058	Pentanema britannica (L.) D.Gut.Larr., Santos-Vicente, Anderb., E.Rico & M.M.Mart.Ort.	0	n	1	0	Asteraceae Bercht. & J.Presl
Iz_4_1109	DJI_0058	Peucedanum oreoselinum (L.) Moench	0	n	1	0	Apiaceae Lindl.
Iz_4_1109	DJI_0058	Peucedanum ruthenicum M.Bieb.	0	n	1	0	Apiaceae Lindl.
Iz_4_1109	DJI_0058	Pilosella echioides (Lumn.) F.W.Schultz & Sch.Bip.	0	n	1	0	Asteraceae Bercht. & J.Presl
Iz_4_1109	DJI_0058	Pilosella officinarum F. Schultz et Sch. Bip.	0	n	1	0	Asteraceae Bercht. & J.Presl
Iz_4_1109	DJI_0058	Pilosella sp.	0		NA	0	Asteraceae Bercht. & J.Presl
Iz_4_1109	DJI_0058	Pinus sylvestris L.	0	n	1	1	Pinaceae Spreng. ex F.Rudolphi
Iz_4_1109	DJI_0058	Poa annua L.	0	n	1	0	Poaceae Barnhart
Iz_4_1109	DJI_0058	Poa nemoralis L.	0	n	1	0	Poaceae Barnhart
Iz_4_1109	DJI_0058	Poa sp.	0		NA	0	Poaceae Barnhart
Iz_4_1109	DJI_0058	Polygonatum odoratum (Mill.) Druce	0	n	1	0	Asparagaceae Juss.
Iz_4_1109	DJI_0058	Polygonum arenarium Waldst. & Kit.	0	n	1	0	Polygonaceae Juss.
Iz_4_1109	DJI_0058	Populus nigra L.	0	n	1	1	Salicaceae Mirb.
Iz_4_1109	DJI_0058	Populus tremula L.	0	n	1	1	Salicaceae Mirb.
Iz_4_1109	DJI_0058	Populus Ã canescens (Aiton) Sm.	0	n	1	1	Salicaceae Mirb.
Iz_4_1109	DJI_0058	Potentilla argentea L.	0	n	1	0	Rosaceae Juss.
Iz_4_1109	DJI_0058	Psammophiliella muralis (L.) Ikonn.	0	n	1	0	Caryophyllaceae Juss.
Iz_4_1109	DJI_0058	Psephellus sumensis (Kalen.) Greuter	0	n	1	0	Asteraceae Bercht. & J.Presl
Iz_4_1109	DJI_0058	Pulsatilla pratensis (L.) Mill. s.l.	0	n	1	0	Ranunculaceae Juss.
Iz_4_1109	DJI_0058	Pyrus communis L.	0	a	0	1	Rosaceae Juss.
Iz_4_1109	DJI_0058	Quercus robur L.	0	n	1	1	Fagaceae Dumort.
Iz_4_1109	DJI_0058	Robinia pseudoacacia L.	0	a	0	1	Fabaceae Lindl.
Iz_4_1109	DJI_0058	Rosa sp.	0		NA	1	Rosaceae Juss.
Iz_4_1109	DJI_0058	Rubus idaeus L.	0	n	1	0	Rosaceae Juss.
Iz_4_1109	DJI_0058	Rumex acetosella L.	0	n	1	0	Polygonaceae Juss.
Iz_4_1109	DJI_0058	Rumex thyrsiflorus Fingerh.	0	n	1	0	Polygonaceae Juss.
Iz_4_1109	DJI_0058	Salix alba L.	0	n	1	1	Salicaceae Mirb.
Iz_4_1109	DJI_0058	Sambucus nigra L.	0	n	1	1	Viburnaceae Raf.
Iz_4_1109	DJI_0058	Secale sylvestre Host	0	n	1	0	Poaceae Barnhart
Iz_4_1109	DJI_0058	Sempervivum ruthenicum Schnittsp. & C.B. Lehm.	0	n	1	0	Crassulaceae J.St.-Hil.
Iz_4_1109	DJI_0058	Setaria viridis (L.) P. Beauv.	0	n	1	0	Poaceae Barnhart
Iz_4_1109	DJI_0058	Silene borysthenica (Gruner) Walters	0	n	1	0	Caryophyllaceae Juss.
Iz_4_1109	DJI_0058	Silene latifolia subsp. alba (Mill.) Greuter & Burdet	0	n	1	0	Caryophyllaceae Juss.
Iz_4_1109	DJI_0058	Solidago virgaurea L.	0	n	1	0	Asteraceae Bercht. & J.Presl
Iz_4_1109	DJI_0058	Sorbus aucuparia L.	0	n	1	1	Rosaceae Juss.
Iz_4_1109	DJI_0058	Stachys recta L.	0	n	1	0	Lamiacea Lindl.
Iz_4_1109	DJI_0058	Tanacetum vulgare L.	0	n	1	0	Asteraceae Bercht. & J.Presl
Iz_4_1109	DJI_0058	Thymus pallasianus Heinr.Braun	0	n	1	0	Lamiacea Lindl.
Iz_4_1109	DJI_0058	Tilia cordata Mill.	0	n	1	1	Malvaceae Juss.
Iz_4_1109	DJI_0058	Trifolium alpestre L.	0	n	1	0	Fabaceae Lindl.
Iz_4_1109	DJI_0058	Trifolium arvense L.	0	n	1	0	Fabaceae Lindl.
Iz_4_1109	DJI_0058	Ulmus glabra Huds.	0	n	1	1	Ulmaceae Mirb.
Iz_4_1109	DJI_0058	Verbascum densiflorum Bertol.	0	n	1	0	Scrophulariaceae Juss.
Iz_4_1109	DJI_0058	Veronica austriaca L.	0	n	1	0	Plantaginaceae Juss.

Iz_4_1109	DJI_0058	Veronica incana L.	0	n	1	0	Plantaginaceae Juss.
Iz_4_1109	DJI_0058	Veronica verna L.	0	n	1	0	Plantaginaceae Juss.
Iz_4_1109	DJI_0058	Vincetoxicum hirundinaria Medik	0	n	1	0	Apocynaceae Juss.
Iz_4_1109	DJI_0006	Acer tataricum L.	0	n	1	1	Sapindaceae Juss.
Iz_4_1109	DJI_0006	Achillea millefolium L. s.l.	0	n	1	0	Asteraceae Bercht. & J.Presl
Iz_4_1109	DJI_0006	Allium oleraceum L.	0	n	1	0	Amaryllidaceae J.St.-Hil.
Iz_4_1109	DJI_0006	Ambrosia artemisiifolia L.	0	a	0	0	Asteraceae Bercht. & J.Presl
Iz_4_1109	DJI_0006	Amorpha fruticosa L.	0	a	0	0	Fabaceae Lindl.
Iz_4_1109	DJI_0006	Anthericum ramosum L.	0	n	1	0	Asparagaceae Juss.
Iz_4_1109	DJI_0006	Artemisia marschalliana Spreng.	0	n	1	0	Asteraceae Bercht. & J.Presl
Iz_4_1109	DJI_0006	Asparagus officinalis L.	0	n	1	0	Asparagaceae Juss.
Iz_4_1109	DJI_0006	Asteraceae sp.	0	n	1	0	Asteraceae Bercht. & J.Presl
Iz_4_1109	DJI_0006	Astragalus cicer L.	0	n	1	0	Fabaceae Lindl.
Iz_4_1109	DJI_0006	Atriplex sp.	0		NA	NA	
Iz_4_1109	DJI_0006	Bassia laniflora (S.G.Gmel.) A.J.Scott	0	n	1	0	Amaranthaceae Juss.
Iz_4_1109	DJI_0006	Berberis vulgaris L.	0	n	1	0	Berberidaceae Juss.
Iz_4_1109	DJI_0006	Betula pendula Roth.	0	n	1	1	Betulaceae Gray
Iz_4_1109	DJI_0006	Betula pendula Roth.	0	n	1	0	Poaceae Barnhart
Iz_4_1109	DJI_0006	Bromus inermis Leyss.	0		NA	NA	
Iz_4_1109	DJI_0006	Bromus squarrosus L.	0	a	0	0	Poaceae Barnhart
Iz_4_1109	DJI_0006	Bromus tectorum L.	0	a	0	0	Poaceae Barnhart
Iz_4_1109	DJI_0006	Calamagrostis epigejos (L.) Roth	0.2	n	1	0	Poaceae Barnhart
Iz_4_1109	DJI_0006	Carduus acanthoides L.	0	n	1	0	Asteraceae Bercht. & J.Presl
Iz_4_1109	DJI_0006	Carex sp.	0	n	1	0	Cyperaceae Juss.
Iz_4_1109	DJI_0006	Centaurea majorovii Dumbadze	0	n	1	0	Asteraceae Bercht. & J.Presl
Iz_4_1109	DJI_0006	Chamaecytisus ruthenicus (Fisch.ex Wol.) Klaskova	0	n	1	0	Fabaceae Lindl.
Iz_4_1109	DJI_0006	Chelidonium majus L.	0	n	1	0	Papaveraceae Juss.
Iz_4_1109	DJI_0006	Chenopodium betaceum Andrz.	0	n	1	0	Amaranthaceae Juss.
Iz_4_1109	DJI_0006	Chenopodium cf. album	0	n	1	0	Amaranthaceae Juss.
Iz_4_1109	DJI_0006	Chenopodium sp.	0		NA	0	Amaranthaceae Juss.
Iz_4_1109	DJI_0006	Chondrilla juncea L.	0	n	1	0	Asteraceae Bercht. & J.Presl
Iz_4_1109	DJI_0006	Cirsium vulgare (Savi) Ten.	0	n	1	0	Asteraceae Bercht. & J.Presl
Iz_4_1109	DJI_0006	Clinopodium acinos (L.) Kuntze	0	n	1	0	Lamiacea Lindl.
Iz_4_1109	DJI_0006	Convallaria majalis L.	0	n	1	0	Asparagaceae Juss.
Iz_4_1109	DJI_0006	Crataegus rhipidophylla Gand.	0	n	1	1	Rosaceae Juss.
Iz_4_1109	DJI_0006	Crepis foetida L.	0	n	1	0	Asteraceae Bercht. & J.Presl
Iz_4_1109	DJI_0006	Dactylis glomerata L.	0	n	1	0	Poaceae Barnhart
Iz_4_1109	DJI_0006	Dianthus campestris M.Bieb.	0	n	1	0	Caryophyllaceae Juss.
Iz_4_1109	DJI_0006	Digitaria sanguinalis (L.) Scop.	0	a	0	0	Poaceae Barnhart
Iz_4_1109	DJI_0006	Elymus repens (L.) Gould	0	n	1	0	Poaceae Barnhart
Iz_4_1109	DJI_0006	Epilobium tetragonum L.	0	n	1	0	Onagraceae Juss.
Iz_4_1109	DJI_0006	Eragrostis minor Host	0	a	0	0	Poaceae Barnhart
Iz_4_1109	DJI_0006	Erigeron annuus (L.) Desf.	0	a	0	0	Asteraceae Bercht. & J.Presl
Iz_4_1109	DJI_0006	Erigeron canadensis L.	1	a	0	0	Asteraceae Bercht. & J.Presl
Iz_4_1109	DJI_0006	Euphorbia seguieriana Neck.	0	n	1	0	Euphorbiaceae Juss.
Iz_4_1109	DJI_0006	Fallopia dumetorum (L.) Holub	0	n	1	0	Polygonaceae Juss.
Iz_4_1109	DJI_0006	Festuca beckeri (Hack.) Trautv.	0	n	1	0	Poaceae Barnhart
Iz_4_1109	DJI_0006	Fragaria vesca L.	0	n	1	0	Rosaceae Juss.
Iz_4_1109	DJI_0006	Frangula alnus Mill.	0	n	1	1	Rhamnaceae Juss.
Iz_4_1109	DJI_0006	Fraxinus pennsylvanica Marshall	0	a	0	1	Oleaceae Hoffmanns. & Link
Iz_4_1109	DJI_0006	Galeopsis bifida Boenn.	0	n	1	0	Lamiacea Lindl.
Iz_4_1109	DJI_0006	Galium verum L.	0	n	1	0	Rubiaceae Juss.
Iz_4_1109	DJI_0006	Genista tinctoria L.	0	n	1	0	Fabaceae Lindl.
Iz_4_1109	DJI_0006	Geranium robertianum L.	0	n	1	0	Geraniaceae Juss.
Iz_4_1109	DJI_0006	Geranium sanquineum L.	0	n	1	0	Geraniaceae Juss.
Iz_4_1109	DJI_0006	Gypsophila paniculata L.	0	n	1	0	Caryophyllaceae Juss.
Iz_4_1109	DJI_0006	Helichrysum arenarium (L.) Moench	0	n	1	0	Asteraceae Bercht. & J.Presl
Iz_4_1109	DJI_0006	Herniaria polygama J.Gay	0	n	1	0	Caryophyllaceae Juss.
Iz_4_1109	DJI_0006	Hieracium robustum Fr.	0	n	1	0	Asteraceae Bercht. & J.Presl
Iz_4_1109	DJI_0006	Hieracium umbellatum L.	0	n	1	NA	Asteraceae Bercht. & J.Presl
Iz_4_1109	DJI_0006	Hylotelephium maximum (L.) Holub	0	n	1	0	Crassulaceae J.St.-Hil.
Iz_4_1109	DJI_0006	Jacobaea borysthenica (DC.) B.Nord. & Greuter	0	n	1	0	Asteraceae Bercht. & J.Presl
Iz_4_1109	DJI_0006	Jasione montana L.	0	n	1	0	Campanulaceae Juss.
Iz_4_1109	DJI_0006	Jurinea cyanoides (L.) Rchb.s.l.	0	n	1	0	Asteraceae Bercht. & J.Presl
Iz_4_1109	DJI_0006	Koeleria glauca (Spreng.) DC.	0	n	1	0	Poaceae Barnhart
Iz_4_1109	DJI_0006	Lactuca serriola L.	0	a	0	0	Asteraceae Bercht. & J.Presl
Iz_4_1109	DJI_0006	Linaria genistifolia (L.) Mill.	0	n	1	0	Plantaginaceae Juss.
Iz_4_1109	DJI_0006	Lomelosia argentea (L.) Greuter & Burdet	0	n	1	0	Caprifoliaceae Juss.
Iz_4_1109	DJI_0006	Melampyrum pratense L.	0	n	1	0	Orobanchaceae Vent.
Iz_4_1109	DJI_0006	Oenothera biennis L.	0	a	0	0	Onagraceae Juss.
Iz_4_1109	DJI_0006	Pentanema britannica (L.) D.Gut.Larr., Santos-Vicente, Anderb., E.Rico & M.M.Mart.Ort.	0	n	1	0	Asteraceae Bercht. & J.Presl
Iz_4_1109	DJI_0006	Peucedanum oreoselinum (L.) Moench	0	n	1	0	Apiaceae Lindl.
Iz_4_1109	DJI_0006	Peucedanum ruthenicum M.Bieb.	0	n	1	0	Apiaceae Lindl.
Iz_4_1109	DJI_0006	Pilosella echioides (Lumn.) F.W.Schultz & Sch.Bip.	0	n	1	0	Asteraceae Bercht. & J.Presl
Iz_4_1109	DJI_0006	Pilosella officinarum F. Schultz et Sch. Bip.	0	n	1	0	Asteraceae Bercht. & J.Presl
Iz_4_1109	DJI_0006	Pilosella sp.	0		NA	0	Asteraceae Bercht. & J.Presl
Iz_4_1109	DJI_0006	Pinus sylvestris L.	0	n	1	1	Pinaceae Spreng. ex F.Rudolphi
Iz_4_1109	DJI_0006	Poa annua L.	0	n	1	0	Poaceae Barnhart
Iz_4_1109	DJI_0006	Poa nemoralis L.	0	n	1	0	Poaceae Barnhart
Iz_4_1109	DJI_0006	Poa sp.	0		NA	0	Poaceae Barnhart
Iz_4_1109	DJI_0006	Polygonatum odoratum (Mill.) Druce	0	n	1	0	Asparagaceae Juss.
Iz_4_1109	DJI_0006	Polygonum arenarium Waldst. & Kit.	0	n	1	0	Polygonaceae Juss.
Iz_4_1109	DJI_0006	Populus nigra L.	0	n	1	1	Salicaceae Mirb.
Iz_4_1109	DJI_0006	Populus tremula L.	0	n	1	1	Salicaceae Mirb.
Iz_4_1109	DJI_0006	Populus Ã canescens (Aiton) Sm.	0	n	1	1	Salicaceae Mirb.
Iz_4_1109	DJI_0006	Potentilla argentea L.	0	n	1	0	Rosaceae Juss.
Iz_4_1109	DJI_0006	Psammophiliella muralis (L.) Ikonn.	0	n	1	0	Caryophyllaceae Juss.
Iz_4_1109	DJI_0006	Psephellus sumensis (Kalen.) Greuter	0	n	1	0	Asteraceae Bercht. & J.Presl
Iz_4_1109	DJI_0006	Pulsatilla pratensis (L.) Mill. s.l.	0	n	1	0	Ranunculaceae Juss.
Iz_4_1109	DJI_0006	Pyrus communis L.	0	a	0	1	Rosaceae Juss.
Iz_4_1109	DJI_0006	Quercus robur L.	0	n	1	1	Fagaceae Dumort.
Iz_4_1109	DJI_0006	Robinia pseudoacacia L.	0	a	0	1	Fabaceae Lindl.
Iz_4_1109	DJI_0006	Rosa sp.	0		NA	1	Rosaceae Juss.
Iz_4_1109	DJI_0006	Rubus idaeus L.	0	n	1	0	Rosaceae Juss.
Iz_4_1109	DJI_0006	Rumex acetosella L.	0	n	1	0	Polygonaceae Juss.
Iz_4_1109	DJI_0006	Rumex thyrsiflorus Fingerh.	0	n	1	0	Polygonaceae Juss.
Iz_4_1109	DJI_0006	Salix alba L.	0	n	1	1	Salicaceae Mirb.
Iz_4_1109	DJI_0006	Sambucus nigra L.	0	n	1	1	Viburnaceae Raf.

Iz_4_1109	DJI_0006	Secale sylvestre Host	0	n	1	0	Poaceae Barnhart
Iz_4_1109	DJI_0006	Sempervivum ruthenicum Schnittsp. & C.B. Lehm.	0	n	1	0	Crassulaceae J.St.-Hil.
Iz_4_1109	DJI_0006	Setaria viridis (L.) P. Beauv.	0	n	1	0	Poaceae Barnhart
Iz_4_1109	DJI_0006	Silene borysthenica (Gruner) Walters	0	n	1	0	Caryophyllaceae Juss.
Iz_4_1109	DJI_0006	Silene latifolia subsp. alba (Mill.) Greuter & Burdet	0	n	1	0	Caryophyllaceae Juss.
Iz_4_1109	DJI_0006	Solidago virgaurea L.	0	n	1	0	Asteraceae Bercht. & J.Presl
Iz_4_1109	DJI_0006	Sorbus aucuparia L.	0	n	1	1	Rosaceae Juss.
Iz_4_1109	DJI_0006	Stachys recta L.	0	n	1	0	Lamiacea Lindl.
Iz_4_1109	DJI_0006	Tanacetum vulgare L.	0	n	1	0	Asteraceae Bercht. & J.Presl
Iz_4_1109	DJI_0006	Thymus pallasianus Heinr.Braun	0	n	1	0	Lamiacea Lindl.
Iz_4_1109	DJI_0006	Tilia cordata Mill.	0	n	1	1	Malvaceae Juss.
Iz_4_1109	DJI_0006	Trifolium alpestre L.	0	n	1	0	Fabaceae Lindl.
Iz_4_1109	DJI_0006	Trifolium arvense L.	0	n	1	0	Fabaceae Lindl.
Iz_4_1109	DJI_0006	Ulmus glabra Huds.	0	n	1	1	Ulmaceae Mirb.
Iz_4_1109	DJI_0006	Verbascum densiflorum Bertol.	0	n	1	0	Scrophulariaceae Juss.
Iz_4_1109	DJI_0006	Veronica austriaca L.	0	n	1	0	Plantaginaceae Juss.
Iz_4_1109	DJI_0006	Veronica incana L.	0	n	1	0	Plantaginaceae Juss.
Iz_4_1109	DJI_0006	Veronica verna L.	0	n	1	0	Plantaginaceae Juss.
Iz_4_1109	DJI_0006	Vincetoxicum hirundinaria Medik	0	n	1	0	Apocynaceae Juss.
Iz_4_1109	DJI_0060	Acer tataricum L.	0	n	1	1	Sapindaceae Juss.
Iz_4_1109	DJI_0060	Achillea millefolium L. s.l.	0	n	1	0	Asteraceae Bercht. & J.Presl
Iz_4_1109	DJI_0060	Allium oleraceum L.	0	n	1	0	Amaryllidaceae J.St.-Hil.
Iz_4_1109	DJI_0060	Ambrosia artemisiifolia L.	0	a	0	0	Asteraceae Bercht. & J.Presl
Iz_4_1109	DJI_0060	Amorpha fruticosa L.	0	a	0	0	Fabaceae Lindl.
Iz_4_1109	DJI_0060	Anthericum ramosum L.	0	n	1	0	Asparagaceae Juss.
Iz_4_1109	DJI_0060	Artemisia marschalliana Spreng.	0	n	1	0	Asteraceae Bercht. & J.Presl
Iz_4_1109	DJI_0060	Asparagus officinalis L.	0	n	1	0	Asparagaceae Juss.
Iz_4_1109	DJI_0060	Asteraceae sp.	0	n	1	0	Asteraceae Bercht. & J.Presl
Iz_4_1109	DJI_0060	Astragalus cicer L.	0	n	1	0	Fabaceae Lindl.
Iz_4_1109	DJI_0060	Atriplex sp.	0		NA	NA	
Iz_4_1109	DJI_0060	Bassia laniflora (S.G.Gmel.) A.J.Scott	0	n	1	0	Amaranthaceae Juss.
Iz_4_1109	DJI_0060	Berberis vulgaris L.	0	n	1	0	Berberidaceae Juss.
Iz_4_1109	DJI_0060	Betula pendula Roth.	0	n	1	1	Betulaceae Gray
Iz_4_1109	DJI_0060	Betula pendula Roth.	0	n	1	0	Poaceae Barnhart
Iz_4_1109	DJI_0060	Bromus inermis Leyss.	0		NA	NA	
Iz_4_1109	DJI_0060	Bromus squarrosus L.	0	a	0	0	Poaceae Barnhart
Iz_4_1109	DJI_0060	Bromus tectorum L.	0	a	0	0	Poaceae Barnhart
Iz_4_1109	DJI_0060	Calamagrostis epigejos (L.) Roth	2	n	1	0	Poaceae Barnhart
Iz_4_1109	DJI_0060	Carduus acanthoides L.	0	n	1	0	Asteraceae Bercht. & J.Presl
Iz_4_1109	DJI_0060	Carex sp.	0	n	1	0	Cyperaceae Juss.
Iz_4_1109	DJI_0060	Centaurea majorovii Dumbadze	0	n	1	0	Asteraceae Bercht. & J.Presl
Iz_4_1109	DJI_0060	Chamaecytisus ruthenicus (Fisch.ex Wol.) Klaskova	0	n	1	0	Fabaceae Lindl.
Iz_4_1109	DJI_0060	Chelidonium majus L.	0	n	1	0	Papaveraceae Juss.
Iz_4_1109	DJI_0060	Chenopodium betaceum Andrz.	0	n	1	0	Amaranthaceae Juss.
Iz_4_1109	DJI_0060	Chenopodium cf. album	0	n	1	0	Amaranthaceae Juss.
Iz_4_1109	DJI_0060	Chenopodium sp.	0		NA	0	Amaranthaceae Juss.
Iz_4_1109	DJI_0060	Chondrilla juncea L.	0	n	1	0	Asteraceae Bercht. & J.Presl
Iz_4_1109	DJI_0060	Cirsium vulgare (Savi) Ten.	0	n	1	0	Asteraceae Bercht. & J.Presl
Iz_4_1109	DJI_0060	Clinopodium acinos (L.) Kuntze	0	n	1	0	Lamiacea Lindl.
Iz_4_1109	DJI_0060	Convallaria majalis L.	0	n	1	0	Asparagaceae Juss.
Iz_4_1109	DJI_0060	Crataegus rhipidophylla Gand.	0	n	1	1	Rosaceae Juss.
Iz_4_1109	DJI_0060	Crepis foetida L.	0	n	1	0	Asteraceae Bercht. & J.Presl
Iz_4_1109	DJI_0060	Dactylis glomerata L.	0	n	1	0	Poaceae Barnhart
Iz_4_1109	DJI_0060	Dianthus campestris M.Bieb.	0	n	1	0	Caryophyllaceae Juss.
Iz_4_1109	DJI_0060	Digitaria sanguinalis (L.) Scop.	0	a	0	0	Poaceae Barnhart
Iz_4_1109	DJI_0060	Elymus repens (L.) Gould	0	n	1	0	Poaceae Barnhart
Iz_4_1109	DJI_0060	Epilobium tetragonum L.	0	n	1	0	Onagraceae Juss.
Iz_4_1109	DJI_0060	Eragrostis minor Host	0	a	0	0	Poaceae Barnhart
Iz_4_1109	DJI_0060	Erigeron annuus (L.) Desf.	0	a	0	0	Asteraceae Bercht. & J.Presl
Iz_4_1109	DJI_0060	Erigeron canadensis L.	5	a	0	0	Asteraceae Bercht. & J.Presl
Iz_4_1109	DJI_0060	Euphorbia seguieriana Neck.	0	n	1	0	Euphorbiaceae Juss.
Iz_4_1109	DJI_0060	Fallopia dumetorum (L.) Holub	2	n	1	0	Polygonaceae Juss.
Iz_4_1109	DJI_0060	Festuca beckeri (Hack.) Trautv.	0	n	1	0	Poaceae Barnhart
Iz_4_1109	DJI_0060	Fragaria vesca L.	0	n	1	0	Rosaceae Juss.
Iz_4_1109	DJI_0060	Frangula alnus Mill.	0	n	1	1	Rhamnaceae Juss.
Iz_4_1109	DJI_0060	Fraxinus pennsylvanica Marshall	0	a	0	1	Oleaceae Hoffmanns. & Link
Iz_4_1109	DJI_0060	Galeopsis bifida Boenn.	0	n	1	0	Lamiacea Lindl.
Iz_4_1109	DJI_0060	Galium verum L.	0	n	1	0	Rubiaceae Juss.
Iz_4_1109	DJI_0060	Genista tinctoria L.	0	n	1	0	Fabaceae Lindl.
Iz_4_1109	DJI_0060	Geranium robertianum L.	0	n	1	0	Geraniaceae Juss.
Iz_4_1109	DJI_0060	Geranium sanquineum L.	0	n	1	0	Geraniaceae Juss.
Iz_4_1109	DJI_0060	Gypsophila paniculata L.	0	n	1	0	Caryophyllaceae Juss.
Iz_4_1109	DJI_0060	Helichrysum arenarium (L.) Moench	0	n	1	0	Asteraceae Bercht. & J.Presl
Iz_4_1109	DJI_0060	Herniaria polygama J.Gay	0	n	1	0	Caryophyllaceae Juss.
Iz_4_1109	DJI_0060	Hieracium robustum Fr.	0	n	1	0	Asteraceae Bercht. & J.Presl
Iz_4_1109	DJI_0060	Hieracium umbellatum L.	0	n	1	NA	Asteraceae Bercht. & J.Presl
Iz_4_1109	DJI_0060	Hylotelephium maximum (L.) Holub	0	n	1	0	Crassulaceae J.St.-Hil.
Iz_4_1109	DJI_0060	Jacobaea borysthenica (DC.) B.Nord. & Greuter	0	n	1	0	Asteraceae Bercht. & J.Presl
Iz_4_1109	DJI_0060	Jasione montana L.	0	n	1	0	Campanulaceae Juss.
Iz_4_1109	DJI_0060	Jurinea cyanoides (L.) Rchb.s.l.	0	n	1	0	Asteraceae Bercht. & J.Presl
Iz_4_1109	DJI_0060	Koeleria glauca (Spreng.) DC.	0	n	1	0	Poaceae Barnhart
Iz_4_1109	DJI_0060	Lactuca serriola L.	0	a	0	0	Asteraceae Bercht. & J.Presl
Iz_4_1109	DJI_0060	Linaria genistifolia (L.) Mill.	0	n	1	0	Plantaginaceae Juss.
Iz_4_1109	DJI_0060	Lomelosia argentea (L.) Greuter & Burdet	0	n	1	0	Caprifoliaceae Juss.
Iz_4_1109	DJI_0060	Melampyrum pratense L.	0	n	1	0	Orobanchaceae Vent.
Iz_4_1109	DJI_0060	Oenothera biennis L.	0	a	0	0	Onagraceae Juss.
Iz_4_1109	DJI_0060	Pentanema britannica (L.) D.Gut.Larr., Santos-Vicente, Anderb., E.Rico & M.M.Mart.Ort.	0	n	1	0	Asteraceae Bercht. & J.Presl
Iz_4_1109	DJI_0060	Peucedanum oreoselinum (L.) Moench	0.2	n	1	0	Apiaceae Lindl.
Iz_4_1109	DJI_0060	Peucedanum ruthenicum M.Bieb.	0	n	1	0	Apiaceae Lindl.
Iz_4_1109	DJI_0060	Pilosella echioides (Lumn.) F.W.Schultz & Sch.Bip.	0	n	1	0	Asteraceae Bercht. & J.Presl
Iz_4_1109	DJI_0060	Pilosella officinarum F. Schultz et Sch. Bip.	0	n	1	0	Asteraceae Bercht. & J.Presl
Iz_4_1109	DJI_0060	Pilosella sp.	0		NA	0	Asteraceae Bercht. & J.Presl
Iz_4_1109	DJI_0060	Pinus sylvestris L.	0	n	1	1	Pinaceae Spreng. ex F.Rudolphi
Iz_4_1109	DJI_0060	Poa annua L.	0	n	1	0	Poaceae Barnhart
Iz_4_1109	DJI_0060	Poa nemoralis L.	0	n	1	0	Poaceae Barnhart
Iz_4_1109	DJI_0060	Poa sp.	0		NA	0	Poaceae Barnhart
Iz_4_1109	DJI_0060	Polygonatum odoratum (Mill.) Druce	0	n	1	0	Asparagaceae Juss.
Iz_4_1109	DJI_0060	Polygonum arenarium Waldst. & Kit.	0	n	1	0	Polygonaceae Juss.

Iz_4_1109	DJI_0060	Populus nigra L.	0	n	1	1	Salicaceae Mirb.
Iz_4_1109	DJI_0060	Populus tremula L.	0	n	1	1	Salicaceae Mirb.
Iz_4_1109	DJI_0060	Populus Ã canescens (Aiton) Sm.	0	n	1	1	Salicaceae Mirb.
Iz_4_1109	DJI_0060	Potentilla argentea L.	0	n	1	0	Rosaceae Juss.
Iz_4_1109	DJI_0060	Psammophiliella muralis (L.) Ikonn.	0	n	1	0	Caryophyllaceae Juss.
Iz_4_1109	DJI_0060	Psephellus sumensis (Kalen.) Greuter	0	n	1	0	Asteraceae Bercht. & J.Presl
Iz_4_1109	DJI_0060	Pulsatilla pratensis (L.) Mill. s.l.	0	n	1	0	Ranunculaceae Juss.
Iz_4_1109	DJI_0060	Pyrus communis L.	0	a	0	1	Rosaceae Juss.
Iz_4_1109	DJI_0060	Quercus robur L.	0	n	1	1	Fagaceae Dumort.
Iz_4_1109	DJI_0060	Robinia pseudoacacia L.	0	a	0	1	Fabaceae Lindl.
Iz_4_1109	DJI_0060	Rosa sp.	0		NA	1	Rosaceae Juss.
Iz_4_1109	DJI_0060	Rubus idaeus L.	0	n	1	0	Rosaceae Juss.
Iz_4_1109	DJI_0060	Rumex acetosella L.	5	n	1	0	Polygonaceae Juss.
Iz_4_1109	DJI_0060	Rumex thyrsiflorus Fingerh.	0	n	1	0	Polygonaceae Juss.
Iz_4_1109	DJI_0060	Salix alba L.	0	n	1	1	Salicaceae Mirb.
Iz_4_1109	DJI_0060	Sambucus nigra L.	0	n	1	1	Viburnaceae Raf.
Iz_4_1109	DJI_0060	Secale sylvestre Host	0	n	1	0	Poaceae Barnhart
Iz_4_1109	DJI_0060	Sempervivum ruthenicum Schnittsp. & C.B. Lehm.	0	n	1	0	Crassulaceae J.St.-Hil.
Iz_4_1109	DJI_0060	Setaria viridis (L.) P. Beauv.	0	n	1	0	Poaceae Barnhart
Iz_4_1109	DJI_0060	Silene borysthenica (Gruner) Walters	0	n	1	0	Caryophyllaceae Juss.
Iz_4_1109	DJI_0060	Silene latifolia subsp. alba (Mill.) Greuter & Burdet	0	n	1	0	Caryophyllaceae Juss.
Iz_4_1109	DJI_0060	Solidago virgaurea L.	0	n	1	0	Asteraceae Bercht. & J.Presl
Iz_4_1109	DJI_0060	Sorbus aucuparia L.	0	n	1	1	Rosaceae Juss.
Iz_4_1109	DJI_0060	Stachys recta L.	0	n	1	0	Lamiacea Lindl.
Iz_4_1109	DJI_0060	Tanacetum vulgare L.	0	n	1	0	Asteraceae Bercht. & J.Presl
Iz_4_1109	DJI_0060	Thymus pallasianus Heinr.Braun	0	n	1	0	Lamiacea Lindl.
Iz_4_1109	DJI_0060	Tilia cordata Mill.	0	n	1	1	Malvaceae Juss.
Iz_4_1109	DJI_0060	Trifolium alpestre L.	0	n	1	0	Fabaceae Lindl.
Iz_4_1109	DJI_0060	Trifolium arvense L.	0	n	1	0	Fabaceae Lindl.
Iz_4_1109	DJI_0060	Ulmus glabra Huds.	0	n	1	1	Ulmaceae Mirb.
Iz_4_1109	DJI_0060	Verbascum densiflorum Bertol.	0	n	1	0	Scrophulariaceae Juss.
Iz_4_1109	DJI_0060	Veronica austriaca L.	0	n	1	0	Plantaginaceae Juss.
Iz_4_1109	DJI_0060	Veronica incana L.	0	n	1	0	Plantaginaceae Juss.
Iz_4_1109	DJI_0060	Veronica verna L.	0	n	1	0	Plantaginaceae Juss.
Iz_4_1109	DJI_0060	Vincetoxicum hirundinaria Medik	0	n	1	0	Apocynaceae Juss.
Iz_4_1109	DJI_0063	Acer tataricum L.	0	n	1	1	Sapindaceae Juss.
Iz_4_1109	DJI_0063	Achillea millefolium L. s.l.	0	n	1	0	Asteraceae Bercht. & J.Presl
Iz_4_1109	DJI_0063	Allium oleraceum L.	0	n	1	0	Amaryllidaceae J.St.-Hil.
Iz_4_1109	DJI_0063	Ambrosia artemisiifolia L.	0	a	0	0	Asteraceae Bercht. & J.Presl
Iz_4_1109	DJI_0063	Amorpha fruticosa L.	0	a	0	0	Fabaceae Lindl.
Iz_4_1109	DJI_0063	Anthericum ramosum L.	0	n	1	0	Asparagaceae Juss.
Iz_4_1109	DJI_0063	Artemisia marschalliana Spreng.	0	n	1	0	Asteraceae Bercht. & J.Presl
Iz_4_1109	DJI_0063	Asparagus officinalis L.	0	n	1	0	Asparagaceae Juss.
Iz_4_1109	DJI_0063	Asteraceae sp.	0	n	1	0	Asteraceae Bercht. & J.Presl
Iz_4_1109	DJI_0063	Astragalus cicer L.	0	n	1	0	Fabaceae Lindl.
Iz_4_1109	DJI_0063	Atriplex sp.	0		NA	NA	
Iz_4_1109	DJI_0063	Bassia laniflora (S.G.Gmel.) A.J.Scott	0	n	1	0	Amaranthaceae Juss.
Iz_4_1109	DJI_0063	Berberis vulgaris L.	0	n	1	0	Berberidaceae Juss.
Iz_4_1109	DJI_0063	Betula pendula Roth.	0	n	1	1	Betulaceae Gray
Iz_4_1109	DJI_0063	Betula pendula Roth.	0	n	1	0	Poaceae Barnhart
Iz_4_1109	DJI_0063	Bromus inermis Leyss.	0		NA	NA	
Iz_4_1109	DJI_0063	Bromus squarrosus L.	0	a	0	0	Poaceae Barnhart
Iz_4_1109	DJI_0063	Bromus tectorum L.	0	a	0	0	Poaceae Barnhart
Iz_4_1109	DJI_0063	Calamagrostis epigejos (L.) Roth	5	n	1	0	Poaceae Barnhart
Iz_4_1109	DJI_0063	Carduus acanthoides L.	0	n	1	0	Asteraceae Bercht. & J.Presl
Iz_4_1109	DJI_0063	Carex sp.	10	n	1	0	Cyperaceae Juss.
Iz_4_1109	DJI_0063	Centaurea majorovii Dumbadze	0	n	1	0	Asteraceae Bercht. & J.Presl
Iz_4_1109	DJI_0063	Chamaecytisus ruthenicus (Fisch.ex Wol.) Klaskova	0	n	1	0	Fabaceae Lindl.
Iz_4_1109	DJI_0063	Chelidonium majus L.	0	n	1	0	Papaveraceae Juss.
Iz_4_1109	DJI_0063	Chenopodium betaceum Andrz.	0	n	1	0	Amaranthaceae Juss.
Iz_4_1109	DJI_0063	Chenopodium cf. album	0	n	1	0	Amaranthaceae Juss.
Iz_4_1109	DJI_0063	Chenopodium sp.	0		NA	0	Amaranthaceae Juss.
Iz_4_1109	DJI_0063	Chondrilla juncea L.	0	n	1	0	Asteraceae Bercht. & J.Presl
Iz_4_1109	DJI_0063	Cirsium vulgare (Savi) Ten.	0	n	1	0	Asteraceae Bercht. & J.Presl
Iz_4_1109	DJI_0063	Clinopodium acinos (L.) Kuntze	0	n	1	0	Lamiacea Lindl.
Iz_4_1109	DJI_0063	Convallaria majalis L.	0	n	1	0	Asparagaceae Juss.
Iz_4_1109	DJI_0063	Crataegus rhipidophylla Gand.	0	n	1	1	Rosaceae Juss.
Iz_4_1109	DJI_0063	Crepis foetida L.	0	n	1	0	Asteraceae Bercht. & J.Presl
Iz_4_1109	DJI_0063	Dactylis glomerata L.	0	n	1	0	Poaceae Barnhart
Iz_4_1109	DJI_0063	Dianthus campestris M.Bieb.	0	n	1	0	Caryophyllaceae Juss.
Iz_4_1109	DJI_0063	Digitaria sanguinalis (L.) Scop.	0	a	0	0	Poaceae Barnhart
Iz_4_1109	DJI_0063	Elymus repens (L.) Gould	0	n	1	0	Poaceae Barnhart
Iz_4_1109	DJI_0063	Epilobium tetragonum L.	0	n	1	0	Onagraceae Juss.
Iz_4_1109	DJI_0063	Eragrostis minor Host	0	a	0	0	Poaceae Barnhart
Iz_4_1109	DJI_0063	Erigeron annuus (L.) Desf.	0.2	a	0	0	Asteraceae Bercht. & J.Presl
Iz_4_1109	DJI_0063	Erigeron canadensis L.	2	a	0	0	Asteraceae Bercht. & J.Presl
Iz_4_1109	DJI_0063	Euphorbia seguieriana Neck.	0	n	1	0	Euphorbiaceae Juss.
Iz_4_1109	DJI_0063	Fallopia dumetorum (L.) Holub	0	n	1	0	Polygonaceae Juss.
Iz_4_1109	DJI_0063	Festuca beckeri (Hack.) Trautv.	0	n	1	0	Poaceae Barnhart
Iz_4_1109	DJI_0063	Fragaria vesca L.	0	n	1	0	Rosaceae Juss.
Iz_4_1109	DJI_0063	Frangula alnus Mill.	0	n	1	1	Rhamnaceae Juss.
Iz_4_1109	DJI_0063	Fraxinus pennsylvanica Marshall	0	a	0	1	Oleaceae Hoffmanns. & Link
Iz_4_1109	DJI_0063	Galeopsis bifida Boenn.	0	n	1	0	Lamiacea Lindl.
Iz_4_1109	DJI_0063	Galium verum L.	0.5	n	1	0	Rubiaceae Juss.
Iz_4_1109	DJI_0063	Genista tinctoria L.	0	n	1	0	Fabaceae Lindl.
Iz_4_1109	DJI_0063	Geranium robertianum L.	0	n	1	0	Geraniaceae Juss.
Iz_4_1109	DJI_0063	Geranium sanquineum L.	0	n	1	0	Geraniaceae Juss.
Iz_4_1109	DJI_0063	Gypsophila paniculata L.	0	n	1	0	Caryophyllaceae Juss.
Iz_4_1109	DJI_0063	Helichrysum arenarium (L.) Moench	0	n	1	0	Asteraceae Bercht. & J.Presl
Iz_4_1109	DJI_0063	Herniaria polygama J.Gay	0	n	1	0	Caryophyllaceae Juss.
Iz_4_1109	DJI_0063	Hieracium robustum Fr.	0	n	1	0	Asteraceae Bercht. & J.Presl
Iz_4_1109	DJI_0063	Hieracium umbellatum L.	0	n	1	NA	Asteraceae Bercht. & J.Presl
Iz_4_1109	DJI_0063	Hylotelephium maximum (L.) Holub	0	n	1	0	Crassulaceae J.St.-Hil.
Iz_4_1109	DJI_0063	Jacobaea borysthenica (DC.) B.Nord. & Greuter	0	n	1	0	Asteraceae Bercht. & J.Presl
Iz_4_1109	DJI_0063	Jasione montana L.	0	n	1	0	Campanulaceae Juss.
Iz_4_1109	DJI_0063	Jurinea cyanoides (L.) Rchb.s.l.	0	n	1	0	Asteraceae Bercht. & J.Presl
Iz_4_1109	DJI_0063	Koeleria glauca (Spreng.) DC.	0	n	1	0	Poaceae Barnhart
Iz_4_1109	DJI_0063	Lactuca serriola L.	0	a	0	0	Asteraceae Bercht. & J.Presl

Iz_4_1109	DJI_0063	Linaria genistifolia (L.) Mill.	0	n	1	0	Plantaginaceae Juss.
Iz_4_1109	DJI_0063	Lomelosia argentea (L.) Greuter & Burdet	0	n	1	0	Caprifoliaceae Juss.
Iz_4_1109	DJI_0063	Melampyrum pratense L.	0	n	1	0	Orobanchaceae Vent.
Iz_4_1109	DJI_0063	Oenothera biennis L.	0	a	0	0	Onagraceae Juss.
Iz_4_1109	DJI_0063	Pentanema britannica (L.) D.Gut.Larr., Santos-Vicente, Anderb., E.Rico & M.M.Mart.Ort.	0	n	1	0	Asteraceae Bercht. & J.Presl
Iz_4_1109	DJI_0063	Peucedanum oreoselinum (L.) Moench	6	n	1	0	Apiaceae Lindl.
Iz_4_1109	DJI_0063	Peucedanum ruthenicum M.Bieb.	0	n	1	0	Apiaceae Lindl.
Iz_4_1109	DJI_0063	Pilosella echioides (Lumn.) F.W.Schultz & Sch.Bip.	0	n	1	0	Asteraceae Bercht. & J.Presl
Iz_4_1109	DJI_0063	Pilosella officinarum F. Schultz et Sch. Bip.	0	n	1	0	Asteraceae Bercht. & J.Presl
Iz_4_1109	DJI_0063	Pilosella sp.	0		NA	0	Asteraceae Bercht. & J.Presl
Iz_4_1109	DJI_0063	Pinus sylvestris L.	1	n	1	1	Pinaceae Spreng. ex F.Rudolphi
Iz_4_1109	DJI_0063	Poa annua L.	0	n	1	0	Poaceae Barnhart
Iz_4_1109	DJI_0063	Poa nemoralis L.	0	n	1	0	Poaceae Barnhart
Iz_4_1109	DJI_0063	Poa sp.	0		NA	0	Poaceae Barnhart
Iz_4_1109	DJI_0063	Polygonatum odoratum (Mill.) Druce	0	n	1	0	Asparagaceae Juss.
Iz_4_1109	DJI_0063	Polygonum arenarium Waldst. & Kit.	0	n	1	0	Polygonaceae Juss.
Iz_4_1109	DJI_0063	Populus nigra L.	0	n	1	1	Salicaceae Mirb.
Iz_4_1109	DJI_0063	Populus tremula L.	0	n	1	1	Salicaceae Mirb.
Iz_4_1109	DJI_0063	Populus Ã canescens (Aiton) Sm.	0	n	1	1	Salicaceae Mirb.
Iz_4_1109	DJI_0063	Potentilla argentea L.	0	n	1	0	Rosaceae Juss.
Iz_4_1109	DJI_0063	Psammophiliella muralis (L.) Ikonn.	0	n	1	0	Caryophyllaceae Juss.
Iz_4_1109	DJI_0063	Psephellus sumensis (Kalen.) Greuter	0	n	1	0	Asteraceae Bercht. & J.Presl
Iz_4_1109	DJI_0063	Pulsatilla pratensis (L.) Mill. s.l.	0	n	1	0	Ranunculaceae Juss.
Iz_4_1109	DJI_0063	Pyrus communis L.	0	a	0	1	Rosaceae Juss.
Iz_4_1109	DJI_0063	Quercus robur L.	0	n	1	1	Fagaceae Dumort.
Iz_4_1109	DJI_0063	Robinia pseudoacacia L.	0	a	0	1	Fabaceae Lindl.
Iz_4_1109	DJI_0063	Rosa sp.	0		NA	1	Rosaceae Juss.
Iz_4_1109	DJI_0063	Rubus idaeus L.	0	n	1	0	Rosaceae Juss.
Iz_4_1109	DJI_0063	Rumex acetosella L.	8	n	1	0	Polygonaceae Juss.
Iz_4_1109	DJI_0063	Rumex thyrsiflorus Fingerh.	0	n	1	0	Polygonaceae Juss.
Iz_4_1109	DJI_0063	Salix alba L.	0	n	1	1	Salicaceae Mirb.
Iz_4_1109	DJI_0063	Sambucus nigra L.	0	n	1	1	Viburnaceae Raf.
Iz_4_1109	DJI_0063	Secale sylvestre Host	0	n	1	0	Poaceae Barnhart
Iz_4_1109	DJI_0063	Sempervivum ruthenicum Schnittsp. & C.B. Lehm.	0	n	1	0	Crassulaceae J.St.-Hil.
Iz_4_1109	DJI_0063	Setaria viridis (L.) P. Beauv.	0	n	1	0	Poaceae Barnhart
Iz_4_1109	DJI_0063	Silene borysthenica (Gruner) Walters	0	n	1	0	Caryophyllaceae Juss.
Iz_4_1109	DJI_0063	Silene latifolia subsp. alba (Mill.) Greuter & Burdet	0	n	1	0	Caryophyllaceae Juss.
Iz_4_1109	DJI_0063	Solidago virgaurea L.	2	n	1	0	Asteraceae Bercht. & J.Presl
Iz_4_1109	DJI_0063	Sorbus aucuparia L.	0	n	1	1	Rosaceae Juss.
Iz_4_1109	DJI_0063	Stachys recta L.	0	n	1	0	Lamiacea Lindl.
Iz_4_1109	DJI_0063	Tanacetum vulgare L.	0	n	1	0	Asteraceae Bercht. & J.Presl
Iz_4_1109	DJI_0063	Thymus pallasianus Heinr.Braun	0	n	1	0	Lamiacea Lindl.
Iz_4_1109	DJI_0063	Tilia cordata Mill.	0	n	1	1	Malvaceae Juss.
Iz_4_1109	DJI_0063	Trifolium alpestre L.	0	n	1	0	Fabaceae Lindl.
Iz_4_1109	DJI_0063	Trifolium arvense L.	0	n	1	0	Fabaceae Lindl.
Iz_4_1109	DJI_0063	Ulmus glabra Huds.	0	n	1	1	Ulmaceae Mirb.
Iz_4_1109	DJI_0063	Verbascum densiflorum Bertol.	0	n	1	0	Scrophulariaceae Juss.
Iz_4_1109	DJI_0063	Veronica austriaca L.	0	n	1	0	Plantaginaceae Juss.
Iz_4_1109	DJI_0063	Veronica incana L.	0	n	1	0	Plantaginaceae Juss.
Iz_4_1109	DJI_0063	Veronica verna L.	0	n	1	0	Plantaginaceae Juss.
Iz_4_1109	DJI_0063	Vincetoxicum hirundinaria Medik	0	n	1	0	Apocynaceae Juss.
Iz_4_1109	DJI_0065	Acer tataricum L.	0	n	1	1	Sapindaceae Juss.
Iz_4_1109	DJI_0065	Achillea millefolium L. s.l.	0	n	1	0	Asteraceae Bercht. & J.Presl
Iz_4_1109	DJI_0065	Allium oleraceum L.	0	n	1	0	Amaryllidaceae J.St.-Hil.
Iz_4_1109	DJI_0065	Ambrosia artemisiifolia L.	0	a	0	0	Asteraceae Bercht. & J.Presl
Iz_4_1109	DJI_0065	Amorpha fruticosa L.	0	a	0	0	Fabaceae Lindl.
Iz_4_1109	DJI_0065	Anthericum ramosum L.	0	n	1	0	Asparagaceae Juss.
Iz_4_1109	DJI_0065	Artemisia marschalliana Spreng.	0	n	1	0	Asteraceae Bercht. & J.Presl
Iz_4_1109	DJI_0065	Asparagus officinalis L.	0	n	1	0	Asparagaceae Juss.
Iz_4_1109	DJI_0065	Asteraceae sp.	0	n	1	0	Asteraceae Bercht. & J.Presl
Iz_4_1109	DJI_0065	Astragalus cicer L.	0	n	1	0	Fabaceae Lindl.
Iz_4_1109	DJI_0065	Atriplex sp.	0		NA	NA	
Iz_4_1109	DJI_0065	Bassia laniflora (S.G.Gmel.) A.J.Scott	0	n	1	0	Amaranthaceae Juss.
Iz_4_1109	DJI_0065	Berberis vulgaris L.	0	n	1	0	Berberidaceae Juss.
Iz_4_1109	DJI_0065	Betula pendula Roth.	0	n	1	1	Betulaceae Gray
Iz_4_1109	DJI_0065	Betula pendula Roth.	0	n	1	0	Poaceae Barnhart
Iz_4_1109	DJI_0065	Bromus inermis Leyss.	0		NA	NA	
Iz_4_1109	DJI_0065	Bromus squarrosus L.	0	a	0	0	Poaceae Barnhart
Iz_4_1109	DJI_0065	Bromus tectorum L.	0	a	0	0	Poaceae Barnhart
Iz_4_1109	DJI_0065	Calamagrostis epigejos (L.) Roth	20	n	1	0	Poaceae Barnhart
Iz_4_1109	DJI_0065	Carduus acanthoides L.	0	n	1	0	Asteraceae Bercht. & J.Presl
Iz_4_1109	DJI_0065	Carex sp.	0	n	1	0	Cyperaceae Juss.
Iz_4_1109	DJI_0065	Centaurea majorovii Dumbadze	0	n	1	0	Asteraceae Bercht. & J.Presl
Iz_4_1109	DJI_0065	Chamaecytisus ruthenicus (Fisch.ex Wol.) Klaskova	0	n	1	0	Fabaceae Lindl.
Iz_4_1109	DJI_0065	Chelidonium majus L.	0	n	1	0	Papaveraceae Juss.
Iz_4_1109	DJI_0065	Chenopodium betaceum Andrz.	0	n	1	0	Amaranthaceae Juss.
Iz_4_1109	DJI_0065	Chenopodium cf. album	0	n	1	0	Amaranthaceae Juss.
Iz_4_1109	DJI_0065	Chenopodium sp.	0		NA	0	Amaranthaceae Juss.
Iz_4_1109	DJI_0065	Chondrilla juncea L.	0.2	n	1	0	Asteraceae Bercht. & J.Presl
Iz_4_1109	DJI_0065	Cirsium vulgare (Savi) Ten.	0	n	1	0	Asteraceae Bercht. & J.Presl
Iz_4_1109	DJI_0065	Clinopodium acinos (L.) Kuntze	0	n	1	0	Lamiacea Lindl.
Iz_4_1109	DJI_0065	Convallaria majalis L.	0	n	1	0	Asparagaceae Juss.
Iz_4_1109	DJI_0065	Crataegus rhipidophylla Gand.	0	n	1	1	Rosaceae Juss.
Iz_4_1109	DJI_0065	Crepis foetida L.	0	n	1	0	Asteraceae Bercht. & J.Presl
Iz_4_1109	DJI_0065	Dactylis glomerata L.	0	n	1	0	Poaceae Barnhart
Iz_4_1109	DJI_0065	Dianthus campestris M.Bieb.	0	n	1	0	Caryophyllaceae Juss.
Iz_4_1109	DJI_0065	Digitaria sanguinalis (L.) Scop.	0	a	0	0	Poaceae Barnhart
Iz_4_1109	DJI_0065	Elymus repens (L.) Gould	0	n	1	0	Poaceae Barnhart
Iz_4_1109	DJI_0065	Epilobium tetragonum L.	0	n	1	0	Onagraceae Juss.
Iz_4_1109	DJI_0065	Eragrostis minor Host	0	a	0	0	Poaceae Barnhart
Iz_4_1109	DJI_0065	Erigeron annuus (L.) Desf.	0	a	0	0	Asteraceae Bercht. & J.Presl
Iz_4_1109	DJI_0065	Erigeron canadensis L.	5	a	0	0	Asteraceae Bercht. & J.Presl
Iz_4_1109	DJI_0065	Euphorbia seguieriana Neck.	0	n	1	0	Euphorbiaceae Juss.
Iz_4_1109	DJI_0065	Fallopia dumetorum (L.) Holub	0	n	1	0	Polygonaceae Juss.
Iz_4_1109	DJI_0065	Festuca beckeri (Hack.) Trautv.	0	n	1	0	Poaceae Barnhart
Iz_4_1109	DJI_0065	Fragaria vesca L.	0	n	1	0	Rosaceae Juss.
Iz_4_1109	DJI_0065	Frangula alnus Mill.	0	n	1	1	Rhamnaceae Juss.
Iz_4_1109	DJI_0065	Fraxinus pennsylvanica Marshall	0	a	0	1	Oleaceae Hoffmanns. & Link

Iz_4_1109	DJI_0065	Galeopsis bifida Boenn.	0	n	1	0	Lamiacea Lindl.
Iz_4_1109	DJI_0065	Galium verum L.	0	n	1	0	Rubiaceae Juss.
Iz_4_1109	DJI_0065	Genista tinctoria L.	0	n	1	0	Fabaceae Lindl.
Iz_4_1109	DJI_0065	Geranium robertianum L.	0	n	1	0	Geraniaceae Juss.
Iz_4_1109	DJI_0065	Geranium sanquineum L.	0	n	1	0	Geraniaceae Juss.
Iz_4_1109	DJI_0065	Gypsophila paniculata L.	0	n	1	0	Caryophyllaceae Juss.
Iz_4_1109	DJI_0065	Helichrysum arenarium (L.) Moench	0	n	1	0	Asteraceae Bercht. & J.Presl
Iz_4_1109	DJI_0065	Herniaria polygama J.Gay	0	n	1	0	Caryophyllaceae Juss.
Iz_4_1109	DJI_0065	Hieracium robustum Fr.	0	n	1	0	Asteraceae Bercht. & J.Presl
Iz_4_1109	DJI_0065	Hieracium umbellatum L.	0	n	1	NA	Asteraceae Bercht. & J.Presl
Iz_4_1109	DJI_0065	Hylotelephium maximum (L.) Holub	0	n	1	0	Crassulaceae J.St.-Hil.
Iz_4_1109	DJI_0065	Jacobaea borysthenica (DC.) B.Nord. & Greuter	0	n	1	0	Asteraceae Bercht. & J.Presl
Iz_4_1109	DJI_0065	Jasione montana L.	0	n	1	0	Campanulaceae Juss.
Iz_4_1109	DJI_0065	Jurinea cyanoides (L.) Rchb.s.l.	0	n	1	0	Asteraceae Bercht. & J.Presl
Iz_4_1109	DJI_0065	Koeleria glauca (Spreng.) DC.	0	n	1	0	Poaceae Barnhart
Iz_4_1109	DJI_0065	Lactuca serriola L.	0	a	0	0	Asteraceae Bercht. & J.Presl
Iz_4_1109	DJI_0065	Linaria genistifolia (L.) Mill.	0	n	1	0	Plantaginaceae Juss.
Iz_4_1109	DJI_0065	Lomelosia argentea (L.) Greuter & Burdet	0	n	1	0	Caprifoliaceae Juss.
Iz_4_1109	DJI_0065	Melampyrum pratense L.	0	n	1	0	Orobanchaceae Vent.
Iz_4_1109	DJI_0065	Oenothera biennis L.	0	a	0	0	Onagraceae Juss.
Iz_4_1109	DJI_0065	Pentanema britannica (L.) D.Gut.Larr., Santos-Vicente, Anderb., E.Rico & M.M.Mart.Ort.	0	n	1	0	Asteraceae Bercht. & J.Presl
Iz_4_1109	DJI_0065	Peucedanum oreoselinum (L.) Moench	0	n	1	0	Apiaceae Lindl.
Iz_4_1109	DJI_0065	Peucedanum ruthenicum M.Bieb.	0	n	1	0	Apiaceae Lindl.
Iz_4_1109	DJI_0065	Pilosella echioides (Lumn.) F.W.Schultz & Sch.Bip.	0	n	1	0	Asteraceae Bercht. & J.Presl
Iz_4_1109	DJI_0065	Pilosella officinarum F. Schultz et Sch. Bip.	0	n	1	0	Asteraceae Bercht. & J.Presl
Iz_4_1109	DJI_0065	Pilosella sp.	0		NA	0	Asteraceae Bercht. & J.Presl
Iz_4_1109	DJI_0065	Pinus sylvestris L.	0	n	1	1	Pinaceae Spreng. ex F.Rudolphi
Iz_4_1109	DJI_0065	Poa annua L.	0	n	1	0	Poaceae Barnhart
Iz_4_1109	DJI_0065	Poa nemoralis L.	0	n	1	0	Poaceae Barnhart
Iz_4_1109	DJI_0065	Poa sp.	0		NA	0	Poaceae Barnhart
Iz_4_1109	DJI_0065	Polygonatum odoratum (Mill.) Druce	0	n	1	0	Asparagaceae Juss.
Iz_4_1109	DJI_0065	Polygonum arenarium Waldst. & Kit.	0	n	1	0	Polygonaceae Juss.
Iz_4_1109	DJI_0065	Populus nigra L.	0	n	1	1	Salicaceae Mirb.
Iz_4_1109	DJI_0065	Populus tremula L.	0	n	1	1	Salicaceae Mirb.
Iz_4_1109	DJI_0065	Populus Ã canescens (Aiton) Sm.	0	n	1	1	Salicaceae Mirb.
Iz_4_1109	DJI_0065	Potentilla argentea L.	0	n	1	0	Rosaceae Juss.
Iz_4_1109	DJI_0065	Psammophiliella muralis (L.) Ikonn.	0	n	1	0	Caryophyllaceae Juss.
Iz_4_1109	DJI_0065	Psephellus sumensis (Kalen.) Greuter	0	n	1	0	Asteraceae Bercht. & J.Presl
Iz_4_1109	DJI_0065	Pulsatilla pratensis (L.) Mill. s.l.	0	n	1	0	Ranunculaceae Juss.
Iz_4_1109	DJI_0065	Pyrus communis L.	0	a	0	1	Rosaceae Juss.
Iz_4_1109	DJI_0065	Quercus robur L.	0	n	1	1	Fagaceae Dumort.
Iz_4_1109	DJI_0065	Robinia pseudoacacia L.	0	a	0	1	Fabaceae Lindl.
Iz_4_1109	DJI_0065	Rosa sp.	0		NA	1	Rosaceae Juss.
Iz_4_1109	DJI_0065	Rubus idaeus L.	0	n	1	0	Rosaceae Juss.
Iz_4_1109	DJI_0065	Rumex acetosella L.	0	n	1	0	Polygonaceae Juss.
Iz_4_1109	DJI_0065	Rumex thyrsiflorus Fingerh.	0	n	1	0	Polygonaceae Juss.
Iz_4_1109	DJI_0065	Salix alba L.	0	n	1	1	Salicaceae Mirb.
Iz_4_1109	DJI_0065	Sambucus nigra L.	0	n	1	1	Viburnaceae Raf.
Iz_4_1109	DJI_0065	Secale sylvestre Host	0	n	1	0	Poaceae Barnhart
Iz_4_1109	DJI_0065	Sempervivum ruthenicum Schnittsp. & C.B. Lehm.	0	n	1	0	Crassulaceae J.St.-Hil.
Iz_4_1109	DJI_0065	Setaria viridis (L.) P. Beauv.	0	n	1	0	Poaceae Barnhart
Iz_4_1109	DJI_0065	Silene borysthenica (Gruner) Walters	0	n	1	0	Caryophyllaceae Juss.
Iz_4_1109	DJI_0065	Silene latifolia subsp. alba (Mill.) Greuter & Burdet	0	n	1	0	Caryophyllaceae Juss.
Iz_4_1109	DJI_0065	Solidago virgaurea L.	0	n	1	0	Asteraceae Bercht. & J.Presl
Iz_4_1109	DJI_0065	Sorbus aucuparia L.	0	n	1	1	Rosaceae Juss.
Iz_4_1109	DJI_0065	Stachys recta L.	0	n	1	0	Lamiacea Lindl.
Iz_4_1109	DJI_0065	Tanacetum vulgare L.	0	n	1	0	Asteraceae Bercht. & J.Presl
Iz_4_1109	DJI_0065	Thymus pallasianus Heinr.Braun	0	n	1	0	Lamiacea Lindl.
Iz_4_1109	DJI_0065	Tilia cordata Mill.	0	n	1	1	Malvaceae Juss.
Iz_4_1109	DJI_0065	Trifolium alpestre L.	0	n	1	0	Fabaceae Lindl.
Iz_4_1109	DJI_0065	Trifolium arvense L.	0	n	1	0	Fabaceae Lindl.
Iz_4_1109	DJI_0065	Ulmus glabra Huds.	0	n	1	1	Ulmaceae Mirb.
Iz_4_1109	DJI_0065	Verbascum densiflorum Bertol.	0	n	1	0	Scrophulariaceae Juss.
Iz_4_1109	DJI_0065	Veronica austriaca L.	0	n	1	0	Plantaginaceae Juss.
Iz_4_1109	DJI_0065	Veronica incana L.	0	n	1	0	Plantaginaceae Juss.
Iz_4_1109	DJI_0065	Veronica verna L.	0	n	1	0	Plantaginaceae Juss.
Iz_4_1109	DJI_0065	Vincetoxicum hirundinaria Medik	0	n	1	0	Apocynaceae Juss.
Iz_4_1109	DJI_0069	Acer tataricum L.	0	n	1	1	Sapindaceae Juss.
Iz_4_1109	DJI_0069	Achillea millefolium L. s.l.	0	n	1	0	Asteraceae Bercht. & J.Presl
Iz_4_1109	DJI_0069	Allium oleraceum L.	0	n	1	0	Amaryllidaceae J.St.-Hil.
Iz_4_1109	DJI_0069	Ambrosia artemisiifolia L.	0	a	0	0	Asteraceae Bercht. & J.Presl
Iz_4_1109	DJI_0069	Amorpha fruticosa L.	0	a	0	0	Fabaceae Lindl.
Iz_4_1109	DJI_0069	Anthericum ramosum L.	0	n	1	0	Asparagaceae Juss.
Iz_4_1109	DJI_0069	Artemisia marschalliana Spreng.	0	n	1	0	Asteraceae Bercht. & J.Presl
Iz_4_1109	DJI_0069	Asparagus officinalis L.	0	n	1	0	Asparagaceae Juss.
Iz_4_1109	DJI_0069	Asteraceae sp.	0	n	1	0	Asteraceae Bercht. & J.Presl
Iz_4_1109	DJI_0069	Astragalus cicer L.	0	n	1	0	Fabaceae Lindl.
Iz_4_1109	DJI_0069	Atriplex sp.	0		NA	NA	
Iz_4_1109	DJI_0069	Bassia laniflora (S.G.Gmel.) A.J.Scott	0	n	1	0	Amaranthaceae Juss.
Iz_4_1109	DJI_0069	Berberis vulgaris L.	0	n	1	0	Berberidaceae Juss.
Iz_4_1109	DJI_0069	Betula pendula Roth.	0	n	1	1	Betulaceae Gray
Iz_4_1109	DJI_0069	Betula pendula Roth.	0	n	1	0	Poaceae Barnhart
Iz_4_1109	DJI_0069	Bromus inermis Leyss.	0		NA	NA	
Iz_4_1109	DJI_0069	Bromus squarrosus L.	0	a	0	0	Poaceae Barnhart
Iz_4_1109	DJI_0069	Bromus tectorum L.	0	a	0	0	Poaceae Barnhart
Iz_4_1109	DJI_0069	Calamagrostis epigejos (L.) Roth	40	n	1	0	Poaceae Barnhart
Iz_4_1109	DJI_0069	Carduus acanthoides L.	0	n	1	0	Asteraceae Bercht. & J.Presl
Iz_4_1109	DJI_0069	Carex sp.	0	n	1	0	Cyperaceae Juss.
Iz_4_1109	DJI_0069	Centaurea majorovii Dumbadze	0	n	1	0	Asteraceae Bercht. & J.Presl
Iz_4_1109	DJI_0069	Chamaecytisus ruthenicus (Fisch.ex Wol.) Klaskova	0	n	1	0	Fabaceae Lindl.
Iz_4_1109	DJI_0069	Chelidonium majus L.	0	n	1	0	Papaveraceae Juss.
Iz_4_1109	DJI_0069	Chenopodium betaceum Andrz.	0	n	1	0	Amaranthaceae Juss.
Iz_4_1109	DJI_0069	Chenopodium cf. album	0	n	1	0	Amaranthaceae Juss.
Iz_4_1109	DJI_0069	Chenopodium sp.	0		NA	0	Amaranthaceae Juss.
Iz_4_1109	DJI_0069	Chondrilla juncea L.	0	n	1	0	Asteraceae Bercht. & J.Presl
Iz_4_1109	DJI_0069	Cirsium vulgare (Savi) Ten.	0	n	1	0	Asteraceae Bercht. & J.Presl
Iz_4_1109	DJI_0069	Clinopodium acinos (L.) Kuntze	0	n	1	0	Lamiacea Lindl.
Iz_4_1109	DJI_0069	Convallaria majalis L.	0	n	1	0	Asparagaceae Juss.

Iz_4_1109	DJI_0069	Crataegus rhipidophylla Gand.	0	n	1	1	Rosaceae Juss.
Iz_4_1109	DJI_0069	Crepis foetida L.	0	n	1	0	Asteraceae Bercht. & J.Presl
Iz_4_1109	DJI_0069	Dactylis glomerata L.	0	n	1	0	Poaceae Barnhart
Iz_4_1109	DJI_0069	Dianthus campestris M.Bieb.	0	n	1	0	Caryophyllaceae Juss.
Iz_4_1109	DJI_0069	Digitaria sanguinalis (L.) Scop.	0	a	0	0	Poaceae Barnhart
Iz_4_1109	DJI_0069	Elymus repens (L.) Gould	0	n	1	0	Poaceae Barnhart
Iz_4_1109	DJI_0069	Epilobium tetragonum L.	0	n	1	0	Onagraceae Juss.
Iz_4_1109	DJI_0069	Eragrostis minor Host	0	a	0	0	Poaceae Barnhart
Iz_4_1109	DJI_0069	Erigeron annuus (L.) Desf.	0	a	0	0	Asteraceae Bercht. & J.Presl
Iz_4_1109	DJI_0069	Erigeron canadensis L.	2	a	0	0	Asteraceae Bercht. & J.Presl
Iz_4_1109	DJI_0069	Euphorbia seguieriana Neck.	0	n	1	0	Euphorbiaceae Juss.
Iz_4_1109	DJI_0069	Fallopia dumetorum (L.) Holub	0	n	1	0	Polygonaceae Juss.
Iz_4_1109	DJI_0069	Festuca beckeri (Hack.) Trautv.	0	n	1	0	Poaceae Barnhart
Iz_4_1109	DJI_0069	Fragaria vesca L.	0	n	1	0	Rosaceae Juss.
Iz_4_1109	DJI_0069	Frangula alnus Mill.	0	n	1	1	Rhamnaceae Juss.
Iz_4_1109	DJI_0069	Fraxinus pennsylvanica Marshall	0	a	0	1	Oleaceae Hoffmanns. & Link
Iz_4_1109	DJI_0069	Galeopsis bifida Boenn.	0	n	1	0	Lamiacea Lindl.
Iz_4_1109	DJI_0069	Galium verum L.	0	n	1	0	Rubiaceae Juss.
Iz_4_1109	DJI_0069	Genista tinctoria L.	0	n	1	0	Fabaceae Lindl.
Iz_4_1109	DJI_0069	Geranium robertianum L.	0	n	1	0	Geraniaceae Juss.
Iz_4_1109	DJI_0069	Geranium sanquineum L.	0	n	1	0	Geraniaceae Juss.
Iz_4_1109	DJI_0069	Gypsophila paniculata L.	0	n	1	0	Caryophyllaceae Juss.
Iz_4_1109	DJI_0069	Helichrysum arenarium (L.) Moench	1	n	1	0	Asteraceae Bercht. & J.Presl
Iz_4_1109	DJI_0069	Herniaria polygama J.Gay	0	n	1	0	Caryophyllaceae Juss.
Iz_4_1109	DJI_0069	Hieracium robustum Fr.	0	n	1	0	Asteraceae Bercht. & J.Presl
Iz_4_1109	DJI_0069	Hieracium umbellatum L.	0	n	1	NA	Asteraceae Bercht. & J.Presl
Iz_4_1109	DJI_0069	Hylotelephium maximum (L.) Holub	0	n	1	0	Crassulaceae J.St.-Hil.
Iz_4_1109	DJI_0069	Jacobaea borysthenica (DC.) B.Nord. & Greuter	0	n	1	0	Asteraceae Bercht. & J.Presl
Iz_4_1109	DJI_0069	Jasione montana L.	0	n	1	0	Campanulaceae Juss.
Iz_4_1109	DJI_0069	Jurinea cyanoides (L.) Rchb.s.l.	0	n	1	0	Asteraceae Bercht. & J.Presl
Iz_4_1109	DJI_0069	Koeleria glauca (Spreng.) DC.	0	n	1	0	Poaceae Barnhart
Iz_4_1109	DJI_0069	Lactuca serriola L.	0	a	0	0	Asteraceae Bercht. & J.Presl
Iz_4_1109	DJI_0069	Linaria genistifolia (L.) Mill.	0	n	1	0	Plantaginaceae Juss.
Iz_4_1109	DJI_0069	Lomelosia argentea (L.) Greuter & Burdet	0	n	1	0	Caprifoliaceae Juss.
Iz_4_1109	DJI_0069	Melampyrum pratense L.	0	n	1	0	Orobanchaceae Vent.
Iz_4_1109	DJI_0069	Oenothera biennis L.	0	a	0	0	Onagraceae Juss.
Iz_4_1109	DJI_0069	Pentanema britannica (L.) D.Gut.Larr., Santos-Vicente, Anderb., E.Rico & M.M.Mart.Ort.	0	n	1	0	Asteraceae Bercht. & J.Presl
Iz_4_1109	DJI_0069	Peucedanum oreoselinum (L.) Moench	0	n	1	0	Apiaceae Lindl.
Iz_4_1109	DJI_0069	Peucedanum ruthenicum M.Bieb.	0	n	1	0	Apiaceae Lindl.
Iz_4_1109	DJI_0069	Pilosella echioides (Lumn.) F.W.Schultz & Sch.Bip.	0	n	1	0	Asteraceae Bercht. & J.Presl
Iz_4_1109	DJI_0069	Pilosella officinarum F. Schultz et Sch. Bip.	0	n	1	0	Asteraceae Bercht. & J.Presl
Iz_4_1109	DJI_0069	Pilosella sp.	0		NA	0	Asteraceae Bercht. & J.Presl
Iz_4_1109	DJI_0069	Pinus sylvestris L.	0	n	1	1	Pinaceae Spreng. ex F.Rudolphi
Iz_4_1109	DJI_0069	Poa annua L.	0	n	1	0	Poaceae Barnhart
Iz_4_1109	DJI_0069	Poa nemoralis L.	0	n	1	0	Poaceae Barnhart
Iz_4_1109	DJI_0069	Poa sp.	0		NA	0	Poaceae Barnhart
Iz_4_1109	DJI_0069	Polygonatum odoratum (Mill.) Druce	0	n	1	0	Asparagaceae Juss.
Iz_4_1109	DJI_0069	Polygonum arenarium Waldst. & Kit.	0	n	1	0	Polygonaceae Juss.
Iz_4_1109	DJI_0069	Populus nigra L.	0	n	1	1	Salicaceae Mirb.
Iz_4_1109	DJI_0069	Populus tremula L.	0	n	1	1	Salicaceae Mirb.
Iz_4_1109	DJI_0069	Populus Ã canescens (Aiton) Sm.	0	n	1	1	Salicaceae Mirb.
Iz_4_1109	DJI_0069	Potentilla argentea L.	0	n	1	0	Rosaceae Juss.
Iz_4_1109	DJI_0069	Psammophiliella muralis (L.) Ikonn.	0	n	1	0	Caryophyllaceae Juss.
Iz_4_1109	DJI_0069	Psephellus sumensis (Kalen.) Greuter	0	n	1	0	Asteraceae Bercht. & J.Presl
Iz_4_1109	DJI_0069	Pulsatilla pratensis (L.) Mill. s.l.	0	n	1	0	Ranunculaceae Juss.
Iz_4_1109	DJI_0069	Pyrus communis L.	0	a	0	1	Rosaceae Juss.
Iz_4_1109	DJI_0069	Quercus robur L.	0	n	1	1	Fagaceae Dumort.
Iz_4_1109	DJI_0069	Robinia pseudoacacia L.	0	a	0	1	Fabaceae Lindl.
Iz_4_1109	DJI_0069	Rosa sp.	0		NA	1	Rosaceae Juss.
Iz_4_1109	DJI_0069	Rubus idaeus L.	0	n	1	0	Rosaceae Juss.
Iz_4_1109	DJI_0069	Rumex acetosella L.	10	n	1	0	Polygonaceae Juss.
Iz_4_1109	DJI_0069	Rumex thyrsiflorus Fingerh.	0	n	1	0	Polygonaceae Juss.
Iz_4_1109	DJI_0069	Salix alba L.	0	n	1	1	Salicaceae Mirb.
Iz_4_1109	DJI_0069	Sambucus nigra L.	0	n	1	1	Viburnaceae Raf.
Iz_4_1109	DJI_0069	Secale sylvestre Host	0	n	1	0	Poaceae Barnhart
Iz_4_1109	DJI_0069	Sempervivum ruthenicum Schnittsp. & C.B. Lehm.	0	n	1	0	Crassulaceae J.St.-Hil.
Iz_4_1109	DJI_0069	Setaria viridis (L.) P. Beauv.	0	n	1	0	Poaceae Barnhart
Iz_4_1109	DJI_0069	Silene borysthenica (Gruner) Walters	0	n	1	0	Caryophyllaceae Juss.
Iz_4_1109	DJI_0069	Silene latifolia subsp. alba (Mill.) Greuter & Burdet	0	n	1	0	Caryophyllaceae Juss.
Iz_4_1109	DJI_0069	Solidago virgaurea L.	0	n	1	0	Asteraceae Bercht. & J.Presl
Iz_4_1109	DJI_0069	Sorbus aucuparia L.	0	n	1	1	Rosaceae Juss.
Iz_4_1109	DJI_0069	Stachys recta L.	0	n	1	0	Lamiacea Lindl.
Iz_4_1109	DJI_0069	Tanacetum vulgare L.	0	n	1	0	Asteraceae Bercht. & J.Presl
Iz_4_1109	DJI_0069	Thymus pallasianus Heinr.Braun	0	n	1	0	Lamiacea Lindl.
Iz_4_1109	DJI_0069	Tilia cordata Mill.	0	n	1	1	Malvaceae Juss.
Iz_4_1109	DJI_0069	Trifolium alpestre L.	0	n	1	0	Fabaceae Lindl.
Iz_4_1109	DJI_0069	Trifolium arvense L.	0	n	1	0	Fabaceae Lindl.
Iz_4_1109	DJI_0069	Ulmus glabra Huds.	0	n	1	1	Ulmaceae Mirb.
Iz_4_1109	DJI_0069	Verbascum densiflorum Bertol.	0	n	1	0	Scrophulariaceae Juss.
Iz_4_1109	DJI_0069	Veronica austriaca L.	0	n	1	0	Plantaginaceae Juss.
Iz_4_1109	DJI_0069	Veronica incana L.	0	n	1	0	Plantaginaceae Juss.
Iz_4_1109	DJI_0069	Veronica verna L.	0	n	1	0	Plantaginaceae Juss.
Iz_4_1109	DJI_0069	Vincetoxicum hirundinaria Medik	0	n	1	0	Apocynaceae Juss.
Iz_4_1109	DJI_0079	Acer tataricum L.	0	n	1	1	Sapindaceae Juss.
Iz_4_1109	DJI_0079	Achillea millefolium L. s.l.	0	n	1	0	Asteraceae Bercht. & J.Presl
Iz_4_1109	DJI_0079	Allium oleraceum L.	0	n	1	0	Amaryllidaceae J.St.-Hil.
Iz_4_1109	DJI_0079	Ambrosia artemisiifolia L.	0	a	0	0	Asteraceae Bercht. & J.Presl
Iz_4_1109	DJI_0079	Amorpha fruticosa L.	0	a	0	0	Fabaceae Lindl.
Iz_4_1109	DJI_0079	Anthericum ramosum L.	0	n	1	0	Asparagaceae Juss.
Iz_4_1109	DJI_0079	Artemisia marschalliana Spreng.	0	n	1	0	Asteraceae Bercht. & J.Presl
Iz_4_1109	DJI_0079	Asparagus officinalis L.	0	n	1	0	Asparagaceae Juss.
Iz_4_1109	DJI_0079	Asteraceae sp.	0	n	1	0	Asteraceae Bercht. & J.Presl
Iz_4_1109	DJI_0079	Astragalus cicer L.	0	n	1	0	Fabaceae Lindl.
Iz_4_1109	DJI_0079	Atriplex sp.	0		NA	NA	
Iz_4_1109	DJI_0079	Bassia laniflora (S.G.Gmel.) A.J.Scott	0	n	1	0	Amaranthaceae Juss.
Iz_4_1109	DJI_0079	Berberis vulgaris L.	0	n	1	0	Berberidaceae Juss.
Iz_4_1109	DJI_0079	Betula pendula Roth.	0	n	1	1	Betulaceae Gray
Iz_4_1109	DJI_0079	Betula pendula Roth.	0	n	1	0	Poaceae Barnhart

Iz_4_1109	DJI_0079	Bromus inermis Leyss.	0		NA	NA	
Iz_4_1109	DJI_0079	Bromus squarrosus L.	0	a	0	0	Poaceae Barnhart
Iz_4_1109	DJI_0079	Bromus tectorum L.	0	a	0	0	Poaceae Barnhart
Iz_4_1109	DJI_0079	Calamagrostis epigejos (L.) Roth	2	n	1	0	Poaceae Barnhart
Iz_4_1109	DJI_0079	Carduus acanthoides L.	0	n	1	0	Asteraceae Bercht. & J.Presl
Iz_4_1109	DJI_0079	Carex sp.	0	n	1	0	Cyperaceae Juss.
Iz_4_1109	DJI_0079	Centaurea majorovii Dumbadze	0	n	1	0	Asteraceae Bercht. & J.Presl
Iz_4_1109	DJI_0079	Chamaecytisus ruthenicus (Fisch.ex Wol.) Klaskova	0	n	1	0	Fabaceae Lindl.
Iz_4_1109	DJI_0079	Chelidonium majus L.	4	n	1	0	Papaveraceae Juss.
Iz_4_1109	DJI_0079	Chenopodium betaceum Andrz.	0	n	1	0	Amaranthaceae Juss.
Iz_4_1109	DJI_0079	Chenopodium cf. album	0	n	1	0	Amaranthaceae Juss.
Iz_4_1109	DJI_0079	Chenopodium sp.	0		NA	0	Amaranthaceae Juss.
Iz_4_1109	DJI_0079	Chondrilla juncea L.	0	n	1	0	Asteraceae Bercht. & J.Presl
Iz_4_1109	DJI_0079	Cirsium vulgare (Savi) Ten.	0	n	1	0	Asteraceae Bercht. & J.Presl
Iz_4_1109	DJI_0079	Clinopodium acinos (L.) Kuntze	0	n	1	0	Lamiacea Lindl.
Iz_4_1109	DJI_0079	Convallaria majalis L.	0	n	1	0	Asparagaceae Juss.
Iz_4_1109	DJI_0079	Crataegus rhipidophylla Gand.	0	n	1	1	Rosaceae Juss.
Iz_4_1109	DJI_0079	Crepis foetida L.	0	n	1	0	Asteraceae Bercht. & J.Presl
Iz_4_1109	DJI_0079	Dactylis glomerata L.	0	n	1	0	Poaceae Barnhart
Iz_4_1109	DJI_0079	Dianthus campestris M.Bieb.	0	n	1	0	Caryophyllaceae Juss.
Iz_4_1109	DJI_0079	Digitaria sanguinalis (L.) Scop.	0	a	0	0	Poaceae Barnhart
Iz_4_1109	DJI_0079	Elymus repens (L.) Gould	0	n	1	0	Poaceae Barnhart
Iz_4_1109	DJI_0079	Epilobium tetragonum L.	0	n	1	0	Onagraceae Juss.
Iz_4_1109	DJI_0079	Eragrostis minor Host	0	a	0	0	Poaceae Barnhart
Iz_4_1109	DJI_0079	Erigeron annuus (L.) Desf.	0	a	0	0	Asteraceae Bercht. & J.Presl
Iz_4_1109	DJI_0079	Erigeron canadensis L.	2	a	0	0	Asteraceae Bercht. & J.Presl
Iz_4_1109	DJI_0079	Euphorbia seguieriana Neck.	0	n	1	0	Euphorbiaceae Juss.
Iz_4_1109	DJI_0079	Fallopia dumetorum (L.) Holub	3	n	1	0	Polygonaceae Juss.
Iz_4_1109	DJI_0079	Festuca beckeri (Hack.) Trautv.	0	n	1	0	Poaceae Barnhart
Iz_4_1109	DJI_0079	Fragaria vesca L.	0	n	1	0	Rosaceae Juss.
Iz_4_1109	DJI_0079	Frangula alnus Mill.	0	n	1	1	Rhamnaceae Juss.
Iz_4_1109	DJI_0079	Fraxinus pennsylvanica Marshall	0	a	0	1	Oleaceae Hoffmanns. & Link
Iz_4_1109	DJI_0079	Galeopsis bifida Boenn.	0	n	1	0	Lamiacea Lindl.
Iz_4_1109	DJI_0079	Galium verum L.	0	n	1	0	Rubiaceae Juss.
Iz_4_1109	DJI_0079	Genista tinctoria L.	0	n	1	0	Fabaceae Lindl.
Iz_4_1109	DJI_0079	Geranium robertianum L.	0	n	1	0	Geraniaceae Juss.
Iz_4_1109	DJI_0079	Geranium sanquineum L.	0	n	1	0	Geraniaceae Juss.
Iz_4_1109	DJI_0079	Gypsophila paniculata L.	0	n	1	0	Caryophyllaceae Juss.
Iz_4_1109	DJI_0079	Helichrysum arenarium (L.) Moench	0	n	1	0	Asteraceae Bercht. & J.Presl
Iz_4_1109	DJI_0079	Herniaria polygama J.Gay	0	n	1	0	Caryophyllaceae Juss.
Iz_4_1109	DJI_0079	Hieracium robustum Fr.	0	n	1	0	Asteraceae Bercht. & J.Presl
Iz_4_1109	DJI_0079	Hieracium umbellatum L.	0	n	1	NA	Asteraceae Bercht. & J.Presl
Iz_4_1109	DJI_0079	Hylotelephium maximum (L.) Holub	0	n	1	0	Crassulaceae J.St.-Hil.
Iz_4_1109	DJI_0079	Jacobaea borysthenica (DC.) B.Nord. & Greuter	0	n	1	0	Asteraceae Bercht. & J.Presl
Iz_4_1109	DJI_0079	Jasione montana L.	0	n	1	0	Campanulaceae Juss.
Iz_4_1109	DJI_0079	Jurinea cyanoides (L.) Rchb.s.l.	0	n	1	0	Asteraceae Bercht. & J.Presl
Iz_4_1109	DJI_0079	Koeleria glauca (Spreng.) DC.	0	n	1	0	Poaceae Barnhart
Iz_4_1109	DJI_0079	Lactuca serriola L.	0	a	0	0	Asteraceae Bercht. & J.Presl
Iz_4_1109	DJI_0079	Linaria genistifolia (L.) Mill.	0	n	1	0	Plantaginaceae Juss.
Iz_4_1109	DJI_0079	Lomelosia argentea (L.) Greuter & Burdet	0	n	1	0	Caprifoliaceae Juss.
Iz_4_1109	DJI_0079	Melampyrum pratense L.	0	n	1	0	Orobanchaceae Vent.
Iz_4_1109	DJI_0079	Oenothera biennis L.	0	a	0	0	Onagraceae Juss.
Iz_4_1109	DJI_0079	Pentanema britannica (L.) D.Gut.Larr., Santos-Vicente, Anderb., E.Rico & M.M.Mart.Ort.	0	n	1	0	Asteraceae Bercht. & J.Presl
Iz_4_1109	DJI_0079	Peucedanum oreoselinum (L.) Moench	0	n	1	0	Apiaceae Lindl.
Iz_4_1109	DJI_0079	Peucedanum ruthenicum M.Bieb.	0	n	1	0	Apiaceae Lindl.
Iz_4_1109	DJI_0079	Pilosella echioides (Lumn.) F.W.Schultz & Sch.Bip.	0	n	1	0	Asteraceae Bercht. & J.Presl
Iz_4_1109	DJI_0079	Pilosella officinarum F. Schultz et Sch. Bip.	0	n	1	0	Asteraceae Bercht. & J.Presl
Iz_4_1109	DJI_0079	Pilosella sp.	0		NA	0	Asteraceae Bercht. & J.Presl
Iz_4_1109	DJI_0079	Pinus sylvestris L.	0	n	1	1	Pinaceae Spreng. ex F.Rudolphi
Iz_4_1109	DJI_0079	Poa annua L.	0	n	1	0	Poaceae Barnhart
Iz_4_1109	DJI_0079	Poa nemoralis L.	0	n	1	0	Poaceae Barnhart
Iz_4_1109	DJI_0079	Poa sp.	0		NA	0	Poaceae Barnhart
Iz_4_1109	DJI_0079	Polygonatum odoratum (Mill.) Druce	0	n	1	0	Asparagaceae Juss.
Iz_4_1109	DJI_0079	Polygonum arenarium Waldst. & Kit.	0	n	1	0	Polygonaceae Juss.
Iz_4_1109	DJI_0079	Populus nigra L.	0	n	1	1	Salicaceae Mirb.
Iz_4_1109	DJI_0079	Populus tremula L.	0	n	1	1	Salicaceae Mirb.
Iz_4_1109	DJI_0079	Populus Ã canescens (Aiton) Sm.	0	n	1	1	Salicaceae Mirb.
Iz_4_1109	DJI_0079	Potentilla argentea L.	0	n	1	0	Rosaceae Juss.
Iz_4_1109	DJI_0079	Psammophiliella muralis (L.) Ikonn.	0	n	1	0	Caryophyllaceae Juss.
Iz_4_1109	DJI_0079	Psephellus sumensis (Kalen.) Greuter	0	n	1	0	Asteraceae Bercht. & J.Presl
Iz_4_1109	DJI_0079	Pulsatilla pratensis (L.) Mill. s.l.	0	n	1	0	Ranunculaceae Juss.
Iz_4_1109	DJI_0079	Pyrus communis L.	0	a	0	1	Rosaceae Juss.
Iz_4_1109	DJI_0079	Quercus robur L.	0	n	1	1	Fagaceae Dumort.
Iz_4_1109	DJI_0079	Robinia pseudoacacia L.	0	a	0	1	Fabaceae Lindl.
Iz_4_1109	DJI_0079	Rosa sp.	0		NA	1	Rosaceae Juss.
Iz_4_1109	DJI_0079	Rubus idaeus L.	0	n	1	0	Rosaceae Juss.
Iz_4_1109	DJI_0079	Rumex acetosella L.	0	n	1	0	Polygonaceae Juss.
Iz_4_1109	DJI_0079	Rumex thyrsiflorus Fingerh.	0	n	1	0	Polygonaceae Juss.
Iz_4_1109	DJI_0079	Salix alba L.	0	n	1	1	Salicaceae Mirb.
Iz_4_1109	DJI_0079	Sambucus nigra L.	0	n	1	1	Viburnaceae Raf.
Iz_4_1109	DJI_0079	Secale sylvestre Host	0	n	1	0	Poaceae Barnhart
Iz_4_1109	DJI_0079	Sempervivum ruthenicum Schnittsp. & C.B. Lehm.	0	n	1	0	Crassulaceae J.St.-Hil.
Iz_4_1109	DJI_0079	Setaria viridis (L.) P. Beauv.	0	n	1	0	Poaceae Barnhart
Iz_4_1109	DJI_0079	Silene borysthenica (Gruner) Walters	0	n	1	0	Caryophyllaceae Juss.
Iz_4_1109	DJI_0079	Silene latifolia subsp. alba (Mill.) Greuter & Burdet	0	n	1	0	Caryophyllaceae Juss.
Iz_4_1109	DJI_0079	Solidago virgaurea L.	0	n	1	0	Asteraceae Bercht. & J.Presl
Iz_4_1109	DJI_0079	Sorbus aucuparia L.	0	n	1	1	Rosaceae Juss.
Iz_4_1109	DJI_0079	Stachys recta L.	0	n	1	0	Lamiacea Lindl.
Iz_4_1109	DJI_0079	Tanacetum vulgare L.	0	n	1	0	Asteraceae Bercht. & J.Presl
Iz_4_1109	DJI_0079	Thymus pallasianus Heinr.Braun	0	n	1	0	Lamiacea Lindl.
Iz_4_1109	DJI_0079	Tilia cordata Mill.	0	n	1	1	Malvaceae Juss.
Iz_4_1109	DJI_0079	Trifolium alpestre L.	0	n	1	0	Fabaceae Lindl.
Iz_4_1109	DJI_0079	Trifolium arvense L.	0	n	1	0	Fabaceae Lindl.
Iz_4_1109	DJI_0079	Ulmus glabra Huds.	0	n	1	1	Ulmaceae Mirb.
Iz_4_1109	DJI_0079	Verbascum densiflorum Bertol.	0	n	1	0	Scrophulariaceae Juss.
Iz_4_1109	DJI_0079	Veronica austriaca L.	0	n	1	0	Plantaginaceae Juss.
Iz_4_1109	DJI_0079	Veronica incana L.	0	n	1	0	Plantaginaceae Juss.
Iz_4_1109	DJI_0079	Veronica verna L.	0	n	1	0	Plantaginaceae Juss.

Iz_4_1109	DJI_0079	Vincetoxicum hirundinaria Medik	0	n	1	0	Apocynaceae Juss.
Iz_4_1109	DJI_0084	Acer tataricum L.	0	n	1	1	Sapindaceae Juss.
Iz_4_1109	DJI_0084	Achillea millefolium L. s.l.	0	n	1	0	Asteraceae Bercht. & J.Presl
Iz_4_1109	DJI_0084	Allium oleraceum L.	0	n	1	0	Amaryllidaceae J.St.-Hil.
Iz_4_1109	DJI_0084	Ambrosia artemisiifolia L.	0	a	0	0	Asteraceae Bercht. & J.Presl
Iz_4_1109	DJI_0084	Amorpha fruticosa L.	0	a	0	0	Fabaceae Lindl.
Iz_4_1109	DJI_0084	Anthericum ramosum L.	0	n	1	0	Asparagaceae Juss.
Iz_4_1109	DJI_0084	Artemisia marschalliana Spreng.	0	n	1	0	Asteraceae Bercht. & J.Presl
Iz_4_1109	DJI_0084	Asparagus officinalis L.	0	n	1	0	Asparagaceae Juss.
Iz_4_1109	DJI_0084	Asteraceae sp.	0	n	1	0	Asteraceae Bercht. & J.Presl
Iz_4_1109	DJI_0084	Astragalus cicer L.	0	n	1	0	Fabaceae Lindl.
Iz_4_1109	DJI_0084	Atriplex sp.	0		NA	NA	
Iz_4_1109	DJI_0084	Bassia laniflora (S.G.Gmel.) A.J.Scott	0	n	1	0	Amaranthaceae Juss.
Iz_4_1109	DJI_0084	Berberis vulgaris L.	0	n	1	0	Berberidaceae Juss.
Iz_4_1109	DJI_0084	Betula pendula Roth.	0	n	1	1	Betulaceae Gray
Iz_4_1109	DJI_0084	Betula pendula Roth.	0	n	1	0	Poaceae Barnhart
Iz_4_1109	DJI_0084	Bromus inermis Leyss.	0		NA	NA	
Iz_4_1109	DJI_0084	Bromus squarrosus L.	0	a	0	0	Poaceae Barnhart
Iz_4_1109	DJI_0084	Bromus tectorum L.	0	a	0	0	Poaceae Barnhart
Iz_4_1109	DJI_0084	Calamagrostis epigejos (L.) Roth	3	n	1	0	Poaceae Barnhart
Iz_4_1109	DJI_0084	Carduus acanthoides L.	0	n	1	0	Asteraceae Bercht. & J.Presl
Iz_4_1109	DJI_0084	Carex sp.	0	n	1	0	Cyperaceae Juss.
Iz_4_1109	DJI_0084	Centaurea majorovii Dumbadze	0	n	1	0	Asteraceae Bercht. & J.Presl
Iz_4_1109	DJI_0084	Chamaecytisus ruthenicus (Fisch.ex Wol.) Klaskova	0	n	1	0	Fabaceae Lindl.
Iz_4_1109	DJI_0084	Chelidonium majus L.	0	n	1	0	Papaveraceae Juss.
Iz_4_1109	DJI_0084	Chenopodium betaceum Andrz.	0	n	1	0	Amaranthaceae Juss.
Iz_4_1109	DJI_0084	Chenopodium cf. album	0	n	1	0	Amaranthaceae Juss.
Iz_4_1109	DJI_0084	Chenopodium sp.	0		NA	0	Amaranthaceae Juss.
Iz_4_1109	DJI_0084	Chondrilla juncea L.	0	n	1	0	Asteraceae Bercht. & J.Presl
Iz_4_1109	DJI_0084	Cirsium vulgare (Savi) Ten.	0	n	1	0	Asteraceae Bercht. & J.Presl
Iz_4_1109	DJI_0084	Clinopodium acinos (L.) Kuntze	0	n	1	0	Lamiacea Lindl.
Iz_4_1109	DJI_0084	Convallaria majalis L.	0	n	1	0	Asparagaceae Juss.
Iz_4_1109	DJI_0084	Crataegus rhipidophylla Gand.	0	n	1	1	Rosaceae Juss.
Iz_4_1109	DJI_0084	Crepis foetida L.	0	n	1	0	Asteraceae Bercht. & J.Presl
Iz_4_1109	DJI_0084	Dactylis glomerata L.	0	n	1	0	Poaceae Barnhart
Iz_4_1109	DJI_0084	Dianthus campestris M.Bieb.	0	n	1	0	Caryophyllaceae Juss.
Iz_4_1109	DJI_0084	Digitaria sanguinalis (L.) Scop.	0	a	0	0	Poaceae Barnhart
Iz_4_1109	DJI_0084	Elymus repens (L.) Gould	0	n	1	0	Poaceae Barnhart
Iz_4_1109	DJI_0084	Epilobium tetragonum L.	0	n	1	0	Onagraceae Juss.
Iz_4_1109	DJI_0084	Eragrostis minor Host	0	a	0	0	Poaceae Barnhart
Iz_4_1109	DJI_0084	Erigeron annuus (L.) Desf.	0	a	0	0	Asteraceae Bercht. & J.Presl
Iz_4_1109	DJI_0084	Erigeron canadensis L.	4	a	0	0	Asteraceae Bercht. & J.Presl
Iz_4_1109	DJI_0084	Euphorbia seguieriana Neck.	0	n	1	0	Euphorbiaceae Juss.
Iz_4_1109	DJI_0084	Fallopia dumetorum (L.) Holub	0	n	1	0	Polygonaceae Juss.
Iz_4_1109	DJI_0084	Festuca beckeri (Hack.) Trautv.	0	n	1	0	Poaceae Barnhart
Iz_4_1109	DJI_0084	Fragaria vesca L.	0	n	1	0	Rosaceae Juss.
Iz_4_1109	DJI_0084	Frangula alnus Mill.	0	n	1	1	Rhamnaceae Juss.
Iz_4_1109	DJI_0084	Fraxinus pennsylvanica Marshall	0	a	0	1	Oleaceae Hoffmanns. & Link
Iz_4_1109	DJI_0084	Galeopsis bifida Boenn.	0	n	1	0	Lamiacea Lindl.
Iz_4_1109	DJI_0084	Galium verum L.	0	n	1	0	Rubiaceae Juss.
Iz_4_1109	DJI_0084	Genista tinctoria L.	0	n	1	0	Fabaceae Lindl.
Iz_4_1109	DJI_0084	Geranium robertianum L.	0	n	1	0	Geraniaceae Juss.
Iz_4_1109	DJI_0084	Geranium sanquineum L.	0	n	1	0	Geraniaceae Juss.
Iz_4_1109	DJI_0084	Gypsophila paniculata L.	0	n	1	0	Caryophyllaceae Juss.
Iz_4_1109	DJI_0084	Helichrysum arenarium (L.) Moench	0	n	1	0	Asteraceae Bercht. & J.Presl
Iz_4_1109	DJI_0084	Herniaria polygama J.Gay	0	n	1	0	Caryophyllaceae Juss.
Iz_4_1109	DJI_0084	Hieracium robustum Fr.	0	n	1	0	Asteraceae Bercht. & J.Presl
Iz_4_1109	DJI_0084	Hieracium umbellatum L.	0	n	1	NA	Asteraceae Bercht. & J.Presl
Iz_4_1109	DJI_0084	Hylotelephium maximum (L.) Holub	0	n	1	0	Crassulaceae J.St.-Hil.
Iz_4_1109	DJI_0084	Jacobaea borysthenica (DC.) B.Nord. & Greuter	0	n	1	0	Asteraceae Bercht. & J.Presl
Iz_4_1109	DJI_0084	Jasione montana L.	0	n	1	0	Campanulaceae Juss.
Iz_4_1109	DJI_0084	Jurinea cyanoides (L.) Rchb.s.l.	0	n	1	0	Asteraceae Bercht. & J.Presl
Iz_4_1109	DJI_0084	Koeleria glauca (Spreng.) DC.	0	n	1	0	Poaceae Barnhart
Iz_4_1109	DJI_0084	Lactuca serriola L.	0	a	0	0	Asteraceae Bercht. & J.Presl
Iz_4_1109	DJI_0084	Linaria genistifolia (L.) Mill.	0	n	1	0	Plantaginaceae Juss.
Iz_4_1109	DJI_0084	Lomelosia argentea (L.) Greuter & Burdet	0	n	1	0	Caprifoliaceae Juss.
Iz_4_1109	DJI_0084	Melampyrum pratense L.	0	n	1	0	Orobanchaceae Vent.
Iz_4_1109	DJI_0084	Oenothera biennis L.	0	a	0	0	Onagraceae Juss.
Iz_4_1109	DJI_0084	Pentanema britannica (L.) D.Gut.Larr., Santos-Vicente, Anderb., E.Rico & M.M.Mart.Ort.	0	n	1	0	Asteraceae Bercht. & J.Presl
Iz_4_1109	DJI_0084	Peucedanum oreoselinum (L.) Moench	0	n	1	0	Apiaceae Lindl.
Iz_4_1109	DJI_0084	Peucedanum ruthenicum M.Bieb.	0	n	1	0	Apiaceae Lindl.
Iz_4_1109	DJI_0084	Pilosella echioides (Lumn.) F.W.Schultz & Sch.Bip.	0	n	1	0	Asteraceae Bercht. & J.Presl
Iz_4_1109	DJI_0084	Pilosella officinarum F. Schultz et Sch. Bip.	0	n	1	0	Asteraceae Bercht. & J.Presl
Iz_4_1109	DJI_0084	Pilosella sp.	0		NA	0	Asteraceae Bercht. & J.Presl
Iz_4_1109	DJI_0084	Pinus sylvestris L.	0.2	n	1	1	Pinaceae Spreng. ex F.Rudolphi
Iz_4_1109	DJI_0084	Poa annua L.	0	n	1	0	Poaceae Barnhart
Iz_4_1109	DJI_0084	Poa nemoralis L.	0	n	1	0	Poaceae Barnhart
Iz_4_1109	DJI_0084	Poa sp.	0		NA	0	Poaceae Barnhart
Iz_4_1109	DJI_0084	Polygonatum odoratum (Mill.) Druce	0	n	1	0	Asparagaceae Juss.
Iz_4_1109	DJI_0084	Polygonum arenarium Waldst. & Kit.	0	n	1	0	Polygonaceae Juss.
Iz_4_1109	DJI_0084	Populus nigra L.	0	n	1	1	Salicaceae Mirb.
Iz_4_1109	DJI_0084	Populus tremula L.	0	n	1	1	Salicaceae Mirb.
Iz_4_1109	DJI_0084	Populus Ã canescens (Aiton) Sm.	0	n	1	1	Salicaceae Mirb.
Iz_4_1109	DJI_0084	Potentilla argentea L.	0	n	1	0	Rosaceae Juss.
Iz_4_1109	DJI_0084	Psammophiliella muralis (L.) Ikonn.	0	n	1	0	Caryophyllaceae Juss.
Iz_4_1109	DJI_0084	Psephellus sumensis (Kalen.) Greuter	0	n	1	0	Asteraceae Bercht. & J.Presl
Iz_4_1109	DJI_0084	Pulsatilla pratensis (L.) Mill. s.l.	0	n	1	0	Ranunculaceae Juss.
Iz_4_1109	DJI_0084	Pyrus communis L.	0	a	0	1	Rosaceae Juss.
Iz_4_1109	DJI_0084	Quercus robur L.	0	n	1	1	Fagaceae Dumort.
Iz_4_1109	DJI_0084	Robinia pseudoacacia L.	0	a	0	1	Fabaceae Lindl.
Iz_4_1109	DJI_0084	Rosa sp.	0		NA	1	Rosaceae Juss.
Iz_4_1109	DJI_0084	Rubus idaeus L.	0	n	1	0	Rosaceae Juss.
Iz_4_1109	DJI_0084	Rumex acetosella L.	8	n	1	0	Polygonaceae Juss.
Iz_4_1109	DJI_0084	Rumex thyrsiflorus Fingerh.	0	n	1	0	Polygonaceae Juss.
Iz_4_1109	DJI_0084	Salix alba L.	0	n	1	1	Salicaceae Mirb.
Iz_4_1109	DJI_0084	Sambucus nigra L.	0	n	1	1	Viburnaceae Raf.
Iz_4_1109	DJI_0084	Secale sylvestre Host	0	n	1	0	Poaceae Barnhart
Iz_4_1109	DJI_0084	Sempervivum ruthenicum Schnittsp. & C.B. Lehm.	0	n	1	0	Crassulaceae J.St.-Hil.

Iz_4_1109	DJI_0084	Setaria viridis (L.) P. Beauv.	0	n	1	0	Poaceae Barnhart
Iz_4_1109	DJI_0084	Silene borysthenica (Gruner) Walters	0	n	1	0	Caryophyllaceae Juss.
Iz_4_1109	DJI_0084	Silene latifolia subsp. alba (Mill.) Greuter & Burdet	0	n	1	0	Caryophyllaceae Juss.
Iz_4_1109	DJI_0084	Solidago virgaurea L.	0	n	1	0	Asteraceae Bercht. & J.Presl
Iz_4_1109	DJI_0084	Sorbus aucuparia L.	0	n	1	1	Rosaceae Juss.
Iz_4_1109	DJI_0084	Stachys recta L.	0	n	1	0	Lamiacea Lindl.
Iz_4_1109	DJI_0084	Tanacetum vulgare L.	0	n	1	0	Asteraceae Bercht. & J.Presl
Iz_4_1109	DJI_0084	Thymus pallasianus Heinr.Braun	0	n	1	0	Lamiacea Lindl.
Iz_4_1109	DJI_0084	Tilia cordata Mill.	0	n	1	1	Malvaceae Juss.
Iz_4_1109	DJI_0084	Trifolium alpestre L.	0	n	1	0	Fabaceae Lindl.
Iz_4_1109	DJI_0084	Trifolium arvense L.	0	n	1	0	Fabaceae Lindl.
Iz_4_1109	DJI_0084	Ulmus glabra Huds.	0	n	1	1	Ulmaceae Mirb.
Iz_4_1109	DJI_0084	Verbascum densiflorum Bertol.	0	n	1	0	Scrophulariaceae Juss.
Iz_4_1109	DJI_0084	Veronica austriaca L.	0	n	1	0	Plantaginaceae Juss.
Iz_4_1109	DJI_0084	Veronica incana L.	0	n	1	0	Plantaginaceae Juss.
Iz_4_1109	DJI_0084	Veronica verna L.	0	n	1	0	Plantaginaceae Juss.
Iz_4_1109	DJI_0084	Vincetoxicum hirundinaria Medik	0	n	1	0	Apocynaceae Juss.
Iz_4_1109	DJI_0087	Acer tataricum L.	0	n	1	1	Sapindaceae Juss.
Iz_4_1109	DJI_0087	Achillea millefolium L. s.l.	0	n	1	0	Asteraceae Bercht. & J.Presl
Iz_4_1109	DJI_0087	Allium oleraceum L.	0	n	1	0	Amaryllidaceae J.St.-Hil.
Iz_4_1109	DJI_0087	Ambrosia artemisiifolia L.	0	a	0	0	Asteraceae Bercht. & J.Presl
Iz_4_1109	DJI_0087	Amorpha fruticosa L.	0	a	0	0	Fabaceae Lindl.
Iz_4_1109	DJI_0087	Anthericum ramosum L.	0	n	1	0	Asparagaceae Juss.
Iz_4_1109	DJI_0087	Artemisia marschalliana Spreng.	0	n	1	0	Asteraceae Bercht. & J.Presl
Iz_4_1109	DJI_0087	Asparagus officinalis L.	0	n	1	0	Asparagaceae Juss.
Iz_4_1109	DJI_0087	Asteraceae sp.	1	n	1	0	Asteraceae Bercht. & J.Presl
Iz_4_1109	DJI_0087	Astragalus cicer L.	0	n	1	0	Fabaceae Lindl.
Iz_4_1109	DJI_0087	Atriplex sp.	0		NA	NA	
Iz_4_1109	DJI_0087	Bassia laniflora (S.G.Gmel.) A.J.Scott	0	n	1	0	Amaranthaceae Juss.
Iz_4_1109	DJI_0087	Berberis vulgaris L.	0	n	1	0	Berberidaceae Juss.
Iz_4_1109	DJI_0087	Betula pendula Roth.	0	n	1	1	Betulaceae Gray
Iz_4_1109	DJI_0087	Betula pendula Roth.	0	n	1	0	Poaceae Barnhart
Iz_4_1109	DJI_0087	Bromus inermis Leyss.	0		NA	NA	
Iz_4_1109	DJI_0087	Bromus squarrosus L.	0	a	0	0	Poaceae Barnhart
Iz_4_1109	DJI_0087	Bromus tectorum L.	0	a	0	0	Poaceae Barnhart
Iz_4_1109	DJI_0087	Calamagrostis epigejos (L.) Roth	4	n	1	0	Poaceae Barnhart
Iz_4_1109	DJI_0087	Carduus acanthoides L.	0	n	1	0	Asteraceae Bercht. & J.Presl
Iz_4_1109	DJI_0087	Carex sp.	0	n	1	0	Cyperaceae Juss.
Iz_4_1109	DJI_0087	Centaurea majorovii Dumbadze	0	n	1	0	Asteraceae Bercht. & J.Presl
Iz_4_1109	DJI_0087	Chamaecytisus ruthenicus (Fisch.ex Wol.) Klaskova	0	n	1	0	Fabaceae Lindl.
Iz_4_1109	DJI_0087	Chelidonium majus L.	1	n	1	0	Papaveraceae Juss.
Iz_4_1109	DJI_0087	Chenopodium betaceum Andrz.	0	n	1	0	Amaranthaceae Juss.
Iz_4_1109	DJI_0087	Chenopodium cf. album	0	n	1	0	Amaranthaceae Juss.
Iz_4_1109	DJI_0087	Chenopodium sp.	0		NA	0	Amaranthaceae Juss.
Iz_4_1109	DJI_0087	Chondrilla juncea L.	0	n	1	0	Asteraceae Bercht. & J.Presl
Iz_4_1109	DJI_0087	Cirsium vulgare (Savi) Ten.	0	n	1	0	Asteraceae Bercht. & J.Presl
Iz_4_1109	DJI_0087	Clinopodium acinos (L.) Kuntze	0	n	1	0	Lamiacea Lindl.
Iz_4_1109	DJI_0087	Convallaria majalis L.	0	n	1	0	Asparagaceae Juss.
Iz_4_1109	DJI_0087	Crataegus rhipidophylla Gand.	0	n	1	1	Rosaceae Juss.
Iz_4_1109	DJI_0087	Crepis foetida L.	0	n	1	0	Asteraceae Bercht. & J.Presl
Iz_4_1109	DJI_0087	Dactylis glomerata L.	0	n	1	0	Poaceae Barnhart
Iz_4_1109	DJI_0087	Dianthus campestris M.Bieb.	0	n	1	0	Caryophyllaceae Juss.
Iz_4_1109	DJI_0087	Digitaria sanguinalis (L.) Scop.	0	a	0	0	Poaceae Barnhart
Iz_4_1109	DJI_0087	Elymus repens (L.) Gould	0	n	1	0	Poaceae Barnhart
Iz_4_1109	DJI_0087	Epilobium tetragonum L.	0	n	1	0	Onagraceae Juss.
Iz_4_1109	DJI_0087	Eragrostis minor Host	0	a	0	0	Poaceae Barnhart
Iz_4_1109	DJI_0087	Erigeron annuus (L.) Desf.	0	a	0	0	Asteraceae Bercht. & J.Presl
Iz_4_1109	DJI_0087	Erigeron canadensis L.	3	a	0	0	Asteraceae Bercht. & J.Presl
Iz_4_1109	DJI_0087	Euphorbia seguieriana Neck.	0	n	1	0	Euphorbiaceae Juss.
Iz_4_1109	DJI_0087	Fallopia dumetorum (L.) Holub	0	n	1	0	Polygonaceae Juss.
Iz_4_1109	DJI_0087	Festuca beckeri (Hack.) Trautv.	0	n	1	0	Poaceae Barnhart
Iz_4_1109	DJI_0087	Fragaria vesca L.	0	n	1	0	Rosaceae Juss.
Iz_4_1109	DJI_0087	Frangula alnus Mill.	0	n	1	1	Rhamnaceae Juss.
Iz_4_1109	DJI_0087	Fraxinus pennsylvanica Marshall	0	a	0	1	Oleaceae Hoffmanns. & Link
Iz_4_1109	DJI_0087	Galeopsis bifida Boenn.	0	n	1	0	Lamiacea Lindl.
Iz_4_1109	DJI_0087	Galium verum L.	0	n	1	0	Rubiaceae Juss.
Iz_4_1109	DJI_0087	Genista tinctoria L.	0	n	1	0	Fabaceae Lindl.
Iz_4_1109	DJI_0087	Geranium robertianum L.	0	n	1	0	Geraniaceae Juss.
Iz_4_1109	DJI_0087	Geranium sanquineum L.	0	n	1	0	Geraniaceae Juss.
Iz_4_1109	DJI_0087	Gypsophila paniculata L.	0	n	1	0	Caryophyllaceae Juss.
Iz_4_1109	DJI_0087	Helichrysum arenarium (L.) Moench	0	n	1	0	Asteraceae Bercht. & J.Presl
Iz_4_1109	DJI_0087	Herniaria polygama J.Gay	0	n	1	0	Caryophyllaceae Juss.
Iz_4_1109	DJI_0087	Hieracium robustum Fr.	0	n	1	0	Asteraceae Bercht. & J.Presl
Iz_4_1109	DJI_0087	Hieracium umbellatum L.	0	n	1	NA	Asteraceae Bercht. & J.Presl
Iz_4_1109	DJI_0087	Hylotelephium maximum (L.) Holub	0	n	1	0	Crassulaceae J.St.-Hil.
Iz_4_1109	DJI_0087	Jacobaea borysthenica (DC.) B.Nord. & Greuter	0	n	1	0	Asteraceae Bercht. & J.Presl
Iz_4_1109	DJI_0087	Jasione montana L.	0	n	1	0	Campanulaceae Juss.
Iz_4_1109	DJI_0087	Jurinea cyanoides (L.) Rchb.s.l.	0	n	1	0	Asteraceae Bercht. & J.Presl
Iz_4_1109	DJI_0087	Koeleria glauca (Spreng.) DC.	0	n	1	0	Poaceae Barnhart
Iz_4_1109	DJI_0087	Lactuca serriola L.	0	a	0	0	Asteraceae Bercht. & J.Presl
Iz_4_1109	DJI_0087	Linaria genistifolia (L.) Mill.	0	n	1	0	Plantaginaceae Juss.
Iz_4_1109	DJI_0087	Lomelosia argentea (L.) Greuter & Burdet	0	n	1	0	Caprifoliaceae Juss.
Iz_4_1109	DJI_0087	Melampyrum pratense L.	0	n	1	0	Orobanchaceae Vent.
Iz_4_1109	DJI_0087	Oenothera biennis L.	0	a	0	0	Onagraceae Juss.
Iz_4_1109	DJI_0087	Pentanema britannica (L.) D.Gut.Larr., Santos-Vicente, Anderb., E.Rico & M.M.Mart.Ort.	0	n	1	0	Asteraceae Bercht. & J.Presl
Iz_4_1109	DJI_0087	Peucedanum oreoselinum (L.) Moench	0.5	n	1	0	Apiaceae Lindl.
Iz_4_1109	DJI_0087	Peucedanum ruthenicum M.Bieb.	0	n	1	0	Apiaceae Lindl.
Iz_4_1109	DJI_0087	Pilosella echioides (Lumn.) F.W.Schultz & Sch.Bip.	0	n	1	0	Asteraceae Bercht. & J.Presl
Iz_4_1109	DJI_0087	Pilosella officinarum F. Schultz et Sch. Bip.	0	n	1	0	Asteraceae Bercht. & J.Presl
Iz_4_1109	DJI_0087	Pilosella sp.	0		NA	0	Asteraceae Bercht. & J.Presl
Iz_4_1109	DJI_0087	Pinus sylvestris L.	0	n	1	1	Pinaceae Spreng. ex F.Rudolphi
Iz_4_1109	DJI_0087	Poa annua L.	0	n	1	0	Poaceae Barnhart
Iz_4_1109	DJI_0087	Poa nemoralis L.	0	n	1	0	Poaceae Barnhart
Iz_4_1109	DJI_0087	Poa sp.	0		NA	0	Poaceae Barnhart
Iz_4_1109	DJI_0087	Polygonatum odoratum (Mill.) Druce	0	n	1	0	Asparagaceae Juss.
Iz_4_1109	DJI_0087	Polygonum arenarium Waldst. & Kit.	0	n	1	0	Polygonaceae Juss.
Iz_4_1109	DJI_0087	Populus nigra L.	0	n	1	1	Salicaceae Mirb.
Iz_4_1109	DJI_0087	Populus tremula L.	0	n	1	1	Salicaceae Mirb.

Iz_4_1109	DJI_0087	Populus Ã canescens (Aiton) Sm.	0	n	1	1	Salicaceae Mirb.
Iz_4_1109	DJI_0087	Potentilla argentea L.	0	n	1	0	Rosaceae Juss.
Iz_4_1109	DJI_0087	Psammophiliella muralis (L.) Ikonn.	0	n	1	0	Caryophyllaceae Juss.
Iz_4_1109	DJI_0087	Psephellus sumensis (Kalen.) Greuter	0	n	1	0	Asteraceae Bercht. & J.Presl
Iz_4_1109	DJI_0087	Pulsatilla pratensis (L.) Mill. s.l.	0	n	1	0	Ranunculaceae Juss.
Iz_4_1109	DJI_0087	Pyrus communis L.	0	a	0	1	Rosaceae Juss.
Iz_4_1109	DJI_0087	Quercus robur L.	1	n	1	1	Fagaceae Dumort.
Iz_4_1109	DJI_0087	Robinia pseudoacacia L.	0	a	0	1	Fabaceae Lindl.
Iz_4_1109	DJI_0087	Rosa sp.	0		NA	1	Rosaceae Juss.
Iz_4_1109	DJI_0087	Rubus idaeus L.	0	n	1	0	Rosaceae Juss.
Iz_4_1109	DJI_0087	Rumex acetosella L.	2	n	1	0	Polygonaceae Juss.
Iz_4_1109	DJI_0087	Rumex thyrsiflorus Fingerh.	0	n	1	0	Polygonaceae Juss.
Iz_4_1109	DJI_0087	Salix alba L.	0	n	1	1	Salicaceae Mirb.
Iz_4_1109	DJI_0087	Sambucus nigra L.	0	n	1	1	Viburnaceae Raf.
Iz_4_1109	DJI_0087	Secale sylvestre Host	0	n	1	0	Poaceae Barnhart
Iz_4_1109	DJI_0087	Sempervivum ruthenicum Schnittsp. & C.B. Lehm.	0	n	1	0	Crassulaceae J.St.-Hil.
Iz_4_1109	DJI_0087	Setaria viridis (L.) P. Beauv.	0	n	1	0	Poaceae Barnhart
Iz_4_1109	DJI_0087	Silene borysthenica (Gruner) Walters	0	n	1	0	Caryophyllaceae Juss.
Iz_4_1109	DJI_0087	Silene latifolia subsp. alba (Mill.) Greuter & Burdet	0	n	1	0	Caryophyllaceae Juss.
Iz_4_1109	DJI_0087	Solidago virgaurea L.	0	n	1	0	Asteraceae Bercht. & J.Presl
Iz_4_1109	DJI_0087	Sorbus aucuparia L.	0	n	1	1	Rosaceae Juss.
Iz_4_1109	DJI_0087	Stachys recta L.	0	n	1	0	Lamiacea Lindl.
Iz_4_1109	DJI_0087	Tanacetum vulgare L.	0	n	1	0	Asteraceae Bercht. & J.Presl
Iz_4_1109	DJI_0087	Thymus pallasianus Heinr.Braun	0	n	1	0	Lamiacea Lindl.
Iz_4_1109	DJI_0087	Tilia cordata Mill.	0	n	1	1	Malvaceae Juss.
Iz_4_1109	DJI_0087	Trifolium alpestre L.	0	n	1	0	Fabaceae Lindl.
Iz_4_1109	DJI_0087	Trifolium arvense L.	0	n	1	0	Fabaceae Lindl.
Iz_4_1109	DJI_0087	Ulmus glabra Huds.	0	n	1	1	Ulmaceae Mirb.
Iz_4_1109	DJI_0087	Verbascum densiflorum Bertol.	0	n	1	0	Scrophulariaceae Juss.
Iz_4_1109	DJI_0087	Veronica austriaca L.	0	n	1	0	Plantaginaceae Juss.
Iz_4_1109	DJI_0087	Veronica incana L.	0	n	1	0	Plantaginaceae Juss.
Iz_4_1109	DJI_0087	Veronica verna L.	0	n	1	0	Plantaginaceae Juss.
Iz_4_1109	DJI_0087	Vincetoxicum hirundinaria Medik	0	n	1	0	Apocynaceae Juss.
Iz_4_1109	DJI_0009	Acer tataricum L.	0	n	1	1	Sapindaceae Juss.
Iz_4_1109	DJI_0009	Achillea millefolium L. s.l.	0	n	1	0	Asteraceae Bercht. & J.Presl
Iz_4_1109	DJI_0009	Allium oleraceum L.	0	n	1	0	Amaryllidaceae J.St.-Hil.
Iz_4_1109	DJI_0009	Ambrosia artemisiifolia L.	0	a	0	0	Asteraceae Bercht. & J.Presl
Iz_4_1109	DJI_0009	Amorpha fruticosa L.	0	a	0	0	Fabaceae Lindl.
Iz_4_1109	DJI_0009	Anthericum ramosum L.	0	n	1	0	Asparagaceae Juss.
Iz_4_1109	DJI_0009	Artemisia marschalliana Spreng.	0	n	1	0	Asteraceae Bercht. & J.Presl
Iz_4_1109	DJI_0009	Asparagus officinalis L.	0	n	1	0	Asparagaceae Juss.
Iz_4_1109	DJI_0009	Asteraceae sp.	0	n	1	0	Asteraceae Bercht. & J.Presl
Iz_4_1109	DJI_0009	Astragalus cicer L.	0	n	1	0	Fabaceae Lindl.
Iz_4_1109	DJI_0009	Atriplex sp.	0		NA	NA	
Iz_4_1109	DJI_0009	Bassia laniflora (S.G.Gmel.) A.J.Scott	0	n	1	0	Amaranthaceae Juss.
Iz_4_1109	DJI_0009	Berberis vulgaris L.	0	n	1	0	Berberidaceae Juss.
Iz_4_1109	DJI_0009	Betula pendula Roth.	0	n	1	1	Betulaceae Gray
Iz_4_1109	DJI_0009	Betula pendula Roth.	0	n	1	0	Poaceae Barnhart
Iz_4_1109	DJI_0009	Bromus inermis Leyss.	0		NA	NA	
Iz_4_1109	DJI_0009	Bromus squarrosus L.	0	a	0	0	Poaceae Barnhart
Iz_4_1109	DJI_0009	Bromus tectorum L.	0	a	0	0	Poaceae Barnhart
Iz_4_1109	DJI_0009	Calamagrostis epigejos (L.) Roth	5	n	1	0	Poaceae Barnhart
Iz_4_1109	DJI_0009	Carduus acanthoides L.	0	n	1	0	Asteraceae Bercht. & J.Presl
Iz_4_1109	DJI_0009	Carex sp.	0	n	1	0	Cyperaceae Juss.
Iz_4_1109	DJI_0009	Centaurea majorovii Dumbadze	0	n	1	0	Asteraceae Bercht. & J.Presl
Iz_4_1109	DJI_0009	Chamaecytisus ruthenicus (Fisch.ex Wol.) Klaskova	0	n	1	0	Fabaceae Lindl.
Iz_4_1109	DJI_0009	Chelidonium majus L.	0	n	1	0	Papaveraceae Juss.
Iz_4_1109	DJI_0009	Chenopodium betaceum Andrz.	0	n	1	0	Amaranthaceae Juss.
Iz_4_1109	DJI_0009	Chenopodium cf. album	0	n	1	0	Amaranthaceae Juss.
Iz_4_1109	DJI_0009	Chenopodium sp.	0		NA	0	Amaranthaceae Juss.
Iz_4_1109	DJI_0009	Chondrilla juncea L.	0	n	1	0	Asteraceae Bercht. & J.Presl
Iz_4_1109	DJI_0009	Cirsium vulgare (Savi) Ten.	0	n	1	0	Asteraceae Bercht. & J.Presl
Iz_4_1109	DJI_0009	Clinopodium acinos (L.) Kuntze	0	n	1	0	Lamiacea Lindl.
Iz_4_1109	DJI_0009	Convallaria majalis L.	0	n	1	0	Asparagaceae Juss.
Iz_4_1109	DJI_0009	Crataegus rhipidophylla Gand.	0	n	1	1	Rosaceae Juss.
Iz_4_1109	DJI_0009	Crepis foetida L.	0	n	1	0	Asteraceae Bercht. & J.Presl
Iz_4_1109	DJI_0009	Dactylis glomerata L.	0	n	1	0	Poaceae Barnhart
Iz_4_1109	DJI_0009	Dianthus campestris M.Bieb.	0	n	1	0	Caryophyllaceae Juss.
Iz_4_1109	DJI_0009	Digitaria sanguinalis (L.) Scop.	0	a	0	0	Poaceae Barnhart
Iz_4_1109	DJI_0009	Elymus repens (L.) Gould	0	n	1	0	Poaceae Barnhart
Iz_4_1109	DJI_0009	Epilobium tetragonum L.	0	n	1	0	Onagraceae Juss.
Iz_4_1109	DJI_0009	Eragrostis minor Host	0	a	0	0	Poaceae Barnhart
Iz_4_1109	DJI_0009	Erigeron annuus (L.) Desf.	0	a	0	0	Asteraceae Bercht. & J.Presl
Iz_4_1109	DJI_0009	Erigeron canadensis L.	2	a	0	0	Asteraceae Bercht. & J.Presl
Iz_4_1109	DJI_0009	Euphorbia seguieriana Neck.	0	n	1	0	Euphorbiaceae Juss.
Iz_4_1109	DJI_0009	Fallopia dumetorum (L.) Holub	0	n	1	0	Polygonaceae Juss.
Iz_4_1109	DJI_0009	Festuca beckeri (Hack.) Trautv.	0	n	1	0	Poaceae Barnhart
Iz_4_1109	DJI_0009	Fragaria vesca L.	0	n	1	0	Rosaceae Juss.
Iz_4_1109	DJI_0009	Frangula alnus Mill.	0	n	1	1	Rhamnaceae Juss.
Iz_4_1109	DJI_0009	Fraxinus pennsylvanica Marshall	0	a	0	1	Oleaceae Hoffmanns. & Link
Iz_4_1109	DJI_0009	Galeopsis bifida Boenn.	0	n	1	0	Lamiacea Lindl.
Iz_4_1109	DJI_0009	Galium verum L.	0	n	1	0	Rubiaceae Juss.
Iz_4_1109	DJI_0009	Genista tinctoria L.	0	n	1	0	Fabaceae Lindl.
Iz_4_1109	DJI_0009	Geranium robertianum L.	0	n	1	0	Geraniaceae Juss.
Iz_4_1109	DJI_0009	Geranium sanquineum L.	0	n	1	0	Geraniaceae Juss.
Iz_4_1109	DJI_0009	Gypsophila paniculata L.	0	n	1	0	Caryophyllaceae Juss.
Iz_4_1109	DJI_0009	Helichrysum arenarium (L.) Moench	0	n	1	0	Asteraceae Bercht. & J.Presl
Iz_4_1109	DJI_0009	Herniaria polygama J.Gay	0	n	1	0	Caryophyllaceae Juss.
Iz_4_1109	DJI_0009	Hieracium robustum Fr.	0	n	1	0	Asteraceae Bercht. & J.Presl
Iz_4_1109	DJI_0009	Hieracium umbellatum L.	0	n	1	NA	Asteraceae Bercht. & J.Presl
Iz_4_1109	DJI_0009	Hylotelephium maximum (L.) Holub	0	n	1	0	Crassulaceae J.St.-Hil.
Iz_4_1109	DJI_0009	Jacobaea borysthenica (DC.) B.Nord. & Greuter	0	n	1	0	Asteraceae Bercht. & J.Presl
Iz_4_1109	DJI_0009	Jasione montana L.	0	n	1	0	Campanulaceae Juss.
Iz_4_1109	DJI_0009	Jurinea cyanoides (L.) Rchb.s.l.	0	n	1	0	Asteraceae Bercht. & J.Presl
Iz_4_1109	DJI_0009	Koeleria glauca (Spreng.) DC.	0	n	1	0	Poaceae Barnhart
Iz_4_1109	DJI_0009	Lactuca serriola L.	0	a	0	0	Asteraceae Bercht. & J.Presl
Iz_4_1109	DJI_0009	Linaria genistifolia (L.) Mill.	0	n	1	0	Plantaginaceae Juss.
Iz_4_1109	DJI_0009	Lomelosia argentea (L.) Greuter & Burdet	0	n	1	0	Caprifoliaceae Juss.

Iz_4_1109	DJI_0009	Melampyrum pratense L.	0	n	1	0	Orobanchaceae Vent.
Iz_4_1109	DJI_0009	Oenothera biennis L.	0	a	0	0	Onagraceae Juss.
Iz_4_1109	DJI_0009	Pentanema britannica (L.) D.Gut.Larr., Santos-Vicente, Anderb., E.Rico & M.M.Mart.Ort.	0	n	1	0	Asteraceae Bercht. & J.Presl
Iz_4_1109	DJI_0009	Peucedanum oreoselinum (L.) Moench	0.2	n	1	0	Apiaceae Lindl.
Iz_4_1109	DJI_0009	Peucedanum ruthenicum M.Bieb.	0	n	1	0	Apiaceae Lindl.
Iz_4_1109	DJI_0009	Pilosella echioides (Lumn.) F.W.Schultz & Sch.Bip.	0	n	1	0	Asteraceae Bercht. & J.Presl
Iz_4_1109	DJI_0009	Pilosella officinarum F. Schultz et Sch. Bip.	0	n	1	0	Asteraceae Bercht. & J.Presl
Iz_4_1109	DJI_0009	Pilosella sp.	0		NA	0	Asteraceae Bercht. & J.Presl
Iz_4_1109	DJI_0009	Pinus sylvestris L.	0	n	1	1	Pinaceae Spreng. ex F.Rudolphi
Iz_4_1109	DJI_0009	Poa annua L.	0	n	1	0	Poaceae Barnhart
Iz_4_1109	DJI_0009	Poa nemoralis L.	0	n	1	0	Poaceae Barnhart
Iz_4_1109	DJI_0009	Poa sp.	0		NA	0	Poaceae Barnhart
Iz_4_1109	DJI_0009	Polygonatum odoratum (Mill.) Druce	0	n	1	0	Asparagaceae Juss.
Iz_4_1109	DJI_0009	Polygonum arenarium Waldst. & Kit.	0	n	1	0	Polygonaceae Juss.
Iz_4_1109	DJI_0009	Populus nigra L.	0	n	1	1	Salicaceae Mirb.
Iz_4_1109	DJI_0009	Populus tremula L.	0	n	1	1	Salicaceae Mirb.
Iz_4_1109	DJI_0009	Populus Ã canescens (Aiton) Sm.	0	n	1	1	Salicaceae Mirb.
Iz_4_1109	DJI_0009	Potentilla argentea L.	0	n	1	0	Rosaceae Juss.
Iz_4_1109	DJI_0009	Psammophiliella muralis (L.) Ikonn.	0	n	1	0	Caryophyllaceae Juss.
Iz_4_1109	DJI_0009	Psephellus sumensis (Kalen.) Greuter	0	n	1	0	Asteraceae Bercht. & J.Presl
Iz_4_1109	DJI_0009	Pulsatilla pratensis (L.) Mill. s.l.	0	n	1	0	Ranunculaceae Juss.
Iz_4_1109	DJI_0009	Pyrus communis L.	0	a	0	1	Rosaceae Juss.
Iz_4_1109	DJI_0009	Quercus robur L.	0	n	1	1	Fagaceae Dumort.
Iz_4_1109	DJI_0009	Robinia pseudoacacia L.	0	a	0	1	Fabaceae Lindl.
Iz_4_1109	DJI_0009	Rosa sp.	0		NA	1	Rosaceae Juss.
Iz_4_1109	DJI_0009	Rubus idaeus L.	0	n	1	0	Rosaceae Juss.
Iz_4_1109	DJI_0009	Rumex acetosella L.	0	n	1	0	Polygonaceae Juss.
Iz_4_1109	DJI_0009	Rumex thyrsiflorus Fingerh.	0	n	1	0	Polygonaceae Juss.
Iz_4_1109	DJI_0009	Salix alba L.	0	n	1	1	Salicaceae Mirb.
Iz_4_1109	DJI_0009	Sambucus nigra L.	0	n	1	1	Viburnaceae Raf.
Iz_4_1109	DJI_0009	Secale sylvestre Host	0	n	1	0	Poaceae Barnhart
Iz_4_1109	DJI_0009	Sempervivum ruthenicum Schnittsp. & C.B. Lehm.	0	n	1	0	Crassulaceae J.St.-Hil.
Iz_4_1109	DJI_0009	Setaria viridis (L.) P. Beauv.	0	n	1	0	Poaceae Barnhart
Iz_4_1109	DJI_0009	Silene borysthenica (Gruner) Walters	0	n	1	0	Caryophyllaceae Juss.
Iz_4_1109	DJI_0009	Silene latifolia subsp. alba (Mill.) Greuter & Burdet	0	n	1	0	Caryophyllaceae Juss.
Iz_4_1109	DJI_0009	Solidago virgaurea L.	0	n	1	0	Asteraceae Bercht. & J.Presl
Iz_4_1109	DJI_0009	Sorbus aucuparia L.	0	n	1	1	Rosaceae Juss.
Iz_4_1109	DJI_0009	Stachys recta L.	0	n	1	0	Lamiacea Lindl.
Iz_4_1109	DJI_0009	Tanacetum vulgare L.	0	n	1	0	Asteraceae Bercht. & J.Presl
Iz_4_1109	DJI_0009	Thymus pallasianus Heinr.Braun	0	n	1	0	Lamiacea Lindl.
Iz_4_1109	DJI_0009	Tilia cordata Mill.	0	n	1	1	Malvaceae Juss.
Iz_4_1109	DJI_0009	Trifolium alpestre L.	0	n	1	0	Fabaceae Lindl.
Iz_4_1109	DJI_0009	Trifolium arvense L.	0	n	1	0	Fabaceae Lindl.
Iz_4_1109	DJI_0009	Ulmus glabra Huds.	0	n	1	1	Ulmaceae Mirb.
Iz_4_1109	DJI_0009	Verbascum densiflorum Bertol.	0	n	1	0	Scrophulariaceae Juss.
Iz_4_1109	DJI_0009	Veronica austriaca L.	0	n	1	0	Plantaginaceae Juss.
Iz_4_1109	DJI_0009	Veronica incana L.	0	n	1	0	Plantaginaceae Juss.
Iz_4_1109	DJI_0009	Veronica verna L.	0	n	1	0	Plantaginaceae Juss.
Iz_4_1109	DJI_0009	Vincetoxicum hirundinaria Medik	0	n	1	0	Apocynaceae Juss.
Iz_4_1109	DJI_0090	Acer tataricum L.	0	n	1	1	Sapindaceae Juss.
Iz_4_1109	DJI_0090	Achillea millefolium L. s.l.	0	n	1	0	Asteraceae Bercht. & J.Presl
Iz_4_1109	DJI_0090	Allium oleraceum L.	0	n	1	0	Amaryllidaceae J.St.-Hil.
Iz_4_1109	DJI_0090	Ambrosia artemisiifolia L.	0	a	0	0	Asteraceae Bercht. & J.Presl
Iz_4_1109	DJI_0090	Amorpha fruticosa L.	0	a	0	0	Fabaceae Lindl.
Iz_4_1109	DJI_0090	Anthericum ramosum L.	0	n	1	0	Asparagaceae Juss.
Iz_4_1109	DJI_0090	Artemisia marschalliana Spreng.	0	n	1	0	Asteraceae Bercht. & J.Presl
Iz_4_1109	DJI_0090	Asparagus officinalis L.	0	n	1	0	Asparagaceae Juss.
Iz_4_1109	DJI_0090	Asteraceae sp.	0	n	1	0	Asteraceae Bercht. & J.Presl
Iz_4_1109	DJI_0090	Astragalus cicer L.	0	n	1	0	Fabaceae Lindl.
Iz_4_1109	DJI_0090	Atriplex sp.	0		NA	NA	
Iz_4_1109	DJI_0090	Bassia laniflora (S.G.Gmel.) A.J.Scott	0	n	1	0	Amaranthaceae Juss.
Iz_4_1109	DJI_0090	Berberis vulgaris L.	0	n	1	0	Berberidaceae Juss.
Iz_4_1109	DJI_0090	Betula pendula Roth.	0	n	1	1	Betulaceae Gray
Iz_4_1109	DJI_0090	Betula pendula Roth.	0	n	1	0	Poaceae Barnhart
Iz_4_1109	DJI_0090	Bromus inermis Leyss.	0		NA	NA	
Iz_4_1109	DJI_0090	Bromus squarrosus L.	0	a	0	0	Poaceae Barnhart
Iz_4_1109	DJI_0090	Bromus tectorum L.	0	a	0	0	Poaceae Barnhart
Iz_4_1109	DJI_0090	Calamagrostis epigejos (L.) Roth	60	n	1	0	Poaceae Barnhart
Iz_4_1109	DJI_0090	Carduus acanthoides L.	0	n	1	0	Asteraceae Bercht. & J.Presl
Iz_4_1109	DJI_0090	Carex sp.	0	n	1	0	Cyperaceae Juss.
Iz_4_1109	DJI_0090	Centaurea majorovii Dumbadze	0	n	1	0	Asteraceae Bercht. & J.Presl
Iz_4_1109	DJI_0090	Chamaecytisus ruthenicus (Fisch.ex Wol.) Klaskova	0	n	1	0	Fabaceae Lindl.
Iz_4_1109	DJI_0090	Chelidonium majus L.	0	n	1	0	Papaveraceae Juss.
Iz_4_1109	DJI_0090	Chenopodium betaceum Andrz.	0	n	1	0	Amaranthaceae Juss.
Iz_4_1109	DJI_0090	Chenopodium cf. album	0	n	1	0	Amaranthaceae Juss.
Iz_4_1109	DJI_0090	Chenopodium sp.	0		NA	0	Amaranthaceae Juss.
Iz_4_1109	DJI_0090	Chondrilla juncea L.	0	n	1	0	Asteraceae Bercht. & J.Presl
Iz_4_1109	DJI_0090	Cirsium vulgare (Savi) Ten.	0	n	1	0	Asteraceae Bercht. & J.Presl
Iz_4_1109	DJI_0090	Clinopodium acinos (L.) Kuntze	0	n	1	0	Lamiacea Lindl.
Iz_4_1109	DJI_0090	Convallaria majalis L.	0	n	1	0	Asparagaceae Juss.
Iz_4_1109	DJI_0090	Crataegus rhipidophylla Gand.	0	n	1	1	Rosaceae Juss.
Iz_4_1109	DJI_0090	Crepis foetida L.	0	n	1	0	Asteraceae Bercht. & J.Presl
Iz_4_1109	DJI_0090	Dactylis glomerata L.	0	n	1	0	Poaceae Barnhart
Iz_4_1109	DJI_0090	Dianthus campestris M.Bieb.	0	n	1	0	Caryophyllaceae Juss.
Iz_4_1109	DJI_0090	Digitaria sanguinalis (L.) Scop.	0	a	0	0	Poaceae Barnhart
Iz_4_1109	DJI_0090	Elymus repens (L.) Gould	0	n	1	0	Poaceae Barnhart
Iz_4_1109	DJI_0090	Epilobium tetragonum L.	0	n	1	0	Onagraceae Juss.
Iz_4_1109	DJI_0090	Eragrostis minor Host	0	a	0	0	Poaceae Barnhart
Iz_4_1109	DJI_0090	Erigeron annuus (L.) Desf.	0	a	0	0	Asteraceae Bercht. & J.Presl
Iz_4_1109	DJI_0090	Erigeron canadensis L.	3	a	0	0	Asteraceae Bercht. & J.Presl
Iz_4_1109	DJI_0090	Euphorbia seguieriana Neck.	0	n	1	0	Euphorbiaceae Juss.
Iz_4_1109	DJI_0090	Fallopia dumetorum (L.) Holub	0	n	1	0	Polygonaceae Juss.
Iz_4_1109	DJI_0090	Festuca beckeri (Hack.) Trautv.	0	n	1	0	Poaceae Barnhart
Iz_4_1109	DJI_0090	Fragaria vesca L.	0	n	1	0	Rosaceae Juss.
Iz_4_1109	DJI_0090	Frangula alnus Mill.	0	n	1	1	Rhamnaceae Juss.
Iz_4_1109	DJI_0090	Fraxinus pennsylvanica Marshall	0	a	0	1	Oleaceae Hoffmanns. & Link
Iz_4_1109	DJI_0090	Galeopsis bifida Boenn.	0	n	1	0	Lamiacea Lindl.
Iz_4_1109	DJI_0090	Galium verum L.	0	n	1	0	Rubiaceae Juss.

Iz_4_1109	DJI_0090	Genista tinctoria L.	0	n	1	0	Fabaceae Lindl.
Iz_4_1109	DJI_0090	Geranium robertianum L.	0	n	1	0	Geraniaceae Juss.
Iz_4_1109	DJI_0090	Geranium sanquineum L.	0	n	1	0	Geraniaceae Juss.
Iz_4_1109	DJI_0090	Gypsophila paniculata L.	0	n	1	0	Caryophyllaceae Juss.
Iz_4_1109	DJI_0090	Helichrysum arenarium (L.) Moench	0	n	1	0	Asteraceae Bercht. & J.Presl
Iz_4_1109	DJI_0090	Herniaria polygama J.Gay	0	n	1	0	Caryophyllaceae Juss.
Iz_4_1109	DJI_0090	Hieracium robustum Fr.	0	n	1	0	Asteraceae Bercht. & J.Presl
Iz_4_1109	DJI_0090	Hieracium umbellatum L.	0	n	1	NA	Asteraceae Bercht. & J.Presl
Iz_4_1109	DJI_0090	Hylotelephium maximum (L.) Holub	0	n	1	0	Crassulaceae J.St.-Hil.
Iz_4_1109	DJI_0090	Jacobaea borysthenica (DC.) B.Nord. & Greuter	0	n	1	0	Asteraceae Bercht. & J.Presl
Iz_4_1109	DJI_0090	Jasione montana L.	0	n	1	0	Campanulaceae Juss.
Iz_4_1109	DJI_0090	Jurinea cyanoides (L.) Rchb.s.l.	0	n	1	0	Asteraceae Bercht. & J.Presl
Iz_4_1109	DJI_0090	Koeleria glauca (Spreng.) DC.	0	n	1	0	Poaceae Barnhart
Iz_4_1109	DJI_0090	Lactuca serriola L.	0	a	0	0	Asteraceae Bercht. & J.Presl
Iz_4_1109	DJI_0090	Linaria genistifolia (L.) Mill.	0	n	1	0	Plantaginaceae Juss.
Iz_4_1109	DJI_0090	Lomelosia argentea (L.) Greuter & Burdet	0	n	1	0	Caprifoliaceae Juss.
Iz_4_1109	DJI_0090	Melampyrum pratense L.	0	n	1	0	Orobanchaceae Vent.
Iz_4_1109	DJI_0090	Oenothera biennis L.	0	a	0	0	Onagraceae Juss.
Iz_4_1109	DJI_0090	Pentanema britannica (L.) D.Gut.Larr., Santos-Vicente, Anderb., E.Rico & M.M.Mart.Ort.	0	n	1	0	Asteraceae Bercht. & J.Presl
Iz_4_1109	DJI_0090	Peucedanum oreoselinum (L.) Moench	0	n	1	0	Apiaceae Lindl.
Iz_4_1109	DJI_0090	Peucedanum ruthenicum M.Bieb.	0	n	1	0	Apiaceae Lindl.
Iz_4_1109	DJI_0090	Pilosella echioides (Lumn.) F.W.Schultz & Sch.Bip.	0	n	1	0	Asteraceae Bercht. & J.Presl
Iz_4_1109	DJI_0090	Pilosella officinarum F. Schultz et Sch. Bip.	0	n	1	0	Asteraceae Bercht. & J.Presl
Iz_4_1109	DJI_0090	Pilosella sp.	0		NA	0	Asteraceae Bercht. & J.Presl
Iz_4_1109	DJI_0090	Pinus sylvestris L.	0	n	1	1	Pinaceae Spreng. ex F.Rudolphi
Iz_4_1109	DJI_0090	Poa annua L.	0	n	1	0	Poaceae Barnhart
Iz_4_1109	DJI_0090	Poa nemoralis L.	0	n	1	0	Poaceae Barnhart
Iz_4_1109	DJI_0090	Poa sp.	0		NA	0	Poaceae Barnhart
Iz_4_1109	DJI_0090	Polygonatum odoratum (Mill.) Druce	0	n	1	0	Asparagaceae Juss.
Iz_4_1109	DJI_0090	Polygonum arenarium Waldst. & Kit.	0	n	1	0	Polygonaceae Juss.
Iz_4_1109	DJI_0090	Populus nigra L.	0	n	1	1	Salicaceae Mirb.
Iz_4_1109	DJI_0090	Populus tremula L.	0	n	1	1	Salicaceae Mirb.
Iz_4_1109	DJI_0090	Populus Ã canescens (Aiton) Sm.	0	n	1	1	Salicaceae Mirb.
Iz_4_1109	DJI_0090	Potentilla argentea L.	0	n	1	0	Rosaceae Juss.
Iz_4_1109	DJI_0090	Psammophiliella muralis (L.) Ikonn.	0	n	1	0	Caryophyllaceae Juss.
Iz_4_1109	DJI_0090	Psephellus sumensis (Kalen.) Greuter	0	n	1	0	Asteraceae Bercht. & J.Presl
Iz_4_1109	DJI_0090	Pulsatilla pratensis (L.) Mill. s.l.	0	n	1	0	Ranunculaceae Juss.
Iz_4_1109	DJI_0090	Pyrus communis L.	0	a	0	1	Rosaceae Juss.
Iz_4_1109	DJI_0090	Quercus robur L.	0	n	1	1	Fagaceae Dumort.
Iz_4_1109	DJI_0090	Robinia pseudoacacia L.	0	a	0	1	Fabaceae Lindl.
Iz_4_1109	DJI_0090	Rosa sp.	0		NA	1	Rosaceae Juss.
Iz_4_1109	DJI_0090	Rubus idaeus L.	0	n	1	0	Rosaceae Juss.
Iz_4_1109	DJI_0090	Rumex acetosella L.	0	n	1	0	Polygonaceae Juss.
Iz_4_1109	DJI_0090	Rumex thyrsiflorus Fingerh.	0	n	1	0	Polygonaceae Juss.
Iz_4_1109	DJI_0090	Salix alba L.	0	n	1	1	Salicaceae Mirb.
Iz_4_1109	DJI_0090	Sambucus nigra L.	0	n	1	1	Viburnaceae Raf.
Iz_4_1109	DJI_0090	Secale sylvestre Host	0	n	1	0	Poaceae Barnhart
Iz_4_1109	DJI_0090	Sempervivum ruthenicum Schnittsp. & C.B. Lehm.	0	n	1	0	Crassulaceae J.St.-Hil.
Iz_4_1109	DJI_0090	Setaria viridis (L.) P. Beauv.	0	n	1	0	Poaceae Barnhart
Iz_4_1109	DJI_0090	Silene borysthenica (Gruner) Walters	0	n	1	0	Caryophyllaceae Juss.
Iz_4_1109	DJI_0090	Silene latifolia subsp. alba (Mill.) Greuter & Burdet	0	n	1	0	Caryophyllaceae Juss.
Iz_4_1109	DJI_0090	Solidago virgaurea L.	0	n	1	0	Asteraceae Bercht. & J.Presl
Iz_4_1109	DJI_0090	Sorbus aucuparia L.	0	n	1	1	Rosaceae Juss.
Iz_4_1109	DJI_0090	Stachys recta L.	0	n	1	0	Lamiacea Lindl.
Iz_4_1109	DJI_0090	Tanacetum vulgare L.	0	n	1	0	Asteraceae Bercht. & J.Presl
Iz_4_1109	DJI_0090	Thymus pallasianus Heinr.Braun	0	n	1	0	Lamiacea Lindl.
Iz_4_1109	DJI_0090	Tilia cordata Mill.	0	n	1	1	Malvaceae Juss.
Iz_4_1109	DJI_0090	Trifolium alpestre L.	0	n	1	0	Fabaceae Lindl.
Iz_4_1109	DJI_0090	Trifolium arvense L.	0	n	1	0	Fabaceae Lindl.
Iz_4_1109	DJI_0090	Ulmus glabra Huds.	0	n	1	1	Ulmaceae Mirb.
Iz_4_1109	DJI_0090	Verbascum densiflorum Bertol.	0	n	1	0	Scrophulariaceae Juss.
Iz_4_1109	DJI_0090	Veronica austriaca L.	0	n	1	0	Plantaginaceae Juss.
Iz_4_1109	DJI_0090	Veronica incana L.	0	n	1	0	Plantaginaceae Juss.
Iz_4_1109	DJI_0090	Veronica verna L.	0	n	1	0	Plantaginaceae Juss.
Iz_4_1109	DJI_0090	Vincetoxicum hirundinaria Medik	0	n	1	0	Apocynaceae Juss.
Iz_4_1109	DJI_0091	Acer tataricum L.	0	n	1	1	Sapindaceae Juss.
Iz_4_1109	DJI_0091	Achillea millefolium L. s.l.	0	n	1	0	Asteraceae Bercht. & J.Presl
Iz_4_1109	DJI_0091	Allium oleraceum L.	0	n	1	0	Amaryllidaceae J.St.-Hil.
Iz_4_1109	DJI_0091	Ambrosia artemisiifolia L.	0	a	0	0	Asteraceae Bercht. & J.Presl
Iz_4_1109	DJI_0091	Amorpha fruticosa L.	0	a	0	0	Fabaceae Lindl.
Iz_4_1109	DJI_0091	Anthericum ramosum L.	0	n	1	0	Asparagaceae Juss.
Iz_4_1109	DJI_0091	Artemisia marschalliana Spreng.	0	n	1	0	Asteraceae Bercht. & J.Presl
Iz_4_1109	DJI_0091	Asparagus officinalis L.	0	n	1	0	Asparagaceae Juss.
Iz_4_1109	DJI_0091	Asteraceae sp.	0	n	1	0	Asteraceae Bercht. & J.Presl
Iz_4_1109	DJI_0091	Astragalus cicer L.	0	n	1	0	Fabaceae Lindl.
Iz_4_1109	DJI_0091	Atriplex sp.	0		NA	NA	
Iz_4_1109	DJI_0091	Bassia laniflora (S.G.Gmel.) A.J.Scott	0	n	1	0	Amaranthaceae Juss.
Iz_4_1109	DJI_0091	Berberis vulgaris L.	0	n	1	0	Berberidaceae Juss.
Iz_4_1109	DJI_0091	Betula pendula Roth.	0	n	1	1	Betulaceae Gray
Iz_4_1109	DJI_0091	Betula pendula Roth.	0	n	1	0	Poaceae Barnhart
Iz_4_1109	DJI_0091	Bromus inermis Leyss.	0		NA	NA	
Iz_4_1109	DJI_0091	Bromus squarrosus L.	0	a	0	0	Poaceae Barnhart
Iz_4_1109	DJI_0091	Bromus tectorum L.	0	a	0	0	Poaceae Barnhart
Iz_4_1109	DJI_0091	Calamagrostis epigejos (L.) Roth	40	n	1	0	Poaceae Barnhart
Iz_4_1109	DJI_0091	Carduus acanthoides L.	0	n	1	0	Asteraceae Bercht. & J.Presl
Iz_4_1109	DJI_0091	Carex sp.	0	n	1	0	Cyperaceae Juss.
Iz_4_1109	DJI_0091	Centaurea majorovii Dumbadze	0	n	1	0	Asteraceae Bercht. & J.Presl
Iz_4_1109	DJI_0091	Chamaecytisus ruthenicus (Fisch.ex Wol.) Klaskova	0	n	1	0	Fabaceae Lindl.
Iz_4_1109	DJI_0091	Chelidonium majus L.	0.2	n	1	0	Papaveraceae Juss.
Iz_4_1109	DJI_0091	Chenopodium betaceum Andrz.	0	n	1	0	Amaranthaceae Juss.
Iz_4_1109	DJI_0091	Chenopodium cf. album	0	n	1	0	Amaranthaceae Juss.
Iz_4_1109	DJI_0091	Chenopodium sp.	0		NA	0	Amaranthaceae Juss.
Iz_4_1109	DJI_0091	Chondrilla juncea L.	0	n	1	0	Asteraceae Bercht. & J.Presl
Iz_4_1109	DJI_0091	Cirsium vulgare (Savi) Ten.	0	n	1	0	Asteraceae Bercht. & J.Presl
Iz_4_1109	DJI_0091	Clinopodium acinos (L.) Kuntze	0	n	1	0	Lamiacea Lindl.
Iz_4_1109	DJI_0091	Convallaria majalis L.	0	n	1	0	Asparagaceae Juss.
Iz_4_1109	DJI_0091	Crataegus rhipidophylla Gand.	0	n	1	1	Rosaceae Juss.
Iz_4_1109	DJI_0091	Crepis foetida L.	0	n	1	0	Asteraceae Bercht. & J.Presl

Iz_4_1109	DJI_0091	Dactylis glomerata L.	0	n	1	0	Poaceae Barnhart
Iz_4_1109	DJI_0091	Dianthus campestris M.Bieb.	0	n	1	0	Caryophyllaceae Juss.
Iz_4_1109	DJI_0091	Digitaria sanguinalis (L.) Scop.	0	a	0	0	Poaceae Barnhart
Iz_4_1109	DJI_0091	Elymus repens (L.) Gould	0	n	1	0	Poaceae Barnhart
Iz_4_1109	DJI_0091	Epilobium tetragonum L.	0	n	1	0	Onagraceae Juss.
Iz_4_1109	DJI_0091	Eragrostis minor Host	0	a	0	0	Poaceae Barnhart
Iz_4_1109	DJI_0091	Erigeron annuus (L.) Desf.	0	a	0	0	Asteraceae Bercht. & J.Presl
Iz_4_1109	DJI_0091	Erigeron canadensis L.	2	a	0	0	Asteraceae Bercht. & J.Presl
Iz_4_1109	DJI_0091	Euphorbia seguieriana Neck.	0	n	1	0	Euphorbiaceae Juss.
Iz_4_1109	DJI_0091	Fallopia dumetorum (L.) Holub	0	n	1	0	Polygonaceae Juss.
Iz_4_1109	DJI_0091	Festuca beckeri (Hack.) Trautv.	0	n	1	0	Poaceae Barnhart
Iz_4_1109	DJI_0091	Fragaria vesca L.	0	n	1	0	Rosaceae Juss.
Iz_4_1109	DJI_0091	Frangula alnus Mill.	0	n	1	1	Rhamnaceae Juss.
Iz_4_1109	DJI_0091	Fraxinus pennsylvanica Marshall	0	a	0	1	Oleaceae Hoffmanns. & Link
Iz_4_1109	DJI_0091	Galeopsis bifida Boenn.	0	n	1	0	Lamiacea Lindl.
Iz_4_1109	DJI_0091	Galium verum L.	0	n	1	0	Rubiaceae Juss.
Iz_4_1109	DJI_0091	Genista tinctoria L.	0	n	1	0	Fabaceae Lindl.
Iz_4_1109	DJI_0091	Geranium robertianum L.	0	n	1	0	Geraniaceae Juss.
Iz_4_1109	DJI_0091	Geranium sanquineum L.	0	n	1	0	Geraniaceae Juss.
Iz_4_1109	DJI_0091	Gypsophila paniculata L.	0	n	1	0	Caryophyllaceae Juss.
Iz_4_1109	DJI_0091	Helichrysum arenarium (L.) Moench	0	n	1	0	Asteraceae Bercht. & J.Presl
Iz_4_1109	DJI_0091	Herniaria polygama J.Gay	0	n	1	0	Caryophyllaceae Juss.
Iz_4_1109	DJI_0091	Hieracium robustum Fr.	0	n	1	0	Asteraceae Bercht. & J.Presl
Iz_4_1109	DJI_0091	Hieracium umbellatum L.	0	n	1	NA	Asteraceae Bercht. & J.Presl
Iz_4_1109	DJI_0091	Hylotelephium maximum (L.) Holub	0	n	1	0	Crassulaceae J.St.-Hil.
Iz_4_1109	DJI_0091	Jacobaea borysthenica (DC.) B.Nord. & Greuter	0	n	1	0	Asteraceae Bercht. & J.Presl
Iz_4_1109	DJI_0091	Jasione montana L.	0	n	1	0	Campanulaceae Juss.
Iz_4_1109	DJI_0091	Jurinea cyanoides (L.) Rchb.s.l.	0	n	1	0	Asteraceae Bercht. & J.Presl
Iz_4_1109	DJI_0091	Koeleria glauca (Spreng.) DC.	0	n	1	0	Poaceae Barnhart
Iz_4_1109	DJI_0091	Lactuca serriola L.	0	a	0	0	Asteraceae Bercht. & J.Presl
Iz_4_1109	DJI_0091	Linaria genistifolia (L.) Mill.	0	n	1	0	Plantaginaceae Juss.
Iz_4_1109	DJI_0091	Lomelosia argentea (L.) Greuter & Burdet	0	n	1	0	Caprifoliaceae Juss.
Iz_4_1109	DJI_0091	Melampyrum pratense L.	0	n	1	0	Orobanchaceae Vent.
Iz_4_1109	DJI_0091	Oenothera biennis L.	0	a	0	0	Onagraceae Juss.
Iz_4_1109	DJI_0091	Pentanema britannica (L.) D.Gut.Larr., Santos-Vicente, Anderb., E.Rico & M.M.Mart.Ort.	0	n	1	0	Asteraceae Bercht. & J.Presl
Iz_4_1109	DJI_0091	Peucedanum oreoselinum (L.) Moench	0	n	1	0	Apiaceae Lindl.
Iz_4_1109	DJI_0091	Peucedanum ruthenicum M.Bieb.	0	n	1	0	Apiaceae Lindl.
Iz_4_1109	DJI_0091	Pilosella echioides (Lumn.) F.W.Schultz & Sch.Bip.	0	n	1	0	Asteraceae Bercht. & J.Presl
Iz_4_1109	DJI_0091	Pilosella officinarum F. Schultz et Sch. Bip.	0	n	1	0	Asteraceae Bercht. & J.Presl
Iz_4_1109	DJI_0091	Pilosella sp.	0		NA	0	Asteraceae Bercht. & J.Presl
Iz_4_1109	DJI_0091	Pinus sylvestris L.	0	n	1	1	Pinaceae Spreng. ex F.Rudolphi
Iz_4_1109	DJI_0091	Poa annua L.	0	n	1	0	Poaceae Barnhart
Iz_4_1109	DJI_0091	Poa nemoralis L.	0	n	1	0	Poaceae Barnhart
Iz_4_1109	DJI_0091	Poa sp.	0		NA	0	Poaceae Barnhart
Iz_4_1109	DJI_0091	Polygonatum odoratum (Mill.) Druce	0	n	1	0	Asparagaceae Juss.
Iz_4_1109	DJI_0091	Polygonum arenarium Waldst. & Kit.	0	n	1	0	Polygonaceae Juss.
Iz_4_1109	DJI_0091	Populus nigra L.	0	n	1	1	Salicaceae Mirb.
Iz_4_1109	DJI_0091	Populus tremula L.	0	n	1	1	Salicaceae Mirb.
Iz_4_1109	DJI_0091	Populus Ã canescens (Aiton) Sm.	0	n	1	1	Salicaceae Mirb.
Iz_4_1109	DJI_0091	Potentilla argentea L.	0	n	1	0	Rosaceae Juss.
Iz_4_1109	DJI_0091	Psammophiliella muralis (L.) Ikonn.	0	n	1	0	Caryophyllaceae Juss.
Iz_4_1109	DJI_0091	Psephellus sumensis (Kalen.) Greuter	0	n	1	0	Asteraceae Bercht. & J.Presl
Iz_4_1109	DJI_0091	Pulsatilla pratensis (L.) Mill. s.l.	0	n	1	0	Ranunculaceae Juss.
Iz_4_1109	DJI_0091	Pyrus communis L.	0	a	0	1	Rosaceae Juss.
Iz_4_1109	DJI_0091	Quercus robur L.	0	n	1	1	Fagaceae Dumort.
Iz_4_1109	DJI_0091	Robinia pseudoacacia L.	0	a	0	1	Fabaceae Lindl.
Iz_4_1109	DJI_0091	Rosa sp.	0		NA	1	Rosaceae Juss.
Iz_4_1109	DJI_0091	Rubus idaeus L.	0	n	1	0	Rosaceae Juss.
Iz_4_1109	DJI_0091	Rumex acetosella L.	0	n	1	0	Polygonaceae Juss.
Iz_4_1109	DJI_0091	Rumex thyrsiflorus Fingerh.	0	n	1	0	Polygonaceae Juss.
Iz_4_1109	DJI_0091	Salix alba L.	0	n	1	1	Salicaceae Mirb.
Iz_4_1109	DJI_0091	Sambucus nigra L.	0	n	1	1	Viburnaceae Raf.
Iz_4_1109	DJI_0091	Secale sylvestre Host	0	n	1	0	Poaceae Barnhart
Iz_4_1109	DJI_0091	Sempervivum ruthenicum Schnittsp. & C.B. Lehm.	0	n	1	0	Crassulaceae J.St.-Hil.
Iz_4_1109	DJI_0091	Setaria viridis (L.) P. Beauv.	0	n	1	0	Poaceae Barnhart
Iz_4_1109	DJI_0091	Silene borysthenica (Gruner) Walters	0	n	1	0	Caryophyllaceae Juss.
Iz_4_1109	DJI_0091	Silene latifolia subsp. alba (Mill.) Greuter & Burdet	0	n	1	0	Caryophyllaceae Juss.
Iz_4_1109	DJI_0091	Solidago virgaurea L.	0	n	1	0	Asteraceae Bercht. & J.Presl
Iz_4_1109	DJI_0091	Sorbus aucuparia L.	0	n	1	1	Rosaceae Juss.
Iz_4_1109	DJI_0091	Stachys recta L.	0	n	1	0	Lamiacea Lindl.
Iz_4_1109	DJI_0091	Tanacetum vulgare L.	0	n	1	0	Asteraceae Bercht. & J.Presl
Iz_4_1109	DJI_0091	Thymus pallasianus Heinr.Braun	0	n	1	0	Lamiacea Lindl.
Iz_4_1109	DJI_0091	Tilia cordata Mill.	0	n	1	1	Malvaceae Juss.
Iz_4_1109	DJI_0091	Trifolium alpestre L.	0	n	1	0	Fabaceae Lindl.
Iz_4_1109	DJI_0091	Trifolium arvense L.	0	n	1	0	Fabaceae Lindl.
Iz_4_1109	DJI_0091	Ulmus glabra Huds.	0	n	1	1	Ulmaceae Mirb.
Iz_4_1109	DJI_0091	Verbascum densiflorum Bertol.	0	n	1	0	Scrophulariaceae Juss.
Iz_4_1109	DJI_0091	Veronica austriaca L.	0	n	1	0	Plantaginaceae Juss.
Iz_4_1109	DJI_0091	Veronica incana L.	0	n	1	0	Plantaginaceae Juss.
Iz_4_1109	DJI_0091	Veronica verna L.	0	n	1	0	Plantaginaceae Juss.
Iz_4_1109	DJI_0091	Vincetoxicum hirundinaria Medik	0	n	1	0	Apocynaceae Juss.
Iz_4_1109	DJI_0096	Acer tataricum L.	0	n	1	1	Sapindaceae Juss.
Iz_4_1109	DJI_0096	Achillea millefolium L. s.l.	0	n	1	0	Asteraceae Bercht. & J.Presl
Iz_4_1109	DJI_0096	Allium oleraceum L.	0	n	1	0	Amaryllidaceae J.St.-Hil.
Iz_4_1109	DJI_0096	Ambrosia artemisiifolia L.	0	a	0	0	Asteraceae Bercht. & J.Presl
Iz_4_1109	DJI_0096	Amorpha fruticosa L.	0	a	0	0	Fabaceae Lindl.
Iz_4_1109	DJI_0096	Anthericum ramosum L.	0	n	1	0	Asparagaceae Juss.
Iz_4_1109	DJI_0096	Artemisia marschalliana Spreng.	0	n	1	0	Asteraceae Bercht. & J.Presl
Iz_4_1109	DJI_0096	Asparagus officinalis L.	0	n	1	0	Asparagaceae Juss.
Iz_4_1109	DJI_0096	Asteraceae sp.	0	n	1	0	Asteraceae Bercht. & J.Presl
Iz_4_1109	DJI_0096	Astragalus cicer L.	0	n	1	0	Fabaceae Lindl.
Iz_4_1109	DJI_0096	Atriplex sp.	0		NA	NA	
Iz_4_1109	DJI_0096	Bassia laniflora (S.G.Gmel.) A.J.Scott	0	n	1	0	Amaranthaceae Juss.
Iz_4_1109	DJI_0096	Berberis vulgaris L.	0	n	1	0	Berberidaceae Juss.
Iz_4_1109	DJI_0096	Betula pendula Roth.	0	n	1	1	Betulaceae Gray
Iz_4_1109	DJI_0096	Betula pendula Roth.	0	n	1	0	Poaceae Barnhart
Iz_4_1109	DJI_0096	Bromus inermis Leyss.	0		NA	NA	
Iz_4_1109	DJI_0096	Bromus squarrosus L.	0	a	0	0	Poaceae Barnhart

Iz_4_1109	DJI_0096	Bromus tectorum L.	0	a	0	0	Poaceae Barnhart
Iz_4_1109	DJI_0096	Calamagrostis epigejos (L.) Roth	60	n	1	0	Poaceae Barnhart
Iz_4_1109	DJI_0096	Carduus acanthoides L.	0	n	1	0	Asteraceae Bercht. & J.Presl
Iz_4_1109	DJI_0096	Carex sp.	0	n	1	0	Cyperaceae Juss.
Iz_4_1109	DJI_0096	Centaurea majorovii Dumbadze	0	n	1	0	Asteraceae Bercht. & J.Presl
Iz_4_1109	DJI_0096	Chamaecytisus ruthenicus (Fisch.ex Wol.) Klaskova	0	n	1	0	Fabaceae Lindl.
Iz_4_1109	DJI_0096	Chelidonium majus L.	0	n	1	0	Papaveraceae Juss.
Iz_4_1109	DJI_0096	Chenopodium betaceum Andrz.	0	n	1	0	Amaranthaceae Juss.
Iz_4_1109	DJI_0096	Chenopodium cf. album	0	n	1	0	Amaranthaceae Juss.
Iz_4_1109	DJI_0096	Chenopodium sp.	0		NA	0	Amaranthaceae Juss.
Iz_4_1109	DJI_0096	Chondrilla juncea L.	0	n	1	0	Asteraceae Bercht. & J.Presl
Iz_4_1109	DJI_0096	Cirsium vulgare (Savi) Ten.	0	n	1	0	Asteraceae Bercht. & J.Presl
Iz_4_1109	DJI_0096	Clinopodium acinos (L.) Kuntze	0	n	1	0	Lamiacea Lindl.
Iz_4_1109	DJI_0096	Convallaria majalis L.	0	n	1	0	Asparagaceae Juss.
Iz_4_1109	DJI_0096	Crataegus rhipidophylla Gand.	0	n	1	1	Rosaceae Juss.
Iz_4_1109	DJI_0096	Crepis foetida L.	0	n	1	0	Asteraceae Bercht. & J.Presl
Iz_4_1109	DJI_0096	Dactylis glomerata L.	0	n	1	0	Poaceae Barnhart
Iz_4_1109	DJI_0096	Dianthus campestris M.Bieb.	0	n	1	0	Caryophyllaceae Juss.
Iz_4_1109	DJI_0096	Digitaria sanguinalis (L.) Scop.	0	a	0	0	Poaceae Barnhart
Iz_4_1109	DJI_0096	Elymus repens (L.) Gould	0	n	1	0	Poaceae Barnhart
Iz_4_1109	DJI_0096	Epilobium tetragonum L.	0	n	1	0	Onagraceae Juss.
Iz_4_1109	DJI_0096	Eragrostis minor Host	0	a	0	0	Poaceae Barnhart
Iz_4_1109	DJI_0096	Erigeron annuus (L.) Desf.	0	a	0	0	Asteraceae Bercht. & J.Presl
Iz_4_1109	DJI_0096	Erigeron canadensis L.	6	a	0	0	Asteraceae Bercht. & J.Presl
Iz_4_1109	DJI_0096	Euphorbia seguieriana Neck.	0	n	1	0	Euphorbiaceae Juss.
Iz_4_1109	DJI_0096	Fallopia dumetorum (L.) Holub	3	n	1	0	Polygonaceae Juss.
Iz_4_1109	DJI_0096	Festuca beckeri (Hack.) Trautv.	0	n	1	0	Poaceae Barnhart
Iz_4_1109	DJI_0096	Fragaria vesca L.	0	n	1	0	Rosaceae Juss.
Iz_4_1109	DJI_0096	Frangula alnus Mill.	0	n	1	1	Rhamnaceae Juss.
Iz_4_1109	DJI_0096	Fraxinus pennsylvanica Marshall	0	a	0	1	Oleaceae Hoffmanns. & Link
Iz_4_1109	DJI_0096	Galeopsis bifida Boenn.	0	n	1	0	Lamiacea Lindl.
Iz_4_1109	DJI_0096	Galium verum L.	0	n	1	0	Rubiaceae Juss.
Iz_4_1109	DJI_0096	Genista tinctoria L.	0	n	1	0	Fabaceae Lindl.
Iz_4_1109	DJI_0096	Geranium robertianum L.	0	n	1	0	Geraniaceae Juss.
Iz_4_1109	DJI_0096	Geranium sanquineum L.	0	n	1	0	Geraniaceae Juss.
Iz_4_1109	DJI_0096	Gypsophila paniculata L.	0	n	1	0	Caryophyllaceae Juss.
Iz_4_1109	DJI_0096	Helichrysum arenarium (L.) Moench	0	n	1	0	Asteraceae Bercht. & J.Presl
Iz_4_1109	DJI_0096	Herniaria polygama J.Gay	0	n	1	0	Caryophyllaceae Juss.
Iz_4_1109	DJI_0096	Hieracium robustum Fr.	0	n	1	0	Asteraceae Bercht. & J.Presl
Iz_4_1109	DJI_0096	Hieracium umbellatum L.	0	n	1	NA	Asteraceae Bercht. & J.Presl
Iz_4_1109	DJI_0096	Hylotelephium maximum (L.) Holub	0	n	1	0	Crassulaceae J.St.-Hil.
Iz_4_1109	DJI_0096	Jacobaea borysthenica (DC.) B.Nord. & Greuter	0	n	1	0	Asteraceae Bercht. & J.Presl
Iz_4_1109	DJI_0096	Jasione montana L.	0	n	1	0	Campanulaceae Juss.
Iz_4_1109	DJI_0096	Jurinea cyanoides (L.) Rchb.s.l.	0	n	1	0	Asteraceae Bercht. & J.Presl
Iz_4_1109	DJI_0096	Koeleria glauca (Spreng.) DC.	0	n	1	0	Poaceae Barnhart
Iz_4_1109	DJI_0096	Lactuca serriola L.	1	a	0	0	Asteraceae Bercht. & J.Presl
Iz_4_1109	DJI_0096	Linaria genistifolia (L.) Mill.	0	n	1	0	Plantaginaceae Juss.
Iz_4_1109	DJI_0096	Lomelosia argentea (L.) Greuter & Burdet	0	n	1	0	Caprifoliaceae Juss.
Iz_4_1109	DJI_0096	Melampyrum pratense L.	0	n	1	0	Orobanchaceae Vent.
Iz_4_1109	DJI_0096	Oenothera biennis L.	0	a	0	0	Onagraceae Juss.
Iz_4_1109	DJI_0096	Pentanema britannica (L.) D.Gut.Larr., Santos-Vicente, Anderb., E.Rico & M.M.Mart.Ort.	0	n	1	0	Asteraceae Bercht. & J.Presl
Iz_4_1109	DJI_0096	Peucedanum oreoselinum (L.) Moench	5	n	1	0	Apiaceae Lindl.
Iz_4_1109	DJI_0096	Peucedanum ruthenicum M.Bieb.	0	n	1	0	Apiaceae Lindl.
Iz_4_1109	DJI_0096	Pilosella echioides (Lumn.) F.W.Schultz & Sch.Bip.	0	n	1	0	Asteraceae Bercht. & J.Presl
Iz_4_1109	DJI_0096	Pilosella officinarum F. Schultz et Sch. Bip.	0	n	1	0	Asteraceae Bercht. & J.Presl
Iz_4_1109	DJI_0096	Pilosella sp.	0		NA	0	Asteraceae Bercht. & J.Presl
Iz_4_1109	DJI_0096	Pinus sylvestris L.	0	n	1	1	Pinaceae Spreng. ex F.Rudolphi
Iz_4_1109	DJI_0096	Poa annua L.	0	n	1	0	Poaceae Barnhart
Iz_4_1109	DJI_0096	Poa nemoralis L.	0	n	1	0	Poaceae Barnhart
Iz_4_1109	DJI_0096	Poa sp.	0		NA	0	Poaceae Barnhart
Iz_4_1109	DJI_0096	Polygonatum odoratum (Mill.) Druce	0	n	1	0	Asparagaceae Juss.
Iz_4_1109	DJI_0096	Polygonum arenarium Waldst. & Kit.	0	n	1	0	Polygonaceae Juss.
Iz_4_1109	DJI_0096	Populus nigra L.	0	n	1	1	Salicaceae Mirb.
Iz_4_1109	DJI_0096	Populus tremula L.	0	n	1	1	Salicaceae Mirb.
Iz_4_1109	DJI_0096	Populus Ã canescens (Aiton) Sm.	0	n	1	1	Salicaceae Mirb.
Iz_4_1109	DJI_0096	Potentilla argentea L.	0	n	1	0	Rosaceae Juss.
Iz_4_1109	DJI_0096	Psammophiliella muralis (L.) Ikonn.	0	n	1	0	Caryophyllaceae Juss.
Iz_4_1109	DJI_0096	Psephellus sumensis (Kalen.) Greuter	0	n	1	0	Asteraceae Bercht. & J.Presl
Iz_4_1109	DJI_0096	Pulsatilla pratensis (L.) Mill. s.l.	0	n	1	0	Ranunculaceae Juss.
Iz_4_1109	DJI_0096	Pyrus communis L.	0	a	0	1	Rosaceae Juss.
Iz_4_1109	DJI_0096	Quercus robur L.	0	n	1	1	Fagaceae Dumort.
Iz_4_1109	DJI_0096	Robinia pseudoacacia L.	0	a	0	1	Fabaceae Lindl.
Iz_4_1109	DJI_0096	Rosa sp.	0		NA	1	Rosaceae Juss.
Iz_4_1109	DJI_0096	Rubus idaeus L.	0	n	1	0	Rosaceae Juss.
Iz_4_1109	DJI_0096	Rumex acetosella L.	0	n	1	0	Polygonaceae Juss.
Iz_4_1109	DJI_0096	Rumex thyrsiflorus Fingerh.	0	n	1	0	Polygonaceae Juss.
Iz_4_1109	DJI_0096	Salix alba L.	0	n	1	1	Salicaceae Mirb.
Iz_4_1109	DJI_0096	Sambucus nigra L.	0	n	1	1	Viburnaceae Raf.
Iz_4_1109	DJI_0096	Secale sylvestre Host	0	n	1	0	Poaceae Barnhart
Iz_4_1109	DJI_0096	Sempervivum ruthenicum Schnittsp. & C.B. Lehm.	0	n	1	0	Crassulaceae J.St.-Hil.
Iz_4_1109	DJI_0096	Setaria viridis (L.) P. Beauv.	0	n	1	0	Poaceae Barnhart
Iz_4_1109	DJI_0096	Silene borysthenica (Gruner) Walters	0	n	1	0	Caryophyllaceae Juss.
Iz_4_1109	DJI_0096	Silene latifolia subsp. alba (Mill.) Greuter & Burdet	0	n	1	0	Caryophyllaceae Juss.
Iz_4_1109	DJI_0096	Solidago virgaurea L.	0	n	1	0	Asteraceae Bercht. & J.Presl
Iz_4_1109	DJI_0096	Sorbus aucuparia L.	0	n	1	1	Rosaceae Juss.
Iz_4_1109	DJI_0096	Stachys recta L.	0	n	1	0	Lamiacea Lindl.
Iz_4_1109	DJI_0096	Tanacetum vulgare L.	0	n	1	0	Asteraceae Bercht. & J.Presl
Iz_4_1109	DJI_0096	Thymus pallasianus Heinr.Braun	0	n	1	0	Lamiacea Lindl.
Iz_4_1109	DJI_0096	Tilia cordata Mill.	0	n	1	1	Malvaceae Juss.
Iz_4_1109	DJI_0096	Trifolium alpestre L.	0	n	1	0	Fabaceae Lindl.
Iz_4_1109	DJI_0096	Trifolium arvense L.	0	n	1	0	Fabaceae Lindl.
Iz_4_1109	DJI_0096	Ulmus glabra Huds.	0	n	1	1	Ulmaceae Mirb.
Iz_4_1109	DJI_0096	Verbascum densiflorum Bertol.	0	n	1	0	Scrophulariaceae Juss.
Iz_4_1109	DJI_0096	Veronica austriaca L.	0	n	1	0	Plantaginaceae Juss.
Iz_4_1109	DJI_0096	Veronica incana L.	0	n	1	0	Plantaginaceae Juss.
Iz_4_1109	DJI_0096	Veronica verna L.	0	n	1	0	Plantaginaceae Juss.
Iz_4_1109	DJI_0096	Vincetoxicum hirundinaria Medik	0	n	1	0	Apocynaceae Juss.
Iz_4_1109	DJI_0103	Acer tataricum L.	0	n	1	1	Sapindaceae Juss.

Iz_4_1109	DJI_0103	Achillea millefolium L. s.l.	0	n	1	0	Asteraceae Bercht. & J.Presl
Iz_4_1109	DJI_0103	Allium oleraceum L.	0	n	1	0	Amaryllidaceae J.St.-Hil.
Iz_4_1109	DJI_0103	Ambrosia artemisiifolia L.	0	a	0	0	Asteraceae Bercht. & J.Presl
Iz_4_1109	DJI_0103	Amorpha fruticosa L.	0	a	0	0	Fabaceae Lindl.
Iz_4_1109	DJI_0103	Anthericum ramosum L.	0	n	1	0	Asparagaceae Juss.
Iz_4_1109	DJI_0103	Artemisia marschalliana Spreng.	0	n	1	0	Asteraceae Bercht. & J.Presl
Iz_4_1109	DJI_0103	Asparagus officinalis L.	0	n	1	0	Asparagaceae Juss.
Iz_4_1109	DJI_0103	Asteraceae sp.	0	n	1	0	Asteraceae Bercht. & J.Presl
Iz_4_1109	DJI_0103	Astragalus cicer L.	0	n	1	0	Fabaceae Lindl.
Iz_4_1109	DJI_0103	Atriplex sp.	0		NA	NA	
Iz_4_1109	DJI_0103	Bassia laniflora (S.G.Gmel.) A.J.Scott	0	n	1	0	Amaranthaceae Juss.
Iz_4_1109	DJI_0103	Berberis vulgaris L.	0	n	1	0	Berberidaceae Juss.
Iz_4_1109	DJI_0103	Betula pendula Roth.	0	n	1	1	Betulaceae Gray
Iz_4_1109	DJI_0103	Betula pendula Roth.	0	n	1	0	Poaceae Barnhart
Iz_4_1109	DJI_0103	Bromus inermis Leyss.	0		NA	NA	
Iz_4_1109	DJI_0103	Bromus squarrosus L.	0	a	0	0	Poaceae Barnhart
Iz_4_1109	DJI_0103	Bromus tectorum L.	0	a	0	0	Poaceae Barnhart
Iz_4_1109	DJI_0103	Calamagrostis epigejos (L.) Roth	0	n	1	0	Poaceae Barnhart
Iz_4_1109	DJI_0103	Carduus acanthoides L.	0	n	1	0	Asteraceae Bercht. & J.Presl
Iz_4_1109	DJI_0103	Carex sp.	0	n	1	0	Cyperaceae Juss.
Iz_4_1109	DJI_0103	Centaurea majorovii Dumbadze	0	n	1	0	Asteraceae Bercht. & J.Presl
Iz_4_1109	DJI_0103	Chamaecytisus ruthenicus (Fisch.ex Wol.) Klaskova	0	n	1	0	Fabaceae Lindl.
Iz_4_1109	DJI_0103	Chelidonium majus L.	0	n	1	0	Papaveraceae Juss.
Iz_4_1109	DJI_0103	Chenopodium betaceum Andrz.	0	n	1	0	Amaranthaceae Juss.
Iz_4_1109	DJI_0103	Chenopodium cf. album	0	n	1	0	Amaranthaceae Juss.
Iz_4_1109	DJI_0103	Chenopodium sp.	0		NA	0	Amaranthaceae Juss.
Iz_4_1109	DJI_0103	Chondrilla juncea L.	0	n	1	0	Asteraceae Bercht. & J.Presl
Iz_4_1109	DJI_0103	Cirsium vulgare (Savi) Ten.	0	n	1	0	Asteraceae Bercht. & J.Presl
Iz_4_1109	DJI_0103	Clinopodium acinos (L.) Kuntze	0	n	1	0	Lamiacea Lindl.
Iz_4_1109	DJI_0103	Convallaria majalis L.	0	n	1	0	Asparagaceae Juss.
Iz_4_1109	DJI_0103	Crataegus rhipidophylla Gand.	0	n	1	1	Rosaceae Juss.
Iz_4_1109	DJI_0103	Crepis foetida L.	0	n	1	0	Asteraceae Bercht. & J.Presl
Iz_4_1109	DJI_0103	Dactylis glomerata L.	0	n	1	0	Poaceae Barnhart
Iz_4_1109	DJI_0103	Dianthus campestris M.Bieb.	0	n	1	0	Caryophyllaceae Juss.
Iz_4_1109	DJI_0103	Digitaria sanguinalis (L.) Scop.	0	a	0	0	Poaceae Barnhart
Iz_4_1109	DJI_0103	Elymus repens (L.) Gould	0	n	1	0	Poaceae Barnhart
Iz_4_1109	DJI_0103	Epilobium tetragonum L.	0	n	1	0	Onagraceae Juss.
Iz_4_1109	DJI_0103	Eragrostis minor Host	0	a	0	0	Poaceae Barnhart
Iz_4_1109	DJI_0103	Erigeron annuus (L.) Desf.	0	a	0	0	Asteraceae Bercht. & J.Presl
Iz_4_1109	DJI_0103	Erigeron canadensis L.	10	a	0	0	Asteraceae Bercht. & J.Presl
Iz_4_1109	DJI_0103	Euphorbia seguieriana Neck.	0	n	1	0	Euphorbiaceae Juss.
Iz_4_1109	DJI_0103	Fallopia dumetorum (L.) Holub	3	n	1	0	Polygonaceae Juss.
Iz_4_1109	DJI_0103	Festuca beckeri (Hack.) Trautv.	0	n	1	0	Poaceae Barnhart
Iz_4_1109	DJI_0103	Fragaria vesca L.	0	n	1	0	Rosaceae Juss.
Iz_4_1109	DJI_0103	Frangula alnus Mill.	0	n	1	1	Rhamnaceae Juss.
Iz_4_1109	DJI_0103	Fraxinus pennsylvanica Marshall	0	a	0	1	Oleaceae Hoffmanns. & Link
Iz_4_1109	DJI_0103	Galeopsis bifida Boenn.	0	n	1	0	Lamiacea Lindl.
Iz_4_1109	DJI_0103	Galium verum L.	0	n	1	0	Rubiaceae Juss.
Iz_4_1109	DJI_0103	Genista tinctoria L.	0	n	1	0	Fabaceae Lindl.
Iz_4_1109	DJI_0103	Geranium robertianum L.	0	n	1	0	Geraniaceae Juss.
Iz_4_1109	DJI_0103	Geranium sanquineum L.	0	n	1	0	Geraniaceae Juss.
Iz_4_1109	DJI_0103	Gypsophila paniculata L.	0	n	1	0	Caryophyllaceae Juss.
Iz_4_1109	DJI_0103	Helichrysum arenarium (L.) Moench	0	n	1	0	Asteraceae Bercht. & J.Presl
Iz_4_1109	DJI_0103	Herniaria polygama J.Gay	0	n	1	0	Caryophyllaceae Juss.
Iz_4_1109	DJI_0103	Hieracium robustum Fr.	0	n	1	0	Asteraceae Bercht. & J.Presl
Iz_4_1109	DJI_0103	Hieracium umbellatum L.	0	n	1	NA	Asteraceae Bercht. & J.Presl
Iz_4_1109	DJI_0103	Hylotelephium maximum (L.) Holub	0	n	1	0	Crassulaceae J.St.-Hil.
Iz_4_1109	DJI_0103	Jacobaea borysthenica (DC.) B.Nord. & Greuter	0	n	1	0	Asteraceae Bercht. & J.Presl
Iz_4_1109	DJI_0103	Jasione montana L.	0	n	1	0	Campanulaceae Juss.
Iz_4_1109	DJI_0103	Jurinea cyanoides (L.) Rchb.s.l.	1	n	1	0	Asteraceae Bercht. & J.Presl
Iz_4_1109	DJI_0103	Koeleria glauca (Spreng.) DC.	0	n	1	0	Poaceae Barnhart
Iz_4_1109	DJI_0103	Lactuca serriola L.	0	a	0	0	Asteraceae Bercht. & J.Presl
Iz_4_1109	DJI_0103	Linaria genistifolia (L.) Mill.	0	n	1	0	Plantaginaceae Juss.
Iz_4_1109	DJI_0103	Lomelosia argentea (L.) Greuter & Burdet	0	n	1	0	Caprifoliaceae Juss.
Iz_4_1109	DJI_0103	Melampyrum pratense L.	0	n	1	0	Orobanchaceae Vent.
Iz_4_1109	DJI_0103	Oenothera biennis L.	0	a	0	0	Onagraceae Juss.
Iz_4_1109	DJI_0103	Pentanema britannica (L.) D.Gut.Larr., Santos-Vicente, Anderb., E.Rico & M.M.Mart.Ort.	0	n	1	0	Asteraceae Bercht. & J.Presl
Iz_4_1109	DJI_0103	Peucedanum oreoselinum (L.) Moench	0	n	1	0	Apiaceae Lindl.
Iz_4_1109	DJI_0103	Peucedanum ruthenicum M.Bieb.	0	n	1	0	Apiaceae Lindl.
Iz_4_1109	DJI_0103	Pilosella echioides (Lumn.) F.W.Schultz & Sch.Bip.	0	n	1	0	Asteraceae Bercht. & J.Presl
Iz_4_1109	DJI_0103	Pilosella officinarum F. Schultz et Sch. Bip.	0	n	1	0	Asteraceae Bercht. & J.Presl
Iz_4_1109	DJI_0103	Pilosella sp.	0		NA	0	Asteraceae Bercht. & J.Presl
Iz_4_1109	DJI_0103	Pinus sylvestris L.	0	n	1	1	Pinaceae Spreng. ex F.Rudolphi
Iz_4_1109	DJI_0103	Poa annua L.	0	n	1	0	Poaceae Barnhart
Iz_4_1109	DJI_0103	Poa nemoralis L.	0	n	1	0	Poaceae Barnhart
Iz_4_1109	DJI_0103	Poa sp.	0		NA	0	Poaceae Barnhart
Iz_4_1109	DJI_0103	Polygonatum odoratum (Mill.) Druce	0	n	1	0	Asparagaceae Juss.
Iz_4_1109	DJI_0103	Polygonum arenarium Waldst. & Kit.	0	n	1	0	Polygonaceae Juss.
Iz_4_1109	DJI_0103	Populus nigra L.	0	n	1	1	Salicaceae Mirb.
Iz_4_1109	DJI_0103	Populus tremula L.	0	n	1	1	Salicaceae Mirb.
Iz_4_1109	DJI_0103	Populus Ã canescens (Aiton) Sm.	0	n	1	1	Salicaceae Mirb.
Iz_4_1109	DJI_0103	Potentilla argentea L.	0	n	1	0	Rosaceae Juss.
Iz_4_1109	DJI_0103	Psammophiliella muralis (L.) Ikonn.	0	n	1	0	Caryophyllaceae Juss.
Iz_4_1109	DJI_0103	Psephellus sumensis (Kalen.) Greuter	0	n	1	0	Asteraceae Bercht. & J.Presl
Iz_4_1109	DJI_0103	Pulsatilla pratensis (L.) Mill. s.l.	0	n	1	0	Ranunculaceae Juss.
Iz_4_1109	DJI_0103	Pyrus communis L.	0	a	0	1	Rosaceae Juss.
Iz_4_1109	DJI_0103	Quercus robur L.	0	n	1	1	Fagaceae Dumort.
Iz_4_1109	DJI_0103	Robinia pseudoacacia L.	0	a	0	1	Fabaceae Lindl.
Iz_4_1109	DJI_0103	Rosa sp.	0		NA	1	Rosaceae Juss.
Iz_4_1109	DJI_0103	Rubus idaeus L.	0	n	1	0	Rosaceae Juss.
Iz_4_1109	DJI_0103	Rumex acetosella L.	8	n	1	0	Polygonaceae Juss.
Iz_4_1109	DJI_0103	Rumex thyrsiflorus Fingerh.	0	n	1	0	Polygonaceae Juss.
Iz_4_1109	DJI_0103	Salix alba L.	0	n	1	1	Salicaceae Mirb.
Iz_4_1109	DJI_0103	Sambucus nigra L.	0	n	1	1	Viburnaceae Raf.
Iz_4_1109	DJI_0103	Secale sylvestre Host	0	n	1	0	Poaceae Barnhart
Iz_4_1109	DJI_0103	Sempervivum ruthenicum Schnittsp. & C.B. Lehm.	0	n	1	0	Crassulaceae J.St.-Hil.
Iz_4_1109	DJI_0103	Setaria viridis (L.) P. Beauv.	0	n	1	0	Poaceae Barnhart
Iz_4_1109	DJI_0103	Silene borysthenica (Gruner) Walters	0	n	1	0	Caryophyllaceae Juss.

Iz_4_1109	DJI_0103	Silene latifolia subsp. alba (Mill.) Greuter & Burdet	0	n	1	0	Caryophyllaceae Juss.
Iz_4_1109	DJI_0103	Solidago virgaurea L.	0	n	1	0	Asteraceae Bercht. & J.Presl
Iz_4_1109	DJI_0103	Sorbus aucuparia L.	0	n	1	1	Rosaceae Juss.
Iz_4_1109	DJI_0103	Stachys recta L.	0	n	1	0	Lamiacea Lindl.
Iz_4_1109	DJI_0103	Tanacetum vulgare L.	0	n	1	0	Asteraceae Bercht. & J.Presl
Iz_4_1109	DJI_0103	Thymus pallasianus Heinr.Braun	0	n	1	0	Lamiacea Lindl.
Iz_4_1109	DJI_0103	Tilia cordata Mill.	0	n	1	1	Malvaceae Juss.
Iz_4_1109	DJI_0103	Trifolium alpestre L.	0	n	1	0	Fabaceae Lindl.
Iz_4_1109	DJI_0103	Trifolium arvense L.	0	n	1	0	Fabaceae Lindl.
Iz_4_1109	DJI_0103	Ulmus glabra Huds.	0	n	1	1	Ulmaceae Mirb.
Iz_4_1109	DJI_0103	Verbascum densiflorum Bertol.	0	n	1	0	Scrophulariaceae Juss.
Iz_4_1109	DJI_0103	Veronica austriaca L.	0	n	1	0	Plantaginaceae Juss.
Iz_4_1109	DJI_0103	Veronica incana L.	0	n	1	0	Plantaginaceae Juss.
Iz_4_1109	DJI_0103	Veronica verna L.	0	n	1	0	Plantaginaceae Juss.
Iz_4_1109	DJI_0103	Vincetoxicum hirundinaria Medik	0	n	1	0	Apocynaceae Juss.
Iz_4_1109	DJI_0111	Acer tataricum L.	0	n	1	1	Sapindaceae Juss.
Iz_4_1109	DJI_0111	Achillea millefolium L. s.l.	0	n	1	0	Asteraceae Bercht. & J.Presl
Iz_4_1109	DJI_0111	Allium oleraceum L.	0	n	1	0	Amaryllidaceae J.St.-Hil.
Iz_4_1109	DJI_0111	Ambrosia artemisiifolia L.	0	a	0	0	Asteraceae Bercht. & J.Presl
Iz_4_1109	DJI_0111	Amorpha fruticosa L.	0	a	0	0	Fabaceae Lindl.
Iz_4_1109	DJI_0111	Anthericum ramosum L.	0	n	1	0	Asparagaceae Juss.
Iz_4_1109	DJI_0111	Artemisia marschalliana Spreng.	0	n	1	0	Asteraceae Bercht. & J.Presl
Iz_4_1109	DJI_0111	Asparagus officinalis L.	0	n	1	0	Asparagaceae Juss.
Iz_4_1109	DJI_0111	Asteraceae sp.	0	n	1	0	Asteraceae Bercht. & J.Presl
Iz_4_1109	DJI_0111	Astragalus cicer L.	0	n	1	0	Fabaceae Lindl.
Iz_4_1109	DJI_0111	Atriplex sp.	0		NA	NA	
Iz_4_1109	DJI_0111	Bassia laniflora (S.G.Gmel.) A.J.Scott	0	n	1	0	Amaranthaceae Juss.
Iz_4_1109	DJI_0111	Berberis vulgaris L.	0	n	1	0	Berberidaceae Juss.
Iz_4_1109	DJI_0111	Betula pendula Roth.	0	n	1	1	Betulaceae Gray
Iz_4_1109	DJI_0111	Betula pendula Roth.	0	n	1	0	Poaceae Barnhart
Iz_4_1109	DJI_0111	Bromus inermis Leyss.	0		NA	NA	
Iz_4_1109	DJI_0111	Bromus squarrosus L.	0	a	0	0	Poaceae Barnhart
Iz_4_1109	DJI_0111	Bromus tectorum L.	0	a	0	0	Poaceae Barnhart
Iz_4_1109	DJI_0111	Calamagrostis epigejos (L.) Roth	0.2	n	1	0	Poaceae Barnhart
Iz_4_1109	DJI_0111	Carduus acanthoides L.	0	n	1	0	Asteraceae Bercht. & J.Presl
Iz_4_1109	DJI_0111	Carex sp.	0	n	1	0	Cyperaceae Juss.
Iz_4_1109	DJI_0111	Centaurea majorovii Dumbadze	0	n	1	0	Asteraceae Bercht. & J.Presl
Iz_4_1109	DJI_0111	Chamaecytisus ruthenicus (Fisch.ex Wol.) Klaskova	0	n	1	0	Fabaceae Lindl.
Iz_4_1109	DJI_0111	Chelidonium majus L.	0	n	1	0	Papaveraceae Juss.
Iz_4_1109	DJI_0111	Chenopodium betaceum Andrz.	0	n	1	0	Amaranthaceae Juss.
Iz_4_1109	DJI_0111	Chenopodium cf. album	0	n	1	0	Amaranthaceae Juss.
Iz_4_1109	DJI_0111	Chenopodium sp.	0		NA	0	Amaranthaceae Juss.
Iz_4_1109	DJI_0111	Chondrilla juncea L.	1	n	1	0	Asteraceae Bercht. & J.Presl
Iz_4_1109	DJI_0111	Cirsium vulgare (Savi) Ten.	0	n	1	0	Asteraceae Bercht. & J.Presl
Iz_4_1109	DJI_0111	Clinopodium acinos (L.) Kuntze	0	n	1	0	Lamiacea Lindl.
Iz_4_1109	DJI_0111	Convallaria majalis L.	0	n	1	0	Asparagaceae Juss.
Iz_4_1109	DJI_0111	Crataegus rhipidophylla Gand.	0	n	1	1	Rosaceae Juss.
Iz_4_1109	DJI_0111	Crepis foetida L.	0	n	1	0	Asteraceae Bercht. & J.Presl
Iz_4_1109	DJI_0111	Dactylis glomerata L.	0	n	1	0	Poaceae Barnhart
Iz_4_1109	DJI_0111	Dianthus campestris M.Bieb.	0	n	1	0	Caryophyllaceae Juss.
Iz_4_1109	DJI_0111	Digitaria sanguinalis (L.) Scop.	0	a	0	0	Poaceae Barnhart
Iz_4_1109	DJI_0111	Elymus repens (L.) Gould	0	n	1	0	Poaceae Barnhart
Iz_4_1109	DJI_0111	Epilobium tetragonum L.	0	n	1	0	Onagraceae Juss.
Iz_4_1109	DJI_0111	Eragrostis minor Host	0	a	0	0	Poaceae Barnhart
Iz_4_1109	DJI_0111	Erigeron annuus (L.) Desf.	0	a	0	0	Asteraceae Bercht. & J.Presl
Iz_4_1109	DJI_0111	Erigeron canadensis L.	6	a	0	0	Asteraceae Bercht. & J.Presl
Iz_4_1109	DJI_0111	Euphorbia seguieriana Neck.	0	n	1	0	Euphorbiaceae Juss.
Iz_4_1109	DJI_0111	Fallopia dumetorum (L.) Holub	0	n	1	0	Polygonaceae Juss.
Iz_4_1109	DJI_0111	Festuca beckeri (Hack.) Trautv.	0	n	1	0	Poaceae Barnhart
Iz_4_1109	DJI_0111	Fragaria vesca L.	0	n	1	0	Rosaceae Juss.
Iz_4_1109	DJI_0111	Frangula alnus Mill.	0	n	1	1	Rhamnaceae Juss.
Iz_4_1109	DJI_0111	Fraxinus pennsylvanica Marshall	0	a	0	1	Oleaceae Hoffmanns. & Link
Iz_4_1109	DJI_0111	Galeopsis bifida Boenn.	0	n	1	0	Lamiacea Lindl.
Iz_4_1109	DJI_0111	Galium verum L.	0	n	1	0	Rubiaceae Juss.
Iz_4_1109	DJI_0111	Genista tinctoria L.	0	n	1	0	Fabaceae Lindl.
Iz_4_1109	DJI_0111	Geranium robertianum L.	0	n	1	0	Geraniaceae Juss.
Iz_4_1109	DJI_0111	Geranium sanquineum L.	0	n	1	0	Geraniaceae Juss.
Iz_4_1109	DJI_0111	Gypsophila paniculata L.	0	n	1	0	Caryophyllaceae Juss.
Iz_4_1109	DJI_0111	Helichrysum arenarium (L.) Moench	0	n	1	0	Asteraceae Bercht. & J.Presl
Iz_4_1109	DJI_0111	Herniaria polygama J.Gay	0	n	1	0	Caryophyllaceae Juss.
Iz_4_1109	DJI_0111	Hieracium robustum Fr.	0	n	1	0	Asteraceae Bercht. & J.Presl
Iz_4_1109	DJI_0111	Hieracium umbellatum L.	0	n	1	NA	Asteraceae Bercht. & J.Presl
Iz_4_1109	DJI_0111	Hylotelephium maximum (L.) Holub	0	n	1	0	Crassulaceae J.St.-Hil.
Iz_4_1109	DJI_0111	Jacobaea borysthenica (DC.) B.Nord. & Greuter	0	n	1	0	Asteraceae Bercht. & J.Presl
Iz_4_1109	DJI_0111	Jasione montana L.	0	n	1	0	Campanulaceae Juss.
Iz_4_1109	DJI_0111	Jurinea cyanoides (L.) Rchb.s.l.	0	n	1	0	Asteraceae Bercht. & J.Presl
Iz_4_1109	DJI_0111	Koeleria glauca (Spreng.) DC.	0	n	1	0	Poaceae Barnhart
Iz_4_1109	DJI_0111	Lactuca serriola L.	0	a	0	0	Asteraceae Bercht. & J.Presl
Iz_4_1109	DJI_0111	Linaria genistifolia (L.) Mill.	0	n	1	0	Plantaginaceae Juss.
Iz_4_1109	DJI_0111	Lomelosia argentea (L.) Greuter & Burdet	0	n	1	0	Caprifoliaceae Juss.
Iz_4_1109	DJI_0111	Melampyrum pratense L.	0	n	1	0	Orobanchaceae Vent.
Iz_4_1109	DJI_0111	Oenothera biennis L.	0	a	0	0	Onagraceae Juss.
Iz_4_1109	DJI_0111	Pentanema britannica (L.) D.Gut.Larr., Santos-Vicente, Anderb., E.Rico & M.M.Mart.Ort.	0	n	1	0	Asteraceae Bercht. & J.Presl
Iz_4_1109	DJI_0111	Peucedanum oreoselinum (L.) Moench	0	n	1	0	Apiaceae Lindl.
Iz_4_1109	DJI_0111	Peucedanum ruthenicum M.Bieb.	0	n	1	0	Apiaceae Lindl.
Iz_4_1109	DJI_0111	Pilosella echioides (Lumn.) F.W.Schultz & Sch.Bip.	0	n	1	0	Asteraceae Bercht. & J.Presl
Iz_4_1109	DJI_0111	Pilosella officinarum F. Schultz et Sch. Bip.	0	n	1	0	Asteraceae Bercht. & J.Presl
Iz_4_1109	DJI_0111	Pilosella sp.	0		NA	0	Asteraceae Bercht. & J.Presl
Iz_4_1109	DJI_0111	Pinus sylvestris L.	0	n	1	1	Pinaceae Spreng. ex F.Rudolphi
Iz_4_1109	DJI_0111	Poa annua L.	0	n	1	0	Poaceae Barnhart
Iz_4_1109	DJI_0111	Poa nemoralis L.	0	n	1	0	Poaceae Barnhart
Iz_4_1109	DJI_0111	Poa sp.	0		NA	0	Poaceae Barnhart
Iz_4_1109	DJI_0111	Polygonatum odoratum (Mill.) Druce	0	n	1	0	Asparagaceae Juss.
Iz_4_1109	DJI_0111	Polygonum arenarium Waldst. & Kit.	0	n	1	0	Polygonaceae Juss.
Iz_4_1109	DJI_0111	Populus nigra L.	0	n	1	1	Salicaceae Mirb.
Iz_4_1109	DJI_0111	Populus tremula L.	0	n	1	1	Salicaceae Mirb.
Iz_4_1109	DJI_0111	Populus Ã canescens (Aiton) Sm.	0	n	1	1	Salicaceae Mirb.
Iz_4_1109	DJI_0111	Potentilla argentea L.	0	n	1	0	Rosaceae Juss.

Iz_4_1109	DJI_0111	Psammophiliella muralis (L.) Ikonn.	0	n	1	0	Caryophyllaceae Juss.
Iz_4_1109	DJI_0111	Psephellus sumensis (Kalen.) Greuter	0	n	1	0	Asteraceae Bercht. & J.Presl
Iz_4_1109	DJI_0111	Pulsatilla pratensis (L.) Mill. s.l.	0	n	1	0	Ranunculaceae Juss.
Iz_4_1109	DJI_0111	Pyrus communis L.	0	a	0	1	Rosaceae Juss.
Iz_4_1109	DJI_0111	Quercus robur L.	0	n	1	1	Fagaceae Dumort.
Iz_4_1109	DJI_0111	Robinia pseudoacacia L.	0	a	0	1	Fabaceae Lindl.
Iz_4_1109	DJI_0111	Rosa sp.	0		NA	1	Rosaceae Juss.
Iz_4_1109	DJI_0111	Rubus idaeus L.	0	n	1	0	Rosaceae Juss.
Iz_4_1109	DJI_0111	Rumex acetosella L.	0	n	1	0	Polygonaceae Juss.
Iz_4_1109	DJI_0111	Rumex thyrsiflorus Fingerh.	0	n	1	0	Polygonaceae Juss.
Iz_4_1109	DJI_0111	Salix alba L.	0	n	1	1	Salicaceae Mirb.
Iz_4_1109	DJI_0111	Sambucus nigra L.	0	n	1	1	Viburnaceae Raf.
Iz_4_1109	DJI_0111	Secale sylvestre Host	0	n	1	0	Poaceae Barnhart
Iz_4_1109	DJI_0111	Sempervivum ruthenicum Schnittsp. & C.B. Lehm.	0	n	1	0	Crassulaceae J.St.-Hil.
Iz_4_1109	DJI_0111	Setaria viridis (L.) P. Beauv.	0	n	1	0	Poaceae Barnhart
Iz_4_1109	DJI_0111	Silene borysthenica (Gruner) Walters	0	n	1	0	Caryophyllaceae Juss.
Iz_4_1109	DJI_0111	Silene latifolia subsp. alba (Mill.) Greuter & Burdet	0	n	1	0	Caryophyllaceae Juss.
Iz_4_1109	DJI_0111	Solidago virgaurea L.	0	n	1	0	Asteraceae Bercht. & J.Presl
Iz_4_1109	DJI_0111	Sorbus aucuparia L.	0	n	1	1	Rosaceae Juss.
Iz_4_1109	DJI_0111	Stachys recta L.	0	n	1	0	Lamiacea Lindl.
Iz_4_1109	DJI_0111	Tanacetum vulgare L.	0	n	1	0	Asteraceae Bercht. & J.Presl
Iz_4_1109	DJI_0111	Thymus pallasianus Heinr.Braun	0	n	1	0	Lamiacea Lindl.
Iz_4_1109	DJI_0111	Tilia cordata Mill.	0	n	1	1	Malvaceae Juss.
Iz_4_1109	DJI_0111	Trifolium alpestre L.	0	n	1	0	Fabaceae Lindl.
Iz_4_1109	DJI_0111	Trifolium arvense L.	0	n	1	0	Fabaceae Lindl.
Iz_4_1109	DJI_0111	Ulmus glabra Huds.	0	n	1	1	Ulmaceae Mirb.
Iz_4_1109	DJI_0111	Verbascum densiflorum Bertol.	0	n	1	0	Scrophulariaceae Juss.
Iz_4_1109	DJI_0111	Veronica austriaca L.	0	n	1	0	Plantaginaceae Juss.
Iz_4_1109	DJI_0111	Veronica incana L.	0	n	1	0	Plantaginaceae Juss.
Iz_4_1109	DJI_0111	Veronica verna L.	0	n	1	0	Plantaginaceae Juss.
Iz_4_1109	DJI_0111	Vincetoxicum hirundinaria Medik	0	n	1	0	Apocynaceae Juss.
Iz_4_1209	DJI_0171	Acer tataricum L.	0	n	1	1	Sapindaceae Juss.
Iz_4_1209	DJI_0171	Achillea millefolium L. s.l.	0	n	1	0	Asteraceae Bercht. & J.Presl
Iz_4_1209	DJI_0171	Allium oleraceum L.	0	n	1	0	Amaryllidaceae J.St.-Hil.
Iz_4_1209	DJI_0171	Ambrosia artemisiifolia L.	0	a	0	0	Asteraceae Bercht. & J.Presl
Iz_4_1209	DJI_0171	Amorpha fruticosa L.	0	a	0	0	Fabaceae Lindl.
Iz_4_1209	DJI_0171	Anthericum ramosum L.	0	n	1	0	Asparagaceae Juss.
Iz_4_1209	DJI_0171	Artemisia marschalliana Spreng.	0	n	1	0	Asteraceae Bercht. & J.Presl
Iz_4_1209	DJI_0171	Asparagus officinalis L.	0	n	1	0	Asparagaceae Juss.
Iz_4_1209	DJI_0171	Astragalus cicer L.	0	n	1	0	Fabaceae Lindl.
Iz_4_1209	DJI_0171	Bassia laniflora (S.G.Gmel.) A.J.Scott	0	n	1	0	Amaranthaceae Juss.
Iz_4_1209	DJI_0171	Berberis vulgaris L.	0	n	1	0	Berberidaceae Juss.
Iz_4_1209	DJI_0171	Betula pendula Roth.	0	n	1	1	Betulaceae Gray
Iz_4_1209	DJI_0171	Betula pendula Roth.	0	n	1	0	Poaceae Barnhart
Iz_4_1209	DJI_0171	Bromus inermis Leyss.	0		NA	NA	
Iz_4_1209	DJI_0171	Bromus squarrosus L.	0	a	0	0	Poaceae Barnhart
Iz_4_1209	DJI_0171	Bromus tectorum L.	0	a	0	0	Poaceae Barnhart
Iz_4_1209	DJI_0171	Calamagrostis epigejos (L.) Roth	0.5	n	1	0	Poaceae Barnhart
Iz_4_1209	DJI_0171	Carduus acanthoides L.	0	n	1	0	Asteraceae Bercht. & J.Presl
Iz_4_1209	DJI_0171	Carex sp.	0	n	1	0	Cyperaceae Juss.
Iz_4_1209	DJI_0171	Centaurea majorovii Dumbadze	0	n	1	0	Asteraceae Bercht. & J.Presl
Iz_4_1209	DJI_0171	Chamaecytisus ruthenicus (Fisch.ex Wol.) Klaskova	0	n	1	0	Fabaceae Lindl.
Iz_4_1209	DJI_0171	Chelidonium majus L.	0	n	1	0	Papaveraceae Juss.
Iz_4_1209	DJI_0171	Chenopodium betaceum Andrz.	0	n	1	0	Amaranthaceae Juss.
Iz_4_1209	DJI_0171	Chenopodium cf. album	0	n	1	0	Amaranthaceae Juss.
Iz_4_1209	DJI_0171	Chenopodium sp.	0		NA	0	Amaranthaceae Juss.
Iz_4_1209	DJI_0171	Chondrilla juncea L.	0	n	1	0	Asteraceae Bercht. & J.Presl
Iz_4_1209	DJI_0171	Cirsium vulgare (Savi) Ten.	0	n	1	0	Asteraceae Bercht. & J.Presl
Iz_4_1209	DJI_0171	Clinopodium acinos (L.) Kuntze	0	n	1	0	Lamiacea Lindl.
Iz_4_1209	DJI_0171	Convallaria majalis L.	0	n	1	0	Asparagaceae Juss.
Iz_4_1209	DJI_0171	Crataegus rhipidophylla Gand.	0	n	1	1	Rosaceae Juss.
Iz_4_1209	DJI_0171	Crepis foetida L.	0	n	1	0	Asteraceae Bercht. & J.Presl
Iz_4_1209	DJI_0171	Dactylis glomerata L.	0	n	1	0	Poaceae Barnhart
Iz_4_1209	DJI_0171	Dianthus campestris M.Bieb.	0	n	1	0	Caryophyllaceae Juss.
Iz_4_1209	DJI_0171	Digitaria sanguinalis (L.) Scop.	0	a	0	0	Poaceae Barnhart
Iz_4_1209	DJI_0171	Elymus repens (L.) Gould	0	n	1	0	Poaceae Barnhart
Iz_4_1209	DJI_0171	Epilobium tetragonum L.	0	n	1	0	Onagraceae Juss.
Iz_4_1209	DJI_0171	Eragrostis minor Host	0	a	0	0	Poaceae Barnhart
Iz_4_1209	DJI_0171	Erigeron canadensis L.	0.2	a	0	0	Asteraceae Bercht. & J.Presl
Iz_4_1209	DJI_0171	Euphorbia seguieriana Neck.	0	n	1	0	Euphorbiaceae Juss.
Iz_4_1209	DJI_0171	Fallopia dumetorum (L.) Holub	0	n	1	0	Polygonaceae Juss.
Iz_4_1209	DJI_0171	Festuca beckeri (Hack.) Trautv.	0	n	1	0	Poaceae Barnhart
Iz_4_1209	DJI_0171	Fragaria vesca L.	0	n	1	0	Rosaceae Juss.
Iz_4_1209	DJI_0171	Frangula alnus Mill.	0	n	1	1	Rhamnaceae Juss.
Iz_4_1209	DJI_0171	Fraxinus pennsylvanica Marshall	0	a	0	1	Oleaceae Hoffmanns. & Link
Iz_4_1209	DJI_0171	Galeopsis bifida Boenn.	0	n	1	0	Lamiacea Lindl.
Iz_4_1209	DJI_0171	Galium verum L.	0	n	1	0	Rubiaceae Juss.
Iz_4_1209	DJI_0171	Genista tinctoria L.	0	n	1	0	Fabaceae Lindl.
Iz_4_1209	DJI_0171	Geranium robertianum L.	0	n	1	0	Geraniaceae Juss.
Iz_4_1209	DJI_0171	Geranium sanquineum L.	0	n	1	0	Geraniaceae Juss.
Iz_4_1209	DJI_0171	Gypsophila paniculata L.	0	n	1	0	Caryophyllaceae Juss.
Iz_4_1209	DJI_0171	Helichrysum arenarium (L.) Moench	0	n	1	0	Asteraceae Bercht. & J.Presl
Iz_4_1209	DJI_0171	Herniaria polygama J.Gay	0	n	1	0	Caryophyllaceae Juss.
Iz_4_1209	DJI_0171	Hieracium robustum Fr.	0	n	1	0	Asteraceae Bercht. & J.Presl
Iz_4_1209	DJI_0171	Hieracium umbellatum L.	0	n	1	NA	Asteraceae Bercht. & J.Presl
Iz_4_1209	DJI_0171	Hylotelephium maximum (L.) Holub	0	n	1	0	Crassulaceae J.St.-Hil.
Iz_4_1209	DJI_0171	Jacobaea borysthenica (DC.) B.Nord. & Greuter	0	n	1	0	Asteraceae Bercht. & J.Presl
Iz_4_1209	DJI_0171	Jasione montana L.	0	n	1	0	Campanulaceae Juss.
Iz_4_1209	DJI_0171	Jurinea cyanoides (L.) Rchb.s.l.	0	n	1	0	Asteraceae Bercht. & J.Presl
Iz_4_1209	DJI_0171	Koeleria glauca (Spreng.) DC.	0	n	1	0	Poaceae Barnhart
Iz_4_1209	DJI_0171	Lactuca serriola L.	0	a	0	0	Asteraceae Bercht. & J.Presl
Iz_4_1209	DJI_0171	Linaria genistifolia (L.) Mill.	0	n	1	0	Plantaginaceae Juss.
Iz_4_1209	DJI_0171	Lomelosia argentea (L.) Greuter & Burdet	0	n	1	0	Caprifoliaceae Juss.
Iz_4_1209	DJI_0171	Melampyrum pratense L.	0	n	1	0	Orobanchaceae Vent.
Iz_4_1209	DJI_0171	Oenothera biennis L.	0	a	0	0	Onagraceae Juss.
Iz_4_1209	DJI_0171	Pentanema britannica (L.) D.Gut.Larr., Santos-Vicente, Anderb., E.Rico & M.M.Mart.Ort.	0	n	1	0	Asteraceae Bercht. & J.Presl
Iz_4_1209	DJI_0171	Peucedanum oreoselinum (L.) Moench	0	n	1	0	Apiaceae Lindl.
Iz_4_1209	DJI_0171	Peucedanum ruthenicum M.Bieb.	0	n	1	0	Apiaceae Lindl.

Iz_4_1209	DJI_0171	Pilosella echioides (Lumn.) F.W.Schultz & Sch.Bip.	0	n	1	0	Asteraceae Bercht. & J.Presl
Iz_4_1209	DJI_0171	Pilosella officinarum F. Schultz et Sch. Bip.	0	n	1	0	Asteraceae Bercht. & J.Presl
Iz_4_1209	DJI_0171	Pilosella sp.	0		NA	0	Asteraceae Bercht. & J.Presl
Iz_4_1209	DJI_0171	Pinus sylvestris L.	0	n	1	1	Pinaceae Spreng. ex F.Rudolphi
Iz_4_1209	DJI_0171	Poa annua L.	0	n	1	0	Poaceae Barnhart
Iz_4_1209	DJI_0171	Poa nemoralis L.	0	n	1	0	Poaceae Barnhart
Iz_4_1209	DJI_0171	Poa sp.	0		NA	0	Poaceae Barnhart
Iz_4_1209	DJI_0171	Polygonatum odoratum (Mill.) Druce	0	n	1	0	Asparagaceae Juss.
Iz_4_1209	DJI_0171	Polygonum arenarium Waldst. & Kit.	0	n	1	0	Polygonaceae Juss.
Iz_4_1209	DJI_0171	Populus nigra L.	0	n	1	1	Salicaceae Mirb.
Iz_4_1209	DJI_0171	Populus tremula L.	0	n	1	1	Salicaceae Mirb.
Iz_4_1209	DJI_0171	Populus Ã canescens (Aiton) Sm.	0	n	1	1	Salicaceae Mirb.
Iz_4_1209	DJI_0171	Potentilla argentea L.	0	n	1	0	Rosaceae Juss.
Iz_4_1209	DJI_0171	Psammophiliella muralis (L.) Ikonn.	0	n	1	0	Caryophyllaceae Juss.
Iz_4_1209	DJI_0171	Psephellus sumensis (Kalen.) Greuter	0	n	1	0	Asteraceae Bercht. & J.Presl
Iz_4_1209	DJI_0171	Pulsatilla pratensis (L.) Mill. s.l.	0	n	1	0	Ranunculaceae Juss.
Iz_4_1209	DJI_0171	Pyrus communis L.	0	a	0	1	Rosaceae Juss.
Iz_4_1209	DJI_0171	Quercus robur L.	0	n	1	1	Fagaceae Dumort.
Iz_4_1209	DJI_0171	Robinia pseudoacacia L.	0	a	0	1	Fabaceae Lindl.
Iz_4_1209	DJI_0171	Rosa sp.	0		NA	1	Rosaceae Juss.
Iz_4_1209	DJI_0171	Rubus idaeus L.	0	n	1	0	Rosaceae Juss.
Iz_4_1209	DJI_0171	Rumex acetosella L.	0	n	1	0	Polygonaceae Juss.
Iz_4_1209	DJI_0171	Rumex thyrsiflorus Fingerh.	0	n	1	0	Polygonaceae Juss.
Iz_4_1209	DJI_0171	Salix alba L.	0	n	1	1	Salicaceae Mirb.
Iz_4_1209	DJI_0171	Sambucus nigra L.	0	n	1	1	Viburnaceae Raf.
Iz_4_1209	DJI_0171	Secale sylvestre Host	0	n	1	0	Poaceae Barnhart
Iz_4_1209	DJI_0171	Sempervivum ruthenicum Schnittsp. & C.B. Lehm.	0	n	1	0	Crassulaceae J.St.-Hil.
Iz_4_1209	DJI_0171	Setaria viridis (L.) P. Beauv.	0	n	1	0	Poaceae Barnhart
Iz_4_1209	DJI_0171	Silene borysthenica (Gruner) Walters	0	n	1	0	Caryophyllaceae Juss.
Iz_4_1209	DJI_0171	Silene latifolia subsp. alba (Mill.) Greuter & Burdet	0	n	1	0	Caryophyllaceae Juss.
Iz_4_1209	DJI_0171	Solidago virgaurea L.	0	n	1	0	Asteraceae Bercht. & J.Presl
Iz_4_1209	DJI_0171	Sorbus aucuparia L.	0	n	1	1	Rosaceae Juss.
Iz_4_1209	DJI_0171	Stachys recta L.	0	n	1	0	Lamiacea Lindl.
Iz_4_1209	DJI_0171	Tanacetum vulgare L.	0	n	1	0	Asteraceae Bercht. & J.Presl
Iz_4_1209	DJI_0171	Thymus pallasianus Heinr.Braun	0	n	1	0	Lamiacea Lindl.
Iz_4_1209	DJI_0171	Tilia cordata Mill.	0	n	1	1	Malvaceae Juss.
Iz_4_1209	DJI_0171	Trifolium alpestre L.	0	n	1	0	Fabaceae Lindl.
Iz_4_1209	DJI_0171	Trifolium arvense L.	0	n	1	0	Fabaceae Lindl.
Iz_4_1209	DJI_0171	Ulmus glabra Huds.	0	n	1	1	Ulmaceae Mirb.
Iz_4_1209	DJI_0171	Verbascum densiflorum Bertol.	0	n	1	0	Scrophulariaceae Juss.
Iz_4_1209	DJI_0171	Veronica austriaca L.	0	n	1	0	Plantaginaceae Juss.
Iz_4_1209	DJI_0171	Veronica incana L.	0	n	1	0	Plantaginaceae Juss.
Iz_4_1209	DJI_0171	Veronica verna L.	0	n	1	0	Plantaginaceae Juss.
Iz_4_1209	DJI_0171	Vincetoxicum hirundinaria Medik	0	n	1	0	Apocynaceae Juss.
Iz_4_1209	DJI_0174	Acer tataricum L.	0	n	1	1	Sapindaceae Juss.
Iz_4_1209	DJI_0174	Achillea millefolium L. s.l.	0	n	1	0	Asteraceae Bercht. & J.Presl
Iz_4_1209	DJI_0174	Allium oleraceum L.	0	n	1	0	Amaryllidaceae J.St.-Hil.
Iz_4_1209	DJI_0174	Ambrosia artemisiifolia L.	0	a	0	0	Asteraceae Bercht. & J.Presl
Iz_4_1209	DJI_0174	Amorpha fruticosa L.	0	a	0	0	Fabaceae Lindl.
Iz_4_1209	DJI_0174	Anthericum ramosum L.	0	n	1	0	Asparagaceae Juss.
Iz_4_1209	DJI_0174	Artemisia marschalliana Spreng.	0	n	1	0	Asteraceae Bercht. & J.Presl
Iz_4_1209	DJI_0174	Asparagus officinalis L.	0	n	1	0	Asparagaceae Juss.
Iz_4_1209	DJI_0174	Astragalus cicer L.	0	n	1	0	Fabaceae Lindl.
Iz_4_1209	DJI_0174	Bassia laniflora (S.G.Gmel.) A.J.Scott	0	n	1	0	Amaranthaceae Juss.
Iz_4_1209	DJI_0174	Berberis vulgaris L.	0	n	1	0	Berberidaceae Juss.
Iz_4_1209	DJI_0174	Betula pendula Roth.	0	n	1	1	Betulaceae Gray
Iz_4_1209	DJI_0174	Betula pendula Roth.	0	n	1	0	Poaceae Barnhart
Iz_4_1209	DJI_0174	Bromus inermis Leyss.	0		NA	NA	
Iz_4_1209	DJI_0174	Bromus squarrosus L.	0	a	0	0	Poaceae Barnhart
Iz_4_1209	DJI_0174	Bromus tectorum L.	0	a	0	0	Poaceae Barnhart
Iz_4_1209	DJI_0174	Calamagrostis epigejos (L.) Roth	0	n	1	0	Poaceae Barnhart
Iz_4_1209	DJI_0174	Carduus acanthoides L.	0	n	1	0	Asteraceae Bercht. & J.Presl
Iz_4_1209	DJI_0174	Carex sp.	0	n	1	0	Cyperaceae Juss.
Iz_4_1209	DJI_0174	Centaurea majorovii Dumbadze	0	n	1	0	Asteraceae Bercht. & J.Presl
Iz_4_1209	DJI_0174	Chamaecytisus ruthenicus (Fisch.ex Wol.) Klaskova	0	n	1	0	Fabaceae Lindl.
Iz_4_1209	DJI_0174	Chelidonium majus L.	0	n	1	0	Papaveraceae Juss.
Iz_4_1209	DJI_0174	Chenopodium betaceum Andrz.	0	n	1	0	Amaranthaceae Juss.
Iz_4_1209	DJI_0174	Chenopodium cf. album	0	n	1	0	Amaranthaceae Juss.
Iz_4_1209	DJI_0174	Chenopodium sp.	0		NA	0	Amaranthaceae Juss.
Iz_4_1209	DJI_0174	Chondrilla juncea L.	0	n	1	0	Asteraceae Bercht. & J.Presl
Iz_4_1209	DJI_0174	Cirsium vulgare (Savi) Ten.	0	n	1	0	Asteraceae Bercht. & J.Presl
Iz_4_1209	DJI_0174	Clinopodium acinos (L.) Kuntze	0	n	1	0	Lamiacea Lindl.
Iz_4_1209	DJI_0174	Convallaria majalis L.	0	n	1	0	Asparagaceae Juss.
Iz_4_1209	DJI_0174	Crataegus rhipidophylla Gand.	0	n	1	1	Rosaceae Juss.
Iz_4_1209	DJI_0174	Crepis foetida L.	0	n	1	0	Asteraceae Bercht. & J.Presl
Iz_4_1209	DJI_0174	Dactylis glomerata L.	0	n	1	0	Poaceae Barnhart
Iz_4_1209	DJI_0174	Dianthus campestris M.Bieb.	0	n	1	0	Caryophyllaceae Juss.
Iz_4_1209	DJI_0174	Digitaria sanguinalis (L.) Scop.	0	a	0	0	Poaceae Barnhart
Iz_4_1209	DJI_0174	Elymus repens (L.) Gould	0	n	1	0	Poaceae Barnhart
Iz_4_1209	DJI_0174	Epilobium tetragonum L.	0	n	1	0	Onagraceae Juss.
Iz_4_1209	DJI_0174	Eragrostis minor Host	0	a	0	0	Poaceae Barnhart
Iz_4_1209	DJI_0174	Erigeron canadensis L.	0	a	0	0	Asteraceae Bercht. & J.Presl
Iz_4_1209	DJI_0174	Euphorbia seguieriana Neck.	0	n	1	0	Euphorbiaceae Juss.
Iz_4_1209	DJI_0174	Fallopia dumetorum (L.) Holub	0	n	1	0	Polygonaceae Juss.
Iz_4_1209	DJI_0174	Festuca beckeri (Hack.) Trautv.	0	n	1	0	Poaceae Barnhart
Iz_4_1209	DJI_0174	Fragaria vesca L.	0	n	1	0	Rosaceae Juss.
Iz_4_1209	DJI_0174	Frangula alnus Mill.	0	n	1	1	Rhamnaceae Juss.
Iz_4_1209	DJI_0174	Fraxinus pennsylvanica Marshall	0	a	0	1	Oleaceae Hoffmanns. & Link
Iz_4_1209	DJI_0174	Galeopsis bifida Boenn.	0	n	1	0	Lamiacea Lindl.
Iz_4_1209	DJI_0174	Galium verum L.	0	n	1	0	Rubiaceae Juss.
Iz_4_1209	DJI_0174	Genista tinctoria L.	0	n	1	0	Fabaceae Lindl.
Iz_4_1209	DJI_0174	Geranium robertianum L.	0	n	1	0	Geraniaceae Juss.
Iz_4_1209	DJI_0174	Geranium sanquineum L.	0	n	1	0	Geraniaceae Juss.
Iz_4_1209	DJI_0174	Gypsophila paniculata L.	0	n	1	0	Caryophyllaceae Juss.
Iz_4_1209	DJI_0174	Helichrysum arenarium (L.) Moench	0	n	1	0	Asteraceae Bercht. & J.Presl
Iz_4_1209	DJI_0174	Herniaria polygama J.Gay	0	n	1	0	Caryophyllaceae Juss.
Iz_4_1209	DJI_0174	Hieracium robustum Fr.	0	n	1	0	Asteraceae Bercht. & J.Presl
Iz_4_1209	DJI_0174	Hieracium umbellatum L.	0.1	n	1	NA	Asteraceae Bercht. & J.Presl

Iz_4_1209	DJI_0174	Hylotelephium maximum (L.) Holub	0	n	1	0	Crassulaceae J.St.-Hil.
Iz_4_1209	DJI_0174	Jacobaea borysthenica (DC.) B.Nord. & Greuter	0	n	1	0	Asteraceae Bercht. & J.Presl
Iz_4_1209	DJI_0174	Jasione montana L.	0	n	1	0	Campanulaceae Juss.
Iz_4_1209	DJI_0174	Jurinea cyanoides (L.) Rchb.s.l.	0	n	1	0	Asteraceae Bercht. & J.Presl
Iz_4_1209	DJI_0174	Koeleria glauca (Spreng.) DC.	0	n	1	0	Poaceae Barnhart
Iz_4_1209	DJI_0174	Lactuca serriola L.	0	a	0	0	Asteraceae Bercht. & J.Presl
Iz_4_1209	DJI_0174	Linaria genistifolia (L.) Mill.	0	n	1	0	Plantaginaceae Juss.
Iz_4_1209	DJI_0174	Lomelosia argentea (L.) Greuter & Burdet	0	n	1	0	Caprifoliaceae Juss.
Iz_4_1209	DJI_0174	Melampyrum pratense L.	0	n	1	0	Orobanchaceae Vent.
Iz_4_1209	DJI_0174	Oenothera biennis L.	0	a	0	0	Onagraceae Juss.
Iz_4_1209	DJI_0174	Pentanema britannica (L.) D.Gut.Larr., Santos-Vicente, Anderb., E.Rico & M.M.Mart.Ort.	0	n	1	0	Asteraceae Bercht. & J.Presl
Iz_4_1209	DJI_0174	Peucedanum oreoselinum (L.) Moench	0	n	1	0	Apiaceae Lindl.
Iz_4_1209	DJI_0174	Peucedanum ruthenicum M.Bieb.	0	n	1	0	Apiaceae Lindl.
Iz_4_1209	DJI_0174	Pilosella echioides (Lumn.) F.W.Schultz & Sch.Bip.	0	n	1	0	Asteraceae Bercht. & J.Presl
Iz_4_1209	DJI_0174	Pilosella officinarum F. Schultz et Sch. Bip.	0	n	1	0	Asteraceae Bercht. & J.Presl
Iz_4_1209	DJI_0174	Pilosella sp.	0		NA	0	Asteraceae Bercht. & J.Presl
Iz_4_1209	DJI_0174	Pinus sylvestris L.	0	n	1	1	Pinaceae Spreng. ex F.Rudolphi
Iz_4_1209	DJI_0174	Poa annua L.	0	n	1	0	Poaceae Barnhart
Iz_4_1209	DJI_0174	Poa nemoralis L.	0	n	1	0	Poaceae Barnhart
Iz_4_1209	DJI_0174	Poa sp.	0		NA	0	Poaceae Barnhart
Iz_4_1209	DJI_0174	Polygonatum odoratum (Mill.) Druce	0	n	1	0	Asparagaceae Juss.
Iz_4_1209	DJI_0174	Polygonum arenarium Waldst. & Kit.	0	n	1	0	Polygonaceae Juss.
Iz_4_1209	DJI_0174	Populus nigra L.	0	n	1	1	Salicaceae Mirb.
Iz_4_1209	DJI_0174	Populus tremula L.	0	n	1	1	Salicaceae Mirb.
Iz_4_1209	DJI_0174	Populus Ã canescens (Aiton) Sm.	0	n	1	1	Salicaceae Mirb.
Iz_4_1209	DJI_0174	Potentilla argentea L.	0	n	1	0	Rosaceae Juss.
Iz_4_1209	DJI_0174	Psammophiliella muralis (L.) Ikonn.	0	n	1	0	Caryophyllaceae Juss.
Iz_4_1209	DJI_0174	Psephellus sumensis (Kalen.) Greuter	0	n	1	0	Asteraceae Bercht. & J.Presl
Iz_4_1209	DJI_0174	Pulsatilla pratensis (L.) Mill. s.l.	0	n	1	0	Ranunculaceae Juss.
Iz_4_1209	DJI_0174	Pyrus communis L.	0	a	0	1	Rosaceae Juss.
Iz_4_1209	DJI_0174	Quercus robur L.	0	n	1	1	Fagaceae Dumort.
Iz_4_1209	DJI_0174	Robinia pseudoacacia L.	0	a	0	1	Fabaceae Lindl.
Iz_4_1209	DJI_0174	Rosa sp.	0		NA	1	Rosaceae Juss.
Iz_4_1209	DJI_0174	Rubus idaeus L.	0	n	1	0	Rosaceae Juss.
Iz_4_1209	DJI_0174	Rumex acetosella L.	0	n	1	0	Polygonaceae Juss.
Iz_4_1209	DJI_0174	Rumex thyrsiflorus Fingerh.	0	n	1	0	Polygonaceae Juss.
Iz_4_1209	DJI_0174	Salix alba L.	0	n	1	1	Salicaceae Mirb.
Iz_4_1209	DJI_0174	Sambucus nigra L.	0	n	1	1	Viburnaceae Raf.
Iz_4_1209	DJI_0174	Secale sylvestre Host	0	n	1	0	Poaceae Barnhart
Iz_4_1209	DJI_0174	Sempervivum ruthenicum Schnittsp. & C.B. Lehm.	0	n	1	0	Crassulaceae J.St.-Hil.
Iz_4_1209	DJI_0174	Setaria viridis (L.) P. Beauv.	0	n	1	0	Poaceae Barnhart
Iz_4_1209	DJI_0174	Silene borysthenica (Gruner) Walters	0	n	1	0	Caryophyllaceae Juss.
Iz_4_1209	DJI_0174	Silene latifolia subsp. alba (Mill.) Greuter & Burdet	0	n	1	0	Caryophyllaceae Juss.
Iz_4_1209	DJI_0174	Solidago virgaurea L.	0	n	1	0	Asteraceae Bercht. & J.Presl
Iz_4_1209	DJI_0174	Sorbus aucuparia L.	0	n	1	1	Rosaceae Juss.
Iz_4_1209	DJI_0174	Stachys recta L.	0	n	1	0	Lamiacea Lindl.
Iz_4_1209	DJI_0174	Tanacetum vulgare L.	0	n	1	0	Asteraceae Bercht. & J.Presl
Iz_4_1209	DJI_0174	Thymus pallasianus Heinr.Braun	0	n	1	0	Lamiacea Lindl.
Iz_4_1209	DJI_0174	Tilia cordata Mill.	0	n	1	1	Malvaceae Juss.
Iz_4_1209	DJI_0174	Trifolium alpestre L.	0	n	1	0	Fabaceae Lindl.
Iz_4_1209	DJI_0174	Trifolium arvense L.	0	n	1	0	Fabaceae Lindl.
Iz_4_1209	DJI_0174	Ulmus glabra Huds.	0	n	1	1	Ulmaceae Mirb.
Iz_4_1209	DJI_0174	Verbascum densiflorum Bertol.	0	n	1	0	Scrophulariaceae Juss.
Iz_4_1209	DJI_0174	Veronica austriaca L.	0	n	1	0	Plantaginaceae Juss.
Iz_4_1209	DJI_0174	Veronica incana L.	0	n	1	0	Plantaginaceae Juss.
Iz_4_1209	DJI_0174	Veronica verna L.	0	n	1	0	Plantaginaceae Juss.
Iz_4_1209	DJI_0174	Vincetoxicum hirundinaria Medik	0	n	1	0	Apocynaceae Juss.
Iz_4_1209	DJI_0178	Acer tataricum L.	0	n	1	1	Sapindaceae Juss.
Iz_4_1209	DJI_0178	Achillea millefolium L. s.l.	0	n	1	0	Asteraceae Bercht. & J.Presl
Iz_4_1209	DJI_0178	Allium oleraceum L.	0	n	1	0	Amaryllidaceae J.St.-Hil.
Iz_4_1209	DJI_0178	Ambrosia artemisiifolia L.	0	a	0	0	Asteraceae Bercht. & J.Presl
Iz_4_1209	DJI_0178	Amorpha fruticosa L.	0	a	0	0	Fabaceae Lindl.
Iz_4_1209	DJI_0178	Anthericum ramosum L.	0	n	1	0	Asparagaceae Juss.
Iz_4_1209	DJI_0178	Artemisia marschalliana Spreng.	0	n	1	0	Asteraceae Bercht. & J.Presl
Iz_4_1209	DJI_0178	Asparagus officinalis L.	0	n	1	0	Asparagaceae Juss.
Iz_4_1209	DJI_0178	Astragalus cicer L.	0	n	1	0	Fabaceae Lindl.
Iz_4_1209	DJI_0178	Bassia laniflora (S.G.Gmel.) A.J.Scott	0	n	1	0	Amaranthaceae Juss.
Iz_4_1209	DJI_0178	Berberis vulgaris L.	0	n	1	0	Berberidaceae Juss.
Iz_4_1209	DJI_0178	Betula pendula Roth.	0	n	1	1	Betulaceae Gray
Iz_4_1209	DJI_0178	Betula pendula Roth.	0	n	1	0	Poaceae Barnhart
Iz_4_1209	DJI_0178	Bromus inermis Leyss.	0		NA	NA	
Iz_4_1209	DJI_0178	Bromus squarrosus L.	0	a	0	0	Poaceae Barnhart
Iz_4_1209	DJI_0178	Bromus tectorum L.	0	a	0	0	Poaceae Barnhart
Iz_4_1209	DJI_0178	Calamagrostis epigejos (L.) Roth	10	n	1	0	Poaceae Barnhart
Iz_4_1209	DJI_0178	Carduus acanthoides L.	0	n	1	0	Asteraceae Bercht. & J.Presl
Iz_4_1209	DJI_0178	Carex sp.	0	n	1	0	Cyperaceae Juss.
Iz_4_1209	DJI_0178	Centaurea majorovii Dumbadze	0	n	1	0	Asteraceae Bercht. & J.Presl
Iz_4_1209	DJI_0178	Chamaecytisus ruthenicus (Fisch.ex Wol.) Klaskova	0	n	1	0	Fabaceae Lindl.
Iz_4_1209	DJI_0178	Chelidonium majus L.	0	n	1	0	Papaveraceae Juss.
Iz_4_1209	DJI_0178	Chenopodium betaceum Andrz.	0	n	1	0	Amaranthaceae Juss.
Iz_4_1209	DJI_0178	Chenopodium cf. album	0	n	1	0	Amaranthaceae Juss.
Iz_4_1209	DJI_0178	Chenopodium sp.	0		NA	0	Amaranthaceae Juss.
Iz_4_1209	DJI_0178	Chondrilla juncea L.	0	n	1	0	Asteraceae Bercht. & J.Presl
Iz_4_1209	DJI_0178	Cirsium vulgare (Savi) Ten.	0	n	1	0	Asteraceae Bercht. & J.Presl
Iz_4_1209	DJI_0178	Clinopodium acinos (L.) Kuntze	0	n	1	0	Lamiacea Lindl.
Iz_4_1209	DJI_0178	Convallaria majalis L.	0	n	1	0	Asparagaceae Juss.
Iz_4_1209	DJI_0178	Crataegus rhipidophylla Gand.	0	n	1	1	Rosaceae Juss.
Iz_4_1209	DJI_0178	Crepis foetida L.	0	n	1	0	Asteraceae Bercht. & J.Presl
Iz_4_1209	DJI_0178	Dactylis glomerata L.	0	n	1	0	Poaceae Barnhart
Iz_4_1209	DJI_0178	Dianthus campestris M.Bieb.	0	n	1	0	Caryophyllaceae Juss.
Iz_4_1209	DJI_0178	Digitaria sanguinalis (L.) Scop.	0	a	0	0	Poaceae Barnhart
Iz_4_1209	DJI_0178	Elymus repens (L.) Gould	0	n	1	0	Poaceae Barnhart
Iz_4_1209	DJI_0178	Epilobium tetragonum L.	0	n	1	0	Onagraceae Juss.
Iz_4_1209	DJI_0178	Eragrostis minor Host	0	a	0	0	Poaceae Barnhart
Iz_4_1209	DJI_0178	Erigeron canadensis L.	2	a	0	0	Asteraceae Bercht. & J.Presl
Iz_4_1209	DJI_0178	Euphorbia seguieriana Neck.	0	n	1	0	Euphorbiaceae Juss.
Iz_4_1209	DJI_0178	Fallopia dumetorum (L.) Holub	0	n	1	0	Polygonaceae Juss.
Iz_4_1209	DJI_0178	Festuca beckeri (Hack.) Trautv.	0	n	1	0	Poaceae Barnhart

Iz_4_1209	DJI_0178	Fragaria vesca L.	0	n	1	0	Rosaceae Juss.
Iz_4_1209	DJI_0178	Frangula alnus Mill.	0	n	1	1	Rhamnaceae Juss.
Iz_4_1209	DJI_0178	Fraxinus pennsylvanica Marshall	0	a	0	1	Oleaceae Hoffmanns. & Link
Iz_4_1209	DJI_0178	Galeopsis bifida Boenn.	0	n	1	0	Lamiacea Lindl.
Iz_4_1209	DJI_0178	Galium verum L.	0	n	1	0	Rubiaceae Juss.
Iz_4_1209	DJI_0178	Genista tinctoria L.	0	n	1	0	Fabaceae Lindl.
Iz_4_1209	DJI_0178	Geranium robertianum L.	0	n	1	0	Geraniaceae Juss.
Iz_4_1209	DJI_0178	Geranium sanquineum L.	0	n	1	0	Geraniaceae Juss.
Iz_4_1209	DJI_0178	Gypsophila paniculata L.	0	n	1	0	Caryophyllaceae Juss.
Iz_4_1209	DJI_0178	Helichrysum arenarium (L.) Moench	0	n	1	0	Asteraceae Bercht. & J.Presl
Iz_4_1209	DJI_0178	Herniaria polygama J.Gay	0	n	1	0	Caryophyllaceae Juss.
Iz_4_1209	DJI_0178	Hieracium robustum Fr.	0	n	1	0	Asteraceae Bercht. & J.Presl
Iz_4_1209	DJI_0178	Hieracium umbellatum L.	0	n	1	NA	Asteraceae Bercht. & J.Presl
Iz_4_1209	DJI_0178	Hylotelephium maximum (L.) Holub	0	n	1	0	Crassulaceae J.St.-Hil.
Iz_4_1209	DJI_0178	Jacobaea borysthenica (DC.) B.Nord. & Greuter	0	n	1	0	Asteraceae Bercht. & J.Presl
Iz_4_1209	DJI_0178	Jasione montana L.	0	n	1	0	Campanulaceae Juss.
Iz_4_1209	DJI_0178	Jurinea cyanoides (L.) Rchb.s.l.	0	n	1	0	Asteraceae Bercht. & J.Presl
Iz_4_1209	DJI_0178	Koeleria glauca (Spreng.) DC.	0	n	1	0	Poaceae Barnhart
Iz_4_1209	DJI_0178	Lactuca serriola L.	0	a	0	0	Asteraceae Bercht. & J.Presl
Iz_4_1209	DJI_0178	Linaria genistifolia (L.) Mill.	0	n	1	0	Plantaginaceae Juss.
Iz_4_1209	DJI_0178	Lomelosia argentea (L.) Greuter & Burdet	0	n	1	0	Caprifoliaceae Juss.
Iz_4_1209	DJI_0178	Melampyrum pratense L.	0	n	1	0	Orobanchaceae Vent.
Iz_4_1209	DJI_0178	Oenothera biennis L.	0	a	0	0	Onagraceae Juss.
Iz_4_1209	DJI_0178	Pentanema britannica (L.) D.Gut.Larr., Santos-Vicente, Anderb., E.Rico & M.M.Mart.Ort.	0	n	1	0	Asteraceae Bercht. & J.Presl
Iz_4_1209	DJI_0178	Peucedanum oreoselinum (L.) Moench	0	n	1	0	Apiaceae Lindl.
Iz_4_1209	DJI_0178	Peucedanum ruthenicum M.Bieb.	0	n	1	0	Apiaceae Lindl.
Iz_4_1209	DJI_0178	Pilosella echioides (Lumn.) F.W.Schultz & Sch.Bip.	0	n	1	0	Asteraceae Bercht. & J.Presl
Iz_4_1209	DJI_0178	Pilosella officinarum F. Schultz et Sch. Bip.	0	n	1	0	Asteraceae Bercht. & J.Presl
Iz_4_1209	DJI_0178	Pilosella sp.	0		NA	0	Asteraceae Bercht. & J.Presl
Iz_4_1209	DJI_0178	Pinus sylvestris L.	0	n	1	1	Pinaceae Spreng. ex F.Rudolphi
Iz_4_1209	DJI_0178	Poa annua L.	0	n	1	0	Poaceae Barnhart
Iz_4_1209	DJI_0178	Poa nemoralis L.	0	n	1	0	Poaceae Barnhart
Iz_4_1209	DJI_0178	Poa sp.	0		NA	0	Poaceae Barnhart
Iz_4_1209	DJI_0178	Polygonatum odoratum (Mill.) Druce	0	n	1	0	Asparagaceae Juss.
Iz_4_1209	DJI_0178	Polygonum arenarium Waldst. & Kit.	0	n	1	0	Polygonaceae Juss.
Iz_4_1209	DJI_0178	Populus nigra L.	0	n	1	1	Salicaceae Mirb.
Iz_4_1209	DJI_0178	Populus tremula L.	0	n	1	1	Salicaceae Mirb.
Iz_4_1209	DJI_0178	Populus Ã canescens (Aiton) Sm.	0	n	1	1	Salicaceae Mirb.
Iz_4_1209	DJI_0178	Potentilla argentea L.	0	n	1	0	Rosaceae Juss.
Iz_4_1209	DJI_0178	Psammophiliella muralis (L.) Ikonn.	0	n	1	0	Caryophyllaceae Juss.
Iz_4_1209	DJI_0178	Psephellus sumensis (Kalen.) Greuter	0	n	1	0	Asteraceae Bercht. & J.Presl
Iz_4_1209	DJI_0178	Pulsatilla pratensis (L.) Mill. s.l.	0	n	1	0	Ranunculaceae Juss.
Iz_4_1209	DJI_0178	Pyrus communis L.	0	a	0	1	Rosaceae Juss.
Iz_4_1209	DJI_0178	Quercus robur L.	0	n	1	1	Fagaceae Dumort.
Iz_4_1209	DJI_0178	Robinia pseudoacacia L.	0	a	0	1	Fabaceae Lindl.
Iz_4_1209	DJI_0178	Rosa sp.	0		NA	1	Rosaceae Juss.
Iz_4_1209	DJI_0178	Rubus idaeus L.	0	n	1	0	Rosaceae Juss.
Iz_4_1209	DJI_0178	Rumex acetosella L.	0	n	1	0	Polygonaceae Juss.
Iz_4_1209	DJI_0178	Rumex thyrsiflorus Fingerh.	0	n	1	0	Polygonaceae Juss.
Iz_4_1209	DJI_0178	Salix alba L.	0	n	1	1	Salicaceae Mirb.
Iz_4_1209	DJI_0178	Sambucus nigra L.	0	n	1	1	Viburnaceae Raf.
Iz_4_1209	DJI_0178	Secale sylvestre Host	0	n	1	0	Poaceae Barnhart
Iz_4_1209	DJI_0178	Sempervivum ruthenicum Schnittsp. & C.B. Lehm.	0	n	1	0	Crassulaceae J.St.-Hil.
Iz_4_1209	DJI_0178	Setaria viridis (L.) P. Beauv.	0	n	1	0	Poaceae Barnhart
Iz_4_1209	DJI_0178	Silene borysthenica (Gruner) Walters	0	n	1	0	Caryophyllaceae Juss.
Iz_4_1209	DJI_0178	Silene latifolia subsp. alba (Mill.) Greuter & Burdet	0	n	1	0	Caryophyllaceae Juss.
Iz_4_1209	DJI_0178	Solidago virgaurea L.	0	n	1	0	Asteraceae Bercht. & J.Presl
Iz_4_1209	DJI_0178	Sorbus aucuparia L.	0	n	1	1	Rosaceae Juss.
Iz_4_1209	DJI_0178	Stachys recta L.	0	n	1	0	Lamiacea Lindl.
Iz_4_1209	DJI_0178	Tanacetum vulgare L.	0	n	1	0	Asteraceae Bercht. & J.Presl
Iz_4_1209	DJI_0178	Thymus pallasianus Heinr.Braun	0	n	1	0	Lamiacea Lindl.
Iz_4_1209	DJI_0178	Tilia cordata Mill.	0	n	1	1	Malvaceae Juss.
Iz_4_1209	DJI_0178	Trifolium alpestre L.	0	n	1	0	Fabaceae Lindl.
Iz_4_1209	DJI_0178	Trifolium arvense L.	0	n	1	0	Fabaceae Lindl.
Iz_4_1209	DJI_0178	Ulmus glabra Huds.	0	n	1	1	Ulmaceae Mirb.
Iz_4_1209	DJI_0178	Verbascum densiflorum Bertol.	0	n	1	0	Scrophulariaceae Juss.
Iz_4_1209	DJI_0178	Veronica austriaca L.	0	n	1	0	Plantaginaceae Juss.
Iz_4_1209	DJI_0178	Veronica incana L.	0	n	1	0	Plantaginaceae Juss.
Iz_4_1209	DJI_0178	Veronica verna L.	0	n	1	0	Plantaginaceae Juss.
Iz_4_1209	DJI_0178	Vincetoxicum hirundinaria Medik	0	n	1	0	Apocynaceae Juss.
Iz_4_1209	DJI_0181	Acer tataricum L.	0	n	1	1	Sapindaceae Juss.
Iz_4_1209	DJI_0181	Achillea millefolium L. s.l.	0	n	1	0	Asteraceae Bercht. & J.Presl
Iz_4_1209	DJI_0181	Allium oleraceum L.	0	n	1	0	Amaryllidaceae J.St.-Hil.
Iz_4_1209	DJI_0181	Ambrosia artemisiifolia L.	0	a	0	0	Asteraceae Bercht. & J.Presl
Iz_4_1209	DJI_0181	Amorpha fruticosa L.	0	a	0	0	Fabaceae Lindl.
Iz_4_1209	DJI_0181	Anthericum ramosum L.	0	n	1	0	Asparagaceae Juss.
Iz_4_1209	DJI_0181	Artemisia marschalliana Spreng.	0	n	1	0	Asteraceae Bercht. & J.Presl
Iz_4_1209	DJI_0181	Asparagus officinalis L.	0	n	1	0	Asparagaceae Juss.
Iz_4_1209	DJI_0181	Astragalus cicer L.	0	n	1	0	Fabaceae Lindl.
Iz_4_1209	DJI_0181	Bassia laniflora (S.G.Gmel.) A.J.Scott	0	n	1	0	Amaranthaceae Juss.
Iz_4_1209	DJI_0181	Berberis vulgaris L.	0	n	1	0	Berberidaceae Juss.
Iz_4_1209	DJI_0181	Betula pendula Roth.	0	n	1	1	Betulaceae Gray
Iz_4_1209	DJI_0181	Betula pendula Roth.	0	n	1	0	Poaceae Barnhart
Iz_4_1209	DJI_0181	Bromus inermis Leyss.	0		NA	NA	
Iz_4_1209	DJI_0181	Bromus squarrosus L.	0	a	0	0	Poaceae Barnhart
Iz_4_1209	DJI_0181	Bromus tectorum L.	0	a	0	0	Poaceae Barnhart
Iz_4_1209	DJI_0181	Calamagrostis epigejos (L.) Roth	50	n	1	0	Poaceae Barnhart
Iz_4_1209	DJI_0181	Carduus acanthoides L.	0	n	1	0	Asteraceae Bercht. & J.Presl
Iz_4_1209	DJI_0181	Carex sp.	0	n	1	0	Cyperaceae Juss.
Iz_4_1209	DJI_0181	Centaurea majorovii Dumbadze	0	n	1	0	Asteraceae Bercht. & J.Presl
Iz_4_1209	DJI_0181	Chamaecytisus ruthenicus (Fisch.ex Wol.) Klaskova	0	n	1	0	Fabaceae Lindl.
Iz_4_1209	DJI_0181	Chelidonium majus L.	0	n	1	0	Papaveraceae Juss.
Iz_4_1209	DJI_0181	Chenopodium betaceum Andrz.	0	n	1	0	Amaranthaceae Juss.
Iz_4_1209	DJI_0181	Chenopodium cf. album	0	n	1	0	Amaranthaceae Juss.
Iz_4_1209	DJI_0181	Chenopodium sp.	0		NA	0	Amaranthaceae Juss.
Iz_4_1209	DJI_0181	Chondrilla juncea L.	0	n	1	0	Asteraceae Bercht. & J.Presl
Iz_4_1209	DJI_0181	Cirsium vulgare (Savi) Ten.	0	n	1	0	Asteraceae Bercht. & J.Presl
Iz_4_1209	DJI_0181	Clinopodium acinos (L.) Kuntze	0	n	1	0	Lamiacea Lindl.

Iz_4_1209	DJI_0181	Convallaria majalis L.	0	n	1	0	Asparagaceae Juss.
Iz_4_1209	DJI_0181	Crataegus rhipidophylla Gand.	0	n	1	1	Rosaceae Juss.
Iz_4_1209	DJI_0181	Crepis foetida L.	0	n	1	0	Asteraceae Bercht. & J.Presl
Iz_4_1209	DJI_0181	Dactylis glomerata L.	0	n	1	0	Poaceae Barnhart
Iz_4_1209	DJI_0181	Dianthus campestris M.Bieb.	0	n	1	0	Caryophyllaceae Juss.
Iz_4_1209	DJI_0181	Digitaria sanguinalis (L.) Scop.	0	a	0	0	Poaceae Barnhart
Iz_4_1209	DJI_0181	Elymus repens (L.) Gould	0	n	1	0	Poaceae Barnhart
Iz_4_1209	DJI_0181	Epilobium tetragonum L.	0	n	1	0	Onagraceae Juss.
Iz_4_1209	DJI_0181	Eragrostis minor Host	0	a	0	0	Poaceae Barnhart
Iz_4_1209	DJI_0181	Erigeron canadensis L.	0	a	0	0	Asteraceae Bercht. & J.Presl
Iz_4_1209	DJI_0181	Euphorbia seguieriana Neck.	0	n	1	0	Euphorbiaceae Juss.
Iz_4_1209	DJI_0181	Fallopia dumetorum (L.) Holub	0	n	1	0	Polygonaceae Juss.
Iz_4_1209	DJI_0181	Festuca beckeri (Hack.) Trautv.	0	n	1	0	Poaceae Barnhart
Iz_4_1209	DJI_0181	Fragaria vesca L.	0	n	1	0	Rosaceae Juss.
Iz_4_1209	DJI_0181	Frangula alnus Mill.	0	n	1	1	Rhamnaceae Juss.
Iz_4_1209	DJI_0181	Fraxinus pennsylvanica Marshall	0	a	0	1	Oleaceae Hoffmanns. & Link
Iz_4_1209	DJI_0181	Galeopsis bifida Boenn.	0	n	1	0	Lamiacea Lindl.
Iz_4_1209	DJI_0181	Galium verum L.	0	n	1	0	Rubiaceae Juss.
Iz_4_1209	DJI_0181	Genista tinctoria L.	0	n	1	0	Fabaceae Lindl.
Iz_4_1209	DJI_0181	Geranium robertianum L.	0	n	1	0	Geraniaceae Juss.
Iz_4_1209	DJI_0181	Geranium sanquineum L.	0	n	1	0	Geraniaceae Juss.
Iz_4_1209	DJI_0181	Gypsophila paniculata L.	0	n	1	0	Caryophyllaceae Juss.
Iz_4_1209	DJI_0181	Helichrysum arenarium (L.) Moench	0	n	1	0	Asteraceae Bercht. & J.Presl
Iz_4_1209	DJI_0181	Herniaria polygama J.Gay	0	n	1	0	Caryophyllaceae Juss.
Iz_4_1209	DJI_0181	Hieracium robustum Fr.	0	n	1	0	Asteraceae Bercht. & J.Presl
Iz_4_1209	DJI_0181	Hieracium umbellatum L.	0	n	1	NA	Asteraceae Bercht. & J.Presl
Iz_4_1209	DJI_0181	Hylotelephium maximum (L.) Holub	0	n	1	0	Crassulaceae J.St.-Hil.
Iz_4_1209	DJI_0181	Jacobaea borysthenica (DC.) B.Nord. & Greuter	0	n	1	0	Asteraceae Bercht. & J.Presl
Iz_4_1209	DJI_0181	Jasione montana L.	0	n	1	0	Campanulaceae Juss.
Iz_4_1209	DJI_0181	Jurinea cyanoides (L.) Rchb.s.l.	0	n	1	0	Asteraceae Bercht. & J.Presl
Iz_4_1209	DJI_0181	Koeleria glauca (Spreng.) DC.	0	n	1	0	Poaceae Barnhart
Iz_4_1209	DJI_0181	Lactuca serriola L.	0	a	0	0	Asteraceae Bercht. & J.Presl
Iz_4_1209	DJI_0181	Linaria genistifolia (L.) Mill.	0	n	1	0	Plantaginaceae Juss.
Iz_4_1209	DJI_0181	Lomelosia argentea (L.) Greuter & Burdet	0	n	1	0	Caprifoliaceae Juss.
Iz_4_1209	DJI_0181	Melampyrum pratense L.	0	n	1	0	Orobanchaceae Vent.
Iz_4_1209	DJI_0181	Oenothera biennis L.	0	a	0	0	Onagraceae Juss.
Iz_4_1209	DJI_0181	Pentanema britannica (L.) D.Gut.Larr., Santos-Vicente, Anderb., E.Rico & M.M.Mart.Ort.	0	n	1	0	Asteraceae Bercht. & J.Presl
Iz_4_1209	DJI_0181	Peucedanum oreoselinum (L.) Moench	0	n	1	0	Apiaceae Lindl.
Iz_4_1209	DJI_0181	Peucedanum ruthenicum M.Bieb.	0	n	1	0	Apiaceae Lindl.
Iz_4_1209	DJI_0181	Pilosella echioides (Lumn.) F.W.Schultz & Sch.Bip.	0	n	1	0	Asteraceae Bercht. & J.Presl
Iz_4_1209	DJI_0181	Pilosella officinarum F. Schultz et Sch. Bip.	0	n	1	0	Asteraceae Bercht. & J.Presl
Iz_4_1209	DJI_0181	Pilosella sp.	0		NA	0	Asteraceae Bercht. & J.Presl
Iz_4_1209	DJI_0181	Pinus sylvestris L.	0	n	1	1	Pinaceae Spreng. ex F.Rudolphi
Iz_4_1209	DJI_0181	Poa annua L.	0	n	1	0	Poaceae Barnhart
Iz_4_1209	DJI_0181	Poa nemoralis L.	0	n	1	0	Poaceae Barnhart
Iz_4_1209	DJI_0181	Poa sp.	0		NA	0	Poaceae Barnhart
Iz_4_1209	DJI_0181	Polygonatum odoratum (Mill.) Druce	0	n	1	0	Asparagaceae Juss.
Iz_4_1209	DJI_0181	Polygonum arenarium Waldst. & Kit.	0	n	1	0	Polygonaceae Juss.
Iz_4_1209	DJI_0181	Populus nigra L.	0	n	1	1	Salicaceae Mirb.
Iz_4_1209	DJI_0181	Populus tremula L.	0	n	1	1	Salicaceae Mirb.
Iz_4_1209	DJI_0181	Populus Ã canescens (Aiton) Sm.	0	n	1	1	Salicaceae Mirb.
Iz_4_1209	DJI_0181	Potentilla argentea L.	0	n	1	0	Rosaceae Juss.
Iz_4_1209	DJI_0181	Psammophiliella muralis (L.) Ikonn.	0	n	1	0	Caryophyllaceae Juss.
Iz_4_1209	DJI_0181	Psephellus sumensis (Kalen.) Greuter	0	n	1	0	Asteraceae Bercht. & J.Presl
Iz_4_1209	DJI_0181	Pulsatilla pratensis (L.) Mill. s.l.	0	n	1	0	Ranunculaceae Juss.
Iz_4_1209	DJI_0181	Pyrus communis L.	0	a	0	1	Rosaceae Juss.
Iz_4_1209	DJI_0181	Quercus robur L.	0	n	1	1	Fagaceae Dumort.
Iz_4_1209	DJI_0181	Robinia pseudoacacia L.	0	a	0	1	Fabaceae Lindl.
Iz_4_1209	DJI_0181	Rosa sp.	0		NA	1	Rosaceae Juss.
Iz_4_1209	DJI_0181	Rubus idaeus L.	0	n	1	0	Rosaceae Juss.
Iz_4_1209	DJI_0181	Rumex acetosella L.	0	n	1	0	Polygonaceae Juss.
Iz_4_1209	DJI_0181	Rumex thyrsiflorus Fingerh.	0	n	1	0	Polygonaceae Juss.
Iz_4_1209	DJI_0181	Salix alba L.	0	n	1	1	Salicaceae Mirb.
Iz_4_1209	DJI_0181	Sambucus nigra L.	0	n	1	1	Viburnaceae Raf.
Iz_4_1209	DJI_0181	Secale sylvestre Host	0	n	1	0	Poaceae Barnhart
Iz_4_1209	DJI_0181	Sempervivum ruthenicum Schnittsp. & C.B. Lehm.	0	n	1	0	Crassulaceae J.St.-Hil.
Iz_4_1209	DJI_0181	Setaria viridis (L.) P. Beauv.	0	n	1	0	Poaceae Barnhart
Iz_4_1209	DJI_0181	Silene borysthenica (Gruner) Walters	0	n	1	0	Caryophyllaceae Juss.
Iz_4_1209	DJI_0181	Silene latifolia subsp. alba (Mill.) Greuter & Burdet	0	n	1	0	Caryophyllaceae Juss.
Iz_4_1209	DJI_0181	Solidago virgaurea L.	0	n	1	0	Asteraceae Bercht. & J.Presl
Iz_4_1209	DJI_0181	Sorbus aucuparia L.	0	n	1	1	Rosaceae Juss.
Iz_4_1209	DJI_0181	Stachys recta L.	0	n	1	0	Lamiacea Lindl.
Iz_4_1209	DJI_0181	Tanacetum vulgare L.	0	n	1	0	Asteraceae Bercht. & J.Presl
Iz_4_1209	DJI_0181	Thymus pallasianus Heinr.Braun	0	n	1	0	Lamiacea Lindl.
Iz_4_1209	DJI_0181	Tilia cordata Mill.	0	n	1	1	Malvaceae Juss.
Iz_4_1209	DJI_0181	Trifolium alpestre L.	0	n	1	0	Fabaceae Lindl.
Iz_4_1209	DJI_0181	Trifolium arvense L.	0	n	1	0	Fabaceae Lindl.
Iz_4_1209	DJI_0181	Ulmus glabra Huds.	0	n	1	1	Ulmaceae Mirb.
Iz_4_1209	DJI_0181	Verbascum densiflorum Bertol.	0	n	1	0	Scrophulariaceae Juss.
Iz_4_1209	DJI_0181	Veronica austriaca L.	0	n	1	0	Plantaginaceae Juss.
Iz_4_1209	DJI_0181	Veronica incana L.	0	n	1	0	Plantaginaceae Juss.
Iz_4_1209	DJI_0181	Veronica verna L.	0	n	1	0	Plantaginaceae Juss.
Iz_4_1209	DJI_0181	Vincetoxicum hirundinaria Medik	0	n	1	0	Apocynaceae Juss.
Iz_4_1209	DJI_0185	Acer tataricum L.	0	n	1	1	Sapindaceae Juss.
Iz_4_1209	DJI_0185	Achillea millefolium L. s.l.	0	n	1	0	Asteraceae Bercht. & J.Presl
Iz_4_1209	DJI_0185	Allium oleraceum L.	0	n	1	0	Amaryllidaceae J.St.-Hil.
Iz_4_1209	DJI_0185	Ambrosia artemisiifolia L.	0	a	0	0	Asteraceae Bercht. & J.Presl
Iz_4_1209	DJI_0185	Amorpha fruticosa L.	0	a	0	0	Fabaceae Lindl.
Iz_4_1209	DJI_0185	Anthericum ramosum L.	0	n	1	0	Asparagaceae Juss.
Iz_4_1209	DJI_0185	Artemisia marschalliana Spreng.	0	n	1	0	Asteraceae Bercht. & J.Presl
Iz_4_1209	DJI_0185	Asparagus officinalis L.	0	n	1	0	Asparagaceae Juss.
Iz_4_1209	DJI_0185	Astragalus cicer L.	0	n	1	0	Fabaceae Lindl.
Iz_4_1209	DJI_0185	Bassia laniflora (S.G.Gmel.) A.J.Scott	0	n	1	0	Amaranthaceae Juss.
Iz_4_1209	DJI_0185	Berberis vulgaris L.	0	n	1	0	Berberidaceae Juss.
Iz_4_1209	DJI_0185	Betula pendula Roth.	0	n	1	1	Betulaceae Gray
Iz_4_1209	DJI_0185	Betula pendula Roth.	0	n	1	0	Poaceae Barnhart
Iz_4_1209	DJI_0185	Bromus inermis Leyss.	0		NA	NA	
Iz_4_1209	DJI_0185	Bromus squarrosus L.	0	a	0	0	Poaceae Barnhart

Iz_4_1209	DJI_0185	Bromus tectorum L.	0	a	0	0	Poaceae Barnhart
Iz_4_1209	DJI_0185	Calamagrostis epigejos (L.) Roth	40	n	1	0	Poaceae Barnhart
Iz_4_1209	DJI_0185	Carduus acanthoides L.	0	n	1	0	Asteraceae Bercht. & J.Presl
Iz_4_1209	DJI_0185	Carex sp.	0	n	1	0	Cyperaceae Juss.
Iz_4_1209	DJI_0185	Centaurea majorovii Dumbadze	0	n	1	0	Asteraceae Bercht. & J.Presl
Iz_4_1209	DJI_0185	Chamaecytisus ruthenicus (Fisch.ex Wol.) Klaskova	0	n	1	0	Fabaceae Lindl.
Iz_4_1209	DJI_0185	Chelidonium majus L.	0	n	1	0	Papaveraceae Juss.
Iz_4_1209	DJI_0185	Chenopodium betaceum Andrz.	0	n	1	0	Amaranthaceae Juss.
Iz_4_1209	DJI_0185	Chenopodium cf. album	0	n	1	0	Amaranthaceae Juss.
Iz_4_1209	DJI_0185	Chenopodium sp.	0		NA	0	Amaranthaceae Juss.
Iz_4_1209	DJI_0185	Chondrilla juncea L.	0	n	1	0	Asteraceae Bercht. & J.Presl
Iz_4_1209	DJI_0185	Cirsium vulgare (Savi) Ten.	0	n	1	0	Asteraceae Bercht. & J.Presl
Iz_4_1209	DJI_0185	Clinopodium acinos (L.) Kuntze	0	n	1	0	Lamiacea Lindl.
Iz_4_1209	DJI_0185	Convallaria majalis L.	0	n	1	0	Asparagaceae Juss.
Iz_4_1209	DJI_0185	Crataegus rhipidophylla Gand.	0	n	1	1	Rosaceae Juss.
Iz_4_1209	DJI_0185	Crepis foetida L.	0	n	1	0	Asteraceae Bercht. & J.Presl
Iz_4_1209	DJI_0185	Dactylis glomerata L.	0	n	1	0	Poaceae Barnhart
Iz_4_1209	DJI_0185	Dianthus campestris M.Bieb.	0	n	1	0	Caryophyllaceae Juss.
Iz_4_1209	DJI_0185	Digitaria sanguinalis (L.) Scop.	0	a	0	0	Poaceae Barnhart
Iz_4_1209	DJI_0185	Elymus repens (L.) Gould	0	n	1	0	Poaceae Barnhart
Iz_4_1209	DJI_0185	Epilobium tetragonum L.	0	n	1	0	Onagraceae Juss.
Iz_4_1209	DJI_0185	Eragrostis minor Host	0	a	0	0	Poaceae Barnhart
Iz_4_1209	DJI_0185	Erigeron canadensis L.	0	a	0	0	Asteraceae Bercht. & J.Presl
Iz_4_1209	DJI_0185	Euphorbia seguieriana Neck.	0	n	1	0	Euphorbiaceae Juss.
Iz_4_1209	DJI_0185	Fallopia dumetorum (L.) Holub	0	n	1	0	Polygonaceae Juss.
Iz_4_1209	DJI_0185	Festuca beckeri (Hack.) Trautv.	0	n	1	0	Poaceae Barnhart
Iz_4_1209	DJI_0185	Fragaria vesca L.	0	n	1	0	Rosaceae Juss.
Iz_4_1209	DJI_0185	Frangula alnus Mill.	0	n	1	1	Rhamnaceae Juss.
Iz_4_1209	DJI_0185	Fraxinus pennsylvanica Marshall	0	a	0	1	Oleaceae Hoffmanns. & Link
Iz_4_1209	DJI_0185	Galeopsis bifida Boenn.	0	n	1	0	Lamiacea Lindl.
Iz_4_1209	DJI_0185	Galium verum L.	0	n	1	0	Rubiaceae Juss.
Iz_4_1209	DJI_0185	Genista tinctoria L.	0	n	1	0	Fabaceae Lindl.
Iz_4_1209	DJI_0185	Geranium robertianum L.	0	n	1	0	Geraniaceae Juss.
Iz_4_1209	DJI_0185	Geranium sanquineum L.	0	n	1	0	Geraniaceae Juss.
Iz_4_1209	DJI_0185	Gypsophila paniculata L.	0	n	1	0	Caryophyllaceae Juss.
Iz_4_1209	DJI_0185	Helichrysum arenarium (L.) Moench	0	n	1	0	Asteraceae Bercht. & J.Presl
Iz_4_1209	DJI_0185	Herniaria polygama J.Gay	0	n	1	0	Caryophyllaceae Juss.
Iz_4_1209	DJI_0185	Hieracium robustum Fr.	0	n	1	0	Asteraceae Bercht. & J.Presl
Iz_4_1209	DJI_0185	Hieracium umbellatum L.	0	n	1	NA	Asteraceae Bercht. & J.Presl
Iz_4_1209	DJI_0185	Hylotelephium maximum (L.) Holub	0	n	1	0	Crassulaceae J.St.-Hil.
Iz_4_1209	DJI_0185	Jacobaea borysthenica (DC.) B.Nord. & Greuter	0	n	1	0	Asteraceae Bercht. & J.Presl
Iz_4_1209	DJI_0185	Jasione montana L.	0	n	1	0	Campanulaceae Juss.
Iz_4_1209	DJI_0185	Jurinea cyanoides (L.) Rchb.s.l.	0	n	1	0	Asteraceae Bercht. & J.Presl
Iz_4_1209	DJI_0185	Koeleria glauca (Spreng.) DC.	0	n	1	0	Poaceae Barnhart
Iz_4_1209	DJI_0185	Lactuca serriola L.	0	a	0	0	Asteraceae Bercht. & J.Presl
Iz_4_1209	DJI_0185	Linaria genistifolia (L.) Mill.	0	n	1	0	Plantaginaceae Juss.
Iz_4_1209	DJI_0185	Lomelosia argentea (L.) Greuter & Burdet	0	n	1	0	Caprifoliaceae Juss.
Iz_4_1209	DJI_0185	Melampyrum pratense L.	0	n	1	0	Orobanchaceae Vent.
Iz_4_1209	DJI_0185	Oenothera biennis L.	0	a	0	0	Onagraceae Juss.
Iz_4_1209	DJI_0185	Pentanema britannica (L.) D.Gut.Larr., Santos-Vicente, Anderb., E.Rico & M.M.Mart.Ort.	0	n	1	0	Asteraceae Bercht. & J.Presl
Iz_4_1209	DJI_0185	Peucedanum oreoselinum (L.) Moench	0	n	1	0	Apiaceae Lindl.
Iz_4_1209	DJI_0185	Peucedanum ruthenicum M.Bieb.	0	n	1	0	Apiaceae Lindl.
Iz_4_1209	DJI_0185	Pilosella echioides (Lumn.) F.W.Schultz & Sch.Bip.	0	n	1	0	Asteraceae Bercht. & J.Presl
Iz_4_1209	DJI_0185	Pilosella officinarum F. Schultz et Sch. Bip.	0	n	1	0	Asteraceae Bercht. & J.Presl
Iz_4_1209	DJI_0185	Pilosella sp.	0		NA	0	Asteraceae Bercht. & J.Presl
Iz_4_1209	DJI_0185	Pinus sylvestris L.	0	n	1	1	Pinaceae Spreng. ex F.Rudolphi
Iz_4_1209	DJI_0185	Poa annua L.	0	n	1	0	Poaceae Barnhart
Iz_4_1209	DJI_0185	Poa nemoralis L.	0	n	1	0	Poaceae Barnhart
Iz_4_1209	DJI_0185	Poa sp.	0		NA	0	Poaceae Barnhart
Iz_4_1209	DJI_0185	Polygonatum odoratum (Mill.) Druce	0	n	1	0	Asparagaceae Juss.
Iz_4_1209	DJI_0185	Polygonum arenarium Waldst. & Kit.	0	n	1	0	Polygonaceae Juss.
Iz_4_1209	DJI_0185	Populus nigra L.	0	n	1	1	Salicaceae Mirb.
Iz_4_1209	DJI_0185	Populus tremula L.	0	n	1	1	Salicaceae Mirb.
Iz_4_1209	DJI_0185	Populus Ã canescens (Aiton) Sm.	0	n	1	1	Salicaceae Mirb.
Iz_4_1209	DJI_0185	Potentilla argentea L.	0	n	1	0	Rosaceae Juss.
Iz_4_1209	DJI_0185	Psammophiliella muralis (L.) Ikonn.	0	n	1	0	Caryophyllaceae Juss.
Iz_4_1209	DJI_0185	Psephellus sumensis (Kalen.) Greuter	0	n	1	0	Asteraceae Bercht. & J.Presl
Iz_4_1209	DJI_0185	Pulsatilla pratensis (L.) Mill. s.l.	0	n	1	0	Ranunculaceae Juss.
Iz_4_1209	DJI_0185	Pyrus communis L.	0	a	0	1	Rosaceae Juss.
Iz_4_1209	DJI_0185	Quercus robur L.	0	n	1	1	Fagaceae Dumort.
Iz_4_1209	DJI_0185	Robinia pseudoacacia L.	0	a	0	1	Fabaceae Lindl.
Iz_4_1209	DJI_0185	Rosa sp.	0		NA	1	Rosaceae Juss.
Iz_4_1209	DJI_0185	Rubus idaeus L.	0	n	1	0	Rosaceae Juss.
Iz_4_1209	DJI_0185	Rumex acetosella L.	0	n	1	0	Polygonaceae Juss.
Iz_4_1209	DJI_0185	Rumex thyrsiflorus Fingerh.	0	n	1	0	Polygonaceae Juss.
Iz_4_1209	DJI_0185	Salix alba L.	0	n	1	1	Salicaceae Mirb.
Iz_4_1209	DJI_0185	Sambucus nigra L.	0	n	1	1	Viburnaceae Raf.
Iz_4_1209	DJI_0185	Secale sylvestre Host	0	n	1	0	Poaceae Barnhart
Iz_4_1209	DJI_0185	Sempervivum ruthenicum Schnittsp. & C.B. Lehm.	0	n	1	0	Crassulaceae J.St.-Hil.
Iz_4_1209	DJI_0185	Setaria viridis (L.) P. Beauv.	0	n	1	0	Poaceae Barnhart
Iz_4_1209	DJI_0185	Silene borysthenica (Gruner) Walters	0	n	1	0	Caryophyllaceae Juss.
Iz_4_1209	DJI_0185	Silene latifolia subsp. alba (Mill.) Greuter & Burdet	0	n	1	0	Caryophyllaceae Juss.
Iz_4_1209	DJI_0185	Solidago virgaurea L.	0	n	1	0	Asteraceae Bercht. & J.Presl
Iz_4_1209	DJI_0185	Sorbus aucuparia L.	0	n	1	1	Rosaceae Juss.
Iz_4_1209	DJI_0185	Stachys recta L.	0	n	1	0	Lamiacea Lindl.
Iz_4_1209	DJI_0185	Tanacetum vulgare L.	0	n	1	0	Asteraceae Bercht. & J.Presl
Iz_4_1209	DJI_0185	Thymus pallasianus Heinr.Braun	0	n	1	0	Lamiacea Lindl.
Iz_4_1209	DJI_0185	Tilia cordata Mill.	0	n	1	1	Malvaceae Juss.
Iz_4_1209	DJI_0185	Trifolium alpestre L.	0	n	1	0	Fabaceae Lindl.
Iz_4_1209	DJI_0185	Trifolium arvense L.	0	n	1	0	Fabaceae Lindl.
Iz_4_1209	DJI_0185	Ulmus glabra Huds.	0	n	1	1	Ulmaceae Mirb.
Iz_4_1209	DJI_0185	Verbascum densiflorum Bertol.	0	n	1	0	Scrophulariaceae Juss.
Iz_4_1209	DJI_0185	Veronica austriaca L.	0	n	1	0	Plantaginaceae Juss.
Iz_4_1209	DJI_0185	Veronica incana L.	0	n	1	0	Plantaginaceae Juss.
Iz_4_1209	DJI_0185	Veronica verna L.	0	n	1	0	Plantaginaceae Juss.
Iz_4_1209	DJI_0185	Vincetoxicum hirundinaria Medik	0	n	1	0	Apocynaceae Juss.
Iz_4_1209	DJI_0188	Acer tataricum L.	0	n	1	1	Sapindaceae Juss.
Iz_4_1209	DJI_0188	Achillea millefolium L. s.l.	0	n	1	0	Asteraceae Bercht. & J.Presl

Iz_4_1209	DJI_0188	Allium oleraceum L.	0	n	1	0	Amaryllidaceae J.St.-Hil.
Iz_4_1209	DJI_0188	Ambrosia artemisiifolia L.	0	a	0	0	Asteraceae Bercht. & J.Presl
Iz_4_1209	DJI_0188	Amorpha fruticosa L.	0	a	0	0	Fabaceae Lindl.
Iz_4_1209	DJI_0188	Anthericum ramosum L.	0	n	1	0	Asparagaceae Juss.
Iz_4_1209	DJI_0188	Artemisia marschalliana Spreng.	0	n	1	0	Asteraceae Bercht. & J.Presl
Iz_4_1209	DJI_0188	Asparagus officinalis L.	0	n	1	0	Asparagaceae Juss.
Iz_4_1209	DJI_0188	Astragalus cicer L.	0	n	1	0	Fabaceae Lindl.
Iz_4_1209	DJI_0188	Bassia laniflora (S.G.Gmel.) A.J.Scott	0	n	1	0	Amaranthaceae Juss.
Iz_4_1209	DJI_0188	Berberis vulgaris L.	0	n	1	0	Berberidaceae Juss.
Iz_4_1209	DJI_0188	Betula pendula Roth.	0	n	1	1	Betulaceae Gray
Iz_4_1209	DJI_0188	Betula pendula Roth.	0	n	1	0	Poaceae Barnhart
Iz_4_1209	DJI_0188	Bromus inermis Leyss.	0		NA	NA	
Iz_4_1209	DJI_0188	Bromus squarrosus L.	0	a	0	0	Poaceae Barnhart
Iz_4_1209	DJI_0188	Bromus tectorum L.	0	a	0	0	Poaceae Barnhart
Iz_4_1209	DJI_0188	Calamagrostis epigejos (L.) Roth	10	n	1	0	Poaceae Barnhart
Iz_4_1209	DJI_0188	Carduus acanthoides L.	0	n	1	0	Asteraceae Bercht. & J.Presl
Iz_4_1209	DJI_0188	Carex sp.	0	n	1	0	Cyperaceae Juss.
Iz_4_1209	DJI_0188	Centaurea majorovii Dumbadze	0	n	1	0	Asteraceae Bercht. & J.Presl
Iz_4_1209	DJI_0188	Chamaecytisus ruthenicus (Fisch.ex Wol.) Klaskova	0	n	1	0	Fabaceae Lindl.
Iz_4_1209	DJI_0188	Chelidonium majus L.	0	n	1	0	Papaveraceae Juss.
Iz_4_1209	DJI_0188	Chenopodium betaceum Andrz.	0	n	1	0	Amaranthaceae Juss.
Iz_4_1209	DJI_0188	Chenopodium cf. album	0	n	1	0	Amaranthaceae Juss.
Iz_4_1209	DJI_0188	Chenopodium sp.	0		NA	0	Amaranthaceae Juss.
Iz_4_1209	DJI_0188	Chondrilla juncea L.	0	n	1	0	Asteraceae Bercht. & J.Presl
Iz_4_1209	DJI_0188	Cirsium vulgare (Savi) Ten.	0	n	1	0	Asteraceae Bercht. & J.Presl
Iz_4_1209	DJI_0188	Clinopodium acinos (L.) Kuntze	0	n	1	0	Lamiacea Lindl.
Iz_4_1209	DJI_0188	Convallaria majalis L.	0	n	1	0	Asparagaceae Juss.
Iz_4_1209	DJI_0188	Crataegus rhipidophylla Gand.	0	n	1	1	Rosaceae Juss.
Iz_4_1209	DJI_0188	Crepis foetida L.	0	n	1	0	Asteraceae Bercht. & J.Presl
Iz_4_1209	DJI_0188	Dactylis glomerata L.	0	n	1	0	Poaceae Barnhart
Iz_4_1209	DJI_0188	Dianthus campestris M.Bieb.	0	n	1	0	Caryophyllaceae Juss.
Iz_4_1209	DJI_0188	Digitaria sanguinalis (L.) Scop.	0	a	0	0	Poaceae Barnhart
Iz_4_1209	DJI_0188	Elymus repens (L.) Gould	0	n	1	0	Poaceae Barnhart
Iz_4_1209	DJI_0188	Epilobium tetragonum L.	0	n	1	0	Onagraceae Juss.
Iz_4_1209	DJI_0188	Eragrostis minor Host	0	a	0	0	Poaceae Barnhart
Iz_4_1209	DJI_0188	Erigeron canadensis L.	15	a	0	0	Asteraceae Bercht. & J.Presl
Iz_4_1209	DJI_0188	Euphorbia seguieriana Neck.	0	n	1	0	Euphorbiaceae Juss.
Iz_4_1209	DJI_0188	Fallopia dumetorum (L.) Holub	0	n	1	0	Polygonaceae Juss.
Iz_4_1209	DJI_0188	Festuca beckeri (Hack.) Trautv.	0	n	1	0	Poaceae Barnhart
Iz_4_1209	DJI_0188	Fragaria vesca L.	0	n	1	0	Rosaceae Juss.
Iz_4_1209	DJI_0188	Frangula alnus Mill.	0	n	1	1	Rhamnaceae Juss.
Iz_4_1209	DJI_0188	Fraxinus pennsylvanica Marshall	0	a	0	1	Oleaceae Hoffmanns. & Link
Iz_4_1209	DJI_0188	Galeopsis bifida Boenn.	0	n	1	0	Lamiacea Lindl.
Iz_4_1209	DJI_0188	Galium verum L.	0	n	1	0	Rubiaceae Juss.
Iz_4_1209	DJI_0188	Genista tinctoria L.	0	n	1	0	Fabaceae Lindl.
Iz_4_1209	DJI_0188	Geranium robertianum L.	0	n	1	0	Geraniaceae Juss.
Iz_4_1209	DJI_0188	Geranium sanquineum L.	0	n	1	0	Geraniaceae Juss.
Iz_4_1209	DJI_0188	Gypsophila paniculata L.	0	n	1	0	Caryophyllaceae Juss.
Iz_4_1209	DJI_0188	Helichrysum arenarium (L.) Moench	0	n	1	0	Asteraceae Bercht. & J.Presl
Iz_4_1209	DJI_0188	Herniaria polygama J.Gay	0	n	1	0	Caryophyllaceae Juss.
Iz_4_1209	DJI_0188	Hieracium robustum Fr.	0	n	1	0	Asteraceae Bercht. & J.Presl
Iz_4_1209	DJI_0188	Hieracium umbellatum L.	0	n	1	NA	Asteraceae Bercht. & J.Presl
Iz_4_1209	DJI_0188	Hylotelephium maximum (L.) Holub	0	n	1	0	Crassulaceae J.St.-Hil.
Iz_4_1209	DJI_0188	Jacobaea borysthenica (DC.) B.Nord. & Greuter	0	n	1	0	Asteraceae Bercht. & J.Presl
Iz_4_1209	DJI_0188	Jasione montana L.	0	n	1	0	Campanulaceae Juss.
Iz_4_1209	DJI_0188	Jurinea cyanoides (L.) Rchb.s.l.	0	n	1	0	Asteraceae Bercht. & J.Presl
Iz_4_1209	DJI_0188	Koeleria glauca (Spreng.) DC.	0	n	1	0	Poaceae Barnhart
Iz_4_1209	DJI_0188	Lactuca serriola L.	0	a	0	0	Asteraceae Bercht. & J.Presl
Iz_4_1209	DJI_0188	Linaria genistifolia (L.) Mill.	0	n	1	0	Plantaginaceae Juss.
Iz_4_1209	DJI_0188	Lomelosia argentea (L.) Greuter & Burdet	0	n	1	0	Caprifoliaceae Juss.
Iz_4_1209	DJI_0188	Melampyrum pratense L.	0	n	1	0	Orobanchaceae Vent.
Iz_4_1209	DJI_0188	Oenothera biennis L.	0	a	0	0	Onagraceae Juss.
Iz_4_1209	DJI_0188	Pentanema britannica (L.) D.Gut.Larr., Santos-Vicente, Anderb., E.Rico & M.M.Mart.Ort.	0	n	1	0	Asteraceae Bercht. & J.Presl
Iz_4_1209	DJI_0188	Peucedanum oreoselinum (L.) Moench	0	n	1	0	Apiaceae Lindl.
Iz_4_1209	DJI_0188	Peucedanum ruthenicum M.Bieb.	0	n	1	0	Apiaceae Lindl.
Iz_4_1209	DJI_0188	Pilosella echioides (Lumn.) F.W.Schultz & Sch.Bip.	0	n	1	0	Asteraceae Bercht. & J.Presl
Iz_4_1209	DJI_0188	Pilosella officinarum F. Schultz et Sch. Bip.	0	n	1	0	Asteraceae Bercht. & J.Presl
Iz_4_1209	DJI_0188	Pilosella sp.	0		NA	0	Asteraceae Bercht. & J.Presl
Iz_4_1209	DJI_0188	Pinus sylvestris L.	0	n	1	1	Pinaceae Spreng. ex F.Rudolphi
Iz_4_1209	DJI_0188	Poa annua L.	0	n	1	0	Poaceae Barnhart
Iz_4_1209	DJI_0188	Poa nemoralis L.	0	n	1	0	Poaceae Barnhart
Iz_4_1209	DJI_0188	Poa sp.	0		NA	0	Poaceae Barnhart
Iz_4_1209	DJI_0188	Polygonatum odoratum (Mill.) Druce	0	n	1	0	Asparagaceae Juss.
Iz_4_1209	DJI_0188	Polygonum arenarium Waldst. & Kit.	0	n	1	0	Polygonaceae Juss.
Iz_4_1209	DJI_0188	Populus nigra L.	0	n	1	1	Salicaceae Mirb.
Iz_4_1209	DJI_0188	Populus tremula L.	0	n	1	1	Salicaceae Mirb.
Iz_4_1209	DJI_0188	Populus Ã canescens (Aiton) Sm.	0	n	1	1	Salicaceae Mirb.
Iz_4_1209	DJI_0188	Potentilla argentea L.	0	n	1	0	Rosaceae Juss.
Iz_4_1209	DJI_0188	Psammophiliella muralis (L.) Ikonn.	0	n	1	0	Caryophyllaceae Juss.
Iz_4_1209	DJI_0188	Psephellus sumensis (Kalen.) Greuter	0	n	1	0	Asteraceae Bercht. & J.Presl
Iz_4_1209	DJI_0188	Pulsatilla pratensis (L.) Mill. s.l.	0	n	1	0	Ranunculaceae Juss.
Iz_4_1209	DJI_0188	Pyrus communis L.	0	a	0	1	Rosaceae Juss.
Iz_4_1209	DJI_0188	Quercus robur L.	0	n	1	1	Fagaceae Dumort.
Iz_4_1209	DJI_0188	Robinia pseudoacacia L.	0	a	0	1	Fabaceae Lindl.
Iz_4_1209	DJI_0188	Rosa sp.	0		NA	1	Rosaceae Juss.
Iz_4_1209	DJI_0188	Rubus idaeus L.	0	n	1	0	Rosaceae Juss.
Iz_4_1209	DJI_0188	Rumex acetosella L.	0	n	1	0	Polygonaceae Juss.
Iz_4_1209	DJI_0188	Rumex thyrsiflorus Fingerh.	0	n	1	0	Polygonaceae Juss.
Iz_4_1209	DJI_0188	Salix alba L.	0	n	1	1	Salicaceae Mirb.
Iz_4_1209	DJI_0188	Sambucus nigra L.	0	n	1	1	Viburnaceae Raf.
Iz_4_1209	DJI_0188	Secale sylvestre Host	0	n	1	0	Poaceae Barnhart
Iz_4_1209	DJI_0188	Sempervivum ruthenicum Schnittsp. & C.B. Lehm.	0	n	1	0	Crassulaceae J.St.-Hil.
Iz_4_1209	DJI_0188	Setaria viridis (L.) P. Beauv.	0	n	1	0	Poaceae Barnhart
Iz_4_1209	DJI_0188	Silene borysthenica (Gruner) Walters	0	n	1	0	Caryophyllaceae Juss.
Iz_4_1209	DJI_0188	Silene latifolia subsp. alba (Mill.) Greuter & Burdet	0	n	1	0	Caryophyllaceae Juss.
Iz_4_1209	DJI_0188	Solidago virgaurea L.	0	n	1	0	Asteraceae Bercht. & J.Presl
Iz_4_1209	DJI_0188	Sorbus aucuparia L.	0	n	1	1	Rosaceae Juss.
Iz_4_1209	DJI_0188	Stachys recta L.	0	n	1	0	Lamiacea Lindl.

Iz_4_1209	DJI_0188	Tanacetum vulgare L.	0	n	1	0	Asteraceae Bercht. & J.Presl
Iz_4_1209	DJI_0188	Thymus pallasianus Heinr.Braun	0	n	1	0	Lamiacea Lindl.
Iz_4_1209	DJI_0188	Tilia cordata Mill.	0	n	1	1	Malvaceae Juss.
Iz_4_1209	DJI_0188	Trifolium alpestre L.	0	n	1	0	Fabaceae Lindl.
Iz_4_1209	DJI_0188	Trifolium arvense L.	0	n	1	0	Fabaceae Lindl.
Iz_4_1209	DJI_0188	Ulmus glabra Huds.	0	n	1	1	Ulmaceae Mirb.
Iz_4_1209	DJI_0188	Verbascum densiflorum Bertol.	0	n	1	0	Scrophulariaceae Juss.
Iz_4_1209	DJI_0188	Veronica austriaca L.	0	n	1	0	Plantaginaceae Juss.
Iz_4_1209	DJI_0188	Veronica incana L.	0	n	1	0	Plantaginaceae Juss.
Iz_4_1209	DJI_0188	Veronica verna L.	0	n	1	0	Plantaginaceae Juss.
Iz_4_1209	DJI_0188	Vincetoxicum hirundinaria Medik	0	n	1	0	Apocynaceae Juss.
Iz_4_1209	DJI_0191	Acer tataricum L.	0	n	1	1	Sapindaceae Juss.
Iz_4_1209	DJI_0191	Achillea millefolium L. s.l.	0	n	1	0	Asteraceae Bercht. & J.Presl
Iz_4_1209	DJI_0191	Allium oleraceum L.	0	n	1	0	Amaryllidaceae J.St.-Hil.
Iz_4_1209	DJI_0191	Ambrosia artemisiifolia L.	0	a	0	0	Asteraceae Bercht. & J.Presl
Iz_4_1209	DJI_0191	Amorpha fruticosa L.	0	a	0	0	Fabaceae Lindl.
Iz_4_1209	DJI_0191	Anthericum ramosum L.	0	n	1	0	Asparagaceae Juss.
Iz_4_1209	DJI_0191	Artemisia marschalliana Spreng.	0	n	1	0	Asteraceae Bercht. & J.Presl
Iz_4_1209	DJI_0191	Asparagus officinalis L.	0	n	1	0	Asparagaceae Juss.
Iz_4_1209	DJI_0191	Astragalus cicer L.	0	n	1	0	Fabaceae Lindl.
Iz_4_1209	DJI_0191	Bassia laniflora (S.G.Gmel.) A.J.Scott	0	n	1	0	Amaranthaceae Juss.
Iz_4_1209	DJI_0191	Berberis vulgaris L.	0	n	1	0	Berberidaceae Juss.
Iz_4_1209	DJI_0191	Betula pendula Roth.	0	n	1	1	Betulaceae Gray
Iz_4_1209	DJI_0191	Betula pendula Roth.	0	n	1	0	Poaceae Barnhart
Iz_4_1209	DJI_0191	Bromus inermis Leyss.	0		NA	NA	
Iz_4_1209	DJI_0191	Bromus squarrosus L.	0	a	0	0	Poaceae Barnhart
Iz_4_1209	DJI_0191	Bromus tectorum L.	0	a	0	0	Poaceae Barnhart
Iz_4_1209	DJI_0191	Calamagrostis epigejos (L.) Roth	30	n	1	0	Poaceae Barnhart
Iz_4_1209	DJI_0191	Carduus acanthoides L.	0	n	1	0	Asteraceae Bercht. & J.Presl
Iz_4_1209	DJI_0191	Carex sp.	0	n	1	0	Cyperaceae Juss.
Iz_4_1209	DJI_0191	Centaurea majorovii Dumbadze	0	n	1	0	Asteraceae Bercht. & J.Presl
Iz_4_1209	DJI_0191	Chamaecytisus ruthenicus (Fisch.ex Wol.) Klaskova	0	n	1	0	Fabaceae Lindl.
Iz_4_1209	DJI_0191	Chelidonium majus L.	0	n	1	0	Papaveraceae Juss.
Iz_4_1209	DJI_0191	Chenopodium betaceum Andrz.	0	n	1	0	Amaranthaceae Juss.
Iz_4_1209	DJI_0191	Chenopodium cf. album	0	n	1	0	Amaranthaceae Juss.
Iz_4_1209	DJI_0191	Chenopodium sp.	0		NA	0	Amaranthaceae Juss.
Iz_4_1209	DJI_0191	Chondrilla juncea L.	0	n	1	0	Asteraceae Bercht. & J.Presl
Iz_4_1209	DJI_0191	Cirsium vulgare (Savi) Ten.	0	n	1	0	Asteraceae Bercht. & J.Presl
Iz_4_1209	DJI_0191	Clinopodium acinos (L.) Kuntze	0	n	1	0	Lamiacea Lindl.
Iz_4_1209	DJI_0191	Convallaria majalis L.	0	n	1	0	Asparagaceae Juss.
Iz_4_1209	DJI_0191	Crataegus rhipidophylla Gand.	0	n	1	1	Rosaceae Juss.
Iz_4_1209	DJI_0191	Crepis foetida L.	0	n	1	0	Asteraceae Bercht. & J.Presl
Iz_4_1209	DJI_0191	Dactylis glomerata L.	0	n	1	0	Poaceae Barnhart
Iz_4_1209	DJI_0191	Dianthus campestris M.Bieb.	0	n	1	0	Caryophyllaceae Juss.
Iz_4_1209	DJI_0191	Digitaria sanguinalis (L.) Scop.	0	a	0	0	Poaceae Barnhart
Iz_4_1209	DJI_0191	Elymus repens (L.) Gould	0	n	1	0	Poaceae Barnhart
Iz_4_1209	DJI_0191	Epilobium tetragonum L.	0	n	1	0	Onagraceae Juss.
Iz_4_1209	DJI_0191	Eragrostis minor Host	0	a	0	0	Poaceae Barnhart
Iz_4_1209	DJI_0191	Erigeron canadensis L.	10	a	0	0	Asteraceae Bercht. & J.Presl
Iz_4_1209	DJI_0191	Euphorbia seguieriana Neck.	0	n	1	0	Euphorbiaceae Juss.
Iz_4_1209	DJI_0191	Fallopia dumetorum (L.) Holub	0	n	1	0	Polygonaceae Juss.
Iz_4_1209	DJI_0191	Festuca beckeri (Hack.) Trautv.	0	n	1	0	Poaceae Barnhart
Iz_4_1209	DJI_0191	Fragaria vesca L.	0	n	1	0	Rosaceae Juss.
Iz_4_1209	DJI_0191	Frangula alnus Mill.	0	n	1	1	Rhamnaceae Juss.
Iz_4_1209	DJI_0191	Fraxinus pennsylvanica Marshall	0	a	0	1	Oleaceae Hoffmanns. & Link
Iz_4_1209	DJI_0191	Galeopsis bifida Boenn.	0	n	1	0	Lamiacea Lindl.
Iz_4_1209	DJI_0191	Galium verum L.	0	n	1	0	Rubiaceae Juss.
Iz_4_1209	DJI_0191	Genista tinctoria L.	0	n	1	0	Fabaceae Lindl.
Iz_4_1209	DJI_0191	Geranium robertianum L.	0	n	1	0	Geraniaceae Juss.
Iz_4_1209	DJI_0191	Geranium sanquineum L.	0	n	1	0	Geraniaceae Juss.
Iz_4_1209	DJI_0191	Gypsophila paniculata L.	0	n	1	0	Caryophyllaceae Juss.
Iz_4_1209	DJI_0191	Helichrysum arenarium (L.) Moench	0	n	1	0	Asteraceae Bercht. & J.Presl
Iz_4_1209	DJI_0191	Herniaria polygama J.Gay	0	n	1	0	Caryophyllaceae Juss.
Iz_4_1209	DJI_0191	Hieracium robustum Fr.	0	n	1	0	Asteraceae Bercht. & J.Presl
Iz_4_1209	DJI_0191	Hieracium umbellatum L.	0	n	1	NA	Asteraceae Bercht. & J.Presl
Iz_4_1209	DJI_0191	Hylotelephium maximum (L.) Holub	0	n	1	0	Crassulaceae J.St.-Hil.
Iz_4_1209	DJI_0191	Jacobaea borysthenica (DC.) B.Nord. & Greuter	0	n	1	0	Asteraceae Bercht. & J.Presl
Iz_4_1209	DJI_0191	Jasione montana L.	0	n	1	0	Campanulaceae Juss.
Iz_4_1209	DJI_0191	Jurinea cyanoides (L.) Rchb.s.l.	0	n	1	0	Asteraceae Bercht. & J.Presl
Iz_4_1209	DJI_0191	Koeleria glauca (Spreng.) DC.	0	n	1	0	Poaceae Barnhart
Iz_4_1209	DJI_0191	Lactuca serriola L.	0	a	0	0	Asteraceae Bercht. & J.Presl
Iz_4_1209	DJI_0191	Linaria genistifolia (L.) Mill.	0	n	1	0	Plantaginaceae Juss.
Iz_4_1209	DJI_0191	Lomelosia argentea (L.) Greuter & Burdet	0	n	1	0	Caprifoliaceae Juss.
Iz_4_1209	DJI_0191	Melampyrum pratense L.	0	n	1	0	Orobanchaceae Vent.
Iz_4_1209	DJI_0191	Oenothera biennis L.	0	a	0	0	Onagraceae Juss.
Iz_4_1209	DJI_0191	Pentanema britannica (L.) D.Gut.Larr., Santos-Vicente, Anderb., E.Rico & M.M.Mart.Ort.	0	n	1	0	Asteraceae Bercht. & J.Presl
Iz_4_1209	DJI_0191	Peucedanum oreoselinum (L.) Moench	0	n	1	0	Apiaceae Lindl.
Iz_4_1209	DJI_0191	Peucedanum ruthenicum M.Bieb.	0	n	1	0	Apiaceae Lindl.
Iz_4_1209	DJI_0191	Pilosella echioides (Lumn.) F.W.Schultz & Sch.Bip.	0	n	1	0	Asteraceae Bercht. & J.Presl
Iz_4_1209	DJI_0191	Pilosella officinarum F. Schultz et Sch. Bip.	0	n	1	0	Asteraceae Bercht. & J.Presl
Iz_4_1209	DJI_0191	Pilosella sp.	0		NA	0	Asteraceae Bercht. & J.Presl
Iz_4_1209	DJI_0191	Pinus sylvestris L.	0	n	1	1	Pinaceae Spreng. ex F.Rudolphi
Iz_4_1209	DJI_0191	Poa annua L.	0	n	1	0	Poaceae Barnhart
Iz_4_1209	DJI_0191	Poa nemoralis L.	0	n	1	0	Poaceae Barnhart
Iz_4_1209	DJI_0191	Poa sp.	0		NA	0	Poaceae Barnhart
Iz_4_1209	DJI_0191	Polygonatum odoratum (Mill.) Druce	0	n	1	0	Asparagaceae Juss.
Iz_4_1209	DJI_0191	Polygonum arenarium Waldst. & Kit.	0	n	1	0	Polygonaceae Juss.
Iz_4_1209	DJI_0191	Populus nigra L.	0	n	1	1	Salicaceae Mirb.
Iz_4_1209	DJI_0191	Populus tremula L.	0	n	1	1	Salicaceae Mirb.
Iz_4_1209	DJI_0191	Populus Ã canescens (Aiton) Sm.	0	n	1	1	Salicaceae Mirb.
Iz_4_1209	DJI_0191	Potentilla argentea L.	0	n	1	0	Rosaceae Juss.
Iz_4_1209	DJI_0191	Psammophiliella muralis (L.) Ikonn.	0	n	1	0	Caryophyllaceae Juss.
Iz_4_1209	DJI_0191	Psephellus sumensis (Kalen.) Greuter	0	n	1	0	Asteraceae Bercht. & J.Presl
Iz_4_1209	DJI_0191	Pulsatilla pratensis (L.) Mill. s.l.	0	n	1	0	Ranunculaceae Juss.
Iz_4_1209	DJI_0191	Pyrus communis L.	0	a	0	1	Rosaceae Juss.
Iz_4_1209	DJI_0191	Quercus robur L.	0	n	1	1	Fagaceae Dumort.
Iz_4_1209	DJI_0191	Robinia pseudoacacia L.	0	a	0	1	Fabaceae Lindl.
Iz_4_1209	DJI_0191	Rosa sp.	0		NA	1	Rosaceae Juss.

Iz_4_1209	DJI_0191	Rubus idaeus L.	0	n	1	0	Rosaceae Juss.
Iz_4_1209	DJI_0191	Rumex acetosella L.	0	n	1	0	Polygonaceae Juss.
Iz_4_1209	DJI_0191	Rumex thyrsiflorus Fingerh.	0	n	1	0	Polygonaceae Juss.
Iz_4_1209	DJI_0191	Salix alba L.	0	n	1	1	Salicaceae Mirb.
Iz_4_1209	DJI_0191	Sambucus nigra L.	0	n	1	1	Viburnaceae Raf.
Iz_4_1209	DJI_0191	Secale sylvestre Host	0	n	1	0	Poaceae Barnhart
Iz_4_1209	DJI_0191	Sempervivum ruthenicum Schnittsp. & C.B. Lehm.	0	n	1	0	Crassulaceae J.St.-Hil.
Iz_4_1209	DJI_0191	Setaria viridis (L.) P. Beauv.	0	n	1	0	Poaceae Barnhart
Iz_4_1209	DJI_0191	Silene borysthenica (Gruner) Walters	0	n	1	0	Caryophyllaceae Juss.
Iz_4_1209	DJI_0191	Silene latifolia subsp. alba (Mill.) Greuter & Burdet	0	n	1	0	Caryophyllaceae Juss.
Iz_4_1209	DJI_0191	Solidago virgaurea L.	0	n	1	0	Asteraceae Bercht. & J.Presl
Iz_4_1209	DJI_0191	Sorbus aucuparia L.	0	n	1	1	Rosaceae Juss.
Iz_4_1209	DJI_0191	Stachys recta L.	0	n	1	0	Lamiacea Lindl.
Iz_4_1209	DJI_0191	Tanacetum vulgare L.	0	n	1	0	Asteraceae Bercht. & J.Presl
Iz_4_1209	DJI_0191	Thymus pallasianus Heinr.Braun	0	n	1	0	Lamiacea Lindl.
Iz_4_1209	DJI_0191	Tilia cordata Mill.	0	n	1	1	Malvaceae Juss.
Iz_4_1209	DJI_0191	Trifolium alpestre L.	0	n	1	0	Fabaceae Lindl.
Iz_4_1209	DJI_0191	Trifolium arvense L.	0	n	1	0	Fabaceae Lindl.
Iz_4_1209	DJI_0191	Ulmus glabra Huds.	0	n	1	1	Ulmaceae Mirb.
Iz_4_1209	DJI_0191	Verbascum densiflorum Bertol.	0	n	1	0	Scrophulariaceae Juss.
Iz_4_1209	DJI_0191	Veronica austriaca L.	0	n	1	0	Plantaginaceae Juss.
Iz_4_1209	DJI_0191	Veronica incana L.	0	n	1	0	Plantaginaceae Juss.
Iz_4_1209	DJI_0191	Veronica verna L.	0	n	1	0	Plantaginaceae Juss.
Iz_4_1209	DJI_0191	Vincetoxicum hirundinaria Medik	0	n	1	0	Apocynaceae Juss.
Iz_4_1209	DJI_0195	Acer tataricum L.	0	n	1	1	Sapindaceae Juss.
Iz_4_1209	DJI_0195	Achillea millefolium L. s.l.	0	n	1	0	Asteraceae Bercht. & J.Presl
Iz_4_1209	DJI_0195	Allium oleraceum L.	0	n	1	0	Amaryllidaceae J.St.-Hil.
Iz_4_1209	DJI_0195	Ambrosia artemisiifolia L.	0	a	0	0	Asteraceae Bercht. & J.Presl
Iz_4_1209	DJI_0195	Amorpha fruticosa L.	0	a	0	0	Fabaceae Lindl.
Iz_4_1209	DJI_0195	Anthericum ramosum L.	0	n	1	0	Asparagaceae Juss.
Iz_4_1209	DJI_0195	Artemisia marschalliana Spreng.	0	n	1	0	Asteraceae Bercht. & J.Presl
Iz_4_1209	DJI_0195	Asparagus officinalis L.	0	n	1	0	Asparagaceae Juss.
Iz_4_1209	DJI_0195	Astragalus cicer L.	0	n	1	0	Fabaceae Lindl.
Iz_4_1209	DJI_0195	Bassia laniflora (S.G.Gmel.) A.J.Scott	0	n	1	0	Amaranthaceae Juss.
Iz_4_1209	DJI_0195	Berberis vulgaris L.	0	n	1	0	Berberidaceae Juss.
Iz_4_1209	DJI_0195	Betula pendula Roth.	0	n	1	1	Betulaceae Gray
Iz_4_1209	DJI_0195	Betula pendula Roth.	0	n	1	0	Poaceae Barnhart
Iz_4_1209	DJI_0195	Bromus inermis Leyss.	0		NA	NA	
Iz_4_1209	DJI_0195	Bromus squarrosus L.	0	a	0	0	Poaceae Barnhart
Iz_4_1209	DJI_0195	Bromus tectorum L.	0	a	0	0	Poaceae Barnhart
Iz_4_1209	DJI_0195	Calamagrostis epigejos (L.) Roth	10	n	1	0	Poaceae Barnhart
Iz_4_1209	DJI_0195	Carduus acanthoides L.	0	n	1	0	Asteraceae Bercht. & J.Presl
Iz_4_1209	DJI_0195	Carex sp.	0	n	1	0	Cyperaceae Juss.
Iz_4_1209	DJI_0195	Centaurea majorovii Dumbadze	0	n	1	0	Asteraceae Bercht. & J.Presl
Iz_4_1209	DJI_0195	Chamaecytisus ruthenicus (Fisch.ex Wol.) Klaskova	0	n	1	0	Fabaceae Lindl.
Iz_4_1209	DJI_0195	Chelidonium majus L.	0	n	1	0	Papaveraceae Juss.
Iz_4_1209	DJI_0195	Chenopodium betaceum Andrz.	0	n	1	0	Amaranthaceae Juss.
Iz_4_1209	DJI_0195	Chenopodium cf. album	0	n	1	0	Amaranthaceae Juss.
Iz_4_1209	DJI_0195	Chenopodium sp.	0		NA	0	Amaranthaceae Juss.
Iz_4_1209	DJI_0195	Chondrilla juncea L.	0	n	1	0	Asteraceae Bercht. & J.Presl
Iz_4_1209	DJI_0195	Cirsium vulgare (Savi) Ten.	0	n	1	0	Asteraceae Bercht. & J.Presl
Iz_4_1209	DJI_0195	Clinopodium acinos (L.) Kuntze	0	n	1	0	Lamiacea Lindl.
Iz_4_1209	DJI_0195	Convallaria majalis L.	0	n	1	0	Asparagaceae Juss.
Iz_4_1209	DJI_0195	Crataegus rhipidophylla Gand.	0	n	1	1	Rosaceae Juss.
Iz_4_1209	DJI_0195	Crepis foetida L.	0	n	1	0	Asteraceae Bercht. & J.Presl
Iz_4_1209	DJI_0195	Dactylis glomerata L.	0	n	1	0	Poaceae Barnhart
Iz_4_1209	DJI_0195	Dianthus campestris M.Bieb.	0	n	1	0	Caryophyllaceae Juss.
Iz_4_1209	DJI_0195	Digitaria sanguinalis (L.) Scop.	0	a	0	0	Poaceae Barnhart
Iz_4_1209	DJI_0195	Elymus repens (L.) Gould	0	n	1	0	Poaceae Barnhart
Iz_4_1209	DJI_0195	Epilobium tetragonum L.	0	n	1	0	Onagraceae Juss.
Iz_4_1209	DJI_0195	Eragrostis minor Host	0	a	0	0	Poaceae Barnhart
Iz_4_1209	DJI_0195	Erigeron canadensis L.	15	a	0	0	Asteraceae Bercht. & J.Presl
Iz_4_1209	DJI_0195	Euphorbia seguieriana Neck.	0	n	1	0	Euphorbiaceae Juss.
Iz_4_1209	DJI_0195	Fallopia dumetorum (L.) Holub	0	n	1	0	Polygonaceae Juss.
Iz_4_1209	DJI_0195	Festuca beckeri (Hack.) Trautv.	0	n	1	0	Poaceae Barnhart
Iz_4_1209	DJI_0195	Fragaria vesca L.	0	n	1	0	Rosaceae Juss.
Iz_4_1209	DJI_0195	Frangula alnus Mill.	0	n	1	1	Rhamnaceae Juss.
Iz_4_1209	DJI_0195	Fraxinus pennsylvanica Marshall	0	a	0	1	Oleaceae Hoffmanns. & Link
Iz_4_1209	DJI_0195	Galeopsis bifida Boenn.	0	n	1	0	Lamiacea Lindl.
Iz_4_1209	DJI_0195	Galium verum L.	0	n	1	0	Rubiaceae Juss.
Iz_4_1209	DJI_0195	Genista tinctoria L.	0	n	1	0	Fabaceae Lindl.
Iz_4_1209	DJI_0195	Geranium robertianum L.	0	n	1	0	Geraniaceae Juss.
Iz_4_1209	DJI_0195	Geranium sanquineum L.	0	n	1	0	Geraniaceae Juss.
Iz_4_1209	DJI_0195	Gypsophila paniculata L.	0	n	1	0	Caryophyllaceae Juss.
Iz_4_1209	DJI_0195	Helichrysum arenarium (L.) Moench	0	n	1	0	Asteraceae Bercht. & J.Presl
Iz_4_1209	DJI_0195	Herniaria polygama J.Gay	0	n	1	0	Caryophyllaceae Juss.
Iz_4_1209	DJI_0195	Hieracium robustum Fr.	0	n	1	0	Asteraceae Bercht. & J.Presl
Iz_4_1209	DJI_0195	Hieracium umbellatum L.	0	n	1	NA	Asteraceae Bercht. & J.Presl
Iz_4_1209	DJI_0195	Hylotelephium maximum (L.) Holub	0	n	1	0	Crassulaceae J.St.-Hil.
Iz_4_1209	DJI_0195	Jacobaea borysthenica (DC.) B.Nord. & Greuter	0	n	1	0	Asteraceae Bercht. & J.Presl
Iz_4_1209	DJI_0195	Jasione montana L.	0	n	1	0	Campanulaceae Juss.
Iz_4_1209	DJI_0195	Jurinea cyanoides (L.) Rchb.s.l.	0	n	1	0	Asteraceae Bercht. & J.Presl
Iz_4_1209	DJI_0195	Koeleria glauca (Spreng.) DC.	0	n	1	0	Poaceae Barnhart
Iz_4_1209	DJI_0195	Lactuca serriola L.	0	a	0	0	Asteraceae Bercht. & J.Presl
Iz_4_1209	DJI_0195	Linaria genistifolia (L.) Mill.	0	n	1	0	Plantaginaceae Juss.
Iz_4_1209	DJI_0195	Lomelosia argentea (L.) Greuter & Burdet	0	n	1	0	Caprifoliaceae Juss.
Iz_4_1209	DJI_0195	Melampyrum pratense L.	0	n	1	0	Orobanchaceae Vent.
Iz_4_1209	DJI_0195	Oenothera biennis L.	0	a	0	0	Onagraceae Juss.
Iz_4_1209	DJI_0195	Pentanema britannica (L.) D.Gut.Larr., Santos-Vicente, Anderb., E.Rico & M.M.Mart.Ort.	0	n	1	0	Asteraceae Bercht. & J.Presl
Iz_4_1209	DJI_0195	Peucedanum oreoselinum (L.) Moench	0	n	1	0	Apiaceae Lindl.
Iz_4_1209	DJI_0195	Peucedanum ruthenicum M.Bieb.	0	n	1	0	Apiaceae Lindl.
Iz_4_1209	DJI_0195	Pilosella echioides (Lumn.) F.W.Schultz & Sch.Bip.	0	n	1	0	Asteraceae Bercht. & J.Presl
Iz_4_1209	DJI_0195	Pilosella officinarum F. Schultz et Sch. Bip.	0	n	1	0	Asteraceae Bercht. & J.Presl
Iz_4_1209	DJI_0195	Pilosella sp.	0		NA	0	Asteraceae Bercht. & J.Presl
Iz_4_1209	DJI_0195	Pinus sylvestris L.	0	n	1	1	Pinaceae Spreng. ex F.Rudolphi
Iz_4_1209	DJI_0195	Poa annua L.	0	n	1	0	Poaceae Barnhart
Iz_4_1209	DJI_0195	Poa nemoralis L.	0	n	1	0	Poaceae Barnhart
Iz_4_1209	DJI_0195	Poa sp.	0		NA	0	Poaceae Barnhart

Iz_4_1209	DJI_0195	Polygonatum odoratum (Mill.) Druce	0	n	1	0	Asparagaceae Juss.
Iz_4_1209	DJI_0195	Polygonum arenarium Waldst. & Kit.	0	n	1	0	Polygonaceae Juss.
Iz_4_1209	DJI_0195	Populus nigra L.	0	n	1	1	Salicaceae Mirb.
Iz_4_1209	DJI_0195	Populus tremula L.	0	n	1	1	Salicaceae Mirb.
Iz_4_1209	DJI_0195	Populus Ã canescens (Aiton) Sm.	0	n	1	1	Salicaceae Mirb.
Iz_4_1209	DJI_0195	Potentilla argentea L.	0	n	1	0	Rosaceae Juss.
Iz_4_1209	DJI_0195	Psammophiliella muralis (L.) Ikonn.	0	n	1	0	Caryophyllaceae Juss.
Iz_4_1209	DJI_0195	Psephellus sumensis (Kalen.) Greuter	0	n	1	0	Asteraceae Bercht. & J.Presl
Iz_4_1209	DJI_0195	Pulsatilla pratensis (L.) Mill. s.l.	0	n	1	0	Ranunculaceae Juss.
Iz_4_1209	DJI_0195	Pyrus communis L.	0	a	0	1	Rosaceae Juss.
Iz_4_1209	DJI_0195	Quercus robur L.	0	n	1	1	Fagaceae Dumort.
Iz_4_1209	DJI_0195	Robinia pseudoacacia L.	0	a	0	1	Fabaceae Lindl.
Iz_4_1209	DJI_0195	Rosa sp.	0		NA	1	Rosaceae Juss.
Iz_4_1209	DJI_0195	Rubus idaeus L.	0	n	1	0	Rosaceae Juss.
Iz_4_1209	DJI_0195	Rumex acetosella L.	0	n	1	0	Polygonaceae Juss.
Iz_4_1209	DJI_0195	Rumex thyrsiflorus Fingerh.	0	n	1	0	Polygonaceae Juss.
Iz_4_1209	DJI_0195	Salix alba L.	0	n	1	1	Salicaceae Mirb.
Iz_4_1209	DJI_0195	Sambucus nigra L.	0	n	1	1	Viburnaceae Raf.
Iz_4_1209	DJI_0195	Secale sylvestre Host	0	n	1	0	Poaceae Barnhart
Iz_4_1209	DJI_0195	Sempervivum ruthenicum Schnittsp. & C.B. Lehm.	0	n	1	0	Crassulaceae J.St.-Hil.
Iz_4_1209	DJI_0195	Setaria viridis (L.) P. Beauv.	0	n	1	0	Poaceae Barnhart
Iz_4_1209	DJI_0195	Silene borysthenica (Gruner) Walters	0	n	1	0	Caryophyllaceae Juss.
Iz_4_1209	DJI_0195	Silene latifolia subsp. alba (Mill.) Greuter & Burdet	0	n	1	0	Caryophyllaceae Juss.
Iz_4_1209	DJI_0195	Solidago virgaurea L.	0	n	1	0	Asteraceae Bercht. & J.Presl
Iz_4_1209	DJI_0195	Sorbus aucuparia L.	0	n	1	1	Rosaceae Juss.
Iz_4_1209	DJI_0195	Stachys recta L.	0	n	1	0	Lamiacea Lindl.
Iz_4_1209	DJI_0195	Tanacetum vulgare L.	0	n	1	0	Asteraceae Bercht. & J.Presl
Iz_4_1209	DJI_0195	Thymus pallasianus Heinr.Braun	0	n	1	0	Lamiacea Lindl.
Iz_4_1209	DJI_0195	Tilia cordata Mill.	0	n	1	1	Malvaceae Juss.
Iz_4_1209	DJI_0195	Trifolium alpestre L.	0	n	1	0	Fabaceae Lindl.
Iz_4_1209	DJI_0195	Trifolium arvense L.	0	n	1	0	Fabaceae Lindl.
Iz_4_1209	DJI_0195	Ulmus glabra Huds.	0	n	1	1	Ulmaceae Mirb.
Iz_4_1209	DJI_0195	Verbascum densiflorum Bertol.	0	n	1	0	Scrophulariaceae Juss.
Iz_4_1209	DJI_0195	Veronica austriaca L.	0	n	1	0	Plantaginaceae Juss.
Iz_4_1209	DJI_0195	Veronica incana L.	0	n	1	0	Plantaginaceae Juss.
Iz_4_1209	DJI_0195	Veronica verna L.	0	n	1	0	Plantaginaceae Juss.
Iz_4_1209	DJI_0195	Vincetoxicum hirundinaria Medik	0	n	1	0	Apocynaceae Juss.
Iz_4_1209	DJI_0199	Acer tataricum L.	0	n	1	1	Sapindaceae Juss.
Iz_4_1209	DJI_0199	Achillea millefolium L. s.l.	0	n	1	0	Asteraceae Bercht. & J.Presl
Iz_4_1209	DJI_0199	Allium oleraceum L.	0	n	1	0	Amaryllidaceae J.St.-Hil.
Iz_4_1209	DJI_0199	Ambrosia artemisiifolia L.	0	a	0	0	Asteraceae Bercht. & J.Presl
Iz_4_1209	DJI_0199	Amorpha fruticosa L.	0	a	0	0	Fabaceae Lindl.
Iz_4_1209	DJI_0199	Anthericum ramosum L.	0	n	1	0	Asparagaceae Juss.
Iz_4_1209	DJI_0199	Artemisia marschalliana Spreng.	0	n	1	0	Asteraceae Bercht. & J.Presl
Iz_4_1209	DJI_0199	Asparagus officinalis L.	0	n	1	0	Asparagaceae Juss.
Iz_4_1209	DJI_0199	Astragalus cicer L.	0	n	1	0	Fabaceae Lindl.
Iz_4_1209	DJI_0199	Bassia laniflora (S.G.Gmel.) A.J.Scott	0	n	1	0	Amaranthaceae Juss.
Iz_4_1209	DJI_0199	Berberis vulgaris L.	0	n	1	0	Berberidaceae Juss.
Iz_4_1209	DJI_0199	Betula pendula Roth.	0	n	1	1	Betulaceae Gray
Iz_4_1209	DJI_0199	Betula pendula Roth.	0	n	1	0	Poaceae Barnhart
Iz_4_1209	DJI_0199	Bromus inermis Leyss.	0		NA	NA	
Iz_4_1209	DJI_0199	Bromus squarrosus L.	0	a	0	0	Poaceae Barnhart
Iz_4_1209	DJI_0199	Bromus tectorum L.	0	a	0	0	Poaceae Barnhart
Iz_4_1209	DJI_0199	Calamagrostis epigejos (L.) Roth	10	n	1	0	Poaceae Barnhart
Iz_4_1209	DJI_0199	Carduus acanthoides L.	0	n	1	0	Asteraceae Bercht. & J.Presl
Iz_4_1209	DJI_0199	Carex sp.	0	n	1	0	Cyperaceae Juss.
Iz_4_1209	DJI_0199	Centaurea majorovii Dumbadze	0	n	1	0	Asteraceae Bercht. & J.Presl
Iz_4_1209	DJI_0199	Chamaecytisus ruthenicus (Fisch.ex Wol.) Klaskova	0	n	1	0	Fabaceae Lindl.
Iz_4_1209	DJI_0199	Chelidonium majus L.	0	n	1	0	Papaveraceae Juss.
Iz_4_1209	DJI_0199	Chenopodium betaceum Andrz.	0	n	1	0	Amaranthaceae Juss.
Iz_4_1209	DJI_0199	Chenopodium cf. album	0	n	1	0	Amaranthaceae Juss.
Iz_4_1209	DJI_0199	Chenopodium sp.	0		NA	0	Amaranthaceae Juss.
Iz_4_1209	DJI_0199	Chondrilla juncea L.	0	n	1	0	Asteraceae Bercht. & J.Presl
Iz_4_1209	DJI_0199	Cirsium vulgare (Savi) Ten.	0	n	1	0	Asteraceae Bercht. & J.Presl
Iz_4_1209	DJI_0199	Clinopodium acinos (L.) Kuntze	0	n	1	0	Lamiacea Lindl.
Iz_4_1209	DJI_0199	Convallaria majalis L.	0	n	1	0	Asparagaceae Juss.
Iz_4_1209	DJI_0199	Crataegus rhipidophylla Gand.	0	n	1	1	Rosaceae Juss.
Iz_4_1209	DJI_0199	Crepis foetida L.	0	n	1	0	Asteraceae Bercht. & J.Presl
Iz_4_1209	DJI_0199	Dactylis glomerata L.	0	n	1	0	Poaceae Barnhart
Iz_4_1209	DJI_0199	Dianthus campestris M.Bieb.	0	n	1	0	Caryophyllaceae Juss.
Iz_4_1209	DJI_0199	Digitaria sanguinalis (L.) Scop.	0	a	0	0	Poaceae Barnhart
Iz_4_1209	DJI_0199	Elymus repens (L.) Gould	0	n	1	0	Poaceae Barnhart
Iz_4_1209	DJI_0199	Epilobium tetragonum L.	0	n	1	0	Onagraceae Juss.
Iz_4_1209	DJI_0199	Eragrostis minor Host	0	a	0	0	Poaceae Barnhart
Iz_4_1209	DJI_0199	Erigeron canadensis L.	0	a	0	0	Asteraceae Bercht. & J.Presl
Iz_4_1209	DJI_0199	Euphorbia seguieriana Neck.	0	n	1	0	Euphorbiaceae Juss.
Iz_4_1209	DJI_0199	Fallopia dumetorum (L.) Holub	0.2	n	1	0	Polygonaceae Juss.
Iz_4_1209	DJI_0199	Festuca beckeri (Hack.) Trautv.	0	n	1	0	Poaceae Barnhart
Iz_4_1209	DJI_0199	Fragaria vesca L.	0	n	1	0	Rosaceae Juss.
Iz_4_1209	DJI_0199	Frangula alnus Mill.	0	n	1	1	Rhamnaceae Juss.
Iz_4_1209	DJI_0199	Fraxinus pennsylvanica Marshall	0	a	0	1	Oleaceae Hoffmanns. & Link
Iz_4_1209	DJI_0199	Galeopsis bifida Boenn.	0	n	1	0	Lamiacea Lindl.
Iz_4_1209	DJI_0199	Galium verum L.	0	n	1	0	Rubiaceae Juss.
Iz_4_1209	DJI_0199	Genista tinctoria L.	0	n	1	0	Fabaceae Lindl.
Iz_4_1209	DJI_0199	Geranium robertianum L.	0	n	1	0	Geraniaceae Juss.
Iz_4_1209	DJI_0199	Geranium sanquineum L.	0	n	1	0	Geraniaceae Juss.
Iz_4_1209	DJI_0199	Gypsophila paniculata L.	0	n	1	0	Caryophyllaceae Juss.
Iz_4_1209	DJI_0199	Helichrysum arenarium (L.) Moench	0	n	1	0	Asteraceae Bercht. & J.Presl
Iz_4_1209	DJI_0199	Herniaria polygama J.Gay	0	n	1	0	Caryophyllaceae Juss.
Iz_4_1209	DJI_0199	Hieracium robustum Fr.	0	n	1	0	Asteraceae Bercht. & J.Presl
Iz_4_1209	DJI_0199	Hieracium umbellatum L.	0	n	1	NA	Asteraceae Bercht. & J.Presl
Iz_4_1209	DJI_0199	Hylotelephium maximum (L.) Holub	0	n	1	0	Crassulaceae J.St.-Hil.
Iz_4_1209	DJI_0199	Jacobaea borysthenica (DC.) B.Nord. & Greuter	0	n	1	0	Asteraceae Bercht. & J.Presl
Iz_4_1209	DJI_0199	Jasione montana L.	0	n	1	0	Campanulaceae Juss.
Iz_4_1209	DJI_0199	Jurinea cyanoides (L.) Rchb.s.l.	0	n	1	0	Asteraceae Bercht. & J.Presl
Iz_4_1209	DJI_0199	Koeleria glauca (Spreng.) DC.	0	n	1	0	Poaceae Barnhart
Iz_4_1209	DJI_0199	Lactuca serriola L.	0	a	0	0	Asteraceae Bercht. & J.Presl
Iz_4_1209	DJI_0199	Linaria genistifolia (L.) Mill.	0	n	1	0	Plantaginaceae Juss.

Iz_4_1209	DJI_0199	Lomelosia argentea (L.) Greuter & Burdet	0	n	1	0	Caprifoliaceae Juss.
Iz_4_1209	DJI_0199	Melampyrum pratense L.	0	n	1	0	Orobanchaceae Vent.
Iz_4_1209	DJI_0199	Oenothera biennis L.	0	a	0	0	Onagraceae Juss.
Iz_4_1209	DJI_0199	Pentanema britannica (L.) D.Gut.Larr., Santos-Vicente, Anderb., E.Rico & M.M.Mart.Ort.	0	n	1	0	Asteraceae Bercht. & J.Presl
Iz_4_1209	DJI_0199	Peucedanum oreoselinum (L.) Moench	0	n	1	0	Apiaceae Lindl.
Iz_4_1209	DJI_0199	Peucedanum ruthenicum M.Bieb.	0	n	1	0	Apiaceae Lindl.
Iz_4_1209	DJI_0199	Pilosella echioides (Lumn.) F.W.Schultz & Sch.Bip.	0	n	1	0	Asteraceae Bercht. & J.Presl
Iz_4_1209	DJI_0199	Pilosella officinarum F. Schultz et Sch. Bip.	0	n	1	0	Asteraceae Bercht. & J.Presl
Iz_4_1209	DJI_0199	Pilosella sp.	0		NA	0	Asteraceae Bercht. & J.Presl
Iz_4_1209	DJI_0199	Pinus sylvestris L.	0	n	1	1	Pinaceae Spreng. ex F.Rudolphi
Iz_4_1209	DJI_0199	Poa annua L.	0	n	1	0	Poaceae Barnhart
Iz_4_1209	DJI_0199	Poa nemoralis L.	0	n	1	0	Poaceae Barnhart
Iz_4_1209	DJI_0199	Poa sp.	0		NA	0	Poaceae Barnhart
Iz_4_1209	DJI_0199	Polygonatum odoratum (Mill.) Druce	0	n	1	0	Asparagaceae Juss.
Iz_4_1209	DJI_0199	Polygonum arenarium Waldst. & Kit.	0	n	1	0	Polygonaceae Juss.
Iz_4_1209	DJI_0199	Populus nigra L.	0	n	1	1	Salicaceae Mirb.
Iz_4_1209	DJI_0199	Populus tremula L.	0	n	1	1	Salicaceae Mirb.
Iz_4_1209	DJI_0199	Populus Ã canescens (Aiton) Sm.	0	n	1	1	Salicaceae Mirb.
Iz_4_1209	DJI_0199	Potentilla argentea L.	0	n	1	0	Rosaceae Juss.
Iz_4_1209	DJI_0199	Psammophiliella muralis (L.) Ikonn.	0	n	1	0	Caryophyllaceae Juss.
Iz_4_1209	DJI_0199	Psephellus sumensis (Kalen.) Greuter	0	n	1	0	Asteraceae Bercht. & J.Presl
Iz_4_1209	DJI_0199	Pulsatilla pratensis (L.) Mill. s.l.	0	n	1	0	Ranunculaceae Juss.
Iz_4_1209	DJI_0199	Pyrus communis L.	0	a	0	1	Rosaceae Juss.
Iz_4_1209	DJI_0199	Quercus robur L.	0	n	1	1	Fagaceae Dumort.
Iz_4_1209	DJI_0199	Robinia pseudoacacia L.	0	a	0	1	Fabaceae Lindl.
Iz_4_1209	DJI_0199	Rosa sp.	0		NA	1	Rosaceae Juss.
Iz_4_1209	DJI_0199	Rubus idaeus L.	0	n	1	0	Rosaceae Juss.
Iz_4_1209	DJI_0199	Rumex acetosella L.	0	n	1	0	Polygonaceae Juss.
Iz_4_1209	DJI_0199	Rumex thyrsiflorus Fingerh.	0	n	1	0	Polygonaceae Juss.
Iz_4_1209	DJI_0199	Salix alba L.	0	n	1	1	Salicaceae Mirb.
Iz_4_1209	DJI_0199	Sambucus nigra L.	0	n	1	1	Viburnaceae Raf.
Iz_4_1209	DJI_0199	Secale sylvestre Host	0	n	1	0	Poaceae Barnhart
Iz_4_1209	DJI_0199	Sempervivum ruthenicum Schnittsp. & C.B. Lehm.	0	n	1	0	Crassulaceae J.St.-Hil.
Iz_4_1209	DJI_0199	Setaria viridis (L.) P. Beauv.	0	n	1	0	Poaceae Barnhart
Iz_4_1209	DJI_0199	Silene borysthenica (Gruner) Walters	0	n	1	0	Caryophyllaceae Juss.
Iz_4_1209	DJI_0199	Silene latifolia subsp. alba (Mill.) Greuter & Burdet	0	n	1	0	Caryophyllaceae Juss.
Iz_4_1209	DJI_0199	Solidago virgaurea L.	0	n	1	0	Asteraceae Bercht. & J.Presl
Iz_4_1209	DJI_0199	Sorbus aucuparia L.	0	n	1	1	Rosaceae Juss.
Iz_4_1209	DJI_0199	Stachys recta L.	0	n	1	0	Lamiacea Lindl.
Iz_4_1209	DJI_0199	Tanacetum vulgare L.	0	n	1	0	Asteraceae Bercht. & J.Presl
Iz_4_1209	DJI_0199	Thymus pallasianus Heinr.Braun	0	n	1	0	Lamiacea Lindl.
Iz_4_1209	DJI_0199	Tilia cordata Mill.	0	n	1	1	Malvaceae Juss.
Iz_4_1209	DJI_0199	Trifolium alpestre L.	0	n	1	0	Fabaceae Lindl.
Iz_4_1209	DJI_0199	Trifolium arvense L.	0	n	1	0	Fabaceae Lindl.
Iz_4_1209	DJI_0199	Ulmus glabra Huds.	0	n	1	1	Ulmaceae Mirb.
Iz_4_1209	DJI_0199	Verbascum densiflorum Bertol.	0	n	1	0	Scrophulariaceae Juss.
Iz_4_1209	DJI_0199	Veronica austriaca L.	0	n	1	0	Plantaginaceae Juss.
Iz_4_1209	DJI_0199	Veronica incana L.	0	n	1	0	Plantaginaceae Juss.
Iz_4_1209	DJI_0199	Veronica verna L.	0	n	1	0	Plantaginaceae Juss.
Iz_4_1209	DJI_0199	Vincetoxicum hirundinaria Medik	0	n	1	0	Apocynaceae Juss.
Iz_4_1209	DJI_0203	Acer tataricum L.	0	n	1	1	Sapindaceae Juss.
Iz_4_1209	DJI_0203	Achillea millefolium L. s.l.	0	n	1	0	Asteraceae Bercht. & J.Presl
Iz_4_1209	DJI_0203	Allium oleraceum L.	0	n	1	0	Amaryllidaceae J.St.-Hil.
Iz_4_1209	DJI_0203	Ambrosia artemisiifolia L.	0	a	0	0	Asteraceae Bercht. & J.Presl
Iz_4_1209	DJI_0203	Amorpha fruticosa L.	0	a	0	0	Fabaceae Lindl.
Iz_4_1209	DJI_0203	Anthericum ramosum L.	0	n	1	0	Asparagaceae Juss.
Iz_4_1209	DJI_0203	Artemisia marschalliana Spreng.	0	n	1	0	Asteraceae Bercht. & J.Presl
Iz_4_1209	DJI_0203	Asparagus officinalis L.	0	n	1	0	Asparagaceae Juss.
Iz_4_1209	DJI_0203	Astragalus cicer L.	0	n	1	0	Fabaceae Lindl.
Iz_4_1209	DJI_0203	Bassia laniflora (S.G.Gmel.) A.J.Scott	0	n	1	0	Amaranthaceae Juss.
Iz_4_1209	DJI_0203	Berberis vulgaris L.	0	n	1	0	Berberidaceae Juss.
Iz_4_1209	DJI_0203	Betula pendula Roth.	0	n	1	1	Betulaceae Gray
Iz_4_1209	DJI_0203	Betula pendula Roth.	0	n	1	0	Poaceae Barnhart
Iz_4_1209	DJI_0203	Bromus inermis Leyss.	0		NA	NA	
Iz_4_1209	DJI_0203	Bromus squarrosus L.	0	a	0	0	Poaceae Barnhart
Iz_4_1209	DJI_0203	Bromus tectorum L.	0	a	0	0	Poaceae Barnhart
Iz_4_1209	DJI_0203	Calamagrostis epigejos (L.) Roth	15	n	1	0	Poaceae Barnhart
Iz_4_1209	DJI_0203	Carduus acanthoides L.	0	n	1	0	Asteraceae Bercht. & J.Presl
Iz_4_1209	DJI_0203	Carex sp.	0	n	1	0	Cyperaceae Juss.
Iz_4_1209	DJI_0203	Centaurea majorovii Dumbadze	0	n	1	0	Asteraceae Bercht. & J.Presl
Iz_4_1209	DJI_0203	Chamaecytisus ruthenicus (Fisch.ex Wol.) Klaskova	0	n	1	0	Fabaceae Lindl.
Iz_4_1209	DJI_0203	Chelidonium majus L.	0	n	1	0	Papaveraceae Juss.
Iz_4_1209	DJI_0203	Chenopodium betaceum Andrz.	0	n	1	0	Amaranthaceae Juss.
Iz_4_1209	DJI_0203	Chenopodium cf. album	0	n	1	0	Amaranthaceae Juss.
Iz_4_1209	DJI_0203	Chenopodium sp.	0		NA	0	Amaranthaceae Juss.
Iz_4_1209	DJI_0203	Chondrilla juncea L.	0	n	1	0	Asteraceae Bercht. & J.Presl
Iz_4_1209	DJI_0203	Cirsium vulgare (Savi) Ten.	0	n	1	0	Asteraceae Bercht. & J.Presl
Iz_4_1209	DJI_0203	Clinopodium acinos (L.) Kuntze	0	n	1	0	Lamiacea Lindl.
Iz_4_1209	DJI_0203	Convallaria majalis L.	0	n	1	0	Asparagaceae Juss.
Iz_4_1209	DJI_0203	Crataegus rhipidophylla Gand.	0	n	1	1	Rosaceae Juss.
Iz_4_1209	DJI_0203	Crepis foetida L.	0	n	1	0	Asteraceae Bercht. & J.Presl
Iz_4_1209	DJI_0203	Dactylis glomerata L.	0	n	1	0	Poaceae Barnhart
Iz_4_1209	DJI_0203	Dianthus campestris M.Bieb.	0	n	1	0	Caryophyllaceae Juss.
Iz_4_1209	DJI_0203	Digitaria sanguinalis (L.) Scop.	0	a	0	0	Poaceae Barnhart
Iz_4_1209	DJI_0203	Elymus repens (L.) Gould	0	n	1	0	Poaceae Barnhart
Iz_4_1209	DJI_0203	Epilobium tetragonum L.	0	n	1	0	Onagraceae Juss.
Iz_4_1209	DJI_0203	Eragrostis minor Host	0	a	0	0	Poaceae Barnhart
Iz_4_1209	DJI_0203	Erigeron canadensis L.	0.2	a	0	0	Asteraceae Bercht. & J.Presl
Iz_4_1209	DJI_0203	Euphorbia seguieriana Neck.	0	n	1	0	Euphorbiaceae Juss.
Iz_4_1209	DJI_0203	Fallopia dumetorum (L.) Holub	0	n	1	0	Polygonaceae Juss.
Iz_4_1209	DJI_0203	Festuca beckeri (Hack.) Trautv.	0	n	1	0	Poaceae Barnhart
Iz_4_1209	DJI_0203	Fragaria vesca L.	0	n	1	0	Rosaceae Juss.
Iz_4_1209	DJI_0203	Frangula alnus Mill.	0	n	1	1	Rhamnaceae Juss.
Iz_4_1209	DJI_0203	Fraxinus pennsylvanica Marshall	0	a	0	1	Oleaceae Hoffmanns. & Link
Iz_4_1209	DJI_0203	Galeopsis bifida Boenn.	0	n	1	0	Lamiacea Lindl.
Iz_4_1209	DJI_0203	Galium verum L.	0	n	1	0	Rubiaceae Juss.
Iz_4_1209	DJI_0203	Genista tinctoria L.	0	n	1	0	Fabaceae Lindl.
Iz_4_1209	DJI_0203	Geranium robertianum L.	0	n	1	0	Geraniaceae Juss.

Iz_4_1209	DJI_0203	Geranium sanquineum L.	0	n	1	0	Geraniaceae Juss.
Iz_4_1209	DJI_0203	Gypsophila paniculata L.	0	n	1	0	Caryophyllaceae Juss.
Iz_4_1209	DJI_0203	Helichrysum arenarium (L.) Moench	0	n	1	0	Asteraceae Bercht. & J.Presl
Iz_4_1209	DJI_0203	Herniaria polygama J.Gay	0	n	1	0	Caryophyllaceae Juss.
Iz_4_1209	DJI_0203	Hieracium robustum Fr.	0	n	1	0	Asteraceae Bercht. & J.Presl
Iz_4_1209	DJI_0203	Hieracium umbellatum L.	0	n	1	NA	Asteraceae Bercht. & J.Presl
Iz_4_1209	DJI_0203	Hylotelephium maximum (L.) Holub	0	n	1	0	Crassulaceae J.St.-Hil.
Iz_4_1209	DJI_0203	Jacobaea borysthenica (DC.) B.Nord. & Greuter	0	n	1	0	Asteraceae Bercht. & J.Presl
Iz_4_1209	DJI_0203	Jasione montana L.	0	n	1	0	Campanulaceae Juss.
Iz_4_1209	DJI_0203	Jurinea cyanoides (L.) Rchb.s.l.	0	n	1	0	Asteraceae Bercht. & J.Presl
Iz_4_1209	DJI_0203	Koeleria glauca (Spreng.) DC.	0	n	1	0	Poaceae Barnhart
Iz_4_1209	DJI_0203	Lactuca serriola L.	0	a	0	0	Asteraceae Bercht. & J.Presl
Iz_4_1209	DJI_0203	Linaria genistifolia (L.) Mill.	0	n	1	0	Plantaginaceae Juss.
Iz_4_1209	DJI_0203	Lomelosia argentea (L.) Greuter & Burdet	0	n	1	0	Caprifoliaceae Juss.
Iz_4_1209	DJI_0203	Melampyrum pratense L.	0	n	1	0	Orobanchaceae Vent.
Iz_4_1209	DJI_0203	Oenothera biennis L.	0	a	0	0	Onagraceae Juss.
Iz_4_1209	DJI_0203	Pentanema britannica (L.) D.Gut.Larr., Santos-Vicente, Anderb., E.Rico & M.M.Mart.Ort.	0	n	1	0	Asteraceae Bercht. & J.Presl
Iz_4_1209	DJI_0203	Peucedanum oreoselinum (L.) Moench	0.5	n	1	0	Apiaceae Lindl.
Iz_4_1209	DJI_0203	Peucedanum ruthenicum M.Bieb.	0	n	1	0	Apiaceae Lindl.
Iz_4_1209	DJI_0203	Pilosella echioides (Lumn.) F.W.Schultz & Sch.Bip.	0	n	1	0	Asteraceae Bercht. & J.Presl
Iz_4_1209	DJI_0203	Pilosella officinarum F. Schultz et Sch. Bip.	0	n	1	0	Asteraceae Bercht. & J.Presl
Iz_4_1209	DJI_0203	Pilosella sp.	0		NA	0	Asteraceae Bercht. & J.Presl
Iz_4_1209	DJI_0203	Pinus sylvestris L.	0	n	1	1	Pinaceae Spreng. ex F.Rudolphi
Iz_4_1209	DJI_0203	Poa annua L.	0	n	1	0	Poaceae Barnhart
Iz_4_1209	DJI_0203	Poa nemoralis L.	0	n	1	0	Poaceae Barnhart
Iz_4_1209	DJI_0203	Poa sp.	0		NA	0	Poaceae Barnhart
Iz_4_1209	DJI_0203	Polygonatum odoratum (Mill.) Druce	0	n	1	0	Asparagaceae Juss.
Iz_4_1209	DJI_0203	Polygonum arenarium Waldst. & Kit.	0	n	1	0	Polygonaceae Juss.
Iz_4_1209	DJI_0203	Populus nigra L.	0	n	1	1	Salicaceae Mirb.
Iz_4_1209	DJI_0203	Populus tremula L.	0	n	1	1	Salicaceae Mirb.
Iz_4_1209	DJI_0203	Populus Ã canescens (Aiton) Sm.	0	n	1	1	Salicaceae Mirb.
Iz_4_1209	DJI_0203	Potentilla argentea L.	0	n	1	0	Rosaceae Juss.
Iz_4_1209	DJI_0203	Psammophiliella muralis (L.) Ikonn.	0	n	1	0	Caryophyllaceae Juss.
Iz_4_1209	DJI_0203	Psephellus sumensis (Kalen.) Greuter	0	n	1	0	Asteraceae Bercht. & J.Presl
Iz_4_1209	DJI_0203	Pulsatilla pratensis (L.) Mill. s.l.	0	n	1	0	Ranunculaceae Juss.
Iz_4_1209	DJI_0203	Pyrus communis L.	0	a	0	1	Rosaceae Juss.
Iz_4_1209	DJI_0203	Quercus robur L.	0	n	1	1	Fagaceae Dumort.
Iz_4_1209	DJI_0203	Robinia pseudoacacia L.	0	a	0	1	Fabaceae Lindl.
Iz_4_1209	DJI_0203	Rosa sp.	0		NA	1	Rosaceae Juss.
Iz_4_1209	DJI_0203	Rubus idaeus L.	0	n	1	0	Rosaceae Juss.
Iz_4_1209	DJI_0203	Rumex acetosella L.	0	n	1	0	Polygonaceae Juss.
Iz_4_1209	DJI_0203	Rumex thyrsiflorus Fingerh.	0	n	1	0	Polygonaceae Juss.
Iz_4_1209	DJI_0203	Salix alba L.	0	n	1	1	Salicaceae Mirb.
Iz_4_1209	DJI_0203	Sambucus nigra L.	0	n	1	1	Viburnaceae Raf.
Iz_4_1209	DJI_0203	Secale sylvestre Host	0	n	1	0	Poaceae Barnhart
Iz_4_1209	DJI_0203	Sempervivum ruthenicum Schnittsp. & C.B. Lehm.	0	n	1	0	Crassulaceae J.St.-Hil.
Iz_4_1209	DJI_0203	Setaria viridis (L.) P. Beauv.	0	n	1	0	Poaceae Barnhart
Iz_4_1209	DJI_0203	Silene borysthenica (Gruner) Walters	0	n	1	0	Caryophyllaceae Juss.
Iz_4_1209	DJI_0203	Silene latifolia subsp. alba (Mill.) Greuter & Burdet	0	n	1	0	Caryophyllaceae Juss.
Iz_4_1209	DJI_0203	Solidago virgaurea L.	0	n	1	0	Asteraceae Bercht. & J.Presl
Iz_4_1209	DJI_0203	Sorbus aucuparia L.	0	n	1	1	Rosaceae Juss.
Iz_4_1209	DJI_0203	Stachys recta L.	0	n	1	0	Lamiacea Lindl.
Iz_4_1209	DJI_0203	Tanacetum vulgare L.	0	n	1	0	Asteraceae Bercht. & J.Presl
Iz_4_1209	DJI_0203	Thymus pallasianus Heinr.Braun	0	n	1	0	Lamiacea Lindl.
Iz_4_1209	DJI_0203	Tilia cordata Mill.	0	n	1	1	Malvaceae Juss.
Iz_4_1209	DJI_0203	Trifolium alpestre L.	0	n	1	0	Fabaceae Lindl.
Iz_4_1209	DJI_0203	Trifolium arvense L.	0	n	1	0	Fabaceae Lindl.
Iz_4_1209	DJI_0203	Ulmus glabra Huds.	0	n	1	1	Ulmaceae Mirb.
Iz_4_1209	DJI_0203	Verbascum densiflorum Bertol.	0	n	1	0	Scrophulariaceae Juss.
Iz_4_1209	DJI_0203	Veronica austriaca L.	0	n	1	0	Plantaginaceae Juss.
Iz_4_1209	DJI_0203	Veronica incana L.	0	n	1	0	Plantaginaceae Juss.
Iz_4_1209	DJI_0203	Veronica verna L.	0	n	1	0	Plantaginaceae Juss.
Iz_4_1209	DJI_0203	Vincetoxicum hirundinaria Medik	0	n	1	0	Apocynaceae Juss.
Iz_4_1209	DJI_0207	Acer tataricum L.	0	n	1	1	Sapindaceae Juss.
Iz_4_1209	DJI_0207	Achillea millefolium L. s.l.	0	n	1	0	Asteraceae Bercht. & J.Presl
Iz_4_1209	DJI_0207	Allium oleraceum L.	0	n	1	0	Amaryllidaceae J.St.-Hil.
Iz_4_1209	DJI_0207	Ambrosia artemisiifolia L.	0	a	0	0	Asteraceae Bercht. & J.Presl
Iz_4_1209	DJI_0207	Amorpha fruticosa L.	0	a	0	0	Fabaceae Lindl.
Iz_4_1209	DJI_0207	Anthericum ramosum L.	0	n	1	0	Asparagaceae Juss.
Iz_4_1209	DJI_0207	Artemisia marschalliana Spreng.	0	n	1	0	Asteraceae Bercht. & J.Presl
Iz_4_1209	DJI_0207	Asparagus officinalis L.	0	n	1	0	Asparagaceae Juss.
Iz_4_1209	DJI_0207	Astragalus cicer L.	0	n	1	0	Fabaceae Lindl.
Iz_4_1209	DJI_0207	Bassia laniflora (S.G.Gmel.) A.J.Scott	0	n	1	0	Amaranthaceae Juss.
Iz_4_1209	DJI_0207	Berberis vulgaris L.	0	n	1	0	Berberidaceae Juss.
Iz_4_1209	DJI_0207	Betula pendula Roth.	0	n	1	1	Betulaceae Gray
Iz_4_1209	DJI_0207	Betula pendula Roth.	0	n	1	0	Poaceae Barnhart
Iz_4_1209	DJI_0207	Bromus inermis Leyss.	0		NA	NA	
Iz_4_1209	DJI_0207	Bromus squarrosus L.	0	a	0	0	Poaceae Barnhart
Iz_4_1209	DJI_0207	Bromus tectorum L.	0	a	0	0	Poaceae Barnhart
Iz_4_1209	DJI_0207	Calamagrostis epigejos (L.) Roth	7	n	1	0	Poaceae Barnhart
Iz_4_1209	DJI_0207	Carduus acanthoides L.	0	n	1	0	Asteraceae Bercht. & J.Presl
Iz_4_1209	DJI_0207	Carex sp.	0	n	1	0	Cyperaceae Juss.
Iz_4_1209	DJI_0207	Centaurea majorovii Dumbadze	0	n	1	0	Asteraceae Bercht. & J.Presl
Iz_4_1209	DJI_0207	Chamaecytisus ruthenicus (Fisch.ex Wol.) Klaskova	0	n	1	0	Fabaceae Lindl.
Iz_4_1209	DJI_0207	Chelidonium majus L.	0	n	1	0	Papaveraceae Juss.
Iz_4_1209	DJI_0207	Chenopodium betaceum Andrz.	0	n	1	0	Amaranthaceae Juss.
Iz_4_1209	DJI_0207	Chenopodium cf. album	0	n	1	0	Amaranthaceae Juss.
Iz_4_1209	DJI_0207	Chenopodium sp.	0		NA	0	Amaranthaceae Juss.
Iz_4_1209	DJI_0207	Chondrilla juncea L.	0	n	1	0	Asteraceae Bercht. & J.Presl
Iz_4_1209	DJI_0207	Cirsium vulgare (Savi) Ten.	0	n	1	0	Asteraceae Bercht. & J.Presl
Iz_4_1209	DJI_0207	Clinopodium acinos (L.) Kuntze	0	n	1	0	Lamiacea Lindl.
Iz_4_1209	DJI_0207	Convallaria majalis L.	0	n	1	0	Asparagaceae Juss.
Iz_4_1209	DJI_0207	Crataegus rhipidophylla Gand.	0	n	1	1	Rosaceae Juss.
Iz_4_1209	DJI_0207	Crepis foetida L.	0	n	1	0	Asteraceae Bercht. & J.Presl
Iz_4_1209	DJI_0207	Dactylis glomerata L.	0	n	1	0	Poaceae Barnhart
Iz_4_1209	DJI_0207	Dianthus campestris M.Bieb.	0	n	1	0	Caryophyllaceae Juss.
Iz_4_1209	DJI_0207	Digitaria sanguinalis (L.) Scop.	0	a	0	0	Poaceae Barnhart
Iz_4_1209	DJI_0207	Elymus repens (L.) Gould	0	n	1	0	Poaceae Barnhart

Iz_4_1209	DJI_0207	Epilobium tetragonum L.	0	n	1	0	Onagraceae Juss.
Iz_4_1209	DJI_0207	Eragrostis minor Host	0	a	0	0	Poaceae Barnhart
Iz_4_1209	DJI_0207	Erigeron canadensis L.	3	a	0	0	Asteraceae Bercht. & J.Presl
Iz_4_1209	DJI_0207	Euphorbia seguieriana Neck.	0	n	1	0	Euphorbiaceae Juss.
Iz_4_1209	DJI_0207	Fallopia dumetorum (L.) Holub	0	n	1	0	Polygonaceae Juss.
Iz_4_1209	DJI_0207	Festuca beckeri (Hack.) Trautv.	0	n	1	0	Poaceae Barnhart
Iz_4_1209	DJI_0207	Fragaria vesca L.	0	n	1	0	Rosaceae Juss.
Iz_4_1209	DJI_0207	Frangula alnus Mill.	0	n	1	1	Rhamnaceae Juss.
Iz_4_1209	DJI_0207	Fraxinus pennsylvanica Marshall	0	a	0	1	Oleaceae Hoffmanns. & Link
Iz_4_1209	DJI_0207	Galeopsis bifida Boenn.	0	n	1	0	Lamiacea Lindl.
Iz_4_1209	DJI_0207	Galium verum L.	0	n	1	0	Rubiaceae Juss.
Iz_4_1209	DJI_0207	Genista tinctoria L.	0	n	1	0	Fabaceae Lindl.
Iz_4_1209	DJI_0207	Geranium robertianum L.	0	n	1	0	Geraniaceae Juss.
Iz_4_1209	DJI_0207	Geranium sanquineum L.	0	n	1	0	Geraniaceae Juss.
Iz_4_1209	DJI_0207	Gypsophila paniculata L.	0	n	1	0	Caryophyllaceae Juss.
Iz_4_1209	DJI_0207	Helichrysum arenarium (L.) Moench	0	n	1	0	Asteraceae Bercht. & J.Presl
Iz_4_1209	DJI_0207	Herniaria polygama J.Gay	0	n	1	0	Caryophyllaceae Juss.
Iz_4_1209	DJI_0207	Hieracium robustum Fr.	0	n	1	0	Asteraceae Bercht. & J.Presl
Iz_4_1209	DJI_0207	Hieracium umbellatum L.	0	n	1	NA	Asteraceae Bercht. & J.Presl
Iz_4_1209	DJI_0207	Hylotelephium maximum (L.) Holub	0	n	1	0	Crassulaceae J.St.-Hil.
Iz_4_1209	DJI_0207	Jacobaea borysthenica (DC.) B.Nord. & Greuter	0	n	1	0	Asteraceae Bercht. & J.Presl
Iz_4_1209	DJI_0207	Jasione montana L.	0	n	1	0	Campanulaceae Juss.
Iz_4_1209	DJI_0207	Jurinea cyanoides (L.) Rchb.s.l.	0	n	1	0	Asteraceae Bercht. & J.Presl
Iz_4_1209	DJI_0207	Koeleria glauca (Spreng.) DC.	0	n	1	0	Poaceae Barnhart
Iz_4_1209	DJI_0207	Lactuca serriola L.	0	a	0	0	Asteraceae Bercht. & J.Presl
Iz_4_1209	DJI_0207	Linaria genistifolia (L.) Mill.	0	n	1	0	Plantaginaceae Juss.
Iz_4_1209	DJI_0207	Lomelosia argentea (L.) Greuter & Burdet	0	n	1	0	Caprifoliaceae Juss.
Iz_4_1209	DJI_0207	Melampyrum pratense L.	0	n	1	0	Orobanchaceae Vent.
Iz_4_1209	DJI_0207	Oenothera biennis L.	0	a	0	0	Onagraceae Juss.
Iz_4_1209	DJI_0207	Pentanema britannica (L.) D.Gut.Larr., Santos-Vicente, Anderb., E.Rico & M.M.Mart.Ort.	0	n	1	0	Asteraceae Bercht. & J.Presl
Iz_4_1209	DJI_0207	Peucedanum oreoselinum (L.) Moench	0	n	1	0	Apiaceae Lindl.
Iz_4_1209	DJI_0207	Peucedanum ruthenicum M.Bieb.	0	n	1	0	Apiaceae Lindl.
Iz_4_1209	DJI_0207	Pilosella echioides (Lumn.) F.W.Schultz & Sch.Bip.	0	n	1	0	Asteraceae Bercht. & J.Presl
Iz_4_1209	DJI_0207	Pilosella officinarum F. Schultz et Sch. Bip.	0	n	1	0	Asteraceae Bercht. & J.Presl
Iz_4_1209	DJI_0207	Pilosella sp.	0		NA	0	Asteraceae Bercht. & J.Presl
Iz_4_1209	DJI_0207	Pinus sylvestris L.	0	n	1	1	Pinaceae Spreng. ex F.Rudolphi
Iz_4_1209	DJI_0207	Poa annua L.	0	n	1	0	Poaceae Barnhart
Iz_4_1209	DJI_0207	Poa nemoralis L.	0	n	1	0	Poaceae Barnhart
Iz_4_1209	DJI_0207	Poa sp.	0		NA	0	Poaceae Barnhart
Iz_4_1209	DJI_0207	Polygonatum odoratum (Mill.) Druce	0	n	1	0	Asparagaceae Juss.
Iz_4_1209	DJI_0207	Polygonum arenarium Waldst. & Kit.	0	n	1	0	Polygonaceae Juss.
Iz_4_1209	DJI_0207	Populus nigra L.	0	n	1	1	Salicaceae Mirb.
Iz_4_1209	DJI_0207	Populus tremula L.	0	n	1	1	Salicaceae Mirb.
Iz_4_1209	DJI_0207	Populus Ã canescens (Aiton) Sm.	0	n	1	1	Salicaceae Mirb.
Iz_4_1209	DJI_0207	Potentilla argentea L.	0	n	1	0	Rosaceae Juss.
Iz_4_1209	DJI_0207	Psammophiliella muralis (L.) Ikonn.	0	n	1	0	Caryophyllaceae Juss.
Iz_4_1209	DJI_0207	Psephellus sumensis (Kalen.) Greuter	0	n	1	0	Asteraceae Bercht. & J.Presl
Iz_4_1209	DJI_0207	Pulsatilla pratensis (L.) Mill. s.l.	0	n	1	0	Ranunculaceae Juss.
Iz_4_1209	DJI_0207	Pyrus communis L.	0	a	0	1	Rosaceae Juss.
Iz_4_1209	DJI_0207	Quercus robur L.	0	n	1	1	Fagaceae Dumort.
Iz_4_1209	DJI_0207	Robinia pseudoacacia L.	0	a	0	1	Fabaceae Lindl.
Iz_4_1209	DJI_0207	Rosa sp.	0		NA	1	Rosaceae Juss.
Iz_4_1209	DJI_0207	Rubus idaeus L.	0	n	1	0	Rosaceae Juss.
Iz_4_1209	DJI_0207	Rumex acetosella L.	0	n	1	0	Polygonaceae Juss.
Iz_4_1209	DJI_0207	Rumex thyrsiflorus Fingerh.	0	n	1	0	Polygonaceae Juss.
Iz_4_1209	DJI_0207	Salix alba L.	0	n	1	1	Salicaceae Mirb.
Iz_4_1209	DJI_0207	Sambucus nigra L.	0	n	1	1	Viburnaceae Raf.
Iz_4_1209	DJI_0207	Secale sylvestre Host	0	n	1	0	Poaceae Barnhart
Iz_4_1209	DJI_0207	Sempervivum ruthenicum Schnittsp. & C.B. Lehm.	0	n	1	0	Crassulaceae J.St.-Hil.
Iz_4_1209	DJI_0207	Setaria viridis (L.) P. Beauv.	0	n	1	0	Poaceae Barnhart
Iz_4_1209	DJI_0207	Silene borysthenica (Gruner) Walters	0	n	1	0	Caryophyllaceae Juss.
Iz_4_1209	DJI_0207	Silene latifolia subsp. alba (Mill.) Greuter & Burdet	0	n	1	0	Caryophyllaceae Juss.
Iz_4_1209	DJI_0207	Solidago virgaurea L.	0	n	1	0	Asteraceae Bercht. & J.Presl
Iz_4_1209	DJI_0207	Sorbus aucuparia L.	0	n	1	1	Rosaceae Juss.
Iz_4_1209	DJI_0207	Stachys recta L.	0	n	1	0	Lamiacea Lindl.
Iz_4_1209	DJI_0207	Tanacetum vulgare L.	0	n	1	0	Asteraceae Bercht. & J.Presl
Iz_4_1209	DJI_0207	Thymus pallasianus Heinr.Braun	0	n	1	0	Lamiacea Lindl.
Iz_4_1209	DJI_0207	Tilia cordata Mill.	0	n	1	1	Malvaceae Juss.
Iz_4_1209	DJI_0207	Trifolium alpestre L.	0	n	1	0	Fabaceae Lindl.
Iz_4_1209	DJI_0207	Trifolium arvense L.	0	n	1	0	Fabaceae Lindl.
Iz_4_1209	DJI_0207	Ulmus glabra Huds.	0	n	1	1	Ulmaceae Mirb.
Iz_4_1209	DJI_0207	Verbascum densiflorum Bertol.	0	n	1	0	Scrophulariaceae Juss.
Iz_4_1209	DJI_0207	Veronica austriaca L.	0	n	1	0	Plantaginaceae Juss.
Iz_4_1209	DJI_0207	Veronica incana L.	0	n	1	0	Plantaginaceae Juss.
Iz_4_1209	DJI_0207	Veronica verna L.	0	n	1	0	Plantaginaceae Juss.
Iz_4_1209	DJI_0207	Vincetoxicum hirundinaria Medik	0	n	1	0	Apocynaceae Juss.
Iz_4_1209	DJI_0210	Acer tataricum L.	0	n	1	1	Sapindaceae Juss.
Iz_4_1209	DJI_0210	Achillea millefolium L. s.l.	0	n	1	0	Asteraceae Bercht. & J.Presl
Iz_4_1209	DJI_0210	Allium oleraceum L.	0	n	1	0	Amaryllidaceae J.St.-Hil.
Iz_4_1209	DJI_0210	Ambrosia artemisiifolia L.	0	a	0	0	Asteraceae Bercht. & J.Presl
Iz_4_1209	DJI_0210	Amorpha fruticosa L.	0	a	0	0	Fabaceae Lindl.
Iz_4_1209	DJI_0210	Anthericum ramosum L.	0	n	1	0	Asparagaceae Juss.
Iz_4_1209	DJI_0210	Artemisia marschalliana Spreng.	0	n	1	0	Asteraceae Bercht. & J.Presl
Iz_4_1209	DJI_0210	Asparagus officinalis L.	0	n	1	0	Asparagaceae Juss.
Iz_4_1209	DJI_0210	Astragalus cicer L.	0	n	1	0	Fabaceae Lindl.
Iz_4_1209	DJI_0210	Bassia laniflora (S.G.Gmel.) A.J.Scott	0	n	1	0	Amaranthaceae Juss.
Iz_4_1209	DJI_0210	Berberis vulgaris L.	0	n	1	0	Berberidaceae Juss.
Iz_4_1209	DJI_0210	Betula pendula Roth.	0	n	1	1	Betulaceae Gray
Iz_4_1209	DJI_0210	Betula pendula Roth.	0	n	1	0	Poaceae Barnhart
Iz_4_1209	DJI_0210	Bromus inermis Leyss.	0		NA	NA	
Iz_4_1209	DJI_0210	Bromus squarrosus L.	0	a	0	0	Poaceae Barnhart
Iz_4_1209	DJI_0210	Bromus tectorum L.	0	a	0	0	Poaceae Barnhart
Iz_4_1209	DJI_0210	Calamagrostis epigejos (L.) Roth	5	n	1	0	Poaceae Barnhart
Iz_4_1209	DJI_0210	Carduus acanthoides L.	0	n	1	0	Asteraceae Bercht. & J.Presl
Iz_4_1209	DJI_0210	Carex sp.	0	n	1	0	Cyperaceae Juss.
Iz_4_1209	DJI_0210	Centaurea majorovii Dumbadze	0	n	1	0	Asteraceae Bercht. & J.Presl
Iz_4_1209	DJI_0210	Chamaecytisus ruthenicus (Fisch.ex Wol.) Klaskova	0	n	1	0	Fabaceae Lindl.
Iz_4_1209	DJI_0210	Chelidonium majus L.	0	n	1	0	Papaveraceae Juss.

Iz_4_1209	DJI_0210	Chenopodium betaceum Andrz.	0	n	1	0	Amaranthaceae Juss.
Iz_4_1209	DJI_0210	Chenopodium cf. album	0	n	1	0	Amaranthaceae Juss.
Iz_4_1209	DJI_0210	Chenopodium sp.	0		NA	0	Amaranthaceae Juss.
Iz_4_1209	DJI_0210	Chondrilla juncea L.	0	n	1	0	Asteraceae Bercht. & J.Presl
Iz_4_1209	DJI_0210	Cirsium vulgare (Savi) Ten.	0	n	1	0	Asteraceae Bercht. & J.Presl
Iz_4_1209	DJI_0210	Clinopodium acinos (L.) Kuntze	0	n	1	0	Lamiacea Lindl.
Iz_4_1209	DJI_0210	Convallaria majalis L.	0	n	1	0	Asparagaceae Juss.
Iz_4_1209	DJI_0210	Crataegus rhipidophylla Gand.	0	n	1	1	Rosaceae Juss.
Iz_4_1209	DJI_0210	Crepis foetida L.	0	n	1	0	Asteraceae Bercht. & J.Presl
Iz_4_1209	DJI_0210	Dactylis glomerata L.	0	n	1	0	Poaceae Barnhart
Iz_4_1209	DJI_0210	Dianthus campestris M.Bieb.	0	n	1	0	Caryophyllaceae Juss.
Iz_4_1209	DJI_0210	Digitaria sanguinalis (L.) Scop.	0	a	0	0	Poaceae Barnhart
Iz_4_1209	DJI_0210	Elymus repens (L.) Gould	0	n	1	0	Poaceae Barnhart
Iz_4_1209	DJI_0210	Epilobium tetragonum L.	0	n	1	0	Onagraceae Juss.
Iz_4_1209	DJI_0210	Eragrostis minor Host	0	a	0	0	Poaceae Barnhart
Iz_4_1209	DJI_0210	Erigeron canadensis L.	6	a	0	0	Asteraceae Bercht. & J.Presl
Iz_4_1209	DJI_0210	Euphorbia seguieriana Neck.	0	n	1	0	Euphorbiaceae Juss.
Iz_4_1209	DJI_0210	Fallopia dumetorum (L.) Holub	0	n	1	0	Polygonaceae Juss.
Iz_4_1209	DJI_0210	Festuca beckeri (Hack.) Trautv.	0	n	1	0	Poaceae Barnhart
Iz_4_1209	DJI_0210	Fragaria vesca L.	0	n	1	0	Rosaceae Juss.
Iz_4_1209	DJI_0210	Frangula alnus Mill.	0	n	1	1	Rhamnaceae Juss.
Iz_4_1209	DJI_0210	Fraxinus pennsylvanica Marshall	0	a	0	1	Oleaceae Hoffmanns. & Link
Iz_4_1209	DJI_0210	Galeopsis bifida Boenn.	0	n	1	0	Lamiacea Lindl.
Iz_4_1209	DJI_0210	Galium verum L.	0	n	1	0	Rubiaceae Juss.
Iz_4_1209	DJI_0210	Genista tinctoria L.	0	n	1	0	Fabaceae Lindl.
Iz_4_1209	DJI_0210	Geranium robertianum L.	0	n	1	0	Geraniaceae Juss.
Iz_4_1209	DJI_0210	Geranium sanquineum L.	0	n	1	0	Geraniaceae Juss.
Iz_4_1209	DJI_0210	Gypsophila paniculata L.	0	n	1	0	Caryophyllaceae Juss.
Iz_4_1209	DJI_0210	Helichrysum arenarium (L.) Moench	0	n	1	0	Asteraceae Bercht. & J.Presl
Iz_4_1209	DJI_0210	Herniaria polygama J.Gay	0	n	1	0	Caryophyllaceae Juss.
Iz_4_1209	DJI_0210	Hieracium robustum Fr.	0	n	1	0	Asteraceae Bercht. & J.Presl
Iz_4_1209	DJI_0210	Hieracium umbellatum L.	0	n	1	NA	Asteraceae Bercht. & J.Presl
Iz_4_1209	DJI_0210	Hylotelephium maximum (L.) Holub	0	n	1	0	Crassulaceae J.St.-Hil.
Iz_4_1209	DJI_0210	Jacobaea borysthenica (DC.) B.Nord. & Greuter	0	n	1	0	Asteraceae Bercht. & J.Presl
Iz_4_1209	DJI_0210	Jasione montana L.	0	n	1	0	Campanulaceae Juss.
Iz_4_1209	DJI_0210	Jurinea cyanoides (L.) Rchb.s.l.	0	n	1	0	Asteraceae Bercht. & J.Presl
Iz_4_1209	DJI_0210	Koeleria glauca (Spreng.) DC.	0	n	1	0	Poaceae Barnhart
Iz_4_1209	DJI_0210	Lactuca serriola L.	0	a	0	0	Asteraceae Bercht. & J.Presl
Iz_4_1209	DJI_0210	Linaria genistifolia (L.) Mill.	0	n	1	0	Plantaginaceae Juss.
Iz_4_1209	DJI_0210	Lomelosia argentea (L.) Greuter & Burdet	0	n	1	0	Caprifoliaceae Juss.
Iz_4_1209	DJI_0210	Melampyrum pratense L.	0	n	1	0	Orobanchaceae Vent.
Iz_4_1209	DJI_0210	Oenothera biennis L.	0	a	0	0	Onagraceae Juss.
Iz_4_1209	DJI_0210	Pentanema britannica (L.) D.Gut.Larr., Santos-Vicente, Anderb., E.Rico & M.M.Mart.Ort.	0	n	1	0	Asteraceae Bercht. & J.Presl
Iz_4_1209	DJI_0210	Peucedanum oreoselinum (L.) Moench	0	n	1	0	Apiaceae Lindl.
Iz_4_1209	DJI_0210	Peucedanum ruthenicum M.Bieb.	0	n	1	0	Apiaceae Lindl.
Iz_4_1209	DJI_0210	Pilosella echioides (Lumn.) F.W.Schultz & Sch.Bip.	0	n	1	0	Asteraceae Bercht. & J.Presl
Iz_4_1209	DJI_0210	Pilosella officinarum F. Schultz et Sch. Bip.	0	n	1	0	Asteraceae Bercht. & J.Presl
Iz_4_1209	DJI_0210	Pilosella sp.	0		NA	0	Asteraceae Bercht. & J.Presl
Iz_4_1209	DJI_0210	Pinus sylvestris L.	0	n	1	1	Pinaceae Spreng. ex F.Rudolphi
Iz_4_1209	DJI_0210	Poa annua L.	0	n	1	0	Poaceae Barnhart
Iz_4_1209	DJI_0210	Poa nemoralis L.	0	n	1	0	Poaceae Barnhart
Iz_4_1209	DJI_0210	Poa sp.	0		NA	0	Poaceae Barnhart
Iz_4_1209	DJI_0210	Polygonatum odoratum (Mill.) Druce	0	n	1	0	Asparagaceae Juss.
Iz_4_1209	DJI_0210	Polygonum arenarium Waldst. & Kit.	0	n	1	0	Polygonaceae Juss.
Iz_4_1209	DJI_0210	Populus nigra L.	0	n	1	1	Salicaceae Mirb.
Iz_4_1209	DJI_0210	Populus tremula L.	0	n	1	1	Salicaceae Mirb.
Iz_4_1209	DJI_0210	Populus Ã canescens (Aiton) Sm.	0	n	1	1	Salicaceae Mirb.
Iz_4_1209	DJI_0210	Potentilla argentea L.	0	n	1	0	Rosaceae Juss.
Iz_4_1209	DJI_0210	Psammophiliella muralis (L.) Ikonn.	0	n	1	0	Caryophyllaceae Juss.
Iz_4_1209	DJI_0210	Psephellus sumensis (Kalen.) Greuter	0	n	1	0	Asteraceae Bercht. & J.Presl
Iz_4_1209	DJI_0210	Pulsatilla pratensis (L.) Mill. s.l.	0	n	1	0	Ranunculaceae Juss.
Iz_4_1209	DJI_0210	Pyrus communis L.	0	a	0	1	Rosaceae Juss.
Iz_4_1209	DJI_0210	Quercus robur L.	0	n	1	1	Fagaceae Dumort.
Iz_4_1209	DJI_0210	Robinia pseudoacacia L.	0	a	0	1	Fabaceae Lindl.
Iz_4_1209	DJI_0210	Rosa sp.	0		NA	1	Rosaceae Juss.
Iz_4_1209	DJI_0210	Rubus idaeus L.	0	n	1	0	Rosaceae Juss.
Iz_4_1209	DJI_0210	Rumex acetosella L.	0	n	1	0	Polygonaceae Juss.
Iz_4_1209	DJI_0210	Rumex thyrsiflorus Fingerh.	0	n	1	0	Polygonaceae Juss.
Iz_4_1209	DJI_0210	Salix alba L.	0	n	1	1	Salicaceae Mirb.
Iz_4_1209	DJI_0210	Sambucus nigra L.	0	n	1	1	Viburnaceae Raf.
Iz_4_1209	DJI_0210	Secale sylvestre Host	0	n	1	0	Poaceae Barnhart
Iz_4_1209	DJI_0210	Sempervivum ruthenicum Schnittsp. & C.B. Lehm.	0	n	1	0	Crassulaceae J.St.-Hil.
Iz_4_1209	DJI_0210	Setaria viridis (L.) P. Beauv.	0	n	1	0	Poaceae Barnhart
Iz_4_1209	DJI_0210	Silene borysthenica (Gruner) Walters	0	n	1	0	Caryophyllaceae Juss.
Iz_4_1209	DJI_0210	Silene latifolia subsp. alba (Mill.) Greuter & Burdet	0	n	1	0	Caryophyllaceae Juss.
Iz_4_1209	DJI_0210	Solidago virgaurea L.	0	n	1	0	Asteraceae Bercht. & J.Presl
Iz_4_1209	DJI_0210	Sorbus aucuparia L.	0	n	1	1	Rosaceae Juss.
Iz_4_1209	DJI_0210	Stachys recta L.	0	n	1	0	Lamiacea Lindl.
Iz_4_1209	DJI_0210	Tanacetum vulgare L.	0	n	1	0	Asteraceae Bercht. & J.Presl
Iz_4_1209	DJI_0210	Thymus pallasianus Heinr.Braun	0	n	1	0	Lamiacea Lindl.
Iz_4_1209	DJI_0210	Tilia cordata Mill.	0	n	1	1	Malvaceae Juss.
Iz_4_1209	DJI_0210	Trifolium alpestre L.	0	n	1	0	Fabaceae Lindl.
Iz_4_1209	DJI_0210	Trifolium arvense L.	0	n	1	0	Fabaceae Lindl.
Iz_4_1209	DJI_0210	Ulmus glabra Huds.	0	n	1	1	Ulmaceae Mirb.
Iz_4_1209	DJI_0210	Verbascum densiflorum Bertol.	0	n	1	0	Scrophulariaceae Juss.
Iz_4_1209	DJI_0210	Veronica austriaca L.	0	n	1	0	Plantaginaceae Juss.
Iz_4_1209	DJI_0210	Veronica incana L.	0	n	1	0	Plantaginaceae Juss.
Iz_4_1209	DJI_0210	Veronica verna L.	0	n	1	0	Plantaginaceae Juss.
Iz_4_1209	DJI_0210	Vincetoxicum hirundinaria Medik	0	n	1	0	Apocynaceae Juss.
Iz_5_1009	DJI_0671	Acer tataricum L.	0	n	1	1	Sapindaceae Juss.
Iz_5_1009	DJI_0671	Achillea millefolium L. s.l.	0	n	1	0	Asteraceae Bercht. & J.Presl
Iz_5_1009	DJI_0671	Allium oleraceum L.	0	n	1	0	Amaryllidaceae J.St.-Hil.
Iz_5_1009	DJI_0671	Ambrosia artemisiifolia L.	0	a	0	0	Asteraceae Bercht. & J.Presl
Iz_5_1009	DJI_0671	Amorpha fruticosa L.	0	a	0	0	Fabaceae Lindl.
Iz_5_1009	DJI_0671	Anthericum ramosum L.	0	n	1	0	Asparagaceae Juss.
Iz_5_1009	DJI_0671	Artemisia marschalliana Spreng.	0	n	1	0	Asteraceae Bercht. & J.Presl
Iz_5_1009	DJI_0671	Asparagus officinalis L.	0	n	1	0	Asparagaceae Juss.
Iz_5_1009	DJI_0671	Astragalus cicer L.	0	n	1	0	Fabaceae Lindl.

Iz_5_1009	DJI_0671	Bassia laniflora (S.G.Gmel.) A.J.Scott	0	n	1	0	Amaranthaceae Juss.
Iz_5_1009	DJI_0671	Berberis vulgaris L.	0	n	1	0	Berberidaceae Juss.
Iz_5_1009	DJI_0671	Betula pendula Roth.	0	n	1	1	Betulaceae Gray
Iz_5_1009	DJI_0671	Betula pendula Roth.	0	n	1	0	Poaceae Barnhart
Iz_5_1009	DJI_0671	Bromus inermis Leyss.	0		NA	NA	
Iz_5_1009	DJI_0671	Bromus squarrosus L.	0	a	0	0	Poaceae Barnhart
Iz_5_1009	DJI_0671	Bromus tectorum L.	0	a	0	0	Poaceae Barnhart
Iz_5_1009	DJI_0671	Calamagrostis epigejos (L.) Roth	1	n	1	0	Poaceae Barnhart
Iz_5_1009	DJI_0671	Carduus acanthoides L.	0	n	1	0	Asteraceae Bercht. & J.Presl
Iz_5_1009	DJI_0671	Carex sp.	0	n	1	0	Cyperaceae Juss.
Iz_5_1009	DJI_0671	Centaurea majorovii Dumbadze	0	n	1	0	Asteraceae Bercht. & J.Presl
Iz_5_1009	DJI_0671	Chamaecytisus ruthenicus (Fisch.ex Wol.) Klaskova	0	n	1	0	Fabaceae Lindl.
Iz_5_1009	DJI_0671	Chelidonium majus L.	0	n	1	0	Papaveraceae Juss.
Iz_5_1009	DJI_0671	Chenopodium betaceum Andrz.	0	n	1	0	Amaranthaceae Juss.
Iz_5_1009	DJI_0671	Chenopodium cf. album	0	n	1	0	Amaranthaceae Juss.
Iz_5_1009	DJI_0671	Chenopodium sp.	0		NA	0	Amaranthaceae Juss.
Iz_5_1009	DJI_0671	Chondrilla juncea L.	0	n	1	0	Asteraceae Bercht. & J.Presl
Iz_5_1009	DJI_0671	Cirsium vulgare (Savi) Ten.	0	n	1	0	Asteraceae Bercht. & J.Presl
Iz_5_1009	DJI_0671	Clinopodium acinos (L.) Kuntze	0	n	1	0	Lamiacea Lindl.
Iz_5_1009	DJI_0671	Convallaria majalis L.	0	n	1	0	Asparagaceae Juss.
Iz_5_1009	DJI_0671	Crataegus rhipidophylla Gand.	0	n	1	1	Rosaceae Juss.
Iz_5_1009	DJI_0671	Crepis foetida L.	0	n	1	0	Asteraceae Bercht. & J.Presl
Iz_5_1009	DJI_0671	Dactylis glomerata L.	0	n	1	0	Poaceae Barnhart
Iz_5_1009	DJI_0671	Dianthus campestris M.Bieb.	0	n	1	0	Caryophyllaceae Juss.
Iz_5_1009	DJI_0671	Digitaria sanguinalis (L.) Scop.	0	a	0	0	Poaceae Barnhart
Iz_5_1009	DJI_0671	Elymus repens (L.) Gould	0	n	1	0	Poaceae Barnhart
Iz_5_1009	DJI_0671	Epilobium tetragonum L.	0	n	1	0	Onagraceae Juss.
Iz_5_1009	DJI_0671	Eragrostis minor Host	0	a	0	0	Poaceae Barnhart
Iz_5_1009	DJI_0671	Erigeron canadensis L.	1	a	0	0	Asteraceae Bercht. & J.Presl
Iz_5_1009	DJI_0671	Euphorbia seguieriana Neck.	0	n	1	0	Euphorbiaceae Juss.
Iz_5_1009	DJI_0671	Fallopia dumetorum (L.) Holub	0	n	1	0	Polygonaceae Juss.
Iz_5_1009	DJI_0671	Festuca beckeri (Hack.) Trautv.	0	n	1	0	Poaceae Barnhart
Iz_5_1009	DJI_0671	Fragaria vesca L.	0	n	1	0	Rosaceae Juss.
Iz_5_1009	DJI_0671	Frangula alnus Mill.	0	n	1	1	Rhamnaceae Juss.
Iz_5_1009	DJI_0671	Fraxinus pennsylvanica Marshall	0	a	0	1	Oleaceae Hoffmanns. & Link
Iz_5_1009	DJI_0671	Galeopsis bifida Boenn.	0	n	1	0	Lamiacea Lindl.
Iz_5_1009	DJI_0671	Galium verum L.	0	n	1	0	Rubiaceae Juss.
Iz_5_1009	DJI_0671	Genista tinctoria L.	0	n	1	0	Fabaceae Lindl.
Iz_5_1009	DJI_0671	Geranium robertianum L.	0	n	1	0	Geraniaceae Juss.
Iz_5_1009	DJI_0671	Geranium sanquineum L.	0	n	1	0	Geraniaceae Juss.
Iz_5_1009	DJI_0671	Gypsophila paniculata L.	0	n	1	0	Caryophyllaceae Juss.
Iz_5_1009	DJI_0671	Helichrysum arenarium (L.) Moench	0	n	1	0	Asteraceae Bercht. & J.Presl
Iz_5_1009	DJI_0671	Herniaria polygama J.Gay	0	n	1	0	Caryophyllaceae Juss.
Iz_5_1009	DJI_0671	Hieracium robustum Fr.	0	n	1	0	Asteraceae Bercht. & J.Presl
Iz_5_1009	DJI_0671	Hieracium umbellatum L.	0	n	1	NA	Asteraceae Bercht. & J.Presl
Iz_5_1009	DJI_0671	Hylotelephium maximum (L.) Holub	0	n	1	0	Crassulaceae J.St.-Hil.
Iz_5_1009	DJI_0671	Jacobaea borysthenica (DC.) B.Nord. & Greuter	0	n	1	0	Asteraceae Bercht. & J.Presl
Iz_5_1009	DJI_0671	Jasione montana L.	0	n	1	0	Campanulaceae Juss.
Iz_5_1009	DJI_0671	Jurinea cyanoides (L.) Rchb.s.l.	0	n	1	0	Asteraceae Bercht. & J.Presl
Iz_5_1009	DJI_0671	Koeleria glauca (Spreng.) DC.	0	n	1	0	Poaceae Barnhart
Iz_5_1009	DJI_0671	Lactuca serriola L.	0	a	0	0	Asteraceae Bercht. & J.Presl
Iz_5_1009	DJI_0671	Linaria genistifolia (L.) Mill.	0	n	1	0	Plantaginaceae Juss.
Iz_5_1009	DJI_0671	Lomelosia argentea (L.) Greuter & Burdet	0	n	1	0	Caprifoliaceae Juss.
Iz_5_1009	DJI_0671	Melampyrum pratense L.	0	n	1	0	Orobanchaceae Vent.
Iz_5_1009	DJI_0671	Oenothera biennis L.	0	a	0	0	Onagraceae Juss.
Iz_5_1009	DJI_0671	Pentanema britannica (L.) D.Gut.Larr., Santos-Vicente, Anderb., E.Rico & M.M.Mart.Ort.	0	n	1	0	Asteraceae Bercht. & J.Presl
Iz_5_1009	DJI_0671	Peucedanum oreoselinum (L.) Moench	0	n	1	0	Apiaceae Lindl.
Iz_5_1009	DJI_0671	Peucedanum ruthenicum M.Bieb.	0	n	1	0	Apiaceae Lindl.
Iz_5_1009	DJI_0671	Pilosella echioides (Lumn.) F.W.Schultz & Sch.Bip.	0	n	1	0	Asteraceae Bercht. & J.Presl
Iz_5_1009	DJI_0671	Pilosella officinarum F. Schultz et Sch. Bip.	0	n	1	0	Asteraceae Bercht. & J.Presl
Iz_5_1009	DJI_0671	Pilosella sp.	0		NA	0	Asteraceae Bercht. & J.Presl
Iz_5_1009	DJI_0671	Pinus sylvestris L.	0	n	1	1	Pinaceae Spreng. ex F.Rudolphi
Iz_5_1009	DJI_0671	Poa annua L.	0	n	1	0	Poaceae Barnhart
Iz_5_1009	DJI_0671	Poa nemoralis L.	0	n	1	0	Poaceae Barnhart
Iz_5_1009	DJI_0671	Poa sp.	0		NA	0	Poaceae Barnhart
Iz_5_1009	DJI_0671	Polygonatum odoratum (Mill.) Druce	0	n	1	0	Asparagaceae Juss.
Iz_5_1009	DJI_0671	Polygonum arenarium Waldst. & Kit.	0	n	1	0	Polygonaceae Juss.
Iz_5_1009	DJI_0671	Populus nigra L.	0	n	1	1	Salicaceae Mirb.
Iz_5_1009	DJI_0671	Populus sp.	0	n	1	1	Salicaceae Mirb.
Iz_5_1009	DJI_0671	Populus tremula L.	0	n	1	1	Salicaceae Mirb.
Iz_5_1009	DJI_0671	Populus Ã canescens (Aiton) Sm.	0	n	1	1	Salicaceae Mirb.
Iz_5_1009	DJI_0671	Potentilla argentea L.	0	n	1	0	Rosaceae Juss.
Iz_5_1009	DJI_0671	Psammophiliella muralis (L.) Ikonn.	0	n	1	0	Caryophyllaceae Juss.
Iz_5_1009	DJI_0671	Psephellus sumensis (Kalen.) Greuter	0	n	1	0	Asteraceae Bercht. & J.Presl
Iz_5_1009	DJI_0671	Pulsatilla pratensis (L.) Mill. s.l.	0	n	1	0	Ranunculaceae Juss.
Iz_5_1009	DJI_0671	Pyrus communis L.	0	a	0	1	Rosaceae Juss.
Iz_5_1009	DJI_0671	Quercus robur L.	0	n	1	1	Fagaceae Dumort.
Iz_5_1009	DJI_0671	Robinia pseudoacacia L.	0	a	0	1	Fabaceae Lindl.
Iz_5_1009	DJI_0671	Rosa sp.	0		NA	1	Rosaceae Juss.
Iz_5_1009	DJI_0671	Rubus idaeus L.	0	n	1	0	Rosaceae Juss.
Iz_5_1009	DJI_0671	Rumex acetosella L.	0	n	1	0	Polygonaceae Juss.
Iz_5_1009	DJI_0671	Rumex thyrsiflorus Fingerh.	0	n	1	0	Polygonaceae Juss.
Iz_5_1009	DJI_0671	Salix alba L.	0	n	1	1	Salicaceae Mirb.
Iz_5_1009	DJI_0671	Sambucus nigra L.	0	n	1	1	Viburnaceae Raf.
Iz_5_1009	DJI_0671	Secale sylvestre Host	0	n	1	0	Poaceae Barnhart
Iz_5_1009	DJI_0671	Sempervivum ruthenicum Schnittsp. & C.B. Lehm.	0	n	1	0	Crassulaceae J.St.-Hil.
Iz_5_1009	DJI_0671	Setaria viridis (L.) P. Beauv.	0	n	1	0	Poaceae Barnhart
Iz_5_1009	DJI_0671	Silene borysthenica (Gruner) Walters	0	n	1	0	Caryophyllaceae Juss.
Iz_5_1009	DJI_0671	Silene latifolia subsp. alba (Mill.) Greuter & Burdet	0	n	1	0	Caryophyllaceae Juss.
Iz_5_1009	DJI_0671	Solidago virgaurea L.	5	n	1	0	Asteraceae Bercht. & J.Presl
Iz_5_1009	DJI_0671	Solidago Ã niederederi (S. canadensis Ã S. virgaurea)	0	hybrid	0	0	Asteraceae Bercht. & J.Presl
Iz_5_1009	DJI_0671	Sorbus aucuparia L.	0	n	1	1	Rosaceae Juss.
Iz_5_1009	DJI_0671	Stachys recta L.	0	n	1	0	Lamiacea Lindl.
Iz_5_1009	DJI_0671	Tanacetum vulgare L.	0	n	1	0	Asteraceae Bercht. & J.Presl
Iz_5_1009	DJI_0671	Thymus pallasianus Heinr.Braun	0	n	1	0	Lamiacea Lindl.
Iz_5_1009	DJI_0671	Tilia cordata Mill.	0	n	1	1	Malvaceae Juss.
Iz_5_1009	DJI_0671	Trifolium alpestre L.	0	n	1	0	Fabaceae Lindl.
Iz_5_1009	DJI_0671	Trifolium arvense L.	0	n	1	0	Fabaceae Lindl.

Iz_5_1009	DJI_0671	Ulmus glabra Huds.	0	n	1	1	Ulmaceae Mirb.
Iz_5_1009	DJI_0671	Verbascum densiflorum Bertol.	0	n	1	0	Scrophulariaceae Juss.
Iz_5_1009	DJI_0671	Veronica austriaca L.	0	n	1	0	Plantaginaceae Juss.
Iz_5_1009	DJI_0671	Veronica incana L.	0	n	1	0	Plantaginaceae Juss.
Iz_5_1009	DJI_0671	Veronica verna L.	0	n	1	0	Plantaginaceae Juss.
Iz_5_1009	DJI_0671	Vincetoxicum hirundinaria Medik	0	n	1	0	Apocynaceae Juss.
Iz_5_1009	DJI_0672	Acer tataricum L.	0	n	1	1	Sapindaceae Juss.
Iz_5_1009	DJI_0672	Achillea millefolium L. s.l.	0	n	1	0	Asteraceae Bercht. & J.Presl
Iz_5_1009	DJI_0672	Allium oleraceum L.	0	n	1	0	Amaryllidaceae J.St.-Hil.
Iz_5_1009	DJI_0672	Ambrosia artemisiifolia L.	0	a	0	0	Asteraceae Bercht. & J.Presl
Iz_5_1009	DJI_0672	Amorpha fruticosa L.	0	a	0	0	Fabaceae Lindl.
Iz_5_1009	DJI_0672	Anthericum ramosum L.	0	n	1	0	Asparagaceae Juss.
Iz_5_1009	DJI_0672	Artemisia marschalliana Spreng.	0	n	1	0	Asteraceae Bercht. & J.Presl
Iz_5_1009	DJI_0672	Asparagus officinalis L.	0	n	1	0	Asparagaceae Juss.
Iz_5_1009	DJI_0672	Astragalus cicer L.	0	n	1	0	Fabaceae Lindl.
Iz_5_1009	DJI_0672	Bassia laniflora (S.G.Gmel.) A.J.Scott	0	n	1	0	Amaranthaceae Juss.
Iz_5_1009	DJI_0672	Berberis vulgaris L.	0	n	1	0	Berberidaceae Juss.
Iz_5_1009	DJI_0672	Betula pendula Roth.	0	n	1	1	Betulaceae Gray
Iz_5_1009	DJI_0672	Betula pendula Roth.	0	n	1	0	Poaceae Barnhart
Iz_5_1009	DJI_0672	Bromus inermis Leyss.	0		NA	NA	
Iz_5_1009	DJI_0672	Bromus squarrosus L.	0	a	0	0	Poaceae Barnhart
Iz_5_1009	DJI_0672	Bromus tectorum L.	0	a	0	0	Poaceae Barnhart
Iz_5_1009	DJI_0672	Calamagrostis epigejos (L.) Roth	0.2	n	1	0	Poaceae Barnhart
Iz_5_1009	DJI_0672	Carduus acanthoides L.	0	n	1	0	Asteraceae Bercht. & J.Presl
Iz_5_1009	DJI_0672	Carex sp.	0	n	1	0	Cyperaceae Juss.
Iz_5_1009	DJI_0672	Centaurea majorovii Dumbadze	0	n	1	0	Asteraceae Bercht. & J.Presl
Iz_5_1009	DJI_0672	Chamaecytisus ruthenicus (Fisch.ex Wol.) Klaskova	0	n	1	0	Fabaceae Lindl.
Iz_5_1009	DJI_0672	Chelidonium majus L.	0	n	1	0	Papaveraceae Juss.
Iz_5_1009	DJI_0672	Chenopodium betaceum Andrz.	0	n	1	0	Amaranthaceae Juss.
Iz_5_1009	DJI_0672	Chenopodium cf. album	0	n	1	0	Amaranthaceae Juss.
Iz_5_1009	DJI_0672	Chenopodium sp.	0		NA	0	Amaranthaceae Juss.
Iz_5_1009	DJI_0672	Chondrilla juncea L.	0	n	1	0	Asteraceae Bercht. & J.Presl
Iz_5_1009	DJI_0672	Cirsium vulgare (Savi) Ten.	0	n	1	0	Asteraceae Bercht. & J.Presl
Iz_5_1009	DJI_0672	Clinopodium acinos (L.) Kuntze	0	n	1	0	Lamiacea Lindl.
Iz_5_1009	DJI_0672	Convallaria majalis L.	0	n	1	0	Asparagaceae Juss.
Iz_5_1009	DJI_0672	Crataegus rhipidophylla Gand.	0	n	1	1	Rosaceae Juss.
Iz_5_1009	DJI_0672	Crepis foetida L.	0	n	1	0	Asteraceae Bercht. & J.Presl
Iz_5_1009	DJI_0672	Dactylis glomerata L.	0	n	1	0	Poaceae Barnhart
Iz_5_1009	DJI_0672	Dianthus campestris M.Bieb.	0	n	1	0	Caryophyllaceae Juss.
Iz_5_1009	DJI_0672	Digitaria sanguinalis (L.) Scop.	0	a	0	0	Poaceae Barnhart
Iz_5_1009	DJI_0672	Elymus repens (L.) Gould	0	n	1	0	Poaceae Barnhart
Iz_5_1009	DJI_0672	Epilobium tetragonum L.	0	n	1	0	Onagraceae Juss.
Iz_5_1009	DJI_0672	Eragrostis minor Host	0	a	0	0	Poaceae Barnhart
Iz_5_1009	DJI_0672	Erigeron canadensis L.	4	a	0	0	Asteraceae Bercht. & J.Presl
Iz_5_1009	DJI_0672	Euphorbia seguieriana Neck.	0	n	1	0	Euphorbiaceae Juss.
Iz_5_1009	DJI_0672	Fallopia dumetorum (L.) Holub	0	n	1	0	Polygonaceae Juss.
Iz_5_1009	DJI_0672	Festuca beckeri (Hack.) Trautv.	0	n	1	0	Poaceae Barnhart
Iz_5_1009	DJI_0672	Fragaria vesca L.	0	n	1	0	Rosaceae Juss.
Iz_5_1009	DJI_0672	Frangula alnus Mill.	0	n	1	1	Rhamnaceae Juss.
Iz_5_1009	DJI_0672	Fraxinus pennsylvanica Marshall	0	a	0	1	Oleaceae Hoffmanns. & Link
Iz_5_1009	DJI_0672	Galeopsis bifida Boenn.	0	n	1	0	Lamiacea Lindl.
Iz_5_1009	DJI_0672	Galium verum L.	0	n	1	0	Rubiaceae Juss.
Iz_5_1009	DJI_0672	Genista tinctoria L.	0	n	1	0	Fabaceae Lindl.
Iz_5_1009	DJI_0672	Geranium robertianum L.	0	n	1	0	Geraniaceae Juss.
Iz_5_1009	DJI_0672	Geranium sanquineum L.	0	n	1	0	Geraniaceae Juss.
Iz_5_1009	DJI_0672	Gypsophila paniculata L.	0	n	1	0	Caryophyllaceae Juss.
Iz_5_1009	DJI_0672	Helichrysum arenarium (L.) Moench	0	n	1	0	Asteraceae Bercht. & J.Presl
Iz_5_1009	DJI_0672	Herniaria polygama J.Gay	0	n	1	0	Caryophyllaceae Juss.
Iz_5_1009	DJI_0672	Hieracium robustum Fr.	0	n	1	0	Asteraceae Bercht. & J.Presl
Iz_5_1009	DJI_0672	Hieracium umbellatum L.	0	n	1	NA	Asteraceae Bercht. & J.Presl
Iz_5_1009	DJI_0672	Hylotelephium maximum (L.) Holub	0	n	1	0	Crassulaceae J.St.-Hil.
Iz_5_1009	DJI_0672	Jacobaea borysthenica (DC.) B.Nord. & Greuter	0	n	1	0	Asteraceae Bercht. & J.Presl
Iz_5_1009	DJI_0672	Jasione montana L.	0	n	1	0	Campanulaceae Juss.
Iz_5_1009	DJI_0672	Jurinea cyanoides (L.) Rchb.s.l.	0	n	1	0	Asteraceae Bercht. & J.Presl
Iz_5_1009	DJI_0672	Koeleria glauca (Spreng.) DC.	0	n	1	0	Poaceae Barnhart
Iz_5_1009	DJI_0672	Lactuca serriola L.	0	a	0	0	Asteraceae Bercht. & J.Presl
Iz_5_1009	DJI_0672	Linaria genistifolia (L.) Mill.	0	n	1	0	Plantaginaceae Juss.
Iz_5_1009	DJI_0672	Lomelosia argentea (L.) Greuter & Burdet	0	n	1	0	Caprifoliaceae Juss.
Iz_5_1009	DJI_0672	Melampyrum pratense L.	0	n	1	0	Orobanchaceae Vent.
Iz_5_1009	DJI_0672	Oenothera biennis L.	0	a	0	0	Onagraceae Juss.
Iz_5_1009	DJI_0672	Pentanema britannica (L.) D.Gut.Larr., Santos-Vicente, Anderb., E.Rico & M.M.Mart.Ort.	0	n	1	0	Asteraceae Bercht. & J.Presl
Iz_5_1009	DJI_0672	Peucedanum oreoselinum (L.) Moench	0	n	1	0	Apiaceae Lindl.
Iz_5_1009	DJI_0672	Peucedanum ruthenicum M.Bieb.	0	n	1	0	Apiaceae Lindl.
Iz_5_1009	DJI_0672	Pilosella echioides (Lumn.) F.W.Schultz & Sch.Bip.	0	n	1	0	Asteraceae Bercht. & J.Presl
Iz_5_1009	DJI_0672	Pilosella officinarum F. Schultz et Sch. Bip.	0	n	1	0	Asteraceae Bercht. & J.Presl
Iz_5_1009	DJI_0672	Pilosella sp.	0		NA	0	Asteraceae Bercht. & J.Presl
Iz_5_1009	DJI_0672	Pinus sylvestris L.	0	n	1	1	Pinaceae Spreng. ex F.Rudolphi
Iz_5_1009	DJI_0672	Poa annua L.	0	n	1	0	Poaceae Barnhart
Iz_5_1009	DJI_0672	Poa nemoralis L.	0	n	1	0	Poaceae Barnhart
Iz_5_1009	DJI_0672	Poa sp.	0		NA	0	Poaceae Barnhart
Iz_5_1009	DJI_0672	Polygonatum odoratum (Mill.) Druce	0	n	1	0	Asparagaceae Juss.
Iz_5_1009	DJI_0672	Polygonum arenarium Waldst. & Kit.	0	n	1	0	Polygonaceae Juss.
Iz_5_1009	DJI_0672	Populus nigra L.	0	n	1	1	Salicaceae Mirb.
Iz_5_1009	DJI_0672	Populus sp.	3	n	1	1	Salicaceae Mirb.
Iz_5_1009	DJI_0672	Populus tremula L.	0	n	1	1	Salicaceae Mirb.
Iz_5_1009	DJI_0672	Populus Ã canescens (Aiton) Sm.	0	n	1	1	Salicaceae Mirb.
Iz_5_1009	DJI_0672	Potentilla argentea L.	0	n	1	0	Rosaceae Juss.
Iz_5_1009	DJI_0672	Psammophiliella muralis (L.) Ikonn.	0	n	1	0	Caryophyllaceae Juss.
Iz_5_1009	DJI_0672	Psephellus sumensis (Kalen.) Greuter	0	n	1	0	Asteraceae Bercht. & J.Presl
Iz_5_1009	DJI_0672	Pulsatilla pratensis (L.) Mill. s.l.	0	n	1	0	Ranunculaceae Juss.
Iz_5_1009	DJI_0672	Pyrus communis L.	0	a	0	1	Rosaceae Juss.
Iz_5_1009	DJI_0672	Quercus robur L.	0	n	1	1	Fagaceae Dumort.
Iz_5_1009	DJI_0672	Robinia pseudoacacia L.	0	a	0	1	Fabaceae Lindl.
Iz_5_1009	DJI_0672	Rosa sp.	0		NA	1	Rosaceae Juss.
Iz_5_1009	DJI_0672	Rubus idaeus L.	0	n	1	0	Rosaceae Juss.
Iz_5_1009	DJI_0672	Rumex acetosella L.	0	n	1	0	Polygonaceae Juss.
Iz_5_1009	DJI_0672	Rumex thyrsiflorus Fingerh.	0	n	1	0	Polygonaceae Juss.
Iz_5_1009	DJI_0672	Salix alba L.	0	n	1	1	Salicaceae Mirb.

Iz_5_1009	DJI_0672	Sambucus nigra L.	0	n	1	1	Viburnaceae Raf.
Iz_5_1009	DJI_0672	Secale sylvestre Host	0	n	1	0	Poaceae Barnhart
Iz_5_1009	DJI_0672	Sempervivum ruthenicum Schnittsp. & C.B. Lehm.	0	n	1	0	Crassulaceae J.St.-Hil.
Iz_5_1009	DJI_0672	Setaria viridis (L.) P. Beauv.	0	n	1	0	Poaceae Barnhart
Iz_5_1009	DJI_0672	Silene borysthenica (Gruner) Walters	0	n	1	0	Caryophyllaceae Juss.
Iz_5_1009	DJI_0672	Silene latifolia subsp. alba (Mill.) Greuter & Burdet	0	n	1	0	Caryophyllaceae Juss.
Iz_5_1009	DJI_0672	Solidago virgaurea L.	4	n	1	0	Asteraceae Bercht. & J.Presl
Iz_5_1009	DJI_0672	Solidago Ã niederederi (S. canadensis Ã S. virgaurea)	0	hybrid	0	0	Asteraceae Bercht. & J.Presl
Iz_5_1009	DJI_0672	Sorbus aucuparia L.	0	n	1	1	Rosaceae Juss.
Iz_5_1009	DJI_0672	Stachys recta L.	0	n	1	0	Lamiacea Lindl.
Iz_5_1009	DJI_0672	Tanacetum vulgare L.	0	n	1	0	Asteraceae Bercht. & J.Presl
Iz_5_1009	DJI_0672	Thymus pallasianus Heinr.Braun	0	n	1	0	Lamiacea Lindl.
Iz_5_1009	DJI_0672	Tilia cordata Mill.	0	n	1	1	Malvaceae Juss.
Iz_5_1009	DJI_0672	Trifolium alpestre L.	0	n	1	0	Fabaceae Lindl.
Iz_5_1009	DJI_0672	Trifolium arvense L.	0	n	1	0	Fabaceae Lindl.
Iz_5_1009	DJI_0672	Ulmus glabra Huds.	0	n	1	1	Ulmaceae Mirb.
Iz_5_1009	DJI_0672	Verbascum densiflorum Bertol.	0	n	1	0	Scrophulariaceae Juss.
Iz_5_1009	DJI_0672	Veronica austriaca L.	0	n	1	0	Plantaginaceae Juss.
Iz_5_1009	DJI_0672	Veronica incana L.	0	n	1	0	Plantaginaceae Juss.
Iz_5_1009	DJI_0672	Veronica verna L.	0	n	1	0	Plantaginaceae Juss.
Iz_5_1009	DJI_0672	Vincetoxicum hirundinaria Medik	0	n	1	0	Apocynaceae Juss.
Iz_5_1009	DJI_0673	Acer tataricum L.	0	n	1	1	Sapindaceae Juss.
Iz_5_1009	DJI_0673	Achillea millefolium L. s.l.	0	n	1	0	Asteraceae Bercht. & J.Presl
Iz_5_1009	DJI_0673	Allium oleraceum L.	0	n	1	0	Amaryllidaceae J.St.-Hil.
Iz_5_1009	DJI_0673	Ambrosia artemisiifolia L.	0	a	0	0	Asteraceae Bercht. & J.Presl
Iz_5_1009	DJI_0673	Amorpha fruticosa L.	0	a	0	0	Fabaceae Lindl.
Iz_5_1009	DJI_0673	Anthericum ramosum L.	0	n	1	0	Asparagaceae Juss.
Iz_5_1009	DJI_0673	Artemisia marschalliana Spreng.	0	n	1	0	Asteraceae Bercht. & J.Presl
Iz_5_1009	DJI_0673	Asparagus officinalis L.	0	n	1	0	Asparagaceae Juss.
Iz_5_1009	DJI_0673	Astragalus cicer L.	0	n	1	0	Fabaceae Lindl.
Iz_5_1009	DJI_0673	Bassia laniflora (S.G.Gmel.) A.J.Scott	0	n	1	0	Amaranthaceae Juss.
Iz_5_1009	DJI_0673	Berberis vulgaris L.	0	n	1	0	Berberidaceae Juss.
Iz_5_1009	DJI_0673	Betula pendula Roth.	0	n	1	1	Betulaceae Gray
Iz_5_1009	DJI_0673	Betula pendula Roth.	0	n	1	0	Poaceae Barnhart
Iz_5_1009	DJI_0673	Bromus inermis Leyss.	0		NA	NA	
Iz_5_1009	DJI_0673	Bromus squarrosus L.	0	a	0	0	Poaceae Barnhart
Iz_5_1009	DJI_0673	Bromus tectorum L.	0	a	0	0	Poaceae Barnhart
Iz_5_1009	DJI_0673	Calamagrostis epigejos (L.) Roth	10	n	1	0	Poaceae Barnhart
Iz_5_1009	DJI_0673	Carduus acanthoides L.	0	n	1	0	Asteraceae Bercht. & J.Presl
Iz_5_1009	DJI_0673	Carex sp.	0	n	1	0	Cyperaceae Juss.
Iz_5_1009	DJI_0673	Centaurea majorovii Dumbadze	0	n	1	0	Asteraceae Bercht. & J.Presl
Iz_5_1009	DJI_0673	Chamaecytisus ruthenicus (Fisch.ex Wol.) Klaskova	0	n	1	0	Fabaceae Lindl.
Iz_5_1009	DJI_0673	Chelidonium majus L.	0	n	1	0	Papaveraceae Juss.
Iz_5_1009	DJI_0673	Chenopodium betaceum Andrz.	0	n	1	0	Amaranthaceae Juss.
Iz_5_1009	DJI_0673	Chenopodium cf. album	0	n	1	0	Amaranthaceae Juss.
Iz_5_1009	DJI_0673	Chenopodium sp.	0		NA	0	Amaranthaceae Juss.
Iz_5_1009	DJI_0673	Chondrilla juncea L.	0	n	1	0	Asteraceae Bercht. & J.Presl
Iz_5_1009	DJI_0673	Cirsium vulgare (Savi) Ten.	0	n	1	0	Asteraceae Bercht. & J.Presl
Iz_5_1009	DJI_0673	Clinopodium acinos (L.) Kuntze	0	n	1	0	Lamiacea Lindl.
Iz_5_1009	DJI_0673	Convallaria majalis L.	0	n	1	0	Asparagaceae Juss.
Iz_5_1009	DJI_0673	Crataegus rhipidophylla Gand.	0	n	1	1	Rosaceae Juss.
Iz_5_1009	DJI_0673	Crepis foetida L.	0	n	1	0	Asteraceae Bercht. & J.Presl
Iz_5_1009	DJI_0673	Dactylis glomerata L.	0	n	1	0	Poaceae Barnhart
Iz_5_1009	DJI_0673	Dianthus campestris M.Bieb.	0	n	1	0	Caryophyllaceae Juss.
Iz_5_1009	DJI_0673	Digitaria sanguinalis (L.) Scop.	0	a	0	0	Poaceae Barnhart
Iz_5_1009	DJI_0673	Elymus repens (L.) Gould	0	n	1	0	Poaceae Barnhart
Iz_5_1009	DJI_0673	Epilobium tetragonum L.	0	n	1	0	Onagraceae Juss.
Iz_5_1009	DJI_0673	Eragrostis minor Host	0	a	0	0	Poaceae Barnhart
Iz_5_1009	DJI_0673	Erigeron canadensis L.	3	a	0	0	Asteraceae Bercht. & J.Presl
Iz_5_1009	DJI_0673	Euphorbia seguieriana Neck.	0	n	1	0	Euphorbiaceae Juss.
Iz_5_1009	DJI_0673	Fallopia dumetorum (L.) Holub	0	n	1	0	Polygonaceae Juss.
Iz_5_1009	DJI_0673	Festuca beckeri (Hack.) Trautv.	0	n	1	0	Poaceae Barnhart
Iz_5_1009	DJI_0673	Fragaria vesca L.	0	n	1	0	Rosaceae Juss.
Iz_5_1009	DJI_0673	Frangula alnus Mill.	0	n	1	1	Rhamnaceae Juss.
Iz_5_1009	DJI_0673	Fraxinus pennsylvanica Marshall	0	a	0	1	Oleaceae Hoffmanns. & Link
Iz_5_1009	DJI_0673	Galeopsis bifida Boenn.	0	n	1	0	Lamiacea Lindl.
Iz_5_1009	DJI_0673	Galium verum L.	0	n	1	0	Rubiaceae Juss.
Iz_5_1009	DJI_0673	Genista tinctoria L.	0	n	1	0	Fabaceae Lindl.
Iz_5_1009	DJI_0673	Geranium robertianum L.	0	n	1	0	Geraniaceae Juss.
Iz_5_1009	DJI_0673	Geranium sanquineum L.	0	n	1	0	Geraniaceae Juss.
Iz_5_1009	DJI_0673	Gypsophila paniculata L.	0	n	1	0	Caryophyllaceae Juss.
Iz_5_1009	DJI_0673	Helichrysum arenarium (L.) Moench	0	n	1	0	Asteraceae Bercht. & J.Presl
Iz_5_1009	DJI_0673	Herniaria polygama J.Gay	0	n	1	0	Caryophyllaceae Juss.
Iz_5_1009	DJI_0673	Hieracium robustum Fr.	0	n	1	0	Asteraceae Bercht. & J.Presl
Iz_5_1009	DJI_0673	Hieracium umbellatum L.	0	n	1	NA	Asteraceae Bercht. & J.Presl
Iz_5_1009	DJI_0673	Hylotelephium maximum (L.) Holub	0	n	1	0	Crassulaceae J.St.-Hil.
Iz_5_1009	DJI_0673	Jacobaea borysthenica (DC.) B.Nord. & Greuter	0	n	1	0	Asteraceae Bercht. & J.Presl
Iz_5_1009	DJI_0673	Jasione montana L.	0	n	1	0	Campanulaceae Juss.
Iz_5_1009	DJI_0673	Jurinea cyanoides (L.) Rchb.s.l.	0	n	1	0	Asteraceae Bercht. & J.Presl
Iz_5_1009	DJI_0673	Koeleria glauca (Spreng.) DC.	1	n	1	0	Poaceae Barnhart
Iz_5_1009	DJI_0673	Lactuca serriola L.	0	a	0	0	Asteraceae Bercht. & J.Presl
Iz_5_1009	DJI_0673	Linaria genistifolia (L.) Mill.	0	n	1	0	Plantaginaceae Juss.
Iz_5_1009	DJI_0673	Lomelosia argentea (L.) Greuter & Burdet	0	n	1	0	Caprifoliaceae Juss.
Iz_5_1009	DJI_0673	Melampyrum pratense L.	0	n	1	0	Orobanchaceae Vent.
Iz_5_1009	DJI_0673	Oenothera biennis L.	0	a	0	0	Onagraceae Juss.
Iz_5_1009	DJI_0673	Pentanema britannica (L.) D.Gut.Larr., Santos-Vicente, Anderb., E.Rico & M.M.Mart.Ort.	0	n	1	0	Asteraceae Bercht. & J.Presl
Iz_5_1009	DJI_0673	Peucedanum oreoselinum (L.) Moench	0	n	1	0	Apiaceae Lindl.
Iz_5_1009	DJI_0673	Peucedanum ruthenicum M.Bieb.	0	n	1	0	Apiaceae Lindl.
Iz_5_1009	DJI_0673	Pilosella echioides (Lumn.) F.W.Schultz & Sch.Bip.	0	n	1	0	Asteraceae Bercht. & J.Presl
Iz_5_1009	DJI_0673	Pilosella officinarum F. Schultz et Sch. Bip.	0	n	1	0	Asteraceae Bercht. & J.Presl
Iz_5_1009	DJI_0673	Pilosella sp.	0		NA	0	Asteraceae Bercht. & J.Presl
Iz_5_1009	DJI_0673	Pinus sylvestris L.	0	n	1	1	Pinaceae Spreng. ex F.Rudolphi
Iz_5_1009	DJI_0673	Poa annua L.	0	n	1	0	Poaceae Barnhart
Iz_5_1009	DJI_0673	Poa nemoralis L.	0	n	1	0	Poaceae Barnhart
Iz_5_1009	DJI_0673	Poa sp.	0		NA	0	Poaceae Barnhart
Iz_5_1009	DJI_0673	Polygonatum odoratum (Mill.) Druce	0	n	1	0	Asparagaceae Juss.
Iz_5_1009	DJI_0673	Polygonum arenarium Waldst. & Kit.	0	n	1	0	Polygonaceae Juss.
Iz_5_1009	DJI_0673	Populus nigra L.	0	n	1	1	Salicaceae Mirb.

Iz_5_1009	DJI_0673	Populus sp.	3	n	1	1	Salicaceae Mirb.
Iz_5_1009	DJI_0673	Populus tremula L.	0	n	1	1	Salicaceae Mirb.
Iz_5_1009	DJI_0673	Populus Ã canescens (Aiton) Sm.	0	n	1	1	Salicaceae Mirb.
Iz_5_1009	DJI_0673	Potentilla argentea L.	0	n	1	0	Rosaceae Juss.
Iz_5_1009	DJI_0673	Psammophiliella muralis (L.) Ikonn.	0	n	1	0	Caryophyllaceae Juss.
Iz_5_1009	DJI_0673	Psephellus sumensis (Kalen.) Greuter	0	n	1	0	Asteraceae Bercht. & J.Presl
Iz_5_1009	DJI_0673	Pulsatilla pratensis (L.) Mill. s.l.	0	n	1	0	Ranunculaceae Juss.
Iz_5_1009	DJI_0673	Pyrus communis L.	0	a	0	1	Rosaceae Juss.
Iz_5_1009	DJI_0673	Quercus robur L.	0	n	1	1	Fagaceae Dumort.
Iz_5_1009	DJI_0673	Robinia pseudoacacia L.	0	a	0	1	Fabaceae Lindl.
Iz_5_1009	DJI_0673	Rosa sp.	0		NA	1	Rosaceae Juss.
Iz_5_1009	DJI_0673	Rubus idaeus L.	0	n	1	0	Rosaceae Juss.
Iz_5_1009	DJI_0673	Rumex acetosella L.	0	n	1	0	Polygonaceae Juss.
Iz_5_1009	DJI_0673	Rumex thyrsiflorus Fingerh.	0	n	1	0	Polygonaceae Juss.
Iz_5_1009	DJI_0673	Salix alba L.	0	n	1	1	Salicaceae Mirb.
Iz_5_1009	DJI_0673	Sambucus nigra L.	0	n	1	1	Viburnaceae Raf.
Iz_5_1009	DJI_0673	Secale sylvestre Host	0	n	1	0	Poaceae Barnhart
Iz_5_1009	DJI_0673	Sempervivum ruthenicum Schnittsp. & C.B. Lehm.	0	n	1	0	Crassulaceae J.St.-Hil.
Iz_5_1009	DJI_0673	Setaria viridis (L.) P. Beauv.	0	n	1	0	Poaceae Barnhart
Iz_5_1009	DJI_0673	Silene borysthenica (Gruner) Walters	0	n	1	0	Caryophyllaceae Juss.
Iz_5_1009	DJI_0673	Silene latifolia subsp. alba (Mill.) Greuter & Burdet	0	n	1	0	Caryophyllaceae Juss.
Iz_5_1009	DJI_0673	Solidago virgaurea L.	3	n	1	0	Asteraceae Bercht. & J.Presl
Iz_5_1009	DJI_0673	Solidago Ã niederederi (S. canadensis Ã S. virgaurea)	0	hybrid	0	0	Asteraceae Bercht. & J.Presl
Iz_5_1009	DJI_0673	Sorbus aucuparia L.	0	n	1	1	Rosaceae Juss.
Iz_5_1009	DJI_0673	Stachys recta L.	0	n	1	0	Lamiacea Lindl.
Iz_5_1009	DJI_0673	Tanacetum vulgare L.	0	n	1	0	Asteraceae Bercht. & J.Presl
Iz_5_1009	DJI_0673	Thymus pallasianus Heinr.Braun	0	n	1	0	Lamiacea Lindl.
Iz_5_1009	DJI_0673	Tilia cordata Mill.	0	n	1	1	Malvaceae Juss.
Iz_5_1009	DJI_0673	Trifolium alpestre L.	0	n	1	0	Fabaceae Lindl.
Iz_5_1009	DJI_0673	Trifolium arvense L.	0	n	1	0	Fabaceae Lindl.
Iz_5_1009	DJI_0673	Ulmus glabra Huds.	0	n	1	1	Ulmaceae Mirb.
Iz_5_1009	DJI_0673	Verbascum densiflorum Bertol.	0	n	1	0	Scrophulariaceae Juss.
Iz_5_1009	DJI_0673	Veronica austriaca L.	0	n	1	0	Plantaginaceae Juss.
Iz_5_1009	DJI_0673	Veronica incana L.	0	n	1	0	Plantaginaceae Juss.
Iz_5_1009	DJI_0673	Veronica verna L.	0	n	1	0	Plantaginaceae Juss.
Iz_5_1009	DJI_0673	Vincetoxicum hirundinaria Medik	0	n	1	0	Apocynaceae Juss.
Iz_5_1009	DJI_0674	Acer tataricum L.	0	n	1	1	Sapindaceae Juss.
Iz_5_1009	DJI_0674	Achillea millefolium L. s.l.	0	n	1	0	Asteraceae Bercht. & J.Presl
Iz_5_1009	DJI_0674	Allium oleraceum L.	0	n	1	0	Amaryllidaceae J.St.-Hil.
Iz_5_1009	DJI_0674	Ambrosia artemisiifolia L.	0	a	0	0	Asteraceae Bercht. & J.Presl
Iz_5_1009	DJI_0674	Amorpha fruticosa L.	0	a	0	0	Fabaceae Lindl.
Iz_5_1009	DJI_0674	Anthericum ramosum L.	0	n	1	0	Asparagaceae Juss.
Iz_5_1009	DJI_0674	Artemisia marschalliana Spreng.	0	n	1	0	Asteraceae Bercht. & J.Presl
Iz_5_1009	DJI_0674	Asparagus officinalis L.	0	n	1	0	Asparagaceae Juss.
Iz_5_1009	DJI_0674	Astragalus cicer L.	0	n	1	0	Fabaceae Lindl.
Iz_5_1009	DJI_0674	Bassia laniflora (S.G.Gmel.) A.J.Scott	0	n	1	0	Amaranthaceae Juss.
Iz_5_1009	DJI_0674	Berberis vulgaris L.	0	n	1	0	Berberidaceae Juss.
Iz_5_1009	DJI_0674	Betula pendula Roth.	0	n	1	1	Betulaceae Gray
Iz_5_1009	DJI_0674	Betula pendula Roth.	0	n	1	0	Poaceae Barnhart
Iz_5_1009	DJI_0674	Bromus inermis Leyss.	0		NA	NA	
Iz_5_1009	DJI_0674	Bromus squarrosus L.	0	a	0	0	Poaceae Barnhart
Iz_5_1009	DJI_0674	Bromus tectorum L.	0	a	0	0	Poaceae Barnhart
Iz_5_1009	DJI_0674	Calamagrostis epigejos (L.) Roth	0	n	1	0	Poaceae Barnhart
Iz_5_1009	DJI_0674	Carduus acanthoides L.	0	n	1	0	Asteraceae Bercht. & J.Presl
Iz_5_1009	DJI_0674	Carex sp.	0	n	1	0	Cyperaceae Juss.
Iz_5_1009	DJI_0674	Centaurea majorovii Dumbadze	0	n	1	0	Asteraceae Bercht. & J.Presl
Iz_5_1009	DJI_0674	Chamaecytisus ruthenicus (Fisch.ex Wol.) Klaskova	0	n	1	0	Fabaceae Lindl.
Iz_5_1009	DJI_0674	Chelidonium majus L.	0	n	1	0	Papaveraceae Juss.
Iz_5_1009	DJI_0674	Chenopodium betaceum Andrz.	0	n	1	0	Amaranthaceae Juss.
Iz_5_1009	DJI_0674	Chenopodium cf. album	0	n	1	0	Amaranthaceae Juss.
Iz_5_1009	DJI_0674	Chenopodium sp.	0		NA	0	Amaranthaceae Juss.
Iz_5_1009	DJI_0674	Chondrilla juncea L.	0	n	1	0	Asteraceae Bercht. & J.Presl
Iz_5_1009	DJI_0674	Cirsium vulgare (Savi) Ten.	0	n	1	0	Asteraceae Bercht. & J.Presl
Iz_5_1009	DJI_0674	Clinopodium acinos (L.) Kuntze	0	n	1	0	Lamiacea Lindl.
Iz_5_1009	DJI_0674	Convallaria majalis L.	0	n	1	0	Asparagaceae Juss.
Iz_5_1009	DJI_0674	Crataegus rhipidophylla Gand.	0	n	1	1	Rosaceae Juss.
Iz_5_1009	DJI_0674	Crepis foetida L.	0	n	1	0	Asteraceae Bercht. & J.Presl
Iz_5_1009	DJI_0674	Dactylis glomerata L.	0	n	1	0	Poaceae Barnhart
Iz_5_1009	DJI_0674	Dianthus campestris M.Bieb.	0	n	1	0	Caryophyllaceae Juss.
Iz_5_1009	DJI_0674	Digitaria sanguinalis (L.) Scop.	0	a	0	0	Poaceae Barnhart
Iz_5_1009	DJI_0674	Elymus repens (L.) Gould	0	n	1	0	Poaceae Barnhart
Iz_5_1009	DJI_0674	Epilobium tetragonum L.	0	n	1	0	Onagraceae Juss.
Iz_5_1009	DJI_0674	Eragrostis minor Host	0	a	0	0	Poaceae Barnhart
Iz_5_1009	DJI_0674	Erigeron canadensis L.	3	a	0	0	Asteraceae Bercht. & J.Presl
Iz_5_1009	DJI_0674	Euphorbia seguieriana Neck.	0	n	1	0	Euphorbiaceae Juss.
Iz_5_1009	DJI_0674	Fallopia dumetorum (L.) Holub	0	n	1	0	Polygonaceae Juss.
Iz_5_1009	DJI_0674	Festuca beckeri (Hack.) Trautv.	0	n	1	0	Poaceae Barnhart
Iz_5_1009	DJI_0674	Fragaria vesca L.	0	n	1	0	Rosaceae Juss.
Iz_5_1009	DJI_0674	Frangula alnus Mill.	0	n	1	1	Rhamnaceae Juss.
Iz_5_1009	DJI_0674	Fraxinus pennsylvanica Marshall	0	a	0	1	Oleaceae Hoffmanns. & Link
Iz_5_1009	DJI_0674	Galeopsis bifida Boenn.	0	n	1	0	Lamiacea Lindl.
Iz_5_1009	DJI_0674	Galium verum L.	0	n	1	0	Rubiaceae Juss.
Iz_5_1009	DJI_0674	Genista tinctoria L.	0	n	1	0	Fabaceae Lindl.
Iz_5_1009	DJI_0674	Geranium robertianum L.	0	n	1	0	Geraniaceae Juss.
Iz_5_1009	DJI_0674	Geranium sanquineum L.	0	n	1	0	Geraniaceae Juss.
Iz_5_1009	DJI_0674	Gypsophila paniculata L.	0	n	1	0	Caryophyllaceae Juss.
Iz_5_1009	DJI_0674	Helichrysum arenarium (L.) Moench	0	n	1	0	Asteraceae Bercht. & J.Presl
Iz_5_1009	DJI_0674	Herniaria polygama J.Gay	0	n	1	0	Caryophyllaceae Juss.
Iz_5_1009	DJI_0674	Hieracium robustum Fr.	0	n	1	0	Asteraceae Bercht. & J.Presl
Iz_5_1009	DJI_0674	Hieracium umbellatum L.	0	n	1	NA	Asteraceae Bercht. & J.Presl
Iz_5_1009	DJI_0674	Hylotelephium maximum (L.) Holub	0	n	1	0	Crassulaceae J.St.-Hil.
Iz_5_1009	DJI_0674	Jacobaea borysthenica (DC.) B.Nord. & Greuter	0	n	1	0	Asteraceae Bercht. & J.Presl
Iz_5_1009	DJI_0674	Jasione montana L.	0	n	1	0	Campanulaceae Juss.
Iz_5_1009	DJI_0674	Jurinea cyanoides (L.) Rchb.s.l.	0	n	1	0	Asteraceae Bercht. & J.Presl
Iz_5_1009	DJI_0674	Koeleria glauca (Spreng.) DC.	1	n	1	0	Poaceae Barnhart
Iz_5_1009	DJI_0674	Lactuca serriola L.	0	a	0	0	Asteraceae Bercht. & J.Presl
Iz_5_1009	DJI_0674	Linaria genistifolia (L.) Mill.	0	n	1	0	Plantaginaceae Juss.
Iz_5_1009	DJI_0674	Lomelosia argentea (L.) Greuter & Burdet	0	n	1	0	Caprifoliaceae Juss.

Iz_5_1009	DJI_0674	Melampyrum pratense L.	0	n	1	0	Orobanchaceae Vent.
Iz_5_1009	DJI_0674	Oenothera biennis L.	0	a	0	0	Onagraceae Juss.
Iz_5_1009	DJI_0674	Pentanema britannica (L.) D.Gut.Larr., Santos-Vicente, Anderb., E.Rico & M.M.Mart.Ort.	0	n	1	0	Asteraceae Bercht. & J.Presl
Iz_5_1009	DJI_0674	Peucedanum oreoselinum (L.) Moench	0	n	1	0	Apiaceae Lindl.
Iz_5_1009	DJI_0674	Peucedanum ruthenicum M.Bieb.	0	n	1	0	Apiaceae Lindl.
Iz_5_1009	DJI_0674	Pilosella echioides (Lumn.) F.W.Schultz & Sch.Bip.	0	n	1	0	Asteraceae Bercht. & J.Presl
Iz_5_1009	DJI_0674	Pilosella officinarum F. Schultz et Sch. Bip.	0	n	1	0	Asteraceae Bercht. & J.Presl
Iz_5_1009	DJI_0674	Pilosella sp.	0		NA	0	Asteraceae Bercht. & J.Presl
Iz_5_1009	DJI_0674	Pinus sylvestris L.	0	n	1	1	Pinaceae Spreng. ex F.Rudolphi
Iz_5_1009	DJI_0674	Poa annua L.	0	n	1	0	Poaceae Barnhart
Iz_5_1009	DJI_0674	Poa nemoralis L.	0	n	1	0	Poaceae Barnhart
Iz_5_1009	DJI_0674	Poa sp.	0		NA	0	Poaceae Barnhart
Iz_5_1009	DJI_0674	Polygonatum odoratum (Mill.) Druce	0	n	1	0	Asparagaceae Juss.
Iz_5_1009	DJI_0674	Polygonum arenarium Waldst. & Kit.	0	n	1	0	Polygonaceae Juss.
Iz_5_1009	DJI_0674	Populus nigra L.	0	n	1	1	Salicaceae Mirb.
Iz_5_1009	DJI_0674	Populus sp.	0	n	1	1	Salicaceae Mirb.
Iz_5_1009	DJI_0674	Populus tremula L.	0	n	1	1	Salicaceae Mirb.
Iz_5_1009	DJI_0674	Populus Ã canescens (Aiton) Sm.	0	n	1	1	Salicaceae Mirb.
Iz_5_1009	DJI_0674	Potentilla argentea L.	0	n	1	0	Rosaceae Juss.
Iz_5_1009	DJI_0674	Psammophiliella muralis (L.) Ikonn.	0	n	1	0	Caryophyllaceae Juss.
Iz_5_1009	DJI_0674	Psephellus sumensis (Kalen.) Greuter	0	n	1	0	Asteraceae Bercht. & J.Presl
Iz_5_1009	DJI_0674	Pulsatilla pratensis (L.) Mill. s.l.	0	n	1	0	Ranunculaceae Juss.
Iz_5_1009	DJI_0674	Pyrus communis L.	0	a	0	1	Rosaceae Juss.
Iz_5_1009	DJI_0674	Quercus robur L.	0	n	1	1	Fagaceae Dumort.
Iz_5_1009	DJI_0674	Robinia pseudoacacia L.	0	a	0	1	Fabaceae Lindl.
Iz_5_1009	DJI_0674	Rosa sp.	0		NA	1	Rosaceae Juss.
Iz_5_1009	DJI_0674	Rubus idaeus L.	0	n	1	0	Rosaceae Juss.
Iz_5_1009	DJI_0674	Rumex acetosella L.	0	n	1	0	Polygonaceae Juss.
Iz_5_1009	DJI_0674	Rumex thyrsiflorus Fingerh.	0	n	1	0	Polygonaceae Juss.
Iz_5_1009	DJI_0674	Salix alba L.	0	n	1	1	Salicaceae Mirb.
Iz_5_1009	DJI_0674	Sambucus nigra L.	0	n	1	1	Viburnaceae Raf.
Iz_5_1009	DJI_0674	Secale sylvestre Host	0	n	1	0	Poaceae Barnhart
Iz_5_1009	DJI_0674	Sempervivum ruthenicum Schnittsp. & C.B. Lehm.	0	n	1	0	Crassulaceae J.St.-Hil.
Iz_5_1009	DJI_0674	Setaria viridis (L.) P. Beauv.	0	n	1	0	Poaceae Barnhart
Iz_5_1009	DJI_0674	Silene borysthenica (Gruner) Walters	0	n	1	0	Caryophyllaceae Juss.
Iz_5_1009	DJI_0674	Silene latifolia subsp. alba (Mill.) Greuter & Burdet	0	n	1	0	Caryophyllaceae Juss.
Iz_5_1009	DJI_0674	Solidago virgaurea L.	2	n	1	0	Asteraceae Bercht. & J.Presl
Iz_5_1009	DJI_0674	Solidago Ã niederederi (S. canadensis Ã S. virgaurea)	0	hybrid	0	0	Asteraceae Bercht. & J.Presl
Iz_5_1009	DJI_0674	Sorbus aucuparia L.	0	n	1	1	Rosaceae Juss.
Iz_5_1009	DJI_0674	Stachys recta L.	0	n	1	0	Lamiacea Lindl.
Iz_5_1009	DJI_0674	Tanacetum vulgare L.	0	n	1	0	Asteraceae Bercht. & J.Presl
Iz_5_1009	DJI_0674	Thymus pallasianus Heinr.Braun	0	n	1	0	Lamiacea Lindl.
Iz_5_1009	DJI_0674	Tilia cordata Mill.	0	n	1	1	Malvaceae Juss.
Iz_5_1009	DJI_0674	Trifolium alpestre L.	0	n	1	0	Fabaceae Lindl.
Iz_5_1009	DJI_0674	Trifolium arvense L.	0	n	1	0	Fabaceae Lindl.
Iz_5_1009	DJI_0674	Ulmus glabra Huds.	0	n	1	1	Ulmaceae Mirb.
Iz_5_1009	DJI_0674	Verbascum densiflorum Bertol.	0	n	1	0	Scrophulariaceae Juss.
Iz_5_1009	DJI_0674	Veronica austriaca L.	0	n	1	0	Plantaginaceae Juss.
Iz_5_1009	DJI_0674	Veronica incana L.	0	n	1	0	Plantaginaceae Juss.
Iz_5_1009	DJI_0674	Veronica verna L.	0	n	1	0	Plantaginaceae Juss.
Iz_5_1009	DJI_0674	Vincetoxicum hirundinaria Medik	0	n	1	0	Apocynaceae Juss.
Iz_5_1009	DJI_0676	Acer tataricum L.	0	n	1	1	Sapindaceae Juss.
Iz_5_1009	DJI_0676	Achillea millefolium L. s.l.	0	n	1	0	Asteraceae Bercht. & J.Presl
Iz_5_1009	DJI_0676	Allium oleraceum L.	0	n	1	0	Amaryllidaceae J.St.-Hil.
Iz_5_1009	DJI_0676	Ambrosia artemisiifolia L.	0	a	0	0	Asteraceae Bercht. & J.Presl
Iz_5_1009	DJI_0676	Amorpha fruticosa L.	0	a	0	0	Fabaceae Lindl.
Iz_5_1009	DJI_0676	Anthericum ramosum L.	0	n	1	0	Asparagaceae Juss.
Iz_5_1009	DJI_0676	Artemisia marschalliana Spreng.	0	n	1	0	Asteraceae Bercht. & J.Presl
Iz_5_1009	DJI_0676	Asparagus officinalis L.	0	n	1	0	Asparagaceae Juss.
Iz_5_1009	DJI_0676	Astragalus cicer L.	0	n	1	0	Fabaceae Lindl.
Iz_5_1009	DJI_0676	Bassia laniflora (S.G.Gmel.) A.J.Scott	0	n	1	0	Amaranthaceae Juss.
Iz_5_1009	DJI_0676	Berberis vulgaris L.	0	n	1	0	Berberidaceae Juss.
Iz_5_1009	DJI_0676	Betula pendula Roth.	0	n	1	1	Betulaceae Gray
Iz_5_1009	DJI_0676	Betula pendula Roth.	0	n	1	0	Poaceae Barnhart
Iz_5_1009	DJI_0676	Bromus inermis Leyss.	0		NA	NA	
Iz_5_1009	DJI_0676	Bromus squarrosus L.	0	a	0	0	Poaceae Barnhart
Iz_5_1009	DJI_0676	Bromus tectorum L.	0	a	0	0	Poaceae Barnhart
Iz_5_1009	DJI_0676	Calamagrostis epigejos (L.) Roth	10	n	1	0	Poaceae Barnhart
Iz_5_1009	DJI_0676	Carduus acanthoides L.	0	n	1	0	Asteraceae Bercht. & J.Presl
Iz_5_1009	DJI_0676	Carex sp.	0	n	1	0	Cyperaceae Juss.
Iz_5_1009	DJI_0676	Centaurea majorovii Dumbadze	0	n	1	0	Asteraceae Bercht. & J.Presl
Iz_5_1009	DJI_0676	Chamaecytisus ruthenicus (Fisch.ex Wol.) Klaskova	0	n	1	0	Fabaceae Lindl.
Iz_5_1009	DJI_0676	Chelidonium majus L.	0	n	1	0	Papaveraceae Juss.
Iz_5_1009	DJI_0676	Chenopodium betaceum Andrz.	0	n	1	0	Amaranthaceae Juss.
Iz_5_1009	DJI_0676	Chenopodium cf. album	0	n	1	0	Amaranthaceae Juss.
Iz_5_1009	DJI_0676	Chenopodium sp.	0		NA	0	Amaranthaceae Juss.
Iz_5_1009	DJI_0676	Chondrilla juncea L.	0	n	1	0	Asteraceae Bercht. & J.Presl
Iz_5_1009	DJI_0676	Cirsium vulgare (Savi) Ten.	0	n	1	0	Asteraceae Bercht. & J.Presl
Iz_5_1009	DJI_0676	Clinopodium acinos (L.) Kuntze	0	n	1	0	Lamiacea Lindl.
Iz_5_1009	DJI_0676	Convallaria majalis L.	0	n	1	0	Asparagaceae Juss.
Iz_5_1009	DJI_0676	Crataegus rhipidophylla Gand.	0	n	1	1	Rosaceae Juss.
Iz_5_1009	DJI_0676	Crepis foetida L.	0	n	1	0	Asteraceae Bercht. & J.Presl
Iz_5_1009	DJI_0676	Dactylis glomerata L.	0	n	1	0	Poaceae Barnhart
Iz_5_1009	DJI_0676	Dianthus campestris M.Bieb.	0	n	1	0	Caryophyllaceae Juss.
Iz_5_1009	DJI_0676	Digitaria sanguinalis (L.) Scop.	0	a	0	0	Poaceae Barnhart
Iz_5_1009	DJI_0676	Elymus repens (L.) Gould	0	n	1	0	Poaceae Barnhart
Iz_5_1009	DJI_0676	Epilobium tetragonum L.	0	n	1	0	Onagraceae Juss.
Iz_5_1009	DJI_0676	Eragrostis minor Host	0	a	0	0	Poaceae Barnhart
Iz_5_1009	DJI_0676	Erigeron canadensis L.	3	a	0	0	Asteraceae Bercht. & J.Presl
Iz_5_1009	DJI_0676	Euphorbia seguieriana Neck.	0	n	1	0	Euphorbiaceae Juss.
Iz_5_1009	DJI_0676	Fallopia dumetorum (L.) Holub	0	n	1	0	Polygonaceae Juss.
Iz_5_1009	DJI_0676	Festuca beckeri (Hack.) Trautv.	0	n	1	0	Poaceae Barnhart
Iz_5_1009	DJI_0676	Fragaria vesca L.	0	n	1	0	Rosaceae Juss.
Iz_5_1009	DJI_0676	Frangula alnus Mill.	0	n	1	1	Rhamnaceae Juss.
Iz_5_1009	DJI_0676	Fraxinus pennsylvanica Marshall	0	a	0	1	Oleaceae Hoffmanns. & Link
Iz_5_1009	DJI_0676	Galeopsis bifida Boenn.	0	n	1	0	Lamiacea Lindl.
Iz_5_1009	DJI_0676	Galium verum L.	0	n	1	0	Rubiaceae Juss.
Iz_5_1009	DJI_0676	Genista tinctoria L.	0	n	1	0	Fabaceae Lindl.

Iz_5_1009	DJI_0676	Geranium robertianum L.	0	n	1	0	Geraniaceae Juss.
Iz_5_1009	DJI_0676	Geranium sanquineum L.	0	n	1	0	Geraniaceae Juss.
Iz_5_1009	DJI_0676	Gypsophila paniculata L.	0	n	1	0	Caryophyllaceae Juss.
Iz_5_1009	DJI_0676	Helichrysum arenarium (L.) Moench	0	n	1	0	Asteraceae Bercht. & J.Presl
Iz_5_1009	DJI_0676	Herniaria polygama J.Gay	0	n	1	0	Caryophyllaceae Juss.
Iz_5_1009	DJI_0676	Hieracium robustum Fr.	0	n	1	0	Asteraceae Bercht. & J.Presl
Iz_5_1009	DJI_0676	Hieracium umbellatum L.	0	n	1	NA	Asteraceae Bercht. & J.Presl
Iz_5_1009	DJI_0676	Hylotelephium maximum (L.) Holub	0	n	1	0	Crassulaceae J.St.-Hil.
Iz_5_1009	DJI_0676	Jacobaea borysthenica (DC.) B.Nord. & Greuter	0	n	1	0	Asteraceae Bercht. & J.Presl
Iz_5_1009	DJI_0676	Jasione montana L.	0	n	1	0	Campanulaceae Juss.
Iz_5_1009	DJI_0676	Jurinea cyanoides (L.) Rchb.s.l.	0	n	1	0	Asteraceae Bercht. & J.Presl
Iz_5_1009	DJI_0676	Koeleria glauca (Spreng.) DC.	0	n	1	0	Poaceae Barnhart
Iz_5_1009	DJI_0676	Lactuca serriola L.	0	a	0	0	Asteraceae Bercht. & J.Presl
Iz_5_1009	DJI_0676	Linaria genistifolia (L.) Mill.	0	n	1	0	Plantaginaceae Juss.
Iz_5_1009	DJI_0676	Lomelosia argentea (L.) Greuter & Burdet	0	n	1	0	Caprifoliaceae Juss.
Iz_5_1009	DJI_0676	Melampyrum pratense L.	0	n	1	0	Orobanchaceae Vent.
Iz_5_1009	DJI_0676	Oenothera biennis L.	0	a	0	0	Onagraceae Juss.
Iz_5_1009	DJI_0676	Pentanema britannica (L.) D.Gut.Larr., Santos-Vicente, Anderb., E.Rico & M.M.Mart.Ort.	0	n	1	0	Asteraceae Bercht. & J.Presl
Iz_5_1009	DJI_0676	Peucedanum oreoselinum (L.) Moench	0	n	1	0	Apiaceae Lindl.
Iz_5_1009	DJI_0676	Peucedanum ruthenicum M.Bieb.	0	n	1	0	Apiaceae Lindl.
Iz_5_1009	DJI_0676	Pilosella echioides (Lumn.) F.W.Schultz & Sch.Bip.	0	n	1	0	Asteraceae Bercht. & J.Presl
Iz_5_1009	DJI_0676	Pilosella officinarum F. Schultz et Sch. Bip.	0	n	1	0	Asteraceae Bercht. & J.Presl
Iz_5_1009	DJI_0676	Pilosella sp.	0		NA	0	Asteraceae Bercht. & J.Presl
Iz_5_1009	DJI_0676	Pinus sylvestris L.	0	n	1	1	Pinaceae Spreng. ex F.Rudolphi
Iz_5_1009	DJI_0676	Poa annua L.	0	n	1	0	Poaceae Barnhart
Iz_5_1009	DJI_0676	Poa nemoralis L.	0	n	1	0	Poaceae Barnhart
Iz_5_1009	DJI_0676	Poa sp.	0		NA	0	Poaceae Barnhart
Iz_5_1009	DJI_0676	Polygonatum odoratum (Mill.) Druce	0	n	1	0	Asparagaceae Juss.
Iz_5_1009	DJI_0676	Polygonum arenarium Waldst. & Kit.	0	n	1	0	Polygonaceae Juss.
Iz_5_1009	DJI_0676	Populus nigra L.	0	n	1	1	Salicaceae Mirb.
Iz_5_1009	DJI_0676	Populus sp.	0	n	1	1	Salicaceae Mirb.
Iz_5_1009	DJI_0676	Populus tremula L.	0	n	1	1	Salicaceae Mirb.
Iz_5_1009	DJI_0676	Populus Ã canescens (Aiton) Sm.	0	n	1	1	Salicaceae Mirb.
Iz_5_1009	DJI_0676	Potentilla argentea L.	0	n	1	0	Rosaceae Juss.
Iz_5_1009	DJI_0676	Psammophiliella muralis (L.) Ikonn.	0	n	1	0	Caryophyllaceae Juss.
Iz_5_1009	DJI_0676	Psephellus sumensis (Kalen.) Greuter	0	n	1	0	Asteraceae Bercht. & J.Presl
Iz_5_1009	DJI_0676	Pulsatilla pratensis (L.) Mill. s.l.	0	n	1	0	Ranunculaceae Juss.
Iz_5_1009	DJI_0676	Pyrus communis L.	0	a	0	1	Rosaceae Juss.
Iz_5_1009	DJI_0676	Quercus robur L.	0	n	1	1	Fagaceae Dumort.
Iz_5_1009	DJI_0676	Robinia pseudoacacia L.	0	a	0	1	Fabaceae Lindl.
Iz_5_1009	DJI_0676	Rosa sp.	0		NA	1	Rosaceae Juss.
Iz_5_1009	DJI_0676	Rubus idaeus L.	0	n	1	0	Rosaceae Juss.
Iz_5_1009	DJI_0676	Rumex acetosella L.	0	n	1	0	Polygonaceae Juss.
Iz_5_1009	DJI_0676	Rumex thyrsiflorus Fingerh.	0	n	1	0	Polygonaceae Juss.
Iz_5_1009	DJI_0676	Salix alba L.	0	n	1	1	Salicaceae Mirb.
Iz_5_1009	DJI_0676	Sambucus nigra L.	0	n	1	1	Viburnaceae Raf.
Iz_5_1009	DJI_0676	Secale sylvestre Host	0	n	1	0	Poaceae Barnhart
Iz_5_1009	DJI_0676	Sempervivum ruthenicum Schnittsp. & C.B. Lehm.	0	n	1	0	Crassulaceae J.St.-Hil.
Iz_5_1009	DJI_0676	Setaria viridis (L.) P. Beauv.	0	n	1	0	Poaceae Barnhart
Iz_5_1009	DJI_0676	Silene borysthenica (Gruner) Walters	0	n	1	0	Caryophyllaceae Juss.
Iz_5_1009	DJI_0676	Silene latifolia subsp. alba (Mill.) Greuter & Burdet	0	n	1	0	Caryophyllaceae Juss.
Iz_5_1009	DJI_0676	Solidago virgaurea L.	0	n	1	0	Asteraceae Bercht. & J.Presl
Iz_5_1009	DJI_0676	Solidago Ã niederederi (S. canadensis Ã S. virgaurea)	0	hybrid	0	0	Asteraceae Bercht. & J.Presl
Iz_5_1009	DJI_0676	Sorbus aucuparia L.	0	n	1	1	Rosaceae Juss.
Iz_5_1009	DJI_0676	Stachys recta L.	0	n	1	0	Lamiacea Lindl.
Iz_5_1009	DJI_0676	Tanacetum vulgare L.	0	n	1	0	Asteraceae Bercht. & J.Presl
Iz_5_1009	DJI_0676	Thymus pallasianus Heinr.Braun	0	n	1	0	Lamiacea Lindl.
Iz_5_1009	DJI_0676	Tilia cordata Mill.	0	n	1	1	Malvaceae Juss.
Iz_5_1009	DJI_0676	Trifolium alpestre L.	0	n	1	0	Fabaceae Lindl.
Iz_5_1009	DJI_0676	Trifolium arvense L.	0	n	1	0	Fabaceae Lindl.
Iz_5_1009	DJI_0676	Ulmus glabra Huds.	0	n	1	1	Ulmaceae Mirb.
Iz_5_1009	DJI_0676	Verbascum densiflorum Bertol.	0	n	1	0	Scrophulariaceae Juss.
Iz_5_1009	DJI_0676	Veronica austriaca L.	0	n	1	0	Plantaginaceae Juss.
Iz_5_1009	DJI_0676	Veronica incana L.	0	n	1	0	Plantaginaceae Juss.
Iz_5_1009	DJI_0676	Veronica verna L.	0	n	1	0	Plantaginaceae Juss.
Iz_5_1009	DJI_0676	Vincetoxicum hirundinaria Medik	0	n	1	0	Apocynaceae Juss.
Iz_5_1009	DJI_0677	Acer tataricum L.	0	n	1	1	Sapindaceae Juss.
Iz_5_1009	DJI_0677	Achillea millefolium L. s.l.	0	n	1	0	Asteraceae Bercht. & J.Presl
Iz_5_1009	DJI_0677	Allium oleraceum L.	0	n	1	0	Amaryllidaceae J.St.-Hil.
Iz_5_1009	DJI_0677	Ambrosia artemisiifolia L.	0	a	0	0	Asteraceae Bercht. & J.Presl
Iz_5_1009	DJI_0677	Amorpha fruticosa L.	0	a	0	0	Fabaceae Lindl.
Iz_5_1009	DJI_0677	Anthericum ramosum L.	0	n	1	0	Asparagaceae Juss.
Iz_5_1009	DJI_0677	Artemisia marschalliana Spreng.	0	n	1	0	Asteraceae Bercht. & J.Presl
Iz_5_1009	DJI_0677	Asparagus officinalis L.	0	n	1	0	Asparagaceae Juss.
Iz_5_1009	DJI_0677	Astragalus cicer L.	0	n	1	0	Fabaceae Lindl.
Iz_5_1009	DJI_0677	Bassia laniflora (S.G.Gmel.) A.J.Scott	0	n	1	0	Amaranthaceae Juss.
Iz_5_1009	DJI_0677	Berberis vulgaris L.	0	n	1	0	Berberidaceae Juss.
Iz_5_1009	DJI_0677	Betula pendula Roth.	0	n	1	1	Betulaceae Gray
Iz_5_1009	DJI_0677	Betula pendula Roth.	0	n	1	0	Poaceae Barnhart
Iz_5_1009	DJI_0677	Bromus inermis Leyss.	0		NA	NA	
Iz_5_1009	DJI_0677	Bromus squarrosus L.	0	a	0	0	Poaceae Barnhart
Iz_5_1009	DJI_0677	Bromus tectorum L.	0	a	0	0	Poaceae Barnhart
Iz_5_1009	DJI_0677	Calamagrostis epigejos (L.) Roth	0	n	1	0	Poaceae Barnhart
Iz_5_1009	DJI_0677	Carduus acanthoides L.	0	n	1	0	Asteraceae Bercht. & J.Presl
Iz_5_1009	DJI_0677	Carex sp.	0	n	1	0	Cyperaceae Juss.
Iz_5_1009	DJI_0677	Centaurea majorovii Dumbadze	0	n	1	0	Asteraceae Bercht. & J.Presl
Iz_5_1009	DJI_0677	Chamaecytisus ruthenicus (Fisch.ex Wol.) Klaskova	0	n	1	0	Fabaceae Lindl.
Iz_5_1009	DJI_0677	Chelidonium majus L.	0	n	1	0	Papaveraceae Juss.
Iz_5_1009	DJI_0677	Chenopodium betaceum Andrz.	0	n	1	0	Amaranthaceae Juss.
Iz_5_1009	DJI_0677	Chenopodium cf. album	0	n	1	0	Amaranthaceae Juss.
Iz_5_1009	DJI_0677	Chenopodium sp.	0		NA	0	Amaranthaceae Juss.
Iz_5_1009	DJI_0677	Chondrilla juncea L.	0	n	1	0	Asteraceae Bercht. & J.Presl
Iz_5_1009	DJI_0677	Cirsium vulgare (Savi) Ten.	0	n	1	0	Asteraceae Bercht. & J.Presl
Iz_5_1009	DJI_0677	Clinopodium acinos (L.) Kuntze	0	n	1	0	Lamiacea Lindl.
Iz_5_1009	DJI_0677	Convallaria majalis L.	0	n	1	0	Asparagaceae Juss.
Iz_5_1009	DJI_0677	Crataegus rhipidophylla Gand.	0	n	1	1	Rosaceae Juss.
Iz_5_1009	DJI_0677	Crepis foetida L.	0	n	1	0	Asteraceae Bercht. & J.Presl
Iz_5_1009	DJI_0677	Dactylis glomerata L.	0	n	1	0	Poaceae Barnhart

Iz_5_1009	DJI_0677	Dianthus campestris M.Bieb.	0	n	1	0	Caryophyllaceae Juss.
Iz_5_1009	DJI_0677	Digitaria sanguinalis (L.) Scop.	0	a	0	0	Poaceae Barnhart
Iz_5_1009	DJI_0677	Elymus repens (L.) Gould	0	n	1	0	Poaceae Barnhart
Iz_5_1009	DJI_0677	Epilobium tetragonum L.	0	n	1	0	Onagraceae Juss.
Iz_5_1009	DJI_0677	Eragrostis minor Host	0	a	0	0	Poaceae Barnhart
Iz_5_1009	DJI_0677	Erigeron canadensis L.	10	a	0	0	Asteraceae Bercht. & J.Presl
Iz_5_1009	DJI_0677	Euphorbia seguieriana Neck.	0	n	1	0	Euphorbiaceae Juss.
Iz_5_1009	DJI_0677	Fallopia dumetorum (L.) Holub	0	n	1	0	Polygonaceae Juss.
Iz_5_1009	DJI_0677	Festuca beckeri (Hack.) Trautv.	0	n	1	0	Poaceae Barnhart
Iz_5_1009	DJI_0677	Fragaria vesca L.	0	n	1	0	Rosaceae Juss.
Iz_5_1009	DJI_0677	Frangula alnus Mill.	0	n	1	1	Rhamnaceae Juss.
Iz_5_1009	DJI_0677	Fraxinus pennsylvanica Marshall	0	a	0	1	Oleaceae Hoffmanns. & Link
Iz_5_1009	DJI_0677	Galeopsis bifida Boenn.	0	n	1	0	Lamiacea Lindl.
Iz_5_1009	DJI_0677	Galium verum L.	0	n	1	0	Rubiaceae Juss.
Iz_5_1009	DJI_0677	Genista tinctoria L.	0	n	1	0	Fabaceae Lindl.
Iz_5_1009	DJI_0677	Geranium robertianum L.	0	n	1	0	Geraniaceae Juss.
Iz_5_1009	DJI_0677	Geranium sanquineum L.	0	n	1	0	Geraniaceae Juss.
Iz_5_1009	DJI_0677	Gypsophila paniculata L.	0	n	1	0	Caryophyllaceae Juss.
Iz_5_1009	DJI_0677	Helichrysum arenarium (L.) Moench	0	n	1	0	Asteraceae Bercht. & J.Presl
Iz_5_1009	DJI_0677	Herniaria polygama J.Gay	0	n	1	0	Caryophyllaceae Juss.
Iz_5_1009	DJI_0677	Hieracium robustum Fr.	0	n	1	0	Asteraceae Bercht. & J.Presl
Iz_5_1009	DJI_0677	Hieracium umbellatum L.	0	n	1	NA	Asteraceae Bercht. & J.Presl
Iz_5_1009	DJI_0677	Hylotelephium maximum (L.) Holub	0	n	1	0	Crassulaceae J.St.-Hil.
Iz_5_1009	DJI_0677	Jacobaea borysthenica (DC.) B.Nord. & Greuter	0	n	1	0	Asteraceae Bercht. & J.Presl
Iz_5_1009	DJI_0677	Jasione montana L.	0	n	1	0	Campanulaceae Juss.
Iz_5_1009	DJI_0677	Jurinea cyanoides (L.) Rchb.s.l.	0	n	1	0	Asteraceae Bercht. & J.Presl
Iz_5_1009	DJI_0677	Koeleria glauca (Spreng.) DC.	0	n	1	0	Poaceae Barnhart
Iz_5_1009	DJI_0677	Lactuca serriola L.	0	a	0	0	Asteraceae Bercht. & J.Presl
Iz_5_1009	DJI_0677	Linaria genistifolia (L.) Mill.	0	n	1	0	Plantaginaceae Juss.
Iz_5_1009	DJI_0677	Lomelosia argentea (L.) Greuter & Burdet	0	n	1	0	Caprifoliaceae Juss.
Iz_5_1009	DJI_0677	Melampyrum pratense L.	0	n	1	0	Orobanchaceae Vent.
Iz_5_1009	DJI_0677	Oenothera biennis L.	0	a	0	0	Onagraceae Juss.
Iz_5_1009	DJI_0677	Pentanema britannica (L.) D.Gut.Larr., Santos-Vicente, Anderb., E.Rico & M.M.Mart.Ort.	0	n	1	0	Asteraceae Bercht. & J.Presl
Iz_5_1009	DJI_0677	Peucedanum oreoselinum (L.) Moench	0	n	1	0	Apiaceae Lindl.
Iz_5_1009	DJI_0677	Peucedanum ruthenicum M.Bieb.	0	n	1	0	Apiaceae Lindl.
Iz_5_1009	DJI_0677	Pilosella echioides (Lumn.) F.W.Schultz & Sch.Bip.	0	n	1	0	Asteraceae Bercht. & J.Presl
Iz_5_1009	DJI_0677	Pilosella officinarum F. Schultz et Sch. Bip.	0	n	1	0	Asteraceae Bercht. & J.Presl
Iz_5_1009	DJI_0677	Pilosella sp.	0		NA	0	Asteraceae Bercht. & J.Presl
Iz_5_1009	DJI_0677	Pinus sylvestris L.	0	n	1	1	Pinaceae Spreng. ex F.Rudolphi
Iz_5_1009	DJI_0677	Poa annua L.	0	n	1	0	Poaceae Barnhart
Iz_5_1009	DJI_0677	Poa nemoralis L.	0	n	1	0	Poaceae Barnhart
Iz_5_1009	DJI_0677	Poa sp.	0		NA	0	Poaceae Barnhart
Iz_5_1009	DJI_0677	Polygonatum odoratum (Mill.) Druce	0	n	1	0	Asparagaceae Juss.
Iz_5_1009	DJI_0677	Polygonum arenarium Waldst. & Kit.	0	n	1	0	Polygonaceae Juss.
Iz_5_1009	DJI_0677	Populus nigra L.	0	n	1	1	Salicaceae Mirb.
Iz_5_1009	DJI_0677	Populus sp.	0	n	1	1	Salicaceae Mirb.
Iz_5_1009	DJI_0677	Populus tremula L.	0	n	1	1	Salicaceae Mirb.
Iz_5_1009	DJI_0677	Populus Ã canescens (Aiton) Sm.	0	n	1	1	Salicaceae Mirb.
Iz_5_1009	DJI_0677	Potentilla argentea L.	0	n	1	0	Rosaceae Juss.
Iz_5_1009	DJI_0677	Psammophiliella muralis (L.) Ikonn.	0	n	1	0	Caryophyllaceae Juss.
Iz_5_1009	DJI_0677	Psephellus sumensis (Kalen.) Greuter	0	n	1	0	Asteraceae Bercht. & J.Presl
Iz_5_1009	DJI_0677	Pulsatilla pratensis (L.) Mill. s.l.	0	n	1	0	Ranunculaceae Juss.
Iz_5_1009	DJI_0677	Pyrus communis L.	0	a	0	1	Rosaceae Juss.
Iz_5_1009	DJI_0677	Quercus robur L.	0	n	1	1	Fagaceae Dumort.
Iz_5_1009	DJI_0677	Robinia pseudoacacia L.	0	a	0	1	Fabaceae Lindl.
Iz_5_1009	DJI_0677	Rosa sp.	0		NA	1	Rosaceae Juss.
Iz_5_1009	DJI_0677	Rubus idaeus L.	0	n	1	0	Rosaceae Juss.
Iz_5_1009	DJI_0677	Rumex acetosella L.	0	n	1	0	Polygonaceae Juss.
Iz_5_1009	DJI_0677	Rumex thyrsiflorus Fingerh.	0	n	1	0	Polygonaceae Juss.
Iz_5_1009	DJI_0677	Salix alba L.	0	n	1	1	Salicaceae Mirb.
Iz_5_1009	DJI_0677	Sambucus nigra L.	0	n	1	1	Viburnaceae Raf.
Iz_5_1009	DJI_0677	Secale sylvestre Host	0	n	1	0	Poaceae Barnhart
Iz_5_1009	DJI_0677	Sempervivum ruthenicum Schnittsp. & C.B. Lehm.	0	n	1	0	Crassulaceae J.St.-Hil.
Iz_5_1009	DJI_0677	Setaria viridis (L.) P. Beauv.	0	n	1	0	Poaceae Barnhart
Iz_5_1009	DJI_0677	Silene borysthenica (Gruner) Walters	0	n	1	0	Caryophyllaceae Juss.
Iz_5_1009	DJI_0677	Silene latifolia subsp. alba (Mill.) Greuter & Burdet	0	n	1	0	Caryophyllaceae Juss.
Iz_5_1009	DJI_0677	Solidago virgaurea L.	1	n	1	0	Asteraceae Bercht. & J.Presl
Iz_5_1009	DJI_0677	Solidago Ã niederederi (S. canadensis Ã S. virgaurea)	0	hybrid	0	0	Asteraceae Bercht. & J.Presl
Iz_5_1009	DJI_0677	Sorbus aucuparia L.	0	n	1	1	Rosaceae Juss.
Iz_5_1009	DJI_0677	Stachys recta L.	0	n	1	0	Lamiacea Lindl.
Iz_5_1009	DJI_0677	Tanacetum vulgare L.	0	n	1	0	Asteraceae Bercht. & J.Presl
Iz_5_1009	DJI_0677	Thymus pallasianus Heinr.Braun	0	n	1	0	Lamiacea Lindl.
Iz_5_1009	DJI_0677	Tilia cordata Mill.	0	n	1	1	Malvaceae Juss.
Iz_5_1009	DJI_0677	Trifolium alpestre L.	0	n	1	0	Fabaceae Lindl.
Iz_5_1009	DJI_0677	Trifolium arvense L.	0	n	1	0	Fabaceae Lindl.
Iz_5_1009	DJI_0677	Ulmus glabra Huds.	0	n	1	1	Ulmaceae Mirb.
Iz_5_1009	DJI_0677	Verbascum densiflorum Bertol.	0	n	1	0	Scrophulariaceae Juss.
Iz_5_1009	DJI_0677	Veronica austriaca L.	0	n	1	0	Plantaginaceae Juss.
Iz_5_1009	DJI_0677	Veronica incana L.	0	n	1	0	Plantaginaceae Juss.
Iz_5_1009	DJI_0677	Veronica verna L.	0	n	1	0	Plantaginaceae Juss.
Iz_5_1009	DJI_0677	Vincetoxicum hirundinaria Medik	0	n	1	0	Apocynaceae Juss.
Iz_5_1009	DJI_0679	Acer tataricum L.	0	n	1	1	Sapindaceae Juss.
Iz_5_1009	DJI_0679	Achillea millefolium L. s.l.	0	n	1	0	Asteraceae Bercht. & J.Presl
Iz_5_1009	DJI_0679	Allium oleraceum L.	0	n	1	0	Amaryllidaceae J.St.-Hil.
Iz_5_1009	DJI_0679	Ambrosia artemisiifolia L.	0	a	0	0	Asteraceae Bercht. & J.Presl
Iz_5_1009	DJI_0679	Amorpha fruticosa L.	0	a	0	0	Fabaceae Lindl.
Iz_5_1009	DJI_0679	Anthericum ramosum L.	0	n	1	0	Asparagaceae Juss.
Iz_5_1009	DJI_0679	Artemisia marschalliana Spreng.	0	n	1	0	Asteraceae Bercht. & J.Presl
Iz_5_1009	DJI_0679	Asparagus officinalis L.	0	n	1	0	Asparagaceae Juss.
Iz_5_1009	DJI_0679	Astragalus cicer L.	0	n	1	0	Fabaceae Lindl.
Iz_5_1009	DJI_0679	Bassia laniflora (S.G.Gmel.) A.J.Scott	0	n	1	0	Amaranthaceae Juss.
Iz_5_1009	DJI_0679	Berberis vulgaris L.	0	n	1	0	Berberidaceae Juss.
Iz_5_1009	DJI_0679	Betula pendula Roth.	0	n	1	1	Betulaceae Gray
Iz_5_1009	DJI_0679	Betula pendula Roth.	0	n	1	0	Poaceae Barnhart
Iz_5_1009	DJI_0679	Bromus inermis Leyss.	0		NA	NA	
Iz_5_1009	DJI_0679	Bromus squarrosus L.	0	a	0	0	Poaceae Barnhart
Iz_5_1009	DJI_0679	Bromus tectorum L.	0	a	0	0	Poaceae Barnhart
Iz_5_1009	DJI_0679	Calamagrostis epigejos (L.) Roth	0.2	n	1	0	Poaceae Barnhart

Iz_5_1009	DJI_0679	Carduus acanthoides L.	0	n	1	0	Asteraceae Bercht. & J.Presl
Iz_5_1009	DJI_0679	Carex sp.	0	n	1	0	Cyperaceae Juss.
Iz_5_1009	DJI_0679	Centaurea majorovii Dumbadze	0	n	1	0	Asteraceae Bercht. & J.Presl
Iz_5_1009	DJI_0679	Chamaecytisus ruthenicus (Fisch.ex Wol.) Klaskova	0	n	1	0	Fabaceae Lindl.
Iz_5_1009	DJI_0679	Chelidonium majus L.	0	n	1	0	Papaveraceae Juss.
Iz_5_1009	DJI_0679	Chenopodium betaceum Andrz.	0	n	1	0	Amaranthaceae Juss.
Iz_5_1009	DJI_0679	Chenopodium cf. album	0	n	1	0	Amaranthaceae Juss.
Iz_5_1009	DJI_0679	Chenopodium sp.	0		NA	0	Amaranthaceae Juss.
Iz_5_1009	DJI_0679	Chondrilla juncea L.	0	n	1	0	Asteraceae Bercht. & J.Presl
Iz_5_1009	DJI_0679	Cirsium vulgare (Savi) Ten.	0	n	1	0	Asteraceae Bercht. & J.Presl
Iz_5_1009	DJI_0679	Clinopodium acinos (L.) Kuntze	0	n	1	0	Lamiacea Lindl.
Iz_5_1009	DJI_0679	Convallaria majalis L.	0	n	1	0	Asparagaceae Juss.
Iz_5_1009	DJI_0679	Crataegus rhipidophylla Gand.	0	n	1	1	Rosaceae Juss.
Iz_5_1009	DJI_0679	Crepis foetida L.	0	n	1	0	Asteraceae Bercht. & J.Presl
Iz_5_1009	DJI_0679	Dactylis glomerata L.	0	n	1	0	Poaceae Barnhart
Iz_5_1009	DJI_0679	Dianthus campestris M.Bieb.	0	n	1	0	Caryophyllaceae Juss.
Iz_5_1009	DJI_0679	Digitaria sanguinalis (L.) Scop.	0	a	0	0	Poaceae Barnhart
Iz_5_1009	DJI_0679	Elymus repens (L.) Gould	0	n	1	0	Poaceae Barnhart
Iz_5_1009	DJI_0679	Epilobium tetragonum L.	0	n	1	0	Onagraceae Juss.
Iz_5_1009	DJI_0679	Eragrostis minor Host	0	a	0	0	Poaceae Barnhart
Iz_5_1009	DJI_0679	Erigeron canadensis L.	10	a	0	0	Asteraceae Bercht. & J.Presl
Iz_5_1009	DJI_0679	Euphorbia seguieriana Neck.	0	n	1	0	Euphorbiaceae Juss.
Iz_5_1009	DJI_0679	Fallopia dumetorum (L.) Holub	0	n	1	0	Polygonaceae Juss.
Iz_5_1009	DJI_0679	Festuca beckeri (Hack.) Trautv.	0	n	1	0	Poaceae Barnhart
Iz_5_1009	DJI_0679	Fragaria vesca L.	0	n	1	0	Rosaceae Juss.
Iz_5_1009	DJI_0679	Frangula alnus Mill.	0	n	1	1	Rhamnaceae Juss.
Iz_5_1009	DJI_0679	Fraxinus pennsylvanica Marshall	0	a	0	1	Oleaceae Hoffmanns. & Link
Iz_5_1009	DJI_0679	Galeopsis bifida Boenn.	0	n	1	0	Lamiacea Lindl.
Iz_5_1009	DJI_0679	Galium verum L.	0	n	1	0	Rubiaceae Juss.
Iz_5_1009	DJI_0679	Genista tinctoria L.	0	n	1	0	Fabaceae Lindl.
Iz_5_1009	DJI_0679	Geranium robertianum L.	0	n	1	0	Geraniaceae Juss.
Iz_5_1009	DJI_0679	Geranium sanquineum L.	0	n	1	0	Geraniaceae Juss.
Iz_5_1009	DJI_0679	Gypsophila paniculata L.	0	n	1	0	Caryophyllaceae Juss.
Iz_5_1009	DJI_0679	Helichrysum arenarium (L.) Moench	0	n	1	0	Asteraceae Bercht. & J.Presl
Iz_5_1009	DJI_0679	Herniaria polygama J.Gay	0	n	1	0	Caryophyllaceae Juss.
Iz_5_1009	DJI_0679	Hieracium robustum Fr.	0	n	1	0	Asteraceae Bercht. & J.Presl
Iz_5_1009	DJI_0679	Hieracium umbellatum L.	0	n	1	NA	Asteraceae Bercht. & J.Presl
Iz_5_1009	DJI_0679	Hylotelephium maximum (L.) Holub	0	n	1	0	Crassulaceae J.St.-Hil.
Iz_5_1009	DJI_0679	Jacobaea borysthenica (DC.) B.Nord. & Greuter	0	n	1	0	Asteraceae Bercht. & J.Presl
Iz_5_1009	DJI_0679	Jasione montana L.	0	n	1	0	Campanulaceae Juss.
Iz_5_1009	DJI_0679	Jurinea cyanoides (L.) Rchb.s.l.	0	n	1	0	Asteraceae Bercht. & J.Presl
Iz_5_1009	DJI_0679	Koeleria glauca (Spreng.) DC.	1	n	1	0	Poaceae Barnhart
Iz_5_1009	DJI_0679	Lactuca serriola L.	0	a	0	0	Asteraceae Bercht. & J.Presl
Iz_5_1009	DJI_0679	Linaria genistifolia (L.) Mill.	0	n	1	0	Plantaginaceae Juss.
Iz_5_1009	DJI_0679	Lomelosia argentea (L.) Greuter & Burdet	0	n	1	0	Caprifoliaceae Juss.
Iz_5_1009	DJI_0679	Melampyrum pratense L.	0	n	1	0	Orobanchaceae Vent.
Iz_5_1009	DJI_0679	Oenothera biennis L.	0	a	0	0	Onagraceae Juss.
Iz_5_1009	DJI_0679	Pentanema britannica (L.) D.Gut.Larr., Santos-Vicente, Anderb., E.Rico & M.M.Mart.Ort.	0	n	1	0	Asteraceae Bercht. & J.Presl
Iz_5_1009	DJI_0679	Peucedanum oreoselinum (L.) Moench	0	n	1	0	Apiaceae Lindl.
Iz_5_1009	DJI_0679	Peucedanum ruthenicum M.Bieb.	0	n	1	0	Apiaceae Lindl.
Iz_5_1009	DJI_0679	Pilosella echioides (Lumn.) F.W.Schultz & Sch.Bip.	0	n	1	0	Asteraceae Bercht. & J.Presl
Iz_5_1009	DJI_0679	Pilosella officinarum F. Schultz et Sch. Bip.	0	n	1	0	Asteraceae Bercht. & J.Presl
Iz_5_1009	DJI_0679	Pilosella sp.	0		NA	0	Asteraceae Bercht. & J.Presl
Iz_5_1009	DJI_0679	Pinus sylvestris L.	0	n	1	1	Pinaceae Spreng. ex F.Rudolphi
Iz_5_1009	DJI_0679	Poa annua L.	0	n	1	0	Poaceae Barnhart
Iz_5_1009	DJI_0679	Poa nemoralis L.	0	n	1	0	Poaceae Barnhart
Iz_5_1009	DJI_0679	Poa sp.	0		NA	0	Poaceae Barnhart
Iz_5_1009	DJI_0679	Polygonatum odoratum (Mill.) Druce	0	n	1	0	Asparagaceae Juss.
Iz_5_1009	DJI_0679	Polygonum arenarium Waldst. & Kit.	0	n	1	0	Polygonaceae Juss.
Iz_5_1009	DJI_0679	Populus nigra L.	0	n	1	1	Salicaceae Mirb.
Iz_5_1009	DJI_0679	Populus sp.	0	n	1	1	Salicaceae Mirb.
Iz_5_1009	DJI_0679	Populus tremula L.	10	n	1	1	Salicaceae Mirb.
Iz_5_1009	DJI_0679	Populus Ã canescens (Aiton) Sm.	0	n	1	1	Salicaceae Mirb.
Iz_5_1009	DJI_0679	Potentilla argentea L.	0	n	1	0	Rosaceae Juss.
Iz_5_1009	DJI_0679	Psammophiliella muralis (L.) Ikonn.	0	n	1	0	Caryophyllaceae Juss.
Iz_5_1009	DJI_0679	Psephellus sumensis (Kalen.) Greuter	0	n	1	0	Asteraceae Bercht. & J.Presl
Iz_5_1009	DJI_0679	Pulsatilla pratensis (L.) Mill. s.l.	0	n	1	0	Ranunculaceae Juss.
Iz_5_1009	DJI_0679	Pyrus communis L.	0	a	0	1	Rosaceae Juss.
Iz_5_1009	DJI_0679	Quercus robur L.	0	n	1	1	Fagaceae Dumort.
Iz_5_1009	DJI_0679	Robinia pseudoacacia L.	0	a	0	1	Fabaceae Lindl.
Iz_5_1009	DJI_0679	Rosa sp.	0		NA	1	Rosaceae Juss.
Iz_5_1009	DJI_0679	Rubus idaeus L.	0	n	1	0	Rosaceae Juss.
Iz_5_1009	DJI_0679	Rumex acetosella L.	0	n	1	0	Polygonaceae Juss.
Iz_5_1009	DJI_0679	Rumex thyrsiflorus Fingerh.	0	n	1	0	Polygonaceae Juss.
Iz_5_1009	DJI_0679	Salix alba L.	0	n	1	1	Salicaceae Mirb.
Iz_5_1009	DJI_0679	Sambucus nigra L.	0	n	1	1	Viburnaceae Raf.
Iz_5_1009	DJI_0679	Secale sylvestre Host	0	n	1	0	Poaceae Barnhart
Iz_5_1009	DJI_0679	Sempervivum ruthenicum Schnittsp. & C.B. Lehm.	0	n	1	0	Crassulaceae J.St.-Hil.
Iz_5_1009	DJI_0679	Setaria viridis (L.) P. Beauv.	0	n	1	0	Poaceae Barnhart
Iz_5_1009	DJI_0679	Silene borysthenica (Gruner) Walters	0	n	1	0	Caryophyllaceae Juss.
Iz_5_1009	DJI_0679	Silene latifolia subsp. alba (Mill.) Greuter & Burdet	0	n	1	0	Caryophyllaceae Juss.
Iz_5_1009	DJI_0679	Solidago virgaurea L.	3	n	1	0	Asteraceae Bercht. & J.Presl
Iz_5_1009	DJI_0679	Solidago Ã niederederi (S. canadensis Ã S. virgaurea)	0	hybrid	0	0	Asteraceae Bercht. & J.Presl
Iz_5_1009	DJI_0679	Sorbus aucuparia L.	0	n	1	1	Rosaceae Juss.
Iz_5_1009	DJI_0679	Stachys recta L.	0	n	1	0	Lamiacea Lindl.
Iz_5_1009	DJI_0679	Tanacetum vulgare L.	0	n	1	0	Asteraceae Bercht. & J.Presl
Iz_5_1009	DJI_0679	Thymus pallasianus Heinr.Braun	0	n	1	0	Lamiacea Lindl.
Iz_5_1009	DJI_0679	Tilia cordata Mill.	0	n	1	1	Malvaceae Juss.
Iz_5_1009	DJI_0679	Trifolium alpestre L.	0	n	1	0	Fabaceae Lindl.
Iz_5_1009	DJI_0679	Trifolium arvense L.	0	n	1	0	Fabaceae Lindl.
Iz_5_1009	DJI_0679	Ulmus glabra Huds.	0	n	1	1	Ulmaceae Mirb.
Iz_5_1009	DJI_0679	Verbascum densiflorum Bertol.	0	n	1	0	Scrophulariaceae Juss.
Iz_5_1009	DJI_0679	Veronica austriaca L.	0	n	1	0	Plantaginaceae Juss.
Iz_5_1009	DJI_0679	Veronica incana L.	0	n	1	0	Plantaginaceae Juss.
Iz_5_1009	DJI_0679	Veronica verna L.	0	n	1	0	Plantaginaceae Juss.
Iz_5_1009	DJI_0679	Vincetoxicum hirundinaria Medik	0	n	1	0	Apocynaceae Juss.
Iz_5_1009	DJI_0685	Acer tataricum L.	0	n	1	1	Sapindaceae Juss.
Iz_5_1009	DJI_0685	Achillea millefolium L. s.l.	0	n	1	0	Asteraceae Bercht. & J.Presl

Iz_5_1009	DJI_0685	Allium oleraceum L.	0	n	1	0	Amaryllidaceae J.St.-Hil.
Iz_5_1009	DJI_0685	Ambrosia artemisiifolia L.	0	a	0	0	Asteraceae Bercht. & J.Presl
Iz_5_1009	DJI_0685	Amorpha fruticosa L.	0	a	0	0	Fabaceae Lindl.
Iz_5_1009	DJI_0685	Anthericum ramosum L.	0	n	1	0	Asparagaceae Juss.
Iz_5_1009	DJI_0685	Artemisia marschalliana Spreng.	0	n	1	0	Asteraceae Bercht. & J.Presl
Iz_5_1009	DJI_0685	Asparagus officinalis L.	0	n	1	0	Asparagaceae Juss.
Iz_5_1009	DJI_0685	Astragalus cicer L.	0	n	1	0	Fabaceae Lindl.
Iz_5_1009	DJI_0685	Bassia laniflora (S.G.Gmel.) A.J.Scott	0	n	1	0	Amaranthaceae Juss.
Iz_5_1009	DJI_0685	Berberis vulgaris L.	0	n	1	0	Berberidaceae Juss.
Iz_5_1009	DJI_0685	Betula pendula Roth.	0	n	1	1	Betulaceae Gray
Iz_5_1009	DJI_0685	Betula pendula Roth.	0	n	1	0	Poaceae Barnhart
Iz_5_1009	DJI_0685	Bromus inermis Leyss.	0		NA	NA	
Iz_5_1009	DJI_0685	Bromus squarrosus L.	0	a	0	0	Poaceae Barnhart
Iz_5_1009	DJI_0685	Bromus tectorum L.	0	a	0	0	Poaceae Barnhart
Iz_5_1009	DJI_0685	Calamagrostis epigejos (L.) Roth	0	n	1	0	Poaceae Barnhart
Iz_5_1009	DJI_0685	Carduus acanthoides L.	0	n	1	0	Asteraceae Bercht. & J.Presl
Iz_5_1009	DJI_0685	Carex sp.	0	n	1	0	Cyperaceae Juss.
Iz_5_1009	DJI_0685	Centaurea majorovii Dumbadze	0	n	1	0	Asteraceae Bercht. & J.Presl
Iz_5_1009	DJI_0685	Chamaecytisus ruthenicus (Fisch.ex Wol.) Klaskova	0	n	1	0	Fabaceae Lindl.
Iz_5_1009	DJI_0685	Chelidonium majus L.	0	n	1	0	Papaveraceae Juss.
Iz_5_1009	DJI_0685	Chenopodium betaceum Andrz.	0	n	1	0	Amaranthaceae Juss.
Iz_5_1009	DJI_0685	Chenopodium cf. album	0	n	1	0	Amaranthaceae Juss.
Iz_5_1009	DJI_0685	Chenopodium sp.	0		NA	0	Amaranthaceae Juss.
Iz_5_1009	DJI_0685	Chondrilla juncea L.	0	n	1	0	Asteraceae Bercht. & J.Presl
Iz_5_1009	DJI_0685	Cirsium vulgare (Savi) Ten.	0	n	1	0	Asteraceae Bercht. & J.Presl
Iz_5_1009	DJI_0685	Clinopodium acinos (L.) Kuntze	0	n	1	0	Lamiacea Lindl.
Iz_5_1009	DJI_0685	Convallaria majalis L.	0	n	1	0	Asparagaceae Juss.
Iz_5_1009	DJI_0685	Crataegus rhipidophylla Gand.	0	n	1	1	Rosaceae Juss.
Iz_5_1009	DJI_0685	Crepis foetida L.	0	n	1	0	Asteraceae Bercht. & J.Presl
Iz_5_1009	DJI_0685	Dactylis glomerata L.	0	n	1	0	Poaceae Barnhart
Iz_5_1009	DJI_0685	Dianthus campestris M.Bieb.	0	n	1	0	Caryophyllaceae Juss.
Iz_5_1009	DJI_0685	Digitaria sanguinalis (L.) Scop.	0	a	0	0	Poaceae Barnhart
Iz_5_1009	DJI_0685	Elymus repens (L.) Gould	0	n	1	0	Poaceae Barnhart
Iz_5_1009	DJI_0685	Epilobium tetragonum L.	0	n	1	0	Onagraceae Juss.
Iz_5_1009	DJI_0685	Eragrostis minor Host	0	a	0	0	Poaceae Barnhart
Iz_5_1009	DJI_0685	Erigeron canadensis L.	10	a	0	0	Asteraceae Bercht. & J.Presl
Iz_5_1009	DJI_0685	Euphorbia seguieriana Neck.	0	n	1	0	Euphorbiaceae Juss.
Iz_5_1009	DJI_0685	Fallopia dumetorum (L.) Holub	0	n	1	0	Polygonaceae Juss.
Iz_5_1009	DJI_0685	Festuca beckeri (Hack.) Trautv.	0	n	1	0	Poaceae Barnhart
Iz_5_1009	DJI_0685	Fragaria vesca L.	0	n	1	0	Rosaceae Juss.
Iz_5_1009	DJI_0685	Frangula alnus Mill.	0	n	1	1	Rhamnaceae Juss.
Iz_5_1009	DJI_0685	Fraxinus pennsylvanica Marshall	0	a	0	1	Oleaceae Hoffmanns. & Link
Iz_5_1009	DJI_0685	Galeopsis bifida Boenn.	0	n	1	0	Lamiacea Lindl.
Iz_5_1009	DJI_0685	Galium verum L.	0	n	1	0	Rubiaceae Juss.
Iz_5_1009	DJI_0685	Genista tinctoria L.	0	n	1	0	Fabaceae Lindl.
Iz_5_1009	DJI_0685	Geranium robertianum L.	0	n	1	0	Geraniaceae Juss.
Iz_5_1009	DJI_0685	Geranium sanquineum L.	0	n	1	0	Geraniaceae Juss.
Iz_5_1009	DJI_0685	Gypsophila paniculata L.	0	n	1	0	Caryophyllaceae Juss.
Iz_5_1009	DJI_0685	Helichrysum arenarium (L.) Moench	0	n	1	0	Asteraceae Bercht. & J.Presl
Iz_5_1009	DJI_0685	Herniaria polygama J.Gay	0	n	1	0	Caryophyllaceae Juss.
Iz_5_1009	DJI_0685	Hieracium robustum Fr.	0	n	1	0	Asteraceae Bercht. & J.Presl
Iz_5_1009	DJI_0685	Hieracium umbellatum L.	0	n	1	NA	Asteraceae Bercht. & J.Presl
Iz_5_1009	DJI_0685	Hylotelephium maximum (L.) Holub	0	n	1	0	Crassulaceae J.St.-Hil.
Iz_5_1009	DJI_0685	Jacobaea borysthenica (DC.) B.Nord. & Greuter	0	n	1	0	Asteraceae Bercht. & J.Presl
Iz_5_1009	DJI_0685	Jasione montana L.	0	n	1	0	Campanulaceae Juss.
Iz_5_1009	DJI_0685	Jurinea cyanoides (L.) Rchb.s.l.	0	n	1	0	Asteraceae Bercht. & J.Presl
Iz_5_1009	DJI_0685	Koeleria glauca (Spreng.) DC.	0	n	1	0	Poaceae Barnhart
Iz_5_1009	DJI_0685	Lactuca serriola L.	0	a	0	0	Asteraceae Bercht. & J.Presl
Iz_5_1009	DJI_0685	Linaria genistifolia (L.) Mill.	0	n	1	0	Plantaginaceae Juss.
Iz_5_1009	DJI_0685	Lomelosia argentea (L.) Greuter & Burdet	0	n	1	0	Caprifoliaceae Juss.
Iz_5_1009	DJI_0685	Melampyrum pratense L.	0	n	1	0	Orobanchaceae Vent.
Iz_5_1009	DJI_0685	Oenothera biennis L.	0	a	0	0	Onagraceae Juss.
Iz_5_1009	DJI_0685	Pentanema britannica (L.) D.Gut.Larr., Santos-Vicente, Anderb., E.Rico & M.M.Mart.Ort.	0	n	1	0	Asteraceae Bercht. & J.Presl
Iz_5_1009	DJI_0685	Peucedanum oreoselinum (L.) Moench	0	n	1	0	Apiaceae Lindl.
Iz_5_1009	DJI_0685	Peucedanum ruthenicum M.Bieb.	0	n	1	0	Apiaceae Lindl.
Iz_5_1009	DJI_0685	Pilosella echioides (Lumn.) F.W.Schultz & Sch.Bip.	0	n	1	0	Asteraceae Bercht. & J.Presl
Iz_5_1009	DJI_0685	Pilosella officinarum F. Schultz et Sch. Bip.	0	n	1	0	Asteraceae Bercht. & J.Presl
Iz_5_1009	DJI_0685	Pilosella sp.	0		NA	0	Asteraceae Bercht. & J.Presl
Iz_5_1009	DJI_0685	Pinus sylvestris L.	0	n	1	1	Pinaceae Spreng. ex F.Rudolphi
Iz_5_1009	DJI_0685	Poa annua L.	0	n	1	0	Poaceae Barnhart
Iz_5_1009	DJI_0685	Poa nemoralis L.	0	n	1	0	Poaceae Barnhart
Iz_5_1009	DJI_0685	Poa sp.	0		NA	0	Poaceae Barnhart
Iz_5_1009	DJI_0685	Polygonatum odoratum (Mill.) Druce	0	n	1	0	Asparagaceae Juss.
Iz_5_1009	DJI_0685	Polygonum arenarium Waldst. & Kit.	0	n	1	0	Polygonaceae Juss.
Iz_5_1009	DJI_0685	Populus nigra L.	0	n	1	1	Salicaceae Mirb.
Iz_5_1009	DJI_0685	Populus sp.	0	n	1	1	Salicaceae Mirb.
Iz_5_1009	DJI_0685	Populus tremula L.	0	n	1	1	Salicaceae Mirb.
Iz_5_1009	DJI_0685	Populus Ã canescens (Aiton) Sm.	0	n	1	1	Salicaceae Mirb.
Iz_5_1009	DJI_0685	Potentilla argentea L.	0	n	1	0	Rosaceae Juss.
Iz_5_1009	DJI_0685	Psammophiliella muralis (L.) Ikonn.	0	n	1	0	Caryophyllaceae Juss.
Iz_5_1009	DJI_0685	Psephellus sumensis (Kalen.) Greuter	0	n	1	0	Asteraceae Bercht. & J.Presl
Iz_5_1009	DJI_0685	Pulsatilla pratensis (L.) Mill. s.l.	0	n	1	0	Ranunculaceae Juss.
Iz_5_1009	DJI_0685	Pyrus communis L.	0	a	0	1	Rosaceae Juss.
Iz_5_1009	DJI_0685	Quercus robur L.	0	n	1	1	Fagaceae Dumort.
Iz_5_1009	DJI_0685	Robinia pseudoacacia L.	0	a	0	1	Fabaceae Lindl.
Iz_5_1009	DJI_0685	Rosa sp.	0		NA	1	Rosaceae Juss.
Iz_5_1009	DJI_0685	Rubus idaeus L.	0	n	1	0	Rosaceae Juss.
Iz_5_1009	DJI_0685	Rumex acetosella L.	0	n	1	0	Polygonaceae Juss.
Iz_5_1009	DJI_0685	Rumex thyrsiflorus Fingerh.	0	n	1	0	Polygonaceae Juss.
Iz_5_1009	DJI_0685	Salix alba L.	0	n	1	1	Salicaceae Mirb.
Iz_5_1009	DJI_0685	Sambucus nigra L.	0	n	1	1	Viburnaceae Raf.
Iz_5_1009	DJI_0685	Secale sylvestre Host	0	n	1	0	Poaceae Barnhart
Iz_5_1009	DJI_0685	Sempervivum ruthenicum Schnittsp. & C.B. Lehm.	0	n	1	0	Crassulaceae J.St.-Hil.
Iz_5_1009	DJI_0685	Setaria viridis (L.) P. Beauv.	0	n	1	0	Poaceae Barnhart
Iz_5_1009	DJI_0685	Silene borysthenica (Gruner) Walters	0	n	1	0	Caryophyllaceae Juss.
Iz_5_1009	DJI_0685	Silene latifolia subsp. alba (Mill.) Greuter & Burdet	0	n	1	0	Caryophyllaceae Juss.
Iz_5_1009	DJI_0685	Solidago virgaurea L.	2	n	1	0	Asteraceae Bercht. & J.Presl
Iz_5_1009	DJI_0685	Solidago Ã niederederi (S. canadensis Ã S. virgaurea)	0	hybrid	0	0	Asteraceae Bercht. & J.Presl

Iz_5_1009	DJI_0685	Sorbus aucuparia L.	0	n	1	1	Rosaceae Juss.
Iz_5_1009	DJI_0685	Stachys recta L.	0	n	1	0	Lamiacea Lindl.
Iz_5_1009	DJI_0685	Tanacetum vulgare L.	0	n	1	0	Asteraceae Bercht. & J.Presl
Iz_5_1009	DJI_0685	Thymus pallasianus Heinr.Braun	0	n	1	0	Lamiacea Lindl.
Iz_5_1009	DJI_0685	Tilia cordata Mill.	0	n	1	1	Malvaceae Juss.
Iz_5_1009	DJI_0685	Trifolium alpestre L.	0	n	1	0	Fabaceae Lindl.
Iz_5_1009	DJI_0685	Trifolium arvense L.	0	n	1	0	Fabaceae Lindl.
Iz_5_1009	DJI_0685	Ulmus glabra Huds.	0	n	1	1	Ulmaceae Mirb.
Iz_5_1009	DJI_0685	Verbascum densiflorum Bertol.	0	n	1	0	Scrophulariaceae Juss.
Iz_5_1009	DJI_0685	Veronica austriaca L.	0	n	1	0	Plantaginaceae Juss.
Iz_5_1009	DJI_0685	Veronica incana L.	0	n	1	0	Plantaginaceae Juss.
Iz_5_1009	DJI_0685	Veronica verna L.	0	n	1	0	Plantaginaceae Juss.
Iz_5_1009	DJI_0685	Vincetoxicum hirundinaria Medik	0	n	1	0	Apocynaceae Juss.
Iz_5_1009	DJI_0686	Acer tataricum L.	0	n	1	1	Sapindaceae Juss.
Iz_5_1009	DJI_0686	Achillea millefolium L. s.l.	0	n	1	0	Asteraceae Bercht. & J.Presl
Iz_5_1009	DJI_0686	Allium oleraceum L.	0	n	1	0	Amaryllidaceae J.St.-Hil.
Iz_5_1009	DJI_0686	Ambrosia artemisiifolia L.	0	a	0	0	Asteraceae Bercht. & J.Presl
Iz_5_1009	DJI_0686	Amorpha fruticosa L.	0	a	0	0	Fabaceae Lindl.
Iz_5_1009	DJI_0686	Anthericum ramosum L.	0	n	1	0	Asparagaceae Juss.
Iz_5_1009	DJI_0686	Artemisia marschalliana Spreng.	0	n	1	0	Asteraceae Bercht. & J.Presl
Iz_5_1009	DJI_0686	Asparagus officinalis L.	0	n	1	0	Asparagaceae Juss.
Iz_5_1009	DJI_0686	Astragalus cicer L.	0	n	1	0	Fabaceae Lindl.
Iz_5_1009	DJI_0686	Bassia laniflora (S.G.Gmel.) A.J.Scott	0	n	1	0	Amaranthaceae Juss.
Iz_5_1009	DJI_0686	Berberis vulgaris L.	0	n	1	0	Berberidaceae Juss.
Iz_5_1009	DJI_0686	Betula pendula Roth.	0	n	1	1	Betulaceae Gray
Iz_5_1009	DJI_0686	Betula pendula Roth.	0	n	1	0	Poaceae Barnhart
Iz_5_1009	DJI_0686	Bromus inermis Leyss.	0		NA	NA	
Iz_5_1009	DJI_0686	Bromus squarrosus L.	0	a	0	0	Poaceae Barnhart
Iz_5_1009	DJI_0686	Bromus tectorum L.	0	a	0	0	Poaceae Barnhart
Iz_5_1009	DJI_0686	Calamagrostis epigejos (L.) Roth	0.5	n	1	0	Poaceae Barnhart
Iz_5_1009	DJI_0686	Carduus acanthoides L.	0	n	1	0	Asteraceae Bercht. & J.Presl
Iz_5_1009	DJI_0686	Carex sp.	0	n	1	0	Cyperaceae Juss.
Iz_5_1009	DJI_0686	Centaurea majorovii Dumbadze	0	n	1	0	Asteraceae Bercht. & J.Presl
Iz_5_1009	DJI_0686	Chamaecytisus ruthenicus (Fisch.ex Wol.) Klaskova	0	n	1	0	Fabaceae Lindl.
Iz_5_1009	DJI_0686	Chelidonium majus L.	0	n	1	0	Papaveraceae Juss.
Iz_5_1009	DJI_0686	Chenopodium betaceum Andrz.	0	n	1	0	Amaranthaceae Juss.
Iz_5_1009	DJI_0686	Chenopodium cf. album	0	n	1	0	Amaranthaceae Juss.
Iz_5_1009	DJI_0686	Chenopodium sp.	0		NA	0	Amaranthaceae Juss.
Iz_5_1009	DJI_0686	Chondrilla juncea L.	0	n	1	0	Asteraceae Bercht. & J.Presl
Iz_5_1009	DJI_0686	Cirsium vulgare (Savi) Ten.	0	n	1	0	Asteraceae Bercht. & J.Presl
Iz_5_1009	DJI_0686	Clinopodium acinos (L.) Kuntze	0	n	1	0	Lamiacea Lindl.
Iz_5_1009	DJI_0686	Convallaria majalis L.	0	n	1	0	Asparagaceae Juss.
Iz_5_1009	DJI_0686	Crataegus rhipidophylla Gand.	0	n	1	1	Rosaceae Juss.
Iz_5_1009	DJI_0686	Crepis foetida L.	0	n	1	0	Asteraceae Bercht. & J.Presl
Iz_5_1009	DJI_0686	Dactylis glomerata L.	0	n	1	0	Poaceae Barnhart
Iz_5_1009	DJI_0686	Dianthus campestris M.Bieb.	0	n	1	0	Caryophyllaceae Juss.
Iz_5_1009	DJI_0686	Digitaria sanguinalis (L.) Scop.	0	a	0	0	Poaceae Barnhart
Iz_5_1009	DJI_0686	Elymus repens (L.) Gould	0	n	1	0	Poaceae Barnhart
Iz_5_1009	DJI_0686	Epilobium tetragonum L.	0	n	1	0	Onagraceae Juss.
Iz_5_1009	DJI_0686	Eragrostis minor Host	0	a	0	0	Poaceae Barnhart
Iz_5_1009	DJI_0686	Erigeron canadensis L.	10	a	0	0	Asteraceae Bercht. & J.Presl
Iz_5_1009	DJI_0686	Euphorbia seguieriana Neck.	0	n	1	0	Euphorbiaceae Juss.
Iz_5_1009	DJI_0686	Fallopia dumetorum (L.) Holub	0	n	1	0	Polygonaceae Juss.
Iz_5_1009	DJI_0686	Festuca beckeri (Hack.) Trautv.	0	n	1	0	Poaceae Barnhart
Iz_5_1009	DJI_0686	Fragaria vesca L.	0	n	1	0	Rosaceae Juss.
Iz_5_1009	DJI_0686	Frangula alnus Mill.	0	n	1	1	Rhamnaceae Juss.
Iz_5_1009	DJI_0686	Fraxinus pennsylvanica Marshall	0	a	0	1	Oleaceae Hoffmanns. & Link
Iz_5_1009	DJI_0686	Galeopsis bifida Boenn.	0	n	1	0	Lamiacea Lindl.
Iz_5_1009	DJI_0686	Galium verum L.	0	n	1	0	Rubiaceae Juss.
Iz_5_1009	DJI_0686	Genista tinctoria L.	0	n	1	0	Fabaceae Lindl.
Iz_5_1009	DJI_0686	Geranium robertianum L.	0	n	1	0	Geraniaceae Juss.
Iz_5_1009	DJI_0686	Geranium sanquineum L.	0	n	1	0	Geraniaceae Juss.
Iz_5_1009	DJI_0686	Gypsophila paniculata L.	0	n	1	0	Caryophyllaceae Juss.
Iz_5_1009	DJI_0686	Helichrysum arenarium (L.) Moench	0	n	1	0	Asteraceae Bercht. & J.Presl
Iz_5_1009	DJI_0686	Herniaria polygama J.Gay	0	n	1	0	Caryophyllaceae Juss.
Iz_5_1009	DJI_0686	Hieracium robustum Fr.	0	n	1	0	Asteraceae Bercht. & J.Presl
Iz_5_1009	DJI_0686	Hieracium umbellatum L.	0	n	1	NA	Asteraceae Bercht. & J.Presl
Iz_5_1009	DJI_0686	Hylotelephium maximum (L.) Holub	0	n	1	0	Crassulaceae J.St.-Hil.
Iz_5_1009	DJI_0686	Jacobaea borysthenica (DC.) B.Nord. & Greuter	0	n	1	0	Asteraceae Bercht. & J.Presl
Iz_5_1009	DJI_0686	Jasione montana L.	0	n	1	0	Campanulaceae Juss.
Iz_5_1009	DJI_0686	Jurinea cyanoides (L.) Rchb.s.l.	0	n	1	0	Asteraceae Bercht. & J.Presl
Iz_5_1009	DJI_0686	Koeleria glauca (Spreng.) DC.	0	n	1	0	Poaceae Barnhart
Iz_5_1009	DJI_0686	Lactuca serriola L.	0	a	0	0	Asteraceae Bercht. & J.Presl
Iz_5_1009	DJI_0686	Linaria genistifolia (L.) Mill.	0	n	1	0	Plantaginaceae Juss.
Iz_5_1009	DJI_0686	Lomelosia argentea (L.) Greuter & Burdet	0	n	1	0	Caprifoliaceae Juss.
Iz_5_1009	DJI_0686	Melampyrum pratense L.	0	n	1	0	Orobanchaceae Vent.
Iz_5_1009	DJI_0686	Oenothera biennis L.	0	a	0	0	Onagraceae Juss.
Iz_5_1009	DJI_0686	Pentanema britannica (L.) D.Gut.Larr., Santos-Vicente, Anderb., E.Rico & M.M.Mart.Ort.	0	n	1	0	Asteraceae Bercht. & J.Presl
Iz_5_1009	DJI_0686	Peucedanum oreoselinum (L.) Moench	0	n	1	0	Apiaceae Lindl.
Iz_5_1009	DJI_0686	Peucedanum ruthenicum M.Bieb.	0	n	1	0	Apiaceae Lindl.
Iz_5_1009	DJI_0686	Pilosella echioides (Lumn.) F.W.Schultz & Sch.Bip.	0	n	1	0	Asteraceae Bercht. & J.Presl
Iz_5_1009	DJI_0686	Pilosella officinarum F. Schultz et Sch. Bip.	0	n	1	0	Asteraceae Bercht. & J.Presl
Iz_5_1009	DJI_0686	Pilosella sp.	0		NA	0	Asteraceae Bercht. & J.Presl
Iz_5_1009	DJI_0686	Pinus sylvestris L.	0	n	1	1	Pinaceae Spreng. ex F.Rudolphi
Iz_5_1009	DJI_0686	Poa annua L.	0	n	1	0	Poaceae Barnhart
Iz_5_1009	DJI_0686	Poa nemoralis L.	0	n	1	0	Poaceae Barnhart
Iz_5_1009	DJI_0686	Poa sp.	0		NA	0	Poaceae Barnhart
Iz_5_1009	DJI_0686	Polygonatum odoratum (Mill.) Druce	0	n	1	0	Asparagaceae Juss.
Iz_5_1009	DJI_0686	Polygonum arenarium Waldst. & Kit.	0	n	1	0	Polygonaceae Juss.
Iz_5_1009	DJI_0686	Populus nigra L.	0	n	1	1	Salicaceae Mirb.
Iz_5_1009	DJI_0686	Populus sp.	0	n	1	1	Salicaceae Mirb.
Iz_5_1009	DJI_0686	Populus tremula L.	0	n	1	1	Salicaceae Mirb.
Iz_5_1009	DJI_0686	Populus Ã canescens (Aiton) Sm.	0	n	1	1	Salicaceae Mirb.
Iz_5_1009	DJI_0686	Potentilla argentea L.	0	n	1	0	Rosaceae Juss.
Iz_5_1009	DJI_0686	Psammophiliella muralis (L.) Ikonn.	0	n	1	0	Caryophyllaceae Juss.
Iz_5_1009	DJI_0686	Psephellus sumensis (Kalen.) Greuter	0	n	1	0	Asteraceae Bercht. & J.Presl
Iz_5_1009	DJI_0686	Pulsatilla pratensis (L.) Mill. s.l.	0	n	1	0	Ranunculaceae Juss.
Iz_5_1009	DJI_0686	Pyrus communis L.	0	a	0	1	Rosaceae Juss.

Iz_5_1009	DJI_0686	Quercus robur L.	0	n	1	1	Fagaceae Dumort.
Iz_5_1009	DJI_0686	Robinia pseudoacacia L.	0	a	0	1	Fabaceae Lindl.
Iz_5_1009	DJI_0686	Rosa sp.	0		NA	1	Rosaceae Juss.
Iz_5_1009	DJI_0686	Rubus idaeus L.	0	n	1	0	Rosaceae Juss.
Iz_5_1009	DJI_0686	Rumex acetosella L.	0	n	1	0	Polygonaceae Juss.
Iz_5_1009	DJI_0686	Rumex thyrsiflorus Fingerh.	0	n	1	0	Polygonaceae Juss.
Iz_5_1009	DJI_0686	Salix alba L.	0	n	1	1	Salicaceae Mirb.
Iz_5_1009	DJI_0686	Sambucus nigra L.	0	n	1	1	Viburnaceae Raf.
Iz_5_1009	DJI_0686	Secale sylvestre Host	0	n	1	0	Poaceae Barnhart
Iz_5_1009	DJI_0686	Sempervivum ruthenicum Schnittsp. & C.B. Lehm.	0	n	1	0	Crassulaceae J.St.-Hil.
Iz_5_1009	DJI_0686	Setaria viridis (L.) P. Beauv.	0	n	1	0	Poaceae Barnhart
Iz_5_1009	DJI_0686	Silene borysthenica (Gruner) Walters	0	n	1	0	Caryophyllaceae Juss.
Iz_5_1009	DJI_0686	Silene latifolia subsp. alba (Mill.) Greuter & Burdet	0	n	1	0	Caryophyllaceae Juss.
Iz_5_1009	DJI_0686	Solidago virgaurea L.	3	n	1	0	Asteraceae Bercht. & J.Presl
Iz_5_1009	DJI_0686	Solidago Ã niederederi (S. canadensis Ã S. virgaurea)	0	hybrid	0	0	Asteraceae Bercht. & J.Presl
Iz_5_1009	DJI_0686	Sorbus aucuparia L.	0	n	1	1	Rosaceae Juss.
Iz_5_1009	DJI_0686	Stachys recta L.	0	n	1	0	Lamiacea Lindl.
Iz_5_1009	DJI_0686	Tanacetum vulgare L.	0	n	1	0	Asteraceae Bercht. & J.Presl
Iz_5_1009	DJI_0686	Thymus pallasianus Heinr.Braun	0	n	1	0	Lamiacea Lindl.
Iz_5_1009	DJI_0686	Tilia cordata Mill.	0	n	1	1	Malvaceae Juss.
Iz_5_1009	DJI_0686	Trifolium alpestre L.	0	n	1	0	Fabaceae Lindl.
Iz_5_1009	DJI_0686	Trifolium arvense L.	0	n	1	0	Fabaceae Lindl.
Iz_5_1009	DJI_0686	Ulmus glabra Huds.	0	n	1	1	Ulmaceae Mirb.
Iz_5_1009	DJI_0686	Verbascum densiflorum Bertol.	0	n	1	0	Scrophulariaceae Juss.
Iz_5_1009	DJI_0686	Veronica austriaca L.	0	n	1	0	Plantaginaceae Juss.
Iz_5_1009	DJI_0686	Veronica incana L.	0	n	1	0	Plantaginaceae Juss.
Iz_5_1009	DJI_0686	Veronica verna L.	0	n	1	0	Plantaginaceae Juss.
Iz_5_1009	DJI_0686	Vincetoxicum hirundinaria Medik	0	n	1	0	Apocynaceae Juss.
Iz_5_1009	DJI_0689	Acer tataricum L.	0	n	1	1	Sapindaceae Juss.
Iz_5_1009	DJI_0689	Achillea millefolium L. s.l.	0	n	1	0	Asteraceae Bercht. & J.Presl
Iz_5_1009	DJI_0689	Allium oleraceum L.	0	n	1	0	Amaryllidaceae J.St.-Hil.
Iz_5_1009	DJI_0689	Ambrosia artemisiifolia L.	0	a	0	0	Asteraceae Bercht. & J.Presl
Iz_5_1009	DJI_0689	Amorpha fruticosa L.	0	a	0	0	Fabaceae Lindl.
Iz_5_1009	DJI_0689	Anthericum ramosum L.	0	n	1	0	Asparagaceae Juss.
Iz_5_1009	DJI_0689	Artemisia marschalliana Spreng.	0	n	1	0	Asteraceae Bercht. & J.Presl
Iz_5_1009	DJI_0689	Asparagus officinalis L.	0	n	1	0	Asparagaceae Juss.
Iz_5_1009	DJI_0689	Astragalus cicer L.	0	n	1	0	Fabaceae Lindl.
Iz_5_1009	DJI_0689	Bassia laniflora (S.G.Gmel.) A.J.Scott	0	n	1	0	Amaranthaceae Juss.
Iz_5_1009	DJI_0689	Berberis vulgaris L.	0	n	1	0	Berberidaceae Juss.
Iz_5_1009	DJI_0689	Betula pendula Roth.	0	n	1	1	Betulaceae Gray
Iz_5_1009	DJI_0689	Betula pendula Roth.	0	n	1	0	Poaceae Barnhart
Iz_5_1009	DJI_0689	Bromus inermis Leyss.	0		NA	NA	
Iz_5_1009	DJI_0689	Bromus squarrosus L.	0	a	0	0	Poaceae Barnhart
Iz_5_1009	DJI_0689	Bromus tectorum L.	0	a	0	0	Poaceae Barnhart
Iz_5_1009	DJI_0689	Calamagrostis epigejos (L.) Roth	35	n	1	0	Poaceae Barnhart
Iz_5_1009	DJI_0689	Carduus acanthoides L.	0	n	1	0	Asteraceae Bercht. & J.Presl
Iz_5_1009	DJI_0689	Carex sp.	0	n	1	0	Cyperaceae Juss.
Iz_5_1009	DJI_0689	Centaurea majorovii Dumbadze	0	n	1	0	Asteraceae Bercht. & J.Presl
Iz_5_1009	DJI_0689	Chamaecytisus ruthenicus (Fisch.ex Wol.) Klaskova	0.5	n	1	0	Fabaceae Lindl.
Iz_5_1009	DJI_0689	Chelidonium majus L.	0	n	1	0	Papaveraceae Juss.
Iz_5_1009	DJI_0689	Chenopodium betaceum Andrz.	0	n	1	0	Amaranthaceae Juss.
Iz_5_1009	DJI_0689	Chenopodium cf. album	0	n	1	0	Amaranthaceae Juss.
Iz_5_1009	DJI_0689	Chenopodium sp.	0		NA	0	Amaranthaceae Juss.
Iz_5_1009	DJI_0689	Chondrilla juncea L.	0	n	1	0	Asteraceae Bercht. & J.Presl
Iz_5_1009	DJI_0689	Cirsium vulgare (Savi) Ten.	0	n	1	0	Asteraceae Bercht. & J.Presl
Iz_5_1009	DJI_0689	Clinopodium acinos (L.) Kuntze	0	n	1	0	Lamiacea Lindl.
Iz_5_1009	DJI_0689	Convallaria majalis L.	0	n	1	0	Asparagaceae Juss.
Iz_5_1009	DJI_0689	Crataegus rhipidophylla Gand.	0	n	1	1	Rosaceae Juss.
Iz_5_1009	DJI_0689	Crepis foetida L.	0	n	1	0	Asteraceae Bercht. & J.Presl
Iz_5_1009	DJI_0689	Dactylis glomerata L.	0	n	1	0	Poaceae Barnhart
Iz_5_1009	DJI_0689	Dianthus campestris M.Bieb.	0	n	1	0	Caryophyllaceae Juss.
Iz_5_1009	DJI_0689	Digitaria sanguinalis (L.) Scop.	0	a	0	0	Poaceae Barnhart
Iz_5_1009	DJI_0689	Elymus repens (L.) Gould	0	n	1	0	Poaceae Barnhart
Iz_5_1009	DJI_0689	Epilobium tetragonum L.	0	n	1	0	Onagraceae Juss.
Iz_5_1009	DJI_0689	Eragrostis minor Host	0	a	0	0	Poaceae Barnhart
Iz_5_1009	DJI_0689	Erigeron canadensis L.	4	a	0	0	Asteraceae Bercht. & J.Presl
Iz_5_1009	DJI_0689	Euphorbia seguieriana Neck.	0	n	1	0	Euphorbiaceae Juss.
Iz_5_1009	DJI_0689	Fallopia dumetorum (L.) Holub	0	n	1	0	Polygonaceae Juss.
Iz_5_1009	DJI_0689	Festuca beckeri (Hack.) Trautv.	0	n	1	0	Poaceae Barnhart
Iz_5_1009	DJI_0689	Fragaria vesca L.	0	n	1	0	Rosaceae Juss.
Iz_5_1009	DJI_0689	Frangula alnus Mill.	0	n	1	1	Rhamnaceae Juss.
Iz_5_1009	DJI_0689	Fraxinus pennsylvanica Marshall	0	a	0	1	Oleaceae Hoffmanns. & Link
Iz_5_1009	DJI_0689	Galeopsis bifida Boenn.	0	n	1	0	Lamiacea Lindl.
Iz_5_1009	DJI_0689	Galium verum L.	0	n	1	0	Rubiaceae Juss.
Iz_5_1009	DJI_0689	Genista tinctoria L.	0	n	1	0	Fabaceae Lindl.
Iz_5_1009	DJI_0689	Geranium robertianum L.	0	n	1	0	Geraniaceae Juss.
Iz_5_1009	DJI_0689	Geranium sanquineum L.	0	n	1	0	Geraniaceae Juss.
Iz_5_1009	DJI_0689	Gypsophila paniculata L.	0	n	1	0	Caryophyllaceae Juss.
Iz_5_1009	DJI_0689	Helichrysum arenarium (L.) Moench	0	n	1	0	Asteraceae Bercht. & J.Presl
Iz_5_1009	DJI_0689	Herniaria polygama J.Gay	0	n	1	0	Caryophyllaceae Juss.
Iz_5_1009	DJI_0689	Hieracium robustum Fr.	0	n	1	0	Asteraceae Bercht. & J.Presl
Iz_5_1009	DJI_0689	Hieracium umbellatum L.	0	n	1	NA	Asteraceae Bercht. & J.Presl
Iz_5_1009	DJI_0689	Hylotelephium maximum (L.) Holub	0	n	1	0	Crassulaceae J.St.-Hil.
Iz_5_1009	DJI_0689	Jacobaea borysthenica (DC.) B.Nord. & Greuter	0	n	1	0	Asteraceae Bercht. & J.Presl
Iz_5_1009	DJI_0689	Jasione montana L.	0	n	1	0	Campanulaceae Juss.
Iz_5_1009	DJI_0689	Jurinea cyanoides (L.) Rchb.s.l.	0	n	1	0	Asteraceae Bercht. & J.Presl
Iz_5_1009	DJI_0689	Koeleria glauca (Spreng.) DC.	0	n	1	0	Poaceae Barnhart
Iz_5_1009	DJI_0689	Lactuca serriola L.	0	a	0	0	Asteraceae Bercht. & J.Presl
Iz_5_1009	DJI_0689	Linaria genistifolia (L.) Mill.	0	n	1	0	Plantaginaceae Juss.
Iz_5_1009	DJI_0689	Lomelosia argentea (L.) Greuter & Burdet	0	n	1	0	Caprifoliaceae Juss.
Iz_5_1009	DJI_0689	Melampyrum pratense L.	0	n	1	0	Orobanchaceae Vent.
Iz_5_1009	DJI_0689	Oenothera biennis L.	0	a	0	0	Onagraceae Juss.
Iz_5_1009	DJI_0689	Pentanema britannica (L.) D.Gut.Larr., Santos-Vicente, Anderb., E.Rico & M.M.Mart.Ort.	0	n	1	0	Asteraceae Bercht. & J.Presl
Iz_5_1009	DJI_0689	Peucedanum oreoselinum (L.) Moench	0	n	1	0	Apiaceae Lindl.
Iz_5_1009	DJI_0689	Peucedanum ruthenicum M.Bieb.	0	n	1	0	Apiaceae Lindl.
Iz_5_1009	DJI_0689	Pilosella echioides (Lumn.) F.W.Schultz & Sch.Bip.	0	n	1	0	Asteraceae Bercht. & J.Presl
Iz_5_1009	DJI_0689	Pilosella officinarum F. Schultz et Sch. Bip.	0	n	1	0	Asteraceae Bercht. & J.Presl
Iz_5_1009	DJI_0689	Pilosella sp.	0		NA	0	Asteraceae Bercht. & J.Presl

Iz_5_1009	DJI_0689	Pinus sylvestris L.	0	n	1	1	Pinaceae Spreng. ex F.Rudolphi
Iz_5_1009	DJI_0689	Poa annua L.	0	n	1	0	Poaceae Barnhart
Iz_5_1009	DJI_0689	Poa nemoralis L.	0	n	1	0	Poaceae Barnhart
Iz_5_1009	DJI_0689	Poa sp.	0		NA	0	Poaceae Barnhart
Iz_5_1009	DJI_0689	Polygonatum odoratum (Mill.) Druce	0	n	1	0	Asparagaceae Juss.
Iz_5_1009	DJI_0689	Polygonum arenarium Waldst. & Kit.	0	n	1	0	Polygonaceae Juss.
Iz_5_1009	DJI_0689	Populus nigra L.	0	n	1	1	Salicaceae Mirb.
Iz_5_1009	DJI_0689	Populus sp.	0	n	1	1	Salicaceae Mirb.
Iz_5_1009	DJI_0689	Populus tremula L.	0	n	1	1	Salicaceae Mirb.
Iz_5_1009	DJI_0689	Populus Ã canescens (Aiton) Sm.	0	n	1	1	Salicaceae Mirb.
Iz_5_1009	DJI_0689	Potentilla argentea L.	0	n	1	0	Rosaceae Juss.
Iz_5_1009	DJI_0689	Psammophiliella muralis (L.) Ikonn.	0	n	1	0	Caryophyllaceae Juss.
Iz_5_1009	DJI_0689	Psephellus sumensis (Kalen.) Greuter	0	n	1	0	Asteraceae Bercht. & J.Presl
Iz_5_1009	DJI_0689	Pulsatilla pratensis (L.) Mill. s.l.	0	n	1	0	Ranunculaceae Juss.
Iz_5_1009	DJI_0689	Pyrus communis L.	0	a	0	1	Rosaceae Juss.
Iz_5_1009	DJI_0689	Quercus robur L.	0	n	1	1	Fagaceae Dumort.
Iz_5_1009	DJI_0689	Robinia pseudoacacia L.	0	a	0	1	Fabaceae Lindl.
Iz_5_1009	DJI_0689	Rosa sp.	0		NA	1	Rosaceae Juss.
Iz_5_1009	DJI_0689	Rubus idaeus L.	0	n	1	0	Rosaceae Juss.
Iz_5_1009	DJI_0689	Rumex acetosella L.	0	n	1	0	Polygonaceae Juss.
Iz_5_1009	DJI_0689	Rumex thyrsiflorus Fingerh.	0	n	1	0	Polygonaceae Juss.
Iz_5_1009	DJI_0689	Salix alba L.	0	n	1	1	Salicaceae Mirb.
Iz_5_1009	DJI_0689	Sambucus nigra L.	0	n	1	1	Viburnaceae Raf.
Iz_5_1009	DJI_0689	Secale sylvestre Host	0	n	1	0	Poaceae Barnhart
Iz_5_1009	DJI_0689	Sempervivum ruthenicum Schnittsp. & C.B. Lehm.	0	n	1	0	Crassulaceae J.St.-Hil.
Iz_5_1009	DJI_0689	Setaria viridis (L.) P. Beauv.	0	n	1	0	Poaceae Barnhart
Iz_5_1009	DJI_0689	Silene borysthenica (Gruner) Walters	0	n	1	0	Caryophyllaceae Juss.
Iz_5_1009	DJI_0689	Silene latifolia subsp. alba (Mill.) Greuter & Burdet	0	n	1	0	Caryophyllaceae Juss.
Iz_5_1009	DJI_0689	Solidago virgaurea L.	0	n	1	0	Asteraceae Bercht. & J.Presl
Iz_5_1009	DJI_0689	Solidago Ã niederederi (S. canadensis Ã S. virgaurea)	0	hybrid	0	0	Asteraceae Bercht. & J.Presl
Iz_5_1009	DJI_0689	Sorbus aucuparia L.	0	n	1	1	Rosaceae Juss.
Iz_5_1009	DJI_0689	Stachys recta L.	0	n	1	0	Lamiacea Lindl.
Iz_5_1009	DJI_0689	Tanacetum vulgare L.	0	n	1	0	Asteraceae Bercht. & J.Presl
Iz_5_1009	DJI_0689	Thymus pallasianus Heinr.Braun	0	n	1	0	Lamiacea Lindl.
Iz_5_1009	DJI_0689	Tilia cordata Mill.	0	n	1	1	Malvaceae Juss.
Iz_5_1009	DJI_0689	Trifolium alpestre L.	0	n	1	0	Fabaceae Lindl.
Iz_5_1009	DJI_0689	Trifolium arvense L.	0	n	1	0	Fabaceae Lindl.
Iz_5_1009	DJI_0689	Ulmus glabra Huds.	0	n	1	1	Ulmaceae Mirb.
Iz_5_1009	DJI_0689	Verbascum densiflorum Bertol.	0	n	1	0	Scrophulariaceae Juss.
Iz_5_1009	DJI_0689	Veronica austriaca L.	0	n	1	0	Plantaginaceae Juss.
Iz_5_1009	DJI_0689	Veronica incana L.	0	n	1	0	Plantaginaceae Juss.
Iz_5_1009	DJI_0689	Veronica verna L.	0	n	1	0	Plantaginaceae Juss.
Iz_5_1009	DJI_0689	Vincetoxicum hirundinaria Medik	0	n	1	0	Apocynaceae Juss.
Iz_5_1009	DJI_0691	Acer tataricum L.	0	n	1	1	Sapindaceae Juss.
Iz_5_1009	DJI_0691	Achillea millefolium L. s.l.	0	n	1	0	Asteraceae Bercht. & J.Presl
Iz_5_1009	DJI_0691	Allium oleraceum L.	0	n	1	0	Amaryllidaceae J.St.-Hil.
Iz_5_1009	DJI_0691	Ambrosia artemisiifolia L.	0	a	0	0	Asteraceae Bercht. & J.Presl
Iz_5_1009	DJI_0691	Amorpha fruticosa L.	0	a	0	0	Fabaceae Lindl.
Iz_5_1009	DJI_0691	Anthericum ramosum L.	0	n	1	0	Asparagaceae Juss.
Iz_5_1009	DJI_0691	Artemisia marschalliana Spreng.	0	n	1	0	Asteraceae Bercht. & J.Presl
Iz_5_1009	DJI_0691	Asparagus officinalis L.	0	n	1	0	Asparagaceae Juss.
Iz_5_1009	DJI_0691	Astragalus cicer L.	0	n	1	0	Fabaceae Lindl.
Iz_5_1009	DJI_0691	Bassia laniflora (S.G.Gmel.) A.J.Scott	0	n	1	0	Amaranthaceae Juss.
Iz_5_1009	DJI_0691	Berberis vulgaris L.	0	n	1	0	Berberidaceae Juss.
Iz_5_1009	DJI_0691	Betula pendula Roth.	0	n	1	1	Betulaceae Gray
Iz_5_1009	DJI_0691	Betula pendula Roth.	0	n	1	0	Poaceae Barnhart
Iz_5_1009	DJI_0691	Bromus inermis Leyss.	0		NA	NA	
Iz_5_1009	DJI_0691	Bromus squarrosus L.	0	a	0	0	Poaceae Barnhart
Iz_5_1009	DJI_0691	Bromus tectorum L.	0	a	0	0	Poaceae Barnhart
Iz_5_1009	DJI_0691	Calamagrostis epigejos (L.) Roth	50	n	1	0	Poaceae Barnhart
Iz_5_1009	DJI_0691	Carduus acanthoides L.	0	n	1	0	Asteraceae Bercht. & J.Presl
Iz_5_1009	DJI_0691	Carex sp.	0	n	1	0	Cyperaceae Juss.
Iz_5_1009	DJI_0691	Centaurea majorovii Dumbadze	0	n	1	0	Asteraceae Bercht. & J.Presl
Iz_5_1009	DJI_0691	Chamaecytisus ruthenicus (Fisch.ex Wol.) Klaskova	0	n	1	0	Fabaceae Lindl.
Iz_5_1009	DJI_0691	Chelidonium majus L.	0	n	1	0	Papaveraceae Juss.
Iz_5_1009	DJI_0691	Chenopodium betaceum Andrz.	0	n	1	0	Amaranthaceae Juss.
Iz_5_1009	DJI_0691	Chenopodium cf. album	0	n	1	0	Amaranthaceae Juss.
Iz_5_1009	DJI_0691	Chenopodium sp.	0		NA	0	Amaranthaceae Juss.
Iz_5_1009	DJI_0691	Chondrilla juncea L.	0	n	1	0	Asteraceae Bercht. & J.Presl
Iz_5_1009	DJI_0691	Cirsium vulgare (Savi) Ten.	0	n	1	0	Asteraceae Bercht. & J.Presl
Iz_5_1009	DJI_0691	Clinopodium acinos (L.) Kuntze	0	n	1	0	Lamiacea Lindl.
Iz_5_1009	DJI_0691	Convallaria majalis L.	0	n	1	0	Asparagaceae Juss.
Iz_5_1009	DJI_0691	Crataegus rhipidophylla Gand.	0	n	1	1	Rosaceae Juss.
Iz_5_1009	DJI_0691	Crepis foetida L.	0	n	1	0	Asteraceae Bercht. & J.Presl
Iz_5_1009	DJI_0691	Dactylis glomerata L.	0	n	1	0	Poaceae Barnhart
Iz_5_1009	DJI_0691	Dianthus campestris M.Bieb.	0	n	1	0	Caryophyllaceae Juss.
Iz_5_1009	DJI_0691	Digitaria sanguinalis (L.) Scop.	0	a	0	0	Poaceae Barnhart
Iz_5_1009	DJI_0691	Elymus repens (L.) Gould	0	n	1	0	Poaceae Barnhart
Iz_5_1009	DJI_0691	Epilobium tetragonum L.	0	n	1	0	Onagraceae Juss.
Iz_5_1009	DJI_0691	Eragrostis minor Host	0	a	0	0	Poaceae Barnhart
Iz_5_1009	DJI_0691	Erigeron canadensis L.	1	a	0	0	Asteraceae Bercht. & J.Presl
Iz_5_1009	DJI_0691	Euphorbia seguieriana Neck.	0	n	1	0	Euphorbiaceae Juss.
Iz_5_1009	DJI_0691	Fallopia dumetorum (L.) Holub	0	n	1	0	Polygonaceae Juss.
Iz_5_1009	DJI_0691	Festuca beckeri (Hack.) Trautv.	0	n	1	0	Poaceae Barnhart
Iz_5_1009	DJI_0691	Fragaria vesca L.	0	n	1	0	Rosaceae Juss.
Iz_5_1009	DJI_0691	Frangula alnus Mill.	0	n	1	1	Rhamnaceae Juss.
Iz_5_1009	DJI_0691	Fraxinus pennsylvanica Marshall	0	a	0	1	Oleaceae Hoffmanns. & Link
Iz_5_1009	DJI_0691	Galeopsis bifida Boenn.	0	n	1	0	Lamiacea Lindl.
Iz_5_1009	DJI_0691	Galium verum L.	0	n	1	0	Rubiaceae Juss.
Iz_5_1009	DJI_0691	Genista tinctoria L.	0	n	1	0	Fabaceae Lindl.
Iz_5_1009	DJI_0691	Geranium robertianum L.	0	n	1	0	Geraniaceae Juss.
Iz_5_1009	DJI_0691	Geranium sanquineum L.	0	n	1	0	Geraniaceae Juss.
Iz_5_1009	DJI_0691	Gypsophila paniculata L.	0	n	1	0	Caryophyllaceae Juss.
Iz_5_1009	DJI_0691	Helichrysum arenarium (L.) Moench	0	n	1	0	Asteraceae Bercht. & J.Presl
Iz_5_1009	DJI_0691	Herniaria polygama J.Gay	0	n	1	0	Caryophyllaceae Juss.
Iz_5_1009	DJI_0691	Hieracium robustum Fr.	0	n	1	0	Asteraceae Bercht. & J.Presl
Iz_5_1009	DJI_0691	Hieracium umbellatum L.	0	n	1	NA	Asteraceae Bercht. & J.Presl
Iz_5_1009	DJI_0691	Hylotelephium maximum (L.) Holub	0	n	1	0	Crassulaceae J.St.-Hil.

Iz_5_1009	DJI_0691	Jacobaea borysthenica (DC.) B.Nord. & Greuter	0	n	1	0	Asteraceae Bercht. & J.Presl
Iz_5_1009	DJI_0691	Jasione montana L.	0	n	1	0	Campanulaceae Juss.
Iz_5_1009	DJI_0691	Jurinea cyanoides (L.) Rchb.s.l.	0	n	1	0	Asteraceae Bercht. & J.Presl
Iz_5_1009	DJI_0691	Koeleria glauca (Spreng.) DC.	0	n	1	0	Poaceae Barnhart
Iz_5_1009	DJI_0691	Lactuca serriola L.	0	a	0	0	Asteraceae Bercht. & J.Presl
Iz_5_1009	DJI_0691	Linaria genistifolia (L.) Mill.	0	n	1	0	Plantaginaceae Juss.
Iz_5_1009	DJI_0691	Lomelosia argentea (L.) Greuter & Burdet	0	n	1	0	Caprifoliaceae Juss.
Iz_5_1009	DJI_0691	Melampyrum pratense L.	0	n	1	0	Orobanchaceae Vent.
Iz_5_1009	DJI_0691	Oenothera biennis L.	0	a	0	0	Onagraceae Juss.
Iz_5_1009	DJI_0691	Pentanema britannica (L.) D.Gut.Larr., Santos-Vicente, Anderb., E.Rico & M.M.Mart.Ort.	0	n	1	0	Asteraceae Bercht. & J.Presl
Iz_5_1009	DJI_0691	Peucedanum oreoselinum (L.) Moench	0	n	1	0	Apiaceae Lindl.
Iz_5_1009	DJI_0691	Peucedanum ruthenicum M.Bieb.	0	n	1	0	Apiaceae Lindl.
Iz_5_1009	DJI_0691	Pilosella echioides (Lumn.) F.W.Schultz & Sch.Bip.	0	n	1	0	Asteraceae Bercht. & J.Presl
Iz_5_1009	DJI_0691	Pilosella officinarum F. Schultz et Sch. Bip.	0	n	1	0	Asteraceae Bercht. & J.Presl
Iz_5_1009	DJI_0691	Pilosella sp.	0		NA	0	Asteraceae Bercht. & J.Presl
Iz_5_1009	DJI_0691	Pinus sylvestris L.	0	n	1	1	Pinaceae Spreng. ex F.Rudolphi
Iz_5_1009	DJI_0691	Poa annua L.	0	n	1	0	Poaceae Barnhart
Iz_5_1009	DJI_0691	Poa nemoralis L.	0	n	1	0	Poaceae Barnhart
Iz_5_1009	DJI_0691	Poa sp.	0		NA	0	Poaceae Barnhart
Iz_5_1009	DJI_0691	Polygonatum odoratum (Mill.) Druce	0	n	1	0	Asparagaceae Juss.
Iz_5_1009	DJI_0691	Polygonum arenarium Waldst. & Kit.	0	n	1	0	Polygonaceae Juss.
Iz_5_1009	DJI_0691	Populus nigra L.	0	n	1	1	Salicaceae Mirb.
Iz_5_1009	DJI_0691	Populus sp.	0	n	1	1	Salicaceae Mirb.
Iz_5_1009	DJI_0691	Populus tremula L.	0	n	1	1	Salicaceae Mirb.
Iz_5_1009	DJI_0691	Populus Ã canescens (Aiton) Sm.	0	n	1	1	Salicaceae Mirb.
Iz_5_1009	DJI_0691	Potentilla argentea L.	0	n	1	0	Rosaceae Juss.
Iz_5_1009	DJI_0691	Psammophiliella muralis (L.) Ikonn.	0	n	1	0	Caryophyllaceae Juss.
Iz_5_1009	DJI_0691	Psephellus sumensis (Kalen.) Greuter	0	n	1	0	Asteraceae Bercht. & J.Presl
Iz_5_1009	DJI_0691	Pulsatilla pratensis (L.) Mill. s.l.	0	n	1	0	Ranunculaceae Juss.
Iz_5_1009	DJI_0691	Pyrus communis L.	0	a	0	1	Rosaceae Juss.
Iz_5_1009	DJI_0691	Quercus robur L.	0	n	1	1	Fagaceae Dumort.
Iz_5_1009	DJI_0691	Robinia pseudoacacia L.	0	a	0	1	Fabaceae Lindl.
Iz_5_1009	DJI_0691	Rosa sp.	0		NA	1	Rosaceae Juss.
Iz_5_1009	DJI_0691	Rubus idaeus L.	0	n	1	0	Rosaceae Juss.
Iz_5_1009	DJI_0691	Rumex acetosella L.	0	n	1	0	Polygonaceae Juss.
Iz_5_1009	DJI_0691	Rumex thyrsiflorus Fingerh.	0	n	1	0	Polygonaceae Juss.
Iz_5_1009	DJI_0691	Salix alba L.	0	n	1	1	Salicaceae Mirb.
Iz_5_1009	DJI_0691	Sambucus nigra L.	0	n	1	1	Viburnaceae Raf.
Iz_5_1009	DJI_0691	Secale sylvestre Host	0	n	1	0	Poaceae Barnhart
Iz_5_1009	DJI_0691	Sempervivum ruthenicum Schnittsp. & C.B. Lehm.	0	n	1	0	Crassulaceae J.St.-Hil.
Iz_5_1009	DJI_0691	Setaria viridis (L.) P. Beauv.	0	n	1	0	Poaceae Barnhart
Iz_5_1009	DJI_0691	Silene borysthenica (Gruner) Walters	0	n	1	0	Caryophyllaceae Juss.
Iz_5_1009	DJI_0691	Silene latifolia subsp. alba (Mill.) Greuter & Burdet	0	n	1	0	Caryophyllaceae Juss.
Iz_5_1009	DJI_0691	Solidago virgaurea L.	2	n	1	0	Asteraceae Bercht. & J.Presl
Iz_5_1009	DJI_0691	Solidago Ã niederederi (S. canadensis Ã S. virgaurea)	0	hybrid	0	0	Asteraceae Bercht. & J.Presl
Iz_5_1009	DJI_0691	Sorbus aucuparia L.	0	n	1	1	Rosaceae Juss.
Iz_5_1009	DJI_0691	Stachys recta L.	0	n	1	0	Lamiacea Lindl.
Iz_5_1009	DJI_0691	Tanacetum vulgare L.	0	n	1	0	Asteraceae Bercht. & J.Presl
Iz_5_1009	DJI_0691	Thymus pallasianus Heinr.Braun	0	n	1	0	Lamiacea Lindl.
Iz_5_1009	DJI_0691	Tilia cordata Mill.	0	n	1	1	Malvaceae Juss.
Iz_5_1009	DJI_0691	Trifolium alpestre L.	0	n	1	0	Fabaceae Lindl.
Iz_5_1009	DJI_0691	Trifolium arvense L.	0	n	1	0	Fabaceae Lindl.
Iz_5_1009	DJI_0691	Ulmus glabra Huds.	0	n	1	1	Ulmaceae Mirb.
Iz_5_1009	DJI_0691	Verbascum densiflorum Bertol.	0	n	1	0	Scrophulariaceae Juss.
Iz_5_1009	DJI_0691	Veronica austriaca L.	0	n	1	0	Plantaginaceae Juss.
Iz_5_1009	DJI_0691	Veronica incana L.	0	n	1	0	Plantaginaceae Juss.
Iz_5_1009	DJI_0691	Veronica verna L.	0	n	1	0	Plantaginaceae Juss.
Iz_5_1009	DJI_0691	Vincetoxicum hirundinaria Medik	0	n	1	0	Apocynaceae Juss.
Iz_5_1009	DJI_0693	Acer tataricum L.	0	n	1	1	Sapindaceae Juss.
Iz_5_1009	DJI_0693	Achillea millefolium L. s.l.	3	n	1	0	Asteraceae Bercht. & J.Presl
Iz_5_1009	DJI_0693	Allium oleraceum L.	0	n	1	0	Amaryllidaceae J.St.-Hil.
Iz_5_1009	DJI_0693	Ambrosia artemisiifolia L.	0	a	0	0	Asteraceae Bercht. & J.Presl
Iz_5_1009	DJI_0693	Amorpha fruticosa L.	0	a	0	0	Fabaceae Lindl.
Iz_5_1009	DJI_0693	Anthericum ramosum L.	0	n	1	0	Asparagaceae Juss.
Iz_5_1009	DJI_0693	Artemisia marschalliana Spreng.	0	n	1	0	Asteraceae Bercht. & J.Presl
Iz_5_1009	DJI_0693	Asparagus officinalis L.	0	n	1	0	Asparagaceae Juss.
Iz_5_1009	DJI_0693	Astragalus cicer L.	0	n	1	0	Fabaceae Lindl.
Iz_5_1009	DJI_0693	Bassia laniflora (S.G.Gmel.) A.J.Scott	0	n	1	0	Amaranthaceae Juss.
Iz_5_1009	DJI_0693	Berberis vulgaris L.	0	n	1	0	Berberidaceae Juss.
Iz_5_1009	DJI_0693	Betula pendula Roth.	0.2	n	1	1	Betulaceae Gray
Iz_5_1009	DJI_0693	Betula pendula Roth.	0.2	n	1	0	Poaceae Barnhart
Iz_5_1009	DJI_0693	Bromus inermis Leyss.	0		NA	NA	
Iz_5_1009	DJI_0693	Bromus squarrosus L.	0	a	0	0	Poaceae Barnhart
Iz_5_1009	DJI_0693	Bromus tectorum L.	0	a	0	0	Poaceae Barnhart
Iz_5_1009	DJI_0693	Calamagrostis epigejos (L.) Roth	50	n	1	0	Poaceae Barnhart
Iz_5_1009	DJI_0693	Carduus acanthoides L.	0	n	1	0	Asteraceae Bercht. & J.Presl
Iz_5_1009	DJI_0693	Carex sp.	0	n	1	0	Cyperaceae Juss.
Iz_5_1009	DJI_0693	Centaurea majorovii Dumbadze	0	n	1	0	Asteraceae Bercht. & J.Presl
Iz_5_1009	DJI_0693	Chamaecytisus ruthenicus (Fisch.ex Wol.) Klaskova	0	n	1	0	Fabaceae Lindl.
Iz_5_1009	DJI_0693	Chelidonium majus L.	0	n	1	0	Papaveraceae Juss.
Iz_5_1009	DJI_0693	Chenopodium betaceum Andrz.	0	n	1	0	Amaranthaceae Juss.
Iz_5_1009	DJI_0693	Chenopodium cf. album	0	n	1	0	Amaranthaceae Juss.
Iz_5_1009	DJI_0693	Chenopodium sp.	0		NA	0	Amaranthaceae Juss.
Iz_5_1009	DJI_0693	Chondrilla juncea L.	0	n	1	0	Asteraceae Bercht. & J.Presl
Iz_5_1009	DJI_0693	Cirsium vulgare (Savi) Ten.	0	n	1	0	Asteraceae Bercht. & J.Presl
Iz_5_1009	DJI_0693	Clinopodium acinos (L.) Kuntze	0	n	1	0	Lamiacea Lindl.
Iz_5_1009	DJI_0693	Convallaria majalis L.	0	n	1	0	Asparagaceae Juss.
Iz_5_1009	DJI_0693	Crataegus rhipidophylla Gand.	0	n	1	1	Rosaceae Juss.
Iz_5_1009	DJI_0693	Crepis foetida L.	0	n	1	0	Asteraceae Bercht. & J.Presl
Iz_5_1009	DJI_0693	Dactylis glomerata L.	0	n	1	0	Poaceae Barnhart
Iz_5_1009	DJI_0693	Dianthus campestris M.Bieb.	0	n	1	0	Caryophyllaceae Juss.
Iz_5_1009	DJI_0693	Digitaria sanguinalis (L.) Scop.	0	a	0	0	Poaceae Barnhart
Iz_5_1009	DJI_0693	Elymus repens (L.) Gould	0	n	1	0	Poaceae Barnhart
Iz_5_1009	DJI_0693	Epilobium tetragonum L.	0	n	1	0	Onagraceae Juss.
Iz_5_1009	DJI_0693	Eragrostis minor Host	0	a	0	0	Poaceae Barnhart
Iz_5_1009	DJI_0693	Erigeron canadensis L.	5	a	0	0	Asteraceae Bercht. & J.Presl
Iz_5_1009	DJI_0693	Euphorbia seguieriana Neck.	0	n	1	0	Euphorbiaceae Juss.
Iz_5_1009	DJI_0693	Fallopia dumetorum (L.) Holub	0	n	1	0	Polygonaceae Juss.

Iz_5_1009	DJI_0693	Festuca beckeri (Hack.) Trautv.	0	n	1	0	Poaceae Barnhart
Iz_5_1009	DJI_0693	Fragaria vesca L.	0	n	1	0	Rosaceae Juss.
Iz_5_1009	DJI_0693	Frangula alnus Mill.	0	n	1	1	Rhamnaceae Juss.
Iz_5_1009	DJI_0693	Fraxinus pennsylvanica Marshall	0	a	0	1	Oleaceae Hoffmanns. & Link
Iz_5_1009	DJI_0693	Galeopsis bifida Boenn.	0	n	1	0	Lamiacea Lindl.
Iz_5_1009	DJI_0693	Galium verum L.	0	n	1	0	Rubiaceae Juss.
Iz_5_1009	DJI_0693	Genista tinctoria L.	0	n	1	0	Fabaceae Lindl.
Iz_5_1009	DJI_0693	Geranium robertianum L.	0	n	1	0	Geraniaceae Juss.
Iz_5_1009	DJI_0693	Geranium sanquineum L.	0	n	1	0	Geraniaceae Juss.
Iz_5_1009	DJI_0693	Gypsophila paniculata L.	0	n	1	0	Caryophyllaceae Juss.
Iz_5_1009	DJI_0693	Helichrysum arenarium (L.) Moench	0	n	1	0	Asteraceae Bercht. & J.Presl
Iz_5_1009	DJI_0693	Herniaria polygama J.Gay	0	n	1	0	Caryophyllaceae Juss.
Iz_5_1009	DJI_0693	Hieracium robustum Fr.	0	n	1	0	Asteraceae Bercht. & J.Presl
Iz_5_1009	DJI_0693	Hieracium umbellatum L.	0	n	1	NA	Asteraceae Bercht. & J.Presl
Iz_5_1009	DJI_0693	Hylotelephium maximum (L.) Holub	0	n	1	0	Crassulaceae J.St.-Hil.
Iz_5_1009	DJI_0693	Jacobaea borysthenica (DC.) B.Nord. & Greuter	0	n	1	0	Asteraceae Bercht. & J.Presl
Iz_5_1009	DJI_0693	Jasione montana L.	0	n	1	0	Campanulaceae Juss.
Iz_5_1009	DJI_0693	Jurinea cyanoides (L.) Rchb.s.l.	0	n	1	0	Asteraceae Bercht. & J.Presl
Iz_5_1009	DJI_0693	Koeleria glauca (Spreng.) DC.	0	n	1	0	Poaceae Barnhart
Iz_5_1009	DJI_0693	Lactuca serriola L.	0	a	0	0	Asteraceae Bercht. & J.Presl
Iz_5_1009	DJI_0693	Linaria genistifolia (L.) Mill.	0	n	1	0	Plantaginaceae Juss.
Iz_5_1009	DJI_0693	Lomelosia argentea (L.) Greuter & Burdet	0	n	1	0	Caprifoliaceae Juss.
Iz_5_1009	DJI_0693	Melampyrum pratense L.	0	n	1	0	Orobanchaceae Vent.
Iz_5_1009	DJI_0693	Oenothera biennis L.	0	a	0	0	Onagraceae Juss.
Iz_5_1009	DJI_0693	Pentanema britannica (L.) D.Gut.Larr., Santos-Vicente, Anderb., E.Rico & M.M.Mart.Ort.	0	n	1	0	Asteraceae Bercht. & J.Presl
Iz_5_1009	DJI_0693	Peucedanum oreoselinum (L.) Moench	0	n	1	0	Apiaceae Lindl.
Iz_5_1009	DJI_0693	Peucedanum ruthenicum M.Bieb.	0	n	1	0	Apiaceae Lindl.
Iz_5_1009	DJI_0693	Pilosella echioides (Lumn.) F.W.Schultz & Sch.Bip.	0	n	1	0	Asteraceae Bercht. & J.Presl
Iz_5_1009	DJI_0693	Pilosella officinarum F. Schultz et Sch. Bip.	1	n	1	0	Asteraceae Bercht. & J.Presl
Iz_5_1009	DJI_0693	Pilosella sp.	0		NA	0	Asteraceae Bercht. & J.Presl
Iz_5_1009	DJI_0693	Pinus sylvestris L.	0	n	1	1	Pinaceae Spreng. ex F.Rudolphi
Iz_5_1009	DJI_0693	Poa annua L.	0	n	1	0	Poaceae Barnhart
Iz_5_1009	DJI_0693	Poa nemoralis L.	0	n	1	0	Poaceae Barnhart
Iz_5_1009	DJI_0693	Poa sp.	0		NA	0	Poaceae Barnhart
Iz_5_1009	DJI_0693	Polygonatum odoratum (Mill.) Druce	0	n	1	0	Asparagaceae Juss.
Iz_5_1009	DJI_0693	Polygonum arenarium Waldst. & Kit.	0	n	1	0	Polygonaceae Juss.
Iz_5_1009	DJI_0693	Populus nigra L.	0	n	1	1	Salicaceae Mirb.
Iz_5_1009	DJI_0693	Populus sp.	0	n	1	1	Salicaceae Mirb.
Iz_5_1009	DJI_0693	Populus tremula L.	0	n	1	1	Salicaceae Mirb.
Iz_5_1009	DJI_0693	Populus Ã canescens (Aiton) Sm.	0	n	1	1	Salicaceae Mirb.
Iz_5_1009	DJI_0693	Potentilla argentea L.	0	n	1	0	Rosaceae Juss.
Iz_5_1009	DJI_0693	Psammophiliella muralis (L.) Ikonn.	0	n	1	0	Caryophyllaceae Juss.
Iz_5_1009	DJI_0693	Psephellus sumensis (Kalen.) Greuter	0	n	1	0	Asteraceae Bercht. & J.Presl
Iz_5_1009	DJI_0693	Pulsatilla pratensis (L.) Mill. s.l.	0	n	1	0	Ranunculaceae Juss.
Iz_5_1009	DJI_0693	Pyrus communis L.	0	a	0	1	Rosaceae Juss.
Iz_5_1009	DJI_0693	Quercus robur L.	0	n	1	1	Fagaceae Dumort.
Iz_5_1009	DJI_0693	Robinia pseudoacacia L.	0	a	0	1	Fabaceae Lindl.
Iz_5_1009	DJI_0693	Rosa sp.	0		NA	1	Rosaceae Juss.
Iz_5_1009	DJI_0693	Rubus idaeus L.	0	n	1	0	Rosaceae Juss.
Iz_5_1009	DJI_0693	Rumex acetosella L.	0	n	1	0	Polygonaceae Juss.
Iz_5_1009	DJI_0693	Rumex thyrsiflorus Fingerh.	0	n	1	0	Polygonaceae Juss.
Iz_5_1009	DJI_0693	Salix alba L.	0	n	1	1	Salicaceae Mirb.
Iz_5_1009	DJI_0693	Sambucus nigra L.	0	n	1	1	Viburnaceae Raf.
Iz_5_1009	DJI_0693	Secale sylvestre Host	0	n	1	0	Poaceae Barnhart
Iz_5_1009	DJI_0693	Sempervivum ruthenicum Schnittsp. & C.B. Lehm.	0	n	1	0	Crassulaceae J.St.-Hil.
Iz_5_1009	DJI_0693	Setaria viridis (L.) P. Beauv.	0	n	1	0	Poaceae Barnhart
Iz_5_1009	DJI_0693	Silene borysthenica (Gruner) Walters	0	n	1	0	Caryophyllaceae Juss.
Iz_5_1009	DJI_0693	Silene latifolia subsp. alba (Mill.) Greuter & Burdet	0	n	1	0	Caryophyllaceae Juss.
Iz_5_1009	DJI_0693	Solidago virgaurea L.	0	n	1	0	Asteraceae Bercht. & J.Presl
Iz_5_1009	DJI_0693	Solidago Ã niederederi (S. canadensis Ã S. virgaurea)	0	hybrid	0	0	Asteraceae Bercht. & J.Presl
Iz_5_1009	DJI_0693	Sorbus aucuparia L.	0	n	1	1	Rosaceae Juss.
Iz_5_1009	DJI_0693	Stachys recta L.	0.5	n	1	0	Lamiacea Lindl.
Iz_5_1009	DJI_0693	Tanacetum vulgare L.	0	n	1	0	Asteraceae Bercht. & J.Presl
Iz_5_1009	DJI_0693	Thymus pallasianus Heinr.Braun	0	n	1	0	Lamiacea Lindl.
Iz_5_1009	DJI_0693	Tilia cordata Mill.	0	n	1	1	Malvaceae Juss.
Iz_5_1009	DJI_0693	Trifolium alpestre L.	0	n	1	0	Fabaceae Lindl.
Iz_5_1009	DJI_0693	Trifolium arvense L.	0	n	1	0	Fabaceae Lindl.
Iz_5_1009	DJI_0693	Ulmus glabra Huds.	0	n	1	1	Ulmaceae Mirb.
Iz_5_1009	DJI_0693	Verbascum densiflorum Bertol.	0	n	1	0	Scrophulariaceae Juss.
Iz_5_1009	DJI_0693	Veronica austriaca L.	0	n	1	0	Plantaginaceae Juss.
Iz_5_1009	DJI_0693	Veronica incana L.	0	n	1	0	Plantaginaceae Juss.
Iz_5_1009	DJI_0693	Veronica verna L.	0	n	1	0	Plantaginaceae Juss.
Iz_5_1009	DJI_0693	Vincetoxicum hirundinaria Medik	0	n	1	0	Apocynaceae Juss.
Iz_5_1009	DJI_0695	Acer tataricum L.	0	n	1	1	Sapindaceae Juss.
Iz_5_1009	DJI_0695	Achillea millefolium L. s.l.	0	n	1	0	Asteraceae Bercht. & J.Presl
Iz_5_1009	DJI_0695	Allium oleraceum L.	0	n	1	0	Amaryllidaceae J.St.-Hil.
Iz_5_1009	DJI_0695	Ambrosia artemisiifolia L.	0	a	0	0	Asteraceae Bercht. & J.Presl
Iz_5_1009	DJI_0695	Amorpha fruticosa L.	0	a	0	0	Fabaceae Lindl.
Iz_5_1009	DJI_0695	Anthericum ramosum L.	0	n	1	0	Asparagaceae Juss.
Iz_5_1009	DJI_0695	Artemisia marschalliana Spreng.	0	n	1	0	Asteraceae Bercht. & J.Presl
Iz_5_1009	DJI_0695	Asparagus officinalis L.	0	n	1	0	Asparagaceae Juss.
Iz_5_1009	DJI_0695	Astragalus cicer L.	0	n	1	0	Fabaceae Lindl.
Iz_5_1009	DJI_0695	Bassia laniflora (S.G.Gmel.) A.J.Scott	0	n	1	0	Amaranthaceae Juss.
Iz_5_1009	DJI_0695	Berberis vulgaris L.	0	n	1	0	Berberidaceae Juss.
Iz_5_1009	DJI_0695	Betula pendula Roth.	0	n	1	1	Betulaceae Gray
Iz_5_1009	DJI_0695	Betula pendula Roth.	0	n	1	0	Poaceae Barnhart
Iz_5_1009	DJI_0695	Bromus inermis Leyss.	0		NA	NA	
Iz_5_1009	DJI_0695	Bromus squarrosus L.	0	a	0	0	Poaceae Barnhart
Iz_5_1009	DJI_0695	Bromus tectorum L.	0	a	0	0	Poaceae Barnhart
Iz_5_1009	DJI_0695	Calamagrostis epigejos (L.) Roth	42	n	1	0	Poaceae Barnhart
Iz_5_1009	DJI_0695	Carduus acanthoides L.	0	n	1	0	Asteraceae Bercht. & J.Presl
Iz_5_1009	DJI_0695	Carex sp.	0	n	1	0	Cyperaceae Juss.
Iz_5_1009	DJI_0695	Centaurea majorovii Dumbadze	0	n	1	0	Asteraceae Bercht. & J.Presl
Iz_5_1009	DJI_0695	Chamaecytisus ruthenicus (Fisch.ex Wol.) Klaskova	0	n	1	0	Fabaceae Lindl.
Iz_5_1009	DJI_0695	Chelidonium majus L.	0	n	1	0	Papaveraceae Juss.
Iz_5_1009	DJI_0695	Chenopodium betaceum Andrz.	0	n	1	0	Amaranthaceae Juss.
Iz_5_1009	DJI_0695	Chenopodium cf. album	0	n	1	0	Amaranthaceae Juss.
Iz_5_1009	DJI_0695	Chenopodium sp.	0		NA	0	Amaranthaceae Juss.

Iz_5_1009	DJI_0695	Chondrilla juncea L.	0	n	1	0	Asteraceae Bercht. & J.Presl
Iz_5_1009	DJI_0695	Cirsium vulgare (Savi) Ten.	0	n	1	0	Asteraceae Bercht. & J.Presl
Iz_5_1009	DJI_0695	Clinopodium acinos (L.) Kuntze	0	n	1	0	Lamiacea Lindl.
Iz_5_1009	DJI_0695	Convallaria majalis L.	0	n	1	0	Asparagaceae Juss.
Iz_5_1009	DJI_0695	Crataegus rhipidophylla Gand.	0	n	1	1	Rosaceae Juss.
Iz_5_1009	DJI_0695	Crepis foetida L.	0	n	1	0	Asteraceae Bercht. & J.Presl
Iz_5_1009	DJI_0695	Dactylis glomerata L.	0	n	1	0	Poaceae Barnhart
Iz_5_1009	DJI_0695	Dianthus campestris M.Bieb.	0	n	1	0	Caryophyllaceae Juss.
Iz_5_1009	DJI_0695	Digitaria sanguinalis (L.) Scop.	0	a	0	0	Poaceae Barnhart
Iz_5_1009	DJI_0695	Elymus repens (L.) Gould	0	n	1	0	Poaceae Barnhart
Iz_5_1009	DJI_0695	Epilobium tetragonum L.	0	n	1	0	Onagraceae Juss.
Iz_5_1009	DJI_0695	Eragrostis minor Host	0	a	0	0	Poaceae Barnhart
Iz_5_1009	DJI_0695	Erigeron canadensis L.	8	a	0	0	Asteraceae Bercht. & J.Presl
Iz_5_1009	DJI_0695	Euphorbia seguieriana Neck.	0	n	1	0	Euphorbiaceae Juss.
Iz_5_1009	DJI_0695	Fallopia dumetorum (L.) Holub	0	n	1	0	Polygonaceae Juss.
Iz_5_1009	DJI_0695	Festuca beckeri (Hack.) Trautv.	0	n	1	0	Poaceae Barnhart
Iz_5_1009	DJI_0695	Fragaria vesca L.	0	n	1	0	Rosaceae Juss.
Iz_5_1009	DJI_0695	Frangula alnus Mill.	0	n	1	1	Rhamnaceae Juss.
Iz_5_1009	DJI_0695	Fraxinus pennsylvanica Marshall	0	a	0	1	Oleaceae Hoffmanns. & Link
Iz_5_1009	DJI_0695	Galeopsis bifida Boenn.	0	n	1	0	Lamiacea Lindl.
Iz_5_1009	DJI_0695	Galium verum L.	0	n	1	0	Rubiaceae Juss.
Iz_5_1009	DJI_0695	Genista tinctoria L.	0	n	1	0	Fabaceae Lindl.
Iz_5_1009	DJI_0695	Geranium robertianum L.	0	n	1	0	Geraniaceae Juss.
Iz_5_1009	DJI_0695	Geranium sanquineum L.	0	n	1	0	Geraniaceae Juss.
Iz_5_1009	DJI_0695	Gypsophila paniculata L.	0	n	1	0	Caryophyllaceae Juss.
Iz_5_1009	DJI_0695	Helichrysum arenarium (L.) Moench	0	n	1	0	Asteraceae Bercht. & J.Presl
Iz_5_1009	DJI_0695	Herniaria polygama J.Gay	0	n	1	0	Caryophyllaceae Juss.
Iz_5_1009	DJI_0695	Hieracium robustum Fr.	0	n	1	0	Asteraceae Bercht. & J.Presl
Iz_5_1009	DJI_0695	Hieracium umbellatum L.	0	n	1	NA	Asteraceae Bercht. & J.Presl
Iz_5_1009	DJI_0695	Hylotelephium maximum (L.) Holub	0	n	1	0	Crassulaceae J.St.-Hil.
Iz_5_1009	DJI_0695	Jacobaea borysthenica (DC.) B.Nord. & Greuter	0	n	1	0	Asteraceae Bercht. & J.Presl
Iz_5_1009	DJI_0695	Jasione montana L.	0	n	1	0	Campanulaceae Juss.
Iz_5_1009	DJI_0695	Jurinea cyanoides (L.) Rchb.s.l.	0	n	1	0	Asteraceae Bercht. & J.Presl
Iz_5_1009	DJI_0695	Koeleria glauca (Spreng.) DC.	0	n	1	0	Poaceae Barnhart
Iz_5_1009	DJI_0695	Lactuca serriola L.	0	a	0	0	Asteraceae Bercht. & J.Presl
Iz_5_1009	DJI_0695	Linaria genistifolia (L.) Mill.	0	n	1	0	Plantaginaceae Juss.
Iz_5_1009	DJI_0695	Lomelosia argentea (L.) Greuter & Burdet	0	n	1	0	Caprifoliaceae Juss.
Iz_5_1009	DJI_0695	Melampyrum pratense L.	0	n	1	0	Orobanchaceae Vent.
Iz_5_1009	DJI_0695	Oenothera biennis L.	0	a	0	0	Onagraceae Juss.
Iz_5_1009	DJI_0695	Pentanema britannica (L.) D.Gut.Larr., Santos-Vicente, Anderb., E.Rico & M.M.Mart.Ort.	0	n	1	0	Asteraceae Bercht. & J.Presl
Iz_5_1009	DJI_0695	Peucedanum oreoselinum (L.) Moench	0	n	1	0	Apiaceae Lindl.
Iz_5_1009	DJI_0695	Peucedanum ruthenicum M.Bieb.	0	n	1	0	Apiaceae Lindl.
Iz_5_1009	DJI_0695	Pilosella echioides (Lumn.) F.W.Schultz & Sch.Bip.	0	n	1	0	Asteraceae Bercht. & J.Presl
Iz_5_1009	DJI_0695	Pilosella officinarum F. Schultz et Sch. Bip.	0	n	1	0	Asteraceae Bercht. & J.Presl
Iz_5_1009	DJI_0695	Pilosella sp.	0		NA	0	Asteraceae Bercht. & J.Presl
Iz_5_1009	DJI_0695	Pinus sylvestris L.	0	n	1	1	Pinaceae Spreng. ex F.Rudolphi
Iz_5_1009	DJI_0695	Poa annua L.	0	n	1	0	Poaceae Barnhart
Iz_5_1009	DJI_0695	Poa nemoralis L.	0	n	1	0	Poaceae Barnhart
Iz_5_1009	DJI_0695	Poa sp.	0		NA	0	Poaceae Barnhart
Iz_5_1009	DJI_0695	Polygonatum odoratum (Mill.) Druce	0	n	1	0	Asparagaceae Juss.
Iz_5_1009	DJI_0695	Polygonum arenarium Waldst. & Kit.	0	n	1	0	Polygonaceae Juss.
Iz_5_1009	DJI_0695	Populus nigra L.	0	n	1	1	Salicaceae Mirb.
Iz_5_1009	DJI_0695	Populus sp.	0	n	1	1	Salicaceae Mirb.
Iz_5_1009	DJI_0695	Populus tremula L.	0	n	1	1	Salicaceae Mirb.
Iz_5_1009	DJI_0695	Populus Ã canescens (Aiton) Sm.	0	n	1	1	Salicaceae Mirb.
Iz_5_1009	DJI_0695	Potentilla argentea L.	0	n	1	0	Rosaceae Juss.
Iz_5_1009	DJI_0695	Psammophiliella muralis (L.) Ikonn.	0	n	1	0	Caryophyllaceae Juss.
Iz_5_1009	DJI_0695	Psephellus sumensis (Kalen.) Greuter	0	n	1	0	Asteraceae Bercht. & J.Presl
Iz_5_1009	DJI_0695	Pulsatilla pratensis (L.) Mill. s.l.	0	n	1	0	Ranunculaceae Juss.
Iz_5_1009	DJI_0695	Pyrus communis L.	0	a	0	1	Rosaceae Juss.
Iz_5_1009	DJI_0695	Quercus robur L.	0	n	1	1	Fagaceae Dumort.
Iz_5_1009	DJI_0695	Robinia pseudoacacia L.	0	a	0	1	Fabaceae Lindl.
Iz_5_1009	DJI_0695	Rosa sp.	0		NA	1	Rosaceae Juss.
Iz_5_1009	DJI_0695	Rubus idaeus L.	0	n	1	0	Rosaceae Juss.
Iz_5_1009	DJI_0695	Rumex acetosella L.	0	n	1	0	Polygonaceae Juss.
Iz_5_1009	DJI_0695	Rumex thyrsiflorus Fingerh.	0	n	1	0	Polygonaceae Juss.
Iz_5_1009	DJI_0695	Salix alba L.	0	n	1	1	Salicaceae Mirb.
Iz_5_1009	DJI_0695	Sambucus nigra L.	0	n	1	1	Viburnaceae Raf.
Iz_5_1009	DJI_0695	Secale sylvestre Host	0	n	1	0	Poaceae Barnhart
Iz_5_1009	DJI_0695	Sempervivum ruthenicum Schnittsp. & C.B. Lehm.	0	n	1	0	Crassulaceae J.St.-Hil.
Iz_5_1009	DJI_0695	Setaria viridis (L.) P. Beauv.	0	n	1	0	Poaceae Barnhart
Iz_5_1009	DJI_0695	Silene borysthenica (Gruner) Walters	0	n	1	0	Caryophyllaceae Juss.
Iz_5_1009	DJI_0695	Silene latifolia subsp. alba (Mill.) Greuter & Burdet	0	n	1	0	Caryophyllaceae Juss.
Iz_5_1009	DJI_0695	Solidago virgaurea L.	0	n	1	0	Asteraceae Bercht. & J.Presl
Iz_5_1009	DJI_0695	Solidago Ã niederederi (S. canadensis Ã S. virgaurea)	2	hybrid	0	0	Asteraceae Bercht. & J.Presl
Iz_5_1009	DJI_0695	Sorbus aucuparia L.	0	n	1	1	Rosaceae Juss.
Iz_5_1009	DJI_0695	Stachys recta L.	0	n	1	0	Lamiacea Lindl.
Iz_5_1009	DJI_0695	Tanacetum vulgare L.	0	n	1	0	Asteraceae Bercht. & J.Presl
Iz_5_1009	DJI_0695	Thymus pallasianus Heinr.Braun	0	n	1	0	Lamiacea Lindl.
Iz_5_1009	DJI_0695	Tilia cordata Mill.	0	n	1	1	Malvaceae Juss.
Iz_5_1009	DJI_0695	Trifolium alpestre L.	0	n	1	0	Fabaceae Lindl.
Iz_5_1009	DJI_0695	Trifolium arvense L.	0	n	1	0	Fabaceae Lindl.
Iz_5_1009	DJI_0695	Ulmus glabra Huds.	0	n	1	1	Ulmaceae Mirb.
Iz_5_1009	DJI_0695	Verbascum densiflorum Bertol.	0	n	1	0	Scrophulariaceae Juss.
Iz_5_1009	DJI_0695	Veronica austriaca L.	0	n	1	0	Plantaginaceae Juss.
Iz_5_1009	DJI_0695	Veronica incana L.	0	n	1	0	Plantaginaceae Juss.
Iz_5_1009	DJI_0695	Veronica verna L.	0	n	1	0	Plantaginaceae Juss.
Iz_5_1009	DJI_0695	Vincetoxicum hirundinaria Medik	0	n	1	0	Apocynaceae Juss.
Iz_5_1009	DJI_0697	Acer tataricum L.	0	n	1	1	Sapindaceae Juss.
Iz_5_1009	DJI_0697	Achillea millefolium L. s.l.	0	n	1	0	Asteraceae Bercht. & J.Presl
Iz_5_1009	DJI_0697	Allium oleraceum L.	0	n	1	0	Amaryllidaceae J.St.-Hil.
Iz_5_1009	DJI_0697	Ambrosia artemisiifolia L.	0	a	0	0	Asteraceae Bercht. & J.Presl
Iz_5_1009	DJI_0697	Amorpha fruticosa L.	0	a	0	0	Fabaceae Lindl.
Iz_5_1009	DJI_0697	Anthericum ramosum L.	0	n	1	0	Asparagaceae Juss.
Iz_5_1009	DJI_0697	Artemisia marschalliana Spreng.	0	n	1	0	Asteraceae Bercht. & J.Presl
Iz_5_1009	DJI_0697	Asparagus officinalis L.	0	n	1	0	Asparagaceae Juss.
Iz_5_1009	DJI_0697	Astragalus cicer L.	0	n	1	0	Fabaceae Lindl.
Iz_5_1009	DJI_0697	Bassia laniflora (S.G.Gmel.) A.J.Scott	0	n	1	0	Amaranthaceae Juss.

Iz_5_1009	DJI_0697	Berberis vulgaris L.	0	n	1	0	Berberidaceae Juss.
Iz_5_1009	DJI_0697	Betula pendula Roth.	0	n	1	1	Betulaceae Gray
Iz_5_1009	DJI_0697	Betula pendula Roth.	0	n	1	0	Poaceae Barnhart
Iz_5_1009	DJI_0697	Bromus inermis Leyss.	0		NA	NA	
Iz_5_1009	DJI_0697	Bromus squarrosus L.	0	a	0	0	Poaceae Barnhart
Iz_5_1009	DJI_0697	Bromus tectorum L.	0	a	0	0	Poaceae Barnhart
Iz_5_1009	DJI_0697	Calamagrostis epigejos (L.) Roth	30	n	1	0	Poaceae Barnhart
Iz_5_1009	DJI_0697	Carduus acanthoides L.	0	n	1	0	Asteraceae Bercht. & J.Presl
Iz_5_1009	DJI_0697	Carex sp.	0	n	1	0	Cyperaceae Juss.
Iz_5_1009	DJI_0697	Centaurea majorovii Dumbadze	0	n	1	0	Asteraceae Bercht. & J.Presl
Iz_5_1009	DJI_0697	Chamaecytisus ruthenicus (Fisch.ex Wol.) Klaskova	0	n	1	0	Fabaceae Lindl.
Iz_5_1009	DJI_0697	Chelidonium majus L.	0	n	1	0	Papaveraceae Juss.
Iz_5_1009	DJI_0697	Chenopodium betaceum Andrz.	0	n	1	0	Amaranthaceae Juss.
Iz_5_1009	DJI_0697	Chenopodium cf. album	0	n	1	0	Amaranthaceae Juss.
Iz_5_1009	DJI_0697	Chenopodium sp.	0		NA	0	Amaranthaceae Juss.
Iz_5_1009	DJI_0697	Chondrilla juncea L.	0	n	1	0	Asteraceae Bercht. & J.Presl
Iz_5_1009	DJI_0697	Cirsium vulgare (Savi) Ten.	0	n	1	0	Asteraceae Bercht. & J.Presl
Iz_5_1009	DJI_0697	Clinopodium acinos (L.) Kuntze	0	n	1	0	Lamiacea Lindl.
Iz_5_1009	DJI_0697	Convallaria majalis L.	0	n	1	0	Asparagaceae Juss.
Iz_5_1009	DJI_0697	Crataegus rhipidophylla Gand.	0	n	1	1	Rosaceae Juss.
Iz_5_1009	DJI_0697	Crepis foetida L.	0	n	1	0	Asteraceae Bercht. & J.Presl
Iz_5_1009	DJI_0697	Dactylis glomerata L.	0	n	1	0	Poaceae Barnhart
Iz_5_1009	DJI_0697	Dianthus campestris M.Bieb.	0	n	1	0	Caryophyllaceae Juss.
Iz_5_1009	DJI_0697	Digitaria sanguinalis (L.) Scop.	0	a	0	0	Poaceae Barnhart
Iz_5_1009	DJI_0697	Elymus repens (L.) Gould	0	n	1	0	Poaceae Barnhart
Iz_5_1009	DJI_0697	Epilobium tetragonum L.	0	n	1	0	Onagraceae Juss.
Iz_5_1009	DJI_0697	Eragrostis minor Host	0	a	0	0	Poaceae Barnhart
Iz_5_1009	DJI_0697	Erigeron canadensis L.	10	a	0	0	Asteraceae Bercht. & J.Presl
Iz_5_1009	DJI_0697	Euphorbia seguieriana Neck.	0	n	1	0	Euphorbiaceae Juss.
Iz_5_1009	DJI_0697	Fallopia dumetorum (L.) Holub	0	n	1	0	Polygonaceae Juss.
Iz_5_1009	DJI_0697	Festuca beckeri (Hack.) Trautv.	0	n	1	0	Poaceae Barnhart
Iz_5_1009	DJI_0697	Fragaria vesca L.	0	n	1	0	Rosaceae Juss.
Iz_5_1009	DJI_0697	Frangula alnus Mill.	0	n	1	1	Rhamnaceae Juss.
Iz_5_1009	DJI_0697	Fraxinus pennsylvanica Marshall	0	a	0	1	Oleaceae Hoffmanns. & Link
Iz_5_1009	DJI_0697	Galeopsis bifida Boenn.	0	n	1	0	Lamiacea Lindl.
Iz_5_1009	DJI_0697	Galium verum L.	0	n	1	0	Rubiaceae Juss.
Iz_5_1009	DJI_0697	Genista tinctoria L.	0	n	1	0	Fabaceae Lindl.
Iz_5_1009	DJI_0697	Geranium robertianum L.	0	n	1	0	Geraniaceae Juss.
Iz_5_1009	DJI_0697	Geranium sanquineum L.	0	n	1	0	Geraniaceae Juss.
Iz_5_1009	DJI_0697	Gypsophila paniculata L.	0	n	1	0	Caryophyllaceae Juss.
Iz_5_1009	DJI_0697	Helichrysum arenarium (L.) Moench	0	n	1	0	Asteraceae Bercht. & J.Presl
Iz_5_1009	DJI_0697	Herniaria polygama J.Gay	0	n	1	0	Caryophyllaceae Juss.
Iz_5_1009	DJI_0697	Hieracium robustum Fr.	0	n	1	0	Asteraceae Bercht. & J.Presl
Iz_5_1009	DJI_0697	Hieracium umbellatum L.	0	n	1	NA	Asteraceae Bercht. & J.Presl
Iz_5_1009	DJI_0697	Hylotelephium maximum (L.) Holub	0	n	1	0	Crassulaceae J.St.-Hil.
Iz_5_1009	DJI_0697	Jacobaea borysthenica (DC.) B.Nord. & Greuter	0	n	1	0	Asteraceae Bercht. & J.Presl
Iz_5_1009	DJI_0697	Jasione montana L.	0	n	1	0	Campanulaceae Juss.
Iz_5_1009	DJI_0697	Jurinea cyanoides (L.) Rchb.s.l.	0	n	1	0	Asteraceae Bercht. & J.Presl
Iz_5_1009	DJI_0697	Koeleria glauca (Spreng.) DC.	0	n	1	0	Poaceae Barnhart
Iz_5_1009	DJI_0697	Lactuca serriola L.	0	a	0	0	Asteraceae Bercht. & J.Presl
Iz_5_1009	DJI_0697	Linaria genistifolia (L.) Mill.	0	n	1	0	Plantaginaceae Juss.
Iz_5_1009	DJI_0697	Lomelosia argentea (L.) Greuter & Burdet	0	n	1	0	Caprifoliaceae Juss.
Iz_5_1009	DJI_0697	Melampyrum pratense L.	0	n	1	0	Orobanchaceae Vent.
Iz_5_1009	DJI_0697	Oenothera biennis L.	0	a	0	0	Onagraceae Juss.
Iz_5_1009	DJI_0697	Pentanema britannica (L.) D.Gut.Larr., Santos-Vicente, Anderb., E.Rico & M.M.Mart.Ort.	0	n	1	0	Asteraceae Bercht. & J.Presl
Iz_5_1009	DJI_0697	Peucedanum oreoselinum (L.) Moench	0	n	1	0	Apiaceae Lindl.
Iz_5_1009	DJI_0697	Peucedanum ruthenicum M.Bieb.	0	n	1	0	Apiaceae Lindl.
Iz_5_1009	DJI_0697	Pilosella echioides (Lumn.) F.W.Schultz & Sch.Bip.	0	n	1	0	Asteraceae Bercht. & J.Presl
Iz_5_1009	DJI_0697	Pilosella officinarum F. Schultz et Sch. Bip.	0	n	1	0	Asteraceae Bercht. & J.Presl
Iz_5_1009	DJI_0697	Pilosella sp.	0		NA	0	Asteraceae Bercht. & J.Presl
Iz_5_1009	DJI_0697	Pinus sylvestris L.	0	n	1	1	Pinaceae Spreng. ex F.Rudolphi
Iz_5_1009	DJI_0697	Poa annua L.	0	n	1	0	Poaceae Barnhart
Iz_5_1009	DJI_0697	Poa nemoralis L.	0	n	1	0	Poaceae Barnhart
Iz_5_1009	DJI_0697	Poa sp.	0		NA	0	Poaceae Barnhart
Iz_5_1009	DJI_0697	Polygonatum odoratum (Mill.) Druce	0	n	1	0	Asparagaceae Juss.
Iz_5_1009	DJI_0697	Polygonum arenarium Waldst. & Kit.	0	n	1	0	Polygonaceae Juss.
Iz_5_1009	DJI_0697	Populus nigra L.	0	n	1	1	Salicaceae Mirb.
Iz_5_1009	DJI_0697	Populus sp.	0	n	1	1	Salicaceae Mirb.
Iz_5_1009	DJI_0697	Populus tremula L.	0	n	1	1	Salicaceae Mirb.
Iz_5_1009	DJI_0697	Populus Ã canescens (Aiton) Sm.	0	n	1	1	Salicaceae Mirb.
Iz_5_1009	DJI_0697	Potentilla argentea L.	0	n	1	0	Rosaceae Juss.
Iz_5_1009	DJI_0697	Psammophiliella muralis (L.) Ikonn.	0	n	1	0	Caryophyllaceae Juss.
Iz_5_1009	DJI_0697	Psephellus sumensis (Kalen.) Greuter	0	n	1	0	Asteraceae Bercht. & J.Presl
Iz_5_1009	DJI_0697	Pulsatilla pratensis (L.) Mill. s.l.	0	n	1	0	Ranunculaceae Juss.
Iz_5_1009	DJI_0697	Pyrus communis L.	0	a	0	1	Rosaceae Juss.
Iz_5_1009	DJI_0697	Quercus robur L.	0	n	1	1	Fagaceae Dumort.
Iz_5_1009	DJI_0697	Robinia pseudoacacia L.	0	a	0	1	Fabaceae Lindl.
Iz_5_1009	DJI_0697	Rosa sp.	0		NA	1	Rosaceae Juss.
Iz_5_1009	DJI_0697	Rubus idaeus L.	0	n	1	0	Rosaceae Juss.
Iz_5_1009	DJI_0697	Rumex acetosella L.	0	n	1	0	Polygonaceae Juss.
Iz_5_1009	DJI_0697	Rumex thyrsiflorus Fingerh.	0	n	1	0	Polygonaceae Juss.
Iz_5_1009	DJI_0697	Salix alba L.	0	n	1	1	Salicaceae Mirb.
Iz_5_1009	DJI_0697	Sambucus nigra L.	0	n	1	1	Viburnaceae Raf.
Iz_5_1009	DJI_0697	Secale sylvestre Host	0	n	1	0	Poaceae Barnhart
Iz_5_1009	DJI_0697	Sempervivum ruthenicum Schnittsp. & C.B. Lehm.	0	n	1	0	Crassulaceae J.St.-Hil.
Iz_5_1009	DJI_0697	Setaria viridis (L.) P. Beauv.	0	n	1	0	Poaceae Barnhart
Iz_5_1009	DJI_0697	Silene borysthenica (Gruner) Walters	0	n	1	0	Caryophyllaceae Juss.
Iz_5_1009	DJI_0697	Silene latifolia subsp. alba (Mill.) Greuter & Burdet	0	n	1	0	Caryophyllaceae Juss.
Iz_5_1009	DJI_0697	Solidago virgaurea L.	0	n	1	0	Asteraceae Bercht. & J.Presl
Iz_5_1009	DJI_0697	Solidago Ã niederederi (S. canadensis Ã S. virgaurea)	0	hybrid	0	0	Asteraceae Bercht. & J.Presl
Iz_5_1009	DJI_0697	Sorbus aucuparia L.	0	n	1	1	Rosaceae Juss.
Iz_5_1009	DJI_0697	Stachys recta L.	0	n	1	0	Lamiacea Lindl.
Iz_5_1009	DJI_0697	Tanacetum vulgare L.	0	n	1	0	Asteraceae Bercht. & J.Presl
Iz_5_1009	DJI_0697	Thymus pallasianus Heinr.Braun	0	n	1	0	Lamiacea Lindl.
Iz_5_1009	DJI_0697	Tilia cordata Mill.	0	n	1	1	Malvaceae Juss.
Iz_5_1009	DJI_0697	Trifolium alpestre L.	0	n	1	0	Fabaceae Lindl.
Iz_5_1009	DJI_0697	Trifolium arvense L.	0	n	1	0	Fabaceae Lindl.
Iz_5_1009	DJI_0697	Ulmus glabra Huds.	0	n	1	1	Ulmaceae Mirb.

Iz_5_1009	DJI_0697	Verbascum densiflorum Bertol.	0	n	1	0	Scrophulariaceae Juss.
Iz_5_1009	DJI_0697	Veronica austriaca L.	0	n	1	0	Plantaginaceae Juss.
Iz_5_1009	DJI_0697	Veronica incana L.	0	n	1	0	Plantaginaceae Juss.
Iz_5_1009	DJI_0697	Veronica verna L.	0	n	1	0	Plantaginaceae Juss.
Iz_5_1009	DJI_0697	Vincetoxicum hirundinaria Medik	0	n	1	0	Apocynaceae Juss.
Iz_5_1009	DJI_0701	Acer tataricum L.	0	n	1	1	Sapindaceae Juss.
Iz_5_1009	DJI_0701	Achillea millefolium L. s.l.	0	n	1	0	Asteraceae Bercht. & J.Presl
Iz_5_1009	DJI_0701	Allium oleraceum L.	0	n	1	0	Amaryllidaceae J.St.-Hil.
Iz_5_1009	DJI_0701	Ambrosia artemisiifolia L.	0	a	0	0	Asteraceae Bercht. & J.Presl
Iz_5_1009	DJI_0701	Amorpha fruticosa L.	0	a	0	0	Fabaceae Lindl.
Iz_5_1009	DJI_0701	Anthericum ramosum L.	0	n	1	0	Asparagaceae Juss.
Iz_5_1009	DJI_0701	Artemisia marschalliana Spreng.	0	n	1	0	Asteraceae Bercht. & J.Presl
Iz_5_1009	DJI_0701	Asparagus officinalis L.	0	n	1	0	Asparagaceae Juss.
Iz_5_1009	DJI_0701	Astragalus cicer L.	0	n	1	0	Fabaceae Lindl.
Iz_5_1009	DJI_0701	Bassia laniflora (S.G.Gmel.) A.J.Scott	0	n	1	0	Amaranthaceae Juss.
Iz_5_1009	DJI_0701	Berberis vulgaris L.	0	n	1	0	Berberidaceae Juss.
Iz_5_1009	DJI_0701	Betula pendula Roth.	0	n	1	1	Betulaceae Gray
Iz_5_1009	DJI_0701	Betula pendula Roth.	0	n	1	0	Poaceae Barnhart
Iz_5_1009	DJI_0701	Bromus inermis Leyss.	0		NA	NA	
Iz_5_1009	DJI_0701	Bromus squarrosus L.	0	a	0	0	Poaceae Barnhart
Iz_5_1009	DJI_0701	Bromus tectorum L.	0	a	0	0	Poaceae Barnhart
Iz_5_1009	DJI_0701	Calamagrostis epigejos (L.) Roth	5	n	1	0	Poaceae Barnhart
Iz_5_1009	DJI_0701	Carduus acanthoides L.	0	n	1	0	Asteraceae Bercht. & J.Presl
Iz_5_1009	DJI_0701	Carex sp.	0	n	1	0	Cyperaceae Juss.
Iz_5_1009	DJI_0701	Centaurea majorovii Dumbadze	0	n	1	0	Asteraceae Bercht. & J.Presl
Iz_5_1009	DJI_0701	Chamaecytisus ruthenicus (Fisch.ex Wol.) Klaskova	0	n	1	0	Fabaceae Lindl.
Iz_5_1009	DJI_0701	Chelidonium majus L.	0	n	1	0	Papaveraceae Juss.
Iz_5_1009	DJI_0701	Chenopodium betaceum Andrz.	0	n	1	0	Amaranthaceae Juss.
Iz_5_1009	DJI_0701	Chenopodium cf. album	0	n	1	0	Amaranthaceae Juss.
Iz_5_1009	DJI_0701	Chenopodium sp.	0		NA	0	Amaranthaceae Juss.
Iz_5_1009	DJI_0701	Chondrilla juncea L.	0	n	1	0	Asteraceae Bercht. & J.Presl
Iz_5_1009	DJI_0701	Cirsium vulgare (Savi) Ten.	0	n	1	0	Asteraceae Bercht. & J.Presl
Iz_5_1009	DJI_0701	Clinopodium acinos (L.) Kuntze	0	n	1	0	Lamiacea Lindl.
Iz_5_1009	DJI_0701	Convallaria majalis L.	0	n	1	0	Asparagaceae Juss.
Iz_5_1009	DJI_0701	Crataegus rhipidophylla Gand.	0	n	1	1	Rosaceae Juss.
Iz_5_1009	DJI_0701	Crepis foetida L.	0	n	1	0	Asteraceae Bercht. & J.Presl
Iz_5_1009	DJI_0701	Dactylis glomerata L.	0	n	1	0	Poaceae Barnhart
Iz_5_1009	DJI_0701	Dianthus campestris M.Bieb.	0	n	1	0	Caryophyllaceae Juss.
Iz_5_1009	DJI_0701	Digitaria sanguinalis (L.) Scop.	0	a	0	0	Poaceae Barnhart
Iz_5_1009	DJI_0701	Elymus repens (L.) Gould	0	n	1	0	Poaceae Barnhart
Iz_5_1009	DJI_0701	Epilobium tetragonum L.	0	n	1	0	Onagraceae Juss.
Iz_5_1009	DJI_0701	Eragrostis minor Host	0	a	0	0	Poaceae Barnhart
Iz_5_1009	DJI_0701	Erigeron canadensis L.	10	a	0	0	Asteraceae Bercht. & J.Presl
Iz_5_1009	DJI_0701	Euphorbia seguieriana Neck.	0	n	1	0	Euphorbiaceae Juss.
Iz_5_1009	DJI_0701	Fallopia dumetorum (L.) Holub	0	n	1	0	Polygonaceae Juss.
Iz_5_1009	DJI_0701	Festuca beckeri (Hack.) Trautv.	0	n	1	0	Poaceae Barnhart
Iz_5_1009	DJI_0701	Fragaria vesca L.	0	n	1	0	Rosaceae Juss.
Iz_5_1009	DJI_0701	Frangula alnus Mill.	0	n	1	1	Rhamnaceae Juss.
Iz_5_1009	DJI_0701	Fraxinus pennsylvanica Marshall	0	a	0	1	Oleaceae Hoffmanns. & Link
Iz_5_1009	DJI_0701	Galeopsis bifida Boenn.	0	n	1	0	Lamiacea Lindl.
Iz_5_1009	DJI_0701	Galium verum L.	0	n	1	0	Rubiaceae Juss.
Iz_5_1009	DJI_0701	Genista tinctoria L.	0	n	1	0	Fabaceae Lindl.
Iz_5_1009	DJI_0701	Geranium robertianum L.	0	n	1	0	Geraniaceae Juss.
Iz_5_1009	DJI_0701	Geranium sanquineum L.	0	n	1	0	Geraniaceae Juss.
Iz_5_1009	DJI_0701	Gypsophila paniculata L.	0	n	1	0	Caryophyllaceae Juss.
Iz_5_1009	DJI_0701	Helichrysum arenarium (L.) Moench	0	n	1	0	Asteraceae Bercht. & J.Presl
Iz_5_1009	DJI_0701	Herniaria polygama J.Gay	0	n	1	0	Caryophyllaceae Juss.
Iz_5_1009	DJI_0701	Hieracium robustum Fr.	0	n	1	0	Asteraceae Bercht. & J.Presl
Iz_5_1009	DJI_0701	Hieracium umbellatum L.	0	n	1	NA	Asteraceae Bercht. & J.Presl
Iz_5_1009	DJI_0701	Hylotelephium maximum (L.) Holub	0	n	1	0	Crassulaceae J.St.-Hil.
Iz_5_1009	DJI_0701	Jacobaea borysthenica (DC.) B.Nord. & Greuter	0	n	1	0	Asteraceae Bercht. & J.Presl
Iz_5_1009	DJI_0701	Jasione montana L.	0	n	1	0	Campanulaceae Juss.
Iz_5_1009	DJI_0701	Jurinea cyanoides (L.) Rchb.s.l.	0	n	1	0	Asteraceae Bercht. & J.Presl
Iz_5_1009	DJI_0701	Koeleria glauca (Spreng.) DC.	0	n	1	0	Poaceae Barnhart
Iz_5_1009	DJI_0701	Lactuca serriola L.	0	a	0	0	Asteraceae Bercht. & J.Presl
Iz_5_1009	DJI_0701	Linaria genistifolia (L.) Mill.	0	n	1	0	Plantaginaceae Juss.
Iz_5_1009	DJI_0701	Lomelosia argentea (L.) Greuter & Burdet	0	n	1	0	Caprifoliaceae Juss.
Iz_5_1009	DJI_0701	Melampyrum pratense L.	0	n	1	0	Orobanchaceae Vent.
Iz_5_1009	DJI_0701	Oenothera biennis L.	0	a	0	0	Onagraceae Juss.
Iz_5_1009	DJI_0701	Pentanema britannica (L.) D.Gut.Larr., Santos-Vicente, Anderb., E.Rico & M.M.Mart.Ort.	0	n	1	0	Asteraceae Bercht. & J.Presl
Iz_5_1009	DJI_0701	Peucedanum oreoselinum (L.) Moench	0.5	n	1	0	Apiaceae Lindl.
Iz_5_1009	DJI_0701	Peucedanum ruthenicum M.Bieb.	0	n	1	0	Apiaceae Lindl.
Iz_5_1009	DJI_0701	Pilosella echioides (Lumn.) F.W.Schultz & Sch.Bip.	0	n	1	0	Asteraceae Bercht. & J.Presl
Iz_5_1009	DJI_0701	Pilosella officinarum F. Schultz et Sch. Bip.	0	n	1	0	Asteraceae Bercht. & J.Presl
Iz_5_1009	DJI_0701	Pilosella sp.	0		NA	0	Asteraceae Bercht. & J.Presl
Iz_5_1009	DJI_0701	Pinus sylvestris L.	0	n	1	1	Pinaceae Spreng. ex F.Rudolphi
Iz_5_1009	DJI_0701	Poa annua L.	0	n	1	0	Poaceae Barnhart
Iz_5_1009	DJI_0701	Poa nemoralis L.	0	n	1	0	Poaceae Barnhart
Iz_5_1009	DJI_0701	Poa sp.	0		NA	0	Poaceae Barnhart
Iz_5_1009	DJI_0701	Polygonatum odoratum (Mill.) Druce	0	n	1	0	Asparagaceae Juss.
Iz_5_1009	DJI_0701	Polygonum arenarium Waldst. & Kit.	0	n	1	0	Polygonaceae Juss.
Iz_5_1009	DJI_0701	Populus nigra L.	0	n	1	1	Salicaceae Mirb.
Iz_5_1009	DJI_0701	Populus sp.	0	n	1	1	Salicaceae Mirb.
Iz_5_1009	DJI_0701	Populus tremula L.	0	n	1	1	Salicaceae Mirb.
Iz_5_1009	DJI_0701	Populus Ã canescens (Aiton) Sm.	0	n	1	1	Salicaceae Mirb.
Iz_5_1009	DJI_0701	Potentilla argentea L.	0	n	1	0	Rosaceae Juss.
Iz_5_1009	DJI_0701	Psammophiliella muralis (L.) Ikonn.	0	n	1	0	Caryophyllaceae Juss.
Iz_5_1009	DJI_0701	Psephellus sumensis (Kalen.) Greuter	0	n	1	0	Asteraceae Bercht. & J.Presl
Iz_5_1009	DJI_0701	Pulsatilla pratensis (L.) Mill. s.l.	0	n	1	0	Ranunculaceae Juss.
Iz_5_1009	DJI_0701	Pyrus communis L.	0	a	0	1	Rosaceae Juss.
Iz_5_1009	DJI_0701	Quercus robur L.	0	n	1	1	Fagaceae Dumort.
Iz_5_1009	DJI_0701	Robinia pseudoacacia L.	0	a	0	1	Fabaceae Lindl.
Iz_5_1009	DJI_0701	Rosa sp.	0		NA	1	Rosaceae Juss.
Iz_5_1009	DJI_0701	Rubus idaeus L.	0	n	1	0	Rosaceae Juss.
Iz_5_1009	DJI_0701	Rumex acetosella L.	0	n	1	0	Polygonaceae Juss.
Iz_5_1009	DJI_0701	Rumex thyrsiflorus Fingerh.	0	n	1	0	Polygonaceae Juss.
Iz_5_1009	DJI_0701	Salix alba L.	0	n	1	1	Salicaceae Mirb.
Iz_5_1009	DJI_0701	Sambucus nigra L.	0	n	1	1	Viburnaceae Raf.

Iz_5_1009	DJI_0701	Secale sylvestre Host	0	n	1	0	Poaceae Barnhart
Iz_5_1009	DJI_0701	Sempervivum ruthenicum Schnittsp. & C.B. Lehm.	0	n	1	0	Crassulaceae J.St.-Hil.
Iz_5_1009	DJI_0701	Setaria viridis (L.) P. Beauv.	0	n	1	0	Poaceae Barnhart
Iz_5_1009	DJI_0701	Silene borysthenica (Gruner) Walters	0	n	1	0	Caryophyllaceae Juss.
Iz_5_1009	DJI_0701	Silene latifolia subsp. alba (Mill.) Greuter & Burdet	0	n	1	0	Caryophyllaceae Juss.
Iz_5_1009	DJI_0701	Solidago virgaurea L.	2	n	1	0	Asteraceae Bercht. & J.Presl
Iz_5_1009	DJI_0701	Solidago Ã niederederi (S. canadensis Ã S. virgaurea)	0	hybrid	0	0	Asteraceae Bercht. & J.Presl
Iz_5_1009	DJI_0701	Sorbus aucuparia L.	0	n	1	1	Rosaceae Juss.
Iz_5_1009	DJI_0701	Stachys recta L.	0	n	1	0	Lamiacea Lindl.
Iz_5_1009	DJI_0701	Tanacetum vulgare L.	0	n	1	0	Asteraceae Bercht. & J.Presl
Iz_5_1009	DJI_0701	Thymus pallasianus Heinr.Braun	0	n	1	0	Lamiacea Lindl.
Iz_5_1009	DJI_0701	Tilia cordata Mill.	0	n	1	1	Malvaceae Juss.
Iz_5_1009	DJI_0701	Trifolium alpestre L.	0	n	1	0	Fabaceae Lindl.
Iz_5_1009	DJI_0701	Trifolium arvense L.	0	n	1	0	Fabaceae Lindl.
Iz_5_1009	DJI_0701	Ulmus glabra Huds.	0	n	1	1	Ulmaceae Mirb.
Iz_5_1009	DJI_0701	Verbascum densiflorum Bertol.	0	n	1	0	Scrophulariaceae Juss.
Iz_5_1009	DJI_0701	Veronica austriaca L.	0	n	1	0	Plantaginaceae Juss.
Iz_5_1009	DJI_0701	Veronica incana L.	0	n	1	0	Plantaginaceae Juss.
Iz_5_1009	DJI_0701	Veronica verna L.	0	n	1	0	Plantaginaceae Juss.
Iz_5_1009	DJI_0701	Vincetoxicum hirundinaria Medik	0	n	1	0	Apocynaceae Juss.
Iz_5_1009	DJI_0703	Acer tataricum L.	0	n	1	1	Sapindaceae Juss.
Iz_5_1009	DJI_0703	Achillea millefolium L. s.l.	0	n	1	0	Asteraceae Bercht. & J.Presl
Iz_5_1009	DJI_0703	Allium oleraceum L.	0	n	1	0	Amaryllidaceae J.St.-Hil.
Iz_5_1009	DJI_0703	Ambrosia artemisiifolia L.	0	a	0	0	Asteraceae Bercht. & J.Presl
Iz_5_1009	DJI_0703	Amorpha fruticosa L.	0	a	0	0	Fabaceae Lindl.
Iz_5_1009	DJI_0703	Anthericum ramosum L.	0	n	1	0	Asparagaceae Juss.
Iz_5_1009	DJI_0703	Artemisia marschalliana Spreng.	0	n	1	0	Asteraceae Bercht. & J.Presl
Iz_5_1009	DJI_0703	Asparagus officinalis L.	0	n	1	0	Asparagaceae Juss.
Iz_5_1009	DJI_0703	Astragalus cicer L.	0	n	1	0	Fabaceae Lindl.
Iz_5_1009	DJI_0703	Bassia laniflora (S.G.Gmel.) A.J.Scott	0	n	1	0	Amaranthaceae Juss.
Iz_5_1009	DJI_0703	Berberis vulgaris L.	0	n	1	0	Berberidaceae Juss.
Iz_5_1009	DJI_0703	Betula pendula Roth.	2	n	1	1	Betulaceae Gray
Iz_5_1009	DJI_0703	Betula pendula Roth.	2	n	1	0	Poaceae Barnhart
Iz_5_1009	DJI_0703	Bromus inermis Leyss.	0		NA	NA	
Iz_5_1009	DJI_0703	Bromus squarrosus L.	0	a	0	0	Poaceae Barnhart
Iz_5_1009	DJI_0703	Bromus tectorum L.	0	a	0	0	Poaceae Barnhart
Iz_5_1009	DJI_0703	Calamagrostis epigejos (L.) Roth	1	n	1	0	Poaceae Barnhart
Iz_5_1009	DJI_0703	Carduus acanthoides L.	0	n	1	0	Asteraceae Bercht. & J.Presl
Iz_5_1009	DJI_0703	Carex sp.	0	n	1	0	Cyperaceae Juss.
Iz_5_1009	DJI_0703	Centaurea majorovii Dumbadze	0	n	1	0	Asteraceae Bercht. & J.Presl
Iz_5_1009	DJI_0703	Chamaecytisus ruthenicus (Fisch.ex Wol.) Klaskova	0	n	1	0	Fabaceae Lindl.
Iz_5_1009	DJI_0703	Chelidonium majus L.	0	n	1	0	Papaveraceae Juss.
Iz_5_1009	DJI_0703	Chenopodium betaceum Andrz.	0	n	1	0	Amaranthaceae Juss.
Iz_5_1009	DJI_0703	Chenopodium cf. album	0	n	1	0	Amaranthaceae Juss.
Iz_5_1009	DJI_0703	Chenopodium sp.	0		NA	0	Amaranthaceae Juss.
Iz_5_1009	DJI_0703	Chondrilla juncea L.	0	n	1	0	Asteraceae Bercht. & J.Presl
Iz_5_1009	DJI_0703	Cirsium vulgare (Savi) Ten.	0	n	1	0	Asteraceae Bercht. & J.Presl
Iz_5_1009	DJI_0703	Clinopodium acinos (L.) Kuntze	0	n	1	0	Lamiacea Lindl.
Iz_5_1009	DJI_0703	Convallaria majalis L.	0	n	1	0	Asparagaceae Juss.
Iz_5_1009	DJI_0703	Crataegus rhipidophylla Gand.	0	n	1	1	Rosaceae Juss.
Iz_5_1009	DJI_0703	Crepis foetida L.	0	n	1	0	Asteraceae Bercht. & J.Presl
Iz_5_1009	DJI_0703	Dactylis glomerata L.	0	n	1	0	Poaceae Barnhart
Iz_5_1009	DJI_0703	Dianthus campestris M.Bieb.	0	n	1	0	Caryophyllaceae Juss.
Iz_5_1009	DJI_0703	Digitaria sanguinalis (L.) Scop.	0	a	0	0	Poaceae Barnhart
Iz_5_1009	DJI_0703	Elymus repens (L.) Gould	0	n	1	0	Poaceae Barnhart
Iz_5_1009	DJI_0703	Epilobium tetragonum L.	0	n	1	0	Onagraceae Juss.
Iz_5_1009	DJI_0703	Eragrostis minor Host	0	a	0	0	Poaceae Barnhart
Iz_5_1009	DJI_0703	Erigeron canadensis L.	12	a	0	0	Asteraceae Bercht. & J.Presl
Iz_5_1009	DJI_0703	Euphorbia seguieriana Neck.	0	n	1	0	Euphorbiaceae Juss.
Iz_5_1009	DJI_0703	Fallopia dumetorum (L.) Holub	0	n	1	0	Polygonaceae Juss.
Iz_5_1009	DJI_0703	Festuca beckeri (Hack.) Trautv.	0	n	1	0	Poaceae Barnhart
Iz_5_1009	DJI_0703	Fragaria vesca L.	0	n	1	0	Rosaceae Juss.
Iz_5_1009	DJI_0703	Frangula alnus Mill.	0	n	1	1	Rhamnaceae Juss.
Iz_5_1009	DJI_0703	Fraxinus pennsylvanica Marshall	0	a	0	1	Oleaceae Hoffmanns. & Link
Iz_5_1009	DJI_0703	Galeopsis bifida Boenn.	0	n	1	0	Lamiacea Lindl.
Iz_5_1009	DJI_0703	Galium verum L.	0	n	1	0	Rubiaceae Juss.
Iz_5_1009	DJI_0703	Genista tinctoria L.	0	n	1	0	Fabaceae Lindl.
Iz_5_1009	DJI_0703	Geranium robertianum L.	0	n	1	0	Geraniaceae Juss.
Iz_5_1009	DJI_0703	Geranium sanquineum L.	0	n	1	0	Geraniaceae Juss.
Iz_5_1009	DJI_0703	Gypsophila paniculata L.	0	n	1	0	Caryophyllaceae Juss.
Iz_5_1009	DJI_0703	Helichrysum arenarium (L.) Moench	0	n	1	0	Asteraceae Bercht. & J.Presl
Iz_5_1009	DJI_0703	Herniaria polygama J.Gay	0	n	1	0	Caryophyllaceae Juss.
Iz_5_1009	DJI_0703	Hieracium robustum Fr.	2	n	1	0	Asteraceae Bercht. & J.Presl
Iz_5_1009	DJI_0703	Hieracium umbellatum L.	0	n	1	NA	Asteraceae Bercht. & J.Presl
Iz_5_1009	DJI_0703	Hylotelephium maximum (L.) Holub	0	n	1	0	Crassulaceae J.St.-Hil.
Iz_5_1009	DJI_0703	Jacobaea borysthenica (DC.) B.Nord. & Greuter	0	n	1	0	Asteraceae Bercht. & J.Presl
Iz_5_1009	DJI_0703	Jasione montana L.	0	n	1	0	Campanulaceae Juss.
Iz_5_1009	DJI_0703	Jurinea cyanoides (L.) Rchb.s.l.	0	n	1	0	Asteraceae Bercht. & J.Presl
Iz_5_1009	DJI_0703	Koeleria glauca (Spreng.) DC.	0	n	1	0	Poaceae Barnhart
Iz_5_1009	DJI_0703	Lactuca serriola L.	0	a	0	0	Asteraceae Bercht. & J.Presl
Iz_5_1009	DJI_0703	Linaria genistifolia (L.) Mill.	0	n	1	0	Plantaginaceae Juss.
Iz_5_1009	DJI_0703	Lomelosia argentea (L.) Greuter & Burdet	0	n	1	0	Caprifoliaceae Juss.
Iz_5_1009	DJI_0703	Melampyrum pratense L.	0	n	1	0	Orobanchaceae Vent.
Iz_5_1009	DJI_0703	Oenothera biennis L.	0	a	0	0	Onagraceae Juss.
Iz_5_1009	DJI_0703	Pentanema britannica (L.) D.Gut.Larr., Santos-Vicente, Anderb., E.Rico & M.M.Mart.Ort.	0	n	1	0	Asteraceae Bercht. & J.Presl
Iz_5_1009	DJI_0703	Peucedanum oreoselinum (L.) Moench	0	n	1	0	Apiaceae Lindl.
Iz_5_1009	DJI_0703	Peucedanum ruthenicum M.Bieb.	0	n	1	0	Apiaceae Lindl.
Iz_5_1009	DJI_0703	Pilosella echioides (Lumn.) F.W.Schultz & Sch.Bip.	0	n	1	0	Asteraceae Bercht. & J.Presl
Iz_5_1009	DJI_0703	Pilosella officinarum F. Schultz et Sch. Bip.	0	n	1	0	Asteraceae Bercht. & J.Presl
Iz_5_1009	DJI_0703	Pilosella sp.	0		NA	0	Asteraceae Bercht. & J.Presl
Iz_5_1009	DJI_0703	Pinus sylvestris L.	0	n	1	1	Pinaceae Spreng. ex F.Rudolphi
Iz_5_1009	DJI_0703	Poa annua L.	0	n	1	0	Poaceae Barnhart
Iz_5_1009	DJI_0703	Poa nemoralis L.	0	n	1	0	Poaceae Barnhart
Iz_5_1009	DJI_0703	Poa sp.	0		NA	0	Poaceae Barnhart
Iz_5_1009	DJI_0703	Polygonatum odoratum (Mill.) Druce	0	n	1	0	Asparagaceae Juss.
Iz_5_1009	DJI_0703	Polygonum arenarium Waldst. & Kit.	0	n	1	0	Polygonaceae Juss.
Iz_5_1009	DJI_0703	Populus nigra L.	0	n	1	1	Salicaceae Mirb.
Iz_5_1009	DJI_0703	Populus sp.	0	n	1	1	Salicaceae Mirb.

Iz_5_1009	DJI_0703	Populus tremula L.	0	n	1	1	Salicaceae Mirb.
Iz_5_1009	DJI_0703	Populus Ã canescens (Aiton) Sm.	0	n	1	1	Salicaceae Mirb.
Iz_5_1009	DJI_0703	Potentilla argentea L.	0	n	1	0	Rosaceae Juss.
Iz_5_1009	DJI_0703	Psammophiliella muralis (L.) Ikonn.	0	n	1	0	Caryophyllaceae Juss.
Iz_5_1009	DJI_0703	Psephellus sumensis (Kalen.) Greuter	0	n	1	0	Asteraceae Bercht. & J.Presl
Iz_5_1009	DJI_0703	Pulsatilla pratensis (L.) Mill. s.l.	0	n	1	0	Ranunculaceae Juss.
Iz_5_1009	DJI_0703	Pyrus communis L.	0	a	0	1	Rosaceae Juss.
Iz_5_1009	DJI_0703	Quercus robur L.	0	n	1	1	Fagaceae Dumort.
Iz_5_1009	DJI_0703	Robinia pseudoacacia L.	0	a	0	1	Fabaceae Lindl.
Iz_5_1009	DJI_0703	Rosa sp.	0		NA	1	Rosaceae Juss.
Iz_5_1009	DJI_0703	Rubus idaeus L.	0	n	1	0	Rosaceae Juss.
Iz_5_1009	DJI_0703	Rumex acetosella L.	0	n	1	0	Polygonaceae Juss.
Iz_5_1009	DJI_0703	Rumex thyrsiflorus Fingerh.	0	n	1	0	Polygonaceae Juss.
Iz_5_1009	DJI_0703	Salix alba L.	0	n	1	1	Salicaceae Mirb.
Iz_5_1009	DJI_0703	Sambucus nigra L.	0	n	1	1	Viburnaceae Raf.
Iz_5_1009	DJI_0703	Secale sylvestre Host	0	n	1	0	Poaceae Barnhart
Iz_5_1009	DJI_0703	Sempervivum ruthenicum Schnittsp. & C.B. Lehm.	0	n	1	0	Crassulaceae J.St.-Hil.
Iz_5_1009	DJI_0703	Setaria viridis (L.) P. Beauv.	0	n	1	0	Poaceae Barnhart
Iz_5_1009	DJI_0703	Silene borysthenica (Gruner) Walters	0	n	1	0	Caryophyllaceae Juss.
Iz_5_1009	DJI_0703	Silene latifolia subsp. alba (Mill.) Greuter & Burdet	0	n	1	0	Caryophyllaceae Juss.
Iz_5_1009	DJI_0703	Solidago virgaurea L.	0	n	1	0	Asteraceae Bercht. & J.Presl
Iz_5_1009	DJI_0703	Solidago Ã niederederi (S. canadensis Ã S. virgaurea)	0	hybrid	0	0	Asteraceae Bercht. & J.Presl
Iz_5_1009	DJI_0703	Sorbus aucuparia L.	0	n	1	1	Rosaceae Juss.
Iz_5_1009	DJI_0703	Stachys recta L.	0	n	1	0	Lamiacea Lindl.
Iz_5_1009	DJI_0703	Tanacetum vulgare L.	0	n	1	0	Asteraceae Bercht. & J.Presl
Iz_5_1009	DJI_0703	Thymus pallasianus Heinr.Braun	0	n	1	0	Lamiacea Lindl.
Iz_5_1009	DJI_0703	Tilia cordata Mill.	0	n	1	1	Malvaceae Juss.
Iz_5_1009	DJI_0703	Trifolium alpestre L.	0	n	1	0	Fabaceae Lindl.
Iz_5_1009	DJI_0703	Trifolium arvense L.	0	n	1	0	Fabaceae Lindl.
Iz_5_1009	DJI_0703	Ulmus glabra Huds.	0	n	1	1	Ulmaceae Mirb.
Iz_5_1009	DJI_0703	Verbascum densiflorum Bertol.	0	n	1	0	Scrophulariaceae Juss.
Iz_5_1009	DJI_0703	Veronica austriaca L.	0	n	1	0	Plantaginaceae Juss.
Iz_5_1009	DJI_0703	Veronica incana L.	0	n	1	0	Plantaginaceae Juss.
Iz_5_1009	DJI_0703	Veronica verna L.	0	n	1	0	Plantaginaceae Juss.
Iz_5_1009	DJI_0703	Vincetoxicum hirundinaria Medik	0	n	1	0	Apocynaceae Juss.
Iz_5_1009	DJI_0707	Acer tataricum L.	0	n	1	1	Sapindaceae Juss.
Iz_5_1009	DJI_0707	Achillea millefolium L. s.l.	0	n	1	0	Asteraceae Bercht. & J.Presl
Iz_5_1009	DJI_0707	Allium oleraceum L.	0	n	1	0	Amaryllidaceae J.St.-Hil.
Iz_5_1009	DJI_0707	Ambrosia artemisiifolia L.	0	a	0	0	Asteraceae Bercht. & J.Presl
Iz_5_1009	DJI_0707	Amorpha fruticosa L.	0	a	0	0	Fabaceae Lindl.
Iz_5_1009	DJI_0707	Anthericum ramosum L.	0	n	1	0	Asparagaceae Juss.
Iz_5_1009	DJI_0707	Artemisia marschalliana Spreng.	0	n	1	0	Asteraceae Bercht. & J.Presl
Iz_5_1009	DJI_0707	Asparagus officinalis L.	0	n	1	0	Asparagaceae Juss.
Iz_5_1009	DJI_0707	Astragalus cicer L.	0	n	1	0	Fabaceae Lindl.
Iz_5_1009	DJI_0707	Bassia laniflora (S.G.Gmel.) A.J.Scott	0	n	1	0	Amaranthaceae Juss.
Iz_5_1009	DJI_0707	Berberis vulgaris L.	0	n	1	0	Berberidaceae Juss.
Iz_5_1009	DJI_0707	Betula pendula Roth.	0	n	1	1	Betulaceae Gray
Iz_5_1009	DJI_0707	Betula pendula Roth.	0	n	1	0	Poaceae Barnhart
Iz_5_1009	DJI_0707	Bromus inermis Leyss.	0		NA	NA	
Iz_5_1009	DJI_0707	Bromus squarrosus L.	0	a	0	0	Poaceae Barnhart
Iz_5_1009	DJI_0707	Bromus tectorum L.	0	a	0	0	Poaceae Barnhart
Iz_5_1009	DJI_0707	Calamagrostis epigejos (L.) Roth	5	n	1	0	Poaceae Barnhart
Iz_5_1009	DJI_0707	Carduus acanthoides L.	0	n	1	0	Asteraceae Bercht. & J.Presl
Iz_5_1009	DJI_0707	Carex sp.	1	n	1	0	Cyperaceae Juss.
Iz_5_1009	DJI_0707	Centaurea majorovii Dumbadze	0	n	1	0	Asteraceae Bercht. & J.Presl
Iz_5_1009	DJI_0707	Chamaecytisus ruthenicus (Fisch.ex Wol.) Klaskova	0	n	1	0	Fabaceae Lindl.
Iz_5_1009	DJI_0707	Chelidonium majus L.	0	n	1	0	Papaveraceae Juss.
Iz_5_1009	DJI_0707	Chenopodium betaceum Andrz.	0	n	1	0	Amaranthaceae Juss.
Iz_5_1009	DJI_0707	Chenopodium cf. album	0	n	1	0	Amaranthaceae Juss.
Iz_5_1009	DJI_0707	Chenopodium sp.	0		NA	0	Amaranthaceae Juss.
Iz_5_1009	DJI_0707	Chondrilla juncea L.	0	n	1	0	Asteraceae Bercht. & J.Presl
Iz_5_1009	DJI_0707	Cirsium vulgare (Savi) Ten.	0	n	1	0	Asteraceae Bercht. & J.Presl
Iz_5_1009	DJI_0707	Clinopodium acinos (L.) Kuntze	0	n	1	0	Lamiacea Lindl.
Iz_5_1009	DJI_0707	Convallaria majalis L.	0	n	1	0	Asparagaceae Juss.
Iz_5_1009	DJI_0707	Crataegus rhipidophylla Gand.	0	n	1	1	Rosaceae Juss.
Iz_5_1009	DJI_0707	Crepis foetida L.	0	n	1	0	Asteraceae Bercht. & J.Presl
Iz_5_1009	DJI_0707	Dactylis glomerata L.	0	n	1	0	Poaceae Barnhart
Iz_5_1009	DJI_0707	Dianthus campestris M.Bieb.	0	n	1	0	Caryophyllaceae Juss.
Iz_5_1009	DJI_0707	Digitaria sanguinalis (L.) Scop.	0	a	0	0	Poaceae Barnhart
Iz_5_1009	DJI_0707	Elymus repens (L.) Gould	0	n	1	0	Poaceae Barnhart
Iz_5_1009	DJI_0707	Epilobium tetragonum L.	0	n	1	0	Onagraceae Juss.
Iz_5_1009	DJI_0707	Eragrostis minor Host	0	a	0	0	Poaceae Barnhart
Iz_5_1009	DJI_0707	Erigeron canadensis L.	15	a	0	0	Asteraceae Bercht. & J.Presl
Iz_5_1009	DJI_0707	Euphorbia seguieriana Neck.	0	n	1	0	Euphorbiaceae Juss.
Iz_5_1009	DJI_0707	Fallopia dumetorum (L.) Holub	0	n	1	0	Polygonaceae Juss.
Iz_5_1009	DJI_0707	Festuca beckeri (Hack.) Trautv.	0	n	1	0	Poaceae Barnhart
Iz_5_1009	DJI_0707	Fragaria vesca L.	0	n	1	0	Rosaceae Juss.
Iz_5_1009	DJI_0707	Frangula alnus Mill.	0	n	1	1	Rhamnaceae Juss.
Iz_5_1009	DJI_0707	Fraxinus pennsylvanica Marshall	0	a	0	1	Oleaceae Hoffmanns. & Link
Iz_5_1009	DJI_0707	Galeopsis bifida Boenn.	0	n	1	0	Lamiacea Lindl.
Iz_5_1009	DJI_0707	Galium verum L.	0	n	1	0	Rubiaceae Juss.
Iz_5_1009	DJI_0707	Genista tinctoria L.	0	n	1	0	Fabaceae Lindl.
Iz_5_1009	DJI_0707	Geranium robertianum L.	0	n	1	0	Geraniaceae Juss.
Iz_5_1009	DJI_0707	Geranium sanquineum L.	0	n	1	0	Geraniaceae Juss.
Iz_5_1009	DJI_0707	Gypsophila paniculata L.	0	n	1	0	Caryophyllaceae Juss.
Iz_5_1009	DJI_0707	Helichrysum arenarium (L.) Moench	0	n	1	0	Asteraceae Bercht. & J.Presl
Iz_5_1009	DJI_0707	Herniaria polygama J.Gay	0	n	1	0	Caryophyllaceae Juss.
Iz_5_1009	DJI_0707	Hieracium robustum Fr.	0	n	1	0	Asteraceae Bercht. & J.Presl
Iz_5_1009	DJI_0707	Hieracium umbellatum L.	0	n	1	NA	Asteraceae Bercht. & J.Presl
Iz_5_1009	DJI_0707	Hylotelephium maximum (L.) Holub	0	n	1	0	Crassulaceae J.St.-Hil.
Iz_5_1009	DJI_0707	Jacobaea borysthenica (DC.) B.Nord. & Greuter	0	n	1	0	Asteraceae Bercht. & J.Presl
Iz_5_1009	DJI_0707	Jasione montana L.	0	n	1	0	Campanulaceae Juss.
Iz_5_1009	DJI_0707	Jurinea cyanoides (L.) Rchb.s.l.	0	n	1	0	Asteraceae Bercht. & J.Presl
Iz_5_1009	DJI_0707	Koeleria glauca (Spreng.) DC.	0	n	1	0	Poaceae Barnhart
Iz_5_1009	DJI_0707	Lactuca serriola L.	0	a	0	0	Asteraceae Bercht. & J.Presl
Iz_5_1009	DJI_0707	Linaria genistifolia (L.) Mill.	0	n	1	0	Plantaginaceae Juss.
Iz_5_1009	DJI_0707	Lomelosia argentea (L.) Greuter & Burdet	0	n	1	0	Caprifoliaceae Juss.
Iz_5_1009	DJI_0707	Melampyrum pratense L.	0	n	1	0	Orobanchaceae Vent.

Iz_5_1009	DJI_0707	Oenothera biennis L.	0	a	0	0	Onagraceae Juss.
Iz_5_1009	DJI_0707	Pentanema britannica (L.) D.Gut.Larr., Santos-Vicente, Anderb., E.Rico & M.M.Mart.Ort.	0	n	1	0	Asteraceae Bercht. & J.Presl
Iz_5_1009	DJI_0707	Peucedanum oreoselinum (L.) Moench	0	n	1	0	Apiaceae Lindl.
Iz_5_1009	DJI_0707	Peucedanum ruthenicum M.Bieb.	0	n	1	0	Apiaceae Lindl.
Iz_5_1009	DJI_0707	Pilosella echioides (Lumn.) F.W.Schultz & Sch.Bip.	0	n	1	0	Asteraceae Bercht. & J.Presl
Iz_5_1009	DJI_0707	Pilosella officinarum F. Schultz et Sch. Bip.	0	n	1	0	Asteraceae Bercht. & J.Presl
Iz_5_1009	DJI_0707	Pilosella sp.	0		NA	0	Asteraceae Bercht. & J.Presl
Iz_5_1009	DJI_0707	Pinus sylvestris L.	0	n	1	1	Pinaceae Spreng. ex F.Rudolphi
Iz_5_1009	DJI_0707	Poa annua L.	0	n	1	0	Poaceae Barnhart
Iz_5_1009	DJI_0707	Poa nemoralis L.	0	n	1	0	Poaceae Barnhart
Iz_5_1009	DJI_0707	Poa sp.	0		NA	0	Poaceae Barnhart
Iz_5_1009	DJI_0707	Polygonatum odoratum (Mill.) Druce	0	n	1	0	Asparagaceae Juss.
Iz_5_1009	DJI_0707	Polygonum arenarium Waldst. & Kit.	0	n	1	0	Polygonaceae Juss.
Iz_5_1009	DJI_0707	Populus nigra L.	0	n	1	1	Salicaceae Mirb.
Iz_5_1009	DJI_0707	Populus sp.	0	n	1	1	Salicaceae Mirb.
Iz_5_1009	DJI_0707	Populus tremula L.	0	n	1	1	Salicaceae Mirb.
Iz_5_1009	DJI_0707	Populus Ã canescens (Aiton) Sm.	0	n	1	1	Salicaceae Mirb.
Iz_5_1009	DJI_0707	Potentilla argentea L.	0	n	1	0	Rosaceae Juss.
Iz_5_1009	DJI_0707	Psammophiliella muralis (L.) Ikonn.	0	n	1	0	Caryophyllaceae Juss.
Iz_5_1009	DJI_0707	Psephellus sumensis (Kalen.) Greuter	0	n	1	0	Asteraceae Bercht. & J.Presl
Iz_5_1009	DJI_0707	Pulsatilla pratensis (L.) Mill. s.l.	0	n	1	0	Ranunculaceae Juss.
Iz_5_1009	DJI_0707	Pyrus communis L.	0	a	0	1	Rosaceae Juss.
Iz_5_1009	DJI_0707	Quercus robur L.	0	n	1	1	Fagaceae Dumort.
Iz_5_1009	DJI_0707	Robinia pseudoacacia L.	0	a	0	1	Fabaceae Lindl.
Iz_5_1009	DJI_0707	Rosa sp.	0		NA	1	Rosaceae Juss.
Iz_5_1009	DJI_0707	Rubus idaeus L.	0	n	1	0	Rosaceae Juss.
Iz_5_1009	DJI_0707	Rumex acetosella L.	5	n	1	0	Polygonaceae Juss.
Iz_5_1009	DJI_0707	Rumex thyrsiflorus Fingerh.	0	n	1	0	Polygonaceae Juss.
Iz_5_1009	DJI_0707	Salix alba L.	0	n	1	1	Salicaceae Mirb.
Iz_5_1009	DJI_0707	Sambucus nigra L.	0	n	1	1	Viburnaceae Raf.
Iz_5_1009	DJI_0707	Secale sylvestre Host	0	n	1	0	Poaceae Barnhart
Iz_5_1009	DJI_0707	Sempervivum ruthenicum Schnittsp. & C.B. Lehm.	0	n	1	0	Crassulaceae J.St.-Hil.
Iz_5_1009	DJI_0707	Setaria viridis (L.) P. Beauv.	0	n	1	0	Poaceae Barnhart
Iz_5_1009	DJI_0707	Silene borysthenica (Gruner) Walters	0	n	1	0	Caryophyllaceae Juss.
Iz_5_1009	DJI_0707	Silene latifolia subsp. alba (Mill.) Greuter & Burdet	0	n	1	0	Caryophyllaceae Juss.
Iz_5_1009	DJI_0707	Solidago virgaurea L.	0	n	1	0	Asteraceae Bercht. & J.Presl
Iz_5_1009	DJI_0707	Solidago Ã niederederi (S. canadensis Ã S. virgaurea)	0	hybrid	0	0	Asteraceae Bercht. & J.Presl
Iz_5_1009	DJI_0707	Sorbus aucuparia L.	0	n	1	1	Rosaceae Juss.
Iz_5_1009	DJI_0707	Stachys recta L.	0	n	1	0	Lamiacea Lindl.
Iz_5_1009	DJI_0707	Tanacetum vulgare L.	0	n	1	0	Asteraceae Bercht. & J.Presl
Iz_5_1009	DJI_0707	Thymus pallasianus Heinr.Braun	0	n	1	0	Lamiacea Lindl.
Iz_5_1009	DJI_0707	Tilia cordata Mill.	0	n	1	1	Malvaceae Juss.
Iz_5_1009	DJI_0707	Trifolium alpestre L.	0	n	1	0	Fabaceae Lindl.
Iz_5_1009	DJI_0707	Trifolium arvense L.	0	n	1	0	Fabaceae Lindl.
Iz_5_1009	DJI_0707	Ulmus glabra Huds.	0	n	1	1	Ulmaceae Mirb.
Iz_5_1009	DJI_0707	Verbascum densiflorum Bertol.	0	n	1	0	Scrophulariaceae Juss.
Iz_5_1009	DJI_0707	Veronica austriaca L.	0	n	1	0	Plantaginaceae Juss.
Iz_5_1009	DJI_0707	Veronica incana L.	0	n	1	0	Plantaginaceae Juss.
Iz_5_1009	DJI_0707	Veronica verna L.	0	n	1	0	Plantaginaceae Juss.
Iz_5_1009	DJI_0707	Vincetoxicum hirundinaria Medik	0	n	1	0	Apocynaceae Juss.
Iz_5_1009	DJI_0710	Acer tataricum L.	0	n	1	1	Sapindaceae Juss.
Iz_5_1009	DJI_0710	Achillea millefolium L. s.l.	0	n	1	0	Asteraceae Bercht. & J.Presl
Iz_5_1009	DJI_0710	Allium oleraceum L.	0	n	1	0	Amaryllidaceae J.St.-Hil.
Iz_5_1009	DJI_0710	Ambrosia artemisiifolia L.	0	a	0	0	Asteraceae Bercht. & J.Presl
Iz_5_1009	DJI_0710	Amorpha fruticosa L.	0	a	0	0	Fabaceae Lindl.
Iz_5_1009	DJI_0710	Anthericum ramosum L.	0	n	1	0	Asparagaceae Juss.
Iz_5_1009	DJI_0710	Artemisia marschalliana Spreng.	0	n	1	0	Asteraceae Bercht. & J.Presl
Iz_5_1009	DJI_0710	Asparagus officinalis L.	0	n	1	0	Asparagaceae Juss.
Iz_5_1009	DJI_0710	Astragalus cicer L.	0	n	1	0	Fabaceae Lindl.
Iz_5_1009	DJI_0710	Bassia laniflora (S.G.Gmel.) A.J.Scott	0	n	1	0	Amaranthaceae Juss.
Iz_5_1009	DJI_0710	Berberis vulgaris L.	0	n	1	0	Berberidaceae Juss.
Iz_5_1009	DJI_0710	Betula pendula Roth.	0	n	1	1	Betulaceae Gray
Iz_5_1009	DJI_0710	Betula pendula Roth.	0	n	1	0	Poaceae Barnhart
Iz_5_1009	DJI_0710	Bromus inermis Leyss.	0		NA	NA	
Iz_5_1009	DJI_0710	Bromus squarrosus L.	0	a	0	0	Poaceae Barnhart
Iz_5_1009	DJI_0710	Bromus tectorum L.	0	a	0	0	Poaceae Barnhart
Iz_5_1009	DJI_0710	Calamagrostis epigejos (L.) Roth	30	n	1	0	Poaceae Barnhart
Iz_5_1009	DJI_0710	Carduus acanthoides L.	0	n	1	0	Asteraceae Bercht. & J.Presl
Iz_5_1009	DJI_0710	Carex sp.	1	n	1	0	Cyperaceae Juss.
Iz_5_1009	DJI_0710	Centaurea majorovii Dumbadze	0	n	1	0	Asteraceae Bercht. & J.Presl
Iz_5_1009	DJI_0710	Chamaecytisus ruthenicus (Fisch.ex Wol.) Klaskova	0	n	1	0	Fabaceae Lindl.
Iz_5_1009	DJI_0710	Chelidonium majus L.	0	n	1	0	Papaveraceae Juss.
Iz_5_1009	DJI_0710	Chenopodium betaceum Andrz.	0	n	1	0	Amaranthaceae Juss.
Iz_5_1009	DJI_0710	Chenopodium cf. album	0	n	1	0	Amaranthaceae Juss.
Iz_5_1009	DJI_0710	Chenopodium sp.	0		NA	0	Amaranthaceae Juss.
Iz_5_1009	DJI_0710	Chondrilla juncea L.	0	n	1	0	Asteraceae Bercht. & J.Presl
Iz_5_1009	DJI_0710	Cirsium vulgare (Savi) Ten.	0	n	1	0	Asteraceae Bercht. & J.Presl
Iz_5_1009	DJI_0710	Clinopodium acinos (L.) Kuntze	0	n	1	0	Lamiacea Lindl.
Iz_5_1009	DJI_0710	Convallaria majalis L.	0	n	1	0	Asparagaceae Juss.
Iz_5_1009	DJI_0710	Crataegus rhipidophylla Gand.	0	n	1	1	Rosaceae Juss.
Iz_5_1009	DJI_0710	Crepis foetida L.	0	n	1	0	Asteraceae Bercht. & J.Presl
Iz_5_1009	DJI_0710	Dactylis glomerata L.	0	n	1	0	Poaceae Barnhart
Iz_5_1009	DJI_0710	Dianthus campestris M.Bieb.	0	n	1	0	Caryophyllaceae Juss.
Iz_5_1009	DJI_0710	Digitaria sanguinalis (L.) Scop.	0	a	0	0	Poaceae Barnhart
Iz_5_1009	DJI_0710	Elymus repens (L.) Gould	0	n	1	0	Poaceae Barnhart
Iz_5_1009	DJI_0710	Epilobium tetragonum L.	0	n	1	0	Onagraceae Juss.
Iz_5_1009	DJI_0710	Eragrostis minor Host	0	a	0	0	Poaceae Barnhart
Iz_5_1009	DJI_0710	Erigeron canadensis L.	1	a	0	0	Asteraceae Bercht. & J.Presl
Iz_5_1009	DJI_0710	Euphorbia seguieriana Neck.	0	n	1	0	Euphorbiaceae Juss.
Iz_5_1009	DJI_0710	Fallopia dumetorum (L.) Holub	0	n	1	0	Polygonaceae Juss.
Iz_5_1009	DJI_0710	Festuca beckeri (Hack.) Trautv.	0	n	1	0	Poaceae Barnhart
Iz_5_1009	DJI_0710	Fragaria vesca L.	0	n	1	0	Rosaceae Juss.
Iz_5_1009	DJI_0710	Frangula alnus Mill.	0	n	1	1	Rhamnaceae Juss.
Iz_5_1009	DJI_0710	Fraxinus pennsylvanica Marshall	0	a	0	1	Oleaceae Hoffmanns. & Link
Iz_5_1009	DJI_0710	Galeopsis bifida Boenn.	0	n	1	0	Lamiacea Lindl.
Iz_5_1009	DJI_0710	Galium verum L.	0	n	1	0	Rubiaceae Juss.
Iz_5_1009	DJI_0710	Genista tinctoria L.	0	n	1	0	Fabaceae Lindl.
Iz_5_1009	DJI_0710	Geranium robertianum L.	0	n	1	0	Geraniaceae Juss.

Iz_5_1009	DJI_0710	Geranium sanquineum L.	0	n	1	0	Geraniaceae Juss.
Iz_5_1009	DJI_0710	Gypsophila paniculata L.	0	n	1	0	Caryophyllaceae Juss.
Iz_5_1009	DJI_0710	Helichrysum arenarium (L.) Moench	0	n	1	0	Asteraceae Bercht. & J.Presl
Iz_5_1009	DJI_0710	Herniaria polygama J.Gay	0	n	1	0	Caryophyllaceae Juss.
Iz_5_1009	DJI_0710	Hieracium robustum Fr.	0	n	1	0	Asteraceae Bercht. & J.Presl
Iz_5_1009	DJI_0710	Hieracium umbellatum L.	0	n	1	NA	Asteraceae Bercht. & J.Presl
Iz_5_1009	DJI_0710	Hylotelephium maximum (L.) Holub	0	n	1	0	Crassulaceae J.St.-Hil.
Iz_5_1009	DJI_0710	Jacobaea borysthenica (DC.) B.Nord. & Greuter	0	n	1	0	Asteraceae Bercht. & J.Presl
Iz_5_1009	DJI_0710	Jasione montana L.	0	n	1	0	Campanulaceae Juss.
Iz_5_1009	DJI_0710	Jurinea cyanoides (L.) Rchb.s.l.	0	n	1	0	Asteraceae Bercht. & J.Presl
Iz_5_1009	DJI_0710	Koeleria glauca (Spreng.) DC.	0	n	1	0	Poaceae Barnhart
Iz_5_1009	DJI_0710	Lactuca serriola L.	0	a	0	0	Asteraceae Bercht. & J.Presl
Iz_5_1009	DJI_0710	Linaria genistifolia (L.) Mill.	0	n	1	0	Plantaginaceae Juss.
Iz_5_1009	DJI_0710	Lomelosia argentea (L.) Greuter & Burdet	0	n	1	0	Caprifoliaceae Juss.
Iz_5_1009	DJI_0710	Melampyrum pratense L.	0	n	1	0	Orobanchaceae Vent.
Iz_5_1009	DJI_0710	Oenothera biennis L.	0	a	0	0	Onagraceae Juss.
Iz_5_1009	DJI_0710	Pentanema britannica (L.) D.Gut.Larr., Santos-Vicente, Anderb., E.Rico & M.M.Mart.Ort.	0	n	1	0	Asteraceae Bercht. & J.Presl
Iz_5_1009	DJI_0710	Peucedanum oreoselinum (L.) Moench	2	n	1	0	Apiaceae Lindl.
Iz_5_1009	DJI_0710	Peucedanum ruthenicum M.Bieb.	0	n	1	0	Apiaceae Lindl.
Iz_5_1009	DJI_0710	Pilosella echioides (Lumn.) F.W.Schultz & Sch.Bip.	0	n	1	0	Asteraceae Bercht. & J.Presl
Iz_5_1009	DJI_0710	Pilosella officinarum F. Schultz et Sch. Bip.	0	n	1	0	Asteraceae Bercht. & J.Presl
Iz_5_1009	DJI_0710	Pilosella sp.	0		NA	0	Asteraceae Bercht. & J.Presl
Iz_5_1009	DJI_0710	Pinus sylvestris L.	0	n	1	1	Pinaceae Spreng. ex F.Rudolphi
Iz_5_1009	DJI_0710	Poa annua L.	0	n	1	0	Poaceae Barnhart
Iz_5_1009	DJI_0710	Poa nemoralis L.	0	n	1	0	Poaceae Barnhart
Iz_5_1009	DJI_0710	Poa sp.	0		NA	0	Poaceae Barnhart
Iz_5_1009	DJI_0710	Polygonatum odoratum (Mill.) Druce	0	n	1	0	Asparagaceae Juss.
Iz_5_1009	DJI_0710	Polygonum arenarium Waldst. & Kit.	0	n	1	0	Polygonaceae Juss.
Iz_5_1009	DJI_0710	Populus nigra L.	0	n	1	1	Salicaceae Mirb.
Iz_5_1009	DJI_0710	Populus sp.	0	n	1	1	Salicaceae Mirb.
Iz_5_1009	DJI_0710	Populus tremula L.	0	n	1	1	Salicaceae Mirb.
Iz_5_1009	DJI_0710	Populus Ã canescens (Aiton) Sm.	0	n	1	1	Salicaceae Mirb.
Iz_5_1009	DJI_0710	Potentilla argentea L.	0	n	1	0	Rosaceae Juss.
Iz_5_1009	DJI_0710	Psammophiliella muralis (L.) Ikonn.	0	n	1	0	Caryophyllaceae Juss.
Iz_5_1009	DJI_0710	Psephellus sumensis (Kalen.) Greuter	0	n	1	0	Asteraceae Bercht. & J.Presl
Iz_5_1009	DJI_0710	Pulsatilla pratensis (L.) Mill. s.l.	0	n	1	0	Ranunculaceae Juss.
Iz_5_1009	DJI_0710	Pyrus communis L.	0	a	0	1	Rosaceae Juss.
Iz_5_1009	DJI_0710	Quercus robur L.	0	n	1	1	Fagaceae Dumort.
Iz_5_1009	DJI_0710	Robinia pseudoacacia L.	0	a	0	1	Fabaceae Lindl.
Iz_5_1009	DJI_0710	Rosa sp.	0		NA	1	Rosaceae Juss.
Iz_5_1009	DJI_0710	Rubus idaeus L.	0	n	1	0	Rosaceae Juss.
Iz_5_1009	DJI_0710	Rumex acetosella L.	0	n	1	0	Polygonaceae Juss.
Iz_5_1009	DJI_0710	Rumex thyrsiflorus Fingerh.	0	n	1	0	Polygonaceae Juss.
Iz_5_1009	DJI_0710	Salix alba L.	0	n	1	1	Salicaceae Mirb.
Iz_5_1009	DJI_0710	Sambucus nigra L.	0	n	1	1	Viburnaceae Raf.
Iz_5_1009	DJI_0710	Secale sylvestre Host	0	n	1	0	Poaceae Barnhart
Iz_5_1009	DJI_0710	Sempervivum ruthenicum Schnittsp. & C.B. Lehm.	0	n	1	0	Crassulaceae J.St.-Hil.
Iz_5_1009	DJI_0710	Setaria viridis (L.) P. Beauv.	0	n	1	0	Poaceae Barnhart
Iz_5_1009	DJI_0710	Silene borysthenica (Gruner) Walters	0	n	1	0	Caryophyllaceae Juss.
Iz_5_1009	DJI_0710	Silene latifolia subsp. alba (Mill.) Greuter & Burdet	0	n	1	0	Caryophyllaceae Juss.
Iz_5_1009	DJI_0710	Solidago virgaurea L.	0	n	1	0	Asteraceae Bercht. & J.Presl
Iz_5_1009	DJI_0710	Solidago Ã niederederi (S. canadensis Ã S. virgaurea)	0	hybrid	0	0	Asteraceae Bercht. & J.Presl
Iz_5_1009	DJI_0710	Sorbus aucuparia L.	0	n	1	1	Rosaceae Juss.
Iz_5_1009	DJI_0710	Stachys recta L.	0	n	1	0	Lamiacea Lindl.
Iz_5_1009	DJI_0710	Tanacetum vulgare L.	0	n	1	0	Asteraceae Bercht. & J.Presl
Iz_5_1009	DJI_0710	Thymus pallasianus Heinr.Braun	0	n	1	0	Lamiacea Lindl.
Iz_5_1009	DJI_0710	Tilia cordata Mill.	0	n	1	1	Malvaceae Juss.
Iz_5_1009	DJI_0710	Trifolium alpestre L.	0	n	1	0	Fabaceae Lindl.
Iz_5_1009	DJI_0710	Trifolium arvense L.	0	n	1	0	Fabaceae Lindl.
Iz_5_1009	DJI_0710	Ulmus glabra Huds.	0	n	1	1	Ulmaceae Mirb.
Iz_5_1009	DJI_0710	Verbascum densiflorum Bertol.	0	n	1	0	Scrophulariaceae Juss.
Iz_5_1009	DJI_0710	Veronica austriaca L.	0	n	1	0	Plantaginaceae Juss.
Iz_5_1009	DJI_0710	Veronica incana L.	0	n	1	0	Plantaginaceae Juss.
Iz_5_1009	DJI_0710	Veronica verna L.	0	n	1	0	Plantaginaceae Juss.
Iz_5_1009	DJI_0710	Vincetoxicum hirundinaria Medik	0	n	1	0	Apocynaceae Juss.
Iz_5_1009	DJI_0713	Acer tataricum L.	0	n	1	1	Sapindaceae Juss.
Iz_5_1009	DJI_0713	Achillea millefolium L. s.l.	0	n	1	0	Asteraceae Bercht. & J.Presl
Iz_5_1009	DJI_0713	Allium oleraceum L.	0	n	1	0	Amaryllidaceae J.St.-Hil.
Iz_5_1009	DJI_0713	Ambrosia artemisiifolia L.	0	a	0	0	Asteraceae Bercht. & J.Presl
Iz_5_1009	DJI_0713	Amorpha fruticosa L.	0	a	0	0	Fabaceae Lindl.
Iz_5_1009	DJI_0713	Anthericum ramosum L.	0	n	1	0	Asparagaceae Juss.
Iz_5_1009	DJI_0713	Artemisia marschalliana Spreng.	0	n	1	0	Asteraceae Bercht. & J.Presl
Iz_5_1009	DJI_0713	Asparagus officinalis L.	0	n	1	0	Asparagaceae Juss.
Iz_5_1009	DJI_0713	Astragalus cicer L.	0	n	1	0	Fabaceae Lindl.
Iz_5_1009	DJI_0713	Bassia laniflora (S.G.Gmel.) A.J.Scott	0	n	1	0	Amaranthaceae Juss.
Iz_5_1009	DJI_0713	Berberis vulgaris L.	0	n	1	0	Berberidaceae Juss.
Iz_5_1009	DJI_0713	Betula pendula Roth.	0	n	1	1	Betulaceae Gray
Iz_5_1009	DJI_0713	Betula pendula Roth.	0	n	1	0	Poaceae Barnhart
Iz_5_1009	DJI_0713	Bromus inermis Leyss.	0		NA	NA	
Iz_5_1009	DJI_0713	Bromus squarrosus L.	0	a	0	0	Poaceae Barnhart
Iz_5_1009	DJI_0713	Bromus tectorum L.	0	a	0	0	Poaceae Barnhart
Iz_5_1009	DJI_0713	Calamagrostis epigejos (L.) Roth	65	n	1	0	Poaceae Barnhart
Iz_5_1009	DJI_0713	Carduus acanthoides L.	0	n	1	0	Asteraceae Bercht. & J.Presl
Iz_5_1009	DJI_0713	Carex sp.	0	n	1	0	Cyperaceae Juss.
Iz_5_1009	DJI_0713	Centaurea majorovii Dumbadze	0	n	1	0	Asteraceae Bercht. & J.Presl
Iz_5_1009	DJI_0713	Chamaecytisus ruthenicus (Fisch.ex Wol.) Klaskova	0	n	1	0	Fabaceae Lindl.
Iz_5_1009	DJI_0713	Chelidonium majus L.	0	n	1	0	Papaveraceae Juss.
Iz_5_1009	DJI_0713	Chenopodium betaceum Andrz.	0	n	1	0	Amaranthaceae Juss.
Iz_5_1009	DJI_0713	Chenopodium cf. album	0	n	1	0	Amaranthaceae Juss.
Iz_5_1009	DJI_0713	Chenopodium sp.	0		NA	0	Amaranthaceae Juss.
Iz_5_1009	DJI_0713	Chondrilla juncea L.	0	n	1	0	Asteraceae Bercht. & J.Presl
Iz_5_1009	DJI_0713	Cirsium vulgare (Savi) Ten.	0	n	1	0	Asteraceae Bercht. & J.Presl
Iz_5_1009	DJI_0713	Clinopodium acinos (L.) Kuntze	0	n	1	0	Lamiacea Lindl.
Iz_5_1009	DJI_0713	Convallaria majalis L.	0	n	1	0	Asparagaceae Juss.
Iz_5_1009	DJI_0713	Crataegus rhipidophylla Gand.	0	n	1	1	Rosaceae Juss.
Iz_5_1009	DJI_0713	Crepis foetida L.	0	n	1	0	Asteraceae Bercht. & J.Presl
Iz_5_1009	DJI_0713	Dactylis glomerata L.	0	n	1	0	Poaceae Barnhart
Iz_5_1009	DJI_0713	Dianthus campestris M.Bieb.	0	n	1	0	Caryophyllaceae Juss.

Iz_5_1009	DJI_0713	Digitaria sanguinalis (L.) Scop.	0	a	0	0	Poaceae Barnhart
Iz_5_1009	DJI_0713	Elymus repens (L.) Gould	0	n	1	0	Poaceae Barnhart
Iz_5_1009	DJI_0713	Epilobium tetragonum L.	0	n	1	0	Onagraceae Juss.
Iz_5_1009	DJI_0713	Eragrostis minor Host	0	a	0	0	Poaceae Barnhart
Iz_5_1009	DJI_0713	Erigeron canadensis L.	3	a	0	0	Asteraceae Bercht. & J.Presl
Iz_5_1009	DJI_0713	Euphorbia seguieriana Neck.	0	n	1	0	Euphorbiaceae Juss.
Iz_5_1009	DJI_0713	Fallopia dumetorum (L.) Holub	0	n	1	0	Polygonaceae Juss.
Iz_5_1009	DJI_0713	Festuca beckeri (Hack.) Trautv.	0	n	1	0	Poaceae Barnhart
Iz_5_1009	DJI_0713	Fragaria vesca L.	0	n	1	0	Rosaceae Juss.
Iz_5_1009	DJI_0713	Frangula alnus Mill.	0	n	1	1	Rhamnaceae Juss.
Iz_5_1009	DJI_0713	Fraxinus pennsylvanica Marshall	0	a	0	1	Oleaceae Hoffmanns. & Link
Iz_5_1009	DJI_0713	Galeopsis bifida Boenn.	0	n	1	0	Lamiacea Lindl.
Iz_5_1009	DJI_0713	Galium verum L.	0	n	1	0	Rubiaceae Juss.
Iz_5_1009	DJI_0713	Genista tinctoria L.	0	n	1	0	Fabaceae Lindl.
Iz_5_1009	DJI_0713	Geranium robertianum L.	0	n	1	0	Geraniaceae Juss.
Iz_5_1009	DJI_0713	Geranium sanquineum L.	0	n	1	0	Geraniaceae Juss.
Iz_5_1009	DJI_0713	Gypsophila paniculata L.	0	n	1	0	Caryophyllaceae Juss.
Iz_5_1009	DJI_0713	Helichrysum arenarium (L.) Moench	0	n	1	0	Asteraceae Bercht. & J.Presl
Iz_5_1009	DJI_0713	Herniaria polygama J.Gay	0	n	1	0	Caryophyllaceae Juss.
Iz_5_1009	DJI_0713	Hieracium robustum Fr.	0	n	1	0	Asteraceae Bercht. & J.Presl
Iz_5_1009	DJI_0713	Hieracium umbellatum L.	0	n	1	NA	Asteraceae Bercht. & J.Presl
Iz_5_1009	DJI_0713	Hylotelephium maximum (L.) Holub	0	n	1	0	Crassulaceae J.St.-Hil.
Iz_5_1009	DJI_0713	Jacobaea borysthenica (DC.) B.Nord. & Greuter	0	n	1	0	Asteraceae Bercht. & J.Presl
Iz_5_1009	DJI_0713	Jasione montana L.	0	n	1	0	Campanulaceae Juss.
Iz_5_1009	DJI_0713	Jurinea cyanoides (L.) Rchb.s.l.	0	n	1	0	Asteraceae Bercht. & J.Presl
Iz_5_1009	DJI_0713	Koeleria glauca (Spreng.) DC.	0	n	1	0	Poaceae Barnhart
Iz_5_1009	DJI_0713	Lactuca serriola L.	0	a	0	0	Asteraceae Bercht. & J.Presl
Iz_5_1009	DJI_0713	Linaria genistifolia (L.) Mill.	0	n	1	0	Plantaginaceae Juss.
Iz_5_1009	DJI_0713	Lomelosia argentea (L.) Greuter & Burdet	0	n	1	0	Caprifoliaceae Juss.
Iz_5_1009	DJI_0713	Melampyrum pratense L.	0	n	1	0	Orobanchaceae Vent.
Iz_5_1009	DJI_0713	Oenothera biennis L.	0	a	0	0	Onagraceae Juss.
Iz_5_1009	DJI_0713	Pentanema britannica (L.) D.Gut.Larr., Santos-Vicente, Anderb., E.Rico & M.M.Mart.Ort.	0	n	1	0	Asteraceae Bercht. & J.Presl
Iz_5_1009	DJI_0713	Peucedanum oreoselinum (L.) Moench	3	n	1	0	Apiaceae Lindl.
Iz_5_1009	DJI_0713	Peucedanum ruthenicum M.Bieb.	0	n	1	0	Apiaceae Lindl.
Iz_5_1009	DJI_0713	Pilosella echioides (Lumn.) F.W.Schultz & Sch.Bip.	0	n	1	0	Asteraceae Bercht. & J.Presl
Iz_5_1009	DJI_0713	Pilosella officinarum F. Schultz et Sch. Bip.	0	n	1	0	Asteraceae Bercht. & J.Presl
Iz_5_1009	DJI_0713	Pilosella sp.	0		NA	0	Asteraceae Bercht. & J.Presl
Iz_5_1009	DJI_0713	Pinus sylvestris L.	0	n	1	1	Pinaceae Spreng. ex F.Rudolphi
Iz_5_1009	DJI_0713	Poa annua L.	0	n	1	0	Poaceae Barnhart
Iz_5_1009	DJI_0713	Poa nemoralis L.	0	n	1	0	Poaceae Barnhart
Iz_5_1009	DJI_0713	Poa sp.	0		NA	0	Poaceae Barnhart
Iz_5_1009	DJI_0713	Polygonatum odoratum (Mill.) Druce	0	n	1	0	Asparagaceae Juss.
Iz_5_1009	DJI_0713	Polygonum arenarium Waldst. & Kit.	0	n	1	0	Polygonaceae Juss.
Iz_5_1009	DJI_0713	Populus nigra L.	0	n	1	1	Salicaceae Mirb.
Iz_5_1009	DJI_0713	Populus sp.	0	n	1	1	Salicaceae Mirb.
Iz_5_1009	DJI_0713	Populus tremula L.	0	n	1	1	Salicaceae Mirb.
Iz_5_1009	DJI_0713	Populus Ã canescens (Aiton) Sm.	0	n	1	1	Salicaceae Mirb.
Iz_5_1009	DJI_0713	Potentilla argentea L.	0	n	1	0	Rosaceae Juss.
Iz_5_1009	DJI_0713	Psammophiliella muralis (L.) Ikonn.	0	n	1	0	Caryophyllaceae Juss.
Iz_5_1009	DJI_0713	Psephellus sumensis (Kalen.) Greuter	0	n	1	0	Asteraceae Bercht. & J.Presl
Iz_5_1009	DJI_0713	Pulsatilla pratensis (L.) Mill. s.l.	0	n	1	0	Ranunculaceae Juss.
Iz_5_1009	DJI_0713	Pyrus communis L.	0	a	0	1	Rosaceae Juss.
Iz_5_1009	DJI_0713	Quercus robur L.	0	n	1	1	Fagaceae Dumort.
Iz_5_1009	DJI_0713	Robinia pseudoacacia L.	0	a	0	1	Fabaceae Lindl.
Iz_5_1009	DJI_0713	Rosa sp.	0		NA	1	Rosaceae Juss.
Iz_5_1009	DJI_0713	Rubus idaeus L.	0	n	1	0	Rosaceae Juss.
Iz_5_1009	DJI_0713	Rumex acetosella L.	0	n	1	0	Polygonaceae Juss.
Iz_5_1009	DJI_0713	Rumex thyrsiflorus Fingerh.	0	n	1	0	Polygonaceae Juss.
Iz_5_1009	DJI_0713	Salix alba L.	0	n	1	1	Salicaceae Mirb.
Iz_5_1009	DJI_0713	Sambucus nigra L.	0	n	1	1	Viburnaceae Raf.
Iz_5_1009	DJI_0713	Secale sylvestre Host	0	n	1	0	Poaceae Barnhart
Iz_5_1009	DJI_0713	Sempervivum ruthenicum Schnittsp. & C.B. Lehm.	0	n	1	0	Crassulaceae J.St.-Hil.
Iz_5_1009	DJI_0713	Setaria viridis (L.) P. Beauv.	0	n	1	0	Poaceae Barnhart
Iz_5_1009	DJI_0713	Silene borysthenica (Gruner) Walters	0	n	1	0	Caryophyllaceae Juss.
Iz_5_1009	DJI_0713	Silene latifolia subsp. alba (Mill.) Greuter & Burdet	0	n	1	0	Caryophyllaceae Juss.
Iz_5_1009	DJI_0713	Solidago virgaurea L.	0	n	1	0	Asteraceae Bercht. & J.Presl
Iz_5_1009	DJI_0713	Solidago Ã niederederi (S. canadensis Ã S. virgaurea)	0	hybrid	0	0	Asteraceae Bercht. & J.Presl
Iz_5_1009	DJI_0713	Sorbus aucuparia L.	0	n	1	1	Rosaceae Juss.
Iz_5_1009	DJI_0713	Stachys recta L.	0	n	1	0	Lamiacea Lindl.
Iz_5_1009	DJI_0713	Tanacetum vulgare L.	0	n	1	0	Asteraceae Bercht. & J.Presl
Iz_5_1009	DJI_0713	Thymus pallasianus Heinr.Braun	0	n	1	0	Lamiacea Lindl.
Iz_5_1009	DJI_0713	Tilia cordata Mill.	0	n	1	1	Malvaceae Juss.
Iz_5_1009	DJI_0713	Trifolium alpestre L.	0	n	1	0	Fabaceae Lindl.
Iz_5_1009	DJI_0713	Trifolium arvense L.	0	n	1	0	Fabaceae Lindl.
Iz_5_1009	DJI_0713	Ulmus glabra Huds.	0	n	1	1	Ulmaceae Mirb.
Iz_5_1009	DJI_0713	Verbascum densiflorum Bertol.	0	n	1	0	Scrophulariaceae Juss.
Iz_5_1009	DJI_0713	Veronica austriaca L.	0	n	1	0	Plantaginaceae Juss.
Iz_5_1009	DJI_0713	Veronica incana L.	0	n	1	0	Plantaginaceae Juss.
Iz_5_1009	DJI_0713	Veronica verna L.	0	n	1	0	Plantaginaceae Juss.
Iz_5_1009	DJI_0713	Vincetoxicum hirundinaria Medik	0	n	1	0	Apocynaceae Juss.
Iz_5_1009	DJI_0715	Acer tataricum L.	0	n	1	1	Sapindaceae Juss.
Iz_5_1009	DJI_0715	Achillea millefolium L. s.l.	0	n	1	0	Asteraceae Bercht. & J.Presl
Iz_5_1009	DJI_0715	Allium oleraceum L.	0	n	1	0	Amaryllidaceae J.St.-Hil.
Iz_5_1009	DJI_0715	Ambrosia artemisiifolia L.	0	a	0	0	Asteraceae Bercht. & J.Presl
Iz_5_1009	DJI_0715	Amorpha fruticosa L.	0	a	0	0	Fabaceae Lindl.
Iz_5_1009	DJI_0715	Anthericum ramosum L.	0	n	1	0	Asparagaceae Juss.
Iz_5_1009	DJI_0715	Artemisia marschalliana Spreng.	0	n	1	0	Asteraceae Bercht. & J.Presl
Iz_5_1009	DJI_0715	Asparagus officinalis L.	0	n	1	0	Asparagaceae Juss.
Iz_5_1009	DJI_0715	Astragalus cicer L.	0	n	1	0	Fabaceae Lindl.
Iz_5_1009	DJI_0715	Bassia laniflora (S.G.Gmel.) A.J.Scott	0	n	1	0	Amaranthaceae Juss.
Iz_5_1009	DJI_0715	Berberis vulgaris L.	0	n	1	0	Berberidaceae Juss.
Iz_5_1009	DJI_0715	Betula pendula Roth.	0	n	1	1	Betulaceae Gray
Iz_5_1009	DJI_0715	Betula pendula Roth.	0	n	1	0	Poaceae Barnhart
Iz_5_1009	DJI_0715	Bromus inermis Leyss.	0		NA	NA	
Iz_5_1009	DJI_0715	Bromus squarrosus L.	0	a	0	0	Poaceae Barnhart
Iz_5_1009	DJI_0715	Bromus tectorum L.	0	a	0	0	Poaceae Barnhart
Iz_5_1009	DJI_0715	Calamagrostis epigejos (L.) Roth	60	n	1	0	Poaceae Barnhart
Iz_5_1009	DJI_0715	Carduus acanthoides L.	0	n	1	0	Asteraceae Bercht. & J.Presl

Iz_5_1009	DJI_0715	Carex sp.	0	n	1	0	Cyperaceae Juss.
Iz_5_1009	DJI_0715	Centaurea majorovii Dumbadze	0	n	1	0	Asteraceae Bercht. & J.Presl
Iz_5_1009	DJI_0715	Chamaecytisus ruthenicus (Fisch.ex Wol.) Klaskova	0	n	1	0	Fabaceae Lindl.
Iz_5_1009	DJI_0715	Chelidonium majus L.	0	n	1	0	Papaveraceae Juss.
Iz_5_1009	DJI_0715	Chenopodium betaceum Andrz.	0	n	1	0	Amaranthaceae Juss.
Iz_5_1009	DJI_0715	Chenopodium cf. album	0	n	1	0	Amaranthaceae Juss.
Iz_5_1009	DJI_0715	Chenopodium sp.	0		NA	0	Amaranthaceae Juss.
Iz_5_1009	DJI_0715	Chondrilla juncea L.	0	n	1	0	Asteraceae Bercht. & J.Presl
Iz_5_1009	DJI_0715	Cirsium vulgare (Savi) Ten.	0	n	1	0	Asteraceae Bercht. & J.Presl
Iz_5_1009	DJI_0715	Clinopodium acinos (L.) Kuntze	0	n	1	0	Lamiacea Lindl.
Iz_5_1009	DJI_0715	Convallaria majalis L.	0	n	1	0	Asparagaceae Juss.
Iz_5_1009	DJI_0715	Crataegus rhipidophylla Gand.	0	n	1	1	Rosaceae Juss.
Iz_5_1009	DJI_0715	Crepis foetida L.	0	n	1	0	Asteraceae Bercht. & J.Presl
Iz_5_1009	DJI_0715	Dactylis glomerata L.	0	n	1	0	Poaceae Barnhart
Iz_5_1009	DJI_0715	Dianthus campestris M.Bieb.	0	n	1	0	Caryophyllaceae Juss.
Iz_5_1009	DJI_0715	Digitaria sanguinalis (L.) Scop.	0	a	0	0	Poaceae Barnhart
Iz_5_1009	DJI_0715	Elymus repens (L.) Gould	0	n	1	0	Poaceae Barnhart
Iz_5_1009	DJI_0715	Epilobium tetragonum L.	0	n	1	0	Onagraceae Juss.
Iz_5_1009	DJI_0715	Eragrostis minor Host	0	a	0	0	Poaceae Barnhart
Iz_5_1009	DJI_0715	Erigeron canadensis L.	3	a	0	0	Asteraceae Bercht. & J.Presl
Iz_5_1009	DJI_0715	Euphorbia seguieriana Neck.	0	n	1	0	Euphorbiaceae Juss.
Iz_5_1009	DJI_0715	Fallopia dumetorum (L.) Holub	0	n	1	0	Polygonaceae Juss.
Iz_5_1009	DJI_0715	Festuca beckeri (Hack.) Trautv.	0	n	1	0	Poaceae Barnhart
Iz_5_1009	DJI_0715	Fragaria vesca L.	0	n	1	0	Rosaceae Juss.
Iz_5_1009	DJI_0715	Frangula alnus Mill.	0	n	1	1	Rhamnaceae Juss.
Iz_5_1009	DJI_0715	Fraxinus pennsylvanica Marshall	0	a	0	1	Oleaceae Hoffmanns. & Link
Iz_5_1009	DJI_0715	Galeopsis bifida Boenn.	0	n	1	0	Lamiacea Lindl.
Iz_5_1009	DJI_0715	Galium verum L.	0	n	1	0	Rubiaceae Juss.
Iz_5_1009	DJI_0715	Genista tinctoria L.	0	n	1	0	Fabaceae Lindl.
Iz_5_1009	DJI_0715	Geranium robertianum L.	0	n	1	0	Geraniaceae Juss.
Iz_5_1009	DJI_0715	Geranium sanquineum L.	0	n	1	0	Geraniaceae Juss.
Iz_5_1009	DJI_0715	Gypsophila paniculata L.	0	n	1	0	Caryophyllaceae Juss.
Iz_5_1009	DJI_0715	Helichrysum arenarium (L.) Moench	0	n	1	0	Asteraceae Bercht. & J.Presl
Iz_5_1009	DJI_0715	Herniaria polygama J.Gay	0	n	1	0	Caryophyllaceae Juss.
Iz_5_1009	DJI_0715	Hieracium robustum Fr.	0	n	1	0	Asteraceae Bercht. & J.Presl
Iz_5_1009	DJI_0715	Hieracium umbellatum L.	0	n	1	NA	Asteraceae Bercht. & J.Presl
Iz_5_1009	DJI_0715	Hylotelephium maximum (L.) Holub	0	n	1	0	Crassulaceae J.St.-Hil.
Iz_5_1009	DJI_0715	Jacobaea borysthenica (DC.) B.Nord. & Greuter	0	n	1	0	Asteraceae Bercht. & J.Presl
Iz_5_1009	DJI_0715	Jasione montana L.	0	n	1	0	Campanulaceae Juss.
Iz_5_1009	DJI_0715	Jurinea cyanoides (L.) Rchb.s.l.	0	n	1	0	Asteraceae Bercht. & J.Presl
Iz_5_1009	DJI_0715	Koeleria glauca (Spreng.) DC.	0	n	1	0	Poaceae Barnhart
Iz_5_1009	DJI_0715	Lactuca serriola L.	0	a	0	0	Asteraceae Bercht. & J.Presl
Iz_5_1009	DJI_0715	Linaria genistifolia (L.) Mill.	0	n	1	0	Plantaginaceae Juss.
Iz_5_1009	DJI_0715	Lomelosia argentea (L.) Greuter & Burdet	0	n	1	0	Caprifoliaceae Juss.
Iz_5_1009	DJI_0715	Melampyrum pratense L.	0	n	1	0	Orobanchaceae Vent.
Iz_5_1009	DJI_0715	Oenothera biennis L.	0	a	0	0	Onagraceae Juss.
Iz_5_1009	DJI_0715	Pentanema britannica (L.) D.Gut.Larr., Santos-Vicente, Anderb., E.Rico & M.M.Mart.Ort.	0	n	1	0	Asteraceae Bercht. & J.Presl
Iz_5_1009	DJI_0715	Peucedanum oreoselinum (L.) Moench	0	n	1	0	Apiaceae Lindl.
Iz_5_1009	DJI_0715	Peucedanum ruthenicum M.Bieb.	0	n	1	0	Apiaceae Lindl.
Iz_5_1009	DJI_0715	Pilosella echioides (Lumn.) F.W.Schultz & Sch.Bip.	0	n	1	0	Asteraceae Bercht. & J.Presl
Iz_5_1009	DJI_0715	Pilosella officinarum F. Schultz et Sch. Bip.	0	n	1	0	Asteraceae Bercht. & J.Presl
Iz_5_1009	DJI_0715	Pilosella sp.	0		NA	0	Asteraceae Bercht. & J.Presl
Iz_5_1009	DJI_0715	Pinus sylvestris L.	0	n	1	1	Pinaceae Spreng. ex F.Rudolphi
Iz_5_1009	DJI_0715	Poa annua L.	0	n	1	0	Poaceae Barnhart
Iz_5_1009	DJI_0715	Poa nemoralis L.	0	n	1	0	Poaceae Barnhart
Iz_5_1009	DJI_0715	Poa sp.	0		NA	0	Poaceae Barnhart
Iz_5_1009	DJI_0715	Polygonatum odoratum (Mill.) Druce	0	n	1	0	Asparagaceae Juss.
Iz_5_1009	DJI_0715	Polygonum arenarium Waldst. & Kit.	0	n	1	0	Polygonaceae Juss.
Iz_5_1009	DJI_0715	Populus nigra L.	0	n	1	1	Salicaceae Mirb.
Iz_5_1009	DJI_0715	Populus sp.	0	n	1	1	Salicaceae Mirb.
Iz_5_1009	DJI_0715	Populus tremula L.	0	n	1	1	Salicaceae Mirb.
Iz_5_1009	DJI_0715	Populus Ã canescens (Aiton) Sm.	0	n	1	1	Salicaceae Mirb.
Iz_5_1009	DJI_0715	Potentilla argentea L.	0	n	1	0	Rosaceae Juss.
Iz_5_1009	DJI_0715	Psammophiliella muralis (L.) Ikonn.	0	n	1	0	Caryophyllaceae Juss.
Iz_5_1009	DJI_0715	Psephellus sumensis (Kalen.) Greuter	0	n	1	0	Asteraceae Bercht. & J.Presl
Iz_5_1009	DJI_0715	Pulsatilla pratensis (L.) Mill. s.l.	0	n	1	0	Ranunculaceae Juss.
Iz_5_1009	DJI_0715	Pyrus communis L.	0	a	0	1	Rosaceae Juss.
Iz_5_1009	DJI_0715	Quercus robur L.	0	n	1	1	Fagaceae Dumort.
Iz_5_1009	DJI_0715	Robinia pseudoacacia L.	0	a	0	1	Fabaceae Lindl.
Iz_5_1009	DJI_0715	Rosa sp.	0		NA	1	Rosaceae Juss.
Iz_5_1009	DJI_0715	Rubus idaeus L.	0	n	1	0	Rosaceae Juss.
Iz_5_1009	DJI_0715	Rumex acetosella L.	0	n	1	0	Polygonaceae Juss.
Iz_5_1009	DJI_0715	Rumex thyrsiflorus Fingerh.	0	n	1	0	Polygonaceae Juss.
Iz_5_1009	DJI_0715	Salix alba L.	0	n	1	1	Salicaceae Mirb.
Iz_5_1009	DJI_0715	Sambucus nigra L.	0	n	1	1	Viburnaceae Raf.
Iz_5_1009	DJI_0715	Secale sylvestre Host	0	n	1	0	Poaceae Barnhart
Iz_5_1009	DJI_0715	Sempervivum ruthenicum Schnittsp. & C.B. Lehm.	0	n	1	0	Crassulaceae J.St.-Hil.
Iz_5_1009	DJI_0715	Setaria viridis (L.) P. Beauv.	0	n	1	0	Poaceae Barnhart
Iz_5_1009	DJI_0715	Silene borysthenica (Gruner) Walters	0	n	1	0	Caryophyllaceae Juss.
Iz_5_1009	DJI_0715	Silene latifolia subsp. alba (Mill.) Greuter & Burdet	0	n	1	0	Caryophyllaceae Juss.
Iz_5_1009	DJI_0715	Solidago virgaurea L.	0	n	1	0	Asteraceae Bercht. & J.Presl
Iz_5_1009	DJI_0715	Solidago Ã niederederi (S. canadensis Ã S. virgaurea)	0	hybrid	0	0	Asteraceae Bercht. & J.Presl
Iz_5_1009	DJI_0715	Sorbus aucuparia L.	0	n	1	1	Rosaceae Juss.
Iz_5_1009	DJI_0715	Stachys recta L.	0	n	1	0	Lamiacea Lindl.
Iz_5_1009	DJI_0715	Tanacetum vulgare L.	0	n	1	0	Asteraceae Bercht. & J.Presl
Iz_5_1009	DJI_0715	Thymus pallasianus Heinr.Braun	0	n	1	0	Lamiacea Lindl.
Iz_5_1009	DJI_0715	Tilia cordata Mill.	0	n	1	1	Malvaceae Juss.
Iz_5_1009	DJI_0715	Trifolium alpestre L.	0	n	1	0	Fabaceae Lindl.
Iz_5_1009	DJI_0715	Trifolium arvense L.	0	n	1	0	Fabaceae Lindl.
Iz_5_1009	DJI_0715	Ulmus glabra Huds.	0	n	1	1	Ulmaceae Mirb.
Iz_5_1009	DJI_0715	Verbascum densiflorum Bertol.	0	n	1	0	Scrophulariaceae Juss.
Iz_5_1009	DJI_0715	Veronica austriaca L.	0	n	1	0	Plantaginaceae Juss.
Iz_5_1009	DJI_0715	Veronica incana L.	0	n	1	0	Plantaginaceae Juss.
Iz_5_1009	DJI_0715	Veronica verna L.	0	n	1	0	Plantaginaceae Juss.
Iz_5_1009	DJI_0715	Vincetoxicum hirundinaria Medik	0	n	1	0	Apocynaceae Juss.
Iz_5_1009	DJI_0717	Acer tataricum L.	0	n	1	1	Sapindaceae Juss.
Iz_5_1009	DJI_0717	Achillea millefolium L. s.l.	0	n	1	0	Asteraceae Bercht. & J.Presl
Iz_5_1009	DJI_0717	Allium oleraceum L.	0	n	1	0	Amaryllidaceae J.St.-Hil.

Iz_5_1009	DJI_0717	Ambrosia artemisiifolia L.	0	a	0	0	Asteraceae Bercht. & J.Presl
Iz_5_1009	DJI_0717	Amorpha fruticosa L.	0	a	0	0	Fabaceae Lindl.
Iz_5_1009	DJI_0717	Anthericum ramosum L.	0	n	1	0	Asparagaceae Juss.
Iz_5_1009	DJI_0717	Artemisia marschalliana Spreng.	0	n	1	0	Asteraceae Bercht. & J.Presl
Iz_5_1009	DJI_0717	Asparagus officinalis L.	0	n	1	0	Asparagaceae Juss.
Iz_5_1009	DJI_0717	Astragalus cicer L.	0	n	1	0	Fabaceae Lindl.
Iz_5_1009	DJI_0717	Bassia laniflora (S.G.Gmel.) A.J.Scott	0	n	1	0	Amaranthaceae Juss.
Iz_5_1009	DJI_0717	Berberis vulgaris L.	0	n	1	0	Berberidaceae Juss.
Iz_5_1009	DJI_0717	Betula pendula Roth.	0	n	1	1	Betulaceae Gray
Iz_5_1009	DJI_0717	Betula pendula Roth.	0	n	1	0	Poaceae Barnhart
Iz_5_1009	DJI_0717	Bromus inermis Leyss.	0		NA	NA	
Iz_5_1009	DJI_0717	Bromus squarrosus L.	0	a	0	0	Poaceae Barnhart
Iz_5_1009	DJI_0717	Bromus tectorum L.	0	a	0	0	Poaceae Barnhart
Iz_5_1009	DJI_0717	Calamagrostis epigejos (L.) Roth	26	n	1	0	Poaceae Barnhart
Iz_5_1009	DJI_0717	Carduus acanthoides L.	0	n	1	0	Asteraceae Bercht. & J.Presl
Iz_5_1009	DJI_0717	Carex sp.	0	n	1	0	Cyperaceae Juss.
Iz_5_1009	DJI_0717	Centaurea majorovii Dumbadze	0	n	1	0	Asteraceae Bercht. & J.Presl
Iz_5_1009	DJI_0717	Chamaecytisus ruthenicus (Fisch.ex Wol.) Klaskova	0	n	1	0	Fabaceae Lindl.
Iz_5_1009	DJI_0717	Chelidonium majus L.	0	n	1	0	Papaveraceae Juss.
Iz_5_1009	DJI_0717	Chenopodium betaceum Andrz.	0	n	1	0	Amaranthaceae Juss.
Iz_5_1009	DJI_0717	Chenopodium cf. album	0	n	1	0	Amaranthaceae Juss.
Iz_5_1009	DJI_0717	Chenopodium sp.	0		NA	0	Amaranthaceae Juss.
Iz_5_1009	DJI_0717	Chondrilla juncea L.	0	n	1	0	Asteraceae Bercht. & J.Presl
Iz_5_1009	DJI_0717	Cirsium vulgare (Savi) Ten.	0	n	1	0	Asteraceae Bercht. & J.Presl
Iz_5_1009	DJI_0717	Clinopodium acinos (L.) Kuntze	0	n	1	0	Lamiacea Lindl.
Iz_5_1009	DJI_0717	Convallaria majalis L.	0	n	1	0	Asparagaceae Juss.
Iz_5_1009	DJI_0717	Crataegus rhipidophylla Gand.	0	n	1	1	Rosaceae Juss.
Iz_5_1009	DJI_0717	Crepis foetida L.	0	n	1	0	Asteraceae Bercht. & J.Presl
Iz_5_1009	DJI_0717	Dactylis glomerata L.	0	n	1	0	Poaceae Barnhart
Iz_5_1009	DJI_0717	Dianthus campestris M.Bieb.	0	n	1	0	Caryophyllaceae Juss.
Iz_5_1009	DJI_0717	Digitaria sanguinalis (L.) Scop.	0	a	0	0	Poaceae Barnhart
Iz_5_1009	DJI_0717	Elymus repens (L.) Gould	0	n	1	0	Poaceae Barnhart
Iz_5_1009	DJI_0717	Epilobium tetragonum L.	0	n	1	0	Onagraceae Juss.
Iz_5_1009	DJI_0717	Eragrostis minor Host	0	a	0	0	Poaceae Barnhart
Iz_5_1009	DJI_0717	Erigeron canadensis L.	2	a	0	0	Asteraceae Bercht. & J.Presl
Iz_5_1009	DJI_0717	Euphorbia seguieriana Neck.	0	n	1	0	Euphorbiaceae Juss.
Iz_5_1009	DJI_0717	Fallopia dumetorum (L.) Holub	0	n	1	0	Polygonaceae Juss.
Iz_5_1009	DJI_0717	Festuca beckeri (Hack.) Trautv.	0	n	1	0	Poaceae Barnhart
Iz_5_1009	DJI_0717	Fragaria vesca L.	0	n	1	0	Rosaceae Juss.
Iz_5_1009	DJI_0717	Frangula alnus Mill.	0	n	1	1	Rhamnaceae Juss.
Iz_5_1009	DJI_0717	Fraxinus pennsylvanica Marshall	0	a	0	1	Oleaceae Hoffmanns. & Link
Iz_5_1009	DJI_0717	Galeopsis bifida Boenn.	0	n	1	0	Lamiacea Lindl.
Iz_5_1009	DJI_0717	Galium verum L.	0	n	1	0	Rubiaceae Juss.
Iz_5_1009	DJI_0717	Genista tinctoria L.	0	n	1	0	Fabaceae Lindl.
Iz_5_1009	DJI_0717	Geranium robertianum L.	0	n	1	0	Geraniaceae Juss.
Iz_5_1009	DJI_0717	Geranium sanquineum L.	0	n	1	0	Geraniaceae Juss.
Iz_5_1009	DJI_0717	Gypsophila paniculata L.	0	n	1	0	Caryophyllaceae Juss.
Iz_5_1009	DJI_0717	Helichrysum arenarium (L.) Moench	0	n	1	0	Asteraceae Bercht. & J.Presl
Iz_5_1009	DJI_0717	Herniaria polygama J.Gay	0	n	1	0	Caryophyllaceae Juss.
Iz_5_1009	DJI_0717	Hieracium robustum Fr.	0	n	1	0	Asteraceae Bercht. & J.Presl
Iz_5_1009	DJI_0717	Hieracium umbellatum L.	0	n	1	NA	Asteraceae Bercht. & J.Presl
Iz_5_1009	DJI_0717	Hylotelephium maximum (L.) Holub	0	n	1	0	Crassulaceae J.St.-Hil.
Iz_5_1009	DJI_0717	Jacobaea borysthenica (DC.) B.Nord. & Greuter	0	n	1	0	Asteraceae Bercht. & J.Presl
Iz_5_1009	DJI_0717	Jasione montana L.	0	n	1	0	Campanulaceae Juss.
Iz_5_1009	DJI_0717	Jurinea cyanoides (L.) Rchb.s.l.	0	n	1	0	Asteraceae Bercht. & J.Presl
Iz_5_1009	DJI_0717	Koeleria glauca (Spreng.) DC.	0	n	1	0	Poaceae Barnhart
Iz_5_1009	DJI_0717	Lactuca serriola L.	0	a	0	0	Asteraceae Bercht. & J.Presl
Iz_5_1009	DJI_0717	Linaria genistifolia (L.) Mill.	0	n	1	0	Plantaginaceae Juss.
Iz_5_1009	DJI_0717	Lomelosia argentea (L.) Greuter & Burdet	0	n	1	0	Caprifoliaceae Juss.
Iz_5_1009	DJI_0717	Melampyrum pratense L.	0	n	1	0	Orobanchaceae Vent.
Iz_5_1009	DJI_0717	Oenothera biennis L.	0	a	0	0	Onagraceae Juss.
Iz_5_1009	DJI_0717	Pentanema britannica (L.) D.Gut.Larr., Santos-Vicente, Anderb., E.Rico & M.M.Mart.Ort.	0	n	1	0	Asteraceae Bercht. & J.Presl
Iz_5_1009	DJI_0717	Peucedanum oreoselinum (L.) Moench	2	n	1	0	Apiaceae Lindl.
Iz_5_1009	DJI_0717	Peucedanum ruthenicum M.Bieb.	0	n	1	0	Apiaceae Lindl.
Iz_5_1009	DJI_0717	Pilosella echioides (Lumn.) F.W.Schultz & Sch.Bip.	0	n	1	0	Asteraceae Bercht. & J.Presl
Iz_5_1009	DJI_0717	Pilosella officinarum F. Schultz et Sch. Bip.	0	n	1	0	Asteraceae Bercht. & J.Presl
Iz_5_1009	DJI_0717	Pilosella sp.	0		NA	0	Asteraceae Bercht. & J.Presl
Iz_5_1009	DJI_0717	Pinus sylvestris L.	0	n	1	1	Pinaceae Spreng. ex F.Rudolphi
Iz_5_1009	DJI_0717	Poa annua L.	0	n	1	0	Poaceae Barnhart
Iz_5_1009	DJI_0717	Poa nemoralis L.	0	n	1	0	Poaceae Barnhart
Iz_5_1009	DJI_0717	Poa sp.	0		NA	0	Poaceae Barnhart
Iz_5_1009	DJI_0717	Polygonatum odoratum (Mill.) Druce	0	n	1	0	Asparagaceae Juss.
Iz_5_1009	DJI_0717	Polygonum arenarium Waldst. & Kit.	0	n	1	0	Polygonaceae Juss.
Iz_5_1009	DJI_0717	Populus nigra L.	0	n	1	1	Salicaceae Mirb.
Iz_5_1009	DJI_0717	Populus sp.	0	n	1	1	Salicaceae Mirb.
Iz_5_1009	DJI_0717	Populus tremula L.	0	n	1	1	Salicaceae Mirb.
Iz_5_1009	DJI_0717	Populus Ã canescens (Aiton) Sm.	0	n	1	1	Salicaceae Mirb.
Iz_5_1009	DJI_0717	Potentilla argentea L.	0	n	1	0	Rosaceae Juss.
Iz_5_1009	DJI_0717	Psammophiliella muralis (L.) Ikonn.	0	n	1	0	Caryophyllaceae Juss.
Iz_5_1009	DJI_0717	Psephellus sumensis (Kalen.) Greuter	0	n	1	0	Asteraceae Bercht. & J.Presl
Iz_5_1009	DJI_0717	Pulsatilla pratensis (L.) Mill. s.l.	0	n	1	0	Ranunculaceae Juss.
Iz_5_1009	DJI_0717	Pyrus communis L.	0	a	0	1	Rosaceae Juss.
Iz_5_1009	DJI_0717	Quercus robur L.	0	n	1	1	Fagaceae Dumort.
Iz_5_1009	DJI_0717	Robinia pseudoacacia L.	0	a	0	1	Fabaceae Lindl.
Iz_5_1009	DJI_0717	Rosa sp.	0		NA	1	Rosaceae Juss.
Iz_5_1009	DJI_0717	Rubus idaeus L.	0	n	1	0	Rosaceae Juss.
Iz_5_1009	DJI_0717	Rumex acetosella L.	0	n	1	0	Polygonaceae Juss.
Iz_5_1009	DJI_0717	Rumex thyrsiflorus Fingerh.	0	n	1	0	Polygonaceae Juss.
Iz_5_1009	DJI_0717	Salix alba L.	0	n	1	1	Salicaceae Mirb.
Iz_5_1009	DJI_0717	Sambucus nigra L.	0	n	1	1	Viburnaceae Raf.
Iz_5_1009	DJI_0717	Secale sylvestre Host	0	n	1	0	Poaceae Barnhart
Iz_5_1009	DJI_0717	Sempervivum ruthenicum Schnittsp. & C.B. Lehm.	0	n	1	0	Crassulaceae J.St.-Hil.
Iz_5_1009	DJI_0717	Setaria viridis (L.) P. Beauv.	0	n	1	0	Poaceae Barnhart
Iz_5_1009	DJI_0717	Silene borysthenica (Gruner) Walters	0	n	1	0	Caryophyllaceae Juss.
Iz_5_1009	DJI_0717	Silene latifolia subsp. alba (Mill.) Greuter & Burdet	0	n	1	0	Caryophyllaceae Juss.
Iz_5_1009	DJI_0717	Solidago virgaurea L.	0	n	1	0	Asteraceae Bercht. & J.Presl
Iz_5_1009	DJI_0717	Solidago Ã niederederi (S. canadensis Ã S. virgaurea)	0	hybrid	0	0	Asteraceae Bercht. & J.Presl
Iz_5_1009	DJI_0717	Sorbus aucuparia L.	0	n	1	1	Rosaceae Juss.

Iz_5_1009	DJI_0717	Stachys recta L.	0	n	1	0	Lamiacea Lindl.
Iz_5_1009	DJI_0717	Tanacetum vulgare L.	0	n	1	0	Asteraceae Bercht. & J.Presl
Iz_5_1009	DJI_0717	Thymus pallasianus Heinr.Braun	0	n	1	0	Lamiacea Lindl.
Iz_5_1009	DJI_0717	Tilia cordata Mill.	0	n	1	1	Malvaceae Juss.
Iz_5_1009	DJI_0717	Trifolium alpestre L.	0	n	1	0	Fabaceae Lindl.
Iz_5_1009	DJI_0717	Trifolium arvense L.	0	n	1	0	Fabaceae Lindl.
Iz_5_1009	DJI_0717	Ulmus glabra Huds.	0	n	1	1	Ulmaceae Mirb.
Iz_5_1009	DJI_0717	Verbascum densiflorum Bertol.	0	n	1	0	Scrophulariaceae Juss.
Iz_5_1009	DJI_0717	Veronica austriaca L.	0	n	1	0	Plantaginaceae Juss.
Iz_5_1009	DJI_0717	Veronica incana L.	0	n	1	0	Plantaginaceae Juss.
Iz_5_1009	DJI_0717	Veronica verna L.	0	n	1	0	Plantaginaceae Juss.
Iz_5_1009	DJI_0717	Vincetoxicum hirundinaria Medik	0	n	1	0	Apocynaceae Juss.
Iz_5_1009	DJI_0725	Acer tataricum L.	0	n	1	1	Sapindaceae Juss.
Iz_5_1009	DJI_0725	Achillea millefolium L. s.l.	0	n	1	0	Asteraceae Bercht. & J.Presl
Iz_5_1009	DJI_0725	Allium oleraceum L.	0	n	1	0	Amaryllidaceae J.St.-Hil.
Iz_5_1009	DJI_0725	Ambrosia artemisiifolia L.	0	a	0	0	Asteraceae Bercht. & J.Presl
Iz_5_1009	DJI_0725	Amorpha fruticosa L.	0	a	0	0	Fabaceae Lindl.
Iz_5_1009	DJI_0725	Anthericum ramosum L.	0	n	1	0	Asparagaceae Juss.
Iz_5_1009	DJI_0725	Artemisia marschalliana Spreng.	0	n	1	0	Asteraceae Bercht. & J.Presl
Iz_5_1009	DJI_0725	Asparagus officinalis L.	0	n	1	0	Asparagaceae Juss.
Iz_5_1009	DJI_0725	Astragalus cicer L.	0	n	1	0	Fabaceae Lindl.
Iz_5_1009	DJI_0725	Bassia laniflora (S.G.Gmel.) A.J.Scott	0	n	1	0	Amaranthaceae Juss.
Iz_5_1009	DJI_0725	Berberis vulgaris L.	0	n	1	0	Berberidaceae Juss.
Iz_5_1009	DJI_0725	Betula pendula Roth.	0	n	1	1	Betulaceae Gray
Iz_5_1009	DJI_0725	Betula pendula Roth.	0	n	1	0	Poaceae Barnhart
Iz_5_1009	DJI_0725	Bromus inermis Leyss.	0		NA	NA	
Iz_5_1009	DJI_0725	Bromus squarrosus L.	0	a	0	0	Poaceae Barnhart
Iz_5_1009	DJI_0725	Bromus tectorum L.	0	a	0	0	Poaceae Barnhart
Iz_5_1009	DJI_0725	Calamagrostis epigejos (L.) Roth	15	n	1	0	Poaceae Barnhart
Iz_5_1009	DJI_0725	Carduus acanthoides L.	0	n	1	0	Asteraceae Bercht. & J.Presl
Iz_5_1009	DJI_0725	Carex sp.	0	n	1	0	Cyperaceae Juss.
Iz_5_1009	DJI_0725	Centaurea majorovii Dumbadze	0	n	1	0	Asteraceae Bercht. & J.Presl
Iz_5_1009	DJI_0725	Chamaecytisus ruthenicus (Fisch.ex Wol.) Klaskova	0	n	1	0	Fabaceae Lindl.
Iz_5_1009	DJI_0725	Chelidonium majus L.	0	n	1	0	Papaveraceae Juss.
Iz_5_1009	DJI_0725	Chenopodium betaceum Andrz.	0	n	1	0	Amaranthaceae Juss.
Iz_5_1009	DJI_0725	Chenopodium cf. album	0	n	1	0	Amaranthaceae Juss.
Iz_5_1009	DJI_0725	Chenopodium sp.	1		NA	0	Amaranthaceae Juss.
Iz_5_1009	DJI_0725	Chondrilla juncea L.	0	n	1	0	Asteraceae Bercht. & J.Presl
Iz_5_1009	DJI_0725	Cirsium vulgare (Savi) Ten.	0	n	1	0	Asteraceae Bercht. & J.Presl
Iz_5_1009	DJI_0725	Clinopodium acinos (L.) Kuntze	0	n	1	0	Lamiacea Lindl.
Iz_5_1009	DJI_0725	Convallaria majalis L.	0	n	1	0	Asparagaceae Juss.
Iz_5_1009	DJI_0725	Crataegus rhipidophylla Gand.	0	n	1	1	Rosaceae Juss.
Iz_5_1009	DJI_0725	Crepis foetida L.	0	n	1	0	Asteraceae Bercht. & J.Presl
Iz_5_1009	DJI_0725	Dactylis glomerata L.	0	n	1	0	Poaceae Barnhart
Iz_5_1009	DJI_0725	Dianthus campestris M.Bieb.	0	n	1	0	Caryophyllaceae Juss.
Iz_5_1009	DJI_0725	Digitaria sanguinalis (L.) Scop.	0	a	0	0	Poaceae Barnhart
Iz_5_1009	DJI_0725	Elymus repens (L.) Gould	0	n	1	0	Poaceae Barnhart
Iz_5_1009	DJI_0725	Epilobium tetragonum L.	0	n	1	0	Onagraceae Juss.
Iz_5_1009	DJI_0725	Eragrostis minor Host	0	a	0	0	Poaceae Barnhart
Iz_5_1009	DJI_0725	Erigeron canadensis L.	0.5	a	0	0	Asteraceae Bercht. & J.Presl
Iz_5_1009	DJI_0725	Euphorbia seguieriana Neck.	0	n	1	0	Euphorbiaceae Juss.
Iz_5_1009	DJI_0725	Fallopia dumetorum (L.) Holub	0	n	1	0	Polygonaceae Juss.
Iz_5_1009	DJI_0725	Festuca beckeri (Hack.) Trautv.	0	n	1	0	Poaceae Barnhart
Iz_5_1009	DJI_0725	Fragaria vesca L.	0	n	1	0	Rosaceae Juss.
Iz_5_1009	DJI_0725	Frangula alnus Mill.	0	n	1	1	Rhamnaceae Juss.
Iz_5_1009	DJI_0725	Fraxinus pennsylvanica Marshall	0	a	0	1	Oleaceae Hoffmanns. & Link
Iz_5_1009	DJI_0725	Galeopsis bifida Boenn.	0	n	1	0	Lamiacea Lindl.
Iz_5_1009	DJI_0725	Galium verum L.	0	n	1	0	Rubiaceae Juss.
Iz_5_1009	DJI_0725	Genista tinctoria L.	0	n	1	0	Fabaceae Lindl.
Iz_5_1009	DJI_0725	Geranium robertianum L.	0	n	1	0	Geraniaceae Juss.
Iz_5_1009	DJI_0725	Geranium sanquineum L.	0	n	1	0	Geraniaceae Juss.
Iz_5_1009	DJI_0725	Gypsophila paniculata L.	0	n	1	0	Caryophyllaceae Juss.
Iz_5_1009	DJI_0725	Helichrysum arenarium (L.) Moench	0	n	1	0	Asteraceae Bercht. & J.Presl
Iz_5_1009	DJI_0725	Herniaria polygama J.Gay	0	n	1	0	Caryophyllaceae Juss.
Iz_5_1009	DJI_0725	Hieracium robustum Fr.	0	n	1	0	Asteraceae Bercht. & J.Presl
Iz_5_1009	DJI_0725	Hieracium umbellatum L.	0	n	1	NA	Asteraceae Bercht. & J.Presl
Iz_5_1009	DJI_0725	Hylotelephium maximum (L.) Holub	0	n	1	0	Crassulaceae J.St.-Hil.
Iz_5_1009	DJI_0725	Jacobaea borysthenica (DC.) B.Nord. & Greuter	0	n	1	0	Asteraceae Bercht. & J.Presl
Iz_5_1009	DJI_0725	Jasione montana L.	0	n	1	0	Campanulaceae Juss.
Iz_5_1009	DJI_0725	Jurinea cyanoides (L.) Rchb.s.l.	0	n	1	0	Asteraceae Bercht. & J.Presl
Iz_5_1009	DJI_0725	Koeleria glauca (Spreng.) DC.	0	n	1	0	Poaceae Barnhart
Iz_5_1009	DJI_0725	Lactuca serriola L.	0	a	0	0	Asteraceae Bercht. & J.Presl
Iz_5_1009	DJI_0725	Linaria genistifolia (L.) Mill.	0	n	1	0	Plantaginaceae Juss.
Iz_5_1009	DJI_0725	Lomelosia argentea (L.) Greuter & Burdet	0	n	1	0	Caprifoliaceae Juss.
Iz_5_1009	DJI_0725	Melampyrum pratense L.	0	n	1	0	Orobanchaceae Vent.
Iz_5_1009	DJI_0725	Oenothera biennis L.	0	a	0	0	Onagraceae Juss.
Iz_5_1009	DJI_0725	Pentanema britannica (L.) D.Gut.Larr., Santos-Vicente, Anderb., E.Rico & M.M.Mart.Ort.	0	n	1	0	Asteraceae Bercht. & J.Presl
Iz_5_1009	DJI_0725	Peucedanum oreoselinum (L.) Moench	0	n	1	0	Apiaceae Lindl.
Iz_5_1009	DJI_0725	Peucedanum ruthenicum M.Bieb.	0	n	1	0	Apiaceae Lindl.
Iz_5_1009	DJI_0725	Pilosella echioides (Lumn.) F.W.Schultz & Sch.Bip.	0	n	1	0	Asteraceae Bercht. & J.Presl
Iz_5_1009	DJI_0725	Pilosella officinarum F. Schultz et Sch. Bip.	0	n	1	0	Asteraceae Bercht. & J.Presl
Iz_5_1009	DJI_0725	Pilosella sp.	0		NA	0	Asteraceae Bercht. & J.Presl
Iz_5_1009	DJI_0725	Pinus sylvestris L.	0	n	1	1	Pinaceae Spreng. ex F.Rudolphi
Iz_5_1009	DJI_0725	Poa annua L.	0	n	1	0	Poaceae Barnhart
Iz_5_1009	DJI_0725	Poa nemoralis L.	0	n	1	0	Poaceae Barnhart
Iz_5_1009	DJI_0725	Poa sp.	0		NA	0	Poaceae Barnhart
Iz_5_1009	DJI_0725	Polygonatum odoratum (Mill.) Druce	0	n	1	0	Asparagaceae Juss.
Iz_5_1009	DJI_0725	Polygonum arenarium Waldst. & Kit.	0	n	1	0	Polygonaceae Juss.
Iz_5_1009	DJI_0725	Populus nigra L.	0	n	1	1	Salicaceae Mirb.
Iz_5_1009	DJI_0725	Populus sp.	0	n	1	1	Salicaceae Mirb.
Iz_5_1009	DJI_0725	Populus tremula L.	0	n	1	1	Salicaceae Mirb.
Iz_5_1009	DJI_0725	Populus Ã canescens (Aiton) Sm.	0	n	1	1	Salicaceae Mirb.
Iz_5_1009	DJI_0725	Potentilla argentea L.	0	n	1	0	Rosaceae Juss.
Iz_5_1009	DJI_0725	Psammophiliella muralis (L.) Ikonn.	0	n	1	0	Caryophyllaceae Juss.
Iz_5_1009	DJI_0725	Psephellus sumensis (Kalen.) Greuter	0	n	1	0	Asteraceae Bercht. & J.Presl
Iz_5_1009	DJI_0725	Pulsatilla pratensis (L.) Mill. s.l.	0	n	1	0	Ranunculaceae Juss.
Iz_5_1009	DJI_0725	Pyrus communis L.	0	a	0	1	Rosaceae Juss.
Iz_5_1009	DJI_0725	Quercus robur L.	0	n	1	1	Fagaceae Dumort.

Iz_5_1009	DJI_0725	Robinia pseudoacacia L.	0	a	0	1	Fabaceae Lindl.
Iz_5_1009	DJI_0725	Rosa sp.	0		NA	1	Rosaceae Juss.
Iz_5_1009	DJI_0725	Rubus idaeus L.	0	n	1	0	Rosaceae Juss.
Iz_5_1009	DJI_0725	Rumex acetosella L.	0	n	1	0	Polygonaceae Juss.
Iz_5_1009	DJI_0725	Rumex thyrsiflorus Fingerh.	0	n	1	0	Polygonaceae Juss.
Iz_5_1009	DJI_0725	Salix alba L.	0	n	1	1	Salicaceae Mirb.
Iz_5_1009	DJI_0725	Sambucus nigra L.	0	n	1	1	Viburnaceae Raf.
Iz_5_1009	DJI_0725	Secale sylvestre Host	0	n	1	0	Poaceae Barnhart
Iz_5_1009	DJI_0725	Sempervivum ruthenicum Schnittsp. & C.B. Lehm.	0	n	1	0	Crassulaceae J.St.-Hil.
Iz_5_1009	DJI_0725	Setaria viridis (L.) P. Beauv.	0	n	1	0	Poaceae Barnhart
Iz_5_1009	DJI_0725	Silene borysthenica (Gruner) Walters	0	n	1	0	Caryophyllaceae Juss.
Iz_5_1009	DJI_0725	Silene latifolia subsp. alba (Mill.) Greuter & Burdet	0	n	1	0	Caryophyllaceae Juss.
Iz_5_1009	DJI_0725	Solidago virgaurea L.	0	n	1	0	Asteraceae Bercht. & J.Presl
Iz_5_1009	DJI_0725	Solidago Ã niederederi (S. canadensis Ã S. virgaurea)	0	hybrid	0	0	Asteraceae Bercht. & J.Presl
Iz_5_1009	DJI_0725	Sorbus aucuparia L.	0	n	1	1	Rosaceae Juss.
Iz_5_1009	DJI_0725	Stachys recta L.	0	n	1	0	Lamiacea Lindl.
Iz_5_1009	DJI_0725	Tanacetum vulgare L.	0	n	1	0	Asteraceae Bercht. & J.Presl
Iz_5_1009	DJI_0725	Thymus pallasianus Heinr.Braun	0	n	1	0	Lamiacea Lindl.
Iz_5_1009	DJI_0725	Tilia cordata Mill.	0	n	1	1	Malvaceae Juss.
Iz_5_1009	DJI_0725	Trifolium alpestre L.	0	n	1	0	Fabaceae Lindl.
Iz_5_1009	DJI_0725	Trifolium arvense L.	0	n	1	0	Fabaceae Lindl.
Iz_5_1009	DJI_0725	Ulmus glabra Huds.	0	n	1	1	Ulmaceae Mirb.
Iz_5_1009	DJI_0725	Verbascum densiflorum Bertol.	0	n	1	0	Scrophulariaceae Juss.
Iz_5_1009	DJI_0725	Veronica austriaca L.	0	n	1	0	Plantaginaceae Juss.
Iz_5_1009	DJI_0725	Veronica incana L.	0	n	1	0	Plantaginaceae Juss.
Iz_5_1009	DJI_0725	Veronica verna L.	0	n	1	0	Plantaginaceae Juss.
Iz_5_1009	DJI_0725	Vincetoxicum hirundinaria Medik	0	n	1	0	Apocynaceae Juss.
Iz_5_1009	DJI_0731	Acer tataricum L.	0	n	1	1	Sapindaceae Juss.
Iz_5_1009	DJI_0731	Achillea millefolium L. s.l.	0	n	1	0	Asteraceae Bercht. & J.Presl
Iz_5_1009	DJI_0731	Allium oleraceum L.	0	n	1	0	Amaryllidaceae J.St.-Hil.
Iz_5_1009	DJI_0731	Ambrosia artemisiifolia L.	0	a	0	0	Asteraceae Bercht. & J.Presl
Iz_5_1009	DJI_0731	Amorpha fruticosa L.	0	a	0	0	Fabaceae Lindl.
Iz_5_1009	DJI_0731	Anthericum ramosum L.	0	n	1	0	Asparagaceae Juss.
Iz_5_1009	DJI_0731	Artemisia marschalliana Spreng.	0	n	1	0	Asteraceae Bercht. & J.Presl
Iz_5_1009	DJI_0731	Asparagus officinalis L.	0	n	1	0	Asparagaceae Juss.
Iz_5_1009	DJI_0731	Astragalus cicer L.	0	n	1	0	Fabaceae Lindl.
Iz_5_1009	DJI_0731	Bassia laniflora (S.G.Gmel.) A.J.Scott	0	n	1	0	Amaranthaceae Juss.
Iz_5_1009	DJI_0731	Berberis vulgaris L.	0	n	1	0	Berberidaceae Juss.
Iz_5_1009	DJI_0731	Betula pendula Roth.	0	n	1	1	Betulaceae Gray
Iz_5_1009	DJI_0731	Betula pendula Roth.	0	n	1	0	Poaceae Barnhart
Iz_5_1009	DJI_0731	Bromus inermis Leyss.	0		NA	NA	
Iz_5_1009	DJI_0731	Bromus squarrosus L.	0	a	0	0	Poaceae Barnhart
Iz_5_1009	DJI_0731	Bromus tectorum L.	0	a	0	0	Poaceae Barnhart
Iz_5_1009	DJI_0731	Calamagrostis epigejos (L.) Roth	60	n	1	0	Poaceae Barnhart
Iz_5_1009	DJI_0731	Carduus acanthoides L.	0	n	1	0	Asteraceae Bercht. & J.Presl
Iz_5_1009	DJI_0731	Carex sp.	0	n	1	0	Cyperaceae Juss.
Iz_5_1009	DJI_0731	Centaurea majorovii Dumbadze	0	n	1	0	Asteraceae Bercht. & J.Presl
Iz_5_1009	DJI_0731	Chamaecytisus ruthenicus (Fisch.ex Wol.) Klaskova	0	n	1	0	Fabaceae Lindl.
Iz_5_1009	DJI_0731	Chelidonium majus L.	0	n	1	0	Papaveraceae Juss.
Iz_5_1009	DJI_0731	Chenopodium betaceum Andrz.	0	n	1	0	Amaranthaceae Juss.
Iz_5_1009	DJI_0731	Chenopodium cf. album	0	n	1	0	Amaranthaceae Juss.
Iz_5_1009	DJI_0731	Chenopodium sp.	0		NA	0	Amaranthaceae Juss.
Iz_5_1009	DJI_0731	Chondrilla juncea L.	0	n	1	0	Asteraceae Bercht. & J.Presl
Iz_5_1009	DJI_0731	Cirsium vulgare (Savi) Ten.	0	n	1	0	Asteraceae Bercht. & J.Presl
Iz_5_1009	DJI_0731	Clinopodium acinos (L.) Kuntze	0	n	1	0	Lamiacea Lindl.
Iz_5_1009	DJI_0731	Convallaria majalis L.	0	n	1	0	Asparagaceae Juss.
Iz_5_1009	DJI_0731	Crataegus rhipidophylla Gand.	0	n	1	1	Rosaceae Juss.
Iz_5_1009	DJI_0731	Crepis foetida L.	0	n	1	0	Asteraceae Bercht. & J.Presl
Iz_5_1009	DJI_0731	Dactylis glomerata L.	0	n	1	0	Poaceae Barnhart
Iz_5_1009	DJI_0731	Dianthus campestris M.Bieb.	0	n	1	0	Caryophyllaceae Juss.
Iz_5_1009	DJI_0731	Digitaria sanguinalis (L.) Scop.	0	a	0	0	Poaceae Barnhart
Iz_5_1009	DJI_0731	Elymus repens (L.) Gould	0	n	1	0	Poaceae Barnhart
Iz_5_1009	DJI_0731	Epilobium tetragonum L.	0	n	1	0	Onagraceae Juss.
Iz_5_1009	DJI_0731	Eragrostis minor Host	0	a	0	0	Poaceae Barnhart
Iz_5_1009	DJI_0731	Erigeron canadensis L.	0	a	0	0	Asteraceae Bercht. & J.Presl
Iz_5_1009	DJI_0731	Euphorbia seguieriana Neck.	0	n	1	0	Euphorbiaceae Juss.
Iz_5_1009	DJI_0731	Fallopia dumetorum (L.) Holub	0	n	1	0	Polygonaceae Juss.
Iz_5_1009	DJI_0731	Festuca beckeri (Hack.) Trautv.	0	n	1	0	Poaceae Barnhart
Iz_5_1009	DJI_0731	Fragaria vesca L.	0	n	1	0	Rosaceae Juss.
Iz_5_1009	DJI_0731	Frangula alnus Mill.	0	n	1	1	Rhamnaceae Juss.
Iz_5_1009	DJI_0731	Fraxinus pennsylvanica Marshall	0	a	0	1	Oleaceae Hoffmanns. & Link
Iz_5_1009	DJI_0731	Galeopsis bifida Boenn.	0	n	1	0	Lamiacea Lindl.
Iz_5_1009	DJI_0731	Galium verum L.	0	n	1	0	Rubiaceae Juss.
Iz_5_1009	DJI_0731	Genista tinctoria L.	0	n	1	0	Fabaceae Lindl.
Iz_5_1009	DJI_0731	Geranium robertianum L.	0	n	1	0	Geraniaceae Juss.
Iz_5_1009	DJI_0731	Geranium sanquineum L.	0	n	1	0	Geraniaceae Juss.
Iz_5_1009	DJI_0731	Gypsophila paniculata L.	0	n	1	0	Caryophyllaceae Juss.
Iz_5_1009	DJI_0731	Helichrysum arenarium (L.) Moench	0	n	1	0	Asteraceae Bercht. & J.Presl
Iz_5_1009	DJI_0731	Herniaria polygama J.Gay	0	n	1	0	Caryophyllaceae Juss.
Iz_5_1009	DJI_0731	Hieracium robustum Fr.	0	n	1	0	Asteraceae Bercht. & J.Presl
Iz_5_1009	DJI_0731	Hieracium umbellatum L.	0	n	1	NA	Asteraceae Bercht. & J.Presl
Iz_5_1009	DJI_0731	Hylotelephium maximum (L.) Holub	0	n	1	0	Crassulaceae J.St.-Hil.
Iz_5_1009	DJI_0731	Jacobaea borysthenica (DC.) B.Nord. & Greuter	0	n	1	0	Asteraceae Bercht. & J.Presl
Iz_5_1009	DJI_0731	Jasione montana L.	0	n	1	0	Campanulaceae Juss.
Iz_5_1009	DJI_0731	Jurinea cyanoides (L.) Rchb.s.l.	0	n	1	0	Asteraceae Bercht. & J.Presl
Iz_5_1009	DJI_0731	Koeleria glauca (Spreng.) DC.	0	n	1	0	Poaceae Barnhart
Iz_5_1009	DJI_0731	Lactuca serriola L.	0	a	0	0	Asteraceae Bercht. & J.Presl
Iz_5_1009	DJI_0731	Linaria genistifolia (L.) Mill.	0	n	1	0	Plantaginaceae Juss.
Iz_5_1009	DJI_0731	Lomelosia argentea (L.) Greuter & Burdet	0	n	1	0	Caprifoliaceae Juss.
Iz_5_1009	DJI_0731	Melampyrum pratense L.	0	n	1	0	Orobanchaceae Vent.
Iz_5_1009	DJI_0731	Oenothera biennis L.	0	a	0	0	Onagraceae Juss.
Iz_5_1009	DJI_0731	Pentanema britannica (L.) D.Gut.Larr., Santos-Vicente, Anderb., E.Rico & M.M.Mart.Ort.	0	n	1	0	Asteraceae Bercht. & J.Presl
Iz_5_1009	DJI_0731	Peucedanum oreoselinum (L.) Moench	2	n	1	0	Apiaceae Lindl.
Iz_5_1009	DJI_0731	Peucedanum ruthenicum M.Bieb.	0	n	1	0	Apiaceae Lindl.
Iz_5_1009	DJI_0731	Pilosella echioides (Lumn.) F.W.Schultz & Sch.Bip.	0	n	1	0	Asteraceae Bercht. & J.Presl
Iz_5_1009	DJI_0731	Pilosella officinarum F. Schultz et Sch. Bip.	0	n	1	0	Asteraceae Bercht. & J.Presl
Iz_5_1009	DJI_0731	Pilosella sp.	0		NA	0	Asteraceae Bercht. & J.Presl
Iz_5_1009	DJI_0731	Pinus sylvestris L.	0	n	1	1	Pinaceae Spreng. ex F.Rudolphi

Iz_5_1009	DJI_0731	Poa annua L.	0	n	1	0	Poaceae Barnhart
Iz_5_1009	DJI_0731	Poa nemoralis L.	0	n	1	0	Poaceae Barnhart
Iz_5_1009	DJI_0731	Poa sp.	0		NA	0	Poaceae Barnhart
Iz_5_1009	DJI_0731	Polygonatum odoratum (Mill.) Druce	3	n	1	0	Asparagaceae Juss.
Iz_5_1009	DJI_0731	Polygonum arenarium Waldst. & Kit.	0	n	1	0	Polygonaceae Juss.
Iz_5_1009	DJI_0731	Populus nigra L.	0	n	1	1	Salicaceae Mirb.
Iz_5_1009	DJI_0731	Populus sp.	0	n	1	1	Salicaceae Mirb.
Iz_5_1009	DJI_0731	Populus tremula L.	0	n	1	1	Salicaceae Mirb.
Iz_5_1009	DJI_0731	Populus Ã canescens (Aiton) Sm.	0	n	1	1	Salicaceae Mirb.
Iz_5_1009	DJI_0731	Potentilla argentea L.	0	n	1	0	Rosaceae Juss.
Iz_5_1009	DJI_0731	Psammophiliella muralis (L.) Ikonn.	0	n	1	0	Caryophyllaceae Juss.
Iz_5_1009	DJI_0731	Psephellus sumensis (Kalen.) Greuter	0	n	1	0	Asteraceae Bercht. & J.Presl
Iz_5_1009	DJI_0731	Pulsatilla pratensis (L.) Mill. s.l.	0	n	1	0	Ranunculaceae Juss.
Iz_5_1009	DJI_0731	Pyrus communis L.	0	a	0	1	Rosaceae Juss.
Iz_5_1009	DJI_0731	Quercus robur L.	0	n	1	1	Fagaceae Dumort.
Iz_5_1009	DJI_0731	Robinia pseudoacacia L.	0	a	0	1	Fabaceae Lindl.
Iz_5_1009	DJI_0731	Rosa sp.	0		NA	1	Rosaceae Juss.
Iz_5_1009	DJI_0731	Rubus idaeus L.	0	n	1	0	Rosaceae Juss.
Iz_5_1009	DJI_0731	Rumex acetosella L.	0	n	1	0	Polygonaceae Juss.
Iz_5_1009	DJI_0731	Rumex thyrsiflorus Fingerh.	0	n	1	0	Polygonaceae Juss.
Iz_5_1009	DJI_0731	Salix alba L.	0	n	1	1	Salicaceae Mirb.
Iz_5_1009	DJI_0731	Sambucus nigra L.	0	n	1	1	Viburnaceae Raf.
Iz_5_1009	DJI_0731	Secale sylvestre Host	0	n	1	0	Poaceae Barnhart
Iz_5_1009	DJI_0731	Sempervivum ruthenicum Schnittsp. & C.B. Lehm.	0	n	1	0	Crassulaceae J.St.-Hil.
Iz_5_1009	DJI_0731	Setaria viridis (L.) P. Beauv.	0	n	1	0	Poaceae Barnhart
Iz_5_1009	DJI_0731	Silene borysthenica (Gruner) Walters	0	n	1	0	Caryophyllaceae Juss.
Iz_5_1009	DJI_0731	Silene latifolia subsp. alba (Mill.) Greuter & Burdet	0	n	1	0	Caryophyllaceae Juss.
Iz_5_1009	DJI_0731	Solidago virgaurea L.	0	n	1	0	Asteraceae Bercht. & J.Presl
Iz_5_1009	DJI_0731	Solidago Ã niederederi (S. canadensis Ã S. virgaurea)	0	hybrid	0	0	Asteraceae Bercht. & J.Presl
Iz_5_1009	DJI_0731	Sorbus aucuparia L.	0	n	1	1	Rosaceae Juss.
Iz_5_1009	DJI_0731	Stachys recta L.	0	n	1	0	Lamiacea Lindl.
Iz_5_1009	DJI_0731	Tanacetum vulgare L.	0	n	1	0	Asteraceae Bercht. & J.Presl
Iz_5_1009	DJI_0731	Thymus pallasianus Heinr.Braun	0	n	1	0	Lamiacea Lindl.
Iz_5_1009	DJI_0731	Tilia cordata Mill.	0	n	1	1	Malvaceae Juss.
Iz_5_1009	DJI_0731	Trifolium alpestre L.	0	n	1	0	Fabaceae Lindl.
Iz_5_1009	DJI_0731	Trifolium arvense L.	0	n	1	0	Fabaceae Lindl.
Iz_5_1009	DJI_0731	Ulmus glabra Huds.	0	n	1	1	Ulmaceae Mirb.
Iz_5_1009	DJI_0731	Verbascum densiflorum Bertol.	0	n	1	0	Scrophulariaceae Juss.
Iz_5_1009	DJI_0731	Veronica austriaca L.	0	n	1	0	Plantaginaceae Juss.
Iz_5_1009	DJI_0731	Veronica incana L.	0	n	1	0	Plantaginaceae Juss.
Iz_5_1009	DJI_0731	Veronica verna L.	0	n	1	0	Plantaginaceae Juss.
Iz_5_1009	DJI_0731	Vincetoxicum hirundinaria Medik	0	n	1	0	Apocynaceae Juss.
Iz_5_1009	DJI_0735	Acer tataricum L.	0	n	1	1	Sapindaceae Juss.
Iz_5_1009	DJI_0735	Achillea millefolium L. s.l.	0	n	1	0	Asteraceae Bercht. & J.Presl
Iz_5_1009	DJI_0735	Allium oleraceum L.	0	n	1	0	Amaryllidaceae J.St.-Hil.
Iz_5_1009	DJI_0735	Ambrosia artemisiifolia L.	0	a	0	0	Asteraceae Bercht. & J.Presl
Iz_5_1009	DJI_0735	Amorpha fruticosa L.	0	a	0	0	Fabaceae Lindl.
Iz_5_1009	DJI_0735	Anthericum ramosum L.	0	n	1	0	Asparagaceae Juss.
Iz_5_1009	DJI_0735	Artemisia marschalliana Spreng.	0	n	1	0	Asteraceae Bercht. & J.Presl
Iz_5_1009	DJI_0735	Asparagus officinalis L.	0	n	1	0	Asparagaceae Juss.
Iz_5_1009	DJI_0735	Astragalus cicer L.	0	n	1	0	Fabaceae Lindl.
Iz_5_1009	DJI_0735	Bassia laniflora (S.G.Gmel.) A.J.Scott	0	n	1	0	Amaranthaceae Juss.
Iz_5_1009	DJI_0735	Berberis vulgaris L.	0	n	1	0	Berberidaceae Juss.
Iz_5_1009	DJI_0735	Betula pendula Roth.	0	n	1	1	Betulaceae Gray
Iz_5_1009	DJI_0735	Betula pendula Roth.	0	n	1	0	Poaceae Barnhart
Iz_5_1009	DJI_0735	Bromus inermis Leyss.	0		NA	NA	
Iz_5_1009	DJI_0735	Bromus squarrosus L.	0	a	0	0	Poaceae Barnhart
Iz_5_1009	DJI_0735	Bromus tectorum L.	0	a	0	0	Poaceae Barnhart
Iz_5_1009	DJI_0735	Calamagrostis epigejos (L.) Roth	35	n	1	0	Poaceae Barnhart
Iz_5_1009	DJI_0735	Carduus acanthoides L.	0	n	1	0	Asteraceae Bercht. & J.Presl
Iz_5_1009	DJI_0735	Carex sp.	0	n	1	0	Cyperaceae Juss.
Iz_5_1009	DJI_0735	Centaurea majorovii Dumbadze	0	n	1	0	Asteraceae Bercht. & J.Presl
Iz_5_1009	DJI_0735	Chamaecytisus ruthenicus (Fisch.ex Wol.) Klaskova	0	n	1	0	Fabaceae Lindl.
Iz_5_1009	DJI_0735	Chelidonium majus L.	0	n	1	0	Papaveraceae Juss.
Iz_5_1009	DJI_0735	Chenopodium betaceum Andrz.	0	n	1	0	Amaranthaceae Juss.
Iz_5_1009	DJI_0735	Chenopodium cf. album	0	n	1	0	Amaranthaceae Juss.
Iz_5_1009	DJI_0735	Chenopodium sp.	0		NA	0	Amaranthaceae Juss.
Iz_5_1009	DJI_0735	Chondrilla juncea L.	0	n	1	0	Asteraceae Bercht. & J.Presl
Iz_5_1009	DJI_0735	Cirsium vulgare (Savi) Ten.	0	n	1	0	Asteraceae Bercht. & J.Presl
Iz_5_1009	DJI_0735	Clinopodium acinos (L.) Kuntze	0	n	1	0	Lamiacea Lindl.
Iz_5_1009	DJI_0735	Convallaria majalis L.	0	n	1	0	Asparagaceae Juss.
Iz_5_1009	DJI_0735	Crataegus rhipidophylla Gand.	0	n	1	1	Rosaceae Juss.
Iz_5_1009	DJI_0735	Crepis foetida L.	0	n	1	0	Asteraceae Bercht. & J.Presl
Iz_5_1009	DJI_0735	Dactylis glomerata L.	0	n	1	0	Poaceae Barnhart
Iz_5_1009	DJI_0735	Dianthus campestris M.Bieb.	0	n	1	0	Caryophyllaceae Juss.
Iz_5_1009	DJI_0735	Digitaria sanguinalis (L.) Scop.	0	a	0	0	Poaceae Barnhart
Iz_5_1009	DJI_0735	Elymus repens (L.) Gould	0	n	1	0	Poaceae Barnhart
Iz_5_1009	DJI_0735	Epilobium tetragonum L.	0	n	1	0	Onagraceae Juss.
Iz_5_1009	DJI_0735	Eragrostis minor Host	0	a	0	0	Poaceae Barnhart
Iz_5_1009	DJI_0735	Erigeron canadensis L.	0	a	0	0	Asteraceae Bercht. & J.Presl
Iz_5_1009	DJI_0735	Euphorbia seguieriana Neck.	0	n	1	0	Euphorbiaceae Juss.
Iz_5_1009	DJI_0735	Fallopia dumetorum (L.) Holub	0	n	1	0	Polygonaceae Juss.
Iz_5_1009	DJI_0735	Festuca beckeri (Hack.) Trautv.	0	n	1	0	Poaceae Barnhart
Iz_5_1009	DJI_0735	Fragaria vesca L.	0	n	1	0	Rosaceae Juss.
Iz_5_1009	DJI_0735	Frangula alnus Mill.	0	n	1	1	Rhamnaceae Juss.
Iz_5_1009	DJI_0735	Fraxinus pennsylvanica Marshall	0	a	0	1	Oleaceae Hoffmanns. & Link
Iz_5_1009	DJI_0735	Galeopsis bifida Boenn.	0	n	1	0	Lamiacea Lindl.
Iz_5_1009	DJI_0735	Galium verum L.	0	n	1	0	Rubiaceae Juss.
Iz_5_1009	DJI_0735	Genista tinctoria L.	0	n	1	0	Fabaceae Lindl.
Iz_5_1009	DJI_0735	Geranium robertianum L.	0	n	1	0	Geraniaceae Juss.
Iz_5_1009	DJI_0735	Geranium sanquineum L.	0	n	1	0	Geraniaceae Juss.
Iz_5_1009	DJI_0735	Gypsophila paniculata L.	0	n	1	0	Caryophyllaceae Juss.
Iz_5_1009	DJI_0735	Helichrysum arenarium (L.) Moench	0	n	1	0	Asteraceae Bercht. & J.Presl
Iz_5_1009	DJI_0735	Herniaria polygama J.Gay	0	n	1	0	Caryophyllaceae Juss.
Iz_5_1009	DJI_0735	Hieracium robustum Fr.	0	n	1	0	Asteraceae Bercht. & J.Presl
Iz_5_1009	DJI_0735	Hieracium umbellatum L.	0	n	1	NA	Asteraceae Bercht. & J.Presl
Iz_5_1009	DJI_0735	Hylotelephium maximum (L.) Holub	0	n	1	0	Crassulaceae J.St.-Hil.
Iz_5_1009	DJI_0735	Jacobaea borysthenica (DC.) B.Nord. & Greuter	0	n	1	0	Asteraceae Bercht. & J.Presl

Iz_5_1009	DJI_0735	Jasione montana L.	0	n	1	0	Campanulaceae Juss.
Iz_5_1009	DJI_0735	Jurinea cyanoides (L.) Rchb.s.l.	0	n	1	0	Asteraceae Bercht. & J.Presl
Iz_5_1009	DJI_0735	Koeleria glauca (Spreng.) DC.	0	n	1	0	Poaceae Barnhart
Iz_5_1009	DJI_0735	Lactuca serriola L.	0	a	0	0	Asteraceae Bercht. & J.Presl
Iz_5_1009	DJI_0735	Linaria genistifolia (L.) Mill.	0	n	1	0	Plantaginaceae Juss.
Iz_5_1009	DJI_0735	Lomelosia argentea (L.) Greuter & Burdet	0	n	1	0	Caprifoliaceae Juss.
Iz_5_1009	DJI_0735	Melampyrum pratense L.	0	n	1	0	Orobanchaceae Vent.
Iz_5_1009	DJI_0735	Oenothera biennis L.	0	a	0	0	Onagraceae Juss.
Iz_5_1009	DJI_0735	Pentanema britannica (L.) D.Gut.Larr., Santos-Vicente, Anderb., E.Rico & M.M.Mart.Ort.	0	n	1	0	Asteraceae Bercht. & J.Presl
Iz_5_1009	DJI_0735	Peucedanum oreoselinum (L.) Moench	0	n	1	0	Apiaceae Lindl.
Iz_5_1009	DJI_0735	Peucedanum ruthenicum M.Bieb.	0	n	1	0	Apiaceae Lindl.
Iz_5_1009	DJI_0735	Pilosella echioides (Lumn.) F.W.Schultz & Sch.Bip.	0	n	1	0	Asteraceae Bercht. & J.Presl
Iz_5_1009	DJI_0735	Pilosella officinarum F. Schultz et Sch. Bip.	0	n	1	0	Asteraceae Bercht. & J.Presl
Iz_5_1009	DJI_0735	Pilosella sp.	0		NA	0	Asteraceae Bercht. & J.Presl
Iz_5_1009	DJI_0735	Pinus sylvestris L.	0	n	1	1	Pinaceae Spreng. ex F.Rudolphi
Iz_5_1009	DJI_0735	Poa annua L.	0	n	1	0	Poaceae Barnhart
Iz_5_1009	DJI_0735	Poa nemoralis L.	0	n	1	0	Poaceae Barnhart
Iz_5_1009	DJI_0735	Poa sp.	0		NA	0	Poaceae Barnhart
Iz_5_1009	DJI_0735	Polygonatum odoratum (Mill.) Druce	0	n	1	0	Asparagaceae Juss.
Iz_5_1009	DJI_0735	Polygonum arenarium Waldst. & Kit.	0	n	1	0	Polygonaceae Juss.
Iz_5_1009	DJI_0735	Populus nigra L.	0	n	1	1	Salicaceae Mirb.
Iz_5_1009	DJI_0735	Populus sp.	0	n	1	1	Salicaceae Mirb.
Iz_5_1009	DJI_0735	Populus tremula L.	0	n	1	1	Salicaceae Mirb.
Iz_5_1009	DJI_0735	Populus Ã canescens (Aiton) Sm.	0	n	1	1	Salicaceae Mirb.
Iz_5_1009	DJI_0735	Potentilla argentea L.	0	n	1	0	Rosaceae Juss.
Iz_5_1009	DJI_0735	Psammophiliella muralis (L.) Ikonn.	0	n	1	0	Caryophyllaceae Juss.
Iz_5_1009	DJI_0735	Psephellus sumensis (Kalen.) Greuter	0	n	1	0	Asteraceae Bercht. & J.Presl
Iz_5_1009	DJI_0735	Pulsatilla pratensis (L.) Mill. s.l.	0	n	1	0	Ranunculaceae Juss.
Iz_5_1009	DJI_0735	Pyrus communis L.	0	a	0	1	Rosaceae Juss.
Iz_5_1009	DJI_0735	Quercus robur L.	0	n	1	1	Fagaceae Dumort.
Iz_5_1009	DJI_0735	Robinia pseudoacacia L.	0	a	0	1	Fabaceae Lindl.
Iz_5_1009	DJI_0735	Rosa sp.	0		NA	1	Rosaceae Juss.
Iz_5_1009	DJI_0735	Rubus idaeus L.	0	n	1	0	Rosaceae Juss.
Iz_5_1009	DJI_0735	Rumex acetosella L.	0	n	1	0	Polygonaceae Juss.
Iz_5_1009	DJI_0735	Rumex thyrsiflorus Fingerh.	0	n	1	0	Polygonaceae Juss.
Iz_5_1009	DJI_0735	Salix alba L.	0	n	1	1	Salicaceae Mirb.
Iz_5_1009	DJI_0735	Sambucus nigra L.	0	n	1	1	Viburnaceae Raf.
Iz_5_1009	DJI_0735	Secale sylvestre Host	0	n	1	0	Poaceae Barnhart
Iz_5_1009	DJI_0735	Sempervivum ruthenicum Schnittsp. & C.B. Lehm.	0	n	1	0	Crassulaceae J.St.-Hil.
Iz_5_1009	DJI_0735	Setaria viridis (L.) P. Beauv.	0	n	1	0	Poaceae Barnhart
Iz_5_1009	DJI_0735	Silene borysthenica (Gruner) Walters	0	n	1	0	Caryophyllaceae Juss.
Iz_5_1009	DJI_0735	Silene latifolia subsp. alba (Mill.) Greuter & Burdet	0	n	1	0	Caryophyllaceae Juss.
Iz_5_1009	DJI_0735	Solidago virgaurea L.	0	n	1	0	Asteraceae Bercht. & J.Presl
Iz_5_1009	DJI_0735	Solidago Ã niederederi (S. canadensis Ã S. virgaurea)	0	hybrid	0	0	Asteraceae Bercht. & J.Presl
Iz_5_1009	DJI_0735	Sorbus aucuparia L.	0	n	1	1	Rosaceae Juss.
Iz_5_1009	DJI_0735	Stachys recta L.	0	n	1	0	Lamiacea Lindl.
Iz_5_1009	DJI_0735	Tanacetum vulgare L.	0	n	1	0	Asteraceae Bercht. & J.Presl
Iz_5_1009	DJI_0735	Thymus pallasianus Heinr.Braun	0	n	1	0	Lamiacea Lindl.
Iz_5_1009	DJI_0735	Tilia cordata Mill.	0	n	1	1	Malvaceae Juss.
Iz_5_1009	DJI_0735	Trifolium alpestre L.	0	n	1	0	Fabaceae Lindl.
Iz_5_1009	DJI_0735	Trifolium arvense L.	0	n	1	0	Fabaceae Lindl.
Iz_5_1009	DJI_0735	Ulmus glabra Huds.	0	n	1	1	Ulmaceae Mirb.
Iz_5_1009	DJI_0735	Verbascum densiflorum Bertol.	0	n	1	0	Scrophulariaceae Juss.
Iz_5_1009	DJI_0735	Veronica austriaca L.	0	n	1	0	Plantaginaceae Juss.
Iz_5_1009	DJI_0735	Veronica incana L.	0	n	1	0	Plantaginaceae Juss.
Iz_5_1009	DJI_0735	Veronica verna L.	0	n	1	0	Plantaginaceae Juss.
Iz_5_1009	DJI_0735	Vincetoxicum hirundinaria Medik	0	n	1	0	Apocynaceae Juss.
Iz_5_1009	DJI_0756	Acer tataricum L.	0	n	1	1	Sapindaceae Juss.
Iz_5_1009	DJI_0756	Achillea millefolium L. s.l.	0	n	1	0	Asteraceae Bercht. & J.Presl
Iz_5_1009	DJI_0756	Allium oleraceum L.	0	n	1	0	Amaryllidaceae J.St.-Hil.
Iz_5_1009	DJI_0756	Ambrosia artemisiifolia L.	0	a	0	0	Asteraceae Bercht. & J.Presl
Iz_5_1009	DJI_0756	Amorpha fruticosa L.	0	a	0	0	Fabaceae Lindl.
Iz_5_1009	DJI_0756	Anthericum ramosum L.	0	n	1	0	Asparagaceae Juss.
Iz_5_1009	DJI_0756	Artemisia marschalliana Spreng.	0	n	1	0	Asteraceae Bercht. & J.Presl
Iz_5_1009	DJI_0756	Asparagus officinalis L.	0	n	1	0	Asparagaceae Juss.
Iz_5_1009	DJI_0756	Astragalus cicer L.	0	n	1	0	Fabaceae Lindl.
Iz_5_1009	DJI_0756	Bassia laniflora (S.G.Gmel.) A.J.Scott	0	n	1	0	Amaranthaceae Juss.
Iz_5_1009	DJI_0756	Berberis vulgaris L.	0	n	1	0	Berberidaceae Juss.
Iz_5_1009	DJI_0756	Betula pendula Roth.	0	n	1	1	Betulaceae Gray
Iz_5_1009	DJI_0756	Betula pendula Roth.	0	n	1	0	Poaceae Barnhart
Iz_5_1009	DJI_0756	Bromus inermis Leyss.	0		NA	NA	
Iz_5_1009	DJI_0756	Bromus squarrosus L.	0	a	0	0	Poaceae Barnhart
Iz_5_1009	DJI_0756	Bromus tectorum L.	0	a	0	0	Poaceae Barnhart
Iz_5_1009	DJI_0756	Calamagrostis epigejos (L.) Roth	3	n	1	0	Poaceae Barnhart
Iz_5_1009	DJI_0756	Carduus acanthoides L.	0	n	1	0	Asteraceae Bercht. & J.Presl
Iz_5_1009	DJI_0756	Carex sp.	0	n	1	0	Cyperaceae Juss.
Iz_5_1009	DJI_0756	Centaurea majorovii Dumbadze	0	n	1	0	Asteraceae Bercht. & J.Presl
Iz_5_1009	DJI_0756	Chamaecytisus ruthenicus (Fisch.ex Wol.) Klaskova	0	n	1	0	Fabaceae Lindl.
Iz_5_1009	DJI_0756	Chelidonium majus L.	0	n	1	0	Papaveraceae Juss.
Iz_5_1009	DJI_0756	Chenopodium betaceum Andrz.	0	n	1	0	Amaranthaceae Juss.
Iz_5_1009	DJI_0756	Chenopodium cf. album	0	n	1	0	Amaranthaceae Juss.
Iz_5_1009	DJI_0756	Chenopodium sp.	0		NA	0	Amaranthaceae Juss.
Iz_5_1009	DJI_0756	Chondrilla juncea L.	0	n	1	0	Asteraceae Bercht. & J.Presl
Iz_5_1009	DJI_0756	Cirsium vulgare (Savi) Ten.	0	n	1	0	Asteraceae Bercht. & J.Presl
Iz_5_1009	DJI_0756	Clinopodium acinos (L.) Kuntze	0	n	1	0	Lamiacea Lindl.
Iz_5_1009	DJI_0756	Convallaria majalis L.	0	n	1	0	Asparagaceae Juss.
Iz_5_1009	DJI_0756	Crataegus rhipidophylla Gand.	0	n	1	1	Rosaceae Juss.
Iz_5_1009	DJI_0756	Crepis foetida L.	0	n	1	0	Asteraceae Bercht. & J.Presl
Iz_5_1009	DJI_0756	Dactylis glomerata L.	0	n	1	0	Poaceae Barnhart
Iz_5_1009	DJI_0756	Dianthus campestris M.Bieb.	0	n	1	0	Caryophyllaceae Juss.
Iz_5_1009	DJI_0756	Digitaria sanguinalis (L.) Scop.	0	a	0	0	Poaceae Barnhart
Iz_5_1009	DJI_0756	Elymus repens (L.) Gould	0	n	1	0	Poaceae Barnhart
Iz_5_1009	DJI_0756	Epilobium tetragonum L.	0	n	1	0	Onagraceae Juss.
Iz_5_1009	DJI_0756	Eragrostis minor Host	0	a	0	0	Poaceae Barnhart
Iz_5_1009	DJI_0756	Erigeron canadensis L.	0.5	a	0	0	Asteraceae Bercht. & J.Presl
Iz_5_1009	DJI_0756	Euphorbia seguieriana Neck.	0	n	1	0	Euphorbiaceae Juss.
Iz_5_1009	DJI_0756	Fallopia dumetorum (L.) Holub	0	n	1	0	Polygonaceae Juss.
Iz_5_1009	DJI_0756	Festuca beckeri (Hack.) Trautv.	0	n	1	0	Poaceae Barnhart

Iz_5_1009	DJI_0756	Fragaria vesca L.	0	n	1	0	Rosaceae Juss.
Iz_5_1009	DJI_0756	Frangula alnus Mill.	0	n	1	1	Rhamnaceae Juss.
Iz_5_1009	DJI_0756	Fraxinus pennsylvanica Marshall	0	a	0	1	Oleaceae Hoffmanns. & Link
Iz_5_1009	DJI_0756	Galeopsis bifida Boenn.	0	n	1	0	Lamiacea Lindl.
Iz_5_1009	DJI_0756	Galium verum L.	0	n	1	0	Rubiaceae Juss.
Iz_5_1009	DJI_0756	Genista tinctoria L.	0	n	1	0	Fabaceae Lindl.
Iz_5_1009	DJI_0756	Geranium robertianum L.	0	n	1	0	Geraniaceae Juss.
Iz_5_1009	DJI_0756	Geranium sanquineum L.	0	n	1	0	Geraniaceae Juss.
Iz_5_1009	DJI_0756	Gypsophila paniculata L.	0	n	1	0	Caryophyllaceae Juss.
Iz_5_1009	DJI_0756	Helichrysum arenarium (L.) Moench	0	n	1	0	Asteraceae Bercht. & J.Presl
Iz_5_1009	DJI_0756	Herniaria polygama J.Gay	0	n	1	0	Caryophyllaceae Juss.
Iz_5_1009	DJI_0756	Hieracium robustum Fr.	0	n	1	0	Asteraceae Bercht. & J.Presl
Iz_5_1009	DJI_0756	Hieracium umbellatum L.	0	n	1	NA	Asteraceae Bercht. & J.Presl
Iz_5_1009	DJI_0756	Hylotelephium maximum (L.) Holub	0	n	1	0	Crassulaceae J.St.-Hil.
Iz_5_1009	DJI_0756	Jacobaea borysthenica (DC.) B.Nord. & Greuter	0	n	1	0	Asteraceae Bercht. & J.Presl
Iz_5_1009	DJI_0756	Jasione montana L.	0	n	1	0	Campanulaceae Juss.
Iz_5_1009	DJI_0756	Jurinea cyanoides (L.) Rchb.s.l.	0	n	1	0	Asteraceae Bercht. & J.Presl
Iz_5_1009	DJI_0756	Koeleria glauca (Spreng.) DC.	0	n	1	0	Poaceae Barnhart
Iz_5_1009	DJI_0756	Lactuca serriola L.	0	a	0	0	Asteraceae Bercht. & J.Presl
Iz_5_1009	DJI_0756	Linaria genistifolia (L.) Mill.	0	n	1	0	Plantaginaceae Juss.
Iz_5_1009	DJI_0756	Lomelosia argentea (L.) Greuter & Burdet	0	n	1	0	Caprifoliaceae Juss.
Iz_5_1009	DJI_0756	Melampyrum pratense L.	0	n	1	0	Orobanchaceae Vent.
Iz_5_1009	DJI_0756	Oenothera biennis L.	0	a	0	0	Onagraceae Juss.
Iz_5_1009	DJI_0756	Pentanema britannica (L.) D.Gut.Larr., Santos-Vicente, Anderb., E.Rico & M.M.Mart.Ort.	0	n	1	0	Asteraceae Bercht. & J.Presl
Iz_5_1009	DJI_0756	Peucedanum oreoselinum (L.) Moench	0	n	1	0	Apiaceae Lindl.
Iz_5_1009	DJI_0756	Peucedanum ruthenicum M.Bieb.	0	n	1	0	Apiaceae Lindl.
Iz_5_1009	DJI_0756	Pilosella echioides (Lumn.) F.W.Schultz & Sch.Bip.	0	n	1	0	Asteraceae Bercht. & J.Presl
Iz_5_1009	DJI_0756	Pilosella officinarum F. Schultz et Sch. Bip.	0	n	1	0	Asteraceae Bercht. & J.Presl
Iz_5_1009	DJI_0756	Pilosella sp.	0		NA	0	Asteraceae Bercht. & J.Presl
Iz_5_1009	DJI_0756	Pinus sylvestris L.	0	n	1	1	Pinaceae Spreng. ex F.Rudolphi
Iz_5_1009	DJI_0756	Poa annua L.	0	n	1	0	Poaceae Barnhart
Iz_5_1009	DJI_0756	Poa nemoralis L.	0	n	1	0	Poaceae Barnhart
Iz_5_1009	DJI_0756	Poa sp.	0		NA	0	Poaceae Barnhart
Iz_5_1009	DJI_0756	Polygonatum odoratum (Mill.) Druce	0	n	1	0	Asparagaceae Juss.
Iz_5_1009	DJI_0756	Polygonum arenarium Waldst. & Kit.	0	n	1	0	Polygonaceae Juss.
Iz_5_1009	DJI_0756	Populus nigra L.	0	n	1	1	Salicaceae Mirb.
Iz_5_1009	DJI_0756	Populus sp.	0	n	1	1	Salicaceae Mirb.
Iz_5_1009	DJI_0756	Populus tremula L.	0	n	1	1	Salicaceae Mirb.
Iz_5_1009	DJI_0756	Populus Ã canescens (Aiton) Sm.	0	n	1	1	Salicaceae Mirb.
Iz_5_1009	DJI_0756	Potentilla argentea L.	0	n	1	0	Rosaceae Juss.
Iz_5_1009	DJI_0756	Psammophiliella muralis (L.) Ikonn.	0	n	1	0	Caryophyllaceae Juss.
Iz_5_1009	DJI_0756	Psephellus sumensis (Kalen.) Greuter	0	n	1	0	Asteraceae Bercht. & J.Presl
Iz_5_1009	DJI_0756	Pulsatilla pratensis (L.) Mill. s.l.	0	n	1	0	Ranunculaceae Juss.
Iz_5_1009	DJI_0756	Pyrus communis L.	0	a	0	1	Rosaceae Juss.
Iz_5_1009	DJI_0756	Quercus robur L.	0	n	1	1	Fagaceae Dumort.
Iz_5_1009	DJI_0756	Robinia pseudoacacia L.	0	a	0	1	Fabaceae Lindl.
Iz_5_1009	DJI_0756	Rosa sp.	0		NA	1	Rosaceae Juss.
Iz_5_1009	DJI_0756	Rubus idaeus L.	0	n	1	0	Rosaceae Juss.
Iz_5_1009	DJI_0756	Rumex acetosella L.	0	n	1	0	Polygonaceae Juss.
Iz_5_1009	DJI_0756	Rumex thyrsiflorus Fingerh.	0	n	1	0	Polygonaceae Juss.
Iz_5_1009	DJI_0756	Salix alba L.	0	n	1	1	Salicaceae Mirb.
Iz_5_1009	DJI_0756	Sambucus nigra L.	0	n	1	1	Viburnaceae Raf.
Iz_5_1009	DJI_0756	Secale sylvestre Host	0	n	1	0	Poaceae Barnhart
Iz_5_1009	DJI_0756	Sempervivum ruthenicum Schnittsp. & C.B. Lehm.	0	n	1	0	Crassulaceae J.St.-Hil.
Iz_5_1009	DJI_0756	Setaria viridis (L.) P. Beauv.	0	n	1	0	Poaceae Barnhart
Iz_5_1009	DJI_0756	Silene borysthenica (Gruner) Walters	0	n	1	0	Caryophyllaceae Juss.
Iz_5_1009	DJI_0756	Silene latifolia subsp. alba (Mill.) Greuter & Burdet	0	n	1	0	Caryophyllaceae Juss.
Iz_5_1009	DJI_0756	Solidago virgaurea L.	0.5	n	1	0	Asteraceae Bercht. & J.Presl
Iz_5_1009	DJI_0756	Solidago Ã niederederi (S. canadensis Ã S. virgaurea)	0	hybrid	0	0	Asteraceae Bercht. & J.Presl
Iz_5_1009	DJI_0756	Sorbus aucuparia L.	0	n	1	1	Rosaceae Juss.
Iz_5_1009	DJI_0756	Stachys recta L.	0	n	1	0	Lamiacea Lindl.
Iz_5_1009	DJI_0756	Tanacetum vulgare L.	0	n	1	0	Asteraceae Bercht. & J.Presl
Iz_5_1009	DJI_0756	Thymus pallasianus Heinr.Braun	0	n	1	0	Lamiacea Lindl.
Iz_5_1009	DJI_0756	Tilia cordata Mill.	0	n	1	1	Malvaceae Juss.
Iz_5_1009	DJI_0756	Trifolium alpestre L.	0	n	1	0	Fabaceae Lindl.
Iz_5_1009	DJI_0756	Trifolium arvense L.	0	n	1	0	Fabaceae Lindl.
Iz_5_1009	DJI_0756	Ulmus glabra Huds.	0	n	1	1	Ulmaceae Mirb.
Iz_5_1009	DJI_0756	Verbascum densiflorum Bertol.	0	n	1	0	Scrophulariaceae Juss.
Iz_5_1009	DJI_0756	Veronica austriaca L.	0	n	1	0	Plantaginaceae Juss.
Iz_5_1009	DJI_0756	Veronica incana L.	0	n	1	0	Plantaginaceae Juss.
Iz_5_1009	DJI_0756	Veronica verna L.	0	n	1	0	Plantaginaceae Juss.
Iz_5_1009	DJI_0756	Vincetoxicum hirundinaria Medik	0	n	1	0	Apocynaceae Juss.
Iz_5_1009	DJI_0761	Acer tataricum L.	0	n	1	1	Sapindaceae Juss.
Iz_5_1009	DJI_0761	Achillea millefolium L. s.l.	0	n	1	0	Asteraceae Bercht. & J.Presl
Iz_5_1009	DJI_0761	Allium oleraceum L.	0	n	1	0	Amaryllidaceae J.St.-Hil.
Iz_5_1009	DJI_0761	Ambrosia artemisiifolia L.	0	a	0	0	Asteraceae Bercht. & J.Presl
Iz_5_1009	DJI_0761	Amorpha fruticosa L.	0	a	0	0	Fabaceae Lindl.
Iz_5_1009	DJI_0761	Anthericum ramosum L.	0	n	1	0	Asparagaceae Juss.
Iz_5_1009	DJI_0761	Artemisia marschalliana Spreng.	0	n	1	0	Asteraceae Bercht. & J.Presl
Iz_5_1009	DJI_0761	Asparagus officinalis L.	0	n	1	0	Asparagaceae Juss.
Iz_5_1009	DJI_0761	Astragalus cicer L.	0	n	1	0	Fabaceae Lindl.
Iz_5_1009	DJI_0761	Bassia laniflora (S.G.Gmel.) A.J.Scott	0	n	1	0	Amaranthaceae Juss.
Iz_5_1009	DJI_0761	Berberis vulgaris L.	0	n	1	0	Berberidaceae Juss.
Iz_5_1009	DJI_0761	Betula pendula Roth.	0	n	1	1	Betulaceae Gray
Iz_5_1009	DJI_0761	Betula pendula Roth.	0	n	1	0	Poaceae Barnhart
Iz_5_1009	DJI_0761	Bromus inermis Leyss.	0		NA	NA	
Iz_5_1009	DJI_0761	Bromus squarrosus L.	0	a	0	0	Poaceae Barnhart
Iz_5_1009	DJI_0761	Bromus tectorum L.	0	a	0	0	Poaceae Barnhart
Iz_5_1009	DJI_0761	Calamagrostis epigejos (L.) Roth	8	n	1	0	Poaceae Barnhart
Iz_5_1009	DJI_0761	Carduus acanthoides L.	0	n	1	0	Asteraceae Bercht. & J.Presl
Iz_5_1009	DJI_0761	Carex sp.	0	n	1	0	Cyperaceae Juss.
Iz_5_1009	DJI_0761	Centaurea majorovii Dumbadze	0	n	1	0	Asteraceae Bercht. & J.Presl
Iz_5_1009	DJI_0761	Chamaecytisus ruthenicus (Fisch.ex Wol.) Klaskova	0	n	1	0	Fabaceae Lindl.
Iz_5_1009	DJI_0761	Chelidonium majus L.	0	n	1	0	Papaveraceae Juss.
Iz_5_1009	DJI_0761	Chenopodium betaceum Andrz.	0	n	1	0	Amaranthaceae Juss.
Iz_5_1009	DJI_0761	Chenopodium cf. album	0	n	1	0	Amaranthaceae Juss.
Iz_5_1009	DJI_0761	Chenopodium sp.	0		NA	0	Amaranthaceae Juss.
Iz_5_1009	DJI_0761	Chondrilla juncea L.	0	n	1	0	Asteraceae Bercht. & J.Presl

Iz_5_1009	DJI_0761	Cirsium vulgare (Savi) Ten.	0	n	1	0	Asteraceae Bercht. & J.Presl
Iz_5_1009	DJI_0761	Clinopodium acinos (L.) Kuntze	0	n	1	0	Lamiacea Lindl.
Iz_5_1009	DJI_0761	Convallaria majalis L.	0	n	1	0	Asparagaceae Juss.
Iz_5_1009	DJI_0761	Crataegus rhipidophylla Gand.	0	n	1	1	Rosaceae Juss.
Iz_5_1009	DJI_0761	Crepis foetida L.	0	n	1	0	Asteraceae Bercht. & J.Presl
Iz_5_1009	DJI_0761	Dactylis glomerata L.	0	n	1	0	Poaceae Barnhart
Iz_5_1009	DJI_0761	Dianthus campestris M.Bieb.	0	n	1	0	Caryophyllaceae Juss.
Iz_5_1009	DJI_0761	Digitaria sanguinalis (L.) Scop.	0	a	0	0	Poaceae Barnhart
Iz_5_1009	DJI_0761	Elymus repens (L.) Gould	0	n	1	0	Poaceae Barnhart
Iz_5_1009	DJI_0761	Epilobium tetragonum L.	0	n	1	0	Onagraceae Juss.
Iz_5_1009	DJI_0761	Eragrostis minor Host	0	a	0	0	Poaceae Barnhart
Iz_5_1009	DJI_0761	Erigeron canadensis L.	0	a	0	0	Asteraceae Bercht. & J.Presl
Iz_5_1009	DJI_0761	Euphorbia seguieriana Neck.	0	n	1	0	Euphorbiaceae Juss.
Iz_5_1009	DJI_0761	Fallopia dumetorum (L.) Holub	0	n	1	0	Polygonaceae Juss.
Iz_5_1009	DJI_0761	Festuca beckeri (Hack.) Trautv.	0	n	1	0	Poaceae Barnhart
Iz_5_1009	DJI_0761	Fragaria vesca L.	0	n	1	0	Rosaceae Juss.
Iz_5_1009	DJI_0761	Frangula alnus Mill.	0	n	1	1	Rhamnaceae Juss.
Iz_5_1009	DJI_0761	Fraxinus pennsylvanica Marshall	0	a	0	1	Oleaceae Hoffmanns. & Link
Iz_5_1009	DJI_0761	Galeopsis bifida Boenn.	0	n	1	0	Lamiacea Lindl.
Iz_5_1009	DJI_0761	Galium verum L.	0	n	1	0	Rubiaceae Juss.
Iz_5_1009	DJI_0761	Genista tinctoria L.	0	n	1	0	Fabaceae Lindl.
Iz_5_1009	DJI_0761	Geranium robertianum L.	0	n	1	0	Geraniaceae Juss.
Iz_5_1009	DJI_0761	Geranium sanguineum L.	0	n	1	0	Geraniaceae Juss.
Iz_5_1009	DJI_0761	Gypsophila paniculata L.	0	n	1	0	Caryophyllaceae Juss.
Iz_5_1009	DJI_0761	Helichrysum arenarium (L.) Moench	0	n	1	0	Asteraceae Bercht. & J.Presl
Iz_5_1009	DJI_0761	Herniaria polygama J.Gay	0	n	1	0	Caryophyllaceae Juss.
Iz_5_1009	DJI_0761	Hieracium robustum Fr.	0	n	1	0	Asteraceae Bercht. & J.Presl
Iz_5_1009	DJI_0761	Hieracium umbellatum L.	0	n	1	NA	Asteraceae Bercht. & J.Presl
Iz_5_1009	DJI_0761	Hylotelephium maximum (L.) Holub	0	n	1	0	Crassulaceae J.St.-Hil.
Iz_5_1009	DJI_0761	Jacobaea borysthenica (DC.) B.Nord. & Greuter	0	n	1	0	Asteraceae Bercht. & J.Presl
Iz_5_1009	DJI_0761	Jasione montana L.	0	n	1	0	Campanulaceae Juss.
Iz_5_1009	DJI_0761	Jurinea cyanoides (L.) Rchb.s.l.	0	n	1	0	Asteraceae Bercht. & J.Presl
Iz_5_1009	DJI_0761	Koeleria glauca (Spreng.) DC.	0	n	1	0	Poaceae Barnhart
Iz_5_1009	DJI_0761	Lactuca serriola L.	0	a	0	0	Asteraceae Bercht. & J.Presl
Iz_5_1009	DJI_0761	Linaria genistifolia (L.) Mill.	0	n	1	0	Plantaginaceae Juss.
Iz_5_1009	DJI_0761	Lomelosia argentea (L.) Greuter & Burdet	0	n	1	0	Caprifoliaceae Juss.
Iz_5_1009	DJI_0761	Melampyrum pratense L.	0	n	1	0	Orobanchaceae Vent.
Iz_5_1009	DJI_0761	Oenothera biennis L.	0	a	0	0	Onagraceae Juss.
Iz_5_1009	DJI_0761	Pentanema britannica (L.) D.Gut.Larr., Santos-Vicente, Anderb., E.Rico & M.M.Mart.Ort.	0	n	1	0	Asteraceae Bercht. & J.Presl
Iz_5_1009	DJI_0761	Peucedanum oreoselinum (L.) Moench	0	n	1	0	Apiaceae Lindl.
Iz_5_1009	DJI_0761	Peucedanum ruthenicum M.Bieb.	0	n	1	0	Apiaceae Lindl.
Iz_5_1009	DJI_0761	Pilosella echioides (Lumn.) F.W.Schultz & Sch.Bip.	0	n	1	0	Asteraceae Bercht. & J.Presl
Iz_5_1009	DJI_0761	Pilosella officinarum F. Schultz et Sch. Bip.	0	n	1	0	Asteraceae Bercht. & J.Presl
Iz_5_1009	DJI_0761	Pilosella sp.	0		NA	0	Asteraceae Bercht. & J.Presl
Iz_5_1009	DJI_0761	Pinus sylvestris L.	0	n	1	1	Pinaceae Spreng. ex F.Rudolphi
Iz_5_1009	DJI_0761	Poa annua L.	0	n	1	0	Poaceae Barnhart
Iz_5_1009	DJI_0761	Poa nemoralis L.	0	n	1	0	Poaceae Barnhart
Iz_5_1009	DJI_0761	Poa sp.	0		NA	0	Poaceae Barnhart
Iz_5_1009	DJI_0761	Polygonatum odoratum (Mill.) Druce	0	n	1	0	Asparagaceae Juss.
Iz_5_1009	DJI_0761	Polygonum arenarium Waldst. & Kit.	0	n	1	0	Polygonaceae Juss.
Iz_5_1009	DJI_0761	Populus nigra L.	0	n	1	1	Salicaceae Mirb.
Iz_5_1009	DJI_0761	Populus sp.	0	n	1	1	Salicaceae Mirb.
Iz_5_1009	DJI_0761	Populus tremula L.	0	n	1	1	Salicaceae Mirb.
Iz_5_1009	DJI_0761	Populus Ã canescens (Aiton) Sm.	0	n	1	1	Salicaceae Mirb.
Iz_5_1009	DJI_0761	Potentilla argentea L.	0	n	1	0	Rosaceae Juss.
Iz_5_1009	DJI_0761	Psammophiliella muralis (L.) Ikonn.	0	n	1	0	Caryophyllaceae Juss.
Iz_5_1009	DJI_0761	Psephellus sumensis (Kalen.) Greuter	0	n	1	0	Asteraceae Bercht. & J.Presl
Iz_5_1009	DJI_0761	Pulsatilla pratensis (L.) Mill. s.l.	0	n	1	0	Ranunculaceae Juss.
Iz_5_1009	DJI_0761	Pyrus communis L.	0	a	0	1	Rosaceae Juss.
Iz_5_1009	DJI_0761	Quercus robur L.	0	n	1	1	Fagaceae Dumort.
Iz_5_1009	DJI_0761	Robinia pseudoacacia L.	0	a	0	1	Fabaceae Lindl.
Iz_5_1009	DJI_0761	Rosa sp.	0		NA	1	Rosaceae Juss.
Iz_5_1009	DJI_0761	Rubus idaeus L.	0	n	1	0	Rosaceae Juss.
Iz_5_1009	DJI_0761	Rumex acetosella L.	0	n	1	0	Polygonaceae Juss.
Iz_5_1009	DJI_0761	Rumex thyrsiflorus Fingerh.	0	n	1	0	Polygonaceae Juss.
Iz_5_1009	DJI_0761	Salix alba L.	0	n	1	1	Salicaceae Mirb.
Iz_5_1009	DJI_0761	Sambucus nigra L.	0	n	1	1	Viburnaceae Raf.
Iz_5_1009	DJI_0761	Secale sylvestre Host	0	n	1	0	Poaceae Barnhart
Iz_5_1009	DJI_0761	Sempervivum ruthenicum Schnittsp. & C.B. Lehm.	0	n	1	0	Crassulaceae J.St.-Hil.
Iz_5_1009	DJI_0761	Setaria viridis (L.) P. Beauv.	0	n	1	0	Poaceae Barnhart
Iz_5_1009	DJI_0761	Silene borysthenica (Gruner) Walters	0	n	1	0	Caryophyllaceae Juss.
Iz_5_1009	DJI_0761	Silene latifolia subsp. alba (Mill.) Greuter & Burdet	0	n	1	0	Caryophyllaceae Juss.
Iz_5_1009	DJI_0761	Solidago virgaurea L.	0.5	n	1	0	Asteraceae Bercht. & J.Presl
Iz_5_1009	DJI_0761	Solidago Ã niederederi (S. canadensis Ã S. virgaurea)	0	hybrid	0	0	Asteraceae Bercht. & J.Presl
Iz_5_1009	DJI_0761	Sorbus aucuparia L.	0	n	1	1	Rosaceae Juss.
Iz_5_1009	DJI_0761	Stachys recta L.	0	n	1	0	Lamiacea Lindl.
Iz_5_1009	DJI_0761	Tanacetum vulgare L.	0	n	1	0	Asteraceae Bercht. & J.Presl
Iz_5_1009	DJI_0761	Thymus pallasianus Heinr.Braun	0	n	1	0	Lamiacea Lindl.
Iz_5_1009	DJI_0761	Tilia cordata Mill.	0	n	1	1	Malvaceae Juss.
Iz_5_1009	DJI_0761	Trifolium alpestre L.	0	n	1	0	Fabaceae Lindl.
Iz_5_1009	DJI_0761	Trifolium arvense L.	0	n	1	0	Fabaceae Lindl.
Iz_5_1009	DJI_0761	Ulmus glabra Huds.	0	n	1	1	Ulmaceae Mirb.
Iz_5_1009	DJI_0761	Verbascum densiflorum Bertol.	0	n	1	0	Scrophulariaceae Juss.
Iz_5_1009	DJI_0761	Veronica austriaca L.	0	n	1	0	Plantaginaceae Juss.
Iz_5_1009	DJI_0761	Veronica incana L.	0	n	1	0	Plantaginaceae Juss.
Iz_5_1009	DJI_0761	Veronica verna L.	0	n	1	0	Plantaginaceae Juss.
Iz_5_1009	DJI_0761	Vincetoxicum hirundinaria Medik	0	n	1	0	Apocynaceae Juss.
Iz_5_1009	DJI_0765	Acer tataricum L.	0	n	1	1	Sapindaceae Juss.
Iz_5_1009	DJI_0765	Achillea millefolium L. s.l.	0	n	1	0	Asteraceae Bercht. & J.Presl
Iz_5_1009	DJI_0765	Allium oleraceum L.	0	n	1	0	Amaryllidaceae J.St.-Hil.
Iz_5_1009	DJI_0765	Ambrosia artemisiifolia L.	0	a	0	0	Asteraceae Bercht. & J.Presl
Iz_5_1009	DJI_0765	Amorpha fruticosa L.	0	a	0	0	Fabaceae Lindl.
Iz_5_1009	DJI_0765	Anthericum ramosum L.	0	n	1	0	Asparagaceae Juss.
Iz_5_1009	DJI_0765	Artemisia marschalliana Spreng.	0	n	1	0	Asteraceae Bercht. & J.Presl
Iz_5_1009	DJI_0765	Asparagus officinalis L.	0	n	1	0	Asparagaceae Juss.
Iz_5_1009	DJI_0765	Astragalus cicer L.	0	n	1	0	Fabaceae Lindl.
Iz_5_1009	DJI_0765	Bassia laniflora (S.G.Gmel.) A.J.Scott	0	n	1	0	Amaranthaceae Juss.
Iz_5_1009	DJI_0765	Berberis vulgaris L.	0	n	1	0	Berberidaceae Juss.

Iz_5_1009	DJI_0765	Betula pendula Roth.	0	n	1	1	Betulaceae Gray
Iz_5_1009	DJI_0765	Betula pendula Roth.	0	n	1	0	Poaceae Barnhart
Iz_5_1009	DJI_0765	Bromus inermis Leyss.	0		NA	NA	
Iz_5_1009	DJI_0765	Bromus squarrosus L.	0	a	0	0	Poaceae Barnhart
Iz_5_1009	DJI_0765	Bromus tectorum L.	0	a	0	0	Poaceae Barnhart
Iz_5_1009	DJI_0765	Calamagrostis epigejos (L.) Roth	3	n	1	0	Poaceae Barnhart
Iz_5_1009	DJI_0765	Carduus acanthoides L.	0	n	1	0	Asteraceae Bercht. & J.Presl
Iz_5_1009	DJI_0765	Carex sp.	0	n	1	0	Cyperaceae Juss.
Iz_5_1009	DJI_0765	Centaurea majorovii Dumbadze	0	n	1	0	Asteraceae Bercht. & J.Presl
Iz_5_1009	DJI_0765	Chamaecytisus ruthenicus (Fisch.ex Wol.) Klaskova	0	n	1	0	Fabaceae Lindl.
Iz_5_1009	DJI_0765	Chelidonium majus L.	0	n	1	0	Papaveraceae Juss.
Iz_5_1009	DJI_0765	Chenopodium betaceum Andrz.	0	n	1	0	Amaranthaceae Juss.
Iz_5_1009	DJI_0765	Chenopodium cf. album	0	n	1	0	Amaranthaceae Juss.
Iz_5_1009	DJI_0765	Chenopodium sp.	0		NA	0	Amaranthaceae Juss.
Iz_5_1009	DJI_0765	Chondrilla juncea L.	0	n	1	0	Asteraceae Bercht. & J.Presl
Iz_5_1009	DJI_0765	Cirsium vulgare (Savi) Ten.	0	n	1	0	Asteraceae Bercht. & J.Presl
Iz_5_1009	DJI_0765	Clinopodium acinos (L.) Kuntze	0	n	1	0	Lamiacea Lindl.
Iz_5_1009	DJI_0765	Convallaria majalis L.	0	n	1	0	Asparagaceae Juss.
Iz_5_1009	DJI_0765	Crataegus rhipidophylla Gand.	0	n	1	1	Rosaceae Juss.
Iz_5_1009	DJI_0765	Crepis foetida L.	0	n	1	0	Asteraceae Bercht. & J.Presl
Iz_5_1009	DJI_0765	Dactylis glomerata L.	0	n	1	0	Poaceae Barnhart
Iz_5_1009	DJI_0765	Dianthus campestris M.Bieb.	0	n	1	0	Caryophyllaceae Juss.
Iz_5_1009	DJI_0765	Digitaria sanguinalis (L.) Scop.	0	a	0	0	Poaceae Barnhart
Iz_5_1009	DJI_0765	Elymus repens (L.) Gould	0	n	1	0	Poaceae Barnhart
Iz_5_1009	DJI_0765	Epilobium tetragonum L.	0	n	1	0	Onagraceae Juss.
Iz_5_1009	DJI_0765	Eragrostis minor Host	0	a	0	0	Poaceae Barnhart
Iz_5_1009	DJI_0765	Erigeron canadensis L.	0	a	0	0	Asteraceae Bercht. & J.Presl
Iz_5_1009	DJI_0765	Euphorbia seguieriana Neck.	0	n	1	0	Euphorbiaceae Juss.
Iz_5_1009	DJI_0765	Fallopia dumetorum (L.) Holub	0	n	1	0	Polygonaceae Juss.
Iz_5_1009	DJI_0765	Festuca beckeri (Hack.) Trautv.	0	n	1	0	Poaceae Barnhart
Iz_5_1009	DJI_0765	Fragaria vesca L.	0	n	1	0	Rosaceae Juss.
Iz_5_1009	DJI_0765	Frangula alnus Mill.	0	n	1	1	Rhamnaceae Juss.
Iz_5_1009	DJI_0765	Fraxinus pennsylvanica Marshall	0	a	0	1	Oleaceae Hoffmanns. & Link
Iz_5_1009	DJI_0765	Galeopsis bifida Boenn.	0	n	1	0	Lamiacea Lindl.
Iz_5_1009	DJI_0765	Galium verum L.	0	n	1	0	Rubiaceae Juss.
Iz_5_1009	DJI_0765	Genista tinctoria L.	0	n	1	0	Fabaceae Lindl.
Iz_5_1009	DJI_0765	Geranium robertianum L.	0	n	1	0	Geraniaceae Juss.
Iz_5_1009	DJI_0765	Geranium sanquineum L.	0	n	1	0	Geraniaceae Juss.
Iz_5_1009	DJI_0765	Gypsophila paniculata L.	0	n	1	0	Caryophyllaceae Juss.
Iz_5_1009	DJI_0765	Helichrysum arenarium (L.) Moench	0	n	1	0	Asteraceae Bercht. & J.Presl
Iz_5_1009	DJI_0765	Herniaria polygama J.Gay	0	n	1	0	Caryophyllaceae Juss.
Iz_5_1009	DJI_0765	Hieracium robustum Fr.	0	n	1	0	Asteraceae Bercht. & J.Presl
Iz_5_1009	DJI_0765	Hieracium umbellatum L.	0	n	1	NA	Asteraceae Bercht. & J.Presl
Iz_5_1009	DJI_0765	Hylotelephium maximum (L.) Holub	0	n	1	0	Crassulaceae J.St.-Hil.
Iz_5_1009	DJI_0765	Jacobaea borysthenica (DC.) B.Nord. & Greuter	0	n	1	0	Asteraceae Bercht. & J.Presl
Iz_5_1009	DJI_0765	Jasione montana L.	0	n	1	0	Campanulaceae Juss.
Iz_5_1009	DJI_0765	Jurinea cyanoides (L.) Rchb.s.l.	0	n	1	0	Asteraceae Bercht. & J.Presl
Iz_5_1009	DJI_0765	Koeleria glauca (Spreng.) DC.	0	n	1	0	Poaceae Barnhart
Iz_5_1009	DJI_0765	Lactuca serriola L.	0	a	0	0	Asteraceae Bercht. & J.Presl
Iz_5_1009	DJI_0765	Linaria genistifolia (L.) Mill.	0	n	1	0	Plantaginaceae Juss.
Iz_5_1009	DJI_0765	Lomelosia argentea (L.) Greuter & Burdet	0	n	1	0	Caprifoliaceae Juss.
Iz_5_1009	DJI_0765	Melampyrum pratense L.	0	n	1	0	Orobanchaceae Vent.
Iz_5_1009	DJI_0765	Oenothera biennis L.	0	a	0	0	Onagraceae Juss.
Iz_5_1009	DJI_0765	Pentanema britannica (L.) D.Gut.Larr., Santos-Vicente, Anderb., E.Rico & M.M.Mart.Ort.	0	n	1	0	Asteraceae Bercht. & J.Presl
Iz_5_1009	DJI_0765	Peucedanum oreoselinum (L.) Moench	0	n	1	0	Apiaceae Lindl.
Iz_5_1009	DJI_0765	Peucedanum ruthenicum M.Bieb.	0	n	1	0	Apiaceae Lindl.
Iz_5_1009	DJI_0765	Pilosella echioides (Lumn.) F.W.Schultz & Sch.Bip.	0	n	1	0	Asteraceae Bercht. & J.Presl
Iz_5_1009	DJI_0765	Pilosella officinarum F. Schultz et Sch. Bip.	0	n	1	0	Asteraceae Bercht. & J.Presl
Iz_5_1009	DJI_0765	Pilosella sp.	0		NA	0	Asteraceae Bercht. & J.Presl
Iz_5_1009	DJI_0765	Pinus sylvestris L.	0	n	1	1	Pinaceae Spreng. ex F.Rudolphi
Iz_5_1009	DJI_0765	Poa annua L.	0	n	1	0	Poaceae Barnhart
Iz_5_1009	DJI_0765	Poa nemoralis L.	0	n	1	0	Poaceae Barnhart
Iz_5_1009	DJI_0765	Poa sp.	0		NA	0	Poaceae Barnhart
Iz_5_1009	DJI_0765	Polygonatum odoratum (Mill.) Druce	1	n	1	0	Asparagaceae Juss.
Iz_5_1009	DJI_0765	Polygonum arenarium Waldst. & Kit.	0	n	1	0	Polygonaceae Juss.
Iz_5_1009	DJI_0765	Populus nigra L.	0	n	1	1	Salicaceae Mirb.
Iz_5_1009	DJI_0765	Populus sp.	0	n	1	1	Salicaceae Mirb.
Iz_5_1009	DJI_0765	Populus tremula L.	0	n	1	1	Salicaceae Mirb.
Iz_5_1009	DJI_0765	Populus Ã canescens (Aiton) Sm.	0	n	1	1	Salicaceae Mirb.
Iz_5_1009	DJI_0765	Potentilla argentea L.	0	n	1	0	Rosaceae Juss.
Iz_5_1009	DJI_0765	Psammophiliella muralis (L.) Ikonn.	0	n	1	0	Caryophyllaceae Juss.
Iz_5_1009	DJI_0765	Psephellus sumensis (Kalen.) Greuter	0	n	1	0	Asteraceae Bercht. & J.Presl
Iz_5_1009	DJI_0765	Pulsatilla pratensis (L.) Mill. s.l.	0	n	1	0	Ranunculaceae Juss.
Iz_5_1009	DJI_0765	Pyrus communis L.	0	a	0	1	Rosaceae Juss.
Iz_5_1009	DJI_0765	Quercus robur L.	0.2	n	1	1	Fagaceae Dumort.
Iz_5_1009	DJI_0765	Robinia pseudoacacia L.	0	a	0	1	Fabaceae Lindl.
Iz_5_1009	DJI_0765	Rosa sp.	0		NA	1	Rosaceae Juss.
Iz_5_1009	DJI_0765	Rubus idaeus L.	0	n	1	0	Rosaceae Juss.
Iz_5_1009	DJI_0765	Rumex acetosella L.	0	n	1	0	Polygonaceae Juss.
Iz_5_1009	DJI_0765	Rumex thyrsiflorus Fingerh.	0	n	1	0	Polygonaceae Juss.
Iz_5_1009	DJI_0765	Salix alba L.	0	n	1	1	Salicaceae Mirb.
Iz_5_1009	DJI_0765	Sambucus nigra L.	0	n	1	1	Viburnaceae Raf.
Iz_5_1009	DJI_0765	Secale sylvestre Host	0	n	1	0	Poaceae Barnhart
Iz_5_1009	DJI_0765	Sempervivum ruthenicum Schnittsp. & C.B. Lehm.	0	n	1	0	Crassulaceae J.St.-Hil.
Iz_5_1009	DJI_0765	Setaria viridis (L.) P. Beauv.	0	n	1	0	Poaceae Barnhart
Iz_5_1009	DJI_0765	Silene borysthenica (Gruner) Walters	0	n	1	0	Caryophyllaceae Juss.
Iz_5_1009	DJI_0765	Silene latifolia subsp. alba (Mill.) Greuter & Burdet	0	n	1	0	Caryophyllaceae Juss.
Iz_5_1009	DJI_0765	Solidago virgaurea L.	0	n	1	0	Asteraceae Bercht. & J.Presl
Iz_5_1009	DJI_0765	Solidago Ã niederederi (S. canadensis Ã S. virgaurea)	0	hybrid	0	0	Asteraceae Bercht. & J.Presl
Iz_5_1009	DJI_0765	Sorbus aucuparia L.	0	n	1	1	Rosaceae Juss.
Iz_5_1009	DJI_0765	Stachys recta L.	0	n	1	0	Lamiacea Lindl.
Iz_5_1009	DJI_0765	Tanacetum vulgare L.	0	n	1	0	Asteraceae Bercht. & J.Presl
Iz_5_1009	DJI_0765	Thymus pallasianus Heinr.Braun	0	n	1	0	Lamiacea Lindl.
Iz_5_1009	DJI_0765	Tilia cordata Mill.	0	n	1	1	Malvaceae Juss.
Iz_5_1009	DJI_0765	Trifolium alpestre L.	0	n	1	0	Fabaceae Lindl.
Iz_5_1009	DJI_0765	Trifolium arvense L.	0	n	1	0	Fabaceae Lindl.
Iz_5_1009	DJI_0765	Ulmus glabra Huds.	0	n	1	1	Ulmaceae Mirb.
Iz_5_1009	DJI_0765	Verbascum densiflorum Bertol.	0	n	1	0	Scrophulariaceae Juss.

Iz_5_1009	DJI_0765	Veronica austriaca L.	0	n	1	0	Plantaginaceae Juss.
Iz_5_1009	DJI_0765	Veronica incana L.	0	n	1	0	Plantaginaceae Juss.
Iz_5_1009	DJI_0765	Veronica verna L.	0	n	1	0	Plantaginaceae Juss.
Iz_5_1009	DJI_0765	Vincetoxicum hirundinaria Medik	0	n	1	0	Apocynaceae Juss.
Iz_5_1009	DJI_0772	Acer tataricum L.	0	n	1	1	Sapindaceae Juss.
Iz_5_1009	DJI_0772	Achillea millefolium L. s.l.	0	n	1	0	Asteraceae Bercht. & J.Presl
Iz_5_1009	DJI_0772	Allium oleraceum L.	0	n	1	0	Amaryllidaceae J.St.-Hil.
Iz_5_1009	DJI_0772	Ambrosia artemisiifolia L.	0	a	0	0	Asteraceae Bercht. & J.Presl
Iz_5_1009	DJI_0772	Amorpha fruticosa L.	0	a	0	0	Fabaceae Lindl.
Iz_5_1009	DJI_0772	Anthericum ramosum L.	0	n	1	0	Asparagaceae Juss.
Iz_5_1009	DJI_0772	Artemisia marschalliana Spreng.	0	n	1	0	Asteraceae Bercht. & J.Presl
Iz_5_1009	DJI_0772	Asparagus officinalis L.	0	n	1	0	Asparagaceae Juss.
Iz_5_1009	DJI_0772	Astragalus cicer L.	0	n	1	0	Fabaceae Lindl.
Iz_5_1009	DJI_0772	Bassia laniflora (S.G.Gmel.) A.J.Scott	0	n	1	0	Amaranthaceae Juss.
Iz_5_1009	DJI_0772	Berberis vulgaris L.	0	n	1	0	Berberidaceae Juss.
Iz_5_1009	DJI_0772	Betula pendula Roth.	0	n	1	1	Betulaceae Gray
Iz_5_1009	DJI_0772	Betula pendula Roth.	0	n	1	0	Poaceae Barnhart
Iz_5_1009	DJI_0772	Bromus inermis Leyss.	0		NA	NA	
Iz_5_1009	DJI_0772	Bromus squarrosus L.	0	a	0	0	Poaceae Barnhart
Iz_5_1009	DJI_0772	Bromus tectorum L.	0	a	0	0	Poaceae Barnhart
Iz_5_1009	DJI_0772	Calamagrostis epigejos (L.) Roth	10	n	1	0	Poaceae Barnhart
Iz_5_1009	DJI_0772	Carduus acanthoides L.	0	n	1	0	Asteraceae Bercht. & J.Presl
Iz_5_1009	DJI_0772	Carex sp.	0	n	1	0	Cyperaceae Juss.
Iz_5_1009	DJI_0772	Centaurea majorovii Dumbadze	0	n	1	0	Asteraceae Bercht. & J.Presl
Iz_5_1009	DJI_0772	Chamaecytisus ruthenicus (Fisch.ex Wol.) Klaskova	3	n	1	0	Fabaceae Lindl.
Iz_5_1009	DJI_0772	Chelidonium majus L.	0	n	1	0	Papaveraceae Juss.
Iz_5_1009	DJI_0772	Chenopodium betaceum Andrz.	0	n	1	0	Amaranthaceae Juss.
Iz_5_1009	DJI_0772	Chenopodium cf. album	0	n	1	0	Amaranthaceae Juss.
Iz_5_1009	DJI_0772	Chenopodium sp.	0		NA	0	Amaranthaceae Juss.
Iz_5_1009	DJI_0772	Chondrilla juncea L.	0	n	1	0	Asteraceae Bercht. & J.Presl
Iz_5_1009	DJI_0772	Cirsium vulgare (Savi) Ten.	0	n	1	0	Asteraceae Bercht. & J.Presl
Iz_5_1009	DJI_0772	Clinopodium acinos (L.) Kuntze	0	n	1	0	Lamiacea Lindl.
Iz_5_1009	DJI_0772	Convallaria majalis L.	0	n	1	0	Asparagaceae Juss.
Iz_5_1009	DJI_0772	Crataegus rhipidophylla Gand.	0	n	1	1	Rosaceae Juss.
Iz_5_1009	DJI_0772	Crepis foetida L.	0	n	1	0	Asteraceae Bercht. & J.Presl
Iz_5_1009	DJI_0772	Dactylis glomerata L.	0	n	1	0	Poaceae Barnhart
Iz_5_1009	DJI_0772	Dianthus campestris M.Bieb.	0	n	1	0	Caryophyllaceae Juss.
Iz_5_1009	DJI_0772	Digitaria sanguinalis (L.) Scop.	0	a	0	0	Poaceae Barnhart
Iz_5_1009	DJI_0772	Elymus repens (L.) Gould	0	n	1	0	Poaceae Barnhart
Iz_5_1009	DJI_0772	Epilobium tetragonum L.	0	n	1	0	Onagraceae Juss.
Iz_5_1009	DJI_0772	Eragrostis minor Host	0	a	0	0	Poaceae Barnhart
Iz_5_1009	DJI_0772	Erigeron canadensis L.	0.5	a	0	0	Asteraceae Bercht. & J.Presl
Iz_5_1009	DJI_0772	Euphorbia seguieriana Neck.	0	n	1	0	Euphorbiaceae Juss.
Iz_5_1009	DJI_0772	Fallopia dumetorum (L.) Holub	0	n	1	0	Polygonaceae Juss.
Iz_5_1009	DJI_0772	Festuca beckeri (Hack.) Trautv.	0	n	1	0	Poaceae Barnhart
Iz_5_1009	DJI_0772	Fragaria vesca L.	0	n	1	0	Rosaceae Juss.
Iz_5_1009	DJI_0772	Frangula alnus Mill.	0	n	1	1	Rhamnaceae Juss.
Iz_5_1009	DJI_0772	Fraxinus pennsylvanica Marshall	0	a	0	1	Oleaceae Hoffmanns. & Link
Iz_5_1009	DJI_0772	Galeopsis bifida Boenn.	0	n	1	0	Lamiacea Lindl.
Iz_5_1009	DJI_0772	Galium verum L.	0	n	1	0	Rubiaceae Juss.
Iz_5_1009	DJI_0772	Genista tinctoria L.	0	n	1	0	Fabaceae Lindl.
Iz_5_1009	DJI_0772	Geranium robertianum L.	0	n	1	0	Geraniaceae Juss.
Iz_5_1009	DJI_0772	Geranium sanquineum L.	0	n	1	0	Geraniaceae Juss.
Iz_5_1009	DJI_0772	Gypsophila paniculata L.	0	n	1	0	Caryophyllaceae Juss.
Iz_5_1009	DJI_0772	Helichrysum arenarium (L.) Moench	0	n	1	0	Asteraceae Bercht. & J.Presl
Iz_5_1009	DJI_0772	Herniaria polygama J.Gay	0	n	1	0	Caryophyllaceae Juss.
Iz_5_1009	DJI_0772	Hieracium robustum Fr.	0	n	1	0	Asteraceae Bercht. & J.Presl
Iz_5_1009	DJI_0772	Hieracium umbellatum L.	0	n	1	NA	Asteraceae Bercht. & J.Presl
Iz_5_1009	DJI_0772	Hylotelephium maximum (L.) Holub	0	n	1	0	Crassulaceae J.St.-Hil.
Iz_5_1009	DJI_0772	Jacobaea borysthenica (DC.) B.Nord. & Greuter	0	n	1	0	Asteraceae Bercht. & J.Presl
Iz_5_1009	DJI_0772	Jasione montana L.	0	n	1	0	Campanulaceae Juss.
Iz_5_1009	DJI_0772	Jurinea cyanoides (L.) Rchb.s.l.	0	n	1	0	Asteraceae Bercht. & J.Presl
Iz_5_1009	DJI_0772	Koeleria glauca (Spreng.) DC.	0	n	1	0	Poaceae Barnhart
Iz_5_1009	DJI_0772	Lactuca serriola L.	0	a	0	0	Asteraceae Bercht. & J.Presl
Iz_5_1009	DJI_0772	Linaria genistifolia (L.) Mill.	0	n	1	0	Plantaginaceae Juss.
Iz_5_1009	DJI_0772	Lomelosia argentea (L.) Greuter & Burdet	0	n	1	0	Caprifoliaceae Juss.
Iz_5_1009	DJI_0772	Melampyrum pratense L.	0	n	1	0	Orobanchaceae Vent.
Iz_5_1009	DJI_0772	Oenothera biennis L.	0	a	0	0	Onagraceae Juss.
Iz_5_1009	DJI_0772	Pentanema britannica (L.) D.Gut.Larr., Santos-Vicente, Anderb., E.Rico & M.M.Mart.Ort.	0	n	1	0	Asteraceae Bercht. & J.Presl
Iz_5_1009	DJI_0772	Peucedanum oreoselinum (L.) Moench	2	n	1	0	Apiaceae Lindl.
Iz_5_1009	DJI_0772	Peucedanum ruthenicum M.Bieb.	0	n	1	0	Apiaceae Lindl.
Iz_5_1009	DJI_0772	Pilosella echioides (Lumn.) F.W.Schultz & Sch.Bip.	0	n	1	0	Asteraceae Bercht. & J.Presl
Iz_5_1009	DJI_0772	Pilosella officinarum F. Schultz et Sch. Bip.	0	n	1	0	Asteraceae Bercht. & J.Presl
Iz_5_1009	DJI_0772	Pilosella sp.	0		NA	0	Asteraceae Bercht. & J.Presl
Iz_5_1009	DJI_0772	Pinus sylvestris L.	0	n	1	1	Pinaceae Spreng. ex F.Rudolphi
Iz_5_1009	DJI_0772	Poa annua L.	0	n	1	0	Poaceae Barnhart
Iz_5_1009	DJI_0772	Poa nemoralis L.	0	n	1	0	Poaceae Barnhart
Iz_5_1009	DJI_0772	Poa sp.	0		NA	0	Poaceae Barnhart
Iz_5_1009	DJI_0772	Polygonatum odoratum (Mill.) Druce	0	n	1	0	Asparagaceae Juss.
Iz_5_1009	DJI_0772	Polygonum arenarium Waldst. & Kit.	0	n	1	0	Polygonaceae Juss.
Iz_5_1009	DJI_0772	Populus nigra L.	0	n	1	1	Salicaceae Mirb.
Iz_5_1009	DJI_0772	Populus sp.	0	n	1	1	Salicaceae Mirb.
Iz_5_1009	DJI_0772	Populus tremula L.	0	n	1	1	Salicaceae Mirb.
Iz_5_1009	DJI_0772	Populus Ã canescens (Aiton) Sm.	0	n	1	1	Salicaceae Mirb.
Iz_5_1009	DJI_0772	Potentilla argentea L.	0	n	1	0	Rosaceae Juss.
Iz_5_1009	DJI_0772	Psammophiliella muralis (L.) Ikonn.	0	n	1	0	Caryophyllaceae Juss.
Iz_5_1009	DJI_0772	Psephellus sumensis (Kalen.) Greuter	0	n	1	0	Asteraceae Bercht. & J.Presl
Iz_5_1009	DJI_0772	Pulsatilla pratensis (L.) Mill. s.l.	0	n	1	0	Ranunculaceae Juss.
Iz_5_1009	DJI_0772	Pyrus communis L.	0	a	0	1	Rosaceae Juss.
Iz_5_1009	DJI_0772	Quercus robur L.	0	n	1	1	Fagaceae Dumort.
Iz_5_1009	DJI_0772	Robinia pseudoacacia L.	0	a	0	1	Fabaceae Lindl.
Iz_5_1009	DJI_0772	Rosa sp.	0		NA	1	Rosaceae Juss.
Iz_5_1009	DJI_0772	Rubus idaeus L.	0	n	1	0	Rosaceae Juss.
Iz_5_1009	DJI_0772	Rumex acetosella L.	0	n	1	0	Polygonaceae Juss.
Iz_5_1009	DJI_0772	Rumex thyrsiflorus Fingerh.	0	n	1	0	Polygonaceae Juss.
Iz_5_1009	DJI_0772	Salix alba L.	0	n	1	1	Salicaceae Mirb.
Iz_5_1009	DJI_0772	Sambucus nigra L.	0	n	1	1	Viburnaceae Raf.
Iz_5_1009	DJI_0772	Secale sylvestre Host	0	n	1	0	Poaceae Barnhart

Iz_5_1009	DJI_0772	Sempervivum ruthenicum Schnittsp. & C.B. Lehm.	0	n	1	0	Crassulaceae J.St.-Hil.
Iz_5_1009	DJI_0772	Setaria viridis (L.) P. Beauv.	0	n	1	0	Poaceae Barnhart
Iz_5_1009	DJI_0772	Silene borysthenica (Gruner) Walters	0	n	1	0	Caryophyllaceae Juss.
Iz_5_1009	DJI_0772	Silene latifolia subsp. alba (Mill.) Greuter & Burdet	0	n	1	0	Caryophyllaceae Juss.
Iz_5_1009	DJI_0772	Solidago virgaurea L.	0.5	n	1	0	Asteraceae Bercht. & J.Presl
Iz_5_1009	DJI_0772	Solidago Ã niederederi (S. canadensis Ã S. virgaurea)	0	hybrid	0	0	Asteraceae Bercht. & J.Presl
Iz_5_1009	DJI_0772	Sorbus aucuparia L.	0	n	1	1	Rosaceae Juss.
Iz_5_1009	DJI_0772	Stachys recta L.	0	n	1	0	Lamiacea Lindl.
Iz_5_1009	DJI_0772	Tanacetum vulgare L.	0	n	1	0	Asteraceae Bercht. & J.Presl
Iz_5_1009	DJI_0772	Thymus pallasianus Heinr.Braun	0	n	1	0	Lamiacea Lindl.
Iz_5_1009	DJI_0772	Tilia cordata Mill.	0	n	1	1	Malvaceae Juss.
Iz_5_1009	DJI_0772	Trifolium alpestre L.	0	n	1	0	Fabaceae Lindl.
Iz_5_1009	DJI_0772	Trifolium arvense L.	0	n	1	0	Fabaceae Lindl.
Iz_5_1009	DJI_0772	Ulmus glabra Huds.	0	n	1	1	Ulmaceae Mirb.
Iz_5_1009	DJI_0772	Verbascum densiflorum Bertol.	0	n	1	0	Scrophulariaceae Juss.
Iz_5_1009	DJI_0772	Veronica austriaca L.	0	n	1	0	Plantaginaceae Juss.
Iz_5_1009	DJI_0772	Veronica incana L.	0	n	1	0	Plantaginaceae Juss.
Iz_5_1009	DJI_0772	Veronica verna L.	0	n	1	0	Plantaginaceae Juss.
Iz_5_1009	DJI_0772	Vincetoxicum hirundinaria Medik	0	n	1	0	Apocynaceae Juss.
Iz_5_1009	DJI_0774	Acer tataricum L.	0	n	1	1	Sapindaceae Juss.
Iz_5_1009	DJI_0774	Achillea millefolium L. s.l.	0	n	1	0	Asteraceae Bercht. & J.Presl
Iz_5_1009	DJI_0774	Allium oleraceum L.	0	n	1	0	Amaryllidaceae J.St.-Hil.
Iz_5_1009	DJI_0774	Ambrosia artemisiifolia L.	0	a	0	0	Asteraceae Bercht. & J.Presl
Iz_5_1009	DJI_0774	Amorpha fruticosa L.	0	a	0	0	Fabaceae Lindl.
Iz_5_1009	DJI_0774	Anthericum ramosum L.	0	n	1	0	Asparagaceae Juss.
Iz_5_1009	DJI_0774	Artemisia marschalliana Spreng.	0	n	1	0	Asteraceae Bercht. & J.Presl
Iz_5_1009	DJI_0774	Asparagus officinalis L.	0	n	1	0	Asparagaceae Juss.
Iz_5_1009	DJI_0774	Astragalus cicer L.	0	n	1	0	Fabaceae Lindl.
Iz_5_1009	DJI_0774	Bassia laniflora (S.G.Gmel.) A.J.Scott	0	n	1	0	Amaranthaceae Juss.
Iz_5_1009	DJI_0774	Berberis vulgaris L.	0	n	1	0	Berberidaceae Juss.
Iz_5_1009	DJI_0774	Betula pendula Roth.	0	n	1	1	Betulaceae Gray
Iz_5_1009	DJI_0774	Betula pendula Roth.	0	n	1	0	Poaceae Barnhart
Iz_5_1009	DJI_0774	Bromus inermis Leyss.	0		NA	NA	
Iz_5_1009	DJI_0774	Bromus squarrosus L.	0	a	0	0	Poaceae Barnhart
Iz_5_1009	DJI_0774	Bromus tectorum L.	0	a	0	0	Poaceae Barnhart
Iz_5_1009	DJI_0774	Calamagrostis epigejos (L.) Roth	40	n	1	0	Poaceae Barnhart
Iz_5_1009	DJI_0774	Carduus acanthoides L.	0	n	1	0	Asteraceae Bercht. & J.Presl
Iz_5_1009	DJI_0774	Carex sp.	0	n	1	0	Cyperaceae Juss.
Iz_5_1009	DJI_0774	Centaurea majorovii Dumbadze	0	n	1	0	Asteraceae Bercht. & J.Presl
Iz_5_1009	DJI_0774	Chamaecytisus ruthenicus (Fisch.ex Wol.) Klaskova	1	n	1	0	Fabaceae Lindl.
Iz_5_1009	DJI_0774	Chelidonium majus L.	0	n	1	0	Papaveraceae Juss.
Iz_5_1009	DJI_0774	Chenopodium betaceum Andrz.	0	n	1	0	Amaranthaceae Juss.
Iz_5_1009	DJI_0774	Chenopodium cf. album	0	n	1	0	Amaranthaceae Juss.
Iz_5_1009	DJI_0774	Chenopodium sp.	0		NA	0	Amaranthaceae Juss.
Iz_5_1009	DJI_0774	Chondrilla juncea L.	0	n	1	0	Asteraceae Bercht. & J.Presl
Iz_5_1009	DJI_0774	Cirsium vulgare (Savi) Ten.	0	n	1	0	Asteraceae Bercht. & J.Presl
Iz_5_1009	DJI_0774	Clinopodium acinos (L.) Kuntze	0	n	1	0	Lamiacea Lindl.
Iz_5_1009	DJI_0774	Convallaria majalis L.	0	n	1	0	Asparagaceae Juss.
Iz_5_1009	DJI_0774	Crataegus rhipidophylla Gand.	0	n	1	1	Rosaceae Juss.
Iz_5_1009	DJI_0774	Crepis foetida L.	0	n	1	0	Asteraceae Bercht. & J.Presl
Iz_5_1009	DJI_0774	Dactylis glomerata L.	0	n	1	0	Poaceae Barnhart
Iz_5_1009	DJI_0774	Dianthus campestris M.Bieb.	0	n	1	0	Caryophyllaceae Juss.
Iz_5_1009	DJI_0774	Digitaria sanguinalis (L.) Scop.	0	a	0	0	Poaceae Barnhart
Iz_5_1009	DJI_0774	Elymus repens (L.) Gould	0	n	1	0	Poaceae Barnhart
Iz_5_1009	DJI_0774	Epilobium tetragonum L.	0	n	1	0	Onagraceae Juss.
Iz_5_1009	DJI_0774	Eragrostis minor Host	0	a	0	0	Poaceae Barnhart
Iz_5_1009	DJI_0774	Erigeron canadensis L.	0	a	0	0	Asteraceae Bercht. & J.Presl
Iz_5_1009	DJI_0774	Euphorbia seguieriana Neck.	0	n	1	0	Euphorbiaceae Juss.
Iz_5_1009	DJI_0774	Fallopia dumetorum (L.) Holub	0	n	1	0	Polygonaceae Juss.
Iz_5_1009	DJI_0774	Festuca beckeri (Hack.) Trautv.	0	n	1	0	Poaceae Barnhart
Iz_5_1009	DJI_0774	Fragaria vesca L.	0	n	1	0	Rosaceae Juss.
Iz_5_1009	DJI_0774	Frangula alnus Mill.	0	n	1	1	Rhamnaceae Juss.
Iz_5_1009	DJI_0774	Fraxinus pennsylvanica Marshall	0	a	0	1	Oleaceae Hoffmanns. & Link
Iz_5_1009	DJI_0774	Galeopsis bifida Boenn.	0	n	1	0	Lamiacea Lindl.
Iz_5_1009	DJI_0774	Galium verum L.	0	n	1	0	Rubiaceae Juss.
Iz_5_1009	DJI_0774	Genista tinctoria L.	0	n	1	0	Fabaceae Lindl.
Iz_5_1009	DJI_0774	Geranium robertianum L.	0	n	1	0	Geraniaceae Juss.
Iz_5_1009	DJI_0774	Geranium sanquineum L.	0	n	1	0	Geraniaceae Juss.
Iz_5_1009	DJI_0774	Gypsophila paniculata L.	0	n	1	0	Caryophyllaceae Juss.
Iz_5_1009	DJI_0774	Helichrysum arenarium (L.) Moench	0	n	1	0	Asteraceae Bercht. & J.Presl
Iz_5_1009	DJI_0774	Herniaria polygama J.Gay	0	n	1	0	Caryophyllaceae Juss.
Iz_5_1009	DJI_0774	Hieracium robustum Fr.	0	n	1	0	Asteraceae Bercht. & J.Presl
Iz_5_1009	DJI_0774	Hieracium umbellatum L.	0	n	1	NA	Asteraceae Bercht. & J.Presl
Iz_5_1009	DJI_0774	Hylotelephium maximum (L.) Holub	0	n	1	0	Crassulaceae J.St.-Hil.
Iz_5_1009	DJI_0774	Jacobaea borysthenica (DC.) B.Nord. & Greuter	0	n	1	0	Asteraceae Bercht. & J.Presl
Iz_5_1009	DJI_0774	Jasione montana L.	0	n	1	0	Campanulaceae Juss.
Iz_5_1009	DJI_0774	Jurinea cyanoides (L.) Rchb.s.l.	0	n	1	0	Asteraceae Bercht. & J.Presl
Iz_5_1009	DJI_0774	Koeleria glauca (Spreng.) DC.	0	n	1	0	Poaceae Barnhart
Iz_5_1009	DJI_0774	Lactuca serriola L.	0	a	0	0	Asteraceae Bercht. & J.Presl
Iz_5_1009	DJI_0774	Linaria genistifolia (L.) Mill.	0	n	1	0	Plantaginaceae Juss.
Iz_5_1009	DJI_0774	Lomelosia argentea (L.) Greuter & Burdet	0	n	1	0	Caprifoliaceae Juss.
Iz_5_1009	DJI_0774	Melampyrum pratense L.	0	n	1	0	Orobanchaceae Vent.
Iz_5_1009	DJI_0774	Oenothera biennis L.	0	a	0	0	Onagraceae Juss.
Iz_5_1009	DJI_0774	Pentanema britannica (L.) D.Gut.Larr., Santos-Vicente, Anderb., E.Rico & M.M.Mart.Ort.	0	n	1	0	Asteraceae Bercht. & J.Presl
Iz_5_1009	DJI_0774	Peucedanum oreoselinum (L.) Moench	3	n	1	0	Apiaceae Lindl.
Iz_5_1009	DJI_0774	Peucedanum ruthenicum M.Bieb.	0	n	1	0	Apiaceae Lindl.
Iz_5_1009	DJI_0774	Pilosella echioides (Lumn.) F.W.Schultz & Sch.Bip.	0	n	1	0	Asteraceae Bercht. & J.Presl
Iz_5_1009	DJI_0774	Pilosella officinarum F. Schultz et Sch. Bip.	0	n	1	0	Asteraceae Bercht. & J.Presl
Iz_5_1009	DJI_0774	Pilosella sp.	0		NA	0	Asteraceae Bercht. & J.Presl
Iz_5_1009	DJI_0774	Pinus sylvestris L.	0	n	1	1	Pinaceae Spreng. ex F.Rudolphi
Iz_5_1009	DJI_0774	Poa annua L.	0	n	1	0	Poaceae Barnhart
Iz_5_1009	DJI_0774	Poa nemoralis L.	0	n	1	0	Poaceae Barnhart
Iz_5_1009	DJI_0774	Poa sp.	0		NA	0	Poaceae Barnhart
Iz_5_1009	DJI_0774	Polygonatum odoratum (Mill.) Druce	0	n	1	0	Asparagaceae Juss.
Iz_5_1009	DJI_0774	Polygonum arenarium Waldst. & Kit.	0	n	1	0	Polygonaceae Juss.
Iz_5_1009	DJI_0774	Populus nigra L.	0	n	1	1	Salicaceae Mirb.
Iz_5_1009	DJI_0774	Populus sp.	0	n	1	1	Salicaceae Mirb.
Iz_5_1009	DJI_0774	Populus tremula L.	0	n	1	1	Salicaceae Mirb.

Iz_5_1009	DJI_0774	Populus Ã canescens (Aiton) Sm.	0	n	1	1	Salicaceae Mirb.
Iz_5_1009	DJI_0774	Potentilla argentea L.	0	n	1	0	Rosaceae Juss.
Iz_5_1009	DJI_0774	Psammophiliella muralis (L.) Ikonn.	0	n	1	0	Caryophyllaceae Juss.
Iz_5_1009	DJI_0774	Psephellus sumensis (Kalen.) Greuter	0	n	1	0	Asteraceae Bercht. & J.Presl
Iz_5_1009	DJI_0774	Pulsatilla pratensis (L.) Mill. s.l.	0	n	1	0	Ranunculaceae Juss.
Iz_5_1009	DJI_0774	Pyrus communis L.	0	a	0	1	Rosaceae Juss.
Iz_5_1009	DJI_0774	Quercus robur L.	0	n	1	1	Fagaceae Dumort.
Iz_5_1009	DJI_0774	Robinia pseudoacacia L.	0	a	0	1	Fabaceae Lindl.
Iz_5_1009	DJI_0774	Rosa sp.	0		NA	1	Rosaceae Juss.
Iz_5_1009	DJI_0774	Rubus idaeus L.	0	n	1	0	Rosaceae Juss.
Iz_5_1009	DJI_0774	Rumex acetosella L.	0	n	1	0	Polygonaceae Juss.
Iz_5_1009	DJI_0774	Rumex thyrsiflorus Fingerh.	0	n	1	0	Polygonaceae Juss.
Iz_5_1009	DJI_0774	Salix alba L.	0	n	1	1	Salicaceae Mirb.
Iz_5_1009	DJI_0774	Sambucus nigra L.	0	n	1	1	Viburnaceae Raf.
Iz_5_1009	DJI_0774	Secale sylvestre Host	0	n	1	0	Poaceae Barnhart
Iz_5_1009	DJI_0774	Sempervivum ruthenicum Schnittsp. & C.B. Lehm.	0	n	1	0	Crassulaceae J.St.-Hil.
Iz_5_1009	DJI_0774	Setaria viridis (L.) P. Beauv.	0	n	1	0	Poaceae Barnhart
Iz_5_1009	DJI_0774	Silene borysthenica (Gruner) Walters	0	n	1	0	Caryophyllaceae Juss.
Iz_5_1009	DJI_0774	Silene latifolia subsp. alba (Mill.) Greuter & Burdet	0	n	1	0	Caryophyllaceae Juss.
Iz_5_1009	DJI_0774	Solidago virgaurea L.	0	n	1	0	Asteraceae Bercht. & J.Presl
Iz_5_1009	DJI_0774	Solidago Ã niederederi (S. canadensis Ã S. virgaurea)	0	hybrid	0	0	Asteraceae Bercht. & J.Presl
Iz_5_1009	DJI_0774	Sorbus aucuparia L.	0	n	1	1	Rosaceae Juss.
Iz_5_1009	DJI_0774	Stachys recta L.	0	n	1	0	Lamiacea Lindl.
Iz_5_1009	DJI_0774	Tanacetum vulgare L.	0	n	1	0	Asteraceae Bercht. & J.Presl
Iz_5_1009	DJI_0774	Thymus pallasianus Heinr.Braun	0	n	1	0	Lamiacea Lindl.
Iz_5_1009	DJI_0774	Tilia cordata Mill.	0	n	1	1	Malvaceae Juss.
Iz_5_1009	DJI_0774	Trifolium alpestre L.	0	n	1	0	Fabaceae Lindl.
Iz_5_1009	DJI_0774	Trifolium arvense L.	0	n	1	0	Fabaceae Lindl.
Iz_5_1009	DJI_0774	Ulmus glabra Huds.	0	n	1	1	Ulmaceae Mirb.
Iz_5_1009	DJI_0774	Verbascum densiflorum Bertol.	0	n	1	0	Scrophulariaceae Juss.
Iz_5_1009	DJI_0774	Veronica austriaca L.	0	n	1	0	Plantaginaceae Juss.
Iz_5_1009	DJI_0774	Veronica incana L.	0	n	1	0	Plantaginaceae Juss.
Iz_5_1009	DJI_0774	Veronica verna L.	0	n	1	0	Plantaginaceae Juss.
Iz_5_1009	DJI_0774	Vincetoxicum hirundinaria Medik	0	n	1	0	Apocynaceae Juss.
Iz_5_1009	DJI_0776	Acer tataricum L.	0	n	1	1	Sapindaceae Juss.
Iz_5_1009	DJI_0776	Achillea millefolium L. s.l.	0	n	1	0	Asteraceae Bercht. & J.Presl
Iz_5_1009	DJI_0776	Allium oleraceum L.	0	n	1	0	Amaryllidaceae J.St.-Hil.
Iz_5_1009	DJI_0776	Ambrosia artemisiifolia L.	0	a	0	0	Asteraceae Bercht. & J.Presl
Iz_5_1009	DJI_0776	Amorpha fruticosa L.	0	a	0	0	Fabaceae Lindl.
Iz_5_1009	DJI_0776	Anthericum ramosum L.	0	n	1	0	Asparagaceae Juss.
Iz_5_1009	DJI_0776	Artemisia marschalliana Spreng.	0	n	1	0	Asteraceae Bercht. & J.Presl
Iz_5_1009	DJI_0776	Asparagus officinalis L.	0	n	1	0	Asparagaceae Juss.
Iz_5_1009	DJI_0776	Astragalus cicer L.	0	n	1	0	Fabaceae Lindl.
Iz_5_1009	DJI_0776	Bassia laniflora (S.G.Gmel.) A.J.Scott	0	n	1	0	Amaranthaceae Juss.
Iz_5_1009	DJI_0776	Berberis vulgaris L.	0	n	1	0	Berberidaceae Juss.
Iz_5_1009	DJI_0776	Betula pendula Roth.	0	n	1	1	Betulaceae Gray
Iz_5_1009	DJI_0776	Betula pendula Roth.	0	n	1	0	Poaceae Barnhart
Iz_5_1009	DJI_0776	Bromus inermis Leyss.	0		NA	NA	
Iz_5_1009	DJI_0776	Bromus squarrosus L.	0	a	0	0	Poaceae Barnhart
Iz_5_1009	DJI_0776	Bromus tectorum L.	0	a	0	0	Poaceae Barnhart
Iz_5_1009	DJI_0776	Calamagrostis epigejos (L.) Roth	25	n	1	0	Poaceae Barnhart
Iz_5_1009	DJI_0776	Carduus acanthoides L.	0	n	1	0	Asteraceae Bercht. & J.Presl
Iz_5_1009	DJI_0776	Carex sp.	0	n	1	0	Cyperaceae Juss.
Iz_5_1009	DJI_0776	Centaurea majorovii Dumbadze	0	n	1	0	Asteraceae Bercht. & J.Presl
Iz_5_1009	DJI_0776	Chamaecytisus ruthenicus (Fisch.ex Wol.) Klaskova	0	n	1	0	Fabaceae Lindl.
Iz_5_1009	DJI_0776	Chelidonium majus L.	0	n	1	0	Papaveraceae Juss.
Iz_5_1009	DJI_0776	Chenopodium betaceum Andrz.	0	n	1	0	Amaranthaceae Juss.
Iz_5_1009	DJI_0776	Chenopodium cf. album	0	n	1	0	Amaranthaceae Juss.
Iz_5_1009	DJI_0776	Chenopodium sp.	0		NA	0	Amaranthaceae Juss.
Iz_5_1009	DJI_0776	Chondrilla juncea L.	0	n	1	0	Asteraceae Bercht. & J.Presl
Iz_5_1009	DJI_0776	Cirsium vulgare (Savi) Ten.	0	n	1	0	Asteraceae Bercht. & J.Presl
Iz_5_1009	DJI_0776	Clinopodium acinos (L.) Kuntze	0	n	1	0	Lamiacea Lindl.
Iz_5_1009	DJI_0776	Convallaria majalis L.	0	n	1	0	Asparagaceae Juss.
Iz_5_1009	DJI_0776	Crataegus rhipidophylla Gand.	0	n	1	1	Rosaceae Juss.
Iz_5_1009	DJI_0776	Crepis foetida L.	0	n	1	0	Asteraceae Bercht. & J.Presl
Iz_5_1009	DJI_0776	Dactylis glomerata L.	0	n	1	0	Poaceae Barnhart
Iz_5_1009	DJI_0776	Dianthus campestris M.Bieb.	0	n	1	0	Caryophyllaceae Juss.
Iz_5_1009	DJI_0776	Digitaria sanguinalis (L.) Scop.	0	a	0	0	Poaceae Barnhart
Iz_5_1009	DJI_0776	Elymus repens (L.) Gould	0	n	1	0	Poaceae Barnhart
Iz_5_1009	DJI_0776	Epilobium tetragonum L.	0	n	1	0	Onagraceae Juss.
Iz_5_1009	DJI_0776	Eragrostis minor Host	0	a	0	0	Poaceae Barnhart
Iz_5_1009	DJI_0776	Erigeron canadensis L.	1	a	0	0	Asteraceae Bercht. & J.Presl
Iz_5_1009	DJI_0776	Euphorbia seguieriana Neck.	0	n	1	0	Euphorbiaceae Juss.
Iz_5_1009	DJI_0776	Fallopia dumetorum (L.) Holub	0	n	1	0	Polygonaceae Juss.
Iz_5_1009	DJI_0776	Festuca beckeri (Hack.) Trautv.	0	n	1	0	Poaceae Barnhart
Iz_5_1009	DJI_0776	Fragaria vesca L.	0	n	1	0	Rosaceae Juss.
Iz_5_1009	DJI_0776	Frangula alnus Mill.	0	n	1	1	Rhamnaceae Juss.
Iz_5_1009	DJI_0776	Fraxinus pennsylvanica Marshall	0	a	0	1	Oleaceae Hoffmanns. & Link
Iz_5_1009	DJI_0776	Galeopsis bifida Boenn.	0	n	1	0	Lamiacea Lindl.
Iz_5_1009	DJI_0776	Galium verum L.	0	n	1	0	Rubiaceae Juss.
Iz_5_1009	DJI_0776	Genista tinctoria L.	0	n	1	0	Fabaceae Lindl.
Iz_5_1009	DJI_0776	Geranium robertianum L.	0	n	1	0	Geraniaceae Juss.
Iz_5_1009	DJI_0776	Geranium sanquineum L.	0	n	1	0	Geraniaceae Juss.
Iz_5_1009	DJI_0776	Gypsophila paniculata L.	0	n	1	0	Caryophyllaceae Juss.
Iz_5_1009	DJI_0776	Helichrysum arenarium (L.) Moench	0	n	1	0	Asteraceae Bercht. & J.Presl
Iz_5_1009	DJI_0776	Herniaria polygama J.Gay	0	n	1	0	Caryophyllaceae Juss.
Iz_5_1009	DJI_0776	Hieracium robustum Fr.	0	n	1	0	Asteraceae Bercht. & J.Presl
Iz_5_1009	DJI_0776	Hieracium umbellatum L.	0	n	1	NA	Asteraceae Bercht. & J.Presl
Iz_5_1009	DJI_0776	Hylotelephium maximum (L.) Holub	0	n	1	0	Crassulaceae J.St.-Hil.
Iz_5_1009	DJI_0776	Jacobaea borysthenica (DC.) B.Nord. & Greuter	0	n	1	0	Asteraceae Bercht. & J.Presl
Iz_5_1009	DJI_0776	Jasione montana L.	0	n	1	0	Campanulaceae Juss.
Iz_5_1009	DJI_0776	Jurinea cyanoides (L.) Rchb.s.l.	0	n	1	0	Asteraceae Bercht. & J.Presl
Iz_5_1009	DJI_0776	Koeleria glauca (Spreng.) DC.	0	n	1	0	Poaceae Barnhart
Iz_5_1009	DJI_0776	Lactuca serriola L.	0	a	0	0	Asteraceae Bercht. & J.Presl
Iz_5_1009	DJI_0776	Linaria genistifolia (L.) Mill.	0	n	1	0	Plantaginaceae Juss.
Iz_5_1009	DJI_0776	Lomelosia argentea (L.) Greuter & Burdet	0	n	1	0	Caprifoliaceae Juss.
Iz_5_1009	DJI_0776	Melampyrum pratense L.	0	n	1	0	Orobanchaceae Vent.
Iz_5_1009	DJI_0776	Oenothera biennis L.	0	a	0	0	Onagraceae Juss.

Iz_5_1009	DJI_0776	Pentanema britannica (L.) D.Gut.Larr., Santos-Vicente, Anderb., E.Rico & M.M.Mart.Ort.	0	n	1	0	Asteraceae Bercht. & J.Presl
Iz_5_1009	DJI_0776	Peucedanum oreoselinum (L.) Moench	4	n	1	0	Apiaceae Lindl.
Iz_5_1009	DJI_0776	Peucedanum ruthenicum M.Bieb.	0	n	1	0	Apiaceae Lindl.
Iz_5_1009	DJI_0776	Pilosella echioides (Lumn.) F.W.Schultz & Sch.Bip.	0	n	1	0	Asteraceae Bercht. & J.Presl
Iz_5_1009	DJI_0776	Pilosella officinarum F. Schultz et Sch. Bip.	0	n	1	0	Asteraceae Bercht. & J.Presl
Iz_5_1009	DJI_0776	Pilosella sp.	0		NA	0	Asteraceae Bercht. & J.Presl
Iz_5_1009	DJI_0776	Pinus sylvestris L.	0	n	1	1	Pinaceae Spreng. ex F.Rudolphi
Iz_5_1009	DJI_0776	Poa annua L.	0	n	1	0	Poaceae Barnhart
Iz_5_1009	DJI_0776	Poa nemoralis L.	0	n	1	0	Poaceae Barnhart
Iz_5_1009	DJI_0776	Poa sp.	0		NA	0	Poaceae Barnhart
Iz_5_1009	DJI_0776	Polygonatum odoratum (Mill.) Druce	0	n	1	0	Asparagaceae Juss.
Iz_5_1009	DJI_0776	Polygonum arenarium Waldst. & Kit.	0	n	1	0	Polygonaceae Juss.
Iz_5_1009	DJI_0776	Populus nigra L.	0	n	1	1	Salicaceae Mirb.
Iz_5_1009	DJI_0776	Populus sp.	0	n	1	1	Salicaceae Mirb.
Iz_5_1009	DJI_0776	Populus tremula L.	0	n	1	1	Salicaceae Mirb.
Iz_5_1009	DJI_0776	Populus Ã canescens (Aiton) Sm.	0	n	1	1	Salicaceae Mirb.
Iz_5_1009	DJI_0776	Potentilla argentea L.	0	n	1	0	Rosaceae Juss.
Iz_5_1009	DJI_0776	Psammophiliella muralis (L.) Ikonn.	0	n	1	0	Caryophyllaceae Juss.
Iz_5_1009	DJI_0776	Psephellus sumensis (Kalen.) Greuter	0	n	1	0	Asteraceae Bercht. & J.Presl
Iz_5_1009	DJI_0776	Pulsatilla pratensis (L.) Mill. s.l.	0	n	1	0	Ranunculaceae Juss.
Iz_5_1009	DJI_0776	Pyrus communis L.	0	a	0	1	Rosaceae Juss.
Iz_5_1009	DJI_0776	Quercus robur L.	0	n	1	1	Fagaceae Dumort.
Iz_5_1009	DJI_0776	Robinia pseudoacacia L.	0	a	0	1	Fabaceae Lindl.
Iz_5_1009	DJI_0776	Rosa sp.	0		NA	1	Rosaceae Juss.
Iz_5_1009	DJI_0776	Rubus idaeus L.	0	n	1	0	Rosaceae Juss.
Iz_5_1009	DJI_0776	Rumex acetosella L.	0	n	1	0	Polygonaceae Juss.
Iz_5_1009	DJI_0776	Rumex thyrsiflorus Fingerh.	0	n	1	0	Polygonaceae Juss.
Iz_5_1009	DJI_0776	Salix alba L.	0	n	1	1	Salicaceae Mirb.
Iz_5_1009	DJI_0776	Sambucus nigra L.	0	n	1	1	Viburnaceae Raf.
Iz_5_1009	DJI_0776	Secale sylvestre Host	0	n	1	0	Poaceae Barnhart
Iz_5_1009	DJI_0776	Sempervivum ruthenicum Schnittsp. & C.B. Lehm.	0	n	1	0	Crassulaceae J.St.-Hil.
Iz_5_1009	DJI_0776	Setaria viridis (L.) P. Beauv.	0	n	1	0	Poaceae Barnhart
Iz_5_1009	DJI_0776	Silene borysthenica (Gruner) Walters	0	n	1	0	Caryophyllaceae Juss.
Iz_5_1009	DJI_0776	Silene latifolia subsp. alba (Mill.) Greuter & Burdet	0	n	1	0	Caryophyllaceae Juss.
Iz_5_1009	DJI_0776	Solidago virgaurea L.	1	n	1	0	Asteraceae Bercht. & J.Presl
Iz_5_1009	DJI_0776	Solidago Ã niederederi (S. canadensis Ã S. virgaurea)	0	hybrid	0	0	Asteraceae Bercht. & J.Presl
Iz_5_1009	DJI_0776	Sorbus aucuparia L.	0	n	1	1	Rosaceae Juss.
Iz_5_1009	DJI_0776	Stachys recta L.	0	n	1	0	Lamiacea Lindl.
Iz_5_1009	DJI_0776	Tanacetum vulgare L.	0	n	1	0	Asteraceae Bercht. & J.Presl
Iz_5_1009	DJI_0776	Thymus pallasianus Heinr.Braun	0	n	1	0	Lamiacea Lindl.
Iz_5_1009	DJI_0776	Tilia cordata Mill.	0	n	1	1	Malvaceae Juss.
Iz_5_1009	DJI_0776	Trifolium alpestre L.	0	n	1	0	Fabaceae Lindl.
Iz_5_1009	DJI_0776	Trifolium arvense L.	0	n	1	0	Fabaceae Lindl.
Iz_5_1009	DJI_0776	Ulmus glabra Huds.	0	n	1	1	Ulmaceae Mirb.
Iz_5_1009	DJI_0776	Verbascum densiflorum Bertol.	0	n	1	0	Scrophulariaceae Juss.
Iz_5_1009	DJI_0776	Veronica austriaca L.	0	n	1	0	Plantaginaceae Juss.
Iz_5_1009	DJI_0776	Veronica incana L.	0	n	1	0	Plantaginaceae Juss.
Iz_5_1009	DJI_0776	Veronica verna L.	0	n	1	0	Plantaginaceae Juss.
Iz_5_1009	DJI_0776	Vincetoxicum hirundinaria Medik	0	n	1	0	Apocynaceae Juss.
Iz_5_1009	DJI_0777	Acer tataricum L.	0	n	1	1	Sapindaceae Juss.
Iz_5_1009	DJI_0777	Achillea millefolium L. s.l.	0	n	1	0	Asteraceae Bercht. & J.Presl
Iz_5_1009	DJI_0777	Allium oleraceum L.	0	n	1	0	Amaryllidaceae J.St.-Hil.
Iz_5_1009	DJI_0777	Ambrosia artemisiifolia L.	0	a	0	0	Asteraceae Bercht. & J.Presl
Iz_5_1009	DJI_0777	Amorpha fruticosa L.	0	a	0	0	Fabaceae Lindl.
Iz_5_1009	DJI_0777	Anthericum ramosum L.	0	n	1	0	Asparagaceae Juss.
Iz_5_1009	DJI_0777	Artemisia marschalliana Spreng.	0	n	1	0	Asteraceae Bercht. & J.Presl
Iz_5_1009	DJI_0777	Asparagus officinalis L.	0	n	1	0	Asparagaceae Juss.
Iz_5_1009	DJI_0777	Astragalus cicer L.	0	n	1	0	Fabaceae Lindl.
Iz_5_1009	DJI_0777	Bassia laniflora (S.G.Gmel.) A.J.Scott	0	n	1	0	Amaranthaceae Juss.
Iz_5_1009	DJI_0777	Berberis vulgaris L.	0	n	1	0	Berberidaceae Juss.
Iz_5_1009	DJI_0777	Betula pendula Roth.	0	n	1	1	Betulaceae Gray
Iz_5_1009	DJI_0777	Betula pendula Roth.	0	n	1	0	Poaceae Barnhart
Iz_5_1009	DJI_0777	Bromus inermis Leyss.	0		NA	NA	
Iz_5_1009	DJI_0777	Bromus squarrosus L.	0	a	0	0	Poaceae Barnhart
Iz_5_1009	DJI_0777	Bromus tectorum L.	0	a	0	0	Poaceae Barnhart
Iz_5_1009	DJI_0777	Calamagrostis epigejos (L.) Roth	10	n	1	0	Poaceae Barnhart
Iz_5_1009	DJI_0777	Carduus acanthoides L.	0	n	1	0	Asteraceae Bercht. & J.Presl
Iz_5_1009	DJI_0777	Carex sp.	0	n	1	0	Cyperaceae Juss.
Iz_5_1009	DJI_0777	Centaurea majorovii Dumbadze	0	n	1	0	Asteraceae Bercht. & J.Presl
Iz_5_1009	DJI_0777	Chamaecytisus ruthenicus (Fisch.ex Wol.) Klaskova	0	n	1	0	Fabaceae Lindl.
Iz_5_1009	DJI_0777	Chelidonium majus L.	0	n	1	0	Papaveraceae Juss.
Iz_5_1009	DJI_0777	Chenopodium betaceum Andrz.	0	n	1	0	Amaranthaceae Juss.
Iz_5_1009	DJI_0777	Chenopodium cf. album	0	n	1	0	Amaranthaceae Juss.
Iz_5_1009	DJI_0777	Chenopodium sp.	0		NA	0	Amaranthaceae Juss.
Iz_5_1009	DJI_0777	Chondrilla juncea L.	0	n	1	0	Asteraceae Bercht. & J.Presl
Iz_5_1009	DJI_0777	Cirsium vulgare (Savi) Ten.	0	n	1	0	Asteraceae Bercht. & J.Presl
Iz_5_1009	DJI_0777	Clinopodium acinos (L.) Kuntze	0	n	1	0	Lamiacea Lindl.
Iz_5_1009	DJI_0777	Convallaria majalis L.	0	n	1	0	Asparagaceae Juss.
Iz_5_1009	DJI_0777	Crataegus rhipidophylla Gand.	0	n	1	1	Rosaceae Juss.
Iz_5_1009	DJI_0777	Crepis foetida L.	0	n	1	0	Asteraceae Bercht. & J.Presl
Iz_5_1009	DJI_0777	Dactylis glomerata L.	0	n	1	0	Poaceae Barnhart
Iz_5_1009	DJI_0777	Dianthus campestris M.Bieb.	0	n	1	0	Caryophyllaceae Juss.
Iz_5_1009	DJI_0777	Digitaria sanguinalis (L.) Scop.	0	a	0	0	Poaceae Barnhart
Iz_5_1009	DJI_0777	Elymus repens (L.) Gould	0	n	1	0	Poaceae Barnhart
Iz_5_1009	DJI_0777	Epilobium tetragonum L.	0	n	1	0	Onagraceae Juss.
Iz_5_1009	DJI_0777	Eragrostis minor Host	0	a	0	0	Poaceae Barnhart
Iz_5_1009	DJI_0777	Erigeron canadensis L.	0.5	a	0	0	Asteraceae Bercht. & J.Presl
Iz_5_1009	DJI_0777	Euphorbia seguieriana Neck.	0	n	1	0	Euphorbiaceae Juss.
Iz_5_1009	DJI_0777	Fallopia dumetorum (L.) Holub	0	n	1	0	Polygonaceae Juss.
Iz_5_1009	DJI_0777	Festuca beckeri (Hack.) Trautv.	0	n	1	0	Poaceae Barnhart
Iz_5_1009	DJI_0777	Fragaria vesca L.	0	n	1	0	Rosaceae Juss.
Iz_5_1009	DJI_0777	Frangula alnus Mill.	0	n	1	1	Rhamnaceae Juss.
Iz_5_1009	DJI_0777	Fraxinus pennsylvanica Marshall	0	a	0	1	Oleaceae Hoffmanns. & Link
Iz_5_1009	DJI_0777	Galeopsis bifida Boenn.	0	n	1	0	Lamiacea Lindl.
Iz_5_1009	DJI_0777	Galium verum L.	0	n	1	0	Rubiaceae Juss.
Iz_5_1009	DJI_0777	Genista tinctoria L.	0	n	1	0	Fabaceae Lindl.
Iz_5_1009	DJI_0777	Geranium robertianum L.	0	n	1	0	Geraniaceae Juss.
Iz_5_1009	DJI_0777	Geranium sanquineum L.	0	n	1	0	Geraniaceae Juss.

Iz_5_1009	DJI_0777	Gypsophila paniculata L.	0	n	1	0	Caryophyllaceae Juss.
Iz_5_1009	DJI_0777	Helichrysum arenarium (L.) Moench	0	n	1	0	Asteraceae Bercht. & J.Presl
Iz_5_1009	DJI_0777	Herniaria polygama J.Gay	0	n	1	0	Caryophyllaceae Juss.
Iz_5_1009	DJI_0777	Hieracium robustum Fr.	0	n	1	0	Asteraceae Bercht. & J.Presl
Iz_5_1009	DJI_0777	Hieracium umbellatum L.	0	n	1	NA	Asteraceae Bercht. & J.Presl
Iz_5_1009	DJI_0777	Hylotelephium maximum (L.) Holub	0	n	1	0	Crassulaceae J.St.-Hil.
Iz_5_1009	DJI_0777	Jacobaea borysthenica (DC.) B.Nord. & Greuter	0	n	1	0	Asteraceae Bercht. & J.Presl
Iz_5_1009	DJI_0777	Jasione montana L.	0	n	1	0	Campanulaceae Juss.
Iz_5_1009	DJI_0777	Jurinea cyanoides (L.) Rchb.s.l.	0	n	1	0	Asteraceae Bercht. & J.Presl
Iz_5_1009	DJI_0777	Koeleria glauca (Spreng.) DC.	0	n	1	0	Poaceae Barnhart
Iz_5_1009	DJI_0777	Lactuca serriola L.	0	a	0	0	Asteraceae Bercht. & J.Presl
Iz_5_1009	DJI_0777	Linaria genistifolia (L.) Mill.	0	n	1	0	Plantaginaceae Juss.
Iz_5_1009	DJI_0777	Lomelosia argentea (L.) Greuter & Burdet	0	n	1	0	Caprifoliaceae Juss.
Iz_5_1009	DJI_0777	Melampyrum pratense L.	0	n	1	0	Orobanchaceae Vent.
Iz_5_1009	DJI_0777	Oenothera biennis L.	0	a	0	0	Onagraceae Juss.
Iz_5_1009	DJI_0777	Pentanema britannica (L.) D.Gut.Larr., Santos-Vicente, Anderb., E.Rico & M.M.Mart.Ort.	0	n	1	0	Asteraceae Bercht. & J.Presl
Iz_5_1009	DJI_0777	Peucedanum oreoselinum (L.) Moench	3	n	1	0	Apiaceae Lindl.
Iz_5_1009	DJI_0777	Peucedanum ruthenicum M.Bieb.	0	n	1	0	Apiaceae Lindl.
Iz_5_1009	DJI_0777	Pilosella echioides (Lumn.) F.W.Schultz & Sch.Bip.	0	n	1	0	Asteraceae Bercht. & J.Presl
Iz_5_1009	DJI_0777	Pilosella officinarum F. Schultz et Sch. Bip.	0	n	1	0	Asteraceae Bercht. & J.Presl
Iz_5_1009	DJI_0777	Pilosella sp.	0		NA	0	Asteraceae Bercht. & J.Presl
Iz_5_1009	DJI_0777	Pinus sylvestris L.	0	n	1	1	Pinaceae Spreng. ex F.Rudolphi
Iz_5_1009	DJI_0777	Poa annua L.	0	n	1	0	Poaceae Barnhart
Iz_5_1009	DJI_0777	Poa nemoralis L.	0	n	1	0	Poaceae Barnhart
Iz_5_1009	DJI_0777	Poa sp.	0		NA	0	Poaceae Barnhart
Iz_5_1009	DJI_0777	Polygonatum odoratum (Mill.) Druce	0	n	1	0	Asparagaceae Juss.
Iz_5_1009	DJI_0777	Polygonum arenarium Waldst. & Kit.	0	n	1	0	Polygonaceae Juss.
Iz_5_1009	DJI_0777	Populus nigra L.	0	n	1	1	Salicaceae Mirb.
Iz_5_1009	DJI_0777	Populus sp.	0	n	1	1	Salicaceae Mirb.
Iz_5_1009	DJI_0777	Populus tremula L.	0	n	1	1	Salicaceae Mirb.
Iz_5_1009	DJI_0777	Populus Ã canescens (Aiton) Sm.	0	n	1	1	Salicaceae Mirb.
Iz_5_1009	DJI_0777	Potentilla argentea L.	0	n	1	0	Rosaceae Juss.
Iz_5_1009	DJI_0777	Psammophiliella muralis (L.) Ikonn.	0	n	1	0	Caryophyllaceae Juss.
Iz_5_1009	DJI_0777	Psephellus sumensis (Kalen.) Greuter	0	n	1	0	Asteraceae Bercht. & J.Presl
Iz_5_1009	DJI_0777	Pulsatilla pratensis (L.) Mill. s.l.	0	n	1	0	Ranunculaceae Juss.
Iz_5_1009	DJI_0777	Pyrus communis L.	0	a	0	1	Rosaceae Juss.
Iz_5_1009	DJI_0777	Quercus robur L.	0	n	1	1	Fagaceae Dumort.
Iz_5_1009	DJI_0777	Robinia pseudoacacia L.	0	a	0	1	Fabaceae Lindl.
Iz_5_1009	DJI_0777	Rosa sp.	0		NA	1	Rosaceae Juss.
Iz_5_1009	DJI_0777	Rubus idaeus L.	0	n	1	0	Rosaceae Juss.
Iz_5_1009	DJI_0777	Rumex acetosella L.	0	n	1	0	Polygonaceae Juss.
Iz_5_1009	DJI_0777	Rumex thyrsiflorus Fingerh.	0	n	1	0	Polygonaceae Juss.
Iz_5_1009	DJI_0777	Salix alba L.	0	n	1	1	Salicaceae Mirb.
Iz_5_1009	DJI_0777	Sambucus nigra L.	0	n	1	1	Viburnaceae Raf.
Iz_5_1009	DJI_0777	Secale sylvestre Host	0	n	1	0	Poaceae Barnhart
Iz_5_1009	DJI_0777	Sempervivum ruthenicum Schnittsp. & C.B. Lehm.	0	n	1	0	Crassulaceae J.St.-Hil.
Iz_5_1009	DJI_0777	Setaria viridis (L.) P. Beauv.	0	n	1	0	Poaceae Barnhart
Iz_5_1009	DJI_0777	Silene borysthenica (Gruner) Walters	0	n	1	0	Caryophyllaceae Juss.
Iz_5_1009	DJI_0777	Silene latifolia subsp. alba (Mill.) Greuter & Burdet	0	n	1	0	Caryophyllaceae Juss.
Iz_5_1009	DJI_0777	Solidago virgaurea L.	1	n	1	0	Asteraceae Bercht. & J.Presl
Iz_5_1009	DJI_0777	Solidago Ã niederederi (S. canadensis Ã S. virgaurea)	0	hybrid	0	0	Asteraceae Bercht. & J.Presl
Iz_5_1009	DJI_0777	Sorbus aucuparia L.	0	n	1	1	Rosaceae Juss.
Iz_5_1009	DJI_0777	Stachys recta L.	0	n	1	0	Lamiacea Lindl.
Iz_5_1009	DJI_0777	Tanacetum vulgare L.	0	n	1	0	Asteraceae Bercht. & J.Presl
Iz_5_1009	DJI_0777	Thymus pallasianus Heinr.Braun	0	n	1	0	Lamiacea Lindl.
Iz_5_1009	DJI_0777	Tilia cordata Mill.	0	n	1	1	Malvaceae Juss.
Iz_5_1009	DJI_0777	Trifolium alpestre L.	0	n	1	0	Fabaceae Lindl.
Iz_5_1009	DJI_0777	Trifolium arvense L.	0	n	1	0	Fabaceae Lindl.
Iz_5_1009	DJI_0777	Ulmus glabra Huds.	0	n	1	1	Ulmaceae Mirb.
Iz_5_1009	DJI_0777	Verbascum densiflorum Bertol.	0	n	1	0	Scrophulariaceae Juss.
Iz_5_1009	DJI_0777	Veronica austriaca L.	0	n	1	0	Plantaginaceae Juss.
Iz_5_1009	DJI_0777	Veronica incana L.	0	n	1	0	Plantaginaceae Juss.
Iz_5_1009	DJI_0777	Veronica verna L.	0	n	1	0	Plantaginaceae Juss.
Iz_5_1009	DJI_0777	Vincetoxicum hirundinaria Medik	0	n	1	0	Apocynaceae Juss.
Iz_5_1009	DJI_0781	Acer tataricum L.	0	n	1	1	Sapindaceae Juss.
Iz_5_1009	DJI_0781	Achillea millefolium L. s.l.	0	n	1	0	Asteraceae Bercht. & J.Presl
Iz_5_1009	DJI_0781	Allium oleraceum L.	0	n	1	0	Amaryllidaceae J.St.-Hil.
Iz_5_1009	DJI_0781	Ambrosia artemisiifolia L.	0	a	0	0	Asteraceae Bercht. & J.Presl
Iz_5_1009	DJI_0781	Amorpha fruticosa L.	0	a	0	0	Fabaceae Lindl.
Iz_5_1009	DJI_0781	Anthericum ramosum L.	0	n	1	0	Asparagaceae Juss.
Iz_5_1009	DJI_0781	Artemisia marschalliana Spreng.	0	n	1	0	Asteraceae Bercht. & J.Presl
Iz_5_1009	DJI_0781	Asparagus officinalis L.	0	n	1	0	Asparagaceae Juss.
Iz_5_1009	DJI_0781	Astragalus cicer L.	0	n	1	0	Fabaceae Lindl.
Iz_5_1009	DJI_0781	Bassia laniflora (S.G.Gmel.) A.J.Scott	0	n	1	0	Amaranthaceae Juss.
Iz_5_1009	DJI_0781	Berberis vulgaris L.	0	n	1	0	Berberidaceae Juss.
Iz_5_1009	DJI_0781	Betula pendula Roth.	0	n	1	1	Betulaceae Gray
Iz_5_1009	DJI_0781	Betula pendula Roth.	0	n	1	0	Poaceae Barnhart
Iz_5_1009	DJI_0781	Bromus inermis Leyss.	0		NA	NA	
Iz_5_1009	DJI_0781	Bromus squarrosus L.	0	a	0	0	Poaceae Barnhart
Iz_5_1009	DJI_0781	Bromus tectorum L.	0	a	0	0	Poaceae Barnhart
Iz_5_1009	DJI_0781	Calamagrostis epigejos (L.) Roth	2	n	1	0	Poaceae Barnhart
Iz_5_1009	DJI_0781	Carduus acanthoides L.	0	n	1	0	Asteraceae Bercht. & J.Presl
Iz_5_1009	DJI_0781	Carex sp.	0	n	1	0	Cyperaceae Juss.
Iz_5_1009	DJI_0781	Centaurea majorovii Dumbadze	0	n	1	0	Asteraceae Bercht. & J.Presl
Iz_5_1009	DJI_0781	Chamaecytisus ruthenicus (Fisch.ex Wol.) Klaskova	0	n	1	0	Fabaceae Lindl.
Iz_5_1009	DJI_0781	Chelidonium majus L.	0	n	1	0	Papaveraceae Juss.
Iz_5_1009	DJI_0781	Chenopodium betaceum Andrz.	0	n	1	0	Amaranthaceae Juss.
Iz_5_1009	DJI_0781	Chenopodium cf. album	0	n	1	0	Amaranthaceae Juss.
Iz_5_1009	DJI_0781	Chenopodium sp.	0		NA	0	Amaranthaceae Juss.
Iz_5_1009	DJI_0781	Chondrilla juncea L.	0	n	1	0	Asteraceae Bercht. & J.Presl
Iz_5_1009	DJI_0781	Cirsium vulgare (Savi) Ten.	0	n	1	0	Asteraceae Bercht. & J.Presl
Iz_5_1009	DJI_0781	Clinopodium acinos (L.) Kuntze	0	n	1	0	Lamiacea Lindl.
Iz_5_1009	DJI_0781	Convallaria majalis L.	0	n	1	0	Asparagaceae Juss.
Iz_5_1009	DJI_0781	Crataegus rhipidophylla Gand.	0	n	1	1	Rosaceae Juss.
Iz_5_1009	DJI_0781	Crepis foetida L.	0	n	1	0	Asteraceae Bercht. & J.Presl
Iz_5_1009	DJI_0781	Dactylis glomerata L.	0	n	1	0	Poaceae Barnhart
Iz_5_1009	DJI_0781	Dianthus campestris M.Bieb.	0	n	1	0	Caryophyllaceae Juss.
Iz_5_1009	DJI_0781	Digitaria sanguinalis (L.) Scop.	0	a	0	0	Poaceae Barnhart

Iz_5_1009	DJI_0781	Elymus repens (L.) Gould	0	n	1	0	Poaceae Barnhart
Iz_5_1009	DJI_0781	Epilobium tetragonum L.	0	n	1	0	Onagraceae Juss.
Iz_5_1009	DJI_0781	Eragrostis minor Host	0	a	0	0	Poaceae Barnhart
Iz_5_1009	DJI_0781	Erigeron canadensis L.	1	a	0	0	Asteraceae Bercht. & J.Presl
Iz_5_1009	DJI_0781	Euphorbia seguieriana Neck.	0	n	1	0	Euphorbiaceae Juss.
Iz_5_1009	DJI_0781	Fallopia dumetorum (L.) Holub	0	n	1	0	Polygonaceae Juss.
Iz_5_1009	DJI_0781	Festuca beckeri (Hack.) Trautv.	0	n	1	0	Poaceae Barnhart
Iz_5_1009	DJI_0781	Fragaria vesca L.	0	n	1	0	Rosaceae Juss.
Iz_5_1009	DJI_0781	Frangula alnus Mill.	0	n	1	1	Rhamnaceae Juss.
Iz_5_1009	DJI_0781	Fraxinus pennsylvanica Marshall	0	a	0	1	Oleaceae Hoffmanns. & Link
Iz_5_1009	DJI_0781	Galeopsis bifida Boenn.	0	n	1	0	Lamiacea Lindl.
Iz_5_1009	DJI_0781	Galium verum L.	0	n	1	0	Rubiaceae Juss.
Iz_5_1009	DJI_0781	Genista tinctoria L.	0	n	1	0	Fabaceae Lindl.
Iz_5_1009	DJI_0781	Geranium robertianum L.	0	n	1	0	Geraniaceae Juss.
Iz_5_1009	DJI_0781	Geranium sanquineum L.	0	n	1	0	Geraniaceae Juss.
Iz_5_1009	DJI_0781	Gypsophila paniculata L.	0	n	1	0	Caryophyllaceae Juss.
Iz_5_1009	DJI_0781	Helichrysum arenarium (L.) Moench	0	n	1	0	Asteraceae Bercht. & J.Presl
Iz_5_1009	DJI_0781	Herniaria polygama J.Gay	0	n	1	0	Caryophyllaceae Juss.
Iz_5_1009	DJI_0781	Hieracium robustum Fr.	0	n	1	0	Asteraceae Bercht. & J.Presl
Iz_5_1009	DJI_0781	Hieracium umbellatum L.	0	n	1	NA	Asteraceae Bercht. & J.Presl
Iz_5_1009	DJI_0781	Hylotelephium maximum (L.) Holub	0	n	1	0	Crassulaceae J.St.-Hil.
Iz_5_1009	DJI_0781	Jacobaea borysthenica (DC.) B.Nord. & Greuter	0	n	1	0	Asteraceae Bercht. & J.Presl
Iz_5_1009	DJI_0781	Jasione montana L.	0	n	1	0	Campanulaceae Juss.
Iz_5_1009	DJI_0781	Jurinea cyanoides (L.) Rchb.s.l.	0	n	1	0	Asteraceae Bercht. & J.Presl
Iz_5_1009	DJI_0781	Koeleria glauca (Spreng.) DC.	0	n	1	0	Poaceae Barnhart
Iz_5_1009	DJI_0781	Lactuca serriola L.	0	a	0	0	Asteraceae Bercht. & J.Presl
Iz_5_1009	DJI_0781	Linaria genistifolia (L.) Mill.	0	n	1	0	Plantaginaceae Juss.
Iz_5_1009	DJI_0781	Lomelosia argentea (L.) Greuter & Burdet	0	n	1	0	Caprifoliaceae Juss.
Iz_5_1009	DJI_0781	Melampyrum pratense L.	0	n	1	0	Orobanchaceae Vent.
Iz_5_1009	DJI_0781	Oenothera biennis L.	0	a	0	0	Onagraceae Juss.
Iz_5_1009	DJI_0781	Pentanema britannica (L.) D.Gut.Larr., Santos-Vicente, Anderb., E.Rico & M.M.Mart.Ort.	0	n	1	0	Asteraceae Bercht. & J.Presl
Iz_5_1009	DJI_0781	Peucedanum oreoselinum (L.) Moench	0	n	1	0	Apiaceae Lindl.
Iz_5_1009	DJI_0781	Peucedanum ruthenicum M.Bieb.	0	n	1	0	Apiaceae Lindl.
Iz_5_1009	DJI_0781	Pilosella echioides (Lumn.) F.W.Schultz & Sch.Bip.	0	n	1	0	Asteraceae Bercht. & J.Presl
Iz_5_1009	DJI_0781	Pilosella officinarum F. Schultz et Sch. Bip.	0	n	1	0	Asteraceae Bercht. & J.Presl
Iz_5_1009	DJI_0781	Pilosella sp.	0		NA	0	Asteraceae Bercht. & J.Presl
Iz_5_1009	DJI_0781	Pinus sylvestris L.	0	n	1	1	Pinaceae Spreng. ex F.Rudolphi
Iz_5_1009	DJI_0781	Poa annua L.	0	n	1	0	Poaceae Barnhart
Iz_5_1009	DJI_0781	Poa nemoralis L.	0	n	1	0	Poaceae Barnhart
Iz_5_1009	DJI_0781	Poa sp.	0		NA	0	Poaceae Barnhart
Iz_5_1009	DJI_0781	Polygonatum odoratum (Mill.) Druce	0	n	1	0	Asparagaceae Juss.
Iz_5_1009	DJI_0781	Polygonum arenarium Waldst. & Kit.	0	n	1	0	Polygonaceae Juss.
Iz_5_1009	DJI_0781	Populus nigra L.	0	n	1	1	Salicaceae Mirb.
Iz_5_1009	DJI_0781	Populus sp.	0	n	1	1	Salicaceae Mirb.
Iz_5_1009	DJI_0781	Populus tremula L.	0	n	1	1	Salicaceae Mirb.
Iz_5_1009	DJI_0781	Populus Ã canescens (Aiton) Sm.	0	n	1	1	Salicaceae Mirb.
Iz_5_1009	DJI_0781	Potentilla argentea L.	0	n	1	0	Rosaceae Juss.
Iz_5_1009	DJI_0781	Psammophiliella muralis (L.) Ikonn.	0	n	1	0	Caryophyllaceae Juss.
Iz_5_1009	DJI_0781	Psephellus sumensis (Kalen.) Greuter	0	n	1	0	Asteraceae Bercht. & J.Presl
Iz_5_1009	DJI_0781	Pulsatilla pratensis (L.) Mill. s.l.	0	n	1	0	Ranunculaceae Juss.
Iz_5_1009	DJI_0781	Pyrus communis L.	0	a	0	1	Rosaceae Juss.
Iz_5_1009	DJI_0781	Quercus robur L.	0	n	1	1	Fagaceae Dumort.
Iz_5_1009	DJI_0781	Robinia pseudoacacia L.	0	a	0	1	Fabaceae Lindl.
Iz_5_1009	DJI_0781	Rosa sp.	0		NA	1	Rosaceae Juss.
Iz_5_1009	DJI_0781	Rubus idaeus L.	0	n	1	0	Rosaceae Juss.
Iz_5_1009	DJI_0781	Rumex acetosella L.	0	n	1	0	Polygonaceae Juss.
Iz_5_1009	DJI_0781	Rumex thyrsiflorus Fingerh.	0	n	1	0	Polygonaceae Juss.
Iz_5_1009	DJI_0781	Salix alba L.	0	n	1	1	Salicaceae Mirb.
Iz_5_1009	DJI_0781	Sambucus nigra L.	0	n	1	1	Viburnaceae Raf.
Iz_5_1009	DJI_0781	Secale sylvestre Host	0	n	1	0	Poaceae Barnhart
Iz_5_1009	DJI_0781	Sempervivum ruthenicum Schnittsp. & C.B. Lehm.	0	n	1	0	Crassulaceae J.St.-Hil.
Iz_5_1009	DJI_0781	Setaria viridis (L.) P. Beauv.	0	n	1	0	Poaceae Barnhart
Iz_5_1009	DJI_0781	Silene borysthenica (Gruner) Walters	0	n	1	0	Caryophyllaceae Juss.
Iz_5_1009	DJI_0781	Silene latifolia subsp. alba (Mill.) Greuter & Burdet	0	n	1	0	Caryophyllaceae Juss.
Iz_5_1009	DJI_0781	Solidago virgaurea L.	6	n	1	0	Asteraceae Bercht. & J.Presl
Iz_5_1009	DJI_0781	Solidago Ã niederederi (S. canadensis Ã S. virgaurea)	0	hybrid	0	0	Asteraceae Bercht. & J.Presl
Iz_5_1009	DJI_0781	Sorbus aucuparia L.	0	n	1	1	Rosaceae Juss.
Iz_5_1009	DJI_0781	Stachys recta L.	0	n	1	0	Lamiacea Lindl.
Iz_5_1009	DJI_0781	Tanacetum vulgare L.	0	n	1	0	Asteraceae Bercht. & J.Presl
Iz_5_1009	DJI_0781	Thymus pallasianus Heinr.Braun	0	n	1	0	Lamiacea Lindl.
Iz_5_1009	DJI_0781	Tilia cordata Mill.	0	n	1	1	Malvaceae Juss.
Iz_5_1009	DJI_0781	Trifolium alpestre L.	0	n	1	0	Fabaceae Lindl.
Iz_5_1009	DJI_0781	Trifolium arvense L.	0	n	1	0	Fabaceae Lindl.
Iz_5_1009	DJI_0781	Ulmus glabra Huds.	0	n	1	1	Ulmaceae Mirb.
Iz_5_1009	DJI_0781	Verbascum densiflorum Bertol.	0	n	1	0	Scrophulariaceae Juss.
Iz_5_1009	DJI_0781	Veronica austriaca L.	0	n	1	0	Plantaginaceae Juss.
Iz_5_1009	DJI_0781	Veronica incana L.	0	n	1	0	Plantaginaceae Juss.
Iz_5_1009	DJI_0781	Veronica verna L.	0	n	1	0	Plantaginaceae Juss.
Iz_5_1009	DJI_0781	Vincetoxicum hirundinaria Medik	0	n	1	0	Apocynaceae Juss.
Iz_5_1009	DJI_0783	Acer tataricum L.	0	n	1	1	Sapindaceae Juss.
Iz_5_1009	DJI_0783	Achillea millefolium L. s.l.	0	n	1	0	Asteraceae Bercht. & J.Presl
Iz_5_1009	DJI_0783	Allium oleraceum L.	0	n	1	0	Amaryllidaceae J.St.-Hil.
Iz_5_1009	DJI_0783	Ambrosia artemisiifolia L.	0	a	0	0	Asteraceae Bercht. & J.Presl
Iz_5_1009	DJI_0783	Amorpha fruticosa L.	0	a	0	0	Fabaceae Lindl.
Iz_5_1009	DJI_0783	Anthericum ramosum L.	0	n	1	0	Asparagaceae Juss.
Iz_5_1009	DJI_0783	Artemisia marschalliana Spreng.	0	n	1	0	Asteraceae Bercht. & J.Presl
Iz_5_1009	DJI_0783	Asparagus officinalis L.	0	n	1	0	Asparagaceae Juss.
Iz_5_1009	DJI_0783	Astragalus cicer L.	0	n	1	0	Fabaceae Lindl.
Iz_5_1009	DJI_0783	Bassia laniflora (S.G.Gmel.) A.J.Scott	0	n	1	0	Amaranthaceae Juss.
Iz_5_1009	DJI_0783	Berberis vulgaris L.	0	n	1	0	Berberidaceae Juss.
Iz_5_1009	DJI_0783	Betula pendula Roth.	0	n	1	1	Betulaceae Gray
Iz_5_1009	DJI_0783	Betula pendula Roth.	0	n	1	0	Poaceae Barnhart
Iz_5_1009	DJI_0783	Bromus inermis Leyss.	0		NA	NA	
Iz_5_1009	DJI_0783	Bromus squarrosus L.	0	a	0	0	Poaceae Barnhart
Iz_5_1009	DJI_0783	Bromus tectorum L.	0	a	0	0	Poaceae Barnhart
Iz_5_1009	DJI_0783	Calamagrostis epigejos (L.) Roth	1	n	1	0	Poaceae Barnhart
Iz_5_1009	DJI_0783	Carduus acanthoides L.	0	n	1	0	Asteraceae Bercht. & J.Presl
Iz_5_1009	DJI_0783	Carex sp.	0	n	1	0	Cyperaceae Juss.

Iz_5_1009	DJI_0783	Centaurea majorovii Dumbadze	0	n	1	0	Asteraceae Bercht. & J.Presl
Iz_5_1009	DJI_0783	Chamaecytisus ruthenicus (Fisch.ex Wol.) Klaskova	0	n	1	0	Fabaceae Lindl.
Iz_5_1009	DJI_0783	Chelidonium majus L.	0	n	1	0	Papaveraceae Juss.
Iz_5_1009	DJI_0783	Chenopodium betaceum Andrz.	0	n	1	0	Amaranthaceae Juss.
Iz_5_1009	DJI_0783	Chenopodium cf. album	0	n	1	0	Amaranthaceae Juss.
Iz_5_1009	DJI_0783	Chenopodium sp.	0		NA	0	Amaranthaceae Juss.
Iz_5_1009	DJI_0783	Chondrilla juncea L.	0	n	1	0	Asteraceae Bercht. & J.Presl
Iz_5_1009	DJI_0783	Cirsium vulgare (Savi) Ten.	0	n	1	0	Asteraceae Bercht. & J.Presl
Iz_5_1009	DJI_0783	Clinopodium acinos (L.) Kuntze	0	n	1	0	Lamiacea Lindl.
Iz_5_1009	DJI_0783	Convallaria majalis L.	0	n	1	0	Asparagaceae Juss.
Iz_5_1009	DJI_0783	Crataegus rhipidophylla Gand.	0	n	1	1	Rosaceae Juss.
Iz_5_1009	DJI_0783	Crepis foetida L.	0	n	1	0	Asteraceae Bercht. & J.Presl
Iz_5_1009	DJI_0783	Dactylis glomerata L.	0	n	1	0	Poaceae Barnhart
Iz_5_1009	DJI_0783	Dianthus campestris M.Bieb.	0	n	1	0	Caryophyllaceae Juss.
Iz_5_1009	DJI_0783	Digitaria sanguinalis (L.) Scop.	0	a	0	0	Poaceae Barnhart
Iz_5_1009	DJI_0783	Elymus repens (L.) Gould	0	n	1	0	Poaceae Barnhart
Iz_5_1009	DJI_0783	Epilobium tetragonum L.	0	n	1	0	Onagraceae Juss.
Iz_5_1009	DJI_0783	Eragrostis minor Host	0	a	0	0	Poaceae Barnhart
Iz_5_1009	DJI_0783	Erigeron canadensis L.	2	a	0	0	Asteraceae Bercht. & J.Presl
Iz_5_1009	DJI_0783	Euphorbia seguieriana Neck.	0	n	1	0	Euphorbiaceae Juss.
Iz_5_1009	DJI_0783	Fallopia dumetorum (L.) Holub	0	n	1	0	Polygonaceae Juss.
Iz_5_1009	DJI_0783	Festuca beckeri (Hack.) Trautv.	0	n	1	0	Poaceae Barnhart
Iz_5_1009	DJI_0783	Fragaria vesca L.	0	n	1	0	Rosaceae Juss.
Iz_5_1009	DJI_0783	Frangula alnus Mill.	0	n	1	1	Rhamnaceae Juss.
Iz_5_1009	DJI_0783	Fraxinus pennsylvanica Marshall	0	a	0	1	Oleaceae Hoffmanns. & Link
Iz_5_1009	DJI_0783	Galeopsis bifida Boenn.	0	n	1	0	Lamiacea Lindl.
Iz_5_1009	DJI_0783	Galium verum L.	0	n	1	0	Rubiaceae Juss.
Iz_5_1009	DJI_0783	Genista tinctoria L.	0	n	1	0	Fabaceae Lindl.
Iz_5_1009	DJI_0783	Geranium robertianum L.	0	n	1	0	Geraniaceae Juss.
Iz_5_1009	DJI_0783	Geranium sanquineum L.	0	n	1	0	Geraniaceae Juss.
Iz_5_1009	DJI_0783	Gypsophila paniculata L.	0	n	1	0	Caryophyllaceae Juss.
Iz_5_1009	DJI_0783	Helichrysum arenarium (L.) Moench	0	n	1	0	Asteraceae Bercht. & J.Presl
Iz_5_1009	DJI_0783	Herniaria polygama J.Gay	0	n	1	0	Caryophyllaceae Juss.
Iz_5_1009	DJI_0783	Hieracium robustum Fr.	0	n	1	0	Asteraceae Bercht. & J.Presl
Iz_5_1009	DJI_0783	Hieracium umbellatum L.	0	n	1	NA	Asteraceae Bercht. & J.Presl
Iz_5_1009	DJI_0783	Hylotelephium maximum (L.) Holub	0	n	1	0	Crassulaceae J.St.-Hil.
Iz_5_1009	DJI_0783	Jacobaea borysthenica (DC.) B.Nord. & Greuter	0	n	1	0	Asteraceae Bercht. & J.Presl
Iz_5_1009	DJI_0783	Jasione montana L.	0	n	1	0	Campanulaceae Juss.
Iz_5_1009	DJI_0783	Jurinea cyanoides (L.) Rchb.s.l.	0	n	1	0	Asteraceae Bercht. & J.Presl
Iz_5_1009	DJI_0783	Koeleria glauca (Spreng.) DC.	0	n	1	0	Poaceae Barnhart
Iz_5_1009	DJI_0783	Lactuca serriola L.	0	a	0	0	Asteraceae Bercht. & J.Presl
Iz_5_1009	DJI_0783	Linaria genistifolia (L.) Mill.	0	n	1	0	Plantaginaceae Juss.
Iz_5_1009	DJI_0783	Lomelosia argentea (L.) Greuter & Burdet	0	n	1	0	Caprifoliaceae Juss.
Iz_5_1009	DJI_0783	Melampyrum pratense L.	0	n	1	0	Orobanchaceae Vent.
Iz_5_1009	DJI_0783	Oenothera biennis L.	0	a	0	0	Onagraceae Juss.
Iz_5_1009	DJI_0783	Pentanema britannica (L.) D.Gut.Larr., Santos-Vicente, Anderb., E.Rico & M.M.Mart.Ort.	0	n	1	0	Asteraceae Bercht. & J.Presl
Iz_5_1009	DJI_0783	Peucedanum oreoselinum (L.) Moench	0	n	1	0	Apiaceae Lindl.
Iz_5_1009	DJI_0783	Peucedanum ruthenicum M.Bieb.	0	n	1	0	Apiaceae Lindl.
Iz_5_1009	DJI_0783	Pilosella echioides (Lumn.) F.W.Schultz & Sch.Bip.	0	n	1	0	Asteraceae Bercht. & J.Presl
Iz_5_1009	DJI_0783	Pilosella officinarum F. Schultz et Sch. Bip.	0	n	1	0	Asteraceae Bercht. & J.Presl
Iz_5_1009	DJI_0783	Pilosella sp.	0		NA	0	Asteraceae Bercht. & J.Presl
Iz_5_1009	DJI_0783	Pinus sylvestris L.	0	n	1	1	Pinaceae Spreng. ex F.Rudolphi
Iz_5_1009	DJI_0783	Poa annua L.	0	n	1	0	Poaceae Barnhart
Iz_5_1009	DJI_0783	Poa nemoralis L.	0	n	1	0	Poaceae Barnhart
Iz_5_1009	DJI_0783	Poa sp.	0		NA	0	Poaceae Barnhart
Iz_5_1009	DJI_0783	Polygonatum odoratum (Mill.) Druce	0	n	1	0	Asparagaceae Juss.
Iz_5_1009	DJI_0783	Polygonum arenarium Waldst. & Kit.	0	n	1	0	Polygonaceae Juss.
Iz_5_1009	DJI_0783	Populus nigra L.	0	n	1	1	Salicaceae Mirb.
Iz_5_1009	DJI_0783	Populus sp.	0	n	1	1	Salicaceae Mirb.
Iz_5_1009	DJI_0783	Populus tremula L.	0	n	1	1	Salicaceae Mirb.
Iz_5_1009	DJI_0783	Populus Ã canescens (Aiton) Sm.	0	n	1	1	Salicaceae Mirb.
Iz_5_1009	DJI_0783	Potentilla argentea L.	0	n	1	0	Rosaceae Juss.
Iz_5_1009	DJI_0783	Psammophiliella muralis (L.) Ikonn.	0	n	1	0	Caryophyllaceae Juss.
Iz_5_1009	DJI_0783	Psephellus sumensis (Kalen.) Greuter	0	n	1	0	Asteraceae Bercht. & J.Presl
Iz_5_1009	DJI_0783	Pulsatilla pratensis (L.) Mill. s.l.	0	n	1	0	Ranunculaceae Juss.
Iz_5_1009	DJI_0783	Pyrus communis L.	0	a	0	1	Rosaceae Juss.
Iz_5_1009	DJI_0783	Quercus robur L.	0	n	1	1	Fagaceae Dumort.
Iz_5_1009	DJI_0783	Robinia pseudoacacia L.	0	a	0	1	Fabaceae Lindl.
Iz_5_1009	DJI_0783	Rosa sp.	0		NA	1	Rosaceae Juss.
Iz_5_1009	DJI_0783	Rubus idaeus L.	0	n	1	0	Rosaceae Juss.
Iz_5_1009	DJI_0783	Rumex acetosella L.	0	n	1	0	Polygonaceae Juss.
Iz_5_1009	DJI_0783	Rumex thyrsiflorus Fingerh.	0	n	1	0	Polygonaceae Juss.
Iz_5_1009	DJI_0783	Salix alba L.	0	n	1	1	Salicaceae Mirb.
Iz_5_1009	DJI_0783	Sambucus nigra L.	0	n	1	1	Viburnaceae Raf.
Iz_5_1009	DJI_0783	Secale sylvestre Host	0	n	1	0	Poaceae Barnhart
Iz_5_1009	DJI_0783	Sempervivum ruthenicum Schnittsp. & C.B. Lehm.	0	n	1	0	Crassulaceae J.St.-Hil.
Iz_5_1009	DJI_0783	Setaria viridis (L.) P. Beauv.	0	n	1	0	Poaceae Barnhart
Iz_5_1009	DJI_0783	Silene borysthenica (Gruner) Walters	0	n	1	0	Caryophyllaceae Juss.
Iz_5_1009	DJI_0783	Silene latifolia subsp. alba (Mill.) Greuter & Burdet	0	n	1	0	Caryophyllaceae Juss.
Iz_5_1009	DJI_0783	Solidago virgaurea L.	0	n	1	0	Asteraceae Bercht. & J.Presl
Iz_5_1009	DJI_0783	Solidago Ã niederederi (S. canadensis Ã S. virgaurea)	0	hybrid	0	0	Asteraceae Bercht. & J.Presl
Iz_5_1009	DJI_0783	Sorbus aucuparia L.	0	n	1	1	Rosaceae Juss.
Iz_5_1009	DJI_0783	Stachys recta L.	0	n	1	0	Lamiacea Lindl.
Iz_5_1009	DJI_0783	Tanacetum vulgare L.	0	n	1	0	Asteraceae Bercht. & J.Presl
Iz_5_1009	DJI_0783	Thymus pallasianus Heinr.Braun	0	n	1	0	Lamiacea Lindl.
Iz_5_1009	DJI_0783	Tilia cordata Mill.	0	n	1	1	Malvaceae Juss.
Iz_5_1009	DJI_0783	Trifolium alpestre L.	0	n	1	0	Fabaceae Lindl.
Iz_5_1009	DJI_0783	Trifolium arvense L.	0	n	1	0	Fabaceae Lindl.
Iz_5_1009	DJI_0783	Ulmus glabra Huds.	0	n	1	1	Ulmaceae Mirb.
Iz_5_1009	DJI_0783	Verbascum densiflorum Bertol.	0	n	1	0	Scrophulariaceae Juss.
Iz_5_1009	DJI_0783	Veronica austriaca L.	0	n	1	0	Plantaginaceae Juss.
Iz_5_1009	DJI_0783	Veronica incana L.	0	n	1	0	Plantaginaceae Juss.
Iz_5_1009	DJI_0783	Veronica verna L.	0	n	1	0	Plantaginaceae Juss.
Iz_5_1009	DJI_0783	Vincetoxicum hirundinaria Medik	0	n	1	0	Apocynaceae Juss.
Iz_5_1009	DJI_0785	Acer tataricum L.	0	n	1	1	Sapindaceae Juss.
Iz_5_1009	DJI_0785	Achillea millefolium L. s.l.	0	n	1	0	Asteraceae Bercht. & J.Presl
Iz_5_1009	DJI_0785	Allium oleraceum L.	0	n	1	0	Amaryllidaceae J.St.-Hil.
Iz_5_1009	DJI_0785	Ambrosia artemisiifolia L.	0	a	0	0	Asteraceae Bercht. & J.Presl

Iz_5_1009	DJI_0785	Amorpha fruticosa L.	0	a	0	0	Fabaceae Lindl.
Iz_5_1009	DJI_0785	Anthericum ramosum L.	0	n	1	0	Asparagaceae Juss.
Iz_5_1009	DJI_0785	Artemisia marschalliana Spreng.	0	n	1	0	Asteraceae Bercht. & J.Presl
Iz_5_1009	DJI_0785	Asparagus officinalis L.	0	n	1	0	Asparagaceae Juss.
Iz_5_1009	DJI_0785	Astragalus cicer L.	0	n	1	0	Fabaceae Lindl.
Iz_5_1009	DJI_0785	Bassia laniflora (S.G.Gmel.) A.J.Scott	0	n	1	0	Amaranthaceae Juss.
Iz_5_1009	DJI_0785	Berberis vulgaris L.	0	n	1	0	Berberidaceae Juss.
Iz_5_1009	DJI_0785	Betula pendula Roth.	0	n	1	1	Betulaceae Gray
Iz_5_1009	DJI_0785	Betula pendula Roth.	0	n	1	0	Poaceae Barnhart
Iz_5_1009	DJI_0785	Bromus inermis Leyss.	0		NA	NA	
Iz_5_1009	DJI_0785	Bromus squarrosus L.	0	a	0	0	Poaceae Barnhart
Iz_5_1009	DJI_0785	Bromus tectorum L.	0	a	0	0	Poaceae Barnhart
Iz_5_1009	DJI_0785	Calamagrostis epigejos (L.) Roth	0.5	n	1	0	Poaceae Barnhart
Iz_5_1009	DJI_0785	Carduus acanthoides L.	0	n	1	0	Asteraceae Bercht. & J.Presl
Iz_5_1009	DJI_0785	Carex sp.	0	n	1	0	Cyperaceae Juss.
Iz_5_1009	DJI_0785	Centaurea majorovii Dumbadze	0	n	1	0	Asteraceae Bercht. & J.Presl
Iz_5_1009	DJI_0785	Chamaecytisus ruthenicus (Fisch.ex Wol.) Klaskova	0	n	1	0	Fabaceae Lindl.
Iz_5_1009	DJI_0785	Chelidonium majus L.	0	n	1	0	Papaveraceae Juss.
Iz_5_1009	DJI_0785	Chenopodium betaceum Andrz.	0	n	1	0	Amaranthaceae Juss.
Iz_5_1009	DJI_0785	Chenopodium cf. album	0	n	1	0	Amaranthaceae Juss.
Iz_5_1009	DJI_0785	Chenopodium sp.	0		NA	0	Amaranthaceae Juss.
Iz_5_1009	DJI_0785	Chondrilla juncea L.	0	n	1	0	Asteraceae Bercht. & J.Presl
Iz_5_1009	DJI_0785	Cirsium vulgare (Savi) Ten.	0	n	1	0	Asteraceae Bercht. & J.Presl
Iz_5_1009	DJI_0785	Clinopodium acinos (L.) Kuntze	0	n	1	0	Lamiacea Lindl.
Iz_5_1009	DJI_0785	Convallaria majalis L.	0	n	1	0	Asparagaceae Juss.
Iz_5_1009	DJI_0785	Crataegus rhipidophylla Gand.	0	n	1	1	Rosaceae Juss.
Iz_5_1009	DJI_0785	Crepis foetida L.	0	n	1	0	Asteraceae Bercht. & J.Presl
Iz_5_1009	DJI_0785	Dactylis glomerata L.	0	n	1	0	Poaceae Barnhart
Iz_5_1009	DJI_0785	Dianthus campestris M.Bieb.	0	n	1	0	Caryophyllaceae Juss.
Iz_5_1009	DJI_0785	Digitaria sanguinalis (L.) Scop.	0	a	0	0	Poaceae Barnhart
Iz_5_1009	DJI_0785	Elymus repens (L.) Gould	0	n	1	0	Poaceae Barnhart
Iz_5_1009	DJI_0785	Epilobium tetragonum L.	0	n	1	0	Onagraceae Juss.
Iz_5_1009	DJI_0785	Eragrostis minor Host	0	a	0	0	Poaceae Barnhart
Iz_5_1009	DJI_0785	Erigeron canadensis L.	4	a	0	0	Asteraceae Bercht. & J.Presl
Iz_5_1009	DJI_0785	Euphorbia seguieriana Neck.	0	n	1	0	Euphorbiaceae Juss.
Iz_5_1009	DJI_0785	Fallopia dumetorum (L.) Holub	0	n	1	0	Polygonaceae Juss.
Iz_5_1009	DJI_0785	Festuca beckeri (Hack.) Trautv.	0	n	1	0	Poaceae Barnhart
Iz_5_1009	DJI_0785	Fragaria vesca L.	0	n	1	0	Rosaceae Juss.
Iz_5_1009	DJI_0785	Frangula alnus Mill.	0	n	1	1	Rhamnaceae Juss.
Iz_5_1009	DJI_0785	Fraxinus pennsylvanica Marshall	0	a	0	1	Oleaceae Hoffmanns. & Link
Iz_5_1009	DJI_0785	Galeopsis bifida Boenn.	0	n	1	0	Lamiacea Lindl.
Iz_5_1009	DJI_0785	Galium verum L.	0	n	1	0	Rubiaceae Juss.
Iz_5_1009	DJI_0785	Genista tinctoria L.	0	n	1	0	Fabaceae Lindl.
Iz_5_1009	DJI_0785	Geranium robertianum L.	0	n	1	0	Geraniaceae Juss.
Iz_5_1009	DJI_0785	Geranium sanquineum L.	0	n	1	0	Geraniaceae Juss.
Iz_5_1009	DJI_0785	Gypsophila paniculata L.	0	n	1	0	Caryophyllaceae Juss.
Iz_5_1009	DJI_0785	Helichrysum arenarium (L.) Moench	0	n	1	0	Asteraceae Bercht. & J.Presl
Iz_5_1009	DJI_0785	Herniaria polygama J.Gay	0	n	1	0	Caryophyllaceae Juss.
Iz_5_1009	DJI_0785	Hieracium robustum Fr.	0	n	1	0	Asteraceae Bercht. & J.Presl
Iz_5_1009	DJI_0785	Hieracium umbellatum L.	0	n	1	NA	Asteraceae Bercht. & J.Presl
Iz_5_1009	DJI_0785	Hylotelephium maximum (L.) Holub	0	n	1	0	Crassulaceae J.St.-Hil.
Iz_5_1009	DJI_0785	Jacobaea borysthenica (DC.) B.Nord. & Greuter	0	n	1	0	Asteraceae Bercht. & J.Presl
Iz_5_1009	DJI_0785	Jasione montana L.	0	n	1	0	Campanulaceae Juss.
Iz_5_1009	DJI_0785	Jurinea cyanoides (L.) Rchb.s.l.	0	n	1	0	Asteraceae Bercht. & J.Presl
Iz_5_1009	DJI_0785	Koeleria glauca (Spreng.) DC.	0	n	1	0	Poaceae Barnhart
Iz_5_1009	DJI_0785	Lactuca serriola L.	0	a	0	0	Asteraceae Bercht. & J.Presl
Iz_5_1009	DJI_0785	Linaria genistifolia (L.) Mill.	0	n	1	0	Plantaginaceae Juss.
Iz_5_1009	DJI_0785	Lomelosia argentea (L.) Greuter & Burdet	0	n	1	0	Caprifoliaceae Juss.
Iz_5_1009	DJI_0785	Melampyrum pratense L.	0	n	1	0	Orobanchaceae Vent.
Iz_5_1009	DJI_0785	Oenothera biennis L.	0	a	0	0	Onagraceae Juss.
Iz_5_1009	DJI_0785	Pentanema britannica (L.) D.Gut.Larr., Santos-Vicente, Anderb., E.Rico & M.M.Mart.Ort.	0	n	1	0	Asteraceae Bercht. & J.Presl
Iz_5_1009	DJI_0785	Peucedanum oreoselinum (L.) Moench	0	n	1	0	Apiaceae Lindl.
Iz_5_1009	DJI_0785	Peucedanum ruthenicum M.Bieb.	0	n	1	0	Apiaceae Lindl.
Iz_5_1009	DJI_0785	Pilosella echioides (Lumn.) F.W.Schultz & Sch.Bip.	0	n	1	0	Asteraceae Bercht. & J.Presl
Iz_5_1009	DJI_0785	Pilosella officinarum F. Schultz et Sch. Bip.	0	n	1	0	Asteraceae Bercht. & J.Presl
Iz_5_1009	DJI_0785	Pilosella sp.	0		NA	0	Asteraceae Bercht. & J.Presl
Iz_5_1009	DJI_0785	Pinus sylvestris L.	0	n	1	1	Pinaceae Spreng. ex F.Rudolphi
Iz_5_1009	DJI_0785	Poa annua L.	0	n	1	0	Poaceae Barnhart
Iz_5_1009	DJI_0785	Poa nemoralis L.	0	n	1	0	Poaceae Barnhart
Iz_5_1009	DJI_0785	Poa sp.	0		NA	0	Poaceae Barnhart
Iz_5_1009	DJI_0785	Polygonatum odoratum (Mill.) Druce	0	n	1	0	Asparagaceae Juss.
Iz_5_1009	DJI_0785	Polygonum arenarium Waldst. & Kit.	0	n	1	0	Polygonaceae Juss.
Iz_5_1009	DJI_0785	Populus nigra L.	0	n	1	1	Salicaceae Mirb.
Iz_5_1009	DJI_0785	Populus sp.	0	n	1	1	Salicaceae Mirb.
Iz_5_1009	DJI_0785	Populus tremula L.	0	n	1	1	Salicaceae Mirb.
Iz_5_1009	DJI_0785	Populus Ã canescens (Aiton) Sm.	0	n	1	1	Salicaceae Mirb.
Iz_5_1009	DJI_0785	Potentilla argentea L.	0	n	1	0	Rosaceae Juss.
Iz_5_1009	DJI_0785	Psammophiliella muralis (L.) Ikonn.	0	n	1	0	Caryophyllaceae Juss.
Iz_5_1009	DJI_0785	Psephellus sumensis (Kalen.) Greuter	0	n	1	0	Asteraceae Bercht. & J.Presl
Iz_5_1009	DJI_0785	Pulsatilla pratensis (L.) Mill. s.l.	0	n	1	0	Ranunculaceae Juss.
Iz_5_1009	DJI_0785	Pyrus communis L.	0	a	0	1	Rosaceae Juss.
Iz_5_1009	DJI_0785	Quercus robur L.	0	n	1	1	Fagaceae Dumort.
Iz_5_1009	DJI_0785	Robinia pseudoacacia L.	0	a	0	1	Fabaceae Lindl.
Iz_5_1009	DJI_0785	Rosa sp.	0		NA	1	Rosaceae Juss.
Iz_5_1009	DJI_0785	Rubus idaeus L.	0	n	1	0	Rosaceae Juss.
Iz_5_1009	DJI_0785	Rumex acetosella L.	1	n	1	0	Polygonaceae Juss.
Iz_5_1009	DJI_0785	Rumex thyrsiflorus Fingerh.	0	n	1	0	Polygonaceae Juss.
Iz_5_1009	DJI_0785	Salix alba L.	0	n	1	1	Salicaceae Mirb.
Iz_5_1009	DJI_0785	Sambucus nigra L.	0	n	1	1	Viburnaceae Raf.
Iz_5_1009	DJI_0785	Secale sylvestre Host	0	n	1	0	Poaceae Barnhart
Iz_5_1009	DJI_0785	Sempervivum ruthenicum Schnittsp. & C.B. Lehm.	0	n	1	0	Crassulaceae J.St.-Hil.
Iz_5_1009	DJI_0785	Setaria viridis (L.) P. Beauv.	0	n	1	0	Poaceae Barnhart
Iz_5_1009	DJI_0785	Silene borysthenica (Gruner) Walters	0	n	1	0	Caryophyllaceae Juss.
Iz_5_1009	DJI_0785	Silene latifolia subsp. alba (Mill.) Greuter & Burdet	0	n	1	0	Caryophyllaceae Juss.
Iz_5_1009	DJI_0785	Solidago virgaurea L.	2	n	1	0	Asteraceae Bercht. & J.Presl
Iz_5_1009	DJI_0785	Solidago Ã niederederi (S. canadensis Ã S. virgaurea)	0	hybrid	0	0	Asteraceae Bercht. & J.Presl
Iz_5_1009	DJI_0785	Sorbus aucuparia L.	0	n	1	1	Rosaceae Juss.
Iz_5_1009	DJI_0785	Stachys recta L.	0	n	1	0	Lamiacea Lindl.

Iz_5_1009	DJI_0785	Tanacetum vulgare L.	0	n	1	0	Asteraceae Bercht. & J.Presl
Iz_5_1009	DJI_0785	Thymus pallasianus Heinr.Braun	0	n	1	0	Lamiacea Lindl.
Iz_5_1009	DJI_0785	Tilia cordata Mill.	0	n	1	1	Malvaceae Juss.
Iz_5_1009	DJI_0785	Trifolium alpestre L.	0	n	1	0	Fabaceae Lindl.
Iz_5_1009	DJI_0785	Trifolium arvense L.	0	n	1	0	Fabaceae Lindl.
Iz_5_1009	DJI_0785	Ulmus glabra Huds.	0	n	1	1	Ulmaceae Mirb.
Iz_5_1009	DJI_0785	Verbascum densiflorum Bertol.	0	n	1	0	Scrophulariaceae Juss.
Iz_5_1009	DJI_0785	Veronica austriaca L.	0	n	1	0	Plantaginaceae Juss.
Iz_5_1009	DJI_0785	Veronica incana L.	0	n	1	0	Plantaginaceae Juss.
Iz_5_1009	DJI_0785	Veronica verna L.	0	n	1	0	Plantaginaceae Juss.
Iz_5_1009	DJI_0785	Vincetoxicum hirundinaria Medik	0	n	1	0	Apocynaceae Juss.
Iz_5_1009	DJI_0789	Acer tataricum L.	0	n	1	1	Sapindaceae Juss.
Iz_5_1009	DJI_0789	Achillea millefolium L. s.l.	0	n	1	0	Asteraceae Bercht. & J.Presl
Iz_5_1009	DJI_0789	Allium oleraceum L.	0	n	1	0	Amaryllidaceae J.St.-Hil.
Iz_5_1009	DJI_0789	Ambrosia artemisiifolia L.	0	a	0	0	Asteraceae Bercht. & J.Presl
Iz_5_1009	DJI_0789	Amorpha fruticosa L.	0	a	0	0	Fabaceae Lindl.
Iz_5_1009	DJI_0789	Anthericum ramosum L.	0	n	1	0	Asparagaceae Juss.
Iz_5_1009	DJI_0789	Artemisia marschalliana Spreng.	0	n	1	0	Asteraceae Bercht. & J.Presl
Iz_5_1009	DJI_0789	Asparagus officinalis L.	0	n	1	0	Asparagaceae Juss.
Iz_5_1009	DJI_0789	Astragalus cicer L.	0	n	1	0	Fabaceae Lindl.
Iz_5_1009	DJI_0789	Bassia laniflora (S.G.Gmel.) A.J.Scott	0	n	1	0	Amaranthaceae Juss.
Iz_5_1009	DJI_0789	Berberis vulgaris L.	0	n	1	0	Berberidaceae Juss.
Iz_5_1009	DJI_0789	Betula pendula Roth.	0	n	1	1	Betulaceae Gray
Iz_5_1009	DJI_0789	Betula pendula Roth.	0	n	1	0	Poaceae Barnhart
Iz_5_1009	DJI_0789	Bromus inermis Leyss.	0		NA	NA	
Iz_5_1009	DJI_0789	Bromus squarrosus L.	0	a	0	0	Poaceae Barnhart
Iz_5_1009	DJI_0789	Bromus tectorum L.	0	a	0	0	Poaceae Barnhart
Iz_5_1009	DJI_0789	Calamagrostis epigejos (L.) Roth	0.2	n	1	0	Poaceae Barnhart
Iz_5_1009	DJI_0789	Carduus acanthoides L.	0	n	1	0	Asteraceae Bercht. & J.Presl
Iz_5_1009	DJI_0789	Carex sp.	0	n	1	0	Cyperaceae Juss.
Iz_5_1009	DJI_0789	Centaurea majorovii Dumbadze	0	n	1	0	Asteraceae Bercht. & J.Presl
Iz_5_1009	DJI_0789	Chamaecytisus ruthenicus (Fisch.ex Wol.) Klaskova	0	n	1	0	Fabaceae Lindl.
Iz_5_1009	DJI_0789	Chelidonium majus L.	0	n	1	0	Papaveraceae Juss.
Iz_5_1009	DJI_0789	Chenopodium betaceum Andrz.	0	n	1	0	Amaranthaceae Juss.
Iz_5_1009	DJI_0789	Chenopodium cf. album	0.5	n	1	0	Amaranthaceae Juss.
Iz_5_1009	DJI_0789	Chenopodium sp.	0		NA	0	Amaranthaceae Juss.
Iz_5_1009	DJI_0789	Chondrilla juncea L.	0	n	1	0	Asteraceae Bercht. & J.Presl
Iz_5_1009	DJI_0789	Cirsium vulgare (Savi) Ten.	0	n	1	0	Asteraceae Bercht. & J.Presl
Iz_5_1009	DJI_0789	Clinopodium acinos (L.) Kuntze	0	n	1	0	Lamiacea Lindl.
Iz_5_1009	DJI_0789	Convallaria majalis L.	0	n	1	0	Asparagaceae Juss.
Iz_5_1009	DJI_0789	Crataegus rhipidophylla Gand.	0	n	1	1	Rosaceae Juss.
Iz_5_1009	DJI_0789	Crepis foetida L.	0	n	1	0	Asteraceae Bercht. & J.Presl
Iz_5_1009	DJI_0789	Dactylis glomerata L.	0	n	1	0	Poaceae Barnhart
Iz_5_1009	DJI_0789	Dianthus campestris M.Bieb.	0	n	1	0	Caryophyllaceae Juss.
Iz_5_1009	DJI_0789	Digitaria sanguinalis (L.) Scop.	0	a	0	0	Poaceae Barnhart
Iz_5_1009	DJI_0789	Elymus repens (L.) Gould	0	n	1	0	Poaceae Barnhart
Iz_5_1009	DJI_0789	Epilobium tetragonum L.	0	n	1	0	Onagraceae Juss.
Iz_5_1009	DJI_0789	Eragrostis minor Host	0	a	0	0	Poaceae Barnhart
Iz_5_1009	DJI_0789	Erigeron canadensis L.	0	a	0	0	Asteraceae Bercht. & J.Presl
Iz_5_1009	DJI_0789	Euphorbia seguieriana Neck.	0	n	1	0	Euphorbiaceae Juss.
Iz_5_1009	DJI_0789	Fallopia dumetorum (L.) Holub	0	n	1	0	Polygonaceae Juss.
Iz_5_1009	DJI_0789	Festuca beckeri (Hack.) Trautv.	0	n	1	0	Poaceae Barnhart
Iz_5_1009	DJI_0789	Fragaria vesca L.	0	n	1	0	Rosaceae Juss.
Iz_5_1009	DJI_0789	Frangula alnus Mill.	0	n	1	1	Rhamnaceae Juss.
Iz_5_1009	DJI_0789	Fraxinus pennsylvanica Marshall	0	a	0	1	Oleaceae Hoffmanns. & Link
Iz_5_1009	DJI_0789	Galeopsis bifida Boenn.	0	n	1	0	Lamiacea Lindl.
Iz_5_1009	DJI_0789	Galium verum L.	0	n	1	0	Rubiaceae Juss.
Iz_5_1009	DJI_0789	Genista tinctoria L.	0	n	1	0	Fabaceae Lindl.
Iz_5_1009	DJI_0789	Geranium robertianum L.	0	n	1	0	Geraniaceae Juss.
Iz_5_1009	DJI_0789	Geranium sanquineum L.	0	n	1	0	Geraniaceae Juss.
Iz_5_1009	DJI_0789	Gypsophila paniculata L.	0	n	1	0	Caryophyllaceae Juss.
Iz_5_1009	DJI_0789	Helichrysum arenarium (L.) Moench	0	n	1	0	Asteraceae Bercht. & J.Presl
Iz_5_1009	DJI_0789	Herniaria polygama J.Gay	0	n	1	0	Caryophyllaceae Juss.
Iz_5_1009	DJI_0789	Hieracium robustum Fr.	0	n	1	0	Asteraceae Bercht. & J.Presl
Iz_5_1009	DJI_0789	Hieracium umbellatum L.	0	n	1	NA	Asteraceae Bercht. & J.Presl
Iz_5_1009	DJI_0789	Hylotelephium maximum (L.) Holub	0	n	1	0	Crassulaceae J.St.-Hil.
Iz_5_1009	DJI_0789	Jacobaea borysthenica (DC.) B.Nord. & Greuter	0	n	1	0	Asteraceae Bercht. & J.Presl
Iz_5_1009	DJI_0789	Jasione montana L.	0	n	1	0	Campanulaceae Juss.
Iz_5_1009	DJI_0789	Jurinea cyanoides (L.) Rchb.s.l.	0	n	1	0	Asteraceae Bercht. & J.Presl
Iz_5_1009	DJI_0789	Koeleria glauca (Spreng.) DC.	0	n	1	0	Poaceae Barnhart
Iz_5_1009	DJI_0789	Lactuca serriola L.	0	a	0	0	Asteraceae Bercht. & J.Presl
Iz_5_1009	DJI_0789	Linaria genistifolia (L.) Mill.	0	n	1	0	Plantaginaceae Juss.
Iz_5_1009	DJI_0789	Lomelosia argentea (L.) Greuter & Burdet	0	n	1	0	Caprifoliaceae Juss.
Iz_5_1009	DJI_0789	Melampyrum pratense L.	0	n	1	0	Orobanchaceae Vent.
Iz_5_1009	DJI_0789	Oenothera biennis L.	0	a	0	0	Onagraceae Juss.
Iz_5_1009	DJI_0789	Pentanema britannica (L.) D.Gut.Larr., Santos-Vicente, Anderb., E.Rico & M.M.Mart.Ort.	0	n	1	0	Asteraceae Bercht. & J.Presl
Iz_5_1009	DJI_0789	Peucedanum oreoselinum (L.) Moench	0	n	1	0	Apiaceae Lindl.
Iz_5_1009	DJI_0789	Peucedanum ruthenicum M.Bieb.	0	n	1	0	Apiaceae Lindl.
Iz_5_1009	DJI_0789	Pilosella echioides (Lumn.) F.W.Schultz & Sch.Bip.	0	n	1	0	Asteraceae Bercht. & J.Presl
Iz_5_1009	DJI_0789	Pilosella officinarum F. Schultz et Sch. Bip.	0	n	1	0	Asteraceae Bercht. & J.Presl
Iz_5_1009	DJI_0789	Pilosella sp.	0		NA	0	Asteraceae Bercht. & J.Presl
Iz_5_1009	DJI_0789	Pinus sylvestris L.	0	n	1	1	Pinaceae Spreng. ex F.Rudolphi
Iz_5_1009	DJI_0789	Poa annua L.	0	n	1	0	Poaceae Barnhart
Iz_5_1009	DJI_0789	Poa nemoralis L.	0	n	1	0	Poaceae Barnhart
Iz_5_1009	DJI_0789	Poa sp.	0		NA	0	Poaceae Barnhart
Iz_5_1009	DJI_0789	Polygonatum odoratum (Mill.) Druce	0	n	1	0	Asparagaceae Juss.
Iz_5_1009	DJI_0789	Polygonum arenarium Waldst. & Kit.	0	n	1	0	Polygonaceae Juss.
Iz_5_1009	DJI_0789	Populus nigra L.	0	n	1	1	Salicaceae Mirb.
Iz_5_1009	DJI_0789	Populus sp.	0	n	1	1	Salicaceae Mirb.
Iz_5_1009	DJI_0789	Populus tremula L.	0	n	1	1	Salicaceae Mirb.
Iz_5_1009	DJI_0789	Populus Ã canescens (Aiton) Sm.	0	n	1	1	Salicaceae Mirb.
Iz_5_1009	DJI_0789	Potentilla argentea L.	0	n	1	0	Rosaceae Juss.
Iz_5_1009	DJI_0789	Psammophiliella muralis (L.) Ikonn.	0	n	1	0	Caryophyllaceae Juss.
Iz_5_1009	DJI_0789	Psephellus sumensis (Kalen.) Greuter	0	n	1	0	Asteraceae Bercht. & J.Presl
Iz_5_1009	DJI_0789	Pulsatilla pratensis (L.) Mill. s.l.	0	n	1	0	Ranunculaceae Juss.
Iz_5_1009	DJI_0789	Pyrus communis L.	0	a	0	1	Rosaceae Juss.
Iz_5_1009	DJI_0789	Quercus robur L.	0	n	1	1	Fagaceae Dumort.
Iz_5_1009	DJI_0789	Robinia pseudoacacia L.	0	a	0	1	Fabaceae Lindl.

Iz_5_1009	DJI_0789	Rosa sp.	0		NA	1	Rosaceae Juss.
Iz_5_1009	DJI_0789	Rubus idaeus L.	0	n	1	0	Rosaceae Juss.
Iz_5_1009	DJI_0789	Rumex acetosella L.	0	n	1	0	Polygonaceae Juss.
Iz_5_1009	DJI_0789	Rumex thyrsiflorus Fingerh.	0	n	1	0	Polygonaceae Juss.
Iz_5_1009	DJI_0789	Salix alba L.	0	n	1	1	Salicaceae Mirb.
Iz_5_1009	DJI_0789	Sambucus nigra L.	0	n	1	1	Viburnaceae Raf.
Iz_5_1009	DJI_0789	Secale sylvestre Host	0	n	1	0	Poaceae Barnhart
Iz_5_1009	DJI_0789	Sempervivum ruthenicum Schnittsp. & C.B. Lehm.	0	n	1	0	Crassulaceae J.St.-Hil.
Iz_5_1009	DJI_0789	Setaria viridis (L.) P. Beauv.	0	n	1	0	Poaceae Barnhart
Iz_5_1009	DJI_0789	Silene borysthenica (Gruner) Walters	0	n	1	0	Caryophyllaceae Juss.
Iz_5_1009	DJI_0789	Silene latifolia subsp. alba (Mill.) Greuter & Burdet	0	n	1	0	Caryophyllaceae Juss.
Iz_5_1009	DJI_0789	Solidago virgaurea L.	0	n	1	0	Asteraceae Bercht. & J.Presl
Iz_5_1009	DJI_0789	Solidago Ã niederederi (S. canadensis Ã S. virgaurea)	0	hybrid	0	0	Asteraceae Bercht. & J.Presl
Iz_5_1009	DJI_0789	Sorbus aucuparia L.	0	n	1	1	Rosaceae Juss.
Iz_5_1009	DJI_0789	Stachys recta L.	0	n	1	0	Lamiacea Lindl.
Iz_5_1009	DJI_0789	Tanacetum vulgare L.	0	n	1	0	Asteraceae Bercht. & J.Presl
Iz_5_1009	DJI_0789	Thymus pallasianus Heinr.Braun	0	n	1	0	Lamiacea Lindl.
Iz_5_1009	DJI_0789	Tilia cordata Mill.	0	n	1	1	Malvaceae Juss.
Iz_5_1009	DJI_0789	Trifolium alpestre L.	0	n	1	0	Fabaceae Lindl.
Iz_5_1009	DJI_0789	Trifolium arvense L.	0	n	1	0	Fabaceae Lindl.
Iz_5_1009	DJI_0789	Ulmus glabra Huds.	0	n	1	1	Ulmaceae Mirb.
Iz_5_1009	DJI_0789	Verbascum densiflorum Bertol.	0	n	1	0	Scrophulariaceae Juss.
Iz_5_1009	DJI_0789	Veronica austriaca L.	0	n	1	0	Plantaginaceae Juss.
Iz_5_1009	DJI_0789	Veronica incana L.	0	n	1	0	Plantaginaceae Juss.
Iz_5_1009	DJI_0789	Veronica verna L.	0	n	1	0	Plantaginaceae Juss.
Iz_5_1009	DJI_0789	Vincetoxicum hirundinaria Medik	0	n	1	0	Apocynaceae Juss.
Iz_5_1009	DJI_0793	Acer tataricum L.	0	n	1	1	Sapindaceae Juss.
Iz_5_1009	DJI_0793	Achillea millefolium L. s.l.	0	n	1	0	Asteraceae Bercht. & J.Presl
Iz_5_1009	DJI_0793	Allium oleraceum L.	0	n	1	0	Amaryllidaceae J.St.-Hil.
Iz_5_1009	DJI_0793	Ambrosia artemisiifolia L.	0	a	0	0	Asteraceae Bercht. & J.Presl
Iz_5_1009	DJI_0793	Amorpha fruticosa L.	0	a	0	0	Fabaceae Lindl.
Iz_5_1009	DJI_0793	Anthericum ramosum L.	0	n	1	0	Asparagaceae Juss.
Iz_5_1009	DJI_0793	Artemisia marschalliana Spreng.	0	n	1	0	Asteraceae Bercht. & J.Presl
Iz_5_1009	DJI_0793	Asparagus officinalis L.	0	n	1	0	Asparagaceae Juss.
Iz_5_1009	DJI_0793	Astragalus cicer L.	0	n	1	0	Fabaceae Lindl.
Iz_5_1009	DJI_0793	Bassia laniflora (S.G.Gmel.) A.J.Scott	0	n	1	0	Amaranthaceae Juss.
Iz_5_1009	DJI_0793	Berberis vulgaris L.	0	n	1	0	Berberidaceae Juss.
Iz_5_1009	DJI_0793	Betula pendula Roth.	0	n	1	1	Betulaceae Gray
Iz_5_1009	DJI_0793	Betula pendula Roth.	0	n	1	0	Poaceae Barnhart
Iz_5_1009	DJI_0793	Bromus inermis Leyss.	0		NA	NA	
Iz_5_1009	DJI_0793	Bromus squarrosus L.	0	a	0	0	Poaceae Barnhart
Iz_5_1009	DJI_0793	Bromus tectorum L.	0	a	0	0	Poaceae Barnhart
Iz_5_1009	DJI_0793	Calamagrostis epigejos (L.) Roth	0.2	n	1	0	Poaceae Barnhart
Iz_5_1009	DJI_0793	Carduus acanthoides L.	0	n	1	0	Asteraceae Bercht. & J.Presl
Iz_5_1009	DJI_0793	Carex sp.	0	n	1	0	Cyperaceae Juss.
Iz_5_1009	DJI_0793	Centaurea majorovii Dumbadze	0	n	1	0	Asteraceae Bercht. & J.Presl
Iz_5_1009	DJI_0793	Chamaecytisus ruthenicus (Fisch.ex Wol.) Klaskova	0	n	1	0	Fabaceae Lindl.
Iz_5_1009	DJI_0793	Chelidonium majus L.	0	n	1	0	Papaveraceae Juss.
Iz_5_1009	DJI_0793	Chenopodium betaceum Andrz.	0	n	1	0	Amaranthaceae Juss.
Iz_5_1009	DJI_0793	Chenopodium cf. album	0.2	n	1	0	Amaranthaceae Juss.
Iz_5_1009	DJI_0793	Chenopodium sp.	0		NA	0	Amaranthaceae Juss.
Iz_5_1009	DJI_0793	Chondrilla juncea L.	0	n	1	0	Asteraceae Bercht. & J.Presl
Iz_5_1009	DJI_0793	Cirsium vulgare (Savi) Ten.	0	n	1	0	Asteraceae Bercht. & J.Presl
Iz_5_1009	DJI_0793	Clinopodium acinos (L.) Kuntze	0	n	1	0	Lamiacea Lindl.
Iz_5_1009	DJI_0793	Convallaria majalis L.	0	n	1	0	Asparagaceae Juss.
Iz_5_1009	DJI_0793	Crataegus rhipidophylla Gand.	0	n	1	1	Rosaceae Juss.
Iz_5_1009	DJI_0793	Crepis foetida L.	0	n	1	0	Asteraceae Bercht. & J.Presl
Iz_5_1009	DJI_0793	Dactylis glomerata L.	0	n	1	0	Poaceae Barnhart
Iz_5_1009	DJI_0793	Dianthus campestris M.Bieb.	0	n	1	0	Caryophyllaceae Juss.
Iz_5_1009	DJI_0793	Digitaria sanguinalis (L.) Scop.	0	a	0	0	Poaceae Barnhart
Iz_5_1009	DJI_0793	Elymus repens (L.) Gould	0	n	1	0	Poaceae Barnhart
Iz_5_1009	DJI_0793	Epilobium tetragonum L.	0	n	1	0	Onagraceae Juss.
Iz_5_1009	DJI_0793	Eragrostis minor Host	0	a	0	0	Poaceae Barnhart
Iz_5_1009	DJI_0793	Erigeron canadensis L.	0	a	0	0	Asteraceae Bercht. & J.Presl
Iz_5_1009	DJI_0793	Euphorbia seguieriana Neck.	0	n	1	0	Euphorbiaceae Juss.
Iz_5_1009	DJI_0793	Fallopia dumetorum (L.) Holub	0.2	n	1	0	Polygonaceae Juss.
Iz_5_1009	DJI_0793	Festuca beckeri (Hack.) Trautv.	0	n	1	0	Poaceae Barnhart
Iz_5_1009	DJI_0793	Fragaria vesca L.	0	n	1	0	Rosaceae Juss.
Iz_5_1009	DJI_0793	Frangula alnus Mill.	0	n	1	1	Rhamnaceae Juss.
Iz_5_1009	DJI_0793	Fraxinus pennsylvanica Marshall	0	a	0	1	Oleaceae Hoffmanns. & Link
Iz_5_1009	DJI_0793	Galeopsis bifida Boenn.	0	n	1	0	Lamiacea Lindl.
Iz_5_1009	DJI_0793	Galium verum L.	0	n	1	0	Rubiaceae Juss.
Iz_5_1009	DJI_0793	Genista tinctoria L.	0	n	1	0	Fabaceae Lindl.
Iz_5_1009	DJI_0793	Geranium robertianum L.	0	n	1	0	Geraniaceae Juss.
Iz_5_1009	DJI_0793	Geranium sanquineum L.	0	n	1	0	Geraniaceae Juss.
Iz_5_1009	DJI_0793	Gypsophila paniculata L.	0	n	1	0	Caryophyllaceae Juss.
Iz_5_1009	DJI_0793	Helichrysum arenarium (L.) Moench	0	n	1	0	Asteraceae Bercht. & J.Presl
Iz_5_1009	DJI_0793	Herniaria polygama J.Gay	0	n	1	0	Caryophyllaceae Juss.
Iz_5_1009	DJI_0793	Hieracium robustum Fr.	0	n	1	0	Asteraceae Bercht. & J.Presl
Iz_5_1009	DJI_0793	Hieracium umbellatum L.	0	n	1	NA	Asteraceae Bercht. & J.Presl
Iz_5_1009	DJI_0793	Hylotelephium maximum (L.) Holub	0	n	1	0	Crassulaceae J.St.-Hil.
Iz_5_1009	DJI_0793	Jacobaea borysthenica (DC.) B.Nord. & Greuter	0	n	1	0	Asteraceae Bercht. & J.Presl
Iz_5_1009	DJI_0793	Jasione montana L.	0	n	1	0	Campanulaceae Juss.
Iz_5_1009	DJI_0793	Jurinea cyanoides (L.) Rchb.s.l.	0	n	1	0	Asteraceae Bercht. & J.Presl
Iz_5_1009	DJI_0793	Koeleria glauca (Spreng.) DC.	0	n	1	0	Poaceae Barnhart
Iz_5_1009	DJI_0793	Lactuca serriola L.	0	a	0	0	Asteraceae Bercht. & J.Presl
Iz_5_1009	DJI_0793	Linaria genistifolia (L.) Mill.	0	n	1	0	Plantaginaceae Juss.
Iz_5_1009	DJI_0793	Lomelosia argentea (L.) Greuter & Burdet	0	n	1	0	Caprifoliaceae Juss.
Iz_5_1009	DJI_0793	Melampyrum pratense L.	0	n	1	0	Orobanchaceae Vent.
Iz_5_1009	DJI_0793	Oenothera biennis L.	0	a	0	0	Onagraceae Juss.
Iz_5_1009	DJI_0793	Pentanema britannica (L.) D.Gut.Larr., Santos-Vicente, Anderb., E.Rico & M.M.Mart.Ort.	0	n	1	0	Asteraceae Bercht. & J.Presl
Iz_5_1009	DJI_0793	Peucedanum oreoselinum (L.) Moench	0	n	1	0	Apiaceae Lindl.
Iz_5_1009	DJI_0793	Peucedanum ruthenicum M.Bieb.	0	n	1	0	Apiaceae Lindl.
Iz_5_1009	DJI_0793	Pilosella echioides (Lumn.) F.W.Schultz & Sch.Bip.	0	n	1	0	Asteraceae Bercht. & J.Presl
Iz_5_1009	DJI_0793	Pilosella officinarum F. Schultz et Sch. Bip.	0	n	1	0	Asteraceae Bercht. & J.Presl
Iz_5_1009	DJI_0793	Pilosella sp.	0		NA	0	Asteraceae Bercht. & J.Presl
Iz_5_1009	DJI_0793	Pinus sylvestris L.	0	n	1	1	Pinaceae Spreng. ex F.Rudolphi
Iz_5_1009	DJI_0793	Poa annua L.	0	n	1	0	Poaceae Barnhart

Iz_5_1009	DJI_0793	Poa nemoralis L.	0	n	1	0	Poaceae Barnhart
Iz_5_1009	DJI_0793	Poa sp.	0		NA	0	Poaceae Barnhart
Iz_5_1009	DJI_0793	Polygonatum odoratum (Mill.) Druce	0	n	1	0	Asparagaceae Juss.
Iz_5_1009	DJI_0793	Polygonum arenarium Waldst. & Kit.	0	n	1	0	Polygonaceae Juss.
Iz_5_1009	DJI_0793	Populus nigra L.	0	n	1	1	Salicaceae Mirb.
Iz_5_1009	DJI_0793	Populus sp.	0	n	1	1	Salicaceae Mirb.
Iz_5_1009	DJI_0793	Populus tremula L.	0	n	1	1	Salicaceae Mirb.
Iz_5_1009	DJI_0793	Populus Ã canescens (Aiton) Sm.	0	n	1	1	Salicaceae Mirb.
Iz_5_1009	DJI_0793	Potentilla argentea L.	0	n	1	0	Rosaceae Juss.
Iz_5_1009	DJI_0793	Psammophiliella muralis (L.) Ikonn.	0	n	1	0	Caryophyllaceae Juss.
Iz_5_1009	DJI_0793	Psephellus sumensis (Kalen.) Greuter	0	n	1	0	Asteraceae Bercht. & J.Presl
Iz_5_1009	DJI_0793	Pulsatilla pratensis (L.) Mill. s.l.	0	n	1	0	Ranunculaceae Juss.
Iz_5_1009	DJI_0793	Pyrus communis L.	0	a	0	1	Rosaceae Juss.
Iz_5_1009	DJI_0793	Quercus robur L.	0	n	1	1	Fagaceae Dumort.
Iz_5_1009	DJI_0793	Robinia pseudoacacia L.	0	a	0	1	Fabaceae Lindl.
Iz_5_1009	DJI_0793	Rosa sp.	0		NA	1	Rosaceae Juss.
Iz_5_1009	DJI_0793	Rubus idaeus L.	0	n	1	0	Rosaceae Juss.
Iz_5_1009	DJI_0793	Rumex acetosella L.	0	n	1	0	Polygonaceae Juss.
Iz_5_1009	DJI_0793	Rumex thyrsiflorus Fingerh.	0	n	1	0	Polygonaceae Juss.
Iz_5_1009	DJI_0793	Salix alba L.	0	n	1	1	Salicaceae Mirb.
Iz_5_1009	DJI_0793	Sambucus nigra L.	0	n	1	1	Viburnaceae Raf.
Iz_5_1009	DJI_0793	Secale sylvestre Host	0	n	1	0	Poaceae Barnhart
Iz_5_1009	DJI_0793	Sempervivum ruthenicum Schnittsp. & C.B. Lehm.	0	n	1	0	Crassulaceae J.St.-Hil.
Iz_5_1009	DJI_0793	Setaria viridis (L.) P. Beauv.	0	n	1	0	Poaceae Barnhart
Iz_5_1009	DJI_0793	Silene borysthenica (Gruner) Walters	0	n	1	0	Caryophyllaceae Juss.
Iz_5_1009	DJI_0793	Silene latifolia subsp. alba (Mill.) Greuter & Burdet	0	n	1	0	Caryophyllaceae Juss.
Iz_5_1009	DJI_0793	Solidago virgaurea L.	0	n	1	0	Asteraceae Bercht. & J.Presl
Iz_5_1009	DJI_0793	Solidago Ã niederederi (S. canadensis Ã S. virgaurea)	0	hybrid	0	0	Asteraceae Bercht. & J.Presl
Iz_5_1009	DJI_0793	Sorbus aucuparia L.	0	n	1	1	Rosaceae Juss.
Iz_5_1009	DJI_0793	Stachys recta L.	0	n	1	0	Lamiacea Lindl.
Iz_5_1009	DJI_0793	Tanacetum vulgare L.	0	n	1	0	Asteraceae Bercht. & J.Presl
Iz_5_1009	DJI_0793	Thymus pallasianus Heinr.Braun	0	n	1	0	Lamiacea Lindl.
Iz_5_1009	DJI_0793	Tilia cordata Mill.	0	n	1	1	Malvaceae Juss.
Iz_5_1009	DJI_0793	Trifolium alpestre L.	0	n	1	0	Fabaceae Lindl.
Iz_5_1009	DJI_0793	Trifolium arvense L.	0	n	1	0	Fabaceae Lindl.
Iz_5_1009	DJI_0793	Ulmus glabra Huds.	0	n	1	1	Ulmaceae Mirb.
Iz_5_1009	DJI_0793	Verbascum densiflorum Bertol.	0	n	1	0	Scrophulariaceae Juss.
Iz_5_1009	DJI_0793	Veronica austriaca L.	0	n	1	0	Plantaginaceae Juss.
Iz_5_1009	DJI_0793	Veronica incana L.	0	n	1	0	Plantaginaceae Juss.
Iz_5_1009	DJI_0793	Veronica verna L.	0	n	1	0	Plantaginaceae Juss.
Iz_5_1009	DJI_0793	Vincetoxicum hirundinaria Medik	0	n	1	0	Apocynaceae Juss.
Iz_5_1009	DJI_0795	Acer tataricum L.	0	n	1	1	Sapindaceae Juss.
Iz_5_1009	DJI_0795	Achillea millefolium L. s.l.	0	n	1	0	Asteraceae Bercht. & J.Presl
Iz_5_1009	DJI_0795	Allium oleraceum L.	0	n	1	0	Amaryllidaceae J.St.-Hil.
Iz_5_1009	DJI_0795	Ambrosia artemisiifolia L.	0	a	0	0	Asteraceae Bercht. & J.Presl
Iz_5_1009	DJI_0795	Amorpha fruticosa L.	0	a	0	0	Fabaceae Lindl.
Iz_5_1009	DJI_0795	Anthericum ramosum L.	0	n	1	0	Asparagaceae Juss.
Iz_5_1009	DJI_0795	Artemisia marschalliana Spreng.	0	n	1	0	Asteraceae Bercht. & J.Presl
Iz_5_1009	DJI_0795	Asparagus officinalis L.	0	n	1	0	Asparagaceae Juss.
Iz_5_1009	DJI_0795	Astragalus cicer L.	0	n	1	0	Fabaceae Lindl.
Iz_5_1009	DJI_0795	Bassia laniflora (S.G.Gmel.) A.J.Scott	0	n	1	0	Amaranthaceae Juss.
Iz_5_1009	DJI_0795	Berberis vulgaris L.	0	n	1	0	Berberidaceae Juss.
Iz_5_1009	DJI_0795	Betula pendula Roth.	0	n	1	1	Betulaceae Gray
Iz_5_1009	DJI_0795	Betula pendula Roth.	0	n	1	0	Poaceae Barnhart
Iz_5_1009	DJI_0795	Bromus inermis Leyss.	0		NA	NA	
Iz_5_1009	DJI_0795	Bromus squarrosus L.	0	a	0	0	Poaceae Barnhart
Iz_5_1009	DJI_0795	Bromus tectorum L.	0	a	0	0	Poaceae Barnhart
Iz_5_1009	DJI_0795	Calamagrostis epigejos (L.) Roth	1	n	1	0	Poaceae Barnhart
Iz_5_1009	DJI_0795	Carduus acanthoides L.	0	n	1	0	Asteraceae Bercht. & J.Presl
Iz_5_1009	DJI_0795	Carex sp.	0	n	1	0	Cyperaceae Juss.
Iz_5_1009	DJI_0795	Centaurea majorovii Dumbadze	0	n	1	0	Asteraceae Bercht. & J.Presl
Iz_5_1009	DJI_0795	Chamaecytisus ruthenicus (Fisch.ex Wol.) Klaskova	0	n	1	0	Fabaceae Lindl.
Iz_5_1009	DJI_0795	Chelidonium majus L.	0	n	1	0	Papaveraceae Juss.
Iz_5_1009	DJI_0795	Chenopodium betaceum Andrz.	0	n	1	0	Amaranthaceae Juss.
Iz_5_1009	DJI_0795	Chenopodium cf. album	0	n	1	0	Amaranthaceae Juss.
Iz_5_1009	DJI_0795	Chenopodium sp.	0		NA	0	Amaranthaceae Juss.
Iz_5_1009	DJI_0795	Chondrilla juncea L.	0	n	1	0	Asteraceae Bercht. & J.Presl
Iz_5_1009	DJI_0795	Cirsium vulgare (Savi) Ten.	0	n	1	0	Asteraceae Bercht. & J.Presl
Iz_5_1009	DJI_0795	Clinopodium acinos (L.) Kuntze	0	n	1	0	Lamiacea Lindl.
Iz_5_1009	DJI_0795	Convallaria majalis L.	0	n	1	0	Asparagaceae Juss.
Iz_5_1009	DJI_0795	Crataegus rhipidophylla Gand.	0	n	1	1	Rosaceae Juss.
Iz_5_1009	DJI_0795	Crepis foetida L.	0	n	1	0	Asteraceae Bercht. & J.Presl
Iz_5_1009	DJI_0795	Dactylis glomerata L.	0	n	1	0	Poaceae Barnhart
Iz_5_1009	DJI_0795	Dianthus campestris M.Bieb.	0	n	1	0	Caryophyllaceae Juss.
Iz_5_1009	DJI_0795	Digitaria sanguinalis (L.) Scop.	0	a	0	0	Poaceae Barnhart
Iz_5_1009	DJI_0795	Elymus repens (L.) Gould	0	n	1	0	Poaceae Barnhart
Iz_5_1009	DJI_0795	Epilobium tetragonum L.	0	n	1	0	Onagraceae Juss.
Iz_5_1009	DJI_0795	Eragrostis minor Host	0	a	0	0	Poaceae Barnhart
Iz_5_1009	DJI_0795	Erigeron canadensis L.	0	a	0	0	Asteraceae Bercht. & J.Presl
Iz_5_1009	DJI_0795	Euphorbia seguieriana Neck.	0	n	1	0	Euphorbiaceae Juss.
Iz_5_1009	DJI_0795	Fallopia dumetorum (L.) Holub	0	n	1	0	Polygonaceae Juss.
Iz_5_1009	DJI_0795	Festuca beckeri (Hack.) Trautv.	0	n	1	0	Poaceae Barnhart
Iz_5_1009	DJI_0795	Fragaria vesca L.	0	n	1	0	Rosaceae Juss.
Iz_5_1009	DJI_0795	Frangula alnus Mill.	0	n	1	1	Rhamnaceae Juss.
Iz_5_1009	DJI_0795	Fraxinus pennsylvanica Marshall	0	a	0	1	Oleaceae Hoffmanns. & Link
Iz_5_1009	DJI_0795	Galeopsis bifida Boenn.	0	n	1	0	Lamiacea Lindl.
Iz_5_1009	DJI_0795	Galium verum L.	0	n	1	0	Rubiaceae Juss.
Iz_5_1009	DJI_0795	Genista tinctoria L.	0	n	1	0	Fabaceae Lindl.
Iz_5_1009	DJI_0795	Geranium robertianum L.	0	n	1	0	Geraniaceae Juss.
Iz_5_1009	DJI_0795	Geranium sanquineum L.	0	n	1	0	Geraniaceae Juss.
Iz_5_1009	DJI_0795	Gypsophila paniculata L.	0	n	1	0	Caryophyllaceae Juss.
Iz_5_1009	DJI_0795	Helichrysum arenarium (L.) Moench	0	n	1	0	Asteraceae Bercht. & J.Presl
Iz_5_1009	DJI_0795	Herniaria polygama J.Gay	0	n	1	0	Caryophyllaceae Juss.
Iz_5_1009	DJI_0795	Hieracium robustum Fr.	0	n	1	0	Asteraceae Bercht. & J.Presl
Iz_5_1009	DJI_0795	Hieracium umbellatum L.	0	n	1	NA	Asteraceae Bercht. & J.Presl
Iz_5_1009	DJI_0795	Hylotelephium maximum (L.) Holub	0	n	1	0	Crassulaceae J.St.-Hil.
Iz_5_1009	DJI_0795	Jacobaea borysthenica (DC.) B.Nord. & Greuter	0	n	1	0	Asteraceae Bercht. & J.Presl
Iz_5_1009	DJI_0795	Jasione montana L.	0	n	1	0	Campanulaceae Juss.

Iz_5_1009	DJI_0795	Jurinea cyanoides (L.) Rchb.s.l.	0	n	1	0	Asteraceae Bercht. & J.Presl
Iz_5_1009	DJI_0795	Koeleria glauca (Spreng.) DC.	0	n	1	0	Poaceae Barnhart
Iz_5_1009	DJI_0795	Lactuca serriola L.	0	a	0	0	Asteraceae Bercht. & J.Presl
Iz_5_1009	DJI_0795	Linaria genistifolia (L.) Mill.	0	n	1	0	Plantaginaceae Juss.
Iz_5_1009	DJI_0795	Lomelosia argentea (L.) Greuter & Burdet	0	n	1	0	Caprifoliaceae Juss.
Iz_5_1009	DJI_0795	Melampyrum pratense L.	0	n	1	0	Orobanchaceae Vent.
Iz_5_1009	DJI_0795	Oenothera biennis L.	0	a	0	0	Onagraceae Juss.
Iz_5_1009	DJI_0795	Pentanema britannica (L.) D.Gut.Larr., Santos-Vicente, Anderb., E.Rico & M.M.Mart.Ort.	0	n	1	0	Asteraceae Bercht. & J.Presl
Iz_5_1009	DJI_0795	Peucedanum oreoselinum (L.) Moench	0	n	1	0	Apiaceae Lindl.
Iz_5_1009	DJI_0795	Peucedanum ruthenicum M.Bieb.	0	n	1	0	Apiaceae Lindl.
Iz_5_1009	DJI_0795	Pilosella echioides (Lumn.) F.W.Schultz & Sch.Bip.	0	n	1	0	Asteraceae Bercht. & J.Presl
Iz_5_1009	DJI_0795	Pilosella officinarum F. Schultz et Sch. Bip.	0	n	1	0	Asteraceae Bercht. & J.Presl
Iz_5_1009	DJI_0795	Pilosella sp.	0		NA	0	Asteraceae Bercht. & J.Presl
Iz_5_1009	DJI_0795	Pinus sylvestris L.	0	n	1	1	Pinaceae Spreng. ex F.Rudolphi
Iz_5_1009	DJI_0795	Poa annua L.	0	n	1	0	Poaceae Barnhart
Iz_5_1009	DJI_0795	Poa nemoralis L.	1	n	1	0	Poaceae Barnhart
Iz_5_1009	DJI_0795	Poa sp.	0		NA	0	Poaceae Barnhart
Iz_5_1009	DJI_0795	Polygonatum odoratum (Mill.) Druce	0	n	1	0	Asparagaceae Juss.
Iz_5_1009	DJI_0795	Polygonum arenarium Waldst. & Kit.	0	n	1	0	Polygonaceae Juss.
Iz_5_1009	DJI_0795	Populus nigra L.	0	n	1	1	Salicaceae Mirb.
Iz_5_1009	DJI_0795	Populus sp.	0	n	1	1	Salicaceae Mirb.
Iz_5_1009	DJI_0795	Populus tremula L.	0	n	1	1	Salicaceae Mirb.
Iz_5_1009	DJI_0795	Populus Ã canescens (Aiton) Sm.	0	n	1	1	Salicaceae Mirb.
Iz_5_1009	DJI_0795	Potentilla argentea L.	0	n	1	0	Rosaceae Juss.
Iz_5_1009	DJI_0795	Psammophiliella muralis (L.) Ikonn.	0	n	1	0	Caryophyllaceae Juss.
Iz_5_1009	DJI_0795	Psephellus sumensis (Kalen.) Greuter	0	n	1	0	Asteraceae Bercht. & J.Presl
Iz_5_1009	DJI_0795	Pulsatilla pratensis (L.) Mill. s.l.	0	n	1	0	Ranunculaceae Juss.
Iz_5_1009	DJI_0795	Pyrus communis L.	0	a	0	1	Rosaceae Juss.
Iz_5_1009	DJI_0795	Quercus robur L.	0	n	1	1	Fagaceae Dumort.
Iz_5_1009	DJI_0795	Robinia pseudoacacia L.	0	a	0	1	Fabaceae Lindl.
Iz_5_1009	DJI_0795	Rosa sp.	0		NA	1	Rosaceae Juss.
Iz_5_1009	DJI_0795	Rubus idaeus L.	0	n	1	0	Rosaceae Juss.
Iz_5_1009	DJI_0795	Rumex acetosella L.	0	n	1	0	Polygonaceae Juss.
Iz_5_1009	DJI_0795	Rumex thyrsiflorus Fingerh.	0	n	1	0	Polygonaceae Juss.
Iz_5_1009	DJI_0795	Salix alba L.	0	n	1	1	Salicaceae Mirb.
Iz_5_1009	DJI_0795	Sambucus nigra L.	0	n	1	1	Viburnaceae Raf.
Iz_5_1009	DJI_0795	Secale sylvestre Host	0	n	1	0	Poaceae Barnhart
Iz_5_1009	DJI_0795	Sempervivum ruthenicum Schnittsp. & C.B. Lehm.	0	n	1	0	Crassulaceae J.St.-Hil.
Iz_5_1009	DJI_0795	Setaria viridis (L.) P. Beauv.	0	n	1	0	Poaceae Barnhart
Iz_5_1009	DJI_0795	Silene borysthenica (Gruner) Walters	0	n	1	0	Caryophyllaceae Juss.
Iz_5_1009	DJI_0795	Silene latifolia subsp. alba (Mill.) Greuter & Burdet	0	n	1	0	Caryophyllaceae Juss.
Iz_5_1009	DJI_0795	Solidago virgaurea L.	0	n	1	0	Asteraceae Bercht. & J.Presl
Iz_5_1009	DJI_0795	Solidago Ã niederederi (S. canadensis Ã S. virgaurea)	0	hybrid	0	0	Asteraceae Bercht. & J.Presl
Iz_5_1009	DJI_0795	Sorbus aucuparia L.	0	n	1	1	Rosaceae Juss.
Iz_5_1009	DJI_0795	Stachys recta L.	0	n	1	0	Lamiacea Lindl.
Iz_5_1009	DJI_0795	Tanacetum vulgare L.	0	n	1	0	Asteraceae Bercht. & J.Presl
Iz_5_1009	DJI_0795	Thymus pallasianus Heinr.Braun	0	n	1	0	Lamiacea Lindl.
Iz_5_1009	DJI_0795	Tilia cordata Mill.	0	n	1	1	Malvaceae Juss.
Iz_5_1009	DJI_0795	Trifolium alpestre L.	0	n	1	0	Fabaceae Lindl.
Iz_5_1009	DJI_0795	Trifolium arvense L.	0	n	1	0	Fabaceae Lindl.
Iz_5_1009	DJI_0795	Ulmus glabra Huds.	0	n	1	1	Ulmaceae Mirb.
Iz_5_1009	DJI_0795	Verbascum densiflorum Bertol.	0	n	1	0	Scrophulariaceae Juss.
Iz_5_1009	DJI_0795	Veronica austriaca L.	0	n	1	0	Plantaginaceae Juss.
Iz_5_1009	DJI_0795	Veronica incana L.	0	n	1	0	Plantaginaceae Juss.
Iz_5_1009	DJI_0795	Veronica verna L.	0	n	1	0	Plantaginaceae Juss.
Iz_5_1009	DJI_0795	Vincetoxicum hirundinaria Medik	0	n	1	0	Apocynaceae Juss.
Iz_5_1009	DJI_0800	Acer tataricum L.	0	n	1	1	Sapindaceae Juss.
Iz_5_1009	DJI_0800	Achillea millefolium L. s.l.	0	n	1	0	Asteraceae Bercht. & J.Presl
Iz_5_1009	DJI_0800	Allium oleraceum L.	0	n	1	0	Amaryllidaceae J.St.-Hil.
Iz_5_1009	DJI_0800	Ambrosia artemisiifolia L.	0	a	0	0	Asteraceae Bercht. & J.Presl
Iz_5_1009	DJI_0800	Amorpha fruticosa L.	0	a	0	0	Fabaceae Lindl.
Iz_5_1009	DJI_0800	Anthericum ramosum L.	0	n	1	0	Asparagaceae Juss.
Iz_5_1009	DJI_0800	Artemisia marschalliana Spreng.	0	n	1	0	Asteraceae Bercht. & J.Presl
Iz_5_1009	DJI_0800	Asparagus officinalis L.	0	n	1	0	Asparagaceae Juss.
Iz_5_1009	DJI_0800	Astragalus cicer L.	0	n	1	0	Fabaceae Lindl.
Iz_5_1009	DJI_0800	Bassia laniflora (S.G.Gmel.) A.J.Scott	0	n	1	0	Amaranthaceae Juss.
Iz_5_1009	DJI_0800	Berberis vulgaris L.	0	n	1	0	Berberidaceae Juss.
Iz_5_1009	DJI_0800	Betula pendula Roth.	0	n	1	1	Betulaceae Gray
Iz_5_1009	DJI_0800	Betula pendula Roth.	0	n	1	0	Poaceae Barnhart
Iz_5_1009	DJI_0800	Bromus inermis Leyss.	0		NA	NA	
Iz_5_1009	DJI_0800	Bromus squarrosus L.	0	a	0	0	Poaceae Barnhart
Iz_5_1009	DJI_0800	Bromus tectorum L.	0	a	0	0	Poaceae Barnhart
Iz_5_1009	DJI_0800	Calamagrostis epigejos (L.) Roth	10	n	1	0	Poaceae Barnhart
Iz_5_1009	DJI_0800	Carduus acanthoides L.	0	n	1	0	Asteraceae Bercht. & J.Presl
Iz_5_1009	DJI_0800	Carex sp.	0	n	1	0	Cyperaceae Juss.
Iz_5_1009	DJI_0800	Centaurea majorovii Dumbadze	0	n	1	0	Asteraceae Bercht. & J.Presl
Iz_5_1009	DJI_0800	Chamaecytisus ruthenicus (Fisch.ex Wol.) Klaskova	0	n	1	0	Fabaceae Lindl.
Iz_5_1009	DJI_0800	Chelidonium majus L.	0	n	1	0	Papaveraceae Juss.
Iz_5_1009	DJI_0800	Chenopodium betaceum Andrz.	0	n	1	0	Amaranthaceae Juss.
Iz_5_1009	DJI_0800	Chenopodium cf. album	0	n	1	0	Amaranthaceae Juss.
Iz_5_1009	DJI_0800	Chenopodium sp.	0		NA	0	Amaranthaceae Juss.
Iz_5_1009	DJI_0800	Chondrilla juncea L.	0	n	1	0	Asteraceae Bercht. & J.Presl
Iz_5_1009	DJI_0800	Cirsium vulgare (Savi) Ten.	0	n	1	0	Asteraceae Bercht. & J.Presl
Iz_5_1009	DJI_0800	Clinopodium acinos (L.) Kuntze	0	n	1	0	Lamiacea Lindl.
Iz_5_1009	DJI_0800	Convallaria majalis L.	0	n	1	0	Asparagaceae Juss.
Iz_5_1009	DJI_0800	Crataegus rhipidophylla Gand.	0	n	1	1	Rosaceae Juss.
Iz_5_1009	DJI_0800	Crepis foetida L.	0	n	1	0	Asteraceae Bercht. & J.Presl
Iz_5_1009	DJI_0800	Dactylis glomerata L.	0	n	1	0	Poaceae Barnhart
Iz_5_1009	DJI_0800	Dianthus campestris M.Bieb.	0	n	1	0	Caryophyllaceae Juss.
Iz_5_1009	DJI_0800	Digitaria sanguinalis (L.) Scop.	0	a	0	0	Poaceae Barnhart
Iz_5_1009	DJI_0800	Elymus repens (L.) Gould	0	n	1	0	Poaceae Barnhart
Iz_5_1009	DJI_0800	Epilobium tetragonum L.	0	n	1	0	Onagraceae Juss.
Iz_5_1009	DJI_0800	Eragrostis minor Host	0	a	0	0	Poaceae Barnhart
Iz_5_1009	DJI_0800	Erigeron canadensis L.	0	a	0	0	Asteraceae Bercht. & J.Presl
Iz_5_1009	DJI_0800	Euphorbia seguieriana Neck.	0	n	1	0	Euphorbiaceae Juss.
Iz_5_1009	DJI_0800	Fallopia dumetorum (L.) Holub	0	n	1	0	Polygonaceae Juss.
Iz_5_1009	DJI_0800	Festuca beckeri (Hack.) Trautv.	0	n	1	0	Poaceae Barnhart
Iz_5_1009	DJI_0800	Fragaria vesca L.	0	n	1	0	Rosaceae Juss.

Iz_5_1009	DJI_0800	Frangula alnus Mill.	0	n	1	1	Rhamnaceae Juss.
Iz_5_1009	DJI_0800	Fraxinus pennsylvanica Marshall	0	a	0	1	Oleaceae Hoffmanns. & Link
Iz_5_1009	DJI_0800	Galeopsis bifida Boenn.	0	n	1	0	Lamiacea Lindl.
Iz_5_1009	DJI_0800	Galium verum L.	0	n	1	0	Rubiaceae Juss.
Iz_5_1009	DJI_0800	Genista tinctoria L.	0	n	1	0	Fabaceae Lindl.
Iz_5_1009	DJI_0800	Geranium robertianum L.	0	n	1	0	Geraniaceae Juss.
Iz_5_1009	DJI_0800	Geranium sanquineum L.	0	n	1	0	Geraniaceae Juss.
Iz_5_1009	DJI_0800	Gypsophila paniculata L.	0	n	1	0	Caryophyllaceae Juss.
Iz_5_1009	DJI_0800	Helichrysum arenarium (L.) Moench	0	n	1	0	Asteraceae Bercht. & J.Presl
Iz_5_1009	DJI_0800	Herniaria polygama J.Gay	0	n	1	0	Caryophyllaceae Juss.
Iz_5_1009	DJI_0800	Hieracium robustum Fr.	0	n	1	0	Asteraceae Bercht. & J.Presl
Iz_5_1009	DJI_0800	Hieracium umbellatum L.	0	n	1	NA	Asteraceae Bercht. & J.Presl
Iz_5_1009	DJI_0800	Hylotelephium maximum (L.) Holub	0	n	1	0	Crassulaceae J.St.-Hil.
Iz_5_1009	DJI_0800	Jacobaea borysthenica (DC.) B.Nord. & Greuter	0	n	1	0	Asteraceae Bercht. & J.Presl
Iz_5_1009	DJI_0800	Jasione montana L.	0	n	1	0	Campanulaceae Juss.
Iz_5_1009	DJI_0800	Jurinea cyanoides (L.) Rchb.s.l.	0	n	1	0	Asteraceae Bercht. & J.Presl
Iz_5_1009	DJI_0800	Koeleria glauca (Spreng.) DC.	0	n	1	0	Poaceae Barnhart
Iz_5_1009	DJI_0800	Lactuca serriola L.	0	a	0	0	Asteraceae Bercht. & J.Presl
Iz_5_1009	DJI_0800	Linaria genistifolia (L.) Mill.	0	n	1	0	Plantaginaceae Juss.
Iz_5_1009	DJI_0800	Lomelosia argentea (L.) Greuter & Burdet	0	n	1	0	Caprifoliaceae Juss.
Iz_5_1009	DJI_0800	Melampyrum pratense L.	0	n	1	0	Orobanchaceae Vent.
Iz_5_1009	DJI_0800	Oenothera biennis L.	0	a	0	0	Onagraceae Juss.
Iz_5_1009	DJI_0800	Pentanema britannica (L.) D.Gut.Larr., Santos-Vicente, Anderb., E.Rico & M.M.Mart.Ort.	0	n	1	0	Asteraceae Bercht. & J.Presl
Iz_5_1009	DJI_0800	Peucedanum oreoselinum (L.) Moench	3	n	1	0	Apiaceae Lindl.
Iz_5_1009	DJI_0800	Peucedanum ruthenicum M.Bieb.	0	n	1	0	Apiaceae Lindl.
Iz_5_1009	DJI_0800	Pilosella echioides (Lumn.) F.W.Schultz & Sch.Bip.	0	n	1	0	Asteraceae Bercht. & J.Presl
Iz_5_1009	DJI_0800	Pilosella officinarum F. Schultz et Sch. Bip.	0	n	1	0	Asteraceae Bercht. & J.Presl
Iz_5_1009	DJI_0800	Pilosella sp.	0		NA	0	Asteraceae Bercht. & J.Presl
Iz_5_1009	DJI_0800	Pinus sylvestris L.	0	n	1	1	Pinaceae Spreng. ex F.Rudolphi
Iz_5_1009	DJI_0800	Poa annua L.	0	n	1	0	Poaceae Barnhart
Iz_5_1009	DJI_0800	Poa nemoralis L.	0	n	1	0	Poaceae Barnhart
Iz_5_1009	DJI_0800	Poa sp.	0		NA	0	Poaceae Barnhart
Iz_5_1009	DJI_0800	Polygonatum odoratum (Mill.) Druce	0	n	1	0	Asparagaceae Juss.
Iz_5_1009	DJI_0800	Polygonum arenarium Waldst. & Kit.	0	n	1	0	Polygonaceae Juss.
Iz_5_1009	DJI_0800	Populus nigra L.	0	n	1	1	Salicaceae Mirb.
Iz_5_1009	DJI_0800	Populus sp.	0	n	1	1	Salicaceae Mirb.
Iz_5_1009	DJI_0800	Populus tremula L.	0	n	1	1	Salicaceae Mirb.
Iz_5_1009	DJI_0800	Populus Ã canescens (Aiton) Sm.	0	n	1	1	Salicaceae Mirb.
Iz_5_1009	DJI_0800	Potentilla argentea L.	0	n	1	0	Rosaceae Juss.
Iz_5_1009	DJI_0800	Psammophiliella muralis (L.) Ikonn.	0	n	1	0	Caryophyllaceae Juss.
Iz_5_1009	DJI_0800	Psephellus sumensis (Kalen.) Greuter	0	n	1	0	Asteraceae Bercht. & J.Presl
Iz_5_1009	DJI_0800	Pulsatilla pratensis (L.) Mill. s.l.	0	n	1	0	Ranunculaceae Juss.
Iz_5_1009	DJI_0800	Pyrus communis L.	0	a	0	1	Rosaceae Juss.
Iz_5_1009	DJI_0800	Quercus robur L.	0	n	1	1	Fagaceae Dumort.
Iz_5_1009	DJI_0800	Robinia pseudoacacia L.	0	a	0	1	Fabaceae Lindl.
Iz_5_1009	DJI_0800	Rosa sp.	0		NA	1	Rosaceae Juss.
Iz_5_1009	DJI_0800	Rubus idaeus L.	0	n	1	0	Rosaceae Juss.
Iz_5_1009	DJI_0800	Rumex acetosella L.	0	n	1	0	Polygonaceae Juss.
Iz_5_1009	DJI_0800	Rumex thyrsiflorus Fingerh.	0	n	1	0	Polygonaceae Juss.
Iz_5_1009	DJI_0800	Salix alba L.	0	n	1	1	Salicaceae Mirb.
Iz_5_1009	DJI_0800	Sambucus nigra L.	0	n	1	1	Viburnaceae Raf.
Iz_5_1009	DJI_0800	Secale sylvestre Host	0	n	1	0	Poaceae Barnhart
Iz_5_1009	DJI_0800	Sempervivum ruthenicum Schnittsp. & C.B. Lehm.	0	n	1	0	Crassulaceae J.St.-Hil.
Iz_5_1009	DJI_0800	Setaria viridis (L.) P. Beauv.	0	n	1	0	Poaceae Barnhart
Iz_5_1009	DJI_0800	Silene borysthenica (Gruner) Walters	0	n	1	0	Caryophyllaceae Juss.
Iz_5_1009	DJI_0800	Silene latifolia subsp. alba (Mill.) Greuter & Burdet	0	n	1	0	Caryophyllaceae Juss.
Iz_5_1009	DJI_0800	Solidago virgaurea L.	0	n	1	0	Asteraceae Bercht. & J.Presl
Iz_5_1009	DJI_0800	Solidago Ã niederederi (S. canadensis Ã S. virgaurea)	0	hybrid	0	0	Asteraceae Bercht. & J.Presl
Iz_5_1009	DJI_0800	Sorbus aucuparia L.	0	n	1	1	Rosaceae Juss.
Iz_5_1009	DJI_0800	Stachys recta L.	0	n	1	0	Lamiacea Lindl.
Iz_5_1009	DJI_0800	Tanacetum vulgare L.	0	n	1	0	Asteraceae Bercht. & J.Presl
Iz_5_1009	DJI_0800	Thymus pallasianus Heinr.Braun	0	n	1	0	Lamiacea Lindl.
Iz_5_1009	DJI_0800	Tilia cordata Mill.	0	n	1	1	Malvaceae Juss.
Iz_5_1009	DJI_0800	Trifolium alpestre L.	0	n	1	0	Fabaceae Lindl.
Iz_5_1009	DJI_0800	Trifolium arvense L.	0	n	1	0	Fabaceae Lindl.
Iz_5_1009	DJI_0800	Ulmus glabra Huds.	0	n	1	1	Ulmaceae Mirb.
Iz_5_1009	DJI_0800	Verbascum densiflorum Bertol.	0	n	1	0	Scrophulariaceae Juss.
Iz_5_1009	DJI_0800	Veronica austriaca L.	0	n	1	0	Plantaginaceae Juss.
Iz_5_1009	DJI_0800	Veronica incana L.	0	n	1	0	Plantaginaceae Juss.
Iz_5_1009	DJI_0800	Veronica verna L.	0	n	1	0	Plantaginaceae Juss.
Iz_5_1009	DJI_0800	Vincetoxicum hirundinaria Medik	0	n	1	0	Apocynaceae Juss.
Iz_5_1009	DJI_0803	Acer tataricum L.	0	n	1	1	Sapindaceae Juss.
Iz_5_1009	DJI_0803	Achillea millefolium L. s.l.	0	n	1	0	Asteraceae Bercht. & J.Presl
Iz_5_1009	DJI_0803	Allium oleraceum L.	0	n	1	0	Amaryllidaceae J.St.-Hil.
Iz_5_1009	DJI_0803	Ambrosia artemisiifolia L.	0	a	0	0	Asteraceae Bercht. & J.Presl
Iz_5_1009	DJI_0803	Amorpha fruticosa L.	0	a	0	0	Fabaceae Lindl.
Iz_5_1009	DJI_0803	Anthericum ramosum L.	0	n	1	0	Asparagaceae Juss.
Iz_5_1009	DJI_0803	Artemisia marschalliana Spreng.	0	n	1	0	Asteraceae Bercht. & J.Presl
Iz_5_1009	DJI_0803	Asparagus officinalis L.	0	n	1	0	Asparagaceae Juss.
Iz_5_1009	DJI_0803	Astragalus cicer L.	0	n	1	0	Fabaceae Lindl.
Iz_5_1009	DJI_0803	Bassia laniflora (S.G.Gmel.) A.J.Scott	0	n	1	0	Amaranthaceae Juss.
Iz_5_1009	DJI_0803	Berberis vulgaris L.	0	n	1	0	Berberidaceae Juss.
Iz_5_1009	DJI_0803	Betula pendula Roth.	0	n	1	1	Betulaceae Gray
Iz_5_1009	DJI_0803	Betula pendula Roth.	0	n	1	0	Poaceae Barnhart
Iz_5_1009	DJI_0803	Bromus inermis Leyss.	0		NA	NA	
Iz_5_1009	DJI_0803	Bromus squarrosus L.	0	a	0	0	Poaceae Barnhart
Iz_5_1009	DJI_0803	Bromus tectorum L.	0	a	0	0	Poaceae Barnhart
Iz_5_1009	DJI_0803	Calamagrostis epigejos (L.) Roth	5	n	1	0	Poaceae Barnhart
Iz_5_1009	DJI_0803	Carduus acanthoides L.	0	n	1	0	Asteraceae Bercht. & J.Presl
Iz_5_1009	DJI_0803	Carex sp.	0	n	1	0	Cyperaceae Juss.
Iz_5_1009	DJI_0803	Centaurea majorovii Dumbadze	0	n	1	0	Asteraceae Bercht. & J.Presl
Iz_5_1009	DJI_0803	Chamaecytisus ruthenicus (Fisch.ex Wol.) Klaskova	0	n	1	0	Fabaceae Lindl.
Iz_5_1009	DJI_0803	Chelidonium majus L.	0	n	1	0	Papaveraceae Juss.
Iz_5_1009	DJI_0803	Chenopodium betaceum Andrz.	0	n	1	0	Amaranthaceae Juss.
Iz_5_1009	DJI_0803	Chenopodium cf. album	0	n	1	0	Amaranthaceae Juss.
Iz_5_1009	DJI_0803	Chenopodium sp.	0		NA	0	Amaranthaceae Juss.
Iz_5_1009	DJI_0803	Chondrilla juncea L.	0	n	1	0	Asteraceae Bercht. & J.Presl
Iz_5_1009	DJI_0803	Cirsium vulgare (Savi) Ten.	0	n	1	0	Asteraceae Bercht. & J.Presl

Iz_5_1009	DJI_0803	Clinopodium acinos (L.) Kuntze	0	n	1	0	Lamiacea Lindl.
Iz_5_1009	DJI_0803	Convallaria majalis L.	0	n	1	0	Asparagaceae Juss.
Iz_5_1009	DJI_0803	Crataegus rhipidophylla Gand.	0	n	1	1	Rosaceae Juss.
Iz_5_1009	DJI_0803	Crepis foetida L.	0	n	1	0	Asteraceae Bercht. & J.Presl
Iz_5_1009	DJI_0803	Dactylis glomerata L.	0	n	1	0	Poaceae Barnhart
Iz_5_1009	DJI_0803	Dianthus campestris M.Bieb.	0	n	1	0	Caryophyllaceae Juss.
Iz_5_1009	DJI_0803	Digitaria sanguinalis (L.) Scop.	0	a	0	0	Poaceae Barnhart
Iz_5_1009	DJI_0803	Elymus repens (L.) Gould	0	n	1	0	Poaceae Barnhart
Iz_5_1009	DJI_0803	Epilobium tetragonum L.	0	n	1	0	Onagraceae Juss.
Iz_5_1009	DJI_0803	Eragrostis minor Host	0	a	0	0	Poaceae Barnhart
Iz_5_1009	DJI_0803	Erigeron canadensis L.	2	a	0	0	Asteraceae Bercht. & J.Presl
Iz_5_1009	DJI_0803	Euphorbia seguieriana Neck.	0	n	1	0	Euphorbiaceae Juss.
Iz_5_1009	DJI_0803	Fallopia dumetorum (L.) Holub	0	n	1	0	Polygonaceae Juss.
Iz_5_1009	DJI_0803	Festuca beckeri (Hack.) Trautv.	0	n	1	0	Poaceae Barnhart
Iz_5_1009	DJI_0803	Fragaria vesca L.	0	n	1	0	Rosaceae Juss.
Iz_5_1009	DJI_0803	Frangula alnus Mill.	0	n	1	1	Rhamnaceae Juss.
Iz_5_1009	DJI_0803	Fraxinus pennsylvanica Marshall	0	a	0	1	Oleaceae Hoffmanns. & Link
Iz_5_1009	DJI_0803	Galeopsis bifida Boenn.	0	n	1	0	Lamiacea Lindl.
Iz_5_1009	DJI_0803	Galium verum L.	0	n	1	0	Rubiaceae Juss.
Iz_5_1009	DJI_0803	Genista tinctoria L.	0	n	1	0	Fabaceae Lindl.
Iz_5_1009	DJI_0803	Geranium robertianum L.	0	n	1	0	Geraniaceae Juss.
Iz_5_1009	DJI_0803	Geranium sanquineum L.	0	n	1	0	Geraniaceae Juss.
Iz_5_1009	DJI_0803	Gypsophila paniculata L.	0	n	1	0	Caryophyllaceae Juss.
Iz_5_1009	DJI_0803	Helichrysum arenarium (L.) Moench	0	n	1	0	Asteraceae Bercht. & J.Presl
Iz_5_1009	DJI_0803	Herniaria polygama J.Gay	0	n	1	0	Caryophyllaceae Juss.
Iz_5_1009	DJI_0803	Hieracium robustum Fr.	0	n	1	0	Asteraceae Bercht. & J.Presl
Iz_5_1009	DJI_0803	Hieracium umbellatum L.	0	n	1	NA	Asteraceae Bercht. & J.Presl
Iz_5_1009	DJI_0803	Hylotelephium maximum (L.) Holub	0	n	1	0	Crassulaceae J.St.-Hil.
Iz_5_1009	DJI_0803	Jacobaea borysthenica (DC.) B.Nord. & Greuter	0	n	1	0	Asteraceae Bercht. & J.Presl
Iz_5_1009	DJI_0803	Jasione montana L.	0	n	1	0	Campanulaceae Juss.
Iz_5_1009	DJI_0803	Jurinea cyanoides (L.) Rchb.s.l.	0	n	1	0	Asteraceae Bercht. & J.Presl
Iz_5_1009	DJI_0803	Koeleria glauca (Spreng.) DC.	0	n	1	0	Poaceae Barnhart
Iz_5_1009	DJI_0803	Lactuca serriola L.	0	a	0	0	Asteraceae Bercht. & J.Presl
Iz_5_1009	DJI_0803	Linaria genistifolia (L.) Mill.	0	n	1	0	Plantaginaceae Juss.
Iz_5_1009	DJI_0803	Lomelosia argentea (L.) Greuter & Burdet	0	n	1	0	Caprifoliaceae Juss.
Iz_5_1009	DJI_0803	Melampyrum pratense L.	0	n	1	0	Orobanchaceae Vent.
Iz_5_1009	DJI_0803	Oenothera biennis L.	0	a	0	0	Onagraceae Juss.
Iz_5_1009	DJI_0803	Pentanema britannica (L.) D.Gut.Larr., Santos-Vicente, Anderb., E.Rico & M.M.Mart.Ort.	0	n	1	0	Asteraceae Bercht. & J.Presl
Iz_5_1009	DJI_0803	Peucedanum oreoselinum (L.) Moench	0	n	1	0	Apiaceae Lindl.
Iz_5_1009	DJI_0803	Peucedanum ruthenicum M.Bieb.	0	n	1	0	Apiaceae Lindl.
Iz_5_1009	DJI_0803	Pilosella echioides (Lumn.) F.W.Schultz & Sch.Bip.	0	n	1	0	Asteraceae Bercht. & J.Presl
Iz_5_1009	DJI_0803	Pilosella officinarum F. Schultz et Sch. Bip.	0	n	1	0	Asteraceae Bercht. & J.Presl
Iz_5_1009	DJI_0803	Pilosella sp.	0		NA	0	Asteraceae Bercht. & J.Presl
Iz_5_1009	DJI_0803	Pinus sylvestris L.	0	n	1	1	Pinaceae Spreng. ex F.Rudolphi
Iz_5_1009	DJI_0803	Poa annua L.	0	n	1	0	Poaceae Barnhart
Iz_5_1009	DJI_0803	Poa nemoralis L.	0	n	1	0	Poaceae Barnhart
Iz_5_1009	DJI_0803	Poa sp.	0		NA	0	Poaceae Barnhart
Iz_5_1009	DJI_0803	Polygonatum odoratum (Mill.) Druce	0	n	1	0	Asparagaceae Juss.
Iz_5_1009	DJI_0803	Polygonum arenarium Waldst. & Kit.	0	n	1	0	Polygonaceae Juss.
Iz_5_1009	DJI_0803	Populus nigra L.	0	n	1	1	Salicaceae Mirb.
Iz_5_1009	DJI_0803	Populus sp.	0	n	1	1	Salicaceae Mirb.
Iz_5_1009	DJI_0803	Populus tremula L.	0	n	1	1	Salicaceae Mirb.
Iz_5_1009	DJI_0803	Populus Ã canescens (Aiton) Sm.	0	n	1	1	Salicaceae Mirb.
Iz_5_1009	DJI_0803	Potentilla argentea L.	0	n	1	0	Rosaceae Juss.
Iz_5_1009	DJI_0803	Psammophiliella muralis (L.) Ikonn.	0	n	1	0	Caryophyllaceae Juss.
Iz_5_1009	DJI_0803	Psephellus sumensis (Kalen.) Greuter	0	n	1	0	Asteraceae Bercht. & J.Presl
Iz_5_1009	DJI_0803	Pulsatilla pratensis (L.) Mill. s.l.	0	n	1	0	Ranunculaceae Juss.
Iz_5_1009	DJI_0803	Pyrus communis L.	0	a	0	1	Rosaceae Juss.
Iz_5_1009	DJI_0803	Quercus robur L.	0	n	1	1	Fagaceae Dumort.
Iz_5_1009	DJI_0803	Robinia pseudoacacia L.	0	a	0	1	Fabaceae Lindl.
Iz_5_1009	DJI_0803	Rosa sp.	0		NA	1	Rosaceae Juss.
Iz_5_1009	DJI_0803	Rubus idaeus L.	0	n	1	0	Rosaceae Juss.
Iz_5_1009	DJI_0803	Rumex acetosella L.	0	n	1	0	Polygonaceae Juss.
Iz_5_1009	DJI_0803	Rumex thyrsiflorus Fingerh.	0	n	1	0	Polygonaceae Juss.
Iz_5_1009	DJI_0803	Salix alba L.	0	n	1	1	Salicaceae Mirb.
Iz_5_1009	DJI_0803	Sambucus nigra L.	0	n	1	1	Viburnaceae Raf.
Iz_5_1009	DJI_0803	Secale sylvestre Host	0	n	1	0	Poaceae Barnhart
Iz_5_1009	DJI_0803	Sempervivum ruthenicum Schnittsp. & C.B. Lehm.	0	n	1	0	Crassulaceae J.St.-Hil.
Iz_5_1009	DJI_0803	Setaria viridis (L.) P. Beauv.	0	n	1	0	Poaceae Barnhart
Iz_5_1009	DJI_0803	Silene borysthenica (Gruner) Walters	0	n	1	0	Caryophyllaceae Juss.
Iz_5_1009	DJI_0803	Silene latifolia subsp. alba (Mill.) Greuter & Burdet	0	n	1	0	Caryophyllaceae Juss.
Iz_5_1009	DJI_0803	Solidago virgaurea L.	0	n	1	0	Asteraceae Bercht. & J.Presl
Iz_5_1009	DJI_0803	Solidago Ã niederederi (S. canadensis Ã S. virgaurea)	0	hybrid	0	0	Asteraceae Bercht. & J.Presl
Iz_5_1009	DJI_0803	Sorbus aucuparia L.	0	n	1	1	Rosaceae Juss.
Iz_5_1009	DJI_0803	Stachys recta L.	0	n	1	0	Lamiacea Lindl.
Iz_5_1009	DJI_0803	Tanacetum vulgare L.	0	n	1	0	Asteraceae Bercht. & J.Presl
Iz_5_1009	DJI_0803	Thymus pallasianus Heinr.Braun	0	n	1	0	Lamiacea Lindl.
Iz_5_1009	DJI_0803	Tilia cordata Mill.	0	n	1	1	Malvaceae Juss.
Iz_5_1009	DJI_0803	Trifolium alpestre L.	0	n	1	0	Fabaceae Lindl.
Iz_5_1009	DJI_0803	Trifolium arvense L.	0	n	1	0	Fabaceae Lindl.
Iz_5_1009	DJI_0803	Ulmus glabra Huds.	0	n	1	1	Ulmaceae Mirb.
Iz_5_1009	DJI_0803	Verbascum densiflorum Bertol.	0	n	1	0	Scrophulariaceae Juss.
Iz_5_1009	DJI_0803	Veronica austriaca L.	0	n	1	0	Plantaginaceae Juss.
Iz_5_1009	DJI_0803	Veronica incana L.	0	n	1	0	Plantaginaceae Juss.
Iz_5_1009	DJI_0803	Veronica verna L.	0	n	1	0	Plantaginaceae Juss.
Iz_5_1009	DJI_0803	Vincetoxicum hirundinaria Medik	0	n	1	0	Apocynaceae Juss.
Iz_5_1009	DJI_0811	Acer tataricum L.	0	n	1	1	Sapindaceae Juss.
Iz_5_1009	DJI_0811	Achillea millefolium L. s.l.	0	n	1	0	Asteraceae Bercht. & J.Presl
Iz_5_1009	DJI_0811	Allium oleraceum L.	0	n	1	0	Amaryllidaceae J.St.-Hil.
Iz_5_1009	DJI_0811	Ambrosia artemisiifolia L.	0	a	0	0	Asteraceae Bercht. & J.Presl
Iz_5_1009	DJI_0811	Amorpha fruticosa L.	0	a	0	0	Fabaceae Lindl.
Iz_5_1009	DJI_0811	Anthericum ramosum L.	0	n	1	0	Asparagaceae Juss.
Iz_5_1009	DJI_0811	Artemisia marschalliana Spreng.	0	n	1	0	Asteraceae Bercht. & J.Presl
Iz_5_1009	DJI_0811	Asparagus officinalis L.	0	n	1	0	Asparagaceae Juss.
Iz_5_1009	DJI_0811	Astragalus cicer L.	0	n	1	0	Fabaceae Lindl.
Iz_5_1009	DJI_0811	Bassia laniflora (S.G.Gmel.) A.J.Scott	0	n	1	0	Amaranthaceae Juss.
Iz_5_1009	DJI_0811	Berberis vulgaris L.	0	n	1	0	Berberidaceae Juss.
Iz_5_1009	DJI_0811	Betula pendula Roth.	0	n	1	1	Betulaceae Gray

Iz_5_1009	DJI_0811	Betula pendula Roth.	0	n	1	0	Poaceae Barnhart
Iz_5_1009	DJI_0811	Bromus inermis Leyss.	0		NA	NA	
Iz_5_1009	DJI_0811	Bromus squarrosus L.	0	a	0	0	Poaceae Barnhart
Iz_5_1009	DJI_0811	Bromus tectorum L.	0	a	0	0	Poaceae Barnhart
Iz_5_1009	DJI_0811	Calamagrostis epigejos (L.) Roth	5	n	1	0	Poaceae Barnhart
Iz_5_1009	DJI_0811	Carduus acanthoides L.	0	n	1	0	Asteraceae Bercht. & J.Presl
Iz_5_1009	DJI_0811	Carex sp.	0	n	1	0	Cyperaceae Juss.
Iz_5_1009	DJI_0811	Centaurea majorovii Dumbadze	0	n	1	0	Asteraceae Bercht. & J.Presl
Iz_5_1009	DJI_0811	Chamaecytisus ruthenicus (Fisch.ex Wol.) Klaskova	0	n	1	0	Fabaceae Lindl.
Iz_5_1009	DJI_0811	Chelidonium majus L.	0	n	1	0	Papaveraceae Juss.
Iz_5_1009	DJI_0811	Chenopodium betaceum Andrz.	0	n	1	0	Amaranthaceae Juss.
Iz_5_1009	DJI_0811	Chenopodium cf. album	0	n	1	0	Amaranthaceae Juss.
Iz_5_1009	DJI_0811	Chenopodium sp.	0		NA	0	Amaranthaceae Juss.
Iz_5_1009	DJI_0811	Chondrilla juncea L.	0	n	1	0	Asteraceae Bercht. & J.Presl
Iz_5_1009	DJI_0811	Cirsium vulgare (Savi) Ten.	0	n	1	0	Asteraceae Bercht. & J.Presl
Iz_5_1009	DJI_0811	Clinopodium acinos (L.) Kuntze	0	n	1	0	Lamiacea Lindl.
Iz_5_1009	DJI_0811	Convallaria majalis L.	0	n	1	0	Asparagaceae Juss.
Iz_5_1009	DJI_0811	Crataegus rhipidophylla Gand.	0	n	1	1	Rosaceae Juss.
Iz_5_1009	DJI_0811	Crepis foetida L.	0	n	1	0	Asteraceae Bercht. & J.Presl
Iz_5_1009	DJI_0811	Dactylis glomerata L.	0	n	1	0	Poaceae Barnhart
Iz_5_1009	DJI_0811	Dianthus campestris M.Bieb.	0	n	1	0	Caryophyllaceae Juss.
Iz_5_1009	DJI_0811	Digitaria sanguinalis (L.) Scop.	0	a	0	0	Poaceae Barnhart
Iz_5_1009	DJI_0811	Elymus repens (L.) Gould	0	n	1	0	Poaceae Barnhart
Iz_5_1009	DJI_0811	Epilobium tetragonum L.	0	n	1	0	Onagraceae Juss.
Iz_5_1009	DJI_0811	Eragrostis minor Host	0	a	0	0	Poaceae Barnhart
Iz_5_1009	DJI_0811	Erigeron canadensis L.	0	a	0	0	Asteraceae Bercht. & J.Presl
Iz_5_1009	DJI_0811	Euphorbia seguieriana Neck.	0	n	1	0	Euphorbiaceae Juss.
Iz_5_1009	DJI_0811	Fallopia dumetorum (L.) Holub	0	n	1	0	Polygonaceae Juss.
Iz_5_1009	DJI_0811	Festuca beckeri (Hack.) Trautv.	0	n	1	0	Poaceae Barnhart
Iz_5_1009	DJI_0811	Fragaria vesca L.	0	n	1	0	Rosaceae Juss.
Iz_5_1009	DJI_0811	Frangula alnus Mill.	0	n	1	1	Rhamnaceae Juss.
Iz_5_1009	DJI_0811	Fraxinus pennsylvanica Marshall	0	a	0	1	Oleaceae Hoffmanns. & Link
Iz_5_1009	DJI_0811	Galeopsis bifida Boenn.	0	n	1	0	Lamiacea Lindl.
Iz_5_1009	DJI_0811	Galium verum L.	0	n	1	0	Rubiaceae Juss.
Iz_5_1009	DJI_0811	Genista tinctoria L.	0	n	1	0	Fabaceae Lindl.
Iz_5_1009	DJI_0811	Geranium robertianum L.	0	n	1	0	Geraniaceae Juss.
Iz_5_1009	DJI_0811	Geranium sanquineum L.	0	n	1	0	Geraniaceae Juss.
Iz_5_1009	DJI_0811	Gypsophila paniculata L.	0	n	1	0	Caryophyllaceae Juss.
Iz_5_1009	DJI_0811	Helichrysum arenarium (L.) Moench	0	n	1	0	Asteraceae Bercht. & J.Presl
Iz_5_1009	DJI_0811	Herniaria polygama J.Gay	0	n	1	0	Caryophyllaceae Juss.
Iz_5_1009	DJI_0811	Hieracium robustum Fr.	0	n	1	0	Asteraceae Bercht. & J.Presl
Iz_5_1009	DJI_0811	Hieracium umbellatum L.	0	n	1	NA	Asteraceae Bercht. & J.Presl
Iz_5_1009	DJI_0811	Hylotelephium maximum (L.) Holub	0	n	1	0	Crassulaceae J.St.-Hil.
Iz_5_1009	DJI_0811	Jacobaea borysthenica (DC.) B.Nord. & Greuter	0	n	1	0	Asteraceae Bercht. & J.Presl
Iz_5_1009	DJI_0811	Jasione montana L.	0	n	1	0	Campanulaceae Juss.
Iz_5_1009	DJI_0811	Jurinea cyanoides (L.) Rchb.s.l.	0	n	1	0	Asteraceae Bercht. & J.Presl
Iz_5_1009	DJI_0811	Koeleria glauca (Spreng.) DC.	0	n	1	0	Poaceae Barnhart
Iz_5_1009	DJI_0811	Lactuca serriola L.	0	a	0	0	Asteraceae Bercht. & J.Presl
Iz_5_1009	DJI_0811	Linaria genistifolia (L.) Mill.	0	n	1	0	Plantaginaceae Juss.
Iz_5_1009	DJI_0811	Lomelosia argentea (L.) Greuter & Burdet	0	n	1	0	Caprifoliaceae Juss.
Iz_5_1009	DJI_0811	Melampyrum pratense L.	0	n	1	0	Orobanchaceae Vent.
Iz_5_1009	DJI_0811	Oenothera biennis L.	0	a	0	0	Onagraceae Juss.
Iz_5_1009	DJI_0811	Pentanema britannica (L.) D.Gut.Larr., Santos-Vicente, Anderb., E.Rico & M.M.Mart.Ort.	0	n	1	0	Asteraceae Bercht. & J.Presl
Iz_5_1009	DJI_0811	Peucedanum oreoselinum (L.) Moench	2	n	1	0	Apiaceae Lindl.
Iz_5_1009	DJI_0811	Peucedanum ruthenicum M.Bieb.	0	n	1	0	Apiaceae Lindl.
Iz_5_1009	DJI_0811	Pilosella echioides (Lumn.) F.W.Schultz & Sch.Bip.	0	n	1	0	Asteraceae Bercht. & J.Presl
Iz_5_1009	DJI_0811	Pilosella officinarum F. Schultz et Sch. Bip.	0	n	1	0	Asteraceae Bercht. & J.Presl
Iz_5_1009	DJI_0811	Pilosella sp.	0		NA	0	Asteraceae Bercht. & J.Presl
Iz_5_1009	DJI_0811	Pinus sylvestris L.	0	n	1	1	Pinaceae Spreng. ex F.Rudolphi
Iz_5_1009	DJI_0811	Poa annua L.	0	n	1	0	Poaceae Barnhart
Iz_5_1009	DJI_0811	Poa nemoralis L.	0	n	1	0	Poaceae Barnhart
Iz_5_1009	DJI_0811	Poa sp.	0		NA	0	Poaceae Barnhart
Iz_5_1009	DJI_0811	Polygonatum odoratum (Mill.) Druce	0	n	1	0	Asparagaceae Juss.
Iz_5_1009	DJI_0811	Polygonum arenarium Waldst. & Kit.	0	n	1	0	Polygonaceae Juss.
Iz_5_1009	DJI_0811	Populus nigra L.	0	n	1	1	Salicaceae Mirb.
Iz_5_1009	DJI_0811	Populus sp.	0	n	1	1	Salicaceae Mirb.
Iz_5_1009	DJI_0811	Populus tremula L.	0	n	1	1	Salicaceae Mirb.
Iz_5_1009	DJI_0811	Populus Ã canescens (Aiton) Sm.	0	n	1	1	Salicaceae Mirb.
Iz_5_1009	DJI_0811	Potentilla argentea L.	0	n	1	0	Rosaceae Juss.
Iz_5_1009	DJI_0811	Psammophiliella muralis (L.) Ikonn.	0	n	1	0	Caryophyllaceae Juss.
Iz_5_1009	DJI_0811	Psephellus sumensis (Kalen.) Greuter	0	n	1	0	Asteraceae Bercht. & J.Presl
Iz_5_1009	DJI_0811	Pulsatilla pratensis (L.) Mill. s.l.	0	n	1	0	Ranunculaceae Juss.
Iz_5_1009	DJI_0811	Pyrus communis L.	0	a	0	1	Rosaceae Juss.
Iz_5_1009	DJI_0811	Quercus robur L.	0	n	1	1	Fagaceae Dumort.
Iz_5_1009	DJI_0811	Robinia pseudoacacia L.	0	a	0	1	Fabaceae Lindl.
Iz_5_1009	DJI_0811	Rosa sp.	0		NA	1	Rosaceae Juss.
Iz_5_1009	DJI_0811	Rubus idaeus L.	0	n	1	0	Rosaceae Juss.
Iz_5_1009	DJI_0811	Rumex acetosella L.	0	n	1	0	Polygonaceae Juss.
Iz_5_1009	DJI_0811	Rumex thyrsiflorus Fingerh.	0	n	1	0	Polygonaceae Juss.
Iz_5_1009	DJI_0811	Salix alba L.	0	n	1	1	Salicaceae Mirb.
Iz_5_1009	DJI_0811	Sambucus nigra L.	0	n	1	1	Viburnaceae Raf.
Iz_5_1009	DJI_0811	Secale sylvestre Host	0	n	1	0	Poaceae Barnhart
Iz_5_1009	DJI_0811	Sempervivum ruthenicum Schnittsp. & C.B. Lehm.	0	n	1	0	Crassulaceae J.St.-Hil.
Iz_5_1009	DJI_0811	Setaria viridis (L.) P. Beauv.	0	n	1	0	Poaceae Barnhart
Iz_5_1009	DJI_0811	Silene borysthenica (Gruner) Walters	0	n	1	0	Caryophyllaceae Juss.
Iz_5_1009	DJI_0811	Silene latifolia subsp. alba (Mill.) Greuter & Burdet	0	n	1	0	Caryophyllaceae Juss.
Iz_5_1009	DJI_0811	Solidago virgaurea L.	0	n	1	0	Asteraceae Bercht. & J.Presl
Iz_5_1009	DJI_0811	Solidago Ã niederederi (S. canadensis Ã S. virgaurea)	0	hybrid	0	0	Asteraceae Bercht. & J.Presl
Iz_5_1009	DJI_0811	Sorbus aucuparia L.	0	n	1	1	Rosaceae Juss.
Iz_5_1009	DJI_0811	Stachys recta L.	0	n	1	0	Lamiacea Lindl.
Iz_5_1009	DJI_0811	Tanacetum vulgare L.	0	n	1	0	Asteraceae Bercht. & J.Presl
Iz_5_1009	DJI_0811	Thymus pallasianus Heinr.Braun	0	n	1	0	Lamiacea Lindl.
Iz_5_1009	DJI_0811	Tilia cordata Mill.	0	n	1	1	Malvaceae Juss.
Iz_5_1009	DJI_0811	Trifolium alpestre L.	0	n	1	0	Fabaceae Lindl.
Iz_5_1009	DJI_0811	Trifolium arvense L.	0	n	1	0	Fabaceae Lindl.
Iz_5_1009	DJI_0811	Ulmus glabra Huds.	0	n	1	1	Ulmaceae Mirb.
Iz_5_1009	DJI_0811	Verbascum densiflorum Bertol.	0	n	1	0	Scrophulariaceae Juss.
Iz_5_1009	DJI_0811	Veronica austriaca L.	0	n	1	0	Plantaginaceae Juss.

Iz_5_1009	DJI_0811	Veronica incana L.	0	n	1	0	Plantaginaceae Juss.
Iz_5_1009	DJI_0811	Veronica verna L.	0	n	1	0	Plantaginaceae Juss.
Iz_5_1009	DJI_0811	Vincetoxicum hirundinaria Medik	0	n	1	0	Apocynaceae Juss.
Iz_5_1009	DJI_0814	Acer tataricum L.	0	n	1	1	Sapindaceae Juss.
Iz_5_1009	DJI_0814	Achillea millefolium L. s.l.	0	n	1	0	Asteraceae Bercht. & J.Presl
Iz_5_1009	DJI_0814	Allium oleraceum L.	0	n	1	0	Amaryllidaceae J.St.-Hil.
Iz_5_1009	DJI_0814	Ambrosia artemisiifolia L.	0	a	0	0	Asteraceae Bercht. & J.Presl
Iz_5_1009	DJI_0814	Amorpha fruticosa L.	0	a	0	0	Fabaceae Lindl.
Iz_5_1009	DJI_0814	Anthericum ramosum L.	0	n	1	0	Asparagaceae Juss.
Iz_5_1009	DJI_0814	Artemisia marschalliana Spreng.	0	n	1	0	Asteraceae Bercht. & J.Presl
Iz_5_1009	DJI_0814	Asparagus officinalis L.	0	n	1	0	Asparagaceae Juss.
Iz_5_1009	DJI_0814	Astragalus cicer L.	0	n	1	0	Fabaceae Lindl.
Iz_5_1009	DJI_0814	Bassia laniflora (S.G.Gmel.) A.J.Scott	0	n	1	0	Amaranthaceae Juss.
Iz_5_1009	DJI_0814	Berberis vulgaris L.	0	n	1	0	Berberidaceae Juss.
Iz_5_1009	DJI_0814	Betula pendula Roth.	0	n	1	1	Betulaceae Gray
Iz_5_1009	DJI_0814	Betula pendula Roth.	0	n	1	0	Poaceae Barnhart
Iz_5_1009	DJI_0814	Bromus inermis Leyss.	0		NA	NA	
Iz_5_1009	DJI_0814	Bromus squarrosus L.	0	a	0	0	Poaceae Barnhart
Iz_5_1009	DJI_0814	Bromus tectorum L.	0	a	0	0	Poaceae Barnhart
Iz_5_1009	DJI_0814	Calamagrostis epigejos (L.) Roth	20	n	1	0	Poaceae Barnhart
Iz_5_1009	DJI_0814	Carduus acanthoides L.	0	n	1	0	Asteraceae Bercht. & J.Presl
Iz_5_1009	DJI_0814	Carex sp.	0	n	1	0	Cyperaceae Juss.
Iz_5_1009	DJI_0814	Centaurea majorovii Dumbadze	0	n	1	0	Asteraceae Bercht. & J.Presl
Iz_5_1009	DJI_0814	Chamaecytisus ruthenicus (Fisch.ex Wol.) Klaskova	3	n	1	0	Fabaceae Lindl.
Iz_5_1009	DJI_0814	Chelidonium majus L.	0	n	1	0	Papaveraceae Juss.
Iz_5_1009	DJI_0814	Chenopodium betaceum Andrz.	0	n	1	0	Amaranthaceae Juss.
Iz_5_1009	DJI_0814	Chenopodium cf. album	0	n	1	0	Amaranthaceae Juss.
Iz_5_1009	DJI_0814	Chenopodium sp.	0		NA	0	Amaranthaceae Juss.
Iz_5_1009	DJI_0814	Chondrilla juncea L.	0	n	1	0	Asteraceae Bercht. & J.Presl
Iz_5_1009	DJI_0814	Cirsium vulgare (Savi) Ten.	0	n	1	0	Asteraceae Bercht. & J.Presl
Iz_5_1009	DJI_0814	Clinopodium acinos (L.) Kuntze	0	n	1	0	Lamiacea Lindl.
Iz_5_1009	DJI_0814	Convallaria majalis L.	0	n	1	0	Asparagaceae Juss.
Iz_5_1009	DJI_0814	Crataegus rhipidophylla Gand.	0	n	1	1	Rosaceae Juss.
Iz_5_1009	DJI_0814	Crepis foetida L.	0	n	1	0	Asteraceae Bercht. & J.Presl
Iz_5_1009	DJI_0814	Dactylis glomerata L.	0	n	1	0	Poaceae Barnhart
Iz_5_1009	DJI_0814	Dianthus campestris M.Bieb.	0	n	1	0	Caryophyllaceae Juss.
Iz_5_1009	DJI_0814	Digitaria sanguinalis (L.) Scop.	0	a	0	0	Poaceae Barnhart
Iz_5_1009	DJI_0814	Elymus repens (L.) Gould	0	n	1	0	Poaceae Barnhart
Iz_5_1009	DJI_0814	Epilobium tetragonum L.	0	n	1	0	Onagraceae Juss.
Iz_5_1009	DJI_0814	Eragrostis minor Host	0	a	0	0	Poaceae Barnhart
Iz_5_1009	DJI_0814	Erigeron canadensis L.	1	a	0	0	Asteraceae Bercht. & J.Presl
Iz_5_1009	DJI_0814	Euphorbia seguieriana Neck.	0	n	1	0	Euphorbiaceae Juss.
Iz_5_1009	DJI_0814	Fallopia dumetorum (L.) Holub	0	n	1	0	Polygonaceae Juss.
Iz_5_1009	DJI_0814	Festuca beckeri (Hack.) Trautv.	0	n	1	0	Poaceae Barnhart
Iz_5_1009	DJI_0814	Fragaria vesca L.	0	n	1	0	Rosaceae Juss.
Iz_5_1009	DJI_0814	Frangula alnus Mill.	0	n	1	1	Rhamnaceae Juss.
Iz_5_1009	DJI_0814	Fraxinus pennsylvanica Marshall	0	a	0	1	Oleaceae Hoffmanns. & Link
Iz_5_1009	DJI_0814	Galeopsis bifida Boenn.	0	n	1	0	Lamiacea Lindl.
Iz_5_1009	DJI_0814	Galium verum L.	0	n	1	0	Rubiaceae Juss.
Iz_5_1009	DJI_0814	Genista tinctoria L.	0	n	1	0	Fabaceae Lindl.
Iz_5_1009	DJI_0814	Geranium robertianum L.	0	n	1	0	Geraniaceae Juss.
Iz_5_1009	DJI_0814	Geranium sanquineum L.	0	n	1	0	Geraniaceae Juss.
Iz_5_1009	DJI_0814	Gypsophila paniculata L.	0	n	1	0	Caryophyllaceae Juss.
Iz_5_1009	DJI_0814	Helichrysum arenarium (L.) Moench	0	n	1	0	Asteraceae Bercht. & J.Presl
Iz_5_1009	DJI_0814	Herniaria polygama J.Gay	0	n	1	0	Caryophyllaceae Juss.
Iz_5_1009	DJI_0814	Hieracium robustum Fr.	0	n	1	0	Asteraceae Bercht. & J.Presl
Iz_5_1009	DJI_0814	Hieracium umbellatum L.	0	n	1	NA	Asteraceae Bercht. & J.Presl
Iz_5_1009	DJI_0814	Hylotelephium maximum (L.) Holub	0	n	1	0	Crassulaceae J.St.-Hil.
Iz_5_1009	DJI_0814	Jacobaea borysthenica (DC.) B.Nord. & Greuter	0	n	1	0	Asteraceae Bercht. & J.Presl
Iz_5_1009	DJI_0814	Jasione montana L.	0	n	1	0	Campanulaceae Juss.
Iz_5_1009	DJI_0814	Jurinea cyanoides (L.) Rchb.s.l.	0	n	1	0	Asteraceae Bercht. & J.Presl
Iz_5_1009	DJI_0814	Koeleria glauca (Spreng.) DC.	0	n	1	0	Poaceae Barnhart
Iz_5_1009	DJI_0814	Lactuca serriola L.	0	a	0	0	Asteraceae Bercht. & J.Presl
Iz_5_1009	DJI_0814	Linaria genistifolia (L.) Mill.	0	n	1	0	Plantaginaceae Juss.
Iz_5_1009	DJI_0814	Lomelosia argentea (L.) Greuter & Burdet	0	n	1	0	Caprifoliaceae Juss.
Iz_5_1009	DJI_0814	Melampyrum pratense L.	0	n	1	0	Orobanchaceae Vent.
Iz_5_1009	DJI_0814	Oenothera biennis L.	0	a	0	0	Onagraceae Juss.
Iz_5_1009	DJI_0814	Pentanema britannica (L.) D.Gut.Larr., Santos-Vicente, Anderb., E.Rico & M.M.Mart.Ort.	0	n	1	0	Asteraceae Bercht. & J.Presl
Iz_5_1009	DJI_0814	Peucedanum oreoselinum (L.) Moench	2	n	1	0	Apiaceae Lindl.
Iz_5_1009	DJI_0814	Peucedanum ruthenicum M.Bieb.	0	n	1	0	Apiaceae Lindl.
Iz_5_1009	DJI_0814	Pilosella echioides (Lumn.) F.W.Schultz & Sch.Bip.	0	n	1	0	Asteraceae Bercht. & J.Presl
Iz_5_1009	DJI_0814	Pilosella officinarum F. Schultz et Sch. Bip.	0	n	1	0	Asteraceae Bercht. & J.Presl
Iz_5_1009	DJI_0814	Pilosella sp.	0		NA	0	Asteraceae Bercht. & J.Presl
Iz_5_1009	DJI_0814	Pinus sylvestris L.	0	n	1	1	Pinaceae Spreng. ex F.Rudolphi
Iz_5_1009	DJI_0814	Poa annua L.	0	n	1	0	Poaceae Barnhart
Iz_5_1009	DJI_0814	Poa nemoralis L.	0	n	1	0	Poaceae Barnhart
Iz_5_1009	DJI_0814	Poa sp.	0		NA	0	Poaceae Barnhart
Iz_5_1009	DJI_0814	Polygonatum odoratum (Mill.) Druce	0	n	1	0	Asparagaceae Juss.
Iz_5_1009	DJI_0814	Polygonum arenarium Waldst. & Kit.	0	n	1	0	Polygonaceae Juss.
Iz_5_1009	DJI_0814	Populus nigra L.	0	n	1	1	Salicaceae Mirb.
Iz_5_1009	DJI_0814	Populus sp.	0	n	1	1	Salicaceae Mirb.
Iz_5_1009	DJI_0814	Populus tremula L.	0	n	1	1	Salicaceae Mirb.
Iz_5_1009	DJI_0814	Populus Ã canescens (Aiton) Sm.	0	n	1	1	Salicaceae Mirb.
Iz_5_1009	DJI_0814	Potentilla argentea L.	0	n	1	0	Rosaceae Juss.
Iz_5_1009	DJI_0814	Psammophiliella muralis (L.) Ikonn.	0	n	1	0	Caryophyllaceae Juss.
Iz_5_1009	DJI_0814	Psephellus sumensis (Kalen.) Greuter	0	n	1	0	Asteraceae Bercht. & J.Presl
Iz_5_1009	DJI_0814	Pulsatilla pratensis (L.) Mill. s.l.	0	n	1	0	Ranunculaceae Juss.
Iz_5_1009	DJI_0814	Pyrus communis L.	0	a	0	1	Rosaceae Juss.
Iz_5_1009	DJI_0814	Quercus robur L.	0	n	1	1	Fagaceae Dumort.
Iz_5_1009	DJI_0814	Robinia pseudoacacia L.	0	a	0	1	Fabaceae Lindl.
Iz_5_1009	DJI_0814	Rosa sp.	0		NA	1	Rosaceae Juss.
Iz_5_1009	DJI_0814	Rubus idaeus L.	0	n	1	0	Rosaceae Juss.
Iz_5_1009	DJI_0814	Rumex acetosella L.	0	n	1	0	Polygonaceae Juss.
Iz_5_1009	DJI_0814	Rumex thyrsiflorus Fingerh.	0	n	1	0	Polygonaceae Juss.
Iz_5_1009	DJI_0814	Salix alba L.	0	n	1	1	Salicaceae Mirb.
Iz_5_1009	DJI_0814	Sambucus nigra L.	0	n	1	1	Viburnaceae Raf.
Iz_5_1009	DJI_0814	Secale sylvestre Host	0	n	1	0	Poaceae Barnhart
Iz_5_1009	DJI_0814	Sempervivum ruthenicum Schnittsp. & C.B. Lehm.	0	n	1	0	Crassulaceae J.St.-Hil.

Iz_5_1009	DJI_0814	Setaria viridis (L.) P. Beauv.	0	n	1	0	Poaceae Barnhart
Iz_5_1009	DJI_0814	Silene borysthenica (Gruner) Walters	0	n	1	0	Caryophyllaceae Juss.
Iz_5_1009	DJI_0814	Silene latifolia subsp. alba (Mill.) Greuter & Burdet	0	n	1	0	Caryophyllaceae Juss.
Iz_5_1009	DJI_0814	Solidago virgaurea L.	0	n	1	0	Asteraceae Bercht. & J.Presl
Iz_5_1009	DJI_0814	Solidago Ã niederederi (S. canadensis Ã S. virgaurea)	0	hybrid	0	0	Asteraceae Bercht. & J.Presl
Iz_5_1009	DJI_0814	Sorbus aucuparia L.	0	n	1	1	Rosaceae Juss.
Iz_5_1009	DJI_0814	Stachys recta L.	0	n	1	0	Lamiacea Lindl.
Iz_5_1009	DJI_0814	Tanacetum vulgare L.	0	n	1	0	Asteraceae Bercht. & J.Presl
Iz_5_1009	DJI_0814	Thymus pallasianus Heinr.Braun	0	n	1	0	Lamiacea Lindl.
Iz_5_1009	DJI_0814	Tilia cordata Mill.	0	n	1	1	Malvaceae Juss.
Iz_5_1009	DJI_0814	Trifolium alpestre L.	0	n	1	0	Fabaceae Lindl.
Iz_5_1009	DJI_0814	Trifolium arvense L.	0	n	1	0	Fabaceae Lindl.
Iz_5_1009	DJI_0814	Ulmus glabra Huds.	0	n	1	1	Ulmaceae Mirb.
Iz_5_1009	DJI_0814	Verbascum densiflorum Bertol.	0	n	1	0	Scrophulariaceae Juss.
Iz_5_1009	DJI_0814	Veronica austriaca L.	0	n	1	0	Plantaginaceae Juss.
Iz_5_1009	DJI_0814	Veronica incana L.	0	n	1	0	Plantaginaceae Juss.
Iz_5_1009	DJI_0814	Veronica verna L.	0	n	1	0	Plantaginaceae Juss.
Iz_5_1009	DJI_0814	Vincetoxicum hirundinaria Medik	0	n	1	0	Apocynaceae Juss.
Iz_5_1009	DJI_0816	Acer tataricum L.	0	n	1	1	Sapindaceae Juss.
Iz_5_1009	DJI_0816	Achillea millefolium L. s.l.	0	n	1	0	Asteraceae Bercht. & J.Presl
Iz_5_1009	DJI_0816	Allium oleraceum L.	0	n	1	0	Amaryllidaceae J.St.-Hil.
Iz_5_1009	DJI_0816	Ambrosia artemisiifolia L.	0	a	0	0	Asteraceae Bercht. & J.Presl
Iz_5_1009	DJI_0816	Amorpha fruticosa L.	0	a	0	0	Fabaceae Lindl.
Iz_5_1009	DJI_0816	Anthericum ramosum L.	0	n	1	0	Asparagaceae Juss.
Iz_5_1009	DJI_0816	Artemisia marschalliana Spreng.	0	n	1	0	Asteraceae Bercht. & J.Presl
Iz_5_1009	DJI_0816	Asparagus officinalis L.	0	n	1	0	Asparagaceae Juss.
Iz_5_1009	DJI_0816	Astragalus cicer L.	0	n	1	0	Fabaceae Lindl.
Iz_5_1009	DJI_0816	Bassia laniflora (S.G.Gmel.) A.J.Scott	0	n	1	0	Amaranthaceae Juss.
Iz_5_1009	DJI_0816	Berberis vulgaris L.	0	n	1	0	Berberidaceae Juss.
Iz_5_1009	DJI_0816	Betula pendula Roth.	0	n	1	1	Betulaceae Gray
Iz_5_1009	DJI_0816	Betula pendula Roth.	0	n	1	0	Poaceae Barnhart
Iz_5_1009	DJI_0816	Bromus inermis Leyss.	0		NA	NA	
Iz_5_1009	DJI_0816	Bromus squarrosus L.	0	a	0	0	Poaceae Barnhart
Iz_5_1009	DJI_0816	Bromus tectorum L.	0	a	0	0	Poaceae Barnhart
Iz_5_1009	DJI_0816	Calamagrostis epigejos (L.) Roth	20	n	1	0	Poaceae Barnhart
Iz_5_1009	DJI_0816	Carduus acanthoides L.	0	n	1	0	Asteraceae Bercht. & J.Presl
Iz_5_1009	DJI_0816	Carex sp.	1	n	1	0	Cyperaceae Juss.
Iz_5_1009	DJI_0816	Centaurea majorovii Dumbadze	0	n	1	0	Asteraceae Bercht. & J.Presl
Iz_5_1009	DJI_0816	Chamaecytisus ruthenicus (Fisch.ex Wol.) Klaskova	0	n	1	0	Fabaceae Lindl.
Iz_5_1009	DJI_0816	Chelidonium majus L.	0	n	1	0	Papaveraceae Juss.
Iz_5_1009	DJI_0816	Chenopodium betaceum Andrz.	0	n	1	0	Amaranthaceae Juss.
Iz_5_1009	DJI_0816	Chenopodium cf. album	0	n	1	0	Amaranthaceae Juss.
Iz_5_1009	DJI_0816	Chenopodium sp.	0		NA	0	Amaranthaceae Juss.
Iz_5_1009	DJI_0816	Chondrilla juncea L.	0	n	1	0	Asteraceae Bercht. & J.Presl
Iz_5_1009	DJI_0816	Cirsium vulgare (Savi) Ten.	0	n	1	0	Asteraceae Bercht. & J.Presl
Iz_5_1009	DJI_0816	Clinopodium acinos (L.) Kuntze	0	n	1	0	Lamiacea Lindl.
Iz_5_1009	DJI_0816	Convallaria majalis L.	0	n	1	0	Asparagaceae Juss.
Iz_5_1009	DJI_0816	Crataegus rhipidophylla Gand.	0	n	1	1	Rosaceae Juss.
Iz_5_1009	DJI_0816	Crepis foetida L.	0	n	1	0	Asteraceae Bercht. & J.Presl
Iz_5_1009	DJI_0816	Dactylis glomerata L.	0	n	1	0	Poaceae Barnhart
Iz_5_1009	DJI_0816	Dianthus campestris M.Bieb.	0	n	1	0	Caryophyllaceae Juss.
Iz_5_1009	DJI_0816	Digitaria sanguinalis (L.) Scop.	0	a	0	0	Poaceae Barnhart
Iz_5_1009	DJI_0816	Elymus repens (L.) Gould	0	n	1	0	Poaceae Barnhart
Iz_5_1009	DJI_0816	Epilobium tetragonum L.	0	n	1	0	Onagraceae Juss.
Iz_5_1009	DJI_0816	Eragrostis minor Host	0	a	0	0	Poaceae Barnhart
Iz_5_1009	DJI_0816	Erigeron canadensis L.	0	a	0	0	Asteraceae Bercht. & J.Presl
Iz_5_1009	DJI_0816	Euphorbia seguieriana Neck.	0	n	1	0	Euphorbiaceae Juss.
Iz_5_1009	DJI_0816	Fallopia dumetorum (L.) Holub	0	n	1	0	Polygonaceae Juss.
Iz_5_1009	DJI_0816	Festuca beckeri (Hack.) Trautv.	0	n	1	0	Poaceae Barnhart
Iz_5_1009	DJI_0816	Fragaria vesca L.	0	n	1	0	Rosaceae Juss.
Iz_5_1009	DJI_0816	Frangula alnus Mill.	0	n	1	1	Rhamnaceae Juss.
Iz_5_1009	DJI_0816	Fraxinus pennsylvanica Marshall	0	a	0	1	Oleaceae Hoffmanns. & Link
Iz_5_1009	DJI_0816	Galeopsis bifida Boenn.	0	n	1	0	Lamiacea Lindl.
Iz_5_1009	DJI_0816	Galium verum L.	0	n	1	0	Rubiaceae Juss.
Iz_5_1009	DJI_0816	Genista tinctoria L.	0	n	1	0	Fabaceae Lindl.
Iz_5_1009	DJI_0816	Geranium robertianum L.	0	n	1	0	Geraniaceae Juss.
Iz_5_1009	DJI_0816	Geranium sanquineum L.	0	n	1	0	Geraniaceae Juss.
Iz_5_1009	DJI_0816	Gypsophila paniculata L.	0	n	1	0	Caryophyllaceae Juss.
Iz_5_1009	DJI_0816	Helichrysum arenarium (L.) Moench	0	n	1	0	Asteraceae Bercht. & J.Presl
Iz_5_1009	DJI_0816	Herniaria polygama J.Gay	0	n	1	0	Caryophyllaceae Juss.
Iz_5_1009	DJI_0816	Hieracium robustum Fr.	0	n	1	0	Asteraceae Bercht. & J.Presl
Iz_5_1009	DJI_0816	Hieracium umbellatum L.	0	n	1	NA	Asteraceae Bercht. & J.Presl
Iz_5_1009	DJI_0816	Hylotelephium maximum (L.) Holub	0	n	1	0	Crassulaceae J.St.-Hil.
Iz_5_1009	DJI_0816	Jacobaea borysthenica (DC.) B.Nord. & Greuter	0	n	1	0	Asteraceae Bercht. & J.Presl
Iz_5_1009	DJI_0816	Jasione montana L.	0	n	1	0	Campanulaceae Juss.
Iz_5_1009	DJI_0816	Jurinea cyanoides (L.) Rchb.s.l.	0	n	1	0	Asteraceae Bercht. & J.Presl
Iz_5_1009	DJI_0816	Koeleria glauca (Spreng.) DC.	0	n	1	0	Poaceae Barnhart
Iz_5_1009	DJI_0816	Lactuca serriola L.	0	a	0	0	Asteraceae Bercht. & J.Presl
Iz_5_1009	DJI_0816	Linaria genistifolia (L.) Mill.	0	n	1	0	Plantaginaceae Juss.
Iz_5_1009	DJI_0816	Lomelosia argentea (L.) Greuter & Burdet	0	n	1	0	Caprifoliaceae Juss.
Iz_5_1009	DJI_0816	Melampyrum pratense L.	0	n	1	0	Orobanchaceae Vent.
Iz_5_1009	DJI_0816	Oenothera biennis L.	0	a	0	0	Onagraceae Juss.
Iz_5_1009	DJI_0816	Pentanema britannica (L.) D.Gut.Larr., Santos-Vicente, Anderb., E.Rico & M.M.Mart.Ort.	0	n	1	0	Asteraceae Bercht. & J.Presl
Iz_5_1009	DJI_0816	Peucedanum oreoselinum (L.) Moench	2	n	1	0	Apiaceae Lindl.
Iz_5_1009	DJI_0816	Peucedanum ruthenicum M.Bieb.	0	n	1	0	Apiaceae Lindl.
Iz_5_1009	DJI_0816	Pilosella echioides (Lumn.) F.W.Schultz & Sch.Bip.	0	n	1	0	Asteraceae Bercht. & J.Presl
Iz_5_1009	DJI_0816	Pilosella officinarum F. Schultz et Sch. Bip.	0	n	1	0	Asteraceae Bercht. & J.Presl
Iz_5_1009	DJI_0816	Pilosella sp.	0		NA	0	Asteraceae Bercht. & J.Presl
Iz_5_1009	DJI_0816	Pinus sylvestris L.	0	n	1	1	Pinaceae Spreng. ex F.Rudolphi
Iz_5_1009	DJI_0816	Poa annua L.	0	n	1	0	Poaceae Barnhart
Iz_5_1009	DJI_0816	Poa nemoralis L.	0	n	1	0	Poaceae Barnhart
Iz_5_1009	DJI_0816	Poa sp.	0		NA	0	Poaceae Barnhart
Iz_5_1009	DJI_0816	Polygonatum odoratum (Mill.) Druce	0	n	1	0	Asparagaceae Juss.
Iz_5_1009	DJI_0816	Polygonum arenarium Waldst. & Kit.	0	n	1	0	Polygonaceae Juss.
Iz_5_1009	DJI_0816	Populus nigra L.	0	n	1	1	Salicaceae Mirb.
Iz_5_1009	DJI_0816	Populus sp.	0	n	1	1	Salicaceae Mirb.
Iz_5_1009	DJI_0816	Populus tremula L.	0	n	1	1	Salicaceae Mirb.
Iz_5_1009	DJI_0816	Populus Ã canescens (Aiton) Sm.	0	n	1	1	Salicaceae Mirb.

Iz_5_1009	DJI_0816	Potentilla argentea L.	0	n	1	0	Rosaceae Juss.
Iz_5_1009	DJI_0816	Psammophiliella muralis (L.) Ikonn.	0	n	1	0	Caryophyllaceae Juss.
Iz_5_1009	DJI_0816	Psephellus sumensis (Kalen.) Greuter	0	n	1	0	Asteraceae Bercht. & J.Presl
Iz_5_1009	DJI_0816	Pulsatilla pratensis (L.) Mill. s.l.	0	n	1	0	Ranunculaceae Juss.
Iz_5_1009	DJI_0816	Pyrus communis L.	0	a	0	1	Rosaceae Juss.
Iz_5_1009	DJI_0816	Quercus robur L.	0	n	1	1	Fagaceae Dumort.
Iz_5_1009	DJI_0816	Robinia pseudoacacia L.	0	a	0	1	Fabaceae Lindl.
Iz_5_1009	DJI_0816	Rosa sp.	0		NA	1	Rosaceae Juss.
Iz_5_1009	DJI_0816	Rubus idaeus L.	0	n	1	0	Rosaceae Juss.
Iz_5_1009	DJI_0816	Rumex acetosella L.	0	n	1	0	Polygonaceae Juss.
Iz_5_1009	DJI_0816	Rumex thyrsiflorus Fingerh.	0	n	1	0	Polygonaceae Juss.
Iz_5_1009	DJI_0816	Salix alba L.	0	n	1	1	Salicaceae Mirb.
Iz_5_1009	DJI_0816	Sambucus nigra L.	0	n	1	1	Viburnaceae Raf.
Iz_5_1009	DJI_0816	Secale sylvestre Host	0	n	1	0	Poaceae Barnhart
Iz_5_1009	DJI_0816	Sempervivum ruthenicum Schnittsp. & C.B. Lehm.	0	n	1	0	Crassulaceae J.St.-Hil.
Iz_5_1009	DJI_0816	Setaria viridis (L.) P. Beauv.	0	n	1	0	Poaceae Barnhart
Iz_5_1009	DJI_0816	Silene borysthenica (Gruner) Walters	0	n	1	0	Caryophyllaceae Juss.
Iz_5_1009	DJI_0816	Silene latifolia subsp. alba (Mill.) Greuter & Burdet	0	n	1	0	Caryophyllaceae Juss.
Iz_5_1009	DJI_0816	Solidago virgaurea L.	4	n	1	0	Asteraceae Bercht. & J.Presl
Iz_5_1009	DJI_0816	Solidago Ã niederederi (S. canadensis Ã S. virgaurea)	0	hybrid	0	0	Asteraceae Bercht. & J.Presl
Iz_5_1009	DJI_0816	Sorbus aucuparia L.	0	n	1	1	Rosaceae Juss.
Iz_5_1009	DJI_0816	Stachys recta L.	0	n	1	0	Lamiacea Lindl.
Iz_5_1009	DJI_0816	Tanacetum vulgare L.	0	n	1	0	Asteraceae Bercht. & J.Presl
Iz_5_1009	DJI_0816	Thymus pallasianus Heinr.Braun	0	n	1	0	Lamiacea Lindl.
Iz_5_1009	DJI_0816	Tilia cordata Mill.	0	n	1	1	Malvaceae Juss.
Iz_5_1009	DJI_0816	Trifolium alpestre L.	0	n	1	0	Fabaceae Lindl.
Iz_5_1009	DJI_0816	Trifolium arvense L.	0	n	1	0	Fabaceae Lindl.
Iz_5_1009	DJI_0816	Ulmus glabra Huds.	0	n	1	1	Ulmaceae Mirb.
Iz_5_1009	DJI_0816	Verbascum densiflorum Bertol.	0	n	1	0	Scrophulariaceae Juss.
Iz_5_1009	DJI_0816	Veronica austriaca L.	0	n	1	0	Plantaginaceae Juss.
Iz_5_1009	DJI_0816	Veronica incana L.	0	n	1	0	Plantaginaceae Juss.
Iz_5_1009	DJI_0816	Veronica verna L.	0	n	1	0	Plantaginaceae Juss.
Iz_5_1009	DJI_0816	Vincetoxicum hirundinaria Medik	0	n	1	0	Apocynaceae Juss.
Iz_5_1009	DJI_0819	Acer tataricum L.	0	n	1	1	Sapindaceae Juss.
Iz_5_1009	DJI_0819	Achillea millefolium L. s.l.	0	n	1	0	Asteraceae Bercht. & J.Presl
Iz_5_1009	DJI_0819	Allium oleraceum L.	0	n	1	0	Amaryllidaceae J.St.-Hil.
Iz_5_1009	DJI_0819	Ambrosia artemisiifolia L.	0	a	0	0	Asteraceae Bercht. & J.Presl
Iz_5_1009	DJI_0819	Amorpha fruticosa L.	0	a	0	0	Fabaceae Lindl.
Iz_5_1009	DJI_0819	Anthericum ramosum L.	0	n	1	0	Asparagaceae Juss.
Iz_5_1009	DJI_0819	Artemisia marschalliana Spreng.	0	n	1	0	Asteraceae Bercht. & J.Presl
Iz_5_1009	DJI_0819	Asparagus officinalis L.	0	n	1	0	Asparagaceae Juss.
Iz_5_1009	DJI_0819	Astragalus cicer L.	0	n	1	0	Fabaceae Lindl.
Iz_5_1009	DJI_0819	Bassia laniflora (S.G.Gmel.) A.J.Scott	0	n	1	0	Amaranthaceae Juss.
Iz_5_1009	DJI_0819	Berberis vulgaris L.	0	n	1	0	Berberidaceae Juss.
Iz_5_1009	DJI_0819	Betula pendula Roth.	0	n	1	1	Betulaceae Gray
Iz_5_1009	DJI_0819	Betula pendula Roth.	0	n	1	0	Poaceae Barnhart
Iz_5_1009	DJI_0819	Bromus inermis Leyss.	0		NA	NA	
Iz_5_1009	DJI_0819	Bromus squarrosus L.	0	a	0	0	Poaceae Barnhart
Iz_5_1009	DJI_0819	Bromus tectorum L.	0	a	0	0	Poaceae Barnhart
Iz_5_1009	DJI_0819	Calamagrostis epigejos (L.) Roth	5	n	1	0	Poaceae Barnhart
Iz_5_1009	DJI_0819	Carduus acanthoides L.	0	n	1	0	Asteraceae Bercht. & J.Presl
Iz_5_1009	DJI_0819	Carex sp.	0	n	1	0	Cyperaceae Juss.
Iz_5_1009	DJI_0819	Centaurea majorovii Dumbadze	0	n	1	0	Asteraceae Bercht. & J.Presl
Iz_5_1009	DJI_0819	Chamaecytisus ruthenicus (Fisch.ex Wol.) Klaskova	1	n	1	0	Fabaceae Lindl.
Iz_5_1009	DJI_0819	Chelidonium majus L.	0	n	1	0	Papaveraceae Juss.
Iz_5_1009	DJI_0819	Chenopodium betaceum Andrz.	0	n	1	0	Amaranthaceae Juss.
Iz_5_1009	DJI_0819	Chenopodium cf. album	0	n	1	0	Amaranthaceae Juss.
Iz_5_1009	DJI_0819	Chenopodium sp.	0		NA	0	Amaranthaceae Juss.
Iz_5_1009	DJI_0819	Chondrilla juncea L.	0	n	1	0	Asteraceae Bercht. & J.Presl
Iz_5_1009	DJI_0819	Cirsium vulgare (Savi) Ten.	0	n	1	0	Asteraceae Bercht. & J.Presl
Iz_5_1009	DJI_0819	Clinopodium acinos (L.) Kuntze	0	n	1	0	Lamiacea Lindl.
Iz_5_1009	DJI_0819	Convallaria majalis L.	0	n	1	0	Asparagaceae Juss.
Iz_5_1009	DJI_0819	Crataegus rhipidophylla Gand.	0	n	1	1	Rosaceae Juss.
Iz_5_1009	DJI_0819	Crepis foetida L.	0	n	1	0	Asteraceae Bercht. & J.Presl
Iz_5_1009	DJI_0819	Dactylis glomerata L.	0	n	1	0	Poaceae Barnhart
Iz_5_1009	DJI_0819	Dianthus campestris M.Bieb.	0	n	1	0	Caryophyllaceae Juss.
Iz_5_1009	DJI_0819	Digitaria sanguinalis (L.) Scop.	0	a	0	0	Poaceae Barnhart
Iz_5_1009	DJI_0819	Elymus repens (L.) Gould	0	n	1	0	Poaceae Barnhart
Iz_5_1009	DJI_0819	Epilobium tetragonum L.	0	n	1	0	Onagraceae Juss.
Iz_5_1009	DJI_0819	Eragrostis minor Host	0	a	0	0	Poaceae Barnhart
Iz_5_1009	DJI_0819	Erigeron canadensis L.	0	a	0	0	Asteraceae Bercht. & J.Presl
Iz_5_1009	DJI_0819	Euphorbia seguieriana Neck.	0	n	1	0	Euphorbiaceae Juss.
Iz_5_1009	DJI_0819	Fallopia dumetorum (L.) Holub	0	n	1	0	Polygonaceae Juss.
Iz_5_1009	DJI_0819	Festuca beckeri (Hack.) Trautv.	0	n	1	0	Poaceae Barnhart
Iz_5_1009	DJI_0819	Fragaria vesca L.	0	n	1	0	Rosaceae Juss.
Iz_5_1009	DJI_0819	Frangula alnus Mill.	0	n	1	1	Rhamnaceae Juss.
Iz_5_1009	DJI_0819	Fraxinus pennsylvanica Marshall	0	a	0	1	Oleaceae Hoffmanns. & Link
Iz_5_1009	DJI_0819	Galeopsis bifida Boenn.	0	n	1	0	Lamiacea Lindl.
Iz_5_1009	DJI_0819	Galium verum L.	0	n	1	0	Rubiaceae Juss.
Iz_5_1009	DJI_0819	Genista tinctoria L.	0	n	1	0	Fabaceae Lindl.
Iz_5_1009	DJI_0819	Geranium robertianum L.	0	n	1	0	Geraniaceae Juss.
Iz_5_1009	DJI_0819	Geranium sanquineum L.	0	n	1	0	Geraniaceae Juss.
Iz_5_1009	DJI_0819	Gypsophila paniculata L.	0	n	1	0	Caryophyllaceae Juss.
Iz_5_1009	DJI_0819	Helichrysum arenarium (L.) Moench	0	n	1	0	Asteraceae Bercht. & J.Presl
Iz_5_1009	DJI_0819	Herniaria polygama J.Gay	0	n	1	0	Caryophyllaceae Juss.
Iz_5_1009	DJI_0819	Hieracium robustum Fr.	0	n	1	0	Asteraceae Bercht. & J.Presl
Iz_5_1009	DJI_0819	Hieracium umbellatum L.	0	n	1	NA	Asteraceae Bercht. & J.Presl
Iz_5_1009	DJI_0819	Hylotelephium maximum (L.) Holub	0	n	1	0	Crassulaceae J.St.-Hil.
Iz_5_1009	DJI_0819	Jacobaea borysthenica (DC.) B.Nord. & Greuter	0	n	1	0	Asteraceae Bercht. & J.Presl
Iz_5_1009	DJI_0819	Jasione montana L.	0	n	1	0	Campanulaceae Juss.
Iz_5_1009	DJI_0819	Jurinea cyanoides (L.) Rchb.s.l.	0	n	1	0	Asteraceae Bercht. & J.Presl
Iz_5_1009	DJI_0819	Koeleria glauca (Spreng.) DC.	0	n	1	0	Poaceae Barnhart
Iz_5_1009	DJI_0819	Lactuca serriola L.	0	a	0	0	Asteraceae Bercht. & J.Presl
Iz_5_1009	DJI_0819	Linaria genistifolia (L.) Mill.	0	n	1	0	Plantaginaceae Juss.
Iz_5_1009	DJI_0819	Lomelosia argentea (L.) Greuter & Burdet	0	n	1	0	Caprifoliaceae Juss.
Iz_5_1009	DJI_0819	Melampyrum pratense L.	0	n	1	0	Orobanchaceae Vent.
Iz_5_1009	DJI_0819	Oenothera biennis L.	0	a	0	0	Onagraceae Juss.
Iz_5_1009	DJI_0819	Pentanema britannica (L.) D.Gut.Larr., Santos-Vicente, Anderb., E.Rico & M.M.Mart.Ort.	0	n	1	0	Asteraceae Bercht. & J.Presl

Iz_5_1009	DJI_0819	Peucedanum oreoselinum (L.) Moench	0	n	1	0	Apiaceae Lindl.
Iz_5_1009	DJI_0819	Peucedanum ruthenicum M.Bieb.	0	n	1	0	Apiaceae Lindl.
Iz_5_1009	DJI_0819	Pilosella echioides (Lumn.) F.W.Schultz & Sch.Bip.	0	n	1	0	Asteraceae Bercht. & J.Presl
Iz_5_1009	DJI_0819	Pilosella officinarum F. Schultz et Sch. Bip.	0	n	1	0	Asteraceae Bercht. & J.Presl
Iz_5_1009	DJI_0819	Pilosella sp.	0		NA	0	Asteraceae Bercht. & J.Presl
Iz_5_1009	DJI_0819	Pinus sylvestris L.	0	n	1	1	Pinaceae Spreng. ex F.Rudolphi
Iz_5_1009	DJI_0819	Poa annua L.	0	n	1	0	Poaceae Barnhart
Iz_5_1009	DJI_0819	Poa nemoralis L.	0	n	1	0	Poaceae Barnhart
Iz_5_1009	DJI_0819	Poa sp.	0		NA	0	Poaceae Barnhart
Iz_5_1009	DJI_0819	Polygonatum odoratum (Mill.) Druce	0	n	1	0	Asparagaceae Juss.
Iz_5_1009	DJI_0819	Polygonum arenarium Waldst. & Kit.	0	n	1	0	Polygonaceae Juss.
Iz_5_1009	DJI_0819	Populus nigra L.	0	n	1	1	Salicaceae Mirb.
Iz_5_1009	DJI_0819	Populus sp.	0	n	1	1	Salicaceae Mirb.
Iz_5_1009	DJI_0819	Populus tremula L.	0	n	1	1	Salicaceae Mirb.
Iz_5_1009	DJI_0819	Populus Ã canescens (Aiton) Sm.	0	n	1	1	Salicaceae Mirb.
Iz_5_1009	DJI_0819	Potentilla argentea L.	0	n	1	0	Rosaceae Juss.
Iz_5_1009	DJI_0819	Psammophiliella muralis (L.) Ikonn.	0	n	1	0	Caryophyllaceae Juss.
Iz_5_1009	DJI_0819	Psephellus sumensis (Kalen.) Greuter	0	n	1	0	Asteraceae Bercht. & J.Presl
Iz_5_1009	DJI_0819	Pulsatilla pratensis (L.) Mill. s.l.	0	n	1	0	Ranunculaceae Juss.
Iz_5_1009	DJI_0819	Pyrus communis L.	0	a	0	1	Rosaceae Juss.
Iz_5_1009	DJI_0819	Quercus robur L.	0	n	1	1	Fagaceae Dumort.
Iz_5_1009	DJI_0819	Robinia pseudoacacia L.	0	a	0	1	Fabaceae Lindl.
Iz_5_1009	DJI_0819	Rosa sp.	0		NA	1	Rosaceae Juss.
Iz_5_1009	DJI_0819	Rubus idaeus L.	0	n	1	0	Rosaceae Juss.
Iz_5_1009	DJI_0819	Rumex acetosella L.	0	n	1	0	Polygonaceae Juss.
Iz_5_1009	DJI_0819	Rumex thyrsiflorus Fingerh.	0	n	1	0	Polygonaceae Juss.
Iz_5_1009	DJI_0819	Salix alba L.	0	n	1	1	Salicaceae Mirb.
Iz_5_1009	DJI_0819	Sambucus nigra L.	0	n	1	1	Viburnaceae Raf.
Iz_5_1009	DJI_0819	Secale sylvestre Host	0	n	1	0	Poaceae Barnhart
Iz_5_1009	DJI_0819	Sempervivum ruthenicum Schnittsp. & C.B. Lehm.	0	n	1	0	Crassulaceae J.St.-Hil.
Iz_5_1009	DJI_0819	Setaria viridis (L.) P. Beauv.	0	n	1	0	Poaceae Barnhart
Iz_5_1009	DJI_0819	Silene borysthenica (Gruner) Walters	0	n	1	0	Caryophyllaceae Juss.
Iz_5_1009	DJI_0819	Silene latifolia subsp. alba (Mill.) Greuter & Burdet	0	n	1	0	Caryophyllaceae Juss.
Iz_5_1009	DJI_0819	Solidago virgaurea L.	0	n	1	0	Asteraceae Bercht. & J.Presl
Iz_5_1009	DJI_0819	Solidago Ã niederederi (S. canadensis Ã S. virgaurea)	0	hybrid	0	0	Asteraceae Bercht. & J.Presl
Iz_5_1009	DJI_0819	Sorbus aucuparia L.	0	n	1	1	Rosaceae Juss.
Iz_5_1009	DJI_0819	Stachys recta L.	0	n	1	0	Lamiacea Lindl.
Iz_5_1009	DJI_0819	Tanacetum vulgare L.	0	n	1	0	Asteraceae Bercht. & J.Presl
Iz_5_1009	DJI_0819	Thymus pallasianus Heinr.Braun	0	n	1	0	Lamiacea Lindl.
Iz_5_1009	DJI_0819	Tilia cordata Mill.	0	n	1	1	Malvaceae Juss.
Iz_5_1009	DJI_0819	Trifolium alpestre L.	0	n	1	0	Fabaceae Lindl.
Iz_5_1009	DJI_0819	Trifolium arvense L.	0	n	1	0	Fabaceae Lindl.
Iz_5_1009	DJI_0819	Ulmus glabra Huds.	0	n	1	1	Ulmaceae Mirb.
Iz_5_1009	DJI_0819	Verbascum densiflorum Bertol.	0	n	1	0	Scrophulariaceae Juss.
Iz_5_1009	DJI_0819	Veronica austriaca L.	0	n	1	0	Plantaginaceae Juss.
Iz_5_1009	DJI_0819	Veronica incana L.	0	n	1	0	Plantaginaceae Juss.
Iz_5_1009	DJI_0819	Veronica verna L.	0	n	1	0	Plantaginaceae Juss.
Iz_5_1009	DJI_0819	Vincetoxicum hirundinaria Medik	0	n	1	0	Apocynaceae Juss.
Iz_5_1009	DJI_0820	Acer tataricum L.	0	n	1	1	Sapindaceae Juss.
Iz_5_1009	DJI_0820	Achillea millefolium L. s.l.	0	n	1	0	Asteraceae Bercht. & J.Presl
Iz_5_1009	DJI_0820	Allium oleraceum L.	0	n	1	0	Amaryllidaceae J.St.-Hil.
Iz_5_1009	DJI_0820	Ambrosia artemisiifolia L.	0	a	0	0	Asteraceae Bercht. & J.Presl
Iz_5_1009	DJI_0820	Amorpha fruticosa L.	0	a	0	0	Fabaceae Lindl.
Iz_5_1009	DJI_0820	Anthericum ramosum L.	0	n	1	0	Asparagaceae Juss.
Iz_5_1009	DJI_0820	Artemisia marschalliana Spreng.	0	n	1	0	Asteraceae Bercht. & J.Presl
Iz_5_1009	DJI_0820	Asparagus officinalis L.	0	n	1	0	Asparagaceae Juss.
Iz_5_1009	DJI_0820	Astragalus cicer L.	0	n	1	0	Fabaceae Lindl.
Iz_5_1009	DJI_0820	Bassia laniflora (S.G.Gmel.) A.J.Scott	0	n	1	0	Amaranthaceae Juss.
Iz_5_1009	DJI_0820	Berberis vulgaris L.	0	n	1	0	Berberidaceae Juss.
Iz_5_1009	DJI_0820	Betula pendula Roth.	0	n	1	1	Betulaceae Gray
Iz_5_1009	DJI_0820	Betula pendula Roth.	0	n	1	0	Poaceae Barnhart
Iz_5_1009	DJI_0820	Bromus inermis Leyss.	0		NA	NA	
Iz_5_1009	DJI_0820	Bromus squarrosus L.	0	a	0	0	Poaceae Barnhart
Iz_5_1009	DJI_0820	Bromus tectorum L.	0	a	0	0	Poaceae Barnhart
Iz_5_1009	DJI_0820	Calamagrostis epigejos (L.) Roth	20	n	1	0	Poaceae Barnhart
Iz_5_1009	DJI_0820	Carduus acanthoides L.	0	n	1	0	Asteraceae Bercht. & J.Presl
Iz_5_1009	DJI_0820	Carex sp.	0	n	1	0	Cyperaceae Juss.
Iz_5_1009	DJI_0820	Centaurea majorovii Dumbadze	0	n	1	0	Asteraceae Bercht. & J.Presl
Iz_5_1009	DJI_0820	Chamaecytisus ruthenicus (Fisch.ex Wol.) Klaskova	0	n	1	0	Fabaceae Lindl.
Iz_5_1009	DJI_0820	Chelidonium majus L.	0	n	1	0	Papaveraceae Juss.
Iz_5_1009	DJI_0820	Chenopodium betaceum Andrz.	0	n	1	0	Amaranthaceae Juss.
Iz_5_1009	DJI_0820	Chenopodium cf. album	0	n	1	0	Amaranthaceae Juss.
Iz_5_1009	DJI_0820	Chenopodium sp.	0		NA	0	Amaranthaceae Juss.
Iz_5_1009	DJI_0820	Chondrilla juncea L.	0	n	1	0	Asteraceae Bercht. & J.Presl
Iz_5_1009	DJI_0820	Cirsium vulgare (Savi) Ten.	0	n	1	0	Asteraceae Bercht. & J.Presl
Iz_5_1009	DJI_0820	Clinopodium acinos (L.) Kuntze	0	n	1	0	Lamiacea Lindl.
Iz_5_1009	DJI_0820	Convallaria majalis L.	0	n	1	0	Asparagaceae Juss.
Iz_5_1009	DJI_0820	Crataegus rhipidophylla Gand.	0	n	1	1	Rosaceae Juss.
Iz_5_1009	DJI_0820	Crepis foetida L.	0	n	1	0	Asteraceae Bercht. & J.Presl
Iz_5_1009	DJI_0820	Dactylis glomerata L.	0	n	1	0	Poaceae Barnhart
Iz_5_1009	DJI_0820	Dianthus campestris M.Bieb.	0	n	1	0	Caryophyllaceae Juss.
Iz_5_1009	DJI_0820	Digitaria sanguinalis (L.) Scop.	0	a	0	0	Poaceae Barnhart
Iz_5_1009	DJI_0820	Elymus repens (L.) Gould	0	n	1	0	Poaceae Barnhart
Iz_5_1009	DJI_0820	Epilobium tetragonum L.	0	n	1	0	Onagraceae Juss.
Iz_5_1009	DJI_0820	Eragrostis minor Host	0	a	0	0	Poaceae Barnhart
Iz_5_1009	DJI_0820	Erigeron canadensis L.	0	a	0	0	Asteraceae Bercht. & J.Presl
Iz_5_1009	DJI_0820	Euphorbia seguieriana Neck.	0	n	1	0	Euphorbiaceae Juss.
Iz_5_1009	DJI_0820	Fallopia dumetorum (L.) Holub	0	n	1	0	Polygonaceae Juss.
Iz_5_1009	DJI_0820	Festuca beckeri (Hack.) Trautv.	0	n	1	0	Poaceae Barnhart
Iz_5_1009	DJI_0820	Fragaria vesca L.	0	n	1	0	Rosaceae Juss.
Iz_5_1009	DJI_0820	Frangula alnus Mill.	0	n	1	1	Rhamnaceae Juss.
Iz_5_1009	DJI_0820	Fraxinus pennsylvanica Marshall	0	a	0	1	Oleaceae Hoffmanns. & Link
Iz_5_1009	DJI_0820	Galeopsis bifida Boenn.	0	n	1	0	Lamiacea Lindl.
Iz_5_1009	DJI_0820	Galium verum L.	0	n	1	0	Rubiaceae Juss.
Iz_5_1009	DJI_0820	Genista tinctoria L.	0	n	1	0	Fabaceae Lindl.
Iz_5_1009	DJI_0820	Geranium robertianum L.	0	n	1	0	Geraniaceae Juss.
Iz_5_1009	DJI_0820	Geranium sanquineum L.	0	n	1	0	Geraniaceae Juss.
Iz_5_1009	DJI_0820	Gypsophila paniculata L.	0	n	1	0	Caryophyllaceae Juss.

Iz_5_1009	DJI_0820	Helichrysum arenarium (L.) Moench	0	n	1	0	Asteraceae Bercht. & J.Presl
Iz_5_1009	DJI_0820	Herniaria polygama J.Gay	0	n	1	0	Caryophyllaceae Juss.
Iz_5_1009	DJI_0820	Hieracium robustum Fr.	0	n	1	0	Asteraceae Bercht. & J.Presl
Iz_5_1009	DJI_0820	Hieracium umbellatum L.	0	n	1	NA	Asteraceae Bercht. & J.Presl
Iz_5_1009	DJI_0820	Hylotelephium maximum (L.) Holub	0	n	1	0	Crassulaceae J.St.-Hil.
Iz_5_1009	DJI_0820	Jacobaea borysthenica (DC.) B.Nord. & Greuter	0	n	1	0	Asteraceae Bercht. & J.Presl
Iz_5_1009	DJI_0820	Jasione montana L.	0	n	1	0	Campanulaceae Juss.
Iz_5_1009	DJI_0820	Jurinea cyanoides (L.) Rchb.s.l.	0	n	1	0	Asteraceae Bercht. & J.Presl
Iz_5_1009	DJI_0820	Koeleria glauca (Spreng.) DC.	0	n	1	0	Poaceae Barnhart
Iz_5_1009	DJI_0820	Lactuca serriola L.	0	a	0	0	Asteraceae Bercht. & J.Presl
Iz_5_1009	DJI_0820	Linaria genistifolia (L.) Mill.	0	n	1	0	Plantaginaceae Juss.
Iz_5_1009	DJI_0820	Lomelosia argentea (L.) Greuter & Burdet	0	n	1	0	Caprifoliaceae Juss.
Iz_5_1009	DJI_0820	Melampyrum pratense L.	0	n	1	0	Orobanchaceae Vent.
Iz_5_1009	DJI_0820	Oenothera biennis L.	0	a	0	0	Onagraceae Juss.
Iz_5_1009	DJI_0820	Pentanema britannica (L.) D.Gut.Larr., Santos-Vicente, Anderb., E.Rico & M.M.Mart.Ort.	0	n	1	0	Asteraceae Bercht. & J.Presl
Iz_5_1009	DJI_0820	Peucedanum oreoselinum (L.) Moench	0	n	1	0	Apiaceae Lindl.
Iz_5_1009	DJI_0820	Peucedanum ruthenicum M.Bieb.	0	n	1	0	Apiaceae Lindl.
Iz_5_1009	DJI_0820	Pilosella echioides (Lumn.) F.W.Schultz & Sch.Bip.	0	n	1	0	Asteraceae Bercht. & J.Presl
Iz_5_1009	DJI_0820	Pilosella officinarum F. Schultz et Sch. Bip.	0	n	1	0	Asteraceae Bercht. & J.Presl
Iz_5_1009	DJI_0820	Pilosella sp.	0		NA	0	Asteraceae Bercht. & J.Presl
Iz_5_1009	DJI_0820	Pinus sylvestris L.	0	n	1	1	Pinaceae Spreng. ex F.Rudolphi
Iz_5_1009	DJI_0820	Poa annua L.	0	n	1	0	Poaceae Barnhart
Iz_5_1009	DJI_0820	Poa nemoralis L.	0	n	1	0	Poaceae Barnhart
Iz_5_1009	DJI_0820	Poa sp.	0		NA	0	Poaceae Barnhart
Iz_5_1009	DJI_0820	Polygonatum odoratum (Mill.) Druce	0	n	1	0	Asparagaceae Juss.
Iz_5_1009	DJI_0820	Polygonum arenarium Waldst. & Kit.	0	n	1	0	Polygonaceae Juss.
Iz_5_1009	DJI_0820	Populus nigra L.	0	n	1	1	Salicaceae Mirb.
Iz_5_1009	DJI_0820	Populus sp.	0	n	1	1	Salicaceae Mirb.
Iz_5_1009	DJI_0820	Populus tremula L.	0	n	1	1	Salicaceae Mirb.
Iz_5_1009	DJI_0820	Populus Ã canescens (Aiton) Sm.	0	n	1	1	Salicaceae Mirb.
Iz_5_1009	DJI_0820	Potentilla argentea L.	0	n	1	0	Rosaceae Juss.
Iz_5_1009	DJI_0820	Psammophiliella muralis (L.) Ikonn.	0	n	1	0	Caryophyllaceae Juss.
Iz_5_1009	DJI_0820	Psephellus sumensis (Kalen.) Greuter	0	n	1	0	Asteraceae Bercht. & J.Presl
Iz_5_1009	DJI_0820	Pulsatilla pratensis (L.) Mill. s.l.	0	n	1	0	Ranunculaceae Juss.
Iz_5_1009	DJI_0820	Pyrus communis L.	0	a	0	1	Rosaceae Juss.
Iz_5_1009	DJI_0820	Quercus robur L.	0	n	1	1	Fagaceae Dumort.
Iz_5_1009	DJI_0820	Robinia pseudoacacia L.	0	a	0	1	Fabaceae Lindl.
Iz_5_1009	DJI_0820	Rosa sp.	0		NA	1	Rosaceae Juss.
Iz_5_1009	DJI_0820	Rubus idaeus L.	0	n	1	0	Rosaceae Juss.
Iz_5_1009	DJI_0820	Rumex acetosella L.	1	n	1	0	Polygonaceae Juss.
Iz_5_1009	DJI_0820	Rumex thyrsiflorus Fingerh.	0	n	1	0	Polygonaceae Juss.
Iz_5_1009	DJI_0820	Salix alba L.	0	n	1	1	Salicaceae Mirb.
Iz_5_1009	DJI_0820	Sambucus nigra L.	0	n	1	1	Viburnaceae Raf.
Iz_5_1009	DJI_0820	Secale sylvestre Host	0	n	1	0	Poaceae Barnhart
Iz_5_1009	DJI_0820	Sempervivum ruthenicum Schnittsp. & C.B. Lehm.	0	n	1	0	Crassulaceae J.St.-Hil.
Iz_5_1009	DJI_0820	Setaria viridis (L.) P. Beauv.	0	n	1	0	Poaceae Barnhart
Iz_5_1009	DJI_0820	Silene borysthenica (Gruner) Walters	0	n	1	0	Caryophyllaceae Juss.
Iz_5_1009	DJI_0820	Silene latifolia subsp. alba (Mill.) Greuter & Burdet	0	n	1	0	Caryophyllaceae Juss.
Iz_5_1009	DJI_0820	Solidago virgaurea L.	0	n	1	0	Asteraceae Bercht. & J.Presl
Iz_5_1009	DJI_0820	Solidago Ã niederederi (S. canadensis Ã S. virgaurea)	0	hybrid	0	0	Asteraceae Bercht. & J.Presl
Iz_5_1009	DJI_0820	Sorbus aucuparia L.	0	n	1	1	Rosaceae Juss.
Iz_5_1009	DJI_0820	Stachys recta L.	0	n	1	0	Lamiacea Lindl.
Iz_5_1009	DJI_0820	Tanacetum vulgare L.	0	n	1	0	Asteraceae Bercht. & J.Presl
Iz_5_1009	DJI_0820	Thymus pallasianus Heinr.Braun	0	n	1	0	Lamiacea Lindl.
Iz_5_1009	DJI_0820	Tilia cordata Mill.	0	n	1	1	Malvaceae Juss.
Iz_5_1009	DJI_0820	Trifolium alpestre L.	0	n	1	0	Fabaceae Lindl.
Iz_5_1009	DJI_0820	Trifolium arvense L.	0	n	1	0	Fabaceae Lindl.
Iz_5_1009	DJI_0820	Ulmus glabra Huds.	0	n	1	1	Ulmaceae Mirb.
Iz_5_1009	DJI_0820	Verbascum densiflorum Bertol.	0	n	1	0	Scrophulariaceae Juss.
Iz_5_1009	DJI_0820	Veronica austriaca L.	0	n	1	0	Plantaginaceae Juss.
Iz_5_1009	DJI_0820	Veronica incana L.	0	n	1	0	Plantaginaceae Juss.
Iz_5_1009	DJI_0820	Veronica verna L.	0	n	1	0	Plantaginaceae Juss.
Iz_5_1009	DJI_0820	Vincetoxicum hirundinaria Medik	0	n	1	0	Apocynaceae Juss.
Iz_5_1009	DJI_0822	Acer tataricum L.	0	n	1	1	Sapindaceae Juss.
Iz_5_1009	DJI_0822	Achillea millefolium L. s.l.	0	n	1	0	Asteraceae Bercht. & J.Presl
Iz_5_1009	DJI_0822	Allium oleraceum L.	0	n	1	0	Amaryllidaceae J.St.-Hil.
Iz_5_1009	DJI_0822	Ambrosia artemisiifolia L.	0	a	0	0	Asteraceae Bercht. & J.Presl
Iz_5_1009	DJI_0822	Amorpha fruticosa L.	0	a	0	0	Fabaceae Lindl.
Iz_5_1009	DJI_0822	Anthericum ramosum L.	0	n	1	0	Asparagaceae Juss.
Iz_5_1009	DJI_0822	Artemisia marschalliana Spreng.	0	n	1	0	Asteraceae Bercht. & J.Presl
Iz_5_1009	DJI_0822	Asparagus officinalis L.	0	n	1	0	Asparagaceae Juss.
Iz_5_1009	DJI_0822	Astragalus cicer L.	0	n	1	0	Fabaceae Lindl.
Iz_5_1009	DJI_0822	Bassia laniflora (S.G.Gmel.) A.J.Scott	0	n	1	0	Amaranthaceae Juss.
Iz_5_1009	DJI_0822	Berberis vulgaris L.	0	n	1	0	Berberidaceae Juss.
Iz_5_1009	DJI_0822	Betula pendula Roth.	0	n	1	1	Betulaceae Gray
Iz_5_1009	DJI_0822	Betula pendula Roth.	0	n	1	0	Poaceae Barnhart
Iz_5_1009	DJI_0822	Bromus inermis Leyss.	0		NA	NA	
Iz_5_1009	DJI_0822	Bromus squarrosus L.	0	a	0	0	Poaceae Barnhart
Iz_5_1009	DJI_0822	Bromus tectorum L.	0	a	0	0	Poaceae Barnhart
Iz_5_1009	DJI_0822	Calamagrostis epigejos (L.) Roth	20	n	1	0	Poaceae Barnhart
Iz_5_1009	DJI_0822	Carduus acanthoides L.	0	n	1	0	Asteraceae Bercht. & J.Presl
Iz_5_1009	DJI_0822	Carex sp.	0	n	1	0	Cyperaceae Juss.
Iz_5_1009	DJI_0822	Centaurea majorovii Dumbadze	0	n	1	0	Asteraceae Bercht. & J.Presl
Iz_5_1009	DJI_0822	Chamaecytisus ruthenicus (Fisch.ex Wol.) Klaskova	0	n	1	0	Fabaceae Lindl.
Iz_5_1009	DJI_0822	Chelidonium majus L.	0	n	1	0	Papaveraceae Juss.
Iz_5_1009	DJI_0822	Chenopodium betaceum Andrz.	0	n	1	0	Amaranthaceae Juss.
Iz_5_1009	DJI_0822	Chenopodium cf. album	0	n	1	0	Amaranthaceae Juss.
Iz_5_1009	DJI_0822	Chenopodium sp.	0		NA	0	Amaranthaceae Juss.
Iz_5_1009	DJI_0822	Chondrilla juncea L.	0	n	1	0	Asteraceae Bercht. & J.Presl
Iz_5_1009	DJI_0822	Cirsium vulgare (Savi) Ten.	0	n	1	0	Asteraceae Bercht. & J.Presl
Iz_5_1009	DJI_0822	Clinopodium acinos (L.) Kuntze	0	n	1	0	Lamiacea Lindl.
Iz_5_1009	DJI_0822	Convallaria majalis L.	0	n	1	0	Asparagaceae Juss.
Iz_5_1009	DJI_0822	Crataegus rhipidophylla Gand.	0	n	1	1	Rosaceae Juss.
Iz_5_1009	DJI_0822	Crepis foetida L.	0	n	1	0	Asteraceae Bercht. & J.Presl
Iz_5_1009	DJI_0822	Dactylis glomerata L.	0	n	1	0	Poaceae Barnhart
Iz_5_1009	DJI_0822	Dianthus campestris M.Bieb.	0	n	1	0	Caryophyllaceae Juss.
Iz_5_1009	DJI_0822	Digitaria sanguinalis (L.) Scop.	0	a	0	0	Poaceae Barnhart
Iz_5_1009	DJI_0822	Elymus repens (L.) Gould	0	n	1	0	Poaceae Barnhart

Iz_5_1009	DJI_0822	Epilobium tetragonum L.	0	n	1	0	Onagraceae Juss.
Iz_5_1009	DJI_0822	Eragrostis minor Host	0	a	0	0	Poaceae Barnhart
Iz_5_1009	DJI_0822	Erigeron canadensis L.	0	a	0	0	Asteraceae Bercht. & J.Presl
Iz_5_1009	DJI_0822	Euphorbia seguieriana Neck.	0	n	1	0	Euphorbiaceae Juss.
Iz_5_1009	DJI_0822	Fallopia dumetorum (L.) Holub	0	n	1	0	Polygonaceae Juss.
Iz_5_1009	DJI_0822	Festuca beckeri (Hack.) Trautv.	0	n	1	0	Poaceae Barnhart
Iz_5_1009	DJI_0822	Fragaria vesca L.	0	n	1	0	Rosaceae Juss.
Iz_5_1009	DJI_0822	Frangula alnus Mill.	0	n	1	1	Rhamnaceae Juss.
Iz_5_1009	DJI_0822	Fraxinus pennsylvanica Marshall	0	a	0	1	Oleaceae Hoffmanns. & Link
Iz_5_1009	DJI_0822	Galeopsis bifida Boenn.	0	n	1	0	Lamiacea Lindl.
Iz_5_1009	DJI_0822	Galium verum L.	0	n	1	0	Rubiaceae Juss.
Iz_5_1009	DJI_0822	Genista tinctoria L.	0	n	1	0	Fabaceae Lindl.
Iz_5_1009	DJI_0822	Geranium robertianum L.	0	n	1	0	Geraniaceae Juss.
Iz_5_1009	DJI_0822	Geranium sanquineum L.	0	n	1	0	Geraniaceae Juss.
Iz_5_1009	DJI_0822	Gypsophila paniculata L.	0	n	1	0	Caryophyllaceae Juss.
Iz_5_1009	DJI_0822	Helichrysum arenarium (L.) Moench	0	n	1	0	Asteraceae Bercht. & J.Presl
Iz_5_1009	DJI_0822	Herniaria polygama J.Gay	0	n	1	0	Caryophyllaceae Juss.
Iz_5_1009	DJI_0822	Hieracium robustum Fr.	0	n	1	0	Asteraceae Bercht. & J.Presl
Iz_5_1009	DJI_0822	Hieracium umbellatum L.	0	n	1	NA	Asteraceae Bercht. & J.Presl
Iz_5_1009	DJI_0822	Hylotelephium maximum (L.) Holub	0	n	1	0	Crassulaceae J.St.-Hil.
Iz_5_1009	DJI_0822	Jacobaea borysthenica (DC.) B.Nord. & Greuter	0	n	1	0	Asteraceae Bercht. & J.Presl
Iz_5_1009	DJI_0822	Jasione montana L.	0	n	1	0	Campanulaceae Juss.
Iz_5_1009	DJI_0822	Jurinea cyanoides (L.) Rchb.s.l.	0	n	1	0	Asteraceae Bercht. & J.Presl
Iz_5_1009	DJI_0822	Koeleria glauca (Spreng.) DC.	0	n	1	0	Poaceae Barnhart
Iz_5_1009	DJI_0822	Lactuca serriola L.	0	a	0	0	Asteraceae Bercht. & J.Presl
Iz_5_1009	DJI_0822	Linaria genistifolia (L.) Mill.	0	n	1	0	Plantaginaceae Juss.
Iz_5_1009	DJI_0822	Lomelosia argentea (L.) Greuter & Burdet	0	n	1	0	Caprifoliaceae Juss.
Iz_5_1009	DJI_0822	Melampyrum pratense L.	0	n	1	0	Orobanchaceae Vent.
Iz_5_1009	DJI_0822	Oenothera biennis L.	0	a	0	0	Onagraceae Juss.
Iz_5_1009	DJI_0822	Pentanema britannica (L.) D.Gut.Larr., Santos-Vicente, Anderb., E.Rico & M.M.Mart.Ort.	0	n	1	0	Asteraceae Bercht. & J.Presl
Iz_5_1009	DJI_0822	Peucedanum oreoselinum (L.) Moench	1	n	1	0	Apiaceae Lindl.
Iz_5_1009	DJI_0822	Peucedanum ruthenicum M.Bieb.	0	n	1	0	Apiaceae Lindl.
Iz_5_1009	DJI_0822	Pilosella echioides (Lumn.) F.W.Schultz & Sch.Bip.	0	n	1	0	Asteraceae Bercht. & J.Presl
Iz_5_1009	DJI_0822	Pilosella officinarum F. Schultz et Sch. Bip.	0	n	1	0	Asteraceae Bercht. & J.Presl
Iz_5_1009	DJI_0822	Pilosella sp.	0		NA	0	Asteraceae Bercht. & J.Presl
Iz_5_1009	DJI_0822	Pinus sylvestris L.	0	n	1	1	Pinaceae Spreng. ex F.Rudolphi
Iz_5_1009	DJI_0822	Poa annua L.	0	n	1	0	Poaceae Barnhart
Iz_5_1009	DJI_0822	Poa nemoralis L.	0	n	1	0	Poaceae Barnhart
Iz_5_1009	DJI_0822	Poa sp.	0		NA	0	Poaceae Barnhart
Iz_5_1009	DJI_0822	Polygonatum odoratum (Mill.) Druce	0	n	1	0	Asparagaceae Juss.
Iz_5_1009	DJI_0822	Polygonum arenarium Waldst. & Kit.	0	n	1	0	Polygonaceae Juss.
Iz_5_1009	DJI_0822	Populus nigra L.	0	n	1	1	Salicaceae Mirb.
Iz_5_1009	DJI_0822	Populus sp.	0	n	1	1	Salicaceae Mirb.
Iz_5_1009	DJI_0822	Populus tremula L.	0	n	1	1	Salicaceae Mirb.
Iz_5_1009	DJI_0822	Populus Ã canescens (Aiton) Sm.	0	n	1	1	Salicaceae Mirb.
Iz_5_1009	DJI_0822	Potentilla argentea L.	0	n	1	0	Rosaceae Juss.
Iz_5_1009	DJI_0822	Psammophiliella muralis (L.) Ikonn.	0	n	1	0	Caryophyllaceae Juss.
Iz_5_1009	DJI_0822	Psephellus sumensis (Kalen.) Greuter	0	n	1	0	Asteraceae Bercht. & J.Presl
Iz_5_1009	DJI_0822	Pulsatilla pratensis (L.) Mill. s.l.	0	n	1	0	Ranunculaceae Juss.
Iz_5_1009	DJI_0822	Pyrus communis L.	0	a	0	1	Rosaceae Juss.
Iz_5_1009	DJI_0822	Quercus robur L.	0	n	1	1	Fagaceae Dumort.
Iz_5_1009	DJI_0822	Robinia pseudoacacia L.	0	a	0	1	Fabaceae Lindl.
Iz_5_1009	DJI_0822	Rosa sp.	0		NA	1	Rosaceae Juss.
Iz_5_1009	DJI_0822	Rubus idaeus L.	0	n	1	0	Rosaceae Juss.
Iz_5_1009	DJI_0822	Rumex acetosella L.	0	n	1	0	Polygonaceae Juss.
Iz_5_1009	DJI_0822	Rumex thyrsiflorus Fingerh.	0	n	1	0	Polygonaceae Juss.
Iz_5_1009	DJI_0822	Salix alba L.	0	n	1	1	Salicaceae Mirb.
Iz_5_1009	DJI_0822	Sambucus nigra L.	0	n	1	1	Viburnaceae Raf.
Iz_5_1009	DJI_0822	Secale sylvestre Host	0	n	1	0	Poaceae Barnhart
Iz_5_1009	DJI_0822	Sempervivum ruthenicum Schnittsp. & C.B. Lehm.	0	n	1	0	Crassulaceae J.St.-Hil.
Iz_5_1009	DJI_0822	Setaria viridis (L.) P. Beauv.	0	n	1	0	Poaceae Barnhart
Iz_5_1009	DJI_0822	Silene borysthenica (Gruner) Walters	0	n	1	0	Caryophyllaceae Juss.
Iz_5_1009	DJI_0822	Silene latifolia subsp. alba (Mill.) Greuter & Burdet	0	n	1	0	Caryophyllaceae Juss.
Iz_5_1009	DJI_0822	Solidago virgaurea L.	1	n	1	0	Asteraceae Bercht. & J.Presl
Iz_5_1009	DJI_0822	Solidago Ã niederederi (S. canadensis Ã S. virgaurea)	0	hybrid	0	0	Asteraceae Bercht. & J.Presl
Iz_5_1009	DJI_0822	Sorbus aucuparia L.	0	n	1	1	Rosaceae Juss.
Iz_5_1009	DJI_0822	Stachys recta L.	0	n	1	0	Lamiacea Lindl.
Iz_5_1009	DJI_0822	Tanacetum vulgare L.	0	n	1	0	Asteraceae Bercht. & J.Presl
Iz_5_1009	DJI_0822	Thymus pallasianus Heinr.Braun	0	n	1	0	Lamiacea Lindl.
Iz_5_1009	DJI_0822	Tilia cordata Mill.	0	n	1	1	Malvaceae Juss.
Iz_5_1009	DJI_0822	Trifolium alpestre L.	0	n	1	0	Fabaceae Lindl.
Iz_5_1009	DJI_0822	Trifolium arvense L.	0	n	1	0	Fabaceae Lindl.
Iz_5_1009	DJI_0822	Ulmus glabra Huds.	0	n	1	1	Ulmaceae Mirb.
Iz_5_1009	DJI_0822	Verbascum densiflorum Bertol.	0	n	1	0	Scrophulariaceae Juss.
Iz_5_1009	DJI_0822	Veronica austriaca L.	0	n	1	0	Plantaginaceae Juss.
Iz_5_1009	DJI_0822	Veronica incana L.	0	n	1	0	Plantaginaceae Juss.
Iz_5_1009	DJI_0822	Veronica verna L.	0	n	1	0	Plantaginaceae Juss.
Iz_5_1009	DJI_0822	Vincetoxicum hirundinaria Medik	0	n	1	0	Apocynaceae Juss.
Iz_5_1009	DJI_0823	Acer tataricum L.	0	n	1	1	Sapindaceae Juss.
Iz_5_1009	DJI_0823	Achillea millefolium L. s.l.	0	n	1	0	Asteraceae Bercht. & J.Presl
Iz_5_1009	DJI_0823	Allium oleraceum L.	0	n	1	0	Amaryllidaceae J.St.-Hil.
Iz_5_1009	DJI_0823	Ambrosia artemisiifolia L.	0	a	0	0	Asteraceae Bercht. & J.Presl
Iz_5_1009	DJI_0823	Amorpha fruticosa L.	0	a	0	0	Fabaceae Lindl.
Iz_5_1009	DJI_0823	Anthericum ramosum L.	0	n	1	0	Asparagaceae Juss.
Iz_5_1009	DJI_0823	Artemisia marschalliana Spreng.	0	n	1	0	Asteraceae Bercht. & J.Presl
Iz_5_1009	DJI_0823	Asparagus officinalis L.	0	n	1	0	Asparagaceae Juss.
Iz_5_1009	DJI_0823	Astragalus cicer L.	0	n	1	0	Fabaceae Lindl.
Iz_5_1009	DJI_0823	Bassia laniflora (S.G.Gmel.) A.J.Scott	0	n	1	0	Amaranthaceae Juss.
Iz_5_1009	DJI_0823	Berberis vulgaris L.	0	n	1	0	Berberidaceae Juss.
Iz_5_1009	DJI_0823	Betula pendula Roth.	0	n	1	1	Betulaceae Gray
Iz_5_1009	DJI_0823	Betula pendula Roth.	0	n	1	0	Poaceae Barnhart
Iz_5_1009	DJI_0823	Bromus inermis Leyss.	0		NA	NA	
Iz_5_1009	DJI_0823	Bromus squarrosus L.	0	a	0	0	Poaceae Barnhart
Iz_5_1009	DJI_0823	Bromus tectorum L.	0	a	0	0	Poaceae Barnhart
Iz_5_1009	DJI_0823	Calamagrostis epigejos (L.) Roth	25	n	1	0	Poaceae Barnhart
Iz_5_1009	DJI_0823	Carduus acanthoides L.	0	n	1	0	Asteraceae Bercht. & J.Presl
Iz_5_1009	DJI_0823	Carex sp.	0	n	1	0	Cyperaceae Juss.
Iz_5_1009	DJI_0823	Centaurea majorovii Dumbadze	0	n	1	0	Asteraceae Bercht. & J.Presl

Iz_5_1009	DJI_0823	Chamaecytisus ruthenicus (Fisch.ex Wol.) Klaskova	0	n	1	0	Fabaceae Lindl.
Iz_5_1009	DJI_0823	Chelidonium majus L.	0	n	1	0	Papaveraceae Juss.
Iz_5_1009	DJI_0823	Chenopodium betaceum Andrz.	0	n	1	0	Amaranthaceae Juss.
Iz_5_1009	DJI_0823	Chenopodium cf. album	0	n	1	0	Amaranthaceae Juss.
Iz_5_1009	DJI_0823	Chenopodium sp.	0		NA	0	Amaranthaceae Juss.
Iz_5_1009	DJI_0823	Chondrilla juncea L.	0	n	1	0	Asteraceae Bercht. & J.Presl
Iz_5_1009	DJI_0823	Cirsium vulgare (Savi) Ten.	0	n	1	0	Asteraceae Bercht. & J.Presl
Iz_5_1009	DJI_0823	Clinopodium acinos (L.) Kuntze	0	n	1	0	Lamiacea Lindl.
Iz_5_1009	DJI_0823	Convallaria majalis L.	0	n	1	0	Asparagaceae Juss.
Iz_5_1009	DJI_0823	Crataegus rhipidophylla Gand.	0	n	1	1	Rosaceae Juss.
Iz_5_1009	DJI_0823	Crepis foetida L.	0	n	1	0	Asteraceae Bercht. & J.Presl
Iz_5_1009	DJI_0823	Dactylis glomerata L.	0	n	1	0	Poaceae Barnhart
Iz_5_1009	DJI_0823	Dianthus campestris M.Bieb.	0	n	1	0	Caryophyllaceae Juss.
Iz_5_1009	DJI_0823	Digitaria sanguinalis (L.) Scop.	0	a	0	0	Poaceae Barnhart
Iz_5_1009	DJI_0823	Elymus repens (L.) Gould	0	n	1	0	Poaceae Barnhart
Iz_5_1009	DJI_0823	Epilobium tetragonum L.	0	n	1	0	Onagraceae Juss.
Iz_5_1009	DJI_0823	Eragrostis minor Host	0	a	0	0	Poaceae Barnhart
Iz_5_1009	DJI_0823	Erigeron canadensis L.	0	a	0	0	Asteraceae Bercht. & J.Presl
Iz_5_1009	DJI_0823	Euphorbia seguieriana Neck.	0	n	1	0	Euphorbiaceae Juss.
Iz_5_1009	DJI_0823	Fallopia dumetorum (L.) Holub	0.5	n	1	0	Polygonaceae Juss.
Iz_5_1009	DJI_0823	Festuca beckeri (Hack.) Trautv.	0	n	1	0	Poaceae Barnhart
Iz_5_1009	DJI_0823	Fragaria vesca L.	0	n	1	0	Rosaceae Juss.
Iz_5_1009	DJI_0823	Frangula alnus Mill.	0	n	1	1	Rhamnaceae Juss.
Iz_5_1009	DJI_0823	Fraxinus pennsylvanica Marshall	0	a	0	1	Oleaceae Hoffmanns. & Link
Iz_5_1009	DJI_0823	Galeopsis bifida Boenn.	0	n	1	0	Lamiacea Lindl.
Iz_5_1009	DJI_0823	Galium verum L.	0	n	1	0	Rubiaceae Juss.
Iz_5_1009	DJI_0823	Genista tinctoria L.	0	n	1	0	Fabaceae Lindl.
Iz_5_1009	DJI_0823	Geranium robertianum L.	0	n	1	0	Geraniaceae Juss.
Iz_5_1009	DJI_0823	Geranium sanquineum L.	0	n	1	0	Geraniaceae Juss.
Iz_5_1009	DJI_0823	Gypsophila paniculata L.	0	n	1	0	Caryophyllaceae Juss.
Iz_5_1009	DJI_0823	Helichrysum arenarium (L.) Moench	0	n	1	0	Asteraceae Bercht. & J.Presl
Iz_5_1009	DJI_0823	Herniaria polygama J.Gay	0	n	1	0	Caryophyllaceae Juss.
Iz_5_1009	DJI_0823	Hieracium robustum Fr.	0	n	1	0	Asteraceae Bercht. & J.Presl
Iz_5_1009	DJI_0823	Hieracium umbellatum L.	0	n	1	NA	Asteraceae Bercht. & J.Presl
Iz_5_1009	DJI_0823	Hylotelephium maximum (L.) Holub	0	n	1	0	Crassulaceae J.St.-Hil.
Iz_5_1009	DJI_0823	Jacobaea borysthenica (DC.) B.Nord. & Greuter	0	n	1	0	Asteraceae Bercht. & J.Presl
Iz_5_1009	DJI_0823	Jasione montana L.	0	n	1	0	Campanulaceae Juss.
Iz_5_1009	DJI_0823	Jurinea cyanoides (L.) Rchb.s.l.	0	n	1	0	Asteraceae Bercht. & J.Presl
Iz_5_1009	DJI_0823	Koeleria glauca (Spreng.) DC.	0	n	1	0	Poaceae Barnhart
Iz_5_1009	DJI_0823	Lactuca serriola L.	0	a	0	0	Asteraceae Bercht. & J.Presl
Iz_5_1009	DJI_0823	Linaria genistifolia (L.) Mill.	2	n	1	0	Plantaginaceae Juss.
Iz_5_1009	DJI_0823	Lomelosia argentea (L.) Greuter & Burdet	0	n	1	0	Caprifoliaceae Juss.
Iz_5_1009	DJI_0823	Melampyrum pratense L.	0	n	1	0	Orobanchaceae Vent.
Iz_5_1009	DJI_0823	Oenothera biennis L.	0	a	0	0	Onagraceae Juss.
Iz_5_1009	DJI_0823	Pentanema britannica (L.) D.Gut.Larr., Santos-Vicente, Anderb., E.Rico & M.M.Mart.Ort.	0	n	1	0	Asteraceae Bercht. & J.Presl
Iz_5_1009	DJI_0823	Peucedanum oreoselinum (L.) Moench	1	n	1	0	Apiaceae Lindl.
Iz_5_1009	DJI_0823	Peucedanum ruthenicum M.Bieb.	0	n	1	0	Apiaceae Lindl.
Iz_5_1009	DJI_0823	Pilosella echioides (Lumn.) F.W.Schultz & Sch.Bip.	0	n	1	0	Asteraceae Bercht. & J.Presl
Iz_5_1009	DJI_0823	Pilosella officinarum F. Schultz et Sch. Bip.	0	n	1	0	Asteraceae Bercht. & J.Presl
Iz_5_1009	DJI_0823	Pilosella sp.	0		NA	0	Asteraceae Bercht. & J.Presl
Iz_5_1009	DJI_0823	Pinus sylvestris L.	0	n	1	1	Pinaceae Spreng. ex F.Rudolphi
Iz_5_1009	DJI_0823	Poa annua L.	0	n	1	0	Poaceae Barnhart
Iz_5_1009	DJI_0823	Poa nemoralis L.	0	n	1	0	Poaceae Barnhart
Iz_5_1009	DJI_0823	Poa sp.	0		NA	0	Poaceae Barnhart
Iz_5_1009	DJI_0823	Polygonatum odoratum (Mill.) Druce	0	n	1	0	Asparagaceae Juss.
Iz_5_1009	DJI_0823	Polygonum arenarium Waldst. & Kit.	0	n	1	0	Polygonaceae Juss.
Iz_5_1009	DJI_0823	Populus nigra L.	0	n	1	1	Salicaceae Mirb.
Iz_5_1009	DJI_0823	Populus sp.	0	n	1	1	Salicaceae Mirb.
Iz_5_1009	DJI_0823	Populus tremula L.	0	n	1	1	Salicaceae Mirb.
Iz_5_1009	DJI_0823	Populus Ã canescens (Aiton) Sm.	0	n	1	1	Salicaceae Mirb.
Iz_5_1009	DJI_0823	Potentilla argentea L.	0	n	1	0	Rosaceae Juss.
Iz_5_1009	DJI_0823	Psammophiliella muralis (L.) Ikonn.	0	n	1	0	Caryophyllaceae Juss.
Iz_5_1009	DJI_0823	Psephellus sumensis (Kalen.) Greuter	0	n	1	0	Asteraceae Bercht. & J.Presl
Iz_5_1009	DJI_0823	Pulsatilla pratensis (L.) Mill. s.l.	0	n	1	0	Ranunculaceae Juss.
Iz_5_1009	DJI_0823	Pyrus communis L.	0	a	0	1	Rosaceae Juss.
Iz_5_1009	DJI_0823	Quercus robur L.	0	n	1	1	Fagaceae Dumort.
Iz_5_1009	DJI_0823	Robinia pseudoacacia L.	0	a	0	1	Fabaceae Lindl.
Iz_5_1009	DJI_0823	Rosa sp.	0		NA	1	Rosaceae Juss.
Iz_5_1009	DJI_0823	Rubus idaeus L.	0	n	1	0	Rosaceae Juss.
Iz_5_1009	DJI_0823	Rumex acetosella L.	0	n	1	0	Polygonaceae Juss.
Iz_5_1009	DJI_0823	Rumex thyrsiflorus Fingerh.	0	n	1	0	Polygonaceae Juss.
Iz_5_1009	DJI_0823	Salix alba L.	0	n	1	1	Salicaceae Mirb.
Iz_5_1009	DJI_0823	Sambucus nigra L.	0	n	1	1	Viburnaceae Raf.
Iz_5_1009	DJI_0823	Secale sylvestre Host	0	n	1	0	Poaceae Barnhart
Iz_5_1009	DJI_0823	Sempervivum ruthenicum Schnittsp. & C.B. Lehm.	0	n	1	0	Crassulaceae J.St.-Hil.
Iz_5_1009	DJI_0823	Setaria viridis (L.) P. Beauv.	0	n	1	0	Poaceae Barnhart
Iz_5_1009	DJI_0823	Silene borysthenica (Gruner) Walters	0	n	1	0	Caryophyllaceae Juss.
Iz_5_1009	DJI_0823	Silene latifolia subsp. alba (Mill.) Greuter & Burdet	0	n	1	0	Caryophyllaceae Juss.
Iz_5_1009	DJI_0823	Solidago virgaurea L.	1	n	1	0	Asteraceae Bercht. & J.Presl
Iz_5_1009	DJI_0823	Solidago Ã niederederi (S. canadensis Ã S. virgaurea)	0	hybrid	0	0	Asteraceae Bercht. & J.Presl
Iz_5_1009	DJI_0823	Sorbus aucuparia L.	0	n	1	1	Rosaceae Juss.
Iz_5_1009	DJI_0823	Stachys recta L.	0	n	1	0	Lamiacea Lindl.
Iz_5_1009	DJI_0823	Tanacetum vulgare L.	0	n	1	0	Asteraceae Bercht. & J.Presl
Iz_5_1009	DJI_0823	Thymus pallasianus Heinr.Braun	0	n	1	0	Lamiacea Lindl.
Iz_5_1009	DJI_0823	Tilia cordata Mill.	0	n	1	1	Malvaceae Juss.
Iz_5_1009	DJI_0823	Trifolium alpestre L.	0	n	1	0	Fabaceae Lindl.
Iz_5_1009	DJI_0823	Trifolium arvense L.	0	n	1	0	Fabaceae Lindl.
Iz_5_1009	DJI_0823	Ulmus glabra Huds.	0	n	1	1	Ulmaceae Mirb.
Iz_5_1009	DJI_0823	Verbascum densiflorum Bertol.	0	n	1	0	Scrophulariaceae Juss.
Iz_5_1009	DJI_0823	Veronica austriaca L.	0	n	1	0	Plantaginaceae Juss.
Iz_5_1009	DJI_0823	Veronica incana L.	0	n	1	0	Plantaginaceae Juss.
Iz_5_1009	DJI_0823	Veronica verna L.	0	n	1	0	Plantaginaceae Juss.
Iz_5_1009	DJI_0823	Vincetoxicum hirundinaria Medik	0	n	1	0	Apocynaceae Juss.
Iz_5_1009	DJI_0832	Acer tataricum L.	0	n	1	1	Sapindaceae Juss.
Iz_5_1009	DJI_0832	Achillea millefolium L. s.l.	0	n	1	0	Asteraceae Bercht. & J.Presl
Iz_5_1009	DJI_0832	Allium oleraceum L.	0	n	1	0	Amaryllidaceae J.St.-Hil.
Iz_5_1009	DJI_0832	Ambrosia artemisiifolia L.	0	a	0	0	Asteraceae Bercht. & J.Presl
Iz_5_1009	DJI_0832	Amorpha fruticosa L.	0	a	0	0	Fabaceae Lindl.

Iz_5_1009	DJI_0832	Anthericum ramosum L.	0	n	1	0	Asparagaceae Juss.
Iz_5_1009	DJI_0832	Artemisia marschalliana Spreng.	0	n	1	0	Asteraceae Bercht. & J.Presl
Iz_5_1009	DJI_0832	Asparagus officinalis L.	0	n	1	0	Asparagaceae Juss.
Iz_5_1009	DJI_0832	Astragalus cicer L.	0	n	1	0	Fabaceae Lindl.
Iz_5_1009	DJI_0832	Bassia laniflora (S.G.Gmel.) A.J.Scott	0	n	1	0	Amaranthaceae Juss.
Iz_5_1009	DJI_0832	Berberis vulgaris L.	0	n	1	0	Berberidaceae Juss.
Iz_5_1009	DJI_0832	Betula pendula Roth.	0	n	1	1	Betulaceae Gray
Iz_5_1009	DJI_0832	Betula pendula Roth.	0	n	1	0	Poaceae Barnhart
Iz_5_1009	DJI_0832	Bromus inermis Leyss.	0		NA	NA	
Iz_5_1009	DJI_0832	Bromus squarrosus L.	0	a	0	0	Poaceae Barnhart
Iz_5_1009	DJI_0832	Bromus tectorum L.	0	a	0	0	Poaceae Barnhart
Iz_5_1009	DJI_0832	Calamagrostis epigejos (L.) Roth	3	n	1	0	Poaceae Barnhart
Iz_5_1009	DJI_0832	Carduus acanthoides L.	0	n	1	0	Asteraceae Bercht. & J.Presl
Iz_5_1009	DJI_0832	Carex sp.	1	n	1	0	Cyperaceae Juss.
Iz_5_1009	DJI_0832	Centaurea majorovii Dumbadze	0	n	1	0	Asteraceae Bercht. & J.Presl
Iz_5_1009	DJI_0832	Chamaecytisus ruthenicus (Fisch.ex Wol.) Klaskova	0	n	1	0	Fabaceae Lindl.
Iz_5_1009	DJI_0832	Chelidonium majus L.	0	n	1	0	Papaveraceae Juss.
Iz_5_1009	DJI_0832	Chenopodium betaceum Andrz.	0	n	1	0	Amaranthaceae Juss.
Iz_5_1009	DJI_0832	Chenopodium cf. album	0	n	1	0	Amaranthaceae Juss.
Iz_5_1009	DJI_0832	Chenopodium sp.	0		NA	0	Amaranthaceae Juss.
Iz_5_1009	DJI_0832	Chondrilla juncea L.	0	n	1	0	Asteraceae Bercht. & J.Presl
Iz_5_1009	DJI_0832	Cirsium vulgare (Savi) Ten.	0	n	1	0	Asteraceae Bercht. & J.Presl
Iz_5_1009	DJI_0832	Clinopodium acinos (L.) Kuntze	0	n	1	0	Lamiacea Lindl.
Iz_5_1009	DJI_0832	Convallaria majalis L.	0	n	1	0	Asparagaceae Juss.
Iz_5_1009	DJI_0832	Crataegus rhipidophylla Gand.	0	n	1	1	Rosaceae Juss.
Iz_5_1009	DJI_0832	Crepis foetida L.	0	n	1	0	Asteraceae Bercht. & J.Presl
Iz_5_1009	DJI_0832	Dactylis glomerata L.	0	n	1	0	Poaceae Barnhart
Iz_5_1009	DJI_0832	Dianthus campestris M.Bieb.	0	n	1	0	Caryophyllaceae Juss.
Iz_5_1009	DJI_0832	Digitaria sanguinalis (L.) Scop.	0	a	0	0	Poaceae Barnhart
Iz_5_1009	DJI_0832	Elymus repens (L.) Gould	0	n	1	0	Poaceae Barnhart
Iz_5_1009	DJI_0832	Epilobium tetragonum L.	0	n	1	0	Onagraceae Juss.
Iz_5_1009	DJI_0832	Eragrostis minor Host	0	a	0	0	Poaceae Barnhart
Iz_5_1009	DJI_0832	Erigeron canadensis L.	0	a	0	0	Asteraceae Bercht. & J.Presl
Iz_5_1009	DJI_0832	Euphorbia seguieriana Neck.	0	n	1	0	Euphorbiaceae Juss.
Iz_5_1009	DJI_0832	Fallopia dumetorum (L.) Holub	0	n	1	0	Polygonaceae Juss.
Iz_5_1009	DJI_0832	Festuca beckeri (Hack.) Trautv.	0	n	1	0	Poaceae Barnhart
Iz_5_1009	DJI_0832	Fragaria vesca L.	0	n	1	0	Rosaceae Juss.
Iz_5_1009	DJI_0832	Frangula alnus Mill.	0	n	1	1	Rhamnaceae Juss.
Iz_5_1009	DJI_0832	Fraxinus pennsylvanica Marshall	0	a	0	1	Oleaceae Hoffmanns. & Link
Iz_5_1009	DJI_0832	Galeopsis bifida Boenn.	0	n	1	0	Lamiacea Lindl.
Iz_5_1009	DJI_0832	Galium verum L.	0	n	1	0	Rubiaceae Juss.
Iz_5_1009	DJI_0832	Genista tinctoria L.	0	n	1	0	Fabaceae Lindl.
Iz_5_1009	DJI_0832	Geranium robertianum L.	0	n	1	0	Geraniaceae Juss.
Iz_5_1009	DJI_0832	Geranium sanquineum L.	0	n	1	0	Geraniaceae Juss.
Iz_5_1009	DJI_0832	Gypsophila paniculata L.	0	n	1	0	Caryophyllaceae Juss.
Iz_5_1009	DJI_0832	Helichrysum arenarium (L.) Moench	0	n	1	0	Asteraceae Bercht. & J.Presl
Iz_5_1009	DJI_0832	Herniaria polygama J.Gay	0	n	1	0	Caryophyllaceae Juss.
Iz_5_1009	DJI_0832	Hieracium robustum Fr.	0	n	1	0	Asteraceae Bercht. & J.Presl
Iz_5_1009	DJI_0832	Hieracium umbellatum L.	0	n	1	NA	Asteraceae Bercht. & J.Presl
Iz_5_1009	DJI_0832	Hylotelephium maximum (L.) Holub	1	n	1	0	Crassulaceae J.St.-Hil.
Iz_5_1009	DJI_0832	Jacobaea borysthenica (DC.) B.Nord. & Greuter	0	n	1	0	Asteraceae Bercht. & J.Presl
Iz_5_1009	DJI_0832	Jasione montana L.	0	n	1	0	Campanulaceae Juss.
Iz_5_1009	DJI_0832	Jurinea cyanoides (L.) Rchb.s.l.	0	n	1	0	Asteraceae Bercht. & J.Presl
Iz_5_1009	DJI_0832	Koeleria glauca (Spreng.) DC.	0	n	1	0	Poaceae Barnhart
Iz_5_1009	DJI_0832	Lactuca serriola L.	0	a	0	0	Asteraceae Bercht. & J.Presl
Iz_5_1009	DJI_0832	Linaria genistifolia (L.) Mill.	0	n	1	0	Plantaginaceae Juss.
Iz_5_1009	DJI_0832	Lomelosia argentea (L.) Greuter & Burdet	0	n	1	0	Caprifoliaceae Juss.
Iz_5_1009	DJI_0832	Melampyrum pratense L.	0	n	1	0	Orobanchaceae Vent.
Iz_5_1009	DJI_0832	Oenothera biennis L.	0	a	0	0	Onagraceae Juss.
Iz_5_1009	DJI_0832	Pentanema britannica (L.) D.Gut.Larr., Santos-Vicente, Anderb., E.Rico & M.M.Mart.Ort.	0	n	1	0	Asteraceae Bercht. & J.Presl
Iz_5_1009	DJI_0832	Peucedanum oreoselinum (L.) Moench	2	n	1	0	Apiaceae Lindl.
Iz_5_1009	DJI_0832	Peucedanum ruthenicum M.Bieb.	0	n	1	0	Apiaceae Lindl.
Iz_5_1009	DJI_0832	Pilosella echioides (Lumn.) F.W.Schultz & Sch.Bip.	0	n	1	0	Asteraceae Bercht. & J.Presl
Iz_5_1009	DJI_0832	Pilosella officinarum F. Schultz et Sch. Bip.	5	n	1	0	Asteraceae Bercht. & J.Presl
Iz_5_1009	DJI_0832	Pilosella sp.	0		NA	0	Asteraceae Bercht. & J.Presl
Iz_5_1009	DJI_0832	Pinus sylvestris L.	0	n	1	1	Pinaceae Spreng. ex F.Rudolphi
Iz_5_1009	DJI_0832	Poa annua L.	0	n	1	0	Poaceae Barnhart
Iz_5_1009	DJI_0832	Poa nemoralis L.	0	n	1	0	Poaceae Barnhart
Iz_5_1009	DJI_0832	Poa sp.	0		NA	0	Poaceae Barnhart
Iz_5_1009	DJI_0832	Polygonatum odoratum (Mill.) Druce	0	n	1	0	Asparagaceae Juss.
Iz_5_1009	DJI_0832	Polygonum arenarium Waldst. & Kit.	0	n	1	0	Polygonaceae Juss.
Iz_5_1009	DJI_0832	Populus nigra L.	0	n	1	1	Salicaceae Mirb.
Iz_5_1009	DJI_0832	Populus sp.	0	n	1	1	Salicaceae Mirb.
Iz_5_1009	DJI_0832	Populus tremula L.	0	n	1	1	Salicaceae Mirb.
Iz_5_1009	DJI_0832	Populus Ã canescens (Aiton) Sm.	0	n	1	1	Salicaceae Mirb.
Iz_5_1009	DJI_0832	Potentilla argentea L.	0	n	1	0	Rosaceae Juss.
Iz_5_1009	DJI_0832	Psammophiliella muralis (L.) Ikonn.	0	n	1	0	Caryophyllaceae Juss.
Iz_5_1009	DJI_0832	Psephellus sumensis (Kalen.) Greuter	0	n	1	0	Asteraceae Bercht. & J.Presl
Iz_5_1009	DJI_0832	Pulsatilla pratensis (L.) Mill. s.l.	0	n	1	0	Ranunculaceae Juss.
Iz_5_1009	DJI_0832	Pyrus communis L.	0	a	0	1	Rosaceae Juss.
Iz_5_1009	DJI_0832	Quercus robur L.	0	n	1	1	Fagaceae Dumort.
Iz_5_1009	DJI_0832	Robinia pseudoacacia L.	0	a	0	1	Fabaceae Lindl.
Iz_5_1009	DJI_0832	Rosa sp.	0		NA	1	Rosaceae Juss.
Iz_5_1009	DJI_0832	Rubus idaeus L.	0	n	1	0	Rosaceae Juss.
Iz_5_1009	DJI_0832	Rumex acetosella L.	0	n	1	0	Polygonaceae Juss.
Iz_5_1009	DJI_0832	Rumex thyrsiflorus Fingerh.	0	n	1	0	Polygonaceae Juss.
Iz_5_1009	DJI_0832	Salix alba L.	0	n	1	1	Salicaceae Mirb.
Iz_5_1009	DJI_0832	Sambucus nigra L.	0	n	1	1	Viburnaceae Raf.
Iz_5_1009	DJI_0832	Secale sylvestre Host	0	n	1	0	Poaceae Barnhart
Iz_5_1009	DJI_0832	Sempervivum ruthenicum Schnittsp. & C.B. Lehm.	0	n	1	0	Crassulaceae J.St.-Hil.
Iz_5_1009	DJI_0832	Setaria viridis (L.) P. Beauv.	0	n	1	0	Poaceae Barnhart
Iz_5_1009	DJI_0832	Silene borysthenica (Gruner) Walters	0	n	1	0	Caryophyllaceae Juss.
Iz_5_1009	DJI_0832	Silene latifolia subsp. alba (Mill.) Greuter & Burdet	0	n	1	0	Caryophyllaceae Juss.
Iz_5_1009	DJI_0832	Solidago virgaurea L.	0	n	1	0	Asteraceae Bercht. & J.Presl
Iz_5_1009	DJI_0832	Solidago Ã niederederi (S. canadensis Ã S. virgaurea)	0	hybrid	0	0	Asteraceae Bercht. & J.Presl
Iz_5_1009	DJI_0832	Sorbus aucuparia L.	0	n	1	1	Rosaceae Juss.
Iz_5_1009	DJI_0832	Stachys recta L.	0	n	1	0	Lamiacea Lindl.
Iz_5_1009	DJI_0832	Tanacetum vulgare L.	0	n	1	0	Asteraceae Bercht. & J.Presl

Iz_5_1009	DJI_0832	Thymus pallasianus Heinr.Braun	0	n	1	0	Lamiacea Lindl.
Iz_5_1009	DJI_0832	Tilia cordata Mill.	0	n	1	1	Malvaceae Juss.
Iz_5_1009	DJI_0832	Trifolium alpestre L.	0	n	1	0	Fabaceae Lindl.
Iz_5_1009	DJI_0832	Trifolium arvense L.	0	n	1	0	Fabaceae Lindl.
Iz_5_1009	DJI_0832	Ulmus glabra Huds.	0	n	1	1	Ulmaceae Mirb.
Iz_5_1009	DJI_0832	Verbascum densiflorum Bertol.	0	n	1	0	Scrophulariaceae Juss.
Iz_5_1009	DJI_0832	Veronica austriaca L.	0	n	1	0	Plantaginaceae Juss.
Iz_5_1009	DJI_0832	Veronica incana L.	0	n	1	0	Plantaginaceae Juss.
Iz_5_1009	DJI_0832	Veronica verna L.	0	n	1	0	Plantaginaceae Juss.
Iz_5_1009	DJI_0832	Vincetoxicum hirundinaria Medik	0	n	1	0	Apocynaceae Juss.
Iz_5_1009	DJI_0838	Acer tataricum L.	0	n	1	1	Sapindaceae Juss.
Iz_5_1009	DJI_0838	Achillea millefolium L. s.l.	0	n	1	0	Asteraceae Bercht. & J.Presl
Iz_5_1009	DJI_0838	Allium oleraceum L.	0	n	1	0	Amaryllidaceae J.St.-Hil.
Iz_5_1009	DJI_0838	Ambrosia artemisiifolia L.	0	a	0	0	Asteraceae Bercht. & J.Presl
Iz_5_1009	DJI_0838	Amorpha fruticosa L.	0	a	0	0	Fabaceae Lindl.
Iz_5_1009	DJI_0838	Anthericum ramosum L.	0	n	1	0	Asparagaceae Juss.
Iz_5_1009	DJI_0838	Artemisia marschalliana Spreng.	0	n	1	0	Asteraceae Bercht. & J.Presl
Iz_5_1009	DJI_0838	Asparagus officinalis L.	0	n	1	0	Asparagaceae Juss.
Iz_5_1009	DJI_0838	Astragalus cicer L.	0	n	1	0	Fabaceae Lindl.
Iz_5_1009	DJI_0838	Bassia laniflora (S.G.Gmel.) A.J.Scott	0	n	1	0	Amaranthaceae Juss.
Iz_5_1009	DJI_0838	Berberis vulgaris L.	0	n	1	0	Berberidaceae Juss.
Iz_5_1009	DJI_0838	Betula pendula Roth.	0	n	1	1	Betulaceae Gray
Iz_5_1009	DJI_0838	Betula pendula Roth.	0	n	1	0	Poaceae Barnhart
Iz_5_1009	DJI_0838	Bromus inermis Leyss.	0		NA	NA	
Iz_5_1009	DJI_0838	Bromus squarrosus L.	0	a	0	0	Poaceae Barnhart
Iz_5_1009	DJI_0838	Bromus tectorum L.	0	a	0	0	Poaceae Barnhart
Iz_5_1009	DJI_0838	Calamagrostis epigejos (L.) Roth	25	n	1	0	Poaceae Barnhart
Iz_5_1009	DJI_0838	Carduus acanthoides L.	0	n	1	0	Asteraceae Bercht. & J.Presl
Iz_5_1009	DJI_0838	Carex sp.	0	n	1	0	Cyperaceae Juss.
Iz_5_1009	DJI_0838	Centaurea majorovii Dumbadze	0	n	1	0	Asteraceae Bercht. & J.Presl
Iz_5_1009	DJI_0838	Chamaecytisus ruthenicus (Fisch.ex Wol.) Klaskova	0	n	1	0	Fabaceae Lindl.
Iz_5_1009	DJI_0838	Chelidonium majus L.	0	n	1	0	Papaveraceae Juss.
Iz_5_1009	DJI_0838	Chenopodium betaceum Andrz.	0	n	1	0	Amaranthaceae Juss.
Iz_5_1009	DJI_0838	Chenopodium cf. album	0	n	1	0	Amaranthaceae Juss.
Iz_5_1009	DJI_0838	Chenopodium sp.	0		NA	0	Amaranthaceae Juss.
Iz_5_1009	DJI_0838	Chondrilla juncea L.	0	n	1	0	Asteraceae Bercht. & J.Presl
Iz_5_1009	DJI_0838	Cirsium vulgare (Savi) Ten.	0	n	1	0	Asteraceae Bercht. & J.Presl
Iz_5_1009	DJI_0838	Clinopodium acinos (L.) Kuntze	0	n	1	0	Lamiacea Lindl.
Iz_5_1009	DJI_0838	Convallaria majalis L.	0	n	1	0	Asparagaceae Juss.
Iz_5_1009	DJI_0838	Crataegus rhipidophylla Gand.	0	n	1	1	Rosaceae Juss.
Iz_5_1009	DJI_0838	Crepis foetida L.	0	n	1	0	Asteraceae Bercht. & J.Presl
Iz_5_1009	DJI_0838	Dactylis glomerata L.	0	n	1	0	Poaceae Barnhart
Iz_5_1009	DJI_0838	Dianthus campestris M.Bieb.	0	n	1	0	Caryophyllaceae Juss.
Iz_5_1009	DJI_0838	Digitaria sanguinalis (L.) Scop.	0	a	0	0	Poaceae Barnhart
Iz_5_1009	DJI_0838	Elymus repens (L.) Gould	0	n	1	0	Poaceae Barnhart
Iz_5_1009	DJI_0838	Epilobium tetragonum L.	0	n	1	0	Onagraceae Juss.
Iz_5_1009	DJI_0838	Eragrostis minor Host	0	a	0	0	Poaceae Barnhart
Iz_5_1009	DJI_0838	Erigeron canadensis L.	0	a	0	0	Asteraceae Bercht. & J.Presl
Iz_5_1009	DJI_0838	Euphorbia seguieriana Neck.	0	n	1	0	Euphorbiaceae Juss.
Iz_5_1009	DJI_0838	Fallopia dumetorum (L.) Holub	0	n	1	0	Polygonaceae Juss.
Iz_5_1009	DJI_0838	Festuca beckeri (Hack.) Trautv.	0	n	1	0	Poaceae Barnhart
Iz_5_1009	DJI_0838	Fragaria vesca L.	0	n	1	0	Rosaceae Juss.
Iz_5_1009	DJI_0838	Frangula alnus Mill.	0	n	1	1	Rhamnaceae Juss.
Iz_5_1009	DJI_0838	Fraxinus pennsylvanica Marshall	0	a	0	1	Oleaceae Hoffmanns. & Link
Iz_5_1009	DJI_0838	Galeopsis bifida Boenn.	0	n	1	0	Lamiacea Lindl.
Iz_5_1009	DJI_0838	Galium verum L.	0	n	1	0	Rubiaceae Juss.
Iz_5_1009	DJI_0838	Genista tinctoria L.	0	n	1	0	Fabaceae Lindl.
Iz_5_1009	DJI_0838	Geranium robertianum L.	0	n	1	0	Geraniaceae Juss.
Iz_5_1009	DJI_0838	Geranium sanquineum L.	0	n	1	0	Geraniaceae Juss.
Iz_5_1009	DJI_0838	Gypsophila paniculata L.	0	n	1	0	Caryophyllaceae Juss.
Iz_5_1009	DJI_0838	Helichrysum arenarium (L.) Moench	0	n	1	0	Asteraceae Bercht. & J.Presl
Iz_5_1009	DJI_0838	Herniaria polygama J.Gay	0	n	1	0	Caryophyllaceae Juss.
Iz_5_1009	DJI_0838	Hieracium robustum Fr.	0	n	1	0	Asteraceae Bercht. & J.Presl
Iz_5_1009	DJI_0838	Hieracium umbellatum L.	0	n	1	NA	Asteraceae Bercht. & J.Presl
Iz_5_1009	DJI_0838	Hylotelephium maximum (L.) Holub	0	n	1	0	Crassulaceae J.St.-Hil.
Iz_5_1009	DJI_0838	Jacobaea borysthenica (DC.) B.Nord. & Greuter	0	n	1	0	Asteraceae Bercht. & J.Presl
Iz_5_1009	DJI_0838	Jasione montana L.	0	n	1	0	Campanulaceae Juss.
Iz_5_1009	DJI_0838	Jurinea cyanoides (L.) Rchb.s.l.	0	n	1	0	Asteraceae Bercht. & J.Presl
Iz_5_1009	DJI_0838	Koeleria glauca (Spreng.) DC.	0	n	1	0	Poaceae Barnhart
Iz_5_1009	DJI_0838	Lactuca serriola L.	0	a	0	0	Asteraceae Bercht. & J.Presl
Iz_5_1009	DJI_0838	Linaria genistifolia (L.) Mill.	0	n	1	0	Plantaginaceae Juss.
Iz_5_1009	DJI_0838	Lomelosia argentea (L.) Greuter & Burdet	0	n	1	0	Caprifoliaceae Juss.
Iz_5_1009	DJI_0838	Melampyrum pratense L.	0	n	1	0	Orobanchaceae Vent.
Iz_5_1009	DJI_0838	Oenothera biennis L.	0	a	0	0	Onagraceae Juss.
Iz_5_1009	DJI_0838	Pentanema britannica (L.) D.Gut.Larr., Santos-Vicente, Anderb., E.Rico & M.M.Mart.Ort.	0	n	1	0	Asteraceae Bercht. & J.Presl
Iz_5_1009	DJI_0838	Peucedanum oreoselinum (L.) Moench	2	n	1	0	Apiaceae Lindl.
Iz_5_1009	DJI_0838	Peucedanum ruthenicum M.Bieb.	0	n	1	0	Apiaceae Lindl.
Iz_5_1009	DJI_0838	Pilosella echioides (Lumn.) F.W.Schultz & Sch.Bip.	0	n	1	0	Asteraceae Bercht. & J.Presl
Iz_5_1009	DJI_0838	Pilosella officinarum F. Schultz et Sch. Bip.	0	n	1	0	Asteraceae Bercht. & J.Presl
Iz_5_1009	DJI_0838	Pilosella sp.	0		NA	0	Asteraceae Bercht. & J.Presl
Iz_5_1009	DJI_0838	Pinus sylvestris L.	0	n	1	1	Pinaceae Spreng. ex F.Rudolphi
Iz_5_1009	DJI_0838	Poa annua L.	0	n	1	0	Poaceae Barnhart
Iz_5_1009	DJI_0838	Poa nemoralis L.	0	n	1	0	Poaceae Barnhart
Iz_5_1009	DJI_0838	Poa sp.	0		NA	0	Poaceae Barnhart
Iz_5_1009	DJI_0838	Polygonatum odoratum (Mill.) Druce	0	n	1	0	Asparagaceae Juss.
Iz_5_1009	DJI_0838	Polygonum arenarium Waldst. & Kit.	0	n	1	0	Polygonaceae Juss.
Iz_5_1009	DJI_0838	Populus nigra L.	0	n	1	1	Salicaceae Mirb.
Iz_5_1009	DJI_0838	Populus sp.	0	n	1	1	Salicaceae Mirb.
Iz_5_1009	DJI_0838	Populus tremula L.	0	n	1	1	Salicaceae Mirb.
Iz_5_1009	DJI_0838	Populus Ã canescens (Aiton) Sm.	0	n	1	1	Salicaceae Mirb.
Iz_5_1009	DJI_0838	Potentilla argentea L.	0	n	1	0	Rosaceae Juss.
Iz_5_1009	DJI_0838	Psammophiliella muralis (L.) Ikonn.	0	n	1	0	Caryophyllaceae Juss.
Iz_5_1009	DJI_0838	Psephellus sumensis (Kalen.) Greuter	0	n	1	0	Asteraceae Bercht. & J.Presl
Iz_5_1009	DJI_0838	Pulsatilla pratensis (L.) Mill. s.l.	0	n	1	0	Ranunculaceae Juss.
Iz_5_1009	DJI_0838	Pyrus communis L.	0	a	0	1	Rosaceae Juss.
Iz_5_1009	DJI_0838	Quercus robur L.	0	n	1	1	Fagaceae Dumort.
Iz_5_1009	DJI_0838	Robinia pseudoacacia L.	0	a	0	1	Fabaceae Lindl.
Iz_5_1009	DJI_0838	Rosa sp.	0		NA	1	Rosaceae Juss.

Iz_5_1009	DJI_0838	Rubus idaeus L.	0	n	1	0	Rosaceae Juss.
Iz_5_1009	DJI_0838	Rumex acetosella L.	0	n	1	0	Polygonaceae Juss.
Iz_5_1009	DJI_0838	Rumex thyrsiflorus Fingerh.	0	n	1	0	Polygonaceae Juss.
Iz_5_1009	DJI_0838	Salix alba L.	0	n	1	1	Salicaceae Mirb.
Iz_5_1009	DJI_0838	Sambucus nigra L.	0	n	1	1	Viburnaceae Raf.
Iz_5_1009	DJI_0838	Secale sylvestre Host	0	n	1	0	Poaceae Barnhart
Iz_5_1009	DJI_0838	Sempervivum ruthenicum Schnittsp. & C.B. Lehm.	0	n	1	0	Crassulaceae J.St.-Hil.
Iz_5_1009	DJI_0838	Setaria viridis (L.) P. Beauv.	0	n	1	0	Poaceae Barnhart
Iz_5_1009	DJI_0838	Silene borysthenica (Gruner) Walters	0	n	1	0	Caryophyllaceae Juss.
Iz_5_1009	DJI_0838	Silene latifolia subsp. alba (Mill.) Greuter & Burdet	0	n	1	0	Caryophyllaceae Juss.
Iz_5_1009	DJI_0838	Solidago virgaurea L.	0	n	1	0	Asteraceae Bercht. & J.Presl
Iz_5_1009	DJI_0838	Solidago Ã niederederi (S. canadensis Ã S. virgaurea)	0	hybrid	0	0	Asteraceae Bercht. & J.Presl
Iz_5_1009	DJI_0838	Sorbus aucuparia L.	0	n	1	1	Rosaceae Juss.
Iz_5_1009	DJI_0838	Stachys recta L.	0	n	1	0	Lamiacea Lindl.
Iz_5_1009	DJI_0838	Tanacetum vulgare L.	0	n	1	0	Asteraceae Bercht. & J.Presl
Iz_5_1009	DJI_0838	Thymus pallasianus Heinr.Braun	0	n	1	0	Lamiacea Lindl.
Iz_5_1009	DJI_0838	Tilia cordata Mill.	0	n	1	1	Malvaceae Juss.
Iz_5_1009	DJI_0838	Trifolium alpestre L.	0	n	1	0	Fabaceae Lindl.
Iz_5_1009	DJI_0838	Trifolium arvense L.	0	n	1	0	Fabaceae Lindl.
Iz_5_1009	DJI_0838	Ulmus glabra Huds.	0	n	1	1	Ulmaceae Mirb.
Iz_5_1009	DJI_0838	Verbascum densiflorum Bertol.	0	n	1	0	Scrophulariaceae Juss.
Iz_5_1009	DJI_0838	Veronica austriaca L.	0	n	1	0	Plantaginaceae Juss.
Iz_5_1009	DJI_0838	Veronica incana L.	0	n	1	0	Plantaginaceae Juss.
Iz_5_1009	DJI_0838	Veronica verna L.	0	n	1	0	Plantaginaceae Juss.
Iz_5_1009	DJI_0838	Vincetoxicum hirundinaria Medik	0	n	1	0	Apocynaceae Juss.
Iz_5_1009	DJI_0841	Acer tataricum L.	0	n	1	1	Sapindaceae Juss.
Iz_5_1009	DJI_0841	Achillea millefolium L. s.l.	0	n	1	0	Asteraceae Bercht. & J.Presl
Iz_5_1009	DJI_0841	Allium oleraceum L.	0	n	1	0	Amaryllidaceae J.St.-Hil.
Iz_5_1009	DJI_0841	Ambrosia artemisiifolia L.	0	a	0	0	Asteraceae Bercht. & J.Presl
Iz_5_1009	DJI_0841	Amorpha fruticosa L.	0	a	0	0	Fabaceae Lindl.
Iz_5_1009	DJI_0841	Anthericum ramosum L.	0	n	1	0	Asparagaceae Juss.
Iz_5_1009	DJI_0841	Artemisia marschalliana Spreng.	0	n	1	0	Asteraceae Bercht. & J.Presl
Iz_5_1009	DJI_0841	Asparagus officinalis L.	0	n	1	0	Asparagaceae Juss.
Iz_5_1009	DJI_0841	Astragalus cicer L.	0	n	1	0	Fabaceae Lindl.
Iz_5_1009	DJI_0841	Bassia laniflora (S.G.Gmel.) A.J.Scott	0	n	1	0	Amaranthaceae Juss.
Iz_5_1009	DJI_0841	Berberis vulgaris L.	0	n	1	0	Berberidaceae Juss.
Iz_5_1009	DJI_0841	Betula pendula Roth.	0	n	1	1	Betulaceae Gray
Iz_5_1009	DJI_0841	Betula pendula Roth.	0	n	1	0	Poaceae Barnhart
Iz_5_1009	DJI_0841	Bromus inermis Leyss.	0		NA	NA	
Iz_5_1009	DJI_0841	Bromus squarrosus L.	0	a	0	0	Poaceae Barnhart
Iz_5_1009	DJI_0841	Bromus tectorum L.	0	a	0	0	Poaceae Barnhart
Iz_5_1009	DJI_0841	Calamagrostis epigejos (L.) Roth	40	n	1	0	Poaceae Barnhart
Iz_5_1009	DJI_0841	Carduus acanthoides L.	0	n	1	0	Asteraceae Bercht. & J.Presl
Iz_5_1009	DJI_0841	Carex sp.	0	n	1	0	Cyperaceae Juss.
Iz_5_1009	DJI_0841	Centaurea majorovii Dumbadze	0	n	1	0	Asteraceae Bercht. & J.Presl
Iz_5_1009	DJI_0841	Chamaecytisus ruthenicus (Fisch.ex Wol.) Klaskova	0	n	1	0	Fabaceae Lindl.
Iz_5_1009	DJI_0841	Chelidonium majus L.	0	n	1	0	Papaveraceae Juss.
Iz_5_1009	DJI_0841	Chenopodium betaceum Andrz.	0	n	1	0	Amaranthaceae Juss.
Iz_5_1009	DJI_0841	Chenopodium cf. album	0	n	1	0	Amaranthaceae Juss.
Iz_5_1009	DJI_0841	Chenopodium sp.	0		NA	0	Amaranthaceae Juss.
Iz_5_1009	DJI_0841	Chondrilla juncea L.	0	n	1	0	Asteraceae Bercht. & J.Presl
Iz_5_1009	DJI_0841	Cirsium vulgare (Savi) Ten.	0	n	1	0	Asteraceae Bercht. & J.Presl
Iz_5_1009	DJI_0841	Clinopodium acinos (L.) Kuntze	0	n	1	0	Lamiacea Lindl.
Iz_5_1009	DJI_0841	Convallaria majalis L.	0	n	1	0	Asparagaceae Juss.
Iz_5_1009	DJI_0841	Crataegus rhipidophylla Gand.	0	n	1	1	Rosaceae Juss.
Iz_5_1009	DJI_0841	Crepis foetida L.	0	n	1	0	Asteraceae Bercht. & J.Presl
Iz_5_1009	DJI_0841	Dactylis glomerata L.	0	n	1	0	Poaceae Barnhart
Iz_5_1009	DJI_0841	Dianthus campestris M.Bieb.	0	n	1	0	Caryophyllaceae Juss.
Iz_5_1009	DJI_0841	Digitaria sanguinalis (L.) Scop.	0	a	0	0	Poaceae Barnhart
Iz_5_1009	DJI_0841	Elymus repens (L.) Gould	0	n	1	0	Poaceae Barnhart
Iz_5_1009	DJI_0841	Epilobium tetragonum L.	0	n	1	0	Onagraceae Juss.
Iz_5_1009	DJI_0841	Eragrostis minor Host	0	a	0	0	Poaceae Barnhart
Iz_5_1009	DJI_0841	Erigeron canadensis L.	0	a	0	0	Asteraceae Bercht. & J.Presl
Iz_5_1009	DJI_0841	Euphorbia seguieriana Neck.	0	n	1	0	Euphorbiaceae Juss.
Iz_5_1009	DJI_0841	Fallopia dumetorum (L.) Holub	0	n	1	0	Polygonaceae Juss.
Iz_5_1009	DJI_0841	Festuca beckeri (Hack.) Trautv.	0	n	1	0	Poaceae Barnhart
Iz_5_1009	DJI_0841	Fragaria vesca L.	0	n	1	0	Rosaceae Juss.
Iz_5_1009	DJI_0841	Frangula alnus Mill.	0	n	1	1	Rhamnaceae Juss.
Iz_5_1009	DJI_0841	Fraxinus pennsylvanica Marshall	0	a	0	1	Oleaceae Hoffmanns. & Link
Iz_5_1009	DJI_0841	Galeopsis bifida Boenn.	0	n	1	0	Lamiacea Lindl.
Iz_5_1009	DJI_0841	Galium verum L.	0	n	1	0	Rubiaceae Juss.
Iz_5_1009	DJI_0841	Genista tinctoria L.	0	n	1	0	Fabaceae Lindl.
Iz_5_1009	DJI_0841	Geranium robertianum L.	0	n	1	0	Geraniaceae Juss.
Iz_5_1009	DJI_0841	Geranium sanquineum L.	0	n	1	0	Geraniaceae Juss.
Iz_5_1009	DJI_0841	Gypsophila paniculata L.	0	n	1	0	Caryophyllaceae Juss.
Iz_5_1009	DJI_0841	Helichrysum arenarium (L.) Moench	0	n	1	0	Asteraceae Bercht. & J.Presl
Iz_5_1009	DJI_0841	Herniaria polygama J.Gay	0	n	1	0	Caryophyllaceae Juss.
Iz_5_1009	DJI_0841	Hieracium robustum Fr.	0	n	1	0	Asteraceae Bercht. & J.Presl
Iz_5_1009	DJI_0841	Hieracium umbellatum L.	0	n	1	NA	Asteraceae Bercht. & J.Presl
Iz_5_1009	DJI_0841	Hylotelephium maximum (L.) Holub	0	n	1	0	Crassulaceae J.St.-Hil.
Iz_5_1009	DJI_0841	Jacobaea borysthenica (DC.) B.Nord. & Greuter	0	n	1	0	Asteraceae Bercht. & J.Presl
Iz_5_1009	DJI_0841	Jasione montana L.	0	n	1	0	Campanulaceae Juss.
Iz_5_1009	DJI_0841	Jurinea cyanoides (L.) Rchb.s.l.	0	n	1	0	Asteraceae Bercht. & J.Presl
Iz_5_1009	DJI_0841	Koeleria glauca (Spreng.) DC.	0	n	1	0	Poaceae Barnhart
Iz_5_1009	DJI_0841	Lactuca serriola L.	0	a	0	0	Asteraceae Bercht. & J.Presl
Iz_5_1009	DJI_0841	Linaria genistifolia (L.) Mill.	0	n	1	0	Plantaginaceae Juss.
Iz_5_1009	DJI_0841	Lomelosia argentea (L.) Greuter & Burdet	0	n	1	0	Caprifoliaceae Juss.
Iz_5_1009	DJI_0841	Melampyrum pratense L.	0	n	1	0	Orobanchaceae Vent.
Iz_5_1009	DJI_0841	Oenothera biennis L.	0	a	0	0	Onagraceae Juss.
Iz_5_1009	DJI_0841	Pentanema britannica (L.) D.Gut.Larr., Santos-Vicente, Anderb., E.Rico & M.M.Mart.Ort.	0	n	1	0	Asteraceae Bercht. & J.Presl
Iz_5_1009	DJI_0841	Peucedanum oreoselinum (L.) Moench	5	n	1	0	Apiaceae Lindl.
Iz_5_1009	DJI_0841	Peucedanum ruthenicum M.Bieb.	0	n	1	0	Apiaceae Lindl.
Iz_5_1009	DJI_0841	Pilosella echioides (Lumn.) F.W.Schultz & Sch.Bip.	0	n	1	0	Asteraceae Bercht. & J.Presl
Iz_5_1009	DJI_0841	Pilosella officinarum F. Schultz et Sch. Bip.	0	n	1	0	Asteraceae Bercht. & J.Presl
Iz_5_1009	DJI_0841	Pilosella sp.	0		NA	0	Asteraceae Bercht. & J.Presl
Iz_5_1009	DJI_0841	Pinus sylvestris L.	0	n	1	1	Pinaceae Spreng. ex F.Rudolphi
Iz_5_1009	DJI_0841	Poa annua L.	0	n	1	0	Poaceae Barnhart
Iz_5_1009	DJI_0841	Poa nemoralis L.	0	n	1	0	Poaceae Barnhart

Iz_5_1009	DJI_0841	Poa sp.	0		NA	0	Poaceae Barnhart
Iz_5_1009	DJI_0841	Polygonatum odoratum (Mill.) Druce	0	n	1	0	Asparagaceae Juss.
Iz_5_1009	DJI_0841	Polygonum arenarium Waldst. & Kit.	0	n	1	0	Polygonaceae Juss.
Iz_5_1009	DJI_0841	Populus nigra L.	0	n	1	1	Salicaceae Mirb.
Iz_5_1009	DJI_0841	Populus sp.	0	n	1	1	Salicaceae Mirb.
Iz_5_1009	DJI_0841	Populus tremula L.	0	n	1	1	Salicaceae Mirb.
Iz_5_1009	DJI_0841	Populus Ã canescens (Aiton) Sm.	0	n	1	1	Salicaceae Mirb.
Iz_5_1009	DJI_0841	Potentilla argentea L.	0	n	1	0	Rosaceae Juss.
Iz_5_1009	DJI_0841	Psammophiliella muralis (L.) Ikonn.	0	n	1	0	Caryophyllaceae Juss.
Iz_5_1009	DJI_0841	Psephellus sumensis (Kalen.) Greuter	0	n	1	0	Asteraceae Bercht. & J.Presl
Iz_5_1009	DJI_0841	Pulsatilla pratensis (L.) Mill. s.l.	0	n	1	0	Ranunculaceae Juss.
Iz_5_1009	DJI_0841	Pyrus communis L.	0	a	0	1	Rosaceae Juss.
Iz_5_1009	DJI_0841	Quercus robur L.	0	n	1	1	Fagaceae Dumort.
Iz_5_1009	DJI_0841	Robinia pseudoacacia L.	0	a	0	1	Fabaceae Lindl.
Iz_5_1009	DJI_0841	Rosa sp.	0		NA	1	Rosaceae Juss.
Iz_5_1009	DJI_0841	Rubus idaeus L.	0	n	1	0	Rosaceae Juss.
Iz_5_1009	DJI_0841	Rumex acetosella L.	0	n	1	0	Polygonaceae Juss.
Iz_5_1009	DJI_0841	Rumex thyrsiflorus Fingerh.	0	n	1	0	Polygonaceae Juss.
Iz_5_1009	DJI_0841	Salix alba L.	0	n	1	1	Salicaceae Mirb.
Iz_5_1009	DJI_0841	Sambucus nigra L.	0	n	1	1	Viburnaceae Raf.
Iz_5_1009	DJI_0841	Secale sylvestre Host	0	n	1	0	Poaceae Barnhart
Iz_5_1009	DJI_0841	Sempervivum ruthenicum Schnittsp. & C.B. Lehm.	0	n	1	0	Crassulaceae J.St.-Hil.
Iz_5_1009	DJI_0841	Setaria viridis (L.) P. Beauv.	0	n	1	0	Poaceae Barnhart
Iz_5_1009	DJI_0841	Silene borysthenica (Gruner) Walters	0	n	1	0	Caryophyllaceae Juss.
Iz_5_1009	DJI_0841	Silene latifolia subsp. alba (Mill.) Greuter & Burdet	0	n	1	0	Caryophyllaceae Juss.
Iz_5_1009	DJI_0841	Solidago virgaurea L.	0	n	1	0	Asteraceae Bercht. & J.Presl
Iz_5_1009	DJI_0841	Solidago Ã niederederi (S. canadensis Ã S. virgaurea)	0	hybrid	0	0	Asteraceae Bercht. & J.Presl
Iz_5_1009	DJI_0841	Sorbus aucuparia L.	0	n	1	1	Rosaceae Juss.
Iz_5_1009	DJI_0841	Stachys recta L.	0	n	1	0	Lamiacea Lindl.
Iz_5_1009	DJI_0841	Tanacetum vulgare L.	0	n	1	0	Asteraceae Bercht. & J.Presl
Iz_5_1009	DJI_0841	Thymus pallasianus Heinr.Braun	0	n	1	0	Lamiacea Lindl.
Iz_5_1009	DJI_0841	Tilia cordata Mill.	0	n	1	1	Malvaceae Juss.
Iz_5_1009	DJI_0841	Trifolium alpestre L.	0	n	1	0	Fabaceae Lindl.
Iz_5_1009	DJI_0841	Trifolium arvense L.	0	n	1	0	Fabaceae Lindl.
Iz_5_1009	DJI_0841	Ulmus glabra Huds.	0	n	1	1	Ulmaceae Mirb.
Iz_5_1009	DJI_0841	Verbascum densiflorum Bertol.	0	n	1	0	Scrophulariaceae Juss.
Iz_5_1009	DJI_0841	Veronica austriaca L.	0	n	1	0	Plantaginaceae Juss.
Iz_5_1009	DJI_0841	Veronica incana L.	0	n	1	0	Plantaginaceae Juss.
Iz_5_1009	DJI_0841	Veronica verna L.	0	n	1	0	Plantaginaceae Juss.
Iz_5_1009	DJI_0841	Vincetoxicum hirundinaria Medik	0	n	1	0	Apocynaceae Juss.
Iz_5_1009	DJI_0843	Acer tataricum L.	0	n	1	1	Sapindaceae Juss.
Iz_5_1009	DJI_0843	Achillea millefolium L. s.l.	0	n	1	0	Asteraceae Bercht. & J.Presl
Iz_5_1009	DJI_0843	Allium oleraceum L.	0	n	1	0	Amaryllidaceae J.St.-Hil.
Iz_5_1009	DJI_0843	Ambrosia artemisiifolia L.	0	a	0	0	Asteraceae Bercht. & J.Presl
Iz_5_1009	DJI_0843	Amorpha fruticosa L.	0	a	0	0	Fabaceae Lindl.
Iz_5_1009	DJI_0843	Anthericum ramosum L.	0	n	1	0	Asparagaceae Juss.
Iz_5_1009	DJI_0843	Artemisia marschalliana Spreng.	0	n	1	0	Asteraceae Bercht. & J.Presl
Iz_5_1009	DJI_0843	Asparagus officinalis L.	0	n	1	0	Asparagaceae Juss.
Iz_5_1009	DJI_0843	Astragalus cicer L.	0	n	1	0	Fabaceae Lindl.
Iz_5_1009	DJI_0843	Bassia laniflora (S.G.Gmel.) A.J.Scott	0	n	1	0	Amaranthaceae Juss.
Iz_5_1009	DJI_0843	Berberis vulgaris L.	0	n	1	0	Berberidaceae Juss.
Iz_5_1009	DJI_0843	Betula pendula Roth.	0	n	1	1	Betulaceae Gray
Iz_5_1009	DJI_0843	Betula pendula Roth.	0	n	1	0	Poaceae Barnhart
Iz_5_1009	DJI_0843	Bromus inermis Leyss.	0		NA	NA	
Iz_5_1009	DJI_0843	Bromus squarrosus L.	0	a	0	0	Poaceae Barnhart
Iz_5_1009	DJI_0843	Bromus tectorum L.	0	a	0	0	Poaceae Barnhart
Iz_5_1009	DJI_0843	Calamagrostis epigejos (L.) Roth	5	n	1	0	Poaceae Barnhart
Iz_5_1009	DJI_0843	Carduus acanthoides L.	0	n	1	0	Asteraceae Bercht. & J.Presl
Iz_5_1009	DJI_0843	Carex sp.	0	n	1	0	Cyperaceae Juss.
Iz_5_1009	DJI_0843	Centaurea majorovii Dumbadze	0	n	1	0	Asteraceae Bercht. & J.Presl
Iz_5_1009	DJI_0843	Chamaecytisus ruthenicus (Fisch.ex Wol.) Klaskova	0	n	1	0	Fabaceae Lindl.
Iz_5_1009	DJI_0843	Chelidonium majus L.	0	n	1	0	Papaveraceae Juss.
Iz_5_1009	DJI_0843	Chenopodium betaceum Andrz.	0	n	1	0	Amaranthaceae Juss.
Iz_5_1009	DJI_0843	Chenopodium cf. album	0	n	1	0	Amaranthaceae Juss.
Iz_5_1009	DJI_0843	Chenopodium sp.	0		NA	0	Amaranthaceae Juss.
Iz_5_1009	DJI_0843	Chondrilla juncea L.	0	n	1	0	Asteraceae Bercht. & J.Presl
Iz_5_1009	DJI_0843	Cirsium vulgare (Savi) Ten.	0	n	1	0	Asteraceae Bercht. & J.Presl
Iz_5_1009	DJI_0843	Clinopodium acinos (L.) Kuntze	0	n	1	0	Lamiacea Lindl.
Iz_5_1009	DJI_0843	Convallaria majalis L.	0	n	1	0	Asparagaceae Juss.
Iz_5_1009	DJI_0843	Crataegus rhipidophylla Gand.	0	n	1	1	Rosaceae Juss.
Iz_5_1009	DJI_0843	Crepis foetida L.	0	n	1	0	Asteraceae Bercht. & J.Presl
Iz_5_1009	DJI_0843	Dactylis glomerata L.	0	n	1	0	Poaceae Barnhart
Iz_5_1009	DJI_0843	Dianthus campestris M.Bieb.	0	n	1	0	Caryophyllaceae Juss.
Iz_5_1009	DJI_0843	Digitaria sanguinalis (L.) Scop.	0	a	0	0	Poaceae Barnhart
Iz_5_1009	DJI_0843	Elymus repens (L.) Gould	0	n	1	0	Poaceae Barnhart
Iz_5_1009	DJI_0843	Epilobium tetragonum L.	0	n	1	0	Onagraceae Juss.
Iz_5_1009	DJI_0843	Eragrostis minor Host	0	a	0	0	Poaceae Barnhart
Iz_5_1009	DJI_0843	Erigeron canadensis L.	0	a	0	0	Asteraceae Bercht. & J.Presl
Iz_5_1009	DJI_0843	Euphorbia seguieriana Neck.	0	n	1	0	Euphorbiaceae Juss.
Iz_5_1009	DJI_0843	Fallopia dumetorum (L.) Holub	0	n	1	0	Polygonaceae Juss.
Iz_5_1009	DJI_0843	Festuca beckeri (Hack.) Trautv.	0	n	1	0	Poaceae Barnhart
Iz_5_1009	DJI_0843	Fragaria vesca L.	0	n	1	0	Rosaceae Juss.
Iz_5_1009	DJI_0843	Frangula alnus Mill.	0	n	1	1	Rhamnaceae Juss.
Iz_5_1009	DJI_0843	Fraxinus pennsylvanica Marshall	0	a	0	1	Oleaceae Hoffmanns. & Link
Iz_5_1009	DJI_0843	Galeopsis bifida Boenn.	0	n	1	0	Lamiacea Lindl.
Iz_5_1009	DJI_0843	Galium verum L.	0	n	1	0	Rubiaceae Juss.
Iz_5_1009	DJI_0843	Genista tinctoria L.	0	n	1	0	Fabaceae Lindl.
Iz_5_1009	DJI_0843	Geranium robertianum L.	0	n	1	0	Geraniaceae Juss.
Iz_5_1009	DJI_0843	Geranium sanquineum L.	0	n	1	0	Geraniaceae Juss.
Iz_5_1009	DJI_0843	Gypsophila paniculata L.	0	n	1	0	Caryophyllaceae Juss.
Iz_5_1009	DJI_0843	Helichrysum arenarium (L.) Moench	0	n	1	0	Asteraceae Bercht. & J.Presl
Iz_5_1009	DJI_0843	Herniaria polygama J.Gay	0	n	1	0	Caryophyllaceae Juss.
Iz_5_1009	DJI_0843	Hieracium robustum Fr.	0	n	1	0	Asteraceae Bercht. & J.Presl
Iz_5_1009	DJI_0843	Hieracium umbellatum L.	0	n	1	NA	Asteraceae Bercht. & J.Presl
Iz_5_1009	DJI_0843	Hylotelephium maximum (L.) Holub	0	n	1	0	Crassulaceae J.St.-Hil.
Iz_5_1009	DJI_0843	Jacobaea borysthenica (DC.) B.Nord. & Greuter	0	n	1	0	Asteraceae Bercht. & J.Presl
Iz_5_1009	DJI_0843	Jasione montana L.	0	n	1	0	Campanulaceae Juss.
Iz_5_1009	DJI_0843	Jurinea cyanoides (L.) Rchb.s.l.	0	n	1	0	Asteraceae Bercht. & J.Presl

Iz_5_1009	DJI_0843	Koeleria glauca (Spreng.) DC.	0	n	1	0	Poaceae Barnhart
Iz_5_1009	DJI_0843	Lactuca serriola L.	0	a	0	0	Asteraceae Bercht. & J.Presl
Iz_5_1009	DJI_0843	Linaria genistifolia (L.) Mill.	0	n	1	0	Plantaginaceae Juss.
Iz_5_1009	DJI_0843	Lomelosia argentea (L.) Greuter & Burdet	0	n	1	0	Caprifoliaceae Juss.
Iz_5_1009	DJI_0843	Melampyrum pratense L.	0	n	1	0	Orobanchaceae Vent.
Iz_5_1009	DJI_0843	Oenothera biennis L.	0	a	0	0	Onagraceae Juss.
Iz_5_1009	DJI_0843	Pentanema britannica (L.) D.Gut.Larr., Santos-Vicente, Anderb., E.Rico & M.M.Mart.Ort.	0	n	1	0	Asteraceae Bercht. & J.Presl
Iz_5_1009	DJI_0843	Peucedanum oreoselinum (L.) Moench	1	n	1	0	Apiaceae Lindl.
Iz_5_1009	DJI_0843	Peucedanum ruthenicum M.Bieb.	0	n	1	0	Apiaceae Lindl.
Iz_5_1009	DJI_0843	Pilosella echioides (Lumn.) F.W.Schultz & Sch.Bip.	0	n	1	0	Asteraceae Bercht. & J.Presl
Iz_5_1009	DJI_0843	Pilosella officinarum F. Schultz et Sch. Bip.	0	n	1	0	Asteraceae Bercht. & J.Presl
Iz_5_1009	DJI_0843	Pilosella sp.	0		NA	0	Asteraceae Bercht. & J.Presl
Iz_5_1009	DJI_0843	Pinus sylvestris L.	0	n	1	1	Pinaceae Spreng. ex F.Rudolphi
Iz_5_1009	DJI_0843	Poa annua L.	0	n	1	0	Poaceae Barnhart
Iz_5_1009	DJI_0843	Poa nemoralis L.	0	n	1	0	Poaceae Barnhart
Iz_5_1009	DJI_0843	Poa sp.	0		NA	0	Poaceae Barnhart
Iz_5_1009	DJI_0843	Polygonatum odoratum (Mill.) Druce	0.5	n	1	0	Asparagaceae Juss.
Iz_5_1009	DJI_0843	Polygonum arenarium Waldst. & Kit.	0	n	1	0	Polygonaceae Juss.
Iz_5_1009	DJI_0843	Populus nigra L.	0	n	1	1	Salicaceae Mirb.
Iz_5_1009	DJI_0843	Populus sp.	0	n	1	1	Salicaceae Mirb.
Iz_5_1009	DJI_0843	Populus tremula L.	0	n	1	1	Salicaceae Mirb.
Iz_5_1009	DJI_0843	Populus Ã canescens (Aiton) Sm.	0	n	1	1	Salicaceae Mirb.
Iz_5_1009	DJI_0843	Potentilla argentea L.	0	n	1	0	Rosaceae Juss.
Iz_5_1009	DJI_0843	Psammophiliella muralis (L.) Ikonn.	0	n	1	0	Caryophyllaceae Juss.
Iz_5_1009	DJI_0843	Psephellus sumensis (Kalen.) Greuter	0	n	1	0	Asteraceae Bercht. & J.Presl
Iz_5_1009	DJI_0843	Pulsatilla pratensis (L.) Mill. s.l.	0	n	1	0	Ranunculaceae Juss.
Iz_5_1009	DJI_0843	Pyrus communis L.	0	a	0	1	Rosaceae Juss.
Iz_5_1009	DJI_0843	Quercus robur L.	0	n	1	1	Fagaceae Dumort.
Iz_5_1009	DJI_0843	Robinia pseudoacacia L.	0	a	0	1	Fabaceae Lindl.
Iz_5_1009	DJI_0843	Rosa sp.	0		NA	1	Rosaceae Juss.
Iz_5_1009	DJI_0843	Rubus idaeus L.	0	n	1	0	Rosaceae Juss.
Iz_5_1009	DJI_0843	Rumex acetosella L.	0	n	1	0	Polygonaceae Juss.
Iz_5_1009	DJI_0843	Rumex thyrsiflorus Fingerh.	0	n	1	0	Polygonaceae Juss.
Iz_5_1009	DJI_0843	Salix alba L.	0	n	1	1	Salicaceae Mirb.
Iz_5_1009	DJI_0843	Sambucus nigra L.	0	n	1	1	Viburnaceae Raf.
Iz_5_1009	DJI_0843	Secale sylvestre Host	0	n	1	0	Poaceae Barnhart
Iz_5_1009	DJI_0843	Sempervivum ruthenicum Schnittsp. & C.B. Lehm.	0	n	1	0	Crassulaceae J.St.-Hil.
Iz_5_1009	DJI_0843	Setaria viridis (L.) P. Beauv.	0	n	1	0	Poaceae Barnhart
Iz_5_1009	DJI_0843	Silene borysthenica (Gruner) Walters	0	n	1	0	Caryophyllaceae Juss.
Iz_5_1009	DJI_0843	Silene latifolia subsp. alba (Mill.) Greuter & Burdet	0	n	1	0	Caryophyllaceae Juss.
Iz_5_1009	DJI_0843	Solidago virgaurea L.	2	n	1	0	Asteraceae Bercht. & J.Presl
Iz_5_1009	DJI_0843	Solidago Ã niederederi (S. canadensis Ã S. virgaurea)	0	hybrid	0	0	Asteraceae Bercht. & J.Presl
Iz_5_1009	DJI_0843	Sorbus aucuparia L.	0	n	1	1	Rosaceae Juss.
Iz_5_1009	DJI_0843	Stachys recta L.	0	n	1	0	Lamiacea Lindl.
Iz_5_1009	DJI_0843	Tanacetum vulgare L.	0	n	1	0	Asteraceae Bercht. & J.Presl
Iz_5_1009	DJI_0843	Thymus pallasianus Heinr.Braun	0	n	1	0	Lamiacea Lindl.
Iz_5_1009	DJI_0843	Tilia cordata Mill.	0	n	1	1	Malvaceae Juss.
Iz_5_1009	DJI_0843	Trifolium alpestre L.	0	n	1	0	Fabaceae Lindl.
Iz_5_1009	DJI_0843	Trifolium arvense L.	0	n	1	0	Fabaceae Lindl.
Iz_5_1009	DJI_0843	Ulmus glabra Huds.	0	n	1	1	Ulmaceae Mirb.
Iz_5_1009	DJI_0843	Verbascum densiflorum Bertol.	0	n	1	0	Scrophulariaceae Juss.
Iz_5_1009	DJI_0843	Veronica austriaca L.	0	n	1	0	Plantaginaceae Juss.
Iz_5_1009	DJI_0843	Veronica incana L.	0	n	1	0	Plantaginaceae Juss.
Iz_5_1009	DJI_0843	Veronica verna L.	0	n	1	0	Plantaginaceae Juss.
Iz_5_1009	DJI_0843	Vincetoxicum hirundinaria Medik	0	n	1	0	Apocynaceae Juss.
Iz_5_1009	DJI_0851	Acer tataricum L.	0	n	1	1	Sapindaceae Juss.
Iz_5_1009	DJI_0851	Achillea millefolium L. s.l.	0	n	1	0	Asteraceae Bercht. & J.Presl
Iz_5_1009	DJI_0851	Allium oleraceum L.	0	n	1	0	Amaryllidaceae J.St.-Hil.
Iz_5_1009	DJI_0851	Ambrosia artemisiifolia L.	0	a	0	0	Asteraceae Bercht. & J.Presl
Iz_5_1009	DJI_0851	Amorpha fruticosa L.	0	a	0	0	Fabaceae Lindl.
Iz_5_1009	DJI_0851	Anthericum ramosum L.	0	n	1	0	Asparagaceae Juss.
Iz_5_1009	DJI_0851	Artemisia marschalliana Spreng.	0	n	1	0	Asteraceae Bercht. & J.Presl
Iz_5_1009	DJI_0851	Asparagus officinalis L.	0	n	1	0	Asparagaceae Juss.
Iz_5_1009	DJI_0851	Astragalus cicer L.	0	n	1	0	Fabaceae Lindl.
Iz_5_1009	DJI_0851	Bassia laniflora (S.G.Gmel.) A.J.Scott	0	n	1	0	Amaranthaceae Juss.
Iz_5_1009	DJI_0851	Berberis vulgaris L.	0	n	1	0	Berberidaceae Juss.
Iz_5_1009	DJI_0851	Betula pendula Roth.	0	n	1	1	Betulaceae Gray
Iz_5_1009	DJI_0851	Betula pendula Roth.	0	n	1	0	Poaceae Barnhart
Iz_5_1009	DJI_0851	Bromus inermis Leyss.	0		NA	NA	
Iz_5_1009	DJI_0851	Bromus squarrosus L.	0	a	0	0	Poaceae Barnhart
Iz_5_1009	DJI_0851	Bromus tectorum L.	0	a	0	0	Poaceae Barnhart
Iz_5_1009	DJI_0851	Calamagrostis epigejos (L.) Roth	20	n	1	0	Poaceae Barnhart
Iz_5_1009	DJI_0851	Carduus acanthoides L.	0	n	1	0	Asteraceae Bercht. & J.Presl
Iz_5_1009	DJI_0851	Carex sp.	0	n	1	0	Cyperaceae Juss.
Iz_5_1009	DJI_0851	Centaurea majorovii Dumbadze	0	n	1	0	Asteraceae Bercht. & J.Presl
Iz_5_1009	DJI_0851	Chamaecytisus ruthenicus (Fisch.ex Wol.) Klaskova	0	n	1	0	Fabaceae Lindl.
Iz_5_1009	DJI_0851	Chelidonium majus L.	0	n	1	0	Papaveraceae Juss.
Iz_5_1009	DJI_0851	Chenopodium betaceum Andrz.	0	n	1	0	Amaranthaceae Juss.
Iz_5_1009	DJI_0851	Chenopodium cf. album	0	n	1	0	Amaranthaceae Juss.
Iz_5_1009	DJI_0851	Chenopodium sp.	0		NA	0	Amaranthaceae Juss.
Iz_5_1009	DJI_0851	Chondrilla juncea L.	0	n	1	0	Asteraceae Bercht. & J.Presl
Iz_5_1009	DJI_0851	Cirsium vulgare (Savi) Ten.	0	n	1	0	Asteraceae Bercht. & J.Presl
Iz_5_1009	DJI_0851	Clinopodium acinos (L.) Kuntze	0	n	1	0	Lamiacea Lindl.
Iz_5_1009	DJI_0851	Convallaria majalis L.	0	n	1	0	Asparagaceae Juss.
Iz_5_1009	DJI_0851	Crataegus rhipidophylla Gand.	0	n	1	1	Rosaceae Juss.
Iz_5_1009	DJI_0851	Crepis foetida L.	0	n	1	0	Asteraceae Bercht. & J.Presl
Iz_5_1009	DJI_0851	Dactylis glomerata L.	0	n	1	0	Poaceae Barnhart
Iz_5_1009	DJI_0851	Dianthus campestris M.Bieb.	0	n	1	0	Caryophyllaceae Juss.
Iz_5_1009	DJI_0851	Digitaria sanguinalis (L.) Scop.	0	a	0	0	Poaceae Barnhart
Iz_5_1009	DJI_0851	Elymus repens (L.) Gould	0	n	1	0	Poaceae Barnhart
Iz_5_1009	DJI_0851	Epilobium tetragonum L.	0	n	1	0	Onagraceae Juss.
Iz_5_1009	DJI_0851	Eragrostis minor Host	0	a	0	0	Poaceae Barnhart
Iz_5_1009	DJI_0851	Erigeron canadensis L.	0	a	0	0	Asteraceae Bercht. & J.Presl
Iz_5_1009	DJI_0851	Euphorbia seguieriana Neck.	0	n	1	0	Euphorbiaceae Juss.
Iz_5_1009	DJI_0851	Fallopia dumetorum (L.) Holub	0	n	1	0	Polygonaceae Juss.
Iz_5_1009	DJI_0851	Festuca beckeri (Hack.) Trautv.	0	n	1	0	Poaceae Barnhart
Iz_5_1009	DJI_0851	Fragaria vesca L.	0	n	1	0	Rosaceae Juss.
Iz_5_1009	DJI_0851	Frangula alnus Mill.	0	n	1	1	Rhamnaceae Juss.

Iz_5_1009	DJI_0851	Fraxinus pennsylvanica Marshall	0	a	0	1	Oleaceae Hoffmanns. & Link
Iz_5_1009	DJI_0851	Galeopsis bifida Boenn.	0	n	1	0	Lamiacea Lindl.
Iz_5_1009	DJI_0851	Galium verum L.	0	n	1	0	Rubiaceae Juss.
Iz_5_1009	DJI_0851	Genista tinctoria L.	0	n	1	0	Fabaceae Lindl.
Iz_5_1009	DJI_0851	Geranium robertianum L.	0	n	1	0	Geraniaceae Juss.
Iz_5_1009	DJI_0851	Geranium sanquineum L.	0	n	1	0	Geraniaceae Juss.
Iz_5_1009	DJI_0851	Gypsophila paniculata L.	0	n	1	0	Caryophyllaceae Juss.
Iz_5_1009	DJI_0851	Helichrysum arenarium (L.) Moench	0	n	1	0	Asteraceae Bercht. & J.Presl
Iz_5_1009	DJI_0851	Herniaria polygama J.Gay	0	n	1	0	Caryophyllaceae Juss.
Iz_5_1009	DJI_0851	Hieracium robustum Fr.	0	n	1	0	Asteraceae Bercht. & J.Presl
Iz_5_1009	DJI_0851	Hieracium umbellatum L.	0	n	1	NA	Asteraceae Bercht. & J.Presl
Iz_5_1009	DJI_0851	Hylotelephium maximum (L.) Holub	0	n	1	0	Crassulaceae J.St.-Hil.
Iz_5_1009	DJI_0851	Jacobaea borysthenica (DC.) B.Nord. & Greuter	0	n	1	0	Asteraceae Bercht. & J.Presl
Iz_5_1009	DJI_0851	Jasione montana L.	0	n	1	0	Campanulaceae Juss.
Iz_5_1009	DJI_0851	Jurinea cyanoides (L.) Rchb.s.l.	0	n	1	0	Asteraceae Bercht. & J.Presl
Iz_5_1009	DJI_0851	Koeleria glauca (Spreng.) DC.	0	n	1	0	Poaceae Barnhart
Iz_5_1009	DJI_0851	Lactuca serriola L.	0	a	0	0	Asteraceae Bercht. & J.Presl
Iz_5_1009	DJI_0851	Linaria genistifolia (L.) Mill.	0	n	1	0	Plantaginaceae Juss.
Iz_5_1009	DJI_0851	Lomelosia argentea (L.) Greuter & Burdet	0	n	1	0	Caprifoliaceae Juss.
Iz_5_1009	DJI_0851	Melampyrum pratense L.	0	n	1	0	Orobanchaceae Vent.
Iz_5_1009	DJI_0851	Oenothera biennis L.	0	a	0	0	Onagraceae Juss.
Iz_5_1009	DJI_0851	Pentanema britannica (L.) D.Gut.Larr., Santos-Vicente, Anderb., E.Rico & M.M.Mart.Ort.	0	n	1	0	Asteraceae Bercht. & J.Presl
Iz_5_1009	DJI_0851	Peucedanum oreoselinum (L.) Moench	0	n	1	0	Apiaceae Lindl.
Iz_5_1009	DJI_0851	Peucedanum ruthenicum M.Bieb.	0	n	1	0	Apiaceae Lindl.
Iz_5_1009	DJI_0851	Pilosella echioides (Lumn.) F.W.Schultz & Sch.Bip.	0	n	1	0	Asteraceae Bercht. & J.Presl
Iz_5_1009	DJI_0851	Pilosella officinarum F. Schultz et Sch. Bip.	0	n	1	0	Asteraceae Bercht. & J.Presl
Iz_5_1009	DJI_0851	Pilosella sp.	0		NA	0	Asteraceae Bercht. & J.Presl
Iz_5_1009	DJI_0851	Pinus sylvestris L.	0	n	1	1	Pinaceae Spreng. ex F.Rudolphi
Iz_5_1009	DJI_0851	Poa annua L.	0	n	1	0	Poaceae Barnhart
Iz_5_1009	DJI_0851	Poa nemoralis L.	0	n	1	0	Poaceae Barnhart
Iz_5_1009	DJI_0851	Poa sp.	0		NA	0	Poaceae Barnhart
Iz_5_1009	DJI_0851	Polygonatum odoratum (Mill.) Druce	0	n	1	0	Asparagaceae Juss.
Iz_5_1009	DJI_0851	Polygonum arenarium Waldst. & Kit.	0	n	1	0	Polygonaceae Juss.
Iz_5_1009	DJI_0851	Populus nigra L.	0	n	1	1	Salicaceae Mirb.
Iz_5_1009	DJI_0851	Populus sp.	0	n	1	1	Salicaceae Mirb.
Iz_5_1009	DJI_0851	Populus tremula L.	0	n	1	1	Salicaceae Mirb.
Iz_5_1009	DJI_0851	Populus Ã canescens (Aiton) Sm.	0	n	1	1	Salicaceae Mirb.
Iz_5_1009	DJI_0851	Potentilla argentea L.	0	n	1	0	Rosaceae Juss.
Iz_5_1009	DJI_0851	Psammophiliella muralis (L.) Ikonn.	0	n	1	0	Caryophyllaceae Juss.
Iz_5_1009	DJI_0851	Psephellus sumensis (Kalen.) Greuter	0	n	1	0	Asteraceae Bercht. & J.Presl
Iz_5_1009	DJI_0851	Pulsatilla pratensis (L.) Mill. s.l.	0	n	1	0	Ranunculaceae Juss.
Iz_5_1009	DJI_0851	Pyrus communis L.	0	a	0	1	Rosaceae Juss.
Iz_5_1009	DJI_0851	Quercus robur L.	0	n	1	1	Fagaceae Dumort.
Iz_5_1009	DJI_0851	Robinia pseudoacacia L.	0	a	0	1	Fabaceae Lindl.
Iz_5_1009	DJI_0851	Rosa sp.	0		NA	1	Rosaceae Juss.
Iz_5_1009	DJI_0851	Rubus idaeus L.	0	n	1	0	Rosaceae Juss.
Iz_5_1009	DJI_0851	Rumex acetosella L.	0	n	1	0	Polygonaceae Juss.
Iz_5_1009	DJI_0851	Rumex thyrsiflorus Fingerh.	0	n	1	0	Polygonaceae Juss.
Iz_5_1009	DJI_0851	Salix alba L.	0	n	1	1	Salicaceae Mirb.
Iz_5_1009	DJI_0851	Sambucus nigra L.	0	n	1	1	Viburnaceae Raf.
Iz_5_1009	DJI_0851	Secale sylvestre Host	0	n	1	0	Poaceae Barnhart
Iz_5_1009	DJI_0851	Sempervivum ruthenicum Schnittsp. & C.B. Lehm.	0	n	1	0	Crassulaceae J.St.-Hil.
Iz_5_1009	DJI_0851	Setaria viridis (L.) P. Beauv.	0	n	1	0	Poaceae Barnhart
Iz_5_1009	DJI_0851	Silene borysthenica (Gruner) Walters	0	n	1	0	Caryophyllaceae Juss.
Iz_5_1009	DJI_0851	Silene latifolia subsp. alba (Mill.) Greuter & Burdet	0	n	1	0	Caryophyllaceae Juss.
Iz_5_1009	DJI_0851	Solidago virgaurea L.	5	n	1	0	Asteraceae Bercht. & J.Presl
Iz_5_1009	DJI_0851	Solidago Ã niederederi (S. canadensis Ã S. virgaurea)	0	hybrid	0	0	Asteraceae Bercht. & J.Presl
Iz_5_1009	DJI_0851	Sorbus aucuparia L.	0	n	1	1	Rosaceae Juss.
Iz_5_1009	DJI_0851	Stachys recta L.	0	n	1	0	Lamiacea Lindl.
Iz_5_1009	DJI_0851	Tanacetum vulgare L.	0	n	1	0	Asteraceae Bercht. & J.Presl
Iz_5_1009	DJI_0851	Thymus pallasianus Heinr.Braun	0	n	1	0	Lamiacea Lindl.
Iz_5_1009	DJI_0851	Tilia cordata Mill.	0	n	1	1	Malvaceae Juss.
Iz_5_1009	DJI_0851	Trifolium alpestre L.	0	n	1	0	Fabaceae Lindl.
Iz_5_1009	DJI_0851	Trifolium arvense L.	0	n	1	0	Fabaceae Lindl.
Iz_5_1009	DJI_0851	Ulmus glabra Huds.	0	n	1	1	Ulmaceae Mirb.
Iz_5_1009	DJI_0851	Verbascum densiflorum Bertol.	0	n	1	0	Scrophulariaceae Juss.
Iz_5_1009	DJI_0851	Veronica austriaca L.	0	n	1	0	Plantaginaceae Juss.
Iz_5_1009	DJI_0851	Veronica incana L.	0	n	1	0	Plantaginaceae Juss.
Iz_5_1009	DJI_0851	Veronica verna L.	0	n	1	0	Plantaginaceae Juss.
Iz_5_1009	DJI_0851	Vincetoxicum hirundinaria Medik	0	n	1	0	Apocynaceae Juss.
Iz_5_1009	DJI_0858	Acer tataricum L.	0	n	1	1	Sapindaceae Juss.
Iz_5_1009	DJI_0858	Achillea millefolium L. s.l.	0	n	1	0	Asteraceae Bercht. & J.Presl
Iz_5_1009	DJI_0858	Allium oleraceum L.	0	n	1	0	Amaryllidaceae J.St.-Hil.
Iz_5_1009	DJI_0858	Ambrosia artemisiifolia L.	0	a	0	0	Asteraceae Bercht. & J.Presl
Iz_5_1009	DJI_0858	Amorpha fruticosa L.	0	a	0	0	Fabaceae Lindl.
Iz_5_1009	DJI_0858	Anthericum ramosum L.	0	n	1	0	Asparagaceae Juss.
Iz_5_1009	DJI_0858	Artemisia marschalliana Spreng.	0	n	1	0	Asteraceae Bercht. & J.Presl
Iz_5_1009	DJI_0858	Asparagus officinalis L.	0	n	1	0	Asparagaceae Juss.
Iz_5_1009	DJI_0858	Astragalus cicer L.	0	n	1	0	Fabaceae Lindl.
Iz_5_1009	DJI_0858	Bassia laniflora (S.G.Gmel.) A.J.Scott	0	n	1	0	Amaranthaceae Juss.
Iz_5_1009	DJI_0858	Berberis vulgaris L.	0	n	1	0	Berberidaceae Juss.
Iz_5_1009	DJI_0858	Betula pendula Roth.	0	n	1	1	Betulaceae Gray
Iz_5_1009	DJI_0858	Betula pendula Roth.	0	n	1	0	Poaceae Barnhart
Iz_5_1009	DJI_0858	Bromus inermis Leyss.	0		NA	NA	
Iz_5_1009	DJI_0858	Bromus squarrosus L.	0	a	0	0	Poaceae Barnhart
Iz_5_1009	DJI_0858	Bromus tectorum L.	0	a	0	0	Poaceae Barnhart
Iz_5_1009	DJI_0858	Calamagrostis epigejos (L.) Roth	20	n	1	0	Poaceae Barnhart
Iz_5_1009	DJI_0858	Carduus acanthoides L.	0	n	1	0	Asteraceae Bercht. & J.Presl
Iz_5_1009	DJI_0858	Carex sp.	0	n	1	0	Cyperaceae Juss.
Iz_5_1009	DJI_0858	Centaurea majorovii Dumbadze	0	n	1	0	Asteraceae Bercht. & J.Presl
Iz_5_1009	DJI_0858	Chamaecytisus ruthenicus (Fisch.ex Wol.) Klaskova	0	n	1	0	Fabaceae Lindl.
Iz_5_1009	DJI_0858	Chelidonium majus L.	0	n	1	0	Papaveraceae Juss.
Iz_5_1009	DJI_0858	Chenopodium betaceum Andrz.	0	n	1	0	Amaranthaceae Juss.
Iz_5_1009	DJI_0858	Chenopodium cf. album	0	n	1	0	Amaranthaceae Juss.
Iz_5_1009	DJI_0858	Chenopodium sp.	0		NA	0	Amaranthaceae Juss.
Iz_5_1009	DJI_0858	Chondrilla juncea L.	0	n	1	0	Asteraceae Bercht. & J.Presl
Iz_5_1009	DJI_0858	Cirsium vulgare (Savi) Ten.	0	n	1	0	Asteraceae Bercht. & J.Presl
Iz_5_1009	DJI_0858	Clinopodium acinos (L.) Kuntze	0	n	1	0	Lamiacea Lindl.

Iz_5_1009	DJI_0858	Convallaria majalis L.	0	n	1	0	Asparagaceae Juss.
Iz_5_1009	DJI_0858	Crataegus rhipidophylla Gand.	0	n	1	1	Rosaceae Juss.
Iz_5_1009	DJI_0858	Crepis foetida L.	0	n	1	0	Asteraceae Bercht. & J.Presl
Iz_5_1009	DJI_0858	Dactylis glomerata L.	0	n	1	0	Poaceae Barnhart
Iz_5_1009	DJI_0858	Dianthus campestris M.Bieb.	0	n	1	0	Caryophyllaceae Juss.
Iz_5_1009	DJI_0858	Digitaria sanguinalis (L.) Scop.	0	a	0	0	Poaceae Barnhart
Iz_5_1009	DJI_0858	Elymus repens (L.) Gould	0	n	1	0	Poaceae Barnhart
Iz_5_1009	DJI_0858	Epilobium tetragonum L.	0	n	1	0	Onagraceae Juss.
Iz_5_1009	DJI_0858	Eragrostis minor Host	0	a	0	0	Poaceae Barnhart
Iz_5_1009	DJI_0858	Erigeron canadensis L.	0	a	0	0	Asteraceae Bercht. & J.Presl
Iz_5_1009	DJI_0858	Euphorbia seguieriana Neck.	0	n	1	0	Euphorbiaceae Juss.
Iz_5_1009	DJI_0858	Fallopia dumetorum (L.) Holub	0	n	1	0	Polygonaceae Juss.
Iz_5_1009	DJI_0858	Festuca beckeri (Hack.) Trautv.	0	n	1	0	Poaceae Barnhart
Iz_5_1009	DJI_0858	Fragaria vesca L.	0	n	1	0	Rosaceae Juss.
Iz_5_1009	DJI_0858	Frangula alnus Mill.	0	n	1	1	Rhamnaceae Juss.
Iz_5_1009	DJI_0858	Fraxinus pennsylvanica Marshall	0	a	0	1	Oleaceae Hoffmanns. & Link
Iz_5_1009	DJI_0858	Galeopsis bifida Boenn.	0	n	1	0	Lamiacea Lindl.
Iz_5_1009	DJI_0858	Galium verum L.	0	n	1	0	Rubiaceae Juss.
Iz_5_1009	DJI_0858	Genista tinctoria L.	0	n	1	0	Fabaceae Lindl.
Iz_5_1009	DJI_0858	Geranium robertianum L.	0	n	1	0	Geraniaceae Juss.
Iz_5_1009	DJI_0858	Geranium sanquineum L.	0	n	1	0	Geraniaceae Juss.
Iz_5_1009	DJI_0858	Gypsophila paniculata L.	0	n	1	0	Caryophyllaceae Juss.
Iz_5_1009	DJI_0858	Helichrysum arenarium (L.) Moench	0	n	1	0	Asteraceae Bercht. & J.Presl
Iz_5_1009	DJI_0858	Herniaria polygama J.Gay	0	n	1	0	Caryophyllaceae Juss.
Iz_5_1009	DJI_0858	Hieracium robustum Fr.	0	n	1	0	Asteraceae Bercht. & J.Presl
Iz_5_1009	DJI_0858	Hieracium umbellatum L.	0	n	1	NA	Asteraceae Bercht. & J.Presl
Iz_5_1009	DJI_0858	Hylotelephium maximum (L.) Holub	0	n	1	0	Crassulaceae J.St.-Hil.
Iz_5_1009	DJI_0858	Jacobaea borysthenica (DC.) B.Nord. & Greuter	0	n	1	0	Asteraceae Bercht. & J.Presl
Iz_5_1009	DJI_0858	Jasione montana L.	0	n	1	0	Campanulaceae Juss.
Iz_5_1009	DJI_0858	Jurinea cyanoides (L.) Rchb.s.l.	0	n	1	0	Asteraceae Bercht. & J.Presl
Iz_5_1009	DJI_0858	Koeleria glauca (Spreng.) DC.	0	n	1	0	Poaceae Barnhart
Iz_5_1009	DJI_0858	Lactuca serriola L.	0	a	0	0	Asteraceae Bercht. & J.Presl
Iz_5_1009	DJI_0858	Linaria genistifolia (L.) Mill.	0	n	1	0	Plantaginaceae Juss.
Iz_5_1009	DJI_0858	Lomelosia argentea (L.) Greuter & Burdet	0	n	1	0	Caprifoliaceae Juss.
Iz_5_1009	DJI_0858	Melampyrum pratense L.	0	n	1	0	Orobanchaceae Vent.
Iz_5_1009	DJI_0858	Oenothera biennis L.	0	a	0	0	Onagraceae Juss.
Iz_5_1009	DJI_0858	Pentanema britannica (L.) D.Gut.Larr., Santos-Vicente, Anderb., E.Rico & M.M.Mart.Ort.	0	n	1	0	Asteraceae Bercht. & J.Presl
Iz_5_1009	DJI_0858	Peucedanum oreoselinum (L.) Moench	2	n	1	0	Apiaceae Lindl.
Iz_5_1009	DJI_0858	Peucedanum ruthenicum M.Bieb.	0	n	1	0	Apiaceae Lindl.
Iz_5_1009	DJI_0858	Pilosella echioides (Lumn.) F.W.Schultz & Sch.Bip.	0	n	1	0	Asteraceae Bercht. & J.Presl
Iz_5_1009	DJI_0858	Pilosella officinarum F. Schultz et Sch. Bip.	0	n	1	0	Asteraceae Bercht. & J.Presl
Iz_5_1009	DJI_0858	Pilosella sp.	0		NA	0	Asteraceae Bercht. & J.Presl
Iz_5_1009	DJI_0858	Pinus sylvestris L.	0	n	1	1	Pinaceae Spreng. ex F.Rudolphi
Iz_5_1009	DJI_0858	Poa annua L.	0	n	1	0	Poaceae Barnhart
Iz_5_1009	DJI_0858	Poa nemoralis L.	0	n	1	0	Poaceae Barnhart
Iz_5_1009	DJI_0858	Poa sp.	0		NA	0	Poaceae Barnhart
Iz_5_1009	DJI_0858	Polygonatum odoratum (Mill.) Druce	0	n	1	0	Asparagaceae Juss.
Iz_5_1009	DJI_0858	Polygonum arenarium Waldst. & Kit.	0	n	1	0	Polygonaceae Juss.
Iz_5_1009	DJI_0858	Populus nigra L.	0	n	1	1	Salicaceae Mirb.
Iz_5_1009	DJI_0858	Populus sp.	0	n	1	1	Salicaceae Mirb.
Iz_5_1009	DJI_0858	Populus tremula L.	0	n	1	1	Salicaceae Mirb.
Iz_5_1009	DJI_0858	Populus Ã canescens (Aiton) Sm.	0	n	1	1	Salicaceae Mirb.
Iz_5_1009	DJI_0858	Potentilla argentea L.	0	n	1	0	Rosaceae Juss.
Iz_5_1009	DJI_0858	Psammophiliella muralis (L.) Ikonn.	0	n	1	0	Caryophyllaceae Juss.
Iz_5_1009	DJI_0858	Psephellus sumensis (Kalen.) Greuter	0	n	1	0	Asteraceae Bercht. & J.Presl
Iz_5_1009	DJI_0858	Pulsatilla pratensis (L.) Mill. s.l.	0	n	1	0	Ranunculaceae Juss.
Iz_5_1009	DJI_0858	Pyrus communis L.	0	a	0	1	Rosaceae Juss.
Iz_5_1009	DJI_0858	Quercus robur L.	0	n	1	1	Fagaceae Dumort.
Iz_5_1009	DJI_0858	Robinia pseudoacacia L.	0	a	0	1	Fabaceae Lindl.
Iz_5_1009	DJI_0858	Rosa sp.	0		NA	1	Rosaceae Juss.
Iz_5_1009	DJI_0858	Rubus idaeus L.	0	n	1	0	Rosaceae Juss.
Iz_5_1009	DJI_0858	Rumex acetosella L.	0	n	1	0	Polygonaceae Juss.
Iz_5_1009	DJI_0858	Rumex thyrsiflorus Fingerh.	0	n	1	0	Polygonaceae Juss.
Iz_5_1009	DJI_0858	Salix alba L.	0	n	1	1	Salicaceae Mirb.
Iz_5_1009	DJI_0858	Sambucus nigra L.	0	n	1	1	Viburnaceae Raf.
Iz_5_1009	DJI_0858	Secale sylvestre Host	0	n	1	0	Poaceae Barnhart
Iz_5_1009	DJI_0858	Sempervivum ruthenicum Schnittsp. & C.B. Lehm.	0	n	1	0	Crassulaceae J.St.-Hil.
Iz_5_1009	DJI_0858	Setaria viridis (L.) P. Beauv.	0	n	1	0	Poaceae Barnhart
Iz_5_1009	DJI_0858	Silene borysthenica (Gruner) Walters	0	n	1	0	Caryophyllaceae Juss.
Iz_5_1009	DJI_0858	Silene latifolia subsp. alba (Mill.) Greuter & Burdet	0	n	1	0	Caryophyllaceae Juss.
Iz_5_1009	DJI_0858	Solidago virgaurea L.	0	n	1	0	Asteraceae Bercht. & J.Presl
Iz_5_1009	DJI_0858	Solidago Ã niederederi (S. canadensis Ã S. virgaurea)	0	hybrid	0	0	Asteraceae Bercht. & J.Presl
Iz_5_1009	DJI_0858	Sorbus aucuparia L.	0	n	1	1	Rosaceae Juss.
Iz_5_1009	DJI_0858	Stachys recta L.	0	n	1	0	Lamiacea Lindl.
Iz_5_1009	DJI_0858	Tanacetum vulgare L.	0	n	1	0	Asteraceae Bercht. & J.Presl
Iz_5_1009	DJI_0858	Thymus pallasianus Heinr.Braun	0	n	1	0	Lamiacea Lindl.
Iz_5_1009	DJI_0858	Tilia cordata Mill.	0	n	1	1	Malvaceae Juss.
Iz_5_1009	DJI_0858	Trifolium alpestre L.	0	n	1	0	Fabaceae Lindl.
Iz_5_1009	DJI_0858	Trifolium arvense L.	0	n	1	0	Fabaceae Lindl.
Iz_5_1009	DJI_0858	Ulmus glabra Huds.	0	n	1	1	Ulmaceae Mirb.
Iz_5_1009	DJI_0858	Verbascum densiflorum Bertol.	0	n	1	0	Scrophulariaceae Juss.
Iz_5_1009	DJI_0858	Veronica austriaca L.	0	n	1	0	Plantaginaceae Juss.
Iz_5_1009	DJI_0858	Veronica incana L.	0	n	1	0	Plantaginaceae Juss.
Iz_5_1009	DJI_0858	Veronica verna L.	0	n	1	0	Plantaginaceae Juss.
Iz_5_1009	DJI_0858	Vincetoxicum hirundinaria Medik	0	n	1	0	Apocynaceae Juss.
Iz_5_1009	DJI_0859	Acer tataricum L.	0	n	1	1	Sapindaceae Juss.
Iz_5_1009	DJI_0859	Achillea millefolium L. s.l.	0	n	1	0	Asteraceae Bercht. & J.Presl
Iz_5_1009	DJI_0859	Allium oleraceum L.	0	n	1	0	Amaryllidaceae J.St.-Hil.
Iz_5_1009	DJI_0859	Ambrosia artemisiifolia L.	0	a	0	0	Asteraceae Bercht. & J.Presl
Iz_5_1009	DJI_0859	Amorpha fruticosa L.	0	a	0	0	Fabaceae Lindl.
Iz_5_1009	DJI_0859	Anthericum ramosum L.	0	n	1	0	Asparagaceae Juss.
Iz_5_1009	DJI_0859	Artemisia marschalliana Spreng.	0	n	1	0	Asteraceae Bercht. & J.Presl
Iz_5_1009	DJI_0859	Asparagus officinalis L.	0	n	1	0	Asparagaceae Juss.
Iz_5_1009	DJI_0859	Astragalus cicer L.	0	n	1	0	Fabaceae Lindl.
Iz_5_1009	DJI_0859	Bassia laniflora (S.G.Gmel.) A.J.Scott	0	n	1	0	Amaranthaceae Juss.
Iz_5_1009	DJI_0859	Berberis vulgaris L.	0	n	1	0	Berberidaceae Juss.
Iz_5_1009	DJI_0859	Betula pendula Roth.	0	n	1	1	Betulaceae Gray
Iz_5_1009	DJI_0859	Betula pendula Roth.	0	n	1	0	Poaceae Barnhart

Iz_5_1009	DJI_0859	Bromus inermis Leyss.	0		NA	NA	
Iz_5_1009	DJI_0859	Bromus squarrosus L.	0	a	0	0	Poaceae Barnhart
Iz_5_1009	DJI_0859	Bromus tectorum L.	0	a	0	0	Poaceae Barnhart
Iz_5_1009	DJI_0859	Calamagrostis epigejos (L.) Roth	60	n	1	0	Poaceae Barnhart
Iz_5_1009	DJI_0859	Carduus acanthoides L.	0	n	1	0	Asteraceae Bercht. & J.Presl
Iz_5_1009	DJI_0859	Carex sp.	0	n	1	0	Cyperaceae Juss.
Iz_5_1009	DJI_0859	Centaurea majorovii Dumbadze	0	n	1	0	Asteraceae Bercht. & J.Presl
Iz_5_1009	DJI_0859	Chamaecytisus ruthenicus (Fisch.ex Wol.) Klaskova	0	n	1	0	Fabaceae Lindl.
Iz_5_1009	DJI_0859	Chelidonium majus L.	0	n	1	0	Papaveraceae Juss.
Iz_5_1009	DJI_0859	Chenopodium betaceum Andrz.	0	n	1	0	Amaranthaceae Juss.
Iz_5_1009	DJI_0859	Chenopodium cf. album	0	n	1	0	Amaranthaceae Juss.
Iz_5_1009	DJI_0859	Chenopodium sp.	0		NA	0	Amaranthaceae Juss.
Iz_5_1009	DJI_0859	Chondrilla juncea L.	0	n	1	0	Asteraceae Bercht. & J.Presl
Iz_5_1009	DJI_0859	Cirsium vulgare (Savi) Ten.	0	n	1	0	Asteraceae Bercht. & J.Presl
Iz_5_1009	DJI_0859	Clinopodium acinos (L.) Kuntze	0	n	1	0	Lamiacea Lindl.
Iz_5_1009	DJI_0859	Convallaria majalis L.	0	n	1	0	Asparagaceae Juss.
Iz_5_1009	DJI_0859	Crataegus rhipidophylla Gand.	0	n	1	1	Rosaceae Juss.
Iz_5_1009	DJI_0859	Crepis foetida L.	0	n	1	0	Asteraceae Bercht. & J.Presl
Iz_5_1009	DJI_0859	Dactylis glomerata L.	0	n	1	0	Poaceae Barnhart
Iz_5_1009	DJI_0859	Dianthus campestris M.Bieb.	0	n	1	0	Caryophyllaceae Juss.
Iz_5_1009	DJI_0859	Digitaria sanguinalis (L.) Scop.	0	a	0	0	Poaceae Barnhart
Iz_5_1009	DJI_0859	Elymus repens (L.) Gould	0	n	1	0	Poaceae Barnhart
Iz_5_1009	DJI_0859	Epilobium tetragonum L.	0	n	1	0	Onagraceae Juss.
Iz_5_1009	DJI_0859	Eragrostis minor Host	0	a	0	0	Poaceae Barnhart
Iz_5_1009	DJI_0859	Erigeron canadensis L.	0	a	0	0	Asteraceae Bercht. & J.Presl
Iz_5_1009	DJI_0859	Euphorbia seguieriana Neck.	0	n	1	0	Euphorbiaceae Juss.
Iz_5_1009	DJI_0859	Fallopia dumetorum (L.) Holub	0	n	1	0	Polygonaceae Juss.
Iz_5_1009	DJI_0859	Festuca beckeri (Hack.) Trautv.	0	n	1	0	Poaceae Barnhart
Iz_5_1009	DJI_0859	Fragaria vesca L.	0	n	1	0	Rosaceae Juss.
Iz_5_1009	DJI_0859	Frangula alnus Mill.	0	n	1	1	Rhamnaceae Juss.
Iz_5_1009	DJI_0859	Fraxinus pennsylvanica Marshall	0	a	0	1	Oleaceae Hoffmanns. & Link
Iz_5_1009	DJI_0859	Galeopsis bifida Boenn.	0	n	1	0	Lamiacea Lindl.
Iz_5_1009	DJI_0859	Galium verum L.	0	n	1	0	Rubiaceae Juss.
Iz_5_1009	DJI_0859	Genista tinctoria L.	0	n	1	0	Fabaceae Lindl.
Iz_5_1009	DJI_0859	Geranium robertianum L.	0	n	1	0	Geraniaceae Juss.
Iz_5_1009	DJI_0859	Geranium sanquineum L.	0	n	1	0	Geraniaceae Juss.
Iz_5_1009	DJI_0859	Gypsophila paniculata L.	0	n	1	0	Caryophyllaceae Juss.
Iz_5_1009	DJI_0859	Helichrysum arenarium (L.) Moench	0	n	1	0	Asteraceae Bercht. & J.Presl
Iz_5_1009	DJI_0859	Herniaria polygama J.Gay	0	n	1	0	Caryophyllaceae Juss.
Iz_5_1009	DJI_0859	Hieracium robustum Fr.	0	n	1	0	Asteraceae Bercht. & J.Presl
Iz_5_1009	DJI_0859	Hieracium umbellatum L.	0	n	1	NA	Asteraceae Bercht. & J.Presl
Iz_5_1009	DJI_0859	Hylotelephium maximum (L.) Holub	0	n	1	0	Crassulaceae J.St.-Hil.
Iz_5_1009	DJI_0859	Jacobaea borysthenica (DC.) B.Nord. & Greuter	0	n	1	0	Asteraceae Bercht. & J.Presl
Iz_5_1009	DJI_0859	Jasione montana L.	0	n	1	0	Campanulaceae Juss.
Iz_5_1009	DJI_0859	Jurinea cyanoides (L.) Rchb.s.l.	0	n	1	0	Asteraceae Bercht. & J.Presl
Iz_5_1009	DJI_0859	Koeleria glauca (Spreng.) DC.	0	n	1	0	Poaceae Barnhart
Iz_5_1009	DJI_0859	Lactuca serriola L.	0	a	0	0	Asteraceae Bercht. & J.Presl
Iz_5_1009	DJI_0859	Linaria genistifolia (L.) Mill.	0	n	1	0	Plantaginaceae Juss.
Iz_5_1009	DJI_0859	Lomelosia argentea (L.) Greuter & Burdet	0	n	1	0	Caprifoliaceae Juss.
Iz_5_1009	DJI_0859	Melampyrum pratense L.	0	n	1	0	Orobanchaceae Vent.
Iz_5_1009	DJI_0859	Oenothera biennis L.	0	a	0	0	Onagraceae Juss.
Iz_5_1009	DJI_0859	Pentanema britannica (L.) D.Gut.Larr., Santos-Vicente, Anderb., E.Rico & M.M.Mart.Ort.	0	n	1	0	Asteraceae Bercht. & J.Presl
Iz_5_1009	DJI_0859	Peucedanum oreoselinum (L.) Moench	0	n	1	0	Apiaceae Lindl.
Iz_5_1009	DJI_0859	Peucedanum ruthenicum M.Bieb.	0	n	1	0	Apiaceae Lindl.
Iz_5_1009	DJI_0859	Pilosella echioides (Lumn.) F.W.Schultz & Sch.Bip.	0	n	1	0	Asteraceae Bercht. & J.Presl
Iz_5_1009	DJI_0859	Pilosella officinarum F. Schultz et Sch. Bip.	0	n	1	0	Asteraceae Bercht. & J.Presl
Iz_5_1009	DJI_0859	Pilosella sp.	0		NA	0	Asteraceae Bercht. & J.Presl
Iz_5_1009	DJI_0859	Pinus sylvestris L.	0	n	1	1	Pinaceae Spreng. ex F.Rudolphi
Iz_5_1009	DJI_0859	Poa annua L.	0	n	1	0	Poaceae Barnhart
Iz_5_1009	DJI_0859	Poa nemoralis L.	0	n	1	0	Poaceae Barnhart
Iz_5_1009	DJI_0859	Poa sp.	0		NA	0	Poaceae Barnhart
Iz_5_1009	DJI_0859	Polygonatum odoratum (Mill.) Druce	0	n	1	0	Asparagaceae Juss.
Iz_5_1009	DJI_0859	Polygonum arenarium Waldst. & Kit.	0	n	1	0	Polygonaceae Juss.
Iz_5_1009	DJI_0859	Populus nigra L.	0	n	1	1	Salicaceae Mirb.
Iz_5_1009	DJI_0859	Populus sp.	0	n	1	1	Salicaceae Mirb.
Iz_5_1009	DJI_0859	Populus tremula L.	0	n	1	1	Salicaceae Mirb.
Iz_5_1009	DJI_0859	Populus Ã canescens (Aiton) Sm.	0	n	1	1	Salicaceae Mirb.
Iz_5_1009	DJI_0859	Potentilla argentea L.	0	n	1	0	Rosaceae Juss.
Iz_5_1009	DJI_0859	Psammophiliella muralis (L.) Ikonn.	0	n	1	0	Caryophyllaceae Juss.
Iz_5_1009	DJI_0859	Psephellus sumensis (Kalen.) Greuter	0	n	1	0	Asteraceae Bercht. & J.Presl
Iz_5_1009	DJI_0859	Pulsatilla pratensis (L.) Mill. s.l.	0	n	1	0	Ranunculaceae Juss.
Iz_5_1009	DJI_0859	Pyrus communis L.	0	a	0	1	Rosaceae Juss.
Iz_5_1009	DJI_0859	Quercus robur L.	0	n	1	1	Fagaceae Dumort.
Iz_5_1009	DJI_0859	Robinia pseudoacacia L.	0	a	0	1	Fabaceae Lindl.
Iz_5_1009	DJI_0859	Rosa sp.	0		NA	1	Rosaceae Juss.
Iz_5_1009	DJI_0859	Rubus idaeus L.	0	n	1	0	Rosaceae Juss.
Iz_5_1009	DJI_0859	Rumex acetosella L.	0	n	1	0	Polygonaceae Juss.
Iz_5_1009	DJI_0859	Rumex thyrsiflorus Fingerh.	0	n	1	0	Polygonaceae Juss.
Iz_5_1009	DJI_0859	Salix alba L.	0	n	1	1	Salicaceae Mirb.
Iz_5_1009	DJI_0859	Sambucus nigra L.	0	n	1	1	Viburnaceae Raf.
Iz_5_1009	DJI_0859	Secale sylvestre Host	0	n	1	0	Poaceae Barnhart
Iz_5_1009	DJI_0859	Sempervivum ruthenicum Schnittsp. & C.B. Lehm.	0	n	1	0	Crassulaceae J.St.-Hil.
Iz_5_1009	DJI_0859	Setaria viridis (L.) P. Beauv.	0	n	1	0	Poaceae Barnhart
Iz_5_1009	DJI_0859	Silene borysthenica (Gruner) Walters	0	n	1	0	Caryophyllaceae Juss.
Iz_5_1009	DJI_0859	Silene latifolia subsp. alba (Mill.) Greuter & Burdet	0	n	1	0	Caryophyllaceae Juss.
Iz_5_1009	DJI_0859	Solidago virgaurea L.	0	n	1	0	Asteraceae Bercht. & J.Presl
Iz_5_1009	DJI_0859	Solidago Ã niederederi (S. canadensis Ã S. virgaurea)	0	hybrid	0	0	Asteraceae Bercht. & J.Presl
Iz_5_1009	DJI_0859	Sorbus aucuparia L.	0	n	1	1	Rosaceae Juss.
Iz_5_1009	DJI_0859	Stachys recta L.	0	n	1	0	Lamiacea Lindl.
Iz_5_1009	DJI_0859	Tanacetum vulgare L.	0	n	1	0	Asteraceae Bercht. & J.Presl
Iz_5_1009	DJI_0859	Thymus pallasianus Heinr.Braun	0	n	1	0	Lamiacea Lindl.
Iz_5_1009	DJI_0859	Tilia cordata Mill.	0	n	1	1	Malvaceae Juss.
Iz_5_1009	DJI_0859	Trifolium alpestre L.	0	n	1	0	Fabaceae Lindl.
Iz_5_1009	DJI_0859	Trifolium arvense L.	0	n	1	0	Fabaceae Lindl.
Iz_5_1009	DJI_0859	Ulmus glabra Huds.	0	n	1	1	Ulmaceae Mirb.
Iz_5_1009	DJI_0859	Verbascum densiflorum Bertol.	0	n	1	0	Scrophulariaceae Juss.
Iz_5_1009	DJI_0859	Veronica austriaca L.	0	n	1	0	Plantaginaceae Juss.
Iz_5_1009	DJI_0859	Veronica incana L.	0	n	1	0	Plantaginaceae Juss.

Iz_5_1009	DJI_0859	Veronica verna L.	0	n	1	0	Plantaginaceae Juss.
Iz_5_1009	DJI_0859	Vincetoxicum hirundinaria Medik	0	n	1	0	Apocynaceae Juss.
Iz_5_1009	DJI_0860	Acer tataricum L.	0	n	1	1	Sapindaceae Juss.
Iz_5_1009	DJI_0860	Achillea millefolium L. s.l.	0	n	1	0	Asteraceae Bercht. & J.Presl
Iz_5_1009	DJI_0860	Allium oleraceum L.	0	n	1	0	Amaryllidaceae J.St.-Hil.
Iz_5_1009	DJI_0860	Ambrosia artemisiifolia L.	0	a	0	0	Asteraceae Bercht. & J.Presl
Iz_5_1009	DJI_0860	Amorpha fruticosa L.	0	a	0	0	Fabaceae Lindl.
Iz_5_1009	DJI_0860	Anthericum ramosum L.	0	n	1	0	Asparagaceae Juss.
Iz_5_1009	DJI_0860	Artemisia marschalliana Spreng.	0	n	1	0	Asteraceae Bercht. & J.Presl
Iz_5_1009	DJI_0860	Asparagus officinalis L.	0	n	1	0	Asparagaceae Juss.
Iz_5_1009	DJI_0860	Astragalus cicer L.	0	n	1	0	Fabaceae Lindl.
Iz_5_1009	DJI_0860	Bassia laniflora (S.G.Gmel.) A.J.Scott	0	n	1	0	Amaranthaceae Juss.
Iz_5_1009	DJI_0860	Berberis vulgaris L.	0	n	1	0	Berberidaceae Juss.
Iz_5_1009	DJI_0860	Betula pendula Roth.	0	n	1	1	Betulaceae Gray
Iz_5_1009	DJI_0860	Betula pendula Roth.	0	n	1	0	Poaceae Barnhart
Iz_5_1009	DJI_0860	Bromus inermis Leyss.	0		NA	NA	
Iz_5_1009	DJI_0860	Bromus squarrosus L.	0	a	0	0	Poaceae Barnhart
Iz_5_1009	DJI_0860	Bromus tectorum L.	0	a	0	0	Poaceae Barnhart
Iz_5_1009	DJI_0860	Calamagrostis epigejos (L.) Roth	60	n	1	0	Poaceae Barnhart
Iz_5_1009	DJI_0860	Carduus acanthoides L.	0	n	1	0	Asteraceae Bercht. & J.Presl
Iz_5_1009	DJI_0860	Carex sp.	0	n	1	0	Cyperaceae Juss.
Iz_5_1009	DJI_0860	Centaurea majorovii Dumbadze	0	n	1	0	Asteraceae Bercht. & J.Presl
Iz_5_1009	DJI_0860	Chamaecytisus ruthenicus (Fisch.ex Wol.) Klaskova	0	n	1	0	Fabaceae Lindl.
Iz_5_1009	DJI_0860	Chelidonium majus L.	0	n	1	0	Papaveraceae Juss.
Iz_5_1009	DJI_0860	Chenopodium betaceum Andrz.	0	n	1	0	Amaranthaceae Juss.
Iz_5_1009	DJI_0860	Chenopodium cf. album	0	n	1	0	Amaranthaceae Juss.
Iz_5_1009	DJI_0860	Chenopodium sp.	0		NA	0	Amaranthaceae Juss.
Iz_5_1009	DJI_0860	Chondrilla juncea L.	0	n	1	0	Asteraceae Bercht. & J.Presl
Iz_5_1009	DJI_0860	Cirsium vulgare (Savi) Ten.	0	n	1	0	Asteraceae Bercht. & J.Presl
Iz_5_1009	DJI_0860	Clinopodium acinos (L.) Kuntze	0	n	1	0	Lamiacea Lindl.
Iz_5_1009	DJI_0860	Convallaria majalis L.	0	n	1	0	Asparagaceae Juss.
Iz_5_1009	DJI_0860	Crataegus rhipidophylla Gand.	0	n	1	1	Rosaceae Juss.
Iz_5_1009	DJI_0860	Crepis foetida L.	0	n	1	0	Asteraceae Bercht. & J.Presl
Iz_5_1009	DJI_0860	Dactylis glomerata L.	0	n	1	0	Poaceae Barnhart
Iz_5_1009	DJI_0860	Dianthus campestris M.Bieb.	0	n	1	0	Caryophyllaceae Juss.
Iz_5_1009	DJI_0860	Digitaria sanguinalis (L.) Scop.	0	a	0	0	Poaceae Barnhart
Iz_5_1009	DJI_0860	Elymus repens (L.) Gould	0	n	1	0	Poaceae Barnhart
Iz_5_1009	DJI_0860	Epilobium tetragonum L.	0	n	1	0	Onagraceae Juss.
Iz_5_1009	DJI_0860	Eragrostis minor Host	0	a	0	0	Poaceae Barnhart
Iz_5_1009	DJI_0860	Erigeron canadensis L.	0	a	0	0	Asteraceae Bercht. & J.Presl
Iz_5_1009	DJI_0860	Euphorbia seguieriana Neck.	0	n	1	0	Euphorbiaceae Juss.
Iz_5_1009	DJI_0860	Fallopia dumetorum (L.) Holub	0	n	1	0	Polygonaceae Juss.
Iz_5_1009	DJI_0860	Festuca beckeri (Hack.) Trautv.	0	n	1	0	Poaceae Barnhart
Iz_5_1009	DJI_0860	Fragaria vesca L.	0	n	1	0	Rosaceae Juss.
Iz_5_1009	DJI_0860	Frangula alnus Mill.	0	n	1	1	Rhamnaceae Juss.
Iz_5_1009	DJI_0860	Fraxinus pennsylvanica Marshall	0	a	0	1	Oleaceae Hoffmanns. & Link
Iz_5_1009	DJI_0860	Galeopsis bifida Boenn.	0	n	1	0	Lamiacea Lindl.
Iz_5_1009	DJI_0860	Galium verum L.	0	n	1	0	Rubiaceae Juss.
Iz_5_1009	DJI_0860	Genista tinctoria L.	0	n	1	0	Fabaceae Lindl.
Iz_5_1009	DJI_0860	Geranium robertianum L.	0	n	1	0	Geraniaceae Juss.
Iz_5_1009	DJI_0860	Geranium sanquineum L.	0	n	1	0	Geraniaceae Juss.
Iz_5_1009	DJI_0860	Gypsophila paniculata L.	0	n	1	0	Caryophyllaceae Juss.
Iz_5_1009	DJI_0860	Helichrysum arenarium (L.) Moench	0	n	1	0	Asteraceae Bercht. & J.Presl
Iz_5_1009	DJI_0860	Herniaria polygama J.Gay	0	n	1	0	Caryophyllaceae Juss.
Iz_5_1009	DJI_0860	Hieracium robustum Fr.	0	n	1	0	Asteraceae Bercht. & J.Presl
Iz_5_1009	DJI_0860	Hieracium umbellatum L.	0	n	1	NA	Asteraceae Bercht. & J.Presl
Iz_5_1009	DJI_0860	Hylotelephium maximum (L.) Holub	0	n	1	0	Crassulaceae J.St.-Hil.
Iz_5_1009	DJI_0860	Jacobaea borysthenica (DC.) B.Nord. & Greuter	0	n	1	0	Asteraceae Bercht. & J.Presl
Iz_5_1009	DJI_0860	Jasione montana L.	0	n	1	0	Campanulaceae Juss.
Iz_5_1009	DJI_0860	Jurinea cyanoides (L.) Rchb.s.l.	0	n	1	0	Asteraceae Bercht. & J.Presl
Iz_5_1009	DJI_0860	Koeleria glauca (Spreng.) DC.	0	n	1	0	Poaceae Barnhart
Iz_5_1009	DJI_0860	Lactuca serriola L.	0	a	0	0	Asteraceae Bercht. & J.Presl
Iz_5_1009	DJI_0860	Linaria genistifolia (L.) Mill.	0	n	1	0	Plantaginaceae Juss.
Iz_5_1009	DJI_0860	Lomelosia argentea (L.) Greuter & Burdet	0	n	1	0	Caprifoliaceae Juss.
Iz_5_1009	DJI_0860	Melampyrum pratense L.	0	n	1	0	Orobanchaceae Vent.
Iz_5_1009	DJI_0860	Oenothera biennis L.	0	a	0	0	Onagraceae Juss.
Iz_5_1009	DJI_0860	Pentanema britannica (L.) D.Gut.Larr., Santos-Vicente, Anderb., E.Rico & M.M.Mart.Ort.	0	n	1	0	Asteraceae Bercht. & J.Presl
Iz_5_1009	DJI_0860	Peucedanum oreoselinum (L.) Moench	0	n	1	0	Apiaceae Lindl.
Iz_5_1009	DJI_0860	Peucedanum ruthenicum M.Bieb.	0	n	1	0	Apiaceae Lindl.
Iz_5_1009	DJI_0860	Pilosella echioides (Lumn.) F.W.Schultz & Sch.Bip.	0	n	1	0	Asteraceae Bercht. & J.Presl
Iz_5_1009	DJI_0860	Pilosella officinarum F. Schultz et Sch. Bip.	0	n	1	0	Asteraceae Bercht. & J.Presl
Iz_5_1009	DJI_0860	Pilosella sp.	0		NA	0	Asteraceae Bercht. & J.Presl
Iz_5_1009	DJI_0860	Pinus sylvestris L.	0	n	1	1	Pinaceae Spreng. ex F.Rudolphi
Iz_5_1009	DJI_0860	Poa annua L.	0	n	1	0	Poaceae Barnhart
Iz_5_1009	DJI_0860	Poa nemoralis L.	0	n	1	0	Poaceae Barnhart
Iz_5_1009	DJI_0860	Poa sp.	0		NA	0	Poaceae Barnhart
Iz_5_1009	DJI_0860	Polygonatum odoratum (Mill.) Druce	0	n	1	0	Asparagaceae Juss.
Iz_5_1009	DJI_0860	Polygonum arenarium Waldst. & Kit.	0	n	1	0	Polygonaceae Juss.
Iz_5_1009	DJI_0860	Populus nigra L.	0	n	1	1	Salicaceae Mirb.
Iz_5_1009	DJI_0860	Populus sp.	0	n	1	1	Salicaceae Mirb.
Iz_5_1009	DJI_0860	Populus tremula L.	0	n	1	1	Salicaceae Mirb.
Iz_5_1009	DJI_0860	Populus Ã canescens (Aiton) Sm.	0	n	1	1	Salicaceae Mirb.
Iz_5_1009	DJI_0860	Potentilla argentea L.	0	n	1	0	Rosaceae Juss.
Iz_5_1009	DJI_0860	Psammophiliella muralis (L.) Ikonn.	0	n	1	0	Caryophyllaceae Juss.
Iz_5_1009	DJI_0860	Psephellus sumensis (Kalen.) Greuter	0	n	1	0	Asteraceae Bercht. & J.Presl
Iz_5_1009	DJI_0860	Pulsatilla pratensis (L.) Mill. s.l.	0	n	1	0	Ranunculaceae Juss.
Iz_5_1009	DJI_0860	Pyrus communis L.	0	a	0	1	Rosaceae Juss.
Iz_5_1009	DJI_0860	Quercus robur L.	0	n	1	1	Fagaceae Dumort.
Iz_5_1009	DJI_0860	Robinia pseudoacacia L.	0	a	0	1	Fabaceae Lindl.
Iz_5_1009	DJI_0860	Rosa sp.	0		NA	1	Rosaceae Juss.
Iz_5_1009	DJI_0860	Rubus idaeus L.	0	n	1	0	Rosaceae Juss.
Iz_5_1009	DJI_0860	Rumex acetosella L.	0	n	1	0	Polygonaceae Juss.
Iz_5_1009	DJI_0860	Rumex thyrsiflorus Fingerh.	0	n	1	0	Polygonaceae Juss.
Iz_5_1009	DJI_0860	Salix alba L.	0	n	1	1	Salicaceae Mirb.
Iz_5_1009	DJI_0860	Sambucus nigra L.	0	n	1	1	Viburnaceae Raf.
Iz_5_1009	DJI_0860	Secale sylvestre Host	0	n	1	0	Poaceae Barnhart
Iz_5_1009	DJI_0860	Sempervivum ruthenicum Schnittsp. & C.B. Lehm.	0	n	1	0	Crassulaceae J.St.-Hil.
Iz_5_1009	DJI_0860	Setaria viridis (L.) P. Beauv.	0	n	1	0	Poaceae Barnhart

Iz_5_1009	DJI_0860	Silene borysthenica (Gruner) Walters	0	n	1	0	Caryophyllaceae Juss.
Iz_5_1009	DJI_0860	Silene latifolia subsp. alba (Mill.) Greuter & Burdet	0	n	1	0	Caryophyllaceae Juss.
Iz_5_1009	DJI_0860	Solidago virgaurea L.	0	n	1	0	Asteraceae Bercht. & J.Presl
Iz_5_1009	DJI_0860	Solidago Ã niederederi (S. canadensis Ã S. virgaurea)	0	hybrid	0	0	Asteraceae Bercht. & J.Presl
Iz_5_1009	DJI_0860	Sorbus aucuparia L.	0	n	1	1	Rosaceae Juss.
Iz_5_1009	DJI_0860	Stachys recta L.	0	n	1	0	Lamiacea Lindl.
Iz_5_1009	DJI_0860	Tanacetum vulgare L.	0	n	1	0	Asteraceae Bercht. & J.Presl
Iz_5_1009	DJI_0860	Thymus pallasianus Heinr.Braun	0	n	1	0	Lamiacea Lindl.
Iz_5_1009	DJI_0860	Tilia cordata Mill.	0	n	1	1	Malvaceae Juss.
Iz_5_1009	DJI_0860	Trifolium alpestre L.	0	n	1	0	Fabaceae Lindl.
Iz_5_1009	DJI_0860	Trifolium arvense L.	0	n	1	0	Fabaceae Lindl.
Iz_5_1009	DJI_0860	Ulmus glabra Huds.	0	n	1	1	Ulmaceae Mirb.
Iz_5_1009	DJI_0860	Verbascum densiflorum Bertol.	0	n	1	0	Scrophulariaceae Juss.
Iz_5_1009	DJI_0860	Veronica austriaca L.	0	n	1	0	Plantaginaceae Juss.
Iz_5_1009	DJI_0860	Veronica incana L.	0	n	1	0	Plantaginaceae Juss.
Iz_5_1009	DJI_0860	Veronica verna L.	0	n	1	0	Plantaginaceae Juss.
Iz_5_1009	DJI_0860	Vincetoxicum hirundinaria Medik	0	n	1	0	Apocynaceae Juss.
Iz_5_1009	DJI_0864	Acer tataricum L.	0	n	1	1	Sapindaceae Juss.
Iz_5_1009	DJI_0864	Achillea millefolium L. s.l.	0	n	1	0	Asteraceae Bercht. & J.Presl
Iz_5_1009	DJI_0864	Allium oleraceum L.	0	n	1	0	Amaryllidaceae J.St.-Hil.
Iz_5_1009	DJI_0864	Ambrosia artemisiifolia L.	0	a	0	0	Asteraceae Bercht. & J.Presl
Iz_5_1009	DJI_0864	Amorpha fruticosa L.	0	a	0	0	Fabaceae Lindl.
Iz_5_1009	DJI_0864	Anthericum ramosum L.	0	n	1	0	Asparagaceae Juss.
Iz_5_1009	DJI_0864	Artemisia marschalliana Spreng.	0	n	1	0	Asteraceae Bercht. & J.Presl
Iz_5_1009	DJI_0864	Asparagus officinalis L.	0	n	1	0	Asparagaceae Juss.
Iz_5_1009	DJI_0864	Astragalus cicer L.	0	n	1	0	Fabaceae Lindl.
Iz_5_1009	DJI_0864	Bassia laniflora (S.G.Gmel.) A.J.Scott	0	n	1	0	Amaranthaceae Juss.
Iz_5_1009	DJI_0864	Berberis vulgaris L.	0	n	1	0	Berberidaceae Juss.
Iz_5_1009	DJI_0864	Betula pendula Roth.	0	n	1	1	Betulaceae Gray
Iz_5_1009	DJI_0864	Betula pendula Roth.	0	n	1	0	Poaceae Barnhart
Iz_5_1009	DJI_0864	Bromus inermis Leyss.	0		NA	NA	
Iz_5_1009	DJI_0864	Bromus squarrosus L.	0	a	0	0	Poaceae Barnhart
Iz_5_1009	DJI_0864	Bromus tectorum L.	0	a	0	0	Poaceae Barnhart
Iz_5_1009	DJI_0864	Calamagrostis epigejos (L.) Roth	8	n	1	0	Poaceae Barnhart
Iz_5_1009	DJI_0864	Carduus acanthoides L.	0	n	1	0	Asteraceae Bercht. & J.Presl
Iz_5_1009	DJI_0864	Carex sp.	0	n	1	0	Cyperaceae Juss.
Iz_5_1009	DJI_0864	Centaurea majorovii Dumbadze	0	n	1	0	Asteraceae Bercht. & J.Presl
Iz_5_1009	DJI_0864	Chamaecytisus ruthenicus (Fisch.ex Wol.) Klaskova	0	n	1	0	Fabaceae Lindl.
Iz_5_1009	DJI_0864	Chelidonium majus L.	0	n	1	0	Papaveraceae Juss.
Iz_5_1009	DJI_0864	Chenopodium betaceum Andrz.	0	n	1	0	Amaranthaceae Juss.
Iz_5_1009	DJI_0864	Chenopodium cf. album	0	n	1	0	Amaranthaceae Juss.
Iz_5_1009	DJI_0864	Chenopodium sp.	0		NA	0	Amaranthaceae Juss.
Iz_5_1009	DJI_0864	Chondrilla juncea L.	0	n	1	0	Asteraceae Bercht. & J.Presl
Iz_5_1009	DJI_0864	Cirsium vulgare (Savi) Ten.	0	n	1	0	Asteraceae Bercht. & J.Presl
Iz_5_1009	DJI_0864	Clinopodium acinos (L.) Kuntze	0	n	1	0	Lamiacea Lindl.
Iz_5_1009	DJI_0864	Convallaria majalis L.	0	n	1	0	Asparagaceae Juss.
Iz_5_1009	DJI_0864	Crataegus rhipidophylla Gand.	0	n	1	1	Rosaceae Juss.
Iz_5_1009	DJI_0864	Crepis foetida L.	0	n	1	0	Asteraceae Bercht. & J.Presl
Iz_5_1009	DJI_0864	Dactylis glomerata L.	0	n	1	0	Poaceae Barnhart
Iz_5_1009	DJI_0864	Dianthus campestris M.Bieb.	0	n	1	0	Caryophyllaceae Juss.
Iz_5_1009	DJI_0864	Digitaria sanguinalis (L.) Scop.	0	a	0	0	Poaceae Barnhart
Iz_5_1009	DJI_0864	Elymus repens (L.) Gould	0	n	1	0	Poaceae Barnhart
Iz_5_1009	DJI_0864	Epilobium tetragonum L.	0	n	1	0	Onagraceae Juss.
Iz_5_1009	DJI_0864	Eragrostis minor Host	0	a	0	0	Poaceae Barnhart
Iz_5_1009	DJI_0864	Erigeron canadensis L.	0	a	0	0	Asteraceae Bercht. & J.Presl
Iz_5_1009	DJI_0864	Euphorbia seguieriana Neck.	0	n	1	0	Euphorbiaceae Juss.
Iz_5_1009	DJI_0864	Fallopia dumetorum (L.) Holub	0	n	1	0	Polygonaceae Juss.
Iz_5_1009	DJI_0864	Festuca beckeri (Hack.) Trautv.	0	n	1	0	Poaceae Barnhart
Iz_5_1009	DJI_0864	Fragaria vesca L.	0	n	1	0	Rosaceae Juss.
Iz_5_1009	DJI_0864	Frangula alnus Mill.	0	n	1	1	Rhamnaceae Juss.
Iz_5_1009	DJI_0864	Fraxinus pennsylvanica Marshall	0	a	0	1	Oleaceae Hoffmanns. & Link
Iz_5_1009	DJI_0864	Galeopsis bifida Boenn.	0	n	1	0	Lamiacea Lindl.
Iz_5_1009	DJI_0864	Galium verum L.	0	n	1	0	Rubiaceae Juss.
Iz_5_1009	DJI_0864	Genista tinctoria L.	0	n	1	0	Fabaceae Lindl.
Iz_5_1009	DJI_0864	Geranium robertianum L.	0	n	1	0	Geraniaceae Juss.
Iz_5_1009	DJI_0864	Geranium sanquineum L.	0	n	1	0	Geraniaceae Juss.
Iz_5_1009	DJI_0864	Gypsophila paniculata L.	0	n	1	0	Caryophyllaceae Juss.
Iz_5_1009	DJI_0864	Helichrysum arenarium (L.) Moench	1	n	1	0	Asteraceae Bercht. & J.Presl
Iz_5_1009	DJI_0864	Herniaria polygama J.Gay	0	n	1	0	Caryophyllaceae Juss.
Iz_5_1009	DJI_0864	Hieracium robustum Fr.	0	n	1	0	Asteraceae Bercht. & J.Presl
Iz_5_1009	DJI_0864	Hieracium umbellatum L.	0	n	1	NA	Asteraceae Bercht. & J.Presl
Iz_5_1009	DJI_0864	Hylotelephium maximum (L.) Holub	0	n	1	0	Crassulaceae J.St.-Hil.
Iz_5_1009	DJI_0864	Jacobaea borysthenica (DC.) B.Nord. & Greuter	0	n	1	0	Asteraceae Bercht. & J.Presl
Iz_5_1009	DJI_0864	Jasione montana L.	0	n	1	0	Campanulaceae Juss.
Iz_5_1009	DJI_0864	Jurinea cyanoides (L.) Rchb.s.l.	0	n	1	0	Asteraceae Bercht. & J.Presl
Iz_5_1009	DJI_0864	Koeleria glauca (Spreng.) DC.	0	n	1	0	Poaceae Barnhart
Iz_5_1009	DJI_0864	Lactuca serriola L.	0	a	0	0	Asteraceae Bercht. & J.Presl
Iz_5_1009	DJI_0864	Linaria genistifolia (L.) Mill.	0	n	1	0	Plantaginaceae Juss.
Iz_5_1009	DJI_0864	Lomelosia argentea (L.) Greuter & Burdet	0	n	1	0	Caprifoliaceae Juss.
Iz_5_1009	DJI_0864	Melampyrum pratense L.	0	n	1	0	Orobanchaceae Vent.
Iz_5_1009	DJI_0864	Oenothera biennis L.	0	a	0	0	Onagraceae Juss.
Iz_5_1009	DJI_0864	Pentanema britannica (L.) D.Gut.Larr., Santos-Vicente, Anderb., E.Rico & M.M.Mart.Ort.	0	n	1	0	Asteraceae Bercht. & J.Presl
Iz_5_1009	DJI_0864	Peucedanum oreoselinum (L.) Moench	0	n	1	0	Apiaceae Lindl.
Iz_5_1009	DJI_0864	Peucedanum ruthenicum M.Bieb.	0	n	1	0	Apiaceae Lindl.
Iz_5_1009	DJI_0864	Pilosella echioides (Lumn.) F.W.Schultz & Sch.Bip.	0	n	1	0	Asteraceae Bercht. & J.Presl
Iz_5_1009	DJI_0864	Pilosella officinarum F. Schultz et Sch. Bip.	0	n	1	0	Asteraceae Bercht. & J.Presl
Iz_5_1009	DJI_0864	Pilosella sp.	0		NA	0	Asteraceae Bercht. & J.Presl
Iz_5_1009	DJI_0864	Pinus sylvestris L.	0	n	1	1	Pinaceae Spreng. ex F.Rudolphi
Iz_5_1009	DJI_0864	Poa annua L.	0	n	1	0	Poaceae Barnhart
Iz_5_1009	DJI_0864	Poa nemoralis L.	0	n	1	0	Poaceae Barnhart
Iz_5_1009	DJI_0864	Poa sp.	0		NA	0	Poaceae Barnhart
Iz_5_1009	DJI_0864	Polygonatum odoratum (Mill.) Druce	0	n	1	0	Asparagaceae Juss.
Iz_5_1009	DJI_0864	Polygonum arenarium Waldst. & Kit.	0	n	1	0	Polygonaceae Juss.
Iz_5_1009	DJI_0864	Populus nigra L.	0	n	1	1	Salicaceae Mirb.
Iz_5_1009	DJI_0864	Populus sp.	0	n	1	1	Salicaceae Mirb.
Iz_5_1009	DJI_0864	Populus tremula L.	0	n	1	1	Salicaceae Mirb.
Iz_5_1009	DJI_0864	Populus Ã canescens (Aiton) Sm.	0	n	1	1	Salicaceae Mirb.
Iz_5_1009	DJI_0864	Potentilla argentea L.	0	n	1	0	Rosaceae Juss.

Iz_5_1009	DJI_0864	Psammophiliella muralis (L.) Ikonn.	0	n	1	0	Caryophyllaceae Juss.
Iz_5_1009	DJI_0864	Psephellus sumensis (Kalen.) Greuter	0	n	1	0	Asteraceae Bercht. & J.Presl
Iz_5_1009	DJI_0864	Pulsatilla pratensis (L.) Mill. s.l.	0	n	1	0	Ranunculaceae Juss.
Iz_5_1009	DJI_0864	Pyrus communis L.	0	a	0	1	Rosaceae Juss.
Iz_5_1009	DJI_0864	Quercus robur L.	0	n	1	1	Fagaceae Dumort.
Iz_5_1009	DJI_0864	Robinia pseudoacacia L.	0	a	0	1	Fabaceae Lindl.
Iz_5_1009	DJI_0864	Rosa sp.	0		NA	1	Rosaceae Juss.
Iz_5_1009	DJI_0864	Rubus idaeus L.	0	n	1	0	Rosaceae Juss.
Iz_5_1009	DJI_0864	Rumex acetosella L.	0	n	1	0	Polygonaceae Juss.
Iz_5_1009	DJI_0864	Rumex thyrsiflorus Fingerh.	0	n	1	0	Polygonaceae Juss.
Iz_5_1009	DJI_0864	Salix alba L.	0	n	1	1	Salicaceae Mirb.
Iz_5_1009	DJI_0864	Sambucus nigra L.	0	n	1	1	Viburnaceae Raf.
Iz_5_1009	DJI_0864	Secale sylvestre Host	0	n	1	0	Poaceae Barnhart
Iz_5_1009	DJI_0864	Sempervivum ruthenicum Schnittsp. & C.B. Lehm.	0	n	1	0	Crassulaceae J.St.-Hil.
Iz_5_1009	DJI_0864	Setaria viridis (L.) P. Beauv.	0	n	1	0	Poaceae Barnhart
Iz_5_1009	DJI_0864	Silene borysthenica (Gruner) Walters	0	n	1	0	Caryophyllaceae Juss.
Iz_5_1009	DJI_0864	Silene latifolia subsp. alba (Mill.) Greuter & Burdet	0	n	1	0	Caryophyllaceae Juss.
Iz_5_1009	DJI_0864	Solidago virgaurea L.	1	n	1	0	Asteraceae Bercht. & J.Presl
Iz_5_1009	DJI_0864	Solidago Ã niederederi (S. canadensis Ã S. virgaurea)	0	hybrid	0	0	Asteraceae Bercht. & J.Presl
Iz_5_1009	DJI_0864	Sorbus aucuparia L.	0	n	1	1	Rosaceae Juss.
Iz_5_1009	DJI_0864	Stachys recta L.	0	n	1	0	Lamiacea Lindl.
Iz_5_1009	DJI_0864	Tanacetum vulgare L.	0	n	1	0	Asteraceae Bercht. & J.Presl
Iz_5_1009	DJI_0864	Thymus pallasianus Heinr.Braun	0	n	1	0	Lamiacea Lindl.
Iz_5_1009	DJI_0864	Tilia cordata Mill.	0	n	1	1	Malvaceae Juss.
Iz_5_1009	DJI_0864	Trifolium alpestre L.	0	n	1	0	Fabaceae Lindl.
Iz_5_1009	DJI_0864	Trifolium arvense L.	0	n	1	0	Fabaceae Lindl.
Iz_5_1009	DJI_0864	Ulmus glabra Huds.	0	n	1	1	Ulmaceae Mirb.
Iz_5_1009	DJI_0864	Verbascum densiflorum Bertol.	0	n	1	0	Scrophulariaceae Juss.
Iz_5_1009	DJI_0864	Veronica austriaca L.	0	n	1	0	Plantaginaceae Juss.
Iz_5_1009	DJI_0864	Veronica incana L.	0	n	1	0	Plantaginaceae Juss.
Iz_5_1009	DJI_0864	Veronica verna L.	0	n	1	0	Plantaginaceae Juss.
Iz_5_1009	DJI_0864	Vincetoxicum hirundinaria Medik	0	n	1	0	Apocynaceae Juss.
Iz_5_1109	DJI_0125	Acer tataricum L.	0	n	1	1	Sapindaceae Juss.
Iz_5_1109	DJI_0125	Achillea millefolium L. s.l.	0	n	1	0	Asteraceae Bercht. & J.Presl
Iz_5_1109	DJI_0125	Allium oleraceum L.	0	n	1	0	Amaryllidaceae J.St.-Hil.
Iz_5_1109	DJI_0125	Ambrosia artemisiifolia L.	0	a	0	0	Asteraceae Bercht. & J.Presl
Iz_5_1109	DJI_0125	Amorpha fruticosa L.	0	a	0	0	Fabaceae Lindl.
Iz_5_1109	DJI_0125	Anthericum ramosum L.	0	n	1	0	Asparagaceae Juss.
Iz_5_1109	DJI_0125	Anthoxanthum repens (Host) Veldkamp	0		NA	NA	
Iz_5_1109	DJI_0125	Artemisia marschalliana Spreng.	0	n	1	0	Asteraceae Bercht. & J.Presl
Iz_5_1109	DJI_0125	Asparagus officinalis L.	0	n	1	0	Asparagaceae Juss.
Iz_5_1109	DJI_0125	Astragalus cicer L.	0	n	1	0	Fabaceae Lindl.
Iz_5_1109	DJI_0125	Bassia laniflora (S.G.Gmel.) A.J.Scott	0	n	1	0	Amaranthaceae Juss.
Iz_5_1109	DJI_0125	Berberis vulgaris L.	0	n	1	0	Berberidaceae Juss.
Iz_5_1109	DJI_0125	Berteroa incana (L.) DC.	0		NA	NA	
Iz_5_1109	DJI_0125	Betula pendula Roth.	0	n	1	1	Betulaceae Gray
Iz_5_1109	DJI_0125	Betula pendula Roth.	0	n	1	0	Poaceae Barnhart
Iz_5_1109	DJI_0125	Bromus inermis Leyss.	0		NA	NA	
Iz_5_1109	DJI_0125	Bromus squarrosus L.	0	a	0	0	Poaceae Barnhart
Iz_5_1109	DJI_0125	Bromus tectorum L.	0	a	0	0	Poaceae Barnhart
Iz_5_1109	DJI_0125	Calamagrostis epigejos (L.) Roth	1	n	1	0	Poaceae Barnhart
Iz_5_1109	DJI_0125	Carduus acanthoides L.	0	n	1	0	Asteraceae Bercht. & J.Presl
Iz_5_1109	DJI_0125	Carex sp.	0	n	1	0	Cyperaceae Juss.
Iz_5_1109	DJI_0125	Centaurea majorovii Dumbadze	0	n	1	0	Asteraceae Bercht. & J.Presl
Iz_5_1109	DJI_0125	Chamaecytisus ruthenicus (Fisch.ex Wol.) Klaskova	0	n	1	0	Fabaceae Lindl.
Iz_5_1109	DJI_0125	Chelidonium majus L.	0.5	n	1	0	Papaveraceae Juss.
Iz_5_1109	DJI_0125	Chenopodium betaceum Andrz.	0	n	1	0	Amaranthaceae Juss.
Iz_5_1109	DJI_0125	Chenopodium cf. album	0	n	1	0	Amaranthaceae Juss.
Iz_5_1109	DJI_0125	Chenopodium sp.	0		NA	0	Amaranthaceae Juss.
Iz_5_1109	DJI_0125	Chondrilla juncea L.	0	n	1	0	Asteraceae Bercht. & J.Presl
Iz_5_1109	DJI_0125	Cirsium vulgare (Savi) Ten.	0	n	1	0	Asteraceae Bercht. & J.Presl
Iz_5_1109	DJI_0125	Clinopodium acinos (L.) Kuntze	0	n	1	0	Lamiacea Lindl.
Iz_5_1109	DJI_0125	Convallaria majalis L.	0	n	1	0	Asparagaceae Juss.
Iz_5_1109	DJI_0125	Crataegus rhipidophylla Gand.	0	n	1	1	Rosaceae Juss.
Iz_5_1109	DJI_0125	Crepis foetida L.	0	n	1	0	Asteraceae Bercht. & J.Presl
Iz_5_1109	DJI_0125	Dactylis glomerata L.	0	n	1	0	Poaceae Barnhart
Iz_5_1109	DJI_0125	Dianthus campestris M.Bieb.	0	n	1	0	Caryophyllaceae Juss.
Iz_5_1109	DJI_0125	Digitaria sanguinalis (L.) Scop.	0	a	0	0	Poaceae Barnhart
Iz_5_1109	DJI_0125	Elymus repens (L.) Gould	0	n	1	0	Poaceae Barnhart
Iz_5_1109	DJI_0125	Epilobium tetragonum L.	0	n	1	0	Onagraceae Juss.
Iz_5_1109	DJI_0125	Eragrostis minor Host	0	a	0	0	Poaceae Barnhart
Iz_5_1109	DJI_0125	Erigeron canadensis L.	0.2	a	0	0	Asteraceae Bercht. & J.Presl
Iz_5_1109	DJI_0125	Euphorbia seguieriana Neck.	0	n	1	0	Euphorbiaceae Juss.
Iz_5_1109	DJI_0125	Fallopia dumetorum (L.) Holub	5	n	1	0	Polygonaceae Juss.
Iz_5_1109	DJI_0125	Festuca beckeri (Hack.) Trautv.	0	n	1	0	Poaceae Barnhart
Iz_5_1109	DJI_0125	Fragaria vesca L.	0	n	1	0	Rosaceae Juss.
Iz_5_1109	DJI_0125	Frangula alnus Mill.	0	n	1	1	Rhamnaceae Juss.
Iz_5_1109	DJI_0125	Fraxinus pennsylvanica Marshall	0	a	0	1	Oleaceae Hoffmanns. & Link
Iz_5_1109	DJI_0125	Galeopsis bifida Boenn.	0	n	1	0	Lamiacea Lindl.
Iz_5_1109	DJI_0125	Galium verum L.	0	n	1	0	Rubiaceae Juss.
Iz_5_1109	DJI_0125	Genista tinctoria L.	0	n	1	0	Fabaceae Lindl.
Iz_5_1109	DJI_0125	Geranium robertianum L.	0	n	1	0	Geraniaceae Juss.
Iz_5_1109	DJI_0125	Geranium sanquineum L.	0	n	1	0	Geraniaceae Juss.
Iz_5_1109	DJI_0125	Gypsophila paniculata L.	0	n	1	0	Caryophyllaceae Juss.
Iz_5_1109	DJI_0125	Helichrysum arenarium (L.) Moench	0	n	1	0	Asteraceae Bercht. & J.Presl
Iz_5_1109	DJI_0125	Herniaria polygama J.Gay	0	n	1	0	Caryophyllaceae Juss.
Iz_5_1109	DJI_0125	Hieracium robustum Fr.	0	n	1	0	Asteraceae Bercht. & J.Presl
Iz_5_1109	DJI_0125	Hieracium umbellatum L.	0	n	1	NA	Asteraceae Bercht. & J.Presl
Iz_5_1109	DJI_0125	Hylotelephium maximum (L.) Holub	0	n	1	0	Crassulaceae J.St.-Hil.
Iz_5_1109	DJI_0125	Jacobaea borysthenica (DC.) B.Nord. & Greuter	0	n	1	0	Asteraceae Bercht. & J.Presl
Iz_5_1109	DJI_0125	Jasione montana L.	0	n	1	0	Campanulaceae Juss.
Iz_5_1109	DJI_0125	Jurinea cyanoides (L.) Rchb.s.l.	0	n	1	0	Asteraceae Bercht. & J.Presl
Iz_5_1109	DJI_0125	Koeleria glauca (Spreng.) DC.	0	n	1	0	Poaceae Barnhart
Iz_5_1109	DJI_0125	Lactuca serriola L.	0	a	0	0	Asteraceae Bercht. & J.Presl
Iz_5_1109	DJI_0125	Linaria genistifolia (L.) Mill.	0	n	1	0	Plantaginaceae Juss.
Iz_5_1109	DJI_0125	Lomelosia argentea (L.) Greuter & Burdet	0	n	1	0	Caprifoliaceae Juss.
Iz_5_1109	DJI_0125	Melampyrum pratense L.	0	n	1	0	Orobanchaceae Vent.
Iz_5_1109	DJI_0125	Oenothera biennis L.	0	a	0	0	Onagraceae Juss.

Iz_5_1109	DJI_0125	Pentanema britannica (L.) D.Gut.Larr., Santos-Vicente, Anderb., E.Rico & M.M.Mart.Ort.	0	n	1	0	Asteraceae Bercht. & J.Presl
Iz_5_1109	DJI_0125	Peucedanum oreoselinum (L.) Moench	0	n	1	0	Apiaceae Lindl.
Iz_5_1109	DJI_0125	Peucedanum ruthenicum M.Bieb.	0	n	1	0	Apiaceae Lindl.
Iz_5_1109	DJI_0125	Pilosella echioides (Lumn.) F.W.Schultz & Sch.Bip.	0	n	1	0	Asteraceae Bercht. & J.Presl
Iz_5_1109	DJI_0125	Pilosella officinarum F. Schultz et Sch. Bip.	0	n	1	0	Asteraceae Bercht. & J.Presl
Iz_5_1109	DJI_0125	Pilosella sp.	0		NA	0	Asteraceae Bercht. & J.Presl
Iz_5_1109	DJI_0125	Pinus sylvestris L.	0	n	1	1	Pinaceae Spreng. ex F.Rudolphi
Iz_5_1109	DJI_0125	Poa annua L.	0	n	1	0	Poaceae Barnhart
Iz_5_1109	DJI_0125	Poa nemoralis L.	0	n	1	0	Poaceae Barnhart
Iz_5_1109	DJI_0125	Poa sp.	0		NA	0	Poaceae Barnhart
Iz_5_1109	DJI_0125	Polygonatum odoratum (Mill.) Druce	0	n	1	0	Asparagaceae Juss.
Iz_5_1109	DJI_0125	Polygonum arenarium Waldst. & Kit.	0	n	1	0	Polygonaceae Juss.
Iz_5_1109	DJI_0125	Populus nigra L.	0	n	1	1	Salicaceae Mirb.
Iz_5_1109	DJI_0125	Populus tremula L.	0	n	1	1	Salicaceae Mirb.
Iz_5_1109	DJI_0125	Populus Ã canescens (Aiton) Sm.	0	n	1	1	Salicaceae Mirb.
Iz_5_1109	DJI_0125	Potentilla argentea L.	0	n	1	0	Rosaceae Juss.
Iz_5_1109	DJI_0125	Psammophiliella muralis (L.) Ikonn.	0	n	1	0	Caryophyllaceae Juss.
Iz_5_1109	DJI_0125	Psephellus sumensis (Kalen.) Greuter	0	n	1	0	Asteraceae Bercht. & J.Presl
Iz_5_1109	DJI_0125	Pulsatilla pratensis (L.) Mill. s.l.	0	n	1	0	Ranunculaceae Juss.
Iz_5_1109	DJI_0125	Pyrus communis L.	0	a	0	1	Rosaceae Juss.
Iz_5_1109	DJI_0125	Quercus robur L.	0	n	1	1	Fagaceae Dumort.
Iz_5_1109	DJI_0125	Robinia pseudoacacia L.	0	a	0	1	Fabaceae Lindl.
Iz_5_1109	DJI_0125	Rosa sp.	0		NA	1	Rosaceae Juss.
Iz_5_1109	DJI_0125	Rubus idaeus L.	0	n	1	0	Rosaceae Juss.
Iz_5_1109	DJI_0125	Rumex acetosella L.	0	n	1	0	Polygonaceae Juss.
Iz_5_1109	DJI_0125	Rumex thyrsiflorus Fingerh.	0	n	1	0	Polygonaceae Juss.
Iz_5_1109	DJI_0125	Salix alba L.	0	n	1	1	Salicaceae Mirb.
Iz_5_1109	DJI_0125	Sambucus nigra L.	0	n	1	1	Viburnaceae Raf.
Iz_5_1109	DJI_0125	Secale sylvestre Host	0	n	1	0	Poaceae Barnhart
Iz_5_1109	DJI_0125	Sempervivum ruthenicum Schnittsp. & C.B. Lehm.	0	n	1	0	Crassulaceae J.St.-Hil.
Iz_5_1109	DJI_0125	Setaria viridis (L.) P. Beauv.	0	n	1	0	Poaceae Barnhart
Iz_5_1109	DJI_0125	Silene borysthenica (Gruner) Walters	0	n	1	0	Caryophyllaceae Juss.
Iz_5_1109	DJI_0125	Silene latifolia subsp. alba (Mill.) Greuter & Burdet	0	n	1	0	Caryophyllaceae Juss.
Iz_5_1109	DJI_0125	Solidago virgaurea L.	0	n	1	0	Asteraceae Bercht. & J.Presl
Iz_5_1109	DJI_0125	Sorbus aucuparia L.	0	n	1	1	Rosaceae Juss.
Iz_5_1109	DJI_0125	Stachys recta L.	0	n	1	0	Lamiacea Lindl.
Iz_5_1109	DJI_0125	Tanacetum vulgare L.	0	n	1	0	Asteraceae Bercht. & J.Presl
Iz_5_1109	DJI_0125	Thymus pallasianus Heinr.Braun	0	n	1	0	Lamiacea Lindl.
Iz_5_1109	DJI_0125	Tilia cordata Mill.	0	n	1	1	Malvaceae Juss.
Iz_5_1109	DJI_0125	Trifolium alpestre L.	0	n	1	0	Fabaceae Lindl.
Iz_5_1109	DJI_0125	Trifolium arvense L.	0	n	1	0	Fabaceae Lindl.
Iz_5_1109	DJI_0125	Ulmus glabra Huds.	0	n	1	1	Ulmaceae Mirb.
Iz_5_1109	DJI_0125	Verbascum cf. phlomoides L.	0		NA	NA	
Iz_5_1109	DJI_0125	Verbascum densiflorum Bertol.	0	n	1	0	Scrophulariaceae Juss.
Iz_5_1109	DJI_0125	Verbascum sp.	0	n	1	0	Scrophulariaceae Juss.
Iz_5_1109	DJI_0125	Veronica austriaca L.	0	n	1	0	Plantaginaceae Juss.
Iz_5_1109	DJI_0125	Veronica incana L.	0	n	1	0	Plantaginaceae Juss.
Iz_5_1109	DJI_0125	Veronica verna L.	0	n	1	0	Plantaginaceae Juss.
Iz_5_1109	DJI_0125	Vincetoxicum hirundinaria Medik	0	n	1	0	Apocynaceae Juss.
Iz_5_1109	DJI_0126	Acer tataricum L.	0	n	1	1	Sapindaceae Juss.
Iz_5_1109	DJI_0126	Achillea millefolium L. s.l.	0	n	1	0	Asteraceae Bercht. & J.Presl
Iz_5_1109	DJI_0126	Allium oleraceum L.	0	n	1	0	Amaryllidaceae J.St.-Hil.
Iz_5_1109	DJI_0126	Ambrosia artemisiifolia L.	0	a	0	0	Asteraceae Bercht. & J.Presl
Iz_5_1109	DJI_0126	Amorpha fruticosa L.	0	a	0	0	Fabaceae Lindl.
Iz_5_1109	DJI_0126	Anthericum ramosum L.	0	n	1	0	Asparagaceae Juss.
Iz_5_1109	DJI_0126	Anthoxanthum repens (Host) Veldkamp	0		NA	NA	
Iz_5_1109	DJI_0126	Artemisia marschalliana Spreng.	0	n	1	0	Asteraceae Bercht. & J.Presl
Iz_5_1109	DJI_0126	Asparagus officinalis L.	0	n	1	0	Asparagaceae Juss.
Iz_5_1109	DJI_0126	Astragalus cicer L.	0	n	1	0	Fabaceae Lindl.
Iz_5_1109	DJI_0126	Bassia laniflora (S.G.Gmel.) A.J.Scott	0	n	1	0	Amaranthaceae Juss.
Iz_5_1109	DJI_0126	Berberis vulgaris L.	0	n	1	0	Berberidaceae Juss.
Iz_5_1109	DJI_0126	Berteroa incana (L.) DC.	0		NA	NA	
Iz_5_1109	DJI_0126	Betula pendula Roth.	0	n	1	1	Betulaceae Gray
Iz_5_1109	DJI_0126	Betula pendula Roth.	0	n	1	0	Poaceae Barnhart
Iz_5_1109	DJI_0126	Bromus inermis Leyss.	0		NA	NA	
Iz_5_1109	DJI_0126	Bromus squarrosus L.	0	a	0	0	Poaceae Barnhart
Iz_5_1109	DJI_0126	Bromus tectorum L.	0	a	0	0	Poaceae Barnhart
Iz_5_1109	DJI_0126	Calamagrostis epigejos (L.) Roth	0.1	n	1	0	Poaceae Barnhart
Iz_5_1109	DJI_0126	Carduus acanthoides L.	0	n	1	0	Asteraceae Bercht. & J.Presl
Iz_5_1109	DJI_0126	Carex sp.	0	n	1	0	Cyperaceae Juss.
Iz_5_1109	DJI_0126	Centaurea majorovii Dumbadze	0	n	1	0	Asteraceae Bercht. & J.Presl
Iz_5_1109	DJI_0126	Chamaecytisus ruthenicus (Fisch.ex Wol.) Klaskova	0	n	1	0	Fabaceae Lindl.
Iz_5_1109	DJI_0126	Chelidonium majus L.	0	n	1	0	Papaveraceae Juss.
Iz_5_1109	DJI_0126	Chenopodium betaceum Andrz.	0	n	1	0	Amaranthaceae Juss.
Iz_5_1109	DJI_0126	Chenopodium cf. album	0	n	1	0	Amaranthaceae Juss.
Iz_5_1109	DJI_0126	Chenopodium sp.	0		NA	0	Amaranthaceae Juss.
Iz_5_1109	DJI_0126	Chondrilla juncea L.	0	n	1	0	Asteraceae Bercht. & J.Presl
Iz_5_1109	DJI_0126	Cirsium vulgare (Savi) Ten.	0	n	1	0	Asteraceae Bercht. & J.Presl
Iz_5_1109	DJI_0126	Clinopodium acinos (L.) Kuntze	0	n	1	0	Lamiacea Lindl.
Iz_5_1109	DJI_0126	Convallaria majalis L.	0	n	1	0	Asparagaceae Juss.
Iz_5_1109	DJI_0126	Crataegus rhipidophylla Gand.	0	n	1	1	Rosaceae Juss.
Iz_5_1109	DJI_0126	Crepis foetida L.	0	n	1	0	Asteraceae Bercht. & J.Presl
Iz_5_1109	DJI_0126	Dactylis glomerata L.	0	n	1	0	Poaceae Barnhart
Iz_5_1109	DJI_0126	Dianthus campestris M.Bieb.	0	n	1	0	Caryophyllaceae Juss.
Iz_5_1109	DJI_0126	Digitaria sanguinalis (L.) Scop.	0	a	0	0	Poaceae Barnhart
Iz_5_1109	DJI_0126	Elymus repens (L.) Gould	0.2	n	1	0	Poaceae Barnhart
Iz_5_1109	DJI_0126	Epilobium tetragonum L.	0	n	1	0	Onagraceae Juss.
Iz_5_1109	DJI_0126	Eragrostis minor Host	0	a	0	0	Poaceae Barnhart
Iz_5_1109	DJI_0126	Erigeron canadensis L.	0.1	a	0	0	Asteraceae Bercht. & J.Presl
Iz_5_1109	DJI_0126	Euphorbia seguieriana Neck.	0	n	1	0	Euphorbiaceae Juss.
Iz_5_1109	DJI_0126	Fallopia dumetorum (L.) Holub	5	n	1	0	Polygonaceae Juss.
Iz_5_1109	DJI_0126	Festuca beckeri (Hack.) Trautv.	0	n	1	0	Poaceae Barnhart
Iz_5_1109	DJI_0126	Fragaria vesca L.	0	n	1	0	Rosaceae Juss.
Iz_5_1109	DJI_0126	Frangula alnus Mill.	0	n	1	1	Rhamnaceae Juss.
Iz_5_1109	DJI_0126	Fraxinus pennsylvanica Marshall	0	a	0	1	Oleaceae Hoffmanns. & Link
Iz_5_1109	DJI_0126	Galeopsis bifida Boenn.	0	n	1	0	Lamiacea Lindl.
Iz_5_1109	DJI_0126	Galium verum L.	0	n	1	0	Rubiaceae Juss.
Iz_5_1109	DJI_0126	Genista tinctoria L.	0	n	1	0	Fabaceae Lindl.

Iz_5_1109	DJI_0126	Geranium robertianum L.	0	n	1	0	Geraniaceae Juss.
Iz_5_1109	DJI_0126	Geranium sanquineum L.	0	n	1	0	Geraniaceae Juss.
Iz_5_1109	DJI_0126	Gypsophila paniculata L.	0	n	1	0	Caryophyllaceae Juss.
Iz_5_1109	DJI_0126	Helichrysum arenarium (L.) Moench	0	n	1	0	Asteraceae Bercht. & J.Presl
Iz_5_1109	DJI_0126	Herniaria polygama J.Gay	0	n	1	0	Caryophyllaceae Juss.
Iz_5_1109	DJI_0126	Hieracium robustum Fr.	0	n	1	0	Asteraceae Bercht. & J.Presl
Iz_5_1109	DJI_0126	Hieracium umbellatum L.	0	n	1	NA	Asteraceae Bercht. & J.Presl
Iz_5_1109	DJI_0126	Hylotelephium maximum (L.) Holub	0	n	1	0	Crassulaceae J.St.-Hil.
Iz_5_1109	DJI_0126	Jacobaea borysthenica (DC.) B.Nord. & Greuter	0	n	1	0	Asteraceae Bercht. & J.Presl
Iz_5_1109	DJI_0126	Jasione montana L.	0	n	1	0	Campanulaceae Juss.
Iz_5_1109	DJI_0126	Jurinea cyanoides (L.) Rchb.s.l.	0	n	1	0	Asteraceae Bercht. & J.Presl
Iz_5_1109	DJI_0126	Koeleria glauca (Spreng.) DC.	0	n	1	0	Poaceae Barnhart
Iz_5_1109	DJI_0126	Lactuca serriola L.	0	a	0	0	Asteraceae Bercht. & J.Presl
Iz_5_1109	DJI_0126	Linaria genistifolia (L.) Mill.	0	n	1	0	Plantaginaceae Juss.
Iz_5_1109	DJI_0126	Lomelosia argentea (L.) Greuter & Burdet	0	n	1	0	Caprifoliaceae Juss.
Iz_5_1109	DJI_0126	Melampyrum pratense L.	0	n	1	0	Orobanchaceae Vent.
Iz_5_1109	DJI_0126	Oenothera biennis L.	0	a	0	0	Onagraceae Juss.
Iz_5_1109	DJI_0126	Pentanema britannica (L.) D.Gut.Larr., Santos-Vicente, Anderb., E.Rico & M.M.Mart.Ort.	0	n	1	0	Asteraceae Bercht. & J.Presl
Iz_5_1109	DJI_0126	Peucedanum oreoselinum (L.) Moench	0	n	1	0	Apiaceae Lindl.
Iz_5_1109	DJI_0126	Peucedanum ruthenicum M.Bieb.	0	n	1	0	Apiaceae Lindl.
Iz_5_1109	DJI_0126	Pilosella echioides (Lumn.) F.W.Schultz & Sch.Bip.	0	n	1	0	Asteraceae Bercht. & J.Presl
Iz_5_1109	DJI_0126	Pilosella officinarum F. Schultz et Sch. Bip.	0	n	1	0	Asteraceae Bercht. & J.Presl
Iz_5_1109	DJI_0126	Pilosella sp.	0		NA	0	Asteraceae Bercht. & J.Presl
Iz_5_1109	DJI_0126	Pinus sylvestris L.	0	n	1	1	Pinaceae Spreng. ex F.Rudolphi
Iz_5_1109	DJI_0126	Poa annua L.	0	n	1	0	Poaceae Barnhart
Iz_5_1109	DJI_0126	Poa nemoralis L.	0	n	1	0	Poaceae Barnhart
Iz_5_1109	DJI_0126	Poa sp.	0		NA	0	Poaceae Barnhart
Iz_5_1109	DJI_0126	Polygonatum odoratum (Mill.) Druce	0	n	1	0	Asparagaceae Juss.
Iz_5_1109	DJI_0126	Polygonum arenarium Waldst. & Kit.	0	n	1	0	Polygonaceae Juss.
Iz_5_1109	DJI_0126	Populus nigra L.	0	n	1	1	Salicaceae Mirb.
Iz_5_1109	DJI_0126	Populus tremula L.	0	n	1	1	Salicaceae Mirb.
Iz_5_1109	DJI_0126	Populus Ã canescens (Aiton) Sm.	0	n	1	1	Salicaceae Mirb.
Iz_5_1109	DJI_0126	Potentilla argentea L.	0	n	1	0	Rosaceae Juss.
Iz_5_1109	DJI_0126	Psammophiliella muralis (L.) Ikonn.	0	n	1	0	Caryophyllaceae Juss.
Iz_5_1109	DJI_0126	Psephellus sumensis (Kalen.) Greuter	0	n	1	0	Asteraceae Bercht. & J.Presl
Iz_5_1109	DJI_0126	Pulsatilla pratensis (L.) Mill. s.l.	0	n	1	0	Ranunculaceae Juss.
Iz_5_1109	DJI_0126	Pyrus communis L.	0	a	0	1	Rosaceae Juss.
Iz_5_1109	DJI_0126	Quercus robur L.	0	n	1	1	Fagaceae Dumort.
Iz_5_1109	DJI_0126	Robinia pseudoacacia L.	0	a	0	1	Fabaceae Lindl.
Iz_5_1109	DJI_0126	Rosa sp.	0		NA	1	Rosaceae Juss.
Iz_5_1109	DJI_0126	Rubus idaeus L.	0	n	1	0	Rosaceae Juss.
Iz_5_1109	DJI_0126	Rumex acetosella L.	0	n	1	0	Polygonaceae Juss.
Iz_5_1109	DJI_0126	Rumex thyrsiflorus Fingerh.	0	n	1	0	Polygonaceae Juss.
Iz_5_1109	DJI_0126	Salix alba L.	0	n	1	1	Salicaceae Mirb.
Iz_5_1109	DJI_0126	Sambucus nigra L.	0	n	1	1	Viburnaceae Raf.
Iz_5_1109	DJI_0126	Secale sylvestre Host	0	n	1	0	Poaceae Barnhart
Iz_5_1109	DJI_0126	Sempervivum ruthenicum Schnittsp. & C.B. Lehm.	0	n	1	0	Crassulaceae J.St.-Hil.
Iz_5_1109	DJI_0126	Setaria viridis (L.) P. Beauv.	0	n	1	0	Poaceae Barnhart
Iz_5_1109	DJI_0126	Silene borysthenica (Gruner) Walters	0	n	1	0	Caryophyllaceae Juss.
Iz_5_1109	DJI_0126	Silene latifolia subsp. alba (Mill.) Greuter & Burdet	0	n	1	0	Caryophyllaceae Juss.
Iz_5_1109	DJI_0126	Solidago virgaurea L.	0	n	1	0	Asteraceae Bercht. & J.Presl
Iz_5_1109	DJI_0126	Sorbus aucuparia L.	0	n	1	1	Rosaceae Juss.
Iz_5_1109	DJI_0126	Stachys recta L.	0	n	1	0	Lamiacea Lindl.
Iz_5_1109	DJI_0126	Tanacetum vulgare L.	0	n	1	0	Asteraceae Bercht. & J.Presl
Iz_5_1109	DJI_0126	Thymus pallasianus Heinr.Braun	0	n	1	0	Lamiacea Lindl.
Iz_5_1109	DJI_0126	Tilia cordata Mill.	0	n	1	1	Malvaceae Juss.
Iz_5_1109	DJI_0126	Trifolium alpestre L.	0	n	1	0	Fabaceae Lindl.
Iz_5_1109	DJI_0126	Trifolium arvense L.	0	n	1	0	Fabaceae Lindl.
Iz_5_1109	DJI_0126	Ulmus glabra Huds.	0	n	1	1	Ulmaceae Mirb.
Iz_5_1109	DJI_0126	Verbascum cf. phlomoides L.	0		NA	NA	
Iz_5_1109	DJI_0126	Verbascum densiflorum Bertol.	0	n	1	0	Scrophulariaceae Juss.
Iz_5_1109	DJI_0126	Verbascum sp.	0	n	1	0	Scrophulariaceae Juss.
Iz_5_1109	DJI_0126	Veronica austriaca L.	0	n	1	0	Plantaginaceae Juss.
Iz_5_1109	DJI_0126	Veronica incana L.	0	n	1	0	Plantaginaceae Juss.
Iz_5_1109	DJI_0126	Veronica verna L.	0	n	1	0	Plantaginaceae Juss.
Iz_5_1109	DJI_0126	Vincetoxicum hirundinaria Medik	0	n	1	0	Apocynaceae Juss.
Iz_5_1109	DJI_0127	Acer tataricum L.	0	n	1	1	Sapindaceae Juss.
Iz_5_1109	DJI_0127	Achillea millefolium L. s.l.	0	n	1	0	Asteraceae Bercht. & J.Presl
Iz_5_1109	DJI_0127	Allium oleraceum L.	0	n	1	0	Amaryllidaceae J.St.-Hil.
Iz_5_1109	DJI_0127	Ambrosia artemisiifolia L.	0	a	0	0	Asteraceae Bercht. & J.Presl
Iz_5_1109	DJI_0127	Amorpha fruticosa L.	0	a	0	0	Fabaceae Lindl.
Iz_5_1109	DJI_0127	Anthericum ramosum L.	0	n	1	0	Asparagaceae Juss.
Iz_5_1109	DJI_0127	Anthoxanthum repens (Host) Veldkamp	0		NA	NA	
Iz_5_1109	DJI_0127	Artemisia marschalliana Spreng.	0	n	1	0	Asteraceae Bercht. & J.Presl
Iz_5_1109	DJI_0127	Asparagus officinalis L.	0	n	1	0	Asparagaceae Juss.
Iz_5_1109	DJI_0127	Astragalus cicer L.	0	n	1	0	Fabaceae Lindl.
Iz_5_1109	DJI_0127	Bassia laniflora (S.G.Gmel.) A.J.Scott	0	n	1	0	Amaranthaceae Juss.
Iz_5_1109	DJI_0127	Berberis vulgaris L.	0	n	1	0	Berberidaceae Juss.
Iz_5_1109	DJI_0127	Berteroa incana (L.) DC.	0		NA	NA	
Iz_5_1109	DJI_0127	Betula pendula Roth.	0	n	1	1	Betulaceae Gray
Iz_5_1109	DJI_0127	Betula pendula Roth.	0	n	1	0	Poaceae Barnhart
Iz_5_1109	DJI_0127	Bromus inermis Leyss.	0		NA	NA	
Iz_5_1109	DJI_0127	Bromus squarrosus L.	0	a	0	0	Poaceae Barnhart
Iz_5_1109	DJI_0127	Bromus tectorum L.	0	a	0	0	Poaceae Barnhart
Iz_5_1109	DJI_0127	Calamagrostis epigejos (L.) Roth	3	n	1	0	Poaceae Barnhart
Iz_5_1109	DJI_0127	Carduus acanthoides L.	0	n	1	0	Asteraceae Bercht. & J.Presl
Iz_5_1109	DJI_0127	Carex sp.	0	n	1	0	Cyperaceae Juss.
Iz_5_1109	DJI_0127	Centaurea majorovii Dumbadze	0	n	1	0	Asteraceae Bercht. & J.Presl
Iz_5_1109	DJI_0127	Chamaecytisus ruthenicus (Fisch.ex Wol.) Klaskova	0	n	1	0	Fabaceae Lindl.
Iz_5_1109	DJI_0127	Chelidonium majus L.	0	n	1	0	Papaveraceae Juss.
Iz_5_1109	DJI_0127	Chenopodium betaceum Andrz.	0	n	1	0	Amaranthaceae Juss.
Iz_5_1109	DJI_0127	Chenopodium cf. album	0	n	1	0	Amaranthaceae Juss.
Iz_5_1109	DJI_0127	Chenopodium sp.	0		NA	0	Amaranthaceae Juss.
Iz_5_1109	DJI_0127	Chondrilla juncea L.	0	n	1	0	Asteraceae Bercht. & J.Presl
Iz_5_1109	DJI_0127	Cirsium vulgare (Savi) Ten.	0	n	1	0	Asteraceae Bercht. & J.Presl
Iz_5_1109	DJI_0127	Clinopodium acinos (L.) Kuntze	0	n	1	0	Lamiacea Lindl.
Iz_5_1109	DJI_0127	Convallaria majalis L.	0	n	1	0	Asparagaceae Juss.
Iz_5_1109	DJI_0127	Crataegus rhipidophylla Gand.	0	n	1	1	Rosaceae Juss.

Iz_5_1109	DJI_0127	Crepis foetida L.	0	n	1	0	Asteraceae Bercht. & J.Presl
Iz_5_1109	DJI_0127	Dactylis glomerata L.	0	n	1	0	Poaceae Barnhart
Iz_5_1109	DJI_0127	Dianthus campestris M.Bieb.	0	n	1	0	Caryophyllaceae Juss.
Iz_5_1109	DJI_0127	Digitaria sanguinalis (L.) Scop.	0	a	0	0	Poaceae Barnhart
Iz_5_1109	DJI_0127	Elymus repens (L.) Gould	0	n	1	0	Poaceae Barnhart
Iz_5_1109	DJI_0127	Epilobium tetragonum L.	0	n	1	0	Onagraceae Juss.
Iz_5_1109	DJI_0127	Eragrostis minor Host	0	a	0	0	Poaceae Barnhart
Iz_5_1109	DJI_0127	Erigeron canadensis L.	0.1	a	0	0	Asteraceae Bercht. & J.Presl
Iz_5_1109	DJI_0127	Euphorbia seguieriana Neck.	0	n	1	0	Euphorbiaceae Juss.
Iz_5_1109	DJI_0127	Fallopia dumetorum (L.) Holub	5	n	1	0	Polygonaceae Juss.
Iz_5_1109	DJI_0127	Festuca beckeri (Hack.) Trautv.	0	n	1	0	Poaceae Barnhart
Iz_5_1109	DJI_0127	Fragaria vesca L.	0	n	1	0	Rosaceae Juss.
Iz_5_1109	DJI_0127	Frangula alnus Mill.	0	n	1	1	Rhamnaceae Juss.
Iz_5_1109	DJI_0127	Fraxinus pennsylvanica Marshall	0	a	0	1	Oleaceae Hoffmanns. & Link
Iz_5_1109	DJI_0127	Galeopsis bifida Boenn.	0	n	1	0	Lamiacea Lindl.
Iz_5_1109	DJI_0127	Galium verum L.	0	n	1	0	Rubiaceae Juss.
Iz_5_1109	DJI_0127	Genista tinctoria L.	0	n	1	0	Fabaceae Lindl.
Iz_5_1109	DJI_0127	Geranium robertianum L.	0	n	1	0	Geraniaceae Juss.
Iz_5_1109	DJI_0127	Geranium sanquineum L.	0	n	1	0	Geraniaceae Juss.
Iz_5_1109	DJI_0127	Gypsophila paniculata L.	0	n	1	0	Caryophyllaceae Juss.
Iz_5_1109	DJI_0127	Helichrysum arenarium (L.) Moench	0	n	1	0	Asteraceae Bercht. & J.Presl
Iz_5_1109	DJI_0127	Herniaria polygama J.Gay	0	n	1	0	Caryophyllaceae Juss.
Iz_5_1109	DJI_0127	Hieracium robustum Fr.	0	n	1	0	Asteraceae Bercht. & J.Presl
Iz_5_1109	DJI_0127	Hieracium umbellatum L.	0	n	1	NA	Asteraceae Bercht. & J.Presl
Iz_5_1109	DJI_0127	Hylotelephium maximum (L.) Holub	0	n	1	0	Crassulaceae J.St.-Hil.
Iz_5_1109	DJI_0127	Jacobaea borysthenica (DC.) B.Nord. & Greuter	0	n	1	0	Asteraceae Bercht. & J.Presl
Iz_5_1109	DJI_0127	Jasione montana L.	0	n	1	0	Campanulaceae Juss.
Iz_5_1109	DJI_0127	Jurinea cyanoides (L.) Rchb.s.l.	0	n	1	0	Asteraceae Bercht. & J.Presl
Iz_5_1109	DJI_0127	Koeleria glauca (Spreng.) DC.	0	n	1	0	Poaceae Barnhart
Iz_5_1109	DJI_0127	Lactuca serriola L.	0.1	a	0	0	Asteraceae Bercht. & J.Presl
Iz_5_1109	DJI_0127	Linaria genistifolia (L.) Mill.	0	n	1	0	Plantaginaceae Juss.
Iz_5_1109	DJI_0127	Lomelosia argentea (L.) Greuter & Burdet	0	n	1	0	Caprifoliaceae Juss.
Iz_5_1109	DJI_0127	Melampyrum pratense L.	0	n	1	0	Orobanchaceae Vent.
Iz_5_1109	DJI_0127	Oenothera biennis L.	0	a	0	0	Onagraceae Juss.
Iz_5_1109	DJI_0127	Pentanema britannica (L.) D.Gut.Larr., Santos-Vicente, Anderb., E.Rico & M.M.Mart.Ort.	0	n	1	0	Asteraceae Bercht. & J.Presl
Iz_5_1109	DJI_0127	Peucedanum oreoselinum (L.) Moench	0	n	1	0	Apiaceae Lindl.
Iz_5_1109	DJI_0127	Peucedanum ruthenicum M.Bieb.	0	n	1	0	Apiaceae Lindl.
Iz_5_1109	DJI_0127	Pilosella echioides (Lumn.) F.W.Schultz & Sch.Bip.	0	n	1	0	Asteraceae Bercht. & J.Presl
Iz_5_1109	DJI_0127	Pilosella officinarum F. Schultz et Sch. Bip.	0	n	1	0	Asteraceae Bercht. & J.Presl
Iz_5_1109	DJI_0127	Pilosella sp.	0		NA	0	Asteraceae Bercht. & J.Presl
Iz_5_1109	DJI_0127	Pinus sylvestris L.	0	n	1	1	Pinaceae Spreng. ex F.Rudolphi
Iz_5_1109	DJI_0127	Poa annua L.	0	n	1	0	Poaceae Barnhart
Iz_5_1109	DJI_0127	Poa nemoralis L.	0	n	1	0	Poaceae Barnhart
Iz_5_1109	DJI_0127	Poa sp.	0		NA	0	Poaceae Barnhart
Iz_5_1109	DJI_0127	Polygonatum odoratum (Mill.) Druce	0	n	1	0	Asparagaceae Juss.
Iz_5_1109	DJI_0127	Polygonum arenarium Waldst. & Kit.	0	n	1	0	Polygonaceae Juss.
Iz_5_1109	DJI_0127	Populus nigra L.	0	n	1	1	Salicaceae Mirb.
Iz_5_1109	DJI_0127	Populus tremula L.	0	n	1	1	Salicaceae Mirb.
Iz_5_1109	DJI_0127	Populus Ã canescens (Aiton) Sm.	0	n	1	1	Salicaceae Mirb.
Iz_5_1109	DJI_0127	Potentilla argentea L.	0	n	1	0	Rosaceae Juss.
Iz_5_1109	DJI_0127	Psammophiliella muralis (L.) Ikonn.	0	n	1	0	Caryophyllaceae Juss.
Iz_5_1109	DJI_0127	Psephellus sumensis (Kalen.) Greuter	0	n	1	0	Asteraceae Bercht. & J.Presl
Iz_5_1109	DJI_0127	Pulsatilla pratensis (L.) Mill. s.l.	0	n	1	0	Ranunculaceae Juss.
Iz_5_1109	DJI_0127	Pyrus communis L.	0	a	0	1	Rosaceae Juss.
Iz_5_1109	DJI_0127	Quercus robur L.	0	n	1	1	Fagaceae Dumort.
Iz_5_1109	DJI_0127	Robinia pseudoacacia L.	0	a	0	1	Fabaceae Lindl.
Iz_5_1109	DJI_0127	Rosa sp.	0		NA	1	Rosaceae Juss.
Iz_5_1109	DJI_0127	Rubus idaeus L.	0	n	1	0	Rosaceae Juss.
Iz_5_1109	DJI_0127	Rumex acetosella L.	0	n	1	0	Polygonaceae Juss.
Iz_5_1109	DJI_0127	Rumex thyrsiflorus Fingerh.	0	n	1	0	Polygonaceae Juss.
Iz_5_1109	DJI_0127	Salix alba L.	0	n	1	1	Salicaceae Mirb.
Iz_5_1109	DJI_0127	Sambucus nigra L.	0	n	1	1	Viburnaceae Raf.
Iz_5_1109	DJI_0127	Secale sylvestre Host	0	n	1	0	Poaceae Barnhart
Iz_5_1109	DJI_0127	Sempervivum ruthenicum Schnittsp. & C.B. Lehm.	0	n	1	0	Crassulaceae J.St.-Hil.
Iz_5_1109	DJI_0127	Setaria viridis (L.) P. Beauv.	0	n	1	0	Poaceae Barnhart
Iz_5_1109	DJI_0127	Silene borysthenica (Gruner) Walters	0	n	1	0	Caryophyllaceae Juss.
Iz_5_1109	DJI_0127	Silene latifolia subsp. alba (Mill.) Greuter & Burdet	0	n	1	0	Caryophyllaceae Juss.
Iz_5_1109	DJI_0127	Solidago virgaurea L.	0	n	1	0	Asteraceae Bercht. & J.Presl
Iz_5_1109	DJI_0127	Sorbus aucuparia L.	0	n	1	1	Rosaceae Juss.
Iz_5_1109	DJI_0127	Stachys recta L.	0	n	1	0	Lamiacea Lindl.
Iz_5_1109	DJI_0127	Tanacetum vulgare L.	0	n	1	0	Asteraceae Bercht. & J.Presl
Iz_5_1109	DJI_0127	Thymus pallasianus Heinr.Braun	0	n	1	0	Lamiacea Lindl.
Iz_5_1109	DJI_0127	Tilia cordata Mill.	0	n	1	1	Malvaceae Juss.
Iz_5_1109	DJI_0127	Trifolium alpestre L.	0	n	1	0	Fabaceae Lindl.
Iz_5_1109	DJI_0127	Trifolium arvense L.	0	n	1	0	Fabaceae Lindl.
Iz_5_1109	DJI_0127	Ulmus glabra Huds.	0	n	1	1	Ulmaceae Mirb.
Iz_5_1109	DJI_0127	Verbascum cf. phlomoides L.	0		NA	NA	
Iz_5_1109	DJI_0127	Verbascum densiflorum Bertol.	0	n	1	0	Scrophulariaceae Juss.
Iz_5_1109	DJI_0127	Verbascum sp.	0	n	1	0	Scrophulariaceae Juss.
Iz_5_1109	DJI_0127	Veronica austriaca L.	0	n	1	0	Plantaginaceae Juss.
Iz_5_1109	DJI_0127	Veronica incana L.	0	n	1	0	Plantaginaceae Juss.
Iz_5_1109	DJI_0127	Veronica verna L.	0	n	1	0	Plantaginaceae Juss.
Iz_5_1109	DJI_0127	Vincetoxicum hirundinaria Medik	0	n	1	0	Apocynaceae Juss.
Iz_5_1109	DJI_0130	Acer tataricum L.	0	n	1	1	Sapindaceae Juss.
Iz_5_1109	DJI_0130	Achillea millefolium L. s.l.	0	n	1	0	Asteraceae Bercht. & J.Presl
Iz_5_1109	DJI_0130	Allium oleraceum L.	0	n	1	0	Amaryllidaceae J.St.-Hil.
Iz_5_1109	DJI_0130	Ambrosia artemisiifolia L.	0	a	0	0	Asteraceae Bercht. & J.Presl
Iz_5_1109	DJI_0130	Amorpha fruticosa L.	0	a	0	0	Fabaceae Lindl.
Iz_5_1109	DJI_0130	Anthericum ramosum L.	0	n	1	0	Asparagaceae Juss.
Iz_5_1109	DJI_0130	Anthoxanthum repens (Host) Veldkamp	0		NA	NA	
Iz_5_1109	DJI_0130	Artemisia marschalliana Spreng.	0	n	1	0	Asteraceae Bercht. & J.Presl
Iz_5_1109	DJI_0130	Asparagus officinalis L.	0	n	1	0	Asparagaceae Juss.
Iz_5_1109	DJI_0130	Astragalus cicer L.	0	n	1	0	Fabaceae Lindl.
Iz_5_1109	DJI_0130	Bassia laniflora (S.G.Gmel.) A.J.Scott	0	n	1	0	Amaranthaceae Juss.
Iz_5_1109	DJI_0130	Berberis vulgaris L.	0	n	1	0	Berberidaceae Juss.
Iz_5_1109	DJI_0130	Berteroa incana (L.) DC.	0		NA	NA	
Iz_5_1109	DJI_0130	Betula pendula Roth.	0	n	1	1	Betulaceae Gray
Iz_5_1109	DJI_0130	Betula pendula Roth.	0	n	1	0	Poaceae Barnhart

Iz_5_1109	DJI_0130	Bromus inermis Leyss.	0		NA	NA	
Iz_5_1109	DJI_0130	Bromus squarrosus L.	0	a	0	0	Poaceae Barnhart
Iz_5_1109	DJI_0130	Bromus tectorum L.	0	a	0	0	Poaceae Barnhart
Iz_5_1109	DJI_0130	Calamagrostis epigejos (L.) Roth	0	n	1	0	Poaceae Barnhart
Iz_5_1109	DJI_0130	Carduus acanthoides L.	0	n	1	0	Asteraceae Bercht. & J.Presl
Iz_5_1109	DJI_0130	Carex sp.	0	n	1	0	Cyperaceae Juss.
Iz_5_1109	DJI_0130	Centaurea majorovii Dumbadze	0	n	1	0	Asteraceae Bercht. & J.Presl
Iz_5_1109	DJI_0130	Chamaecytisus ruthenicus (Fisch.ex Wol.) Klaskova	0	n	1	0	Fabaceae Lindl.
Iz_5_1109	DJI_0130	Chelidonium majus L.	0	n	1	0	Papaveraceae Juss.
Iz_5_1109	DJI_0130	Chenopodium betaceum Andrz.	0	n	1	0	Amaranthaceae Juss.
Iz_5_1109	DJI_0130	Chenopodium cf. album	0	n	1	0	Amaranthaceae Juss.
Iz_5_1109	DJI_0130	Chenopodium sp.	0		NA	0	Amaranthaceae Juss.
Iz_5_1109	DJI_0130	Chondrilla juncea L.	0	n	1	0	Asteraceae Bercht. & J.Presl
Iz_5_1109	DJI_0130	Cirsium vulgare (Savi) Ten.	0	n	1	0	Asteraceae Bercht. & J.Presl
Iz_5_1109	DJI_0130	Clinopodium acinos (L.) Kuntze	0	n	1	0	Lamiacea Lindl.
Iz_5_1109	DJI_0130	Convallaria majalis L.	0	n	1	0	Asparagaceae Juss.
Iz_5_1109	DJI_0130	Crataegus rhipidophylla Gand.	0	n	1	1	Rosaceae Juss.
Iz_5_1109	DJI_0130	Crepis foetida L.	0	n	1	0	Asteraceae Bercht. & J.Presl
Iz_5_1109	DJI_0130	Dactylis glomerata L.	0	n	1	0	Poaceae Barnhart
Iz_5_1109	DJI_0130	Dianthus campestris M.Bieb.	0	n	1	0	Caryophyllaceae Juss.
Iz_5_1109	DJI_0130	Digitaria sanguinalis (L.) Scop.	0	a	0	0	Poaceae Barnhart
Iz_5_1109	DJI_0130	Elymus repens (L.) Gould	0	n	1	0	Poaceae Barnhart
Iz_5_1109	DJI_0130	Epilobium tetragonum L.	0	n	1	0	Onagraceae Juss.
Iz_5_1109	DJI_0130	Eragrostis minor Host	0	a	0	0	Poaceae Barnhart
Iz_5_1109	DJI_0130	Erigeron canadensis L.	0.2	a	0	0	Asteraceae Bercht. & J.Presl
Iz_5_1109	DJI_0130	Euphorbia seguieriana Neck.	0	n	1	0	Euphorbiaceae Juss.
Iz_5_1109	DJI_0130	Fallopia dumetorum (L.) Holub	2	n	1	0	Polygonaceae Juss.
Iz_5_1109	DJI_0130	Festuca beckeri (Hack.) Trautv.	0	n	1	0	Poaceae Barnhart
Iz_5_1109	DJI_0130	Fragaria vesca L.	0	n	1	0	Rosaceae Juss.
Iz_5_1109	DJI_0130	Frangula alnus Mill.	0	n	1	1	Rhamnaceae Juss.
Iz_5_1109	DJI_0130	Fraxinus pennsylvanica Marshall	0	a	0	1	Oleaceae Hoffmanns. & Link
Iz_5_1109	DJI_0130	Galeopsis bifida Boenn.	0	n	1	0	Lamiacea Lindl.
Iz_5_1109	DJI_0130	Galium verum L.	0	n	1	0	Rubiaceae Juss.
Iz_5_1109	DJI_0130	Genista tinctoria L.	0	n	1	0	Fabaceae Lindl.
Iz_5_1109	DJI_0130	Geranium robertianum L.	0	n	1	0	Geraniaceae Juss.
Iz_5_1109	DJI_0130	Geranium sanquineum L.	0	n	1	0	Geraniaceae Juss.
Iz_5_1109	DJI_0130	Gypsophila paniculata L.	0	n	1	0	Caryophyllaceae Juss.
Iz_5_1109	DJI_0130	Helichrysum arenarium (L.) Moench	0	n	1	0	Asteraceae Bercht. & J.Presl
Iz_5_1109	DJI_0130	Herniaria polygama J.Gay	0	n	1	0	Caryophyllaceae Juss.
Iz_5_1109	DJI_0130	Hieracium robustum Fr.	0	n	1	0	Asteraceae Bercht. & J.Presl
Iz_5_1109	DJI_0130	Hieracium umbellatum L.	0	n	1	NA	Asteraceae Bercht. & J.Presl
Iz_5_1109	DJI_0130	Hylotelephium maximum (L.) Holub	0	n	1	0	Crassulaceae J.St.-Hil.
Iz_5_1109	DJI_0130	Jacobaea borysthenica (DC.) B.Nord. & Greuter	0	n	1	0	Asteraceae Bercht. & J.Presl
Iz_5_1109	DJI_0130	Jasione montana L.	0	n	1	0	Campanulaceae Juss.
Iz_5_1109	DJI_0130	Jurinea cyanoides (L.) Rchb.s.l.	0	n	1	0	Asteraceae Bercht. & J.Presl
Iz_5_1109	DJI_0130	Koeleria glauca (Spreng.) DC.	0	n	1	0	Poaceae Barnhart
Iz_5_1109	DJI_0130	Lactuca serriola L.	0	a	0	0	Asteraceae Bercht. & J.Presl
Iz_5_1109	DJI_0130	Linaria genistifolia (L.) Mill.	0	n	1	0	Plantaginaceae Juss.
Iz_5_1109	DJI_0130	Lomelosia argentea (L.) Greuter & Burdet	0	n	1	0	Caprifoliaceae Juss.
Iz_5_1109	DJI_0130	Melampyrum pratense L.	0	n	1	0	Orobanchaceae Vent.
Iz_5_1109	DJI_0130	Oenothera biennis L.	0	a	0	0	Onagraceae Juss.
Iz_5_1109	DJI_0130	Pentanema britannica (L.) D.Gut.Larr., Santos-Vicente, Anderb., E.Rico & M.M.Mart.Ort.	0	n	1	0	Asteraceae Bercht. & J.Presl
Iz_5_1109	DJI_0130	Peucedanum oreoselinum (L.) Moench	0	n	1	0	Apiaceae Lindl.
Iz_5_1109	DJI_0130	Peucedanum ruthenicum M.Bieb.	0	n	1	0	Apiaceae Lindl.
Iz_5_1109	DJI_0130	Pilosella echioides (Lumn.) F.W.Schultz & Sch.Bip.	0	n	1	0	Asteraceae Bercht. & J.Presl
Iz_5_1109	DJI_0130	Pilosella officinarum F. Schultz et Sch. Bip.	0	n	1	0	Asteraceae Bercht. & J.Presl
Iz_5_1109	DJI_0130	Pilosella sp.	0		NA	0	Asteraceae Bercht. & J.Presl
Iz_5_1109	DJI_0130	Pinus sylvestris L.	0	n	1	1	Pinaceae Spreng. ex F.Rudolphi
Iz_5_1109	DJI_0130	Poa annua L.	0	n	1	0	Poaceae Barnhart
Iz_5_1109	DJI_0130	Poa nemoralis L.	0	n	1	0	Poaceae Barnhart
Iz_5_1109	DJI_0130	Poa sp.	0		NA	0	Poaceae Barnhart
Iz_5_1109	DJI_0130	Polygonatum odoratum (Mill.) Druce	0	n	1	0	Asparagaceae Juss.
Iz_5_1109	DJI_0130	Polygonum arenarium Waldst. & Kit.	0	n	1	0	Polygonaceae Juss.
Iz_5_1109	DJI_0130	Populus nigra L.	0	n	1	1	Salicaceae Mirb.
Iz_5_1109	DJI_0130	Populus tremula L.	0	n	1	1	Salicaceae Mirb.
Iz_5_1109	DJI_0130	Populus Ã canescens (Aiton) Sm.	0	n	1	1	Salicaceae Mirb.
Iz_5_1109	DJI_0130	Potentilla argentea L.	0	n	1	0	Rosaceae Juss.
Iz_5_1109	DJI_0130	Psammophiliella muralis (L.) Ikonn.	0	n	1	0	Caryophyllaceae Juss.
Iz_5_1109	DJI_0130	Psephellus sumensis (Kalen.) Greuter	0	n	1	0	Asteraceae Bercht. & J.Presl
Iz_5_1109	DJI_0130	Pulsatilla pratensis (L.) Mill. s.l.	0	n	1	0	Ranunculaceae Juss.
Iz_5_1109	DJI_0130	Pyrus communis L.	0	a	0	1	Rosaceae Juss.
Iz_5_1109	DJI_0130	Quercus robur L.	0	n	1	1	Fagaceae Dumort.
Iz_5_1109	DJI_0130	Robinia pseudoacacia L.	0	a	0	1	Fabaceae Lindl.
Iz_5_1109	DJI_0130	Rosa sp.	0		NA	1	Rosaceae Juss.
Iz_5_1109	DJI_0130	Rubus idaeus L.	0	n	1	0	Rosaceae Juss.
Iz_5_1109	DJI_0130	Rumex acetosella L.	0.5	n	1	0	Polygonaceae Juss.
Iz_5_1109	DJI_0130	Rumex thyrsiflorus Fingerh.	0	n	1	0	Polygonaceae Juss.
Iz_5_1109	DJI_0130	Salix alba L.	0	n	1	1	Salicaceae Mirb.
Iz_5_1109	DJI_0130	Sambucus nigra L.	0	n	1	1	Viburnaceae Raf.
Iz_5_1109	DJI_0130	Secale sylvestre Host	0	n	1	0	Poaceae Barnhart
Iz_5_1109	DJI_0130	Sempervivum ruthenicum Schnittsp. & C.B. Lehm.	0	n	1	0	Crassulaceae J.St.-Hil.
Iz_5_1109	DJI_0130	Setaria viridis (L.) P. Beauv.	0	n	1	0	Poaceae Barnhart
Iz_5_1109	DJI_0130	Silene borysthenica (Gruner) Walters	0	n	1	0	Caryophyllaceae Juss.
Iz_5_1109	DJI_0130	Silene latifolia subsp. alba (Mill.) Greuter & Burdet	0	n	1	0	Caryophyllaceae Juss.
Iz_5_1109	DJI_0130	Solidago virgaurea L.	0	n	1	0	Asteraceae Bercht. & J.Presl
Iz_5_1109	DJI_0130	Sorbus aucuparia L.	0	n	1	1	Rosaceae Juss.
Iz_5_1109	DJI_0130	Stachys recta L.	0	n	1	0	Lamiacea Lindl.
Iz_5_1109	DJI_0130	Tanacetum vulgare L.	0	n	1	0	Asteraceae Bercht. & J.Presl
Iz_5_1109	DJI_0130	Thymus pallasianus Heinr.Braun	0	n	1	0	Lamiacea Lindl.
Iz_5_1109	DJI_0130	Tilia cordata Mill.	0	n	1	1	Malvaceae Juss.
Iz_5_1109	DJI_0130	Trifolium alpestre L.	0	n	1	0	Fabaceae Lindl.
Iz_5_1109	DJI_0130	Trifolium arvense L.	0	n	1	0	Fabaceae Lindl.
Iz_5_1109	DJI_0130	Ulmus glabra Huds.	0	n	1	1	Ulmaceae Mirb.
Iz_5_1109	DJI_0130	Verbascum cf. phlomoides L.	0		NA	NA	
Iz_5_1109	DJI_0130	Verbascum densiflorum Bertol.	0	n	1	0	Scrophulariaceae Juss.
Iz_5_1109	DJI_0130	Verbascum sp.	0	n	1	0	Scrophulariaceae Juss.
Iz_5_1109	DJI_0130	Veronica austriaca L.	0	n	1	0	Plantaginaceae Juss.
Iz_5_1109	DJI_0130	Veronica incana L.	0	n	1	0	Plantaginaceae Juss.

Iz_5_1109	DJI_0130	Veronica verna L.	0	n	1	0	Plantaginaceae Juss.
Iz_5_1109	DJI_0130	Vincetoxicum hirundinaria Medik	0	n	1	0	Apocynaceae Juss.
Iz_5_1109	DJI_0132	Acer tataricum L.	0	n	1	1	Sapindaceae Juss.
Iz_5_1109	DJI_0132	Achillea millefolium L. s.l.	0	n	1	0	Asteraceae Bercht. & J.Presl
Iz_5_1109	DJI_0132	Allium oleraceum L.	0	n	1	0	Amaryllidaceae J.St.-Hil.
Iz_5_1109	DJI_0132	Ambrosia artemisiifolia L.	0	a	0	0	Asteraceae Bercht. & J.Presl
Iz_5_1109	DJI_0132	Amorpha fruticosa L.	0	a	0	0	Fabaceae Lindl.
Iz_5_1109	DJI_0132	Anthericum ramosum L.	0	n	1	0	Asparagaceae Juss.
Iz_5_1109	DJI_0132	Anthoxanthum repens (Host) Veldkamp	0		NA	NA	
Iz_5_1109	DJI_0132	Artemisia marschalliana Spreng.	0	n	1	0	Asteraceae Bercht. & J.Presl
Iz_5_1109	DJI_0132	Asparagus officinalis L.	0	n	1	0	Asparagaceae Juss.
Iz_5_1109	DJI_0132	Astragalus cicer L.	0	n	1	0	Fabaceae Lindl.
Iz_5_1109	DJI_0132	Bassia laniflora (S.G.Gmel.) A.J.Scott	0	n	1	0	Amaranthaceae Juss.
Iz_5_1109	DJI_0132	Berberis vulgaris L.	0	n	1	0	Berberidaceae Juss.
Iz_5_1109	DJI_0132	Berteroa incana (L.) DC.	0		NA	NA	
Iz_5_1109	DJI_0132	Betula pendula Roth.	0	n	1	1	Betulaceae Gray
Iz_5_1109	DJI_0132	Betula pendula Roth.	0	n	1	0	Poaceae Barnhart
Iz_5_1109	DJI_0132	Bromus inermis Leyss.	0		NA	NA	
Iz_5_1109	DJI_0132	Bromus squarrosus L.	0	a	0	0	Poaceae Barnhart
Iz_5_1109	DJI_0132	Bromus tectorum L.	0	a	0	0	Poaceae Barnhart
Iz_5_1109	DJI_0132	Calamagrostis epigejos (L.) Roth	0.1	n	1	0	Poaceae Barnhart
Iz_5_1109	DJI_0132	Carduus acanthoides L.	0.1	n	1	0	Asteraceae Bercht. & J.Presl
Iz_5_1109	DJI_0132	Carex sp.	0	n	1	0	Cyperaceae Juss.
Iz_5_1109	DJI_0132	Centaurea majorovii Dumbadze	0	n	1	0	Asteraceae Bercht. & J.Presl
Iz_5_1109	DJI_0132	Chamaecytisus ruthenicus (Fisch.ex Wol.) Klaskova	0	n	1	0	Fabaceae Lindl.
Iz_5_1109	DJI_0132	Chelidonium majus L.	0	n	1	0	Papaveraceae Juss.
Iz_5_1109	DJI_0132	Chenopodium betaceum Andrz.	0	n	1	0	Amaranthaceae Juss.
Iz_5_1109	DJI_0132	Chenopodium cf. album	0	n	1	0	Amaranthaceae Juss.
Iz_5_1109	DJI_0132	Chenopodium sp.	0		NA	0	Amaranthaceae Juss.
Iz_5_1109	DJI_0132	Chondrilla juncea L.	0	n	1	0	Asteraceae Bercht. & J.Presl
Iz_5_1109	DJI_0132	Cirsium vulgare (Savi) Ten.	0	n	1	0	Asteraceae Bercht. & J.Presl
Iz_5_1109	DJI_0132	Clinopodium acinos (L.) Kuntze	0	n	1	0	Lamiacea Lindl.
Iz_5_1109	DJI_0132	Convallaria majalis L.	0	n	1	0	Asparagaceae Juss.
Iz_5_1109	DJI_0132	Crataegus rhipidophylla Gand.	0	n	1	1	Rosaceae Juss.
Iz_5_1109	DJI_0132	Crepis foetida L.	0	n	1	0	Asteraceae Bercht. & J.Presl
Iz_5_1109	DJI_0132	Dactylis glomerata L.	0	n	1	0	Poaceae Barnhart
Iz_5_1109	DJI_0132	Dianthus campestris M.Bieb.	0	n	1	0	Caryophyllaceae Juss.
Iz_5_1109	DJI_0132	Digitaria sanguinalis (L.) Scop.	0	a	0	0	Poaceae Barnhart
Iz_5_1109	DJI_0132	Elymus repens (L.) Gould	0	n	1	0	Poaceae Barnhart
Iz_5_1109	DJI_0132	Epilobium tetragonum L.	0	n	1	0	Onagraceae Juss.
Iz_5_1109	DJI_0132	Eragrostis minor Host	0	a	0	0	Poaceae Barnhart
Iz_5_1109	DJI_0132	Erigeron canadensis L.	0.2	a	0	0	Asteraceae Bercht. & J.Presl
Iz_5_1109	DJI_0132	Euphorbia seguieriana Neck.	0	n	1	0	Euphorbiaceae Juss.
Iz_5_1109	DJI_0132	Fallopia dumetorum (L.) Holub	25	n	1	0	Polygonaceae Juss.
Iz_5_1109	DJI_0132	Festuca beckeri (Hack.) Trautv.	0	n	1	0	Poaceae Barnhart
Iz_5_1109	DJI_0132	Fragaria vesca L.	0	n	1	0	Rosaceae Juss.
Iz_5_1109	DJI_0132	Frangula alnus Mill.	0	n	1	1	Rhamnaceae Juss.
Iz_5_1109	DJI_0132	Fraxinus pennsylvanica Marshall	0	a	0	1	Oleaceae Hoffmanns. & Link
Iz_5_1109	DJI_0132	Galeopsis bifida Boenn.	0	n	1	0	Lamiacea Lindl.
Iz_5_1109	DJI_0132	Galium verum L.	0	n	1	0	Rubiaceae Juss.
Iz_5_1109	DJI_0132	Genista tinctoria L.	0	n	1	0	Fabaceae Lindl.
Iz_5_1109	DJI_0132	Geranium robertianum L.	0	n	1	0	Geraniaceae Juss.
Iz_5_1109	DJI_0132	Geranium sanquineum L.	0	n	1	0	Geraniaceae Juss.
Iz_5_1109	DJI_0132	Gypsophila paniculata L.	0	n	1	0	Caryophyllaceae Juss.
Iz_5_1109	DJI_0132	Helichrysum arenarium (L.) Moench	0	n	1	0	Asteraceae Bercht. & J.Presl
Iz_5_1109	DJI_0132	Herniaria polygama J.Gay	0	n	1	0	Caryophyllaceae Juss.
Iz_5_1109	DJI_0132	Hieracium robustum Fr.	0	n	1	0	Asteraceae Bercht. & J.Presl
Iz_5_1109	DJI_0132	Hieracium umbellatum L.	0	n	1	NA	Asteraceae Bercht. & J.Presl
Iz_5_1109	DJI_0132	Hylotelephium maximum (L.) Holub	0	n	1	0	Crassulaceae J.St.-Hil.
Iz_5_1109	DJI_0132	Jacobaea borysthenica (DC.) B.Nord. & Greuter	0	n	1	0	Asteraceae Bercht. & J.Presl
Iz_5_1109	DJI_0132	Jasione montana L.	0	n	1	0	Campanulaceae Juss.
Iz_5_1109	DJI_0132	Jurinea cyanoides (L.) Rchb.s.l.	0	n	1	0	Asteraceae Bercht. & J.Presl
Iz_5_1109	DJI_0132	Koeleria glauca (Spreng.) DC.	0	n	1	0	Poaceae Barnhart
Iz_5_1109	DJI_0132	Lactuca serriola L.	0	a	0	0	Asteraceae Bercht. & J.Presl
Iz_5_1109	DJI_0132	Linaria genistifolia (L.) Mill.	0	n	1	0	Plantaginaceae Juss.
Iz_5_1109	DJI_0132	Lomelosia argentea (L.) Greuter & Burdet	0	n	1	0	Caprifoliaceae Juss.
Iz_5_1109	DJI_0132	Melampyrum pratense L.	0	n	1	0	Orobanchaceae Vent.
Iz_5_1109	DJI_0132	Oenothera biennis L.	0	a	0	0	Onagraceae Juss.
Iz_5_1109	DJI_0132	Pentanema britannica (L.) D.Gut.Larr., Santos-Vicente, Anderb., E.Rico & M.M.Mart.Ort.	0	n	1	0	Asteraceae Bercht. & J.Presl
Iz_5_1109	DJI_0132	Peucedanum oreoselinum (L.) Moench	0	n	1	0	Apiaceae Lindl.
Iz_5_1109	DJI_0132	Peucedanum ruthenicum M.Bieb.	0	n	1	0	Apiaceae Lindl.
Iz_5_1109	DJI_0132	Pilosella echioides (Lumn.) F.W.Schultz & Sch.Bip.	0	n	1	0	Asteraceae Bercht. & J.Presl
Iz_5_1109	DJI_0132	Pilosella officinarum F. Schultz et Sch. Bip.	0	n	1	0	Asteraceae Bercht. & J.Presl
Iz_5_1109	DJI_0132	Pilosella sp.	0		NA	0	Asteraceae Bercht. & J.Presl
Iz_5_1109	DJI_0132	Pinus sylvestris L.	0	n	1	1	Pinaceae Spreng. ex F.Rudolphi
Iz_5_1109	DJI_0132	Poa annua L.	0	n	1	0	Poaceae Barnhart
Iz_5_1109	DJI_0132	Poa nemoralis L.	0	n	1	0	Poaceae Barnhart
Iz_5_1109	DJI_0132	Poa sp.	0		NA	0	Poaceae Barnhart
Iz_5_1109	DJI_0132	Polygonatum odoratum (Mill.) Druce	0	n	1	0	Asparagaceae Juss.
Iz_5_1109	DJI_0132	Polygonum arenarium Waldst. & Kit.	0	n	1	0	Polygonaceae Juss.
Iz_5_1109	DJI_0132	Populus nigra L.	0	n	1	1	Salicaceae Mirb.
Iz_5_1109	DJI_0132	Populus tremula L.	0	n	1	1	Salicaceae Mirb.
Iz_5_1109	DJI_0132	Populus Ã canescens (Aiton) Sm.	0	n	1	1	Salicaceae Mirb.
Iz_5_1109	DJI_0132	Potentilla argentea L.	0	n	1	0	Rosaceae Juss.
Iz_5_1109	DJI_0132	Psammophiliella muralis (L.) Ikonn.	0	n	1	0	Caryophyllaceae Juss.
Iz_5_1109	DJI_0132	Psephellus sumensis (Kalen.) Greuter	0	n	1	0	Asteraceae Bercht. & J.Presl
Iz_5_1109	DJI_0132	Pulsatilla pratensis (L.) Mill. s.l.	0	n	1	0	Ranunculaceae Juss.
Iz_5_1109	DJI_0132	Pyrus communis L.	0	a	0	1	Rosaceae Juss.
Iz_5_1109	DJI_0132	Quercus robur L.	0	n	1	1	Fagaceae Dumort.
Iz_5_1109	DJI_0132	Robinia pseudoacacia L.	0	a	0	1	Fabaceae Lindl.
Iz_5_1109	DJI_0132	Rosa sp.	0		NA	1	Rosaceae Juss.
Iz_5_1109	DJI_0132	Rubus idaeus L.	0	n	1	0	Rosaceae Juss.
Iz_5_1109	DJI_0132	Rumex acetosella L.	0	n	1	0	Polygonaceae Juss.
Iz_5_1109	DJI_0132	Rumex thyrsiflorus Fingerh.	0	n	1	0	Polygonaceae Juss.
Iz_5_1109	DJI_0132	Salix alba L.	0	n	1	1	Salicaceae Mirb.
Iz_5_1109	DJI_0132	Sambucus nigra L.	0	n	1	1	Viburnaceae Raf.
Iz_5_1109	DJI_0132	Secale sylvestre Host	0	n	1	0	Poaceae Barnhart
Iz_5_1109	DJI_0132	Sempervivum ruthenicum Schnittsp. & C.B. Lehm.	0	n	1	0	Crassulaceae J.St.-Hil.

Iz_5_1109	DJI_0132	Setaria viridis (L.) P. Beauv.	0	n	1	0	Poaceae Barnhart
Iz_5_1109	DJI_0132	Silene borysthenica (Gruner) Walters	0	n	1	0	Caryophyllaceae Juss.
Iz_5_1109	DJI_0132	Silene latifolia subsp. alba (Mill.) Greuter & Burdet	0	n	1	0	Caryophyllaceae Juss.
Iz_5_1109	DJI_0132	Solidago virgaurea L.	0	n	1	0	Asteraceae Bercht. & J.Presl
Iz_5_1109	DJI_0132	Sorbus aucuparia L.	0	n	1	1	Rosaceae Juss.
Iz_5_1109	DJI_0132	Stachys recta L.	0	n	1	0	Lamiacea Lindl.
Iz_5_1109	DJI_0132	Tanacetum vulgare L.	0	n	1	0	Asteraceae Bercht. & J.Presl
Iz_5_1109	DJI_0132	Thymus pallasianus Heinr.Braun	0	n	1	0	Lamiacea Lindl.
Iz_5_1109	DJI_0132	Tilia cordata Mill.	0	n	1	1	Malvaceae Juss.
Iz_5_1109	DJI_0132	Trifolium alpestre L.	0	n	1	0	Fabaceae Lindl.
Iz_5_1109	DJI_0132	Trifolium arvense L.	0	n	1	0	Fabaceae Lindl.
Iz_5_1109	DJI_0132	Ulmus glabra Huds.	0	n	1	1	Ulmaceae Mirb.
Iz_5_1109	DJI_0132	Verbascum cf. phlomoides L.	0		NA	NA	
Iz_5_1109	DJI_0132	Verbascum densiflorum Bertol.	0	n	1	0	Scrophulariaceae Juss.
Iz_5_1109	DJI_0132	Verbascum sp.	0	n	1	0	Scrophulariaceae Juss.
Iz_5_1109	DJI_0132	Veronica austriaca L.	0	n	1	0	Plantaginaceae Juss.
Iz_5_1109	DJI_0132	Veronica incana L.	0	n	1	0	Plantaginaceae Juss.
Iz_5_1109	DJI_0132	Veronica verna L.	0	n	1	0	Plantaginaceae Juss.
Iz_5_1109	DJI_0132	Vincetoxicum hirundinaria Medik	0	n	1	0	Apocynaceae Juss.
Iz_5_1109	DJI_0134	Acer tataricum L.	0	n	1	1	Sapindaceae Juss.
Iz_5_1109	DJI_0134	Achillea millefolium L. s.l.	0	n	1	0	Asteraceae Bercht. & J.Presl
Iz_5_1109	DJI_0134	Allium oleraceum L.	0	n	1	0	Amaryllidaceae J.St.-Hil.
Iz_5_1109	DJI_0134	Ambrosia artemisiifolia L.	0	a	0	0	Asteraceae Bercht. & J.Presl
Iz_5_1109	DJI_0134	Amorpha fruticosa L.	0	a	0	0	Fabaceae Lindl.
Iz_5_1109	DJI_0134	Anthericum ramosum L.	0	n	1	0	Asparagaceae Juss.
Iz_5_1109	DJI_0134	Anthoxanthum repens (Host) Veldkamp	0		NA	NA	
Iz_5_1109	DJI_0134	Artemisia marschalliana Spreng.	0	n	1	0	Asteraceae Bercht. & J.Presl
Iz_5_1109	DJI_0134	Asparagus officinalis L.	0	n	1	0	Asparagaceae Juss.
Iz_5_1109	DJI_0134	Astragalus cicer L.	0	n	1	0	Fabaceae Lindl.
Iz_5_1109	DJI_0134	Bassia laniflora (S.G.Gmel.) A.J.Scott	0	n	1	0	Amaranthaceae Juss.
Iz_5_1109	DJI_0134	Berberis vulgaris L.	0	n	1	0	Berberidaceae Juss.
Iz_5_1109	DJI_0134	Berteroa incana (L.) DC.	0		NA	NA	
Iz_5_1109	DJI_0134	Betula pendula Roth.	0	n	1	1	Betulaceae Gray
Iz_5_1109	DJI_0134	Betula pendula Roth.	0	n	1	0	Poaceae Barnhart
Iz_5_1109	DJI_0134	Bromus inermis Leyss.	0		NA	NA	
Iz_5_1109	DJI_0134	Bromus squarrosus L.	0	a	0	0	Poaceae Barnhart
Iz_5_1109	DJI_0134	Bromus tectorum L.	0	a	0	0	Poaceae Barnhart
Iz_5_1109	DJI_0134	Calamagrostis epigejos (L.) Roth	0	n	1	0	Poaceae Barnhart
Iz_5_1109	DJI_0134	Carduus acanthoides L.	0	n	1	0	Asteraceae Bercht. & J.Presl
Iz_5_1109	DJI_0134	Carex sp.	0	n	1	0	Cyperaceae Juss.
Iz_5_1109	DJI_0134	Centaurea majorovii Dumbadze	0	n	1	0	Asteraceae Bercht. & J.Presl
Iz_5_1109	DJI_0134	Chamaecytisus ruthenicus (Fisch.ex Wol.) Klaskova	0	n	1	0	Fabaceae Lindl.
Iz_5_1109	DJI_0134	Chelidonium majus L.	0	n	1	0	Papaveraceae Juss.
Iz_5_1109	DJI_0134	Chenopodium betaceum Andrz.	0	n	1	0	Amaranthaceae Juss.
Iz_5_1109	DJI_0134	Chenopodium cf. album	0	n	1	0	Amaranthaceae Juss.
Iz_5_1109	DJI_0134	Chenopodium sp.	0		NA	0	Amaranthaceae Juss.
Iz_5_1109	DJI_0134	Chondrilla juncea L.	0	n	1	0	Asteraceae Bercht. & J.Presl
Iz_5_1109	DJI_0134	Cirsium vulgare (Savi) Ten.	0	n	1	0	Asteraceae Bercht. & J.Presl
Iz_5_1109	DJI_0134	Clinopodium acinos (L.) Kuntze	0	n	1	0	Lamiacea Lindl.
Iz_5_1109	DJI_0134	Convallaria majalis L.	0	n	1	0	Asparagaceae Juss.
Iz_5_1109	DJI_0134	Crataegus rhipidophylla Gand.	0	n	1	1	Rosaceae Juss.
Iz_5_1109	DJI_0134	Crepis foetida L.	0	n	1	0	Asteraceae Bercht. & J.Presl
Iz_5_1109	DJI_0134	Dactylis glomerata L.	0	n	1	0	Poaceae Barnhart
Iz_5_1109	DJI_0134	Dianthus campestris M.Bieb.	0	n	1	0	Caryophyllaceae Juss.
Iz_5_1109	DJI_0134	Digitaria sanguinalis (L.) Scop.	0	a	0	0	Poaceae Barnhart
Iz_5_1109	DJI_0134	Elymus repens (L.) Gould	0	n	1	0	Poaceae Barnhart
Iz_5_1109	DJI_0134	Epilobium tetragonum L.	0	n	1	0	Onagraceae Juss.
Iz_5_1109	DJI_0134	Eragrostis minor Host	0	a	0	0	Poaceae Barnhart
Iz_5_1109	DJI_0134	Erigeron canadensis L.	0.2	a	0	0	Asteraceae Bercht. & J.Presl
Iz_5_1109	DJI_0134	Euphorbia seguieriana Neck.	0	n	1	0	Euphorbiaceae Juss.
Iz_5_1109	DJI_0134	Fallopia dumetorum (L.) Holub	10	n	1	0	Polygonaceae Juss.
Iz_5_1109	DJI_0134	Festuca beckeri (Hack.) Trautv.	0	n	1	0	Poaceae Barnhart
Iz_5_1109	DJI_0134	Fragaria vesca L.	0	n	1	0	Rosaceae Juss.
Iz_5_1109	DJI_0134	Frangula alnus Mill.	0	n	1	1	Rhamnaceae Juss.
Iz_5_1109	DJI_0134	Fraxinus pennsylvanica Marshall	0	a	0	1	Oleaceae Hoffmanns. & Link
Iz_5_1109	DJI_0134	Galeopsis bifida Boenn.	0	n	1	0	Lamiacea Lindl.
Iz_5_1109	DJI_0134	Galium verum L.	0	n	1	0	Rubiaceae Juss.
Iz_5_1109	DJI_0134	Genista tinctoria L.	0	n	1	0	Fabaceae Lindl.
Iz_5_1109	DJI_0134	Geranium robertianum L.	0	n	1	0	Geraniaceae Juss.
Iz_5_1109	DJI_0134	Geranium sanquineum L.	0	n	1	0	Geraniaceae Juss.
Iz_5_1109	DJI_0134	Gypsophila paniculata L.	0	n	1	0	Caryophyllaceae Juss.
Iz_5_1109	DJI_0134	Helichrysum arenarium (L.) Moench	0	n	1	0	Asteraceae Bercht. & J.Presl
Iz_5_1109	DJI_0134	Herniaria polygama J.Gay	0	n	1	0	Caryophyllaceae Juss.
Iz_5_1109	DJI_0134	Hieracium robustum Fr.	0	n	1	0	Asteraceae Bercht. & J.Presl
Iz_5_1109	DJI_0134	Hieracium umbellatum L.	0	n	1	NA	Asteraceae Bercht. & J.Presl
Iz_5_1109	DJI_0134	Hylotelephium maximum (L.) Holub	0	n	1	0	Crassulaceae J.St.-Hil.
Iz_5_1109	DJI_0134	Jacobaea borysthenica (DC.) B.Nord. & Greuter	0	n	1	0	Asteraceae Bercht. & J.Presl
Iz_5_1109	DJI_0134	Jasione montana L.	0	n	1	0	Campanulaceae Juss.
Iz_5_1109	DJI_0134	Jurinea cyanoides (L.) Rchb.s.l.	0	n	1	0	Asteraceae Bercht. & J.Presl
Iz_5_1109	DJI_0134	Koeleria glauca (Spreng.) DC.	0	n	1	0	Poaceae Barnhart
Iz_5_1109	DJI_0134	Lactuca serriola L.	0	a	0	0	Asteraceae Bercht. & J.Presl
Iz_5_1109	DJI_0134	Linaria genistifolia (L.) Mill.	0	n	1	0	Plantaginaceae Juss.
Iz_5_1109	DJI_0134	Lomelosia argentea (L.) Greuter & Burdet	0	n	1	0	Caprifoliaceae Juss.
Iz_5_1109	DJI_0134	Melampyrum pratense L.	0	n	1	0	Orobanchaceae Vent.
Iz_5_1109	DJI_0134	Oenothera biennis L.	0	a	0	0	Onagraceae Juss.
Iz_5_1109	DJI_0134	Pentanema britannica (L.) D.Gut.Larr., Santos-Vicente, Anderb., E.Rico & M.M.Mart.Ort.	0	n	1	0	Asteraceae Bercht. & J.Presl
Iz_5_1109	DJI_0134	Peucedanum oreoselinum (L.) Moench	2	n	1	0	Apiaceae Lindl.
Iz_5_1109	DJI_0134	Peucedanum ruthenicum M.Bieb.	0	n	1	0	Apiaceae Lindl.
Iz_5_1109	DJI_0134	Pilosella echioides (Lumn.) F.W.Schultz & Sch.Bip.	0	n	1	0	Asteraceae Bercht. & J.Presl
Iz_5_1109	DJI_0134	Pilosella officinarum F. Schultz et Sch. Bip.	0	n	1	0	Asteraceae Bercht. & J.Presl
Iz_5_1109	DJI_0134	Pilosella sp.	0		NA	0	Asteraceae Bercht. & J.Presl
Iz_5_1109	DJI_0134	Pinus sylvestris L.	0	n	1	1	Pinaceae Spreng. ex F.Rudolphi
Iz_5_1109	DJI_0134	Poa annua L.	0	n	1	0	Poaceae Barnhart
Iz_5_1109	DJI_0134	Poa nemoralis L.	0	n	1	0	Poaceae Barnhart
Iz_5_1109	DJI_0134	Poa sp.	0		NA	0	Poaceae Barnhart
Iz_5_1109	DJI_0134	Polygonatum odoratum (Mill.) Druce	0	n	1	0	Asparagaceae Juss.
Iz_5_1109	DJI_0134	Polygonum arenarium Waldst. & Kit.	0	n	1	0	Polygonaceae Juss.
Iz_5_1109	DJI_0134	Populus nigra L.	0	n	1	1	Salicaceae Mirb.

Iz_5_1109	DJI_0134	Populus tremula L.	0	n	1	1	Salicaceae Mirb.
Iz_5_1109	DJI_0134	Populus Ã canescens (Aiton) Sm.	0	n	1	1	Salicaceae Mirb.
Iz_5_1109	DJI_0134	Potentilla argentea L.	0	n	1	0	Rosaceae Juss.
Iz_5_1109	DJI_0134	Psammophiliella muralis (L.) Ikonn.	0	n	1	0	Caryophyllaceae Juss.
Iz_5_1109	DJI_0134	Psephellus sumensis (Kalen.) Greuter	0	n	1	0	Asteraceae Bercht. & J.Presl
Iz_5_1109	DJI_0134	Pulsatilla pratensis (L.) Mill. s.l.	0	n	1	0	Ranunculaceae Juss.
Iz_5_1109	DJI_0134	Pyrus communis L.	0	a	0	1	Rosaceae Juss.
Iz_5_1109	DJI_0134	Quercus robur L.	0	n	1	1	Fagaceae Dumort.
Iz_5_1109	DJI_0134	Robinia pseudoacacia L.	0	a	0	1	Fabaceae Lindl.
Iz_5_1109	DJI_0134	Rosa sp.	0		NA	1	Rosaceae Juss.
Iz_5_1109	DJI_0134	Rubus idaeus L.	0	n	1	0	Rosaceae Juss.
Iz_5_1109	DJI_0134	Rumex acetosella L.	15	n	1	0	Polygonaceae Juss.
Iz_5_1109	DJI_0134	Rumex thyrsiflorus Fingerh.	0	n	1	0	Polygonaceae Juss.
Iz_5_1109	DJI_0134	Salix alba L.	0	n	1	1	Salicaceae Mirb.
Iz_5_1109	DJI_0134	Sambucus nigra L.	0	n	1	1	Viburnaceae Raf.
Iz_5_1109	DJI_0134	Secale sylvestre Host	0	n	1	0	Poaceae Barnhart
Iz_5_1109	DJI_0134	Sempervivum ruthenicum Schnittsp. & C.B. Lehm.	0	n	1	0	Crassulaceae J.St.-Hil.
Iz_5_1109	DJI_0134	Setaria viridis (L.) P. Beauv.	0	n	1	0	Poaceae Barnhart
Iz_5_1109	DJI_0134	Silene borysthenica (Gruner) Walters	0	n	1	0	Caryophyllaceae Juss.
Iz_5_1109	DJI_0134	Silene latifolia subsp. alba (Mill.) Greuter & Burdet	0	n	1	0	Caryophyllaceae Juss.
Iz_5_1109	DJI_0134	Solidago virgaurea L.	0	n	1	0	Asteraceae Bercht. & J.Presl
Iz_5_1109	DJI_0134	Sorbus aucuparia L.	0	n	1	1	Rosaceae Juss.
Iz_5_1109	DJI_0134	Stachys recta L.	0	n	1	0	Lamiacea Lindl.
Iz_5_1109	DJI_0134	Tanacetum vulgare L.	0	n	1	0	Asteraceae Bercht. & J.Presl
Iz_5_1109	DJI_0134	Thymus pallasianus Heinr.Braun	0	n	1	0	Lamiacea Lindl.
Iz_5_1109	DJI_0134	Tilia cordata Mill.	0	n	1	1	Malvaceae Juss.
Iz_5_1109	DJI_0134	Trifolium alpestre L.	0	n	1	0	Fabaceae Lindl.
Iz_5_1109	DJI_0134	Trifolium arvense L.	0	n	1	0	Fabaceae Lindl.
Iz_5_1109	DJI_0134	Ulmus glabra Huds.	0	n	1	1	Ulmaceae Mirb.
Iz_5_1109	DJI_0134	Verbascum cf. phlomoides L.	0		NA	NA	
Iz_5_1109	DJI_0134	Verbascum densiflorum Bertol.	0	n	1	0	Scrophulariaceae Juss.
Iz_5_1109	DJI_0134	Verbascum sp.	0	n	1	0	Scrophulariaceae Juss.
Iz_5_1109	DJI_0134	Veronica austriaca L.	0	n	1	0	Plantaginaceae Juss.
Iz_5_1109	DJI_0134	Veronica incana L.	0	n	1	0	Plantaginaceae Juss.
Iz_5_1109	DJI_0134	Veronica verna L.	0	n	1	0	Plantaginaceae Juss.
Iz_5_1109	DJI_0134	Vincetoxicum hirundinaria Medik	0	n	1	0	Apocynaceae Juss.
Iz_5_1109	DJI_0136	Acer tataricum L.	0	n	1	1	Sapindaceae Juss.
Iz_5_1109	DJI_0136	Achillea millefolium L. s.l.	0	n	1	0	Asteraceae Bercht. & J.Presl
Iz_5_1109	DJI_0136	Allium oleraceum L.	0	n	1	0	Amaryllidaceae J.St.-Hil.
Iz_5_1109	DJI_0136	Ambrosia artemisiifolia L.	0	a	0	0	Asteraceae Bercht. & J.Presl
Iz_5_1109	DJI_0136	Amorpha fruticosa L.	0	a	0	0	Fabaceae Lindl.
Iz_5_1109	DJI_0136	Anthericum ramosum L.	0	n	1	0	Asparagaceae Juss.
Iz_5_1109	DJI_0136	Anthoxanthum repens (Host) Veldkamp	0		NA	NA	
Iz_5_1109	DJI_0136	Artemisia marschalliana Spreng.	0	n	1	0	Asteraceae Bercht. & J.Presl
Iz_5_1109	DJI_0136	Asparagus officinalis L.	0	n	1	0	Asparagaceae Juss.
Iz_5_1109	DJI_0136	Astragalus cicer L.	0	n	1	0	Fabaceae Lindl.
Iz_5_1109	DJI_0136	Bassia laniflora (S.G.Gmel.) A.J.Scott	0	n	1	0	Amaranthaceae Juss.
Iz_5_1109	DJI_0136	Berberis vulgaris L.	0	n	1	0	Berberidaceae Juss.
Iz_5_1109	DJI_0136	Berteroa incana (L.) DC.	0		NA	NA	
Iz_5_1109	DJI_0136	Betula pendula Roth.	0	n	1	1	Betulaceae Gray
Iz_5_1109	DJI_0136	Betula pendula Roth.	0	n	1	0	Poaceae Barnhart
Iz_5_1109	DJI_0136	Bromus inermis Leyss.	0		NA	NA	
Iz_5_1109	DJI_0136	Bromus squarrosus L.	0	a	0	0	Poaceae Barnhart
Iz_5_1109	DJI_0136	Bromus tectorum L.	0	a	0	0	Poaceae Barnhart
Iz_5_1109	DJI_0136	Calamagrostis epigejos (L.) Roth	0	n	1	0	Poaceae Barnhart
Iz_5_1109	DJI_0136	Carduus acanthoides L.	0	n	1	0	Asteraceae Bercht. & J.Presl
Iz_5_1109	DJI_0136	Carex sp.	0	n	1	0	Cyperaceae Juss.
Iz_5_1109	DJI_0136	Centaurea majorovii Dumbadze	0	n	1	0	Asteraceae Bercht. & J.Presl
Iz_5_1109	DJI_0136	Chamaecytisus ruthenicus (Fisch.ex Wol.) Klaskova	0	n	1	0	Fabaceae Lindl.
Iz_5_1109	DJI_0136	Chelidonium majus L.	0	n	1	0	Papaveraceae Juss.
Iz_5_1109	DJI_0136	Chenopodium betaceum Andrz.	0	n	1	0	Amaranthaceae Juss.
Iz_5_1109	DJI_0136	Chenopodium cf. album	0	n	1	0	Amaranthaceae Juss.
Iz_5_1109	DJI_0136	Chenopodium sp.	0		NA	0	Amaranthaceae Juss.
Iz_5_1109	DJI_0136	Chondrilla juncea L.	0	n	1	0	Asteraceae Bercht. & J.Presl
Iz_5_1109	DJI_0136	Cirsium vulgare (Savi) Ten.	0	n	1	0	Asteraceae Bercht. & J.Presl
Iz_5_1109	DJI_0136	Clinopodium acinos (L.) Kuntze	0	n	1	0	Lamiacea Lindl.
Iz_5_1109	DJI_0136	Convallaria majalis L.	0	n	1	0	Asparagaceae Juss.
Iz_5_1109	DJI_0136	Crataegus rhipidophylla Gand.	0	n	1	1	Rosaceae Juss.
Iz_5_1109	DJI_0136	Crepis foetida L.	0	n	1	0	Asteraceae Bercht. & J.Presl
Iz_5_1109	DJI_0136	Dactylis glomerata L.	0	n	1	0	Poaceae Barnhart
Iz_5_1109	DJI_0136	Dianthus campestris M.Bieb.	0	n	1	0	Caryophyllaceae Juss.
Iz_5_1109	DJI_0136	Digitaria sanguinalis (L.) Scop.	0	a	0	0	Poaceae Barnhart
Iz_5_1109	DJI_0136	Elymus repens (L.) Gould	0	n	1	0	Poaceae Barnhart
Iz_5_1109	DJI_0136	Epilobium tetragonum L.	0	n	1	0	Onagraceae Juss.
Iz_5_1109	DJI_0136	Eragrostis minor Host	0	a	0	0	Poaceae Barnhart
Iz_5_1109	DJI_0136	Erigeron canadensis L.	0.1	a	0	0	Asteraceae Bercht. & J.Presl
Iz_5_1109	DJI_0136	Euphorbia seguieriana Neck.	0	n	1	0	Euphorbiaceae Juss.
Iz_5_1109	DJI_0136	Fallopia dumetorum (L.) Holub	0	n	1	0	Polygonaceae Juss.
Iz_5_1109	DJI_0136	Festuca beckeri (Hack.) Trautv.	0	n	1	0	Poaceae Barnhart
Iz_5_1109	DJI_0136	Fragaria vesca L.	0	n	1	0	Rosaceae Juss.
Iz_5_1109	DJI_0136	Frangula alnus Mill.	0	n	1	1	Rhamnaceae Juss.
Iz_5_1109	DJI_0136	Fraxinus pennsylvanica Marshall	0	a	0	1	Oleaceae Hoffmanns. & Link
Iz_5_1109	DJI_0136	Galeopsis bifida Boenn.	0	n	1	0	Lamiacea Lindl.
Iz_5_1109	DJI_0136	Galium verum L.	0	n	1	0	Rubiaceae Juss.
Iz_5_1109	DJI_0136	Genista tinctoria L.	0	n	1	0	Fabaceae Lindl.
Iz_5_1109	DJI_0136	Geranium robertianum L.	0	n	1	0	Geraniaceae Juss.
Iz_5_1109	DJI_0136	Geranium sanquineum L.	0	n	1	0	Geraniaceae Juss.
Iz_5_1109	DJI_0136	Gypsophila paniculata L.	0	n	1	0	Caryophyllaceae Juss.
Iz_5_1109	DJI_0136	Helichrysum arenarium (L.) Moench	0	n	1	0	Asteraceae Bercht. & J.Presl
Iz_5_1109	DJI_0136	Herniaria polygama J.Gay	0	n	1	0	Caryophyllaceae Juss.
Iz_5_1109	DJI_0136	Hieracium robustum Fr.	0	n	1	0	Asteraceae Bercht. & J.Presl
Iz_5_1109	DJI_0136	Hieracium umbellatum L.	0	n	1	NA	Asteraceae Bercht. & J.Presl
Iz_5_1109	DJI_0136	Hylotelephium maximum (L.) Holub	0	n	1	0	Crassulaceae J.St.-Hil.
Iz_5_1109	DJI_0136	Jacobaea borysthenica (DC.) B.Nord. & Greuter	0	n	1	0	Asteraceae Bercht. & J.Presl
Iz_5_1109	DJI_0136	Jasione montana L.	0	n	1	0	Campanulaceae Juss.
Iz_5_1109	DJI_0136	Jurinea cyanoides (L.) Rchb.s.l.	0	n	1	0	Asteraceae Bercht. & J.Presl
Iz_5_1109	DJI_0136	Koeleria glauca (Spreng.) DC.	0	n	1	0	Poaceae Barnhart
Iz_5_1109	DJI_0136	Lactuca serriola L.	0	a	0	0	Asteraceae Bercht. & J.Presl

Iz_5_1109	DJI_0136	Linaria genistifolia (L.) Mill.	0	n	1	0	Plantaginaceae Juss.
Iz_5_1109	DJI_0136	Lomelosia argentea (L.) Greuter & Burdet	0	n	1	0	Caprifoliaceae Juss.
Iz_5_1109	DJI_0136	Melampyrum pratense L.	0	n	1	0	Orobanchaceae Vent.
Iz_5_1109	DJI_0136	Oenothera biennis L.	0	a	0	0	Onagraceae Juss.
Iz_5_1109	DJI_0136	Pentanema britannica (L.) D.Gut.Larr., Santos-Vicente, Anderb., E.Rico & M.M.Mart.Ort.	0	n	1	0	Asteraceae Bercht. & J.Presl
Iz_5_1109	DJI_0136	Peucedanum oreoselinum (L.) Moench	0	n	1	0	Apiaceae Lindl.
Iz_5_1109	DJI_0136	Peucedanum ruthenicum M.Bieb.	0	n	1	0	Apiaceae Lindl.
Iz_5_1109	DJI_0136	Pilosella echioides (Lumn.) F.W.Schultz & Sch.Bip.	0	n	1	0	Asteraceae Bercht. & J.Presl
Iz_5_1109	DJI_0136	Pilosella officinarum F. Schultz et Sch. Bip.	0	n	1	0	Asteraceae Bercht. & J.Presl
Iz_5_1109	DJI_0136	Pilosella sp.	0		NA	0	Asteraceae Bercht. & J.Presl
Iz_5_1109	DJI_0136	Pinus sylvestris L.	0	n	1	1	Pinaceae Spreng. ex F.Rudolphi
Iz_5_1109	DJI_0136	Poa annua L.	0	n	1	0	Poaceae Barnhart
Iz_5_1109	DJI_0136	Poa nemoralis L.	0	n	1	0	Poaceae Barnhart
Iz_5_1109	DJI_0136	Poa sp.	0		NA	0	Poaceae Barnhart
Iz_5_1109	DJI_0136	Polygonatum odoratum (Mill.) Druce	0	n	1	0	Asparagaceae Juss.
Iz_5_1109	DJI_0136	Polygonum arenarium Waldst. & Kit.	0	n	1	0	Polygonaceae Juss.
Iz_5_1109	DJI_0136	Populus nigra L.	0	n	1	1	Salicaceae Mirb.
Iz_5_1109	DJI_0136	Populus tremula L.	0	n	1	1	Salicaceae Mirb.
Iz_5_1109	DJI_0136	Populus Ã canescens (Aiton) Sm.	0	n	1	1	Salicaceae Mirb.
Iz_5_1109	DJI_0136	Potentilla argentea L.	0	n	1	0	Rosaceae Juss.
Iz_5_1109	DJI_0136	Psammophiliella muralis (L.) Ikonn.	0	n	1	0	Caryophyllaceae Juss.
Iz_5_1109	DJI_0136	Psephellus sumensis (Kalen.) Greuter	0	n	1	0	Asteraceae Bercht. & J.Presl
Iz_5_1109	DJI_0136	Pulsatilla pratensis (L.) Mill. s.l.	0	n	1	0	Ranunculaceae Juss.
Iz_5_1109	DJI_0136	Pyrus communis L.	0	a	0	1	Rosaceae Juss.
Iz_5_1109	DJI_0136	Quercus robur L.	0	n	1	1	Fagaceae Dumort.
Iz_5_1109	DJI_0136	Robinia pseudoacacia L.	0	a	0	1	Fabaceae Lindl.
Iz_5_1109	DJI_0136	Rosa sp.	0		NA	1	Rosaceae Juss.
Iz_5_1109	DJI_0136	Rubus idaeus L.	0	n	1	0	Rosaceae Juss.
Iz_5_1109	DJI_0136	Rumex acetosella L.	0.1	n	1	0	Polygonaceae Juss.
Iz_5_1109	DJI_0136	Rumex thyrsiflorus Fingerh.	0	n	1	0	Polygonaceae Juss.
Iz_5_1109	DJI_0136	Salix alba L.	0	n	1	1	Salicaceae Mirb.
Iz_5_1109	DJI_0136	Sambucus nigra L.	0	n	1	1	Viburnaceae Raf.
Iz_5_1109	DJI_0136	Secale sylvestre Host	0	n	1	0	Poaceae Barnhart
Iz_5_1109	DJI_0136	Sempervivum ruthenicum Schnittsp. & C.B. Lehm.	0	n	1	0	Crassulaceae J.St.-Hil.
Iz_5_1109	DJI_0136	Setaria viridis (L.) P. Beauv.	0	n	1	0	Poaceae Barnhart
Iz_5_1109	DJI_0136	Silene borysthenica (Gruner) Walters	0	n	1	0	Caryophyllaceae Juss.
Iz_5_1109	DJI_0136	Silene latifolia subsp. alba (Mill.) Greuter & Burdet	0	n	1	0	Caryophyllaceae Juss.
Iz_5_1109	DJI_0136	Solidago virgaurea L.	0	n	1	0	Asteraceae Bercht. & J.Presl
Iz_5_1109	DJI_0136	Sorbus aucuparia L.	0	n	1	1	Rosaceae Juss.
Iz_5_1109	DJI_0136	Stachys recta L.	0	n	1	0	Lamiacea Lindl.
Iz_5_1109	DJI_0136	Tanacetum vulgare L.	0	n	1	0	Asteraceae Bercht. & J.Presl
Iz_5_1109	DJI_0136	Thymus pallasianus Heinr.Braun	0	n	1	0	Lamiacea Lindl.
Iz_5_1109	DJI_0136	Tilia cordata Mill.	0	n	1	1	Malvaceae Juss.
Iz_5_1109	DJI_0136	Trifolium alpestre L.	0	n	1	0	Fabaceae Lindl.
Iz_5_1109	DJI_0136	Trifolium arvense L.	0	n	1	0	Fabaceae Lindl.
Iz_5_1109	DJI_0136	Ulmus glabra Huds.	0	n	1	1	Ulmaceae Mirb.
Iz_5_1109	DJI_0136	Verbascum cf. phlomoides L.	0		NA	NA	
Iz_5_1109	DJI_0136	Verbascum densiflorum Bertol.	0	n	1	0	Scrophulariaceae Juss.
Iz_5_1109	DJI_0136	Verbascum sp.	0	n	1	0	Scrophulariaceae Juss.
Iz_5_1109	DJI_0136	Veronica austriaca L.	0	n	1	0	Plantaginaceae Juss.
Iz_5_1109	DJI_0136	Veronica incana L.	0	n	1	0	Plantaginaceae Juss.
Iz_5_1109	DJI_0136	Veronica verna L.	0	n	1	0	Plantaginaceae Juss.
Iz_5_1109	DJI_0136	Vincetoxicum hirundinaria Medik	0	n	1	0	Apocynaceae Juss.
Iz_5_1109	DJI_0138	Acer tataricum L.	0	n	1	1	Sapindaceae Juss.
Iz_5_1109	DJI_0138	Achillea millefolium L. s.l.	0	n	1	0	Asteraceae Bercht. & J.Presl
Iz_5_1109	DJI_0138	Allium oleraceum L.	0	n	1	0	Amaryllidaceae J.St.-Hil.
Iz_5_1109	DJI_0138	Ambrosia artemisiifolia L.	0	a	0	0	Asteraceae Bercht. & J.Presl
Iz_5_1109	DJI_0138	Amorpha fruticosa L.	0	a	0	0	Fabaceae Lindl.
Iz_5_1109	DJI_0138	Anthericum ramosum L.	0	n	1	0	Asparagaceae Juss.
Iz_5_1109	DJI_0138	Anthoxanthum repens (Host) Veldkamp	0		NA	NA	
Iz_5_1109	DJI_0138	Artemisia marschalliana Spreng.	0	n	1	0	Asteraceae Bercht. & J.Presl
Iz_5_1109	DJI_0138	Asparagus officinalis L.	0	n	1	0	Asparagaceae Juss.
Iz_5_1109	DJI_0138	Astragalus cicer L.	0	n	1	0	Fabaceae Lindl.
Iz_5_1109	DJI_0138	Bassia laniflora (S.G.Gmel.) A.J.Scott	0	n	1	0	Amaranthaceae Juss.
Iz_5_1109	DJI_0138	Berberis vulgaris L.	0	n	1	0	Berberidaceae Juss.
Iz_5_1109	DJI_0138	Berteroa incana (L.) DC.	0		NA	NA	
Iz_5_1109	DJI_0138	Betula pendula Roth.	0	n	1	1	Betulaceae Gray
Iz_5_1109	DJI_0138	Betula pendula Roth.	0	n	1	0	Poaceae Barnhart
Iz_5_1109	DJI_0138	Bromus inermis Leyss.	0		NA	NA	
Iz_5_1109	DJI_0138	Bromus squarrosus L.	0	a	0	0	Poaceae Barnhart
Iz_5_1109	DJI_0138	Bromus tectorum L.	0	a	0	0	Poaceae Barnhart
Iz_5_1109	DJI_0138	Calamagrostis epigejos (L.) Roth	0	n	1	0	Poaceae Barnhart
Iz_5_1109	DJI_0138	Carduus acanthoides L.	0	n	1	0	Asteraceae Bercht. & J.Presl
Iz_5_1109	DJI_0138	Carex sp.	0	n	1	0	Cyperaceae Juss.
Iz_5_1109	DJI_0138	Centaurea majorovii Dumbadze	0	n	1	0	Asteraceae Bercht. & J.Presl
Iz_5_1109	DJI_0138	Chamaecytisus ruthenicus (Fisch.ex Wol.) Klaskova	0	n	1	0	Fabaceae Lindl.
Iz_5_1109	DJI_0138	Chelidonium majus L.	0	n	1	0	Papaveraceae Juss.
Iz_5_1109	DJI_0138	Chenopodium betaceum Andrz.	0	n	1	0	Amaranthaceae Juss.
Iz_5_1109	DJI_0138	Chenopodium cf. album	0	n	1	0	Amaranthaceae Juss.
Iz_5_1109	DJI_0138	Chenopodium sp.	0		NA	0	Amaranthaceae Juss.
Iz_5_1109	DJI_0138	Chondrilla juncea L.	0	n	1	0	Asteraceae Bercht. & J.Presl
Iz_5_1109	DJI_0138	Cirsium vulgare (Savi) Ten.	0	n	1	0	Asteraceae Bercht. & J.Presl
Iz_5_1109	DJI_0138	Clinopodium acinos (L.) Kuntze	0	n	1	0	Lamiacea Lindl.
Iz_5_1109	DJI_0138	Convallaria majalis L.	0	n	1	0	Asparagaceae Juss.
Iz_5_1109	DJI_0138	Crataegus rhipidophylla Gand.	0	n	1	1	Rosaceae Juss.
Iz_5_1109	DJI_0138	Crepis foetida L.	0	n	1	0	Asteraceae Bercht. & J.Presl
Iz_5_1109	DJI_0138	Dactylis glomerata L.	0	n	1	0	Poaceae Barnhart
Iz_5_1109	DJI_0138	Dianthus campestris M.Bieb.	0	n	1	0	Caryophyllaceae Juss.
Iz_5_1109	DJI_0138	Digitaria sanguinalis (L.) Scop.	0	a	0	0	Poaceae Barnhart
Iz_5_1109	DJI_0138	Elymus repens (L.) Gould	0	n	1	0	Poaceae Barnhart
Iz_5_1109	DJI_0138	Epilobium tetragonum L.	0	n	1	0	Onagraceae Juss.
Iz_5_1109	DJI_0138	Eragrostis minor Host	0	a	0	0	Poaceae Barnhart
Iz_5_1109	DJI_0138	Erigeron canadensis L.	0.1	a	0	0	Asteraceae Bercht. & J.Presl
Iz_5_1109	DJI_0138	Euphorbia seguieriana Neck.	0	n	1	0	Euphorbiaceae Juss.
Iz_5_1109	DJI_0138	Fallopia dumetorum (L.) Holub	0	n	1	0	Polygonaceae Juss.
Iz_5_1109	DJI_0138	Festuca beckeri (Hack.) Trautv.	0	n	1	0	Poaceae Barnhart
Iz_5_1109	DJI_0138	Fragaria vesca L.	0	n	1	0	Rosaceae Juss.
Iz_5_1109	DJI_0138	Frangula alnus Mill.	0	n	1	1	Rhamnaceae Juss.

Iz_5_1109	DJI_0138	Fraxinus pennsylvanica Marshall	0	a	0	1	Oleaceae Hoffmanns. & Link
Iz_5_1109	DJI_0138	Galeopsis bifida Boenn.	0	n	1	0	Lamiacea Lindl.
Iz_5_1109	DJI_0138	Galium verum L.	0	n	1	0	Rubiaceae Juss.
Iz_5_1109	DJI_0138	Genista tinctoria L.	0	n	1	0	Fabaceae Lindl.
Iz_5_1109	DJI_0138	Geranium robertianum L.	0	n	1	0	Geraniaceae Juss.
Iz_5_1109	DJI_0138	Geranium sanquineum L.	0	n	1	0	Geraniaceae Juss.
Iz_5_1109	DJI_0138	Gypsophila paniculata L.	0	n	1	0	Caryophyllaceae Juss.
Iz_5_1109	DJI_0138	Helichrysum arenarium (L.) Moench	0	n	1	0	Asteraceae Bercht. & J.Presl
Iz_5_1109	DJI_0138	Herniaria polygama J.Gay	0	n	1	0	Caryophyllaceae Juss.
Iz_5_1109	DJI_0138	Hieracium robustum Fr.	0	n	1	0	Asteraceae Bercht. & J.Presl
Iz_5_1109	DJI_0138	Hieracium umbellatum L.	0	n	1	NA	Asteraceae Bercht. & J.Presl
Iz_5_1109	DJI_0138	Hylotelephium maximum (L.) Holub	0	n	1	0	Crassulaceae J.St.-Hil.
Iz_5_1109	DJI_0138	Jacobaea borysthenica (DC.) B.Nord. & Greuter	0	n	1	0	Asteraceae Bercht. & J.Presl
Iz_5_1109	DJI_0138	Jasione montana L.	0	n	1	0	Campanulaceae Juss.
Iz_5_1109	DJI_0138	Jurinea cyanoides (L.) Rchb.s.l.	0	n	1	0	Asteraceae Bercht. & J.Presl
Iz_5_1109	DJI_0138	Koeleria glauca (Spreng.) DC.	0	n	1	0	Poaceae Barnhart
Iz_5_1109	DJI_0138	Lactuca serriola L.	0	a	0	0	Asteraceae Bercht. & J.Presl
Iz_5_1109	DJI_0138	Linaria genistifolia (L.) Mill.	0	n	1	0	Plantaginaceae Juss.
Iz_5_1109	DJI_0138	Lomelosia argentea (L.) Greuter & Burdet	0	n	1	0	Caprifoliaceae Juss.
Iz_5_1109	DJI_0138	Melampyrum pratense L.	0	n	1	0	Orobanchaceae Vent.
Iz_5_1109	DJI_0138	Oenothera biennis L.	0	a	0	0	Onagraceae Juss.
Iz_5_1109	DJI_0138	Pentanema britannica (L.) D.Gut.Larr., Santos-Vicente, Anderb., E.Rico & M.M.Mart.Ort.	0	n	1	0	Asteraceae Bercht. & J.Presl
Iz_5_1109	DJI_0138	Peucedanum oreoselinum (L.) Moench	0	n	1	0	Apiaceae Lindl.
Iz_5_1109	DJI_0138	Peucedanum ruthenicum M.Bieb.	0	n	1	0	Apiaceae Lindl.
Iz_5_1109	DJI_0138	Pilosella echioides (Lumn.) F.W.Schultz & Sch.Bip.	0	n	1	0	Asteraceae Bercht. & J.Presl
Iz_5_1109	DJI_0138	Pilosella officinarum F. Schultz et Sch. Bip.	0	n	1	0	Asteraceae Bercht. & J.Presl
Iz_5_1109	DJI_0138	Pilosella sp.	0		NA	0	Asteraceae Bercht. & J.Presl
Iz_5_1109	DJI_0138	Pinus sylvestris L.	0	n	1	1	Pinaceae Spreng. ex F.Rudolphi
Iz_5_1109	DJI_0138	Poa annua L.	0	n	1	0	Poaceae Barnhart
Iz_5_1109	DJI_0138	Poa nemoralis L.	0	n	1	0	Poaceae Barnhart
Iz_5_1109	DJI_0138	Poa sp.	0		NA	0	Poaceae Barnhart
Iz_5_1109	DJI_0138	Polygonatum odoratum (Mill.) Druce	0	n	1	0	Asparagaceae Juss.
Iz_5_1109	DJI_0138	Polygonum arenarium Waldst. & Kit.	0	n	1	0	Polygonaceae Juss.
Iz_5_1109	DJI_0138	Populus nigra L.	0	n	1	1	Salicaceae Mirb.
Iz_5_1109	DJI_0138	Populus tremula L.	0	n	1	1	Salicaceae Mirb.
Iz_5_1109	DJI_0138	Populus Ã canescens (Aiton) Sm.	0	n	1	1	Salicaceae Mirb.
Iz_5_1109	DJI_0138	Potentilla argentea L.	0	n	1	0	Rosaceae Juss.
Iz_5_1109	DJI_0138	Psammophiliella muralis (L.) Ikonn.	0	n	1	0	Caryophyllaceae Juss.
Iz_5_1109	DJI_0138	Psephellus sumensis (Kalen.) Greuter	0	n	1	0	Asteraceae Bercht. & J.Presl
Iz_5_1109	DJI_0138	Pulsatilla pratensis (L.) Mill. s.l.	0	n	1	0	Ranunculaceae Juss.
Iz_5_1109	DJI_0138	Pyrus communis L.	0	a	0	1	Rosaceae Juss.
Iz_5_1109	DJI_0138	Quercus robur L.	0	n	1	1	Fagaceae Dumort.
Iz_5_1109	DJI_0138	Robinia pseudoacacia L.	0	a	0	1	Fabaceae Lindl.
Iz_5_1109	DJI_0138	Rosa sp.	0		NA	1	Rosaceae Juss.
Iz_5_1109	DJI_0138	Rubus idaeus L.	0	n	1	0	Rosaceae Juss.
Iz_5_1109	DJI_0138	Rumex acetosella L.	0	n	1	0	Polygonaceae Juss.
Iz_5_1109	DJI_0138	Rumex thyrsiflorus Fingerh.	0	n	1	0	Polygonaceae Juss.
Iz_5_1109	DJI_0138	Salix alba L.	0	n	1	1	Salicaceae Mirb.
Iz_5_1109	DJI_0138	Sambucus nigra L.	0	n	1	1	Viburnaceae Raf.
Iz_5_1109	DJI_0138	Secale sylvestre Host	0	n	1	0	Poaceae Barnhart
Iz_5_1109	DJI_0138	Sempervivum ruthenicum Schnittsp. & C.B. Lehm.	0	n	1	0	Crassulaceae J.St.-Hil.
Iz_5_1109	DJI_0138	Setaria viridis (L.) P. Beauv.	0	n	1	0	Poaceae Barnhart
Iz_5_1109	DJI_0138	Silene borysthenica (Gruner) Walters	0	n	1	0	Caryophyllaceae Juss.
Iz_5_1109	DJI_0138	Silene latifolia subsp. alba (Mill.) Greuter & Burdet	0	n	1	0	Caryophyllaceae Juss.
Iz_5_1109	DJI_0138	Solidago virgaurea L.	0	n	1	0	Asteraceae Bercht. & J.Presl
Iz_5_1109	DJI_0138	Sorbus aucuparia L.	0	n	1	1	Rosaceae Juss.
Iz_5_1109	DJI_0138	Stachys recta L.	0	n	1	0	Lamiacea Lindl.
Iz_5_1109	DJI_0138	Tanacetum vulgare L.	0	n	1	0	Asteraceae Bercht. & J.Presl
Iz_5_1109	DJI_0138	Thymus pallasianus Heinr.Braun	0	n	1	0	Lamiacea Lindl.
Iz_5_1109	DJI_0138	Tilia cordata Mill.	0	n	1	1	Malvaceae Juss.
Iz_5_1109	DJI_0138	Trifolium alpestre L.	0	n	1	0	Fabaceae Lindl.
Iz_5_1109	DJI_0138	Trifolium arvense L.	0	n	1	0	Fabaceae Lindl.
Iz_5_1109	DJI_0138	Ulmus glabra Huds.	0	n	1	1	Ulmaceae Mirb.
Iz_5_1109	DJI_0138	Verbascum cf. phlomoides L.	0		NA	NA	
Iz_5_1109	DJI_0138	Verbascum densiflorum Bertol.	0	n	1	0	Scrophulariaceae Juss.
Iz_5_1109	DJI_0138	Verbascum sp.	0	n	1	0	Scrophulariaceae Juss.
Iz_5_1109	DJI_0138	Veronica austriaca L.	0	n	1	0	Plantaginaceae Juss.
Iz_5_1109	DJI_0138	Veronica incana L.	0	n	1	0	Plantaginaceae Juss.
Iz_5_1109	DJI_0138	Veronica verna L.	0	n	1	0	Plantaginaceae Juss.
Iz_5_1109	DJI_0138	Vincetoxicum hirundinaria Medik	0	n	1	0	Apocynaceae Juss.
Iz_5_1109	DJI_0140	Acer tataricum L.	0	n	1	1	Sapindaceae Juss.
Iz_5_1109	DJI_0140	Achillea millefolium L. s.l.	0	n	1	0	Asteraceae Bercht. & J.Presl
Iz_5_1109	DJI_0140	Allium oleraceum L.	0	n	1	0	Amaryllidaceae J.St.-Hil.
Iz_5_1109	DJI_0140	Ambrosia artemisiifolia L.	0	a	0	0	Asteraceae Bercht. & J.Presl
Iz_5_1109	DJI_0140	Amorpha fruticosa L.	0	a	0	0	Fabaceae Lindl.
Iz_5_1109	DJI_0140	Anthericum ramosum L.	0	n	1	0	Asparagaceae Juss.
Iz_5_1109	DJI_0140	Anthoxanthum repens (Host) Veldkamp	0		NA	NA	
Iz_5_1109	DJI_0140	Artemisia marschalliana Spreng.	0	n	1	0	Asteraceae Bercht. & J.Presl
Iz_5_1109	DJI_0140	Asparagus officinalis L.	0	n	1	0	Asparagaceae Juss.
Iz_5_1109	DJI_0140	Astragalus cicer L.	0	n	1	0	Fabaceae Lindl.
Iz_5_1109	DJI_0140	Bassia laniflora (S.G.Gmel.) A.J.Scott	0	n	1	0	Amaranthaceae Juss.
Iz_5_1109	DJI_0140	Berberis vulgaris L.	0	n	1	0	Berberidaceae Juss.
Iz_5_1109	DJI_0140	Berteroa incana (L.) DC.	0		NA	NA	
Iz_5_1109	DJI_0140	Betula pendula Roth.	0	n	1	1	Betulaceae Gray
Iz_5_1109	DJI_0140	Betula pendula Roth.	0	n	1	0	Poaceae Barnhart
Iz_5_1109	DJI_0140	Bromus inermis Leyss.	0		NA	NA	
Iz_5_1109	DJI_0140	Bromus squarrosus L.	0	a	0	0	Poaceae Barnhart
Iz_5_1109	DJI_0140	Bromus tectorum L.	0	a	0	0	Poaceae Barnhart
Iz_5_1109	DJI_0140	Calamagrostis epigejos (L.) Roth	0	n	1	0	Poaceae Barnhart
Iz_5_1109	DJI_0140	Carduus acanthoides L.	0	n	1	0	Asteraceae Bercht. & J.Presl
Iz_5_1109	DJI_0140	Carex sp.	0	n	1	0	Cyperaceae Juss.
Iz_5_1109	DJI_0140	Centaurea majorovii Dumbadze	0	n	1	0	Asteraceae Bercht. & J.Presl
Iz_5_1109	DJI_0140	Chamaecytisus ruthenicus (Fisch.ex Wol.) Klaskova	0	n	1	0	Fabaceae Lindl.
Iz_5_1109	DJI_0140	Chelidonium majus L.	0	n	1	0	Papaveraceae Juss.
Iz_5_1109	DJI_0140	Chenopodium betaceum Andrz.	0	n	1	0	Amaranthaceae Juss.
Iz_5_1109	DJI_0140	Chenopodium cf. album	0	n	1	0	Amaranthaceae Juss.
Iz_5_1109	DJI_0140	Chenopodium sp.	0		NA	0	Amaranthaceae Juss.
Iz_5_1109	DJI_0140	Chondrilla juncea L.	0	n	1	0	Asteraceae Bercht. & J.Presl

Iz_5_1109	DJI_0140	Cirsium vulgare (Savi) Ten.	0	n	1	0	Asteraceae Bercht. & J.Presl
Iz_5_1109	DJI_0140	Clinopodium acinos (L.) Kuntze	0	n	1	0	Lamiacea Lindl.
Iz_5_1109	DJI_0140	Convallaria majalis L.	0	n	1	0	Asparagaceae Juss.
Iz_5_1109	DJI_0140	Crataegus rhipidophylla Gand.	0	n	1	1	Rosaceae Juss.
Iz_5_1109	DJI_0140	Crepis foetida L.	0	n	1	0	Asteraceae Bercht. & J.Presl
Iz_5_1109	DJI_0140	Dactylis glomerata L.	0	n	1	0	Poaceae Barnhart
Iz_5_1109	DJI_0140	Dianthus campestris M.Bieb.	0	n	1	0	Caryophyllaceae Juss.
Iz_5_1109	DJI_0140	Digitaria sanguinalis (L.) Scop.	0	a	0	0	Poaceae Barnhart
Iz_5_1109	DJI_0140	Elymus repens (L.) Gould	0	n	1	0	Poaceae Barnhart
Iz_5_1109	DJI_0140	Epilobium tetragonum L.	0	n	1	0	Onagraceae Juss.
Iz_5_1109	DJI_0140	Eragrostis minor Host	0	a	0	0	Poaceae Barnhart
Iz_5_1109	DJI_0140	Erigeron canadensis L.	2	a	0	0	Asteraceae Bercht. & J.Presl
Iz_5_1109	DJI_0140	Euphorbia seguieriana Neck.	0	n	1	0	Euphorbiaceae Juss.
Iz_5_1109	DJI_0140	Fallopia dumetorum (L.) Holub	0.1	n	1	0	Polygonaceae Juss.
Iz_5_1109	DJI_0140	Festuca beckeri (Hack.) Trautv.	0	n	1	0	Poaceae Barnhart
Iz_5_1109	DJI_0140	Fragaria vesca L.	0	n	1	0	Rosaceae Juss.
Iz_5_1109	DJI_0140	Frangula alnus Mill.	0	n	1	1	Rhamnaceae Juss.
Iz_5_1109	DJI_0140	Fraxinus pennsylvanica Marshall	0	a	0	1	Oleaceae Hoffmanns. & Link
Iz_5_1109	DJI_0140	Galeopsis bifida Boenn.	0	n	1	0	Lamiacea Lindl.
Iz_5_1109	DJI_0140	Galium verum L.	0	n	1	0	Rubiaceae Juss.
Iz_5_1109	DJI_0140	Genista tinctoria L.	0	n	1	0	Fabaceae Lindl.
Iz_5_1109	DJI_0140	Geranium robertianum L.	0	n	1	0	Geraniaceae Juss.
Iz_5_1109	DJI_0140	Geranium sanquineum L.	0	n	1	0	Geraniaceae Juss.
Iz_5_1109	DJI_0140	Gypsophila paniculata L.	0	n	1	0	Caryophyllaceae Juss.
Iz_5_1109	DJI_0140	Helichrysum arenarium (L.) Moench	0	n	1	0	Asteraceae Bercht. & J.Presl
Iz_5_1109	DJI_0140	Herniaria polygama J.Gay	0	n	1	0	Caryophyllaceae Juss.
Iz_5_1109	DJI_0140	Hieracium robustum Fr.	0	n	1	0	Asteraceae Bercht. & J.Presl
Iz_5_1109	DJI_0140	Hieracium umbellatum L.	0	n	1	NA	Asteraceae Bercht. & J.Presl
Iz_5_1109	DJI_0140	Hylotelephium maximum (L.) Holub	0	n	1	0	Crassulaceae J.St.-Hil.
Iz_5_1109	DJI_0140	Jacobaea borysthenica (DC.) B.Nord. & Greuter	0	n	1	0	Asteraceae Bercht. & J.Presl
Iz_5_1109	DJI_0140	Jasione montana L.	0	n	1	0	Campanulaceae Juss.
Iz_5_1109	DJI_0140	Jurinea cyanoides (L.) Rchb.s.l.	0	n	1	0	Asteraceae Bercht. & J.Presl
Iz_5_1109	DJI_0140	Koeleria glauca (Spreng.) DC.	0	n	1	0	Poaceae Barnhart
Iz_5_1109	DJI_0140	Lactuca serriola L.	0	a	0	0	Asteraceae Bercht. & J.Presl
Iz_5_1109	DJI_0140	Linaria genistifolia (L.) Mill.	0	n	1	0	Plantaginaceae Juss.
Iz_5_1109	DJI_0140	Lomelosia argentea (L.) Greuter & Burdet	0	n	1	0	Caprifoliaceae Juss.
Iz_5_1109	DJI_0140	Melampyrum pratense L.	0	n	1	0	Orobanchaceae Vent.
Iz_5_1109	DJI_0140	Oenothera biennis L.	0	a	0	0	Onagraceae Juss.
Iz_5_1109	DJI_0140	Pentanema britannica (L.) D.Gut.Larr., Santos-Vicente, Anderb., E.Rico & M.M.Mart.Ort.	0	n	1	0	Asteraceae Bercht. & J.Presl
Iz_5_1109	DJI_0140	Peucedanum oreoselinum (L.) Moench	0	n	1	0	Apiaceae Lindl.
Iz_5_1109	DJI_0140	Peucedanum ruthenicum M.Bieb.	0	n	1	0	Apiaceae Lindl.
Iz_5_1109	DJI_0140	Pilosella echioides (Lumn.) F.W.Schultz & Sch.Bip.	0	n	1	0	Asteraceae Bercht. & J.Presl
Iz_5_1109	DJI_0140	Pilosella officinarum F. Schultz et Sch. Bip.	0	n	1	0	Asteraceae Bercht. & J.Presl
Iz_5_1109	DJI_0140	Pilosella sp.	0		NA	0	Asteraceae Bercht. & J.Presl
Iz_5_1109	DJI_0140	Pinus sylvestris L.	0	n	1	1	Pinaceae Spreng. ex F.Rudolphi
Iz_5_1109	DJI_0140	Poa annua L.	0	n	1	0	Poaceae Barnhart
Iz_5_1109	DJI_0140	Poa nemoralis L.	0	n	1	0	Poaceae Barnhart
Iz_5_1109	DJI_0140	Poa sp.	0		NA	0	Poaceae Barnhart
Iz_5_1109	DJI_0140	Polygonatum odoratum (Mill.) Druce	0	n	1	0	Asparagaceae Juss.
Iz_5_1109	DJI_0140	Polygonum arenarium Waldst. & Kit.	0	n	1	0	Polygonaceae Juss.
Iz_5_1109	DJI_0140	Populus nigra L.	0	n	1	1	Salicaceae Mirb.
Iz_5_1109	DJI_0140	Populus tremula L.	0	n	1	1	Salicaceae Mirb.
Iz_5_1109	DJI_0140	Populus Ã canescens (Aiton) Sm.	0	n	1	1	Salicaceae Mirb.
Iz_5_1109	DJI_0140	Potentilla argentea L.	0	n	1	0	Rosaceae Juss.
Iz_5_1109	DJI_0140	Psammophiliella muralis (L.) Ikonn.	0	n	1	0	Caryophyllaceae Juss.
Iz_5_1109	DJI_0140	Psephellus sumensis (Kalen.) Greuter	0	n	1	0	Asteraceae Bercht. & J.Presl
Iz_5_1109	DJI_0140	Pulsatilla pratensis (L.) Mill. s.l.	0	n	1	0	Ranunculaceae Juss.
Iz_5_1109	DJI_0140	Pyrus communis L.	0	a	0	1	Rosaceae Juss.
Iz_5_1109	DJI_0140	Quercus robur L.	0	n	1	1	Fagaceae Dumort.
Iz_5_1109	DJI_0140	Robinia pseudoacacia L.	0	a	0	1	Fabaceae Lindl.
Iz_5_1109	DJI_0140	Rosa sp.	0		NA	1	Rosaceae Juss.
Iz_5_1109	DJI_0140	Rubus idaeus L.	0	n	1	0	Rosaceae Juss.
Iz_5_1109	DJI_0140	Rumex acetosella L.	3	n	1	0	Polygonaceae Juss.
Iz_5_1109	DJI_0140	Rumex thyrsiflorus Fingerh.	0	n	1	0	Polygonaceae Juss.
Iz_5_1109	DJI_0140	Salix alba L.	0	n	1	1	Salicaceae Mirb.
Iz_5_1109	DJI_0140	Sambucus nigra L.	0	n	1	1	Viburnaceae Raf.
Iz_5_1109	DJI_0140	Secale sylvestre Host	0	n	1	0	Poaceae Barnhart
Iz_5_1109	DJI_0140	Sempervivum ruthenicum Schnittsp. & C.B. Lehm.	0	n	1	0	Crassulaceae J.St.-Hil.
Iz_5_1109	DJI_0140	Setaria viridis (L.) P. Beauv.	0	n	1	0	Poaceae Barnhart
Iz_5_1109	DJI_0140	Silene borysthenica (Gruner) Walters	0	n	1	0	Caryophyllaceae Juss.
Iz_5_1109	DJI_0140	Silene latifolia subsp. alba (Mill.) Greuter & Burdet	0	n	1	0	Caryophyllaceae Juss.
Iz_5_1109	DJI_0140	Solidago virgaurea L.	0	n	1	0	Asteraceae Bercht. & J.Presl
Iz_5_1109	DJI_0140	Sorbus aucuparia L.	0	n	1	1	Rosaceae Juss.
Iz_5_1109	DJI_0140	Stachys recta L.	0	n	1	0	Lamiacea Lindl.
Iz_5_1109	DJI_0140	Tanacetum vulgare L.	0	n	1	0	Asteraceae Bercht. & J.Presl
Iz_5_1109	DJI_0140	Thymus pallasianus Heinr.Braun	0	n	1	0	Lamiacea Lindl.
Iz_5_1109	DJI_0140	Tilia cordata Mill.	0	n	1	1	Malvaceae Juss.
Iz_5_1109	DJI_0140	Trifolium alpestre L.	0	n	1	0	Fabaceae Lindl.
Iz_5_1109	DJI_0140	Trifolium arvense L.	0	n	1	0	Fabaceae Lindl.
Iz_5_1109	DJI_0140	Ulmus glabra Huds.	0	n	1	1	Ulmaceae Mirb.
Iz_5_1109	DJI_0140	Verbascum cf. phlomoides L.	0		NA	NA	
Iz_5_1109	DJI_0140	Verbascum densiflorum Bertol.	0	n	1	0	Scrophulariaceae Juss.
Iz_5_1109	DJI_0140	Verbascum sp.	0	n	1	0	Scrophulariaceae Juss.
Iz_5_1109	DJI_0140	Veronica austriaca L.	0	n	1	0	Plantaginaceae Juss.
Iz_5_1109	DJI_0140	Veronica incana L.	0	n	1	0	Plantaginaceae Juss.
Iz_5_1109	DJI_0140	Veronica verna L.	0	n	1	0	Plantaginaceae Juss.
Iz_5_1109	DJI_0140	Vincetoxicum hirundinaria Medik	0	n	1	0	Apocynaceae Juss.
Iz_5_1109	DJI_0143	Acer tataricum L.	0	n	1	1	Sapindaceae Juss.
Iz_5_1109	DJI_0143	Achillea millefolium L. s.l.	0	n	1	0	Asteraceae Bercht. & J.Presl
Iz_5_1109	DJI_0143	Allium oleraceum L.	0	n	1	0	Amaryllidaceae J.St.-Hil.
Iz_5_1109	DJI_0143	Ambrosia artemisiifolia L.	0	a	0	0	Asteraceae Bercht. & J.Presl
Iz_5_1109	DJI_0143	Amorpha fruticosa L.	0	a	0	0	Fabaceae Lindl.
Iz_5_1109	DJI_0143	Anthericum ramosum L.	0	n	1	0	Asparagaceae Juss.
Iz_5_1109	DJI_0143	Anthoxanthum repens (Host) Veldkamp	0		NA	NA	
Iz_5_1109	DJI_0143	Artemisia marschalliana Spreng.	0	n	1	0	Asteraceae Bercht. & J.Presl
Iz_5_1109	DJI_0143	Asparagus officinalis L.	0	n	1	0	Asparagaceae Juss.
Iz_5_1109	DJI_0143	Astragalus cicer L.	0	n	1	0	Fabaceae Lindl.
Iz_5_1109	DJI_0143	Bassia laniflora (S.G.Gmel.) A.J.Scott	0	n	1	0	Amaranthaceae Juss.

Iz_5_1109	DJI_0143	Berberis vulgaris L.	0	n	1	0	Berberidaceae Juss.
Iz_5_1109	DJI_0143	Berteroa incana (L.) DC.	0.1		NA	NA	
Iz_5_1109	DJI_0143	Betula pendula Roth.	0	n	1	1	Betulaceae Gray
Iz_5_1109	DJI_0143	Betula pendula Roth.	0	n	1	0	Poaceae Barnhart
Iz_5_1109	DJI_0143	Bromus inermis Leyss.	0		NA	NA	
Iz_5_1109	DJI_0143	Bromus squarrosus L.	0	a	0	0	Poaceae Barnhart
Iz_5_1109	DJI_0143	Bromus tectorum L.	0	a	0	0	Poaceae Barnhart
Iz_5_1109	DJI_0143	Calamagrostis epigejos (L.) Roth	0.1	n	1	0	Poaceae Barnhart
Iz_5_1109	DJI_0143	Carduus acanthoides L.	0	n	1	0	Asteraceae Bercht. & J.Presl
Iz_5_1109	DJI_0143	Carex sp.	0	n	1	0	Cyperaceae Juss.
Iz_5_1109	DJI_0143	Centaurea majorovii Dumbadze	0	n	1	0	Asteraceae Bercht. & J.Presl
Iz_5_1109	DJI_0143	Chamaecytisus ruthenicus (Fisch.ex Wol.) Klaskova	0	n	1	0	Fabaceae Lindl.
Iz_5_1109	DJI_0143	Chelidonium majus L.	0	n	1	0	Papaveraceae Juss.
Iz_5_1109	DJI_0143	Chenopodium betaceum Andrz.	0	n	1	0	Amaranthaceae Juss.
Iz_5_1109	DJI_0143	Chenopodium cf. album	0	n	1	0	Amaranthaceae Juss.
Iz_5_1109	DJI_0143	Chenopodium sp.	0		NA	0	Amaranthaceae Juss.
Iz_5_1109	DJI_0143	Chondrilla juncea L.	0	n	1	0	Asteraceae Bercht. & J.Presl
Iz_5_1109	DJI_0143	Cirsium vulgare (Savi) Ten.	0	n	1	0	Asteraceae Bercht. & J.Presl
Iz_5_1109	DJI_0143	Clinopodium acinos (L.) Kuntze	0	n	1	0	Lamiacea Lindl.
Iz_5_1109	DJI_0143	Convallaria majalis L.	0	n	1	0	Asparagaceae Juss.
Iz_5_1109	DJI_0143	Crataegus rhipidophylla Gand.	0	n	1	1	Rosaceae Juss.
Iz_5_1109	DJI_0143	Crepis foetida L.	0	n	1	0	Asteraceae Bercht. & J.Presl
Iz_5_1109	DJI_0143	Dactylis glomerata L.	0	n	1	0	Poaceae Barnhart
Iz_5_1109	DJI_0143	Dianthus campestris M.Bieb.	0	n	1	0	Caryophyllaceae Juss.
Iz_5_1109	DJI_0143	Digitaria sanguinalis (L.) Scop.	0	a	0	0	Poaceae Barnhart
Iz_5_1109	DJI_0143	Elymus repens (L.) Gould	0	n	1	0	Poaceae Barnhart
Iz_5_1109	DJI_0143	Epilobium tetragonum L.	0	n	1	0	Onagraceae Juss.
Iz_5_1109	DJI_0143	Eragrostis minor Host	0	a	0	0	Poaceae Barnhart
Iz_5_1109	DJI_0143	Erigeron canadensis L.	1	a	0	0	Asteraceae Bercht. & J.Presl
Iz_5_1109	DJI_0143	Euphorbia seguieriana Neck.	0	n	1	0	Euphorbiaceae Juss.
Iz_5_1109	DJI_0143	Fallopia dumetorum (L.) Holub	0.1	n	1	0	Polygonaceae Juss.
Iz_5_1109	DJI_0143	Festuca beckeri (Hack.) Trautv.	0	n	1	0	Poaceae Barnhart
Iz_5_1109	DJI_0143	Fragaria vesca L.	0	n	1	0	Rosaceae Juss.
Iz_5_1109	DJI_0143	Frangula alnus Mill.	0	n	1	1	Rhamnaceae Juss.
Iz_5_1109	DJI_0143	Fraxinus pennsylvanica Marshall	0	a	0	1	Oleaceae Hoffmanns. & Link
Iz_5_1109	DJI_0143	Galeopsis bifida Boenn.	0	n	1	0	Lamiacea Lindl.
Iz_5_1109	DJI_0143	Galium verum L.	0	n	1	0	Rubiaceae Juss.
Iz_5_1109	DJI_0143	Genista tinctoria L.	0	n	1	0	Fabaceae Lindl.
Iz_5_1109	DJI_0143	Geranium robertianum L.	0	n	1	0	Geraniaceae Juss.
Iz_5_1109	DJI_0143	Geranium sanquineum L.	0	n	1	0	Geraniaceae Juss.
Iz_5_1109	DJI_0143	Gypsophila paniculata L.	0	n	1	0	Caryophyllaceae Juss.
Iz_5_1109	DJI_0143	Helichrysum arenarium (L.) Moench	0	n	1	0	Asteraceae Bercht. & J.Presl
Iz_5_1109	DJI_0143	Herniaria polygama J.Gay	0	n	1	0	Caryophyllaceae Juss.
Iz_5_1109	DJI_0143	Hieracium robustum Fr.	0	n	1	0	Asteraceae Bercht. & J.Presl
Iz_5_1109	DJI_0143	Hieracium umbellatum L.	0	n	1	NA	Asteraceae Bercht. & J.Presl
Iz_5_1109	DJI_0143	Hylotelephium maximum (L.) Holub	0	n	1	0	Crassulaceae J.St.-Hil.
Iz_5_1109	DJI_0143	Jacobaea borysthenica (DC.) B.Nord. & Greuter	0	n	1	0	Asteraceae Bercht. & J.Presl
Iz_5_1109	DJI_0143	Jasione montana L.	0	n	1	0	Campanulaceae Juss.
Iz_5_1109	DJI_0143	Jurinea cyanoides (L.) Rchb.s.l.	0	n	1	0	Asteraceae Bercht. & J.Presl
Iz_5_1109	DJI_0143	Koeleria glauca (Spreng.) DC.	0	n	1	0	Poaceae Barnhart
Iz_5_1109	DJI_0143	Lactuca serriola L.	0	a	0	0	Asteraceae Bercht. & J.Presl
Iz_5_1109	DJI_0143	Linaria genistifolia (L.) Mill.	0	n	1	0	Plantaginaceae Juss.
Iz_5_1109	DJI_0143	Lomelosia argentea (L.) Greuter & Burdet	0	n	1	0	Caprifoliaceae Juss.
Iz_5_1109	DJI_0143	Melampyrum pratense L.	0	n	1	0	Orobanchaceae Vent.
Iz_5_1109	DJI_0143	Oenothera biennis L.	0	a	0	0	Onagraceae Juss.
Iz_5_1109	DJI_0143	Pentanema britannica (L.) D.Gut.Larr., Santos-Vicente, Anderb., E.Rico & M.M.Mart.Ort.	0	n	1	0	Asteraceae Bercht. & J.Presl
Iz_5_1109	DJI_0143	Peucedanum oreoselinum (L.) Moench	0	n	1	0	Apiaceae Lindl.
Iz_5_1109	DJI_0143	Peucedanum ruthenicum M.Bieb.	0	n	1	0	Apiaceae Lindl.
Iz_5_1109	DJI_0143	Pilosella echioides (Lumn.) F.W.Schultz & Sch.Bip.	0	n	1	0	Asteraceae Bercht. & J.Presl
Iz_5_1109	DJI_0143	Pilosella officinarum F. Schultz et Sch. Bip.	0	n	1	0	Asteraceae Bercht. & J.Presl
Iz_5_1109	DJI_0143	Pilosella sp.	0		NA	0	Asteraceae Bercht. & J.Presl
Iz_5_1109	DJI_0143	Pinus sylvestris L.	0	n	1	1	Pinaceae Spreng. ex F.Rudolphi
Iz_5_1109	DJI_0143	Poa annua L.	0	n	1	0	Poaceae Barnhart
Iz_5_1109	DJI_0143	Poa nemoralis L.	0	n	1	0	Poaceae Barnhart
Iz_5_1109	DJI_0143	Poa sp.	0		NA	0	Poaceae Barnhart
Iz_5_1109	DJI_0143	Polygonatum odoratum (Mill.) Druce	0	n	1	0	Asparagaceae Juss.
Iz_5_1109	DJI_0143	Polygonum arenarium Waldst. & Kit.	0	n	1	0	Polygonaceae Juss.
Iz_5_1109	DJI_0143	Populus nigra L.	0	n	1	1	Salicaceae Mirb.
Iz_5_1109	DJI_0143	Populus tremula L.	0	n	1	1	Salicaceae Mirb.
Iz_5_1109	DJI_0143	Populus Ã canescens (Aiton) Sm.	0	n	1	1	Salicaceae Mirb.
Iz_5_1109	DJI_0143	Potentilla argentea L.	0	n	1	0	Rosaceae Juss.
Iz_5_1109	DJI_0143	Psammophiliella muralis (L.) Ikonn.	0	n	1	0	Caryophyllaceae Juss.
Iz_5_1109	DJI_0143	Psephellus sumensis (Kalen.) Greuter	0	n	1	0	Asteraceae Bercht. & J.Presl
Iz_5_1109	DJI_0143	Pulsatilla pratensis (L.) Mill. s.l.	0	n	1	0	Ranunculaceae Juss.
Iz_5_1109	DJI_0143	Pyrus communis L.	0	a	0	1	Rosaceae Juss.
Iz_5_1109	DJI_0143	Quercus robur L.	0	n	1	1	Fagaceae Dumort.
Iz_5_1109	DJI_0143	Robinia pseudoacacia L.	0	a	0	1	Fabaceae Lindl.
Iz_5_1109	DJI_0143	Rosa sp.	0		NA	1	Rosaceae Juss.
Iz_5_1109	DJI_0143	Rubus idaeus L.	0	n	1	0	Rosaceae Juss.
Iz_5_1109	DJI_0143	Rumex acetosella L.	0	n	1	0	Polygonaceae Juss.
Iz_5_1109	DJI_0143	Rumex thyrsiflorus Fingerh.	0	n	1	0	Polygonaceae Juss.
Iz_5_1109	DJI_0143	Salix alba L.	0	n	1	1	Salicaceae Mirb.
Iz_5_1109	DJI_0143	Sambucus nigra L.	0	n	1	1	Viburnaceae Raf.
Iz_5_1109	DJI_0143	Secale sylvestre Host	0	n	1	0	Poaceae Barnhart
Iz_5_1109	DJI_0143	Sempervivum ruthenicum Schnittsp. & C.B. Lehm.	0	n	1	0	Crassulaceae J.St.-Hil.
Iz_5_1109	DJI_0143	Setaria viridis (L.) P. Beauv.	0	n	1	0	Poaceae Barnhart
Iz_5_1109	DJI_0143	Silene borysthenica (Gruner) Walters	0	n	1	0	Caryophyllaceae Juss.
Iz_5_1109	DJI_0143	Silene latifolia subsp. alba (Mill.) Greuter & Burdet	0	n	1	0	Caryophyllaceae Juss.
Iz_5_1109	DJI_0143	Solidago virgaurea L.	0.2	n	1	0	Asteraceae Bercht. & J.Presl
Iz_5_1109	DJI_0143	Sorbus aucuparia L.	0	n	1	1	Rosaceae Juss.
Iz_5_1109	DJI_0143	Stachys recta L.	0	n	1	0	Lamiacea Lindl.
Iz_5_1109	DJI_0143	Tanacetum vulgare L.	0	n	1	0	Asteraceae Bercht. & J.Presl
Iz_5_1109	DJI_0143	Thymus pallasianus Heinr.Braun	0	n	1	0	Lamiacea Lindl.
Iz_5_1109	DJI_0143	Tilia cordata Mill.	0	n	1	1	Malvaceae Juss.
Iz_5_1109	DJI_0143	Trifolium alpestre L.	0	n	1	0	Fabaceae Lindl.
Iz_5_1109	DJI_0143	Trifolium arvense L.	0	n	1	0	Fabaceae Lindl.
Iz_5_1109	DJI_0143	Ulmus glabra Huds.	0	n	1	1	Ulmaceae Mirb.
Iz_5_1109	DJI_0143	Verbascum cf. phlomoides L.	0		NA	NA	

Iz_5_1109	DJI_0143	Verbascum densiflorum Bertol.	0	n	1	0	Scrophulariaceae Juss.
Iz_5_1109	DJI_0143	Verbascum sp.	0	n	1	0	Scrophulariaceae Juss.
Iz_5_1109	DJI_0143	Veronica austriaca L.	0	n	1	0	Plantaginaceae Juss.
Iz_5_1109	DJI_0143	Veronica incana L.	0	n	1	0	Plantaginaceae Juss.
Iz_5_1109	DJI_0143	Veronica verna L.	0	n	1	0	Plantaginaceae Juss.
Iz_5_1109	DJI_0143	Vincetoxicum hirundinaria Medik	0	n	1	0	Apocynaceae Juss.
Iz_5_1109	DJI_0146	Acer tataricum L.	0	n	1	1	Sapindaceae Juss.
Iz_5_1109	DJI_0146	Achillea millefolium L. s.l.	0	n	1	0	Asteraceae Bercht. & J.Presl
Iz_5_1109	DJI_0146	Allium oleraceum L.	0	n	1	0	Amaryllidaceae J.St.-Hil.
Iz_5_1109	DJI_0146	Ambrosia artemisiifolia L.	0	a	0	0	Asteraceae Bercht. & J.Presl
Iz_5_1109	DJI_0146	Amorpha fruticosa L.	0	a	0	0	Fabaceae Lindl.
Iz_5_1109	DJI_0146	Anthericum ramosum L.	0	n	1	0	Asparagaceae Juss.
Iz_5_1109	DJI_0146	Anthoxanthum repens (Host) Veldkamp	0		NA	NA	
Iz_5_1109	DJI_0146	Artemisia marschalliana Spreng.	0	n	1	0	Asteraceae Bercht. & J.Presl
Iz_5_1109	DJI_0146	Asparagus officinalis L.	0	n	1	0	Asparagaceae Juss.
Iz_5_1109	DJI_0146	Astragalus cicer L.	0	n	1	0	Fabaceae Lindl.
Iz_5_1109	DJI_0146	Bassia laniflora (S.G.Gmel.) A.J.Scott	0	n	1	0	Amaranthaceae Juss.
Iz_5_1109	DJI_0146	Berberis vulgaris L.	0	n	1	0	Berberidaceae Juss.
Iz_5_1109	DJI_0146	Berteroa incana (L.) DC.	0		NA	NA	
Iz_5_1109	DJI_0146	Betula pendula Roth.	0	n	1	1	Betulaceae Gray
Iz_5_1109	DJI_0146	Betula pendula Roth.	0	n	1	0	Poaceae Barnhart
Iz_5_1109	DJI_0146	Bromus inermis Leyss.	0		NA	NA	
Iz_5_1109	DJI_0146	Bromus squarrosus L.	0	a	0	0	Poaceae Barnhart
Iz_5_1109	DJI_0146	Bromus tectorum L.	0	a	0	0	Poaceae Barnhart
Iz_5_1109	DJI_0146	Calamagrostis epigejos (L.) Roth	0	n	1	0	Poaceae Barnhart
Iz_5_1109	DJI_0146	Carduus acanthoides L.	0	n	1	0	Asteraceae Bercht. & J.Presl
Iz_5_1109	DJI_0146	Carex sp.	0	n	1	0	Cyperaceae Juss.
Iz_5_1109	DJI_0146	Centaurea majorovii Dumbadze	0	n	1	0	Asteraceae Bercht. & J.Presl
Iz_5_1109	DJI_0146	Chamaecytisus ruthenicus (Fisch.ex Wol.) Klaskova	0	n	1	0	Fabaceae Lindl.
Iz_5_1109	DJI_0146	Chelidonium majus L.	0	n	1	0	Papaveraceae Juss.
Iz_5_1109	DJI_0146	Chenopodium betaceum Andrz.	0	n	1	0	Amaranthaceae Juss.
Iz_5_1109	DJI_0146	Chenopodium cf. album	0	n	1	0	Amaranthaceae Juss.
Iz_5_1109	DJI_0146	Chenopodium sp.	0		NA	0	Amaranthaceae Juss.
Iz_5_1109	DJI_0146	Chondrilla juncea L.	0	n	1	0	Asteraceae Bercht. & J.Presl
Iz_5_1109	DJI_0146	Cirsium vulgare (Savi) Ten.	0	n	1	0	Asteraceae Bercht. & J.Presl
Iz_5_1109	DJI_0146	Clinopodium acinos (L.) Kuntze	0	n	1	0	Lamiacea Lindl.
Iz_5_1109	DJI_0146	Convallaria majalis L.	0	n	1	0	Asparagaceae Juss.
Iz_5_1109	DJI_0146	Crataegus rhipidophylla Gand.	0	n	1	1	Rosaceae Juss.
Iz_5_1109	DJI_0146	Crepis foetida L.	0	n	1	0	Asteraceae Bercht. & J.Presl
Iz_5_1109	DJI_0146	Dactylis glomerata L.	0	n	1	0	Poaceae Barnhart
Iz_5_1109	DJI_0146	Dianthus campestris M.Bieb.	0	n	1	0	Caryophyllaceae Juss.
Iz_5_1109	DJI_0146	Digitaria sanguinalis (L.) Scop.	0	a	0	0	Poaceae Barnhart
Iz_5_1109	DJI_0146	Elymus repens (L.) Gould	0	n	1	0	Poaceae Barnhart
Iz_5_1109	DJI_0146	Epilobium tetragonum L.	0	n	1	0	Onagraceae Juss.
Iz_5_1109	DJI_0146	Eragrostis minor Host	0	a	0	0	Poaceae Barnhart
Iz_5_1109	DJI_0146	Erigeron canadensis L.	0.2	a	0	0	Asteraceae Bercht. & J.Presl
Iz_5_1109	DJI_0146	Euphorbia seguieriana Neck.	0	n	1	0	Euphorbiaceae Juss.
Iz_5_1109	DJI_0146	Fallopia dumetorum (L.) Holub	0	n	1	0	Polygonaceae Juss.
Iz_5_1109	DJI_0146	Festuca beckeri (Hack.) Trautv.	0	n	1	0	Poaceae Barnhart
Iz_5_1109	DJI_0146	Fragaria vesca L.	0	n	1	0	Rosaceae Juss.
Iz_5_1109	DJI_0146	Frangula alnus Mill.	0	n	1	1	Rhamnaceae Juss.
Iz_5_1109	DJI_0146	Fraxinus pennsylvanica Marshall	0	a	0	1	Oleaceae Hoffmanns. & Link
Iz_5_1109	DJI_0146	Galeopsis bifida Boenn.	0	n	1	0	Lamiacea Lindl.
Iz_5_1109	DJI_0146	Galium verum L.	0	n	1	0	Rubiaceae Juss.
Iz_5_1109	DJI_0146	Genista tinctoria L.	0	n	1	0	Fabaceae Lindl.
Iz_5_1109	DJI_0146	Geranium robertianum L.	0	n	1	0	Geraniaceae Juss.
Iz_5_1109	DJI_0146	Geranium sanquineum L.	0	n	1	0	Geraniaceae Juss.
Iz_5_1109	DJI_0146	Gypsophila paniculata L.	0	n	1	0	Caryophyllaceae Juss.
Iz_5_1109	DJI_0146	Helichrysum arenarium (L.) Moench	0	n	1	0	Asteraceae Bercht. & J.Presl
Iz_5_1109	DJI_0146	Herniaria polygama J.Gay	0	n	1	0	Caryophyllaceae Juss.
Iz_5_1109	DJI_0146	Hieracium robustum Fr.	0	n	1	0	Asteraceae Bercht. & J.Presl
Iz_5_1109	DJI_0146	Hieracium umbellatum L.	0	n	1	NA	Asteraceae Bercht. & J.Presl
Iz_5_1109	DJI_0146	Hylotelephium maximum (L.) Holub	0	n	1	0	Crassulaceae J.St.-Hil.
Iz_5_1109	DJI_0146	Jacobaea borysthenica (DC.) B.Nord. & Greuter	0	n	1	0	Asteraceae Bercht. & J.Presl
Iz_5_1109	DJI_0146	Jasione montana L.	0	n	1	0	Campanulaceae Juss.
Iz_5_1109	DJI_0146	Jurinea cyanoides (L.) Rchb.s.l.	0	n	1	0	Asteraceae Bercht. & J.Presl
Iz_5_1109	DJI_0146	Koeleria glauca (Spreng.) DC.	0	n	1	0	Poaceae Barnhart
Iz_5_1109	DJI_0146	Lactuca serriola L.	0	a	0	0	Asteraceae Bercht. & J.Presl
Iz_5_1109	DJI_0146	Linaria genistifolia (L.) Mill.	0	n	1	0	Plantaginaceae Juss.
Iz_5_1109	DJI_0146	Lomelosia argentea (L.) Greuter & Burdet	0	n	1	0	Caprifoliaceae Juss.
Iz_5_1109	DJI_0146	Melampyrum pratense L.	0	n	1	0	Orobanchaceae Vent.
Iz_5_1109	DJI_0146	Oenothera biennis L.	0	a	0	0	Onagraceae Juss.
Iz_5_1109	DJI_0146	Pentanema britannica (L.) D.Gut.Larr., Santos-Vicente, Anderb., E.Rico & M.M.Mart.Ort.	0	n	1	0	Asteraceae Bercht. & J.Presl
Iz_5_1109	DJI_0146	Peucedanum oreoselinum (L.) Moench	0	n	1	0	Apiaceae Lindl.
Iz_5_1109	DJI_0146	Peucedanum ruthenicum M.Bieb.	0	n	1	0	Apiaceae Lindl.
Iz_5_1109	DJI_0146	Pilosella echioides (Lumn.) F.W.Schultz & Sch.Bip.	0	n	1	0	Asteraceae Bercht. & J.Presl
Iz_5_1109	DJI_0146	Pilosella officinarum F. Schultz et Sch. Bip.	0	n	1	0	Asteraceae Bercht. & J.Presl
Iz_5_1109	DJI_0146	Pilosella sp.	0		NA	0	Asteraceae Bercht. & J.Presl
Iz_5_1109	DJI_0146	Pinus sylvestris L.	0	n	1	1	Pinaceae Spreng. ex F.Rudolphi
Iz_5_1109	DJI_0146	Poa annua L.	0	n	1	0	Poaceae Barnhart
Iz_5_1109	DJI_0146	Poa nemoralis L.	0	n	1	0	Poaceae Barnhart
Iz_5_1109	DJI_0146	Poa sp.	0		NA	0	Poaceae Barnhart
Iz_5_1109	DJI_0146	Polygonatum odoratum (Mill.) Druce	0	n	1	0	Asparagaceae Juss.
Iz_5_1109	DJI_0146	Polygonum arenarium Waldst. & Kit.	0	n	1	0	Polygonaceae Juss.
Iz_5_1109	DJI_0146	Populus nigra L.	0	n	1	1	Salicaceae Mirb.
Iz_5_1109	DJI_0146	Populus tremula L.	0	n	1	1	Salicaceae Mirb.
Iz_5_1109	DJI_0146	Populus Ã canescens (Aiton) Sm.	0	n	1	1	Salicaceae Mirb.
Iz_5_1109	DJI_0146	Potentilla argentea L.	0	n	1	0	Rosaceae Juss.
Iz_5_1109	DJI_0146	Psammophiliella muralis (L.) Ikonn.	0	n	1	0	Caryophyllaceae Juss.
Iz_5_1109	DJI_0146	Psephellus sumensis (Kalen.) Greuter	0	n	1	0	Asteraceae Bercht. & J.Presl
Iz_5_1109	DJI_0146	Pulsatilla pratensis (L.) Mill. s.l.	0	n	1	0	Ranunculaceae Juss.
Iz_5_1109	DJI_0146	Pyrus communis L.	0	a	0	1	Rosaceae Juss.
Iz_5_1109	DJI_0146	Quercus robur L.	0	n	1	1	Fagaceae Dumort.
Iz_5_1109	DJI_0146	Robinia pseudoacacia L.	0	a	0	1	Fabaceae Lindl.
Iz_5_1109	DJI_0146	Rosa sp.	0		NA	1	Rosaceae Juss.
Iz_5_1109	DJI_0146	Rubus idaeus L.	0	n	1	0	Rosaceae Juss.
Iz_5_1109	DJI_0146	Rumex acetosella L.	0	n	1	0	Polygonaceae Juss.
Iz_5_1109	DJI_0146	Rumex thyrsiflorus Fingerh.	0	n	1	0	Polygonaceae Juss.

Iz_5_1109	DJI_0146	Salix alba L.	0	n	1	1	Salicaceae Mirb.
Iz_5_1109	DJI_0146	Sambucus nigra L.	0	n	1	1	Viburnaceae Raf.
Iz_5_1109	DJI_0146	Secale sylvestre Host	0	n	1	0	Poaceae Barnhart
Iz_5_1109	DJI_0146	Sempervivum ruthenicum Schnittsp. & C.B. Lehm.	0	n	1	0	Crassulaceae J.St.-Hil.
Iz_5_1109	DJI_0146	Setaria viridis (L.) P. Beauv.	0	n	1	0	Poaceae Barnhart
Iz_5_1109	DJI_0146	Silene borysthenica (Gruner) Walters	0	n	1	0	Caryophyllaceae Juss.
Iz_5_1109	DJI_0146	Silene latifolia subsp. alba (Mill.) Greuter & Burdet	0	n	1	0	Caryophyllaceae Juss.
Iz_5_1109	DJI_0146	Solidago virgaurea L.	0	n	1	0	Asteraceae Bercht. & J.Presl
Iz_5_1109	DJI_0146	Sorbus aucuparia L.	0	n	1	1	Rosaceae Juss.
Iz_5_1109	DJI_0146	Stachys recta L.	0	n	1	0	Lamiacea Lindl.
Iz_5_1109	DJI_0146	Tanacetum vulgare L.	0	n	1	0	Asteraceae Bercht. & J.Presl
Iz_5_1109	DJI_0146	Thymus pallasianus Heinr.Braun	0	n	1	0	Lamiacea Lindl.
Iz_5_1109	DJI_0146	Tilia cordata Mill.	0	n	1	1	Malvaceae Juss.
Iz_5_1109	DJI_0146	Trifolium alpestre L.	0	n	1	0	Fabaceae Lindl.
Iz_5_1109	DJI_0146	Trifolium arvense L.	0	n	1	0	Fabaceae Lindl.
Iz_5_1109	DJI_0146	Ulmus glabra Huds.	0	n	1	1	Ulmaceae Mirb.
Iz_5_1109	DJI_0146	Verbascum cf. phlomoides L.	0		NA	NA	
Iz_5_1109	DJI_0146	Verbascum densiflorum Bertol.	0	n	1	0	Scrophulariaceae Juss.
Iz_5_1109	DJI_0146	Verbascum sp.	0	n	1	0	Scrophulariaceae Juss.
Iz_5_1109	DJI_0146	Veronica austriaca L.	0	n	1	0	Plantaginaceae Juss.
Iz_5_1109	DJI_0146	Veronica incana L.	0	n	1	0	Plantaginaceae Juss.
Iz_5_1109	DJI_0146	Veronica verna L.	0	n	1	0	Plantaginaceae Juss.
Iz_5_1109	DJI_0146	Vincetoxicum hirundinaria Medik	0	n	1	0	Apocynaceae Juss.
Iz_5_1109	DJI_0148	Acer tataricum L.	0	n	1	1	Sapindaceae Juss.
Iz_5_1109	DJI_0148	Achillea millefolium L. s.l.	0	n	1	0	Asteraceae Bercht. & J.Presl
Iz_5_1109	DJI_0148	Allium oleraceum L.	0	n	1	0	Amaryllidaceae J.St.-Hil.
Iz_5_1109	DJI_0148	Ambrosia artemisiifolia L.	0	a	0	0	Asteraceae Bercht. & J.Presl
Iz_5_1109	DJI_0148	Amorpha fruticosa L.	0	a	0	0	Fabaceae Lindl.
Iz_5_1109	DJI_0148	Anthericum ramosum L.	0	n	1	0	Asparagaceae Juss.
Iz_5_1109	DJI_0148	Anthoxanthum repens (Host) Veldkamp	0		NA	NA	
Iz_5_1109	DJI_0148	Artemisia marschalliana Spreng.	0	n	1	0	Asteraceae Bercht. & J.Presl
Iz_5_1109	DJI_0148	Asparagus officinalis L.	0	n	1	0	Asparagaceae Juss.
Iz_5_1109	DJI_0148	Astragalus cicer L.	0	n	1	0	Fabaceae Lindl.
Iz_5_1109	DJI_0148	Bassia laniflora (S.G.Gmel.) A.J.Scott	0	n	1	0	Amaranthaceae Juss.
Iz_5_1109	DJI_0148	Berberis vulgaris L.	0	n	1	0	Berberidaceae Juss.
Iz_5_1109	DJI_0148	Berteroa incana (L.) DC.	1		NA	NA	
Iz_5_1109	DJI_0148	Betula pendula Roth.	0	n	1	1	Betulaceae Gray
Iz_5_1109	DJI_0148	Betula pendula Roth.	0	n	1	0	Poaceae Barnhart
Iz_5_1109	DJI_0148	Bromus inermis Leyss.	0		NA	NA	
Iz_5_1109	DJI_0148	Bromus squarrosus L.	0	a	0	0	Poaceae Barnhart
Iz_5_1109	DJI_0148	Bromus tectorum L.	0	a	0	0	Poaceae Barnhart
Iz_5_1109	DJI_0148	Calamagrostis epigejos (L.) Roth	3	n	1	0	Poaceae Barnhart
Iz_5_1109	DJI_0148	Carduus acanthoides L.	0	n	1	0	Asteraceae Bercht. & J.Presl
Iz_5_1109	DJI_0148	Carex sp.	0	n	1	0	Cyperaceae Juss.
Iz_5_1109	DJI_0148	Centaurea majorovii Dumbadze	0	n	1	0	Asteraceae Bercht. & J.Presl
Iz_5_1109	DJI_0148	Chamaecytisus ruthenicus (Fisch.ex Wol.) Klaskova	0	n	1	0	Fabaceae Lindl.
Iz_5_1109	DJI_0148	Chelidonium majus L.	0	n	1	0	Papaveraceae Juss.
Iz_5_1109	DJI_0148	Chenopodium betaceum Andrz.	0	n	1	0	Amaranthaceae Juss.
Iz_5_1109	DJI_0148	Chenopodium cf. album	0	n	1	0	Amaranthaceae Juss.
Iz_5_1109	DJI_0148	Chenopodium sp.	0		NA	0	Amaranthaceae Juss.
Iz_5_1109	DJI_0148	Chondrilla juncea L.	0.5	n	1	0	Asteraceae Bercht. & J.Presl
Iz_5_1109	DJI_0148	Cirsium vulgare (Savi) Ten.	0	n	1	0	Asteraceae Bercht. & J.Presl
Iz_5_1109	DJI_0148	Clinopodium acinos (L.) Kuntze	0	n	1	0	Lamiacea Lindl.
Iz_5_1109	DJI_0148	Convallaria majalis L.	0	n	1	0	Asparagaceae Juss.
Iz_5_1109	DJI_0148	Crataegus rhipidophylla Gand.	0	n	1	1	Rosaceae Juss.
Iz_5_1109	DJI_0148	Crepis foetida L.	0	n	1	0	Asteraceae Bercht. & J.Presl
Iz_5_1109	DJI_0148	Dactylis glomerata L.	0	n	1	0	Poaceae Barnhart
Iz_5_1109	DJI_0148	Dianthus campestris M.Bieb.	0	n	1	0	Caryophyllaceae Juss.
Iz_5_1109	DJI_0148	Digitaria sanguinalis (L.) Scop.	0	a	0	0	Poaceae Barnhart
Iz_5_1109	DJI_0148	Elymus repens (L.) Gould	0	n	1	0	Poaceae Barnhart
Iz_5_1109	DJI_0148	Epilobium tetragonum L.	0	n	1	0	Onagraceae Juss.
Iz_5_1109	DJI_0148	Eragrostis minor Host	0	a	0	0	Poaceae Barnhart
Iz_5_1109	DJI_0148	Erigeron canadensis L.	2	a	0	0	Asteraceae Bercht. & J.Presl
Iz_5_1109	DJI_0148	Euphorbia seguieriana Neck.	0	n	1	0	Euphorbiaceae Juss.
Iz_5_1109	DJI_0148	Fallopia dumetorum (L.) Holub	0	n	1	0	Polygonaceae Juss.
Iz_5_1109	DJI_0148	Festuca beckeri (Hack.) Trautv.	0	n	1	0	Poaceae Barnhart
Iz_5_1109	DJI_0148	Fragaria vesca L.	0	n	1	0	Rosaceae Juss.
Iz_5_1109	DJI_0148	Frangula alnus Mill.	0	n	1	1	Rhamnaceae Juss.
Iz_5_1109	DJI_0148	Fraxinus pennsylvanica Marshall	0	a	0	1	Oleaceae Hoffmanns. & Link
Iz_5_1109	DJI_0148	Galeopsis bifida Boenn.	0	n	1	0	Lamiacea Lindl.
Iz_5_1109	DJI_0148	Galium verum L.	0	n	1	0	Rubiaceae Juss.
Iz_5_1109	DJI_0148	Genista tinctoria L.	0	n	1	0	Fabaceae Lindl.
Iz_5_1109	DJI_0148	Geranium robertianum L.	0	n	1	0	Geraniaceae Juss.
Iz_5_1109	DJI_0148	Geranium sanquineum L.	0	n	1	0	Geraniaceae Juss.
Iz_5_1109	DJI_0148	Gypsophila paniculata L.	0	n	1	0	Caryophyllaceae Juss.
Iz_5_1109	DJI_0148	Helichrysum arenarium (L.) Moench	0	n	1	0	Asteraceae Bercht. & J.Presl
Iz_5_1109	DJI_0148	Herniaria polygama J.Gay	0	n	1	0	Caryophyllaceae Juss.
Iz_5_1109	DJI_0148	Hieracium robustum Fr.	0	n	1	0	Asteraceae Bercht. & J.Presl
Iz_5_1109	DJI_0148	Hieracium umbellatum L.	0	n	1	NA	Asteraceae Bercht. & J.Presl
Iz_5_1109	DJI_0148	Hylotelephium maximum (L.) Holub	0	n	1	0	Crassulaceae J.St.-Hil.
Iz_5_1109	DJI_0148	Jacobaea borysthenica (DC.) B.Nord. & Greuter	2	n	1	0	Asteraceae Bercht. & J.Presl
Iz_5_1109	DJI_0148	Jasione montana L.	0	n	1	0	Campanulaceae Juss.
Iz_5_1109	DJI_0148	Jurinea cyanoides (L.) Rchb.s.l.	0	n	1	0	Asteraceae Bercht. & J.Presl
Iz_5_1109	DJI_0148	Koeleria glauca (Spreng.) DC.	0	n	1	0	Poaceae Barnhart
Iz_5_1109	DJI_0148	Lactuca serriola L.	0.1	a	0	0	Asteraceae Bercht. & J.Presl
Iz_5_1109	DJI_0148	Linaria genistifolia (L.) Mill.	0	n	1	0	Plantaginaceae Juss.
Iz_5_1109	DJI_0148	Lomelosia argentea (L.) Greuter & Burdet	0	n	1	0	Caprifoliaceae Juss.
Iz_5_1109	DJI_0148	Melampyrum pratense L.	0	n	1	0	Orobanchaceae Vent.
Iz_5_1109	DJI_0148	Oenothera biennis L.	0	a	0	0	Onagraceae Juss.
Iz_5_1109	DJI_0148	Pentanema britannica (L.) D.Gut.Larr., Santos-Vicente, Anderb., E.Rico & M.M.Mart.Ort.	0	n	1	0	Asteraceae Bercht. & J.Presl
Iz_5_1109	DJI_0148	Peucedanum oreoselinum (L.) Moench	3	n	1	0	Apiaceae Lindl.
Iz_5_1109	DJI_0148	Peucedanum ruthenicum M.Bieb.	0	n	1	0	Apiaceae Lindl.
Iz_5_1109	DJI_0148	Pilosella echioides (Lumn.) F.W.Schultz & Sch.Bip.	0	n	1	0	Asteraceae Bercht. & J.Presl
Iz_5_1109	DJI_0148	Pilosella officinarum F. Schultz et Sch. Bip.	0	n	1	0	Asteraceae Bercht. & J.Presl
Iz_5_1109	DJI_0148	Pilosella sp.	0		NA	0	Asteraceae Bercht. & J.Presl
Iz_5_1109	DJI_0148	Pinus sylvestris L.	0	n	1	1	Pinaceae Spreng. ex F.Rudolphi
Iz_5_1109	DJI_0148	Poa annua L.	0	n	1	0	Poaceae Barnhart
Iz_5_1109	DJI_0148	Poa nemoralis L.	0	n	1	0	Poaceae Barnhart

Iz_5_1109	DJI_0148	Poa sp.	0		NA	0	Poaceae Barnhart
Iz_5_1109	DJI_0148	Polygonatum odoratum (Mill.) Druce	0	n	1	0	Asparagaceae Juss.
Iz_5_1109	DJI_0148	Polygonum arenarium Waldst. & Kit.	0	n	1	0	Polygonaceae Juss.
Iz_5_1109	DJI_0148	Populus nigra L.	0	n	1	1	Salicaceae Mirb.
Iz_5_1109	DJI_0148	Populus tremula L.	0	n	1	1	Salicaceae Mirb.
Iz_5_1109	DJI_0148	Populus Ã canescens (Aiton) Sm.	0	n	1	1	Salicaceae Mirb.
Iz_5_1109	DJI_0148	Potentilla argentea L.	0	n	1	0	Rosaceae Juss.
Iz_5_1109	DJI_0148	Psammophiliella muralis (L.) Ikonn.	0	n	1	0	Caryophyllaceae Juss.
Iz_5_1109	DJI_0148	Psephellus sumensis (Kalen.) Greuter	3	n	1	0	Asteraceae Bercht. & J.Presl
Iz_5_1109	DJI_0148	Pulsatilla pratensis (L.) Mill. s.l.	0	n	1	0	Ranunculaceae Juss.
Iz_5_1109	DJI_0148	Pyrus communis L.	0	a	0	1	Rosaceae Juss.
Iz_5_1109	DJI_0148	Quercus robur L.	0	n	1	1	Fagaceae Dumort.
Iz_5_1109	DJI_0148	Robinia pseudoacacia L.	0	a	0	1	Fabaceae Lindl.
Iz_5_1109	DJI_0148	Rosa sp.	0		NA	1	Rosaceae Juss.
Iz_5_1109	DJI_0148	Rubus idaeus L.	0	n	1	0	Rosaceae Juss.
Iz_5_1109	DJI_0148	Rumex acetosella L.	0.1	n	1	0	Polygonaceae Juss.
Iz_5_1109	DJI_0148	Rumex thyrsiflorus Fingerh.	0	n	1	0	Polygonaceae Juss.
Iz_5_1109	DJI_0148	Salix alba L.	0	n	1	1	Salicaceae Mirb.
Iz_5_1109	DJI_0148	Sambucus nigra L.	0	n	1	1	Viburnaceae Raf.
Iz_5_1109	DJI_0148	Secale sylvestre Host	0	n	1	0	Poaceae Barnhart
Iz_5_1109	DJI_0148	Sempervivum ruthenicum Schnittsp. & C.B. Lehm.	0	n	1	0	Crassulaceae J.St.-Hil.
Iz_5_1109	DJI_0148	Setaria viridis (L.) P. Beauv.	0	n	1	0	Poaceae Barnhart
Iz_5_1109	DJI_0148	Silene borysthenica (Gruner) Walters	0	n	1	0	Caryophyllaceae Juss.
Iz_5_1109	DJI_0148	Silene latifolia subsp. alba (Mill.) Greuter & Burdet	0	n	1	0	Caryophyllaceae Juss.
Iz_5_1109	DJI_0148	Solidago virgaurea L.	1	n	1	0	Asteraceae Bercht. & J.Presl
Iz_5_1109	DJI_0148	Sorbus aucuparia L.	0	n	1	1	Rosaceae Juss.
Iz_5_1109	DJI_0148	Stachys recta L.	0	n	1	0	Lamiacea Lindl.
Iz_5_1109	DJI_0148	Tanacetum vulgare L.	0	n	1	0	Asteraceae Bercht. & J.Presl
Iz_5_1109	DJI_0148	Thymus pallasianus Heinr.Braun	0	n	1	0	Lamiacea Lindl.
Iz_5_1109	DJI_0148	Tilia cordata Mill.	0	n	1	1	Malvaceae Juss.
Iz_5_1109	DJI_0148	Trifolium alpestre L.	0	n	1	0	Fabaceae Lindl.
Iz_5_1109	DJI_0148	Trifolium arvense L.	0	n	1	0	Fabaceae Lindl.
Iz_5_1109	DJI_0148	Ulmus glabra Huds.	0	n	1	1	Ulmaceae Mirb.
Iz_5_1109	DJI_0148	Verbascum cf. phlomoides L.	1		NA	NA	
Iz_5_1109	DJI_0148	Verbascum densiflorum Bertol.	0	n	1	0	Scrophulariaceae Juss.
Iz_5_1109	DJI_0148	Verbascum sp.	0	n	1	0	Scrophulariaceae Juss.
Iz_5_1109	DJI_0148	Veronica austriaca L.	0	n	1	0	Plantaginaceae Juss.
Iz_5_1109	DJI_0148	Veronica incana L.	0	n	1	0	Plantaginaceae Juss.
Iz_5_1109	DJI_0148	Veronica verna L.	0	n	1	0	Plantaginaceae Juss.
Iz_5_1109	DJI_0148	Vincetoxicum hirundinaria Medik	0	n	1	0	Apocynaceae Juss.
Iz_5_1109	DJI_0152	Acer tataricum L.	0	n	1	1	Sapindaceae Juss.
Iz_5_1109	DJI_0152	Achillea millefolium L. s.l.	0	n	1	0	Asteraceae Bercht. & J.Presl
Iz_5_1109	DJI_0152	Allium oleraceum L.	0	n	1	0	Amaryllidaceae J.St.-Hil.
Iz_5_1109	DJI_0152	Ambrosia artemisiifolia L.	0	a	0	0	Asteraceae Bercht. & J.Presl
Iz_5_1109	DJI_0152	Amorpha fruticosa L.	0	a	0	0	Fabaceae Lindl.
Iz_5_1109	DJI_0152	Anthericum ramosum L.	0	n	1	0	Asparagaceae Juss.
Iz_5_1109	DJI_0152	Anthoxanthum repens (Host) Veldkamp	0		NA	NA	
Iz_5_1109	DJI_0152	Artemisia marschalliana Spreng.	3	n	1	0	Asteraceae Bercht. & J.Presl
Iz_5_1109	DJI_0152	Asparagus officinalis L.	0	n	1	0	Asparagaceae Juss.
Iz_5_1109	DJI_0152	Astragalus cicer L.	0	n	1	0	Fabaceae Lindl.
Iz_5_1109	DJI_0152	Bassia laniflora (S.G.Gmel.) A.J.Scott	0	n	1	0	Amaranthaceae Juss.
Iz_5_1109	DJI_0152	Berberis vulgaris L.	0	n	1	0	Berberidaceae Juss.
Iz_5_1109	DJI_0152	Berteroa incana (L.) DC.	0		NA	NA	
Iz_5_1109	DJI_0152	Betula pendula Roth.	0	n	1	1	Betulaceae Gray
Iz_5_1109	DJI_0152	Betula pendula Roth.	0	n	1	0	Poaceae Barnhart
Iz_5_1109	DJI_0152	Bromus inermis Leyss.	0		NA	NA	
Iz_5_1109	DJI_0152	Bromus squarrosus L.	0	a	0	0	Poaceae Barnhart
Iz_5_1109	DJI_0152	Bromus tectorum L.	0	a	0	0	Poaceae Barnhart
Iz_5_1109	DJI_0152	Calamagrostis epigejos (L.) Roth	3	n	1	0	Poaceae Barnhart
Iz_5_1109	DJI_0152	Carduus acanthoides L.	0	n	1	0	Asteraceae Bercht. & J.Presl
Iz_5_1109	DJI_0152	Carex sp.	0	n	1	0	Cyperaceae Juss.
Iz_5_1109	DJI_0152	Centaurea majorovii Dumbadze	0	n	1	0	Asteraceae Bercht. & J.Presl
Iz_5_1109	DJI_0152	Chamaecytisus ruthenicus (Fisch.ex Wol.) Klaskova	0	n	1	0	Fabaceae Lindl.
Iz_5_1109	DJI_0152	Chelidonium majus L.	0	n	1	0	Papaveraceae Juss.
Iz_5_1109	DJI_0152	Chenopodium betaceum Andrz.	0	n	1	0	Amaranthaceae Juss.
Iz_5_1109	DJI_0152	Chenopodium cf. album	0	n	1	0	Amaranthaceae Juss.
Iz_5_1109	DJI_0152	Chenopodium sp.	0		NA	0	Amaranthaceae Juss.
Iz_5_1109	DJI_0152	Chondrilla juncea L.	0	n	1	0	Asteraceae Bercht. & J.Presl
Iz_5_1109	DJI_0152	Cirsium vulgare (Savi) Ten.	0	n	1	0	Asteraceae Bercht. & J.Presl
Iz_5_1109	DJI_0152	Clinopodium acinos (L.) Kuntze	0	n	1	0	Lamiacea Lindl.
Iz_5_1109	DJI_0152	Convallaria majalis L.	0	n	1	0	Asparagaceae Juss.
Iz_5_1109	DJI_0152	Crataegus rhipidophylla Gand.	0	n	1	1	Rosaceae Juss.
Iz_5_1109	DJI_0152	Crepis foetida L.	0	n	1	0	Asteraceae Bercht. & J.Presl
Iz_5_1109	DJI_0152	Dactylis glomerata L.	0	n	1	0	Poaceae Barnhart
Iz_5_1109	DJI_0152	Dianthus campestris M.Bieb.	0	n	1	0	Caryophyllaceae Juss.
Iz_5_1109	DJI_0152	Digitaria sanguinalis (L.) Scop.	0	a	0	0	Poaceae Barnhart
Iz_5_1109	DJI_0152	Elymus repens (L.) Gould	0	n	1	0	Poaceae Barnhart
Iz_5_1109	DJI_0152	Epilobium tetragonum L.	0	n	1	0	Onagraceae Juss.
Iz_5_1109	DJI_0152	Eragrostis minor Host	0	a	0	0	Poaceae Barnhart
Iz_5_1109	DJI_0152	Erigeron canadensis L.	7	a	0	0	Asteraceae Bercht. & J.Presl
Iz_5_1109	DJI_0152	Euphorbia seguieriana Neck.	0	n	1	0	Euphorbiaceae Juss.
Iz_5_1109	DJI_0152	Fallopia dumetorum (L.) Holub	0	n	1	0	Polygonaceae Juss.
Iz_5_1109	DJI_0152	Festuca beckeri (Hack.) Trautv.	0	n	1	0	Poaceae Barnhart
Iz_5_1109	DJI_0152	Fragaria vesca L.	0	n	1	0	Rosaceae Juss.
Iz_5_1109	DJI_0152	Frangula alnus Mill.	0	n	1	1	Rhamnaceae Juss.
Iz_5_1109	DJI_0152	Fraxinus pennsylvanica Marshall	0	a	0	1	Oleaceae Hoffmanns. & Link
Iz_5_1109	DJI_0152	Galeopsis bifida Boenn.	0	n	1	0	Lamiacea Lindl.
Iz_5_1109	DJI_0152	Galium verum L.	0	n	1	0	Rubiaceae Juss.
Iz_5_1109	DJI_0152	Genista tinctoria L.	0	n	1	0	Fabaceae Lindl.
Iz_5_1109	DJI_0152	Geranium robertianum L.	0	n	1	0	Geraniaceae Juss.
Iz_5_1109	DJI_0152	Geranium sanquineum L.	0	n	1	0	Geraniaceae Juss.
Iz_5_1109	DJI_0152	Gypsophila paniculata L.	0	n	1	0	Caryophyllaceae Juss.
Iz_5_1109	DJI_0152	Helichrysum arenarium (L.) Moench	0	n	1	0	Asteraceae Bercht. & J.Presl
Iz_5_1109	DJI_0152	Herniaria polygama J.Gay	0	n	1	0	Caryophyllaceae Juss.
Iz_5_1109	DJI_0152	Hieracium robustum Fr.	0	n	1	0	Asteraceae Bercht. & J.Presl
Iz_5_1109	DJI_0152	Hieracium umbellatum L.	0	n	1	NA	Asteraceae Bercht. & J.Presl
Iz_5_1109	DJI_0152	Hylotelephium maximum (L.) Holub	0	n	1	0	Crassulaceae J.St.-Hil.
Iz_5_1109	DJI_0152	Jacobaea borysthenica (DC.) B.Nord. & Greuter	0	n	1	0	Asteraceae Bercht. & J.Presl

Iz_5_1109	DJI_0152	Jasione montana L.	0	n	1	0	Campanulaceae Juss.
Iz_5_1109	DJI_0152	Jurinea cyanoides (L.) Rchb.s.l.	0	n	1	0	Asteraceae Bercht. & J.Presl
Iz_5_1109	DJI_0152	Koeleria glauca (Spreng.) DC.	0	n	1	0	Poaceae Barnhart
Iz_5_1109	DJI_0152	Lactuca serriola L.	0	a	0	0	Asteraceae Bercht. & J.Presl
Iz_5_1109	DJI_0152	Linaria genistifolia (L.) Mill.	0	n	1	0	Plantaginaceae Juss.
Iz_5_1109	DJI_0152	Lomelosia argentea (L.) Greuter & Burdet	0	n	1	0	Caprifoliaceae Juss.
Iz_5_1109	DJI_0152	Melampyrum pratense L.	0	n	1	0	Orobanchaceae Vent.
Iz_5_1109	DJI_0152	Oenothera biennis L.	0	a	0	0	Onagraceae Juss.
Iz_5_1109	DJI_0152	Pentanema britannica (L.) D.Gut.Larr., Santos-Vicente, Anderb., E.Rico & M.M.Mart.Ort.	0	n	1	0	Asteraceae Bercht. & J.Presl
Iz_5_1109	DJI_0152	Peucedanum oreoselinum (L.) Moench	0	n	1	0	Apiaceae Lindl.
Iz_5_1109	DJI_0152	Peucedanum ruthenicum M.Bieb.	0	n	1	0	Apiaceae Lindl.
Iz_5_1109	DJI_0152	Pilosella echioides (Lumn.) F.W.Schultz & Sch.Bip.	0	n	1	0	Asteraceae Bercht. & J.Presl
Iz_5_1109	DJI_0152	Pilosella officinarum F. Schultz et Sch. Bip.	0	n	1	0	Asteraceae Bercht. & J.Presl
Iz_5_1109	DJI_0152	Pilosella sp.	0		NA	0	Asteraceae Bercht. & J.Presl
Iz_5_1109	DJI_0152	Pinus sylvestris L.	0.5	n	1	1	Pinaceae Spreng. ex F.Rudolphi
Iz_5_1109	DJI_0152	Poa annua L.	0	n	1	0	Poaceae Barnhart
Iz_5_1109	DJI_0152	Poa nemoralis L.	0	n	1	0	Poaceae Barnhart
Iz_5_1109	DJI_0152	Poa sp.	0		NA	0	Poaceae Barnhart
Iz_5_1109	DJI_0152	Polygonatum odoratum (Mill.) Druce	0	n	1	0	Asparagaceae Juss.
Iz_5_1109	DJI_0152	Polygonum arenarium Waldst. & Kit.	0	n	1	0	Polygonaceae Juss.
Iz_5_1109	DJI_0152	Populus nigra L.	0	n	1	1	Salicaceae Mirb.
Iz_5_1109	DJI_0152	Populus tremula L.	0	n	1	1	Salicaceae Mirb.
Iz_5_1109	DJI_0152	Populus Ã canescens (Aiton) Sm.	0	n	1	1	Salicaceae Mirb.
Iz_5_1109	DJI_0152	Potentilla argentea L.	0	n	1	0	Rosaceae Juss.
Iz_5_1109	DJI_0152	Psammophiliella muralis (L.) Ikonn.	0	n	1	0	Caryophyllaceae Juss.
Iz_5_1109	DJI_0152	Psephellus sumensis (Kalen.) Greuter	0	n	1	0	Asteraceae Bercht. & J.Presl
Iz_5_1109	DJI_0152	Pulsatilla pratensis (L.) Mill. s.l.	0	n	1	0	Ranunculaceae Juss.
Iz_5_1109	DJI_0152	Pyrus communis L.	0	a	0	1	Rosaceae Juss.
Iz_5_1109	DJI_0152	Quercus robur L.	2	n	1	1	Fagaceae Dumort.
Iz_5_1109	DJI_0152	Robinia pseudoacacia L.	7	a	0	1	Fabaceae Lindl.
Iz_5_1109	DJI_0152	Rosa sp.	0		NA	1	Rosaceae Juss.
Iz_5_1109	DJI_0152	Rubus idaeus L.	0	n	1	0	Rosaceae Juss.
Iz_5_1109	DJI_0152	Rumex acetosella L.	0	n	1	0	Polygonaceae Juss.
Iz_5_1109	DJI_0152	Rumex thyrsiflorus Fingerh.	0	n	1	0	Polygonaceae Juss.
Iz_5_1109	DJI_0152	Salix alba L.	0	n	1	1	Salicaceae Mirb.
Iz_5_1109	DJI_0152	Sambucus nigra L.	0	n	1	1	Viburnaceae Raf.
Iz_5_1109	DJI_0152	Secale sylvestre Host	0	n	1	0	Poaceae Barnhart
Iz_5_1109	DJI_0152	Sempervivum ruthenicum Schnittsp. & C.B. Lehm.	0	n	1	0	Crassulaceae J.St.-Hil.
Iz_5_1109	DJI_0152	Setaria viridis (L.) P. Beauv.	0	n	1	0	Poaceae Barnhart
Iz_5_1109	DJI_0152	Silene borysthenica (Gruner) Walters	0	n	1	0	Caryophyllaceae Juss.
Iz_5_1109	DJI_0152	Silene latifolia subsp. alba (Mill.) Greuter & Burdet	0	n	1	0	Caryophyllaceae Juss.
Iz_5_1109	DJI_0152	Solidago virgaurea L.	1	n	1	0	Asteraceae Bercht. & J.Presl
Iz_5_1109	DJI_0152	Sorbus aucuparia L.	0	n	1	1	Rosaceae Juss.
Iz_5_1109	DJI_0152	Stachys recta L.	0	n	1	0	Lamiacea Lindl.
Iz_5_1109	DJI_0152	Tanacetum vulgare L.	0	n	1	0	Asteraceae Bercht. & J.Presl
Iz_5_1109	DJI_0152	Thymus pallasianus Heinr.Braun	0	n	1	0	Lamiacea Lindl.
Iz_5_1109	DJI_0152	Tilia cordata Mill.	0	n	1	1	Malvaceae Juss.
Iz_5_1109	DJI_0152	Trifolium alpestre L.	0	n	1	0	Fabaceae Lindl.
Iz_5_1109	DJI_0152	Trifolium arvense L.	0	n	1	0	Fabaceae Lindl.
Iz_5_1109	DJI_0152	Ulmus glabra Huds.	0	n	1	1	Ulmaceae Mirb.
Iz_5_1109	DJI_0152	Verbascum cf. phlomoides L.	0		NA	NA	
Iz_5_1109	DJI_0152	Verbascum densiflorum Bertol.	0	n	1	0	Scrophulariaceae Juss.
Iz_5_1109	DJI_0152	Verbascum sp.	0	n	1	0	Scrophulariaceae Juss.
Iz_5_1109	DJI_0152	Veronica austriaca L.	0	n	1	0	Plantaginaceae Juss.
Iz_5_1109	DJI_0152	Veronica incana L.	0	n	1	0	Plantaginaceae Juss.
Iz_5_1109	DJI_0152	Veronica verna L.	0	n	1	0	Plantaginaceae Juss.
Iz_5_1109	DJI_0152	Vincetoxicum hirundinaria Medik	0	n	1	0	Apocynaceae Juss.
Iz_5_1109	DJI_0154	Acer tataricum L.	0	n	1	1	Sapindaceae Juss.
Iz_5_1109	DJI_0154	Achillea millefolium L. s.l.	0	n	1	0	Asteraceae Bercht. & J.Presl
Iz_5_1109	DJI_0154	Allium oleraceum L.	0	n	1	0	Amaryllidaceae J.St.-Hil.
Iz_5_1109	DJI_0154	Ambrosia artemisiifolia L.	0	a	0	0	Asteraceae Bercht. & J.Presl
Iz_5_1109	DJI_0154	Amorpha fruticosa L.	0	a	0	0	Fabaceae Lindl.
Iz_5_1109	DJI_0154	Anthericum ramosum L.	0	n	1	0	Asparagaceae Juss.
Iz_5_1109	DJI_0154	Anthoxanthum repens (Host) Veldkamp	0		NA	NA	
Iz_5_1109	DJI_0154	Artemisia marschalliana Spreng.	7	n	1	0	Asteraceae Bercht. & J.Presl
Iz_5_1109	DJI_0154	Asparagus officinalis L.	0	n	1	0	Asparagaceae Juss.
Iz_5_1109	DJI_0154	Astragalus cicer L.	0	n	1	0	Fabaceae Lindl.
Iz_5_1109	DJI_0154	Bassia laniflora (S.G.Gmel.) A.J.Scott	0	n	1	0	Amaranthaceae Juss.
Iz_5_1109	DJI_0154	Berberis vulgaris L.	0	n	1	0	Berberidaceae Juss.
Iz_5_1109	DJI_0154	Berteroa incana (L.) DC.	0		NA	NA	
Iz_5_1109	DJI_0154	Betula pendula Roth.	0	n	1	1	Betulaceae Gray
Iz_5_1109	DJI_0154	Betula pendula Roth.	0	n	1	0	Poaceae Barnhart
Iz_5_1109	DJI_0154	Bromus inermis Leyss.	0		NA	NA	
Iz_5_1109	DJI_0154	Bromus squarrosus L.	0	a	0	0	Poaceae Barnhart
Iz_5_1109	DJI_0154	Bromus tectorum L.	0	a	0	0	Poaceae Barnhart
Iz_5_1109	DJI_0154	Calamagrostis epigejos (L.) Roth	3	n	1	0	Poaceae Barnhart
Iz_5_1109	DJI_0154	Carduus acanthoides L.	0	n	1	0	Asteraceae Bercht. & J.Presl
Iz_5_1109	DJI_0154	Carex sp.	0	n	1	0	Cyperaceae Juss.
Iz_5_1109	DJI_0154	Centaurea majorovii Dumbadze	0	n	1	0	Asteraceae Bercht. & J.Presl
Iz_5_1109	DJI_0154	Chamaecytisus ruthenicus (Fisch.ex Wol.) Klaskova	0	n	1	0	Fabaceae Lindl.
Iz_5_1109	DJI_0154	Chelidonium majus L.	0	n	1	0	Papaveraceae Juss.
Iz_5_1109	DJI_0154	Chenopodium betaceum Andrz.	0	n	1	0	Amaranthaceae Juss.
Iz_5_1109	DJI_0154	Chenopodium cf. album	0	n	1	0	Amaranthaceae Juss.
Iz_5_1109	DJI_0154	Chenopodium sp.	0		NA	0	Amaranthaceae Juss.
Iz_5_1109	DJI_0154	Chondrilla juncea L.	0.5	n	1	0	Asteraceae Bercht. & J.Presl
Iz_5_1109	DJI_0154	Cirsium vulgare (Savi) Ten.	0	n	1	0	Asteraceae Bercht. & J.Presl
Iz_5_1109	DJI_0154	Clinopodium acinos (L.) Kuntze	0	n	1	0	Lamiacea Lindl.
Iz_5_1109	DJI_0154	Convallaria majalis L.	0	n	1	0	Asparagaceae Juss.
Iz_5_1109	DJI_0154	Crataegus rhipidophylla Gand.	0	n	1	1	Rosaceae Juss.
Iz_5_1109	DJI_0154	Crepis foetida L.	0	n	1	0	Asteraceae Bercht. & J.Presl
Iz_5_1109	DJI_0154	Dactylis glomerata L.	0	n	1	0	Poaceae Barnhart
Iz_5_1109	DJI_0154	Dianthus campestris M.Bieb.	0	n	1	0	Caryophyllaceae Juss.
Iz_5_1109	DJI_0154	Digitaria sanguinalis (L.) Scop.	0	a	0	0	Poaceae Barnhart
Iz_5_1109	DJI_0154	Elymus repens (L.) Gould	0	n	1	0	Poaceae Barnhart
Iz_5_1109	DJI_0154	Epilobium tetragonum L.	0	n	1	0	Onagraceae Juss.
Iz_5_1109	DJI_0154	Eragrostis minor Host	0	a	0	0	Poaceae Barnhart
Iz_5_1109	DJI_0154	Erigeron canadensis L.	5	a	0	0	Asteraceae Bercht. & J.Presl
Iz_5_1109	DJI_0154	Euphorbia seguieriana Neck.	0	n	1	0	Euphorbiaceae Juss.

Iz_5_1109	DJI_0154	Fallopia dumetorum (L.) Holub	0	n	1	0	Polygonaceae Juss.
Iz_5_1109	DJI_0154	Festuca beckeri (Hack.) Trautv.	0	n	1	0	Poaceae Barnhart
Iz_5_1109	DJI_0154	Fragaria vesca L.	0	n	1	0	Rosaceae Juss.
Iz_5_1109	DJI_0154	Frangula alnus Mill.	0	n	1	1	Rhamnaceae Juss.
Iz_5_1109	DJI_0154	Fraxinus pennsylvanica Marshall	0	a	0	1	Oleaceae Hoffmanns. & Link
Iz_5_1109	DJI_0154	Galeopsis bifida Boenn.	0	n	1	0	Lamiacea Lindl.
Iz_5_1109	DJI_0154	Galium verum L.	0	n	1	0	Rubiaceae Juss.
Iz_5_1109	DJI_0154	Genista tinctoria L.	0	n	1	0	Fabaceae Lindl.
Iz_5_1109	DJI_0154	Geranium robertianum L.	0	n	1	0	Geraniaceae Juss.
Iz_5_1109	DJI_0154	Geranium sanquineum L.	0	n	1	0	Geraniaceae Juss.
Iz_5_1109	DJI_0154	Gypsophila paniculata L.	0	n	1	0	Caryophyllaceae Juss.
Iz_5_1109	DJI_0154	Helichrysum arenarium (L.) Moench	0	n	1	0	Asteraceae Bercht. & J.Presl
Iz_5_1109	DJI_0154	Herniaria polygama J.Gay	0	n	1	0	Caryophyllaceae Juss.
Iz_5_1109	DJI_0154	Hieracium robustum Fr.	0	n	1	0	Asteraceae Bercht. & J.Presl
Iz_5_1109	DJI_0154	Hieracium umbellatum L.	0	n	1	NA	Asteraceae Bercht. & J.Presl
Iz_5_1109	DJI_0154	Hylotelephium maximum (L.) Holub	0	n	1	0	Crassulaceae J.St.-Hil.
Iz_5_1109	DJI_0154	Jacobaea borysthenica (DC.) B.Nord. & Greuter	0	n	1	0	Asteraceae Bercht. & J.Presl
Iz_5_1109	DJI_0154	Jasione montana L.	0	n	1	0	Campanulaceae Juss.
Iz_5_1109	DJI_0154	Jurinea cyanoides (L.) Rchb.s.l.	0	n	1	0	Asteraceae Bercht. & J.Presl
Iz_5_1109	DJI_0154	Koeleria glauca (Spreng.) DC.	0	n	1	0	Poaceae Barnhart
Iz_5_1109	DJI_0154	Lactuca serriola L.	0	a	0	0	Asteraceae Bercht. & J.Presl
Iz_5_1109	DJI_0154	Linaria genistifolia (L.) Mill.	0	n	1	0	Plantaginaceae Juss.
Iz_5_1109	DJI_0154	Lomelosia argentea (L.) Greuter & Burdet	0	n	1	0	Caprifoliaceae Juss.
Iz_5_1109	DJI_0154	Melampyrum pratense L.	0	n	1	0	Orobanchaceae Vent.
Iz_5_1109	DJI_0154	Oenothera biennis L.	0	a	0	0	Onagraceae Juss.
Iz_5_1109	DJI_0154	Pentanema britannica (L.) D.Gut.Larr., Santos-Vicente, Anderb., E.Rico & M.M.Mart.Ort.	0	n	1	0	Asteraceae Bercht. & J.Presl
Iz_5_1109	DJI_0154	Peucedanum oreoselinum (L.) Moench	0	n	1	0	Apiaceae Lindl.
Iz_5_1109	DJI_0154	Peucedanum ruthenicum M.Bieb.	0	n	1	0	Apiaceae Lindl.
Iz_5_1109	DJI_0154	Pilosella echioides (Lumn.) F.W.Schultz & Sch.Bip.	0	n	1	0	Asteraceae Bercht. & J.Presl
Iz_5_1109	DJI_0154	Pilosella officinarum F. Schultz et Sch. Bip.	0	n	1	0	Asteraceae Bercht. & J.Presl
Iz_5_1109	DJI_0154	Pilosella sp.	0		NA	0	Asteraceae Bercht. & J.Presl
Iz_5_1109	DJI_0154	Pinus sylvestris L.	0	n	1	1	Pinaceae Spreng. ex F.Rudolphi
Iz_5_1109	DJI_0154	Poa annua L.	0	n	1	0	Poaceae Barnhart
Iz_5_1109	DJI_0154	Poa nemoralis L.	0	n	1	0	Poaceae Barnhart
Iz_5_1109	DJI_0154	Poa sp.	0		NA	0	Poaceae Barnhart
Iz_5_1109	DJI_0154	Polygonatum odoratum (Mill.) Druce	0	n	1	0	Asparagaceae Juss.
Iz_5_1109	DJI_0154	Polygonum arenarium Waldst. & Kit.	0	n	1	0	Polygonaceae Juss.
Iz_5_1109	DJI_0154	Populus nigra L.	0	n	1	1	Salicaceae Mirb.
Iz_5_1109	DJI_0154	Populus tremula L.	0	n	1	1	Salicaceae Mirb.
Iz_5_1109	DJI_0154	Populus Ã canescens (Aiton) Sm.	0	n	1	1	Salicaceae Mirb.
Iz_5_1109	DJI_0154	Potentilla argentea L.	0	n	1	0	Rosaceae Juss.
Iz_5_1109	DJI_0154	Psammophiliella muralis (L.) Ikonn.	0	n	1	0	Caryophyllaceae Juss.
Iz_5_1109	DJI_0154	Psephellus sumensis (Kalen.) Greuter	0	n	1	0	Asteraceae Bercht. & J.Presl
Iz_5_1109	DJI_0154	Pulsatilla pratensis (L.) Mill. s.l.	0	n	1	0	Ranunculaceae Juss.
Iz_5_1109	DJI_0154	Pyrus communis L.	0	a	0	1	Rosaceae Juss.
Iz_5_1109	DJI_0154	Quercus robur L.	0	n	1	1	Fagaceae Dumort.
Iz_5_1109	DJI_0154	Robinia pseudoacacia L.	0	a	0	1	Fabaceae Lindl.
Iz_5_1109	DJI_0154	Rosa sp.	0		NA	1	Rosaceae Juss.
Iz_5_1109	DJI_0154	Rubus idaeus L.	0	n	1	0	Rosaceae Juss.
Iz_5_1109	DJI_0154	Rumex acetosella L.	1	n	1	0	Polygonaceae Juss.
Iz_5_1109	DJI_0154	Rumex thyrsiflorus Fingerh.	0	n	1	0	Polygonaceae Juss.
Iz_5_1109	DJI_0154	Salix alba L.	0	n	1	1	Salicaceae Mirb.
Iz_5_1109	DJI_0154	Sambucus nigra L.	0	n	1	1	Viburnaceae Raf.
Iz_5_1109	DJI_0154	Secale sylvestre Host	0	n	1	0	Poaceae Barnhart
Iz_5_1109	DJI_0154	Sempervivum ruthenicum Schnittsp. & C.B. Lehm.	0	n	1	0	Crassulaceae J.St.-Hil.
Iz_5_1109	DJI_0154	Setaria viridis (L.) P. Beauv.	0	n	1	0	Poaceae Barnhart
Iz_5_1109	DJI_0154	Silene borysthenica (Gruner) Walters	0	n	1	0	Caryophyllaceae Juss.
Iz_5_1109	DJI_0154	Silene latifolia subsp. alba (Mill.) Greuter & Burdet	0	n	1	0	Caryophyllaceae Juss.
Iz_5_1109	DJI_0154	Solidago virgaurea L.	0	n	1	0	Asteraceae Bercht. & J.Presl
Iz_5_1109	DJI_0154	Sorbus aucuparia L.	0	n	1	1	Rosaceae Juss.
Iz_5_1109	DJI_0154	Stachys recta L.	0	n	1	0	Lamiacea Lindl.
Iz_5_1109	DJI_0154	Tanacetum vulgare L.	0	n	1	0	Asteraceae Bercht. & J.Presl
Iz_5_1109	DJI_0154	Thymus pallasianus Heinr.Braun	0	n	1	0	Lamiacea Lindl.
Iz_5_1109	DJI_0154	Tilia cordata Mill.	0	n	1	1	Malvaceae Juss.
Iz_5_1109	DJI_0154	Trifolium alpestre L.	0	n	1	0	Fabaceae Lindl.
Iz_5_1109	DJI_0154	Trifolium arvense L.	0	n	1	0	Fabaceae Lindl.
Iz_5_1109	DJI_0154	Ulmus glabra Huds.	0	n	1	1	Ulmaceae Mirb.
Iz_5_1109	DJI_0154	Verbascum cf. phlomoides L.	0		NA	NA	
Iz_5_1109	DJI_0154	Verbascum densiflorum Bertol.	0	n	1	0	Scrophulariaceae Juss.
Iz_5_1109	DJI_0154	Verbascum sp.	1	n	1	0	Scrophulariaceae Juss.
Iz_5_1109	DJI_0154	Veronica austriaca L.	0	n	1	0	Plantaginaceae Juss.
Iz_5_1109	DJI_0154	Veronica incana L.	0	n	1	0	Plantaginaceae Juss.
Iz_5_1109	DJI_0154	Veronica verna L.	0	n	1	0	Plantaginaceae Juss.
Iz_5_1109	DJI_0154	Vincetoxicum hirundinaria Medik	0	n	1	0	Apocynaceae Juss.
Iz_5_1109	DJI_0156	Acer tataricum L.	0	n	1	1	Sapindaceae Juss.
Iz_5_1109	DJI_0156	Achillea millefolium L. s.l.	0	n	1	0	Asteraceae Bercht. & J.Presl
Iz_5_1109	DJI_0156	Allium oleraceum L.	0	n	1	0	Amaryllidaceae J.St.-Hil.
Iz_5_1109	DJI_0156	Ambrosia artemisiifolia L.	0	a	0	0	Asteraceae Bercht. & J.Presl
Iz_5_1109	DJI_0156	Amorpha fruticosa L.	0	a	0	0	Fabaceae Lindl.
Iz_5_1109	DJI_0156	Anthericum ramosum L.	0	n	1	0	Asparagaceae Juss.
Iz_5_1109	DJI_0156	Anthoxanthum repens (Host) Veldkamp	0		NA	NA	
Iz_5_1109	DJI_0156	Artemisia marschalliana Spreng.	0	n	1	0	Asteraceae Bercht. & J.Presl
Iz_5_1109	DJI_0156	Asparagus officinalis L.	0	n	1	0	Asparagaceae Juss.
Iz_5_1109	DJI_0156	Astragalus cicer L.	0	n	1	0	Fabaceae Lindl.
Iz_5_1109	DJI_0156	Bassia laniflora (S.G.Gmel.) A.J.Scott	0	n	1	0	Amaranthaceae Juss.
Iz_5_1109	DJI_0156	Berberis vulgaris L.	0	n	1	0	Berberidaceae Juss.
Iz_5_1109	DJI_0156	Berteroa incana (L.) DC.	0		NA	NA	
Iz_5_1109	DJI_0156	Betula pendula Roth.	0	n	1	1	Betulaceae Gray
Iz_5_1109	DJI_0156	Betula pendula Roth.	0	n	1	0	Poaceae Barnhart
Iz_5_1109	DJI_0156	Bromus inermis Leyss.	0		NA	NA	
Iz_5_1109	DJI_0156	Bromus squarrosus L.	0	a	0	0	Poaceae Barnhart
Iz_5_1109	DJI_0156	Bromus tectorum L.	0	a	0	0	Poaceae Barnhart
Iz_5_1109	DJI_0156	Calamagrostis epigejos (L.) Roth	75	n	1	0	Poaceae Barnhart
Iz_5_1109	DJI_0156	Carduus acanthoides L.	0	n	1	0	Asteraceae Bercht. & J.Presl
Iz_5_1109	DJI_0156	Carex sp.	0	n	1	0	Cyperaceae Juss.
Iz_5_1109	DJI_0156	Centaurea majorovii Dumbadze	0	n	1	0	Asteraceae Bercht. & J.Presl
Iz_5_1109	DJI_0156	Chamaecytisus ruthenicus (Fisch.ex Wol.) Klaskova	0	n	1	0	Fabaceae Lindl.
Iz_5_1109	DJI_0156	Chelidonium majus L.	0	n	1	0	Papaveraceae Juss.

Iz_5_1109	DJI_0156	Chenopodium betaceum Andrz.	0	n	1	0	Amaranthaceae Juss.
Iz_5_1109	DJI_0156	Chenopodium cf. album	0	n	1	0	Amaranthaceae Juss.
Iz_5_1109	DJI_0156	Chenopodium sp.	0		NA	0	Amaranthaceae Juss.
Iz_5_1109	DJI_0156	Chondrilla juncea L.	0	n	1	0	Asteraceae Bercht. & J.Presl
Iz_5_1109	DJI_0156	Cirsium vulgare (Savi) Ten.	0	n	1	0	Asteraceae Bercht. & J.Presl
Iz_5_1109	DJI_0156	Clinopodium acinos (L.) Kuntze	0	n	1	0	Lamiacea Lindl.
Iz_5_1109	DJI_0156	Convallaria majalis L.	0	n	1	0	Asparagaceae Juss.
Iz_5_1109	DJI_0156	Crataegus rhipidophylla Gand.	0	n	1	1	Rosaceae Juss.
Iz_5_1109	DJI_0156	Crepis foetida L.	0	n	1	0	Asteraceae Bercht. & J.Presl
Iz_5_1109	DJI_0156	Dactylis glomerata L.	0	n	1	0	Poaceae Barnhart
Iz_5_1109	DJI_0156	Dianthus campestris M.Bieb.	0	n	1	0	Caryophyllaceae Juss.
Iz_5_1109	DJI_0156	Digitaria sanguinalis (L.) Scop.	0	a	0	0	Poaceae Barnhart
Iz_5_1109	DJI_0156	Elymus repens (L.) Gould	0	n	1	0	Poaceae Barnhart
Iz_5_1109	DJI_0156	Epilobium tetragonum L.	0	n	1	0	Onagraceae Juss.
Iz_5_1109	DJI_0156	Eragrostis minor Host	0	a	0	0	Poaceae Barnhart
Iz_5_1109	DJI_0156	Erigeron canadensis L.	2	a	0	0	Asteraceae Bercht. & J.Presl
Iz_5_1109	DJI_0156	Euphorbia seguieriana Neck.	0	n	1	0	Euphorbiaceae Juss.
Iz_5_1109	DJI_0156	Fallopia dumetorum (L.) Holub	0	n	1	0	Polygonaceae Juss.
Iz_5_1109	DJI_0156	Festuca beckeri (Hack.) Trautv.	0	n	1	0	Poaceae Barnhart
Iz_5_1109	DJI_0156	Fragaria vesca L.	0	n	1	0	Rosaceae Juss.
Iz_5_1109	DJI_0156	Frangula alnus Mill.	0	n	1	1	Rhamnaceae Juss.
Iz_5_1109	DJI_0156	Fraxinus pennsylvanica Marshall	0	a	0	1	Oleaceae Hoffmanns. & Link
Iz_5_1109	DJI_0156	Galeopsis bifida Boenn.	0	n	1	0	Lamiacea Lindl.
Iz_5_1109	DJI_0156	Galium verum L.	0	n	1	0	Rubiaceae Juss.
Iz_5_1109	DJI_0156	Genista tinctoria L.	0	n	1	0	Fabaceae Lindl.
Iz_5_1109	DJI_0156	Geranium robertianum L.	0	n	1	0	Geraniaceae Juss.
Iz_5_1109	DJI_0156	Geranium sanquineum L.	0	n	1	0	Geraniaceae Juss.
Iz_5_1109	DJI_0156	Gypsophila paniculata L.	0	n	1	0	Caryophyllaceae Juss.
Iz_5_1109	DJI_0156	Helichrysum arenarium (L.) Moench	0	n	1	0	Asteraceae Bercht. & J.Presl
Iz_5_1109	DJI_0156	Herniaria polygama J.Gay	0	n	1	0	Caryophyllaceae Juss.
Iz_5_1109	DJI_0156	Hieracium robustum Fr.	0	n	1	0	Asteraceae Bercht. & J.Presl
Iz_5_1109	DJI_0156	Hieracium umbellatum L.	0	n	1	NA	Asteraceae Bercht. & J.Presl
Iz_5_1109	DJI_0156	Hylotelephium maximum (L.) Holub	0	n	1	0	Crassulaceae J.St.-Hil.
Iz_5_1109	DJI_0156	Jacobaea borysthenica (DC.) B.Nord. & Greuter	0	n	1	0	Asteraceae Bercht. & J.Presl
Iz_5_1109	DJI_0156	Jasione montana L.	0	n	1	0	Campanulaceae Juss.
Iz_5_1109	DJI_0156	Jurinea cyanoides (L.) Rchb.s.l.	0	n	1	0	Asteraceae Bercht. & J.Presl
Iz_5_1109	DJI_0156	Koeleria glauca (Spreng.) DC.	0	n	1	0	Poaceae Barnhart
Iz_5_1109	DJI_0156	Lactuca serriola L.	0	a	0	0	Asteraceae Bercht. & J.Presl
Iz_5_1109	DJI_0156	Linaria genistifolia (L.) Mill.	0	n	1	0	Plantaginaceae Juss.
Iz_5_1109	DJI_0156	Lomelosia argentea (L.) Greuter & Burdet	0	n	1	0	Caprifoliaceae Juss.
Iz_5_1109	DJI_0156	Melampyrum pratense L.	0	n	1	0	Orobanchaceae Vent.
Iz_5_1109	DJI_0156	Oenothera biennis L.	0	a	0	0	Onagraceae Juss.
Iz_5_1109	DJI_0156	Pentanema britannica (L.) D.Gut.Larr., Santos-Vicente, Anderb., E.Rico & M.M.Mart.Ort.	0	n	1	0	Asteraceae Bercht. & J.Presl
Iz_5_1109	DJI_0156	Peucedanum oreoselinum (L.) Moench	0	n	1	0	Apiaceae Lindl.
Iz_5_1109	DJI_0156	Peucedanum ruthenicum M.Bieb.	0	n	1	0	Apiaceae Lindl.
Iz_5_1109	DJI_0156	Pilosella echioides (Lumn.) F.W.Schultz & Sch.Bip.	0	n	1	0	Asteraceae Bercht. & J.Presl
Iz_5_1109	DJI_0156	Pilosella officinarum F. Schultz et Sch. Bip.	0	n	1	0	Asteraceae Bercht. & J.Presl
Iz_5_1109	DJI_0156	Pilosella sp.	0		NA	0	Asteraceae Bercht. & J.Presl
Iz_5_1109	DJI_0156	Pinus sylvestris L.	0	n	1	1	Pinaceae Spreng. ex F.Rudolphi
Iz_5_1109	DJI_0156	Poa annua L.	0	n	1	0	Poaceae Barnhart
Iz_5_1109	DJI_0156	Poa nemoralis L.	0	n	1	0	Poaceae Barnhart
Iz_5_1109	DJI_0156	Poa sp.	0		NA	0	Poaceae Barnhart
Iz_5_1109	DJI_0156	Polygonatum odoratum (Mill.) Druce	0	n	1	0	Asparagaceae Juss.
Iz_5_1109	DJI_0156	Polygonum arenarium Waldst. & Kit.	0	n	1	0	Polygonaceae Juss.
Iz_5_1109	DJI_0156	Populus nigra L.	0	n	1	1	Salicaceae Mirb.
Iz_5_1109	DJI_0156	Populus tremula L.	0	n	1	1	Salicaceae Mirb.
Iz_5_1109	DJI_0156	Populus Ã canescens (Aiton) Sm.	0	n	1	1	Salicaceae Mirb.
Iz_5_1109	DJI_0156	Potentilla argentea L.	0	n	1	0	Rosaceae Juss.
Iz_5_1109	DJI_0156	Psammophiliella muralis (L.) Ikonn.	0	n	1	0	Caryophyllaceae Juss.
Iz_5_1109	DJI_0156	Psephellus sumensis (Kalen.) Greuter	0	n	1	0	Asteraceae Bercht. & J.Presl
Iz_5_1109	DJI_0156	Pulsatilla pratensis (L.) Mill. s.l.	0	n	1	0	Ranunculaceae Juss.
Iz_5_1109	DJI_0156	Pyrus communis L.	0	a	0	1	Rosaceae Juss.
Iz_5_1109	DJI_0156	Quercus robur L.	0	n	1	1	Fagaceae Dumort.
Iz_5_1109	DJI_0156	Robinia pseudoacacia L.	0	a	0	1	Fabaceae Lindl.
Iz_5_1109	DJI_0156	Rosa sp.	0		NA	1	Rosaceae Juss.
Iz_5_1109	DJI_0156	Rubus idaeus L.	0	n	1	0	Rosaceae Juss.
Iz_5_1109	DJI_0156	Rumex acetosella L.	0	n	1	0	Polygonaceae Juss.
Iz_5_1109	DJI_0156	Rumex thyrsiflorus Fingerh.	0	n	1	0	Polygonaceae Juss.
Iz_5_1109	DJI_0156	Salix alba L.	0	n	1	1	Salicaceae Mirb.
Iz_5_1109	DJI_0156	Sambucus nigra L.	0	n	1	1	Viburnaceae Raf.
Iz_5_1109	DJI_0156	Secale sylvestre Host	0	n	1	0	Poaceae Barnhart
Iz_5_1109	DJI_0156	Sempervivum ruthenicum Schnittsp. & C.B. Lehm.	0	n	1	0	Crassulaceae J.St.-Hil.
Iz_5_1109	DJI_0156	Setaria viridis (L.) P. Beauv.	0	n	1	0	Poaceae Barnhart
Iz_5_1109	DJI_0156	Silene borysthenica (Gruner) Walters	0	n	1	0	Caryophyllaceae Juss.
Iz_5_1109	DJI_0156	Silene latifolia subsp. alba (Mill.) Greuter & Burdet	0	n	1	0	Caryophyllaceae Juss.
Iz_5_1109	DJI_0156	Solidago virgaurea L.	0	n	1	0	Asteraceae Bercht. & J.Presl
Iz_5_1109	DJI_0156	Sorbus aucuparia L.	0	n	1	1	Rosaceae Juss.
Iz_5_1109	DJI_0156	Stachys recta L.	0	n	1	0	Lamiacea Lindl.
Iz_5_1109	DJI_0156	Tanacetum vulgare L.	0	n	1	0	Asteraceae Bercht. & J.Presl
Iz_5_1109	DJI_0156	Thymus pallasianus Heinr.Braun	0	n	1	0	Lamiacea Lindl.
Iz_5_1109	DJI_0156	Tilia cordata Mill.	0	n	1	1	Malvaceae Juss.
Iz_5_1109	DJI_0156	Trifolium alpestre L.	0	n	1	0	Fabaceae Lindl.
Iz_5_1109	DJI_0156	Trifolium arvense L.	0	n	1	0	Fabaceae Lindl.
Iz_5_1109	DJI_0156	Ulmus glabra Huds.	0	n	1	1	Ulmaceae Mirb.
Iz_5_1109	DJI_0156	Verbascum cf. phlomoides L.	0		NA	NA	
Iz_5_1109	DJI_0156	Verbascum densiflorum Bertol.	0	n	1	0	Scrophulariaceae Juss.
Iz_5_1109	DJI_0156	Verbascum sp.	1	n	1	0	Scrophulariaceae Juss.
Iz_5_1109	DJI_0156	Veronica austriaca L.	0	n	1	0	Plantaginaceae Juss.
Iz_5_1109	DJI_0156	Veronica incana L.	0	n	1	0	Plantaginaceae Juss.
Iz_5_1109	DJI_0156	Veronica verna L.	0	n	1	0	Plantaginaceae Juss.
Iz_5_1109	DJI_0156	Vincetoxicum hirundinaria Medik	0	n	1	0	Apocynaceae Juss.
Iz_5_1109	DJI_0162	Acer tataricum L.	0	n	1	1	Sapindaceae Juss.
Iz_5_1109	DJI_0162	Achillea millefolium L. s.l.	0	n	1	0	Asteraceae Bercht. & J.Presl
Iz_5_1109	DJI_0162	Allium oleraceum L.	0	n	1	0	Amaryllidaceae J.St.-Hil.
Iz_5_1109	DJI_0162	Ambrosia artemisiifolia L.	0	a	0	0	Asteraceae Bercht. & J.Presl
Iz_5_1109	DJI_0162	Amorpha fruticosa L.	0	a	0	0	Fabaceae Lindl.
Iz_5_1109	DJI_0162	Anthericum ramosum L.	0	n	1	0	Asparagaceae Juss.
Iz_5_1109	DJI_0162	Anthoxanthum repens (Host) Veldkamp	0		NA	NA	

Iz_5_1109	DJI_0162	Artemisia marschalliana Spreng.	2	n	1	0	Asteraceae Bercht. & J.Presl
Iz_5_1109	DJI_0162	Asparagus officinalis L.	0	n	1	0	Asparagaceae Juss.
Iz_5_1109	DJI_0162	Astragalus cicer L.	0	n	1	0	Fabaceae Lindl.
Iz_5_1109	DJI_0162	Bassia laniflora (S.G.Gmel.) A.J.Scott	0	n	1	0	Amaranthaceae Juss.
Iz_5_1109	DJI_0162	Berberis vulgaris L.	0	n	1	0	Berberidaceae Juss.
Iz_5_1109	DJI_0162	Berteroa incana (L.) DC.	0		NA	NA	
Iz_5_1109	DJI_0162	Betula pendula Roth.	0	n	1	1	Betulaceae Gray
Iz_5_1109	DJI_0162	Betula pendula Roth.	0	n	1	0	Poaceae Barnhart
Iz_5_1109	DJI_0162	Bromus inermis Leyss.	0		NA	NA	
Iz_5_1109	DJI_0162	Bromus squarrosus L.	0	a	0	0	Poaceae Barnhart
Iz_5_1109	DJI_0162	Bromus tectorum L.	0	a	0	0	Poaceae Barnhart
Iz_5_1109	DJI_0162	Calamagrostis epigejos (L.) Roth	2	n	1	0	Poaceae Barnhart
Iz_5_1109	DJI_0162	Carduus acanthoides L.	0	n	1	0	Asteraceae Bercht. & J.Presl
Iz_5_1109	DJI_0162	Carex sp.	0	n	1	0	Cyperaceae Juss.
Iz_5_1109	DJI_0162	Centaurea majorovii Dumbadze	0	n	1	0	Asteraceae Bercht. & J.Presl
Iz_5_1109	DJI_0162	Chamaecytisus ruthenicus (Fisch.ex Wol.) Klaskova	0	n	1	0	Fabaceae Lindl.
Iz_5_1109	DJI_0162	Chelidonium majus L.	0	n	1	0	Papaveraceae Juss.
Iz_5_1109	DJI_0162	Chenopodium betaceum Andrz.	0	n	1	0	Amaranthaceae Juss.
Iz_5_1109	DJI_0162	Chenopodium cf. album	0	n	1	0	Amaranthaceae Juss.
Iz_5_1109	DJI_0162	Chenopodium sp.	0		NA	0	Amaranthaceae Juss.
Iz_5_1109	DJI_0162	Chondrilla juncea L.	0.5	n	1	0	Asteraceae Bercht. & J.Presl
Iz_5_1109	DJI_0162	Cirsium vulgare (Savi) Ten.	0	n	1	0	Asteraceae Bercht. & J.Presl
Iz_5_1109	DJI_0162	Clinopodium acinos (L.) Kuntze	0	n	1	0	Lamiacea Lindl.
Iz_5_1109	DJI_0162	Convallaria majalis L.	0	n	1	0	Asparagaceae Juss.
Iz_5_1109	DJI_0162	Crataegus rhipidophylla Gand.	0	n	1	1	Rosaceae Juss.
Iz_5_1109	DJI_0162	Crepis foetida L.	0	n	1	0	Asteraceae Bercht. & J.Presl
Iz_5_1109	DJI_0162	Dactylis glomerata L.	0	n	1	0	Poaceae Barnhart
Iz_5_1109	DJI_0162	Dianthus campestris M.Bieb.	0	n	1	0	Caryophyllaceae Juss.
Iz_5_1109	DJI_0162	Digitaria sanguinalis (L.) Scop.	0	a	0	0	Poaceae Barnhart
Iz_5_1109	DJI_0162	Elymus repens (L.) Gould	0	n	1	0	Poaceae Barnhart
Iz_5_1109	DJI_0162	Epilobium tetragonum L.	0	n	1	0	Onagraceae Juss.
Iz_5_1109	DJI_0162	Eragrostis minor Host	0	a	0	0	Poaceae Barnhart
Iz_5_1109	DJI_0162	Erigeron canadensis L.	3	a	0	0	Asteraceae Bercht. & J.Presl
Iz_5_1109	DJI_0162	Euphorbia seguieriana Neck.	0	n	1	0	Euphorbiaceae Juss.
Iz_5_1109	DJI_0162	Fallopia dumetorum (L.) Holub	0	n	1	0	Polygonaceae Juss.
Iz_5_1109	DJI_0162	Festuca beckeri (Hack.) Trautv.	0	n	1	0	Poaceae Barnhart
Iz_5_1109	DJI_0162	Fragaria vesca L.	0	n	1	0	Rosaceae Juss.
Iz_5_1109	DJI_0162	Frangula alnus Mill.	0	n	1	1	Rhamnaceae Juss.
Iz_5_1109	DJI_0162	Fraxinus pennsylvanica Marshall	0	a	0	1	Oleaceae Hoffmanns. & Link
Iz_5_1109	DJI_0162	Galeopsis bifida Boenn.	0	n	1	0	Lamiacea Lindl.
Iz_5_1109	DJI_0162	Galium verum L.	0	n	1	0	Rubiaceae Juss.
Iz_5_1109	DJI_0162	Genista tinctoria L.	0	n	1	0	Fabaceae Lindl.
Iz_5_1109	DJI_0162	Geranium robertianum L.	0	n	1	0	Geraniaceae Juss.
Iz_5_1109	DJI_0162	Geranium sanquineum L.	0	n	1	0	Geraniaceae Juss.
Iz_5_1109	DJI_0162	Gypsophila paniculata L.	0	n	1	0	Caryophyllaceae Juss.
Iz_5_1109	DJI_0162	Helichrysum arenarium (L.) Moench	0	n	1	0	Asteraceae Bercht. & J.Presl
Iz_5_1109	DJI_0162	Herniaria polygama J.Gay	0	n	1	0	Caryophyllaceae Juss.
Iz_5_1109	DJI_0162	Hieracium robustum Fr.	0	n	1	0	Asteraceae Bercht. & J.Presl
Iz_5_1109	DJI_0162	Hieracium umbellatum L.	0	n	1	NA	Asteraceae Bercht. & J.Presl
Iz_5_1109	DJI_0162	Hylotelephium maximum (L.) Holub	0	n	1	0	Crassulaceae J.St.-Hil.
Iz_5_1109	DJI_0162	Jacobaea borysthenica (DC.) B.Nord. & Greuter	0	n	1	0	Asteraceae Bercht. & J.Presl
Iz_5_1109	DJI_0162	Jasione montana L.	0	n	1	0	Campanulaceae Juss.
Iz_5_1109	DJI_0162	Jurinea cyanoides (L.) Rchb.s.l.	0	n	1	0	Asteraceae Bercht. & J.Presl
Iz_5_1109	DJI_0162	Koeleria glauca (Spreng.) DC.	0	n	1	0	Poaceae Barnhart
Iz_5_1109	DJI_0162	Lactuca serriola L.	0	a	0	0	Asteraceae Bercht. & J.Presl
Iz_5_1109	DJI_0162	Linaria genistifolia (L.) Mill.	0	n	1	0	Plantaginaceae Juss.
Iz_5_1109	DJI_0162	Lomelosia argentea (L.) Greuter & Burdet	0	n	1	0	Caprifoliaceae Juss.
Iz_5_1109	DJI_0162	Melampyrum pratense L.	0	n	1	0	Orobanchaceae Vent.
Iz_5_1109	DJI_0162	Oenothera biennis L.	0	a	0	0	Onagraceae Juss.
Iz_5_1109	DJI_0162	Pentanema britannica (L.) D.Gut.Larr., Santos-Vicente, Anderb., E.Rico & M.M.Mart.Ort.	0	n	1	0	Asteraceae Bercht. & J.Presl
Iz_5_1109	DJI_0162	Peucedanum oreoselinum (L.) Moench	0	n	1	0	Apiaceae Lindl.
Iz_5_1109	DJI_0162	Peucedanum ruthenicum M.Bieb.	0	n	1	0	Apiaceae Lindl.
Iz_5_1109	DJI_0162	Pilosella echioides (Lumn.) F.W.Schultz & Sch.Bip.	0	n	1	0	Asteraceae Bercht. & J.Presl
Iz_5_1109	DJI_0162	Pilosella officinarum F. Schultz et Sch. Bip.	0	n	1	0	Asteraceae Bercht. & J.Presl
Iz_5_1109	DJI_0162	Pilosella sp.	0		NA	0	Asteraceae Bercht. & J.Presl
Iz_5_1109	DJI_0162	Pinus sylvestris L.	0	n	1	1	Pinaceae Spreng. ex F.Rudolphi
Iz_5_1109	DJI_0162	Poa annua L.	0	n	1	0	Poaceae Barnhart
Iz_5_1109	DJI_0162	Poa nemoralis L.	0	n	1	0	Poaceae Barnhart
Iz_5_1109	DJI_0162	Poa sp.	0		NA	0	Poaceae Barnhart
Iz_5_1109	DJI_0162	Polygonatum odoratum (Mill.) Druce	0	n	1	0	Asparagaceae Juss.
Iz_5_1109	DJI_0162	Polygonum arenarium Waldst. & Kit.	0	n	1	0	Polygonaceae Juss.
Iz_5_1109	DJI_0162	Populus nigra L.	0	n	1	1	Salicaceae Mirb.
Iz_5_1109	DJI_0162	Populus tremula L.	0	n	1	1	Salicaceae Mirb.
Iz_5_1109	DJI_0162	Populus Ã canescens (Aiton) Sm.	0	n	1	1	Salicaceae Mirb.
Iz_5_1109	DJI_0162	Potentilla argentea L.	0	n	1	0	Rosaceae Juss.
Iz_5_1109	DJI_0162	Psammophiliella muralis (L.) Ikonn.	0	n	1	0	Caryophyllaceae Juss.
Iz_5_1109	DJI_0162	Psephellus sumensis (Kalen.) Greuter	0	n	1	0	Asteraceae Bercht. & J.Presl
Iz_5_1109	DJI_0162	Pulsatilla pratensis (L.) Mill. s.l.	0	n	1	0	Ranunculaceae Juss.
Iz_5_1109	DJI_0162	Pyrus communis L.	0	a	0	1	Rosaceae Juss.
Iz_5_1109	DJI_0162	Quercus robur L.	10	n	1	1	Fagaceae Dumort.
Iz_5_1109	DJI_0162	Robinia pseudoacacia L.	0	a	0	1	Fabaceae Lindl.
Iz_5_1109	DJI_0162	Rosa sp.	0		NA	1	Rosaceae Juss.
Iz_5_1109	DJI_0162	Rubus idaeus L.	0	n	1	0	Rosaceae Juss.
Iz_5_1109	DJI_0162	Rumex acetosella L.	1	n	1	0	Polygonaceae Juss.
Iz_5_1109	DJI_0162	Rumex thyrsiflorus Fingerh.	0	n	1	0	Polygonaceae Juss.
Iz_5_1109	DJI_0162	Salix alba L.	0	n	1	1	Salicaceae Mirb.
Iz_5_1109	DJI_0162	Sambucus nigra L.	0	n	1	1	Viburnaceae Raf.
Iz_5_1109	DJI_0162	Secale sylvestre Host	0	n	1	0	Poaceae Barnhart
Iz_5_1109	DJI_0162	Sempervivum ruthenicum Schnittsp. & C.B. Lehm.	0	n	1	0	Crassulaceae J.St.-Hil.
Iz_5_1109	DJI_0162	Setaria viridis (L.) P. Beauv.	0	n	1	0	Poaceae Barnhart
Iz_5_1109	DJI_0162	Silene borysthenica (Gruner) Walters	0	n	1	0	Caryophyllaceae Juss.
Iz_5_1109	DJI_0162	Silene latifolia subsp. alba (Mill.) Greuter & Burdet	0	n	1	0	Caryophyllaceae Juss.
Iz_5_1109	DJI_0162	Solidago virgaurea L.	1	n	1	0	Asteraceae Bercht. & J.Presl
Iz_5_1109	DJI_0162	Sorbus aucuparia L.	0	n	1	1	Rosaceae Juss.
Iz_5_1109	DJI_0162	Stachys recta L.	0	n	1	0	Lamiacea Lindl.
Iz_5_1109	DJI_0162	Tanacetum vulgare L.	0	n	1	0	Asteraceae Bercht. & J.Presl
Iz_5_1109	DJI_0162	Thymus pallasianus Heinr.Braun	0	n	1	0	Lamiacea Lindl.
Iz_5_1109	DJI_0162	Tilia cordata Mill.	0	n	1	1	Malvaceae Juss.

Iz_5_1109	DJI_0162	Trifolium alpestre L.	0	n	1	0	Fabaceae Lindl.
Iz_5_1109	DJI_0162	Trifolium arvense L.	0	n	1	0	Fabaceae Lindl.
Iz_5_1109	DJI_0162	Ulmus glabra Huds.	0	n	1	1	Ulmaceae Mirb.
Iz_5_1109	DJI_0162	Verbascum cf. phlomoides L.	5		NA	NA	
Iz_5_1109	DJI_0162	Verbascum densiflorum Bertol.	0	n	1	0	Scrophulariaceae Juss.
Iz_5_1109	DJI_0162	Verbascum sp.	0	n	1	0	Scrophulariaceae Juss.
Iz_5_1109	DJI_0162	Veronica austriaca L.	0	n	1	0	Plantaginaceae Juss.
Iz_5_1109	DJI_0162	Veronica incana L.	0	n	1	0	Plantaginaceae Juss.
Iz_5_1109	DJI_0162	Veronica verna L.	0	n	1	0	Plantaginaceae Juss.
Iz_5_1109	DJI_0162	Vincetoxicum hirundinaria Medik	0	n	1	0	Apocynaceae Juss.
Iz_5_1109	DJI_0167	Acer tataricum L.	0	n	1	1	Sapindaceae Juss.
Iz_5_1109	DJI_0167	Achillea millefolium L. s.l.	0	n	1	0	Asteraceae Bercht. & J.Presl
Iz_5_1109	DJI_0167	Allium oleraceum L.	0	n	1	0	Amaryllidaceae J.St.-Hil.
Iz_5_1109	DJI_0167	Ambrosia artemisiifolia L.	0	a	0	0	Asteraceae Bercht. & J.Presl
Iz_5_1109	DJI_0167	Amorpha fruticosa L.	7	a	0	0	Fabaceae Lindl.
Iz_5_1109	DJI_0167	Anthericum ramosum L.	0	n	1	0	Asparagaceae Juss.
Iz_5_1109	DJI_0167	Anthoxanthum repens (Host) Veldkamp	0		NA	NA	
Iz_5_1109	DJI_0167	Artemisia marschalliana Spreng.	3	n	1	0	Asteraceae Bercht. & J.Presl
Iz_5_1109	DJI_0167	Asparagus officinalis L.	0	n	1	0	Asparagaceae Juss.
Iz_5_1109	DJI_0167	Astragalus cicer L.	0	n	1	0	Fabaceae Lindl.
Iz_5_1109	DJI_0167	Bassia laniflora (S.G.Gmel.) A.J.Scott	0	n	1	0	Amaranthaceae Juss.
Iz_5_1109	DJI_0167	Berberis vulgaris L.	0	n	1	0	Berberidaceae Juss.
Iz_5_1109	DJI_0167	Berteroa incana (L.) DC.	0		NA	NA	
Iz_5_1109	DJI_0167	Betula pendula Roth.	0	n	1	1	Betulaceae Gray
Iz_5_1109	DJI_0167	Betula pendula Roth.	0	n	1	0	Poaceae Barnhart
Iz_5_1109	DJI_0167	Bromus inermis Leyss.	0		NA	NA	
Iz_5_1109	DJI_0167	Bromus squarrosus L.	0	a	0	0	Poaceae Barnhart
Iz_5_1109	DJI_0167	Bromus tectorum L.	0	a	0	0	Poaceae Barnhart
Iz_5_1109	DJI_0167	Calamagrostis epigejos (L.) Roth	1	n	1	0	Poaceae Barnhart
Iz_5_1109	DJI_0167	Carduus acanthoides L.	0	n	1	0	Asteraceae Bercht. & J.Presl
Iz_5_1109	DJI_0167	Carex sp.	0	n	1	0	Cyperaceae Juss.
Iz_5_1109	DJI_0167	Centaurea majorovii Dumbadze	0	n	1	0	Asteraceae Bercht. & J.Presl
Iz_5_1109	DJI_0167	Chamaecytisus ruthenicus (Fisch.ex Wol.) Klaskova	0	n	1	0	Fabaceae Lindl.
Iz_5_1109	DJI_0167	Chelidonium majus L.	0	n	1	0	Papaveraceae Juss.
Iz_5_1109	DJI_0167	Chenopodium betaceum Andrz.	0	n	1	0	Amaranthaceae Juss.
Iz_5_1109	DJI_0167	Chenopodium cf. album	0	n	1	0	Amaranthaceae Juss.
Iz_5_1109	DJI_0167	Chenopodium sp.	0		NA	0	Amaranthaceae Juss.
Iz_5_1109	DJI_0167	Chondrilla juncea L.	1	n	1	0	Asteraceae Bercht. & J.Presl
Iz_5_1109	DJI_0167	Cirsium vulgare (Savi) Ten.	0	n	1	0	Asteraceae Bercht. & J.Presl
Iz_5_1109	DJI_0167	Clinopodium acinos (L.) Kuntze	0	n	1	0	Lamiacea Lindl.
Iz_5_1109	DJI_0167	Convallaria majalis L.	0	n	1	0	Asparagaceae Juss.
Iz_5_1109	DJI_0167	Crataegus rhipidophylla Gand.	0	n	1	1	Rosaceae Juss.
Iz_5_1109	DJI_0167	Crepis foetida L.	0	n	1	0	Asteraceae Bercht. & J.Presl
Iz_5_1109	DJI_0167	Dactylis glomerata L.	0	n	1	0	Poaceae Barnhart
Iz_5_1109	DJI_0167	Dianthus campestris M.Bieb.	0	n	1	0	Caryophyllaceae Juss.
Iz_5_1109	DJI_0167	Digitaria sanguinalis (L.) Scop.	0	a	0	0	Poaceae Barnhart
Iz_5_1109	DJI_0167	Elymus repens (L.) Gould	0	n	1	0	Poaceae Barnhart
Iz_5_1109	DJI_0167	Epilobium tetragonum L.	0	n	1	0	Onagraceae Juss.
Iz_5_1109	DJI_0167	Eragrostis minor Host	0	a	0	0	Poaceae Barnhart
Iz_5_1109	DJI_0167	Erigeron canadensis L.	7	a	0	0	Asteraceae Bercht. & J.Presl
Iz_5_1109	DJI_0167	Euphorbia seguieriana Neck.	0	n	1	0	Euphorbiaceae Juss.
Iz_5_1109	DJI_0167	Fallopia dumetorum (L.) Holub	0	n	1	0	Polygonaceae Juss.
Iz_5_1109	DJI_0167	Festuca beckeri (Hack.) Trautv.	0.5	n	1	0	Poaceae Barnhart
Iz_5_1109	DJI_0167	Fragaria vesca L.	0	n	1	0	Rosaceae Juss.
Iz_5_1109	DJI_0167	Frangula alnus Mill.	0	n	1	1	Rhamnaceae Juss.
Iz_5_1109	DJI_0167	Fraxinus pennsylvanica Marshall	0	a	0	1	Oleaceae Hoffmanns. & Link
Iz_5_1109	DJI_0167	Galeopsis bifida Boenn.	0	n	1	0	Lamiacea Lindl.
Iz_5_1109	DJI_0167	Galium verum L.	0	n	1	0	Rubiaceae Juss.
Iz_5_1109	DJI_0167	Genista tinctoria L.	0	n	1	0	Fabaceae Lindl.
Iz_5_1109	DJI_0167	Geranium robertianum L.	0	n	1	0	Geraniaceae Juss.
Iz_5_1109	DJI_0167	Geranium sanquineum L.	0	n	1	0	Geraniaceae Juss.
Iz_5_1109	DJI_0167	Gypsophila paniculata L.	0	n	1	0	Caryophyllaceae Juss.
Iz_5_1109	DJI_0167	Helichrysum arenarium (L.) Moench	0	n	1	0	Asteraceae Bercht. & J.Presl
Iz_5_1109	DJI_0167	Herniaria polygama J.Gay	0	n	1	0	Caryophyllaceae Juss.
Iz_5_1109	DJI_0167	Hieracium robustum Fr.	0	n	1	0	Asteraceae Bercht. & J.Presl
Iz_5_1109	DJI_0167	Hieracium umbellatum L.	0	n	1	NA	Asteraceae Bercht. & J.Presl
Iz_5_1109	DJI_0167	Hylotelephium maximum (L.) Holub	0	n	1	0	Crassulaceae J.St.-Hil.
Iz_5_1109	DJI_0167	Jacobaea borysthenica (DC.) B.Nord. & Greuter	0	n	1	0	Asteraceae Bercht. & J.Presl
Iz_5_1109	DJI_0167	Jasione montana L.	0	n	1	0	Campanulaceae Juss.
Iz_5_1109	DJI_0167	Jurinea cyanoides (L.) Rchb.s.l.	0	n	1	0	Asteraceae Bercht. & J.Presl
Iz_5_1109	DJI_0167	Koeleria glauca (Spreng.) DC.	0	n	1	0	Poaceae Barnhart
Iz_5_1109	DJI_0167	Lactuca serriola L.	0	a	0	0	Asteraceae Bercht. & J.Presl
Iz_5_1109	DJI_0167	Linaria genistifolia (L.) Mill.	0	n	1	0	Plantaginaceae Juss.
Iz_5_1109	DJI_0167	Lomelosia argentea (L.) Greuter & Burdet	0	n	1	0	Caprifoliaceae Juss.
Iz_5_1109	DJI_0167	Melampyrum pratense L.	0	n	1	0	Orobanchaceae Vent.
Iz_5_1109	DJI_0167	Oenothera biennis L.	0	a	0	0	Onagraceae Juss.
Iz_5_1109	DJI_0167	Pentanema britannica (L.) D.Gut.Larr., Santos-Vicente, Anderb., E.Rico & M.M.Mart.Ort.	0	n	1	0	Asteraceae Bercht. & J.Presl
Iz_5_1109	DJI_0167	Peucedanum oreoselinum (L.) Moench	0	n	1	0	Apiaceae Lindl.
Iz_5_1109	DJI_0167	Peucedanum ruthenicum M.Bieb.	0	n	1	0	Apiaceae Lindl.
Iz_5_1109	DJI_0167	Pilosella echioides (Lumn.) F.W.Schultz & Sch.Bip.	0	n	1	0	Asteraceae Bercht. & J.Presl
Iz_5_1109	DJI_0167	Pilosella officinarum F. Schultz et Sch. Bip.	0	n	1	0	Asteraceae Bercht. & J.Presl
Iz_5_1109	DJI_0167	Pilosella sp.	0		NA	0	Asteraceae Bercht. & J.Presl
Iz_5_1109	DJI_0167	Pinus sylvestris L.	0	n	1	1	Pinaceae Spreng. ex F.Rudolphi
Iz_5_1109	DJI_0167	Poa annua L.	0	n	1	0	Poaceae Barnhart
Iz_5_1109	DJI_0167	Poa nemoralis L.	0	n	1	0	Poaceae Barnhart
Iz_5_1109	DJI_0167	Poa sp.	0		NA	0	Poaceae Barnhart
Iz_5_1109	DJI_0167	Polygonatum odoratum (Mill.) Druce	0	n	1	0	Asparagaceae Juss.
Iz_5_1109	DJI_0167	Polygonum arenarium Waldst. & Kit.	0	n	1	0	Polygonaceae Juss.
Iz_5_1109	DJI_0167	Populus nigra L.	0	n	1	1	Salicaceae Mirb.
Iz_5_1109	DJI_0167	Populus tremula L.	0	n	1	1	Salicaceae Mirb.
Iz_5_1109	DJI_0167	Populus Ã canescens (Aiton) Sm.	0	n	1	1	Salicaceae Mirb.
Iz_5_1109	DJI_0167	Potentilla argentea L.	0	n	1	0	Rosaceae Juss.
Iz_5_1109	DJI_0167	Psammophiliella muralis (L.) Ikonn.	0	n	1	0	Caryophyllaceae Juss.
Iz_5_1109	DJI_0167	Psephellus sumensis (Kalen.) Greuter	0	n	1	0	Asteraceae Bercht. & J.Presl
Iz_5_1109	DJI_0167	Pulsatilla pratensis (L.) Mill. s.l.	0	n	1	0	Ranunculaceae Juss.
Iz_5_1109	DJI_0167	Pyrus communis L.	0	a	0	1	Rosaceae Juss.
Iz_5_1109	DJI_0167	Quercus robur L.	3	n	1	1	Fagaceae Dumort.
Iz_5_1109	DJI_0167	Robinia pseudoacacia L.	0	a	0	1	Fabaceae Lindl.

Iz_5_1109	DJI_0167	Rosa sp.	0		NA	1	Rosaceae Juss.
Iz_5_1109	DJI_0167	Rubus idaeus L.	0	n	1	0	Rosaceae Juss.
Iz_5_1109	DJI_0167	Rumex acetosella L.	0.2	n	1	0	Polygonaceae Juss.
Iz_5_1109	DJI_0167	Rumex thyrsiflorus Fingerh.	0	n	1	0	Polygonaceae Juss.
Iz_5_1109	DJI_0167	Salix alba L.	0	n	1	1	Salicaceae Mirb.
Iz_5_1109	DJI_0167	Sambucus nigra L.	0	n	1	1	Viburnaceae Raf.
Iz_5_1109	DJI_0167	Secale sylvestre Host	0	n	1	0	Poaceae Barnhart
Iz_5_1109	DJI_0167	Sempervivum ruthenicum Schnittsp. & C.B. Lehm.	0	n	1	0	Crassulaceae J.St.-Hil.
Iz_5_1109	DJI_0167	Setaria viridis (L.) P. Beauv.	0	n	1	0	Poaceae Barnhart
Iz_5_1109	DJI_0167	Silene borysthenica (Gruner) Walters	0	n	1	0	Caryophyllaceae Juss.
Iz_5_1109	DJI_0167	Silene latifolia subsp. alba (Mill.) Greuter & Burdet	0	n	1	0	Caryophyllaceae Juss.
Iz_5_1109	DJI_0167	Solidago virgaurea L.	1	n	1	0	Asteraceae Bercht. & J.Presl
Iz_5_1109	DJI_0167	Sorbus aucuparia L.	0	n	1	1	Rosaceae Juss.
Iz_5_1109	DJI_0167	Stachys recta L.	0	n	1	0	Lamiacea Lindl.
Iz_5_1109	DJI_0167	Tanacetum vulgare L.	0	n	1	0	Asteraceae Bercht. & J.Presl
Iz_5_1109	DJI_0167	Thymus pallasianus Heinr.Braun	0	n	1	0	Lamiacea Lindl.
Iz_5_1109	DJI_0167	Tilia cordata Mill.	0	n	1	1	Malvaceae Juss.
Iz_5_1109	DJI_0167	Trifolium alpestre L.	0	n	1	0	Fabaceae Lindl.
Iz_5_1109	DJI_0167	Trifolium arvense L.	0	n	1	0	Fabaceae Lindl.
Iz_5_1109	DJI_0167	Ulmus glabra Huds.	0	n	1	1	Ulmaceae Mirb.
Iz_5_1109	DJI_0167	Verbascum cf. phlomoides L.	1		NA	NA	
Iz_5_1109	DJI_0167	Verbascum densiflorum Bertol.	0	n	1	0	Scrophulariaceae Juss.
Iz_5_1109	DJI_0167	Verbascum sp.	0	n	1	0	Scrophulariaceae Juss.
Iz_5_1109	DJI_0167	Veronica austriaca L.	0	n	1	0	Plantaginaceae Juss.
Iz_5_1109	DJI_0167	Veronica incana L.	0	n	1	0	Plantaginaceae Juss.
Iz_5_1109	DJI_0167	Veronica verna L.	0	n	1	0	Plantaginaceae Juss.
Iz_5_1109	DJI_0167	Vincetoxicum hirundinaria Medik	0	n	1	0	Apocynaceae Juss.
Iz_5_1109	DJI_0190	Acer tataricum L.	0	n	1	1	Sapindaceae Juss.
Iz_5_1109	DJI_0190	Achillea millefolium L. s.l.	0	n	1	0	Asteraceae Bercht. & J.Presl
Iz_5_1109	DJI_0190	Allium oleraceum L.	0	n	1	0	Amaryllidaceae J.St.-Hil.
Iz_5_1109	DJI_0190	Ambrosia artemisiifolia L.	0	a	0	0	Asteraceae Bercht. & J.Presl
Iz_5_1109	DJI_0190	Amorpha fruticosa L.	0	a	0	0	Fabaceae Lindl.
Iz_5_1109	DJI_0190	Anthericum ramosum L.	0	n	1	0	Asparagaceae Juss.
Iz_5_1109	DJI_0190	Anthoxanthum repens (Host) Veldkamp	0		NA	NA	
Iz_5_1109	DJI_0190	Artemisia marschalliana Spreng.	0	n	1	0	Asteraceae Bercht. & J.Presl
Iz_5_1109	DJI_0190	Asparagus officinalis L.	0	n	1	0	Asparagaceae Juss.
Iz_5_1109	DJI_0190	Astragalus cicer L.	0	n	1	0	Fabaceae Lindl.
Iz_5_1109	DJI_0190	Bassia laniflora (S.G.Gmel.) A.J.Scott	0	n	1	0	Amaranthaceae Juss.
Iz_5_1109	DJI_0190	Berberis vulgaris L.	0	n	1	0	Berberidaceae Juss.
Iz_5_1109	DJI_0190	Berteroa incana (L.) DC.	0		NA	NA	
Iz_5_1109	DJI_0190	Betula pendula Roth.	0	n	1	1	Betulaceae Gray
Iz_5_1109	DJI_0190	Betula pendula Roth.	0	n	1	0	Poaceae Barnhart
Iz_5_1109	DJI_0190	Bromus inermis Leyss.	0		NA	NA	
Iz_5_1109	DJI_0190	Bromus squarrosus L.	0	a	0	0	Poaceae Barnhart
Iz_5_1109	DJI_0190	Bromus tectorum L.	0	a	0	0	Poaceae Barnhart
Iz_5_1109	DJI_0190	Calamagrostis epigejos (L.) Roth	0	n	1	0	Poaceae Barnhart
Iz_5_1109	DJI_0190	Carduus acanthoides L.	0	n	1	0	Asteraceae Bercht. & J.Presl
Iz_5_1109	DJI_0190	Carex sp.	0	n	1	0	Cyperaceae Juss.
Iz_5_1109	DJI_0190	Centaurea majorovii Dumbadze	0	n	1	0	Asteraceae Bercht. & J.Presl
Iz_5_1109	DJI_0190	Chamaecytisus ruthenicus (Fisch.ex Wol.) Klaskova	0	n	1	0	Fabaceae Lindl.
Iz_5_1109	DJI_0190	Chelidonium majus L.	0	n	1	0	Papaveraceae Juss.
Iz_5_1109	DJI_0190	Chenopodium betaceum Andrz.	0	n	1	0	Amaranthaceae Juss.
Iz_5_1109	DJI_0190	Chenopodium cf. album	0	n	1	0	Amaranthaceae Juss.
Iz_5_1109	DJI_0190	Chenopodium sp.	0		NA	0	Amaranthaceae Juss.
Iz_5_1109	DJI_0190	Chondrilla juncea L.	0	n	1	0	Asteraceae Bercht. & J.Presl
Iz_5_1109	DJI_0190	Cirsium vulgare (Savi) Ten.	0	n	1	0	Asteraceae Bercht. & J.Presl
Iz_5_1109	DJI_0190	Clinopodium acinos (L.) Kuntze	0	n	1	0	Lamiacea Lindl.
Iz_5_1109	DJI_0190	Convallaria majalis L.	0	n	1	0	Asparagaceae Juss.
Iz_5_1109	DJI_0190	Crataegus rhipidophylla Gand.	0	n	1	1	Rosaceae Juss.
Iz_5_1109	DJI_0190	Crepis foetida L.	0	n	1	0	Asteraceae Bercht. & J.Presl
Iz_5_1109	DJI_0190	Dactylis glomerata L.	0	n	1	0	Poaceae Barnhart
Iz_5_1109	DJI_0190	Dianthus campestris M.Bieb.	0	n	1	0	Caryophyllaceae Juss.
Iz_5_1109	DJI_0190	Digitaria sanguinalis (L.) Scop.	0	a	0	0	Poaceae Barnhart
Iz_5_1109	DJI_0190	Elymus repens (L.) Gould	0	n	1	0	Poaceae Barnhart
Iz_5_1109	DJI_0190	Epilobium tetragonum L.	0	n	1	0	Onagraceae Juss.
Iz_5_1109	DJI_0190	Eragrostis minor Host	0	a	0	0	Poaceae Barnhart
Iz_5_1109	DJI_0190	Erigeron canadensis L.	0	a	0	0	Asteraceae Bercht. & J.Presl
Iz_5_1109	DJI_0190	Euphorbia seguieriana Neck.	0	n	1	0	Euphorbiaceae Juss.
Iz_5_1109	DJI_0190	Fallopia dumetorum (L.) Holub	0	n	1	0	Polygonaceae Juss.
Iz_5_1109	DJI_0190	Festuca beckeri (Hack.) Trautv.	0	n	1	0	Poaceae Barnhart
Iz_5_1109	DJI_0190	Fragaria vesca L.	0	n	1	0	Rosaceae Juss.
Iz_5_1109	DJI_0190	Frangula alnus Mill.	0	n	1	1	Rhamnaceae Juss.
Iz_5_1109	DJI_0190	Fraxinus pennsylvanica Marshall	0	a	0	1	Oleaceae Hoffmanns. & Link
Iz_5_1109	DJI_0190	Galeopsis bifida Boenn.	0	n	1	0	Lamiacea Lindl.
Iz_5_1109	DJI_0190	Galium verum L.	0	n	1	0	Rubiaceae Juss.
Iz_5_1109	DJI_0190	Genista tinctoria L.	0	n	1	0	Fabaceae Lindl.
Iz_5_1109	DJI_0190	Geranium robertianum L.	0	n	1	0	Geraniaceae Juss.
Iz_5_1109	DJI_0190	Geranium sanguineum L.	0	n	1	0	Geraniaceae Juss.
Iz_5_1109	DJI_0190	Gypsophila paniculata L.	0	n	1	0	Caryophyllaceae Juss.
Iz_5_1109	DJI_0190	Helichrysum arenarium (L.) Moench	0	n	1	0	Asteraceae Bercht. & J.Presl
Iz_5_1109	DJI_0190	Herniaria polygama J.Gay	0	n	1	0	Caryophyllaceae Juss.
Iz_5_1109	DJI_0190	Hieracium robustum Fr.	0	n	1	0	Asteraceae Bercht. & J.Presl
Iz_5_1109	DJI_0190	Hieracium umbellatum L.	0	n	1	NA	Asteraceae Bercht. & J.Presl
Iz_5_1109	DJI_0190	Hylotelephium maximum (L.) Holub	0	n	1	0	Crassulaceae J.St.-Hil.
Iz_5_1109	DJI_0190	Jacobaea borysthenica (DC.) B.Nord. & Greuter	0	n	1	0	Asteraceae Bercht. & J.Presl
Iz_5_1109	DJI_0190	Jasione montana L.	0	n	1	0	Campanulaceae Juss.
Iz_5_1109	DJI_0190	Jurinea cyanoides (L.) Rchb.s.l.	0	n	1	0	Asteraceae Bercht. & J.Presl
Iz_5_1109	DJI_0190	Koeleria glauca (Spreng.) DC.	0	n	1	0	Poaceae Barnhart
Iz_5_1109	DJI_0190	Lactuca serriola L.	0	a	0	0	Asteraceae Bercht. & J.Presl
Iz_5_1109	DJI_0190	Linaria genistifolia (L.) Mill.	0	n	1	0	Plantaginaceae Juss.
Iz_5_1109	DJI_0190	Lomelosia argentea (L.) Greuter & Burdet	0	n	1	0	Caprifoliaceae Juss.
Iz_5_1109	DJI_0190	Melampyrum pratense L.	0	n	1	0	Orobanchaceae Vent.
Iz_5_1109	DJI_0190	Oenothera biennis L.	0	a	0	0	Onagraceae Juss.
Iz_5_1109	DJI_0190	Pentanema britannica (L.) D.Gut.Larr., Santos-Vicente, Anderb., E.Rico & M.M.Mart.Ort.	0	n	1	0	Asteraceae Bercht. & J.Presl
Iz_5_1109	DJI_0190	Peucedanum oreoselinum (L.) Moench	0.1	n	1	0	Apiaceae Lindl.
Iz_5_1109	DJI_0190	Peucedanum ruthenicum M.Bieb.	0	n	1	0	Apiaceae Lindl.
Iz_5_1109	DJI_0190	Pilosella echioides (Lumn.) F.W.Schultz & Sch.Bip.	0	n	1	0	Asteraceae Bercht. & J.Presl
Iz_5_1109	DJI_0190	Pilosella officinarum F. Schultz et Sch. Bip.	0	n	1	0	Asteraceae Bercht. & J.Presl

Iz_5_1109	DJI_0190	Pilosella sp.	0		NA	0	Asteraceae Bercht. & J.Presl
Iz_5_1109	DJI_0190	Pinus sylvestris L.	0	n	1	1	Pinaceae Spreng. ex F.Rudolphi
Iz_5_1109	DJI_0190	Poa annua L.	0	n	1	0	Poaceae Barnhart
Iz_5_1109	DJI_0190	Poa nemoralis L.	0	n	1	0	Poaceae Barnhart
Iz_5_1109	DJI_0190	Poa sp.	0		NA	0	Poaceae Barnhart
Iz_5_1109	DJI_0190	Polygonatum odoratum (Mill.) Druce	0	n	1	0	Asparagaceae Juss.
Iz_5_1109	DJI_0190	Polygonum arenarium Waldst. & Kit.	0	n	1	0	Polygonaceae Juss.
Iz_5_1109	DJI_0190	Populus nigra L.	0	n	1	1	Salicaceae Mirb.
Iz_5_1109	DJI_0190	Populus tremula L.	0	n	1	1	Salicaceae Mirb.
Iz_5_1109	DJI_0190	Populus Ã canescens (Aiton) Sm.	0	n	1	1	Salicaceae Mirb.
Iz_5_1109	DJI_0190	Potentilla argentea L.	0	n	1	0	Rosaceae Juss.
Iz_5_1109	DJI_0190	Psammophiliella muralis (L.) Ikonn.	0	n	1	0	Caryophyllaceae Juss.
Iz_5_1109	DJI_0190	Psephellus sumensis (Kalen.) Greuter	0	n	1	0	Asteraceae Bercht. & J.Presl
Iz_5_1109	DJI_0190	Pulsatilla pratensis (L.) Mill. s.l.	0	n	1	0	Ranunculaceae Juss.
Iz_5_1109	DJI_0190	Pyrus communis L.	0	a	0	1	Rosaceae Juss.
Iz_5_1109	DJI_0190	Quercus robur L.	0	n	1	1	Fagaceae Dumort.
Iz_5_1109	DJI_0190	Robinia pseudoacacia L.	0	a	0	1	Fabaceae Lindl.
Iz_5_1109	DJI_0190	Rosa sp.	0		NA	1	Rosaceae Juss.
Iz_5_1109	DJI_0190	Rubus idaeus L.	0	n	1	0	Rosaceae Juss.
Iz_5_1109	DJI_0190	Rumex acetosella L.	0	n	1	0	Polygonaceae Juss.
Iz_5_1109	DJI_0190	Rumex thyrsiflorus Fingerh.	0	n	1	0	Polygonaceae Juss.
Iz_5_1109	DJI_0190	Salix alba L.	0	n	1	1	Salicaceae Mirb.
Iz_5_1109	DJI_0190	Sambucus nigra L.	0	n	1	1	Viburnaceae Raf.
Iz_5_1109	DJI_0190	Secale sylvestre Host	0	n	1	0	Poaceae Barnhart
Iz_5_1109	DJI_0190	Sempervivum ruthenicum Schnittsp. & C.B. Lehm.	0	n	1	0	Crassulaceae J.St.-Hil.
Iz_5_1109	DJI_0190	Setaria viridis (L.) P. Beauv.	0	n	1	0	Poaceae Barnhart
Iz_5_1109	DJI_0190	Silene borysthenica (Gruner) Walters	0	n	1	0	Caryophyllaceae Juss.
Iz_5_1109	DJI_0190	Silene latifolia subsp. alba (Mill.) Greuter & Burdet	0	n	1	0	Caryophyllaceae Juss.
Iz_5_1109	DJI_0190	Solidago virgaurea L.	0	n	1	0	Asteraceae Bercht. & J.Presl
Iz_5_1109	DJI_0190	Sorbus aucuparia L.	0	n	1	1	Rosaceae Juss.
Iz_5_1109	DJI_0190	Stachys recta L.	0	n	1	0	Lamiacea Lindl.
Iz_5_1109	DJI_0190	Tanacetum vulgare L.	0	n	1	0	Asteraceae Bercht. & J.Presl
Iz_5_1109	DJI_0190	Thymus pallasianus Heinr.Braun	0	n	1	0	Lamiacea Lindl.
Iz_5_1109	DJI_0190	Tilia cordata Mill.	0	n	1	1	Malvaceae Juss.
Iz_5_1109	DJI_0190	Trifolium alpestre L.	0	n	1	0	Fabaceae Lindl.
Iz_5_1109	DJI_0190	Trifolium arvense L.	0	n	1	0	Fabaceae Lindl.
Iz_5_1109	DJI_0190	Ulmus glabra Huds.	0	n	1	1	Ulmaceae Mirb.
Iz_5_1109	DJI_0190	Verbascum cf. phlomoides L.	0		NA	NA	
Iz_5_1109	DJI_0190	Verbascum densiflorum Bertol.	0	n	1	0	Scrophulariaceae Juss.
Iz_5_1109	DJI_0190	Verbascum sp.	0	n	1	0	Scrophulariaceae Juss.
Iz_5_1109	DJI_0190	Veronica austriaca L.	0	n	1	0	Plantaginaceae Juss.
Iz_5_1109	DJI_0190	Veronica incana L.	0	n	1	0	Plantaginaceae Juss.
Iz_5_1109	DJI_0190	Veronica verna L.	0	n	1	0	Plantaginaceae Juss.
Iz_5_1109	DJI_0190	Vincetoxicum hirundinaria Medik	0	n	1	0	Apocynaceae Juss.
Iz_5_1109	DJI_0191	Acer tataricum L.	0	n	1	1	Sapindaceae Juss.
Iz_5_1109	DJI_0191	Achillea millefolium L. s.l.	0	n	1	0	Asteraceae Bercht. & J.Presl
Iz_5_1109	DJI_0191	Allium oleraceum L.	0	n	1	0	Amaryllidaceae J.St.-Hil.
Iz_5_1109	DJI_0191	Ambrosia artemisiifolia L.	0	a	0	0	Asteraceae Bercht. & J.Presl
Iz_5_1109	DJI_0191	Amorpha fruticosa L.	0	a	0	0	Fabaceae Lindl.
Iz_5_1109	DJI_0191	Anthericum ramosum L.	0	n	1	0	Asparagaceae Juss.
Iz_5_1109	DJI_0191	Anthoxanthum repens (Host) Veldkamp	0		NA	NA	
Iz_5_1109	DJI_0191	Artemisia marschalliana Spreng.	0	n	1	0	Asteraceae Bercht. & J.Presl
Iz_5_1109	DJI_0191	Asparagus officinalis L.	0	n	1	0	Asparagaceae Juss.
Iz_5_1109	DJI_0191	Astragalus cicer L.	0	n	1	0	Fabaceae Lindl.
Iz_5_1109	DJI_0191	Bassia laniflora (S.G.Gmel.) A.J.Scott	0	n	1	0	Amaranthaceae Juss.
Iz_5_1109	DJI_0191	Berberis vulgaris L.	0	n	1	0	Berberidaceae Juss.
Iz_5_1109	DJI_0191	Berteroa incana (L.) DC.	0		NA	NA	
Iz_5_1109	DJI_0191	Betula pendula Roth.	0	n	1	1	Betulaceae Gray
Iz_5_1109	DJI_0191	Betula pendula Roth.	0	n	1	0	Poaceae Barnhart
Iz_5_1109	DJI_0191	Bromus inermis Leyss.	0		NA	NA	
Iz_5_1109	DJI_0191	Bromus squarrosus L.	0	a	0	0	Poaceae Barnhart
Iz_5_1109	DJI_0191	Bromus tectorum L.	0	a	0	0	Poaceae Barnhart
Iz_5_1109	DJI_0191	Calamagrostis epigejos (L.) Roth	0	n	1	0	Poaceae Barnhart
Iz_5_1109	DJI_0191	Carduus acanthoides L.	0	n	1	0	Asteraceae Bercht. & J.Presl
Iz_5_1109	DJI_0191	Carex sp.	0	n	1	0	Cyperaceae Juss.
Iz_5_1109	DJI_0191	Centaurea majorovii Dumbadze	0	n	1	0	Asteraceae Bercht. & J.Presl
Iz_5_1109	DJI_0191	Chamaecytisus ruthenicus (Fisch.ex Wol.) Klaskova	0	n	1	0	Fabaceae Lindl.
Iz_5_1109	DJI_0191	Chelidonium majus L.	0	n	1	0	Papaveraceae Juss.
Iz_5_1109	DJI_0191	Chenopodium betaceum Andrz.	0	n	1	0	Amaranthaceae Juss.
Iz_5_1109	DJI_0191	Chenopodium cf. album	0	n	1	0	Amaranthaceae Juss.
Iz_5_1109	DJI_0191	Chenopodium sp.	0		NA	0	Amaranthaceae Juss.
Iz_5_1109	DJI_0191	Chondrilla juncea L.	0	n	1	0	Asteraceae Bercht. & J.Presl
Iz_5_1109	DJI_0191	Cirsium vulgare (Savi) Ten.	0	n	1	0	Asteraceae Bercht. & J.Presl
Iz_5_1109	DJI_0191	Clinopodium acinos (L.) Kuntze	0	n	1	0	Lamiacea Lindl.
Iz_5_1109	DJI_0191	Convallaria majalis L.	0	n	1	0	Asparagaceae Juss.
Iz_5_1109	DJI_0191	Crataegus rhipidophylla Gand.	0	n	1	1	Rosaceae Juss.
Iz_5_1109	DJI_0191	Crepis foetida L.	0	n	1	0	Asteraceae Bercht. & J.Presl
Iz_5_1109	DJI_0191	Dactylis glomerata L.	0	n	1	0	Poaceae Barnhart
Iz_5_1109	DJI_0191	Dianthus campestris M.Bieb.	0	n	1	0	Caryophyllaceae Juss.
Iz_5_1109	DJI_0191	Digitaria sanguinalis (L.) Scop.	0	a	0	0	Poaceae Barnhart
Iz_5_1109	DJI_0191	Elymus repens (L.) Gould	0	n	1	0	Poaceae Barnhart
Iz_5_1109	DJI_0191	Epilobium tetragonum L.	0	n	1	0	Onagraceae Juss.
Iz_5_1109	DJI_0191	Eragrostis minor Host	0	a	0	0	Poaceae Barnhart
Iz_5_1109	DJI_0191	Erigeron canadensis L.	2	a	0	0	Asteraceae Bercht. & J.Presl
Iz_5_1109	DJI_0191	Euphorbia seguieriana Neck.	0	n	1	0	Euphorbiaceae Juss.
Iz_5_1109	DJI_0191	Fallopia dumetorum (L.) Holub	0	n	1	0	Polygonaceae Juss.
Iz_5_1109	DJI_0191	Festuca beckeri (Hack.) Trautv.	0	n	1	0	Poaceae Barnhart
Iz_5_1109	DJI_0191	Fragaria vesca L.	0	n	1	0	Rosaceae Juss.
Iz_5_1109	DJI_0191	Frangula alnus Mill.	0	n	1	1	Rhamnaceae Juss.
Iz_5_1109	DJI_0191	Fraxinus pennsylvanica Marshall	0	a	0	1	Oleaceae Hoffmanns. & Link
Iz_5_1109	DJI_0191	Galeopsis bifida Boenn.	0	n	1	0	Lamiacea Lindl.
Iz_5_1109	DJI_0191	Galium verum L.	0	n	1	0	Rubiaceae Juss.
Iz_5_1109	DJI_0191	Genista tinctoria L.	0	n	1	0	Fabaceae Lindl.
Iz_5_1109	DJI_0191	Geranium robertianum L.	0	n	1	0	Geraniaceae Juss.
Iz_5_1109	DJI_0191	Geranium sanquineum L.	0	n	1	0	Geraniaceae Juss.
Iz_5_1109	DJI_0191	Gypsophila paniculata L.	0	n	1	0	Caryophyllaceae Juss.
Iz_5_1109	DJI_0191	Helichrysum arenarium (L.) Moench	0	n	1	0	Asteraceae Bercht. & J.Presl
Iz_5_1109	DJI_0191	Herniaria polygama J.Gay	0	n	1	0	Caryophyllaceae Juss.

Iz_5_1109	DJI_0191	Hieracium robustum Fr.	0	n	1	0	Asteraceae Bercht. & J.Presl
Iz_5_1109	DJI_0191	Hieracium umbellatum L.	0	n	1	NA	Asteraceae Bercht. & J.Presl
Iz_5_1109	DJI_0191	Hylotelephium maximum (L.) Holub	0	n	1	0	Crassulaceae J.St.-Hil.
Iz_5_1109	DJI_0191	Jacobaea borysthenica (DC.) B.Nord. & Greuter	0	n	1	0	Asteraceae Bercht. & J.Presl
Iz_5_1109	DJI_0191	Jasione montana L.	0	n	1	0	Campanulaceae Juss.
Iz_5_1109	DJI_0191	Jurinea cyanoides (L.) Rchb.s.l.	0	n	1	0	Asteraceae Bercht. & J.Presl
Iz_5_1109	DJI_0191	Koeleria glauca (Spreng.) DC.	0	n	1	0	Poaceae Barnhart
Iz_5_1109	DJI_0191	Lactuca serriola L.	0	a	0	0	Asteraceae Bercht. & J.Presl
Iz_5_1109	DJI_0191	Linaria genistifolia (L.) Mill.	0	n	1	0	Plantaginaceae Juss.
Iz_5_1109	DJI_0191	Lomelosia argentea (L.) Greuter & Burdet	0	n	1	0	Caprifoliaceae Juss.
Iz_5_1109	DJI_0191	Melampyrum pratense L.	0	n	1	0	Orobanchaceae Vent.
Iz_5_1109	DJI_0191	Oenothera biennis L.	0	a	0	0	Onagraceae Juss.
Iz_5_1109	DJI_0191	Pentanema britannica (L.) D.Gut.Larr., Santos-Vicente, Anderb., E.Rico & M.M.Mart.Ort.	0	n	1	0	Asteraceae Bercht. & J.Presl
Iz_5_1109	DJI_0191	Peucedanum oreoselinum (L.) Moench	0	n	1	0	Apiaceae Lindl.
Iz_5_1109	DJI_0191	Peucedanum ruthenicum M.Bieb.	0	n	1	0	Apiaceae Lindl.
Iz_5_1109	DJI_0191	Pilosella echioides (Lumn.) F.W.Schultz & Sch.Bip.	0	n	1	0	Asteraceae Bercht. & J.Presl
Iz_5_1109	DJI_0191	Pilosella officinarum F. Schultz et Sch. Bip.	0	n	1	0	Asteraceae Bercht. & J.Presl
Iz_5_1109	DJI_0191	Pilosella sp.	0		NA	0	Asteraceae Bercht. & J.Presl
Iz_5_1109	DJI_0191	Pinus sylvestris L.	0	n	1	1	Pinaceae Spreng. ex F.Rudolphi
Iz_5_1109	DJI_0191	Poa annua L.	0	n	1	0	Poaceae Barnhart
Iz_5_1109	DJI_0191	Poa nemoralis L.	0	n	1	0	Poaceae Barnhart
Iz_5_1109	DJI_0191	Poa sp.	0		NA	0	Poaceae Barnhart
Iz_5_1109	DJI_0191	Polygonatum odoratum (Mill.) Druce	0	n	1	0	Asparagaceae Juss.
Iz_5_1109	DJI_0191	Polygonum arenarium Waldst. & Kit.	0	n	1	0	Polygonaceae Juss.
Iz_5_1109	DJI_0191	Populus nigra L.	0	n	1	1	Salicaceae Mirb.
Iz_5_1109	DJI_0191	Populus tremula L.	0	n	1	1	Salicaceae Mirb.
Iz_5_1109	DJI_0191	Populus Ã canescens (Aiton) Sm.	0	n	1	1	Salicaceae Mirb.
Iz_5_1109	DJI_0191	Potentilla argentea L.	0	n	1	0	Rosaceae Juss.
Iz_5_1109	DJI_0191	Psammophiliella muralis (L.) Ikonn.	0	n	1	0	Caryophyllaceae Juss.
Iz_5_1109	DJI_0191	Psephellus sumensis (Kalen.) Greuter	0	n	1	0	Asteraceae Bercht. & J.Presl
Iz_5_1109	DJI_0191	Pulsatilla pratensis (L.) Mill. s.l.	0	n	1	0	Ranunculaceae Juss.
Iz_5_1109	DJI_0191	Pyrus communis L.	0	a	0	1	Rosaceae Juss.
Iz_5_1109	DJI_0191	Quercus robur L.	0	n	1	1	Fagaceae Dumort.
Iz_5_1109	DJI_0191	Robinia pseudoacacia L.	0	a	0	1	Fabaceae Lindl.
Iz_5_1109	DJI_0191	Rosa sp.	0		NA	1	Rosaceae Juss.
Iz_5_1109	DJI_0191	Rubus idaeus L.	0	n	1	0	Rosaceae Juss.
Iz_5_1109	DJI_0191	Rumex acetosella L.	0	n	1	0	Polygonaceae Juss.
Iz_5_1109	DJI_0191	Rumex thyrsiflorus Fingerh.	0	n	1	0	Polygonaceae Juss.
Iz_5_1109	DJI_0191	Salix alba L.	0	n	1	1	Salicaceae Mirb.
Iz_5_1109	DJI_0191	Sambucus nigra L.	0	n	1	1	Viburnaceae Raf.
Iz_5_1109	DJI_0191	Secale sylvestre Host	0	n	1	0	Poaceae Barnhart
Iz_5_1109	DJI_0191	Sempervivum ruthenicum Schnittsp. & C.B. Lehm.	0	n	1	0	Crassulaceae J.St.-Hil.
Iz_5_1109	DJI_0191	Setaria viridis (L.) P. Beauv.	0	n	1	0	Poaceae Barnhart
Iz_5_1109	DJI_0191	Silene borysthenica (Gruner) Walters	0	n	1	0	Caryophyllaceae Juss.
Iz_5_1109	DJI_0191	Silene latifolia subsp. alba (Mill.) Greuter & Burdet	0	n	1	0	Caryophyllaceae Juss.
Iz_5_1109	DJI_0191	Solidago virgaurea L.	0	n	1	0	Asteraceae Bercht. & J.Presl
Iz_5_1109	DJI_0191	Sorbus aucuparia L.	0	n	1	1	Rosaceae Juss.
Iz_5_1109	DJI_0191	Stachys recta L.	0	n	1	0	Lamiacea Lindl.
Iz_5_1109	DJI_0191	Tanacetum vulgare L.	0	n	1	0	Asteraceae Bercht. & J.Presl
Iz_5_1109	DJI_0191	Thymus pallasianus Heinr.Braun	0	n	1	0	Lamiacea Lindl.
Iz_5_1109	DJI_0191	Tilia cordata Mill.	0	n	1	1	Malvaceae Juss.
Iz_5_1109	DJI_0191	Trifolium alpestre L.	0	n	1	0	Fabaceae Lindl.
Iz_5_1109	DJI_0191	Trifolium arvense L.	0	n	1	0	Fabaceae Lindl.
Iz_5_1109	DJI_0191	Ulmus glabra Huds.	0	n	1	1	Ulmaceae Mirb.
Iz_5_1109	DJI_0191	Verbascum cf. phlomoides L.	0		NA	NA	
Iz_5_1109	DJI_0191	Verbascum densiflorum Bertol.	0	n	1	0	Scrophulariaceae Juss.
Iz_5_1109	DJI_0191	Verbascum sp.	0	n	1	0	Scrophulariaceae Juss.
Iz_5_1109	DJI_0191	Veronica austriaca L.	0	n	1	0	Plantaginaceae Juss.
Iz_5_1109	DJI_0191	Veronica incana L.	0	n	1	0	Plantaginaceae Juss.
Iz_5_1109	DJI_0191	Veronica verna L.	0	n	1	0	Plantaginaceae Juss.
Iz_5_1109	DJI_0191	Vincetoxicum hirundinaria Medik	0	n	1	0	Apocynaceae Juss.
Iz_5_1109	DJI_0192	Acer tataricum L.	0	n	1	1	Sapindaceae Juss.
Iz_5_1109	DJI_0192	Achillea millefolium L. s.l.	0	n	1	0	Asteraceae Bercht. & J.Presl
Iz_5_1109	DJI_0192	Allium oleraceum L.	0	n	1	0	Amaryllidaceae J.St.-Hil.
Iz_5_1109	DJI_0192	Ambrosia artemisiifolia L.	0	a	0	0	Asteraceae Bercht. & J.Presl
Iz_5_1109	DJI_0192	Amorpha fruticosa L.	0	a	0	0	Fabaceae Lindl.
Iz_5_1109	DJI_0192	Anthericum ramosum L.	0	n	1	0	Asparagaceae Juss.
Iz_5_1109	DJI_0192	Anthoxanthum repens (Host) Veldkamp	0		NA	NA	
Iz_5_1109	DJI_0192	Artemisia marschalliana Spreng.	0	n	1	0	Asteraceae Bercht. & J.Presl
Iz_5_1109	DJI_0192	Asparagus officinalis L.	0	n	1	0	Asparagaceae Juss.
Iz_5_1109	DJI_0192	Astragalus cicer L.	0	n	1	0	Fabaceae Lindl.
Iz_5_1109	DJI_0192	Bassia laniflora (S.G.Gmel.) A.J.Scott	0	n	1	0	Amaranthaceae Juss.
Iz_5_1109	DJI_0192	Berberis vulgaris L.	0	n	1	0	Berberidaceae Juss.
Iz_5_1109	DJI_0192	Berteroa incana (L.) DC.	0		NA	NA	
Iz_5_1109	DJI_0192	Betula pendula Roth.	0	n	1	1	Betulaceae Gray
Iz_5_1109	DJI_0192	Betula pendula Roth.	0	n	1	0	Poaceae Barnhart
Iz_5_1109	DJI_0192	Bromus inermis Leyss.	0		NA	NA	
Iz_5_1109	DJI_0192	Bromus squarrosus L.	0	a	0	0	Poaceae Barnhart
Iz_5_1109	DJI_0192	Bromus tectorum L.	0	a	0	0	Poaceae Barnhart
Iz_5_1109	DJI_0192	Calamagrostis epigejos (L.) Roth	0	n	1	0	Poaceae Barnhart
Iz_5_1109	DJI_0192	Carduus acanthoides L.	0	n	1	0	Asteraceae Bercht. & J.Presl
Iz_5_1109	DJI_0192	Carex sp.	0	n	1	0	Cyperaceae Juss.
Iz_5_1109	DJI_0192	Centaurea majorovii Dumbadze	0	n	1	0	Asteraceae Bercht. & J.Presl
Iz_5_1109	DJI_0192	Chamaecytisus ruthenicus (Fisch.ex Wol.) Klaskova	0	n	1	0	Fabaceae Lindl.
Iz_5_1109	DJI_0192	Chelidonium majus L.	0	n	1	0	Papaveraceae Juss.
Iz_5_1109	DJI_0192	Chenopodium betaceum Andrz.	0	n	1	0	Amaranthaceae Juss.
Iz_5_1109	DJI_0192	Chenopodium cf. album	0	n	1	0	Amaranthaceae Juss.
Iz_5_1109	DJI_0192	Chenopodium sp.	0		NA	0	Amaranthaceae Juss.
Iz_5_1109	DJI_0192	Chondrilla juncea L.	0	n	1	0	Asteraceae Bercht. & J.Presl
Iz_5_1109	DJI_0192	Cirsium vulgare (Savi) Ten.	0	n	1	0	Asteraceae Bercht. & J.Presl
Iz_5_1109	DJI_0192	Clinopodium acinos (L.) Kuntze	0	n	1	0	Lamiacea Lindl.
Iz_5_1109	DJI_0192	Convallaria majalis L.	0	n	1	0	Asparagaceae Juss.
Iz_5_1109	DJI_0192	Crataegus rhipidophylla Gand.	0	n	1	1	Rosaceae Juss.
Iz_5_1109	DJI_0192	Crepis foetida L.	0	n	1	0	Asteraceae Bercht. & J.Presl
Iz_5_1109	DJI_0192	Dactylis glomerata L.	0	n	1	0	Poaceae Barnhart
Iz_5_1109	DJI_0192	Dianthus campestris M.Bieb.	0	n	1	0	Caryophyllaceae Juss.
Iz_5_1109	DJI_0192	Digitaria sanguinalis (L.) Scop.	0	a	0	0	Poaceae Barnhart
Iz_5_1109	DJI_0192	Elymus repens (L.) Gould	0	n	1	0	Poaceae Barnhart

Iz_5_1109	DJI_0192	Epilobium tetragonum L.	0	n	1	0	Onagraceae Juss.
Iz_5_1109	DJI_0192	Eragrostis minor Host	0	a	0	0	Poaceae Barnhart
Iz_5_1109	DJI_0192	Erigeron canadensis L.	3	a	0	0	Asteraceae Bercht. & J.Presl
Iz_5_1109	DJI_0192	Euphorbia seguieriana Neck.	0	n	1	0	Euphorbiaceae Juss.
Iz_5_1109	DJI_0192	Fallopia dumetorum (L.) Holub	0	n	1	0	Polygonaceae Juss.
Iz_5_1109	DJI_0192	Festuca beckeri (Hack.) Trautv.	0	n	1	0	Poaceae Barnhart
Iz_5_1109	DJI_0192	Fragaria vesca L.	0	n	1	0	Rosaceae Juss.
Iz_5_1109	DJI_0192	Frangula alnus Mill.	0	n	1	1	Rhamnaceae Juss.
Iz_5_1109	DJI_0192	Fraxinus pennsylvanica Marshall	0	a	0	1	Oleaceae Hoffmanns. & Link
Iz_5_1109	DJI_0192	Galeopsis bifida Boenn.	0	n	1	0	Lamiacea Lindl.
Iz_5_1109	DJI_0192	Galium verum L.	0	n	1	0	Rubiaceae Juss.
Iz_5_1109	DJI_0192	Genista tinctoria L.	0	n	1	0	Fabaceae Lindl.
Iz_5_1109	DJI_0192	Geranium robertianum L.	0	n	1	0	Geraniaceae Juss.
Iz_5_1109	DJI_0192	Geranium sanquineum L.	0	n	1	0	Geraniaceae Juss.
Iz_5_1109	DJI_0192	Gypsophila paniculata L.	0	n	1	0	Caryophyllaceae Juss.
Iz_5_1109	DJI_0192	Helichrysum arenarium (L.) Moench	0	n	1	0	Asteraceae Bercht. & J.Presl
Iz_5_1109	DJI_0192	Herniaria polygama J.Gay	0	n	1	0	Caryophyllaceae Juss.
Iz_5_1109	DJI_0192	Hieracium robustum Fr.	0	n	1	0	Asteraceae Bercht. & J.Presl
Iz_5_1109	DJI_0192	Hieracium umbellatum L.	0	n	1	NA	Asteraceae Bercht. & J.Presl
Iz_5_1109	DJI_0192	Hylotelephium maximum (L.) Holub	0	n	1	0	Crassulaceae J.St.-Hil.
Iz_5_1109	DJI_0192	Jacobaea borysthenica (DC.) B.Nord. & Greuter	0	n	1	0	Asteraceae Bercht. & J.Presl
Iz_5_1109	DJI_0192	Jasione montana L.	0	n	1	0	Campanulaceae Juss.
Iz_5_1109	DJI_0192	Jurinea cyanoides (L.) Rchb.s.l.	0	n	1	0	Asteraceae Bercht. & J.Presl
Iz_5_1109	DJI_0192	Koeleria glauca (Spreng.) DC.	0	n	1	0	Poaceae Barnhart
Iz_5_1109	DJI_0192	Lactuca serriola L.	0	a	0	0	Asteraceae Bercht. & J.Presl
Iz_5_1109	DJI_0192	Linaria genistifolia (L.) Mill.	0	n	1	0	Plantaginaceae Juss.
Iz_5_1109	DJI_0192	Lomelosia argentea (L.) Greuter & Burdet	0	n	1	0	Caprifoliaceae Juss.
Iz_5_1109	DJI_0192	Melampyrum pratense L.	0	n	1	0	Orobanchaceae Vent.
Iz_5_1109	DJI_0192	Oenothera biennis L.	0	a	0	0	Onagraceae Juss.
Iz_5_1109	DJI_0192	Pentanema britannica (L.) D.Gut.Larr., Santos-Vicente, Anderb., E.Rico & M.M.Mart.Ort.	0	n	1	0	Asteraceae Bercht. & J.Presl
Iz_5_1109	DJI_0192	Peucedanum oreoselinum (L.) Moench	0	n	1	0	Apiaceae Lindl.
Iz_5_1109	DJI_0192	Peucedanum ruthenicum M.Bieb.	0	n	1	0	Apiaceae Lindl.
Iz_5_1109	DJI_0192	Pilosella echioides (Lumn.) F.W.Schultz & Sch.Bip.	0	n	1	0	Asteraceae Bercht. & J.Presl
Iz_5_1109	DJI_0192	Pilosella officinarum F. Schultz et Sch. Bip.	0	n	1	0	Asteraceae Bercht. & J.Presl
Iz_5_1109	DJI_0192	Pilosella sp.	0		NA	0	Asteraceae Bercht. & J.Presl
Iz_5_1109	DJI_0192	Pinus sylvestris L.	0	n	1	1	Pinaceae Spreng. ex F.Rudolphi
Iz_5_1109	DJI_0192	Poa annua L.	0	n	1	0	Poaceae Barnhart
Iz_5_1109	DJI_0192	Poa nemoralis L.	0	n	1	0	Poaceae Barnhart
Iz_5_1109	DJI_0192	Poa sp.	0		NA	0	Poaceae Barnhart
Iz_5_1109	DJI_0192	Polygonatum odoratum (Mill.) Druce	0	n	1	0	Asparagaceae Juss.
Iz_5_1109	DJI_0192	Polygonum arenarium Waldst. & Kit.	0	n	1	0	Polygonaceae Juss.
Iz_5_1109	DJI_0192	Populus nigra L.	0	n	1	1	Salicaceae Mirb.
Iz_5_1109	DJI_0192	Populus tremula L.	0	n	1	1	Salicaceae Mirb.
Iz_5_1109	DJI_0192	Populus Ã canescens (Aiton) Sm.	0	n	1	1	Salicaceae Mirb.
Iz_5_1109	DJI_0192	Potentilla argentea L.	0	n	1	0	Rosaceae Juss.
Iz_5_1109	DJI_0192	Psammophiliella muralis (L.) Ikonn.	0	n	1	0	Caryophyllaceae Juss.
Iz_5_1109	DJI_0192	Psephellus sumensis (Kalen.) Greuter	0	n	1	0	Asteraceae Bercht. & J.Presl
Iz_5_1109	DJI_0192	Pulsatilla pratensis (L.) Mill. s.l.	0	n	1	0	Ranunculaceae Juss.
Iz_5_1109	DJI_0192	Pyrus communis L.	0	a	0	1	Rosaceae Juss.
Iz_5_1109	DJI_0192	Quercus robur L.	0	n	1	1	Fagaceae Dumort.
Iz_5_1109	DJI_0192	Robinia pseudoacacia L.	0	a	0	1	Fabaceae Lindl.
Iz_5_1109	DJI_0192	Rosa sp.	0		NA	1	Rosaceae Juss.
Iz_5_1109	DJI_0192	Rubus idaeus L.	0	n	1	0	Rosaceae Juss.
Iz_5_1109	DJI_0192	Rumex acetosella L.	0	n	1	0	Polygonaceae Juss.
Iz_5_1109	DJI_0192	Rumex thyrsiflorus Fingerh.	0	n	1	0	Polygonaceae Juss.
Iz_5_1109	DJI_0192	Salix alba L.	0	n	1	1	Salicaceae Mirb.
Iz_5_1109	DJI_0192	Sambucus nigra L.	0	n	1	1	Viburnaceae Raf.
Iz_5_1109	DJI_0192	Secale sylvestre Host	0	n	1	0	Poaceae Barnhart
Iz_5_1109	DJI_0192	Sempervivum ruthenicum Schnittsp. & C.B. Lehm.	0	n	1	0	Crassulaceae J.St.-Hil.
Iz_5_1109	DJI_0192	Setaria viridis (L.) P. Beauv.	0	n	1	0	Poaceae Barnhart
Iz_5_1109	DJI_0192	Silene borysthenica (Gruner) Walters	0	n	1	0	Caryophyllaceae Juss.
Iz_5_1109	DJI_0192	Silene latifolia subsp. alba (Mill.) Greuter & Burdet	0	n	1	0	Caryophyllaceae Juss.
Iz_5_1109	DJI_0192	Solidago virgaurea L.	0	n	1	0	Asteraceae Bercht. & J.Presl
Iz_5_1109	DJI_0192	Sorbus aucuparia L.	0	n	1	1	Rosaceae Juss.
Iz_5_1109	DJI_0192	Stachys recta L.	0	n	1	0	Lamiacea Lindl.
Iz_5_1109	DJI_0192	Tanacetum vulgare L.	0	n	1	0	Asteraceae Bercht. & J.Presl
Iz_5_1109	DJI_0192	Thymus pallasianus Heinr.Braun	0	n	1	0	Lamiacea Lindl.
Iz_5_1109	DJI_0192	Tilia cordata Mill.	0	n	1	1	Malvaceae Juss.
Iz_5_1109	DJI_0192	Trifolium alpestre L.	0	n	1	0	Fabaceae Lindl.
Iz_5_1109	DJI_0192	Trifolium arvense L.	0	n	1	0	Fabaceae Lindl.
Iz_5_1109	DJI_0192	Ulmus glabra Huds.	0	n	1	1	Ulmaceae Mirb.
Iz_5_1109	DJI_0192	Verbascum cf. phlomoides L.	0		NA	NA	
Iz_5_1109	DJI_0192	Verbascum densiflorum Bertol.	0	n	1	0	Scrophulariaceae Juss.
Iz_5_1109	DJI_0192	Verbascum sp.	0	n	1	0	Scrophulariaceae Juss.
Iz_5_1109	DJI_0192	Veronica austriaca L.	0	n	1	0	Plantaginaceae Juss.
Iz_5_1109	DJI_0192	Veronica incana L.	0	n	1	0	Plantaginaceae Juss.
Iz_5_1109	DJI_0192	Veronica verna L.	0	n	1	0	Plantaginaceae Juss.
Iz_5_1109	DJI_0192	Vincetoxicum hirundinaria Medik	0	n	1	0	Apocynaceae Juss.
Iz_5_1109	DJI_0193	Acer tataricum L.	0	n	1	1	Sapindaceae Juss.
Iz_5_1109	DJI_0193	Achillea millefolium L. s.l.	0	n	1	0	Asteraceae Bercht. & J.Presl
Iz_5_1109	DJI_0193	Allium oleraceum L.	0	n	1	0	Amaryllidaceae J.St.-Hil.
Iz_5_1109	DJI_0193	Ambrosia artemisiifolia L.	0	a	0	0	Asteraceae Bercht. & J.Presl
Iz_5_1109	DJI_0193	Amorpha fruticosa L.	0	a	0	0	Fabaceae Lindl.
Iz_5_1109	DJI_0193	Anthericum ramosum L.	0	n	1	0	Asparagaceae Juss.
Iz_5_1109	DJI_0193	Anthoxanthum repens (Host) Veldkamp	0		NA	NA	
Iz_5_1109	DJI_0193	Artemisia marschalliana Spreng.	0	n	1	0	Asteraceae Bercht. & J.Presl
Iz_5_1109	DJI_0193	Asparagus officinalis L.	0	n	1	0	Asparagaceae Juss.
Iz_5_1109	DJI_0193	Astragalus cicer L.	0	n	1	0	Fabaceae Lindl.
Iz_5_1109	DJI_0193	Bassia laniflora (S.G.Gmel.) A.J.Scott	0	n	1	0	Amaranthaceae Juss.
Iz_5_1109	DJI_0193	Berberis vulgaris L.	0	n	1	0	Berberidaceae Juss.
Iz_5_1109	DJI_0193	Berteroa incana (L.) DC.	0		NA	NA	
Iz_5_1109	DJI_0193	Betula pendula Roth.	0	n	1	1	Betulaceae Gray
Iz_5_1109	DJI_0193	Betula pendula Roth.	0	n	1	0	Poaceae Barnhart
Iz_5_1109	DJI_0193	Bromus inermis Leyss.	0		NA	NA	
Iz_5_1109	DJI_0193	Bromus squarrosus L.	0	a	0	0	Poaceae Barnhart
Iz_5_1109	DJI_0193	Bromus tectorum L.	0	a	0	0	Poaceae Barnhart
Iz_5_1109	DJI_0193	Calamagrostis epigejos (L.) Roth	0	n	1	0	Poaceae Barnhart
Iz_5_1109	DJI_0193	Carduus acanthoides L.	0	n	1	0	Asteraceae Bercht. & J.Presl

Iz_5_1109	DJI_0193	Carex sp.	0	n	1	0	Cyperaceae Juss.
Iz_5_1109	DJI_0193	Centaurea majorovii Dumbadze	0	n	1	0	Asteraceae Bercht. & J.Presl
Iz_5_1109	DJI_0193	Chamaecytisus ruthenicus (Fisch.ex Wol.) Klaskova	0	n	1	0	Fabaceae Lindl.
Iz_5_1109	DJI_0193	Chelidonium majus L.	0	n	1	0	Papaveraceae Juss.
Iz_5_1109	DJI_0193	Chenopodium betaceum Andrz.	0	n	1	0	Amaranthaceae Juss.
Iz_5_1109	DJI_0193	Chenopodium cf. album	0	n	1	0	Amaranthaceae Juss.
Iz_5_1109	DJI_0193	Chenopodium sp.	0		NA	0	Amaranthaceae Juss.
Iz_5_1109	DJI_0193	Chondrilla juncea L.	0	n	1	0	Asteraceae Bercht. & J.Presl
Iz_5_1109	DJI_0193	Cirsium vulgare (Savi) Ten.	0	n	1	0	Asteraceae Bercht. & J.Presl
Iz_5_1109	DJI_0193	Clinopodium acinos (L.) Kuntze	0	n	1	0	Lamiacea Lindl.
Iz_5_1109	DJI_0193	Convallaria majalis L.	0	n	1	0	Asparagaceae Juss.
Iz_5_1109	DJI_0193	Crataegus rhipidophylla Gand.	0	n	1	1	Rosaceae Juss.
Iz_5_1109	DJI_0193	Crepis foetida L.	0	n	1	0	Asteraceae Bercht. & J.Presl
Iz_5_1109	DJI_0193	Dactylis glomerata L.	0	n	1	0	Poaceae Barnhart
Iz_5_1109	DJI_0193	Dianthus campestris M.Bieb.	0	n	1	0	Caryophyllaceae Juss.
Iz_5_1109	DJI_0193	Digitaria sanguinalis (L.) Scop.	0	a	0	0	Poaceae Barnhart
Iz_5_1109	DJI_0193	Elymus repens (L.) Gould	0	n	1	0	Poaceae Barnhart
Iz_5_1109	DJI_0193	Epilobium tetragonum L.	0	n	1	0	Onagraceae Juss.
Iz_5_1109	DJI_0193	Eragrostis minor Host	0	a	0	0	Poaceae Barnhart
Iz_5_1109	DJI_0193	Erigeron canadensis L.	0.2	a	0	0	Asteraceae Bercht. & J.Presl
Iz_5_1109	DJI_0193	Euphorbia seguieriana Neck.	0	n	1	0	Euphorbiaceae Juss.
Iz_5_1109	DJI_0193	Fallopia dumetorum (L.) Holub	0	n	1	0	Polygonaceae Juss.
Iz_5_1109	DJI_0193	Festuca beckeri (Hack.) Trautv.	0	n	1	0	Poaceae Barnhart
Iz_5_1109	DJI_0193	Fragaria vesca L.	0	n	1	0	Rosaceae Juss.
Iz_5_1109	DJI_0193	Frangula alnus Mill.	0	n	1	1	Rhamnaceae Juss.
Iz_5_1109	DJI_0193	Fraxinus pennsylvanica Marshall	0	a	0	1	Oleaceae Hoffmanns. & Link
Iz_5_1109	DJI_0193	Galeopsis bifida Boenn.	0	n	1	0	Lamiacea Lindl.
Iz_5_1109	DJI_0193	Galium verum L.	0	n	1	0	Rubiaceae Juss.
Iz_5_1109	DJI_0193	Genista tinctoria L.	0	n	1	0	Fabaceae Lindl.
Iz_5_1109	DJI_0193	Geranium robertianum L.	0	n	1	0	Geraniaceae Juss.
Iz_5_1109	DJI_0193	Geranium sanquineum L.	0	n	1	0	Geraniaceae Juss.
Iz_5_1109	DJI_0193	Gypsophila paniculata L.	0	n	1	0	Caryophyllaceae Juss.
Iz_5_1109	DJI_0193	Helichrysum arenarium (L.) Moench	0	n	1	0	Asteraceae Bercht. & J.Presl
Iz_5_1109	DJI_0193	Herniaria polygama J.Gay	0	n	1	0	Caryophyllaceae Juss.
Iz_5_1109	DJI_0193	Hieracium robustum Fr.	0	n	1	0	Asteraceae Bercht. & J.Presl
Iz_5_1109	DJI_0193	Hieracium umbellatum L.	0	n	1	NA	Asteraceae Bercht. & J.Presl
Iz_5_1109	DJI_0193	Hylotelephium maximum (L.) Holub	0	n	1	0	Crassulaceae J.St.-Hil.
Iz_5_1109	DJI_0193	Jacobaea borysthenica (DC.) B.Nord. & Greuter	0	n	1	0	Asteraceae Bercht. & J.Presl
Iz_5_1109	DJI_0193	Jasione montana L.	0	n	1	0	Campanulaceae Juss.
Iz_5_1109	DJI_0193	Jurinea cyanoides (L.) Rchb.s.l.	0	n	1	0	Asteraceae Bercht. & J.Presl
Iz_5_1109	DJI_0193	Koeleria glauca (Spreng.) DC.	0	n	1	0	Poaceae Barnhart
Iz_5_1109	DJI_0193	Lactuca serriola L.	0	a	0	0	Asteraceae Bercht. & J.Presl
Iz_5_1109	DJI_0193	Linaria genistifolia (L.) Mill.	0	n	1	0	Plantaginaceae Juss.
Iz_5_1109	DJI_0193	Lomelosia argentea (L.) Greuter & Burdet	0	n	1	0	Caprifoliaceae Juss.
Iz_5_1109	DJI_0193	Melampyrum pratense L.	0	n	1	0	Orobanchaceae Vent.
Iz_5_1109	DJI_0193	Oenothera biennis L.	0	a	0	0	Onagraceae Juss.
Iz_5_1109	DJI_0193	Pentanema britannica (L.) D.Gut.Larr., Santos-Vicente, Anderb., E.Rico & M.M.Mart.Ort.	0	n	1	0	Asteraceae Bercht. & J.Presl
Iz_5_1109	DJI_0193	Peucedanum oreoselinum (L.) Moench	0	n	1	0	Apiaceae Lindl.
Iz_5_1109	DJI_0193	Peucedanum ruthenicum M.Bieb.	0	n	1	0	Apiaceae Lindl.
Iz_5_1109	DJI_0193	Pilosella echioides (Lumn.) F.W.Schultz & Sch.Bip.	0	n	1	0	Asteraceae Bercht. & J.Presl
Iz_5_1109	DJI_0193	Pilosella officinarum F. Schultz et Sch. Bip.	0	n	1	0	Asteraceae Bercht. & J.Presl
Iz_5_1109	DJI_0193	Pilosella sp.	0		NA	0	Asteraceae Bercht. & J.Presl
Iz_5_1109	DJI_0193	Pinus sylvestris L.	0	n	1	1	Pinaceae Spreng. ex F.Rudolphi
Iz_5_1109	DJI_0193	Poa annua L.	0	n	1	0	Poaceae Barnhart
Iz_5_1109	DJI_0193	Poa nemoralis L.	0	n	1	0	Poaceae Barnhart
Iz_5_1109	DJI_0193	Poa sp.	0		NA	0	Poaceae Barnhart
Iz_5_1109	DJI_0193	Polygonatum odoratum (Mill.) Druce	0	n	1	0	Asparagaceae Juss.
Iz_5_1109	DJI_0193	Polygonum arenarium Waldst. & Kit.	0	n	1	0	Polygonaceae Juss.
Iz_5_1109	DJI_0193	Populus nigra L.	0	n	1	1	Salicaceae Mirb.
Iz_5_1109	DJI_0193	Populus tremula L.	0	n	1	1	Salicaceae Mirb.
Iz_5_1109	DJI_0193	Populus Ã canescens (Aiton) Sm.	0	n	1	1	Salicaceae Mirb.
Iz_5_1109	DJI_0193	Potentilla argentea L.	0	n	1	0	Rosaceae Juss.
Iz_5_1109	DJI_0193	Psammophiliella muralis (L.) Ikonn.	0	n	1	0	Caryophyllaceae Juss.
Iz_5_1109	DJI_0193	Psephellus sumensis (Kalen.) Greuter	0	n	1	0	Asteraceae Bercht. & J.Presl
Iz_5_1109	DJI_0193	Pulsatilla pratensis (L.) Mill. s.l.	0	n	1	0	Ranunculaceae Juss.
Iz_5_1109	DJI_0193	Pyrus communis L.	0	a	0	1	Rosaceae Juss.
Iz_5_1109	DJI_0193	Quercus robur L.	0	n	1	1	Fagaceae Dumort.
Iz_5_1109	DJI_0193	Robinia pseudoacacia L.	0	a	0	1	Fabaceae Lindl.
Iz_5_1109	DJI_0193	Rosa sp.	0		NA	1	Rosaceae Juss.
Iz_5_1109	DJI_0193	Rubus idaeus L.	0	n	1	0	Rosaceae Juss.
Iz_5_1109	DJI_0193	Rumex acetosella L.	0	n	1	0	Polygonaceae Juss.
Iz_5_1109	DJI_0193	Rumex thyrsiflorus Fingerh.	0	n	1	0	Polygonaceae Juss.
Iz_5_1109	DJI_0193	Salix alba L.	0	n	1	1	Salicaceae Mirb.
Iz_5_1109	DJI_0193	Sambucus nigra L.	0	n	1	1	Viburnaceae Raf.
Iz_5_1109	DJI_0193	Secale sylvestre Host	0	n	1	0	Poaceae Barnhart
Iz_5_1109	DJI_0193	Sempervivum ruthenicum Schnittsp. & C.B. Lehm.	0	n	1	0	Crassulaceae J.St.-Hil.
Iz_5_1109	DJI_0193	Setaria viridis (L.) P. Beauv.	0	n	1	0	Poaceae Barnhart
Iz_5_1109	DJI_0193	Silene borysthenica (Gruner) Walters	0	n	1	0	Caryophyllaceae Juss.
Iz_5_1109	DJI_0193	Silene latifolia subsp. alba (Mill.) Greuter & Burdet	0	n	1	0	Caryophyllaceae Juss.
Iz_5_1109	DJI_0193	Solidago virgaurea L.	0	n	1	0	Asteraceae Bercht. & J.Presl
Iz_5_1109	DJI_0193	Sorbus aucuparia L.	0	n	1	1	Rosaceae Juss.
Iz_5_1109	DJI_0193	Stachys recta L.	0	n	1	0	Lamiacea Lindl.
Iz_5_1109	DJI_0193	Tanacetum vulgare L.	0	n	1	0	Asteraceae Bercht. & J.Presl
Iz_5_1109	DJI_0193	Thymus pallasianus Heinr.Braun	0	n	1	0	Lamiacea Lindl.
Iz_5_1109	DJI_0193	Tilia cordata Mill.	0	n	1	1	Malvaceae Juss.
Iz_5_1109	DJI_0193	Trifolium alpestre L.	0	n	1	0	Fabaceae Lindl.
Iz_5_1109	DJI_0193	Trifolium arvense L.	0	n	1	0	Fabaceae Lindl.
Iz_5_1109	DJI_0193	Ulmus glabra Huds.	0	n	1	1	Ulmaceae Mirb.
Iz_5_1109	DJI_0193	Verbascum cf. phlomoides L.	0		NA	NA	
Iz_5_1109	DJI_0193	Verbascum densiflorum Bertol.	0	n	1	0	Scrophulariaceae Juss.
Iz_5_1109	DJI_0193	Verbascum sp.	0	n	1	0	Scrophulariaceae Juss.
Iz_5_1109	DJI_0193	Veronica austriaca L.	0	n	1	0	Plantaginaceae Juss.
Iz_5_1109	DJI_0193	Veronica incana L.	0	n	1	0	Plantaginaceae Juss.
Iz_5_1109	DJI_0193	Veronica verna L.	0	n	1	0	Plantaginaceae Juss.
Iz_5_1109	DJI_0193	Vincetoxicum hirundinaria Medik	0	n	1	0	Apocynaceae Juss.
Iz_5_1109	DJI_0195	Acer tataricum L.	0	n	1	1	Sapindaceae Juss.
Iz_5_1109	DJI_0195	Achillea millefolium L. s.l.	0	n	1	0	Asteraceae Bercht. & J.Presl
Iz_5_1109	DJI_0195	Allium oleraceum L.	0	n	1	0	Amaryllidaceae J.St.-Hil.

Iz_5_1109	DJI_0195	Ambrosia artemisiifolia L.	0	a	0	0	Asteraceae Bercht. & J.Presl
Iz_5_1109	DJI_0195	Amorpha fruticosa L.	0	a	0	0	Fabaceae Lindl.
Iz_5_1109	DJI_0195	Anthericum ramosum L.	0	n	1	0	Asparagaceae Juss.
Iz_5_1109	DJI_0195	Anthoxanthum repens (Host) Veldkamp	0		NA	NA	
Iz_5_1109	DJI_0195	Artemisia marschalliana Spreng.	0	n	1	0	Asteraceae Bercht. & J.Presl
Iz_5_1109	DJI_0195	Asparagus officinalis L.	0	n	1	0	Asparagaceae Juss.
Iz_5_1109	DJI_0195	Astragalus cicer L.	0	n	1	0	Fabaceae Lindl.
Iz_5_1109	DJI_0195	Bassia laniflora (S.G.Gmel.) A.J.Scott	0	n	1	0	Amaranthaceae Juss.
Iz_5_1109	DJI_0195	Berberis vulgaris L.	0	n	1	0	Berberidaceae Juss.
Iz_5_1109	DJI_0195	Berteroa incana (L.) DC.	0		NA	NA	
Iz_5_1109	DJI_0195	Betula pendula Roth.	0	n	1	1	Betulaceae Gray
Iz_5_1109	DJI_0195	Betula pendula Roth.	0	n	1	0	Poaceae Barnhart
Iz_5_1109	DJI_0195	Bromus inermis Leyss.	0		NA	NA	
Iz_5_1109	DJI_0195	Bromus squarrosus L.	0	a	0	0	Poaceae Barnhart
Iz_5_1109	DJI_0195	Bromus tectorum L.	0	a	0	0	Poaceae Barnhart
Iz_5_1109	DJI_0195	Calamagrostis epigejos (L.) Roth	0	n	1	0	Poaceae Barnhart
Iz_5_1109	DJI_0195	Carduus acanthoides L.	0	n	1	0	Asteraceae Bercht. & J.Presl
Iz_5_1109	DJI_0195	Carex sp.	0	n	1	0	Cyperaceae Juss.
Iz_5_1109	DJI_0195	Centaurea majorovii Dumbadze	0	n	1	0	Asteraceae Bercht. & J.Presl
Iz_5_1109	DJI_0195	Chamaecytisus ruthenicus (Fisch.ex Wol.) Klaskova	0	n	1	0	Fabaceae Lindl.
Iz_5_1109	DJI_0195	Chelidonium majus L.	0	n	1	0	Papaveraceae Juss.
Iz_5_1109	DJI_0195	Chenopodium betaceum Andrz.	0	n	1	0	Amaranthaceae Juss.
Iz_5_1109	DJI_0195	Chenopodium cf. album	0	n	1	0	Amaranthaceae Juss.
Iz_5_1109	DJI_0195	Chenopodium sp.	0		NA	0	Amaranthaceae Juss.
Iz_5_1109	DJI_0195	Chondrilla juncea L.	0	n	1	0	Asteraceae Bercht. & J.Presl
Iz_5_1109	DJI_0195	Cirsium vulgare (Savi) Ten.	0	n	1	0	Asteraceae Bercht. & J.Presl
Iz_5_1109	DJI_0195	Clinopodium acinos (L.) Kuntze	0	n	1	0	Lamiacea Lindl.
Iz_5_1109	DJI_0195	Convallaria majalis L.	0	n	1	0	Asparagaceae Juss.
Iz_5_1109	DJI_0195	Crataegus rhipidophylla Gand.	0	n	1	1	Rosaceae Juss.
Iz_5_1109	DJI_0195	Crepis foetida L.	0	n	1	0	Asteraceae Bercht. & J.Presl
Iz_5_1109	DJI_0195	Dactylis glomerata L.	0	n	1	0	Poaceae Barnhart
Iz_5_1109	DJI_0195	Dianthus campestris M.Bieb.	0	n	1	0	Caryophyllaceae Juss.
Iz_5_1109	DJI_0195	Digitaria sanguinalis (L.) Scop.	0	a	0	0	Poaceae Barnhart
Iz_5_1109	DJI_0195	Elymus repens (L.) Gould	0	n	1	0	Poaceae Barnhart
Iz_5_1109	DJI_0195	Epilobium tetragonum L.	0	n	1	0	Onagraceae Juss.
Iz_5_1109	DJI_0195	Eragrostis minor Host	0	a	0	0	Poaceae Barnhart
Iz_5_1109	DJI_0195	Erigeron canadensis L.	3	a	0	0	Asteraceae Bercht. & J.Presl
Iz_5_1109	DJI_0195	Euphorbia seguieriana Neck.	0	n	1	0	Euphorbiaceae Juss.
Iz_5_1109	DJI_0195	Fallopia dumetorum (L.) Holub	0	n	1	0	Polygonaceae Juss.
Iz_5_1109	DJI_0195	Festuca beckeri (Hack.) Trautv.	0	n	1	0	Poaceae Barnhart
Iz_5_1109	DJI_0195	Fragaria vesca L.	0	n	1	0	Rosaceae Juss.
Iz_5_1109	DJI_0195	Frangula alnus Mill.	0	n	1	1	Rhamnaceae Juss.
Iz_5_1109	DJI_0195	Fraxinus pennsylvanica Marshall	0	a	0	1	Oleaceae Hoffmanns. & Link
Iz_5_1109	DJI_0195	Galeopsis bifida Boenn.	0	n	1	0	Lamiacea Lindl.
Iz_5_1109	DJI_0195	Galium verum L.	0	n	1	0	Rubiaceae Juss.
Iz_5_1109	DJI_0195	Genista tinctoria L.	0	n	1	0	Fabaceae Lindl.
Iz_5_1109	DJI_0195	Geranium robertianum L.	0	n	1	0	Geraniaceae Juss.
Iz_5_1109	DJI_0195	Geranium sanquineum L.	0	n	1	0	Geraniaceae Juss.
Iz_5_1109	DJI_0195	Gypsophila paniculata L.	0	n	1	0	Caryophyllaceae Juss.
Iz_5_1109	DJI_0195	Helichrysum arenarium (L.) Moench	0	n	1	0	Asteraceae Bercht. & J.Presl
Iz_5_1109	DJI_0195	Herniaria polygama J.Gay	0	n	1	0	Caryophyllaceae Juss.
Iz_5_1109	DJI_0195	Hieracium robustum Fr.	0	n	1	0	Asteraceae Bercht. & J.Presl
Iz_5_1109	DJI_0195	Hieracium umbellatum L.	0	n	1	NA	Asteraceae Bercht. & J.Presl
Iz_5_1109	DJI_0195	Hylotelephium maximum (L.) Holub	0	n	1	0	Crassulaceae J.St.-Hil.
Iz_5_1109	DJI_0195	Jacobaea borysthenica (DC.) B.Nord. & Greuter	0	n	1	0	Asteraceae Bercht. & J.Presl
Iz_5_1109	DJI_0195	Jasione montana L.	0	n	1	0	Campanulaceae Juss.
Iz_5_1109	DJI_0195	Jurinea cyanoides (L.) Rchb.s.l.	0	n	1	0	Asteraceae Bercht. & J.Presl
Iz_5_1109	DJI_0195	Koeleria glauca (Spreng.) DC.	0	n	1	0	Poaceae Barnhart
Iz_5_1109	DJI_0195	Lactuca serriola L.	0	a	0	0	Asteraceae Bercht. & J.Presl
Iz_5_1109	DJI_0195	Linaria genistifolia (L.) Mill.	0	n	1	0	Plantaginaceae Juss.
Iz_5_1109	DJI_0195	Lomelosia argentea (L.) Greuter & Burdet	0	n	1	0	Caprifoliaceae Juss.
Iz_5_1109	DJI_0195	Melampyrum pratense L.	0	n	1	0	Orobanchaceae Vent.
Iz_5_1109	DJI_0195	Oenothera biennis L.	0	a	0	0	Onagraceae Juss.
Iz_5_1109	DJI_0195	Pentanema britannica (L.) D.Gut.Larr., Santos-Vicente, Anderb., E.Rico & M.M.Mart.Ort.	0	n	1	0	Asteraceae Bercht. & J.Presl
Iz_5_1109	DJI_0195	Peucedanum oreoselinum (L.) Moench	0	n	1	0	Apiaceae Lindl.
Iz_5_1109	DJI_0195	Peucedanum ruthenicum M.Bieb.	0	n	1	0	Apiaceae Lindl.
Iz_5_1109	DJI_0195	Pilosella echioides (Lumn.) F.W.Schultz & Sch.Bip.	0	n	1	0	Asteraceae Bercht. & J.Presl
Iz_5_1109	DJI_0195	Pilosella officinarum F. Schultz et Sch. Bip.	0	n	1	0	Asteraceae Bercht. & J.Presl
Iz_5_1109	DJI_0195	Pilosella sp.	0		NA	0	Asteraceae Bercht. & J.Presl
Iz_5_1109	DJI_0195	Pinus sylvestris L.	0	n	1	1	Pinaceae Spreng. ex F.Rudolphi
Iz_5_1109	DJI_0195	Poa annua L.	0	n	1	0	Poaceae Barnhart
Iz_5_1109	DJI_0195	Poa nemoralis L.	0	n	1	0	Poaceae Barnhart
Iz_5_1109	DJI_0195	Poa sp.	0		NA	0	Poaceae Barnhart
Iz_5_1109	DJI_0195	Polygonatum odoratum (Mill.) Druce	0	n	1	0	Asparagaceae Juss.
Iz_5_1109	DJI_0195	Polygonum arenarium Waldst. & Kit.	0	n	1	0	Polygonaceae Juss.
Iz_5_1109	DJI_0195	Populus nigra L.	0	n	1	1	Salicaceae Mirb.
Iz_5_1109	DJI_0195	Populus tremula L.	0	n	1	1	Salicaceae Mirb.
Iz_5_1109	DJI_0195	Populus Ã canescens (Aiton) Sm.	0	n	1	1	Salicaceae Mirb.
Iz_5_1109	DJI_0195	Potentilla argentea L.	0	n	1	0	Rosaceae Juss.
Iz_5_1109	DJI_0195	Psammophiliella muralis (L.) Ikonn.	0	n	1	0	Caryophyllaceae Juss.
Iz_5_1109	DJI_0195	Psephellus sumensis (Kalen.) Greuter	0	n	1	0	Asteraceae Bercht. & J.Presl
Iz_5_1109	DJI_0195	Pulsatilla pratensis (L.) Mill. s.l.	0	n	1	0	Ranunculaceae Juss.
Iz_5_1109	DJI_0195	Pyrus communis L.	0	a	0	1	Rosaceae Juss.
Iz_5_1109	DJI_0195	Quercus robur L.	0	n	1	1	Fagaceae Dumort.
Iz_5_1109	DJI_0195	Robinia pseudoacacia L.	0	a	0	1	Fabaceae Lindl.
Iz_5_1109	DJI_0195	Rosa sp.	0		NA	1	Rosaceae Juss.
Iz_5_1109	DJI_0195	Rubus idaeus L.	0	n	1	0	Rosaceae Juss.
Iz_5_1109	DJI_0195	Rumex acetosella L.	0	n	1	0	Polygonaceae Juss.
Iz_5_1109	DJI_0195	Rumex thyrsiflorus Fingerh.	0	n	1	0	Polygonaceae Juss.
Iz_5_1109	DJI_0195	Salix alba L.	0	n	1	1	Salicaceae Mirb.
Iz_5_1109	DJI_0195	Sambucus nigra L.	0	n	1	1	Viburnaceae Raf.
Iz_5_1109	DJI_0195	Secale sylvestre Host	0	n	1	0	Poaceae Barnhart
Iz_5_1109	DJI_0195	Sempervivum ruthenicum Schnittsp. & C.B. Lehm.	0	n	1	0	Crassulaceae J.St.-Hil.
Iz_5_1109	DJI_0195	Setaria viridis (L.) P. Beauv.	0	n	1	0	Poaceae Barnhart
Iz_5_1109	DJI_0195	Silene borysthenica (Gruner) Walters	0	n	1	0	Caryophyllaceae Juss.
Iz_5_1109	DJI_0195	Silene latifolia subsp. alba (Mill.) Greuter & Burdet	0	n	1	0	Caryophyllaceae Juss.
Iz_5_1109	DJI_0195	Solidago virgaurea L.	0	n	1	0	Asteraceae Bercht. & J.Presl
Iz_5_1109	DJI_0195	Sorbus aucuparia L.	0	n	1	1	Rosaceae Juss.

Iz_5_1109	DJI_0195	Stachys recta L.	0	n	1	0	Lamiacea Lindl.
Iz_5_1109	DJI_0195	Tanacetum vulgare L.	0	n	1	0	Asteraceae Bercht. & J.Presl
Iz_5_1109	DJI_0195	Thymus pallasianus Heinr.Braun	0	n	1	0	Lamiacea Lindl.
Iz_5_1109	DJI_0195	Tilia cordata Mill.	0	n	1	1	Malvaceae Juss.
Iz_5_1109	DJI_0195	Trifolium alpestre L.	0	n	1	0	Fabaceae Lindl.
Iz_5_1109	DJI_0195	Trifolium arvense L.	0	n	1	0	Fabaceae Lindl.
Iz_5_1109	DJI_0195	Ulmus glabra Huds.	0	n	1	1	Ulmaceae Mirb.
Iz_5_1109	DJI_0195	Verbascum cf. phlomoides L.	0		NA	NA	
Iz_5_1109	DJI_0195	Verbascum densiflorum Bertol.	0	n	1	0	Scrophulariaceae Juss.
Iz_5_1109	DJI_0195	Verbascum sp.	0	n	1	0	Scrophulariaceae Juss.
Iz_5_1109	DJI_0195	Veronica austriaca L.	0	n	1	0	Plantaginaceae Juss.
Iz_5_1109	DJI_0195	Veronica incana L.	0	n	1	0	Plantaginaceae Juss.
Iz_5_1109	DJI_0195	Veronica verna L.	0	n	1	0	Plantaginaceae Juss.
Iz_5_1109	DJI_0195	Vincetoxicum hirundinaria Medik	0	n	1	0	Apocynaceae Juss.
Iz_5_1109	DJI_0199	Acer tataricum L.	0	n	1	1	Sapindaceae Juss.
Iz_5_1109	DJI_0199	Achillea millefolium L. s.l.	0	n	1	0	Asteraceae Bercht. & J.Presl
Iz_5_1109	DJI_0199	Allium oleraceum L.	0	n	1	0	Amaryllidaceae J.St.-Hil.
Iz_5_1109	DJI_0199	Ambrosia artemisiifolia L.	0	a	0	0	Asteraceae Bercht. & J.Presl
Iz_5_1109	DJI_0199	Amorpha fruticosa L.	0	a	0	0	Fabaceae Lindl.
Iz_5_1109	DJI_0199	Anthericum ramosum L.	0	n	1	0	Asparagaceae Juss.
Iz_5_1109	DJI_0199	Anthoxanthum repens (Host) Veldkamp	0		NA	NA	
Iz_5_1109	DJI_0199	Artemisia marschalliana Spreng.	0	n	1	0	Asteraceae Bercht. & J.Presl
Iz_5_1109	DJI_0199	Asparagus officinalis L.	0	n	1	0	Asparagaceae Juss.
Iz_5_1109	DJI_0199	Astragalus cicer L.	0	n	1	0	Fabaceae Lindl.
Iz_5_1109	DJI_0199	Bassia laniflora (S.G.Gmel.) A.J.Scott	0	n	1	0	Amaranthaceae Juss.
Iz_5_1109	DJI_0199	Berberis vulgaris L.	0	n	1	0	Berberidaceae Juss.
Iz_5_1109	DJI_0199	Berteroa incana (L.) DC.	0		NA	NA	
Iz_5_1109	DJI_0199	Betula pendula Roth.	0	n	1	1	Betulaceae Gray
Iz_5_1109	DJI_0199	Betula pendula Roth.	0	n	1	0	Poaceae Barnhart
Iz_5_1109	DJI_0199	Bromus inermis Leyss.	0		NA	NA	
Iz_5_1109	DJI_0199	Bromus squarrosus L.	0	a	0	0	Poaceae Barnhart
Iz_5_1109	DJI_0199	Bromus tectorum L.	0	a	0	0	Poaceae Barnhart
Iz_5_1109	DJI_0199	Calamagrostis epigejos (L.) Roth	5	n	1	0	Poaceae Barnhart
Iz_5_1109	DJI_0199	Carduus acanthoides L.	0	n	1	0	Asteraceae Bercht. & J.Presl
Iz_5_1109	DJI_0199	Carex sp.	0	n	1	0	Cyperaceae Juss.
Iz_5_1109	DJI_0199	Centaurea majorovii Dumbadze	0	n	1	0	Asteraceae Bercht. & J.Presl
Iz_5_1109	DJI_0199	Chamaecytisus ruthenicus (Fisch.ex Wol.) Klaskova	0	n	1	0	Fabaceae Lindl.
Iz_5_1109	DJI_0199	Chelidonium majus L.	1	n	1	0	Papaveraceae Juss.
Iz_5_1109	DJI_0199	Chenopodium betaceum Andrz.	0	n	1	0	Amaranthaceae Juss.
Iz_5_1109	DJI_0199	Chenopodium cf. album	0	n	1	0	Amaranthaceae Juss.
Iz_5_1109	DJI_0199	Chenopodium sp.	0		NA	0	Amaranthaceae Juss.
Iz_5_1109	DJI_0199	Chondrilla juncea L.	0	n	1	0	Asteraceae Bercht. & J.Presl
Iz_5_1109	DJI_0199	Cirsium vulgare (Savi) Ten.	0	n	1	0	Asteraceae Bercht. & J.Presl
Iz_5_1109	DJI_0199	Clinopodium acinos (L.) Kuntze	0	n	1	0	Lamiacea Lindl.
Iz_5_1109	DJI_0199	Convallaria majalis L.	0	n	1	0	Asparagaceae Juss.
Iz_5_1109	DJI_0199	Crataegus rhipidophylla Gand.	0	n	1	1	Rosaceae Juss.
Iz_5_1109	DJI_0199	Crepis foetida L.	0	n	1	0	Asteraceae Bercht. & J.Presl
Iz_5_1109	DJI_0199	Dactylis glomerata L.	0	n	1	0	Poaceae Barnhart
Iz_5_1109	DJI_0199	Dianthus campestris M.Bieb.	0	n	1	0	Caryophyllaceae Juss.
Iz_5_1109	DJI_0199	Digitaria sanguinalis (L.) Scop.	0	a	0	0	Poaceae Barnhart
Iz_5_1109	DJI_0199	Elymus repens (L.) Gould	0	n	1	0	Poaceae Barnhart
Iz_5_1109	DJI_0199	Epilobium tetragonum L.	0	n	1	0	Onagraceae Juss.
Iz_5_1109	DJI_0199	Eragrostis minor Host	0	a	0	0	Poaceae Barnhart
Iz_5_1109	DJI_0199	Erigeron canadensis L.	2	a	0	0	Asteraceae Bercht. & J.Presl
Iz_5_1109	DJI_0199	Euphorbia seguieriana Neck.	0	n	1	0	Euphorbiaceae Juss.
Iz_5_1109	DJI_0199	Fallopia dumetorum (L.) Holub	0	n	1	0	Polygonaceae Juss.
Iz_5_1109	DJI_0199	Festuca beckeri (Hack.) Trautv.	0	n	1	0	Poaceae Barnhart
Iz_5_1109	DJI_0199	Fragaria vesca L.	0	n	1	0	Rosaceae Juss.
Iz_5_1109	DJI_0199	Frangula alnus Mill.	0	n	1	1	Rhamnaceae Juss.
Iz_5_1109	DJI_0199	Fraxinus pennsylvanica Marshall	0	a	0	1	Oleaceae Hoffmanns. & Link
Iz_5_1109	DJI_0199	Galeopsis bifida Boenn.	0	n	1	0	Lamiacea Lindl.
Iz_5_1109	DJI_0199	Galium verum L.	0	n	1	0	Rubiaceae Juss.
Iz_5_1109	DJI_0199	Genista tinctoria L.	0	n	1	0	Fabaceae Lindl.
Iz_5_1109	DJI_0199	Geranium robertianum L.	0	n	1	0	Geraniaceae Juss.
Iz_5_1109	DJI_0199	Geranium sanquineum L.	0	n	1	0	Geraniaceae Juss.
Iz_5_1109	DJI_0199	Gypsophila paniculata L.	0	n	1	0	Caryophyllaceae Juss.
Iz_5_1109	DJI_0199	Helichrysum arenarium (L.) Moench	0	n	1	0	Asteraceae Bercht. & J.Presl
Iz_5_1109	DJI_0199	Herniaria polygama J.Gay	0	n	1	0	Caryophyllaceae Juss.
Iz_5_1109	DJI_0199	Hieracium robustum Fr.	0	n	1	0	Asteraceae Bercht. & J.Presl
Iz_5_1109	DJI_0199	Hieracium umbellatum L.	0	n	1	NA	Asteraceae Bercht. & J.Presl
Iz_5_1109	DJI_0199	Hylotelephium maximum (L.) Holub	0	n	1	0	Crassulaceae J.St.-Hil.
Iz_5_1109	DJI_0199	Jacobaea borysthenica (DC.) B.Nord. & Greuter	0	n	1	0	Asteraceae Bercht. & J.Presl
Iz_5_1109	DJI_0199	Jasione montana L.	0	n	1	0	Campanulaceae Juss.
Iz_5_1109	DJI_0199	Jurinea cyanoides (L.) Rchb.s.l.	0	n	1	0	Asteraceae Bercht. & J.Presl
Iz_5_1109	DJI_0199	Koeleria glauca (Spreng.) DC.	0	n	1	0	Poaceae Barnhart
Iz_5_1109	DJI_0199	Lactuca serriola L.	0	a	0	0	Asteraceae Bercht. & J.Presl
Iz_5_1109	DJI_0199	Linaria genistifolia (L.) Mill.	0	n	1	0	Plantaginaceae Juss.
Iz_5_1109	DJI_0199	Lomelosia argentea (L.) Greuter & Burdet	0	n	1	0	Caprifoliaceae Juss.
Iz_5_1109	DJI_0199	Melampyrum pratense L.	0	n	1	0	Orobanchaceae Vent.
Iz_5_1109	DJI_0199	Oenothera biennis L.	0	a	0	0	Onagraceae Juss.
Iz_5_1109	DJI_0199	Pentanema britannica (L.) D.Gut.Larr., Santos-Vicente, Anderb., E.Rico & M.M.Mart.Ort.	0	n	1	0	Asteraceae Bercht. & J.Presl
Iz_5_1109	DJI_0199	Peucedanum oreoselinum (L.) Moench	0	n	1	0	Apiaceae Lindl.
Iz_5_1109	DJI_0199	Peucedanum ruthenicum M.Bieb.	0	n	1	0	Apiaceae Lindl.
Iz_5_1109	DJI_0199	Pilosella echioides (Lumn.) F.W.Schultz & Sch.Bip.	0	n	1	0	Asteraceae Bercht. & J.Presl
Iz_5_1109	DJI_0199	Pilosella officinarum F. Schultz et Sch. Bip.	0	n	1	0	Asteraceae Bercht. & J.Presl
Iz_5_1109	DJI_0199	Pilosella sp.	0		NA	0	Asteraceae Bercht. & J.Presl
Iz_5_1109	DJI_0199	Pinus sylvestris L.	0	n	1	1	Pinaceae Spreng. ex F.Rudolphi
Iz_5_1109	DJI_0199	Poa annua L.	0	n	1	0	Poaceae Barnhart
Iz_5_1109	DJI_0199	Poa nemoralis L.	0	n	1	0	Poaceae Barnhart
Iz_5_1109	DJI_0199	Poa sp.	0		NA	0	Poaceae Barnhart
Iz_5_1109	DJI_0199	Polygonatum odoratum (Mill.) Druce	0	n	1	0	Asparagaceae Juss.
Iz_5_1109	DJI_0199	Polygonum arenarium Waldst. & Kit.	0	n	1	0	Polygonaceae Juss.
Iz_5_1109	DJI_0199	Populus nigra L.	0	n	1	1	Salicaceae Mirb.
Iz_5_1109	DJI_0199	Populus tremula L.	0	n	1	1	Salicaceae Mirb.
Iz_5_1109	DJI_0199	Populus Ã canescens (Aiton) Sm.	0	n	1	1	Salicaceae Mirb.
Iz_5_1109	DJI_0199	Potentilla argentea L.	0	n	1	0	Rosaceae Juss.
Iz_5_1109	DJI_0199	Psammophiliella muralis (L.) Ikonn.	0	n	1	0	Caryophyllaceae Juss.
Iz_5_1109	DJI_0199	Psephellus sumensis (Kalen.) Greuter	0	n	1	0	Asteraceae Bercht. & J.Presl

Iz_5_1109	DJI_0199	Pulsatilla pratensis (L.) Mill. s.l.	0	n	1	0	Ranunculaceae Juss.
Iz_5_1109	DJI_0199	Pyrus communis L.	0	a	0	1	Rosaceae Juss.
Iz_5_1109	DJI_0199	Quercus robur L.	0	n	1	1	Fagaceae Dumort.
Iz_5_1109	DJI_0199	Robinia pseudoacacia L.	0	a	0	1	Fabaceae Lindl.
Iz_5_1109	DJI_0199	Rosa sp.	0		NA	1	Rosaceae Juss.
Iz_5_1109	DJI_0199	Rubus idaeus L.	0	n	1	0	Rosaceae Juss.
Iz_5_1109	DJI_0199	Rumex acetosella L.	0	n	1	0	Polygonaceae Juss.
Iz_5_1109	DJI_0199	Rumex thyrsiflorus Fingerh.	0	n	1	0	Polygonaceae Juss.
Iz_5_1109	DJI_0199	Salix alba L.	0	n	1	1	Salicaceae Mirb.
Iz_5_1109	DJI_0199	Sambucus nigra L.	0	n	1	1	Viburnaceae Raf.
Iz_5_1109	DJI_0199	Secale sylvestre Host	0	n	1	0	Poaceae Barnhart
Iz_5_1109	DJI_0199	Sempervivum ruthenicum Schnittsp. & C.B. Lehm.	0	n	1	0	Crassulaceae J.St.-Hil.
Iz_5_1109	DJI_0199	Setaria viridis (L.) P. Beauv.	0	n	1	0	Poaceae Barnhart
Iz_5_1109	DJI_0199	Silene borysthenica (Gruner) Walters	0	n	1	0	Caryophyllaceae Juss.
Iz_5_1109	DJI_0199	Silene latifolia subsp. alba (Mill.) Greuter & Burdet	0	n	1	0	Caryophyllaceae Juss.
Iz_5_1109	DJI_0199	Solidago virgaurea L.	0	n	1	0	Asteraceae Bercht. & J.Presl
Iz_5_1109	DJI_0199	Sorbus aucuparia L.	0	n	1	1	Rosaceae Juss.
Iz_5_1109	DJI_0199	Stachys recta L.	0	n	1	0	Lamiacea Lindl.
Iz_5_1109	DJI_0199	Tanacetum vulgare L.	0	n	1	0	Asteraceae Bercht. & J.Presl
Iz_5_1109	DJI_0199	Thymus pallasianus Heinr.Braun	0	n	1	0	Lamiacea Lindl.
Iz_5_1109	DJI_0199	Tilia cordata Mill.	0	n	1	1	Malvaceae Juss.
Iz_5_1109	DJI_0199	Trifolium alpestre L.	0	n	1	0	Fabaceae Lindl.
Iz_5_1109	DJI_0199	Trifolium arvense L.	0	n	1	0	Fabaceae Lindl.
Iz_5_1109	DJI_0199	Ulmus glabra Huds.	0	n	1	1	Ulmaceae Mirb.
Iz_5_1109	DJI_0199	Verbascum cf. phlomoides L.	0		NA	NA	
Iz_5_1109	DJI_0199	Verbascum densiflorum Bertol.	0	n	1	0	Scrophulariaceae Juss.
Iz_5_1109	DJI_0199	Verbascum sp.	0	n	1	0	Scrophulariaceae Juss.
Iz_5_1109	DJI_0199	Veronica austriaca L.	0	n	1	0	Plantaginaceae Juss.
Iz_5_1109	DJI_0199	Veronica incana L.	0	n	1	0	Plantaginaceae Juss.
Iz_5_1109	DJI_0199	Veronica verna L.	0	n	1	0	Plantaginaceae Juss.
Iz_5_1109	DJI_0199	Vincetoxicum hirundinaria Medik	0	n	1	0	Apocynaceae Juss.
Iz_5_1109	DJI_0204	Acer tataricum L.	0	n	1	1	Sapindaceae Juss.
Iz_5_1109	DJI_0204	Achillea millefolium L. s.l.	0	n	1	0	Asteraceae Bercht. & J.Presl
Iz_5_1109	DJI_0204	Allium oleraceum L.	0	n	1	0	Amaryllidaceae J.St.-Hil.
Iz_5_1109	DJI_0204	Ambrosia artemisiifolia L.	0	a	0	0	Asteraceae Bercht. & J.Presl
Iz_5_1109	DJI_0204	Amorpha fruticosa L.	0	a	0	0	Fabaceae Lindl.
Iz_5_1109	DJI_0204	Anthericum ramosum L.	0	n	1	0	Asparagaceae Juss.
Iz_5_1109	DJI_0204	Anthoxanthum repens (Host) Veldkamp	0		NA	NA	
Iz_5_1109	DJI_0204	Artemisia marschalliana Spreng.	0	n	1	0	Asteraceae Bercht. & J.Presl
Iz_5_1109	DJI_0204	Asparagus officinalis L.	0	n	1	0	Asparagaceae Juss.
Iz_5_1109	DJI_0204	Astragalus cicer L.	0	n	1	0	Fabaceae Lindl.
Iz_5_1109	DJI_0204	Bassia laniflora (S.G.Gmel.) A.J.Scott	0	n	1	0	Amaranthaceae Juss.
Iz_5_1109	DJI_0204	Berberis vulgaris L.	0	n	1	0	Berberidaceae Juss.
Iz_5_1109	DJI_0204	Berteroa incana (L.) DC.	0		NA	NA	
Iz_5_1109	DJI_0204	Betula pendula Roth.	0	n	1	1	Betulaceae Gray
Iz_5_1109	DJI_0204	Betula pendula Roth.	0	n	1	0	Poaceae Barnhart
Iz_5_1109	DJI_0204	Bromus inermis Leyss.	0		NA	NA	
Iz_5_1109	DJI_0204	Bromus squarrosus L.	0	a	0	0	Poaceae Barnhart
Iz_5_1109	DJI_0204	Bromus tectorum L.	0	a	0	0	Poaceae Barnhart
Iz_5_1109	DJI_0204	Calamagrostis epigejos (L.) Roth	3	n	1	0	Poaceae Barnhart
Iz_5_1109	DJI_0204	Carduus acanthoides L.	0	n	1	0	Asteraceae Bercht. & J.Presl
Iz_5_1109	DJI_0204	Carex sp.	0	n	1	0	Cyperaceae Juss.
Iz_5_1109	DJI_0204	Centaurea majorovii Dumbadze	0	n	1	0	Asteraceae Bercht. & J.Presl
Iz_5_1109	DJI_0204	Chamaecytisus ruthenicus (Fisch.ex Wol.) Klaskova	0	n	1	0	Fabaceae Lindl.
Iz_5_1109	DJI_0204	Chelidonium majus L.	0.5	n	1	0	Papaveraceae Juss.
Iz_5_1109	DJI_0204	Chenopodium betaceum Andrz.	0	n	1	0	Amaranthaceae Juss.
Iz_5_1109	DJI_0204	Chenopodium cf. album	0	n	1	0	Amaranthaceae Juss.
Iz_5_1109	DJI_0204	Chenopodium sp.	0		NA	0	Amaranthaceae Juss.
Iz_5_1109	DJI_0204	Chondrilla juncea L.	0	n	1	0	Asteraceae Bercht. & J.Presl
Iz_5_1109	DJI_0204	Cirsium vulgare (Savi) Ten.	0	n	1	0	Asteraceae Bercht. & J.Presl
Iz_5_1109	DJI_0204	Clinopodium acinos (L.) Kuntze	0	n	1	0	Lamiacea Lindl.
Iz_5_1109	DJI_0204	Convallaria majalis L.	0	n	1	0	Asparagaceae Juss.
Iz_5_1109	DJI_0204	Crataegus rhipidophylla Gand.	0	n	1	1	Rosaceae Juss.
Iz_5_1109	DJI_0204	Crepis foetida L.	0	n	1	0	Asteraceae Bercht. & J.Presl
Iz_5_1109	DJI_0204	Dactylis glomerata L.	0	n	1	0	Poaceae Barnhart
Iz_5_1109	DJI_0204	Dianthus campestris M.Bieb.	0	n	1	0	Caryophyllaceae Juss.
Iz_5_1109	DJI_0204	Digitaria sanguinalis (L.) Scop.	0	a	0	0	Poaceae Barnhart
Iz_5_1109	DJI_0204	Elymus repens (L.) Gould	0	n	1	0	Poaceae Barnhart
Iz_5_1109	DJI_0204	Epilobium tetragonum L.	0	n	1	0	Onagraceae Juss.
Iz_5_1109	DJI_0204	Eragrostis minor Host	0	a	0	0	Poaceae Barnhart
Iz_5_1109	DJI_0204	Erigeron canadensis L.	3	a	0	0	Asteraceae Bercht. & J.Presl
Iz_5_1109	DJI_0204	Euphorbia seguieriana Neck.	0	n	1	0	Euphorbiaceae Juss.
Iz_5_1109	DJI_0204	Fallopia dumetorum (L.) Holub	0	n	1	0	Polygonaceae Juss.
Iz_5_1109	DJI_0204	Festuca beckeri (Hack.) Trautv.	0	n	1	0	Poaceae Barnhart
Iz_5_1109	DJI_0204	Fragaria vesca L.	0	n	1	0	Rosaceae Juss.
Iz_5_1109	DJI_0204	Frangula alnus Mill.	0	n	1	1	Rhamnaceae Juss.
Iz_5_1109	DJI_0204	Fraxinus pennsylvanica Marshall	0	a	0	1	Oleaceae Hoffmanns. & Link
Iz_5_1109	DJI_0204	Galeopsis bifida Boenn.	0	n	1	0	Lamiacea Lindl.
Iz_5_1109	DJI_0204	Galium verum L.	0	n	1	0	Rubiaceae Juss.
Iz_5_1109	DJI_0204	Genista tinctoria L.	0	n	1	0	Fabaceae Lindl.
Iz_5_1109	DJI_0204	Geranium robertianum L.	0	n	1	0	Geraniaceae Juss.
Iz_5_1109	DJI_0204	Geranium sanquineum L.	0	n	1	0	Geraniaceae Juss.
Iz_5_1109	DJI_0204	Gypsophila paniculata L.	0	n	1	0	Caryophyllaceae Juss.
Iz_5_1109	DJI_0204	Helichrysum arenarium (L.) Moench	0	n	1	0	Asteraceae Bercht. & J.Presl
Iz_5_1109	DJI_0204	Herniaria polygama J.Gay	0	n	1	0	Caryophyllaceae Juss.
Iz_5_1109	DJI_0204	Hieracium robustum Fr.	0	n	1	0	Asteraceae Bercht. & J.Presl
Iz_5_1109	DJI_0204	Hieracium umbellatum L.	0	n	1	NA	Asteraceae Bercht. & J.Presl
Iz_5_1109	DJI_0204	Hylotelephium maximum (L.) Holub	0	n	1	0	Crassulaceae J.St.-Hil.
Iz_5_1109	DJI_0204	Jacobaea borysthenica (DC.) B.Nord. & Greuter	0	n	1	0	Asteraceae Bercht. & J.Presl
Iz_5_1109	DJI_0204	Jasione montana L.	0	n	1	0	Campanulaceae Juss.
Iz_5_1109	DJI_0204	Jurinea cyanoides (L.) Rchb.s.l.	0	n	1	0	Asteraceae Bercht. & J.Presl
Iz_5_1109	DJI_0204	Koeleria glauca (Spreng.) DC.	0	n	1	0	Poaceae Barnhart
Iz_5_1109	DJI_0204	Lactuca serriola L.	0	a	0	0	Asteraceae Bercht. & J.Presl
Iz_5_1109	DJI_0204	Linaria genistifolia (L.) Mill.	0	n	1	0	Plantaginaceae Juss.
Iz_5_1109	DJI_0204	Lomelosia argentea (L.) Greuter & Burdet	0	n	1	0	Caprifoliaceae Juss.
Iz_5_1109	DJI_0204	Melampyrum pratense L.	0	n	1	0	Orobanchaceae Vent.
Iz_5_1109	DJI_0204	Oenothera biennis L.	0	a	0	0	Onagraceae Juss.
Iz_5_1109	DJI_0204	Pentanema britannica (L.) D.Gut.Larr., Santos-Vicente, Anderb., E.Rico & M.M.Mart.Ort.	0	n	1	0	Asteraceae Bercht. & J.Presl

Iz_5_1109	DJI_0204	Peucedanum oreoselinum (L.) Moench	0	n	1	0	Apiaceae Lindl.
Iz_5_1109	DJI_0204	Peucedanum ruthenicum M.Bieb.	0	n	1	0	Apiaceae Lindl.
Iz_5_1109	DJI_0204	Pilosella echioides (Lumn.) F.W.Schultz & Sch.Bip.	0	n	1	0	Asteraceae Bercht. & J.Presl
Iz_5_1109	DJI_0204	Pilosella officinarum F. Schultz et Sch. Bip.	0	n	1	0	Asteraceae Bercht. & J.Presl
Iz_5_1109	DJI_0204	Pilosella sp.	0		NA	0	Asteraceae Bercht. & J.Presl
Iz_5_1109	DJI_0204	Pinus sylvestris L.	0	n	1	1	Pinaceae Spreng. ex F.Rudolphi
Iz_5_1109	DJI_0204	Poa annua L.	0	n	1	0	Poaceae Barnhart
Iz_5_1109	DJI_0204	Poa nemoralis L.	0	n	1	0	Poaceae Barnhart
Iz_5_1109	DJI_0204	Poa sp.	0		NA	0	Poaceae Barnhart
Iz_5_1109	DJI_0204	Polygonatum odoratum (Mill.) Druce	0	n	1	0	Asparagaceae Juss.
Iz_5_1109	DJI_0204	Polygonum arenarium Waldst. & Kit.	0	n	1	0	Polygonaceae Juss.
Iz_5_1109	DJI_0204	Populus nigra L.	0	n	1	1	Salicaceae Mirb.
Iz_5_1109	DJI_0204	Populus tremula L.	0	n	1	1	Salicaceae Mirb.
Iz_5_1109	DJI_0204	Populus Ã canescens (Aiton) Sm.	0	n	1	1	Salicaceae Mirb.
Iz_5_1109	DJI_0204	Potentilla argentea L.	0	n	1	0	Rosaceae Juss.
Iz_5_1109	DJI_0204	Psammophiliella muralis (L.) Ikonn.	0	n	1	0	Caryophyllaceae Juss.
Iz_5_1109	DJI_0204	Psephellus sumensis (Kalen.) Greuter	0	n	1	0	Asteraceae Bercht. & J.Presl
Iz_5_1109	DJI_0204	Pulsatilla pratensis (L.) Mill. s.l.	0	n	1	0	Ranunculaceae Juss.
Iz_5_1109	DJI_0204	Pyrus communis L.	0	a	0	1	Rosaceae Juss.
Iz_5_1109	DJI_0204	Quercus robur L.	0	n	1	1	Fagaceae Dumort.
Iz_5_1109	DJI_0204	Robinia pseudoacacia L.	0	a	0	1	Fabaceae Lindl.
Iz_5_1109	DJI_0204	Rosa sp.	0		NA	1	Rosaceae Juss.
Iz_5_1109	DJI_0204	Rubus idaeus L.	0	n	1	0	Rosaceae Juss.
Iz_5_1109	DJI_0204	Rumex acetosella L.	0	n	1	0	Polygonaceae Juss.
Iz_5_1109	DJI_0204	Rumex thyrsiflorus Fingerh.	0	n	1	0	Polygonaceae Juss.
Iz_5_1109	DJI_0204	Salix alba L.	0	n	1	1	Salicaceae Mirb.
Iz_5_1109	DJI_0204	Sambucus nigra L.	0	n	1	1	Viburnaceae Raf.
Iz_5_1109	DJI_0204	Secale sylvestre Host	0	n	1	0	Poaceae Barnhart
Iz_5_1109	DJI_0204	Sempervivum ruthenicum Schnittsp. & C.B. Lehm.	0	n	1	0	Crassulaceae J.St.-Hil.
Iz_5_1109	DJI_0204	Setaria viridis (L.) P. Beauv.	0	n	1	0	Poaceae Barnhart
Iz_5_1109	DJI_0204	Silene borysthenica (Gruner) Walters	0	n	1	0	Caryophyllaceae Juss.
Iz_5_1109	DJI_0204	Silene latifolia subsp. alba (Mill.) Greuter & Burdet	0	n	1	0	Caryophyllaceae Juss.
Iz_5_1109	DJI_0204	Solidago virgaurea L.	0	n	1	0	Asteraceae Bercht. & J.Presl
Iz_5_1109	DJI_0204	Sorbus aucuparia L.	0	n	1	1	Rosaceae Juss.
Iz_5_1109	DJI_0204	Stachys recta L.	0	n	1	0	Lamiacea Lindl.
Iz_5_1109	DJI_0204	Tanacetum vulgare L.	0	n	1	0	Asteraceae Bercht. & J.Presl
Iz_5_1109	DJI_0204	Thymus pallasianus Heinr.Braun	0	n	1	0	Lamiacea Lindl.
Iz_5_1109	DJI_0204	Tilia cordata Mill.	0	n	1	1	Malvaceae Juss.
Iz_5_1109	DJI_0204	Trifolium alpestre L.	0	n	1	0	Fabaceae Lindl.
Iz_5_1109	DJI_0204	Trifolium arvense L.	0	n	1	0	Fabaceae Lindl.
Iz_5_1109	DJI_0204	Ulmus glabra Huds.	0	n	1	1	Ulmaceae Mirb.
Iz_5_1109	DJI_0204	Verbascum cf. phlomoides L.	0		NA	NA	
Iz_5_1109	DJI_0204	Verbascum densiflorum Bertol.	0	n	1	0	Scrophulariaceae Juss.
Iz_5_1109	DJI_0204	Verbascum sp.	0	n	1	0	Scrophulariaceae Juss.
Iz_5_1109	DJI_0204	Veronica austriaca L.	0	n	1	0	Plantaginaceae Juss.
Iz_5_1109	DJI_0204	Veronica incana L.	0	n	1	0	Plantaginaceae Juss.
Iz_5_1109	DJI_0204	Veronica verna L.	0	n	1	0	Plantaginaceae Juss.
Iz_5_1109	DJI_0204	Vincetoxicum hirundinaria Medik	0	n	1	0	Apocynaceae Juss.
Iz_5_1109	DJI_0207	Acer tataricum L.	0	n	1	1	Sapindaceae Juss.
Iz_5_1109	DJI_0207	Achillea millefolium L. s.l.	0	n	1	0	Asteraceae Bercht. & J.Presl
Iz_5_1109	DJI_0207	Allium oleraceum L.	0	n	1	0	Amaryllidaceae J.St.-Hil.
Iz_5_1109	DJI_0207	Ambrosia artemisiifolia L.	0	a	0	0	Asteraceae Bercht. & J.Presl
Iz_5_1109	DJI_0207	Amorpha fruticosa L.	0	a	0	0	Fabaceae Lindl.
Iz_5_1109	DJI_0207	Anthericum ramosum L.	0	n	1	0	Asparagaceae Juss.
Iz_5_1109	DJI_0207	Anthoxanthum repens (Host) Veldkamp	0		NA	NA	
Iz_5_1109	DJI_0207	Artemisia marschalliana Spreng.	0	n	1	0	Asteraceae Bercht. & J.Presl
Iz_5_1109	DJI_0207	Asparagus officinalis L.	0	n	1	0	Asparagaceae Juss.
Iz_5_1109	DJI_0207	Astragalus cicer L.	0	n	1	0	Fabaceae Lindl.
Iz_5_1109	DJI_0207	Bassia laniflora (S.G.Gmel.) A.J.Scott	0	n	1	0	Amaranthaceae Juss.
Iz_5_1109	DJI_0207	Berberis vulgaris L.	0	n	1	0	Berberidaceae Juss.
Iz_5_1109	DJI_0207	Berteroa incana (L.) DC.	0		NA	NA	
Iz_5_1109	DJI_0207	Betula pendula Roth.	0	n	1	1	Betulaceae Gray
Iz_5_1109	DJI_0207	Betula pendula Roth.	0	n	1	0	Poaceae Barnhart
Iz_5_1109	DJI_0207	Bromus inermis Leyss.	0		NA	NA	
Iz_5_1109	DJI_0207	Bromus squarrosus L.	0	a	0	0	Poaceae Barnhart
Iz_5_1109	DJI_0207	Bromus tectorum L.	0	a	0	0	Poaceae Barnhart
Iz_5_1109	DJI_0207	Calamagrostis epigejos (L.) Roth	15	n	1	0	Poaceae Barnhart
Iz_5_1109	DJI_0207	Carduus acanthoides L.	0	n	1	0	Asteraceae Bercht. & J.Presl
Iz_5_1109	DJI_0207	Carex sp.	0	n	1	0	Cyperaceae Juss.
Iz_5_1109	DJI_0207	Centaurea majorovii Dumbadze	0	n	1	0	Asteraceae Bercht. & J.Presl
Iz_5_1109	DJI_0207	Chamaecytisus ruthenicus (Fisch.ex Wol.) Klaskova	0	n	1	0	Fabaceae Lindl.
Iz_5_1109	DJI_0207	Chelidonium majus L.	0	n	1	0	Papaveraceae Juss.
Iz_5_1109	DJI_0207	Chenopodium betaceum Andrz.	0	n	1	0	Amaranthaceae Juss.
Iz_5_1109	DJI_0207	Chenopodium cf. album	0	n	1	0	Amaranthaceae Juss.
Iz_5_1109	DJI_0207	Chenopodium sp.	0		NA	0	Amaranthaceae Juss.
Iz_5_1109	DJI_0207	Chondrilla juncea L.	0	n	1	0	Asteraceae Bercht. & J.Presl
Iz_5_1109	DJI_0207	Cirsium vulgare (Savi) Ten.	0	n	1	0	Asteraceae Bercht. & J.Presl
Iz_5_1109	DJI_0207	Clinopodium acinos (L.) Kuntze	0	n	1	0	Lamiacea Lindl.
Iz_5_1109	DJI_0207	Convallaria majalis L.	0	n	1	0	Asparagaceae Juss.
Iz_5_1109	DJI_0207	Crataegus rhipidophylla Gand.	0	n	1	1	Rosaceae Juss.
Iz_5_1109	DJI_0207	Crepis foetida L.	0	n	1	0	Asteraceae Bercht. & J.Presl
Iz_5_1109	DJI_0207	Dactylis glomerata L.	0	n	1	0	Poaceae Barnhart
Iz_5_1109	DJI_0207	Dianthus campestris M.Bieb.	0	n	1	0	Caryophyllaceae Juss.
Iz_5_1109	DJI_0207	Digitaria sanguinalis (L.) Scop.	0	a	0	0	Poaceae Barnhart
Iz_5_1109	DJI_0207	Elymus repens (L.) Gould	0	n	1	0	Poaceae Barnhart
Iz_5_1109	DJI_0207	Epilobium tetragonum L.	0	n	1	0	Onagraceae Juss.
Iz_5_1109	DJI_0207	Eragrostis minor Host	0	a	0	0	Poaceae Barnhart
Iz_5_1109	DJI_0207	Erigeron canadensis L.	5	a	0	0	Asteraceae Bercht. & J.Presl
Iz_5_1109	DJI_0207	Euphorbia seguieriana Neck.	0	n	1	0	Euphorbiaceae Juss.
Iz_5_1109	DJI_0207	Fallopia dumetorum (L.) Holub	0	n	1	0	Polygonaceae Juss.
Iz_5_1109	DJI_0207	Festuca beckeri (Hack.) Trautv.	0	n	1	0	Poaceae Barnhart
Iz_5_1109	DJI_0207	Fragaria vesca L.	0	n	1	0	Rosaceae Juss.
Iz_5_1109	DJI_0207	Frangula alnus Mill.	0	n	1	1	Rhamnaceae Juss.
Iz_5_1109	DJI_0207	Fraxinus pennsylvanica Marshall	0	a	0	1	Oleaceae Hoffmanns. & Link
Iz_5_1109	DJI_0207	Galeopsis bifida Boenn.	0	n	1	0	Lamiacea Lindl.
Iz_5_1109	DJI_0207	Galium verum L.	0	n	1	0	Rubiaceae Juss.
Iz_5_1109	DJI_0207	Genista tinctoria L.	0	n	1	0	Fabaceae Lindl.
Iz_5_1109	DJI_0207	Geranium robertianum L.	0	n	1	0	Geraniaceae Juss.

Iz_5_1109	DJI_0207	Geranium sanquineum L.	0	n	1	0	Geraniaceae Juss.
Iz_5_1109	DJI_0207	Gypsophila paniculata L.	0	n	1	0	Caryophyllaceae Juss.
Iz_5_1109	DJI_0207	Helichrysum arenarium (L.) Moench	0	n	1	0	Asteraceae Bercht. & J.Presl
Iz_5_1109	DJI_0207	Herniaria polygama J.Gay	0	n	1	0	Caryophyllaceae Juss.
Iz_5_1109	DJI_0207	Hieracium robustum Fr.	0	n	1	0	Asteraceae Bercht. & J.Presl
Iz_5_1109	DJI_0207	Hieracium umbellatum L.	0	n	1	NA	Asteraceae Bercht. & J.Presl
Iz_5_1109	DJI_0207	Hylotelephium maximum (L.) Holub	0	n	1	0	Crassulaceae J.St.-Hil.
Iz_5_1109	DJI_0207	Jacobaea borysthenica (DC.) B.Nord. & Greuter	0	n	1	0	Asteraceae Bercht. & J.Presl
Iz_5_1109	DJI_0207	Jasione montana L.	0	n	1	0	Campanulaceae Juss.
Iz_5_1109	DJI_0207	Jurinea cyanoides (L.) Rchb.s.l.	0	n	1	0	Asteraceae Bercht. & J.Presl
Iz_5_1109	DJI_0207	Koeleria glauca (Spreng.) DC.	0	n	1	0	Poaceae Barnhart
Iz_5_1109	DJI_0207	Lactuca serriola L.	0	a	0	0	Asteraceae Bercht. & J.Presl
Iz_5_1109	DJI_0207	Linaria genistifolia (L.) Mill.	0	n	1	0	Plantaginaceae Juss.
Iz_5_1109	DJI_0207	Lomelosia argentea (L.) Greuter & Burdet	0	n	1	0	Caprifoliaceae Juss.
Iz_5_1109	DJI_0207	Melampyrum pratense L.	0	n	1	0	Orobanchaceae Vent.
Iz_5_1109	DJI_0207	Oenothera biennis L.	0	a	0	0	Onagraceae Juss.
Iz_5_1109	DJI_0207	Pentanema britannica (L.) D.Gut.Larr., Santos-Vicente, Anderb., E.Rico & M.M.Mart.Ort.	0	n	1	0	Asteraceae Bercht. & J.Presl
Iz_5_1109	DJI_0207	Peucedanum oreoselinum (L.) Moench	0	n	1	0	Apiaceae Lindl.
Iz_5_1109	DJI_0207	Peucedanum ruthenicum M.Bieb.	0	n	1	0	Apiaceae Lindl.
Iz_5_1109	DJI_0207	Pilosella echioides (Lumn.) F.W.Schultz & Sch.Bip.	0	n	1	0	Asteraceae Bercht. & J.Presl
Iz_5_1109	DJI_0207	Pilosella officinarum F. Schultz et Sch. Bip.	0	n	1	0	Asteraceae Bercht. & J.Presl
Iz_5_1109	DJI_0207	Pilosella sp.	0.5		NA	0	Asteraceae Bercht. & J.Presl
Iz_5_1109	DJI_0207	Pinus sylvestris L.	0	n	1	1	Pinaceae Spreng. ex F.Rudolphi
Iz_5_1109	DJI_0207	Poa annua L.	0	n	1	0	Poaceae Barnhart
Iz_5_1109	DJI_0207	Poa nemoralis L.	0	n	1	0	Poaceae Barnhart
Iz_5_1109	DJI_0207	Poa sp.	0		NA	0	Poaceae Barnhart
Iz_5_1109	DJI_0207	Polygonatum odoratum (Mill.) Druce	0	n	1	0	Asparagaceae Juss.
Iz_5_1109	DJI_0207	Polygonum arenarium Waldst. & Kit.	0	n	1	0	Polygonaceae Juss.
Iz_5_1109	DJI_0207	Populus nigra L.	0	n	1	1	Salicaceae Mirb.
Iz_5_1109	DJI_0207	Populus tremula L.	0	n	1	1	Salicaceae Mirb.
Iz_5_1109	DJI_0207	Populus Ã canescens (Aiton) Sm.	0	n	1	1	Salicaceae Mirb.
Iz_5_1109	DJI_0207	Potentilla argentea L.	0	n	1	0	Rosaceae Juss.
Iz_5_1109	DJI_0207	Psammophiliella muralis (L.) Ikonn.	0	n	1	0	Caryophyllaceae Juss.
Iz_5_1109	DJI_0207	Psephellus sumensis (Kalen.) Greuter	0	n	1	0	Asteraceae Bercht. & J.Presl
Iz_5_1109	DJI_0207	Pulsatilla pratensis (L.) Mill. s.l.	0	n	1	0	Ranunculaceae Juss.
Iz_5_1109	DJI_0207	Pyrus communis L.	0	a	0	1	Rosaceae Juss.
Iz_5_1109	DJI_0207	Quercus robur L.	0	n	1	1	Fagaceae Dumort.
Iz_5_1109	DJI_0207	Robinia pseudoacacia L.	0	a	0	1	Fabaceae Lindl.
Iz_5_1109	DJI_0207	Rosa sp.	0		NA	1	Rosaceae Juss.
Iz_5_1109	DJI_0207	Rubus idaeus L.	0	n	1	0	Rosaceae Juss.
Iz_5_1109	DJI_0207	Rumex acetosella L.	0	n	1	0	Polygonaceae Juss.
Iz_5_1109	DJI_0207	Rumex thyrsiflorus Fingerh.	0	n	1	0	Polygonaceae Juss.
Iz_5_1109	DJI_0207	Salix alba L.	0	n	1	1	Salicaceae Mirb.
Iz_5_1109	DJI_0207	Sambucus nigra L.	0	n	1	1	Viburnaceae Raf.
Iz_5_1109	DJI_0207	Secale sylvestre Host	0	n	1	0	Poaceae Barnhart
Iz_5_1109	DJI_0207	Sempervivum ruthenicum Schnittsp. & C.B. Lehm.	0	n	1	0	Crassulaceae J.St.-Hil.
Iz_5_1109	DJI_0207	Setaria viridis (L.) P. Beauv.	0	n	1	0	Poaceae Barnhart
Iz_5_1109	DJI_0207	Silene borysthenica (Gruner) Walters	0	n	1	0	Caryophyllaceae Juss.
Iz_5_1109	DJI_0207	Silene latifolia subsp. alba (Mill.) Greuter & Burdet	0	n	1	0	Caryophyllaceae Juss.
Iz_5_1109	DJI_0207	Solidago virgaurea L.	0	n	1	0	Asteraceae Bercht. & J.Presl
Iz_5_1109	DJI_0207	Sorbus aucuparia L.	0	n	1	1	Rosaceae Juss.
Iz_5_1109	DJI_0207	Stachys recta L.	0	n	1	0	Lamiacea Lindl.
Iz_5_1109	DJI_0207	Tanacetum vulgare L.	0	n	1	0	Asteraceae Bercht. & J.Presl
Iz_5_1109	DJI_0207	Thymus pallasianus Heinr.Braun	0	n	1	0	Lamiacea Lindl.
Iz_5_1109	DJI_0207	Tilia cordata Mill.	0	n	1	1	Malvaceae Juss.
Iz_5_1109	DJI_0207	Trifolium alpestre L.	0	n	1	0	Fabaceae Lindl.
Iz_5_1109	DJI_0207	Trifolium arvense L.	0	n	1	0	Fabaceae Lindl.
Iz_5_1109	DJI_0207	Ulmus glabra Huds.	0	n	1	1	Ulmaceae Mirb.
Iz_5_1109	DJI_0207	Verbascum cf. phlomoides L.	0		NA	NA	
Iz_5_1109	DJI_0207	Verbascum densiflorum Bertol.	0	n	1	0	Scrophulariaceae Juss.
Iz_5_1109	DJI_0207	Verbascum sp.	0	n	1	0	Scrophulariaceae Juss.
Iz_5_1109	DJI_0207	Veronica austriaca L.	0	n	1	0	Plantaginaceae Juss.
Iz_5_1109	DJI_0207	Veronica incana L.	0	n	1	0	Plantaginaceae Juss.
Iz_5_1109	DJI_0207	Veronica verna L.	0	n	1	0	Plantaginaceae Juss.
Iz_5_1109	DJI_0207	Vincetoxicum hirundinaria Medik	0	n	1	0	Apocynaceae Juss.
Iz_5_1109	DJI_0210	Acer tataricum L.	0	n	1	1	Sapindaceae Juss.
Iz_5_1109	DJI_0210	Achillea millefolium L. s.l.	0	n	1	0	Asteraceae Bercht. & J.Presl
Iz_5_1109	DJI_0210	Allium oleraceum L.	0	n	1	0	Amaryllidaceae J.St.-Hil.
Iz_5_1109	DJI_0210	Ambrosia artemisiifolia L.	0	a	0	0	Asteraceae Bercht. & J.Presl
Iz_5_1109	DJI_0210	Amorpha fruticosa L.	0	a	0	0	Fabaceae Lindl.
Iz_5_1109	DJI_0210	Anthericum ramosum L.	0	n	1	0	Asparagaceae Juss.
Iz_5_1109	DJI_0210	Anthoxanthum repens (Host) Veldkamp	0		NA	NA	
Iz_5_1109	DJI_0210	Artemisia marschalliana Spreng.	0	n	1	0	Asteraceae Bercht. & J.Presl
Iz_5_1109	DJI_0210	Asparagus officinalis L.	0	n	1	0	Asparagaceae Juss.
Iz_5_1109	DJI_0210	Astragalus cicer L.	0	n	1	0	Fabaceae Lindl.
Iz_5_1109	DJI_0210	Bassia laniflora (S.G.Gmel.) A.J.Scott	0	n	1	0	Amaranthaceae Juss.
Iz_5_1109	DJI_0210	Berberis vulgaris L.	0	n	1	0	Berberidaceae Juss.
Iz_5_1109	DJI_0210	Berteroa incana (L.) DC.	0		NA	NA	
Iz_5_1109	DJI_0210	Betula pendula Roth.	0	n	1	1	Betulaceae Gray
Iz_5_1109	DJI_0210	Betula pendula Roth.	0	n	1	0	Poaceae Barnhart
Iz_5_1109	DJI_0210	Bromus inermis Leyss.	0		NA	NA	
Iz_5_1109	DJI_0210	Bromus squarrosus L.	0	a	0	0	Poaceae Barnhart
Iz_5_1109	DJI_0210	Bromus tectorum L.	0	a	0	0	Poaceae Barnhart
Iz_5_1109	DJI_0210	Calamagrostis epigejos (L.) Roth	10	n	1	0	Poaceae Barnhart
Iz_5_1109	DJI_0210	Carduus acanthoides L.	0	n	1	0	Asteraceae Bercht. & J.Presl
Iz_5_1109	DJI_0210	Carex sp.	2	n	1	0	Cyperaceae Juss.
Iz_5_1109	DJI_0210	Centaurea majorovii Dumbadze	0	n	1	0	Asteraceae Bercht. & J.Presl
Iz_5_1109	DJI_0210	Chamaecytisus ruthenicus (Fisch.ex Wol.) Klaskova	0	n	1	0	Fabaceae Lindl.
Iz_5_1109	DJI_0210	Chelidonium majus L.	0	n	1	0	Papaveraceae Juss.
Iz_5_1109	DJI_0210	Chenopodium betaceum Andrz.	0	n	1	0	Amaranthaceae Juss.
Iz_5_1109	DJI_0210	Chenopodium cf. album	0	n	1	0	Amaranthaceae Juss.
Iz_5_1109	DJI_0210	Chenopodium sp.	0		NA	0	Amaranthaceae Juss.
Iz_5_1109	DJI_0210	Chondrilla juncea L.	0	n	1	0	Asteraceae Bercht. & J.Presl
Iz_5_1109	DJI_0210	Cirsium vulgare (Savi) Ten.	0	n	1	0	Asteraceae Bercht. & J.Presl
Iz_5_1109	DJI_0210	Clinopodium acinos (L.) Kuntze	0	n	1	0	Lamiacea Lindl.
Iz_5_1109	DJI_0210	Convallaria majalis L.	0	n	1	0	Asparagaceae Juss.
Iz_5_1109	DJI_0210	Crataegus rhipidophylla Gand.	0	n	1	1	Rosaceae Juss.
Iz_5_1109	DJI_0210	Crepis foetida L.	0	n	1	0	Asteraceae Bercht. & J.Presl

Iz_5_1109	DJI_0210	Dactylis glomerata L.	0	n	1	0	Poaceae Barnhart
Iz_5_1109	DJI_0210	Dianthus campestris M.Bieb.	0	n	1	0	Caryophyllaceae Juss.
Iz_5_1109	DJI_0210	Digitaria sanguinalis (L.) Scop.	0	a	0	0	Poaceae Barnhart
Iz_5_1109	DJI_0210	Elymus repens (L.) Gould	0	n	1	0	Poaceae Barnhart
Iz_5_1109	DJI_0210	Epilobium tetragonum L.	0	n	1	0	Onagraceae Juss.
Iz_5_1109	DJI_0210	Eragrostis minor Host	0	a	0	0	Poaceae Barnhart
Iz_5_1109	DJI_0210	Erigeron canadensis L.	5	a	0	0	Asteraceae Bercht. & J.Presl
Iz_5_1109	DJI_0210	Euphorbia seguieriana Neck.	0	n	1	0	Euphorbiaceae Juss.
Iz_5_1109	DJI_0210	Fallopia dumetorum (L.) Holub	0	n	1	0	Polygonaceae Juss.
Iz_5_1109	DJI_0210	Festuca beckeri (Hack.) Trautv.	0	n	1	0	Poaceae Barnhart
Iz_5_1109	DJI_0210	Fragaria vesca L.	0	n	1	0	Rosaceae Juss.
Iz_5_1109	DJI_0210	Frangula alnus Mill.	0	n	1	1	Rhamnaceae Juss.
Iz_5_1109	DJI_0210	Fraxinus pennsylvanica Marshall	0	a	0	1	Oleaceae Hoffmanns. & Link
Iz_5_1109	DJI_0210	Galeopsis bifida Boenn.	0	n	1	0	Lamiacea Lindl.
Iz_5_1109	DJI_0210	Galium verum L.	0	n	1	0	Rubiaceae Juss.
Iz_5_1109	DJI_0210	Genista tinctoria L.	0	n	1	0	Fabaceae Lindl.
Iz_5_1109	DJI_0210	Geranium robertianum L.	0	n	1	0	Geraniaceae Juss.
Iz_5_1109	DJI_0210	Geranium sanquineum L.	0	n	1	0	Geraniaceae Juss.
Iz_5_1109	DJI_0210	Gypsophila paniculata L.	0	n	1	0	Caryophyllaceae Juss.
Iz_5_1109	DJI_0210	Helichrysum arenarium (L.) Moench	0	n	1	0	Asteraceae Bercht. & J.Presl
Iz_5_1109	DJI_0210	Herniaria polygama J.Gay	0	n	1	0	Caryophyllaceae Juss.
Iz_5_1109	DJI_0210	Hieracium robustum Fr.	0	n	1	0	Asteraceae Bercht. & J.Presl
Iz_5_1109	DJI_0210	Hieracium umbellatum L.	0	n	1	NA	Asteraceae Bercht. & J.Presl
Iz_5_1109	DJI_0210	Hylotelephium maximum (L.) Holub	0	n	1	0	Crassulaceae J.St.-Hil.
Iz_5_1109	DJI_0210	Jacobaea borysthenica (DC.) B.Nord. & Greuter	0	n	1	0	Asteraceae Bercht. & J.Presl
Iz_5_1109	DJI_0210	Jasione montana L.	0	n	1	0	Campanulaceae Juss.
Iz_5_1109	DJI_0210	Jurinea cyanoides (L.) Rchb.s.l.	0	n	1	0	Asteraceae Bercht. & J.Presl
Iz_5_1109	DJI_0210	Koeleria glauca (Spreng.) DC.	0	n	1	0	Poaceae Barnhart
Iz_5_1109	DJI_0210	Lactuca serriola L.	0	a	0	0	Asteraceae Bercht. & J.Presl
Iz_5_1109	DJI_0210	Linaria genistifolia (L.) Mill.	0	n	1	0	Plantaginaceae Juss.
Iz_5_1109	DJI_0210	Lomelosia argentea (L.) Greuter & Burdet	0	n	1	0	Caprifoliaceae Juss.
Iz_5_1109	DJI_0210	Melampyrum pratense L.	0	n	1	0	Orobanchaceae Vent.
Iz_5_1109	DJI_0210	Oenothera biennis L.	0	a	0	0	Onagraceae Juss.
Iz_5_1109	DJI_0210	Pentanema britannica (L.) D.Gut.Larr., Santos-Vicente, Anderb., E.Rico & M.M.Mart.Ort.	0	n	1	0	Asteraceae Bercht. & J.Presl
Iz_5_1109	DJI_0210	Peucedanum oreoselinum (L.) Moench	1	n	1	0	Apiaceae Lindl.
Iz_5_1109	DJI_0210	Peucedanum ruthenicum M.Bieb.	0	n	1	0	Apiaceae Lindl.
Iz_5_1109	DJI_0210	Pilosella echioides (Lumn.) F.W.Schultz & Sch.Bip.	0	n	1	0	Asteraceae Bercht. & J.Presl
Iz_5_1109	DJI_0210	Pilosella officinarum F. Schultz et Sch. Bip.	0	n	1	0	Asteraceae Bercht. & J.Presl
Iz_5_1109	DJI_0210	Pilosella sp.	0.5		NA	0	Asteraceae Bercht. & J.Presl
Iz_5_1109	DJI_0210	Pinus sylvestris L.	0	n	1	1	Pinaceae Spreng. ex F.Rudolphi
Iz_5_1109	DJI_0210	Poa annua L.	0	n	1	0	Poaceae Barnhart
Iz_5_1109	DJI_0210	Poa nemoralis L.	0	n	1	0	Poaceae Barnhart
Iz_5_1109	DJI_0210	Poa sp.	0		NA	0	Poaceae Barnhart
Iz_5_1109	DJI_0210	Polygonatum odoratum (Mill.) Druce	0	n	1	0	Asparagaceae Juss.
Iz_5_1109	DJI_0210	Polygonum arenarium Waldst. & Kit.	0	n	1	0	Polygonaceae Juss.
Iz_5_1109	DJI_0210	Populus nigra L.	0	n	1	1	Salicaceae Mirb.
Iz_5_1109	DJI_0210	Populus tremula L.	0	n	1	1	Salicaceae Mirb.
Iz_5_1109	DJI_0210	Populus Ã canescens (Aiton) Sm.	0	n	1	1	Salicaceae Mirb.
Iz_5_1109	DJI_0210	Potentilla argentea L.	0	n	1	0	Rosaceae Juss.
Iz_5_1109	DJI_0210	Psammophiliella muralis (L.) Ikonn.	0	n	1	0	Caryophyllaceae Juss.
Iz_5_1109	DJI_0210	Psephellus sumensis (Kalen.) Greuter	0	n	1	0	Asteraceae Bercht. & J.Presl
Iz_5_1109	DJI_0210	Pulsatilla pratensis (L.) Mill. s.l.	0	n	1	0	Ranunculaceae Juss.
Iz_5_1109	DJI_0210	Pyrus communis L.	0	a	0	1	Rosaceae Juss.
Iz_5_1109	DJI_0210	Quercus robur L.	0	n	1	1	Fagaceae Dumort.
Iz_5_1109	DJI_0210	Robinia pseudoacacia L.	0	a	0	1	Fabaceae Lindl.
Iz_5_1109	DJI_0210	Rosa sp.	0		NA	1	Rosaceae Juss.
Iz_5_1109	DJI_0210	Rubus idaeus L.	0	n	1	0	Rosaceae Juss.
Iz_5_1109	DJI_0210	Rumex acetosella L.	2	n	1	0	Polygonaceae Juss.
Iz_5_1109	DJI_0210	Rumex thyrsiflorus Fingerh.	0	n	1	0	Polygonaceae Juss.
Iz_5_1109	DJI_0210	Salix alba L.	0	n	1	1	Salicaceae Mirb.
Iz_5_1109	DJI_0210	Sambucus nigra L.	0	n	1	1	Viburnaceae Raf.
Iz_5_1109	DJI_0210	Secale sylvestre Host	0	n	1	0	Poaceae Barnhart
Iz_5_1109	DJI_0210	Sempervivum ruthenicum Schnittsp. & C.B. Lehm.	0	n	1	0	Crassulaceae J.St.-Hil.
Iz_5_1109	DJI_0210	Setaria viridis (L.) P. Beauv.	0	n	1	0	Poaceae Barnhart
Iz_5_1109	DJI_0210	Silene borysthenica (Gruner) Walters	0	n	1	0	Caryophyllaceae Juss.
Iz_5_1109	DJI_0210	Silene latifolia subsp. alba (Mill.) Greuter & Burdet	0	n	1	0	Caryophyllaceae Juss.
Iz_5_1109	DJI_0210	Solidago virgaurea L.	0	n	1	0	Asteraceae Bercht. & J.Presl
Iz_5_1109	DJI_0210	Sorbus aucuparia L.	0	n	1	1	Rosaceae Juss.
Iz_5_1109	DJI_0210	Stachys recta L.	0	n	1	0	Lamiacea Lindl.
Iz_5_1109	DJI_0210	Tanacetum vulgare L.	0	n	1	0	Asteraceae Bercht. & J.Presl
Iz_5_1109	DJI_0210	Thymus pallasianus Heinr.Braun	0	n	1	0	Lamiacea Lindl.
Iz_5_1109	DJI_0210	Tilia cordata Mill.	0	n	1	1	Malvaceae Juss.
Iz_5_1109	DJI_0210	Trifolium alpestre L.	0	n	1	0	Fabaceae Lindl.
Iz_5_1109	DJI_0210	Trifolium arvense L.	0	n	1	0	Fabaceae Lindl.
Iz_5_1109	DJI_0210	Ulmus glabra Huds.	0	n	1	1	Ulmaceae Mirb.
Iz_5_1109	DJI_0210	Verbascum cf. phlomoides L.	0		NA	NA	
Iz_5_1109	DJI_0210	Verbascum densiflorum Bertol.	0	n	1	0	Scrophulariaceae Juss.
Iz_5_1109	DJI_0210	Verbascum sp.	0	n	1	0	Scrophulariaceae Juss.
Iz_5_1109	DJI_0210	Veronica austriaca L.	0	n	1	0	Plantaginaceae Juss.
Iz_5_1109	DJI_0210	Veronica incana L.	0	n	1	0	Plantaginaceae Juss.
Iz_5_1109	DJI_0210	Veronica verna L.	0	n	1	0	Plantaginaceae Juss.
Iz_5_1109	DJI_0210	Vincetoxicum hirundinaria Medik	0	n	1	0	Apocynaceae Juss.
Iz_5_1109	DJI_0213	Acer tataricum L.	0	n	1	1	Sapindaceae Juss.
Iz_5_1109	DJI_0213	Achillea millefolium L. s.l.	0	n	1	0	Asteraceae Bercht. & J.Presl
Iz_5_1109	DJI_0213	Allium oleraceum L.	0	n	1	0	Amaryllidaceae J.St.-Hil.
Iz_5_1109	DJI_0213	Ambrosia artemisiifolia L.	0	a	0	0	Asteraceae Bercht. & J.Presl
Iz_5_1109	DJI_0213	Amorpha fruticosa L.	0	a	0	0	Fabaceae Lindl.
Iz_5_1109	DJI_0213	Anthericum ramosum L.	0	n	1	0	Asparagaceae Juss.
Iz_5_1109	DJI_0213	Anthoxanthum repens (Host) Veldkamp	0		NA	NA	
Iz_5_1109	DJI_0213	Artemisia marschalliana Spreng.	0	n	1	0	Asteraceae Bercht. & J.Presl
Iz_5_1109	DJI_0213	Asparagus officinalis L.	0	n	1	0	Asparagaceae Juss.
Iz_5_1109	DJI_0213	Astragalus cicer L.	0	n	1	0	Fabaceae Lindl.
Iz_5_1109	DJI_0213	Bassia laniflora (S.G.Gmel.) A.J.Scott	0	n	1	0	Amaranthaceae Juss.
Iz_5_1109	DJI_0213	Berberis vulgaris L.	0	n	1	0	Berberidaceae Juss.
Iz_5_1109	DJI_0213	Berteroa incana (L.) DC.	0		NA	NA	
Iz_5_1109	DJI_0213	Betula pendula Roth.	0	n	1	1	Betulaceae Gray
Iz_5_1109	DJI_0213	Betula pendula Roth.	0	n	1	0	Poaceae Barnhart
Iz_5_1109	DJI_0213	Bromus inermis Leyss.	0		NA	NA	

Iz_5_1109	DJI_0213	Bromus squarrosus L.	0	a	0	0	Poaceae Barnhart
Iz_5_1109	DJI_0213	Bromus tectorum L.	0	a	0	0	Poaceae Barnhart
Iz_5_1109	DJI_0213	Calamagrostis epigejos (L.) Roth	3	n	1	0	Poaceae Barnhart
Iz_5_1109	DJI_0213	Carduus acanthoides L.	0	n	1	0	Asteraceae Bercht. & J.Presl
Iz_5_1109	DJI_0213	Carex sp.	2.5	n	1	0	Cyperaceae Juss.
Iz_5_1109	DJI_0213	Centaurea majorovii Dumbadze	0	n	1	0	Asteraceae Bercht. & J.Presl
Iz_5_1109	DJI_0213	Chamaecytisus ruthenicus (Fisch.ex Wol.) Klaskova	0	n	1	0	Fabaceae Lindl.
Iz_5_1109	DJI_0213	Chelidonium majus L.	0	n	1	0	Papaveraceae Juss.
Iz_5_1109	DJI_0213	Chenopodium betaceum Andrz.	0	n	1	0	Amaranthaceae Juss.
Iz_5_1109	DJI_0213	Chenopodium cf. album	0	n	1	0	Amaranthaceae Juss.
Iz_5_1109	DJI_0213	Chenopodium sp.	0		NA	0	Amaranthaceae Juss.
Iz_5_1109	DJI_0213	Chondrilla juncea L.	0	n	1	0	Asteraceae Bercht. & J.Presl
Iz_5_1109	DJI_0213	Cirsium vulgare (Savi) Ten.	0	n	1	0	Asteraceae Bercht. & J.Presl
Iz_5_1109	DJI_0213	Clinopodium acinos (L.) Kuntze	0	n	1	0	Lamiacea Lindl.
Iz_5_1109	DJI_0213	Convallaria majalis L.	0	n	1	0	Asparagaceae Juss.
Iz_5_1109	DJI_0213	Crataegus rhipidophylla Gand.	0	n	1	1	Rosaceae Juss.
Iz_5_1109	DJI_0213	Crepis foetida L.	0	n	1	0	Asteraceae Bercht. & J.Presl
Iz_5_1109	DJI_0213	Dactylis glomerata L.	0	n	1	0	Poaceae Barnhart
Iz_5_1109	DJI_0213	Dianthus campestris M.Bieb.	0	n	1	0	Caryophyllaceae Juss.
Iz_5_1109	DJI_0213	Digitaria sanguinalis (L.) Scop.	0	a	0	0	Poaceae Barnhart
Iz_5_1109	DJI_0213	Elymus repens (L.) Gould	0	n	1	0	Poaceae Barnhart
Iz_5_1109	DJI_0213	Epilobium tetragonum L.	0	n	1	0	Onagraceae Juss.
Iz_5_1109	DJI_0213	Eragrostis minor Host	0	a	0	0	Poaceae Barnhart
Iz_5_1109	DJI_0213	Erigeron canadensis L.	3	a	0	0	Asteraceae Bercht. & J.Presl
Iz_5_1109	DJI_0213	Euphorbia seguieriana Neck.	0	n	1	0	Euphorbiaceae Juss.
Iz_5_1109	DJI_0213	Fallopia dumetorum (L.) Holub	0	n	1	0	Polygonaceae Juss.
Iz_5_1109	DJI_0213	Festuca beckeri (Hack.) Trautv.	0	n	1	0	Poaceae Barnhart
Iz_5_1109	DJI_0213	Fragaria vesca L.	0	n	1	0	Rosaceae Juss.
Iz_5_1109	DJI_0213	Frangula alnus Mill.	0	n	1	1	Rhamnaceae Juss.
Iz_5_1109	DJI_0213	Fraxinus pennsylvanica Marshall	0	a	0	1	Oleaceae Hoffmanns. & Link
Iz_5_1109	DJI_0213	Galeopsis bifida Boenn.	0	n	1	0	Lamiacea Lindl.
Iz_5_1109	DJI_0213	Galium verum L.	0	n	1	0	Rubiaceae Juss.
Iz_5_1109	DJI_0213	Genista tinctoria L.	0	n	1	0	Fabaceae Lindl.
Iz_5_1109	DJI_0213	Geranium robertianum L.	0	n	1	0	Geraniaceae Juss.
Iz_5_1109	DJI_0213	Geranium sanquineum L.	0	n	1	0	Geraniaceae Juss.
Iz_5_1109	DJI_0213	Gypsophila paniculata L.	0	n	1	0	Caryophyllaceae Juss.
Iz_5_1109	DJI_0213	Helichrysum arenarium (L.) Moench	0	n	1	0	Asteraceae Bercht. & J.Presl
Iz_5_1109	DJI_0213	Herniaria polygama J.Gay	0	n	1	0	Caryophyllaceae Juss.
Iz_5_1109	DJI_0213	Hieracium robustum Fr.	0	n	1	0	Asteraceae Bercht. & J.Presl
Iz_5_1109	DJI_0213	Hieracium umbellatum L.	0	n	1	NA	Asteraceae Bercht. & J.Presl
Iz_5_1109	DJI_0213	Hylotelephium maximum (L.) Holub	0	n	1	0	Crassulaceae J.St.-Hil.
Iz_5_1109	DJI_0213	Jacobaea borysthenica (DC.) B.Nord. & Greuter	0	n	1	0	Asteraceae Bercht. & J.Presl
Iz_5_1109	DJI_0213	Jasione montana L.	0	n	1	0	Campanulaceae Juss.
Iz_5_1109	DJI_0213	Jurinea cyanoides (L.) Rchb.s.l.	0	n	1	0	Asteraceae Bercht. & J.Presl
Iz_5_1109	DJI_0213	Koeleria glauca (Spreng.) DC.	0	n	1	0	Poaceae Barnhart
Iz_5_1109	DJI_0213	Lactuca serriola L.	0	a	0	0	Asteraceae Bercht. & J.Presl
Iz_5_1109	DJI_0213	Linaria genistifolia (L.) Mill.	0	n	1	0	Plantaginaceae Juss.
Iz_5_1109	DJI_0213	Lomelosia argentea (L.) Greuter & Burdet	0	n	1	0	Caprifoliaceae Juss.
Iz_5_1109	DJI_0213	Melampyrum pratense L.	0	n	1	0	Orobanchaceae Vent.
Iz_5_1109	DJI_0213	Oenothera biennis L.	0	a	0	0	Onagraceae Juss.
Iz_5_1109	DJI_0213	Pentanema britannica (L.) D.Gut.Larr., Santos-Vicente, Anderb., E.Rico & M.M.Mart.Ort.	0	n	1	0	Asteraceae Bercht. & J.Presl
Iz_5_1109	DJI_0213	Peucedanum oreoselinum (L.) Moench	1	n	1	0	Apiaceae Lindl.
Iz_5_1109	DJI_0213	Peucedanum ruthenicum M.Bieb.	0	n	1	0	Apiaceae Lindl.
Iz_5_1109	DJI_0213	Pilosella echioides (Lumn.) F.W.Schultz & Sch.Bip.	0	n	1	0	Asteraceae Bercht. & J.Presl
Iz_5_1109	DJI_0213	Pilosella officinarum F. Schultz et Sch. Bip.	0	n	1	0	Asteraceae Bercht. & J.Presl
Iz_5_1109	DJI_0213	Pilosella sp.	0		NA	0	Asteraceae Bercht. & J.Presl
Iz_5_1109	DJI_0213	Pinus sylvestris L.	0	n	1	1	Pinaceae Spreng. ex F.Rudolphi
Iz_5_1109	DJI_0213	Poa annua L.	0	n	1	0	Poaceae Barnhart
Iz_5_1109	DJI_0213	Poa nemoralis L.	0	n	1	0	Poaceae Barnhart
Iz_5_1109	DJI_0213	Poa sp.	0		NA	0	Poaceae Barnhart
Iz_5_1109	DJI_0213	Polygonatum odoratum (Mill.) Druce	0	n	1	0	Asparagaceae Juss.
Iz_5_1109	DJI_0213	Polygonum arenarium Waldst. & Kit.	0	n	1	0	Polygonaceae Juss.
Iz_5_1109	DJI_0213	Populus nigra L.	0	n	1	1	Salicaceae Mirb.
Iz_5_1109	DJI_0213	Populus tremula L.	0	n	1	1	Salicaceae Mirb.
Iz_5_1109	DJI_0213	Populus Ã canescens (Aiton) Sm.	0	n	1	1	Salicaceae Mirb.
Iz_5_1109	DJI_0213	Potentilla argentea L.	0	n	1	0	Rosaceae Juss.
Iz_5_1109	DJI_0213	Psammophiliella muralis (L.) Ikonn.	0	n	1	0	Caryophyllaceae Juss.
Iz_5_1109	DJI_0213	Psephellus sumensis (Kalen.) Greuter	0	n	1	0	Asteraceae Bercht. & J.Presl
Iz_5_1109	DJI_0213	Pulsatilla pratensis (L.) Mill. s.l.	0	n	1	0	Ranunculaceae Juss.
Iz_5_1109	DJI_0213	Pyrus communis L.	0	a	0	1	Rosaceae Juss.
Iz_5_1109	DJI_0213	Quercus robur L.	0	n	1	1	Fagaceae Dumort.
Iz_5_1109	DJI_0213	Robinia pseudoacacia L.	0	a	0	1	Fabaceae Lindl.
Iz_5_1109	DJI_0213	Rosa sp.	0		NA	1	Rosaceae Juss.
Iz_5_1109	DJI_0213	Rubus idaeus L.	0	n	1	0	Rosaceae Juss.
Iz_5_1109	DJI_0213	Rumex acetosella L.	3	n	1	0	Polygonaceae Juss.
Iz_5_1109	DJI_0213	Rumex thyrsiflorus Fingerh.	0	n	1	0	Polygonaceae Juss.
Iz_5_1109	DJI_0213	Salix alba L.	0	n	1	1	Salicaceae Mirb.
Iz_5_1109	DJI_0213	Sambucus nigra L.	0	n	1	1	Viburnaceae Raf.
Iz_5_1109	DJI_0213	Secale sylvestre Host	0	n	1	0	Poaceae Barnhart
Iz_5_1109	DJI_0213	Sempervivum ruthenicum Schnittsp. & C.B. Lehm.	0	n	1	0	Crassulaceae J.St.-Hil.
Iz_5_1109	DJI_0213	Setaria viridis (L.) P. Beauv.	0	n	1	0	Poaceae Barnhart
Iz_5_1109	DJI_0213	Silene borysthenica (Gruner) Walters	0	n	1	0	Caryophyllaceae Juss.
Iz_5_1109	DJI_0213	Silene latifolia subsp. alba (Mill.) Greuter & Burdet	0	n	1	0	Caryophyllaceae Juss.
Iz_5_1109	DJI_0213	Solidago virgaurea L.	0	n	1	0	Asteraceae Bercht. & J.Presl
Iz_5_1109	DJI_0213	Sorbus aucuparia L.	0	n	1	1	Rosaceae Juss.
Iz_5_1109	DJI_0213	Stachys recta L.	0	n	1	0	Lamiacea Lindl.
Iz_5_1109	DJI_0213	Tanacetum vulgare L.	0	n	1	0	Asteraceae Bercht. & J.Presl
Iz_5_1109	DJI_0213	Thymus pallasianus Heinr.Braun	0	n	1	0	Lamiacea Lindl.
Iz_5_1109	DJI_0213	Tilia cordata Mill.	0	n	1	1	Malvaceae Juss.
Iz_5_1109	DJI_0213	Trifolium alpestre L.	0	n	1	0	Fabaceae Lindl.
Iz_5_1109	DJI_0213	Trifolium arvense L.	0	n	1	0	Fabaceae Lindl.
Iz_5_1109	DJI_0213	Ulmus glabra Huds.	0	n	1	1	Ulmaceae Mirb.
Iz_5_1109	DJI_0213	Verbascum cf. phlomoides L.	0		NA	NA	
Iz_5_1109	DJI_0213	Verbascum densiflorum Bertol.	0	n	1	0	Scrophulariaceae Juss.
Iz_5_1109	DJI_0213	Verbascum sp.	0	n	1	0	Scrophulariaceae Juss.
Iz_5_1109	DJI_0213	Veronica austriaca L.	0	n	1	0	Plantaginaceae Juss.
Iz_5_1109	DJI_0213	Veronica incana L.	0	n	1	0	Plantaginaceae Juss.
Iz_5_1109	DJI_0213	Veronica verna L.	0	n	1	0	Plantaginaceae Juss.

Iz_5_1109	DJI_0213	Vincetoxicum hirundinaria Medik	0	n	1	0	Apocynaceae Juss.
Iz_5_1109	DJI_0217	Acer tataricum L.	0	n	1	1	Sapindaceae Juss.
Iz_5_1109	DJI_0217	Achillea millefolium L. s.l.	0	n	1	0	Asteraceae Bercht. & J.Presl
Iz_5_1109	DJI_0217	Allium oleraceum L.	0	n	1	0	Amaryllidaceae J.St.-Hil.
Iz_5_1109	DJI_0217	Ambrosia artemisiifolia L.	0	a	0	0	Asteraceae Bercht. & J.Presl
Iz_5_1109	DJI_0217	Amorpha fruticosa L.	0	a	0	0	Fabaceae Lindl.
Iz_5_1109	DJI_0217	Anthericum ramosum L.	0	n	1	0	Asparagaceae Juss.
Iz_5_1109	DJI_0217	Anthoxanthum repens (Host) Veldkamp	0		NA	NA	
Iz_5_1109	DJI_0217	Artemisia marschalliana Spreng.	0	n	1	0	Asteraceae Bercht. & J.Presl
Iz_5_1109	DJI_0217	Asparagus officinalis L.	0	n	1	0	Asparagaceae Juss.
Iz_5_1109	DJI_0217	Astragalus cicer L.	0	n	1	0	Fabaceae Lindl.
Iz_5_1109	DJI_0217	Bassia laniflora (S.G.Gmel.) A.J.Scott	0	n	1	0	Amaranthaceae Juss.
Iz_5_1109	DJI_0217	Berberis vulgaris L.	0	n	1	0	Berberidaceae Juss.
Iz_5_1109	DJI_0217	Berteroa incana (L.) DC.	0		NA	NA	
Iz_5_1109	DJI_0217	Betula pendula Roth.	0	n	1	1	Betulaceae Gray
Iz_5_1109	DJI_0217	Betula pendula Roth.	0	n	1	0	Poaceae Barnhart
Iz_5_1109	DJI_0217	Bromus inermis Leyss.	0		NA	NA	
Iz_5_1109	DJI_0217	Bromus squarrosus L.	0	a	0	0	Poaceae Barnhart
Iz_5_1109	DJI_0217	Bromus tectorum L.	0	a	0	0	Poaceae Barnhart
Iz_5_1109	DJI_0217	Calamagrostis epigejos (L.) Roth	1	n	1	0	Poaceae Barnhart
Iz_5_1109	DJI_0217	Carduus acanthoides L.	0	n	1	0	Asteraceae Bercht. & J.Presl
Iz_5_1109	DJI_0217	Carex sp.	0	n	1	0	Cyperaceae Juss.
Iz_5_1109	DJI_0217	Centaurea majorovii Dumbadze	0	n	1	0	Asteraceae Bercht. & J.Presl
Iz_5_1109	DJI_0217	Chamaecytisus ruthenicus (Fisch.ex Wol.) Klaskova	0	n	1	0	Fabaceae Lindl.
Iz_5_1109	DJI_0217	Chelidonium majus L.	0	n	1	0	Papaveraceae Juss.
Iz_5_1109	DJI_0217	Chenopodium betaceum Andrz.	0	n	1	0	Amaranthaceae Juss.
Iz_5_1109	DJI_0217	Chenopodium cf. album	0	n	1	0	Amaranthaceae Juss.
Iz_5_1109	DJI_0217	Chenopodium sp.	0.2		NA	0	Amaranthaceae Juss.
Iz_5_1109	DJI_0217	Chondrilla juncea L.	0	n	1	0	Asteraceae Bercht. & J.Presl
Iz_5_1109	DJI_0217	Cirsium vulgare (Savi) Ten.	0	n	1	0	Asteraceae Bercht. & J.Presl
Iz_5_1109	DJI_0217	Clinopodium acinos (L.) Kuntze	0	n	1	0	Lamiacea Lindl.
Iz_5_1109	DJI_0217	Convallaria majalis L.	0	n	1	0	Asparagaceae Juss.
Iz_5_1109	DJI_0217	Crataegus rhipidophylla Gand.	0	n	1	1	Rosaceae Juss.
Iz_5_1109	DJI_0217	Crepis foetida L.	0	n	1	0	Asteraceae Bercht. & J.Presl
Iz_5_1109	DJI_0217	Dactylis glomerata L.	0	n	1	0	Poaceae Barnhart
Iz_5_1109	DJI_0217	Dianthus campestris M.Bieb.	0	n	1	0	Caryophyllaceae Juss.
Iz_5_1109	DJI_0217	Digitaria sanguinalis (L.) Scop.	0	a	0	0	Poaceae Barnhart
Iz_5_1109	DJI_0217	Elymus repens (L.) Gould	0	n	1	0	Poaceae Barnhart
Iz_5_1109	DJI_0217	Epilobium tetragonum L.	0	n	1	0	Onagraceae Juss.
Iz_5_1109	DJI_0217	Eragrostis minor Host	0	a	0	0	Poaceae Barnhart
Iz_5_1109	DJI_0217	Erigeron canadensis L.	0	a	0	0	Asteraceae Bercht. & J.Presl
Iz_5_1109	DJI_0217	Euphorbia seguieriana Neck.	0	n	1	0	Euphorbiaceae Juss.
Iz_5_1109	DJI_0217	Fallopia dumetorum (L.) Holub	3	n	1	0	Polygonaceae Juss.
Iz_5_1109	DJI_0217	Festuca beckeri (Hack.) Trautv.	0	n	1	0	Poaceae Barnhart
Iz_5_1109	DJI_0217	Fragaria vesca L.	0	n	1	0	Rosaceae Juss.
Iz_5_1109	DJI_0217	Frangula alnus Mill.	0	n	1	1	Rhamnaceae Juss.
Iz_5_1109	DJI_0217	Fraxinus pennsylvanica Marshall	0	a	0	1	Oleaceae Hoffmanns. & Link
Iz_5_1109	DJI_0217	Galeopsis bifida Boenn.	0	n	1	0	Lamiacea Lindl.
Iz_5_1109	DJI_0217	Galium verum L.	0	n	1	0	Rubiaceae Juss.
Iz_5_1109	DJI_0217	Genista tinctoria L.	0	n	1	0	Fabaceae Lindl.
Iz_5_1109	DJI_0217	Geranium robertianum L.	0	n	1	0	Geraniaceae Juss.
Iz_5_1109	DJI_0217	Geranium sanquineum L.	0	n	1	0	Geraniaceae Juss.
Iz_5_1109	DJI_0217	Gypsophila paniculata L.	0	n	1	0	Caryophyllaceae Juss.
Iz_5_1109	DJI_0217	Helichrysum arenarium (L.) Moench	0	n	1	0	Asteraceae Bercht. & J.Presl
Iz_5_1109	DJI_0217	Herniaria polygama J.Gay	0	n	1	0	Caryophyllaceae Juss.
Iz_5_1109	DJI_0217	Hieracium robustum Fr.	0	n	1	0	Asteraceae Bercht. & J.Presl
Iz_5_1109	DJI_0217	Hieracium umbellatum L.	0	n	1	NA	Asteraceae Bercht. & J.Presl
Iz_5_1109	DJI_0217	Hylotelephium maximum (L.) Holub	0	n	1	0	Crassulaceae J.St.-Hil.
Iz_5_1109	DJI_0217	Jacobaea borysthenica (DC.) B.Nord. & Greuter	0	n	1	0	Asteraceae Bercht. & J.Presl
Iz_5_1109	DJI_0217	Jasione montana L.	0	n	1	0	Campanulaceae Juss.
Iz_5_1109	DJI_0217	Jurinea cyanoides (L.) Rchb.s.l.	0	n	1	0	Asteraceae Bercht. & J.Presl
Iz_5_1109	DJI_0217	Koeleria glauca (Spreng.) DC.	0	n	1	0	Poaceae Barnhart
Iz_5_1109	DJI_0217	Lactuca serriola L.	0	a	0	0	Asteraceae Bercht. & J.Presl
Iz_5_1109	DJI_0217	Linaria genistifolia (L.) Mill.	0	n	1	0	Plantaginaceae Juss.
Iz_5_1109	DJI_0217	Lomelosia argentea (L.) Greuter & Burdet	0	n	1	0	Caprifoliaceae Juss.
Iz_5_1109	DJI_0217	Melampyrum pratense L.	0	n	1	0	Orobanchaceae Vent.
Iz_5_1109	DJI_0217	Oenothera biennis L.	0	a	0	0	Onagraceae Juss.
Iz_5_1109	DJI_0217	Pentanema britannica (L.) D.Gut.Larr., Santos-Vicente, Anderb., E.Rico & M.M.Mart.Ort.	0	n	1	0	Asteraceae Bercht. & J.Presl
Iz_5_1109	DJI_0217	Peucedanum oreoselinum (L.) Moench	0	n	1	0	Apiaceae Lindl.
Iz_5_1109	DJI_0217	Peucedanum ruthenicum M.Bieb.	0	n	1	0	Apiaceae Lindl.
Iz_5_1109	DJI_0217	Pilosella echioides (Lumn.) F.W.Schultz & Sch.Bip.	0	n	1	0	Asteraceae Bercht. & J.Presl
Iz_5_1109	DJI_0217	Pilosella officinarum F. Schultz et Sch. Bip.	0	n	1	0	Asteraceae Bercht. & J.Presl
Iz_5_1109	DJI_0217	Pilosella sp.	0		NA	0	Asteraceae Bercht. & J.Presl
Iz_5_1109	DJI_0217	Pinus sylvestris L.	0	n	1	1	Pinaceae Spreng. ex F.Rudolphi
Iz_5_1109	DJI_0217	Poa annua L.	0	n	1	0	Poaceae Barnhart
Iz_5_1109	DJI_0217	Poa nemoralis L.	0	n	1	0	Poaceae Barnhart
Iz_5_1109	DJI_0217	Poa sp.	0		NA	0	Poaceae Barnhart
Iz_5_1109	DJI_0217	Polygonatum odoratum (Mill.) Druce	0	n	1	0	Asparagaceae Juss.
Iz_5_1109	DJI_0217	Polygonum arenarium Waldst. & Kit.	0	n	1	0	Polygonaceae Juss.
Iz_5_1109	DJI_0217	Populus nigra L.	0	n	1	1	Salicaceae Mirb.
Iz_5_1109	DJI_0217	Populus tremula L.	0	n	1	1	Salicaceae Mirb.
Iz_5_1109	DJI_0217	Populus Ã canescens (Aiton) Sm.	0	n	1	1	Salicaceae Mirb.
Iz_5_1109	DJI_0217	Potentilla argentea L.	0	n	1	0	Rosaceae Juss.
Iz_5_1109	DJI_0217	Psammophiliella muralis (L.) Ikonn.	0	n	1	0	Caryophyllaceae Juss.
Iz_5_1109	DJI_0217	Psephellus sumensis (Kalen.) Greuter	0	n	1	0	Asteraceae Bercht. & J.Presl
Iz_5_1109	DJI_0217	Pulsatilla pratensis (L.) Mill. s.l.	0	n	1	0	Ranunculaceae Juss.
Iz_5_1109	DJI_0217	Pyrus communis L.	0	a	0	1	Rosaceae Juss.
Iz_5_1109	DJI_0217	Quercus robur L.	0	n	1	1	Fagaceae Dumort.
Iz_5_1109	DJI_0217	Robinia pseudoacacia L.	0	a	0	1	Fabaceae Lindl.
Iz_5_1109	DJI_0217	Rosa sp.	0		NA	1	Rosaceae Juss.
Iz_5_1109	DJI_0217	Rubus idaeus L.	0	n	1	0	Rosaceae Juss.
Iz_5_1109	DJI_0217	Rumex acetosella L.	8	n	1	0	Polygonaceae Juss.
Iz_5_1109	DJI_0217	Rumex thyrsiflorus Fingerh.	0	n	1	0	Polygonaceae Juss.
Iz_5_1109	DJI_0217	Salix alba L.	0	n	1	1	Salicaceae Mirb.
Iz_5_1109	DJI_0217	Sambucus nigra L.	0	n	1	1	Viburnaceae Raf.
Iz_5_1109	DJI_0217	Secale sylvestre Host	0	n	1	0	Poaceae Barnhart
Iz_5_1109	DJI_0217	Sempervivum ruthenicum Schnittsp. & C.B. Lehm.	0	n	1	0	Crassulaceae J.St.-Hil.
Iz_5_1109	DJI_0217	Setaria viridis (L.) P. Beauv.	0	n	1	0	Poaceae Barnhart

Iz_5_1109	DJI_0217	Silene borysthenica (Gruner) Walters	0	n	1	0	Caryophyllaceae Juss.
Iz_5_1109	DJI_0217	Silene latifolia subsp. alba (Mill.) Greuter & Burdet	0	n	1	0	Caryophyllaceae Juss.
Iz_5_1109	DJI_0217	Solidago virgaurea L.	0	n	1	0	Asteraceae Bercht. & J.Presl
Iz_5_1109	DJI_0217	Sorbus aucuparia L.	0	n	1	1	Rosaceae Juss.
Iz_5_1109	DJI_0217	Stachys recta L.	0	n	1	0	Lamiacea Lindl.
Iz_5_1109	DJI_0217	Tanacetum vulgare L.	0	n	1	0	Asteraceae Bercht. & J.Presl
Iz_5_1109	DJI_0217	Thymus pallasianus Heinr.Braun	0	n	1	0	Lamiacea Lindl.
Iz_5_1109	DJI_0217	Tilia cordata Mill.	0	n	1	1	Malvaceae Juss.
Iz_5_1109	DJI_0217	Trifolium alpestre L.	0	n	1	0	Fabaceae Lindl.
Iz_5_1109	DJI_0217	Trifolium arvense L.	0	n	1	0	Fabaceae Lindl.
Iz_5_1109	DJI_0217	Ulmus glabra Huds.	0	n	1	1	Ulmaceae Mirb.
Iz_5_1109	DJI_0217	Verbascum cf. phlomoides L.	0		NA	NA	
Iz_5_1109	DJI_0217	Verbascum densiflorum Bertol.	0	n	1	0	Scrophulariaceae Juss.
Iz_5_1109	DJI_0217	Verbascum sp.	0	n	1	0	Scrophulariaceae Juss.
Iz_5_1109	DJI_0217	Veronica austriaca L.	0	n	1	0	Plantaginaceae Juss.
Iz_5_1109	DJI_0217	Veronica incana L.	0	n	1	0	Plantaginaceae Juss.
Iz_5_1109	DJI_0217	Veronica verna L.	0	n	1	0	Plantaginaceae Juss.
Iz_5_1109	DJI_0217	Vincetoxicum hirundinaria Medik	0	n	1	0	Apocynaceae Juss.
Iz_5_1109	DJI_0220	Acer tataricum L.	0	n	1	1	Sapindaceae Juss.
Iz_5_1109	DJI_0220	Achillea millefolium L. s.l.	0	n	1	0	Asteraceae Bercht. & J.Presl
Iz_5_1109	DJI_0220	Allium oleraceum L.	0	n	1	0	Amaryllidaceae J.St.-Hil.
Iz_5_1109	DJI_0220	Ambrosia artemisiifolia L.	0	a	0	0	Asteraceae Bercht. & J.Presl
Iz_5_1109	DJI_0220	Amorpha fruticosa L.	0	a	0	0	Fabaceae Lindl.
Iz_5_1109	DJI_0220	Anthericum ramosum L.	0	n	1	0	Asparagaceae Juss.
Iz_5_1109	DJI_0220	Anthoxanthum repens (Host) Veldkamp	0		NA	NA	
Iz_5_1109	DJI_0220	Artemisia marschalliana Spreng.	0	n	1	0	Asteraceae Bercht. & J.Presl
Iz_5_1109	DJI_0220	Asparagus officinalis L.	0	n	1	0	Asparagaceae Juss.
Iz_5_1109	DJI_0220	Astragalus cicer L.	0	n	1	0	Fabaceae Lindl.
Iz_5_1109	DJI_0220	Bassia laniflora (S.G.Gmel.) A.J.Scott	0	n	1	0	Amaranthaceae Juss.
Iz_5_1109	DJI_0220	Berberis vulgaris L.	0	n	1	0	Berberidaceae Juss.
Iz_5_1109	DJI_0220	Berteroa incana (L.) DC.	0		NA	NA	
Iz_5_1109	DJI_0220	Betula pendula Roth.	0	n	1	1	Betulaceae Gray
Iz_5_1109	DJI_0220	Betula pendula Roth.	0	n	1	0	Poaceae Barnhart
Iz_5_1109	DJI_0220	Bromus inermis Leyss.	0		NA	NA	
Iz_5_1109	DJI_0220	Bromus squarrosus L.	0	a	0	0	Poaceae Barnhart
Iz_5_1109	DJI_0220	Bromus tectorum L.	0	a	0	0	Poaceae Barnhart
Iz_5_1109	DJI_0220	Calamagrostis epigejos (L.) Roth	2	n	1	0	Poaceae Barnhart
Iz_5_1109	DJI_0220	Carduus acanthoides L.	0	n	1	0	Asteraceae Bercht. & J.Presl
Iz_5_1109	DJI_0220	Carex sp.	0	n	1	0	Cyperaceae Juss.
Iz_5_1109	DJI_0220	Centaurea majorovii Dumbadze	0	n	1	0	Asteraceae Bercht. & J.Presl
Iz_5_1109	DJI_0220	Chamaecytisus ruthenicus (Fisch.ex Wol.) Klaskova	0	n	1	0	Fabaceae Lindl.
Iz_5_1109	DJI_0220	Chelidonium majus L.	0	n	1	0	Papaveraceae Juss.
Iz_5_1109	DJI_0220	Chenopodium betaceum Andrz.	0	n	1	0	Amaranthaceae Juss.
Iz_5_1109	DJI_0220	Chenopodium cf. album	0	n	1	0	Amaranthaceae Juss.
Iz_5_1109	DJI_0220	Chenopodium sp.	1		NA	0	Amaranthaceae Juss.
Iz_5_1109	DJI_0220	Chondrilla juncea L.	0	n	1	0	Asteraceae Bercht. & J.Presl
Iz_5_1109	DJI_0220	Cirsium vulgare (Savi) Ten.	0	n	1	0	Asteraceae Bercht. & J.Presl
Iz_5_1109	DJI_0220	Clinopodium acinos (L.) Kuntze	0	n	1	0	Lamiacea Lindl.
Iz_5_1109	DJI_0220	Convallaria majalis L.	0	n	1	0	Asparagaceae Juss.
Iz_5_1109	DJI_0220	Crataegus rhipidophylla Gand.	0	n	1	1	Rosaceae Juss.
Iz_5_1109	DJI_0220	Crepis foetida L.	0	n	1	0	Asteraceae Bercht. & J.Presl
Iz_5_1109	DJI_0220	Dactylis glomerata L.	0	n	1	0	Poaceae Barnhart
Iz_5_1109	DJI_0220	Dianthus campestris M.Bieb.	0	n	1	0	Caryophyllaceae Juss.
Iz_5_1109	DJI_0220	Digitaria sanguinalis (L.) Scop.	0	a	0	0	Poaceae Barnhart
Iz_5_1109	DJI_0220	Elymus repens (L.) Gould	0	n	1	0	Poaceae Barnhart
Iz_5_1109	DJI_0220	Epilobium tetragonum L.	0	n	1	0	Onagraceae Juss.
Iz_5_1109	DJI_0220	Eragrostis minor Host	0	a	0	0	Poaceae Barnhart
Iz_5_1109	DJI_0220	Erigeron canadensis L.	3	a	0	0	Asteraceae Bercht. & J.Presl
Iz_5_1109	DJI_0220	Euphorbia seguieriana Neck.	0	n	1	0	Euphorbiaceae Juss.
Iz_5_1109	DJI_0220	Fallopia dumetorum (L.) Holub	3	n	1	0	Polygonaceae Juss.
Iz_5_1109	DJI_0220	Festuca beckeri (Hack.) Trautv.	0	n	1	0	Poaceae Barnhart
Iz_5_1109	DJI_0220	Fragaria vesca L.	0	n	1	0	Rosaceae Juss.
Iz_5_1109	DJI_0220	Frangula alnus Mill.	0	n	1	1	Rhamnaceae Juss.
Iz_5_1109	DJI_0220	Fraxinus pennsylvanica Marshall	0	a	0	1	Oleaceae Hoffmanns. & Link
Iz_5_1109	DJI_0220	Galeopsis bifida Boenn.	0	n	1	0	Lamiacea Lindl.
Iz_5_1109	DJI_0220	Galium verum L.	0	n	1	0	Rubiaceae Juss.
Iz_5_1109	DJI_0220	Genista tinctoria L.	0	n	1	0	Fabaceae Lindl.
Iz_5_1109	DJI_0220	Geranium robertianum L.	0	n	1	0	Geraniaceae Juss.
Iz_5_1109	DJI_0220	Geranium sanquineum L.	0	n	1	0	Geraniaceae Juss.
Iz_5_1109	DJI_0220	Gypsophila paniculata L.	0	n	1	0	Caryophyllaceae Juss.
Iz_5_1109	DJI_0220	Helichrysum arenarium (L.) Moench	0	n	1	0	Asteraceae Bercht. & J.Presl
Iz_5_1109	DJI_0220	Herniaria polygama J.Gay	0	n	1	0	Caryophyllaceae Juss.
Iz_5_1109	DJI_0220	Hieracium robustum Fr.	0	n	1	0	Asteraceae Bercht. & J.Presl
Iz_5_1109	DJI_0220	Hieracium umbellatum L.	0	n	1	NA	Asteraceae Bercht. & J.Presl
Iz_5_1109	DJI_0220	Hylotelephium maximum (L.) Holub	0	n	1	0	Crassulaceae J.St.-Hil.
Iz_5_1109	DJI_0220	Jacobaea borysthenica (DC.) B.Nord. & Greuter	0	n	1	0	Asteraceae Bercht. & J.Presl
Iz_5_1109	DJI_0220	Jasione montana L.	0	n	1	0	Campanulaceae Juss.
Iz_5_1109	DJI_0220	Jurinea cyanoides (L.) Rchb.s.l.	1	n	1	0	Asteraceae Bercht. & J.Presl
Iz_5_1109	DJI_0220	Koeleria glauca (Spreng.) DC.	0	n	1	0	Poaceae Barnhart
Iz_5_1109	DJI_0220	Lactuca serriola L.	0	a	0	0	Asteraceae Bercht. & J.Presl
Iz_5_1109	DJI_0220	Linaria genistifolia (L.) Mill.	0	n	1	0	Plantaginaceae Juss.
Iz_5_1109	DJI_0220	Lomelosia argentea (L.) Greuter & Burdet	0	n	1	0	Caprifoliaceae Juss.
Iz_5_1109	DJI_0220	Melampyrum pratense L.	0	n	1	0	Orobanchaceae Vent.
Iz_5_1109	DJI_0220	Oenothera biennis L.	0	a	0	0	Onagraceae Juss.
Iz_5_1109	DJI_0220	Pentanema britannica (L.) D.Gut.Larr., Santos-Vicente, Anderb., E.Rico & M.M.Mart.Ort.	0	n	1	0	Asteraceae Bercht. & J.Presl
Iz_5_1109	DJI_0220	Peucedanum oreoselinum (L.) Moench	0	n	1	0	Apiaceae Lindl.
Iz_5_1109	DJI_0220	Peucedanum ruthenicum M.Bieb.	0	n	1	0	Apiaceae Lindl.
Iz_5_1109	DJI_0220	Pilosella echioides (Lumn.) F.W.Schultz & Sch.Bip.	0	n	1	0	Asteraceae Bercht. & J.Presl
Iz_5_1109	DJI_0220	Pilosella officinarum F. Schultz et Sch. Bip.	0	n	1	0	Asteraceae Bercht. & J.Presl
Iz_5_1109	DJI_0220	Pilosella sp.	0		NA	0	Asteraceae Bercht. & J.Presl
Iz_5_1109	DJI_0220	Pinus sylvestris L.	0	n	1	1	Pinaceae Spreng. ex F.Rudolphi
Iz_5_1109	DJI_0220	Poa annua L.	0	n	1	0	Poaceae Barnhart
Iz_5_1109	DJI_0220	Poa nemoralis L.	0	n	1	0	Poaceae Barnhart
Iz_5_1109	DJI_0220	Poa sp.	0		NA	0	Poaceae Barnhart
Iz_5_1109	DJI_0220	Polygonatum odoratum (Mill.) Druce	0	n	1	0	Asparagaceae Juss.
Iz_5_1109	DJI_0220	Polygonum arenarium Waldst. & Kit.	0	n	1	0	Polygonaceae Juss.
Iz_5_1109	DJI_0220	Populus nigra L.	0	n	1	1	Salicaceae Mirb.
Iz_5_1109	DJI_0220	Populus tremula L.	0	n	1	1	Salicaceae Mirb.

Iz_5_1109	DJI_0220	Populus Ã canescens (Aiton) Sm.	0	n	1	1	Salicaceae Mirb.
Iz_5_1109	DJI_0220	Potentilla argentea L.	0	n	1	0	Rosaceae Juss.
Iz_5_1109	DJI_0220	Psammophiliella muralis (L.) Ikonn.	0	n	1	0	Caryophyllaceae Juss.
Iz_5_1109	DJI_0220	Psephellus sumensis (Kalen.) Greuter	0	n	1	0	Asteraceae Bercht. & J.Presl
Iz_5_1109	DJI_0220	Pulsatilla pratensis (L.) Mill. s.l.	0	n	1	0	Ranunculaceae Juss.
Iz_5_1109	DJI_0220	Pyrus communis L.	0	a	0	1	Rosaceae Juss.
Iz_5_1109	DJI_0220	Quercus robur L.	0	n	1	1	Fagaceae Dumort.
Iz_5_1109	DJI_0220	Robinia pseudoacacia L.	0	a	0	1	Fabaceae Lindl.
Iz_5_1109	DJI_0220	Rosa sp.	0		NA	1	Rosaceae Juss.
Iz_5_1109	DJI_0220	Rubus idaeus L.	0	n	1	0	Rosaceae Juss.
Iz_5_1109	DJI_0220	Rumex acetosella L.	5	n	1	0	Polygonaceae Juss.
Iz_5_1109	DJI_0220	Rumex thyrsiflorus Fingerh.	0	n	1	0	Polygonaceae Juss.
Iz_5_1109	DJI_0220	Salix alba L.	0	n	1	1	Salicaceae Mirb.
Iz_5_1109	DJI_0220	Sambucus nigra L.	0	n	1	1	Viburnaceae Raf.
Iz_5_1109	DJI_0220	Secale sylvestre Host	0	n	1	0	Poaceae Barnhart
Iz_5_1109	DJI_0220	Sempervivum ruthenicum Schnittsp. & C.B. Lehm.	0	n	1	0	Crassulaceae J.St.-Hil.
Iz_5_1109	DJI_0220	Setaria viridis (L.) P. Beauv.	0	n	1	0	Poaceae Barnhart
Iz_5_1109	DJI_0220	Silene borysthenica (Gruner) Walters	0	n	1	0	Caryophyllaceae Juss.
Iz_5_1109	DJI_0220	Silene latifolia subsp. alba (Mill.) Greuter & Burdet	0	n	1	0	Caryophyllaceae Juss.
Iz_5_1109	DJI_0220	Solidago virgaurea L.	0	n	1	0	Asteraceae Bercht. & J.Presl
Iz_5_1109	DJI_0220	Sorbus aucuparia L.	0	n	1	1	Rosaceae Juss.
Iz_5_1109	DJI_0220	Stachys recta L.	0	n	1	0	Lamiacea Lindl.
Iz_5_1109	DJI_0220	Tanacetum vulgare L.	0	n	1	0	Asteraceae Bercht. & J.Presl
Iz_5_1109	DJI_0220	Thymus pallasianus Heinr.Braun	0	n	1	0	Lamiacea Lindl.
Iz_5_1109	DJI_0220	Tilia cordata Mill.	0	n	1	1	Malvaceae Juss.
Iz_5_1109	DJI_0220	Trifolium alpestre L.	0	n	1	0	Fabaceae Lindl.
Iz_5_1109	DJI_0220	Trifolium arvense L.	0	n	1	0	Fabaceae Lindl.
Iz_5_1109	DJI_0220	Ulmus glabra Huds.	0	n	1	1	Ulmaceae Mirb.
Iz_5_1109	DJI_0220	Verbascum cf. phlomoides L.	0		NA	NA	
Iz_5_1109	DJI_0220	Verbascum densiflorum Bertol.	0	n	1	0	Scrophulariaceae Juss.
Iz_5_1109	DJI_0220	Verbascum sp.	0	n	1	0	Scrophulariaceae Juss.
Iz_5_1109	DJI_0220	Veronica austriaca L.	0	n	1	0	Plantaginaceae Juss.
Iz_5_1109	DJI_0220	Veronica incana L.	0	n	1	0	Plantaginaceae Juss.
Iz_5_1109	DJI_0220	Veronica verna L.	0	n	1	0	Plantaginaceae Juss.
Iz_5_1109	DJI_0220	Vincetoxicum hirundinaria Medik	0	n	1	0	Apocynaceae Juss.
Iz_5_1109	DJI_0223	Acer tataricum L.	0	n	1	1	Sapindaceae Juss.
Iz_5_1109	DJI_0223	Achillea millefolium L. s.l.	0	n	1	0	Asteraceae Bercht. & J.Presl
Iz_5_1109	DJI_0223	Allium oleraceum L.	0	n	1	0	Amaryllidaceae J.St.-Hil.
Iz_5_1109	DJI_0223	Ambrosia artemisiifolia L.	0	a	0	0	Asteraceae Bercht. & J.Presl
Iz_5_1109	DJI_0223	Amorpha fruticosa L.	0	a	0	0	Fabaceae Lindl.
Iz_5_1109	DJI_0223	Anthericum ramosum L.	0	n	1	0	Asparagaceae Juss.
Iz_5_1109	DJI_0223	Anthoxanthum repens (Host) Veldkamp	0		NA	NA	
Iz_5_1109	DJI_0223	Artemisia marschalliana Spreng.	0	n	1	0	Asteraceae Bercht. & J.Presl
Iz_5_1109	DJI_0223	Asparagus officinalis L.	0	n	1	0	Asparagaceae Juss.
Iz_5_1109	DJI_0223	Astragalus cicer L.	0	n	1	0	Fabaceae Lindl.
Iz_5_1109	DJI_0223	Bassia laniflora (S.G.Gmel.) A.J.Scott	0	n	1	0	Amaranthaceae Juss.
Iz_5_1109	DJI_0223	Berberis vulgaris L.	0	n	1	0	Berberidaceae Juss.
Iz_5_1109	DJI_0223	Berteroa incana (L.) DC.	0		NA	NA	
Iz_5_1109	DJI_0223	Betula pendula Roth.	0	n	1	1	Betulaceae Gray
Iz_5_1109	DJI_0223	Betula pendula Roth.	0	n	1	0	Poaceae Barnhart
Iz_5_1109	DJI_0223	Bromus inermis Leyss.	0		NA	NA	
Iz_5_1109	DJI_0223	Bromus squarrosus L.	0	a	0	0	Poaceae Barnhart
Iz_5_1109	DJI_0223	Bromus tectorum L.	0	a	0	0	Poaceae Barnhart
Iz_5_1109	DJI_0223	Calamagrostis epigejos (L.) Roth	5	n	1	0	Poaceae Barnhart
Iz_5_1109	DJI_0223	Carduus acanthoides L.	0	n	1	0	Asteraceae Bercht. & J.Presl
Iz_5_1109	DJI_0223	Carex sp.	0	n	1	0	Cyperaceae Juss.
Iz_5_1109	DJI_0223	Centaurea majorovii Dumbadze	0	n	1	0	Asteraceae Bercht. & J.Presl
Iz_5_1109	DJI_0223	Chamaecytisus ruthenicus (Fisch.ex Wol.) Klaskova	0	n	1	0	Fabaceae Lindl.
Iz_5_1109	DJI_0223	Chelidonium majus L.	0	n	1	0	Papaveraceae Juss.
Iz_5_1109	DJI_0223	Chenopodium betaceum Andrz.	0	n	1	0	Amaranthaceae Juss.
Iz_5_1109	DJI_0223	Chenopodium cf. album	0	n	1	0	Amaranthaceae Juss.
Iz_5_1109	DJI_0223	Chenopodium sp.	2		NA	0	Amaranthaceae Juss.
Iz_5_1109	DJI_0223	Chondrilla juncea L.	0	n	1	0	Asteraceae Bercht. & J.Presl
Iz_5_1109	DJI_0223	Cirsium vulgare (Savi) Ten.	0	n	1	0	Asteraceae Bercht. & J.Presl
Iz_5_1109	DJI_0223	Clinopodium acinos (L.) Kuntze	0	n	1	0	Lamiacea Lindl.
Iz_5_1109	DJI_0223	Convallaria majalis L.	0	n	1	0	Asparagaceae Juss.
Iz_5_1109	DJI_0223	Crataegus rhipidophylla Gand.	0	n	1	1	Rosaceae Juss.
Iz_5_1109	DJI_0223	Crepis foetida L.	0	n	1	0	Asteraceae Bercht. & J.Presl
Iz_5_1109	DJI_0223	Dactylis glomerata L.	0	n	1	0	Poaceae Barnhart
Iz_5_1109	DJI_0223	Dianthus campestris M.Bieb.	0	n	1	0	Caryophyllaceae Juss.
Iz_5_1109	DJI_0223	Digitaria sanguinalis (L.) Scop.	0	a	0	0	Poaceae Barnhart
Iz_5_1109	DJI_0223	Elymus repens (L.) Gould	0	n	1	0	Poaceae Barnhart
Iz_5_1109	DJI_0223	Epilobium tetragonum L.	0	n	1	0	Onagraceae Juss.
Iz_5_1109	DJI_0223	Eragrostis minor Host	0	a	0	0	Poaceae Barnhart
Iz_5_1109	DJI_0223	Erigeron canadensis L.	7	a	0	0	Asteraceae Bercht. & J.Presl
Iz_5_1109	DJI_0223	Euphorbia seguieriana Neck.	0	n	1	0	Euphorbiaceae Juss.
Iz_5_1109	DJI_0223	Fallopia dumetorum (L.) Holub	0	n	1	0	Polygonaceae Juss.
Iz_5_1109	DJI_0223	Festuca beckeri (Hack.) Trautv.	0	n	1	0	Poaceae Barnhart
Iz_5_1109	DJI_0223	Fragaria vesca L.	0	n	1	0	Rosaceae Juss.
Iz_5_1109	DJI_0223	Frangula alnus Mill.	0	n	1	1	Rhamnaceae Juss.
Iz_5_1109	DJI_0223	Fraxinus pennsylvanica Marshall	0	a	0	1	Oleaceae Hoffmanns. & Link
Iz_5_1109	DJI_0223	Galeopsis bifida Boenn.	0	n	1	0	Lamiacea Lindl.
Iz_5_1109	DJI_0223	Galium verum L.	0	n	1	0	Rubiaceae Juss.
Iz_5_1109	DJI_0223	Genista tinctoria L.	0	n	1	0	Fabaceae Lindl.
Iz_5_1109	DJI_0223	Geranium robertianum L.	0	n	1	0	Geraniaceae Juss.
Iz_5_1109	DJI_0223	Geranium sanquineum L.	0	n	1	0	Geraniaceae Juss.
Iz_5_1109	DJI_0223	Gypsophila paniculata L.	0	n	1	0	Caryophyllaceae Juss.
Iz_5_1109	DJI_0223	Helichrysum arenarium (L.) Moench	0	n	1	0	Asteraceae Bercht. & J.Presl
Iz_5_1109	DJI_0223	Herniaria polygama J.Gay	0	n	1	0	Caryophyllaceae Juss.
Iz_5_1109	DJI_0223	Hieracium robustum Fr.	0	n	1	0	Asteraceae Bercht. & J.Presl
Iz_5_1109	DJI_0223	Hieracium umbellatum L.	0	n	1	NA	Asteraceae Bercht. & J.Presl
Iz_5_1109	DJI_0223	Hylotelephium maximum (L.) Holub	0	n	1	0	Crassulaceae J.St.-Hil.
Iz_5_1109	DJI_0223	Jacobaea borysthenica (DC.) B.Nord. & Greuter	0	n	1	0	Asteraceae Bercht. & J.Presl
Iz_5_1109	DJI_0223	Jasione montana L.	0	n	1	0	Campanulaceae Juss.
Iz_5_1109	DJI_0223	Jurinea cyanoides (L.) Rchb.s.l.	0	n	1	0	Asteraceae Bercht. & J.Presl
Iz_5_1109	DJI_0223	Koeleria glauca (Spreng.) DC.	0	n	1	0	Poaceae Barnhart
Iz_5_1109	DJI_0223	Lactuca serriola L.	0	a	0	0	Asteraceae Bercht. & J.Presl
Iz_5_1109	DJI_0223	Linaria genistifolia (L.) Mill.	0	n	1	0	Plantaginaceae Juss.

Iz_5_1109	DJI_0223	Lomelosia argentea (L.) Greuter & Burdet	0	n	1	0	Caprifoliaceae Juss.
Iz_5_1109	DJI_0223	Melampyrum pratense L.	0	n	1	0	Orobanchaceae Vent.
Iz_5_1109	DJI_0223	Oenothera biennis L.	0	a	0	0	Onagraceae Juss.
Iz_5_1109	DJI_0223	Pentanema britannica (L.) D.Gut.Larr., Santos-Vicente, Anderb., E.Rico & M.M.Mart.Ort.	0	n	1	0	Asteraceae Bercht. & J.Presl
Iz_5_1109	DJI_0223	Peucedanum oreoselinum (L.) Moench	0	n	1	0	Apiaceae Lindl.
Iz_5_1109	DJI_0223	Peucedanum ruthenicum M.Bieb.	0	n	1	0	Apiaceae Lindl.
Iz_5_1109	DJI_0223	Pilosella echioides (Lumn.) F.W.Schultz & Sch.Bip.	0	n	1	0	Asteraceae Bercht. & J.Presl
Iz_5_1109	DJI_0223	Pilosella officinarum F. Schultz et Sch. Bip.	0	n	1	0	Asteraceae Bercht. & J.Presl
Iz_5_1109	DJI_0223	Pilosella sp.	0		NA	0	Asteraceae Bercht. & J.Presl
Iz_5_1109	DJI_0223	Pinus sylvestris L.	0	n	1	1	Pinaceae Spreng. ex F.Rudolphi
Iz_5_1109	DJI_0223	Poa annua L.	0	n	1	0	Poaceae Barnhart
Iz_5_1109	DJI_0223	Poa nemoralis L.	0	n	1	0	Poaceae Barnhart
Iz_5_1109	DJI_0223	Poa sp.	0		NA	0	Poaceae Barnhart
Iz_5_1109	DJI_0223	Polygonatum odoratum (Mill.) Druce	0	n	1	0	Asparagaceae Juss.
Iz_5_1109	DJI_0223	Polygonum arenarium Waldst. & Kit.	0	n	1	0	Polygonaceae Juss.
Iz_5_1109	DJI_0223	Populus nigra L.	0	n	1	1	Salicaceae Mirb.
Iz_5_1109	DJI_0223	Populus tremula L.	0	n	1	1	Salicaceae Mirb.
Iz_5_1109	DJI_0223	Populus Ã canescens (Aiton) Sm.	0	n	1	1	Salicaceae Mirb.
Iz_5_1109	DJI_0223	Potentilla argentea L.	0	n	1	0	Rosaceae Juss.
Iz_5_1109	DJI_0223	Psammophiliella muralis (L.) Ikonn.	0	n	1	0	Caryophyllaceae Juss.
Iz_5_1109	DJI_0223	Psephellus sumensis (Kalen.) Greuter	0	n	1	0	Asteraceae Bercht. & J.Presl
Iz_5_1109	DJI_0223	Pulsatilla pratensis (L.) Mill. s.l.	0	n	1	0	Ranunculaceae Juss.
Iz_5_1109	DJI_0223	Pyrus communis L.	0	a	0	1	Rosaceae Juss.
Iz_5_1109	DJI_0223	Quercus robur L.	0	n	1	1	Fagaceae Dumort.
Iz_5_1109	DJI_0223	Robinia pseudoacacia L.	0	a	0	1	Fabaceae Lindl.
Iz_5_1109	DJI_0223	Rosa sp.	0		NA	1	Rosaceae Juss.
Iz_5_1109	DJI_0223	Rubus idaeus L.	0	n	1	0	Rosaceae Juss.
Iz_5_1109	DJI_0223	Rumex acetosella L.	0	n	1	0	Polygonaceae Juss.
Iz_5_1109	DJI_0223	Rumex thyrsiflorus Fingerh.	0	n	1	0	Polygonaceae Juss.
Iz_5_1109	DJI_0223	Salix alba L.	0	n	1	1	Salicaceae Mirb.
Iz_5_1109	DJI_0223	Sambucus nigra L.	0	n	1	1	Viburnaceae Raf.
Iz_5_1109	DJI_0223	Secale sylvestre Host	0	n	1	0	Poaceae Barnhart
Iz_5_1109	DJI_0223	Sempervivum ruthenicum Schnittsp. & C.B. Lehm.	0	n	1	0	Crassulaceae J.St.-Hil.
Iz_5_1109	DJI_0223	Setaria viridis (L.) P. Beauv.	0	n	1	0	Poaceae Barnhart
Iz_5_1109	DJI_0223	Silene borysthenica (Gruner) Walters	0	n	1	0	Caryophyllaceae Juss.
Iz_5_1109	DJI_0223	Silene latifolia subsp. alba (Mill.) Greuter & Burdet	0	n	1	0	Caryophyllaceae Juss.
Iz_5_1109	DJI_0223	Solidago virgaurea L.	0	n	1	0	Asteraceae Bercht. & J.Presl
Iz_5_1109	DJI_0223	Sorbus aucuparia L.	0	n	1	1	Rosaceae Juss.
Iz_5_1109	DJI_0223	Stachys recta L.	0	n	1	0	Lamiacea Lindl.
Iz_5_1109	DJI_0223	Tanacetum vulgare L.	0	n	1	0	Asteraceae Bercht. & J.Presl
Iz_5_1109	DJI_0223	Thymus pallasianus Heinr.Braun	0	n	1	0	Lamiacea Lindl.
Iz_5_1109	DJI_0223	Tilia cordata Mill.	0	n	1	1	Malvaceae Juss.
Iz_5_1109	DJI_0223	Trifolium alpestre L.	0	n	1	0	Fabaceae Lindl.
Iz_5_1109	DJI_0223	Trifolium arvense L.	0	n	1	0	Fabaceae Lindl.
Iz_5_1109	DJI_0223	Ulmus glabra Huds.	0	n	1	1	Ulmaceae Mirb.
Iz_5_1109	DJI_0223	Verbascum cf. phlomoides L.	0		NA	NA	
Iz_5_1109	DJI_0223	Verbascum densiflorum Bertol.	0	n	1	0	Scrophulariaceae Juss.
Iz_5_1109	DJI_0223	Verbascum sp.	0	n	1	0	Scrophulariaceae Juss.
Iz_5_1109	DJI_0223	Veronica austriaca L.	0	n	1	0	Plantaginaceae Juss.
Iz_5_1109	DJI_0223	Veronica incana L.	0	n	1	0	Plantaginaceae Juss.
Iz_5_1109	DJI_0223	Veronica verna L.	0	n	1	0	Plantaginaceae Juss.
Iz_5_1109	DJI_0223	Vincetoxicum hirundinaria Medik	0	n	1	0	Apocynaceae Juss.
Iz_5_1209	DJI_0242	Acer tataricum L.	0	n	1	1	Sapindaceae Juss.
Iz_5_1209	DJI_0242	Achillea millefolium L. s.l.	0	n	1	0	Asteraceae Bercht. & J.Presl
Iz_5_1209	DJI_0242	Allium oleraceum L.	0	n	1	0	Amaryllidaceae J.St.-Hil.
Iz_5_1209	DJI_0242	Ambrosia artemisiifolia L.	0	a	0	0	Asteraceae Bercht. & J.Presl
Iz_5_1209	DJI_0242	Amorpha fruticosa L.	0	a	0	0	Fabaceae Lindl.
Iz_5_1209	DJI_0242	Anthericum ramosum L.	0	n	1	0	Asparagaceae Juss.
Iz_5_1209	DJI_0242	Artemisia marschalliana Spreng.	0	n	1	0	Asteraceae Bercht. & J.Presl
Iz_5_1209	DJI_0242	Asparagus officinalis L.	0	n	1	0	Asparagaceae Juss.
Iz_5_1209	DJI_0242	Astragalus cicer L.	0	n	1	0	Fabaceae Lindl.
Iz_5_1209	DJI_0242	Bassia laniflora (S.G.Gmel.) A.J.Scott	0	n	1	0	Amaranthaceae Juss.
Iz_5_1209	DJI_0242	Berberis vulgaris L.	0	n	1	0	Berberidaceae Juss.
Iz_5_1209	DJI_0242	Betula pendula Roth.	0	n	1	1	Betulaceae Gray
Iz_5_1209	DJI_0242	Betula pendula Roth.	0	n	1	0	Poaceae Barnhart
Iz_5_1209	DJI_0242	Bromus inermis Leyss.	0		NA	NA	
Iz_5_1209	DJI_0242	Bromus squarrosus L.	0	a	0	0	Poaceae Barnhart
Iz_5_1209	DJI_0242	Bromus tectorum L.	0	a	0	0	Poaceae Barnhart
Iz_5_1209	DJI_0242	Calamagrostis epigejos (L.) Roth	50	n	1	0	Poaceae Barnhart
Iz_5_1209	DJI_0242	Carduus acanthoides L.	0	n	1	0	Asteraceae Bercht. & J.Presl
Iz_5_1209	DJI_0242	Carex sp.	0	n	1	0	Cyperaceae Juss.
Iz_5_1209	DJI_0242	Centaurea majorovii Dumbadze	0	n	1	0	Asteraceae Bercht. & J.Presl
Iz_5_1209	DJI_0242	Chamaecytisus ruthenicus (Fisch.ex Wol.) Klaskova	0	n	1	0	Fabaceae Lindl.
Iz_5_1209	DJI_0242	Chelidonium majus L.	0	n	1	0	Papaveraceae Juss.
Iz_5_1209	DJI_0242	Chenopodium betaceum Andrz.	0	n	1	0	Amaranthaceae Juss.
Iz_5_1209	DJI_0242	Chenopodium cf. album	0	n	1	0	Amaranthaceae Juss.
Iz_5_1209	DJI_0242	Chenopodium sp.	0		NA	0	Amaranthaceae Juss.
Iz_5_1209	DJI_0242	Chondrilla juncea L.	0	n	1	0	Asteraceae Bercht. & J.Presl
Iz_5_1209	DJI_0242	Cirsium vulgare (Savi) Ten.	0	n	1	0	Asteraceae Bercht. & J.Presl
Iz_5_1209	DJI_0242	Clinopodium acinos (L.) Kuntze	0	n	1	0	Lamiacea Lindl.
Iz_5_1209	DJI_0242	Convallaria majalis L.	0	n	1	0	Asparagaceae Juss.
Iz_5_1209	DJI_0242	Crataegus rhipidophylla Gand.	0	n	1	1	Rosaceae Juss.
Iz_5_1209	DJI_0242	Crepis foetida L.	0	n	1	0	Asteraceae Bercht. & J.Presl
Iz_5_1209	DJI_0242	Dactylis glomerata L.	0	n	1	0	Poaceae Barnhart
Iz_5_1209	DJI_0242	Dianthus campestris M.Bieb.	0	n	1	0	Caryophyllaceae Juss.
Iz_5_1209	DJI_0242	Digitaria sanguinalis (L.) Scop.	0	a	0	0	Poaceae Barnhart
Iz_5_1209	DJI_0242	Elymus repens (L.) Gould	0	n	1	0	Poaceae Barnhart
Iz_5_1209	DJI_0242	Epilobium tetragonum L.	0	n	1	0	Onagraceae Juss.
Iz_5_1209	DJI_0242	Eragrostis minor Host	0	a	0	0	Poaceae Barnhart
Iz_5_1209	DJI_0242	Erigeron canadensis L.	0	a	0	0	Asteraceae Bercht. & J.Presl
Iz_5_1209	DJI_0242	Euphorbia seguieriana Neck.	0	n	1	0	Euphorbiaceae Juss.
Iz_5_1209	DJI_0242	Fallopia dumetorum (L.) Holub	0	n	1	0	Polygonaceae Juss.
Iz_5_1209	DJI_0242	Festuca beckeri (Hack.) Trautv.	0	n	1	0	Poaceae Barnhart
Iz_5_1209	DJI_0242	Fragaria vesca L.	0	n	1	0	Rosaceae Juss.
Iz_5_1209	DJI_0242	Frangula alnus Mill.	0	n	1	1	Rhamnaceae Juss.
Iz_5_1209	DJI_0242	Fraxinus pennsylvanica Marshall	0	a	0	1	Oleaceae Hoffmanns. & Link
Iz_5_1209	DJI_0242	Galeopsis bifida Boenn.	0	n	1	0	Lamiacea Lindl.
Iz_5_1209	DJI_0242	Galium verum L.	0	n	1	0	Rubiaceae Juss.

Iz_5_1209	DJI_0242	Genista tinctoria L.	0	n	1	0	Fabaceae Lindl.
Iz_5_1209	DJI_0242	Geranium robertianum L.	0	n	1	0	Geraniaceae Juss.
Iz_5_1209	DJI_0242	Geranium sanquineum L.	0	n	1	0	Geraniaceae Juss.
Iz_5_1209	DJI_0242	Gypsophila paniculata L.	0	n	1	0	Caryophyllaceae Juss.
Iz_5_1209	DJI_0242	Helichrysum arenarium (L.) Moench	0	n	1	0	Asteraceae Bercht. & J.Presl
Iz_5_1209	DJI_0242	Herniaria polygama J.Gay	0	n	1	0	Caryophyllaceae Juss.
Iz_5_1209	DJI_0242	Hieracium robustum Fr.	0	n	1	0	Asteraceae Bercht. & J.Presl
Iz_5_1209	DJI_0242	Hieracium umbellatum L.	0	n	1	NA	Asteraceae Bercht. & J.Presl
Iz_5_1209	DJI_0242	Hylotelephium maximum (L.) Holub	0	n	1	0	Crassulaceae J.St.-Hil.
Iz_5_1209	DJI_0242	Jacobaea borysthenica (DC.) B.Nord. & Greuter	0	n	1	0	Asteraceae Bercht. & J.Presl
Iz_5_1209	DJI_0242	Jasione montana L.	0	n	1	0	Campanulaceae Juss.
Iz_5_1209	DJI_0242	Jurinea cyanoides (L.) Rchb.s.l.	0	n	1	0	Asteraceae Bercht. & J.Presl
Iz_5_1209	DJI_0242	Koeleria glauca (Spreng.) DC.	0	n	1	0	Poaceae Barnhart
Iz_5_1209	DJI_0242	Lactuca serriola L.	0	a	0	0	Asteraceae Bercht. & J.Presl
Iz_5_1209	DJI_0242	Linaria genistifolia (L.) Mill.	0	n	1	0	Plantaginaceae Juss.
Iz_5_1209	DJI_0242	Lomelosia argentea (L.) Greuter & Burdet	0	n	1	0	Caprifoliaceae Juss.
Iz_5_1209	DJI_0242	Melampyrum pratense L.	0	n	1	0	Orobanchaceae Vent.
Iz_5_1209	DJI_0242	Oenothera biennis L.	0	a	0	0	Onagraceae Juss.
Iz_5_1209	DJI_0242	Pentanema britannica (L.) D.Gut.Larr., Santos-Vicente, Anderb., E.Rico & M.M.Mart.Ort.	0	n	1	0	Asteraceae Bercht. & J.Presl
Iz_5_1209	DJI_0242	Peucedanum oreoselinum (L.) Moench	0	n	1	0	Apiaceae Lindl.
Iz_5_1209	DJI_0242	Peucedanum ruthenicum M.Bieb.	0	n	1	0	Apiaceae Lindl.
Iz_5_1209	DJI_0242	Pilosella echioides (Lumn.) F.W.Schultz & Sch.Bip.	0	n	1	0	Asteraceae Bercht. & J.Presl
Iz_5_1209	DJI_0242	Pilosella officinarum F. Schultz et Sch. Bip.	0	n	1	0	Asteraceae Bercht. & J.Presl
Iz_5_1209	DJI_0242	Pilosella sp.	0		NA	0	Asteraceae Bercht. & J.Presl
Iz_5_1209	DJI_0242	Pinus sylvestris L.	0	n	1	1	Pinaceae Spreng. ex F.Rudolphi
Iz_5_1209	DJI_0242	Poa annua L.	0	n	1	0	Poaceae Barnhart
Iz_5_1209	DJI_0242	Poa nemoralis L.	0	n	1	0	Poaceae Barnhart
Iz_5_1209	DJI_0242	Poa sp.	0		NA	0	Poaceae Barnhart
Iz_5_1209	DJI_0242	Polygonatum odoratum (Mill.) Druce	0	n	1	0	Asparagaceae Juss.
Iz_5_1209	DJI_0242	Polygonum arenarium Waldst. & Kit.	0	n	1	0	Polygonaceae Juss.
Iz_5_1209	DJI_0242	Populus nigra L.	0	n	1	1	Salicaceae Mirb.
Iz_5_1209	DJI_0242	Populus tremula L.	0	n	1	1	Salicaceae Mirb.
Iz_5_1209	DJI_0242	Populus Ã canescens (Aiton) Sm.	0	n	1	1	Salicaceae Mirb.
Iz_5_1209	DJI_0242	Potentilla argentea L.	0	n	1	0	Rosaceae Juss.
Iz_5_1209	DJI_0242	Psammophiliella muralis (L.) Ikonn.	0	n	1	0	Caryophyllaceae Juss.
Iz_5_1209	DJI_0242	Psephellus sumensis (Kalen.) Greuter	0	n	1	0	Asteraceae Bercht. & J.Presl
Iz_5_1209	DJI_0242	Pulsatilla pratensis (L.) Mill. s.l.	0	n	1	0	Ranunculaceae Juss.
Iz_5_1209	DJI_0242	Pyrus communis L.	0	a	0	1	Rosaceae Juss.
Iz_5_1209	DJI_0242	Quercus robur L.	0	n	1	1	Fagaceae Dumort.
Iz_5_1209	DJI_0242	Robinia pseudoacacia L.	0	a	0	1	Fabaceae Lindl.
Iz_5_1209	DJI_0242	Rosa sp.	0		NA	1	Rosaceae Juss.
Iz_5_1209	DJI_0242	Rubus idaeus L.	0	n	1	0	Rosaceae Juss.
Iz_5_1209	DJI_0242	Rumex acetosella L.	0	n	1	0	Polygonaceae Juss.
Iz_5_1209	DJI_0242	Rumex thyrsiflorus Fingerh.	0	n	1	0	Polygonaceae Juss.
Iz_5_1209	DJI_0242	Salix alba L.	0	n	1	1	Salicaceae Mirb.
Iz_5_1209	DJI_0242	Sambucus nigra L.	0	n	1	1	Viburnaceae Raf.
Iz_5_1209	DJI_0242	Secale sylvestre Host	0	n	1	0	Poaceae Barnhart
Iz_5_1209	DJI_0242	Sempervivum ruthenicum Schnittsp. & C.B. Lehm.	0	n	1	0	Crassulaceae J.St.-Hil.
Iz_5_1209	DJI_0242	Setaria viridis (L.) P. Beauv.	0	n	1	0	Poaceae Barnhart
Iz_5_1209	DJI_0242	Silene borysthenica (Gruner) Walters	0	n	1	0	Caryophyllaceae Juss.
Iz_5_1209	DJI_0242	Silene latifolia subsp. alba (Mill.) Greuter & Burdet	0	n	1	0	Caryophyllaceae Juss.
Iz_5_1209	DJI_0242	Solidago virgaurea L.	0	n	1	0	Asteraceae Bercht. & J.Presl
Iz_5_1209	DJI_0242	Sorbus aucuparia L.	0	n	1	1	Rosaceae Juss.
Iz_5_1209	DJI_0242	Stachys recta L.	0	n	1	0	Lamiacea Lindl.
Iz_5_1209	DJI_0242	Tanacetum vulgare L.	0	n	1	0	Asteraceae Bercht. & J.Presl
Iz_5_1209	DJI_0242	Thymus pallasianus Heinr.Braun	0	n	1	0	Lamiacea Lindl.
Iz_5_1209	DJI_0242	Tilia cordata Mill.	0	n	1	1	Malvaceae Juss.
Iz_5_1209	DJI_0242	Trifolium alpestre L.	0	n	1	0	Fabaceae Lindl.
Iz_5_1209	DJI_0242	Trifolium arvense L.	0	n	1	0	Fabaceae Lindl.
Iz_5_1209	DJI_0242	Ulmus glabra Huds.	0	n	1	1	Ulmaceae Mirb.
Iz_5_1209	DJI_0242	Verbascum densiflorum Bertol.	0	n	1	0	Scrophulariaceae Juss.
Iz_5_1209	DJI_0242	Veronica austriaca L.	0	n	1	0	Plantaginaceae Juss.
Iz_5_1209	DJI_0242	Veronica incana L.	0	n	1	0	Plantaginaceae Juss.
Iz_5_1209	DJI_0242	Veronica verna L.	0	n	1	0	Plantaginaceae Juss.
Iz_5_1209	DJI_0242	Vincetoxicum hirundinaria Medik	0	n	1	0	Apocynaceae Juss.
Iz_5_1209	DJI_0245	Acer tataricum L.	0	n	1	1	Sapindaceae Juss.
Iz_5_1209	DJI_0245	Achillea millefolium L. s.l.	0	n	1	0	Asteraceae Bercht. & J.Presl
Iz_5_1209	DJI_0245	Allium oleraceum L.	0	n	1	0	Amaryllidaceae J.St.-Hil.
Iz_5_1209	DJI_0245	Ambrosia artemisiifolia L.	0	a	0	0	Asteraceae Bercht. & J.Presl
Iz_5_1209	DJI_0245	Amorpha fruticosa L.	0	a	0	0	Fabaceae Lindl.
Iz_5_1209	DJI_0245	Anthericum ramosum L.	0	n	1	0	Asparagaceae Juss.
Iz_5_1209	DJI_0245	Artemisia marschalliana Spreng.	0	n	1	0	Asteraceae Bercht. & J.Presl
Iz_5_1209	DJI_0245	Asparagus officinalis L.	0	n	1	0	Asparagaceae Juss.
Iz_5_1209	DJI_0245	Astragalus cicer L.	0	n	1	0	Fabaceae Lindl.
Iz_5_1209	DJI_0245	Bassia laniflora (S.G.Gmel.) A.J.Scott	0	n	1	0	Amaranthaceae Juss.
Iz_5_1209	DJI_0245	Berberis vulgaris L.	0	n	1	0	Berberidaceae Juss.
Iz_5_1209	DJI_0245	Betula pendula Roth.	0	n	1	1	Betulaceae Gray
Iz_5_1209	DJI_0245	Betula pendula Roth.	0	n	1	0	Poaceae Barnhart
Iz_5_1209	DJI_0245	Bromus inermis Leyss.	0		NA	NA	
Iz_5_1209	DJI_0245	Bromus squarrosus L.	0	a	0	0	Poaceae Barnhart
Iz_5_1209	DJI_0245	Bromus tectorum L.	0	a	0	0	Poaceae Barnhart
Iz_5_1209	DJI_0245	Calamagrostis epigejos (L.) Roth	1	n	1	0	Poaceae Barnhart
Iz_5_1209	DJI_0245	Carduus acanthoides L.	0	n	1	0	Asteraceae Bercht. & J.Presl
Iz_5_1209	DJI_0245	Carex sp.	0	n	1	0	Cyperaceae Juss.
Iz_5_1209	DJI_0245	Centaurea majorovii Dumbadze	0	n	1	0	Asteraceae Bercht. & J.Presl
Iz_5_1209	DJI_0245	Chamaecytisus ruthenicus (Fisch.ex Wol.) Klaskova	0	n	1	0	Fabaceae Lindl.
Iz_5_1209	DJI_0245	Chelidonium majus L.	0	n	1	0	Papaveraceae Juss.
Iz_5_1209	DJI_0245	Chenopodium betaceum Andrz.	0	n	1	0	Amaranthaceae Juss.
Iz_5_1209	DJI_0245	Chenopodium cf. album	1	n	1	0	Amaranthaceae Juss.
Iz_5_1209	DJI_0245	Chenopodium sp.	0		NA	0	Amaranthaceae Juss.
Iz_5_1209	DJI_0245	Chondrilla juncea L.	0	n	1	0	Asteraceae Bercht. & J.Presl
Iz_5_1209	DJI_0245	Cirsium vulgare (Savi) Ten.	0	n	1	0	Asteraceae Bercht. & J.Presl
Iz_5_1209	DJI_0245	Clinopodium acinos (L.) Kuntze	0	n	1	0	Lamiacea Lindl.
Iz_5_1209	DJI_0245	Convallaria majalis L.	0	n	1	0	Asparagaceae Juss.
Iz_5_1209	DJI_0245	Crataegus rhipidophylla Gand.	0	n	1	1	Rosaceae Juss.
Iz_5_1209	DJI_0245	Crepis foetida L.	0	n	1	0	Asteraceae Bercht. & J.Presl
Iz_5_1209	DJI_0245	Dactylis glomerata L.	0	n	1	0	Poaceae Barnhart
Iz_5_1209	DJI_0245	Dianthus campestris M.Bieb.	0	n	1	0	Caryophyllaceae Juss.

Iz_5_1209	DJI_0245	Digitaria sanguinalis (L.) Scop.	0	a	0	0	Poaceae Barnhart
Iz_5_1209	DJI_0245	Elymus repens (L.) Gould	0	n	1	0	Poaceae Barnhart
Iz_5_1209	DJI_0245	Epilobium tetragonum L.	0	n	1	0	Onagraceae Juss.
Iz_5_1209	DJI_0245	Eragrostis minor Host	0	a	0	0	Poaceae Barnhart
Iz_5_1209	DJI_0245	Erigeron canadensis L.	10	a	0	0	Asteraceae Bercht. & J.Presl
Iz_5_1209	DJI_0245	Euphorbia seguieriana Neck.	0	n	1	0	Euphorbiaceae Juss.
Iz_5_1209	DJI_0245	Fallopia dumetorum (L.) Holub	0	n	1	0	Polygonaceae Juss.
Iz_5_1209	DJI_0245	Festuca beckeri (Hack.) Trautv.	0	n	1	0	Poaceae Barnhart
Iz_5_1209	DJI_0245	Fragaria vesca L.	0	n	1	0	Rosaceae Juss.
Iz_5_1209	DJI_0245	Frangula alnus Mill.	0	n	1	1	Rhamnaceae Juss.
Iz_5_1209	DJI_0245	Fraxinus pennsylvanica Marshall	0	a	0	1	Oleaceae Hoffmanns. & Link
Iz_5_1209	DJI_0245	Galeopsis bifida Boenn.	0	n	1	0	Lamiacea Lindl.
Iz_5_1209	DJI_0245	Galium verum L.	0	n	1	0	Rubiaceae Juss.
Iz_5_1209	DJI_0245	Genista tinctoria L.	0	n	1	0	Fabaceae Lindl.
Iz_5_1209	DJI_0245	Geranium robertianum L.	0	n	1	0	Geraniaceae Juss.
Iz_5_1209	DJI_0245	Geranium sanquineum L.	0	n	1	0	Geraniaceae Juss.
Iz_5_1209	DJI_0245	Gypsophila paniculata L.	0	n	1	0	Caryophyllaceae Juss.
Iz_5_1209	DJI_0245	Helichrysum arenarium (L.) Moench	0	n	1	0	Asteraceae Bercht. & J.Presl
Iz_5_1209	DJI_0245	Herniaria polygama J.Gay	0	n	1	0	Caryophyllaceae Juss.
Iz_5_1209	DJI_0245	Hieracium robustum Fr.	0	n	1	0	Asteraceae Bercht. & J.Presl
Iz_5_1209	DJI_0245	Hieracium umbellatum L.	0	n	1	NA	Asteraceae Bercht. & J.Presl
Iz_5_1209	DJI_0245	Hylotelephium maximum (L.) Holub	0	n	1	0	Crassulaceae J.St.-Hil.
Iz_5_1209	DJI_0245	Jacobaea borysthenica (DC.) B.Nord. & Greuter	0	n	1	0	Asteraceae Bercht. & J.Presl
Iz_5_1209	DJI_0245	Jasione montana L.	0	n	1	0	Campanulaceae Juss.
Iz_5_1209	DJI_0245	Jurinea cyanoides (L.) Rchb.s.l.	0	n	1	0	Asteraceae Bercht. & J.Presl
Iz_5_1209	DJI_0245	Koeleria glauca (Spreng.) DC.	0	n	1	0	Poaceae Barnhart
Iz_5_1209	DJI_0245	Lactuca serriola L.	0	a	0	0	Asteraceae Bercht. & J.Presl
Iz_5_1209	DJI_0245	Linaria genistifolia (L.) Mill.	0	n	1	0	Plantaginaceae Juss.
Iz_5_1209	DJI_0245	Lomelosia argentea (L.) Greuter & Burdet	0	n	1	0	Caprifoliaceae Juss.
Iz_5_1209	DJI_0245	Melampyrum pratense L.	0	n	1	0	Orobanchaceae Vent.
Iz_5_1209	DJI_0245	Oenothera biennis L.	0	a	0	0	Onagraceae Juss.
Iz_5_1209	DJI_0245	Pentanema britannica (L.) D.Gut.Larr., Santos-Vicente, Anderb., E.Rico & M.M.Mart.Ort.	0	n	1	0	Asteraceae Bercht. & J.Presl
Iz_5_1209	DJI_0245	Peucedanum oreoselinum (L.) Moench	0	n	1	0	Apiaceae Lindl.
Iz_5_1209	DJI_0245	Peucedanum ruthenicum M.Bieb.	0	n	1	0	Apiaceae Lindl.
Iz_5_1209	DJI_0245	Pilosella echioides (Lumn.) F.W.Schultz & Sch.Bip.	0	n	1	0	Asteraceae Bercht. & J.Presl
Iz_5_1209	DJI_0245	Pilosella officinarum F. Schultz et Sch. Bip.	0	n	1	0	Asteraceae Bercht. & J.Presl
Iz_5_1209	DJI_0245	Pilosella sp.	0		NA	0	Asteraceae Bercht. & J.Presl
Iz_5_1209	DJI_0245	Pinus sylvestris L.	0	n	1	1	Pinaceae Spreng. ex F.Rudolphi
Iz_5_1209	DJI_0245	Poa annua L.	0	n	1	0	Poaceae Barnhart
Iz_5_1209	DJI_0245	Poa nemoralis L.	0	n	1	0	Poaceae Barnhart
Iz_5_1209	DJI_0245	Poa sp.	0		NA	0	Poaceae Barnhart
Iz_5_1209	DJI_0245	Polygonatum odoratum (Mill.) Druce	0	n	1	0	Asparagaceae Juss.
Iz_5_1209	DJI_0245	Polygonum arenarium Waldst. & Kit.	0	n	1	0	Polygonaceae Juss.
Iz_5_1209	DJI_0245	Populus nigra L.	0	n	1	1	Salicaceae Mirb.
Iz_5_1209	DJI_0245	Populus tremula L.	0	n	1	1	Salicaceae Mirb.
Iz_5_1209	DJI_0245	Populus Ã canescens (Aiton) Sm.	0	n	1	1	Salicaceae Mirb.
Iz_5_1209	DJI_0245	Potentilla argentea L.	0	n	1	0	Rosaceae Juss.
Iz_5_1209	DJI_0245	Psammophiliella muralis (L.) Ikonn.	0	n	1	0	Caryophyllaceae Juss.
Iz_5_1209	DJI_0245	Psephellus sumensis (Kalen.) Greuter	0	n	1	0	Asteraceae Bercht. & J.Presl
Iz_5_1209	DJI_0245	Pulsatilla pratensis (L.) Mill. s.l.	0	n	1	0	Ranunculaceae Juss.
Iz_5_1209	DJI_0245	Pyrus communis L.	0	a	0	1	Rosaceae Juss.
Iz_5_1209	DJI_0245	Quercus robur L.	0	n	1	1	Fagaceae Dumort.
Iz_5_1209	DJI_0245	Robinia pseudoacacia L.	0	a	0	1	Fabaceae Lindl.
Iz_5_1209	DJI_0245	Rosa sp.	0		NA	1	Rosaceae Juss.
Iz_5_1209	DJI_0245	Rubus idaeus L.	0	n	1	0	Rosaceae Juss.
Iz_5_1209	DJI_0245	Rumex acetosella L.	0	n	1	0	Polygonaceae Juss.
Iz_5_1209	DJI_0245	Rumex thyrsiflorus Fingerh.	0	n	1	0	Polygonaceae Juss.
Iz_5_1209	DJI_0245	Salix alba L.	0	n	1	1	Salicaceae Mirb.
Iz_5_1209	DJI_0245	Sambucus nigra L.	0	n	1	1	Viburnaceae Raf.
Iz_5_1209	DJI_0245	Secale sylvestre Host	0	n	1	0	Poaceae Barnhart
Iz_5_1209	DJI_0245	Sempervivum ruthenicum Schnittsp. & C.B. Lehm.	0	n	1	0	Crassulaceae J.St.-Hil.
Iz_5_1209	DJI_0245	Setaria viridis (L.) P. Beauv.	0	n	1	0	Poaceae Barnhart
Iz_5_1209	DJI_0245	Silene borysthenica (Gruner) Walters	0	n	1	0	Caryophyllaceae Juss.
Iz_5_1209	DJI_0245	Silene latifolia subsp. alba (Mill.) Greuter & Burdet	0	n	1	0	Caryophyllaceae Juss.
Iz_5_1209	DJI_0245	Solidago virgaurea L.	0	n	1	0	Asteraceae Bercht. & J.Presl
Iz_5_1209	DJI_0245	Sorbus aucuparia L.	0	n	1	1	Rosaceae Juss.
Iz_5_1209	DJI_0245	Stachys recta L.	0	n	1	0	Lamiacea Lindl.
Iz_5_1209	DJI_0245	Tanacetum vulgare L.	0	n	1	0	Asteraceae Bercht. & J.Presl
Iz_5_1209	DJI_0245	Thymus pallasianus Heinr.Braun	0	n	1	0	Lamiacea Lindl.
Iz_5_1209	DJI_0245	Tilia cordata Mill.	0	n	1	1	Malvaceae Juss.
Iz_5_1209	DJI_0245	Trifolium alpestre L.	0	n	1	0	Fabaceae Lindl.
Iz_5_1209	DJI_0245	Trifolium arvense L.	0	n	1	0	Fabaceae Lindl.
Iz_5_1209	DJI_0245	Ulmus glabra Huds.	0	n	1	1	Ulmaceae Mirb.
Iz_5_1209	DJI_0245	Verbascum densiflorum Bertol.	0	n	1	0	Scrophulariaceae Juss.
Iz_5_1209	DJI_0245	Veronica austriaca L.	0	n	1	0	Plantaginaceae Juss.
Iz_5_1209	DJI_0245	Veronica incana L.	0	n	1	0	Plantaginaceae Juss.
Iz_5_1209	DJI_0245	Veronica verna L.	0	n	1	0	Plantaginaceae Juss.
Iz_5_1209	DJI_0245	Vincetoxicum hirundinaria Medik	0	n	1	0	Apocynaceae Juss.
Iz_5_1209	DJI_0249	Acer tataricum L.	0	n	1	1	Sapindaceae Juss.
Iz_5_1209	DJI_0249	Achillea millefolium L. s.l.	0	n	1	0	Asteraceae Bercht. & J.Presl
Iz_5_1209	DJI_0249	Allium oleraceum L.	0	n	1	0	Amaryllidaceae J.St.-Hil.
Iz_5_1209	DJI_0249	Ambrosia artemisiifolia L.	0	a	0	0	Asteraceae Bercht. & J.Presl
Iz_5_1209	DJI_0249	Amorpha fruticosa L.	0	a	0	0	Fabaceae Lindl.
Iz_5_1209	DJI_0249	Anthericum ramosum L.	0	n	1	0	Asparagaceae Juss.
Iz_5_1209	DJI_0249	Artemisia marschalliana Spreng.	0	n	1	0	Asteraceae Bercht. & J.Presl
Iz_5_1209	DJI_0249	Asparagus officinalis L.	0	n	1	0	Asparagaceae Juss.
Iz_5_1209	DJI_0249	Astragalus cicer L.	0	n	1	0	Fabaceae Lindl.
Iz_5_1209	DJI_0249	Bassia laniflora (S.G.Gmel.) A.J.Scott	0	n	1	0	Amaranthaceae Juss.
Iz_5_1209	DJI_0249	Berberis vulgaris L.	0	n	1	0	Berberidaceae Juss.
Iz_5_1209	DJI_0249	Betula pendula Roth.	0	n	1	1	Betulaceae Gray
Iz_5_1209	DJI_0249	Betula pendula Roth.	0	n	1	0	Poaceae Barnhart
Iz_5_1209	DJI_0249	Bromus inermis Leyss.	0		NA	NA	
Iz_5_1209	DJI_0249	Bromus squarrosus L.	0	a	0	0	Poaceae Barnhart
Iz_5_1209	DJI_0249	Bromus tectorum L.	0	a	0	0	Poaceae Barnhart
Iz_5_1209	DJI_0249	Calamagrostis epigejos (L.) Roth	10	n	1	0	Poaceae Barnhart
Iz_5_1209	DJI_0249	Carduus acanthoides L.	0	n	1	0	Asteraceae Bercht. & J.Presl
Iz_5_1209	DJI_0249	Carex sp.	0	n	1	0	Cyperaceae Juss.
Iz_5_1209	DJI_0249	Centaurea majorovii Dumbadze	0	n	1	0	Asteraceae Bercht. & J.Presl

Iz_5_1209	DJI_0249	Chamaecytisus ruthenicus (Fisch.ex Wol.) Klaskova	0	n	1	0	Fabaceae Lindl.
Iz_5_1209	DJI_0249	Chelidonium majus L.	0	n	1	0	Papaveraceae Juss.
Iz_5_1209	DJI_0249	Chenopodium betaceum Andrz.	0	n	1	0	Amaranthaceae Juss.
Iz_5_1209	DJI_0249	Chenopodium cf. album	3	n	1	0	Amaranthaceae Juss.
Iz_5_1209	DJI_0249	Chenopodium sp.	0		NA	0	Amaranthaceae Juss.
Iz_5_1209	DJI_0249	Chondrilla juncea L.	0	n	1	0	Asteraceae Bercht. & J.Presl
Iz_5_1209	DJI_0249	Cirsium vulgare (Savi) Ten.	0	n	1	0	Asteraceae Bercht. & J.Presl
Iz_5_1209	DJI_0249	Clinopodium acinos (L.) Kuntze	0	n	1	0	Lamiacea Lindl.
Iz_5_1209	DJI_0249	Convallaria majalis L.	0	n	1	0	Asparagaceae Juss.
Iz_5_1209	DJI_0249	Crataegus rhipidophylla Gand.	0	n	1	1	Rosaceae Juss.
Iz_5_1209	DJI_0249	Crepis foetida L.	0	n	1	0	Asteraceae Bercht. & J.Presl
Iz_5_1209	DJI_0249	Dactylis glomerata L.	0	n	1	0	Poaceae Barnhart
Iz_5_1209	DJI_0249	Dianthus campestris M.Bieb.	0	n	1	0	Caryophyllaceae Juss.
Iz_5_1209	DJI_0249	Digitaria sanguinalis (L.) Scop.	0	a	0	0	Poaceae Barnhart
Iz_5_1209	DJI_0249	Elymus repens (L.) Gould	0	n	1	0	Poaceae Barnhart
Iz_5_1209	DJI_0249	Epilobium tetragonum L.	0	n	1	0	Onagraceae Juss.
Iz_5_1209	DJI_0249	Eragrostis minor Host	0	a	0	0	Poaceae Barnhart
Iz_5_1209	DJI_0249	Erigeron canadensis L.	0	a	0	0	Asteraceae Bercht. & J.Presl
Iz_5_1209	DJI_0249	Euphorbia seguieriana Neck.	0	n	1	0	Euphorbiaceae Juss.
Iz_5_1209	DJI_0249	Fallopia dumetorum (L.) Holub	0	n	1	0	Polygonaceae Juss.
Iz_5_1209	DJI_0249	Festuca beckeri (Hack.) Trautv.	0	n	1	0	Poaceae Barnhart
Iz_5_1209	DJI_0249	Fragaria vesca L.	0	n	1	0	Rosaceae Juss.
Iz_5_1209	DJI_0249	Frangula alnus Mill.	0	n	1	1	Rhamnaceae Juss.
Iz_5_1209	DJI_0249	Fraxinus pennsylvanica Marshall	0	a	0	1	Oleaceae Hoffmanns. & Link
Iz_5_1209	DJI_0249	Galeopsis bifida Boenn.	0	n	1	0	Lamiacea Lindl.
Iz_5_1209	DJI_0249	Galium verum L.	0	n	1	0	Rubiaceae Juss.
Iz_5_1209	DJI_0249	Genista tinctoria L.	0	n	1	0	Fabaceae Lindl.
Iz_5_1209	DJI_0249	Geranium robertianum L.	0	n	1	0	Geraniaceae Juss.
Iz_5_1209	DJI_0249	Geranium sanquineum L.	0	n	1	0	Geraniaceae Juss.
Iz_5_1209	DJI_0249	Gypsophila paniculata L.	0	n	1	0	Caryophyllaceae Juss.
Iz_5_1209	DJI_0249	Helichrysum arenarium (L.) Moench	0	n	1	0	Asteraceae Bercht. & J.Presl
Iz_5_1209	DJI_0249	Herniaria polygama J.Gay	0	n	1	0	Caryophyllaceae Juss.
Iz_5_1209	DJI_0249	Hieracium robustum Fr.	0	n	1	0	Asteraceae Bercht. & J.Presl
Iz_5_1209	DJI_0249	Hieracium umbellatum L.	0	n	1	NA	Asteraceae Bercht. & J.Presl
Iz_5_1209	DJI_0249	Hylotelephium maximum (L.) Holub	0	n	1	0	Crassulaceae J.St.-Hil.
Iz_5_1209	DJI_0249	Jacobaea borysthenica (DC.) B.Nord. & Greuter	0	n	1	0	Asteraceae Bercht. & J.Presl
Iz_5_1209	DJI_0249	Jasione montana L.	0	n	1	0	Campanulaceae Juss.
Iz_5_1209	DJI_0249	Jurinea cyanoides (L.) Rchb.s.l.	0	n	1	0	Asteraceae Bercht. & J.Presl
Iz_5_1209	DJI_0249	Koeleria glauca (Spreng.) DC.	0	n	1	0	Poaceae Barnhart
Iz_5_1209	DJI_0249	Lactuca serriola L.	0	a	0	0	Asteraceae Bercht. & J.Presl
Iz_5_1209	DJI_0249	Linaria genistifolia (L.) Mill.	0	n	1	0	Plantaginaceae Juss.
Iz_5_1209	DJI_0249	Lomelosia argentea (L.) Greuter & Burdet	0	n	1	0	Caprifoliaceae Juss.
Iz_5_1209	DJI_0249	Melampyrum pratense L.	0	n	1	0	Orobanchaceae Vent.
Iz_5_1209	DJI_0249	Oenothera biennis L.	0	a	0	0	Onagraceae Juss.
Iz_5_1209	DJI_0249	Pentanema britannica (L.) D.Gut.Larr., Santos-Vicente, Anderb., E.Rico & M.M.Mart.Ort.	0	n	1	0	Asteraceae Bercht. & J.Presl
Iz_5_1209	DJI_0249	Peucedanum oreoselinum (L.) Moench	0	n	1	0	Apiaceae Lindl.
Iz_5_1209	DJI_0249	Peucedanum ruthenicum M.Bieb.	0	n	1	0	Apiaceae Lindl.
Iz_5_1209	DJI_0249	Pilosella echioides (Lumn.) F.W.Schultz & Sch.Bip.	0	n	1	0	Asteraceae Bercht. & J.Presl
Iz_5_1209	DJI_0249	Pilosella officinarum F. Schultz et Sch. Bip.	0	n	1	0	Asteraceae Bercht. & J.Presl
Iz_5_1209	DJI_0249	Pilosella sp.	0		NA	0	Asteraceae Bercht. & J.Presl
Iz_5_1209	DJI_0249	Pinus sylvestris L.	0	n	1	1	Pinaceae Spreng. ex F.Rudolphi
Iz_5_1209	DJI_0249	Poa annua L.	0	n	1	0	Poaceae Barnhart
Iz_5_1209	DJI_0249	Poa nemoralis L.	0	n	1	0	Poaceae Barnhart
Iz_5_1209	DJI_0249	Poa sp.	0		NA	0	Poaceae Barnhart
Iz_5_1209	DJI_0249	Polygonatum odoratum (Mill.) Druce	0	n	1	0	Asparagaceae Juss.
Iz_5_1209	DJI_0249	Polygonum arenarium Waldst. & Kit.	0	n	1	0	Polygonaceae Juss.
Iz_5_1209	DJI_0249	Populus nigra L.	0	n	1	1	Salicaceae Mirb.
Iz_5_1209	DJI_0249	Populus tremula L.	0	n	1	1	Salicaceae Mirb.
Iz_5_1209	DJI_0249	Populus Ã canescens (Aiton) Sm.	0	n	1	1	Salicaceae Mirb.
Iz_5_1209	DJI_0249	Potentilla argentea L.	0	n	1	0	Rosaceae Juss.
Iz_5_1209	DJI_0249	Psammophiliella muralis (L.) Ikonn.	0	n	1	0	Caryophyllaceae Juss.
Iz_5_1209	DJI_0249	Psephellus sumensis (Kalen.) Greuter	0	n	1	0	Asteraceae Bercht. & J.Presl
Iz_5_1209	DJI_0249	Pulsatilla pratensis (L.) Mill. s.l.	0	n	1	0	Ranunculaceae Juss.
Iz_5_1209	DJI_0249	Pyrus communis L.	0	a	0	1	Rosaceae Juss.
Iz_5_1209	DJI_0249	Quercus robur L.	0	n	1	1	Fagaceae Dumort.
Iz_5_1209	DJI_0249	Robinia pseudoacacia L.	0	a	0	1	Fabaceae Lindl.
Iz_5_1209	DJI_0249	Rosa sp.	0		NA	1	Rosaceae Juss.
Iz_5_1209	DJI_0249	Rubus idaeus L.	0	n	1	0	Rosaceae Juss.
Iz_5_1209	DJI_0249	Rumex acetosella L.	0	n	1	0	Polygonaceae Juss.
Iz_5_1209	DJI_0249	Rumex thyrsiflorus Fingerh.	0	n	1	0	Polygonaceae Juss.
Iz_5_1209	DJI_0249	Salix alba L.	0	n	1	1	Salicaceae Mirb.
Iz_5_1209	DJI_0249	Sambucus nigra L.	0	n	1	1	Viburnaceae Raf.
Iz_5_1209	DJI_0249	Secale sylvestre Host	0	n	1	0	Poaceae Barnhart
Iz_5_1209	DJI_0249	Sempervivum ruthenicum Schnittsp. & C.B. Lehm.	0	n	1	0	Crassulaceae J.St.-Hil.
Iz_5_1209	DJI_0249	Setaria viridis (L.) P. Beauv.	0	n	1	0	Poaceae Barnhart
Iz_5_1209	DJI_0249	Silene borysthenica (Gruner) Walters	0	n	1	0	Caryophyllaceae Juss.
Iz_5_1209	DJI_0249	Silene latifolia subsp. alba (Mill.) Greuter & Burdet	0	n	1	0	Caryophyllaceae Juss.
Iz_5_1209	DJI_0249	Solidago virgaurea L.	0	n	1	0	Asteraceae Bercht. & J.Presl
Iz_5_1209	DJI_0249	Sorbus aucuparia L.	0	n	1	1	Rosaceae Juss.
Iz_5_1209	DJI_0249	Stachys recta L.	0	n	1	0	Lamiacea Lindl.
Iz_5_1209	DJI_0249	Tanacetum vulgare L.	0	n	1	0	Asteraceae Bercht. & J.Presl
Iz_5_1209	DJI_0249	Thymus pallasianus Heinr.Braun	0	n	1	0	Lamiacea Lindl.
Iz_5_1209	DJI_0249	Tilia cordata Mill.	0	n	1	1	Malvaceae Juss.
Iz_5_1209	DJI_0249	Trifolium alpestre L.	0	n	1	0	Fabaceae Lindl.
Iz_5_1209	DJI_0249	Trifolium arvense L.	0	n	1	0	Fabaceae Lindl.
Iz_5_1209	DJI_0249	Ulmus glabra Huds.	0	n	1	1	Ulmaceae Mirb.
Iz_5_1209	DJI_0249	Verbascum densiflorum Bertol.	0	n	1	0	Scrophulariaceae Juss.
Iz_5_1209	DJI_0249	Veronica austriaca L.	0	n	1	0	Plantaginaceae Juss.
Iz_5_1209	DJI_0249	Veronica incana L.	0	n	1	0	Plantaginaceae Juss.
Iz_5_1209	DJI_0249	Veronica verna L.	0	n	1	0	Plantaginaceae Juss.
Iz_5_1209	DJI_0249	Vincetoxicum hirundinaria Medik	0	n	1	0	Apocynaceae Juss.
Iz_5_1209	DJI_0253	Acer tataricum L.	0	n	1	1	Sapindaceae Juss.
Iz_5_1209	DJI_0253	Achillea millefolium L. s.l.	0	n	1	0	Asteraceae Bercht. & J.Presl
Iz_5_1209	DJI_0253	Allium oleraceum L.	0	n	1	0	Amaryllidaceae J.St.-Hil.
Iz_5_1209	DJI_0253	Ambrosia artemisiifolia L.	0	a	0	0	Asteraceae Bercht. & J.Presl
Iz_5_1209	DJI_0253	Amorpha fruticosa L.	0	a	0	0	Fabaceae Lindl.
Iz_5_1209	DJI_0253	Anthericum ramosum L.	0	n	1	0	Asparagaceae Juss.
Iz_5_1209	DJI_0253	Artemisia marschalliana Spreng.	0	n	1	0	Asteraceae Bercht. & J.Presl

Iz_5_1209	DJI_0253	Asparagus officinalis L.	0	n	1	0	Asparagaceae Juss.
Iz_5_1209	DJI_0253	Astragalus cicer L.	0	n	1	0	Fabaceae Lindl.
Iz_5_1209	DJI_0253	Bassia laniflora (S.G.Gmel.) A.J.Scott	0	n	1	0	Amaranthaceae Juss.
Iz_5_1209	DJI_0253	Berberis vulgaris L.	0	n	1	0	Berberidaceae Juss.
Iz_5_1209	DJI_0253	Betula pendula Roth.	0	n	1	1	Betulaceae Gray
Iz_5_1209	DJI_0253	Betula pendula Roth.	0	n	1	0	Poaceae Barnhart
Iz_5_1209	DJI_0253	Bromus inermis Leyss.	0		NA	NA	
Iz_5_1209	DJI_0253	Bromus squarrosus L.	0	a	0	0	Poaceae Barnhart
Iz_5_1209	DJI_0253	Bromus tectorum L.	0	a	0	0	Poaceae Barnhart
Iz_5_1209	DJI_0253	Calamagrostis epigejos (L.) Roth	20	n	1	0	Poaceae Barnhart
Iz_5_1209	DJI_0253	Carduus acanthoides L.	0	n	1	0	Asteraceae Bercht. & J.Presl
Iz_5_1209	DJI_0253	Carex sp.	0	n	1	0	Cyperaceae Juss.
Iz_5_1209	DJI_0253	Centaurea majorovii Dumbadze	0	n	1	0	Asteraceae Bercht. & J.Presl
Iz_5_1209	DJI_0253	Chamaecytisus ruthenicus (Fisch.ex Wol.) Klaskova	0	n	1	0	Fabaceae Lindl.
Iz_5_1209	DJI_0253	Chelidonium majus L.	0	n	1	0	Papaveraceae Juss.
Iz_5_1209	DJI_0253	Chenopodium betaceum Andrz.	0	n	1	0	Amaranthaceae Juss.
Iz_5_1209	DJI_0253	Chenopodium cf. album	0	n	1	0	Amaranthaceae Juss.
Iz_5_1209	DJI_0253	Chenopodium sp.	0		NA	0	Amaranthaceae Juss.
Iz_5_1209	DJI_0253	Chondrilla juncea L.	0	n	1	0	Asteraceae Bercht. & J.Presl
Iz_5_1209	DJI_0253	Cirsium vulgare (Savi) Ten.	0	n	1	0	Asteraceae Bercht. & J.Presl
Iz_5_1209	DJI_0253	Clinopodium acinos (L.) Kuntze	0	n	1	0	Lamiacea Lindl.
Iz_5_1209	DJI_0253	Convallaria majalis L.	0	n	1	0	Asparagaceae Juss.
Iz_5_1209	DJI_0253	Crataegus rhipidophylla Gand.	0	n	1	1	Rosaceae Juss.
Iz_5_1209	DJI_0253	Crepis foetida L.	0	n	1	0	Asteraceae Bercht. & J.Presl
Iz_5_1209	DJI_0253	Dactylis glomerata L.	0	n	1	0	Poaceae Barnhart
Iz_5_1209	DJI_0253	Dianthus campestris M.Bieb.	0	n	1	0	Caryophyllaceae Juss.
Iz_5_1209	DJI_0253	Digitaria sanguinalis (L.) Scop.	0	a	0	0	Poaceae Barnhart
Iz_5_1209	DJI_0253	Elymus repens (L.) Gould	0	n	1	0	Poaceae Barnhart
Iz_5_1209	DJI_0253	Epilobium tetragonum L.	0	n	1	0	Onagraceae Juss.
Iz_5_1209	DJI_0253	Eragrostis minor Host	0	a	0	0	Poaceae Barnhart
Iz_5_1209	DJI_0253	Erigeron canadensis L.	2	a	0	0	Asteraceae Bercht. & J.Presl
Iz_5_1209	DJI_0253	Euphorbia seguieriana Neck.	0	n	1	0	Euphorbiaceae Juss.
Iz_5_1209	DJI_0253	Fallopia dumetorum (L.) Holub	0	n	1	0	Polygonaceae Juss.
Iz_5_1209	DJI_0253	Festuca beckeri (Hack.) Trautv.	0	n	1	0	Poaceae Barnhart
Iz_5_1209	DJI_0253	Fragaria vesca L.	0	n	1	0	Rosaceae Juss.
Iz_5_1209	DJI_0253	Frangula alnus Mill.	0	n	1	1	Rhamnaceae Juss.
Iz_5_1209	DJI_0253	Fraxinus pennsylvanica Marshall	0	a	0	1	Oleaceae Hoffmanns. & Link
Iz_5_1209	DJI_0253	Galeopsis bifida Boenn.	0	n	1	0	Lamiacea Lindl.
Iz_5_1209	DJI_0253	Galium verum L.	0	n	1	0	Rubiaceae Juss.
Iz_5_1209	DJI_0253	Genista tinctoria L.	0	n	1	0	Fabaceae Lindl.
Iz_5_1209	DJI_0253	Geranium robertianum L.	0	n	1	0	Geraniaceae Juss.
Iz_5_1209	DJI_0253	Geranium sanquineum L.	0	n	1	0	Geraniaceae Juss.
Iz_5_1209	DJI_0253	Gypsophila paniculata L.	0	n	1	0	Caryophyllaceae Juss.
Iz_5_1209	DJI_0253	Helichrysum arenarium (L.) Moench	0	n	1	0	Asteraceae Bercht. & J.Presl
Iz_5_1209	DJI_0253	Herniaria polygama J.Gay	0	n	1	0	Caryophyllaceae Juss.
Iz_5_1209	DJI_0253	Hieracium robustum Fr.	0	n	1	0	Asteraceae Bercht. & J.Presl
Iz_5_1209	DJI_0253	Hieracium umbellatum L.	0	n	1	NA	Asteraceae Bercht. & J.Presl
Iz_5_1209	DJI_0253	Hylotelephium maximum (L.) Holub	0	n	1	0	Crassulaceae J.St.-Hil.
Iz_5_1209	DJI_0253	Jacobaea borysthenica (DC.) B.Nord. & Greuter	0	n	1	0	Asteraceae Bercht. & J.Presl
Iz_5_1209	DJI_0253	Jasione montana L.	0	n	1	0	Campanulaceae Juss.
Iz_5_1209	DJI_0253	Jurinea cyanoides (L.) Rchb.s.l.	0	n	1	0	Asteraceae Bercht. & J.Presl
Iz_5_1209	DJI_0253	Koeleria glauca (Spreng.) DC.	0	n	1	0	Poaceae Barnhart
Iz_5_1209	DJI_0253	Lactuca serriola L.	0	a	0	0	Asteraceae Bercht. & J.Presl
Iz_5_1209	DJI_0253	Linaria genistifolia (L.) Mill.	0	n	1	0	Plantaginaceae Juss.
Iz_5_1209	DJI_0253	Lomelosia argentea (L.) Greuter & Burdet	0	n	1	0	Caprifoliaceae Juss.
Iz_5_1209	DJI_0253	Melampyrum pratense L.	0	n	1	0	Orobanchaceae Vent.
Iz_5_1209	DJI_0253	Oenothera biennis L.	0	a	0	0	Onagraceae Juss.
Iz_5_1209	DJI_0253	Pentanema britannica (L.) D.Gut.Larr., Santos-Vicente, Anderb., E.Rico & M.M.Mart.Ort.	0	n	1	0	Asteraceae Bercht. & J.Presl
Iz_5_1209	DJI_0253	Peucedanum oreoselinum (L.) Moench	0	n	1	0	Apiaceae Lindl.
Iz_5_1209	DJI_0253	Peucedanum ruthenicum M.Bieb.	0	n	1	0	Apiaceae Lindl.
Iz_5_1209	DJI_0253	Pilosella echioides (Lumn.) F.W.Schultz & Sch.Bip.	0	n	1	0	Asteraceae Bercht. & J.Presl
Iz_5_1209	DJI_0253	Pilosella officinarum F. Schultz et Sch. Bip.	0	n	1	0	Asteraceae Bercht. & J.Presl
Iz_5_1209	DJI_0253	Pilosella sp.	0		NA	0	Asteraceae Bercht. & J.Presl
Iz_5_1209	DJI_0253	Pinus sylvestris L.	0	n	1	1	Pinaceae Spreng. ex F.Rudolphi
Iz_5_1209	DJI_0253	Poa annua L.	0	n	1	0	Poaceae Barnhart
Iz_5_1209	DJI_0253	Poa nemoralis L.	0	n	1	0	Poaceae Barnhart
Iz_5_1209	DJI_0253	Poa sp.	0		NA	0	Poaceae Barnhart
Iz_5_1209	DJI_0253	Polygonatum odoratum (Mill.) Druce	0	n	1	0	Asparagaceae Juss.
Iz_5_1209	DJI_0253	Polygonum arenarium Waldst. & Kit.	0	n	1	0	Polygonaceae Juss.
Iz_5_1209	DJI_0253	Populus nigra L.	0	n	1	1	Salicaceae Mirb.
Iz_5_1209	DJI_0253	Populus tremula L.	0	n	1	1	Salicaceae Mirb.
Iz_5_1209	DJI_0253	Populus Ã canescens (Aiton) Sm.	0	n	1	1	Salicaceae Mirb.
Iz_5_1209	DJI_0253	Potentilla argentea L.	0	n	1	0	Rosaceae Juss.
Iz_5_1209	DJI_0253	Psammophiliella muralis (L.) Ikonn.	0	n	1	0	Caryophyllaceae Juss.
Iz_5_1209	DJI_0253	Psephellus sumensis (Kalen.) Greuter	0	n	1	0	Asteraceae Bercht. & J.Presl
Iz_5_1209	DJI_0253	Pulsatilla pratensis (L.) Mill. s.l.	0	n	1	0	Ranunculaceae Juss.
Iz_5_1209	DJI_0253	Pyrus communis L.	0	a	0	1	Rosaceae Juss.
Iz_5_1209	DJI_0253	Quercus robur L.	0	n	1	1	Fagaceae Dumort.
Iz_5_1209	DJI_0253	Robinia pseudoacacia L.	0	a	0	1	Fabaceae Lindl.
Iz_5_1209	DJI_0253	Rosa sp.	0		NA	1	Rosaceae Juss.
Iz_5_1209	DJI_0253	Rubus idaeus L.	0	n	1	0	Rosaceae Juss.
Iz_5_1209	DJI_0253	Rumex acetosella L.	0	n	1	0	Polygonaceae Juss.
Iz_5_1209	DJI_0253	Rumex thyrsiflorus Fingerh.	0	n	1	0	Polygonaceae Juss.
Iz_5_1209	DJI_0253	Salix alba L.	0	n	1	1	Salicaceae Mirb.
Iz_5_1209	DJI_0253	Sambucus nigra L.	0	n	1	1	Viburnaceae Raf.
Iz_5_1209	DJI_0253	Secale sylvestre Host	0	n	1	0	Poaceae Barnhart
Iz_5_1209	DJI_0253	Sempervivum ruthenicum Schnittsp. & C.B. Lehm.	0	n	1	0	Crassulaceae J.St.-Hil.
Iz_5_1209	DJI_0253	Setaria viridis (L.) P. Beauv.	0	n	1	0	Poaceae Barnhart
Iz_5_1209	DJI_0253	Silene borysthenica (Gruner) Walters	0	n	1	0	Caryophyllaceae Juss.
Iz_5_1209	DJI_0253	Silene latifolia subsp. alba (Mill.) Greuter & Burdet	0	n	1	0	Caryophyllaceae Juss.
Iz_5_1209	DJI_0253	Solidago virgaurea L.	0	n	1	0	Asteraceae Bercht. & J.Presl
Iz_5_1209	DJI_0253	Sorbus aucuparia L.	0	n	1	1	Rosaceae Juss.
Iz_5_1209	DJI_0253	Stachys recta L.	0	n	1	0	Lamiacea Lindl.
Iz_5_1209	DJI_0253	Tanacetum vulgare L.	0	n	1	0	Asteraceae Bercht. & J.Presl
Iz_5_1209	DJI_0253	Thymus pallasianus Heinr.Braun	0	n	1	0	Lamiacea Lindl.
Iz_5_1209	DJI_0253	Tilia cordata Mill.	0	n	1	1	Malvaceae Juss.
Iz_5_1209	DJI_0253	Trifolium alpestre L.	0	n	1	0	Fabaceae Lindl.
Iz_5_1209	DJI_0253	Trifolium arvense L.	0	n	1	0	Fabaceae Lindl.

Iz_5_1209	DJI_0253	Ulmus glabra Huds.	0	n	1	1	Ulmaceae Mirb.
Iz_5_1209	DJI_0253	Verbascum densiflorum Bertol.	0	n	1	0	Scrophulariaceae Juss.
Iz_5_1209	DJI_0253	Veronica austriaca L.	0	n	1	0	Plantaginaceae Juss.
Iz_5_1209	DJI_0253	Veronica incana L.	0	n	1	0	Plantaginaceae Juss.
Iz_5_1209	DJI_0253	Veronica verna L.	0	n	1	0	Plantaginaceae Juss.
Iz_5_1209	DJI_0253	Vincetoxicum hirundinaria Medik	0	n	1	0	Apocynaceae Juss.
Iz_5_1209	DJI_0256	Acer tataricum L.	0	n	1	1	Sapindaceae Juss.
Iz_5_1209	DJI_0256	Achillea millefolium L. s.l.	0	n	1	0	Asteraceae Bercht. & J.Presl
Iz_5_1209	DJI_0256	Allium oleraceum L.	0	n	1	0	Amaryllidaceae J.St.-Hil.
Iz_5_1209	DJI_0256	Ambrosia artemisiifolia L.	0	a	0	0	Asteraceae Bercht. & J.Presl
Iz_5_1209	DJI_0256	Amorpha fruticosa L.	0	a	0	0	Fabaceae Lindl.
Iz_5_1209	DJI_0256	Anthericum ramosum L.	0	n	1	0	Asparagaceae Juss.
Iz_5_1209	DJI_0256	Artemisia marschalliana Spreng.	0	n	1	0	Asteraceae Bercht. & J.Presl
Iz_5_1209	DJI_0256	Asparagus officinalis L.	0	n	1	0	Asparagaceae Juss.
Iz_5_1209	DJI_0256	Astragalus cicer L.	0	n	1	0	Fabaceae Lindl.
Iz_5_1209	DJI_0256	Bassia laniflora (S.G.Gmel.) A.J.Scott	0	n	1	0	Amaranthaceae Juss.
Iz_5_1209	DJI_0256	Berberis vulgaris L.	0	n	1	0	Berberidaceae Juss.
Iz_5_1209	DJI_0256	Betula pendula Roth.	0	n	1	1	Betulaceae Gray
Iz_5_1209	DJI_0256	Betula pendula Roth.	0	n	1	0	Poaceae Barnhart
Iz_5_1209	DJI_0256	Bromus inermis Leyss.	0		NA	NA	
Iz_5_1209	DJI_0256	Bromus squarrosus L.	0	a	0	0	Poaceae Barnhart
Iz_5_1209	DJI_0256	Bromus tectorum L.	0	a	0	0	Poaceae Barnhart
Iz_5_1209	DJI_0256	Calamagrostis epigejos (L.) Roth	50	n	1	0	Poaceae Barnhart
Iz_5_1209	DJI_0256	Carduus acanthoides L.	0	n	1	0	Asteraceae Bercht. & J.Presl
Iz_5_1209	DJI_0256	Carex sp.	0	n	1	0	Cyperaceae Juss.
Iz_5_1209	DJI_0256	Centaurea majorovii Dumbadze	0	n	1	0	Asteraceae Bercht. & J.Presl
Iz_5_1209	DJI_0256	Chamaecytisus ruthenicus (Fisch.ex Wol.) Klaskova	0	n	1	0	Fabaceae Lindl.
Iz_5_1209	DJI_0256	Chelidonium majus L.	0	n	1	0	Papaveraceae Juss.
Iz_5_1209	DJI_0256	Chenopodium betaceum Andrz.	0	n	1	0	Amaranthaceae Juss.
Iz_5_1209	DJI_0256	Chenopodium cf. album	0	n	1	0	Amaranthaceae Juss.
Iz_5_1209	DJI_0256	Chenopodium sp.	0		NA	0	Amaranthaceae Juss.
Iz_5_1209	DJI_0256	Chondrilla juncea L.	0	n	1	0	Asteraceae Bercht. & J.Presl
Iz_5_1209	DJI_0256	Cirsium vulgare (Savi) Ten.	0	n	1	0	Asteraceae Bercht. & J.Presl
Iz_5_1209	DJI_0256	Clinopodium acinos (L.) Kuntze	0	n	1	0	Lamiacea Lindl.
Iz_5_1209	DJI_0256	Convallaria majalis L.	0	n	1	0	Asparagaceae Juss.
Iz_5_1209	DJI_0256	Crataegus rhipidophylla Gand.	0	n	1	1	Rosaceae Juss.
Iz_5_1209	DJI_0256	Crepis foetida L.	0	n	1	0	Asteraceae Bercht. & J.Presl
Iz_5_1209	DJI_0256	Dactylis glomerata L.	0	n	1	0	Poaceae Barnhart
Iz_5_1209	DJI_0256	Dianthus campestris M.Bieb.	0	n	1	0	Caryophyllaceae Juss.
Iz_5_1209	DJI_0256	Digitaria sanguinalis (L.) Scop.	0	a	0	0	Poaceae Barnhart
Iz_5_1209	DJI_0256	Elymus repens (L.) Gould	0	n	1	0	Poaceae Barnhart
Iz_5_1209	DJI_0256	Epilobium tetragonum L.	0	n	1	0	Onagraceae Juss.
Iz_5_1209	DJI_0256	Eragrostis minor Host	0	a	0	0	Poaceae Barnhart
Iz_5_1209	DJI_0256	Erigeron canadensis L.	0	a	0	0	Asteraceae Bercht. & J.Presl
Iz_5_1209	DJI_0256	Euphorbia seguieriana Neck.	0	n	1	0	Euphorbiaceae Juss.
Iz_5_1209	DJI_0256	Fallopia dumetorum (L.) Holub	0	n	1	0	Polygonaceae Juss.
Iz_5_1209	DJI_0256	Festuca beckeri (Hack.) Trautv.	0	n	1	0	Poaceae Barnhart
Iz_5_1209	DJI_0256	Fragaria vesca L.	0	n	1	0	Rosaceae Juss.
Iz_5_1209	DJI_0256	Frangula alnus Mill.	0	n	1	1	Rhamnaceae Juss.
Iz_5_1209	DJI_0256	Fraxinus pennsylvanica Marshall	0	a	0	1	Oleaceae Hoffmanns. & Link
Iz_5_1209	DJI_0256	Galeopsis bifida Boenn.	0	n	1	0	Lamiacea Lindl.
Iz_5_1209	DJI_0256	Galium verum L.	0	n	1	0	Rubiaceae Juss.
Iz_5_1209	DJI_0256	Genista tinctoria L.	0	n	1	0	Fabaceae Lindl.
Iz_5_1209	DJI_0256	Geranium robertianum L.	0	n	1	0	Geraniaceae Juss.
Iz_5_1209	DJI_0256	Geranium sanquineum L.	0	n	1	0	Geraniaceae Juss.
Iz_5_1209	DJI_0256	Gypsophila paniculata L.	0	n	1	0	Caryophyllaceae Juss.
Iz_5_1209	DJI_0256	Helichrysum arenarium (L.) Moench	0	n	1	0	Asteraceae Bercht. & J.Presl
Iz_5_1209	DJI_0256	Herniaria polygama J.Gay	0	n	1	0	Caryophyllaceae Juss.
Iz_5_1209	DJI_0256	Hieracium robustum Fr.	0	n	1	0	Asteraceae Bercht. & J.Presl
Iz_5_1209	DJI_0256	Hieracium umbellatum L.	0	n	1	NA	Asteraceae Bercht. & J.Presl
Iz_5_1209	DJI_0256	Hylotelephium maximum (L.) Holub	0	n	1	0	Crassulaceae J.St.-Hil.
Iz_5_1209	DJI_0256	Jacobaea borysthenica (DC.) B.Nord. & Greuter	0	n	1	0	Asteraceae Bercht. & J.Presl
Iz_5_1209	DJI_0256	Jasione montana L.	0	n	1	0	Campanulaceae Juss.
Iz_5_1209	DJI_0256	Jurinea cyanoides (L.) Rchb.s.l.	0	n	1	0	Asteraceae Bercht. & J.Presl
Iz_5_1209	DJI_0256	Koeleria glauca (Spreng.) DC.	0	n	1	0	Poaceae Barnhart
Iz_5_1209	DJI_0256	Lactuca serriola L.	0	a	0	0	Asteraceae Bercht. & J.Presl
Iz_5_1209	DJI_0256	Linaria genistifolia (L.) Mill.	0	n	1	0	Plantaginaceae Juss.
Iz_5_1209	DJI_0256	Lomelosia argentea (L.) Greuter & Burdet	0	n	1	0	Caprifoliaceae Juss.
Iz_5_1209	DJI_0256	Melampyrum pratense L.	0	n	1	0	Orobanchaceae Vent.
Iz_5_1209	DJI_0256	Oenothera biennis L.	0	a	0	0	Onagraceae Juss.
Iz_5_1209	DJI_0256	Pentanema britannica (L.) D.Gut.Larr., Santos-Vicente, Anderb., E.Rico & M.M.Mart.Ort.	0	n	1	0	Asteraceae Bercht. & J.Presl
Iz_5_1209	DJI_0256	Peucedanum oreoselinum (L.) Moench	0	n	1	0	Apiaceae Lindl.
Iz_5_1209	DJI_0256	Peucedanum ruthenicum M.Bieb.	0	n	1	0	Apiaceae Lindl.
Iz_5_1209	DJI_0256	Pilosella echioides (Lumn.) F.W.Schultz & Sch.Bip.	0	n	1	0	Asteraceae Bercht. & J.Presl
Iz_5_1209	DJI_0256	Pilosella officinarum F. Schultz et Sch. Bip.	0	n	1	0	Asteraceae Bercht. & J.Presl
Iz_5_1209	DJI_0256	Pilosella sp.	0		NA	0	Asteraceae Bercht. & J.Presl
Iz_5_1209	DJI_0256	Pinus sylvestris L.	0	n	1	1	Pinaceae Spreng. ex F.Rudolphi
Iz_5_1209	DJI_0256	Poa annua L.	0	n	1	0	Poaceae Barnhart
Iz_5_1209	DJI_0256	Poa nemoralis L.	0	n	1	0	Poaceae Barnhart
Iz_5_1209	DJI_0256	Poa sp.	0		NA	0	Poaceae Barnhart
Iz_5_1209	DJI_0256	Polygonatum odoratum (Mill.) Druce	0	n	1	0	Asparagaceae Juss.
Iz_5_1209	DJI_0256	Polygonum arenarium Waldst. & Kit.	0	n	1	0	Polygonaceae Juss.
Iz_5_1209	DJI_0256	Populus nigra L.	0	n	1	1	Salicaceae Mirb.
Iz_5_1209	DJI_0256	Populus tremula L.	0	n	1	1	Salicaceae Mirb.
Iz_5_1209	DJI_0256	Populus Ã canescens (Aiton) Sm.	0	n	1	1	Salicaceae Mirb.
Iz_5_1209	DJI_0256	Potentilla argentea L.	0	n	1	0	Rosaceae Juss.
Iz_5_1209	DJI_0256	Psammophiliella muralis (L.) Ikonn.	0	n	1	0	Caryophyllaceae Juss.
Iz_5_1209	DJI_0256	Psephellus sumensis (Kalen.) Greuter	0	n	1	0	Asteraceae Bercht. & J.Presl
Iz_5_1209	DJI_0256	Pulsatilla pratensis (L.) Mill. s.l.	0	n	1	0	Ranunculaceae Juss.
Iz_5_1209	DJI_0256	Pyrus communis L.	0	a	0	1	Rosaceae Juss.
Iz_5_1209	DJI_0256	Quercus robur L.	0	n	1	1	Fagaceae Dumort.
Iz_5_1209	DJI_0256	Robinia pseudoacacia L.	0	a	0	1	Fabaceae Lindl.
Iz_5_1209	DJI_0256	Rosa sp.	0		NA	1	Rosaceae Juss.
Iz_5_1209	DJI_0256	Rubus idaeus L.	0	n	1	0	Rosaceae Juss.
Iz_5_1209	DJI_0256	Rumex acetosella L.	0	n	1	0	Polygonaceae Juss.
Iz_5_1209	DJI_0256	Rumex thyrsiflorus Fingerh.	0	n	1	0	Polygonaceae Juss.
Iz_5_1209	DJI_0256	Salix alba L.	0	n	1	1	Salicaceae Mirb.
Iz_5_1209	DJI_0256	Sambucus nigra L.	0	n	1	1	Viburnaceae Raf.

Iz_5_1209	DJI_0256	Secale sylvestre Host	0	n	1	0	Poaceae Barnhart
Iz_5_1209	DJI_0256	Sempervivum ruthenicum Schnittsp. & C.B. Lehm.	0	n	1	0	Crassulaceae J.St.-Hil.
Iz_5_1209	DJI_0256	Setaria viridis (L.) P. Beauv.	0	n	1	0	Poaceae Barnhart
Iz_5_1209	DJI_0256	Silene borysthenica (Gruner) Walters	0	n	1	0	Caryophyllaceae Juss.
Iz_5_1209	DJI_0256	Silene latifolia subsp. alba (Mill.) Greuter & Burdet	0	n	1	0	Caryophyllaceae Juss.
Iz_5_1209	DJI_0256	Solidago virgaurea L.	0.5	n	1	0	Asteraceae Bercht. & J.Presl
Iz_5_1209	DJI_0256	Sorbus aucuparia L.	0	n	1	1	Rosaceae Juss.
Iz_5_1209	DJI_0256	Stachys recta L.	0	n	1	0	Lamiacea Lindl.
Iz_5_1209	DJI_0256	Tanacetum vulgare L.	0	n	1	0	Asteraceae Bercht. & J.Presl
Iz_5_1209	DJI_0256	Thymus pallasianus Heinr.Braun	0	n	1	0	Lamiacea Lindl.
Iz_5_1209	DJI_0256	Tilia cordata Mill.	0	n	1	1	Malvaceae Juss.
Iz_5_1209	DJI_0256	Trifolium alpestre L.	0	n	1	0	Fabaceae Lindl.
Iz_5_1209	DJI_0256	Trifolium arvense L.	0	n	1	0	Fabaceae Lindl.
Iz_5_1209	DJI_0256	Ulmus glabra Huds.	0	n	1	1	Ulmaceae Mirb.
Iz_5_1209	DJI_0256	Verbascum densiflorum Bertol.	0	n	1	0	Scrophulariaceae Juss.
Iz_5_1209	DJI_0256	Veronica austriaca L.	0	n	1	0	Plantaginaceae Juss.
Iz_5_1209	DJI_0256	Veronica incana L.	0	n	1	0	Plantaginaceae Juss.
Iz_5_1209	DJI_0256	Veronica verna L.	0	n	1	0	Plantaginaceae Juss.
Iz_5_1209	DJI_0256	Vincetoxicum hirundinaria Medik	0	n	1	0	Apocynaceae Juss.
Iz_5_1209	DJI_0259	Acer tataricum L.	0	n	1	1	Sapindaceae Juss.
Iz_5_1209	DJI_0259	Achillea millefolium L. s.l.	0	n	1	0	Asteraceae Bercht. & J.Presl
Iz_5_1209	DJI_0259	Allium oleraceum L.	0	n	1	0	Amaryllidaceae J.St.-Hil.
Iz_5_1209	DJI_0259	Ambrosia artemisiifolia L.	0	a	0	0	Asteraceae Bercht. & J.Presl
Iz_5_1209	DJI_0259	Amorpha fruticosa L.	0	a	0	0	Fabaceae Lindl.
Iz_5_1209	DJI_0259	Anthericum ramosum L.	0	n	1	0	Asparagaceae Juss.
Iz_5_1209	DJI_0259	Artemisia marschalliana Spreng.	0	n	1	0	Asteraceae Bercht. & J.Presl
Iz_5_1209	DJI_0259	Asparagus officinalis L.	0	n	1	0	Asparagaceae Juss.
Iz_5_1209	DJI_0259	Astragalus cicer L.	0	n	1	0	Fabaceae Lindl.
Iz_5_1209	DJI_0259	Bassia laniflora (S.G.Gmel.) A.J.Scott	0	n	1	0	Amaranthaceae Juss.
Iz_5_1209	DJI_0259	Berberis vulgaris L.	0	n	1	0	Berberidaceae Juss.
Iz_5_1209	DJI_0259	Betula pendula Roth.	0	n	1	1	Betulaceae Gray
Iz_5_1209	DJI_0259	Betula pendula Roth.	0	n	1	0	Poaceae Barnhart
Iz_5_1209	DJI_0259	Bromus inermis Leyss.	0		NA	NA	
Iz_5_1209	DJI_0259	Bromus squarrosus L.	0	a	0	0	Poaceae Barnhart
Iz_5_1209	DJI_0259	Bromus tectorum L.	0	a	0	0	Poaceae Barnhart
Iz_5_1209	DJI_0259	Calamagrostis epigejos (L.) Roth	25	n	1	0	Poaceae Barnhart
Iz_5_1209	DJI_0259	Carduus acanthoides L.	0	n	1	0	Asteraceae Bercht. & J.Presl
Iz_5_1209	DJI_0259	Carex sp.	0	n	1	0	Cyperaceae Juss.
Iz_5_1209	DJI_0259	Centaurea majorovii Dumbadze	0	n	1	0	Asteraceae Bercht. & J.Presl
Iz_5_1209	DJI_0259	Chamaecytisus ruthenicus (Fisch.ex Wol.) Klaskova	0	n	1	0	Fabaceae Lindl.
Iz_5_1209	DJI_0259	Chelidonium majus L.	0	n	1	0	Papaveraceae Juss.
Iz_5_1209	DJI_0259	Chenopodium betaceum Andrz.	0	n	1	0	Amaranthaceae Juss.
Iz_5_1209	DJI_0259	Chenopodium cf. album	0	n	1	0	Amaranthaceae Juss.
Iz_5_1209	DJI_0259	Chenopodium sp.	0		NA	0	Amaranthaceae Juss.
Iz_5_1209	DJI_0259	Chondrilla juncea L.	0	n	1	0	Asteraceae Bercht. & J.Presl
Iz_5_1209	DJI_0259	Cirsium vulgare (Savi) Ten.	0	n	1	0	Asteraceae Bercht. & J.Presl
Iz_5_1209	DJI_0259	Clinopodium acinos (L.) Kuntze	0	n	1	0	Lamiacea Lindl.
Iz_5_1209	DJI_0259	Convallaria majalis L.	0	n	1	0	Asparagaceae Juss.
Iz_5_1209	DJI_0259	Crataegus rhipidophylla Gand.	0	n	1	1	Rosaceae Juss.
Iz_5_1209	DJI_0259	Crepis foetida L.	0	n	1	0	Asteraceae Bercht. & J.Presl
Iz_5_1209	DJI_0259	Dactylis glomerata L.	0	n	1	0	Poaceae Barnhart
Iz_5_1209	DJI_0259	Dianthus campestris M.Bieb.	0	n	1	0	Caryophyllaceae Juss.
Iz_5_1209	DJI_0259	Digitaria sanguinalis (L.) Scop.	0	a	0	0	Poaceae Barnhart
Iz_5_1209	DJI_0259	Elymus repens (L.) Gould	0	n	1	0	Poaceae Barnhart
Iz_5_1209	DJI_0259	Epilobium tetragonum L.	0	n	1	0	Onagraceae Juss.
Iz_5_1209	DJI_0259	Eragrostis minor Host	0	a	0	0	Poaceae Barnhart
Iz_5_1209	DJI_0259	Erigeron canadensis L.	6	a	0	0	Asteraceae Bercht. & J.Presl
Iz_5_1209	DJI_0259	Euphorbia seguieriana Neck.	0	n	1	0	Euphorbiaceae Juss.
Iz_5_1209	DJI_0259	Fallopia dumetorum (L.) Holub	0	n	1	0	Polygonaceae Juss.
Iz_5_1209	DJI_0259	Festuca beckeri (Hack.) Trautv.	0	n	1	0	Poaceae Barnhart
Iz_5_1209	DJI_0259	Fragaria vesca L.	0	n	1	0	Rosaceae Juss.
Iz_5_1209	DJI_0259	Frangula alnus Mill.	0	n	1	1	Rhamnaceae Juss.
Iz_5_1209	DJI_0259	Fraxinus pennsylvanica Marshall	0	a	0	1	Oleaceae Hoffmanns. & Link
Iz_5_1209	DJI_0259	Galeopsis bifida Boenn.	0	n	1	0	Lamiacea Lindl.
Iz_5_1209	DJI_0259	Galium verum L.	0	n	1	0	Rubiaceae Juss.
Iz_5_1209	DJI_0259	Genista tinctoria L.	0	n	1	0	Fabaceae Lindl.
Iz_5_1209	DJI_0259	Geranium robertianum L.	0	n	1	0	Geraniaceae Juss.
Iz_5_1209	DJI_0259	Geranium sanquineum L.	0	n	1	0	Geraniaceae Juss.
Iz_5_1209	DJI_0259	Gypsophila paniculata L.	0	n	1	0	Caryophyllaceae Juss.
Iz_5_1209	DJI_0259	Helichrysum arenarium (L.) Moench	0	n	1	0	Asteraceae Bercht. & J.Presl
Iz_5_1209	DJI_0259	Herniaria polygama J.Gay	0	n	1	0	Caryophyllaceae Juss.
Iz_5_1209	DJI_0259	Hieracium robustum Fr.	0	n	1	0	Asteraceae Bercht. & J.Presl
Iz_5_1209	DJI_0259	Hieracium umbellatum L.	0	n	1	NA	Asteraceae Bercht. & J.Presl
Iz_5_1209	DJI_0259	Hylotelephium maximum (L.) Holub	0	n	1	0	Crassulaceae J.St.-Hil.
Iz_5_1209	DJI_0259	Jacobaea borysthenica (DC.) B.Nord. & Greuter	0	n	1	0	Asteraceae Bercht. & J.Presl
Iz_5_1209	DJI_0259	Jasione montana L.	0	n	1	0	Campanulaceae Juss.
Iz_5_1209	DJI_0259	Jurinea cyanoides (L.) Rchb.s.l.	0	n	1	0	Asteraceae Bercht. & J.Presl
Iz_5_1209	DJI_0259	Koeleria glauca (Spreng.) DC.	0	n	1	0	Poaceae Barnhart
Iz_5_1209	DJI_0259	Lactuca serriola L.	0	a	0	0	Asteraceae Bercht. & J.Presl
Iz_5_1209	DJI_0259	Linaria genistifolia (L.) Mill.	0	n	1	0	Plantaginaceae Juss.
Iz_5_1209	DJI_0259	Lomelosia argentea (L.) Greuter & Burdet	0	n	1	0	Caprifoliaceae Juss.
Iz_5_1209	DJI_0259	Melampyrum pratense L.	0	n	1	0	Orobanchaceae Vent.
Iz_5_1209	DJI_0259	Oenothera biennis L.	0	a	0	0	Onagraceae Juss.
Iz_5_1209	DJI_0259	Pentanema britannica (L.) D.Gut.Larr., Santos-Vicente, Anderb., E.Rico & M.M.Mart.Ort.	0	n	1	0	Asteraceae Bercht. & J.Presl
Iz_5_1209	DJI_0259	Peucedanum oreoselinum (L.) Moench	0	n	1	0	Apiaceae Lindl.
Iz_5_1209	DJI_0259	Peucedanum ruthenicum M.Bieb.	0	n	1	0	Apiaceae Lindl.
Iz_5_1209	DJI_0259	Pilosella echioides (Lumn.) F.W.Schultz & Sch.Bip.	0.5	n	1	0	Asteraceae Bercht. & J.Presl
Iz_5_1209	DJI_0259	Pilosella officinarum F. Schultz et Sch. Bip.	0	n	1	0	Asteraceae Bercht. & J.Presl
Iz_5_1209	DJI_0259	Pilosella sp.	0		NA	0	Asteraceae Bercht. & J.Presl
Iz_5_1209	DJI_0259	Pinus sylvestris L.	0	n	1	1	Pinaceae Spreng. ex F.Rudolphi
Iz_5_1209	DJI_0259	Poa annua L.	0	n	1	0	Poaceae Barnhart
Iz_5_1209	DJI_0259	Poa nemoralis L.	0	n	1	0	Poaceae Barnhart
Iz_5_1209	DJI_0259	Poa sp.	0		NA	0	Poaceae Barnhart
Iz_5_1209	DJI_0259	Polygonatum odoratum (Mill.) Druce	0	n	1	0	Asparagaceae Juss.
Iz_5_1209	DJI_0259	Polygonum arenarium Waldst. & Kit.	0	n	1	0	Polygonaceae Juss.
Iz_5_1209	DJI_0259	Populus nigra L.	0	n	1	1	Salicaceae Mirb.
Iz_5_1209	DJI_0259	Populus tremula L.	0	n	1	1	Salicaceae Mirb.
Iz_5_1209	DJI_0259	Populus Ã canescens (Aiton) Sm.	0	n	1	1	Salicaceae Mirb.

Iz_5_1209	DJI_0259	Potentilla argentea L.	0	n	1	0	Rosaceae Juss.
Iz_5_1209	DJI_0259	Psammophiliella muralis (L.) Ikonn.	0	n	1	0	Caryophyllaceae Juss.
Iz_5_1209	DJI_0259	Psephellus sumensis (Kalen.) Greuter	0	n	1	0	Asteraceae Bercht. & J.Presl
Iz_5_1209	DJI_0259	Pulsatilla pratensis (L.) Mill. s.l.	0	n	1	0	Ranunculaceae Juss.
Iz_5_1209	DJI_0259	Pyrus communis L.	0	a	0	1	Rosaceae Juss.
Iz_5_1209	DJI_0259	Quercus robur L.	0	n	1	1	Fagaceae Dumort.
Iz_5_1209	DJI_0259	Robinia pseudoacacia L.	0	a	0	1	Fabaceae Lindl.
Iz_5_1209	DJI_0259	Rosa sp.	0		NA	1	Rosaceae Juss.
Iz_5_1209	DJI_0259	Rubus idaeus L.	0	n	1	0	Rosaceae Juss.
Iz_5_1209	DJI_0259	Rumex acetosella L.	0	n	1	0	Polygonaceae Juss.
Iz_5_1209	DJI_0259	Rumex thyrsiflorus Fingerh.	0	n	1	0	Polygonaceae Juss.
Iz_5_1209	DJI_0259	Salix alba L.	0	n	1	1	Salicaceae Mirb.
Iz_5_1209	DJI_0259	Sambucus nigra L.	0	n	1	1	Viburnaceae Raf.
Iz_5_1209	DJI_0259	Secale sylvestre Host	0	n	1	0	Poaceae Barnhart
Iz_5_1209	DJI_0259	Sempervivum ruthenicum Schnittsp. & C.B. Lehm.	0	n	1	0	Crassulaceae J.St.-Hil.
Iz_5_1209	DJI_0259	Setaria viridis (L.) P. Beauv.	0	n	1	0	Poaceae Barnhart
Iz_5_1209	DJI_0259	Silene borysthenica (Gruner) Walters	0	n	1	0	Caryophyllaceae Juss.
Iz_5_1209	DJI_0259	Silene latifolia subsp. alba (Mill.) Greuter & Burdet	0	n	1	0	Caryophyllaceae Juss.
Iz_5_1209	DJI_0259	Solidago virgaurea L.	0	n	1	0	Asteraceae Bercht. & J.Presl
Iz_5_1209	DJI_0259	Sorbus aucuparia L.	0	n	1	1	Rosaceae Juss.
Iz_5_1209	DJI_0259	Stachys recta L.	0	n	1	0	Lamiacea Lindl.
Iz_5_1209	DJI_0259	Tanacetum vulgare L.	0	n	1	0	Asteraceae Bercht. & J.Presl
Iz_5_1209	DJI_0259	Thymus pallasianus Heinr.Braun	0	n	1	0	Lamiacea Lindl.
Iz_5_1209	DJI_0259	Tilia cordata Mill.	0	n	1	1	Malvaceae Juss.
Iz_5_1209	DJI_0259	Trifolium alpestre L.	0	n	1	0	Fabaceae Lindl.
Iz_5_1209	DJI_0259	Trifolium arvense L.	0	n	1	0	Fabaceae Lindl.
Iz_5_1209	DJI_0259	Ulmus glabra Huds.	0	n	1	1	Ulmaceae Mirb.
Iz_5_1209	DJI_0259	Verbascum densiflorum Bertol.	0	n	1	0	Scrophulariaceae Juss.
Iz_5_1209	DJI_0259	Veronica austriaca L.	0	n	1	0	Plantaginaceae Juss.
Iz_5_1209	DJI_0259	Veronica incana L.	0	n	1	0	Plantaginaceae Juss.
Iz_5_1209	DJI_0259	Veronica verna L.	0	n	1	0	Plantaginaceae Juss.
Iz_5_1209	DJI_0259	Vincetoxicum hirundinaria Medik	0	n	1	0	Apocynaceae Juss.
Iz_5_1209	DJI_0262	Acer tataricum L.	0	n	1	1	Sapindaceae Juss.
Iz_5_1209	DJI_0262	Achillea millefolium L. s.l.	0	n	1	0	Asteraceae Bercht. & J.Presl
Iz_5_1209	DJI_0262	Allium oleraceum L.	0	n	1	0	Amaryllidaceae J.St.-Hil.
Iz_5_1209	DJI_0262	Ambrosia artemisiifolia L.	0	a	0	0	Asteraceae Bercht. & J.Presl
Iz_5_1209	DJI_0262	Amorpha fruticosa L.	0	a	0	0	Fabaceae Lindl.
Iz_5_1209	DJI_0262	Anthericum ramosum L.	0	n	1	0	Asparagaceae Juss.
Iz_5_1209	DJI_0262	Artemisia marschalliana Spreng.	0	n	1	0	Asteraceae Bercht. & J.Presl
Iz_5_1209	DJI_0262	Asparagus officinalis L.	0	n	1	0	Asparagaceae Juss.
Iz_5_1209	DJI_0262	Astragalus cicer L.	0	n	1	0	Fabaceae Lindl.
Iz_5_1209	DJI_0262	Bassia laniflora (S.G.Gmel.) A.J.Scott	0	n	1	0	Amaranthaceae Juss.
Iz_5_1209	DJI_0262	Berberis vulgaris L.	0	n	1	0	Berberidaceae Juss.
Iz_5_1209	DJI_0262	Betula pendula Roth.	0	n	1	1	Betulaceae Gray
Iz_5_1209	DJI_0262	Betula pendula Roth.	0	n	1	0	Poaceae Barnhart
Iz_5_1209	DJI_0262	Bromus inermis Leyss.	0		NA	NA	
Iz_5_1209	DJI_0262	Bromus squarrosus L.	0	a	0	0	Poaceae Barnhart
Iz_5_1209	DJI_0262	Bromus tectorum L.	0	a	0	0	Poaceae Barnhart
Iz_5_1209	DJI_0262	Calamagrostis epigejos (L.) Roth	20	n	1	0	Poaceae Barnhart
Iz_5_1209	DJI_0262	Carduus acanthoides L.	0	n	1	0	Asteraceae Bercht. & J.Presl
Iz_5_1209	DJI_0262	Carex sp.	0	n	1	0	Cyperaceae Juss.
Iz_5_1209	DJI_0262	Centaurea majorovii Dumbadze	0	n	1	0	Asteraceae Bercht. & J.Presl
Iz_5_1209	DJI_0262	Chamaecytisus ruthenicus (Fisch.ex Wol.) Klaskova	0	n	1	0	Fabaceae Lindl.
Iz_5_1209	DJI_0262	Chelidonium majus L.	0	n	1	0	Papaveraceae Juss.
Iz_5_1209	DJI_0262	Chenopodium betaceum Andrz.	0	n	1	0	Amaranthaceae Juss.
Iz_5_1209	DJI_0262	Chenopodium cf. album	0	n	1	0	Amaranthaceae Juss.
Iz_5_1209	DJI_0262	Chenopodium sp.	0		NA	0	Amaranthaceae Juss.
Iz_5_1209	DJI_0262	Chondrilla juncea L.	0	n	1	0	Asteraceae Bercht. & J.Presl
Iz_5_1209	DJI_0262	Cirsium vulgare (Savi) Ten.	0	n	1	0	Asteraceae Bercht. & J.Presl
Iz_5_1209	DJI_0262	Clinopodium acinos (L.) Kuntze	0	n	1	0	Lamiacea Lindl.
Iz_5_1209	DJI_0262	Convallaria majalis L.	0	n	1	0	Asparagaceae Juss.
Iz_5_1209	DJI_0262	Crataegus rhipidophylla Gand.	0	n	1	1	Rosaceae Juss.
Iz_5_1209	DJI_0262	Crepis foetida L.	0	n	1	0	Asteraceae Bercht. & J.Presl
Iz_5_1209	DJI_0262	Dactylis glomerata L.	0	n	1	0	Poaceae Barnhart
Iz_5_1209	DJI_0262	Dianthus campestris M.Bieb.	0	n	1	0	Caryophyllaceae Juss.
Iz_5_1209	DJI_0262	Digitaria sanguinalis (L.) Scop.	0	a	0	0	Poaceae Barnhart
Iz_5_1209	DJI_0262	Elymus repens (L.) Gould	0	n	1	0	Poaceae Barnhart
Iz_5_1209	DJI_0262	Epilobium tetragonum L.	0	n	1	0	Onagraceae Juss.
Iz_5_1209	DJI_0262	Eragrostis minor Host	0	a	0	0	Poaceae Barnhart
Iz_5_1209	DJI_0262	Erigeron canadensis L.	15	a	0	0	Asteraceae Bercht. & J.Presl
Iz_5_1209	DJI_0262	Euphorbia seguieriana Neck.	0	n	1	0	Euphorbiaceae Juss.
Iz_5_1209	DJI_0262	Fallopia dumetorum (L.) Holub	0	n	1	0	Polygonaceae Juss.
Iz_5_1209	DJI_0262	Festuca beckeri (Hack.) Trautv.	0	n	1	0	Poaceae Barnhart
Iz_5_1209	DJI_0262	Fragaria vesca L.	0	n	1	0	Rosaceae Juss.
Iz_5_1209	DJI_0262	Frangula alnus Mill.	0	n	1	1	Rhamnaceae Juss.
Iz_5_1209	DJI_0262	Fraxinus pennsylvanica Marshall	0	a	0	1	Oleaceae Hoffmanns. & Link
Iz_5_1209	DJI_0262	Galeopsis bifida Boenn.	0	n	1	0	Lamiacea Lindl.
Iz_5_1209	DJI_0262	Galium verum L.	0	n	1	0	Rubiaceae Juss.
Iz_5_1209	DJI_0262	Genista tinctoria L.	0	n	1	0	Fabaceae Lindl.
Iz_5_1209	DJI_0262	Geranium robertianum L.	0	n	1	0	Geraniaceae Juss.
Iz_5_1209	DJI_0262	Geranium sanquineum L.	0	n	1	0	Geraniaceae Juss.
Iz_5_1209	DJI_0262	Gypsophila paniculata L.	0	n	1	0	Caryophyllaceae Juss.
Iz_5_1209	DJI_0262	Helichrysum arenarium (L.) Moench	0	n	1	0	Asteraceae Bercht. & J.Presl
Iz_5_1209	DJI_0262	Herniaria polygama J.Gay	0	n	1	0	Caryophyllaceae Juss.
Iz_5_1209	DJI_0262	Hieracium robustum Fr.	0	n	1	0	Asteraceae Bercht. & J.Presl
Iz_5_1209	DJI_0262	Hieracium umbellatum L.	0	n	1	NA	Asteraceae Bercht. & J.Presl
Iz_5_1209	DJI_0262	Hylotelephium maximum (L.) Holub	0	n	1	0	Crassulaceae J.St.-Hil.
Iz_5_1209	DJI_0262	Jacobaea borysthenica (DC.) B.Nord. & Greuter	0	n	1	0	Asteraceae Bercht. & J.Presl
Iz_5_1209	DJI_0262	Jasione montana L.	0	n	1	0	Campanulaceae Juss.
Iz_5_1209	DJI_0262	Jurinea cyanoides (L.) Rchb.s.l.	0	n	1	0	Asteraceae Bercht. & J.Presl
Iz_5_1209	DJI_0262	Koeleria glauca (Spreng.) DC.	0	n	1	0	Poaceae Barnhart
Iz_5_1209	DJI_0262	Lactuca serriola L.	0	a	0	0	Asteraceae Bercht. & J.Presl
Iz_5_1209	DJI_0262	Linaria genistifolia (L.) Mill.	0	n	1	0	Plantaginaceae Juss.
Iz_5_1209	DJI_0262	Lomelosia argentea (L.) Greuter & Burdet	0	n	1	0	Caprifoliaceae Juss.
Iz_5_1209	DJI_0262	Melampyrum pratense L.	0	n	1	0	Orobanchaceae Vent.
Iz_5_1209	DJI_0262	Oenothera biennis L.	0	a	0	0	Onagraceae Juss.
Iz_5_1209	DJI_0262	Pentanema britannica (L.) D.Gut.Larr., Santos-Vicente, Anderb., E.Rico & M.M.Mart.Ort.	0	n	1	0	Asteraceae Bercht. & J.Presl
Iz_5_1209	DJI_0262	Peucedanum oreoselinum (L.) Moench	1	n	1	0	Apiaceae Lindl.

Iz_5_1209	DJI_0262	Peucedanum ruthenicum M.Bieb.	0	n	1	0	Apiaceae Lindl.
Iz_5_1209	DJI_0262	Pilosella echioides (Lumn.) F.W.Schultz & Sch.Bip.	0	n	1	0	Asteraceae Bercht. & J.Presl
Iz_5_1209	DJI_0262	Pilosella officinarum F. Schultz et Sch. Bip.	0	n	1	0	Asteraceae Bercht. & J.Presl
Iz_5_1209	DJI_0262	Pilosella sp.	0		NA	0	Asteraceae Bercht. & J.Presl
Iz_5_1209	DJI_0262	Pinus sylvestris L.	0	n	1	1	Pinaceae Spreng. ex F.Rudolphi
Iz_5_1209	DJI_0262	Poa annua L.	0	n	1	0	Poaceae Barnhart
Iz_5_1209	DJI_0262	Poa nemoralis L.	0	n	1	0	Poaceae Barnhart
Iz_5_1209	DJI_0262	Poa sp.	0		NA	0	Poaceae Barnhart
Iz_5_1209	DJI_0262	Polygonatum odoratum (Mill.) Druce	0	n	1	0	Asparagaceae Juss.
Iz_5_1209	DJI_0262	Polygonum arenarium Waldst. & Kit.	0	n	1	0	Polygonaceae Juss.
Iz_5_1209	DJI_0262	Populus nigra L.	0	n	1	1	Salicaceae Mirb.
Iz_5_1209	DJI_0262	Populus tremula L.	0	n	1	1	Salicaceae Mirb.
Iz_5_1209	DJI_0262	Populus Ã canescens (Aiton) Sm.	0	n	1	1	Salicaceae Mirb.
Iz_5_1209	DJI_0262	Potentilla argentea L.	0	n	1	0	Rosaceae Juss.
Iz_5_1209	DJI_0262	Psammophiliella muralis (L.) Ikonn.	0	n	1	0	Caryophyllaceae Juss.
Iz_5_1209	DJI_0262	Psephellus sumensis (Kalen.) Greuter	0	n	1	0	Asteraceae Bercht. & J.Presl
Iz_5_1209	DJI_0262	Pulsatilla pratensis (L.) Mill. s.l.	0	n	1	0	Ranunculaceae Juss.
Iz_5_1209	DJI_0262	Pyrus communis L.	0	a	0	1	Rosaceae Juss.
Iz_5_1209	DJI_0262	Quercus robur L.	0	n	1	1	Fagaceae Dumort.
Iz_5_1209	DJI_0262	Robinia pseudoacacia L.	0	a	0	1	Fabaceae Lindl.
Iz_5_1209	DJI_0262	Rosa sp.	0		NA	1	Rosaceae Juss.
Iz_5_1209	DJI_0262	Rubus idaeus L.	0	n	1	0	Rosaceae Juss.
Iz_5_1209	DJI_0262	Rumex acetosella L.	0	n	1	0	Polygonaceae Juss.
Iz_5_1209	DJI_0262	Rumex thyrsiflorus Fingerh.	0	n	1	0	Polygonaceae Juss.
Iz_5_1209	DJI_0262	Salix alba L.	0	n	1	1	Salicaceae Mirb.
Iz_5_1209	DJI_0262	Sambucus nigra L.	0	n	1	1	Viburnaceae Raf.
Iz_5_1209	DJI_0262	Secale sylvestre Host	0	n	1	0	Poaceae Barnhart
Iz_5_1209	DJI_0262	Sempervivum ruthenicum Schnittsp. & C.B. Lehm.	0	n	1	0	Crassulaceae J.St.-Hil.
Iz_5_1209	DJI_0262	Setaria viridis (L.) P. Beauv.	0	n	1	0	Poaceae Barnhart
Iz_5_1209	DJI_0262	Silene borysthenica (Gruner) Walters	0	n	1	0	Caryophyllaceae Juss.
Iz_5_1209	DJI_0262	Silene latifolia subsp. alba (Mill.) Greuter & Burdet	0	n	1	0	Caryophyllaceae Juss.
Iz_5_1209	DJI_0262	Solidago virgaurea L.	0	n	1	0	Asteraceae Bercht. & J.Presl
Iz_5_1209	DJI_0262	Sorbus aucuparia L.	0	n	1	1	Rosaceae Juss.
Iz_5_1209	DJI_0262	Stachys recta L.	0	n	1	0	Lamiacea Lindl.
Iz_5_1209	DJI_0262	Tanacetum vulgare L.	0	n	1	0	Asteraceae Bercht. & J.Presl
Iz_5_1209	DJI_0262	Thymus pallasianus Heinr.Braun	0	n	1	0	Lamiacea Lindl.
Iz_5_1209	DJI_0262	Tilia cordata Mill.	0	n	1	1	Malvaceae Juss.
Iz_5_1209	DJI_0262	Trifolium alpestre L.	0	n	1	0	Fabaceae Lindl.
Iz_5_1209	DJI_0262	Trifolium arvense L.	0	n	1	0	Fabaceae Lindl.
Iz_5_1209	DJI_0262	Ulmus glabra Huds.	0	n	1	1	Ulmaceae Mirb.
Iz_5_1209	DJI_0262	Verbascum densiflorum Bertol.	0	n	1	0	Scrophulariaceae Juss.
Iz_5_1209	DJI_0262	Veronica austriaca L.	0	n	1	0	Plantaginaceae Juss.
Iz_5_1209	DJI_0262	Veronica incana L.	0	n	1	0	Plantaginaceae Juss.
Iz_5_1209	DJI_0262	Veronica verna L.	0	n	1	0	Plantaginaceae Juss.
Iz_5_1209	DJI_0262	Vincetoxicum hirundinaria Medik	0	n	1	0	Apocynaceae Juss.
Iz_5_1209	DJI_0265	Acer tataricum L.	0	n	1	1	Sapindaceae Juss.
Iz_5_1209	DJI_0265	Achillea millefolium L. s.l.	0	n	1	0	Asteraceae Bercht. & J.Presl
Iz_5_1209	DJI_0265	Allium oleraceum L.	0	n	1	0	Amaryllidaceae J.St.-Hil.
Iz_5_1209	DJI_0265	Ambrosia artemisiifolia L.	0	a	0	0	Asteraceae Bercht. & J.Presl
Iz_5_1209	DJI_0265	Amorpha fruticosa L.	0	a	0	0	Fabaceae Lindl.
Iz_5_1209	DJI_0265	Anthericum ramosum L.	0	n	1	0	Asparagaceae Juss.
Iz_5_1209	DJI_0265	Artemisia marschalliana Spreng.	0	n	1	0	Asteraceae Bercht. & J.Presl
Iz_5_1209	DJI_0265	Asparagus officinalis L.	0	n	1	0	Asparagaceae Juss.
Iz_5_1209	DJI_0265	Astragalus cicer L.	0	n	1	0	Fabaceae Lindl.
Iz_5_1209	DJI_0265	Bassia laniflora (S.G.Gmel.) A.J.Scott	0	n	1	0	Amaranthaceae Juss.
Iz_5_1209	DJI_0265	Berberis vulgaris L.	0	n	1	0	Berberidaceae Juss.
Iz_5_1209	DJI_0265	Betula pendula Roth.	0	n	1	1	Betulaceae Gray
Iz_5_1209	DJI_0265	Betula pendula Roth.	0	n	1	0	Poaceae Barnhart
Iz_5_1209	DJI_0265	Bromus inermis Leyss.	0		NA	NA	
Iz_5_1209	DJI_0265	Bromus squarrosus L.	0	a	0	0	Poaceae Barnhart
Iz_5_1209	DJI_0265	Bromus tectorum L.	0	a	0	0	Poaceae Barnhart
Iz_5_1209	DJI_0265	Calamagrostis epigejos (L.) Roth	15	n	1	0	Poaceae Barnhart
Iz_5_1209	DJI_0265	Carduus acanthoides L.	0	n	1	0	Asteraceae Bercht. & J.Presl
Iz_5_1209	DJI_0265	Carex sp.	0	n	1	0	Cyperaceae Juss.
Iz_5_1209	DJI_0265	Centaurea majorovii Dumbadze	0	n	1	0	Asteraceae Bercht. & J.Presl
Iz_5_1209	DJI_0265	Chamaecytisus ruthenicus (Fisch.ex Wol.) Klaskova	0	n	1	0	Fabaceae Lindl.
Iz_5_1209	DJI_0265	Chelidonium majus L.	0	n	1	0	Papaveraceae Juss.
Iz_5_1209	DJI_0265	Chenopodium betaceum Andrz.	0	n	1	0	Amaranthaceae Juss.
Iz_5_1209	DJI_0265	Chenopodium cf. album	0	n	1	0	Amaranthaceae Juss.
Iz_5_1209	DJI_0265	Chenopodium sp.	0		NA	0	Amaranthaceae Juss.
Iz_5_1209	DJI_0265	Chondrilla juncea L.	0	n	1	0	Asteraceae Bercht. & J.Presl
Iz_5_1209	DJI_0265	Cirsium vulgare (Savi) Ten.	0	n	1	0	Asteraceae Bercht. & J.Presl
Iz_5_1209	DJI_0265	Clinopodium acinos (L.) Kuntze	0	n	1	0	Lamiacea Lindl.
Iz_5_1209	DJI_0265	Convallaria majalis L.	0	n	1	0	Asparagaceae Juss.
Iz_5_1209	DJI_0265	Crataegus rhipidophylla Gand.	0	n	1	1	Rosaceae Juss.
Iz_5_1209	DJI_0265	Crepis foetida L.	0	n	1	0	Asteraceae Bercht. & J.Presl
Iz_5_1209	DJI_0265	Dactylis glomerata L.	0	n	1	0	Poaceae Barnhart
Iz_5_1209	DJI_0265	Dianthus campestris M.Bieb.	0	n	1	0	Caryophyllaceae Juss.
Iz_5_1209	DJI_0265	Digitaria sanguinalis (L.) Scop.	0	a	0	0	Poaceae Barnhart
Iz_5_1209	DJI_0265	Elymus repens (L.) Gould	0	n	1	0	Poaceae Barnhart
Iz_5_1209	DJI_0265	Epilobium tetragonum L.	0	n	1	0	Onagraceae Juss.
Iz_5_1209	DJI_0265	Eragrostis minor Host	0	a	0	0	Poaceae Barnhart
Iz_5_1209	DJI_0265	Erigeron canadensis L.	10	a	0	0	Asteraceae Bercht. & J.Presl
Iz_5_1209	DJI_0265	Euphorbia seguieriana Neck.	0	n	1	0	Euphorbiaceae Juss.
Iz_5_1209	DJI_0265	Fallopia dumetorum (L.) Holub	0	n	1	0	Polygonaceae Juss.
Iz_5_1209	DJI_0265	Festuca beckeri (Hack.) Trautv.	0	n	1	0	Poaceae Barnhart
Iz_5_1209	DJI_0265	Fragaria vesca L.	0	n	1	0	Rosaceae Juss.
Iz_5_1209	DJI_0265	Frangula alnus Mill.	0	n	1	1	Rhamnaceae Juss.
Iz_5_1209	DJI_0265	Fraxinus pennsylvanica Marshall	0	a	0	1	Oleaceae Hoffmanns. & Link
Iz_5_1209	DJI_0265	Galeopsis bifida Boenn.	0	n	1	0	Lamiacea Lindl.
Iz_5_1209	DJI_0265	Galium verum L.	0	n	1	0	Rubiaceae Juss.
Iz_5_1209	DJI_0265	Genista tinctoria L.	0	n	1	0	Fabaceae Lindl.
Iz_5_1209	DJI_0265	Geranium robertianum L.	0	n	1	0	Geraniaceae Juss.
Iz_5_1209	DJI_0265	Geranium sanquineum L.	0	n	1	0	Geraniaceae Juss.
Iz_5_1209	DJI_0265	Gypsophila paniculata L.	0	n	1	0	Caryophyllaceae Juss.
Iz_5_1209	DJI_0265	Helichrysum arenarium (L.) Moench	0	n	1	0	Asteraceae Bercht. & J.Presl
Iz_5_1209	DJI_0265	Herniaria polygama J.Gay	0	n	1	0	Caryophyllaceae Juss.
Iz_5_1209	DJI_0265	Hieracium robustum Fr.	0	n	1	0	Asteraceae Bercht. & J.Presl

Iz_5_1209	DJI_0265	Hieracium umbellatum L.	0	n	1	NA	Asteraceae Bercht. & J.Presl
Iz_5_1209	DJI_0265	Hylotelephium maximum (L.) Holub	0	n	1	0	Crassulaceae J.St.-Hil.
Iz_5_1209	DJI_0265	Jacobaea borysthenica (DC.) B.Nord. & Greuter	0	n	1	0	Asteraceae Bercht. & J.Presl
Iz_5_1209	DJI_0265	Jasione montana L.	0	n	1	0	Campanulaceae Juss.
Iz_5_1209	DJI_0265	Jurinea cyanoides (L.) Rchb.s.l.	0	n	1	0	Asteraceae Bercht. & J.Presl
Iz_5_1209	DJI_0265	Koeleria glauca (Spreng.) DC.	0	n	1	0	Poaceae Barnhart
Iz_5_1209	DJI_0265	Lactuca serriola L.	0	a	0	0	Asteraceae Bercht. & J.Presl
Iz_5_1209	DJI_0265	Linaria genistifolia (L.) Mill.	0	n	1	0	Plantaginaceae Juss.
Iz_5_1209	DJI_0265	Lomelosia argentea (L.) Greuter & Burdet	0	n	1	0	Caprifoliaceae Juss.
Iz_5_1209	DJI_0265	Melampyrum pratense L.	0	n	1	0	Orobanchaceae Vent.
Iz_5_1209	DJI_0265	Oenothera biennis L.	0	a	0	0	Onagraceae Juss.
Iz_5_1209	DJI_0265	Pentanema britannica (L.) D.Gut.Larr., Santos-Vicente, Anderb., E.Rico & M.M.Mart.Ort.	0	n	1	0	Asteraceae Bercht. & J.Presl
Iz_5_1209	DJI_0265	Peucedanum oreoselinum (L.) Moench	2	n	1	0	Apiaceae Lindl.
Iz_5_1209	DJI_0265	Peucedanum ruthenicum M.Bieb.	0	n	1	0	Apiaceae Lindl.
Iz_5_1209	DJI_0265	Pilosella echioides (Lumn.) F.W.Schultz & Sch.Bip.	0	n	1	0	Asteraceae Bercht. & J.Presl
Iz_5_1209	DJI_0265	Pilosella officinarum F. Schultz et Sch. Bip.	0	n	1	0	Asteraceae Bercht. & J.Presl
Iz_5_1209	DJI_0265	Pilosella sp.	0		NA	0	Asteraceae Bercht. & J.Presl
Iz_5_1209	DJI_0265	Pinus sylvestris L.	0	n	1	1	Pinaceae Spreng. ex F.Rudolphi
Iz_5_1209	DJI_0265	Poa annua L.	0	n	1	0	Poaceae Barnhart
Iz_5_1209	DJI_0265	Poa nemoralis L.	0	n	1	0	Poaceae Barnhart
Iz_5_1209	DJI_0265	Poa sp.	0		NA	0	Poaceae Barnhart
Iz_5_1209	DJI_0265	Polygonatum odoratum (Mill.) Druce	0	n	1	0	Asparagaceae Juss.
Iz_5_1209	DJI_0265	Polygonum arenarium Waldst. & Kit.	0	n	1	0	Polygonaceae Juss.
Iz_5_1209	DJI_0265	Populus nigra L.	0	n	1	1	Salicaceae Mirb.
Iz_5_1209	DJI_0265	Populus tremula L.	0	n	1	1	Salicaceae Mirb.
Iz_5_1209	DJI_0265	Populus Ã canescens (Aiton) Sm.	0	n	1	1	Salicaceae Mirb.
Iz_5_1209	DJI_0265	Potentilla argentea L.	0	n	1	0	Rosaceae Juss.
Iz_5_1209	DJI_0265	Psammophiliella muralis (L.) Ikonn.	0	n	1	0	Caryophyllaceae Juss.
Iz_5_1209	DJI_0265	Psephellus sumensis (Kalen.) Greuter	0	n	1	0	Asteraceae Bercht. & J.Presl
Iz_5_1209	DJI_0265	Pulsatilla pratensis (L.) Mill. s.l.	0	n	1	0	Ranunculaceae Juss.
Iz_5_1209	DJI_0265	Pyrus communis L.	0	a	0	1	Rosaceae Juss.
Iz_5_1209	DJI_0265	Quercus robur L.	0	n	1	1	Fagaceae Dumort.
Iz_5_1209	DJI_0265	Robinia pseudoacacia L.	0	a	0	1	Fabaceae Lindl.
Iz_5_1209	DJI_0265	Rosa sp.	0		NA	1	Rosaceae Juss.
Iz_5_1209	DJI_0265	Rubus idaeus L.	0	n	1	0	Rosaceae Juss.
Iz_5_1209	DJI_0265	Rumex acetosella L.	0	n	1	0	Polygonaceae Juss.
Iz_5_1209	DJI_0265	Rumex thyrsiflorus Fingerh.	0	n	1	0	Polygonaceae Juss.
Iz_5_1209	DJI_0265	Salix alba L.	0	n	1	1	Salicaceae Mirb.
Iz_5_1209	DJI_0265	Sambucus nigra L.	0	n	1	1	Viburnaceae Raf.
Iz_5_1209	DJI_0265	Secale sylvestre Host	0	n	1	0	Poaceae Barnhart
Iz_5_1209	DJI_0265	Sempervivum ruthenicum Schnittsp. & C.B. Lehm.	0	n	1	0	Crassulaceae J.St.-Hil.
Iz_5_1209	DJI_0265	Setaria viridis (L.) P. Beauv.	0	n	1	0	Poaceae Barnhart
Iz_5_1209	DJI_0265	Silene borysthenica (Gruner) Walters	0	n	1	0	Caryophyllaceae Juss.
Iz_5_1209	DJI_0265	Silene latifolia subsp. alba (Mill.) Greuter & Burdet	0	n	1	0	Caryophyllaceae Juss.
Iz_5_1209	DJI_0265	Solidago virgaurea L.	0	n	1	0	Asteraceae Bercht. & J.Presl
Iz_5_1209	DJI_0265	Sorbus aucuparia L.	0	n	1	1	Rosaceae Juss.
Iz_5_1209	DJI_0265	Stachys recta L.	0	n	1	0	Lamiacea Lindl.
Iz_5_1209	DJI_0265	Tanacetum vulgare L.	0	n	1	0	Asteraceae Bercht. & J.Presl
Iz_5_1209	DJI_0265	Thymus pallasianus Heinr.Braun	0	n	1	0	Lamiacea Lindl.
Iz_5_1209	DJI_0265	Tilia cordata Mill.	0	n	1	1	Malvaceae Juss.
Iz_5_1209	DJI_0265	Trifolium alpestre L.	0	n	1	0	Fabaceae Lindl.
Iz_5_1209	DJI_0265	Trifolium arvense L.	0	n	1	0	Fabaceae Lindl.
Iz_5_1209	DJI_0265	Ulmus glabra Huds.	0	n	1	1	Ulmaceae Mirb.
Iz_5_1209	DJI_0265	Verbascum densiflorum Bertol.	0	n	1	0	Scrophulariaceae Juss.
Iz_5_1209	DJI_0265	Veronica austriaca L.	0	n	1	0	Plantaginaceae Juss.
Iz_5_1209	DJI_0265	Veronica incana L.	0	n	1	0	Plantaginaceae Juss.
Iz_5_1209	DJI_0265	Veronica verna L.	0	n	1	0	Plantaginaceae Juss.
Iz_5_1209	DJI_0265	Vincetoxicum hirundinaria Medik	0	n	1	0	Apocynaceae Juss.
Iz_5_1209	DJI_0270	Acer tataricum L.	0	n	1	1	Sapindaceae Juss.
Iz_5_1209	DJI_0270	Achillea millefolium L. s.l.	0	n	1	0	Asteraceae Bercht. & J.Presl
Iz_5_1209	DJI_0270	Allium oleraceum L.	0	n	1	0	Amaryllidaceae J.St.-Hil.
Iz_5_1209	DJI_0270	Ambrosia artemisiifolia L.	0	a	0	0	Asteraceae Bercht. & J.Presl
Iz_5_1209	DJI_0270	Amorpha fruticosa L.	0	a	0	0	Fabaceae Lindl.
Iz_5_1209	DJI_0270	Anthericum ramosum L.	0	n	1	0	Asparagaceae Juss.
Iz_5_1209	DJI_0270	Artemisia marschalliana Spreng.	0	n	1	0	Asteraceae Bercht. & J.Presl
Iz_5_1209	DJI_0270	Asparagus officinalis L.	0	n	1	0	Asparagaceae Juss.
Iz_5_1209	DJI_0270	Astragalus cicer L.	0	n	1	0	Fabaceae Lindl.
Iz_5_1209	DJI_0270	Bassia laniflora (S.G.Gmel.) A.J.Scott	0	n	1	0	Amaranthaceae Juss.
Iz_5_1209	DJI_0270	Berberis vulgaris L.	0	n	1	0	Berberidaceae Juss.
Iz_5_1209	DJI_0270	Betula pendula Roth.	0	n	1	1	Betulaceae Gray
Iz_5_1209	DJI_0270	Betula pendula Roth.	0	n	1	0	Poaceae Barnhart
Iz_5_1209	DJI_0270	Bromus inermis Leyss.	0		NA	NA	
Iz_5_1209	DJI_0270	Bromus squarrosus L.	0	a	0	0	Poaceae Barnhart
Iz_5_1209	DJI_0270	Bromus tectorum L.	0	a	0	0	Poaceae Barnhart
Iz_5_1209	DJI_0270	Calamagrostis epigejos (L.) Roth	5	n	1	0	Poaceae Barnhart
Iz_5_1209	DJI_0270	Carduus acanthoides L.	0	n	1	0	Asteraceae Bercht. & J.Presl
Iz_5_1209	DJI_0270	Carex sp.	0	n	1	0	Cyperaceae Juss.
Iz_5_1209	DJI_0270	Centaurea majorovii Dumbadze	0	n	1	0	Asteraceae Bercht. & J.Presl
Iz_5_1209	DJI_0270	Chamaecytisus ruthenicus (Fisch.ex Wol.) Klaskova	0	n	1	0	Fabaceae Lindl.
Iz_5_1209	DJI_0270	Chelidonium majus L.	0	n	1	0	Papaveraceae Juss.
Iz_5_1209	DJI_0270	Chenopodium betaceum Andrz.	0	n	1	0	Amaranthaceae Juss.
Iz_5_1209	DJI_0270	Chenopodium cf. album	0	n	1	0	Amaranthaceae Juss.
Iz_5_1209	DJI_0270	Chenopodium sp.	0		NA	0	Amaranthaceae Juss.
Iz_5_1209	DJI_0270	Chondrilla juncea L.	0	n	1	0	Asteraceae Bercht. & J.Presl
Iz_5_1209	DJI_0270	Cirsium vulgare (Savi) Ten.	0	n	1	0	Asteraceae Bercht. & J.Presl
Iz_5_1209	DJI_0270	Clinopodium acinos (L.) Kuntze	0	n	1	0	Lamiacea Lindl.
Iz_5_1209	DJI_0270	Convallaria majalis L.	0	n	1	0	Asparagaceae Juss.
Iz_5_1209	DJI_0270	Crataegus rhipidophylla Gand.	0	n	1	1	Rosaceae Juss.
Iz_5_1209	DJI_0270	Crepis foetida L.	0	n	1	0	Asteraceae Bercht. & J.Presl
Iz_5_1209	DJI_0270	Dactylis glomerata L.	0	n	1	0	Poaceae Barnhart
Iz_5_1209	DJI_0270	Dianthus campestris M.Bieb.	0	n	1	0	Caryophyllaceae Juss.
Iz_5_1209	DJI_0270	Digitaria sanguinalis (L.) Scop.	0	a	0	0	Poaceae Barnhart
Iz_5_1209	DJI_0270	Elymus repens (L.) Gould	0	n	1	0	Poaceae Barnhart
Iz_5_1209	DJI_0270	Epilobium tetragonum L.	0	n	1	0	Onagraceae Juss.
Iz_5_1209	DJI_0270	Eragrostis minor Host	0	a	0	0	Poaceae Barnhart
Iz_5_1209	DJI_0270	Erigeron canadensis L.	3	a	0	0	Asteraceae Bercht. & J.Presl
Iz_5_1209	DJI_0270	Euphorbia seguieriana Neck.	0	n	1	0	Euphorbiaceae Juss.
Iz_5_1209	DJI_0270	Fallopia dumetorum (L.) Holub	0	n	1	0	Polygonaceae Juss.

Iz_5_1209	DJI_0270	Festuca beckeri (Hack.) Trautv.	0	n	1	0	Poaceae Barnhart
Iz_5_1209	DJI_0270	Fragaria vesca L.	0	n	1	0	Rosaceae Juss.
Iz_5_1209	DJI_0270	Frangula alnus Mill.	0	n	1	1	Rhamnaceae Juss.
Iz_5_1209	DJI_0270	Fraxinus pennsylvanica Marshall	0	a	0	1	Oleaceae Hoffmanns. & Link
Iz_5_1209	DJI_0270	Galeopsis bifida Boenn.	0	n	1	0	Lamiacea Lindl.
Iz_5_1209	DJI_0270	Galium verum L.	0	n	1	0	Rubiaceae Juss.
Iz_5_1209	DJI_0270	Genista tinctoria L.	0	n	1	0	Fabaceae Lindl.
Iz_5_1209	DJI_0270	Geranium robertianum L.	0	n	1	0	Geraniaceae Juss.
Iz_5_1209	DJI_0270	Geranium sanquineum L.	0	n	1	0	Geraniaceae Juss.
Iz_5_1209	DJI_0270	Gypsophila paniculata L.	0	n	1	0	Caryophyllaceae Juss.
Iz_5_1209	DJI_0270	Helichrysum arenarium (L.) Moench	0	n	1	0	Asteraceae Bercht. & J.Presl
Iz_5_1209	DJI_0270	Herniaria polygama J.Gay	0	n	1	0	Caryophyllaceae Juss.
Iz_5_1209	DJI_0270	Hieracium robustum Fr.	0	n	1	0	Asteraceae Bercht. & J.Presl
Iz_5_1209	DJI_0270	Hieracium umbellatum L.	0	n	1	NA	Asteraceae Bercht. & J.Presl
Iz_5_1209	DJI_0270	Hylotelephium maximum (L.) Holub	0	n	1	0	Crassulaceae J.St.-Hil.
Iz_5_1209	DJI_0270	Jacobaea borysthenica (DC.) B.Nord. & Greuter	0	n	1	0	Asteraceae Bercht. & J.Presl
Iz_5_1209	DJI_0270	Jasione montana L.	0	n	1	0	Campanulaceae Juss.
Iz_5_1209	DJI_0270	Jurinea cyanoides (L.) Rchb.s.l.	0	n	1	0	Asteraceae Bercht. & J.Presl
Iz_5_1209	DJI_0270	Koeleria glauca (Spreng.) DC.	0	n	1	0	Poaceae Barnhart
Iz_5_1209	DJI_0270	Lactuca serriola L.	0	a	0	0	Asteraceae Bercht. & J.Presl
Iz_5_1209	DJI_0270	Linaria genistifolia (L.) Mill.	0	n	1	0	Plantaginaceae Juss.
Iz_5_1209	DJI_0270	Lomelosia argentea (L.) Greuter & Burdet	0	n	1	0	Caprifoliaceae Juss.
Iz_5_1209	DJI_0270	Melampyrum pratense L.	0	n	1	0	Orobanchaceae Vent.
Iz_5_1209	DJI_0270	Oenothera biennis L.	0	a	0	0	Onagraceae Juss.
Iz_5_1209	DJI_0270	Pentanema britannica (L.) D.Gut.Larr., Santos-Vicente, Anderb., E.Rico & M.M.Mart.Ort.	0	n	1	0	Asteraceae Bercht. & J.Presl
Iz_5_1209	DJI_0270	Peucedanum oreoselinum (L.) Moench	0	n	1	0	Apiaceae Lindl.
Iz_5_1209	DJI_0270	Peucedanum ruthenicum M.Bieb.	0	n	1	0	Apiaceae Lindl.
Iz_5_1209	DJI_0270	Pilosella echioides (Lumn.) F.W.Schultz & Sch.Bip.	0	n	1	0	Asteraceae Bercht. & J.Presl
Iz_5_1209	DJI_0270	Pilosella officinarum F. Schultz et Sch. Bip.	0	n	1	0	Asteraceae Bercht. & J.Presl
Iz_5_1209	DJI_0270	Pilosella sp.	0		NA	0	Asteraceae Bercht. & J.Presl
Iz_5_1209	DJI_0270	Pinus sylvestris L.	0	n	1	1	Pinaceae Spreng. ex F.Rudolphi
Iz_5_1209	DJI_0270	Poa annua L.	0	n	1	0	Poaceae Barnhart
Iz_5_1209	DJI_0270	Poa nemoralis L.	0	n	1	0	Poaceae Barnhart
Iz_5_1209	DJI_0270	Poa sp.	0		NA	0	Poaceae Barnhart
Iz_5_1209	DJI_0270	Polygonatum odoratum (Mill.) Druce	0	n	1	0	Asparagaceae Juss.
Iz_5_1209	DJI_0270	Polygonum arenarium Waldst. & Kit.	0	n	1	0	Polygonaceae Juss.
Iz_5_1209	DJI_0270	Populus nigra L.	0	n	1	1	Salicaceae Mirb.
Iz_5_1209	DJI_0270	Populus tremula L.	0	n	1	1	Salicaceae Mirb.
Iz_5_1209	DJI_0270	Populus Ã canescens (Aiton) Sm.	0	n	1	1	Salicaceae Mirb.
Iz_5_1209	DJI_0270	Potentilla argentea L.	0	n	1	0	Rosaceae Juss.
Iz_5_1209	DJI_0270	Psammophiliella muralis (L.) Ikonn.	0	n	1	0	Caryophyllaceae Juss.
Iz_5_1209	DJI_0270	Psephellus sumensis (Kalen.) Greuter	0	n	1	0	Asteraceae Bercht. & J.Presl
Iz_5_1209	DJI_0270	Pulsatilla pratensis (L.) Mill. s.l.	0	n	1	0	Ranunculaceae Juss.
Iz_5_1209	DJI_0270	Pyrus communis L.	0	a	0	1	Rosaceae Juss.
Iz_5_1209	DJI_0270	Quercus robur L.	0	n	1	1	Fagaceae Dumort.
Iz_5_1209	DJI_0270	Robinia pseudoacacia L.	0	a	0	1	Fabaceae Lindl.
Iz_5_1209	DJI_0270	Rosa sp.	0		NA	1	Rosaceae Juss.
Iz_5_1209	DJI_0270	Rubus idaeus L.	0	n	1	0	Rosaceae Juss.
Iz_5_1209	DJI_0270	Rumex acetosella L.	0	n	1	0	Polygonaceae Juss.
Iz_5_1209	DJI_0270	Rumex thyrsiflorus Fingerh.	0	n	1	0	Polygonaceae Juss.
Iz_5_1209	DJI_0270	Salix alba L.	0	n	1	1	Salicaceae Mirb.
Iz_5_1209	DJI_0270	Sambucus nigra L.	0	n	1	1	Viburnaceae Raf.
Iz_5_1209	DJI_0270	Secale sylvestre Host	0	n	1	0	Poaceae Barnhart
Iz_5_1209	DJI_0270	Sempervivum ruthenicum Schnittsp. & C.B. Lehm.	0	n	1	0	Crassulaceae J.St.-Hil.
Iz_5_1209	DJI_0270	Setaria viridis (L.) P. Beauv.	0	n	1	0	Poaceae Barnhart
Iz_5_1209	DJI_0270	Silene borysthenica (Gruner) Walters	0	n	1	0	Caryophyllaceae Juss.
Iz_5_1209	DJI_0270	Silene latifolia subsp. alba (Mill.) Greuter & Burdet	0	n	1	0	Caryophyllaceae Juss.
Iz_5_1209	DJI_0270	Solidago virgaurea L.	0.5	n	1	0	Asteraceae Bercht. & J.Presl
Iz_5_1209	DJI_0270	Sorbus aucuparia L.	0	n	1	1	Rosaceae Juss.
Iz_5_1209	DJI_0270	Stachys recta L.	0	n	1	0	Lamiacea Lindl.
Iz_5_1209	DJI_0270	Tanacetum vulgare L.	0	n	1	0	Asteraceae Bercht. & J.Presl
Iz_5_1209	DJI_0270	Thymus pallasianus Heinr.Braun	0	n	1	0	Lamiacea Lindl.
Iz_5_1209	DJI_0270	Tilia cordata Mill.	0	n	1	1	Malvaceae Juss.
Iz_5_1209	DJI_0270	Trifolium alpestre L.	0	n	1	0	Fabaceae Lindl.
Iz_5_1209	DJI_0270	Trifolium arvense L.	0	n	1	0	Fabaceae Lindl.
Iz_5_1209	DJI_0270	Ulmus glabra Huds.	0	n	1	1	Ulmaceae Mirb.
Iz_5_1209	DJI_0270	Verbascum densiflorum Bertol.	0	n	1	0	Scrophulariaceae Juss.
Iz_5_1209	DJI_0270	Veronica austriaca L.	0	n	1	0	Plantaginaceae Juss.
Iz_5_1209	DJI_0270	Veronica incana L.	0	n	1	0	Plantaginaceae Juss.
Iz_5_1209	DJI_0270	Veronica verna L.	0	n	1	0	Plantaginaceae Juss.
Iz_5_1209	DJI_0270	Vincetoxicum hirundinaria Medik	0	n	1	0	Apocynaceae Juss.
Iz_5_1209	DJI_0272	Acer tataricum L.	0	n	1	1	Sapindaceae Juss.
Iz_5_1209	DJI_0272	Achillea millefolium L. s.l.	0	n	1	0	Asteraceae Bercht. & J.Presl
Iz_5_1209	DJI_0272	Allium oleraceum L.	0	n	1	0	Amaryllidaceae J.St.-Hil.
Iz_5_1209	DJI_0272	Ambrosia artemisiifolia L.	0	a	0	0	Asteraceae Bercht. & J.Presl
Iz_5_1209	DJI_0272	Amorpha fruticosa L.	0	a	0	0	Fabaceae Lindl.
Iz_5_1209	DJI_0272	Anthericum ramosum L.	0	n	1	0	Asparagaceae Juss.
Iz_5_1209	DJI_0272	Artemisia marschalliana Spreng.	0	n	1	0	Asteraceae Bercht. & J.Presl
Iz_5_1209	DJI_0272	Asparagus officinalis L.	0	n	1	0	Asparagaceae Juss.
Iz_5_1209	DJI_0272	Astragalus cicer L.	0	n	1	0	Fabaceae Lindl.
Iz_5_1209	DJI_0272	Bassia laniflora (S.G.Gmel.) A.J.Scott	0	n	1	0	Amaranthaceae Juss.
Iz_5_1209	DJI_0272	Berberis vulgaris L.	0	n	1	0	Berberidaceae Juss.
Iz_5_1209	DJI_0272	Betula pendula Roth.	0	n	1	1	Betulaceae Gray
Iz_5_1209	DJI_0272	Betula pendula Roth.	0	n	1	0	Poaceae Barnhart
Iz_5_1209	DJI_0272	Bromus inermis Leyss.	0		NA	NA	
Iz_5_1209	DJI_0272	Bromus squarrosus L.	0	a	0	0	Poaceae Barnhart
Iz_5_1209	DJI_0272	Bromus tectorum L.	0	a	0	0	Poaceae Barnhart
Iz_5_1209	DJI_0272	Calamagrostis epigejos (L.) Roth	1	n	1	0	Poaceae Barnhart
Iz_5_1209	DJI_0272	Carduus acanthoides L.	0	n	1	0	Asteraceae Bercht. & J.Presl
Iz_5_1209	DJI_0272	Carex sp.	0	n	1	0	Cyperaceae Juss.
Iz_5_1209	DJI_0272	Centaurea majorovii Dumbadze	0	n	1	0	Asteraceae Bercht. & J.Presl
Iz_5_1209	DJI_0272	Chamaecytisus ruthenicus (Fisch.ex Wol.) Klaskova	0	n	1	0	Fabaceae Lindl.
Iz_5_1209	DJI_0272	Chelidonium majus L.	0	n	1	0	Papaveraceae Juss.
Iz_5_1209	DJI_0272	Chenopodium betaceum Andrz.	0	n	1	0	Amaranthaceae Juss.
Iz_5_1209	DJI_0272	Chenopodium cf. album	0	n	1	0	Amaranthaceae Juss.
Iz_5_1209	DJI_0272	Chenopodium sp.	0		NA	0	Amaranthaceae Juss.
Iz_5_1209	DJI_0272	Chondrilla juncea L.	0	n	1	0	Asteraceae Bercht. & J.Presl
Iz_5_1209	DJI_0272	Cirsium vulgare (Savi) Ten.	0	n	1	0	Asteraceae Bercht. & J.Presl

Iz_5_1209	DJI_0272	Clinopodium acinos (L.) Kuntze	0	n	1	0	Lamiacea Lindl.
Iz_5_1209	DJI_0272	Convallaria majalis L.	0	n	1	0	Asparagaceae Juss.
Iz_5_1209	DJI_0272	Crataegus rhipidophylla Gand.	0	n	1	1	Rosaceae Juss.
Iz_5_1209	DJI_0272	Crepis foetida L.	0	n	1	0	Asteraceae Bercht. & J.Presl
Iz_5_1209	DJI_0272	Dactylis glomerata L.	0	n	1	0	Poaceae Barnhart
Iz_5_1209	DJI_0272	Dianthus campestris M.Bieb.	0	n	1	0	Caryophyllaceae Juss.
Iz_5_1209	DJI_0272	Digitaria sanguinalis (L.) Scop.	0	a	0	0	Poaceae Barnhart
Iz_5_1209	DJI_0272	Elymus repens (L.) Gould	0	n	1	0	Poaceae Barnhart
Iz_5_1209	DJI_0272	Epilobium tetragonum L.	0	n	1	0	Onagraceae Juss.
Iz_5_1209	DJI_0272	Eragrostis minor Host	0	a	0	0	Poaceae Barnhart
Iz_5_1209	DJI_0272	Erigeron canadensis L.	6	a	0	0	Asteraceae Bercht. & J.Presl
Iz_5_1209	DJI_0272	Euphorbia seguieriana Neck.	0	n	1	0	Euphorbiaceae Juss.
Iz_5_1209	DJI_0272	Fallopia dumetorum (L.) Holub	0	n	1	0	Polygonaceae Juss.
Iz_5_1209	DJI_0272	Festuca beckeri (Hack.) Trautv.	0	n	1	0	Poaceae Barnhart
Iz_5_1209	DJI_0272	Fragaria vesca L.	0	n	1	0	Rosaceae Juss.
Iz_5_1209	DJI_0272	Frangula alnus Mill.	0	n	1	1	Rhamnaceae Juss.
Iz_5_1209	DJI_0272	Fraxinus pennsylvanica Marshall	0	a	0	1	Oleaceae Hoffmanns. & Link
Iz_5_1209	DJI_0272	Galeopsis bifida Boenn.	0	n	1	0	Lamiacea Lindl.
Iz_5_1209	DJI_0272	Galium verum L.	0	n	1	0	Rubiaceae Juss.
Iz_5_1209	DJI_0272	Genista tinctoria L.	0	n	1	0	Fabaceae Lindl.
Iz_5_1209	DJI_0272	Geranium robertianum L.	0	n	1	0	Geraniaceae Juss.
Iz_5_1209	DJI_0272	Geranium sanquineum L.	0	n	1	0	Geraniaceae Juss.
Iz_5_1209	DJI_0272	Gypsophila paniculata L.	0	n	1	0	Caryophyllaceae Juss.
Iz_5_1209	DJI_0272	Helichrysum arenarium (L.) Moench	0	n	1	0	Asteraceae Bercht. & J.Presl
Iz_5_1209	DJI_0272	Herniaria polygama J.Gay	0	n	1	0	Caryophyllaceae Juss.
Iz_5_1209	DJI_0272	Hieracium robustum Fr.	0	n	1	0	Asteraceae Bercht. & J.Presl
Iz_5_1209	DJI_0272	Hieracium umbellatum L.	0	n	1	NA	Asteraceae Bercht. & J.Presl
Iz_5_1209	DJI_0272	Hylotelephium maximum (L.) Holub	0	n	1	0	Crassulaceae J.St.-Hil.
Iz_5_1209	DJI_0272	Jacobaea borysthenica (DC.) B.Nord. & Greuter	0	n	1	0	Asteraceae Bercht. & J.Presl
Iz_5_1209	DJI_0272	Jasione montana L.	0	n	1	0	Campanulaceae Juss.
Iz_5_1209	DJI_0272	Jurinea cyanoides (L.) Rchb.s.l.	0	n	1	0	Asteraceae Bercht. & J.Presl
Iz_5_1209	DJI_0272	Koeleria glauca (Spreng.) DC.	0	n	1	0	Poaceae Barnhart
Iz_5_1209	DJI_0272	Lactuca serriola L.	0	a	0	0	Asteraceae Bercht. & J.Presl
Iz_5_1209	DJI_0272	Linaria genistifolia (L.) Mill.	0	n	1	0	Plantaginaceae Juss.
Iz_5_1209	DJI_0272	Lomelosia argentea (L.) Greuter & Burdet	0	n	1	0	Caprifoliaceae Juss.
Iz_5_1209	DJI_0272	Melampyrum pratense L.	0	n	1	0	Orobanchaceae Vent.
Iz_5_1209	DJI_0272	Oenothera biennis L.	0	a	0	0	Onagraceae Juss.
Iz_5_1209	DJI_0272	Pentanema britannica (L.) D.Gut.Larr., Santos-Vicente, Anderb., E.Rico & M.M.Mart.Ort.	0	n	1	0	Asteraceae Bercht. & J.Presl
Iz_5_1209	DJI_0272	Peucedanum oreoselinum (L.) Moench	0	n	1	0	Apiaceae Lindl.
Iz_5_1209	DJI_0272	Peucedanum ruthenicum M.Bieb.	0	n	1	0	Apiaceae Lindl.
Iz_5_1209	DJI_0272	Pilosella echioides (Lumn.) F.W.Schultz & Sch.Bip.	0	n	1	0	Asteraceae Bercht. & J.Presl
Iz_5_1209	DJI_0272	Pilosella officinarum F. Schultz et Sch. Bip.	0	n	1	0	Asteraceae Bercht. & J.Presl
Iz_5_1209	DJI_0272	Pilosella sp.	0		NA	0	Asteraceae Bercht. & J.Presl
Iz_5_1209	DJI_0272	Pinus sylvestris L.	0	n	1	1	Pinaceae Spreng. ex F.Rudolphi
Iz_5_1209	DJI_0272	Poa annua L.	0	n	1	0	Poaceae Barnhart
Iz_5_1209	DJI_0272	Poa nemoralis L.	0	n	1	0	Poaceae Barnhart
Iz_5_1209	DJI_0272	Poa sp.	0		NA	0	Poaceae Barnhart
Iz_5_1209	DJI_0272	Polygonatum odoratum (Mill.) Druce	0	n	1	0	Asparagaceae Juss.
Iz_5_1209	DJI_0272	Polygonum arenarium Waldst. & Kit.	0	n	1	0	Polygonaceae Juss.
Iz_5_1209	DJI_0272	Populus nigra L.	0	n	1	1	Salicaceae Mirb.
Iz_5_1209	DJI_0272	Populus tremula L.	0	n	1	1	Salicaceae Mirb.
Iz_5_1209	DJI_0272	Populus Ã canescens (Aiton) Sm.	0	n	1	1	Salicaceae Mirb.
Iz_5_1209	DJI_0272	Potentilla argentea L.	0	n	1	0	Rosaceae Juss.
Iz_5_1209	DJI_0272	Psammophiliella muralis (L.) Ikonn.	0	n	1	0	Caryophyllaceae Juss.
Iz_5_1209	DJI_0272	Psephellus sumensis (Kalen.) Greuter	0	n	1	0	Asteraceae Bercht. & J.Presl
Iz_5_1209	DJI_0272	Pulsatilla pratensis (L.) Mill. s.l.	0	n	1	0	Ranunculaceae Juss.
Iz_5_1209	DJI_0272	Pyrus communis L.	0	a	0	1	Rosaceae Juss.
Iz_5_1209	DJI_0272	Quercus robur L.	0	n	1	1	Fagaceae Dumort.
Iz_5_1209	DJI_0272	Robinia pseudoacacia L.	0	a	0	1	Fabaceae Lindl.
Iz_5_1209	DJI_0272	Rosa sp.	0		NA	1	Rosaceae Juss.
Iz_5_1209	DJI_0272	Rubus idaeus L.	0	n	1	0	Rosaceae Juss.
Iz_5_1209	DJI_0272	Rumex acetosella L.	0	n	1	0	Polygonaceae Juss.
Iz_5_1209	DJI_0272	Rumex thyrsiflorus Fingerh.	0	n	1	0	Polygonaceae Juss.
Iz_5_1209	DJI_0272	Salix alba L.	0	n	1	1	Salicaceae Mirb.
Iz_5_1209	DJI_0272	Sambucus nigra L.	0	n	1	1	Viburnaceae Raf.
Iz_5_1209	DJI_0272	Secale sylvestre Host	0	n	1	0	Poaceae Barnhart
Iz_5_1209	DJI_0272	Sempervivum ruthenicum Schnittsp. & C.B. Lehm.	0	n	1	0	Crassulaceae J.St.-Hil.
Iz_5_1209	DJI_0272	Setaria viridis (L.) P. Beauv.	0	n	1	0	Poaceae Barnhart
Iz_5_1209	DJI_0272	Silene borysthenica (Gruner) Walters	0	n	1	0	Caryophyllaceae Juss.
Iz_5_1209	DJI_0272	Silene latifolia subsp. alba (Mill.) Greuter & Burdet	0	n	1	0	Caryophyllaceae Juss.
Iz_5_1209	DJI_0272	Solidago virgaurea L.	0.2	n	1	0	Asteraceae Bercht. & J.Presl
Iz_5_1209	DJI_0272	Sorbus aucuparia L.	0	n	1	1	Rosaceae Juss.
Iz_5_1209	DJI_0272	Stachys recta L.	0	n	1	0	Lamiacea Lindl.
Iz_5_1209	DJI_0272	Tanacetum vulgare L.	0	n	1	0	Asteraceae Bercht. & J.Presl
Iz_5_1209	DJI_0272	Thymus pallasianus Heinr.Braun	0	n	1	0	Lamiacea Lindl.
Iz_5_1209	DJI_0272	Tilia cordata Mill.	0	n	1	1	Malvaceae Juss.
Iz_5_1209	DJI_0272	Trifolium alpestre L.	0	n	1	0	Fabaceae Lindl.
Iz_5_1209	DJI_0272	Trifolium arvense L.	0	n	1	0	Fabaceae Lindl.
Iz_5_1209	DJI_0272	Ulmus glabra Huds.	0	n	1	1	Ulmaceae Mirb.
Iz_5_1209	DJI_0272	Verbascum densiflorum Bertol.	0	n	1	0	Scrophulariaceae Juss.
Iz_5_1209	DJI_0272	Veronica austriaca L.	0	n	1	0	Plantaginaceae Juss.
Iz_5_1209	DJI_0272	Veronica incana L.	0	n	1	0	Plantaginaceae Juss.
Iz_5_1209	DJI_0272	Veronica verna L.	0	n	1	0	Plantaginaceae Juss.
Iz_5_1209	DJI_0272	Vincetoxicum hirundinaria Medik	0	n	1	0	Apocynaceae Juss.
Iz_5_1209	DJI_0276	Acer tataricum L.	0	n	1	1	Sapindaceae Juss.
Iz_5_1209	DJI_0276	Achillea millefolium L. s.l.	0	n	1	0	Asteraceae Bercht. & J.Presl
Iz_5_1209	DJI_0276	Allium oleraceum L.	0	n	1	0	Amaryllidaceae J.St.-Hil.
Iz_5_1209	DJI_0276	Ambrosia artemisiifolia L.	0	a	0	0	Asteraceae Bercht. & J.Presl
Iz_5_1209	DJI_0276	Amorpha fruticosa L.	0	a	0	0	Fabaceae Lindl.
Iz_5_1209	DJI_0276	Anthericum ramosum L.	0	n	1	0	Asparagaceae Juss.
Iz_5_1209	DJI_0276	Artemisia marschalliana Spreng.	0	n	1	0	Asteraceae Bercht. & J.Presl
Iz_5_1209	DJI_0276	Asparagus officinalis L.	0	n	1	0	Asparagaceae Juss.
Iz_5_1209	DJI_0276	Astragalus cicer L.	0	n	1	0	Fabaceae Lindl.
Iz_5_1209	DJI_0276	Bassia laniflora (S.G.Gmel.) A.J.Scott	0	n	1	0	Amaranthaceae Juss.
Iz_5_1209	DJI_0276	Berberis vulgaris L.	0	n	1	0	Berberidaceae Juss.
Iz_5_1209	DJI_0276	Betula pendula Roth.	0	n	1	1	Betulaceae Gray
Iz_5_1209	DJI_0276	Betula pendula Roth.	0	n	1	0	Poaceae Barnhart
Iz_5_1209	DJI_0276	Bromus inermis Leyss.	0		NA	NA	

Iz_5_1209	DJI_0276	Bromus squarrosus L.	0	a	0	0	Poaceae Barnhart
Iz_5_1209	DJI_0276	Bromus tectorum L.	0	a	0	0	Poaceae Barnhart
Iz_5_1209	DJI_0276	Calamagrostis epigejos (L.) Roth	10	n	1	0	Poaceae Barnhart
Iz_5_1209	DJI_0276	Carduus acanthoides L.	0	n	1	0	Asteraceae Bercht. & J.Presl
Iz_5_1209	DJI_0276	Carex sp.	0	n	1	0	Cyperaceae Juss.
Iz_5_1209	DJI_0276	Centaurea majorovii Dumbadze	0	n	1	0	Asteraceae Bercht. & J.Presl
Iz_5_1209	DJI_0276	Chamaecytisus ruthenicus (Fisch.ex Wol.) Klaskova	0	n	1	0	Fabaceae Lindl.
Iz_5_1209	DJI_0276	Chelidonium majus L.	0	n	1	0	Papaveraceae Juss.
Iz_5_1209	DJI_0276	Chenopodium betaceum Andrz.	0	n	1	0	Amaranthaceae Juss.
Iz_5_1209	DJI_0276	Chenopodium cf. album	0	n	1	0	Amaranthaceae Juss.
Iz_5_1209	DJI_0276	Chenopodium sp.	0		NA	0	Amaranthaceae Juss.
Iz_5_1209	DJI_0276	Chondrilla juncea L.	0	n	1	0	Asteraceae Bercht. & J.Presl
Iz_5_1209	DJI_0276	Cirsium vulgare (Savi) Ten.	0	n	1	0	Asteraceae Bercht. & J.Presl
Iz_5_1209	DJI_0276	Clinopodium acinos (L.) Kuntze	0	n	1	0	Lamiacea Lindl.
Iz_5_1209	DJI_0276	Convallaria majalis L.	0	n	1	0	Asparagaceae Juss.
Iz_5_1209	DJI_0276	Crataegus rhipidophylla Gand.	0	n	1	1	Rosaceae Juss.
Iz_5_1209	DJI_0276	Crepis foetida L.	0	n	1	0	Asteraceae Bercht. & J.Presl
Iz_5_1209	DJI_0276	Dactylis glomerata L.	0	n	1	0	Poaceae Barnhart
Iz_5_1209	DJI_0276	Dianthus campestris M.Bieb.	0	n	1	0	Caryophyllaceae Juss.
Iz_5_1209	DJI_0276	Digitaria sanguinalis (L.) Scop.	0	a	0	0	Poaceae Barnhart
Iz_5_1209	DJI_0276	Elymus repens (L.) Gould	0	n	1	0	Poaceae Barnhart
Iz_5_1209	DJI_0276	Epilobium tetragonum L.	0	n	1	0	Onagraceae Juss.
Iz_5_1209	DJI_0276	Eragrostis minor Host	0	a	0	0	Poaceae Barnhart
Iz_5_1209	DJI_0276	Erigeron canadensis L.	4	a	0	0	Asteraceae Bercht. & J.Presl
Iz_5_1209	DJI_0276	Euphorbia seguieriana Neck.	0	n	1	0	Euphorbiaceae Juss.
Iz_5_1209	DJI_0276	Fallopia dumetorum (L.) Holub	0	n	1	0	Polygonaceae Juss.
Iz_5_1209	DJI_0276	Festuca beckeri (Hack.) Trautv.	0	n	1	0	Poaceae Barnhart
Iz_5_1209	DJI_0276	Fragaria vesca L.	0	n	1	0	Rosaceae Juss.
Iz_5_1209	DJI_0276	Frangula alnus Mill.	0	n	1	1	Rhamnaceae Juss.
Iz_5_1209	DJI_0276	Fraxinus pennsylvanica Marshall	0	a	0	1	Oleaceae Hoffmanns. & Link
Iz_5_1209	DJI_0276	Galeopsis bifida Boenn.	0	n	1	0	Lamiacea Lindl.
Iz_5_1209	DJI_0276	Galium verum L.	0	n	1	0	Rubiaceae Juss.
Iz_5_1209	DJI_0276	Genista tinctoria L.	0	n	1	0	Fabaceae Lindl.
Iz_5_1209	DJI_0276	Geranium robertianum L.	0	n	1	0	Geraniaceae Juss.
Iz_5_1209	DJI_0276	Geranium sanquineum L.	0	n	1	0	Geraniaceae Juss.
Iz_5_1209	DJI_0276	Gypsophila paniculata L.	0	n	1	0	Caryophyllaceae Juss.
Iz_5_1209	DJI_0276	Helichrysum arenarium (L.) Moench	0	n	1	0	Asteraceae Bercht. & J.Presl
Iz_5_1209	DJI_0276	Herniaria polygama J.Gay	0	n	1	0	Caryophyllaceae Juss.
Iz_5_1209	DJI_0276	Hieracium robustum Fr.	0	n	1	0	Asteraceae Bercht. & J.Presl
Iz_5_1209	DJI_0276	Hieracium umbellatum L.	0	n	1	NA	Asteraceae Bercht. & J.Presl
Iz_5_1209	DJI_0276	Hylotelephium maximum (L.) Holub	0	n	1	0	Crassulaceae J.St.-Hil.
Iz_5_1209	DJI_0276	Jacobaea borysthenica (DC.) B.Nord. & Greuter	0	n	1	0	Asteraceae Bercht. & J.Presl
Iz_5_1209	DJI_0276	Jasione montana L.	0	n	1	0	Campanulaceae Juss.
Iz_5_1209	DJI_0276	Jurinea cyanoides (L.) Rchb.s.l.	0	n	1	0	Asteraceae Bercht. & J.Presl
Iz_5_1209	DJI_0276	Koeleria glauca (Spreng.) DC.	0	n	1	0	Poaceae Barnhart
Iz_5_1209	DJI_0276	Lactuca serriola L.	0	a	0	0	Asteraceae Bercht. & J.Presl
Iz_5_1209	DJI_0276	Linaria genistifolia (L.) Mill.	0	n	1	0	Plantaginaceae Juss.
Iz_5_1209	DJI_0276	Lomelosia argentea (L.) Greuter & Burdet	0	n	1	0	Caprifoliaceae Juss.
Iz_5_1209	DJI_0276	Melampyrum pratense L.	0	n	1	0	Orobanchaceae Vent.
Iz_5_1209	DJI_0276	Oenothera biennis L.	0	a	0	0	Onagraceae Juss.
Iz_5_1209	DJI_0276	Pentanema britannica (L.) D.Gut.Larr., Santos-Vicente, Anderb., E.Rico & M.M.Mart.Ort.	0	n	1	0	Asteraceae Bercht. & J.Presl
Iz_5_1209	DJI_0276	Peucedanum oreoselinum (L.) Moench	0	n	1	0	Apiaceae Lindl.
Iz_5_1209	DJI_0276	Peucedanum ruthenicum M.Bieb.	0	n	1	0	Apiaceae Lindl.
Iz_5_1209	DJI_0276	Pilosella echioides (Lumn.) F.W.Schultz & Sch.Bip.	0	n	1	0	Asteraceae Bercht. & J.Presl
Iz_5_1209	DJI_0276	Pilosella officinarum F. Schultz et Sch. Bip.	0	n	1	0	Asteraceae Bercht. & J.Presl
Iz_5_1209	DJI_0276	Pilosella sp.	0		NA	0	Asteraceae Bercht. & J.Presl
Iz_5_1209	DJI_0276	Pinus sylvestris L.	0	n	1	1	Pinaceae Spreng. ex F.Rudolphi
Iz_5_1209	DJI_0276	Poa annua L.	0	n	1	0	Poaceae Barnhart
Iz_5_1209	DJI_0276	Poa nemoralis L.	0	n	1	0	Poaceae Barnhart
Iz_5_1209	DJI_0276	Poa sp.	0		NA	0	Poaceae Barnhart
Iz_5_1209	DJI_0276	Polygonatum odoratum (Mill.) Druce	0	n	1	0	Asparagaceae Juss.
Iz_5_1209	DJI_0276	Polygonum arenarium Waldst. & Kit.	0	n	1	0	Polygonaceae Juss.
Iz_5_1209	DJI_0276	Populus nigra L.	0	n	1	1	Salicaceae Mirb.
Iz_5_1209	DJI_0276	Populus tremula L.	2	n	1	1	Salicaceae Mirb.
Iz_5_1209	DJI_0276	Populus Ã canescens (Aiton) Sm.	0	n	1	1	Salicaceae Mirb.
Iz_5_1209	DJI_0276	Potentilla argentea L.	0	n	1	0	Rosaceae Juss.
Iz_5_1209	DJI_0276	Psammophiliella muralis (L.) Ikonn.	0	n	1	0	Caryophyllaceae Juss.
Iz_5_1209	DJI_0276	Psephellus sumensis (Kalen.) Greuter	0	n	1	0	Asteraceae Bercht. & J.Presl
Iz_5_1209	DJI_0276	Pulsatilla pratensis (L.) Mill. s.l.	0	n	1	0	Ranunculaceae Juss.
Iz_5_1209	DJI_0276	Pyrus communis L.	0	a	0	1	Rosaceae Juss.
Iz_5_1209	DJI_0276	Quercus robur L.	0	n	1	1	Fagaceae Dumort.
Iz_5_1209	DJI_0276	Robinia pseudoacacia L.	0	a	0	1	Fabaceae Lindl.
Iz_5_1209	DJI_0276	Rosa sp.	0		NA	1	Rosaceae Juss.
Iz_5_1209	DJI_0276	Rubus idaeus L.	0	n	1	0	Rosaceae Juss.
Iz_5_1209	DJI_0276	Rumex acetosella L.	0	n	1	0	Polygonaceae Juss.
Iz_5_1209	DJI_0276	Rumex thyrsiflorus Fingerh.	0	n	1	0	Polygonaceae Juss.
Iz_5_1209	DJI_0276	Salix alba L.	0	n	1	1	Salicaceae Mirb.
Iz_5_1209	DJI_0276	Sambucus nigra L.	0	n	1	1	Viburnaceae Raf.
Iz_5_1209	DJI_0276	Secale sylvestre Host	0	n	1	0	Poaceae Barnhart
Iz_5_1209	DJI_0276	Sempervivum ruthenicum Schnittsp. & C.B. Lehm.	0	n	1	0	Crassulaceae J.St.-Hil.
Iz_5_1209	DJI_0276	Setaria viridis (L.) P. Beauv.	0	n	1	0	Poaceae Barnhart
Iz_5_1209	DJI_0276	Silene borysthenica (Gruner) Walters	0	n	1	0	Caryophyllaceae Juss.
Iz_5_1209	DJI_0276	Silene latifolia subsp. alba (Mill.) Greuter & Burdet	0	n	1	0	Caryophyllaceae Juss.
Iz_5_1209	DJI_0276	Solidago virgaurea L.	0	n	1	0	Asteraceae Bercht. & J.Presl
Iz_5_1209	DJI_0276	Sorbus aucuparia L.	0	n	1	1	Rosaceae Juss.
Iz_5_1209	DJI_0276	Stachys recta L.	0	n	1	0	Lamiacea Lindl.
Iz_5_1209	DJI_0276	Tanacetum vulgare L.	0	n	1	0	Asteraceae Bercht. & J.Presl
Iz_5_1209	DJI_0276	Thymus pallasianus Heinr.Braun	0	n	1	0	Lamiacea Lindl.
Iz_5_1209	DJI_0276	Tilia cordata Mill.	0	n	1	1	Malvaceae Juss.
Iz_5_1209	DJI_0276	Trifolium alpestre L.	0	n	1	0	Fabaceae Lindl.
Iz_5_1209	DJI_0276	Trifolium arvense L.	0	n	1	0	Fabaceae Lindl.
Iz_5_1209	DJI_0276	Ulmus glabra Huds.	0	n	1	1	Ulmaceae Mirb.
Iz_5_1209	DJI_0276	Verbascum densiflorum Bertol.	0	n	1	0	Scrophulariaceae Juss.
Iz_5_1209	DJI_0276	Veronica austriaca L.	0	n	1	0	Plantaginaceae Juss.
Iz_5_1209	DJI_0276	Veronica incana L.	0	n	1	0	Plantaginaceae Juss.
Iz_5_1209	DJI_0276	Veronica verna L.	0	n	1	0	Plantaginaceae Juss.
Iz_5_1209	DJI_0276	Vincetoxicum hirundinaria Medik	0	n	1	0	Apocynaceae Juss.
Iz_5_1209	DJI_0281	Acer tataricum L.	0	n	1	1	Sapindaceae Juss.

Iz_5_1209	DJI_0281	Achillea millefolium L. s.l.	0	n	1	0	Asteraceae Bercht. & J.Presl
Iz_5_1209	DJI_0281	Allium oleraceum L.	0	n	1	0	Amaryllidaceae J.St.-Hil.
Iz_5_1209	DJI_0281	Ambrosia artemisiifolia L.	0	a	0	0	Asteraceae Bercht. & J.Presl
Iz_5_1209	DJI_0281	Amorpha fruticosa L.	0	a	0	0	Fabaceae Lindl.
Iz_5_1209	DJI_0281	Anthericum ramosum L.	0	n	1	0	Asparagaceae Juss.
Iz_5_1209	DJI_0281	Artemisia marschalliana Spreng.	0	n	1	0	Asteraceae Bercht. & J.Presl
Iz_5_1209	DJI_0281	Asparagus officinalis L.	0	n	1	0	Asparagaceae Juss.
Iz_5_1209	DJI_0281	Astragalus cicer L.	0	n	1	0	Fabaceae Lindl.
Iz_5_1209	DJI_0281	Bassia laniflora (S.G.Gmel.) A.J.Scott	0	n	1	0	Amaranthaceae Juss.
Iz_5_1209	DJI_0281	Berberis vulgaris L.	0	n	1	0	Berberidaceae Juss.
Iz_5_1209	DJI_0281	Betula pendula Roth.	0	n	1	1	Betulaceae Gray
Iz_5_1209	DJI_0281	Betula pendula Roth.	0	n	1	0	Poaceae Barnhart
Iz_5_1209	DJI_0281	Bromus inermis Leyss.	0		NA	NA	
Iz_5_1209	DJI_0281	Bromus squarrosus L.	0	a	0	0	Poaceae Barnhart
Iz_5_1209	DJI_0281	Bromus tectorum L.	0	a	0	0	Poaceae Barnhart
Iz_5_1209	DJI_0281	Calamagrostis epigejos (L.) Roth	5	n	1	0	Poaceae Barnhart
Iz_5_1209	DJI_0281	Carduus acanthoides L.	0	n	1	0	Asteraceae Bercht. & J.Presl
Iz_5_1209	DJI_0281	Carex sp.	0	n	1	0	Cyperaceae Juss.
Iz_5_1209	DJI_0281	Centaurea majorovii Dumbadze	0	n	1	0	Asteraceae Bercht. & J.Presl
Iz_5_1209	DJI_0281	Chamaecytisus ruthenicus (Fisch.ex Wol.) Klaskova	0	n	1	0	Fabaceae Lindl.
Iz_5_1209	DJI_0281	Chelidonium majus L.	0	n	1	0	Papaveraceae Juss.
Iz_5_1209	DJI_0281	Chenopodium betaceum Andrz.	0	n	1	0	Amaranthaceae Juss.
Iz_5_1209	DJI_0281	Chenopodium cf. album	0	n	1	0	Amaranthaceae Juss.
Iz_5_1209	DJI_0281	Chenopodium sp.	0		NA	0	Amaranthaceae Juss.
Iz_5_1209	DJI_0281	Chondrilla juncea L.	0	n	1	0	Asteraceae Bercht. & J.Presl
Iz_5_1209	DJI_0281	Cirsium vulgare (Savi) Ten.	0	n	1	0	Asteraceae Bercht. & J.Presl
Iz_5_1209	DJI_0281	Clinopodium acinos (L.) Kuntze	0	n	1	0	Lamiacea Lindl.
Iz_5_1209	DJI_0281	Convallaria majalis L.	0	n	1	0	Asparagaceae Juss.
Iz_5_1209	DJI_0281	Crataegus rhipidophylla Gand.	0	n	1	1	Rosaceae Juss.
Iz_5_1209	DJI_0281	Crepis foetida L.	0	n	1	0	Asteraceae Bercht. & J.Presl
Iz_5_1209	DJI_0281	Dactylis glomerata L.	0	n	1	0	Poaceae Barnhart
Iz_5_1209	DJI_0281	Dianthus campestris M.Bieb.	0	n	1	0	Caryophyllaceae Juss.
Iz_5_1209	DJI_0281	Digitaria sanguinalis (L.) Scop.	0	a	0	0	Poaceae Barnhart
Iz_5_1209	DJI_0281	Elymus repens (L.) Gould	0	n	1	0	Poaceae Barnhart
Iz_5_1209	DJI_0281	Epilobium tetragonum L.	0	n	1	0	Onagraceae Juss.
Iz_5_1209	DJI_0281	Eragrostis minor Host	0	a	0	0	Poaceae Barnhart
Iz_5_1209	DJI_0281	Erigeron canadensis L.	1	a	0	0	Asteraceae Bercht. & J.Presl
Iz_5_1209	DJI_0281	Euphorbia seguieriana Neck.	0	n	1	0	Euphorbiaceae Juss.
Iz_5_1209	DJI_0281	Fallopia dumetorum (L.) Holub	0	n	1	0	Polygonaceae Juss.
Iz_5_1209	DJI_0281	Festuca beckeri (Hack.) Trautv.	0	n	1	0	Poaceae Barnhart
Iz_5_1209	DJI_0281	Fragaria vesca L.	0	n	1	0	Rosaceae Juss.
Iz_5_1209	DJI_0281	Frangula alnus Mill.	0	n	1	1	Rhamnaceae Juss.
Iz_5_1209	DJI_0281	Fraxinus pennsylvanica Marshall	0	a	0	1	Oleaceae Hoffmanns. & Link
Iz_5_1209	DJI_0281	Galeopsis bifida Boenn.	0	n	1	0	Lamiacea Lindl.
Iz_5_1209	DJI_0281	Galium verum L.	0	n	1	0	Rubiaceae Juss.
Iz_5_1209	DJI_0281	Genista tinctoria L.	0	n	1	0	Fabaceae Lindl.
Iz_5_1209	DJI_0281	Geranium robertianum L.	0	n	1	0	Geraniaceae Juss.
Iz_5_1209	DJI_0281	Geranium sanquineum L.	0	n	1	0	Geraniaceae Juss.
Iz_5_1209	DJI_0281	Gypsophila paniculata L.	0	n	1	0	Caryophyllaceae Juss.
Iz_5_1209	DJI_0281	Helichrysum arenarium (L.) Moench	0	n	1	0	Asteraceae Bercht. & J.Presl
Iz_5_1209	DJI_0281	Herniaria polygama J.Gay	0	n	1	0	Caryophyllaceae Juss.
Iz_5_1209	DJI_0281	Hieracium robustum Fr.	0	n	1	0	Asteraceae Bercht. & J.Presl
Iz_5_1209	DJI_0281	Hieracium umbellatum L.	0	n	1	NA	Asteraceae Bercht. & J.Presl
Iz_5_1209	DJI_0281	Hylotelephium maximum (L.) Holub	0	n	1	0	Crassulaceae J.St.-Hil.
Iz_5_1209	DJI_0281	Jacobaea borysthenica (DC.) B.Nord. & Greuter	0	n	1	0	Asteraceae Bercht. & J.Presl
Iz_5_1209	DJI_0281	Jasione montana L.	0	n	1	0	Campanulaceae Juss.
Iz_5_1209	DJI_0281	Jurinea cyanoides (L.) Rchb.s.l.	0	n	1	0	Asteraceae Bercht. & J.Presl
Iz_5_1209	DJI_0281	Koeleria glauca (Spreng.) DC.	0	n	1	0	Poaceae Barnhart
Iz_5_1209	DJI_0281	Lactuca serriola L.	0	a	0	0	Asteraceae Bercht. & J.Presl
Iz_5_1209	DJI_0281	Linaria genistifolia (L.) Mill.	0	n	1	0	Plantaginaceae Juss.
Iz_5_1209	DJI_0281	Lomelosia argentea (L.) Greuter & Burdet	0	n	1	0	Caprifoliaceae Juss.
Iz_5_1209	DJI_0281	Melampyrum pratense L.	0	n	1	0	Orobanchaceae Vent.
Iz_5_1209	DJI_0281	Oenothera biennis L.	0	a	0	0	Onagraceae Juss.
Iz_5_1209	DJI_0281	Pentanema britannica (L.) D.Gut.Larr., Santos-Vicente, Anderb., E.Rico & M.M.Mart.Ort.	0	n	1	0	Asteraceae Bercht. & J.Presl
Iz_5_1209	DJI_0281	Peucedanum oreoselinum (L.) Moench	0	n	1	0	Apiaceae Lindl.
Iz_5_1209	DJI_0281	Peucedanum ruthenicum M.Bieb.	0	n	1	0	Apiaceae Lindl.
Iz_5_1209	DJI_0281	Pilosella echioides (Lumn.) F.W.Schultz & Sch.Bip.	0	n	1	0	Asteraceae Bercht. & J.Presl
Iz_5_1209	DJI_0281	Pilosella officinarum F. Schultz et Sch. Bip.	0	n	1	0	Asteraceae Bercht. & J.Presl
Iz_5_1209	DJI_0281	Pilosella sp.	0		NA	0	Asteraceae Bercht. & J.Presl
Iz_5_1209	DJI_0281	Pinus sylvestris L.	0	n	1	1	Pinaceae Spreng. ex F.Rudolphi
Iz_5_1209	DJI_0281	Poa annua L.	0	n	1	0	Poaceae Barnhart
Iz_5_1209	DJI_0281	Poa nemoralis L.	0	n	1	0	Poaceae Barnhart
Iz_5_1209	DJI_0281	Poa sp.	0		NA	0	Poaceae Barnhart
Iz_5_1209	DJI_0281	Polygonatum odoratum (Mill.) Druce	0	n	1	0	Asparagaceae Juss.
Iz_5_1209	DJI_0281	Polygonum arenarium Waldst. & Kit.	0	n	1	0	Polygonaceae Juss.
Iz_5_1209	DJI_0281	Populus nigra L.	0	n	1	1	Salicaceae Mirb.
Iz_5_1209	DJI_0281	Populus tremula L.	0	n	1	1	Salicaceae Mirb.
Iz_5_1209	DJI_0281	Populus Ã canescens (Aiton) Sm.	0	n	1	1	Salicaceae Mirb.
Iz_5_1209	DJI_0281	Potentilla argentea L.	0	n	1	0	Rosaceae Juss.
Iz_5_1209	DJI_0281	Psammophiliella muralis (L.) Ikonn.	0	n	1	0	Caryophyllaceae Juss.
Iz_5_1209	DJI_0281	Psephellus sumensis (Kalen.) Greuter	0	n	1	0	Asteraceae Bercht. & J.Presl
Iz_5_1209	DJI_0281	Pulsatilla pratensis (L.) Mill. s.l.	0	n	1	0	Ranunculaceae Juss.
Iz_5_1209	DJI_0281	Pyrus communis L.	0	a	0	1	Rosaceae Juss.
Iz_5_1209	DJI_0281	Quercus robur L.	0	n	1	1	Fagaceae Dumort.
Iz_5_1209	DJI_0281	Robinia pseudoacacia L.	0	a	0	1	Fabaceae Lindl.
Iz_5_1209	DJI_0281	Rosa sp.	0		NA	1	Rosaceae Juss.
Iz_5_1209	DJI_0281	Rubus idaeus L.	0	n	1	0	Rosaceae Juss.
Iz_5_1209	DJI_0281	Rumex acetosella L.	0	n	1	0	Polygonaceae Juss.
Iz_5_1209	DJI_0281	Rumex thyrsiflorus Fingerh.	0	n	1	0	Polygonaceae Juss.
Iz_5_1209	DJI_0281	Salix alba L.	0	n	1	1	Salicaceae Mirb.
Iz_5_1209	DJI_0281	Sambucus nigra L.	0	n	1	1	Viburnaceae Raf.
Iz_5_1209	DJI_0281	Secale sylvestre Host	0	n	1	0	Poaceae Barnhart
Iz_5_1209	DJI_0281	Sempervivum ruthenicum Schnittsp. & C.B. Lehm.	0	n	1	0	Crassulaceae J.St.-Hil.
Iz_5_1209	DJI_0281	Setaria viridis (L.) P. Beauv.	0	n	1	0	Poaceae Barnhart
Iz_5_1209	DJI_0281	Silene borysthenica (Gruner) Walters	0	n	1	0	Caryophyllaceae Juss.
Iz_5_1209	DJI_0281	Silene latifolia subsp. alba (Mill.) Greuter & Burdet	0	n	1	0	Caryophyllaceae Juss.
Iz_5_1209	DJI_0281	Solidago virgaurea L.	0	n	1	0	Asteraceae Bercht. & J.Presl
Iz_5_1209	DJI_0281	Sorbus aucuparia L.	0	n	1	1	Rosaceae Juss.

Iz_5_1209	DJI_0281	Stachys recta L.	0	n	1	0	Lamiacea Lindl.
Iz_5_1209	DJI_0281	Tanacetum vulgare L.	0	n	1	0	Asteraceae Bercht. & J.Presl
Iz_5_1209	DJI_0281	Thymus pallasianus Heinr.Braun	0	n	1	0	Lamiacea Lindl.
Iz_5_1209	DJI_0281	Tilia cordata Mill.	0	n	1	1	Malvaceae Juss.
Iz_5_1209	DJI_0281	Trifolium alpestre L.	0	n	1	0	Fabaceae Lindl.
Iz_5_1209	DJI_0281	Trifolium arvense L.	0	n	1	0	Fabaceae Lindl.
Iz_5_1209	DJI_0281	Ulmus glabra Huds.	0	n	1	1	Ulmaceae Mirb.
Iz_5_1209	DJI_0281	Verbascum densiflorum Bertol.	0	n	1	0	Scrophulariaceae Juss.
Iz_5_1209	DJI_0281	Veronica austriaca L.	0	n	1	0	Plantaginaceae Juss.
Iz_5_1209	DJI_0281	Veronica incana L.	0	n	1	0	Plantaginaceae Juss.
Iz_5_1209	DJI_0281	Veronica verna L.	0	n	1	0	Plantaginaceae Juss.
Iz_5_1209	DJI_0281	Vincetoxicum hirundinaria Medik	0	n	1	0	Apocynaceae Juss.
Iz_5_1209	DJI_0284	Acer tataricum L.	0	n	1	1	Sapindaceae Juss.
Iz_5_1209	DJI_0284	Achillea millefolium L. s.l.	0	n	1	0	Asteraceae Bercht. & J.Presl
Iz_5_1209	DJI_0284	Allium oleraceum L.	0	n	1	0	Amaryllidaceae J.St.-Hil.
Iz_5_1209	DJI_0284	Ambrosia artemisiifolia L.	0	a	0	0	Asteraceae Bercht. & J.Presl
Iz_5_1209	DJI_0284	Amorpha fruticosa L.	0	a	0	0	Fabaceae Lindl.
Iz_5_1209	DJI_0284	Anthericum ramosum L.	0	n	1	0	Asparagaceae Juss.
Iz_5_1209	DJI_0284	Artemisia marschalliana Spreng.	0	n	1	0	Asteraceae Bercht. & J.Presl
Iz_5_1209	DJI_0284	Asparagus officinalis L.	0	n	1	0	Asparagaceae Juss.
Iz_5_1209	DJI_0284	Astragalus cicer L.	0	n	1	0	Fabaceae Lindl.
Iz_5_1209	DJI_0284	Bassia laniflora (S.G.Gmel.) A.J.Scott	0	n	1	0	Amaranthaceae Juss.
Iz_5_1209	DJI_0284	Berberis vulgaris L.	0	n	1	0	Berberidaceae Juss.
Iz_5_1209	DJI_0284	Betula pendula Roth.	0	n	1	1	Betulaceae Gray
Iz_5_1209	DJI_0284	Betula pendula Roth.	0	n	1	0	Poaceae Barnhart
Iz_5_1209	DJI_0284	Bromus inermis Leyss.	0		NA	NA	
Iz_5_1209	DJI_0284	Bromus squarrosus L.	0	a	0	0	Poaceae Barnhart
Iz_5_1209	DJI_0284	Bromus tectorum L.	0	a	0	0	Poaceae Barnhart
Iz_5_1209	DJI_0284	Calamagrostis epigejos (L.) Roth	5	n	1	0	Poaceae Barnhart
Iz_5_1209	DJI_0284	Carduus acanthoides L.	0	n	1	0	Asteraceae Bercht. & J.Presl
Iz_5_1209	DJI_0284	Carex sp.	0	n	1	0	Cyperaceae Juss.
Iz_5_1209	DJI_0284	Centaurea majorovii Dumbadze	0	n	1	0	Asteraceae Bercht. & J.Presl
Iz_5_1209	DJI_0284	Chamaecytisus ruthenicus (Fisch.ex Wol.) Klaskova	0	n	1	0	Fabaceae Lindl.
Iz_5_1209	DJI_0284	Chelidonium majus L.	0	n	1	0	Papaveraceae Juss.
Iz_5_1209	DJI_0284	Chenopodium betaceum Andrz.	0	n	1	0	Amaranthaceae Juss.
Iz_5_1209	DJI_0284	Chenopodium cf. album	0	n	1	0	Amaranthaceae Juss.
Iz_5_1209	DJI_0284	Chenopodium sp.	0		NA	0	Amaranthaceae Juss.
Iz_5_1209	DJI_0284	Chondrilla juncea L.	0	n	1	0	Asteraceae Bercht. & J.Presl
Iz_5_1209	DJI_0284	Cirsium vulgare (Savi) Ten.	0	n	1	0	Asteraceae Bercht. & J.Presl
Iz_5_1209	DJI_0284	Clinopodium acinos (L.) Kuntze	0	n	1	0	Lamiacea Lindl.
Iz_5_1209	DJI_0284	Convallaria majalis L.	0	n	1	0	Asparagaceae Juss.
Iz_5_1209	DJI_0284	Crataegus rhipidophylla Gand.	0	n	1	1	Rosaceae Juss.
Iz_5_1209	DJI_0284	Crepis foetida L.	0	n	1	0	Asteraceae Bercht. & J.Presl
Iz_5_1209	DJI_0284	Dactylis glomerata L.	0	n	1	0	Poaceae Barnhart
Iz_5_1209	DJI_0284	Dianthus campestris M.Bieb.	0	n	1	0	Caryophyllaceae Juss.
Iz_5_1209	DJI_0284	Digitaria sanguinalis (L.) Scop.	0	a	0	0	Poaceae Barnhart
Iz_5_1209	DJI_0284	Elymus repens (L.) Gould	0	n	1	0	Poaceae Barnhart
Iz_5_1209	DJI_0284	Epilobium tetragonum L.	0	n	1	0	Onagraceae Juss.
Iz_5_1209	DJI_0284	Eragrostis minor Host	0	a	0	0	Poaceae Barnhart
Iz_5_1209	DJI_0284	Erigeron canadensis L.	0	a	0	0	Asteraceae Bercht. & J.Presl
Iz_5_1209	DJI_0284	Euphorbia seguieriana Neck.	0	n	1	0	Euphorbiaceae Juss.
Iz_5_1209	DJI_0284	Fallopia dumetorum (L.) Holub	0	n	1	0	Polygonaceae Juss.
Iz_5_1209	DJI_0284	Festuca beckeri (Hack.) Trautv.	0	n	1	0	Poaceae Barnhart
Iz_5_1209	DJI_0284	Fragaria vesca L.	0	n	1	0	Rosaceae Juss.
Iz_5_1209	DJI_0284	Frangula alnus Mill.	0	n	1	1	Rhamnaceae Juss.
Iz_5_1209	DJI_0284	Fraxinus pennsylvanica Marshall	0	a	0	1	Oleaceae Hoffmanns. & Link
Iz_5_1209	DJI_0284	Galeopsis bifida Boenn.	0	n	1	0	Lamiacea Lindl.
Iz_5_1209	DJI_0284	Galium verum L.	0	n	1	0	Rubiaceae Juss.
Iz_5_1209	DJI_0284	Genista tinctoria L.	0	n	1	0	Fabaceae Lindl.
Iz_5_1209	DJI_0284	Geranium robertianum L.	0	n	1	0	Geraniaceae Juss.
Iz_5_1209	DJI_0284	Geranium sanquineum L.	0	n	1	0	Geraniaceae Juss.
Iz_5_1209	DJI_0284	Gypsophila paniculata L.	0	n	1	0	Caryophyllaceae Juss.
Iz_5_1209	DJI_0284	Helichrysum arenarium (L.) Moench	0	n	1	0	Asteraceae Bercht. & J.Presl
Iz_5_1209	DJI_0284	Herniaria polygama J.Gay	0	n	1	0	Caryophyllaceae Juss.
Iz_5_1209	DJI_0284	Hieracium robustum Fr.	0	n	1	0	Asteraceae Bercht. & J.Presl
Iz_5_1209	DJI_0284	Hieracium umbellatum L.	0	n	1	NA	Asteraceae Bercht. & J.Presl
Iz_5_1209	DJI_0284	Hylotelephium maximum (L.) Holub	0	n	1	0	Crassulaceae J.St.-Hil.
Iz_5_1209	DJI_0284	Jacobaea borysthenica (DC.) B.Nord. & Greuter	0	n	1	0	Asteraceae Bercht. & J.Presl
Iz_5_1209	DJI_0284	Jasione montana L.	0	n	1	0	Campanulaceae Juss.
Iz_5_1209	DJI_0284	Jurinea cyanoides (L.) Rchb.s.l.	0	n	1	0	Asteraceae Bercht. & J.Presl
Iz_5_1209	DJI_0284	Koeleria glauca (Spreng.) DC.	0	n	1	0	Poaceae Barnhart
Iz_5_1209	DJI_0284	Lactuca serriola L.	0	a	0	0	Asteraceae Bercht. & J.Presl
Iz_5_1209	DJI_0284	Linaria genistifolia (L.) Mill.	0	n	1	0	Plantaginaceae Juss.
Iz_5_1209	DJI_0284	Lomelosia argentea (L.) Greuter & Burdet	0	n	1	0	Caprifoliaceae Juss.
Iz_5_1209	DJI_0284	Melampyrum pratense L.	0	n	1	0	Orobanchaceae Vent.
Iz_5_1209	DJI_0284	Oenothera biennis L.	0	a	0	0	Onagraceae Juss.
Iz_5_1209	DJI_0284	Pentanema britannica (L.) D.Gut.Larr., Santos-Vicente, Anderb., E.Rico & M.M.Mart.Ort.	0	n	1	0	Asteraceae Bercht. & J.Presl
Iz_5_1209	DJI_0284	Peucedanum oreoselinum (L.) Moench	0	n	1	0	Apiaceae Lindl.
Iz_5_1209	DJI_0284	Peucedanum ruthenicum M.Bieb.	0	n	1	0	Apiaceae Lindl.
Iz_5_1209	DJI_0284	Pilosella echioides (Lumn.) F.W.Schultz & Sch.Bip.	0	n	1	0	Asteraceae Bercht. & J.Presl
Iz_5_1209	DJI_0284	Pilosella officinarum F. Schultz et Sch. Bip.	0	n	1	0	Asteraceae Bercht. & J.Presl
Iz_5_1209	DJI_0284	Pilosella sp.	0		NA	0	Asteraceae Bercht. & J.Presl
Iz_5_1209	DJI_0284	Pinus sylvestris L.	0	n	1	1	Pinaceae Spreng. ex F.Rudolphi
Iz_5_1209	DJI_0284	Poa annua L.	0	n	1	0	Poaceae Barnhart
Iz_5_1209	DJI_0284	Poa nemoralis L.	0	n	1	0	Poaceae Barnhart
Iz_5_1209	DJI_0284	Poa sp.	0		NA	0	Poaceae Barnhart
Iz_5_1209	DJI_0284	Polygonatum odoratum (Mill.) Druce	0	n	1	0	Asparagaceae Juss.
Iz_5_1209	DJI_0284	Polygonum arenarium Waldst. & Kit.	0	n	1	0	Polygonaceae Juss.
Iz_5_1209	DJI_0284	Populus nigra L.	0	n	1	1	Salicaceae Mirb.
Iz_5_1209	DJI_0284	Populus tremula L.	0	n	1	1	Salicaceae Mirb.
Iz_5_1209	DJI_0284	Populus Ã canescens (Aiton) Sm.	0	n	1	1	Salicaceae Mirb.
Iz_5_1209	DJI_0284	Potentilla argentea L.	0	n	1	0	Rosaceae Juss.
Iz_5_1209	DJI_0284	Psammophiliella muralis (L.) Ikonn.	0	n	1	0	Caryophyllaceae Juss.
Iz_5_1209	DJI_0284	Psephellus sumensis (Kalen.) Greuter	0	n	1	0	Asteraceae Bercht. & J.Presl
Iz_5_1209	DJI_0284	Pulsatilla pratensis (L.) Mill. s.l.	0	n	1	0	Ranunculaceae Juss.
Iz_5_1209	DJI_0284	Pyrus communis L.	0	a	0	1	Rosaceae Juss.
Iz_5_1209	DJI_0284	Quercus robur L.	0	n	1	1	Fagaceae Dumort.
Iz_5_1209	DJI_0284	Robinia pseudoacacia L.	0	a	0	1	Fabaceae Lindl.

Iz_5_1209	DJI_0284	Rosa sp.	0		NA	1	Rosaceae Juss.
Iz_5_1209	DJI_0284	Rubus idaeus L.	0	n	1	0	Rosaceae Juss.
Iz_5_1209	DJI_0284	Rumex acetosella L.	0	n	1	0	Polygonaceae Juss.
Iz_5_1209	DJI_0284	Rumex thyrsiflorus Fingerh.	0	n	1	0	Polygonaceae Juss.
Iz_5_1209	DJI_0284	Salix alba L.	0	n	1	1	Salicaceae Mirb.
Iz_5_1209	DJI_0284	Sambucus nigra L.	0	n	1	1	Viburnaceae Raf.
Iz_5_1209	DJI_0284	Secale sylvestre Host	0	n	1	0	Poaceae Barnhart
Iz_5_1209	DJI_0284	Sempervivum ruthenicum Schnittsp. & C.B. Lehm.	0	n	1	0	Crassulaceae J.St.-Hil.
Iz_5_1209	DJI_0284	Setaria viridis (L.) P. Beauv.	0	n	1	0	Poaceae Barnhart
Iz_5_1209	DJI_0284	Silene borysthenica (Gruner) Walters	0	n	1	0	Caryophyllaceae Juss.
Iz_5_1209	DJI_0284	Silene latifolia subsp. alba (Mill.) Greuter & Burdet	0	n	1	0	Caryophyllaceae Juss.
Iz_5_1209	DJI_0284	Solidago virgaurea L.	0.2	n	1	0	Asteraceae Bercht. & J.Presl
Iz_5_1209	DJI_0284	Sorbus aucuparia L.	0	n	1	1	Rosaceae Juss.
Iz_5_1209	DJI_0284	Stachys recta L.	0	n	1	0	Lamiacea Lindl.
Iz_5_1209	DJI_0284	Tanacetum vulgare L.	0	n	1	0	Asteraceae Bercht. & J.Presl
Iz_5_1209	DJI_0284	Thymus pallasianus Heinr.Braun	0	n	1	0	Lamiacea Lindl.
Iz_5_1209	DJI_0284	Tilia cordata Mill.	0	n	1	1	Malvaceae Juss.
Iz_5_1209	DJI_0284	Trifolium alpestre L.	0	n	1	0	Fabaceae Lindl.
Iz_5_1209	DJI_0284	Trifolium arvense L.	0	n	1	0	Fabaceae Lindl.
Iz_5_1209	DJI_0284	Ulmus glabra Huds.	0	n	1	1	Ulmaceae Mirb.
Iz_5_1209	DJI_0284	Verbascum densiflorum Bertol.	0	n	1	0	Scrophulariaceae Juss.
Iz_5_1209	DJI_0284	Veronica austriaca L.	0	n	1	0	Plantaginaceae Juss.
Iz_5_1209	DJI_0284	Veronica incana L.	0	n	1	0	Plantaginaceae Juss.
Iz_5_1209	DJI_0284	Veronica verna L.	0	n	1	0	Plantaginaceae Juss.
Iz_5_1209	DJI_0284	Vincetoxicum hirundinaria Medik	0	n	1	0	Apocynaceae Juss.
Iz_5_1209	DJI_0286	Acer tataricum L.	0	n	1	1	Sapindaceae Juss.
Iz_5_1209	DJI_0286	Achillea millefolium L. s.l.	0	n	1	0	Asteraceae Bercht. & J.Presl
Iz_5_1209	DJI_0286	Allium oleraceum L.	0	n	1	0	Amaryllidaceae J.St.-Hil.
Iz_5_1209	DJI_0286	Ambrosia artemisiifolia L.	0	a	0	0	Asteraceae Bercht. & J.Presl
Iz_5_1209	DJI_0286	Amorpha fruticosa L.	0	a	0	0	Fabaceae Lindl.
Iz_5_1209	DJI_0286	Anthericum ramosum L.	0	n	1	0	Asparagaceae Juss.
Iz_5_1209	DJI_0286	Artemisia marschalliana Spreng.	0	n	1	0	Asteraceae Bercht. & J.Presl
Iz_5_1209	DJI_0286	Asparagus officinalis L.	0	n	1	0	Asparagaceae Juss.
Iz_5_1209	DJI_0286	Astragalus cicer L.	0	n	1	0	Fabaceae Lindl.
Iz_5_1209	DJI_0286	Bassia laniflora (S.G.Gmel.) A.J.Scott	0	n	1	0	Amaranthaceae Juss.
Iz_5_1209	DJI_0286	Berberis vulgaris L.	0	n	1	0	Berberidaceae Juss.
Iz_5_1209	DJI_0286	Betula pendula Roth.	0	n	1	1	Betulaceae Gray
Iz_5_1209	DJI_0286	Betula pendula Roth.	0	n	1	0	Poaceae Barnhart
Iz_5_1209	DJI_0286	Bromus inermis Leyss.	0		NA	NA	
Iz_5_1209	DJI_0286	Bromus squarrosus L.	0	a	0	0	Poaceae Barnhart
Iz_5_1209	DJI_0286	Bromus tectorum L.	0	a	0	0	Poaceae Barnhart
Iz_5_1209	DJI_0286	Calamagrostis epigejos (L.) Roth	5	n	1	0	Poaceae Barnhart
Iz_5_1209	DJI_0286	Carduus acanthoides L.	0	n	1	0	Asteraceae Bercht. & J.Presl
Iz_5_1209	DJI_0286	Carex sp.	0	n	1	0	Cyperaceae Juss.
Iz_5_1209	DJI_0286	Centaurea majorovii Dumbadze	0	n	1	0	Asteraceae Bercht. & J.Presl
Iz_5_1209	DJI_0286	Chamaecytisus ruthenicus (Fisch.ex Wol.) Klaskova	0	n	1	0	Fabaceae Lindl.
Iz_5_1209	DJI_0286	Chelidonium majus L.	0	n	1	0	Papaveraceae Juss.
Iz_5_1209	DJI_0286	Chenopodium betaceum Andrz.	0	n	1	0	Amaranthaceae Juss.
Iz_5_1209	DJI_0286	Chenopodium cf. album	0	n	1	0	Amaranthaceae Juss.
Iz_5_1209	DJI_0286	Chenopodium sp.	0		NA	0	Amaranthaceae Juss.
Iz_5_1209	DJI_0286	Chondrilla juncea L.	0	n	1	0	Asteraceae Bercht. & J.Presl
Iz_5_1209	DJI_0286	Cirsium vulgare (Savi) Ten.	0	n	1	0	Asteraceae Bercht. & J.Presl
Iz_5_1209	DJI_0286	Clinopodium acinos (L.) Kuntze	0	n	1	0	Lamiacea Lindl.
Iz_5_1209	DJI_0286	Convallaria majalis L.	0	n	1	0	Asparagaceae Juss.
Iz_5_1209	DJI_0286	Crataegus rhipidophylla Gand.	0	n	1	1	Rosaceae Juss.
Iz_5_1209	DJI_0286	Crepis foetida L.	0	n	1	0	Asteraceae Bercht. & J.Presl
Iz_5_1209	DJI_0286	Dactylis glomerata L.	0	n	1	0	Poaceae Barnhart
Iz_5_1209	DJI_0286	Dianthus campestris M.Bieb.	0	n	1	0	Caryophyllaceae Juss.
Iz_5_1209	DJI_0286	Digitaria sanguinalis (L.) Scop.	0	a	0	0	Poaceae Barnhart
Iz_5_1209	DJI_0286	Elymus repens (L.) Gould	0	n	1	0	Poaceae Barnhart
Iz_5_1209	DJI_0286	Epilobium tetragonum L.	0	n	1	0	Onagraceae Juss.
Iz_5_1209	DJI_0286	Eragrostis minor Host	0	a	0	0	Poaceae Barnhart
Iz_5_1209	DJI_0286	Erigeron canadensis L.	0.5	a	0	0	Asteraceae Bercht. & J.Presl
Iz_5_1209	DJI_0286	Euphorbia seguieriana Neck.	0	n	1	0	Euphorbiaceae Juss.
Iz_5_1209	DJI_0286	Fallopia dumetorum (L.) Holub	0	n	1	0	Polygonaceae Juss.
Iz_5_1209	DJI_0286	Festuca beckeri (Hack.) Trautv.	0	n	1	0	Poaceae Barnhart
Iz_5_1209	DJI_0286	Fragaria vesca L.	0	n	1	0	Rosaceae Juss.
Iz_5_1209	DJI_0286	Frangula alnus Mill.	0	n	1	1	Rhamnaceae Juss.
Iz_5_1209	DJI_0286	Fraxinus pennsylvanica Marshall	0	a	0	1	Oleaceae Hoffmanns. & Link
Iz_5_1209	DJI_0286	Galeopsis bifida Boenn.	0	n	1	0	Lamiacea Lindl.
Iz_5_1209	DJI_0286	Galium verum L.	0	n	1	0	Rubiaceae Juss.
Iz_5_1209	DJI_0286	Genista tinctoria L.	0	n	1	0	Fabaceae Lindl.
Iz_5_1209	DJI_0286	Geranium robertianum L.	0	n	1	0	Geraniaceae Juss.
Iz_5_1209	DJI_0286	Geranium sanquineum L.	0	n	1	0	Geraniaceae Juss.
Iz_5_1209	DJI_0286	Gypsophila paniculata L.	0	n	1	0	Caryophyllaceae Juss.
Iz_5_1209	DJI_0286	Helichrysum arenarium (L.) Moench	0	n	1	0	Asteraceae Bercht. & J.Presl
Iz_5_1209	DJI_0286	Herniaria polygama J.Gay	0	n	1	0	Caryophyllaceae Juss.
Iz_5_1209	DJI_0286	Hieracium robustum Fr.	0	n	1	0	Asteraceae Bercht. & J.Presl
Iz_5_1209	DJI_0286	Hieracium umbellatum L.	0	n	1	NA	Asteraceae Bercht. & J.Presl
Iz_5_1209	DJI_0286	Hylotelephium maximum (L.) Holub	0	n	1	0	Crassulaceae J.St.-Hil.
Iz_5_1209	DJI_0286	Jacobaea borysthenica (DC.) B.Nord. & Greuter	0	n	1	0	Asteraceae Bercht. & J.Presl
Iz_5_1209	DJI_0286	Jasione montana L.	0	n	1	0	Campanulaceae Juss.
Iz_5_1209	DJI_0286	Jurinea cyanoides (L.) Rchb.s.l.	0	n	1	0	Asteraceae Bercht. & J.Presl
Iz_5_1209	DJI_0286	Koeleria glauca (Spreng.) DC.	0	n	1	0	Poaceae Barnhart
Iz_5_1209	DJI_0286	Lactuca serriola L.	0	a	0	0	Asteraceae Bercht. & J.Presl
Iz_5_1209	DJI_0286	Linaria genistifolia (L.) Mill.	0	n	1	0	Plantaginaceae Juss.
Iz_5_1209	DJI_0286	Lomelosia argentea (L.) Greuter & Burdet	0	n	1	0	Caprifoliaceae Juss.
Iz_5_1209	DJI_0286	Melampyrum pratense L.	0	n	1	0	Orobanchaceae Vent.
Iz_5_1209	DJI_0286	Oenothera biennis L.	0	a	0	0	Onagraceae Juss.
Iz_5_1209	DJI_0286	Pentanema britannica (L.) D.Gut.Larr., Santos-Vicente, Anderb., E.Rico & M.M.Mart.Ort.	0	n	1	0	Asteraceae Bercht. & J.Presl
Iz_5_1209	DJI_0286	Peucedanum oreoselinum (L.) Moench	0	n	1	0	Apiaceae Lindl.
Iz_5_1209	DJI_0286	Peucedanum ruthenicum M.Bieb.	0	n	1	0	Apiaceae Lindl.
Iz_5_1209	DJI_0286	Pilosella echioides (Lumn.) F.W.Schultz & Sch.Bip.	0	n	1	0	Asteraceae Bercht. & J.Presl
Iz_5_1209	DJI_0286	Pilosella officinarum F. Schultz et Sch. Bip.	0	n	1	0	Asteraceae Bercht. & J.Presl
Iz_5_1209	DJI_0286	Pilosella sp.	0		NA	0	Asteraceae Bercht. & J.Presl
Iz_5_1209	DJI_0286	Pinus sylvestris L.	0	n	1	1	Pinaceae Spreng. ex F.Rudolphi
Iz_5_1209	DJI_0286	Poa annua L.	0	n	1	0	Poaceae Barnhart
Iz_5_1209	DJI_0286	Poa nemoralis L.	0	n	1	0	Poaceae Barnhart

Iz_5_1209	DJI_0286	Poa sp.	0		NA	0	Poaceae Barnhart
Iz_5_1209	DJI_0286	Polygonatum odoratum (Mill.) Druce	0	n	1	0	Asparagaceae Juss.
Iz_5_1209	DJI_0286	Polygonum arenarium Waldst. & Kit.	0	n	1	0	Polygonaceae Juss.
Iz_5_1209	DJI_0286	Populus nigra L.	0	n	1	1	Salicaceae Mirb.
Iz_5_1209	DJI_0286	Populus tremula L.	0	n	1	1	Salicaceae Mirb.
Iz_5_1209	DJI_0286	Populus Ã canescens (Aiton) Sm.	0	n	1	1	Salicaceae Mirb.
Iz_5_1209	DJI_0286	Potentilla argentea L.	0	n	1	0	Rosaceae Juss.
Iz_5_1209	DJI_0286	Psammophiliella muralis (L.) Ikonn.	0	n	1	0	Caryophyllaceae Juss.
Iz_5_1209	DJI_0286	Psephellus sumensis (Kalen.) Greuter	0	n	1	0	Asteraceae Bercht. & J.Presl
Iz_5_1209	DJI_0286	Pulsatilla pratensis (L.) Mill. s.l.	0	n	1	0	Ranunculaceae Juss.
Iz_5_1209	DJI_0286	Pyrus communis L.	0	a	0	1	Rosaceae Juss.
Iz_5_1209	DJI_0286	Quercus robur L.	0	n	1	1	Fagaceae Dumort.
Iz_5_1209	DJI_0286	Robinia pseudoacacia L.	0	a	0	1	Fabaceae Lindl.
Iz_5_1209	DJI_0286	Rosa sp.	0		NA	1	Rosaceae Juss.
Iz_5_1209	DJI_0286	Rubus idaeus L.	0	n	1	0	Rosaceae Juss.
Iz_5_1209	DJI_0286	Rumex acetosella L.	0	n	1	0	Polygonaceae Juss.
Iz_5_1209	DJI_0286	Rumex thyrsiflorus Fingerh.	0	n	1	0	Polygonaceae Juss.
Iz_5_1209	DJI_0286	Salix alba L.	0	n	1	1	Salicaceae Mirb.
Iz_5_1209	DJI_0286	Sambucus nigra L.	0	n	1	1	Viburnaceae Raf.
Iz_5_1209	DJI_0286	Secale sylvestre Host	0	n	1	0	Poaceae Barnhart
Iz_5_1209	DJI_0286	Sempervivum ruthenicum Schnittsp. & C.B. Lehm.	0	n	1	0	Crassulaceae J.St.-Hil.
Iz_5_1209	DJI_0286	Setaria viridis (L.) P. Beauv.	0	n	1	0	Poaceae Barnhart
Iz_5_1209	DJI_0286	Silene borysthenica (Gruner) Walters	0	n	1	0	Caryophyllaceae Juss.
Iz_5_1209	DJI_0286	Silene latifolia subsp. alba (Mill.) Greuter & Burdet	0	n	1	0	Caryophyllaceae Juss.
Iz_5_1209	DJI_0286	Solidago virgaurea L.	1	n	1	0	Asteraceae Bercht. & J.Presl
Iz_5_1209	DJI_0286	Sorbus aucuparia L.	0	n	1	1	Rosaceae Juss.
Iz_5_1209	DJI_0286	Stachys recta L.	0	n	1	0	Lamiacea Lindl.
Iz_5_1209	DJI_0286	Tanacetum vulgare L.	0	n	1	0	Asteraceae Bercht. & J.Presl
Iz_5_1209	DJI_0286	Thymus pallasianus Heinr.Braun	0	n	1	0	Lamiacea Lindl.
Iz_5_1209	DJI_0286	Tilia cordata Mill.	0	n	1	1	Malvaceae Juss.
Iz_5_1209	DJI_0286	Trifolium alpestre L.	0	n	1	0	Fabaceae Lindl.
Iz_5_1209	DJI_0286	Trifolium arvense L.	0	n	1	0	Fabaceae Lindl.
Iz_5_1209	DJI_0286	Ulmus glabra Huds.	0	n	1	1	Ulmaceae Mirb.
Iz_5_1209	DJI_0286	Verbascum densiflorum Bertol.	0	n	1	0	Scrophulariaceae Juss.
Iz_5_1209	DJI_0286	Veronica austriaca L.	0	n	1	0	Plantaginaceae Juss.
Iz_5_1209	DJI_0286	Veronica incana L.	0	n	1	0	Plantaginaceae Juss.
Iz_5_1209	DJI_0286	Veronica verna L.	0	n	1	0	Plantaginaceae Juss.
Iz_5_1209	DJI_0286	Vincetoxicum hirundinaria Medik	0	n	1	0	Apocynaceae Juss.
Iz_5_1209	DJI_0289	Acer tataricum L.	0	n	1	1	Sapindaceae Juss.
Iz_5_1209	DJI_0289	Achillea millefolium L. s.l.	0	n	1	0	Asteraceae Bercht. & J.Presl
Iz_5_1209	DJI_0289	Allium oleraceum L.	0	n	1	0	Amaryllidaceae J.St.-Hil.
Iz_5_1209	DJI_0289	Ambrosia artemisiifolia L.	0	a	0	0	Asteraceae Bercht. & J.Presl
Iz_5_1209	DJI_0289	Amorpha fruticosa L.	0	a	0	0	Fabaceae Lindl.
Iz_5_1209	DJI_0289	Anthericum ramosum L.	0	n	1	0	Asparagaceae Juss.
Iz_5_1209	DJI_0289	Artemisia marschalliana Spreng.	0	n	1	0	Asteraceae Bercht. & J.Presl
Iz_5_1209	DJI_0289	Asparagus officinalis L.	0	n	1	0	Asparagaceae Juss.
Iz_5_1209	DJI_0289	Astragalus cicer L.	0	n	1	0	Fabaceae Lindl.
Iz_5_1209	DJI_0289	Bassia laniflora (S.G.Gmel.) A.J.Scott	0	n	1	0	Amaranthaceae Juss.
Iz_5_1209	DJI_0289	Berberis vulgaris L.	0	n	1	0	Berberidaceae Juss.
Iz_5_1209	DJI_0289	Betula pendula Roth.	0	n	1	1	Betulaceae Gray
Iz_5_1209	DJI_0289	Betula pendula Roth.	0	n	1	0	Poaceae Barnhart
Iz_5_1209	DJI_0289	Bromus inermis Leyss.	0		NA	NA	
Iz_5_1209	DJI_0289	Bromus squarrosus L.	0	a	0	0	Poaceae Barnhart
Iz_5_1209	DJI_0289	Bromus tectorum L.	0	a	0	0	Poaceae Barnhart
Iz_5_1209	DJI_0289	Calamagrostis epigejos (L.) Roth	15	n	1	0	Poaceae Barnhart
Iz_5_1209	DJI_0289	Carduus acanthoides L.	0	n	1	0	Asteraceae Bercht. & J.Presl
Iz_5_1209	DJI_0289	Carex sp.	0	n	1	0	Cyperaceae Juss.
Iz_5_1209	DJI_0289	Centaurea majorovii Dumbadze	0	n	1	0	Asteraceae Bercht. & J.Presl
Iz_5_1209	DJI_0289	Chamaecytisus ruthenicus (Fisch.ex Wol.) Klaskova	0	n	1	0	Fabaceae Lindl.
Iz_5_1209	DJI_0289	Chelidonium majus L.	0	n	1	0	Papaveraceae Juss.
Iz_5_1209	DJI_0289	Chenopodium betaceum Andrz.	0	n	1	0	Amaranthaceae Juss.
Iz_5_1209	DJI_0289	Chenopodium cf. album	0	n	1	0	Amaranthaceae Juss.
Iz_5_1209	DJI_0289	Chenopodium sp.	0		NA	0	Amaranthaceae Juss.
Iz_5_1209	DJI_0289	Chondrilla juncea L.	0	n	1	0	Asteraceae Bercht. & J.Presl
Iz_5_1209	DJI_0289	Cirsium vulgare (Savi) Ten.	0	n	1	0	Asteraceae Bercht. & J.Presl
Iz_5_1209	DJI_0289	Clinopodium acinos (L.) Kuntze	0	n	1	0	Lamiacea Lindl.
Iz_5_1209	DJI_0289	Convallaria majalis L.	0	n	1	0	Asparagaceae Juss.
Iz_5_1209	DJI_0289	Crataegus rhipidophylla Gand.	0	n	1	1	Rosaceae Juss.
Iz_5_1209	DJI_0289	Crepis foetida L.	0	n	1	0	Asteraceae Bercht. & J.Presl
Iz_5_1209	DJI_0289	Dactylis glomerata L.	0	n	1	0	Poaceae Barnhart
Iz_5_1209	DJI_0289	Dianthus campestris M.Bieb.	0	n	1	0	Caryophyllaceae Juss.
Iz_5_1209	DJI_0289	Digitaria sanguinalis (L.) Scop.	0	a	0	0	Poaceae Barnhart
Iz_5_1209	DJI_0289	Elymus repens (L.) Gould	0	n	1	0	Poaceae Barnhart
Iz_5_1209	DJI_0289	Epilobium tetragonum L.	0	n	1	0	Onagraceae Juss.
Iz_5_1209	DJI_0289	Eragrostis minor Host	0	a	0	0	Poaceae Barnhart
Iz_5_1209	DJI_0289	Erigeron canadensis L.	0	a	0	0	Asteraceae Bercht. & J.Presl
Iz_5_1209	DJI_0289	Euphorbia seguieriana Neck.	0	n	1	0	Euphorbiaceae Juss.
Iz_5_1209	DJI_0289	Fallopia dumetorum (L.) Holub	0	n	1	0	Polygonaceae Juss.
Iz_5_1209	DJI_0289	Festuca beckeri (Hack.) Trautv.	0	n	1	0	Poaceae Barnhart
Iz_5_1209	DJI_0289	Fragaria vesca L.	0	n	1	0	Rosaceae Juss.
Iz_5_1209	DJI_0289	Frangula alnus Mill.	0	n	1	1	Rhamnaceae Juss.
Iz_5_1209	DJI_0289	Fraxinus pennsylvanica Marshall	0	a	0	1	Oleaceae Hoffmanns. & Link
Iz_5_1209	DJI_0289	Galeopsis bifida Boenn.	0	n	1	0	Lamiacea Lindl.
Iz_5_1209	DJI_0289	Galium verum L.	0	n	1	0	Rubiaceae Juss.
Iz_5_1209	DJI_0289	Genista tinctoria L.	0	n	1	0	Fabaceae Lindl.
Iz_5_1209	DJI_0289	Geranium robertianum L.	0	n	1	0	Geraniaceae Juss.
Iz_5_1209	DJI_0289	Geranium sanquineum L.	0	n	1	0	Geraniaceae Juss.
Iz_5_1209	DJI_0289	Gypsophila paniculata L.	0	n	1	0	Caryophyllaceae Juss.
Iz_5_1209	DJI_0289	Helichrysum arenarium (L.) Moench	0	n	1	0	Asteraceae Bercht. & J.Presl
Iz_5_1209	DJI_0289	Herniaria polygama J.Gay	0	n	1	0	Caryophyllaceae Juss.
Iz_5_1209	DJI_0289	Hieracium robustum Fr.	0	n	1	0	Asteraceae Bercht. & J.Presl
Iz_5_1209	DJI_0289	Hieracium umbellatum L.	0	n	1	NA	Asteraceae Bercht. & J.Presl
Iz_5_1209	DJI_0289	Hylotelephium maximum (L.) Holub	0	n	1	0	Crassulaceae J.St.-Hil.
Iz_5_1209	DJI_0289	Jacobaea borysthenica (DC.) B.Nord. & Greuter	0	n	1	0	Asteraceae Bercht. & J.Presl
Iz_5_1209	DJI_0289	Jasione montana L.	0	n	1	0	Campanulaceae Juss.
Iz_5_1209	DJI_0289	Jurinea cyanoides (L.) Rchb.s.l.	0	n	1	0	Asteraceae Bercht. & J.Presl
Iz_5_1209	DJI_0289	Koeleria glauca (Spreng.) DC.	0	n	1	0	Poaceae Barnhart
Iz_5_1209	DJI_0289	Lactuca serriola L.	0	a	0	0	Asteraceae Bercht. & J.Presl

Iz_5_1209	DJI_0289	Linaria genistifolia (L.) Mill.	0	n	1	0	Plantaginaceae Juss.
Iz_5_1209	DJI_0289	Lomelosia argentea (L.) Greuter & Burdet	0	n	1	0	Caprifoliaceae Juss.
Iz_5_1209	DJI_0289	Melampyrum pratense L.	0	n	1	0	Orobanchaceae Vent.
Iz_5_1209	DJI_0289	Oenothera biennis L.	0	a	0	0	Onagraceae Juss.
Iz_5_1209	DJI_0289	Pentanema britannica (L.) D.Gut.Larr., Santos-Vicente, Anderb., E.Rico & M.M.Mart.Ort.	0	n	1	0	Asteraceae Bercht. & J.Presl
Iz_5_1209	DJI_0289	Peucedanum oreoselinum (L.) Moench	0	n	1	0	Apiaceae Lindl.
Iz_5_1209	DJI_0289	Peucedanum ruthenicum M.Bieb.	0	n	1	0	Apiaceae Lindl.
Iz_5_1209	DJI_0289	Pilosella echioides (Lumn.) F.W.Schultz & Sch.Bip.	0	n	1	0	Asteraceae Bercht. & J.Presl
Iz_5_1209	DJI_0289	Pilosella officinarum F. Schultz et Sch. Bip.	0	n	1	0	Asteraceae Bercht. & J.Presl
Iz_5_1209	DJI_0289	Pilosella sp.	0		NA	0	Asteraceae Bercht. & J.Presl
Iz_5_1209	DJI_0289	Pinus sylvestris L.	0	n	1	1	Pinaceae Spreng. ex F.Rudolphi
Iz_5_1209	DJI_0289	Poa annua L.	0	n	1	0	Poaceae Barnhart
Iz_5_1209	DJI_0289	Poa nemoralis L.	0	n	1	0	Poaceae Barnhart
Iz_5_1209	DJI_0289	Poa sp.	0		NA	0	Poaceae Barnhart
Iz_5_1209	DJI_0289	Polygonatum odoratum (Mill.) Druce	0	n	1	0	Asparagaceae Juss.
Iz_5_1209	DJI_0289	Polygonum arenarium Waldst. & Kit.	0	n	1	0	Polygonaceae Juss.
Iz_5_1209	DJI_0289	Populus nigra L.	0	n	1	1	Salicaceae Mirb.
Iz_5_1209	DJI_0289	Populus tremula L.	1	n	1	1	Salicaceae Mirb.
Iz_5_1209	DJI_0289	Populus Ã canescens (Aiton) Sm.	0	n	1	1	Salicaceae Mirb.
Iz_5_1209	DJI_0289	Potentilla argentea L.	0	n	1	0	Rosaceae Juss.
Iz_5_1209	DJI_0289	Psammophiliella muralis (L.) Ikonn.	0	n	1	0	Caryophyllaceae Juss.
Iz_5_1209	DJI_0289	Psephellus sumensis (Kalen.) Greuter	0	n	1	0	Asteraceae Bercht. & J.Presl
Iz_5_1209	DJI_0289	Pulsatilla pratensis (L.) Mill. s.l.	0	n	1	0	Ranunculaceae Juss.
Iz_5_1209	DJI_0289	Pyrus communis L.	0	a	0	1	Rosaceae Juss.
Iz_5_1209	DJI_0289	Quercus robur L.	0	n	1	1	Fagaceae Dumort.
Iz_5_1209	DJI_0289	Robinia pseudoacacia L.	0	a	0	1	Fabaceae Lindl.
Iz_5_1209	DJI_0289	Rosa sp.	0		NA	1	Rosaceae Juss.
Iz_5_1209	DJI_0289	Rubus idaeus L.	0	n	1	0	Rosaceae Juss.
Iz_5_1209	DJI_0289	Rumex acetosella L.	0	n	1	0	Polygonaceae Juss.
Iz_5_1209	DJI_0289	Rumex thyrsiflorus Fingerh.	0	n	1	0	Polygonaceae Juss.
Iz_5_1209	DJI_0289	Salix alba L.	0	n	1	1	Salicaceae Mirb.
Iz_5_1209	DJI_0289	Sambucus nigra L.	0	n	1	1	Viburnaceae Raf.
Iz_5_1209	DJI_0289	Secale sylvestre Host	0	n	1	0	Poaceae Barnhart
Iz_5_1209	DJI_0289	Sempervivum ruthenicum Schnittsp. & C.B. Lehm.	0	n	1	0	Crassulaceae J.St.-Hil.
Iz_5_1209	DJI_0289	Setaria viridis (L.) P. Beauv.	0	n	1	0	Poaceae Barnhart
Iz_5_1209	DJI_0289	Silene borysthenica (Gruner) Walters	0	n	1	0	Caryophyllaceae Juss.
Iz_5_1209	DJI_0289	Silene latifolia subsp. alba (Mill.) Greuter & Burdet	0	n	1	0	Caryophyllaceae Juss.
Iz_5_1209	DJI_0289	Solidago virgaurea L.	0	n	1	0	Asteraceae Bercht. & J.Presl
Iz_5_1209	DJI_0289	Sorbus aucuparia L.	0	n	1	1	Rosaceae Juss.
Iz_5_1209	DJI_0289	Stachys recta L.	0	n	1	0	Lamiacea Lindl.
Iz_5_1209	DJI_0289	Tanacetum vulgare L.	0	n	1	0	Asteraceae Bercht. & J.Presl
Iz_5_1209	DJI_0289	Thymus pallasianus Heinr.Braun	0	n	1	0	Lamiacea Lindl.
Iz_5_1209	DJI_0289	Tilia cordata Mill.	0	n	1	1	Malvaceae Juss.
Iz_5_1209	DJI_0289	Trifolium alpestre L.	0	n	1	0	Fabaceae Lindl.
Iz_5_1209	DJI_0289	Trifolium arvense L.	0	n	1	0	Fabaceae Lindl.
Iz_5_1209	DJI_0289	Ulmus glabra Huds.	0	n	1	1	Ulmaceae Mirb.
Iz_5_1209	DJI_0289	Verbascum densiflorum Bertol.	0	n	1	0	Scrophulariaceae Juss.
Iz_5_1209	DJI_0289	Veronica austriaca L.	0	n	1	0	Plantaginaceae Juss.
Iz_5_1209	DJI_0289	Veronica incana L.	0	n	1	0	Plantaginaceae Juss.
Iz_5_1209	DJI_0289	Veronica verna L.	0	n	1	0	Plantaginaceae Juss.
Iz_5_1209	DJI_0289	Vincetoxicum hirundinaria Medik	0	n	1	0	Apocynaceae Juss.
Iz_5_1209	DJI_0291	Acer tataricum L.	0	n	1	1	Sapindaceae Juss.
Iz_5_1209	DJI_0291	Achillea millefolium L. s.l.	0	n	1	0	Asteraceae Bercht. & J.Presl
Iz_5_1209	DJI_0291	Allium oleraceum L.	0	n	1	0	Amaryllidaceae J.St.-Hil.
Iz_5_1209	DJI_0291	Ambrosia artemisiifolia L.	0	a	0	0	Asteraceae Bercht. & J.Presl
Iz_5_1209	DJI_0291	Amorpha fruticosa L.	0	a	0	0	Fabaceae Lindl.
Iz_5_1209	DJI_0291	Anthericum ramosum L.	0	n	1	0	Asparagaceae Juss.
Iz_5_1209	DJI_0291	Artemisia marschalliana Spreng.	0	n	1	0	Asteraceae Bercht. & J.Presl
Iz_5_1209	DJI_0291	Asparagus officinalis L.	0	n	1	0	Asparagaceae Juss.
Iz_5_1209	DJI_0291	Astragalus cicer L.	0	n	1	0	Fabaceae Lindl.
Iz_5_1209	DJI_0291	Bassia laniflora (S.G.Gmel.) A.J.Scott	0	n	1	0	Amaranthaceae Juss.
Iz_5_1209	DJI_0291	Berberis vulgaris L.	0	n	1	0	Berberidaceae Juss.
Iz_5_1209	DJI_0291	Betula pendula Roth.	0	n	1	1	Betulaceae Gray
Iz_5_1209	DJI_0291	Betula pendula Roth.	0	n	1	0	Poaceae Barnhart
Iz_5_1209	DJI_0291	Bromus inermis Leyss.	0		NA	NA	
Iz_5_1209	DJI_0291	Bromus squarrosus L.	0	a	0	0	Poaceae Barnhart
Iz_5_1209	DJI_0291	Bromus tectorum L.	0	a	0	0	Poaceae Barnhart
Iz_5_1209	DJI_0291	Calamagrostis epigejos (L.) Roth	7	n	1	0	Poaceae Barnhart
Iz_5_1209	DJI_0291	Carduus acanthoides L.	0	n	1	0	Asteraceae Bercht. & J.Presl
Iz_5_1209	DJI_0291	Carex sp.	0	n	1	0	Cyperaceae Juss.
Iz_5_1209	DJI_0291	Centaurea majorovii Dumbadze	0	n	1	0	Asteraceae Bercht. & J.Presl
Iz_5_1209	DJI_0291	Chamaecytisus ruthenicus (Fisch.ex Wol.) Klaskova	0	n	1	0	Fabaceae Lindl.
Iz_5_1209	DJI_0291	Chelidonium majus L.	0	n	1	0	Papaveraceae Juss.
Iz_5_1209	DJI_0291	Chenopodium betaceum Andrz.	0	n	1	0	Amaranthaceae Juss.
Iz_5_1209	DJI_0291	Chenopodium cf. album	0	n	1	0	Amaranthaceae Juss.
Iz_5_1209	DJI_0291	Chenopodium sp.	0		NA	0	Amaranthaceae Juss.
Iz_5_1209	DJI_0291	Chondrilla juncea L.	0	n	1	0	Asteraceae Bercht. & J.Presl
Iz_5_1209	DJI_0291	Cirsium vulgare (Savi) Ten.	0	n	1	0	Asteraceae Bercht. & J.Presl
Iz_5_1209	DJI_0291	Clinopodium acinos (L.) Kuntze	0	n	1	0	Lamiacea Lindl.
Iz_5_1209	DJI_0291	Convallaria majalis L.	0	n	1	0	Asparagaceae Juss.
Iz_5_1209	DJI_0291	Crataegus rhipidophylla Gand.	0	n	1	1	Rosaceae Juss.
Iz_5_1209	DJI_0291	Crepis foetida L.	0	n	1	0	Asteraceae Bercht. & J.Presl
Iz_5_1209	DJI_0291	Dactylis glomerata L.	0	n	1	0	Poaceae Barnhart
Iz_5_1209	DJI_0291	Dianthus campestris M.Bieb.	0	n	1	0	Caryophyllaceae Juss.
Iz_5_1209	DJI_0291	Digitaria sanguinalis (L.) Scop.	0	a	0	0	Poaceae Barnhart
Iz_5_1209	DJI_0291	Elymus repens (L.) Gould	0	n	1	0	Poaceae Barnhart
Iz_5_1209	DJI_0291	Epilobium tetragonum L.	0	n	1	0	Onagraceae Juss.
Iz_5_1209	DJI_0291	Eragrostis minor Host	0	a	0	0	Poaceae Barnhart
Iz_5_1209	DJI_0291	Erigeron canadensis L.	2	a	0	0	Asteraceae Bercht. & J.Presl
Iz_5_1209	DJI_0291	Euphorbia seguieriana Neck.	0	n	1	0	Euphorbiaceae Juss.
Iz_5_1209	DJI_0291	Fallopia dumetorum (L.) Holub	0	n	1	0	Polygonaceae Juss.
Iz_5_1209	DJI_0291	Festuca beckeri (Hack.) Trautv.	0	n	1	0	Poaceae Barnhart
Iz_5_1209	DJI_0291	Fragaria vesca L.	0	n	1	0	Rosaceae Juss.
Iz_5_1209	DJI_0291	Frangula alnus Mill.	0	n	1	1	Rhamnaceae Juss.
Iz_5_1209	DJI_0291	Fraxinus pennsylvanica Marshall	0	a	0	1	Oleaceae Hoffmanns. & Link
Iz_5_1209	DJI_0291	Galeopsis bifida Boenn.	0	n	1	0	Lamiacea Lindl.
Iz_5_1209	DJI_0291	Galium verum L.	0	n	1	0	Rubiaceae Juss.
Iz_5_1209	DJI_0291	Genista tinctoria L.	0	n	1	0	Fabaceae Lindl.

Iz_5_1209	DJI_0291	Geranium robertianum L.	0	n	1	0	Geraniaceae Juss.
Iz_5_1209	DJI_0291	Geranium sanquineum L.	0	n	1	0	Geraniaceae Juss.
Iz_5_1209	DJI_0291	Gypsophila paniculata L.	0	n	1	0	Caryophyllaceae Juss.
Iz_5_1209	DJI_0291	Helichrysum arenarium (L.) Moench	0	n	1	0	Asteraceae Bercht. & J.Presl
Iz_5_1209	DJI_0291	Herniaria polygama J.Gay	0	n	1	0	Caryophyllaceae Juss.
Iz_5_1209	DJI_0291	Hieracium robustum Fr.	0	n	1	0	Asteraceae Bercht. & J.Presl
Iz_5_1209	DJI_0291	Hieracium umbellatum L.	0	n	1	NA	Asteraceae Bercht. & J.Presl
Iz_5_1209	DJI_0291	Hylotelephium maximum (L.) Holub	0	n	1	0	Crassulaceae J.St.-Hil.
Iz_5_1209	DJI_0291	Jacobaea borysthenica (DC.) B.Nord. & Greuter	0	n	1	0	Asteraceae Bercht. & J.Presl
Iz_5_1209	DJI_0291	Jasione montana L.	0	n	1	0	Campanulaceae Juss.
Iz_5_1209	DJI_0291	Jurinea cyanoides (L.) Rchb.s.l.	0	n	1	0	Asteraceae Bercht. & J.Presl
Iz_5_1209	DJI_0291	Koeleria glauca (Spreng.) DC.	0	n	1	0	Poaceae Barnhart
Iz_5_1209	DJI_0291	Lactuca serriola L.	0	a	0	0	Asteraceae Bercht. & J.Presl
Iz_5_1209	DJI_0291	Linaria genistifolia (L.) Mill.	0	n	1	0	Plantaginaceae Juss.
Iz_5_1209	DJI_0291	Lomelosia argentea (L.) Greuter & Burdet	0	n	1	0	Caprifoliaceae Juss.
Iz_5_1209	DJI_0291	Melampyrum pratense L.	0	n	1	0	Orobanchaceae Vent.
Iz_5_1209	DJI_0291	Oenothera biennis L.	0	a	0	0	Onagraceae Juss.
Iz_5_1209	DJI_0291	Pentanema britannica (L.) D.Gut.Larr., Santos-Vicente, Anderb., E.Rico & M.M.Mart.Ort.	0	n	1	0	Asteraceae Bercht. & J.Presl
Iz_5_1209	DJI_0291	Peucedanum oreoselinum (L.) Moench	1	n	1	0	Apiaceae Lindl.
Iz_5_1209	DJI_0291	Peucedanum ruthenicum M.Bieb.	0	n	1	0	Apiaceae Lindl.
Iz_5_1209	DJI_0291	Pilosella echioides (Lumn.) F.W.Schultz & Sch.Bip.	0	n	1	0	Asteraceae Bercht. & J.Presl
Iz_5_1209	DJI_0291	Pilosella officinarum F. Schultz et Sch. Bip.	0	n	1	0	Asteraceae Bercht. & J.Presl
Iz_5_1209	DJI_0291	Pilosella sp.	0		NA	0	Asteraceae Bercht. & J.Presl
Iz_5_1209	DJI_0291	Pinus sylvestris L.	0	n	1	1	Pinaceae Spreng. ex F.Rudolphi
Iz_5_1209	DJI_0291	Poa annua L.	0	n	1	0	Poaceae Barnhart
Iz_5_1209	DJI_0291	Poa nemoralis L.	0	n	1	0	Poaceae Barnhart
Iz_5_1209	DJI_0291	Poa sp.	0		NA	0	Poaceae Barnhart
Iz_5_1209	DJI_0291	Polygonatum odoratum (Mill.) Druce	0	n	1	0	Asparagaceae Juss.
Iz_5_1209	DJI_0291	Polygonum arenarium Waldst. & Kit.	0	n	1	0	Polygonaceae Juss.
Iz_5_1209	DJI_0291	Populus nigra L.	0	n	1	1	Salicaceae Mirb.
Iz_5_1209	DJI_0291	Populus tremula L.	0	n	1	1	Salicaceae Mirb.
Iz_5_1209	DJI_0291	Populus Ã canescens (Aiton) Sm.	0	n	1	1	Salicaceae Mirb.
Iz_5_1209	DJI_0291	Potentilla argentea L.	0	n	1	0	Rosaceae Juss.
Iz_5_1209	DJI_0291	Psammophiliella muralis (L.) Ikonn.	0	n	1	0	Caryophyllaceae Juss.
Iz_5_1209	DJI_0291	Psephellus sumensis (Kalen.) Greuter	0	n	1	0	Asteraceae Bercht. & J.Presl
Iz_5_1209	DJI_0291	Pulsatilla pratensis (L.) Mill. s.l.	0	n	1	0	Ranunculaceae Juss.
Iz_5_1209	DJI_0291	Pyrus communis L.	1	a	0	1	Rosaceae Juss.
Iz_5_1209	DJI_0291	Quercus robur L.	0	n	1	1	Fagaceae Dumort.
Iz_5_1209	DJI_0291	Robinia pseudoacacia L.	0	a	0	1	Fabaceae Lindl.
Iz_5_1209	DJI_0291	Rosa sp.	0		NA	1	Rosaceae Juss.
Iz_5_1209	DJI_0291	Rubus idaeus L.	0	n	1	0	Rosaceae Juss.
Iz_5_1209	DJI_0291	Rumex acetosella L.	0	n	1	0	Polygonaceae Juss.
Iz_5_1209	DJI_0291	Rumex thyrsiflorus Fingerh.	0	n	1	0	Polygonaceae Juss.
Iz_5_1209	DJI_0291	Salix alba L.	0	n	1	1	Salicaceae Mirb.
Iz_5_1209	DJI_0291	Sambucus nigra L.	0	n	1	1	Viburnaceae Raf.
Iz_5_1209	DJI_0291	Secale sylvestre Host	0	n	1	0	Poaceae Barnhart
Iz_5_1209	DJI_0291	Sempervivum ruthenicum Schnittsp. & C.B. Lehm.	0	n	1	0	Crassulaceae J.St.-Hil.
Iz_5_1209	DJI_0291	Setaria viridis (L.) P. Beauv.	0	n	1	0	Poaceae Barnhart
Iz_5_1209	DJI_0291	Silene borysthenica (Gruner) Walters	0	n	1	0	Caryophyllaceae Juss.
Iz_5_1209	DJI_0291	Silene latifolia subsp. alba (Mill.) Greuter & Burdet	0	n	1	0	Caryophyllaceae Juss.
Iz_5_1209	DJI_0291	Solidago virgaurea L.	0	n	1	0	Asteraceae Bercht. & J.Presl
Iz_5_1209	DJI_0291	Sorbus aucuparia L.	0	n	1	1	Rosaceae Juss.
Iz_5_1209	DJI_0291	Stachys recta L.	0	n	1	0	Lamiacea Lindl.
Iz_5_1209	DJI_0291	Tanacetum vulgare L.	0	n	1	0	Asteraceae Bercht. & J.Presl
Iz_5_1209	DJI_0291	Thymus pallasianus Heinr.Braun	0	n	1	0	Lamiacea Lindl.
Iz_5_1209	DJI_0291	Tilia cordata Mill.	0	n	1	1	Malvaceae Juss.
Iz_5_1209	DJI_0291	Trifolium alpestre L.	0	n	1	0	Fabaceae Lindl.
Iz_5_1209	DJI_0291	Trifolium arvense L.	0	n	1	0	Fabaceae Lindl.
Iz_5_1209	DJI_0291	Ulmus glabra Huds.	0	n	1	1	Ulmaceae Mirb.
Iz_5_1209	DJI_0291	Verbascum densiflorum Bertol.	0	n	1	0	Scrophulariaceae Juss.
Iz_5_1209	DJI_0291	Veronica austriaca L.	0	n	1	0	Plantaginaceae Juss.
Iz_5_1209	DJI_0291	Veronica incana L.	0	n	1	0	Plantaginaceae Juss.
Iz_5_1209	DJI_0291	Veronica verna L.	0	n	1	0	Plantaginaceae Juss.
Iz_5_1209	DJI_0291	Vincetoxicum hirundinaria Medik	0	n	1	0	Apocynaceae Juss.
Iz_6_1009	DJI_0303	Acer tataricum L.	0	n	1	1	Sapindaceae Juss.
Iz_6_1009	DJI_0303	Achillea millefolium L. s.l.	0	n	1	0	Asteraceae Bercht. & J.Presl
Iz_6_1009	DJI_0303	Allium oleraceum L.	0	n	1	0	Amaryllidaceae J.St.-Hil.
Iz_6_1009	DJI_0303	Ambrosia artemisiifolia L.	0	a	0	0	Asteraceae Bercht. & J.Presl
Iz_6_1009	DJI_0303	Amorpha fruticosa L.	0	a	0	0	Fabaceae Lindl.
Iz_6_1009	DJI_0303	Anthericum ramosum L.	0	n	1	0	Asparagaceae Juss.
Iz_6_1009	DJI_0303	Artemisia marschalliana Spreng.	0	n	1	0	Asteraceae Bercht. & J.Presl
Iz_6_1009	DJI_0303	Asparagus officinalis L.	0	n	1	0	Asparagaceae Juss.
Iz_6_1009	DJI_0303	Astragalus cicer L.	0	n	1	0	Fabaceae Lindl.
Iz_6_1009	DJI_0303	Bassia laniflora (S.G.Gmel.) A.J.Scott	0	n	1	0	Amaranthaceae Juss.
Iz_6_1009	DJI_0303	Berberis vulgaris L.	0	n	1	0	Berberidaceae Juss.
Iz_6_1009	DJI_0303	Betula pendula Roth.	0	n	1	1	Betulaceae Gray
Iz_6_1009	DJI_0303	Betula pendula Roth.	0	n	1	0	Poaceae Barnhart
Iz_6_1009	DJI_0303	Bromus inermis Leyss.	0		NA	NA	
Iz_6_1009	DJI_0303	Bromus squarrosus L.	0	a	0	0	Poaceae Barnhart
Iz_6_1009	DJI_0303	Bromus tectorum L.	0	a	0	0	Poaceae Barnhart
Iz_6_1009	DJI_0303	Calamagrostis epigejos (L.) Roth	15	n	1	0	Poaceae Barnhart
Iz_6_1009	DJI_0303	Carduus acanthoides L.	0	n	1	0	Asteraceae Bercht. & J.Presl
Iz_6_1009	DJI_0303	Carex sp.	0	n	1	0	Cyperaceae Juss.
Iz_6_1009	DJI_0303	Centaurea majorovii Dumbadze	0	n	1	0	Asteraceae Bercht. & J.Presl
Iz_6_1009	DJI_0303	Chamaecytisus ruthenicus (Fisch.ex Wol.) Klaskova	0	n	1	0	Fabaceae Lindl.
Iz_6_1009	DJI_0303	Chelidonium majus L.	0	n	1	0	Papaveraceae Juss.
Iz_6_1009	DJI_0303	Chenopodium betaceum Andrz.	0	n	1	0	Amaranthaceae Juss.
Iz_6_1009	DJI_0303	Chenopodium cf. album	0	n	1	0	Amaranthaceae Juss.
Iz_6_1009	DJI_0303	Chenopodium sp.	0		NA	0	Amaranthaceae Juss.
Iz_6_1009	DJI_0303	Chondrilla juncea L.	0	n	1	0	Asteraceae Bercht. & J.Presl
Iz_6_1009	DJI_0303	Cirsium vulgare (Savi) Ten.	0	n	1	0	Asteraceae Bercht. & J.Presl
Iz_6_1009	DJI_0303	Clinopodium acinos (L.) Kuntze	0	n	1	0	Lamiacea Lindl.
Iz_6_1009	DJI_0303	Convallaria majalis L.	0	n	1	0	Asparagaceae Juss.
Iz_6_1009	DJI_0303	Crataegus rhipidophylla Gand.	0	n	1	1	Rosaceae Juss.
Iz_6_1009	DJI_0303	Crepis foetida L.	0	n	1	0	Asteraceae Bercht. & J.Presl
Iz_6_1009	DJI_0303	Dactylis glomerata L.	0	n	1	0	Poaceae Barnhart
Iz_6_1009	DJI_0303	Dianthus campestris M.Bieb.	0	n	1	0	Caryophyllaceae Juss.
Iz_6_1009	DJI_0303	Digitaria sanguinalis (L.) Scop.	0	a	0	0	Poaceae Barnhart

Iz_6_1009	DJI_0303	Elymus repens (L.) Gould	0	n	1	0	Poaceae Barnhart
Iz_6_1009	DJI_0303	Epilobium tetragonum L.	0	n	1	0	Onagraceae Juss.
Iz_6_1009	DJI_0303	Eragrostis minor Host	0	a	0	0	Poaceae Barnhart
Iz_6_1009	DJI_0303	Erigeron canadensis L.	10	a	0	0	Asteraceae Bercht. & J.Presl
Iz_6_1009	DJI_0303	Euphorbia seguieriana Neck.	0	n	1	0	Euphorbiaceae Juss.
Iz_6_1009	DJI_0303	Fallopia dumetorum (L.) Holub	0	n	1	0	Polygonaceae Juss.
Iz_6_1009	DJI_0303	Festuca beckeri (Hack.) Trautv.	0	n	1	0	Poaceae Barnhart
Iz_6_1009	DJI_0303	Fragaria vesca L.	0	n	1	0	Rosaceae Juss.
Iz_6_1009	DJI_0303	Frangula alnus Mill.	0	n	1	1	Rhamnaceae Juss.
Iz_6_1009	DJI_0303	Fraxinus pennsylvanica Marshall	0	a	0	1	Oleaceae Hoffmanns. & Link
Iz_6_1009	DJI_0303	Galeopsis bifida Boenn.	0	n	1	0	Lamiacea Lindl.
Iz_6_1009	DJI_0303	Galium verum L.	0	n	1	0	Rubiaceae Juss.
Iz_6_1009	DJI_0303	Genista tinctoria L.	0	n	1	0	Fabaceae Lindl.
Iz_6_1009	DJI_0303	Geranium robertianum L.	0	n	1	0	Geraniaceae Juss.
Iz_6_1009	DJI_0303	Geranium sanquineum L.	0	n	1	0	Geraniaceae Juss.
Iz_6_1009	DJI_0303	Gypsophila paniculata L.	0	n	1	0	Caryophyllaceae Juss.
Iz_6_1009	DJI_0303	Helichrysum arenarium (L.) Moench	0	n	1	0	Asteraceae Bercht. & J.Presl
Iz_6_1009	DJI_0303	Herniaria polygama J.Gay	0	n	1	0	Caryophyllaceae Juss.
Iz_6_1009	DJI_0303	Hieracium robustum Fr.	0	n	1	0	Asteraceae Bercht. & J.Presl
Iz_6_1009	DJI_0303	Hieracium umbellatum L.	0	n	1	NA	Asteraceae Bercht. & J.Presl
Iz_6_1009	DJI_0303	Hylotelephium maximum (L.) Holub	0	n	1	0	Crassulaceae J.St.-Hil.
Iz_6_1009	DJI_0303	Jacobaea borysthenica (DC.) B.Nord. & Greuter	0	n	1	0	Asteraceae Bercht. & J.Presl
Iz_6_1009	DJI_0303	Jasione montana L.	0	n	1	0	Campanulaceae Juss.
Iz_6_1009	DJI_0303	Jurinea cyanoides (L.) Rchb.s.l.	0	n	1	0	Asteraceae Bercht. & J.Presl
Iz_6_1009	DJI_0303	Koeleria glauca (Spreng.) DC.	0	n	1	0	Poaceae Barnhart
Iz_6_1009	DJI_0303	Lactuca serriola L.	0	a	0	0	Asteraceae Bercht. & J.Presl
Iz_6_1009	DJI_0303	Linaria genistifolia (L.) Mill.	0	n	1	0	Plantaginaceae Juss.
Iz_6_1009	DJI_0303	Lomelosia argentea (L.) Greuter & Burdet	0	n	1	0	Caprifoliaceae Juss.
Iz_6_1009	DJI_0303	Melampyrum pratense L.	0	n	1	0	Orobanchaceae Vent.
Iz_6_1009	DJI_0303	Oenothera biennis L.	0	a	0	0	Onagraceae Juss.
Iz_6_1009	DJI_0303	Pentanema britannica (L.) D.Gut.Larr., Santos-Vicente, Anderb., E.Rico & M.M.Mart.Ort.	0	n	1	0	Asteraceae Bercht. & J.Presl
Iz_6_1009	DJI_0303	Peucedanum oreoselinum (L.) Moench	0	n	1	0	Apiaceae Lindl.
Iz_6_1009	DJI_0303	Peucedanum ruthenicum M.Bieb.	0	n	1	0	Apiaceae Lindl.
Iz_6_1009	DJI_0303	Pilosella echioides (Lumn.) F.W.Schultz & Sch.Bip.	0	n	1	0	Asteraceae Bercht. & J.Presl
Iz_6_1009	DJI_0303	Pilosella officinarum F. Schultz et Sch. Bip.	0	n	1	0	Asteraceae Bercht. & J.Presl
Iz_6_1009	DJI_0303	Pilosella sp.	0		NA	0	Asteraceae Bercht. & J.Presl
Iz_6_1009	DJI_0303	Pinus sylvestris L.	0	n	1	1	Pinaceae Spreng. ex F.Rudolphi
Iz_6_1009	DJI_0303	Poa annua L.	0	n	1	0	Poaceae Barnhart
Iz_6_1009	DJI_0303	Poa nemoralis L.	0	n	1	0	Poaceae Barnhart
Iz_6_1009	DJI_0303	Poa sp.	0		NA	0	Poaceae Barnhart
Iz_6_1009	DJI_0303	Polygonatum odoratum (Mill.) Druce	0	n	1	0	Asparagaceae Juss.
Iz_6_1009	DJI_0303	Polygonum arenarium Waldst. & Kit.	0	n	1	0	Polygonaceae Juss.
Iz_6_1009	DJI_0303	Populus nigra L.	0	n	1	1	Salicaceae Mirb.
Iz_6_1009	DJI_0303	Populus tremula L.	0	n	1	1	Salicaceae Mirb.
Iz_6_1009	DJI_0303	Populus Ã canescens (Aiton) Sm.	0	n	1	1	Salicaceae Mirb.
Iz_6_1009	DJI_0303	Potentilla argentea L.	0	n	1	0	Rosaceae Juss.
Iz_6_1009	DJI_0303	Psammophiliella muralis (L.) Ikonn.	0	n	1	0	Caryophyllaceae Juss.
Iz_6_1009	DJI_0303	Psephellus sumensis (Kalen.) Greuter	0	n	1	0	Asteraceae Bercht. & J.Presl
Iz_6_1009	DJI_0303	Pulsatilla pratensis (L.) Mill. s.l.	0	n	1	0	Ranunculaceae Juss.
Iz_6_1009	DJI_0303	Pyrus communis L.	0	a	0	1	Rosaceae Juss.
Iz_6_1009	DJI_0303	Quercus robur L.	0	n	1	1	Fagaceae Dumort.
Iz_6_1009	DJI_0303	Robinia pseudoacacia L.	0	a	0	1	Fabaceae Lindl.
Iz_6_1009	DJI_0303	Rosa sp.	0		NA	1	Rosaceae Juss.
Iz_6_1009	DJI_0303	Rubus idaeus L.	0	n	1	0	Rosaceae Juss.
Iz_6_1009	DJI_0303	Rumex acetosella L.	0	n	1	0	Polygonaceae Juss.
Iz_6_1009	DJI_0303	Rumex thyrsiflorus Fingerh.	0	n	1	0	Polygonaceae Juss.
Iz_6_1009	DJI_0303	Salix alba L.	0	n	1	1	Salicaceae Mirb.
Iz_6_1009	DJI_0303	Sambucus nigra L.	0	n	1	1	Viburnaceae Raf.
Iz_6_1009	DJI_0303	Secale sylvestre Host	0	n	1	0	Poaceae Barnhart
Iz_6_1009	DJI_0303	Sempervivum ruthenicum Schnittsp. & C.B. Lehm.	0	n	1	0	Crassulaceae J.St.-Hil.
Iz_6_1009	DJI_0303	Setaria viridis (L.) P. Beauv.	0	n	1	0	Poaceae Barnhart
Iz_6_1009	DJI_0303	Silene borysthenica (Gruner) Walters	0	n	1	0	Caryophyllaceae Juss.
Iz_6_1009	DJI_0303	Silene latifolia subsp. alba (Mill.) Greuter & Burdet	0	n	1	0	Caryophyllaceae Juss.
Iz_6_1009	DJI_0303	Solidago virgaurea L.	0	n	1	0	Asteraceae Bercht. & J.Presl
Iz_6_1009	DJI_0303	Sorbus aucuparia L.	0	n	1	1	Rosaceae Juss.
Iz_6_1009	DJI_0303	Stachys recta L.	0	n	1	0	Lamiacea Lindl.
Iz_6_1009	DJI_0303	Tanacetum vulgare L.	0	n	1	0	Asteraceae Bercht. & J.Presl
Iz_6_1009	DJI_0303	Thymus pallasianus Heinr.Braun	0	n	1	0	Lamiacea Lindl.
Iz_6_1009	DJI_0303	Tilia cordata Mill.	0	n	1	1	Malvaceae Juss.
Iz_6_1009	DJI_0303	Trifolium alpestre L.	0	n	1	0	Fabaceae Lindl.
Iz_6_1009	DJI_0303	Trifolium arvense L.	0	n	1	0	Fabaceae Lindl.
Iz_6_1009	DJI_0303	Ulmus glabra Huds.	0	n	1	1	Ulmaceae Mirb.
Iz_6_1009	DJI_0303	Verbascum densiflorum Bertol.	0	n	1	0	Scrophulariaceae Juss.
Iz_6_1009	DJI_0303	Veronica austriaca L.	0	n	1	0	Plantaginaceae Juss.
Iz_6_1009	DJI_0303	Veronica incana L.	0	n	1	0	Plantaginaceae Juss.
Iz_6_1009	DJI_0303	Veronica verna L.	0	n	1	0	Plantaginaceae Juss.
Iz_6_1009	DJI_0303	Vincetoxicum hirundinaria Medik	0	n	1	0	Apocynaceae Juss.
Iz_6_1009	DJI_0307	Acer tataricum L.	0	n	1	1	Sapindaceae Juss.
Iz_6_1009	DJI_0307	Achillea millefolium L. s.l.	0	n	1	0	Asteraceae Bercht. & J.Presl
Iz_6_1009	DJI_0307	Allium oleraceum L.	0	n	1	0	Amaryllidaceae J.St.-Hil.
Iz_6_1009	DJI_0307	Ambrosia artemisiifolia L.	0	a	0	0	Asteraceae Bercht. & J.Presl
Iz_6_1009	DJI_0307	Amorpha fruticosa L.	0	a	0	0	Fabaceae Lindl.
Iz_6_1009	DJI_0307	Anthericum ramosum L.	0	n	1	0	Asparagaceae Juss.
Iz_6_1009	DJI_0307	Artemisia marschalliana Spreng.	0	n	1	0	Asteraceae Bercht. & J.Presl
Iz_6_1009	DJI_0307	Asparagus officinalis L.	0	n	1	0	Asparagaceae Juss.
Iz_6_1009	DJI_0307	Astragalus cicer L.	0	n	1	0	Fabaceae Lindl.
Iz_6_1009	DJI_0307	Bassia laniflora (S.G.Gmel.) A.J.Scott	0	n	1	0	Amaranthaceae Juss.
Iz_6_1009	DJI_0307	Berberis vulgaris L.	0	n	1	0	Berberidaceae Juss.
Iz_6_1009	DJI_0307	Betula pendula Roth.	0	n	1	1	Betulaceae Gray
Iz_6_1009	DJI_0307	Betula pendula Roth.	0	n	1	0	Poaceae Barnhart
Iz_6_1009	DJI_0307	Bromus inermis Leyss.	0		NA	NA	
Iz_6_1009	DJI_0307	Bromus squarrosus L.	0	a	0	0	Poaceae Barnhart
Iz_6_1009	DJI_0307	Bromus tectorum L.	0	a	0	0	Poaceae Barnhart
Iz_6_1009	DJI_0307	Calamagrostis epigejos (L.) Roth	8	n	1	0	Poaceae Barnhart
Iz_6_1009	DJI_0307	Carduus acanthoides L.	0	n	1	0	Asteraceae Bercht. & J.Presl
Iz_6_1009	DJI_0307	Carex sp.	0	n	1	0	Cyperaceae Juss.
Iz_6_1009	DJI_0307	Centaurea majorovii Dumbadze	0	n	1	0	Asteraceae Bercht. & J.Presl
Iz_6_1009	DJI_0307	Chamaecytisus ruthenicus (Fisch.ex Wol.) Klaskova	0	n	1	0	Fabaceae Lindl.

Iz_6_1009	DJI_0307	Chelidonium majus L.	0	n	1	0	Papaveraceae Juss.
Iz_6_1009	DJI_0307	Chenopodium betaceum Andrz.	0	n	1	0	Amaranthaceae Juss.
Iz_6_1009	DJI_0307	Chenopodium cf. album	0	n	1	0	Amaranthaceae Juss.
Iz_6_1009	DJI_0307	Chenopodium sp.	0		NA	0	Amaranthaceae Juss.
Iz_6_1009	DJI_0307	Chondrilla juncea L.	0	n	1	0	Asteraceae Bercht. & J.Presl
Iz_6_1009	DJI_0307	Cirsium vulgare (Savi) Ten.	0	n	1	0	Asteraceae Bercht. & J.Presl
Iz_6_1009	DJI_0307	Clinopodium acinos (L.) Kuntze	0	n	1	0	Lamiacea Lindl.
Iz_6_1009	DJI_0307	Convallaria majalis L.	0	n	1	0	Asparagaceae Juss.
Iz_6_1009	DJI_0307	Crataegus rhipidophylla Gand.	0	n	1	1	Rosaceae Juss.
Iz_6_1009	DJI_0307	Crepis foetida L.	0	n	1	0	Asteraceae Bercht. & J.Presl
Iz_6_1009	DJI_0307	Dactylis glomerata L.	0	n	1	0	Poaceae Barnhart
Iz_6_1009	DJI_0307	Dianthus campestris M.Bieb.	0	n	1	0	Caryophyllaceae Juss.
Iz_6_1009	DJI_0307	Digitaria sanguinalis (L.) Scop.	0	a	0	0	Poaceae Barnhart
Iz_6_1009	DJI_0307	Elymus repens (L.) Gould	0	n	1	0	Poaceae Barnhart
Iz_6_1009	DJI_0307	Epilobium tetragonum L.	0	n	1	0	Onagraceae Juss.
Iz_6_1009	DJI_0307	Eragrostis minor Host	0	a	0	0	Poaceae Barnhart
Iz_6_1009	DJI_0307	Erigeron canadensis L.	0.5	a	0	0	Asteraceae Bercht. & J.Presl
Iz_6_1009	DJI_0307	Euphorbia seguieriana Neck.	0	n	1	0	Euphorbiaceae Juss.
Iz_6_1009	DJI_0307	Fallopia dumetorum (L.) Holub	0	n	1	0	Polygonaceae Juss.
Iz_6_1009	DJI_0307	Festuca beckeri (Hack.) Trautv.	0	n	1	0	Poaceae Barnhart
Iz_6_1009	DJI_0307	Fragaria vesca L.	0	n	1	0	Rosaceae Juss.
Iz_6_1009	DJI_0307	Frangula alnus Mill.	0	n	1	1	Rhamnaceae Juss.
Iz_6_1009	DJI_0307	Fraxinus pennsylvanica Marshall	0	a	0	1	Oleaceae Hoffmanns. & Link
Iz_6_1009	DJI_0307	Galeopsis bifida Boenn.	0	n	1	0	Lamiacea Lindl.
Iz_6_1009	DJI_0307	Galium verum L.	0	n	1	0	Rubiaceae Juss.
Iz_6_1009	DJI_0307	Genista tinctoria L.	0	n	1	0	Fabaceae Lindl.
Iz_6_1009	DJI_0307	Geranium robertianum L.	0	n	1	0	Geraniaceae Juss.
Iz_6_1009	DJI_0307	Geranium sanquineum L.	0	n	1	0	Geraniaceae Juss.
Iz_6_1009	DJI_0307	Gypsophila paniculata L.	0	n	1	0	Caryophyllaceae Juss.
Iz_6_1009	DJI_0307	Helichrysum arenarium (L.) Moench	0	n	1	0	Asteraceae Bercht. & J.Presl
Iz_6_1009	DJI_0307	Herniaria polygama J.Gay	0	n	1	0	Caryophyllaceae Juss.
Iz_6_1009	DJI_0307	Hieracium robustum Fr.	0	n	1	0	Asteraceae Bercht. & J.Presl
Iz_6_1009	DJI_0307	Hieracium umbellatum L.	0	n	1	NA	Asteraceae Bercht. & J.Presl
Iz_6_1009	DJI_0307	Hylotelephium maximum (L.) Holub	0	n	1	0	Crassulaceae J.St.-Hil.
Iz_6_1009	DJI_0307	Jacobaea borysthenica (DC.) B.Nord. & Greuter	0	n	1	0	Asteraceae Bercht. & J.Presl
Iz_6_1009	DJI_0307	Jasione montana L.	0	n	1	0	Campanulaceae Juss.
Iz_6_1009	DJI_0307	Jurinea cyanoides (L.) Rchb.s.l.	0	n	1	0	Asteraceae Bercht. & J.Presl
Iz_6_1009	DJI_0307	Koeleria glauca (Spreng.) DC.	0	n	1	0	Poaceae Barnhart
Iz_6_1009	DJI_0307	Lactuca serriola L.	0	a	0	0	Asteraceae Bercht. & J.Presl
Iz_6_1009	DJI_0307	Linaria genistifolia (L.) Mill.	0	n	1	0	Plantaginaceae Juss.
Iz_6_1009	DJI_0307	Lomelosia argentea (L.) Greuter & Burdet	0	n	1	0	Caprifoliaceae Juss.
Iz_6_1009	DJI_0307	Melampyrum pratense L.	0	n	1	0	Orobanchaceae Vent.
Iz_6_1009	DJI_0307	Oenothera biennis L.	0	a	0	0	Onagraceae Juss.
Iz_6_1009	DJI_0307	Pentanema britannica (L.) D.Gut.Larr., Santos-Vicente, Anderb., E.Rico & M.M.Mart.Ort.	0	n	1	0	Asteraceae Bercht. & J.Presl
Iz_6_1009	DJI_0307	Peucedanum oreoselinum (L.) Moench	0	n	1	0	Apiaceae Lindl.
Iz_6_1009	DJI_0307	Peucedanum ruthenicum M.Bieb.	0	n	1	0	Apiaceae Lindl.
Iz_6_1009	DJI_0307	Pilosella echioides (Lumn.) F.W.Schultz & Sch.Bip.	0	n	1	0	Asteraceae Bercht. & J.Presl
Iz_6_1009	DJI_0307	Pilosella officinarum F. Schultz et Sch. Bip.	0	n	1	0	Asteraceae Bercht. & J.Presl
Iz_6_1009	DJI_0307	Pilosella sp.	0		NA	0	Asteraceae Bercht. & J.Presl
Iz_6_1009	DJI_0307	Pinus sylvestris L.	0	n	1	1	Pinaceae Spreng. ex F.Rudolphi
Iz_6_1009	DJI_0307	Poa annua L.	0	n	1	0	Poaceae Barnhart
Iz_6_1009	DJI_0307	Poa nemoralis L.	0	n	1	0	Poaceae Barnhart
Iz_6_1009	DJI_0307	Poa sp.	0		NA	0	Poaceae Barnhart
Iz_6_1009	DJI_0307	Polygonatum odoratum (Mill.) Druce	0	n	1	0	Asparagaceae Juss.
Iz_6_1009	DJI_0307	Polygonum arenarium Waldst. & Kit.	0	n	1	0	Polygonaceae Juss.
Iz_6_1009	DJI_0307	Populus nigra L.	0	n	1	1	Salicaceae Mirb.
Iz_6_1009	DJI_0307	Populus tremula L.	0	n	1	1	Salicaceae Mirb.
Iz_6_1009	DJI_0307	Populus Ã canescens (Aiton) Sm.	0	n	1	1	Salicaceae Mirb.
Iz_6_1009	DJI_0307	Potentilla argentea L.	0	n	1	0	Rosaceae Juss.
Iz_6_1009	DJI_0307	Psammophiliella muralis (L.) Ikonn.	0	n	1	0	Caryophyllaceae Juss.
Iz_6_1009	DJI_0307	Psephellus sumensis (Kalen.) Greuter	0	n	1	0	Asteraceae Bercht. & J.Presl
Iz_6_1009	DJI_0307	Pulsatilla pratensis (L.) Mill. s.l.	0	n	1	0	Ranunculaceae Juss.
Iz_6_1009	DJI_0307	Pyrus communis L.	0	a	0	1	Rosaceae Juss.
Iz_6_1009	DJI_0307	Quercus robur L.	0	n	1	1	Fagaceae Dumort.
Iz_6_1009	DJI_0307	Robinia pseudoacacia L.	0	a	0	1	Fabaceae Lindl.
Iz_6_1009	DJI_0307	Rosa sp.	0		NA	1	Rosaceae Juss.
Iz_6_1009	DJI_0307	Rubus idaeus L.	0	n	1	0	Rosaceae Juss.
Iz_6_1009	DJI_0307	Rumex acetosella L.	0	n	1	0	Polygonaceae Juss.
Iz_6_1009	DJI_0307	Rumex thyrsiflorus Fingerh.	0	n	1	0	Polygonaceae Juss.
Iz_6_1009	DJI_0307	Salix alba L.	0	n	1	1	Salicaceae Mirb.
Iz_6_1009	DJI_0307	Sambucus nigra L.	0	n	1	1	Viburnaceae Raf.
Iz_6_1009	DJI_0307	Secale sylvestre Host	0	n	1	0	Poaceae Barnhart
Iz_6_1009	DJI_0307	Sempervivum ruthenicum Schnittsp. & C.B. Lehm.	0	n	1	0	Crassulaceae J.St.-Hil.
Iz_6_1009	DJI_0307	Setaria viridis (L.) P. Beauv.	0	n	1	0	Poaceae Barnhart
Iz_6_1009	DJI_0307	Silene borysthenica (Gruner) Walters	0	n	1	0	Caryophyllaceae Juss.
Iz_6_1009	DJI_0307	Silene latifolia subsp. alba (Mill.) Greuter & Burdet	0	n	1	0	Caryophyllaceae Juss.
Iz_6_1009	DJI_0307	Solidago virgaurea L.	0	n	1	0	Asteraceae Bercht. & J.Presl
Iz_6_1009	DJI_0307	Sorbus aucuparia L.	0	n	1	1	Rosaceae Juss.
Iz_6_1009	DJI_0307	Stachys recta L.	0	n	1	0	Lamiacea Lindl.
Iz_6_1009	DJI_0307	Tanacetum vulgare L.	0	n	1	0	Asteraceae Bercht. & J.Presl
Iz_6_1009	DJI_0307	Thymus pallasianus Heinr.Braun	0	n	1	0	Lamiacea Lindl.
Iz_6_1009	DJI_0307	Tilia cordata Mill.	0	n	1	1	Malvaceae Juss.
Iz_6_1009	DJI_0307	Trifolium alpestre L.	0	n	1	0	Fabaceae Lindl.
Iz_6_1009	DJI_0307	Trifolium arvense L.	0	n	1	0	Fabaceae Lindl.
Iz_6_1009	DJI_0307	Ulmus glabra Huds.	0	n	1	1	Ulmaceae Mirb.
Iz_6_1009	DJI_0307	Verbascum densiflorum Bertol.	0	n	1	0	Scrophulariaceae Juss.
Iz_6_1009	DJI_0307	Veronica austriaca L.	0	n	1	0	Plantaginaceae Juss.
Iz_6_1009	DJI_0307	Veronica incana L.	0	n	1	0	Plantaginaceae Juss.
Iz_6_1009	DJI_0307	Veronica verna L.	0	n	1	0	Plantaginaceae Juss.
Iz_6_1009	DJI_0307	Vincetoxicum hirundinaria Medik	0	n	1	0	Apocynaceae Juss.
Iz_6_1009	DJI_0310	Acer tataricum L.	0	n	1	1	Sapindaceae Juss.
Iz_6_1009	DJI_0310	Achillea millefolium L. s.l.	0	n	1	0	Asteraceae Bercht. & J.Presl
Iz_6_1009	DJI_0310	Allium oleraceum L.	0	n	1	0	Amaryllidaceae J.St.-Hil.
Iz_6_1009	DJI_0310	Ambrosia artemisiifolia L.	0	a	0	0	Asteraceae Bercht. & J.Presl
Iz_6_1009	DJI_0310	Amorpha fruticosa L.	0	a	0	0	Fabaceae Lindl.
Iz_6_1009	DJI_0310	Anthericum ramosum L.	0	n	1	0	Asparagaceae Juss.
Iz_6_1009	DJI_0310	Artemisia marschalliana Spreng.	0	n	1	0	Asteraceae Bercht. & J.Presl
Iz_6_1009	DJI_0310	Asparagus officinalis L.	0	n	1	0	Asparagaceae Juss.

Iz_6_1009	DJI_0310	Astragalus cicer L.	0	n	1	0	Fabaceae Lindl.
Iz_6_1009	DJI_0310	Bassia laniflora (S.G.Gmel.) A.J.Scott	0	n	1	0	Amaranthaceae Juss.
Iz_6_1009	DJI_0310	Berberis vulgaris L.	0	n	1	0	Berberidaceae Juss.
Iz_6_1009	DJI_0310	Betula pendula Roth.	0	n	1	1	Betulaceae Gray
Iz_6_1009	DJI_0310	Betula pendula Roth.	0	n	1	0	Poaceae Barnhart
Iz_6_1009	DJI_0310	Bromus inermis Leyss.	0		NA	NA	
Iz_6_1009	DJI_0310	Bromus squarrosus L.	0	a	0	0	Poaceae Barnhart
Iz_6_1009	DJI_0310	Bromus tectorum L.	0	a	0	0	Poaceae Barnhart
Iz_6_1009	DJI_0310	Calamagrostis epigejos (L.) Roth	2	n	1	0	Poaceae Barnhart
Iz_6_1009	DJI_0310	Carduus acanthoides L.	0	n	1	0	Asteraceae Bercht. & J.Presl
Iz_6_1009	DJI_0310	Carex sp.	0	n	1	0	Cyperaceae Juss.
Iz_6_1009	DJI_0310	Centaurea majorovii Dumbadze	0	n	1	0	Asteraceae Bercht. & J.Presl
Iz_6_1009	DJI_0310	Chamaecytisus ruthenicus (Fisch.ex Wol.) Klaskova	0	n	1	0	Fabaceae Lindl.
Iz_6_1009	DJI_0310	Chelidonium majus L.	0	n	1	0	Papaveraceae Juss.
Iz_6_1009	DJI_0310	Chenopodium betaceum Andrz.	0	n	1	0	Amaranthaceae Juss.
Iz_6_1009	DJI_0310	Chenopodium cf. album	0	n	1	0	Amaranthaceae Juss.
Iz_6_1009	DJI_0310	Chenopodium sp.	0		NA	0	Amaranthaceae Juss.
Iz_6_1009	DJI_0310	Chondrilla juncea L.	0	n	1	0	Asteraceae Bercht. & J.Presl
Iz_6_1009	DJI_0310	Cirsium vulgare (Savi) Ten.	0	n	1	0	Asteraceae Bercht. & J.Presl
Iz_6_1009	DJI_0310	Clinopodium acinos (L.) Kuntze	0	n	1	0	Lamiacea Lindl.
Iz_6_1009	DJI_0310	Convallaria majalis L.	0	n	1	0	Asparagaceae Juss.
Iz_6_1009	DJI_0310	Crataegus rhipidophylla Gand.	0	n	1	1	Rosaceae Juss.
Iz_6_1009	DJI_0310	Crepis foetida L.	0	n	1	0	Asteraceae Bercht. & J.Presl
Iz_6_1009	DJI_0310	Dactylis glomerata L.	0	n	1	0	Poaceae Barnhart
Iz_6_1009	DJI_0310	Dianthus campestris M.Bieb.	0	n	1	0	Caryophyllaceae Juss.
Iz_6_1009	DJI_0310	Digitaria sanguinalis (L.) Scop.	0	a	0	0	Poaceae Barnhart
Iz_6_1009	DJI_0310	Elymus repens (L.) Gould	0	n	1	0	Poaceae Barnhart
Iz_6_1009	DJI_0310	Epilobium tetragonum L.	0	n	1	0	Onagraceae Juss.
Iz_6_1009	DJI_0310	Eragrostis minor Host	0	a	0	0	Poaceae Barnhart
Iz_6_1009	DJI_0310	Erigeron canadensis L.	10	a	0	0	Asteraceae Bercht. & J.Presl
Iz_6_1009	DJI_0310	Euphorbia seguieriana Neck.	0	n	1	0	Euphorbiaceae Juss.
Iz_6_1009	DJI_0310	Fallopia dumetorum (L.) Holub	0	n	1	0	Polygonaceae Juss.
Iz_6_1009	DJI_0310	Festuca beckeri (Hack.) Trautv.	0	n	1	0	Poaceae Barnhart
Iz_6_1009	DJI_0310	Fragaria vesca L.	0	n	1	0	Rosaceae Juss.
Iz_6_1009	DJI_0310	Frangula alnus Mill.	0	n	1	1	Rhamnaceae Juss.
Iz_6_1009	DJI_0310	Fraxinus pennsylvanica Marshall	0	a	0	1	Oleaceae Hoffmanns. & Link
Iz_6_1009	DJI_0310	Galeopsis bifida Boenn.	0	n	1	0	Lamiacea Lindl.
Iz_6_1009	DJI_0310	Galium verum L.	0	n	1	0	Rubiaceae Juss.
Iz_6_1009	DJI_0310	Genista tinctoria L.	0	n	1	0	Fabaceae Lindl.
Iz_6_1009	DJI_0310	Geranium robertianum L.	0	n	1	0	Geraniaceae Juss.
Iz_6_1009	DJI_0310	Geranium sanquineum L.	0	n	1	0	Geraniaceae Juss.
Iz_6_1009	DJI_0310	Gypsophila paniculata L.	0	n	1	0	Caryophyllaceae Juss.
Iz_6_1009	DJI_0310	Helichrysum arenarium (L.) Moench	0	n	1	0	Asteraceae Bercht. & J.Presl
Iz_6_1009	DJI_0310	Herniaria polygama J.Gay	0	n	1	0	Caryophyllaceae Juss.
Iz_6_1009	DJI_0310	Hieracium robustum Fr.	0	n	1	0	Asteraceae Bercht. & J.Presl
Iz_6_1009	DJI_0310	Hieracium umbellatum L.	0	n	1	NA	Asteraceae Bercht. & J.Presl
Iz_6_1009	DJI_0310	Hylotelephium maximum (L.) Holub	0	n	1	0	Crassulaceae J.St.-Hil.
Iz_6_1009	DJI_0310	Jacobaea borysthenica (DC.) B.Nord. & Greuter	0	n	1	0	Asteraceae Bercht. & J.Presl
Iz_6_1009	DJI_0310	Jasione montana L.	0	n	1	0	Campanulaceae Juss.
Iz_6_1009	DJI_0310	Jurinea cyanoides (L.) Rchb.s.l.	0	n	1	0	Asteraceae Bercht. & J.Presl
Iz_6_1009	DJI_0310	Koeleria glauca (Spreng.) DC.	0	n	1	0	Poaceae Barnhart
Iz_6_1009	DJI_0310	Lactuca serriola L.	0	a	0	0	Asteraceae Bercht. & J.Presl
Iz_6_1009	DJI_0310	Linaria genistifolia (L.) Mill.	0	n	1	0	Plantaginaceae Juss.
Iz_6_1009	DJI_0310	Lomelosia argentea (L.) Greuter & Burdet	0	n	1	0	Caprifoliaceae Juss.
Iz_6_1009	DJI_0310	Melampyrum pratense L.	0	n	1	0	Orobanchaceae Vent.
Iz_6_1009	DJI_0310	Oenothera biennis L.	0	a	0	0	Onagraceae Juss.
Iz_6_1009	DJI_0310	Pentanema britannica (L.) D.Gut.Larr., Santos-Vicente, Anderb., E.Rico & M.M.Mart.Ort.	0	n	1	0	Asteraceae Bercht. & J.Presl
Iz_6_1009	DJI_0310	Peucedanum oreoselinum (L.) Moench	0	n	1	0	Apiaceae Lindl.
Iz_6_1009	DJI_0310	Peucedanum ruthenicum M.Bieb.	0	n	1	0	Apiaceae Lindl.
Iz_6_1009	DJI_0310	Pilosella echioides (Lumn.) F.W.Schultz & Sch.Bip.	0	n	1	0	Asteraceae Bercht. & J.Presl
Iz_6_1009	DJI_0310	Pilosella officinarum F. Schultz et Sch. Bip.	0	n	1	0	Asteraceae Bercht. & J.Presl
Iz_6_1009	DJI_0310	Pilosella sp.	0		NA	0	Asteraceae Bercht. & J.Presl
Iz_6_1009	DJI_0310	Pinus sylvestris L.	0	n	1	1	Pinaceae Spreng. ex F.Rudolphi
Iz_6_1009	DJI_0310	Poa annua L.	0	n	1	0	Poaceae Barnhart
Iz_6_1009	DJI_0310	Poa nemoralis L.	0	n	1	0	Poaceae Barnhart
Iz_6_1009	DJI_0310	Poa sp.	0		NA	0	Poaceae Barnhart
Iz_6_1009	DJI_0310	Polygonatum odoratum (Mill.) Druce	0	n	1	0	Asparagaceae Juss.
Iz_6_1009	DJI_0310	Polygonum arenarium Waldst. & Kit.	0	n	1	0	Polygonaceae Juss.
Iz_6_1009	DJI_0310	Populus nigra L.	0	n	1	1	Salicaceae Mirb.
Iz_6_1009	DJI_0310	Populus tremula L.	0	n	1	1	Salicaceae Mirb.
Iz_6_1009	DJI_0310	Populus Ã canescens (Aiton) Sm.	0	n	1	1	Salicaceae Mirb.
Iz_6_1009	DJI_0310	Potentilla argentea L.	0	n	1	0	Rosaceae Juss.
Iz_6_1009	DJI_0310	Psammophiliella muralis (L.) Ikonn.	0	n	1	0	Caryophyllaceae Juss.
Iz_6_1009	DJI_0310	Psephellus sumensis (Kalen.) Greuter	0	n	1	0	Asteraceae Bercht. & J.Presl
Iz_6_1009	DJI_0310	Pulsatilla pratensis (L.) Mill. s.l.	0	n	1	0	Ranunculaceae Juss.
Iz_6_1009	DJI_0310	Pyrus communis L.	0	a	0	1	Rosaceae Juss.
Iz_6_1009	DJI_0310	Quercus robur L.	0	n	1	1	Fagaceae Dumort.
Iz_6_1009	DJI_0310	Robinia pseudoacacia L.	0	a	0	1	Fabaceae Lindl.
Iz_6_1009	DJI_0310	Rosa sp.	0		NA	1	Rosaceae Juss.
Iz_6_1009	DJI_0310	Rubus idaeus L.	0	n	1	0	Rosaceae Juss.
Iz_6_1009	DJI_0310	Rumex acetosella L.	0	n	1	0	Polygonaceae Juss.
Iz_6_1009	DJI_0310	Rumex thyrsiflorus Fingerh.	0	n	1	0	Polygonaceae Juss.
Iz_6_1009	DJI_0310	Salix alba L.	0	n	1	1	Salicaceae Mirb.
Iz_6_1009	DJI_0310	Sambucus nigra L.	0	n	1	1	Viburnaceae Raf.
Iz_6_1009	DJI_0310	Secale sylvestre Host	0	n	1	0	Poaceae Barnhart
Iz_6_1009	DJI_0310	Sempervivum ruthenicum Schnittsp. & C.B. Lehm.	0	n	1	0	Crassulaceae J.St.-Hil.
Iz_6_1009	DJI_0310	Setaria viridis (L.) P. Beauv.	0	n	1	0	Poaceae Barnhart
Iz_6_1009	DJI_0310	Silene borysthenica (Gruner) Walters	0	n	1	0	Caryophyllaceae Juss.
Iz_6_1009	DJI_0310	Silene latifolia subsp. alba (Mill.) Greuter & Burdet	0	n	1	0	Caryophyllaceae Juss.
Iz_6_1009	DJI_0310	Solidago virgaurea L.	0	n	1	0	Asteraceae Bercht. & J.Presl
Iz_6_1009	DJI_0310	Sorbus aucuparia L.	0	n	1	1	Rosaceae Juss.
Iz_6_1009	DJI_0310	Stachys recta L.	0	n	1	0	Lamiacea Lindl.
Iz_6_1009	DJI_0310	Tanacetum vulgare L.	0	n	1	0	Asteraceae Bercht. & J.Presl
Iz_6_1009	DJI_0310	Thymus pallasianus Heinr.Braun	0	n	1	0	Lamiacea Lindl.
Iz_6_1009	DJI_0310	Tilia cordata Mill.	0	n	1	1	Malvaceae Juss.
Iz_6_1009	DJI_0310	Trifolium alpestre L.	0	n	1	0	Fabaceae Lindl.
Iz_6_1009	DJI_0310	Trifolium arvense L.	0	n	1	0	Fabaceae Lindl.
Iz_6_1009	DJI_0310	Ulmus glabra Huds.	0	n	1	1	Ulmaceae Mirb.

Iz_6_1009	DJI_0310	Verbascum densiflorum Bertol.	0	n	1	0	Scrophulariaceae Juss.
Iz_6_1009	DJI_0310	Veronica austriaca L.	0	n	1	0	Plantaginaceae Juss.
Iz_6_1009	DJI_0310	Veronica incana L.	0	n	1	0	Plantaginaceae Juss.
Iz_6_1009	DJI_0310	Veronica verna L.	0	n	1	0	Plantaginaceae Juss.
Iz_6_1009	DJI_0310	Vincetoxicum hirundinaria Medik	0	n	1	0	Apocynaceae Juss.
Iz_6_1009	DJI_0311	Acer tataricum L.	0	n	1	1	Sapindaceae Juss.
Iz_6_1009	DJI_0311	Achillea millefolium L. s.l.	0	n	1	0	Asteraceae Bercht. & J.Presl
Iz_6_1009	DJI_0311	Allium oleraceum L.	0	n	1	0	Amaryllidaceae J.St.-Hil.
Iz_6_1009	DJI_0311	Ambrosia artemisiifolia L.	0	a	0	0	Asteraceae Bercht. & J.Presl
Iz_6_1009	DJI_0311	Amorpha fruticosa L.	0	a	0	0	Fabaceae Lindl.
Iz_6_1009	DJI_0311	Anthericum ramosum L.	0	n	1	0	Asparagaceae Juss.
Iz_6_1009	DJI_0311	Artemisia marschalliana Spreng.	0	n	1	0	Asteraceae Bercht. & J.Presl
Iz_6_1009	DJI_0311	Asparagus officinalis L.	0	n	1	0	Asparagaceae Juss.
Iz_6_1009	DJI_0311	Astragalus cicer L.	0	n	1	0	Fabaceae Lindl.
Iz_6_1009	DJI_0311	Bassia laniflora (S.G.Gmel.) A.J.Scott	0	n	1	0	Amaranthaceae Juss.
Iz_6_1009	DJI_0311	Berberis vulgaris L.	0	n	1	0	Berberidaceae Juss.
Iz_6_1009	DJI_0311	Betula pendula Roth.	0	n	1	1	Betulaceae Gray
Iz_6_1009	DJI_0311	Betula pendula Roth.	0	n	1	0	Poaceae Barnhart
Iz_6_1009	DJI_0311	Bromus inermis Leyss.	0		NA	NA	
Iz_6_1009	DJI_0311	Bromus squarrosus L.	0	a	0	0	Poaceae Barnhart
Iz_6_1009	DJI_0311	Bromus tectorum L.	0	a	0	0	Poaceae Barnhart
Iz_6_1009	DJI_0311	Calamagrostis epigejos (L.) Roth	3	n	1	0	Poaceae Barnhart
Iz_6_1009	DJI_0311	Carduus acanthoides L.	0	n	1	0	Asteraceae Bercht. & J.Presl
Iz_6_1009	DJI_0311	Carex sp.	0	n	1	0	Cyperaceae Juss.
Iz_6_1009	DJI_0311	Centaurea majorovii Dumbadze	0	n	1	0	Asteraceae Bercht. & J.Presl
Iz_6_1009	DJI_0311	Chamaecytisus ruthenicus (Fisch.ex Wol.) Klaskova	0	n	1	0	Fabaceae Lindl.
Iz_6_1009	DJI_0311	Chelidonium majus L.	0	n	1	0	Papaveraceae Juss.
Iz_6_1009	DJI_0311	Chenopodium betaceum Andrz.	0	n	1	0	Amaranthaceae Juss.
Iz_6_1009	DJI_0311	Chenopodium cf. album	0	n	1	0	Amaranthaceae Juss.
Iz_6_1009	DJI_0311	Chenopodium sp.	0		NA	0	Amaranthaceae Juss.
Iz_6_1009	DJI_0311	Chondrilla juncea L.	0	n	1	0	Asteraceae Bercht. & J.Presl
Iz_6_1009	DJI_0311	Cirsium vulgare (Savi) Ten.	0	n	1	0	Asteraceae Bercht. & J.Presl
Iz_6_1009	DJI_0311	Clinopodium acinos (L.) Kuntze	0	n	1	0	Lamiacea Lindl.
Iz_6_1009	DJI_0311	Convallaria majalis L.	0	n	1	0	Asparagaceae Juss.
Iz_6_1009	DJI_0311	Crataegus rhipidophylla Gand.	0	n	1	1	Rosaceae Juss.
Iz_6_1009	DJI_0311	Crepis foetida L.	0	n	1	0	Asteraceae Bercht. & J.Presl
Iz_6_1009	DJI_0311	Dactylis glomerata L.	0	n	1	0	Poaceae Barnhart
Iz_6_1009	DJI_0311	Dianthus campestris M.Bieb.	0	n	1	0	Caryophyllaceae Juss.
Iz_6_1009	DJI_0311	Digitaria sanguinalis (L.) Scop.	0	a	0	0	Poaceae Barnhart
Iz_6_1009	DJI_0311	Elymus repens (L.) Gould	0	n	1	0	Poaceae Barnhart
Iz_6_1009	DJI_0311	Epilobium tetragonum L.	0	n	1	0	Onagraceae Juss.
Iz_6_1009	DJI_0311	Eragrostis minor Host	0	a	0	0	Poaceae Barnhart
Iz_6_1009	DJI_0311	Erigeron canadensis L.	10	a	0	0	Asteraceae Bercht. & J.Presl
Iz_6_1009	DJI_0311	Euphorbia seguieriana Neck.	0	n	1	0	Euphorbiaceae Juss.
Iz_6_1009	DJI_0311	Fallopia dumetorum (L.) Holub	0	n	1	0	Polygonaceae Juss.
Iz_6_1009	DJI_0311	Festuca beckeri (Hack.) Trautv.	0	n	1	0	Poaceae Barnhart
Iz_6_1009	DJI_0311	Fragaria vesca L.	0	n	1	0	Rosaceae Juss.
Iz_6_1009	DJI_0311	Frangula alnus Mill.	0	n	1	1	Rhamnaceae Juss.
Iz_6_1009	DJI_0311	Fraxinus pennsylvanica Marshall	0	a	0	1	Oleaceae Hoffmanns. & Link
Iz_6_1009	DJI_0311	Galeopsis bifida Boenn.	0	n	1	0	Lamiacea Lindl.
Iz_6_1009	DJI_0311	Galium verum L.	0	n	1	0	Rubiaceae Juss.
Iz_6_1009	DJI_0311	Genista tinctoria L.	0	n	1	0	Fabaceae Lindl.
Iz_6_1009	DJI_0311	Geranium robertianum L.	0	n	1	0	Geraniaceae Juss.
Iz_6_1009	DJI_0311	Geranium sanquineum L.	0	n	1	0	Geraniaceae Juss.
Iz_6_1009	DJI_0311	Gypsophila paniculata L.	0	n	1	0	Caryophyllaceae Juss.
Iz_6_1009	DJI_0311	Helichrysum arenarium (L.) Moench	0	n	1	0	Asteraceae Bercht. & J.Presl
Iz_6_1009	DJI_0311	Herniaria polygama J.Gay	0	n	1	0	Caryophyllaceae Juss.
Iz_6_1009	DJI_0311	Hieracium robustum Fr.	0	n	1	0	Asteraceae Bercht. & J.Presl
Iz_6_1009	DJI_0311	Hieracium umbellatum L.	0	n	1	NA	Asteraceae Bercht. & J.Presl
Iz_6_1009	DJI_0311	Hylotelephium maximum (L.) Holub	0	n	1	0	Crassulaceae J.St.-Hil.
Iz_6_1009	DJI_0311	Jacobaea borysthenica (DC.) B.Nord. & Greuter	0	n	1	0	Asteraceae Bercht. & J.Presl
Iz_6_1009	DJI_0311	Jasione montana L.	0	n	1	0	Campanulaceae Juss.
Iz_6_1009	DJI_0311	Jurinea cyanoides (L.) Rchb.s.l.	0	n	1	0	Asteraceae Bercht. & J.Presl
Iz_6_1009	DJI_0311	Koeleria glauca (Spreng.) DC.	0	n	1	0	Poaceae Barnhart
Iz_6_1009	DJI_0311	Lactuca serriola L.	0	a	0	0	Asteraceae Bercht. & J.Presl
Iz_6_1009	DJI_0311	Linaria genistifolia (L.) Mill.	0	n	1	0	Plantaginaceae Juss.
Iz_6_1009	DJI_0311	Lomelosia argentea (L.) Greuter & Burdet	0	n	1	0	Caprifoliaceae Juss.
Iz_6_1009	DJI_0311	Melampyrum pratense L.	0	n	1	0	Orobanchaceae Vent.
Iz_6_1009	DJI_0311	Oenothera biennis L.	0	a	0	0	Onagraceae Juss.
Iz_6_1009	DJI_0311	Pentanema britannica (L.) D.Gut.Larr., Santos-Vicente, Anderb., E.Rico & M.M.Mart.Ort.	0	n	1	0	Asteraceae Bercht. & J.Presl
Iz_6_1009	DJI_0311	Peucedanum oreoselinum (L.) Moench	0	n	1	0	Apiaceae Lindl.
Iz_6_1009	DJI_0311	Peucedanum ruthenicum M.Bieb.	0	n	1	0	Apiaceae Lindl.
Iz_6_1009	DJI_0311	Pilosella echioides (Lumn.) F.W.Schultz & Sch.Bip.	0	n	1	0	Asteraceae Bercht. & J.Presl
Iz_6_1009	DJI_0311	Pilosella officinarum F. Schultz et Sch. Bip.	0	n	1	0	Asteraceae Bercht. & J.Presl
Iz_6_1009	DJI_0311	Pilosella sp.	0		NA	0	Asteraceae Bercht. & J.Presl
Iz_6_1009	DJI_0311	Pinus sylvestris L.	0	n	1	1	Pinaceae Spreng. ex F.Rudolphi
Iz_6_1009	DJI_0311	Poa annua L.	0	n	1	0	Poaceae Barnhart
Iz_6_1009	DJI_0311	Poa nemoralis L.	0	n	1	0	Poaceae Barnhart
Iz_6_1009	DJI_0311	Poa sp.	0		NA	0	Poaceae Barnhart
Iz_6_1009	DJI_0311	Polygonatum odoratum (Mill.) Druce	0	n	1	0	Asparagaceae Juss.
Iz_6_1009	DJI_0311	Polygonum arenarium Waldst. & Kit.	0	n	1	0	Polygonaceae Juss.
Iz_6_1009	DJI_0311	Populus nigra L.	0	n	1	1	Salicaceae Mirb.
Iz_6_1009	DJI_0311	Populus tremula L.	0	n	1	1	Salicaceae Mirb.
Iz_6_1009	DJI_0311	Populus Ã canescens (Aiton) Sm.	0	n	1	1	Salicaceae Mirb.
Iz_6_1009	DJI_0311	Potentilla argentea L.	0	n	1	0	Rosaceae Juss.
Iz_6_1009	DJI_0311	Psammophiliella muralis (L.) Ikonn.	0	n	1	0	Caryophyllaceae Juss.
Iz_6_1009	DJI_0311	Psephellus sumensis (Kalen.) Greuter	0	n	1	0	Asteraceae Bercht. & J.Presl
Iz_6_1009	DJI_0311	Pulsatilla pratensis (L.) Mill. s.l.	0	n	1	0	Ranunculaceae Juss.
Iz_6_1009	DJI_0311	Pyrus communis L.	0	a	0	1	Rosaceae Juss.
Iz_6_1009	DJI_0311	Quercus robur L.	0	n	1	1	Fagaceae Dumort.
Iz_6_1009	DJI_0311	Robinia pseudoacacia L.	0	a	0	1	Fabaceae Lindl.
Iz_6_1009	DJI_0311	Rosa sp.	0		NA	1	Rosaceae Juss.
Iz_6_1009	DJI_0311	Rubus idaeus L.	0	n	1	0	Rosaceae Juss.
Iz_6_1009	DJI_0311	Rumex acetosella L.	0	n	1	0	Polygonaceae Juss.
Iz_6_1009	DJI_0311	Rumex thyrsiflorus Fingerh.	0	n	1	0	Polygonaceae Juss.
Iz_6_1009	DJI_0311	Salix alba L.	0	n	1	1	Salicaceae Mirb.
Iz_6_1009	DJI_0311	Sambucus nigra L.	0	n	1	1	Viburnaceae Raf.
Iz_6_1009	DJI_0311	Secale sylvestre Host	0	n	1	0	Poaceae Barnhart

Iz_6_1009	DJI_0311	Sempervivum ruthenicum Schnittsp. & C.B. Lehm.	0	n	1	0	Crassulaceae J.St.-Hil.
Iz_6_1009	DJI_0311	Setaria viridis (L.) P. Beauv.	0	n	1	0	Poaceae Barnhart
Iz_6_1009	DJI_0311	Silene borysthenica (Gruner) Walters	0	n	1	0	Caryophyllaceae Juss.
Iz_6_1009	DJI_0311	Silene latifolia subsp. alba (Mill.) Greuter & Burdet	0	n	1	0	Caryophyllaceae Juss.
Iz_6_1009	DJI_0311	Solidago virgaurea L.	0	n	1	0	Asteraceae Bercht. & J.Presl
Iz_6_1009	DJI_0311	Sorbus aucuparia L.	0	n	1	1	Rosaceae Juss.
Iz_6_1009	DJI_0311	Stachys recta L.	0	n	1	0	Lamiacea Lindl.
Iz_6_1009	DJI_0311	Tanacetum vulgare L.	0	n	1	0	Asteraceae Bercht. & J.Presl
Iz_6_1009	DJI_0311	Thymus pallasianus Heinr.Braun	0	n	1	0	Lamiacea Lindl.
Iz_6_1009	DJI_0311	Tilia cordata Mill.	0	n	1	1	Malvaceae Juss.
Iz_6_1009	DJI_0311	Trifolium alpestre L.	0	n	1	0	Fabaceae Lindl.
Iz_6_1009	DJI_0311	Trifolium arvense L.	0	n	1	0	Fabaceae Lindl.
Iz_6_1009	DJI_0311	Ulmus glabra Huds.	0	n	1	1	Ulmaceae Mirb.
Iz_6_1009	DJI_0311	Verbascum densiflorum Bertol.	0	n	1	0	Scrophulariaceae Juss.
Iz_6_1009	DJI_0311	Veronica austriaca L.	0	n	1	0	Plantaginaceae Juss.
Iz_6_1009	DJI_0311	Veronica incana L.	0	n	1	0	Plantaginaceae Juss.
Iz_6_1009	DJI_0311	Veronica verna L.	0	n	1	0	Plantaginaceae Juss.
Iz_6_1009	DJI_0311	Vincetoxicum hirundinaria Medik	0	n	1	0	Apocynaceae Juss.
Iz_6_1009	DJI_0312	Acer tataricum L.	0	n	1	1	Sapindaceae Juss.
Iz_6_1009	DJI_0312	Achillea millefolium L. s.l.	0	n	1	0	Asteraceae Bercht. & J.Presl
Iz_6_1009	DJI_0312	Allium oleraceum L.	0	n	1	0	Amaryllidaceae J.St.-Hil.
Iz_6_1009	DJI_0312	Ambrosia artemisiifolia L.	0	a	0	0	Asteraceae Bercht. & J.Presl
Iz_6_1009	DJI_0312	Amorpha fruticosa L.	0	a	0	0	Fabaceae Lindl.
Iz_6_1009	DJI_0312	Anthericum ramosum L.	0	n	1	0	Asparagaceae Juss.
Iz_6_1009	DJI_0312	Artemisia marschalliana Spreng.	0	n	1	0	Asteraceae Bercht. & J.Presl
Iz_6_1009	DJI_0312	Asparagus officinalis L.	0	n	1	0	Asparagaceae Juss.
Iz_6_1009	DJI_0312	Astragalus cicer L.	0	n	1	0	Fabaceae Lindl.
Iz_6_1009	DJI_0312	Bassia laniflora (S.G.Gmel.) A.J.Scott	0	n	1	0	Amaranthaceae Juss.
Iz_6_1009	DJI_0312	Berberis vulgaris L.	0	n	1	0	Berberidaceae Juss.
Iz_6_1009	DJI_0312	Betula pendula Roth.	0	n	1	1	Betulaceae Gray
Iz_6_1009	DJI_0312	Betula pendula Roth.	0	n	1	0	Poaceae Barnhart
Iz_6_1009	DJI_0312	Bromus inermis Leyss.	0		NA	NA	
Iz_6_1009	DJI_0312	Bromus squarrosus L.	0	a	0	0	Poaceae Barnhart
Iz_6_1009	DJI_0312	Bromus tectorum L.	0	a	0	0	Poaceae Barnhart
Iz_6_1009	DJI_0312	Calamagrostis epigejos (L.) Roth	10	n	1	0	Poaceae Barnhart
Iz_6_1009	DJI_0312	Carduus acanthoides L.	0	n	1	0	Asteraceae Bercht. & J.Presl
Iz_6_1009	DJI_0312	Carex sp.	0	n	1	0	Cyperaceae Juss.
Iz_6_1009	DJI_0312	Centaurea majorovii Dumbadze	0	n	1	0	Asteraceae Bercht. & J.Presl
Iz_6_1009	DJI_0312	Chamaecytisus ruthenicus (Fisch.ex Wol.) Klaskova	0	n	1	0	Fabaceae Lindl.
Iz_6_1009	DJI_0312	Chelidonium majus L.	0	n	1	0	Papaveraceae Juss.
Iz_6_1009	DJI_0312	Chenopodium betaceum Andrz.	0	n	1	0	Amaranthaceae Juss.
Iz_6_1009	DJI_0312	Chenopodium cf. album	0	n	1	0	Amaranthaceae Juss.
Iz_6_1009	DJI_0312	Chenopodium sp.	0		NA	0	Amaranthaceae Juss.
Iz_6_1009	DJI_0312	Chondrilla juncea L.	0	n	1	0	Asteraceae Bercht. & J.Presl
Iz_6_1009	DJI_0312	Cirsium vulgare (Savi) Ten.	0	n	1	0	Asteraceae Bercht. & J.Presl
Iz_6_1009	DJI_0312	Clinopodium acinos (L.) Kuntze	0	n	1	0	Lamiacea Lindl.
Iz_6_1009	DJI_0312	Convallaria majalis L.	0	n	1	0	Asparagaceae Juss.
Iz_6_1009	DJI_0312	Crataegus rhipidophylla Gand.	0	n	1	1	Rosaceae Juss.
Iz_6_1009	DJI_0312	Crepis foetida L.	0	n	1	0	Asteraceae Bercht. & J.Presl
Iz_6_1009	DJI_0312	Dactylis glomerata L.	0	n	1	0	Poaceae Barnhart
Iz_6_1009	DJI_0312	Dianthus campestris M.Bieb.	0	n	1	0	Caryophyllaceae Juss.
Iz_6_1009	DJI_0312	Digitaria sanguinalis (L.) Scop.	0	a	0	0	Poaceae Barnhart
Iz_6_1009	DJI_0312	Elymus repens (L.) Gould	0	n	1	0	Poaceae Barnhart
Iz_6_1009	DJI_0312	Epilobium tetragonum L.	0	n	1	0	Onagraceae Juss.
Iz_6_1009	DJI_0312	Eragrostis minor Host	0	a	0	0	Poaceae Barnhart
Iz_6_1009	DJI_0312	Erigeron canadensis L.	5	a	0	0	Asteraceae Bercht. & J.Presl
Iz_6_1009	DJI_0312	Euphorbia seguieriana Neck.	0	n	1	0	Euphorbiaceae Juss.
Iz_6_1009	DJI_0312	Fallopia dumetorum (L.) Holub	0	n	1	0	Polygonaceae Juss.
Iz_6_1009	DJI_0312	Festuca beckeri (Hack.) Trautv.	0	n	1	0	Poaceae Barnhart
Iz_6_1009	DJI_0312	Fragaria vesca L.	0	n	1	0	Rosaceae Juss.
Iz_6_1009	DJI_0312	Frangula alnus Mill.	0	n	1	1	Rhamnaceae Juss.
Iz_6_1009	DJI_0312	Fraxinus pennsylvanica Marshall	0	a	0	1	Oleaceae Hoffmanns. & Link
Iz_6_1009	DJI_0312	Galeopsis bifida Boenn.	0	n	1	0	Lamiacea Lindl.
Iz_6_1009	DJI_0312	Galium verum L.	0	n	1	0	Rubiaceae Juss.
Iz_6_1009	DJI_0312	Genista tinctoria L.	0	n	1	0	Fabaceae Lindl.
Iz_6_1009	DJI_0312	Geranium robertianum L.	0	n	1	0	Geraniaceae Juss.
Iz_6_1009	DJI_0312	Geranium sanquineum L.	0	n	1	0	Geraniaceae Juss.
Iz_6_1009	DJI_0312	Gypsophila paniculata L.	0	n	1	0	Caryophyllaceae Juss.
Iz_6_1009	DJI_0312	Helichrysum arenarium (L.) Moench	0	n	1	0	Asteraceae Bercht. & J.Presl
Iz_6_1009	DJI_0312	Herniaria polygama J.Gay	0	n	1	0	Caryophyllaceae Juss.
Iz_6_1009	DJI_0312	Hieracium robustum Fr.	0	n	1	0	Asteraceae Bercht. & J.Presl
Iz_6_1009	DJI_0312	Hieracium umbellatum L.	0	n	1	NA	Asteraceae Bercht. & J.Presl
Iz_6_1009	DJI_0312	Hylotelephium maximum (L.) Holub	0	n	1	0	Crassulaceae J.St.-Hil.
Iz_6_1009	DJI_0312	Jacobaea borysthenica (DC.) B.Nord. & Greuter	0	n	1	0	Asteraceae Bercht. & J.Presl
Iz_6_1009	DJI_0312	Jasione montana L.	0	n	1	0	Campanulaceae Juss.
Iz_6_1009	DJI_0312	Jurinea cyanoides (L.) Rchb.s.l.	0	n	1	0	Asteraceae Bercht. & J.Presl
Iz_6_1009	DJI_0312	Koeleria glauca (Spreng.) DC.	0	n	1	0	Poaceae Barnhart
Iz_6_1009	DJI_0312	Lactuca serriola L.	0	a	0	0	Asteraceae Bercht. & J.Presl
Iz_6_1009	DJI_0312	Linaria genistifolia (L.) Mill.	0	n	1	0	Plantaginaceae Juss.
Iz_6_1009	DJI_0312	Lomelosia argentea (L.) Greuter & Burdet	0	n	1	0	Caprifoliaceae Juss.
Iz_6_1009	DJI_0312	Melampyrum pratense L.	0	n	1	0	Orobanchaceae Vent.
Iz_6_1009	DJI_0312	Oenothera biennis L.	0	a	0	0	Onagraceae Juss.
Iz_6_1009	DJI_0312	Pentanema britannica (L.) D.Gut.Larr., Santos-Vicente, Anderb., E.Rico & M.M.Mart.Ort.	0	n	1	0	Asteraceae Bercht. & J.Presl
Iz_6_1009	DJI_0312	Peucedanum oreoselinum (L.) Moench	0	n	1	0	Apiaceae Lindl.
Iz_6_1009	DJI_0312	Peucedanum ruthenicum M.Bieb.	0	n	1	0	Apiaceae Lindl.
Iz_6_1009	DJI_0312	Pilosella echioides (Lumn.) F.W.Schultz & Sch.Bip.	0	n	1	0	Asteraceae Bercht. & J.Presl
Iz_6_1009	DJI_0312	Pilosella officinarum F. Schultz et Sch. Bip.	0	n	1	0	Asteraceae Bercht. & J.Presl
Iz_6_1009	DJI_0312	Pilosella sp.	0		NA	0	Asteraceae Bercht. & J.Presl
Iz_6_1009	DJI_0312	Pinus sylvestris L.	0	n	1	1	Pinaceae Spreng. ex F.Rudolphi
Iz_6_1009	DJI_0312	Poa annua L.	0	n	1	0	Poaceae Barnhart
Iz_6_1009	DJI_0312	Poa nemoralis L.	0	n	1	0	Poaceae Barnhart
Iz_6_1009	DJI_0312	Poa sp.	0		NA	0	Poaceae Barnhart
Iz_6_1009	DJI_0312	Polygonatum odoratum (Mill.) Druce	0	n	1	0	Asparagaceae Juss.
Iz_6_1009	DJI_0312	Polygonum arenarium Waldst. & Kit.	0	n	1	0	Polygonaceae Juss.
Iz_6_1009	DJI_0312	Populus nigra L.	0	n	1	1	Salicaceae Mirb.
Iz_6_1009	DJI_0312	Populus tremula L.	0	n	1	1	Salicaceae Mirb.
Iz_6_1009	DJI_0312	Populus Ã canescens (Aiton) Sm.	0	n	1	1	Salicaceae Mirb.
Iz_6_1009	DJI_0312	Potentilla argentea L.	0	n	1	0	Rosaceae Juss.

Iz_6_1009	DJI_0312	Psammophiliella muralis (L.) Ikonn.	0	n	1	0	Caryophyllaceae Juss.
Iz_6_1009	DJI_0312	Psephellus sumensis (Kalen.) Greuter	0	n	1	0	Asteraceae Bercht. & J.Presl
Iz_6_1009	DJI_0312	Pulsatilla pratensis (L.) Mill. s.l.	0	n	1	0	Ranunculaceae Juss.
Iz_6_1009	DJI_0312	Pyrus communis L.	0	a	0	1	Rosaceae Juss.
Iz_6_1009	DJI_0312	Quercus robur L.	0	n	1	1	Fagaceae Dumort.
Iz_6_1009	DJI_0312	Robinia pseudoacacia L.	0	a	0	1	Fabaceae Lindl.
Iz_6_1009	DJI_0312	Rosa sp.	0		NA	1	Rosaceae Juss.
Iz_6_1009	DJI_0312	Rubus idaeus L.	0	n	1	0	Rosaceae Juss.
Iz_6_1009	DJI_0312	Rumex acetosella L.	0	n	1	0	Polygonaceae Juss.
Iz_6_1009	DJI_0312	Rumex thyrsiflorus Fingerh.	0	n	1	0	Polygonaceae Juss.
Iz_6_1009	DJI_0312	Salix alba L.	0	n	1	1	Salicaceae Mirb.
Iz_6_1009	DJI_0312	Sambucus nigra L.	0	n	1	1	Viburnaceae Raf.
Iz_6_1009	DJI_0312	Secale sylvestre Host	0	n	1	0	Poaceae Barnhart
Iz_6_1009	DJI_0312	Sempervivum ruthenicum Schnittsp. & C.B. Lehm.	0	n	1	0	Crassulaceae J.St.-Hil.
Iz_6_1009	DJI_0312	Setaria viridis (L.) P. Beauv.	0	n	1	0	Poaceae Barnhart
Iz_6_1009	DJI_0312	Silene borysthenica (Gruner) Walters	0	n	1	0	Caryophyllaceae Juss.
Iz_6_1009	DJI_0312	Silene latifolia subsp. alba (Mill.) Greuter & Burdet	0	n	1	0	Caryophyllaceae Juss.
Iz_6_1009	DJI_0312	Solidago virgaurea L.	0	n	1	0	Asteraceae Bercht. & J.Presl
Iz_6_1009	DJI_0312	Sorbus aucuparia L.	0	n	1	1	Rosaceae Juss.
Iz_6_1009	DJI_0312	Stachys recta L.	0	n	1	0	Lamiacea Lindl.
Iz_6_1009	DJI_0312	Tanacetum vulgare L.	0	n	1	0	Asteraceae Bercht. & J.Presl
Iz_6_1009	DJI_0312	Thymus pallasianus Heinr.Braun	0	n	1	0	Lamiacea Lindl.
Iz_6_1009	DJI_0312	Tilia cordata Mill.	0	n	1	1	Malvaceae Juss.
Iz_6_1009	DJI_0312	Trifolium alpestre L.	0	n	1	0	Fabaceae Lindl.
Iz_6_1009	DJI_0312	Trifolium arvense L.	0	n	1	0	Fabaceae Lindl.
Iz_6_1009	DJI_0312	Ulmus glabra Huds.	0	n	1	1	Ulmaceae Mirb.
Iz_6_1009	DJI_0312	Verbascum densiflorum Bertol.	0	n	1	0	Scrophulariaceae Juss.
Iz_6_1009	DJI_0312	Veronica austriaca L.	0	n	1	0	Plantaginaceae Juss.
Iz_6_1009	DJI_0312	Veronica incana L.	0	n	1	0	Plantaginaceae Juss.
Iz_6_1009	DJI_0312	Veronica verna L.	0	n	1	0	Plantaginaceae Juss.
Iz_6_1009	DJI_0312	Vincetoxicum hirundinaria Medik	0	n	1	0	Apocynaceae Juss.
Iz_6_1009	DJI_0314	Acer tataricum L.	0	n	1	1	Sapindaceae Juss.
Iz_6_1009	DJI_0314	Achillea millefolium L. s.l.	0	n	1	0	Asteraceae Bercht. & J.Presl
Iz_6_1009	DJI_0314	Allium oleraceum L.	0	n	1	0	Amaryllidaceae J.St.-Hil.
Iz_6_1009	DJI_0314	Ambrosia artemisiifolia L.	0	a	0	0	Asteraceae Bercht. & J.Presl
Iz_6_1009	DJI_0314	Amorpha fruticosa L.	0	a	0	0	Fabaceae Lindl.
Iz_6_1009	DJI_0314	Anthericum ramosum L.	0	n	1	0	Asparagaceae Juss.
Iz_6_1009	DJI_0314	Artemisia marschalliana Spreng.	0	n	1	0	Asteraceae Bercht. & J.Presl
Iz_6_1009	DJI_0314	Asparagus officinalis L.	0	n	1	0	Asparagaceae Juss.
Iz_6_1009	DJI_0314	Astragalus cicer L.	0	n	1	0	Fabaceae Lindl.
Iz_6_1009	DJI_0314	Bassia laniflora (S.G.Gmel.) A.J.Scott	0	n	1	0	Amaranthaceae Juss.
Iz_6_1009	DJI_0314	Berberis vulgaris L.	0	n	1	0	Berberidaceae Juss.
Iz_6_1009	DJI_0314	Betula pendula Roth.	0	n	1	1	Betulaceae Gray
Iz_6_1009	DJI_0314	Betula pendula Roth.	0	n	1	0	Poaceae Barnhart
Iz_6_1009	DJI_0314	Bromus inermis Leyss.	0		NA	NA	
Iz_6_1009	DJI_0314	Bromus squarrosus L.	0	a	0	0	Poaceae Barnhart
Iz_6_1009	DJI_0314	Bromus tectorum L.	0	a	0	0	Poaceae Barnhart
Iz_6_1009	DJI_0314	Calamagrostis epigejos (L.) Roth	0.1	n	1	0	Poaceae Barnhart
Iz_6_1009	DJI_0314	Carduus acanthoides L.	0	n	1	0	Asteraceae Bercht. & J.Presl
Iz_6_1009	DJI_0314	Carex sp.	0	n	1	0	Cyperaceae Juss.
Iz_6_1009	DJI_0314	Centaurea majorovii Dumbadze	0	n	1	0	Asteraceae Bercht. & J.Presl
Iz_6_1009	DJI_0314	Chamaecytisus ruthenicus (Fisch.ex Wol.) Klaskova	0	n	1	0	Fabaceae Lindl.
Iz_6_1009	DJI_0314	Chelidonium majus L.	0	n	1	0	Papaveraceae Juss.
Iz_6_1009	DJI_0314	Chenopodium betaceum Andrz.	0	n	1	0	Amaranthaceae Juss.
Iz_6_1009	DJI_0314	Chenopodium cf. album	0	n	1	0	Amaranthaceae Juss.
Iz_6_1009	DJI_0314	Chenopodium sp.	0		NA	0	Amaranthaceae Juss.
Iz_6_1009	DJI_0314	Chondrilla juncea L.	0	n	1	0	Asteraceae Bercht. & J.Presl
Iz_6_1009	DJI_0314	Cirsium vulgare (Savi) Ten.	0	n	1	0	Asteraceae Bercht. & J.Presl
Iz_6_1009	DJI_0314	Clinopodium acinos (L.) Kuntze	0	n	1	0	Lamiacea Lindl.
Iz_6_1009	DJI_0314	Convallaria majalis L.	0	n	1	0	Asparagaceae Juss.
Iz_6_1009	DJI_0314	Crataegus rhipidophylla Gand.	0	n	1	1	Rosaceae Juss.
Iz_6_1009	DJI_0314	Crepis foetida L.	0	n	1	0	Asteraceae Bercht. & J.Presl
Iz_6_1009	DJI_0314	Dactylis glomerata L.	0	n	1	0	Poaceae Barnhart
Iz_6_1009	DJI_0314	Dianthus campestris M.Bieb.	0	n	1	0	Caryophyllaceae Juss.
Iz_6_1009	DJI_0314	Digitaria sanguinalis (L.) Scop.	0	a	0	0	Poaceae Barnhart
Iz_6_1009	DJI_0314	Elymus repens (L.) Gould	0	n	1	0	Poaceae Barnhart
Iz_6_1009	DJI_0314	Epilobium tetragonum L.	0	n	1	0	Onagraceae Juss.
Iz_6_1009	DJI_0314	Eragrostis minor Host	0	a	0	0	Poaceae Barnhart
Iz_6_1009	DJI_0314	Erigeron canadensis L.	5	a	0	0	Asteraceae Bercht. & J.Presl
Iz_6_1009	DJI_0314	Euphorbia seguieriana Neck.	0	n	1	0	Euphorbiaceae Juss.
Iz_6_1009	DJI_0314	Fallopia dumetorum (L.) Holub	0	n	1	0	Polygonaceae Juss.
Iz_6_1009	DJI_0314	Festuca beckeri (Hack.) Trautv.	0	n	1	0	Poaceae Barnhart
Iz_6_1009	DJI_0314	Fragaria vesca L.	0	n	1	0	Rosaceae Juss.
Iz_6_1009	DJI_0314	Frangula alnus Mill.	0	n	1	1	Rhamnaceae Juss.
Iz_6_1009	DJI_0314	Fraxinus pennsylvanica Marshall	0	a	0	1	Oleaceae Hoffmanns. & Link
Iz_6_1009	DJI_0314	Galeopsis bifida Boenn.	0	n	1	0	Lamiacea Lindl.
Iz_6_1009	DJI_0314	Galium verum L.	0	n	1	0	Rubiaceae Juss.
Iz_6_1009	DJI_0314	Genista tinctoria L.	0	n	1	0	Fabaceae Lindl.
Iz_6_1009	DJI_0314	Geranium robertianum L.	0	n	1	0	Geraniaceae Juss.
Iz_6_1009	DJI_0314	Geranium sanquineum L.	0	n	1	0	Geraniaceae Juss.
Iz_6_1009	DJI_0314	Gypsophila paniculata L.	0	n	1	0	Caryophyllaceae Juss.
Iz_6_1009	DJI_0314	Helichrysum arenarium (L.) Moench	0	n	1	0	Asteraceae Bercht. & J.Presl
Iz_6_1009	DJI_0314	Herniaria polygama J.Gay	0	n	1	0	Caryophyllaceae Juss.
Iz_6_1009	DJI_0314	Hieracium robustum Fr.	0	n	1	0	Asteraceae Bercht. & J.Presl
Iz_6_1009	DJI_0314	Hieracium umbellatum L.	0	n	1	NA	Asteraceae Bercht. & J.Presl
Iz_6_1009	DJI_0314	Hylotelephium maximum (L.) Holub	0	n	1	0	Crassulaceae J.St.-Hil.
Iz_6_1009	DJI_0314	Jacobaea borysthenica (DC.) B.Nord. & Greuter	0	n	1	0	Asteraceae Bercht. & J.Presl
Iz_6_1009	DJI_0314	Jasione montana L.	0	n	1	0	Campanulaceae Juss.
Iz_6_1009	DJI_0314	Jurinea cyanoides (L.) Rchb.s.l.	0	n	1	0	Asteraceae Bercht. & J.Presl
Iz_6_1009	DJI_0314	Koeleria glauca (Spreng.) DC.	0	n	1	0	Poaceae Barnhart
Iz_6_1009	DJI_0314	Lactuca serriola L.	0	a	0	0	Asteraceae Bercht. & J.Presl
Iz_6_1009	DJI_0314	Linaria genistifolia (L.) Mill.	0	n	1	0	Plantaginaceae Juss.
Iz_6_1009	DJI_0314	Lomelosia argentea (L.) Greuter & Burdet	0	n	1	0	Caprifoliaceae Juss.
Iz_6_1009	DJI_0314	Melampyrum pratense L.	0	n	1	0	Orobanchaceae Vent.
Iz_6_1009	DJI_0314	Oenothera biennis L.	0	a	0	0	Onagraceae Juss.
Iz_6_1009	DJI_0314	Pentanema britannica (L.) D.Gut.Larr., Santos-Vicente, Anderb., E.Rico & M.M.Mart.Ort.	0	n	1	0	Asteraceae Bercht. & J.Presl
Iz_6_1009	DJI_0314	Peucedanum oreoselinum (L.) Moench	0	n	1	0	Apiaceae Lindl.
Iz_6_1009	DJI_0314	Peucedanum ruthenicum M.Bieb.	0	n	1	0	Apiaceae Lindl.

Iz_6_1009	DJI_0314	Pilosella echioides (Lumn.) F.W.Schultz & Sch.Bip.	0	n	1	0	Asteraceae Bercht. & J.Presl
Iz_6_1009	DJI_0314	Pilosella officinarum F. Schultz et Sch. Bip.	0	n	1	0	Asteraceae Bercht. & J.Presl
Iz_6_1009	DJI_0314	Pilosella sp.	0		NA	0	Asteraceae Bercht. & J.Presl
Iz_6_1009	DJI_0314	Pinus sylvestris L.	0	n	1	1	Pinaceae Spreng. ex F.Rudolphi
Iz_6_1009	DJI_0314	Poa annua L.	0	n	1	0	Poaceae Barnhart
Iz_6_1009	DJI_0314	Poa nemoralis L.	0	n	1	0	Poaceae Barnhart
Iz_6_1009	DJI_0314	Poa sp.	0		NA	0	Poaceae Barnhart
Iz_6_1009	DJI_0314	Polygonatum odoratum (Mill.) Druce	0	n	1	0	Asparagaceae Juss.
Iz_6_1009	DJI_0314	Polygonum arenarium Waldst. & Kit.	0	n	1	0	Polygonaceae Juss.
Iz_6_1009	DJI_0314	Populus nigra L.	0	n	1	1	Salicaceae Mirb.
Iz_6_1009	DJI_0314	Populus tremula L.	0	n	1	1	Salicaceae Mirb.
Iz_6_1009	DJI_0314	Populus Ã canescens (Aiton) Sm.	0	n	1	1	Salicaceae Mirb.
Iz_6_1009	DJI_0314	Potentilla argentea L.	0	n	1	0	Rosaceae Juss.
Iz_6_1009	DJI_0314	Psammophiliella muralis (L.) Ikonn.	0	n	1	0	Caryophyllaceae Juss.
Iz_6_1009	DJI_0314	Psephellus sumensis (Kalen.) Greuter	0	n	1	0	Asteraceae Bercht. & J.Presl
Iz_6_1009	DJI_0314	Pulsatilla pratensis (L.) Mill. s.l.	0	n	1	0	Ranunculaceae Juss.
Iz_6_1009	DJI_0314	Pyrus communis L.	0	a	0	1	Rosaceae Juss.
Iz_6_1009	DJI_0314	Quercus robur L.	0	n	1	1	Fagaceae Dumort.
Iz_6_1009	DJI_0314	Robinia pseudoacacia L.	0	a	0	1	Fabaceae Lindl.
Iz_6_1009	DJI_0314	Rosa sp.	0		NA	1	Rosaceae Juss.
Iz_6_1009	DJI_0314	Rubus idaeus L.	0	n	1	0	Rosaceae Juss.
Iz_6_1009	DJI_0314	Rumex acetosella L.	0	n	1	0	Polygonaceae Juss.
Iz_6_1009	DJI_0314	Rumex thyrsiflorus Fingerh.	0	n	1	0	Polygonaceae Juss.
Iz_6_1009	DJI_0314	Salix alba L.	0	n	1	1	Salicaceae Mirb.
Iz_6_1009	DJI_0314	Sambucus nigra L.	0	n	1	1	Viburnaceae Raf.
Iz_6_1009	DJI_0314	Secale sylvestre Host	0	n	1	0	Poaceae Barnhart
Iz_6_1009	DJI_0314	Sempervivum ruthenicum Schnittsp. & C.B. Lehm.	0	n	1	0	Crassulaceae J.St.-Hil.
Iz_6_1009	DJI_0314	Setaria viridis (L.) P. Beauv.	0	n	1	0	Poaceae Barnhart
Iz_6_1009	DJI_0314	Silene borysthenica (Gruner) Walters	0	n	1	0	Caryophyllaceae Juss.
Iz_6_1009	DJI_0314	Silene latifolia subsp. alba (Mill.) Greuter & Burdet	0	n	1	0	Caryophyllaceae Juss.
Iz_6_1009	DJI_0314	Solidago virgaurea L.	0	n	1	0	Asteraceae Bercht. & J.Presl
Iz_6_1009	DJI_0314	Sorbus aucuparia L.	0	n	1	1	Rosaceae Juss.
Iz_6_1009	DJI_0314	Stachys recta L.	0	n	1	0	Lamiacea Lindl.
Iz_6_1009	DJI_0314	Tanacetum vulgare L.	0	n	1	0	Asteraceae Bercht. & J.Presl
Iz_6_1009	DJI_0314	Thymus pallasianus Heinr.Braun	0	n	1	0	Lamiacea Lindl.
Iz_6_1009	DJI_0314	Tilia cordata Mill.	0	n	1	1	Malvaceae Juss.
Iz_6_1009	DJI_0314	Trifolium alpestre L.	0	n	1	0	Fabaceae Lindl.
Iz_6_1009	DJI_0314	Trifolium arvense L.	0	n	1	0	Fabaceae Lindl.
Iz_6_1009	DJI_0314	Ulmus glabra Huds.	0	n	1	1	Ulmaceae Mirb.
Iz_6_1009	DJI_0314	Verbascum densiflorum Bertol.	0	n	1	0	Scrophulariaceae Juss.
Iz_6_1009	DJI_0314	Veronica austriaca L.	0	n	1	0	Plantaginaceae Juss.
Iz_6_1009	DJI_0314	Veronica incana L.	0	n	1	0	Plantaginaceae Juss.
Iz_6_1009	DJI_0314	Veronica verna L.	0	n	1	0	Plantaginaceae Juss.
Iz_6_1009	DJI_0314	Vincetoxicum hirundinaria Medik	0	n	1	0	Apocynaceae Juss.
Iz_6_1009	DJI_0317	Acer tataricum L.	0	n	1	1	Sapindaceae Juss.
Iz_6_1009	DJI_0317	Achillea millefolium L. s.l.	0	n	1	0	Asteraceae Bercht. & J.Presl
Iz_6_1009	DJI_0317	Allium oleraceum L.	0	n	1	0	Amaryllidaceae J.St.-Hil.
Iz_6_1009	DJI_0317	Ambrosia artemisiifolia L.	0	a	0	0	Asteraceae Bercht. & J.Presl
Iz_6_1009	DJI_0317	Amorpha fruticosa L.	0	a	0	0	Fabaceae Lindl.
Iz_6_1009	DJI_0317	Anthericum ramosum L.	0	n	1	0	Asparagaceae Juss.
Iz_6_1009	DJI_0317	Artemisia marschalliana Spreng.	0	n	1	0	Asteraceae Bercht. & J.Presl
Iz_6_1009	DJI_0317	Asparagus officinalis L.	0	n	1	0	Asparagaceae Juss.
Iz_6_1009	DJI_0317	Astragalus cicer L.	0	n	1	0	Fabaceae Lindl.
Iz_6_1009	DJI_0317	Bassia laniflora (S.G.Gmel.) A.J.Scott	0	n	1	0	Amaranthaceae Juss.
Iz_6_1009	DJI_0317	Berberis vulgaris L.	0	n	1	0	Berberidaceae Juss.
Iz_6_1009	DJI_0317	Betula pendula Roth.	0	n	1	1	Betulaceae Gray
Iz_6_1009	DJI_0317	Betula pendula Roth.	0	n	1	0	Poaceae Barnhart
Iz_6_1009	DJI_0317	Bromus inermis Leyss.	0		NA	NA	
Iz_6_1009	DJI_0317	Bromus squarrosus L.	0	a	0	0	Poaceae Barnhart
Iz_6_1009	DJI_0317	Bromus tectorum L.	0	a	0	0	Poaceae Barnhart
Iz_6_1009	DJI_0317	Calamagrostis epigejos (L.) Roth	0.1	n	1	0	Poaceae Barnhart
Iz_6_1009	DJI_0317	Carduus acanthoides L.	0	n	1	0	Asteraceae Bercht. & J.Presl
Iz_6_1009	DJI_0317	Carex sp.	0	n	1	0	Cyperaceae Juss.
Iz_6_1009	DJI_0317	Centaurea majorovii Dumbadze	0	n	1	0	Asteraceae Bercht. & J.Presl
Iz_6_1009	DJI_0317	Chamaecytisus ruthenicus (Fisch.ex Wol.) Klaskova	0	n	1	0	Fabaceae Lindl.
Iz_6_1009	DJI_0317	Chelidonium majus L.	0	n	1	0	Papaveraceae Juss.
Iz_6_1009	DJI_0317	Chenopodium betaceum Andrz.	0	n	1	0	Amaranthaceae Juss.
Iz_6_1009	DJI_0317	Chenopodium cf. album	0	n	1	0	Amaranthaceae Juss.
Iz_6_1009	DJI_0317	Chenopodium sp.	0		NA	0	Amaranthaceae Juss.
Iz_6_1009	DJI_0317	Chondrilla juncea L.	0	n	1	0	Asteraceae Bercht. & J.Presl
Iz_6_1009	DJI_0317	Cirsium vulgare (Savi) Ten.	0	n	1	0	Asteraceae Bercht. & J.Presl
Iz_6_1009	DJI_0317	Clinopodium acinos (L.) Kuntze	0	n	1	0	Lamiacea Lindl.
Iz_6_1009	DJI_0317	Convallaria majalis L.	0	n	1	0	Asparagaceae Juss.
Iz_6_1009	DJI_0317	Crataegus rhipidophylla Gand.	0	n	1	1	Rosaceae Juss.
Iz_6_1009	DJI_0317	Crepis foetida L.	0	n	1	0	Asteraceae Bercht. & J.Presl
Iz_6_1009	DJI_0317	Dactylis glomerata L.	0	n	1	0	Poaceae Barnhart
Iz_6_1009	DJI_0317	Dianthus campestris M.Bieb.	0	n	1	0	Caryophyllaceae Juss.
Iz_6_1009	DJI_0317	Digitaria sanguinalis (L.) Scop.	0	a	0	0	Poaceae Barnhart
Iz_6_1009	DJI_0317	Elymus repens (L.) Gould	0	n	1	0	Poaceae Barnhart
Iz_6_1009	DJI_0317	Epilobium tetragonum L.	0	n	1	0	Onagraceae Juss.
Iz_6_1009	DJI_0317	Eragrostis minor Host	0	a	0	0	Poaceae Barnhart
Iz_6_1009	DJI_0317	Erigeron canadensis L.	6	a	0	0	Asteraceae Bercht. & J.Presl
Iz_6_1009	DJI_0317	Euphorbia seguieriana Neck.	0	n	1	0	Euphorbiaceae Juss.
Iz_6_1009	DJI_0317	Fallopia dumetorum (L.) Holub	0	n	1	0	Polygonaceae Juss.
Iz_6_1009	DJI_0317	Festuca beckeri (Hack.) Trautv.	0	n	1	0	Poaceae Barnhart
Iz_6_1009	DJI_0317	Fragaria vesca L.	0	n	1	0	Rosaceae Juss.
Iz_6_1009	DJI_0317	Frangula alnus Mill.	0	n	1	1	Rhamnaceae Juss.
Iz_6_1009	DJI_0317	Fraxinus pennsylvanica Marshall	0	a	0	1	Oleaceae Hoffmanns. & Link
Iz_6_1009	DJI_0317	Galeopsis bifida Boenn.	0	n	1	0	Lamiacea Lindl.
Iz_6_1009	DJI_0317	Galium verum L.	0	n	1	0	Rubiaceae Juss.
Iz_6_1009	DJI_0317	Genista tinctoria L.	0	n	1	0	Fabaceae Lindl.
Iz_6_1009	DJI_0317	Geranium robertianum L.	0	n	1	0	Geraniaceae Juss.
Iz_6_1009	DJI_0317	Geranium sanquineum L.	0	n	1	0	Geraniaceae Juss.
Iz_6_1009	DJI_0317	Gypsophila paniculata L.	0	n	1	0	Caryophyllaceae Juss.
Iz_6_1009	DJI_0317	Helichrysum arenarium (L.) Moench	0	n	1	0	Asteraceae Bercht. & J.Presl
Iz_6_1009	DJI_0317	Herniaria polygama J.Gay	0	n	1	0	Caryophyllaceae Juss.
Iz_6_1009	DJI_0317	Hieracium robustum Fr.	0	n	1	0	Asteraceae Bercht. & J.Presl
Iz_6_1009	DJI_0317	Hieracium umbellatum L.	0	n	1	NA	Asteraceae Bercht. & J.Presl

Iz_6_1009	DJI_0317	Hylotelephium maximum (L.) Holub	0	n	1	0	Crassulaceae J.St.-Hil.
Iz_6_1009	DJI_0317	Jacobaea borysthenica (DC.) B.Nord. & Greuter	0	n	1	0	Asteraceae Bercht. & J.Presl
Iz_6_1009	DJI_0317	Jasione montana L.	0	n	1	0	Campanulaceae Juss.
Iz_6_1009	DJI_0317	Jurinea cyanoides (L.) Rchb.s.l.	0	n	1	0	Asteraceae Bercht. & J.Presl
Iz_6_1009	DJI_0317	Koeleria glauca (Spreng.) DC.	0	n	1	0	Poaceae Barnhart
Iz_6_1009	DJI_0317	Lactuca serriola L.	0	a	0	0	Asteraceae Bercht. & J.Presl
Iz_6_1009	DJI_0317	Linaria genistifolia (L.) Mill.	0	n	1	0	Plantaginaceae Juss.
Iz_6_1009	DJI_0317	Lomelosia argentea (L.) Greuter & Burdet	0	n	1	0	Caprifoliaceae Juss.
Iz_6_1009	DJI_0317	Melampyrum pratense L.	0	n	1	0	Orobanchaceae Vent.
Iz_6_1009	DJI_0317	Oenothera biennis L.	0	a	0	0	Onagraceae Juss.
Iz_6_1009	DJI_0317	Pentanema britannica (L.) D.Gut.Larr., Santos-Vicente, Anderb., E.Rico & M.M.Mart.Ort.	0	n	1	0	Asteraceae Bercht. & J.Presl
Iz_6_1009	DJI_0317	Peucedanum oreoselinum (L.) Moench	0	n	1	0	Apiaceae Lindl.
Iz_6_1009	DJI_0317	Peucedanum ruthenicum M.Bieb.	0	n	1	0	Apiaceae Lindl.
Iz_6_1009	DJI_0317	Pilosella echioides (Lumn.) F.W.Schultz & Sch.Bip.	0	n	1	0	Asteraceae Bercht. & J.Presl
Iz_6_1009	DJI_0317	Pilosella officinarum F. Schultz et Sch. Bip.	0	n	1	0	Asteraceae Bercht. & J.Presl
Iz_6_1009	DJI_0317	Pilosella sp.	0		NA	0	Asteraceae Bercht. & J.Presl
Iz_6_1009	DJI_0317	Pinus sylvestris L.	0	n	1	1	Pinaceae Spreng. ex F.Rudolphi
Iz_6_1009	DJI_0317	Poa annua L.	0	n	1	0	Poaceae Barnhart
Iz_6_1009	DJI_0317	Poa nemoralis L.	0	n	1	0	Poaceae Barnhart
Iz_6_1009	DJI_0317	Poa sp.	0		NA	0	Poaceae Barnhart
Iz_6_1009	DJI_0317	Polygonatum odoratum (Mill.) Druce	0	n	1	0	Asparagaceae Juss.
Iz_6_1009	DJI_0317	Polygonum arenarium Waldst. & Kit.	0	n	1	0	Polygonaceae Juss.
Iz_6_1009	DJI_0317	Populus nigra L.	0	n	1	1	Salicaceae Mirb.
Iz_6_1009	DJI_0317	Populus tremula L.	0	n	1	1	Salicaceae Mirb.
Iz_6_1009	DJI_0317	Populus Ã canescens (Aiton) Sm.	0	n	1	1	Salicaceae Mirb.
Iz_6_1009	DJI_0317	Potentilla argentea L.	0	n	1	0	Rosaceae Juss.
Iz_6_1009	DJI_0317	Psammophiliella muralis (L.) Ikonn.	0	n	1	0	Caryophyllaceae Juss.
Iz_6_1009	DJI_0317	Psephellus sumensis (Kalen.) Greuter	0	n	1	0	Asteraceae Bercht. & J.Presl
Iz_6_1009	DJI_0317	Pulsatilla pratensis (L.) Mill. s.l.	0	n	1	0	Ranunculaceae Juss.
Iz_6_1009	DJI_0317	Pyrus communis L.	0	a	0	1	Rosaceae Juss.
Iz_6_1009	DJI_0317	Quercus robur L.	0	n	1	1	Fagaceae Dumort.
Iz_6_1009	DJI_0317	Robinia pseudoacacia L.	0	a	0	1	Fabaceae Lindl.
Iz_6_1009	DJI_0317	Rosa sp.	0		NA	1	Rosaceae Juss.
Iz_6_1009	DJI_0317	Rubus idaeus L.	0	n	1	0	Rosaceae Juss.
Iz_6_1009	DJI_0317	Rumex acetosella L.	0	n	1	0	Polygonaceae Juss.
Iz_6_1009	DJI_0317	Rumex thyrsiflorus Fingerh.	0	n	1	0	Polygonaceae Juss.
Iz_6_1009	DJI_0317	Salix alba L.	0	n	1	1	Salicaceae Mirb.
Iz_6_1009	DJI_0317	Sambucus nigra L.	0	n	1	1	Viburnaceae Raf.
Iz_6_1009	DJI_0317	Secale sylvestre Host	0	n	1	0	Poaceae Barnhart
Iz_6_1009	DJI_0317	Sempervivum ruthenicum Schnittsp. & C.B. Lehm.	0	n	1	0	Crassulaceae J.St.-Hil.
Iz_6_1009	DJI_0317	Setaria viridis (L.) P. Beauv.	0	n	1	0	Poaceae Barnhart
Iz_6_1009	DJI_0317	Silene borysthenica (Gruner) Walters	0	n	1	0	Caryophyllaceae Juss.
Iz_6_1009	DJI_0317	Silene latifolia subsp. alba (Mill.) Greuter & Burdet	0	n	1	0	Caryophyllaceae Juss.
Iz_6_1009	DJI_0317	Solidago virgaurea L.	0	n	1	0	Asteraceae Bercht. & J.Presl
Iz_6_1009	DJI_0317	Sorbus aucuparia L.	0	n	1	1	Rosaceae Juss.
Iz_6_1009	DJI_0317	Stachys recta L.	0	n	1	0	Lamiacea Lindl.
Iz_6_1009	DJI_0317	Tanacetum vulgare L.	0	n	1	0	Asteraceae Bercht. & J.Presl
Iz_6_1009	DJI_0317	Thymus pallasianus Heinr.Braun	0	n	1	0	Lamiacea Lindl.
Iz_6_1009	DJI_0317	Tilia cordata Mill.	0	n	1	1	Malvaceae Juss.
Iz_6_1009	DJI_0317	Trifolium alpestre L.	0	n	1	0	Fabaceae Lindl.
Iz_6_1009	DJI_0317	Trifolium arvense L.	0	n	1	0	Fabaceae Lindl.
Iz_6_1009	DJI_0317	Ulmus glabra Huds.	0	n	1	1	Ulmaceae Mirb.
Iz_6_1009	DJI_0317	Verbascum densiflorum Bertol.	0	n	1	0	Scrophulariaceae Juss.
Iz_6_1009	DJI_0317	Veronica austriaca L.	0	n	1	0	Plantaginaceae Juss.
Iz_6_1009	DJI_0317	Veronica incana L.	0	n	1	0	Plantaginaceae Juss.
Iz_6_1009	DJI_0317	Veronica verna L.	0	n	1	0	Plantaginaceae Juss.
Iz_6_1009	DJI_0317	Vincetoxicum hirundinaria Medik	0	n	1	0	Apocynaceae Juss.
Iz_6_1009	DJI_0320	Acer tataricum L.	0	n	1	1	Sapindaceae Juss.
Iz_6_1009	DJI_0320	Achillea millefolium L. s.l.	0	n	1	0	Asteraceae Bercht. & J.Presl
Iz_6_1009	DJI_0320	Allium oleraceum L.	0	n	1	0	Amaryllidaceae J.St.-Hil.
Iz_6_1009	DJI_0320	Ambrosia artemisiifolia L.	0	a	0	0	Asteraceae Bercht. & J.Presl
Iz_6_1009	DJI_0320	Amorpha fruticosa L.	0	a	0	0	Fabaceae Lindl.
Iz_6_1009	DJI_0320	Anthericum ramosum L.	0	n	1	0	Asparagaceae Juss.
Iz_6_1009	DJI_0320	Artemisia marschalliana Spreng.	0	n	1	0	Asteraceae Bercht. & J.Presl
Iz_6_1009	DJI_0320	Asparagus officinalis L.	0	n	1	0	Asparagaceae Juss.
Iz_6_1009	DJI_0320	Astragalus cicer L.	0	n	1	0	Fabaceae Lindl.
Iz_6_1009	DJI_0320	Bassia laniflora (S.G.Gmel.) A.J.Scott	0	n	1	0	Amaranthaceae Juss.
Iz_6_1009	DJI_0320	Berberis vulgaris L.	0	n	1	0	Berberidaceae Juss.
Iz_6_1009	DJI_0320	Betula pendula Roth.	0	n	1	1	Betulaceae Gray
Iz_6_1009	DJI_0320	Betula pendula Roth.	0	n	1	0	Poaceae Barnhart
Iz_6_1009	DJI_0320	Bromus inermis Leyss.	0		NA	NA	
Iz_6_1009	DJI_0320	Bromus squarrosus L.	0	a	0	0	Poaceae Barnhart
Iz_6_1009	DJI_0320	Bromus tectorum L.	0	a	0	0	Poaceae Barnhart
Iz_6_1009	DJI_0320	Calamagrostis epigejos (L.) Roth	0.1	n	1	0	Poaceae Barnhart
Iz_6_1009	DJI_0320	Carduus acanthoides L.	0	n	1	0	Asteraceae Bercht. & J.Presl
Iz_6_1009	DJI_0320	Carex sp.	0	n	1	0	Cyperaceae Juss.
Iz_6_1009	DJI_0320	Centaurea majorovii Dumbadze	0	n	1	0	Asteraceae Bercht. & J.Presl
Iz_6_1009	DJI_0320	Chamaecytisus ruthenicus (Fisch.ex Wol.) Klaskova	0	n	1	0	Fabaceae Lindl.
Iz_6_1009	DJI_0320	Chelidonium majus L.	0	n	1	0	Papaveraceae Juss.
Iz_6_1009	DJI_0320	Chenopodium betaceum Andrz.	0	n	1	0	Amaranthaceae Juss.
Iz_6_1009	DJI_0320	Chenopodium cf. album	0	n	1	0	Amaranthaceae Juss.
Iz_6_1009	DJI_0320	Chenopodium sp.	0		NA	0	Amaranthaceae Juss.
Iz_6_1009	DJI_0320	Chondrilla juncea L.	0	n	1	0	Asteraceae Bercht. & J.Presl
Iz_6_1009	DJI_0320	Cirsium vulgare (Savi) Ten.	0	n	1	0	Asteraceae Bercht. & J.Presl
Iz_6_1009	DJI_0320	Clinopodium acinos (L.) Kuntze	0	n	1	0	Lamiacea Lindl.
Iz_6_1009	DJI_0320	Convallaria majalis L.	0	n	1	0	Asparagaceae Juss.
Iz_6_1009	DJI_0320	Crataegus rhipidophylla Gand.	0	n	1	1	Rosaceae Juss.
Iz_6_1009	DJI_0320	Crepis foetida L.	0	n	1	0	Asteraceae Bercht. & J.Presl
Iz_6_1009	DJI_0320	Dactylis glomerata L.	0	n	1	0	Poaceae Barnhart
Iz_6_1009	DJI_0320	Dianthus campestris M.Bieb.	0	n	1	0	Caryophyllaceae Juss.
Iz_6_1009	DJI_0320	Digitaria sanguinalis (L.) Scop.	0	a	0	0	Poaceae Barnhart
Iz_6_1009	DJI_0320	Elymus repens (L.) Gould	0	n	1	0	Poaceae Barnhart
Iz_6_1009	DJI_0320	Epilobium tetragonum L.	0	n	1	0	Onagraceae Juss.
Iz_6_1009	DJI_0320	Eragrostis minor Host	0	a	0	0	Poaceae Barnhart
Iz_6_1009	DJI_0320	Erigeron canadensis L.	10	a	0	0	Asteraceae Bercht. & J.Presl
Iz_6_1009	DJI_0320	Euphorbia seguieriana Neck.	0	n	1	0	Euphorbiaceae Juss.
Iz_6_1009	DJI_0320	Fallopia dumetorum (L.) Holub	0	n	1	0	Polygonaceae Juss.
Iz_6_1009	DJI_0320	Festuca beckeri (Hack.) Trautv.	0	n	1	0	Poaceae Barnhart

Iz_6_1009	DJI_0320	Fragaria vesca L.	0	n	1	0	Rosaceae Juss.
Iz_6_1009	DJI_0320	Frangula alnus Mill.	0	n	1	1	Rhamnaceae Juss.
Iz_6_1009	DJI_0320	Fraxinus pennsylvanica Marshall	0	a	0	1	Oleaceae Hoffmanns. & Link
Iz_6_1009	DJI_0320	Galeopsis bifida Boenn.	0	n	1	0	Lamiacea Lindl.
Iz_6_1009	DJI_0320	Galium verum L.	0	n	1	0	Rubiaceae Juss.
Iz_6_1009	DJI_0320	Genista tinctoria L.	0	n	1	0	Fabaceae Lindl.
Iz_6_1009	DJI_0320	Geranium robertianum L.	0	n	1	0	Geraniaceae Juss.
Iz_6_1009	DJI_0320	Geranium sanquineum L.	0	n	1	0	Geraniaceae Juss.
Iz_6_1009	DJI_0320	Gypsophila paniculata L.	0	n	1	0	Caryophyllaceae Juss.
Iz_6_1009	DJI_0320	Helichrysum arenarium (L.) Moench	0	n	1	0	Asteraceae Bercht. & J.Presl
Iz_6_1009	DJI_0320	Herniaria polygama J.Gay	0	n	1	0	Caryophyllaceae Juss.
Iz_6_1009	DJI_0320	Hieracium robustum Fr.	0	n	1	0	Asteraceae Bercht. & J.Presl
Iz_6_1009	DJI_0320	Hieracium umbellatum L.	0	n	1	NA	Asteraceae Bercht. & J.Presl
Iz_6_1009	DJI_0320	Hylotelephium maximum (L.) Holub	0	n	1	0	Crassulaceae J.St.-Hil.
Iz_6_1009	DJI_0320	Jacobaea borysthenica (DC.) B.Nord. & Greuter	0	n	1	0	Asteraceae Bercht. & J.Presl
Iz_6_1009	DJI_0320	Jasione montana L.	0	n	1	0	Campanulaceae Juss.
Iz_6_1009	DJI_0320	Jurinea cyanoides (L.) Rchb.s.l.	0	n	1	0	Asteraceae Bercht. & J.Presl
Iz_6_1009	DJI_0320	Koeleria glauca (Spreng.) DC.	0	n	1	0	Poaceae Barnhart
Iz_6_1009	DJI_0320	Lactuca serriola L.	0	a	0	0	Asteraceae Bercht. & J.Presl
Iz_6_1009	DJI_0320	Linaria genistifolia (L.) Mill.	0	n	1	0	Plantaginaceae Juss.
Iz_6_1009	DJI_0320	Lomelosia argentea (L.) Greuter & Burdet	0	n	1	0	Caprifoliaceae Juss.
Iz_6_1009	DJI_0320	Melampyrum pratense L.	0	n	1	0	Orobanchaceae Vent.
Iz_6_1009	DJI_0320	Oenothera biennis L.	0	a	0	0	Onagraceae Juss.
Iz_6_1009	DJI_0320	Pentanema britannica (L.) D.Gut.Larr., Santos-Vicente, Anderb., E.Rico & M.M.Mart.Ort.	0	n	1	0	Asteraceae Bercht. & J.Presl
Iz_6_1009	DJI_0320	Peucedanum oreoselinum (L.) Moench	0	n	1	0	Apiaceae Lindl.
Iz_6_1009	DJI_0320	Peucedanum ruthenicum M.Bieb.	0	n	1	0	Apiaceae Lindl.
Iz_6_1009	DJI_0320	Pilosella echioides (Lumn.) F.W.Schultz & Sch.Bip.	0	n	1	0	Asteraceae Bercht. & J.Presl
Iz_6_1009	DJI_0320	Pilosella officinarum F. Schultz et Sch. Bip.	0	n	1	0	Asteraceae Bercht. & J.Presl
Iz_6_1009	DJI_0320	Pilosella sp.	0		NA	0	Asteraceae Bercht. & J.Presl
Iz_6_1009	DJI_0320	Pinus sylvestris L.	0	n	1	1	Pinaceae Spreng. ex F.Rudolphi
Iz_6_1009	DJI_0320	Poa annua L.	0	n	1	0	Poaceae Barnhart
Iz_6_1009	DJI_0320	Poa nemoralis L.	0	n	1	0	Poaceae Barnhart
Iz_6_1009	DJI_0320	Poa sp.	0		NA	0	Poaceae Barnhart
Iz_6_1009	DJI_0320	Polygonatum odoratum (Mill.) Druce	0	n	1	0	Asparagaceae Juss.
Iz_6_1009	DJI_0320	Polygonum arenarium Waldst. & Kit.	0	n	1	0	Polygonaceae Juss.
Iz_6_1009	DJI_0320	Populus nigra L.	0	n	1	1	Salicaceae Mirb.
Iz_6_1009	DJI_0320	Populus tremula L.	0	n	1	1	Salicaceae Mirb.
Iz_6_1009	DJI_0320	Populus Ã canescens (Aiton) Sm.	0	n	1	1	Salicaceae Mirb.
Iz_6_1009	DJI_0320	Potentilla argentea L.	0	n	1	0	Rosaceae Juss.
Iz_6_1009	DJI_0320	Psammophiliella muralis (L.) Ikonn.	0	n	1	0	Caryophyllaceae Juss.
Iz_6_1009	DJI_0320	Psephellus sumensis (Kalen.) Greuter	0	n	1	0	Asteraceae Bercht. & J.Presl
Iz_6_1009	DJI_0320	Pulsatilla pratensis (L.) Mill. s.l.	0	n	1	0	Ranunculaceae Juss.
Iz_6_1009	DJI_0320	Pyrus communis L.	0	a	0	1	Rosaceae Juss.
Iz_6_1009	DJI_0320	Quercus robur L.	0	n	1	1	Fagaceae Dumort.
Iz_6_1009	DJI_0320	Robinia pseudoacacia L.	0	a	0	1	Fabaceae Lindl.
Iz_6_1009	DJI_0320	Rosa sp.	0		NA	1	Rosaceae Juss.
Iz_6_1009	DJI_0320	Rubus idaeus L.	0	n	1	0	Rosaceae Juss.
Iz_6_1009	DJI_0320	Rumex acetosella L.	0	n	1	0	Polygonaceae Juss.
Iz_6_1009	DJI_0320	Rumex thyrsiflorus Fingerh.	0	n	1	0	Polygonaceae Juss.
Iz_6_1009	DJI_0320	Salix alba L.	0	n	1	1	Salicaceae Mirb.
Iz_6_1009	DJI_0320	Sambucus nigra L.	0	n	1	1	Viburnaceae Raf.
Iz_6_1009	DJI_0320	Secale sylvestre Host	0	n	1	0	Poaceae Barnhart
Iz_6_1009	DJI_0320	Sempervivum ruthenicum Schnittsp. & C.B. Lehm.	0	n	1	0	Crassulaceae J.St.-Hil.
Iz_6_1009	DJI_0320	Setaria viridis (L.) P. Beauv.	0	n	1	0	Poaceae Barnhart
Iz_6_1009	DJI_0320	Silene borysthenica (Gruner) Walters	0	n	1	0	Caryophyllaceae Juss.
Iz_6_1009	DJI_0320	Silene latifolia subsp. alba (Mill.) Greuter & Burdet	0	n	1	0	Caryophyllaceae Juss.
Iz_6_1009	DJI_0320	Solidago virgaurea L.	0	n	1	0	Asteraceae Bercht. & J.Presl
Iz_6_1009	DJI_0320	Sorbus aucuparia L.	0	n	1	1	Rosaceae Juss.
Iz_6_1009	DJI_0320	Stachys recta L.	0	n	1	0	Lamiacea Lindl.
Iz_6_1009	DJI_0320	Tanacetum vulgare L.	0	n	1	0	Asteraceae Bercht. & J.Presl
Iz_6_1009	DJI_0320	Thymus pallasianus Heinr.Braun	0	n	1	0	Lamiacea Lindl.
Iz_6_1009	DJI_0320	Tilia cordata Mill.	0	n	1	1	Malvaceae Juss.
Iz_6_1009	DJI_0320	Trifolium alpestre L.	0	n	1	0	Fabaceae Lindl.
Iz_6_1009	DJI_0320	Trifolium arvense L.	0	n	1	0	Fabaceae Lindl.
Iz_6_1009	DJI_0320	Ulmus glabra Huds.	0	n	1	1	Ulmaceae Mirb.
Iz_6_1009	DJI_0320	Verbascum densiflorum Bertol.	0	n	1	0	Scrophulariaceae Juss.
Iz_6_1009	DJI_0320	Veronica austriaca L.	0	n	1	0	Plantaginaceae Juss.
Iz_6_1009	DJI_0320	Veronica incana L.	0	n	1	0	Plantaginaceae Juss.
Iz_6_1009	DJI_0320	Veronica verna L.	0	n	1	0	Plantaginaceae Juss.
Iz_6_1009	DJI_0320	Vincetoxicum hirundinaria Medik	0	n	1	0	Apocynaceae Juss.
Iz_6_1009	DJI_0324	Acer tataricum L.	0	n	1	1	Sapindaceae Juss.
Iz_6_1009	DJI_0324	Achillea millefolium L. s.l.	0	n	1	0	Asteraceae Bercht. & J.Presl
Iz_6_1009	DJI_0324	Allium oleraceum L.	0	n	1	0	Amaryllidaceae J.St.-Hil.
Iz_6_1009	DJI_0324	Ambrosia artemisiifolia L.	0	a	0	0	Asteraceae Bercht. & J.Presl
Iz_6_1009	DJI_0324	Amorpha fruticosa L.	0	a	0	0	Fabaceae Lindl.
Iz_6_1009	DJI_0324	Anthericum ramosum L.	0	n	1	0	Asparagaceae Juss.
Iz_6_1009	DJI_0324	Artemisia marschalliana Spreng.	0	n	1	0	Asteraceae Bercht. & J.Presl
Iz_6_1009	DJI_0324	Asparagus officinalis L.	0	n	1	0	Asparagaceae Juss.
Iz_6_1009	DJI_0324	Astragalus cicer L.	0	n	1	0	Fabaceae Lindl.
Iz_6_1009	DJI_0324	Bassia laniflora (S.G.Gmel.) A.J.Scott	0	n	1	0	Amaranthaceae Juss.
Iz_6_1009	DJI_0324	Berberis vulgaris L.	0	n	1	0	Berberidaceae Juss.
Iz_6_1009	DJI_0324	Betula pendula Roth.	0	n	1	1	Betulaceae Gray
Iz_6_1009	DJI_0324	Betula pendula Roth.	0	n	1	0	Poaceae Barnhart
Iz_6_1009	DJI_0324	Bromus inermis Leyss.	0		NA	NA	
Iz_6_1009	DJI_0324	Bromus squarrosus L.	0	a	0	0	Poaceae Barnhart
Iz_6_1009	DJI_0324	Bromus tectorum L.	0	a	0	0	Poaceae Barnhart
Iz_6_1009	DJI_0324	Calamagrostis epigejos (L.) Roth	30	n	1	0	Poaceae Barnhart
Iz_6_1009	DJI_0324	Carduus acanthoides L.	0	n	1	0	Asteraceae Bercht. & J.Presl
Iz_6_1009	DJI_0324	Carex sp.	0	n	1	0	Cyperaceae Juss.
Iz_6_1009	DJI_0324	Centaurea majorovii Dumbadze	0	n	1	0	Asteraceae Bercht. & J.Presl
Iz_6_1009	DJI_0324	Chamaecytisus ruthenicus (Fisch.ex Wol.) Klaskova	0	n	1	0	Fabaceae Lindl.
Iz_6_1009	DJI_0324	Chelidonium majus L.	0	n	1	0	Papaveraceae Juss.
Iz_6_1009	DJI_0324	Chenopodium betaceum Andrz.	0	n	1	0	Amaranthaceae Juss.
Iz_6_1009	DJI_0324	Chenopodium cf. album	0	n	1	0	Amaranthaceae Juss.
Iz_6_1009	DJI_0324	Chenopodium sp.	0		NA	0	Amaranthaceae Juss.
Iz_6_1009	DJI_0324	Chondrilla juncea L.	0	n	1	0	Asteraceae Bercht. & J.Presl
Iz_6_1009	DJI_0324	Cirsium vulgare (Savi) Ten.	0	n	1	0	Asteraceae Bercht. & J.Presl
Iz_6_1009	DJI_0324	Clinopodium acinos (L.) Kuntze	0	n	1	0	Lamiacea Lindl.

Iz_6_1009	DJI_0324	Convallaria majalis L.	0	n	1	0	Asparagaceae Juss.
Iz_6_1009	DJI_0324	Crataegus rhipidophylla Gand.	0	n	1	1	Rosaceae Juss.
Iz_6_1009	DJI_0324	Crepis foetida L.	0	n	1	0	Asteraceae Bercht. & J.Presl
Iz_6_1009	DJI_0324	Dactylis glomerata L.	0	n	1	0	Poaceae Barnhart
Iz_6_1009	DJI_0324	Dianthus campestris M.Bieb.	0	n	1	0	Caryophyllaceae Juss.
Iz_6_1009	DJI_0324	Digitaria sanguinalis (L.) Scop.	0	a	0	0	Poaceae Barnhart
Iz_6_1009	DJI_0324	Elymus repens (L.) Gould	0	n	1	0	Poaceae Barnhart
Iz_6_1009	DJI_0324	Epilobium tetragonum L.	0	n	1	0	Onagraceae Juss.
Iz_6_1009	DJI_0324	Eragrostis minor Host	0	a	0	0	Poaceae Barnhart
Iz_6_1009	DJI_0324	Erigeron canadensis L.	2	a	0	0	Asteraceae Bercht. & J.Presl
Iz_6_1009	DJI_0324	Euphorbia seguieriana Neck.	0	n	1	0	Euphorbiaceae Juss.
Iz_6_1009	DJI_0324	Fallopia dumetorum (L.) Holub	0	n	1	0	Polygonaceae Juss.
Iz_6_1009	DJI_0324	Festuca beckeri (Hack.) Trautv.	0	n	1	0	Poaceae Barnhart
Iz_6_1009	DJI_0324	Fragaria vesca L.	0	n	1	0	Rosaceae Juss.
Iz_6_1009	DJI_0324	Frangula alnus Mill.	0	n	1	1	Rhamnaceae Juss.
Iz_6_1009	DJI_0324	Fraxinus pennsylvanica Marshall	0	a	0	1	Oleaceae Hoffmanns. & Link
Iz_6_1009	DJI_0324	Galeopsis bifida Boenn.	0	n	1	0	Lamiacea Lindl.
Iz_6_1009	DJI_0324	Galium verum L.	0	n	1	0	Rubiaceae Juss.
Iz_6_1009	DJI_0324	Genista tinctoria L.	0	n	1	0	Fabaceae Lindl.
Iz_6_1009	DJI_0324	Geranium robertianum L.	0	n	1	0	Geraniaceae Juss.
Iz_6_1009	DJI_0324	Geranium sanquineum L.	0	n	1	0	Geraniaceae Juss.
Iz_6_1009	DJI_0324	Gypsophila paniculata L.	0	n	1	0	Caryophyllaceae Juss.
Iz_6_1009	DJI_0324	Helichrysum arenarium (L.) Moench	0	n	1	0	Asteraceae Bercht. & J.Presl
Iz_6_1009	DJI_0324	Herniaria polygama J.Gay	0	n	1	0	Caryophyllaceae Juss.
Iz_6_1009	DJI_0324	Hieracium robustum Fr.	0	n	1	0	Asteraceae Bercht. & J.Presl
Iz_6_1009	DJI_0324	Hieracium umbellatum L.	0	n	1	NA	Asteraceae Bercht. & J.Presl
Iz_6_1009	DJI_0324	Hylotelephium maximum (L.) Holub	0	n	1	0	Crassulaceae J.St.-Hil.
Iz_6_1009	DJI_0324	Jacobaea borysthenica (DC.) B.Nord. & Greuter	0	n	1	0	Asteraceae Bercht. & J.Presl
Iz_6_1009	DJI_0324	Jasione montana L.	0	n	1	0	Campanulaceae Juss.
Iz_6_1009	DJI_0324	Jurinea cyanoides (L.) Rchb.s.l.	0	n	1	0	Asteraceae Bercht. & J.Presl
Iz_6_1009	DJI_0324	Koeleria glauca (Spreng.) DC.	0	n	1	0	Poaceae Barnhart
Iz_6_1009	DJI_0324	Lactuca serriola L.	0	a	0	0	Asteraceae Bercht. & J.Presl
Iz_6_1009	DJI_0324	Linaria genistifolia (L.) Mill.	0	n	1	0	Plantaginaceae Juss.
Iz_6_1009	DJI_0324	Lomelosia argentea (L.) Greuter & Burdet	0	n	1	0	Caprifoliaceae Juss.
Iz_6_1009	DJI_0324	Melampyrum pratense L.	0	n	1	0	Orobanchaceae Vent.
Iz_6_1009	DJI_0324	Oenothera biennis L.	0	a	0	0	Onagraceae Juss.
Iz_6_1009	DJI_0324	Pentanema britannica (L.) D.Gut.Larr., Santos-Vicente, Anderb., E.Rico & M.M.Mart.Ort.	0	n	1	0	Asteraceae Bercht. & J.Presl
Iz_6_1009	DJI_0324	Peucedanum oreoselinum (L.) Moench	1	n	1	0	Apiaceae Lindl.
Iz_6_1009	DJI_0324	Peucedanum ruthenicum M.Bieb.	0	n	1	0	Apiaceae Lindl.
Iz_6_1009	DJI_0324	Pilosella echioides (Lumn.) F.W.Schultz & Sch.Bip.	0	n	1	0	Asteraceae Bercht. & J.Presl
Iz_6_1009	DJI_0324	Pilosella officinarum F. Schultz et Sch. Bip.	0	n	1	0	Asteraceae Bercht. & J.Presl
Iz_6_1009	DJI_0324	Pilosella sp.	0		NA	0	Asteraceae Bercht. & J.Presl
Iz_6_1009	DJI_0324	Pinus sylvestris L.	0	n	1	1	Pinaceae Spreng. ex F.Rudolphi
Iz_6_1009	DJI_0324	Poa annua L.	0	n	1	0	Poaceae Barnhart
Iz_6_1009	DJI_0324	Poa nemoralis L.	0	n	1	0	Poaceae Barnhart
Iz_6_1009	DJI_0324	Poa sp.	0		NA	0	Poaceae Barnhart
Iz_6_1009	DJI_0324	Polygonatum odoratum (Mill.) Druce	0	n	1	0	Asparagaceae Juss.
Iz_6_1009	DJI_0324	Polygonum arenarium Waldst. & Kit.	0	n	1	0	Polygonaceae Juss.
Iz_6_1009	DJI_0324	Populus nigra L.	0	n	1	1	Salicaceae Mirb.
Iz_6_1009	DJI_0324	Populus tremula L.	0	n	1	1	Salicaceae Mirb.
Iz_6_1009	DJI_0324	Populus Ã canescens (Aiton) Sm.	0	n	1	1	Salicaceae Mirb.
Iz_6_1009	DJI_0324	Potentilla argentea L.	0	n	1	0	Rosaceae Juss.
Iz_6_1009	DJI_0324	Psammophiliella muralis (L.) Ikonn.	0	n	1	0	Caryophyllaceae Juss.
Iz_6_1009	DJI_0324	Psephellus sumensis (Kalen.) Greuter	0	n	1	0	Asteraceae Bercht. & J.Presl
Iz_6_1009	DJI_0324	Pulsatilla pratensis (L.) Mill. s.l.	0	n	1	0	Ranunculaceae Juss.
Iz_6_1009	DJI_0324	Pyrus communis L.	0	a	0	1	Rosaceae Juss.
Iz_6_1009	DJI_0324	Quercus robur L.	0	n	1	1	Fagaceae Dumort.
Iz_6_1009	DJI_0324	Robinia pseudoacacia L.	0	a	0	1	Fabaceae Lindl.
Iz_6_1009	DJI_0324	Rosa sp.	0		NA	1	Rosaceae Juss.
Iz_6_1009	DJI_0324	Rubus idaeus L.	0	n	1	0	Rosaceae Juss.
Iz_6_1009	DJI_0324	Rumex acetosella L.	0	n	1	0	Polygonaceae Juss.
Iz_6_1009	DJI_0324	Rumex thyrsiflorus Fingerh.	0	n	1	0	Polygonaceae Juss.
Iz_6_1009	DJI_0324	Salix alba L.	0	n	1	1	Salicaceae Mirb.
Iz_6_1009	DJI_0324	Sambucus nigra L.	0	n	1	1	Viburnaceae Raf.
Iz_6_1009	DJI_0324	Secale sylvestre Host	0	n	1	0	Poaceae Barnhart
Iz_6_1009	DJI_0324	Sempervivum ruthenicum Schnittsp. & C.B. Lehm.	0	n	1	0	Crassulaceae J.St.-Hil.
Iz_6_1009	DJI_0324	Setaria viridis (L.) P. Beauv.	0	n	1	0	Poaceae Barnhart
Iz_6_1009	DJI_0324	Silene borysthenica (Gruner) Walters	0	n	1	0	Caryophyllaceae Juss.
Iz_6_1009	DJI_0324	Silene latifolia subsp. alba (Mill.) Greuter & Burdet	0	n	1	0	Caryophyllaceae Juss.
Iz_6_1009	DJI_0324	Solidago virgaurea L.	0	n	1	0	Asteraceae Bercht. & J.Presl
Iz_6_1009	DJI_0324	Sorbus aucuparia L.	0	n	1	1	Rosaceae Juss.
Iz_6_1009	DJI_0324	Stachys recta L.	0	n	1	0	Lamiacea Lindl.
Iz_6_1009	DJI_0324	Tanacetum vulgare L.	0	n	1	0	Asteraceae Bercht. & J.Presl
Iz_6_1009	DJI_0324	Thymus pallasianus Heinr.Braun	0	n	1	0	Lamiacea Lindl.
Iz_6_1009	DJI_0324	Tilia cordata Mill.	0	n	1	1	Malvaceae Juss.
Iz_6_1009	DJI_0324	Trifolium alpestre L.	0	n	1	0	Fabaceae Lindl.
Iz_6_1009	DJI_0324	Trifolium arvense L.	0	n	1	0	Fabaceae Lindl.
Iz_6_1009	DJI_0324	Ulmus glabra Huds.	0	n	1	1	Ulmaceae Mirb.
Iz_6_1009	DJI_0324	Verbascum densiflorum Bertol.	0	n	1	0	Scrophulariaceae Juss.
Iz_6_1009	DJI_0324	Veronica austriaca L.	0	n	1	0	Plantaginaceae Juss.
Iz_6_1009	DJI_0324	Veronica incana L.	0	n	1	0	Plantaginaceae Juss.
Iz_6_1009	DJI_0324	Veronica verna L.	0	n	1	0	Plantaginaceae Juss.
Iz_6_1009	DJI_0324	Vincetoxicum hirundinaria Medik	0	n	1	0	Apocynaceae Juss.
Iz_6_1009	DJI_0326	Acer tataricum L.	0	n	1	1	Sapindaceae Juss.
Iz_6_1009	DJI_0326	Achillea millefolium L. s.l.	0	n	1	0	Asteraceae Bercht. & J.Presl
Iz_6_1009	DJI_0326	Allium oleraceum L.	0	n	1	0	Amaryllidaceae J.St.-Hil.
Iz_6_1009	DJI_0326	Ambrosia artemisiifolia L.	0	a	0	0	Asteraceae Bercht. & J.Presl
Iz_6_1009	DJI_0326	Amorpha fruticosa L.	0	a	0	0	Fabaceae Lindl.
Iz_6_1009	DJI_0326	Anthericum ramosum L.	0	n	1	0	Asparagaceae Juss.
Iz_6_1009	DJI_0326	Artemisia marschalliana Spreng.	0	n	1	0	Asteraceae Bercht. & J.Presl
Iz_6_1009	DJI_0326	Asparagus officinalis L.	0	n	1	0	Asparagaceae Juss.
Iz_6_1009	DJI_0326	Astragalus cicer L.	0	n	1	0	Fabaceae Lindl.
Iz_6_1009	DJI_0326	Bassia laniflora (S.G.Gmel.) A.J.Scott	0	n	1	0	Amaranthaceae Juss.
Iz_6_1009	DJI_0326	Berberis vulgaris L.	0	n	1	0	Berberidaceae Juss.
Iz_6_1009	DJI_0326	Betula pendula Roth.	0	n	1	1	Betulaceae Gray
Iz_6_1009	DJI_0326	Betula pendula Roth.	0	n	1	0	Poaceae Barnhart
Iz_6_1009	DJI_0326	Bromus inermis Leyss.	0		NA	NA	
Iz_6_1009	DJI_0326	Bromus squarrosus L.	0	a	0	0	Poaceae Barnhart

Iz_6_1009	DJI_0326	Bromus tectorum L.	0	a	0	0	Poaceae Barnhart
Iz_6_1009	DJI_0326	Calamagrostis epigejos (L.) Roth	40	n	1	0	Poaceae Barnhart
Iz_6_1009	DJI_0326	Carduus acanthoides L.	0	n	1	0	Asteraceae Bercht. & J.Presl
Iz_6_1009	DJI_0326	Carex sp.	0	n	1	0	Cyperaceae Juss.
Iz_6_1009	DJI_0326	Centaurea majorovii Dumbadze	0	n	1	0	Asteraceae Bercht. & J.Presl
Iz_6_1009	DJI_0326	Chamaecytisus ruthenicus (Fisch.ex Wol.) Klaskova	0	n	1	0	Fabaceae Lindl.
Iz_6_1009	DJI_0326	Chelidonium majus L.	0	n	1	0	Papaveraceae Juss.
Iz_6_1009	DJI_0326	Chenopodium betaceum Andrz.	0	n	1	0	Amaranthaceae Juss.
Iz_6_1009	DJI_0326	Chenopodium cf. album	0.5	n	1	0	Amaranthaceae Juss.
Iz_6_1009	DJI_0326	Chenopodium sp.	0		NA	0	Amaranthaceae Juss.
Iz_6_1009	DJI_0326	Chondrilla juncea L.	0	n	1	0	Asteraceae Bercht. & J.Presl
Iz_6_1009	DJI_0326	Cirsium vulgare (Savi) Ten.	0	n	1	0	Asteraceae Bercht. & J.Presl
Iz_6_1009	DJI_0326	Clinopodium acinos (L.) Kuntze	0	n	1	0	Lamiacea Lindl.
Iz_6_1009	DJI_0326	Convallaria majalis L.	0	n	1	0	Asparagaceae Juss.
Iz_6_1009	DJI_0326	Crataegus rhipidophylla Gand.	0	n	1	1	Rosaceae Juss.
Iz_6_1009	DJI_0326	Crepis foetida L.	0	n	1	0	Asteraceae Bercht. & J.Presl
Iz_6_1009	DJI_0326	Dactylis glomerata L.	0	n	1	0	Poaceae Barnhart
Iz_6_1009	DJI_0326	Dianthus campestris M.Bieb.	0	n	1	0	Caryophyllaceae Juss.
Iz_6_1009	DJI_0326	Digitaria sanguinalis (L.) Scop.	0	a	0	0	Poaceae Barnhart
Iz_6_1009	DJI_0326	Elymus repens (L.) Gould	0	n	1	0	Poaceae Barnhart
Iz_6_1009	DJI_0326	Epilobium tetragonum L.	0	n	1	0	Onagraceae Juss.
Iz_6_1009	DJI_0326	Eragrostis minor Host	0	a	0	0	Poaceae Barnhart
Iz_6_1009	DJI_0326	Erigeron canadensis L.	3	a	0	0	Asteraceae Bercht. & J.Presl
Iz_6_1009	DJI_0326	Euphorbia seguieriana Neck.	0	n	1	0	Euphorbiaceae Juss.
Iz_6_1009	DJI_0326	Fallopia dumetorum (L.) Holub	0	n	1	0	Polygonaceae Juss.
Iz_6_1009	DJI_0326	Festuca beckeri (Hack.) Trautv.	0	n	1	0	Poaceae Barnhart
Iz_6_1009	DJI_0326	Fragaria vesca L.	0	n	1	0	Rosaceae Juss.
Iz_6_1009	DJI_0326	Frangula alnus Mill.	0	n	1	1	Rhamnaceae Juss.
Iz_6_1009	DJI_0326	Fraxinus pennsylvanica Marshall	0	a	0	1	Oleaceae Hoffmanns. & Link
Iz_6_1009	DJI_0326	Galeopsis bifida Boenn.	0	n	1	0	Lamiacea Lindl.
Iz_6_1009	DJI_0326	Galium verum L.	0	n	1	0	Rubiaceae Juss.
Iz_6_1009	DJI_0326	Genista tinctoria L.	0	n	1	0	Fabaceae Lindl.
Iz_6_1009	DJI_0326	Geranium robertianum L.	0	n	1	0	Geraniaceae Juss.
Iz_6_1009	DJI_0326	Geranium sanquineum L.	0	n	1	0	Geraniaceae Juss.
Iz_6_1009	DJI_0326	Gypsophila paniculata L.	0	n	1	0	Caryophyllaceae Juss.
Iz_6_1009	DJI_0326	Helichrysum arenarium (L.) Moench	0	n	1	0	Asteraceae Bercht. & J.Presl
Iz_6_1009	DJI_0326	Herniaria polygama J.Gay	0	n	1	0	Caryophyllaceae Juss.
Iz_6_1009	DJI_0326	Hieracium robustum Fr.	0	n	1	0	Asteraceae Bercht. & J.Presl
Iz_6_1009	DJI_0326	Hieracium umbellatum L.	0	n	1	NA	Asteraceae Bercht. & J.Presl
Iz_6_1009	DJI_0326	Hylotelephium maximum (L.) Holub	0	n	1	0	Crassulaceae J.St.-Hil.
Iz_6_1009	DJI_0326	Jacobaea borysthenica (DC.) B.Nord. & Greuter	0	n	1	0	Asteraceae Bercht. & J.Presl
Iz_6_1009	DJI_0326	Jasione montana L.	0	n	1	0	Campanulaceae Juss.
Iz_6_1009	DJI_0326	Jurinea cyanoides (L.) Rchb.s.l.	0	n	1	0	Asteraceae Bercht. & J.Presl
Iz_6_1009	DJI_0326	Koeleria glauca (Spreng.) DC.	0	n	1	0	Poaceae Barnhart
Iz_6_1009	DJI_0326	Lactuca serriola L.	0	a	0	0	Asteraceae Bercht. & J.Presl
Iz_6_1009	DJI_0326	Linaria genistifolia (L.) Mill.	0	n	1	0	Plantaginaceae Juss.
Iz_6_1009	DJI_0326	Lomelosia argentea (L.) Greuter & Burdet	0	n	1	0	Caprifoliaceae Juss.
Iz_6_1009	DJI_0326	Melampyrum pratense L.	0	n	1	0	Orobanchaceae Vent.
Iz_6_1009	DJI_0326	Oenothera biennis L.	0	a	0	0	Onagraceae Juss.
Iz_6_1009	DJI_0326	Pentanema britannica (L.) D.Gut.Larr., Santos-Vicente, Anderb., E.Rico & M.M.Mart.Ort.	0	n	1	0	Asteraceae Bercht. & J.Presl
Iz_6_1009	DJI_0326	Peucedanum oreoselinum (L.) Moench	1.5	n	1	0	Apiaceae Lindl.
Iz_6_1009	DJI_0326	Peucedanum ruthenicum M.Bieb.	0	n	1	0	Apiaceae Lindl.
Iz_6_1009	DJI_0326	Pilosella echioides (Lumn.) F.W.Schultz & Sch.Bip.	0	n	1	0	Asteraceae Bercht. & J.Presl
Iz_6_1009	DJI_0326	Pilosella officinarum F. Schultz et Sch. Bip.	0	n	1	0	Asteraceae Bercht. & J.Presl
Iz_6_1009	DJI_0326	Pilosella sp.	0		NA	0	Asteraceae Bercht. & J.Presl
Iz_6_1009	DJI_0326	Pinus sylvestris L.	0	n	1	1	Pinaceae Spreng. ex F.Rudolphi
Iz_6_1009	DJI_0326	Poa annua L.	0	n	1	0	Poaceae Barnhart
Iz_6_1009	DJI_0326	Poa nemoralis L.	0	n	1	0	Poaceae Barnhart
Iz_6_1009	DJI_0326	Poa sp.	0		NA	0	Poaceae Barnhart
Iz_6_1009	DJI_0326	Polygonatum odoratum (Mill.) Druce	0	n	1	0	Asparagaceae Juss.
Iz_6_1009	DJI_0326	Polygonum arenarium Waldst. & Kit.	0	n	1	0	Polygonaceae Juss.
Iz_6_1009	DJI_0326	Populus nigra L.	0	n	1	1	Salicaceae Mirb.
Iz_6_1009	DJI_0326	Populus tremula L.	0	n	1	1	Salicaceae Mirb.
Iz_6_1009	DJI_0326	Populus Ã canescens (Aiton) Sm.	0	n	1	1	Salicaceae Mirb.
Iz_6_1009	DJI_0326	Potentilla argentea L.	0	n	1	0	Rosaceae Juss.
Iz_6_1009	DJI_0326	Psammophiliella muralis (L.) Ikonn.	0	n	1	0	Caryophyllaceae Juss.
Iz_6_1009	DJI_0326	Psephellus sumensis (Kalen.) Greuter	0	n	1	0	Asteraceae Bercht. & J.Presl
Iz_6_1009	DJI_0326	Pulsatilla pratensis (L.) Mill. s.l.	0	n	1	0	Ranunculaceae Juss.
Iz_6_1009	DJI_0326	Pyrus communis L.	0	a	0	1	Rosaceae Juss.
Iz_6_1009	DJI_0326	Quercus robur L.	0	n	1	1	Fagaceae Dumort.
Iz_6_1009	DJI_0326	Robinia pseudoacacia L.	0	a	0	1	Fabaceae Lindl.
Iz_6_1009	DJI_0326	Rosa sp.	0		NA	1	Rosaceae Juss.
Iz_6_1009	DJI_0326	Rubus idaeus L.	0	n	1	0	Rosaceae Juss.
Iz_6_1009	DJI_0326	Rumex acetosella L.	0	n	1	0	Polygonaceae Juss.
Iz_6_1009	DJI_0326	Rumex thyrsiflorus Fingerh.	0	n	1	0	Polygonaceae Juss.
Iz_6_1009	DJI_0326	Salix alba L.	0	n	1	1	Salicaceae Mirb.
Iz_6_1009	DJI_0326	Sambucus nigra L.	0	n	1	1	Viburnaceae Raf.
Iz_6_1009	DJI_0326	Secale sylvestre Host	0	n	1	0	Poaceae Barnhart
Iz_6_1009	DJI_0326	Sempervivum ruthenicum Schnittsp. & C.B. Lehm.	0	n	1	0	Crassulaceae J.St.-Hil.
Iz_6_1009	DJI_0326	Setaria viridis (L.) P. Beauv.	0	n	1	0	Poaceae Barnhart
Iz_6_1009	DJI_0326	Silene borysthenica (Gruner) Walters	0	n	1	0	Caryophyllaceae Juss.
Iz_6_1009	DJI_0326	Silene latifolia subsp. alba (Mill.) Greuter & Burdet	0	n	1	0	Caryophyllaceae Juss.
Iz_6_1009	DJI_0326	Solidago virgaurea L.	0	n	1	0	Asteraceae Bercht. & J.Presl
Iz_6_1009	DJI_0326	Sorbus aucuparia L.	0	n	1	1	Rosaceae Juss.
Iz_6_1009	DJI_0326	Stachys recta L.	0	n	1	0	Lamiacea Lindl.
Iz_6_1009	DJI_0326	Tanacetum vulgare L.	0	n	1	0	Asteraceae Bercht. & J.Presl
Iz_6_1009	DJI_0326	Thymus pallasianus Heinr.Braun	0	n	1	0	Lamiacea Lindl.
Iz_6_1009	DJI_0326	Tilia cordata Mill.	0	n	1	1	Malvaceae Juss.
Iz_6_1009	DJI_0326	Trifolium alpestre L.	0	n	1	0	Fabaceae Lindl.
Iz_6_1009	DJI_0326	Trifolium arvense L.	0	n	1	0	Fabaceae Lindl.
Iz_6_1009	DJI_0326	Ulmus glabra Huds.	0	n	1	1	Ulmaceae Mirb.
Iz_6_1009	DJI_0326	Verbascum densiflorum Bertol.	0	n	1	0	Scrophulariaceae Juss.
Iz_6_1009	DJI_0326	Veronica austriaca L.	0	n	1	0	Plantaginaceae Juss.
Iz_6_1009	DJI_0326	Veronica incana L.	0	n	1	0	Plantaginaceae Juss.
Iz_6_1009	DJI_0326	Veronica verna L.	0	n	1	0	Plantaginaceae Juss.
Iz_6_1009	DJI_0326	Vincetoxicum hirundinaria Medik	0	n	1	0	Apocynaceae Juss.
Iz_6_1009	DJI_0335	Acer tataricum L.	0	n	1	1	Sapindaceae Juss.
Iz_6_1009	DJI_0335	Achillea millefolium L. s.l.	0	n	1	0	Asteraceae Bercht. & J.Presl

Iz_6_1009	DJI_0335	Allium oleraceum L.	0	n	1	0	Amaryllidaceae J.St.-Hil.
Iz_6_1009	DJI_0335	Ambrosia artemisiifolia L.	0	a	0	0	Asteraceae Bercht. & J.Presl
Iz_6_1009	DJI_0335	Amorpha fruticosa L.	0	a	0	0	Fabaceae Lindl.
Iz_6_1009	DJI_0335	Anthericum ramosum L.	0	n	1	0	Asparagaceae Juss.
Iz_6_1009	DJI_0335	Artemisia marschalliana Spreng.	0	n	1	0	Asteraceae Bercht. & J.Presl
Iz_6_1009	DJI_0335	Asparagus officinalis L.	0	n	1	0	Asparagaceae Juss.
Iz_6_1009	DJI_0335	Astragalus cicer L.	0	n	1	0	Fabaceae Lindl.
Iz_6_1009	DJI_0335	Bassia laniflora (S.G.Gmel.) A.J.Scott	0	n	1	0	Amaranthaceae Juss.
Iz_6_1009	DJI_0335	Berberis vulgaris L.	0	n	1	0	Berberidaceae Juss.
Iz_6_1009	DJI_0335	Betula pendula Roth.	0	n	1	1	Betulaceae Gray
Iz_6_1009	DJI_0335	Betula pendula Roth.	0	n	1	0	Poaceae Barnhart
Iz_6_1009	DJI_0335	Bromus inermis Leyss.	0		NA	NA	
Iz_6_1009	DJI_0335	Bromus squarrosus L.	0	a	0	0	Poaceae Barnhart
Iz_6_1009	DJI_0335	Bromus tectorum L.	0	a	0	0	Poaceae Barnhart
Iz_6_1009	DJI_0335	Calamagrostis epigejos (L.) Roth	0	n	1	0	Poaceae Barnhart
Iz_6_1009	DJI_0335	Carduus acanthoides L.	0	n	1	0	Asteraceae Bercht. & J.Presl
Iz_6_1009	DJI_0335	Carex sp.	0	n	1	0	Cyperaceae Juss.
Iz_6_1009	DJI_0335	Centaurea majorovii Dumbadze	0	n	1	0	Asteraceae Bercht. & J.Presl
Iz_6_1009	DJI_0335	Chamaecytisus ruthenicus (Fisch.ex Wol.) Klaskova	0	n	1	0	Fabaceae Lindl.
Iz_6_1009	DJI_0335	Chelidonium majus L.	5	n	1	0	Papaveraceae Juss.
Iz_6_1009	DJI_0335	Chenopodium betaceum Andrz.	0	n	1	0	Amaranthaceae Juss.
Iz_6_1009	DJI_0335	Chenopodium cf. album	0	n	1	0	Amaranthaceae Juss.
Iz_6_1009	DJI_0335	Chenopodium sp.	0		NA	0	Amaranthaceae Juss.
Iz_6_1009	DJI_0335	Chondrilla juncea L.	0	n	1	0	Asteraceae Bercht. & J.Presl
Iz_6_1009	DJI_0335	Cirsium vulgare (Savi) Ten.	0	n	1	0	Asteraceae Bercht. & J.Presl
Iz_6_1009	DJI_0335	Clinopodium acinos (L.) Kuntze	0	n	1	0	Lamiacea Lindl.
Iz_6_1009	DJI_0335	Convallaria majalis L.	0	n	1	0	Asparagaceae Juss.
Iz_6_1009	DJI_0335	Crataegus rhipidophylla Gand.	0	n	1	1	Rosaceae Juss.
Iz_6_1009	DJI_0335	Crepis foetida L.	0	n	1	0	Asteraceae Bercht. & J.Presl
Iz_6_1009	DJI_0335	Dactylis glomerata L.	0	n	1	0	Poaceae Barnhart
Iz_6_1009	DJI_0335	Dianthus campestris M.Bieb.	0	n	1	0	Caryophyllaceae Juss.
Iz_6_1009	DJI_0335	Digitaria sanguinalis (L.) Scop.	0	a	0	0	Poaceae Barnhart
Iz_6_1009	DJI_0335	Elymus repens (L.) Gould	0	n	1	0	Poaceae Barnhart
Iz_6_1009	DJI_0335	Epilobium tetragonum L.	0	n	1	0	Onagraceae Juss.
Iz_6_1009	DJI_0335	Eragrostis minor Host	0	a	0	0	Poaceae Barnhart
Iz_6_1009	DJI_0335	Erigeron canadensis L.	2	a	0	0	Asteraceae Bercht. & J.Presl
Iz_6_1009	DJI_0335	Euphorbia seguieriana Neck.	0	n	1	0	Euphorbiaceae Juss.
Iz_6_1009	DJI_0335	Fallopia dumetorum (L.) Holub	0	n	1	0	Polygonaceae Juss.
Iz_6_1009	DJI_0335	Festuca beckeri (Hack.) Trautv.	0	n	1	0	Poaceae Barnhart
Iz_6_1009	DJI_0335	Fragaria vesca L.	0	n	1	0	Rosaceae Juss.
Iz_6_1009	DJI_0335	Frangula alnus Mill.	0	n	1	1	Rhamnaceae Juss.
Iz_6_1009	DJI_0335	Fraxinus pennsylvanica Marshall	0	a	0	1	Oleaceae Hoffmanns. & Link
Iz_6_1009	DJI_0335	Galeopsis bifida Boenn.	0	n	1	0	Lamiacea Lindl.
Iz_6_1009	DJI_0335	Galium verum L.	0	n	1	0	Rubiaceae Juss.
Iz_6_1009	DJI_0335	Genista tinctoria L.	0	n	1	0	Fabaceae Lindl.
Iz_6_1009	DJI_0335	Geranium robertianum L.	3	n	1	0	Geraniaceae Juss.
Iz_6_1009	DJI_0335	Geranium sanquineum L.	0	n	1	0	Geraniaceae Juss.
Iz_6_1009	DJI_0335	Gypsophila paniculata L.	0	n	1	0	Caryophyllaceae Juss.
Iz_6_1009	DJI_0335	Helichrysum arenarium (L.) Moench	0	n	1	0	Asteraceae Bercht. & J.Presl
Iz_6_1009	DJI_0335	Herniaria polygama J.Gay	0	n	1	0	Caryophyllaceae Juss.
Iz_6_1009	DJI_0335	Hieracium robustum Fr.	0	n	1	0	Asteraceae Bercht. & J.Presl
Iz_6_1009	DJI_0335	Hieracium umbellatum L.	0	n	1	NA	Asteraceae Bercht. & J.Presl
Iz_6_1009	DJI_0335	Hylotelephium maximum (L.) Holub	0	n	1	0	Crassulaceae J.St.-Hil.
Iz_6_1009	DJI_0335	Jacobaea borysthenica (DC.) B.Nord. & Greuter	0	n	1	0	Asteraceae Bercht. & J.Presl
Iz_6_1009	DJI_0335	Jasione montana L.	0	n	1	0	Campanulaceae Juss.
Iz_6_1009	DJI_0335	Jurinea cyanoides (L.) Rchb.s.l.	0	n	1	0	Asteraceae Bercht. & J.Presl
Iz_6_1009	DJI_0335	Koeleria glauca (Spreng.) DC.	0	n	1	0	Poaceae Barnhart
Iz_6_1009	DJI_0335	Lactuca serriola L.	0	a	0	0	Asteraceae Bercht. & J.Presl
Iz_6_1009	DJI_0335	Linaria genistifolia (L.) Mill.	0	n	1	0	Plantaginaceae Juss.
Iz_6_1009	DJI_0335	Lomelosia argentea (L.) Greuter & Burdet	0	n	1	0	Caprifoliaceae Juss.
Iz_6_1009	DJI_0335	Melampyrum pratense L.	0	n	1	0	Orobanchaceae Vent.
Iz_6_1009	DJI_0335	Oenothera biennis L.	0	a	0	0	Onagraceae Juss.
Iz_6_1009	DJI_0335	Pentanema britannica (L.) D.Gut.Larr., Santos-Vicente, Anderb., E.Rico & M.M.Mart.Ort.	0	n	1	0	Asteraceae Bercht. & J.Presl
Iz_6_1009	DJI_0335	Peucedanum oreoselinum (L.) Moench	0	n	1	0	Apiaceae Lindl.
Iz_6_1009	DJI_0335	Peucedanum ruthenicum M.Bieb.	0	n	1	0	Apiaceae Lindl.
Iz_6_1009	DJI_0335	Pilosella echioides (Lumn.) F.W.Schultz & Sch.Bip.	0	n	1	0	Asteraceae Bercht. & J.Presl
Iz_6_1009	DJI_0335	Pilosella officinarum F. Schultz et Sch. Bip.	0	n	1	0	Asteraceae Bercht. & J.Presl
Iz_6_1009	DJI_0335	Pilosella sp.	0		NA	0	Asteraceae Bercht. & J.Presl
Iz_6_1009	DJI_0335	Pinus sylvestris L.	0	n	1	1	Pinaceae Spreng. ex F.Rudolphi
Iz_6_1009	DJI_0335	Poa annua L.	0	n	1	0	Poaceae Barnhart
Iz_6_1009	DJI_0335	Poa nemoralis L.	0	n	1	0	Poaceae Barnhart
Iz_6_1009	DJI_0335	Poa sp.	0		NA	0	Poaceae Barnhart
Iz_6_1009	DJI_0335	Polygonatum odoratum (Mill.) Druce	0	n	1	0	Asparagaceae Juss.
Iz_6_1009	DJI_0335	Polygonum arenarium Waldst. & Kit.	0	n	1	0	Polygonaceae Juss.
Iz_6_1009	DJI_0335	Populus nigra L.	0	n	1	1	Salicaceae Mirb.
Iz_6_1009	DJI_0335	Populus tremula L.	0	n	1	1	Salicaceae Mirb.
Iz_6_1009	DJI_0335	Populus Ã canescens (Aiton) Sm.	0	n	1	1	Salicaceae Mirb.
Iz_6_1009	DJI_0335	Potentilla argentea L.	0	n	1	0	Rosaceae Juss.
Iz_6_1009	DJI_0335	Psammophiliella muralis (L.) Ikonn.	0	n	1	0	Caryophyllaceae Juss.
Iz_6_1009	DJI_0335	Psephellus sumensis (Kalen.) Greuter	0	n	1	0	Asteraceae Bercht. & J.Presl
Iz_6_1009	DJI_0335	Pulsatilla pratensis (L.) Mill. s.l.	0	n	1	0	Ranunculaceae Juss.
Iz_6_1009	DJI_0335	Pyrus communis L.	0	a	0	1	Rosaceae Juss.
Iz_6_1009	DJI_0335	Quercus robur L.	0	n	1	1	Fagaceae Dumort.
Iz_6_1009	DJI_0335	Robinia pseudoacacia L.	0	a	0	1	Fabaceae Lindl.
Iz_6_1009	DJI_0335	Rosa sp.	0		NA	1	Rosaceae Juss.
Iz_6_1009	DJI_0335	Rubus idaeus L.	0	n	1	0	Rosaceae Juss.
Iz_6_1009	DJI_0335	Rumex acetosella L.	0	n	1	0	Polygonaceae Juss.
Iz_6_1009	DJI_0335	Rumex thyrsiflorus Fingerh.	0	n	1	0	Polygonaceae Juss.
Iz_6_1009	DJI_0335	Salix alba L.	0	n	1	1	Salicaceae Mirb.
Iz_6_1009	DJI_0335	Sambucus nigra L.	0	n	1	1	Viburnaceae Raf.
Iz_6_1009	DJI_0335	Secale sylvestre Host	0	n	1	0	Poaceae Barnhart
Iz_6_1009	DJI_0335	Sempervivum ruthenicum Schnittsp. & C.B. Lehm.	0	n	1	0	Crassulaceae J.St.-Hil.
Iz_6_1009	DJI_0335	Setaria viridis (L.) P. Beauv.	0	n	1	0	Poaceae Barnhart
Iz_6_1009	DJI_0335	Silene borysthenica (Gruner) Walters	0	n	1	0	Caryophyllaceae Juss.
Iz_6_1009	DJI_0335	Silene latifolia subsp. alba (Mill.) Greuter & Burdet	0	n	1	0	Caryophyllaceae Juss.
Iz_6_1009	DJI_0335	Solidago virgaurea L.	0	n	1	0	Asteraceae Bercht. & J.Presl
Iz_6_1009	DJI_0335	Sorbus aucuparia L.	0	n	1	1	Rosaceae Juss.
Iz_6_1009	DJI_0335	Stachys recta L.	0	n	1	0	Lamiacea Lindl.

Iz_6_1009	DJI_0335	Tanacetum vulgare L.	0	n	1	0	Asteraceae Bercht. & J.Presl
Iz_6_1009	DJI_0335	Thymus pallasianus Heinr.Braun	0	n	1	0	Lamiacea Lindl.
Iz_6_1009	DJI_0335	Tilia cordata Mill.	0	n	1	1	Malvaceae Juss.
Iz_6_1009	DJI_0335	Trifolium alpestre L.	0	n	1	0	Fabaceae Lindl.
Iz_6_1009	DJI_0335	Trifolium arvense L.	0	n	1	0	Fabaceae Lindl.
Iz_6_1009	DJI_0335	Ulmus glabra Huds.	0	n	1	1	Ulmaceae Mirb.
Iz_6_1009	DJI_0335	Verbascum densiflorum Bertol.	0	n	1	0	Scrophulariaceae Juss.
Iz_6_1009	DJI_0335	Veronica austriaca L.	0	n	1	0	Plantaginaceae Juss.
Iz_6_1009	DJI_0335	Veronica incana L.	0	n	1	0	Plantaginaceae Juss.
Iz_6_1009	DJI_0335	Veronica verna L.	0	n	1	0	Plantaginaceae Juss.
Iz_6_1009	DJI_0335	Vincetoxicum hirundinaria Medik	0	n	1	0	Apocynaceae Juss.
Iz_6_1009	DJI_0338	Acer tataricum L.	0	n	1	1	Sapindaceae Juss.
Iz_6_1009	DJI_0338	Achillea millefolium L. s.l.	0	n	1	0	Asteraceae Bercht. & J.Presl
Iz_6_1009	DJI_0338	Allium oleraceum L.	0	n	1	0	Amaryllidaceae J.St.-Hil.
Iz_6_1009	DJI_0338	Ambrosia artemisiifolia L.	0	a	0	0	Asteraceae Bercht. & J.Presl
Iz_6_1009	DJI_0338	Amorpha fruticosa L.	0	a	0	0	Fabaceae Lindl.
Iz_6_1009	DJI_0338	Anthericum ramosum L.	0	n	1	0	Asparagaceae Juss.
Iz_6_1009	DJI_0338	Artemisia marschalliana Spreng.	0	n	1	0	Asteraceae Bercht. & J.Presl
Iz_6_1009	DJI_0338	Asparagus officinalis L.	0	n	1	0	Asparagaceae Juss.
Iz_6_1009	DJI_0338	Astragalus cicer L.	0	n	1	0	Fabaceae Lindl.
Iz_6_1009	DJI_0338	Bassia laniflora (S.G.Gmel.) A.J.Scott	0	n	1	0	Amaranthaceae Juss.
Iz_6_1009	DJI_0338	Berberis vulgaris L.	0	n	1	0	Berberidaceae Juss.
Iz_6_1009	DJI_0338	Betula pendula Roth.	0	n	1	1	Betulaceae Gray
Iz_6_1009	DJI_0338	Betula pendula Roth.	0	n	1	0	Poaceae Barnhart
Iz_6_1009	DJI_0338	Bromus inermis Leyss.	0		NA	NA	
Iz_6_1009	DJI_0338	Bromus squarrosus L.	0	a	0	0	Poaceae Barnhart
Iz_6_1009	DJI_0338	Bromus tectorum L.	0	a	0	0	Poaceae Barnhart
Iz_6_1009	DJI_0338	Calamagrostis epigejos (L.) Roth	4	n	1	0	Poaceae Barnhart
Iz_6_1009	DJI_0338	Carduus acanthoides L.	0	n	1	0	Asteraceae Bercht. & J.Presl
Iz_6_1009	DJI_0338	Carex sp.	0	n	1	0	Cyperaceae Juss.
Iz_6_1009	DJI_0338	Centaurea majorovii Dumbadze	0	n	1	0	Asteraceae Bercht. & J.Presl
Iz_6_1009	DJI_0338	Chamaecytisus ruthenicus (Fisch.ex Wol.) Klaskova	0	n	1	0	Fabaceae Lindl.
Iz_6_1009	DJI_0338	Chelidonium majus L.	0	n	1	0	Papaveraceae Juss.
Iz_6_1009	DJI_0338	Chenopodium betaceum Andrz.	0	n	1	0	Amaranthaceae Juss.
Iz_6_1009	DJI_0338	Chenopodium cf. album	0	n	1	0	Amaranthaceae Juss.
Iz_6_1009	DJI_0338	Chenopodium sp.	0		NA	0	Amaranthaceae Juss.
Iz_6_1009	DJI_0338	Chondrilla juncea L.	0	n	1	0	Asteraceae Bercht. & J.Presl
Iz_6_1009	DJI_0338	Cirsium vulgare (Savi) Ten.	0	n	1	0	Asteraceae Bercht. & J.Presl
Iz_6_1009	DJI_0338	Clinopodium acinos (L.) Kuntze	0	n	1	0	Lamiacea Lindl.
Iz_6_1009	DJI_0338	Convallaria majalis L.	0	n	1	0	Asparagaceae Juss.
Iz_6_1009	DJI_0338	Crataegus rhipidophylla Gand.	0	n	1	1	Rosaceae Juss.
Iz_6_1009	DJI_0338	Crepis foetida L.	0	n	1	0	Asteraceae Bercht. & J.Presl
Iz_6_1009	DJI_0338	Dactylis glomerata L.	0	n	1	0	Poaceae Barnhart
Iz_6_1009	DJI_0338	Dianthus campestris M.Bieb.	0	n	1	0	Caryophyllaceae Juss.
Iz_6_1009	DJI_0338	Digitaria sanguinalis (L.) Scop.	0	a	0	0	Poaceae Barnhart
Iz_6_1009	DJI_0338	Elymus repens (L.) Gould	0	n	1	0	Poaceae Barnhart
Iz_6_1009	DJI_0338	Epilobium tetragonum L.	0	n	1	0	Onagraceae Juss.
Iz_6_1009	DJI_0338	Eragrostis minor Host	0	a	0	0	Poaceae Barnhart
Iz_6_1009	DJI_0338	Erigeron canadensis L.	3	a	0	0	Asteraceae Bercht. & J.Presl
Iz_6_1009	DJI_0338	Euphorbia seguieriana Neck.	0	n	1	0	Euphorbiaceae Juss.
Iz_6_1009	DJI_0338	Fallopia dumetorum (L.) Holub	0	n	1	0	Polygonaceae Juss.
Iz_6_1009	DJI_0338	Festuca beckeri (Hack.) Trautv.	0	n	1	0	Poaceae Barnhart
Iz_6_1009	DJI_0338	Fragaria vesca L.	0	n	1	0	Rosaceae Juss.
Iz_6_1009	DJI_0338	Frangula alnus Mill.	0	n	1	1	Rhamnaceae Juss.
Iz_6_1009	DJI_0338	Fraxinus pennsylvanica Marshall	0	a	0	1	Oleaceae Hoffmanns. & Link
Iz_6_1009	DJI_0338	Galeopsis bifida Boenn.	0	n	1	0	Lamiacea Lindl.
Iz_6_1009	DJI_0338	Galium verum L.	0	n	1	0	Rubiaceae Juss.
Iz_6_1009	DJI_0338	Genista tinctoria L.	0	n	1	0	Fabaceae Lindl.
Iz_6_1009	DJI_0338	Geranium robertianum L.	0	n	1	0	Geraniaceae Juss.
Iz_6_1009	DJI_0338	Geranium sanquineum L.	0	n	1	0	Geraniaceae Juss.
Iz_6_1009	DJI_0338	Gypsophila paniculata L.	0	n	1	0	Caryophyllaceae Juss.
Iz_6_1009	DJI_0338	Helichrysum arenarium (L.) Moench	0	n	1	0	Asteraceae Bercht. & J.Presl
Iz_6_1009	DJI_0338	Herniaria polygama J.Gay	0	n	1	0	Caryophyllaceae Juss.
Iz_6_1009	DJI_0338	Hieracium robustum Fr.	0	n	1	0	Asteraceae Bercht. & J.Presl
Iz_6_1009	DJI_0338	Hieracium umbellatum L.	0	n	1	NA	Asteraceae Bercht. & J.Presl
Iz_6_1009	DJI_0338	Hylotelephium maximum (L.) Holub	0	n	1	0	Crassulaceae J.St.-Hil.
Iz_6_1009	DJI_0338	Jacobaea borysthenica (DC.) B.Nord. & Greuter	0	n	1	0	Asteraceae Bercht. & J.Presl
Iz_6_1009	DJI_0338	Jasione montana L.	0	n	1	0	Campanulaceae Juss.
Iz_6_1009	DJI_0338	Jurinea cyanoides (L.) Rchb.s.l.	0	n	1	0	Asteraceae Bercht. & J.Presl
Iz_6_1009	DJI_0338	Koeleria glauca (Spreng.) DC.	0	n	1	0	Poaceae Barnhart
Iz_6_1009	DJI_0338	Lactuca serriola L.	0	a	0	0	Asteraceae Bercht. & J.Presl
Iz_6_1009	DJI_0338	Linaria genistifolia (L.) Mill.	0	n	1	0	Plantaginaceae Juss.
Iz_6_1009	DJI_0338	Lomelosia argentea (L.) Greuter & Burdet	0	n	1	0	Caprifoliaceae Juss.
Iz_6_1009	DJI_0338	Melampyrum pratense L.	0	n	1	0	Orobanchaceae Vent.
Iz_6_1009	DJI_0338	Oenothera biennis L.	0	a	0	0	Onagraceae Juss.
Iz_6_1009	DJI_0338	Pentanema britannica (L.) D.Gut.Larr., Santos-Vicente, Anderb., E.Rico & M.M.Mart.Ort.	0	n	1	0	Asteraceae Bercht. & J.Presl
Iz_6_1009	DJI_0338	Peucedanum oreoselinum (L.) Moench	0	n	1	0	Apiaceae Lindl.
Iz_6_1009	DJI_0338	Peucedanum ruthenicum M.Bieb.	0	n	1	0	Apiaceae Lindl.
Iz_6_1009	DJI_0338	Pilosella echioides (Lumn.) F.W.Schultz & Sch.Bip.	0	n	1	0	Asteraceae Bercht. & J.Presl
Iz_6_1009	DJI_0338	Pilosella officinarum F. Schultz et Sch. Bip.	0	n	1	0	Asteraceae Bercht. & J.Presl
Iz_6_1009	DJI_0338	Pilosella sp.	0		NA	0	Asteraceae Bercht. & J.Presl
Iz_6_1009	DJI_0338	Pinus sylvestris L.	0	n	1	1	Pinaceae Spreng. ex F.Rudolphi
Iz_6_1009	DJI_0338	Poa annua L.	0	n	1	0	Poaceae Barnhart
Iz_6_1009	DJI_0338	Poa nemoralis L.	0	n	1	0	Poaceae Barnhart
Iz_6_1009	DJI_0338	Poa sp.	0		NA	0	Poaceae Barnhart
Iz_6_1009	DJI_0338	Polygonatum odoratum (Mill.) Druce	0	n	1	0	Asparagaceae Juss.
Iz_6_1009	DJI_0338	Polygonum arenarium Waldst. & Kit.	0	n	1	0	Polygonaceae Juss.
Iz_6_1009	DJI_0338	Populus nigra L.	0	n	1	1	Salicaceae Mirb.
Iz_6_1009	DJI_0338	Populus tremula L.	0	n	1	1	Salicaceae Mirb.
Iz_6_1009	DJI_0338	Populus Ã canescens (Aiton) Sm.	0	n	1	1	Salicaceae Mirb.
Iz_6_1009	DJI_0338	Potentilla argentea L.	0	n	1	0	Rosaceae Juss.
Iz_6_1009	DJI_0338	Psammophiliella muralis (L.) Ikonn.	0	n	1	0	Caryophyllaceae Juss.
Iz_6_1009	DJI_0338	Psephellus sumensis (Kalen.) Greuter	0	n	1	0	Asteraceae Bercht. & J.Presl
Iz_6_1009	DJI_0338	Pulsatilla pratensis (L.) Mill. s.l.	0	n	1	0	Ranunculaceae Juss.
Iz_6_1009	DJI_0338	Pyrus communis L.	0	a	0	1	Rosaceae Juss.
Iz_6_1009	DJI_0338	Quercus robur L.	0	n	1	1	Fagaceae Dumort.
Iz_6_1009	DJI_0338	Robinia pseudoacacia L.	0	a	0	1	Fabaceae Lindl.
Iz_6_1009	DJI_0338	Rosa sp.	0		NA	1	Rosaceae Juss.

Iz_6_1009	DJI_0338	Rubus idaeus L.	0	n	1	0	Rosaceae Juss.
Iz_6_1009	DJI_0338	Rumex acetosella L.	0	n	1	0	Polygonaceae Juss.
Iz_6_1009	DJI_0338	Rumex thyrsiflorus Fingerh.	0	n	1	0	Polygonaceae Juss.
Iz_6_1009	DJI_0338	Salix alba L.	0	n	1	1	Salicaceae Mirb.
Iz_6_1009	DJI_0338	Sambucus nigra L.	0	n	1	1	Viburnaceae Raf.
Iz_6_1009	DJI_0338	Secale sylvestre Host	0	n	1	0	Poaceae Barnhart
Iz_6_1009	DJI_0338	Sempervivum ruthenicum Schnittsp. & C.B. Lehm.	0	n	1	0	Crassulaceae J.St.-Hil.
Iz_6_1009	DJI_0338	Setaria viridis (L.) P. Beauv.	0	n	1	0	Poaceae Barnhart
Iz_6_1009	DJI_0338	Silene borysthenica (Gruner) Walters	0	n	1	0	Caryophyllaceae Juss.
Iz_6_1009	DJI_0338	Silene latifolia subsp. alba (Mill.) Greuter & Burdet	0	n	1	0	Caryophyllaceae Juss.
Iz_6_1009	DJI_0338	Solidago virgaurea L.	0.5	n	1	0	Asteraceae Bercht. & J.Presl
Iz_6_1009	DJI_0338	Sorbus aucuparia L.	0	n	1	1	Rosaceae Juss.
Iz_6_1009	DJI_0338	Stachys recta L.	0	n	1	0	Lamiacea Lindl.
Iz_6_1009	DJI_0338	Tanacetum vulgare L.	0	n	1	0	Asteraceae Bercht. & J.Presl
Iz_6_1009	DJI_0338	Thymus pallasianus Heinr.Braun	0	n	1	0	Lamiacea Lindl.
Iz_6_1009	DJI_0338	Tilia cordata Mill.	0	n	1	1	Malvaceae Juss.
Iz_6_1009	DJI_0338	Trifolium alpestre L.	0	n	1	0	Fabaceae Lindl.
Iz_6_1009	DJI_0338	Trifolium arvense L.	0	n	1	0	Fabaceae Lindl.
Iz_6_1009	DJI_0338	Ulmus glabra Huds.	0	n	1	1	Ulmaceae Mirb.
Iz_6_1009	DJI_0338	Verbascum densiflorum Bertol.	0	n	1	0	Scrophulariaceae Juss.
Iz_6_1009	DJI_0338	Veronica austriaca L.	0	n	1	0	Plantaginaceae Juss.
Iz_6_1009	DJI_0338	Veronica incana L.	0	n	1	0	Plantaginaceae Juss.
Iz_6_1009	DJI_0338	Veronica verna L.	0	n	1	0	Plantaginaceae Juss.
Iz_6_1009	DJI_0338	Vincetoxicum hirundinaria Medik	0	n	1	0	Apocynaceae Juss.
Iz_6_1009	DJI_0341	Acer tataricum L.	0	n	1	1	Sapindaceae Juss.
Iz_6_1009	DJI_0341	Achillea millefolium L. s.l.	0	n	1	0	Asteraceae Bercht. & J.Presl
Iz_6_1009	DJI_0341	Allium oleraceum L.	0	n	1	0	Amaryllidaceae J.St.-Hil.
Iz_6_1009	DJI_0341	Ambrosia artemisiifolia L.	0	a	0	0	Asteraceae Bercht. & J.Presl
Iz_6_1009	DJI_0341	Amorpha fruticosa L.	0	a	0	0	Fabaceae Lindl.
Iz_6_1009	DJI_0341	Anthericum ramosum L.	0	n	1	0	Asparagaceae Juss.
Iz_6_1009	DJI_0341	Artemisia marschalliana Spreng.	0	n	1	0	Asteraceae Bercht. & J.Presl
Iz_6_1009	DJI_0341	Asparagus officinalis L.	0	n	1	0	Asparagaceae Juss.
Iz_6_1009	DJI_0341	Astragalus cicer L.	0	n	1	0	Fabaceae Lindl.
Iz_6_1009	DJI_0341	Bassia laniflora (S.G.Gmel.) A.J.Scott	0	n	1	0	Amaranthaceae Juss.
Iz_6_1009	DJI_0341	Berberis vulgaris L.	0	n	1	0	Berberidaceae Juss.
Iz_6_1009	DJI_0341	Betula pendula Roth.	0	n	1	1	Betulaceae Gray
Iz_6_1009	DJI_0341	Betula pendula Roth.	0	n	1	0	Poaceae Barnhart
Iz_6_1009	DJI_0341	Bromus inermis Leyss.	0		NA	NA	
Iz_6_1009	DJI_0341	Bromus squarrosus L.	0	a	0	0	Poaceae Barnhart
Iz_6_1009	DJI_0341	Bromus tectorum L.	0	a	0	0	Poaceae Barnhart
Iz_6_1009	DJI_0341	Calamagrostis epigejos (L.) Roth	70	n	1	0	Poaceae Barnhart
Iz_6_1009	DJI_0341	Carduus acanthoides L.	0	n	1	0	Asteraceae Bercht. & J.Presl
Iz_6_1009	DJI_0341	Carex sp.	0	n	1	0	Cyperaceae Juss.
Iz_6_1009	DJI_0341	Centaurea majorovii Dumbadze	0	n	1	0	Asteraceae Bercht. & J.Presl
Iz_6_1009	DJI_0341	Chamaecytisus ruthenicus (Fisch.ex Wol.) Klaskova	0	n	1	0	Fabaceae Lindl.
Iz_6_1009	DJI_0341	Chelidonium majus L.	0	n	1	0	Papaveraceae Juss.
Iz_6_1009	DJI_0341	Chenopodium betaceum Andrz.	0	n	1	0	Amaranthaceae Juss.
Iz_6_1009	DJI_0341	Chenopodium cf. album	0	n	1	0	Amaranthaceae Juss.
Iz_6_1009	DJI_0341	Chenopodium sp.	0		NA	0	Amaranthaceae Juss.
Iz_6_1009	DJI_0341	Chondrilla juncea L.	0	n	1	0	Asteraceae Bercht. & J.Presl
Iz_6_1009	DJI_0341	Cirsium vulgare (Savi) Ten.	0	n	1	0	Asteraceae Bercht. & J.Presl
Iz_6_1009	DJI_0341	Clinopodium acinos (L.) Kuntze	0	n	1	0	Lamiacea Lindl.
Iz_6_1009	DJI_0341	Convallaria majalis L.	0	n	1	0	Asparagaceae Juss.
Iz_6_1009	DJI_0341	Crataegus rhipidophylla Gand.	0	n	1	1	Rosaceae Juss.
Iz_6_1009	DJI_0341	Crepis foetida L.	0	n	1	0	Asteraceae Bercht. & J.Presl
Iz_6_1009	DJI_0341	Dactylis glomerata L.	0	n	1	0	Poaceae Barnhart
Iz_6_1009	DJI_0341	Dianthus campestris M.Bieb.	0	n	1	0	Caryophyllaceae Juss.
Iz_6_1009	DJI_0341	Digitaria sanguinalis (L.) Scop.	0	a	0	0	Poaceae Barnhart
Iz_6_1009	DJI_0341	Elymus repens (L.) Gould	0	n	1	0	Poaceae Barnhart
Iz_6_1009	DJI_0341	Epilobium tetragonum L.	0	n	1	0	Onagraceae Juss.
Iz_6_1009	DJI_0341	Eragrostis minor Host	0	a	0	0	Poaceae Barnhart
Iz_6_1009	DJI_0341	Erigeron canadensis L.	0	a	0	0	Asteraceae Bercht. & J.Presl
Iz_6_1009	DJI_0341	Euphorbia seguieriana Neck.	0	n	1	0	Euphorbiaceae Juss.
Iz_6_1009	DJI_0341	Fallopia dumetorum (L.) Holub	0	n	1	0	Polygonaceae Juss.
Iz_6_1009	DJI_0341	Festuca beckeri (Hack.) Trautv.	0	n	1	0	Poaceae Barnhart
Iz_6_1009	DJI_0341	Fragaria vesca L.	0	n	1	0	Rosaceae Juss.
Iz_6_1009	DJI_0341	Frangula alnus Mill.	0	n	1	1	Rhamnaceae Juss.
Iz_6_1009	DJI_0341	Fraxinus pennsylvanica Marshall	0	a	0	1	Oleaceae Hoffmanns. & Link
Iz_6_1009	DJI_0341	Galeopsis bifida Boenn.	0	n	1	0	Lamiacea Lindl.
Iz_6_1009	DJI_0341	Galium verum L.	0	n	1	0	Rubiaceae Juss.
Iz_6_1009	DJI_0341	Genista tinctoria L.	0	n	1	0	Fabaceae Lindl.
Iz_6_1009	DJI_0341	Geranium robertianum L.	0	n	1	0	Geraniaceae Juss.
Iz_6_1009	DJI_0341	Geranium sanquineum L.	0	n	1	0	Geraniaceae Juss.
Iz_6_1009	DJI_0341	Gypsophila paniculata L.	0	n	1	0	Caryophyllaceae Juss.
Iz_6_1009	DJI_0341	Helichrysum arenarium (L.) Moench	0	n	1	0	Asteraceae Bercht. & J.Presl
Iz_6_1009	DJI_0341	Herniaria polygama J.Gay	0	n	1	0	Caryophyllaceae Juss.
Iz_6_1009	DJI_0341	Hieracium robustum Fr.	0	n	1	0	Asteraceae Bercht. & J.Presl
Iz_6_1009	DJI_0341	Hieracium umbellatum L.	0	n	1	NA	Asteraceae Bercht. & J.Presl
Iz_6_1009	DJI_0341	Hylotelephium maximum (L.) Holub	0	n	1	0	Crassulaceae J.St.-Hil.
Iz_6_1009	DJI_0341	Jacobaea borysthenica (DC.) B.Nord. & Greuter	0	n	1	0	Asteraceae Bercht. & J.Presl
Iz_6_1009	DJI_0341	Jasione montana L.	0	n	1	0	Campanulaceae Juss.
Iz_6_1009	DJI_0341	Jurinea cyanoides (L.) Rchb.s.l.	0	n	1	0	Asteraceae Bercht. & J.Presl
Iz_6_1009	DJI_0341	Koeleria glauca (Spreng.) DC.	0	n	1	0	Poaceae Barnhart
Iz_6_1009	DJI_0341	Lactuca serriola L.	0	a	0	0	Asteraceae Bercht. & J.Presl
Iz_6_1009	DJI_0341	Linaria genistifolia (L.) Mill.	0	n	1	0	Plantaginaceae Juss.
Iz_6_1009	DJI_0341	Lomelosia argentea (L.) Greuter & Burdet	0	n	1	0	Caprifoliaceae Juss.
Iz_6_1009	DJI_0341	Melampyrum pratense L.	0	n	1	0	Orobanchaceae Vent.
Iz_6_1009	DJI_0341	Oenothera biennis L.	0	a	0	0	Onagraceae Juss.
Iz_6_1009	DJI_0341	Pentanema britannica (L.) D.Gut.Larr., Santos-Vicente, Anderb., E.Rico & M.M.Mart.Ort.	0	n	1	0	Asteraceae Bercht. & J.Presl
Iz_6_1009	DJI_0341	Peucedanum oreoselinum (L.) Moench	0	n	1	0	Apiaceae Lindl.
Iz_6_1009	DJI_0341	Peucedanum ruthenicum M.Bieb.	0	n	1	0	Apiaceae Lindl.
Iz_6_1009	DJI_0341	Pilosella echioides (Lumn.) F.W.Schultz & Sch.Bip.	0	n	1	0	Asteraceae Bercht. & J.Presl
Iz_6_1009	DJI_0341	Pilosella officinarum F. Schultz et Sch. Bip.	0	n	1	0	Asteraceae Bercht. & J.Presl
Iz_6_1009	DJI_0341	Pilosella sp.	0		NA	0	Asteraceae Bercht. & J.Presl
Iz_6_1009	DJI_0341	Pinus sylvestris L.	0	n	1	1	Pinaceae Spreng. ex F.Rudolphi
Iz_6_1009	DJI_0341	Poa annua L.	0	n	1	0	Poaceae Barnhart
Iz_6_1009	DJI_0341	Poa nemoralis L.	0	n	1	0	Poaceae Barnhart
Iz_6_1009	DJI_0341	Poa sp.	0		NA	0	Poaceae Barnhart

Iz_6_1009	DJI_0341	Polygonatum odoratum (Mill.) Druce	0	n	1	0	Asparagaceae Juss.
Iz_6_1009	DJI_0341	Polygonum arenarium Waldst. & Kit.	0	n	1	0	Polygonaceae Juss.
Iz_6_1009	DJI_0341	Populus nigra L.	0	n	1	1	Salicaceae Mirb.
Iz_6_1009	DJI_0341	Populus tremula L.	0	n	1	1	Salicaceae Mirb.
Iz_6_1009	DJI_0341	Populus Ã canescens (Aiton) Sm.	0	n	1	1	Salicaceae Mirb.
Iz_6_1009	DJI_0341	Potentilla argentea L.	0	n	1	0	Rosaceae Juss.
Iz_6_1009	DJI_0341	Psammophiliella muralis (L.) Ikonn.	0	n	1	0	Caryophyllaceae Juss.
Iz_6_1009	DJI_0341	Psephellus sumensis (Kalen.) Greuter	0	n	1	0	Asteraceae Bercht. & J.Presl
Iz_6_1009	DJI_0341	Pulsatilla pratensis (L.) Mill. s.l.	0	n	1	0	Ranunculaceae Juss.
Iz_6_1009	DJI_0341	Pyrus communis L.	0	a	0	1	Rosaceae Juss.
Iz_6_1009	DJI_0341	Quercus robur L.	0	n	1	1	Fagaceae Dumort.
Iz_6_1009	DJI_0341	Robinia pseudoacacia L.	0	a	0	1	Fabaceae Lindl.
Iz_6_1009	DJI_0341	Rosa sp.	0		NA	1	Rosaceae Juss.
Iz_6_1009	DJI_0341	Rubus idaeus L.	0	n	1	0	Rosaceae Juss.
Iz_6_1009	DJI_0341	Rumex acetosella L.	0	n	1	0	Polygonaceae Juss.
Iz_6_1009	DJI_0341	Rumex thyrsiflorus Fingerh.	0	n	1	0	Polygonaceae Juss.
Iz_6_1009	DJI_0341	Salix alba L.	0	n	1	1	Salicaceae Mirb.
Iz_6_1009	DJI_0341	Sambucus nigra L.	0	n	1	1	Viburnaceae Raf.
Iz_6_1009	DJI_0341	Secale sylvestre Host	0	n	1	0	Poaceae Barnhart
Iz_6_1009	DJI_0341	Sempervivum ruthenicum Schnittsp. & C.B. Lehm.	0	n	1	0	Crassulaceae J.St.-Hil.
Iz_6_1009	DJI_0341	Setaria viridis (L.) P. Beauv.	0	n	1	0	Poaceae Barnhart
Iz_6_1009	DJI_0341	Silene borysthenica (Gruner) Walters	0	n	1	0	Caryophyllaceae Juss.
Iz_6_1009	DJI_0341	Silene latifolia subsp. alba (Mill.) Greuter & Burdet	0	n	1	0	Caryophyllaceae Juss.
Iz_6_1009	DJI_0341	Solidago virgaurea L.	0	n	1	0	Asteraceae Bercht. & J.Presl
Iz_6_1009	DJI_0341	Sorbus aucuparia L.	0	n	1	1	Rosaceae Juss.
Iz_6_1009	DJI_0341	Stachys recta L.	0	n	1	0	Lamiacea Lindl.
Iz_6_1009	DJI_0341	Tanacetum vulgare L.	0	n	1	0	Asteraceae Bercht. & J.Presl
Iz_6_1009	DJI_0341	Thymus pallasianus Heinr.Braun	0	n	1	0	Lamiacea Lindl.
Iz_6_1009	DJI_0341	Tilia cordata Mill.	0	n	1	1	Malvaceae Juss.
Iz_6_1009	DJI_0341	Trifolium alpestre L.	0	n	1	0	Fabaceae Lindl.
Iz_6_1009	DJI_0341	Trifolium arvense L.	0	n	1	0	Fabaceae Lindl.
Iz_6_1009	DJI_0341	Ulmus glabra Huds.	0	n	1	1	Ulmaceae Mirb.
Iz_6_1009	DJI_0341	Verbascum densiflorum Bertol.	0	n	1	0	Scrophulariaceae Juss.
Iz_6_1009	DJI_0341	Veronica austriaca L.	0	n	1	0	Plantaginaceae Juss.
Iz_6_1009	DJI_0341	Veronica incana L.	0	n	1	0	Plantaginaceae Juss.
Iz_6_1009	DJI_0341	Veronica verna L.	0	n	1	0	Plantaginaceae Juss.
Iz_6_1009	DJI_0341	Vincetoxicum hirundinaria Medik	0	n	1	0	Apocynaceae Juss.
Iz_6_1009	DJI_0343	Acer tataricum L.	0	n	1	1	Sapindaceae Juss.
Iz_6_1009	DJI_0343	Achillea millefolium L. s.l.	0	n	1	0	Asteraceae Bercht. & J.Presl
Iz_6_1009	DJI_0343	Allium oleraceum L.	0	n	1	0	Amaryllidaceae J.St.-Hil.
Iz_6_1009	DJI_0343	Ambrosia artemisiifolia L.	0	a	0	0	Asteraceae Bercht. & J.Presl
Iz_6_1009	DJI_0343	Amorpha fruticosa L.	0	a	0	0	Fabaceae Lindl.
Iz_6_1009	DJI_0343	Anthericum ramosum L.	0	n	1	0	Asparagaceae Juss.
Iz_6_1009	DJI_0343	Artemisia marschalliana Spreng.	0	n	1	0	Asteraceae Bercht. & J.Presl
Iz_6_1009	DJI_0343	Asparagus officinalis L.	0	n	1	0	Asparagaceae Juss.
Iz_6_1009	DJI_0343	Astragalus cicer L.	0	n	1	0	Fabaceae Lindl.
Iz_6_1009	DJI_0343	Bassia laniflora (S.G.Gmel.) A.J.Scott	0	n	1	0	Amaranthaceae Juss.
Iz_6_1009	DJI_0343	Berberis vulgaris L.	0	n	1	0	Berberidaceae Juss.
Iz_6_1009	DJI_0343	Betula pendula Roth.	0	n	1	1	Betulaceae Gray
Iz_6_1009	DJI_0343	Betula pendula Roth.	0	n	1	0	Poaceae Barnhart
Iz_6_1009	DJI_0343	Bromus inermis Leyss.	0		NA	NA	
Iz_6_1009	DJI_0343	Bromus squarrosus L.	0	a	0	0	Poaceae Barnhart
Iz_6_1009	DJI_0343	Bromus tectorum L.	0	a	0	0	Poaceae Barnhart
Iz_6_1009	DJI_0343	Calamagrostis epigejos (L.) Roth	15	n	1	0	Poaceae Barnhart
Iz_6_1009	DJI_0343	Carduus acanthoides L.	0	n	1	0	Asteraceae Bercht. & J.Presl
Iz_6_1009	DJI_0343	Carex sp.	0	n	1	0	Cyperaceae Juss.
Iz_6_1009	DJI_0343	Centaurea majorovii Dumbadze	0	n	1	0	Asteraceae Bercht. & J.Presl
Iz_6_1009	DJI_0343	Chamaecytisus ruthenicus (Fisch.ex Wol.) Klaskova	0	n	1	0	Fabaceae Lindl.
Iz_6_1009	DJI_0343	Chelidonium majus L.	0	n	1	0	Papaveraceae Juss.
Iz_6_1009	DJI_0343	Chenopodium betaceum Andrz.	0	n	1	0	Amaranthaceae Juss.
Iz_6_1009	DJI_0343	Chenopodium cf. album	0	n	1	0	Amaranthaceae Juss.
Iz_6_1009	DJI_0343	Chenopodium sp.	0		NA	0	Amaranthaceae Juss.
Iz_6_1009	DJI_0343	Chondrilla juncea L.	0	n	1	0	Asteraceae Bercht. & J.Presl
Iz_6_1009	DJI_0343	Cirsium vulgare (Savi) Ten.	0	n	1	0	Asteraceae Bercht. & J.Presl
Iz_6_1009	DJI_0343	Clinopodium acinos (L.) Kuntze	0	n	1	0	Lamiacea Lindl.
Iz_6_1009	DJI_0343	Convallaria majalis L.	0	n	1	0	Asparagaceae Juss.
Iz_6_1009	DJI_0343	Crataegus rhipidophylla Gand.	0	n	1	1	Rosaceae Juss.
Iz_6_1009	DJI_0343	Crepis foetida L.	0	n	1	0	Asteraceae Bercht. & J.Presl
Iz_6_1009	DJI_0343	Dactylis glomerata L.	0	n	1	0	Poaceae Barnhart
Iz_6_1009	DJI_0343	Dianthus campestris M.Bieb.	0	n	1	0	Caryophyllaceae Juss.
Iz_6_1009	DJI_0343	Digitaria sanguinalis (L.) Scop.	0	a	0	0	Poaceae Barnhart
Iz_6_1009	DJI_0343	Elymus repens (L.) Gould	0	n	1	0	Poaceae Barnhart
Iz_6_1009	DJI_0343	Epilobium tetragonum L.	0	n	1	0	Onagraceae Juss.
Iz_6_1009	DJI_0343	Eragrostis minor Host	0	a	0	0	Poaceae Barnhart
Iz_6_1009	DJI_0343	Erigeron canadensis L.	2	a	0	0	Asteraceae Bercht. & J.Presl
Iz_6_1009	DJI_0343	Euphorbia seguieriana Neck.	0	n	1	0	Euphorbiaceae Juss.
Iz_6_1009	DJI_0343	Fallopia dumetorum (L.) Holub	0	n	1	0	Polygonaceae Juss.
Iz_6_1009	DJI_0343	Festuca beckeri (Hack.) Trautv.	0	n	1	0	Poaceae Barnhart
Iz_6_1009	DJI_0343	Fragaria vesca L.	0	n	1	0	Rosaceae Juss.
Iz_6_1009	DJI_0343	Frangula alnus Mill.	0	n	1	1	Rhamnaceae Juss.
Iz_6_1009	DJI_0343	Fraxinus pennsylvanica Marshall	0	a	0	1	Oleaceae Hoffmanns. & Link
Iz_6_1009	DJI_0343	Galeopsis bifida Boenn.	0	n	1	0	Lamiacea Lindl.
Iz_6_1009	DJI_0343	Galium verum L.	0	n	1	0	Rubiaceae Juss.
Iz_6_1009	DJI_0343	Genista tinctoria L.	0	n	1	0	Fabaceae Lindl.
Iz_6_1009	DJI_0343	Geranium robertianum L.	0	n	1	0	Geraniaceae Juss.
Iz_6_1009	DJI_0343	Geranium sanquineum L.	0	n	1	0	Geraniaceae Juss.
Iz_6_1009	DJI_0343	Gypsophila paniculata L.	0	n	1	0	Caryophyllaceae Juss.
Iz_6_1009	DJI_0343	Helichrysum arenarium (L.) Moench	0	n	1	0	Asteraceae Bercht. & J.Presl
Iz_6_1009	DJI_0343	Herniaria polygama J.Gay	0	n	1	0	Caryophyllaceae Juss.
Iz_6_1009	DJI_0343	Hieracium robustum Fr.	0	n	1	0	Asteraceae Bercht. & J.Presl
Iz_6_1009	DJI_0343	Hieracium umbellatum L.	0	n	1	NA	Asteraceae Bercht. & J.Presl
Iz_6_1009	DJI_0343	Hylotelephium maximum (L.) Holub	0	n	1	0	Crassulaceae J.St.-Hil.
Iz_6_1009	DJI_0343	Jacobaea borysthenica (DC.) B.Nord. & Greuter	0	n	1	0	Asteraceae Bercht. & J.Presl
Iz_6_1009	DJI_0343	Jasione montana L.	0	n	1	0	Campanulaceae Juss.
Iz_6_1009	DJI_0343	Jurinea cyanoides (L.) Rchb.s.l.	0	n	1	0	Asteraceae Bercht. & J.Presl
Iz_6_1009	DJI_0343	Koeleria glauca (Spreng.) DC.	0	n	1	0	Poaceae Barnhart
Iz_6_1009	DJI_0343	Lactuca serriola L.	0	a	0	0	Asteraceae Bercht. & J.Presl
Iz_6_1009	DJI_0343	Linaria genistifolia (L.) Mill.	0	n	1	0	Plantaginaceae Juss.

Iz_6_1009	DJI_0343	Lomelosia argentea (L.) Greuter & Burdet	0	n	1	0	Caprifoliaceae Juss.
Iz_6_1009	DJI_0343	Melampyrum pratense L.	0	n	1	0	Orobanchaceae Vent.
Iz_6_1009	DJI_0343	Oenothera biennis L.	0	a	0	0	Onagraceae Juss.
Iz_6_1009	DJI_0343	Pentanema britannica (L.) D.Gut.Larr., Santos-Vicente, Anderb., E.Rico & M.M.Mart.Ort.	0	n	1	0	Asteraceae Bercht. & J.Presl
Iz_6_1009	DJI_0343	Peucedanum oreoselinum (L.) Moench	0	n	1	0	Apiaceae Lindl.
Iz_6_1009	DJI_0343	Peucedanum ruthenicum M.Bieb.	0	n	1	0	Apiaceae Lindl.
Iz_6_1009	DJI_0343	Pilosella echioides (Lumn.) F.W.Schultz & Sch.Bip.	0	n	1	0	Asteraceae Bercht. & J.Presl
Iz_6_1009	DJI_0343	Pilosella officinarum F. Schultz et Sch. Bip.	0	n	1	0	Asteraceae Bercht. & J.Presl
Iz_6_1009	DJI_0343	Pilosella sp.	0		NA	0	Asteraceae Bercht. & J.Presl
Iz_6_1009	DJI_0343	Pinus sylvestris L.	0	n	1	1	Pinaceae Spreng. ex F.Rudolphi
Iz_6_1009	DJI_0343	Poa annua L.	0	n	1	0	Poaceae Barnhart
Iz_6_1009	DJI_0343	Poa nemoralis L.	0	n	1	0	Poaceae Barnhart
Iz_6_1009	DJI_0343	Poa sp.	0		NA	0	Poaceae Barnhart
Iz_6_1009	DJI_0343	Polygonatum odoratum (Mill.) Druce	0	n	1	0	Asparagaceae Juss.
Iz_6_1009	DJI_0343	Polygonum arenarium Waldst. & Kit.	0	n	1	0	Polygonaceae Juss.
Iz_6_1009	DJI_0343	Populus nigra L.	0	n	1	1	Salicaceae Mirb.
Iz_6_1009	DJI_0343	Populus tremula L.	0	n	1	1	Salicaceae Mirb.
Iz_6_1009	DJI_0343	Populus Ã canescens (Aiton) Sm.	0	n	1	1	Salicaceae Mirb.
Iz_6_1009	DJI_0343	Potentilla argentea L.	0	n	1	0	Rosaceae Juss.
Iz_6_1009	DJI_0343	Psammophiliella muralis (L.) Ikonn.	0	n	1	0	Caryophyllaceae Juss.
Iz_6_1009	DJI_0343	Psephellus sumensis (Kalen.) Greuter	0	n	1	0	Asteraceae Bercht. & J.Presl
Iz_6_1009	DJI_0343	Pulsatilla pratensis (L.) Mill. s.l.	0	n	1	0	Ranunculaceae Juss.
Iz_6_1009	DJI_0343	Pyrus communis L.	0	a	0	1	Rosaceae Juss.
Iz_6_1009	DJI_0343	Quercus robur L.	0	n	1	1	Fagaceae Dumort.
Iz_6_1009	DJI_0343	Robinia pseudoacacia L.	0	a	0	1	Fabaceae Lindl.
Iz_6_1009	DJI_0343	Rosa sp.	0		NA	1	Rosaceae Juss.
Iz_6_1009	DJI_0343	Rubus idaeus L.	0	n	1	0	Rosaceae Juss.
Iz_6_1009	DJI_0343	Rumex acetosella L.	0	n	1	0	Polygonaceae Juss.
Iz_6_1009	DJI_0343	Rumex thyrsiflorus Fingerh.	0	n	1	0	Polygonaceae Juss.
Iz_6_1009	DJI_0343	Salix alba L.	0	n	1	1	Salicaceae Mirb.
Iz_6_1009	DJI_0343	Sambucus nigra L.	0	n	1	1	Viburnaceae Raf.
Iz_6_1009	DJI_0343	Secale sylvestre Host	0	n	1	0	Poaceae Barnhart
Iz_6_1009	DJI_0343	Sempervivum ruthenicum Schnittsp. & C.B. Lehm.	0	n	1	0	Crassulaceae J.St.-Hil.
Iz_6_1009	DJI_0343	Setaria viridis (L.) P. Beauv.	0	n	1	0	Poaceae Barnhart
Iz_6_1009	DJI_0343	Silene borysthenica (Gruner) Walters	0	n	1	0	Caryophyllaceae Juss.
Iz_6_1009	DJI_0343	Silene latifolia subsp. alba (Mill.) Greuter & Burdet	0	n	1	0	Caryophyllaceae Juss.
Iz_6_1009	DJI_0343	Solidago virgaurea L.	0	n	1	0	Asteraceae Bercht. & J.Presl
Iz_6_1009	DJI_0343	Sorbus aucuparia L.	0	n	1	1	Rosaceae Juss.
Iz_6_1009	DJI_0343	Stachys recta L.	0	n	1	0	Lamiacea Lindl.
Iz_6_1009	DJI_0343	Tanacetum vulgare L.	0	n	1	0	Asteraceae Bercht. & J.Presl
Iz_6_1009	DJI_0343	Thymus pallasianus Heinr.Braun	0	n	1	0	Lamiacea Lindl.
Iz_6_1009	DJI_0343	Tilia cordata Mill.	0	n	1	1	Malvaceae Juss.
Iz_6_1009	DJI_0343	Trifolium alpestre L.	0	n	1	0	Fabaceae Lindl.
Iz_6_1009	DJI_0343	Trifolium arvense L.	0	n	1	0	Fabaceae Lindl.
Iz_6_1009	DJI_0343	Ulmus glabra Huds.	0	n	1	1	Ulmaceae Mirb.
Iz_6_1009	DJI_0343	Verbascum densiflorum Bertol.	0	n	1	0	Scrophulariaceae Juss.
Iz_6_1009	DJI_0343	Veronica austriaca L.	0	n	1	0	Plantaginaceae Juss.
Iz_6_1009	DJI_0343	Veronica incana L.	0	n	1	0	Plantaginaceae Juss.
Iz_6_1009	DJI_0343	Veronica verna L.	0	n	1	0	Plantaginaceae Juss.
Iz_6_1009	DJI_0343	Vincetoxicum hirundinaria Medik	0	n	1	0	Apocynaceae Juss.
Iz_6_1009	DJI_0345	Acer tataricum L.	0	n	1	1	Sapindaceae Juss.
Iz_6_1009	DJI_0345	Achillea millefolium L. s.l.	0	n	1	0	Asteraceae Bercht. & J.Presl
Iz_6_1009	DJI_0345	Allium oleraceum L.	0	n	1	0	Amaryllidaceae J.St.-Hil.
Iz_6_1009	DJI_0345	Ambrosia artemisiifolia L.	0	a	0	0	Asteraceae Bercht. & J.Presl
Iz_6_1009	DJI_0345	Amorpha fruticosa L.	0	a	0	0	Fabaceae Lindl.
Iz_6_1009	DJI_0345	Anthericum ramosum L.	0	n	1	0	Asparagaceae Juss.
Iz_6_1009	DJI_0345	Artemisia marschalliana Spreng.	0	n	1	0	Asteraceae Bercht. & J.Presl
Iz_6_1009	DJI_0345	Asparagus officinalis L.	0	n	1	0	Asparagaceae Juss.
Iz_6_1009	DJI_0345	Astragalus cicer L.	0	n	1	0	Fabaceae Lindl.
Iz_6_1009	DJI_0345	Bassia laniflora (S.G.Gmel.) A.J.Scott	0	n	1	0	Amaranthaceae Juss.
Iz_6_1009	DJI_0345	Berberis vulgaris L.	0	n	1	0	Berberidaceae Juss.
Iz_6_1009	DJI_0345	Betula pendula Roth.	0	n	1	1	Betulaceae Gray
Iz_6_1009	DJI_0345	Betula pendula Roth.	0	n	1	0	Poaceae Barnhart
Iz_6_1009	DJI_0345	Bromus inermis Leyss.	0		NA	NA	
Iz_6_1009	DJI_0345	Bromus squarrosus L.	0	a	0	0	Poaceae Barnhart
Iz_6_1009	DJI_0345	Bromus tectorum L.	0	a	0	0	Poaceae Barnhart
Iz_6_1009	DJI_0345	Calamagrostis epigejos (L.) Roth	2	n	1	0	Poaceae Barnhart
Iz_6_1009	DJI_0345	Carduus acanthoides L.	0	n	1	0	Asteraceae Bercht. & J.Presl
Iz_6_1009	DJI_0345	Carex sp.	0	n	1	0	Cyperaceae Juss.
Iz_6_1009	DJI_0345	Centaurea majorovii Dumbadze	0	n	1	0	Asteraceae Bercht. & J.Presl
Iz_6_1009	DJI_0345	Chamaecytisus ruthenicus (Fisch.ex Wol.) Klaskova	0	n	1	0	Fabaceae Lindl.
Iz_6_1009	DJI_0345	Chelidonium majus L.	0	n	1	0	Papaveraceae Juss.
Iz_6_1009	DJI_0345	Chenopodium betaceum Andrz.	0	n	1	0	Amaranthaceae Juss.
Iz_6_1009	DJI_0345	Chenopodium cf. album	0	n	1	0	Amaranthaceae Juss.
Iz_6_1009	DJI_0345	Chenopodium sp.	0		NA	0	Amaranthaceae Juss.
Iz_6_1009	DJI_0345	Chondrilla juncea L.	0	n	1	0	Asteraceae Bercht. & J.Presl
Iz_6_1009	DJI_0345	Cirsium vulgare (Savi) Ten.	0	n	1	0	Asteraceae Bercht. & J.Presl
Iz_6_1009	DJI_0345	Clinopodium acinos (L.) Kuntze	0	n	1	0	Lamiacea Lindl.
Iz_6_1009	DJI_0345	Convallaria majalis L.	0	n	1	0	Asparagaceae Juss.
Iz_6_1009	DJI_0345	Crataegus rhipidophylla Gand.	0	n	1	1	Rosaceae Juss.
Iz_6_1009	DJI_0345	Crepis foetida L.	0	n	1	0	Asteraceae Bercht. & J.Presl
Iz_6_1009	DJI_0345	Dactylis glomerata L.	0	n	1	0	Poaceae Barnhart
Iz_6_1009	DJI_0345	Dianthus campestris M.Bieb.	0	n	1	0	Caryophyllaceae Juss.
Iz_6_1009	DJI_0345	Digitaria sanguinalis (L.) Scop.	0	a	0	0	Poaceae Barnhart
Iz_6_1009	DJI_0345	Elymus repens (L.) Gould	0	n	1	0	Poaceae Barnhart
Iz_6_1009	DJI_0345	Epilobium tetragonum L.	0	n	1	0	Onagraceae Juss.
Iz_6_1009	DJI_0345	Eragrostis minor Host	0	a	0	0	Poaceae Barnhart
Iz_6_1009	DJI_0345	Erigeron canadensis L.	0.5	a	0	0	Asteraceae Bercht. & J.Presl
Iz_6_1009	DJI_0345	Euphorbia seguieriana Neck.	0	n	1	0	Euphorbiaceae Juss.
Iz_6_1009	DJI_0345	Fallopia dumetorum (L.) Holub	0	n	1	0	Polygonaceae Juss.
Iz_6_1009	DJI_0345	Festuca beckeri (Hack.) Trautv.	0	n	1	0	Poaceae Barnhart
Iz_6_1009	DJI_0345	Fragaria vesca L.	0	n	1	0	Rosaceae Juss.
Iz_6_1009	DJI_0345	Frangula alnus Mill.	0	n	1	1	Rhamnaceae Juss.
Iz_6_1009	DJI_0345	Fraxinus pennsylvanica Marshall	0	a	0	1	Oleaceae Hoffmanns. & Link
Iz_6_1009	DJI_0345	Galeopsis bifida Boenn.	0	n	1	0	Lamiacea Lindl.
Iz_6_1009	DJI_0345	Galium verum L.	0	n	1	0	Rubiaceae Juss.
Iz_6_1009	DJI_0345	Genista tinctoria L.	0	n	1	0	Fabaceae Lindl.
Iz_6_1009	DJI_0345	Geranium robertianum L.	0	n	1	0	Geraniaceae Juss.

Iz_6_1009	DJI_0345	Geranium sanquineum L.	0	n	1	0	Geraniaceae Juss.
Iz_6_1009	DJI_0345	Gypsophila paniculata L.	0	n	1	0	Caryophyllaceae Juss.
Iz_6_1009	DJI_0345	Helichrysum arenarium (L.) Moench	0	n	1	0	Asteraceae Bercht. & J.Presl
Iz_6_1009	DJI_0345	Herniaria polygama J.Gay	0	n	1	0	Caryophyllaceae Juss.
Iz_6_1009	DJI_0345	Hieracium robustum Fr.	0	n	1	0	Asteraceae Bercht. & J.Presl
Iz_6_1009	DJI_0345	Hieracium umbellatum L.	0	n	1	NA	Asteraceae Bercht. & J.Presl
Iz_6_1009	DJI_0345	Hylotelephium maximum (L.) Holub	0	n	1	0	Crassulaceae J.St.-Hil.
Iz_6_1009	DJI_0345	Jacobaea borysthenica (DC.) B.Nord. & Greuter	0	n	1	0	Asteraceae Bercht. & J.Presl
Iz_6_1009	DJI_0345	Jasione montana L.	0	n	1	0	Campanulaceae Juss.
Iz_6_1009	DJI_0345	Jurinea cyanoides (L.) Rchb.s.l.	0	n	1	0	Asteraceae Bercht. & J.Presl
Iz_6_1009	DJI_0345	Koeleria glauca (Spreng.) DC.	0	n	1	0	Poaceae Barnhart
Iz_6_1009	DJI_0345	Lactuca serriola L.	0	a	0	0	Asteraceae Bercht. & J.Presl
Iz_6_1009	DJI_0345	Linaria genistifolia (L.) Mill.	0	n	1	0	Plantaginaceae Juss.
Iz_6_1009	DJI_0345	Lomelosia argentea (L.) Greuter & Burdet	0	n	1	0	Caprifoliaceae Juss.
Iz_6_1009	DJI_0345	Melampyrum pratense L.	0	n	1	0	Orobanchaceae Vent.
Iz_6_1009	DJI_0345	Oenothera biennis L.	0	a	0	0	Onagraceae Juss.
Iz_6_1009	DJI_0345	Pentanema britannica (L.) D.Gut.Larr., Santos-Vicente, Anderb., E.Rico & M.M.Mart.Ort.	0	n	1	0	Asteraceae Bercht. & J.Presl
Iz_6_1009	DJI_0345	Peucedanum oreoselinum (L.) Moench	0	n	1	0	Apiaceae Lindl.
Iz_6_1009	DJI_0345	Peucedanum ruthenicum M.Bieb.	0	n	1	0	Apiaceae Lindl.
Iz_6_1009	DJI_0345	Pilosella echioides (Lumn.) F.W.Schultz & Sch.Bip.	0	n	1	0	Asteraceae Bercht. & J.Presl
Iz_6_1009	DJI_0345	Pilosella officinarum F. Schultz et Sch. Bip.	0	n	1	0	Asteraceae Bercht. & J.Presl
Iz_6_1009	DJI_0345	Pilosella sp.	0		NA	0	Asteraceae Bercht. & J.Presl
Iz_6_1009	DJI_0345	Pinus sylvestris L.	0	n	1	1	Pinaceae Spreng. ex F.Rudolphi
Iz_6_1009	DJI_0345	Poa annua L.	0	n	1	0	Poaceae Barnhart
Iz_6_1009	DJI_0345	Poa nemoralis L.	0	n	1	0	Poaceae Barnhart
Iz_6_1009	DJI_0345	Poa sp.	0		NA	0	Poaceae Barnhart
Iz_6_1009	DJI_0345	Polygonatum odoratum (Mill.) Druce	0	n	1	0	Asparagaceae Juss.
Iz_6_1009	DJI_0345	Polygonum arenarium Waldst. & Kit.	0	n	1	0	Polygonaceae Juss.
Iz_6_1009	DJI_0345	Populus nigra L.	0	n	1	1	Salicaceae Mirb.
Iz_6_1009	DJI_0345	Populus tremula L.	0	n	1	1	Salicaceae Mirb.
Iz_6_1009	DJI_0345	Populus Ã canescens (Aiton) Sm.	0	n	1	1	Salicaceae Mirb.
Iz_6_1009	DJI_0345	Potentilla argentea L.	0	n	1	0	Rosaceae Juss.
Iz_6_1009	DJI_0345	Psammophiliella muralis (L.) Ikonn.	0	n	1	0	Caryophyllaceae Juss.
Iz_6_1009	DJI_0345	Psephellus sumensis (Kalen.) Greuter	0	n	1	0	Asteraceae Bercht. & J.Presl
Iz_6_1009	DJI_0345	Pulsatilla pratensis (L.) Mill. s.l.	0	n	1	0	Ranunculaceae Juss.
Iz_6_1009	DJI_0345	Pyrus communis L.	0	a	0	1	Rosaceae Juss.
Iz_6_1009	DJI_0345	Quercus robur L.	0	n	1	1	Fagaceae Dumort.
Iz_6_1009	DJI_0345	Robinia pseudoacacia L.	0	a	0	1	Fabaceae Lindl.
Iz_6_1009	DJI_0345	Rosa sp.	0		NA	1	Rosaceae Juss.
Iz_6_1009	DJI_0345	Rubus idaeus L.	0	n	1	0	Rosaceae Juss.
Iz_6_1009	DJI_0345	Rumex acetosella L.	0	n	1	0	Polygonaceae Juss.
Iz_6_1009	DJI_0345	Rumex thyrsiflorus Fingerh.	0	n	1	0	Polygonaceae Juss.
Iz_6_1009	DJI_0345	Salix alba L.	0	n	1	1	Salicaceae Mirb.
Iz_6_1009	DJI_0345	Sambucus nigra L.	0	n	1	1	Viburnaceae Raf.
Iz_6_1009	DJI_0345	Secale sylvestre Host	0	n	1	0	Poaceae Barnhart
Iz_6_1009	DJI_0345	Sempervivum ruthenicum Schnittsp. & C.B. Lehm.	0	n	1	0	Crassulaceae J.St.-Hil.
Iz_6_1009	DJI_0345	Setaria viridis (L.) P. Beauv.	0	n	1	0	Poaceae Barnhart
Iz_6_1009	DJI_0345	Silene borysthenica (Gruner) Walters	0	n	1	0	Caryophyllaceae Juss.
Iz_6_1009	DJI_0345	Silene latifolia subsp. alba (Mill.) Greuter & Burdet	0	n	1	0	Caryophyllaceae Juss.
Iz_6_1009	DJI_0345	Solidago virgaurea L.	0	n	1	0	Asteraceae Bercht. & J.Presl
Iz_6_1009	DJI_0345	Sorbus aucuparia L.	0	n	1	1	Rosaceae Juss.
Iz_6_1009	DJI_0345	Stachys recta L.	0	n	1	0	Lamiacea Lindl.
Iz_6_1009	DJI_0345	Tanacetum vulgare L.	0	n	1	0	Asteraceae Bercht. & J.Presl
Iz_6_1009	DJI_0345	Thymus pallasianus Heinr.Braun	0	n	1	0	Lamiacea Lindl.
Iz_6_1009	DJI_0345	Tilia cordata Mill.	0	n	1	1	Malvaceae Juss.
Iz_6_1009	DJI_0345	Trifolium alpestre L.	0	n	1	0	Fabaceae Lindl.
Iz_6_1009	DJI_0345	Trifolium arvense L.	0	n	1	0	Fabaceae Lindl.
Iz_6_1009	DJI_0345	Ulmus glabra Huds.	0	n	1	1	Ulmaceae Mirb.
Iz_6_1009	DJI_0345	Verbascum densiflorum Bertol.	0	n	1	0	Scrophulariaceae Juss.
Iz_6_1009	DJI_0345	Veronica austriaca L.	0	n	1	0	Plantaginaceae Juss.
Iz_6_1009	DJI_0345	Veronica incana L.	0	n	1	0	Plantaginaceae Juss.
Iz_6_1009	DJI_0345	Veronica verna L.	0	n	1	0	Plantaginaceae Juss.
Iz_6_1009	DJI_0345	Vincetoxicum hirundinaria Medik	0	n	1	0	Apocynaceae Juss.
Iz_6_1009	DJI_0347	Acer tataricum L.	0	n	1	1	Sapindaceae Juss.
Iz_6_1009	DJI_0347	Achillea millefolium L. s.l.	0	n	1	0	Asteraceae Bercht. & J.Presl
Iz_6_1009	DJI_0347	Allium oleraceum L.	0	n	1	0	Amaryllidaceae J.St.-Hil.
Iz_6_1009	DJI_0347	Ambrosia artemisiifolia L.	0	a	0	0	Asteraceae Bercht. & J.Presl
Iz_6_1009	DJI_0347	Amorpha fruticosa L.	0	a	0	0	Fabaceae Lindl.
Iz_6_1009	DJI_0347	Anthericum ramosum L.	0	n	1	0	Asparagaceae Juss.
Iz_6_1009	DJI_0347	Artemisia marschalliana Spreng.	0	n	1	0	Asteraceae Bercht. & J.Presl
Iz_6_1009	DJI_0347	Asparagus officinalis L.	0	n	1	0	Asparagaceae Juss.
Iz_6_1009	DJI_0347	Astragalus cicer L.	0	n	1	0	Fabaceae Lindl.
Iz_6_1009	DJI_0347	Bassia laniflora (S.G.Gmel.) A.J.Scott	0	n	1	0	Amaranthaceae Juss.
Iz_6_1009	DJI_0347	Berberis vulgaris L.	0	n	1	0	Berberidaceae Juss.
Iz_6_1009	DJI_0347	Betula pendula Roth.	0	n	1	1	Betulaceae Gray
Iz_6_1009	DJI_0347	Betula pendula Roth.	0	n	1	0	Poaceae Barnhart
Iz_6_1009	DJI_0347	Bromus inermis Leyss.	0		NA	NA	
Iz_6_1009	DJI_0347	Bromus squarrosus L.	0	a	0	0	Poaceae Barnhart
Iz_6_1009	DJI_0347	Bromus tectorum L.	0	a	0	0	Poaceae Barnhart
Iz_6_1009	DJI_0347	Calamagrostis epigejos (L.) Roth	60	n	1	0	Poaceae Barnhart
Iz_6_1009	DJI_0347	Carduus acanthoides L.	0	n	1	0	Asteraceae Bercht. & J.Presl
Iz_6_1009	DJI_0347	Carex sp.	0	n	1	0	Cyperaceae Juss.
Iz_6_1009	DJI_0347	Centaurea majorovii Dumbadze	0	n	1	0	Asteraceae Bercht. & J.Presl
Iz_6_1009	DJI_0347	Chamaecytisus ruthenicus (Fisch.ex Wol.) Klaskova	0	n	1	0	Fabaceae Lindl.
Iz_6_1009	DJI_0347	Chelidonium majus L.	0	n	1	0	Papaveraceae Juss.
Iz_6_1009	DJI_0347	Chenopodium betaceum Andrz.	0	n	1	0	Amaranthaceae Juss.
Iz_6_1009	DJI_0347	Chenopodium cf. album	0	n	1	0	Amaranthaceae Juss.
Iz_6_1009	DJI_0347	Chenopodium sp.	0		NA	0	Amaranthaceae Juss.
Iz_6_1009	DJI_0347	Chondrilla juncea L.	0	n	1	0	Asteraceae Bercht. & J.Presl
Iz_6_1009	DJI_0347	Cirsium vulgare (Savi) Ten.	0	n	1	0	Asteraceae Bercht. & J.Presl
Iz_6_1009	DJI_0347	Clinopodium acinos (L.) Kuntze	0	n	1	0	Lamiacea Lindl.
Iz_6_1009	DJI_0347	Convallaria majalis L.	0	n	1	0	Asparagaceae Juss.
Iz_6_1009	DJI_0347	Crataegus rhipidophylla Gand.	0	n	1	1	Rosaceae Juss.
Iz_6_1009	DJI_0347	Crepis foetida L.	0	n	1	0	Asteraceae Bercht. & J.Presl
Iz_6_1009	DJI_0347	Dactylis glomerata L.	0	n	1	0	Poaceae Barnhart
Iz_6_1009	DJI_0347	Dianthus campestris M.Bieb.	0	n	1	0	Caryophyllaceae Juss.
Iz_6_1009	DJI_0347	Digitaria sanguinalis (L.) Scop.	0	a	0	0	Poaceae Barnhart
Iz_6_1009	DJI_0347	Elymus repens (L.) Gould	0	n	1	0	Poaceae Barnhart

Iz_6_1009	DJI_0347	Epilobium tetragonum L.	0	n	1	0	Onagraceae Juss.
Iz_6_1009	DJI_0347	Eragrostis minor Host	0	a	0	0	Poaceae Barnhart
Iz_6_1009	DJI_0347	Erigeron canadensis L.	1	a	0	0	Asteraceae Bercht. & J.Presl
Iz_6_1009	DJI_0347	Euphorbia seguieriana Neck.	0	n	1	0	Euphorbiaceae Juss.
Iz_6_1009	DJI_0347	Fallopia dumetorum (L.) Holub	0	n	1	0	Polygonaceae Juss.
Iz_6_1009	DJI_0347	Festuca beckeri (Hack.) Trautv.	0	n	1	0	Poaceae Barnhart
Iz_6_1009	DJI_0347	Fragaria vesca L.	0	n	1	0	Rosaceae Juss.
Iz_6_1009	DJI_0347	Frangula alnus Mill.	0	n	1	1	Rhamnaceae Juss.
Iz_6_1009	DJI_0347	Fraxinus pennsylvanica Marshall	0	a	0	1	Oleaceae Hoffmanns. & Link
Iz_6_1009	DJI_0347	Galeopsis bifida Boenn.	0	n	1	0	Lamiacea Lindl.
Iz_6_1009	DJI_0347	Galium verum L.	0	n	1	0	Rubiaceae Juss.
Iz_6_1009	DJI_0347	Genista tinctoria L.	0	n	1	0	Fabaceae Lindl.
Iz_6_1009	DJI_0347	Geranium robertianum L.	0	n	1	0	Geraniaceae Juss.
Iz_6_1009	DJI_0347	Geranium sanquineum L.	0	n	1	0	Geraniaceae Juss.
Iz_6_1009	DJI_0347	Gypsophila paniculata L.	0	n	1	0	Caryophyllaceae Juss.
Iz_6_1009	DJI_0347	Helichrysum arenarium (L.) Moench	0	n	1	0	Asteraceae Bercht. & J.Presl
Iz_6_1009	DJI_0347	Herniaria polygama J.Gay	0	n	1	0	Caryophyllaceae Juss.
Iz_6_1009	DJI_0347	Hieracium robustum Fr.	0	n	1	0	Asteraceae Bercht. & J.Presl
Iz_6_1009	DJI_0347	Hieracium umbellatum L.	0	n	1	NA	Asteraceae Bercht. & J.Presl
Iz_6_1009	DJI_0347	Hylotelephium maximum (L.) Holub	0	n	1	0	Crassulaceae J.St.-Hil.
Iz_6_1009	DJI_0347	Jacobaea borysthenica (DC.) B.Nord. & Greuter	0	n	1	0	Asteraceae Bercht. & J.Presl
Iz_6_1009	DJI_0347	Jasione montana L.	0	n	1	0	Campanulaceae Juss.
Iz_6_1009	DJI_0347	Jurinea cyanoides (L.) Rchb.s.l.	0	n	1	0	Asteraceae Bercht. & J.Presl
Iz_6_1009	DJI_0347	Koeleria glauca (Spreng.) DC.	0	n	1	0	Poaceae Barnhart
Iz_6_1009	DJI_0347	Lactuca serriola L.	0	a	0	0	Asteraceae Bercht. & J.Presl
Iz_6_1009	DJI_0347	Linaria genistifolia (L.) Mill.	0	n	1	0	Plantaginaceae Juss.
Iz_6_1009	DJI_0347	Lomelosia argentea (L.) Greuter & Burdet	0	n	1	0	Caprifoliaceae Juss.
Iz_6_1009	DJI_0347	Melampyrum pratense L.	0	n	1	0	Orobanchaceae Vent.
Iz_6_1009	DJI_0347	Oenothera biennis L.	0	a	0	0	Onagraceae Juss.
Iz_6_1009	DJI_0347	Pentanema britannica (L.) D.Gut.Larr., Santos-Vicente, Anderb., E.Rico & M.M.Mart.Ort.	0	n	1	0	Asteraceae Bercht. & J.Presl
Iz_6_1009	DJI_0347	Peucedanum oreoselinum (L.) Moench	0	n	1	0	Apiaceae Lindl.
Iz_6_1009	DJI_0347	Peucedanum ruthenicum M.Bieb.	0	n	1	0	Apiaceae Lindl.
Iz_6_1009	DJI_0347	Pilosella echioides (Lumn.) F.W.Schultz & Sch.Bip.	0	n	1	0	Asteraceae Bercht. & J.Presl
Iz_6_1009	DJI_0347	Pilosella officinarum F. Schultz et Sch. Bip.	0	n	1	0	Asteraceae Bercht. & J.Presl
Iz_6_1009	DJI_0347	Pilosella sp.	0		NA	0	Asteraceae Bercht. & J.Presl
Iz_6_1009	DJI_0347	Pinus sylvestris L.	0	n	1	1	Pinaceae Spreng. ex F.Rudolphi
Iz_6_1009	DJI_0347	Poa annua L.	0	n	1	0	Poaceae Barnhart
Iz_6_1009	DJI_0347	Poa nemoralis L.	0	n	1	0	Poaceae Barnhart
Iz_6_1009	DJI_0347	Poa sp.	0		NA	0	Poaceae Barnhart
Iz_6_1009	DJI_0347	Polygonatum odoratum (Mill.) Druce	0	n	1	0	Asparagaceae Juss.
Iz_6_1009	DJI_0347	Polygonum arenarium Waldst. & Kit.	0	n	1	0	Polygonaceae Juss.
Iz_6_1009	DJI_0347	Populus nigra L.	0	n	1	1	Salicaceae Mirb.
Iz_6_1009	DJI_0347	Populus tremula L.	0	n	1	1	Salicaceae Mirb.
Iz_6_1009	DJI_0347	Populus Ã canescens (Aiton) Sm.	0	n	1	1	Salicaceae Mirb.
Iz_6_1009	DJI_0347	Potentilla argentea L.	0	n	1	0	Rosaceae Juss.
Iz_6_1009	DJI_0347	Psammophiliella muralis (L.) Ikonn.	0	n	1	0	Caryophyllaceae Juss.
Iz_6_1009	DJI_0347	Psephellus sumensis (Kalen.) Greuter	0	n	1	0	Asteraceae Bercht. & J.Presl
Iz_6_1009	DJI_0347	Pulsatilla pratensis (L.) Mill. s.l.	0	n	1	0	Ranunculaceae Juss.
Iz_6_1009	DJI_0347	Pyrus communis L.	0	a	0	1	Rosaceae Juss.
Iz_6_1009	DJI_0347	Quercus robur L.	0	n	1	1	Fagaceae Dumort.
Iz_6_1009	DJI_0347	Robinia pseudoacacia L.	0	a	0	1	Fabaceae Lindl.
Iz_6_1009	DJI_0347	Rosa sp.	0		NA	1	Rosaceae Juss.
Iz_6_1009	DJI_0347	Rubus idaeus L.	0	n	1	0	Rosaceae Juss.
Iz_6_1009	DJI_0347	Rumex acetosella L.	0	n	1	0	Polygonaceae Juss.
Iz_6_1009	DJI_0347	Rumex thyrsiflorus Fingerh.	0	n	1	0	Polygonaceae Juss.
Iz_6_1009	DJI_0347	Salix alba L.	0	n	1	1	Salicaceae Mirb.
Iz_6_1009	DJI_0347	Sambucus nigra L.	0	n	1	1	Viburnaceae Raf.
Iz_6_1009	DJI_0347	Secale sylvestre Host	0	n	1	0	Poaceae Barnhart
Iz_6_1009	DJI_0347	Sempervivum ruthenicum Schnittsp. & C.B. Lehm.	0	n	1	0	Crassulaceae J.St.-Hil.
Iz_6_1009	DJI_0347	Setaria viridis (L.) P. Beauv.	0	n	1	0	Poaceae Barnhart
Iz_6_1009	DJI_0347	Silene borysthenica (Gruner) Walters	0	n	1	0	Caryophyllaceae Juss.
Iz_6_1009	DJI_0347	Silene latifolia subsp. alba (Mill.) Greuter & Burdet	0	n	1	0	Caryophyllaceae Juss.
Iz_6_1009	DJI_0347	Solidago virgaurea L.	0	n	1	0	Asteraceae Bercht. & J.Presl
Iz_6_1009	DJI_0347	Sorbus aucuparia L.	0	n	1	1	Rosaceae Juss.
Iz_6_1009	DJI_0347	Stachys recta L.	0	n	1	0	Lamiacea Lindl.
Iz_6_1009	DJI_0347	Tanacetum vulgare L.	0	n	1	0	Asteraceae Bercht. & J.Presl
Iz_6_1009	DJI_0347	Thymus pallasianus Heinr.Braun	0	n	1	0	Lamiacea Lindl.
Iz_6_1009	DJI_0347	Tilia cordata Mill.	0	n	1	1	Malvaceae Juss.
Iz_6_1009	DJI_0347	Trifolium alpestre L.	0	n	1	0	Fabaceae Lindl.
Iz_6_1009	DJI_0347	Trifolium arvense L.	0	n	1	0	Fabaceae Lindl.
Iz_6_1009	DJI_0347	Ulmus glabra Huds.	0	n	1	1	Ulmaceae Mirb.
Iz_6_1009	DJI_0347	Verbascum densiflorum Bertol.	0	n	1	0	Scrophulariaceae Juss.
Iz_6_1009	DJI_0347	Veronica austriaca L.	0	n	1	0	Plantaginaceae Juss.
Iz_6_1009	DJI_0347	Veronica incana L.	0	n	1	0	Plantaginaceae Juss.
Iz_6_1009	DJI_0347	Veronica verna L.	0	n	1	0	Plantaginaceae Juss.
Iz_6_1009	DJI_0347	Vincetoxicum hirundinaria Medik	0	n	1	0	Apocynaceae Juss.
Iz_6_1009	DJI_0349	Acer tataricum L.	0	n	1	1	Sapindaceae Juss.
Iz_6_1009	DJI_0349	Achillea millefolium L. s.l.	0	n	1	0	Asteraceae Bercht. & J.Presl
Iz_6_1009	DJI_0349	Allium oleraceum L.	0	n	1	0	Amaryllidaceae J.St.-Hil.
Iz_6_1009	DJI_0349	Ambrosia artemisiifolia L.	0	a	0	0	Asteraceae Bercht. & J.Presl
Iz_6_1009	DJI_0349	Amorpha fruticosa L.	0	a	0	0	Fabaceae Lindl.
Iz_6_1009	DJI_0349	Anthericum ramosum L.	0	n	1	0	Asparagaceae Juss.
Iz_6_1009	DJI_0349	Artemisia marschalliana Spreng.	0	n	1	0	Asteraceae Bercht. & J.Presl
Iz_6_1009	DJI_0349	Asparagus officinalis L.	0	n	1	0	Asparagaceae Juss.
Iz_6_1009	DJI_0349	Astragalus cicer L.	0	n	1	0	Fabaceae Lindl.
Iz_6_1009	DJI_0349	Bassia laniflora (S.G.Gmel.) A.J.Scott	0	n	1	0	Amaranthaceae Juss.
Iz_6_1009	DJI_0349	Berberis vulgaris L.	0	n	1	0	Berberidaceae Juss.
Iz_6_1009	DJI_0349	Betula pendula Roth.	0	n	1	1	Betulaceae Gray
Iz_6_1009	DJI_0349	Betula pendula Roth.	0	n	1	0	Poaceae Barnhart
Iz_6_1009	DJI_0349	Bromus inermis Leyss.	0		NA	NA	
Iz_6_1009	DJI_0349	Bromus squarrosus L.	0	a	0	0	Poaceae Barnhart
Iz_6_1009	DJI_0349	Bromus tectorum L.	0	a	0	0	Poaceae Barnhart
Iz_6_1009	DJI_0349	Calamagrostis epigejos (L.) Roth	20	n	1	0	Poaceae Barnhart
Iz_6_1009	DJI_0349	Carduus acanthoides L.	0	n	1	0	Asteraceae Bercht. & J.Presl
Iz_6_1009	DJI_0349	Carex sp.	0	n	1	0	Cyperaceae Juss.
Iz_6_1009	DJI_0349	Centaurea majorovii Dumbadze	0	n	1	0	Asteraceae Bercht. & J.Presl
Iz_6_1009	DJI_0349	Chamaecytisus ruthenicus (Fisch.ex Wol.) Klaskova	0	n	1	0	Fabaceae Lindl.
Iz_6_1009	DJI_0349	Chelidonium majus L.	0	n	1	0	Papaveraceae Juss.

Iz_6_1009	DJI_0349	Chenopodium betaceum Andrz.	0	n	1	0	Amaranthaceae Juss.
Iz_6_1009	DJI_0349	Chenopodium cf. album	0	n	1	0	Amaranthaceae Juss.
Iz_6_1009	DJI_0349	Chenopodium sp.	0		NA	0	Amaranthaceae Juss.
Iz_6_1009	DJI_0349	Chondrilla juncea L.	0	n	1	0	Asteraceae Bercht. & J.Presl
Iz_6_1009	DJI_0349	Cirsium vulgare (Savi) Ten.	0	n	1	0	Asteraceae Bercht. & J.Presl
Iz_6_1009	DJI_0349	Clinopodium acinos (L.) Kuntze	0	n	1	0	Lamiacea Lindl.
Iz_6_1009	DJI_0349	Convallaria majalis L.	0	n	1	0	Asparagaceae Juss.
Iz_6_1009	DJI_0349	Crataegus rhipidophylla Gand.	0	n	1	1	Rosaceae Juss.
Iz_6_1009	DJI_0349	Crepis foetida L.	0	n	1	0	Asteraceae Bercht. & J.Presl
Iz_6_1009	DJI_0349	Dactylis glomerata L.	0	n	1	0	Poaceae Barnhart
Iz_6_1009	DJI_0349	Dianthus campestris M.Bieb.	0	n	1	0	Caryophyllaceae Juss.
Iz_6_1009	DJI_0349	Digitaria sanguinalis (L.) Scop.	0	a	0	0	Poaceae Barnhart
Iz_6_1009	DJI_0349	Elymus repens (L.) Gould	0	n	1	0	Poaceae Barnhart
Iz_6_1009	DJI_0349	Epilobium tetragonum L.	0	n	1	0	Onagraceae Juss.
Iz_6_1009	DJI_0349	Eragrostis minor Host	0	a	0	0	Poaceae Barnhart
Iz_6_1009	DJI_0349	Erigeron canadensis L.	6	a	0	0	Asteraceae Bercht. & J.Presl
Iz_6_1009	DJI_0349	Euphorbia seguieriana Neck.	0	n	1	0	Euphorbiaceae Juss.
Iz_6_1009	DJI_0349	Fallopia dumetorum (L.) Holub	0	n	1	0	Polygonaceae Juss.
Iz_6_1009	DJI_0349	Festuca beckeri (Hack.) Trautv.	0	n	1	0	Poaceae Barnhart
Iz_6_1009	DJI_0349	Fragaria vesca L.	0	n	1	0	Rosaceae Juss.
Iz_6_1009	DJI_0349	Frangula alnus Mill.	0	n	1	1	Rhamnaceae Juss.
Iz_6_1009	DJI_0349	Fraxinus pennsylvanica Marshall	0	a	0	1	Oleaceae Hoffmanns. & Link
Iz_6_1009	DJI_0349	Galeopsis bifida Boenn.	0	n	1	0	Lamiacea Lindl.
Iz_6_1009	DJI_0349	Galium verum L.	0	n	1	0	Rubiaceae Juss.
Iz_6_1009	DJI_0349	Genista tinctoria L.	0	n	1	0	Fabaceae Lindl.
Iz_6_1009	DJI_0349	Geranium robertianum L.	0	n	1	0	Geraniaceae Juss.
Iz_6_1009	DJI_0349	Geranium sanquineum L.	0	n	1	0	Geraniaceae Juss.
Iz_6_1009	DJI_0349	Gypsophila paniculata L.	0	n	1	0	Caryophyllaceae Juss.
Iz_6_1009	DJI_0349	Helichrysum arenarium (L.) Moench	0	n	1	0	Asteraceae Bercht. & J.Presl
Iz_6_1009	DJI_0349	Herniaria polygama J.Gay	0	n	1	0	Caryophyllaceae Juss.
Iz_6_1009	DJI_0349	Hieracium robustum Fr.	0	n	1	0	Asteraceae Bercht. & J.Presl
Iz_6_1009	DJI_0349	Hieracium umbellatum L.	0	n	1	NA	Asteraceae Bercht. & J.Presl
Iz_6_1009	DJI_0349	Hylotelephium maximum (L.) Holub	0	n	1	0	Crassulaceae J.St.-Hil.
Iz_6_1009	DJI_0349	Jacobaea borysthenica (DC.) B.Nord. & Greuter	0	n	1	0	Asteraceae Bercht. & J.Presl
Iz_6_1009	DJI_0349	Jasione montana L.	0	n	1	0	Campanulaceae Juss.
Iz_6_1009	DJI_0349	Jurinea cyanoides (L.) Rchb.s.l.	0	n	1	0	Asteraceae Bercht. & J.Presl
Iz_6_1009	DJI_0349	Koeleria glauca (Spreng.) DC.	0	n	1	0	Poaceae Barnhart
Iz_6_1009	DJI_0349	Lactuca serriola L.	0	a	0	0	Asteraceae Bercht. & J.Presl
Iz_6_1009	DJI_0349	Linaria genistifolia (L.) Mill.	0	n	1	0	Plantaginaceae Juss.
Iz_6_1009	DJI_0349	Lomelosia argentea (L.) Greuter & Burdet	0	n	1	0	Caprifoliaceae Juss.
Iz_6_1009	DJI_0349	Melampyrum pratense L.	0	n	1	0	Orobanchaceae Vent.
Iz_6_1009	DJI_0349	Oenothera biennis L.	0	a	0	0	Onagraceae Juss.
Iz_6_1009	DJI_0349	Pentanema britannica (L.) D.Gut.Larr., Santos-Vicente, Anderb., E.Rico & M.M.Mart.Ort.	0	n	1	0	Asteraceae Bercht. & J.Presl
Iz_6_1009	DJI_0349	Peucedanum oreoselinum (L.) Moench	0	n	1	0	Apiaceae Lindl.
Iz_6_1009	DJI_0349	Peucedanum ruthenicum M.Bieb.	0	n	1	0	Apiaceae Lindl.
Iz_6_1009	DJI_0349	Pilosella echioides (Lumn.) F.W.Schultz & Sch.Bip.	0	n	1	0	Asteraceae Bercht. & J.Presl
Iz_6_1009	DJI_0349	Pilosella officinarum F. Schultz et Sch. Bip.	0	n	1	0	Asteraceae Bercht. & J.Presl
Iz_6_1009	DJI_0349	Pilosella sp.	0		NA	0	Asteraceae Bercht. & J.Presl
Iz_6_1009	DJI_0349	Pinus sylvestris L.	0	n	1	1	Pinaceae Spreng. ex F.Rudolphi
Iz_6_1009	DJI_0349	Poa annua L.	0	n	1	0	Poaceae Barnhart
Iz_6_1009	DJI_0349	Poa nemoralis L.	0	n	1	0	Poaceae Barnhart
Iz_6_1009	DJI_0349	Poa sp.	0		NA	0	Poaceae Barnhart
Iz_6_1009	DJI_0349	Polygonatum odoratum (Mill.) Druce	0	n	1	0	Asparagaceae Juss.
Iz_6_1009	DJI_0349	Polygonum arenarium Waldst. & Kit.	0	n	1	0	Polygonaceae Juss.
Iz_6_1009	DJI_0349	Populus nigra L.	0	n	1	1	Salicaceae Mirb.
Iz_6_1009	DJI_0349	Populus tremula L.	0	n	1	1	Salicaceae Mirb.
Iz_6_1009	DJI_0349	Populus Ã canescens (Aiton) Sm.	0	n	1	1	Salicaceae Mirb.
Iz_6_1009	DJI_0349	Potentilla argentea L.	0	n	1	0	Rosaceae Juss.
Iz_6_1009	DJI_0349	Psammophiliella muralis (L.) Ikonn.	0	n	1	0	Caryophyllaceae Juss.
Iz_6_1009	DJI_0349	Psephellus sumensis (Kalen.) Greuter	0	n	1	0	Asteraceae Bercht. & J.Presl
Iz_6_1009	DJI_0349	Pulsatilla pratensis (L.) Mill. s.l.	0	n	1	0	Ranunculaceae Juss.
Iz_6_1009	DJI_0349	Pyrus communis L.	0	a	0	1	Rosaceae Juss.
Iz_6_1009	DJI_0349	Quercus robur L.	0	n	1	1	Fagaceae Dumort.
Iz_6_1009	DJI_0349	Robinia pseudoacacia L.	0	a	0	1	Fabaceae Lindl.
Iz_6_1009	DJI_0349	Rosa sp.	0		NA	1	Rosaceae Juss.
Iz_6_1009	DJI_0349	Rubus idaeus L.	0	n	1	0	Rosaceae Juss.
Iz_6_1009	DJI_0349	Rumex acetosella L.	0	n	1	0	Polygonaceae Juss.
Iz_6_1009	DJI_0349	Rumex thyrsiflorus Fingerh.	0	n	1	0	Polygonaceae Juss.
Iz_6_1009	DJI_0349	Salix alba L.	0	n	1	1	Salicaceae Mirb.
Iz_6_1009	DJI_0349	Sambucus nigra L.	0	n	1	1	Viburnaceae Raf.
Iz_6_1009	DJI_0349	Secale sylvestre Host	0	n	1	0	Poaceae Barnhart
Iz_6_1009	DJI_0349	Sempervivum ruthenicum Schnittsp. & C.B. Lehm.	0	n	1	0	Crassulaceae J.St.-Hil.
Iz_6_1009	DJI_0349	Setaria viridis (L.) P. Beauv.	0	n	1	0	Poaceae Barnhart
Iz_6_1009	DJI_0349	Silene borysthenica (Gruner) Walters	0	n	1	0	Caryophyllaceae Juss.
Iz_6_1009	DJI_0349	Silene latifolia subsp. alba (Mill.) Greuter & Burdet	0	n	1	0	Caryophyllaceae Juss.
Iz_6_1009	DJI_0349	Solidago virgaurea L.	0	n	1	0	Asteraceae Bercht. & J.Presl
Iz_6_1009	DJI_0349	Sorbus aucuparia L.	0	n	1	1	Rosaceae Juss.
Iz_6_1009	DJI_0349	Stachys recta L.	0	n	1	0	Lamiacea Lindl.
Iz_6_1009	DJI_0349	Tanacetum vulgare L.	0	n	1	0	Asteraceae Bercht. & J.Presl
Iz_6_1009	DJI_0349	Thymus pallasianus Heinr.Braun	0	n	1	0	Lamiacea Lindl.
Iz_6_1009	DJI_0349	Tilia cordata Mill.	0	n	1	1	Malvaceae Juss.
Iz_6_1009	DJI_0349	Trifolium alpestre L.	0	n	1	0	Fabaceae Lindl.
Iz_6_1009	DJI_0349	Trifolium arvense L.	0	n	1	0	Fabaceae Lindl.
Iz_6_1009	DJI_0349	Ulmus glabra Huds.	0	n	1	1	Ulmaceae Mirb.
Iz_6_1009	DJI_0349	Verbascum densiflorum Bertol.	0	n	1	0	Scrophulariaceae Juss.
Iz_6_1009	DJI_0349	Veronica austriaca L.	0	n	1	0	Plantaginaceae Juss.
Iz_6_1009	DJI_0349	Veronica incana L.	0	n	1	0	Plantaginaceae Juss.
Iz_6_1009	DJI_0349	Veronica verna L.	0	n	1	0	Plantaginaceae Juss.
Iz_6_1009	DJI_0349	Vincetoxicum hirundinaria Medik	0	n	1	0	Apocynaceae Juss.
Iz_6_1009	DJI_0355	Acer tataricum L.	0	n	1	1	Sapindaceae Juss.
Iz_6_1009	DJI_0355	Achillea millefolium L. s.l.	0	n	1	0	Asteraceae Bercht. & J.Presl
Iz_6_1009	DJI_0355	Allium oleraceum L.	0	n	1	0	Amaryllidaceae J.St.-Hil.
Iz_6_1009	DJI_0355	Ambrosia artemisiifolia L.	0	a	0	0	Asteraceae Bercht. & J.Presl
Iz_6_1009	DJI_0355	Amorpha fruticosa L.	0	a	0	0	Fabaceae Lindl.
Iz_6_1009	DJI_0355	Anthericum ramosum L.	0	n	1	0	Asparagaceae Juss.
Iz_6_1009	DJI_0355	Artemisia marschalliana Spreng.	0	n	1	0	Asteraceae Bercht. & J.Presl
Iz_6_1009	DJI_0355	Asparagus officinalis L.	0	n	1	0	Asparagaceae Juss.
Iz_6_1009	DJI_0355	Astragalus cicer L.	0	n	1	0	Fabaceae Lindl.

Iz_6_1009	DJI_0355	Bassia laniflora (S.G.Gmel.) A.J.Scott	0	n	1	0	Amaranthaceae Juss.
Iz_6_1009	DJI_0355	Berberis vulgaris L.	0	n	1	0	Berberidaceae Juss.
Iz_6_1009	DJI_0355	Betula pendula Roth.	0	n	1	1	Betulaceae Gray
Iz_6_1009	DJI_0355	Betula pendula Roth.	0	n	1	0	Poaceae Barnhart
Iz_6_1009	DJI_0355	Bromus inermis Leyss.	0		NA	NA	
Iz_6_1009	DJI_0355	Bromus squarrosus L.	0	a	0	0	Poaceae Barnhart
Iz_6_1009	DJI_0355	Bromus tectorum L.	0	a	0	0	Poaceae Barnhart
Iz_6_1009	DJI_0355	Calamagrostis epigejos (L.) Roth	10	n	1	0	Poaceae Barnhart
Iz_6_1009	DJI_0355	Carduus acanthoides L.	0	n	1	0	Asteraceae Bercht. & J.Presl
Iz_6_1009	DJI_0355	Carex sp.	0	n	1	0	Cyperaceae Juss.
Iz_6_1009	DJI_0355	Centaurea majorovii Dumbadze	0	n	1	0	Asteraceae Bercht. & J.Presl
Iz_6_1009	DJI_0355	Chamaecytisus ruthenicus (Fisch.ex Wol.) Klaskova	0	n	1	0	Fabaceae Lindl.
Iz_6_1009	DJI_0355	Chelidonium majus L.	0	n	1	0	Papaveraceae Juss.
Iz_6_1009	DJI_0355	Chenopodium betaceum Andrz.	0	n	1	0	Amaranthaceae Juss.
Iz_6_1009	DJI_0355	Chenopodium cf. album	0	n	1	0	Amaranthaceae Juss.
Iz_6_1009	DJI_0355	Chenopodium sp.	0		NA	0	Amaranthaceae Juss.
Iz_6_1009	DJI_0355	Chondrilla juncea L.	0	n	1	0	Asteraceae Bercht. & J.Presl
Iz_6_1009	DJI_0355	Cirsium vulgare (Savi) Ten.	0	n	1	0	Asteraceae Bercht. & J.Presl
Iz_6_1009	DJI_0355	Clinopodium acinos (L.) Kuntze	0	n	1	0	Lamiacea Lindl.
Iz_6_1009	DJI_0355	Convallaria majalis L.	0	n	1	0	Asparagaceae Juss.
Iz_6_1009	DJI_0355	Crataegus rhipidophylla Gand.	0	n	1	1	Rosaceae Juss.
Iz_6_1009	DJI_0355	Crepis foetida L.	0	n	1	0	Asteraceae Bercht. & J.Presl
Iz_6_1009	DJI_0355	Dactylis glomerata L.	0	n	1	0	Poaceae Barnhart
Iz_6_1009	DJI_0355	Dianthus campestris M.Bieb.	0	n	1	0	Caryophyllaceae Juss.
Iz_6_1009	DJI_0355	Digitaria sanguinalis (L.) Scop.	0	a	0	0	Poaceae Barnhart
Iz_6_1009	DJI_0355	Elymus repens (L.) Gould	0	n	1	0	Poaceae Barnhart
Iz_6_1009	DJI_0355	Epilobium tetragonum L.	0	n	1	0	Onagraceae Juss.
Iz_6_1009	DJI_0355	Eragrostis minor Host	0	a	0	0	Poaceae Barnhart
Iz_6_1009	DJI_0355	Erigeron canadensis L.	6	a	0	0	Asteraceae Bercht. & J.Presl
Iz_6_1009	DJI_0355	Euphorbia seguieriana Neck.	0	n	1	0	Euphorbiaceae Juss.
Iz_6_1009	DJI_0355	Fallopia dumetorum (L.) Holub	0	n	1	0	Polygonaceae Juss.
Iz_6_1009	DJI_0355	Festuca beckeri (Hack.) Trautv.	0	n	1	0	Poaceae Barnhart
Iz_6_1009	DJI_0355	Fragaria vesca L.	0	n	1	0	Rosaceae Juss.
Iz_6_1009	DJI_0355	Frangula alnus Mill.	0	n	1	1	Rhamnaceae Juss.
Iz_6_1009	DJI_0355	Fraxinus pennsylvanica Marshall	0	a	0	1	Oleaceae Hoffmanns. & Link
Iz_6_1009	DJI_0355	Galeopsis bifida Boenn.	0	n	1	0	Lamiacea Lindl.
Iz_6_1009	DJI_0355	Galium verum L.	0	n	1	0	Rubiaceae Juss.
Iz_6_1009	DJI_0355	Genista tinctoria L.	0	n	1	0	Fabaceae Lindl.
Iz_6_1009	DJI_0355	Geranium robertianum L.	0	n	1	0	Geraniaceae Juss.
Iz_6_1009	DJI_0355	Geranium sanquineum L.	0	n	1	0	Geraniaceae Juss.
Iz_6_1009	DJI_0355	Gypsophila paniculata L.	0	n	1	0	Caryophyllaceae Juss.
Iz_6_1009	DJI_0355	Helichrysum arenarium (L.) Moench	0	n	1	0	Asteraceae Bercht. & J.Presl
Iz_6_1009	DJI_0355	Herniaria polygama J.Gay	0	n	1	0	Caryophyllaceae Juss.
Iz_6_1009	DJI_0355	Hieracium robustum Fr.	0	n	1	0	Asteraceae Bercht. & J.Presl
Iz_6_1009	DJI_0355	Hieracium umbellatum L.	0	n	1	NA	Asteraceae Bercht. & J.Presl
Iz_6_1009	DJI_0355	Hylotelephium maximum (L.) Holub	0	n	1	0	Crassulaceae J.St.-Hil.
Iz_6_1009	DJI_0355	Jacobaea borysthenica (DC.) B.Nord. & Greuter	0	n	1	0	Asteraceae Bercht. & J.Presl
Iz_6_1009	DJI_0355	Jasione montana L.	0	n	1	0	Campanulaceae Juss.
Iz_6_1009	DJI_0355	Jurinea cyanoides (L.) Rchb.s.l.	0	n	1	0	Asteraceae Bercht. & J.Presl
Iz_6_1009	DJI_0355	Koeleria glauca (Spreng.) DC.	0	n	1	0	Poaceae Barnhart
Iz_6_1009	DJI_0355	Lactuca serriola L.	0	a	0	0	Asteraceae Bercht. & J.Presl
Iz_6_1009	DJI_0355	Linaria genistifolia (L.) Mill.	0	n	1	0	Plantaginaceae Juss.
Iz_6_1009	DJI_0355	Lomelosia argentea (L.) Greuter & Burdet	0	n	1	0	Caprifoliaceae Juss.
Iz_6_1009	DJI_0355	Melampyrum pratense L.	0	n	1	0	Orobanchaceae Vent.
Iz_6_1009	DJI_0355	Oenothera biennis L.	0	a	0	0	Onagraceae Juss.
Iz_6_1009	DJI_0355	Pentanema britannica (L.) D.Gut.Larr., Santos-Vicente, Anderb., E.Rico & M.M.Mart.Ort.	0	n	1	0	Asteraceae Bercht. & J.Presl
Iz_6_1009	DJI_0355	Peucedanum oreoselinum (L.) Moench	0	n	1	0	Apiaceae Lindl.
Iz_6_1009	DJI_0355	Peucedanum ruthenicum M.Bieb.	0	n	1	0	Apiaceae Lindl.
Iz_6_1009	DJI_0355	Pilosella echioides (Lumn.) F.W.Schultz & Sch.Bip.	0	n	1	0	Asteraceae Bercht. & J.Presl
Iz_6_1009	DJI_0355	Pilosella officinarum F. Schultz et Sch. Bip.	0	n	1	0	Asteraceae Bercht. & J.Presl
Iz_6_1009	DJI_0355	Pilosella sp.	0		NA	0	Asteraceae Bercht. & J.Presl
Iz_6_1009	DJI_0355	Pinus sylvestris L.	0	n	1	1	Pinaceae Spreng. ex F.Rudolphi
Iz_6_1009	DJI_0355	Poa annua L.	0	n	1	0	Poaceae Barnhart
Iz_6_1009	DJI_0355	Poa nemoralis L.	0	n	1	0	Poaceae Barnhart
Iz_6_1009	DJI_0355	Poa sp.	0		NA	0	Poaceae Barnhart
Iz_6_1009	DJI_0355	Polygonatum odoratum (Mill.) Druce	0	n	1	0	Asparagaceae Juss.
Iz_6_1009	DJI_0355	Polygonum arenarium Waldst. & Kit.	0	n	1	0	Polygonaceae Juss.
Iz_6_1009	DJI_0355	Populus nigra L.	0	n	1	1	Salicaceae Mirb.
Iz_6_1009	DJI_0355	Populus tremula L.	0	n	1	1	Salicaceae Mirb.
Iz_6_1009	DJI_0355	Populus Ã canescens (Aiton) Sm.	0	n	1	1	Salicaceae Mirb.
Iz_6_1009	DJI_0355	Potentilla argentea L.	0	n	1	0	Rosaceae Juss.
Iz_6_1009	DJI_0355	Psammophiliella muralis (L.) Ikonn.	0	n	1	0	Caryophyllaceae Juss.
Iz_6_1009	DJI_0355	Psephellus sumensis (Kalen.) Greuter	0	n	1	0	Asteraceae Bercht. & J.Presl
Iz_6_1009	DJI_0355	Pulsatilla pratensis (L.) Mill. s.l.	0	n	1	0	Ranunculaceae Juss.
Iz_6_1009	DJI_0355	Pyrus communis L.	0	a	0	1	Rosaceae Juss.
Iz_6_1009	DJI_0355	Quercus robur L.	0	n	1	1	Fagaceae Dumort.
Iz_6_1009	DJI_0355	Robinia pseudoacacia L.	0	a	0	1	Fabaceae Lindl.
Iz_6_1009	DJI_0355	Rosa sp.	0		NA	1	Rosaceae Juss.
Iz_6_1009	DJI_0355	Rubus idaeus L.	0	n	1	0	Rosaceae Juss.
Iz_6_1009	DJI_0355	Rumex acetosella L.	0	n	1	0	Polygonaceae Juss.
Iz_6_1009	DJI_0355	Rumex thyrsiflorus Fingerh.	0	n	1	0	Polygonaceae Juss.
Iz_6_1009	DJI_0355	Salix alba L.	0	n	1	1	Salicaceae Mirb.
Iz_6_1009	DJI_0355	Sambucus nigra L.	0	n	1	1	Viburnaceae Raf.
Iz_6_1009	DJI_0355	Secale sylvestre Host	0	n	1	0	Poaceae Barnhart
Iz_6_1009	DJI_0355	Sempervivum ruthenicum Schnittsp. & C.B. Lehm.	0	n	1	0	Crassulaceae J.St.-Hil.
Iz_6_1009	DJI_0355	Setaria viridis (L.) P. Beauv.	0	n	1	0	Poaceae Barnhart
Iz_6_1009	DJI_0355	Silene borysthenica (Gruner) Walters	0	n	1	0	Caryophyllaceae Juss.
Iz_6_1009	DJI_0355	Silene latifolia subsp. alba (Mill.) Greuter & Burdet	0	n	1	0	Caryophyllaceae Juss.
Iz_6_1009	DJI_0355	Solidago virgaurea L.	0	n	1	0	Asteraceae Bercht. & J.Presl
Iz_6_1009	DJI_0355	Sorbus aucuparia L.	0	n	1	1	Rosaceae Juss.
Iz_6_1009	DJI_0355	Stachys recta L.	0	n	1	0	Lamiacea Lindl.
Iz_6_1009	DJI_0355	Tanacetum vulgare L.	0	n	1	0	Asteraceae Bercht. & J.Presl
Iz_6_1009	DJI_0355	Thymus pallasianus Heinr.Braun	0	n	1	0	Lamiacea Lindl.
Iz_6_1009	DJI_0355	Tilia cordata Mill.	0	n	1	1	Malvaceae Juss.
Iz_6_1009	DJI_0355	Trifolium alpestre L.	0	n	1	0	Fabaceae Lindl.
Iz_6_1009	DJI_0355	Trifolium arvense L.	0	n	1	0	Fabaceae Lindl.
Iz_6_1009	DJI_0355	Ulmus glabra Huds.	0	n	1	1	Ulmaceae Mirb.
Iz_6_1009	DJI_0355	Verbascum densiflorum Bertol.	0	n	1	0	Scrophulariaceae Juss.

Iz_6_1009	DJI_0355	Veronica austriaca L.	0	n	1	0	Plantaginaceae Juss.
Iz_6_1009	DJI_0355	Veronica incana L.	0	n	1	0	Plantaginaceae Juss.
Iz_6_1009	DJI_0355	Veronica verna L.	0	n	1	0	Plantaginaceae Juss.
Iz_6_1009	DJI_0355	Vincetoxicum hirundinaria Medik	0	n	1	0	Apocynaceae Juss.
Iz_6_1009	DJI_0358	Acer tataricum L.	0	n	1	1	Sapindaceae Juss.
Iz_6_1009	DJI_0358	Achillea millefolium L. s.l.	0	n	1	0	Asteraceae Bercht. & J.Presl
Iz_6_1009	DJI_0358	Allium oleraceum L.	0	n	1	0	Amaryllidaceae J.St.-Hil.
Iz_6_1009	DJI_0358	Ambrosia artemisiifolia L.	0	a	0	0	Asteraceae Bercht. & J.Presl
Iz_6_1009	DJI_0358	Amorpha fruticosa L.	0	a	0	0	Fabaceae Lindl.
Iz_6_1009	DJI_0358	Anthericum ramosum L.	0	n	1	0	Asparagaceae Juss.
Iz_6_1009	DJI_0358	Artemisia marschalliana Spreng.	0	n	1	0	Asteraceae Bercht. & J.Presl
Iz_6_1009	DJI_0358	Asparagus officinalis L.	0	n	1	0	Asparagaceae Juss.
Iz_6_1009	DJI_0358	Astragalus cicer L.	0	n	1	0	Fabaceae Lindl.
Iz_6_1009	DJI_0358	Bassia laniflora (S.G.Gmel.) A.J.Scott	0	n	1	0	Amaranthaceae Juss.
Iz_6_1009	DJI_0358	Berberis vulgaris L.	0	n	1	0	Berberidaceae Juss.
Iz_6_1009	DJI_0358	Betula pendula Roth.	0	n	1	1	Betulaceae Gray
Iz_6_1009	DJI_0358	Betula pendula Roth.	0	n	1	0	Poaceae Barnhart
Iz_6_1009	DJI_0358	Bromus inermis Leyss.	0		NA	NA	
Iz_6_1009	DJI_0358	Bromus squarrosus L.	0	a	0	0	Poaceae Barnhart
Iz_6_1009	DJI_0358	Bromus tectorum L.	0	a	0	0	Poaceae Barnhart
Iz_6_1009	DJI_0358	Calamagrostis epigejos (L.) Roth	8	n	1	0	Poaceae Barnhart
Iz_6_1009	DJI_0358	Carduus acanthoides L.	0	n	1	0	Asteraceae Bercht. & J.Presl
Iz_6_1009	DJI_0358	Carex sp.	0	n	1	0	Cyperaceae Juss.
Iz_6_1009	DJI_0358	Centaurea majorovii Dumbadze	0	n	1	0	Asteraceae Bercht. & J.Presl
Iz_6_1009	DJI_0358	Chamaecytisus ruthenicus (Fisch.ex Wol.) Klaskova	0	n	1	0	Fabaceae Lindl.
Iz_6_1009	DJI_0358	Chelidonium majus L.	0	n	1	0	Papaveraceae Juss.
Iz_6_1009	DJI_0358	Chenopodium betaceum Andrz.	0	n	1	0	Amaranthaceae Juss.
Iz_6_1009	DJI_0358	Chenopodium cf. album	0	n	1	0	Amaranthaceae Juss.
Iz_6_1009	DJI_0358	Chenopodium sp.	0		NA	0	Amaranthaceae Juss.
Iz_6_1009	DJI_0358	Chondrilla juncea L.	0	n	1	0	Asteraceae Bercht. & J.Presl
Iz_6_1009	DJI_0358	Cirsium vulgare (Savi) Ten.	0	n	1	0	Asteraceae Bercht. & J.Presl
Iz_6_1009	DJI_0358	Clinopodium acinos (L.) Kuntze	0	n	1	0	Lamiacea Lindl.
Iz_6_1009	DJI_0358	Convallaria majalis L.	0	n	1	0	Asparagaceae Juss.
Iz_6_1009	DJI_0358	Crataegus rhipidophylla Gand.	0	n	1	1	Rosaceae Juss.
Iz_6_1009	DJI_0358	Crepis foetida L.	0	n	1	0	Asteraceae Bercht. & J.Presl
Iz_6_1009	DJI_0358	Dactylis glomerata L.	0	n	1	0	Poaceae Barnhart
Iz_6_1009	DJI_0358	Dianthus campestris M.Bieb.	0	n	1	0	Caryophyllaceae Juss.
Iz_6_1009	DJI_0358	Digitaria sanguinalis (L.) Scop.	0	a	0	0	Poaceae Barnhart
Iz_6_1009	DJI_0358	Elymus repens (L.) Gould	0	n	1	0	Poaceae Barnhart
Iz_6_1009	DJI_0358	Epilobium tetragonum L.	0	n	1	0	Onagraceae Juss.
Iz_6_1009	DJI_0358	Eragrostis minor Host	0	a	0	0	Poaceae Barnhart
Iz_6_1009	DJI_0358	Erigeron canadensis L.	10	a	0	0	Asteraceae Bercht. & J.Presl
Iz_6_1009	DJI_0358	Euphorbia seguieriana Neck.	0	n	1	0	Euphorbiaceae Juss.
Iz_6_1009	DJI_0358	Fallopia dumetorum (L.) Holub	0	n	1	0	Polygonaceae Juss.
Iz_6_1009	DJI_0358	Festuca beckeri (Hack.) Trautv.	0	n	1	0	Poaceae Barnhart
Iz_6_1009	DJI_0358	Fragaria vesca L.	0	n	1	0	Rosaceae Juss.
Iz_6_1009	DJI_0358	Frangula alnus Mill.	0	n	1	1	Rhamnaceae Juss.
Iz_6_1009	DJI_0358	Fraxinus pennsylvanica Marshall	0	a	0	1	Oleaceae Hoffmanns. & Link
Iz_6_1009	DJI_0358	Galeopsis bifida Boenn.	0	n	1	0	Lamiacea Lindl.
Iz_6_1009	DJI_0358	Galium verum L.	0	n	1	0	Rubiaceae Juss.
Iz_6_1009	DJI_0358	Genista tinctoria L.	0	n	1	0	Fabaceae Lindl.
Iz_6_1009	DJI_0358	Geranium robertianum L.	0	n	1	0	Geraniaceae Juss.
Iz_6_1009	DJI_0358	Geranium sanquineum L.	0	n	1	0	Geraniaceae Juss.
Iz_6_1009	DJI_0358	Gypsophila paniculata L.	0	n	1	0	Caryophyllaceae Juss.
Iz_6_1009	DJI_0358	Helichrysum arenarium (L.) Moench	0	n	1	0	Asteraceae Bercht. & J.Presl
Iz_6_1009	DJI_0358	Herniaria polygama J.Gay	0	n	1	0	Caryophyllaceae Juss.
Iz_6_1009	DJI_0358	Hieracium robustum Fr.	0	n	1	0	Asteraceae Bercht. & J.Presl
Iz_6_1009	DJI_0358	Hieracium umbellatum L.	0	n	1	NA	Asteraceae Bercht. & J.Presl
Iz_6_1009	DJI_0358	Hylotelephium maximum (L.) Holub	0	n	1	0	Crassulaceae J.St.-Hil.
Iz_6_1009	DJI_0358	Jacobaea borysthenica (DC.) B.Nord. & Greuter	0	n	1	0	Asteraceae Bercht. & J.Presl
Iz_6_1009	DJI_0358	Jasione montana L.	0	n	1	0	Campanulaceae Juss.
Iz_6_1009	DJI_0358	Jurinea cyanoides (L.) Rchb.s.l.	0	n	1	0	Asteraceae Bercht. & J.Presl
Iz_6_1009	DJI_0358	Koeleria glauca (Spreng.) DC.	0	n	1	0	Poaceae Barnhart
Iz_6_1009	DJI_0358	Lactuca serriola L.	0	a	0	0	Asteraceae Bercht. & J.Presl
Iz_6_1009	DJI_0358	Linaria genistifolia (L.) Mill.	0	n	1	0	Plantaginaceae Juss.
Iz_6_1009	DJI_0358	Lomelosia argentea (L.) Greuter & Burdet	0	n	1	0	Caprifoliaceae Juss.
Iz_6_1009	DJI_0358	Melampyrum pratense L.	0	n	1	0	Orobanchaceae Vent.
Iz_6_1009	DJI_0358	Oenothera biennis L.	0	a	0	0	Onagraceae Juss.
Iz_6_1009	DJI_0358	Pentanema britannica (L.) D.Gut.Larr., Santos-Vicente, Anderb., E.Rico & M.M.Mart.Ort.	0	n	1	0	Asteraceae Bercht. & J.Presl
Iz_6_1009	DJI_0358	Peucedanum oreoselinum (L.) Moench	0	n	1	0	Apiaceae Lindl.
Iz_6_1009	DJI_0358	Peucedanum ruthenicum M.Bieb.	0	n	1	0	Apiaceae Lindl.
Iz_6_1009	DJI_0358	Pilosella echioides (Lumn.) F.W.Schultz & Sch.Bip.	0	n	1	0	Asteraceae Bercht. & J.Presl
Iz_6_1009	DJI_0358	Pilosella officinarum F. Schultz et Sch. Bip.	0	n	1	0	Asteraceae Bercht. & J.Presl
Iz_6_1009	DJI_0358	Pilosella sp.	0		NA	0	Asteraceae Bercht. & J.Presl
Iz_6_1009	DJI_0358	Pinus sylvestris L.	0	n	1	1	Pinaceae Spreng. ex F.Rudolphi
Iz_6_1009	DJI_0358	Poa annua L.	0	n	1	0	Poaceae Barnhart
Iz_6_1009	DJI_0358	Poa nemoralis L.	0	n	1	0	Poaceae Barnhart
Iz_6_1009	DJI_0358	Poa sp.	0		NA	0	Poaceae Barnhart
Iz_6_1009	DJI_0358	Polygonatum odoratum (Mill.) Druce	0	n	1	0	Asparagaceae Juss.
Iz_6_1009	DJI_0358	Polygonum arenarium Waldst. & Kit.	0	n	1	0	Polygonaceae Juss.
Iz_6_1009	DJI_0358	Populus nigra L.	0	n	1	1	Salicaceae Mirb.
Iz_6_1009	DJI_0358	Populus tremula L.	0	n	1	1	Salicaceae Mirb.
Iz_6_1009	DJI_0358	Populus Ã canescens (Aiton) Sm.	0	n	1	1	Salicaceae Mirb.
Iz_6_1009	DJI_0358	Potentilla argentea L.	0	n	1	0	Rosaceae Juss.
Iz_6_1009	DJI_0358	Psammophiliella muralis (L.) Ikonn.	0	n	1	0	Caryophyllaceae Juss.
Iz_6_1009	DJI_0358	Psephellus sumensis (Kalen.) Greuter	0	n	1	0	Asteraceae Bercht. & J.Presl
Iz_6_1009	DJI_0358	Pulsatilla pratensis (L.) Mill. s.l.	0	n	1	0	Ranunculaceae Juss.
Iz_6_1009	DJI_0358	Pyrus communis L.	0	a	0	1	Rosaceae Juss.
Iz_6_1009	DJI_0358	Quercus robur L.	0	n	1	1	Fagaceae Dumort.
Iz_6_1009	DJI_0358	Robinia pseudoacacia L.	0	a	0	1	Fabaceae Lindl.
Iz_6_1009	DJI_0358	Rosa sp.	0		NA	1	Rosaceae Juss.
Iz_6_1009	DJI_0358	Rubus idaeus L.	0	n	1	0	Rosaceae Juss.
Iz_6_1009	DJI_0358	Rumex acetosella L.	0.5	n	1	0	Polygonaceae Juss.
Iz_6_1009	DJI_0358	Rumex thyrsiflorus Fingerh.	0	n	1	0	Polygonaceae Juss.
Iz_6_1009	DJI_0358	Salix alba L.	0	n	1	1	Salicaceae Mirb.
Iz_6_1009	DJI_0358	Sambucus nigra L.	0	n	1	1	Viburnaceae Raf.
Iz_6_1009	DJI_0358	Secale sylvestre Host	0	n	1	0	Poaceae Barnhart
Iz_6_1009	DJI_0358	Sempervivum ruthenicum Schnittsp. & C.B. Lehm.	0	n	1	0	Crassulaceae J.St.-Hil.

Iz_6_1009	DJI_0358	Setaria viridis (L.) P. Beauv.	0	n	1	0	Poaceae Barnhart
Iz_6_1009	DJI_0358	Silene borysthenica (Gruner) Walters	0	n	1	0	Caryophyllaceae Juss.
Iz_6_1009	DJI_0358	Silene latifolia subsp. alba (Mill.) Greuter & Burdet	0	n	1	0	Caryophyllaceae Juss.
Iz_6_1009	DJI_0358	Solidago virgaurea L.	0	n	1	0	Asteraceae Bercht. & J.Presl
Iz_6_1009	DJI_0358	Sorbus aucuparia L.	0	n	1	1	Rosaceae Juss.
Iz_6_1009	DJI_0358	Stachys recta L.	0	n	1	0	Lamiacea Lindl.
Iz_6_1009	DJI_0358	Tanacetum vulgare L.	0	n	1	0	Asteraceae Bercht. & J.Presl
Iz_6_1009	DJI_0358	Thymus pallasianus Heinr.Braun	0	n	1	0	Lamiacea Lindl.
Iz_6_1009	DJI_0358	Tilia cordata Mill.	0	n	1	1	Malvaceae Juss.
Iz_6_1009	DJI_0358	Trifolium alpestre L.	0	n	1	0	Fabaceae Lindl.
Iz_6_1009	DJI_0358	Trifolium arvense L.	0	n	1	0	Fabaceae Lindl.
Iz_6_1009	DJI_0358	Ulmus glabra Huds.	0	n	1	1	Ulmaceae Mirb.
Iz_6_1009	DJI_0358	Verbascum densiflorum Bertol.	0	n	1	0	Scrophulariaceae Juss.
Iz_6_1009	DJI_0358	Veronica austriaca L.	0	n	1	0	Plantaginaceae Juss.
Iz_6_1009	DJI_0358	Veronica incana L.	0	n	1	0	Plantaginaceae Juss.
Iz_6_1009	DJI_0358	Veronica verna L.	0	n	1	0	Plantaginaceae Juss.
Iz_6_1009	DJI_0358	Vincetoxicum hirundinaria Medik	0	n	1	0	Apocynaceae Juss.
Iz_6_1009	DJI_0361	Acer tataricum L.	0	n	1	1	Sapindaceae Juss.
Iz_6_1009	DJI_0361	Achillea millefolium L. s.l.	0	n	1	0	Asteraceae Bercht. & J.Presl
Iz_6_1009	DJI_0361	Allium oleraceum L.	0	n	1	0	Amaryllidaceae J.St.-Hil.
Iz_6_1009	DJI_0361	Ambrosia artemisiifolia L.	0	a	0	0	Asteraceae Bercht. & J.Presl
Iz_6_1009	DJI_0361	Amorpha fruticosa L.	0	a	0	0	Fabaceae Lindl.
Iz_6_1009	DJI_0361	Anthericum ramosum L.	0	n	1	0	Asparagaceae Juss.
Iz_6_1009	DJI_0361	Artemisia marschalliana Spreng.	0	n	1	0	Asteraceae Bercht. & J.Presl
Iz_6_1009	DJI_0361	Asparagus officinalis L.	0	n	1	0	Asparagaceae Juss.
Iz_6_1009	DJI_0361	Astragalus cicer L.	0	n	1	0	Fabaceae Lindl.
Iz_6_1009	DJI_0361	Bassia laniflora (S.G.Gmel.) A.J.Scott	0	n	1	0	Amaranthaceae Juss.
Iz_6_1009	DJI_0361	Berberis vulgaris L.	0	n	1	0	Berberidaceae Juss.
Iz_6_1009	DJI_0361	Betula pendula Roth.	0	n	1	1	Betulaceae Gray
Iz_6_1009	DJI_0361	Betula pendula Roth.	0	n	1	0	Poaceae Barnhart
Iz_6_1009	DJI_0361	Bromus inermis Leyss.	0		NA	NA	
Iz_6_1009	DJI_0361	Bromus squarrosus L.	0	a	0	0	Poaceae Barnhart
Iz_6_1009	DJI_0361	Bromus tectorum L.	0	a	0	0	Poaceae Barnhart
Iz_6_1009	DJI_0361	Calamagrostis epigejos (L.) Roth	5	n	1	0	Poaceae Barnhart
Iz_6_1009	DJI_0361	Carduus acanthoides L.	0	n	1	0	Asteraceae Bercht. & J.Presl
Iz_6_1009	DJI_0361	Carex sp.	0	n	1	0	Cyperaceae Juss.
Iz_6_1009	DJI_0361	Centaurea majorovii Dumbadze	0	n	1	0	Asteraceae Bercht. & J.Presl
Iz_6_1009	DJI_0361	Chamaecytisus ruthenicus (Fisch.ex Wol.) Klaskova	0	n	1	0	Fabaceae Lindl.
Iz_6_1009	DJI_0361	Chelidonium majus L.	0.5	n	1	0	Papaveraceae Juss.
Iz_6_1009	DJI_0361	Chenopodium betaceum Andrz.	0	n	1	0	Amaranthaceae Juss.
Iz_6_1009	DJI_0361	Chenopodium cf. album	0	n	1	0	Amaranthaceae Juss.
Iz_6_1009	DJI_0361	Chenopodium sp.	0		NA	0	Amaranthaceae Juss.
Iz_6_1009	DJI_0361	Chondrilla juncea L.	0	n	1	0	Asteraceae Bercht. & J.Presl
Iz_6_1009	DJI_0361	Cirsium vulgare (Savi) Ten.	0	n	1	0	Asteraceae Bercht. & J.Presl
Iz_6_1009	DJI_0361	Clinopodium acinos (L.) Kuntze	0	n	1	0	Lamiacea Lindl.
Iz_6_1009	DJI_0361	Convallaria majalis L.	0	n	1	0	Asparagaceae Juss.
Iz_6_1009	DJI_0361	Crataegus rhipidophylla Gand.	0	n	1	1	Rosaceae Juss.
Iz_6_1009	DJI_0361	Crepis foetida L.	0	n	1	0	Asteraceae Bercht. & J.Presl
Iz_6_1009	DJI_0361	Dactylis glomerata L.	0	n	1	0	Poaceae Barnhart
Iz_6_1009	DJI_0361	Dianthus campestris M.Bieb.	0	n	1	0	Caryophyllaceae Juss.
Iz_6_1009	DJI_0361	Digitaria sanguinalis (L.) Scop.	0	a	0	0	Poaceae Barnhart
Iz_6_1009	DJI_0361	Elymus repens (L.) Gould	0	n	1	0	Poaceae Barnhart
Iz_6_1009	DJI_0361	Epilobium tetragonum L.	0	n	1	0	Onagraceae Juss.
Iz_6_1009	DJI_0361	Eragrostis minor Host	0	a	0	0	Poaceae Barnhart
Iz_6_1009	DJI_0361	Erigeron canadensis L.	5	a	0	0	Asteraceae Bercht. & J.Presl
Iz_6_1009	DJI_0361	Euphorbia seguieriana Neck.	0	n	1	0	Euphorbiaceae Juss.
Iz_6_1009	DJI_0361	Fallopia dumetorum (L.) Holub	0	n	1	0	Polygonaceae Juss.
Iz_6_1009	DJI_0361	Festuca beckeri (Hack.) Trautv.	0	n	1	0	Poaceae Barnhart
Iz_6_1009	DJI_0361	Fragaria vesca L.	0	n	1	0	Rosaceae Juss.
Iz_6_1009	DJI_0361	Frangula alnus Mill.	0	n	1	1	Rhamnaceae Juss.
Iz_6_1009	DJI_0361	Fraxinus pennsylvanica Marshall	0	a	0	1	Oleaceae Hoffmanns. & Link
Iz_6_1009	DJI_0361	Galeopsis bifida Boenn.	0	n	1	0	Lamiacea Lindl.
Iz_6_1009	DJI_0361	Galium verum L.	0	n	1	0	Rubiaceae Juss.
Iz_6_1009	DJI_0361	Genista tinctoria L.	0	n	1	0	Fabaceae Lindl.
Iz_6_1009	DJI_0361	Geranium robertianum L.	0	n	1	0	Geraniaceae Juss.
Iz_6_1009	DJI_0361	Geranium sanquineum L.	0	n	1	0	Geraniaceae Juss.
Iz_6_1009	DJI_0361	Gypsophila paniculata L.	0	n	1	0	Caryophyllaceae Juss.
Iz_6_1009	DJI_0361	Helichrysum arenarium (L.) Moench	0	n	1	0	Asteraceae Bercht. & J.Presl
Iz_6_1009	DJI_0361	Herniaria polygama J.Gay	0	n	1	0	Caryophyllaceae Juss.
Iz_6_1009	DJI_0361	Hieracium robustum Fr.	0	n	1	0	Asteraceae Bercht. & J.Presl
Iz_6_1009	DJI_0361	Hieracium umbellatum L.	0	n	1	NA	Asteraceae Bercht. & J.Presl
Iz_6_1009	DJI_0361	Hylotelephium maximum (L.) Holub	0	n	1	0	Crassulaceae J.St.-Hil.
Iz_6_1009	DJI_0361	Jacobaea borysthenica (DC.) B.Nord. & Greuter	0	n	1	0	Asteraceae Bercht. & J.Presl
Iz_6_1009	DJI_0361	Jasione montana L.	0	n	1	0	Campanulaceae Juss.
Iz_6_1009	DJI_0361	Jurinea cyanoides (L.) Rchb.s.l.	0	n	1	0	Asteraceae Bercht. & J.Presl
Iz_6_1009	DJI_0361	Koeleria glauca (Spreng.) DC.	0	n	1	0	Poaceae Barnhart
Iz_6_1009	DJI_0361	Lactuca serriola L.	0	a	0	0	Asteraceae Bercht. & J.Presl
Iz_6_1009	DJI_0361	Linaria genistifolia (L.) Mill.	0	n	1	0	Plantaginaceae Juss.
Iz_6_1009	DJI_0361	Lomelosia argentea (L.) Greuter & Burdet	0	n	1	0	Caprifoliaceae Juss.
Iz_6_1009	DJI_0361	Melampyrum pratense L.	0	n	1	0	Orobanchaceae Vent.
Iz_6_1009	DJI_0361	Oenothera biennis L.	0	a	0	0	Onagraceae Juss.
Iz_6_1009	DJI_0361	Pentanema britannica (L.) D.Gut.Larr., Santos-Vicente, Anderb., E.Rico & M.M.Mart.Ort.	0	n	1	0	Asteraceae Bercht. & J.Presl
Iz_6_1009	DJI_0361	Peucedanum oreoselinum (L.) Moench	0	n	1	0	Apiaceae Lindl.
Iz_6_1009	DJI_0361	Peucedanum ruthenicum M.Bieb.	0	n	1	0	Apiaceae Lindl.
Iz_6_1009	DJI_0361	Pilosella echioides (Lumn.) F.W.Schultz & Sch.Bip.	0	n	1	0	Asteraceae Bercht. & J.Presl
Iz_6_1009	DJI_0361	Pilosella officinarum F. Schultz et Sch. Bip.	0	n	1	0	Asteraceae Bercht. & J.Presl
Iz_6_1009	DJI_0361	Pilosella sp.	0		NA	0	Asteraceae Bercht. & J.Presl
Iz_6_1009	DJI_0361	Pinus sylvestris L.	0	n	1	1	Pinaceae Spreng. ex F.Rudolphi
Iz_6_1009	DJI_0361	Poa annua L.	0	n	1	0	Poaceae Barnhart
Iz_6_1009	DJI_0361	Poa nemoralis L.	0	n	1	0	Poaceae Barnhart
Iz_6_1009	DJI_0361	Poa sp.	0		NA	0	Poaceae Barnhart
Iz_6_1009	DJI_0361	Polygonatum odoratum (Mill.) Druce	0	n	1	0	Asparagaceae Juss.
Iz_6_1009	DJI_0361	Polygonum arenarium Waldst. & Kit.	0	n	1	0	Polygonaceae Juss.
Iz_6_1009	DJI_0361	Populus nigra L.	0	n	1	1	Salicaceae Mirb.
Iz_6_1009	DJI_0361	Populus tremula L.	0	n	1	1	Salicaceae Mirb.
Iz_6_1009	DJI_0361	Populus Ã canescens (Aiton) Sm.	0	n	1	1	Salicaceae Mirb.
Iz_6_1009	DJI_0361	Potentilla argentea L.	0	n	1	0	Rosaceae Juss.
Iz_6_1009	DJI_0361	Psammophiliella muralis (L.) Ikonn.	0	n	1	0	Caryophyllaceae Juss.

Iz_6_1009	DJI_0361	Psephellus sumensis (Kalen.) Greuter	0	n	1	0	Asteraceae Bercht. & J.Presl
Iz_6_1009	DJI_0361	Pulsatilla pratensis (L.) Mill. s.l.	0	n	1	0	Ranunculaceae Juss.
Iz_6_1009	DJI_0361	Pyrus communis L.	0	a	0	1	Rosaceae Juss.
Iz_6_1009	DJI_0361	Quercus robur L.	0	n	1	1	Fagaceae Dumort.
Iz_6_1009	DJI_0361	Robinia pseudoacacia L.	0	a	0	1	Fabaceae Lindl.
Iz_6_1009	DJI_0361	Rosa sp.	0		NA	1	Rosaceae Juss.
Iz_6_1009	DJI_0361	Rubus idaeus L.	0	n	1	0	Rosaceae Juss.
Iz_6_1009	DJI_0361	Rumex acetosella L.	0	n	1	0	Polygonaceae Juss.
Iz_6_1009	DJI_0361	Rumex thyrsiflorus Fingerh.	0	n	1	0	Polygonaceae Juss.
Iz_6_1009	DJI_0361	Salix alba L.	0	n	1	1	Salicaceae Mirb.
Iz_6_1009	DJI_0361	Sambucus nigra L.	0	n	1	1	Viburnaceae Raf.
Iz_6_1009	DJI_0361	Secale sylvestre Host	0	n	1	0	Poaceae Barnhart
Iz_6_1009	DJI_0361	Sempervivum ruthenicum Schnittsp. & C.B. Lehm.	0	n	1	0	Crassulaceae J.St.-Hil.
Iz_6_1009	DJI_0361	Setaria viridis (L.) P. Beauv.	0	n	1	0	Poaceae Barnhart
Iz_6_1009	DJI_0361	Silene borysthenica (Gruner) Walters	0	n	1	0	Caryophyllaceae Juss.
Iz_6_1009	DJI_0361	Silene latifolia subsp. alba (Mill.) Greuter & Burdet	0	n	1	0	Caryophyllaceae Juss.
Iz_6_1009	DJI_0361	Solidago virgaurea L.	0	n	1	0	Asteraceae Bercht. & J.Presl
Iz_6_1009	DJI_0361	Sorbus aucuparia L.	0	n	1	1	Rosaceae Juss.
Iz_6_1009	DJI_0361	Stachys recta L.	0	n	1	0	Lamiacea Lindl.
Iz_6_1009	DJI_0361	Tanacetum vulgare L.	0	n	1	0	Asteraceae Bercht. & J.Presl
Iz_6_1009	DJI_0361	Thymus pallasianus Heinr.Braun	0	n	1	0	Lamiacea Lindl.
Iz_6_1009	DJI_0361	Tilia cordata Mill.	0	n	1	1	Malvaceae Juss.
Iz_6_1009	DJI_0361	Trifolium alpestre L.	0	n	1	0	Fabaceae Lindl.
Iz_6_1009	DJI_0361	Trifolium arvense L.	0	n	1	0	Fabaceae Lindl.
Iz_6_1009	DJI_0361	Ulmus glabra Huds.	0	n	1	1	Ulmaceae Mirb.
Iz_6_1009	DJI_0361	Verbascum densiflorum Bertol.	0	n	1	0	Scrophulariaceae Juss.
Iz_6_1009	DJI_0361	Veronica austriaca L.	0	n	1	0	Plantaginaceae Juss.
Iz_6_1009	DJI_0361	Veronica incana L.	0	n	1	0	Plantaginaceae Juss.
Iz_6_1009	DJI_0361	Veronica verna L.	0	n	1	0	Plantaginaceae Juss.
Iz_6_1009	DJI_0361	Vincetoxicum hirundinaria Medik	0	n	1	0	Apocynaceae Juss.
Iz_6_1009	DJI_0367	Acer tataricum L.	0	n	1	1	Sapindaceae Juss.
Iz_6_1009	DJI_0367	Achillea millefolium L. s.l.	0	n	1	0	Asteraceae Bercht. & J.Presl
Iz_6_1009	DJI_0367	Allium oleraceum L.	0	n	1	0	Amaryllidaceae J.St.-Hil.
Iz_6_1009	DJI_0367	Ambrosia artemisiifolia L.	0	a	0	0	Asteraceae Bercht. & J.Presl
Iz_6_1009	DJI_0367	Amorpha fruticosa L.	0	a	0	0	Fabaceae Lindl.
Iz_6_1009	DJI_0367	Anthericum ramosum L.	0	n	1	0	Asparagaceae Juss.
Iz_6_1009	DJI_0367	Artemisia marschalliana Spreng.	0	n	1	0	Asteraceae Bercht. & J.Presl
Iz_6_1009	DJI_0367	Asparagus officinalis L.	0	n	1	0	Asparagaceae Juss.
Iz_6_1009	DJI_0367	Astragalus cicer L.	0	n	1	0	Fabaceae Lindl.
Iz_6_1009	DJI_0367	Bassia laniflora (S.G.Gmel.) A.J.Scott	0	n	1	0	Amaranthaceae Juss.
Iz_6_1009	DJI_0367	Berberis vulgaris L.	0	n	1	0	Berberidaceae Juss.
Iz_6_1009	DJI_0367	Betula pendula Roth.	0	n	1	1	Betulaceae Gray
Iz_6_1009	DJI_0367	Betula pendula Roth.	0	n	1	0	Poaceae Barnhart
Iz_6_1009	DJI_0367	Bromus inermis Leyss.	0		NA	NA	
Iz_6_1009	DJI_0367	Bromus squarrosus L.	0	a	0	0	Poaceae Barnhart
Iz_6_1009	DJI_0367	Bromus tectorum L.	0	a	0	0	Poaceae Barnhart
Iz_6_1009	DJI_0367	Calamagrostis epigejos (L.) Roth	0.2	n	1	0	Poaceae Barnhart
Iz_6_1009	DJI_0367	Carduus acanthoides L.	0	n	1	0	Asteraceae Bercht. & J.Presl
Iz_6_1009	DJI_0367	Carex sp.	0	n	1	0	Cyperaceae Juss.
Iz_6_1009	DJI_0367	Centaurea majorovii Dumbadze	0	n	1	0	Asteraceae Bercht. & J.Presl
Iz_6_1009	DJI_0367	Chamaecytisus ruthenicus (Fisch.ex Wol.) Klaskova	0	n	1	0	Fabaceae Lindl.
Iz_6_1009	DJI_0367	Chelidonium majus L.	0	n	1	0	Papaveraceae Juss.
Iz_6_1009	DJI_0367	Chenopodium betaceum Andrz.	0	n	1	0	Amaranthaceae Juss.
Iz_6_1009	DJI_0367	Chenopodium cf. album	0	n	1	0	Amaranthaceae Juss.
Iz_6_1009	DJI_0367	Chenopodium sp.	0		NA	0	Amaranthaceae Juss.
Iz_6_1009	DJI_0367	Chondrilla juncea L.	0	n	1	0	Asteraceae Bercht. & J.Presl
Iz_6_1009	DJI_0367	Cirsium vulgare (Savi) Ten.	0	n	1	0	Asteraceae Bercht. & J.Presl
Iz_6_1009	DJI_0367	Clinopodium acinos (L.) Kuntze	0	n	1	0	Lamiacea Lindl.
Iz_6_1009	DJI_0367	Convallaria majalis L.	0	n	1	0	Asparagaceae Juss.
Iz_6_1009	DJI_0367	Crataegus rhipidophylla Gand.	0	n	1	1	Rosaceae Juss.
Iz_6_1009	DJI_0367	Crepis foetida L.	0	n	1	0	Asteraceae Bercht. & J.Presl
Iz_6_1009	DJI_0367	Dactylis glomerata L.	0	n	1	0	Poaceae Barnhart
Iz_6_1009	DJI_0367	Dianthus campestris M.Bieb.	0	n	1	0	Caryophyllaceae Juss.
Iz_6_1009	DJI_0367	Digitaria sanguinalis (L.) Scop.	0	a	0	0	Poaceae Barnhart
Iz_6_1009	DJI_0367	Elymus repens (L.) Gould	0	n	1	0	Poaceae Barnhart
Iz_6_1009	DJI_0367	Epilobium tetragonum L.	0	n	1	0	Onagraceae Juss.
Iz_6_1009	DJI_0367	Eragrostis minor Host	0	a	0	0	Poaceae Barnhart
Iz_6_1009	DJI_0367	Erigeron canadensis L.	15	a	0	0	Asteraceae Bercht. & J.Presl
Iz_6_1009	DJI_0367	Euphorbia seguieriana Neck.	0	n	1	0	Euphorbiaceae Juss.
Iz_6_1009	DJI_0367	Fallopia dumetorum (L.) Holub	0	n	1	0	Polygonaceae Juss.
Iz_6_1009	DJI_0367	Festuca beckeri (Hack.) Trautv.	0	n	1	0	Poaceae Barnhart
Iz_6_1009	DJI_0367	Fragaria vesca L.	0	n	1	0	Rosaceae Juss.
Iz_6_1009	DJI_0367	Frangula alnus Mill.	0	n	1	1	Rhamnaceae Juss.
Iz_6_1009	DJI_0367	Fraxinus pennsylvanica Marshall	0	a	0	1	Oleaceae Hoffmanns. & Link
Iz_6_1009	DJI_0367	Galeopsis bifida Boenn.	0	n	1	0	Lamiacea Lindl.
Iz_6_1009	DJI_0367	Galium verum L.	0	n	1	0	Rubiaceae Juss.
Iz_6_1009	DJI_0367	Genista tinctoria L.	0	n	1	0	Fabaceae Lindl.
Iz_6_1009	DJI_0367	Geranium robertianum L.	0	n	1	0	Geraniaceae Juss.
Iz_6_1009	DJI_0367	Geranium sanquineum L.	0	n	1	0	Geraniaceae Juss.
Iz_6_1009	DJI_0367	Gypsophila paniculata L.	0	n	1	0	Caryophyllaceae Juss.
Iz_6_1009	DJI_0367	Helichrysum arenarium (L.) Moench	0	n	1	0	Asteraceae Bercht. & J.Presl
Iz_6_1009	DJI_0367	Herniaria polygama J.Gay	0	n	1	0	Caryophyllaceae Juss.
Iz_6_1009	DJI_0367	Hieracium robustum Fr.	0	n	1	0	Asteraceae Bercht. & J.Presl
Iz_6_1009	DJI_0367	Hieracium umbellatum L.	0	n	1	NA	Asteraceae Bercht. & J.Presl
Iz_6_1009	DJI_0367	Hylotelephium maximum (L.) Holub	0	n	1	0	Crassulaceae J.St.-Hil.
Iz_6_1009	DJI_0367	Jacobaea borysthenica (DC.) B.Nord. & Greuter	0	n	1	0	Asteraceae Bercht. & J.Presl
Iz_6_1009	DJI_0367	Jasione montana L.	0	n	1	0	Campanulaceae Juss.
Iz_6_1009	DJI_0367	Jurinea cyanoides (L.) Rchb.s.l.	0	n	1	0	Asteraceae Bercht. & J.Presl
Iz_6_1009	DJI_0367	Koeleria glauca (Spreng.) DC.	0	n	1	0	Poaceae Barnhart
Iz_6_1009	DJI_0367	Lactuca serriola L.	0	a	0	0	Asteraceae Bercht. & J.Presl
Iz_6_1009	DJI_0367	Linaria genistifolia (L.) Mill.	0	n	1	0	Plantaginaceae Juss.
Iz_6_1009	DJI_0367	Lomelosia argentea (L.) Greuter & Burdet	0	n	1	0	Caprifoliaceae Juss.
Iz_6_1009	DJI_0367	Melampyrum pratense L.	0	n	1	0	Orobanchaceae Vent.
Iz_6_1009	DJI_0367	Oenothera biennis L.	0	a	0	0	Onagraceae Juss.
Iz_6_1009	DJI_0367	Pentanema britannica (L.) D.Gut.Larr., Santos-Vicente, Anderb., E.Rico & M.M.Mart.Ort.	0	n	1	0	Asteraceae Bercht. & J.Presl
Iz_6_1009	DJI_0367	Peucedanum oreoselinum (L.) Moench	0	n	1	0	Apiaceae Lindl.
Iz_6_1009	DJI_0367	Peucedanum ruthenicum M.Bieb.	0	n	1	0	Apiaceae Lindl.
Iz_6_1009	DJI_0367	Pilosella echioides (Lumn.) F.W.Schultz & Sch.Bip.	0	n	1	0	Asteraceae Bercht. & J.Presl

Iz_6_1009	DJI_0367	Pilosella officinarum F. Schultz et Sch. Bip.	0	n	1	0	Asteraceae Bercht. & J.Presl
Iz_6_1009	DJI_0367	Pilosella sp.	0		NA	0	Asteraceae Bercht. & J.Presl
Iz_6_1009	DJI_0367	Pinus sylvestris L.	0	n	1	1	Pinaceae Spreng. ex F.Rudolphi
Iz_6_1009	DJI_0367	Poa annua L.	0	n	1	0	Poaceae Barnhart
Iz_6_1009	DJI_0367	Poa nemoralis L.	0	n	1	0	Poaceae Barnhart
Iz_6_1009	DJI_0367	Poa sp.	0		NA	0	Poaceae Barnhart
Iz_6_1009	DJI_0367	Polygonatum odoratum (Mill.) Druce	0	n	1	0	Asparagaceae Juss.
Iz_6_1009	DJI_0367	Polygonum arenarium Waldst. & Kit.	0	n	1	0	Polygonaceae Juss.
Iz_6_1009	DJI_0367	Populus nigra L.	0	n	1	1	Salicaceae Mirb.
Iz_6_1009	DJI_0367	Populus tremula L.	0	n	1	1	Salicaceae Mirb.
Iz_6_1009	DJI_0367	Populus Ã canescens (Aiton) Sm.	0	n	1	1	Salicaceae Mirb.
Iz_6_1009	DJI_0367	Potentilla argentea L.	0	n	1	0	Rosaceae Juss.
Iz_6_1009	DJI_0367	Psammophiliella muralis (L.) Ikonn.	0	n	1	0	Caryophyllaceae Juss.
Iz_6_1009	DJI_0367	Psephellus sumensis (Kalen.) Greuter	0	n	1	0	Asteraceae Bercht. & J.Presl
Iz_6_1009	DJI_0367	Pulsatilla pratensis (L.) Mill. s.l.	0	n	1	0	Ranunculaceae Juss.
Iz_6_1009	DJI_0367	Pyrus communis L.	0	a	0	1	Rosaceae Juss.
Iz_6_1009	DJI_0367	Quercus robur L.	0	n	1	1	Fagaceae Dumort.
Iz_6_1009	DJI_0367	Robinia pseudoacacia L.	0	a	0	1	Fabaceae Lindl.
Iz_6_1009	DJI_0367	Rosa sp.	0		NA	1	Rosaceae Juss.
Iz_6_1009	DJI_0367	Rubus idaeus L.	0	n	1	0	Rosaceae Juss.
Iz_6_1009	DJI_0367	Rumex acetosella L.	0	n	1	0	Polygonaceae Juss.
Iz_6_1009	DJI_0367	Rumex thyrsiflorus Fingerh.	0	n	1	0	Polygonaceae Juss.
Iz_6_1009	DJI_0367	Salix alba L.	0	n	1	1	Salicaceae Mirb.
Iz_6_1009	DJI_0367	Sambucus nigra L.	0	n	1	1	Viburnaceae Raf.
Iz_6_1009	DJI_0367	Secale sylvestre Host	0	n	1	0	Poaceae Barnhart
Iz_6_1009	DJI_0367	Sempervivum ruthenicum Schnittsp. & C.B. Lehm.	0	n	1	0	Crassulaceae J.St.-Hil.
Iz_6_1009	DJI_0367	Setaria viridis (L.) P. Beauv.	0	n	1	0	Poaceae Barnhart
Iz_6_1009	DJI_0367	Silene borysthenica (Gruner) Walters	0	n	1	0	Caryophyllaceae Juss.
Iz_6_1009	DJI_0367	Silene latifolia subsp. alba (Mill.) Greuter & Burdet	0	n	1	0	Caryophyllaceae Juss.
Iz_6_1009	DJI_0367	Solidago virgaurea L.	0	n	1	0	Asteraceae Bercht. & J.Presl
Iz_6_1009	DJI_0367	Sorbus aucuparia L.	0	n	1	1	Rosaceae Juss.
Iz_6_1009	DJI_0367	Stachys recta L.	0	n	1	0	Lamiacea Lindl.
Iz_6_1009	DJI_0367	Tanacetum vulgare L.	0	n	1	0	Asteraceae Bercht. & J.Presl
Iz_6_1009	DJI_0367	Thymus pallasianus Heinr.Braun	0	n	1	0	Lamiacea Lindl.
Iz_6_1009	DJI_0367	Tilia cordata Mill.	0	n	1	1	Malvaceae Juss.
Iz_6_1009	DJI_0367	Trifolium alpestre L.	0	n	1	0	Fabaceae Lindl.
Iz_6_1009	DJI_0367	Trifolium arvense L.	0	n	1	0	Fabaceae Lindl.
Iz_6_1009	DJI_0367	Ulmus glabra Huds.	0	n	1	1	Ulmaceae Mirb.
Iz_6_1009	DJI_0367	Verbascum densiflorum Bertol.	0	n	1	0	Scrophulariaceae Juss.
Iz_6_1009	DJI_0367	Veronica austriaca L.	0	n	1	0	Plantaginaceae Juss.
Iz_6_1009	DJI_0367	Veronica incana L.	0	n	1	0	Plantaginaceae Juss.
Iz_6_1009	DJI_0367	Veronica verna L.	0	n	1	0	Plantaginaceae Juss.
Iz_6_1009	DJI_0367	Vincetoxicum hirundinaria Medik	0	n	1	0	Apocynaceae Juss.
Iz_6_1009	DJI_0372	Acer tataricum L.	0	n	1	1	Sapindaceae Juss.
Iz_6_1009	DJI_0372	Achillea millefolium L. s.l.	0	n	1	0	Asteraceae Bercht. & J.Presl
Iz_6_1009	DJI_0372	Allium oleraceum L.	0	n	1	0	Amaryllidaceae J.St.-Hil.
Iz_6_1009	DJI_0372	Ambrosia artemisiifolia L.	0	a	0	0	Asteraceae Bercht. & J.Presl
Iz_6_1009	DJI_0372	Amorpha fruticosa L.	0	a	0	0	Fabaceae Lindl.
Iz_6_1009	DJI_0372	Anthericum ramosum L.	0	n	1	0	Asparagaceae Juss.
Iz_6_1009	DJI_0372	Artemisia marschalliana Spreng.	0	n	1	0	Asteraceae Bercht. & J.Presl
Iz_6_1009	DJI_0372	Asparagus officinalis L.	0	n	1	0	Asparagaceae Juss.
Iz_6_1009	DJI_0372	Astragalus cicer L.	0	n	1	0	Fabaceae Lindl.
Iz_6_1009	DJI_0372	Bassia laniflora (S.G.Gmel.) A.J.Scott	0	n	1	0	Amaranthaceae Juss.
Iz_6_1009	DJI_0372	Berberis vulgaris L.	0	n	1	0	Berberidaceae Juss.
Iz_6_1009	DJI_0372	Betula pendula Roth.	0	n	1	1	Betulaceae Gray
Iz_6_1009	DJI_0372	Betula pendula Roth.	0	n	1	0	Poaceae Barnhart
Iz_6_1009	DJI_0372	Bromus inermis Leyss.	0		NA	NA	
Iz_6_1009	DJI_0372	Bromus squarrosus L.	0	a	0	0	Poaceae Barnhart
Iz_6_1009	DJI_0372	Bromus tectorum L.	0	a	0	0	Poaceae Barnhart
Iz_6_1009	DJI_0372	Calamagrostis epigejos (L.) Roth	30	n	1	0	Poaceae Barnhart
Iz_6_1009	DJI_0372	Carduus acanthoides L.	0	n	1	0	Asteraceae Bercht. & J.Presl
Iz_6_1009	DJI_0372	Carex sp.	0	n	1	0	Cyperaceae Juss.
Iz_6_1009	DJI_0372	Centaurea majorovii Dumbadze	0	n	1	0	Asteraceae Bercht. & J.Presl
Iz_6_1009	DJI_0372	Chamaecytisus ruthenicus (Fisch.ex Wol.) Klaskova	0	n	1	0	Fabaceae Lindl.
Iz_6_1009	DJI_0372	Chelidonium majus L.	0	n	1	0	Papaveraceae Juss.
Iz_6_1009	DJI_0372	Chenopodium betaceum Andrz.	0	n	1	0	Amaranthaceae Juss.
Iz_6_1009	DJI_0372	Chenopodium cf. album	0	n	1	0	Amaranthaceae Juss.
Iz_6_1009	DJI_0372	Chenopodium sp.	0		NA	0	Amaranthaceae Juss.
Iz_6_1009	DJI_0372	Chondrilla juncea L.	0	n	1	0	Asteraceae Bercht. & J.Presl
Iz_6_1009	DJI_0372	Cirsium vulgare (Savi) Ten.	0	n	1	0	Asteraceae Bercht. & J.Presl
Iz_6_1009	DJI_0372	Clinopodium acinos (L.) Kuntze	0	n	1	0	Lamiacea Lindl.
Iz_6_1009	DJI_0372	Convallaria majalis L.	0	n	1	0	Asparagaceae Juss.
Iz_6_1009	DJI_0372	Crataegus rhipidophylla Gand.	0	n	1	1	Rosaceae Juss.
Iz_6_1009	DJI_0372	Crepis foetida L.	0	n	1	0	Asteraceae Bercht. & J.Presl
Iz_6_1009	DJI_0372	Dactylis glomerata L.	0	n	1	0	Poaceae Barnhart
Iz_6_1009	DJI_0372	Dianthus campestris M.Bieb.	0	n	1	0	Caryophyllaceae Juss.
Iz_6_1009	DJI_0372	Digitaria sanguinalis (L.) Scop.	0	a	0	0	Poaceae Barnhart
Iz_6_1009	DJI_0372	Elymus repens (L.) Gould	0	n	1	0	Poaceae Barnhart
Iz_6_1009	DJI_0372	Epilobium tetragonum L.	0	n	1	0	Onagraceae Juss.
Iz_6_1009	DJI_0372	Eragrostis minor Host	0	a	0	0	Poaceae Barnhart
Iz_6_1009	DJI_0372	Erigeron canadensis L.	5	a	0	0	Asteraceae Bercht. & J.Presl
Iz_6_1009	DJI_0372	Euphorbia seguieriana Neck.	0	n	1	0	Euphorbiaceae Juss.
Iz_6_1009	DJI_0372	Fallopia dumetorum (L.) Holub	0	n	1	0	Polygonaceae Juss.
Iz_6_1009	DJI_0372	Festuca beckeri (Hack.) Trautv.	0	n	1	0	Poaceae Barnhart
Iz_6_1009	DJI_0372	Fragaria vesca L.	0	n	1	0	Rosaceae Juss.
Iz_6_1009	DJI_0372	Frangula alnus Mill.	0	n	1	1	Rhamnaceae Juss.
Iz_6_1009	DJI_0372	Fraxinus pennsylvanica Marshall	0	a	0	1	Oleaceae Hoffmanns. & Link
Iz_6_1009	DJI_0372	Galeopsis bifida Boenn.	0	n	1	0	Lamiacea Lindl.
Iz_6_1009	DJI_0372	Galium verum L.	0	n	1	0	Rubiaceae Juss.
Iz_6_1009	DJI_0372	Genista tinctoria L.	0	n	1	0	Fabaceae Lindl.
Iz_6_1009	DJI_0372	Geranium robertianum L.	0	n	1	0	Geraniaceae Juss.
Iz_6_1009	DJI_0372	Geranium sanquineum L.	0	n	1	0	Geraniaceae Juss.
Iz_6_1009	DJI_0372	Gypsophila paniculata L.	0	n	1	0	Caryophyllaceae Juss.
Iz_6_1009	DJI_0372	Helichrysum arenarium (L.) Moench	0	n	1	0	Asteraceae Bercht. & J.Presl
Iz_6_1009	DJI_0372	Herniaria polygama J.Gay	0	n	1	0	Caryophyllaceae Juss.
Iz_6_1009	DJI_0372	Hieracium robustum Fr.	0	n	1	0	Asteraceae Bercht. & J.Presl
Iz_6_1009	DJI_0372	Hieracium umbellatum L.	0	n	1	NA	Asteraceae Bercht. & J.Presl
Iz_6_1009	DJI_0372	Hylotelephium maximum (L.) Holub	0	n	1	0	Crassulaceae J.St.-Hil.

Iz_6_1009	DJI_0372	Jacobaea borysthenica (DC.) B.Nord. & Greuter	0	n	1	0	Asteraceae Bercht. & J.Presl
Iz_6_1009	DJI_0372	Jasione montana L.	0	n	1	0	Campanulaceae Juss.
Iz_6_1009	DJI_0372	Jurinea cyanoides (L.) Rchb.s.l.	0	n	1	0	Asteraceae Bercht. & J.Presl
Iz_6_1009	DJI_0372	Koeleria glauca (Spreng.) DC.	0	n	1	0	Poaceae Barnhart
Iz_6_1009	DJI_0372	Lactuca serriola L.	0	a	0	0	Asteraceae Bercht. & J.Presl
Iz_6_1009	DJI_0372	Linaria genistifolia (L.) Mill.	0	n	1	0	Plantaginaceae Juss.
Iz_6_1009	DJI_0372	Lomelosia argentea (L.) Greuter & Burdet	0	n	1	0	Caprifoliaceae Juss.
Iz_6_1009	DJI_0372	Melampyrum pratense L.	0	n	1	0	Orobanchaceae Vent.
Iz_6_1009	DJI_0372	Oenothera biennis L.	0	a	0	0	Onagraceae Juss.
Iz_6_1009	DJI_0372	Pentanema britannica (L.) D.Gut.Larr., Santos-Vicente, Anderb., E.Rico & M.M.Mart.Ort.	0	n	1	0	Asteraceae Bercht. & J.Presl
Iz_6_1009	DJI_0372	Peucedanum oreoselinum (L.) Moench	0	n	1	0	Apiaceae Lindl.
Iz_6_1009	DJI_0372	Peucedanum ruthenicum M.Bieb.	0	n	1	0	Apiaceae Lindl.
Iz_6_1009	DJI_0372	Pilosella echioides (Lumn.) F.W.Schultz & Sch.Bip.	0	n	1	0	Asteraceae Bercht. & J.Presl
Iz_6_1009	DJI_0372	Pilosella officinarum F. Schultz et Sch. Bip.	0	n	1	0	Asteraceae Bercht. & J.Presl
Iz_6_1009	DJI_0372	Pilosella sp.	0		NA	0	Asteraceae Bercht. & J.Presl
Iz_6_1009	DJI_0372	Pinus sylvestris L.	0	n	1	1	Pinaceae Spreng. ex F.Rudolphi
Iz_6_1009	DJI_0372	Poa annua L.	0	n	1	0	Poaceae Barnhart
Iz_6_1009	DJI_0372	Poa nemoralis L.	0	n	1	0	Poaceae Barnhart
Iz_6_1009	DJI_0372	Poa sp.	0		NA	0	Poaceae Barnhart
Iz_6_1009	DJI_0372	Polygonatum odoratum (Mill.) Druce	0	n	1	0	Asparagaceae Juss.
Iz_6_1009	DJI_0372	Polygonum arenarium Waldst. & Kit.	0	n	1	0	Polygonaceae Juss.
Iz_6_1009	DJI_0372	Populus nigra L.	0	n	1	1	Salicaceae Mirb.
Iz_6_1009	DJI_0372	Populus tremula L.	0	n	1	1	Salicaceae Mirb.
Iz_6_1009	DJI_0372	Populus Ã canescens (Aiton) Sm.	0	n	1	1	Salicaceae Mirb.
Iz_6_1009	DJI_0372	Potentilla argentea L.	0	n	1	0	Rosaceae Juss.
Iz_6_1009	DJI_0372	Psammophiliella muralis (L.) Ikonn.	0	n	1	0	Caryophyllaceae Juss.
Iz_6_1009	DJI_0372	Psephellus sumensis (Kalen.) Greuter	0	n	1	0	Asteraceae Bercht. & J.Presl
Iz_6_1009	DJI_0372	Pulsatilla pratensis (L.) Mill. s.l.	0	n	1	0	Ranunculaceae Juss.
Iz_6_1009	DJI_0372	Pyrus communis L.	0	a	0	1	Rosaceae Juss.
Iz_6_1009	DJI_0372	Quercus robur L.	0	n	1	1	Fagaceae Dumort.
Iz_6_1009	DJI_0372	Robinia pseudoacacia L.	0	a	0	1	Fabaceae Lindl.
Iz_6_1009	DJI_0372	Rosa sp.	0		NA	1	Rosaceae Juss.
Iz_6_1009	DJI_0372	Rubus idaeus L.	0	n	1	0	Rosaceae Juss.
Iz_6_1009	DJI_0372	Rumex acetosella L.	0.5	n	1	0	Polygonaceae Juss.
Iz_6_1009	DJI_0372	Rumex thyrsiflorus Fingerh.	0	n	1	0	Polygonaceae Juss.
Iz_6_1009	DJI_0372	Salix alba L.	0	n	1	1	Salicaceae Mirb.
Iz_6_1009	DJI_0372	Sambucus nigra L.	0	n	1	1	Viburnaceae Raf.
Iz_6_1009	DJI_0372	Secale sylvestre Host	0	n	1	0	Poaceae Barnhart
Iz_6_1009	DJI_0372	Sempervivum ruthenicum Schnittsp. & C.B. Lehm.	0	n	1	0	Crassulaceae J.St.-Hil.
Iz_6_1009	DJI_0372	Setaria viridis (L.) P. Beauv.	0	n	1	0	Poaceae Barnhart
Iz_6_1009	DJI_0372	Silene borysthenica (Gruner) Walters	0	n	1	0	Caryophyllaceae Juss.
Iz_6_1009	DJI_0372	Silene latifolia subsp. alba (Mill.) Greuter & Burdet	0	n	1	0	Caryophyllaceae Juss.
Iz_6_1009	DJI_0372	Solidago virgaurea L.	0	n	1	0	Asteraceae Bercht. & J.Presl
Iz_6_1009	DJI_0372	Sorbus aucuparia L.	0	n	1	1	Rosaceae Juss.
Iz_6_1009	DJI_0372	Stachys recta L.	0	n	1	0	Lamiacea Lindl.
Iz_6_1009	DJI_0372	Tanacetum vulgare L.	0	n	1	0	Asteraceae Bercht. & J.Presl
Iz_6_1009	DJI_0372	Thymus pallasianus Heinr.Braun	0	n	1	0	Lamiacea Lindl.
Iz_6_1009	DJI_0372	Tilia cordata Mill.	0	n	1	1	Malvaceae Juss.
Iz_6_1009	DJI_0372	Trifolium alpestre L.	0	n	1	0	Fabaceae Lindl.
Iz_6_1009	DJI_0372	Trifolium arvense L.	0	n	1	0	Fabaceae Lindl.
Iz_6_1009	DJI_0372	Ulmus glabra Huds.	0	n	1	1	Ulmaceae Mirb.
Iz_6_1009	DJI_0372	Verbascum densiflorum Bertol.	0	n	1	0	Scrophulariaceae Juss.
Iz_6_1009	DJI_0372	Veronica austriaca L.	0	n	1	0	Plantaginaceae Juss.
Iz_6_1009	DJI_0372	Veronica incana L.	0	n	1	0	Plantaginaceae Juss.
Iz_6_1009	DJI_0372	Veronica verna L.	0	n	1	0	Plantaginaceae Juss.
Iz_6_1009	DJI_0372	Vincetoxicum hirundinaria Medik	0	n	1	0	Apocynaceae Juss.
Iz_6_1009	DJI_0374	Acer tataricum L.	0	n	1	1	Sapindaceae Juss.
Iz_6_1009	DJI_0374	Achillea millefolium L. s.l.	0	n	1	0	Asteraceae Bercht. & J.Presl
Iz_6_1009	DJI_0374	Allium oleraceum L.	0	n	1	0	Amaryllidaceae J.St.-Hil.
Iz_6_1009	DJI_0374	Ambrosia artemisiifolia L.	0	a	0	0	Asteraceae Bercht. & J.Presl
Iz_6_1009	DJI_0374	Amorpha fruticosa L.	0	a	0	0	Fabaceae Lindl.
Iz_6_1009	DJI_0374	Anthericum ramosum L.	0	n	1	0	Asparagaceae Juss.
Iz_6_1009	DJI_0374	Artemisia marschalliana Spreng.	0	n	1	0	Asteraceae Bercht. & J.Presl
Iz_6_1009	DJI_0374	Asparagus officinalis L.	0	n	1	0	Asparagaceae Juss.
Iz_6_1009	DJI_0374	Astragalus cicer L.	0	n	1	0	Fabaceae Lindl.
Iz_6_1009	DJI_0374	Bassia laniflora (S.G.Gmel.) A.J.Scott	0	n	1	0	Amaranthaceae Juss.
Iz_6_1009	DJI_0374	Berberis vulgaris L.	0	n	1	0	Berberidaceae Juss.
Iz_6_1009	DJI_0374	Betula pendula Roth.	0	n	1	1	Betulaceae Gray
Iz_6_1009	DJI_0374	Betula pendula Roth.	0	n	1	0	Poaceae Barnhart
Iz_6_1009	DJI_0374	Bromus inermis Leyss.	0		NA	NA	
Iz_6_1009	DJI_0374	Bromus squarrosus L.	0	a	0	0	Poaceae Barnhart
Iz_6_1009	DJI_0374	Bromus tectorum L.	0	a	0	0	Poaceae Barnhart
Iz_6_1009	DJI_0374	Calamagrostis epigejos (L.) Roth	40	n	1	0	Poaceae Barnhart
Iz_6_1009	DJI_0374	Carduus acanthoides L.	0	n	1	0	Asteraceae Bercht. & J.Presl
Iz_6_1009	DJI_0374	Carex sp.	0	n	1	0	Cyperaceae Juss.
Iz_6_1009	DJI_0374	Centaurea majorovii Dumbadze	0	n	1	0	Asteraceae Bercht. & J.Presl
Iz_6_1009	DJI_0374	Chamaecytisus ruthenicus (Fisch.ex Wol.) Klaskova	0	n	1	0	Fabaceae Lindl.
Iz_6_1009	DJI_0374	Chelidonium majus L.	0	n	1	0	Papaveraceae Juss.
Iz_6_1009	DJI_0374	Chenopodium betaceum Andrz.	0	n	1	0	Amaranthaceae Juss.
Iz_6_1009	DJI_0374	Chenopodium cf. album	0	n	1	0	Amaranthaceae Juss.
Iz_6_1009	DJI_0374	Chenopodium sp.	0		NA	0	Amaranthaceae Juss.
Iz_6_1009	DJI_0374	Chondrilla juncea L.	0	n	1	0	Asteraceae Bercht. & J.Presl
Iz_6_1009	DJI_0374	Cirsium vulgare (Savi) Ten.	0	n	1	0	Asteraceae Bercht. & J.Presl
Iz_6_1009	DJI_0374	Clinopodium acinos (L.) Kuntze	0	n	1	0	Lamiacea Lindl.
Iz_6_1009	DJI_0374	Convallaria majalis L.	0	n	1	0	Asparagaceae Juss.
Iz_6_1009	DJI_0374	Crataegus rhipidophylla Gand.	0	n	1	1	Rosaceae Juss.
Iz_6_1009	DJI_0374	Crepis foetida L.	0	n	1	0	Asteraceae Bercht. & J.Presl
Iz_6_1009	DJI_0374	Dactylis glomerata L.	0	n	1	0	Poaceae Barnhart
Iz_6_1009	DJI_0374	Dianthus campestris M.Bieb.	0	n	1	0	Caryophyllaceae Juss.
Iz_6_1009	DJI_0374	Digitaria sanguinalis (L.) Scop.	0	a	0	0	Poaceae Barnhart
Iz_6_1009	DJI_0374	Elymus repens (L.) Gould	0	n	1	0	Poaceae Barnhart
Iz_6_1009	DJI_0374	Epilobium tetragonum L.	0	n	1	0	Onagraceae Juss.
Iz_6_1009	DJI_0374	Eragrostis minor Host	0	a	0	0	Poaceae Barnhart
Iz_6_1009	DJI_0374	Erigeron canadensis L.	10	a	0	0	Asteraceae Bercht. & J.Presl
Iz_6_1009	DJI_0374	Euphorbia seguieriana Neck.	0	n	1	0	Euphorbiaceae Juss.
Iz_6_1009	DJI_0374	Fallopia dumetorum (L.) Holub	0	n	1	0	Polygonaceae Juss.
Iz_6_1009	DJI_0374	Festuca beckeri (Hack.) Trautv.	0	n	1	0	Poaceae Barnhart
Iz_6_1009	DJI_0374	Fragaria vesca L.	0	n	1	0	Rosaceae Juss.

Iz_6_1009	DJI_0374	Frangula alnus Mill.	0	n	1	1	Rhamnaceae Juss.
Iz_6_1009	DJI_0374	Fraxinus pennsylvanica Marshall	0	a	0	1	Oleaceae Hoffmanns. & Link
Iz_6_1009	DJI_0374	Galeopsis bifida Boenn.	0	n	1	0	Lamiacea Lindl.
Iz_6_1009	DJI_0374	Galium verum L.	0	n	1	0	Rubiaceae Juss.
Iz_6_1009	DJI_0374	Genista tinctoria L.	0	n	1	0	Fabaceae Lindl.
Iz_6_1009	DJI_0374	Geranium robertianum L.	0	n	1	0	Geraniaceae Juss.
Iz_6_1009	DJI_0374	Geranium sanquineum L.	0	n	1	0	Geraniaceae Juss.
Iz_6_1009	DJI_0374	Gypsophila paniculata L.	0	n	1	0	Caryophyllaceae Juss.
Iz_6_1009	DJI_0374	Helichrysum arenarium (L.) Moench	0	n	1	0	Asteraceae Bercht. & J.Presl
Iz_6_1009	DJI_0374	Herniaria polygama J.Gay	0	n	1	0	Caryophyllaceae Juss.
Iz_6_1009	DJI_0374	Hieracium robustum Fr.	0	n	1	0	Asteraceae Bercht. & J.Presl
Iz_6_1009	DJI_0374	Hieracium umbellatum L.	0	n	1	NA	Asteraceae Bercht. & J.Presl
Iz_6_1009	DJI_0374	Hylotelephium maximum (L.) Holub	0	n	1	0	Crassulaceae J.St.-Hil.
Iz_6_1009	DJI_0374	Jacobaea borysthenica (DC.) B.Nord. & Greuter	0	n	1	0	Asteraceae Bercht. & J.Presl
Iz_6_1009	DJI_0374	Jasione montana L.	0	n	1	0	Campanulaceae Juss.
Iz_6_1009	DJI_0374	Jurinea cyanoides (L.) Rchb.s.l.	0	n	1	0	Asteraceae Bercht. & J.Presl
Iz_6_1009	DJI_0374	Koeleria glauca (Spreng.) DC.	0	n	1	0	Poaceae Barnhart
Iz_6_1009	DJI_0374	Lactuca serriola L.	0	a	0	0	Asteraceae Bercht. & J.Presl
Iz_6_1009	DJI_0374	Linaria genistifolia (L.) Mill.	0	n	1	0	Plantaginaceae Juss.
Iz_6_1009	DJI_0374	Lomelosia argentea (L.) Greuter & Burdet	0	n	1	0	Caprifoliaceae Juss.
Iz_6_1009	DJI_0374	Melampyrum pratense L.	0	n	1	0	Orobanchaceae Vent.
Iz_6_1009	DJI_0374	Oenothera biennis L.	0	a	0	0	Onagraceae Juss.
Iz_6_1009	DJI_0374	Pentanema britannica (L.) D.Gut.Larr., Santos-Vicente, Anderb., E.Rico & M.M.Mart.Ort.	0	n	1	0	Asteraceae Bercht. & J.Presl
Iz_6_1009	DJI_0374	Peucedanum oreoselinum (L.) Moench	0	n	1	0	Apiaceae Lindl.
Iz_6_1009	DJI_0374	Peucedanum ruthenicum M.Bieb.	0	n	1	0	Apiaceae Lindl.
Iz_6_1009	DJI_0374	Pilosella echioides (Lumn.) F.W.Schultz & Sch.Bip.	0	n	1	0	Asteraceae Bercht. & J.Presl
Iz_6_1009	DJI_0374	Pilosella officinarum F. Schultz et Sch. Bip.	0	n	1	0	Asteraceae Bercht. & J.Presl
Iz_6_1009	DJI_0374	Pilosella sp.	0		NA	0	Asteraceae Bercht. & J.Presl
Iz_6_1009	DJI_0374	Pinus sylvestris L.	0	n	1	1	Pinaceae Spreng. ex F.Rudolphi
Iz_6_1009	DJI_0374	Poa annua L.	0	n	1	0	Poaceae Barnhart
Iz_6_1009	DJI_0374	Poa nemoralis L.	0	n	1	0	Poaceae Barnhart
Iz_6_1009	DJI_0374	Poa sp.	0		NA	0	Poaceae Barnhart
Iz_6_1009	DJI_0374	Polygonatum odoratum (Mill.) Druce	0	n	1	0	Asparagaceae Juss.
Iz_6_1009	DJI_0374	Polygonum arenarium Waldst. & Kit.	0	n	1	0	Polygonaceae Juss.
Iz_6_1009	DJI_0374	Populus nigra L.	0	n	1	1	Salicaceae Mirb.
Iz_6_1009	DJI_0374	Populus tremula L.	0	n	1	1	Salicaceae Mirb.
Iz_6_1009	DJI_0374	Populus Ã canescens (Aiton) Sm.	0	n	1	1	Salicaceae Mirb.
Iz_6_1009	DJI_0374	Potentilla argentea L.	0	n	1	0	Rosaceae Juss.
Iz_6_1009	DJI_0374	Psammophiliella muralis (L.) Ikonn.	0	n	1	0	Caryophyllaceae Juss.
Iz_6_1009	DJI_0374	Psephellus sumensis (Kalen.) Greuter	0	n	1	0	Asteraceae Bercht. & J.Presl
Iz_6_1009	DJI_0374	Pulsatilla pratensis (L.) Mill. s.l.	0	n	1	0	Ranunculaceae Juss.
Iz_6_1009	DJI_0374	Pyrus communis L.	0	a	0	1	Rosaceae Juss.
Iz_6_1009	DJI_0374	Quercus robur L.	0	n	1	1	Fagaceae Dumort.
Iz_6_1009	DJI_0374	Robinia pseudoacacia L.	0	a	0	1	Fabaceae Lindl.
Iz_6_1009	DJI_0374	Rosa sp.	0		NA	1	Rosaceae Juss.
Iz_6_1009	DJI_0374	Rubus idaeus L.	0	n	1	0	Rosaceae Juss.
Iz_6_1009	DJI_0374	Rumex acetosella L.	0	n	1	0	Polygonaceae Juss.
Iz_6_1009	DJI_0374	Rumex thyrsiflorus Fingerh.	0	n	1	0	Polygonaceae Juss.
Iz_6_1009	DJI_0374	Salix alba L.	0	n	1	1	Salicaceae Mirb.
Iz_6_1009	DJI_0374	Sambucus nigra L.	0	n	1	1	Viburnaceae Raf.
Iz_6_1009	DJI_0374	Secale sylvestre Host	0	n	1	0	Poaceae Barnhart
Iz_6_1009	DJI_0374	Sempervivum ruthenicum Schnittsp. & C.B. Lehm.	0	n	1	0	Crassulaceae J.St.-Hil.
Iz_6_1009	DJI_0374	Setaria viridis (L.) P. Beauv.	0	n	1	0	Poaceae Barnhart
Iz_6_1009	DJI_0374	Silene borysthenica (Gruner) Walters	0	n	1	0	Caryophyllaceae Juss.
Iz_6_1009	DJI_0374	Silene latifolia subsp. alba (Mill.) Greuter & Burdet	0	n	1	0	Caryophyllaceae Juss.
Iz_6_1009	DJI_0374	Solidago virgaurea L.	0	n	1	0	Asteraceae Bercht. & J.Presl
Iz_6_1009	DJI_0374	Sorbus aucuparia L.	0	n	1	1	Rosaceae Juss.
Iz_6_1009	DJI_0374	Stachys recta L.	0	n	1	0	Lamiacea Lindl.
Iz_6_1009	DJI_0374	Tanacetum vulgare L.	0	n	1	0	Asteraceae Bercht. & J.Presl
Iz_6_1009	DJI_0374	Thymus pallasianus Heinr.Braun	0	n	1	0	Lamiacea Lindl.
Iz_6_1009	DJI_0374	Tilia cordata Mill.	0	n	1	1	Malvaceae Juss.
Iz_6_1009	DJI_0374	Trifolium alpestre L.	0	n	1	0	Fabaceae Lindl.
Iz_6_1009	DJI_0374	Trifolium arvense L.	0	n	1	0	Fabaceae Lindl.
Iz_6_1009	DJI_0374	Ulmus glabra Huds.	0	n	1	1	Ulmaceae Mirb.
Iz_6_1009	DJI_0374	Verbascum densiflorum Bertol.	0	n	1	0	Scrophulariaceae Juss.
Iz_6_1009	DJI_0374	Veronica austriaca L.	0	n	1	0	Plantaginaceae Juss.
Iz_6_1009	DJI_0374	Veronica incana L.	0	n	1	0	Plantaginaceae Juss.
Iz_6_1009	DJI_0374	Veronica verna L.	0	n	1	0	Plantaginaceae Juss.
Iz_6_1009	DJI_0374	Vincetoxicum hirundinaria Medik	0	n	1	0	Apocynaceae Juss.
Iz_6_1009	DJI_0382	Acer tataricum L.	0	n	1	1	Sapindaceae Juss.
Iz_6_1009	DJI_0382	Achillea millefolium L. s.l.	0	n	1	0	Asteraceae Bercht. & J.Presl
Iz_6_1009	DJI_0382	Allium oleraceum L.	0	n	1	0	Amaryllidaceae J.St.-Hil.
Iz_6_1009	DJI_0382	Ambrosia artemisiifolia L.	0	a	0	0	Asteraceae Bercht. & J.Presl
Iz_6_1009	DJI_0382	Amorpha fruticosa L.	0	a	0	0	Fabaceae Lindl.
Iz_6_1009	DJI_0382	Anthericum ramosum L.	0	n	1	0	Asparagaceae Juss.
Iz_6_1009	DJI_0382	Artemisia marschalliana Spreng.	0	n	1	0	Asteraceae Bercht. & J.Presl
Iz_6_1009	DJI_0382	Asparagus officinalis L.	0	n	1	0	Asparagaceae Juss.
Iz_6_1009	DJI_0382	Astragalus cicer L.	0	n	1	0	Fabaceae Lindl.
Iz_6_1009	DJI_0382	Bassia laniflora (S.G.Gmel.) A.J.Scott	0	n	1	0	Amaranthaceae Juss.
Iz_6_1009	DJI_0382	Berberis vulgaris L.	0	n	1	0	Berberidaceae Juss.
Iz_6_1009	DJI_0382	Betula pendula Roth.	0	n	1	1	Betulaceae Gray
Iz_6_1009	DJI_0382	Betula pendula Roth.	0	n	1	0	Poaceae Barnhart
Iz_6_1009	DJI_0382	Bromus inermis Leyss.	0		NA	NA	
Iz_6_1009	DJI_0382	Bromus squarrosus L.	0	a	0	0	Poaceae Barnhart
Iz_6_1009	DJI_0382	Bromus tectorum L.	0	a	0	0	Poaceae Barnhart
Iz_6_1009	DJI_0382	Calamagrostis epigejos (L.) Roth	20	n	1	0	Poaceae Barnhart
Iz_6_1009	DJI_0382	Carduus acanthoides L.	0	n	1	0	Asteraceae Bercht. & J.Presl
Iz_6_1009	DJI_0382	Carex sp.	0	n	1	0	Cyperaceae Juss.
Iz_6_1009	DJI_0382	Centaurea majorovii Dumbadze	0	n	1	0	Asteraceae Bercht. & J.Presl
Iz_6_1009	DJI_0382	Chamaecytisus ruthenicus (Fisch.ex Wol.) Klaskova	0	n	1	0	Fabaceae Lindl.
Iz_6_1009	DJI_0382	Chelidonium majus L.	0	n	1	0	Papaveraceae Juss.
Iz_6_1009	DJI_0382	Chenopodium betaceum Andrz.	0	n	1	0	Amaranthaceae Juss.
Iz_6_1009	DJI_0382	Chenopodium cf. album	0	n	1	0	Amaranthaceae Juss.
Iz_6_1009	DJI_0382	Chenopodium sp.	0		NA	0	Amaranthaceae Juss.
Iz_6_1009	DJI_0382	Chondrilla juncea L.	0	n	1	0	Asteraceae Bercht. & J.Presl
Iz_6_1009	DJI_0382	Cirsium vulgare (Savi) Ten.	0	n	1	0	Asteraceae Bercht. & J.Presl
Iz_6_1009	DJI_0382	Clinopodium acinos (L.) Kuntze	0	n	1	0	Lamiacea Lindl.
Iz_6_1009	DJI_0382	Convallaria majalis L.	0	n	1	0	Asparagaceae Juss.

Iz_6_1009	DJI_0382	Crataegus rhipidophylla Gand.	0	n	1	1	Rosaceae Juss.
Iz_6_1009	DJI_0382	Crepis foetida L.	0	n	1	0	Asteraceae Bercht. & J.Presl
Iz_6_1009	DJI_0382	Dactylis glomerata L.	0	n	1	0	Poaceae Barnhart
Iz_6_1009	DJI_0382	Dianthus campestris M.Bieb.	0	n	1	0	Caryophyllaceae Juss.
Iz_6_1009	DJI_0382	Digitaria sanguinalis (L.) Scop.	0	a	0	0	Poaceae Barnhart
Iz_6_1009	DJI_0382	Elymus repens (L.) Gould	0	n	1	0	Poaceae Barnhart
Iz_6_1009	DJI_0382	Epilobium tetragonum L.	0	n	1	0	Onagraceae Juss.
Iz_6_1009	DJI_0382	Eragrostis minor Host	0	a	0	0	Poaceae Barnhart
Iz_6_1009	DJI_0382	Erigeron canadensis L.	5	a	0	0	Asteraceae Bercht. & J.Presl
Iz_6_1009	DJI_0382	Euphorbia seguieriana Neck.	0	n	1	0	Euphorbiaceae Juss.
Iz_6_1009	DJI_0382	Fallopia dumetorum (L.) Holub	0	n	1	0	Polygonaceae Juss.
Iz_6_1009	DJI_0382	Festuca beckeri (Hack.) Trautv.	0	n	1	0	Poaceae Barnhart
Iz_6_1009	DJI_0382	Fragaria vesca L.	0	n	1	0	Rosaceae Juss.
Iz_6_1009	DJI_0382	Frangula alnus Mill.	0	n	1	1	Rhamnaceae Juss.
Iz_6_1009	DJI_0382	Fraxinus pennsylvanica Marshall	0	a	0	1	Oleaceae Hoffmanns. & Link
Iz_6_1009	DJI_0382	Galeopsis bifida Boenn.	0	n	1	0	Lamiacea Lindl.
Iz_6_1009	DJI_0382	Galium verum L.	0	n	1	0	Rubiaceae Juss.
Iz_6_1009	DJI_0382	Genista tinctoria L.	0	n	1	0	Fabaceae Lindl.
Iz_6_1009	DJI_0382	Geranium robertianum L.	0	n	1	0	Geraniaceae Juss.
Iz_6_1009	DJI_0382	Geranium sanquineum L.	0	n	1	0	Geraniaceae Juss.
Iz_6_1009	DJI_0382	Gypsophila paniculata L.	0	n	1	0	Caryophyllaceae Juss.
Iz_6_1009	DJI_0382	Helichrysum arenarium (L.) Moench	0	n	1	0	Asteraceae Bercht. & J.Presl
Iz_6_1009	DJI_0382	Herniaria polygama J.Gay	0	n	1	0	Caryophyllaceae Juss.
Iz_6_1009	DJI_0382	Hieracium robustum Fr.	0	n	1	0	Asteraceae Bercht. & J.Presl
Iz_6_1009	DJI_0382	Hieracium umbellatum L.	0	n	1	NA	Asteraceae Bercht. & J.Presl
Iz_6_1009	DJI_0382	Hylotelephium maximum (L.) Holub	0	n	1	0	Crassulaceae J.St.-Hil.
Iz_6_1009	DJI_0382	Jacobaea borysthenica (DC.) B.Nord. & Greuter	0	n	1	0	Asteraceae Bercht. & J.Presl
Iz_6_1009	DJI_0382	Jasione montana L.	0	n	1	0	Campanulaceae Juss.
Iz_6_1009	DJI_0382	Jurinea cyanoides (L.) Rchb.s.l.	0	n	1	0	Asteraceae Bercht. & J.Presl
Iz_6_1009	DJI_0382	Koeleria glauca (Spreng.) DC.	0	n	1	0	Poaceae Barnhart
Iz_6_1009	DJI_0382	Lactuca serriola L.	0	a	0	0	Asteraceae Bercht. & J.Presl
Iz_6_1009	DJI_0382	Linaria genistifolia (L.) Mill.	0	n	1	0	Plantaginaceae Juss.
Iz_6_1009	DJI_0382	Lomelosia argentea (L.) Greuter & Burdet	0	n	1	0	Caprifoliaceae Juss.
Iz_6_1009	DJI_0382	Melampyrum pratense L.	0	n	1	0	Orobanchaceae Vent.
Iz_6_1009	DJI_0382	Oenothera biennis L.	0	a	0	0	Onagraceae Juss.
Iz_6_1009	DJI_0382	Pentanema britannica (L.) D.Gut.Larr., Santos-Vicente, Anderb., E.Rico & M.M.Mart.Ort.	0	n	1	0	Asteraceae Bercht. & J.Presl
Iz_6_1009	DJI_0382	Peucedanum oreoselinum (L.) Moench	2	n	1	0	Apiaceae Lindl.
Iz_6_1009	DJI_0382	Peucedanum ruthenicum M.Bieb.	0	n	1	0	Apiaceae Lindl.
Iz_6_1009	DJI_0382	Pilosella echioides (Lumn.) F.W.Schultz & Sch.Bip.	0	n	1	0	Asteraceae Bercht. & J.Presl
Iz_6_1009	DJI_0382	Pilosella officinarum F. Schultz et Sch. Bip.	0	n	1	0	Asteraceae Bercht. & J.Presl
Iz_6_1009	DJI_0382	Pilosella sp.	0		NA	0	Asteraceae Bercht. & J.Presl
Iz_6_1009	DJI_0382	Pinus sylvestris L.	0	n	1	1	Pinaceae Spreng. ex F.Rudolphi
Iz_6_1009	DJI_0382	Poa annua L.	0	n	1	0	Poaceae Barnhart
Iz_6_1009	DJI_0382	Poa nemoralis L.	1	n	1	0	Poaceae Barnhart
Iz_6_1009	DJI_0382	Poa sp.	0		NA	0	Poaceae Barnhart
Iz_6_1009	DJI_0382	Polygonatum odoratum (Mill.) Druce	0	n	1	0	Asparagaceae Juss.
Iz_6_1009	DJI_0382	Polygonum arenarium Waldst. & Kit.	0	n	1	0	Polygonaceae Juss.
Iz_6_1009	DJI_0382	Populus nigra L.	0	n	1	1	Salicaceae Mirb.
Iz_6_1009	DJI_0382	Populus tremula L.	0	n	1	1	Salicaceae Mirb.
Iz_6_1009	DJI_0382	Populus Ã canescens (Aiton) Sm.	0	n	1	1	Salicaceae Mirb.
Iz_6_1009	DJI_0382	Potentilla argentea L.	0	n	1	0	Rosaceae Juss.
Iz_6_1009	DJI_0382	Psammophiliella muralis (L.) Ikonn.	0	n	1	0	Caryophyllaceae Juss.
Iz_6_1009	DJI_0382	Psephellus sumensis (Kalen.) Greuter	0	n	1	0	Asteraceae Bercht. & J.Presl
Iz_6_1009	DJI_0382	Pulsatilla pratensis (L.) Mill. s.l.	0	n	1	0	Ranunculaceae Juss.
Iz_6_1009	DJI_0382	Pyrus communis L.	0	a	0	1	Rosaceae Juss.
Iz_6_1009	DJI_0382	Quercus robur L.	0	n	1	1	Fagaceae Dumort.
Iz_6_1009	DJI_0382	Robinia pseudoacacia L.	0	a	0	1	Fabaceae Lindl.
Iz_6_1009	DJI_0382	Rosa sp.	0		NA	1	Rosaceae Juss.
Iz_6_1009	DJI_0382	Rubus idaeus L.	0	n	1	0	Rosaceae Juss.
Iz_6_1009	DJI_0382	Rumex acetosella L.	0	n	1	0	Polygonaceae Juss.
Iz_6_1009	DJI_0382	Rumex thyrsiflorus Fingerh.	0	n	1	0	Polygonaceae Juss.
Iz_6_1009	DJI_0382	Salix alba L.	0	n	1	1	Salicaceae Mirb.
Iz_6_1009	DJI_0382	Sambucus nigra L.	0	n	1	1	Viburnaceae Raf.
Iz_6_1009	DJI_0382	Secale sylvestre Host	0	n	1	0	Poaceae Barnhart
Iz_6_1009	DJI_0382	Sempervivum ruthenicum Schnittsp. & C.B. Lehm.	0	n	1	0	Crassulaceae J.St.-Hil.
Iz_6_1009	DJI_0382	Setaria viridis (L.) P. Beauv.	0	n	1	0	Poaceae Barnhart
Iz_6_1009	DJI_0382	Silene borysthenica (Gruner) Walters	0	n	1	0	Caryophyllaceae Juss.
Iz_6_1009	DJI_0382	Silene latifolia subsp. alba (Mill.) Greuter & Burdet	0	n	1	0	Caryophyllaceae Juss.
Iz_6_1009	DJI_0382	Solidago virgaurea L.	0	n	1	0	Asteraceae Bercht. & J.Presl
Iz_6_1009	DJI_0382	Sorbus aucuparia L.	0	n	1	1	Rosaceae Juss.
Iz_6_1009	DJI_0382	Stachys recta L.	0	n	1	0	Lamiacea Lindl.
Iz_6_1009	DJI_0382	Tanacetum vulgare L.	0	n	1	0	Asteraceae Bercht. & J.Presl
Iz_6_1009	DJI_0382	Thymus pallasianus Heinr.Braun	0	n	1	0	Lamiacea Lindl.
Iz_6_1009	DJI_0382	Tilia cordata Mill.	0	n	1	1	Malvaceae Juss.
Iz_6_1009	DJI_0382	Trifolium alpestre L.	0	n	1	0	Fabaceae Lindl.
Iz_6_1009	DJI_0382	Trifolium arvense L.	0	n	1	0	Fabaceae Lindl.
Iz_6_1009	DJI_0382	Ulmus glabra Huds.	0	n	1	1	Ulmaceae Mirb.
Iz_6_1009	DJI_0382	Verbascum densiflorum Bertol.	0	n	1	0	Scrophulariaceae Juss.
Iz_6_1009	DJI_0382	Veronica austriaca L.	0	n	1	0	Plantaginaceae Juss.
Iz_6_1009	DJI_0382	Veronica incana L.	0	n	1	0	Plantaginaceae Juss.
Iz_6_1009	DJI_0382	Veronica verna L.	0	n	1	0	Plantaginaceae Juss.
Iz_6_1009	DJI_0382	Vincetoxicum hirundinaria Medik	0	n	1	0	Apocynaceae Juss.
Iz_6_1009	DJI_0385	Acer tataricum L.	0	n	1	1	Sapindaceae Juss.
Iz_6_1009	DJI_0385	Achillea millefolium L. s.l.	0	n	1	0	Asteraceae Bercht. & J.Presl
Iz_6_1009	DJI_0385	Allium oleraceum L.	0	n	1	0	Amaryllidaceae J.St.-Hil.
Iz_6_1009	DJI_0385	Ambrosia artemisiifolia L.	0	a	0	0	Asteraceae Bercht. & J.Presl
Iz_6_1009	DJI_0385	Amorpha fruticosa L.	0	a	0	0	Fabaceae Lindl.
Iz_6_1009	DJI_0385	Anthericum ramosum L.	0	n	1	0	Asparagaceae Juss.
Iz_6_1009	DJI_0385	Artemisia marschalliana Spreng.	0	n	1	0	Asteraceae Bercht. & J.Presl
Iz_6_1009	DJI_0385	Asparagus officinalis L.	0	n	1	0	Asparagaceae Juss.
Iz_6_1009	DJI_0385	Astragalus cicer L.	0	n	1	0	Fabaceae Lindl.
Iz_6_1009	DJI_0385	Bassia laniflora (S.G.Gmel.) A.J.Scott	0	n	1	0	Amaranthaceae Juss.
Iz_6_1009	DJI_0385	Berberis vulgaris L.	0	n	1	0	Berberidaceae Juss.
Iz_6_1009	DJI_0385	Betula pendula Roth.	0	n	1	1	Betulaceae Gray
Iz_6_1009	DJI_0385	Betula pendula Roth.	0	n	1	0	Poaceae Barnhart
Iz_6_1009	DJI_0385	Bromus inermis Leyss.	0		NA	NA	
Iz_6_1009	DJI_0385	Bromus squarrosus L.	0	a	0	0	Poaceae Barnhart
Iz_6_1009	DJI_0385	Bromus tectorum L.	0	a	0	0	Poaceae Barnhart

Iz_6_1009	DJI_0385	Calamagrostis epigejos (L.) Roth	85	n	1	0	Poaceae Barnhart
Iz_6_1009	DJI_0385	Carduus acanthoides L.	0	n	1	0	Asteraceae Bercht. & J.Presl
Iz_6_1009	DJI_0385	Carex sp.	0	n	1	0	Cyperaceae Juss.
Iz_6_1009	DJI_0385	Centaurea majorovii Dumbadze	0	n	1	0	Asteraceae Bercht. & J.Presl
Iz_6_1009	DJI_0385	Chamaecytisus ruthenicus (Fisch.ex Wol.) Klaskova	0	n	1	0	Fabaceae Lindl.
Iz_6_1009	DJI_0385	Chelidonium majus L.	0	n	1	0	Papaveraceae Juss.
Iz_6_1009	DJI_0385	Chenopodium betaceum Andrz.	0	n	1	0	Amaranthaceae Juss.
Iz_6_1009	DJI_0385	Chenopodium cf. album	0	n	1	0	Amaranthaceae Juss.
Iz_6_1009	DJI_0385	Chenopodium sp.	0		NA	0	Amaranthaceae Juss.
Iz_6_1009	DJI_0385	Chondrilla juncea L.	0	n	1	0	Asteraceae Bercht. & J.Presl
Iz_6_1009	DJI_0385	Cirsium vulgare (Savi) Ten.	0	n	1	0	Asteraceae Bercht. & J.Presl
Iz_6_1009	DJI_0385	Clinopodium acinos (L.) Kuntze	0	n	1	0	Lamiacea Lindl.
Iz_6_1009	DJI_0385	Convallaria majalis L.	0	n	1	0	Asparagaceae Juss.
Iz_6_1009	DJI_0385	Crataegus rhipidophylla Gand.	0	n	1	1	Rosaceae Juss.
Iz_6_1009	DJI_0385	Crepis foetida L.	0	n	1	0	Asteraceae Bercht. & J.Presl
Iz_6_1009	DJI_0385	Dactylis glomerata L.	0	n	1	0	Poaceae Barnhart
Iz_6_1009	DJI_0385	Dianthus campestris M.Bieb.	0	n	1	0	Caryophyllaceae Juss.
Iz_6_1009	DJI_0385	Digitaria sanguinalis (L.) Scop.	0	a	0	0	Poaceae Barnhart
Iz_6_1009	DJI_0385	Elymus repens (L.) Gould	0	n	1	0	Poaceae Barnhart
Iz_6_1009	DJI_0385	Epilobium tetragonum L.	0	n	1	0	Onagraceae Juss.
Iz_6_1009	DJI_0385	Eragrostis minor Host	0	a	0	0	Poaceae Barnhart
Iz_6_1009	DJI_0385	Erigeron canadensis L.	0	a	0	0	Asteraceae Bercht. & J.Presl
Iz_6_1009	DJI_0385	Euphorbia seguieriana Neck.	0	n	1	0	Euphorbiaceae Juss.
Iz_6_1009	DJI_0385	Fallopia dumetorum (L.) Holub	0	n	1	0	Polygonaceae Juss.
Iz_6_1009	DJI_0385	Festuca beckeri (Hack.) Trautv.	0	n	1	0	Poaceae Barnhart
Iz_6_1009	DJI_0385	Fragaria vesca L.	0	n	1	0	Rosaceae Juss.
Iz_6_1009	DJI_0385	Frangula alnus Mill.	0	n	1	1	Rhamnaceae Juss.
Iz_6_1009	DJI_0385	Fraxinus pennsylvanica Marshall	0	a	0	1	Oleaceae Hoffmanns. & Link
Iz_6_1009	DJI_0385	Galeopsis bifida Boenn.	0	n	1	0	Lamiacea Lindl.
Iz_6_1009	DJI_0385	Galium verum L.	0	n	1	0	Rubiaceae Juss.
Iz_6_1009	DJI_0385	Genista tinctoria L.	0	n	1	0	Fabaceae Lindl.
Iz_6_1009	DJI_0385	Geranium robertianum L.	0	n	1	0	Geraniaceae Juss.
Iz_6_1009	DJI_0385	Geranium sanquineum L.	0	n	1	0	Geraniaceae Juss.
Iz_6_1009	DJI_0385	Gypsophila paniculata L.	0	n	1	0	Caryophyllaceae Juss.
Iz_6_1009	DJI_0385	Helichrysum arenarium (L.) Moench	0	n	1	0	Asteraceae Bercht. & J.Presl
Iz_6_1009	DJI_0385	Herniaria polygama J.Gay	0	n	1	0	Caryophyllaceae Juss.
Iz_6_1009	DJI_0385	Hieracium robustum Fr.	0	n	1	0	Asteraceae Bercht. & J.Presl
Iz_6_1009	DJI_0385	Hieracium umbellatum L.	0	n	1	NA	Asteraceae Bercht. & J.Presl
Iz_6_1009	DJI_0385	Hylotelephium maximum (L.) Holub	0	n	1	0	Crassulaceae J.St.-Hil.
Iz_6_1009	DJI_0385	Jacobaea borysthenica (DC.) B.Nord. & Greuter	0	n	1	0	Asteraceae Bercht. & J.Presl
Iz_6_1009	DJI_0385	Jasione montana L.	0	n	1	0	Campanulaceae Juss.
Iz_6_1009	DJI_0385	Jurinea cyanoides (L.) Rchb.s.l.	0	n	1	0	Asteraceae Bercht. & J.Presl
Iz_6_1009	DJI_0385	Koeleria glauca (Spreng.) DC.	0	n	1	0	Poaceae Barnhart
Iz_6_1009	DJI_0385	Lactuca serriola L.	0	a	0	0	Asteraceae Bercht. & J.Presl
Iz_6_1009	DJI_0385	Linaria genistifolia (L.) Mill.	0	n	1	0	Plantaginaceae Juss.
Iz_6_1009	DJI_0385	Lomelosia argentea (L.) Greuter & Burdet	0	n	1	0	Caprifoliaceae Juss.
Iz_6_1009	DJI_0385	Melampyrum pratense L.	0	n	1	0	Orobanchaceae Vent.
Iz_6_1009	DJI_0385	Oenothera biennis L.	0	a	0	0	Onagraceae Juss.
Iz_6_1009	DJI_0385	Pentanema britannica (L.) D.Gut.Larr., Santos-Vicente, Anderb., E.Rico & M.M.Mart.Ort.	0	n	1	0	Asteraceae Bercht. & J.Presl
Iz_6_1009	DJI_0385	Peucedanum oreoselinum (L.) Moench	2	n	1	0	Apiaceae Lindl.
Iz_6_1009	DJI_0385	Peucedanum ruthenicum M.Bieb.	0	n	1	0	Apiaceae Lindl.
Iz_6_1009	DJI_0385	Pilosella echioides (Lumn.) F.W.Schultz & Sch.Bip.	0	n	1	0	Asteraceae Bercht. & J.Presl
Iz_6_1009	DJI_0385	Pilosella officinarum F. Schultz et Sch. Bip.	0	n	1	0	Asteraceae Bercht. & J.Presl
Iz_6_1009	DJI_0385	Pilosella sp.	0		NA	0	Asteraceae Bercht. & J.Presl
Iz_6_1009	DJI_0385	Pinus sylvestris L.	0	n	1	1	Pinaceae Spreng. ex F.Rudolphi
Iz_6_1009	DJI_0385	Poa annua L.	0	n	1	0	Poaceae Barnhart
Iz_6_1009	DJI_0385	Poa nemoralis L.	0	n	1	0	Poaceae Barnhart
Iz_6_1009	DJI_0385	Poa sp.	0		NA	0	Poaceae Barnhart
Iz_6_1009	DJI_0385	Polygonatum odoratum (Mill.) Druce	0	n	1	0	Asparagaceae Juss.
Iz_6_1009	DJI_0385	Polygonum arenarium Waldst. & Kit.	0	n	1	0	Polygonaceae Juss.
Iz_6_1009	DJI_0385	Populus nigra L.	0	n	1	1	Salicaceae Mirb.
Iz_6_1009	DJI_0385	Populus tremula L.	0	n	1	1	Salicaceae Mirb.
Iz_6_1009	DJI_0385	Populus Ã canescens (Aiton) Sm.	0	n	1	1	Salicaceae Mirb.
Iz_6_1009	DJI_0385	Potentilla argentea L.	0	n	1	0	Rosaceae Juss.
Iz_6_1009	DJI_0385	Psammophiliella muralis (L.) Ikonn.	0	n	1	0	Caryophyllaceae Juss.
Iz_6_1009	DJI_0385	Psephellus sumensis (Kalen.) Greuter	0	n	1	0	Asteraceae Bercht. & J.Presl
Iz_6_1009	DJI_0385	Pulsatilla pratensis (L.) Mill. s.l.	0	n	1	0	Ranunculaceae Juss.
Iz_6_1009	DJI_0385	Pyrus communis L.	0	a	0	1	Rosaceae Juss.
Iz_6_1009	DJI_0385	Quercus robur L.	0	n	1	1	Fagaceae Dumort.
Iz_6_1009	DJI_0385	Robinia pseudoacacia L.	0	a	0	1	Fabaceae Lindl.
Iz_6_1009	DJI_0385	Rosa sp.	0		NA	1	Rosaceae Juss.
Iz_6_1009	DJI_0385	Rubus idaeus L.	0	n	1	0	Rosaceae Juss.
Iz_6_1009	DJI_0385	Rumex acetosella L.	0	n	1	0	Polygonaceae Juss.
Iz_6_1009	DJI_0385	Rumex thyrsiflorus Fingerh.	0	n	1	0	Polygonaceae Juss.
Iz_6_1009	DJI_0385	Salix alba L.	0	n	1	1	Salicaceae Mirb.
Iz_6_1009	DJI_0385	Sambucus nigra L.	0	n	1	1	Viburnaceae Raf.
Iz_6_1009	DJI_0385	Secale sylvestre Host	0	n	1	0	Poaceae Barnhart
Iz_6_1009	DJI_0385	Sempervivum ruthenicum Schnittsp. & C.B. Lehm.	0	n	1	0	Crassulaceae J.St.-Hil.
Iz_6_1009	DJI_0385	Setaria viridis (L.) P. Beauv.	0	n	1	0	Poaceae Barnhart
Iz_6_1009	DJI_0385	Silene borysthenica (Gruner) Walters	0	n	1	0	Caryophyllaceae Juss.
Iz_6_1009	DJI_0385	Silene latifolia subsp. alba (Mill.) Greuter & Burdet	0	n	1	0	Caryophyllaceae Juss.
Iz_6_1009	DJI_0385	Solidago virgaurea L.	0	n	1	0	Asteraceae Bercht. & J.Presl
Iz_6_1009	DJI_0385	Sorbus aucuparia L.	0	n	1	1	Rosaceae Juss.
Iz_6_1009	DJI_0385	Stachys recta L.	0	n	1	0	Lamiacea Lindl.
Iz_6_1009	DJI_0385	Tanacetum vulgare L.	0	n	1	0	Asteraceae Bercht. & J.Presl
Iz_6_1009	DJI_0385	Thymus pallasianus Heinr.Braun	0	n	1	0	Lamiacea Lindl.
Iz_6_1009	DJI_0385	Tilia cordata Mill.	0	n	1	1	Malvaceae Juss.
Iz_6_1009	DJI_0385	Trifolium alpestre L.	0	n	1	0	Fabaceae Lindl.
Iz_6_1009	DJI_0385	Trifolium arvense L.	0	n	1	0	Fabaceae Lindl.
Iz_6_1009	DJI_0385	Ulmus glabra Huds.	0	n	1	1	Ulmaceae Mirb.
Iz_6_1009	DJI_0385	Verbascum densiflorum Bertol.	0	n	1	0	Scrophulariaceae Juss.
Iz_6_1009	DJI_0385	Veronica austriaca L.	0	n	1	0	Plantaginaceae Juss.
Iz_6_1009	DJI_0385	Veronica incana L.	0	n	1	0	Plantaginaceae Juss.
Iz_6_1009	DJI_0385	Veronica verna L.	0	n	1	0	Plantaginaceae Juss.
Iz_6_1009	DJI_0385	Vincetoxicum hirundinaria Medik	0	n	1	0	Apocynaceae Juss.
Iz_6_1009	DJI_0395	Acer tataricum L.	0	n	1	1	Sapindaceae Juss.
Iz_6_1009	DJI_0395	Achillea millefolium L. s.l.	0	n	1	0	Asteraceae Bercht. & J.Presl
Iz_6_1009	DJI_0395	Allium oleraceum L.	0	n	1	0	Amaryllidaceae J.St.-Hil.

Iz_6_1009	DJI_0395	Ambrosia artemisiifolia L.	0	a	0	0	Asteraceae Bercht. & J.Presl
Iz_6_1009	DJI_0395	Amorpha fruticosa L.	0	a	0	0	Fabaceae Lindl.
Iz_6_1009	DJI_0395	Anthericum ramosum L.	0	n	1	0	Asparagaceae Juss.
Iz_6_1009	DJI_0395	Artemisia marschalliana Spreng.	0	n	1	0	Asteraceae Bercht. & J.Presl
Iz_6_1009	DJI_0395	Asparagus officinalis L.	0	n	1	0	Asparagaceae Juss.
Iz_6_1009	DJI_0395	Astragalus cicer L.	0	n	1	0	Fabaceae Lindl.
Iz_6_1009	DJI_0395	Bassia laniflora (S.G.Gmel.) A.J.Scott	0	n	1	0	Amaranthaceae Juss.
Iz_6_1009	DJI_0395	Berberis vulgaris L.	0	n	1	0	Berberidaceae Juss.
Iz_6_1009	DJI_0395	Betula pendula Roth.	0	n	1	1	Betulaceae Gray
Iz_6_1009	DJI_0395	Betula pendula Roth.	0	n	1	0	Poaceae Barnhart
Iz_6_1009	DJI_0395	Bromus inermis Leyss.	0		NA	NA	
Iz_6_1009	DJI_0395	Bromus squarrosus L.	0	a	0	0	Poaceae Barnhart
Iz_6_1009	DJI_0395	Bromus tectorum L.	0	a	0	0	Poaceae Barnhart
Iz_6_1009	DJI_0395	Calamagrostis epigejos (L.) Roth	30	n	1	0	Poaceae Barnhart
Iz_6_1009	DJI_0395	Carduus acanthoides L.	0	n	1	0	Asteraceae Bercht. & J.Presl
Iz_6_1009	DJI_0395	Carex sp.	0	n	1	0	Cyperaceae Juss.
Iz_6_1009	DJI_0395	Centaurea majorovii Dumbadze	0	n	1	0	Asteraceae Bercht. & J.Presl
Iz_6_1009	DJI_0395	Chamaecytisus ruthenicus (Fisch.ex Wol.) Klaskova	0	n	1	0	Fabaceae Lindl.
Iz_6_1009	DJI_0395	Chelidonium majus L.	2	n	1	0	Papaveraceae Juss.
Iz_6_1009	DJI_0395	Chenopodium betaceum Andrz.	0	n	1	0	Amaranthaceae Juss.
Iz_6_1009	DJI_0395	Chenopodium cf. album	0	n	1	0	Amaranthaceae Juss.
Iz_6_1009	DJI_0395	Chenopodium sp.	0		NA	0	Amaranthaceae Juss.
Iz_6_1009	DJI_0395	Chondrilla juncea L.	0	n	1	0	Asteraceae Bercht. & J.Presl
Iz_6_1009	DJI_0395	Cirsium vulgare (Savi) Ten.	0	n	1	0	Asteraceae Bercht. & J.Presl
Iz_6_1009	DJI_0395	Clinopodium acinos (L.) Kuntze	0	n	1	0	Lamiacea Lindl.
Iz_6_1009	DJI_0395	Convallaria majalis L.	0	n	1	0	Asparagaceae Juss.
Iz_6_1009	DJI_0395	Crataegus rhipidophylla Gand.	0	n	1	1	Rosaceae Juss.
Iz_6_1009	DJI_0395	Crepis foetida L.	0	n	1	0	Asteraceae Bercht. & J.Presl
Iz_6_1009	DJI_0395	Dactylis glomerata L.	0	n	1	0	Poaceae Barnhart
Iz_6_1009	DJI_0395	Dianthus campestris M.Bieb.	0	n	1	0	Caryophyllaceae Juss.
Iz_6_1009	DJI_0395	Digitaria sanguinalis (L.) Scop.	0	a	0	0	Poaceae Barnhart
Iz_6_1009	DJI_0395	Elymus repens (L.) Gould	0	n	1	0	Poaceae Barnhart
Iz_6_1009	DJI_0395	Epilobium tetragonum L.	0	n	1	0	Onagraceae Juss.
Iz_6_1009	DJI_0395	Eragrostis minor Host	0	a	0	0	Poaceae Barnhart
Iz_6_1009	DJI_0395	Erigeron canadensis L.	2	a	0	0	Asteraceae Bercht. & J.Presl
Iz_6_1009	DJI_0395	Euphorbia seguieriana Neck.	0	n	1	0	Euphorbiaceae Juss.
Iz_6_1009	DJI_0395	Fallopia dumetorum (L.) Holub	0.5	n	1	0	Polygonaceae Juss.
Iz_6_1009	DJI_0395	Festuca beckeri (Hack.) Trautv.	0	n	1	0	Poaceae Barnhart
Iz_6_1009	DJI_0395	Fragaria vesca L.	0	n	1	0	Rosaceae Juss.
Iz_6_1009	DJI_0395	Frangula alnus Mill.	0	n	1	1	Rhamnaceae Juss.
Iz_6_1009	DJI_0395	Fraxinus pennsylvanica Marshall	0	a	0	1	Oleaceae Hoffmanns. & Link
Iz_6_1009	DJI_0395	Galeopsis bifida Boenn.	0	n	1	0	Lamiacea Lindl.
Iz_6_1009	DJI_0395	Galium verum L.	0	n	1	0	Rubiaceae Juss.
Iz_6_1009	DJI_0395	Genista tinctoria L.	0	n	1	0	Fabaceae Lindl.
Iz_6_1009	DJI_0395	Geranium robertianum L.	0.5	n	1	0	Geraniaceae Juss.
Iz_6_1009	DJI_0395	Geranium sanquineum L.	0	n	1	0	Geraniaceae Juss.
Iz_6_1009	DJI_0395	Gypsophila paniculata L.	0	n	1	0	Caryophyllaceae Juss.
Iz_6_1009	DJI_0395	Helichrysum arenarium (L.) Moench	0	n	1	0	Asteraceae Bercht. & J.Presl
Iz_6_1009	DJI_0395	Herniaria polygama J.Gay	0	n	1	0	Caryophyllaceae Juss.
Iz_6_1009	DJI_0395	Hieracium robustum Fr.	0	n	1	0	Asteraceae Bercht. & J.Presl
Iz_6_1009	DJI_0395	Hieracium umbellatum L.	0	n	1	NA	Asteraceae Bercht. & J.Presl
Iz_6_1009	DJI_0395	Hylotelephium maximum (L.) Holub	0	n	1	0	Crassulaceae J.St.-Hil.
Iz_6_1009	DJI_0395	Jacobaea borysthenica (DC.) B.Nord. & Greuter	0	n	1	0	Asteraceae Bercht. & J.Presl
Iz_6_1009	DJI_0395	Jasione montana L.	0	n	1	0	Campanulaceae Juss.
Iz_6_1009	DJI_0395	Jurinea cyanoides (L.) Rchb.s.l.	0	n	1	0	Asteraceae Bercht. & J.Presl
Iz_6_1009	DJI_0395	Koeleria glauca (Spreng.) DC.	0	n	1	0	Poaceae Barnhart
Iz_6_1009	DJI_0395	Lactuca serriola L.	0	a	0	0	Asteraceae Bercht. & J.Presl
Iz_6_1009	DJI_0395	Linaria genistifolia (L.) Mill.	0	n	1	0	Plantaginaceae Juss.
Iz_6_1009	DJI_0395	Lomelosia argentea (L.) Greuter & Burdet	0	n	1	0	Caprifoliaceae Juss.
Iz_6_1009	DJI_0395	Melampyrum pratense L.	0	n	1	0	Orobanchaceae Vent.
Iz_6_1009	DJI_0395	Oenothera biennis L.	0	a	0	0	Onagraceae Juss.
Iz_6_1009	DJI_0395	Pentanema britannica (L.) D.Gut.Larr., Santos-Vicente, Anderb., E.Rico & M.M.Mart.Ort.	0	n	1	0	Asteraceae Bercht. & J.Presl
Iz_6_1009	DJI_0395	Peucedanum oreoselinum (L.) Moench	0	n	1	0	Apiaceae Lindl.
Iz_6_1009	DJI_0395	Peucedanum ruthenicum M.Bieb.	0	n	1	0	Apiaceae Lindl.
Iz_6_1009	DJI_0395	Pilosella echioides (Lumn.) F.W.Schultz & Sch.Bip.	0	n	1	0	Asteraceae Bercht. & J.Presl
Iz_6_1009	DJI_0395	Pilosella officinarum F. Schultz et Sch. Bip.	0	n	1	0	Asteraceae Bercht. & J.Presl
Iz_6_1009	DJI_0395	Pilosella sp.	0		NA	0	Asteraceae Bercht. & J.Presl
Iz_6_1009	DJI_0395	Pinus sylvestris L.	0	n	1	1	Pinaceae Spreng. ex F.Rudolphi
Iz_6_1009	DJI_0395	Poa annua L.	0	n	1	0	Poaceae Barnhart
Iz_6_1009	DJI_0395	Poa nemoralis L.	0	n	1	0	Poaceae Barnhart
Iz_6_1009	DJI_0395	Poa sp.	0		NA	0	Poaceae Barnhart
Iz_6_1009	DJI_0395	Polygonatum odoratum (Mill.) Druce	0	n	1	0	Asparagaceae Juss.
Iz_6_1009	DJI_0395	Polygonum arenarium Waldst. & Kit.	0	n	1	0	Polygonaceae Juss.
Iz_6_1009	DJI_0395	Populus nigra L.	0	n	1	1	Salicaceae Mirb.
Iz_6_1009	DJI_0395	Populus tremula L.	0	n	1	1	Salicaceae Mirb.
Iz_6_1009	DJI_0395	Populus Ã canescens (Aiton) Sm.	0	n	1	1	Salicaceae Mirb.
Iz_6_1009	DJI_0395	Potentilla argentea L.	0	n	1	0	Rosaceae Juss.
Iz_6_1009	DJI_0395	Psammophiliella muralis (L.) Ikonn.	0	n	1	0	Caryophyllaceae Juss.
Iz_6_1009	DJI_0395	Psephellus sumensis (Kalen.) Greuter	0	n	1	0	Asteraceae Bercht. & J.Presl
Iz_6_1009	DJI_0395	Pulsatilla pratensis (L.) Mill. s.l.	0	n	1	0	Ranunculaceae Juss.
Iz_6_1009	DJI_0395	Pyrus communis L.	0	a	0	1	Rosaceae Juss.
Iz_6_1009	DJI_0395	Quercus robur L.	0	n	1	1	Fagaceae Dumort.
Iz_6_1009	DJI_0395	Robinia pseudoacacia L.	0	a	0	1	Fabaceae Lindl.
Iz_6_1009	DJI_0395	Rosa sp.	0		NA	1	Rosaceae Juss.
Iz_6_1009	DJI_0395	Rubus idaeus L.	0	n	1	0	Rosaceae Juss.
Iz_6_1009	DJI_0395	Rumex acetosella L.	0	n	1	0	Polygonaceae Juss.
Iz_6_1009	DJI_0395	Rumex thyrsiflorus Fingerh.	0	n	1	0	Polygonaceae Juss.
Iz_6_1009	DJI_0395	Salix alba L.	0	n	1	1	Salicaceae Mirb.
Iz_6_1009	DJI_0395	Sambucus nigra L.	0	n	1	1	Viburnaceae Raf.
Iz_6_1009	DJI_0395	Secale sylvestre Host	0	n	1	0	Poaceae Barnhart
Iz_6_1009	DJI_0395	Sempervivum ruthenicum Schnittsp. & C.B. Lehm.	0	n	1	0	Crassulaceae J.St.-Hil.
Iz_6_1009	DJI_0395	Setaria viridis (L.) P. Beauv.	0	n	1	0	Poaceae Barnhart
Iz_6_1009	DJI_0395	Silene borysthenica (Gruner) Walters	0	n	1	0	Caryophyllaceae Juss.
Iz_6_1009	DJI_0395	Silene latifolia subsp. alba (Mill.) Greuter & Burdet	0	n	1	0	Caryophyllaceae Juss.
Iz_6_1009	DJI_0395	Solidago virgaurea L.	0	n	1	0	Asteraceae Bercht. & J.Presl
Iz_6_1009	DJI_0395	Sorbus aucuparia L.	0	n	1	1	Rosaceae Juss.
Iz_6_1009	DJI_0395	Stachys recta L.	0	n	1	0	Lamiacea Lindl.
Iz_6_1009	DJI_0395	Tanacetum vulgare L.	0	n	1	0	Asteraceae Bercht. & J.Presl

Iz_6_1009	DJI_0395	Thymus pallasianus Heinr.Braun	0	n	1	0	Lamiacea Lindl.
Iz_6_1009	DJI_0395	Tilia cordata Mill.	0	n	1	1	Malvaceae Juss.
Iz_6_1009	DJI_0395	Trifolium alpestre L.	0	n	1	0	Fabaceae Lindl.
Iz_6_1009	DJI_0395	Trifolium arvense L.	0	n	1	0	Fabaceae Lindl.
Iz_6_1009	DJI_0395	Ulmus glabra Huds.	0	n	1	1	Ulmaceae Mirb.
Iz_6_1009	DJI_0395	Verbascum densiflorum Bertol.	0	n	1	0	Scrophulariaceae Juss.
Iz_6_1009	DJI_0395	Veronica austriaca L.	0	n	1	0	Plantaginaceae Juss.
Iz_6_1009	DJI_0395	Veronica incana L.	0	n	1	0	Plantaginaceae Juss.
Iz_6_1009	DJI_0395	Veronica verna L.	0	n	1	0	Plantaginaceae Juss.
Iz_6_1009	DJI_0395	Vincetoxicum hirundinaria Medik	0	n	1	0	Apocynaceae Juss.
Iz_6_1009	DJI_0406	Acer tataricum L.	0	n	1	1	Sapindaceae Juss.
Iz_6_1009	DJI_0406	Achillea millefolium L. s.l.	0	n	1	0	Asteraceae Bercht. & J.Presl
Iz_6_1009	DJI_0406	Allium oleraceum L.	0	n	1	0	Amaryllidaceae J.St.-Hil.
Iz_6_1009	DJI_0406	Ambrosia artemisiifolia L.	0	a	0	0	Asteraceae Bercht. & J.Presl
Iz_6_1009	DJI_0406	Amorpha fruticosa L.	0	a	0	0	Fabaceae Lindl.
Iz_6_1009	DJI_0406	Anthericum ramosum L.	0	n	1	0	Asparagaceae Juss.
Iz_6_1009	DJI_0406	Artemisia marschalliana Spreng.	0	n	1	0	Asteraceae Bercht. & J.Presl
Iz_6_1009	DJI_0406	Asparagus officinalis L.	0	n	1	0	Asparagaceae Juss.
Iz_6_1009	DJI_0406	Astragalus cicer L.	0	n	1	0	Fabaceae Lindl.
Iz_6_1009	DJI_0406	Bassia laniflora (S.G.Gmel.) A.J.Scott	0	n	1	0	Amaranthaceae Juss.
Iz_6_1009	DJI_0406	Berberis vulgaris L.	0	n	1	0	Berberidaceae Juss.
Iz_6_1009	DJI_0406	Betula pendula Roth.	0	n	1	1	Betulaceae Gray
Iz_6_1009	DJI_0406	Betula pendula Roth.	0	n	1	0	Poaceae Barnhart
Iz_6_1009	DJI_0406	Bromus inermis Leyss.	0		NA	NA	
Iz_6_1009	DJI_0406	Bromus squarrosus L.	0	a	0	0	Poaceae Barnhart
Iz_6_1009	DJI_0406	Bromus tectorum L.	0	a	0	0	Poaceae Barnhart
Iz_6_1009	DJI_0406	Calamagrostis epigejos (L.) Roth	2	n	1	0	Poaceae Barnhart
Iz_6_1009	DJI_0406	Carduus acanthoides L.	0	n	1	0	Asteraceae Bercht. & J.Presl
Iz_6_1009	DJI_0406	Carex sp.	0	n	1	0	Cyperaceae Juss.
Iz_6_1009	DJI_0406	Centaurea majorovii Dumbadze	0	n	1	0	Asteraceae Bercht. & J.Presl
Iz_6_1009	DJI_0406	Chamaecytisus ruthenicus (Fisch.ex Wol.) Klaskova	0	n	1	0	Fabaceae Lindl.
Iz_6_1009	DJI_0406	Chelidonium majus L.	5	n	1	0	Papaveraceae Juss.
Iz_6_1009	DJI_0406	Chenopodium betaceum Andrz.	0	n	1	0	Amaranthaceae Juss.
Iz_6_1009	DJI_0406	Chenopodium cf. album	0	n	1	0	Amaranthaceae Juss.
Iz_6_1009	DJI_0406	Chenopodium sp.	0		NA	0	Amaranthaceae Juss.
Iz_6_1009	DJI_0406	Chondrilla juncea L.	0	n	1	0	Asteraceae Bercht. & J.Presl
Iz_6_1009	DJI_0406	Cirsium vulgare (Savi) Ten.	0	n	1	0	Asteraceae Bercht. & J.Presl
Iz_6_1009	DJI_0406	Clinopodium acinos (L.) Kuntze	0	n	1	0	Lamiacea Lindl.
Iz_6_1009	DJI_0406	Convallaria majalis L.	0	n	1	0	Asparagaceae Juss.
Iz_6_1009	DJI_0406	Crataegus rhipidophylla Gand.	0	n	1	1	Rosaceae Juss.
Iz_6_1009	DJI_0406	Crepis foetida L.	0	n	1	0	Asteraceae Bercht. & J.Presl
Iz_6_1009	DJI_0406	Dactylis glomerata L.	0	n	1	0	Poaceae Barnhart
Iz_6_1009	DJI_0406	Dianthus campestris M.Bieb.	0	n	1	0	Caryophyllaceae Juss.
Iz_6_1009	DJI_0406	Digitaria sanguinalis (L.) Scop.	0	a	0	0	Poaceae Barnhart
Iz_6_1009	DJI_0406	Elymus repens (L.) Gould	0	n	1	0	Poaceae Barnhart
Iz_6_1009	DJI_0406	Epilobium tetragonum L.	0	n	1	0	Onagraceae Juss.
Iz_6_1009	DJI_0406	Eragrostis minor Host	0	a	0	0	Poaceae Barnhart
Iz_6_1009	DJI_0406	Erigeron canadensis L.	0.5	a	0	0	Asteraceae Bercht. & J.Presl
Iz_6_1009	DJI_0406	Euphorbia seguieriana Neck.	0	n	1	0	Euphorbiaceae Juss.
Iz_6_1009	DJI_0406	Fallopia dumetorum (L.) Holub	0	n	1	0	Polygonaceae Juss.
Iz_6_1009	DJI_0406	Festuca beckeri (Hack.) Trautv.	0	n	1	0	Poaceae Barnhart
Iz_6_1009	DJI_0406	Fragaria vesca L.	0	n	1	0	Rosaceae Juss.
Iz_6_1009	DJI_0406	Frangula alnus Mill.	0	n	1	1	Rhamnaceae Juss.
Iz_6_1009	DJI_0406	Fraxinus pennsylvanica Marshall	0	a	0	1	Oleaceae Hoffmanns. & Link
Iz_6_1009	DJI_0406	Galeopsis bifida Boenn.	0	n	1	0	Lamiacea Lindl.
Iz_6_1009	DJI_0406	Galium verum L.	0	n	1	0	Rubiaceae Juss.
Iz_6_1009	DJI_0406	Genista tinctoria L.	0	n	1	0	Fabaceae Lindl.
Iz_6_1009	DJI_0406	Geranium robertianum L.	0	n	1	0	Geraniaceae Juss.
Iz_6_1009	DJI_0406	Geranium sanquineum L.	0	n	1	0	Geraniaceae Juss.
Iz_6_1009	DJI_0406	Gypsophila paniculata L.	0	n	1	0	Caryophyllaceae Juss.
Iz_6_1009	DJI_0406	Helichrysum arenarium (L.) Moench	0	n	1	0	Asteraceae Bercht. & J.Presl
Iz_6_1009	DJI_0406	Herniaria polygama J.Gay	0	n	1	0	Caryophyllaceae Juss.
Iz_6_1009	DJI_0406	Hieracium robustum Fr.	0	n	1	0	Asteraceae Bercht. & J.Presl
Iz_6_1009	DJI_0406	Hieracium umbellatum L.	0	n	1	NA	Asteraceae Bercht. & J.Presl
Iz_6_1009	DJI_0406	Hylotelephium maximum (L.) Holub	0	n	1	0	Crassulaceae J.St.-Hil.
Iz_6_1009	DJI_0406	Jacobaea borysthenica (DC.) B.Nord. & Greuter	0	n	1	0	Asteraceae Bercht. & J.Presl
Iz_6_1009	DJI_0406	Jasione montana L.	0	n	1	0	Campanulaceae Juss.
Iz_6_1009	DJI_0406	Jurinea cyanoides (L.) Rchb.s.l.	0	n	1	0	Asteraceae Bercht. & J.Presl
Iz_6_1009	DJI_0406	Koeleria glauca (Spreng.) DC.	0	n	1	0	Poaceae Barnhart
Iz_6_1009	DJI_0406	Lactuca serriola L.	0	a	0	0	Asteraceae Bercht. & J.Presl
Iz_6_1009	DJI_0406	Linaria genistifolia (L.) Mill.	0	n	1	0	Plantaginaceae Juss.
Iz_6_1009	DJI_0406	Lomelosia argentea (L.) Greuter & Burdet	0	n	1	0	Caprifoliaceae Juss.
Iz_6_1009	DJI_0406	Melampyrum pratense L.	0	n	1	0	Orobanchaceae Vent.
Iz_6_1009	DJI_0406	Oenothera biennis L.	0	a	0	0	Onagraceae Juss.
Iz_6_1009	DJI_0406	Pentanema britannica (L.) D.Gut.Larr., Santos-Vicente, Anderb., E.Rico & M.M.Mart.Ort.	0	n	1	0	Asteraceae Bercht. & J.Presl
Iz_6_1009	DJI_0406	Peucedanum oreoselinum (L.) Moench	0	n	1	0	Apiaceae Lindl.
Iz_6_1009	DJI_0406	Peucedanum ruthenicum M.Bieb.	0	n	1	0	Apiaceae Lindl.
Iz_6_1009	DJI_0406	Pilosella echioides (Lumn.) F.W.Schultz & Sch.Bip.	0	n	1	0	Asteraceae Bercht. & J.Presl
Iz_6_1009	DJI_0406	Pilosella officinarum F. Schultz et Sch. Bip.	0	n	1	0	Asteraceae Bercht. & J.Presl
Iz_6_1009	DJI_0406	Pilosella sp.	0		NA	0	Asteraceae Bercht. & J.Presl
Iz_6_1009	DJI_0406	Pinus sylvestris L.	0	n	1	1	Pinaceae Spreng. ex F.Rudolphi
Iz_6_1009	DJI_0406	Poa annua L.	0	n	1	0	Poaceae Barnhart
Iz_6_1009	DJI_0406	Poa nemoralis L.	0	n	1	0	Poaceae Barnhart
Iz_6_1009	DJI_0406	Poa sp.	0		NA	0	Poaceae Barnhart
Iz_6_1009	DJI_0406	Polygonatum odoratum (Mill.) Druce	0	n	1	0	Asparagaceae Juss.
Iz_6_1009	DJI_0406	Polygonum arenarium Waldst. & Kit.	0	n	1	0	Polygonaceae Juss.
Iz_6_1009	DJI_0406	Populus nigra L.	0	n	1	1	Salicaceae Mirb.
Iz_6_1009	DJI_0406	Populus tremula L.	0	n	1	1	Salicaceae Mirb.
Iz_6_1009	DJI_0406	Populus Ã canescens (Aiton) Sm.	0	n	1	1	Salicaceae Mirb.
Iz_6_1009	DJI_0406	Potentilla argentea L.	0	n	1	0	Rosaceae Juss.
Iz_6_1009	DJI_0406	Psammophiliella muralis (L.) Ikonn.	0	n	1	0	Caryophyllaceae Juss.
Iz_6_1009	DJI_0406	Psephellus sumensis (Kalen.) Greuter	0	n	1	0	Asteraceae Bercht. & J.Presl
Iz_6_1009	DJI_0406	Pulsatilla pratensis (L.) Mill. s.l.	0	n	1	0	Ranunculaceae Juss.
Iz_6_1009	DJI_0406	Pyrus communis L.	0	a	0	1	Rosaceae Juss.
Iz_6_1009	DJI_0406	Quercus robur L.	4	n	1	1	Fagaceae Dumort.
Iz_6_1009	DJI_0406	Robinia pseudoacacia L.	0	a	0	1	Fabaceae Lindl.
Iz_6_1009	DJI_0406	Rosa sp.	0		NA	1	Rosaceae Juss.
Iz_6_1009	DJI_0406	Rubus idaeus L.	0	n	1	0	Rosaceae Juss.

Iz_6_1009	DJI_0406	Rumex acetosella L.	0	n	1	0	Polygonaceae Juss.
Iz_6_1009	DJI_0406	Rumex thyrsiflorus Fingerh.	0	n	1	0	Polygonaceae Juss.
Iz_6_1009	DJI_0406	Salix alba L.	0	n	1	1	Salicaceae Mirb.
Iz_6_1009	DJI_0406	Sambucus nigra L.	0	n	1	1	Viburnaceae Raf.
Iz_6_1009	DJI_0406	Secale sylvestre Host	0	n	1	0	Poaceae Barnhart
Iz_6_1009	DJI_0406	Sempervivum ruthenicum Schnittsp. & C.B. Lehm.	0	n	1	0	Crassulaceae J.St.-Hil.
Iz_6_1009	DJI_0406	Setaria viridis (L.) P. Beauv.	0	n	1	0	Poaceae Barnhart
Iz_6_1009	DJI_0406	Silene borysthenica (Gruner) Walters	0	n	1	0	Caryophyllaceae Juss.
Iz_6_1009	DJI_0406	Silene latifolia subsp. alba (Mill.) Greuter & Burdet	0	n	1	0	Caryophyllaceae Juss.
Iz_6_1009	DJI_0406	Solidago virgaurea L.	0	n	1	0	Asteraceae Bercht. & J.Presl
Iz_6_1009	DJI_0406	Sorbus aucuparia L.	0	n	1	1	Rosaceae Juss.
Iz_6_1009	DJI_0406	Stachys recta L.	0	n	1	0	Lamiacea Lindl.
Iz_6_1009	DJI_0406	Tanacetum vulgare L.	0	n	1	0	Asteraceae Bercht. & J.Presl
Iz_6_1009	DJI_0406	Thymus pallasianus Heinr.Braun	0	n	1	0	Lamiacea Lindl.
Iz_6_1009	DJI_0406	Tilia cordata Mill.	0	n	1	1	Malvaceae Juss.
Iz_6_1009	DJI_0406	Trifolium alpestre L.	0	n	1	0	Fabaceae Lindl.
Iz_6_1009	DJI_0406	Trifolium arvense L.	0	n	1	0	Fabaceae Lindl.
Iz_6_1009	DJI_0406	Ulmus glabra Huds.	0	n	1	1	Ulmaceae Mirb.
Iz_6_1009	DJI_0406	Verbascum densiflorum Bertol.	0	n	1	0	Scrophulariaceae Juss.
Iz_6_1009	DJI_0406	Veronica austriaca L.	0	n	1	0	Plantaginaceae Juss.
Iz_6_1009	DJI_0406	Veronica incana L.	0	n	1	0	Plantaginaceae Juss.
Iz_6_1009	DJI_0406	Veronica verna L.	0	n	1	0	Plantaginaceae Juss.
Iz_6_1009	DJI_0406	Vincetoxicum hirundinaria Medik	0	n	1	0	Apocynaceae Juss.
Iz_6_1109	DJI_0234	Acer tataricum L.	0	n	1	1	Sapindaceae Juss.
Iz_6_1109	DJI_0234	Achillea millefolium L. s.l.	0	n	1	0	Asteraceae Bercht. & J.Presl
Iz_6_1109	DJI_0234	Allium oleraceum L.	0	n	1	0	Amaryllidaceae J.St.-Hil.
Iz_6_1109	DJI_0234	Ambrosia artemisiifolia L.	0	a	0	0	Asteraceae Bercht. & J.Presl
Iz_6_1109	DJI_0234	Amorpha fruticosa L.	0	a	0	0	Fabaceae Lindl.
Iz_6_1109	DJI_0234	Anthericum ramosum L.	0	n	1	0	Asparagaceae Juss.
Iz_6_1109	DJI_0234	Artemisia marschalliana Spreng.	0	n	1	0	Asteraceae Bercht. & J.Presl
Iz_6_1109	DJI_0234	Asparagus officinalis L.	0	n	1	0	Asparagaceae Juss.
Iz_6_1109	DJI_0234	Astragalus cicer L.	0	n	1	0	Fabaceae Lindl.
Iz_6_1109	DJI_0234	Bassia laniflora (S.G.Gmel.) A.J.Scott	0	n	1	0	Amaranthaceae Juss.
Iz_6_1109	DJI_0234	Berberis vulgaris L.	0	n	1	0	Berberidaceae Juss.
Iz_6_1109	DJI_0234	Betula pendula Roth.	0	n	1	1	Betulaceae Gray
Iz_6_1109	DJI_0234	Betula pendula Roth.	0	n	1	0	Poaceae Barnhart
Iz_6_1109	DJI_0234	Bromus inermis Leyss.	0		NA	NA	
Iz_6_1109	DJI_0234	Bromus squarrosus L.	0	a	0	0	Poaceae Barnhart
Iz_6_1109	DJI_0234	Bromus tectorum L.	0	a	0	0	Poaceae Barnhart
Iz_6_1109	DJI_0234	Calamagrostis epigejos (L.) Roth	70	n	1	0	Poaceae Barnhart
Iz_6_1109	DJI_0234	Carduus acanthoides L.	0	n	1	0	Asteraceae Bercht. & J.Presl
Iz_6_1109	DJI_0234	Carex sp.	2	n	1	0	Cyperaceae Juss.
Iz_6_1109	DJI_0234	Centaurea majorovii Dumbadze	0	n	1	0	Asteraceae Bercht. & J.Presl
Iz_6_1109	DJI_0234	Chamaecytisus ruthenicus (Fisch.ex Wol.) Klaskova	0	n	1	0	Fabaceae Lindl.
Iz_6_1109	DJI_0234	Chelidonium majus L.	0	n	1	0	Papaveraceae Juss.
Iz_6_1109	DJI_0234	Chenopodium betaceum Andrz.	0	n	1	0	Amaranthaceae Juss.
Iz_6_1109	DJI_0234	Chenopodium cf. album	0	n	1	0	Amaranthaceae Juss.
Iz_6_1109	DJI_0234	Chenopodium sp.	0		NA	0	Amaranthaceae Juss.
Iz_6_1109	DJI_0234	Chondrilla juncea L.	0	n	1	0	Asteraceae Bercht. & J.Presl
Iz_6_1109	DJI_0234	Cirsium vulgare (Savi) Ten.	0	n	1	0	Asteraceae Bercht. & J.Presl
Iz_6_1109	DJI_0234	Clinopodium acinos (L.) Kuntze	0	n	1	0	Lamiacea Lindl.
Iz_6_1109	DJI_0234	Convallaria majalis L.	0	n	1	0	Asparagaceae Juss.
Iz_6_1109	DJI_0234	Crataegus rhipidophylla Gand.	0	n	1	1	Rosaceae Juss.
Iz_6_1109	DJI_0234	Crepis foetida L.	0	n	1	0	Asteraceae Bercht. & J.Presl
Iz_6_1109	DJI_0234	Dactylis glomerata L.	0	n	1	0	Poaceae Barnhart
Iz_6_1109	DJI_0234	Dianthus campestris M.Bieb.	0	n	1	0	Caryophyllaceae Juss.
Iz_6_1109	DJI_0234	Digitaria sanguinalis (L.) Scop.	0	a	0	0	Poaceae Barnhart
Iz_6_1109	DJI_0234	Elymus repens (L.) Gould	0	n	1	0	Poaceae Barnhart
Iz_6_1109	DJI_0234	Epilobium tetragonum L.	0	n	1	0	Onagraceae Juss.
Iz_6_1109	DJI_0234	Eragrostis minor Host	0	a	0	0	Poaceae Barnhart
Iz_6_1109	DJI_0234	Erigeron annuus (L.) Desf.	0	a	0	0	Asteraceae Bercht. & J.Presl
Iz_6_1109	DJI_0234	Erigeron canadensis L.	5	a	0	0	Asteraceae Bercht. & J.Presl
Iz_6_1109	DJI_0234	Euphorbia seguieriana Neck.	0	n	1	0	Euphorbiaceae Juss.
Iz_6_1109	DJI_0234	Fallopia dumetorum (L.) Holub	0	n	1	0	Polygonaceae Juss.
Iz_6_1109	DJI_0234	Festuca beckeri (Hack.) Trautv.	0	n	1	0	Poaceae Barnhart
Iz_6_1109	DJI_0234	Fragaria vesca L.	0	n	1	0	Rosaceae Juss.
Iz_6_1109	DJI_0234	Frangula alnus Mill.	0	n	1	1	Rhamnaceae Juss.
Iz_6_1109	DJI_0234	Fraxinus pennsylvanica Marshall	0	a	0	1	Oleaceae Hoffmanns. & Link
Iz_6_1109	DJI_0234	Galeopsis bifida Boenn.	0	n	1	0	Lamiacea Lindl.
Iz_6_1109	DJI_0234	Galium verum L.	0	n	1	0	Rubiaceae Juss.
Iz_6_1109	DJI_0234	Genista tinctoria L.	0	n	1	0	Fabaceae Lindl.
Iz_6_1109	DJI_0234	Geranium robertianum L.	0	n	1	0	Geraniaceae Juss.
Iz_6_1109	DJI_0234	Geranium sanquineum L.	0	n	1	0	Geraniaceae Juss.
Iz_6_1109	DJI_0234	Gypsophila paniculata L.	0	n	1	0	Caryophyllaceae Juss.
Iz_6_1109	DJI_0234	Helichrysum arenarium (L.) Moench	0	n	1	0	Asteraceae Bercht. & J.Presl
Iz_6_1109	DJI_0234	Herniaria polygama J.Gay	0	n	1	0	Caryophyllaceae Juss.
Iz_6_1109	DJI_0234	Hieracium robustum Fr.	0	n	1	0	Asteraceae Bercht. & J.Presl
Iz_6_1109	DJI_0234	Hieracium umbellatum L.	0	n	1	NA	Asteraceae Bercht. & J.Presl
Iz_6_1109	DJI_0234	Hylotelephium maximum (L.) Holub	0	n	1	0	Crassulaceae J.St.-Hil.
Iz_6_1109	DJI_0234	Jacobaea borysthenica (DC.) B.Nord. & Greuter	0	n	1	0	Asteraceae Bercht. & J.Presl
Iz_6_1109	DJI_0234	Jasione montana L.	0	n	1	0	Campanulaceae Juss.
Iz_6_1109	DJI_0234	Jurinea cyanoides (L.) Rchb.s.l.	0	n	1	0	Asteraceae Bercht. & J.Presl
Iz_6_1109	DJI_0234	Koeleria glauca (Spreng.) DC.	0	n	1	0	Poaceae Barnhart
Iz_6_1109	DJI_0234	Lactuca serriola L.	0	a	0	0	Asteraceae Bercht. & J.Presl
Iz_6_1109	DJI_0234	Linaria genistifolia (L.) Mill.	0	n	1	0	Plantaginaceae Juss.
Iz_6_1109	DJI_0234	Lomelosia argentea (L.) Greuter & Burdet	0	n	1	0	Caprifoliaceae Juss.
Iz_6_1109	DJI_0234	Melampyrum pratense L.	0	n	1	0	Orobanchaceae Vent.
Iz_6_1109	DJI_0234	Oenothera biennis L.	0	a	0	0	Onagraceae Juss.
Iz_6_1109	DJI_0234	Pentanema britannica (L.) D.Gut.Larr., Santos-Vicente, Anderb., E.Rico & M.M.Mart.Ort.	0	n	1	0	Asteraceae Bercht. & J.Presl
Iz_6_1109	DJI_0234	Peucedanum oreoselinum (L.) Moench	0	n	1	0	Apiaceae Lindl.
Iz_6_1109	DJI_0234	Peucedanum ruthenicum M.Bieb.	0	n	1	0	Apiaceae Lindl.
Iz_6_1109	DJI_0234	Pilosella echioides (Lumn.) F.W.Schultz & Sch.Bip.	0	n	1	0	Asteraceae Bercht. & J.Presl
Iz_6_1109	DJI_0234	Pilosella officinarum F. Schultz et Sch. Bip.	0	n	1	0	Asteraceae Bercht. & J.Presl
Iz_6_1109	DJI_0234	Pilosella sp.	0		NA	0	Asteraceae Bercht. & J.Presl
Iz_6_1109	DJI_0234	Pinus sylvestris L.	0	n	1	1	Pinaceae Spreng. ex F.Rudolphi
Iz_6_1109	DJI_0234	Poa annua L.	0	n	1	0	Poaceae Barnhart
Iz_6_1109	DJI_0234	Poa nemoralis L.	0	n	1	0	Poaceae Barnhart
Iz_6_1109	DJI_0234	Poa sp.	0		NA	0	Poaceae Barnhart

Iz_6_1109	DJI_0234	Polygonatum odoratum (Mill.) Druce	0	n	1	0	Asparagaceae Juss.
Iz_6_1109	DJI_0234	Polygonum arenarium Waldst. & Kit.	0	n	1	0	Polygonaceae Juss.
Iz_6_1109	DJI_0234	Populus nigra L.	0	n	1	1	Salicaceae Mirb.
Iz_6_1109	DJI_0234	Populus tremula L.	0	n	1	1	Salicaceae Mirb.
Iz_6_1109	DJI_0234	Populus Ã canescens (Aiton) Sm.	0	n	1	1	Salicaceae Mirb.
Iz_6_1109	DJI_0234	Potentilla argentea L.	0	n	1	0	Rosaceae Juss.
Iz_6_1109	DJI_0234	Psammophiliella muralis (L.) Ikonn.	0	n	1	0	Caryophyllaceae Juss.
Iz_6_1109	DJI_0234	Psephellus sumensis (Kalen.) Greuter	0	n	1	0	Asteraceae Bercht. & J.Presl
Iz_6_1109	DJI_0234	Pulsatilla pratensis (L.) Mill. s.l.	0	n	1	0	Ranunculaceae Juss.
Iz_6_1109	DJI_0234	Pyrus communis L.	0	a	0	1	Rosaceae Juss.
Iz_6_1109	DJI_0234	Quercus robur L.	0	n	1	1	Fagaceae Dumort.
Iz_6_1109	DJI_0234	Robinia pseudoacacia L.	0	a	0	1	Fabaceae Lindl.
Iz_6_1109	DJI_0234	Rosa sp.	0		NA	1	Rosaceae Juss.
Iz_6_1109	DJI_0234	Rubus idaeus L.	0	n	1	0	Rosaceae Juss.
Iz_6_1109	DJI_0234	Rumex acetosella L.	0	n	1	0	Polygonaceae Juss.
Iz_6_1109	DJI_0234	Rumex thyrsiflorus Fingerh.	0	n	1	0	Polygonaceae Juss.
Iz_6_1109	DJI_0234	Salix alba L.	0	n	1	1	Salicaceae Mirb.
Iz_6_1109	DJI_0234	Sambucus nigra L.	0	n	1	1	Viburnaceae Raf.
Iz_6_1109	DJI_0234	Secale sylvestre Host	0	n	1	0	Poaceae Barnhart
Iz_6_1109	DJI_0234	Sempervivum ruthenicum Schnittsp. & C.B. Lehm.	0	n	1	0	Crassulaceae J.St.-Hil.
Iz_6_1109	DJI_0234	Setaria viridis (L.) P. Beauv.	0	n	1	0	Poaceae Barnhart
Iz_6_1109	DJI_0234	Silene borysthenica (Gruner) Walters	0	n	1	0	Caryophyllaceae Juss.
Iz_6_1109	DJI_0234	Silene latifolia subsp. alba (Mill.) Greuter & Burdet	0	n	1	0	Caryophyllaceae Juss.
Iz_6_1109	DJI_0234	Solidago virgaurea L.	0	n	1	0	Asteraceae Bercht. & J.Presl
Iz_6_1109	DJI_0234	Sorbus aucuparia L.	0	n	1	1	Rosaceae Juss.
Iz_6_1109	DJI_0234	Species 11.09_6_0254	0		NA	0	
Iz_6_1109	DJI_0234	Stachys recta L.	0	n	1	0	Lamiacea Lindl.
Iz_6_1109	DJI_0234	Tanacetum vulgare L.	0	n	1	0	Asteraceae Bercht. & J.Presl
Iz_6_1109	DJI_0234	Thymus pallasianus Heinr.Braun	0	n	1	0	Lamiacea Lindl.
Iz_6_1109	DJI_0234	Tilia cordata Mill.	0	n	1	1	Malvaceae Juss.
Iz_6_1109	DJI_0234	Trifolium alpestre L.	0	n	1	0	Fabaceae Lindl.
Iz_6_1109	DJI_0234	Trifolium arvense L.	0	n	1	0	Fabaceae Lindl.
Iz_6_1109	DJI_0234	Ulmus glabra Huds.	0	n	1	1	Ulmaceae Mirb.
Iz_6_1109	DJI_0234	Verbascum densiflorum Bertol.	0	n	1	0	Scrophulariaceae Juss.
Iz_6_1109	DJI_0234	Veronica austriaca L.	0	n	1	0	Plantaginaceae Juss.
Iz_6_1109	DJI_0234	Veronica incana L.	0	n	1	0	Plantaginaceae Juss.
Iz_6_1109	DJI_0234	Veronica verna L.	0	n	1	0	Plantaginaceae Juss.
Iz_6_1109	DJI_0234	Vincetoxicum hirundinaria Medik	0	n	1	0	Apocynaceae Juss.
Iz_6_1109	DJI_0238	Acer tataricum L.	0	n	1	1	Sapindaceae Juss.
Iz_6_1109	DJI_0238	Achillea millefolium L. s.l.	0	n	1	0	Asteraceae Bercht. & J.Presl
Iz_6_1109	DJI_0238	Allium oleraceum L.	0	n	1	0	Amaryllidaceae J.St.-Hil.
Iz_6_1109	DJI_0238	Ambrosia artemisiifolia L.	0	a	0	0	Asteraceae Bercht. & J.Presl
Iz_6_1109	DJI_0238	Amorpha fruticosa L.	0	a	0	0	Fabaceae Lindl.
Iz_6_1109	DJI_0238	Anthericum ramosum L.	0	n	1	0	Asparagaceae Juss.
Iz_6_1109	DJI_0238	Artemisia marschalliana Spreng.	0	n	1	0	Asteraceae Bercht. & J.Presl
Iz_6_1109	DJI_0238	Asparagus officinalis L.	0	n	1	0	Asparagaceae Juss.
Iz_6_1109	DJI_0238	Astragalus cicer L.	0	n	1	0	Fabaceae Lindl.
Iz_6_1109	DJI_0238	Bassia laniflora (S.G.Gmel.) A.J.Scott	0	n	1	0	Amaranthaceae Juss.
Iz_6_1109	DJI_0238	Berberis vulgaris L.	0	n	1	0	Berberidaceae Juss.
Iz_6_1109	DJI_0238	Betula pendula Roth.	0	n	1	1	Betulaceae Gray
Iz_6_1109	DJI_0238	Betula pendula Roth.	0	n	1	0	Poaceae Barnhart
Iz_6_1109	DJI_0238	Bromus inermis Leyss.	0		NA	NA	
Iz_6_1109	DJI_0238	Bromus squarrosus L.	0	a	0	0	Poaceae Barnhart
Iz_6_1109	DJI_0238	Bromus tectorum L.	0	a	0	0	Poaceae Barnhart
Iz_6_1109	DJI_0238	Calamagrostis epigejos (L.) Roth	3	n	1	0	Poaceae Barnhart
Iz_6_1109	DJI_0238	Carduus acanthoides L.	0	n	1	0	Asteraceae Bercht. & J.Presl
Iz_6_1109	DJI_0238	Carex sp.	0	n	1	0	Cyperaceae Juss.
Iz_6_1109	DJI_0238	Centaurea majorovii Dumbadze	0	n	1	0	Asteraceae Bercht. & J.Presl
Iz_6_1109	DJI_0238	Chamaecytisus ruthenicus (Fisch.ex Wol.) Klaskova	0	n	1	0	Fabaceae Lindl.
Iz_6_1109	DJI_0238	Chelidonium majus L.	0	n	1	0	Papaveraceae Juss.
Iz_6_1109	DJI_0238	Chenopodium betaceum Andrz.	0	n	1	0	Amaranthaceae Juss.
Iz_6_1109	DJI_0238	Chenopodium cf. album	0	n	1	0	Amaranthaceae Juss.
Iz_6_1109	DJI_0238	Chenopodium sp.	0		NA	0	Amaranthaceae Juss.
Iz_6_1109	DJI_0238	Chondrilla juncea L.	0	n	1	0	Asteraceae Bercht. & J.Presl
Iz_6_1109	DJI_0238	Cirsium vulgare (Savi) Ten.	0	n	1	0	Asteraceae Bercht. & J.Presl
Iz_6_1109	DJI_0238	Clinopodium acinos (L.) Kuntze	0	n	1	0	Lamiacea Lindl.
Iz_6_1109	DJI_0238	Convallaria majalis L.	0	n	1	0	Asparagaceae Juss.
Iz_6_1109	DJI_0238	Crataegus rhipidophylla Gand.	0	n	1	1	Rosaceae Juss.
Iz_6_1109	DJI_0238	Crepis foetida L.	0	n	1	0	Asteraceae Bercht. & J.Presl
Iz_6_1109	DJI_0238	Dactylis glomerata L.	0	n	1	0	Poaceae Barnhart
Iz_6_1109	DJI_0238	Dianthus campestris M.Bieb.	0	n	1	0	Caryophyllaceae Juss.
Iz_6_1109	DJI_0238	Digitaria sanguinalis (L.) Scop.	0	a	0	0	Poaceae Barnhart
Iz_6_1109	DJI_0238	Elymus repens (L.) Gould	0	n	1	0	Poaceae Barnhart
Iz_6_1109	DJI_0238	Epilobium tetragonum L.	0	n	1	0	Onagraceae Juss.
Iz_6_1109	DJI_0238	Eragrostis minor Host	0	a	0	0	Poaceae Barnhart
Iz_6_1109	DJI_0238	Erigeron annuus (L.) Desf.	0	a	0	0	Asteraceae Bercht. & J.Presl
Iz_6_1109	DJI_0238	Erigeron canadensis L.	8	a	0	0	Asteraceae Bercht. & J.Presl
Iz_6_1109	DJI_0238	Euphorbia seguieriana Neck.	0	n	1	0	Euphorbiaceae Juss.
Iz_6_1109	DJI_0238	Fallopia dumetorum (L.) Holub	0	n	1	0	Polygonaceae Juss.
Iz_6_1109	DJI_0238	Festuca beckeri (Hack.) Trautv.	0	n	1	0	Poaceae Barnhart
Iz_6_1109	DJI_0238	Fragaria vesca L.	0	n	1	0	Rosaceae Juss.
Iz_6_1109	DJI_0238	Frangula alnus Mill.	0	n	1	1	Rhamnaceae Juss.
Iz_6_1109	DJI_0238	Fraxinus pennsylvanica Marshall	0	a	0	1	Oleaceae Hoffmanns. & Link
Iz_6_1109	DJI_0238	Galeopsis bifida Boenn.	0	n	1	0	Lamiacea Lindl.
Iz_6_1109	DJI_0238	Galium verum L.	0	n	1	0	Rubiaceae Juss.
Iz_6_1109	DJI_0238	Genista tinctoria L.	0	n	1	0	Fabaceae Lindl.
Iz_6_1109	DJI_0238	Geranium robertianum L.	0	n	1	0	Geraniaceae Juss.
Iz_6_1109	DJI_0238	Geranium sanquineum L.	0	n	1	0	Geraniaceae Juss.
Iz_6_1109	DJI_0238	Gypsophila paniculata L.	0	n	1	0	Caryophyllaceae Juss.
Iz_6_1109	DJI_0238	Helichrysum arenarium (L.) Moench	0	n	1	0	Asteraceae Bercht. & J.Presl
Iz_6_1109	DJI_0238	Herniaria polygama J.Gay	0	n	1	0	Caryophyllaceae Juss.
Iz_6_1109	DJI_0238	Hieracium robustum Fr.	0	n	1	0	Asteraceae Bercht. & J.Presl
Iz_6_1109	DJI_0238	Hieracium umbellatum L.	0	n	1	NA	Asteraceae Bercht. & J.Presl
Iz_6_1109	DJI_0238	Hylotelephium maximum (L.) Holub	0	n	1	0	Crassulaceae J.St.-Hil.
Iz_6_1109	DJI_0238	Jacobaea borysthenica (DC.) B.Nord. & Greuter	0	n	1	0	Asteraceae Bercht. & J.Presl
Iz_6_1109	DJI_0238	Jasione montana L.	0	n	1	0	Campanulaceae Juss.
Iz_6_1109	DJI_0238	Jurinea cyanoides (L.) Rchb.s.l.	0	n	1	0	Asteraceae Bercht. & J.Presl
Iz_6_1109	DJI_0238	Koeleria glauca (Spreng.) DC.	0	n	1	0	Poaceae Barnhart

Iz_6_1109	DJI_0238	Lactuca serriola L.	0	a	0	0	Asteraceae Bercht. & J.Presl
Iz_6_1109	DJI_0238	Linaria genistifolia (L.) Mill.	0	n	1	0	Plantaginaceae Juss.
Iz_6_1109	DJI_0238	Lomelosia argentea (L.) Greuter & Burdet	0	n	1	0	Caprifoliaceae Juss.
Iz_6_1109	DJI_0238	Melampyrum pratense L.	0	n	1	0	Orobanchaceae Vent.
Iz_6_1109	DJI_0238	Oenothera biennis L.	0	a	0	0	Onagraceae Juss.
Iz_6_1109	DJI_0238	Pentanema britannica (L.) D.Gut.Larr., Santos-Vicente, Anderb., E.Rico & M.M.Mart.Ort.	0	n	1	0	Asteraceae Bercht. & J.Presl
Iz_6_1109	DJI_0238	Peucedanum oreoselinum (L.) Moench	0	n	1	0	Apiaceae Lindl.
Iz_6_1109	DJI_0238	Peucedanum ruthenicum M.Bieb.	0	n	1	0	Apiaceae Lindl.
Iz_6_1109	DJI_0238	Pilosella echioides (Lumn.) F.W.Schultz & Sch.Bip.	0	n	1	0	Asteraceae Bercht. & J.Presl
Iz_6_1109	DJI_0238	Pilosella officinarum F. Schultz et Sch. Bip.	0	n	1	0	Asteraceae Bercht. & J.Presl
Iz_6_1109	DJI_0238	Pilosella sp.	0		NA	0	Asteraceae Bercht. & J.Presl
Iz_6_1109	DJI_0238	Pinus sylvestris L.	0	n	1	1	Pinaceae Spreng. ex F.Rudolphi
Iz_6_1109	DJI_0238	Poa annua L.	0	n	1	0	Poaceae Barnhart
Iz_6_1109	DJI_0238	Poa nemoralis L.	0	n	1	0	Poaceae Barnhart
Iz_6_1109	DJI_0238	Poa sp.	0		NA	0	Poaceae Barnhart
Iz_6_1109	DJI_0238	Polygonatum odoratum (Mill.) Druce	0	n	1	0	Asparagaceae Juss.
Iz_6_1109	DJI_0238	Polygonum arenarium Waldst. & Kit.	0	n	1	0	Polygonaceae Juss.
Iz_6_1109	DJI_0238	Populus nigra L.	0	n	1	1	Salicaceae Mirb.
Iz_6_1109	DJI_0238	Populus tremula L.	0	n	1	1	Salicaceae Mirb.
Iz_6_1109	DJI_0238	Populus Ã canescens (Aiton) Sm.	0	n	1	1	Salicaceae Mirb.
Iz_6_1109	DJI_0238	Potentilla argentea L.	0	n	1	0	Rosaceae Juss.
Iz_6_1109	DJI_0238	Psammophiliella muralis (L.) Ikonn.	0	n	1	0	Caryophyllaceae Juss.
Iz_6_1109	DJI_0238	Psephellus sumensis (Kalen.) Greuter	0	n	1	0	Asteraceae Bercht. & J.Presl
Iz_6_1109	DJI_0238	Pulsatilla pratensis (L.) Mill. s.l.	0	n	1	0	Ranunculaceae Juss.
Iz_6_1109	DJI_0238	Pyrus communis L.	0	a	0	1	Rosaceae Juss.
Iz_6_1109	DJI_0238	Quercus robur L.	0	n	1	1	Fagaceae Dumort.
Iz_6_1109	DJI_0238	Robinia pseudoacacia L.	0	a	0	1	Fabaceae Lindl.
Iz_6_1109	DJI_0238	Rosa sp.	0		NA	1	Rosaceae Juss.
Iz_6_1109	DJI_0238	Rubus idaeus L.	0	n	1	0	Rosaceae Juss.
Iz_6_1109	DJI_0238	Rumex acetosella L.	0	n	1	0	Polygonaceae Juss.
Iz_6_1109	DJI_0238	Rumex thyrsiflorus Fingerh.	0	n	1	0	Polygonaceae Juss.
Iz_6_1109	DJI_0238	Salix alba L.	0	n	1	1	Salicaceae Mirb.
Iz_6_1109	DJI_0238	Sambucus nigra L.	0	n	1	1	Viburnaceae Raf.
Iz_6_1109	DJI_0238	Secale sylvestre Host	0	n	1	0	Poaceae Barnhart
Iz_6_1109	DJI_0238	Sempervivum ruthenicum Schnittsp. & C.B. Lehm.	0	n	1	0	Crassulaceae J.St.-Hil.
Iz_6_1109	DJI_0238	Setaria viridis (L.) P. Beauv.	0	n	1	0	Poaceae Barnhart
Iz_6_1109	DJI_0238	Silene borysthenica (Gruner) Walters	0	n	1	0	Caryophyllaceae Juss.
Iz_6_1109	DJI_0238	Silene latifolia subsp. alba (Mill.) Greuter & Burdet	0	n	1	0	Caryophyllaceae Juss.
Iz_6_1109	DJI_0238	Solidago virgaurea L.	0	n	1	0	Asteraceae Bercht. & J.Presl
Iz_6_1109	DJI_0238	Sorbus aucuparia L.	0	n	1	1	Rosaceae Juss.
Iz_6_1109	DJI_0238	Species 11.09_6_0254	0		NA	0	
Iz_6_1109	DJI_0238	Stachys recta L.	0	n	1	0	Lamiacea Lindl.
Iz_6_1109	DJI_0238	Tanacetum vulgare L.	0	n	1	0	Asteraceae Bercht. & J.Presl
Iz_6_1109	DJI_0238	Thymus pallasianus Heinr.Braun	0	n	1	0	Lamiacea Lindl.
Iz_6_1109	DJI_0238	Tilia cordata Mill.	0	n	1	1	Malvaceae Juss.
Iz_6_1109	DJI_0238	Trifolium alpestre L.	0	n	1	0	Fabaceae Lindl.
Iz_6_1109	DJI_0238	Trifolium arvense L.	0	n	1	0	Fabaceae Lindl.
Iz_6_1109	DJI_0238	Ulmus glabra Huds.	0	n	1	1	Ulmaceae Mirb.
Iz_6_1109	DJI_0238	Verbascum densiflorum Bertol.	0	n	1	0	Scrophulariaceae Juss.
Iz_6_1109	DJI_0238	Veronica austriaca L.	0	n	1	0	Plantaginaceae Juss.
Iz_6_1109	DJI_0238	Veronica incana L.	0	n	1	0	Plantaginaceae Juss.
Iz_6_1109	DJI_0238	Veronica verna L.	0	n	1	0	Plantaginaceae Juss.
Iz_6_1109	DJI_0238	Vincetoxicum hirundinaria Medik	0	n	1	0	Apocynaceae Juss.
Iz_6_1109	DJI_0239	Acer tataricum L.	0	n	1	1	Sapindaceae Juss.
Iz_6_1109	DJI_0239	Achillea millefolium L. s.l.	0	n	1	0	Asteraceae Bercht. & J.Presl
Iz_6_1109	DJI_0239	Allium oleraceum L.	0	n	1	0	Amaryllidaceae J.St.-Hil.
Iz_6_1109	DJI_0239	Ambrosia artemisiifolia L.	0	a	0	0	Asteraceae Bercht. & J.Presl
Iz_6_1109	DJI_0239	Amorpha fruticosa L.	0	a	0	0	Fabaceae Lindl.
Iz_6_1109	DJI_0239	Anthericum ramosum L.	0	n	1	0	Asparagaceae Juss.
Iz_6_1109	DJI_0239	Artemisia marschalliana Spreng.	0	n	1	0	Asteraceae Bercht. & J.Presl
Iz_6_1109	DJI_0239	Asparagus officinalis L.	0	n	1	0	Asparagaceae Juss.
Iz_6_1109	DJI_0239	Astragalus cicer L.	0	n	1	0	Fabaceae Lindl.
Iz_6_1109	DJI_0239	Bassia laniflora (S.G.Gmel.) A.J.Scott	0	n	1	0	Amaranthaceae Juss.
Iz_6_1109	DJI_0239	Berberis vulgaris L.	0	n	1	0	Berberidaceae Juss.
Iz_6_1109	DJI_0239	Betula pendula Roth.	0	n	1	1	Betulaceae Gray
Iz_6_1109	DJI_0239	Betula pendula Roth.	0	n	1	0	Poaceae Barnhart
Iz_6_1109	DJI_0239	Bromus inermis Leyss.	0		NA	NA	
Iz_6_1109	DJI_0239	Bromus squarrosus L.	0	a	0	0	Poaceae Barnhart
Iz_6_1109	DJI_0239	Bromus tectorum L.	0	a	0	0	Poaceae Barnhart
Iz_6_1109	DJI_0239	Calamagrostis epigejos (L.) Roth	3	n	1	0	Poaceae Barnhart
Iz_6_1109	DJI_0239	Carduus acanthoides L.	0	n	1	0	Asteraceae Bercht. & J.Presl
Iz_6_1109	DJI_0239	Carex sp.	0	n	1	0	Cyperaceae Juss.
Iz_6_1109	DJI_0239	Centaurea majorovii Dumbadze	0	n	1	0	Asteraceae Bercht. & J.Presl
Iz_6_1109	DJI_0239	Chamaecytisus ruthenicus (Fisch.ex Wol.) Klaskova	0	n	1	0	Fabaceae Lindl.
Iz_6_1109	DJI_0239	Chelidonium majus L.	0	n	1	0	Papaveraceae Juss.
Iz_6_1109	DJI_0239	Chenopodium betaceum Andrz.	0	n	1	0	Amaranthaceae Juss.
Iz_6_1109	DJI_0239	Chenopodium cf. album	0	n	1	0	Amaranthaceae Juss.
Iz_6_1109	DJI_0239	Chenopodium sp.	0		NA	0	Amaranthaceae Juss.
Iz_6_1109	DJI_0239	Chondrilla juncea L.	0	n	1	0	Asteraceae Bercht. & J.Presl
Iz_6_1109	DJI_0239	Cirsium vulgare (Savi) Ten.	0	n	1	0	Asteraceae Bercht. & J.Presl
Iz_6_1109	DJI_0239	Clinopodium acinos (L.) Kuntze	0	n	1	0	Lamiacea Lindl.
Iz_6_1109	DJI_0239	Convallaria majalis L.	0	n	1	0	Asparagaceae Juss.
Iz_6_1109	DJI_0239	Crataegus rhipidophylla Gand.	0	n	1	1	Rosaceae Juss.
Iz_6_1109	DJI_0239	Crepis foetida L.	0	n	1	0	Asteraceae Bercht. & J.Presl
Iz_6_1109	DJI_0239	Dactylis glomerata L.	0	n	1	0	Poaceae Barnhart
Iz_6_1109	DJI_0239	Dianthus campestris M.Bieb.	0	n	1	0	Caryophyllaceae Juss.
Iz_6_1109	DJI_0239	Digitaria sanguinalis (L.) Scop.	0	a	0	0	Poaceae Barnhart
Iz_6_1109	DJI_0239	Elymus repens (L.) Gould	0	n	1	0	Poaceae Barnhart
Iz_6_1109	DJI_0239	Epilobium tetragonum L.	0	n	1	0	Onagraceae Juss.
Iz_6_1109	DJI_0239	Eragrostis minor Host	0	a	0	0	Poaceae Barnhart
Iz_6_1109	DJI_0239	Erigeron annuus (L.) Desf.	0	a	0	0	Asteraceae Bercht. & J.Presl
Iz_6_1109	DJI_0239	Erigeron canadensis L.	5	a	0	0	Asteraceae Bercht. & J.Presl
Iz_6_1109	DJI_0239	Euphorbia seguieriana Neck.	0	n	1	0	Euphorbiaceae Juss.
Iz_6_1109	DJI_0239	Fallopia dumetorum (L.) Holub	0	n	1	0	Polygonaceae Juss.
Iz_6_1109	DJI_0239	Festuca beckeri (Hack.) Trautv.	0	n	1	0	Poaceae Barnhart
Iz_6_1109	DJI_0239	Fragaria vesca L.	0	n	1	0	Rosaceae Juss.
Iz_6_1109	DJI_0239	Frangula alnus Mill.	0	n	1	1	Rhamnaceae Juss.
Iz_6_1109	DJI_0239	Fraxinus pennsylvanica Marshall	0	a	0	1	Oleaceae Hoffmanns. & Link

Iz_6_1109	DJI_0239	Galeopsis bifida Boenn.	0	n	1	0	Lamiacea Lindl.
Iz_6_1109	DJI_0239	Galium verum L.	0	n	1	0	Rubiaceae Juss.
Iz_6_1109	DJI_0239	Genista tinctoria L.	0	n	1	0	Fabaceae Lindl.
Iz_6_1109	DJI_0239	Geranium robertianum L.	0	n	1	0	Geraniaceae Juss.
Iz_6_1109	DJI_0239	Geranium sanquineum L.	0	n	1	0	Geraniaceae Juss.
Iz_6_1109	DJI_0239	Gypsophila paniculata L.	0	n	1	0	Caryophyllaceae Juss.
Iz_6_1109	DJI_0239	Helichrysum arenarium (L.) Moench	0	n	1	0	Asteraceae Bercht. & J.Presl
Iz_6_1109	DJI_0239	Herniaria polygama J.Gay	0	n	1	0	Caryophyllaceae Juss.
Iz_6_1109	DJI_0239	Hieracium robustum Fr.	0	n	1	0	Asteraceae Bercht. & J.Presl
Iz_6_1109	DJI_0239	Hieracium umbellatum L.	0	n	1	NA	Asteraceae Bercht. & J.Presl
Iz_6_1109	DJI_0239	Hylotelephium maximum (L.) Holub	0	n	1	0	Crassulaceae J.St.-Hil.
Iz_6_1109	DJI_0239	Jacobaea borysthenica (DC.) B.Nord. & Greuter	0	n	1	0	Asteraceae Bercht. & J.Presl
Iz_6_1109	DJI_0239	Jasione montana L.	0	n	1	0	Campanulaceae Juss.
Iz_6_1109	DJI_0239	Jurinea cyanoides (L.) Rchb.s.l.	0	n	1	0	Asteraceae Bercht. & J.Presl
Iz_6_1109	DJI_0239	Koeleria glauca (Spreng.) DC.	0	n	1	0	Poaceae Barnhart
Iz_6_1109	DJI_0239	Lactuca serriola L.	0	a	0	0	Asteraceae Bercht. & J.Presl
Iz_6_1109	DJI_0239	Linaria genistifolia (L.) Mill.	0	n	1	0	Plantaginaceae Juss.
Iz_6_1109	DJI_0239	Lomelosia argentea (L.) Greuter & Burdet	0	n	1	0	Caprifoliaceae Juss.
Iz_6_1109	DJI_0239	Melampyrum pratense L.	0	n	1	0	Orobanchaceae Vent.
Iz_6_1109	DJI_0239	Oenothera biennis L.	0	a	0	0	Onagraceae Juss.
Iz_6_1109	DJI_0239	Pentanema britannica (L.) D.Gut.Larr., Santos-Vicente, Anderb., E.Rico & M.M.Mart.Ort.	0	n	1	0	Asteraceae Bercht. & J.Presl
Iz_6_1109	DJI_0239	Peucedanum oreoselinum (L.) Moench	0	n	1	0	Apiaceae Lindl.
Iz_6_1109	DJI_0239	Peucedanum ruthenicum M.Bieb.	0	n	1	0	Apiaceae Lindl.
Iz_6_1109	DJI_0239	Pilosella echioides (Lumn.) F.W.Schultz & Sch.Bip.	0	n	1	0	Asteraceae Bercht. & J.Presl
Iz_6_1109	DJI_0239	Pilosella officinarum F. Schultz et Sch. Bip.	0	n	1	0	Asteraceae Bercht. & J.Presl
Iz_6_1109	DJI_0239	Pilosella sp.	2		NA	0	Asteraceae Bercht. & J.Presl
Iz_6_1109	DJI_0239	Pinus sylvestris L.	0	n	1	1	Pinaceae Spreng. ex F.Rudolphi
Iz_6_1109	DJI_0239	Poa annua L.	0	n	1	0	Poaceae Barnhart
Iz_6_1109	DJI_0239	Poa nemoralis L.	0	n	1	0	Poaceae Barnhart
Iz_6_1109	DJI_0239	Poa sp.	0		NA	0	Poaceae Barnhart
Iz_6_1109	DJI_0239	Polygonatum odoratum (Mill.) Druce	0	n	1	0	Asparagaceae Juss.
Iz_6_1109	DJI_0239	Polygonum arenarium Waldst. & Kit.	0	n	1	0	Polygonaceae Juss.
Iz_6_1109	DJI_0239	Populus nigra L.	0	n	1	1	Salicaceae Mirb.
Iz_6_1109	DJI_0239	Populus tremula L.	0	n	1	1	Salicaceae Mirb.
Iz_6_1109	DJI_0239	Populus Ã canescens (Aiton) Sm.	0	n	1	1	Salicaceae Mirb.
Iz_6_1109	DJI_0239	Potentilla argentea L.	0	n	1	0	Rosaceae Juss.
Iz_6_1109	DJI_0239	Psammophiliella muralis (L.) Ikonn.	0	n	1	0	Caryophyllaceae Juss.
Iz_6_1109	DJI_0239	Psephellus sumensis (Kalen.) Greuter	0	n	1	0	Asteraceae Bercht. & J.Presl
Iz_6_1109	DJI_0239	Pulsatilla pratensis (L.) Mill. s.l.	0	n	1	0	Ranunculaceae Juss.
Iz_6_1109	DJI_0239	Pyrus communis L.	0	a	0	1	Rosaceae Juss.
Iz_6_1109	DJI_0239	Quercus robur L.	0	n	1	1	Fagaceae Dumort.
Iz_6_1109	DJI_0239	Robinia pseudoacacia L.	0	a	0	1	Fabaceae Lindl.
Iz_6_1109	DJI_0239	Rosa sp.	0		NA	1	Rosaceae Juss.
Iz_6_1109	DJI_0239	Rubus idaeus L.	0	n	1	0	Rosaceae Juss.
Iz_6_1109	DJI_0239	Rumex acetosella L.	0	n	1	0	Polygonaceae Juss.
Iz_6_1109	DJI_0239	Rumex thyrsiflorus Fingerh.	0	n	1	0	Polygonaceae Juss.
Iz_6_1109	DJI_0239	Salix alba L.	0	n	1	1	Salicaceae Mirb.
Iz_6_1109	DJI_0239	Sambucus nigra L.	0	n	1	1	Viburnaceae Raf.
Iz_6_1109	DJI_0239	Secale sylvestre Host	0	n	1	0	Poaceae Barnhart
Iz_6_1109	DJI_0239	Sempervivum ruthenicum Schnittsp. & C.B. Lehm.	0	n	1	0	Crassulaceae J.St.-Hil.
Iz_6_1109	DJI_0239	Setaria viridis (L.) P. Beauv.	0	n	1	0	Poaceae Barnhart
Iz_6_1109	DJI_0239	Silene borysthenica (Gruner) Walters	0	n	1	0	Caryophyllaceae Juss.
Iz_6_1109	DJI_0239	Silene latifolia subsp. alba (Mill.) Greuter & Burdet	0	n	1	0	Caryophyllaceae Juss.
Iz_6_1109	DJI_0239	Solidago virgaurea L.	0	n	1	0	Asteraceae Bercht. & J.Presl
Iz_6_1109	DJI_0239	Sorbus aucuparia L.	0	n	1	1	Rosaceae Juss.
Iz_6_1109	DJI_0239	Species 11.09_6_0254	0		NA	0	
Iz_6_1109	DJI_0239	Stachys recta L.	0	n	1	0	Lamiacea Lindl.
Iz_6_1109	DJI_0239	Tanacetum vulgare L.	0	n	1	0	Asteraceae Bercht. & J.Presl
Iz_6_1109	DJI_0239	Thymus pallasianus Heinr.Braun	0	n	1	0	Lamiacea Lindl.
Iz_6_1109	DJI_0239	Tilia cordata Mill.	0	n	1	1	Malvaceae Juss.
Iz_6_1109	DJI_0239	Trifolium alpestre L.	0	n	1	0	Fabaceae Lindl.
Iz_6_1109	DJI_0239	Trifolium arvense L.	0	n	1	0	Fabaceae Lindl.
Iz_6_1109	DJI_0239	Ulmus glabra Huds.	0	n	1	1	Ulmaceae Mirb.
Iz_6_1109	DJI_0239	Verbascum densiflorum Bertol.	0	n	1	0	Scrophulariaceae Juss.
Iz_6_1109	DJI_0239	Veronica austriaca L.	0	n	1	0	Plantaginaceae Juss.
Iz_6_1109	DJI_0239	Veronica incana L.	0	n	1	0	Plantaginaceae Juss.
Iz_6_1109	DJI_0239	Veronica verna L.	0	n	1	0	Plantaginaceae Juss.
Iz_6_1109	DJI_0239	Vincetoxicum hirundinaria Medik	0	n	1	0	Apocynaceae Juss.
Iz_6_1109	DJI_0242	Acer tataricum L.	0	n	1	1	Sapindaceae Juss.
Iz_6_1109	DJI_0242	Achillea millefolium L. s.l.	0	n	1	0	Asteraceae Bercht. & J.Presl
Iz_6_1109	DJI_0242	Allium oleraceum L.	0	n	1	0	Amaryllidaceae J.St.-Hil.
Iz_6_1109	DJI_0242	Ambrosia artemisiifolia L.	0	a	0	0	Asteraceae Bercht. & J.Presl
Iz_6_1109	DJI_0242	Amorpha fruticosa L.	0	a	0	0	Fabaceae Lindl.
Iz_6_1109	DJI_0242	Anthericum ramosum L.	0	n	1	0	Asparagaceae Juss.
Iz_6_1109	DJI_0242	Artemisia marschalliana Spreng.	0	n	1	0	Asteraceae Bercht. & J.Presl
Iz_6_1109	DJI_0242	Asparagus officinalis L.	0	n	1	0	Asparagaceae Juss.
Iz_6_1109	DJI_0242	Astragalus cicer L.	0	n	1	0	Fabaceae Lindl.
Iz_6_1109	DJI_0242	Bassia laniflora (S.G.Gmel.) A.J.Scott	0	n	1	0	Amaranthaceae Juss.
Iz_6_1109	DJI_0242	Berberis vulgaris L.	0	n	1	0	Berberidaceae Juss.
Iz_6_1109	DJI_0242	Betula pendula Roth.	0	n	1	1	Betulaceae Gray
Iz_6_1109	DJI_0242	Betula pendula Roth.	0	n	1	0	Poaceae Barnhart
Iz_6_1109	DJI_0242	Bromus inermis Leyss.	0		NA	NA	
Iz_6_1109	DJI_0242	Bromus squarrosus L.	0	a	0	0	Poaceae Barnhart
Iz_6_1109	DJI_0242	Bromus tectorum L.	0	a	0	0	Poaceae Barnhart
Iz_6_1109	DJI_0242	Calamagrostis epigejos (L.) Roth	2	n	1	0	Poaceae Barnhart
Iz_6_1109	DJI_0242	Carduus acanthoides L.	0	n	1	0	Asteraceae Bercht. & J.Presl
Iz_6_1109	DJI_0242	Carex sp.	0	n	1	0	Cyperaceae Juss.
Iz_6_1109	DJI_0242	Centaurea majorovii Dumbadze	0	n	1	0	Asteraceae Bercht. & J.Presl
Iz_6_1109	DJI_0242	Chamaecytisus ruthenicus (Fisch.ex Wol.) Klaskova	0	n	1	0	Fabaceae Lindl.
Iz_6_1109	DJI_0242	Chelidonium majus L.	0	n	1	0	Papaveraceae Juss.
Iz_6_1109	DJI_0242	Chenopodium betaceum Andrz.	0	n	1	0	Amaranthaceae Juss.
Iz_6_1109	DJI_0242	Chenopodium cf. album	0	n	1	0	Amaranthaceae Juss.
Iz_6_1109	DJI_0242	Chenopodium sp.	0		NA	0	Amaranthaceae Juss.
Iz_6_1109	DJI_0242	Chondrilla juncea L.	0	n	1	0	Asteraceae Bercht. & J.Presl
Iz_6_1109	DJI_0242	Cirsium vulgare (Savi) Ten.	0	n	1	0	Asteraceae Bercht. & J.Presl
Iz_6_1109	DJI_0242	Clinopodium acinos (L.) Kuntze	0	n	1	0	Lamiacea Lindl.
Iz_6_1109	DJI_0242	Convallaria majalis L.	0	n	1	0	Asparagaceae Juss.
Iz_6_1109	DJI_0242	Crataegus rhipidophylla Gand.	0	n	1	1	Rosaceae Juss.

Iz_6_1109	DJI_0242	Crepis foetida L.	0	n	1	0	Asteraceae Bercht. & J.Presl
Iz_6_1109	DJI_0242	Dactylis glomerata L.	0	n	1	0	Poaceae Barnhart
Iz_6_1109	DJI_0242	Dianthus campestris M.Bieb.	0	n	1	0	Caryophyllaceae Juss.
Iz_6_1109	DJI_0242	Digitaria sanguinalis (L.) Scop.	0	a	0	0	Poaceae Barnhart
Iz_6_1109	DJI_0242	Elymus repens (L.) Gould	0	n	1	0	Poaceae Barnhart
Iz_6_1109	DJI_0242	Epilobium tetragonum L.	0	n	1	0	Onagraceae Juss.
Iz_6_1109	DJI_0242	Eragrostis minor Host	0	a	0	0	Poaceae Barnhart
Iz_6_1109	DJI_0242	Erigeron annuus (L.) Desf.	0	a	0	0	Asteraceae Bercht. & J.Presl
Iz_6_1109	DJI_0242	Erigeron canadensis L.	10	a	0	0	Asteraceae Bercht. & J.Presl
Iz_6_1109	DJI_0242	Euphorbia seguieriana Neck.	0	n	1	0	Euphorbiaceae Juss.
Iz_6_1109	DJI_0242	Fallopia dumetorum (L.) Holub	0	n	1	0	Polygonaceae Juss.
Iz_6_1109	DJI_0242	Festuca beckeri (Hack.) Trautv.	0	n	1	0	Poaceae Barnhart
Iz_6_1109	DJI_0242	Fragaria vesca L.	0	n	1	0	Rosaceae Juss.
Iz_6_1109	DJI_0242	Frangula alnus Mill.	0	n	1	1	Rhamnaceae Juss.
Iz_6_1109	DJI_0242	Fraxinus pennsylvanica Marshall	0	a	0	1	Oleaceae Hoffmanns. & Link
Iz_6_1109	DJI_0242	Galeopsis bifida Boenn.	0	n	1	0	Lamiacea Lindl.
Iz_6_1109	DJI_0242	Galium verum L.	0	n	1	0	Rubiaceae Juss.
Iz_6_1109	DJI_0242	Genista tinctoria L.	0	n	1	0	Fabaceae Lindl.
Iz_6_1109	DJI_0242	Geranium robertianum L.	0	n	1	0	Geraniaceae Juss.
Iz_6_1109	DJI_0242	Geranium sanquineum L.	0	n	1	0	Geraniaceae Juss.
Iz_6_1109	DJI_0242	Gypsophila paniculata L.	0	n	1	0	Caryophyllaceae Juss.
Iz_6_1109	DJI_0242	Helichrysum arenarium (L.) Moench	0	n	1	0	Asteraceae Bercht. & J.Presl
Iz_6_1109	DJI_0242	Herniaria polygama J.Gay	0	n	1	0	Caryophyllaceae Juss.
Iz_6_1109	DJI_0242	Hieracium robustum Fr.	0	n	1	0	Asteraceae Bercht. & J.Presl
Iz_6_1109	DJI_0242	Hieracium umbellatum L.	0	n	1	NA	Asteraceae Bercht. & J.Presl
Iz_6_1109	DJI_0242	Hylotelephium maximum (L.) Holub	0	n	1	0	Crassulaceae J.St.-Hil.
Iz_6_1109	DJI_0242	Jacobaea borysthenica (DC.) B.Nord. & Greuter	0	n	1	0	Asteraceae Bercht. & J.Presl
Iz_6_1109	DJI_0242	Jasione montana L.	0	n	1	0	Campanulaceae Juss.
Iz_6_1109	DJI_0242	Jurinea cyanoides (L.) Rchb.s.l.	0	n	1	0	Asteraceae Bercht. & J.Presl
Iz_6_1109	DJI_0242	Koeleria glauca (Spreng.) DC.	0	n	1	0	Poaceae Barnhart
Iz_6_1109	DJI_0242	Lactuca serriola L.	0	a	0	0	Asteraceae Bercht. & J.Presl
Iz_6_1109	DJI_0242	Linaria genistifolia (L.) Mill.	0	n	1	0	Plantaginaceae Juss.
Iz_6_1109	DJI_0242	Lomelosia argentea (L.) Greuter & Burdet	0	n	1	0	Caprifoliaceae Juss.
Iz_6_1109	DJI_0242	Melampyrum pratense L.	0	n	1	0	Orobanchaceae Vent.
Iz_6_1109	DJI_0242	Oenothera biennis L.	0	a	0	0	Onagraceae Juss.
Iz_6_1109	DJI_0242	Pentanema britannica (L.) D.Gut.Larr., Santos-Vicente, Anderb., E.Rico & M.M.Mart.Ort.	0	n	1	0	Asteraceae Bercht. & J.Presl
Iz_6_1109	DJI_0242	Peucedanum oreoselinum (L.) Moench	0	n	1	0	Apiaceae Lindl.
Iz_6_1109	DJI_0242	Peucedanum ruthenicum M.Bieb.	0	n	1	0	Apiaceae Lindl.
Iz_6_1109	DJI_0242	Pilosella echioides (Lumn.) F.W.Schultz & Sch.Bip.	0	n	1	0	Asteraceae Bercht. & J.Presl
Iz_6_1109	DJI_0242	Pilosella officinarum F. Schultz et Sch. Bip.	0	n	1	0	Asteraceae Bercht. & J.Presl
Iz_6_1109	DJI_0242	Pilosella sp.	0		NA	0	Asteraceae Bercht. & J.Presl
Iz_6_1109	DJI_0242	Pinus sylvestris L.	0	n	1	1	Pinaceae Spreng. ex F.Rudolphi
Iz_6_1109	DJI_0242	Poa annua L.	0	n	1	0	Poaceae Barnhart
Iz_6_1109	DJI_0242	Poa nemoralis L.	0	n	1	0	Poaceae Barnhart
Iz_6_1109	DJI_0242	Poa sp.	0		NA	0	Poaceae Barnhart
Iz_6_1109	DJI_0242	Polygonatum odoratum (Mill.) Druce	0	n	1	0	Asparagaceae Juss.
Iz_6_1109	DJI_0242	Polygonum arenarium Waldst. & Kit.	0	n	1	0	Polygonaceae Juss.
Iz_6_1109	DJI_0242	Populus nigra L.	0	n	1	1	Salicaceae Mirb.
Iz_6_1109	DJI_0242	Populus tremula L.	0	n	1	1	Salicaceae Mirb.
Iz_6_1109	DJI_0242	Populus Ã canescens (Aiton) Sm.	0	n	1	1	Salicaceae Mirb.
Iz_6_1109	DJI_0242	Potentilla argentea L.	0	n	1	0	Rosaceae Juss.
Iz_6_1109	DJI_0242	Psammophiliella muralis (L.) Ikonn.	0	n	1	0	Caryophyllaceae Juss.
Iz_6_1109	DJI_0242	Psephellus sumensis (Kalen.) Greuter	0	n	1	0	Asteraceae Bercht. & J.Presl
Iz_6_1109	DJI_0242	Pulsatilla pratensis (L.) Mill. s.l.	0	n	1	0	Ranunculaceae Juss.
Iz_6_1109	DJI_0242	Pyrus communis L.	0	a	0	1	Rosaceae Juss.
Iz_6_1109	DJI_0242	Quercus robur L.	0	n	1	1	Fagaceae Dumort.
Iz_6_1109	DJI_0242	Robinia pseudoacacia L.	0	a	0	1	Fabaceae Lindl.
Iz_6_1109	DJI_0242	Rosa sp.	0		NA	1	Rosaceae Juss.
Iz_6_1109	DJI_0242	Rubus idaeus L.	0	n	1	0	Rosaceae Juss.
Iz_6_1109	DJI_0242	Rumex acetosella L.	0	n	1	0	Polygonaceae Juss.
Iz_6_1109	DJI_0242	Rumex thyrsiflorus Fingerh.	0	n	1	0	Polygonaceae Juss.
Iz_6_1109	DJI_0242	Salix alba L.	0	n	1	1	Salicaceae Mirb.
Iz_6_1109	DJI_0242	Sambucus nigra L.	0	n	1	1	Viburnaceae Raf.
Iz_6_1109	DJI_0242	Secale sylvestre Host	0	n	1	0	Poaceae Barnhart
Iz_6_1109	DJI_0242	Sempervivum ruthenicum Schnittsp. & C.B. Lehm.	0	n	1	0	Crassulaceae J.St.-Hil.
Iz_6_1109	DJI_0242	Setaria viridis (L.) P. Beauv.	0	n	1	0	Poaceae Barnhart
Iz_6_1109	DJI_0242	Silene borysthenica (Gruner) Walters	0	n	1	0	Caryophyllaceae Juss.
Iz_6_1109	DJI_0242	Silene latifolia subsp. alba (Mill.) Greuter & Burdet	0	n	1	0	Caryophyllaceae Juss.
Iz_6_1109	DJI_0242	Solidago virgaurea L.	0	n	1	0	Asteraceae Bercht. & J.Presl
Iz_6_1109	DJI_0242	Sorbus aucuparia L.	0	n	1	1	Rosaceae Juss.
Iz_6_1109	DJI_0242	Species 11.09_6_0254	0		NA	0	
Iz_6_1109	DJI_0242	Stachys recta L.	0	n	1	0	Lamiacea Lindl.
Iz_6_1109	DJI_0242	Tanacetum vulgare L.	0	n	1	0	Asteraceae Bercht. & J.Presl
Iz_6_1109	DJI_0242	Thymus pallasianus Heinr.Braun	0	n	1	0	Lamiacea Lindl.
Iz_6_1109	DJI_0242	Tilia cordata Mill.	0	n	1	1	Malvaceae Juss.
Iz_6_1109	DJI_0242	Trifolium alpestre L.	0	n	1	0	Fabaceae Lindl.
Iz_6_1109	DJI_0242	Trifolium arvense L.	0	n	1	0	Fabaceae Lindl.
Iz_6_1109	DJI_0242	Ulmus glabra Huds.	0	n	1	1	Ulmaceae Mirb.
Iz_6_1109	DJI_0242	Verbascum densiflorum Bertol.	0	n	1	0	Scrophulariaceae Juss.
Iz_6_1109	DJI_0242	Veronica austriaca L.	0	n	1	0	Plantaginaceae Juss.
Iz_6_1109	DJI_0242	Veronica incana L.	0	n	1	0	Plantaginaceae Juss.
Iz_6_1109	DJI_0242	Veronica verna L.	0	n	1	0	Plantaginaceae Juss.
Iz_6_1109	DJI_0242	Vincetoxicum hirundinaria Medik	0	n	1	0	Apocynaceae Juss.
Iz_6_1109	DJI_0244	Acer tataricum L.	0	n	1	1	Sapindaceae Juss.
Iz_6_1109	DJI_0244	Achillea millefolium L. s.l.	0	n	1	0	Asteraceae Bercht. & J.Presl
Iz_6_1109	DJI_0244	Allium oleraceum L.	0	n	1	0	Amaryllidaceae J.St.-Hil.
Iz_6_1109	DJI_0244	Ambrosia artemisiifolia L.	0	a	0	0	Asteraceae Bercht. & J.Presl
Iz_6_1109	DJI_0244	Amorpha fruticosa L.	0	a	0	0	Fabaceae Lindl.
Iz_6_1109	DJI_0244	Anthericum ramosum L.	0	n	1	0	Asparagaceae Juss.
Iz_6_1109	DJI_0244	Artemisia marschalliana Spreng.	0	n	1	0	Asteraceae Bercht. & J.Presl
Iz_6_1109	DJI_0244	Asparagus officinalis L.	0	n	1	0	Asparagaceae Juss.
Iz_6_1109	DJI_0244	Astragalus cicer L.	0	n	1	0	Fabaceae Lindl.
Iz_6_1109	DJI_0244	Bassia laniflora (S.G.Gmel.) A.J.Scott	0	n	1	0	Amaranthaceae Juss.
Iz_6_1109	DJI_0244	Berberis vulgaris L.	0	n	1	0	Berberidaceae Juss.
Iz_6_1109	DJI_0244	Betula pendula Roth.	0	n	1	1	Betulaceae Gray
Iz_6_1109	DJI_0244	Betula pendula Roth.	0	n	1	0	Poaceae Barnhart
Iz_6_1109	DJI_0244	Bromus inermis Leyss.	0		NA	NA	
Iz_6_1109	DJI_0244	Bromus squarrosus L.	0	a	0	0	Poaceae Barnhart

Iz_6_1109	DJI_0244	Bromus tectorum L.	0	a	0	0	Poaceae Barnhart
Iz_6_1109	DJI_0244	Calamagrostis epigejos (L.) Roth	5	n	1	0	Poaceae Barnhart
Iz_6_1109	DJI_0244	Carduus acanthoides L.	0	n	1	0	Asteraceae Bercht. & J.Presl
Iz_6_1109	DJI_0244	Carex sp.	0	n	1	0	Cyperaceae Juss.
Iz_6_1109	DJI_0244	Centaurea majorovii Dumbadze	0	n	1	0	Asteraceae Bercht. & J.Presl
Iz_6_1109	DJI_0244	Chamaecytisus ruthenicus (Fisch.ex Wol.) Klaskova	0	n	1	0	Fabaceae Lindl.
Iz_6_1109	DJI_0244	Chelidonium majus L.	0	n	1	0	Papaveraceae Juss.
Iz_6_1109	DJI_0244	Chenopodium betaceum Andrz.	0	n	1	0	Amaranthaceae Juss.
Iz_6_1109	DJI_0244	Chenopodium cf. album	0	n	1	0	Amaranthaceae Juss.
Iz_6_1109	DJI_0244	Chenopodium sp.	0		NA	0	Amaranthaceae Juss.
Iz_6_1109	DJI_0244	Chondrilla juncea L.	0	n	1	0	Asteraceae Bercht. & J.Presl
Iz_6_1109	DJI_0244	Cirsium vulgare (Savi) Ten.	0	n	1	0	Asteraceae Bercht. & J.Presl
Iz_6_1109	DJI_0244	Clinopodium acinos (L.) Kuntze	0	n	1	0	Lamiacea Lindl.
Iz_6_1109	DJI_0244	Convallaria majalis L.	0	n	1	0	Asparagaceae Juss.
Iz_6_1109	DJI_0244	Crataegus rhipidophylla Gand.	0	n	1	1	Rosaceae Juss.
Iz_6_1109	DJI_0244	Crepis foetida L.	0	n	1	0	Asteraceae Bercht. & J.Presl
Iz_6_1109	DJI_0244	Dactylis glomerata L.	0	n	1	0	Poaceae Barnhart
Iz_6_1109	DJI_0244	Dianthus campestris M.Bieb.	0	n	1	0	Caryophyllaceae Juss.
Iz_6_1109	DJI_0244	Digitaria sanguinalis (L.) Scop.	0	a	0	0	Poaceae Barnhart
Iz_6_1109	DJI_0244	Elymus repens (L.) Gould	0	n	1	0	Poaceae Barnhart
Iz_6_1109	DJI_0244	Epilobium tetragonum L.	0	n	1	0	Onagraceae Juss.
Iz_6_1109	DJI_0244	Eragrostis minor Host	0	a	0	0	Poaceae Barnhart
Iz_6_1109	DJI_0244	Erigeron annuus (L.) Desf.	0	a	0	0	Asteraceae Bercht. & J.Presl
Iz_6_1109	DJI_0244	Erigeron canadensis L.	5	a	0	0	Asteraceae Bercht. & J.Presl
Iz_6_1109	DJI_0244	Euphorbia seguieriana Neck.	0	n	1	0	Euphorbiaceae Juss.
Iz_6_1109	DJI_0244	Fallopia dumetorum (L.) Holub	0	n	1	0	Polygonaceae Juss.
Iz_6_1109	DJI_0244	Festuca beckeri (Hack.) Trautv.	0	n	1	0	Poaceae Barnhart
Iz_6_1109	DJI_0244	Fragaria vesca L.	0	n	1	0	Rosaceae Juss.
Iz_6_1109	DJI_0244	Frangula alnus Mill.	0	n	1	1	Rhamnaceae Juss.
Iz_6_1109	DJI_0244	Fraxinus pennsylvanica Marshall	0	a	0	1	Oleaceae Hoffmanns. & Link
Iz_6_1109	DJI_0244	Galeopsis bifida Boenn.	0	n	1	0	Lamiacea Lindl.
Iz_6_1109	DJI_0244	Galium verum L.	0	n	1	0	Rubiaceae Juss.
Iz_6_1109	DJI_0244	Genista tinctoria L.	0	n	1	0	Fabaceae Lindl.
Iz_6_1109	DJI_0244	Geranium robertianum L.	0	n	1	0	Geraniaceae Juss.
Iz_6_1109	DJI_0244	Geranium sanquineum L.	0	n	1	0	Geraniaceae Juss.
Iz_6_1109	DJI_0244	Gypsophila paniculata L.	0	n	1	0	Caryophyllaceae Juss.
Iz_6_1109	DJI_0244	Helichrysum arenarium (L.) Moench	0	n	1	0	Asteraceae Bercht. & J.Presl
Iz_6_1109	DJI_0244	Herniaria polygama J.Gay	0	n	1	0	Caryophyllaceae Juss.
Iz_6_1109	DJI_0244	Hieracium robustum Fr.	0	n	1	0	Asteraceae Bercht. & J.Presl
Iz_6_1109	DJI_0244	Hieracium umbellatum L.	0	n	1	NA	Asteraceae Bercht. & J.Presl
Iz_6_1109	DJI_0244	Hylotelephium maximum (L.) Holub	0	n	1	0	Crassulaceae J.St.-Hil.
Iz_6_1109	DJI_0244	Jacobaea borysthenica (DC.) B.Nord. & Greuter	0	n	1	0	Asteraceae Bercht. & J.Presl
Iz_6_1109	DJI_0244	Jasione montana L.	0	n	1	0	Campanulaceae Juss.
Iz_6_1109	DJI_0244	Jurinea cyanoides (L.) Rchb.s.l.	0	n	1	0	Asteraceae Bercht. & J.Presl
Iz_6_1109	DJI_0244	Koeleria glauca (Spreng.) DC.	0	n	1	0	Poaceae Barnhart
Iz_6_1109	DJI_0244	Lactuca serriola L.	0	a	0	0	Asteraceae Bercht. & J.Presl
Iz_6_1109	DJI_0244	Linaria genistifolia (L.) Mill.	0	n	1	0	Plantaginaceae Juss.
Iz_6_1109	DJI_0244	Lomelosia argentea (L.) Greuter & Burdet	0	n	1	0	Caprifoliaceae Juss.
Iz_6_1109	DJI_0244	Melampyrum pratense L.	0	n	1	0	Orobanchaceae Vent.
Iz_6_1109	DJI_0244	Oenothera biennis L.	0	a	0	0	Onagraceae Juss.
Iz_6_1109	DJI_0244	Pentanema britannica (L.) D.Gut.Larr., Santos-Vicente, Anderb., E.Rico & M.M.Mart.Ort.	0	n	1	0	Asteraceae Bercht. & J.Presl
Iz_6_1109	DJI_0244	Peucedanum oreoselinum (L.) Moench	0	n	1	0	Apiaceae Lindl.
Iz_6_1109	DJI_0244	Peucedanum ruthenicum M.Bieb.	0	n	1	0	Apiaceae Lindl.
Iz_6_1109	DJI_0244	Pilosella echioides (Lumn.) F.W.Schultz & Sch.Bip.	0	n	1	0	Asteraceae Bercht. & J.Presl
Iz_6_1109	DJI_0244	Pilosella officinarum F. Schultz et Sch. Bip.	0	n	1	0	Asteraceae Bercht. & J.Presl
Iz_6_1109	DJI_0244	Pilosella sp.	1		NA	0	Asteraceae Bercht. & J.Presl
Iz_6_1109	DJI_0244	Pinus sylvestris L.	0	n	1	1	Pinaceae Spreng. ex F.Rudolphi
Iz_6_1109	DJI_0244	Poa annua L.	0	n	1	0	Poaceae Barnhart
Iz_6_1109	DJI_0244	Poa nemoralis L.	0	n	1	0	Poaceae Barnhart
Iz_6_1109	DJI_0244	Poa sp.	0		NA	0	Poaceae Barnhart
Iz_6_1109	DJI_0244	Polygonatum odoratum (Mill.) Druce	0	n	1	0	Asparagaceae Juss.
Iz_6_1109	DJI_0244	Polygonum arenarium Waldst. & Kit.	0	n	1	0	Polygonaceae Juss.
Iz_6_1109	DJI_0244	Populus nigra L.	0	n	1	1	Salicaceae Mirb.
Iz_6_1109	DJI_0244	Populus tremula L.	0	n	1	1	Salicaceae Mirb.
Iz_6_1109	DJI_0244	Populus Ã canescens (Aiton) Sm.	0	n	1	1	Salicaceae Mirb.
Iz_6_1109	DJI_0244	Potentilla argentea L.	0	n	1	0	Rosaceae Juss.
Iz_6_1109	DJI_0244	Psammophiliella muralis (L.) Ikonn.	0	n	1	0	Caryophyllaceae Juss.
Iz_6_1109	DJI_0244	Psephellus sumensis (Kalen.) Greuter	0	n	1	0	Asteraceae Bercht. & J.Presl
Iz_6_1109	DJI_0244	Pulsatilla pratensis (L.) Mill. s.l.	0	n	1	0	Ranunculaceae Juss.
Iz_6_1109	DJI_0244	Pyrus communis L.	0	a	0	1	Rosaceae Juss.
Iz_6_1109	DJI_0244	Quercus robur L.	0	n	1	1	Fagaceae Dumort.
Iz_6_1109	DJI_0244	Robinia pseudoacacia L.	0	a	0	1	Fabaceae Lindl.
Iz_6_1109	DJI_0244	Rosa sp.	0		NA	1	Rosaceae Juss.
Iz_6_1109	DJI_0244	Rubus idaeus L.	0	n	1	0	Rosaceae Juss.
Iz_6_1109	DJI_0244	Rumex acetosella L.	0	n	1	0	Polygonaceae Juss.
Iz_6_1109	DJI_0244	Rumex thyrsiflorus Fingerh.	0	n	1	0	Polygonaceae Juss.
Iz_6_1109	DJI_0244	Salix alba L.	0	n	1	1	Salicaceae Mirb.
Iz_6_1109	DJI_0244	Sambucus nigra L.	0	n	1	1	Viburnaceae Raf.
Iz_6_1109	DJI_0244	Secale sylvestre Host	0	n	1	0	Poaceae Barnhart
Iz_6_1109	DJI_0244	Sempervivum ruthenicum Schnittsp. & C.B. Lehm.	0	n	1	0	Crassulaceae J.St.-Hil.
Iz_6_1109	DJI_0244	Setaria viridis (L.) P. Beauv.	0	n	1	0	Poaceae Barnhart
Iz_6_1109	DJI_0244	Silene borysthenica (Gruner) Walters	0	n	1	0	Caryophyllaceae Juss.
Iz_6_1109	DJI_0244	Silene latifolia subsp. alba (Mill.) Greuter & Burdet	0	n	1	0	Caryophyllaceae Juss.
Iz_6_1109	DJI_0244	Solidago virgaurea L.	0	n	1	0	Asteraceae Bercht. & J.Presl
Iz_6_1109	DJI_0244	Sorbus aucuparia L.	0	n	1	1	Rosaceae Juss.
Iz_6_1109	DJI_0244	Species 11.09_6_0254	0		NA	0	
Iz_6_1109	DJI_0244	Stachys recta L.	0	n	1	0	Lamiacea Lindl.
Iz_6_1109	DJI_0244	Tanacetum vulgare L.	0	n	1	0	Asteraceae Bercht. & J.Presl
Iz_6_1109	DJI_0244	Thymus pallasianus Heinr.Braun	0	n	1	0	Lamiacea Lindl.
Iz_6_1109	DJI_0244	Tilia cordata Mill.	0	n	1	1	Malvaceae Juss.
Iz_6_1109	DJI_0244	Trifolium alpestre L.	0	n	1	0	Fabaceae Lindl.
Iz_6_1109	DJI_0244	Trifolium arvense L.	0	n	1	0	Fabaceae Lindl.
Iz_6_1109	DJI_0244	Ulmus glabra Huds.	0	n	1	1	Ulmaceae Mirb.
Iz_6_1109	DJI_0244	Verbascum densiflorum Bertol.	0	n	1	0	Scrophulariaceae Juss.
Iz_6_1109	DJI_0244	Veronica austriaca L.	0	n	1	0	Plantaginaceae Juss.
Iz_6_1109	DJI_0244	Veronica incana L.	0	n	1	0	Plantaginaceae Juss.
Iz_6_1109	DJI_0244	Veronica verna L.	0	n	1	0	Plantaginaceae Juss.
Iz_6_1109	DJI_0244	Vincetoxicum hirundinaria Medik	0	n	1	0	Apocynaceae Juss.

Iz_6_1109	DJI_0247	Acer tataricum L.	0	n	1	1	Sapindaceae Juss.
Iz_6_1109	DJI_0247	Achillea millefolium L. s.l.	0	n	1	0	Asteraceae Bercht. & J.Presl
Iz_6_1109	DJI_0247	Allium oleraceum L.	0	n	1	0	Amaryllidaceae J.St.-Hil.
Iz_6_1109	DJI_0247	Ambrosia artemisiifolia L.	0	a	0	0	Asteraceae Bercht. & J.Presl
Iz_6_1109	DJI_0247	Amorpha fruticosa L.	0	a	0	0	Fabaceae Lindl.
Iz_6_1109	DJI_0247	Anthericum ramosum L.	0	n	1	0	Asparagaceae Juss.
Iz_6_1109	DJI_0247	Artemisia marschalliana Spreng.	0	n	1	0	Asteraceae Bercht. & J.Presl
Iz_6_1109	DJI_0247	Asparagus officinalis L.	0	n	1	0	Asparagaceae Juss.
Iz_6_1109	DJI_0247	Astragalus cicer L.	0	n	1	0	Fabaceae Lindl.
Iz_6_1109	DJI_0247	Bassia laniflora (S.G.Gmel.) A.J.Scott	0	n	1	0	Amaranthaceae Juss.
Iz_6_1109	DJI_0247	Berberis vulgaris L.	0	n	1	0	Berberidaceae Juss.
Iz_6_1109	DJI_0247	Betula pendula Roth.	0	n	1	1	Betulaceae Gray
Iz_6_1109	DJI_0247	Betula pendula Roth.	0	n	1	0	Poaceae Barnhart
Iz_6_1109	DJI_0247	Bromus inermis Leyss.	0		NA	NA	
Iz_6_1109	DJI_0247	Bromus squarrosus L.	0	a	0	0	Poaceae Barnhart
Iz_6_1109	DJI_0247	Bromus tectorum L.	0	a	0	0	Poaceae Barnhart
Iz_6_1109	DJI_0247	Calamagrostis epigejos (L.) Roth	8	n	1	0	Poaceae Barnhart
Iz_6_1109	DJI_0247	Carduus acanthoides L.	0	n	1	0	Asteraceae Bercht. & J.Presl
Iz_6_1109	DJI_0247	Carex sp.	2	n	1	0	Cyperaceae Juss.
Iz_6_1109	DJI_0247	Centaurea majorovii Dumbadze	0	n	1	0	Asteraceae Bercht. & J.Presl
Iz_6_1109	DJI_0247	Chamaecytisus ruthenicus (Fisch.ex Wol.) Klaskova	0	n	1	0	Fabaceae Lindl.
Iz_6_1109	DJI_0247	Chelidonium majus L.	0	n	1	0	Papaveraceae Juss.
Iz_6_1109	DJI_0247	Chenopodium betaceum Andrz.	0	n	1	0	Amaranthaceae Juss.
Iz_6_1109	DJI_0247	Chenopodium cf. album	0	n	1	0	Amaranthaceae Juss.
Iz_6_1109	DJI_0247	Chenopodium sp.	0		NA	0	Amaranthaceae Juss.
Iz_6_1109	DJI_0247	Chondrilla juncea L.	0	n	1	0	Asteraceae Bercht. & J.Presl
Iz_6_1109	DJI_0247	Cirsium vulgare (Savi) Ten.	0	n	1	0	Asteraceae Bercht. & J.Presl
Iz_6_1109	DJI_0247	Clinopodium acinos (L.) Kuntze	0	n	1	0	Lamiacea Lindl.
Iz_6_1109	DJI_0247	Convallaria majalis L.	0	n	1	0	Asparagaceae Juss.
Iz_6_1109	DJI_0247	Crataegus rhipidophylla Gand.	0	n	1	1	Rosaceae Juss.
Iz_6_1109	DJI_0247	Crepis foetida L.	0	n	1	0	Asteraceae Bercht. & J.Presl
Iz_6_1109	DJI_0247	Dactylis glomerata L.	0	n	1	0	Poaceae Barnhart
Iz_6_1109	DJI_0247	Dianthus campestris M.Bieb.	0	n	1	0	Caryophyllaceae Juss.
Iz_6_1109	DJI_0247	Digitaria sanguinalis (L.) Scop.	0	a	0	0	Poaceae Barnhart
Iz_6_1109	DJI_0247	Elymus repens (L.) Gould	0	n	1	0	Poaceae Barnhart
Iz_6_1109	DJI_0247	Epilobium tetragonum L.	0	n	1	0	Onagraceae Juss.
Iz_6_1109	DJI_0247	Eragrostis minor Host	0	a	0	0	Poaceae Barnhart
Iz_6_1109	DJI_0247	Erigeron annuus (L.) Desf.	0	a	0	0	Asteraceae Bercht. & J.Presl
Iz_6_1109	DJI_0247	Erigeron canadensis L.	7	a	0	0	Asteraceae Bercht. & J.Presl
Iz_6_1109	DJI_0247	Euphorbia seguieriana Neck.	0	n	1	0	Euphorbiaceae Juss.
Iz_6_1109	DJI_0247	Fallopia dumetorum (L.) Holub	0	n	1	0	Polygonaceae Juss.
Iz_6_1109	DJI_0247	Festuca beckeri (Hack.) Trautv.	0	n	1	0	Poaceae Barnhart
Iz_6_1109	DJI_0247	Fragaria vesca L.	0	n	1	0	Rosaceae Juss.
Iz_6_1109	DJI_0247	Frangula alnus Mill.	0	n	1	1	Rhamnaceae Juss.
Iz_6_1109	DJI_0247	Fraxinus pennsylvanica Marshall	0	a	0	1	Oleaceae Hoffmanns. & Link
Iz_6_1109	DJI_0247	Galeopsis bifida Boenn.	0	n	1	0	Lamiacea Lindl.
Iz_6_1109	DJI_0247	Galium verum L.	0	n	1	0	Rubiaceae Juss.
Iz_6_1109	DJI_0247	Genista tinctoria L.	0	n	1	0	Fabaceae Lindl.
Iz_6_1109	DJI_0247	Geranium robertianum L.	0	n	1	0	Geraniaceae Juss.
Iz_6_1109	DJI_0247	Geranium sanquineum L.	0	n	1	0	Geraniaceae Juss.
Iz_6_1109	DJI_0247	Gypsophila paniculata L.	0	n	1	0	Caryophyllaceae Juss.
Iz_6_1109	DJI_0247	Helichrysum arenarium (L.) Moench	0	n	1	0	Asteraceae Bercht. & J.Presl
Iz_6_1109	DJI_0247	Herniaria polygama J.Gay	0	n	1	0	Caryophyllaceae Juss.
Iz_6_1109	DJI_0247	Hieracium robustum Fr.	0	n	1	0	Asteraceae Bercht. & J.Presl
Iz_6_1109	DJI_0247	Hieracium umbellatum L.	0	n	1	NA	Asteraceae Bercht. & J.Presl
Iz_6_1109	DJI_0247	Hylotelephium maximum (L.) Holub	0	n	1	0	Crassulaceae J.St.-Hil.
Iz_6_1109	DJI_0247	Jacobaea borysthenica (DC.) B.Nord. & Greuter	0	n	1	0	Asteraceae Bercht. & J.Presl
Iz_6_1109	DJI_0247	Jasione montana L.	0	n	1	0	Campanulaceae Juss.
Iz_6_1109	DJI_0247	Jurinea cyanoides (L.) Rchb.s.l.	0	n	1	0	Asteraceae Bercht. & J.Presl
Iz_6_1109	DJI_0247	Koeleria glauca (Spreng.) DC.	0	n	1	0	Poaceae Barnhart
Iz_6_1109	DJI_0247	Lactuca serriola L.	0	a	0	0	Asteraceae Bercht. & J.Presl
Iz_6_1109	DJI_0247	Linaria genistifolia (L.) Mill.	0	n	1	0	Plantaginaceae Juss.
Iz_6_1109	DJI_0247	Lomelosia argentea (L.) Greuter & Burdet	0	n	1	0	Caprifoliaceae Juss.
Iz_6_1109	DJI_0247	Melampyrum pratense L.	0	n	1	0	Orobanchaceae Vent.
Iz_6_1109	DJI_0247	Oenothera biennis L.	0	a	0	0	Onagraceae Juss.
Iz_6_1109	DJI_0247	Pentanema britannica (L.) D.Gut.Larr., Santos-Vicente, Anderb., E.Rico & M.M.Mart.Ort.	0	n	1	0	Asteraceae Bercht. & J.Presl
Iz_6_1109	DJI_0247	Peucedanum oreoselinum (L.) Moench	0	n	1	0	Apiaceae Lindl.
Iz_6_1109	DJI_0247	Peucedanum ruthenicum M.Bieb.	0	n	1	0	Apiaceae Lindl.
Iz_6_1109	DJI_0247	Pilosella echioides (Lumn.) F.W.Schultz & Sch.Bip.	0	n	1	0	Asteraceae Bercht. & J.Presl
Iz_6_1109	DJI_0247	Pilosella officinarum F. Schultz et Sch. Bip.	0	n	1	0	Asteraceae Bercht. & J.Presl
Iz_6_1109	DJI_0247	Pilosella sp.	0		NA	0	Asteraceae Bercht. & J.Presl
Iz_6_1109	DJI_0247	Pinus sylvestris L.	0	n	1	1	Pinaceae Spreng. ex F.Rudolphi
Iz_6_1109	DJI_0247	Poa annua L.	0	n	1	0	Poaceae Barnhart
Iz_6_1109	DJI_0247	Poa nemoralis L.	0	n	1	0	Poaceae Barnhart
Iz_6_1109	DJI_0247	Poa sp.	0		NA	0	Poaceae Barnhart
Iz_6_1109	DJI_0247	Polygonatum odoratum (Mill.) Druce	0	n	1	0	Asparagaceae Juss.
Iz_6_1109	DJI_0247	Polygonum arenarium Waldst. & Kit.	0	n	1	0	Polygonaceae Juss.
Iz_6_1109	DJI_0247	Populus nigra L.	0	n	1	1	Salicaceae Mirb.
Iz_6_1109	DJI_0247	Populus tremula L.	0	n	1	1	Salicaceae Mirb.
Iz_6_1109	DJI_0247	Populus Ã canescens (Aiton) Sm.	0	n	1	1	Salicaceae Mirb.
Iz_6_1109	DJI_0247	Potentilla argentea L.	0	n	1	0	Rosaceae Juss.
Iz_6_1109	DJI_0247	Psammophiliella muralis (L.) Ikonn.	0	n	1	0	Caryophyllaceae Juss.
Iz_6_1109	DJI_0247	Psephellus sumensis (Kalen.) Greuter	0	n	1	0	Asteraceae Bercht. & J.Presl
Iz_6_1109	DJI_0247	Pulsatilla pratensis (L.) Mill. s.l.	0	n	1	0	Ranunculaceae Juss.
Iz_6_1109	DJI_0247	Pyrus communis L.	0	a	0	1	Rosaceae Juss.
Iz_6_1109	DJI_0247	Quercus robur L.	0	n	1	1	Fagaceae Dumort.
Iz_6_1109	DJI_0247	Robinia pseudoacacia L.	0	a	0	1	Fabaceae Lindl.
Iz_6_1109	DJI_0247	Rosa sp.	0		NA	1	Rosaceae Juss.
Iz_6_1109	DJI_0247	Rubus idaeus L.	0	n	1	0	Rosaceae Juss.
Iz_6_1109	DJI_0247	Rumex acetosella L.	0	n	1	0	Polygonaceae Juss.
Iz_6_1109	DJI_0247	Rumex thyrsiflorus Fingerh.	0	n	1	0	Polygonaceae Juss.
Iz_6_1109	DJI_0247	Salix alba L.	0	n	1	1	Salicaceae Mirb.
Iz_6_1109	DJI_0247	Sambucus nigra L.	0	n	1	1	Viburnaceae Raf.
Iz_6_1109	DJI_0247	Secale sylvestre Host	0	n	1	0	Poaceae Barnhart
Iz_6_1109	DJI_0247	Sempervivum ruthenicum Schnittsp. & C.B. Lehm.	0	n	1	0	Crassulaceae J.St.-Hil.
Iz_6_1109	DJI_0247	Setaria viridis (L.) P. Beauv.	0	n	1	0	Poaceae Barnhart
Iz_6_1109	DJI_0247	Silene borysthenica (Gruner) Walters	0	n	1	0	Caryophyllaceae Juss.
Iz_6_1109	DJI_0247	Silene latifolia subsp. alba (Mill.) Greuter & Burdet	0	n	1	0	Caryophyllaceae Juss.

Iz_6_1109	DJI_0247	Solidago virgaurea L.	0	n	1	0	Asteraceae Bercht. & J.Presl
Iz_6_1109	DJI_0247	Sorbus aucuparia L.	0	n	1	1	Rosaceae Juss.
Iz_6_1109	DJI_0247	Species 11.09_6_0254	0		NA	0	
Iz_6_1109	DJI_0247	Stachys recta L.	0	n	1	0	Lamiacea Lindl.
Iz_6_1109	DJI_0247	Tanacetum vulgare L.	0	n	1	0	Asteraceae Bercht. & J.Presl
Iz_6_1109	DJI_0247	Thymus pallasianus Heinr.Braun	0	n	1	0	Lamiacea Lindl.
Iz_6_1109	DJI_0247	Tilia cordata Mill.	0	n	1	1	Malvaceae Juss.
Iz_6_1109	DJI_0247	Trifolium alpestre L.	0	n	1	0	Fabaceae Lindl.
Iz_6_1109	DJI_0247	Trifolium arvense L.	0	n	1	0	Fabaceae Lindl.
Iz_6_1109	DJI_0247	Ulmus glabra Huds.	0	n	1	1	Ulmaceae Mirb.
Iz_6_1109	DJI_0247	Verbascum densiflorum Bertol.	0	n	1	0	Scrophulariaceae Juss.
Iz_6_1109	DJI_0247	Veronica austriaca L.	0	n	1	0	Plantaginaceae Juss.
Iz_6_1109	DJI_0247	Veronica incana L.	0	n	1	0	Plantaginaceae Juss.
Iz_6_1109	DJI_0247	Veronica verna L.	0	n	1	0	Plantaginaceae Juss.
Iz_6_1109	DJI_0247	Vincetoxicum hirundinaria Medik	0	n	1	0	Apocynaceae Juss.
Iz_6_1109	DJI_0248	Acer tataricum L.	0	n	1	1	Sapindaceae Juss.
Iz_6_1109	DJI_0248	Achillea millefolium L. s.l.	0	n	1	0	Asteraceae Bercht. & J.Presl
Iz_6_1109	DJI_0248	Allium oleraceum L.	0	n	1	0	Amaryllidaceae J.St.-Hil.
Iz_6_1109	DJI_0248	Ambrosia artemisiifolia L.	0	a	0	0	Asteraceae Bercht. & J.Presl
Iz_6_1109	DJI_0248	Amorpha fruticosa L.	0	a	0	0	Fabaceae Lindl.
Iz_6_1109	DJI_0248	Anthericum ramosum L.	0	n	1	0	Asparagaceae Juss.
Iz_6_1109	DJI_0248	Artemisia marschalliana Spreng.	0	n	1	0	Asteraceae Bercht. & J.Presl
Iz_6_1109	DJI_0248	Asparagus officinalis L.	0	n	1	0	Asparagaceae Juss.
Iz_6_1109	DJI_0248	Astragalus cicer L.	0	n	1	0	Fabaceae Lindl.
Iz_6_1109	DJI_0248	Bassia laniflora (S.G.Gmel.) A.J.Scott	0	n	1	0	Amaranthaceae Juss.
Iz_6_1109	DJI_0248	Berberis vulgaris L.	0	n	1	0	Berberidaceae Juss.
Iz_6_1109	DJI_0248	Betula pendula Roth.	0	n	1	1	Betulaceae Gray
Iz_6_1109	DJI_0248	Betula pendula Roth.	0	n	1	0	Poaceae Barnhart
Iz_6_1109	DJI_0248	Bromus inermis Leyss.	0		NA	NA	
Iz_6_1109	DJI_0248	Bromus squarrosus L.	0	a	0	0	Poaceae Barnhart
Iz_6_1109	DJI_0248	Bromus tectorum L.	0	a	0	0	Poaceae Barnhart
Iz_6_1109	DJI_0248	Calamagrostis epigejos (L.) Roth	10	n	1	0	Poaceae Barnhart
Iz_6_1109	DJI_0248	Carduus acanthoides L.	0	n	1	0	Asteraceae Bercht. & J.Presl
Iz_6_1109	DJI_0248	Carex sp.	0	n	1	0	Cyperaceae Juss.
Iz_6_1109	DJI_0248	Centaurea majorovii Dumbadze	0	n	1	0	Asteraceae Bercht. & J.Presl
Iz_6_1109	DJI_0248	Chamaecytisus ruthenicus (Fisch.ex Wol.) Klaskova	0	n	1	0	Fabaceae Lindl.
Iz_6_1109	DJI_0248	Chelidonium majus L.	0	n	1	0	Papaveraceae Juss.
Iz_6_1109	DJI_0248	Chenopodium betaceum Andrz.	0	n	1	0	Amaranthaceae Juss.
Iz_6_1109	DJI_0248	Chenopodium cf. album	0	n	1	0	Amaranthaceae Juss.
Iz_6_1109	DJI_0248	Chenopodium sp.	0		NA	0	Amaranthaceae Juss.
Iz_6_1109	DJI_0248	Chondrilla juncea L.	0	n	1	0	Asteraceae Bercht. & J.Presl
Iz_6_1109	DJI_0248	Cirsium vulgare (Savi) Ten.	0	n	1	0	Asteraceae Bercht. & J.Presl
Iz_6_1109	DJI_0248	Clinopodium acinos (L.) Kuntze	0	n	1	0	Lamiacea Lindl.
Iz_6_1109	DJI_0248	Convallaria majalis L.	0	n	1	0	Asparagaceae Juss.
Iz_6_1109	DJI_0248	Crataegus rhipidophylla Gand.	0	n	1	1	Rosaceae Juss.
Iz_6_1109	DJI_0248	Crepis foetida L.	0	n	1	0	Asteraceae Bercht. & J.Presl
Iz_6_1109	DJI_0248	Dactylis glomerata L.	0	n	1	0	Poaceae Barnhart
Iz_6_1109	DJI_0248	Dianthus campestris M.Bieb.	0	n	1	0	Caryophyllaceae Juss.
Iz_6_1109	DJI_0248	Digitaria sanguinalis (L.) Scop.	0	a	0	0	Poaceae Barnhart
Iz_6_1109	DJI_0248	Elymus repens (L.) Gould	0	n	1	0	Poaceae Barnhart
Iz_6_1109	DJI_0248	Epilobium tetragonum L.	0	n	1	0	Onagraceae Juss.
Iz_6_1109	DJI_0248	Eragrostis minor Host	0	a	0	0	Poaceae Barnhart
Iz_6_1109	DJI_0248	Erigeron annuus (L.) Desf.	0	a	0	0	Asteraceae Bercht. & J.Presl
Iz_6_1109	DJI_0248	Erigeron canadensis L.	10	a	0	0	Asteraceae Bercht. & J.Presl
Iz_6_1109	DJI_0248	Euphorbia seguieriana Neck.	0	n	1	0	Euphorbiaceae Juss.
Iz_6_1109	DJI_0248	Fallopia dumetorum (L.) Holub	0	n	1	0	Polygonaceae Juss.
Iz_6_1109	DJI_0248	Festuca beckeri (Hack.) Trautv.	0	n	1	0	Poaceae Barnhart
Iz_6_1109	DJI_0248	Fragaria vesca L.	0	n	1	0	Rosaceae Juss.
Iz_6_1109	DJI_0248	Frangula alnus Mill.	0	n	1	1	Rhamnaceae Juss.
Iz_6_1109	DJI_0248	Fraxinus pennsylvanica Marshall	0	a	0	1	Oleaceae Hoffmanns. & Link
Iz_6_1109	DJI_0248	Galeopsis bifida Boenn.	0	n	1	0	Lamiacea Lindl.
Iz_6_1109	DJI_0248	Galium verum L.	0	n	1	0	Rubiaceae Juss.
Iz_6_1109	DJI_0248	Genista tinctoria L.	0	n	1	0	Fabaceae Lindl.
Iz_6_1109	DJI_0248	Geranium robertianum L.	0	n	1	0	Geraniaceae Juss.
Iz_6_1109	DJI_0248	Geranium sanquineum L.	0	n	1	0	Geraniaceae Juss.
Iz_6_1109	DJI_0248	Gypsophila paniculata L.	0	n	1	0	Caryophyllaceae Juss.
Iz_6_1109	DJI_0248	Helichrysum arenarium (L.) Moench	0	n	1	0	Asteraceae Bercht. & J.Presl
Iz_6_1109	DJI_0248	Herniaria polygama J.Gay	0	n	1	0	Caryophyllaceae Juss.
Iz_6_1109	DJI_0248	Hieracium robustum Fr.	0	n	1	0	Asteraceae Bercht. & J.Presl
Iz_6_1109	DJI_0248	Hieracium umbellatum L.	0	n	1	NA	Asteraceae Bercht. & J.Presl
Iz_6_1109	DJI_0248	Hylotelephium maximum (L.) Holub	0	n	1	0	Crassulaceae J.St.-Hil.
Iz_6_1109	DJI_0248	Jacobaea borysthenica (DC.) B.Nord. & Greuter	0	n	1	0	Asteraceae Bercht. & J.Presl
Iz_6_1109	DJI_0248	Jasione montana L.	0	n	1	0	Campanulaceae Juss.
Iz_6_1109	DJI_0248	Jurinea cyanoides (L.) Rchb.s.l.	0	n	1	0	Asteraceae Bercht. & J.Presl
Iz_6_1109	DJI_0248	Koeleria glauca (Spreng.) DC.	0	n	1	0	Poaceae Barnhart
Iz_6_1109	DJI_0248	Lactuca serriola L.	0	a	0	0	Asteraceae Bercht. & J.Presl
Iz_6_1109	DJI_0248	Linaria genistifolia (L.) Mill.	0	n	1	0	Plantaginaceae Juss.
Iz_6_1109	DJI_0248	Lomelosia argentea (L.) Greuter & Burdet	0	n	1	0	Caprifoliaceae Juss.
Iz_6_1109	DJI_0248	Melampyrum pratense L.	0	n	1	0	Orobanchaceae Vent.
Iz_6_1109	DJI_0248	Oenothera biennis L.	0	a	0	0	Onagraceae Juss.
Iz_6_1109	DJI_0248	Pentanema britannica (L.) D.Gut.Larr., Santos-Vicente, Anderb., E.Rico & M.M.Mart.Ort.	0	n	1	0	Asteraceae Bercht. & J.Presl
Iz_6_1109	DJI_0248	Peucedanum oreoselinum (L.) Moench	0	n	1	0	Apiaceae Lindl.
Iz_6_1109	DJI_0248	Peucedanum ruthenicum M.Bieb.	0	n	1	0	Apiaceae Lindl.
Iz_6_1109	DJI_0248	Pilosella echioides (Lumn.) F.W.Schultz & Sch.Bip.	0	n	1	0	Asteraceae Bercht. & J.Presl
Iz_6_1109	DJI_0248	Pilosella officinarum F. Schultz et Sch. Bip.	0	n	1	0	Asteraceae Bercht. & J.Presl
Iz_6_1109	DJI_0248	Pilosella sp.	0		NA	0	Asteraceae Bercht. & J.Presl
Iz_6_1109	DJI_0248	Pinus sylvestris L.	0	n	1	1	Pinaceae Spreng. ex F.Rudolphi
Iz_6_1109	DJI_0248	Poa annua L.	0	n	1	0	Poaceae Barnhart
Iz_6_1109	DJI_0248	Poa nemoralis L.	0	n	1	0	Poaceae Barnhart
Iz_6_1109	DJI_0248	Poa sp.	0		NA	0	Poaceae Barnhart
Iz_6_1109	DJI_0248	Polygonatum odoratum (Mill.) Druce	0	n	1	0	Asparagaceae Juss.
Iz_6_1109	DJI_0248	Polygonum arenarium Waldst. & Kit.	0	n	1	0	Polygonaceae Juss.
Iz_6_1109	DJI_0248	Populus nigra L.	0	n	1	1	Salicaceae Mirb.
Iz_6_1109	DJI_0248	Populus tremula L.	0	n	1	1	Salicaceae Mirb.
Iz_6_1109	DJI_0248	Populus Ã canescens (Aiton) Sm.	0	n	1	1	Salicaceae Mirb.
Iz_6_1109	DJI_0248	Potentilla argentea L.	0	n	1	0	Rosaceae Juss.
Iz_6_1109	DJI_0248	Psammophiliella muralis (L.) Ikonn.	0	n	1	0	Caryophyllaceae Juss.
Iz_6_1109	DJI_0248	Psephellus sumensis (Kalen.) Greuter	0	n	1	0	Asteraceae Bercht. & J.Presl

Iz_6_1109	DJI_0248	Pulsatilla pratensis (L.) Mill. s.l.	0	n	1	0	Ranunculaceae Juss.
Iz_6_1109	DJI_0248	Pyrus communis L.	0	a	0	1	Rosaceae Juss.
Iz_6_1109	DJI_0248	Quercus robur L.	0	n	1	1	Fagaceae Dumort.
Iz_6_1109	DJI_0248	Robinia pseudoacacia L.	0	a	0	1	Fabaceae Lindl.
Iz_6_1109	DJI_0248	Rosa sp.	0		NA	1	Rosaceae Juss.
Iz_6_1109	DJI_0248	Rubus idaeus L.	0	n	1	0	Rosaceae Juss.
Iz_6_1109	DJI_0248	Rumex acetosella L.	0	n	1	0	Polygonaceae Juss.
Iz_6_1109	DJI_0248	Rumex thyrsiflorus Fingerh.	0	n	1	0	Polygonaceae Juss.
Iz_6_1109	DJI_0248	Salix alba L.	0	n	1	1	Salicaceae Mirb.
Iz_6_1109	DJI_0248	Sambucus nigra L.	0	n	1	1	Viburnaceae Raf.
Iz_6_1109	DJI_0248	Secale sylvestre Host	0	n	1	0	Poaceae Barnhart
Iz_6_1109	DJI_0248	Sempervivum ruthenicum Schnittsp. & C.B. Lehm.	0	n	1	0	Crassulaceae J.St.-Hil.
Iz_6_1109	DJI_0248	Setaria viridis (L.) P. Beauv.	0	n	1	0	Poaceae Barnhart
Iz_6_1109	DJI_0248	Silene borysthenica (Gruner) Walters	0	n	1	0	Caryophyllaceae Juss.
Iz_6_1109	DJI_0248	Silene latifolia subsp. alba (Mill.) Greuter & Burdet	0	n	1	0	Caryophyllaceae Juss.
Iz_6_1109	DJI_0248	Solidago virgaurea L.	0	n	1	0	Asteraceae Bercht. & J.Presl
Iz_6_1109	DJI_0248	Sorbus aucuparia L.	0	n	1	1	Rosaceae Juss.
Iz_6_1109	DJI_0248	Species 11.09_6_0254	0		NA	0	
Iz_6_1109	DJI_0248	Stachys recta L.	0	n	1	0	Lamiacea Lindl.
Iz_6_1109	DJI_0248	Tanacetum vulgare L.	0	n	1	0	Asteraceae Bercht. & J.Presl
Iz_6_1109	DJI_0248	Thymus pallasianus Heinr.Braun	0	n	1	0	Lamiacea Lindl.
Iz_6_1109	DJI_0248	Tilia cordata Mill.	0	n	1	1	Malvaceae Juss.
Iz_6_1109	DJI_0248	Trifolium alpestre L.	0	n	1	0	Fabaceae Lindl.
Iz_6_1109	DJI_0248	Trifolium arvense L.	0	n	1	0	Fabaceae Lindl.
Iz_6_1109	DJI_0248	Ulmus glabra Huds.	0	n	1	1	Ulmaceae Mirb.
Iz_6_1109	DJI_0248	Verbascum densiflorum Bertol.	0	n	1	0	Scrophulariaceae Juss.
Iz_6_1109	DJI_0248	Veronica austriaca L.	0	n	1	0	Plantaginaceae Juss.
Iz_6_1109	DJI_0248	Veronica incana L.	0	n	1	0	Plantaginaceae Juss.
Iz_6_1109	DJI_0248	Veronica verna L.	0	n	1	0	Plantaginaceae Juss.
Iz_6_1109	DJI_0248	Vincetoxicum hirundinaria Medik	0	n	1	0	Apocynaceae Juss.
Iz_6_1109	DJI_0250	Acer tataricum L.	0	n	1	1	Sapindaceae Juss.
Iz_6_1109	DJI_0250	Achillea millefolium L. s.l.	0	n	1	0	Asteraceae Bercht. & J.Presl
Iz_6_1109	DJI_0250	Allium oleraceum L.	0	n	1	0	Amaryllidaceae J.St.-Hil.
Iz_6_1109	DJI_0250	Ambrosia artemisiifolia L.	0	a	0	0	Asteraceae Bercht. & J.Presl
Iz_6_1109	DJI_0250	Amorpha fruticosa L.	0	a	0	0	Fabaceae Lindl.
Iz_6_1109	DJI_0250	Anthericum ramosum L.	0	n	1	0	Asparagaceae Juss.
Iz_6_1109	DJI_0250	Artemisia marschalliana Spreng.	0	n	1	0	Asteraceae Bercht. & J.Presl
Iz_6_1109	DJI_0250	Asparagus officinalis L.	0	n	1	0	Asparagaceae Juss.
Iz_6_1109	DJI_0250	Astragalus cicer L.	0	n	1	0	Fabaceae Lindl.
Iz_6_1109	DJI_0250	Bassia laniflora (S.G.Gmel.) A.J.Scott	0	n	1	0	Amaranthaceae Juss.
Iz_6_1109	DJI_0250	Berberis vulgaris L.	0	n	1	0	Berberidaceae Juss.
Iz_6_1109	DJI_0250	Betula pendula Roth.	0	n	1	1	Betulaceae Gray
Iz_6_1109	DJI_0250	Betula pendula Roth.	0	n	1	0	Poaceae Barnhart
Iz_6_1109	DJI_0250	Bromus inermis Leyss.	0		NA	NA	
Iz_6_1109	DJI_0250	Bromus squarrosus L.	0	a	0	0	Poaceae Barnhart
Iz_6_1109	DJI_0250	Bromus tectorum L.	0	a	0	0	Poaceae Barnhart
Iz_6_1109	DJI_0250	Calamagrostis epigejos (L.) Roth	60	n	1	0	Poaceae Barnhart
Iz_6_1109	DJI_0250	Carduus acanthoides L.	0	n	1	0	Asteraceae Bercht. & J.Presl
Iz_6_1109	DJI_0250	Carex sp.	0	n	1	0	Cyperaceae Juss.
Iz_6_1109	DJI_0250	Centaurea majorovii Dumbadze	0	n	1	0	Asteraceae Bercht. & J.Presl
Iz_6_1109	DJI_0250	Chamaecytisus ruthenicus (Fisch.ex Wol.) Klaskova	0	n	1	0	Fabaceae Lindl.
Iz_6_1109	DJI_0250	Chelidonium majus L.	0	n	1	0	Papaveraceae Juss.
Iz_6_1109	DJI_0250	Chenopodium betaceum Andrz.	0	n	1	0	Amaranthaceae Juss.
Iz_6_1109	DJI_0250	Chenopodium cf. album	0	n	1	0	Amaranthaceae Juss.
Iz_6_1109	DJI_0250	Chenopodium sp.	0		NA	0	Amaranthaceae Juss.
Iz_6_1109	DJI_0250	Chondrilla juncea L.	0	n	1	0	Asteraceae Bercht. & J.Presl
Iz_6_1109	DJI_0250	Cirsium vulgare (Savi) Ten.	0	n	1	0	Asteraceae Bercht. & J.Presl
Iz_6_1109	DJI_0250	Clinopodium acinos (L.) Kuntze	0	n	1	0	Lamiacea Lindl.
Iz_6_1109	DJI_0250	Convallaria majalis L.	0	n	1	0	Asparagaceae Juss.
Iz_6_1109	DJI_0250	Crataegus rhipidophylla Gand.	0	n	1	1	Rosaceae Juss.
Iz_6_1109	DJI_0250	Crepis foetida L.	0	n	1	0	Asteraceae Bercht. & J.Presl
Iz_6_1109	DJI_0250	Dactylis glomerata L.	0	n	1	0	Poaceae Barnhart
Iz_6_1109	DJI_0250	Dianthus campestris M.Bieb.	0	n	1	0	Caryophyllaceae Juss.
Iz_6_1109	DJI_0250	Digitaria sanguinalis (L.) Scop.	0	a	0	0	Poaceae Barnhart
Iz_6_1109	DJI_0250	Elymus repens (L.) Gould	0	n	1	0	Poaceae Barnhart
Iz_6_1109	DJI_0250	Epilobium tetragonum L.	0	n	1	0	Onagraceae Juss.
Iz_6_1109	DJI_0250	Eragrostis minor Host	0	a	0	0	Poaceae Barnhart
Iz_6_1109	DJI_0250	Erigeron annuus (L.) Desf.	0	a	0	0	Asteraceae Bercht. & J.Presl
Iz_6_1109	DJI_0250	Erigeron canadensis L.	5	a	0	0	Asteraceae Bercht. & J.Presl
Iz_6_1109	DJI_0250	Euphorbia seguieriana Neck.	0	n	1	0	Euphorbiaceae Juss.
Iz_6_1109	DJI_0250	Fallopia dumetorum (L.) Holub	0	n	1	0	Polygonaceae Juss.
Iz_6_1109	DJI_0250	Festuca beckeri (Hack.) Trautv.	0	n	1	0	Poaceae Barnhart
Iz_6_1109	DJI_0250	Fragaria vesca L.	0	n	1	0	Rosaceae Juss.
Iz_6_1109	DJI_0250	Frangula alnus Mill.	0	n	1	1	Rhamnaceae Juss.
Iz_6_1109	DJI_0250	Fraxinus pennsylvanica Marshall	0	a	0	1	Oleaceae Hoffmanns. & Link
Iz_6_1109	DJI_0250	Galeopsis bifida Boenn.	0	n	1	0	Lamiacea Lindl.
Iz_6_1109	DJI_0250	Galium verum L.	0	n	1	0	Rubiaceae Juss.
Iz_6_1109	DJI_0250	Genista tinctoria L.	0	n	1	0	Fabaceae Lindl.
Iz_6_1109	DJI_0250	Geranium robertianum L.	0	n	1	0	Geraniaceae Juss.
Iz_6_1109	DJI_0250	Geranium sanquineum L.	0	n	1	0	Geraniaceae Juss.
Iz_6_1109	DJI_0250	Gypsophila paniculata L.	0	n	1	0	Caryophyllaceae Juss.
Iz_6_1109	DJI_0250	Helichrysum arenarium (L.) Moench	0	n	1	0	Asteraceae Bercht. & J.Presl
Iz_6_1109	DJI_0250	Herniaria polygama J.Gay	0	n	1	0	Caryophyllaceae Juss.
Iz_6_1109	DJI_0250	Hieracium robustum Fr.	0	n	1	0	Asteraceae Bercht. & J.Presl
Iz_6_1109	DJI_0250	Hieracium umbellatum L.	0	n	1	NA	Asteraceae Bercht. & J.Presl
Iz_6_1109	DJI_0250	Hylotelephium maximum (L.) Holub	0	n	1	0	Crassulaceae J.St.-Hil.
Iz_6_1109	DJI_0250	Jacobaea borysthenica (DC.) B.Nord. & Greuter	0	n	1	0	Asteraceae Bercht. & J.Presl
Iz_6_1109	DJI_0250	Jasione montana L.	0	n	1	0	Campanulaceae Juss.
Iz_6_1109	DJI_0250	Jurinea cyanoides (L.) Rchb.s.l.	0	n	1	0	Asteraceae Bercht. & J.Presl
Iz_6_1109	DJI_0250	Koeleria glauca (Spreng.) DC.	0	n	1	0	Poaceae Barnhart
Iz_6_1109	DJI_0250	Lactuca serriola L.	0	a	0	0	Asteraceae Bercht. & J.Presl
Iz_6_1109	DJI_0250	Linaria genistifolia (L.) Mill.	0	n	1	0	Plantaginaceae Juss.
Iz_6_1109	DJI_0250	Lomelosia argentea (L.) Greuter & Burdet	0	n	1	0	Caprifoliaceae Juss.
Iz_6_1109	DJI_0250	Melampyrum pratense L.	0	n	1	0	Orobanchaceae Vent.
Iz_6_1109	DJI_0250	Oenothera biennis L.	0	a	0	0	Onagraceae Juss.
Iz_6_1109	DJI_0250	Pentanema britannica (L.) D.Gut.Larr., Santos-Vicente, Anderb., E.Rico & M.M.Mart.Ort.	0	n	1	0	Asteraceae Bercht. & J.Presl
Iz_6_1109	DJI_0250	Peucedanum oreoselinum (L.) Moench	0	n	1	0	Apiaceae Lindl.
Iz_6_1109	DJI_0250	Peucedanum ruthenicum M.Bieb.	0	n	1	0	Apiaceae Lindl.

Iz_6_1109	DJI_0250	Pilosella echioides (Lumn.) F.W.Schultz & Sch.Bip.	0	n	1	0	Asteraceae Bercht. & J.Presl
Iz_6_1109	DJI_0250	Pilosella officinarum F. Schultz et Sch. Bip.	0	n	1	0	Asteraceae Bercht. & J.Presl
Iz_6_1109	DJI_0250	Pilosella sp.	0		NA	0	Asteraceae Bercht. & J.Presl
Iz_6_1109	DJI_0250	Pinus sylvestris L.	0	n	1	1	Pinaceae Spreng. ex F.Rudolphi
Iz_6_1109	DJI_0250	Poa annua L.	0	n	1	0	Poaceae Barnhart
Iz_6_1109	DJI_0250	Poa nemoralis L.	0	n	1	0	Poaceae Barnhart
Iz_6_1109	DJI_0250	Poa sp.	0		NA	0	Poaceae Barnhart
Iz_6_1109	DJI_0250	Polygonatum odoratum (Mill.) Druce	0	n	1	0	Asparagaceae Juss.
Iz_6_1109	DJI_0250	Polygonum arenarium Waldst. & Kit.	0	n	1	0	Polygonaceae Juss.
Iz_6_1109	DJI_0250	Populus nigra L.	0	n	1	1	Salicaceae Mirb.
Iz_6_1109	DJI_0250	Populus tremula L.	0	n	1	1	Salicaceae Mirb.
Iz_6_1109	DJI_0250	Populus Ã canescens (Aiton) Sm.	0	n	1	1	Salicaceae Mirb.
Iz_6_1109	DJI_0250	Potentilla argentea L.	0	n	1	0	Rosaceae Juss.
Iz_6_1109	DJI_0250	Psammophiliella muralis (L.) Ikonn.	0	n	1	0	Caryophyllaceae Juss.
Iz_6_1109	DJI_0250	Psephellus sumensis (Kalen.) Greuter	0	n	1	0	Asteraceae Bercht. & J.Presl
Iz_6_1109	DJI_0250	Pulsatilla pratensis (L.) Mill. s.l.	0	n	1	0	Ranunculaceae Juss.
Iz_6_1109	DJI_0250	Pyrus communis L.	0	a	0	1	Rosaceae Juss.
Iz_6_1109	DJI_0250	Quercus robur L.	0	n	1	1	Fagaceae Dumort.
Iz_6_1109	DJI_0250	Robinia pseudoacacia L.	0	a	0	1	Fabaceae Lindl.
Iz_6_1109	DJI_0250	Rosa sp.	0		NA	1	Rosaceae Juss.
Iz_6_1109	DJI_0250	Rubus idaeus L.	0	n	1	0	Rosaceae Juss.
Iz_6_1109	DJI_0250	Rumex acetosella L.	0	n	1	0	Polygonaceae Juss.
Iz_6_1109	DJI_0250	Rumex thyrsiflorus Fingerh.	0	n	1	0	Polygonaceae Juss.
Iz_6_1109	DJI_0250	Salix alba L.	0	n	1	1	Salicaceae Mirb.
Iz_6_1109	DJI_0250	Sambucus nigra L.	0	n	1	1	Viburnaceae Raf.
Iz_6_1109	DJI_0250	Secale sylvestre Host	0	n	1	0	Poaceae Barnhart
Iz_6_1109	DJI_0250	Sempervivum ruthenicum Schnittsp. & C.B. Lehm.	0	n	1	0	Crassulaceae J.St.-Hil.
Iz_6_1109	DJI_0250	Setaria viridis (L.) P. Beauv.	0	n	1	0	Poaceae Barnhart
Iz_6_1109	DJI_0250	Silene borysthenica (Gruner) Walters	0	n	1	0	Caryophyllaceae Juss.
Iz_6_1109	DJI_0250	Silene latifolia subsp. alba (Mill.) Greuter & Burdet	0	n	1	0	Caryophyllaceae Juss.
Iz_6_1109	DJI_0250	Solidago virgaurea L.	0	n	1	0	Asteraceae Bercht. & J.Presl
Iz_6_1109	DJI_0250	Sorbus aucuparia L.	0	n	1	1	Rosaceae Juss.
Iz_6_1109	DJI_0250	Species 11.09_6_0254	0		NA	0	
Iz_6_1109	DJI_0250	Stachys recta L.	0	n	1	0	Lamiacea Lindl.
Iz_6_1109	DJI_0250	Tanacetum vulgare L.	0	n	1	0	Asteraceae Bercht. & J.Presl
Iz_6_1109	DJI_0250	Thymus pallasianus Heinr.Braun	0	n	1	0	Lamiacea Lindl.
Iz_6_1109	DJI_0250	Tilia cordata Mill.	0	n	1	1	Malvaceae Juss.
Iz_6_1109	DJI_0250	Trifolium alpestre L.	0	n	1	0	Fabaceae Lindl.
Iz_6_1109	DJI_0250	Trifolium arvense L.	0	n	1	0	Fabaceae Lindl.
Iz_6_1109	DJI_0250	Ulmus glabra Huds.	0	n	1	1	Ulmaceae Mirb.
Iz_6_1109	DJI_0250	Verbascum densiflorum Bertol.	0	n	1	0	Scrophulariaceae Juss.
Iz_6_1109	DJI_0250	Veronica austriaca L.	0	n	1	0	Plantaginaceae Juss.
Iz_6_1109	DJI_0250	Veronica incana L.	0	n	1	0	Plantaginaceae Juss.
Iz_6_1109	DJI_0250	Veronica verna L.	0	n	1	0	Plantaginaceae Juss.
Iz_6_1109	DJI_0250	Vincetoxicum hirundinaria Medik	0	n	1	0	Apocynaceae Juss.
Iz_6_1109	DJI_0252	Acer tataricum L.	0	n	1	1	Sapindaceae Juss.
Iz_6_1109	DJI_0252	Achillea millefolium L. s.l.	0	n	1	0	Asteraceae Bercht. & J.Presl
Iz_6_1109	DJI_0252	Allium oleraceum L.	0	n	1	0	Amaryllidaceae J.St.-Hil.
Iz_6_1109	DJI_0252	Ambrosia artemisiifolia L.	0	a	0	0	Asteraceae Bercht. & J.Presl
Iz_6_1109	DJI_0252	Amorpha fruticosa L.	0	a	0	0	Fabaceae Lindl.
Iz_6_1109	DJI_0252	Anthericum ramosum L.	0	n	1	0	Asparagaceae Juss.
Iz_6_1109	DJI_0252	Artemisia marschalliana Spreng.	0	n	1	0	Asteraceae Bercht. & J.Presl
Iz_6_1109	DJI_0252	Asparagus officinalis L.	0	n	1	0	Asparagaceae Juss.
Iz_6_1109	DJI_0252	Astragalus cicer L.	0	n	1	0	Fabaceae Lindl.
Iz_6_1109	DJI_0252	Bassia laniflora (S.G.Gmel.) A.J.Scott	0	n	1	0	Amaranthaceae Juss.
Iz_6_1109	DJI_0252	Berberis vulgaris L.	0	n	1	0	Berberidaceae Juss.
Iz_6_1109	DJI_0252	Betula pendula Roth.	0	n	1	1	Betulaceae Gray
Iz_6_1109	DJI_0252	Betula pendula Roth.	0	n	1	0	Poaceae Barnhart
Iz_6_1109	DJI_0252	Bromus inermis Leyss.	0		NA	NA	
Iz_6_1109	DJI_0252	Bromus squarrosus L.	0	a	0	0	Poaceae Barnhart
Iz_6_1109	DJI_0252	Bromus tectorum L.	0	a	0	0	Poaceae Barnhart
Iz_6_1109	DJI_0252	Calamagrostis epigejos (L.) Roth	15	n	1	0	Poaceae Barnhart
Iz_6_1109	DJI_0252	Carduus acanthoides L.	0	n	1	0	Asteraceae Bercht. & J.Presl
Iz_6_1109	DJI_0252	Carex sp.	1	n	1	0	Cyperaceae Juss.
Iz_6_1109	DJI_0252	Centaurea majorovii Dumbadze	0	n	1	0	Asteraceae Bercht. & J.Presl
Iz_6_1109	DJI_0252	Chamaecytisus ruthenicus (Fisch.ex Wol.) Klaskova	0	n	1	0	Fabaceae Lindl.
Iz_6_1109	DJI_0252	Chelidonium majus L.	0	n	1	0	Papaveraceae Juss.
Iz_6_1109	DJI_0252	Chenopodium betaceum Andrz.	0	n	1	0	Amaranthaceae Juss.
Iz_6_1109	DJI_0252	Chenopodium cf. album	0	n	1	0	Amaranthaceae Juss.
Iz_6_1109	DJI_0252	Chenopodium sp.	0		NA	0	Amaranthaceae Juss.
Iz_6_1109	DJI_0252	Chondrilla juncea L.	0	n	1	0	Asteraceae Bercht. & J.Presl
Iz_6_1109	DJI_0252	Cirsium vulgare (Savi) Ten.	0	n	1	0	Asteraceae Bercht. & J.Presl
Iz_6_1109	DJI_0252	Clinopodium acinos (L.) Kuntze	0	n	1	0	Lamiacea Lindl.
Iz_6_1109	DJI_0252	Convallaria majalis L.	0	n	1	0	Asparagaceae Juss.
Iz_6_1109	DJI_0252	Crataegus rhipidophylla Gand.	0	n	1	1	Rosaceae Juss.
Iz_6_1109	DJI_0252	Crepis foetida L.	0	n	1	0	Asteraceae Bercht. & J.Presl
Iz_6_1109	DJI_0252	Dactylis glomerata L.	0	n	1	0	Poaceae Barnhart
Iz_6_1109	DJI_0252	Dianthus campestris M.Bieb.	0	n	1	0	Caryophyllaceae Juss.
Iz_6_1109	DJI_0252	Digitaria sanguinalis (L.) Scop.	0	a	0	0	Poaceae Barnhart
Iz_6_1109	DJI_0252	Elymus repens (L.) Gould	0	n	1	0	Poaceae Barnhart
Iz_6_1109	DJI_0252	Epilobium tetragonum L.	0	n	1	0	Onagraceae Juss.
Iz_6_1109	DJI_0252	Eragrostis minor Host	0	a	0	0	Poaceae Barnhart
Iz_6_1109	DJI_0252	Erigeron annuus (L.) Desf.	0	a	0	0	Asteraceae Bercht. & J.Presl
Iz_6_1109	DJI_0252	Erigeron canadensis L.	10	a	0	0	Asteraceae Bercht. & J.Presl
Iz_6_1109	DJI_0252	Euphorbia seguieriana Neck.	0	n	1	0	Euphorbiaceae Juss.
Iz_6_1109	DJI_0252	Fallopia dumetorum (L.) Holub	0	n	1	0	Polygonaceae Juss.
Iz_6_1109	DJI_0252	Festuca beckeri (Hack.) Trautv.	0	n	1	0	Poaceae Barnhart
Iz_6_1109	DJI_0252	Fragaria vesca L.	0	n	1	0	Rosaceae Juss.
Iz_6_1109	DJI_0252	Frangula alnus Mill.	0	n	1	1	Rhamnaceae Juss.
Iz_6_1109	DJI_0252	Fraxinus pennsylvanica Marshall	0	a	0	1	Oleaceae Hoffmanns. & Link
Iz_6_1109	DJI_0252	Galeopsis bifida Boenn.	0	n	1	0	Lamiacea Lindl.
Iz_6_1109	DJI_0252	Galium verum L.	0	n	1	0	Rubiaceae Juss.
Iz_6_1109	DJI_0252	Genista tinctoria L.	0	n	1	0	Fabaceae Lindl.
Iz_6_1109	DJI_0252	Geranium robertianum L.	0	n	1	0	Geraniaceae Juss.
Iz_6_1109	DJI_0252	Geranium sanquineum L.	0	n	1	0	Geraniaceae Juss.
Iz_6_1109	DJI_0252	Gypsophila paniculata L.	0	n	1	0	Caryophyllaceae Juss.
Iz_6_1109	DJI_0252	Helichrysum arenarium (L.) Moench	0	n	1	0	Asteraceae Bercht. & J.Presl
Iz_6_1109	DJI_0252	Herniaria polygama J.Gay	0	n	1	0	Caryophyllaceae Juss.

Iz_6_1109	DJI_0252	Hieracium robustum Fr.	0	n	1	0	Asteraceae Bercht. & J.Presl
Iz_6_1109	DJI_0252	Hieracium umbellatum L.	0	n	1	NA	Asteraceae Bercht. & J.Presl
Iz_6_1109	DJI_0252	Hylotelephium maximum (L.) Holub	0	n	1	0	Crassulaceae J.St.-Hil.
Iz_6_1109	DJI_0252	Jacobaea borysthenica (DC.) B.Nord. & Greuter	0	n	1	0	Asteraceae Bercht. & J.Presl
Iz_6_1109	DJI_0252	Jasione montana L.	0	n	1	0	Campanulaceae Juss.
Iz_6_1109	DJI_0252	Jurinea cyanoides (L.) Rchb.s.l.	0	n	1	0	Asteraceae Bercht. & J.Presl
Iz_6_1109	DJI_0252	Koeleria glauca (Spreng.) DC.	0	n	1	0	Poaceae Barnhart
Iz_6_1109	DJI_0252	Lactuca serriola L.	0	a	0	0	Asteraceae Bercht. & J.Presl
Iz_6_1109	DJI_0252	Linaria genistifolia (L.) Mill.	0	n	1	0	Plantaginaceae Juss.
Iz_6_1109	DJI_0252	Lomelosia argentea (L.) Greuter & Burdet	0	n	1	0	Caprifoliaceae Juss.
Iz_6_1109	DJI_0252	Melampyrum pratense L.	0	n	1	0	Orobanchaceae Vent.
Iz_6_1109	DJI_0252	Oenothera biennis L.	0	a	0	0	Onagraceae Juss.
Iz_6_1109	DJI_0252	Pentanema britannica (L.) D.Gut.Larr., Santos-Vicente, Anderb., E.Rico & M.M.Mart.Ort.	0	n	1	0	Asteraceae Bercht. & J.Presl
Iz_6_1109	DJI_0252	Peucedanum oreoselinum (L.) Moench	0	n	1	0	Apiaceae Lindl.
Iz_6_1109	DJI_0252	Peucedanum ruthenicum M.Bieb.	0	n	1	0	Apiaceae Lindl.
Iz_6_1109	DJI_0252	Pilosella echioides (Lumn.) F.W.Schultz & Sch.Bip.	0	n	1	0	Asteraceae Bercht. & J.Presl
Iz_6_1109	DJI_0252	Pilosella officinarum F. Schultz et Sch. Bip.	0	n	1	0	Asteraceae Bercht. & J.Presl
Iz_6_1109	DJI_0252	Pilosella sp.	0		NA	0	Asteraceae Bercht. & J.Presl
Iz_6_1109	DJI_0252	Pinus sylvestris L.	0	n	1	1	Pinaceae Spreng. ex F.Rudolphi
Iz_6_1109	DJI_0252	Poa annua L.	0	n	1	0	Poaceae Barnhart
Iz_6_1109	DJI_0252	Poa nemoralis L.	0	n	1	0	Poaceae Barnhart
Iz_6_1109	DJI_0252	Poa sp.	0		NA	0	Poaceae Barnhart
Iz_6_1109	DJI_0252	Polygonatum odoratum (Mill.) Druce	0	n	1	0	Asparagaceae Juss.
Iz_6_1109	DJI_0252	Polygonum arenarium Waldst. & Kit.	0	n	1	0	Polygonaceae Juss.
Iz_6_1109	DJI_0252	Populus nigra L.	0	n	1	1	Salicaceae Mirb.
Iz_6_1109	DJI_0252	Populus tremula L.	0	n	1	1	Salicaceae Mirb.
Iz_6_1109	DJI_0252	Populus Ã canescens (Aiton) Sm.	0	n	1	1	Salicaceae Mirb.
Iz_6_1109	DJI_0252	Potentilla argentea L.	0	n	1	0	Rosaceae Juss.
Iz_6_1109	DJI_0252	Psammophiliella muralis (L.) Ikonn.	0	n	1	0	Caryophyllaceae Juss.
Iz_6_1109	DJI_0252	Psephellus sumensis (Kalen.) Greuter	0	n	1	0	Asteraceae Bercht. & J.Presl
Iz_6_1109	DJI_0252	Pulsatilla pratensis (L.) Mill. s.l.	0	n	1	0	Ranunculaceae Juss.
Iz_6_1109	DJI_0252	Pyrus communis L.	0	a	0	1	Rosaceae Juss.
Iz_6_1109	DJI_0252	Quercus robur L.	0	n	1	1	Fagaceae Dumort.
Iz_6_1109	DJI_0252	Robinia pseudoacacia L.	0	a	0	1	Fabaceae Lindl.
Iz_6_1109	DJI_0252	Rosa sp.	0		NA	1	Rosaceae Juss.
Iz_6_1109	DJI_0252	Rubus idaeus L.	0	n	1	0	Rosaceae Juss.
Iz_6_1109	DJI_0252	Rumex acetosella L.	0	n	1	0	Polygonaceae Juss.
Iz_6_1109	DJI_0252	Rumex thyrsiflorus Fingerh.	0	n	1	0	Polygonaceae Juss.
Iz_6_1109	DJI_0252	Salix alba L.	0	n	1	1	Salicaceae Mirb.
Iz_6_1109	DJI_0252	Sambucus nigra L.	0	n	1	1	Viburnaceae Raf.
Iz_6_1109	DJI_0252	Secale sylvestre Host	0	n	1	0	Poaceae Barnhart
Iz_6_1109	DJI_0252	Sempervivum ruthenicum Schnittsp. & C.B. Lehm.	0	n	1	0	Crassulaceae J.St.-Hil.
Iz_6_1109	DJI_0252	Setaria viridis (L.) P. Beauv.	0	n	1	0	Poaceae Barnhart
Iz_6_1109	DJI_0252	Silene borysthenica (Gruner) Walters	0	n	1	0	Caryophyllaceae Juss.
Iz_6_1109	DJI_0252	Silene latifolia subsp. alba (Mill.) Greuter & Burdet	0	n	1	0	Caryophyllaceae Juss.
Iz_6_1109	DJI_0252	Solidago virgaurea L.	0	n	1	0	Asteraceae Bercht. & J.Presl
Iz_6_1109	DJI_0252	Sorbus aucuparia L.	0	n	1	1	Rosaceae Juss.
Iz_6_1109	DJI_0252	Species 11.09_6_0254	0		NA	0	
Iz_6_1109	DJI_0252	Stachys recta L.	0	n	1	0	Lamiacea Lindl.
Iz_6_1109	DJI_0252	Tanacetum vulgare L.	0	n	1	0	Asteraceae Bercht. & J.Presl
Iz_6_1109	DJI_0252	Thymus pallasianus Heinr.Braun	0	n	1	0	Lamiacea Lindl.
Iz_6_1109	DJI_0252	Tilia cordata Mill.	0	n	1	1	Malvaceae Juss.
Iz_6_1109	DJI_0252	Trifolium alpestre L.	0	n	1	0	Fabaceae Lindl.
Iz_6_1109	DJI_0252	Trifolium arvense L.	0	n	1	0	Fabaceae Lindl.
Iz_6_1109	DJI_0252	Ulmus glabra Huds.	0	n	1	1	Ulmaceae Mirb.
Iz_6_1109	DJI_0252	Verbascum densiflorum Bertol.	0	n	1	0	Scrophulariaceae Juss.
Iz_6_1109	DJI_0252	Veronica austriaca L.	0	n	1	0	Plantaginaceae Juss.
Iz_6_1109	DJI_0252	Veronica incana L.	0	n	1	0	Plantaginaceae Juss.
Iz_6_1109	DJI_0252	Veronica verna L.	0	n	1	0	Plantaginaceae Juss.
Iz_6_1109	DJI_0252	Vincetoxicum hirundinaria Medik	0	n	1	0	Apocynaceae Juss.
Iz_6_1109	DJI_0254	Acer tataricum L.	0	n	1	1	Sapindaceae Juss.
Iz_6_1109	DJI_0254	Achillea millefolium L. s.l.	0	n	1	0	Asteraceae Bercht. & J.Presl
Iz_6_1109	DJI_0254	Allium oleraceum L.	0	n	1	0	Amaryllidaceae J.St.-Hil.
Iz_6_1109	DJI_0254	Ambrosia artemisiifolia L.	0	a	0	0	Asteraceae Bercht. & J.Presl
Iz_6_1109	DJI_0254	Amorpha fruticosa L.	0	a	0	0	Fabaceae Lindl.
Iz_6_1109	DJI_0254	Anthericum ramosum L.	0	n	1	0	Asparagaceae Juss.
Iz_6_1109	DJI_0254	Artemisia marschalliana Spreng.	0	n	1	0	Asteraceae Bercht. & J.Presl
Iz_6_1109	DJI_0254	Asparagus officinalis L.	0	n	1	0	Asparagaceae Juss.
Iz_6_1109	DJI_0254	Astragalus cicer L.	0	n	1	0	Fabaceae Lindl.
Iz_6_1109	DJI_0254	Bassia laniflora (S.G.Gmel.) A.J.Scott	0	n	1	0	Amaranthaceae Juss.
Iz_6_1109	DJI_0254	Berberis vulgaris L.	0	n	1	0	Berberidaceae Juss.
Iz_6_1109	DJI_0254	Betula pendula Roth.	0	n	1	1	Betulaceae Gray
Iz_6_1109	DJI_0254	Betula pendula Roth.	0	n	1	0	Poaceae Barnhart
Iz_6_1109	DJI_0254	Bromus inermis Leyss.	0		NA	NA	
Iz_6_1109	DJI_0254	Bromus squarrosus L.	0	a	0	0	Poaceae Barnhart
Iz_6_1109	DJI_0254	Bromus tectorum L.	0	a	0	0	Poaceae Barnhart
Iz_6_1109	DJI_0254	Calamagrostis epigejos (L.) Roth	75	n	1	0	Poaceae Barnhart
Iz_6_1109	DJI_0254	Carduus acanthoides L.	0	n	1	0	Asteraceae Bercht. & J.Presl
Iz_6_1109	DJI_0254	Carex sp.	5	n	1	0	Cyperaceae Juss.
Iz_6_1109	DJI_0254	Centaurea majorovii Dumbadze	0	n	1	0	Asteraceae Bercht. & J.Presl
Iz_6_1109	DJI_0254	Chamaecytisus ruthenicus (Fisch.ex Wol.) Klaskova	0	n	1	0	Fabaceae Lindl.
Iz_6_1109	DJI_0254	Chelidonium majus L.	0	n	1	0	Papaveraceae Juss.
Iz_6_1109	DJI_0254	Chenopodium betaceum Andrz.	0	n	1	0	Amaranthaceae Juss.
Iz_6_1109	DJI_0254	Chenopodium cf. album	0	n	1	0	Amaranthaceae Juss.
Iz_6_1109	DJI_0254	Chenopodium sp.	0		NA	0	Amaranthaceae Juss.
Iz_6_1109	DJI_0254	Chondrilla juncea L.	0	n	1	0	Asteraceae Bercht. & J.Presl
Iz_6_1109	DJI_0254	Cirsium vulgare (Savi) Ten.	0	n	1	0	Asteraceae Bercht. & J.Presl
Iz_6_1109	DJI_0254	Clinopodium acinos (L.) Kuntze	0	n	1	0	Lamiacea Lindl.
Iz_6_1109	DJI_0254	Convallaria majalis L.	0	n	1	0	Asparagaceae Juss.
Iz_6_1109	DJI_0254	Crataegus rhipidophylla Gand.	0	n	1	1	Rosaceae Juss.
Iz_6_1109	DJI_0254	Crepis foetida L.	0	n	1	0	Asteraceae Bercht. & J.Presl
Iz_6_1109	DJI_0254	Dactylis glomerata L.	0	n	1	0	Poaceae Barnhart
Iz_6_1109	DJI_0254	Dianthus campestris M.Bieb.	0	n	1	0	Caryophyllaceae Juss.
Iz_6_1109	DJI_0254	Digitaria sanguinalis (L.) Scop.	0	a	0	0	Poaceae Barnhart
Iz_6_1109	DJI_0254	Elymus repens (L.) Gould	0	n	1	0	Poaceae Barnhart
Iz_6_1109	DJI_0254	Epilobium tetragonum L.	0	n	1	0	Onagraceae Juss.
Iz_6_1109	DJI_0254	Eragrostis minor Host	0	a	0	0	Poaceae Barnhart
Iz_6_1109	DJI_0254	Erigeron annuus (L.) Desf.	0	a	0	0	Asteraceae Bercht. & J.Presl

Iz_6_1109	DJI_0254	Erigeron canadensis L.	0	a	0	0	Asteraceae Bercht. & J.Presl
Iz_6_1109	DJI_0254	Euphorbia seguieriana Neck.	0	n	1	0	Euphorbiaceae Juss.
Iz_6_1109	DJI_0254	Fallopia dumetorum (L.) Holub	0	n	1	0	Polygonaceae Juss.
Iz_6_1109	DJI_0254	Festuca beckeri (Hack.) Trautv.	0	n	1	0	Poaceae Barnhart
Iz_6_1109	DJI_0254	Fragaria vesca L.	0	n	1	0	Rosaceae Juss.
Iz_6_1109	DJI_0254	Frangula alnus Mill.	0	n	1	1	Rhamnaceae Juss.
Iz_6_1109	DJI_0254	Fraxinus pennsylvanica Marshall	0	a	0	1	Oleaceae Hoffmanns. & Link
Iz_6_1109	DJI_0254	Galeopsis bifida Boenn.	0	n	1	0	Lamiacea Lindl.
Iz_6_1109	DJI_0254	Galium verum L.	0	n	1	0	Rubiaceae Juss.
Iz_6_1109	DJI_0254	Genista tinctoria L.	0	n	1	0	Fabaceae Lindl.
Iz_6_1109	DJI_0254	Geranium robertianum L.	0	n	1	0	Geraniaceae Juss.
Iz_6_1109	DJI_0254	Geranium sanquineum L.	0	n	1	0	Geraniaceae Juss.
Iz_6_1109	DJI_0254	Gypsophila paniculata L.	0	n	1	0	Caryophyllaceae Juss.
Iz_6_1109	DJI_0254	Helichrysum arenarium (L.) Moench	0	n	1	0	Asteraceae Bercht. & J.Presl
Iz_6_1109	DJI_0254	Herniaria polygama J.Gay	0	n	1	0	Caryophyllaceae Juss.
Iz_6_1109	DJI_0254	Hieracium robustum Fr.	0	n	1	0	Asteraceae Bercht. & J.Presl
Iz_6_1109	DJI_0254	Hieracium umbellatum L.	0	n	1	NA	Asteraceae Bercht. & J.Presl
Iz_6_1109	DJI_0254	Hylotelephium maximum (L.) Holub	0	n	1	0	Crassulaceae J.St.-Hil.
Iz_6_1109	DJI_0254	Jacobaea borysthenica (DC.) B.Nord. & Greuter	0	n	1	0	Asteraceae Bercht. & J.Presl
Iz_6_1109	DJI_0254	Jasione montana L.	0	n	1	0	Campanulaceae Juss.
Iz_6_1109	DJI_0254	Jurinea cyanoides (L.) Rchb.s.l.	0	n	1	0	Asteraceae Bercht. & J.Presl
Iz_6_1109	DJI_0254	Koeleria glauca (Spreng.) DC.	0	n	1	0	Poaceae Barnhart
Iz_6_1109	DJI_0254	Lactuca serriola L.	0	a	0	0	Asteraceae Bercht. & J.Presl
Iz_6_1109	DJI_0254	Linaria genistifolia (L.) Mill.	0	n	1	0	Plantaginaceae Juss.
Iz_6_1109	DJI_0254	Lomelosia argentea (L.) Greuter & Burdet	0	n	1	0	Caprifoliaceae Juss.
Iz_6_1109	DJI_0254	Melampyrum pratense L.	0	n	1	0	Orobanchaceae Vent.
Iz_6_1109	DJI_0254	Oenothera biennis L.	0	a	0	0	Onagraceae Juss.
Iz_6_1109	DJI_0254	Pentanema britannica (L.) D.Gut.Larr., Santos-Vicente, Anderb., E.Rico & M.M.Mart.Ort.	0	n	1	0	Asteraceae Bercht. & J.Presl
Iz_6_1109	DJI_0254	Peucedanum oreoselinum (L.) Moench	0	n	1	0	Apiaceae Lindl.
Iz_6_1109	DJI_0254	Peucedanum ruthenicum M.Bieb.	0	n	1	0	Apiaceae Lindl.
Iz_6_1109	DJI_0254	Pilosella echioides (Lumn.) F.W.Schultz & Sch.Bip.	0	n	1	0	Asteraceae Bercht. & J.Presl
Iz_6_1109	DJI_0254	Pilosella officinarum F. Schultz et Sch. Bip.	0	n	1	0	Asteraceae Bercht. & J.Presl
Iz_6_1109	DJI_0254	Pilosella sp.	0		NA	0	Asteraceae Bercht. & J.Presl
Iz_6_1109	DJI_0254	Pinus sylvestris L.	0	n	1	1	Pinaceae Spreng. ex F.Rudolphi
Iz_6_1109	DJI_0254	Poa annua L.	0	n	1	0	Poaceae Barnhart
Iz_6_1109	DJI_0254	Poa nemoralis L.	0	n	1	0	Poaceae Barnhart
Iz_6_1109	DJI_0254	Poa sp.	0		NA	0	Poaceae Barnhart
Iz_6_1109	DJI_0254	Polygonatum odoratum (Mill.) Druce	0	n	1	0	Asparagaceae Juss.
Iz_6_1109	DJI_0254	Polygonum arenarium Waldst. & Kit.	0	n	1	0	Polygonaceae Juss.
Iz_6_1109	DJI_0254	Populus nigra L.	0	n	1	1	Salicaceae Mirb.
Iz_6_1109	DJI_0254	Populus tremula L.	0	n	1	1	Salicaceae Mirb.
Iz_6_1109	DJI_0254	Populus Ã canescens (Aiton) Sm.	0	n	1	1	Salicaceae Mirb.
Iz_6_1109	DJI_0254	Potentilla argentea L.	0	n	1	0	Rosaceae Juss.
Iz_6_1109	DJI_0254	Psammophiliella muralis (L.) Ikonn.	0	n	1	0	Caryophyllaceae Juss.
Iz_6_1109	DJI_0254	Psephellus sumensis (Kalen.) Greuter	0	n	1	0	Asteraceae Bercht. & J.Presl
Iz_6_1109	DJI_0254	Pulsatilla pratensis (L.) Mill. s.l.	0	n	1	0	Ranunculaceae Juss.
Iz_6_1109	DJI_0254	Pyrus communis L.	0	a	0	1	Rosaceae Juss.
Iz_6_1109	DJI_0254	Quercus robur L.	0	n	1	1	Fagaceae Dumort.
Iz_6_1109	DJI_0254	Robinia pseudoacacia L.	0	a	0	1	Fabaceae Lindl.
Iz_6_1109	DJI_0254	Rosa sp.	0		NA	1	Rosaceae Juss.
Iz_6_1109	DJI_0254	Rubus idaeus L.	0	n	1	0	Rosaceae Juss.
Iz_6_1109	DJI_0254	Rumex acetosella L.	0	n	1	0	Polygonaceae Juss.
Iz_6_1109	DJI_0254	Rumex thyrsiflorus Fingerh.	0	n	1	0	Polygonaceae Juss.
Iz_6_1109	DJI_0254	Salix alba L.	0	n	1	1	Salicaceae Mirb.
Iz_6_1109	DJI_0254	Sambucus nigra L.	0	n	1	1	Viburnaceae Raf.
Iz_6_1109	DJI_0254	Secale sylvestre Host	0	n	1	0	Poaceae Barnhart
Iz_6_1109	DJI_0254	Sempervivum ruthenicum Schnittsp. & C.B. Lehm.	0	n	1	0	Crassulaceae J.St.-Hil.
Iz_6_1109	DJI_0254	Setaria viridis (L.) P. Beauv.	0	n	1	0	Poaceae Barnhart
Iz_6_1109	DJI_0254	Silene borysthenica (Gruner) Walters	0	n	1	0	Caryophyllaceae Juss.
Iz_6_1109	DJI_0254	Silene latifolia subsp. alba (Mill.) Greuter & Burdet	0	n	1	0	Caryophyllaceae Juss.
Iz_6_1109	DJI_0254	Solidago virgaurea L.	0	n	1	0	Asteraceae Bercht. & J.Presl
Iz_6_1109	DJI_0254	Sorbus aucuparia L.	0	n	1	1	Rosaceae Juss.
Iz_6_1109	DJI_0254	Species 11.09_6_0254	1		NA	0	
Iz_6_1109	DJI_0254	Stachys recta L.	0	n	1	0	Lamiacea Lindl.
Iz_6_1109	DJI_0254	Tanacetum vulgare L.	0	n	1	0	Asteraceae Bercht. & J.Presl
Iz_6_1109	DJI_0254	Thymus pallasianus Heinr.Braun	0	n	1	0	Lamiacea Lindl.
Iz_6_1109	DJI_0254	Tilia cordata Mill.	0	n	1	1	Malvaceae Juss.
Iz_6_1109	DJI_0254	Trifolium alpestre L.	0	n	1	0	Fabaceae Lindl.
Iz_6_1109	DJI_0254	Trifolium arvense L.	0	n	1	0	Fabaceae Lindl.
Iz_6_1109	DJI_0254	Ulmus glabra Huds.	0	n	1	1	Ulmaceae Mirb.
Iz_6_1109	DJI_0254	Verbascum densiflorum Bertol.	0	n	1	0	Scrophulariaceae Juss.
Iz_6_1109	DJI_0254	Veronica austriaca L.	0	n	1	0	Plantaginaceae Juss.
Iz_6_1109	DJI_0254	Veronica incana L.	0	n	1	0	Plantaginaceae Juss.
Iz_6_1109	DJI_0254	Veronica verna L.	0	n	1	0	Plantaginaceae Juss.
Iz_6_1109	DJI_0254	Vincetoxicum hirundinaria Medik	0	n	1	0	Apocynaceae Juss.
Iz_6_1109	DJI_0256	Acer tataricum L.	0	n	1	1	Sapindaceae Juss.
Iz_6_1109	DJI_0256	Achillea millefolium L. s.l.	0	n	1	0	Asteraceae Bercht. & J.Presl
Iz_6_1109	DJI_0256	Allium oleraceum L.	0	n	1	0	Amaryllidaceae J.St.-Hil.
Iz_6_1109	DJI_0256	Ambrosia artemisiifolia L.	0	a	0	0	Asteraceae Bercht. & J.Presl
Iz_6_1109	DJI_0256	Amorpha fruticosa L.	0	a	0	0	Fabaceae Lindl.
Iz_6_1109	DJI_0256	Anthericum ramosum L.	0	n	1	0	Asparagaceae Juss.
Iz_6_1109	DJI_0256	Artemisia marschalliana Spreng.	0	n	1	0	Asteraceae Bercht. & J.Presl
Iz_6_1109	DJI_0256	Asparagus officinalis L.	0	n	1	0	Asparagaceae Juss.
Iz_6_1109	DJI_0256	Astragalus cicer L.	0	n	1	0	Fabaceae Lindl.
Iz_6_1109	DJI_0256	Bassia laniflora (S.G.Gmel.) A.J.Scott	0	n	1	0	Amaranthaceae Juss.
Iz_6_1109	DJI_0256	Berberis vulgaris L.	0	n	1	0	Berberidaceae Juss.
Iz_6_1109	DJI_0256	Betula pendula Roth.	0	n	1	1	Betulaceae Gray
Iz_6_1109	DJI_0256	Betula pendula Roth.	0	n	1	0	Poaceae Barnhart
Iz_6_1109	DJI_0256	Bromus inermis Leyss.	0		NA	NA	
Iz_6_1109	DJI_0256	Bromus squarrosus L.	0	a	0	0	Poaceae Barnhart
Iz_6_1109	DJI_0256	Bromus tectorum L.	0	a	0	0	Poaceae Barnhart
Iz_6_1109	DJI_0256	Calamagrostis epigejos (L.) Roth	25	n	1	0	Poaceae Barnhart
Iz_6_1109	DJI_0256	Carduus acanthoides L.	0	n	1	0	Asteraceae Bercht. & J.Presl
Iz_6_1109	DJI_0256	Carex sp.	10	n	1	0	Cyperaceae Juss.
Iz_6_1109	DJI_0256	Centaurea majorovii Dumbadze	0	n	1	0	Asteraceae Bercht. & J.Presl
Iz_6_1109	DJI_0256	Chamaecytisus ruthenicus (Fisch.ex Wol.) Klaskova	0	n	1	0	Fabaceae Lindl.
Iz_6_1109	DJI_0256	Chelidonium majus L.	0	n	1	0	Papaveraceae Juss.
Iz_6_1109	DJI_0256	Chenopodium betaceum Andrz.	0	n	1	0	Amaranthaceae Juss.

Iz_6_1109	DJI_0256	Chenopodium cf. album	0	n	1	0	Amaranthaceae Juss.
Iz_6_1109	DJI_0256	Chenopodium sp.	0		NA	0	Amaranthaceae Juss.
Iz_6_1109	DJI_0256	Chondrilla juncea L.	0	n	1	0	Asteraceae Bercht. & J.Presl
Iz_6_1109	DJI_0256	Cirsium vulgare (Savi) Ten.	0	n	1	0	Asteraceae Bercht. & J.Presl
Iz_6_1109	DJI_0256	Clinopodium acinos (L.) Kuntze	0	n	1	0	Lamiacea Lindl.
Iz_6_1109	DJI_0256	Convallaria majalis L.	0	n	1	0	Asparagaceae Juss.
Iz_6_1109	DJI_0256	Crataegus rhipidophylla Gand.	0	n	1	1	Rosaceae Juss.
Iz_6_1109	DJI_0256	Crepis foetida L.	0	n	1	0	Asteraceae Bercht. & J.Presl
Iz_6_1109	DJI_0256	Dactylis glomerata L.	0	n	1	0	Poaceae Barnhart
Iz_6_1109	DJI_0256	Dianthus campestris M.Bieb.	0	n	1	0	Caryophyllaceae Juss.
Iz_6_1109	DJI_0256	Digitaria sanguinalis (L.) Scop.	0	a	0	0	Poaceae Barnhart
Iz_6_1109	DJI_0256	Elymus repens (L.) Gould	0	n	1	0	Poaceae Barnhart
Iz_6_1109	DJI_0256	Epilobium tetragonum L.	0	n	1	0	Onagraceae Juss.
Iz_6_1109	DJI_0256	Eragrostis minor Host	0	a	0	0	Poaceae Barnhart
Iz_6_1109	DJI_0256	Erigeron annuus (L.) Desf.	0	a	0	0	Asteraceae Bercht. & J.Presl
Iz_6_1109	DJI_0256	Erigeron canadensis L.	10	a	0	0	Asteraceae Bercht. & J.Presl
Iz_6_1109	DJI_0256	Euphorbia seguieriana Neck.	0	n	1	0	Euphorbiaceae Juss.
Iz_6_1109	DJI_0256	Fallopia dumetorum (L.) Holub	0	n	1	0	Polygonaceae Juss.
Iz_6_1109	DJI_0256	Festuca beckeri (Hack.) Trautv.	0	n	1	0	Poaceae Barnhart
Iz_6_1109	DJI_0256	Fragaria vesca L.	0	n	1	0	Rosaceae Juss.
Iz_6_1109	DJI_0256	Frangula alnus Mill.	0	n	1	1	Rhamnaceae Juss.
Iz_6_1109	DJI_0256	Fraxinus pennsylvanica Marshall	0	a	0	1	Oleaceae Hoffmanns. & Link
Iz_6_1109	DJI_0256	Galeopsis bifida Boenn.	0	n	1	0	Lamiacea Lindl.
Iz_6_1109	DJI_0256	Galium verum L.	0	n	1	0	Rubiaceae Juss.
Iz_6_1109	DJI_0256	Genista tinctoria L.	0	n	1	0	Fabaceae Lindl.
Iz_6_1109	DJI_0256	Geranium robertianum L.	0	n	1	0	Geraniaceae Juss.
Iz_6_1109	DJI_0256	Geranium sanquineum L.	0	n	1	0	Geraniaceae Juss.
Iz_6_1109	DJI_0256	Gypsophila paniculata L.	0	n	1	0	Caryophyllaceae Juss.
Iz_6_1109	DJI_0256	Helichrysum arenarium (L.) Moench	0	n	1	0	Asteraceae Bercht. & J.Presl
Iz_6_1109	DJI_0256	Herniaria polygama J.Gay	0	n	1	0	Caryophyllaceae Juss.
Iz_6_1109	DJI_0256	Hieracium robustum Fr.	0	n	1	0	Asteraceae Bercht. & J.Presl
Iz_6_1109	DJI_0256	Hieracium umbellatum L.	0	n	1	NA	Asteraceae Bercht. & J.Presl
Iz_6_1109	DJI_0256	Hylotelephium maximum (L.) Holub	0	n	1	0	Crassulaceae J.St.-Hil.
Iz_6_1109	DJI_0256	Jacobaea borysthenica (DC.) B.Nord. & Greuter	0	n	1	0	Asteraceae Bercht. & J.Presl
Iz_6_1109	DJI_0256	Jasione montana L.	0	n	1	0	Campanulaceae Juss.
Iz_6_1109	DJI_0256	Jurinea cyanoides (L.) Rchb.s.l.	0	n	1	0	Asteraceae Bercht. & J.Presl
Iz_6_1109	DJI_0256	Koeleria glauca (Spreng.) DC.	0	n	1	0	Poaceae Barnhart
Iz_6_1109	DJI_0256	Lactuca serriola L.	0	a	0	0	Asteraceae Bercht. & J.Presl
Iz_6_1109	DJI_0256	Linaria genistifolia (L.) Mill.	0	n	1	0	Plantaginaceae Juss.
Iz_6_1109	DJI_0256	Lomelosia argentea (L.) Greuter & Burdet	0	n	1	0	Caprifoliaceae Juss.
Iz_6_1109	DJI_0256	Melampyrum pratense L.	0	n	1	0	Orobanchaceae Vent.
Iz_6_1109	DJI_0256	Oenothera biennis L.	0	a	0	0	Onagraceae Juss.
Iz_6_1109	DJI_0256	Pentanema britannica (L.) D.Gut.Larr., Santos-Vicente, Anderb., E.Rico & M.M.Mart.Ort.	0	n	1	0	Asteraceae Bercht. & J.Presl
Iz_6_1109	DJI_0256	Peucedanum oreoselinum (L.) Moench	0	n	1	0	Apiaceae Lindl.
Iz_6_1109	DJI_0256	Peucedanum ruthenicum M.Bieb.	0	n	1	0	Apiaceae Lindl.
Iz_6_1109	DJI_0256	Pilosella echioides (Lumn.) F.W.Schultz & Sch.Bip.	0	n	1	0	Asteraceae Bercht. & J.Presl
Iz_6_1109	DJI_0256	Pilosella officinarum F. Schultz et Sch. Bip.	0	n	1	0	Asteraceae Bercht. & J.Presl
Iz_6_1109	DJI_0256	Pilosella sp.	0		NA	0	Asteraceae Bercht. & J.Presl
Iz_6_1109	DJI_0256	Pinus sylvestris L.	0	n	1	1	Pinaceae Spreng. ex F.Rudolphi
Iz_6_1109	DJI_0256	Poa annua L.	0	n	1	0	Poaceae Barnhart
Iz_6_1109	DJI_0256	Poa nemoralis L.	0	n	1	0	Poaceae Barnhart
Iz_6_1109	DJI_0256	Poa sp.	0		NA	0	Poaceae Barnhart
Iz_6_1109	DJI_0256	Polygonatum odoratum (Mill.) Druce	0	n	1	0	Asparagaceae Juss.
Iz_6_1109	DJI_0256	Polygonum arenarium Waldst. & Kit.	0	n	1	0	Polygonaceae Juss.
Iz_6_1109	DJI_0256	Populus nigra L.	0	n	1	1	Salicaceae Mirb.
Iz_6_1109	DJI_0256	Populus tremula L.	0	n	1	1	Salicaceae Mirb.
Iz_6_1109	DJI_0256	Populus Ã canescens (Aiton) Sm.	0	n	1	1	Salicaceae Mirb.
Iz_6_1109	DJI_0256	Potentilla argentea L.	0	n	1	0	Rosaceae Juss.
Iz_6_1109	DJI_0256	Psammophiliella muralis (L.) Ikonn.	0	n	1	0	Caryophyllaceae Juss.
Iz_6_1109	DJI_0256	Psephellus sumensis (Kalen.) Greuter	0	n	1	0	Asteraceae Bercht. & J.Presl
Iz_6_1109	DJI_0256	Pulsatilla pratensis (L.) Mill. s.l.	0	n	1	0	Ranunculaceae Juss.
Iz_6_1109	DJI_0256	Pyrus communis L.	0	a	0	1	Rosaceae Juss.
Iz_6_1109	DJI_0256	Quercus robur L.	0	n	1	1	Fagaceae Dumort.
Iz_6_1109	DJI_0256	Robinia pseudoacacia L.	0	a	0	1	Fabaceae Lindl.
Iz_6_1109	DJI_0256	Rosa sp.	0		NA	1	Rosaceae Juss.
Iz_6_1109	DJI_0256	Rubus idaeus L.	0	n	1	0	Rosaceae Juss.
Iz_6_1109	DJI_0256	Rumex acetosella L.	0	n	1	0	Polygonaceae Juss.
Iz_6_1109	DJI_0256	Rumex thyrsiflorus Fingerh.	0	n	1	0	Polygonaceae Juss.
Iz_6_1109	DJI_0256	Salix alba L.	0	n	1	1	Salicaceae Mirb.
Iz_6_1109	DJI_0256	Sambucus nigra L.	0	n	1	1	Viburnaceae Raf.
Iz_6_1109	DJI_0256	Secale sylvestre Host	0	n	1	0	Poaceae Barnhart
Iz_6_1109	DJI_0256	Sempervivum ruthenicum Schnittsp. & C.B. Lehm.	0	n	1	0	Crassulaceae J.St.-Hil.
Iz_6_1109	DJI_0256	Setaria viridis (L.) P. Beauv.	0	n	1	0	Poaceae Barnhart
Iz_6_1109	DJI_0256	Silene borysthenica (Gruner) Walters	0	n	1	0	Caryophyllaceae Juss.
Iz_6_1109	DJI_0256	Silene latifolia subsp. alba (Mill.) Greuter & Burdet	0	n	1	0	Caryophyllaceae Juss.
Iz_6_1109	DJI_0256	Solidago virgaurea L.	0	n	1	0	Asteraceae Bercht. & J.Presl
Iz_6_1109	DJI_0256	Sorbus aucuparia L.	0	n	1	1	Rosaceae Juss.
Iz_6_1109	DJI_0256	Species 11.09_6_0254	0		NA	0	
Iz_6_1109	DJI_0256	Stachys recta L.	0	n	1	0	Lamiacea Lindl.
Iz_6_1109	DJI_0256	Tanacetum vulgare L.	0	n	1	0	Asteraceae Bercht. & J.Presl
Iz_6_1109	DJI_0256	Thymus pallasianus Heinr.Braun	0	n	1	0	Lamiacea Lindl.
Iz_6_1109	DJI_0256	Tilia cordata Mill.	0	n	1	1	Malvaceae Juss.
Iz_6_1109	DJI_0256	Trifolium alpestre L.	0	n	1	0	Fabaceae Lindl.
Iz_6_1109	DJI_0256	Trifolium arvense L.	0	n	1	0	Fabaceae Lindl.
Iz_6_1109	DJI_0256	Ulmus glabra Huds.	0	n	1	1	Ulmaceae Mirb.
Iz_6_1109	DJI_0256	Verbascum densiflorum Bertol.	0	n	1	0	Scrophulariaceae Juss.
Iz_6_1109	DJI_0256	Veronica austriaca L.	0	n	1	0	Plantaginaceae Juss.
Iz_6_1109	DJI_0256	Veronica incana L.	0	n	1	0	Plantaginaceae Juss.
Iz_6_1109	DJI_0256	Veronica verna L.	0	n	1	0	Plantaginaceae Juss.
Iz_6_1109	DJI_0256	Vincetoxicum hirundinaria Medik	0	n	1	0	Apocynaceae Juss.
Iz_6_1109	DJI_0259	Acer tataricum L.	0	n	1	1	Sapindaceae Juss.
Iz_6_1109	DJI_0259	Achillea millefolium L. s.l.	0	n	1	0	Asteraceae Bercht. & J.Presl
Iz_6_1109	DJI_0259	Allium oleraceum L.	0	n	1	0	Amaryllidaceae J.St.-Hil.
Iz_6_1109	DJI_0259	Ambrosia artemisiifolia L.	0	a	0	0	Asteraceae Bercht. & J.Presl
Iz_6_1109	DJI_0259	Amorpha fruticosa L.	0	a	0	0	Fabaceae Lindl.
Iz_6_1109	DJI_0259	Anthericum ramosum L.	0	n	1	0	Asparagaceae Juss.
Iz_6_1109	DJI_0259	Artemisia marschalliana Spreng.	0	n	1	0	Asteraceae Bercht. & J.Presl
Iz_6_1109	DJI_0259	Asparagus officinalis L.	0	n	1	0	Asparagaceae Juss.

Iz_6_1109	DJI_0259	Astragalus cicer L.	0	n	1	0	Fabaceae Lindl.
Iz_6_1109	DJI_0259	Bassia laniflora (S.G.Gmel.) A.J.Scott	0	n	1	0	Amaranthaceae Juss.
Iz_6_1109	DJI_0259	Berberis vulgaris L.	0	n	1	0	Berberidaceae Juss.
Iz_6_1109	DJI_0259	Betula pendula Roth.	0	n	1	1	Betulaceae Gray
Iz_6_1109	DJI_0259	Betula pendula Roth.	0	n	1	0	Poaceae Barnhart
Iz_6_1109	DJI_0259	Bromus inermis Leyss.	0		NA	NA	
Iz_6_1109	DJI_0259	Bromus squarrosus L.	0	a	0	0	Poaceae Barnhart
Iz_6_1109	DJI_0259	Bromus tectorum L.	0	a	0	0	Poaceae Barnhart
Iz_6_1109	DJI_0259	Calamagrostis epigejos (L.) Roth	15	n	1	0	Poaceae Barnhart
Iz_6_1109	DJI_0259	Carduus acanthoides L.	0	n	1	0	Asteraceae Bercht. & J.Presl
Iz_6_1109	DJI_0259	Carex sp.	8	n	1	0	Cyperaceae Juss.
Iz_6_1109	DJI_0259	Centaurea majorovii Dumbadze	0	n	1	0	Asteraceae Bercht. & J.Presl
Iz_6_1109	DJI_0259	Chamaecytisus ruthenicus (Fisch.ex Wol.) Klaskova	0	n	1	0	Fabaceae Lindl.
Iz_6_1109	DJI_0259	Chelidonium majus L.	0	n	1	0	Papaveraceae Juss.
Iz_6_1109	DJI_0259	Chenopodium betaceum Andrz.	0	n	1	0	Amaranthaceae Juss.
Iz_6_1109	DJI_0259	Chenopodium cf. album	0	n	1	0	Amaranthaceae Juss.
Iz_6_1109	DJI_0259	Chenopodium sp.	0		NA	0	Amaranthaceae Juss.
Iz_6_1109	DJI_0259	Chondrilla juncea L.	0	n	1	0	Asteraceae Bercht. & J.Presl
Iz_6_1109	DJI_0259	Cirsium vulgare (Savi) Ten.	0	n	1	0	Asteraceae Bercht. & J.Presl
Iz_6_1109	DJI_0259	Clinopodium acinos (L.) Kuntze	0	n	1	0	Lamiacea Lindl.
Iz_6_1109	DJI_0259	Convallaria majalis L.	0	n	1	0	Asparagaceae Juss.
Iz_6_1109	DJI_0259	Crataegus rhipidophylla Gand.	0	n	1	1	Rosaceae Juss.
Iz_6_1109	DJI_0259	Crepis foetida L.	0	n	1	0	Asteraceae Bercht. & J.Presl
Iz_6_1109	DJI_0259	Dactylis glomerata L.	0	n	1	0	Poaceae Barnhart
Iz_6_1109	DJI_0259	Dianthus campestris M.Bieb.	0	n	1	0	Caryophyllaceae Juss.
Iz_6_1109	DJI_0259	Digitaria sanguinalis (L.) Scop.	0	a	0	0	Poaceae Barnhart
Iz_6_1109	DJI_0259	Elymus repens (L.) Gould	0	n	1	0	Poaceae Barnhart
Iz_6_1109	DJI_0259	Epilobium tetragonum L.	0	n	1	0	Onagraceae Juss.
Iz_6_1109	DJI_0259	Eragrostis minor Host	0	a	0	0	Poaceae Barnhart
Iz_6_1109	DJI_0259	Erigeron annuus (L.) Desf.	0	a	0	0	Asteraceae Bercht. & J.Presl
Iz_6_1109	DJI_0259	Erigeron canadensis L.	10	a	0	0	Asteraceae Bercht. & J.Presl
Iz_6_1109	DJI_0259	Euphorbia seguieriana Neck.	0	n	1	0	Euphorbiaceae Juss.
Iz_6_1109	DJI_0259	Fallopia dumetorum (L.) Holub	0	n	1	0	Polygonaceae Juss.
Iz_6_1109	DJI_0259	Festuca beckeri (Hack.) Trautv.	0	n	1	0	Poaceae Barnhart
Iz_6_1109	DJI_0259	Fragaria vesca L.	0	n	1	0	Rosaceae Juss.
Iz_6_1109	DJI_0259	Frangula alnus Mill.	0	n	1	1	Rhamnaceae Juss.
Iz_6_1109	DJI_0259	Fraxinus pennsylvanica Marshall	0	a	0	1	Oleaceae Hoffmanns. & Link
Iz_6_1109	DJI_0259	Galeopsis bifida Boenn.	0	n	1	0	Lamiacea Lindl.
Iz_6_1109	DJI_0259	Galium verum L.	0	n	1	0	Rubiaceae Juss.
Iz_6_1109	DJI_0259	Genista tinctoria L.	0	n	1	0	Fabaceae Lindl.
Iz_6_1109	DJI_0259	Geranium robertianum L.	0	n	1	0	Geraniaceae Juss.
Iz_6_1109	DJI_0259	Geranium sanquineum L.	0	n	1	0	Geraniaceae Juss.
Iz_6_1109	DJI_0259	Gypsophila paniculata L.	0	n	1	0	Caryophyllaceae Juss.
Iz_6_1109	DJI_0259	Helichrysum arenarium (L.) Moench	0	n	1	0	Asteraceae Bercht. & J.Presl
Iz_6_1109	DJI_0259	Herniaria polygama J.Gay	0	n	1	0	Caryophyllaceae Juss.
Iz_6_1109	DJI_0259	Hieracium robustum Fr.	0	n	1	0	Asteraceae Bercht. & J.Presl
Iz_6_1109	DJI_0259	Hieracium umbellatum L.	0	n	1	NA	Asteraceae Bercht. & J.Presl
Iz_6_1109	DJI_0259	Hylotelephium maximum (L.) Holub	0	n	1	0	Crassulaceae J.St.-Hil.
Iz_6_1109	DJI_0259	Jacobaea borysthenica (DC.) B.Nord. & Greuter	0	n	1	0	Asteraceae Bercht. & J.Presl
Iz_6_1109	DJI_0259	Jasione montana L.	0	n	1	0	Campanulaceae Juss.
Iz_6_1109	DJI_0259	Jurinea cyanoides (L.) Rchb.s.l.	0	n	1	0	Asteraceae Bercht. & J.Presl
Iz_6_1109	DJI_0259	Koeleria glauca (Spreng.) DC.	0	n	1	0	Poaceae Barnhart
Iz_6_1109	DJI_0259	Lactuca serriola L.	0	a	0	0	Asteraceae Bercht. & J.Presl
Iz_6_1109	DJI_0259	Linaria genistifolia (L.) Mill.	0	n	1	0	Plantaginaceae Juss.
Iz_6_1109	DJI_0259	Lomelosia argentea (L.) Greuter & Burdet	0	n	1	0	Caprifoliaceae Juss.
Iz_6_1109	DJI_0259	Melampyrum pratense L.	0	n	1	0	Orobanchaceae Vent.
Iz_6_1109	DJI_0259	Oenothera biennis L.	0	a	0	0	Onagraceae Juss.
Iz_6_1109	DJI_0259	Pentanema britannica (L.) D.Gut.Larr., Santos-Vicente, Anderb., E.Rico & M.M.Mart.Ort.	0	n	1	0	Asteraceae Bercht. & J.Presl
Iz_6_1109	DJI_0259	Peucedanum oreoselinum (L.) Moench	0	n	1	0	Apiaceae Lindl.
Iz_6_1109	DJI_0259	Peucedanum ruthenicum M.Bieb.	0	n	1	0	Apiaceae Lindl.
Iz_6_1109	DJI_0259	Pilosella echioides (Lumn.) F.W.Schultz & Sch.Bip.	0	n	1	0	Asteraceae Bercht. & J.Presl
Iz_6_1109	DJI_0259	Pilosella officinarum F. Schultz et Sch. Bip.	0	n	1	0	Asteraceae Bercht. & J.Presl
Iz_6_1109	DJI_0259	Pilosella sp.	0		NA	0	Asteraceae Bercht. & J.Presl
Iz_6_1109	DJI_0259	Pinus sylvestris L.	0	n	1	1	Pinaceae Spreng. ex F.Rudolphi
Iz_6_1109	DJI_0259	Poa annua L.	0	n	1	0	Poaceae Barnhart
Iz_6_1109	DJI_0259	Poa nemoralis L.	0	n	1	0	Poaceae Barnhart
Iz_6_1109	DJI_0259	Poa sp.	0		NA	0	Poaceae Barnhart
Iz_6_1109	DJI_0259	Polygonatum odoratum (Mill.) Druce	0	n	1	0	Asparagaceae Juss.
Iz_6_1109	DJI_0259	Polygonum arenarium Waldst. & Kit.	0	n	1	0	Polygonaceae Juss.
Iz_6_1109	DJI_0259	Populus nigra L.	0	n	1	1	Salicaceae Mirb.
Iz_6_1109	DJI_0259	Populus tremula L.	5	n	1	1	Salicaceae Mirb.
Iz_6_1109	DJI_0259	Populus Ã canescens (Aiton) Sm.	0	n	1	1	Salicaceae Mirb.
Iz_6_1109	DJI_0259	Potentilla argentea L.	0	n	1	0	Rosaceae Juss.
Iz_6_1109	DJI_0259	Psammophiliella muralis (L.) Ikonn.	0	n	1	0	Caryophyllaceae Juss.
Iz_6_1109	DJI_0259	Psephellus sumensis (Kalen.) Greuter	0	n	1	0	Asteraceae Bercht. & J.Presl
Iz_6_1109	DJI_0259	Pulsatilla pratensis (L.) Mill. s.l.	0	n	1	0	Ranunculaceae Juss.
Iz_6_1109	DJI_0259	Pyrus communis L.	0	a	0	1	Rosaceae Juss.
Iz_6_1109	DJI_0259	Quercus robur L.	0	n	1	1	Fagaceae Dumort.
Iz_6_1109	DJI_0259	Robinia pseudoacacia L.	0	a	0	1	Fabaceae Lindl.
Iz_6_1109	DJI_0259	Rosa sp.	0		NA	1	Rosaceae Juss.
Iz_6_1109	DJI_0259	Rubus idaeus L.	0	n	1	0	Rosaceae Juss.
Iz_6_1109	DJI_0259	Rumex acetosella L.	0	n	1	0	Polygonaceae Juss.
Iz_6_1109	DJI_0259	Rumex thyrsiflorus Fingerh.	0	n	1	0	Polygonaceae Juss.
Iz_6_1109	DJI_0259	Salix alba L.	0	n	1	1	Salicaceae Mirb.
Iz_6_1109	DJI_0259	Sambucus nigra L.	0	n	1	1	Viburnaceae Raf.
Iz_6_1109	DJI_0259	Secale sylvestre Host	0	n	1	0	Poaceae Barnhart
Iz_6_1109	DJI_0259	Sempervivum ruthenicum Schnittsp. & C.B. Lehm.	0	n	1	0	Crassulaceae J.St.-Hil.
Iz_6_1109	DJI_0259	Setaria viridis (L.) P. Beauv.	0	n	1	0	Poaceae Barnhart
Iz_6_1109	DJI_0259	Silene borysthenica (Gruner) Walters	0	n	1	0	Caryophyllaceae Juss.
Iz_6_1109	DJI_0259	Silene latifolia subsp. alba (Mill.) Greuter & Burdet	0	n	1	0	Caryophyllaceae Juss.
Iz_6_1109	DJI_0259	Solidago virgaurea L.	0	n	1	0	Asteraceae Bercht. & J.Presl
Iz_6_1109	DJI_0259	Sorbus aucuparia L.	0	n	1	1	Rosaceae Juss.
Iz_6_1109	DJI_0259	Species 11.09_6_0254	0		NA	0	
Iz_6_1109	DJI_0259	Stachys recta L.	0	n	1	0	Lamiacea Lindl.
Iz_6_1109	DJI_0259	Tanacetum vulgare L.	0	n	1	0	Asteraceae Bercht. & J.Presl
Iz_6_1109	DJI_0259	Thymus pallasianus Heinr.Braun	0	n	1	0	Lamiacea Lindl.
Iz_6_1109	DJI_0259	Tilia cordata Mill.	0	n	1	1	Malvaceae Juss.
Iz_6_1109	DJI_0259	Trifolium alpestre L.	0	n	1	0	Fabaceae Lindl.

Iz_6_1109	DJI_0259	Trifolium arvense L.	0	n	1	0	Fabaceae Lindl.
Iz_6_1109	DJI_0259	Ulmus glabra Huds.	0	n	1	1	Ulmaceae Mirb.
Iz_6_1109	DJI_0259	Verbascum densiflorum Bertol.	0	n	1	0	Scrophulariaceae Juss.
Iz_6_1109	DJI_0259	Veronica austriaca L.	0	n	1	0	Plantaginaceae Juss.
Iz_6_1109	DJI_0259	Veronica incana L.	0	n	1	0	Plantaginaceae Juss.
Iz_6_1109	DJI_0259	Veronica verna L.	0	n	1	0	Plantaginaceae Juss.
Iz_6_1109	DJI_0259	Vincetoxicum hirundinaria Medik	0	n	1	0	Apocynaceae Juss.
Iz_6_1109	DJI_0261	Acer tataricum L.	0	n	1	1	Sapindaceae Juss.
Iz_6_1109	DJI_0261	Achillea millefolium L. s.l.	0	n	1	0	Asteraceae Bercht. & J.Presl
Iz_6_1109	DJI_0261	Allium oleraceum L.	0	n	1	0	Amaryllidaceae J.St.-Hil.
Iz_6_1109	DJI_0261	Ambrosia artemisiifolia L.	0	a	0	0	Asteraceae Bercht. & J.Presl
Iz_6_1109	DJI_0261	Amorpha fruticosa L.	0	a	0	0	Fabaceae Lindl.
Iz_6_1109	DJI_0261	Anthericum ramosum L.	0	n	1	0	Asparagaceae Juss.
Iz_6_1109	DJI_0261	Artemisia marschalliana Spreng.	0	n	1	0	Asteraceae Bercht. & J.Presl
Iz_6_1109	DJI_0261	Asparagus officinalis L.	0	n	1	0	Asparagaceae Juss.
Iz_6_1109	DJI_0261	Astragalus cicer L.	0	n	1	0	Fabaceae Lindl.
Iz_6_1109	DJI_0261	Bassia laniflora (S.G.Gmel.) A.J.Scott	0	n	1	0	Amaranthaceae Juss.
Iz_6_1109	DJI_0261	Berberis vulgaris L.	0	n	1	0	Berberidaceae Juss.
Iz_6_1109	DJI_0261	Betula pendula Roth.	0	n	1	1	Betulaceae Gray
Iz_6_1109	DJI_0261	Betula pendula Roth.	0	n	1	0	Poaceae Barnhart
Iz_6_1109	DJI_0261	Bromus inermis Leyss.	0		NA	NA	
Iz_6_1109	DJI_0261	Bromus squarrosus L.	0	a	0	0	Poaceae Barnhart
Iz_6_1109	DJI_0261	Bromus tectorum L.	0	a	0	0	Poaceae Barnhart
Iz_6_1109	DJI_0261	Calamagrostis epigejos (L.) Roth	80	n	1	0	Poaceae Barnhart
Iz_6_1109	DJI_0261	Carduus acanthoides L.	0	n	1	0	Asteraceae Bercht. & J.Presl
Iz_6_1109	DJI_0261	Carex sp.	0	n	1	0	Cyperaceae Juss.
Iz_6_1109	DJI_0261	Centaurea majorovii Dumbadze	0	n	1	0	Asteraceae Bercht. & J.Presl
Iz_6_1109	DJI_0261	Chamaecytisus ruthenicus (Fisch.ex Wol.) Klaskova	0	n	1	0	Fabaceae Lindl.
Iz_6_1109	DJI_0261	Chelidonium majus L.	0	n	1	0	Papaveraceae Juss.
Iz_6_1109	DJI_0261	Chenopodium betaceum Andrz.	0	n	1	0	Amaranthaceae Juss.
Iz_6_1109	DJI_0261	Chenopodium cf. album	0	n	1	0	Amaranthaceae Juss.
Iz_6_1109	DJI_0261	Chenopodium sp.	0		NA	0	Amaranthaceae Juss.
Iz_6_1109	DJI_0261	Chondrilla juncea L.	0	n	1	0	Asteraceae Bercht. & J.Presl
Iz_6_1109	DJI_0261	Cirsium vulgare (Savi) Ten.	0	n	1	0	Asteraceae Bercht. & J.Presl
Iz_6_1109	DJI_0261	Clinopodium acinos (L.) Kuntze	0	n	1	0	Lamiacea Lindl.
Iz_6_1109	DJI_0261	Convallaria majalis L.	0	n	1	0	Asparagaceae Juss.
Iz_6_1109	DJI_0261	Crataegus rhipidophylla Gand.	0	n	1	1	Rosaceae Juss.
Iz_6_1109	DJI_0261	Crepis foetida L.	0	n	1	0	Asteraceae Bercht. & J.Presl
Iz_6_1109	DJI_0261	Dactylis glomerata L.	0	n	1	0	Poaceae Barnhart
Iz_6_1109	DJI_0261	Dianthus campestris M.Bieb.	0	n	1	0	Caryophyllaceae Juss.
Iz_6_1109	DJI_0261	Digitaria sanguinalis (L.) Scop.	0	a	0	0	Poaceae Barnhart
Iz_6_1109	DJI_0261	Elymus repens (L.) Gould	0	n	1	0	Poaceae Barnhart
Iz_6_1109	DJI_0261	Epilobium tetragonum L.	0	n	1	0	Onagraceae Juss.
Iz_6_1109	DJI_0261	Eragrostis minor Host	0	a	0	0	Poaceae Barnhart
Iz_6_1109	DJI_0261	Erigeron annuus (L.) Desf.	0	a	0	0	Asteraceae Bercht. & J.Presl
Iz_6_1109	DJI_0261	Erigeron canadensis L.	1	a	0	0	Asteraceae Bercht. & J.Presl
Iz_6_1109	DJI_0261	Euphorbia seguieriana Neck.	0	n	1	0	Euphorbiaceae Juss.
Iz_6_1109	DJI_0261	Fallopia dumetorum (L.) Holub	0	n	1	0	Polygonaceae Juss.
Iz_6_1109	DJI_0261	Festuca beckeri (Hack.) Trautv.	0	n	1	0	Poaceae Barnhart
Iz_6_1109	DJI_0261	Fragaria vesca L.	0	n	1	0	Rosaceae Juss.
Iz_6_1109	DJI_0261	Frangula alnus Mill.	0	n	1	1	Rhamnaceae Juss.
Iz_6_1109	DJI_0261	Fraxinus pennsylvanica Marshall	0	a	0	1	Oleaceae Hoffmanns. & Link
Iz_6_1109	DJI_0261	Galeopsis bifida Boenn.	0	n	1	0	Lamiacea Lindl.
Iz_6_1109	DJI_0261	Galium verum L.	0	n	1	0	Rubiaceae Juss.
Iz_6_1109	DJI_0261	Genista tinctoria L.	0	n	1	0	Fabaceae Lindl.
Iz_6_1109	DJI_0261	Geranium robertianum L.	0	n	1	0	Geraniaceae Juss.
Iz_6_1109	DJI_0261	Geranium sanquineum L.	0	n	1	0	Geraniaceae Juss.
Iz_6_1109	DJI_0261	Gypsophila paniculata L.	0	n	1	0	Caryophyllaceae Juss.
Iz_6_1109	DJI_0261	Helichrysum arenarium (L.) Moench	0	n	1	0	Asteraceae Bercht. & J.Presl
Iz_6_1109	DJI_0261	Herniaria polygama J.Gay	0	n	1	0	Caryophyllaceae Juss.
Iz_6_1109	DJI_0261	Hieracium robustum Fr.	0	n	1	0	Asteraceae Bercht. & J.Presl
Iz_6_1109	DJI_0261	Hieracium umbellatum L.	0	n	1	NA	Asteraceae Bercht. & J.Presl
Iz_6_1109	DJI_0261	Hylotelephium maximum (L.) Holub	0	n	1	0	Crassulaceae J.St.-Hil.
Iz_6_1109	DJI_0261	Jacobaea borysthenica (DC.) B.Nord. & Greuter	0	n	1	0	Asteraceae Bercht. & J.Presl
Iz_6_1109	DJI_0261	Jasione montana L.	0	n	1	0	Campanulaceae Juss.
Iz_6_1109	DJI_0261	Jurinea cyanoides (L.) Rchb.s.l.	0	n	1	0	Asteraceae Bercht. & J.Presl
Iz_6_1109	DJI_0261	Koeleria glauca (Spreng.) DC.	0	n	1	0	Poaceae Barnhart
Iz_6_1109	DJI_0261	Lactuca serriola L.	0	a	0	0	Asteraceae Bercht. & J.Presl
Iz_6_1109	DJI_0261	Linaria genistifolia (L.) Mill.	0	n	1	0	Plantaginaceae Juss.
Iz_6_1109	DJI_0261	Lomelosia argentea (L.) Greuter & Burdet	0	n	1	0	Caprifoliaceae Juss.
Iz_6_1109	DJI_0261	Melampyrum pratense L.	0	n	1	0	Orobanchaceae Vent.
Iz_6_1109	DJI_0261	Oenothera biennis L.	0	a	0	0	Onagraceae Juss.
Iz_6_1109	DJI_0261	Pentanema britannica (L.) D.Gut.Larr., Santos-Vicente, Anderb., E.Rico & M.M.Mart.Ort.	0	n	1	0	Asteraceae Bercht. & J.Presl
Iz_6_1109	DJI_0261	Peucedanum oreoselinum (L.) Moench	0	n	1	0	Apiaceae Lindl.
Iz_6_1109	DJI_0261	Peucedanum ruthenicum M.Bieb.	0	n	1	0	Apiaceae Lindl.
Iz_6_1109	DJI_0261	Pilosella echioides (Lumn.) F.W.Schultz & Sch.Bip.	0	n	1	0	Asteraceae Bercht. & J.Presl
Iz_6_1109	DJI_0261	Pilosella officinarum F. Schultz et Sch. Bip.	0	n	1	0	Asteraceae Bercht. & J.Presl
Iz_6_1109	DJI_0261	Pilosella sp.	0		NA	0	Asteraceae Bercht. & J.Presl
Iz_6_1109	DJI_0261	Pinus sylvestris L.	0	n	1	1	Pinaceae Spreng. ex F.Rudolphi
Iz_6_1109	DJI_0261	Poa annua L.	0	n	1	0	Poaceae Barnhart
Iz_6_1109	DJI_0261	Poa nemoralis L.	0	n	1	0	Poaceae Barnhart
Iz_6_1109	DJI_0261	Poa sp.	0		NA	0	Poaceae Barnhart
Iz_6_1109	DJI_0261	Polygonatum odoratum (Mill.) Druce	0	n	1	0	Asparagaceae Juss.
Iz_6_1109	DJI_0261	Polygonum arenarium Waldst. & Kit.	0	n	1	0	Polygonaceae Juss.
Iz_6_1109	DJI_0261	Populus nigra L.	0	n	1	1	Salicaceae Mirb.
Iz_6_1109	DJI_0261	Populus tremula L.	0	n	1	1	Salicaceae Mirb.
Iz_6_1109	DJI_0261	Populus Ã canescens (Aiton) Sm.	0	n	1	1	Salicaceae Mirb.
Iz_6_1109	DJI_0261	Potentilla argentea L.	0	n	1	0	Rosaceae Juss.
Iz_6_1109	DJI_0261	Psammophiliella muralis (L.) Ikonn.	0	n	1	0	Caryophyllaceae Juss.
Iz_6_1109	DJI_0261	Psephellus sumensis (Kalen.) Greuter	0	n	1	0	Asteraceae Bercht. & J.Presl
Iz_6_1109	DJI_0261	Pulsatilla pratensis (L.) Mill. s.l.	0	n	1	0	Ranunculaceae Juss.
Iz_6_1109	DJI_0261	Pyrus communis L.	0	a	0	1	Rosaceae Juss.
Iz_6_1109	DJI_0261	Quercus robur L.	0	n	1	1	Fagaceae Dumort.
Iz_6_1109	DJI_0261	Robinia pseudoacacia L.	0	a	0	1	Fabaceae Lindl.
Iz_6_1109	DJI_0261	Rosa sp.	0		NA	1	Rosaceae Juss.
Iz_6_1109	DJI_0261	Rubus idaeus L.	0	n	1	0	Rosaceae Juss.
Iz_6_1109	DJI_0261	Rumex acetosella L.	0	n	1	0	Polygonaceae Juss.
Iz_6_1109	DJI_0261	Rumex thyrsiflorus Fingerh.	0	n	1	0	Polygonaceae Juss.

Iz_6_1109	DJI_0261	Salix alba L.	0	n	1	1	Salicaceae Mirb.
Iz_6_1109	DJI_0261	Sambucus nigra L.	0	n	1	1	Viburnaceae Raf.
Iz_6_1109	DJI_0261	Secale sylvestre Host	0	n	1	0	Poaceae Barnhart
Iz_6_1109	DJI_0261	Sempervivum ruthenicum Schnittsp. & C.B. Lehm.	0	n	1	0	Crassulaceae J.St.-Hil.
Iz_6_1109	DJI_0261	Setaria viridis (L.) P. Beauv.	0	n	1	0	Poaceae Barnhart
Iz_6_1109	DJI_0261	Silene borysthenica (Gruner) Walters	0	n	1	0	Caryophyllaceae Juss.
Iz_6_1109	DJI_0261	Silene latifolia subsp. alba (Mill.) Greuter & Burdet	0	n	1	0	Caryophyllaceae Juss.
Iz_6_1109	DJI_0261	Solidago virgaurea L.	0	n	1	0	Asteraceae Bercht. & J.Presl
Iz_6_1109	DJI_0261	Sorbus aucuparia L.	0	n	1	1	Rosaceae Juss.
Iz_6_1109	DJI_0261	Species 11.09_6_0254	0		NA	0	
Iz_6_1109	DJI_0261	Stachys recta L.	0	n	1	0	Lamiacea Lindl.
Iz_6_1109	DJI_0261	Tanacetum vulgare L.	0	n	1	0	Asteraceae Bercht. & J.Presl
Iz_6_1109	DJI_0261	Thymus pallasianus Heinr.Braun	0	n	1	0	Lamiacea Lindl.
Iz_6_1109	DJI_0261	Tilia cordata Mill.	0	n	1	1	Malvaceae Juss.
Iz_6_1109	DJI_0261	Trifolium alpestre L.	0	n	1	0	Fabaceae Lindl.
Iz_6_1109	DJI_0261	Trifolium arvense L.	0	n	1	0	Fabaceae Lindl.
Iz_6_1109	DJI_0261	Ulmus glabra Huds.	0	n	1	1	Ulmaceae Mirb.
Iz_6_1109	DJI_0261	Verbascum densiflorum Bertol.	0	n	1	0	Scrophulariaceae Juss.
Iz_6_1109	DJI_0261	Veronica austriaca L.	0	n	1	0	Plantaginaceae Juss.
Iz_6_1109	DJI_0261	Veronica incana L.	0	n	1	0	Plantaginaceae Juss.
Iz_6_1109	DJI_0261	Veronica verna L.	0	n	1	0	Plantaginaceae Juss.
Iz_6_1109	DJI_0261	Vincetoxicum hirundinaria Medik	0	n	1	0	Apocynaceae Juss.
Iz_6_1109	DJI_0267	Acer tataricum L.	0	n	1	1	Sapindaceae Juss.
Iz_6_1109	DJI_0267	Achillea millefolium L. s.l.	0	n	1	0	Asteraceae Bercht. & J.Presl
Iz_6_1109	DJI_0267	Allium oleraceum L.	0	n	1	0	Amaryllidaceae J.St.-Hil.
Iz_6_1109	DJI_0267	Ambrosia artemisiifolia L.	0	a	0	0	Asteraceae Bercht. & J.Presl
Iz_6_1109	DJI_0267	Amorpha fruticosa L.	0	a	0	0	Fabaceae Lindl.
Iz_6_1109	DJI_0267	Anthericum ramosum L.	0	n	1	0	Asparagaceae Juss.
Iz_6_1109	DJI_0267	Artemisia marschalliana Spreng.	0	n	1	0	Asteraceae Bercht. & J.Presl
Iz_6_1109	DJI_0267	Asparagus officinalis L.	0	n	1	0	Asparagaceae Juss.
Iz_6_1109	DJI_0267	Astragalus cicer L.	0	n	1	0	Fabaceae Lindl.
Iz_6_1109	DJI_0267	Bassia laniflora (S.G.Gmel.) A.J.Scott	0	n	1	0	Amaranthaceae Juss.
Iz_6_1109	DJI_0267	Berberis vulgaris L.	0	n	1	0	Berberidaceae Juss.
Iz_6_1109	DJI_0267	Betula pendula Roth.	0	n	1	1	Betulaceae Gray
Iz_6_1109	DJI_0267	Betula pendula Roth.	0	n	1	0	Poaceae Barnhart
Iz_6_1109	DJI_0267	Bromus inermis Leyss.	0		NA	NA	
Iz_6_1109	DJI_0267	Bromus squarrosus L.	0	a	0	0	Poaceae Barnhart
Iz_6_1109	DJI_0267	Bromus tectorum L.	0	a	0	0	Poaceae Barnhart
Iz_6_1109	DJI_0267	Calamagrostis epigejos (L.) Roth	80	n	1	0	Poaceae Barnhart
Iz_6_1109	DJI_0267	Carduus acanthoides L.	0	n	1	0	Asteraceae Bercht. & J.Presl
Iz_6_1109	DJI_0267	Carex sp.	0	n	1	0	Cyperaceae Juss.
Iz_6_1109	DJI_0267	Centaurea majorovii Dumbadze	0	n	1	0	Asteraceae Bercht. & J.Presl
Iz_6_1109	DJI_0267	Chamaecytisus ruthenicus (Fisch.ex Wol.) Klaskova	0	n	1	0	Fabaceae Lindl.
Iz_6_1109	DJI_0267	Chelidonium majus L.	0	n	1	0	Papaveraceae Juss.
Iz_6_1109	DJI_0267	Chenopodium betaceum Andrz.	0	n	1	0	Amaranthaceae Juss.
Iz_6_1109	DJI_0267	Chenopodium cf. album	0	n	1	0	Amaranthaceae Juss.
Iz_6_1109	DJI_0267	Chenopodium sp.	0		NA	0	Amaranthaceae Juss.
Iz_6_1109	DJI_0267	Chondrilla juncea L.	0	n	1	0	Asteraceae Bercht. & J.Presl
Iz_6_1109	DJI_0267	Cirsium vulgare (Savi) Ten.	0	n	1	0	Asteraceae Bercht. & J.Presl
Iz_6_1109	DJI_0267	Clinopodium acinos (L.) Kuntze	0	n	1	0	Lamiacea Lindl.
Iz_6_1109	DJI_0267	Convallaria majalis L.	0	n	1	0	Asparagaceae Juss.
Iz_6_1109	DJI_0267	Crataegus rhipidophylla Gand.	0	n	1	1	Rosaceae Juss.
Iz_6_1109	DJI_0267	Crepis foetida L.	0	n	1	0	Asteraceae Bercht. & J.Presl
Iz_6_1109	DJI_0267	Dactylis glomerata L.	0	n	1	0	Poaceae Barnhart
Iz_6_1109	DJI_0267	Dianthus campestris M.Bieb.	0	n	1	0	Caryophyllaceae Juss.
Iz_6_1109	DJI_0267	Digitaria sanguinalis (L.) Scop.	0	a	0	0	Poaceae Barnhart
Iz_6_1109	DJI_0267	Elymus repens (L.) Gould	0	n	1	0	Poaceae Barnhart
Iz_6_1109	DJI_0267	Epilobium tetragonum L.	0	n	1	0	Onagraceae Juss.
Iz_6_1109	DJI_0267	Eragrostis minor Host	0	a	0	0	Poaceae Barnhart
Iz_6_1109	DJI_0267	Erigeron annuus (L.) Desf.	0	a	0	0	Asteraceae Bercht. & J.Presl
Iz_6_1109	DJI_0267	Erigeron canadensis L.	0	a	0	0	Asteraceae Bercht. & J.Presl
Iz_6_1109	DJI_0267	Euphorbia seguieriana Neck.	0	n	1	0	Euphorbiaceae Juss.
Iz_6_1109	DJI_0267	Fallopia dumetorum (L.) Holub	0	n	1	0	Polygonaceae Juss.
Iz_6_1109	DJI_0267	Festuca beckeri (Hack.) Trautv.	0	n	1	0	Poaceae Barnhart
Iz_6_1109	DJI_0267	Fragaria vesca L.	0	n	1	0	Rosaceae Juss.
Iz_6_1109	DJI_0267	Frangula alnus Mill.	0	n	1	1	Rhamnaceae Juss.
Iz_6_1109	DJI_0267	Fraxinus pennsylvanica Marshall	0	a	0	1	Oleaceae Hoffmanns. & Link
Iz_6_1109	DJI_0267	Galeopsis bifida Boenn.	0	n	1	0	Lamiacea Lindl.
Iz_6_1109	DJI_0267	Galium verum L.	0	n	1	0	Rubiaceae Juss.
Iz_6_1109	DJI_0267	Genista tinctoria L.	0	n	1	0	Fabaceae Lindl.
Iz_6_1109	DJI_0267	Geranium robertianum L.	0	n	1	0	Geraniaceae Juss.
Iz_6_1109	DJI_0267	Geranium sanquineum L.	0	n	1	0	Geraniaceae Juss.
Iz_6_1109	DJI_0267	Gypsophila paniculata L.	0	n	1	0	Caryophyllaceae Juss.
Iz_6_1109	DJI_0267	Helichrysum arenarium (L.) Moench	0	n	1	0	Asteraceae Bercht. & J.Presl
Iz_6_1109	DJI_0267	Herniaria polygama J.Gay	0	n	1	0	Caryophyllaceae Juss.
Iz_6_1109	DJI_0267	Hieracium robustum Fr.	0	n	1	0	Asteraceae Bercht. & J.Presl
Iz_6_1109	DJI_0267	Hieracium umbellatum L.	1	n	1	NA	Asteraceae Bercht. & J.Presl
Iz_6_1109	DJI_0267	Hylotelephium maximum (L.) Holub	0	n	1	0	Crassulaceae J.St.-Hil.
Iz_6_1109	DJI_0267	Jacobaea borysthenica (DC.) B.Nord. & Greuter	0	n	1	0	Asteraceae Bercht. & J.Presl
Iz_6_1109	DJI_0267	Jasione montana L.	0	n	1	0	Campanulaceae Juss.
Iz_6_1109	DJI_0267	Jurinea cyanoides (L.) Rchb.s.l.	0	n	1	0	Asteraceae Bercht. & J.Presl
Iz_6_1109	DJI_0267	Koeleria glauca (Spreng.) DC.	0	n	1	0	Poaceae Barnhart
Iz_6_1109	DJI_0267	Lactuca serriola L.	0	a	0	0	Asteraceae Bercht. & J.Presl
Iz_6_1109	DJI_0267	Linaria genistifolia (L.) Mill.	0	n	1	0	Plantaginaceae Juss.
Iz_6_1109	DJI_0267	Lomelosia argentea (L.) Greuter & Burdet	0	n	1	0	Caprifoliaceae Juss.
Iz_6_1109	DJI_0267	Melampyrum pratense L.	0	n	1	0	Orobanchaceae Vent.
Iz_6_1109	DJI_0267	Oenothera biennis L.	0	a	0	0	Onagraceae Juss.
Iz_6_1109	DJI_0267	Pentanema britannica (L.) D.Gut.Larr., Santos-Vicente, Anderb., E.Rico & M.M.Mart.Ort.	0	n	1	0	Asteraceae Bercht. & J.Presl
Iz_6_1109	DJI_0267	Peucedanum oreoselinum (L.) Moench	0	n	1	0	Apiaceae Lindl.
Iz_6_1109	DJI_0267	Peucedanum ruthenicum M.Bieb.	0	n	1	0	Apiaceae Lindl.
Iz_6_1109	DJI_0267	Pilosella echioides (Lumn.) F.W.Schultz & Sch.Bip.	0	n	1	0	Asteraceae Bercht. & J.Presl
Iz_6_1109	DJI_0267	Pilosella officinarum F. Schultz et Sch. Bip.	0	n	1	0	Asteraceae Bercht. & J.Presl
Iz_6_1109	DJI_0267	Pilosella sp.	0		NA	0	Asteraceae Bercht. & J.Presl
Iz_6_1109	DJI_0267	Pinus sylvestris L.	0	n	1	1	Pinaceae Spreng. ex F.Rudolphi
Iz_6_1109	DJI_0267	Poa annua L.	0	n	1	0	Poaceae Barnhart
Iz_6_1109	DJI_0267	Poa nemoralis L.	0	n	1	0	Poaceae Barnhart
Iz_6_1109	DJI_0267	Poa sp.	0		NA	0	Poaceae Barnhart
Iz_6_1109	DJI_0267	Polygonatum odoratum (Mill.) Druce	0	n	1	0	Asparagaceae Juss.

Iz_6_1109	DJI_0267	Polygonum arenarium Waldst. & Kit.	0	n	1	0	Polygonaceae Juss.
Iz_6_1109	DJI_0267	Populus nigra L.	0	n	1	1	Salicaceae Mirb.
Iz_6_1109	DJI_0267	Populus tremula L.	0	n	1	1	Salicaceae Mirb.
Iz_6_1109	DJI_0267	Populus Ã canescens (Aiton) Sm.	0	n	1	1	Salicaceae Mirb.
Iz_6_1109	DJI_0267	Potentilla argentea L.	0	n	1	0	Rosaceae Juss.
Iz_6_1109	DJI_0267	Psammophiliella muralis (L.) Ikonn.	0	n	1	0	Caryophyllaceae Juss.
Iz_6_1109	DJI_0267	Psephellus sumensis (Kalen.) Greuter	0	n	1	0	Asteraceae Bercht. & J.Presl
Iz_6_1109	DJI_0267	Pulsatilla pratensis (L.) Mill. s.l.	0	n	1	0	Ranunculaceae Juss.
Iz_6_1109	DJI_0267	Pyrus communis L.	0	a	0	1	Rosaceae Juss.
Iz_6_1109	DJI_0267	Quercus robur L.	0	n	1	1	Fagaceae Dumort.
Iz_6_1109	DJI_0267	Robinia pseudoacacia L.	0	a	0	1	Fabaceae Lindl.
Iz_6_1109	DJI_0267	Rosa sp.	0		NA	1	Rosaceae Juss.
Iz_6_1109	DJI_0267	Rubus idaeus L.	0	n	1	0	Rosaceae Juss.
Iz_6_1109	DJI_0267	Rumex acetosella L.	0	n	1	0	Polygonaceae Juss.
Iz_6_1109	DJI_0267	Rumex thyrsiflorus Fingerh.	0	n	1	0	Polygonaceae Juss.
Iz_6_1109	DJI_0267	Salix alba L.	0	n	1	1	Salicaceae Mirb.
Iz_6_1109	DJI_0267	Sambucus nigra L.	0	n	1	1	Viburnaceae Raf.
Iz_6_1109	DJI_0267	Secale sylvestre Host	0	n	1	0	Poaceae Barnhart
Iz_6_1109	DJI_0267	Sempervivum ruthenicum Schnittsp. & C.B. Lehm.	0	n	1	0	Crassulaceae J.St.-Hil.
Iz_6_1109	DJI_0267	Setaria viridis (L.) P. Beauv.	0	n	1	0	Poaceae Barnhart
Iz_6_1109	DJI_0267	Silene borysthenica (Gruner) Walters	0	n	1	0	Caryophyllaceae Juss.
Iz_6_1109	DJI_0267	Silene latifolia subsp. alba (Mill.) Greuter & Burdet	0	n	1	0	Caryophyllaceae Juss.
Iz_6_1109	DJI_0267	Solidago virgaurea L.	0	n	1	0	Asteraceae Bercht. & J.Presl
Iz_6_1109	DJI_0267	Sorbus aucuparia L.	0	n	1	1	Rosaceae Juss.
Iz_6_1109	DJI_0267	Species 11.09_6_0254	0		NA	0	
Iz_6_1109	DJI_0267	Stachys recta L.	0	n	1	0	Lamiacea Lindl.
Iz_6_1109	DJI_0267	Tanacetum vulgare L.	0	n	1	0	Asteraceae Bercht. & J.Presl
Iz_6_1109	DJI_0267	Thymus pallasianus Heinr.Braun	0	n	1	0	Lamiacea Lindl.
Iz_6_1109	DJI_0267	Tilia cordata Mill.	0	n	1	1	Malvaceae Juss.
Iz_6_1109	DJI_0267	Trifolium alpestre L.	0	n	1	0	Fabaceae Lindl.
Iz_6_1109	DJI_0267	Trifolium arvense L.	0	n	1	0	Fabaceae Lindl.
Iz_6_1109	DJI_0267	Ulmus glabra Huds.	0	n	1	1	Ulmaceae Mirb.
Iz_6_1109	DJI_0267	Verbascum densiflorum Bertol.	0	n	1	0	Scrophulariaceae Juss.
Iz_6_1109	DJI_0267	Veronica austriaca L.	0	n	1	0	Plantaginaceae Juss.
Iz_6_1109	DJI_0267	Veronica incana L.	0	n	1	0	Plantaginaceae Juss.
Iz_6_1109	DJI_0267	Veronica verna L.	0	n	1	0	Plantaginaceae Juss.
Iz_6_1109	DJI_0267	Vincetoxicum hirundinaria Medik	0	n	1	0	Apocynaceae Juss.
Iz_6_1109	DJI_0272	Acer tataricum L.	0	n	1	1	Sapindaceae Juss.
Iz_6_1109	DJI_0272	Achillea millefolium L. s.l.	0	n	1	0	Asteraceae Bercht. & J.Presl
Iz_6_1109	DJI_0272	Allium oleraceum L.	0	n	1	0	Amaryllidaceae J.St.-Hil.
Iz_6_1109	DJI_0272	Ambrosia artemisiifolia L.	0	a	0	0	Asteraceae Bercht. & J.Presl
Iz_6_1109	DJI_0272	Amorpha fruticosa L.	0	a	0	0	Fabaceae Lindl.
Iz_6_1109	DJI_0272	Anthericum ramosum L.	0	n	1	0	Asparagaceae Juss.
Iz_6_1109	DJI_0272	Artemisia marschalliana Spreng.	0	n	1	0	Asteraceae Bercht. & J.Presl
Iz_6_1109	DJI_0272	Asparagus officinalis L.	0	n	1	0	Asparagaceae Juss.
Iz_6_1109	DJI_0272	Astragalus cicer L.	0	n	1	0	Fabaceae Lindl.
Iz_6_1109	DJI_0272	Bassia laniflora (S.G.Gmel.) A.J.Scott	0	n	1	0	Amaranthaceae Juss.
Iz_6_1109	DJI_0272	Berberis vulgaris L.	0	n	1	0	Berberidaceae Juss.
Iz_6_1109	DJI_0272	Betula pendula Roth.	0	n	1	1	Betulaceae Gray
Iz_6_1109	DJI_0272	Betula pendula Roth.	0	n	1	0	Poaceae Barnhart
Iz_6_1109	DJI_0272	Bromus inermis Leyss.	0		NA	NA	
Iz_6_1109	DJI_0272	Bromus squarrosus L.	0	a	0	0	Poaceae Barnhart
Iz_6_1109	DJI_0272	Bromus tectorum L.	0	a	0	0	Poaceae Barnhart
Iz_6_1109	DJI_0272	Calamagrostis epigejos (L.) Roth	20	n	1	0	Poaceae Barnhart
Iz_6_1109	DJI_0272	Carduus acanthoides L.	0	n	1	0	Asteraceae Bercht. & J.Presl
Iz_6_1109	DJI_0272	Carex sp.	0	n	1	0	Cyperaceae Juss.
Iz_6_1109	DJI_0272	Centaurea majorovii Dumbadze	0	n	1	0	Asteraceae Bercht. & J.Presl
Iz_6_1109	DJI_0272	Chamaecytisus ruthenicus (Fisch.ex Wol.) Klaskova	0	n	1	0	Fabaceae Lindl.
Iz_6_1109	DJI_0272	Chelidonium majus L.	0	n	1	0	Papaveraceae Juss.
Iz_6_1109	DJI_0272	Chenopodium betaceum Andrz.	0	n	1	0	Amaranthaceae Juss.
Iz_6_1109	DJI_0272	Chenopodium cf. album	0	n	1	0	Amaranthaceae Juss.
Iz_6_1109	DJI_0272	Chenopodium sp.	0		NA	0	Amaranthaceae Juss.
Iz_6_1109	DJI_0272	Chondrilla juncea L.	0	n	1	0	Asteraceae Bercht. & J.Presl
Iz_6_1109	DJI_0272	Cirsium vulgare (Savi) Ten.	0	n	1	0	Asteraceae Bercht. & J.Presl
Iz_6_1109	DJI_0272	Clinopodium acinos (L.) Kuntze	0	n	1	0	Lamiacea Lindl.
Iz_6_1109	DJI_0272	Convallaria majalis L.	0	n	1	0	Asparagaceae Juss.
Iz_6_1109	DJI_0272	Crataegus rhipidophylla Gand.	0	n	1	1	Rosaceae Juss.
Iz_6_1109	DJI_0272	Crepis foetida L.	0	n	1	0	Asteraceae Bercht. & J.Presl
Iz_6_1109	DJI_0272	Dactylis glomerata L.	0	n	1	0	Poaceae Barnhart
Iz_6_1109	DJI_0272	Dianthus campestris M.Bieb.	0	n	1	0	Caryophyllaceae Juss.
Iz_6_1109	DJI_0272	Digitaria sanguinalis (L.) Scop.	0	a	0	0	Poaceae Barnhart
Iz_6_1109	DJI_0272	Elymus repens (L.) Gould	0	n	1	0	Poaceae Barnhart
Iz_6_1109	DJI_0272	Epilobium tetragonum L.	0	n	1	0	Onagraceae Juss.
Iz_6_1109	DJI_0272	Eragrostis minor Host	0	a	0	0	Poaceae Barnhart
Iz_6_1109	DJI_0272	Erigeron annuus (L.) Desf.	0	a	0	0	Asteraceae Bercht. & J.Presl
Iz_6_1109	DJI_0272	Erigeron canadensis L.	5	a	0	0	Asteraceae Bercht. & J.Presl
Iz_6_1109	DJI_0272	Euphorbia seguieriana Neck.	0	n	1	0	Euphorbiaceae Juss.
Iz_6_1109	DJI_0272	Fallopia dumetorum (L.) Holub	0	n	1	0	Polygonaceae Juss.
Iz_6_1109	DJI_0272	Festuca beckeri (Hack.) Trautv.	0	n	1	0	Poaceae Barnhart
Iz_6_1109	DJI_0272	Fragaria vesca L.	0	n	1	0	Rosaceae Juss.
Iz_6_1109	DJI_0272	Frangula alnus Mill.	0	n	1	1	Rhamnaceae Juss.
Iz_6_1109	DJI_0272	Fraxinus pennsylvanica Marshall	0	a	0	1	Oleaceae Hoffmanns. & Link
Iz_6_1109	DJI_0272	Galeopsis bifida Boenn.	0	n	1	0	Lamiacea Lindl.
Iz_6_1109	DJI_0272	Galium verum L.	0	n	1	0	Rubiaceae Juss.
Iz_6_1109	DJI_0272	Genista tinctoria L.	0	n	1	0	Fabaceae Lindl.
Iz_6_1109	DJI_0272	Geranium robertianum L.	0	n	1	0	Geraniaceae Juss.
Iz_6_1109	DJI_0272	Geranium sanquineum L.	0	n	1	0	Geraniaceae Juss.
Iz_6_1109	DJI_0272	Gypsophila paniculata L.	0	n	1	0	Caryophyllaceae Juss.
Iz_6_1109	DJI_0272	Helichrysum arenarium (L.) Moench	0	n	1	0	Asteraceae Bercht. & J.Presl
Iz_6_1109	DJI_0272	Herniaria polygama J.Gay	0	n	1	0	Caryophyllaceae Juss.
Iz_6_1109	DJI_0272	Hieracium robustum Fr.	0	n	1	0	Asteraceae Bercht. & J.Presl
Iz_6_1109	DJI_0272	Hieracium umbellatum L.	0	n	1	NA	Asteraceae Bercht. & J.Presl
Iz_6_1109	DJI_0272	Hylotelephium maximum (L.) Holub	0	n	1	0	Crassulaceae J.St.-Hil.
Iz_6_1109	DJI_0272	Jacobaea borysthenica (DC.) B.Nord. & Greuter	0	n	1	0	Asteraceae Bercht. & J.Presl
Iz_6_1109	DJI_0272	Jasione montana L.	0	n	1	0	Campanulaceae Juss.
Iz_6_1109	DJI_0272	Jurinea cyanoides (L.) Rchb.s.l.	0	n	1	0	Asteraceae Bercht. & J.Presl
Iz_6_1109	DJI_0272	Koeleria glauca (Spreng.) DC.	0	n	1	0	Poaceae Barnhart
Iz_6_1109	DJI_0272	Lactuca serriola L.	0	a	0	0	Asteraceae Bercht. & J.Presl

Iz_6_1109	DJI_0272	Linaria genistifolia (L.) Mill.	0	n	1	0	Plantaginaceae Juss.
Iz_6_1109	DJI_0272	Lomelosia argentea (L.) Greuter & Burdet	0	n	1	0	Caprifoliaceae Juss.
Iz_6_1109	DJI_0272	Melampyrum pratense L.	0	n	1	0	Orobanchaceae Vent.
Iz_6_1109	DJI_0272	Oenothera biennis L.	0	a	0	0	Onagraceae Juss.
Iz_6_1109	DJI_0272	Pentanema britannica (L.) D.Gut.Larr., Santos-Vicente, Anderb., E.Rico & M.M.Mart.Ort.	0	n	1	0	Asteraceae Bercht. & J.Presl
Iz_6_1109	DJI_0272	Peucedanum oreoselinum (L.) Moench	0	n	1	0	Apiaceae Lindl.
Iz_6_1109	DJI_0272	Peucedanum ruthenicum M.Bieb.	0	n	1	0	Apiaceae Lindl.
Iz_6_1109	DJI_0272	Pilosella echioides (Lumn.) F.W.Schultz & Sch.Bip.	0	n	1	0	Asteraceae Bercht. & J.Presl
Iz_6_1109	DJI_0272	Pilosella officinarum F. Schultz et Sch. Bip.	0	n	1	0	Asteraceae Bercht. & J.Presl
Iz_6_1109	DJI_0272	Pilosella sp.	0		NA	0	Asteraceae Bercht. & J.Presl
Iz_6_1109	DJI_0272	Pinus sylvestris L.	0	n	1	1	Pinaceae Spreng. ex F.Rudolphi
Iz_6_1109	DJI_0272	Poa annua L.	0	n	1	0	Poaceae Barnhart
Iz_6_1109	DJI_0272	Poa nemoralis L.	0	n	1	0	Poaceae Barnhart
Iz_6_1109	DJI_0272	Poa sp.	0		NA	0	Poaceae Barnhart
Iz_6_1109	DJI_0272	Polygonatum odoratum (Mill.) Druce	0	n	1	0	Asparagaceae Juss.
Iz_6_1109	DJI_0272	Polygonum arenarium Waldst. & Kit.	0	n	1	0	Polygonaceae Juss.
Iz_6_1109	DJI_0272	Populus nigra L.	0	n	1	1	Salicaceae Mirb.
Iz_6_1109	DJI_0272	Populus tremula L.	0	n	1	1	Salicaceae Mirb.
Iz_6_1109	DJI_0272	Populus Ã canescens (Aiton) Sm.	0	n	1	1	Salicaceae Mirb.
Iz_6_1109	DJI_0272	Potentilla argentea L.	0	n	1	0	Rosaceae Juss.
Iz_6_1109	DJI_0272	Psammophiliella muralis (L.) Ikonn.	0	n	1	0	Caryophyllaceae Juss.
Iz_6_1109	DJI_0272	Psephellus sumensis (Kalen.) Greuter	0	n	1	0	Asteraceae Bercht. & J.Presl
Iz_6_1109	DJI_0272	Pulsatilla pratensis (L.) Mill. s.l.	0	n	1	0	Ranunculaceae Juss.
Iz_6_1109	DJI_0272	Pyrus communis L.	0	a	0	1	Rosaceae Juss.
Iz_6_1109	DJI_0272	Quercus robur L.	0	n	1	1	Fagaceae Dumort.
Iz_6_1109	DJI_0272	Robinia pseudoacacia L.	0	a	0	1	Fabaceae Lindl.
Iz_6_1109	DJI_0272	Rosa sp.	0		NA	1	Rosaceae Juss.
Iz_6_1109	DJI_0272	Rubus idaeus L.	0	n	1	0	Rosaceae Juss.
Iz_6_1109	DJI_0272	Rumex acetosella L.	0	n	1	0	Polygonaceae Juss.
Iz_6_1109	DJI_0272	Rumex thyrsiflorus Fingerh.	0	n	1	0	Polygonaceae Juss.
Iz_6_1109	DJI_0272	Salix alba L.	0	n	1	1	Salicaceae Mirb.
Iz_6_1109	DJI_0272	Sambucus nigra L.	0	n	1	1	Viburnaceae Raf.
Iz_6_1109	DJI_0272	Secale sylvestre Host	0	n	1	0	Poaceae Barnhart
Iz_6_1109	DJI_0272	Sempervivum ruthenicum Schnittsp. & C.B. Lehm.	0	n	1	0	Crassulaceae J.St.-Hil.
Iz_6_1109	DJI_0272	Setaria viridis (L.) P. Beauv.	0	n	1	0	Poaceae Barnhart
Iz_6_1109	DJI_0272	Silene borysthenica (Gruner) Walters	0	n	1	0	Caryophyllaceae Juss.
Iz_6_1109	DJI_0272	Silene latifolia subsp. alba (Mill.) Greuter & Burdet	0	n	1	0	Caryophyllaceae Juss.
Iz_6_1109	DJI_0272	Solidago virgaurea L.	0	n	1	0	Asteraceae Bercht. & J.Presl
Iz_6_1109	DJI_0272	Sorbus aucuparia L.	0	n	1	1	Rosaceae Juss.
Iz_6_1109	DJI_0272	Species 11.09_6_0254	0		NA	0	
Iz_6_1109	DJI_0272	Stachys recta L.	0	n	1	0	Lamiacea Lindl.
Iz_6_1109	DJI_0272	Tanacetum vulgare L.	0	n	1	0	Asteraceae Bercht. & J.Presl
Iz_6_1109	DJI_0272	Thymus pallasianus Heinr.Braun	0	n	1	0	Lamiacea Lindl.
Iz_6_1109	DJI_0272	Tilia cordata Mill.	0	n	1	1	Malvaceae Juss.
Iz_6_1109	DJI_0272	Trifolium alpestre L.	0	n	1	0	Fabaceae Lindl.
Iz_6_1109	DJI_0272	Trifolium arvense L.	0	n	1	0	Fabaceae Lindl.
Iz_6_1109	DJI_0272	Ulmus glabra Huds.	0	n	1	1	Ulmaceae Mirb.
Iz_6_1109	DJI_0272	Verbascum densiflorum Bertol.	0	n	1	0	Scrophulariaceae Juss.
Iz_6_1109	DJI_0272	Veronica austriaca L.	0	n	1	0	Plantaginaceae Juss.
Iz_6_1109	DJI_0272	Veronica incana L.	0	n	1	0	Plantaginaceae Juss.
Iz_6_1109	DJI_0272	Veronica verna L.	0	n	1	0	Plantaginaceae Juss.
Iz_6_1109	DJI_0272	Vincetoxicum hirundinaria Medik	0	n	1	0	Apocynaceae Juss.
Iz_6_1109	DJI_0275	Acer tataricum L.	0	n	1	1	Sapindaceae Juss.
Iz_6_1109	DJI_0275	Achillea millefolium L. s.l.	0	n	1	0	Asteraceae Bercht. & J.Presl
Iz_6_1109	DJI_0275	Allium oleraceum L.	0	n	1	0	Amaryllidaceae J.St.-Hil.
Iz_6_1109	DJI_0275	Ambrosia artemisiifolia L.	0	a	0	0	Asteraceae Bercht. & J.Presl
Iz_6_1109	DJI_0275	Amorpha fruticosa L.	0	a	0	0	Fabaceae Lindl.
Iz_6_1109	DJI_0275	Anthericum ramosum L.	0	n	1	0	Asparagaceae Juss.
Iz_6_1109	DJI_0275	Artemisia marschalliana Spreng.	0	n	1	0	Asteraceae Bercht. & J.Presl
Iz_6_1109	DJI_0275	Asparagus officinalis L.	0	n	1	0	Asparagaceae Juss.
Iz_6_1109	DJI_0275	Astragalus cicer L.	0	n	1	0	Fabaceae Lindl.
Iz_6_1109	DJI_0275	Bassia laniflora (S.G.Gmel.) A.J.Scott	0	n	1	0	Amaranthaceae Juss.
Iz_6_1109	DJI_0275	Berberis vulgaris L.	0	n	1	0	Berberidaceae Juss.
Iz_6_1109	DJI_0275	Betula pendula Roth.	0	n	1	1	Betulaceae Gray
Iz_6_1109	DJI_0275	Betula pendula Roth.	0	n	1	0	Poaceae Barnhart
Iz_6_1109	DJI_0275	Bromus inermis Leyss.	0		NA	NA	
Iz_6_1109	DJI_0275	Bromus squarrosus L.	0	a	0	0	Poaceae Barnhart
Iz_6_1109	DJI_0275	Bromus tectorum L.	0	a	0	0	Poaceae Barnhart
Iz_6_1109	DJI_0275	Calamagrostis epigejos (L.) Roth	25	n	1	0	Poaceae Barnhart
Iz_6_1109	DJI_0275	Carduus acanthoides L.	0	n	1	0	Asteraceae Bercht. & J.Presl
Iz_6_1109	DJI_0275	Carex sp.	5	n	1	0	Cyperaceae Juss.
Iz_6_1109	DJI_0275	Centaurea majorovii Dumbadze	0	n	1	0	Asteraceae Bercht. & J.Presl
Iz_6_1109	DJI_0275	Chamaecytisus ruthenicus (Fisch.ex Wol.) Klaskova	0	n	1	0	Fabaceae Lindl.
Iz_6_1109	DJI_0275	Chelidonium majus L.	0	n	1	0	Papaveraceae Juss.
Iz_6_1109	DJI_0275	Chenopodium betaceum Andrz.	0	n	1	0	Amaranthaceae Juss.
Iz_6_1109	DJI_0275	Chenopodium cf. album	0	n	1	0	Amaranthaceae Juss.
Iz_6_1109	DJI_0275	Chenopodium sp.	0		NA	0	Amaranthaceae Juss.
Iz_6_1109	DJI_0275	Chondrilla juncea L.	0	n	1	0	Asteraceae Bercht. & J.Presl
Iz_6_1109	DJI_0275	Cirsium vulgare (Savi) Ten.	0	n	1	0	Asteraceae Bercht. & J.Presl
Iz_6_1109	DJI_0275	Clinopodium acinos (L.) Kuntze	0	n	1	0	Lamiacea Lindl.
Iz_6_1109	DJI_0275	Convallaria majalis L.	0	n	1	0	Asparagaceae Juss.
Iz_6_1109	DJI_0275	Crataegus rhipidophylla Gand.	0	n	1	1	Rosaceae Juss.
Iz_6_1109	DJI_0275	Crepis foetida L.	0	n	1	0	Asteraceae Bercht. & J.Presl
Iz_6_1109	DJI_0275	Dactylis glomerata L.	0	n	1	0	Poaceae Barnhart
Iz_6_1109	DJI_0275	Dianthus campestris M.Bieb.	0	n	1	0	Caryophyllaceae Juss.
Iz_6_1109	DJI_0275	Digitaria sanguinalis (L.) Scop.	0	a	0	0	Poaceae Barnhart
Iz_6_1109	DJI_0275	Elymus repens (L.) Gould	0	n	1	0	Poaceae Barnhart
Iz_6_1109	DJI_0275	Epilobium tetragonum L.	0	n	1	0	Onagraceae Juss.
Iz_6_1109	DJI_0275	Eragrostis minor Host	0	a	0	0	Poaceae Barnhart
Iz_6_1109	DJI_0275	Erigeron annuus (L.) Desf.	0	a	0	0	Asteraceae Bercht. & J.Presl
Iz_6_1109	DJI_0275	Erigeron canadensis L.	0	a	0	0	Asteraceae Bercht. & J.Presl
Iz_6_1109	DJI_0275	Euphorbia seguieriana Neck.	0	n	1	0	Euphorbiaceae Juss.
Iz_6_1109	DJI_0275	Fallopia dumetorum (L.) Holub	0	n	1	0	Polygonaceae Juss.
Iz_6_1109	DJI_0275	Festuca beckeri (Hack.) Trautv.	0	n	1	0	Poaceae Barnhart
Iz_6_1109	DJI_0275	Fragaria vesca L.	0	n	1	0	Rosaceae Juss.
Iz_6_1109	DJI_0275	Frangula alnus Mill.	0	n	1	1	Rhamnaceae Juss.
Iz_6_1109	DJI_0275	Fraxinus pennsylvanica Marshall	0	a	0	1	Oleaceae Hoffmanns. & Link
Iz_6_1109	DJI_0275	Galeopsis bifida Boenn.	0	n	1	0	Lamiacea Lindl.

Iz_6_1109	DJI_0275	Galium verum L.	0	n	1	0	Rubiaceae Juss.
Iz_6_1109	DJI_0275	Genista tinctoria L.	0	n	1	0	Fabaceae Lindl.
Iz_6_1109	DJI_0275	Geranium robertianum L.	0	n	1	0	Geraniaceae Juss.
Iz_6_1109	DJI_0275	Geranium sanquineum L.	0	n	1	0	Geraniaceae Juss.
Iz_6_1109	DJI_0275	Gypsophila paniculata L.	0	n	1	0	Caryophyllaceae Juss.
Iz_6_1109	DJI_0275	Helichrysum arenarium (L.) Moench	0	n	1	0	Asteraceae Bercht. & J.Presl
Iz_6_1109	DJI_0275	Herniaria polygama J.Gay	0	n	1	0	Caryophyllaceae Juss.
Iz_6_1109	DJI_0275	Hieracium robustum Fr.	0	n	1	0	Asteraceae Bercht. & J.Presl
Iz_6_1109	DJI_0275	Hieracium umbellatum L.	0	n	1	NA	Asteraceae Bercht. & J.Presl
Iz_6_1109	DJI_0275	Hylotelephium maximum (L.) Holub	0	n	1	0	Crassulaceae J.St.-Hil.
Iz_6_1109	DJI_0275	Jacobaea borysthenica (DC.) B.Nord. & Greuter	0	n	1	0	Asteraceae Bercht. & J.Presl
Iz_6_1109	DJI_0275	Jasione montana L.	0	n	1	0	Campanulaceae Juss.
Iz_6_1109	DJI_0275	Jurinea cyanoides (L.) Rchb.s.l.	0	n	1	0	Asteraceae Bercht. & J.Presl
Iz_6_1109	DJI_0275	Koeleria glauca (Spreng.) DC.	0	n	1	0	Poaceae Barnhart
Iz_6_1109	DJI_0275	Lactuca serriola L.	0	a	0	0	Asteraceae Bercht. & J.Presl
Iz_6_1109	DJI_0275	Linaria genistifolia (L.) Mill.	0	n	1	0	Plantaginaceae Juss.
Iz_6_1109	DJI_0275	Lomelosia argentea (L.) Greuter & Burdet	0	n	1	0	Caprifoliaceae Juss.
Iz_6_1109	DJI_0275	Melampyrum pratense L.	0	n	1	0	Orobanchaceae Vent.
Iz_6_1109	DJI_0275	Oenothera biennis L.	0	a	0	0	Onagraceae Juss.
Iz_6_1109	DJI_0275	Pentanema britannica (L.) D.Gut.Larr., Santos-Vicente, Anderb., E.Rico & M.M.Mart.Ort.	0	n	1	0	Asteraceae Bercht. & J.Presl
Iz_6_1109	DJI_0275	Peucedanum oreoselinum (L.) Moench	0	n	1	0	Apiaceae Lindl.
Iz_6_1109	DJI_0275	Peucedanum ruthenicum M.Bieb.	0	n	1	0	Apiaceae Lindl.
Iz_6_1109	DJI_0275	Pilosella echioides (Lumn.) F.W.Schultz & Sch.Bip.	0	n	1	0	Asteraceae Bercht. & J.Presl
Iz_6_1109	DJI_0275	Pilosella officinarum F. Schultz et Sch. Bip.	0	n	1	0	Asteraceae Bercht. & J.Presl
Iz_6_1109	DJI_0275	Pilosella sp.	0		NA	0	Asteraceae Bercht. & J.Presl
Iz_6_1109	DJI_0275	Pinus sylvestris L.	0	n	1	1	Pinaceae Spreng. ex F.Rudolphi
Iz_6_1109	DJI_0275	Poa annua L.	0	n	1	0	Poaceae Barnhart
Iz_6_1109	DJI_0275	Poa nemoralis L.	0	n	1	0	Poaceae Barnhart
Iz_6_1109	DJI_0275	Poa sp.	0		NA	0	Poaceae Barnhart
Iz_6_1109	DJI_0275	Polygonatum odoratum (Mill.) Druce	0	n	1	0	Asparagaceae Juss.
Iz_6_1109	DJI_0275	Polygonum arenarium Waldst. & Kit.	0	n	1	0	Polygonaceae Juss.
Iz_6_1109	DJI_0275	Populus nigra L.	0	n	1	1	Salicaceae Mirb.
Iz_6_1109	DJI_0275	Populus tremula L.	0	n	1	1	Salicaceae Mirb.
Iz_6_1109	DJI_0275	Populus Ã canescens (Aiton) Sm.	0	n	1	1	Salicaceae Mirb.
Iz_6_1109	DJI_0275	Potentilla argentea L.	0	n	1	0	Rosaceae Juss.
Iz_6_1109	DJI_0275	Psammophiliella muralis (L.) Ikonn.	0	n	1	0	Caryophyllaceae Juss.
Iz_6_1109	DJI_0275	Psephellus sumensis (Kalen.) Greuter	0	n	1	0	Asteraceae Bercht. & J.Presl
Iz_6_1109	DJI_0275	Pulsatilla pratensis (L.) Mill. s.l.	0	n	1	0	Ranunculaceae Juss.
Iz_6_1109	DJI_0275	Pyrus communis L.	0	a	0	1	Rosaceae Juss.
Iz_6_1109	DJI_0275	Quercus robur L.	0	n	1	1	Fagaceae Dumort.
Iz_6_1109	DJI_0275	Robinia pseudoacacia L.	0	a	0	1	Fabaceae Lindl.
Iz_6_1109	DJI_0275	Rosa sp.	0		NA	1	Rosaceae Juss.
Iz_6_1109	DJI_0275	Rubus idaeus L.	0	n	1	0	Rosaceae Juss.
Iz_6_1109	DJI_0275	Rumex acetosella L.	0	n	1	0	Polygonaceae Juss.
Iz_6_1109	DJI_0275	Rumex thyrsiflorus Fingerh.	0	n	1	0	Polygonaceae Juss.
Iz_6_1109	DJI_0275	Salix alba L.	0	n	1	1	Salicaceae Mirb.
Iz_6_1109	DJI_0275	Sambucus nigra L.	0	n	1	1	Viburnaceae Raf.
Iz_6_1109	DJI_0275	Secale sylvestre Host	0	n	1	0	Poaceae Barnhart
Iz_6_1109	DJI_0275	Sempervivum ruthenicum Schnittsp. & C.B. Lehm.	0	n	1	0	Crassulaceae J.St.-Hil.
Iz_6_1109	DJI_0275	Setaria viridis (L.) P. Beauv.	0	n	1	0	Poaceae Barnhart
Iz_6_1109	DJI_0275	Silene borysthenica (Gruner) Walters	0	n	1	0	Caryophyllaceae Juss.
Iz_6_1109	DJI_0275	Silene latifolia subsp. alba (Mill.) Greuter & Burdet	0	n	1	0	Caryophyllaceae Juss.
Iz_6_1109	DJI_0275	Solidago virgaurea L.	0	n	1	0	Asteraceae Bercht. & J.Presl
Iz_6_1109	DJI_0275	Sorbus aucuparia L.	0	n	1	1	Rosaceae Juss.
Iz_6_1109	DJI_0275	Species 11.09_6_0254	0		NA	0	
Iz_6_1109	DJI_0275	Stachys recta L.	0	n	1	0	Lamiacea Lindl.
Iz_6_1109	DJI_0275	Tanacetum vulgare L.	0	n	1	0	Asteraceae Bercht. & J.Presl
Iz_6_1109	DJI_0275	Thymus pallasianus Heinr.Braun	0	n	1	0	Lamiacea Lindl.
Iz_6_1109	DJI_0275	Tilia cordata Mill.	0	n	1	1	Malvaceae Juss.
Iz_6_1109	DJI_0275	Trifolium alpestre L.	0	n	1	0	Fabaceae Lindl.
Iz_6_1109	DJI_0275	Trifolium arvense L.	0	n	1	0	Fabaceae Lindl.
Iz_6_1109	DJI_0275	Ulmus glabra Huds.	0	n	1	1	Ulmaceae Mirb.
Iz_6_1109	DJI_0275	Verbascum densiflorum Bertol.	0	n	1	0	Scrophulariaceae Juss.
Iz_6_1109	DJI_0275	Veronica austriaca L.	0	n	1	0	Plantaginaceae Juss.
Iz_6_1109	DJI_0275	Veronica incana L.	0	n	1	0	Plantaginaceae Juss.
Iz_6_1109	DJI_0275	Veronica verna L.	0	n	1	0	Plantaginaceae Juss.
Iz_6_1109	DJI_0275	Vincetoxicum hirundinaria Medik	0	n	1	0	Apocynaceae Juss.
Iz_6_1109	DJI_0278	Acer tataricum L.	0	n	1	1	Sapindaceae Juss.
Iz_6_1109	DJI_0278	Achillea millefolium L. s.l.	0	n	1	0	Asteraceae Bercht. & J.Presl
Iz_6_1109	DJI_0278	Allium oleraceum L.	0	n	1	0	Amaryllidaceae J.St.-Hil.
Iz_6_1109	DJI_0278	Ambrosia artemisiifolia L.	0	a	0	0	Asteraceae Bercht. & J.Presl
Iz_6_1109	DJI_0278	Amorpha fruticosa L.	0	a	0	0	Fabaceae Lindl.
Iz_6_1109	DJI_0278	Anthericum ramosum L.	0	n	1	0	Asparagaceae Juss.
Iz_6_1109	DJI_0278	Artemisia marschalliana Spreng.	0	n	1	0	Asteraceae Bercht. & J.Presl
Iz_6_1109	DJI_0278	Asparagus officinalis L.	0	n	1	0	Asparagaceae Juss.
Iz_6_1109	DJI_0278	Astragalus cicer L.	0	n	1	0	Fabaceae Lindl.
Iz_6_1109	DJI_0278	Bassia laniflora (S.G.Gmel.) A.J.Scott	0	n	1	0	Amaranthaceae Juss.
Iz_6_1109	DJI_0278	Berberis vulgaris L.	0	n	1	0	Berberidaceae Juss.
Iz_6_1109	DJI_0278	Betula pendula Roth.	0	n	1	1	Betulaceae Gray
Iz_6_1109	DJI_0278	Betula pendula Roth.	0	n	1	0	Poaceae Barnhart
Iz_6_1109	DJI_0278	Bromus inermis Leyss.	0		NA	NA	
Iz_6_1109	DJI_0278	Bromus squarrosus L.	0	a	0	0	Poaceae Barnhart
Iz_6_1109	DJI_0278	Bromus tectorum L.	0	a	0	0	Poaceae Barnhart
Iz_6_1109	DJI_0278	Calamagrostis epigejos (L.) Roth	25	n	1	0	Poaceae Barnhart
Iz_6_1109	DJI_0278	Carduus acanthoides L.	0	n	1	0	Asteraceae Bercht. & J.Presl
Iz_6_1109	DJI_0278	Carex sp.	0	n	1	0	Cyperaceae Juss.
Iz_6_1109	DJI_0278	Centaurea majorovii Dumbadze	0	n	1	0	Asteraceae Bercht. & J.Presl
Iz_6_1109	DJI_0278	Chamaecytisus ruthenicus (Fisch.ex Wol.) Klaskova	0	n	1	0	Fabaceae Lindl.
Iz_6_1109	DJI_0278	Chelidonium majus L.	0	n	1	0	Papaveraceae Juss.
Iz_6_1109	DJI_0278	Chenopodium betaceum Andrz.	0	n	1	0	Amaranthaceae Juss.
Iz_6_1109	DJI_0278	Chenopodium cf. album	0	n	1	0	Amaranthaceae Juss.
Iz_6_1109	DJI_0278	Chenopodium sp.	0		NA	0	Amaranthaceae Juss.
Iz_6_1109	DJI_0278	Chondrilla juncea L.	0	n	1	0	Asteraceae Bercht. & J.Presl
Iz_6_1109	DJI_0278	Cirsium vulgare (Savi) Ten.	0	n	1	0	Asteraceae Bercht. & J.Presl
Iz_6_1109	DJI_0278	Clinopodium acinos (L.) Kuntze	0	n	1	0	Lamiacea Lindl.
Iz_6_1109	DJI_0278	Convallaria majalis L.	0	n	1	0	Asparagaceae Juss.
Iz_6_1109	DJI_0278	Crataegus rhipidophylla Gand.	0	n	1	1	Rosaceae Juss.
Iz_6_1109	DJI_0278	Crepis foetida L.	0	n	1	0	Asteraceae Bercht. & J.Presl

Iz_6_1109	DJI_0278	Dactylis glomerata L.	0	n	1	0	Poaceae Barnhart
Iz_6_1109	DJI_0278	Dianthus campestris M.Bieb.	0	n	1	0	Caryophyllaceae Juss.
Iz_6_1109	DJI_0278	Digitaria sanguinalis (L.) Scop.	0	a	0	0	Poaceae Barnhart
Iz_6_1109	DJI_0278	Elymus repens (L.) Gould	0	n	1	0	Poaceae Barnhart
Iz_6_1109	DJI_0278	Epilobium tetragonum L.	0	n	1	0	Onagraceae Juss.
Iz_6_1109	DJI_0278	Eragrostis minor Host	0	a	0	0	Poaceae Barnhart
Iz_6_1109	DJI_0278	Erigeron annuus (L.) Desf.	0	a	0	0	Asteraceae Bercht. & J.Presl
Iz_6_1109	DJI_0278	Erigeron canadensis L.	10	a	0	0	Asteraceae Bercht. & J.Presl
Iz_6_1109	DJI_0278	Euphorbia seguieriana Neck.	0	n	1	0	Euphorbiaceae Juss.
Iz_6_1109	DJI_0278	Fallopia dumetorum (L.) Holub	0	n	1	0	Polygonaceae Juss.
Iz_6_1109	DJI_0278	Festuca beckeri (Hack.) Trautv.	0	n	1	0	Poaceae Barnhart
Iz_6_1109	DJI_0278	Fragaria vesca L.	0	n	1	0	Rosaceae Juss.
Iz_6_1109	DJI_0278	Frangula alnus Mill.	0	n	1	1	Rhamnaceae Juss.
Iz_6_1109	DJI_0278	Fraxinus pennsylvanica Marshall	0	a	0	1	Oleaceae Hoffmanns. & Link
Iz_6_1109	DJI_0278	Galeopsis bifida Boenn.	0	n	1	0	Lamiacea Lindl.
Iz_6_1109	DJI_0278	Galium verum L.	0	n	1	0	Rubiaceae Juss.
Iz_6_1109	DJI_0278	Genista tinctoria L.	0	n	1	0	Fabaceae Lindl.
Iz_6_1109	DJI_0278	Geranium robertianum L.	0	n	1	0	Geraniaceae Juss.
Iz_6_1109	DJI_0278	Geranium sanquineum L.	0	n	1	0	Geraniaceae Juss.
Iz_6_1109	DJI_0278	Gypsophila paniculata L.	0	n	1	0	Caryophyllaceae Juss.
Iz_6_1109	DJI_0278	Helichrysum arenarium (L.) Moench	0	n	1	0	Asteraceae Bercht. & J.Presl
Iz_6_1109	DJI_0278	Herniaria polygama J.Gay	0	n	1	0	Caryophyllaceae Juss.
Iz_6_1109	DJI_0278	Hieracium robustum Fr.	0	n	1	0	Asteraceae Bercht. & J.Presl
Iz_6_1109	DJI_0278	Hieracium umbellatum L.	0	n	1	NA	Asteraceae Bercht. & J.Presl
Iz_6_1109	DJI_0278	Hylotelephium maximum (L.) Holub	0	n	1	0	Crassulaceae J.St.-Hil.
Iz_6_1109	DJI_0278	Jacobaea borysthenica (DC.) B.Nord. & Greuter	0	n	1	0	Asteraceae Bercht. & J.Presl
Iz_6_1109	DJI_0278	Jasione montana L.	0	n	1	0	Campanulaceae Juss.
Iz_6_1109	DJI_0278	Jurinea cyanoides (L.) Rchb.s.l.	0	n	1	0	Asteraceae Bercht. & J.Presl
Iz_6_1109	DJI_0278	Koeleria glauca (Spreng.) DC.	0	n	1	0	Poaceae Barnhart
Iz_6_1109	DJI_0278	Lactuca serriola L.	0	a	0	0	Asteraceae Bercht. & J.Presl
Iz_6_1109	DJI_0278	Linaria genistifolia (L.) Mill.	0	n	1	0	Plantaginaceae Juss.
Iz_6_1109	DJI_0278	Lomelosia argentea (L.) Greuter & Burdet	0	n	1	0	Caprifoliaceae Juss.
Iz_6_1109	DJI_0278	Melampyrum pratense L.	0	n	1	0	Orobanchaceae Vent.
Iz_6_1109	DJI_0278	Oenothera biennis L.	0	a	0	0	Onagraceae Juss.
Iz_6_1109	DJI_0278	Pentanema britannica (L.) D.Gut.Larr., Santos-Vicente, Anderb., E.Rico & M.M.Mart.Ort.	0	n	1	0	Asteraceae Bercht. & J.Presl
Iz_6_1109	DJI_0278	Peucedanum oreoselinum (L.) Moench	0	n	1	0	Apiaceae Lindl.
Iz_6_1109	DJI_0278	Peucedanum ruthenicum M.Bieb.	0	n	1	0	Apiaceae Lindl.
Iz_6_1109	DJI_0278	Pilosella echioides (Lumn.) F.W.Schultz & Sch.Bip.	0	n	1	0	Asteraceae Bercht. & J.Presl
Iz_6_1109	DJI_0278	Pilosella officinarum F. Schultz et Sch. Bip.	0	n	1	0	Asteraceae Bercht. & J.Presl
Iz_6_1109	DJI_0278	Pilosella sp.	0		NA	0	Asteraceae Bercht. & J.Presl
Iz_6_1109	DJI_0278	Pinus sylvestris L.	0	n	1	1	Pinaceae Spreng. ex F.Rudolphi
Iz_6_1109	DJI_0278	Poa annua L.	0	n	1	0	Poaceae Barnhart
Iz_6_1109	DJI_0278	Poa nemoralis L.	0	n	1	0	Poaceae Barnhart
Iz_6_1109	DJI_0278	Poa sp.	0		NA	0	Poaceae Barnhart
Iz_6_1109	DJI_0278	Polygonatum odoratum (Mill.) Druce	0	n	1	0	Asparagaceae Juss.
Iz_6_1109	DJI_0278	Polygonum arenarium Waldst. & Kit.	0	n	1	0	Polygonaceae Juss.
Iz_6_1109	DJI_0278	Populus nigra L.	0	n	1	1	Salicaceae Mirb.
Iz_6_1109	DJI_0278	Populus tremula L.	0	n	1	1	Salicaceae Mirb.
Iz_6_1109	DJI_0278	Populus Ã canescens (Aiton) Sm.	0	n	1	1	Salicaceae Mirb.
Iz_6_1109	DJI_0278	Potentilla argentea L.	0	n	1	0	Rosaceae Juss.
Iz_6_1109	DJI_0278	Psammophiliella muralis (L.) Ikonn.	0	n	1	0	Caryophyllaceae Juss.
Iz_6_1109	DJI_0278	Psephellus sumensis (Kalen.) Greuter	0	n	1	0	Asteraceae Bercht. & J.Presl
Iz_6_1109	DJI_0278	Pulsatilla pratensis (L.) Mill. s.l.	0	n	1	0	Ranunculaceae Juss.
Iz_6_1109	DJI_0278	Pyrus communis L.	0	a	0	1	Rosaceae Juss.
Iz_6_1109	DJI_0278	Quercus robur L.	0	n	1	1	Fagaceae Dumort.
Iz_6_1109	DJI_0278	Robinia pseudoacacia L.	0	a	0	1	Fabaceae Lindl.
Iz_6_1109	DJI_0278	Rosa sp.	0		NA	1	Rosaceae Juss.
Iz_6_1109	DJI_0278	Rubus idaeus L.	0	n	1	0	Rosaceae Juss.
Iz_6_1109	DJI_0278	Rumex acetosella L.	0	n	1	0	Polygonaceae Juss.
Iz_6_1109	DJI_0278	Rumex thyrsiflorus Fingerh.	0	n	1	0	Polygonaceae Juss.
Iz_6_1109	DJI_0278	Salix alba L.	0	n	1	1	Salicaceae Mirb.
Iz_6_1109	DJI_0278	Sambucus nigra L.	0	n	1	1	Viburnaceae Raf.
Iz_6_1109	DJI_0278	Secale sylvestre Host	0	n	1	0	Poaceae Barnhart
Iz_6_1109	DJI_0278	Sempervivum ruthenicum Schnittsp. & C.B. Lehm.	0	n	1	0	Crassulaceae J.St.-Hil.
Iz_6_1109	DJI_0278	Setaria viridis (L.) P. Beauv.	0	n	1	0	Poaceae Barnhart
Iz_6_1109	DJI_0278	Silene borysthenica (Gruner) Walters	0	n	1	0	Caryophyllaceae Juss.
Iz_6_1109	DJI_0278	Silene latifolia subsp. alba (Mill.) Greuter & Burdet	0	n	1	0	Caryophyllaceae Juss.
Iz_6_1109	DJI_0278	Solidago virgaurea L.	0	n	1	0	Asteraceae Bercht. & J.Presl
Iz_6_1109	DJI_0278	Sorbus aucuparia L.	0	n	1	1	Rosaceae Juss.
Iz_6_1109	DJI_0278	Species 11.09_6_0254	0		NA	0	
Iz_6_1109	DJI_0278	Stachys recta L.	0	n	1	0	Lamiacea Lindl.
Iz_6_1109	DJI_0278	Tanacetum vulgare L.	0	n	1	0	Asteraceae Bercht. & J.Presl
Iz_6_1109	DJI_0278	Thymus pallasianus Heinr.Braun	0	n	1	0	Lamiacea Lindl.
Iz_6_1109	DJI_0278	Tilia cordata Mill.	0	n	1	1	Malvaceae Juss.
Iz_6_1109	DJI_0278	Trifolium alpestre L.	0	n	1	0	Fabaceae Lindl.
Iz_6_1109	DJI_0278	Trifolium arvense L.	0	n	1	0	Fabaceae Lindl.
Iz_6_1109	DJI_0278	Ulmus glabra Huds.	0	n	1	1	Ulmaceae Mirb.
Iz_6_1109	DJI_0278	Verbascum densiflorum Bertol.	0	n	1	0	Scrophulariaceae Juss.
Iz_6_1109	DJI_0278	Veronica austriaca L.	0	n	1	0	Plantaginaceae Juss.
Iz_6_1109	DJI_0278	Veronica incana L.	0	n	1	0	Plantaginaceae Juss.
Iz_6_1109	DJI_0278	Veronica verna L.	0	n	1	0	Plantaginaceae Juss.
Iz_6_1109	DJI_0278	Vincetoxicum hirundinaria Medik	0	n	1	0	Apocynaceae Juss.
Iz_6_1109	DJI_0280	Acer tataricum L.	0	n	1	1	Sapindaceae Juss.
Iz_6_1109	DJI_0280	Achillea millefolium L. s.l.	0	n	1	0	Asteraceae Bercht. & J.Presl
Iz_6_1109	DJI_0280	Allium oleraceum L.	0	n	1	0	Amaryllidaceae J.St.-Hil.
Iz_6_1109	DJI_0280	Ambrosia artemisiifolia L.	0	a	0	0	Asteraceae Bercht. & J.Presl
Iz_6_1109	DJI_0280	Amorpha fruticosa L.	0	a	0	0	Fabaceae Lindl.
Iz_6_1109	DJI_0280	Anthericum ramosum L.	0	n	1	0	Asparagaceae Juss.
Iz_6_1109	DJI_0280	Artemisia marschalliana Spreng.	0	n	1	0	Asteraceae Bercht. & J.Presl
Iz_6_1109	DJI_0280	Asparagus officinalis L.	0	n	1	0	Asparagaceae Juss.
Iz_6_1109	DJI_0280	Astragalus cicer L.	0	n	1	0	Fabaceae Lindl.
Iz_6_1109	DJI_0280	Bassia laniflora (S.G.Gmel.) A.J.Scott	0	n	1	0	Amaranthaceae Juss.
Iz_6_1109	DJI_0280	Berberis vulgaris L.	0	n	1	0	Berberidaceae Juss.
Iz_6_1109	DJI_0280	Betula pendula Roth.	0	n	1	1	Betulaceae Gray
Iz_6_1109	DJI_0280	Betula pendula Roth.	0	n	1	0	Poaceae Barnhart
Iz_6_1109	DJI_0280	Bromus inermis Leyss.	0		NA	NA	
Iz_6_1109	DJI_0280	Bromus squarrosus L.	0	a	0	0	Poaceae Barnhart
Iz_6_1109	DJI_0280	Bromus tectorum L.	0	a	0	0	Poaceae Barnhart

Iz_6_1109	DJI_0280	Calamagrostis epigejos (L.) Roth	75	n	1	0	Poaceae Barnhart
Iz_6_1109	DJI_0280	Carduus acanthoides L.	0	n	1	0	Asteraceae Bercht. & J.Presl
Iz_6_1109	DJI_0280	Carex sp.	0	n	1	0	Cyperaceae Juss.
Iz_6_1109	DJI_0280	Centaurea majorovii Dumbadze	0	n	1	0	Asteraceae Bercht. & J.Presl
Iz_6_1109	DJI_0280	Chamaecytisus ruthenicus (Fisch.ex Wol.) Klaskova	0	n	1	0	Fabaceae Lindl.
Iz_6_1109	DJI_0280	Chelidonium majus L.	0	n	1	0	Papaveraceae Juss.
Iz_6_1109	DJI_0280	Chenopodium betaceum Andrz.	0	n	1	0	Amaranthaceae Juss.
Iz_6_1109	DJI_0280	Chenopodium cf. album	0	n	1	0	Amaranthaceae Juss.
Iz_6_1109	DJI_0280	Chenopodium sp.	0		NA	0	Amaranthaceae Juss.
Iz_6_1109	DJI_0280	Chondrilla juncea L.	0	n	1	0	Asteraceae Bercht. & J.Presl
Iz_6_1109	DJI_0280	Cirsium vulgare (Savi) Ten.	0	n	1	0	Asteraceae Bercht. & J.Presl
Iz_6_1109	DJI_0280	Clinopodium acinos (L.) Kuntze	0	n	1	0	Lamiacea Lindl.
Iz_6_1109	DJI_0280	Convallaria majalis L.	0	n	1	0	Asparagaceae Juss.
Iz_6_1109	DJI_0280	Crataegus rhipidophylla Gand.	0	n	1	1	Rosaceae Juss.
Iz_6_1109	DJI_0280	Crepis foetida L.	0	n	1	0	Asteraceae Bercht. & J.Presl
Iz_6_1109	DJI_0280	Dactylis glomerata L.	0	n	1	0	Poaceae Barnhart
Iz_6_1109	DJI_0280	Dianthus campestris M.Bieb.	0	n	1	0	Caryophyllaceae Juss.
Iz_6_1109	DJI_0280	Digitaria sanguinalis (L.) Scop.	0	a	0	0	Poaceae Barnhart
Iz_6_1109	DJI_0280	Elymus repens (L.) Gould	0	n	1	0	Poaceae Barnhart
Iz_6_1109	DJI_0280	Epilobium tetragonum L.	0	n	1	0	Onagraceae Juss.
Iz_6_1109	DJI_0280	Eragrostis minor Host	0	a	0	0	Poaceae Barnhart
Iz_6_1109	DJI_0280	Erigeron annuus (L.) Desf.	0	a	0	0	Asteraceae Bercht. & J.Presl
Iz_6_1109	DJI_0280	Erigeron canadensis L.	1	a	0	0	Asteraceae Bercht. & J.Presl
Iz_6_1109	DJI_0280	Euphorbia seguieriana Neck.	0	n	1	0	Euphorbiaceae Juss.
Iz_6_1109	DJI_0280	Fallopia dumetorum (L.) Holub	0	n	1	0	Polygonaceae Juss.
Iz_6_1109	DJI_0280	Festuca beckeri (Hack.) Trautv.	0	n	1	0	Poaceae Barnhart
Iz_6_1109	DJI_0280	Fragaria vesca L.	0	n	1	0	Rosaceae Juss.
Iz_6_1109	DJI_0280	Frangula alnus Mill.	0	n	1	1	Rhamnaceae Juss.
Iz_6_1109	DJI_0280	Fraxinus pennsylvanica Marshall	0	a	0	1	Oleaceae Hoffmanns. & Link
Iz_6_1109	DJI_0280	Galeopsis bifida Boenn.	0	n	1	0	Lamiacea Lindl.
Iz_6_1109	DJI_0280	Galium verum L.	0	n	1	0	Rubiaceae Juss.
Iz_6_1109	DJI_0280	Genista tinctoria L.	0	n	1	0	Fabaceae Lindl.
Iz_6_1109	DJI_0280	Geranium robertianum L.	0	n	1	0	Geraniaceae Juss.
Iz_6_1109	DJI_0280	Geranium sanquineum L.	0	n	1	0	Geraniaceae Juss.
Iz_6_1109	DJI_0280	Gypsophila paniculata L.	0	n	1	0	Caryophyllaceae Juss.
Iz_6_1109	DJI_0280	Helichrysum arenarium (L.) Moench	0	n	1	0	Asteraceae Bercht. & J.Presl
Iz_6_1109	DJI_0280	Herniaria polygama J.Gay	0	n	1	0	Caryophyllaceae Juss.
Iz_6_1109	DJI_0280	Hieracium robustum Fr.	0	n	1	0	Asteraceae Bercht. & J.Presl
Iz_6_1109	DJI_0280	Hieracium umbellatum L.	0	n	1	NA	Asteraceae Bercht. & J.Presl
Iz_6_1109	DJI_0280	Hylotelephium maximum (L.) Holub	0	n	1	0	Crassulaceae J.St.-Hil.
Iz_6_1109	DJI_0280	Jacobaea borysthenica (DC.) B.Nord. & Greuter	0	n	1	0	Asteraceae Bercht. & J.Presl
Iz_6_1109	DJI_0280	Jasione montana L.	0	n	1	0	Campanulaceae Juss.
Iz_6_1109	DJI_0280	Jurinea cyanoides (L.) Rchb.s.l.	0	n	1	0	Asteraceae Bercht. & J.Presl
Iz_6_1109	DJI_0280	Koeleria glauca (Spreng.) DC.	0	n	1	0	Poaceae Barnhart
Iz_6_1109	DJI_0280	Lactuca serriola L.	0	a	0	0	Asteraceae Bercht. & J.Presl
Iz_6_1109	DJI_0280	Linaria genistifolia (L.) Mill.	0	n	1	0	Plantaginaceae Juss.
Iz_6_1109	DJI_0280	Lomelosia argentea (L.) Greuter & Burdet	0	n	1	0	Caprifoliaceae Juss.
Iz_6_1109	DJI_0280	Melampyrum pratense L.	0	n	1	0	Orobanchaceae Vent.
Iz_6_1109	DJI_0280	Oenothera biennis L.	0	a	0	0	Onagraceae Juss.
Iz_6_1109	DJI_0280	Pentanema britannica (L.) D.Gut.Larr., Santos-Vicente, Anderb., E.Rico & M.M.Mart.Ort.	0	n	1	0	Asteraceae Bercht. & J.Presl
Iz_6_1109	DJI_0280	Peucedanum oreoselinum (L.) Moench	0	n	1	0	Apiaceae Lindl.
Iz_6_1109	DJI_0280	Peucedanum ruthenicum M.Bieb.	0	n	1	0	Apiaceae Lindl.
Iz_6_1109	DJI_0280	Pilosella echioides (Lumn.) F.W.Schultz & Sch.Bip.	0	n	1	0	Asteraceae Bercht. & J.Presl
Iz_6_1109	DJI_0280	Pilosella officinarum F. Schultz et Sch. Bip.	0	n	1	0	Asteraceae Bercht. & J.Presl
Iz_6_1109	DJI_0280	Pilosella sp.	0		NA	0	Asteraceae Bercht. & J.Presl
Iz_6_1109	DJI_0280	Pinus sylvestris L.	0	n	1	1	Pinaceae Spreng. ex F.Rudolphi
Iz_6_1109	DJI_0280	Poa annua L.	0	n	1	0	Poaceae Barnhart
Iz_6_1109	DJI_0280	Poa nemoralis L.	2	n	1	0	Poaceae Barnhart
Iz_6_1109	DJI_0280	Poa sp.	0		NA	0	Poaceae Barnhart
Iz_6_1109	DJI_0280	Polygonatum odoratum (Mill.) Druce	0	n	1	0	Asparagaceae Juss.
Iz_6_1109	DJI_0280	Polygonum arenarium Waldst. & Kit.	0	n	1	0	Polygonaceae Juss.
Iz_6_1109	DJI_0280	Populus nigra L.	0	n	1	1	Salicaceae Mirb.
Iz_6_1109	DJI_0280	Populus tremula L.	0	n	1	1	Salicaceae Mirb.
Iz_6_1109	DJI_0280	Populus Ã canescens (Aiton) Sm.	0	n	1	1	Salicaceae Mirb.
Iz_6_1109	DJI_0280	Potentilla argentea L.	0	n	1	0	Rosaceae Juss.
Iz_6_1109	DJI_0280	Psammophiliella muralis (L.) Ikonn.	0	n	1	0	Caryophyllaceae Juss.
Iz_6_1109	DJI_0280	Psephellus sumensis (Kalen.) Greuter	0	n	1	0	Asteraceae Bercht. & J.Presl
Iz_6_1109	DJI_0280	Pulsatilla pratensis (L.) Mill. s.l.	0	n	1	0	Ranunculaceae Juss.
Iz_6_1109	DJI_0280	Pyrus communis L.	0	a	0	1	Rosaceae Juss.
Iz_6_1109	DJI_0280	Quercus robur L.	0	n	1	1	Fagaceae Dumort.
Iz_6_1109	DJI_0280	Robinia pseudoacacia L.	0	a	0	1	Fabaceae Lindl.
Iz_6_1109	DJI_0280	Rosa sp.	0		NA	1	Rosaceae Juss.
Iz_6_1109	DJI_0280	Rubus idaeus L.	0	n	1	0	Rosaceae Juss.
Iz_6_1109	DJI_0280	Rumex acetosella L.	0	n	1	0	Polygonaceae Juss.
Iz_6_1109	DJI_0280	Rumex thyrsiflorus Fingerh.	0	n	1	0	Polygonaceae Juss.
Iz_6_1109	DJI_0280	Salix alba L.	0	n	1	1	Salicaceae Mirb.
Iz_6_1109	DJI_0280	Sambucus nigra L.	0	n	1	1	Viburnaceae Raf.
Iz_6_1109	DJI_0280	Secale sylvestre Host	0	n	1	0	Poaceae Barnhart
Iz_6_1109	DJI_0280	Sempervivum ruthenicum Schnittsp. & C.B. Lehm.	0	n	1	0	Crassulaceae J.St.-Hil.
Iz_6_1109	DJI_0280	Setaria viridis (L.) P. Beauv.	0	n	1	0	Poaceae Barnhart
Iz_6_1109	DJI_0280	Silene borysthenica (Gruner) Walters	0	n	1	0	Caryophyllaceae Juss.
Iz_6_1109	DJI_0280	Silene latifolia subsp. alba (Mill.) Greuter & Burdet	0	n	1	0	Caryophyllaceae Juss.
Iz_6_1109	DJI_0280	Solidago virgaurea L.	0	n	1	0	Asteraceae Bercht. & J.Presl
Iz_6_1109	DJI_0280	Sorbus aucuparia L.	0	n	1	1	Rosaceae Juss.
Iz_6_1109	DJI_0280	Species 11.09_6_0254	0		NA	0	
Iz_6_1109	DJI_0280	Stachys recta L.	0	n	1	0	Lamiacea Lindl.
Iz_6_1109	DJI_0280	Tanacetum vulgare L.	0	n	1	0	Asteraceae Bercht. & J.Presl
Iz_6_1109	DJI_0280	Thymus pallasianus Heinr.Braun	0	n	1	0	Lamiacea Lindl.
Iz_6_1109	DJI_0280	Tilia cordata Mill.	0	n	1	1	Malvaceae Juss.
Iz_6_1109	DJI_0280	Trifolium alpestre L.	0	n	1	0	Fabaceae Lindl.
Iz_6_1109	DJI_0280	Trifolium arvense L.	0	n	1	0	Fabaceae Lindl.
Iz_6_1109	DJI_0280	Ulmus glabra Huds.	0	n	1	1	Ulmaceae Mirb.
Iz_6_1109	DJI_0280	Verbascum densiflorum Bertol.	0	n	1	0	Scrophulariaceae Juss.
Iz_6_1109	DJI_0280	Veronica austriaca L.	0	n	1	0	Plantaginaceae Juss.
Iz_6_1109	DJI_0280	Veronica incana L.	0	n	1	0	Plantaginaceae Juss.
Iz_6_1109	DJI_0280	Veronica verna L.	0	n	1	0	Plantaginaceae Juss.
Iz_6_1109	DJI_0280	Vincetoxicum hirundinaria Medik	0	n	1	0	Apocynaceae Juss.
Iz_6_1109	DJI_0283	Acer tataricum L.	0	n	1	1	Sapindaceae Juss.

Iz_6_1109	DJI_0283	Achillea millefolium L. s.l.	0	n	1	0	Asteraceae Bercht. & J.Presl
Iz_6_1109	DJI_0283	Allium oleraceum L.	0	n	1	0	Amaryllidaceae J.St.-Hil.
Iz_6_1109	DJI_0283	Ambrosia artemisiifolia L.	0	a	0	0	Asteraceae Bercht. & J.Presl
Iz_6_1109	DJI_0283	Amorpha fruticosa L.	0	a	0	0	Fabaceae Lindl.
Iz_6_1109	DJI_0283	Anthericum ramosum L.	0	n	1	0	Asparagaceae Juss.
Iz_6_1109	DJI_0283	Artemisia marschalliana Spreng.	0	n	1	0	Asteraceae Bercht. & J.Presl
Iz_6_1109	DJI_0283	Asparagus officinalis L.	0	n	1	0	Asparagaceae Juss.
Iz_6_1109	DJI_0283	Astragalus cicer L.	0	n	1	0	Fabaceae Lindl.
Iz_6_1109	DJI_0283	Bassia laniflora (S.G.Gmel.) A.J.Scott	0	n	1	0	Amaranthaceae Juss.
Iz_6_1109	DJI_0283	Berberis vulgaris L.	0	n	1	0	Berberidaceae Juss.
Iz_6_1109	DJI_0283	Betula pendula Roth.	0	n	1	1	Betulaceae Gray
Iz_6_1109	DJI_0283	Betula pendula Roth.	0	n	1	0	Poaceae Barnhart
Iz_6_1109	DJI_0283	Bromus inermis Leyss.	0		NA	NA	
Iz_6_1109	DJI_0283	Bromus squarrosus L.	0	a	0	0	Poaceae Barnhart
Iz_6_1109	DJI_0283	Bromus tectorum L.	0	a	0	0	Poaceae Barnhart
Iz_6_1109	DJI_0283	Calamagrostis epigejos (L.) Roth	85	n	1	0	Poaceae Barnhart
Iz_6_1109	DJI_0283	Carduus acanthoides L.	0	n	1	0	Asteraceae Bercht. & J.Presl
Iz_6_1109	DJI_0283	Carex sp.	0	n	1	0	Cyperaceae Juss.
Iz_6_1109	DJI_0283	Centaurea majorovii Dumbadze	0	n	1	0	Asteraceae Bercht. & J.Presl
Iz_6_1109	DJI_0283	Chamaecytisus ruthenicus (Fisch.ex Wol.) Klaskova	0	n	1	0	Fabaceae Lindl.
Iz_6_1109	DJI_0283	Chelidonium majus L.	0	n	1	0	Papaveraceae Juss.
Iz_6_1109	DJI_0283	Chenopodium betaceum Andrz.	0	n	1	0	Amaranthaceae Juss.
Iz_6_1109	DJI_0283	Chenopodium cf. album	0	n	1	0	Amaranthaceae Juss.
Iz_6_1109	DJI_0283	Chenopodium sp.	0		NA	0	Amaranthaceae Juss.
Iz_6_1109	DJI_0283	Chondrilla juncea L.	0	n	1	0	Asteraceae Bercht. & J.Presl
Iz_6_1109	DJI_0283	Cirsium vulgare (Savi) Ten.	0	n	1	0	Asteraceae Bercht. & J.Presl
Iz_6_1109	DJI_0283	Clinopodium acinos (L.) Kuntze	0	n	1	0	Lamiacea Lindl.
Iz_6_1109	DJI_0283	Convallaria majalis L.	0	n	1	0	Asparagaceae Juss.
Iz_6_1109	DJI_0283	Crataegus rhipidophylla Gand.	0	n	1	1	Rosaceae Juss.
Iz_6_1109	DJI_0283	Crepis foetida L.	0	n	1	0	Asteraceae Bercht. & J.Presl
Iz_6_1109	DJI_0283	Dactylis glomerata L.	0	n	1	0	Poaceae Barnhart
Iz_6_1109	DJI_0283	Dianthus campestris M.Bieb.	0	n	1	0	Caryophyllaceae Juss.
Iz_6_1109	DJI_0283	Digitaria sanguinalis (L.) Scop.	0	a	0	0	Poaceae Barnhart
Iz_6_1109	DJI_0283	Elymus repens (L.) Gould	0	n	1	0	Poaceae Barnhart
Iz_6_1109	DJI_0283	Epilobium tetragonum L.	0	n	1	0	Onagraceae Juss.
Iz_6_1109	DJI_0283	Eragrostis minor Host	0	a	0	0	Poaceae Barnhart
Iz_6_1109	DJI_0283	Erigeron annuus (L.) Desf.	0	a	0	0	Asteraceae Bercht. & J.Presl
Iz_6_1109	DJI_0283	Erigeron canadensis L.	0	a	0	0	Asteraceae Bercht. & J.Presl
Iz_6_1109	DJI_0283	Euphorbia seguieriana Neck.	0	n	1	0	Euphorbiaceae Juss.
Iz_6_1109	DJI_0283	Fallopia dumetorum (L.) Holub	0	n	1	0	Polygonaceae Juss.
Iz_6_1109	DJI_0283	Festuca beckeri (Hack.) Trautv.	0	n	1	0	Poaceae Barnhart
Iz_6_1109	DJI_0283	Fragaria vesca L.	0	n	1	0	Rosaceae Juss.
Iz_6_1109	DJI_0283	Frangula alnus Mill.	0	n	1	1	Rhamnaceae Juss.
Iz_6_1109	DJI_0283	Fraxinus pennsylvanica Marshall	0	a	0	1	Oleaceae Hoffmanns. & Link
Iz_6_1109	DJI_0283	Galeopsis bifida Boenn.	0	n	1	0	Lamiacea Lindl.
Iz_6_1109	DJI_0283	Galium verum L.	0	n	1	0	Rubiaceae Juss.
Iz_6_1109	DJI_0283	Genista tinctoria L.	0	n	1	0	Fabaceae Lindl.
Iz_6_1109	DJI_0283	Geranium robertianum L.	0	n	1	0	Geraniaceae Juss.
Iz_6_1109	DJI_0283	Geranium sanquineum L.	0	n	1	0	Geraniaceae Juss.
Iz_6_1109	DJI_0283	Gypsophila paniculata L.	0	n	1	0	Caryophyllaceae Juss.
Iz_6_1109	DJI_0283	Helichrysum arenarium (L.) Moench	0	n	1	0	Asteraceae Bercht. & J.Presl
Iz_6_1109	DJI_0283	Herniaria polygama J.Gay	0	n	1	0	Caryophyllaceae Juss.
Iz_6_1109	DJI_0283	Hieracium robustum Fr.	0	n	1	0	Asteraceae Bercht. & J.Presl
Iz_6_1109	DJI_0283	Hieracium umbellatum L.	0	n	1	NA	Asteraceae Bercht. & J.Presl
Iz_6_1109	DJI_0283	Hylotelephium maximum (L.) Holub	0	n	1	0	Crassulaceae J.St.-Hil.
Iz_6_1109	DJI_0283	Jacobaea borysthenica (DC.) B.Nord. & Greuter	0	n	1	0	Asteraceae Bercht. & J.Presl
Iz_6_1109	DJI_0283	Jasione montana L.	0	n	1	0	Campanulaceae Juss.
Iz_6_1109	DJI_0283	Jurinea cyanoides (L.) Rchb.s.l.	0	n	1	0	Asteraceae Bercht. & J.Presl
Iz_6_1109	DJI_0283	Koeleria glauca (Spreng.) DC.	0	n	1	0	Poaceae Barnhart
Iz_6_1109	DJI_0283	Lactuca serriola L.	0	a	0	0	Asteraceae Bercht. & J.Presl
Iz_6_1109	DJI_0283	Linaria genistifolia (L.) Mill.	0	n	1	0	Plantaginaceae Juss.
Iz_6_1109	DJI_0283	Lomelosia argentea (L.) Greuter & Burdet	0	n	1	0	Caprifoliaceae Juss.
Iz_6_1109	DJI_0283	Melampyrum pratense L.	0	n	1	0	Orobanchaceae Vent.
Iz_6_1109	DJI_0283	Oenothera biennis L.	0	a	0	0	Onagraceae Juss.
Iz_6_1109	DJI_0283	Pentanema britannica (L.) D.Gut.Larr., Santos-Vicente, Anderb., E.Rico & M.M.Mart.Ort.	0	n	1	0	Asteraceae Bercht. & J.Presl
Iz_6_1109	DJI_0283	Peucedanum oreoselinum (L.) Moench	0	n	1	0	Apiaceae Lindl.
Iz_6_1109	DJI_0283	Peucedanum ruthenicum M.Bieb.	0	n	1	0	Apiaceae Lindl.
Iz_6_1109	DJI_0283	Pilosella echioides (Lumn.) F.W.Schultz & Sch.Bip.	0	n	1	0	Asteraceae Bercht. & J.Presl
Iz_6_1109	DJI_0283	Pilosella officinarum F. Schultz et Sch. Bip.	0	n	1	0	Asteraceae Bercht. & J.Presl
Iz_6_1109	DJI_0283	Pilosella sp.	0		NA	0	Asteraceae Bercht. & J.Presl
Iz_6_1109	DJI_0283	Pinus sylvestris L.	0	n	1	1	Pinaceae Spreng. ex F.Rudolphi
Iz_6_1109	DJI_0283	Poa annua L.	0	n	1	0	Poaceae Barnhart
Iz_6_1109	DJI_0283	Poa nemoralis L.	0	n	1	0	Poaceae Barnhart
Iz_6_1109	DJI_0283	Poa sp.	0		NA	0	Poaceae Barnhart
Iz_6_1109	DJI_0283	Polygonatum odoratum (Mill.) Druce	0	n	1	0	Asparagaceae Juss.
Iz_6_1109	DJI_0283	Polygonum arenarium Waldst. & Kit.	0	n	1	0	Polygonaceae Juss.
Iz_6_1109	DJI_0283	Populus nigra L.	0	n	1	1	Salicaceae Mirb.
Iz_6_1109	DJI_0283	Populus tremula L.	0	n	1	1	Salicaceae Mirb.
Iz_6_1109	DJI_0283	Populus Ã canescens (Aiton) Sm.	0	n	1	1	Salicaceae Mirb.
Iz_6_1109	DJI_0283	Potentilla argentea L.	0	n	1	0	Rosaceae Juss.
Iz_6_1109	DJI_0283	Psammophiliella muralis (L.) Ikonn.	0	n	1	0	Caryophyllaceae Juss.
Iz_6_1109	DJI_0283	Psephellus sumensis (Kalen.) Greuter	0	n	1	0	Asteraceae Bercht. & J.Presl
Iz_6_1109	DJI_0283	Pulsatilla pratensis (L.) Mill. s.l.	0	n	1	0	Ranunculaceae Juss.
Iz_6_1109	DJI_0283	Pyrus communis L.	0	a	0	1	Rosaceae Juss.
Iz_6_1109	DJI_0283	Quercus robur L.	0	n	1	1	Fagaceae Dumort.
Iz_6_1109	DJI_0283	Robinia pseudoacacia L.	0	a	0	1	Fabaceae Lindl.
Iz_6_1109	DJI_0283	Rosa sp.	0		NA	1	Rosaceae Juss.
Iz_6_1109	DJI_0283	Rubus idaeus L.	0	n	1	0	Rosaceae Juss.
Iz_6_1109	DJI_0283	Rumex acetosella L.	0	n	1	0	Polygonaceae Juss.
Iz_6_1109	DJI_0283	Rumex thyrsiflorus Fingerh.	0	n	1	0	Polygonaceae Juss.
Iz_6_1109	DJI_0283	Salix alba L.	0	n	1	1	Salicaceae Mirb.
Iz_6_1109	DJI_0283	Sambucus nigra L.	0	n	1	1	Viburnaceae Raf.
Iz_6_1109	DJI_0283	Secale sylvestre Host	0	n	1	0	Poaceae Barnhart
Iz_6_1109	DJI_0283	Sempervivum ruthenicum Schnittsp. & C.B. Lehm.	0	n	1	0	Crassulaceae J.St.-Hil.
Iz_6_1109	DJI_0283	Setaria viridis (L.) P. Beauv.	0	n	1	0	Poaceae Barnhart
Iz_6_1109	DJI_0283	Silene borysthenica (Gruner) Walters	0	n	1	0	Caryophyllaceae Juss.
Iz_6_1109	DJI_0283	Silene latifolia subsp. alba (Mill.) Greuter & Burdet	0	n	1	0	Caryophyllaceae Juss.
Iz_6_1109	DJI_0283	Solidago virgaurea L.	0	n	1	0	Asteraceae Bercht. & J.Presl

Iz_6_1109	DJI_0283	Sorbus aucuparia L.	0	n	1	1	Rosaceae Juss.
Iz_6_1109	DJI_0283	Species 11.09_6_0254	0		NA	0	
Iz_6_1109	DJI_0283	Stachys recta L.	0	n	1	0	Lamiacea Lindl.
Iz_6_1109	DJI_0283	Tanacetum vulgare L.	0	n	1	0	Asteraceae Bercht. & J.Presl
Iz_6_1109	DJI_0283	Thymus pallasianus Heinr.Braun	0	n	1	0	Lamiacea Lindl.
Iz_6_1109	DJI_0283	Tilia cordata Mill.	0	n	1	1	Malvaceae Juss.
Iz_6_1109	DJI_0283	Trifolium alpestre L.	0	n	1	0	Fabaceae Lindl.
Iz_6_1109	DJI_0283	Trifolium arvense L.	0	n	1	0	Fabaceae Lindl.
Iz_6_1109	DJI_0283	Ulmus glabra Huds.	0	n	1	1	Ulmaceae Mirb.
Iz_6_1109	DJI_0283	Verbascum densiflorum Bertol.	0	n	1	0	Scrophulariaceae Juss.
Iz_6_1109	DJI_0283	Veronica austriaca L.	0	n	1	0	Plantaginaceae Juss.
Iz_6_1109	DJI_0283	Veronica incana L.	0	n	1	0	Plantaginaceae Juss.
Iz_6_1109	DJI_0283	Veronica verna L.	0	n	1	0	Plantaginaceae Juss.
Iz_6_1109	DJI_0283	Vincetoxicum hirundinaria Medik	0	n	1	0	Apocynaceae Juss.
Iz_6_1109	DJI_0286	Acer tataricum L.	0	n	1	1	Sapindaceae Juss.
Iz_6_1109	DJI_0286	Achillea millefolium L. s.l.	0	n	1	0	Asteraceae Bercht. & J.Presl
Iz_6_1109	DJI_0286	Allium oleraceum L.	0	n	1	0	Amaryllidaceae J.St.-Hil.
Iz_6_1109	DJI_0286	Ambrosia artemisiifolia L.	0	a	0	0	Asteraceae Bercht. & J.Presl
Iz_6_1109	DJI_0286	Amorpha fruticosa L.	0	a	0	0	Fabaceae Lindl.
Iz_6_1109	DJI_0286	Anthericum ramosum L.	0	n	1	0	Asparagaceae Juss.
Iz_6_1109	DJI_0286	Artemisia marschalliana Spreng.	0	n	1	0	Asteraceae Bercht. & J.Presl
Iz_6_1109	DJI_0286	Asparagus officinalis L.	0	n	1	0	Asparagaceae Juss.
Iz_6_1109	DJI_0286	Astragalus cicer L.	0	n	1	0	Fabaceae Lindl.
Iz_6_1109	DJI_0286	Bassia laniflora (S.G.Gmel.) A.J.Scott	0	n	1	0	Amaranthaceae Juss.
Iz_6_1109	DJI_0286	Berberis vulgaris L.	0	n	1	0	Berberidaceae Juss.
Iz_6_1109	DJI_0286	Betula pendula Roth.	0	n	1	1	Betulaceae Gray
Iz_6_1109	DJI_0286	Betula pendula Roth.	0	n	1	0	Poaceae Barnhart
Iz_6_1109	DJI_0286	Bromus inermis Leyss.	0		NA	NA	
Iz_6_1109	DJI_0286	Bromus squarrosus L.	0	a	0	0	Poaceae Barnhart
Iz_6_1109	DJI_0286	Bromus tectorum L.	0	a	0	0	Poaceae Barnhart
Iz_6_1109	DJI_0286	Calamagrostis epigejos (L.) Roth	20	n	1	0	Poaceae Barnhart
Iz_6_1109	DJI_0286	Carduus acanthoides L.	0	n	1	0	Asteraceae Bercht. & J.Presl
Iz_6_1109	DJI_0286	Carex sp.	0	n	1	0	Cyperaceae Juss.
Iz_6_1109	DJI_0286	Centaurea majorovii Dumbadze	0	n	1	0	Asteraceae Bercht. & J.Presl
Iz_6_1109	DJI_0286	Chamaecytisus ruthenicus (Fisch.ex Wol.) Klaskova	0	n	1	0	Fabaceae Lindl.
Iz_6_1109	DJI_0286	Chelidonium majus L.	0	n	1	0	Papaveraceae Juss.
Iz_6_1109	DJI_0286	Chenopodium betaceum Andrz.	0	n	1	0	Amaranthaceae Juss.
Iz_6_1109	DJI_0286	Chenopodium cf. album	0	n	1	0	Amaranthaceae Juss.
Iz_6_1109	DJI_0286	Chenopodium sp.	0		NA	0	Amaranthaceae Juss.
Iz_6_1109	DJI_0286	Chondrilla juncea L.	0	n	1	0	Asteraceae Bercht. & J.Presl
Iz_6_1109	DJI_0286	Cirsium vulgare (Savi) Ten.	0	n	1	0	Asteraceae Bercht. & J.Presl
Iz_6_1109	DJI_0286	Clinopodium acinos (L.) Kuntze	0	n	1	0	Lamiacea Lindl.
Iz_6_1109	DJI_0286	Convallaria majalis L.	0	n	1	0	Asparagaceae Juss.
Iz_6_1109	DJI_0286	Crataegus rhipidophylla Gand.	0	n	1	1	Rosaceae Juss.
Iz_6_1109	DJI_0286	Crepis foetida L.	0	n	1	0	Asteraceae Bercht. & J.Presl
Iz_6_1109	DJI_0286	Dactylis glomerata L.	0	n	1	0	Poaceae Barnhart
Iz_6_1109	DJI_0286	Dianthus campestris M.Bieb.	0	n	1	0	Caryophyllaceae Juss.
Iz_6_1109	DJI_0286	Digitaria sanguinalis (L.) Scop.	0	a	0	0	Poaceae Barnhart
Iz_6_1109	DJI_0286	Elymus repens (L.) Gould	0	n	1	0	Poaceae Barnhart
Iz_6_1109	DJI_0286	Epilobium tetragonum L.	0	n	1	0	Onagraceae Juss.
Iz_6_1109	DJI_0286	Eragrostis minor Host	0	a	0	0	Poaceae Barnhart
Iz_6_1109	DJI_0286	Erigeron annuus (L.) Desf.	0	a	0	0	Asteraceae Bercht. & J.Presl
Iz_6_1109	DJI_0286	Erigeron canadensis L.	20	a	0	0	Asteraceae Bercht. & J.Presl
Iz_6_1109	DJI_0286	Euphorbia seguieriana Neck.	0	n	1	0	Euphorbiaceae Juss.
Iz_6_1109	DJI_0286	Fallopia dumetorum (L.) Holub	0	n	1	0	Polygonaceae Juss.
Iz_6_1109	DJI_0286	Festuca beckeri (Hack.) Trautv.	0	n	1	0	Poaceae Barnhart
Iz_6_1109	DJI_0286	Fragaria vesca L.	0	n	1	0	Rosaceae Juss.
Iz_6_1109	DJI_0286	Frangula alnus Mill.	0	n	1	1	Rhamnaceae Juss.
Iz_6_1109	DJI_0286	Fraxinus pennsylvanica Marshall	0	a	0	1	Oleaceae Hoffmanns. & Link
Iz_6_1109	DJI_0286	Galeopsis bifida Boenn.	0	n	1	0	Lamiacea Lindl.
Iz_6_1109	DJI_0286	Galium verum L.	0	n	1	0	Rubiaceae Juss.
Iz_6_1109	DJI_0286	Genista tinctoria L.	0	n	1	0	Fabaceae Lindl.
Iz_6_1109	DJI_0286	Geranium robertianum L.	0	n	1	0	Geraniaceae Juss.
Iz_6_1109	DJI_0286	Geranium sanquineum L.	0	n	1	0	Geraniaceae Juss.
Iz_6_1109	DJI_0286	Gypsophila paniculata L.	0	n	1	0	Caryophyllaceae Juss.
Iz_6_1109	DJI_0286	Helichrysum arenarium (L.) Moench	0	n	1	0	Asteraceae Bercht. & J.Presl
Iz_6_1109	DJI_0286	Herniaria polygama J.Gay	0	n	1	0	Caryophyllaceae Juss.
Iz_6_1109	DJI_0286	Hieracium robustum Fr.	0	n	1	0	Asteraceae Bercht. & J.Presl
Iz_6_1109	DJI_0286	Hieracium umbellatum L.	0	n	1	NA	Asteraceae Bercht. & J.Presl
Iz_6_1109	DJI_0286	Hylotelephium maximum (L.) Holub	0	n	1	0	Crassulaceae J.St.-Hil.
Iz_6_1109	DJI_0286	Jacobaea borysthenica (DC.) B.Nord. & Greuter	0	n	1	0	Asteraceae Bercht. & J.Presl
Iz_6_1109	DJI_0286	Jasione montana L.	0	n	1	0	Campanulaceae Juss.
Iz_6_1109	DJI_0286	Jurinea cyanoides (L.) Rchb.s.l.	0	n	1	0	Asteraceae Bercht. & J.Presl
Iz_6_1109	DJI_0286	Koeleria glauca (Spreng.) DC.	0	n	1	0	Poaceae Barnhart
Iz_6_1109	DJI_0286	Lactuca serriola L.	0	a	0	0	Asteraceae Bercht. & J.Presl
Iz_6_1109	DJI_0286	Linaria genistifolia (L.) Mill.	0	n	1	0	Plantaginaceae Juss.
Iz_6_1109	DJI_0286	Lomelosia argentea (L.) Greuter & Burdet	0	n	1	0	Caprifoliaceae Juss.
Iz_6_1109	DJI_0286	Melampyrum pratense L.	0	n	1	0	Orobanchaceae Vent.
Iz_6_1109	DJI_0286	Oenothera biennis L.	0	a	0	0	Onagraceae Juss.
Iz_6_1109	DJI_0286	Pentanema britannica (L.) D.Gut.Larr., Santos-Vicente, Anderb., E.Rico & M.M.Mart.Ort.	0	n	1	0	Asteraceae Bercht. & J.Presl
Iz_6_1109	DJI_0286	Peucedanum oreoselinum (L.) Moench	0	n	1	0	Apiaceae Lindl.
Iz_6_1109	DJI_0286	Peucedanum ruthenicum M.Bieb.	0	n	1	0	Apiaceae Lindl.
Iz_6_1109	DJI_0286	Pilosella echioides (Lumn.) F.W.Schultz & Sch.Bip.	0	n	1	0	Asteraceae Bercht. & J.Presl
Iz_6_1109	DJI_0286	Pilosella officinarum F. Schultz et Sch. Bip.	0	n	1	0	Asteraceae Bercht. & J.Presl
Iz_6_1109	DJI_0286	Pilosella sp.	0		NA	0	Asteraceae Bercht. & J.Presl
Iz_6_1109	DJI_0286	Pinus sylvestris L.	0	n	1	1	Pinaceae Spreng. ex F.Rudolphi
Iz_6_1109	DJI_0286	Poa annua L.	0	n	1	0	Poaceae Barnhart
Iz_6_1109	DJI_0286	Poa nemoralis L.	0	n	1	0	Poaceae Barnhart
Iz_6_1109	DJI_0286	Poa sp.	0		NA	0	Poaceae Barnhart
Iz_6_1109	DJI_0286	Polygonatum odoratum (Mill.) Druce	0	n	1	0	Asparagaceae Juss.
Iz_6_1109	DJI_0286	Polygonum arenarium Waldst. & Kit.	0	n	1	0	Polygonaceae Juss.
Iz_6_1109	DJI_0286	Populus nigra L.	0	n	1	1	Salicaceae Mirb.
Iz_6_1109	DJI_0286	Populus tremula L.	0	n	1	1	Salicaceae Mirb.
Iz_6_1109	DJI_0286	Populus Ã canescens (Aiton) Sm.	0	n	1	1	Salicaceae Mirb.
Iz_6_1109	DJI_0286	Potentilla argentea L.	0	n	1	0	Rosaceae Juss.
Iz_6_1109	DJI_0286	Psammophiliella muralis (L.) Ikonn.	0	n	1	0	Caryophyllaceae Juss.
Iz_6_1109	DJI_0286	Psephellus sumensis (Kalen.) Greuter	0	n	1	0	Asteraceae Bercht. & J.Presl
Iz_6_1109	DJI_0286	Pulsatilla pratensis (L.) Mill. s.l.	0	n	1	0	Ranunculaceae Juss.

Iz_6_1109	DJI_0286	Pyrus communis L.	0	a	0	1	Rosaceae Juss.
Iz_6_1109	DJI_0286	Quercus robur L.	0	n	1	1	Fagaceae Dumort.
Iz_6_1109	DJI_0286	Robinia pseudoacacia L.	0	a	0	1	Fabaceae Lindl.
Iz_6_1109	DJI_0286	Rosa sp.	0		NA	1	Rosaceae Juss.
Iz_6_1109	DJI_0286	Rubus idaeus L.	0	n	1	0	Rosaceae Juss.
Iz_6_1109	DJI_0286	Rumex acetosella L.	0	n	1	0	Polygonaceae Juss.
Iz_6_1109	DJI_0286	Rumex thyrsiflorus Fingerh.	0	n	1	0	Polygonaceae Juss.
Iz_6_1109	DJI_0286	Salix alba L.	0	n	1	1	Salicaceae Mirb.
Iz_6_1109	DJI_0286	Sambucus nigra L.	0	n	1	1	Viburnaceae Raf.
Iz_6_1109	DJI_0286	Secale sylvestre Host	0	n	1	0	Poaceae Barnhart
Iz_6_1109	DJI_0286	Sempervivum ruthenicum Schnittsp. & C.B. Lehm.	0	n	1	0	Crassulaceae J.St.-Hil.
Iz_6_1109	DJI_0286	Setaria viridis (L.) P. Beauv.	0	n	1	0	Poaceae Barnhart
Iz_6_1109	DJI_0286	Silene borysthenica (Gruner) Walters	0	n	1	0	Caryophyllaceae Juss.
Iz_6_1109	DJI_0286	Silene latifolia subsp. alba (Mill.) Greuter & Burdet	0	n	1	0	Caryophyllaceae Juss.
Iz_6_1109	DJI_0286	Solidago virgaurea L.	0	n	1	0	Asteraceae Bercht. & J.Presl
Iz_6_1109	DJI_0286	Sorbus aucuparia L.	0	n	1	1	Rosaceae Juss.
Iz_6_1109	DJI_0286	Species 11.09_6_0254	0		NA	0	
Iz_6_1109	DJI_0286	Stachys recta L.	0	n	1	0	Lamiacea Lindl.
Iz_6_1109	DJI_0286	Tanacetum vulgare L.	0	n	1	0	Asteraceae Bercht. & J.Presl
Iz_6_1109	DJI_0286	Thymus pallasianus Heinr.Braun	0	n	1	0	Lamiacea Lindl.
Iz_6_1109	DJI_0286	Tilia cordata Mill.	0	n	1	1	Malvaceae Juss.
Iz_6_1109	DJI_0286	Trifolium alpestre L.	0	n	1	0	Fabaceae Lindl.
Iz_6_1109	DJI_0286	Trifolium arvense L.	0	n	1	0	Fabaceae Lindl.
Iz_6_1109	DJI_0286	Ulmus glabra Huds.	0	n	1	1	Ulmaceae Mirb.
Iz_6_1109	DJI_0286	Verbascum densiflorum Bertol.	0	n	1	0	Scrophulariaceae Juss.
Iz_6_1109	DJI_0286	Veronica austriaca L.	0	n	1	0	Plantaginaceae Juss.
Iz_6_1109	DJI_0286	Veronica incana L.	0	n	1	0	Plantaginaceae Juss.
Iz_6_1109	DJI_0286	Veronica verna L.	0	n	1	0	Plantaginaceae Juss.
Iz_6_1109	DJI_0286	Vincetoxicum hirundinaria Medik	0	n	1	0	Apocynaceae Juss.
Iz_6_1109	DJI_0289	Acer tataricum L.	0	n	1	1	Sapindaceae Juss.
Iz_6_1109	DJI_0289	Achillea millefolium L. s.l.	0	n	1	0	Asteraceae Bercht. & J.Presl
Iz_6_1109	DJI_0289	Allium oleraceum L.	0	n	1	0	Amaryllidaceae J.St.-Hil.
Iz_6_1109	DJI_0289	Ambrosia artemisiifolia L.	0	a	0	0	Asteraceae Bercht. & J.Presl
Iz_6_1109	DJI_0289	Amorpha fruticosa L.	0	a	0	0	Fabaceae Lindl.
Iz_6_1109	DJI_0289	Anthericum ramosum L.	0	n	1	0	Asparagaceae Juss.
Iz_6_1109	DJI_0289	Artemisia marschalliana Spreng.	0	n	1	0	Asteraceae Bercht. & J.Presl
Iz_6_1109	DJI_0289	Asparagus officinalis L.	0	n	1	0	Asparagaceae Juss.
Iz_6_1109	DJI_0289	Astragalus cicer L.	0	n	1	0	Fabaceae Lindl.
Iz_6_1109	DJI_0289	Bassia laniflora (S.G.Gmel.) A.J.Scott	0	n	1	0	Amaranthaceae Juss.
Iz_6_1109	DJI_0289	Berberis vulgaris L.	0	n	1	0	Berberidaceae Juss.
Iz_6_1109	DJI_0289	Betula pendula Roth.	0	n	1	1	Betulaceae Gray
Iz_6_1109	DJI_0289	Betula pendula Roth.	0	n	1	0	Poaceae Barnhart
Iz_6_1109	DJI_0289	Bromus inermis Leyss.	0		NA	NA	
Iz_6_1109	DJI_0289	Bromus squarrosus L.	0	a	0	0	Poaceae Barnhart
Iz_6_1109	DJI_0289	Bromus tectorum L.	0	a	0	0	Poaceae Barnhart
Iz_6_1109	DJI_0289	Calamagrostis epigejos (L.) Roth	20	n	1	0	Poaceae Barnhart
Iz_6_1109	DJI_0289	Carduus acanthoides L.	0	n	1	0	Asteraceae Bercht. & J.Presl
Iz_6_1109	DJI_0289	Carex sp.	0	n	1	0	Cyperaceae Juss.
Iz_6_1109	DJI_0289	Centaurea majorovii Dumbadze	0	n	1	0	Asteraceae Bercht. & J.Presl
Iz_6_1109	DJI_0289	Chamaecytisus ruthenicus (Fisch.ex Wol.) Klaskova	0	n	1	0	Fabaceae Lindl.
Iz_6_1109	DJI_0289	Chelidonium majus L.	0	n	1	0	Papaveraceae Juss.
Iz_6_1109	DJI_0289	Chenopodium betaceum Andrz.	0	n	1	0	Amaranthaceae Juss.
Iz_6_1109	DJI_0289	Chenopodium cf. album	0	n	1	0	Amaranthaceae Juss.
Iz_6_1109	DJI_0289	Chenopodium sp.	0		NA	0	Amaranthaceae Juss.
Iz_6_1109	DJI_0289	Chondrilla juncea L.	0	n	1	0	Asteraceae Bercht. & J.Presl
Iz_6_1109	DJI_0289	Cirsium vulgare (Savi) Ten.	0	n	1	0	Asteraceae Bercht. & J.Presl
Iz_6_1109	DJI_0289	Clinopodium acinos (L.) Kuntze	0	n	1	0	Lamiacea Lindl.
Iz_6_1109	DJI_0289	Convallaria majalis L.	0	n	1	0	Asparagaceae Juss.
Iz_6_1109	DJI_0289	Crataegus rhipidophylla Gand.	0	n	1	1	Rosaceae Juss.
Iz_6_1109	DJI_0289	Crepis foetida L.	0	n	1	0	Asteraceae Bercht. & J.Presl
Iz_6_1109	DJI_0289	Dactylis glomerata L.	0	n	1	0	Poaceae Barnhart
Iz_6_1109	DJI_0289	Dianthus campestris M.Bieb.	0	n	1	0	Caryophyllaceae Juss.
Iz_6_1109	DJI_0289	Digitaria sanguinalis (L.) Scop.	0	a	0	0	Poaceae Barnhart
Iz_6_1109	DJI_0289	Elymus repens (L.) Gould	0	n	1	0	Poaceae Barnhart
Iz_6_1109	DJI_0289	Epilobium tetragonum L.	0	n	1	0	Onagraceae Juss.
Iz_6_1109	DJI_0289	Eragrostis minor Host	0	a	0	0	Poaceae Barnhart
Iz_6_1109	DJI_0289	Erigeron annuus (L.) Desf.	0	a	0	0	Asteraceae Bercht. & J.Presl
Iz_6_1109	DJI_0289	Erigeron canadensis L.	8	a	0	0	Asteraceae Bercht. & J.Presl
Iz_6_1109	DJI_0289	Euphorbia seguieriana Neck.	0	n	1	0	Euphorbiaceae Juss.
Iz_6_1109	DJI_0289	Fallopia dumetorum (L.) Holub	0	n	1	0	Polygonaceae Juss.
Iz_6_1109	DJI_0289	Festuca beckeri (Hack.) Trautv.	0	n	1	0	Poaceae Barnhart
Iz_6_1109	DJI_0289	Fragaria vesca L.	0	n	1	0	Rosaceae Juss.
Iz_6_1109	DJI_0289	Frangula alnus Mill.	0	n	1	1	Rhamnaceae Juss.
Iz_6_1109	DJI_0289	Fraxinus pennsylvanica Marshall	0	a	0	1	Oleaceae Hoffmanns. & Link
Iz_6_1109	DJI_0289	Galeopsis bifida Boenn.	0	n	1	0	Lamiacea Lindl.
Iz_6_1109	DJI_0289	Galium verum L.	0	n	1	0	Rubiaceae Juss.
Iz_6_1109	DJI_0289	Genista tinctoria L.	0	n	1	0	Fabaceae Lindl.
Iz_6_1109	DJI_0289	Geranium robertianum L.	0	n	1	0	Geraniaceae Juss.
Iz_6_1109	DJI_0289	Geranium sanquineum L.	0	n	1	0	Geraniaceae Juss.
Iz_6_1109	DJI_0289	Gypsophila paniculata L.	0	n	1	0	Caryophyllaceae Juss.
Iz_6_1109	DJI_0289	Helichrysum arenarium (L.) Moench	0	n	1	0	Asteraceae Bercht. & J.Presl
Iz_6_1109	DJI_0289	Herniaria polygama J.Gay	0	n	1	0	Caryophyllaceae Juss.
Iz_6_1109	DJI_0289	Hieracium robustum Fr.	0	n	1	0	Asteraceae Bercht. & J.Presl
Iz_6_1109	DJI_0289	Hieracium umbellatum L.	0	n	1	NA	Asteraceae Bercht. & J.Presl
Iz_6_1109	DJI_0289	Hylotelephium maximum (L.) Holub	0	n	1	0	Crassulaceae J.St.-Hil.
Iz_6_1109	DJI_0289	Jacobaea borysthenica (DC.) B.Nord. & Greuter	0	n	1	0	Asteraceae Bercht. & J.Presl
Iz_6_1109	DJI_0289	Jasione montana L.	0	n	1	0	Campanulaceae Juss.
Iz_6_1109	DJI_0289	Jurinea cyanoides (L.) Rchb.s.l.	0	n	1	0	Asteraceae Bercht. & J.Presl
Iz_6_1109	DJI_0289	Koeleria glauca (Spreng.) DC.	0	n	1	0	Poaceae Barnhart
Iz_6_1109	DJI_0289	Lactuca serriola L.	0	a	0	0	Asteraceae Bercht. & J.Presl
Iz_6_1109	DJI_0289	Linaria genistifolia (L.) Mill.	0	n	1	0	Plantaginaceae Juss.
Iz_6_1109	DJI_0289	Lomelosia argentea (L.) Greuter & Burdet	0	n	1	0	Caprifoliaceae Juss.
Iz_6_1109	DJI_0289	Melampyrum pratense L.	0	n	1	0	Orobanchaceae Vent.
Iz_6_1109	DJI_0289	Oenothera biennis L.	0	a	0	0	Onagraceae Juss.
Iz_6_1109	DJI_0289	Pentanema britannica (L.) D.Gut.Larr., Santos-Vicente, Anderb., E.Rico & M.M.Mart.Ort.	0	n	1	0	Asteraceae Bercht. & J.Presl
Iz_6_1109	DJI_0289	Peucedanum oreoselinum (L.) Moench	0	n	1	0	Apiaceae Lindl.
Iz_6_1109	DJI_0289	Peucedanum ruthenicum M.Bieb.	0	n	1	0	Apiaceae Lindl.
Iz_6_1109	DJI_0289	Pilosella echioides (Lumn.) F.W.Schultz & Sch.Bip.	0	n	1	0	Asteraceae Bercht. & J.Presl

Iz_6_1109	DJI_0289	Pilosella officinarum F. Schultz et Sch. Bip.	0	n	1	0	Asteraceae Bercht. & J.Presl
Iz_6_1109	DJI_0289	Pilosella sp.	0		NA	0	Asteraceae Bercht. & J.Presl
Iz_6_1109	DJI_0289	Pinus sylvestris L.	0	n	1	1	Pinaceae Spreng. ex F.Rudolphi
Iz_6_1109	DJI_0289	Poa annua L.	0	n	1	0	Poaceae Barnhart
Iz_6_1109	DJI_0289	Poa nemoralis L.	0	n	1	0	Poaceae Barnhart
Iz_6_1109	DJI_0289	Poa sp.	0		NA	0	Poaceae Barnhart
Iz_6_1109	DJI_0289	Polygonatum odoratum (Mill.) Druce	0	n	1	0	Asparagaceae Juss.
Iz_6_1109	DJI_0289	Polygonum arenarium Waldst. & Kit.	0	n	1	0	Polygonaceae Juss.
Iz_6_1109	DJI_0289	Populus nigra L.	0	n	1	1	Salicaceae Mirb.
Iz_6_1109	DJI_0289	Populus tremula L.	0	n	1	1	Salicaceae Mirb.
Iz_6_1109	DJI_0289	Populus Ã canescens (Aiton) Sm.	0	n	1	1	Salicaceae Mirb.
Iz_6_1109	DJI_0289	Potentilla argentea L.	0	n	1	0	Rosaceae Juss.
Iz_6_1109	DJI_0289	Psammophiliella muralis (L.) Ikonn.	0	n	1	0	Caryophyllaceae Juss.
Iz_6_1109	DJI_0289	Psephellus sumensis (Kalen.) Greuter	0	n	1	0	Asteraceae Bercht. & J.Presl
Iz_6_1109	DJI_0289	Pulsatilla pratensis (L.) Mill. s.l.	0	n	1	0	Ranunculaceae Juss.
Iz_6_1109	DJI_0289	Pyrus communis L.	0	a	0	1	Rosaceae Juss.
Iz_6_1109	DJI_0289	Quercus robur L.	0	n	1	1	Fagaceae Dumort.
Iz_6_1109	DJI_0289	Robinia pseudoacacia L.	0	a	0	1	Fabaceae Lindl.
Iz_6_1109	DJI_0289	Rosa sp.	0		NA	1	Rosaceae Juss.
Iz_6_1109	DJI_0289	Rubus idaeus L.	0	n	1	0	Rosaceae Juss.
Iz_6_1109	DJI_0289	Rumex acetosella L.	0	n	1	0	Polygonaceae Juss.
Iz_6_1109	DJI_0289	Rumex thyrsiflorus Fingerh.	0	n	1	0	Polygonaceae Juss.
Iz_6_1109	DJI_0289	Salix alba L.	0	n	1	1	Salicaceae Mirb.
Iz_6_1109	DJI_0289	Sambucus nigra L.	0	n	1	1	Viburnaceae Raf.
Iz_6_1109	DJI_0289	Secale sylvestre Host	0	n	1	0	Poaceae Barnhart
Iz_6_1109	DJI_0289	Sempervivum ruthenicum Schnittsp. & C.B. Lehm.	0	n	1	0	Crassulaceae J.St.-Hil.
Iz_6_1109	DJI_0289	Setaria viridis (L.) P. Beauv.	0	n	1	0	Poaceae Barnhart
Iz_6_1109	DJI_0289	Silene borysthenica (Gruner) Walters	0	n	1	0	Caryophyllaceae Juss.
Iz_6_1109	DJI_0289	Silene latifolia subsp. alba (Mill.) Greuter & Burdet	0	n	1	0	Caryophyllaceae Juss.
Iz_6_1109	DJI_0289	Solidago virgaurea L.	0	n	1	0	Asteraceae Bercht. & J.Presl
Iz_6_1109	DJI_0289	Sorbus aucuparia L.	0	n	1	1	Rosaceae Juss.
Iz_6_1109	DJI_0289	Species 11.09_6_0254	0		NA	0	
Iz_6_1109	DJI_0289	Stachys recta L.	0	n	1	0	Lamiacea Lindl.
Iz_6_1109	DJI_0289	Tanacetum vulgare L.	0	n	1	0	Asteraceae Bercht. & J.Presl
Iz_6_1109	DJI_0289	Thymus pallasianus Heinr.Braun	0	n	1	0	Lamiacea Lindl.
Iz_6_1109	DJI_0289	Tilia cordata Mill.	0	n	1	1	Malvaceae Juss.
Iz_6_1109	DJI_0289	Trifolium alpestre L.	0	n	1	0	Fabaceae Lindl.
Iz_6_1109	DJI_0289	Trifolium arvense L.	0	n	1	0	Fabaceae Lindl.
Iz_6_1109	DJI_0289	Ulmus glabra Huds.	0	n	1	1	Ulmaceae Mirb.
Iz_6_1109	DJI_0289	Verbascum densiflorum Bertol.	0	n	1	0	Scrophulariaceae Juss.
Iz_6_1109	DJI_0289	Veronica austriaca L.	0	n	1	0	Plantaginaceae Juss.
Iz_6_1109	DJI_0289	Veronica incana L.	0	n	1	0	Plantaginaceae Juss.
Iz_6_1109	DJI_0289	Veronica verna L.	0	n	1	0	Plantaginaceae Juss.
Iz_6_1109	DJI_0289	Vincetoxicum hirundinaria Medik	0	n	1	0	Apocynaceae Juss.
Iz_6_1109	DJI_0292	Acer tataricum L.	0	n	1	1	Sapindaceae Juss.
Iz_6_1109	DJI_0292	Achillea millefolium L. s.l.	0	n	1	0	Asteraceae Bercht. & J.Presl
Iz_6_1109	DJI_0292	Allium oleraceum L.	0	n	1	0	Amaryllidaceae J.St.-Hil.
Iz_6_1109	DJI_0292	Ambrosia artemisiifolia L.	0	a	0	0	Asteraceae Bercht. & J.Presl
Iz_6_1109	DJI_0292	Amorpha fruticosa L.	0	a	0	0	Fabaceae Lindl.
Iz_6_1109	DJI_0292	Anthericum ramosum L.	0	n	1	0	Asparagaceae Juss.
Iz_6_1109	DJI_0292	Artemisia marschalliana Spreng.	0	n	1	0	Asteraceae Bercht. & J.Presl
Iz_6_1109	DJI_0292	Asparagus officinalis L.	0	n	1	0	Asparagaceae Juss.
Iz_6_1109	DJI_0292	Astragalus cicer L.	0	n	1	0	Fabaceae Lindl.
Iz_6_1109	DJI_0292	Bassia laniflora (S.G.Gmel.) A.J.Scott	0	n	1	0	Amaranthaceae Juss.
Iz_6_1109	DJI_0292	Berberis vulgaris L.	0	n	1	0	Berberidaceae Juss.
Iz_6_1109	DJI_0292	Betula pendula Roth.	0	n	1	1	Betulaceae Gray
Iz_6_1109	DJI_0292	Betula pendula Roth.	0	n	1	0	Poaceae Barnhart
Iz_6_1109	DJI_0292	Bromus inermis Leyss.	0		NA	NA	
Iz_6_1109	DJI_0292	Bromus squarrosus L.	0	a	0	0	Poaceae Barnhart
Iz_6_1109	DJI_0292	Bromus tectorum L.	0	a	0	0	Poaceae Barnhart
Iz_6_1109	DJI_0292	Calamagrostis epigejos (L.) Roth	25	n	1	0	Poaceae Barnhart
Iz_6_1109	DJI_0292	Carduus acanthoides L.	0	n	1	0	Asteraceae Bercht. & J.Presl
Iz_6_1109	DJI_0292	Carex sp.	0	n	1	0	Cyperaceae Juss.
Iz_6_1109	DJI_0292	Centaurea majorovii Dumbadze	0	n	1	0	Asteraceae Bercht. & J.Presl
Iz_6_1109	DJI_0292	Chamaecytisus ruthenicus (Fisch.ex Wol.) Klaskova	0	n	1	0	Fabaceae Lindl.
Iz_6_1109	DJI_0292	Chelidonium majus L.	0	n	1	0	Papaveraceae Juss.
Iz_6_1109	DJI_0292	Chenopodium betaceum Andrz.	0	n	1	0	Amaranthaceae Juss.
Iz_6_1109	DJI_0292	Chenopodium cf. album	0	n	1	0	Amaranthaceae Juss.
Iz_6_1109	DJI_0292	Chenopodium sp.	0		NA	0	Amaranthaceae Juss.
Iz_6_1109	DJI_0292	Chondrilla juncea L.	0	n	1	0	Asteraceae Bercht. & J.Presl
Iz_6_1109	DJI_0292	Cirsium vulgare (Savi) Ten.	0	n	1	0	Asteraceae Bercht. & J.Presl
Iz_6_1109	DJI_0292	Clinopodium acinos (L.) Kuntze	0	n	1	0	Lamiacea Lindl.
Iz_6_1109	DJI_0292	Convallaria majalis L.	0	n	1	0	Asparagaceae Juss.
Iz_6_1109	DJI_0292	Crataegus rhipidophylla Gand.	0	n	1	1	Rosaceae Juss.
Iz_6_1109	DJI_0292	Crepis foetida L.	0	n	1	0	Asteraceae Bercht. & J.Presl
Iz_6_1109	DJI_0292	Dactylis glomerata L.	0	n	1	0	Poaceae Barnhart
Iz_6_1109	DJI_0292	Dianthus campestris M.Bieb.	0	n	1	0	Caryophyllaceae Juss.
Iz_6_1109	DJI_0292	Digitaria sanguinalis (L.) Scop.	0	a	0	0	Poaceae Barnhart
Iz_6_1109	DJI_0292	Elymus repens (L.) Gould	0	n	1	0	Poaceae Barnhart
Iz_6_1109	DJI_0292	Epilobium tetragonum L.	0	n	1	0	Onagraceae Juss.
Iz_6_1109	DJI_0292	Eragrostis minor Host	0	a	0	0	Poaceae Barnhart
Iz_6_1109	DJI_0292	Erigeron annuus (L.) Desf.	0	a	0	0	Asteraceae Bercht. & J.Presl
Iz_6_1109	DJI_0292	Erigeron canadensis L.	10	a	0	0	Asteraceae Bercht. & J.Presl
Iz_6_1109	DJI_0292	Euphorbia seguieriana Neck.	0	n	1	0	Euphorbiaceae Juss.
Iz_6_1109	DJI_0292	Fallopia dumetorum (L.) Holub	0	n	1	0	Polygonaceae Juss.
Iz_6_1109	DJI_0292	Festuca beckeri (Hack.) Trautv.	0	n	1	0	Poaceae Barnhart
Iz_6_1109	DJI_0292	Fragaria vesca L.	0	n	1	0	Rosaceae Juss.
Iz_6_1109	DJI_0292	Frangula alnus Mill.	0	n	1	1	Rhamnaceae Juss.
Iz_6_1109	DJI_0292	Fraxinus pennsylvanica Marshall	0	a	0	1	Oleaceae Hoffmanns. & Link
Iz_6_1109	DJI_0292	Galeopsis bifida Boenn.	0	n	1	0	Lamiacea Lindl.
Iz_6_1109	DJI_0292	Galium verum L.	0	n	1	0	Rubiaceae Juss.
Iz_6_1109	DJI_0292	Genista tinctoria L.	0	n	1	0	Fabaceae Lindl.
Iz_6_1109	DJI_0292	Geranium robertianum L.	0	n	1	0	Geraniaceae Juss.
Iz_6_1109	DJI_0292	Geranium sanquineum L.	0	n	1	0	Geraniaceae Juss.
Iz_6_1109	DJI_0292	Gypsophila paniculata L.	0	n	1	0	Caryophyllaceae Juss.
Iz_6_1109	DJI_0292	Helichrysum arenarium (L.) Moench	0	n	1	0	Asteraceae Bercht. & J.Presl
Iz_6_1109	DJI_0292	Herniaria polygama J.Gay	0	n	1	0	Caryophyllaceae Juss.
Iz_6_1109	DJI_0292	Hieracium robustum Fr.	0	n	1	0	Asteraceae Bercht. & J.Presl

Iz_6_1109	DJI_0292	Hieracium umbellatum L.	0	n	1	NA	Asteraceae Bercht. & J.Presl
Iz_6_1109	DJI_0292	Hylotelephium maximum (L.) Holub	0	n	1	0	Crassulaceae J.St.-Hil.
Iz_6_1109	DJI_0292	Jacobaea borysthenica (DC.) B.Nord. & Greuter	0	n	1	0	Asteraceae Bercht. & J.Presl
Iz_6_1109	DJI_0292	Jasione montana L.	0	n	1	0	Campanulaceae Juss.
Iz_6_1109	DJI_0292	Jurinea cyanoides (L.) Rchb.s.l.	0	n	1	0	Asteraceae Bercht. & J.Presl
Iz_6_1109	DJI_0292	Koeleria glauca (Spreng.) DC.	0	n	1	0	Poaceae Barnhart
Iz_6_1109	DJI_0292	Lactuca serriola L.	0	a	0	0	Asteraceae Bercht. & J.Presl
Iz_6_1109	DJI_0292	Linaria genistifolia (L.) Mill.	0	n	1	0	Plantaginaceae Juss.
Iz_6_1109	DJI_0292	Lomelosia argentea (L.) Greuter & Burdet	0	n	1	0	Caprifoliaceae Juss.
Iz_6_1109	DJI_0292	Melampyrum pratense L.	0	n	1	0	Orobanchaceae Vent.
Iz_6_1109	DJI_0292	Oenothera biennis L.	0	a	0	0	Onagraceae Juss.
Iz_6_1109	DJI_0292	Pentanema britannica (L.) D.Gut.Larr., Santos-Vicente, Anderb., E.Rico & M.M.Mart.Ort.	0	n	1	0	Asteraceae Bercht. & J.Presl
Iz_6_1109	DJI_0292	Peucedanum oreoselinum (L.) Moench	0	n	1	0	Apiaceae Lindl.
Iz_6_1109	DJI_0292	Peucedanum ruthenicum M.Bieb.	0	n	1	0	Apiaceae Lindl.
Iz_6_1109	DJI_0292	Pilosella echioides (Lumn.) F.W.Schultz & Sch.Bip.	0	n	1	0	Asteraceae Bercht. & J.Presl
Iz_6_1109	DJI_0292	Pilosella officinarum F. Schultz et Sch. Bip.	0	n	1	0	Asteraceae Bercht. & J.Presl
Iz_6_1109	DJI_0292	Pilosella sp.	0		NA	0	Asteraceae Bercht. & J.Presl
Iz_6_1109	DJI_0292	Pinus sylvestris L.	0	n	1	1	Pinaceae Spreng. ex F.Rudolphi
Iz_6_1109	DJI_0292	Poa annua L.	0	n	1	0	Poaceae Barnhart
Iz_6_1109	DJI_0292	Poa nemoralis L.	0	n	1	0	Poaceae Barnhart
Iz_6_1109	DJI_0292	Poa sp.	0		NA	0	Poaceae Barnhart
Iz_6_1109	DJI_0292	Polygonatum odoratum (Mill.) Druce	0	n	1	0	Asparagaceae Juss.
Iz_6_1109	DJI_0292	Polygonum arenarium Waldst. & Kit.	0	n	1	0	Polygonaceae Juss.
Iz_6_1109	DJI_0292	Populus nigra L.	0	n	1	1	Salicaceae Mirb.
Iz_6_1109	DJI_0292	Populus tremula L.	0	n	1	1	Salicaceae Mirb.
Iz_6_1109	DJI_0292	Populus Ã canescens (Aiton) Sm.	0	n	1	1	Salicaceae Mirb.
Iz_6_1109	DJI_0292	Potentilla argentea L.	0	n	1	0	Rosaceae Juss.
Iz_6_1109	DJI_0292	Psammophiliella muralis (L.) Ikonn.	0	n	1	0	Caryophyllaceae Juss.
Iz_6_1109	DJI_0292	Psephellus sumensis (Kalen.) Greuter	0	n	1	0	Asteraceae Bercht. & J.Presl
Iz_6_1109	DJI_0292	Pulsatilla pratensis (L.) Mill. s.l.	0	n	1	0	Ranunculaceae Juss.
Iz_6_1109	DJI_0292	Pyrus communis L.	0	a	0	1	Rosaceae Juss.
Iz_6_1109	DJI_0292	Quercus robur L.	0	n	1	1	Fagaceae Dumort.
Iz_6_1109	DJI_0292	Robinia pseudoacacia L.	0	a	0	1	Fabaceae Lindl.
Iz_6_1109	DJI_0292	Rosa sp.	0		NA	1	Rosaceae Juss.
Iz_6_1109	DJI_0292	Rubus idaeus L.	0	n	1	0	Rosaceae Juss.
Iz_6_1109	DJI_0292	Rumex acetosella L.	0	n	1	0	Polygonaceae Juss.
Iz_6_1109	DJI_0292	Rumex thyrsiflorus Fingerh.	0	n	1	0	Polygonaceae Juss.
Iz_6_1109	DJI_0292	Salix alba L.	0	n	1	1	Salicaceae Mirb.
Iz_6_1109	DJI_0292	Sambucus nigra L.	0	n	1	1	Viburnaceae Raf.
Iz_6_1109	DJI_0292	Secale sylvestre Host	0	n	1	0	Poaceae Barnhart
Iz_6_1109	DJI_0292	Sempervivum ruthenicum Schnittsp. & C.B. Lehm.	0	n	1	0	Crassulaceae J.St.-Hil.
Iz_6_1109	DJI_0292	Setaria viridis (L.) P. Beauv.	0	n	1	0	Poaceae Barnhart
Iz_6_1109	DJI_0292	Silene borysthenica (Gruner) Walters	0	n	1	0	Caryophyllaceae Juss.
Iz_6_1109	DJI_0292	Silene latifolia subsp. alba (Mill.) Greuter & Burdet	0	n	1	0	Caryophyllaceae Juss.
Iz_6_1109	DJI_0292	Solidago virgaurea L.	0	n	1	0	Asteraceae Bercht. & J.Presl
Iz_6_1109	DJI_0292	Sorbus aucuparia L.	0	n	1	1	Rosaceae Juss.
Iz_6_1109	DJI_0292	Species 11.09_6_0254	0		NA	0	
Iz_6_1109	DJI_0292	Stachys recta L.	0	n	1	0	Lamiacea Lindl.
Iz_6_1109	DJI_0292	Tanacetum vulgare L.	0	n	1	0	Asteraceae Bercht. & J.Presl
Iz_6_1109	DJI_0292	Thymus pallasianus Heinr.Braun	0	n	1	0	Lamiacea Lindl.
Iz_6_1109	DJI_0292	Tilia cordata Mill.	0	n	1	1	Malvaceae Juss.
Iz_6_1109	DJI_0292	Trifolium alpestre L.	0	n	1	0	Fabaceae Lindl.
Iz_6_1109	DJI_0292	Trifolium arvense L.	0	n	1	0	Fabaceae Lindl.
Iz_6_1109	DJI_0292	Ulmus glabra Huds.	0	n	1	1	Ulmaceae Mirb.
Iz_6_1109	DJI_0292	Verbascum densiflorum Bertol.	0	n	1	0	Scrophulariaceae Juss.
Iz_6_1109	DJI_0292	Veronica austriaca L.	0	n	1	0	Plantaginaceae Juss.
Iz_6_1109	DJI_0292	Veronica incana L.	0	n	1	0	Plantaginaceae Juss.
Iz_6_1109	DJI_0292	Veronica verna L.	0	n	1	0	Plantaginaceae Juss.
Iz_6_1109	DJI_0292	Vincetoxicum hirundinaria Medik	0	n	1	0	Apocynaceae Juss.
Iz_6_1209	DJI_0300	Acer tataricum L.	0	n	1	1	Sapindaceae Juss.
Iz_6_1209	DJI_0300	Achillea millefolium L. s.l.	0	n	1	0	Asteraceae Bercht. & J.Presl
Iz_6_1209	DJI_0300	Allium oleraceum L.	0	n	1	0	Amaryllidaceae J.St.-Hil.
Iz_6_1209	DJI_0300	Ambrosia artemisiifolia L.	0	a	0	0	Asteraceae Bercht. & J.Presl
Iz_6_1209	DJI_0300	Amorpha fruticosa L.	0	a	0	0	Fabaceae Lindl.
Iz_6_1209	DJI_0300	Anthericum ramosum L.	0	n	1	0	Asparagaceae Juss.
Iz_6_1209	DJI_0300	Artemisia austriaca Jacq.	0		NA	NA	
Iz_6_1209	DJI_0300	Artemisia marschalliana Spreng.	0	n	1	0	Asteraceae Bercht. & J.Presl
Iz_6_1209	DJI_0300	Asparagus officinalis L.	0	n	1	0	Asparagaceae Juss.
Iz_6_1209	DJI_0300	Astragalus cicer L.	0	n	1	0	Fabaceae Lindl.
Iz_6_1209	DJI_0300	Bassia laniflora (S.G.Gmel.) A.J.Scott	0	n	1	0	Amaranthaceae Juss.
Iz_6_1209	DJI_0300	Berberis vulgaris L.	0	n	1	0	Berberidaceae Juss.
Iz_6_1209	DJI_0300	Betula pendula Roth.	0	n	1	1	Betulaceae Gray
Iz_6_1209	DJI_0300	Betula pendula Roth.	0	n	1	0	Poaceae Barnhart
Iz_6_1209	DJI_0300	Bromus inermis Leyss.	0		NA	NA	
Iz_6_1209	DJI_0300	Bromus squarrosus L.	0	a	0	0	Poaceae Barnhart
Iz_6_1209	DJI_0300	Bromus tectorum L.	0	a	0	0	Poaceae Barnhart
Iz_6_1209	DJI_0300	Calamagrostis epigejos (L.) Roth	3	n	1	0	Poaceae Barnhart
Iz_6_1209	DJI_0300	Carduus acanthoides L.	0	n	1	0	Asteraceae Bercht. & J.Presl
Iz_6_1209	DJI_0300	Carex sp.	0	n	1	0	Cyperaceae Juss.
Iz_6_1209	DJI_0300	Centaurea majorovii Dumbadze	0	n	1	0	Asteraceae Bercht. & J.Presl
Iz_6_1209	DJI_0300	Chamaecytisus ruthenicus (Fisch.ex Wol.) Klaskova	0	n	1	0	Fabaceae Lindl.
Iz_6_1209	DJI_0300	Chelidonium majus L.	0	n	1	0	Papaveraceae Juss.
Iz_6_1209	DJI_0300	Chenopodium betaceum Andrz.	0	n	1	0	Amaranthaceae Juss.
Iz_6_1209	DJI_0300	Chenopodium cf. album	0	n	1	0	Amaranthaceae Juss.
Iz_6_1209	DJI_0300	Chenopodium sp.	0		NA	0	Amaranthaceae Juss.
Iz_6_1209	DJI_0300	Chondrilla juncea L.	0	n	1	0	Asteraceae Bercht. & J.Presl
Iz_6_1209	DJI_0300	Cirsium vulgare (Savi) Ten.	0	n	1	0	Asteraceae Bercht. & J.Presl
Iz_6_1209	DJI_0300	Clinopodium acinos (L.) Kuntze	0	n	1	0	Lamiacea Lindl.
Iz_6_1209	DJI_0300	Convallaria majalis L.	0	n	1	0	Asparagaceae Juss.
Iz_6_1209	DJI_0300	Crataegus rhipidophylla Gand.	0	n	1	1	Rosaceae Juss.
Iz_6_1209	DJI_0300	Crepis foetida L.	0	n	1	0	Asteraceae Bercht. & J.Presl
Iz_6_1209	DJI_0300	Dactylis glomerata L.	0	n	1	0	Poaceae Barnhart
Iz_6_1209	DJI_0300	Dianthus campestris M.Bieb.	0	n	1	0	Caryophyllaceae Juss.
Iz_6_1209	DJI_0300	Digitaria sanguinalis (L.) Scop.	0	a	0	0	Poaceae Barnhart
Iz_6_1209	DJI_0300	Elymus repens (L.) Gould	0	n	1	0	Poaceae Barnhart
Iz_6_1209	DJI_0300	Epilobium tetragonum L.	0	n	1	0	Onagraceae Juss.
Iz_6_1209	DJI_0300	Eragrostis minor Host	0	a	0	0	Poaceae Barnhart
Iz_6_1209	DJI_0300	Erigeron canadensis L.	5	a	0	0	Asteraceae Bercht. & J.Presl

Iz_6_1209	DJI_0300	Euphorbia seguieriana Neck.	0	n	1	0	Euphorbiaceae Juss.
Iz_6_1209	DJI_0300	Fallopia dumetorum (L.) Holub	0	n	1	0	Polygonaceae Juss.
Iz_6_1209	DJI_0300	Festuca beckeri (Hack.) Trautv.	0	n	1	0	Poaceae Barnhart
Iz_6_1209	DJI_0300	Fragaria vesca L.	0	n	1	0	Rosaceae Juss.
Iz_6_1209	DJI_0300	Frangula alnus Mill.	0	n	1	1	Rhamnaceae Juss.
Iz_6_1209	DJI_0300	Fraxinus pennsylvanica Marshall	0	a	0	1	Oleaceae Hoffmanns. & Link
Iz_6_1209	DJI_0300	Galeopsis bifida Boenn.	0	n	1	0	Lamiacea Lindl.
Iz_6_1209	DJI_0300	Galium verum L.	0	n	1	0	Rubiaceae Juss.
Iz_6_1209	DJI_0300	Genista tinctoria L.	0	n	1	0	Fabaceae Lindl.
Iz_6_1209	DJI_0300	Geranium robertianum L.	0	n	1	0	Geraniaceae Juss.
Iz_6_1209	DJI_0300	Geranium sanquineum L.	0	n	1	0	Geraniaceae Juss.
Iz_6_1209	DJI_0300	Gypsophila paniculata L.	0	n	1	0	Caryophyllaceae Juss.
Iz_6_1209	DJI_0300	Helichrysum arenarium (L.) Moench	2	n	1	0	Asteraceae Bercht. & J.Presl
Iz_6_1209	DJI_0300	Herniaria polygama J.Gay	0	n	1	0	Caryophyllaceae Juss.
Iz_6_1209	DJI_0300	Hieracium robustum Fr.	0	n	1	0	Asteraceae Bercht. & J.Presl
Iz_6_1209	DJI_0300	Hieracium umbellatum L.	0	n	1	NA	Asteraceae Bercht. & J.Presl
Iz_6_1209	DJI_0300	Hylotelephium maximum (L.) Holub	0	n	1	0	Crassulaceae J.St.-Hil.
Iz_6_1209	DJI_0300	Jacobaea borysthenica (DC.) B.Nord. & Greuter	0	n	1	0	Asteraceae Bercht. & J.Presl
Iz_6_1209	DJI_0300	Jasione montana L.	0	n	1	0	Campanulaceae Juss.
Iz_6_1209	DJI_0300	Jurinea cyanoides (L.) Rchb.s.l.	0	n	1	0	Asteraceae Bercht. & J.Presl
Iz_6_1209	DJI_0300	Koeleria glauca (Spreng.) DC.	2	n	1	0	Poaceae Barnhart
Iz_6_1209	DJI_0300	Lactuca serriola L.	0	a	0	0	Asteraceae Bercht. & J.Presl
Iz_6_1209	DJI_0300	Linaria genistifolia (L.) Mill.	0	n	1	0	Plantaginaceae Juss.
Iz_6_1209	DJI_0300	Lomelosia argentea (L.) Greuter & Burdet	0	n	1	0	Caprifoliaceae Juss.
Iz_6_1209	DJI_0300	Melampyrum pratense L.	0	n	1	0	Orobanchaceae Vent.
Iz_6_1209	DJI_0300	Oenothera biennis L.	0	a	0	0	Onagraceae Juss.
Iz_6_1209	DJI_0300	Pentanema britannica (L.) D.Gut.Larr., Santos-Vicente, Anderb., E.Rico & M.M.Mart.Ort.	0	n	1	0	Asteraceae Bercht. & J.Presl
Iz_6_1209	DJI_0300	Peucedanum oreoselinum (L.) Moench	0	n	1	0	Apiaceae Lindl.
Iz_6_1209	DJI_0300	Peucedanum ruthenicum M.Bieb.	0	n	1	0	Apiaceae Lindl.
Iz_6_1209	DJI_0300	Pilosella echioides (Lumn.) F.W.Schultz & Sch.Bip.	0	n	1	0	Asteraceae Bercht. & J.Presl
Iz_6_1209	DJI_0300	Pilosella officinarum F. Schultz et Sch. Bip.	0	n	1	0	Asteraceae Bercht. & J.Presl
Iz_6_1209	DJI_0300	Pilosella sp.	0		NA	0	Asteraceae Bercht. & J.Presl
Iz_6_1209	DJI_0300	Pinus sylvestris L.	0	n	1	1	Pinaceae Spreng. ex F.Rudolphi
Iz_6_1209	DJI_0300	Poa annua L.	0	n	1	0	Poaceae Barnhart
Iz_6_1209	DJI_0300	Poa nemoralis L.	0	n	1	0	Poaceae Barnhart
Iz_6_1209	DJI_0300	Poa sp.	0		NA	0	Poaceae Barnhart
Iz_6_1209	DJI_0300	Polygonatum odoratum (Mill.) Druce	0	n	1	0	Asparagaceae Juss.
Iz_6_1209	DJI_0300	Polygonum arenarium Waldst. & Kit.	0	n	1	0	Polygonaceae Juss.
Iz_6_1209	DJI_0300	Populus nigra L.	0	n	1	1	Salicaceae Mirb.
Iz_6_1209	DJI_0300	Populus tremula L.	0	n	1	1	Salicaceae Mirb.
Iz_6_1209	DJI_0300	Populus Ã canescens (Aiton) Sm.	0	n	1	1	Salicaceae Mirb.
Iz_6_1209	DJI_0300	Potentilla argentea L.	0	n	1	0	Rosaceae Juss.
Iz_6_1209	DJI_0300	Psammophiliella muralis (L.) Ikonn.	0	n	1	0	Caryophyllaceae Juss.
Iz_6_1209	DJI_0300	Psephellus sumensis (Kalen.) Greuter	0	n	1	0	Asteraceae Bercht. & J.Presl
Iz_6_1209	DJI_0300	Pulsatilla pratensis (L.) Mill. s.l.	0	n	1	0	Ranunculaceae Juss.
Iz_6_1209	DJI_0300	Pyrus communis L.	0	a	0	1	Rosaceae Juss.
Iz_6_1209	DJI_0300	Quercus robur L.	0	n	1	1	Fagaceae Dumort.
Iz_6_1209	DJI_0300	Robinia pseudoacacia L.	0	a	0	1	Fabaceae Lindl.
Iz_6_1209	DJI_0300	Rosa sp.	0		NA	1	Rosaceae Juss.
Iz_6_1209	DJI_0300	Rubus idaeus L.	0	n	1	0	Rosaceae Juss.
Iz_6_1209	DJI_0300	Rumex acetosella L.	1	n	1	0	Polygonaceae Juss.
Iz_6_1209	DJI_0300	Rumex thyrsiflorus Fingerh.	0	n	1	0	Polygonaceae Juss.
Iz_6_1209	DJI_0300	Salix alba L.	0	n	1	1	Salicaceae Mirb.
Iz_6_1209	DJI_0300	Sambucus nigra L.	0	n	1	1	Viburnaceae Raf.
Iz_6_1209	DJI_0300	Secale sylvestre Host	0	n	1	0	Poaceae Barnhart
Iz_6_1209	DJI_0300	Sempervivum ruthenicum Schnittsp. & C.B. Lehm.	0	n	1	0	Crassulaceae J.St.-Hil.
Iz_6_1209	DJI_0300	Setaria viridis (L.) P. Beauv.	0	n	1	0	Poaceae Barnhart
Iz_6_1209	DJI_0300	Silene borysthenica (Gruner) Walters	0	n	1	0	Caryophyllaceae Juss.
Iz_6_1209	DJI_0300	Silene latifolia subsp. alba (Mill.) Greuter & Burdet	0	n	1	0	Caryophyllaceae Juss.
Iz_6_1209	DJI_0300	Solidago virgaurea L.	1	n	1	0	Asteraceae Bercht. & J.Presl
Iz_6_1209	DJI_0300	Sorbus aucuparia L.	0	n	1	1	Rosaceae Juss.
Iz_6_1209	DJI_0300	Stachys recta L.	0	n	1	0	Lamiacea Lindl.
Iz_6_1209	DJI_0300	Tanacetum vulgare L.	0	n	1	0	Asteraceae Bercht. & J.Presl
Iz_6_1209	DJI_0300	Thymus pallasianus Heinr.Braun	0	n	1	0	Lamiacea Lindl.
Iz_6_1209	DJI_0300	Tilia cordata Mill.	0	n	1	1	Malvaceae Juss.
Iz_6_1209	DJI_0300	Trifolium alpestre L.	0	n	1	0	Fabaceae Lindl.
Iz_6_1209	DJI_0300	Trifolium arvense L.	0	n	1	0	Fabaceae Lindl.
Iz_6_1209	DJI_0300	Ulmus glabra Huds.	0	n	1	1	Ulmaceae Mirb.
Iz_6_1209	DJI_0300	Verbascum densiflorum Bertol.	0	n	1	0	Scrophulariaceae Juss.
Iz_6_1209	DJI_0300	Veronica austriaca L.	0	n	1	0	Plantaginaceae Juss.
Iz_6_1209	DJI_0300	Veronica incana L.	0	n	1	0	Plantaginaceae Juss.
Iz_6_1209	DJI_0300	Veronica sp.	0		NA	NA	
Iz_6_1209	DJI_0300	Veronica verna L.	0	n	1	0	Plantaginaceae Juss.
Iz_6_1209	DJI_0300	Vincetoxicum hirundinaria Medik	0	n	1	0	Apocynaceae Juss.
Iz_6_1209	DJI_0303	Acer tataricum L.	0	n	1	1	Sapindaceae Juss.
Iz_6_1209	DJI_0303	Achillea millefolium L. s.l.	0	n	1	0	Asteraceae Bercht. & J.Presl
Iz_6_1209	DJI_0303	Allium oleraceum L.	0	n	1	0	Amaryllidaceae J.St.-Hil.
Iz_6_1209	DJI_0303	Ambrosia artemisiifolia L.	0	a	0	0	Asteraceae Bercht. & J.Presl
Iz_6_1209	DJI_0303	Amorpha fruticosa L.	0	a	0	0	Fabaceae Lindl.
Iz_6_1209	DJI_0303	Anthericum ramosum L.	0	n	1	0	Asparagaceae Juss.
Iz_6_1209	DJI_0303	Artemisia austriaca Jacq.	0		NA	NA	
Iz_6_1209	DJI_0303	Artemisia marschalliana Spreng.	0	n	1	0	Asteraceae Bercht. & J.Presl
Iz_6_1209	DJI_0303	Asparagus officinalis L.	0	n	1	0	Asparagaceae Juss.
Iz_6_1209	DJI_0303	Astragalus cicer L.	0	n	1	0	Fabaceae Lindl.
Iz_6_1209	DJI_0303	Bassia laniflora (S.G.Gmel.) A.J.Scott	0	n	1	0	Amaranthaceae Juss.
Iz_6_1209	DJI_0303	Berberis vulgaris L.	0	n	1	0	Berberidaceae Juss.
Iz_6_1209	DJI_0303	Betula pendula Roth.	0	n	1	1	Betulaceae Gray
Iz_6_1209	DJI_0303	Betula pendula Roth.	0	n	1	0	Poaceae Barnhart
Iz_6_1209	DJI_0303	Bromus inermis Leyss.	0		NA	NA	
Iz_6_1209	DJI_0303	Bromus squarrosus L.	0	a	0	0	Poaceae Barnhart
Iz_6_1209	DJI_0303	Bromus tectorum L.	0	a	0	0	Poaceae Barnhart
Iz_6_1209	DJI_0303	Calamagrostis epigejos (L.) Roth	3	n	1	0	Poaceae Barnhart
Iz_6_1209	DJI_0303	Carduus acanthoides L.	0	n	1	0	Asteraceae Bercht. & J.Presl
Iz_6_1209	DJI_0303	Carex sp.	0	n	1	0	Cyperaceae Juss.
Iz_6_1209	DJI_0303	Centaurea majorovii Dumbadze	0	n	1	0	Asteraceae Bercht. & J.Presl
Iz_6_1209	DJI_0303	Chamaecytisus ruthenicus (Fisch.ex Wol.) Klaskova	0	n	1	0	Fabaceae Lindl.
Iz_6_1209	DJI_0303	Chelidonium majus L.	0	n	1	0	Papaveraceae Juss.
Iz_6_1209	DJI_0303	Chenopodium betaceum Andrz.	0	n	1	0	Amaranthaceae Juss.

Iz_6_1209	DJI_0303	Chenopodium cf. album	0	n	1	0	Amaranthaceae Juss.
Iz_6_1209	DJI_0303	Chenopodium sp.	0		NA	0	Amaranthaceae Juss.
Iz_6_1209	DJI_0303	Chondrilla juncea L.	0	n	1	0	Asteraceae Bercht. & J.Presl
Iz_6_1209	DJI_0303	Cirsium vulgare (Savi) Ten.	0	n	1	0	Asteraceae Bercht. & J.Presl
Iz_6_1209	DJI_0303	Clinopodium acinos (L.) Kuntze	0	n	1	0	Lamiacea Lindl.
Iz_6_1209	DJI_0303	Convallaria majalis L.	0	n	1	0	Asparagaceae Juss.
Iz_6_1209	DJI_0303	Crataegus rhipidophylla Gand.	0	n	1	1	Rosaceae Juss.
Iz_6_1209	DJI_0303	Crepis foetida L.	0	n	1	0	Asteraceae Bercht. & J.Presl
Iz_6_1209	DJI_0303	Dactylis glomerata L.	0	n	1	0	Poaceae Barnhart
Iz_6_1209	DJI_0303	Dianthus campestris M.Bieb.	0	n	1	0	Caryophyllaceae Juss.
Iz_6_1209	DJI_0303	Digitaria sanguinalis (L.) Scop.	0	a	0	0	Poaceae Barnhart
Iz_6_1209	DJI_0303	Elymus repens (L.) Gould	0	n	1	0	Poaceae Barnhart
Iz_6_1209	DJI_0303	Epilobium tetragonum L.	0	n	1	0	Onagraceae Juss.
Iz_6_1209	DJI_0303	Eragrostis minor Host	0	a	0	0	Poaceae Barnhart
Iz_6_1209	DJI_0303	Erigeron canadensis L.	0	a	0	0	Asteraceae Bercht. & J.Presl
Iz_6_1209	DJI_0303	Euphorbia seguieriana Neck.	0	n	1	0	Euphorbiaceae Juss.
Iz_6_1209	DJI_0303	Fallopia dumetorum (L.) Holub	0	n	1	0	Polygonaceae Juss.
Iz_6_1209	DJI_0303	Festuca beckeri (Hack.) Trautv.	0	n	1	0	Poaceae Barnhart
Iz_6_1209	DJI_0303	Fragaria vesca L.	0	n	1	0	Rosaceae Juss.
Iz_6_1209	DJI_0303	Frangula alnus Mill.	0	n	1	1	Rhamnaceae Juss.
Iz_6_1209	DJI_0303	Fraxinus pennsylvanica Marshall	0	a	0	1	Oleaceae Hoffmanns. & Link
Iz_6_1209	DJI_0303	Galeopsis bifida Boenn.	0	n	1	0	Lamiacea Lindl.
Iz_6_1209	DJI_0303	Galium verum L.	0	n	1	0	Rubiaceae Juss.
Iz_6_1209	DJI_0303	Genista tinctoria L.	0	n	1	0	Fabaceae Lindl.
Iz_6_1209	DJI_0303	Geranium robertianum L.	0	n	1	0	Geraniaceae Juss.
Iz_6_1209	DJI_0303	Geranium sanquineum L.	0	n	1	0	Geraniaceae Juss.
Iz_6_1209	DJI_0303	Gypsophila paniculata L.	0	n	1	0	Caryophyllaceae Juss.
Iz_6_1209	DJI_0303	Helichrysum arenarium (L.) Moench	1	n	1	0	Asteraceae Bercht. & J.Presl
Iz_6_1209	DJI_0303	Herniaria polygama J.Gay	0	n	1	0	Caryophyllaceae Juss.
Iz_6_1209	DJI_0303	Hieracium robustum Fr.	0	n	1	0	Asteraceae Bercht. & J.Presl
Iz_6_1209	DJI_0303	Hieracium umbellatum L.	0	n	1	NA	Asteraceae Bercht. & J.Presl
Iz_6_1209	DJI_0303	Hylotelephium maximum (L.) Holub	0	n	1	0	Crassulaceae J.St.-Hil.
Iz_6_1209	DJI_0303	Jacobaea borysthenica (DC.) B.Nord. & Greuter	0	n	1	0	Asteraceae Bercht. & J.Presl
Iz_6_1209	DJI_0303	Jasione montana L.	0	n	1	0	Campanulaceae Juss.
Iz_6_1209	DJI_0303	Jurinea cyanoides (L.) Rchb.s.l.	0	n	1	0	Asteraceae Bercht. & J.Presl
Iz_6_1209	DJI_0303	Koeleria glauca (Spreng.) DC.	0	n	1	0	Poaceae Barnhart
Iz_6_1209	DJI_0303	Lactuca serriola L.	0	a	0	0	Asteraceae Bercht. & J.Presl
Iz_6_1209	DJI_0303	Linaria genistifolia (L.) Mill.	0	n	1	0	Plantaginaceae Juss.
Iz_6_1209	DJI_0303	Lomelosia argentea (L.) Greuter & Burdet	0	n	1	0	Caprifoliaceae Juss.
Iz_6_1209	DJI_0303	Melampyrum pratense L.	0	n	1	0	Orobanchaceae Vent.
Iz_6_1209	DJI_0303	Oenothera biennis L.	0	a	0	0	Onagraceae Juss.
Iz_6_1209	DJI_0303	Pentanema britannica (L.) D.Gut.Larr., Santos-Vicente, Anderb., E.Rico & M.M.Mart.Ort.	0	n	1	0	Asteraceae Bercht. & J.Presl
Iz_6_1209	DJI_0303	Peucedanum oreoselinum (L.) Moench	0	n	1	0	Apiaceae Lindl.
Iz_6_1209	DJI_0303	Peucedanum ruthenicum M.Bieb.	0	n	1	0	Apiaceae Lindl.
Iz_6_1209	DJI_0303	Pilosella echioides (Lumn.) F.W.Schultz & Sch.Bip.	0	n	1	0	Asteraceae Bercht. & J.Presl
Iz_6_1209	DJI_0303	Pilosella officinarum F. Schultz et Sch. Bip.	0	n	1	0	Asteraceae Bercht. & J.Presl
Iz_6_1209	DJI_0303	Pilosella sp.	0		NA	0	Asteraceae Bercht. & J.Presl
Iz_6_1209	DJI_0303	Pinus sylvestris L.	0	n	1	1	Pinaceae Spreng. ex F.Rudolphi
Iz_6_1209	DJI_0303	Poa annua L.	0	n	1	0	Poaceae Barnhart
Iz_6_1209	DJI_0303	Poa nemoralis L.	0	n	1	0	Poaceae Barnhart
Iz_6_1209	DJI_0303	Poa sp.	0		NA	0	Poaceae Barnhart
Iz_6_1209	DJI_0303	Polygonatum odoratum (Mill.) Druce	0	n	1	0	Asparagaceae Juss.
Iz_6_1209	DJI_0303	Polygonum arenarium Waldst. & Kit.	0	n	1	0	Polygonaceae Juss.
Iz_6_1209	DJI_0303	Populus nigra L.	0	n	1	1	Salicaceae Mirb.
Iz_6_1209	DJI_0303	Populus tremula L.	0.2	n	1	1	Salicaceae Mirb.
Iz_6_1209	DJI_0303	Populus Ã canescens (Aiton) Sm.	0	n	1	1	Salicaceae Mirb.
Iz_6_1209	DJI_0303	Potentilla argentea L.	0	n	1	0	Rosaceae Juss.
Iz_6_1209	DJI_0303	Psammophiliella muralis (L.) Ikonn.	0	n	1	0	Caryophyllaceae Juss.
Iz_6_1209	DJI_0303	Psephellus sumensis (Kalen.) Greuter	0	n	1	0	Asteraceae Bercht. & J.Presl
Iz_6_1209	DJI_0303	Pulsatilla pratensis (L.) Mill. s.l.	0	n	1	0	Ranunculaceae Juss.
Iz_6_1209	DJI_0303	Pyrus communis L.	0	a	0	1	Rosaceae Juss.
Iz_6_1209	DJI_0303	Quercus robur L.	0	n	1	1	Fagaceae Dumort.
Iz_6_1209	DJI_0303	Robinia pseudoacacia L.	0	a	0	1	Fabaceae Lindl.
Iz_6_1209	DJI_0303	Rosa sp.	0		NA	1	Rosaceae Juss.
Iz_6_1209	DJI_0303	Rubus idaeus L.	0	n	1	0	Rosaceae Juss.
Iz_6_1209	DJI_0303	Rumex acetosella L.	0	n	1	0	Polygonaceae Juss.
Iz_6_1209	DJI_0303	Rumex thyrsiflorus Fingerh.	0	n	1	0	Polygonaceae Juss.
Iz_6_1209	DJI_0303	Salix alba L.	0	n	1	1	Salicaceae Mirb.
Iz_6_1209	DJI_0303	Sambucus nigra L.	0	n	1	1	Viburnaceae Raf.
Iz_6_1209	DJI_0303	Secale sylvestre Host	0	n	1	0	Poaceae Barnhart
Iz_6_1209	DJI_0303	Sempervivum ruthenicum Schnittsp. & C.B. Lehm.	0	n	1	0	Crassulaceae J.St.-Hil.
Iz_6_1209	DJI_0303	Setaria viridis (L.) P. Beauv.	0	n	1	0	Poaceae Barnhart
Iz_6_1209	DJI_0303	Silene borysthenica (Gruner) Walters	0	n	1	0	Caryophyllaceae Juss.
Iz_6_1209	DJI_0303	Silene latifolia subsp. alba (Mill.) Greuter & Burdet	0	n	1	0	Caryophyllaceae Juss.
Iz_6_1209	DJI_0303	Solidago virgaurea L.	0	n	1	0	Asteraceae Bercht. & J.Presl
Iz_6_1209	DJI_0303	Sorbus aucuparia L.	0	n	1	1	Rosaceae Juss.
Iz_6_1209	DJI_0303	Stachys recta L.	0	n	1	0	Lamiacea Lindl.
Iz_6_1209	DJI_0303	Tanacetum vulgare L.	0	n	1	0	Asteraceae Bercht. & J.Presl
Iz_6_1209	DJI_0303	Thymus pallasianus Heinr.Braun	0	n	1	0	Lamiacea Lindl.
Iz_6_1209	DJI_0303	Tilia cordata Mill.	0	n	1	1	Malvaceae Juss.
Iz_6_1209	DJI_0303	Trifolium alpestre L.	0	n	1	0	Fabaceae Lindl.
Iz_6_1209	DJI_0303	Trifolium arvense L.	0	n	1	0	Fabaceae Lindl.
Iz_6_1209	DJI_0303	Ulmus glabra Huds.	0	n	1	1	Ulmaceae Mirb.
Iz_6_1209	DJI_0303	Verbascum densiflorum Bertol.	0	n	1	0	Scrophulariaceae Juss.
Iz_6_1209	DJI_0303	Veronica austriaca L.	0	n	1	0	Plantaginaceae Juss.
Iz_6_1209	DJI_0303	Veronica incana L.	0	n	1	0	Plantaginaceae Juss.
Iz_6_1209	DJI_0303	Veronica sp.	0		NA	NA	
Iz_6_1209	DJI_0303	Veronica verna L.	0	n	1	0	Plantaginaceae Juss.
Iz_6_1209	DJI_0303	Vincetoxicum hirundinaria Medik	0	n	1	0	Apocynaceae Juss.
Iz_6_1209	DJI_0306	Acer tataricum L.	0	n	1	1	Sapindaceae Juss.
Iz_6_1209	DJI_0306	Achillea millefolium L. s.l.	0	n	1	0	Asteraceae Bercht. & J.Presl
Iz_6_1209	DJI_0306	Allium oleraceum L.	0	n	1	0	Amaryllidaceae J.St.-Hil.
Iz_6_1209	DJI_0306	Ambrosia artemisiifolia L.	0	a	0	0	Asteraceae Bercht. & J.Presl
Iz_6_1209	DJI_0306	Amorpha fruticosa L.	0	a	0	0	Fabaceae Lindl.
Iz_6_1209	DJI_0306	Anthericum ramosum L.	0	n	1	0	Asparagaceae Juss.
Iz_6_1209	DJI_0306	Artemisia austriaca Jacq.	0		NA	NA	
Iz_6_1209	DJI_0306	Artemisia marschalliana Spreng.	0	n	1	0	Asteraceae Bercht. & J.Presl
Iz_6_1209	DJI_0306	Asparagus officinalis L.	0	n	1	0	Asparagaceae Juss.

Iz_6_1209	DJI_0306	Astragalus cicer L.	0	n	1	0	Fabaceae Lindl.
Iz_6_1209	DJI_0306	Bassia laniflora (S.G.Gmel.) A.J.Scott	0	n	1	0	Amaranthaceae Juss.
Iz_6_1209	DJI_0306	Berberis vulgaris L.	0	n	1	0	Berberidaceae Juss.
Iz_6_1209	DJI_0306	Betula pendula Roth.	0	n	1	1	Betulaceae Gray
Iz_6_1209	DJI_0306	Betula pendula Roth.	0	n	1	0	Poaceae Barnhart
Iz_6_1209	DJI_0306	Bromus inermis Leyss.	0		NA	NA	
Iz_6_1209	DJI_0306	Bromus squarrosus L.	0	a	0	0	Poaceae Barnhart
Iz_6_1209	DJI_0306	Bromus tectorum L.	0	a	0	0	Poaceae Barnhart
Iz_6_1209	DJI_0306	Calamagrostis epigejos (L.) Roth	8	n	1	0	Poaceae Barnhart
Iz_6_1209	DJI_0306	Carduus acanthoides L.	0	n	1	0	Asteraceae Bercht. & J.Presl
Iz_6_1209	DJI_0306	Carex sp.	0	n	1	0	Cyperaceae Juss.
Iz_6_1209	DJI_0306	Centaurea majorovii Dumbadze	0	n	1	0	Asteraceae Bercht. & J.Presl
Iz_6_1209	DJI_0306	Chamaecytisus ruthenicus (Fisch.ex Wol.) Klaskova	0	n	1	0	Fabaceae Lindl.
Iz_6_1209	DJI_0306	Chelidonium majus L.	0	n	1	0	Papaveraceae Juss.
Iz_6_1209	DJI_0306	Chenopodium betaceum Andrz.	0	n	1	0	Amaranthaceae Juss.
Iz_6_1209	DJI_0306	Chenopodium cf. album	0	n	1	0	Amaranthaceae Juss.
Iz_6_1209	DJI_0306	Chenopodium sp.	0		NA	0	Amaranthaceae Juss.
Iz_6_1209	DJI_0306	Chondrilla juncea L.	0	n	1	0	Asteraceae Bercht. & J.Presl
Iz_6_1209	DJI_0306	Cirsium vulgare (Savi) Ten.	0	n	1	0	Asteraceae Bercht. & J.Presl
Iz_6_1209	DJI_0306	Clinopodium acinos (L.) Kuntze	0	n	1	0	Lamiacea Lindl.
Iz_6_1209	DJI_0306	Convallaria majalis L.	0	n	1	0	Asparagaceae Juss.
Iz_6_1209	DJI_0306	Crataegus rhipidophylla Gand.	0	n	1	1	Rosaceae Juss.
Iz_6_1209	DJI_0306	Crepis foetida L.	0	n	1	0	Asteraceae Bercht. & J.Presl
Iz_6_1209	DJI_0306	Dactylis glomerata L.	0	n	1	0	Poaceae Barnhart
Iz_6_1209	DJI_0306	Dianthus campestris M.Bieb.	0	n	1	0	Caryophyllaceae Juss.
Iz_6_1209	DJI_0306	Digitaria sanguinalis (L.) Scop.	0	a	0	0	Poaceae Barnhart
Iz_6_1209	DJI_0306	Elymus repens (L.) Gould	0	n	1	0	Poaceae Barnhart
Iz_6_1209	DJI_0306	Epilobium tetragonum L.	0	n	1	0	Onagraceae Juss.
Iz_6_1209	DJI_0306	Eragrostis minor Host	0	a	0	0	Poaceae Barnhart
Iz_6_1209	DJI_0306	Erigeron canadensis L.	2	a	0	0	Asteraceae Bercht. & J.Presl
Iz_6_1209	DJI_0306	Euphorbia seguieriana Neck.	0	n	1	0	Euphorbiaceae Juss.
Iz_6_1209	DJI_0306	Fallopia dumetorum (L.) Holub	0	n	1	0	Polygonaceae Juss.
Iz_6_1209	DJI_0306	Festuca beckeri (Hack.) Trautv.	0	n	1	0	Poaceae Barnhart
Iz_6_1209	DJI_0306	Fragaria vesca L.	0	n	1	0	Rosaceae Juss.
Iz_6_1209	DJI_0306	Frangula alnus Mill.	0	n	1	1	Rhamnaceae Juss.
Iz_6_1209	DJI_0306	Fraxinus pennsylvanica Marshall	0	a	0	1	Oleaceae Hoffmanns. & Link
Iz_6_1209	DJI_0306	Galeopsis bifida Boenn.	0	n	1	0	Lamiacea Lindl.
Iz_6_1209	DJI_0306	Galium verum L.	0	n	1	0	Rubiaceae Juss.
Iz_6_1209	DJI_0306	Genista tinctoria L.	0	n	1	0	Fabaceae Lindl.
Iz_6_1209	DJI_0306	Geranium robertianum L.	0	n	1	0	Geraniaceae Juss.
Iz_6_1209	DJI_0306	Geranium sanquineum L.	0	n	1	0	Geraniaceae Juss.
Iz_6_1209	DJI_0306	Gypsophila paniculata L.	0	n	1	0	Caryophyllaceae Juss.
Iz_6_1209	DJI_0306	Helichrysum arenarium (L.) Moench	0	n	1	0	Asteraceae Bercht. & J.Presl
Iz_6_1209	DJI_0306	Herniaria polygama J.Gay	0	n	1	0	Caryophyllaceae Juss.
Iz_6_1209	DJI_0306	Hieracium robustum Fr.	0	n	1	0	Asteraceae Bercht. & J.Presl
Iz_6_1209	DJI_0306	Hieracium umbellatum L.	0	n	1	NA	Asteraceae Bercht. & J.Presl
Iz_6_1209	DJI_0306	Hylotelephium maximum (L.) Holub	0	n	1	0	Crassulaceae J.St.-Hil.
Iz_6_1209	DJI_0306	Jacobaea borysthenica (DC.) B.Nord. & Greuter	0	n	1	0	Asteraceae Bercht. & J.Presl
Iz_6_1209	DJI_0306	Jasione montana L.	0	n	1	0	Campanulaceae Juss.
Iz_6_1209	DJI_0306	Jurinea cyanoides (L.) Rchb.s.l.	0	n	1	0	Asteraceae Bercht. & J.Presl
Iz_6_1209	DJI_0306	Koeleria glauca (Spreng.) DC.	0	n	1	0	Poaceae Barnhart
Iz_6_1209	DJI_0306	Lactuca serriola L.	0	a	0	0	Asteraceae Bercht. & J.Presl
Iz_6_1209	DJI_0306	Linaria genistifolia (L.) Mill.	0	n	1	0	Plantaginaceae Juss.
Iz_6_1209	DJI_0306	Lomelosia argentea (L.) Greuter & Burdet	0	n	1	0	Caprifoliaceae Juss.
Iz_6_1209	DJI_0306	Melampyrum pratense L.	0	n	1	0	Orobanchaceae Vent.
Iz_6_1209	DJI_0306	Oenothera biennis L.	0	a	0	0	Onagraceae Juss.
Iz_6_1209	DJI_0306	Pentanema britannica (L.) D.Gut.Larr., Santos-Vicente, Anderb., E.Rico & M.M.Mart.Ort.	0	n	1	0	Asteraceae Bercht. & J.Presl
Iz_6_1209	DJI_0306	Peucedanum oreoselinum (L.) Moench	0	n	1	0	Apiaceae Lindl.
Iz_6_1209	DJI_0306	Peucedanum ruthenicum M.Bieb.	0	n	1	0	Apiaceae Lindl.
Iz_6_1209	DJI_0306	Pilosella echioides (Lumn.) F.W.Schultz & Sch.Bip.	0	n	1	0	Asteraceae Bercht. & J.Presl
Iz_6_1209	DJI_0306	Pilosella officinarum F. Schultz et Sch. Bip.	0	n	1	0	Asteraceae Bercht. & J.Presl
Iz_6_1209	DJI_0306	Pilosella sp.	0		NA	0	Asteraceae Bercht. & J.Presl
Iz_6_1209	DJI_0306	Pinus sylvestris L.	0	n	1	1	Pinaceae Spreng. ex F.Rudolphi
Iz_6_1209	DJI_0306	Poa annua L.	0	n	1	0	Poaceae Barnhart
Iz_6_1209	DJI_0306	Poa nemoralis L.	0	n	1	0	Poaceae Barnhart
Iz_6_1209	DJI_0306	Poa sp.	0		NA	0	Poaceae Barnhart
Iz_6_1209	DJI_0306	Polygonatum odoratum (Mill.) Druce	0	n	1	0	Asparagaceae Juss.
Iz_6_1209	DJI_0306	Polygonum arenarium Waldst. & Kit.	0	n	1	0	Polygonaceae Juss.
Iz_6_1209	DJI_0306	Populus nigra L.	0	n	1	1	Salicaceae Mirb.
Iz_6_1209	DJI_0306	Populus tremula L.	0	n	1	1	Salicaceae Mirb.
Iz_6_1209	DJI_0306	Populus Ã canescens (Aiton) Sm.	0	n	1	1	Salicaceae Mirb.
Iz_6_1209	DJI_0306	Potentilla argentea L.	0	n	1	0	Rosaceae Juss.
Iz_6_1209	DJI_0306	Psammophiliella muralis (L.) Ikonn.	0	n	1	0	Caryophyllaceae Juss.
Iz_6_1209	DJI_0306	Psephellus sumensis (Kalen.) Greuter	0	n	1	0	Asteraceae Bercht. & J.Presl
Iz_6_1209	DJI_0306	Pulsatilla pratensis (L.) Mill. s.l.	0	n	1	0	Ranunculaceae Juss.
Iz_6_1209	DJI_0306	Pyrus communis L.	0	a	0	1	Rosaceae Juss.
Iz_6_1209	DJI_0306	Quercus robur L.	0	n	1	1	Fagaceae Dumort.
Iz_6_1209	DJI_0306	Robinia pseudoacacia L.	0	a	0	1	Fabaceae Lindl.
Iz_6_1209	DJI_0306	Rosa sp.	0		NA	1	Rosaceae Juss.
Iz_6_1209	DJI_0306	Rubus idaeus L.	0	n	1	0	Rosaceae Juss.
Iz_6_1209	DJI_0306	Rumex acetosella L.	1	n	1	0	Polygonaceae Juss.
Iz_6_1209	DJI_0306	Rumex thyrsiflorus Fingerh.	0	n	1	0	Polygonaceae Juss.
Iz_6_1209	DJI_0306	Salix alba L.	0	n	1	1	Salicaceae Mirb.
Iz_6_1209	DJI_0306	Sambucus nigra L.	0	n	1	1	Viburnaceae Raf.
Iz_6_1209	DJI_0306	Secale sylvestre Host	0	n	1	0	Poaceae Barnhart
Iz_6_1209	DJI_0306	Sempervivum ruthenicum Schnittsp. & C.B. Lehm.	0	n	1	0	Crassulaceae J.St.-Hil.
Iz_6_1209	DJI_0306	Setaria viridis (L.) P. Beauv.	0	n	1	0	Poaceae Barnhart
Iz_6_1209	DJI_0306	Silene borysthenica (Gruner) Walters	0	n	1	0	Caryophyllaceae Juss.
Iz_6_1209	DJI_0306	Silene latifolia subsp. alba (Mill.) Greuter & Burdet	0	n	1	0	Caryophyllaceae Juss.
Iz_6_1209	DJI_0306	Solidago virgaurea L.	0	n	1	0	Asteraceae Bercht. & J.Presl
Iz_6_1209	DJI_0306	Sorbus aucuparia L.	0	n	1	1	Rosaceae Juss.
Iz_6_1209	DJI_0306	Stachys recta L.	0	n	1	0	Lamiacea Lindl.
Iz_6_1209	DJI_0306	Tanacetum vulgare L.	0	n	1	0	Asteraceae Bercht. & J.Presl
Iz_6_1209	DJI_0306	Thymus pallasianus Heinr.Braun	0	n	1	0	Lamiacea Lindl.
Iz_6_1209	DJI_0306	Tilia cordata Mill.	0	n	1	1	Malvaceae Juss.
Iz_6_1209	DJI_0306	Trifolium alpestre L.	0	n	1	0	Fabaceae Lindl.
Iz_6_1209	DJI_0306	Trifolium arvense L.	0	n	1	0	Fabaceae Lindl.
Iz_6_1209	DJI_0306	Ulmus glabra Huds.	0	n	1	1	Ulmaceae Mirb.

Iz_6_1209	DJI_0306	Verbascum densiflorum Bertol.	0	n	1	0	Scrophulariaceae Juss.
Iz_6_1209	DJI_0306	Veronica austriaca L.	0	n	1	0	Plantaginaceae Juss.
Iz_6_1209	DJI_0306	Veronica incana L.	0	n	1	0	Plantaginaceae Juss.
Iz_6_1209	DJI_0306	Veronica sp.	0		NA	NA	
Iz_6_1209	DJI_0306	Veronica verna L.	0	n	1	0	Plantaginaceae Juss.
Iz_6_1209	DJI_0306	Vincetoxicum hirundinaria Medik	0	n	1	0	Apocynaceae Juss.
Iz_6_1209	DJI_0309	Acer tataricum L.	0	n	1	1	Sapindaceae Juss.
Iz_6_1209	DJI_0309	Achillea millefolium L. s.l.	0	n	1	0	Asteraceae Bercht. & J.Presl
Iz_6_1209	DJI_0309	Allium oleraceum L.	0	n	1	0	Amaryllidaceae J.St.-Hil.
Iz_6_1209	DJI_0309	Ambrosia artemisiifolia L.	0	a	0	0	Asteraceae Bercht. & J.Presl
Iz_6_1209	DJI_0309	Amorpha fruticosa L.	0	a	0	0	Fabaceae Lindl.
Iz_6_1209	DJI_0309	Anthericum ramosum L.	0	n	1	0	Asparagaceae Juss.
Iz_6_1209	DJI_0309	Artemisia austriaca Jacq.	0		NA	NA	
Iz_6_1209	DJI_0309	Artemisia marschalliana Spreng.	0	n	1	0	Asteraceae Bercht. & J.Presl
Iz_6_1209	DJI_0309	Asparagus officinalis L.	0	n	1	0	Asparagaceae Juss.
Iz_6_1209	DJI_0309	Astragalus cicer L.	0	n	1	0	Fabaceae Lindl.
Iz_6_1209	DJI_0309	Bassia laniflora (S.G.Gmel.) A.J.Scott	0	n	1	0	Amaranthaceae Juss.
Iz_6_1209	DJI_0309	Berberis vulgaris L.	0	n	1	0	Berberidaceae Juss.
Iz_6_1209	DJI_0309	Betula pendula Roth.	0	n	1	1	Betulaceae Gray
Iz_6_1209	DJI_0309	Betula pendula Roth.	0	n	1	0	Poaceae Barnhart
Iz_6_1209	DJI_0309	Bromus inermis Leyss.	0		NA	NA	
Iz_6_1209	DJI_0309	Bromus squarrosus L.	0	a	0	0	Poaceae Barnhart
Iz_6_1209	DJI_0309	Bromus tectorum L.	0	a	0	0	Poaceae Barnhart
Iz_6_1209	DJI_0309	Calamagrostis epigejos (L.) Roth	5	n	1	0	Poaceae Barnhart
Iz_6_1209	DJI_0309	Carduus acanthoides L.	0	n	1	0	Asteraceae Bercht. & J.Presl
Iz_6_1209	DJI_0309	Carex sp.	0	n	1	0	Cyperaceae Juss.
Iz_6_1209	DJI_0309	Centaurea majorovii Dumbadze	0	n	1	0	Asteraceae Bercht. & J.Presl
Iz_6_1209	DJI_0309	Chamaecytisus ruthenicus (Fisch.ex Wol.) Klaskova	0	n	1	0	Fabaceae Lindl.
Iz_6_1209	DJI_0309	Chelidonium majus L.	0	n	1	0	Papaveraceae Juss.
Iz_6_1209	DJI_0309	Chenopodium betaceum Andrz.	0	n	1	0	Amaranthaceae Juss.
Iz_6_1209	DJI_0309	Chenopodium cf. album	0	n	1	0	Amaranthaceae Juss.
Iz_6_1209	DJI_0309	Chenopodium sp.	0		NA	0	Amaranthaceae Juss.
Iz_6_1209	DJI_0309	Chondrilla juncea L.	0	n	1	0	Asteraceae Bercht. & J.Presl
Iz_6_1209	DJI_0309	Cirsium vulgare (Savi) Ten.	0	n	1	0	Asteraceae Bercht. & J.Presl
Iz_6_1209	DJI_0309	Clinopodium acinos (L.) Kuntze	0	n	1	0	Lamiacea Lindl.
Iz_6_1209	DJI_0309	Convallaria majalis L.	0	n	1	0	Asparagaceae Juss.
Iz_6_1209	DJI_0309	Crataegus rhipidophylla Gand.	0	n	1	1	Rosaceae Juss.
Iz_6_1209	DJI_0309	Crepis foetida L.	0	n	1	0	Asteraceae Bercht. & J.Presl
Iz_6_1209	DJI_0309	Dactylis glomerata L.	0	n	1	0	Poaceae Barnhart
Iz_6_1209	DJI_0309	Dianthus campestris M.Bieb.	0	n	1	0	Caryophyllaceae Juss.
Iz_6_1209	DJI_0309	Digitaria sanguinalis (L.) Scop.	0	a	0	0	Poaceae Barnhart
Iz_6_1209	DJI_0309	Elymus repens (L.) Gould	0	n	1	0	Poaceae Barnhart
Iz_6_1209	DJI_0309	Epilobium tetragonum L.	0	n	1	0	Onagraceae Juss.
Iz_6_1209	DJI_0309	Eragrostis minor Host	0	a	0	0	Poaceae Barnhart
Iz_6_1209	DJI_0309	Erigeron canadensis L.	3	a	0	0	Asteraceae Bercht. & J.Presl
Iz_6_1209	DJI_0309	Euphorbia seguieriana Neck.	0	n	1	0	Euphorbiaceae Juss.
Iz_6_1209	DJI_0309	Fallopia dumetorum (L.) Holub	0	n	1	0	Polygonaceae Juss.
Iz_6_1209	DJI_0309	Festuca beckeri (Hack.) Trautv.	0	n	1	0	Poaceae Barnhart
Iz_6_1209	DJI_0309	Fragaria vesca L.	0	n	1	0	Rosaceae Juss.
Iz_6_1209	DJI_0309	Frangula alnus Mill.	0	n	1	1	Rhamnaceae Juss.
Iz_6_1209	DJI_0309	Fraxinus pennsylvanica Marshall	0	a	0	1	Oleaceae Hoffmanns. & Link
Iz_6_1209	DJI_0309	Galeopsis bifida Boenn.	0	n	1	0	Lamiacea Lindl.
Iz_6_1209	DJI_0309	Galium verum L.	0	n	1	0	Rubiaceae Juss.
Iz_6_1209	DJI_0309	Genista tinctoria L.	0	n	1	0	Fabaceae Lindl.
Iz_6_1209	DJI_0309	Geranium robertianum L.	0	n	1	0	Geraniaceae Juss.
Iz_6_1209	DJI_0309	Geranium sanquineum L.	0	n	1	0	Geraniaceae Juss.
Iz_6_1209	DJI_0309	Gypsophila paniculata L.	0	n	1	0	Caryophyllaceae Juss.
Iz_6_1209	DJI_0309	Helichrysum arenarium (L.) Moench	0	n	1	0	Asteraceae Bercht. & J.Presl
Iz_6_1209	DJI_0309	Herniaria polygama J.Gay	0	n	1	0	Caryophyllaceae Juss.
Iz_6_1209	DJI_0309	Hieracium robustum Fr.	0	n	1	0	Asteraceae Bercht. & J.Presl
Iz_6_1209	DJI_0309	Hieracium umbellatum L.	0	n	1	NA	Asteraceae Bercht. & J.Presl
Iz_6_1209	DJI_0309	Hylotelephium maximum (L.) Holub	0	n	1	0	Crassulaceae J.St.-Hil.
Iz_6_1209	DJI_0309	Jacobaea borysthenica (DC.) B.Nord. & Greuter	0	n	1	0	Asteraceae Bercht. & J.Presl
Iz_6_1209	DJI_0309	Jasione montana L.	0	n	1	0	Campanulaceae Juss.
Iz_6_1209	DJI_0309	Jurinea cyanoides (L.) Rchb.s.l.	0	n	1	0	Asteraceae Bercht. & J.Presl
Iz_6_1209	DJI_0309	Koeleria glauca (Spreng.) DC.	0	n	1	0	Poaceae Barnhart
Iz_6_1209	DJI_0309	Lactuca serriola L.	0	a	0	0	Asteraceae Bercht. & J.Presl
Iz_6_1209	DJI_0309	Linaria genistifolia (L.) Mill.	0	n	1	0	Plantaginaceae Juss.
Iz_6_1209	DJI_0309	Lomelosia argentea (L.) Greuter & Burdet	0	n	1	0	Caprifoliaceae Juss.
Iz_6_1209	DJI_0309	Melampyrum pratense L.	0	n	1	0	Orobanchaceae Vent.
Iz_6_1209	DJI_0309	Oenothera biennis L.	0	a	0	0	Onagraceae Juss.
Iz_6_1209	DJI_0309	Pentanema britannica (L.) D.Gut.Larr., Santos-Vicente, Anderb., E.Rico & M.M.Mart.Ort.	0	n	1	0	Asteraceae Bercht. & J.Presl
Iz_6_1209	DJI_0309	Peucedanum oreoselinum (L.) Moench	0	n	1	0	Apiaceae Lindl.
Iz_6_1209	DJI_0309	Peucedanum ruthenicum M.Bieb.	0	n	1	0	Apiaceae Lindl.
Iz_6_1209	DJI_0309	Pilosella echioides (Lumn.) F.W.Schultz & Sch.Bip.	0	n	1	0	Asteraceae Bercht. & J.Presl
Iz_6_1209	DJI_0309	Pilosella officinarum F. Schultz et Sch. Bip.	0	n	1	0	Asteraceae Bercht. & J.Presl
Iz_6_1209	DJI_0309	Pilosella sp.	0		NA	0	Asteraceae Bercht. & J.Presl
Iz_6_1209	DJI_0309	Pinus sylvestris L.	0	n	1	1	Pinaceae Spreng. ex F.Rudolphi
Iz_6_1209	DJI_0309	Poa annua L.	0	n	1	0	Poaceae Barnhart
Iz_6_1209	DJI_0309	Poa nemoralis L.	0	n	1	0	Poaceae Barnhart
Iz_6_1209	DJI_0309	Poa sp.	0		NA	0	Poaceae Barnhart
Iz_6_1209	DJI_0309	Polygonatum odoratum (Mill.) Druce	0	n	1	0	Asparagaceae Juss.
Iz_6_1209	DJI_0309	Polygonum arenarium Waldst. & Kit.	0	n	1	0	Polygonaceae Juss.
Iz_6_1209	DJI_0309	Populus nigra L.	0	n	1	1	Salicaceae Mirb.
Iz_6_1209	DJI_0309	Populus tremula L.	0	n	1	1	Salicaceae Mirb.
Iz_6_1209	DJI_0309	Populus Ã canescens (Aiton) Sm.	0	n	1	1	Salicaceae Mirb.
Iz_6_1209	DJI_0309	Potentilla argentea L.	0	n	1	0	Rosaceae Juss.
Iz_6_1209	DJI_0309	Psammophiliella muralis (L.) Ikonn.	0	n	1	0	Caryophyllaceae Juss.
Iz_6_1209	DJI_0309	Psephellus sumensis (Kalen.) Greuter	0	n	1	0	Asteraceae Bercht. & J.Presl
Iz_6_1209	DJI_0309	Pulsatilla pratensis (L.) Mill. s.l.	0	n	1	0	Ranunculaceae Juss.
Iz_6_1209	DJI_0309	Pyrus communis L.	0	a	0	1	Rosaceae Juss.
Iz_6_1209	DJI_0309	Quercus robur L.	0	n	1	1	Fagaceae Dumort.
Iz_6_1209	DJI_0309	Robinia pseudoacacia L.	0	a	0	1	Fabaceae Lindl.
Iz_6_1209	DJI_0309	Rosa sp.	0		NA	1	Rosaceae Juss.
Iz_6_1209	DJI_0309	Rubus idaeus L.	0	n	1	0	Rosaceae Juss.
Iz_6_1209	DJI_0309	Rumex acetosella L.	0	n	1	0	Polygonaceae Juss.
Iz_6_1209	DJI_0309	Rumex thyrsiflorus Fingerh.	0	n	1	0	Polygonaceae Juss.
Iz_6_1209	DJI_0309	Salix alba L.	0	n	1	1	Salicaceae Mirb.

Iz_6_1209	DJI_0309	Sambucus nigra L.	0	n	1	1	Viburnaceae Raf.
Iz_6_1209	DJI_0309	Secale sylvestre Host	0	n	1	0	Poaceae Barnhart
Iz_6_1209	DJI_0309	Sempervivum ruthenicum Schnittsp. & C.B. Lehm.	0	n	1	0	Crassulaceae J.St.-Hil.
Iz_6_1209	DJI_0309	Setaria viridis (L.) P. Beauv.	0	n	1	0	Poaceae Barnhart
Iz_6_1209	DJI_0309	Silene borysthenica (Gruner) Walters	0	n	1	0	Caryophyllaceae Juss.
Iz_6_1209	DJI_0309	Silene latifolia subsp. alba (Mill.) Greuter & Burdet	0	n	1	0	Caryophyllaceae Juss.
Iz_6_1209	DJI_0309	Solidago virgaurea L.	0	n	1	0	Asteraceae Bercht. & J.Presl
Iz_6_1209	DJI_0309	Sorbus aucuparia L.	0	n	1	1	Rosaceae Juss.
Iz_6_1209	DJI_0309	Stachys recta L.	0	n	1	0	Lamiacea Lindl.
Iz_6_1209	DJI_0309	Tanacetum vulgare L.	0	n	1	0	Asteraceae Bercht. & J.Presl
Iz_6_1209	DJI_0309	Thymus pallasianus Heinr.Braun	0	n	1	0	Lamiacea Lindl.
Iz_6_1209	DJI_0309	Tilia cordata Mill.	0	n	1	1	Malvaceae Juss.
Iz_6_1209	DJI_0309	Trifolium alpestre L.	0	n	1	0	Fabaceae Lindl.
Iz_6_1209	DJI_0309	Trifolium arvense L.	0	n	1	0	Fabaceae Lindl.
Iz_6_1209	DJI_0309	Ulmus glabra Huds.	0	n	1	1	Ulmaceae Mirb.
Iz_6_1209	DJI_0309	Verbascum densiflorum Bertol.	0	n	1	0	Scrophulariaceae Juss.
Iz_6_1209	DJI_0309	Veronica austriaca L.	0	n	1	0	Plantaginaceae Juss.
Iz_6_1209	DJI_0309	Veronica incana L.	0	n	1	0	Plantaginaceae Juss.
Iz_6_1209	DJI_0309	Veronica sp.	0		NA	NA	
Iz_6_1209	DJI_0309	Veronica verna L.	0	n	1	0	Plantaginaceae Juss.
Iz_6_1209	DJI_0309	Vincetoxicum hirundinaria Medik	0	n	1	0	Apocynaceae Juss.
Iz_6_1209	DJI_0311	Acer tataricum L.	0	n	1	1	Sapindaceae Juss.
Iz_6_1209	DJI_0311	Achillea millefolium L. s.l.	0	n	1	0	Asteraceae Bercht. & J.Presl
Iz_6_1209	DJI_0311	Allium oleraceum L.	0	n	1	0	Amaryllidaceae J.St.-Hil.
Iz_6_1209	DJI_0311	Ambrosia artemisiifolia L.	0	a	0	0	Asteraceae Bercht. & J.Presl
Iz_6_1209	DJI_0311	Amorpha fruticosa L.	0	a	0	0	Fabaceae Lindl.
Iz_6_1209	DJI_0311	Anthericum ramosum L.	0	n	1	0	Asparagaceae Juss.
Iz_6_1209	DJI_0311	Artemisia austriaca Jacq.	0		NA	NA	
Iz_6_1209	DJI_0311	Artemisia marschalliana Spreng.	0	n	1	0	Asteraceae Bercht. & J.Presl
Iz_6_1209	DJI_0311	Asparagus officinalis L.	0	n	1	0	Asparagaceae Juss.
Iz_6_1209	DJI_0311	Astragalus cicer L.	0	n	1	0	Fabaceae Lindl.
Iz_6_1209	DJI_0311	Bassia laniflora (S.G.Gmel.) A.J.Scott	0	n	1	0	Amaranthaceae Juss.
Iz_6_1209	DJI_0311	Berberis vulgaris L.	0	n	1	0	Berberidaceae Juss.
Iz_6_1209	DJI_0311	Betula pendula Roth.	0	n	1	1	Betulaceae Gray
Iz_6_1209	DJI_0311	Betula pendula Roth.	0	n	1	0	Poaceae Barnhart
Iz_6_1209	DJI_0311	Bromus inermis Leyss.	0		NA	NA	
Iz_6_1209	DJI_0311	Bromus squarrosus L.	0	a	0	0	Poaceae Barnhart
Iz_6_1209	DJI_0311	Bromus tectorum L.	0	a	0	0	Poaceae Barnhart
Iz_6_1209	DJI_0311	Calamagrostis epigejos (L.) Roth	6	n	1	0	Poaceae Barnhart
Iz_6_1209	DJI_0311	Carduus acanthoides L.	0	n	1	0	Asteraceae Bercht. & J.Presl
Iz_6_1209	DJI_0311	Carex sp.	0	n	1	0	Cyperaceae Juss.
Iz_6_1209	DJI_0311	Centaurea majorovii Dumbadze	0	n	1	0	Asteraceae Bercht. & J.Presl
Iz_6_1209	DJI_0311	Chamaecytisus ruthenicus (Fisch.ex Wol.) Klaskova	0	n	1	0	Fabaceae Lindl.
Iz_6_1209	DJI_0311	Chelidonium majus L.	0	n	1	0	Papaveraceae Juss.
Iz_6_1209	DJI_0311	Chenopodium betaceum Andrz.	0	n	1	0	Amaranthaceae Juss.
Iz_6_1209	DJI_0311	Chenopodium cf. album	0	n	1	0	Amaranthaceae Juss.
Iz_6_1209	DJI_0311	Chenopodium sp.	0		NA	0	Amaranthaceae Juss.
Iz_6_1209	DJI_0311	Chondrilla juncea L.	0	n	1	0	Asteraceae Bercht. & J.Presl
Iz_6_1209	DJI_0311	Cirsium vulgare (Savi) Ten.	0	n	1	0	Asteraceae Bercht. & J.Presl
Iz_6_1209	DJI_0311	Clinopodium acinos (L.) Kuntze	0	n	1	0	Lamiacea Lindl.
Iz_6_1209	DJI_0311	Convallaria majalis L.	0	n	1	0	Asparagaceae Juss.
Iz_6_1209	DJI_0311	Crataegus rhipidophylla Gand.	0	n	1	1	Rosaceae Juss.
Iz_6_1209	DJI_0311	Crepis foetida L.	0	n	1	0	Asteraceae Bercht. & J.Presl
Iz_6_1209	DJI_0311	Dactylis glomerata L.	0	n	1	0	Poaceae Barnhart
Iz_6_1209	DJI_0311	Dianthus campestris M.Bieb.	0	n	1	0	Caryophyllaceae Juss.
Iz_6_1209	DJI_0311	Digitaria sanguinalis (L.) Scop.	0	a	0	0	Poaceae Barnhart
Iz_6_1209	DJI_0311	Elymus repens (L.) Gould	0	n	1	0	Poaceae Barnhart
Iz_6_1209	DJI_0311	Epilobium tetragonum L.	0	n	1	0	Onagraceae Juss.
Iz_6_1209	DJI_0311	Eragrostis minor Host	0	a	0	0	Poaceae Barnhart
Iz_6_1209	DJI_0311	Erigeron canadensis L.	0.2	a	0	0	Asteraceae Bercht. & J.Presl
Iz_6_1209	DJI_0311	Euphorbia seguieriana Neck.	0	n	1	0	Euphorbiaceae Juss.
Iz_6_1209	DJI_0311	Fallopia dumetorum (L.) Holub	0	n	1	0	Polygonaceae Juss.
Iz_6_1209	DJI_0311	Festuca beckeri (Hack.) Trautv.	0	n	1	0	Poaceae Barnhart
Iz_6_1209	DJI_0311	Fragaria vesca L.	0	n	1	0	Rosaceae Juss.
Iz_6_1209	DJI_0311	Frangula alnus Mill.	0	n	1	1	Rhamnaceae Juss.
Iz_6_1209	DJI_0311	Fraxinus pennsylvanica Marshall	0	a	0	1	Oleaceae Hoffmanns. & Link
Iz_6_1209	DJI_0311	Galeopsis bifida Boenn.	0	n	1	0	Lamiacea Lindl.
Iz_6_1209	DJI_0311	Galium verum L.	0	n	1	0	Rubiaceae Juss.
Iz_6_1209	DJI_0311	Genista tinctoria L.	0	n	1	0	Fabaceae Lindl.
Iz_6_1209	DJI_0311	Geranium robertianum L.	0	n	1	0	Geraniaceae Juss.
Iz_6_1209	DJI_0311	Geranium sanquineum L.	0	n	1	0	Geraniaceae Juss.
Iz_6_1209	DJI_0311	Gypsophila paniculata L.	0	n	1	0	Caryophyllaceae Juss.
Iz_6_1209	DJI_0311	Helichrysum arenarium (L.) Moench	0	n	1	0	Asteraceae Bercht. & J.Presl
Iz_6_1209	DJI_0311	Herniaria polygama J.Gay	0	n	1	0	Caryophyllaceae Juss.
Iz_6_1209	DJI_0311	Hieracium robustum Fr.	0	n	1	0	Asteraceae Bercht. & J.Presl
Iz_6_1209	DJI_0311	Hieracium umbellatum L.	0	n	1	NA	Asteraceae Bercht. & J.Presl
Iz_6_1209	DJI_0311	Hylotelephium maximum (L.) Holub	0	n	1	0	Crassulaceae J.St.-Hil.
Iz_6_1209	DJI_0311	Jacobaea borysthenica (DC.) B.Nord. & Greuter	0	n	1	0	Asteraceae Bercht. & J.Presl
Iz_6_1209	DJI_0311	Jasione montana L.	0	n	1	0	Campanulaceae Juss.
Iz_6_1209	DJI_0311	Jurinea cyanoides (L.) Rchb.s.l.	0	n	1	0	Asteraceae Bercht. & J.Presl
Iz_6_1209	DJI_0311	Koeleria glauca (Spreng.) DC.	1	n	1	0	Poaceae Barnhart
Iz_6_1209	DJI_0311	Lactuca serriola L.	0	a	0	0	Asteraceae Bercht. & J.Presl
Iz_6_1209	DJI_0311	Linaria genistifolia (L.) Mill.	0	n	1	0	Plantaginaceae Juss.
Iz_6_1209	DJI_0311	Lomelosia argentea (L.) Greuter & Burdet	0	n	1	0	Caprifoliaceae Juss.
Iz_6_1209	DJI_0311	Melampyrum pratense L.	0	n	1	0	Orobanchaceae Vent.
Iz_6_1209	DJI_0311	Oenothera biennis L.	0	a	0	0	Onagraceae Juss.
Iz_6_1209	DJI_0311	Pentanema britannica (L.) D.Gut.Larr., Santos-Vicente, Anderb., E.Rico & M.M.Mart.Ort.	0	n	1	0	Asteraceae Bercht. & J.Presl
Iz_6_1209	DJI_0311	Peucedanum oreoselinum (L.) Moench	0	n	1	0	Apiaceae Lindl.
Iz_6_1209	DJI_0311	Peucedanum ruthenicum M.Bieb.	0	n	1	0	Apiaceae Lindl.
Iz_6_1209	DJI_0311	Pilosella echioides (Lumn.) F.W.Schultz & Sch.Bip.	0	n	1	0	Asteraceae Bercht. & J.Presl
Iz_6_1209	DJI_0311	Pilosella officinarum F. Schultz et Sch. Bip.	0	n	1	0	Asteraceae Bercht. & J.Presl
Iz_6_1209	DJI_0311	Pilosella sp.	0		NA	0	Asteraceae Bercht. & J.Presl
Iz_6_1209	DJI_0311	Pinus sylvestris L.	0	n	1	1	Pinaceae Spreng. ex F.Rudolphi
Iz_6_1209	DJI_0311	Poa annua L.	0	n	1	0	Poaceae Barnhart
Iz_6_1209	DJI_0311	Poa nemoralis L.	0	n	1	0	Poaceae Barnhart
Iz_6_1209	DJI_0311	Poa sp.	0		NA	0	Poaceae Barnhart
Iz_6_1209	DJI_0311	Polygonatum odoratum (Mill.) Druce	0	n	1	0	Asparagaceae Juss.
Iz_6_1209	DJI_0311	Polygonum arenarium Waldst. & Kit.	0	n	1	0	Polygonaceae Juss.

Iz_6_1209	DJI_0311	Populus nigra L.	0	n	1	1	Salicaceae Mirb.
Iz_6_1209	DJI_0311	Populus tremula L.	5	n	1	1	Salicaceae Mirb.
Iz_6_1209	DJI_0311	Populus Ã canescens (Aiton) Sm.	0	n	1	1	Salicaceae Mirb.
Iz_6_1209	DJI_0311	Potentilla argentea L.	0	n	1	0	Rosaceae Juss.
Iz_6_1209	DJI_0311	Psammophiliella muralis (L.) Ikonn.	0	n	1	0	Caryophyllaceae Juss.
Iz_6_1209	DJI_0311	Psephellus sumensis (Kalen.) Greuter	0	n	1	0	Asteraceae Bercht. & J.Presl
Iz_6_1209	DJI_0311	Pulsatilla pratensis (L.) Mill. s.l.	0	n	1	0	Ranunculaceae Juss.
Iz_6_1209	DJI_0311	Pyrus communis L.	0	a	0	1	Rosaceae Juss.
Iz_6_1209	DJI_0311	Quercus robur L.	0	n	1	1	Fagaceae Dumort.
Iz_6_1209	DJI_0311	Robinia pseudoacacia L.	0	a	0	1	Fabaceae Lindl.
Iz_6_1209	DJI_0311	Rosa sp.	0		NA	1	Rosaceae Juss.
Iz_6_1209	DJI_0311	Rubus idaeus L.	0	n	1	0	Rosaceae Juss.
Iz_6_1209	DJI_0311	Rumex acetosella L.	0	n	1	0	Polygonaceae Juss.
Iz_6_1209	DJI_0311	Rumex thyrsiflorus Fingerh.	0	n	1	0	Polygonaceae Juss.
Iz_6_1209	DJI_0311	Salix alba L.	0	n	1	1	Salicaceae Mirb.
Iz_6_1209	DJI_0311	Sambucus nigra L.	0	n	1	1	Viburnaceae Raf.
Iz_6_1209	DJI_0311	Secale sylvestre Host	0	n	1	0	Poaceae Barnhart
Iz_6_1209	DJI_0311	Sempervivum ruthenicum Schnittsp. & C.B. Lehm.	0	n	1	0	Crassulaceae J.St.-Hil.
Iz_6_1209	DJI_0311	Setaria viridis (L.) P. Beauv.	0	n	1	0	Poaceae Barnhart
Iz_6_1209	DJI_0311	Silene borysthenica (Gruner) Walters	0	n	1	0	Caryophyllaceae Juss.
Iz_6_1209	DJI_0311	Silene latifolia subsp. alba (Mill.) Greuter & Burdet	0	n	1	0	Caryophyllaceae Juss.
Iz_6_1209	DJI_0311	Solidago virgaurea L.	2	n	1	0	Asteraceae Bercht. & J.Presl
Iz_6_1209	DJI_0311	Sorbus aucuparia L.	0	n	1	1	Rosaceae Juss.
Iz_6_1209	DJI_0311	Stachys recta L.	0	n	1	0	Lamiacea Lindl.
Iz_6_1209	DJI_0311	Tanacetum vulgare L.	0	n	1	0	Asteraceae Bercht. & J.Presl
Iz_6_1209	DJI_0311	Thymus pallasianus Heinr.Braun	0	n	1	0	Lamiacea Lindl.
Iz_6_1209	DJI_0311	Tilia cordata Mill.	0	n	1	1	Malvaceae Juss.
Iz_6_1209	DJI_0311	Trifolium alpestre L.	0	n	1	0	Fabaceae Lindl.
Iz_6_1209	DJI_0311	Trifolium arvense L.	0	n	1	0	Fabaceae Lindl.
Iz_6_1209	DJI_0311	Ulmus glabra Huds.	0	n	1	1	Ulmaceae Mirb.
Iz_6_1209	DJI_0311	Verbascum densiflorum Bertol.	0	n	1	0	Scrophulariaceae Juss.
Iz_6_1209	DJI_0311	Veronica austriaca L.	0	n	1	0	Plantaginaceae Juss.
Iz_6_1209	DJI_0311	Veronica incana L.	0	n	1	0	Plantaginaceae Juss.
Iz_6_1209	DJI_0311	Veronica sp.	0		NA	NA	
Iz_6_1209	DJI_0311	Veronica verna L.	0	n	1	0	Plantaginaceae Juss.
Iz_6_1209	DJI_0311	Vincetoxicum hirundinaria Medik	0	n	1	0	Apocynaceae Juss.
Iz_6_1209	DJI_0327	Acer tataricum L.	0	n	1	1	Sapindaceae Juss.
Iz_6_1209	DJI_0327	Achillea millefolium L. s.l.	0	n	1	0	Asteraceae Bercht. & J.Presl
Iz_6_1209	DJI_0327	Allium oleraceum L.	0	n	1	0	Amaryllidaceae J.St.-Hil.
Iz_6_1209	DJI_0327	Ambrosia artemisiifolia L.	0	a	0	0	Asteraceae Bercht. & J.Presl
Iz_6_1209	DJI_0327	Amorpha fruticosa L.	0	a	0	0	Fabaceae Lindl.
Iz_6_1209	DJI_0327	Anthericum ramosum L.	0	n	1	0	Asparagaceae Juss.
Iz_6_1209	DJI_0327	Artemisia austriaca Jacq.	0.5		NA	NA	
Iz_6_1209	DJI_0327	Artemisia marschalliana Spreng.	0	n	1	0	Asteraceae Bercht. & J.Presl
Iz_6_1209	DJI_0327	Asparagus officinalis L.	0	n	1	0	Asparagaceae Juss.
Iz_6_1209	DJI_0327	Astragalus cicer L.	0	n	1	0	Fabaceae Lindl.
Iz_6_1209	DJI_0327	Bassia laniflora (S.G.Gmel.) A.J.Scott	0	n	1	0	Amaranthaceae Juss.
Iz_6_1209	DJI_0327	Berberis vulgaris L.	0	n	1	0	Berberidaceae Juss.
Iz_6_1209	DJI_0327	Betula pendula Roth.	0	n	1	1	Betulaceae Gray
Iz_6_1209	DJI_0327	Betula pendula Roth.	0	n	1	0	Poaceae Barnhart
Iz_6_1209	DJI_0327	Bromus inermis Leyss.	0		NA	NA	
Iz_6_1209	DJI_0327	Bromus squarrosus L.	0	a	0	0	Poaceae Barnhart
Iz_6_1209	DJI_0327	Bromus tectorum L.	0	a	0	0	Poaceae Barnhart
Iz_6_1209	DJI_0327	Calamagrostis epigejos (L.) Roth	2	n	1	0	Poaceae Barnhart
Iz_6_1209	DJI_0327	Carduus acanthoides L.	0	n	1	0	Asteraceae Bercht. & J.Presl
Iz_6_1209	DJI_0327	Carex sp.	0	n	1	0	Cyperaceae Juss.
Iz_6_1209	DJI_0327	Centaurea majorovii Dumbadze	0	n	1	0	Asteraceae Bercht. & J.Presl
Iz_6_1209	DJI_0327	Chamaecytisus ruthenicus (Fisch.ex Wol.) Klaskova	0	n	1	0	Fabaceae Lindl.
Iz_6_1209	DJI_0327	Chelidonium majus L.	0	n	1	0	Papaveraceae Juss.
Iz_6_1209	DJI_0327	Chenopodium betaceum Andrz.	0	n	1	0	Amaranthaceae Juss.
Iz_6_1209	DJI_0327	Chenopodium cf. album	0	n	1	0	Amaranthaceae Juss.
Iz_6_1209	DJI_0327	Chenopodium sp.	0		NA	0	Amaranthaceae Juss.
Iz_6_1209	DJI_0327	Chondrilla juncea L.	0	n	1	0	Asteraceae Bercht. & J.Presl
Iz_6_1209	DJI_0327	Cirsium vulgare (Savi) Ten.	0	n	1	0	Asteraceae Bercht. & J.Presl
Iz_6_1209	DJI_0327	Clinopodium acinos (L.) Kuntze	0	n	1	0	Lamiacea Lindl.
Iz_6_1209	DJI_0327	Convallaria majalis L.	0	n	1	0	Asparagaceae Juss.
Iz_6_1209	DJI_0327	Crataegus rhipidophylla Gand.	0	n	1	1	Rosaceae Juss.
Iz_6_1209	DJI_0327	Crepis foetida L.	0	n	1	0	Asteraceae Bercht. & J.Presl
Iz_6_1209	DJI_0327	Dactylis glomerata L.	0	n	1	0	Poaceae Barnhart
Iz_6_1209	DJI_0327	Dianthus campestris M.Bieb.	0.2	n	1	0	Caryophyllaceae Juss.
Iz_6_1209	DJI_0327	Digitaria sanguinalis (L.) Scop.	0	a	0	0	Poaceae Barnhart
Iz_6_1209	DJI_0327	Elymus repens (L.) Gould	0	n	1	0	Poaceae Barnhart
Iz_6_1209	DJI_0327	Epilobium tetragonum L.	0	n	1	0	Onagraceae Juss.
Iz_6_1209	DJI_0327	Eragrostis minor Host	0	a	0	0	Poaceae Barnhart
Iz_6_1209	DJI_0327	Erigeron canadensis L.	1	a	0	0	Asteraceae Bercht. & J.Presl
Iz_6_1209	DJI_0327	Euphorbia seguieriana Neck.	0	n	1	0	Euphorbiaceae Juss.
Iz_6_1209	DJI_0327	Fallopia dumetorum (L.) Holub	0	n	1	0	Polygonaceae Juss.
Iz_6_1209	DJI_0327	Festuca beckeri (Hack.) Trautv.	0	n	1	0	Poaceae Barnhart
Iz_6_1209	DJI_0327	Fragaria vesca L.	0	n	1	0	Rosaceae Juss.
Iz_6_1209	DJI_0327	Frangula alnus Mill.	0	n	1	1	Rhamnaceae Juss.
Iz_6_1209	DJI_0327	Fraxinus pennsylvanica Marshall	0	a	0	1	Oleaceae Hoffmanns. & Link
Iz_6_1209	DJI_0327	Galeopsis bifida Boenn.	0	n	1	0	Lamiacea Lindl.
Iz_6_1209	DJI_0327	Galium verum L.	0	n	1	0	Rubiaceae Juss.
Iz_6_1209	DJI_0327	Genista tinctoria L.	0	n	1	0	Fabaceae Lindl.
Iz_6_1209	DJI_0327	Geranium robertianum L.	0	n	1	0	Geraniaceae Juss.
Iz_6_1209	DJI_0327	Geranium sanquineum L.	0	n	1	0	Geraniaceae Juss.
Iz_6_1209	DJI_0327	Gypsophila paniculata L.	0	n	1	0	Caryophyllaceae Juss.
Iz_6_1209	DJI_0327	Helichrysum arenarium (L.) Moench	0	n	1	0	Asteraceae Bercht. & J.Presl
Iz_6_1209	DJI_0327	Herniaria polygama J.Gay	0	n	1	0	Caryophyllaceae Juss.
Iz_6_1209	DJI_0327	Hieracium robustum Fr.	0	n	1	0	Asteraceae Bercht. & J.Presl
Iz_6_1209	DJI_0327	Hieracium umbellatum L.	0	n	1	NA	Asteraceae Bercht. & J.Presl
Iz_6_1209	DJI_0327	Hylotelephium maximum (L.) Holub	3	n	1	0	Crassulaceae J.St.-Hil.
Iz_6_1209	DJI_0327	Jacobaea borysthenica (DC.) B.Nord. & Greuter	0	n	1	0	Asteraceae Bercht. & J.Presl
Iz_6_1209	DJI_0327	Jasione montana L.	0	n	1	0	Campanulaceae Juss.
Iz_6_1209	DJI_0327	Jurinea cyanoides (L.) Rchb.s.l.	0	n	1	0	Asteraceae Bercht. & J.Presl
Iz_6_1209	DJI_0327	Koeleria glauca (Spreng.) DC.	0	n	1	0	Poaceae Barnhart
Iz_6_1209	DJI_0327	Lactuca serriola L.	0	a	0	0	Asteraceae Bercht. & J.Presl
Iz_6_1209	DJI_0327	Linaria genistifolia (L.) Mill.	0	n	1	0	Plantaginaceae Juss.

Iz_6_1209	DJI_0327	Lomelosia argentea (L.) Greuter & Burdet	1	n	1	0	Caprifoliaceae Juss.
Iz_6_1209	DJI_0327	Melampyrum pratense L.	0	n	1	0	Orobanchaceae Vent.
Iz_6_1209	DJI_0327	Oenothera biennis L.	0	a	0	0	Onagraceae Juss.
Iz_6_1209	DJI_0327	Pentanema britannica (L.) D.Gut.Larr., Santos-Vicente, Anderb., E.Rico & M.M.Mart.Ort.	0	n	1	0	Asteraceae Bercht. & J.Presl
Iz_6_1209	DJI_0327	Peucedanum oreoselinum (L.) Moench	1	n	1	0	Apiaceae Lindl.
Iz_6_1209	DJI_0327	Peucedanum ruthenicum M.Bieb.	0	n	1	0	Apiaceae Lindl.
Iz_6_1209	DJI_0327	Pilosella echioides (Lumn.) F.W.Schultz & Sch.Bip.	0	n	1	0	Asteraceae Bercht. & J.Presl
Iz_6_1209	DJI_0327	Pilosella officinarum F. Schultz et Sch. Bip.	3	n	1	0	Asteraceae Bercht. & J.Presl
Iz_6_1209	DJI_0327	Pilosella sp.	0		NA	0	Asteraceae Bercht. & J.Presl
Iz_6_1209	DJI_0327	Pinus sylvestris L.	0	n	1	1	Pinaceae Spreng. ex F.Rudolphi
Iz_6_1209	DJI_0327	Poa annua L.	0	n	1	0	Poaceae Barnhart
Iz_6_1209	DJI_0327	Poa nemoralis L.	0	n	1	0	Poaceae Barnhart
Iz_6_1209	DJI_0327	Poa sp.	0		NA	0	Poaceae Barnhart
Iz_6_1209	DJI_0327	Polygonatum odoratum (Mill.) Druce	0	n	1	0	Asparagaceae Juss.
Iz_6_1209	DJI_0327	Polygonum arenarium Waldst. & Kit.	0	n	1	0	Polygonaceae Juss.
Iz_6_1209	DJI_0327	Populus nigra L.	0	n	1	1	Salicaceae Mirb.
Iz_6_1209	DJI_0327	Populus tremula L.	8	n	1	1	Salicaceae Mirb.
Iz_6_1209	DJI_0327	Populus Ã canescens (Aiton) Sm.	0	n	1	1	Salicaceae Mirb.
Iz_6_1209	DJI_0327	Potentilla argentea L.	0	n	1	0	Rosaceae Juss.
Iz_6_1209	DJI_0327	Psammophiliella muralis (L.) Ikonn.	0	n	1	0	Caryophyllaceae Juss.
Iz_6_1209	DJI_0327	Psephellus sumensis (Kalen.) Greuter	0.5	n	1	0	Asteraceae Bercht. & J.Presl
Iz_6_1209	DJI_0327	Pulsatilla pratensis (L.) Mill. s.l.	0	n	1	0	Ranunculaceae Juss.
Iz_6_1209	DJI_0327	Pyrus communis L.	0	a	0	1	Rosaceae Juss.
Iz_6_1209	DJI_0327	Quercus robur L.	0	n	1	1	Fagaceae Dumort.
Iz_6_1209	DJI_0327	Robinia pseudoacacia L.	0	a	0	1	Fabaceae Lindl.
Iz_6_1209	DJI_0327	Rosa sp.	0		NA	1	Rosaceae Juss.
Iz_6_1209	DJI_0327	Rubus idaeus L.	0	n	1	0	Rosaceae Juss.
Iz_6_1209	DJI_0327	Rumex acetosella L.	1	n	1	0	Polygonaceae Juss.
Iz_6_1209	DJI_0327	Rumex thyrsiflorus Fingerh.	0	n	1	0	Polygonaceae Juss.
Iz_6_1209	DJI_0327	Salix alba L.	0	n	1	1	Salicaceae Mirb.
Iz_6_1209	DJI_0327	Sambucus nigra L.	0	n	1	1	Viburnaceae Raf.
Iz_6_1209	DJI_0327	Secale sylvestre Host	0	n	1	0	Poaceae Barnhart
Iz_6_1209	DJI_0327	Sempervivum ruthenicum Schnittsp. & C.B. Lehm.	0	n	1	0	Crassulaceae J.St.-Hil.
Iz_6_1209	DJI_0327	Setaria viridis (L.) P. Beauv.	0	n	1	0	Poaceae Barnhart
Iz_6_1209	DJI_0327	Silene borysthenica (Gruner) Walters	0	n	1	0	Caryophyllaceae Juss.
Iz_6_1209	DJI_0327	Silene latifolia subsp. alba (Mill.) Greuter & Burdet	0	n	1	0	Caryophyllaceae Juss.
Iz_6_1209	DJI_0327	Solidago virgaurea L.	0	n	1	0	Asteraceae Bercht. & J.Presl
Iz_6_1209	DJI_0327	Sorbus aucuparia L.	0	n	1	1	Rosaceae Juss.
Iz_6_1209	DJI_0327	Stachys recta L.	4	n	1	0	Lamiacea Lindl.
Iz_6_1209	DJI_0327	Tanacetum vulgare L.	0	n	1	0	Asteraceae Bercht. & J.Presl
Iz_6_1209	DJI_0327	Thymus pallasianus Heinr.Braun	0	n	1	0	Lamiacea Lindl.
Iz_6_1209	DJI_0327	Tilia cordata Mill.	0	n	1	1	Malvaceae Juss.
Iz_6_1209	DJI_0327	Trifolium alpestre L.	0	n	1	0	Fabaceae Lindl.
Iz_6_1209	DJI_0327	Trifolium arvense L.	0	n	1	0	Fabaceae Lindl.
Iz_6_1209	DJI_0327	Ulmus glabra Huds.	0	n	1	1	Ulmaceae Mirb.
Iz_6_1209	DJI_0327	Verbascum densiflorum Bertol.	0	n	1	0	Scrophulariaceae Juss.
Iz_6_1209	DJI_0327	Veronica austriaca L.	0	n	1	0	Plantaginaceae Juss.
Iz_6_1209	DJI_0327	Veronica incana L.	0	n	1	0	Plantaginaceae Juss.
Iz_6_1209	DJI_0327	Veronica sp.	2		NA	NA	
Iz_6_1209	DJI_0327	Veronica verna L.	0	n	1	0	Plantaginaceae Juss.
Iz_6_1209	DJI_0327	Vincetoxicum hirundinaria Medik	0	n	1	0	Apocynaceae Juss.
Iz_6_1209	DJI_0330	Acer tataricum L.	0	n	1	1	Sapindaceae Juss.
Iz_6_1209	DJI_0330	Achillea millefolium L. s.l.	0	n	1	0	Asteraceae Bercht. & J.Presl
Iz_6_1209	DJI_0330	Allium oleraceum L.	0	n	1	0	Amaryllidaceae J.St.-Hil.
Iz_6_1209	DJI_0330	Ambrosia artemisiifolia L.	0	a	0	0	Asteraceae Bercht. & J.Presl
Iz_6_1209	DJI_0330	Amorpha fruticosa L.	0	a	0	0	Fabaceae Lindl.
Iz_6_1209	DJI_0330	Anthericum ramosum L.	0	n	1	0	Asparagaceae Juss.
Iz_6_1209	DJI_0330	Artemisia austriaca Jacq.	0		NA	NA	
Iz_6_1209	DJI_0330	Artemisia marschalliana Spreng.	0	n	1	0	Asteraceae Bercht. & J.Presl
Iz_6_1209	DJI_0330	Asparagus officinalis L.	0	n	1	0	Asparagaceae Juss.
Iz_6_1209	DJI_0330	Astragalus cicer L.	0	n	1	0	Fabaceae Lindl.
Iz_6_1209	DJI_0330	Bassia laniflora (S.G.Gmel.) A.J.Scott	0	n	1	0	Amaranthaceae Juss.
Iz_6_1209	DJI_0330	Berberis vulgaris L.	0	n	1	0	Berberidaceae Juss.
Iz_6_1209	DJI_0330	Betula pendula Roth.	0	n	1	1	Betulaceae Gray
Iz_6_1209	DJI_0330	Betula pendula Roth.	0	n	1	0	Poaceae Barnhart
Iz_6_1209	DJI_0330	Bromus inermis Leyss.	0		NA	NA	
Iz_6_1209	DJI_0330	Bromus squarrosus L.	0	a	0	0	Poaceae Barnhart
Iz_6_1209	DJI_0330	Bromus tectorum L.	0	a	0	0	Poaceae Barnhart
Iz_6_1209	DJI_0330	Calamagrostis epigejos (L.) Roth	4	n	1	0	Poaceae Barnhart
Iz_6_1209	DJI_0330	Carduus acanthoides L.	0	n	1	0	Asteraceae Bercht. & J.Presl
Iz_6_1209	DJI_0330	Carex sp.	0	n	1	0	Cyperaceae Juss.
Iz_6_1209	DJI_0330	Centaurea majorovii Dumbadze	0	n	1	0	Asteraceae Bercht. & J.Presl
Iz_6_1209	DJI_0330	Chamaecytisus ruthenicus (Fisch.ex Wol.) Klaskova	0	n	1	0	Fabaceae Lindl.
Iz_6_1209	DJI_0330	Chelidonium majus L.	0	n	1	0	Papaveraceae Juss.
Iz_6_1209	DJI_0330	Chenopodium betaceum Andrz.	0	n	1	0	Amaranthaceae Juss.
Iz_6_1209	DJI_0330	Chenopodium cf. album	0	n	1	0	Amaranthaceae Juss.
Iz_6_1209	DJI_0330	Chenopodium sp.	0		NA	0	Amaranthaceae Juss.
Iz_6_1209	DJI_0330	Chondrilla juncea L.	0	n	1	0	Asteraceae Bercht. & J.Presl
Iz_6_1209	DJI_0330	Cirsium vulgare (Savi) Ten.	0	n	1	0	Asteraceae Bercht. & J.Presl
Iz_6_1209	DJI_0330	Clinopodium acinos (L.) Kuntze	0	n	1	0	Lamiacea Lindl.
Iz_6_1209	DJI_0330	Convallaria majalis L.	0	n	1	0	Asparagaceae Juss.
Iz_6_1209	DJI_0330	Crataegus rhipidophylla Gand.	0	n	1	1	Rosaceae Juss.
Iz_6_1209	DJI_0330	Crepis foetida L.	0	n	1	0	Asteraceae Bercht. & J.Presl
Iz_6_1209	DJI_0330	Dactylis glomerata L.	0	n	1	0	Poaceae Barnhart
Iz_6_1209	DJI_0330	Dianthus campestris M.Bieb.	0	n	1	0	Caryophyllaceae Juss.
Iz_6_1209	DJI_0330	Digitaria sanguinalis (L.) Scop.	0	a	0	0	Poaceae Barnhart
Iz_6_1209	DJI_0330	Elymus repens (L.) Gould	0	n	1	0	Poaceae Barnhart
Iz_6_1209	DJI_0330	Epilobium tetragonum L.	0	n	1	0	Onagraceae Juss.
Iz_6_1209	DJI_0330	Eragrostis minor Host	0	a	0	0	Poaceae Barnhart
Iz_6_1209	DJI_0330	Erigeron canadensis L.	2	a	0	0	Asteraceae Bercht. & J.Presl
Iz_6_1209	DJI_0330	Euphorbia seguieriana Neck.	0	n	1	0	Euphorbiaceae Juss.
Iz_6_1209	DJI_0330	Fallopia dumetorum (L.) Holub	0	n	1	0	Polygonaceae Juss.
Iz_6_1209	DJI_0330	Festuca beckeri (Hack.) Trautv.	0	n	1	0	Poaceae Barnhart
Iz_6_1209	DJI_0330	Fragaria vesca L.	0	n	1	0	Rosaceae Juss.
Iz_6_1209	DJI_0330	Frangula alnus Mill.	0	n	1	1	Rhamnaceae Juss.
Iz_6_1209	DJI_0330	Fraxinus pennsylvanica Marshall	0	a	0	1	Oleaceae Hoffmanns. & Link
Iz_6_1209	DJI_0330	Galeopsis bifida Boenn.	0	n	1	0	Lamiacea Lindl.
Iz_6_1209	DJI_0330	Galium verum L.	0	n	1	0	Rubiaceae Juss.

Iz_6_1209	DJI_0330	Genista tinctoria L.	0	n	1	0	Fabaceae Lindl.
Iz_6_1209	DJI_0330	Geranium robertianum L.	0	n	1	0	Geraniaceae Juss.
Iz_6_1209	DJI_0330	Geranium sanquineum L.	0	n	1	0	Geraniaceae Juss.
Iz_6_1209	DJI_0330	Gypsophila paniculata L.	0	n	1	0	Caryophyllaceae Juss.
Iz_6_1209	DJI_0330	Helichrysum arenarium (L.) Moench	0	n	1	0	Asteraceae Bercht. & J.Presl
Iz_6_1209	DJI_0330	Herniaria polygama J.Gay	0	n	1	0	Caryophyllaceae Juss.
Iz_6_1209	DJI_0330	Hieracium robustum Fr.	0	n	1	0	Asteraceae Bercht. & J.Presl
Iz_6_1209	DJI_0330	Hieracium umbellatum L.	0	n	1	NA	Asteraceae Bercht. & J.Presl
Iz_6_1209	DJI_0330	Hylotelephium maximum (L.) Holub	0	n	1	0	Crassulaceae J.St.-Hil.
Iz_6_1209	DJI_0330	Jacobaea borysthenica (DC.) B.Nord. & Greuter	0	n	1	0	Asteraceae Bercht. & J.Presl
Iz_6_1209	DJI_0330	Jasione montana L.	0	n	1	0	Campanulaceae Juss.
Iz_6_1209	DJI_0330	Jurinea cyanoides (L.) Rchb.s.l.	0	n	1	0	Asteraceae Bercht. & J.Presl
Iz_6_1209	DJI_0330	Koeleria glauca (Spreng.) DC.	0	n	1	0	Poaceae Barnhart
Iz_6_1209	DJI_0330	Lactuca serriola L.	0	a	0	0	Asteraceae Bercht. & J.Presl
Iz_6_1209	DJI_0330	Linaria genistifolia (L.) Mill.	0	n	1	0	Plantaginaceae Juss.
Iz_6_1209	DJI_0330	Lomelosia argentea (L.) Greuter & Burdet	0	n	1	0	Caprifoliaceae Juss.
Iz_6_1209	DJI_0330	Melampyrum pratense L.	0	n	1	0	Orobanchaceae Vent.
Iz_6_1209	DJI_0330	Oenothera biennis L.	0	a	0	0	Onagraceae Juss.
Iz_6_1209	DJI_0330	Pentanema britannica (L.) D.Gut.Larr., Santos-Vicente, Anderb., E.Rico & M.M.Mart.Ort.	0	n	1	0	Asteraceae Bercht. & J.Presl
Iz_6_1209	DJI_0330	Peucedanum oreoselinum (L.) Moench	0	n	1	0	Apiaceae Lindl.
Iz_6_1209	DJI_0330	Peucedanum ruthenicum M.Bieb.	0	n	1	0	Apiaceae Lindl.
Iz_6_1209	DJI_0330	Pilosella echioides (Lumn.) F.W.Schultz & Sch.Bip.	0	n	1	0	Asteraceae Bercht. & J.Presl
Iz_6_1209	DJI_0330	Pilosella officinarum F. Schultz et Sch. Bip.	0	n	1	0	Asteraceae Bercht. & J.Presl
Iz_6_1209	DJI_0330	Pilosella sp.	0		NA	0	Asteraceae Bercht. & J.Presl
Iz_6_1209	DJI_0330	Pinus sylvestris L.	0	n	1	1	Pinaceae Spreng. ex F.Rudolphi
Iz_6_1209	DJI_0330	Poa annua L.	0	n	1	0	Poaceae Barnhart
Iz_6_1209	DJI_0330	Poa nemoralis L.	0	n	1	0	Poaceae Barnhart
Iz_6_1209	DJI_0330	Poa sp.	0		NA	0	Poaceae Barnhart
Iz_6_1209	DJI_0330	Polygonatum odoratum (Mill.) Druce	0	n	1	0	Asparagaceae Juss.
Iz_6_1209	DJI_0330	Polygonum arenarium Waldst. & Kit.	0	n	1	0	Polygonaceae Juss.
Iz_6_1209	DJI_0330	Populus nigra L.	0	n	1	1	Salicaceae Mirb.
Iz_6_1209	DJI_0330	Populus tremula L.	0	n	1	1	Salicaceae Mirb.
Iz_6_1209	DJI_0330	Populus Ã canescens (Aiton) Sm.	0	n	1	1	Salicaceae Mirb.
Iz_6_1209	DJI_0330	Potentilla argentea L.	0	n	1	0	Rosaceae Juss.
Iz_6_1209	DJI_0330	Psammophiliella muralis (L.) Ikonn.	0	n	1	0	Caryophyllaceae Juss.
Iz_6_1209	DJI_0330	Psephellus sumensis (Kalen.) Greuter	0	n	1	0	Asteraceae Bercht. & J.Presl
Iz_6_1209	DJI_0330	Pulsatilla pratensis (L.) Mill. s.l.	0	n	1	0	Ranunculaceae Juss.
Iz_6_1209	DJI_0330	Pyrus communis L.	0	a	0	1	Rosaceae Juss.
Iz_6_1209	DJI_0330	Quercus robur L.	0	n	1	1	Fagaceae Dumort.
Iz_6_1209	DJI_0330	Robinia pseudoacacia L.	0	a	0	1	Fabaceae Lindl.
Iz_6_1209	DJI_0330	Rosa sp.	0		NA	1	Rosaceae Juss.
Iz_6_1209	DJI_0330	Rubus idaeus L.	0	n	1	0	Rosaceae Juss.
Iz_6_1209	DJI_0330	Rumex acetosella L.	0	n	1	0	Polygonaceae Juss.
Iz_6_1209	DJI_0330	Rumex thyrsiflorus Fingerh.	0	n	1	0	Polygonaceae Juss.
Iz_6_1209	DJI_0330	Salix alba L.	0	n	1	1	Salicaceae Mirb.
Iz_6_1209	DJI_0330	Sambucus nigra L.	0	n	1	1	Viburnaceae Raf.
Iz_6_1209	DJI_0330	Secale sylvestre Host	0	n	1	0	Poaceae Barnhart
Iz_6_1209	DJI_0330	Sempervivum ruthenicum Schnittsp. & C.B. Lehm.	0	n	1	0	Crassulaceae J.St.-Hil.
Iz_6_1209	DJI_0330	Setaria viridis (L.) P. Beauv.	0	n	1	0	Poaceae Barnhart
Iz_6_1209	DJI_0330	Silene borysthenica (Gruner) Walters	0	n	1	0	Caryophyllaceae Juss.
Iz_6_1209	DJI_0330	Silene latifolia subsp. alba (Mill.) Greuter & Burdet	0	n	1	0	Caryophyllaceae Juss.
Iz_6_1209	DJI_0330	Solidago virgaurea L.	0	n	1	0	Asteraceae Bercht. & J.Presl
Iz_6_1209	DJI_0330	Sorbus aucuparia L.	0	n	1	1	Rosaceae Juss.
Iz_6_1209	DJI_0330	Stachys recta L.	0	n	1	0	Lamiacea Lindl.
Iz_6_1209	DJI_0330	Tanacetum vulgare L.	0	n	1	0	Asteraceae Bercht. & J.Presl
Iz_6_1209	DJI_0330	Thymus pallasianus Heinr.Braun	0	n	1	0	Lamiacea Lindl.
Iz_6_1209	DJI_0330	Tilia cordata Mill.	0	n	1	1	Malvaceae Juss.
Iz_6_1209	DJI_0330	Trifolium alpestre L.	0	n	1	0	Fabaceae Lindl.
Iz_6_1209	DJI_0330	Trifolium arvense L.	0	n	1	0	Fabaceae Lindl.
Iz_6_1209	DJI_0330	Ulmus glabra Huds.	0	n	1	1	Ulmaceae Mirb.
Iz_6_1209	DJI_0330	Verbascum densiflorum Bertol.	0	n	1	0	Scrophulariaceae Juss.
Iz_6_1209	DJI_0330	Veronica austriaca L.	0	n	1	0	Plantaginaceae Juss.
Iz_6_1209	DJI_0330	Veronica incana L.	0	n	1	0	Plantaginaceae Juss.
Iz_6_1209	DJI_0330	Veronica sp.	0		NA	NA	
Iz_6_1209	DJI_0330	Veronica verna L.	0	n	1	0	Plantaginaceae Juss.
Iz_6_1209	DJI_0330	Vincetoxicum hirundinaria Medik	0	n	1	0	Apocynaceae Juss.
Iz_6_1209	DJI_0333	Acer tataricum L.	0	n	1	1	Sapindaceae Juss.
Iz_6_1209	DJI_0333	Achillea millefolium L. s.l.	0	n	1	0	Asteraceae Bercht. & J.Presl
Iz_6_1209	DJI_0333	Allium oleraceum L.	0	n	1	0	Amaryllidaceae J.St.-Hil.
Iz_6_1209	DJI_0333	Ambrosia artemisiifolia L.	0	a	0	0	Asteraceae Bercht. & J.Presl
Iz_6_1209	DJI_0333	Amorpha fruticosa L.	0	a	0	0	Fabaceae Lindl.
Iz_6_1209	DJI_0333	Anthericum ramosum L.	0	n	1	0	Asparagaceae Juss.
Iz_6_1209	DJI_0333	Artemisia austriaca Jacq.	0		NA	NA	
Iz_6_1209	DJI_0333	Artemisia marschalliana Spreng.	0	n	1	0	Asteraceae Bercht. & J.Presl
Iz_6_1209	DJI_0333	Asparagus officinalis L.	0	n	1	0	Asparagaceae Juss.
Iz_6_1209	DJI_0333	Astragalus cicer L.	0	n	1	0	Fabaceae Lindl.
Iz_6_1209	DJI_0333	Bassia laniflora (S.G.Gmel.) A.J.Scott	0	n	1	0	Amaranthaceae Juss.
Iz_6_1209	DJI_0333	Berberis vulgaris L.	0	n	1	0	Berberidaceae Juss.
Iz_6_1209	DJI_0333	Betula pendula Roth.	0	n	1	1	Betulaceae Gray
Iz_6_1209	DJI_0333	Betula pendula Roth.	0	n	1	0	Poaceae Barnhart
Iz_6_1209	DJI_0333	Bromus inermis Leyss.	0		NA	NA	
Iz_6_1209	DJI_0333	Bromus squarrosus L.	0	a	0	0	Poaceae Barnhart
Iz_6_1209	DJI_0333	Bromus tectorum L.	0	a	0	0	Poaceae Barnhart
Iz_6_1209	DJI_0333	Calamagrostis epigejos (L.) Roth	10	n	1	0	Poaceae Barnhart
Iz_6_1209	DJI_0333	Carduus acanthoides L.	0	n	1	0	Asteraceae Bercht. & J.Presl
Iz_6_1209	DJI_0333	Carex sp.	0	n	1	0	Cyperaceae Juss.
Iz_6_1209	DJI_0333	Centaurea majorovii Dumbadze	0	n	1	0	Asteraceae Bercht. & J.Presl
Iz_6_1209	DJI_0333	Chamaecytisus ruthenicus (Fisch.ex Wol.) Klaskova	0	n	1	0	Fabaceae Lindl.
Iz_6_1209	DJI_0333	Chelidonium majus L.	0	n	1	0	Papaveraceae Juss.
Iz_6_1209	DJI_0333	Chenopodium betaceum Andrz.	0	n	1	0	Amaranthaceae Juss.
Iz_6_1209	DJI_0333	Chenopodium cf. album	0	n	1	0	Amaranthaceae Juss.
Iz_6_1209	DJI_0333	Chenopodium sp.	0		NA	0	Amaranthaceae Juss.
Iz_6_1209	DJI_0333	Chondrilla juncea L.	0	n	1	0	Asteraceae Bercht. & J.Presl
Iz_6_1209	DJI_0333	Cirsium vulgare (Savi) Ten.	0	n	1	0	Asteraceae Bercht. & J.Presl
Iz_6_1209	DJI_0333	Clinopodium acinos (L.) Kuntze	0	n	1	0	Lamiacea Lindl.
Iz_6_1209	DJI_0333	Convallaria majalis L.	0	n	1	0	Asparagaceae Juss.
Iz_6_1209	DJI_0333	Crataegus rhipidophylla Gand.	0	n	1	1	Rosaceae Juss.
Iz_6_1209	DJI_0333	Crepis foetida L.	0	n	1	0	Asteraceae Bercht. & J.Presl

Iz_6_1209	DJI_0333	Dactylis glomerata L.	0	n	1	0	Poaceae Barnhart
Iz_6_1209	DJI_0333	Dianthus campestris M.Bieb.	0	n	1	0	Caryophyllaceae Juss.
Iz_6_1209	DJI_0333	Digitaria sanguinalis (L.) Scop.	0	a	0	0	Poaceae Barnhart
Iz_6_1209	DJI_0333	Elymus repens (L.) Gould	0	n	1	0	Poaceae Barnhart
Iz_6_1209	DJI_0333	Epilobium tetragonum L.	0	n	1	0	Onagraceae Juss.
Iz_6_1209	DJI_0333	Eragrostis minor Host	0	a	0	0	Poaceae Barnhart
Iz_6_1209	DJI_0333	Erigeron canadensis L.	4	a	0	0	Asteraceae Bercht. & J.Presl
Iz_6_1209	DJI_0333	Euphorbia seguieriana Neck.	0	n	1	0	Euphorbiaceae Juss.
Iz_6_1209	DJI_0333	Fallopia dumetorum (L.) Holub	0	n	1	0	Polygonaceae Juss.
Iz_6_1209	DJI_0333	Festuca beckeri (Hack.) Trautv.	0	n	1	0	Poaceae Barnhart
Iz_6_1209	DJI_0333	Fragaria vesca L.	0	n	1	0	Rosaceae Juss.
Iz_6_1209	DJI_0333	Frangula alnus Mill.	0	n	1	1	Rhamnaceae Juss.
Iz_6_1209	DJI_0333	Fraxinus pennsylvanica Marshall	0	a	0	1	Oleaceae Hoffmanns. & Link
Iz_6_1209	DJI_0333	Galeopsis bifida Boenn.	0	n	1	0	Lamiacea Lindl.
Iz_6_1209	DJI_0333	Galium verum L.	0	n	1	0	Rubiaceae Juss.
Iz_6_1209	DJI_0333	Genista tinctoria L.	0	n	1	0	Fabaceae Lindl.
Iz_6_1209	DJI_0333	Geranium robertianum L.	0	n	1	0	Geraniaceae Juss.
Iz_6_1209	DJI_0333	Geranium sanquineum L.	0	n	1	0	Geraniaceae Juss.
Iz_6_1209	DJI_0333	Gypsophila paniculata L.	0	n	1	0	Caryophyllaceae Juss.
Iz_6_1209	DJI_0333	Helichrysum arenarium (L.) Moench	0	n	1	0	Asteraceae Bercht. & J.Presl
Iz_6_1209	DJI_0333	Herniaria polygama J.Gay	0	n	1	0	Caryophyllaceae Juss.
Iz_6_1209	DJI_0333	Hieracium robustum Fr.	0	n	1	0	Asteraceae Bercht. & J.Presl
Iz_6_1209	DJI_0333	Hieracium umbellatum L.	0	n	1	NA	Asteraceae Bercht. & J.Presl
Iz_6_1209	DJI_0333	Hylotelephium maximum (L.) Holub	0	n	1	0	Crassulaceae J.St.-Hil.
Iz_6_1209	DJI_0333	Jacobaea borysthenica (DC.) B.Nord. & Greuter	0	n	1	0	Asteraceae Bercht. & J.Presl
Iz_6_1209	DJI_0333	Jasione montana L.	0	n	1	0	Campanulaceae Juss.
Iz_6_1209	DJI_0333	Jurinea cyanoides (L.) Rchb.s.l.	0	n	1	0	Asteraceae Bercht. & J.Presl
Iz_6_1209	DJI_0333	Koeleria glauca (Spreng.) DC.	0	n	1	0	Poaceae Barnhart
Iz_6_1209	DJI_0333	Lactuca serriola L.	0	a	0	0	Asteraceae Bercht. & J.Presl
Iz_6_1209	DJI_0333	Linaria genistifolia (L.) Mill.	0	n	1	0	Plantaginaceae Juss.
Iz_6_1209	DJI_0333	Lomelosia argentea (L.) Greuter & Burdet	0	n	1	0	Caprifoliaceae Juss.
Iz_6_1209	DJI_0333	Melampyrum pratense L.	0	n	1	0	Orobanchaceae Vent.
Iz_6_1209	DJI_0333	Oenothera biennis L.	0	a	0	0	Onagraceae Juss.
Iz_6_1209	DJI_0333	Pentanema britannica (L.) D.Gut.Larr., Santos-Vicente, Anderb., E.Rico & M.M.Mart.Ort.	0	n	1	0	Asteraceae Bercht. & J.Presl
Iz_6_1209	DJI_0333	Peucedanum oreoselinum (L.) Moench	0	n	1	0	Apiaceae Lindl.
Iz_6_1209	DJI_0333	Peucedanum ruthenicum M.Bieb.	0	n	1	0	Apiaceae Lindl.
Iz_6_1209	DJI_0333	Pilosella echioides (Lumn.) F.W.Schultz & Sch.Bip.	0	n	1	0	Asteraceae Bercht. & J.Presl
Iz_6_1209	DJI_0333	Pilosella officinarum F. Schultz et Sch. Bip.	0	n	1	0	Asteraceae Bercht. & J.Presl
Iz_6_1209	DJI_0333	Pilosella sp.	0		NA	0	Asteraceae Bercht. & J.Presl
Iz_6_1209	DJI_0333	Pinus sylvestris L.	0	n	1	1	Pinaceae Spreng. ex F.Rudolphi
Iz_6_1209	DJI_0333	Poa annua L.	0	n	1	0	Poaceae Barnhart
Iz_6_1209	DJI_0333	Poa nemoralis L.	0	n	1	0	Poaceae Barnhart
Iz_6_1209	DJI_0333	Poa sp.	0		NA	0	Poaceae Barnhart
Iz_6_1209	DJI_0333	Polygonatum odoratum (Mill.) Druce	0	n	1	0	Asparagaceae Juss.
Iz_6_1209	DJI_0333	Polygonum arenarium Waldst. & Kit.	0	n	1	0	Polygonaceae Juss.
Iz_6_1209	DJI_0333	Populus nigra L.	0	n	1	1	Salicaceae Mirb.
Iz_6_1209	DJI_0333	Populus tremula L.	0	n	1	1	Salicaceae Mirb.
Iz_6_1209	DJI_0333	Populus Ã canescens (Aiton) Sm.	0	n	1	1	Salicaceae Mirb.
Iz_6_1209	DJI_0333	Potentilla argentea L.	0	n	1	0	Rosaceae Juss.
Iz_6_1209	DJI_0333	Psammophiliella muralis (L.) Ikonn.	0	n	1	0	Caryophyllaceae Juss.
Iz_6_1209	DJI_0333	Psephellus sumensis (Kalen.) Greuter	0	n	1	0	Asteraceae Bercht. & J.Presl
Iz_6_1209	DJI_0333	Pulsatilla pratensis (L.) Mill. s.l.	0	n	1	0	Ranunculaceae Juss.
Iz_6_1209	DJI_0333	Pyrus communis L.	0	a	0	1	Rosaceae Juss.
Iz_6_1209	DJI_0333	Quercus robur L.	0	n	1	1	Fagaceae Dumort.
Iz_6_1209	DJI_0333	Robinia pseudoacacia L.	0	a	0	1	Fabaceae Lindl.
Iz_6_1209	DJI_0333	Rosa sp.	0		NA	1	Rosaceae Juss.
Iz_6_1209	DJI_0333	Rubus idaeus L.	0	n	1	0	Rosaceae Juss.
Iz_6_1209	DJI_0333	Rumex acetosella L.	0	n	1	0	Polygonaceae Juss.
Iz_6_1209	DJI_0333	Rumex thyrsiflorus Fingerh.	0	n	1	0	Polygonaceae Juss.
Iz_6_1209	DJI_0333	Salix alba L.	0	n	1	1	Salicaceae Mirb.
Iz_6_1209	DJI_0333	Sambucus nigra L.	0	n	1	1	Viburnaceae Raf.
Iz_6_1209	DJI_0333	Secale sylvestre Host	0	n	1	0	Poaceae Barnhart
Iz_6_1209	DJI_0333	Sempervivum ruthenicum Schnittsp. & C.B. Lehm.	0	n	1	0	Crassulaceae J.St.-Hil.
Iz_6_1209	DJI_0333	Setaria viridis (L.) P. Beauv.	0	n	1	0	Poaceae Barnhart
Iz_6_1209	DJI_0333	Silene borysthenica (Gruner) Walters	0	n	1	0	Caryophyllaceae Juss.
Iz_6_1209	DJI_0333	Silene latifolia subsp. alba (Mill.) Greuter & Burdet	0	n	1	0	Caryophyllaceae Juss.
Iz_6_1209	DJI_0333	Solidago virgaurea L.	0	n	1	0	Asteraceae Bercht. & J.Presl
Iz_6_1209	DJI_0333	Sorbus aucuparia L.	0	n	1	1	Rosaceae Juss.
Iz_6_1209	DJI_0333	Stachys recta L.	0	n	1	0	Lamiacea Lindl.
Iz_6_1209	DJI_0333	Tanacetum vulgare L.	0	n	1	0	Asteraceae Bercht. & J.Presl
Iz_6_1209	DJI_0333	Thymus pallasianus Heinr.Braun	0	n	1	0	Lamiacea Lindl.
Iz_6_1209	DJI_0333	Tilia cordata Mill.	0	n	1	1	Malvaceae Juss.
Iz_6_1209	DJI_0333	Trifolium alpestre L.	0	n	1	0	Fabaceae Lindl.
Iz_6_1209	DJI_0333	Trifolium arvense L.	0	n	1	0	Fabaceae Lindl.
Iz_6_1209	DJI_0333	Ulmus glabra Huds.	0	n	1	1	Ulmaceae Mirb.
Iz_6_1209	DJI_0333	Verbascum densiflorum Bertol.	0	n	1	0	Scrophulariaceae Juss.
Iz_6_1209	DJI_0333	Veronica austriaca L.	0	n	1	0	Plantaginaceae Juss.
Iz_6_1209	DJI_0333	Veronica incana L.	0	n	1	0	Plantaginaceae Juss.
Iz_6_1209	DJI_0333	Veronica sp.	0		NA	NA	
Iz_6_1209	DJI_0333	Veronica verna L.	0	n	1	0	Plantaginaceae Juss.
Iz_6_1209	DJI_0333	Vincetoxicum hirundinaria Medik	0	n	1	0	Apocynaceae Juss.
Iz_6_1209	DJI_0336	Acer tataricum L.	0	n	1	1	Sapindaceae Juss.
Iz_6_1209	DJI_0336	Achillea millefolium L. s.l.	0	n	1	0	Asteraceae Bercht. & J.Presl
Iz_6_1209	DJI_0336	Allium oleraceum L.	0	n	1	0	Amaryllidaceae J.St.-Hil.
Iz_6_1209	DJI_0336	Ambrosia artemisiifolia L.	0	a	0	0	Asteraceae Bercht. & J.Presl
Iz_6_1209	DJI_0336	Amorpha fruticosa L.	0	a	0	0	Fabaceae Lindl.
Iz_6_1209	DJI_0336	Anthericum ramosum L.	0	n	1	0	Asparagaceae Juss.
Iz_6_1209	DJI_0336	Artemisia austriaca Jacq.	0		NA	NA	
Iz_6_1209	DJI_0336	Artemisia marschalliana Spreng.	0	n	1	0	Asteraceae Bercht. & J.Presl
Iz_6_1209	DJI_0336	Asparagus officinalis L.	0	n	1	0	Asparagaceae Juss.
Iz_6_1209	DJI_0336	Astragalus cicer L.	0	n	1	0	Fabaceae Lindl.
Iz_6_1209	DJI_0336	Bassia laniflora (S.G.Gmel.) A.J.Scott	0	n	1	0	Amaranthaceae Juss.
Iz_6_1209	DJI_0336	Berberis vulgaris L.	0	n	1	0	Berberidaceae Juss.
Iz_6_1209	DJI_0336	Betula pendula Roth.	0	n	1	1	Betulaceae Gray
Iz_6_1209	DJI_0336	Betula pendula Roth.	0	n	1	0	Poaceae Barnhart
Iz_6_1209	DJI_0336	Bromus inermis Leyss.	0		NA	NA	
Iz_6_1209	DJI_0336	Bromus squarrosus L.	0	a	0	0	Poaceae Barnhart
Iz_6_1209	DJI_0336	Bromus tectorum L.	0	a	0	0	Poaceae Barnhart

Iz_6_1209	DJI_0336	Calamagrostis epigejos (L.) Roth	0.5	n	1	0	Poaceae Barnhart
Iz_6_1209	DJI_0336	Carduus acanthoides L.	0	n	1	0	Asteraceae Bercht. & J.Presl
Iz_6_1209	DJI_0336	Carex sp.	0	n	1	0	Cyperaceae Juss.
Iz_6_1209	DJI_0336	Centaurea majorovii Dumbadze	0	n	1	0	Asteraceae Bercht. & J.Presl
Iz_6_1209	DJI_0336	Chamaecytisus ruthenicus (Fisch.ex Wol.) Klaskova	0	n	1	0	Fabaceae Lindl.
Iz_6_1209	DJI_0336	Chelidonium majus L.	0	n	1	0	Papaveraceae Juss.
Iz_6_1209	DJI_0336	Chenopodium betaceum Andrz.	0	n	1	0	Amaranthaceae Juss.
Iz_6_1209	DJI_0336	Chenopodium cf. album	0	n	1	0	Amaranthaceae Juss.
Iz_6_1209	DJI_0336	Chenopodium sp.	0		NA	0	Amaranthaceae Juss.
Iz_6_1209	DJI_0336	Chondrilla juncea L.	0	n	1	0	Asteraceae Bercht. & J.Presl
Iz_6_1209	DJI_0336	Cirsium vulgare (Savi) Ten.	0	n	1	0	Asteraceae Bercht. & J.Presl
Iz_6_1209	DJI_0336	Clinopodium acinos (L.) Kuntze	0	n	1	0	Lamiacea Lindl.
Iz_6_1209	DJI_0336	Convallaria majalis L.	0	n	1	0	Asparagaceae Juss.
Iz_6_1209	DJI_0336	Crataegus rhipidophylla Gand.	0	n	1	1	Rosaceae Juss.
Iz_6_1209	DJI_0336	Crepis foetida L.	0	n	1	0	Asteraceae Bercht. & J.Presl
Iz_6_1209	DJI_0336	Dactylis glomerata L.	0	n	1	0	Poaceae Barnhart
Iz_6_1209	DJI_0336	Dianthus campestris M.Bieb.	0	n	1	0	Caryophyllaceae Juss.
Iz_6_1209	DJI_0336	Digitaria sanguinalis (L.) Scop.	0	a	0	0	Poaceae Barnhart
Iz_6_1209	DJI_0336	Elymus repens (L.) Gould	0	n	1	0	Poaceae Barnhart
Iz_6_1209	DJI_0336	Epilobium tetragonum L.	0	n	1	0	Onagraceae Juss.
Iz_6_1209	DJI_0336	Eragrostis minor Host	0	a	0	0	Poaceae Barnhart
Iz_6_1209	DJI_0336	Erigeron canadensis L.	5	a	0	0	Asteraceae Bercht. & J.Presl
Iz_6_1209	DJI_0336	Euphorbia seguieriana Neck.	0	n	1	0	Euphorbiaceae Juss.
Iz_6_1209	DJI_0336	Fallopia dumetorum (L.) Holub	0	n	1	0	Polygonaceae Juss.
Iz_6_1209	DJI_0336	Festuca beckeri (Hack.) Trautv.	0	n	1	0	Poaceae Barnhart
Iz_6_1209	DJI_0336	Fragaria vesca L.	0	n	1	0	Rosaceae Juss.
Iz_6_1209	DJI_0336	Frangula alnus Mill.	0	n	1	1	Rhamnaceae Juss.
Iz_6_1209	DJI_0336	Fraxinus pennsylvanica Marshall	0	a	0	1	Oleaceae Hoffmanns. & Link
Iz_6_1209	DJI_0336	Galeopsis bifida Boenn.	0	n	1	0	Lamiacea Lindl.
Iz_6_1209	DJI_0336	Galium verum L.	0	n	1	0	Rubiaceae Juss.
Iz_6_1209	DJI_0336	Genista tinctoria L.	0	n	1	0	Fabaceae Lindl.
Iz_6_1209	DJI_0336	Geranium robertianum L.	0	n	1	0	Geraniaceae Juss.
Iz_6_1209	DJI_0336	Geranium sanquineum L.	0	n	1	0	Geraniaceae Juss.
Iz_6_1209	DJI_0336	Gypsophila paniculata L.	0	n	1	0	Caryophyllaceae Juss.
Iz_6_1209	DJI_0336	Helichrysum arenarium (L.) Moench	0	n	1	0	Asteraceae Bercht. & J.Presl
Iz_6_1209	DJI_0336	Herniaria polygama J.Gay	0	n	1	0	Caryophyllaceae Juss.
Iz_6_1209	DJI_0336	Hieracium robustum Fr.	0	n	1	0	Asteraceae Bercht. & J.Presl
Iz_6_1209	DJI_0336	Hieracium umbellatum L.	0	n	1	NA	Asteraceae Bercht. & J.Presl
Iz_6_1209	DJI_0336	Hylotelephium maximum (L.) Holub	0	n	1	0	Crassulaceae J.St.-Hil.
Iz_6_1209	DJI_0336	Jacobaea borysthenica (DC.) B.Nord. & Greuter	0	n	1	0	Asteraceae Bercht. & J.Presl
Iz_6_1209	DJI_0336	Jasione montana L.	0	n	1	0	Campanulaceae Juss.
Iz_6_1209	DJI_0336	Jurinea cyanoides (L.) Rchb.s.l.	0	n	1	0	Asteraceae Bercht. & J.Presl
Iz_6_1209	DJI_0336	Koeleria glauca (Spreng.) DC.	0	n	1	0	Poaceae Barnhart
Iz_6_1209	DJI_0336	Lactuca serriola L.	0	a	0	0	Asteraceae Bercht. & J.Presl
Iz_6_1209	DJI_0336	Linaria genistifolia (L.) Mill.	0	n	1	0	Plantaginaceae Juss.
Iz_6_1209	DJI_0336	Lomelosia argentea (L.) Greuter & Burdet	0	n	1	0	Caprifoliaceae Juss.
Iz_6_1209	DJI_0336	Melampyrum pratense L.	0	n	1	0	Orobanchaceae Vent.
Iz_6_1209	DJI_0336	Oenothera biennis L.	0	a	0	0	Onagraceae Juss.
Iz_6_1209	DJI_0336	Pentanema britannica (L.) D.Gut.Larr., Santos-Vicente, Anderb., E.Rico & M.M.Mart.Ort.	0	n	1	0	Asteraceae Bercht. & J.Presl
Iz_6_1209	DJI_0336	Peucedanum oreoselinum (L.) Moench	0	n	1	0	Apiaceae Lindl.
Iz_6_1209	DJI_0336	Peucedanum ruthenicum M.Bieb.	0	n	1	0	Apiaceae Lindl.
Iz_6_1209	DJI_0336	Pilosella echioides (Lumn.) F.W.Schultz & Sch.Bip.	0	n	1	0	Asteraceae Bercht. & J.Presl
Iz_6_1209	DJI_0336	Pilosella officinarum F. Schultz et Sch. Bip.	0	n	1	0	Asteraceae Bercht. & J.Presl
Iz_6_1209	DJI_0336	Pilosella sp.	0		NA	0	Asteraceae Bercht. & J.Presl
Iz_6_1209	DJI_0336	Pinus sylvestris L.	0	n	1	1	Pinaceae Spreng. ex F.Rudolphi
Iz_6_1209	DJI_0336	Poa annua L.	0	n	1	0	Poaceae Barnhart
Iz_6_1209	DJI_0336	Poa nemoralis L.	0	n	1	0	Poaceae Barnhart
Iz_6_1209	DJI_0336	Poa sp.	0		NA	0	Poaceae Barnhart
Iz_6_1209	DJI_0336	Polygonatum odoratum (Mill.) Druce	0	n	1	0	Asparagaceae Juss.
Iz_6_1209	DJI_0336	Polygonum arenarium Waldst. & Kit.	0	n	1	0	Polygonaceae Juss.
Iz_6_1209	DJI_0336	Populus nigra L.	0	n	1	1	Salicaceae Mirb.
Iz_6_1209	DJI_0336	Populus tremula L.	0	n	1	1	Salicaceae Mirb.
Iz_6_1209	DJI_0336	Populus Ã canescens (Aiton) Sm.	0	n	1	1	Salicaceae Mirb.
Iz_6_1209	DJI_0336	Potentilla argentea L.	0	n	1	0	Rosaceae Juss.
Iz_6_1209	DJI_0336	Psammophiliella muralis (L.) Ikonn.	0	n	1	0	Caryophyllaceae Juss.
Iz_6_1209	DJI_0336	Psephellus sumensis (Kalen.) Greuter	0	n	1	0	Asteraceae Bercht. & J.Presl
Iz_6_1209	DJI_0336	Pulsatilla pratensis (L.) Mill. s.l.	0	n	1	0	Ranunculaceae Juss.
Iz_6_1209	DJI_0336	Pyrus communis L.	0	a	0	1	Rosaceae Juss.
Iz_6_1209	DJI_0336	Quercus robur L.	0	n	1	1	Fagaceae Dumort.
Iz_6_1209	DJI_0336	Robinia pseudoacacia L.	0	a	0	1	Fabaceae Lindl.
Iz_6_1209	DJI_0336	Rosa sp.	0		NA	1	Rosaceae Juss.
Iz_6_1209	DJI_0336	Rubus idaeus L.	0	n	1	0	Rosaceae Juss.
Iz_6_1209	DJI_0336	Rumex acetosella L.	0	n	1	0	Polygonaceae Juss.
Iz_6_1209	DJI_0336	Rumex thyrsiflorus Fingerh.	0	n	1	0	Polygonaceae Juss.
Iz_6_1209	DJI_0336	Salix alba L.	0	n	1	1	Salicaceae Mirb.
Iz_6_1209	DJI_0336	Sambucus nigra L.	0	n	1	1	Viburnaceae Raf.
Iz_6_1209	DJI_0336	Secale sylvestre Host	0	n	1	0	Poaceae Barnhart
Iz_6_1209	DJI_0336	Sempervivum ruthenicum Schnittsp. & C.B. Lehm.	0	n	1	0	Crassulaceae J.St.-Hil.
Iz_6_1209	DJI_0336	Setaria viridis (L.) P. Beauv.	0	n	1	0	Poaceae Barnhart
Iz_6_1209	DJI_0336	Silene borysthenica (Gruner) Walters	0	n	1	0	Caryophyllaceae Juss.
Iz_6_1209	DJI_0336	Silene latifolia subsp. alba (Mill.) Greuter & Burdet	0	n	1	0	Caryophyllaceae Juss.
Iz_6_1209	DJI_0336	Solidago virgaurea L.	0	n	1	0	Asteraceae Bercht. & J.Presl
Iz_6_1209	DJI_0336	Sorbus aucuparia L.	0	n	1	1	Rosaceae Juss.
Iz_6_1209	DJI_0336	Stachys recta L.	0	n	1	0	Lamiacea Lindl.
Iz_6_1209	DJI_0336	Tanacetum vulgare L.	0	n	1	0	Asteraceae Bercht. & J.Presl
Iz_6_1209	DJI_0336	Thymus pallasianus Heinr.Braun	0	n	1	0	Lamiacea Lindl.
Iz_6_1209	DJI_0336	Tilia cordata Mill.	0	n	1	1	Malvaceae Juss.
Iz_6_1209	DJI_0336	Trifolium alpestre L.	0	n	1	0	Fabaceae Lindl.
Iz_6_1209	DJI_0336	Trifolium arvense L.	0	n	1	0	Fabaceae Lindl.
Iz_6_1209	DJI_0336	Ulmus glabra Huds.	0	n	1	1	Ulmaceae Mirb.
Iz_6_1209	DJI_0336	Verbascum densiflorum Bertol.	0	n	1	0	Scrophulariaceae Juss.
Iz_6_1209	DJI_0336	Veronica austriaca L.	0	n	1	0	Plantaginaceae Juss.
Iz_6_1209	DJI_0336	Veronica incana L.	0	n	1	0	Plantaginaceae Juss.
Iz_6_1209	DJI_0336	Veronica sp.	0		NA	NA	
Iz_6_1209	DJI_0336	Veronica verna L.	0	n	1	0	Plantaginaceae Juss.
Iz_6_1209	DJI_0336	Vincetoxicum hirundinaria Medik	0	n	1	0	Apocynaceae Juss.
Iz_6_1209	DJI_0341	Acer tataricum L.	0	n	1	1	Sapindaceae Juss.
Iz_6_1209	DJI_0341	Achillea millefolium L. s.l.	0	n	1	0	Asteraceae Bercht. & J.Presl

Iz_6_1209	DJI_0341	Allium oleraceum L.	0	n	1	0	Amaryllidaceae J.St.-Hil.
Iz_6_1209	DJI_0341	Ambrosia artemisiifolia L.	0	a	0	0	Asteraceae Bercht. & J.Presl
Iz_6_1209	DJI_0341	Amorpha fruticosa L.	0	a	0	0	Fabaceae Lindl.
Iz_6_1209	DJI_0341	Anthericum ramosum L.	0	n	1	0	Asparagaceae Juss.
Iz_6_1209	DJI_0341	Artemisia austriaca Jacq.	0		NA	NA	
Iz_6_1209	DJI_0341	Artemisia marschalliana Spreng.	0	n	1	0	Asteraceae Bercht. & J.Presl
Iz_6_1209	DJI_0341	Asparagus officinalis L.	0	n	1	0	Asparagaceae Juss.
Iz_6_1209	DJI_0341	Astragalus cicer L.	0	n	1	0	Fabaceae Lindl.
Iz_6_1209	DJI_0341	Bassia laniflora (S.G.Gmel.) A.J.Scott	0	n	1	0	Amaranthaceae Juss.
Iz_6_1209	DJI_0341	Berberis vulgaris L.	0	n	1	0	Berberidaceae Juss.
Iz_6_1209	DJI_0341	Betula pendula Roth.	0	n	1	1	Betulaceae Gray
Iz_6_1209	DJI_0341	Betula pendula Roth.	0	n	1	0	Poaceae Barnhart
Iz_6_1209	DJI_0341	Bromus inermis Leyss.	0		NA	NA	
Iz_6_1209	DJI_0341	Bromus squarrosus L.	0	a	0	0	Poaceae Barnhart
Iz_6_1209	DJI_0341	Bromus tectorum L.	0	a	0	0	Poaceae Barnhart
Iz_6_1209	DJI_0341	Calamagrostis epigejos (L.) Roth	0	n	1	0	Poaceae Barnhart
Iz_6_1209	DJI_0341	Carduus acanthoides L.	0	n	1	0	Asteraceae Bercht. & J.Presl
Iz_6_1209	DJI_0341	Carex sp.	0	n	1	0	Cyperaceae Juss.
Iz_6_1209	DJI_0341	Centaurea majorovii Dumbadze	0	n	1	0	Asteraceae Bercht. & J.Presl
Iz_6_1209	DJI_0341	Chamaecytisus ruthenicus (Fisch.ex Wol.) Klaskova	0	n	1	0	Fabaceae Lindl.
Iz_6_1209	DJI_0341	Chelidonium majus L.	0	n	1	0	Papaveraceae Juss.
Iz_6_1209	DJI_0341	Chenopodium betaceum Andrz.	0	n	1	0	Amaranthaceae Juss.
Iz_6_1209	DJI_0341	Chenopodium cf. album	0	n	1	0	Amaranthaceae Juss.
Iz_6_1209	DJI_0341	Chenopodium sp.	0		NA	0	Amaranthaceae Juss.
Iz_6_1209	DJI_0341	Chondrilla juncea L.	0	n	1	0	Asteraceae Bercht. & J.Presl
Iz_6_1209	DJI_0341	Cirsium vulgare (Savi) Ten.	0	n	1	0	Asteraceae Bercht. & J.Presl
Iz_6_1209	DJI_0341	Clinopodium acinos (L.) Kuntze	0	n	1	0	Lamiacea Lindl.
Iz_6_1209	DJI_0341	Convallaria majalis L.	0	n	1	0	Asparagaceae Juss.
Iz_6_1209	DJI_0341	Crataegus rhipidophylla Gand.	0	n	1	1	Rosaceae Juss.
Iz_6_1209	DJI_0341	Crepis foetida L.	0	n	1	0	Asteraceae Bercht. & J.Presl
Iz_6_1209	DJI_0341	Dactylis glomerata L.	0	n	1	0	Poaceae Barnhart
Iz_6_1209	DJI_0341	Dianthus campestris M.Bieb.	0	n	1	0	Caryophyllaceae Juss.
Iz_6_1209	DJI_0341	Digitaria sanguinalis (L.) Scop.	0	a	0	0	Poaceae Barnhart
Iz_6_1209	DJI_0341	Elymus repens (L.) Gould	0	n	1	0	Poaceae Barnhart
Iz_6_1209	DJI_0341	Epilobium tetragonum L.	0	n	1	0	Onagraceae Juss.
Iz_6_1209	DJI_0341	Eragrostis minor Host	0	a	0	0	Poaceae Barnhart
Iz_6_1209	DJI_0341	Erigeron canadensis L.	5	a	0	0	Asteraceae Bercht. & J.Presl
Iz_6_1209	DJI_0341	Euphorbia seguieriana Neck.	0	n	1	0	Euphorbiaceae Juss.
Iz_6_1209	DJI_0341	Fallopia dumetorum (L.) Holub	0	n	1	0	Polygonaceae Juss.
Iz_6_1209	DJI_0341	Festuca beckeri (Hack.) Trautv.	0	n	1	0	Poaceae Barnhart
Iz_6_1209	DJI_0341	Fragaria vesca L.	0	n	1	0	Rosaceae Juss.
Iz_6_1209	DJI_0341	Frangula alnus Mill.	0	n	1	1	Rhamnaceae Juss.
Iz_6_1209	DJI_0341	Fraxinus pennsylvanica Marshall	0	a	0	1	Oleaceae Hoffmanns. & Link
Iz_6_1209	DJI_0341	Galeopsis bifida Boenn.	0	n	1	0	Lamiacea Lindl.
Iz_6_1209	DJI_0341	Galium verum L.	0	n	1	0	Rubiaceae Juss.
Iz_6_1209	DJI_0341	Genista tinctoria L.	0	n	1	0	Fabaceae Lindl.
Iz_6_1209	DJI_0341	Geranium robertianum L.	0	n	1	0	Geraniaceae Juss.
Iz_6_1209	DJI_0341	Geranium sanquineum L.	0	n	1	0	Geraniaceae Juss.
Iz_6_1209	DJI_0341	Gypsophila paniculata L.	0	n	1	0	Caryophyllaceae Juss.
Iz_6_1209	DJI_0341	Helichrysum arenarium (L.) Moench	0	n	1	0	Asteraceae Bercht. & J.Presl
Iz_6_1209	DJI_0341	Herniaria polygama J.Gay	0	n	1	0	Caryophyllaceae Juss.
Iz_6_1209	DJI_0341	Hieracium robustum Fr.	0	n	1	0	Asteraceae Bercht. & J.Presl
Iz_6_1209	DJI_0341	Hieracium umbellatum L.	0	n	1	NA	Asteraceae Bercht. & J.Presl
Iz_6_1209	DJI_0341	Hylotelephium maximum (L.) Holub	0	n	1	0	Crassulaceae J.St.-Hil.
Iz_6_1209	DJI_0341	Jacobaea borysthenica (DC.) B.Nord. & Greuter	0	n	1	0	Asteraceae Bercht. & J.Presl
Iz_6_1209	DJI_0341	Jasione montana L.	0	n	1	0	Campanulaceae Juss.
Iz_6_1209	DJI_0341	Jurinea cyanoides (L.) Rchb.s.l.	0	n	1	0	Asteraceae Bercht. & J.Presl
Iz_6_1209	DJI_0341	Koeleria glauca (Spreng.) DC.	0	n	1	0	Poaceae Barnhart
Iz_6_1209	DJI_0341	Lactuca serriola L.	0	a	0	0	Asteraceae Bercht. & J.Presl
Iz_6_1209	DJI_0341	Linaria genistifolia (L.) Mill.	0	n	1	0	Plantaginaceae Juss.
Iz_6_1209	DJI_0341	Lomelosia argentea (L.) Greuter & Burdet	0	n	1	0	Caprifoliaceae Juss.
Iz_6_1209	DJI_0341	Melampyrum pratense L.	0	n	1	0	Orobanchaceae Vent.
Iz_6_1209	DJI_0341	Oenothera biennis L.	0	a	0	0	Onagraceae Juss.
Iz_6_1209	DJI_0341	Pentanema britannica (L.) D.Gut.Larr., Santos-Vicente, Anderb., E.Rico & M.M.Mart.Ort.	0	n	1	0	Asteraceae Bercht. & J.Presl
Iz_6_1209	DJI_0341	Peucedanum oreoselinum (L.) Moench	0	n	1	0	Apiaceae Lindl.
Iz_6_1209	DJI_0341	Peucedanum ruthenicum M.Bieb.	0	n	1	0	Apiaceae Lindl.
Iz_6_1209	DJI_0341	Pilosella echioides (Lumn.) F.W.Schultz & Sch.Bip.	0	n	1	0	Asteraceae Bercht. & J.Presl
Iz_6_1209	DJI_0341	Pilosella officinarum F. Schultz et Sch. Bip.	0	n	1	0	Asteraceae Bercht. & J.Presl
Iz_6_1209	DJI_0341	Pilosella sp.	0		NA	0	Asteraceae Bercht. & J.Presl
Iz_6_1209	DJI_0341	Pinus sylvestris L.	0	n	1	1	Pinaceae Spreng. ex F.Rudolphi
Iz_6_1209	DJI_0341	Poa annua L.	0	n	1	0	Poaceae Barnhart
Iz_6_1209	DJI_0341	Poa nemoralis L.	0	n	1	0	Poaceae Barnhart
Iz_6_1209	DJI_0341	Poa sp.	0		NA	0	Poaceae Barnhart
Iz_6_1209	DJI_0341	Polygonatum odoratum (Mill.) Druce	0	n	1	0	Asparagaceae Juss.
Iz_6_1209	DJI_0341	Polygonum arenarium Waldst. & Kit.	0	n	1	0	Polygonaceae Juss.
Iz_6_1209	DJI_0341	Populus nigra L.	0	n	1	1	Salicaceae Mirb.
Iz_6_1209	DJI_0341	Populus tremula L.	2	n	1	1	Salicaceae Mirb.
Iz_6_1209	DJI_0341	Populus Ã canescens (Aiton) Sm.	0	n	1	1	Salicaceae Mirb.
Iz_6_1209	DJI_0341	Potentilla argentea L.	0	n	1	0	Rosaceae Juss.
Iz_6_1209	DJI_0341	Psammophiliella muralis (L.) Ikonn.	0	n	1	0	Caryophyllaceae Juss.
Iz_6_1209	DJI_0341	Psephellus sumensis (Kalen.) Greuter	0	n	1	0	Asteraceae Bercht. & J.Presl
Iz_6_1209	DJI_0341	Pulsatilla pratensis (L.) Mill. s.l.	0	n	1	0	Ranunculaceae Juss.
Iz_6_1209	DJI_0341	Pyrus communis L.	0	a	0	1	Rosaceae Juss.
Iz_6_1209	DJI_0341	Quercus robur L.	0	n	1	1	Fagaceae Dumort.
Iz_6_1209	DJI_0341	Robinia pseudoacacia L.	0	a	0	1	Fabaceae Lindl.
Iz_6_1209	DJI_0341	Rosa sp.	0		NA	1	Rosaceae Juss.
Iz_6_1209	DJI_0341	Rubus idaeus L.	0	n	1	0	Rosaceae Juss.
Iz_6_1209	DJI_0341	Rumex acetosella L.	0	n	1	0	Polygonaceae Juss.
Iz_6_1209	DJI_0341	Rumex thyrsiflorus Fingerh.	0	n	1	0	Polygonaceae Juss.
Iz_6_1209	DJI_0341	Salix alba L.	0	n	1	1	Salicaceae Mirb.
Iz_6_1209	DJI_0341	Sambucus nigra L.	0	n	1	1	Viburnaceae Raf.
Iz_6_1209	DJI_0341	Secale sylvestre Host	0	n	1	0	Poaceae Barnhart
Iz_6_1209	DJI_0341	Sempervivum ruthenicum Schnittsp. & C.B. Lehm.	0	n	1	0	Crassulaceae J.St.-Hil.
Iz_6_1209	DJI_0341	Setaria viridis (L.) P. Beauv.	0	n	1	0	Poaceae Barnhart
Iz_6_1209	DJI_0341	Silene borysthenica (Gruner) Walters	0	n	1	0	Caryophyllaceae Juss.
Iz_6_1209	DJI_0341	Silene latifolia subsp. alba (Mill.) Greuter & Burdet	0	n	1	0	Caryophyllaceae Juss.
Iz_6_1209	DJI_0341	Solidago virgaurea L.	0	n	1	0	Asteraceae Bercht. & J.Presl
Iz_6_1209	DJI_0341	Sorbus aucuparia L.	0	n	1	1	Rosaceae Juss.

Iz_6_1209	DJI_0341	Stachys recta L.	0	n	1	0	Lamiacea Lindl.
Iz_6_1209	DJI_0341	Tanacetum vulgare L.	0	n	1	0	Asteraceae Bercht. & J.Presl
Iz_6_1209	DJI_0341	Thymus pallasianus Heinr.Braun	0	n	1	0	Lamiacea Lindl.
Iz_6_1209	DJI_0341	Tilia cordata Mill.	0	n	1	1	Malvaceae Juss.
Iz_6_1209	DJI_0341	Trifolium alpestre L.	0	n	1	0	Fabaceae Lindl.
Iz_6_1209	DJI_0341	Trifolium arvense L.	0	n	1	0	Fabaceae Lindl.
Iz_6_1209	DJI_0341	Ulmus glabra Huds.	0	n	1	1	Ulmaceae Mirb.
Iz_6_1209	DJI_0341	Verbascum densiflorum Bertol.	0	n	1	0	Scrophulariaceae Juss.
Iz_6_1209	DJI_0341	Veronica austriaca L.	0	n	1	0	Plantaginaceae Juss.
Iz_6_1209	DJI_0341	Veronica incana L.	0	n	1	0	Plantaginaceae Juss.
Iz_6_1209	DJI_0341	Veronica sp.	0		NA	NA	
Iz_6_1209	DJI_0341	Veronica verna L.	0	n	1	0	Plantaginaceae Juss.
Iz_6_1209	DJI_0341	Vincetoxicum hirundinaria Medik	0	n	1	0	Apocynaceae Juss.
Iz_6_1209	DJI_0346	Acer tataricum L.	0	n	1	1	Sapindaceae Juss.
Iz_6_1209	DJI_0346	Achillea millefolium L. s.l.	0	n	1	0	Asteraceae Bercht. & J.Presl
Iz_6_1209	DJI_0346	Allium oleraceum L.	0	n	1	0	Amaryllidaceae J.St.-Hil.
Iz_6_1209	DJI_0346	Ambrosia artemisiifolia L.	0	a	0	0	Asteraceae Bercht. & J.Presl
Iz_6_1209	DJI_0346	Amorpha fruticosa L.	0	a	0	0	Fabaceae Lindl.
Iz_6_1209	DJI_0346	Anthericum ramosum L.	0	n	1	0	Asparagaceae Juss.
Iz_6_1209	DJI_0346	Artemisia austriaca Jacq.	0		NA	NA	
Iz_6_1209	DJI_0346	Artemisia marschalliana Spreng.	0	n	1	0	Asteraceae Bercht. & J.Presl
Iz_6_1209	DJI_0346	Asparagus officinalis L.	0	n	1	0	Asparagaceae Juss.
Iz_6_1209	DJI_0346	Astragalus cicer L.	0	n	1	0	Fabaceae Lindl.
Iz_6_1209	DJI_0346	Bassia laniflora (S.G.Gmel.) A.J.Scott	0	n	1	0	Amaranthaceae Juss.
Iz_6_1209	DJI_0346	Berberis vulgaris L.	0	n	1	0	Berberidaceae Juss.
Iz_6_1209	DJI_0346	Betula pendula Roth.	0	n	1	1	Betulaceae Gray
Iz_6_1209	DJI_0346	Betula pendula Roth.	0	n	1	0	Poaceae Barnhart
Iz_6_1209	DJI_0346	Bromus inermis Leyss.	0		NA	NA	
Iz_6_1209	DJI_0346	Bromus squarrosus L.	0	a	0	0	Poaceae Barnhart
Iz_6_1209	DJI_0346	Bromus tectorum L.	0	a	0	0	Poaceae Barnhart
Iz_6_1209	DJI_0346	Calamagrostis epigejos (L.) Roth	4	n	1	0	Poaceae Barnhart
Iz_6_1209	DJI_0346	Carduus acanthoides L.	0	n	1	0	Asteraceae Bercht. & J.Presl
Iz_6_1209	DJI_0346	Carex sp.	0	n	1	0	Cyperaceae Juss.
Iz_6_1209	DJI_0346	Centaurea majorovii Dumbadze	0	n	1	0	Asteraceae Bercht. & J.Presl
Iz_6_1209	DJI_0346	Chamaecytisus ruthenicus (Fisch.ex Wol.) Klaskova	0	n	1	0	Fabaceae Lindl.
Iz_6_1209	DJI_0346	Chelidonium majus L.	0	n	1	0	Papaveraceae Juss.
Iz_6_1209	DJI_0346	Chenopodium betaceum Andrz.	0	n	1	0	Amaranthaceae Juss.
Iz_6_1209	DJI_0346	Chenopodium cf. album	0	n	1	0	Amaranthaceae Juss.
Iz_6_1209	DJI_0346	Chenopodium sp.	0		NA	0	Amaranthaceae Juss.
Iz_6_1209	DJI_0346	Chondrilla juncea L.	0	n	1	0	Asteraceae Bercht. & J.Presl
Iz_6_1209	DJI_0346	Cirsium vulgare (Savi) Ten.	0	n	1	0	Asteraceae Bercht. & J.Presl
Iz_6_1209	DJI_0346	Clinopodium acinos (L.) Kuntze	0	n	1	0	Lamiacea Lindl.
Iz_6_1209	DJI_0346	Convallaria majalis L.	0	n	1	0	Asparagaceae Juss.
Iz_6_1209	DJI_0346	Crataegus rhipidophylla Gand.	0	n	1	1	Rosaceae Juss.
Iz_6_1209	DJI_0346	Crepis foetida L.	0	n	1	0	Asteraceae Bercht. & J.Presl
Iz_6_1209	DJI_0346	Dactylis glomerata L.	0	n	1	0	Poaceae Barnhart
Iz_6_1209	DJI_0346	Dianthus campestris M.Bieb.	0	n	1	0	Caryophyllaceae Juss.
Iz_6_1209	DJI_0346	Digitaria sanguinalis (L.) Scop.	0	a	0	0	Poaceae Barnhart
Iz_6_1209	DJI_0346	Elymus repens (L.) Gould	0	n	1	0	Poaceae Barnhart
Iz_6_1209	DJI_0346	Epilobium tetragonum L.	0	n	1	0	Onagraceae Juss.
Iz_6_1209	DJI_0346	Eragrostis minor Host	0	a	0	0	Poaceae Barnhart
Iz_6_1209	DJI_0346	Erigeron canadensis L.	0.5	a	0	0	Asteraceae Bercht. & J.Presl
Iz_6_1209	DJI_0346	Euphorbia seguieriana Neck.	0	n	1	0	Euphorbiaceae Juss.
Iz_6_1209	DJI_0346	Fallopia dumetorum (L.) Holub	0	n	1	0	Polygonaceae Juss.
Iz_6_1209	DJI_0346	Festuca beckeri (Hack.) Trautv.	0	n	1	0	Poaceae Barnhart
Iz_6_1209	DJI_0346	Fragaria vesca L.	0	n	1	0	Rosaceae Juss.
Iz_6_1209	DJI_0346	Frangula alnus Mill.	0	n	1	1	Rhamnaceae Juss.
Iz_6_1209	DJI_0346	Fraxinus pennsylvanica Marshall	0	a	0	1	Oleaceae Hoffmanns. & Link
Iz_6_1209	DJI_0346	Galeopsis bifida Boenn.	0	n	1	0	Lamiacea Lindl.
Iz_6_1209	DJI_0346	Galium verum L.	0	n	1	0	Rubiaceae Juss.
Iz_6_1209	DJI_0346	Genista tinctoria L.	0	n	1	0	Fabaceae Lindl.
Iz_6_1209	DJI_0346	Geranium robertianum L.	0	n	1	0	Geraniaceae Juss.
Iz_6_1209	DJI_0346	Geranium sanquineum L.	0	n	1	0	Geraniaceae Juss.
Iz_6_1209	DJI_0346	Gypsophila paniculata L.	0	n	1	0	Caryophyllaceae Juss.
Iz_6_1209	DJI_0346	Helichrysum arenarium (L.) Moench	0	n	1	0	Asteraceae Bercht. & J.Presl
Iz_6_1209	DJI_0346	Herniaria polygama J.Gay	0	n	1	0	Caryophyllaceae Juss.
Iz_6_1209	DJI_0346	Hieracium robustum Fr.	0	n	1	0	Asteraceae Bercht. & J.Presl
Iz_6_1209	DJI_0346	Hieracium umbellatum L.	0	n	1	NA	Asteraceae Bercht. & J.Presl
Iz_6_1209	DJI_0346	Hylotelephium maximum (L.) Holub	0	n	1	0	Crassulaceae J.St.-Hil.
Iz_6_1209	DJI_0346	Jacobaea borysthenica (DC.) B.Nord. & Greuter	0	n	1	0	Asteraceae Bercht. & J.Presl
Iz_6_1209	DJI_0346	Jasione montana L.	0	n	1	0	Campanulaceae Juss.
Iz_6_1209	DJI_0346	Jurinea cyanoides (L.) Rchb.s.l.	0	n	1	0	Asteraceae Bercht. & J.Presl
Iz_6_1209	DJI_0346	Koeleria glauca (Spreng.) DC.	0	n	1	0	Poaceae Barnhart
Iz_6_1209	DJI_0346	Lactuca serriola L.	0	a	0	0	Asteraceae Bercht. & J.Presl
Iz_6_1209	DJI_0346	Linaria genistifolia (L.) Mill.	0	n	1	0	Plantaginaceae Juss.
Iz_6_1209	DJI_0346	Lomelosia argentea (L.) Greuter & Burdet	0	n	1	0	Caprifoliaceae Juss.
Iz_6_1209	DJI_0346	Melampyrum pratense L.	0	n	1	0	Orobanchaceae Vent.
Iz_6_1209	DJI_0346	Oenothera biennis L.	0	a	0	0	Onagraceae Juss.
Iz_6_1209	DJI_0346	Pentanema britannica (L.) D.Gut.Larr., Santos-Vicente, Anderb., E.Rico & M.M.Mart.Ort.	0	n	1	0	Asteraceae Bercht. & J.Presl
Iz_6_1209	DJI_0346	Peucedanum oreoselinum (L.) Moench	0	n	1	0	Apiaceae Lindl.
Iz_6_1209	DJI_0346	Peucedanum ruthenicum M.Bieb.	0	n	1	0	Apiaceae Lindl.
Iz_6_1209	DJI_0346	Pilosella echioides (Lumn.) F.W.Schultz & Sch.Bip.	0	n	1	0	Asteraceae Bercht. & J.Presl
Iz_6_1209	DJI_0346	Pilosella officinarum F. Schultz et Sch. Bip.	0	n	1	0	Asteraceae Bercht. & J.Presl
Iz_6_1209	DJI_0346	Pilosella sp.	0		NA	0	Asteraceae Bercht. & J.Presl
Iz_6_1209	DJI_0346	Pinus sylvestris L.	0	n	1	1	Pinaceae Spreng. ex F.Rudolphi
Iz_6_1209	DJI_0346	Poa annua L.	0	n	1	0	Poaceae Barnhart
Iz_6_1209	DJI_0346	Poa nemoralis L.	0	n	1	0	Poaceae Barnhart
Iz_6_1209	DJI_0346	Poa sp.	0		NA	0	Poaceae Barnhart
Iz_6_1209	DJI_0346	Polygonatum odoratum (Mill.) Druce	0	n	1	0	Asparagaceae Juss.
Iz_6_1209	DJI_0346	Polygonum arenarium Waldst. & Kit.	0	n	1	0	Polygonaceae Juss.
Iz_6_1209	DJI_0346	Populus nigra L.	0	n	1	1	Salicaceae Mirb.
Iz_6_1209	DJI_0346	Populus tremula L.	0	n	1	1	Salicaceae Mirb.
Iz_6_1209	DJI_0346	Populus Ã canescens (Aiton) Sm.	0	n	1	1	Salicaceae Mirb.
Iz_6_1209	DJI_0346	Potentilla argentea L.	0	n	1	0	Rosaceae Juss.
Iz_6_1209	DJI_0346	Psammophiliella muralis (L.) Ikonn.	0	n	1	0	Caryophyllaceae Juss.
Iz_6_1209	DJI_0346	Psephellus sumensis (Kalen.) Greuter	0	n	1	0	Asteraceae Bercht. & J.Presl
Iz_6_1209	DJI_0346	Pulsatilla pratensis (L.) Mill. s.l.	0	n	1	0	Ranunculaceae Juss.
Iz_6_1209	DJI_0346	Pyrus communis L.	0	a	0	1	Rosaceae Juss.

Iz_6_1209	DJI_0346	Quercus robur L.	0	n	1	1	Fagaceae Dumort.
Iz_6_1209	DJI_0346	Robinia pseudoacacia L.	0	a	0	1	Fabaceae Lindl.
Iz_6_1209	DJI_0346	Rosa sp.	0		NA	1	Rosaceae Juss.
Iz_6_1209	DJI_0346	Rubus idaeus L.	0	n	1	0	Rosaceae Juss.
Iz_6_1209	DJI_0346	Rumex acetosella L.	0	n	1	0	Polygonaceae Juss.
Iz_6_1209	DJI_0346	Rumex thyrsiflorus Fingerh.	0	n	1	0	Polygonaceae Juss.
Iz_6_1209	DJI_0346	Salix alba L.	0	n	1	1	Salicaceae Mirb.
Iz_6_1209	DJI_0346	Sambucus nigra L.	0	n	1	1	Viburnaceae Raf.
Iz_6_1209	DJI_0346	Secale sylvestre Host	0	n	1	0	Poaceae Barnhart
Iz_6_1209	DJI_0346	Sempervivum ruthenicum Schnittsp. & C.B. Lehm.	0	n	1	0	Crassulaceae J.St.-Hil.
Iz_6_1209	DJI_0346	Setaria viridis (L.) P. Beauv.	0	n	1	0	Poaceae Barnhart
Iz_6_1209	DJI_0346	Silene borysthenica (Gruner) Walters	0	n	1	0	Caryophyllaceae Juss.
Iz_6_1209	DJI_0346	Silene latifolia subsp. alba (Mill.) Greuter & Burdet	0	n	1	0	Caryophyllaceae Juss.
Iz_6_1209	DJI_0346	Solidago virgaurea L.	0	n	1	0	Asteraceae Bercht. & J.Presl
Iz_6_1209	DJI_0346	Sorbus aucuparia L.	0	n	1	1	Rosaceae Juss.
Iz_6_1209	DJI_0346	Stachys recta L.	0	n	1	0	Lamiacea Lindl.
Iz_6_1209	DJI_0346	Tanacetum vulgare L.	0	n	1	0	Asteraceae Bercht. & J.Presl
Iz_6_1209	DJI_0346	Thymus pallasianus Heinr.Braun	0	n	1	0	Lamiacea Lindl.
Iz_6_1209	DJI_0346	Tilia cordata Mill.	0	n	1	1	Malvaceae Juss.
Iz_6_1209	DJI_0346	Trifolium alpestre L.	0	n	1	0	Fabaceae Lindl.
Iz_6_1209	DJI_0346	Trifolium arvense L.	0	n	1	0	Fabaceae Lindl.
Iz_6_1209	DJI_0346	Ulmus glabra Huds.	0	n	1	1	Ulmaceae Mirb.
Iz_6_1209	DJI_0346	Verbascum densiflorum Bertol.	0	n	1	0	Scrophulariaceae Juss.
Iz_6_1209	DJI_0346	Veronica austriaca L.	0	n	1	0	Plantaginaceae Juss.
Iz_6_1209	DJI_0346	Veronica incana L.	0	n	1	0	Plantaginaceae Juss.
Iz_6_1209	DJI_0346	Veronica sp.	0		NA	NA	
Iz_6_1209	DJI_0346	Veronica verna L.	0	n	1	0	Plantaginaceae Juss.
Iz_6_1209	DJI_0346	Vincetoxicum hirundinaria Medik	0	n	1	0	Apocynaceae Juss.
Iz_6_1209	DJI_0349	Acer tataricum L.	0	n	1	1	Sapindaceae Juss.
Iz_6_1209	DJI_0349	Achillea millefolium L. s.l.	0	n	1	0	Asteraceae Bercht. & J.Presl
Iz_6_1209	DJI_0349	Allium oleraceum L.	0	n	1	0	Amaryllidaceae J.St.-Hil.
Iz_6_1209	DJI_0349	Ambrosia artemisiifolia L.	0	a	0	0	Asteraceae Bercht. & J.Presl
Iz_6_1209	DJI_0349	Amorpha fruticosa L.	0	a	0	0	Fabaceae Lindl.
Iz_6_1209	DJI_0349	Anthericum ramosum L.	0	n	1	0	Asparagaceae Juss.
Iz_6_1209	DJI_0349	Artemisia austriaca Jacq.	0		NA	NA	
Iz_6_1209	DJI_0349	Artemisia marschalliana Spreng.	0	n	1	0	Asteraceae Bercht. & J.Presl
Iz_6_1209	DJI_0349	Asparagus officinalis L.	0	n	1	0	Asparagaceae Juss.
Iz_6_1209	DJI_0349	Astragalus cicer L.	0	n	1	0	Fabaceae Lindl.
Iz_6_1209	DJI_0349	Bassia laniflora (S.G.Gmel.) A.J.Scott	0	n	1	0	Amaranthaceae Juss.
Iz_6_1209	DJI_0349	Berberis vulgaris L.	0	n	1	0	Berberidaceae Juss.
Iz_6_1209	DJI_0349	Betula pendula Roth.	0	n	1	1	Betulaceae Gray
Iz_6_1209	DJI_0349	Betula pendula Roth.	0	n	1	0	Poaceae Barnhart
Iz_6_1209	DJI_0349	Bromus inermis Leyss.	0		NA	NA	
Iz_6_1209	DJI_0349	Bromus squarrosus L.	0	a	0	0	Poaceae Barnhart
Iz_6_1209	DJI_0349	Bromus tectorum L.	0	a	0	0	Poaceae Barnhart
Iz_6_1209	DJI_0349	Calamagrostis epigejos (L.) Roth	5	n	1	0	Poaceae Barnhart
Iz_6_1209	DJI_0349	Carduus acanthoides L.	0	n	1	0	Asteraceae Bercht. & J.Presl
Iz_6_1209	DJI_0349	Carex sp.	0	n	1	0	Cyperaceae Juss.
Iz_6_1209	DJI_0349	Centaurea majorovii Dumbadze	0	n	1	0	Asteraceae Bercht. & J.Presl
Iz_6_1209	DJI_0349	Chamaecytisus ruthenicus (Fisch.ex Wol.) Klaskova	0	n	1	0	Fabaceae Lindl.
Iz_6_1209	DJI_0349	Chelidonium majus L.	0	n	1	0	Papaveraceae Juss.
Iz_6_1209	DJI_0349	Chenopodium betaceum Andrz.	0	n	1	0	Amaranthaceae Juss.
Iz_6_1209	DJI_0349	Chenopodium cf. album	0	n	1	0	Amaranthaceae Juss.
Iz_6_1209	DJI_0349	Chenopodium sp.	0		NA	0	Amaranthaceae Juss.
Iz_6_1209	DJI_0349	Chondrilla juncea L.	0	n	1	0	Asteraceae Bercht. & J.Presl
Iz_6_1209	DJI_0349	Cirsium vulgare (Savi) Ten.	0	n	1	0	Asteraceae Bercht. & J.Presl
Iz_6_1209	DJI_0349	Clinopodium acinos (L.) Kuntze	0	n	1	0	Lamiacea Lindl.
Iz_6_1209	DJI_0349	Convallaria majalis L.	0	n	1	0	Asparagaceae Juss.
Iz_6_1209	DJI_0349	Crataegus rhipidophylla Gand.	0	n	1	1	Rosaceae Juss.
Iz_6_1209	DJI_0349	Crepis foetida L.	0	n	1	0	Asteraceae Bercht. & J.Presl
Iz_6_1209	DJI_0349	Dactylis glomerata L.	0	n	1	0	Poaceae Barnhart
Iz_6_1209	DJI_0349	Dianthus campestris M.Bieb.	0	n	1	0	Caryophyllaceae Juss.
Iz_6_1209	DJI_0349	Digitaria sanguinalis (L.) Scop.	0	a	0	0	Poaceae Barnhart
Iz_6_1209	DJI_0349	Elymus repens (L.) Gould	0	n	1	0	Poaceae Barnhart
Iz_6_1209	DJI_0349	Epilobium tetragonum L.	0	n	1	0	Onagraceae Juss.
Iz_6_1209	DJI_0349	Eragrostis minor Host	0	a	0	0	Poaceae Barnhart
Iz_6_1209	DJI_0349	Erigeron canadensis L.	0.2	a	0	0	Asteraceae Bercht. & J.Presl
Iz_6_1209	DJI_0349	Euphorbia seguieriana Neck.	0	n	1	0	Euphorbiaceae Juss.
Iz_6_1209	DJI_0349	Fallopia dumetorum (L.) Holub	0	n	1	0	Polygonaceae Juss.
Iz_6_1209	DJI_0349	Festuca beckeri (Hack.) Trautv.	0	n	1	0	Poaceae Barnhart
Iz_6_1209	DJI_0349	Fragaria vesca L.	0	n	1	0	Rosaceae Juss.
Iz_6_1209	DJI_0349	Frangula alnus Mill.	0	n	1	1	Rhamnaceae Juss.
Iz_6_1209	DJI_0349	Fraxinus pennsylvanica Marshall	0	a	0	1	Oleaceae Hoffmanns. & Link
Iz_6_1209	DJI_0349	Galeopsis bifida Boenn.	0	n	1	0	Lamiacea Lindl.
Iz_6_1209	DJI_0349	Galium verum L.	0	n	1	0	Rubiaceae Juss.
Iz_6_1209	DJI_0349	Genista tinctoria L.	0	n	1	0	Fabaceae Lindl.
Iz_6_1209	DJI_0349	Geranium robertianum L.	0	n	1	0	Geraniaceae Juss.
Iz_6_1209	DJI_0349	Geranium sanquineum L.	0	n	1	0	Geraniaceae Juss.
Iz_6_1209	DJI_0349	Gypsophila paniculata L.	0	n	1	0	Caryophyllaceae Juss.
Iz_6_1209	DJI_0349	Helichrysum arenarium (L.) Moench	0	n	1	0	Asteraceae Bercht. & J.Presl
Iz_6_1209	DJI_0349	Herniaria polygama J.Gay	0	n	1	0	Caryophyllaceae Juss.
Iz_6_1209	DJI_0349	Hieracium robustum Fr.	0	n	1	0	Asteraceae Bercht. & J.Presl
Iz_6_1209	DJI_0349	Hieracium umbellatum L.	0	n	1	NA	Asteraceae Bercht. & J.Presl
Iz_6_1209	DJI_0349	Hylotelephium maximum (L.) Holub	0	n	1	0	Crassulaceae J.St.-Hil.
Iz_6_1209	DJI_0349	Jacobaea borysthenica (DC.) B.Nord. & Greuter	0	n	1	0	Asteraceae Bercht. & J.Presl
Iz_6_1209	DJI_0349	Jasione montana L.	0	n	1	0	Campanulaceae Juss.
Iz_6_1209	DJI_0349	Jurinea cyanoides (L.) Rchb.s.l.	0	n	1	0	Asteraceae Bercht. & J.Presl
Iz_6_1209	DJI_0349	Koeleria glauca (Spreng.) DC.	0	n	1	0	Poaceae Barnhart
Iz_6_1209	DJI_0349	Lactuca serriola L.	0	a	0	0	Asteraceae Bercht. & J.Presl
Iz_6_1209	DJI_0349	Linaria genistifolia (L.) Mill.	0	n	1	0	Plantaginaceae Juss.
Iz_6_1209	DJI_0349	Lomelosia argentea (L.) Greuter & Burdet	0	n	1	0	Caprifoliaceae Juss.
Iz_6_1209	DJI_0349	Melampyrum pratense L.	0	n	1	0	Orobanchaceae Vent.
Iz_6_1209	DJI_0349	Oenothera biennis L.	0	a	0	0	Onagraceae Juss.
Iz_6_1209	DJI_0349	Pentanema britannica (L.) D.Gut.Larr., Santos-Vicente, Anderb., E.Rico & M.M.Mart.Ort.	0	n	1	0	Asteraceae Bercht. & J.Presl
Iz_6_1209	DJI_0349	Peucedanum oreoselinum (L.) Moench	0	n	1	0	Apiaceae Lindl.
Iz_6_1209	DJI_0349	Peucedanum ruthenicum M.Bieb.	0	n	1	0	Apiaceae Lindl.
Iz_6_1209	DJI_0349	Pilosella echioides (Lumn.) F.W.Schultz & Sch.Bip.	0	n	1	0	Asteraceae Bercht. & J.Presl
Iz_6_1209	DJI_0349	Pilosella officinarum F. Schultz et Sch. Bip.	0	n	1	0	Asteraceae Bercht. & J.Presl

Iz_6_1209	DJI_0349	Pilosella sp.	0		NA	0	Asteraceae Bercht. & J.Presl
Iz_6_1209	DJI_0349	Pinus sylvestris L.	0	n	1	1	Pinaceae Spreng. ex F.Rudolphi
Iz_6_1209	DJI_0349	Poa annua L.	0	n	1	0	Poaceae Barnhart
Iz_6_1209	DJI_0349	Poa nemoralis L.	0	n	1	0	Poaceae Barnhart
Iz_6_1209	DJI_0349	Poa sp.	0		NA	0	Poaceae Barnhart
Iz_6_1209	DJI_0349	Polygonatum odoratum (Mill.) Druce	0	n	1	0	Asparagaceae Juss.
Iz_6_1209	DJI_0349	Polygonum arenarium Waldst. & Kit.	0	n	1	0	Polygonaceae Juss.
Iz_6_1209	DJI_0349	Populus nigra L.	0	n	1	1	Salicaceae Mirb.
Iz_6_1209	DJI_0349	Populus tremula L.	0	n	1	1	Salicaceae Mirb.
Iz_6_1209	DJI_0349	Populus Ã canescens (Aiton) Sm.	0	n	1	1	Salicaceae Mirb.
Iz_6_1209	DJI_0349	Potentilla argentea L.	0	n	1	0	Rosaceae Juss.
Iz_6_1209	DJI_0349	Psammophiliella muralis (L.) Ikonn.	0	n	1	0	Caryophyllaceae Juss.
Iz_6_1209	DJI_0349	Psephellus sumensis (Kalen.) Greuter	0	n	1	0	Asteraceae Bercht. & J.Presl
Iz_6_1209	DJI_0349	Pulsatilla pratensis (L.) Mill. s.l.	0	n	1	0	Ranunculaceae Juss.
Iz_6_1209	DJI_0349	Pyrus communis L.	0	a	0	1	Rosaceae Juss.
Iz_6_1209	DJI_0349	Quercus robur L.	0	n	1	1	Fagaceae Dumort.
Iz_6_1209	DJI_0349	Robinia pseudoacacia L.	0	a	0	1	Fabaceae Lindl.
Iz_6_1209	DJI_0349	Rosa sp.	0		NA	1	Rosaceae Juss.
Iz_6_1209	DJI_0349	Rubus idaeus L.	0	n	1	0	Rosaceae Juss.
Iz_6_1209	DJI_0349	Rumex acetosella L.	0	n	1	0	Polygonaceae Juss.
Iz_6_1209	DJI_0349	Rumex thyrsiflorus Fingerh.	0	n	1	0	Polygonaceae Juss.
Iz_6_1209	DJI_0349	Salix alba L.	0	n	1	1	Salicaceae Mirb.
Iz_6_1209	DJI_0349	Sambucus nigra L.	0	n	1	1	Viburnaceae Raf.
Iz_6_1209	DJI_0349	Secale sylvestre Host	0	n	1	0	Poaceae Barnhart
Iz_6_1209	DJI_0349	Sempervivum ruthenicum Schnittsp. & C.B. Lehm.	0	n	1	0	Crassulaceae J.St.-Hil.
Iz_6_1209	DJI_0349	Setaria viridis (L.) P. Beauv.	0	n	1	0	Poaceae Barnhart
Iz_6_1209	DJI_0349	Silene borysthenica (Gruner) Walters	0	n	1	0	Caryophyllaceae Juss.
Iz_6_1209	DJI_0349	Silene latifolia subsp. alba (Mill.) Greuter & Burdet	0	n	1	0	Caryophyllaceae Juss.
Iz_6_1209	DJI_0349	Solidago virgaurea L.	0	n	1	0	Asteraceae Bercht. & J.Presl
Iz_6_1209	DJI_0349	Sorbus aucuparia L.	0	n	1	1	Rosaceae Juss.
Iz_6_1209	DJI_0349	Stachys recta L.	0	n	1	0	Lamiacea Lindl.
Iz_6_1209	DJI_0349	Tanacetum vulgare L.	0	n	1	0	Asteraceae Bercht. & J.Presl
Iz_6_1209	DJI_0349	Thymus pallasianus Heinr.Braun	0	n	1	0	Lamiacea Lindl.
Iz_6_1209	DJI_0349	Tilia cordata Mill.	0	n	1	1	Malvaceae Juss.
Iz_6_1209	DJI_0349	Trifolium alpestre L.	0	n	1	0	Fabaceae Lindl.
Iz_6_1209	DJI_0349	Trifolium arvense L.	0	n	1	0	Fabaceae Lindl.
Iz_6_1209	DJI_0349	Ulmus glabra Huds.	0	n	1	1	Ulmaceae Mirb.
Iz_6_1209	DJI_0349	Verbascum densiflorum Bertol.	0	n	1	0	Scrophulariaceae Juss.
Iz_6_1209	DJI_0349	Veronica austriaca L.	0	n	1	0	Plantaginaceae Juss.
Iz_6_1209	DJI_0349	Veronica incana L.	0	n	1	0	Plantaginaceae Juss.
Iz_6_1209	DJI_0349	Veronica sp.	0		NA	NA	
Iz_6_1209	DJI_0349	Veronica verna L.	0	n	1	0	Plantaginaceae Juss.
Iz_6_1209	DJI_0349	Vincetoxicum hirundinaria Medik	0	n	1	0	Apocynaceae Juss.
Iz_6_1209	DJI_0352	Acer tataricum L.	0	n	1	1	Sapindaceae Juss.
Iz_6_1209	DJI_0352	Achillea millefolium L. s.l.	0	n	1	0	Asteraceae Bercht. & J.Presl
Iz_6_1209	DJI_0352	Allium oleraceum L.	0	n	1	0	Amaryllidaceae J.St.-Hil.
Iz_6_1209	DJI_0352	Ambrosia artemisiifolia L.	0	a	0	0	Asteraceae Bercht. & J.Presl
Iz_6_1209	DJI_0352	Amorpha fruticosa L.	0	a	0	0	Fabaceae Lindl.
Iz_6_1209	DJI_0352	Anthericum ramosum L.	0	n	1	0	Asparagaceae Juss.
Iz_6_1209	DJI_0352	Artemisia austriaca Jacq.	0		NA	NA	
Iz_6_1209	DJI_0352	Artemisia marschalliana Spreng.	0	n	1	0	Asteraceae Bercht. & J.Presl
Iz_6_1209	DJI_0352	Asparagus officinalis L.	0	n	1	0	Asparagaceae Juss.
Iz_6_1209	DJI_0352	Astragalus cicer L.	0	n	1	0	Fabaceae Lindl.
Iz_6_1209	DJI_0352	Bassia laniflora (S.G.Gmel.) A.J.Scott	0	n	1	0	Amaranthaceae Juss.
Iz_6_1209	DJI_0352	Berberis vulgaris L.	0	n	1	0	Berberidaceae Juss.
Iz_6_1209	DJI_0352	Betula pendula Roth.	0	n	1	1	Betulaceae Gray
Iz_6_1209	DJI_0352	Betula pendula Roth.	0	n	1	0	Poaceae Barnhart
Iz_6_1209	DJI_0352	Bromus inermis Leyss.	0		NA	NA	
Iz_6_1209	DJI_0352	Bromus squarrosus L.	0	a	0	0	Poaceae Barnhart
Iz_6_1209	DJI_0352	Bromus tectorum L.	0	a	0	0	Poaceae Barnhart
Iz_6_1209	DJI_0352	Calamagrostis epigejos (L.) Roth	0.1	n	1	0	Poaceae Barnhart
Iz_6_1209	DJI_0352	Carduus acanthoides L.	0	n	1	0	Asteraceae Bercht. & J.Presl
Iz_6_1209	DJI_0352	Carex sp.	0	n	1	0	Cyperaceae Juss.
Iz_6_1209	DJI_0352	Centaurea majorovii Dumbadze	0	n	1	0	Asteraceae Bercht. & J.Presl
Iz_6_1209	DJI_0352	Chamaecytisus ruthenicus (Fisch.ex Wol.) Klaskova	0	n	1	0	Fabaceae Lindl.
Iz_6_1209	DJI_0352	Chelidonium majus L.	0	n	1	0	Papaveraceae Juss.
Iz_6_1209	DJI_0352	Chenopodium betaceum Andrz.	0	n	1	0	Amaranthaceae Juss.
Iz_6_1209	DJI_0352	Chenopodium cf. album	0	n	1	0	Amaranthaceae Juss.
Iz_6_1209	DJI_0352	Chenopodium sp.	0		NA	0	Amaranthaceae Juss.
Iz_6_1209	DJI_0352	Chondrilla juncea L.	0	n	1	0	Asteraceae Bercht. & J.Presl
Iz_6_1209	DJI_0352	Cirsium vulgare (Savi) Ten.	0	n	1	0	Asteraceae Bercht. & J.Presl
Iz_6_1209	DJI_0352	Clinopodium acinos (L.) Kuntze	0	n	1	0	Lamiacea Lindl.
Iz_6_1209	DJI_0352	Convallaria majalis L.	0	n	1	0	Asparagaceae Juss.
Iz_6_1209	DJI_0352	Crataegus rhipidophylla Gand.	0	n	1	1	Rosaceae Juss.
Iz_6_1209	DJI_0352	Crepis foetida L.	0	n	1	0	Asteraceae Bercht. & J.Presl
Iz_6_1209	DJI_0352	Dactylis glomerata L.	0	n	1	0	Poaceae Barnhart
Iz_6_1209	DJI_0352	Dianthus campestris M.Bieb.	0	n	1	0	Caryophyllaceae Juss.
Iz_6_1209	DJI_0352	Digitaria sanguinalis (L.) Scop.	0	a	0	0	Poaceae Barnhart
Iz_6_1209	DJI_0352	Elymus repens (L.) Gould	0	n	1	0	Poaceae Barnhart
Iz_6_1209	DJI_0352	Epilobium tetragonum L.	0	n	1	0	Onagraceae Juss.
Iz_6_1209	DJI_0352	Eragrostis minor Host	0	a	0	0	Poaceae Barnhart
Iz_6_1209	DJI_0352	Erigeron canadensis L.	1	a	0	0	Asteraceae Bercht. & J.Presl
Iz_6_1209	DJI_0352	Euphorbia seguieriana Neck.	0	n	1	0	Euphorbiaceae Juss.
Iz_6_1209	DJI_0352	Fallopia dumetorum (L.) Holub	0	n	1	0	Polygonaceae Juss.
Iz_6_1209	DJI_0352	Festuca beckeri (Hack.) Trautv.	0	n	1	0	Poaceae Barnhart
Iz_6_1209	DJI_0352	Fragaria vesca L.	0	n	1	0	Rosaceae Juss.
Iz_6_1209	DJI_0352	Frangula alnus Mill.	0	n	1	1	Rhamnaceae Juss.
Iz_6_1209	DJI_0352	Fraxinus pennsylvanica Marshall	0	a	0	1	Oleaceae Hoffmanns. & Link
Iz_6_1209	DJI_0352	Galeopsis bifida Boenn.	0	n	1	0	Lamiacea Lindl.
Iz_6_1209	DJI_0352	Galium verum L.	0	n	1	0	Rubiaceae Juss.
Iz_6_1209	DJI_0352	Genista tinctoria L.	0	n	1	0	Fabaceae Lindl.
Iz_6_1209	DJI_0352	Geranium robertianum L.	0	n	1	0	Geraniaceae Juss.
Iz_6_1209	DJI_0352	Geranium sanquineum L.	0	n	1	0	Geraniaceae Juss.
Iz_6_1209	DJI_0352	Gypsophila paniculata L.	0	n	1	0	Caryophyllaceae Juss.
Iz_6_1209	DJI_0352	Helichrysum arenarium (L.) Moench	0	n	1	0	Asteraceae Bercht. & J.Presl
Iz_6_1209	DJI_0352	Herniaria polygama J.Gay	0	n	1	0	Caryophyllaceae Juss.
Iz_6_1209	DJI_0352	Hieracium robustum Fr.	0	n	1	0	Asteraceae Bercht. & J.Presl
Iz_6_1209	DJI_0352	Hieracium umbellatum L.	0	n	1	NA	Asteraceae Bercht. & J.Presl

Iz_6_1209	DJI_0352	Hylotelephium maximum (L.) Holub	0	n	1	0	Crassulaceae J.St.-Hil.
Iz_6_1209	DJI_0352	Jacobaea borysthenica (DC.) B.Nord. & Greuter	0	n	1	0	Asteraceae Bercht. & J.Presl
Iz_6_1209	DJI_0352	Jasione montana L.	0	n	1	0	Campanulaceae Juss.
Iz_6_1209	DJI_0352	Jurinea cyanoides (L.) Rchb.s.l.	0	n	1	0	Asteraceae Bercht. & J.Presl
Iz_6_1209	DJI_0352	Koeleria glauca (Spreng.) DC.	0	n	1	0	Poaceae Barnhart
Iz_6_1209	DJI_0352	Lactuca serriola L.	0	a	0	0	Asteraceae Bercht. & J.Presl
Iz_6_1209	DJI_0352	Linaria genistifolia (L.) Mill.	0	n	1	0	Plantaginaceae Juss.
Iz_6_1209	DJI_0352	Lomelosia argentea (L.) Greuter & Burdet	0	n	1	0	Caprifoliaceae Juss.
Iz_6_1209	DJI_0352	Melampyrum pratense L.	0	n	1	0	Orobanchaceae Vent.
Iz_6_1209	DJI_0352	Oenothera biennis L.	0	a	0	0	Onagraceae Juss.
Iz_6_1209	DJI_0352	Pentanema britannica (L.) D.Gut.Larr., Santos-Vicente, Anderb., E.Rico & M.M.Mart.Ort.	0	n	1	0	Asteraceae Bercht. & J.Presl
Iz_6_1209	DJI_0352	Peucedanum oreoselinum (L.) Moench	0	n	1	0	Apiaceae Lindl.
Iz_6_1209	DJI_0352	Peucedanum ruthenicum M.Bieb.	0	n	1	0	Apiaceae Lindl.
Iz_6_1209	DJI_0352	Pilosella echioides (Lumn.) F.W.Schultz & Sch.Bip.	0	n	1	0	Asteraceae Bercht. & J.Presl
Iz_6_1209	DJI_0352	Pilosella officinarum F. Schultz et Sch. Bip.	0	n	1	0	Asteraceae Bercht. & J.Presl
Iz_6_1209	DJI_0352	Pilosella sp.	0		NA	0	Asteraceae Bercht. & J.Presl
Iz_6_1209	DJI_0352	Pinus sylvestris L.	0	n	1	1	Pinaceae Spreng. ex F.Rudolphi
Iz_6_1209	DJI_0352	Poa annua L.	0	n	1	0	Poaceae Barnhart
Iz_6_1209	DJI_0352	Poa nemoralis L.	0	n	1	0	Poaceae Barnhart
Iz_6_1209	DJI_0352	Poa sp.	0		NA	0	Poaceae Barnhart
Iz_6_1209	DJI_0352	Polygonatum odoratum (Mill.) Druce	0	n	1	0	Asparagaceae Juss.
Iz_6_1209	DJI_0352	Polygonum arenarium Waldst. & Kit.	0	n	1	0	Polygonaceae Juss.
Iz_6_1209	DJI_0352	Populus nigra L.	0	n	1	1	Salicaceae Mirb.
Iz_6_1209	DJI_0352	Populus tremula L.	0	n	1	1	Salicaceae Mirb.
Iz_6_1209	DJI_0352	Populus Ã canescens (Aiton) Sm.	0	n	1	1	Salicaceae Mirb.
Iz_6_1209	DJI_0352	Potentilla argentea L.	0	n	1	0	Rosaceae Juss.
Iz_6_1209	DJI_0352	Psammophiliella muralis (L.) Ikonn.	0	n	1	0	Caryophyllaceae Juss.
Iz_6_1209	DJI_0352	Psephellus sumensis (Kalen.) Greuter	0	n	1	0	Asteraceae Bercht. & J.Presl
Iz_6_1209	DJI_0352	Pulsatilla pratensis (L.) Mill. s.l.	0	n	1	0	Ranunculaceae Juss.
Iz_6_1209	DJI_0352	Pyrus communis L.	0	a	0	1	Rosaceae Juss.
Iz_6_1209	DJI_0352	Quercus robur L.	0	n	1	1	Fagaceae Dumort.
Iz_6_1209	DJI_0352	Robinia pseudoacacia L.	0	a	0	1	Fabaceae Lindl.
Iz_6_1209	DJI_0352	Rosa sp.	0		NA	1	Rosaceae Juss.
Iz_6_1209	DJI_0352	Rubus idaeus L.	0	n	1	0	Rosaceae Juss.
Iz_6_1209	DJI_0352	Rumex acetosella L.	0	n	1	0	Polygonaceae Juss.
Iz_6_1209	DJI_0352	Rumex thyrsiflorus Fingerh.	0	n	1	0	Polygonaceae Juss.
Iz_6_1209	DJI_0352	Salix alba L.	0	n	1	1	Salicaceae Mirb.
Iz_6_1209	DJI_0352	Sambucus nigra L.	0	n	1	1	Viburnaceae Raf.
Iz_6_1209	DJI_0352	Secale sylvestre Host	0	n	1	0	Poaceae Barnhart
Iz_6_1209	DJI_0352	Sempervivum ruthenicum Schnittsp. & C.B. Lehm.	0	n	1	0	Crassulaceae J.St.-Hil.
Iz_6_1209	DJI_0352	Setaria viridis (L.) P. Beauv.	0	n	1	0	Poaceae Barnhart
Iz_6_1209	DJI_0352	Silene borysthenica (Gruner) Walters	0	n	1	0	Caryophyllaceae Juss.
Iz_6_1209	DJI_0352	Silene latifolia subsp. alba (Mill.) Greuter & Burdet	0	n	1	0	Caryophyllaceae Juss.
Iz_6_1209	DJI_0352	Solidago virgaurea L.	0	n	1	0	Asteraceae Bercht. & J.Presl
Iz_6_1209	DJI_0352	Sorbus aucuparia L.	0	n	1	1	Rosaceae Juss.
Iz_6_1209	DJI_0352	Stachys recta L.	0	n	1	0	Lamiacea Lindl.
Iz_6_1209	DJI_0352	Tanacetum vulgare L.	0	n	1	0	Asteraceae Bercht. & J.Presl
Iz_6_1209	DJI_0352	Thymus pallasianus Heinr.Braun	0	n	1	0	Lamiacea Lindl.
Iz_6_1209	DJI_0352	Tilia cordata Mill.	0	n	1	1	Malvaceae Juss.
Iz_6_1209	DJI_0352	Trifolium alpestre L.	0	n	1	0	Fabaceae Lindl.
Iz_6_1209	DJI_0352	Trifolium arvense L.	0	n	1	0	Fabaceae Lindl.
Iz_6_1209	DJI_0352	Ulmus glabra Huds.	0	n	1	1	Ulmaceae Mirb.
Iz_6_1209	DJI_0352	Verbascum densiflorum Bertol.	0	n	1	0	Scrophulariaceae Juss.
Iz_6_1209	DJI_0352	Veronica austriaca L.	0	n	1	0	Plantaginaceae Juss.
Iz_6_1209	DJI_0352	Veronica incana L.	0	n	1	0	Plantaginaceae Juss.
Iz_6_1209	DJI_0352	Veronica sp.	0		NA	NA	
Iz_6_1209	DJI_0352	Veronica verna L.	0	n	1	0	Plantaginaceae Juss.
Iz_6_1209	DJI_0352	Vincetoxicum hirundinaria Medik	0	n	1	0	Apocynaceae Juss.
Iz_6_1209	DJI_0355	Acer tataricum L.	0	n	1	1	Sapindaceae Juss.
Iz_6_1209	DJI_0355	Achillea millefolium L. s.l.	0	n	1	0	Asteraceae Bercht. & J.Presl
Iz_6_1209	DJI_0355	Allium oleraceum L.	0	n	1	0	Amaryllidaceae J.St.-Hil.
Iz_6_1209	DJI_0355	Ambrosia artemisiifolia L.	0	a	0	0	Asteraceae Bercht. & J.Presl
Iz_6_1209	DJI_0355	Amorpha fruticosa L.	0	a	0	0	Fabaceae Lindl.
Iz_6_1209	DJI_0355	Anthericum ramosum L.	0	n	1	0	Asparagaceae Juss.
Iz_6_1209	DJI_0355	Artemisia austriaca Jacq.	0		NA	NA	
Iz_6_1209	DJI_0355	Artemisia marschalliana Spreng.	0	n	1	0	Asteraceae Bercht. & J.Presl
Iz_6_1209	DJI_0355	Asparagus officinalis L.	0	n	1	0	Asparagaceae Juss.
Iz_6_1209	DJI_0355	Astragalus cicer L.	0	n	1	0	Fabaceae Lindl.
Iz_6_1209	DJI_0355	Bassia laniflora (S.G.Gmel.) A.J.Scott	0	n	1	0	Amaranthaceae Juss.
Iz_6_1209	DJI_0355	Berberis vulgaris L.	0	n	1	0	Berberidaceae Juss.
Iz_6_1209	DJI_0355	Betula pendula Roth.	0	n	1	1	Betulaceae Gray
Iz_6_1209	DJI_0355	Betula pendula Roth.	0	n	1	0	Poaceae Barnhart
Iz_6_1209	DJI_0355	Bromus inermis Leyss.	0		NA	NA	
Iz_6_1209	DJI_0355	Bromus squarrosus L.	0	a	0	0	Poaceae Barnhart
Iz_6_1209	DJI_0355	Bromus tectorum L.	0	a	0	0	Poaceae Barnhart
Iz_6_1209	DJI_0355	Calamagrostis epigejos (L.) Roth	5	n	1	0	Poaceae Barnhart
Iz_6_1209	DJI_0355	Carduus acanthoides L.	0	n	1	0	Asteraceae Bercht. & J.Presl
Iz_6_1209	DJI_0355	Carex sp.	0	n	1	0	Cyperaceae Juss.
Iz_6_1209	DJI_0355	Centaurea majorovii Dumbadze	0	n	1	0	Asteraceae Bercht. & J.Presl
Iz_6_1209	DJI_0355	Chamaecytisus ruthenicus (Fisch.ex Wol.) Klaskova	0	n	1	0	Fabaceae Lindl.
Iz_6_1209	DJI_0355	Chelidonium majus L.	0	n	1	0	Papaveraceae Juss.
Iz_6_1209	DJI_0355	Chenopodium betaceum Andrz.	0	n	1	0	Amaranthaceae Juss.
Iz_6_1209	DJI_0355	Chenopodium cf. album	0	n	1	0	Amaranthaceae Juss.
Iz_6_1209	DJI_0355	Chenopodium sp.	0		NA	0	Amaranthaceae Juss.
Iz_6_1209	DJI_0355	Chondrilla juncea L.	0	n	1	0	Asteraceae Bercht. & J.Presl
Iz_6_1209	DJI_0355	Cirsium vulgare (Savi) Ten.	0	n	1	0	Asteraceae Bercht. & J.Presl
Iz_6_1209	DJI_0355	Clinopodium acinos (L.) Kuntze	0	n	1	0	Lamiacea Lindl.
Iz_6_1209	DJI_0355	Convallaria majalis L.	0	n	1	0	Asparagaceae Juss.
Iz_6_1209	DJI_0355	Crataegus rhipidophylla Gand.	0	n	1	1	Rosaceae Juss.
Iz_6_1209	DJI_0355	Crepis foetida L.	0	n	1	0	Asteraceae Bercht. & J.Presl
Iz_6_1209	DJI_0355	Dactylis glomerata L.	0	n	1	0	Poaceae Barnhart
Iz_6_1209	DJI_0355	Dianthus campestris M.Bieb.	0	n	1	0	Caryophyllaceae Juss.
Iz_6_1209	DJI_0355	Digitaria sanguinalis (L.) Scop.	0	a	0	0	Poaceae Barnhart
Iz_6_1209	DJI_0355	Elymus repens (L.) Gould	0	n	1	0	Poaceae Barnhart
Iz_6_1209	DJI_0355	Epilobium tetragonum L.	0	n	1	0	Onagraceae Juss.
Iz_6_1209	DJI_0355	Eragrostis minor Host	0	a	0	0	Poaceae Barnhart
Iz_6_1209	DJI_0355	Erigeron canadensis L.	1	a	0	0	Asteraceae Bercht. & J.Presl
Iz_6_1209	DJI_0355	Euphorbia seguieriana Neck.	0	n	1	0	Euphorbiaceae Juss.

Iz_6_1209	DJI_0355	Fallopia dumetorum (L.) Holub	0	n	1	0	Polygonaceae Juss.
Iz_6_1209	DJI_0355	Festuca beckeri (Hack.) Trautv.	0	n	1	0	Poaceae Barnhart
Iz_6_1209	DJI_0355	Fragaria vesca L.	0	n	1	0	Rosaceae Juss.
Iz_6_1209	DJI_0355	Frangula alnus Mill.	0	n	1	1	Rhamnaceae Juss.
Iz_6_1209	DJI_0355	Fraxinus pennsylvanica Marshall	0	a	0	1	Oleaceae Hoffmanns. & Link
Iz_6_1209	DJI_0355	Galeopsis bifida Boenn.	0	n	1	0	Lamiacea Lindl.
Iz_6_1209	DJI_0355	Galium verum L.	0	n	1	0	Rubiaceae Juss.
Iz_6_1209	DJI_0355	Genista tinctoria L.	0	n	1	0	Fabaceae Lindl.
Iz_6_1209	DJI_0355	Geranium robertianum L.	0	n	1	0	Geraniaceae Juss.
Iz_6_1209	DJI_0355	Geranium sanquineum L.	0	n	1	0	Geraniaceae Juss.
Iz_6_1209	DJI_0355	Gypsophila paniculata L.	0	n	1	0	Caryophyllaceae Juss.
Iz_6_1209	DJI_0355	Helichrysum arenarium (L.) Moench	0	n	1	0	Asteraceae Bercht. & J.Presl
Iz_6_1209	DJI_0355	Herniaria polygama J.Gay	0	n	1	0	Caryophyllaceae Juss.
Iz_6_1209	DJI_0355	Hieracium robustum Fr.	0	n	1	0	Asteraceae Bercht. & J.Presl
Iz_6_1209	DJI_0355	Hieracium umbellatum L.	0	n	1	NA	Asteraceae Bercht. & J.Presl
Iz_6_1209	DJI_0355	Hylotelephium maximum (L.) Holub	0	n	1	0	Crassulaceae J.St.-Hil.
Iz_6_1209	DJI_0355	Jacobaea borysthenica (DC.) B.Nord. & Greuter	0	n	1	0	Asteraceae Bercht. & J.Presl
Iz_6_1209	DJI_0355	Jasione montana L.	0	n	1	0	Campanulaceae Juss.
Iz_6_1209	DJI_0355	Jurinea cyanoides (L.) Rchb.s.l.	0	n	1	0	Asteraceae Bercht. & J.Presl
Iz_6_1209	DJI_0355	Koeleria glauca (Spreng.) DC.	0	n	1	0	Poaceae Barnhart
Iz_6_1209	DJI_0355	Lactuca serriola L.	0	a	0	0	Asteraceae Bercht. & J.Presl
Iz_6_1209	DJI_0355	Linaria genistifolia (L.) Mill.	0	n	1	0	Plantaginaceae Juss.
Iz_6_1209	DJI_0355	Lomelosia argentea (L.) Greuter & Burdet	0	n	1	0	Caprifoliaceae Juss.
Iz_6_1209	DJI_0355	Melampyrum pratense L.	0	n	1	0	Orobanchaceae Vent.
Iz_6_1209	DJI_0355	Oenothera biennis L.	0	a	0	0	Onagraceae Juss.
Iz_6_1209	DJI_0355	Pentanema britannica (L.) D.Gut.Larr., Santos-Vicente, Anderb., E.Rico & M.M.Mart.Ort.	0	n	1	0	Asteraceae Bercht. & J.Presl
Iz_6_1209	DJI_0355	Peucedanum oreoselinum (L.) Moench	0	n	1	0	Apiaceae Lindl.
Iz_6_1209	DJI_0355	Peucedanum ruthenicum M.Bieb.	0	n	1	0	Apiaceae Lindl.
Iz_6_1209	DJI_0355	Pilosella echioides (Lumn.) F.W.Schultz & Sch.Bip.	0	n	1	0	Asteraceae Bercht. & J.Presl
Iz_6_1209	DJI_0355	Pilosella officinarum F. Schultz et Sch. Bip.	0	n	1	0	Asteraceae Bercht. & J.Presl
Iz_6_1209	DJI_0355	Pilosella sp.	0		NA	0	Asteraceae Bercht. & J.Presl
Iz_6_1209	DJI_0355	Pinus sylvestris L.	0	n	1	1	Pinaceae Spreng. ex F.Rudolphi
Iz_6_1209	DJI_0355	Poa annua L.	0	n	1	0	Poaceae Barnhart
Iz_6_1209	DJI_0355	Poa nemoralis L.	0	n	1	0	Poaceae Barnhart
Iz_6_1209	DJI_0355	Poa sp.	0		NA	0	Poaceae Barnhart
Iz_6_1209	DJI_0355	Polygonatum odoratum (Mill.) Druce	0	n	1	0	Asparagaceae Juss.
Iz_6_1209	DJI_0355	Polygonum arenarium Waldst. & Kit.	0	n	1	0	Polygonaceae Juss.
Iz_6_1209	DJI_0355	Populus nigra L.	0	n	1	1	Salicaceae Mirb.
Iz_6_1209	DJI_0355	Populus tremula L.	0	n	1	1	Salicaceae Mirb.
Iz_6_1209	DJI_0355	Populus Ã canescens (Aiton) Sm.	0	n	1	1	Salicaceae Mirb.
Iz_6_1209	DJI_0355	Potentilla argentea L.	0	n	1	0	Rosaceae Juss.
Iz_6_1209	DJI_0355	Psammophiliella muralis (L.) Ikonn.	0	n	1	0	Caryophyllaceae Juss.
Iz_6_1209	DJI_0355	Psephellus sumensis (Kalen.) Greuter	0	n	1	0	Asteraceae Bercht. & J.Presl
Iz_6_1209	DJI_0355	Pulsatilla pratensis (L.) Mill. s.l.	0	n	1	0	Ranunculaceae Juss.
Iz_6_1209	DJI_0355	Pyrus communis L.	0	a	0	1	Rosaceae Juss.
Iz_6_1209	DJI_0355	Quercus robur L.	0	n	1	1	Fagaceae Dumort.
Iz_6_1209	DJI_0355	Robinia pseudoacacia L.	0	a	0	1	Fabaceae Lindl.
Iz_6_1209	DJI_0355	Rosa sp.	0		NA	1	Rosaceae Juss.
Iz_6_1209	DJI_0355	Rubus idaeus L.	0	n	1	0	Rosaceae Juss.
Iz_6_1209	DJI_0355	Rumex acetosella L.	0	n	1	0	Polygonaceae Juss.
Iz_6_1209	DJI_0355	Rumex thyrsiflorus Fingerh.	0	n	1	0	Polygonaceae Juss.
Iz_6_1209	DJI_0355	Salix alba L.	0	n	1	1	Salicaceae Mirb.
Iz_6_1209	DJI_0355	Sambucus nigra L.	0	n	1	1	Viburnaceae Raf.
Iz_6_1209	DJI_0355	Secale sylvestre Host	0	n	1	0	Poaceae Barnhart
Iz_6_1209	DJI_0355	Sempervivum ruthenicum Schnittsp. & C.B. Lehm.	0	n	1	0	Crassulaceae J.St.-Hil.
Iz_6_1209	DJI_0355	Setaria viridis (L.) P. Beauv.	0	n	1	0	Poaceae Barnhart
Iz_6_1209	DJI_0355	Silene borysthenica (Gruner) Walters	0	n	1	0	Caryophyllaceae Juss.
Iz_6_1209	DJI_0355	Silene latifolia subsp. alba (Mill.) Greuter & Burdet	0	n	1	0	Caryophyllaceae Juss.
Iz_6_1209	DJI_0355	Solidago virgaurea L.	0	n	1	0	Asteraceae Bercht. & J.Presl
Iz_6_1209	DJI_0355	Sorbus aucuparia L.	0	n	1	1	Rosaceae Juss.
Iz_6_1209	DJI_0355	Stachys recta L.	0	n	1	0	Lamiacea Lindl.
Iz_6_1209	DJI_0355	Tanacetum vulgare L.	0	n	1	0	Asteraceae Bercht. & J.Presl
Iz_6_1209	DJI_0355	Thymus pallasianus Heinr.Braun	0	n	1	0	Lamiacea Lindl.
Iz_6_1209	DJI_0355	Tilia cordata Mill.	0	n	1	1	Malvaceae Juss.
Iz_6_1209	DJI_0355	Trifolium alpestre L.	0	n	1	0	Fabaceae Lindl.
Iz_6_1209	DJI_0355	Trifolium arvense L.	0	n	1	0	Fabaceae Lindl.
Iz_6_1209	DJI_0355	Ulmus glabra Huds.	0	n	1	1	Ulmaceae Mirb.
Iz_6_1209	DJI_0355	Verbascum densiflorum Bertol.	0	n	1	0	Scrophulariaceae Juss.
Iz_6_1209	DJI_0355	Veronica austriaca L.	0	n	1	0	Plantaginaceae Juss.
Iz_6_1209	DJI_0355	Veronica incana L.	0	n	1	0	Plantaginaceae Juss.
Iz_6_1209	DJI_0355	Veronica sp.	0		NA	NA	
Iz_6_1209	DJI_0355	Veronica verna L.	0	n	1	0	Plantaginaceae Juss.
Iz_6_1209	DJI_0355	Vincetoxicum hirundinaria Medik	0	n	1	0	Apocynaceae Juss.
Iz_6_1209	DJI_0358	Acer tataricum L.	0	n	1	1	Sapindaceae Juss.
Iz_6_1209	DJI_0358	Achillea millefolium L. s.l.	0	n	1	0	Asteraceae Bercht. & J.Presl
Iz_6_1209	DJI_0358	Allium oleraceum L.	0	n	1	0	Amaryllidaceae J.St.-Hil.
Iz_6_1209	DJI_0358	Ambrosia artemisiifolia L.	0	a	0	0	Asteraceae Bercht. & J.Presl
Iz_6_1209	DJI_0358	Amorpha fruticosa L.	0	a	0	0	Fabaceae Lindl.
Iz_6_1209	DJI_0358	Anthericum ramosum L.	0	n	1	0	Asparagaceae Juss.
Iz_6_1209	DJI_0358	Artemisia austriaca Jacq.	0		NA	NA	
Iz_6_1209	DJI_0358	Artemisia marschalliana Spreng.	0	n	1	0	Asteraceae Bercht. & J.Presl
Iz_6_1209	DJI_0358	Asparagus officinalis L.	0	n	1	0	Asparagaceae Juss.
Iz_6_1209	DJI_0358	Astragalus cicer L.	0	n	1	0	Fabaceae Lindl.
Iz_6_1209	DJI_0358	Bassia laniflora (S.G.Gmel.) A.J.Scott	0	n	1	0	Amaranthaceae Juss.
Iz_6_1209	DJI_0358	Berberis vulgaris L.	0	n	1	0	Berberidaceae Juss.
Iz_6_1209	DJI_0358	Betula pendula Roth.	0	n	1	1	Betulaceae Gray
Iz_6_1209	DJI_0358	Betula pendula Roth.	0	n	1	0	Poaceae Barnhart
Iz_6_1209	DJI_0358	Bromus inermis Leyss.	0		NA	NA	
Iz_6_1209	DJI_0358	Bromus squarrosus L.	0	a	0	0	Poaceae Barnhart
Iz_6_1209	DJI_0358	Bromus tectorum L.	0	a	0	0	Poaceae Barnhart
Iz_6_1209	DJI_0358	Calamagrostis epigejos (L.) Roth	5	n	1	0	Poaceae Barnhart
Iz_6_1209	DJI_0358	Carduus acanthoides L.	0	n	1	0	Asteraceae Bercht. & J.Presl
Iz_6_1209	DJI_0358	Carex sp.	0	n	1	0	Cyperaceae Juss.
Iz_6_1209	DJI_0358	Centaurea majorovii Dumbadze	0	n	1	0	Asteraceae Bercht. & J.Presl
Iz_6_1209	DJI_0358	Chamaecytisus ruthenicus (Fisch.ex Wol.) Klaskova	0	n	1	0	Fabaceae Lindl.
Iz_6_1209	DJI_0358	Chelidonium majus L.	0	n	1	0	Papaveraceae Juss.
Iz_6_1209	DJI_0358	Chenopodium betaceum Andrz.	0	n	1	0	Amaranthaceae Juss.
Iz_6_1209	DJI_0358	Chenopodium cf. album	0	n	1	0	Amaranthaceae Juss.

Iz_6_1209	DJI_0358	Chenopodium sp.	0		NA	0	Amaranthaceae Juss.
Iz_6_1209	DJI_0358	Chondrilla juncea L.	0	n	1	0	Asteraceae Bercht. & J.Presl
Iz_6_1209	DJI_0358	Cirsium vulgare (Savi) Ten.	0	n	1	0	Asteraceae Bercht. & J.Presl
Iz_6_1209	DJI_0358	Clinopodium acinos (L.) Kuntze	0	n	1	0	Lamiacea Lindl.
Iz_6_1209	DJI_0358	Convallaria majalis L.	0	n	1	0	Asparagaceae Juss.
Iz_6_1209	DJI_0358	Crataegus rhipidophylla Gand.	0	n	1	1	Rosaceae Juss.
Iz_6_1209	DJI_0358	Crepis foetida L.	0	n	1	0	Asteraceae Bercht. & J.Presl
Iz_6_1209	DJI_0358	Dactylis glomerata L.	0	n	1	0	Poaceae Barnhart
Iz_6_1209	DJI_0358	Dianthus campestris M.Bieb.	0	n	1	0	Caryophyllaceae Juss.
Iz_6_1209	DJI_0358	Digitaria sanguinalis (L.) Scop.	0	a	0	0	Poaceae Barnhart
Iz_6_1209	DJI_0358	Elymus repens (L.) Gould	0	n	1	0	Poaceae Barnhart
Iz_6_1209	DJI_0358	Epilobium tetragonum L.	0	n	1	0	Onagraceae Juss.
Iz_6_1209	DJI_0358	Eragrostis minor Host	0	a	0	0	Poaceae Barnhart
Iz_6_1209	DJI_0358	Erigeron canadensis L.	7	a	0	0	Asteraceae Bercht. & J.Presl
Iz_6_1209	DJI_0358	Euphorbia seguieriana Neck.	0	n	1	0	Euphorbiaceae Juss.
Iz_6_1209	DJI_0358	Fallopia dumetorum (L.) Holub	0	n	1	0	Polygonaceae Juss.
Iz_6_1209	DJI_0358	Festuca beckeri (Hack.) Trautv.	0	n	1	0	Poaceae Barnhart
Iz_6_1209	DJI_0358	Fragaria vesca L.	0	n	1	0	Rosaceae Juss.
Iz_6_1209	DJI_0358	Frangula alnus Mill.	0	n	1	1	Rhamnaceae Juss.
Iz_6_1209	DJI_0358	Fraxinus pennsylvanica Marshall	0	a	0	1	Oleaceae Hoffmanns. & Link
Iz_6_1209	DJI_0358	Galeopsis bifida Boenn.	0	n	1	0	Lamiacea Lindl.
Iz_6_1209	DJI_0358	Galium verum L.	0	n	1	0	Rubiaceae Juss.
Iz_6_1209	DJI_0358	Genista tinctoria L.	0	n	1	0	Fabaceae Lindl.
Iz_6_1209	DJI_0358	Geranium robertianum L.	0	n	1	0	Geraniaceae Juss.
Iz_6_1209	DJI_0358	Geranium sanquineum L.	0	n	1	0	Geraniaceae Juss.
Iz_6_1209	DJI_0358	Gypsophila paniculata L.	0	n	1	0	Caryophyllaceae Juss.
Iz_6_1209	DJI_0358	Helichrysum arenarium (L.) Moench	0	n	1	0	Asteraceae Bercht. & J.Presl
Iz_6_1209	DJI_0358	Herniaria polygama J.Gay	0	n	1	0	Caryophyllaceae Juss.
Iz_6_1209	DJI_0358	Hieracium robustum Fr.	0	n	1	0	Asteraceae Bercht. & J.Presl
Iz_6_1209	DJI_0358	Hieracium umbellatum L.	0	n	1	NA	Asteraceae Bercht. & J.Presl
Iz_6_1209	DJI_0358	Hylotelephium maximum (L.) Holub	0	n	1	0	Crassulaceae J.St.-Hil.
Iz_6_1209	DJI_0358	Jacobaea borysthenica (DC.) B.Nord. & Greuter	0	n	1	0	Asteraceae Bercht. & J.Presl
Iz_6_1209	DJI_0358	Jasione montana L.	0	n	1	0	Campanulaceae Juss.
Iz_6_1209	DJI_0358	Jurinea cyanoides (L.) Rchb.s.l.	0	n	1	0	Asteraceae Bercht. & J.Presl
Iz_6_1209	DJI_0358	Koeleria glauca (Spreng.) DC.	0	n	1	0	Poaceae Barnhart
Iz_6_1209	DJI_0358	Lactuca serriola L.	0	a	0	0	Asteraceae Bercht. & J.Presl
Iz_6_1209	DJI_0358	Linaria genistifolia (L.) Mill.	0	n	1	0	Plantaginaceae Juss.
Iz_6_1209	DJI_0358	Lomelosia argentea (L.) Greuter & Burdet	0	n	1	0	Caprifoliaceae Juss.
Iz_6_1209	DJI_0358	Melampyrum pratense L.	0	n	1	0	Orobanchaceae Vent.
Iz_6_1209	DJI_0358	Oenothera biennis L.	0	a	0	0	Onagraceae Juss.
Iz_6_1209	DJI_0358	Pentanema britannica (L.) D.Gut.Larr., Santos-Vicente, Anderb., E.Rico & M.M.Mart.Ort.	0	n	1	0	Asteraceae Bercht. & J.Presl
Iz_6_1209	DJI_0358	Peucedanum oreoselinum (L.) Moench	0	n	1	0	Apiaceae Lindl.
Iz_6_1209	DJI_0358	Peucedanum ruthenicum M.Bieb.	0	n	1	0	Apiaceae Lindl.
Iz_6_1209	DJI_0358	Pilosella echioides (Lumn.) F.W.Schultz & Sch.Bip.	0	n	1	0	Asteraceae Bercht. & J.Presl
Iz_6_1209	DJI_0358	Pilosella officinarum F. Schultz et Sch. Bip.	0	n	1	0	Asteraceae Bercht. & J.Presl
Iz_6_1209	DJI_0358	Pilosella sp.	0		NA	0	Asteraceae Bercht. & J.Presl
Iz_6_1209	DJI_0358	Pinus sylvestris L.	0	n	1	1	Pinaceae Spreng. ex F.Rudolphi
Iz_6_1209	DJI_0358	Poa annua L.	0	n	1	0	Poaceae Barnhart
Iz_6_1209	DJI_0358	Poa nemoralis L.	0	n	1	0	Poaceae Barnhart
Iz_6_1209	DJI_0358	Poa sp.	0		NA	0	Poaceae Barnhart
Iz_6_1209	DJI_0358	Polygonatum odoratum (Mill.) Druce	0	n	1	0	Asparagaceae Juss.
Iz_6_1209	DJI_0358	Polygonum arenarium Waldst. & Kit.	0	n	1	0	Polygonaceae Juss.
Iz_6_1209	DJI_0358	Populus nigra L.	0	n	1	1	Salicaceae Mirb.
Iz_6_1209	DJI_0358	Populus tremula L.	0	n	1	1	Salicaceae Mirb.
Iz_6_1209	DJI_0358	Populus Ã canescens (Aiton) Sm.	0	n	1	1	Salicaceae Mirb.
Iz_6_1209	DJI_0358	Potentilla argentea L.	0	n	1	0	Rosaceae Juss.
Iz_6_1209	DJI_0358	Psammophiliella muralis (L.) Ikonn.	0	n	1	0	Caryophyllaceae Juss.
Iz_6_1209	DJI_0358	Psephellus sumensis (Kalen.) Greuter	0	n	1	0	Asteraceae Bercht. & J.Presl
Iz_6_1209	DJI_0358	Pulsatilla pratensis (L.) Mill. s.l.	0	n	1	0	Ranunculaceae Juss.
Iz_6_1209	DJI_0358	Pyrus communis L.	0	a	0	1	Rosaceae Juss.
Iz_6_1209	DJI_0358	Quercus robur L.	0	n	1	1	Fagaceae Dumort.
Iz_6_1209	DJI_0358	Robinia pseudoacacia L.	0	a	0	1	Fabaceae Lindl.
Iz_6_1209	DJI_0358	Rosa sp.	0		NA	1	Rosaceae Juss.
Iz_6_1209	DJI_0358	Rubus idaeus L.	0	n	1	0	Rosaceae Juss.
Iz_6_1209	DJI_0358	Rumex acetosella L.	0	n	1	0	Polygonaceae Juss.
Iz_6_1209	DJI_0358	Rumex thyrsiflorus Fingerh.	0	n	1	0	Polygonaceae Juss.
Iz_6_1209	DJI_0358	Salix alba L.	0	n	1	1	Salicaceae Mirb.
Iz_6_1209	DJI_0358	Sambucus nigra L.	0	n	1	1	Viburnaceae Raf.
Iz_6_1209	DJI_0358	Secale sylvestre Host	0	n	1	0	Poaceae Barnhart
Iz_6_1209	DJI_0358	Sempervivum ruthenicum Schnittsp. & C.B. Lehm.	0	n	1	0	Crassulaceae J.St.-Hil.
Iz_6_1209	DJI_0358	Setaria viridis (L.) P. Beauv.	0	n	1	0	Poaceae Barnhart
Iz_6_1209	DJI_0358	Silene borysthenica (Gruner) Walters	0	n	1	0	Caryophyllaceae Juss.
Iz_6_1209	DJI_0358	Silene latifolia subsp. alba (Mill.) Greuter & Burdet	0	n	1	0	Caryophyllaceae Juss.
Iz_6_1209	DJI_0358	Solidago virgaurea L.	0	n	1	0	Asteraceae Bercht. & J.Presl
Iz_6_1209	DJI_0358	Sorbus aucuparia L.	0	n	1	1	Rosaceae Juss.
Iz_6_1209	DJI_0358	Stachys recta L.	0	n	1	0	Lamiacea Lindl.
Iz_6_1209	DJI_0358	Tanacetum vulgare L.	0	n	1	0	Asteraceae Bercht. & J.Presl
Iz_6_1209	DJI_0358	Thymus pallasianus Heinr.Braun	0	n	1	0	Lamiacea Lindl.
Iz_6_1209	DJI_0358	Tilia cordata Mill.	0	n	1	1	Malvaceae Juss.
Iz_6_1209	DJI_0358	Trifolium alpestre L.	0	n	1	0	Fabaceae Lindl.
Iz_6_1209	DJI_0358	Trifolium arvense L.	0	n	1	0	Fabaceae Lindl.
Iz_6_1209	DJI_0358	Ulmus glabra Huds.	0	n	1	1	Ulmaceae Mirb.
Iz_6_1209	DJI_0358	Verbascum densiflorum Bertol.	0	n	1	0	Scrophulariaceae Juss.
Iz_6_1209	DJI_0358	Veronica austriaca L.	0	n	1	0	Plantaginaceae Juss.
Iz_6_1209	DJI_0358	Veronica incana L.	0	n	1	0	Plantaginaceae Juss.
Iz_6_1209	DJI_0358	Veronica sp.	0		NA	NA	
Iz_6_1209	DJI_0358	Veronica verna L.	0	n	1	0	Plantaginaceae Juss.
Iz_6_1209	DJI_0358	Vincetoxicum hirundinaria Medik	0	n	1	0	Apocynaceae Juss.
Iz_7_1009	DJI_0438	Acer tataricum L.	0	n	1	1	Sapindaceae Juss.
Iz_7_1009	DJI_0438	Achillea millefolium L. s.l.	0	n	1	0	Asteraceae Bercht. & J.Presl
Iz_7_1009	DJI_0438	Allium oleraceum L.	0	n	1	0	Amaryllidaceae J.St.-Hil.
Iz_7_1009	DJI_0438	Ambrosia artemisiifolia L.	0	a	0	0	Asteraceae Bercht. & J.Presl
Iz_7_1009	DJI_0438	Amorpha fruticosa L.	0	a	0	0	Fabaceae Lindl.
Iz_7_1009	DJI_0438	Anthericum ramosum L.	0	n	1	0	Asparagaceae Juss.
Iz_7_1009	DJI_0438	Artemisia marschalliana Spreng.	0	n	1	0	Asteraceae Bercht. & J.Presl
Iz_7_1009	DJI_0438	Asparagus officinalis L.	0	n	1	0	Asparagaceae Juss.
Iz_7_1009	DJI_0438	Astragalus cicer L.	0	n	1	0	Fabaceae Lindl.
Iz_7_1009	DJI_0438	Bassia laniflora (S.G.Gmel.) A.J.Scott	0	n	1	0	Amaranthaceae Juss.

Iz_7_1009	DJI_0438	Berberis vulgaris L.	0	n	1	0	Berberidaceae Juss.
Iz_7_1009	DJI_0438	Betula pendula Roth.	0	n	1	1	Betulaceae Gray
Iz_7_1009	DJI_0438	Betula pendula Roth.	0	n	1	0	Poaceae Barnhart
Iz_7_1009	DJI_0438	Bromus inermis Leyss.	0		NA	NA	
Iz_7_1009	DJI_0438	Bromus squarrosus L.	0	a	0	0	Poaceae Barnhart
Iz_7_1009	DJI_0438	Bromus tectorum L.	0	a	0	0	Poaceae Barnhart
Iz_7_1009	DJI_0438	Calamagrostis epigejos (L.) Roth	10	n	1	0	Poaceae Barnhart
Iz_7_1009	DJI_0438	Carduus acanthoides L.	0	n	1	0	Asteraceae Bercht. & J.Presl
Iz_7_1009	DJI_0438	Carex sp.	0	n	1	0	Cyperaceae Juss.
Iz_7_1009	DJI_0438	Centaurea majorovii Dumbadze	0	n	1	0	Asteraceae Bercht. & J.Presl
Iz_7_1009	DJI_0438	Chamaecytisus ruthenicus (Fisch.ex Wol.) Klaskova	0	n	1	0	Fabaceae Lindl.
Iz_7_1009	DJI_0438	Chelidonium majus L.	0	n	1	0	Papaveraceae Juss.
Iz_7_1009	DJI_0438	Chenopodium betaceum Andrz.	0	n	1	0	Amaranthaceae Juss.
Iz_7_1009	DJI_0438	Chenopodium cf. album	3	n	1	0	Amaranthaceae Juss.
Iz_7_1009	DJI_0438	Chenopodium sp.	0		NA	0	Amaranthaceae Juss.
Iz_7_1009	DJI_0438	Chondrilla juncea L.	0	n	1	0	Asteraceae Bercht. & J.Presl
Iz_7_1009	DJI_0438	Cirsium vulgare (Savi) Ten.	0	n	1	0	Asteraceae Bercht. & J.Presl
Iz_7_1009	DJI_0438	Clinopodium acinos (L.) Kuntze	0	n	1	0	Lamiacea Lindl.
Iz_7_1009	DJI_0438	Convallaria majalis L.	0	n	1	0	Asparagaceae Juss.
Iz_7_1009	DJI_0438	Crataegus rhipidophylla Gand.	0	n	1	1	Rosaceae Juss.
Iz_7_1009	DJI_0438	Crepis foetida L.	0	n	1	0	Asteraceae Bercht. & J.Presl
Iz_7_1009	DJI_0438	Dactylis glomerata L.	0	n	1	0	Poaceae Barnhart
Iz_7_1009	DJI_0438	Dianthus campestris M.Bieb.	0	n	1	0	Caryophyllaceae Juss.
Iz_7_1009	DJI_0438	Digitaria sanguinalis (L.) Scop.	0	a	0	0	Poaceae Barnhart
Iz_7_1009	DJI_0438	Elymus repens (L.) Gould	0	n	1	0	Poaceae Barnhart
Iz_7_1009	DJI_0438	Epilobium tetragonum L.	0	n	1	0	Onagraceae Juss.
Iz_7_1009	DJI_0438	Eragrostis minor Host	0	a	0	0	Poaceae Barnhart
Iz_7_1009	DJI_0438	Erigeron canadensis L.	1	a	0	0	Asteraceae Bercht. & J.Presl
Iz_7_1009	DJI_0438	Euphorbia seguieriana Neck.	0	n	1	0	Euphorbiaceae Juss.
Iz_7_1009	DJI_0438	Fallopia dumetorum (L.) Holub	0	n	1	0	Polygonaceae Juss.
Iz_7_1009	DJI_0438	Festuca beckeri (Hack.) Trautv.	0	n	1	0	Poaceae Barnhart
Iz_7_1009	DJI_0438	Fragaria vesca L.	0	n	1	0	Rosaceae Juss.
Iz_7_1009	DJI_0438	Frangula alnus Mill.	0	n	1	1	Rhamnaceae Juss.
Iz_7_1009	DJI_0438	Fraxinus pennsylvanica Marshall	0	a	0	1	Oleaceae Hoffmanns. & Link
Iz_7_1009	DJI_0438	Galeopsis bifida Boenn.	0	n	1	0	Lamiacea Lindl.
Iz_7_1009	DJI_0438	Galium verum L.	0	n	1	0	Rubiaceae Juss.
Iz_7_1009	DJI_0438	Genista tinctoria L.	0	n	1	0	Fabaceae Lindl.
Iz_7_1009	DJI_0438	Geranium robertianum L.	0	n	1	0	Geraniaceae Juss.
Iz_7_1009	DJI_0438	Geranium sanquineum L.	0	n	1	0	Geraniaceae Juss.
Iz_7_1009	DJI_0438	Gypsophila paniculata L.	0	n	1	0	Caryophyllaceae Juss.
Iz_7_1009	DJI_0438	Helichrysum arenarium (L.) Moench	0	n	1	0	Asteraceae Bercht. & J.Presl
Iz_7_1009	DJI_0438	Herniaria polygama J.Gay	0	n	1	0	Caryophyllaceae Juss.
Iz_7_1009	DJI_0438	Hieracium robustum Fr.	0	n	1	0	Asteraceae Bercht. & J.Presl
Iz_7_1009	DJI_0438	Hieracium umbellatum L.	0	n	1	NA	Asteraceae Bercht. & J.Presl
Iz_7_1009	DJI_0438	Hylotelephium maximum (L.) Holub	0	n	1	0	Crassulaceae J.St.-Hil.
Iz_7_1009	DJI_0438	Jacobaea borysthenica (DC.) B.Nord. & Greuter	0	n	1	0	Asteraceae Bercht. & J.Presl
Iz_7_1009	DJI_0438	Jasione montana L.	0	n	1	0	Campanulaceae Juss.
Iz_7_1009	DJI_0438	Jurinea cyanoides (L.) Rchb.s.l.	0	n	1	0	Asteraceae Bercht. & J.Presl
Iz_7_1009	DJI_0438	Koeleria glauca (Spreng.) DC.	0	n	1	0	Poaceae Barnhart
Iz_7_1009	DJI_0438	Lactuca serriola L.	0	a	0	0	Asteraceae Bercht. & J.Presl
Iz_7_1009	DJI_0438	Linaria genistifolia (L.) Mill.	0	n	1	0	Plantaginaceae Juss.
Iz_7_1009	DJI_0438	Lomelosia argentea (L.) Greuter & Burdet	0	n	1	0	Caprifoliaceae Juss.
Iz_7_1009	DJI_0438	Melampyrum pratense L.	0	n	1	0	Orobanchaceae Vent.
Iz_7_1009	DJI_0438	Oenothera biennis L.	0	a	0	0	Onagraceae Juss.
Iz_7_1009	DJI_0438	Pentanema britannica (L.) D.Gut.Larr., Santos-Vicente, Anderb., E.Rico & M.M.Mart.Ort.	5	n	1	0	Asteraceae Bercht. & J.Presl
Iz_7_1009	DJI_0438	Peucedanum oreoselinum (L.) Moench	0	n	1	0	Apiaceae Lindl.
Iz_7_1009	DJI_0438	Peucedanum ruthenicum M.Bieb.	0	n	1	0	Apiaceae Lindl.
Iz_7_1009	DJI_0438	Pilosella echioides (Lumn.) F.W.Schultz & Sch.Bip.	0	n	1	0	Asteraceae Bercht. & J.Presl
Iz_7_1009	DJI_0438	Pilosella officinarum F. Schultz et Sch. Bip.	0	n	1	0	Asteraceae Bercht. & J.Presl
Iz_7_1009	DJI_0438	Pilosella sp.	0		NA	0	Asteraceae Bercht. & J.Presl
Iz_7_1009	DJI_0438	Pinus sylvestris L.	0	n	1	1	Pinaceae Spreng. ex F.Rudolphi
Iz_7_1009	DJI_0438	Poa annua L.	0	n	1	0	Poaceae Barnhart
Iz_7_1009	DJI_0438	Poa nemoralis L.	2	n	1	0	Poaceae Barnhart
Iz_7_1009	DJI_0438	Poa sp.	0		NA	0	Poaceae Barnhart
Iz_7_1009	DJI_0438	Polygonatum odoratum (Mill.) Druce	0	n	1	0	Asparagaceae Juss.
Iz_7_1009	DJI_0438	Polygonum arenarium Waldst. & Kit.	0	n	1	0	Polygonaceae Juss.
Iz_7_1009	DJI_0438	Populus nigra L.	0	n	1	1	Salicaceae Mirb.
Iz_7_1009	DJI_0438	Populus tremula L.	0	n	1	1	Salicaceae Mirb.
Iz_7_1009	DJI_0438	Populus Ã canescens (Aiton) Sm.	0	n	1	1	Salicaceae Mirb.
Iz_7_1009	DJI_0438	Potentilla argentea L.	0	n	1	0	Rosaceae Juss.
Iz_7_1009	DJI_0438	Psammophiliella muralis (L.) Ikonn.	0	n	1	0	Caryophyllaceae Juss.
Iz_7_1009	DJI_0438	Psephellus sumensis (Kalen.) Greuter	5	n	1	0	Asteraceae Bercht. & J.Presl
Iz_7_1009	DJI_0438	Pulsatilla pratensis (L.) Mill. s.l.	0	n	1	0	Ranunculaceae Juss.
Iz_7_1009	DJI_0438	Pyrus communis L.	0	a	0	1	Rosaceae Juss.
Iz_7_1009	DJI_0438	Quercus robur L.	0	n	1	1	Fagaceae Dumort.
Iz_7_1009	DJI_0438	Robinia pseudoacacia L.	0	a	0	1	Fabaceae Lindl.
Iz_7_1009	DJI_0438	Rosa sp.	0		NA	1	Rosaceae Juss.
Iz_7_1009	DJI_0438	Rubus idaeus L.	0	n	1	0	Rosaceae Juss.
Iz_7_1009	DJI_0438	Rumex acetosella L.	0	n	1	0	Polygonaceae Juss.
Iz_7_1009	DJI_0438	Rumex thyrsiflorus Fingerh.	0	n	1	0	Polygonaceae Juss.
Iz_7_1009	DJI_0438	Salix alba L.	0	n	1	1	Salicaceae Mirb.
Iz_7_1009	DJI_0438	Sambucus nigra L.	0	n	1	1	Viburnaceae Raf.
Iz_7_1009	DJI_0438	Secale sylvestre Host	0	n	1	0	Poaceae Barnhart
Iz_7_1009	DJI_0438	Sempervivum ruthenicum Schnittsp. & C.B. Lehm.	0	n	1	0	Crassulaceae J.St.-Hil.
Iz_7_1009	DJI_0438	Setaria viridis (L.) P. Beauv.	0	n	1	0	Poaceae Barnhart
Iz_7_1009	DJI_0438	Silene borysthenica (Gruner) Walters	0	n	1	0	Caryophyllaceae Juss.
Iz_7_1009	DJI_0438	Silene latifolia subsp. alba (Mill.) Greuter & Burdet	0	n	1	0	Caryophyllaceae Juss.
Iz_7_1009	DJI_0438	Solidago virgaurea L.	0	n	1	0	Asteraceae Bercht. & J.Presl
Iz_7_1009	DJI_0438	Sorbus aucuparia L.	0	n	1	1	Rosaceae Juss.
Iz_7_1009	DJI_0438	Stachys recta L.	0	n	1	0	Lamiacea Lindl.
Iz_7_1009	DJI_0438	Tanacetum vulgare L.	0	n	1	0	Asteraceae Bercht. & J.Presl
Iz_7_1009	DJI_0438	Thymus pallasianus Heinr.Braun	0	n	1	0	Lamiacea Lindl.
Iz_7_1009	DJI_0438	Tilia cordata Mill.	0	n	1	1	Malvaceae Juss.
Iz_7_1009	DJI_0438	Trifolium alpestre L.	0	n	1	0	Fabaceae Lindl.
Iz_7_1009	DJI_0438	Trifolium arvense L.	0	n	1	0	Fabaceae Lindl.
Iz_7_1009	DJI_0438	Ulmus glabra Huds.	0	n	1	1	Ulmaceae Mirb.
Iz_7_1009	DJI_0438	Verbascum densiflorum Bertol.	0	n	1	0	Scrophulariaceae Juss.
Iz_7_1009	DJI_0438	Veronica austriaca L.	0	n	1	0	Plantaginaceae Juss.

Iz_7_1009	DJI_0438	Veronica incana L.	0	n	1	0	Plantaginaceae Juss.
Iz_7_1009	DJI_0438	Veronica verna L.	0	n	1	0	Plantaginaceae Juss.
Iz_7_1009	DJI_0438	Vincetoxicum hirundinaria Medik	0	n	1	0	Apocynaceae Juss.
Iz_7_1009	DJI_0447	Acer tataricum L.	0	n	1	1	Sapindaceae Juss.
Iz_7_1009	DJI_0447	Achillea millefolium L. s.l.	0	n	1	0	Asteraceae Bercht. & J.Presl
Iz_7_1009	DJI_0447	Allium oleraceum L.	0	n	1	0	Amaryllidaceae J.St.-Hil.
Iz_7_1009	DJI_0447	Ambrosia artemisiifolia L.	0	a	0	0	Asteraceae Bercht. & J.Presl
Iz_7_1009	DJI_0447	Amorpha fruticosa L.	0	a	0	0	Fabaceae Lindl.
Iz_7_1009	DJI_0447	Anthericum ramosum L.	0	n	1	0	Asparagaceae Juss.
Iz_7_1009	DJI_0447	Artemisia marschalliana Spreng.	0	n	1	0	Asteraceae Bercht. & J.Presl
Iz_7_1009	DJI_0447	Asparagus officinalis L.	0	n	1	0	Asparagaceae Juss.
Iz_7_1009	DJI_0447	Astragalus cicer L.	0	n	1	0	Fabaceae Lindl.
Iz_7_1009	DJI_0447	Bassia laniflora (S.G.Gmel.) A.J.Scott	0	n	1	0	Amaranthaceae Juss.
Iz_7_1009	DJI_0447	Berberis vulgaris L.	0	n	1	0	Berberidaceae Juss.
Iz_7_1009	DJI_0447	Betula pendula Roth.	0	n	1	1	Betulaceae Gray
Iz_7_1009	DJI_0447	Betula pendula Roth.	0	n	1	0	Poaceae Barnhart
Iz_7_1009	DJI_0447	Bromus inermis Leyss.	0		NA	NA	
Iz_7_1009	DJI_0447	Bromus squarrosus L.	0	a	0	0	Poaceae Barnhart
Iz_7_1009	DJI_0447	Bromus tectorum L.	0	a	0	0	Poaceae Barnhart
Iz_7_1009	DJI_0447	Calamagrostis epigejos (L.) Roth	0	n	1	0	Poaceae Barnhart
Iz_7_1009	DJI_0447	Carduus acanthoides L.	0	n	1	0	Asteraceae Bercht. & J.Presl
Iz_7_1009	DJI_0447	Carex sp.	0	n	1	0	Cyperaceae Juss.
Iz_7_1009	DJI_0447	Centaurea majorovii Dumbadze	0	n	1	0	Asteraceae Bercht. & J.Presl
Iz_7_1009	DJI_0447	Chamaecytisus ruthenicus (Fisch.ex Wol.) Klaskova	0	n	1	0	Fabaceae Lindl.
Iz_7_1009	DJI_0447	Chelidonium majus L.	0	n	1	0	Papaveraceae Juss.
Iz_7_1009	DJI_0447	Chenopodium betaceum Andrz.	0	n	1	0	Amaranthaceae Juss.
Iz_7_1009	DJI_0447	Chenopodium cf. album	5	n	1	0	Amaranthaceae Juss.
Iz_7_1009	DJI_0447	Chenopodium sp.	0		NA	0	Amaranthaceae Juss.
Iz_7_1009	DJI_0447	Chondrilla juncea L.	0	n	1	0	Asteraceae Bercht. & J.Presl
Iz_7_1009	DJI_0447	Cirsium vulgare (Savi) Ten.	0	n	1	0	Asteraceae Bercht. & J.Presl
Iz_7_1009	DJI_0447	Clinopodium acinos (L.) Kuntze	0	n	1	0	Lamiacea Lindl.
Iz_7_1009	DJI_0447	Convallaria majalis L.	0	n	1	0	Asparagaceae Juss.
Iz_7_1009	DJI_0447	Crataegus rhipidophylla Gand.	0	n	1	1	Rosaceae Juss.
Iz_7_1009	DJI_0447	Crepis foetida L.	0	n	1	0	Asteraceae Bercht. & J.Presl
Iz_7_1009	DJI_0447	Dactylis glomerata L.	0	n	1	0	Poaceae Barnhart
Iz_7_1009	DJI_0447	Dianthus campestris M.Bieb.	0	n	1	0	Caryophyllaceae Juss.
Iz_7_1009	DJI_0447	Digitaria sanguinalis (L.) Scop.	0	a	0	0	Poaceae Barnhart
Iz_7_1009	DJI_0447	Elymus repens (L.) Gould	0	n	1	0	Poaceae Barnhart
Iz_7_1009	DJI_0447	Epilobium tetragonum L.	0	n	1	0	Onagraceae Juss.
Iz_7_1009	DJI_0447	Eragrostis minor Host	0	a	0	0	Poaceae Barnhart
Iz_7_1009	DJI_0447	Erigeron canadensis L.	5	a	0	0	Asteraceae Bercht. & J.Presl
Iz_7_1009	DJI_0447	Euphorbia seguieriana Neck.	0	n	1	0	Euphorbiaceae Juss.
Iz_7_1009	DJI_0447	Fallopia dumetorum (L.) Holub	0	n	1	0	Polygonaceae Juss.
Iz_7_1009	DJI_0447	Festuca beckeri (Hack.) Trautv.	0	n	1	0	Poaceae Barnhart
Iz_7_1009	DJI_0447	Fragaria vesca L.	0	n	1	0	Rosaceae Juss.
Iz_7_1009	DJI_0447	Frangula alnus Mill.	0	n	1	1	Rhamnaceae Juss.
Iz_7_1009	DJI_0447	Fraxinus pennsylvanica Marshall	0	a	0	1	Oleaceae Hoffmanns. & Link
Iz_7_1009	DJI_0447	Galeopsis bifida Boenn.	0	n	1	0	Lamiacea Lindl.
Iz_7_1009	DJI_0447	Galium verum L.	0	n	1	0	Rubiaceae Juss.
Iz_7_1009	DJI_0447	Genista tinctoria L.	0	n	1	0	Fabaceae Lindl.
Iz_7_1009	DJI_0447	Geranium robertianum L.	0	n	1	0	Geraniaceae Juss.
Iz_7_1009	DJI_0447	Geranium sanquineum L.	0	n	1	0	Geraniaceae Juss.
Iz_7_1009	DJI_0447	Gypsophila paniculata L.	0	n	1	0	Caryophyllaceae Juss.
Iz_7_1009	DJI_0447	Helichrysum arenarium (L.) Moench	0	n	1	0	Asteraceae Bercht. & J.Presl
Iz_7_1009	DJI_0447	Herniaria polygama J.Gay	0	n	1	0	Caryophyllaceae Juss.
Iz_7_1009	DJI_0447	Hieracium robustum Fr.	0	n	1	0	Asteraceae Bercht. & J.Presl
Iz_7_1009	DJI_0447	Hieracium umbellatum L.	0	n	1	NA	Asteraceae Bercht. & J.Presl
Iz_7_1009	DJI_0447	Hylotelephium maximum (L.) Holub	0	n	1	0	Crassulaceae J.St.-Hil.
Iz_7_1009	DJI_0447	Jacobaea borysthenica (DC.) B.Nord. & Greuter	0	n	1	0	Asteraceae Bercht. & J.Presl
Iz_7_1009	DJI_0447	Jasione montana L.	0	n	1	0	Campanulaceae Juss.
Iz_7_1009	DJI_0447	Jurinea cyanoides (L.) Rchb.s.l.	0	n	1	0	Asteraceae Bercht. & J.Presl
Iz_7_1009	DJI_0447	Koeleria glauca (Spreng.) DC.	0	n	1	0	Poaceae Barnhart
Iz_7_1009	DJI_0447	Lactuca serriola L.	0	a	0	0	Asteraceae Bercht. & J.Presl
Iz_7_1009	DJI_0447	Linaria genistifolia (L.) Mill.	0	n	1	0	Plantaginaceae Juss.
Iz_7_1009	DJI_0447	Lomelosia argentea (L.) Greuter & Burdet	0	n	1	0	Caprifoliaceae Juss.
Iz_7_1009	DJI_0447	Melampyrum pratense L.	0	n	1	0	Orobanchaceae Vent.
Iz_7_1009	DJI_0447	Oenothera biennis L.	0	a	0	0	Onagraceae Juss.
Iz_7_1009	DJI_0447	Pentanema britannica (L.) D.Gut.Larr., Santos-Vicente, Anderb., E.Rico & M.M.Mart.Ort.	0	n	1	0	Asteraceae Bercht. & J.Presl
Iz_7_1009	DJI_0447	Peucedanum oreoselinum (L.) Moench	0	n	1	0	Apiaceae Lindl.
Iz_7_1009	DJI_0447	Peucedanum ruthenicum M.Bieb.	0	n	1	0	Apiaceae Lindl.
Iz_7_1009	DJI_0447	Pilosella echioides (Lumn.) F.W.Schultz & Sch.Bip.	0	n	1	0	Asteraceae Bercht. & J.Presl
Iz_7_1009	DJI_0447	Pilosella officinarum F. Schultz et Sch. Bip.	0	n	1	0	Asteraceae Bercht. & J.Presl
Iz_7_1009	DJI_0447	Pilosella sp.	0		NA	0	Asteraceae Bercht. & J.Presl
Iz_7_1009	DJI_0447	Pinus sylvestris L.	0	n	1	1	Pinaceae Spreng. ex F.Rudolphi
Iz_7_1009	DJI_0447	Poa annua L.	0	n	1	0	Poaceae Barnhart
Iz_7_1009	DJI_0447	Poa nemoralis L.	0	n	1	0	Poaceae Barnhart
Iz_7_1009	DJI_0447	Poa sp.	0		NA	0	Poaceae Barnhart
Iz_7_1009	DJI_0447	Polygonatum odoratum (Mill.) Druce	0	n	1	0	Asparagaceae Juss.
Iz_7_1009	DJI_0447	Polygonum arenarium Waldst. & Kit.	0	n	1	0	Polygonaceae Juss.
Iz_7_1009	DJI_0447	Populus nigra L.	0	n	1	1	Salicaceae Mirb.
Iz_7_1009	DJI_0447	Populus tremula L.	0	n	1	1	Salicaceae Mirb.
Iz_7_1009	DJI_0447	Populus Ã canescens (Aiton) Sm.	0	n	1	1	Salicaceae Mirb.
Iz_7_1009	DJI_0447	Potentilla argentea L.	0	n	1	0	Rosaceae Juss.
Iz_7_1009	DJI_0447	Psammophiliella muralis (L.) Ikonn.	0	n	1	0	Caryophyllaceae Juss.
Iz_7_1009	DJI_0447	Psephellus sumensis (Kalen.) Greuter	0	n	1	0	Asteraceae Bercht. & J.Presl
Iz_7_1009	DJI_0447	Pulsatilla pratensis (L.) Mill. s.l.	0	n	1	0	Ranunculaceae Juss.
Iz_7_1009	DJI_0447	Pyrus communis L.	0	a	0	1	Rosaceae Juss.
Iz_7_1009	DJI_0447	Quercus robur L.	0	n	1	1	Fagaceae Dumort.
Iz_7_1009	DJI_0447	Robinia pseudoacacia L.	0	a	0	1	Fabaceae Lindl.
Iz_7_1009	DJI_0447	Rosa sp.	0		NA	1	Rosaceae Juss.
Iz_7_1009	DJI_0447	Rubus idaeus L.	0	n	1	0	Rosaceae Juss.
Iz_7_1009	DJI_0447	Rumex acetosella L.	0	n	1	0	Polygonaceae Juss.
Iz_7_1009	DJI_0447	Rumex thyrsiflorus Fingerh.	0	n	1	0	Polygonaceae Juss.
Iz_7_1009	DJI_0447	Salix alba L.	0	n	1	1	Salicaceae Mirb.
Iz_7_1009	DJI_0447	Sambucus nigra L.	0	n	1	1	Viburnaceae Raf.
Iz_7_1009	DJI_0447	Secale sylvestre Host	0	n	1	0	Poaceae Barnhart
Iz_7_1009	DJI_0447	Sempervivum ruthenicum Schnittsp. & C.B. Lehm.	0	n	1	0	Crassulaceae J.St.-Hil.
Iz_7_1009	DJI_0447	Setaria viridis (L.) P. Beauv.	0	n	1	0	Poaceae Barnhart

Iz_7_1009	DJI_0447	Silene borysthenica (Gruner) Walters	0	n	1	0	Caryophyllaceae Juss.
Iz_7_1009	DJI_0447	Silene latifolia subsp. alba (Mill.) Greuter & Burdet	0	n	1	0	Caryophyllaceae Juss.
Iz_7_1009	DJI_0447	Solidago virgaurea L.	0	n	1	0	Asteraceae Bercht. & J.Presl
Iz_7_1009	DJI_0447	Sorbus aucuparia L.	0	n	1	1	Rosaceae Juss.
Iz_7_1009	DJI_0447	Stachys recta L.	0	n	1	0	Lamiacea Lindl.
Iz_7_1009	DJI_0447	Tanacetum vulgare L.	0	n	1	0	Asteraceae Bercht. & J.Presl
Iz_7_1009	DJI_0447	Thymus pallasianus Heinr.Braun	0	n	1	0	Lamiacea Lindl.
Iz_7_1009	DJI_0447	Tilia cordata Mill.	0	n	1	1	Malvaceae Juss.
Iz_7_1009	DJI_0447	Trifolium alpestre L.	0	n	1	0	Fabaceae Lindl.
Iz_7_1009	DJI_0447	Trifolium arvense L.	0	n	1	0	Fabaceae Lindl.
Iz_7_1009	DJI_0447	Ulmus glabra Huds.	0	n	1	1	Ulmaceae Mirb.
Iz_7_1009	DJI_0447	Verbascum densiflorum Bertol.	0	n	1	0	Scrophulariaceae Juss.
Iz_7_1009	DJI_0447	Veronica austriaca L.	0	n	1	0	Plantaginaceae Juss.
Iz_7_1009	DJI_0447	Veronica incana L.	0	n	1	0	Plantaginaceae Juss.
Iz_7_1009	DJI_0447	Veronica verna L.	0	n	1	0	Plantaginaceae Juss.
Iz_7_1009	DJI_0447	Vincetoxicum hirundinaria Medik	0	n	1	0	Apocynaceae Juss.
Iz_7_1009	DJI_0448	Acer tataricum L.	0	n	1	1	Sapindaceae Juss.
Iz_7_1009	DJI_0448	Achillea millefolium L. s.l.	0	n	1	0	Asteraceae Bercht. & J.Presl
Iz_7_1009	DJI_0448	Allium oleraceum L.	0	n	1	0	Amaryllidaceae J.St.-Hil.
Iz_7_1009	DJI_0448	Ambrosia artemisiifolia L.	0	a	0	0	Asteraceae Bercht. & J.Presl
Iz_7_1009	DJI_0448	Amorpha fruticosa L.	0	a	0	0	Fabaceae Lindl.
Iz_7_1009	DJI_0448	Anthericum ramosum L.	0	n	1	0	Asparagaceae Juss.
Iz_7_1009	DJI_0448	Artemisia marschalliana Spreng.	0	n	1	0	Asteraceae Bercht. & J.Presl
Iz_7_1009	DJI_0448	Asparagus officinalis L.	0	n	1	0	Asparagaceae Juss.
Iz_7_1009	DJI_0448	Astragalus cicer L.	0	n	1	0	Fabaceae Lindl.
Iz_7_1009	DJI_0448	Bassia laniflora (S.G.Gmel.) A.J.Scott	0	n	1	0	Amaranthaceae Juss.
Iz_7_1009	DJI_0448	Berberis vulgaris L.	0	n	1	0	Berberidaceae Juss.
Iz_7_1009	DJI_0448	Betula pendula Roth.	0	n	1	1	Betulaceae Gray
Iz_7_1009	DJI_0448	Betula pendula Roth.	0	n	1	0	Poaceae Barnhart
Iz_7_1009	DJI_0448	Bromus inermis Leyss.	0		NA	NA	
Iz_7_1009	DJI_0448	Bromus squarrosus L.	0	a	0	0	Poaceae Barnhart
Iz_7_1009	DJI_0448	Bromus tectorum L.	0	a	0	0	Poaceae Barnhart
Iz_7_1009	DJI_0448	Calamagrostis epigejos (L.) Roth	2	n	1	0	Poaceae Barnhart
Iz_7_1009	DJI_0448	Carduus acanthoides L.	0	n	1	0	Asteraceae Bercht. & J.Presl
Iz_7_1009	DJI_0448	Carex sp.	0	n	1	0	Cyperaceae Juss.
Iz_7_1009	DJI_0448	Centaurea majorovii Dumbadze	0	n	1	0	Asteraceae Bercht. & J.Presl
Iz_7_1009	DJI_0448	Chamaecytisus ruthenicus (Fisch.ex Wol.) Klaskova	0	n	1	0	Fabaceae Lindl.
Iz_7_1009	DJI_0448	Chelidonium majus L.	0	n	1	0	Papaveraceae Juss.
Iz_7_1009	DJI_0448	Chenopodium betaceum Andrz.	0	n	1	0	Amaranthaceae Juss.
Iz_7_1009	DJI_0448	Chenopodium cf. album	3	n	1	0	Amaranthaceae Juss.
Iz_7_1009	DJI_0448	Chenopodium sp.	0		NA	0	Amaranthaceae Juss.
Iz_7_1009	DJI_0448	Chondrilla juncea L.	0	n	1	0	Asteraceae Bercht. & J.Presl
Iz_7_1009	DJI_0448	Cirsium vulgare (Savi) Ten.	0	n	1	0	Asteraceae Bercht. & J.Presl
Iz_7_1009	DJI_0448	Clinopodium acinos (L.) Kuntze	0	n	1	0	Lamiacea Lindl.
Iz_7_1009	DJI_0448	Convallaria majalis L.	0	n	1	0	Asparagaceae Juss.
Iz_7_1009	DJI_0448	Crataegus rhipidophylla Gand.	0	n	1	1	Rosaceae Juss.
Iz_7_1009	DJI_0448	Crepis foetida L.	0	n	1	0	Asteraceae Bercht. & J.Presl
Iz_7_1009	DJI_0448	Dactylis glomerata L.	0	n	1	0	Poaceae Barnhart
Iz_7_1009	DJI_0448	Dianthus campestris M.Bieb.	0	n	1	0	Caryophyllaceae Juss.
Iz_7_1009	DJI_0448	Digitaria sanguinalis (L.) Scop.	0	a	0	0	Poaceae Barnhart
Iz_7_1009	DJI_0448	Elymus repens (L.) Gould	0	n	1	0	Poaceae Barnhart
Iz_7_1009	DJI_0448	Epilobium tetragonum L.	0	n	1	0	Onagraceae Juss.
Iz_7_1009	DJI_0448	Eragrostis minor Host	0	a	0	0	Poaceae Barnhart
Iz_7_1009	DJI_0448	Erigeron canadensis L.	10	a	0	0	Asteraceae Bercht. & J.Presl
Iz_7_1009	DJI_0448	Euphorbia seguieriana Neck.	0	n	1	0	Euphorbiaceae Juss.
Iz_7_1009	DJI_0448	Fallopia dumetorum (L.) Holub	0	n	1	0	Polygonaceae Juss.
Iz_7_1009	DJI_0448	Festuca beckeri (Hack.) Trautv.	0	n	1	0	Poaceae Barnhart
Iz_7_1009	DJI_0448	Fragaria vesca L.	0	n	1	0	Rosaceae Juss.
Iz_7_1009	DJI_0448	Frangula alnus Mill.	0	n	1	1	Rhamnaceae Juss.
Iz_7_1009	DJI_0448	Fraxinus pennsylvanica Marshall	0	a	0	1	Oleaceae Hoffmanns. & Link
Iz_7_1009	DJI_0448	Galeopsis bifida Boenn.	0	n	1	0	Lamiacea Lindl.
Iz_7_1009	DJI_0448	Galium verum L.	0	n	1	0	Rubiaceae Juss.
Iz_7_1009	DJI_0448	Genista tinctoria L.	0	n	1	0	Fabaceae Lindl.
Iz_7_1009	DJI_0448	Geranium robertianum L.	0	n	1	0	Geraniaceae Juss.
Iz_7_1009	DJI_0448	Geranium sanquineum L.	0	n	1	0	Geraniaceae Juss.
Iz_7_1009	DJI_0448	Gypsophila paniculata L.	0	n	1	0	Caryophyllaceae Juss.
Iz_7_1009	DJI_0448	Helichrysum arenarium (L.) Moench	0	n	1	0	Asteraceae Bercht. & J.Presl
Iz_7_1009	DJI_0448	Herniaria polygama J.Gay	0	n	1	0	Caryophyllaceae Juss.
Iz_7_1009	DJI_0448	Hieracium robustum Fr.	0	n	1	0	Asteraceae Bercht. & J.Presl
Iz_7_1009	DJI_0448	Hieracium umbellatum L.	0	n	1	NA	Asteraceae Bercht. & J.Presl
Iz_7_1009	DJI_0448	Hylotelephium maximum (L.) Holub	0	n	1	0	Crassulaceae J.St.-Hil.
Iz_7_1009	DJI_0448	Jacobaea borysthenica (DC.) B.Nord. & Greuter	0	n	1	0	Asteraceae Bercht. & J.Presl
Iz_7_1009	DJI_0448	Jasione montana L.	0	n	1	0	Campanulaceae Juss.
Iz_7_1009	DJI_0448	Jurinea cyanoides (L.) Rchb.s.l.	0	n	1	0	Asteraceae Bercht. & J.Presl
Iz_7_1009	DJI_0448	Koeleria glauca (Spreng.) DC.	0	n	1	0	Poaceae Barnhart
Iz_7_1009	DJI_0448	Lactuca serriola L.	0	a	0	0	Asteraceae Bercht. & J.Presl
Iz_7_1009	DJI_0448	Linaria genistifolia (L.) Mill.	0	n	1	0	Plantaginaceae Juss.
Iz_7_1009	DJI_0448	Lomelosia argentea (L.) Greuter & Burdet	0	n	1	0	Caprifoliaceae Juss.
Iz_7_1009	DJI_0448	Melampyrum pratense L.	0	n	1	0	Orobanchaceae Vent.
Iz_7_1009	DJI_0448	Oenothera biennis L.	0	a	0	0	Onagraceae Juss.
Iz_7_1009	DJI_0448	Pentanema britannica (L.) D.Gut.Larr., Santos-Vicente, Anderb., E.Rico & M.M.Mart.Ort.	0	n	1	0	Asteraceae Bercht. & J.Presl
Iz_7_1009	DJI_0448	Peucedanum oreoselinum (L.) Moench	0	n	1	0	Apiaceae Lindl.
Iz_7_1009	DJI_0448	Peucedanum ruthenicum M.Bieb.	0	n	1	0	Apiaceae Lindl.
Iz_7_1009	DJI_0448	Pilosella echioides (Lumn.) F.W.Schultz & Sch.Bip.	0	n	1	0	Asteraceae Bercht. & J.Presl
Iz_7_1009	DJI_0448	Pilosella officinarum F. Schultz et Sch. Bip.	0	n	1	0	Asteraceae Bercht. & J.Presl
Iz_7_1009	DJI_0448	Pilosella sp.	0		NA	0	Asteraceae Bercht. & J.Presl
Iz_7_1009	DJI_0448	Pinus sylvestris L.	0	n	1	1	Pinaceae Spreng. ex F.Rudolphi
Iz_7_1009	DJI_0448	Poa annua L.	0	n	1	0	Poaceae Barnhart
Iz_7_1009	DJI_0448	Poa nemoralis L.	0	n	1	0	Poaceae Barnhart
Iz_7_1009	DJI_0448	Poa sp.	0		NA	0	Poaceae Barnhart
Iz_7_1009	DJI_0448	Polygonatum odoratum (Mill.) Druce	0	n	1	0	Asparagaceae Juss.
Iz_7_1009	DJI_0448	Polygonum arenarium Waldst. & Kit.	0	n	1	0	Polygonaceae Juss.
Iz_7_1009	DJI_0448	Populus nigra L.	0	n	1	1	Salicaceae Mirb.
Iz_7_1009	DJI_0448	Populus tremula L.	0	n	1	1	Salicaceae Mirb.
Iz_7_1009	DJI_0448	Populus Ã canescens (Aiton) Sm.	0	n	1	1	Salicaceae Mirb.
Iz_7_1009	DJI_0448	Potentilla argentea L.	0	n	1	0	Rosaceae Juss.
Iz_7_1009	DJI_0448	Psammophiliella muralis (L.) Ikonn.	0	n	1	0	Caryophyllaceae Juss.
Iz_7_1009	DJI_0448	Psephellus sumensis (Kalen.) Greuter	0	n	1	0	Asteraceae Bercht. & J.Presl

Iz_7_1009	DJI_0448	Pulsatilla pratensis (L.) Mill. s.l.	0	n	1	0	Ranunculaceae Juss.
Iz_7_1009	DJI_0448	Pyrus communis L.	0	a	0	1	Rosaceae Juss.
Iz_7_1009	DJI_0448	Quercus robur L.	0	n	1	1	Fagaceae Dumort.
Iz_7_1009	DJI_0448	Robinia pseudoacacia L.	0	a	0	1	Fabaceae Lindl.
Iz_7_1009	DJI_0448	Rosa sp.	0		NA	1	Rosaceae Juss.
Iz_7_1009	DJI_0448	Rubus idaeus L.	0	n	1	0	Rosaceae Juss.
Iz_7_1009	DJI_0448	Rumex acetosella L.	0	n	1	0	Polygonaceae Juss.
Iz_7_1009	DJI_0448	Rumex thyrsiflorus Fingerh.	0	n	1	0	Polygonaceae Juss.
Iz_7_1009	DJI_0448	Salix alba L.	0	n	1	1	Salicaceae Mirb.
Iz_7_1009	DJI_0448	Sambucus nigra L.	0	n	1	1	Viburnaceae Raf.
Iz_7_1009	DJI_0448	Secale sylvestre Host	0	n	1	0	Poaceae Barnhart
Iz_7_1009	DJI_0448	Sempervivum ruthenicum Schnittsp. & C.B. Lehm.	0	n	1	0	Crassulaceae J.St.-Hil.
Iz_7_1009	DJI_0448	Setaria viridis (L.) P. Beauv.	0	n	1	0	Poaceae Barnhart
Iz_7_1009	DJI_0448	Silene borysthenica (Gruner) Walters	0	n	1	0	Caryophyllaceae Juss.
Iz_7_1009	DJI_0448	Silene latifolia subsp. alba (Mill.) Greuter & Burdet	0	n	1	0	Caryophyllaceae Juss.
Iz_7_1009	DJI_0448	Solidago virgaurea L.	0	n	1	0	Asteraceae Bercht. & J.Presl
Iz_7_1009	DJI_0448	Sorbus aucuparia L.	0	n	1	1	Rosaceae Juss.
Iz_7_1009	DJI_0448	Stachys recta L.	0	n	1	0	Lamiacea Lindl.
Iz_7_1009	DJI_0448	Tanacetum vulgare L.	0	n	1	0	Asteraceae Bercht. & J.Presl
Iz_7_1009	DJI_0448	Thymus pallasianus Heinr.Braun	0	n	1	0	Lamiacea Lindl.
Iz_7_1009	DJI_0448	Tilia cordata Mill.	0	n	1	1	Malvaceae Juss.
Iz_7_1009	DJI_0448	Trifolium alpestre L.	0	n	1	0	Fabaceae Lindl.
Iz_7_1009	DJI_0448	Trifolium arvense L.	0	n	1	0	Fabaceae Lindl.
Iz_7_1009	DJI_0448	Ulmus glabra Huds.	0	n	1	1	Ulmaceae Mirb.
Iz_7_1009	DJI_0448	Verbascum densiflorum Bertol.	0	n	1	0	Scrophulariaceae Juss.
Iz_7_1009	DJI_0448	Veronica austriaca L.	0	n	1	0	Plantaginaceae Juss.
Iz_7_1009	DJI_0448	Veronica incana L.	0	n	1	0	Plantaginaceae Juss.
Iz_7_1009	DJI_0448	Veronica verna L.	0	n	1	0	Plantaginaceae Juss.
Iz_7_1009	DJI_0448	Vincetoxicum hirundinaria Medik	0	n	1	0	Apocynaceae Juss.
Iz_7_1009	DJI_0450	Acer tataricum L.	0	n	1	1	Sapindaceae Juss.
Iz_7_1009	DJI_0450	Achillea millefolium L. s.l.	0	n	1	0	Asteraceae Bercht. & J.Presl
Iz_7_1009	DJI_0450	Allium oleraceum L.	0	n	1	0	Amaryllidaceae J.St.-Hil.
Iz_7_1009	DJI_0450	Ambrosia artemisiifolia L.	0	a	0	0	Asteraceae Bercht. & J.Presl
Iz_7_1009	DJI_0450	Amorpha fruticosa L.	0	a	0	0	Fabaceae Lindl.
Iz_7_1009	DJI_0450	Anthericum ramosum L.	0	n	1	0	Asparagaceae Juss.
Iz_7_1009	DJI_0450	Artemisia marschalliana Spreng.	0	n	1	0	Asteraceae Bercht. & J.Presl
Iz_7_1009	DJI_0450	Asparagus officinalis L.	0	n	1	0	Asparagaceae Juss.
Iz_7_1009	DJI_0450	Astragalus cicer L.	0	n	1	0	Fabaceae Lindl.
Iz_7_1009	DJI_0450	Bassia laniflora (S.G.Gmel.) A.J.Scott	0	n	1	0	Amaranthaceae Juss.
Iz_7_1009	DJI_0450	Berberis vulgaris L.	0	n	1	0	Berberidaceae Juss.
Iz_7_1009	DJI_0450	Betula pendula Roth.	0	n	1	1	Betulaceae Gray
Iz_7_1009	DJI_0450	Betula pendula Roth.	0	n	1	0	Poaceae Barnhart
Iz_7_1009	DJI_0450	Bromus inermis Leyss.	0		NA	NA	
Iz_7_1009	DJI_0450	Bromus squarrosus L.	0	a	0	0	Poaceae Barnhart
Iz_7_1009	DJI_0450	Bromus tectorum L.	0	a	0	0	Poaceae Barnhart
Iz_7_1009	DJI_0450	Calamagrostis epigejos (L.) Roth	0	n	1	0	Poaceae Barnhart
Iz_7_1009	DJI_0450	Carduus acanthoides L.	0	n	1	0	Asteraceae Bercht. & J.Presl
Iz_7_1009	DJI_0450	Carex sp.	0	n	1	0	Cyperaceae Juss.
Iz_7_1009	DJI_0450	Centaurea majorovii Dumbadze	0	n	1	0	Asteraceae Bercht. & J.Presl
Iz_7_1009	DJI_0450	Chamaecytisus ruthenicus (Fisch.ex Wol.) Klaskova	0	n	1	0	Fabaceae Lindl.
Iz_7_1009	DJI_0450	Chelidonium majus L.	0	n	1	0	Papaveraceae Juss.
Iz_7_1009	DJI_0450	Chenopodium betaceum Andrz.	0	n	1	0	Amaranthaceae Juss.
Iz_7_1009	DJI_0450	Chenopodium cf. album	0	n	1	0	Amaranthaceae Juss.
Iz_7_1009	DJI_0450	Chenopodium sp.	0		NA	0	Amaranthaceae Juss.
Iz_7_1009	DJI_0450	Chondrilla juncea L.	0	n	1	0	Asteraceae Bercht. & J.Presl
Iz_7_1009	DJI_0450	Cirsium vulgare (Savi) Ten.	0	n	1	0	Asteraceae Bercht. & J.Presl
Iz_7_1009	DJI_0450	Clinopodium acinos (L.) Kuntze	0	n	1	0	Lamiacea Lindl.
Iz_7_1009	DJI_0450	Convallaria majalis L.	0	n	1	0	Asparagaceae Juss.
Iz_7_1009	DJI_0450	Crataegus rhipidophylla Gand.	0	n	1	1	Rosaceae Juss.
Iz_7_1009	DJI_0450	Crepis foetida L.	0	n	1	0	Asteraceae Bercht. & J.Presl
Iz_7_1009	DJI_0450	Dactylis glomerata L.	0	n	1	0	Poaceae Barnhart
Iz_7_1009	DJI_0450	Dianthus campestris M.Bieb.	0	n	1	0	Caryophyllaceae Juss.
Iz_7_1009	DJI_0450	Digitaria sanguinalis (L.) Scop.	0	a	0	0	Poaceae Barnhart
Iz_7_1009	DJI_0450	Elymus repens (L.) Gould	0	n	1	0	Poaceae Barnhart
Iz_7_1009	DJI_0450	Epilobium tetragonum L.	0	n	1	0	Onagraceae Juss.
Iz_7_1009	DJI_0450	Eragrostis minor Host	0	a	0	0	Poaceae Barnhart
Iz_7_1009	DJI_0450	Erigeron canadensis L.	15	a	0	0	Asteraceae Bercht. & J.Presl
Iz_7_1009	DJI_0450	Euphorbia seguieriana Neck.	0	n	1	0	Euphorbiaceae Juss.
Iz_7_1009	DJI_0450	Fallopia dumetorum (L.) Holub	0	n	1	0	Polygonaceae Juss.
Iz_7_1009	DJI_0450	Festuca beckeri (Hack.) Trautv.	0	n	1	0	Poaceae Barnhart
Iz_7_1009	DJI_0450	Fragaria vesca L.	0	n	1	0	Rosaceae Juss.
Iz_7_1009	DJI_0450	Frangula alnus Mill.	0	n	1	1	Rhamnaceae Juss.
Iz_7_1009	DJI_0450	Fraxinus pennsylvanica Marshall	0	a	0	1	Oleaceae Hoffmanns. & Link
Iz_7_1009	DJI_0450	Galeopsis bifida Boenn.	0	n	1	0	Lamiacea Lindl.
Iz_7_1009	DJI_0450	Galium verum L.	0	n	1	0	Rubiaceae Juss.
Iz_7_1009	DJI_0450	Genista tinctoria L.	0	n	1	0	Fabaceae Lindl.
Iz_7_1009	DJI_0450	Geranium robertianum L.	0	n	1	0	Geraniaceae Juss.
Iz_7_1009	DJI_0450	Geranium sanquineum L.	0	n	1	0	Geraniaceae Juss.
Iz_7_1009	DJI_0450	Gypsophila paniculata L.	0	n	1	0	Caryophyllaceae Juss.
Iz_7_1009	DJI_0450	Helichrysum arenarium (L.) Moench	0	n	1	0	Asteraceae Bercht. & J.Presl
Iz_7_1009	DJI_0450	Herniaria polygama J.Gay	0	n	1	0	Caryophyllaceae Juss.
Iz_7_1009	DJI_0450	Hieracium robustum Fr.	0	n	1	0	Asteraceae Bercht. & J.Presl
Iz_7_1009	DJI_0450	Hieracium umbellatum L.	0	n	1	NA	Asteraceae Bercht. & J.Presl
Iz_7_1009	DJI_0450	Hylotelephium maximum (L.) Holub	0	n	1	0	Crassulaceae J.St.-Hil.
Iz_7_1009	DJI_0450	Jacobaea borysthenica (DC.) B.Nord. & Greuter	0	n	1	0	Asteraceae Bercht. & J.Presl
Iz_7_1009	DJI_0450	Jasione montana L.	0	n	1	0	Campanulaceae Juss.
Iz_7_1009	DJI_0450	Jurinea cyanoides (L.) Rchb.s.l.	0	n	1	0	Asteraceae Bercht. & J.Presl
Iz_7_1009	DJI_0450	Koeleria glauca (Spreng.) DC.	0	n	1	0	Poaceae Barnhart
Iz_7_1009	DJI_0450	Lactuca serriola L.	0	a	0	0	Asteraceae Bercht. & J.Presl
Iz_7_1009	DJI_0450	Linaria genistifolia (L.) Mill.	0	n	1	0	Plantaginaceae Juss.
Iz_7_1009	DJI_0450	Lomelosia argentea (L.) Greuter & Burdet	0	n	1	0	Caprifoliaceae Juss.
Iz_7_1009	DJI_0450	Melampyrum pratense L.	0	n	1	0	Orobanchaceae Vent.
Iz_7_1009	DJI_0450	Oenothera biennis L.	0	a	0	0	Onagraceae Juss.
Iz_7_1009	DJI_0450	Pentanema britannica (L.) D.Gut.Larr., Santos-Vicente, Anderb., E.Rico & M.M.Mart.Ort.	0	n	1	0	Asteraceae Bercht. & J.Presl
Iz_7_1009	DJI_0450	Peucedanum oreoselinum (L.) Moench	0	n	1	0	Apiaceae Lindl.
Iz_7_1009	DJI_0450	Peucedanum ruthenicum M.Bieb.	0	n	1	0	Apiaceae Lindl.
Iz_7_1009	DJI_0450	Pilosella echioides (Lumn.) F.W.Schultz & Sch.Bip.	0	n	1	0	Asteraceae Bercht. & J.Presl
Iz_7_1009	DJI_0450	Pilosella officinarum F. Schultz et Sch. Bip.	0	n	1	0	Asteraceae Bercht. & J.Presl

Iz_7_1009	DJI_0450	Pilosella sp.	0		NA	0	Asteraceae Bercht. & J.Presl
Iz_7_1009	DJI_0450	Pinus sylvestris L.	0	n	1	1	Pinaceae Spreng. ex F.Rudolphi
Iz_7_1009	DJI_0450	Poa annua L.	0	n	1	0	Poaceae Barnhart
Iz_7_1009	DJI_0450	Poa nemoralis L.	0	n	1	0	Poaceae Barnhart
Iz_7_1009	DJI_0450	Poa sp.	0		NA	0	Poaceae Barnhart
Iz_7_1009	DJI_0450	Polygonatum odoratum (Mill.) Druce	0	n	1	0	Asparagaceae Juss.
Iz_7_1009	DJI_0450	Polygonum arenarium Waldst. & Kit.	0	n	1	0	Polygonaceae Juss.
Iz_7_1009	DJI_0450	Populus nigra L.	0	n	1	1	Salicaceae Mirb.
Iz_7_1009	DJI_0450	Populus tremula L.	0	n	1	1	Salicaceae Mirb.
Iz_7_1009	DJI_0450	Populus Ã canescens (Aiton) Sm.	0	n	1	1	Salicaceae Mirb.
Iz_7_1009	DJI_0450	Potentilla argentea L.	0	n	1	0	Rosaceae Juss.
Iz_7_1009	DJI_0450	Psammophiliella muralis (L.) Ikonn.	0	n	1	0	Caryophyllaceae Juss.
Iz_7_1009	DJI_0450	Psephellus sumensis (Kalen.) Greuter	0	n	1	0	Asteraceae Bercht. & J.Presl
Iz_7_1009	DJI_0450	Pulsatilla pratensis (L.) Mill. s.l.	0	n	1	0	Ranunculaceae Juss.
Iz_7_1009	DJI_0450	Pyrus communis L.	0	a	0	1	Rosaceae Juss.
Iz_7_1009	DJI_0450	Quercus robur L.	0	n	1	1	Fagaceae Dumort.
Iz_7_1009	DJI_0450	Robinia pseudoacacia L.	0	a	0	1	Fabaceae Lindl.
Iz_7_1009	DJI_0450	Rosa sp.	0		NA	1	Rosaceae Juss.
Iz_7_1009	DJI_0450	Rubus idaeus L.	0	n	1	0	Rosaceae Juss.
Iz_7_1009	DJI_0450	Rumex acetosella L.	0	n	1	0	Polygonaceae Juss.
Iz_7_1009	DJI_0450	Rumex thyrsiflorus Fingerh.	0	n	1	0	Polygonaceae Juss.
Iz_7_1009	DJI_0450	Salix alba L.	0	n	1	1	Salicaceae Mirb.
Iz_7_1009	DJI_0450	Sambucus nigra L.	0	n	1	1	Viburnaceae Raf.
Iz_7_1009	DJI_0450	Secale sylvestre Host	0	n	1	0	Poaceae Barnhart
Iz_7_1009	DJI_0450	Sempervivum ruthenicum Schnittsp. & C.B. Lehm.	0	n	1	0	Crassulaceae J.St.-Hil.
Iz_7_1009	DJI_0450	Setaria viridis (L.) P. Beauv.	0	n	1	0	Poaceae Barnhart
Iz_7_1009	DJI_0450	Silene borysthenica (Gruner) Walters	0	n	1	0	Caryophyllaceae Juss.
Iz_7_1009	DJI_0450	Silene latifolia subsp. alba (Mill.) Greuter & Burdet	0	n	1	0	Caryophyllaceae Juss.
Iz_7_1009	DJI_0450	Solidago virgaurea L.	0	n	1	0	Asteraceae Bercht. & J.Presl
Iz_7_1009	DJI_0450	Sorbus aucuparia L.	0	n	1	1	Rosaceae Juss.
Iz_7_1009	DJI_0450	Stachys recta L.	0	n	1	0	Lamiacea Lindl.
Iz_7_1009	DJI_0450	Tanacetum vulgare L.	0	n	1	0	Asteraceae Bercht. & J.Presl
Iz_7_1009	DJI_0450	Thymus pallasianus Heinr.Braun	0	n	1	0	Lamiacea Lindl.
Iz_7_1009	DJI_0450	Tilia cordata Mill.	0	n	1	1	Malvaceae Juss.
Iz_7_1009	DJI_0450	Trifolium alpestre L.	0	n	1	0	Fabaceae Lindl.
Iz_7_1009	DJI_0450	Trifolium arvense L.	0	n	1	0	Fabaceae Lindl.
Iz_7_1009	DJI_0450	Ulmus glabra Huds.	0	n	1	1	Ulmaceae Mirb.
Iz_7_1009	DJI_0450	Verbascum densiflorum Bertol.	0	n	1	0	Scrophulariaceae Juss.
Iz_7_1009	DJI_0450	Veronica austriaca L.	0	n	1	0	Plantaginaceae Juss.
Iz_7_1009	DJI_0450	Veronica incana L.	0	n	1	0	Plantaginaceae Juss.
Iz_7_1009	DJI_0450	Veronica verna L.	0	n	1	0	Plantaginaceae Juss.
Iz_7_1009	DJI_0450	Vincetoxicum hirundinaria Medik	0	n	1	0	Apocynaceae Juss.
Iz_7_1009	DJI_0452	Acer tataricum L.	0	n	1	1	Sapindaceae Juss.
Iz_7_1009	DJI_0452	Achillea millefolium L. s.l.	0	n	1	0	Asteraceae Bercht. & J.Presl
Iz_7_1009	DJI_0452	Allium oleraceum L.	0	n	1	0	Amaryllidaceae J.St.-Hil.
Iz_7_1009	DJI_0452	Ambrosia artemisiifolia L.	0	a	0	0	Asteraceae Bercht. & J.Presl
Iz_7_1009	DJI_0452	Amorpha fruticosa L.	0	a	0	0	Fabaceae Lindl.
Iz_7_1009	DJI_0452	Anthericum ramosum L.	0	n	1	0	Asparagaceae Juss.
Iz_7_1009	DJI_0452	Artemisia marschalliana Spreng.	0	n	1	0	Asteraceae Bercht. & J.Presl
Iz_7_1009	DJI_0452	Asparagus officinalis L.	0	n	1	0	Asparagaceae Juss.
Iz_7_1009	DJI_0452	Astragalus cicer L.	0	n	1	0	Fabaceae Lindl.
Iz_7_1009	DJI_0452	Bassia laniflora (S.G.Gmel.) A.J.Scott	0	n	1	0	Amaranthaceae Juss.
Iz_7_1009	DJI_0452	Berberis vulgaris L.	0	n	1	0	Berberidaceae Juss.
Iz_7_1009	DJI_0452	Betula pendula Roth.	0	n	1	1	Betulaceae Gray
Iz_7_1009	DJI_0452	Betula pendula Roth.	0	n	1	0	Poaceae Barnhart
Iz_7_1009	DJI_0452	Bromus inermis Leyss.	0		NA	NA	
Iz_7_1009	DJI_0452	Bromus squarrosus L.	0	a	0	0	Poaceae Barnhart
Iz_7_1009	DJI_0452	Bromus tectorum L.	0	a	0	0	Poaceae Barnhart
Iz_7_1009	DJI_0452	Calamagrostis epigejos (L.) Roth	0	n	1	0	Poaceae Barnhart
Iz_7_1009	DJI_0452	Carduus acanthoides L.	0	n	1	0	Asteraceae Bercht. & J.Presl
Iz_7_1009	DJI_0452	Carex sp.	0	n	1	0	Cyperaceae Juss.
Iz_7_1009	DJI_0452	Centaurea majorovii Dumbadze	0	n	1	0	Asteraceae Bercht. & J.Presl
Iz_7_1009	DJI_0452	Chamaecytisus ruthenicus (Fisch.ex Wol.) Klaskova	0	n	1	0	Fabaceae Lindl.
Iz_7_1009	DJI_0452	Chelidonium majus L.	0	n	1	0	Papaveraceae Juss.
Iz_7_1009	DJI_0452	Chenopodium betaceum Andrz.	0	n	1	0	Amaranthaceae Juss.
Iz_7_1009	DJI_0452	Chenopodium cf. album	0	n	1	0	Amaranthaceae Juss.
Iz_7_1009	DJI_0452	Chenopodium sp.	0		NA	0	Amaranthaceae Juss.
Iz_7_1009	DJI_0452	Chondrilla juncea L.	0	n	1	0	Asteraceae Bercht. & J.Presl
Iz_7_1009	DJI_0452	Cirsium vulgare (Savi) Ten.	0	n	1	0	Asteraceae Bercht. & J.Presl
Iz_7_1009	DJI_0452	Clinopodium acinos (L.) Kuntze	0	n	1	0	Lamiacea Lindl.
Iz_7_1009	DJI_0452	Convallaria majalis L.	0	n	1	0	Asparagaceae Juss.
Iz_7_1009	DJI_0452	Crataegus rhipidophylla Gand.	0	n	1	1	Rosaceae Juss.
Iz_7_1009	DJI_0452	Crepis foetida L.	0	n	1	0	Asteraceae Bercht. & J.Presl
Iz_7_1009	DJI_0452	Dactylis glomerata L.	0	n	1	0	Poaceae Barnhart
Iz_7_1009	DJI_0452	Dianthus campestris M.Bieb.	0	n	1	0	Caryophyllaceae Juss.
Iz_7_1009	DJI_0452	Digitaria sanguinalis (L.) Scop.	0	a	0	0	Poaceae Barnhart
Iz_7_1009	DJI_0452	Elymus repens (L.) Gould	0	n	1	0	Poaceae Barnhart
Iz_7_1009	DJI_0452	Epilobium tetragonum L.	0	n	1	0	Onagraceae Juss.
Iz_7_1009	DJI_0452	Eragrostis minor Host	0	a	0	0	Poaceae Barnhart
Iz_7_1009	DJI_0452	Erigeron canadensis L.	20	a	0	0	Asteraceae Bercht. & J.Presl
Iz_7_1009	DJI_0452	Euphorbia seguieriana Neck.	0	n	1	0	Euphorbiaceae Juss.
Iz_7_1009	DJI_0452	Fallopia dumetorum (L.) Holub	0	n	1	0	Polygonaceae Juss.
Iz_7_1009	DJI_0452	Festuca beckeri (Hack.) Trautv.	0	n	1	0	Poaceae Barnhart
Iz_7_1009	DJI_0452	Fragaria vesca L.	0	n	1	0	Rosaceae Juss.
Iz_7_1009	DJI_0452	Frangula alnus Mill.	0	n	1	1	Rhamnaceae Juss.
Iz_7_1009	DJI_0452	Fraxinus pennsylvanica Marshall	0	a	0	1	Oleaceae Hoffmanns. & Link
Iz_7_1009	DJI_0452	Galeopsis bifida Boenn.	0	n	1	0	Lamiacea Lindl.
Iz_7_1009	DJI_0452	Galium verum L.	0	n	1	0	Rubiaceae Juss.
Iz_7_1009	DJI_0452	Genista tinctoria L.	0	n	1	0	Fabaceae Lindl.
Iz_7_1009	DJI_0452	Geranium robertianum L.	0	n	1	0	Geraniaceae Juss.
Iz_7_1009	DJI_0452	Geranium sanquineum L.	0	n	1	0	Geraniaceae Juss.
Iz_7_1009	DJI_0452	Gypsophila paniculata L.	0	n	1	0	Caryophyllaceae Juss.
Iz_7_1009	DJI_0452	Helichrysum arenarium (L.) Moench	0	n	1	0	Asteraceae Bercht. & J.Presl
Iz_7_1009	DJI_0452	Herniaria polygama J.Gay	0	n	1	0	Caryophyllaceae Juss.
Iz_7_1009	DJI_0452	Hieracium robustum Fr.	0	n	1	0	Asteraceae Bercht. & J.Presl
Iz_7_1009	DJI_0452	Hieracium umbellatum L.	0	n	1	NA	Asteraceae Bercht. & J.Presl
Iz_7_1009	DJI_0452	Hylotelephium maximum (L.) Holub	0	n	1	0	Crassulaceae J.St.-Hil.
Iz_7_1009	DJI_0452	Jacobaea borysthenica (DC.) B.Nord. & Greuter	0	n	1	0	Asteraceae Bercht. & J.Presl

Iz_7_1009	DJI_0452	Jasione montana L.	0	n	1	0	Campanulaceae Juss.
Iz_7_1009	DJI_0452	Jurinea cyanoides (L.) Rchb.s.l.	0	n	1	0	Asteraceae Bercht. & J.Presl
Iz_7_1009	DJI_0452	Koeleria glauca (Spreng.) DC.	0	n	1	0	Poaceae Barnhart
Iz_7_1009	DJI_0452	Lactuca serriola L.	0	a	0	0	Asteraceae Bercht. & J.Presl
Iz_7_1009	DJI_0452	Linaria genistifolia (L.) Mill.	0	n	1	0	Plantaginaceae Juss.
Iz_7_1009	DJI_0452	Lomelosia argentea (L.) Greuter & Burdet	0	n	1	0	Caprifoliaceae Juss.
Iz_7_1009	DJI_0452	Melampyrum pratense L.	0	n	1	0	Orobanchaceae Vent.
Iz_7_1009	DJI_0452	Oenothera biennis L.	0	a	0	0	Onagraceae Juss.
Iz_7_1009	DJI_0452	Pentanema britannica (L.) D.Gut.Larr., Santos-Vicente, Anderb., E.Rico & M.M.Mart.Ort.	0	n	1	0	Asteraceae Bercht. & J.Presl
Iz_7_1009	DJI_0452	Peucedanum oreoselinum (L.) Moench	0	n	1	0	Apiaceae Lindl.
Iz_7_1009	DJI_0452	Peucedanum ruthenicum M.Bieb.	0	n	1	0	Apiaceae Lindl.
Iz_7_1009	DJI_0452	Pilosella echioides (Lumn.) F.W.Schultz & Sch.Bip.	0	n	1	0	Asteraceae Bercht. & J.Presl
Iz_7_1009	DJI_0452	Pilosella officinarum F. Schultz et Sch. Bip.	0	n	1	0	Asteraceae Bercht. & J.Presl
Iz_7_1009	DJI_0452	Pilosella sp.	0		NA	0	Asteraceae Bercht. & J.Presl
Iz_7_1009	DJI_0452	Pinus sylvestris L.	0	n	1	1	Pinaceae Spreng. ex F.Rudolphi
Iz_7_1009	DJI_0452	Poa annua L.	0	n	1	0	Poaceae Barnhart
Iz_7_1009	DJI_0452	Poa nemoralis L.	0	n	1	0	Poaceae Barnhart
Iz_7_1009	DJI_0452	Poa sp.	0		NA	0	Poaceae Barnhart
Iz_7_1009	DJI_0452	Polygonatum odoratum (Mill.) Druce	0	n	1	0	Asparagaceae Juss.
Iz_7_1009	DJI_0452	Polygonum arenarium Waldst. & Kit.	0	n	1	0	Polygonaceae Juss.
Iz_7_1009	DJI_0452	Populus nigra L.	0	n	1	1	Salicaceae Mirb.
Iz_7_1009	DJI_0452	Populus tremula L.	0	n	1	1	Salicaceae Mirb.
Iz_7_1009	DJI_0452	Populus Ã canescens (Aiton) Sm.	0	n	1	1	Salicaceae Mirb.
Iz_7_1009	DJI_0452	Potentilla argentea L.	0	n	1	0	Rosaceae Juss.
Iz_7_1009	DJI_0452	Psammophiliella muralis (L.) Ikonn.	0	n	1	0	Caryophyllaceae Juss.
Iz_7_1009	DJI_0452	Psephellus sumensis (Kalen.) Greuter	0	n	1	0	Asteraceae Bercht. & J.Presl
Iz_7_1009	DJI_0452	Pulsatilla pratensis (L.) Mill. s.l.	0	n	1	0	Ranunculaceae Juss.
Iz_7_1009	DJI_0452	Pyrus communis L.	0	a	0	1	Rosaceae Juss.
Iz_7_1009	DJI_0452	Quercus robur L.	0	n	1	1	Fagaceae Dumort.
Iz_7_1009	DJI_0452	Robinia pseudoacacia L.	0	a	0	1	Fabaceae Lindl.
Iz_7_1009	DJI_0452	Rosa sp.	0		NA	1	Rosaceae Juss.
Iz_7_1009	DJI_0452	Rubus idaeus L.	0	n	1	0	Rosaceae Juss.
Iz_7_1009	DJI_0452	Rumex acetosella L.	0	n	1	0	Polygonaceae Juss.
Iz_7_1009	DJI_0452	Rumex thyrsiflorus Fingerh.	0	n	1	0	Polygonaceae Juss.
Iz_7_1009	DJI_0452	Salix alba L.	0	n	1	1	Salicaceae Mirb.
Iz_7_1009	DJI_0452	Sambucus nigra L.	0	n	1	1	Viburnaceae Raf.
Iz_7_1009	DJI_0452	Secale sylvestre Host	0	n	1	0	Poaceae Barnhart
Iz_7_1009	DJI_0452	Sempervivum ruthenicum Schnittsp. & C.B. Lehm.	0	n	1	0	Crassulaceae J.St.-Hil.
Iz_7_1009	DJI_0452	Setaria viridis (L.) P. Beauv.	0	n	1	0	Poaceae Barnhart
Iz_7_1009	DJI_0452	Silene borysthenica (Gruner) Walters	0	n	1	0	Caryophyllaceae Juss.
Iz_7_1009	DJI_0452	Silene latifolia subsp. alba (Mill.) Greuter & Burdet	0	n	1	0	Caryophyllaceae Juss.
Iz_7_1009	DJI_0452	Solidago virgaurea L.	0	n	1	0	Asteraceae Bercht. & J.Presl
Iz_7_1009	DJI_0452	Sorbus aucuparia L.	0	n	1	1	Rosaceae Juss.
Iz_7_1009	DJI_0452	Stachys recta L.	0	n	1	0	Lamiacea Lindl.
Iz_7_1009	DJI_0452	Tanacetum vulgare L.	0	n	1	0	Asteraceae Bercht. & J.Presl
Iz_7_1009	DJI_0452	Thymus pallasianus Heinr.Braun	0	n	1	0	Lamiacea Lindl.
Iz_7_1009	DJI_0452	Tilia cordata Mill.	0	n	1	1	Malvaceae Juss.
Iz_7_1009	DJI_0452	Trifolium alpestre L.	0	n	1	0	Fabaceae Lindl.
Iz_7_1009	DJI_0452	Trifolium arvense L.	0	n	1	0	Fabaceae Lindl.
Iz_7_1009	DJI_0452	Ulmus glabra Huds.	0	n	1	1	Ulmaceae Mirb.
Iz_7_1009	DJI_0452	Verbascum densiflorum Bertol.	0	n	1	0	Scrophulariaceae Juss.
Iz_7_1009	DJI_0452	Veronica austriaca L.	0	n	1	0	Plantaginaceae Juss.
Iz_7_1009	DJI_0452	Veronica incana L.	0	n	1	0	Plantaginaceae Juss.
Iz_7_1009	DJI_0452	Veronica verna L.	0	n	1	0	Plantaginaceae Juss.
Iz_7_1009	DJI_0452	Vincetoxicum hirundinaria Medik	0	n	1	0	Apocynaceae Juss.
Iz_7_1009	DJI_0455	Acer tataricum L.	0	n	1	1	Sapindaceae Juss.
Iz_7_1009	DJI_0455	Achillea millefolium L. s.l.	0	n	1	0	Asteraceae Bercht. & J.Presl
Iz_7_1009	DJI_0455	Allium oleraceum L.	0	n	1	0	Amaryllidaceae J.St.-Hil.
Iz_7_1009	DJI_0455	Ambrosia artemisiifolia L.	0	a	0	0	Asteraceae Bercht. & J.Presl
Iz_7_1009	DJI_0455	Amorpha fruticosa L.	0	a	0	0	Fabaceae Lindl.
Iz_7_1009	DJI_0455	Anthericum ramosum L.	0	n	1	0	Asparagaceae Juss.
Iz_7_1009	DJI_0455	Artemisia marschalliana Spreng.	0	n	1	0	Asteraceae Bercht. & J.Presl
Iz_7_1009	DJI_0455	Asparagus officinalis L.	0	n	1	0	Asparagaceae Juss.
Iz_7_1009	DJI_0455	Astragalus cicer L.	0	n	1	0	Fabaceae Lindl.
Iz_7_1009	DJI_0455	Bassia laniflora (S.G.Gmel.) A.J.Scott	0	n	1	0	Amaranthaceae Juss.
Iz_7_1009	DJI_0455	Berberis vulgaris L.	0	n	1	0	Berberidaceae Juss.
Iz_7_1009	DJI_0455	Betula pendula Roth.	0	n	1	1	Betulaceae Gray
Iz_7_1009	DJI_0455	Betula pendula Roth.	0	n	1	0	Poaceae Barnhart
Iz_7_1009	DJI_0455	Bromus inermis Leyss.	0		NA	NA	
Iz_7_1009	DJI_0455	Bromus squarrosus L.	0	a	0	0	Poaceae Barnhart
Iz_7_1009	DJI_0455	Bromus tectorum L.	0	a	0	0	Poaceae Barnhart
Iz_7_1009	DJI_0455	Calamagrostis epigejos (L.) Roth	0	n	1	0	Poaceae Barnhart
Iz_7_1009	DJI_0455	Carduus acanthoides L.	0	n	1	0	Asteraceae Bercht. & J.Presl
Iz_7_1009	DJI_0455	Carex sp.	0	n	1	0	Cyperaceae Juss.
Iz_7_1009	DJI_0455	Centaurea majorovii Dumbadze	0	n	1	0	Asteraceae Bercht. & J.Presl
Iz_7_1009	DJI_0455	Chamaecytisus ruthenicus (Fisch.ex Wol.) Klaskova	0	n	1	0	Fabaceae Lindl.
Iz_7_1009	DJI_0455	Chelidonium majus L.	0	n	1	0	Papaveraceae Juss.
Iz_7_1009	DJI_0455	Chenopodium betaceum Andrz.	0	n	1	0	Amaranthaceae Juss.
Iz_7_1009	DJI_0455	Chenopodium cf. album	0	n	1	0	Amaranthaceae Juss.
Iz_7_1009	DJI_0455	Chenopodium sp.	0		NA	0	Amaranthaceae Juss.
Iz_7_1009	DJI_0455	Chondrilla juncea L.	0	n	1	0	Asteraceae Bercht. & J.Presl
Iz_7_1009	DJI_0455	Cirsium vulgare (Savi) Ten.	0	n	1	0	Asteraceae Bercht. & J.Presl
Iz_7_1009	DJI_0455	Clinopodium acinos (L.) Kuntze	0	n	1	0	Lamiacea Lindl.
Iz_7_1009	DJI_0455	Convallaria majalis L.	0	n	1	0	Asparagaceae Juss.
Iz_7_1009	DJI_0455	Crataegus rhipidophylla Gand.	0	n	1	1	Rosaceae Juss.
Iz_7_1009	DJI_0455	Crepis foetida L.	0	n	1	0	Asteraceae Bercht. & J.Presl
Iz_7_1009	DJI_0455	Dactylis glomerata L.	0	n	1	0	Poaceae Barnhart
Iz_7_1009	DJI_0455	Dianthus campestris M.Bieb.	0	n	1	0	Caryophyllaceae Juss.
Iz_7_1009	DJI_0455	Digitaria sanguinalis (L.) Scop.	0	a	0	0	Poaceae Barnhart
Iz_7_1009	DJI_0455	Elymus repens (L.) Gould	0	n	1	0	Poaceae Barnhart
Iz_7_1009	DJI_0455	Epilobium tetragonum L.	0	n	1	0	Onagraceae Juss.
Iz_7_1009	DJI_0455	Eragrostis minor Host	0	a	0	0	Poaceae Barnhart
Iz_7_1009	DJI_0455	Erigeron canadensis L.	15	a	0	0	Asteraceae Bercht. & J.Presl
Iz_7_1009	DJI_0455	Euphorbia seguieriana Neck.	0	n	1	0	Euphorbiaceae Juss.
Iz_7_1009	DJI_0455	Fallopia dumetorum (L.) Holub	0	n	1	0	Polygonaceae Juss.
Iz_7_1009	DJI_0455	Festuca beckeri (Hack.) Trautv.	0	n	1	0	Poaceae Barnhart
Iz_7_1009	DJI_0455	Fragaria vesca L.	0	n	1	0	Rosaceae Juss.
Iz_7_1009	DJI_0455	Frangula alnus Mill.	0	n	1	1	Rhamnaceae Juss.

Iz_7_1009	DJI_0455	Fraxinus pennsylvanica Marshall	0	a	0	1	Oleaceae Hoffmanns. & Link
Iz_7_1009	DJI_0455	Galeopsis bifida Boenn.	0	n	1	0	Lamiacea Lindl.
Iz_7_1009	DJI_0455	Galium verum L.	0	n	1	0	Rubiaceae Juss.
Iz_7_1009	DJI_0455	Genista tinctoria L.	0	n	1	0	Fabaceae Lindl.
Iz_7_1009	DJI_0455	Geranium robertianum L.	0	n	1	0	Geraniaceae Juss.
Iz_7_1009	DJI_0455	Geranium sanquineum L.	0	n	1	0	Geraniaceae Juss.
Iz_7_1009	DJI_0455	Gypsophila paniculata L.	0	n	1	0	Caryophyllaceae Juss.
Iz_7_1009	DJI_0455	Helichrysum arenarium (L.) Moench	0	n	1	0	Asteraceae Bercht. & J.Presl
Iz_7_1009	DJI_0455	Herniaria polygama J.Gay	0	n	1	0	Caryophyllaceae Juss.
Iz_7_1009	DJI_0455	Hieracium robustum Fr.	0	n	1	0	Asteraceae Bercht. & J.Presl
Iz_7_1009	DJI_0455	Hieracium umbellatum L.	0	n	1	NA	Asteraceae Bercht. & J.Presl
Iz_7_1009	DJI_0455	Hylotelephium maximum (L.) Holub	0	n	1	0	Crassulaceae J.St.-Hil.
Iz_7_1009	DJI_0455	Jacobaea borysthenica (DC.) B.Nord. & Greuter	0	n	1	0	Asteraceae Bercht. & J.Presl
Iz_7_1009	DJI_0455	Jasione montana L.	0	n	1	0	Campanulaceae Juss.
Iz_7_1009	DJI_0455	Jurinea cyanoides (L.) Rchb.s.l.	0	n	1	0	Asteraceae Bercht. & J.Presl
Iz_7_1009	DJI_0455	Koeleria glauca (Spreng.) DC.	0	n	1	0	Poaceae Barnhart
Iz_7_1009	DJI_0455	Lactuca serriola L.	0	a	0	0	Asteraceae Bercht. & J.Presl
Iz_7_1009	DJI_0455	Linaria genistifolia (L.) Mill.	0	n	1	0	Plantaginaceae Juss.
Iz_7_1009	DJI_0455	Lomelosia argentea (L.) Greuter & Burdet	0	n	1	0	Caprifoliaceae Juss.
Iz_7_1009	DJI_0455	Melampyrum pratense L.	0	n	1	0	Orobanchaceae Vent.
Iz_7_1009	DJI_0455	Oenothera biennis L.	0	a	0	0	Onagraceae Juss.
Iz_7_1009	DJI_0455	Pentanema britannica (L.) D.Gut.Larr., Santos-Vicente, Anderb., E.Rico & M.M.Mart.Ort.	0	n	1	0	Asteraceae Bercht. & J.Presl
Iz_7_1009	DJI_0455	Peucedanum oreoselinum (L.) Moench	0	n	1	0	Apiaceae Lindl.
Iz_7_1009	DJI_0455	Peucedanum ruthenicum M.Bieb.	0	n	1	0	Apiaceae Lindl.
Iz_7_1009	DJI_0455	Pilosella echioides (Lumn.) F.W.Schultz & Sch.Bip.	0	n	1	0	Asteraceae Bercht. & J.Presl
Iz_7_1009	DJI_0455	Pilosella officinarum F. Schultz et Sch. Bip.	0	n	1	0	Asteraceae Bercht. & J.Presl
Iz_7_1009	DJI_0455	Pilosella sp.	0		NA	0	Asteraceae Bercht. & J.Presl
Iz_7_1009	DJI_0455	Pinus sylvestris L.	0	n	1	1	Pinaceae Spreng. ex F.Rudolphi
Iz_7_1009	DJI_0455	Poa annua L.	0	n	1	0	Poaceae Barnhart
Iz_7_1009	DJI_0455	Poa nemoralis L.	0	n	1	0	Poaceae Barnhart
Iz_7_1009	DJI_0455	Poa sp.	0		NA	0	Poaceae Barnhart
Iz_7_1009	DJI_0455	Polygonatum odoratum (Mill.) Druce	0	n	1	0	Asparagaceae Juss.
Iz_7_1009	DJI_0455	Polygonum arenarium Waldst. & Kit.	0	n	1	0	Polygonaceae Juss.
Iz_7_1009	DJI_0455	Populus nigra L.	0	n	1	1	Salicaceae Mirb.
Iz_7_1009	DJI_0455	Populus tremula L.	0	n	1	1	Salicaceae Mirb.
Iz_7_1009	DJI_0455	Populus Ã canescens (Aiton) Sm.	0	n	1	1	Salicaceae Mirb.
Iz_7_1009	DJI_0455	Potentilla argentea L.	0	n	1	0	Rosaceae Juss.
Iz_7_1009	DJI_0455	Psammophiliella muralis (L.) Ikonn.	0	n	1	0	Caryophyllaceae Juss.
Iz_7_1009	DJI_0455	Psephellus sumensis (Kalen.) Greuter	0	n	1	0	Asteraceae Bercht. & J.Presl
Iz_7_1009	DJI_0455	Pulsatilla pratensis (L.) Mill. s.l.	0	n	1	0	Ranunculaceae Juss.
Iz_7_1009	DJI_0455	Pyrus communis L.	0	a	0	1	Rosaceae Juss.
Iz_7_1009	DJI_0455	Quercus robur L.	0	n	1	1	Fagaceae Dumort.
Iz_7_1009	DJI_0455	Robinia pseudoacacia L.	0	a	0	1	Fabaceae Lindl.
Iz_7_1009	DJI_0455	Rosa sp.	0		NA	1	Rosaceae Juss.
Iz_7_1009	DJI_0455	Rubus idaeus L.	0	n	1	0	Rosaceae Juss.
Iz_7_1009	DJI_0455	Rumex acetosella L.	0	n	1	0	Polygonaceae Juss.
Iz_7_1009	DJI_0455	Rumex thyrsiflorus Fingerh.	0	n	1	0	Polygonaceae Juss.
Iz_7_1009	DJI_0455	Salix alba L.	0	n	1	1	Salicaceae Mirb.
Iz_7_1009	DJI_0455	Sambucus nigra L.	0	n	1	1	Viburnaceae Raf.
Iz_7_1009	DJI_0455	Secale sylvestre Host	0	n	1	0	Poaceae Barnhart
Iz_7_1009	DJI_0455	Sempervivum ruthenicum Schnittsp. & C.B. Lehm.	0	n	1	0	Crassulaceae J.St.-Hil.
Iz_7_1009	DJI_0455	Setaria viridis (L.) P. Beauv.	0	n	1	0	Poaceae Barnhart
Iz_7_1009	DJI_0455	Silene borysthenica (Gruner) Walters	0	n	1	0	Caryophyllaceae Juss.
Iz_7_1009	DJI_0455	Silene latifolia subsp. alba (Mill.) Greuter & Burdet	0	n	1	0	Caryophyllaceae Juss.
Iz_7_1009	DJI_0455	Solidago virgaurea L.	0	n	1	0	Asteraceae Bercht. & J.Presl
Iz_7_1009	DJI_0455	Sorbus aucuparia L.	0	n	1	1	Rosaceae Juss.
Iz_7_1009	DJI_0455	Stachys recta L.	0	n	1	0	Lamiacea Lindl.
Iz_7_1009	DJI_0455	Tanacetum vulgare L.	0	n	1	0	Asteraceae Bercht. & J.Presl
Iz_7_1009	DJI_0455	Thymus pallasianus Heinr.Braun	0	n	1	0	Lamiacea Lindl.
Iz_7_1009	DJI_0455	Tilia cordata Mill.	0	n	1	1	Malvaceae Juss.
Iz_7_1009	DJI_0455	Trifolium alpestre L.	0	n	1	0	Fabaceae Lindl.
Iz_7_1009	DJI_0455	Trifolium arvense L.	0	n	1	0	Fabaceae Lindl.
Iz_7_1009	DJI_0455	Ulmus glabra Huds.	0	n	1	1	Ulmaceae Mirb.
Iz_7_1009	DJI_0455	Verbascum densiflorum Bertol.	0	n	1	0	Scrophulariaceae Juss.
Iz_7_1009	DJI_0455	Veronica austriaca L.	0	n	1	0	Plantaginaceae Juss.
Iz_7_1009	DJI_0455	Veronica incana L.	0	n	1	0	Plantaginaceae Juss.
Iz_7_1009	DJI_0455	Veronica verna L.	0	n	1	0	Plantaginaceae Juss.
Iz_7_1009	DJI_0455	Vincetoxicum hirundinaria Medik	0	n	1	0	Apocynaceae Juss.
Iz_7_1009	DJI_0458	Acer tataricum L.	0	n	1	1	Sapindaceae Juss.
Iz_7_1009	DJI_0458	Achillea millefolium L. s.l.	0	n	1	0	Asteraceae Bercht. & J.Presl
Iz_7_1009	DJI_0458	Allium oleraceum L.	0	n	1	0	Amaryllidaceae J.St.-Hil.
Iz_7_1009	DJI_0458	Ambrosia artemisiifolia L.	0	a	0	0	Asteraceae Bercht. & J.Presl
Iz_7_1009	DJI_0458	Amorpha fruticosa L.	0	a	0	0	Fabaceae Lindl.
Iz_7_1009	DJI_0458	Anthericum ramosum L.	0	n	1	0	Asparagaceae Juss.
Iz_7_1009	DJI_0458	Artemisia marschalliana Spreng.	0	n	1	0	Asteraceae Bercht. & J.Presl
Iz_7_1009	DJI_0458	Asparagus officinalis L.	0	n	1	0	Asparagaceae Juss.
Iz_7_1009	DJI_0458	Astragalus cicer L.	0	n	1	0	Fabaceae Lindl.
Iz_7_1009	DJI_0458	Bassia laniflora (S.G.Gmel.) A.J.Scott	0	n	1	0	Amaranthaceae Juss.
Iz_7_1009	DJI_0458	Berberis vulgaris L.	0	n	1	0	Berberidaceae Juss.
Iz_7_1009	DJI_0458	Betula pendula Roth.	0	n	1	1	Betulaceae Gray
Iz_7_1009	DJI_0458	Betula pendula Roth.	0	n	1	0	Poaceae Barnhart
Iz_7_1009	DJI_0458	Bromus inermis Leyss.	0		NA	NA	
Iz_7_1009	DJI_0458	Bromus squarrosus L.	0	a	0	0	Poaceae Barnhart
Iz_7_1009	DJI_0458	Bromus tectorum L.	0	a	0	0	Poaceae Barnhart
Iz_7_1009	DJI_0458	Calamagrostis epigejos (L.) Roth	5	n	1	0	Poaceae Barnhart
Iz_7_1009	DJI_0458	Carduus acanthoides L.	0	n	1	0	Asteraceae Bercht. & J.Presl
Iz_7_1009	DJI_0458	Carex sp.	0	n	1	0	Cyperaceae Juss.
Iz_7_1009	DJI_0458	Centaurea majorovii Dumbadze	0	n	1	0	Asteraceae Bercht. & J.Presl
Iz_7_1009	DJI_0458	Chamaecytisus ruthenicus (Fisch.ex Wol.) Klaskova	0	n	1	0	Fabaceae Lindl.
Iz_7_1009	DJI_0458	Chelidonium majus L.	0	n	1	0	Papaveraceae Juss.
Iz_7_1009	DJI_0458	Chenopodium betaceum Andrz.	0	n	1	0	Amaranthaceae Juss.
Iz_7_1009	DJI_0458	Chenopodium cf. album	0	n	1	0	Amaranthaceae Juss.
Iz_7_1009	DJI_0458	Chenopodium sp.	0		NA	0	Amaranthaceae Juss.
Iz_7_1009	DJI_0458	Chondrilla juncea L.	0	n	1	0	Asteraceae Bercht. & J.Presl
Iz_7_1009	DJI_0458	Cirsium vulgare (Savi) Ten.	0	n	1	0	Asteraceae Bercht. & J.Presl
Iz_7_1009	DJI_0458	Clinopodium acinos (L.) Kuntze	0	n	1	0	Lamiacea Lindl.
Iz_7_1009	DJI_0458	Convallaria majalis L.	0	n	1	0	Asparagaceae Juss.
Iz_7_1009	DJI_0458	Crataegus rhipidophylla Gand.	0	n	1	1	Rosaceae Juss.

Iz_7_1009	DJI_0458	Crepis foetida L.	0	n	1	0	Asteraceae Bercht. & J.Presl
Iz_7_1009	DJI_0458	Dactylis glomerata L.	0	n	1	0	Poaceae Barnhart
Iz_7_1009	DJI_0458	Dianthus campestris M.Bieb.	0	n	1	0	Caryophyllaceae Juss.
Iz_7_1009	DJI_0458	Digitaria sanguinalis (L.) Scop.	0	a	0	0	Poaceae Barnhart
Iz_7_1009	DJI_0458	Elymus repens (L.) Gould	0	n	1	0	Poaceae Barnhart
Iz_7_1009	DJI_0458	Epilobium tetragonum L.	0	n	1	0	Onagraceae Juss.
Iz_7_1009	DJI_0458	Eragrostis minor Host	0	a	0	0	Poaceae Barnhart
Iz_7_1009	DJI_0458	Erigeron canadensis L.	20	a	0	0	Asteraceae Bercht. & J.Presl
Iz_7_1009	DJI_0458	Euphorbia seguieriana Neck.	0	n	1	0	Euphorbiaceae Juss.
Iz_7_1009	DJI_0458	Fallopia dumetorum (L.) Holub	0	n	1	0	Polygonaceae Juss.
Iz_7_1009	DJI_0458	Festuca beckeri (Hack.) Trautv.	0	n	1	0	Poaceae Barnhart
Iz_7_1009	DJI_0458	Fragaria vesca L.	0	n	1	0	Rosaceae Juss.
Iz_7_1009	DJI_0458	Frangula alnus Mill.	0	n	1	1	Rhamnaceae Juss.
Iz_7_1009	DJI_0458	Fraxinus pennsylvanica Marshall	0	a	0	1	Oleaceae Hoffmanns. & Link
Iz_7_1009	DJI_0458	Galeopsis bifida Boenn.	0	n	1	0	Lamiacea Lindl.
Iz_7_1009	DJI_0458	Galium verum L.	0	n	1	0	Rubiaceae Juss.
Iz_7_1009	DJI_0458	Genista tinctoria L.	0	n	1	0	Fabaceae Lindl.
Iz_7_1009	DJI_0458	Geranium robertianum L.	0	n	1	0	Geraniaceae Juss.
Iz_7_1009	DJI_0458	Geranium sanquineum L.	0	n	1	0	Geraniaceae Juss.
Iz_7_1009	DJI_0458	Gypsophila paniculata L.	0	n	1	0	Caryophyllaceae Juss.
Iz_7_1009	DJI_0458	Helichrysum arenarium (L.) Moench	0	n	1	0	Asteraceae Bercht. & J.Presl
Iz_7_1009	DJI_0458	Herniaria polygama J.Gay	0	n	1	0	Caryophyllaceae Juss.
Iz_7_1009	DJI_0458	Hieracium robustum Fr.	0	n	1	0	Asteraceae Bercht. & J.Presl
Iz_7_1009	DJI_0458	Hieracium umbellatum L.	0	n	1	NA	Asteraceae Bercht. & J.Presl
Iz_7_1009	DJI_0458	Hylotelephium maximum (L.) Holub	0	n	1	0	Crassulaceae J.St.-Hil.
Iz_7_1009	DJI_0458	Jacobaea borysthenica (DC.) B.Nord. & Greuter	0	n	1	0	Asteraceae Bercht. & J.Presl
Iz_7_1009	DJI_0458	Jasione montana L.	0	n	1	0	Campanulaceae Juss.
Iz_7_1009	DJI_0458	Jurinea cyanoides (L.) Rchb.s.l.	0	n	1	0	Asteraceae Bercht. & J.Presl
Iz_7_1009	DJI_0458	Koeleria glauca (Spreng.) DC.	0	n	1	0	Poaceae Barnhart
Iz_7_1009	DJI_0458	Lactuca serriola L.	0	a	0	0	Asteraceae Bercht. & J.Presl
Iz_7_1009	DJI_0458	Linaria genistifolia (L.) Mill.	0	n	1	0	Plantaginaceae Juss.
Iz_7_1009	DJI_0458	Lomelosia argentea (L.) Greuter & Burdet	0	n	1	0	Caprifoliaceae Juss.
Iz_7_1009	DJI_0458	Melampyrum pratense L.	0	n	1	0	Orobanchaceae Vent.
Iz_7_1009	DJI_0458	Oenothera biennis L.	0	a	0	0	Onagraceae Juss.
Iz_7_1009	DJI_0458	Pentanema britannica (L.) D.Gut.Larr., Santos-Vicente, Anderb., E.Rico & M.M.Mart.Ort.	0	n	1	0	Asteraceae Bercht. & J.Presl
Iz_7_1009	DJI_0458	Peucedanum oreoselinum (L.) Moench	0	n	1	0	Apiaceae Lindl.
Iz_7_1009	DJI_0458	Peucedanum ruthenicum M.Bieb.	0	n	1	0	Apiaceae Lindl.
Iz_7_1009	DJI_0458	Pilosella echioides (Lumn.) F.W.Schultz & Sch.Bip.	0	n	1	0	Asteraceae Bercht. & J.Presl
Iz_7_1009	DJI_0458	Pilosella officinarum F. Schultz et Sch. Bip.	0	n	1	0	Asteraceae Bercht. & J.Presl
Iz_7_1009	DJI_0458	Pilosella sp.	0		NA	0	Asteraceae Bercht. & J.Presl
Iz_7_1009	DJI_0458	Pinus sylvestris L.	0	n	1	1	Pinaceae Spreng. ex F.Rudolphi
Iz_7_1009	DJI_0458	Poa annua L.	0	n	1	0	Poaceae Barnhart
Iz_7_1009	DJI_0458	Poa nemoralis L.	0	n	1	0	Poaceae Barnhart
Iz_7_1009	DJI_0458	Poa sp.	0		NA	0	Poaceae Barnhart
Iz_7_1009	DJI_0458	Polygonatum odoratum (Mill.) Druce	0	n	1	0	Asparagaceae Juss.
Iz_7_1009	DJI_0458	Polygonum arenarium Waldst. & Kit.	0	n	1	0	Polygonaceae Juss.
Iz_7_1009	DJI_0458	Populus nigra L.	0	n	1	1	Salicaceae Mirb.
Iz_7_1009	DJI_0458	Populus tremula L.	0	n	1	1	Salicaceae Mirb.
Iz_7_1009	DJI_0458	Populus Ã canescens (Aiton) Sm.	0	n	1	1	Salicaceae Mirb.
Iz_7_1009	DJI_0458	Potentilla argentea L.	0	n	1	0	Rosaceae Juss.
Iz_7_1009	DJI_0458	Psammophiliella muralis (L.) Ikonn.	0	n	1	0	Caryophyllaceae Juss.
Iz_7_1009	DJI_0458	Psephellus sumensis (Kalen.) Greuter	0	n	1	0	Asteraceae Bercht. & J.Presl
Iz_7_1009	DJI_0458	Pulsatilla pratensis (L.) Mill. s.l.	0	n	1	0	Ranunculaceae Juss.
Iz_7_1009	DJI_0458	Pyrus communis L.	0	a	0	1	Rosaceae Juss.
Iz_7_1009	DJI_0458	Quercus robur L.	0	n	1	1	Fagaceae Dumort.
Iz_7_1009	DJI_0458	Robinia pseudoacacia L.	0	a	0	1	Fabaceae Lindl.
Iz_7_1009	DJI_0458	Rosa sp.	0		NA	1	Rosaceae Juss.
Iz_7_1009	DJI_0458	Rubus idaeus L.	0	n	1	0	Rosaceae Juss.
Iz_7_1009	DJI_0458	Rumex acetosella L.	0	n	1	0	Polygonaceae Juss.
Iz_7_1009	DJI_0458	Rumex thyrsiflorus Fingerh.	0	n	1	0	Polygonaceae Juss.
Iz_7_1009	DJI_0458	Salix alba L.	0	n	1	1	Salicaceae Mirb.
Iz_7_1009	DJI_0458	Sambucus nigra L.	0	n	1	1	Viburnaceae Raf.
Iz_7_1009	DJI_0458	Secale sylvestre Host	0	n	1	0	Poaceae Barnhart
Iz_7_1009	DJI_0458	Sempervivum ruthenicum Schnittsp. & C.B. Lehm.	0	n	1	0	Crassulaceae J.St.-Hil.
Iz_7_1009	DJI_0458	Setaria viridis (L.) P. Beauv.	0	n	1	0	Poaceae Barnhart
Iz_7_1009	DJI_0458	Silene borysthenica (Gruner) Walters	0	n	1	0	Caryophyllaceae Juss.
Iz_7_1009	DJI_0458	Silene latifolia subsp. alba (Mill.) Greuter & Burdet	0	n	1	0	Caryophyllaceae Juss.
Iz_7_1009	DJI_0458	Solidago virgaurea L.	0	n	1	0	Asteraceae Bercht. & J.Presl
Iz_7_1009	DJI_0458	Sorbus aucuparia L.	0	n	1	1	Rosaceae Juss.
Iz_7_1009	DJI_0458	Stachys recta L.	0	n	1	0	Lamiacea Lindl.
Iz_7_1009	DJI_0458	Tanacetum vulgare L.	0	n	1	0	Asteraceae Bercht. & J.Presl
Iz_7_1009	DJI_0458	Thymus pallasianus Heinr.Braun	0	n	1	0	Lamiacea Lindl.
Iz_7_1009	DJI_0458	Tilia cordata Mill.	0	n	1	1	Malvaceae Juss.
Iz_7_1009	DJI_0458	Trifolium alpestre L.	0	n	1	0	Fabaceae Lindl.
Iz_7_1009	DJI_0458	Trifolium arvense L.	0	n	1	0	Fabaceae Lindl.
Iz_7_1009	DJI_0458	Ulmus glabra Huds.	0	n	1	1	Ulmaceae Mirb.
Iz_7_1009	DJI_0458	Verbascum densiflorum Bertol.	0	n	1	0	Scrophulariaceae Juss.
Iz_7_1009	DJI_0458	Veronica austriaca L.	0	n	1	0	Plantaginaceae Juss.
Iz_7_1009	DJI_0458	Veronica incana L.	0	n	1	0	Plantaginaceae Juss.
Iz_7_1009	DJI_0458	Veronica verna L.	0	n	1	0	Plantaginaceae Juss.
Iz_7_1009	DJI_0458	Vincetoxicum hirundinaria Medik	0	n	1	0	Apocynaceae Juss.
Iz_7_1009	DJI_0462	Acer tataricum L.	0	n	1	1	Sapindaceae Juss.
Iz_7_1009	DJI_0462	Achillea millefolium L. s.l.	0	n	1	0	Asteraceae Bercht. & J.Presl
Iz_7_1009	DJI_0462	Allium oleraceum L.	0	n	1	0	Amaryllidaceae J.St.-Hil.
Iz_7_1009	DJI_0462	Ambrosia artemisiifolia L.	0	a	0	0	Asteraceae Bercht. & J.Presl
Iz_7_1009	DJI_0462	Amorpha fruticosa L.	0	a	0	0	Fabaceae Lindl.
Iz_7_1009	DJI_0462	Anthericum ramosum L.	0	n	1	0	Asparagaceae Juss.
Iz_7_1009	DJI_0462	Artemisia marschalliana Spreng.	0	n	1	0	Asteraceae Bercht. & J.Presl
Iz_7_1009	DJI_0462	Asparagus officinalis L.	0	n	1	0	Asparagaceae Juss.
Iz_7_1009	DJI_0462	Astragalus cicer L.	0	n	1	0	Fabaceae Lindl.
Iz_7_1009	DJI_0462	Bassia laniflora (S.G.Gmel.) A.J.Scott	0	n	1	0	Amaranthaceae Juss.
Iz_7_1009	DJI_0462	Berberis vulgaris L.	0	n	1	0	Berberidaceae Juss.
Iz_7_1009	DJI_0462	Betula pendula Roth.	0	n	1	1	Betulaceae Gray
Iz_7_1009	DJI_0462	Betula pendula Roth.	0	n	1	0	Poaceae Barnhart
Iz_7_1009	DJI_0462	Bromus inermis Leyss.	0		NA	NA	
Iz_7_1009	DJI_0462	Bromus squarrosus L.	0	a	0	0	Poaceae Barnhart
Iz_7_1009	DJI_0462	Bromus tectorum L.	0	a	0	0	Poaceae Barnhart
Iz_7_1009	DJI_0462	Calamagrostis epigejos (L.) Roth	0	n	1	0	Poaceae Barnhart

Iz_7_1009	DJI_0462	Carduus acanthoides L.	0	n	1	0	Asteraceae Bercht. & J.Presl
Iz_7_1009	DJI_0462	Carex sp.	0	n	1	0	Cyperaceae Juss.
Iz_7_1009	DJI_0462	Centaurea majorovii Dumbadze	0	n	1	0	Asteraceae Bercht. & J.Presl
Iz_7_1009	DJI_0462	Chamaecytisus ruthenicus (Fisch.ex Wol.) Klaskova	0	n	1	0	Fabaceae Lindl.
Iz_7_1009	DJI_0462	Chelidonium majus L.	0	n	1	0	Papaveraceae Juss.
Iz_7_1009	DJI_0462	Chenopodium betaceum Andrz.	0	n	1	0	Amaranthaceae Juss.
Iz_7_1009	DJI_0462	Chenopodium cf. album	0	n	1	0	Amaranthaceae Juss.
Iz_7_1009	DJI_0462	Chenopodium sp.	0		NA	0	Amaranthaceae Juss.
Iz_7_1009	DJI_0462	Chondrilla juncea L.	0	n	1	0	Asteraceae Bercht. & J.Presl
Iz_7_1009	DJI_0462	Cirsium vulgare (Savi) Ten.	0	n	1	0	Asteraceae Bercht. & J.Presl
Iz_7_1009	DJI_0462	Clinopodium acinos (L.) Kuntze	0	n	1	0	Lamiacea Lindl.
Iz_7_1009	DJI_0462	Convallaria majalis L.	0	n	1	0	Asparagaceae Juss.
Iz_7_1009	DJI_0462	Crataegus rhipidophylla Gand.	0	n	1	1	Rosaceae Juss.
Iz_7_1009	DJI_0462	Crepis foetida L.	0	n	1	0	Asteraceae Bercht. & J.Presl
Iz_7_1009	DJI_0462	Dactylis glomerata L.	0	n	1	0	Poaceae Barnhart
Iz_7_1009	DJI_0462	Dianthus campestris M.Bieb.	0	n	1	0	Caryophyllaceae Juss.
Iz_7_1009	DJI_0462	Digitaria sanguinalis (L.) Scop.	0	a	0	0	Poaceae Barnhart
Iz_7_1009	DJI_0462	Elymus repens (L.) Gould	0	n	1	0	Poaceae Barnhart
Iz_7_1009	DJI_0462	Epilobium tetragonum L.	0	n	1	0	Onagraceae Juss.
Iz_7_1009	DJI_0462	Eragrostis minor Host	0	a	0	0	Poaceae Barnhart
Iz_7_1009	DJI_0462	Erigeron canadensis L.	5	a	0	0	Asteraceae Bercht. & J.Presl
Iz_7_1009	DJI_0462	Euphorbia seguieriana Neck.	0	n	1	0	Euphorbiaceae Juss.
Iz_7_1009	DJI_0462	Fallopia dumetorum (L.) Holub	0	n	1	0	Polygonaceae Juss.
Iz_7_1009	DJI_0462	Festuca beckeri (Hack.) Trautv.	0	n	1	0	Poaceae Barnhart
Iz_7_1009	DJI_0462	Fragaria vesca L.	0	n	1	0	Rosaceae Juss.
Iz_7_1009	DJI_0462	Frangula alnus Mill.	0	n	1	1	Rhamnaceae Juss.
Iz_7_1009	DJI_0462	Fraxinus pennsylvanica Marshall	0	a	0	1	Oleaceae Hoffmanns. & Link
Iz_7_1009	DJI_0462	Galeopsis bifida Boenn.	0	n	1	0	Lamiacea Lindl.
Iz_7_1009	DJI_0462	Galium verum L.	0	n	1	0	Rubiaceae Juss.
Iz_7_1009	DJI_0462	Genista tinctoria L.	0	n	1	0	Fabaceae Lindl.
Iz_7_1009	DJI_0462	Geranium robertianum L.	0	n	1	0	Geraniaceae Juss.
Iz_7_1009	DJI_0462	Geranium sanquineum L.	0	n	1	0	Geraniaceae Juss.
Iz_7_1009	DJI_0462	Gypsophila paniculata L.	0	n	1	0	Caryophyllaceae Juss.
Iz_7_1009	DJI_0462	Helichrysum arenarium (L.) Moench	0	n	1	0	Asteraceae Bercht. & J.Presl
Iz_7_1009	DJI_0462	Herniaria polygama J.Gay	0	n	1	0	Caryophyllaceae Juss.
Iz_7_1009	DJI_0462	Hieracium robustum Fr.	0	n	1	0	Asteraceae Bercht. & J.Presl
Iz_7_1009	DJI_0462	Hieracium umbellatum L.	0	n	1	NA	Asteraceae Bercht. & J.Presl
Iz_7_1009	DJI_0462	Hylotelephium maximum (L.) Holub	0	n	1	0	Crassulaceae J.St.-Hil.
Iz_7_1009	DJI_0462	Jacobaea borysthenica (DC.) B.Nord. & Greuter	0	n	1	0	Asteraceae Bercht. & J.Presl
Iz_7_1009	DJI_0462	Jasione montana L.	0	n	1	0	Campanulaceae Juss.
Iz_7_1009	DJI_0462	Jurinea cyanoides (L.) Rchb.s.l.	0	n	1	0	Asteraceae Bercht. & J.Presl
Iz_7_1009	DJI_0462	Koeleria glauca (Spreng.) DC.	0	n	1	0	Poaceae Barnhart
Iz_7_1009	DJI_0462	Lactuca serriola L.	0	a	0	0	Asteraceae Bercht. & J.Presl
Iz_7_1009	DJI_0462	Linaria genistifolia (L.) Mill.	0	n	1	0	Plantaginaceae Juss.
Iz_7_1009	DJI_0462	Lomelosia argentea (L.) Greuter & Burdet	0	n	1	0	Caprifoliaceae Juss.
Iz_7_1009	DJI_0462	Melampyrum pratense L.	0	n	1	0	Orobanchaceae Vent.
Iz_7_1009	DJI_0462	Oenothera biennis L.	0	a	0	0	Onagraceae Juss.
Iz_7_1009	DJI_0462	Pentanema britannica (L.) D.Gut.Larr., Santos-Vicente, Anderb., E.Rico & M.M.Mart.Ort.	0	n	1	0	Asteraceae Bercht. & J.Presl
Iz_7_1009	DJI_0462	Peucedanum oreoselinum (L.) Moench	0	n	1	0	Apiaceae Lindl.
Iz_7_1009	DJI_0462	Peucedanum ruthenicum M.Bieb.	0	n	1	0	Apiaceae Lindl.
Iz_7_1009	DJI_0462	Pilosella echioides (Lumn.) F.W.Schultz & Sch.Bip.	0	n	1	0	Asteraceae Bercht. & J.Presl
Iz_7_1009	DJI_0462	Pilosella officinarum F. Schultz et Sch. Bip.	0	n	1	0	Asteraceae Bercht. & J.Presl
Iz_7_1009	DJI_0462	Pilosella sp.	0		NA	0	Asteraceae Bercht. & J.Presl
Iz_7_1009	DJI_0462	Pinus sylvestris L.	0	n	1	1	Pinaceae Spreng. ex F.Rudolphi
Iz_7_1009	DJI_0462	Poa annua L.	0	n	1	0	Poaceae Barnhart
Iz_7_1009	DJI_0462	Poa nemoralis L.	0	n	1	0	Poaceae Barnhart
Iz_7_1009	DJI_0462	Poa sp.	0		NA	0	Poaceae Barnhart
Iz_7_1009	DJI_0462	Polygonatum odoratum (Mill.) Druce	0	n	1	0	Asparagaceae Juss.
Iz_7_1009	DJI_0462	Polygonum arenarium Waldst. & Kit.	0	n	1	0	Polygonaceae Juss.
Iz_7_1009	DJI_0462	Populus nigra L.	0	n	1	1	Salicaceae Mirb.
Iz_7_1009	DJI_0462	Populus tremula L.	0	n	1	1	Salicaceae Mirb.
Iz_7_1009	DJI_0462	Populus Ã canescens (Aiton) Sm.	0	n	1	1	Salicaceae Mirb.
Iz_7_1009	DJI_0462	Potentilla argentea L.	0	n	1	0	Rosaceae Juss.
Iz_7_1009	DJI_0462	Psammophiliella muralis (L.) Ikonn.	0	n	1	0	Caryophyllaceae Juss.
Iz_7_1009	DJI_0462	Psephellus sumensis (Kalen.) Greuter	0	n	1	0	Asteraceae Bercht. & J.Presl
Iz_7_1009	DJI_0462	Pulsatilla pratensis (L.) Mill. s.l.	0	n	1	0	Ranunculaceae Juss.
Iz_7_1009	DJI_0462	Pyrus communis L.	0	a	0	1	Rosaceae Juss.
Iz_7_1009	DJI_0462	Quercus robur L.	0	n	1	1	Fagaceae Dumort.
Iz_7_1009	DJI_0462	Robinia pseudoacacia L.	0	a	0	1	Fabaceae Lindl.
Iz_7_1009	DJI_0462	Rosa sp.	0		NA	1	Rosaceae Juss.
Iz_7_1009	DJI_0462	Rubus idaeus L.	0	n	1	0	Rosaceae Juss.
Iz_7_1009	DJI_0462	Rumex acetosella L.	0	n	1	0	Polygonaceae Juss.
Iz_7_1009	DJI_0462	Rumex thyrsiflorus Fingerh.	0	n	1	0	Polygonaceae Juss.
Iz_7_1009	DJI_0462	Salix alba L.	0	n	1	1	Salicaceae Mirb.
Iz_7_1009	DJI_0462	Sambucus nigra L.	0	n	1	1	Viburnaceae Raf.
Iz_7_1009	DJI_0462	Secale sylvestre Host	0	n	1	0	Poaceae Barnhart
Iz_7_1009	DJI_0462	Sempervivum ruthenicum Schnittsp. & C.B. Lehm.	0	n	1	0	Crassulaceae J.St.-Hil.
Iz_7_1009	DJI_0462	Setaria viridis (L.) P. Beauv.	0	n	1	0	Poaceae Barnhart
Iz_7_1009	DJI_0462	Silene borysthenica (Gruner) Walters	0	n	1	0	Caryophyllaceae Juss.
Iz_7_1009	DJI_0462	Silene latifolia subsp. alba (Mill.) Greuter & Burdet	0	n	1	0	Caryophyllaceae Juss.
Iz_7_1009	DJI_0462	Solidago virgaurea L.	0	n	1	0	Asteraceae Bercht. & J.Presl
Iz_7_1009	DJI_0462	Sorbus aucuparia L.	0	n	1	1	Rosaceae Juss.
Iz_7_1009	DJI_0462	Stachys recta L.	0	n	1	0	Lamiacea Lindl.
Iz_7_1009	DJI_0462	Tanacetum vulgare L.	0	n	1	0	Asteraceae Bercht. & J.Presl
Iz_7_1009	DJI_0462	Thymus pallasianus Heinr.Braun	0	n	1	0	Lamiacea Lindl.
Iz_7_1009	DJI_0462	Tilia cordata Mill.	0	n	1	1	Malvaceae Juss.
Iz_7_1009	DJI_0462	Trifolium alpestre L.	0	n	1	0	Fabaceae Lindl.
Iz_7_1009	DJI_0462	Trifolium arvense L.	0	n	1	0	Fabaceae Lindl.
Iz_7_1009	DJI_0462	Ulmus glabra Huds.	0	n	1	1	Ulmaceae Mirb.
Iz_7_1009	DJI_0462	Verbascum densiflorum Bertol.	0	n	1	0	Scrophulariaceae Juss.
Iz_7_1009	DJI_0462	Veronica austriaca L.	0	n	1	0	Plantaginaceae Juss.
Iz_7_1009	DJI_0462	Veronica incana L.	0	n	1	0	Plantaginaceae Juss.
Iz_7_1009	DJI_0462	Veronica verna L.	0	n	1	0	Plantaginaceae Juss.
Iz_7_1009	DJI_0462	Vincetoxicum hirundinaria Medik	0	n	1	0	Apocynaceae Juss.
Iz_7_1009	DJI_0466	Acer tataricum L.	0	n	1	1	Sapindaceae Juss.
Iz_7_1009	DJI_0466	Achillea millefolium L. s.l.	0	n	1	0	Asteraceae Bercht. & J.Presl
Iz_7_1009	DJI_0466	Allium oleraceum L.	0	n	1	0	Amaryllidaceae J.St.-Hil.
Iz_7_1009	DJI_0466	Ambrosia artemisiifolia L.	0	a	0	0	Asteraceae Bercht. & J.Presl

Iz_7_1009	DJI_0466	Amorpha fruticosa L.	0	a	0	0	Fabaceae Lindl.
Iz_7_1009	DJI_0466	Anthericum ramosum L.	0	n	1	0	Asparagaceae Juss.
Iz_7_1009	DJI_0466	Artemisia marschalliana Spreng.	0	n	1	0	Asteraceae Bercht. & J.Presl
Iz_7_1009	DJI_0466	Asparagus officinalis L.	0	n	1	0	Asparagaceae Juss.
Iz_7_1009	DJI_0466	Astragalus cicer L.	0	n	1	0	Fabaceae Lindl.
Iz_7_1009	DJI_0466	Bassia laniflora (S.G.Gmel.) A.J.Scott	0	n	1	0	Amaranthaceae Juss.
Iz_7_1009	DJI_0466	Berberis vulgaris L.	0	n	1	0	Berberidaceae Juss.
Iz_7_1009	DJI_0466	Betula pendula Roth.	0	n	1	1	Betulaceae Gray
Iz_7_1009	DJI_0466	Betula pendula Roth.	0	n	1	0	Poaceae Barnhart
Iz_7_1009	DJI_0466	Bromus inermis Leyss.	0		NA	NA	
Iz_7_1009	DJI_0466	Bromus squarrosus L.	0	a	0	0	Poaceae Barnhart
Iz_7_1009	DJI_0466	Bromus tectorum L.	0	a	0	0	Poaceae Barnhart
Iz_7_1009	DJI_0466	Calamagrostis epigejos (L.) Roth	0	n	1	0	Poaceae Barnhart
Iz_7_1009	DJI_0466	Carduus acanthoides L.	0	n	1	0	Asteraceae Bercht. & J.Presl
Iz_7_1009	DJI_0466	Carex sp.	0	n	1	0	Cyperaceae Juss.
Iz_7_1009	DJI_0466	Centaurea majorovii Dumbadze	0	n	1	0	Asteraceae Bercht. & J.Presl
Iz_7_1009	DJI_0466	Chamaecytisus ruthenicus (Fisch.ex Wol.) Klaskova	0	n	1	0	Fabaceae Lindl.
Iz_7_1009	DJI_0466	Chelidonium majus L.	0	n	1	0	Papaveraceae Juss.
Iz_7_1009	DJI_0466	Chenopodium betaceum Andrz.	0	n	1	0	Amaranthaceae Juss.
Iz_7_1009	DJI_0466	Chenopodium cf. album	0	n	1	0	Amaranthaceae Juss.
Iz_7_1009	DJI_0466	Chenopodium sp.	0		NA	0	Amaranthaceae Juss.
Iz_7_1009	DJI_0466	Chondrilla juncea L.	0	n	1	0	Asteraceae Bercht. & J.Presl
Iz_7_1009	DJI_0466	Cirsium vulgare (Savi) Ten.	0	n	1	0	Asteraceae Bercht. & J.Presl
Iz_7_1009	DJI_0466	Clinopodium acinos (L.) Kuntze	0	n	1	0	Lamiacea Lindl.
Iz_7_1009	DJI_0466	Convallaria majalis L.	0	n	1	0	Asparagaceae Juss.
Iz_7_1009	DJI_0466	Crataegus rhipidophylla Gand.	0	n	1	1	Rosaceae Juss.
Iz_7_1009	DJI_0466	Crepis foetida L.	0	n	1	0	Asteraceae Bercht. & J.Presl
Iz_7_1009	DJI_0466	Dactylis glomerata L.	0	n	1	0	Poaceae Barnhart
Iz_7_1009	DJI_0466	Dianthus campestris M.Bieb.	0	n	1	0	Caryophyllaceae Juss.
Iz_7_1009	DJI_0466	Digitaria sanguinalis (L.) Scop.	0	a	0	0	Poaceae Barnhart
Iz_7_1009	DJI_0466	Elymus repens (L.) Gould	0	n	1	0	Poaceae Barnhart
Iz_7_1009	DJI_0466	Epilobium tetragonum L.	0	n	1	0	Onagraceae Juss.
Iz_7_1009	DJI_0466	Eragrostis minor Host	0	a	0	0	Poaceae Barnhart
Iz_7_1009	DJI_0466	Erigeron canadensis L.	3	a	0	0	Asteraceae Bercht. & J.Presl
Iz_7_1009	DJI_0466	Euphorbia seguieriana Neck.	0	n	1	0	Euphorbiaceae Juss.
Iz_7_1009	DJI_0466	Fallopia dumetorum (L.) Holub	0	n	1	0	Polygonaceae Juss.
Iz_7_1009	DJI_0466	Festuca beckeri (Hack.) Trautv.	0	n	1	0	Poaceae Barnhart
Iz_7_1009	DJI_0466	Fragaria vesca L.	0	n	1	0	Rosaceae Juss.
Iz_7_1009	DJI_0466	Frangula alnus Mill.	0	n	1	1	Rhamnaceae Juss.
Iz_7_1009	DJI_0466	Fraxinus pennsylvanica Marshall	0	a	0	1	Oleaceae Hoffmanns. & Link
Iz_7_1009	DJI_0466	Galeopsis bifida Boenn.	0	n	1	0	Lamiacea Lindl.
Iz_7_1009	DJI_0466	Galium verum L.	0	n	1	0	Rubiaceae Juss.
Iz_7_1009	DJI_0466	Genista tinctoria L.	0	n	1	0	Fabaceae Lindl.
Iz_7_1009	DJI_0466	Geranium robertianum L.	0	n	1	0	Geraniaceae Juss.
Iz_7_1009	DJI_0466	Geranium sanquineum L.	0	n	1	0	Geraniaceae Juss.
Iz_7_1009	DJI_0466	Gypsophila paniculata L.	0	n	1	0	Caryophyllaceae Juss.
Iz_7_1009	DJI_0466	Helichrysum arenarium (L.) Moench	0	n	1	0	Asteraceae Bercht. & J.Presl
Iz_7_1009	DJI_0466	Herniaria polygama J.Gay	0	n	1	0	Caryophyllaceae Juss.
Iz_7_1009	DJI_0466	Hieracium robustum Fr.	0	n	1	0	Asteraceae Bercht. & J.Presl
Iz_7_1009	DJI_0466	Hieracium umbellatum L.	0	n	1	NA	Asteraceae Bercht. & J.Presl
Iz_7_1009	DJI_0466	Hylotelephium maximum (L.) Holub	0	n	1	0	Crassulaceae J.St.-Hil.
Iz_7_1009	DJI_0466	Jacobaea borysthenica (DC.) B.Nord. & Greuter	0	n	1	0	Asteraceae Bercht. & J.Presl
Iz_7_1009	DJI_0466	Jasione montana L.	0	n	1	0	Campanulaceae Juss.
Iz_7_1009	DJI_0466	Jurinea cyanoides (L.) Rchb.s.l.	0	n	1	0	Asteraceae Bercht. & J.Presl
Iz_7_1009	DJI_0466	Koeleria glauca (Spreng.) DC.	0	n	1	0	Poaceae Barnhart
Iz_7_1009	DJI_0466	Lactuca serriola L.	0	a	0	0	Asteraceae Bercht. & J.Presl
Iz_7_1009	DJI_0466	Linaria genistifolia (L.) Mill.	0	n	1	0	Plantaginaceae Juss.
Iz_7_1009	DJI_0466	Lomelosia argentea (L.) Greuter & Burdet	0	n	1	0	Caprifoliaceae Juss.
Iz_7_1009	DJI_0466	Melampyrum pratense L.	0	n	1	0	Orobanchaceae Vent.
Iz_7_1009	DJI_0466	Oenothera biennis L.	0	a	0	0	Onagraceae Juss.
Iz_7_1009	DJI_0466	Pentanema britannica (L.) D.Gut.Larr., Santos-Vicente, Anderb., E.Rico & M.M.Mart.Ort.	0	n	1	0	Asteraceae Bercht. & J.Presl
Iz_7_1009	DJI_0466	Peucedanum oreoselinum (L.) Moench	0	n	1	0	Apiaceae Lindl.
Iz_7_1009	DJI_0466	Peucedanum ruthenicum M.Bieb.	0	n	1	0	Apiaceae Lindl.
Iz_7_1009	DJI_0466	Pilosella echioides (Lumn.) F.W.Schultz & Sch.Bip.	0	n	1	0	Asteraceae Bercht. & J.Presl
Iz_7_1009	DJI_0466	Pilosella officinarum F. Schultz et Sch. Bip.	0	n	1	0	Asteraceae Bercht. & J.Presl
Iz_7_1009	DJI_0466	Pilosella sp.	0		NA	0	Asteraceae Bercht. & J.Presl
Iz_7_1009	DJI_0466	Pinus sylvestris L.	0	n	1	1	Pinaceae Spreng. ex F.Rudolphi
Iz_7_1009	DJI_0466	Poa annua L.	0	n	1	0	Poaceae Barnhart
Iz_7_1009	DJI_0466	Poa nemoralis L.	0	n	1	0	Poaceae Barnhart
Iz_7_1009	DJI_0466	Poa sp.	0		NA	0	Poaceae Barnhart
Iz_7_1009	DJI_0466	Polygonatum odoratum (Mill.) Druce	0	n	1	0	Asparagaceae Juss.
Iz_7_1009	DJI_0466	Polygonum arenarium Waldst. & Kit.	0	n	1	0	Polygonaceae Juss.
Iz_7_1009	DJI_0466	Populus nigra L.	0	n	1	1	Salicaceae Mirb.
Iz_7_1009	DJI_0466	Populus tremula L.	0	n	1	1	Salicaceae Mirb.
Iz_7_1009	DJI_0466	Populus Ã canescens (Aiton) Sm.	0	n	1	1	Salicaceae Mirb.
Iz_7_1009	DJI_0466	Potentilla argentea L.	0	n	1	0	Rosaceae Juss.
Iz_7_1009	DJI_0466	Psammophiliella muralis (L.) Ikonn.	0	n	1	0	Caryophyllaceae Juss.
Iz_7_1009	DJI_0466	Psephellus sumensis (Kalen.) Greuter	0	n	1	0	Asteraceae Bercht. & J.Presl
Iz_7_1009	DJI_0466	Pulsatilla pratensis (L.) Mill. s.l.	0	n	1	0	Ranunculaceae Juss.
Iz_7_1009	DJI_0466	Pyrus communis L.	0	a	0	1	Rosaceae Juss.
Iz_7_1009	DJI_0466	Quercus robur L.	0	n	1	1	Fagaceae Dumort.
Iz_7_1009	DJI_0466	Robinia pseudoacacia L.	0	a	0	1	Fabaceae Lindl.
Iz_7_1009	DJI_0466	Rosa sp.	0		NA	1	Rosaceae Juss.
Iz_7_1009	DJI_0466	Rubus idaeus L.	0	n	1	0	Rosaceae Juss.
Iz_7_1009	DJI_0466	Rumex acetosella L.	1	n	1	0	Polygonaceae Juss.
Iz_7_1009	DJI_0466	Rumex thyrsiflorus Fingerh.	0	n	1	0	Polygonaceae Juss.
Iz_7_1009	DJI_0466	Salix alba L.	0	n	1	1	Salicaceae Mirb.
Iz_7_1009	DJI_0466	Sambucus nigra L.	0	n	1	1	Viburnaceae Raf.
Iz_7_1009	DJI_0466	Secale sylvestre Host	0	n	1	0	Poaceae Barnhart
Iz_7_1009	DJI_0466	Sempervivum ruthenicum Schnittsp. & C.B. Lehm.	0	n	1	0	Crassulaceae J.St.-Hil.
Iz_7_1009	DJI_0466	Setaria viridis (L.) P. Beauv.	0	n	1	0	Poaceae Barnhart
Iz_7_1009	DJI_0466	Silene borysthenica (Gruner) Walters	0	n	1	0	Caryophyllaceae Juss.
Iz_7_1009	DJI_0466	Silene latifolia subsp. alba (Mill.) Greuter & Burdet	0	n	1	0	Caryophyllaceae Juss.
Iz_7_1009	DJI_0466	Solidago virgaurea L.	0	n	1	0	Asteraceae Bercht. & J.Presl
Iz_7_1009	DJI_0466	Sorbus aucuparia L.	0	n	1	1	Rosaceae Juss.
Iz_7_1009	DJI_0466	Stachys recta L.	0	n	1	0	Lamiacea Lindl.
Iz_7_1009	DJI_0466	Tanacetum vulgare L.	0	n	1	0	Asteraceae Bercht. & J.Presl
Iz_7_1009	DJI_0466	Thymus pallasianus Heinr.Braun	0	n	1	0	Lamiacea Lindl.

Iz_7_1009	DJI_0466	Tilia cordata Mill.	0	n	1	1	Malvaceae Juss.
Iz_7_1009	DJI_0466	Trifolium alpestre L.	0	n	1	0	Fabaceae Lindl.
Iz_7_1009	DJI_0466	Trifolium arvense L.	0	n	1	0	Fabaceae Lindl.
Iz_7_1009	DJI_0466	Ulmus glabra Huds.	0	n	1	1	Ulmaceae Mirb.
Iz_7_1009	DJI_0466	Verbascum densiflorum Bertol.	0	n	1	0	Scrophulariaceae Juss.
Iz_7_1009	DJI_0466	Veronica austriaca L.	0	n	1	0	Plantaginaceae Juss.
Iz_7_1009	DJI_0466	Veronica incana L.	0	n	1	0	Plantaginaceae Juss.
Iz_7_1009	DJI_0466	Veronica verna L.	0	n	1	0	Plantaginaceae Juss.
Iz_7_1009	DJI_0466	Vincetoxicum hirundinaria Medik	0	n	1	0	Apocynaceae Juss.
Iz_7_1009	DJI_0470	Acer tataricum L.	0	n	1	1	Sapindaceae Juss.
Iz_7_1009	DJI_0470	Achillea millefolium L. s.l.	0	n	1	0	Asteraceae Bercht. & J.Presl
Iz_7_1009	DJI_0470	Allium oleraceum L.	0	n	1	0	Amaryllidaceae J.St.-Hil.
Iz_7_1009	DJI_0470	Ambrosia artemisiifolia L.	0	a	0	0	Asteraceae Bercht. & J.Presl
Iz_7_1009	DJI_0470	Amorpha fruticosa L.	0	a	0	0	Fabaceae Lindl.
Iz_7_1009	DJI_0470	Anthericum ramosum L.	0	n	1	0	Asparagaceae Juss.
Iz_7_1009	DJI_0470	Artemisia marschalliana Spreng.	0	n	1	0	Asteraceae Bercht. & J.Presl
Iz_7_1009	DJI_0470	Asparagus officinalis L.	0	n	1	0	Asparagaceae Juss.
Iz_7_1009	DJI_0470	Astragalus cicer L.	0	n	1	0	Fabaceae Lindl.
Iz_7_1009	DJI_0470	Bassia laniflora (S.G.Gmel.) A.J.Scott	0	n	1	0	Amaranthaceae Juss.
Iz_7_1009	DJI_0470	Berberis vulgaris L.	0	n	1	0	Berberidaceae Juss.
Iz_7_1009	DJI_0470	Betula pendula Roth.	0	n	1	1	Betulaceae Gray
Iz_7_1009	DJI_0470	Betula pendula Roth.	0	n	1	0	Poaceae Barnhart
Iz_7_1009	DJI_0470	Bromus inermis Leyss.	0		NA	NA	
Iz_7_1009	DJI_0470	Bromus squarrosus L.	0	a	0	0	Poaceae Barnhart
Iz_7_1009	DJI_0470	Bromus tectorum L.	0	a	0	0	Poaceae Barnhart
Iz_7_1009	DJI_0470	Calamagrostis epigejos (L.) Roth	0	n	1	0	Poaceae Barnhart
Iz_7_1009	DJI_0470	Carduus acanthoides L.	0	n	1	0	Asteraceae Bercht. & J.Presl
Iz_7_1009	DJI_0470	Carex sp.	0	n	1	0	Cyperaceae Juss.
Iz_7_1009	DJI_0470	Centaurea majorovii Dumbadze	0	n	1	0	Asteraceae Bercht. & J.Presl
Iz_7_1009	DJI_0470	Chamaecytisus ruthenicus (Fisch.ex Wol.) Klaskova	0	n	1	0	Fabaceae Lindl.
Iz_7_1009	DJI_0470	Chelidonium majus L.	0	n	1	0	Papaveraceae Juss.
Iz_7_1009	DJI_0470	Chenopodium betaceum Andrz.	0	n	1	0	Amaranthaceae Juss.
Iz_7_1009	DJI_0470	Chenopodium cf. album	0	n	1	0	Amaranthaceae Juss.
Iz_7_1009	DJI_0470	Chenopodium sp.	0		NA	0	Amaranthaceae Juss.
Iz_7_1009	DJI_0470	Chondrilla juncea L.	0	n	1	0	Asteraceae Bercht. & J.Presl
Iz_7_1009	DJI_0470	Cirsium vulgare (Savi) Ten.	0	n	1	0	Asteraceae Bercht. & J.Presl
Iz_7_1009	DJI_0470	Clinopodium acinos (L.) Kuntze	0	n	1	0	Lamiacea Lindl.
Iz_7_1009	DJI_0470	Convallaria majalis L.	0	n	1	0	Asparagaceae Juss.
Iz_7_1009	DJI_0470	Crataegus rhipidophylla Gand.	0	n	1	1	Rosaceae Juss.
Iz_7_1009	DJI_0470	Crepis foetida L.	0	n	1	0	Asteraceae Bercht. & J.Presl
Iz_7_1009	DJI_0470	Dactylis glomerata L.	0	n	1	0	Poaceae Barnhart
Iz_7_1009	DJI_0470	Dianthus campestris M.Bieb.	0	n	1	0	Caryophyllaceae Juss.
Iz_7_1009	DJI_0470	Digitaria sanguinalis (L.) Scop.	0	a	0	0	Poaceae Barnhart
Iz_7_1009	DJI_0470	Elymus repens (L.) Gould	0	n	1	0	Poaceae Barnhart
Iz_7_1009	DJI_0470	Epilobium tetragonum L.	0	n	1	0	Onagraceae Juss.
Iz_7_1009	DJI_0470	Eragrostis minor Host	0	a	0	0	Poaceae Barnhart
Iz_7_1009	DJI_0470	Erigeron canadensis L.	10	a	0	0	Asteraceae Bercht. & J.Presl
Iz_7_1009	DJI_0470	Euphorbia seguieriana Neck.	0	n	1	0	Euphorbiaceae Juss.
Iz_7_1009	DJI_0470	Fallopia dumetorum (L.) Holub	0	n	1	0	Polygonaceae Juss.
Iz_7_1009	DJI_0470	Festuca beckeri (Hack.) Trautv.	0	n	1	0	Poaceae Barnhart
Iz_7_1009	DJI_0470	Fragaria vesca L.	0	n	1	0	Rosaceae Juss.
Iz_7_1009	DJI_0470	Frangula alnus Mill.	0	n	1	1	Rhamnaceae Juss.
Iz_7_1009	DJI_0470	Fraxinus pennsylvanica Marshall	0	a	0	1	Oleaceae Hoffmanns. & Link
Iz_7_1009	DJI_0470	Galeopsis bifida Boenn.	0	n	1	0	Lamiacea Lindl.
Iz_7_1009	DJI_0470	Galium verum L.	0	n	1	0	Rubiaceae Juss.
Iz_7_1009	DJI_0470	Genista tinctoria L.	0	n	1	0	Fabaceae Lindl.
Iz_7_1009	DJI_0470	Geranium robertianum L.	0	n	1	0	Geraniaceae Juss.
Iz_7_1009	DJI_0470	Geranium sanquineum L.	0	n	1	0	Geraniaceae Juss.
Iz_7_1009	DJI_0470	Gypsophila paniculata L.	0	n	1	0	Caryophyllaceae Juss.
Iz_7_1009	DJI_0470	Helichrysum arenarium (L.) Moench	0	n	1	0	Asteraceae Bercht. & J.Presl
Iz_7_1009	DJI_0470	Herniaria polygama J.Gay	0	n	1	0	Caryophyllaceae Juss.
Iz_7_1009	DJI_0470	Hieracium robustum Fr.	0	n	1	0	Asteraceae Bercht. & J.Presl
Iz_7_1009	DJI_0470	Hieracium umbellatum L.	0	n	1	NA	Asteraceae Bercht. & J.Presl
Iz_7_1009	DJI_0470	Hylotelephium maximum (L.) Holub	0	n	1	0	Crassulaceae J.St.-Hil.
Iz_7_1009	DJI_0470	Jacobaea borysthenica (DC.) B.Nord. & Greuter	0	n	1	0	Asteraceae Bercht. & J.Presl
Iz_7_1009	DJI_0470	Jasione montana L.	0	n	1	0	Campanulaceae Juss.
Iz_7_1009	DJI_0470	Jurinea cyanoides (L.) Rchb.s.l.	0	n	1	0	Asteraceae Bercht. & J.Presl
Iz_7_1009	DJI_0470	Koeleria glauca (Spreng.) DC.	0	n	1	0	Poaceae Barnhart
Iz_7_1009	DJI_0470	Lactuca serriola L.	0	a	0	0	Asteraceae Bercht. & J.Presl
Iz_7_1009	DJI_0470	Linaria genistifolia (L.) Mill.	0	n	1	0	Plantaginaceae Juss.
Iz_7_1009	DJI_0470	Lomelosia argentea (L.) Greuter & Burdet	0	n	1	0	Caprifoliaceae Juss.
Iz_7_1009	DJI_0470	Melampyrum pratense L.	0	n	1	0	Orobanchaceae Vent.
Iz_7_1009	DJI_0470	Oenothera biennis L.	0	a	0	0	Onagraceae Juss.
Iz_7_1009	DJI_0470	Pentanema britannica (L.) D.Gut.Larr., Santos-Vicente, Anderb., E.Rico & M.M.Mart.Ort.	0	n	1	0	Asteraceae Bercht. & J.Presl
Iz_7_1009	DJI_0470	Peucedanum oreoselinum (L.) Moench	0	n	1	0	Apiaceae Lindl.
Iz_7_1009	DJI_0470	Peucedanum ruthenicum M.Bieb.	0	n	1	0	Apiaceae Lindl.
Iz_7_1009	DJI_0470	Pilosella echioides (Lumn.) F.W.Schultz & Sch.Bip.	0	n	1	0	Asteraceae Bercht. & J.Presl
Iz_7_1009	DJI_0470	Pilosella officinarum F. Schultz et Sch. Bip.	0	n	1	0	Asteraceae Bercht. & J.Presl
Iz_7_1009	DJI_0470	Pilosella sp.	0		NA	0	Asteraceae Bercht. & J.Presl
Iz_7_1009	DJI_0470	Pinus sylvestris L.	0	n	1	1	Pinaceae Spreng. ex F.Rudolphi
Iz_7_1009	DJI_0470	Poa annua L.	0	n	1	0	Poaceae Barnhart
Iz_7_1009	DJI_0470	Poa nemoralis L.	0	n	1	0	Poaceae Barnhart
Iz_7_1009	DJI_0470	Poa sp.	0		NA	0	Poaceae Barnhart
Iz_7_1009	DJI_0470	Polygonatum odoratum (Mill.) Druce	0	n	1	0	Asparagaceae Juss.
Iz_7_1009	DJI_0470	Polygonum arenarium Waldst. & Kit.	0	n	1	0	Polygonaceae Juss.
Iz_7_1009	DJI_0470	Populus nigra L.	0	n	1	1	Salicaceae Mirb.
Iz_7_1009	DJI_0470	Populus tremula L.	0	n	1	1	Salicaceae Mirb.
Iz_7_1009	DJI_0470	Populus Ã canescens (Aiton) Sm.	0	n	1	1	Salicaceae Mirb.
Iz_7_1009	DJI_0470	Potentilla argentea L.	0	n	1	0	Rosaceae Juss.
Iz_7_1009	DJI_0470	Psammophiliella muralis (L.) Ikonn.	0	n	1	0	Caryophyllaceae Juss.
Iz_7_1009	DJI_0470	Psephellus sumensis (Kalen.) Greuter	0	n	1	0	Asteraceae Bercht. & J.Presl
Iz_7_1009	DJI_0470	Pulsatilla pratensis (L.) Mill. s.l.	0	n	1	0	Ranunculaceae Juss.
Iz_7_1009	DJI_0470	Pyrus communis L.	0	a	0	1	Rosaceae Juss.
Iz_7_1009	DJI_0470	Quercus robur L.	0	n	1	1	Fagaceae Dumort.
Iz_7_1009	DJI_0470	Robinia pseudoacacia L.	0	a	0	1	Fabaceae Lindl.
Iz_7_1009	DJI_0470	Rosa sp.	0		NA	1	Rosaceae Juss.
Iz_7_1009	DJI_0470	Rubus idaeus L.	0	n	1	0	Rosaceae Juss.
Iz_7_1009	DJI_0470	Rumex acetosella L.	0	n	1	0	Polygonaceae Juss.

Iz_7_1009	DJI_0470	Rumex thyrsiflorus Fingerh.	0	n	1	0	Polygonaceae Juss.
Iz_7_1009	DJI_0470	Salix alba L.	0	n	1	1	Salicaceae Mirb.
Iz_7_1009	DJI_0470	Sambucus nigra L.	0	n	1	1	Viburnaceae Raf.
Iz_7_1009	DJI_0470	Secale sylvestre Host	0	n	1	0	Poaceae Barnhart
Iz_7_1009	DJI_0470	Sempervivum ruthenicum Schnittsp. & C.B. Lehm.	0	n	1	0	Crassulaceae J.St.-Hil.
Iz_7_1009	DJI_0470	Setaria viridis (L.) P. Beauv.	0	n	1	0	Poaceae Barnhart
Iz_7_1009	DJI_0470	Silene borysthenica (Gruner) Walters	0	n	1	0	Caryophyllaceae Juss.
Iz_7_1009	DJI_0470	Silene latifolia subsp. alba (Mill.) Greuter & Burdet	0	n	1	0	Caryophyllaceae Juss.
Iz_7_1009	DJI_0470	Solidago virgaurea L.	0	n	1	0	Asteraceae Bercht. & J.Presl
Iz_7_1009	DJI_0470	Sorbus aucuparia L.	0	n	1	1	Rosaceae Juss.
Iz_7_1009	DJI_0470	Stachys recta L.	0	n	1	0	Lamiacea Lindl.
Iz_7_1009	DJI_0470	Tanacetum vulgare L.	0	n	1	0	Asteraceae Bercht. & J.Presl
Iz_7_1009	DJI_0470	Thymus pallasianus Heinr.Braun	0	n	1	0	Lamiacea Lindl.
Iz_7_1009	DJI_0470	Tilia cordata Mill.	0	n	1	1	Malvaceae Juss.
Iz_7_1009	DJI_0470	Trifolium alpestre L.	0	n	1	0	Fabaceae Lindl.
Iz_7_1009	DJI_0470	Trifolium arvense L.	0	n	1	0	Fabaceae Lindl.
Iz_7_1009	DJI_0470	Ulmus glabra Huds.	0	n	1	1	Ulmaceae Mirb.
Iz_7_1009	DJI_0470	Verbascum densiflorum Bertol.	0	n	1	0	Scrophulariaceae Juss.
Iz_7_1009	DJI_0470	Veronica austriaca L.	0	n	1	0	Plantaginaceae Juss.
Iz_7_1009	DJI_0470	Veronica incana L.	0	n	1	0	Plantaginaceae Juss.
Iz_7_1009	DJI_0470	Veronica verna L.	0	n	1	0	Plantaginaceae Juss.
Iz_7_1009	DJI_0470	Vincetoxicum hirundinaria Medik	0	n	1	0	Apocynaceae Juss.
Iz_7_1009	DJI_0479	Acer tataricum L.	0	n	1	1	Sapindaceae Juss.
Iz_7_1009	DJI_0479	Achillea millefolium L. s.l.	0	n	1	0	Asteraceae Bercht. & J.Presl
Iz_7_1009	DJI_0479	Allium oleraceum L.	0	n	1	0	Amaryllidaceae J.St.-Hil.
Iz_7_1009	DJI_0479	Ambrosia artemisiifolia L.	0	a	0	0	Asteraceae Bercht. & J.Presl
Iz_7_1009	DJI_0479	Amorpha fruticosa L.	0	a	0	0	Fabaceae Lindl.
Iz_7_1009	DJI_0479	Anthericum ramosum L.	0	n	1	0	Asparagaceae Juss.
Iz_7_1009	DJI_0479	Artemisia marschalliana Spreng.	0	n	1	0	Asteraceae Bercht. & J.Presl
Iz_7_1009	DJI_0479	Asparagus officinalis L.	0	n	1	0	Asparagaceae Juss.
Iz_7_1009	DJI_0479	Astragalus cicer L.	0	n	1	0	Fabaceae Lindl.
Iz_7_1009	DJI_0479	Bassia laniflora (S.G.Gmel.) A.J.Scott	0	n	1	0	Amaranthaceae Juss.
Iz_7_1009	DJI_0479	Berberis vulgaris L.	0	n	1	0	Berberidaceae Juss.
Iz_7_1009	DJI_0479	Betula pendula Roth.	0	n	1	1	Betulaceae Gray
Iz_7_1009	DJI_0479	Betula pendula Roth.	0	n	1	0	Poaceae Barnhart
Iz_7_1009	DJI_0479	Bromus inermis Leyss.	0		NA	NA	
Iz_7_1009	DJI_0479	Bromus squarrosus L.	0	a	0	0	Poaceae Barnhart
Iz_7_1009	DJI_0479	Bromus tectorum L.	0	a	0	0	Poaceae Barnhart
Iz_7_1009	DJI_0479	Calamagrostis epigejos (L.) Roth	2	n	1	0	Poaceae Barnhart
Iz_7_1009	DJI_0479	Carduus acanthoides L.	0	n	1	0	Asteraceae Bercht. & J.Presl
Iz_7_1009	DJI_0479	Carex sp.	0	n	1	0	Cyperaceae Juss.
Iz_7_1009	DJI_0479	Centaurea majorovii Dumbadze	0	n	1	0	Asteraceae Bercht. & J.Presl
Iz_7_1009	DJI_0479	Chamaecytisus ruthenicus (Fisch.ex Wol.) Klaskova	0	n	1	0	Fabaceae Lindl.
Iz_7_1009	DJI_0479	Chelidonium majus L.	0	n	1	0	Papaveraceae Juss.
Iz_7_1009	DJI_0479	Chenopodium betaceum Andrz.	0	n	1	0	Amaranthaceae Juss.
Iz_7_1009	DJI_0479	Chenopodium cf. album	0	n	1	0	Amaranthaceae Juss.
Iz_7_1009	DJI_0479	Chenopodium sp.	0		NA	0	Amaranthaceae Juss.
Iz_7_1009	DJI_0479	Chondrilla juncea L.	0	n	1	0	Asteraceae Bercht. & J.Presl
Iz_7_1009	DJI_0479	Cirsium vulgare (Savi) Ten.	0	n	1	0	Asteraceae Bercht. & J.Presl
Iz_7_1009	DJI_0479	Clinopodium acinos (L.) Kuntze	0	n	1	0	Lamiacea Lindl.
Iz_7_1009	DJI_0479	Convallaria majalis L.	0	n	1	0	Asparagaceae Juss.
Iz_7_1009	DJI_0479	Crataegus rhipidophylla Gand.	0	n	1	1	Rosaceae Juss.
Iz_7_1009	DJI_0479	Crepis foetida L.	0	n	1	0	Asteraceae Bercht. & J.Presl
Iz_7_1009	DJI_0479	Dactylis glomerata L.	0	n	1	0	Poaceae Barnhart
Iz_7_1009	DJI_0479	Dianthus campestris M.Bieb.	0	n	1	0	Caryophyllaceae Juss.
Iz_7_1009	DJI_0479	Digitaria sanguinalis (L.) Scop.	0	a	0	0	Poaceae Barnhart
Iz_7_1009	DJI_0479	Elymus repens (L.) Gould	0	n	1	0	Poaceae Barnhart
Iz_7_1009	DJI_0479	Epilobium tetragonum L.	0	n	1	0	Onagraceae Juss.
Iz_7_1009	DJI_0479	Eragrostis minor Host	0	a	0	0	Poaceae Barnhart
Iz_7_1009	DJI_0479	Erigeron canadensis L.	20	a	0	0	Asteraceae Bercht. & J.Presl
Iz_7_1009	DJI_0479	Euphorbia seguieriana Neck.	0	n	1	0	Euphorbiaceae Juss.
Iz_7_1009	DJI_0479	Fallopia dumetorum (L.) Holub	0	n	1	0	Polygonaceae Juss.
Iz_7_1009	DJI_0479	Festuca beckeri (Hack.) Trautv.	0	n	1	0	Poaceae Barnhart
Iz_7_1009	DJI_0479	Fragaria vesca L.	0	n	1	0	Rosaceae Juss.
Iz_7_1009	DJI_0479	Frangula alnus Mill.	0	n	1	1	Rhamnaceae Juss.
Iz_7_1009	DJI_0479	Fraxinus pennsylvanica Marshall	0	a	0	1	Oleaceae Hoffmanns. & Link
Iz_7_1009	DJI_0479	Galeopsis bifida Boenn.	0	n	1	0	Lamiacea Lindl.
Iz_7_1009	DJI_0479	Galium verum L.	0	n	1	0	Rubiaceae Juss.
Iz_7_1009	DJI_0479	Genista tinctoria L.	0	n	1	0	Fabaceae Lindl.
Iz_7_1009	DJI_0479	Geranium robertianum L.	0	n	1	0	Geraniaceae Juss.
Iz_7_1009	DJI_0479	Geranium sanquineum L.	0	n	1	0	Geraniaceae Juss.
Iz_7_1009	DJI_0479	Gypsophila paniculata L.	0	n	1	0	Caryophyllaceae Juss.
Iz_7_1009	DJI_0479	Helichrysum arenarium (L.) Moench	0	n	1	0	Asteraceae Bercht. & J.Presl
Iz_7_1009	DJI_0479	Herniaria polygama J.Gay	0	n	1	0	Caryophyllaceae Juss.
Iz_7_1009	DJI_0479	Hieracium robustum Fr.	0	n	1	0	Asteraceae Bercht. & J.Presl
Iz_7_1009	DJI_0479	Hieracium umbellatum L.	0	n	1	NA	Asteraceae Bercht. & J.Presl
Iz_7_1009	DJI_0479	Hylotelephium maximum (L.) Holub	0	n	1	0	Crassulaceae J.St.-Hil.
Iz_7_1009	DJI_0479	Jacobaea borysthenica (DC.) B.Nord. & Greuter	0	n	1	0	Asteraceae Bercht. & J.Presl
Iz_7_1009	DJI_0479	Jasione montana L.	0	n	1	0	Campanulaceae Juss.
Iz_7_1009	DJI_0479	Jurinea cyanoides (L.) Rchb.s.l.	0	n	1	0	Asteraceae Bercht. & J.Presl
Iz_7_1009	DJI_0479	Koeleria glauca (Spreng.) DC.	0	n	1	0	Poaceae Barnhart
Iz_7_1009	DJI_0479	Lactuca serriola L.	0	a	0	0	Asteraceae Bercht. & J.Presl
Iz_7_1009	DJI_0479	Linaria genistifolia (L.) Mill.	0	n	1	0	Plantaginaceae Juss.
Iz_7_1009	DJI_0479	Lomelosia argentea (L.) Greuter & Burdet	0	n	1	0	Caprifoliaceae Juss.
Iz_7_1009	DJI_0479	Melampyrum pratense L.	0	n	1	0	Orobanchaceae Vent.
Iz_7_1009	DJI_0479	Oenothera biennis L.	0	a	0	0	Onagraceae Juss.
Iz_7_1009	DJI_0479	Pentanema britannica (L.) D.Gut.Larr., Santos-Vicente, Anderb., E.Rico & M.M.Mart.Ort.	0	n	1	0	Asteraceae Bercht. & J.Presl
Iz_7_1009	DJI_0479	Peucedanum oreoselinum (L.) Moench	0	n	1	0	Apiaceae Lindl.
Iz_7_1009	DJI_0479	Peucedanum ruthenicum M.Bieb.	0	n	1	0	Apiaceae Lindl.
Iz_7_1009	DJI_0479	Pilosella echioides (Lumn.) F.W.Schultz & Sch.Bip.	0	n	1	0	Asteraceae Bercht. & J.Presl
Iz_7_1009	DJI_0479	Pilosella officinarum F. Schultz et Sch. Bip.	0	n	1	0	Asteraceae Bercht. & J.Presl
Iz_7_1009	DJI_0479	Pilosella sp.	0		NA	0	Asteraceae Bercht. & J.Presl
Iz_7_1009	DJI_0479	Pinus sylvestris L.	0	n	1	1	Pinaceae Spreng. ex F.Rudolphi
Iz_7_1009	DJI_0479	Poa annua L.	0	n	1	0	Poaceae Barnhart
Iz_7_1009	DJI_0479	Poa nemoralis L.	0	n	1	0	Poaceae Barnhart
Iz_7_1009	DJI_0479	Poa sp.	0		NA	0	Poaceae Barnhart
Iz_7_1009	DJI_0479	Polygonatum odoratum (Mill.) Druce	0	n	1	0	Asparagaceae Juss.
Iz_7_1009	DJI_0479	Polygonum arenarium Waldst. & Kit.	0	n	1	0	Polygonaceae Juss.

Iz_7_1009	DJI_0479	Populus nigra L.	0	n	1	1	Salicaceae Mirb.
Iz_7_1009	DJI_0479	Populus tremula L.	0	n	1	1	Salicaceae Mirb.
Iz_7_1009	DJI_0479	Populus Ã canescens (Aiton) Sm.	0	n	1	1	Salicaceae Mirb.
Iz_7_1009	DJI_0479	Potentilla argentea L.	0	n	1	0	Rosaceae Juss.
Iz_7_1009	DJI_0479	Psammophiliella muralis (L.) Ikonn.	0	n	1	0	Caryophyllaceae Juss.
Iz_7_1009	DJI_0479	Psephellus sumensis (Kalen.) Greuter	0	n	1	0	Asteraceae Bercht. & J.Presl
Iz_7_1009	DJI_0479	Pulsatilla pratensis (L.) Mill. s.l.	0	n	1	0	Ranunculaceae Juss.
Iz_7_1009	DJI_0479	Pyrus communis L.	0	a	0	1	Rosaceae Juss.
Iz_7_1009	DJI_0479	Quercus robur L.	0	n	1	1	Fagaceae Dumort.
Iz_7_1009	DJI_0479	Robinia pseudoacacia L.	0	a	0	1	Fabaceae Lindl.
Iz_7_1009	DJI_0479	Rosa sp.	0		NA	1	Rosaceae Juss.
Iz_7_1009	DJI_0479	Rubus idaeus L.	0	n	1	0	Rosaceae Juss.
Iz_7_1009	DJI_0479	Rumex acetosella L.	0	n	1	0	Polygonaceae Juss.
Iz_7_1009	DJI_0479	Rumex thyrsiflorus Fingerh.	0	n	1	0	Polygonaceae Juss.
Iz_7_1009	DJI_0479	Salix alba L.	0	n	1	1	Salicaceae Mirb.
Iz_7_1009	DJI_0479	Sambucus nigra L.	0	n	1	1	Viburnaceae Raf.
Iz_7_1009	DJI_0479	Secale sylvestre Host	0	n	1	0	Poaceae Barnhart
Iz_7_1009	DJI_0479	Sempervivum ruthenicum Schnittsp. & C.B. Lehm.	0	n	1	0	Crassulaceae J.St.-Hil.
Iz_7_1009	DJI_0479	Setaria viridis (L.) P. Beauv.	0	n	1	0	Poaceae Barnhart
Iz_7_1009	DJI_0479	Silene borysthenica (Gruner) Walters	0	n	1	0	Caryophyllaceae Juss.
Iz_7_1009	DJI_0479	Silene latifolia subsp. alba (Mill.) Greuter & Burdet	0	n	1	0	Caryophyllaceae Juss.
Iz_7_1009	DJI_0479	Solidago virgaurea L.	0	n	1	0	Asteraceae Bercht. & J.Presl
Iz_7_1009	DJI_0479	Sorbus aucuparia L.	0	n	1	1	Rosaceae Juss.
Iz_7_1009	DJI_0479	Stachys recta L.	0	n	1	0	Lamiacea Lindl.
Iz_7_1009	DJI_0479	Tanacetum vulgare L.	0	n	1	0	Asteraceae Bercht. & J.Presl
Iz_7_1009	DJI_0479	Thymus pallasianus Heinr.Braun	0	n	1	0	Lamiacea Lindl.
Iz_7_1009	DJI_0479	Tilia cordata Mill.	0	n	1	1	Malvaceae Juss.
Iz_7_1009	DJI_0479	Trifolium alpestre L.	0	n	1	0	Fabaceae Lindl.
Iz_7_1009	DJI_0479	Trifolium arvense L.	0	n	1	0	Fabaceae Lindl.
Iz_7_1009	DJI_0479	Ulmus glabra Huds.	0	n	1	1	Ulmaceae Mirb.
Iz_7_1009	DJI_0479	Verbascum densiflorum Bertol.	0	n	1	0	Scrophulariaceae Juss.
Iz_7_1009	DJI_0479	Veronica austriaca L.	0	n	1	0	Plantaginaceae Juss.
Iz_7_1009	DJI_0479	Veronica incana L.	0	n	1	0	Plantaginaceae Juss.
Iz_7_1009	DJI_0479	Veronica verna L.	0	n	1	0	Plantaginaceae Juss.
Iz_7_1009	DJI_0479	Vincetoxicum hirundinaria Medik	0	n	1	0	Apocynaceae Juss.
Iz_7_1009	DJI_0485	Acer tataricum L.	0	n	1	1	Sapindaceae Juss.
Iz_7_1009	DJI_0485	Achillea millefolium L. s.l.	0	n	1	0	Asteraceae Bercht. & J.Presl
Iz_7_1009	DJI_0485	Allium oleraceum L.	0	n	1	0	Amaryllidaceae J.St.-Hil.
Iz_7_1009	DJI_0485	Ambrosia artemisiifolia L.	0	a	0	0	Asteraceae Bercht. & J.Presl
Iz_7_1009	DJI_0485	Amorpha fruticosa L.	0	a	0	0	Fabaceae Lindl.
Iz_7_1009	DJI_0485	Anthericum ramosum L.	0	n	1	0	Asparagaceae Juss.
Iz_7_1009	DJI_0485	Artemisia marschalliana Spreng.	0	n	1	0	Asteraceae Bercht. & J.Presl
Iz_7_1009	DJI_0485	Asparagus officinalis L.	0	n	1	0	Asparagaceae Juss.
Iz_7_1009	DJI_0485	Astragalus cicer L.	0	n	1	0	Fabaceae Lindl.
Iz_7_1009	DJI_0485	Bassia laniflora (S.G.Gmel.) A.J.Scott	0	n	1	0	Amaranthaceae Juss.
Iz_7_1009	DJI_0485	Berberis vulgaris L.	0	n	1	0	Berberidaceae Juss.
Iz_7_1009	DJI_0485	Betula pendula Roth.	0	n	1	1	Betulaceae Gray
Iz_7_1009	DJI_0485	Betula pendula Roth.	0	n	1	0	Poaceae Barnhart
Iz_7_1009	DJI_0485	Bromus inermis Leyss.	0		NA	NA	
Iz_7_1009	DJI_0485	Bromus squarrosus L.	0	a	0	0	Poaceae Barnhart
Iz_7_1009	DJI_0485	Bromus tectorum L.	0	a	0	0	Poaceae Barnhart
Iz_7_1009	DJI_0485	Calamagrostis epigejos (L.) Roth	5	n	1	0	Poaceae Barnhart
Iz_7_1009	DJI_0485	Carduus acanthoides L.	0	n	1	0	Asteraceae Bercht. & J.Presl
Iz_7_1009	DJI_0485	Carex sp.	0	n	1	0	Cyperaceae Juss.
Iz_7_1009	DJI_0485	Centaurea majorovii Dumbadze	0	n	1	0	Asteraceae Bercht. & J.Presl
Iz_7_1009	DJI_0485	Chamaecytisus ruthenicus (Fisch.ex Wol.) Klaskova	0	n	1	0	Fabaceae Lindl.
Iz_7_1009	DJI_0485	Chelidonium majus L.	0	n	1	0	Papaveraceae Juss.
Iz_7_1009	DJI_0485	Chenopodium betaceum Andrz.	0	n	1	0	Amaranthaceae Juss.
Iz_7_1009	DJI_0485	Chenopodium cf. album	0	n	1	0	Amaranthaceae Juss.
Iz_7_1009	DJI_0485	Chenopodium sp.	0		NA	0	Amaranthaceae Juss.
Iz_7_1009	DJI_0485	Chondrilla juncea L.	0	n	1	0	Asteraceae Bercht. & J.Presl
Iz_7_1009	DJI_0485	Cirsium vulgare (Savi) Ten.	0	n	1	0	Asteraceae Bercht. & J.Presl
Iz_7_1009	DJI_0485	Clinopodium acinos (L.) Kuntze	0	n	1	0	Lamiacea Lindl.
Iz_7_1009	DJI_0485	Convallaria majalis L.	0	n	1	0	Asparagaceae Juss.
Iz_7_1009	DJI_0485	Crataegus rhipidophylla Gand.	0	n	1	1	Rosaceae Juss.
Iz_7_1009	DJI_0485	Crepis foetida L.	0	n	1	0	Asteraceae Bercht. & J.Presl
Iz_7_1009	DJI_0485	Dactylis glomerata L.	0	n	1	0	Poaceae Barnhart
Iz_7_1009	DJI_0485	Dianthus campestris M.Bieb.	0	n	1	0	Caryophyllaceae Juss.
Iz_7_1009	DJI_0485	Digitaria sanguinalis (L.) Scop.	0	a	0	0	Poaceae Barnhart
Iz_7_1009	DJI_0485	Elymus repens (L.) Gould	0	n	1	0	Poaceae Barnhart
Iz_7_1009	DJI_0485	Epilobium tetragonum L.	0	n	1	0	Onagraceae Juss.
Iz_7_1009	DJI_0485	Eragrostis minor Host	0	a	0	0	Poaceae Barnhart
Iz_7_1009	DJI_0485	Erigeron canadensis L.	40	a	0	0	Asteraceae Bercht. & J.Presl
Iz_7_1009	DJI_0485	Euphorbia seguieriana Neck.	0	n	1	0	Euphorbiaceae Juss.
Iz_7_1009	DJI_0485	Fallopia dumetorum (L.) Holub	0	n	1	0	Polygonaceae Juss.
Iz_7_1009	DJI_0485	Festuca beckeri (Hack.) Trautv.	0	n	1	0	Poaceae Barnhart
Iz_7_1009	DJI_0485	Fragaria vesca L.	0	n	1	0	Rosaceae Juss.
Iz_7_1009	DJI_0485	Frangula alnus Mill.	0	n	1	1	Rhamnaceae Juss.
Iz_7_1009	DJI_0485	Fraxinus pennsylvanica Marshall	0	a	0	1	Oleaceae Hoffmanns. & Link
Iz_7_1009	DJI_0485	Galeopsis bifida Boenn.	0	n	1	0	Lamiacea Lindl.
Iz_7_1009	DJI_0485	Galium verum L.	0	n	1	0	Rubiaceae Juss.
Iz_7_1009	DJI_0485	Genista tinctoria L.	0	n	1	0	Fabaceae Lindl.
Iz_7_1009	DJI_0485	Geranium robertianum L.	0	n	1	0	Geraniaceae Juss.
Iz_7_1009	DJI_0485	Geranium sanquineum L.	0	n	1	0	Geraniaceae Juss.
Iz_7_1009	DJI_0485	Gypsophila paniculata L.	0	n	1	0	Caryophyllaceae Juss.
Iz_7_1009	DJI_0485	Helichrysum arenarium (L.) Moench	0	n	1	0	Asteraceae Bercht. & J.Presl
Iz_7_1009	DJI_0485	Herniaria polygama J.Gay	0	n	1	0	Caryophyllaceae Juss.
Iz_7_1009	DJI_0485	Hieracium robustum Fr.	0	n	1	0	Asteraceae Bercht. & J.Presl
Iz_7_1009	DJI_0485	Hieracium umbellatum L.	0	n	1	NA	Asteraceae Bercht. & J.Presl
Iz_7_1009	DJI_0485	Hylotelephium maximum (L.) Holub	0	n	1	0	Crassulaceae J.St.-Hil.
Iz_7_1009	DJI_0485	Jacobaea borysthenica (DC.) B.Nord. & Greuter	0	n	1	0	Asteraceae Bercht. & J.Presl
Iz_7_1009	DJI_0485	Jasione montana L.	0	n	1	0	Campanulaceae Juss.
Iz_7_1009	DJI_0485	Jurinea cyanoides (L.) Rchb.s.l.	0	n	1	0	Asteraceae Bercht. & J.Presl
Iz_7_1009	DJI_0485	Koeleria glauca (Spreng.) DC.	0	n	1	0	Poaceae Barnhart
Iz_7_1009	DJI_0485	Lactuca serriola L.	0	a	0	0	Asteraceae Bercht. & J.Presl
Iz_7_1009	DJI_0485	Linaria genistifolia (L.) Mill.	0	n	1	0	Plantaginaceae Juss.
Iz_7_1009	DJI_0485	Lomelosia argentea (L.) Greuter & Burdet	0	n	1	0	Caprifoliaceae Juss.
Iz_7_1009	DJI_0485	Melampyrum pratense L.	0	n	1	0	Orobanchaceae Vent.

Iz_7_1009	DJI_0485	Oenothera biennis L.	0	a	0	0	Onagraceae Juss.
Iz_7_1009	DJI_0485	Pentanema britannica (L.) D.Gut.Larr., Santos-Vicente, Anderb., E.Rico & M.M.Mart.Ort.	0	n	1	0	Asteraceae Bercht. & J.Presl
Iz_7_1009	DJI_0485	Peucedanum oreoselinum (L.) Moench	0	n	1	0	Apiaceae Lindl.
Iz_7_1009	DJI_0485	Peucedanum ruthenicum M.Bieb.	0	n	1	0	Apiaceae Lindl.
Iz_7_1009	DJI_0485	Pilosella echioides (Lumn.) F.W.Schultz & Sch.Bip.	0	n	1	0	Asteraceae Bercht. & J.Presl
Iz_7_1009	DJI_0485	Pilosella officinarum F. Schultz et Sch. Bip.	0	n	1	0	Asteraceae Bercht. & J.Presl
Iz_7_1009	DJI_0485	Pilosella sp.	0		NA	0	Asteraceae Bercht. & J.Presl
Iz_7_1009	DJI_0485	Pinus sylvestris L.	0	n	1	1	Pinaceae Spreng. ex F.Rudolphi
Iz_7_1009	DJI_0485	Poa annua L.	0	n	1	0	Poaceae Barnhart
Iz_7_1009	DJI_0485	Poa nemoralis L.	0	n	1	0	Poaceae Barnhart
Iz_7_1009	DJI_0485	Poa sp.	0		NA	0	Poaceae Barnhart
Iz_7_1009	DJI_0485	Polygonatum odoratum (Mill.) Druce	0	n	1	0	Asparagaceae Juss.
Iz_7_1009	DJI_0485	Polygonum arenarium Waldst. & Kit.	0	n	1	0	Polygonaceae Juss.
Iz_7_1009	DJI_0485	Populus nigra L.	0	n	1	1	Salicaceae Mirb.
Iz_7_1009	DJI_0485	Populus tremula L.	0	n	1	1	Salicaceae Mirb.
Iz_7_1009	DJI_0485	Populus Ã canescens (Aiton) Sm.	0	n	1	1	Salicaceae Mirb.
Iz_7_1009	DJI_0485	Potentilla argentea L.	0	n	1	0	Rosaceae Juss.
Iz_7_1009	DJI_0485	Psammophiliella muralis (L.) Ikonn.	0	n	1	0	Caryophyllaceae Juss.
Iz_7_1009	DJI_0485	Psephellus sumensis (Kalen.) Greuter	0	n	1	0	Asteraceae Bercht. & J.Presl
Iz_7_1009	DJI_0485	Pulsatilla pratensis (L.) Mill. s.l.	0	n	1	0	Ranunculaceae Juss.
Iz_7_1009	DJI_0485	Pyrus communis L.	0	a	0	1	Rosaceae Juss.
Iz_7_1009	DJI_0485	Quercus robur L.	0	n	1	1	Fagaceae Dumort.
Iz_7_1009	DJI_0485	Robinia pseudoacacia L.	0	a	0	1	Fabaceae Lindl.
Iz_7_1009	DJI_0485	Rosa sp.	0		NA	1	Rosaceae Juss.
Iz_7_1009	DJI_0485	Rubus idaeus L.	0	n	1	0	Rosaceae Juss.
Iz_7_1009	DJI_0485	Rumex acetosella L.	0	n	1	0	Polygonaceae Juss.
Iz_7_1009	DJI_0485	Rumex thyrsiflorus Fingerh.	0	n	1	0	Polygonaceae Juss.
Iz_7_1009	DJI_0485	Salix alba L.	0	n	1	1	Salicaceae Mirb.
Iz_7_1009	DJI_0485	Sambucus nigra L.	0	n	1	1	Viburnaceae Raf.
Iz_7_1009	DJI_0485	Secale sylvestre Host	0	n	1	0	Poaceae Barnhart
Iz_7_1009	DJI_0485	Sempervivum ruthenicum Schnittsp. & C.B. Lehm.	0	n	1	0	Crassulaceae J.St.-Hil.
Iz_7_1009	DJI_0485	Setaria viridis (L.) P. Beauv.	0	n	1	0	Poaceae Barnhart
Iz_7_1009	DJI_0485	Silene borysthenica (Gruner) Walters	0	n	1	0	Caryophyllaceae Juss.
Iz_7_1009	DJI_0485	Silene latifolia subsp. alba (Mill.) Greuter & Burdet	0	n	1	0	Caryophyllaceae Juss.
Iz_7_1009	DJI_0485	Solidago virgaurea L.	0	n	1	0	Asteraceae Bercht. & J.Presl
Iz_7_1009	DJI_0485	Sorbus aucuparia L.	0	n	1	1	Rosaceae Juss.
Iz_7_1009	DJI_0485	Stachys recta L.	0	n	1	0	Lamiacea Lindl.
Iz_7_1009	DJI_0485	Tanacetum vulgare L.	0	n	1	0	Asteraceae Bercht. & J.Presl
Iz_7_1009	DJI_0485	Thymus pallasianus Heinr.Braun	0	n	1	0	Lamiacea Lindl.
Iz_7_1009	DJI_0485	Tilia cordata Mill.	0	n	1	1	Malvaceae Juss.
Iz_7_1009	DJI_0485	Trifolium alpestre L.	0	n	1	0	Fabaceae Lindl.
Iz_7_1009	DJI_0485	Trifolium arvense L.	0	n	1	0	Fabaceae Lindl.
Iz_7_1009	DJI_0485	Ulmus glabra Huds.	0	n	1	1	Ulmaceae Mirb.
Iz_7_1009	DJI_0485	Verbascum densiflorum Bertol.	0	n	1	0	Scrophulariaceae Juss.
Iz_7_1009	DJI_0485	Veronica austriaca L.	0	n	1	0	Plantaginaceae Juss.
Iz_7_1009	DJI_0485	Veronica incana L.	0	n	1	0	Plantaginaceae Juss.
Iz_7_1009	DJI_0485	Veronica verna L.	0	n	1	0	Plantaginaceae Juss.
Iz_7_1009	DJI_0485	Vincetoxicum hirundinaria Medik	0	n	1	0	Apocynaceae Juss.
Iz_7_1009	DJI_0489	Acer tataricum L.	0	n	1	1	Sapindaceae Juss.
Iz_7_1009	DJI_0489	Achillea millefolium L. s.l.	0	n	1	0	Asteraceae Bercht. & J.Presl
Iz_7_1009	DJI_0489	Allium oleraceum L.	0	n	1	0	Amaryllidaceae J.St.-Hil.
Iz_7_1009	DJI_0489	Ambrosia artemisiifolia L.	0	a	0	0	Asteraceae Bercht. & J.Presl
Iz_7_1009	DJI_0489	Amorpha fruticosa L.	0	a	0	0	Fabaceae Lindl.
Iz_7_1009	DJI_0489	Anthericum ramosum L.	0	n	1	0	Asparagaceae Juss.
Iz_7_1009	DJI_0489	Artemisia marschalliana Spreng.	0	n	1	0	Asteraceae Bercht. & J.Presl
Iz_7_1009	DJI_0489	Asparagus officinalis L.	0	n	1	0	Asparagaceae Juss.
Iz_7_1009	DJI_0489	Astragalus cicer L.	0	n	1	0	Fabaceae Lindl.
Iz_7_1009	DJI_0489	Bassia laniflora (S.G.Gmel.) A.J.Scott	0	n	1	0	Amaranthaceae Juss.
Iz_7_1009	DJI_0489	Berberis vulgaris L.	0	n	1	0	Berberidaceae Juss.
Iz_7_1009	DJI_0489	Betula pendula Roth.	0	n	1	1	Betulaceae Gray
Iz_7_1009	DJI_0489	Betula pendula Roth.	0	n	1	0	Poaceae Barnhart
Iz_7_1009	DJI_0489	Bromus inermis Leyss.	0		NA	NA	
Iz_7_1009	DJI_0489	Bromus squarrosus L.	0	a	0	0	Poaceae Barnhart
Iz_7_1009	DJI_0489	Bromus tectorum L.	0	a	0	0	Poaceae Barnhart
Iz_7_1009	DJI_0489	Calamagrostis epigejos (L.) Roth	1	n	1	0	Poaceae Barnhart
Iz_7_1009	DJI_0489	Carduus acanthoides L.	0	n	1	0	Asteraceae Bercht. & J.Presl
Iz_7_1009	DJI_0489	Carex sp.	0	n	1	0	Cyperaceae Juss.
Iz_7_1009	DJI_0489	Centaurea majorovii Dumbadze	0	n	1	0	Asteraceae Bercht. & J.Presl
Iz_7_1009	DJI_0489	Chamaecytisus ruthenicus (Fisch.ex Wol.) Klaskova	0	n	1	0	Fabaceae Lindl.
Iz_7_1009	DJI_0489	Chelidonium majus L.	0	n	1	0	Papaveraceae Juss.
Iz_7_1009	DJI_0489	Chenopodium betaceum Andrz.	0	n	1	0	Amaranthaceae Juss.
Iz_7_1009	DJI_0489	Chenopodium cf. album	0	n	1	0	Amaranthaceae Juss.
Iz_7_1009	DJI_0489	Chenopodium sp.	0		NA	0	Amaranthaceae Juss.
Iz_7_1009	DJI_0489	Chondrilla juncea L.	0	n	1	0	Asteraceae Bercht. & J.Presl
Iz_7_1009	DJI_0489	Cirsium vulgare (Savi) Ten.	0	n	1	0	Asteraceae Bercht. & J.Presl
Iz_7_1009	DJI_0489	Clinopodium acinos (L.) Kuntze	0	n	1	0	Lamiacea Lindl.
Iz_7_1009	DJI_0489	Convallaria majalis L.	0	n	1	0	Asparagaceae Juss.
Iz_7_1009	DJI_0489	Crataegus rhipidophylla Gand.	0	n	1	1	Rosaceae Juss.
Iz_7_1009	DJI_0489	Crepis foetida L.	0	n	1	0	Asteraceae Bercht. & J.Presl
Iz_7_1009	DJI_0489	Dactylis glomerata L.	0	n	1	0	Poaceae Barnhart
Iz_7_1009	DJI_0489	Dianthus campestris M.Bieb.	0	n	1	0	Caryophyllaceae Juss.
Iz_7_1009	DJI_0489	Digitaria sanguinalis (L.) Scop.	0	a	0	0	Poaceae Barnhart
Iz_7_1009	DJI_0489	Elymus repens (L.) Gould	0	n	1	0	Poaceae Barnhart
Iz_7_1009	DJI_0489	Epilobium tetragonum L.	0	n	1	0	Onagraceae Juss.
Iz_7_1009	DJI_0489	Eragrostis minor Host	0	a	0	0	Poaceae Barnhart
Iz_7_1009	DJI_0489	Erigeron canadensis L.	40	a	0	0	Asteraceae Bercht. & J.Presl
Iz_7_1009	DJI_0489	Euphorbia seguieriana Neck.	0	n	1	0	Euphorbiaceae Juss.
Iz_7_1009	DJI_0489	Fallopia dumetorum (L.) Holub	0	n	1	0	Polygonaceae Juss.
Iz_7_1009	DJI_0489	Festuca beckeri (Hack.) Trautv.	0	n	1	0	Poaceae Barnhart
Iz_7_1009	DJI_0489	Fragaria vesca L.	0	n	1	0	Rosaceae Juss.
Iz_7_1009	DJI_0489	Frangula alnus Mill.	0	n	1	1	Rhamnaceae Juss.
Iz_7_1009	DJI_0489	Fraxinus pennsylvanica Marshall	0	a	0	1	Oleaceae Hoffmanns. & Link
Iz_7_1009	DJI_0489	Galeopsis bifida Boenn.	0	n	1	0	Lamiacea Lindl.
Iz_7_1009	DJI_0489	Galium verum L.	0	n	1	0	Rubiaceae Juss.
Iz_7_1009	DJI_0489	Genista tinctoria L.	0	n	1	0	Fabaceae Lindl.
Iz_7_1009	DJI_0489	Geranium robertianum L.	0	n	1	0	Geraniaceae Juss.
Iz_7_1009	DJI_0489	Geranium sanquineum L.	0	n	1	0	Geraniaceae Juss.
Iz_7_1009	DJI_0489	Gypsophila paniculata L.	0	n	1	0	Caryophyllaceae Juss.

Iz_7_1009	DJI_0489	Helichrysum arenarium (L.) Moench	0	n	1	0	Asteraceae Bercht. & J.Presl
Iz_7_1009	DJI_0489	Herniaria polygama J.Gay	0	n	1	0	Caryophyllaceae Juss.
Iz_7_1009	DJI_0489	Hieracium robustum Fr.	0	n	1	0	Asteraceae Bercht. & J.Presl
Iz_7_1009	DJI_0489	Hieracium umbellatum L.	0	n	1	NA	Asteraceae Bercht. & J.Presl
Iz_7_1009	DJI_0489	Hylotelephium maximum (L.) Holub	0	n	1	0	Crassulaceae J.St.-Hil.
Iz_7_1009	DJI_0489	Jacobaea borysthenica (DC.) B.Nord. & Greuter	0	n	1	0	Asteraceae Bercht. & J.Presl
Iz_7_1009	DJI_0489	Jasione montana L.	0	n	1	0	Campanulaceae Juss.
Iz_7_1009	DJI_0489	Jurinea cyanoides (L.) Rchb.s.l.	0	n	1	0	Asteraceae Bercht. & J.Presl
Iz_7_1009	DJI_0489	Koeleria glauca (Spreng.) DC.	0	n	1	0	Poaceae Barnhart
Iz_7_1009	DJI_0489	Lactuca serriola L.	0	a	0	0	Asteraceae Bercht. & J.Presl
Iz_7_1009	DJI_0489	Linaria genistifolia (L.) Mill.	0	n	1	0	Plantaginaceae Juss.
Iz_7_1009	DJI_0489	Lomelosia argentea (L.) Greuter & Burdet	0	n	1	0	Caprifoliaceae Juss.
Iz_7_1009	DJI_0489	Melampyrum pratense L.	0	n	1	0	Orobanchaceae Vent.
Iz_7_1009	DJI_0489	Oenothera biennis L.	0	a	0	0	Onagraceae Juss.
Iz_7_1009	DJI_0489	Pentanema britannica (L.) D.Gut.Larr., Santos-Vicente, Anderb., E.Rico & M.M.Mart.Ort.	0	n	1	0	Asteraceae Bercht. & J.Presl
Iz_7_1009	DJI_0489	Peucedanum oreoselinum (L.) Moench	0	n	1	0	Apiaceae Lindl.
Iz_7_1009	DJI_0489	Peucedanum ruthenicum M.Bieb.	0	n	1	0	Apiaceae Lindl.
Iz_7_1009	DJI_0489	Pilosella echioides (Lumn.) F.W.Schultz & Sch.Bip.	0	n	1	0	Asteraceae Bercht. & J.Presl
Iz_7_1009	DJI_0489	Pilosella officinarum F. Schultz et Sch. Bip.	0	n	1	0	Asteraceae Bercht. & J.Presl
Iz_7_1009	DJI_0489	Pilosella sp.	0		NA	0	Asteraceae Bercht. & J.Presl
Iz_7_1009	DJI_0489	Pinus sylvestris L.	0	n	1	1	Pinaceae Spreng. ex F.Rudolphi
Iz_7_1009	DJI_0489	Poa annua L.	0	n	1	0	Poaceae Barnhart
Iz_7_1009	DJI_0489	Poa nemoralis L.	0	n	1	0	Poaceae Barnhart
Iz_7_1009	DJI_0489	Poa sp.	0		NA	0	Poaceae Barnhart
Iz_7_1009	DJI_0489	Polygonatum odoratum (Mill.) Druce	0	n	1	0	Asparagaceae Juss.
Iz_7_1009	DJI_0489	Polygonum arenarium Waldst. & Kit.	0	n	1	0	Polygonaceae Juss.
Iz_7_1009	DJI_0489	Populus nigra L.	0	n	1	1	Salicaceae Mirb.
Iz_7_1009	DJI_0489	Populus tremula L.	0	n	1	1	Salicaceae Mirb.
Iz_7_1009	DJI_0489	Populus Ã canescens (Aiton) Sm.	0	n	1	1	Salicaceae Mirb.
Iz_7_1009	DJI_0489	Potentilla argentea L.	0	n	1	0	Rosaceae Juss.
Iz_7_1009	DJI_0489	Psammophiliella muralis (L.) Ikonn.	0	n	1	0	Caryophyllaceae Juss.
Iz_7_1009	DJI_0489	Psephellus sumensis (Kalen.) Greuter	0	n	1	0	Asteraceae Bercht. & J.Presl
Iz_7_1009	DJI_0489	Pulsatilla pratensis (L.) Mill. s.l.	0	n	1	0	Ranunculaceae Juss.
Iz_7_1009	DJI_0489	Pyrus communis L.	0	a	0	1	Rosaceae Juss.
Iz_7_1009	DJI_0489	Quercus robur L.	0	n	1	1	Fagaceae Dumort.
Iz_7_1009	DJI_0489	Robinia pseudoacacia L.	0	a	0	1	Fabaceae Lindl.
Iz_7_1009	DJI_0489	Rosa sp.	0		NA	1	Rosaceae Juss.
Iz_7_1009	DJI_0489	Rubus idaeus L.	0	n	1	0	Rosaceae Juss.
Iz_7_1009	DJI_0489	Rumex acetosella L.	0	n	1	0	Polygonaceae Juss.
Iz_7_1009	DJI_0489	Rumex thyrsiflorus Fingerh.	0	n	1	0	Polygonaceae Juss.
Iz_7_1009	DJI_0489	Salix alba L.	0	n	1	1	Salicaceae Mirb.
Iz_7_1009	DJI_0489	Sambucus nigra L.	0	n	1	1	Viburnaceae Raf.
Iz_7_1009	DJI_0489	Secale sylvestre Host	0	n	1	0	Poaceae Barnhart
Iz_7_1009	DJI_0489	Sempervivum ruthenicum Schnittsp. & C.B. Lehm.	0	n	1	0	Crassulaceae J.St.-Hil.
Iz_7_1009	DJI_0489	Setaria viridis (L.) P. Beauv.	0	n	1	0	Poaceae Barnhart
Iz_7_1009	DJI_0489	Silene borysthenica (Gruner) Walters	0	n	1	0	Caryophyllaceae Juss.
Iz_7_1009	DJI_0489	Silene latifolia subsp. alba (Mill.) Greuter & Burdet	0	n	1	0	Caryophyllaceae Juss.
Iz_7_1009	DJI_0489	Solidago virgaurea L.	0.5	n	1	0	Asteraceae Bercht. & J.Presl
Iz_7_1009	DJI_0489	Sorbus aucuparia L.	0	n	1	1	Rosaceae Juss.
Iz_7_1009	DJI_0489	Stachys recta L.	0	n	1	0	Lamiacea Lindl.
Iz_7_1009	DJI_0489	Tanacetum vulgare L.	0	n	1	0	Asteraceae Bercht. & J.Presl
Iz_7_1009	DJI_0489	Thymus pallasianus Heinr.Braun	0	n	1	0	Lamiacea Lindl.
Iz_7_1009	DJI_0489	Tilia cordata Mill.	0	n	1	1	Malvaceae Juss.
Iz_7_1009	DJI_0489	Trifolium alpestre L.	0	n	1	0	Fabaceae Lindl.
Iz_7_1009	DJI_0489	Trifolium arvense L.	0	n	1	0	Fabaceae Lindl.
Iz_7_1009	DJI_0489	Ulmus glabra Huds.	0	n	1	1	Ulmaceae Mirb.
Iz_7_1009	DJI_0489	Verbascum densiflorum Bertol.	0	n	1	0	Scrophulariaceae Juss.
Iz_7_1009	DJI_0489	Veronica austriaca L.	0	n	1	0	Plantaginaceae Juss.
Iz_7_1009	DJI_0489	Veronica incana L.	0	n	1	0	Plantaginaceae Juss.
Iz_7_1009	DJI_0489	Veronica verna L.	0	n	1	0	Plantaginaceae Juss.
Iz_7_1009	DJI_0489	Vincetoxicum hirundinaria Medik	0	n	1	0	Apocynaceae Juss.
Iz_7_1009	DJI_0497	Acer tataricum L.	0	n	1	1	Sapindaceae Juss.
Iz_7_1009	DJI_0497	Achillea millefolium L. s.l.	0	n	1	0	Asteraceae Bercht. & J.Presl
Iz_7_1009	DJI_0497	Allium oleraceum L.	0	n	1	0	Amaryllidaceae J.St.-Hil.
Iz_7_1009	DJI_0497	Ambrosia artemisiifolia L.	0	a	0	0	Asteraceae Bercht. & J.Presl
Iz_7_1009	DJI_0497	Amorpha fruticosa L.	0	a	0	0	Fabaceae Lindl.
Iz_7_1009	DJI_0497	Anthericum ramosum L.	0	n	1	0	Asparagaceae Juss.
Iz_7_1009	DJI_0497	Artemisia marschalliana Spreng.	0	n	1	0	Asteraceae Bercht. & J.Presl
Iz_7_1009	DJI_0497	Asparagus officinalis L.	0	n	1	0	Asparagaceae Juss.
Iz_7_1009	DJI_0497	Astragalus cicer L.	0	n	1	0	Fabaceae Lindl.
Iz_7_1009	DJI_0497	Bassia laniflora (S.G.Gmel.) A.J.Scott	0	n	1	0	Amaranthaceae Juss.
Iz_7_1009	DJI_0497	Berberis vulgaris L.	0	n	1	0	Berberidaceae Juss.
Iz_7_1009	DJI_0497	Betula pendula Roth.	0	n	1	1	Betulaceae Gray
Iz_7_1009	DJI_0497	Betula pendula Roth.	0	n	1	0	Poaceae Barnhart
Iz_7_1009	DJI_0497	Bromus inermis Leyss.	0		NA	NA	
Iz_7_1009	DJI_0497	Bromus squarrosus L.	0	a	0	0	Poaceae Barnhart
Iz_7_1009	DJI_0497	Bromus tectorum L.	0	a	0	0	Poaceae Barnhart
Iz_7_1009	DJI_0497	Calamagrostis epigejos (L.) Roth	0	n	1	0	Poaceae Barnhart
Iz_7_1009	DJI_0497	Carduus acanthoides L.	0	n	1	0	Asteraceae Bercht. & J.Presl
Iz_7_1009	DJI_0497	Carex sp.	0	n	1	0	Cyperaceae Juss.
Iz_7_1009	DJI_0497	Centaurea majorovii Dumbadze	0	n	1	0	Asteraceae Bercht. & J.Presl
Iz_7_1009	DJI_0497	Chamaecytisus ruthenicus (Fisch.ex Wol.) Klaskova	0	n	1	0	Fabaceae Lindl.
Iz_7_1009	DJI_0497	Chelidonium majus L.	0	n	1	0	Papaveraceae Juss.
Iz_7_1009	DJI_0497	Chenopodium betaceum Andrz.	0	n	1	0	Amaranthaceae Juss.
Iz_7_1009	DJI_0497	Chenopodium cf. album	0	n	1	0	Amaranthaceae Juss.
Iz_7_1009	DJI_0497	Chenopodium sp.	0		NA	0	Amaranthaceae Juss.
Iz_7_1009	DJI_0497	Chondrilla juncea L.	0	n	1	0	Asteraceae Bercht. & J.Presl
Iz_7_1009	DJI_0497	Cirsium vulgare (Savi) Ten.	0	n	1	0	Asteraceae Bercht. & J.Presl
Iz_7_1009	DJI_0497	Clinopodium acinos (L.) Kuntze	0	n	1	0	Lamiacea Lindl.
Iz_7_1009	DJI_0497	Convallaria majalis L.	0	n	1	0	Asparagaceae Juss.
Iz_7_1009	DJI_0497	Crataegus rhipidophylla Gand.	0	n	1	1	Rosaceae Juss.
Iz_7_1009	DJI_0497	Crepis foetida L.	0	n	1	0	Asteraceae Bercht. & J.Presl
Iz_7_1009	DJI_0497	Dactylis glomerata L.	0	n	1	0	Poaceae Barnhart
Iz_7_1009	DJI_0497	Dianthus campestris M.Bieb.	0	n	1	0	Caryophyllaceae Juss.
Iz_7_1009	DJI_0497	Digitaria sanguinalis (L.) Scop.	0	a	0	0	Poaceae Barnhart
Iz_7_1009	DJI_0497	Elymus repens (L.) Gould	0	n	1	0	Poaceae Barnhart
Iz_7_1009	DJI_0497	Epilobium tetragonum L.	0	n	1	0	Onagraceae Juss.
Iz_7_1009	DJI_0497	Eragrostis minor Host	0	a	0	0	Poaceae Barnhart

Iz_7_1009	DJI_0497	Erigeron canadensis L.	40	a	0	0	Asteraceae Bercht. & J.Presl
Iz_7_1009	DJI_0497	Euphorbia seguieriana Neck.	0	n	1	0	Euphorbiaceae Juss.
Iz_7_1009	DJI_0497	Fallopia dumetorum (L.) Holub	0	n	1	0	Polygonaceae Juss.
Iz_7_1009	DJI_0497	Festuca beckeri (Hack.) Trautv.	0	n	1	0	Poaceae Barnhart
Iz_7_1009	DJI_0497	Fragaria vesca L.	0	n	1	0	Rosaceae Juss.
Iz_7_1009	DJI_0497	Frangula alnus Mill.	0	n	1	1	Rhamnaceae Juss.
Iz_7_1009	DJI_0497	Fraxinus pennsylvanica Marshall	0	a	0	1	Oleaceae Hoffmanns. & Link
Iz_7_1009	DJI_0497	Galeopsis bifida Boenn.	0	n	1	0	Lamiacea Lindl.
Iz_7_1009	DJI_0497	Galium verum L.	0	n	1	0	Rubiaceae Juss.
Iz_7_1009	DJI_0497	Genista tinctoria L.	0	n	1	0	Fabaceae Lindl.
Iz_7_1009	DJI_0497	Geranium robertianum L.	0	n	1	0	Geraniaceae Juss.
Iz_7_1009	DJI_0497	Geranium sanquineum L.	0	n	1	0	Geraniaceae Juss.
Iz_7_1009	DJI_0497	Gypsophila paniculata L.	0	n	1	0	Caryophyllaceae Juss.
Iz_7_1009	DJI_0497	Helichrysum arenarium (L.) Moench	0	n	1	0	Asteraceae Bercht. & J.Presl
Iz_7_1009	DJI_0497	Herniaria polygama J.Gay	0	n	1	0	Caryophyllaceae Juss.
Iz_7_1009	DJI_0497	Hieracium robustum Fr.	0	n	1	0	Asteraceae Bercht. & J.Presl
Iz_7_1009	DJI_0497	Hieracium umbellatum L.	0	n	1	NA	Asteraceae Bercht. & J.Presl
Iz_7_1009	DJI_0497	Hylotelephium maximum (L.) Holub	0	n	1	0	Crassulaceae J.St.-Hil.
Iz_7_1009	DJI_0497	Jacobaea borysthenica (DC.) B.Nord. & Greuter	0	n	1	0	Asteraceae Bercht. & J.Presl
Iz_7_1009	DJI_0497	Jasione montana L.	0	n	1	0	Campanulaceae Juss.
Iz_7_1009	DJI_0497	Jurinea cyanoides (L.) Rchb.s.l.	0	n	1	0	Asteraceae Bercht. & J.Presl
Iz_7_1009	DJI_0497	Koeleria glauca (Spreng.) DC.	0	n	1	0	Poaceae Barnhart
Iz_7_1009	DJI_0497	Lactuca serriola L.	0	a	0	0	Asteraceae Bercht. & J.Presl
Iz_7_1009	DJI_0497	Linaria genistifolia (L.) Mill.	0	n	1	0	Plantaginaceae Juss.
Iz_7_1009	DJI_0497	Lomelosia argentea (L.) Greuter & Burdet	0	n	1	0	Caprifoliaceae Juss.
Iz_7_1009	DJI_0497	Melampyrum pratense L.	0	n	1	0	Orobanchaceae Vent.
Iz_7_1009	DJI_0497	Oenothera biennis L.	0	a	0	0	Onagraceae Juss.
Iz_7_1009	DJI_0497	Pentanema britannica (L.) D.Gut.Larr., Santos-Vicente, Anderb., E.Rico & M.M.Mart.Ort.	0	n	1	0	Asteraceae Bercht. & J.Presl
Iz_7_1009	DJI_0497	Peucedanum oreoselinum (L.) Moench	0	n	1	0	Apiaceae Lindl.
Iz_7_1009	DJI_0497	Peucedanum ruthenicum M.Bieb.	0	n	1	0	Apiaceae Lindl.
Iz_7_1009	DJI_0497	Pilosella echioides (Lumn.) F.W.Schultz & Sch.Bip.	0	n	1	0	Asteraceae Bercht. & J.Presl
Iz_7_1009	DJI_0497	Pilosella officinarum F. Schultz et Sch. Bip.	0	n	1	0	Asteraceae Bercht. & J.Presl
Iz_7_1009	DJI_0497	Pilosella sp.	0		NA	0	Asteraceae Bercht. & J.Presl
Iz_7_1009	DJI_0497	Pinus sylvestris L.	0	n	1	1	Pinaceae Spreng. ex F.Rudolphi
Iz_7_1009	DJI_0497	Poa annua L.	0	n	1	0	Poaceae Barnhart
Iz_7_1009	DJI_0497	Poa nemoralis L.	0	n	1	0	Poaceae Barnhart
Iz_7_1009	DJI_0497	Poa sp.	0		NA	0	Poaceae Barnhart
Iz_7_1009	DJI_0497	Polygonatum odoratum (Mill.) Druce	0	n	1	0	Asparagaceae Juss.
Iz_7_1009	DJI_0497	Polygonum arenarium Waldst. & Kit.	0	n	1	0	Polygonaceae Juss.
Iz_7_1009	DJI_0497	Populus nigra L.	0	n	1	1	Salicaceae Mirb.
Iz_7_1009	DJI_0497	Populus tremula L.	0	n	1	1	Salicaceae Mirb.
Iz_7_1009	DJI_0497	Populus Ã canescens (Aiton) Sm.	0	n	1	1	Salicaceae Mirb.
Iz_7_1009	DJI_0497	Potentilla argentea L.	0	n	1	0	Rosaceae Juss.
Iz_7_1009	DJI_0497	Psammophiliella muralis (L.) Ikonn.	0	n	1	0	Caryophyllaceae Juss.
Iz_7_1009	DJI_0497	Psephellus sumensis (Kalen.) Greuter	0	n	1	0	Asteraceae Bercht. & J.Presl
Iz_7_1009	DJI_0497	Pulsatilla pratensis (L.) Mill. s.l.	0	n	1	0	Ranunculaceae Juss.
Iz_7_1009	DJI_0497	Pyrus communis L.	0	a	0	1	Rosaceae Juss.
Iz_7_1009	DJI_0497	Quercus robur L.	0	n	1	1	Fagaceae Dumort.
Iz_7_1009	DJI_0497	Robinia pseudoacacia L.	0	a	0	1	Fabaceae Lindl.
Iz_7_1009	DJI_0497	Rosa sp.	0		NA	1	Rosaceae Juss.
Iz_7_1009	DJI_0497	Rubus idaeus L.	0	n	1	0	Rosaceae Juss.
Iz_7_1009	DJI_0497	Rumex acetosella L.	0	n	1	0	Polygonaceae Juss.
Iz_7_1009	DJI_0497	Rumex thyrsiflorus Fingerh.	0	n	1	0	Polygonaceae Juss.
Iz_7_1009	DJI_0497	Salix alba L.	0	n	1	1	Salicaceae Mirb.
Iz_7_1009	DJI_0497	Sambucus nigra L.	0	n	1	1	Viburnaceae Raf.
Iz_7_1009	DJI_0497	Secale sylvestre Host	0	n	1	0	Poaceae Barnhart
Iz_7_1009	DJI_0497	Sempervivum ruthenicum Schnittsp. & C.B. Lehm.	0	n	1	0	Crassulaceae J.St.-Hil.
Iz_7_1009	DJI_0497	Setaria viridis (L.) P. Beauv.	0	n	1	0	Poaceae Barnhart
Iz_7_1009	DJI_0497	Silene borysthenica (Gruner) Walters	0	n	1	0	Caryophyllaceae Juss.
Iz_7_1009	DJI_0497	Silene latifolia subsp. alba (Mill.) Greuter & Burdet	0	n	1	0	Caryophyllaceae Juss.
Iz_7_1009	DJI_0497	Solidago virgaurea L.	0	n	1	0	Asteraceae Bercht. & J.Presl
Iz_7_1009	DJI_0497	Sorbus aucuparia L.	0	n	1	1	Rosaceae Juss.
Iz_7_1009	DJI_0497	Stachys recta L.	0	n	1	0	Lamiacea Lindl.
Iz_7_1009	DJI_0497	Tanacetum vulgare L.	0	n	1	0	Asteraceae Bercht. & J.Presl
Iz_7_1009	DJI_0497	Thymus pallasianus Heinr.Braun	0	n	1	0	Lamiacea Lindl.
Iz_7_1009	DJI_0497	Tilia cordata Mill.	0	n	1	1	Malvaceae Juss.
Iz_7_1009	DJI_0497	Trifolium alpestre L.	0	n	1	0	Fabaceae Lindl.
Iz_7_1009	DJI_0497	Trifolium arvense L.	0	n	1	0	Fabaceae Lindl.
Iz_7_1009	DJI_0497	Ulmus glabra Huds.	0	n	1	1	Ulmaceae Mirb.
Iz_7_1009	DJI_0497	Verbascum densiflorum Bertol.	0	n	1	0	Scrophulariaceae Juss.
Iz_7_1009	DJI_0497	Veronica austriaca L.	0	n	1	0	Plantaginaceae Juss.
Iz_7_1009	DJI_0497	Veronica incana L.	0	n	1	0	Plantaginaceae Juss.
Iz_7_1009	DJI_0497	Veronica verna L.	0	n	1	0	Plantaginaceae Juss.
Iz_7_1009	DJI_0497	Vincetoxicum hirundinaria Medik	0	n	1	0	Apocynaceae Juss.
Iz_7_1009	DJI_0499	Acer tataricum L.	0	n	1	1	Sapindaceae Juss.
Iz_7_1009	DJI_0499	Achillea millefolium L. s.l.	0	n	1	0	Asteraceae Bercht. & J.Presl
Iz_7_1009	DJI_0499	Allium oleraceum L.	0	n	1	0	Amaryllidaceae J.St.-Hil.
Iz_7_1009	DJI_0499	Ambrosia artemisiifolia L.	0	a	0	0	Asteraceae Bercht. & J.Presl
Iz_7_1009	DJI_0499	Amorpha fruticosa L.	0	a	0	0	Fabaceae Lindl.
Iz_7_1009	DJI_0499	Anthericum ramosum L.	0	n	1	0	Asparagaceae Juss.
Iz_7_1009	DJI_0499	Artemisia marschalliana Spreng.	0	n	1	0	Asteraceae Bercht. & J.Presl
Iz_7_1009	DJI_0499	Asparagus officinalis L.	0	n	1	0	Asparagaceae Juss.
Iz_7_1009	DJI_0499	Astragalus cicer L.	0	n	1	0	Fabaceae Lindl.
Iz_7_1009	DJI_0499	Bassia laniflora (S.G.Gmel.) A.J.Scott	0	n	1	0	Amaranthaceae Juss.
Iz_7_1009	DJI_0499	Berberis vulgaris L.	0	n	1	0	Berberidaceae Juss.
Iz_7_1009	DJI_0499	Betula pendula Roth.	0	n	1	1	Betulaceae Gray
Iz_7_1009	DJI_0499	Betula pendula Roth.	0	n	1	0	Poaceae Barnhart
Iz_7_1009	DJI_0499	Bromus inermis Leyss.	0		NA	NA	
Iz_7_1009	DJI_0499	Bromus squarrosus L.	0	a	0	0	Poaceae Barnhart
Iz_7_1009	DJI_0499	Bromus tectorum L.	0	a	0	0	Poaceae Barnhart
Iz_7_1009	DJI_0499	Calamagrostis epigejos (L.) Roth	0	n	1	0	Poaceae Barnhart
Iz_7_1009	DJI_0499	Carduus acanthoides L.	0	n	1	0	Asteraceae Bercht. & J.Presl
Iz_7_1009	DJI_0499	Carex sp.	0	n	1	0	Cyperaceae Juss.
Iz_7_1009	DJI_0499	Centaurea majorovii Dumbadze	0	n	1	0	Asteraceae Bercht. & J.Presl
Iz_7_1009	DJI_0499	Chamaecytisus ruthenicus (Fisch.ex Wol.) Klaskova	0	n	1	0	Fabaceae Lindl.
Iz_7_1009	DJI_0499	Chelidonium majus L.	0	n	1	0	Papaveraceae Juss.
Iz_7_1009	DJI_0499	Chenopodium betaceum Andrz.	0	n	1	0	Amaranthaceae Juss.
Iz_7_1009	DJI_0499	Chenopodium cf. album	0	n	1	0	Amaranthaceae Juss.

Iz_7_1009	DJI_0499	Chenopodium sp.	0		NA	0	Amaranthaceae Juss.
Iz_7_1009	DJI_0499	Chondrilla juncea L.	0	n	1	0	Asteraceae Bercht. & J.Presl
Iz_7_1009	DJI_0499	Cirsium vulgare (Savi) Ten.	0	n	1	0	Asteraceae Bercht. & J.Presl
Iz_7_1009	DJI_0499	Clinopodium acinos (L.) Kuntze	0	n	1	0	Lamiacea Lindl.
Iz_7_1009	DJI_0499	Convallaria majalis L.	0	n	1	0	Asparagaceae Juss.
Iz_7_1009	DJI_0499	Crataegus rhipidophylla Gand.	0	n	1	1	Rosaceae Juss.
Iz_7_1009	DJI_0499	Crepis foetida L.	0	n	1	0	Asteraceae Bercht. & J.Presl
Iz_7_1009	DJI_0499	Dactylis glomerata L.	0	n	1	0	Poaceae Barnhart
Iz_7_1009	DJI_0499	Dianthus campestris M.Bieb.	0	n	1	0	Caryophyllaceae Juss.
Iz_7_1009	DJI_0499	Digitaria sanguinalis (L.) Scop.	0	a	0	0	Poaceae Barnhart
Iz_7_1009	DJI_0499	Elymus repens (L.) Gould	0	n	1	0	Poaceae Barnhart
Iz_7_1009	DJI_0499	Epilobium tetragonum L.	0	n	1	0	Onagraceae Juss.
Iz_7_1009	DJI_0499	Eragrostis minor Host	0	a	0	0	Poaceae Barnhart
Iz_7_1009	DJI_0499	Erigeron canadensis L.	40	a	0	0	Asteraceae Bercht. & J.Presl
Iz_7_1009	DJI_0499	Euphorbia seguieriana Neck.	0	n	1	0	Euphorbiaceae Juss.
Iz_7_1009	DJI_0499	Fallopia dumetorum (L.) Holub	0	n	1	0	Polygonaceae Juss.
Iz_7_1009	DJI_0499	Festuca beckeri (Hack.) Trautv.	0	n	1	0	Poaceae Barnhart
Iz_7_1009	DJI_0499	Fragaria vesca L.	0	n	1	0	Rosaceae Juss.
Iz_7_1009	DJI_0499	Frangula alnus Mill.	0	n	1	1	Rhamnaceae Juss.
Iz_7_1009	DJI_0499	Fraxinus pennsylvanica Marshall	0	a	0	1	Oleaceae Hoffmanns. & Link
Iz_7_1009	DJI_0499	Galeopsis bifida Boenn.	0	n	1	0	Lamiacea Lindl.
Iz_7_1009	DJI_0499	Galium verum L.	0	n	1	0	Rubiaceae Juss.
Iz_7_1009	DJI_0499	Genista tinctoria L.	0	n	1	0	Fabaceae Lindl.
Iz_7_1009	DJI_0499	Geranium robertianum L.	0	n	1	0	Geraniaceae Juss.
Iz_7_1009	DJI_0499	Geranium sanquineum L.	0	n	1	0	Geraniaceae Juss.
Iz_7_1009	DJI_0499	Gypsophila paniculata L.	0	n	1	0	Caryophyllaceae Juss.
Iz_7_1009	DJI_0499	Helichrysum arenarium (L.) Moench	0	n	1	0	Asteraceae Bercht. & J.Presl
Iz_7_1009	DJI_0499	Herniaria polygama J.Gay	0	n	1	0	Caryophyllaceae Juss.
Iz_7_1009	DJI_0499	Hieracium robustum Fr.	0	n	1	0	Asteraceae Bercht. & J.Presl
Iz_7_1009	DJI_0499	Hieracium umbellatum L.	0	n	1	NA	Asteraceae Bercht. & J.Presl
Iz_7_1009	DJI_0499	Hylotelephium maximum (L.) Holub	0	n	1	0	Crassulaceae J.St.-Hil.
Iz_7_1009	DJI_0499	Jacobaea borysthenica (DC.) B.Nord. & Greuter	0	n	1	0	Asteraceae Bercht. & J.Presl
Iz_7_1009	DJI_0499	Jasione montana L.	0	n	1	0	Campanulaceae Juss.
Iz_7_1009	DJI_0499	Jurinea cyanoides (L.) Rchb.s.l.	0	n	1	0	Asteraceae Bercht. & J.Presl
Iz_7_1009	DJI_0499	Koeleria glauca (Spreng.) DC.	0	n	1	0	Poaceae Barnhart
Iz_7_1009	DJI_0499	Lactuca serriola L.	0	a	0	0	Asteraceae Bercht. & J.Presl
Iz_7_1009	DJI_0499	Linaria genistifolia (L.) Mill.	0	n	1	0	Plantaginaceae Juss.
Iz_7_1009	DJI_0499	Lomelosia argentea (L.) Greuter & Burdet	0	n	1	0	Caprifoliaceae Juss.
Iz_7_1009	DJI_0499	Melampyrum pratense L.	0	n	1	0	Orobanchaceae Vent.
Iz_7_1009	DJI_0499	Oenothera biennis L.	0	a	0	0	Onagraceae Juss.
Iz_7_1009	DJI_0499	Pentanema britannica (L.) D.Gut.Larr., Santos-Vicente, Anderb., E.Rico & M.M.Mart.Ort.	0	n	1	0	Asteraceae Bercht. & J.Presl
Iz_7_1009	DJI_0499	Peucedanum oreoselinum (L.) Moench	0	n	1	0	Apiaceae Lindl.
Iz_7_1009	DJI_0499	Peucedanum ruthenicum M.Bieb.	0	n	1	0	Apiaceae Lindl.
Iz_7_1009	DJI_0499	Pilosella echioides (Lumn.) F.W.Schultz & Sch.Bip.	0	n	1	0	Asteraceae Bercht. & J.Presl
Iz_7_1009	DJI_0499	Pilosella officinarum F. Schultz et Sch. Bip.	0	n	1	0	Asteraceae Bercht. & J.Presl
Iz_7_1009	DJI_0499	Pilosella sp.	0		NA	0	Asteraceae Bercht. & J.Presl
Iz_7_1009	DJI_0499	Pinus sylvestris L.	0	n	1	1	Pinaceae Spreng. ex F.Rudolphi
Iz_7_1009	DJI_0499	Poa annua L.	0	n	1	0	Poaceae Barnhart
Iz_7_1009	DJI_0499	Poa nemoralis L.	0	n	1	0	Poaceae Barnhart
Iz_7_1009	DJI_0499	Poa sp.	0		NA	0	Poaceae Barnhart
Iz_7_1009	DJI_0499	Polygonatum odoratum (Mill.) Druce	0	n	1	0	Asparagaceae Juss.
Iz_7_1009	DJI_0499	Polygonum arenarium Waldst. & Kit.	0	n	1	0	Polygonaceae Juss.
Iz_7_1009	DJI_0499	Populus nigra L.	0	n	1	1	Salicaceae Mirb.
Iz_7_1009	DJI_0499	Populus tremula L.	0	n	1	1	Salicaceae Mirb.
Iz_7_1009	DJI_0499	Populus Ã canescens (Aiton) Sm.	0	n	1	1	Salicaceae Mirb.
Iz_7_1009	DJI_0499	Potentilla argentea L.	0	n	1	0	Rosaceae Juss.
Iz_7_1009	DJI_0499	Psammophiliella muralis (L.) Ikonn.	0	n	1	0	Caryophyllaceae Juss.
Iz_7_1009	DJI_0499	Psephellus sumensis (Kalen.) Greuter	0	n	1	0	Asteraceae Bercht. & J.Presl
Iz_7_1009	DJI_0499	Pulsatilla pratensis (L.) Mill. s.l.	0	n	1	0	Ranunculaceae Juss.
Iz_7_1009	DJI_0499	Pyrus communis L.	0	a	0	1	Rosaceae Juss.
Iz_7_1009	DJI_0499	Quercus robur L.	0	n	1	1	Fagaceae Dumort.
Iz_7_1009	DJI_0499	Robinia pseudoacacia L.	0	a	0	1	Fabaceae Lindl.
Iz_7_1009	DJI_0499	Rosa sp.	0		NA	1	Rosaceae Juss.
Iz_7_1009	DJI_0499	Rubus idaeus L.	0	n	1	0	Rosaceae Juss.
Iz_7_1009	DJI_0499	Rumex acetosella L.	0	n	1	0	Polygonaceae Juss.
Iz_7_1009	DJI_0499	Rumex thyrsiflorus Fingerh.	0	n	1	0	Polygonaceae Juss.
Iz_7_1009	DJI_0499	Salix alba L.	0	n	1	1	Salicaceae Mirb.
Iz_7_1009	DJI_0499	Sambucus nigra L.	0	n	1	1	Viburnaceae Raf.
Iz_7_1009	DJI_0499	Secale sylvestre Host	0	n	1	0	Poaceae Barnhart
Iz_7_1009	DJI_0499	Sempervivum ruthenicum Schnittsp. & C.B. Lehm.	0	n	1	0	Crassulaceae J.St.-Hil.
Iz_7_1009	DJI_0499	Setaria viridis (L.) P. Beauv.	0	n	1	0	Poaceae Barnhart
Iz_7_1009	DJI_0499	Silene borysthenica (Gruner) Walters	0	n	1	0	Caryophyllaceae Juss.
Iz_7_1009	DJI_0499	Silene latifolia subsp. alba (Mill.) Greuter & Burdet	0	n	1	0	Caryophyllaceae Juss.
Iz_7_1009	DJI_0499	Solidago virgaurea L.	0	n	1	0	Asteraceae Bercht. & J.Presl
Iz_7_1009	DJI_0499	Sorbus aucuparia L.	0	n	1	1	Rosaceae Juss.
Iz_7_1009	DJI_0499	Stachys recta L.	0	n	1	0	Lamiacea Lindl.
Iz_7_1009	DJI_0499	Tanacetum vulgare L.	0	n	1	0	Asteraceae Bercht. & J.Presl
Iz_7_1009	DJI_0499	Thymus pallasianus Heinr.Braun	0	n	1	0	Lamiacea Lindl.
Iz_7_1009	DJI_0499	Tilia cordata Mill.	0	n	1	1	Malvaceae Juss.
Iz_7_1009	DJI_0499	Trifolium alpestre L.	0	n	1	0	Fabaceae Lindl.
Iz_7_1009	DJI_0499	Trifolium arvense L.	0	n	1	0	Fabaceae Lindl.
Iz_7_1009	DJI_0499	Ulmus glabra Huds.	0	n	1	1	Ulmaceae Mirb.
Iz_7_1009	DJI_0499	Verbascum densiflorum Bertol.	0	n	1	0	Scrophulariaceae Juss.
Iz_7_1009	DJI_0499	Veronica austriaca L.	0	n	1	0	Plantaginaceae Juss.
Iz_7_1009	DJI_0499	Veronica incana L.	0	n	1	0	Plantaginaceae Juss.
Iz_7_1009	DJI_0499	Veronica verna L.	0	n	1	0	Plantaginaceae Juss.
Iz_7_1009	DJI_0499	Vincetoxicum hirundinaria Medik	0	n	1	0	Apocynaceae Juss.
Iz_7_1009	DJI_0507	Acer tataricum L.	0	n	1	1	Sapindaceae Juss.
Iz_7_1009	DJI_0507	Achillea millefolium L. s.l.	0	n	1	0	Asteraceae Bercht. & J.Presl
Iz_7_1009	DJI_0507	Allium oleraceum L.	0	n	1	0	Amaryllidaceae J.St.-Hil.
Iz_7_1009	DJI_0507	Ambrosia artemisiifolia L.	0	a	0	0	Asteraceae Bercht. & J.Presl
Iz_7_1009	DJI_0507	Amorpha fruticosa L.	0	a	0	0	Fabaceae Lindl.
Iz_7_1009	DJI_0507	Anthericum ramosum L.	0	n	1	0	Asparagaceae Juss.
Iz_7_1009	DJI_0507	Artemisia marschalliana Spreng.	0	n	1	0	Asteraceae Bercht. & J.Presl
Iz_7_1009	DJI_0507	Asparagus officinalis L.	0	n	1	0	Asparagaceae Juss.
Iz_7_1009	DJI_0507	Astragalus cicer L.	0	n	1	0	Fabaceae Lindl.
Iz_7_1009	DJI_0507	Bassia laniflora (S.G.Gmel.) A.J.Scott	0	n	1	0	Amaranthaceae Juss.
Iz_7_1009	DJI_0507	Berberis vulgaris L.	0	n	1	0	Berberidaceae Juss.

Iz_7_1009	DJI_0507	Betula pendula Roth.	0	n	1	1	Betulaceae Gray
Iz_7_1009	DJI_0507	Betula pendula Roth.	0	n	1	0	Poaceae Barnhart
Iz_7_1009	DJI_0507	Bromus inermis Leyss.	0		NA	NA	
Iz_7_1009	DJI_0507	Bromus squarrosus L.	0	a	0	0	Poaceae Barnhart
Iz_7_1009	DJI_0507	Bromus tectorum L.	0	a	0	0	Poaceae Barnhart
Iz_7_1009	DJI_0507	Calamagrostis epigejos (L.) Roth	0	n	1	0	Poaceae Barnhart
Iz_7_1009	DJI_0507	Carduus acanthoides L.	0	n	1	0	Asteraceae Bercht. & J.Presl
Iz_7_1009	DJI_0507	Carex sp.	0	n	1	0	Cyperaceae Juss.
Iz_7_1009	DJI_0507	Centaurea majorovii Dumbadze	0	n	1	0	Asteraceae Bercht. & J.Presl
Iz_7_1009	DJI_0507	Chamaecytisus ruthenicus (Fisch.ex Wol.) Klaskova	0	n	1	0	Fabaceae Lindl.
Iz_7_1009	DJI_0507	Chelidonium majus L.	0	n	1	0	Papaveraceae Juss.
Iz_7_1009	DJI_0507	Chenopodium betaceum Andrz.	0	n	1	0	Amaranthaceae Juss.
Iz_7_1009	DJI_0507	Chenopodium cf. album	0	n	1	0	Amaranthaceae Juss.
Iz_7_1009	DJI_0507	Chenopodium sp.	0		NA	0	Amaranthaceae Juss.
Iz_7_1009	DJI_0507	Chondrilla juncea L.	0	n	1	0	Asteraceae Bercht. & J.Presl
Iz_7_1009	DJI_0507	Cirsium vulgare (Savi) Ten.	0	n	1	0	Asteraceae Bercht. & J.Presl
Iz_7_1009	DJI_0507	Clinopodium acinos (L.) Kuntze	0	n	1	0	Lamiacea Lindl.
Iz_7_1009	DJI_0507	Convallaria majalis L.	0	n	1	0	Asparagaceae Juss.
Iz_7_1009	DJI_0507	Crataegus rhipidophylla Gand.	0	n	1	1	Rosaceae Juss.
Iz_7_1009	DJI_0507	Crepis foetida L.	0	n	1	0	Asteraceae Bercht. & J.Presl
Iz_7_1009	DJI_0507	Dactylis glomerata L.	0	n	1	0	Poaceae Barnhart
Iz_7_1009	DJI_0507	Dianthus campestris M.Bieb.	0	n	1	0	Caryophyllaceae Juss.
Iz_7_1009	DJI_0507	Digitaria sanguinalis (L.) Scop.	0	a	0	0	Poaceae Barnhart
Iz_7_1009	DJI_0507	Elymus repens (L.) Gould	0	n	1	0	Poaceae Barnhart
Iz_7_1009	DJI_0507	Epilobium tetragonum L.	0	n	1	0	Onagraceae Juss.
Iz_7_1009	DJI_0507	Eragrostis minor Host	0	a	0	0	Poaceae Barnhart
Iz_7_1009	DJI_0507	Erigeron canadensis L.	40	a	0	0	Asteraceae Bercht. & J.Presl
Iz_7_1009	DJI_0507	Euphorbia seguieriana Neck.	0	n	1	0	Euphorbiaceae Juss.
Iz_7_1009	DJI_0507	Fallopia dumetorum (L.) Holub	0	n	1	0	Polygonaceae Juss.
Iz_7_1009	DJI_0507	Festuca beckeri (Hack.) Trautv.	0	n	1	0	Poaceae Barnhart
Iz_7_1009	DJI_0507	Fragaria vesca L.	0	n	1	0	Rosaceae Juss.
Iz_7_1009	DJI_0507	Frangula alnus Mill.	0	n	1	1	Rhamnaceae Juss.
Iz_7_1009	DJI_0507	Fraxinus pennsylvanica Marshall	0	a	0	1	Oleaceae Hoffmanns. & Link
Iz_7_1009	DJI_0507	Galeopsis bifida Boenn.	0	n	1	0	Lamiacea Lindl.
Iz_7_1009	DJI_0507	Galium verum L.	0	n	1	0	Rubiaceae Juss.
Iz_7_1009	DJI_0507	Genista tinctoria L.	0	n	1	0	Fabaceae Lindl.
Iz_7_1009	DJI_0507	Geranium robertianum L.	0	n	1	0	Geraniaceae Juss.
Iz_7_1009	DJI_0507	Geranium sanquineum L.	0	n	1	0	Geraniaceae Juss.
Iz_7_1009	DJI_0507	Gypsophila paniculata L.	0	n	1	0	Caryophyllaceae Juss.
Iz_7_1009	DJI_0507	Helichrysum arenarium (L.) Moench	0	n	1	0	Asteraceae Bercht. & J.Presl
Iz_7_1009	DJI_0507	Herniaria polygama J.Gay	0	n	1	0	Caryophyllaceae Juss.
Iz_7_1009	DJI_0507	Hieracium robustum Fr.	0	n	1	0	Asteraceae Bercht. & J.Presl
Iz_7_1009	DJI_0507	Hieracium umbellatum L.	0	n	1	NA	Asteraceae Bercht. & J.Presl
Iz_7_1009	DJI_0507	Hylotelephium maximum (L.) Holub	0	n	1	0	Crassulaceae J.St.-Hil.
Iz_7_1009	DJI_0507	Jacobaea borysthenica (DC.) B.Nord. & Greuter	0	n	1	0	Asteraceae Bercht. & J.Presl
Iz_7_1009	DJI_0507	Jasione montana L.	0	n	1	0	Campanulaceae Juss.
Iz_7_1009	DJI_0507	Jurinea cyanoides (L.) Rchb.s.l.	0	n	1	0	Asteraceae Bercht. & J.Presl
Iz_7_1009	DJI_0507	Koeleria glauca (Spreng.) DC.	0	n	1	0	Poaceae Barnhart
Iz_7_1009	DJI_0507	Lactuca serriola L.	0	a	0	0	Asteraceae Bercht. & J.Presl
Iz_7_1009	DJI_0507	Linaria genistifolia (L.) Mill.	0	n	1	0	Plantaginaceae Juss.
Iz_7_1009	DJI_0507	Lomelosia argentea (L.) Greuter & Burdet	0	n	1	0	Caprifoliaceae Juss.
Iz_7_1009	DJI_0507	Melampyrum pratense L.	0	n	1	0	Orobanchaceae Vent.
Iz_7_1009	DJI_0507	Oenothera biennis L.	0	a	0	0	Onagraceae Juss.
Iz_7_1009	DJI_0507	Pentanema britannica (L.) D.Gut.Larr., Santos-Vicente, Anderb., E.Rico & M.M.Mart.Ort.	0	n	1	0	Asteraceae Bercht. & J.Presl
Iz_7_1009	DJI_0507	Peucedanum oreoselinum (L.) Moench	0	n	1	0	Apiaceae Lindl.
Iz_7_1009	DJI_0507	Peucedanum ruthenicum M.Bieb.	0	n	1	0	Apiaceae Lindl.
Iz_7_1009	DJI_0507	Pilosella echioides (Lumn.) F.W.Schultz & Sch.Bip.	0	n	1	0	Asteraceae Bercht. & J.Presl
Iz_7_1009	DJI_0507	Pilosella officinarum F. Schultz et Sch. Bip.	0	n	1	0	Asteraceae Bercht. & J.Presl
Iz_7_1009	DJI_0507	Pilosella sp.	0		NA	0	Asteraceae Bercht. & J.Presl
Iz_7_1009	DJI_0507	Pinus sylvestris L.	0	n	1	1	Pinaceae Spreng. ex F.Rudolphi
Iz_7_1009	DJI_0507	Poa annua L.	0	n	1	0	Poaceae Barnhart
Iz_7_1009	DJI_0507	Poa nemoralis L.	0	n	1	0	Poaceae Barnhart
Iz_7_1009	DJI_0507	Poa sp.	0		NA	0	Poaceae Barnhart
Iz_7_1009	DJI_0507	Polygonatum odoratum (Mill.) Druce	0	n	1	0	Asparagaceae Juss.
Iz_7_1009	DJI_0507	Polygonum arenarium Waldst. & Kit.	0	n	1	0	Polygonaceae Juss.
Iz_7_1009	DJI_0507	Populus nigra L.	0	n	1	1	Salicaceae Mirb.
Iz_7_1009	DJI_0507	Populus tremula L.	0	n	1	1	Salicaceae Mirb.
Iz_7_1009	DJI_0507	Populus Ã canescens (Aiton) Sm.	0	n	1	1	Salicaceae Mirb.
Iz_7_1009	DJI_0507	Potentilla argentea L.	0	n	1	0	Rosaceae Juss.
Iz_7_1009	DJI_0507	Psammophiliella muralis (L.) Ikonn.	0	n	1	0	Caryophyllaceae Juss.
Iz_7_1009	DJI_0507	Psephellus sumensis (Kalen.) Greuter	0	n	1	0	Asteraceae Bercht. & J.Presl
Iz_7_1009	DJI_0507	Pulsatilla pratensis (L.) Mill. s.l.	0	n	1	0	Ranunculaceae Juss.
Iz_7_1009	DJI_0507	Pyrus communis L.	0	a	0	1	Rosaceae Juss.
Iz_7_1009	DJI_0507	Quercus robur L.	0	n	1	1	Fagaceae Dumort.
Iz_7_1009	DJI_0507	Robinia pseudoacacia L.	0	a	0	1	Fabaceae Lindl.
Iz_7_1009	DJI_0507	Rosa sp.	0		NA	1	Rosaceae Juss.
Iz_7_1009	DJI_0507	Rubus idaeus L.	0	n	1	0	Rosaceae Juss.
Iz_7_1009	DJI_0507	Rumex acetosella L.	0	n	1	0	Polygonaceae Juss.
Iz_7_1009	DJI_0507	Rumex thyrsiflorus Fingerh.	0	n	1	0	Polygonaceae Juss.
Iz_7_1009	DJI_0507	Salix alba L.	0	n	1	1	Salicaceae Mirb.
Iz_7_1009	DJI_0507	Sambucus nigra L.	0	n	1	1	Viburnaceae Raf.
Iz_7_1009	DJI_0507	Secale sylvestre Host	0	n	1	0	Poaceae Barnhart
Iz_7_1009	DJI_0507	Sempervivum ruthenicum Schnittsp. & C.B. Lehm.	0	n	1	0	Crassulaceae J.St.-Hil.
Iz_7_1009	DJI_0507	Setaria viridis (L.) P. Beauv.	0	n	1	0	Poaceae Barnhart
Iz_7_1009	DJI_0507	Silene borysthenica (Gruner) Walters	0	n	1	0	Caryophyllaceae Juss.
Iz_7_1009	DJI_0507	Silene latifolia subsp. alba (Mill.) Greuter & Burdet	0	n	1	0	Caryophyllaceae Juss.
Iz_7_1009	DJI_0507	Solidago virgaurea L.	0	n	1	0	Asteraceae Bercht. & J.Presl
Iz_7_1009	DJI_0507	Sorbus aucuparia L.	0	n	1	1	Rosaceae Juss.
Iz_7_1009	DJI_0507	Stachys recta L.	0	n	1	0	Lamiacea Lindl.
Iz_7_1009	DJI_0507	Tanacetum vulgare L.	0	n	1	0	Asteraceae Bercht. & J.Presl
Iz_7_1009	DJI_0507	Thymus pallasianus Heinr.Braun	0	n	1	0	Lamiacea Lindl.
Iz_7_1009	DJI_0507	Tilia cordata Mill.	0	n	1	1	Malvaceae Juss.
Iz_7_1009	DJI_0507	Trifolium alpestre L.	0	n	1	0	Fabaceae Lindl.
Iz_7_1009	DJI_0507	Trifolium arvense L.	0	n	1	0	Fabaceae Lindl.
Iz_7_1009	DJI_0507	Ulmus glabra Huds.	0	n	1	1	Ulmaceae Mirb.
Iz_7_1009	DJI_0507	Verbascum densiflorum Bertol.	0	n	1	0	Scrophulariaceae Juss.
Iz_7_1009	DJI_0507	Veronica austriaca L.	0	n	1	0	Plantaginaceae Juss.
Iz_7_1009	DJI_0507	Veronica incana L.	0	n	1	0	Plantaginaceae Juss.

Iz_7_1009	DJI_0507	Veronica verna L.	0	n	1	0	Plantaginaceae Juss.
Iz_7_1009	DJI_0507	Vincetoxicum hirundinaria Medik	0	n	1	0	Apocynaceae Juss.
Iz_7_1109	DJI_0315	Acer tataricum L.	0	n	1	1	Sapindaceae Juss.
Iz_7_1109	DJI_0315	Achillea millefolium L. s.l.	0	n	1	0	Asteraceae Bercht. & J.Presl
Iz_7_1109	DJI_0315	Allium oleraceum L.	0	n	1	0	Amaryllidaceae J.St.-Hil.
Iz_7_1109	DJI_0315	Ambrosia artemisiifolia L.	0	a	0	0	Asteraceae Bercht. & J.Presl
Iz_7_1109	DJI_0315	Amorpha fruticosa L.	0	a	0	0	Fabaceae Lindl.
Iz_7_1109	DJI_0315	Anthericum ramosum L.	0	n	1	0	Asparagaceae Juss.
Iz_7_1109	DJI_0315	Artemisia marschalliana Spreng.	0	n	1	0	Asteraceae Bercht. & J.Presl
Iz_7_1109	DJI_0315	Asparagus officinalis L.	0	n	1	0	Asparagaceae Juss.
Iz_7_1109	DJI_0315	Astragalus cicer L.	0	n	1	0	Fabaceae Lindl.
Iz_7_1109	DJI_0315	Bassia laniflora (S.G.Gmel.) A.J.Scott	0	n	1	0	Amaranthaceae Juss.
Iz_7_1109	DJI_0315	Berberis vulgaris L.	0	n	1	0	Berberidaceae Juss.
Iz_7_1109	DJI_0315	Betula pendula Roth.	0	n	1	1	Betulaceae Gray
Iz_7_1109	DJI_0315	Betula pendula Roth.	0	n	1	0	Poaceae Barnhart
Iz_7_1109	DJI_0315	Bromus inermis Leyss.	0		NA	NA	
Iz_7_1109	DJI_0315	Bromus squarrosus L.	0	a	0	0	Poaceae Barnhart
Iz_7_1109	DJI_0315	Bromus tectorum L.	0	a	0	0	Poaceae Barnhart
Iz_7_1109	DJI_0315	Calamagrostis epigejos (L.) Roth	10	n	1	0	Poaceae Barnhart
Iz_7_1109	DJI_0315	Carduus acanthoides L.	0	n	1	0	Asteraceae Bercht. & J.Presl
Iz_7_1109	DJI_0315	Carex sp.	0	n	1	0	Cyperaceae Juss.
Iz_7_1109	DJI_0315	Centaurea majorovii Dumbadze	0	n	1	0	Asteraceae Bercht. & J.Presl
Iz_7_1109	DJI_0315	Chamaecytisus ruthenicus (Fisch.ex Wol.) Klaskova	0	n	1	0	Fabaceae Lindl.
Iz_7_1109	DJI_0315	Chelidonium majus L.	0	n	1	0	Papaveraceae Juss.
Iz_7_1109	DJI_0315	Chenopodium betaceum Andrz.	0	n	1	0	Amaranthaceae Juss.
Iz_7_1109	DJI_0315	Chenopodium cf. album	0	n	1	0	Amaranthaceae Juss.
Iz_7_1109	DJI_0315	Chenopodium sp.	0		NA	0	Amaranthaceae Juss.
Iz_7_1109	DJI_0315	Chondrilla juncea L.	0	n	1	0	Asteraceae Bercht. & J.Presl
Iz_7_1109	DJI_0315	Cirsium vulgare (Savi) Ten.	0	n	1	0	Asteraceae Bercht. & J.Presl
Iz_7_1109	DJI_0315	Clinopodium acinos (L.) Kuntze	0	n	1	0	Lamiacea Lindl.
Iz_7_1109	DJI_0315	Convallaria majalis L.	0	n	1	0	Asparagaceae Juss.
Iz_7_1109	DJI_0315	Crataegus rhipidophylla Gand.	0	n	1	1	Rosaceae Juss.
Iz_7_1109	DJI_0315	Crepis foetida L.	0	n	1	0	Asteraceae Bercht. & J.Presl
Iz_7_1109	DJI_0315	Dactylis glomerata L.	0	n	1	0	Poaceae Barnhart
Iz_7_1109	DJI_0315	Dianthus campestris M.Bieb.	0	n	1	0	Caryophyllaceae Juss.
Iz_7_1109	DJI_0315	Digitaria sanguinalis (L.) Scop.	0	a	0	0	Poaceae Barnhart
Iz_7_1109	DJI_0315	Elymus repens (L.) Gould	0	n	1	0	Poaceae Barnhart
Iz_7_1109	DJI_0315	Epilobium tetragonum L.	0	n	1	0	Onagraceae Juss.
Iz_7_1109	DJI_0315	Eragrostis minor Host	0	a	0	0	Poaceae Barnhart
Iz_7_1109	DJI_0315	Erigeron canadensis L.	8	a	0	0	Asteraceae Bercht. & J.Presl
Iz_7_1109	DJI_0315	Euphorbia seguieriana Neck.	0	n	1	0	Euphorbiaceae Juss.
Iz_7_1109	DJI_0315	Fallopia dumetorum (L.) Holub	0	n	1	0	Polygonaceae Juss.
Iz_7_1109	DJI_0315	Festuca beckeri (Hack.) Trautv.	0	n	1	0	Poaceae Barnhart
Iz_7_1109	DJI_0315	Fragaria vesca L.	0	n	1	0	Rosaceae Juss.
Iz_7_1109	DJI_0315	Frangula alnus Mill.	0	n	1	1	Rhamnaceae Juss.
Iz_7_1109	DJI_0315	Fraxinus pennsylvanica Marshall	0	a	0	1	Oleaceae Hoffmanns. & Link
Iz_7_1109	DJI_0315	Galeopsis bifida Boenn.	0	n	1	0	Lamiacea Lindl.
Iz_7_1109	DJI_0315	Galium verum L.	0	n	1	0	Rubiaceae Juss.
Iz_7_1109	DJI_0315	Genista tinctoria L.	0	n	1	0	Fabaceae Lindl.
Iz_7_1109	DJI_0315	Geranium robertianum L.	0	n	1	0	Geraniaceae Juss.
Iz_7_1109	DJI_0315	Geranium sanquineum L.	0	n	1	0	Geraniaceae Juss.
Iz_7_1109	DJI_0315	Gypsophila paniculata L.	0	n	1	0	Caryophyllaceae Juss.
Iz_7_1109	DJI_0315	Helichrysum arenarium (L.) Moench	0	n	1	0	Asteraceae Bercht. & J.Presl
Iz_7_1109	DJI_0315	Herniaria polygama J.Gay	0	n	1	0	Caryophyllaceae Juss.
Iz_7_1109	DJI_0315	Hieracium robustum Fr.	1	n	1	0	Asteraceae Bercht. & J.Presl
Iz_7_1109	DJI_0315	Hieracium umbellatum L.	0	n	1	NA	Asteraceae Bercht. & J.Presl
Iz_7_1109	DJI_0315	Hylotelephium maximum (L.) Holub	0	n	1	0	Crassulaceae J.St.-Hil.
Iz_7_1109	DJI_0315	Jacobaea borysthenica (DC.) B.Nord. & Greuter	0	n	1	0	Asteraceae Bercht. & J.Presl
Iz_7_1109	DJI_0315	Jasione montana L.	0	n	1	0	Campanulaceae Juss.
Iz_7_1109	DJI_0315	Jurinea cyanoides (L.) Rchb.s.l.	0	n	1	0	Asteraceae Bercht. & J.Presl
Iz_7_1109	DJI_0315	Koeleria glauca (Spreng.) DC.	0	n	1	0	Poaceae Barnhart
Iz_7_1109	DJI_0315	Lactuca serriola L.	0	a	0	0	Asteraceae Bercht. & J.Presl
Iz_7_1109	DJI_0315	Linaria genistifolia (L.) Mill.	0	n	1	0	Plantaginaceae Juss.
Iz_7_1109	DJI_0315	Lomelosia argentea (L.) Greuter & Burdet	0	n	1	0	Caprifoliaceae Juss.
Iz_7_1109	DJI_0315	Melampyrum pratense L.	0	n	1	0	Orobanchaceae Vent.
Iz_7_1109	DJI_0315	Oenothera biennis L.	0	a	0	0	Onagraceae Juss.
Iz_7_1109	DJI_0315	Pentanema britannica (L.) D.Gut.Larr., Santos-Vicente, Anderb., E.Rico & M.M.Mart.Ort.	0	n	1	0	Asteraceae Bercht. & J.Presl
Iz_7_1109	DJI_0315	Peucedanum oreoselinum (L.) Moench	0	n	1	0	Apiaceae Lindl.
Iz_7_1109	DJI_0315	Peucedanum ruthenicum M.Bieb.	0	n	1	0	Apiaceae Lindl.
Iz_7_1109	DJI_0315	Pilosella echioides (Lumn.) F.W.Schultz & Sch.Bip.	0	n	1	0	Asteraceae Bercht. & J.Presl
Iz_7_1109	DJI_0315	Pilosella officinarum F. Schultz et Sch. Bip.	0	n	1	0	Asteraceae Bercht. & J.Presl
Iz_7_1109	DJI_0315	Pilosella sp.	1		NA	0	Asteraceae Bercht. & J.Presl
Iz_7_1109	DJI_0315	Pinus sylvestris L.	0	n	1	1	Pinaceae Spreng. ex F.Rudolphi
Iz_7_1109	DJI_0315	Poa annua L.	0	n	1	0	Poaceae Barnhart
Iz_7_1109	DJI_0315	Poa nemoralis L.	0	n	1	0	Poaceae Barnhart
Iz_7_1109	DJI_0315	Poa sp.	0		NA	0	Poaceae Barnhart
Iz_7_1109	DJI_0315	Polygonatum odoratum (Mill.) Druce	0	n	1	0	Asparagaceae Juss.
Iz_7_1109	DJI_0315	Polygonum arenarium Waldst. & Kit.	0	n	1	0	Polygonaceae Juss.
Iz_7_1109	DJI_0315	Populus nigra L.	0	n	1	1	Salicaceae Mirb.
Iz_7_1109	DJI_0315	Populus tremula L.	0	n	1	1	Salicaceae Mirb.
Iz_7_1109	DJI_0315	Populus Ã canescens (Aiton) Sm.	0	n	1	1	Salicaceae Mirb.
Iz_7_1109	DJI_0315	Potentilla argentea L.	0	n	1	0	Rosaceae Juss.
Iz_7_1109	DJI_0315	Psammophiliella muralis (L.) Ikonn.	0	n	1	0	Caryophyllaceae Juss.
Iz_7_1109	DJI_0315	Psephellus sumensis (Kalen.) Greuter	0	n	1	0	Asteraceae Bercht. & J.Presl
Iz_7_1109	DJI_0315	Pulsatilla pratensis (L.) Mill. s.l.	0	n	1	0	Ranunculaceae Juss.
Iz_7_1109	DJI_0315	Pyrus communis L.	0	a	0	1	Rosaceae Juss.
Iz_7_1109	DJI_0315	Quercus robur L.	0	n	1	1	Fagaceae Dumort.
Iz_7_1109	DJI_0315	Robinia pseudoacacia L.	0	a	0	1	Fabaceae Lindl.
Iz_7_1109	DJI_0315	Rosa sp.	0		NA	1	Rosaceae Juss.
Iz_7_1109	DJI_0315	Rubus idaeus L.	0	n	1	0	Rosaceae Juss.
Iz_7_1109	DJI_0315	Rumex acetosella L.	1	n	1	0	Polygonaceae Juss.
Iz_7_1109	DJI_0315	Rumex thyrsiflorus Fingerh.	0	n	1	0	Polygonaceae Juss.
Iz_7_1109	DJI_0315	Salix alba L.	0	n	1	1	Salicaceae Mirb.
Iz_7_1109	DJI_0315	Sambucus nigra L.	0	n	1	1	Viburnaceae Raf.
Iz_7_1109	DJI_0315	Secale sylvestre Host	0	n	1	0	Poaceae Barnhart
Iz_7_1109	DJI_0315	Sempervivum ruthenicum Schnittsp. & C.B. Lehm.	0	n	1	0	Crassulaceae J.St.-Hil.
Iz_7_1109	DJI_0315	Setaria viridis (L.) P. Beauv.	0	n	1	0	Poaceae Barnhart
Iz_7_1109	DJI_0315	Silene borysthenica (Gruner) Walters	0	n	1	0	Caryophyllaceae Juss.

Iz_7_1109	DJI_0315	Silene latifolia subsp. alba (Mill.) Greuter & Burdet	0	n	1	0	Caryophyllaceae Juss.
Iz_7_1109	DJI_0315	Solidago virgaurea L.	0	n	1	0	Asteraceae Bercht. & J.Presl
Iz_7_1109	DJI_0315	Sonchus oleraceus L.	0	a	0	0	Asteraceae Bercht. & J.Presl
Iz_7_1109	DJI_0315	Sorbus aucuparia L.	0	n	1	1	Rosaceae Juss.
Iz_7_1109	DJI_0315	Stachys recta L.	0	n	1	0	Lamiacea Lindl.
Iz_7_1109	DJI_0315	Tanacetum vulgare L.	0	n	1	0	Asteraceae Bercht. & J.Presl
Iz_7_1109	DJI_0315	Thymus pallasianus Heinr.Braun	0	n	1	0	Lamiacea Lindl.
Iz_7_1109	DJI_0315	Tilia cordata Mill.	0	n	1	1	Malvaceae Juss.
Iz_7_1109	DJI_0315	Trifolium alpestre L.	0	n	1	0	Fabaceae Lindl.
Iz_7_1109	DJI_0315	Trifolium arvense L.	0	n	1	0	Fabaceae Lindl.
Iz_7_1109	DJI_0315	Ulmus glabra Huds.	0	n	1	1	Ulmaceae Mirb.
Iz_7_1109	DJI_0315	Verbascum densiflorum Bertol.	0	n	1	0	Scrophulariaceae Juss.
Iz_7_1109	DJI_0315	Veronica austriaca L.	0	n	1	0	Plantaginaceae Juss.
Iz_7_1109	DJI_0315	Veronica incana L.	0	n	1	0	Plantaginaceae Juss.
Iz_7_1109	DJI_0315	Veronica verna L.	0	n	1	0	Plantaginaceae Juss.
Iz_7_1109	DJI_0315	Vincetoxicum hirundinaria Medik	0	n	1	0	Apocynaceae Juss.
Iz_7_1109	DJI_0321	Acer tataricum L.	0	n	1	1	Sapindaceae Juss.
Iz_7_1109	DJI_0321	Achillea millefolium L. s.l.	0	n	1	0	Asteraceae Bercht. & J.Presl
Iz_7_1109	DJI_0321	Allium oleraceum L.	0	n	1	0	Amaryllidaceae J.St.-Hil.
Iz_7_1109	DJI_0321	Ambrosia artemisiifolia L.	0	a	0	0	Asteraceae Bercht. & J.Presl
Iz_7_1109	DJI_0321	Amorpha fruticosa L.	0	a	0	0	Fabaceae Lindl.
Iz_7_1109	DJI_0321	Anthericum ramosum L.	0	n	1	0	Asparagaceae Juss.
Iz_7_1109	DJI_0321	Artemisia marschalliana Spreng.	0	n	1	0	Asteraceae Bercht. & J.Presl
Iz_7_1109	DJI_0321	Asparagus officinalis L.	0	n	1	0	Asparagaceae Juss.
Iz_7_1109	DJI_0321	Astragalus cicer L.	0	n	1	0	Fabaceae Lindl.
Iz_7_1109	DJI_0321	Bassia laniflora (S.G.Gmel.) A.J.Scott	0	n	1	0	Amaranthaceae Juss.
Iz_7_1109	DJI_0321	Berberis vulgaris L.	0	n	1	0	Berberidaceae Juss.
Iz_7_1109	DJI_0321	Betula pendula Roth.	0	n	1	1	Betulaceae Gray
Iz_7_1109	DJI_0321	Betula pendula Roth.	0	n	1	0	Poaceae Barnhart
Iz_7_1109	DJI_0321	Bromus inermis Leyss.	0		NA	NA	
Iz_7_1109	DJI_0321	Bromus squarrosus L.	0	a	0	0	Poaceae Barnhart
Iz_7_1109	DJI_0321	Bromus tectorum L.	0	a	0	0	Poaceae Barnhart
Iz_7_1109	DJI_0321	Calamagrostis epigejos (L.) Roth	0.2	n	1	0	Poaceae Barnhart
Iz_7_1109	DJI_0321	Carduus acanthoides L.	0	n	1	0	Asteraceae Bercht. & J.Presl
Iz_7_1109	DJI_0321	Carex sp.	0	n	1	0	Cyperaceae Juss.
Iz_7_1109	DJI_0321	Centaurea majorovii Dumbadze	0	n	1	0	Asteraceae Bercht. & J.Presl
Iz_7_1109	DJI_0321	Chamaecytisus ruthenicus (Fisch.ex Wol.) Klaskova	0	n	1	0	Fabaceae Lindl.
Iz_7_1109	DJI_0321	Chelidonium majus L.	0	n	1	0	Papaveraceae Juss.
Iz_7_1109	DJI_0321	Chenopodium betaceum Andrz.	0	n	1	0	Amaranthaceae Juss.
Iz_7_1109	DJI_0321	Chenopodium cf. album	0	n	1	0	Amaranthaceae Juss.
Iz_7_1109	DJI_0321	Chenopodium sp.	0		NA	0	Amaranthaceae Juss.
Iz_7_1109	DJI_0321	Chondrilla juncea L.	0	n	1	0	Asteraceae Bercht. & J.Presl
Iz_7_1109	DJI_0321	Cirsium vulgare (Savi) Ten.	0	n	1	0	Asteraceae Bercht. & J.Presl
Iz_7_1109	DJI_0321	Clinopodium acinos (L.) Kuntze	0	n	1	0	Lamiacea Lindl.
Iz_7_1109	DJI_0321	Convallaria majalis L.	0	n	1	0	Asparagaceae Juss.
Iz_7_1109	DJI_0321	Crataegus rhipidophylla Gand.	0	n	1	1	Rosaceae Juss.
Iz_7_1109	DJI_0321	Crepis foetida L.	0	n	1	0	Asteraceae Bercht. & J.Presl
Iz_7_1109	DJI_0321	Dactylis glomerata L.	0	n	1	0	Poaceae Barnhart
Iz_7_1109	DJI_0321	Dianthus campestris M.Bieb.	0	n	1	0	Caryophyllaceae Juss.
Iz_7_1109	DJI_0321	Digitaria sanguinalis (L.) Scop.	0	a	0	0	Poaceae Barnhart
Iz_7_1109	DJI_0321	Elymus repens (L.) Gould	0	n	1	0	Poaceae Barnhart
Iz_7_1109	DJI_0321	Epilobium tetragonum L.	0	n	1	0	Onagraceae Juss.
Iz_7_1109	DJI_0321	Eragrostis minor Host	0	a	0	0	Poaceae Barnhart
Iz_7_1109	DJI_0321	Erigeron canadensis L.	50	a	0	0	Asteraceae Bercht. & J.Presl
Iz_7_1109	DJI_0321	Euphorbia seguieriana Neck.	0	n	1	0	Euphorbiaceae Juss.
Iz_7_1109	DJI_0321	Fallopia dumetorum (L.) Holub	0	n	1	0	Polygonaceae Juss.
Iz_7_1109	DJI_0321	Festuca beckeri (Hack.) Trautv.	0	n	1	0	Poaceae Barnhart
Iz_7_1109	DJI_0321	Fragaria vesca L.	0	n	1	0	Rosaceae Juss.
Iz_7_1109	DJI_0321	Frangula alnus Mill.	0	n	1	1	Rhamnaceae Juss.
Iz_7_1109	DJI_0321	Fraxinus pennsylvanica Marshall	0	a	0	1	Oleaceae Hoffmanns. & Link
Iz_7_1109	DJI_0321	Galeopsis bifida Boenn.	0	n	1	0	Lamiacea Lindl.
Iz_7_1109	DJI_0321	Galium verum L.	0	n	1	0	Rubiaceae Juss.
Iz_7_1109	DJI_0321	Genista tinctoria L.	0	n	1	0	Fabaceae Lindl.
Iz_7_1109	DJI_0321	Geranium robertianum L.	0	n	1	0	Geraniaceae Juss.
Iz_7_1109	DJI_0321	Geranium sanquineum L.	0	n	1	0	Geraniaceae Juss.
Iz_7_1109	DJI_0321	Gypsophila paniculata L.	0	n	1	0	Caryophyllaceae Juss.
Iz_7_1109	DJI_0321	Helichrysum arenarium (L.) Moench	0	n	1	0	Asteraceae Bercht. & J.Presl
Iz_7_1109	DJI_0321	Herniaria polygama J.Gay	0	n	1	0	Caryophyllaceae Juss.
Iz_7_1109	DJI_0321	Hieracium robustum Fr.	0	n	1	0	Asteraceae Bercht. & J.Presl
Iz_7_1109	DJI_0321	Hieracium umbellatum L.	0	n	1	NA	Asteraceae Bercht. & J.Presl
Iz_7_1109	DJI_0321	Hylotelephium maximum (L.) Holub	0	n	1	0	Crassulaceae J.St.-Hil.
Iz_7_1109	DJI_0321	Jacobaea borysthenica (DC.) B.Nord. & Greuter	0	n	1	0	Asteraceae Bercht. & J.Presl
Iz_7_1109	DJI_0321	Jasione montana L.	0	n	1	0	Campanulaceae Juss.
Iz_7_1109	DJI_0321	Jurinea cyanoides (L.) Rchb.s.l.	0	n	1	0	Asteraceae Bercht. & J.Presl
Iz_7_1109	DJI_0321	Koeleria glauca (Spreng.) DC.	0	n	1	0	Poaceae Barnhart
Iz_7_1109	DJI_0321	Lactuca serriola L.	0	a	0	0	Asteraceae Bercht. & J.Presl
Iz_7_1109	DJI_0321	Linaria genistifolia (L.) Mill.	0	n	1	0	Plantaginaceae Juss.
Iz_7_1109	DJI_0321	Lomelosia argentea (L.) Greuter & Burdet	0	n	1	0	Caprifoliaceae Juss.
Iz_7_1109	DJI_0321	Melampyrum pratense L.	0	n	1	0	Orobanchaceae Vent.
Iz_7_1109	DJI_0321	Oenothera biennis L.	0	a	0	0	Onagraceae Juss.
Iz_7_1109	DJI_0321	Pentanema britannica (L.) D.Gut.Larr., Santos-Vicente, Anderb., E.Rico & M.M.Mart.Ort.	0	n	1	0	Asteraceae Bercht. & J.Presl
Iz_7_1109	DJI_0321	Peucedanum oreoselinum (L.) Moench	0	n	1	0	Apiaceae Lindl.
Iz_7_1109	DJI_0321	Peucedanum ruthenicum M.Bieb.	0	n	1	0	Apiaceae Lindl.
Iz_7_1109	DJI_0321	Pilosella echioides (Lumn.) F.W.Schultz & Sch.Bip.	0	n	1	0	Asteraceae Bercht. & J.Presl
Iz_7_1109	DJI_0321	Pilosella officinarum F. Schultz et Sch. Bip.	0	n	1	0	Asteraceae Bercht. & J.Presl
Iz_7_1109	DJI_0321	Pilosella sp.	0		NA	0	Asteraceae Bercht. & J.Presl
Iz_7_1109	DJI_0321	Pinus sylvestris L.	0	n	1	1	Pinaceae Spreng. ex F.Rudolphi
Iz_7_1109	DJI_0321	Poa annua L.	0	n	1	0	Poaceae Barnhart
Iz_7_1109	DJI_0321	Poa nemoralis L.	0	n	1	0	Poaceae Barnhart
Iz_7_1109	DJI_0321	Poa sp.	0		NA	0	Poaceae Barnhart
Iz_7_1109	DJI_0321	Polygonatum odoratum (Mill.) Druce	0	n	1	0	Asparagaceae Juss.
Iz_7_1109	DJI_0321	Polygonum arenarium Waldst. & Kit.	0	n	1	0	Polygonaceae Juss.
Iz_7_1109	DJI_0321	Populus nigra L.	0	n	1	1	Salicaceae Mirb.
Iz_7_1109	DJI_0321	Populus tremula L.	0	n	1	1	Salicaceae Mirb.
Iz_7_1109	DJI_0321	Populus Ã canescens (Aiton) Sm.	0	n	1	1	Salicaceae Mirb.
Iz_7_1109	DJI_0321	Potentilla argentea L.	0	n	1	0	Rosaceae Juss.
Iz_7_1109	DJI_0321	Psammophiliella muralis (L.) Ikonn.	0	n	1	0	Caryophyllaceae Juss.
Iz_7_1109	DJI_0321	Psephellus sumensis (Kalen.) Greuter	0	n	1	0	Asteraceae Bercht. & J.Presl

Iz_7_1109	DJI_0321	Pulsatilla pratensis (L.) Mill. s.l.	0	n	1	0	Ranunculaceae Juss.
Iz_7_1109	DJI_0321	Pyrus communis L.	0	a	0	1	Rosaceae Juss.
Iz_7_1109	DJI_0321	Quercus robur L.	0	n	1	1	Fagaceae Dumort.
Iz_7_1109	DJI_0321	Robinia pseudoacacia L.	0	a	0	1	Fabaceae Lindl.
Iz_7_1109	DJI_0321	Rosa sp.	0		NA	1	Rosaceae Juss.
Iz_7_1109	DJI_0321	Rubus idaeus L.	0	n	1	0	Rosaceae Juss.
Iz_7_1109	DJI_0321	Rumex acetosella L.	0	n	1	0	Polygonaceae Juss.
Iz_7_1109	DJI_0321	Rumex thyrsiflorus Fingerh.	0	n	1	0	Polygonaceae Juss.
Iz_7_1109	DJI_0321	Salix alba L.	0	n	1	1	Salicaceae Mirb.
Iz_7_1109	DJI_0321	Sambucus nigra L.	0	n	1	1	Viburnaceae Raf.
Iz_7_1109	DJI_0321	Secale sylvestre Host	0	n	1	0	Poaceae Barnhart
Iz_7_1109	DJI_0321	Sempervivum ruthenicum Schnittsp. & C.B. Lehm.	0	n	1	0	Crassulaceae J.St.-Hil.
Iz_7_1109	DJI_0321	Setaria viridis (L.) P. Beauv.	0	n	1	0	Poaceae Barnhart
Iz_7_1109	DJI_0321	Silene borysthenica (Gruner) Walters	0	n	1	0	Caryophyllaceae Juss.
Iz_7_1109	DJI_0321	Silene latifolia subsp. alba (Mill.) Greuter & Burdet	0	n	1	0	Caryophyllaceae Juss.
Iz_7_1109	DJI_0321	Solidago virgaurea L.	0	n	1	0	Asteraceae Bercht. & J.Presl
Iz_7_1109	DJI_0321	Sonchus oleraceus L.	0	a	0	0	Asteraceae Bercht. & J.Presl
Iz_7_1109	DJI_0321	Sorbus aucuparia L.	1	n	1	1	Rosaceae Juss.
Iz_7_1109	DJI_0321	Stachys recta L.	0	n	1	0	Lamiacea Lindl.
Iz_7_1109	DJI_0321	Tanacetum vulgare L.	0	n	1	0	Asteraceae Bercht. & J.Presl
Iz_7_1109	DJI_0321	Thymus pallasianus Heinr.Braun	0	n	1	0	Lamiacea Lindl.
Iz_7_1109	DJI_0321	Tilia cordata Mill.	0	n	1	1	Malvaceae Juss.
Iz_7_1109	DJI_0321	Trifolium alpestre L.	0	n	1	0	Fabaceae Lindl.
Iz_7_1109	DJI_0321	Trifolium arvense L.	0	n	1	0	Fabaceae Lindl.
Iz_7_1109	DJI_0321	Ulmus glabra Huds.	0	n	1	1	Ulmaceae Mirb.
Iz_7_1109	DJI_0321	Verbascum densiflorum Bertol.	0	n	1	0	Scrophulariaceae Juss.
Iz_7_1109	DJI_0321	Veronica austriaca L.	0	n	1	0	Plantaginaceae Juss.
Iz_7_1109	DJI_0321	Veronica incana L.	0	n	1	0	Plantaginaceae Juss.
Iz_7_1109	DJI_0321	Veronica verna L.	0	n	1	0	Plantaginaceae Juss.
Iz_7_1109	DJI_0321	Vincetoxicum hirundinaria Medik	0	n	1	0	Apocynaceae Juss.
Iz_7_1109	DJI_0325	Acer tataricum L.	0	n	1	1	Sapindaceae Juss.
Iz_7_1109	DJI_0325	Achillea millefolium L. s.l.	0	n	1	0	Asteraceae Bercht. & J.Presl
Iz_7_1109	DJI_0325	Allium oleraceum L.	0	n	1	0	Amaryllidaceae J.St.-Hil.
Iz_7_1109	DJI_0325	Ambrosia artemisiifolia L.	0	a	0	0	Asteraceae Bercht. & J.Presl
Iz_7_1109	DJI_0325	Amorpha fruticosa L.	0	a	0	0	Fabaceae Lindl.
Iz_7_1109	DJI_0325	Anthericum ramosum L.	0	n	1	0	Asparagaceae Juss.
Iz_7_1109	DJI_0325	Artemisia marschalliana Spreng.	0	n	1	0	Asteraceae Bercht. & J.Presl
Iz_7_1109	DJI_0325	Asparagus officinalis L.	0	n	1	0	Asparagaceae Juss.
Iz_7_1109	DJI_0325	Astragalus cicer L.	0	n	1	0	Fabaceae Lindl.
Iz_7_1109	DJI_0325	Bassia laniflora (S.G.Gmel.) A.J.Scott	0	n	1	0	Amaranthaceae Juss.
Iz_7_1109	DJI_0325	Berberis vulgaris L.	0	n	1	0	Berberidaceae Juss.
Iz_7_1109	DJI_0325	Betula pendula Roth.	0	n	1	1	Betulaceae Gray
Iz_7_1109	DJI_0325	Betula pendula Roth.	0	n	1	0	Poaceae Barnhart
Iz_7_1109	DJI_0325	Bromus inermis Leyss.	0		NA	NA	
Iz_7_1109	DJI_0325	Bromus squarrosus L.	0	a	0	0	Poaceae Barnhart
Iz_7_1109	DJI_0325	Bromus tectorum L.	0	a	0	0	Poaceae Barnhart
Iz_7_1109	DJI_0325	Calamagrostis epigejos (L.) Roth	1	n	1	0	Poaceae Barnhart
Iz_7_1109	DJI_0325	Carduus acanthoides L.	0	n	1	0	Asteraceae Bercht. & J.Presl
Iz_7_1109	DJI_0325	Carex sp.	0	n	1	0	Cyperaceae Juss.
Iz_7_1109	DJI_0325	Centaurea majorovii Dumbadze	0	n	1	0	Asteraceae Bercht. & J.Presl
Iz_7_1109	DJI_0325	Chamaecytisus ruthenicus (Fisch.ex Wol.) Klaskova	0	n	1	0	Fabaceae Lindl.
Iz_7_1109	DJI_0325	Chelidonium majus L.	0	n	1	0	Papaveraceae Juss.
Iz_7_1109	DJI_0325	Chenopodium betaceum Andrz.	0	n	1	0	Amaranthaceae Juss.
Iz_7_1109	DJI_0325	Chenopodium cf. album	0	n	1	0	Amaranthaceae Juss.
Iz_7_1109	DJI_0325	Chenopodium sp.	0		NA	0	Amaranthaceae Juss.
Iz_7_1109	DJI_0325	Chondrilla juncea L.	0	n	1	0	Asteraceae Bercht. & J.Presl
Iz_7_1109	DJI_0325	Cirsium vulgare (Savi) Ten.	0	n	1	0	Asteraceae Bercht. & J.Presl
Iz_7_1109	DJI_0325	Clinopodium acinos (L.) Kuntze	0	n	1	0	Lamiacea Lindl.
Iz_7_1109	DJI_0325	Convallaria majalis L.	0	n	1	0	Asparagaceae Juss.
Iz_7_1109	DJI_0325	Crataegus rhipidophylla Gand.	0	n	1	1	Rosaceae Juss.
Iz_7_1109	DJI_0325	Crepis foetida L.	0	n	1	0	Asteraceae Bercht. & J.Presl
Iz_7_1109	DJI_0325	Dactylis glomerata L.	0	n	1	0	Poaceae Barnhart
Iz_7_1109	DJI_0325	Dianthus campestris M.Bieb.	0	n	1	0	Caryophyllaceae Juss.
Iz_7_1109	DJI_0325	Digitaria sanguinalis (L.) Scop.	0	a	0	0	Poaceae Barnhart
Iz_7_1109	DJI_0325	Elymus repens (L.) Gould	0	n	1	0	Poaceae Barnhart
Iz_7_1109	DJI_0325	Epilobium tetragonum L.	0	n	1	0	Onagraceae Juss.
Iz_7_1109	DJI_0325	Eragrostis minor Host	0	a	0	0	Poaceae Barnhart
Iz_7_1109	DJI_0325	Erigeron canadensis L.	60	a	0	0	Asteraceae Bercht. & J.Presl
Iz_7_1109	DJI_0325	Euphorbia seguieriana Neck.	0	n	1	0	Euphorbiaceae Juss.
Iz_7_1109	DJI_0325	Fallopia dumetorum (L.) Holub	0	n	1	0	Polygonaceae Juss.
Iz_7_1109	DJI_0325	Festuca beckeri (Hack.) Trautv.	0	n	1	0	Poaceae Barnhart
Iz_7_1109	DJI_0325	Fragaria vesca L.	0	n	1	0	Rosaceae Juss.
Iz_7_1109	DJI_0325	Frangula alnus Mill.	0	n	1	1	Rhamnaceae Juss.
Iz_7_1109	DJI_0325	Fraxinus pennsylvanica Marshall	0	a	0	1	Oleaceae Hoffmanns. & Link
Iz_7_1109	DJI_0325	Galeopsis bifida Boenn.	0	n	1	0	Lamiacea Lindl.
Iz_7_1109	DJI_0325	Galium verum L.	0	n	1	0	Rubiaceae Juss.
Iz_7_1109	DJI_0325	Genista tinctoria L.	0	n	1	0	Fabaceae Lindl.
Iz_7_1109	DJI_0325	Geranium robertianum L.	0	n	1	0	Geraniaceae Juss.
Iz_7_1109	DJI_0325	Geranium sanquineum L.	0	n	1	0	Geraniaceae Juss.
Iz_7_1109	DJI_0325	Gypsophila paniculata L.	0	n	1	0	Caryophyllaceae Juss.
Iz_7_1109	DJI_0325	Helichrysum arenarium (L.) Moench	0	n	1	0	Asteraceae Bercht. & J.Presl
Iz_7_1109	DJI_0325	Herniaria polygama J.Gay	0	n	1	0	Caryophyllaceae Juss.
Iz_7_1109	DJI_0325	Hieracium robustum Fr.	0	n	1	0	Asteraceae Bercht. & J.Presl
Iz_7_1109	DJI_0325	Hieracium umbellatum L.	0	n	1	NA	Asteraceae Bercht. & J.Presl
Iz_7_1109	DJI_0325	Hylotelephium maximum (L.) Holub	0	n	1	0	Crassulaceae J.St.-Hil.
Iz_7_1109	DJI_0325	Jacobaea borysthenica (DC.) B.Nord. & Greuter	0	n	1	0	Asteraceae Bercht. & J.Presl
Iz_7_1109	DJI_0325	Jasione montana L.	0	n	1	0	Campanulaceae Juss.
Iz_7_1109	DJI_0325	Jurinea cyanoides (L.) Rchb.s.l.	0	n	1	0	Asteraceae Bercht. & J.Presl
Iz_7_1109	DJI_0325	Koeleria glauca (Spreng.) DC.	0	n	1	0	Poaceae Barnhart
Iz_7_1109	DJI_0325	Lactuca serriola L.	0	a	0	0	Asteraceae Bercht. & J.Presl
Iz_7_1109	DJI_0325	Linaria genistifolia (L.) Mill.	0	n	1	0	Plantaginaceae Juss.
Iz_7_1109	DJI_0325	Lomelosia argentea (L.) Greuter & Burdet	0	n	1	0	Caprifoliaceae Juss.
Iz_7_1109	DJI_0325	Melampyrum pratense L.	0	n	1	0	Orobanchaceae Vent.
Iz_7_1109	DJI_0325	Oenothera biennis L.	0	a	0	0	Onagraceae Juss.
Iz_7_1109	DJI_0325	Pentanema britannica (L.) D.Gut.Larr., Santos-Vicente, Anderb., E.Rico & M.M.Mart.Ort.	0	n	1	0	Asteraceae Bercht. & J.Presl
Iz_7_1109	DJI_0325	Peucedanum oreoselinum (L.) Moench	0	n	1	0	Apiaceae Lindl.
Iz_7_1109	DJI_0325	Peucedanum ruthenicum M.Bieb.	0	n	1	0	Apiaceae Lindl.
Iz_7_1109	DJI_0325	Pilosella echioides (Lumn.) F.W.Schultz & Sch.Bip.	0	n	1	0	Asteraceae Bercht. & J.Presl

Iz_7_1109	DJI_0325	Pilosella officinarum F. Schultz et Sch. Bip.	0	n	1	0	Asteraceae Bercht. & J.Presl
Iz_7_1109	DJI_0325	Pilosella sp.	0		NA	0	Asteraceae Bercht. & J.Presl
Iz_7_1109	DJI_0325	Pinus sylvestris L.	0	n	1	1	Pinaceae Spreng. ex F.Rudolphi
Iz_7_1109	DJI_0325	Poa annua L.	0	n	1	0	Poaceae Barnhart
Iz_7_1109	DJI_0325	Poa nemoralis L.	0	n	1	0	Poaceae Barnhart
Iz_7_1109	DJI_0325	Poa sp.	0		NA	0	Poaceae Barnhart
Iz_7_1109	DJI_0325	Polygonatum odoratum (Mill.) Druce	0	n	1	0	Asparagaceae Juss.
Iz_7_1109	DJI_0325	Polygonum arenarium Waldst. & Kit.	0	n	1	0	Polygonaceae Juss.
Iz_7_1109	DJI_0325	Populus nigra L.	0	n	1	1	Salicaceae Mirb.
Iz_7_1109	DJI_0325	Populus tremula L.	0	n	1	1	Salicaceae Mirb.
Iz_7_1109	DJI_0325	Populus Ã canescens (Aiton) Sm.	0	n	1	1	Salicaceae Mirb.
Iz_7_1109	DJI_0325	Potentilla argentea L.	0	n	1	0	Rosaceae Juss.
Iz_7_1109	DJI_0325	Psammophiliella muralis (L.) Ikonn.	0	n	1	0	Caryophyllaceae Juss.
Iz_7_1109	DJI_0325	Psephellus sumensis (Kalen.) Greuter	0	n	1	0	Asteraceae Bercht. & J.Presl
Iz_7_1109	DJI_0325	Pulsatilla pratensis (L.) Mill. s.l.	0	n	1	0	Ranunculaceae Juss.
Iz_7_1109	DJI_0325	Pyrus communis L.	0	a	0	1	Rosaceae Juss.
Iz_7_1109	DJI_0325	Quercus robur L.	0	n	1	1	Fagaceae Dumort.
Iz_7_1109	DJI_0325	Robinia pseudoacacia L.	0	a	0	1	Fabaceae Lindl.
Iz_7_1109	DJI_0325	Rosa sp.	0		NA	1	Rosaceae Juss.
Iz_7_1109	DJI_0325	Rubus idaeus L.	0	n	1	0	Rosaceae Juss.
Iz_7_1109	DJI_0325	Rumex acetosella L.	0	n	1	0	Polygonaceae Juss.
Iz_7_1109	DJI_0325	Rumex thyrsiflorus Fingerh.	0	n	1	0	Polygonaceae Juss.
Iz_7_1109	DJI_0325	Salix alba L.	0	n	1	1	Salicaceae Mirb.
Iz_7_1109	DJI_0325	Sambucus nigra L.	0	n	1	1	Viburnaceae Raf.
Iz_7_1109	DJI_0325	Secale sylvestre Host	0	n	1	0	Poaceae Barnhart
Iz_7_1109	DJI_0325	Sempervivum ruthenicum Schnittsp. & C.B. Lehm.	0	n	1	0	Crassulaceae J.St.-Hil.
Iz_7_1109	DJI_0325	Setaria viridis (L.) P. Beauv.	0	n	1	0	Poaceae Barnhart
Iz_7_1109	DJI_0325	Silene borysthenica (Gruner) Walters	0	n	1	0	Caryophyllaceae Juss.
Iz_7_1109	DJI_0325	Silene latifolia subsp. alba (Mill.) Greuter & Burdet	0	n	1	0	Caryophyllaceae Juss.
Iz_7_1109	DJI_0325	Solidago virgaurea L.	0	n	1	0	Asteraceae Bercht. & J.Presl
Iz_7_1109	DJI_0325	Sonchus oleraceus L.	0	a	0	0	Asteraceae Bercht. & J.Presl
Iz_7_1109	DJI_0325	Sorbus aucuparia L.	0	n	1	1	Rosaceae Juss.
Iz_7_1109	DJI_0325	Stachys recta L.	0	n	1	0	Lamiacea Lindl.
Iz_7_1109	DJI_0325	Tanacetum vulgare L.	0	n	1	0	Asteraceae Bercht. & J.Presl
Iz_7_1109	DJI_0325	Thymus pallasianus Heinr.Braun	0	n	1	0	Lamiacea Lindl.
Iz_7_1109	DJI_0325	Tilia cordata Mill.	0	n	1	1	Malvaceae Juss.
Iz_7_1109	DJI_0325	Trifolium alpestre L.	0	n	1	0	Fabaceae Lindl.
Iz_7_1109	DJI_0325	Trifolium arvense L.	0	n	1	0	Fabaceae Lindl.
Iz_7_1109	DJI_0325	Ulmus glabra Huds.	0	n	1	1	Ulmaceae Mirb.
Iz_7_1109	DJI_0325	Verbascum densiflorum Bertol.	0	n	1	0	Scrophulariaceae Juss.
Iz_7_1109	DJI_0325	Veronica austriaca L.	0	n	1	0	Plantaginaceae Juss.
Iz_7_1109	DJI_0325	Veronica incana L.	0	n	1	0	Plantaginaceae Juss.
Iz_7_1109	DJI_0325	Veronica verna L.	0	n	1	0	Plantaginaceae Juss.
Iz_7_1109	DJI_0325	Vincetoxicum hirundinaria Medik	0	n	1	0	Apocynaceae Juss.
Iz_7_1109	DJI_0328	Acer tataricum L.	0	n	1	1	Sapindaceae Juss.
Iz_7_1109	DJI_0328	Achillea millefolium L. s.l.	0	n	1	0	Asteraceae Bercht. & J.Presl
Iz_7_1109	DJI_0328	Allium oleraceum L.	0	n	1	0	Amaryllidaceae J.St.-Hil.
Iz_7_1109	DJI_0328	Ambrosia artemisiifolia L.	0	a	0	0	Asteraceae Bercht. & J.Presl
Iz_7_1109	DJI_0328	Amorpha fruticosa L.	0	a	0	0	Fabaceae Lindl.
Iz_7_1109	DJI_0328	Anthericum ramosum L.	0	n	1	0	Asparagaceae Juss.
Iz_7_1109	DJI_0328	Artemisia marschalliana Spreng.	0	n	1	0	Asteraceae Bercht. & J.Presl
Iz_7_1109	DJI_0328	Asparagus officinalis L.	0	n	1	0	Asparagaceae Juss.
Iz_7_1109	DJI_0328	Astragalus cicer L.	0	n	1	0	Fabaceae Lindl.
Iz_7_1109	DJI_0328	Bassia laniflora (S.G.Gmel.) A.J.Scott	0	n	1	0	Amaranthaceae Juss.
Iz_7_1109	DJI_0328	Berberis vulgaris L.	0	n	1	0	Berberidaceae Juss.
Iz_7_1109	DJI_0328	Betula pendula Roth.	0.2	n	1	1	Betulaceae Gray
Iz_7_1109	DJI_0328	Betula pendula Roth.	0.2	n	1	0	Poaceae Barnhart
Iz_7_1109	DJI_0328	Bromus inermis Leyss.	0		NA	NA	
Iz_7_1109	DJI_0328	Bromus squarrosus L.	0	a	0	0	Poaceae Barnhart
Iz_7_1109	DJI_0328	Bromus tectorum L.	0	a	0	0	Poaceae Barnhart
Iz_7_1109	DJI_0328	Calamagrostis epigejos (L.) Roth	0.2	n	1	0	Poaceae Barnhart
Iz_7_1109	DJI_0328	Carduus acanthoides L.	0	n	1	0	Asteraceae Bercht. & J.Presl
Iz_7_1109	DJI_0328	Carex sp.	0	n	1	0	Cyperaceae Juss.
Iz_7_1109	DJI_0328	Centaurea majorovii Dumbadze	0	n	1	0	Asteraceae Bercht. & J.Presl
Iz_7_1109	DJI_0328	Chamaecytisus ruthenicus (Fisch.ex Wol.) Klaskova	0	n	1	0	Fabaceae Lindl.
Iz_7_1109	DJI_0328	Chelidonium majus L.	0	n	1	0	Papaveraceae Juss.
Iz_7_1109	DJI_0328	Chenopodium betaceum Andrz.	0	n	1	0	Amaranthaceae Juss.
Iz_7_1109	DJI_0328	Chenopodium cf. album	0	n	1	0	Amaranthaceae Juss.
Iz_7_1109	DJI_0328	Chenopodium sp.	0		NA	0	Amaranthaceae Juss.
Iz_7_1109	DJI_0328	Chondrilla juncea L.	0	n	1	0	Asteraceae Bercht. & J.Presl
Iz_7_1109	DJI_0328	Cirsium vulgare (Savi) Ten.	0	n	1	0	Asteraceae Bercht. & J.Presl
Iz_7_1109	DJI_0328	Clinopodium acinos (L.) Kuntze	0	n	1	0	Lamiacea Lindl.
Iz_7_1109	DJI_0328	Convallaria majalis L.	0	n	1	0	Asparagaceae Juss.
Iz_7_1109	DJI_0328	Crataegus rhipidophylla Gand.	0	n	1	1	Rosaceae Juss.
Iz_7_1109	DJI_0328	Crepis foetida L.	0	n	1	0	Asteraceae Bercht. & J.Presl
Iz_7_1109	DJI_0328	Dactylis glomerata L.	0	n	1	0	Poaceae Barnhart
Iz_7_1109	DJI_0328	Dianthus campestris M.Bieb.	0	n	1	0	Caryophyllaceae Juss.
Iz_7_1109	DJI_0328	Digitaria sanguinalis (L.) Scop.	0	a	0	0	Poaceae Barnhart
Iz_7_1109	DJI_0328	Elymus repens (L.) Gould	0	n	1	0	Poaceae Barnhart
Iz_7_1109	DJI_0328	Epilobium tetragonum L.	0	n	1	0	Onagraceae Juss.
Iz_7_1109	DJI_0328	Eragrostis minor Host	0	a	0	0	Poaceae Barnhart
Iz_7_1109	DJI_0328	Erigeron canadensis L.	17	a	0	0	Asteraceae Bercht. & J.Presl
Iz_7_1109	DJI_0328	Euphorbia seguieriana Neck.	0	n	1	0	Euphorbiaceae Juss.
Iz_7_1109	DJI_0328	Fallopia dumetorum (L.) Holub	0	n	1	0	Polygonaceae Juss.
Iz_7_1109	DJI_0328	Festuca beckeri (Hack.) Trautv.	0	n	1	0	Poaceae Barnhart
Iz_7_1109	DJI_0328	Fragaria vesca L.	0	n	1	0	Rosaceae Juss.
Iz_7_1109	DJI_0328	Frangula alnus Mill.	0	n	1	1	Rhamnaceae Juss.
Iz_7_1109	DJI_0328	Fraxinus pennsylvanica Marshall	0	a	0	1	Oleaceae Hoffmanns. & Link
Iz_7_1109	DJI_0328	Galeopsis bifida Boenn.	0	n	1	0	Lamiacea Lindl.
Iz_7_1109	DJI_0328	Galium verum L.	0	n	1	0	Rubiaceae Juss.
Iz_7_1109	DJI_0328	Genista tinctoria L.	0	n	1	0	Fabaceae Lindl.
Iz_7_1109	DJI_0328	Geranium robertianum L.	0	n	1	0	Geraniaceae Juss.
Iz_7_1109	DJI_0328	Geranium sanquineum L.	0	n	1	0	Geraniaceae Juss.
Iz_7_1109	DJI_0328	Gypsophila paniculata L.	0	n	1	0	Caryophyllaceae Juss.
Iz_7_1109	DJI_0328	Helichrysum arenarium (L.) Moench	0	n	1	0	Asteraceae Bercht. & J.Presl
Iz_7_1109	DJI_0328	Herniaria polygama J.Gay	0	n	1	0	Caryophyllaceae Juss.
Iz_7_1109	DJI_0328	Hieracium robustum Fr.	0	n	1	0	Asteraceae Bercht. & J.Presl
Iz_7_1109	DJI_0328	Hieracium umbellatum L.	0	n	1	NA	Asteraceae Bercht. & J.Presl

Iz_7_1109	DJI_0328	Hylotelephium maximum (L.) Holub	0	n	1	0	Crassulaceae J.St.-Hil.
Iz_7_1109	DJI_0328	Jacobaea borysthenica (DC.) B.Nord. & Greuter	0	n	1	0	Asteraceae Bercht. & J.Presl
Iz_7_1109	DJI_0328	Jasione montana L.	0	n	1	0	Campanulaceae Juss.
Iz_7_1109	DJI_0328	Jurinea cyanoides (L.) Rchb.s.l.	0	n	1	0	Asteraceae Bercht. & J.Presl
Iz_7_1109	DJI_0328	Koeleria glauca (Spreng.) DC.	0	n	1	0	Poaceae Barnhart
Iz_7_1109	DJI_0328	Lactuca serriola L.	0	a	0	0	Asteraceae Bercht. & J.Presl
Iz_7_1109	DJI_0328	Linaria genistifolia (L.) Mill.	0	n	1	0	Plantaginaceae Juss.
Iz_7_1109	DJI_0328	Lomelosia argentea (L.) Greuter & Burdet	0	n	1	0	Caprifoliaceae Juss.
Iz_7_1109	DJI_0328	Melampyrum pratense L.	0	n	1	0	Orobanchaceae Vent.
Iz_7_1109	DJI_0328	Oenothera biennis L.	0	a	0	0	Onagraceae Juss.
Iz_7_1109	DJI_0328	Pentanema britannica (L.) D.Gut.Larr., Santos-Vicente, Anderb., E.Rico & M.M.Mart.Ort.	0	n	1	0	Asteraceae Bercht. & J.Presl
Iz_7_1109	DJI_0328	Peucedanum oreoselinum (L.) Moench	0	n	1	0	Apiaceae Lindl.
Iz_7_1109	DJI_0328	Peucedanum ruthenicum M.Bieb.	0	n	1	0	Apiaceae Lindl.
Iz_7_1109	DJI_0328	Pilosella echioides (Lumn.) F.W.Schultz & Sch.Bip.	0	n	1	0	Asteraceae Bercht. & J.Presl
Iz_7_1109	DJI_0328	Pilosella officinarum F. Schultz et Sch. Bip.	0	n	1	0	Asteraceae Bercht. & J.Presl
Iz_7_1109	DJI_0328	Pilosella sp.	0		NA	0	Asteraceae Bercht. & J.Presl
Iz_7_1109	DJI_0328	Pinus sylvestris L.	0	n	1	1	Pinaceae Spreng. ex F.Rudolphi
Iz_7_1109	DJI_0328	Poa annua L.	0	n	1	0	Poaceae Barnhart
Iz_7_1109	DJI_0328	Poa nemoralis L.	0	n	1	0	Poaceae Barnhart
Iz_7_1109	DJI_0328	Poa sp.	0		NA	0	Poaceae Barnhart
Iz_7_1109	DJI_0328	Polygonatum odoratum (Mill.) Druce	0	n	1	0	Asparagaceae Juss.
Iz_7_1109	DJI_0328	Polygonum arenarium Waldst. & Kit.	0	n	1	0	Polygonaceae Juss.
Iz_7_1109	DJI_0328	Populus nigra L.	0	n	1	1	Salicaceae Mirb.
Iz_7_1109	DJI_0328	Populus tremula L.	0	n	1	1	Salicaceae Mirb.
Iz_7_1109	DJI_0328	Populus Ã canescens (Aiton) Sm.	0	n	1	1	Salicaceae Mirb.
Iz_7_1109	DJI_0328	Potentilla argentea L.	0	n	1	0	Rosaceae Juss.
Iz_7_1109	DJI_0328	Psammophiliella muralis (L.) Ikonn.	0	n	1	0	Caryophyllaceae Juss.
Iz_7_1109	DJI_0328	Psephellus sumensis (Kalen.) Greuter	0	n	1	0	Asteraceae Bercht. & J.Presl
Iz_7_1109	DJI_0328	Pulsatilla pratensis (L.) Mill. s.l.	0	n	1	0	Ranunculaceae Juss.
Iz_7_1109	DJI_0328	Pyrus communis L.	0	a	0	1	Rosaceae Juss.
Iz_7_1109	DJI_0328	Quercus robur L.	0	n	1	1	Fagaceae Dumort.
Iz_7_1109	DJI_0328	Robinia pseudoacacia L.	0	a	0	1	Fabaceae Lindl.
Iz_7_1109	DJI_0328	Rosa sp.	0		NA	1	Rosaceae Juss.
Iz_7_1109	DJI_0328	Rubus idaeus L.	0	n	1	0	Rosaceae Juss.
Iz_7_1109	DJI_0328	Rumex acetosella L.	0	n	1	0	Polygonaceae Juss.
Iz_7_1109	DJI_0328	Rumex thyrsiflorus Fingerh.	0	n	1	0	Polygonaceae Juss.
Iz_7_1109	DJI_0328	Salix alba L.	0	n	1	1	Salicaceae Mirb.
Iz_7_1109	DJI_0328	Sambucus nigra L.	0	n	1	1	Viburnaceae Raf.
Iz_7_1109	DJI_0328	Secale sylvestre Host	0	n	1	0	Poaceae Barnhart
Iz_7_1109	DJI_0328	Sempervivum ruthenicum Schnittsp. & C.B. Lehm.	0	n	1	0	Crassulaceae J.St.-Hil.
Iz_7_1109	DJI_0328	Setaria viridis (L.) P. Beauv.	0	n	1	0	Poaceae Barnhart
Iz_7_1109	DJI_0328	Silene borysthenica (Gruner) Walters	0	n	1	0	Caryophyllaceae Juss.
Iz_7_1109	DJI_0328	Silene latifolia subsp. alba (Mill.) Greuter & Burdet	0	n	1	0	Caryophyllaceae Juss.
Iz_7_1109	DJI_0328	Solidago virgaurea L.	0	n	1	0	Asteraceae Bercht. & J.Presl
Iz_7_1109	DJI_0328	Sonchus oleraceus L.	0	a	0	0	Asteraceae Bercht. & J.Presl
Iz_7_1109	DJI_0328	Sorbus aucuparia L.	0	n	1	1	Rosaceae Juss.
Iz_7_1109	DJI_0328	Stachys recta L.	0	n	1	0	Lamiacea Lindl.
Iz_7_1109	DJI_0328	Tanacetum vulgare L.	0	n	1	0	Asteraceae Bercht. & J.Presl
Iz_7_1109	DJI_0328	Thymus pallasianus Heinr.Braun	0	n	1	0	Lamiacea Lindl.
Iz_7_1109	DJI_0328	Tilia cordata Mill.	0	n	1	1	Malvaceae Juss.
Iz_7_1109	DJI_0328	Trifolium alpestre L.	0	n	1	0	Fabaceae Lindl.
Iz_7_1109	DJI_0328	Trifolium arvense L.	0	n	1	0	Fabaceae Lindl.
Iz_7_1109	DJI_0328	Ulmus glabra Huds.	0	n	1	1	Ulmaceae Mirb.
Iz_7_1109	DJI_0328	Verbascum densiflorum Bertol.	0	n	1	0	Scrophulariaceae Juss.
Iz_7_1109	DJI_0328	Veronica austriaca L.	0	n	1	0	Plantaginaceae Juss.
Iz_7_1109	DJI_0328	Veronica incana L.	0	n	1	0	Plantaginaceae Juss.
Iz_7_1109	DJI_0328	Veronica verna L.	0	n	1	0	Plantaginaceae Juss.
Iz_7_1109	DJI_0328	Vincetoxicum hirundinaria Medik	0	n	1	0	Apocynaceae Juss.
Iz_7_1109	DJI_0331	Acer tataricum L.	0	n	1	1	Sapindaceae Juss.
Iz_7_1109	DJI_0331	Achillea millefolium L. s.l.	0	n	1	0	Asteraceae Bercht. & J.Presl
Iz_7_1109	DJI_0331	Allium oleraceum L.	0	n	1	0	Amaryllidaceae J.St.-Hil.
Iz_7_1109	DJI_0331	Ambrosia artemisiifolia L.	0	a	0	0	Asteraceae Bercht. & J.Presl
Iz_7_1109	DJI_0331	Amorpha fruticosa L.	0	a	0	0	Fabaceae Lindl.
Iz_7_1109	DJI_0331	Anthericum ramosum L.	0	n	1	0	Asparagaceae Juss.
Iz_7_1109	DJI_0331	Artemisia marschalliana Spreng.	0	n	1	0	Asteraceae Bercht. & J.Presl
Iz_7_1109	DJI_0331	Asparagus officinalis L.	0	n	1	0	Asparagaceae Juss.
Iz_7_1109	DJI_0331	Astragalus cicer L.	0	n	1	0	Fabaceae Lindl.
Iz_7_1109	DJI_0331	Bassia laniflora (S.G.Gmel.) A.J.Scott	0	n	1	0	Amaranthaceae Juss.
Iz_7_1109	DJI_0331	Berberis vulgaris L.	0	n	1	0	Berberidaceae Juss.
Iz_7_1109	DJI_0331	Betula pendula Roth.	0	n	1	1	Betulaceae Gray
Iz_7_1109	DJI_0331	Betula pendula Roth.	0	n	1	0	Poaceae Barnhart
Iz_7_1109	DJI_0331	Bromus inermis Leyss.	0		NA	NA	
Iz_7_1109	DJI_0331	Bromus squarrosus L.	0	a	0	0	Poaceae Barnhart
Iz_7_1109	DJI_0331	Bromus tectorum L.	0	a	0	0	Poaceae Barnhart
Iz_7_1109	DJI_0331	Calamagrostis epigejos (L.) Roth	2	n	1	0	Poaceae Barnhart
Iz_7_1109	DJI_0331	Carduus acanthoides L.	0	n	1	0	Asteraceae Bercht. & J.Presl
Iz_7_1109	DJI_0331	Carex sp.	0	n	1	0	Cyperaceae Juss.
Iz_7_1109	DJI_0331	Centaurea majorovii Dumbadze	0	n	1	0	Asteraceae Bercht. & J.Presl
Iz_7_1109	DJI_0331	Chamaecytisus ruthenicus (Fisch.ex Wol.) Klaskova	0	n	1	0	Fabaceae Lindl.
Iz_7_1109	DJI_0331	Chelidonium majus L.	0.5	n	1	0	Papaveraceae Juss.
Iz_7_1109	DJI_0331	Chenopodium betaceum Andrz.	0	n	1	0	Amaranthaceae Juss.
Iz_7_1109	DJI_0331	Chenopodium cf. album	0	n	1	0	Amaranthaceae Juss.
Iz_7_1109	DJI_0331	Chenopodium sp.	0		NA	0	Amaranthaceae Juss.
Iz_7_1109	DJI_0331	Chondrilla juncea L.	0	n	1	0	Asteraceae Bercht. & J.Presl
Iz_7_1109	DJI_0331	Cirsium vulgare (Savi) Ten.	0	n	1	0	Asteraceae Bercht. & J.Presl
Iz_7_1109	DJI_0331	Clinopodium acinos (L.) Kuntze	0	n	1	0	Lamiacea Lindl.
Iz_7_1109	DJI_0331	Convallaria majalis L.	0	n	1	0	Asparagaceae Juss.
Iz_7_1109	DJI_0331	Crataegus rhipidophylla Gand.	0	n	1	1	Rosaceae Juss.
Iz_7_1109	DJI_0331	Crepis foetida L.	0	n	1	0	Asteraceae Bercht. & J.Presl
Iz_7_1109	DJI_0331	Dactylis glomerata L.	0	n	1	0	Poaceae Barnhart
Iz_7_1109	DJI_0331	Dianthus campestris M.Bieb.	0	n	1	0	Caryophyllaceae Juss.
Iz_7_1109	DJI_0331	Digitaria sanguinalis (L.) Scop.	0	a	0	0	Poaceae Barnhart
Iz_7_1109	DJI_0331	Elymus repens (L.) Gould	0	n	1	0	Poaceae Barnhart
Iz_7_1109	DJI_0331	Epilobium tetragonum L.	0	n	1	0	Onagraceae Juss.
Iz_7_1109	DJI_0331	Eragrostis minor Host	0	a	0	0	Poaceae Barnhart
Iz_7_1109	DJI_0331	Erigeron canadensis L.	2	a	0	0	Asteraceae Bercht. & J.Presl
Iz_7_1109	DJI_0331	Euphorbia seguieriana Neck.	0	n	1	0	Euphorbiaceae Juss.
Iz_7_1109	DJI_0331	Fallopia dumetorum (L.) Holub	0	n	1	0	Polygonaceae Juss.

Iz_7_1109	DJI_0331	Festuca beckeri (Hack.) Trautv.	0	n	1	0	Poaceae Barnhart
Iz_7_1109	DJI_0331	Fragaria vesca L.	0	n	1	0	Rosaceae Juss.
Iz_7_1109	DJI_0331	Frangula alnus Mill.	0	n	1	1	Rhamnaceae Juss.
Iz_7_1109	DJI_0331	Fraxinus pennsylvanica Marshall	0	a	0	1	Oleaceae Hoffmanns. & Link
Iz_7_1109	DJI_0331	Galeopsis bifida Boenn.	0	n	1	0	Lamiacea Lindl.
Iz_7_1109	DJI_0331	Galium verum L.	0	n	1	0	Rubiaceae Juss.
Iz_7_1109	DJI_0331	Genista tinctoria L.	0	n	1	0	Fabaceae Lindl.
Iz_7_1109	DJI_0331	Geranium robertianum L.	0	n	1	0	Geraniaceae Juss.
Iz_7_1109	DJI_0331	Geranium sanquineum L.	0	n	1	0	Geraniaceae Juss.
Iz_7_1109	DJI_0331	Gypsophila paniculata L.	0	n	1	0	Caryophyllaceae Juss.
Iz_7_1109	DJI_0331	Helichrysum arenarium (L.) Moench	0	n	1	0	Asteraceae Bercht. & J.Presl
Iz_7_1109	DJI_0331	Herniaria polygama J.Gay	0	n	1	0	Caryophyllaceae Juss.
Iz_7_1109	DJI_0331	Hieracium robustum Fr.	0	n	1	0	Asteraceae Bercht. & J.Presl
Iz_7_1109	DJI_0331	Hieracium umbellatum L.	0	n	1	NA	Asteraceae Bercht. & J.Presl
Iz_7_1109	DJI_0331	Hylotelephium maximum (L.) Holub	0	n	1	0	Crassulaceae J.St.-Hil.
Iz_7_1109	DJI_0331	Jacobaea borysthenica (DC.) B.Nord. & Greuter	0	n	1	0	Asteraceae Bercht. & J.Presl
Iz_7_1109	DJI_0331	Jasione montana L.	0	n	1	0	Campanulaceae Juss.
Iz_7_1109	DJI_0331	Jurinea cyanoides (L.) Rchb.s.l.	0	n	1	0	Asteraceae Bercht. & J.Presl
Iz_7_1109	DJI_0331	Koeleria glauca (Spreng.) DC.	0	n	1	0	Poaceae Barnhart
Iz_7_1109	DJI_0331	Lactuca serriola L.	0	a	0	0	Asteraceae Bercht. & J.Presl
Iz_7_1109	DJI_0331	Linaria genistifolia (L.) Mill.	0	n	1	0	Plantaginaceae Juss.
Iz_7_1109	DJI_0331	Lomelosia argentea (L.) Greuter & Burdet	0	n	1	0	Caprifoliaceae Juss.
Iz_7_1109	DJI_0331	Melampyrum pratense L.	0	n	1	0	Orobanchaceae Vent.
Iz_7_1109	DJI_0331	Oenothera biennis L.	0	a	0	0	Onagraceae Juss.
Iz_7_1109	DJI_0331	Pentanema britannica (L.) D.Gut.Larr., Santos-Vicente, Anderb., E.Rico & M.M.Mart.Ort.	0	n	1	0	Asteraceae Bercht. & J.Presl
Iz_7_1109	DJI_0331	Peucedanum oreoselinum (L.) Moench	0	n	1	0	Apiaceae Lindl.
Iz_7_1109	DJI_0331	Peucedanum ruthenicum M.Bieb.	0	n	1	0	Apiaceae Lindl.
Iz_7_1109	DJI_0331	Pilosella echioides (Lumn.) F.W.Schultz & Sch.Bip.	0	n	1	0	Asteraceae Bercht. & J.Presl
Iz_7_1109	DJI_0331	Pilosella officinarum F. Schultz et Sch. Bip.	0	n	1	0	Asteraceae Bercht. & J.Presl
Iz_7_1109	DJI_0331	Pilosella sp.	0		NA	0	Asteraceae Bercht. & J.Presl
Iz_7_1109	DJI_0331	Pinus sylvestris L.	0	n	1	1	Pinaceae Spreng. ex F.Rudolphi
Iz_7_1109	DJI_0331	Poa annua L.	0	n	1	0	Poaceae Barnhart
Iz_7_1109	DJI_0331	Poa nemoralis L.	0	n	1	0	Poaceae Barnhart
Iz_7_1109	DJI_0331	Poa sp.	0		NA	0	Poaceae Barnhart
Iz_7_1109	DJI_0331	Polygonatum odoratum (Mill.) Druce	0	n	1	0	Asparagaceae Juss.
Iz_7_1109	DJI_0331	Polygonum arenarium Waldst. & Kit.	0	n	1	0	Polygonaceae Juss.
Iz_7_1109	DJI_0331	Populus nigra L.	0	n	1	1	Salicaceae Mirb.
Iz_7_1109	DJI_0331	Populus tremula L.	0	n	1	1	Salicaceae Mirb.
Iz_7_1109	DJI_0331	Populus Ã canescens (Aiton) Sm.	0	n	1	1	Salicaceae Mirb.
Iz_7_1109	DJI_0331	Potentilla argentea L.	0	n	1	0	Rosaceae Juss.
Iz_7_1109	DJI_0331	Psammophiliella muralis (L.) Ikonn.	0	n	1	0	Caryophyllaceae Juss.
Iz_7_1109	DJI_0331	Psephellus sumensis (Kalen.) Greuter	0	n	1	0	Asteraceae Bercht. & J.Presl
Iz_7_1109	DJI_0331	Pulsatilla pratensis (L.) Mill. s.l.	0	n	1	0	Ranunculaceae Juss.
Iz_7_1109	DJI_0331	Pyrus communis L.	0	a	0	1	Rosaceae Juss.
Iz_7_1109	DJI_0331	Quercus robur L.	0	n	1	1	Fagaceae Dumort.
Iz_7_1109	DJI_0331	Robinia pseudoacacia L.	0	a	0	1	Fabaceae Lindl.
Iz_7_1109	DJI_0331	Rosa sp.	0		NA	1	Rosaceae Juss.
Iz_7_1109	DJI_0331	Rubus idaeus L.	0	n	1	0	Rosaceae Juss.
Iz_7_1109	DJI_0331	Rumex acetosella L.	0	n	1	0	Polygonaceae Juss.
Iz_7_1109	DJI_0331	Rumex thyrsiflorus Fingerh.	0	n	1	0	Polygonaceae Juss.
Iz_7_1109	DJI_0331	Salix alba L.	0	n	1	1	Salicaceae Mirb.
Iz_7_1109	DJI_0331	Sambucus nigra L.	0	n	1	1	Viburnaceae Raf.
Iz_7_1109	DJI_0331	Secale sylvestre Host	0	n	1	0	Poaceae Barnhart
Iz_7_1109	DJI_0331	Sempervivum ruthenicum Schnittsp. & C.B. Lehm.	0	n	1	0	Crassulaceae J.St.-Hil.
Iz_7_1109	DJI_0331	Setaria viridis (L.) P. Beauv.	0	n	1	0	Poaceae Barnhart
Iz_7_1109	DJI_0331	Silene borysthenica (Gruner) Walters	0	n	1	0	Caryophyllaceae Juss.
Iz_7_1109	DJI_0331	Silene latifolia subsp. alba (Mill.) Greuter & Burdet	0	n	1	0	Caryophyllaceae Juss.
Iz_7_1109	DJI_0331	Solidago virgaurea L.	0	n	1	0	Asteraceae Bercht. & J.Presl
Iz_7_1109	DJI_0331	Sonchus oleraceus L.	0	a	0	0	Asteraceae Bercht. & J.Presl
Iz_7_1109	DJI_0331	Sorbus aucuparia L.	0	n	1	1	Rosaceae Juss.
Iz_7_1109	DJI_0331	Stachys recta L.	0	n	1	0	Lamiacea Lindl.
Iz_7_1109	DJI_0331	Tanacetum vulgare L.	0	n	1	0	Asteraceae Bercht. & J.Presl
Iz_7_1109	DJI_0331	Thymus pallasianus Heinr.Braun	0	n	1	0	Lamiacea Lindl.
Iz_7_1109	DJI_0331	Tilia cordata Mill.	0	n	1	1	Malvaceae Juss.
Iz_7_1109	DJI_0331	Trifolium alpestre L.	0	n	1	0	Fabaceae Lindl.
Iz_7_1109	DJI_0331	Trifolium arvense L.	0	n	1	0	Fabaceae Lindl.
Iz_7_1109	DJI_0331	Ulmus glabra Huds.	0	n	1	1	Ulmaceae Mirb.
Iz_7_1109	DJI_0331	Verbascum densiflorum Bertol.	0	n	1	0	Scrophulariaceae Juss.
Iz_7_1109	DJI_0331	Veronica austriaca L.	0	n	1	0	Plantaginaceae Juss.
Iz_7_1109	DJI_0331	Veronica incana L.	0	n	1	0	Plantaginaceae Juss.
Iz_7_1109	DJI_0331	Veronica verna L.	0	n	1	0	Plantaginaceae Juss.
Iz_7_1109	DJI_0331	Vincetoxicum hirundinaria Medik	0	n	1	0	Apocynaceae Juss.
Iz_7_1109	DJI_0334	Acer tataricum L.	0	n	1	1	Sapindaceae Juss.
Iz_7_1109	DJI_0334	Achillea millefolium L. s.l.	0	n	1	0	Asteraceae Bercht. & J.Presl
Iz_7_1109	DJI_0334	Allium oleraceum L.	0	n	1	0	Amaryllidaceae J.St.-Hil.
Iz_7_1109	DJI_0334	Ambrosia artemisiifolia L.	0	a	0	0	Asteraceae Bercht. & J.Presl
Iz_7_1109	DJI_0334	Amorpha fruticosa L.	0	a	0	0	Fabaceae Lindl.
Iz_7_1109	DJI_0334	Anthericum ramosum L.	0	n	1	0	Asparagaceae Juss.
Iz_7_1109	DJI_0334	Artemisia marschalliana Spreng.	0	n	1	0	Asteraceae Bercht. & J.Presl
Iz_7_1109	DJI_0334	Asparagus officinalis L.	0	n	1	0	Asparagaceae Juss.
Iz_7_1109	DJI_0334	Astragalus cicer L.	0	n	1	0	Fabaceae Lindl.
Iz_7_1109	DJI_0334	Bassia laniflora (S.G.Gmel.) A.J.Scott	0	n	1	0	Amaranthaceae Juss.
Iz_7_1109	DJI_0334	Berberis vulgaris L.	0	n	1	0	Berberidaceae Juss.
Iz_7_1109	DJI_0334	Betula pendula Roth.	0	n	1	1	Betulaceae Gray
Iz_7_1109	DJI_0334	Betula pendula Roth.	0	n	1	0	Poaceae Barnhart
Iz_7_1109	DJI_0334	Bromus inermis Leyss.	0		NA	NA	
Iz_7_1109	DJI_0334	Bromus squarrosus L.	0	a	0	0	Poaceae Barnhart
Iz_7_1109	DJI_0334	Bromus tectorum L.	0	a	0	0	Poaceae Barnhart
Iz_7_1109	DJI_0334	Calamagrostis epigejos (L.) Roth	1	n	1	0	Poaceae Barnhart
Iz_7_1109	DJI_0334	Carduus acanthoides L.	0	n	1	0	Asteraceae Bercht. & J.Presl
Iz_7_1109	DJI_0334	Carex sp.	0	n	1	0	Cyperaceae Juss.
Iz_7_1109	DJI_0334	Centaurea majorovii Dumbadze	0	n	1	0	Asteraceae Bercht. & J.Presl
Iz_7_1109	DJI_0334	Chamaecytisus ruthenicus (Fisch.ex Wol.) Klaskova	0	n	1	0	Fabaceae Lindl.
Iz_7_1109	DJI_0334	Chelidonium majus L.	0	n	1	0	Papaveraceae Juss.
Iz_7_1109	DJI_0334	Chenopodium betaceum Andrz.	0	n	1	0	Amaranthaceae Juss.
Iz_7_1109	DJI_0334	Chenopodium cf. album	0	n	1	0	Amaranthaceae Juss.
Iz_7_1109	DJI_0334	Chenopodium sp.	0		NA	0	Amaranthaceae Juss.
Iz_7_1109	DJI_0334	Chondrilla juncea L.	0	n	1	0	Asteraceae Bercht. & J.Presl

Iz_7_1109	DJI_0334	Cirsium vulgare (Savi) Ten.	0	n	1	0	Asteraceae Bercht. & J.Presl
Iz_7_1109	DJI_0334	Clinopodium acinos (L.) Kuntze	0	n	1	0	Lamiacea Lindl.
Iz_7_1109	DJI_0334	Convallaria majalis L.	0	n	1	0	Asparagaceae Juss.
Iz_7_1109	DJI_0334	Crataegus rhipidophylla Gand.	0	n	1	1	Rosaceae Juss.
Iz_7_1109	DJI_0334	Crepis foetida L.	0	n	1	0	Asteraceae Bercht. & J.Presl
Iz_7_1109	DJI_0334	Dactylis glomerata L.	0	n	1	0	Poaceae Barnhart
Iz_7_1109	DJI_0334	Dianthus campestris M.Bieb.	0	n	1	0	Caryophyllaceae Juss.
Iz_7_1109	DJI_0334	Digitaria sanguinalis (L.) Scop.	0	a	0	0	Poaceae Barnhart
Iz_7_1109	DJI_0334	Elymus repens (L.) Gould	0	n	1	0	Poaceae Barnhart
Iz_7_1109	DJI_0334	Epilobium tetragonum L.	0	n	1	0	Onagraceae Juss.
Iz_7_1109	DJI_0334	Eragrostis minor Host	0	a	0	0	Poaceae Barnhart
Iz_7_1109	DJI_0334	Erigeron canadensis L.	2	a	0	0	Asteraceae Bercht. & J.Presl
Iz_7_1109	DJI_0334	Euphorbia seguieriana Neck.	0	n	1	0	Euphorbiaceae Juss.
Iz_7_1109	DJI_0334	Fallopia dumetorum (L.) Holub	0	n	1	0	Polygonaceae Juss.
Iz_7_1109	DJI_0334	Festuca beckeri (Hack.) Trautv.	0	n	1	0	Poaceae Barnhart
Iz_7_1109	DJI_0334	Fragaria vesca L.	0	n	1	0	Rosaceae Juss.
Iz_7_1109	DJI_0334	Frangula alnus Mill.	0	n	1	1	Rhamnaceae Juss.
Iz_7_1109	DJI_0334	Fraxinus pennsylvanica Marshall	0	a	0	1	Oleaceae Hoffmanns. & Link
Iz_7_1109	DJI_0334	Galeopsis bifida Boenn.	0	n	1	0	Lamiacea Lindl.
Iz_7_1109	DJI_0334	Galium verum L.	0	n	1	0	Rubiaceae Juss.
Iz_7_1109	DJI_0334	Genista tinctoria L.	0	n	1	0	Fabaceae Lindl.
Iz_7_1109	DJI_0334	Geranium robertianum L.	0	n	1	0	Geraniaceae Juss.
Iz_7_1109	DJI_0334	Geranium sanquineum L.	0	n	1	0	Geraniaceae Juss.
Iz_7_1109	DJI_0334	Gypsophila paniculata L.	0	n	1	0	Caryophyllaceae Juss.
Iz_7_1109	DJI_0334	Helichrysum arenarium (L.) Moench	0	n	1	0	Asteraceae Bercht. & J.Presl
Iz_7_1109	DJI_0334	Herniaria polygama J.Gay	0	n	1	0	Caryophyllaceae Juss.
Iz_7_1109	DJI_0334	Hieracium robustum Fr.	0	n	1	0	Asteraceae Bercht. & J.Presl
Iz_7_1109	DJI_0334	Hieracium umbellatum L.	0	n	1	NA	Asteraceae Bercht. & J.Presl
Iz_7_1109	DJI_0334	Hylotelephium maximum (L.) Holub	0	n	1	0	Crassulaceae J.St.-Hil.
Iz_7_1109	DJI_0334	Jacobaea borysthenica (DC.) B.Nord. & Greuter	0	n	1	0	Asteraceae Bercht. & J.Presl
Iz_7_1109	DJI_0334	Jasione montana L.	0	n	1	0	Campanulaceae Juss.
Iz_7_1109	DJI_0334	Jurinea cyanoides (L.) Rchb.s.l.	0	n	1	0	Asteraceae Bercht. & J.Presl
Iz_7_1109	DJI_0334	Koeleria glauca (Spreng.) DC.	0	n	1	0	Poaceae Barnhart
Iz_7_1109	DJI_0334	Lactuca serriola L.	0	a	0	0	Asteraceae Bercht. & J.Presl
Iz_7_1109	DJI_0334	Linaria genistifolia (L.) Mill.	0	n	1	0	Plantaginaceae Juss.
Iz_7_1109	DJI_0334	Lomelosia argentea (L.) Greuter & Burdet	0	n	1	0	Caprifoliaceae Juss.
Iz_7_1109	DJI_0334	Melampyrum pratense L.	0	n	1	0	Orobanchaceae Vent.
Iz_7_1109	DJI_0334	Oenothera biennis L.	0	a	0	0	Onagraceae Juss.
Iz_7_1109	DJI_0334	Pentanema britannica (L.) D.Gut.Larr., Santos-Vicente, Anderb., E.Rico & M.M.Mart.Ort.	0	n	1	0	Asteraceae Bercht. & J.Presl
Iz_7_1109	DJI_0334	Peucedanum oreoselinum (L.) Moench	0	n	1	0	Apiaceae Lindl.
Iz_7_1109	DJI_0334	Peucedanum ruthenicum M.Bieb.	0	n	1	0	Apiaceae Lindl.
Iz_7_1109	DJI_0334	Pilosella echioides (Lumn.) F.W.Schultz & Sch.Bip.	0	n	1	0	Asteraceae Bercht. & J.Presl
Iz_7_1109	DJI_0334	Pilosella officinarum F. Schultz et Sch. Bip.	0	n	1	0	Asteraceae Bercht. & J.Presl
Iz_7_1109	DJI_0334	Pilosella sp.	0		NA	0	Asteraceae Bercht. & J.Presl
Iz_7_1109	DJI_0334	Pinus sylvestris L.	0	n	1	1	Pinaceae Spreng. ex F.Rudolphi
Iz_7_1109	DJI_0334	Poa annua L.	0	n	1	0	Poaceae Barnhart
Iz_7_1109	DJI_0334	Poa nemoralis L.	0	n	1	0	Poaceae Barnhart
Iz_7_1109	DJI_0334	Poa sp.	0		NA	0	Poaceae Barnhart
Iz_7_1109	DJI_0334	Polygonatum odoratum (Mill.) Druce	0	n	1	0	Asparagaceae Juss.
Iz_7_1109	DJI_0334	Polygonum arenarium Waldst. & Kit.	0	n	1	0	Polygonaceae Juss.
Iz_7_1109	DJI_0334	Populus nigra L.	0	n	1	1	Salicaceae Mirb.
Iz_7_1109	DJI_0334	Populus tremula L.	0	n	1	1	Salicaceae Mirb.
Iz_7_1109	DJI_0334	Populus Ã canescens (Aiton) Sm.	0	n	1	1	Salicaceae Mirb.
Iz_7_1109	DJI_0334	Potentilla argentea L.	0	n	1	0	Rosaceae Juss.
Iz_7_1109	DJI_0334	Psammophiliella muralis (L.) Ikonn.	0	n	1	0	Caryophyllaceae Juss.
Iz_7_1109	DJI_0334	Psephellus sumensis (Kalen.) Greuter	0	n	1	0	Asteraceae Bercht. & J.Presl
Iz_7_1109	DJI_0334	Pulsatilla pratensis (L.) Mill. s.l.	0	n	1	0	Ranunculaceae Juss.
Iz_7_1109	DJI_0334	Pyrus communis L.	0	a	0	1	Rosaceae Juss.
Iz_7_1109	DJI_0334	Quercus robur L.	0	n	1	1	Fagaceae Dumort.
Iz_7_1109	DJI_0334	Robinia pseudoacacia L.	0	a	0	1	Fabaceae Lindl.
Iz_7_1109	DJI_0334	Rosa sp.	0		NA	1	Rosaceae Juss.
Iz_7_1109	DJI_0334	Rubus idaeus L.	0	n	1	0	Rosaceae Juss.
Iz_7_1109	DJI_0334	Rumex acetosella L.	0.5	n	1	0	Polygonaceae Juss.
Iz_7_1109	DJI_0334	Rumex thyrsiflorus Fingerh.	0	n	1	0	Polygonaceae Juss.
Iz_7_1109	DJI_0334	Salix alba L.	0	n	1	1	Salicaceae Mirb.
Iz_7_1109	DJI_0334	Sambucus nigra L.	0	n	1	1	Viburnaceae Raf.
Iz_7_1109	DJI_0334	Secale sylvestre Host	0	n	1	0	Poaceae Barnhart
Iz_7_1109	DJI_0334	Sempervivum ruthenicum Schnittsp. & C.B. Lehm.	0	n	1	0	Crassulaceae J.St.-Hil.
Iz_7_1109	DJI_0334	Setaria viridis (L.) P. Beauv.	0	n	1	0	Poaceae Barnhart
Iz_7_1109	DJI_0334	Silene borysthenica (Gruner) Walters	0	n	1	0	Caryophyllaceae Juss.
Iz_7_1109	DJI_0334	Silene latifolia subsp. alba (Mill.) Greuter & Burdet	0	n	1	0	Caryophyllaceae Juss.
Iz_7_1109	DJI_0334	Solidago virgaurea L.	0	n	1	0	Asteraceae Bercht. & J.Presl
Iz_7_1109	DJI_0334	Sonchus oleraceus L.	0	a	0	0	Asteraceae Bercht. & J.Presl
Iz_7_1109	DJI_0334	Sorbus aucuparia L.	0	n	1	1	Rosaceae Juss.
Iz_7_1109	DJI_0334	Stachys recta L.	0	n	1	0	Lamiacea Lindl.
Iz_7_1109	DJI_0334	Tanacetum vulgare L.	0	n	1	0	Asteraceae Bercht. & J.Presl
Iz_7_1109	DJI_0334	Thymus pallasianus Heinr.Braun	0	n	1	0	Lamiacea Lindl.
Iz_7_1109	DJI_0334	Tilia cordata Mill.	0	n	1	1	Malvaceae Juss.
Iz_7_1109	DJI_0334	Trifolium alpestre L.	0	n	1	0	Fabaceae Lindl.
Iz_7_1109	DJI_0334	Trifolium arvense L.	0	n	1	0	Fabaceae Lindl.
Iz_7_1109	DJI_0334	Ulmus glabra Huds.	0	n	1	1	Ulmaceae Mirb.
Iz_7_1109	DJI_0334	Verbascum densiflorum Bertol.	0	n	1	0	Scrophulariaceae Juss.
Iz_7_1109	DJI_0334	Veronica austriaca L.	0	n	1	0	Plantaginaceae Juss.
Iz_7_1109	DJI_0334	Veronica incana L.	0	n	1	0	Plantaginaceae Juss.
Iz_7_1109	DJI_0334	Veronica verna L.	0	n	1	0	Plantaginaceae Juss.
Iz_7_1109	DJI_0334	Vincetoxicum hirundinaria Medik	0	n	1	0	Apocynaceae Juss.
Iz_7_1109	DJI_0341	Acer tataricum L.	0	n	1	1	Sapindaceae Juss.
Iz_7_1109	DJI_0341	Achillea millefolium L. s.l.	0	n	1	0	Asteraceae Bercht. & J.Presl
Iz_7_1109	DJI_0341	Allium oleraceum L.	0	n	1	0	Amaryllidaceae J.St.-Hil.
Iz_7_1109	DJI_0341	Ambrosia artemisiifolia L.	0	a	0	0	Asteraceae Bercht. & J.Presl
Iz_7_1109	DJI_0341	Amorpha fruticosa L.	0	a	0	0	Fabaceae Lindl.
Iz_7_1109	DJI_0341	Anthericum ramosum L.	0	n	1	0	Asparagaceae Juss.
Iz_7_1109	DJI_0341	Artemisia marschalliana Spreng.	0	n	1	0	Asteraceae Bercht. & J.Presl
Iz_7_1109	DJI_0341	Asparagus officinalis L.	0	n	1	0	Asparagaceae Juss.
Iz_7_1109	DJI_0341	Astragalus cicer L.	0	n	1	0	Fabaceae Lindl.
Iz_7_1109	DJI_0341	Bassia laniflora (S.G.Gmel.) A.J.Scott	0	n	1	0	Amaranthaceae Juss.
Iz_7_1109	DJI_0341	Berberis vulgaris L.	0	n	1	0	Berberidaceae Juss.
Iz_7_1109	DJI_0341	Betula pendula Roth.	0	n	1	1	Betulaceae Gray

Iz_7_1109	DJI_0341	Betula pendula Roth.	0	n	1	0	Poaceae Barnhart
Iz_7_1109	DJI_0341	Bromus inermis Leyss.	0		NA	NA	
Iz_7_1109	DJI_0341	Bromus squarrosus L.	0	a	0	0	Poaceae Barnhart
Iz_7_1109	DJI_0341	Bromus tectorum L.	0	a	0	0	Poaceae Barnhart
Iz_7_1109	DJI_0341	Calamagrostis epigejos (L.) Roth	2	n	1	0	Poaceae Barnhart
Iz_7_1109	DJI_0341	Carduus acanthoides L.	0	n	1	0	Asteraceae Bercht. & J.Presl
Iz_7_1109	DJI_0341	Carex sp.	0	n	1	0	Cyperaceae Juss.
Iz_7_1109	DJI_0341	Centaurea majorovii Dumbadze	0	n	1	0	Asteraceae Bercht. & J.Presl
Iz_7_1109	DJI_0341	Chamaecytisus ruthenicus (Fisch.ex Wol.) Klaskova	0	n	1	0	Fabaceae Lindl.
Iz_7_1109	DJI_0341	Chelidonium majus L.	0	n	1	0	Papaveraceae Juss.
Iz_7_1109	DJI_0341	Chenopodium betaceum Andrz.	0	n	1	0	Amaranthaceae Juss.
Iz_7_1109	DJI_0341	Chenopodium cf. album	0	n	1	0	Amaranthaceae Juss.
Iz_7_1109	DJI_0341	Chenopodium sp.	0		NA	0	Amaranthaceae Juss.
Iz_7_1109	DJI_0341	Chondrilla juncea L.	0	n	1	0	Asteraceae Bercht. & J.Presl
Iz_7_1109	DJI_0341	Cirsium vulgare (Savi) Ten.	0	n	1	0	Asteraceae Bercht. & J.Presl
Iz_7_1109	DJI_0341	Clinopodium acinos (L.) Kuntze	0	n	1	0	Lamiacea Lindl.
Iz_7_1109	DJI_0341	Convallaria majalis L.	0	n	1	0	Asparagaceae Juss.
Iz_7_1109	DJI_0341	Crataegus rhipidophylla Gand.	0	n	1	1	Rosaceae Juss.
Iz_7_1109	DJI_0341	Crepis foetida L.	0	n	1	0	Asteraceae Bercht. & J.Presl
Iz_7_1109	DJI_0341	Dactylis glomerata L.	0	n	1	0	Poaceae Barnhart
Iz_7_1109	DJI_0341	Dianthus campestris M.Bieb.	0	n	1	0	Caryophyllaceae Juss.
Iz_7_1109	DJI_0341	Digitaria sanguinalis (L.) Scop.	0	a	0	0	Poaceae Barnhart
Iz_7_1109	DJI_0341	Elymus repens (L.) Gould	0	n	1	0	Poaceae Barnhart
Iz_7_1109	DJI_0341	Epilobium tetragonum L.	0	n	1	0	Onagraceae Juss.
Iz_7_1109	DJI_0341	Eragrostis minor Host	0	a	0	0	Poaceae Barnhart
Iz_7_1109	DJI_0341	Erigeron canadensis L.	15	a	0	0	Asteraceae Bercht. & J.Presl
Iz_7_1109	DJI_0341	Euphorbia seguieriana Neck.	0	n	1	0	Euphorbiaceae Juss.
Iz_7_1109	DJI_0341	Fallopia dumetorum (L.) Holub	0	n	1	0	Polygonaceae Juss.
Iz_7_1109	DJI_0341	Festuca beckeri (Hack.) Trautv.	0	n	1	0	Poaceae Barnhart
Iz_7_1109	DJI_0341	Fragaria vesca L.	0	n	1	0	Rosaceae Juss.
Iz_7_1109	DJI_0341	Frangula alnus Mill.	0	n	1	1	Rhamnaceae Juss.
Iz_7_1109	DJI_0341	Fraxinus pennsylvanica Marshall	0	a	0	1	Oleaceae Hoffmanns. & Link
Iz_7_1109	DJI_0341	Galeopsis bifida Boenn.	0	n	1	0	Lamiacea Lindl.
Iz_7_1109	DJI_0341	Galium verum L.	0	n	1	0	Rubiaceae Juss.
Iz_7_1109	DJI_0341	Genista tinctoria L.	0	n	1	0	Fabaceae Lindl.
Iz_7_1109	DJI_0341	Geranium robertianum L.	0	n	1	0	Geraniaceae Juss.
Iz_7_1109	DJI_0341	Geranium sanquineum L.	0	n	1	0	Geraniaceae Juss.
Iz_7_1109	DJI_0341	Gypsophila paniculata L.	0	n	1	0	Caryophyllaceae Juss.
Iz_7_1109	DJI_0341	Helichrysum arenarium (L.) Moench	0	n	1	0	Asteraceae Bercht. & J.Presl
Iz_7_1109	DJI_0341	Herniaria polygama J.Gay	0	n	1	0	Caryophyllaceae Juss.
Iz_7_1109	DJI_0341	Hieracium robustum Fr.	0	n	1	0	Asteraceae Bercht. & J.Presl
Iz_7_1109	DJI_0341	Hieracium umbellatum L.	0	n	1	NA	Asteraceae Bercht. & J.Presl
Iz_7_1109	DJI_0341	Hylotelephium maximum (L.) Holub	0	n	1	0	Crassulaceae J.St.-Hil.
Iz_7_1109	DJI_0341	Jacobaea borysthenica (DC.) B.Nord. & Greuter	0	n	1	0	Asteraceae Bercht. & J.Presl
Iz_7_1109	DJI_0341	Jasione montana L.	0	n	1	0	Campanulaceae Juss.
Iz_7_1109	DJI_0341	Jurinea cyanoides (L.) Rchb.s.l.	0	n	1	0	Asteraceae Bercht. & J.Presl
Iz_7_1109	DJI_0341	Koeleria glauca (Spreng.) DC.	0	n	1	0	Poaceae Barnhart
Iz_7_1109	DJI_0341	Lactuca serriola L.	0	a	0	0	Asteraceae Bercht. & J.Presl
Iz_7_1109	DJI_0341	Linaria genistifolia (L.) Mill.	0	n	1	0	Plantaginaceae Juss.
Iz_7_1109	DJI_0341	Lomelosia argentea (L.) Greuter & Burdet	0	n	1	0	Caprifoliaceae Juss.
Iz_7_1109	DJI_0341	Melampyrum pratense L.	0	n	1	0	Orobanchaceae Vent.
Iz_7_1109	DJI_0341	Oenothera biennis L.	0	a	0	0	Onagraceae Juss.
Iz_7_1109	DJI_0341	Pentanema britannica (L.) D.Gut.Larr., Santos-Vicente, Anderb., E.Rico & M.M.Mart.Ort.	0	n	1	0	Asteraceae Bercht. & J.Presl
Iz_7_1109	DJI_0341	Peucedanum oreoselinum (L.) Moench	0	n	1	0	Apiaceae Lindl.
Iz_7_1109	DJI_0341	Peucedanum ruthenicum M.Bieb.	0	n	1	0	Apiaceae Lindl.
Iz_7_1109	DJI_0341	Pilosella echioides (Lumn.) F.W.Schultz & Sch.Bip.	0	n	1	0	Asteraceae Bercht. & J.Presl
Iz_7_1109	DJI_0341	Pilosella officinarum F. Schultz et Sch. Bip.	0	n	1	0	Asteraceae Bercht. & J.Presl
Iz_7_1109	DJI_0341	Pilosella sp.	0		NA	0	Asteraceae Bercht. & J.Presl
Iz_7_1109	DJI_0341	Pinus sylvestris L.	0	n	1	1	Pinaceae Spreng. ex F.Rudolphi
Iz_7_1109	DJI_0341	Poa annua L.	0	n	1	0	Poaceae Barnhart
Iz_7_1109	DJI_0341	Poa nemoralis L.	0	n	1	0	Poaceae Barnhart
Iz_7_1109	DJI_0341	Poa sp.	0		NA	0	Poaceae Barnhart
Iz_7_1109	DJI_0341	Polygonatum odoratum (Mill.) Druce	0	n	1	0	Asparagaceae Juss.
Iz_7_1109	DJI_0341	Polygonum arenarium Waldst. & Kit.	0	n	1	0	Polygonaceae Juss.
Iz_7_1109	DJI_0341	Populus nigra L.	0	n	1	1	Salicaceae Mirb.
Iz_7_1109	DJI_0341	Populus tremula L.	0	n	1	1	Salicaceae Mirb.
Iz_7_1109	DJI_0341	Populus Ã canescens (Aiton) Sm.	0	n	1	1	Salicaceae Mirb.
Iz_7_1109	DJI_0341	Potentilla argentea L.	0	n	1	0	Rosaceae Juss.
Iz_7_1109	DJI_0341	Psammophiliella muralis (L.) Ikonn.	0	n	1	0	Caryophyllaceae Juss.
Iz_7_1109	DJI_0341	Psephellus sumensis (Kalen.) Greuter	0	n	1	0	Asteraceae Bercht. & J.Presl
Iz_7_1109	DJI_0341	Pulsatilla pratensis (L.) Mill. s.l.	0	n	1	0	Ranunculaceae Juss.
Iz_7_1109	DJI_0341	Pyrus communis L.	0	a	0	1	Rosaceae Juss.
Iz_7_1109	DJI_0341	Quercus robur L.	0	n	1	1	Fagaceae Dumort.
Iz_7_1109	DJI_0341	Robinia pseudoacacia L.	0	a	0	1	Fabaceae Lindl.
Iz_7_1109	DJI_0341	Rosa sp.	0		NA	1	Rosaceae Juss.
Iz_7_1109	DJI_0341	Rubus idaeus L.	0	n	1	0	Rosaceae Juss.
Iz_7_1109	DJI_0341	Rumex acetosella L.	0	n	1	0	Polygonaceae Juss.
Iz_7_1109	DJI_0341	Rumex thyrsiflorus Fingerh.	0	n	1	0	Polygonaceae Juss.
Iz_7_1109	DJI_0341	Salix alba L.	0	n	1	1	Salicaceae Mirb.
Iz_7_1109	DJI_0341	Sambucus nigra L.	0	n	1	1	Viburnaceae Raf.
Iz_7_1109	DJI_0341	Secale sylvestre Host	0	n	1	0	Poaceae Barnhart
Iz_7_1109	DJI_0341	Sempervivum ruthenicum Schnittsp. & C.B. Lehm.	0	n	1	0	Crassulaceae J.St.-Hil.
Iz_7_1109	DJI_0341	Setaria viridis (L.) P. Beauv.	0	n	1	0	Poaceae Barnhart
Iz_7_1109	DJI_0341	Silene borysthenica (Gruner) Walters	0	n	1	0	Caryophyllaceae Juss.
Iz_7_1109	DJI_0341	Silene latifolia subsp. alba (Mill.) Greuter & Burdet	0	n	1	0	Caryophyllaceae Juss.
Iz_7_1109	DJI_0341	Solidago virgaurea L.	0	n	1	0	Asteraceae Bercht. & J.Presl
Iz_7_1109	DJI_0341	Sonchus oleraceus L.	0	a	0	0	Asteraceae Bercht. & J.Presl
Iz_7_1109	DJI_0341	Sorbus aucuparia L.	0	n	1	1	Rosaceae Juss.
Iz_7_1109	DJI_0341	Stachys recta L.	0	n	1	0	Lamiacea Lindl.
Iz_7_1109	DJI_0341	Tanacetum vulgare L.	0	n	1	0	Asteraceae Bercht. & J.Presl
Iz_7_1109	DJI_0341	Thymus pallasianus Heinr.Braun	0	n	1	0	Lamiacea Lindl.
Iz_7_1109	DJI_0341	Tilia cordata Mill.	0	n	1	1	Malvaceae Juss.
Iz_7_1109	DJI_0341	Trifolium alpestre L.	0	n	1	0	Fabaceae Lindl.
Iz_7_1109	DJI_0341	Trifolium arvense L.	0	n	1	0	Fabaceae Lindl.
Iz_7_1109	DJI_0341	Ulmus glabra Huds.	0	n	1	1	Ulmaceae Mirb.
Iz_7_1109	DJI_0341	Verbascum densiflorum Bertol.	0	n	1	0	Scrophulariaceae Juss.
Iz_7_1109	DJI_0341	Veronica austriaca L.	0	n	1	0	Plantaginaceae Juss.
Iz_7_1109	DJI_0341	Veronica incana L.	0	n	1	0	Plantaginaceae Juss.

Iz_7_1109	DJI_0341	Veronica verna L.	0	n	1	0	Plantaginaceae Juss.
Iz_7_1109	DJI_0341	Vincetoxicum hirundinaria Medik	0	n	1	0	Apocynaceae Juss.
Iz_7_1109	DJI_0345	Acer tataricum L.	0	n	1	1	Sapindaceae Juss.
Iz_7_1109	DJI_0345	Achillea millefolium L. s.l.	0	n	1	0	Asteraceae Bercht. & J.Presl
Iz_7_1109	DJI_0345	Allium oleraceum L.	0	n	1	0	Amaryllidaceae J.St.-Hil.
Iz_7_1109	DJI_0345	Ambrosia artemisiifolia L.	0	a	0	0	Asteraceae Bercht. & J.Presl
Iz_7_1109	DJI_0345	Amorpha fruticosa L.	0	a	0	0	Fabaceae Lindl.
Iz_7_1109	DJI_0345	Anthericum ramosum L.	0	n	1	0	Asparagaceae Juss.
Iz_7_1109	DJI_0345	Artemisia marschalliana Spreng.	0	n	1	0	Asteraceae Bercht. & J.Presl
Iz_7_1109	DJI_0345	Asparagus officinalis L.	0	n	1	0	Asparagaceae Juss.
Iz_7_1109	DJI_0345	Astragalus cicer L.	0	n	1	0	Fabaceae Lindl.
Iz_7_1109	DJI_0345	Bassia laniflora (S.G.Gmel.) A.J.Scott	0	n	1	0	Amaranthaceae Juss.
Iz_7_1109	DJI_0345	Berberis vulgaris L.	0	n	1	0	Berberidaceae Juss.
Iz_7_1109	DJI_0345	Betula pendula Roth.	0	n	1	1	Betulaceae Gray
Iz_7_1109	DJI_0345	Betula pendula Roth.	0	n	1	0	Poaceae Barnhart
Iz_7_1109	DJI_0345	Bromus inermis Leyss.	0		NA	NA	
Iz_7_1109	DJI_0345	Bromus squarrosus L.	0	a	0	0	Poaceae Barnhart
Iz_7_1109	DJI_0345	Bromus tectorum L.	0	a	0	0	Poaceae Barnhart
Iz_7_1109	DJI_0345	Calamagrostis epigejos (L.) Roth	0.5	n	1	0	Poaceae Barnhart
Iz_7_1109	DJI_0345	Carduus acanthoides L.	0	n	1	0	Asteraceae Bercht. & J.Presl
Iz_7_1109	DJI_0345	Carex sp.	0	n	1	0	Cyperaceae Juss.
Iz_7_1109	DJI_0345	Centaurea majorovii Dumbadze	0	n	1	0	Asteraceae Bercht. & J.Presl
Iz_7_1109	DJI_0345	Chamaecytisus ruthenicus (Fisch.ex Wol.) Klaskova	0	n	1	0	Fabaceae Lindl.
Iz_7_1109	DJI_0345	Chelidonium majus L.	0	n	1	0	Papaveraceae Juss.
Iz_7_1109	DJI_0345	Chenopodium betaceum Andrz.	0	n	1	0	Amaranthaceae Juss.
Iz_7_1109	DJI_0345	Chenopodium cf. album	0	n	1	0	Amaranthaceae Juss.
Iz_7_1109	DJI_0345	Chenopodium sp.	0		NA	0	Amaranthaceae Juss.
Iz_7_1109	DJI_0345	Chondrilla juncea L.	0	n	1	0	Asteraceae Bercht. & J.Presl
Iz_7_1109	DJI_0345	Cirsium vulgare (Savi) Ten.	0	n	1	0	Asteraceae Bercht. & J.Presl
Iz_7_1109	DJI_0345	Clinopodium acinos (L.) Kuntze	0	n	1	0	Lamiacea Lindl.
Iz_7_1109	DJI_0345	Convallaria majalis L.	0	n	1	0	Asparagaceae Juss.
Iz_7_1109	DJI_0345	Crataegus rhipidophylla Gand.	0	n	1	1	Rosaceae Juss.
Iz_7_1109	DJI_0345	Crepis foetida L.	0	n	1	0	Asteraceae Bercht. & J.Presl
Iz_7_1109	DJI_0345	Dactylis glomerata L.	0	n	1	0	Poaceae Barnhart
Iz_7_1109	DJI_0345	Dianthus campestris M.Bieb.	0	n	1	0	Caryophyllaceae Juss.
Iz_7_1109	DJI_0345	Digitaria sanguinalis (L.) Scop.	0	a	0	0	Poaceae Barnhart
Iz_7_1109	DJI_0345	Elymus repens (L.) Gould	0	n	1	0	Poaceae Barnhart
Iz_7_1109	DJI_0345	Epilobium tetragonum L.	0	n	1	0	Onagraceae Juss.
Iz_7_1109	DJI_0345	Eragrostis minor Host	0	a	0	0	Poaceae Barnhart
Iz_7_1109	DJI_0345	Erigeron canadensis L.	15	a	0	0	Asteraceae Bercht. & J.Presl
Iz_7_1109	DJI_0345	Euphorbia seguieriana Neck.	0	n	1	0	Euphorbiaceae Juss.
Iz_7_1109	DJI_0345	Fallopia dumetorum (L.) Holub	0	n	1	0	Polygonaceae Juss.
Iz_7_1109	DJI_0345	Festuca beckeri (Hack.) Trautv.	0	n	1	0	Poaceae Barnhart
Iz_7_1109	DJI_0345	Fragaria vesca L.	0	n	1	0	Rosaceae Juss.
Iz_7_1109	DJI_0345	Frangula alnus Mill.	0	n	1	1	Rhamnaceae Juss.
Iz_7_1109	DJI_0345	Fraxinus pennsylvanica Marshall	0	a	0	1	Oleaceae Hoffmanns. & Link
Iz_7_1109	DJI_0345	Galeopsis bifida Boenn.	0	n	1	0	Lamiacea Lindl.
Iz_7_1109	DJI_0345	Galium verum L.	0	n	1	0	Rubiaceae Juss.
Iz_7_1109	DJI_0345	Genista tinctoria L.	0	n	1	0	Fabaceae Lindl.
Iz_7_1109	DJI_0345	Geranium robertianum L.	0	n	1	0	Geraniaceae Juss.
Iz_7_1109	DJI_0345	Geranium sanquineum L.	0	n	1	0	Geraniaceae Juss.
Iz_7_1109	DJI_0345	Gypsophila paniculata L.	0	n	1	0	Caryophyllaceae Juss.
Iz_7_1109	DJI_0345	Helichrysum arenarium (L.) Moench	0	n	1	0	Asteraceae Bercht. & J.Presl
Iz_7_1109	DJI_0345	Herniaria polygama J.Gay	0	n	1	0	Caryophyllaceae Juss.
Iz_7_1109	DJI_0345	Hieracium robustum Fr.	0	n	1	0	Asteraceae Bercht. & J.Presl
Iz_7_1109	DJI_0345	Hieracium umbellatum L.	0	n	1	NA	Asteraceae Bercht. & J.Presl
Iz_7_1109	DJI_0345	Hylotelephium maximum (L.) Holub	0	n	1	0	Crassulaceae J.St.-Hil.
Iz_7_1109	DJI_0345	Jacobaea borysthenica (DC.) B.Nord. & Greuter	0	n	1	0	Asteraceae Bercht. & J.Presl
Iz_7_1109	DJI_0345	Jasione montana L.	0	n	1	0	Campanulaceae Juss.
Iz_7_1109	DJI_0345	Jurinea cyanoides (L.) Rchb.s.l.	0	n	1	0	Asteraceae Bercht. & J.Presl
Iz_7_1109	DJI_0345	Koeleria glauca (Spreng.) DC.	0	n	1	0	Poaceae Barnhart
Iz_7_1109	DJI_0345	Lactuca serriola L.	0	a	0	0	Asteraceae Bercht. & J.Presl
Iz_7_1109	DJI_0345	Linaria genistifolia (L.) Mill.	0	n	1	0	Plantaginaceae Juss.
Iz_7_1109	DJI_0345	Lomelosia argentea (L.) Greuter & Burdet	0	n	1	0	Caprifoliaceae Juss.
Iz_7_1109	DJI_0345	Melampyrum pratense L.	0	n	1	0	Orobanchaceae Vent.
Iz_7_1109	DJI_0345	Oenothera biennis L.	0	a	0	0	Onagraceae Juss.
Iz_7_1109	DJI_0345	Pentanema britannica (L.) D.Gut.Larr., Santos-Vicente, Anderb., E.Rico & M.M.Mart.Ort.	0	n	1	0	Asteraceae Bercht. & J.Presl
Iz_7_1109	DJI_0345	Peucedanum oreoselinum (L.) Moench	0	n	1	0	Apiaceae Lindl.
Iz_7_1109	DJI_0345	Peucedanum ruthenicum M.Bieb.	0	n	1	0	Apiaceae Lindl.
Iz_7_1109	DJI_0345	Pilosella echioides (Lumn.) F.W.Schultz & Sch.Bip.	0	n	1	0	Asteraceae Bercht. & J.Presl
Iz_7_1109	DJI_0345	Pilosella officinarum F. Schultz et Sch. Bip.	0	n	1	0	Asteraceae Bercht. & J.Presl
Iz_7_1109	DJI_0345	Pilosella sp.	0		NA	0	Asteraceae Bercht. & J.Presl
Iz_7_1109	DJI_0345	Pinus sylvestris L.	0	n	1	1	Pinaceae Spreng. ex F.Rudolphi
Iz_7_1109	DJI_0345	Poa annua L.	0	n	1	0	Poaceae Barnhart
Iz_7_1109	DJI_0345	Poa nemoralis L.	0	n	1	0	Poaceae Barnhart
Iz_7_1109	DJI_0345	Poa sp.	0		NA	0	Poaceae Barnhart
Iz_7_1109	DJI_0345	Polygonatum odoratum (Mill.) Druce	0	n	1	0	Asparagaceae Juss.
Iz_7_1109	DJI_0345	Polygonum arenarium Waldst. & Kit.	0	n	1	0	Polygonaceae Juss.
Iz_7_1109	DJI_0345	Populus nigra L.	0	n	1	1	Salicaceae Mirb.
Iz_7_1109	DJI_0345	Populus tremula L.	0	n	1	1	Salicaceae Mirb.
Iz_7_1109	DJI_0345	Populus Ã canescens (Aiton) Sm.	0	n	1	1	Salicaceae Mirb.
Iz_7_1109	DJI_0345	Potentilla argentea L.	0	n	1	0	Rosaceae Juss.
Iz_7_1109	DJI_0345	Psammophiliella muralis (L.) Ikonn.	0	n	1	0	Caryophyllaceae Juss.
Iz_7_1109	DJI_0345	Psephellus sumensis (Kalen.) Greuter	0	n	1	0	Asteraceae Bercht. & J.Presl
Iz_7_1109	DJI_0345	Pulsatilla pratensis (L.) Mill. s.l.	0	n	1	0	Ranunculaceae Juss.
Iz_7_1109	DJI_0345	Pyrus communis L.	0	a	0	1	Rosaceae Juss.
Iz_7_1109	DJI_0345	Quercus robur L.	0	n	1	1	Fagaceae Dumort.
Iz_7_1109	DJI_0345	Robinia pseudoacacia L.	0	a	0	1	Fabaceae Lindl.
Iz_7_1109	DJI_0345	Rosa sp.	0		NA	1	Rosaceae Juss.
Iz_7_1109	DJI_0345	Rubus idaeus L.	0	n	1	0	Rosaceae Juss.
Iz_7_1109	DJI_0345	Rumex acetosella L.	0	n	1	0	Polygonaceae Juss.
Iz_7_1109	DJI_0345	Rumex thyrsiflorus Fingerh.	0	n	1	0	Polygonaceae Juss.
Iz_7_1109	DJI_0345	Salix alba L.	0	n	1	1	Salicaceae Mirb.
Iz_7_1109	DJI_0345	Sambucus nigra L.	0	n	1	1	Viburnaceae Raf.
Iz_7_1109	DJI_0345	Secale sylvestre Host	0	n	1	0	Poaceae Barnhart
Iz_7_1109	DJI_0345	Sempervivum ruthenicum Schnittsp. & C.B. Lehm.	0	n	1	0	Crassulaceae J.St.-Hil.
Iz_7_1109	DJI_0345	Setaria viridis (L.) P. Beauv.	0	n	1	0	Poaceae Barnhart
Iz_7_1109	DJI_0345	Silene borysthenica (Gruner) Walters	0	n	1	0	Caryophyllaceae Juss.

Iz_7_1109	DJI_0345	Silene latifolia subsp. alba (Mill.) Greuter & Burdet	0	n	1	0	Caryophyllaceae Juss.
Iz_7_1109	DJI_0345	Solidago virgaurea L.	0	n	1	0	Asteraceae Bercht. & J.Presl
Iz_7_1109	DJI_0345	Sonchus oleraceus L.	0	a	0	0	Asteraceae Bercht. & J.Presl
Iz_7_1109	DJI_0345	Sorbus aucuparia L.	0	n	1	1	Rosaceae Juss.
Iz_7_1109	DJI_0345	Stachys recta L.	0	n	1	0	Lamiacea Lindl.
Iz_7_1109	DJI_0345	Tanacetum vulgare L.	0	n	1	0	Asteraceae Bercht. & J.Presl
Iz_7_1109	DJI_0345	Thymus pallasianus Heinr.Braun	0	n	1	0	Lamiacea Lindl.
Iz_7_1109	DJI_0345	Tilia cordata Mill.	0	n	1	1	Malvaceae Juss.
Iz_7_1109	DJI_0345	Trifolium alpestre L.	0	n	1	0	Fabaceae Lindl.
Iz_7_1109	DJI_0345	Trifolium arvense L.	0	n	1	0	Fabaceae Lindl.
Iz_7_1109	DJI_0345	Ulmus glabra Huds.	0	n	1	1	Ulmaceae Mirb.
Iz_7_1109	DJI_0345	Verbascum densiflorum Bertol.	0	n	1	0	Scrophulariaceae Juss.
Iz_7_1109	DJI_0345	Veronica austriaca L.	0	n	1	0	Plantaginaceae Juss.
Iz_7_1109	DJI_0345	Veronica incana L.	0	n	1	0	Plantaginaceae Juss.
Iz_7_1109	DJI_0345	Veronica verna L.	0	n	1	0	Plantaginaceae Juss.
Iz_7_1109	DJI_0345	Vincetoxicum hirundinaria Medik	0	n	1	0	Apocynaceae Juss.
Iz_7_1109	DJI_0349	Acer tataricum L.	0	n	1	1	Sapindaceae Juss.
Iz_7_1109	DJI_0349	Achillea millefolium L. s.l.	0	n	1	0	Asteraceae Bercht. & J.Presl
Iz_7_1109	DJI_0349	Allium oleraceum L.	0	n	1	0	Amaryllidaceae J.St.-Hil.
Iz_7_1109	DJI_0349	Ambrosia artemisiifolia L.	0	a	0	0	Asteraceae Bercht. & J.Presl
Iz_7_1109	DJI_0349	Amorpha fruticosa L.	0	a	0	0	Fabaceae Lindl.
Iz_7_1109	DJI_0349	Anthericum ramosum L.	0	n	1	0	Asparagaceae Juss.
Iz_7_1109	DJI_0349	Artemisia marschalliana Spreng.	0	n	1	0	Asteraceae Bercht. & J.Presl
Iz_7_1109	DJI_0349	Asparagus officinalis L.	0	n	1	0	Asparagaceae Juss.
Iz_7_1109	DJI_0349	Astragalus cicer L.	0	n	1	0	Fabaceae Lindl.
Iz_7_1109	DJI_0349	Bassia laniflora (S.G.Gmel.) A.J.Scott	0	n	1	0	Amaranthaceae Juss.
Iz_7_1109	DJI_0349	Berberis vulgaris L.	0	n	1	0	Berberidaceae Juss.
Iz_7_1109	DJI_0349	Betula pendula Roth.	0	n	1	1	Betulaceae Gray
Iz_7_1109	DJI_0349	Betula pendula Roth.	0	n	1	0	Poaceae Barnhart
Iz_7_1109	DJI_0349	Bromus inermis Leyss.	0		NA	NA	
Iz_7_1109	DJI_0349	Bromus squarrosus L.	0	a	0	0	Poaceae Barnhart
Iz_7_1109	DJI_0349	Bromus tectorum L.	0	a	0	0	Poaceae Barnhart
Iz_7_1109	DJI_0349	Calamagrostis epigejos (L.) Roth	6	n	1	0	Poaceae Barnhart
Iz_7_1109	DJI_0349	Carduus acanthoides L.	0	n	1	0	Asteraceae Bercht. & J.Presl
Iz_7_1109	DJI_0349	Carex sp.	0	n	1	0	Cyperaceae Juss.
Iz_7_1109	DJI_0349	Centaurea majorovii Dumbadze	0	n	1	0	Asteraceae Bercht. & J.Presl
Iz_7_1109	DJI_0349	Chamaecytisus ruthenicus (Fisch.ex Wol.) Klaskova	0	n	1	0	Fabaceae Lindl.
Iz_7_1109	DJI_0349	Chelidonium majus L.	0	n	1	0	Papaveraceae Juss.
Iz_7_1109	DJI_0349	Chenopodium betaceum Andrz.	0	n	1	0	Amaranthaceae Juss.
Iz_7_1109	DJI_0349	Chenopodium cf. album	0	n	1	0	Amaranthaceae Juss.
Iz_7_1109	DJI_0349	Chenopodium sp.	0		NA	0	Amaranthaceae Juss.
Iz_7_1109	DJI_0349	Chondrilla juncea L.	0	n	1	0	Asteraceae Bercht. & J.Presl
Iz_7_1109	DJI_0349	Cirsium vulgare (Savi) Ten.	0	n	1	0	Asteraceae Bercht. & J.Presl
Iz_7_1109	DJI_0349	Clinopodium acinos (L.) Kuntze	0	n	1	0	Lamiacea Lindl.
Iz_7_1109	DJI_0349	Convallaria majalis L.	0	n	1	0	Asparagaceae Juss.
Iz_7_1109	DJI_0349	Crataegus rhipidophylla Gand.	0	n	1	1	Rosaceae Juss.
Iz_7_1109	DJI_0349	Crepis foetida L.	0	n	1	0	Asteraceae Bercht. & J.Presl
Iz_7_1109	DJI_0349	Dactylis glomerata L.	0	n	1	0	Poaceae Barnhart
Iz_7_1109	DJI_0349	Dianthus campestris M.Bieb.	0	n	1	0	Caryophyllaceae Juss.
Iz_7_1109	DJI_0349	Digitaria sanguinalis (L.) Scop.	0	a	0	0	Poaceae Barnhart
Iz_7_1109	DJI_0349	Elymus repens (L.) Gould	0	n	1	0	Poaceae Barnhart
Iz_7_1109	DJI_0349	Epilobium tetragonum L.	0	n	1	0	Onagraceae Juss.
Iz_7_1109	DJI_0349	Eragrostis minor Host	0	a	0	0	Poaceae Barnhart
Iz_7_1109	DJI_0349	Erigeron canadensis L.	10	a	0	0	Asteraceae Bercht. & J.Presl
Iz_7_1109	DJI_0349	Euphorbia seguieriana Neck.	0	n	1	0	Euphorbiaceae Juss.
Iz_7_1109	DJI_0349	Fallopia dumetorum (L.) Holub	0	n	1	0	Polygonaceae Juss.
Iz_7_1109	DJI_0349	Festuca beckeri (Hack.) Trautv.	0	n	1	0	Poaceae Barnhart
Iz_7_1109	DJI_0349	Fragaria vesca L.	0	n	1	0	Rosaceae Juss.
Iz_7_1109	DJI_0349	Frangula alnus Mill.	0	n	1	1	Rhamnaceae Juss.
Iz_7_1109	DJI_0349	Fraxinus pennsylvanica Marshall	0	a	0	1	Oleaceae Hoffmanns. & Link
Iz_7_1109	DJI_0349	Galeopsis bifida Boenn.	0	n	1	0	Lamiacea Lindl.
Iz_7_1109	DJI_0349	Galium verum L.	0	n	1	0	Rubiaceae Juss.
Iz_7_1109	DJI_0349	Genista tinctoria L.	0	n	1	0	Fabaceae Lindl.
Iz_7_1109	DJI_0349	Geranium robertianum L.	0	n	1	0	Geraniaceae Juss.
Iz_7_1109	DJI_0349	Geranium sanquineum L.	0	n	1	0	Geraniaceae Juss.
Iz_7_1109	DJI_0349	Gypsophila paniculata L.	0	n	1	0	Caryophyllaceae Juss.
Iz_7_1109	DJI_0349	Helichrysum arenarium (L.) Moench	0	n	1	0	Asteraceae Bercht. & J.Presl
Iz_7_1109	DJI_0349	Herniaria polygama J.Gay	0	n	1	0	Caryophyllaceae Juss.
Iz_7_1109	DJI_0349	Hieracium robustum Fr.	0	n	1	0	Asteraceae Bercht. & J.Presl
Iz_7_1109	DJI_0349	Hieracium umbellatum L.	0	n	1	NA	Asteraceae Bercht. & J.Presl
Iz_7_1109	DJI_0349	Hylotelephium maximum (L.) Holub	0	n	1	0	Crassulaceae J.St.-Hil.
Iz_7_1109	DJI_0349	Jacobaea borysthenica (DC.) B.Nord. & Greuter	0	n	1	0	Asteraceae Bercht. & J.Presl
Iz_7_1109	DJI_0349	Jasione montana L.	0	n	1	0	Campanulaceae Juss.
Iz_7_1109	DJI_0349	Jurinea cyanoides (L.) Rchb.s.l.	0	n	1	0	Asteraceae Bercht. & J.Presl
Iz_7_1109	DJI_0349	Koeleria glauca (Spreng.) DC.	0	n	1	0	Poaceae Barnhart
Iz_7_1109	DJI_0349	Lactuca serriola L.	0	a	0	0	Asteraceae Bercht. & J.Presl
Iz_7_1109	DJI_0349	Linaria genistifolia (L.) Mill.	0	n	1	0	Plantaginaceae Juss.
Iz_7_1109	DJI_0349	Lomelosia argentea (L.) Greuter & Burdet	0	n	1	0	Caprifoliaceae Juss.
Iz_7_1109	DJI_0349	Melampyrum pratense L.	0	n	1	0	Orobanchaceae Vent.
Iz_7_1109	DJI_0349	Oenothera biennis L.	0	a	0	0	Onagraceae Juss.
Iz_7_1109	DJI_0349	Pentanema britannica (L.) D.Gut.Larr., Santos-Vicente, Anderb., E.Rico & M.M.Mart.Ort.	0	n	1	0	Asteraceae Bercht. & J.Presl
Iz_7_1109	DJI_0349	Peucedanum oreoselinum (L.) Moench	0	n	1	0	Apiaceae Lindl.
Iz_7_1109	DJI_0349	Peucedanum ruthenicum M.Bieb.	0	n	1	0	Apiaceae Lindl.
Iz_7_1109	DJI_0349	Pilosella echioides (Lumn.) F.W.Schultz & Sch.Bip.	0	n	1	0	Asteraceae Bercht. & J.Presl
Iz_7_1109	DJI_0349	Pilosella officinarum F. Schultz et Sch. Bip.	0	n	1	0	Asteraceae Bercht. & J.Presl
Iz_7_1109	DJI_0349	Pilosella sp.	0		NA	0	Asteraceae Bercht. & J.Presl
Iz_7_1109	DJI_0349	Pinus sylvestris L.	0	n	1	1	Pinaceae Spreng. ex F.Rudolphi
Iz_7_1109	DJI_0349	Poa annua L.	0	n	1	0	Poaceae Barnhart
Iz_7_1109	DJI_0349	Poa nemoralis L.	0	n	1	0	Poaceae Barnhart
Iz_7_1109	DJI_0349	Poa sp.	0		NA	0	Poaceae Barnhart
Iz_7_1109	DJI_0349	Polygonatum odoratum (Mill.) Druce	0	n	1	0	Asparagaceae Juss.
Iz_7_1109	DJI_0349	Polygonum arenarium Waldst. & Kit.	0	n	1	0	Polygonaceae Juss.
Iz_7_1109	DJI_0349	Populus nigra L.	0	n	1	1	Salicaceae Mirb.
Iz_7_1109	DJI_0349	Populus tremula L.	0	n	1	1	Salicaceae Mirb.
Iz_7_1109	DJI_0349	Populus Ã canescens (Aiton) Sm.	0	n	1	1	Salicaceae Mirb.
Iz_7_1109	DJI_0349	Potentilla argentea L.	0	n	1	0	Rosaceae Juss.
Iz_7_1109	DJI_0349	Psammophiliella muralis (L.) Ikonn.	0	n	1	0	Caryophyllaceae Juss.
Iz_7_1109	DJI_0349	Psephellus sumensis (Kalen.) Greuter	0	n	1	0	Asteraceae Bercht. & J.Presl

Iz_7_1109	DJI_0349	Pulsatilla pratensis (L.) Mill. s.l.	0	n	1	0	Ranunculaceae Juss.
Iz_7_1109	DJI_0349	Pyrus communis L.	0	a	0	1	Rosaceae Juss.
Iz_7_1109	DJI_0349	Quercus robur L.	0	n	1	1	Fagaceae Dumort.
Iz_7_1109	DJI_0349	Robinia pseudoacacia L.	0	a	0	1	Fabaceae Lindl.
Iz_7_1109	DJI_0349	Rosa sp.	0		NA	1	Rosaceae Juss.
Iz_7_1109	DJI_0349	Rubus idaeus L.	0	n	1	0	Rosaceae Juss.
Iz_7_1109	DJI_0349	Rumex acetosella L.	0	n	1	0	Polygonaceae Juss.
Iz_7_1109	DJI_0349	Rumex thyrsiflorus Fingerh.	0	n	1	0	Polygonaceae Juss.
Iz_7_1109	DJI_0349	Salix alba L.	0	n	1	1	Salicaceae Mirb.
Iz_7_1109	DJI_0349	Sambucus nigra L.	0	n	1	1	Viburnaceae Raf.
Iz_7_1109	DJI_0349	Secale sylvestre Host	0	n	1	0	Poaceae Barnhart
Iz_7_1109	DJI_0349	Sempervivum ruthenicum Schnittsp. & C.B. Lehm.	0	n	1	0	Crassulaceae J.St.-Hil.
Iz_7_1109	DJI_0349	Setaria viridis (L.) P. Beauv.	0	n	1	0	Poaceae Barnhart
Iz_7_1109	DJI_0349	Silene borysthenica (Gruner) Walters	0	n	1	0	Caryophyllaceae Juss.
Iz_7_1109	DJI_0349	Silene latifolia subsp. alba (Mill.) Greuter & Burdet	0	n	1	0	Caryophyllaceae Juss.
Iz_7_1109	DJI_0349	Solidago virgaurea L.	0	n	1	0	Asteraceae Bercht. & J.Presl
Iz_7_1109	DJI_0349	Sonchus oleraceus L.	0	a	0	0	Asteraceae Bercht. & J.Presl
Iz_7_1109	DJI_0349	Sorbus aucuparia L.	0	n	1	1	Rosaceae Juss.
Iz_7_1109	DJI_0349	Stachys recta L.	0	n	1	0	Lamiacea Lindl.
Iz_7_1109	DJI_0349	Tanacetum vulgare L.	0	n	1	0	Asteraceae Bercht. & J.Presl
Iz_7_1109	DJI_0349	Thymus pallasianus Heinr.Braun	0	n	1	0	Lamiacea Lindl.
Iz_7_1109	DJI_0349	Tilia cordata Mill.	0	n	1	1	Malvaceae Juss.
Iz_7_1109	DJI_0349	Trifolium alpestre L.	0	n	1	0	Fabaceae Lindl.
Iz_7_1109	DJI_0349	Trifolium arvense L.	0	n	1	0	Fabaceae Lindl.
Iz_7_1109	DJI_0349	Ulmus glabra Huds.	0	n	1	1	Ulmaceae Mirb.
Iz_7_1109	DJI_0349	Verbascum densiflorum Bertol.	0	n	1	0	Scrophulariaceae Juss.
Iz_7_1109	DJI_0349	Veronica austriaca L.	0	n	1	0	Plantaginaceae Juss.
Iz_7_1109	DJI_0349	Veronica incana L.	0	n	1	0	Plantaginaceae Juss.
Iz_7_1109	DJI_0349	Veronica verna L.	0	n	1	0	Plantaginaceae Juss.
Iz_7_1109	DJI_0349	Vincetoxicum hirundinaria Medik	0	n	1	0	Apocynaceae Juss.
Iz_7_1109	DJI_0352	Acer tataricum L.	0	n	1	1	Sapindaceae Juss.
Iz_7_1109	DJI_0352	Achillea millefolium L. s.l.	0	n	1	0	Asteraceae Bercht. & J.Presl
Iz_7_1109	DJI_0352	Allium oleraceum L.	0	n	1	0	Amaryllidaceae J.St.-Hil.
Iz_7_1109	DJI_0352	Ambrosia artemisiifolia L.	0	a	0	0	Asteraceae Bercht. & J.Presl
Iz_7_1109	DJI_0352	Amorpha fruticosa L.	0	a	0	0	Fabaceae Lindl.
Iz_7_1109	DJI_0352	Anthericum ramosum L.	0	n	1	0	Asparagaceae Juss.
Iz_7_1109	DJI_0352	Artemisia marschalliana Spreng.	0	n	1	0	Asteraceae Bercht. & J.Presl
Iz_7_1109	DJI_0352	Asparagus officinalis L.	0	n	1	0	Asparagaceae Juss.
Iz_7_1109	DJI_0352	Astragalus cicer L.	0	n	1	0	Fabaceae Lindl.
Iz_7_1109	DJI_0352	Bassia laniflora (S.G.Gmel.) A.J.Scott	0	n	1	0	Amaranthaceae Juss.
Iz_7_1109	DJI_0352	Berberis vulgaris L.	0	n	1	0	Berberidaceae Juss.
Iz_7_1109	DJI_0352	Betula pendula Roth.	0	n	1	1	Betulaceae Gray
Iz_7_1109	DJI_0352	Betula pendula Roth.	0	n	1	0	Poaceae Barnhart
Iz_7_1109	DJI_0352	Bromus inermis Leyss.	0		NA	NA	
Iz_7_1109	DJI_0352	Bromus squarrosus L.	0	a	0	0	Poaceae Barnhart
Iz_7_1109	DJI_0352	Bromus tectorum L.	0	a	0	0	Poaceae Barnhart
Iz_7_1109	DJI_0352	Calamagrostis epigejos (L.) Roth	1	n	1	0	Poaceae Barnhart
Iz_7_1109	DJI_0352	Carduus acanthoides L.	0	n	1	0	Asteraceae Bercht. & J.Presl
Iz_7_1109	DJI_0352	Carex sp.	0	n	1	0	Cyperaceae Juss.
Iz_7_1109	DJI_0352	Centaurea majorovii Dumbadze	0	n	1	0	Asteraceae Bercht. & J.Presl
Iz_7_1109	DJI_0352	Chamaecytisus ruthenicus (Fisch.ex Wol.) Klaskova	0	n	1	0	Fabaceae Lindl.
Iz_7_1109	DJI_0352	Chelidonium majus L.	0	n	1	0	Papaveraceae Juss.
Iz_7_1109	DJI_0352	Chenopodium betaceum Andrz.	0	n	1	0	Amaranthaceae Juss.
Iz_7_1109	DJI_0352	Chenopodium cf. album	0	n	1	0	Amaranthaceae Juss.
Iz_7_1109	DJI_0352	Chenopodium sp.	0		NA	0	Amaranthaceae Juss.
Iz_7_1109	DJI_0352	Chondrilla juncea L.	0	n	1	0	Asteraceae Bercht. & J.Presl
Iz_7_1109	DJI_0352	Cirsium vulgare (Savi) Ten.	0	n	1	0	Asteraceae Bercht. & J.Presl
Iz_7_1109	DJI_0352	Clinopodium acinos (L.) Kuntze	0	n	1	0	Lamiacea Lindl.
Iz_7_1109	DJI_0352	Convallaria majalis L.	0	n	1	0	Asparagaceae Juss.
Iz_7_1109	DJI_0352	Crataegus rhipidophylla Gand.	0	n	1	1	Rosaceae Juss.
Iz_7_1109	DJI_0352	Crepis foetida L.	0	n	1	0	Asteraceae Bercht. & J.Presl
Iz_7_1109	DJI_0352	Dactylis glomerata L.	0	n	1	0	Poaceae Barnhart
Iz_7_1109	DJI_0352	Dianthus campestris M.Bieb.	0	n	1	0	Caryophyllaceae Juss.
Iz_7_1109	DJI_0352	Digitaria sanguinalis (L.) Scop.	0	a	0	0	Poaceae Barnhart
Iz_7_1109	DJI_0352	Elymus repens (L.) Gould	0	n	1	0	Poaceae Barnhart
Iz_7_1109	DJI_0352	Epilobium tetragonum L.	0	n	1	0	Onagraceae Juss.
Iz_7_1109	DJI_0352	Eragrostis minor Host	0	a	0	0	Poaceae Barnhart
Iz_7_1109	DJI_0352	Erigeron canadensis L.	10	a	0	0	Asteraceae Bercht. & J.Presl
Iz_7_1109	DJI_0352	Euphorbia seguieriana Neck.	0	n	1	0	Euphorbiaceae Juss.
Iz_7_1109	DJI_0352	Fallopia dumetorum (L.) Holub	0	n	1	0	Polygonaceae Juss.
Iz_7_1109	DJI_0352	Festuca beckeri (Hack.) Trautv.	0	n	1	0	Poaceae Barnhart
Iz_7_1109	DJI_0352	Fragaria vesca L.	0	n	1	0	Rosaceae Juss.
Iz_7_1109	DJI_0352	Frangula alnus Mill.	0	n	1	1	Rhamnaceae Juss.
Iz_7_1109	DJI_0352	Fraxinus pennsylvanica Marshall	0	a	0	1	Oleaceae Hoffmanns. & Link
Iz_7_1109	DJI_0352	Galeopsis bifida Boenn.	0	n	1	0	Lamiacea Lindl.
Iz_7_1109	DJI_0352	Galium verum L.	0	n	1	0	Rubiaceae Juss.
Iz_7_1109	DJI_0352	Genista tinctoria L.	0	n	1	0	Fabaceae Lindl.
Iz_7_1109	DJI_0352	Geranium robertianum L.	0	n	1	0	Geraniaceae Juss.
Iz_7_1109	DJI_0352	Geranium sanquineum L.	0	n	1	0	Geraniaceae Juss.
Iz_7_1109	DJI_0352	Gypsophila paniculata L.	0	n	1	0	Caryophyllaceae Juss.
Iz_7_1109	DJI_0352	Helichrysum arenarium (L.) Moench	0	n	1	0	Asteraceae Bercht. & J.Presl
Iz_7_1109	DJI_0352	Herniaria polygama J.Gay	0	n	1	0	Caryophyllaceae Juss.
Iz_7_1109	DJI_0352	Hieracium robustum Fr.	0	n	1	0	Asteraceae Bercht. & J.Presl
Iz_7_1109	DJI_0352	Hieracium umbellatum L.	0	n	1	NA	Asteraceae Bercht. & J.Presl
Iz_7_1109	DJI_0352	Hylotelephium maximum (L.) Holub	0	n	1	0	Crassulaceae J.St.-Hil.
Iz_7_1109	DJI_0352	Jacobaea borysthenica (DC.) B.Nord. & Greuter	0	n	1	0	Asteraceae Bercht. & J.Presl
Iz_7_1109	DJI_0352	Jasione montana L.	0	n	1	0	Campanulaceae Juss.
Iz_7_1109	DJI_0352	Jurinea cyanoides (L.) Rchb.s.l.	0	n	1	0	Asteraceae Bercht. & J.Presl
Iz_7_1109	DJI_0352	Koeleria glauca (Spreng.) DC.	0	n	1	0	Poaceae Barnhart
Iz_7_1109	DJI_0352	Lactuca serriola L.	0	a	0	0	Asteraceae Bercht. & J.Presl
Iz_7_1109	DJI_0352	Linaria genistifolia (L.) Mill.	0	n	1	0	Plantaginaceae Juss.
Iz_7_1109	DJI_0352	Lomelosia argentea (L.) Greuter & Burdet	0	n	1	0	Caprifoliaceae Juss.
Iz_7_1109	DJI_0352	Melampyrum pratense L.	0	n	1	0	Orobanchaceae Vent.
Iz_7_1109	DJI_0352	Oenothera biennis L.	0	a	0	0	Onagraceae Juss.
Iz_7_1109	DJI_0352	Pentanema britannica (L.) D.Gut.Larr., Santos-Vicente, Anderb., E.Rico & M.M.Mart.Ort.	0	n	1	0	Asteraceae Bercht. & J.Presl
Iz_7_1109	DJI_0352	Peucedanum oreoselinum (L.) Moench	0	n	1	0	Apiaceae Lindl.
Iz_7_1109	DJI_0352	Peucedanum ruthenicum M.Bieb.	0	n	1	0	Apiaceae Lindl.
Iz_7_1109	DJI_0352	Pilosella echioides (Lumn.) F.W.Schultz & Sch.Bip.	0	n	1	0	Asteraceae Bercht. & J.Presl

Iz_7_1109	DJI_0352	Pilosella officinarum F. Schultz et Sch. Bip.	0	n	1	0	Asteraceae Bercht. & J.Presl
Iz_7_1109	DJI_0352	Pilosella sp.	0		NA	0	Asteraceae Bercht. & J.Presl
Iz_7_1109	DJI_0352	Pinus sylvestris L.	0	n	1	1	Pinaceae Spreng. ex F.Rudolphi
Iz_7_1109	DJI_0352	Poa annua L.	0	n	1	0	Poaceae Barnhart
Iz_7_1109	DJI_0352	Poa nemoralis L.	0	n	1	0	Poaceae Barnhart
Iz_7_1109	DJI_0352	Poa sp.	0		NA	0	Poaceae Barnhart
Iz_7_1109	DJI_0352	Polygonatum odoratum (Mill.) Druce	0	n	1	0	Asparagaceae Juss.
Iz_7_1109	DJI_0352	Polygonum arenarium Waldst. & Kit.	0	n	1	0	Polygonaceae Juss.
Iz_7_1109	DJI_0352	Populus nigra L.	0	n	1	1	Salicaceae Mirb.
Iz_7_1109	DJI_0352	Populus tremula L.	0	n	1	1	Salicaceae Mirb.
Iz_7_1109	DJI_0352	Populus Ã canescens (Aiton) Sm.	0	n	1	1	Salicaceae Mirb.
Iz_7_1109	DJI_0352	Potentilla argentea L.	0	n	1	0	Rosaceae Juss.
Iz_7_1109	DJI_0352	Psammophiliella muralis (L.) Ikonn.	0	n	1	0	Caryophyllaceae Juss.
Iz_7_1109	DJI_0352	Psephellus sumensis (Kalen.) Greuter	0	n	1	0	Asteraceae Bercht. & J.Presl
Iz_7_1109	DJI_0352	Pulsatilla pratensis (L.) Mill. s.l.	0	n	1	0	Ranunculaceae Juss.
Iz_7_1109	DJI_0352	Pyrus communis L.	0	a	0	1	Rosaceae Juss.
Iz_7_1109	DJI_0352	Quercus robur L.	0	n	1	1	Fagaceae Dumort.
Iz_7_1109	DJI_0352	Robinia pseudoacacia L.	0	a	0	1	Fabaceae Lindl.
Iz_7_1109	DJI_0352	Rosa sp.	0		NA	1	Rosaceae Juss.
Iz_7_1109	DJI_0352	Rubus idaeus L.	0	n	1	0	Rosaceae Juss.
Iz_7_1109	DJI_0352	Rumex acetosella L.	0	n	1	0	Polygonaceae Juss.
Iz_7_1109	DJI_0352	Rumex thyrsiflorus Fingerh.	0	n	1	0	Polygonaceae Juss.
Iz_7_1109	DJI_0352	Salix alba L.	0	n	1	1	Salicaceae Mirb.
Iz_7_1109	DJI_0352	Sambucus nigra L.	0	n	1	1	Viburnaceae Raf.
Iz_7_1109	DJI_0352	Secale sylvestre Host	0	n	1	0	Poaceae Barnhart
Iz_7_1109	DJI_0352	Sempervivum ruthenicum Schnittsp. & C.B. Lehm.	0	n	1	0	Crassulaceae J.St.-Hil.
Iz_7_1109	DJI_0352	Setaria viridis (L.) P. Beauv.	0	n	1	0	Poaceae Barnhart
Iz_7_1109	DJI_0352	Silene borysthenica (Gruner) Walters	0	n	1	0	Caryophyllaceae Juss.
Iz_7_1109	DJI_0352	Silene latifolia subsp. alba (Mill.) Greuter & Burdet	0	n	1	0	Caryophyllaceae Juss.
Iz_7_1109	DJI_0352	Solidago virgaurea L.	0	n	1	0	Asteraceae Bercht. & J.Presl
Iz_7_1109	DJI_0352	Sonchus oleraceus L.	0	a	0	0	Asteraceae Bercht. & J.Presl
Iz_7_1109	DJI_0352	Sorbus aucuparia L.	0	n	1	1	Rosaceae Juss.
Iz_7_1109	DJI_0352	Stachys recta L.	0	n	1	0	Lamiacea Lindl.
Iz_7_1109	DJI_0352	Tanacetum vulgare L.	0	n	1	0	Asteraceae Bercht. & J.Presl
Iz_7_1109	DJI_0352	Thymus pallasianus Heinr.Braun	0	n	1	0	Lamiacea Lindl.
Iz_7_1109	DJI_0352	Tilia cordata Mill.	0	n	1	1	Malvaceae Juss.
Iz_7_1109	DJI_0352	Trifolium alpestre L.	0	n	1	0	Fabaceae Lindl.
Iz_7_1109	DJI_0352	Trifolium arvense L.	0	n	1	0	Fabaceae Lindl.
Iz_7_1109	DJI_0352	Ulmus glabra Huds.	0	n	1	1	Ulmaceae Mirb.
Iz_7_1109	DJI_0352	Verbascum densiflorum Bertol.	0	n	1	0	Scrophulariaceae Juss.
Iz_7_1109	DJI_0352	Veronica austriaca L.	0	n	1	0	Plantaginaceae Juss.
Iz_7_1109	DJI_0352	Veronica incana L.	0	n	1	0	Plantaginaceae Juss.
Iz_7_1109	DJI_0352	Veronica verna L.	0	n	1	0	Plantaginaceae Juss.
Iz_7_1109	DJI_0352	Vincetoxicum hirundinaria Medik	0	n	1	0	Apocynaceae Juss.
Iz_7_1109	DJI_0356	Acer tataricum L.	0	n	1	1	Sapindaceae Juss.
Iz_7_1109	DJI_0356	Achillea millefolium L. s.l.	0	n	1	0	Asteraceae Bercht. & J.Presl
Iz_7_1109	DJI_0356	Allium oleraceum L.	0	n	1	0	Amaryllidaceae J.St.-Hil.
Iz_7_1109	DJI_0356	Ambrosia artemisiifolia L.	0	a	0	0	Asteraceae Bercht. & J.Presl
Iz_7_1109	DJI_0356	Amorpha fruticosa L.	0	a	0	0	Fabaceae Lindl.
Iz_7_1109	DJI_0356	Anthericum ramosum L.	0	n	1	0	Asparagaceae Juss.
Iz_7_1109	DJI_0356	Artemisia marschalliana Spreng.	0	n	1	0	Asteraceae Bercht. & J.Presl
Iz_7_1109	DJI_0356	Asparagus officinalis L.	0	n	1	0	Asparagaceae Juss.
Iz_7_1109	DJI_0356	Astragalus cicer L.	0	n	1	0	Fabaceae Lindl.
Iz_7_1109	DJI_0356	Bassia laniflora (S.G.Gmel.) A.J.Scott	0	n	1	0	Amaranthaceae Juss.
Iz_7_1109	DJI_0356	Berberis vulgaris L.	0	n	1	0	Berberidaceae Juss.
Iz_7_1109	DJI_0356	Betula pendula Roth.	0	n	1	1	Betulaceae Gray
Iz_7_1109	DJI_0356	Betula pendula Roth.	0	n	1	0	Poaceae Barnhart
Iz_7_1109	DJI_0356	Bromus inermis Leyss.	0		NA	NA	
Iz_7_1109	DJI_0356	Bromus squarrosus L.	0	a	0	0	Poaceae Barnhart
Iz_7_1109	DJI_0356	Bromus tectorum L.	0	a	0	0	Poaceae Barnhart
Iz_7_1109	DJI_0356	Calamagrostis epigejos (L.) Roth	10	n	1	0	Poaceae Barnhart
Iz_7_1109	DJI_0356	Carduus acanthoides L.	0	n	1	0	Asteraceae Bercht. & J.Presl
Iz_7_1109	DJI_0356	Carex sp.	0	n	1	0	Cyperaceae Juss.
Iz_7_1109	DJI_0356	Centaurea majorovii Dumbadze	0	n	1	0	Asteraceae Bercht. & J.Presl
Iz_7_1109	DJI_0356	Chamaecytisus ruthenicus (Fisch.ex Wol.) Klaskova	0	n	1	0	Fabaceae Lindl.
Iz_7_1109	DJI_0356	Chelidonium majus L.	0	n	1	0	Papaveraceae Juss.
Iz_7_1109	DJI_0356	Chenopodium betaceum Andrz.	0	n	1	0	Amaranthaceae Juss.
Iz_7_1109	DJI_0356	Chenopodium cf. album	0	n	1	0	Amaranthaceae Juss.
Iz_7_1109	DJI_0356	Chenopodium sp.	0		NA	0	Amaranthaceae Juss.
Iz_7_1109	DJI_0356	Chondrilla juncea L.	0	n	1	0	Asteraceae Bercht. & J.Presl
Iz_7_1109	DJI_0356	Cirsium vulgare (Savi) Ten.	0	n	1	0	Asteraceae Bercht. & J.Presl
Iz_7_1109	DJI_0356	Clinopodium acinos (L.) Kuntze	0	n	1	0	Lamiacea Lindl.
Iz_7_1109	DJI_0356	Convallaria majalis L.	0	n	1	0	Asparagaceae Juss.
Iz_7_1109	DJI_0356	Crataegus rhipidophylla Gand.	0	n	1	1	Rosaceae Juss.
Iz_7_1109	DJI_0356	Crepis foetida L.	0	n	1	0	Asteraceae Bercht. & J.Presl
Iz_7_1109	DJI_0356	Dactylis glomerata L.	0	n	1	0	Poaceae Barnhart
Iz_7_1109	DJI_0356	Dianthus campestris M.Bieb.	0	n	1	0	Caryophyllaceae Juss.
Iz_7_1109	DJI_0356	Digitaria sanguinalis (L.) Scop.	0	a	0	0	Poaceae Barnhart
Iz_7_1109	DJI_0356	Elymus repens (L.) Gould	0	n	1	0	Poaceae Barnhart
Iz_7_1109	DJI_0356	Epilobium tetragonum L.	0	n	1	0	Onagraceae Juss.
Iz_7_1109	DJI_0356	Eragrostis minor Host	0	a	0	0	Poaceae Barnhart
Iz_7_1109	DJI_0356	Erigeron canadensis L.	15	a	0	0	Asteraceae Bercht. & J.Presl
Iz_7_1109	DJI_0356	Euphorbia seguieriana Neck.	0	n	1	0	Euphorbiaceae Juss.
Iz_7_1109	DJI_0356	Fallopia dumetorum (L.) Holub	0	n	1	0	Polygonaceae Juss.
Iz_7_1109	DJI_0356	Festuca beckeri (Hack.) Trautv.	0	n	1	0	Poaceae Barnhart
Iz_7_1109	DJI_0356	Fragaria vesca L.	0	n	1	0	Rosaceae Juss.
Iz_7_1109	DJI_0356	Frangula alnus Mill.	0	n	1	1	Rhamnaceae Juss.
Iz_7_1109	DJI_0356	Fraxinus pennsylvanica Marshall	0	a	0	1	Oleaceae Hoffmanns. & Link
Iz_7_1109	DJI_0356	Galeopsis bifida Boenn.	0	n	1	0	Lamiacea Lindl.
Iz_7_1109	DJI_0356	Galium verum L.	0	n	1	0	Rubiaceae Juss.
Iz_7_1109	DJI_0356	Genista tinctoria L.	0	n	1	0	Fabaceae Lindl.
Iz_7_1109	DJI_0356	Geranium robertianum L.	0	n	1	0	Geraniaceae Juss.
Iz_7_1109	DJI_0356	Geranium sanquineum L.	0	n	1	0	Geraniaceae Juss.
Iz_7_1109	DJI_0356	Gypsophila paniculata L.	0	n	1	0	Caryophyllaceae Juss.
Iz_7_1109	DJI_0356	Helichrysum arenarium (L.) Moench	0	n	1	0	Asteraceae Bercht. & J.Presl
Iz_7_1109	DJI_0356	Herniaria polygama J.Gay	0	n	1	0	Caryophyllaceae Juss.
Iz_7_1109	DJI_0356	Hieracium robustum Fr.	0	n	1	0	Asteraceae Bercht. & J.Presl
Iz_7_1109	DJI_0356	Hieracium umbellatum L.	0	n	1	NA	Asteraceae Bercht. & J.Presl

Iz_7_1109	DJI_0356	Hylotelephium maximum (L.) Holub	0	n	1	0	Crassulaceae J.St.-Hil.
Iz_7_1109	DJI_0356	Jacobaea borysthenica (DC.) B.Nord. & Greuter	0	n	1	0	Asteraceae Bercht. & J.Presl
Iz_7_1109	DJI_0356	Jasione montana L.	0	n	1	0	Campanulaceae Juss.
Iz_7_1109	DJI_0356	Jurinea cyanoides (L.) Rchb.s.l.	0	n	1	0	Asteraceae Bercht. & J.Presl
Iz_7_1109	DJI_0356	Koeleria glauca (Spreng.) DC.	0	n	1	0	Poaceae Barnhart
Iz_7_1109	DJI_0356	Lactuca serriola L.	0	a	0	0	Asteraceae Bercht. & J.Presl
Iz_7_1109	DJI_0356	Linaria genistifolia (L.) Mill.	0	n	1	0	Plantaginaceae Juss.
Iz_7_1109	DJI_0356	Lomelosia argentea (L.) Greuter & Burdet	0	n	1	0	Caprifoliaceae Juss.
Iz_7_1109	DJI_0356	Melampyrum pratense L.	0	n	1	0	Orobanchaceae Vent.
Iz_7_1109	DJI_0356	Oenothera biennis L.	0	a	0	0	Onagraceae Juss.
Iz_7_1109	DJI_0356	Pentanema britannica (L.) D.Gut.Larr., Santos-Vicente, Anderb., E.Rico & M.M.Mart.Ort.	0	n	1	0	Asteraceae Bercht. & J.Presl
Iz_7_1109	DJI_0356	Peucedanum oreoselinum (L.) Moench	0	n	1	0	Apiaceae Lindl.
Iz_7_1109	DJI_0356	Peucedanum ruthenicum M.Bieb.	0	n	1	0	Apiaceae Lindl.
Iz_7_1109	DJI_0356	Pilosella echioides (Lumn.) F.W.Schultz & Sch.Bip.	0	n	1	0	Asteraceae Bercht. & J.Presl
Iz_7_1109	DJI_0356	Pilosella officinarum F. Schultz et Sch. Bip.	0	n	1	0	Asteraceae Bercht. & J.Presl
Iz_7_1109	DJI_0356	Pilosella sp.	0		NA	0	Asteraceae Bercht. & J.Presl
Iz_7_1109	DJI_0356	Pinus sylvestris L.	0	n	1	1	Pinaceae Spreng. ex F.Rudolphi
Iz_7_1109	DJI_0356	Poa annua L.	0	n	1	0	Poaceae Barnhart
Iz_7_1109	DJI_0356	Poa nemoralis L.	0	n	1	0	Poaceae Barnhart
Iz_7_1109	DJI_0356	Poa sp.	0		NA	0	Poaceae Barnhart
Iz_7_1109	DJI_0356	Polygonatum odoratum (Mill.) Druce	0	n	1	0	Asparagaceae Juss.
Iz_7_1109	DJI_0356	Polygonum arenarium Waldst. & Kit.	0	n	1	0	Polygonaceae Juss.
Iz_7_1109	DJI_0356	Populus nigra L.	0	n	1	1	Salicaceae Mirb.
Iz_7_1109	DJI_0356	Populus tremula L.	0	n	1	1	Salicaceae Mirb.
Iz_7_1109	DJI_0356	Populus Ã canescens (Aiton) Sm.	0	n	1	1	Salicaceae Mirb.
Iz_7_1109	DJI_0356	Potentilla argentea L.	0	n	1	0	Rosaceae Juss.
Iz_7_1109	DJI_0356	Psammophiliella muralis (L.) Ikonn.	0	n	1	0	Caryophyllaceae Juss.
Iz_7_1109	DJI_0356	Psephellus sumensis (Kalen.) Greuter	0	n	1	0	Asteraceae Bercht. & J.Presl
Iz_7_1109	DJI_0356	Pulsatilla pratensis (L.) Mill. s.l.	0	n	1	0	Ranunculaceae Juss.
Iz_7_1109	DJI_0356	Pyrus communis L.	0	a	0	1	Rosaceae Juss.
Iz_7_1109	DJI_0356	Quercus robur L.	0	n	1	1	Fagaceae Dumort.
Iz_7_1109	DJI_0356	Robinia pseudoacacia L.	0	a	0	1	Fabaceae Lindl.
Iz_7_1109	DJI_0356	Rosa sp.	0		NA	1	Rosaceae Juss.
Iz_7_1109	DJI_0356	Rubus idaeus L.	0	n	1	0	Rosaceae Juss.
Iz_7_1109	DJI_0356	Rumex acetosella L.	0	n	1	0	Polygonaceae Juss.
Iz_7_1109	DJI_0356	Rumex thyrsiflorus Fingerh.	0	n	1	0	Polygonaceae Juss.
Iz_7_1109	DJI_0356	Salix alba L.	0	n	1	1	Salicaceae Mirb.
Iz_7_1109	DJI_0356	Sambucus nigra L.	0	n	1	1	Viburnaceae Raf.
Iz_7_1109	DJI_0356	Secale sylvestre Host	0	n	1	0	Poaceae Barnhart
Iz_7_1109	DJI_0356	Sempervivum ruthenicum Schnittsp. & C.B. Lehm.	0	n	1	0	Crassulaceae J.St.-Hil.
Iz_7_1109	DJI_0356	Setaria viridis (L.) P. Beauv.	0	n	1	0	Poaceae Barnhart
Iz_7_1109	DJI_0356	Silene borysthenica (Gruner) Walters	0	n	1	0	Caryophyllaceae Juss.
Iz_7_1109	DJI_0356	Silene latifolia subsp. alba (Mill.) Greuter & Burdet	0	n	1	0	Caryophyllaceae Juss.
Iz_7_1109	DJI_0356	Solidago virgaurea L.	0	n	1	0	Asteraceae Bercht. & J.Presl
Iz_7_1109	DJI_0356	Sonchus oleraceus L.	0	a	0	0	Asteraceae Bercht. & J.Presl
Iz_7_1109	DJI_0356	Sorbus aucuparia L.	0	n	1	1	Rosaceae Juss.
Iz_7_1109	DJI_0356	Stachys recta L.	0	n	1	0	Lamiacea Lindl.
Iz_7_1109	DJI_0356	Tanacetum vulgare L.	0	n	1	0	Asteraceae Bercht. & J.Presl
Iz_7_1109	DJI_0356	Thymus pallasianus Heinr.Braun	0	n	1	0	Lamiacea Lindl.
Iz_7_1109	DJI_0356	Tilia cordata Mill.	0	n	1	1	Malvaceae Juss.
Iz_7_1109	DJI_0356	Trifolium alpestre L.	0	n	1	0	Fabaceae Lindl.
Iz_7_1109	DJI_0356	Trifolium arvense L.	0	n	1	0	Fabaceae Lindl.
Iz_7_1109	DJI_0356	Ulmus glabra Huds.	0	n	1	1	Ulmaceae Mirb.
Iz_7_1109	DJI_0356	Verbascum densiflorum Bertol.	0	n	1	0	Scrophulariaceae Juss.
Iz_7_1109	DJI_0356	Veronica austriaca L.	0	n	1	0	Plantaginaceae Juss.
Iz_7_1109	DJI_0356	Veronica incana L.	0	n	1	0	Plantaginaceae Juss.
Iz_7_1109	DJI_0356	Veronica verna L.	0	n	1	0	Plantaginaceae Juss.
Iz_7_1109	DJI_0356	Vincetoxicum hirundinaria Medik	0	n	1	0	Apocynaceae Juss.
Iz_7_1109	DJI_0359	Acer tataricum L.	0	n	1	1	Sapindaceae Juss.
Iz_7_1109	DJI_0359	Achillea millefolium L. s.l.	0	n	1	0	Asteraceae Bercht. & J.Presl
Iz_7_1109	DJI_0359	Allium oleraceum L.	0	n	1	0	Amaryllidaceae J.St.-Hil.
Iz_7_1109	DJI_0359	Ambrosia artemisiifolia L.	0	a	0	0	Asteraceae Bercht. & J.Presl
Iz_7_1109	DJI_0359	Amorpha fruticosa L.	0	a	0	0	Fabaceae Lindl.
Iz_7_1109	DJI_0359	Anthericum ramosum L.	0	n	1	0	Asparagaceae Juss.
Iz_7_1109	DJI_0359	Artemisia marschalliana Spreng.	0	n	1	0	Asteraceae Bercht. & J.Presl
Iz_7_1109	DJI_0359	Asparagus officinalis L.	0	n	1	0	Asparagaceae Juss.
Iz_7_1109	DJI_0359	Astragalus cicer L.	0	n	1	0	Fabaceae Lindl.
Iz_7_1109	DJI_0359	Bassia laniflora (S.G.Gmel.) A.J.Scott	0	n	1	0	Amaranthaceae Juss.
Iz_7_1109	DJI_0359	Berberis vulgaris L.	0	n	1	0	Berberidaceae Juss.
Iz_7_1109	DJI_0359	Betula pendula Roth.	0	n	1	1	Betulaceae Gray
Iz_7_1109	DJI_0359	Betula pendula Roth.	0	n	1	0	Poaceae Barnhart
Iz_7_1109	DJI_0359	Bromus inermis Leyss.	0		NA	NA	
Iz_7_1109	DJI_0359	Bromus squarrosus L.	0	a	0	0	Poaceae Barnhart
Iz_7_1109	DJI_0359	Bromus tectorum L.	0	a	0	0	Poaceae Barnhart
Iz_7_1109	DJI_0359	Calamagrostis epigejos (L.) Roth	20	n	1	0	Poaceae Barnhart
Iz_7_1109	DJI_0359	Carduus acanthoides L.	0	n	1	0	Asteraceae Bercht. & J.Presl
Iz_7_1109	DJI_0359	Carex sp.	0	n	1	0	Cyperaceae Juss.
Iz_7_1109	DJI_0359	Centaurea majorovii Dumbadze	0	n	1	0	Asteraceae Bercht. & J.Presl
Iz_7_1109	DJI_0359	Chamaecytisus ruthenicus (Fisch.ex Wol.) Klaskova	0	n	1	0	Fabaceae Lindl.
Iz_7_1109	DJI_0359	Chelidonium majus L.	0	n	1	0	Papaveraceae Juss.
Iz_7_1109	DJI_0359	Chenopodium betaceum Andrz.	0	n	1	0	Amaranthaceae Juss.
Iz_7_1109	DJI_0359	Chenopodium cf. album	0	n	1	0	Amaranthaceae Juss.
Iz_7_1109	DJI_0359	Chenopodium sp.	0		NA	0	Amaranthaceae Juss.
Iz_7_1109	DJI_0359	Chondrilla juncea L.	0	n	1	0	Asteraceae Bercht. & J.Presl
Iz_7_1109	DJI_0359	Cirsium vulgare (Savi) Ten.	0	n	1	0	Asteraceae Bercht. & J.Presl
Iz_7_1109	DJI_0359	Clinopodium acinos (L.) Kuntze	0	n	1	0	Lamiacea Lindl.
Iz_7_1109	DJI_0359	Convallaria majalis L.	0	n	1	0	Asparagaceae Juss.
Iz_7_1109	DJI_0359	Crataegus rhipidophylla Gand.	0	n	1	1	Rosaceae Juss.
Iz_7_1109	DJI_0359	Crepis foetida L.	0	n	1	0	Asteraceae Bercht. & J.Presl
Iz_7_1109	DJI_0359	Dactylis glomerata L.	0	n	1	0	Poaceae Barnhart
Iz_7_1109	DJI_0359	Dianthus campestris M.Bieb.	0	n	1	0	Caryophyllaceae Juss.
Iz_7_1109	DJI_0359	Digitaria sanguinalis (L.) Scop.	0	a	0	0	Poaceae Barnhart
Iz_7_1109	DJI_0359	Elymus repens (L.) Gould	0	n	1	0	Poaceae Barnhart
Iz_7_1109	DJI_0359	Epilobium tetragonum L.	0	n	1	0	Onagraceae Juss.
Iz_7_1109	DJI_0359	Eragrostis minor Host	0	a	0	0	Poaceae Barnhart
Iz_7_1109	DJI_0359	Erigeron canadensis L.	10	a	0	0	Asteraceae Bercht. & J.Presl
Iz_7_1109	DJI_0359	Euphorbia seguieriana Neck.	0	n	1	0	Euphorbiaceae Juss.
Iz_7_1109	DJI_0359	Fallopia dumetorum (L.) Holub	2	n	1	0	Polygonaceae Juss.

Iz_7_1109	DJI_0359	Festuca beckeri (Hack.) Trautv.	0	n	1	0	Poaceae Barnhart
Iz_7_1109	DJI_0359	Fragaria vesca L.	0	n	1	0	Rosaceae Juss.
Iz_7_1109	DJI_0359	Frangula alnus Mill.	0	n	1	1	Rhamnaceae Juss.
Iz_7_1109	DJI_0359	Fraxinus pennsylvanica Marshall	0	a	0	1	Oleaceae Hoffmanns. & Link
Iz_7_1109	DJI_0359	Galeopsis bifida Boenn.	0	n	1	0	Lamiacea Lindl.
Iz_7_1109	DJI_0359	Galium verum L.	0	n	1	0	Rubiaceae Juss.
Iz_7_1109	DJI_0359	Genista tinctoria L.	0	n	1	0	Fabaceae Lindl.
Iz_7_1109	DJI_0359	Geranium robertianum L.	0	n	1	0	Geraniaceae Juss.
Iz_7_1109	DJI_0359	Geranium sanquineum L.	0	n	1	0	Geraniaceae Juss.
Iz_7_1109	DJI_0359	Gypsophila paniculata L.	0	n	1	0	Caryophyllaceae Juss.
Iz_7_1109	DJI_0359	Helichrysum arenarium (L.) Moench	0	n	1	0	Asteraceae Bercht. & J.Presl
Iz_7_1109	DJI_0359	Herniaria polygama J.Gay	0	n	1	0	Caryophyllaceae Juss.
Iz_7_1109	DJI_0359	Hieracium robustum Fr.	0	n	1	0	Asteraceae Bercht. & J.Presl
Iz_7_1109	DJI_0359	Hieracium umbellatum L.	0	n	1	NA	Asteraceae Bercht. & J.Presl
Iz_7_1109	DJI_0359	Hylotelephium maximum (L.) Holub	0	n	1	0	Crassulaceae J.St.-Hil.
Iz_7_1109	DJI_0359	Jacobaea borysthenica (DC.) B.Nord. & Greuter	0	n	1	0	Asteraceae Bercht. & J.Presl
Iz_7_1109	DJI_0359	Jasione montana L.	0	n	1	0	Campanulaceae Juss.
Iz_7_1109	DJI_0359	Jurinea cyanoides (L.) Rchb.s.l.	0	n	1	0	Asteraceae Bercht. & J.Presl
Iz_7_1109	DJI_0359	Koeleria glauca (Spreng.) DC.	0	n	1	0	Poaceae Barnhart
Iz_7_1109	DJI_0359	Lactuca serriola L.	0	a	0	0	Asteraceae Bercht. & J.Presl
Iz_7_1109	DJI_0359	Linaria genistifolia (L.) Mill.	0	n	1	0	Plantaginaceae Juss.
Iz_7_1109	DJI_0359	Lomelosia argentea (L.) Greuter & Burdet	0	n	1	0	Caprifoliaceae Juss.
Iz_7_1109	DJI_0359	Melampyrum pratense L.	0	n	1	0	Orobanchaceae Vent.
Iz_7_1109	DJI_0359	Oenothera biennis L.	0	a	0	0	Onagraceae Juss.
Iz_7_1109	DJI_0359	Pentanema britannica (L.) D.Gut.Larr., Santos-Vicente, Anderb., E.Rico & M.M.Mart.Ort.	0	n	1	0	Asteraceae Bercht. & J.Presl
Iz_7_1109	DJI_0359	Peucedanum oreoselinum (L.) Moench	0	n	1	0	Apiaceae Lindl.
Iz_7_1109	DJI_0359	Peucedanum ruthenicum M.Bieb.	0	n	1	0	Apiaceae Lindl.
Iz_7_1109	DJI_0359	Pilosella echioides (Lumn.) F.W.Schultz & Sch.Bip.	0	n	1	0	Asteraceae Bercht. & J.Presl
Iz_7_1109	DJI_0359	Pilosella officinarum F. Schultz et Sch. Bip.	0	n	1	0	Asteraceae Bercht. & J.Presl
Iz_7_1109	DJI_0359	Pilosella sp.	0		NA	0	Asteraceae Bercht. & J.Presl
Iz_7_1109	DJI_0359	Pinus sylvestris L.	0	n	1	1	Pinaceae Spreng. ex F.Rudolphi
Iz_7_1109	DJI_0359	Poa annua L.	0	n	1	0	Poaceae Barnhart
Iz_7_1109	DJI_0359	Poa nemoralis L.	0	n	1	0	Poaceae Barnhart
Iz_7_1109	DJI_0359	Poa sp.	0		NA	0	Poaceae Barnhart
Iz_7_1109	DJI_0359	Polygonatum odoratum (Mill.) Druce	0	n	1	0	Asparagaceae Juss.
Iz_7_1109	DJI_0359	Polygonum arenarium Waldst. & Kit.	0	n	1	0	Polygonaceae Juss.
Iz_7_1109	DJI_0359	Populus nigra L.	0	n	1	1	Salicaceae Mirb.
Iz_7_1109	DJI_0359	Populus tremula L.	0	n	1	1	Salicaceae Mirb.
Iz_7_1109	DJI_0359	Populus Ã canescens (Aiton) Sm.	0	n	1	1	Salicaceae Mirb.
Iz_7_1109	DJI_0359	Potentilla argentea L.	0	n	1	0	Rosaceae Juss.
Iz_7_1109	DJI_0359	Psammophiliella muralis (L.) Ikonn.	0	n	1	0	Caryophyllaceae Juss.
Iz_7_1109	DJI_0359	Psephellus sumensis (Kalen.) Greuter	0	n	1	0	Asteraceae Bercht. & J.Presl
Iz_7_1109	DJI_0359	Pulsatilla pratensis (L.) Mill. s.l.	0	n	1	0	Ranunculaceae Juss.
Iz_7_1109	DJI_0359	Pyrus communis L.	0	a	0	1	Rosaceae Juss.
Iz_7_1109	DJI_0359	Quercus robur L.	0	n	1	1	Fagaceae Dumort.
Iz_7_1109	DJI_0359	Robinia pseudoacacia L.	0	a	0	1	Fabaceae Lindl.
Iz_7_1109	DJI_0359	Rosa sp.	0		NA	1	Rosaceae Juss.
Iz_7_1109	DJI_0359	Rubus idaeus L.	0	n	1	0	Rosaceae Juss.
Iz_7_1109	DJI_0359	Rumex acetosella L.	0	n	1	0	Polygonaceae Juss.
Iz_7_1109	DJI_0359	Rumex thyrsiflorus Fingerh.	0	n	1	0	Polygonaceae Juss.
Iz_7_1109	DJI_0359	Salix alba L.	0	n	1	1	Salicaceae Mirb.
Iz_7_1109	DJI_0359	Sambucus nigra L.	0	n	1	1	Viburnaceae Raf.
Iz_7_1109	DJI_0359	Secale sylvestre Host	0	n	1	0	Poaceae Barnhart
Iz_7_1109	DJI_0359	Sempervivum ruthenicum Schnittsp. & C.B. Lehm.	0	n	1	0	Crassulaceae J.St.-Hil.
Iz_7_1109	DJI_0359	Setaria viridis (L.) P. Beauv.	0	n	1	0	Poaceae Barnhart
Iz_7_1109	DJI_0359	Silene borysthenica (Gruner) Walters	0	n	1	0	Caryophyllaceae Juss.
Iz_7_1109	DJI_0359	Silene latifolia subsp. alba (Mill.) Greuter & Burdet	0	n	1	0	Caryophyllaceae Juss.
Iz_7_1109	DJI_0359	Solidago virgaurea L.	0	n	1	0	Asteraceae Bercht. & J.Presl
Iz_7_1109	DJI_0359	Sonchus oleraceus L.	0	a	0	0	Asteraceae Bercht. & J.Presl
Iz_7_1109	DJI_0359	Sorbus aucuparia L.	0	n	1	1	Rosaceae Juss.
Iz_7_1109	DJI_0359	Stachys recta L.	0	n	1	0	Lamiacea Lindl.
Iz_7_1109	DJI_0359	Tanacetum vulgare L.	0	n	1	0	Asteraceae Bercht. & J.Presl
Iz_7_1109	DJI_0359	Thymus pallasianus Heinr.Braun	0	n	1	0	Lamiacea Lindl.
Iz_7_1109	DJI_0359	Tilia cordata Mill.	0	n	1	1	Malvaceae Juss.
Iz_7_1109	DJI_0359	Trifolium alpestre L.	0	n	1	0	Fabaceae Lindl.
Iz_7_1109	DJI_0359	Trifolium arvense L.	0	n	1	0	Fabaceae Lindl.
Iz_7_1109	DJI_0359	Ulmus glabra Huds.	0	n	1	1	Ulmaceae Mirb.
Iz_7_1109	DJI_0359	Verbascum densiflorum Bertol.	0	n	1	0	Scrophulariaceae Juss.
Iz_7_1109	DJI_0359	Veronica austriaca L.	0	n	1	0	Plantaginaceae Juss.
Iz_7_1109	DJI_0359	Veronica incana L.	0	n	1	0	Plantaginaceae Juss.
Iz_7_1109	DJI_0359	Veronica verna L.	0	n	1	0	Plantaginaceae Juss.
Iz_7_1109	DJI_0359	Vincetoxicum hirundinaria Medik	0	n	1	0	Apocynaceae Juss.
Iz_7_1109	DJI_0338	Acer tataricum L.	0	n	1	1	Sapindaceae Juss.
Iz_7_1109	DJI_0338	Achillea millefolium L. s.l.	0	n	1	0	Asteraceae Bercht. & J.Presl
Iz_7_1109	DJI_0338	Allium oleraceum L.	0	n	1	0	Amaryllidaceae J.St.-Hil.
Iz_7_1109	DJI_0338	Ambrosia artemisiifolia L.	0	a	0	0	Asteraceae Bercht. & J.Presl
Iz_7_1109	DJI_0338	Amorpha fruticosa L.	0	a	0	0	Fabaceae Lindl.
Iz_7_1109	DJI_0338	Anthericum ramosum L.	0	n	1	0	Asparagaceae Juss.
Iz_7_1109	DJI_0338	Artemisia marschalliana Spreng.	0	n	1	0	Asteraceae Bercht. & J.Presl
Iz_7_1109	DJI_0338	Asparagus officinalis L.	0	n	1	0	Asparagaceae Juss.
Iz_7_1109	DJI_0338	Astragalus cicer L.	0	n	1	0	Fabaceae Lindl.
Iz_7_1109	DJI_0338	Bassia laniflora (S.G.Gmel.) A.J.Scott	0	n	1	0	Amaranthaceae Juss.
Iz_7_1109	DJI_0338	Berberis vulgaris L.	0	n	1	0	Berberidaceae Juss.
Iz_7_1109	DJI_0338	Betula pendula Roth.	0	n	1	1	Betulaceae Gray
Iz_7_1109	DJI_0338	Betula pendula Roth.	0	n	1	0	Poaceae Barnhart
Iz_7_1109	DJI_0338	Bromus inermis Leyss.	0		NA	NA	
Iz_7_1109	DJI_0338	Bromus squarrosus L.	0	a	0	0	Poaceae Barnhart
Iz_7_1109	DJI_0338	Bromus tectorum L.	0	a	0	0	Poaceae Barnhart
Iz_7_1109	DJI_0338	Calamagrostis epigejos (L.) Roth	1	n	1	0	Poaceae Barnhart
Iz_7_1109	DJI_0338	Carduus acanthoides L.	0	n	1	0	Asteraceae Bercht. & J.Presl
Iz_7_1109	DJI_0338	Carex sp.	0.5	n	1	0	Cyperaceae Juss.
Iz_7_1109	DJI_0338	Centaurea majorovii Dumbadze	0	n	1	0	Asteraceae Bercht. & J.Presl
Iz_7_1109	DJI_0338	Chamaecytisus ruthenicus (Fisch.ex Wol.) Klaskova	0	n	1	0	Fabaceae Lindl.
Iz_7_1109	DJI_0338	Chelidonium majus L.	0	n	1	0	Papaveraceae Juss.
Iz_7_1109	DJI_0338	Chenopodium betaceum Andrz.	0	n	1	0	Amaranthaceae Juss.
Iz_7_1109	DJI_0338	Chenopodium cf. album	0	n	1	0	Amaranthaceae Juss.
Iz_7_1109	DJI_0338	Chenopodium sp.	0		NA	0	Amaranthaceae Juss.
Iz_7_1109	DJI_0338	Chondrilla juncea L.	0	n	1	0	Asteraceae Bercht. & J.Presl

Iz_7_1109	DJI_0338	Cirsium vulgare (Savi) Ten.	0	n	1	0	Asteraceae Bercht. & J.Presl
Iz_7_1109	DJI_0338	Clinopodium acinos (L.) Kuntze	0	n	1	0	Lamiacea Lindl.
Iz_7_1109	DJI_0338	Convallaria majalis L.	0	n	1	0	Asparagaceae Juss.
Iz_7_1109	DJI_0338	Crataegus rhipidophylla Gand.	0	n	1	1	Rosaceae Juss.
Iz_7_1109	DJI_0338	Crepis foetida L.	0	n	1	0	Asteraceae Bercht. & J.Presl
Iz_7_1109	DJI_0338	Dactylis glomerata L.	0	n	1	0	Poaceae Barnhart
Iz_7_1109	DJI_0338	Dianthus campestris M.Bieb.	0	n	1	0	Caryophyllaceae Juss.
Iz_7_1109	DJI_0338	Digitaria sanguinalis (L.) Scop.	0	a	0	0	Poaceae Barnhart
Iz_7_1109	DJI_0338	Elymus repens (L.) Gould	0	n	1	0	Poaceae Barnhart
Iz_7_1109	DJI_0338	Epilobium tetragonum L.	0	n	1	0	Onagraceae Juss.
Iz_7_1109	DJI_0338	Eragrostis minor Host	0	a	0	0	Poaceae Barnhart
Iz_7_1109	DJI_0338	Erigeron canadensis L.	5	a	0	0	Asteraceae Bercht. & J.Presl
Iz_7_1109	DJI_0338	Euphorbia seguieriana Neck.	0	n	1	0	Euphorbiaceae Juss.
Iz_7_1109	DJI_0338	Fallopia dumetorum (L.) Holub	0	n	1	0	Polygonaceae Juss.
Iz_7_1109	DJI_0338	Festuca beckeri (Hack.) Trautv.	0	n	1	0	Poaceae Barnhart
Iz_7_1109	DJI_0338	Fragaria vesca L.	0	n	1	0	Rosaceae Juss.
Iz_7_1109	DJI_0338	Frangula alnus Mill.	0	n	1	1	Rhamnaceae Juss.
Iz_7_1109	DJI_0338	Fraxinus pennsylvanica Marshall	0	a	0	1	Oleaceae Hoffmanns. & Link
Iz_7_1109	DJI_0338	Galeopsis bifida Boenn.	0	n	1	0	Lamiacea Lindl.
Iz_7_1109	DJI_0338	Galium verum L.	0	n	1	0	Rubiaceae Juss.
Iz_7_1109	DJI_0338	Genista tinctoria L.	0	n	1	0	Fabaceae Lindl.
Iz_7_1109	DJI_0338	Geranium robertianum L.	0	n	1	0	Geraniaceae Juss.
Iz_7_1109	DJI_0338	Geranium sanquineum L.	0	n	1	0	Geraniaceae Juss.
Iz_7_1109	DJI_0338	Gypsophila paniculata L.	0	n	1	0	Caryophyllaceae Juss.
Iz_7_1109	DJI_0338	Helichrysum arenarium (L.) Moench	0	n	1	0	Asteraceae Bercht. & J.Presl
Iz_7_1109	DJI_0338	Herniaria polygama J.Gay	0	n	1	0	Caryophyllaceae Juss.
Iz_7_1109	DJI_0338	Hieracium robustum Fr.	0	n	1	0	Asteraceae Bercht. & J.Presl
Iz_7_1109	DJI_0338	Hieracium umbellatum L.	0	n	1	NA	Asteraceae Bercht. & J.Presl
Iz_7_1109	DJI_0338	Hylotelephium maximum (L.) Holub	0	n	1	0	Crassulaceae J.St.-Hil.
Iz_7_1109	DJI_0338	Jacobaea borysthenica (DC.) B.Nord. & Greuter	0	n	1	0	Asteraceae Bercht. & J.Presl
Iz_7_1109	DJI_0338	Jasione montana L.	0	n	1	0	Campanulaceae Juss.
Iz_7_1109	DJI_0338	Jurinea cyanoides (L.) Rchb.s.l.	0	n	1	0	Asteraceae Bercht. & J.Presl
Iz_7_1109	DJI_0338	Koeleria glauca (Spreng.) DC.	0	n	1	0	Poaceae Barnhart
Iz_7_1109	DJI_0338	Lactuca serriola L.	0	a	0	0	Asteraceae Bercht. & J.Presl
Iz_7_1109	DJI_0338	Linaria genistifolia (L.) Mill.	0	n	1	0	Plantaginaceae Juss.
Iz_7_1109	DJI_0338	Lomelosia argentea (L.) Greuter & Burdet	0	n	1	0	Caprifoliaceae Juss.
Iz_7_1109	DJI_0338	Melampyrum pratense L.	0	n	1	0	Orobanchaceae Vent.
Iz_7_1109	DJI_0338	Oenothera biennis L.	0	a	0	0	Onagraceae Juss.
Iz_7_1109	DJI_0338	Pentanema britannica (L.) D.Gut.Larr., Santos-Vicente, Anderb., E.Rico & M.M.Mart.Ort.	0	n	1	0	Asteraceae Bercht. & J.Presl
Iz_7_1109	DJI_0338	Peucedanum oreoselinum (L.) Moench	0	n	1	0	Apiaceae Lindl.
Iz_7_1109	DJI_0338	Peucedanum ruthenicum M.Bieb.	0	n	1	0	Apiaceae Lindl.
Iz_7_1109	DJI_0338	Pilosella echioides (Lumn.) F.W.Schultz & Sch.Bip.	0	n	1	0	Asteraceae Bercht. & J.Presl
Iz_7_1109	DJI_0338	Pilosella officinarum F. Schultz et Sch. Bip.	0	n	1	0	Asteraceae Bercht. & J.Presl
Iz_7_1109	DJI_0338	Pilosella sp.	0		NA	0	Asteraceae Bercht. & J.Presl
Iz_7_1109	DJI_0338	Pinus sylvestris L.	0	n	1	1	Pinaceae Spreng. ex F.Rudolphi
Iz_7_1109	DJI_0338	Poa annua L.	0	n	1	0	Poaceae Barnhart
Iz_7_1109	DJI_0338	Poa nemoralis L.	0	n	1	0	Poaceae Barnhart
Iz_7_1109	DJI_0338	Poa sp.	0		NA	0	Poaceae Barnhart
Iz_7_1109	DJI_0338	Polygonatum odoratum (Mill.) Druce	0	n	1	0	Asparagaceae Juss.
Iz_7_1109	DJI_0338	Polygonum arenarium Waldst. & Kit.	0	n	1	0	Polygonaceae Juss.
Iz_7_1109	DJI_0338	Populus nigra L.	0	n	1	1	Salicaceae Mirb.
Iz_7_1109	DJI_0338	Populus tremula L.	0	n	1	1	Salicaceae Mirb.
Iz_7_1109	DJI_0338	Populus Ã canescens (Aiton) Sm.	0	n	1	1	Salicaceae Mirb.
Iz_7_1109	DJI_0338	Potentilla argentea L.	0	n	1	0	Rosaceae Juss.
Iz_7_1109	DJI_0338	Psammophiliella muralis (L.) Ikonn.	0	n	1	0	Caryophyllaceae Juss.
Iz_7_1109	DJI_0338	Psephellus sumensis (Kalen.) Greuter	0	n	1	0	Asteraceae Bercht. & J.Presl
Iz_7_1109	DJI_0338	Pulsatilla pratensis (L.) Mill. s.l.	0	n	1	0	Ranunculaceae Juss.
Iz_7_1109	DJI_0338	Pyrus communis L.	0	a	0	1	Rosaceae Juss.
Iz_7_1109	DJI_0338	Quercus robur L.	0	n	1	1	Fagaceae Dumort.
Iz_7_1109	DJI_0338	Robinia pseudoacacia L.	0	a	0	1	Fabaceae Lindl.
Iz_7_1109	DJI_0338	Rosa sp.	0		NA	1	Rosaceae Juss.
Iz_7_1109	DJI_0338	Rubus idaeus L.	0	n	1	0	Rosaceae Juss.
Iz_7_1109	DJI_0338	Rumex acetosella L.	0	n	1	0	Polygonaceae Juss.
Iz_7_1109	DJI_0338	Rumex thyrsiflorus Fingerh.	0	n	1	0	Polygonaceae Juss.
Iz_7_1109	DJI_0338	Salix alba L.	0	n	1	1	Salicaceae Mirb.
Iz_7_1109	DJI_0338	Sambucus nigra L.	0	n	1	1	Viburnaceae Raf.
Iz_7_1109	DJI_0338	Secale sylvestre Host	0	n	1	0	Poaceae Barnhart
Iz_7_1109	DJI_0338	Sempervivum ruthenicum Schnittsp. & C.B. Lehm.	0	n	1	0	Crassulaceae J.St.-Hil.
Iz_7_1109	DJI_0338	Setaria viridis (L.) P. Beauv.	0	n	1	0	Poaceae Barnhart
Iz_7_1109	DJI_0338	Silene borysthenica (Gruner) Walters	0	n	1	0	Caryophyllaceae Juss.
Iz_7_1109	DJI_0338	Silene latifolia subsp. alba (Mill.) Greuter & Burdet	0	n	1	0	Caryophyllaceae Juss.
Iz_7_1109	DJI_0338	Solidago virgaurea L.	0	n	1	0	Asteraceae Bercht. & J.Presl
Iz_7_1109	DJI_0338	Sonchus oleraceus L.	0	a	0	0	Asteraceae Bercht. & J.Presl
Iz_7_1109	DJI_0338	Sorbus aucuparia L.	0	n	1	1	Rosaceae Juss.
Iz_7_1109	DJI_0338	Stachys recta L.	0	n	1	0	Lamiacea Lindl.
Iz_7_1109	DJI_0338	Tanacetum vulgare L.	0	n	1	0	Asteraceae Bercht. & J.Presl
Iz_7_1109	DJI_0338	Thymus pallasianus Heinr.Braun	0	n	1	0	Lamiacea Lindl.
Iz_7_1109	DJI_0338	Tilia cordata Mill.	0	n	1	1	Malvaceae Juss.
Iz_7_1109	DJI_0338	Trifolium alpestre L.	0	n	1	0	Fabaceae Lindl.
Iz_7_1109	DJI_0338	Trifolium arvense L.	0	n	1	0	Fabaceae Lindl.
Iz_7_1109	DJI_0338	Ulmus glabra Huds.	0	n	1	1	Ulmaceae Mirb.
Iz_7_1109	DJI_0338	Verbascum densiflorum Bertol.	0	n	1	0	Scrophulariaceae Juss.
Iz_7_1109	DJI_0338	Veronica austriaca L.	0	n	1	0	Plantaginaceae Juss.
Iz_7_1109	DJI_0338	Veronica incana L.	0	n	1	0	Plantaginaceae Juss.
Iz_7_1109	DJI_0338	Veronica verna L.	0	n	1	0	Plantaginaceae Juss.
Iz_7_1109	DJI_0338	Vincetoxicum hirundinaria Medik	0	n	1	0	Apocynaceae Juss.
Iz_7_1209	DJI_0392	Acer tataricum L.	0	n	1	1	Sapindaceae Juss.
Iz_7_1209	DJI_0392	Achillea millefolium L. s.l.	0	n	1	0	Asteraceae Bercht. & J.Presl
Iz_7_1209	DJI_0392	Allium oleraceum L.	0	n	1	0	Amaryllidaceae J.St.-Hil.
Iz_7_1209	DJI_0392	Ambrosia artemisiifolia L.	0	a	0	0	Asteraceae Bercht. & J.Presl
Iz_7_1209	DJI_0392	Amorpha fruticosa L.	0	a	0	0	Fabaceae Lindl.
Iz_7_1209	DJI_0392	Anthericum ramosum L.	0	n	1	0	Asparagaceae Juss.
Iz_7_1209	DJI_0392	Artemisia marschalliana Spreng.	0	n	1	0	Asteraceae Bercht. & J.Presl
Iz_7_1209	DJI_0392	Asparagus officinalis L.	0	n	1	0	Asparagaceae Juss.
Iz_7_1209	DJI_0392	Astragalus cicer L.	0	n	1	0	Fabaceae Lindl.
Iz_7_1209	DJI_0392	Bassia laniflora (S.G.Gmel.) A.J.Scott	0	n	1	0	Amaranthaceae Juss.
Iz_7_1209	DJI_0392	Berberis vulgaris L.	0	n	1	0	Berberidaceae Juss.
Iz_7_1209	DJI_0392	Betula pendula Roth.	0	n	1	1	Betulaceae Gray

Iz_7_1209	DJI_0392	Betula pendula Roth.	0	n	1	0	Poaceae Barnhart
Iz_7_1209	DJI_0392	Bromus inermis Leyss.	0		NA	NA	
Iz_7_1209	DJI_0392	Bromus squarrosus L.	0	a	0	0	Poaceae Barnhart
Iz_7_1209	DJI_0392	Bromus tectorum L.	0	a	0	0	Poaceae Barnhart
Iz_7_1209	DJI_0392	Calamagrostis epigejos (L.) Roth	4	n	1	0	Poaceae Barnhart
Iz_7_1209	DJI_0392	Carduus acanthoides L.	0	n	1	0	Asteraceae Bercht. & J.Presl
Iz_7_1209	DJI_0392	Carex sp.	0	n	1	0	Cyperaceae Juss.
Iz_7_1209	DJI_0392	Centaurea majorovii Dumbadze	0	n	1	0	Asteraceae Bercht. & J.Presl
Iz_7_1209	DJI_0392	Chamaecytisus ruthenicus (Fisch.ex Wol.) Klaskova	0	n	1	0	Fabaceae Lindl.
Iz_7_1209	DJI_0392	Chelidonium majus L.	0	n	1	0	Papaveraceae Juss.
Iz_7_1209	DJI_0392	Chenopodium betaceum Andrz.	0	n	1	0	Amaranthaceae Juss.
Iz_7_1209	DJI_0392	Chenopodium cf. album	0	n	1	0	Amaranthaceae Juss.
Iz_7_1209	DJI_0392	Chenopodium sp.	0		NA	0	Amaranthaceae Juss.
Iz_7_1209	DJI_0392	Chondrilla juncea L.	0	n	1	0	Asteraceae Bercht. & J.Presl
Iz_7_1209	DJI_0392	Cirsium vulgare (Savi) Ten.	0	n	1	0	Asteraceae Bercht. & J.Presl
Iz_7_1209	DJI_0392	Clinopodium acinos (L.) Kuntze	0	n	1	0	Lamiacea Lindl.
Iz_7_1209	DJI_0392	Convallaria majalis L.	0	n	1	0	Asparagaceae Juss.
Iz_7_1209	DJI_0392	Crataegus rhipidophylla Gand.	0	n	1	1	Rosaceae Juss.
Iz_7_1209	DJI_0392	Crepis foetida L.	0	n	1	0	Asteraceae Bercht. & J.Presl
Iz_7_1209	DJI_0392	Dactylis glomerata L.	0	n	1	0	Poaceae Barnhart
Iz_7_1209	DJI_0392	Dianthus campestris M.Bieb.	0	n	1	0	Caryophyllaceae Juss.
Iz_7_1209	DJI_0392	Digitaria sanguinalis (L.) Scop.	0	a	0	0	Poaceae Barnhart
Iz_7_1209	DJI_0392	Elymus repens (L.) Gould	0	n	1	0	Poaceae Barnhart
Iz_7_1209	DJI_0392	Epilobium tetragonum L.	0	n	1	0	Onagraceae Juss.
Iz_7_1209	DJI_0392	Eragrostis minor Host	0	a	0	0	Poaceae Barnhart
Iz_7_1209	DJI_0392	Erigeron canadensis L.	5	a	0	0	Asteraceae Bercht. & J.Presl
Iz_7_1209	DJI_0392	Euphorbia seguieriana Neck.	0	n	1	0	Euphorbiaceae Juss.
Iz_7_1209	DJI_0392	Fallopia dumetorum (L.) Holub	0	n	1	0	Polygonaceae Juss.
Iz_7_1209	DJI_0392	Festuca beckeri (Hack.) Trautv.	0	n	1	0	Poaceae Barnhart
Iz_7_1209	DJI_0392	Fragaria vesca L.	0	n	1	0	Rosaceae Juss.
Iz_7_1209	DJI_0392	Frangula alnus Mill.	0	n	1	1	Rhamnaceae Juss.
Iz_7_1209	DJI_0392	Fraxinus pennsylvanica Marshall	0	a	0	1	Oleaceae Hoffmanns. & Link
Iz_7_1209	DJI_0392	Galeopsis bifida Boenn.	0	n	1	0	Lamiacea Lindl.
Iz_7_1209	DJI_0392	Galium verum L.	0	n	1	0	Rubiaceae Juss.
Iz_7_1209	DJI_0392	Genista tinctoria L.	0	n	1	0	Fabaceae Lindl.
Iz_7_1209	DJI_0392	Geranium robertianum L.	0	n	1	0	Geraniaceae Juss.
Iz_7_1209	DJI_0392	Geranium sanquineum L.	0	n	1	0	Geraniaceae Juss.
Iz_7_1209	DJI_0392	Gypsophila paniculata L.	0	n	1	0	Caryophyllaceae Juss.
Iz_7_1209	DJI_0392	Helichrysum arenarium (L.) Moench	0	n	1	0	Asteraceae Bercht. & J.Presl
Iz_7_1209	DJI_0392	Herniaria polygama J.Gay	0	n	1	0	Caryophyllaceae Juss.
Iz_7_1209	DJI_0392	Hieracium robustum Fr.	0	n	1	0	Asteraceae Bercht. & J.Presl
Iz_7_1209	DJI_0392	Hieracium umbellatum L.	0	n	1	NA	Asteraceae Bercht. & J.Presl
Iz_7_1209	DJI_0392	Hylotelephium maximum (L.) Holub	0	n	1	0	Crassulaceae J.St.-Hil.
Iz_7_1209	DJI_0392	Jacobaea borysthenica (DC.) B.Nord. & Greuter	0	n	1	0	Asteraceae Bercht. & J.Presl
Iz_7_1209	DJI_0392	Jasione montana L.	0	n	1	0	Campanulaceae Juss.
Iz_7_1209	DJI_0392	Jurinea cyanoides (L.) Rchb.s.l.	0	n	1	0	Asteraceae Bercht. & J.Presl
Iz_7_1209	DJI_0392	Koeleria glauca (Spreng.) DC.	0	n	1	0	Poaceae Barnhart
Iz_7_1209	DJI_0392	Lactuca serriola L.	0	a	0	0	Asteraceae Bercht. & J.Presl
Iz_7_1209	DJI_0392	Linaria genistifolia (L.) Mill.	0	n	1	0	Plantaginaceae Juss.
Iz_7_1209	DJI_0392	Lomelosia argentea (L.) Greuter & Burdet	0	n	1	0	Caprifoliaceae Juss.
Iz_7_1209	DJI_0392	Melampyrum pratense L.	0	n	1	0	Orobanchaceae Vent.
Iz_7_1209	DJI_0392	Oenothera biennis L.	0	a	0	0	Onagraceae Juss.
Iz_7_1209	DJI_0392	Pentanema britannica (L.) D.Gut.Larr., Santos-Vicente, Anderb., E.Rico & M.M.Mart.Ort.	0	n	1	0	Asteraceae Bercht. & J.Presl
Iz_7_1209	DJI_0392	Peucedanum oreoselinum (L.) Moench	0	n	1	0	Apiaceae Lindl.
Iz_7_1209	DJI_0392	Peucedanum ruthenicum M.Bieb.	0	n	1	0	Apiaceae Lindl.
Iz_7_1209	DJI_0392	Pilosella echioides (Lumn.) F.W.Schultz & Sch.Bip.	0	n	1	0	Asteraceae Bercht. & J.Presl
Iz_7_1209	DJI_0392	Pilosella officinarum F. Schultz et Sch. Bip.	0	n	1	0	Asteraceae Bercht. & J.Presl
Iz_7_1209	DJI_0392	Pilosella sp.	0		NA	0	Asteraceae Bercht. & J.Presl
Iz_7_1209	DJI_0392	Pinus sylvestris L.	0	n	1	1	Pinaceae Spreng. ex F.Rudolphi
Iz_7_1209	DJI_0392	Poa annua L.	0	n	1	0	Poaceae Barnhart
Iz_7_1209	DJI_0392	Poa nemoralis L.	0	n	1	0	Poaceae Barnhart
Iz_7_1209	DJI_0392	Poa sp.	0		NA	0	Poaceae Barnhart
Iz_7_1209	DJI_0392	Polygonatum odoratum (Mill.) Druce	0	n	1	0	Asparagaceae Juss.
Iz_7_1209	DJI_0392	Polygonum arenarium Waldst. & Kit.	0	n	1	0	Polygonaceae Juss.
Iz_7_1209	DJI_0392	Populus nigra L.	0	n	1	1	Salicaceae Mirb.
Iz_7_1209	DJI_0392	Populus tremula L.	0	n	1	1	Salicaceae Mirb.
Iz_7_1209	DJI_0392	Populus Ã canescens (Aiton) Sm.	0	n	1	1	Salicaceae Mirb.
Iz_7_1209	DJI_0392	Potentilla argentea L.	0	n	1	0	Rosaceae Juss.
Iz_7_1209	DJI_0392	Psammophiliella muralis (L.) Ikonn.	0	n	1	0	Caryophyllaceae Juss.
Iz_7_1209	DJI_0392	Psephellus sumensis (Kalen.) Greuter	0	n	1	0	Asteraceae Bercht. & J.Presl
Iz_7_1209	DJI_0392	Pulsatilla pratensis (L.) Mill. s.l.	0	n	1	0	Ranunculaceae Juss.
Iz_7_1209	DJI_0392	Pyrus communis L.	0	a	0	1	Rosaceae Juss.
Iz_7_1209	DJI_0392	Quercus robur L.	0	n	1	1	Fagaceae Dumort.
Iz_7_1209	DJI_0392	Robinia pseudoacacia L.	0	a	0	1	Fabaceae Lindl.
Iz_7_1209	DJI_0392	Rosa sp.	0		NA	1	Rosaceae Juss.
Iz_7_1209	DJI_0392	Rubus idaeus L.	0	n	1	0	Rosaceae Juss.
Iz_7_1209	DJI_0392	Rumex acetosella L.	0	n	1	0	Polygonaceae Juss.
Iz_7_1209	DJI_0392	Rumex thyrsiflorus Fingerh.	0	n	1	0	Polygonaceae Juss.
Iz_7_1209	DJI_0392	Salix alba L.	0	n	1	1	Salicaceae Mirb.
Iz_7_1209	DJI_0392	Sambucus nigra L.	0	n	1	1	Viburnaceae Raf.
Iz_7_1209	DJI_0392	Secale sylvestre Host	0	n	1	0	Poaceae Barnhart
Iz_7_1209	DJI_0392	Sempervivum ruthenicum Schnittsp. & C.B. Lehm.	0	n	1	0	Crassulaceae J.St.-Hil.
Iz_7_1209	DJI_0392	Setaria viridis (L.) P. Beauv.	0	n	1	0	Poaceae Barnhart
Iz_7_1209	DJI_0392	Silene borysthenica (Gruner) Walters	0	n	1	0	Caryophyllaceae Juss.
Iz_7_1209	DJI_0392	Silene latifolia subsp. alba (Mill.) Greuter & Burdet	0	n	1	0	Caryophyllaceae Juss.
Iz_7_1209	DJI_0392	Solidago virgaurea L.	0	n	1	0	Asteraceae Bercht. & J.Presl
Iz_7_1209	DJI_0392	Sorbus aucuparia L.	0	n	1	1	Rosaceae Juss.
Iz_7_1209	DJI_0392	Stachys recta L.	0	n	1	0	Lamiacea Lindl.
Iz_7_1209	DJI_0392	Tanacetum vulgare L.	0	n	1	0	Asteraceae Bercht. & J.Presl
Iz_7_1209	DJI_0392	Thymus pallasianus Heinr.Braun	0	n	1	0	Lamiacea Lindl.
Iz_7_1209	DJI_0392	Tilia cordata Mill.	0	n	1	1	Malvaceae Juss.
Iz_7_1209	DJI_0392	Trifolium alpestre L.	0	n	1	0	Fabaceae Lindl.
Iz_7_1209	DJI_0392	Trifolium arvense L.	0	n	1	0	Fabaceae Lindl.
Iz_7_1209	DJI_0392	Ulmus glabra Huds.	0	n	1	1	Ulmaceae Mirb.
Iz_7_1209	DJI_0392	Verbascum densiflorum Bertol.	0	n	1	0	Scrophulariaceae Juss.
Iz_7_1209	DJI_0392	Veronica austriaca L.	0	n	1	0	Plantaginaceae Juss.
Iz_7_1209	DJI_0392	Veronica incana L.	0	n	1	0	Plantaginaceae Juss.
Iz_7_1209	DJI_0392	Veronica verna L.	0	n	1	0	Plantaginaceae Juss.

Iz_7_1209	DJI_0392	Vincetoxicum hirundinaria Medik	0	n	1	0	Apocynaceae Juss.
Iz_7_1209	DJI_0395	Acer tataricum L.	0	n	1	1	Sapindaceae Juss.
Iz_7_1209	DJI_0395	Achillea millefolium L. s.l.	0	n	1	0	Asteraceae Bercht. & J.Presl
Iz_7_1209	DJI_0395	Allium oleraceum L.	0	n	1	0	Amaryllidaceae J.St.-Hil.
Iz_7_1209	DJI_0395	Ambrosia artemisiifolia L.	0	a	0	0	Asteraceae Bercht. & J.Presl
Iz_7_1209	DJI_0395	Amorpha fruticosa L.	0	a	0	0	Fabaceae Lindl.
Iz_7_1209	DJI_0395	Anthericum ramosum L.	0	n	1	0	Asparagaceae Juss.
Iz_7_1209	DJI_0395	Artemisia marschalliana Spreng.	0	n	1	0	Asteraceae Bercht. & J.Presl
Iz_7_1209	DJI_0395	Asparagus officinalis L.	0	n	1	0	Asparagaceae Juss.
Iz_7_1209	DJI_0395	Astragalus cicer L.	0	n	1	0	Fabaceae Lindl.
Iz_7_1209	DJI_0395	Bassia laniflora (S.G.Gmel.) A.J.Scott	0	n	1	0	Amaranthaceae Juss.
Iz_7_1209	DJI_0395	Berberis vulgaris L.	0	n	1	0	Berberidaceae Juss.
Iz_7_1209	DJI_0395	Betula pendula Roth.	0	n	1	1	Betulaceae Gray
Iz_7_1209	DJI_0395	Betula pendula Roth.	0	n	1	0	Poaceae Barnhart
Iz_7_1209	DJI_0395	Bromus inermis Leyss.	0		NA	NA	
Iz_7_1209	DJI_0395	Bromus squarrosus L.	0	a	0	0	Poaceae Barnhart
Iz_7_1209	DJI_0395	Bromus tectorum L.	0	a	0	0	Poaceae Barnhart
Iz_7_1209	DJI_0395	Calamagrostis epigejos (L.) Roth	3	n	1	0	Poaceae Barnhart
Iz_7_1209	DJI_0395	Carduus acanthoides L.	0	n	1	0	Asteraceae Bercht. & J.Presl
Iz_7_1209	DJI_0395	Carex sp.	2	n	1	0	Cyperaceae Juss.
Iz_7_1209	DJI_0395	Centaurea majorovii Dumbadze	0	n	1	0	Asteraceae Bercht. & J.Presl
Iz_7_1209	DJI_0395	Chamaecytisus ruthenicus (Fisch.ex Wol.) Klaskova	0	n	1	0	Fabaceae Lindl.
Iz_7_1209	DJI_0395	Chelidonium majus L.	0	n	1	0	Papaveraceae Juss.
Iz_7_1209	DJI_0395	Chenopodium betaceum Andrz.	0	n	1	0	Amaranthaceae Juss.
Iz_7_1209	DJI_0395	Chenopodium cf. album	0	n	1	0	Amaranthaceae Juss.
Iz_7_1209	DJI_0395	Chenopodium sp.	0		NA	0	Amaranthaceae Juss.
Iz_7_1209	DJI_0395	Chondrilla juncea L.	0	n	1	0	Asteraceae Bercht. & J.Presl
Iz_7_1209	DJI_0395	Cirsium vulgare (Savi) Ten.	0	n	1	0	Asteraceae Bercht. & J.Presl
Iz_7_1209	DJI_0395	Clinopodium acinos (L.) Kuntze	0	n	1	0	Lamiacea Lindl.
Iz_7_1209	DJI_0395	Convallaria majalis L.	0	n	1	0	Asparagaceae Juss.
Iz_7_1209	DJI_0395	Crataegus rhipidophylla Gand.	0	n	1	1	Rosaceae Juss.
Iz_7_1209	DJI_0395	Crepis foetida L.	0	n	1	0	Asteraceae Bercht. & J.Presl
Iz_7_1209	DJI_0395	Dactylis glomerata L.	0	n	1	0	Poaceae Barnhart
Iz_7_1209	DJI_0395	Dianthus campestris M.Bieb.	0	n	1	0	Caryophyllaceae Juss.
Iz_7_1209	DJI_0395	Digitaria sanguinalis (L.) Scop.	0	a	0	0	Poaceae Barnhart
Iz_7_1209	DJI_0395	Elymus repens (L.) Gould	0	n	1	0	Poaceae Barnhart
Iz_7_1209	DJI_0395	Epilobium tetragonum L.	0	n	1	0	Onagraceae Juss.
Iz_7_1209	DJI_0395	Eragrostis minor Host	0	a	0	0	Poaceae Barnhart
Iz_7_1209	DJI_0395	Erigeron canadensis L.	4	a	0	0	Asteraceae Bercht. & J.Presl
Iz_7_1209	DJI_0395	Euphorbia seguieriana Neck.	0	n	1	0	Euphorbiaceae Juss.
Iz_7_1209	DJI_0395	Fallopia dumetorum (L.) Holub	0	n	1	0	Polygonaceae Juss.
Iz_7_1209	DJI_0395	Festuca beckeri (Hack.) Trautv.	0	n	1	0	Poaceae Barnhart
Iz_7_1209	DJI_0395	Fragaria vesca L.	0	n	1	0	Rosaceae Juss.
Iz_7_1209	DJI_0395	Frangula alnus Mill.	0	n	1	1	Rhamnaceae Juss.
Iz_7_1209	DJI_0395	Fraxinus pennsylvanica Marshall	0	a	0	1	Oleaceae Hoffmanns. & Link
Iz_7_1209	DJI_0395	Galeopsis bifida Boenn.	0	n	1	0	Lamiacea Lindl.
Iz_7_1209	DJI_0395	Galium verum L.	0	n	1	0	Rubiaceae Juss.
Iz_7_1209	DJI_0395	Genista tinctoria L.	0	n	1	0	Fabaceae Lindl.
Iz_7_1209	DJI_0395	Geranium robertianum L.	0	n	1	0	Geraniaceae Juss.
Iz_7_1209	DJI_0395	Geranium sanquineum L.	0	n	1	0	Geraniaceae Juss.
Iz_7_1209	DJI_0395	Gypsophila paniculata L.	0	n	1	0	Caryophyllaceae Juss.
Iz_7_1209	DJI_0395	Helichrysum arenarium (L.) Moench	0	n	1	0	Asteraceae Bercht. & J.Presl
Iz_7_1209	DJI_0395	Herniaria polygama J.Gay	0	n	1	0	Caryophyllaceae Juss.
Iz_7_1209	DJI_0395	Hieracium robustum Fr.	0	n	1	0	Asteraceae Bercht. & J.Presl
Iz_7_1209	DJI_0395	Hieracium umbellatum L.	0	n	1	NA	Asteraceae Bercht. & J.Presl
Iz_7_1209	DJI_0395	Hylotelephium maximum (L.) Holub	0	n	1	0	Crassulaceae J.St.-Hil.
Iz_7_1209	DJI_0395	Jacobaea borysthenica (DC.) B.Nord. & Greuter	0	n	1	0	Asteraceae Bercht. & J.Presl
Iz_7_1209	DJI_0395	Jasione montana L.	0	n	1	0	Campanulaceae Juss.
Iz_7_1209	DJI_0395	Jurinea cyanoides (L.) Rchb.s.l.	0	n	1	0	Asteraceae Bercht. & J.Presl
Iz_7_1209	DJI_0395	Koeleria glauca (Spreng.) DC.	0	n	1	0	Poaceae Barnhart
Iz_7_1209	DJI_0395	Lactuca serriola L.	0	a	0	0	Asteraceae Bercht. & J.Presl
Iz_7_1209	DJI_0395	Linaria genistifolia (L.) Mill.	0	n	1	0	Plantaginaceae Juss.
Iz_7_1209	DJI_0395	Lomelosia argentea (L.) Greuter & Burdet	0	n	1	0	Caprifoliaceae Juss.
Iz_7_1209	DJI_0395	Melampyrum pratense L.	0	n	1	0	Orobanchaceae Vent.
Iz_7_1209	DJI_0395	Oenothera biennis L.	0	a	0	0	Onagraceae Juss.
Iz_7_1209	DJI_0395	Pentanema britannica (L.) D.Gut.Larr., Santos-Vicente, Anderb., E.Rico & M.M.Mart.Ort.	0	n	1	0	Asteraceae Bercht. & J.Presl
Iz_7_1209	DJI_0395	Peucedanum oreoselinum (L.) Moench	0	n	1	0	Apiaceae Lindl.
Iz_7_1209	DJI_0395	Peucedanum ruthenicum M.Bieb.	0	n	1	0	Apiaceae Lindl.
Iz_7_1209	DJI_0395	Pilosella echioides (Lumn.) F.W.Schultz & Sch.Bip.	0	n	1	0	Asteraceae Bercht. & J.Presl
Iz_7_1209	DJI_0395	Pilosella officinarum F. Schultz et Sch. Bip.	0	n	1	0	Asteraceae Bercht. & J.Presl
Iz_7_1209	DJI_0395	Pilosella sp.	0		NA	0	Asteraceae Bercht. & J.Presl
Iz_7_1209	DJI_0395	Pinus sylvestris L.	0	n	1	1	Pinaceae Spreng. ex F.Rudolphi
Iz_7_1209	DJI_0395	Poa annua L.	0	n	1	0	Poaceae Barnhart
Iz_7_1209	DJI_0395	Poa nemoralis L.	0	n	1	0	Poaceae Barnhart
Iz_7_1209	DJI_0395	Poa sp.	0		NA	0	Poaceae Barnhart
Iz_7_1209	DJI_0395	Polygonatum odoratum (Mill.) Druce	0	n	1	0	Asparagaceae Juss.
Iz_7_1209	DJI_0395	Polygonum arenarium Waldst. & Kit.	0	n	1	0	Polygonaceae Juss.
Iz_7_1209	DJI_0395	Populus nigra L.	0	n	1	1	Salicaceae Mirb.
Iz_7_1209	DJI_0395	Populus tremula L.	1	n	1	1	Salicaceae Mirb.
Iz_7_1209	DJI_0395	Populus Ã canescens (Aiton) Sm.	0	n	1	1	Salicaceae Mirb.
Iz_7_1209	DJI_0395	Potentilla argentea L.	0	n	1	0	Rosaceae Juss.
Iz_7_1209	DJI_0395	Psammophiliella muralis (L.) Ikonn.	0	n	1	0	Caryophyllaceae Juss.
Iz_7_1209	DJI_0395	Psephellus sumensis (Kalen.) Greuter	0	n	1	0	Asteraceae Bercht. & J.Presl
Iz_7_1209	DJI_0395	Pulsatilla pratensis (L.) Mill. s.l.	0	n	1	0	Ranunculaceae Juss.
Iz_7_1209	DJI_0395	Pyrus communis L.	0	a	0	1	Rosaceae Juss.
Iz_7_1209	DJI_0395	Quercus robur L.	0	n	1	1	Fagaceae Dumort.
Iz_7_1209	DJI_0395	Robinia pseudoacacia L.	0	a	0	1	Fabaceae Lindl.
Iz_7_1209	DJI_0395	Rosa sp.	0		NA	1	Rosaceae Juss.
Iz_7_1209	DJI_0395	Rubus idaeus L.	0	n	1	0	Rosaceae Juss.
Iz_7_1209	DJI_0395	Rumex acetosella L.	0	n	1	0	Polygonaceae Juss.
Iz_7_1209	DJI_0395	Rumex thyrsiflorus Fingerh.	0	n	1	0	Polygonaceae Juss.
Iz_7_1209	DJI_0395	Salix alba L.	0	n	1	1	Salicaceae Mirb.
Iz_7_1209	DJI_0395	Sambucus nigra L.	0	n	1	1	Viburnaceae Raf.
Iz_7_1209	DJI_0395	Secale sylvestre Host	0	n	1	0	Poaceae Barnhart
Iz_7_1209	DJI_0395	Sempervivum ruthenicum Schnittsp. & C.B. Lehm.	0	n	1	0	Crassulaceae J.St.-Hil.
Iz_7_1209	DJI_0395	Setaria viridis (L.) P. Beauv.	0	n	1	0	Poaceae Barnhart
Iz_7_1209	DJI_0395	Silene borysthenica (Gruner) Walters	0	n	1	0	Caryophyllaceae Juss.
Iz_7_1209	DJI_0395	Silene latifolia subsp. alba (Mill.) Greuter & Burdet	0	n	1	0	Caryophyllaceae Juss.

Iz_7_1209	DJI_0395	Solidago virgaurea L.	0	n	1	0	Asteraceae Bercht. & J.Presl
Iz_7_1209	DJI_0395	Sorbus aucuparia L.	0	n	1	1	Rosaceae Juss.
Iz_7_1209	DJI_0395	Stachys recta L.	0	n	1	0	Lamiacea Lindl.
Iz_7_1209	DJI_0395	Tanacetum vulgare L.	0	n	1	0	Asteraceae Bercht. & J.Presl
Iz_7_1209	DJI_0395	Thymus pallasianus Heinr.Braun	0	n	1	0	Lamiacea Lindl.
Iz_7_1209	DJI_0395	Tilia cordata Mill.	0	n	1	1	Malvaceae Juss.
Iz_7_1209	DJI_0395	Trifolium alpestre L.	0	n	1	0	Fabaceae Lindl.
Iz_7_1209	DJI_0395	Trifolium arvense L.	0	n	1	0	Fabaceae Lindl.
Iz_7_1209	DJI_0395	Ulmus glabra Huds.	0	n	1	1	Ulmaceae Mirb.
Iz_7_1209	DJI_0395	Verbascum densiflorum Bertol.	0	n	1	0	Scrophulariaceae Juss.
Iz_7_1209	DJI_0395	Veronica austriaca L.	0	n	1	0	Plantaginaceae Juss.
Iz_7_1209	DJI_0395	Veronica incana L.	0	n	1	0	Plantaginaceae Juss.
Iz_7_1209	DJI_0395	Veronica verna L.	0	n	1	0	Plantaginaceae Juss.
Iz_7_1209	DJI_0395	Vincetoxicum hirundinaria Medik	0	n	1	0	Apocynaceae Juss.
Iz_7_1209	DJI_0398	Acer tataricum L.	0	n	1	1	Sapindaceae Juss.
Iz_7_1209	DJI_0398	Achillea millefolium L. s.l.	0	n	1	0	Asteraceae Bercht. & J.Presl
Iz_7_1209	DJI_0398	Allium oleraceum L.	0	n	1	0	Amaryllidaceae J.St.-Hil.
Iz_7_1209	DJI_0398	Ambrosia artemisiifolia L.	0	a	0	0	Asteraceae Bercht. & J.Presl
Iz_7_1209	DJI_0398	Amorpha fruticosa L.	0	a	0	0	Fabaceae Lindl.
Iz_7_1209	DJI_0398	Anthericum ramosum L.	0	n	1	0	Asparagaceae Juss.
Iz_7_1209	DJI_0398	Artemisia marschalliana Spreng.	0	n	1	0	Asteraceae Bercht. & J.Presl
Iz_7_1209	DJI_0398	Asparagus officinalis L.	0	n	1	0	Asparagaceae Juss.
Iz_7_1209	DJI_0398	Astragalus cicer L.	0	n	1	0	Fabaceae Lindl.
Iz_7_1209	DJI_0398	Bassia laniflora (S.G.Gmel.) A.J.Scott	0	n	1	0	Amaranthaceae Juss.
Iz_7_1209	DJI_0398	Berberis vulgaris L.	0	n	1	0	Berberidaceae Juss.
Iz_7_1209	DJI_0398	Betula pendula Roth.	0	n	1	1	Betulaceae Gray
Iz_7_1209	DJI_0398	Betula pendula Roth.	0	n	1	0	Poaceae Barnhart
Iz_7_1209	DJI_0398	Bromus inermis Leyss.	0		NA	NA	
Iz_7_1209	DJI_0398	Bromus squarrosus L.	0	a	0	0	Poaceae Barnhart
Iz_7_1209	DJI_0398	Bromus tectorum L.	0	a	0	0	Poaceae Barnhart
Iz_7_1209	DJI_0398	Calamagrostis epigejos (L.) Roth	15	n	1	0	Poaceae Barnhart
Iz_7_1209	DJI_0398	Carduus acanthoides L.	0	n	1	0	Asteraceae Bercht. & J.Presl
Iz_7_1209	DJI_0398	Carex sp.	0	n	1	0	Cyperaceae Juss.
Iz_7_1209	DJI_0398	Centaurea majorovii Dumbadze	0	n	1	0	Asteraceae Bercht. & J.Presl
Iz_7_1209	DJI_0398	Chamaecytisus ruthenicus (Fisch.ex Wol.) Klaskova	1	n	1	0	Fabaceae Lindl.
Iz_7_1209	DJI_0398	Chelidonium majus L.	0	n	1	0	Papaveraceae Juss.
Iz_7_1209	DJI_0398	Chenopodium betaceum Andrz.	0	n	1	0	Amaranthaceae Juss.
Iz_7_1209	DJI_0398	Chenopodium cf. album	0	n	1	0	Amaranthaceae Juss.
Iz_7_1209	DJI_0398	Chenopodium sp.	0		NA	0	Amaranthaceae Juss.
Iz_7_1209	DJI_0398	Chondrilla juncea L.	0	n	1	0	Asteraceae Bercht. & J.Presl
Iz_7_1209	DJI_0398	Cirsium vulgare (Savi) Ten.	0	n	1	0	Asteraceae Bercht. & J.Presl
Iz_7_1209	DJI_0398	Clinopodium acinos (L.) Kuntze	0	n	1	0	Lamiacea Lindl.
Iz_7_1209	DJI_0398	Convallaria majalis L.	0	n	1	0	Asparagaceae Juss.
Iz_7_1209	DJI_0398	Crataegus rhipidophylla Gand.	0	n	1	1	Rosaceae Juss.
Iz_7_1209	DJI_0398	Crepis foetida L.	0	n	1	0	Asteraceae Bercht. & J.Presl
Iz_7_1209	DJI_0398	Dactylis glomerata L.	0	n	1	0	Poaceae Barnhart
Iz_7_1209	DJI_0398	Dianthus campestris M.Bieb.	0	n	1	0	Caryophyllaceae Juss.
Iz_7_1209	DJI_0398	Digitaria sanguinalis (L.) Scop.	0	a	0	0	Poaceae Barnhart
Iz_7_1209	DJI_0398	Elymus repens (L.) Gould	0	n	1	0	Poaceae Barnhart
Iz_7_1209	DJI_0398	Epilobium tetragonum L.	0	n	1	0	Onagraceae Juss.
Iz_7_1209	DJI_0398	Eragrostis minor Host	0	a	0	0	Poaceae Barnhart
Iz_7_1209	DJI_0398	Erigeron canadensis L.	5	a	0	0	Asteraceae Bercht. & J.Presl
Iz_7_1209	DJI_0398	Euphorbia seguieriana Neck.	0	n	1	0	Euphorbiaceae Juss.
Iz_7_1209	DJI_0398	Fallopia dumetorum (L.) Holub	0	n	1	0	Polygonaceae Juss.
Iz_7_1209	DJI_0398	Festuca beckeri (Hack.) Trautv.	0	n	1	0	Poaceae Barnhart
Iz_7_1209	DJI_0398	Fragaria vesca L.	0	n	1	0	Rosaceae Juss.
Iz_7_1209	DJI_0398	Frangula alnus Mill.	0	n	1	1	Rhamnaceae Juss.
Iz_7_1209	DJI_0398	Fraxinus pennsylvanica Marshall	0	a	0	1	Oleaceae Hoffmanns. & Link
Iz_7_1209	DJI_0398	Galeopsis bifida Boenn.	0	n	1	0	Lamiacea Lindl.
Iz_7_1209	DJI_0398	Galium verum L.	0	n	1	0	Rubiaceae Juss.
Iz_7_1209	DJI_0398	Genista tinctoria L.	0	n	1	0	Fabaceae Lindl.
Iz_7_1209	DJI_0398	Geranium robertianum L.	0	n	1	0	Geraniaceae Juss.
Iz_7_1209	DJI_0398	Geranium sanquineum L.	0	n	1	0	Geraniaceae Juss.
Iz_7_1209	DJI_0398	Gypsophila paniculata L.	0	n	1	0	Caryophyllaceae Juss.
Iz_7_1209	DJI_0398	Helichrysum arenarium (L.) Moench	0.2	n	1	0	Asteraceae Bercht. & J.Presl
Iz_7_1209	DJI_0398	Herniaria polygama J.Gay	0	n	1	0	Caryophyllaceae Juss.
Iz_7_1209	DJI_0398	Hieracium robustum Fr.	0	n	1	0	Asteraceae Bercht. & J.Presl
Iz_7_1209	DJI_0398	Hieracium umbellatum L.	0	n	1	NA	Asteraceae Bercht. & J.Presl
Iz_7_1209	DJI_0398	Hylotelephium maximum (L.) Holub	0	n	1	0	Crassulaceae J.St.-Hil.
Iz_7_1209	DJI_0398	Jacobaea borysthenica (DC.) B.Nord. & Greuter	0	n	1	0	Asteraceae Bercht. & J.Presl
Iz_7_1209	DJI_0398	Jasione montana L.	0	n	1	0	Campanulaceae Juss.
Iz_7_1209	DJI_0398	Jurinea cyanoides (L.) Rchb.s.l.	0	n	1	0	Asteraceae Bercht. & J.Presl
Iz_7_1209	DJI_0398	Koeleria glauca (Spreng.) DC.	0	n	1	0	Poaceae Barnhart
Iz_7_1209	DJI_0398	Lactuca serriola L.	0	a	0	0	Asteraceae Bercht. & J.Presl
Iz_7_1209	DJI_0398	Linaria genistifolia (L.) Mill.	0	n	1	0	Plantaginaceae Juss.
Iz_7_1209	DJI_0398	Lomelosia argentea (L.) Greuter & Burdet	0	n	1	0	Caprifoliaceae Juss.
Iz_7_1209	DJI_0398	Melampyrum pratense L.	0	n	1	0	Orobanchaceae Vent.
Iz_7_1209	DJI_0398	Oenothera biennis L.	0	a	0	0	Onagraceae Juss.
Iz_7_1209	DJI_0398	Pentanema britannica (L.) D.Gut.Larr., Santos-Vicente, Anderb., E.Rico & M.M.Mart.Ort.	0	n	1	0	Asteraceae Bercht. & J.Presl
Iz_7_1209	DJI_0398	Peucedanum oreoselinum (L.) Moench	0	n	1	0	Apiaceae Lindl.
Iz_7_1209	DJI_0398	Peucedanum ruthenicum M.Bieb.	0	n	1	0	Apiaceae Lindl.
Iz_7_1209	DJI_0398	Pilosella echioides (Lumn.) F.W.Schultz & Sch.Bip.	0	n	1	0	Asteraceae Bercht. & J.Presl
Iz_7_1209	DJI_0398	Pilosella officinarum F. Schultz et Sch. Bip.	0	n	1	0	Asteraceae Bercht. & J.Presl
Iz_7_1209	DJI_0398	Pilosella sp.	0		NA	0	Asteraceae Bercht. & J.Presl
Iz_7_1209	DJI_0398	Pinus sylvestris L.	0	n	1	1	Pinaceae Spreng. ex F.Rudolphi
Iz_7_1209	DJI_0398	Poa annua L.	0	n	1	0	Poaceae Barnhart
Iz_7_1209	DJI_0398	Poa nemoralis L.	0	n	1	0	Poaceae Barnhart
Iz_7_1209	DJI_0398	Poa sp.	0		NA	0	Poaceae Barnhart
Iz_7_1209	DJI_0398	Polygonatum odoratum (Mill.) Druce	0	n	1	0	Asparagaceae Juss.
Iz_7_1209	DJI_0398	Polygonum arenarium Waldst. & Kit.	0	n	1	0	Polygonaceae Juss.
Iz_7_1209	DJI_0398	Populus nigra L.	0	n	1	1	Salicaceae Mirb.
Iz_7_1209	DJI_0398	Populus tremula L.	2	n	1	1	Salicaceae Mirb.
Iz_7_1209	DJI_0398	Populus Ã canescens (Aiton) Sm.	0	n	1	1	Salicaceae Mirb.
Iz_7_1209	DJI_0398	Potentilla argentea L.	0	n	1	0	Rosaceae Juss.
Iz_7_1209	DJI_0398	Psammophiliella muralis (L.) Ikonn.	0	n	1	0	Caryophyllaceae Juss.
Iz_7_1209	DJI_0398	Psephellus sumensis (Kalen.) Greuter	0	n	1	0	Asteraceae Bercht. & J.Presl
Iz_7_1209	DJI_0398	Pulsatilla pratensis (L.) Mill. s.l.	0	n	1	0	Ranunculaceae Juss.
Iz_7_1209	DJI_0398	Pyrus communis L.	0	a	0	1	Rosaceae Juss.

Iz_7_1209	DJI_0398	Quercus robur L.	0	n	1	1	Fagaceae Dumort.
Iz_7_1209	DJI_0398	Robinia pseudoacacia L.	0	a	0	1	Fabaceae Lindl.
Iz_7_1209	DJI_0398	Rosa sp.	0		NA	1	Rosaceae Juss.
Iz_7_1209	DJI_0398	Rubus idaeus L.	0	n	1	0	Rosaceae Juss.
Iz_7_1209	DJI_0398	Rumex acetosella L.	0	n	1	0	Polygonaceae Juss.
Iz_7_1209	DJI_0398	Rumex thyrsiflorus Fingerh.	0	n	1	0	Polygonaceae Juss.
Iz_7_1209	DJI_0398	Salix alba L.	0	n	1	1	Salicaceae Mirb.
Iz_7_1209	DJI_0398	Sambucus nigra L.	0	n	1	1	Viburnaceae Raf.
Iz_7_1209	DJI_0398	Secale sylvestre Host	0	n	1	0	Poaceae Barnhart
Iz_7_1209	DJI_0398	Sempervivum ruthenicum Schnittsp. & C.B. Lehm.	0	n	1	0	Crassulaceae J.St.-Hil.
Iz_7_1209	DJI_0398	Setaria viridis (L.) P. Beauv.	0	n	1	0	Poaceae Barnhart
Iz_7_1209	DJI_0398	Silene borysthenica (Gruner) Walters	0	n	1	0	Caryophyllaceae Juss.
Iz_7_1209	DJI_0398	Silene latifolia subsp. alba (Mill.) Greuter & Burdet	0	n	1	0	Caryophyllaceae Juss.
Iz_7_1209	DJI_0398	Solidago virgaurea L.	0	n	1	0	Asteraceae Bercht. & J.Presl
Iz_7_1209	DJI_0398	Sorbus aucuparia L.	0	n	1	1	Rosaceae Juss.
Iz_7_1209	DJI_0398	Stachys recta L.	0	n	1	0	Lamiacea Lindl.
Iz_7_1209	DJI_0398	Tanacetum vulgare L.	0	n	1	0	Asteraceae Bercht. & J.Presl
Iz_7_1209	DJI_0398	Thymus pallasianus Heinr.Braun	0	n	1	0	Lamiacea Lindl.
Iz_7_1209	DJI_0398	Tilia cordata Mill.	0	n	1	1	Malvaceae Juss.
Iz_7_1209	DJI_0398	Trifolium alpestre L.	0	n	1	0	Fabaceae Lindl.
Iz_7_1209	DJI_0398	Trifolium arvense L.	0	n	1	0	Fabaceae Lindl.
Iz_7_1209	DJI_0398	Ulmus glabra Huds.	0	n	1	1	Ulmaceae Mirb.
Iz_7_1209	DJI_0398	Verbascum densiflorum Bertol.	0	n	1	0	Scrophulariaceae Juss.
Iz_7_1209	DJI_0398	Veronica austriaca L.	0	n	1	0	Plantaginaceae Juss.
Iz_7_1209	DJI_0398	Veronica incana L.	0	n	1	0	Plantaginaceae Juss.
Iz_7_1209	DJI_0398	Veronica verna L.	0	n	1	0	Plantaginaceae Juss.
Iz_7_1209	DJI_0398	Vincetoxicum hirundinaria Medik	0	n	1	0	Apocynaceae Juss.
Iz_7_1209	DJI_0405	Acer tataricum L.	0	n	1	1	Sapindaceae Juss.
Iz_7_1209	DJI_0405	Achillea millefolium L. s.l.	0	n	1	0	Asteraceae Bercht. & J.Presl
Iz_7_1209	DJI_0405	Allium oleraceum L.	0	n	1	0	Amaryllidaceae J.St.-Hil.
Iz_7_1209	DJI_0405	Ambrosia artemisiifolia L.	0	a	0	0	Asteraceae Bercht. & J.Presl
Iz_7_1209	DJI_0405	Amorpha fruticosa L.	0	a	0	0	Fabaceae Lindl.
Iz_7_1209	DJI_0405	Anthericum ramosum L.	0	n	1	0	Asparagaceae Juss.
Iz_7_1209	DJI_0405	Artemisia marschalliana Spreng.	0	n	1	0	Asteraceae Bercht. & J.Presl
Iz_7_1209	DJI_0405	Asparagus officinalis L.	0	n	1	0	Asparagaceae Juss.
Iz_7_1209	DJI_0405	Astragalus cicer L.	0	n	1	0	Fabaceae Lindl.
Iz_7_1209	DJI_0405	Bassia laniflora (S.G.Gmel.) A.J.Scott	0	n	1	0	Amaranthaceae Juss.
Iz_7_1209	DJI_0405	Berberis vulgaris L.	0	n	1	0	Berberidaceae Juss.
Iz_7_1209	DJI_0405	Betula pendula Roth.	0	n	1	1	Betulaceae Gray
Iz_7_1209	DJI_0405	Betula pendula Roth.	0	n	1	0	Poaceae Barnhart
Iz_7_1209	DJI_0405	Bromus inermis Leyss.	0		NA	NA	
Iz_7_1209	DJI_0405	Bromus squarrosus L.	0	a	0	0	Poaceae Barnhart
Iz_7_1209	DJI_0405	Bromus tectorum L.	0	a	0	0	Poaceae Barnhart
Iz_7_1209	DJI_0405	Calamagrostis epigejos (L.) Roth	0.5	n	1	0	Poaceae Barnhart
Iz_7_1209	DJI_0405	Carduus acanthoides L.	0	n	1	0	Asteraceae Bercht. & J.Presl
Iz_7_1209	DJI_0405	Carex sp.	0	n	1	0	Cyperaceae Juss.
Iz_7_1209	DJI_0405	Centaurea majorovii Dumbadze	0	n	1	0	Asteraceae Bercht. & J.Presl
Iz_7_1209	DJI_0405	Chamaecytisus ruthenicus (Fisch.ex Wol.) Klaskova	0	n	1	0	Fabaceae Lindl.
Iz_7_1209	DJI_0405	Chelidonium majus L.	0	n	1	0	Papaveraceae Juss.
Iz_7_1209	DJI_0405	Chenopodium betaceum Andrz.	0	n	1	0	Amaranthaceae Juss.
Iz_7_1209	DJI_0405	Chenopodium cf. album	0	n	1	0	Amaranthaceae Juss.
Iz_7_1209	DJI_0405	Chenopodium sp.	0		NA	0	Amaranthaceae Juss.
Iz_7_1209	DJI_0405	Chondrilla juncea L.	0.2	n	1	0	Asteraceae Bercht. & J.Presl
Iz_7_1209	DJI_0405	Cirsium vulgare (Savi) Ten.	0	n	1	0	Asteraceae Bercht. & J.Presl
Iz_7_1209	DJI_0405	Clinopodium acinos (L.) Kuntze	0	n	1	0	Lamiacea Lindl.
Iz_7_1209	DJI_0405	Convallaria majalis L.	0	n	1	0	Asparagaceae Juss.
Iz_7_1209	DJI_0405	Crataegus rhipidophylla Gand.	0	n	1	1	Rosaceae Juss.
Iz_7_1209	DJI_0405	Crepis foetida L.	0	n	1	0	Asteraceae Bercht. & J.Presl
Iz_7_1209	DJI_0405	Dactylis glomerata L.	0	n	1	0	Poaceae Barnhart
Iz_7_1209	DJI_0405	Dianthus campestris M.Bieb.	0	n	1	0	Caryophyllaceae Juss.
Iz_7_1209	DJI_0405	Digitaria sanguinalis (L.) Scop.	0	a	0	0	Poaceae Barnhart
Iz_7_1209	DJI_0405	Elymus repens (L.) Gould	0	n	1	0	Poaceae Barnhart
Iz_7_1209	DJI_0405	Epilobium tetragonum L.	0	n	1	0	Onagraceae Juss.
Iz_7_1209	DJI_0405	Eragrostis minor Host	0	a	0	0	Poaceae Barnhart
Iz_7_1209	DJI_0405	Erigeron canadensis L.	10	a	0	0	Asteraceae Bercht. & J.Presl
Iz_7_1209	DJI_0405	Euphorbia seguieriana Neck.	0	n	1	0	Euphorbiaceae Juss.
Iz_7_1209	DJI_0405	Fallopia dumetorum (L.) Holub	0	n	1	0	Polygonaceae Juss.
Iz_7_1209	DJI_0405	Festuca beckeri (Hack.) Trautv.	0	n	1	0	Poaceae Barnhart
Iz_7_1209	DJI_0405	Fragaria vesca L.	0	n	1	0	Rosaceae Juss.
Iz_7_1209	DJI_0405	Frangula alnus Mill.	0	n	1	1	Rhamnaceae Juss.
Iz_7_1209	DJI_0405	Fraxinus pennsylvanica Marshall	0	a	0	1	Oleaceae Hoffmanns. & Link
Iz_7_1209	DJI_0405	Galeopsis bifida Boenn.	0	n	1	0	Lamiacea Lindl.
Iz_7_1209	DJI_0405	Galium verum L.	0	n	1	0	Rubiaceae Juss.
Iz_7_1209	DJI_0405	Genista tinctoria L.	0	n	1	0	Fabaceae Lindl.
Iz_7_1209	DJI_0405	Geranium robertianum L.	0	n	1	0	Geraniaceae Juss.
Iz_7_1209	DJI_0405	Geranium sanquineum L.	0	n	1	0	Geraniaceae Juss.
Iz_7_1209	DJI_0405	Gypsophila paniculata L.	0	n	1	0	Caryophyllaceae Juss.
Iz_7_1209	DJI_0405	Helichrysum arenarium (L.) Moench	0.5	n	1	0	Asteraceae Bercht. & J.Presl
Iz_7_1209	DJI_0405	Herniaria polygama J.Gay	0	n	1	0	Caryophyllaceae Juss.
Iz_7_1209	DJI_0405	Hieracium robustum Fr.	0	n	1	0	Asteraceae Bercht. & J.Presl
Iz_7_1209	DJI_0405	Hieracium umbellatum L.	0	n	1	NA	Asteraceae Bercht. & J.Presl
Iz_7_1209	DJI_0405	Hylotelephium maximum (L.) Holub	0	n	1	0	Crassulaceae J.St.-Hil.
Iz_7_1209	DJI_0405	Jacobaea borysthenica (DC.) B.Nord. & Greuter	0	n	1	0	Asteraceae Bercht. & J.Presl
Iz_7_1209	DJI_0405	Jasione montana L.	0	n	1	0	Campanulaceae Juss.
Iz_7_1209	DJI_0405	Jurinea cyanoides (L.) Rchb.s.l.	0	n	1	0	Asteraceae Bercht. & J.Presl
Iz_7_1209	DJI_0405	Koeleria glauca (Spreng.) DC.	0	n	1	0	Poaceae Barnhart
Iz_7_1209	DJI_0405	Lactuca serriola L.	0	a	0	0	Asteraceae Bercht. & J.Presl
Iz_7_1209	DJI_0405	Linaria genistifolia (L.) Mill.	0	n	1	0	Plantaginaceae Juss.
Iz_7_1209	DJI_0405	Lomelosia argentea (L.) Greuter & Burdet	0	n	1	0	Caprifoliaceae Juss.
Iz_7_1209	DJI_0405	Melampyrum pratense L.	0	n	1	0	Orobanchaceae Vent.
Iz_7_1209	DJI_0405	Oenothera biennis L.	0	a	0	0	Onagraceae Juss.
Iz_7_1209	DJI_0405	Pentanema britannica (L.) D.Gut.Larr., Santos-Vicente, Anderb., E.Rico & M.M.Mart.Ort.	0	n	1	0	Asteraceae Bercht. & J.Presl
Iz_7_1209	DJI_0405	Peucedanum oreoselinum (L.) Moench	0	n	1	0	Apiaceae Lindl.
Iz_7_1209	DJI_0405	Peucedanum ruthenicum M.Bieb.	0	n	1	0	Apiaceae Lindl.
Iz_7_1209	DJI_0405	Pilosella echioides (Lumn.) F.W.Schultz & Sch.Bip.	0	n	1	0	Asteraceae Bercht. & J.Presl
Iz_7_1209	DJI_0405	Pilosella officinarum F. Schultz et Sch. Bip.	0	n	1	0	Asteraceae Bercht. & J.Presl
Iz_7_1209	DJI_0405	Pilosella sp.	0		NA	0	Asteraceae Bercht. & J.Presl
Iz_7_1209	DJI_0405	Pinus sylvestris L.	0	n	1	1	Pinaceae Spreng. ex F.Rudolphi

Iz_7_1209	DJI_0405	Poa annua L.	0	n	1	0	Poaceae Barnhart
Iz_7_1209	DJI_0405	Poa nemoralis L.	0	n	1	0	Poaceae Barnhart
Iz_7_1209	DJI_0405	Poa sp.	0		NA	0	Poaceae Barnhart
Iz_7_1209	DJI_0405	Polygonatum odoratum (Mill.) Druce	0	n	1	0	Asparagaceae Juss.
Iz_7_1209	DJI_0405	Polygonum arenarium Waldst. & Kit.	0	n	1	0	Polygonaceae Juss.
Iz_7_1209	DJI_0405	Populus nigra L.	0	n	1	1	Salicaceae Mirb.
Iz_7_1209	DJI_0405	Populus tremula L.	0	n	1	1	Salicaceae Mirb.
Iz_7_1209	DJI_0405	Populus Ã canescens (Aiton) Sm.	0	n	1	1	Salicaceae Mirb.
Iz_7_1209	DJI_0405	Potentilla argentea L.	0	n	1	0	Rosaceae Juss.
Iz_7_1209	DJI_0405	Psammophiliella muralis (L.) Ikonn.	0	n	1	0	Caryophyllaceae Juss.
Iz_7_1209	DJI_0405	Psephellus sumensis (Kalen.) Greuter	0	n	1	0	Asteraceae Bercht. & J.Presl
Iz_7_1209	DJI_0405	Pulsatilla pratensis (L.) Mill. s.l.	0	n	1	0	Ranunculaceae Juss.
Iz_7_1209	DJI_0405	Pyrus communis L.	0	a	0	1	Rosaceae Juss.
Iz_7_1209	DJI_0405	Quercus robur L.	0	n	1	1	Fagaceae Dumort.
Iz_7_1209	DJI_0405	Robinia pseudoacacia L.	0	a	0	1	Fabaceae Lindl.
Iz_7_1209	DJI_0405	Rosa sp.	0		NA	1	Rosaceae Juss.
Iz_7_1209	DJI_0405	Rubus idaeus L.	0	n	1	0	Rosaceae Juss.
Iz_7_1209	DJI_0405	Rumex acetosella L.	0	n	1	0	Polygonaceae Juss.
Iz_7_1209	DJI_0405	Rumex thyrsiflorus Fingerh.	0	n	1	0	Polygonaceae Juss.
Iz_7_1209	DJI_0405	Salix alba L.	0	n	1	1	Salicaceae Mirb.
Iz_7_1209	DJI_0405	Sambucus nigra L.	0	n	1	1	Viburnaceae Raf.
Iz_7_1209	DJI_0405	Secale sylvestre Host	0	n	1	0	Poaceae Barnhart
Iz_7_1209	DJI_0405	Sempervivum ruthenicum Schnittsp. & C.B. Lehm.	0	n	1	0	Crassulaceae J.St.-Hil.
Iz_7_1209	DJI_0405	Setaria viridis (L.) P. Beauv.	0	n	1	0	Poaceae Barnhart
Iz_7_1209	DJI_0405	Silene borysthenica (Gruner) Walters	0	n	1	0	Caryophyllaceae Juss.
Iz_7_1209	DJI_0405	Silene latifolia subsp. alba (Mill.) Greuter & Burdet	0	n	1	0	Caryophyllaceae Juss.
Iz_7_1209	DJI_0405	Solidago virgaurea L.	0	n	1	0	Asteraceae Bercht. & J.Presl
Iz_7_1209	DJI_0405	Sorbus aucuparia L.	0	n	1	1	Rosaceae Juss.
Iz_7_1209	DJI_0405	Stachys recta L.	0	n	1	0	Lamiacea Lindl.
Iz_7_1209	DJI_0405	Tanacetum vulgare L.	0	n	1	0	Asteraceae Bercht. & J.Presl
Iz_7_1209	DJI_0405	Thymus pallasianus Heinr.Braun	0	n	1	0	Lamiacea Lindl.
Iz_7_1209	DJI_0405	Tilia cordata Mill.	0	n	1	1	Malvaceae Juss.
Iz_7_1209	DJI_0405	Trifolium alpestre L.	0	n	1	0	Fabaceae Lindl.
Iz_7_1209	DJI_0405	Trifolium arvense L.	0	n	1	0	Fabaceae Lindl.
Iz_7_1209	DJI_0405	Ulmus glabra Huds.	0	n	1	1	Ulmaceae Mirb.
Iz_7_1209	DJI_0405	Verbascum densiflorum Bertol.	0	n	1	0	Scrophulariaceae Juss.
Iz_7_1209	DJI_0405	Veronica austriaca L.	0	n	1	0	Plantaginaceae Juss.
Iz_7_1209	DJI_0405	Veronica incana L.	0	n	1	0	Plantaginaceae Juss.
Iz_7_1209	DJI_0405	Veronica verna L.	0	n	1	0	Plantaginaceae Juss.
Iz_7_1209	DJI_0405	Vincetoxicum hirundinaria Medik	0	n	1	0	Apocynaceae Juss.
Iz_7_1209	DJI_0409	Acer tataricum L.	0	n	1	1	Sapindaceae Juss.
Iz_7_1209	DJI_0409	Achillea millefolium L. s.l.	0	n	1	0	Asteraceae Bercht. & J.Presl
Iz_7_1209	DJI_0409	Allium oleraceum L.	0	n	1	0	Amaryllidaceae J.St.-Hil.
Iz_7_1209	DJI_0409	Ambrosia artemisiifolia L.	0	a	0	0	Asteraceae Bercht. & J.Presl
Iz_7_1209	DJI_0409	Amorpha fruticosa L.	0	a	0	0	Fabaceae Lindl.
Iz_7_1209	DJI_0409	Anthericum ramosum L.	0	n	1	0	Asparagaceae Juss.
Iz_7_1209	DJI_0409	Artemisia marschalliana Spreng.	0	n	1	0	Asteraceae Bercht. & J.Presl
Iz_7_1209	DJI_0409	Asparagus officinalis L.	0	n	1	0	Asparagaceae Juss.
Iz_7_1209	DJI_0409	Astragalus cicer L.	0	n	1	0	Fabaceae Lindl.
Iz_7_1209	DJI_0409	Bassia laniflora (S.G.Gmel.) A.J.Scott	0	n	1	0	Amaranthaceae Juss.
Iz_7_1209	DJI_0409	Berberis vulgaris L.	0	n	1	0	Berberidaceae Juss.
Iz_7_1209	DJI_0409	Betula pendula Roth.	0	n	1	1	Betulaceae Gray
Iz_7_1209	DJI_0409	Betula pendula Roth.	0	n	1	0	Poaceae Barnhart
Iz_7_1209	DJI_0409	Bromus inermis Leyss.	0		NA	NA	
Iz_7_1209	DJI_0409	Bromus squarrosus L.	0	a	0	0	Poaceae Barnhart
Iz_7_1209	DJI_0409	Bromus tectorum L.	0	a	0	0	Poaceae Barnhart
Iz_7_1209	DJI_0409	Calamagrostis epigejos (L.) Roth	10	n	1	0	Poaceae Barnhart
Iz_7_1209	DJI_0409	Carduus acanthoides L.	0	n	1	0	Asteraceae Bercht. & J.Presl
Iz_7_1209	DJI_0409	Carex sp.	0	n	1	0	Cyperaceae Juss.
Iz_7_1209	DJI_0409	Centaurea majorovii Dumbadze	0	n	1	0	Asteraceae Bercht. & J.Presl
Iz_7_1209	DJI_0409	Chamaecytisus ruthenicus (Fisch.ex Wol.) Klaskova	0	n	1	0	Fabaceae Lindl.
Iz_7_1209	DJI_0409	Chelidonium majus L.	0	n	1	0	Papaveraceae Juss.
Iz_7_1209	DJI_0409	Chenopodium betaceum Andrz.	0	n	1	0	Amaranthaceae Juss.
Iz_7_1209	DJI_0409	Chenopodium cf. album	0	n	1	0	Amaranthaceae Juss.
Iz_7_1209	DJI_0409	Chenopodium sp.	0		NA	0	Amaranthaceae Juss.
Iz_7_1209	DJI_0409	Chondrilla juncea L.	0	n	1	0	Asteraceae Bercht. & J.Presl
Iz_7_1209	DJI_0409	Cirsium vulgare (Savi) Ten.	0	n	1	0	Asteraceae Bercht. & J.Presl
Iz_7_1209	DJI_0409	Clinopodium acinos (L.) Kuntze	0	n	1	0	Lamiacea Lindl.
Iz_7_1209	DJI_0409	Convallaria majalis L.	0	n	1	0	Asparagaceae Juss.
Iz_7_1209	DJI_0409	Crataegus rhipidophylla Gand.	0	n	1	1	Rosaceae Juss.
Iz_7_1209	DJI_0409	Crepis foetida L.	0	n	1	0	Asteraceae Bercht. & J.Presl
Iz_7_1209	DJI_0409	Dactylis glomerata L.	0	n	1	0	Poaceae Barnhart
Iz_7_1209	DJI_0409	Dianthus campestris M.Bieb.	0	n	1	0	Caryophyllaceae Juss.
Iz_7_1209	DJI_0409	Digitaria sanguinalis (L.) Scop.	0	a	0	0	Poaceae Barnhart
Iz_7_1209	DJI_0409	Elymus repens (L.) Gould	0	n	1	0	Poaceae Barnhart
Iz_7_1209	DJI_0409	Epilobium tetragonum L.	0	n	1	0	Onagraceae Juss.
Iz_7_1209	DJI_0409	Eragrostis minor Host	0	a	0	0	Poaceae Barnhart
Iz_7_1209	DJI_0409	Erigeron canadensis L.	0.2	a	0	0	Asteraceae Bercht. & J.Presl
Iz_7_1209	DJI_0409	Euphorbia seguieriana Neck.	0	n	1	0	Euphorbiaceae Juss.
Iz_7_1209	DJI_0409	Fallopia dumetorum (L.) Holub	0	n	1	0	Polygonaceae Juss.
Iz_7_1209	DJI_0409	Festuca beckeri (Hack.) Trautv.	0	n	1	0	Poaceae Barnhart
Iz_7_1209	DJI_0409	Fragaria vesca L.	0	n	1	0	Rosaceae Juss.
Iz_7_1209	DJI_0409	Frangula alnus Mill.	0	n	1	1	Rhamnaceae Juss.
Iz_7_1209	DJI_0409	Fraxinus pennsylvanica Marshall	0	a	0	1	Oleaceae Hoffmanns. & Link
Iz_7_1209	DJI_0409	Galeopsis bifida Boenn.	0	n	1	0	Lamiacea Lindl.
Iz_7_1209	DJI_0409	Galium verum L.	0	n	1	0	Rubiaceae Juss.
Iz_7_1209	DJI_0409	Genista tinctoria L.	0	n	1	0	Fabaceae Lindl.
Iz_7_1209	DJI_0409	Geranium robertianum L.	0	n	1	0	Geraniaceae Juss.
Iz_7_1209	DJI_0409	Geranium sanquineum L.	0	n	1	0	Geraniaceae Juss.
Iz_7_1209	DJI_0409	Gypsophila paniculata L.	0	n	1	0	Caryophyllaceae Juss.
Iz_7_1209	DJI_0409	Helichrysum arenarium (L.) Moench	0	n	1	0	Asteraceae Bercht. & J.Presl
Iz_7_1209	DJI_0409	Herniaria polygama J.Gay	0	n	1	0	Caryophyllaceae Juss.
Iz_7_1209	DJI_0409	Hieracium robustum Fr.	0	n	1	0	Asteraceae Bercht. & J.Presl
Iz_7_1209	DJI_0409	Hieracium umbellatum L.	0	n	1	NA	Asteraceae Bercht. & J.Presl
Iz_7_1209	DJI_0409	Hylotelephium maximum (L.) Holub	0	n	1	0	Crassulaceae J.St.-Hil.
Iz_7_1209	DJI_0409	Jacobaea borysthenica (DC.) B.Nord. & Greuter	0	n	1	0	Asteraceae Bercht. & J.Presl
Iz_7_1209	DJI_0409	Jasione montana L.	0	n	1	0	Campanulaceae Juss.
Iz_7_1209	DJI_0409	Jurinea cyanoides (L.) Rchb.s.l.	0	n	1	0	Asteraceae Bercht. & J.Presl

Iz_7_1209	DJI_0409	Koeleria glauca (Spreng.) DC.	0	n	1	0	Poaceae Barnhart
Iz_7_1209	DJI_0409	Lactuca serriola L.	0	a	0	0	Asteraceae Bercht. & J.Presl
Iz_7_1209	DJI_0409	Linaria genistifolia (L.) Mill.	0	n	1	0	Plantaginaceae Juss.
Iz_7_1209	DJI_0409	Lomelosia argentea (L.) Greuter & Burdet	0	n	1	0	Caprifoliaceae Juss.
Iz_7_1209	DJI_0409	Melampyrum pratense L.	0	n	1	0	Orobanchaceae Vent.
Iz_7_1209	DJI_0409	Oenothera biennis L.	0	a	0	0	Onagraceae Juss.
Iz_7_1209	DJI_0409	Pentanema britannica (L.) D.Gut.Larr., Santos-Vicente, Anderb., E.Rico & M.M.Mart.Ort.	0	n	1	0	Asteraceae Bercht. & J.Presl
Iz_7_1209	DJI_0409	Peucedanum oreoselinum (L.) Moench	0	n	1	0	Apiaceae Lindl.
Iz_7_1209	DJI_0409	Peucedanum ruthenicum M.Bieb.	0	n	1	0	Apiaceae Lindl.
Iz_7_1209	DJI_0409	Pilosella echioides (Lumn.) F.W.Schultz & Sch.Bip.	0	n	1	0	Asteraceae Bercht. & J.Presl
Iz_7_1209	DJI_0409	Pilosella officinarum F. Schultz et Sch. Bip.	0	n	1	0	Asteraceae Bercht. & J.Presl
Iz_7_1209	DJI_0409	Pilosella sp.	0		NA	0	Asteraceae Bercht. & J.Presl
Iz_7_1209	DJI_0409	Pinus sylvestris L.	0	n	1	1	Pinaceae Spreng. ex F.Rudolphi
Iz_7_1209	DJI_0409	Poa annua L.	0	n	1	0	Poaceae Barnhart
Iz_7_1209	DJI_0409	Poa nemoralis L.	0	n	1	0	Poaceae Barnhart
Iz_7_1209	DJI_0409	Poa sp.	0		NA	0	Poaceae Barnhart
Iz_7_1209	DJI_0409	Polygonatum odoratum (Mill.) Druce	0	n	1	0	Asparagaceae Juss.
Iz_7_1209	DJI_0409	Polygonum arenarium Waldst. & Kit.	0	n	1	0	Polygonaceae Juss.
Iz_7_1209	DJI_0409	Populus nigra L.	0	n	1	1	Salicaceae Mirb.
Iz_7_1209	DJI_0409	Populus tremula L.	0	n	1	1	Salicaceae Mirb.
Iz_7_1209	DJI_0409	Populus Ã canescens (Aiton) Sm.	0	n	1	1	Salicaceae Mirb.
Iz_7_1209	DJI_0409	Potentilla argentea L.	0	n	1	0	Rosaceae Juss.
Iz_7_1209	DJI_0409	Psammophiliella muralis (L.) Ikonn.	0	n	1	0	Caryophyllaceae Juss.
Iz_7_1209	DJI_0409	Psephellus sumensis (Kalen.) Greuter	0	n	1	0	Asteraceae Bercht. & J.Presl
Iz_7_1209	DJI_0409	Pulsatilla pratensis (L.) Mill. s.l.	0	n	1	0	Ranunculaceae Juss.
Iz_7_1209	DJI_0409	Pyrus communis L.	0	a	0	1	Rosaceae Juss.
Iz_7_1209	DJI_0409	Quercus robur L.	0	n	1	1	Fagaceae Dumort.
Iz_7_1209	DJI_0409	Robinia pseudoacacia L.	0	a	0	1	Fabaceae Lindl.
Iz_7_1209	DJI_0409	Rosa sp.	0		NA	1	Rosaceae Juss.
Iz_7_1209	DJI_0409	Rubus idaeus L.	0	n	1	0	Rosaceae Juss.
Iz_7_1209	DJI_0409	Rumex acetosella L.	0	n	1	0	Polygonaceae Juss.
Iz_7_1209	DJI_0409	Rumex thyrsiflorus Fingerh.	0	n	1	0	Polygonaceae Juss.
Iz_7_1209	DJI_0409	Salix alba L.	0	n	1	1	Salicaceae Mirb.
Iz_7_1209	DJI_0409	Sambucus nigra L.	0	n	1	1	Viburnaceae Raf.
Iz_7_1209	DJI_0409	Secale sylvestre Host	0	n	1	0	Poaceae Barnhart
Iz_7_1209	DJI_0409	Sempervivum ruthenicum Schnittsp. & C.B. Lehm.	0	n	1	0	Crassulaceae J.St.-Hil.
Iz_7_1209	DJI_0409	Setaria viridis (L.) P. Beauv.	0	n	1	0	Poaceae Barnhart
Iz_7_1209	DJI_0409	Silene borysthenica (Gruner) Walters	0	n	1	0	Caryophyllaceae Juss.
Iz_7_1209	DJI_0409	Silene latifolia subsp. alba (Mill.) Greuter & Burdet	0	n	1	0	Caryophyllaceae Juss.
Iz_7_1209	DJI_0409	Solidago virgaurea L.	1	n	1	0	Asteraceae Bercht. & J.Presl
Iz_7_1209	DJI_0409	Sorbus aucuparia L.	0	n	1	1	Rosaceae Juss.
Iz_7_1209	DJI_0409	Stachys recta L.	0	n	1	0	Lamiacea Lindl.
Iz_7_1209	DJI_0409	Tanacetum vulgare L.	0	n	1	0	Asteraceae Bercht. & J.Presl
Iz_7_1209	DJI_0409	Thymus pallasianus Heinr.Braun	0	n	1	0	Lamiacea Lindl.
Iz_7_1209	DJI_0409	Tilia cordata Mill.	0	n	1	1	Malvaceae Juss.
Iz_7_1209	DJI_0409	Trifolium alpestre L.	0	n	1	0	Fabaceae Lindl.
Iz_7_1209	DJI_0409	Trifolium arvense L.	0	n	1	0	Fabaceae Lindl.
Iz_7_1209	DJI_0409	Ulmus glabra Huds.	0	n	1	1	Ulmaceae Mirb.
Iz_7_1209	DJI_0409	Verbascum densiflorum Bertol.	0	n	1	0	Scrophulariaceae Juss.
Iz_7_1209	DJI_0409	Veronica austriaca L.	0	n	1	0	Plantaginaceae Juss.
Iz_7_1209	DJI_0409	Veronica incana L.	0	n	1	0	Plantaginaceae Juss.
Iz_7_1209	DJI_0409	Veronica verna L.	0	n	1	0	Plantaginaceae Juss.
Iz_7_1209	DJI_0409	Vincetoxicum hirundinaria Medik	0	n	1	0	Apocynaceae Juss.
Iz_7_1209	DJI_0413	Acer tataricum L.	0	n	1	1	Sapindaceae Juss.
Iz_7_1209	DJI_0413	Achillea millefolium L. s.l.	0	n	1	0	Asteraceae Bercht. & J.Presl
Iz_7_1209	DJI_0413	Allium oleraceum L.	0	n	1	0	Amaryllidaceae J.St.-Hil.
Iz_7_1209	DJI_0413	Ambrosia artemisiifolia L.	0	a	0	0	Asteraceae Bercht. & J.Presl
Iz_7_1209	DJI_0413	Amorpha fruticosa L.	0	a	0	0	Fabaceae Lindl.
Iz_7_1209	DJI_0413	Anthericum ramosum L.	0	n	1	0	Asparagaceae Juss.
Iz_7_1209	DJI_0413	Artemisia marschalliana Spreng.	0	n	1	0	Asteraceae Bercht. & J.Presl
Iz_7_1209	DJI_0413	Asparagus officinalis L.	0	n	1	0	Asparagaceae Juss.
Iz_7_1209	DJI_0413	Astragalus cicer L.	0	n	1	0	Fabaceae Lindl.
Iz_7_1209	DJI_0413	Bassia laniflora (S.G.Gmel.) A.J.Scott	0	n	1	0	Amaranthaceae Juss.
Iz_7_1209	DJI_0413	Berberis vulgaris L.	0	n	1	0	Berberidaceae Juss.
Iz_7_1209	DJI_0413	Betula pendula Roth.	0	n	1	1	Betulaceae Gray
Iz_7_1209	DJI_0413	Betula pendula Roth.	0	n	1	0	Poaceae Barnhart
Iz_7_1209	DJI_0413	Bromus inermis Leyss.	0		NA	NA	
Iz_7_1209	DJI_0413	Bromus squarrosus L.	0	a	0	0	Poaceae Barnhart
Iz_7_1209	DJI_0413	Bromus tectorum L.	0	a	0	0	Poaceae Barnhart
Iz_7_1209	DJI_0413	Calamagrostis epigejos (L.) Roth	0.2	n	1	0	Poaceae Barnhart
Iz_7_1209	DJI_0413	Carduus acanthoides L.	0	n	1	0	Asteraceae Bercht. & J.Presl
Iz_7_1209	DJI_0413	Carex sp.	0	n	1	0	Cyperaceae Juss.
Iz_7_1209	DJI_0413	Centaurea majorovii Dumbadze	0	n	1	0	Asteraceae Bercht. & J.Presl
Iz_7_1209	DJI_0413	Chamaecytisus ruthenicus (Fisch.ex Wol.) Klaskova	0	n	1	0	Fabaceae Lindl.
Iz_7_1209	DJI_0413	Chelidonium majus L.	0	n	1	0	Papaveraceae Juss.
Iz_7_1209	DJI_0413	Chenopodium betaceum Andrz.	0	n	1	0	Amaranthaceae Juss.
Iz_7_1209	DJI_0413	Chenopodium cf. album	0	n	1	0	Amaranthaceae Juss.
Iz_7_1209	DJI_0413	Chenopodium sp.	0		NA	0	Amaranthaceae Juss.
Iz_7_1209	DJI_0413	Chondrilla juncea L.	0	n	1	0	Asteraceae Bercht. & J.Presl
Iz_7_1209	DJI_0413	Cirsium vulgare (Savi) Ten.	0	n	1	0	Asteraceae Bercht. & J.Presl
Iz_7_1209	DJI_0413	Clinopodium acinos (L.) Kuntze	0	n	1	0	Lamiacea Lindl.
Iz_7_1209	DJI_0413	Convallaria majalis L.	0	n	1	0	Asparagaceae Juss.
Iz_7_1209	DJI_0413	Crataegus rhipidophylla Gand.	0	n	1	1	Rosaceae Juss.
Iz_7_1209	DJI_0413	Crepis foetida L.	0	n	1	0	Asteraceae Bercht. & J.Presl
Iz_7_1209	DJI_0413	Dactylis glomerata L.	0	n	1	0	Poaceae Barnhart
Iz_7_1209	DJI_0413	Dianthus campestris M.Bieb.	0	n	1	0	Caryophyllaceae Juss.
Iz_7_1209	DJI_0413	Digitaria sanguinalis (L.) Scop.	0	a	0	0	Poaceae Barnhart
Iz_7_1209	DJI_0413	Elymus repens (L.) Gould	0	n	1	0	Poaceae Barnhart
Iz_7_1209	DJI_0413	Epilobium tetragonum L.	0	n	1	0	Onagraceae Juss.
Iz_7_1209	DJI_0413	Eragrostis minor Host	0	a	0	0	Poaceae Barnhart
Iz_7_1209	DJI_0413	Erigeron canadensis L.	10	a	0	0	Asteraceae Bercht. & J.Presl
Iz_7_1209	DJI_0413	Euphorbia seguieriana Neck.	0	n	1	0	Euphorbiaceae Juss.
Iz_7_1209	DJI_0413	Fallopia dumetorum (L.) Holub	0	n	1	0	Polygonaceae Juss.
Iz_7_1209	DJI_0413	Festuca beckeri (Hack.) Trautv.	0	n	1	0	Poaceae Barnhart
Iz_7_1209	DJI_0413	Fragaria vesca L.	0	n	1	0	Rosaceae Juss.
Iz_7_1209	DJI_0413	Frangula alnus Mill.	0	n	1	1	Rhamnaceae Juss.
Iz_7_1209	DJI_0413	Fraxinus pennsylvanica Marshall	0	a	0	1	Oleaceae Hoffmanns. & Link
Iz_7_1209	DJI_0413	Galeopsis bifida Boenn.	0	n	1	0	Lamiacea Lindl.

Iz_7_1209	DJI_0413	Galium verum L.	0	n	1	0	Rubiaceae Juss.
Iz_7_1209	DJI_0413	Genista tinctoria L.	0	n	1	0	Fabaceae Lindl.
Iz_7_1209	DJI_0413	Geranium robertianum L.	0	n	1	0	Geraniaceae Juss.
Iz_7_1209	DJI_0413	Geranium sanquineum L.	0	n	1	0	Geraniaceae Juss.
Iz_7_1209	DJI_0413	Gypsophila paniculata L.	0	n	1	0	Caryophyllaceae Juss.
Iz_7_1209	DJI_0413	Helichrysum arenarium (L.) Moench	0	n	1	0	Asteraceae Bercht. & J.Presl
Iz_7_1209	DJI_0413	Herniaria polygama J.Gay	0	n	1	0	Caryophyllaceae Juss.
Iz_7_1209	DJI_0413	Hieracium robustum Fr.	0	n	1	0	Asteraceae Bercht. & J.Presl
Iz_7_1209	DJI_0413	Hieracium umbellatum L.	0	n	1	NA	Asteraceae Bercht. & J.Presl
Iz_7_1209	DJI_0413	Hylotelephium maximum (L.) Holub	0	n	1	0	Crassulaceae J.St.-Hil.
Iz_7_1209	DJI_0413	Jacobaea borysthenica (DC.) B.Nord. & Greuter	0	n	1	0	Asteraceae Bercht. & J.Presl
Iz_7_1209	DJI_0413	Jasione montana L.	0	n	1	0	Campanulaceae Juss.
Iz_7_1209	DJI_0413	Jurinea cyanoides (L.) Rchb.s.l.	0	n	1	0	Asteraceae Bercht. & J.Presl
Iz_7_1209	DJI_0413	Koeleria glauca (Spreng.) DC.	0	n	1	0	Poaceae Barnhart
Iz_7_1209	DJI_0413	Lactuca serriola L.	0	a	0	0	Asteraceae Bercht. & J.Presl
Iz_7_1209	DJI_0413	Linaria genistifolia (L.) Mill.	0	n	1	0	Plantaginaceae Juss.
Iz_7_1209	DJI_0413	Lomelosia argentea (L.) Greuter & Burdet	0	n	1	0	Caprifoliaceae Juss.
Iz_7_1209	DJI_0413	Melampyrum pratense L.	0	n	1	0	Orobanchaceae Vent.
Iz_7_1209	DJI_0413	Oenothera biennis L.	0	a	0	0	Onagraceae Juss.
Iz_7_1209	DJI_0413	Pentanema britannica (L.) D.Gut.Larr., Santos-Vicente, Anderb., E.Rico & M.M.Mart.Ort.	0	n	1	0	Asteraceae Bercht. & J.Presl
Iz_7_1209	DJI_0413	Peucedanum oreoselinum (L.) Moench	0	n	1	0	Apiaceae Lindl.
Iz_7_1209	DJI_0413	Peucedanum ruthenicum M.Bieb.	0	n	1	0	Apiaceae Lindl.
Iz_7_1209	DJI_0413	Pilosella echioides (Lumn.) F.W.Schultz & Sch.Bip.	0	n	1	0	Asteraceae Bercht. & J.Presl
Iz_7_1209	DJI_0413	Pilosella officinarum F. Schultz et Sch. Bip.	0	n	1	0	Asteraceae Bercht. & J.Presl
Iz_7_1209	DJI_0413	Pilosella sp.	0		NA	0	Asteraceae Bercht. & J.Presl
Iz_7_1209	DJI_0413	Pinus sylvestris L.	0	n	1	1	Pinaceae Spreng. ex F.Rudolphi
Iz_7_1209	DJI_0413	Poa annua L.	0	n	1	0	Poaceae Barnhart
Iz_7_1209	DJI_0413	Poa nemoralis L.	0	n	1	0	Poaceae Barnhart
Iz_7_1209	DJI_0413	Poa sp.	0		NA	0	Poaceae Barnhart
Iz_7_1209	DJI_0413	Polygonatum odoratum (Mill.) Druce	0	n	1	0	Asparagaceae Juss.
Iz_7_1209	DJI_0413	Polygonum arenarium Waldst. & Kit.	0	n	1	0	Polygonaceae Juss.
Iz_7_1209	DJI_0413	Populus nigra L.	0	n	1	1	Salicaceae Mirb.
Iz_7_1209	DJI_0413	Populus tremula L.	0	n	1	1	Salicaceae Mirb.
Iz_7_1209	DJI_0413	Populus Ã canescens (Aiton) Sm.	0	n	1	1	Salicaceae Mirb.
Iz_7_1209	DJI_0413	Potentilla argentea L.	0	n	1	0	Rosaceae Juss.
Iz_7_1209	DJI_0413	Psammophiliella muralis (L.) Ikonn.	0	n	1	0	Caryophyllaceae Juss.
Iz_7_1209	DJI_0413	Psephellus sumensis (Kalen.) Greuter	0	n	1	0	Asteraceae Bercht. & J.Presl
Iz_7_1209	DJI_0413	Pulsatilla pratensis (L.) Mill. s.l.	0	n	1	0	Ranunculaceae Juss.
Iz_7_1209	DJI_0413	Pyrus communis L.	0	a	0	1	Rosaceae Juss.
Iz_7_1209	DJI_0413	Quercus robur L.	0	n	1	1	Fagaceae Dumort.
Iz_7_1209	DJI_0413	Robinia pseudoacacia L.	0	a	0	1	Fabaceae Lindl.
Iz_7_1209	DJI_0413	Rosa sp.	0		NA	1	Rosaceae Juss.
Iz_7_1209	DJI_0413	Rubus idaeus L.	0	n	1	0	Rosaceae Juss.
Iz_7_1209	DJI_0413	Rumex acetosella L.	0	n	1	0	Polygonaceae Juss.
Iz_7_1209	DJI_0413	Rumex thyrsiflorus Fingerh.	0	n	1	0	Polygonaceae Juss.
Iz_7_1209	DJI_0413	Salix alba L.	0	n	1	1	Salicaceae Mirb.
Iz_7_1209	DJI_0413	Sambucus nigra L.	0	n	1	1	Viburnaceae Raf.
Iz_7_1209	DJI_0413	Secale sylvestre Host	0	n	1	0	Poaceae Barnhart
Iz_7_1209	DJI_0413	Sempervivum ruthenicum Schnittsp. & C.B. Lehm.	0	n	1	0	Crassulaceae J.St.-Hil.
Iz_7_1209	DJI_0413	Setaria viridis (L.) P. Beauv.	0	n	1	0	Poaceae Barnhart
Iz_7_1209	DJI_0413	Silene borysthenica (Gruner) Walters	0	n	1	0	Caryophyllaceae Juss.
Iz_7_1209	DJI_0413	Silene latifolia subsp. alba (Mill.) Greuter & Burdet	0	n	1	0	Caryophyllaceae Juss.
Iz_7_1209	DJI_0413	Solidago virgaurea L.	0	n	1	0	Asteraceae Bercht. & J.Presl
Iz_7_1209	DJI_0413	Sorbus aucuparia L.	0	n	1	1	Rosaceae Juss.
Iz_7_1209	DJI_0413	Stachys recta L.	0	n	1	0	Lamiacea Lindl.
Iz_7_1209	DJI_0413	Tanacetum vulgare L.	0	n	1	0	Asteraceae Bercht. & J.Presl
Iz_7_1209	DJI_0413	Thymus pallasianus Heinr.Braun	0	n	1	0	Lamiacea Lindl.
Iz_7_1209	DJI_0413	Tilia cordata Mill.	0	n	1	1	Malvaceae Juss.
Iz_7_1209	DJI_0413	Trifolium alpestre L.	0	n	1	0	Fabaceae Lindl.
Iz_7_1209	DJI_0413	Trifolium arvense L.	0	n	1	0	Fabaceae Lindl.
Iz_7_1209	DJI_0413	Ulmus glabra Huds.	0	n	1	1	Ulmaceae Mirb.
Iz_7_1209	DJI_0413	Verbascum densiflorum Bertol.	0	n	1	0	Scrophulariaceae Juss.
Iz_7_1209	DJI_0413	Veronica austriaca L.	0	n	1	0	Plantaginaceae Juss.
Iz_7_1209	DJI_0413	Veronica incana L.	0	n	1	0	Plantaginaceae Juss.
Iz_7_1209	DJI_0413	Veronica verna L.	0	n	1	0	Plantaginaceae Juss.
Iz_7_1209	DJI_0413	Vincetoxicum hirundinaria Medik	0	n	1	0	Apocynaceae Juss.
Iz_7_1209	DJI_0416	Acer tataricum L.	0	n	1	1	Sapindaceae Juss.
Iz_7_1209	DJI_0416	Achillea millefolium L. s.l.	0	n	1	0	Asteraceae Bercht. & J.Presl
Iz_7_1209	DJI_0416	Allium oleraceum L.	0	n	1	0	Amaryllidaceae J.St.-Hil.
Iz_7_1209	DJI_0416	Ambrosia artemisiifolia L.	0	a	0	0	Asteraceae Bercht. & J.Presl
Iz_7_1209	DJI_0416	Amorpha fruticosa L.	0	a	0	0	Fabaceae Lindl.
Iz_7_1209	DJI_0416	Anthericum ramosum L.	0	n	1	0	Asparagaceae Juss.
Iz_7_1209	DJI_0416	Artemisia marschalliana Spreng.	0	n	1	0	Asteraceae Bercht. & J.Presl
Iz_7_1209	DJI_0416	Asparagus officinalis L.	0	n	1	0	Asparagaceae Juss.
Iz_7_1209	DJI_0416	Astragalus cicer L.	0	n	1	0	Fabaceae Lindl.
Iz_7_1209	DJI_0416	Bassia laniflora (S.G.Gmel.) A.J.Scott	0	n	1	0	Amaranthaceae Juss.
Iz_7_1209	DJI_0416	Berberis vulgaris L.	0	n	1	0	Berberidaceae Juss.
Iz_7_1209	DJI_0416	Betula pendula Roth.	0	n	1	1	Betulaceae Gray
Iz_7_1209	DJI_0416	Betula pendula Roth.	0	n	1	0	Poaceae Barnhart
Iz_7_1209	DJI_0416	Bromus inermis Leyss.	0		NA	NA	
Iz_7_1209	DJI_0416	Bromus squarrosus L.	0	a	0	0	Poaceae Barnhart
Iz_7_1209	DJI_0416	Bromus tectorum L.	0	a	0	0	Poaceae Barnhart
Iz_7_1209	DJI_0416	Calamagrostis epigejos (L.) Roth	5	n	1	0	Poaceae Barnhart
Iz_7_1209	DJI_0416	Carduus acanthoides L.	0	n	1	0	Asteraceae Bercht. & J.Presl
Iz_7_1209	DJI_0416	Carex sp.	0	n	1	0	Cyperaceae Juss.
Iz_7_1209	DJI_0416	Centaurea majorovii Dumbadze	0	n	1	0	Asteraceae Bercht. & J.Presl
Iz_7_1209	DJI_0416	Chamaecytisus ruthenicus (Fisch.ex Wol.) Klaskova	0	n	1	0	Fabaceae Lindl.
Iz_7_1209	DJI_0416	Chelidonium majus L.	0	n	1	0	Papaveraceae Juss.
Iz_7_1209	DJI_0416	Chenopodium betaceum Andrz.	0	n	1	0	Amaranthaceae Juss.
Iz_7_1209	DJI_0416	Chenopodium cf. album	0	n	1	0	Amaranthaceae Juss.
Iz_7_1209	DJI_0416	Chenopodium sp.	0		NA	0	Amaranthaceae Juss.
Iz_7_1209	DJI_0416	Chondrilla juncea L.	0	n	1	0	Asteraceae Bercht. & J.Presl
Iz_7_1209	DJI_0416	Cirsium vulgare (Savi) Ten.	0	n	1	0	Asteraceae Bercht. & J.Presl
Iz_7_1209	DJI_0416	Clinopodium acinos (L.) Kuntze	0	n	1	0	Lamiacea Lindl.
Iz_7_1209	DJI_0416	Convallaria majalis L.	0	n	1	0	Asparagaceae Juss.
Iz_7_1209	DJI_0416	Crataegus rhipidophylla Gand.	0	n	1	1	Rosaceae Juss.
Iz_7_1209	DJI_0416	Crepis foetida L.	0	n	1	0	Asteraceae Bercht. & J.Presl
Iz_7_1209	DJI_0416	Dactylis glomerata L.	0	n	1	0	Poaceae Barnhart

Iz_7_1209	DJI_0416	Dianthus campestris M.Bieb.	0	n	1	0	Caryophyllaceae Juss.
Iz_7_1209	DJI_0416	Digitaria sanguinalis (L.) Scop.	0	a	0	0	Poaceae Barnhart
Iz_7_1209	DJI_0416	Elymus repens (L.) Gould	0	n	1	0	Poaceae Barnhart
Iz_7_1209	DJI_0416	Epilobium tetragonum L.	0	n	1	0	Onagraceae Juss.
Iz_7_1209	DJI_0416	Eragrostis minor Host	0	a	0	0	Poaceae Barnhart
Iz_7_1209	DJI_0416	Erigeron canadensis L.	0.5	a	0	0	Asteraceae Bercht. & J.Presl
Iz_7_1209	DJI_0416	Euphorbia seguieriana Neck.	0	n	1	0	Euphorbiaceae Juss.
Iz_7_1209	DJI_0416	Fallopia dumetorum (L.) Holub	0	n	1	0	Polygonaceae Juss.
Iz_7_1209	DJI_0416	Festuca beckeri (Hack.) Trautv.	0	n	1	0	Poaceae Barnhart
Iz_7_1209	DJI_0416	Fragaria vesca L.	0	n	1	0	Rosaceae Juss.
Iz_7_1209	DJI_0416	Frangula alnus Mill.	0	n	1	1	Rhamnaceae Juss.
Iz_7_1209	DJI_0416	Fraxinus pennsylvanica Marshall	0	a	0	1	Oleaceae Hoffmanns. & Link
Iz_7_1209	DJI_0416	Galeopsis bifida Boenn.	0	n	1	0	Lamiacea Lindl.
Iz_7_1209	DJI_0416	Galium verum L.	0	n	1	0	Rubiaceae Juss.
Iz_7_1209	DJI_0416	Genista tinctoria L.	0	n	1	0	Fabaceae Lindl.
Iz_7_1209	DJI_0416	Geranium robertianum L.	0	n	1	0	Geraniaceae Juss.
Iz_7_1209	DJI_0416	Geranium sanquineum L.	0	n	1	0	Geraniaceae Juss.
Iz_7_1209	DJI_0416	Gypsophila paniculata L.	0	n	1	0	Caryophyllaceae Juss.
Iz_7_1209	DJI_0416	Helichrysum arenarium (L.) Moench	1.5	n	1	0	Asteraceae Bercht. & J.Presl
Iz_7_1209	DJI_0416	Herniaria polygama J.Gay	0	n	1	0	Caryophyllaceae Juss.
Iz_7_1209	DJI_0416	Hieracium robustum Fr.	0	n	1	0	Asteraceae Bercht. & J.Presl
Iz_7_1209	DJI_0416	Hieracium umbellatum L.	0	n	1	NA	Asteraceae Bercht. & J.Presl
Iz_7_1209	DJI_0416	Hylotelephium maximum (L.) Holub	0	n	1	0	Crassulaceae J.St.-Hil.
Iz_7_1209	DJI_0416	Jacobaea borysthenica (DC.) B.Nord. & Greuter	0	n	1	0	Asteraceae Bercht. & J.Presl
Iz_7_1209	DJI_0416	Jasione montana L.	0	n	1	0	Campanulaceae Juss.
Iz_7_1209	DJI_0416	Jurinea cyanoides (L.) Rchb.s.l.	0	n	1	0	Asteraceae Bercht. & J.Presl
Iz_7_1209	DJI_0416	Koeleria glauca (Spreng.) DC.	0	n	1	0	Poaceae Barnhart
Iz_7_1209	DJI_0416	Lactuca serriola L.	0	a	0	0	Asteraceae Bercht. & J.Presl
Iz_7_1209	DJI_0416	Linaria genistifolia (L.) Mill.	0	n	1	0	Plantaginaceae Juss.
Iz_7_1209	DJI_0416	Lomelosia argentea (L.) Greuter & Burdet	0	n	1	0	Caprifoliaceae Juss.
Iz_7_1209	DJI_0416	Melampyrum pratense L.	0	n	1	0	Orobanchaceae Vent.
Iz_7_1209	DJI_0416	Oenothera biennis L.	0	a	0	0	Onagraceae Juss.
Iz_7_1209	DJI_0416	Pentanema britannica (L.) D.Gut.Larr., Santos-Vicente, Anderb., E.Rico & M.M.Mart.Ort.	0	n	1	0	Asteraceae Bercht. & J.Presl
Iz_7_1209	DJI_0416	Peucedanum oreoselinum (L.) Moench	0	n	1	0	Apiaceae Lindl.
Iz_7_1209	DJI_0416	Peucedanum ruthenicum M.Bieb.	0	n	1	0	Apiaceae Lindl.
Iz_7_1209	DJI_0416	Pilosella echioides (Lumn.) F.W.Schultz & Sch.Bip.	0	n	1	0	Asteraceae Bercht. & J.Presl
Iz_7_1209	DJI_0416	Pilosella officinarum F. Schultz et Sch. Bip.	0	n	1	0	Asteraceae Bercht. & J.Presl
Iz_7_1209	DJI_0416	Pilosella sp.	0		NA	0	Asteraceae Bercht. & J.Presl
Iz_7_1209	DJI_0416	Pinus sylvestris L.	0.5	n	1	1	Pinaceae Spreng. ex F.Rudolphi
Iz_7_1209	DJI_0416	Poa annua L.	0	n	1	0	Poaceae Barnhart
Iz_7_1209	DJI_0416	Poa nemoralis L.	0	n	1	0	Poaceae Barnhart
Iz_7_1209	DJI_0416	Poa sp.	0		NA	0	Poaceae Barnhart
Iz_7_1209	DJI_0416	Polygonatum odoratum (Mill.) Druce	0	n	1	0	Asparagaceae Juss.
Iz_7_1209	DJI_0416	Polygonum arenarium Waldst. & Kit.	0	n	1	0	Polygonaceae Juss.
Iz_7_1209	DJI_0416	Populus nigra L.	0	n	1	1	Salicaceae Mirb.
Iz_7_1209	DJI_0416	Populus tremula L.	0	n	1	1	Salicaceae Mirb.
Iz_7_1209	DJI_0416	Populus Ã canescens (Aiton) Sm.	0	n	1	1	Salicaceae Mirb.
Iz_7_1209	DJI_0416	Potentilla argentea L.	0	n	1	0	Rosaceae Juss.
Iz_7_1209	DJI_0416	Psammophiliella muralis (L.) Ikonn.	0	n	1	0	Caryophyllaceae Juss.
Iz_7_1209	DJI_0416	Psephellus sumensis (Kalen.) Greuter	0	n	1	0	Asteraceae Bercht. & J.Presl
Iz_7_1209	DJI_0416	Pulsatilla pratensis (L.) Mill. s.l.	0	n	1	0	Ranunculaceae Juss.
Iz_7_1209	DJI_0416	Pyrus communis L.	0	a	0	1	Rosaceae Juss.
Iz_7_1209	DJI_0416	Quercus robur L.	0	n	1	1	Fagaceae Dumort.
Iz_7_1209	DJI_0416	Robinia pseudoacacia L.	0	a	0	1	Fabaceae Lindl.
Iz_7_1209	DJI_0416	Rosa sp.	0		NA	1	Rosaceae Juss.
Iz_7_1209	DJI_0416	Rubus idaeus L.	0	n	1	0	Rosaceae Juss.
Iz_7_1209	DJI_0416	Rumex acetosella L.	0	n	1	0	Polygonaceae Juss.
Iz_7_1209	DJI_0416	Rumex thyrsiflorus Fingerh.	0	n	1	0	Polygonaceae Juss.
Iz_7_1209	DJI_0416	Salix alba L.	0	n	1	1	Salicaceae Mirb.
Iz_7_1209	DJI_0416	Sambucus nigra L.	0	n	1	1	Viburnaceae Raf.
Iz_7_1209	DJI_0416	Secale sylvestre Host	0	n	1	0	Poaceae Barnhart
Iz_7_1209	DJI_0416	Sempervivum ruthenicum Schnittsp. & C.B. Lehm.	0	n	1	0	Crassulaceae J.St.-Hil.
Iz_7_1209	DJI_0416	Setaria viridis (L.) P. Beauv.	0	n	1	0	Poaceae Barnhart
Iz_7_1209	DJI_0416	Silene borysthenica (Gruner) Walters	0	n	1	0	Caryophyllaceae Juss.
Iz_7_1209	DJI_0416	Silene latifolia subsp. alba (Mill.) Greuter & Burdet	0	n	1	0	Caryophyllaceae Juss.
Iz_7_1209	DJI_0416	Solidago virgaurea L.	1	n	1	0	Asteraceae Bercht. & J.Presl
Iz_7_1209	DJI_0416	Sorbus aucuparia L.	0	n	1	1	Rosaceae Juss.
Iz_7_1209	DJI_0416	Stachys recta L.	0	n	1	0	Lamiacea Lindl.
Iz_7_1209	DJI_0416	Tanacetum vulgare L.	0	n	1	0	Asteraceae Bercht. & J.Presl
Iz_7_1209	DJI_0416	Thymus pallasianus Heinr.Braun	0	n	1	0	Lamiacea Lindl.
Iz_7_1209	DJI_0416	Tilia cordata Mill.	0	n	1	1	Malvaceae Juss.
Iz_7_1209	DJI_0416	Trifolium alpestre L.	0	n	1	0	Fabaceae Lindl.
Iz_7_1209	DJI_0416	Trifolium arvense L.	0	n	1	0	Fabaceae Lindl.
Iz_7_1209	DJI_0416	Ulmus glabra Huds.	0	n	1	1	Ulmaceae Mirb.
Iz_7_1209	DJI_0416	Verbascum densiflorum Bertol.	0	n	1	0	Scrophulariaceae Juss.
Iz_7_1209	DJI_0416	Veronica austriaca L.	0	n	1	0	Plantaginaceae Juss.
Iz_7_1209	DJI_0416	Veronica incana L.	0	n	1	0	Plantaginaceae Juss.
Iz_7_1209	DJI_0416	Veronica verna L.	0	n	1	0	Plantaginaceae Juss.
Iz_7_1209	DJI_0416	Vincetoxicum hirundinaria Medik	0	n	1	0	Apocynaceae Juss.
Iz_7_1209	DJI_0419	Acer tataricum L.	0	n	1	1	Sapindaceae Juss.
Iz_7_1209	DJI_0419	Achillea millefolium L. s.l.	0	n	1	0	Asteraceae Bercht. & J.Presl
Iz_7_1209	DJI_0419	Allium oleraceum L.	0	n	1	0	Amaryllidaceae J.St.-Hil.
Iz_7_1209	DJI_0419	Ambrosia artemisiifolia L.	0	a	0	0	Asteraceae Bercht. & J.Presl
Iz_7_1209	DJI_0419	Amorpha fruticosa L.	0	a	0	0	Fabaceae Lindl.
Iz_7_1209	DJI_0419	Anthericum ramosum L.	0	n	1	0	Asparagaceae Juss.
Iz_7_1209	DJI_0419	Artemisia marschalliana Spreng.	0	n	1	0	Asteraceae Bercht. & J.Presl
Iz_7_1209	DJI_0419	Asparagus officinalis L.	0	n	1	0	Asparagaceae Juss.
Iz_7_1209	DJI_0419	Astragalus cicer L.	0	n	1	0	Fabaceae Lindl.
Iz_7_1209	DJI_0419	Bassia laniflora (S.G.Gmel.) A.J.Scott	0	n	1	0	Amaranthaceae Juss.
Iz_7_1209	DJI_0419	Berberis vulgaris L.	0	n	1	0	Berberidaceae Juss.
Iz_7_1209	DJI_0419	Betula pendula Roth.	0	n	1	1	Betulaceae Gray
Iz_7_1209	DJI_0419	Betula pendula Roth.	0	n	1	0	Poaceae Barnhart
Iz_7_1209	DJI_0419	Bromus inermis Leyss.	0		NA	NA	
Iz_7_1209	DJI_0419	Bromus squarrosus L.	0	a	0	0	Poaceae Barnhart
Iz_7_1209	DJI_0419	Bromus tectorum L.	0	a	0	0	Poaceae Barnhart
Iz_7_1209	DJI_0419	Calamagrostis epigejos (L.) Roth	10	n	1	0	Poaceae Barnhart
Iz_7_1209	DJI_0419	Carduus acanthoides L.	0	n	1	0	Asteraceae Bercht. & J.Presl
Iz_7_1209	DJI_0419	Carex sp.	0	n	1	0	Cyperaceae Juss.

Iz_7_1209	DJI_0419	Centaurea majorovii Dumbadze	0	n	1	0	Asteraceae Bercht. & J.Presl
Iz_7_1209	DJI_0419	Chamaecytisus ruthenicus (Fisch.ex Wol.) Klaskova	0	n	1	0	Fabaceae Lindl.
Iz_7_1209	DJI_0419	Chelidonium majus L.	0	n	1	0	Papaveraceae Juss.
Iz_7_1209	DJI_0419	Chenopodium betaceum Andrz.	0	n	1	0	Amaranthaceae Juss.
Iz_7_1209	DJI_0419	Chenopodium cf. album	0	n	1	0	Amaranthaceae Juss.
Iz_7_1209	DJI_0419	Chenopodium sp.	0		NA	0	Amaranthaceae Juss.
Iz_7_1209	DJI_0419	Chondrilla juncea L.	0	n	1	0	Asteraceae Bercht. & J.Presl
Iz_7_1209	DJI_0419	Cirsium vulgare (Savi) Ten.	0	n	1	0	Asteraceae Bercht. & J.Presl
Iz_7_1209	DJI_0419	Clinopodium acinos (L.) Kuntze	0	n	1	0	Lamiacea Lindl.
Iz_7_1209	DJI_0419	Convallaria majalis L.	0	n	1	0	Asparagaceae Juss.
Iz_7_1209	DJI_0419	Crataegus rhipidophylla Gand.	0	n	1	1	Rosaceae Juss.
Iz_7_1209	DJI_0419	Crepis foetida L.	0	n	1	0	Asteraceae Bercht. & J.Presl
Iz_7_1209	DJI_0419	Dactylis glomerata L.	0	n	1	0	Poaceae Barnhart
Iz_7_1209	DJI_0419	Dianthus campestris M.Bieb.	0	n	1	0	Caryophyllaceae Juss.
Iz_7_1209	DJI_0419	Digitaria sanguinalis (L.) Scop.	0	a	0	0	Poaceae Barnhart
Iz_7_1209	DJI_0419	Elymus repens (L.) Gould	0	n	1	0	Poaceae Barnhart
Iz_7_1209	DJI_0419	Epilobium tetragonum L.	0	n	1	0	Onagraceae Juss.
Iz_7_1209	DJI_0419	Eragrostis minor Host	0	a	0	0	Poaceae Barnhart
Iz_7_1209	DJI_0419	Erigeron canadensis L.	0.2	a	0	0	Asteraceae Bercht. & J.Presl
Iz_7_1209	DJI_0419	Euphorbia seguieriana Neck.	0	n	1	0	Euphorbiaceae Juss.
Iz_7_1209	DJI_0419	Fallopia dumetorum (L.) Holub	0	n	1	0	Polygonaceae Juss.
Iz_7_1209	DJI_0419	Festuca beckeri (Hack.) Trautv.	0	n	1	0	Poaceae Barnhart
Iz_7_1209	DJI_0419	Fragaria vesca L.	0	n	1	0	Rosaceae Juss.
Iz_7_1209	DJI_0419	Frangula alnus Mill.	0	n	1	1	Rhamnaceae Juss.
Iz_7_1209	DJI_0419	Fraxinus pennsylvanica Marshall	0	a	0	1	Oleaceae Hoffmanns. & Link
Iz_7_1209	DJI_0419	Galeopsis bifida Boenn.	0	n	1	0	Lamiacea Lindl.
Iz_7_1209	DJI_0419	Galium verum L.	0	n	1	0	Rubiaceae Juss.
Iz_7_1209	DJI_0419	Genista tinctoria L.	0	n	1	0	Fabaceae Lindl.
Iz_7_1209	DJI_0419	Geranium robertianum L.	0	n	1	0	Geraniaceae Juss.
Iz_7_1209	DJI_0419	Geranium sanquineum L.	0	n	1	0	Geraniaceae Juss.
Iz_7_1209	DJI_0419	Gypsophila paniculata L.	0	n	1	0	Caryophyllaceae Juss.
Iz_7_1209	DJI_0419	Helichrysum arenarium (L.) Moench	0	n	1	0	Asteraceae Bercht. & J.Presl
Iz_7_1209	DJI_0419	Herniaria polygama J.Gay	0	n	1	0	Caryophyllaceae Juss.
Iz_7_1209	DJI_0419	Hieracium robustum Fr.	0	n	1	0	Asteraceae Bercht. & J.Presl
Iz_7_1209	DJI_0419	Hieracium umbellatum L.	0	n	1	NA	Asteraceae Bercht. & J.Presl
Iz_7_1209	DJI_0419	Hylotelephium maximum (L.) Holub	0	n	1	0	Crassulaceae J.St.-Hil.
Iz_7_1209	DJI_0419	Jacobaea borysthenica (DC.) B.Nord. & Greuter	0	n	1	0	Asteraceae Bercht. & J.Presl
Iz_7_1209	DJI_0419	Jasione montana L.	0	n	1	0	Campanulaceae Juss.
Iz_7_1209	DJI_0419	Jurinea cyanoides (L.) Rchb.s.l.	0	n	1	0	Asteraceae Bercht. & J.Presl
Iz_7_1209	DJI_0419	Koeleria glauca (Spreng.) DC.	0	n	1	0	Poaceae Barnhart
Iz_7_1209	DJI_0419	Lactuca serriola L.	0	a	0	0	Asteraceae Bercht. & J.Presl
Iz_7_1209	DJI_0419	Linaria genistifolia (L.) Mill.	0	n	1	0	Plantaginaceae Juss.
Iz_7_1209	DJI_0419	Lomelosia argentea (L.) Greuter & Burdet	0	n	1	0	Caprifoliaceae Juss.
Iz_7_1209	DJI_0419	Melampyrum pratense L.	0	n	1	0	Orobanchaceae Vent.
Iz_7_1209	DJI_0419	Oenothera biennis L.	0	a	0	0	Onagraceae Juss.
Iz_7_1209	DJI_0419	Pentanema britannica (L.) D.Gut.Larr., Santos-Vicente, Anderb., E.Rico & M.M.Mart.Ort.	0	n	1	0	Asteraceae Bercht. & J.Presl
Iz_7_1209	DJI_0419	Peucedanum oreoselinum (L.) Moench	0	n	1	0	Apiaceae Lindl.
Iz_7_1209	DJI_0419	Peucedanum ruthenicum M.Bieb.	0	n	1	0	Apiaceae Lindl.
Iz_7_1209	DJI_0419	Pilosella echioides (Lumn.) F.W.Schultz & Sch.Bip.	0	n	1	0	Asteraceae Bercht. & J.Presl
Iz_7_1209	DJI_0419	Pilosella officinarum F. Schultz et Sch. Bip.	0	n	1	0	Asteraceae Bercht. & J.Presl
Iz_7_1209	DJI_0419	Pilosella sp.	0		NA	0	Asteraceae Bercht. & J.Presl
Iz_7_1209	DJI_0419	Pinus sylvestris L.	0	n	1	1	Pinaceae Spreng. ex F.Rudolphi
Iz_7_1209	DJI_0419	Poa annua L.	0	n	1	0	Poaceae Barnhart
Iz_7_1209	DJI_0419	Poa nemoralis L.	0	n	1	0	Poaceae Barnhart
Iz_7_1209	DJI_0419	Poa sp.	0		NA	0	Poaceae Barnhart
Iz_7_1209	DJI_0419	Polygonatum odoratum (Mill.) Druce	0	n	1	0	Asparagaceae Juss.
Iz_7_1209	DJI_0419	Polygonum arenarium Waldst. & Kit.	0	n	1	0	Polygonaceae Juss.
Iz_7_1209	DJI_0419	Populus nigra L.	0	n	1	1	Salicaceae Mirb.
Iz_7_1209	DJI_0419	Populus tremula L.	0	n	1	1	Salicaceae Mirb.
Iz_7_1209	DJI_0419	Populus Ã canescens (Aiton) Sm.	0	n	1	1	Salicaceae Mirb.
Iz_7_1209	DJI_0419	Potentilla argentea L.	0	n	1	0	Rosaceae Juss.
Iz_7_1209	DJI_0419	Psammophiliella muralis (L.) Ikonn.	0	n	1	0	Caryophyllaceae Juss.
Iz_7_1209	DJI_0419	Psephellus sumensis (Kalen.) Greuter	0	n	1	0	Asteraceae Bercht. & J.Presl
Iz_7_1209	DJI_0419	Pulsatilla pratensis (L.) Mill. s.l.	0	n	1	0	Ranunculaceae Juss.
Iz_7_1209	DJI_0419	Pyrus communis L.	0	a	0	1	Rosaceae Juss.
Iz_7_1209	DJI_0419	Quercus robur L.	0	n	1	1	Fagaceae Dumort.
Iz_7_1209	DJI_0419	Robinia pseudoacacia L.	0	a	0	1	Fabaceae Lindl.
Iz_7_1209	DJI_0419	Rosa sp.	0		NA	1	Rosaceae Juss.
Iz_7_1209	DJI_0419	Rubus idaeus L.	0	n	1	0	Rosaceae Juss.
Iz_7_1209	DJI_0419	Rumex acetosella L.	0	n	1	0	Polygonaceae Juss.
Iz_7_1209	DJI_0419	Rumex thyrsiflorus Fingerh.	0	n	1	0	Polygonaceae Juss.
Iz_7_1209	DJI_0419	Salix alba L.	0	n	1	1	Salicaceae Mirb.
Iz_7_1209	DJI_0419	Sambucus nigra L.	0	n	1	1	Viburnaceae Raf.
Iz_7_1209	DJI_0419	Secale sylvestre Host	0	n	1	0	Poaceae Barnhart
Iz_7_1209	DJI_0419	Sempervivum ruthenicum Schnittsp. & C.B. Lehm.	0	n	1	0	Crassulaceae J.St.-Hil.
Iz_7_1209	DJI_0419	Setaria viridis (L.) P. Beauv.	0	n	1	0	Poaceae Barnhart
Iz_7_1209	DJI_0419	Silene borysthenica (Gruner) Walters	0	n	1	0	Caryophyllaceae Juss.
Iz_7_1209	DJI_0419	Silene latifolia subsp. alba (Mill.) Greuter & Burdet	0	n	1	0	Caryophyllaceae Juss.
Iz_7_1209	DJI_0419	Solidago virgaurea L.	0	n	1	0	Asteraceae Bercht. & J.Presl
Iz_7_1209	DJI_0419	Sorbus aucuparia L.	0	n	1	1	Rosaceae Juss.
Iz_7_1209	DJI_0419	Stachys recta L.	0	n	1	0	Lamiacea Lindl.
Iz_7_1209	DJI_0419	Tanacetum vulgare L.	0	n	1	0	Asteraceae Bercht. & J.Presl
Iz_7_1209	DJI_0419	Thymus pallasianus Heinr.Braun	0	n	1	0	Lamiacea Lindl.
Iz_7_1209	DJI_0419	Tilia cordata Mill.	0	n	1	1	Malvaceae Juss.
Iz_7_1209	DJI_0419	Trifolium alpestre L.	0	n	1	0	Fabaceae Lindl.
Iz_7_1209	DJI_0419	Trifolium arvense L.	0	n	1	0	Fabaceae Lindl.
Iz_7_1209	DJI_0419	Ulmus glabra Huds.	0	n	1	1	Ulmaceae Mirb.
Iz_7_1209	DJI_0419	Verbascum densiflorum Bertol.	0	n	1	0	Scrophulariaceae Juss.
Iz_7_1209	DJI_0419	Veronica austriaca L.	0	n	1	0	Plantaginaceae Juss.
Iz_7_1209	DJI_0419	Veronica incana L.	0	n	1	0	Plantaginaceae Juss.
Iz_7_1209	DJI_0419	Veronica verna L.	0	n	1	0	Plantaginaceae Juss.
Iz_7_1209	DJI_0419	Vincetoxicum hirundinaria Medik	0	n	1	0	Apocynaceae Juss.
Iz_7_1209	DJI_0424	Acer tataricum L.	0	n	1	1	Sapindaceae Juss.
Iz_7_1209	DJI_0424	Achillea millefolium L. s.l.	0	n	1	0	Asteraceae Bercht. & J.Presl
Iz_7_1209	DJI_0424	Allium oleraceum L.	0	n	1	0	Amaryllidaceae J.St.-Hil.
Iz_7_1209	DJI_0424	Ambrosia artemisiifolia L.	0	a	0	0	Asteraceae Bercht. & J.Presl
Iz_7_1209	DJI_0424	Amorpha fruticosa L.	0	a	0	0	Fabaceae Lindl.
Iz_7_1209	DJI_0424	Anthericum ramosum L.	0	n	1	0	Asparagaceae Juss.

Iz_7_1209	DJI_0424	Artemisia marschalliana Spreng.	0	n	1	0	Asteraceae Bercht. & J.Presl
Iz_7_1209	DJI_0424	Asparagus officinalis L.	0	n	1	0	Asparagaceae Juss.
Iz_7_1209	DJI_0424	Astragalus cicer L.	0	n	1	0	Fabaceae Lindl.
Iz_7_1209	DJI_0424	Bassia laniflora (S.G.Gmel.) A.J.Scott	0	n	1	0	Amaranthaceae Juss.
Iz_7_1209	DJI_0424	Berberis vulgaris L.	0	n	1	0	Berberidaceae Juss.
Iz_7_1209	DJI_0424	Betula pendula Roth.	0	n	1	1	Betulaceae Gray
Iz_7_1209	DJI_0424	Betula pendula Roth.	0	n	1	0	Poaceae Barnhart
Iz_7_1209	DJI_0424	Bromus inermis Leyss.	0		NA	NA	
Iz_7_1209	DJI_0424	Bromus squarrosus L.	0	a	0	0	Poaceae Barnhart
Iz_7_1209	DJI_0424	Bromus tectorum L.	0	a	0	0	Poaceae Barnhart
Iz_7_1209	DJI_0424	Calamagrostis epigejos (L.) Roth	3	n	1	0	Poaceae Barnhart
Iz_7_1209	DJI_0424	Carduus acanthoides L.	0	n	1	0	Asteraceae Bercht. & J.Presl
Iz_7_1209	DJI_0424	Carex sp.	0	n	1	0	Cyperaceae Juss.
Iz_7_1209	DJI_0424	Centaurea majorovii Dumbadze	0	n	1	0	Asteraceae Bercht. & J.Presl
Iz_7_1209	DJI_0424	Chamaecytisus ruthenicus (Fisch.ex Wol.) Klaskova	0	n	1	0	Fabaceae Lindl.
Iz_7_1209	DJI_0424	Chelidonium majus L.	0	n	1	0	Papaveraceae Juss.
Iz_7_1209	DJI_0424	Chenopodium betaceum Andrz.	0	n	1	0	Amaranthaceae Juss.
Iz_7_1209	DJI_0424	Chenopodium cf. album	0	n	1	0	Amaranthaceae Juss.
Iz_7_1209	DJI_0424	Chenopodium sp.	0		NA	0	Amaranthaceae Juss.
Iz_7_1209	DJI_0424	Chondrilla juncea L.	0	n	1	0	Asteraceae Bercht. & J.Presl
Iz_7_1209	DJI_0424	Cirsium vulgare (Savi) Ten.	0	n	1	0	Asteraceae Bercht. & J.Presl
Iz_7_1209	DJI_0424	Clinopodium acinos (L.) Kuntze	0	n	1	0	Lamiacea Lindl.
Iz_7_1209	DJI_0424	Convallaria majalis L.	0	n	1	0	Asparagaceae Juss.
Iz_7_1209	DJI_0424	Crataegus rhipidophylla Gand.	0	n	1	1	Rosaceae Juss.
Iz_7_1209	DJI_0424	Crepis foetida L.	0	n	1	0	Asteraceae Bercht. & J.Presl
Iz_7_1209	DJI_0424	Dactylis glomerata L.	0	n	1	0	Poaceae Barnhart
Iz_7_1209	DJI_0424	Dianthus campestris M.Bieb.	0	n	1	0	Caryophyllaceae Juss.
Iz_7_1209	DJI_0424	Digitaria sanguinalis (L.) Scop.	0	a	0	0	Poaceae Barnhart
Iz_7_1209	DJI_0424	Elymus repens (L.) Gould	0	n	1	0	Poaceae Barnhart
Iz_7_1209	DJI_0424	Epilobium tetragonum L.	0	n	1	0	Onagraceae Juss.
Iz_7_1209	DJI_0424	Eragrostis minor Host	0	a	0	0	Poaceae Barnhart
Iz_7_1209	DJI_0424	Erigeron canadensis L.	3	a	0	0	Asteraceae Bercht. & J.Presl
Iz_7_1209	DJI_0424	Euphorbia seguieriana Neck.	0	n	1	0	Euphorbiaceae Juss.
Iz_7_1209	DJI_0424	Fallopia dumetorum (L.) Holub	0	n	1	0	Polygonaceae Juss.
Iz_7_1209	DJI_0424	Festuca beckeri (Hack.) Trautv.	0	n	1	0	Poaceae Barnhart
Iz_7_1209	DJI_0424	Fragaria vesca L.	0	n	1	0	Rosaceae Juss.
Iz_7_1209	DJI_0424	Frangula alnus Mill.	0	n	1	1	Rhamnaceae Juss.
Iz_7_1209	DJI_0424	Fraxinus pennsylvanica Marshall	0	a	0	1	Oleaceae Hoffmanns. & Link
Iz_7_1209	DJI_0424	Galeopsis bifida Boenn.	0	n	1	0	Lamiacea Lindl.
Iz_7_1209	DJI_0424	Galium verum L.	0	n	1	0	Rubiaceae Juss.
Iz_7_1209	DJI_0424	Genista tinctoria L.	0	n	1	0	Fabaceae Lindl.
Iz_7_1209	DJI_0424	Geranium robertianum L.	0	n	1	0	Geraniaceae Juss.
Iz_7_1209	DJI_0424	Geranium sanquineum L.	0	n	1	0	Geraniaceae Juss.
Iz_7_1209	DJI_0424	Gypsophila paniculata L.	0	n	1	0	Caryophyllaceae Juss.
Iz_7_1209	DJI_0424	Helichrysum arenarium (L.) Moench	0	n	1	0	Asteraceae Bercht. & J.Presl
Iz_7_1209	DJI_0424	Herniaria polygama J.Gay	0	n	1	0	Caryophyllaceae Juss.
Iz_7_1209	DJI_0424	Hieracium robustum Fr.	0	n	1	0	Asteraceae Bercht. & J.Presl
Iz_7_1209	DJI_0424	Hieracium umbellatum L.	0	n	1	NA	Asteraceae Bercht. & J.Presl
Iz_7_1209	DJI_0424	Hylotelephium maximum (L.) Holub	0	n	1	0	Crassulaceae J.St.-Hil.
Iz_7_1209	DJI_0424	Jacobaea borysthenica (DC.) B.Nord. & Greuter	0	n	1	0	Asteraceae Bercht. & J.Presl
Iz_7_1209	DJI_0424	Jasione montana L.	0	n	1	0	Campanulaceae Juss.
Iz_7_1209	DJI_0424	Jurinea cyanoides (L.) Rchb.s.l.	0	n	1	0	Asteraceae Bercht. & J.Presl
Iz_7_1209	DJI_0424	Koeleria glauca (Spreng.) DC.	0	n	1	0	Poaceae Barnhart
Iz_7_1209	DJI_0424	Lactuca serriola L.	0	a	0	0	Asteraceae Bercht. & J.Presl
Iz_7_1209	DJI_0424	Linaria genistifolia (L.) Mill.	0	n	1	0	Plantaginaceae Juss.
Iz_7_1209	DJI_0424	Lomelosia argentea (L.) Greuter & Burdet	0	n	1	0	Caprifoliaceae Juss.
Iz_7_1209	DJI_0424	Melampyrum pratense L.	0	n	1	0	Orobanchaceae Vent.
Iz_7_1209	DJI_0424	Oenothera biennis L.	0	a	0	0	Onagraceae Juss.
Iz_7_1209	DJI_0424	Pentanema britannica (L.) D.Gut.Larr., Santos-Vicente, Anderb., E.Rico & M.M.Mart.Ort.	0	n	1	0	Asteraceae Bercht. & J.Presl
Iz_7_1209	DJI_0424	Peucedanum oreoselinum (L.) Moench	0	n	1	0	Apiaceae Lindl.
Iz_7_1209	DJI_0424	Peucedanum ruthenicum M.Bieb.	0	n	1	0	Apiaceae Lindl.
Iz_7_1209	DJI_0424	Pilosella echioides (Lumn.) F.W.Schultz & Sch.Bip.	0	n	1	0	Asteraceae Bercht. & J.Presl
Iz_7_1209	DJI_0424	Pilosella officinarum F. Schultz et Sch. Bip.	0	n	1	0	Asteraceae Bercht. & J.Presl
Iz_7_1209	DJI_0424	Pilosella sp.	0		NA	0	Asteraceae Bercht. & J.Presl
Iz_7_1209	DJI_0424	Pinus sylvestris L.	0	n	1	1	Pinaceae Spreng. ex F.Rudolphi
Iz_7_1209	DJI_0424	Poa annua L.	0	n	1	0	Poaceae Barnhart
Iz_7_1209	DJI_0424	Poa nemoralis L.	0	n	1	0	Poaceae Barnhart
Iz_7_1209	DJI_0424	Poa sp.	0		NA	0	Poaceae Barnhart
Iz_7_1209	DJI_0424	Polygonatum odoratum (Mill.) Druce	0	n	1	0	Asparagaceae Juss.
Iz_7_1209	DJI_0424	Polygonum arenarium Waldst. & Kit.	0	n	1	0	Polygonaceae Juss.
Iz_7_1209	DJI_0424	Populus nigra L.	0	n	1	1	Salicaceae Mirb.
Iz_7_1209	DJI_0424	Populus tremula L.	0	n	1	1	Salicaceae Mirb.
Iz_7_1209	DJI_0424	Populus Ã canescens (Aiton) Sm.	0	n	1	1	Salicaceae Mirb.
Iz_7_1209	DJI_0424	Potentilla argentea L.	0	n	1	0	Rosaceae Juss.
Iz_7_1209	DJI_0424	Psammophiliella muralis (L.) Ikonn.	0	n	1	0	Caryophyllaceae Juss.
Iz_7_1209	DJI_0424	Psephellus sumensis (Kalen.) Greuter	0	n	1	0	Asteraceae Bercht. & J.Presl
Iz_7_1209	DJI_0424	Pulsatilla pratensis (L.) Mill. s.l.	0	n	1	0	Ranunculaceae Juss.
Iz_7_1209	DJI_0424	Pyrus communis L.	0	a	0	1	Rosaceae Juss.
Iz_7_1209	DJI_0424	Quercus robur L.	0	n	1	1	Fagaceae Dumort.
Iz_7_1209	DJI_0424	Robinia pseudoacacia L.	0	a	0	1	Fabaceae Lindl.
Iz_7_1209	DJI_0424	Rosa sp.	0		NA	1	Rosaceae Juss.
Iz_7_1209	DJI_0424	Rubus idaeus L.	0	n	1	0	Rosaceae Juss.
Iz_7_1209	DJI_0424	Rumex acetosella L.	0	n	1	0	Polygonaceae Juss.
Iz_7_1209	DJI_0424	Rumex thyrsiflorus Fingerh.	0	n	1	0	Polygonaceae Juss.
Iz_7_1209	DJI_0424	Salix alba L.	0	n	1	1	Salicaceae Mirb.
Iz_7_1209	DJI_0424	Sambucus nigra L.	0	n	1	1	Viburnaceae Raf.
Iz_7_1209	DJI_0424	Secale sylvestre Host	0	n	1	0	Poaceae Barnhart
Iz_7_1209	DJI_0424	Sempervivum ruthenicum Schnittsp. & C.B. Lehm.	0	n	1	0	Crassulaceae J.St.-Hil.
Iz_7_1209	DJI_0424	Setaria viridis (L.) P. Beauv.	0	n	1	0	Poaceae Barnhart
Iz_7_1209	DJI_0424	Silene borysthenica (Gruner) Walters	0	n	1	0	Caryophyllaceae Juss.
Iz_7_1209	DJI_0424	Silene latifolia subsp. alba (Mill.) Greuter & Burdet	0	n	1	0	Caryophyllaceae Juss.
Iz_7_1209	DJI_0424	Solidago virgaurea L.	0	n	1	0	Asteraceae Bercht. & J.Presl
Iz_7_1209	DJI_0424	Sorbus aucuparia L.	0	n	1	1	Rosaceae Juss.
Iz_7_1209	DJI_0424	Stachys recta L.	0	n	1	0	Lamiacea Lindl.
Iz_7_1209	DJI_0424	Tanacetum vulgare L.	0	n	1	0	Asteraceae Bercht. & J.Presl
Iz_7_1209	DJI_0424	Thymus pallasianus Heinr.Braun	0	n	1	0	Lamiacea Lindl.
Iz_7_1209	DJI_0424	Tilia cordata Mill.	0	n	1	1	Malvaceae Juss.
Iz_7_1209	DJI_0424	Trifolium alpestre L.	0	n	1	0	Fabaceae Lindl.

Iz_7_1209	DJI_0424	Trifolium arvense L.	0	n	1	0	Fabaceae Lindl.
Iz_7_1209	DJI_0424	Ulmus glabra Huds.	0	n	1	1	Ulmaceae Mirb.
Iz_7_1209	DJI_0424	Verbascum densiflorum Bertol.	0	n	1	0	Scrophulariaceae Juss.
Iz_7_1209	DJI_0424	Veronica austriaca L.	0	n	1	0	Plantaginaceae Juss.
Iz_7_1209	DJI_0424	Veronica incana L.	0	n	1	0	Plantaginaceae Juss.
Iz_7_1209	DJI_0424	Veronica verna L.	0	n	1	0	Plantaginaceae Juss.
Iz_7_1209	DJI_0424	Vincetoxicum hirundinaria Medik	0	n	1	0	Apocynaceae Juss.
Iz_7_1209	DJI_0427	Acer tataricum L.	0	n	1	1	Sapindaceae Juss.
Iz_7_1209	DJI_0427	Achillea millefolium L. s.l.	0	n	1	0	Asteraceae Bercht. & J.Presl
Iz_7_1209	DJI_0427	Allium oleraceum L.	0	n	1	0	Amaryllidaceae J.St.-Hil.
Iz_7_1209	DJI_0427	Ambrosia artemisiifolia L.	0	a	0	0	Asteraceae Bercht. & J.Presl
Iz_7_1209	DJI_0427	Amorpha fruticosa L.	0	a	0	0	Fabaceae Lindl.
Iz_7_1209	DJI_0427	Anthericum ramosum L.	0	n	1	0	Asparagaceae Juss.
Iz_7_1209	DJI_0427	Artemisia marschalliana Spreng.	0	n	1	0	Asteraceae Bercht. & J.Presl
Iz_7_1209	DJI_0427	Asparagus officinalis L.	0	n	1	0	Asparagaceae Juss.
Iz_7_1209	DJI_0427	Astragalus cicer L.	0	n	1	0	Fabaceae Lindl.
Iz_7_1209	DJI_0427	Bassia laniflora (S.G.Gmel.) A.J.Scott	0	n	1	0	Amaranthaceae Juss.
Iz_7_1209	DJI_0427	Berberis vulgaris L.	0	n	1	0	Berberidaceae Juss.
Iz_7_1209	DJI_0427	Betula pendula Roth.	0	n	1	1	Betulaceae Gray
Iz_7_1209	DJI_0427	Betula pendula Roth.	0	n	1	0	Poaceae Barnhart
Iz_7_1209	DJI_0427	Bromus inermis Leyss.	0		NA	NA	
Iz_7_1209	DJI_0427	Bromus squarrosus L.	0	a	0	0	Poaceae Barnhart
Iz_7_1209	DJI_0427	Bromus tectorum L.	0	a	0	0	Poaceae Barnhart
Iz_7_1209	DJI_0427	Calamagrostis epigejos (L.) Roth	3	n	1	0	Poaceae Barnhart
Iz_7_1209	DJI_0427	Carduus acanthoides L.	0	n	1	0	Asteraceae Bercht. & J.Presl
Iz_7_1209	DJI_0427	Carex sp.	0	n	1	0	Cyperaceae Juss.
Iz_7_1209	DJI_0427	Centaurea majorovii Dumbadze	0	n	1	0	Asteraceae Bercht. & J.Presl
Iz_7_1209	DJI_0427	Chamaecytisus ruthenicus (Fisch.ex Wol.) Klaskova	0	n	1	0	Fabaceae Lindl.
Iz_7_1209	DJI_0427	Chelidonium majus L.	0	n	1	0	Papaveraceae Juss.
Iz_7_1209	DJI_0427	Chenopodium betaceum Andrz.	0	n	1	0	Amaranthaceae Juss.
Iz_7_1209	DJI_0427	Chenopodium cf. album	0	n	1	0	Amaranthaceae Juss.
Iz_7_1209	DJI_0427	Chenopodium sp.	0		NA	0	Amaranthaceae Juss.
Iz_7_1209	DJI_0427	Chondrilla juncea L.	0	n	1	0	Asteraceae Bercht. & J.Presl
Iz_7_1209	DJI_0427	Cirsium vulgare (Savi) Ten.	0	n	1	0	Asteraceae Bercht. & J.Presl
Iz_7_1209	DJI_0427	Clinopodium acinos (L.) Kuntze	0	n	1	0	Lamiacea Lindl.
Iz_7_1209	DJI_0427	Convallaria majalis L.	0	n	1	0	Asparagaceae Juss.
Iz_7_1209	DJI_0427	Crataegus rhipidophylla Gand.	0	n	1	1	Rosaceae Juss.
Iz_7_1209	DJI_0427	Crepis foetida L.	0	n	1	0	Asteraceae Bercht. & J.Presl
Iz_7_1209	DJI_0427	Dactylis glomerata L.	0	n	1	0	Poaceae Barnhart
Iz_7_1209	DJI_0427	Dianthus campestris M.Bieb.	0	n	1	0	Caryophyllaceae Juss.
Iz_7_1209	DJI_0427	Digitaria sanguinalis (L.) Scop.	0	a	0	0	Poaceae Barnhart
Iz_7_1209	DJI_0427	Elymus repens (L.) Gould	0	n	1	0	Poaceae Barnhart
Iz_7_1209	DJI_0427	Epilobium tetragonum L.	0	n	1	0	Onagraceae Juss.
Iz_7_1209	DJI_0427	Eragrostis minor Host	0	a	0	0	Poaceae Barnhart
Iz_7_1209	DJI_0427	Erigeron canadensis L.	1	a	0	0	Asteraceae Bercht. & J.Presl
Iz_7_1209	DJI_0427	Euphorbia seguieriana Neck.	0	n	1	0	Euphorbiaceae Juss.
Iz_7_1209	DJI_0427	Fallopia dumetorum (L.) Holub	0	n	1	0	Polygonaceae Juss.
Iz_7_1209	DJI_0427	Festuca beckeri (Hack.) Trautv.	0	n	1	0	Poaceae Barnhart
Iz_7_1209	DJI_0427	Fragaria vesca L.	0	n	1	0	Rosaceae Juss.
Iz_7_1209	DJI_0427	Frangula alnus Mill.	0	n	1	1	Rhamnaceae Juss.
Iz_7_1209	DJI_0427	Fraxinus pennsylvanica Marshall	0	a	0	1	Oleaceae Hoffmanns. & Link
Iz_7_1209	DJI_0427	Galeopsis bifida Boenn.	0	n	1	0	Lamiacea Lindl.
Iz_7_1209	DJI_0427	Galium verum L.	0	n	1	0	Rubiaceae Juss.
Iz_7_1209	DJI_0427	Genista tinctoria L.	0	n	1	0	Fabaceae Lindl.
Iz_7_1209	DJI_0427	Geranium robertianum L.	0	n	1	0	Geraniaceae Juss.
Iz_7_1209	DJI_0427	Geranium sanquineum L.	0	n	1	0	Geraniaceae Juss.
Iz_7_1209	DJI_0427	Gypsophila paniculata L.	0	n	1	0	Caryophyllaceae Juss.
Iz_7_1209	DJI_0427	Helichrysum arenarium (L.) Moench	0	n	1	0	Asteraceae Bercht. & J.Presl
Iz_7_1209	DJI_0427	Herniaria polygama J.Gay	0	n	1	0	Caryophyllaceae Juss.
Iz_7_1209	DJI_0427	Hieracium robustum Fr.	0	n	1	0	Asteraceae Bercht. & J.Presl
Iz_7_1209	DJI_0427	Hieracium umbellatum L.	0	n	1	NA	Asteraceae Bercht. & J.Presl
Iz_7_1209	DJI_0427	Hylotelephium maximum (L.) Holub	0	n	1	0	Crassulaceae J.St.-Hil.
Iz_7_1209	DJI_0427	Jacobaea borysthenica (DC.) B.Nord. & Greuter	0	n	1	0	Asteraceae Bercht. & J.Presl
Iz_7_1209	DJI_0427	Jasione montana L.	0	n	1	0	Campanulaceae Juss.
Iz_7_1209	DJI_0427	Jurinea cyanoides (L.) Rchb.s.l.	0	n	1	0	Asteraceae Bercht. & J.Presl
Iz_7_1209	DJI_0427	Koeleria glauca (Spreng.) DC.	0	n	1	0	Poaceae Barnhart
Iz_7_1209	DJI_0427	Lactuca serriola L.	0	a	0	0	Asteraceae Bercht. & J.Presl
Iz_7_1209	DJI_0427	Linaria genistifolia (L.) Mill.	0	n	1	0	Plantaginaceae Juss.
Iz_7_1209	DJI_0427	Lomelosia argentea (L.) Greuter & Burdet	0	n	1	0	Caprifoliaceae Juss.
Iz_7_1209	DJI_0427	Melampyrum pratense L.	0	n	1	0	Orobanchaceae Vent.
Iz_7_1209	DJI_0427	Oenothera biennis L.	0	a	0	0	Onagraceae Juss.
Iz_7_1209	DJI_0427	Pentanema britannica (L.) D.Gut.Larr., Santos-Vicente, Anderb., E.Rico & M.M.Mart.Ort.	0	n	1	0	Asteraceae Bercht. & J.Presl
Iz_7_1209	DJI_0427	Peucedanum oreoselinum (L.) Moench	0	n	1	0	Apiaceae Lindl.
Iz_7_1209	DJI_0427	Peucedanum ruthenicum M.Bieb.	0	n	1	0	Apiaceae Lindl.
Iz_7_1209	DJI_0427	Pilosella echioides (Lumn.) F.W.Schultz & Sch.Bip.	0	n	1	0	Asteraceae Bercht. & J.Presl
Iz_7_1209	DJI_0427	Pilosella officinarum F. Schultz et Sch. Bip.	0	n	1	0	Asteraceae Bercht. & J.Presl
Iz_7_1209	DJI_0427	Pilosella sp.	0		NA	0	Asteraceae Bercht. & J.Presl
Iz_7_1209	DJI_0427	Pinus sylvestris L.	0	n	1	1	Pinaceae Spreng. ex F.Rudolphi
Iz_7_1209	DJI_0427	Poa annua L.	0	n	1	0	Poaceae Barnhart
Iz_7_1209	DJI_0427	Poa nemoralis L.	0	n	1	0	Poaceae Barnhart
Iz_7_1209	DJI_0427	Poa sp.	0		NA	0	Poaceae Barnhart
Iz_7_1209	DJI_0427	Polygonatum odoratum (Mill.) Druce	0	n	1	0	Asparagaceae Juss.
Iz_7_1209	DJI_0427	Polygonum arenarium Waldst. & Kit.	0	n	1	0	Polygonaceae Juss.
Iz_7_1209	DJI_0427	Populus nigra L.	0	n	1	1	Salicaceae Mirb.
Iz_7_1209	DJI_0427	Populus tremula L.	0	n	1	1	Salicaceae Mirb.
Iz_7_1209	DJI_0427	Populus Ã canescens (Aiton) Sm.	0	n	1	1	Salicaceae Mirb.
Iz_7_1209	DJI_0427	Potentilla argentea L.	0	n	1	0	Rosaceae Juss.
Iz_7_1209	DJI_0427	Psammophiliella muralis (L.) Ikonn.	0	n	1	0	Caryophyllaceae Juss.
Iz_7_1209	DJI_0427	Psephellus sumensis (Kalen.) Greuter	0	n	1	0	Asteraceae Bercht. & J.Presl
Iz_7_1209	DJI_0427	Pulsatilla pratensis (L.) Mill. s.l.	0	n	1	0	Ranunculaceae Juss.
Iz_7_1209	DJI_0427	Pyrus communis L.	0	a	0	1	Rosaceae Juss.
Iz_7_1209	DJI_0427	Quercus robur L.	0	n	1	1	Fagaceae Dumort.
Iz_7_1209	DJI_0427	Robinia pseudoacacia L.	0	a	0	1	Fabaceae Lindl.
Iz_7_1209	DJI_0427	Rosa sp.	0		NA	1	Rosaceae Juss.
Iz_7_1209	DJI_0427	Rubus idaeus L.	0	n	1	0	Rosaceae Juss.
Iz_7_1209	DJI_0427	Rumex acetosella L.	0	n	1	0	Polygonaceae Juss.
Iz_7_1209	DJI_0427	Rumex thyrsiflorus Fingerh.	0	n	1	0	Polygonaceae Juss.
Iz_7_1209	DJI_0427	Salix alba L.	0	n	1	1	Salicaceae Mirb.

Iz_7_1209	DJI_0427	Sambucus nigra L.	0	n	1	1	Viburnaceae Raf.
Iz_7_1209	DJI_0427	Secale sylvestre Host	0	n	1	0	Poaceae Barnhart
Iz_7_1209	DJI_0427	Sempervivum ruthenicum Schnittsp. & C.B. Lehm.	0	n	1	0	Crassulaceae J.St.-Hil.
Iz_7_1209	DJI_0427	Setaria viridis (L.) P. Beauv.	0	n	1	0	Poaceae Barnhart
Iz_7_1209	DJI_0427	Silene borysthenica (Gruner) Walters	0	n	1	0	Caryophyllaceae Juss.
Iz_7_1209	DJI_0427	Silene latifolia subsp. alba (Mill.) Greuter & Burdet	0	n	1	0	Caryophyllaceae Juss.
Iz_7_1209	DJI_0427	Solidago virgaurea L.	0.3	n	1	0	Asteraceae Bercht. & J.Presl
Iz_7_1209	DJI_0427	Sorbus aucuparia L.	0	n	1	1	Rosaceae Juss.
Iz_7_1209	DJI_0427	Stachys recta L.	0	n	1	0	Lamiacea Lindl.
Iz_7_1209	DJI_0427	Tanacetum vulgare L.	0	n	1	0	Asteraceae Bercht. & J.Presl
Iz_7_1209	DJI_0427	Thymus pallasianus Heinr.Braun	0	n	1	0	Lamiacea Lindl.
Iz_7_1209	DJI_0427	Tilia cordata Mill.	0	n	1	1	Malvaceae Juss.
Iz_7_1209	DJI_0427	Trifolium alpestre L.	0	n	1	0	Fabaceae Lindl.
Iz_7_1209	DJI_0427	Trifolium arvense L.	0	n	1	0	Fabaceae Lindl.
Iz_7_1209	DJI_0427	Ulmus glabra Huds.	0	n	1	1	Ulmaceae Mirb.
Iz_7_1209	DJI_0427	Verbascum densiflorum Bertol.	0	n	1	0	Scrophulariaceae Juss.
Iz_7_1209	DJI_0427	Veronica austriaca L.	0	n	1	0	Plantaginaceae Juss.
Iz_7_1209	DJI_0427	Veronica incana L.	0	n	1	0	Plantaginaceae Juss.
Iz_7_1209	DJI_0427	Veronica verna L.	0	n	1	0	Plantaginaceae Juss.
Iz_7_1209	DJI_0427	Vincetoxicum hirundinaria Medik	0	n	1	0	Apocynaceae Juss.
Iz_7_1209	DJI_0431	Acer tataricum L.	0	n	1	1	Sapindaceae Juss.
Iz_7_1209	DJI_0431	Achillea millefolium L. s.l.	0	n	1	0	Asteraceae Bercht. & J.Presl
Iz_7_1209	DJI_0431	Allium oleraceum L.	0	n	1	0	Amaryllidaceae J.St.-Hil.
Iz_7_1209	DJI_0431	Ambrosia artemisiifolia L.	0	a	0	0	Asteraceae Bercht. & J.Presl
Iz_7_1209	DJI_0431	Amorpha fruticosa L.	0	a	0	0	Fabaceae Lindl.
Iz_7_1209	DJI_0431	Anthericum ramosum L.	0	n	1	0	Asparagaceae Juss.
Iz_7_1209	DJI_0431	Artemisia marschalliana Spreng.	0	n	1	0	Asteraceae Bercht. & J.Presl
Iz_7_1209	DJI_0431	Asparagus officinalis L.	0	n	1	0	Asparagaceae Juss.
Iz_7_1209	DJI_0431	Astragalus cicer L.	0	n	1	0	Fabaceae Lindl.
Iz_7_1209	DJI_0431	Bassia laniflora (S.G.Gmel.) A.J.Scott	0	n	1	0	Amaranthaceae Juss.
Iz_7_1209	DJI_0431	Berberis vulgaris L.	0	n	1	0	Berberidaceae Juss.
Iz_7_1209	DJI_0431	Betula pendula Roth.	0	n	1	1	Betulaceae Gray
Iz_7_1209	DJI_0431	Betula pendula Roth.	0	n	1	0	Poaceae Barnhart
Iz_7_1209	DJI_0431	Bromus inermis Leyss.	0		NA	NA	
Iz_7_1209	DJI_0431	Bromus squarrosus L.	0	a	0	0	Poaceae Barnhart
Iz_7_1209	DJI_0431	Bromus tectorum L.	0	a	0	0	Poaceae Barnhart
Iz_7_1209	DJI_0431	Calamagrostis epigejos (L.) Roth	8	n	1	0	Poaceae Barnhart
Iz_7_1209	DJI_0431	Carduus acanthoides L.	0	n	1	0	Asteraceae Bercht. & J.Presl
Iz_7_1209	DJI_0431	Carex sp.	0	n	1	0	Cyperaceae Juss.
Iz_7_1209	DJI_0431	Centaurea majorovii Dumbadze	0	n	1	0	Asteraceae Bercht. & J.Presl
Iz_7_1209	DJI_0431	Chamaecytisus ruthenicus (Fisch.ex Wol.) Klaskova	1	n	1	0	Fabaceae Lindl.
Iz_7_1209	DJI_0431	Chelidonium majus L.	0	n	1	0	Papaveraceae Juss.
Iz_7_1209	DJI_0431	Chenopodium betaceum Andrz.	0	n	1	0	Amaranthaceae Juss.
Iz_7_1209	DJI_0431	Chenopodium cf. album	0	n	1	0	Amaranthaceae Juss.
Iz_7_1209	DJI_0431	Chenopodium sp.	0		NA	0	Amaranthaceae Juss.
Iz_7_1209	DJI_0431	Chondrilla juncea L.	0	n	1	0	Asteraceae Bercht. & J.Presl
Iz_7_1209	DJI_0431	Cirsium vulgare (Savi) Ten.	0	n	1	0	Asteraceae Bercht. & J.Presl
Iz_7_1209	DJI_0431	Clinopodium acinos (L.) Kuntze	0	n	1	0	Lamiacea Lindl.
Iz_7_1209	DJI_0431	Convallaria majalis L.	0	n	1	0	Asparagaceae Juss.
Iz_7_1209	DJI_0431	Crataegus rhipidophylla Gand.	0	n	1	1	Rosaceae Juss.
Iz_7_1209	DJI_0431	Crepis foetida L.	0	n	1	0	Asteraceae Bercht. & J.Presl
Iz_7_1209	DJI_0431	Dactylis glomerata L.	0	n	1	0	Poaceae Barnhart
Iz_7_1209	DJI_0431	Dianthus campestris M.Bieb.	0	n	1	0	Caryophyllaceae Juss.
Iz_7_1209	DJI_0431	Digitaria sanguinalis (L.) Scop.	0	a	0	0	Poaceae Barnhart
Iz_7_1209	DJI_0431	Elymus repens (L.) Gould	0	n	1	0	Poaceae Barnhart
Iz_7_1209	DJI_0431	Epilobium tetragonum L.	0	n	1	0	Onagraceae Juss.
Iz_7_1209	DJI_0431	Eragrostis minor Host	0	a	0	0	Poaceae Barnhart
Iz_7_1209	DJI_0431	Erigeron canadensis L.	6	a	0	0	Asteraceae Bercht. & J.Presl
Iz_7_1209	DJI_0431	Euphorbia seguieriana Neck.	0	n	1	0	Euphorbiaceae Juss.
Iz_7_1209	DJI_0431	Fallopia dumetorum (L.) Holub	0	n	1	0	Polygonaceae Juss.
Iz_7_1209	DJI_0431	Festuca beckeri (Hack.) Trautv.	0	n	1	0	Poaceae Barnhart
Iz_7_1209	DJI_0431	Fragaria vesca L.	0	n	1	0	Rosaceae Juss.
Iz_7_1209	DJI_0431	Frangula alnus Mill.	0	n	1	1	Rhamnaceae Juss.
Iz_7_1209	DJI_0431	Fraxinus pennsylvanica Marshall	0	a	0	1	Oleaceae Hoffmanns. & Link
Iz_7_1209	DJI_0431	Galeopsis bifida Boenn.	0	n	1	0	Lamiacea Lindl.
Iz_7_1209	DJI_0431	Galium verum L.	0	n	1	0	Rubiaceae Juss.
Iz_7_1209	DJI_0431	Genista tinctoria L.	0	n	1	0	Fabaceae Lindl.
Iz_7_1209	DJI_0431	Geranium robertianum L.	0	n	1	0	Geraniaceae Juss.
Iz_7_1209	DJI_0431	Geranium sanquineum L.	0	n	1	0	Geraniaceae Juss.
Iz_7_1209	DJI_0431	Gypsophila paniculata L.	0	n	1	0	Caryophyllaceae Juss.
Iz_7_1209	DJI_0431	Helichrysum arenarium (L.) Moench	0	n	1	0	Asteraceae Bercht. & J.Presl
Iz_7_1209	DJI_0431	Herniaria polygama J.Gay	0	n	1	0	Caryophyllaceae Juss.
Iz_7_1209	DJI_0431	Hieracium robustum Fr.	0	n	1	0	Asteraceae Bercht. & J.Presl
Iz_7_1209	DJI_0431	Hieracium umbellatum L.	0	n	1	NA	Asteraceae Bercht. & J.Presl
Iz_7_1209	DJI_0431	Hylotelephium maximum (L.) Holub	0	n	1	0	Crassulaceae J.St.-Hil.
Iz_7_1209	DJI_0431	Jacobaea borysthenica (DC.) B.Nord. & Greuter	0	n	1	0	Asteraceae Bercht. & J.Presl
Iz_7_1209	DJI_0431	Jasione montana L.	0	n	1	0	Campanulaceae Juss.
Iz_7_1209	DJI_0431	Jurinea cyanoides (L.) Rchb.s.l.	0	n	1	0	Asteraceae Bercht. & J.Presl
Iz_7_1209	DJI_0431	Koeleria glauca (Spreng.) DC.	0	n	1	0	Poaceae Barnhart
Iz_7_1209	DJI_0431	Lactuca serriola L.	0	a	0	0	Asteraceae Bercht. & J.Presl
Iz_7_1209	DJI_0431	Linaria genistifolia (L.) Mill.	0	n	1	0	Plantaginaceae Juss.
Iz_7_1209	DJI_0431	Lomelosia argentea (L.) Greuter & Burdet	0	n	1	0	Caprifoliaceae Juss.
Iz_7_1209	DJI_0431	Melampyrum pratense L.	0	n	1	0	Orobanchaceae Vent.
Iz_7_1209	DJI_0431	Oenothera biennis L.	0	a	0	0	Onagraceae Juss.
Iz_7_1209	DJI_0431	Pentanema britannica (L.) D.Gut.Larr., Santos-Vicente, Anderb., E.Rico & M.M.Mart.Ort.	0	n	1	0	Asteraceae Bercht. & J.Presl
Iz_7_1209	DJI_0431	Peucedanum oreoselinum (L.) Moench	0	n	1	0	Apiaceae Lindl.
Iz_7_1209	DJI_0431	Peucedanum ruthenicum M.Bieb.	0	n	1	0	Apiaceae Lindl.
Iz_7_1209	DJI_0431	Pilosella echioides (Lumn.) F.W.Schultz & Sch.Bip.	0	n	1	0	Asteraceae Bercht. & J.Presl
Iz_7_1209	DJI_0431	Pilosella officinarum F. Schultz et Sch. Bip.	0	n	1	0	Asteraceae Bercht. & J.Presl
Iz_7_1209	DJI_0431	Pilosella sp.	0		NA	0	Asteraceae Bercht. & J.Presl
Iz_7_1209	DJI_0431	Pinus sylvestris L.	0	n	1	1	Pinaceae Spreng. ex F.Rudolphi
Iz_7_1209	DJI_0431	Poa annua L.	0	n	1	0	Poaceae Barnhart
Iz_7_1209	DJI_0431	Poa nemoralis L.	0	n	1	0	Poaceae Barnhart
Iz_7_1209	DJI_0431	Poa sp.	0		NA	0	Poaceae Barnhart
Iz_7_1209	DJI_0431	Polygonatum odoratum (Mill.) Druce	0	n	1	0	Asparagaceae Juss.
Iz_7_1209	DJI_0431	Polygonum arenarium Waldst. & Kit.	0	n	1	0	Polygonaceae Juss.
Iz_7_1209	DJI_0431	Populus nigra L.	0	n	1	1	Salicaceae Mirb.
Iz_7_1209	DJI_0431	Populus tremula L.	2	n	1	1	Salicaceae Mirb.

Iz_7_1209	DJI_0431	Populus Ã canescens (Aiton) Sm.	0	n	1	1	Salicaceae Mirb.
Iz_7_1209	DJI_0431	Potentilla argentea L.	0	n	1	0	Rosaceae Juss.
Iz_7_1209	DJI_0431	Psammophiliella muralis (L.) Ikonn.	0	n	1	0	Caryophyllaceae Juss.
Iz_7_1209	DJI_0431	Psephellus sumensis (Kalen.) Greuter	0	n	1	0	Asteraceae Bercht. & J.Presl
Iz_7_1209	DJI_0431	Pulsatilla pratensis (L.) Mill. s.l.	0	n	1	0	Ranunculaceae Juss.
Iz_7_1209	DJI_0431	Pyrus communis L.	0	a	0	1	Rosaceae Juss.
Iz_7_1209	DJI_0431	Quercus robur L.	0	n	1	1	Fagaceae Dumort.
Iz_7_1209	DJI_0431	Robinia pseudoacacia L.	0	a	0	1	Fabaceae Lindl.
Iz_7_1209	DJI_0431	Rosa sp.	0		NA	1	Rosaceae Juss.
Iz_7_1209	DJI_0431	Rubus idaeus L.	0	n	1	0	Rosaceae Juss.
Iz_7_1209	DJI_0431	Rumex acetosella L.	0	n	1	0	Polygonaceae Juss.
Iz_7_1209	DJI_0431	Rumex thyrsiflorus Fingerh.	0	n	1	0	Polygonaceae Juss.
Iz_7_1209	DJI_0431	Salix alba L.	0	n	1	1	Salicaceae Mirb.
Iz_7_1209	DJI_0431	Sambucus nigra L.	0	n	1	1	Viburnaceae Raf.
Iz_7_1209	DJI_0431	Secale sylvestre Host	0	n	1	0	Poaceae Barnhart
Iz_7_1209	DJI_0431	Sempervivum ruthenicum Schnittsp. & C.B. Lehm.	0	n	1	0	Crassulaceae J.St.-Hil.
Iz_7_1209	DJI_0431	Setaria viridis (L.) P. Beauv.	0	n	1	0	Poaceae Barnhart
Iz_7_1209	DJI_0431	Silene borysthenica (Gruner) Walters	0	n	1	0	Caryophyllaceae Juss.
Iz_7_1209	DJI_0431	Silene latifolia subsp. alba (Mill.) Greuter & Burdet	0	n	1	0	Caryophyllaceae Juss.
Iz_7_1209	DJI_0431	Solidago virgaurea L.	0	n	1	0	Asteraceae Bercht. & J.Presl
Iz_7_1209	DJI_0431	Sorbus aucuparia L.	0	n	1	1	Rosaceae Juss.
Iz_7_1209	DJI_0431	Stachys recta L.	0	n	1	0	Lamiacea Lindl.
Iz_7_1209	DJI_0431	Tanacetum vulgare L.	0	n	1	0	Asteraceae Bercht. & J.Presl
Iz_7_1209	DJI_0431	Thymus pallasianus Heinr.Braun	0	n	1	0	Lamiacea Lindl.
Iz_7_1209	DJI_0431	Tilia cordata Mill.	0	n	1	1	Malvaceae Juss.
Iz_7_1209	DJI_0431	Trifolium alpestre L.	0	n	1	0	Fabaceae Lindl.
Iz_7_1209	DJI_0431	Trifolium arvense L.	0	n	1	0	Fabaceae Lindl.
Iz_7_1209	DJI_0431	Ulmus glabra Huds.	0	n	1	1	Ulmaceae Mirb.
Iz_7_1209	DJI_0431	Verbascum densiflorum Bertol.	0	n	1	0	Scrophulariaceae Juss.
Iz_7_1209	DJI_0431	Veronica austriaca L.	0	n	1	0	Plantaginaceae Juss.
Iz_7_1209	DJI_0431	Veronica incana L.	0	n	1	0	Plantaginaceae Juss.
Iz_7_1209	DJI_0431	Veronica verna L.	0	n	1	0	Plantaginaceae Juss.
Iz_7_1209	DJI_0431	Vincetoxicum hirundinaria Medik	0	n	1	0	Apocynaceae Juss.
Iz_7_1209	DJI_0439	Acer tataricum L.	0	n	1	1	Sapindaceae Juss.
Iz_7_1209	DJI_0439	Achillea millefolium L. s.l.	0	n	1	0	Asteraceae Bercht. & J.Presl
Iz_7_1209	DJI_0439	Allium oleraceum L.	0	n	1	0	Amaryllidaceae J.St.-Hil.
Iz_7_1209	DJI_0439	Ambrosia artemisiifolia L.	0	a	0	0	Asteraceae Bercht. & J.Presl
Iz_7_1209	DJI_0439	Amorpha fruticosa L.	0	a	0	0	Fabaceae Lindl.
Iz_7_1209	DJI_0439	Anthericum ramosum L.	0	n	1	0	Asparagaceae Juss.
Iz_7_1209	DJI_0439	Artemisia marschalliana Spreng.	0	n	1	0	Asteraceae Bercht. & J.Presl
Iz_7_1209	DJI_0439	Asparagus officinalis L.	0	n	1	0	Asparagaceae Juss.
Iz_7_1209	DJI_0439	Astragalus cicer L.	0	n	1	0	Fabaceae Lindl.
Iz_7_1209	DJI_0439	Bassia laniflora (S.G.Gmel.) A.J.Scott	0	n	1	0	Amaranthaceae Juss.
Iz_7_1209	DJI_0439	Berberis vulgaris L.	0	n	1	0	Berberidaceae Juss.
Iz_7_1209	DJI_0439	Betula pendula Roth.	0	n	1	1	Betulaceae Gray
Iz_7_1209	DJI_0439	Betula pendula Roth.	0	n	1	0	Poaceae Barnhart
Iz_7_1209	DJI_0439	Bromus inermis Leyss.	0		NA	NA	
Iz_7_1209	DJI_0439	Bromus squarrosus L.	0	a	0	0	Poaceae Barnhart
Iz_7_1209	DJI_0439	Bromus tectorum L.	0	a	0	0	Poaceae Barnhart
Iz_7_1209	DJI_0439	Calamagrostis epigejos (L.) Roth	3	n	1	0	Poaceae Barnhart
Iz_7_1209	DJI_0439	Carduus acanthoides L.	0	n	1	0	Asteraceae Bercht. & J.Presl
Iz_7_1209	DJI_0439	Carex sp.	0	n	1	0	Cyperaceae Juss.
Iz_7_1209	DJI_0439	Centaurea majorovii Dumbadze	0	n	1	0	Asteraceae Bercht. & J.Presl
Iz_7_1209	DJI_0439	Chamaecytisus ruthenicus (Fisch.ex Wol.) Klaskova	0	n	1	0	Fabaceae Lindl.
Iz_7_1209	DJI_0439	Chelidonium majus L.	0	n	1	0	Papaveraceae Juss.
Iz_7_1209	DJI_0439	Chenopodium betaceum Andrz.	0	n	1	0	Amaranthaceae Juss.
Iz_7_1209	DJI_0439	Chenopodium cf. album	0	n	1	0	Amaranthaceae Juss.
Iz_7_1209	DJI_0439	Chenopodium sp.	0		NA	0	Amaranthaceae Juss.
Iz_7_1209	DJI_0439	Chondrilla juncea L.	0	n	1	0	Asteraceae Bercht. & J.Presl
Iz_7_1209	DJI_0439	Cirsium vulgare (Savi) Ten.	0	n	1	0	Asteraceae Bercht. & J.Presl
Iz_7_1209	DJI_0439	Clinopodium acinos (L.) Kuntze	0	n	1	0	Lamiacea Lindl.
Iz_7_1209	DJI_0439	Convallaria majalis L.	0	n	1	0	Asparagaceae Juss.
Iz_7_1209	DJI_0439	Crataegus rhipidophylla Gand.	0	n	1	1	Rosaceae Juss.
Iz_7_1209	DJI_0439	Crepis foetida L.	0	n	1	0	Asteraceae Bercht. & J.Presl
Iz_7_1209	DJI_0439	Dactylis glomerata L.	0	n	1	0	Poaceae Barnhart
Iz_7_1209	DJI_0439	Dianthus campestris M.Bieb.	0	n	1	0	Caryophyllaceae Juss.
Iz_7_1209	DJI_0439	Digitaria sanguinalis (L.) Scop.	0	a	0	0	Poaceae Barnhart
Iz_7_1209	DJI_0439	Elymus repens (L.) Gould	0	n	1	0	Poaceae Barnhart
Iz_7_1209	DJI_0439	Epilobium tetragonum L.	0	n	1	0	Onagraceae Juss.
Iz_7_1209	DJI_0439	Eragrostis minor Host	0	a	0	0	Poaceae Barnhart
Iz_7_1209	DJI_0439	Erigeron canadensis L.	5	a	0	0	Asteraceae Bercht. & J.Presl
Iz_7_1209	DJI_0439	Euphorbia seguieriana Neck.	0	n	1	0	Euphorbiaceae Juss.
Iz_7_1209	DJI_0439	Fallopia dumetorum (L.) Holub	0	n	1	0	Polygonaceae Juss.
Iz_7_1209	DJI_0439	Festuca beckeri (Hack.) Trautv.	0	n	1	0	Poaceae Barnhart
Iz_7_1209	DJI_0439	Fragaria vesca L.	0	n	1	0	Rosaceae Juss.
Iz_7_1209	DJI_0439	Frangula alnus Mill.	0	n	1	1	Rhamnaceae Juss.
Iz_7_1209	DJI_0439	Fraxinus pennsylvanica Marshall	0	a	0	1	Oleaceae Hoffmanns. & Link
Iz_7_1209	DJI_0439	Galeopsis bifida Boenn.	0	n	1	0	Lamiacea Lindl.
Iz_7_1209	DJI_0439	Galium verum L.	0	n	1	0	Rubiaceae Juss.
Iz_7_1209	DJI_0439	Genista tinctoria L.	0	n	1	0	Fabaceae Lindl.
Iz_7_1209	DJI_0439	Geranium robertianum L.	0	n	1	0	Geraniaceae Juss.
Iz_7_1209	DJI_0439	Geranium sanquineum L.	0	n	1	0	Geraniaceae Juss.
Iz_7_1209	DJI_0439	Gypsophila paniculata L.	0	n	1	0	Caryophyllaceae Juss.
Iz_7_1209	DJI_0439	Helichrysum arenarium (L.) Moench	0	n	1	0	Asteraceae Bercht. & J.Presl
Iz_7_1209	DJI_0439	Herniaria polygama J.Gay	0	n	1	0	Caryophyllaceae Juss.
Iz_7_1209	DJI_0439	Hieracium robustum Fr.	0	n	1	0	Asteraceae Bercht. & J.Presl
Iz_7_1209	DJI_0439	Hieracium umbellatum L.	0	n	1	NA	Asteraceae Bercht. & J.Presl
Iz_7_1209	DJI_0439	Hylotelephium maximum (L.) Holub	0	n	1	0	Crassulaceae J.St.-Hil.
Iz_7_1209	DJI_0439	Jacobaea borysthenica (DC.) B.Nord. & Greuter	0	n	1	0	Asteraceae Bercht. & J.Presl
Iz_7_1209	DJI_0439	Jasione montana L.	0	n	1	0	Campanulaceae Juss.
Iz_7_1209	DJI_0439	Jurinea cyanoides (L.) Rchb.s.l.	0	n	1	0	Asteraceae Bercht. & J.Presl
Iz_7_1209	DJI_0439	Koeleria glauca (Spreng.) DC.	0	n	1	0	Poaceae Barnhart
Iz_7_1209	DJI_0439	Lactuca serriola L.	0	a	0	0	Asteraceae Bercht. & J.Presl
Iz_7_1209	DJI_0439	Linaria genistifolia (L.) Mill.	0	n	1	0	Plantaginaceae Juss.
Iz_7_1209	DJI_0439	Lomelosia argentea (L.) Greuter & Burdet	0	n	1	0	Caprifoliaceae Juss.
Iz_7_1209	DJI_0439	Melampyrum pratense L.	0	n	1	0	Orobanchaceae Vent.
Iz_7_1209	DJI_0439	Oenothera biennis L.	0	a	0	0	Onagraceae Juss.
Iz_7_1209	DJI_0439	Pentanema britannica (L.) D.Gut.Larr., Santos-Vicente, Anderb., E.Rico & M.M.Mart.Ort.	0	n	1	0	Asteraceae Bercht. & J.Presl

Iz_7_1209	DJI_0439	Peucedanum oreoselinum (L.) Moench	0	n	1	0	Apiaceae Lindl.
Iz_7_1209	DJI_0439	Peucedanum ruthenicum M.Bieb.	0	n	1	0	Apiaceae Lindl.
Iz_7_1209	DJI_0439	Pilosella echioides (Lumn.) F.W.Schultz & Sch.Bip.	0	n	1	0	Asteraceae Bercht. & J.Presl
Iz_7_1209	DJI_0439	Pilosella officinarum F. Schultz et Sch. Bip.	0	n	1	0	Asteraceae Bercht. & J.Presl
Iz_7_1209	DJI_0439	Pilosella sp.	0		NA	0	Asteraceae Bercht. & J.Presl
Iz_7_1209	DJI_0439	Pinus sylvestris L.	0	n	1	1	Pinaceae Spreng. ex F.Rudolphi
Iz_7_1209	DJI_0439	Poa annua L.	0	n	1	0	Poaceae Barnhart
Iz_7_1209	DJI_0439	Poa nemoralis L.	0	n	1	0	Poaceae Barnhart
Iz_7_1209	DJI_0439	Poa sp.	0		NA	0	Poaceae Barnhart
Iz_7_1209	DJI_0439	Polygonatum odoratum (Mill.) Druce	0	n	1	0	Asparagaceae Juss.
Iz_7_1209	DJI_0439	Polygonum arenarium Waldst. & Kit.	0	n	1	0	Polygonaceae Juss.
Iz_7_1209	DJI_0439	Populus nigra L.	0	n	1	1	Salicaceae Mirb.
Iz_7_1209	DJI_0439	Populus tremula L.	0	n	1	1	Salicaceae Mirb.
Iz_7_1209	DJI_0439	Populus Ã canescens (Aiton) Sm.	0	n	1	1	Salicaceae Mirb.
Iz_7_1209	DJI_0439	Potentilla argentea L.	0	n	1	0	Rosaceae Juss.
Iz_7_1209	DJI_0439	Psammophiliella muralis (L.) Ikonn.	0	n	1	0	Caryophyllaceae Juss.
Iz_7_1209	DJI_0439	Psephellus sumensis (Kalen.) Greuter	0	n	1	0	Asteraceae Bercht. & J.Presl
Iz_7_1209	DJI_0439	Pulsatilla pratensis (L.) Mill. s.l.	0	n	1	0	Ranunculaceae Juss.
Iz_7_1209	DJI_0439	Pyrus communis L.	0	a	0	1	Rosaceae Juss.
Iz_7_1209	DJI_0439	Quercus robur L.	0	n	1	1	Fagaceae Dumort.
Iz_7_1209	DJI_0439	Robinia pseudoacacia L.	0	a	0	1	Fabaceae Lindl.
Iz_7_1209	DJI_0439	Rosa sp.	0		NA	1	Rosaceae Juss.
Iz_7_1209	DJI_0439	Rubus idaeus L.	0	n	1	0	Rosaceae Juss.
Iz_7_1209	DJI_0439	Rumex acetosella L.	0	n	1	0	Polygonaceae Juss.
Iz_7_1209	DJI_0439	Rumex thyrsiflorus Fingerh.	0	n	1	0	Polygonaceae Juss.
Iz_7_1209	DJI_0439	Salix alba L.	0	n	1	1	Salicaceae Mirb.
Iz_7_1209	DJI_0439	Sambucus nigra L.	0	n	1	1	Viburnaceae Raf.
Iz_7_1209	DJI_0439	Secale sylvestre Host	0	n	1	0	Poaceae Barnhart
Iz_7_1209	DJI_0439	Sempervivum ruthenicum Schnittsp. & C.B. Lehm.	0	n	1	0	Crassulaceae J.St.-Hil.
Iz_7_1209	DJI_0439	Setaria viridis (L.) P. Beauv.	0	n	1	0	Poaceae Barnhart
Iz_7_1209	DJI_0439	Silene borysthenica (Gruner) Walters	0	n	1	0	Caryophyllaceae Juss.
Iz_7_1209	DJI_0439	Silene latifolia subsp. alba (Mill.) Greuter & Burdet	0	n	1	0	Caryophyllaceae Juss.
Iz_7_1209	DJI_0439	Solidago virgaurea L.	0	n	1	0	Asteraceae Bercht. & J.Presl
Iz_7_1209	DJI_0439	Sorbus aucuparia L.	0	n	1	1	Rosaceae Juss.
Iz_7_1209	DJI_0439	Stachys recta L.	0	n	1	0	Lamiacea Lindl.
Iz_7_1209	DJI_0439	Tanacetum vulgare L.	0	n	1	0	Asteraceae Bercht. & J.Presl
Iz_7_1209	DJI_0439	Thymus pallasianus Heinr.Braun	0	n	1	0	Lamiacea Lindl.
Iz_7_1209	DJI_0439	Tilia cordata Mill.	0	n	1	1	Malvaceae Juss.
Iz_7_1209	DJI_0439	Trifolium alpestre L.	0	n	1	0	Fabaceae Lindl.
Iz_7_1209	DJI_0439	Trifolium arvense L.	0	n	1	0	Fabaceae Lindl.
Iz_7_1209	DJI_0439	Ulmus glabra Huds.	0	n	1	1	Ulmaceae Mirb.
Iz_7_1209	DJI_0439	Verbascum densiflorum Bertol.	0	n	1	0	Scrophulariaceae Juss.
Iz_7_1209	DJI_0439	Veronica austriaca L.	0	n	1	0	Plantaginaceae Juss.
Iz_7_1209	DJI_0439	Veronica incana L.	0	n	1	0	Plantaginaceae Juss.
Iz_7_1209	DJI_0439	Veronica verna L.	0	n	1	0	Plantaginaceae Juss.
Iz_7_1209	DJI_0439	Vincetoxicum hirundinaria Medik	0	n	1	0	Apocynaceae Juss.
Iz_7_1209	DJI_0442	Acer tataricum L.	0	n	1	1	Sapindaceae Juss.
Iz_7_1209	DJI_0442	Achillea millefolium L. s.l.	0	n	1	0	Asteraceae Bercht. & J.Presl
Iz_7_1209	DJI_0442	Allium oleraceum L.	0	n	1	0	Amaryllidaceae J.St.-Hil.
Iz_7_1209	DJI_0442	Ambrosia artemisiifolia L.	0	a	0	0	Asteraceae Bercht. & J.Presl
Iz_7_1209	DJI_0442	Amorpha fruticosa L.	0	a	0	0	Fabaceae Lindl.
Iz_7_1209	DJI_0442	Anthericum ramosum L.	0	n	1	0	Asparagaceae Juss.
Iz_7_1209	DJI_0442	Artemisia marschalliana Spreng.	0	n	1	0	Asteraceae Bercht. & J.Presl
Iz_7_1209	DJI_0442	Asparagus officinalis L.	0	n	1	0	Asparagaceae Juss.
Iz_7_1209	DJI_0442	Astragalus cicer L.	0	n	1	0	Fabaceae Lindl.
Iz_7_1209	DJI_0442	Bassia laniflora (S.G.Gmel.) A.J.Scott	0	n	1	0	Amaranthaceae Juss.
Iz_7_1209	DJI_0442	Berberis vulgaris L.	0	n	1	0	Berberidaceae Juss.
Iz_7_1209	DJI_0442	Betula pendula Roth.	0	n	1	1	Betulaceae Gray
Iz_7_1209	DJI_0442	Betula pendula Roth.	0	n	1	0	Poaceae Barnhart
Iz_7_1209	DJI_0442	Bromus inermis Leyss.	0		NA	NA	
Iz_7_1209	DJI_0442	Bromus squarrosus L.	0	a	0	0	Poaceae Barnhart
Iz_7_1209	DJI_0442	Bromus tectorum L.	0	a	0	0	Poaceae Barnhart
Iz_7_1209	DJI_0442	Calamagrostis epigejos (L.) Roth	4	n	1	0	Poaceae Barnhart
Iz_7_1209	DJI_0442	Carduus acanthoides L.	0	n	1	0	Asteraceae Bercht. & J.Presl
Iz_7_1209	DJI_0442	Carex sp.	0	n	1	0	Cyperaceae Juss.
Iz_7_1209	DJI_0442	Centaurea majorovii Dumbadze	0	n	1	0	Asteraceae Bercht. & J.Presl
Iz_7_1209	DJI_0442	Chamaecytisus ruthenicus (Fisch.ex Wol.) Klaskova	0	n	1	0	Fabaceae Lindl.
Iz_7_1209	DJI_0442	Chelidonium majus L.	0	n	1	0	Papaveraceae Juss.
Iz_7_1209	DJI_0442	Chenopodium betaceum Andrz.	0	n	1	0	Amaranthaceae Juss.
Iz_7_1209	DJI_0442	Chenopodium cf. album	0	n	1	0	Amaranthaceae Juss.
Iz_7_1209	DJI_0442	Chenopodium sp.	0		NA	0	Amaranthaceae Juss.
Iz_7_1209	DJI_0442	Chondrilla juncea L.	0	n	1	0	Asteraceae Bercht. & J.Presl
Iz_7_1209	DJI_0442	Cirsium vulgare (Savi) Ten.	0	n	1	0	Asteraceae Bercht. & J.Presl
Iz_7_1209	DJI_0442	Clinopodium acinos (L.) Kuntze	0	n	1	0	Lamiacea Lindl.
Iz_7_1209	DJI_0442	Convallaria majalis L.	0	n	1	0	Asparagaceae Juss.
Iz_7_1209	DJI_0442	Crataegus rhipidophylla Gand.	0	n	1	1	Rosaceae Juss.
Iz_7_1209	DJI_0442	Crepis foetida L.	0	n	1	0	Asteraceae Bercht. & J.Presl
Iz_7_1209	DJI_0442	Dactylis glomerata L.	0	n	1	0	Poaceae Barnhart
Iz_7_1209	DJI_0442	Dianthus campestris M.Bieb.	0	n	1	0	Caryophyllaceae Juss.
Iz_7_1209	DJI_0442	Digitaria sanguinalis (L.) Scop.	0	a	0	0	Poaceae Barnhart
Iz_7_1209	DJI_0442	Elymus repens (L.) Gould	0	n	1	0	Poaceae Barnhart
Iz_7_1209	DJI_0442	Epilobium tetragonum L.	0	n	1	0	Onagraceae Juss.
Iz_7_1209	DJI_0442	Eragrostis minor Host	0	a	0	0	Poaceae Barnhart
Iz_7_1209	DJI_0442	Erigeron canadensis L.	0.5	a	0	0	Asteraceae Bercht. & J.Presl
Iz_7_1209	DJI_0442	Euphorbia seguieriana Neck.	0	n	1	0	Euphorbiaceae Juss.
Iz_7_1209	DJI_0442	Fallopia dumetorum (L.) Holub	0	n	1	0	Polygonaceae Juss.
Iz_7_1209	DJI_0442	Festuca beckeri (Hack.) Trautv.	0	n	1	0	Poaceae Barnhart
Iz_7_1209	DJI_0442	Fragaria vesca L.	0	n	1	0	Rosaceae Juss.
Iz_7_1209	DJI_0442	Frangula alnus Mill.	0	n	1	1	Rhamnaceae Juss.
Iz_7_1209	DJI_0442	Fraxinus pennsylvanica Marshall	0	a	0	1	Oleaceae Hoffmanns. & Link
Iz_7_1209	DJI_0442	Galeopsis bifida Boenn.	0	n	1	0	Lamiacea Lindl.
Iz_7_1209	DJI_0442	Galium verum L.	0	n	1	0	Rubiaceae Juss.
Iz_7_1209	DJI_0442	Genista tinctoria L.	0	n	1	0	Fabaceae Lindl.
Iz_7_1209	DJI_0442	Geranium robertianum L.	0	n	1	0	Geraniaceae Juss.
Iz_7_1209	DJI_0442	Geranium sanquineum L.	0	n	1	0	Geraniaceae Juss.
Iz_7_1209	DJI_0442	Gypsophila paniculata L.	0	n	1	0	Caryophyllaceae Juss.
Iz_7_1209	DJI_0442	Helichrysum arenarium (L.) Moench	0	n	1	0	Asteraceae Bercht. & J.Presl
Iz_7_1209	DJI_0442	Herniaria polygama J.Gay	0	n	1	0	Caryophyllaceae Juss.

Iz_7_1209	DJI_0442	Hieracium robustum Fr.	0	n	1	0	Asteraceae Bercht. & J.Presl
Iz_7_1209	DJI_0442	Hieracium umbellatum L.	0	n	1	NA	Asteraceae Bercht. & J.Presl
Iz_7_1209	DJI_0442	Hylotelephium maximum (L.) Holub	0	n	1	0	Crassulaceae J.St.-Hil.
Iz_7_1209	DJI_0442	Jacobaea borysthenica (DC.) B.Nord. & Greuter	0	n	1	0	Asteraceae Bercht. & J.Presl
Iz_7_1209	DJI_0442	Jasione montana L.	0	n	1	0	Campanulaceae Juss.
Iz_7_1209	DJI_0442	Jurinea cyanoides (L.) Rchb.s.l.	0	n	1	0	Asteraceae Bercht. & J.Presl
Iz_7_1209	DJI_0442	Koeleria glauca (Spreng.) DC.	0	n	1	0	Poaceae Barnhart
Iz_7_1209	DJI_0442	Lactuca serriola L.	0	a	0	0	Asteraceae Bercht. & J.Presl
Iz_7_1209	DJI_0442	Linaria genistifolia (L.) Mill.	0	n	1	0	Plantaginaceae Juss.
Iz_7_1209	DJI_0442	Lomelosia argentea (L.) Greuter & Burdet	0	n	1	0	Caprifoliaceae Juss.
Iz_7_1209	DJI_0442	Melampyrum pratense L.	0	n	1	0	Orobanchaceae Vent.
Iz_7_1209	DJI_0442	Oenothera biennis L.	0	a	0	0	Onagraceae Juss.
Iz_7_1209	DJI_0442	Pentanema britannica (L.) D.Gut.Larr., Santos-Vicente, Anderb., E.Rico & M.M.Mart.Ort.	0	n	1	0	Asteraceae Bercht. & J.Presl
Iz_7_1209	DJI_0442	Peucedanum oreoselinum (L.) Moench	0	n	1	0	Apiaceae Lindl.
Iz_7_1209	DJI_0442	Peucedanum ruthenicum M.Bieb.	0	n	1	0	Apiaceae Lindl.
Iz_7_1209	DJI_0442	Pilosella echioides (Lumn.) F.W.Schultz & Sch.Bip.	0	n	1	0	Asteraceae Bercht. & J.Presl
Iz_7_1209	DJI_0442	Pilosella officinarum F. Schultz et Sch. Bip.	0	n	1	0	Asteraceae Bercht. & J.Presl
Iz_7_1209	DJI_0442	Pilosella sp.	0		NA	0	Asteraceae Bercht. & J.Presl
Iz_7_1209	DJI_0442	Pinus sylvestris L.	0	n	1	1	Pinaceae Spreng. ex F.Rudolphi
Iz_7_1209	DJI_0442	Poa annua L.	0	n	1	0	Poaceae Barnhart
Iz_7_1209	DJI_0442	Poa nemoralis L.	0	n	1	0	Poaceae Barnhart
Iz_7_1209	DJI_0442	Poa sp.	0		NA	0	Poaceae Barnhart
Iz_7_1209	DJI_0442	Polygonatum odoratum (Mill.) Druce	0	n	1	0	Asparagaceae Juss.
Iz_7_1209	DJI_0442	Polygonum arenarium Waldst. & Kit.	0	n	1	0	Polygonaceae Juss.
Iz_7_1209	DJI_0442	Populus nigra L.	0	n	1	1	Salicaceae Mirb.
Iz_7_1209	DJI_0442	Populus tremula L.	0	n	1	1	Salicaceae Mirb.
Iz_7_1209	DJI_0442	Populus Ã canescens (Aiton) Sm.	0	n	1	1	Salicaceae Mirb.
Iz_7_1209	DJI_0442	Potentilla argentea L.	0	n	1	0	Rosaceae Juss.
Iz_7_1209	DJI_0442	Psammophiliella muralis (L.) Ikonn.	0	n	1	0	Caryophyllaceae Juss.
Iz_7_1209	DJI_0442	Psephellus sumensis (Kalen.) Greuter	0	n	1	0	Asteraceae Bercht. & J.Presl
Iz_7_1209	DJI_0442	Pulsatilla pratensis (L.) Mill. s.l.	0	n	1	0	Ranunculaceae Juss.
Iz_7_1209	DJI_0442	Pyrus communis L.	0	a	0	1	Rosaceae Juss.
Iz_7_1209	DJI_0442	Quercus robur L.	0	n	1	1	Fagaceae Dumort.
Iz_7_1209	DJI_0442	Robinia pseudoacacia L.	0	a	0	1	Fabaceae Lindl.
Iz_7_1209	DJI_0442	Rosa sp.	0		NA	1	Rosaceae Juss.
Iz_7_1209	DJI_0442	Rubus idaeus L.	0	n	1	0	Rosaceae Juss.
Iz_7_1209	DJI_0442	Rumex acetosella L.	0	n	1	0	Polygonaceae Juss.
Iz_7_1209	DJI_0442	Rumex thyrsiflorus Fingerh.	0	n	1	0	Polygonaceae Juss.
Iz_7_1209	DJI_0442	Salix alba L.	0	n	1	1	Salicaceae Mirb.
Iz_7_1209	DJI_0442	Sambucus nigra L.	0	n	1	1	Viburnaceae Raf.
Iz_7_1209	DJI_0442	Secale sylvestre Host	0	n	1	0	Poaceae Barnhart
Iz_7_1209	DJI_0442	Sempervivum ruthenicum Schnittsp. & C.B. Lehm.	0	n	1	0	Crassulaceae J.St.-Hil.
Iz_7_1209	DJI_0442	Setaria viridis (L.) P. Beauv.	0	n	1	0	Poaceae Barnhart
Iz_7_1209	DJI_0442	Silene borysthenica (Gruner) Walters	0	n	1	0	Caryophyllaceae Juss.
Iz_7_1209	DJI_0442	Silene latifolia subsp. alba (Mill.) Greuter & Burdet	0	n	1	0	Caryophyllaceae Juss.
Iz_7_1209	DJI_0442	Solidago virgaurea L.	0	n	1	0	Asteraceae Bercht. & J.Presl
Iz_7_1209	DJI_0442	Sorbus aucuparia L.	0	n	1	1	Rosaceae Juss.
Iz_7_1209	DJI_0442	Stachys recta L.	0	n	1	0	Lamiacea Lindl.
Iz_7_1209	DJI_0442	Tanacetum vulgare L.	0	n	1	0	Asteraceae Bercht. & J.Presl
Iz_7_1209	DJI_0442	Thymus pallasianus Heinr.Braun	0	n	1	0	Lamiacea Lindl.
Iz_7_1209	DJI_0442	Tilia cordata Mill.	0	n	1	1	Malvaceae Juss.
Iz_7_1209	DJI_0442	Trifolium alpestre L.	0	n	1	0	Fabaceae Lindl.
Iz_7_1209	DJI_0442	Trifolium arvense L.	0	n	1	0	Fabaceae Lindl.
Iz_7_1209	DJI_0442	Ulmus glabra Huds.	0	n	1	1	Ulmaceae Mirb.
Iz_7_1209	DJI_0442	Verbascum densiflorum Bertol.	0	n	1	0	Scrophulariaceae Juss.
Iz_7_1209	DJI_0442	Veronica austriaca L.	0	n	1	0	Plantaginaceae Juss.
Iz_7_1209	DJI_0442	Veronica incana L.	0	n	1	0	Plantaginaceae Juss.
Iz_7_1209	DJI_0442	Veronica verna L.	0	n	1	0	Plantaginaceae Juss.
Iz_7_1209	DJI_0442	Vincetoxicum hirundinaria Medik	0	n	1	0	Apocynaceae Juss.
Iz_8_1009	DJI_0894	Acer tataricum L.	0	n	1	1	Sapindaceae Juss.
Iz_8_1009	DJI_0894	Achillea millefolium L. s.l.	0	n	1	0	Asteraceae Bercht. & J.Presl
Iz_8_1009	DJI_0894	Allium oleraceum L.	0	n	1	0	Amaryllidaceae J.St.-Hil.
Iz_8_1009	DJI_0894	Ambrosia artemisiifolia L.	0	a	0	0	Asteraceae Bercht. & J.Presl
Iz_8_1009	DJI_0894	Amorpha fruticosa L.	0	a	0	0	Fabaceae Lindl.
Iz_8_1009	DJI_0894	Anthericum ramosum L.	0	n	1	0	Asparagaceae Juss.
Iz_8_1009	DJI_0894	Artemisia marschalliana Spreng.	0	n	1	0	Asteraceae Bercht. & J.Presl
Iz_8_1009	DJI_0894	Asparagus officinalis L.	0	n	1	0	Asparagaceae Juss.
Iz_8_1009	DJI_0894	Astragalus cicer L.	0	n	1	0	Fabaceae Lindl.
Iz_8_1009	DJI_0894	Bassia laniflora (S.G.Gmel.) A.J.Scott	0	n	1	0	Amaranthaceae Juss.
Iz_8_1009	DJI_0894	Berberis vulgaris L.	0	n	1	0	Berberidaceae Juss.
Iz_8_1009	DJI_0894	Betula pendula Roth.	0	n	1	1	Betulaceae Gray
Iz_8_1009	DJI_0894	Betula pendula Roth.	0	n	1	0	Poaceae Barnhart
Iz_8_1009	DJI_0894	Bromus inermis Leyss.	0		NA	NA	
Iz_8_1009	DJI_0894	Bromus squarrosus L.	0	a	0	0	Poaceae Barnhart
Iz_8_1009	DJI_0894	Bromus tectorum L.	0	a	0	0	Poaceae Barnhart
Iz_8_1009	DJI_0894	Calamagrostis epigejos (L.) Roth	5	n	1	0	Poaceae Barnhart
Iz_8_1009	DJI_0894	Carduus acanthoides L.	0	n	1	0	Asteraceae Bercht. & J.Presl
Iz_8_1009	DJI_0894	Carex sp.	5	n	1	0	Cyperaceae Juss.
Iz_8_1009	DJI_0894	Centaurea majorovii Dumbadze	0	n	1	0	Asteraceae Bercht. & J.Presl
Iz_8_1009	DJI_0894	Chamaecytisus ruthenicus (Fisch.ex Wol.) Klaskova	0	n	1	0	Fabaceae Lindl.
Iz_8_1009	DJI_0894	Chelidonium majus L.	0	n	1	0	Papaveraceae Juss.
Iz_8_1009	DJI_0894	Chenopodium betaceum Andrz.	0	n	1	0	Amaranthaceae Juss.
Iz_8_1009	DJI_0894	Chenopodium cf. album	0	n	1	0	Amaranthaceae Juss.
Iz_8_1009	DJI_0894	Chenopodium sp.	0		NA	0	Amaranthaceae Juss.
Iz_8_1009	DJI_0894	Chondrilla juncea L.	0	n	1	0	Asteraceae Bercht. & J.Presl
Iz_8_1009	DJI_0894	Cirsium vulgare (Savi) Ten.	0	n	1	0	Asteraceae Bercht. & J.Presl
Iz_8_1009	DJI_0894	Clinopodium acinos (L.) Kuntze	0	n	1	0	Lamiacea Lindl.
Iz_8_1009	DJI_0894	Convallaria majalis L.	0	n	1	0	Asparagaceae Juss.
Iz_8_1009	DJI_0894	Crataegus rhipidophylla Gand.	0	n	1	1	Rosaceae Juss.
Iz_8_1009	DJI_0894	Crepis foetida L.	0	n	1	0	Asteraceae Bercht. & J.Presl
Iz_8_1009	DJI_0894	Dactylis glomerata L.	0	n	1	0	Poaceae Barnhart
Iz_8_1009	DJI_0894	Dianthus campestris M.Bieb.	0	n	1	0	Caryophyllaceae Juss.
Iz_8_1009	DJI_0894	Digitaria sanguinalis (L.) Scop.	0	a	0	0	Poaceae Barnhart
Iz_8_1009	DJI_0894	Elymus repens (L.) Gould	0	n	1	0	Poaceae Barnhart
Iz_8_1009	DJI_0894	Epilobium tetragonum L.	0	n	1	0	Onagraceae Juss.
Iz_8_1009	DJI_0894	Eragrostis minor Host	0	a	0	0	Poaceae Barnhart
Iz_8_1009	DJI_0894	Erigeron canadensis L.	10	a	0	0	Asteraceae Bercht. & J.Presl
Iz_8_1009	DJI_0894	Euphorbia seguieriana Neck.	0	n	1	0	Euphorbiaceae Juss.

Iz_8_1009	DJI_0894	Fallopia dumetorum (L.) Holub	0	n	1	0	Polygonaceae Juss.
Iz_8_1009	DJI_0894	Festuca beckeri (Hack.) Trautv.	0	n	1	0	Poaceae Barnhart
Iz_8_1009	DJI_0894	Fragaria vesca L.	0	n	1	0	Rosaceae Juss.
Iz_8_1009	DJI_0894	Frangula alnus Mill.	0	n	1	1	Rhamnaceae Juss.
Iz_8_1009	DJI_0894	Fraxinus pennsylvanica Marshall	0	a	0	1	Oleaceae Hoffmanns. & Link
Iz_8_1009	DJI_0894	Galeopsis bifida Boenn.	0	n	1	0	Lamiacea Lindl.
Iz_8_1009	DJI_0894	Galium verum L.	0	n	1	0	Rubiaceae Juss.
Iz_8_1009	DJI_0894	Genista tinctoria L.	0	n	1	0	Fabaceae Lindl.
Iz_8_1009	DJI_0894	Geranium robertianum L.	0	n	1	0	Geraniaceae Juss.
Iz_8_1009	DJI_0894	Geranium sanquineum L.	0	n	1	0	Geraniaceae Juss.
Iz_8_1009	DJI_0894	Gypsophila paniculata L.	0	n	1	0	Caryophyllaceae Juss.
Iz_8_1009	DJI_0894	Helichrysum arenarium (L.) Moench	0	n	1	0	Asteraceae Bercht. & J.Presl
Iz_8_1009	DJI_0894	Herniaria polygama J.Gay	0	n	1	0	Caryophyllaceae Juss.
Iz_8_1009	DJI_0894	Hieracium robustum Fr.	0	n	1	0	Asteraceae Bercht. & J.Presl
Iz_8_1009	DJI_0894	Hieracium umbellatum L.	0	n	1	NA	Asteraceae Bercht. & J.Presl
Iz_8_1009	DJI_0894	Hylotelephium maximum (L.) Holub	0	n	1	0	Crassulaceae J.St.-Hil.
Iz_8_1009	DJI_0894	Jacobaea borysthenica (DC.) B.Nord. & Greuter	0	n	1	0	Asteraceae Bercht. & J.Presl
Iz_8_1009	DJI_0894	Jasione montana L.	0	n	1	0	Campanulaceae Juss.
Iz_8_1009	DJI_0894	Jurinea cyanoides (L.) Rchb.s.l.	0	n	1	0	Asteraceae Bercht. & J.Presl
Iz_8_1009	DJI_0894	Koeleria glauca (Spreng.) DC.	0	n	1	0	Poaceae Barnhart
Iz_8_1009	DJI_0894	Lactuca serriola L.	0	a	0	0	Asteraceae Bercht. & J.Presl
Iz_8_1009	DJI_0894	Linaria genistifolia (L.) Mill.	0	n	1	0	Plantaginaceae Juss.
Iz_8_1009	DJI_0894	Lomelosia argentea (L.) Greuter & Burdet	0	n	1	0	Caprifoliaceae Juss.
Iz_8_1009	DJI_0894	Melampyrum pratense L.	0	n	1	0	Orobanchaceae Vent.
Iz_8_1009	DJI_0894	Oenothera biennis L.	0	a	0	0	Onagraceae Juss.
Iz_8_1009	DJI_0894	Pentanema britannica (L.) D.Gut.Larr., Santos-Vicente, Anderb., E.Rico & M.M.Mart.Ort.	0	n	1	0	Asteraceae Bercht. & J.Presl
Iz_8_1009	DJI_0894	Peucedanum oreoselinum (L.) Moench	1	n	1	0	Apiaceae Lindl.
Iz_8_1009	DJI_0894	Peucedanum ruthenicum M.Bieb.	0	n	1	0	Apiaceae Lindl.
Iz_8_1009	DJI_0894	Pilosella echioides (Lumn.) F.W.Schultz & Sch.Bip.	0	n	1	0	Asteraceae Bercht. & J.Presl
Iz_8_1009	DJI_0894	Pilosella officinarum F. Schultz et Sch. Bip.	0	n	1	0	Asteraceae Bercht. & J.Presl
Iz_8_1009	DJI_0894	Pilosella sp.	0		NA	0	Asteraceae Bercht. & J.Presl
Iz_8_1009	DJI_0894	Pinus sylvestris L.	0	n	1	1	Pinaceae Spreng. ex F.Rudolphi
Iz_8_1009	DJI_0894	Poa annua L.	0	n	1	0	Poaceae Barnhart
Iz_8_1009	DJI_0894	Poa nemoralis L.	0	n	1	0	Poaceae Barnhart
Iz_8_1009	DJI_0894	Poa sp.	0		NA	0	Poaceae Barnhart
Iz_8_1009	DJI_0894	Polygonatum odoratum (Mill.) Druce	0	n	1	0	Asparagaceae Juss.
Iz_8_1009	DJI_0894	Polygonum arenarium Waldst. & Kit.	0	n	1	0	Polygonaceae Juss.
Iz_8_1009	DJI_0894	Populus nigra L.	0	n	1	1	Salicaceae Mirb.
Iz_8_1009	DJI_0894	Populus tremula L.	0	n	1	1	Salicaceae Mirb.
Iz_8_1009	DJI_0894	Populus Ã canescens (Aiton) Sm.	0	n	1	1	Salicaceae Mirb.
Iz_8_1009	DJI_0894	Potentilla argentea L.	0	n	1	0	Rosaceae Juss.
Iz_8_1009	DJI_0894	Psammophiliella muralis (L.) Ikonn.	0	n	1	0	Caryophyllaceae Juss.
Iz_8_1009	DJI_0894	Psephellus sumensis (Kalen.) Greuter	0	n	1	0	Asteraceae Bercht. & J.Presl
Iz_8_1009	DJI_0894	Pulsatilla pratensis (L.) Mill. s.l.	0	n	1	0	Ranunculaceae Juss.
Iz_8_1009	DJI_0894	Pyrus communis L.	0	a	0	1	Rosaceae Juss.
Iz_8_1009	DJI_0894	Quercus robur L.	0	n	1	1	Fagaceae Dumort.
Iz_8_1009	DJI_0894	Robinia pseudoacacia L.	0	a	0	1	Fabaceae Lindl.
Iz_8_1009	DJI_0894	Rosa sp.	0		NA	1	Rosaceae Juss.
Iz_8_1009	DJI_0894	Rubus idaeus L.	0	n	1	0	Rosaceae Juss.
Iz_8_1009	DJI_0894	Rumex acetosella L.	0	n	1	0	Polygonaceae Juss.
Iz_8_1009	DJI_0894	Rumex thyrsiflorus Fingerh.	0	n	1	0	Polygonaceae Juss.
Iz_8_1009	DJI_0894	Salix alba L.	0	n	1	1	Salicaceae Mirb.
Iz_8_1009	DJI_0894	Sambucus nigra L.	0	n	1	1	Viburnaceae Raf.
Iz_8_1009	DJI_0894	Secale sylvestre Host	0	n	1	0	Poaceae Barnhart
Iz_8_1009	DJI_0894	Sempervivum ruthenicum Schnittsp. & C.B. Lehm.	0	n	1	0	Crassulaceae J.St.-Hil.
Iz_8_1009	DJI_0894	Setaria viridis (L.) P. Beauv.	0	n	1	0	Poaceae Barnhart
Iz_8_1009	DJI_0894	Silene borysthenica (Gruner) Walters	0	n	1	0	Caryophyllaceae Juss.
Iz_8_1009	DJI_0894	Silene latifolia subsp. alba (Mill.) Greuter & Burdet	0	n	1	0	Caryophyllaceae Juss.
Iz_8_1009	DJI_0894	Solidago virgaurea L.	0	n	1	0	Asteraceae Bercht. & J.Presl
Iz_8_1009	DJI_0894	Sorbus aucuparia L.	0	n	1	1	Rosaceae Juss.
Iz_8_1009	DJI_0894	Stachys recta L.	0	n	1	0	Lamiacea Lindl.
Iz_8_1009	DJI_0894	Tanacetum vulgare L.	0	n	1	0	Asteraceae Bercht. & J.Presl
Iz_8_1009	DJI_0894	Thymus pallasianus Heinr.Braun	0	n	1	0	Lamiacea Lindl.
Iz_8_1009	DJI_0894	Tilia cordata Mill.	0	n	1	1	Malvaceae Juss.
Iz_8_1009	DJI_0894	Trifolium alpestre L.	0	n	1	0	Fabaceae Lindl.
Iz_8_1009	DJI_0894	Trifolium arvense L.	0	n	1	0	Fabaceae Lindl.
Iz_8_1009	DJI_0894	Ulmus glabra Huds.	0	n	1	1	Ulmaceae Mirb.
Iz_8_1009	DJI_0894	Verbascum densiflorum Bertol.	0	n	1	0	Scrophulariaceae Juss.
Iz_8_1009	DJI_0894	Veronica austriaca L.	0	n	1	0	Plantaginaceae Juss.
Iz_8_1009	DJI_0894	Veronica incana L.	0	n	1	0	Plantaginaceae Juss.
Iz_8_1009	DJI_0894	Veronica verna L.	0	n	1	0	Plantaginaceae Juss.
Iz_8_1009	DJI_0894	Vincetoxicum hirundinaria Medik	0	n	1	0	Apocynaceae Juss.
Iz_8_1009	DJI_0895	Acer tataricum L.	0	n	1	1	Sapindaceae Juss.
Iz_8_1009	DJI_0895	Achillea millefolium L. s.l.	0	n	1	0	Asteraceae Bercht. & J.Presl
Iz_8_1009	DJI_0895	Allium oleraceum L.	0	n	1	0	Amaryllidaceae J.St.-Hil.
Iz_8_1009	DJI_0895	Ambrosia artemisiifolia L.	0	a	0	0	Asteraceae Bercht. & J.Presl
Iz_8_1009	DJI_0895	Amorpha fruticosa L.	0	a	0	0	Fabaceae Lindl.
Iz_8_1009	DJI_0895	Anthericum ramosum L.	0	n	1	0	Asparagaceae Juss.
Iz_8_1009	DJI_0895	Artemisia marschalliana Spreng.	0	n	1	0	Asteraceae Bercht. & J.Presl
Iz_8_1009	DJI_0895	Asparagus officinalis L.	0	n	1	0	Asparagaceae Juss.
Iz_8_1009	DJI_0895	Astragalus cicer L.	0	n	1	0	Fabaceae Lindl.
Iz_8_1009	DJI_0895	Bassia laniflora (S.G.Gmel.) A.J.Scott	0	n	1	0	Amaranthaceae Juss.
Iz_8_1009	DJI_0895	Berberis vulgaris L.	0	n	1	0	Berberidaceae Juss.
Iz_8_1009	DJI_0895	Betula pendula Roth.	0	n	1	1	Betulaceae Gray
Iz_8_1009	DJI_0895	Betula pendula Roth.	0	n	1	0	Poaceae Barnhart
Iz_8_1009	DJI_0895	Bromus inermis Leyss.	0		NA	NA	
Iz_8_1009	DJI_0895	Bromus squarrosus L.	0	a	0	0	Poaceae Barnhart
Iz_8_1009	DJI_0895	Bromus tectorum L.	0	a	0	0	Poaceae Barnhart
Iz_8_1009	DJI_0895	Calamagrostis epigejos (L.) Roth	6	n	1	0	Poaceae Barnhart
Iz_8_1009	DJI_0895	Carduus acanthoides L.	0	n	1	0	Asteraceae Bercht. & J.Presl
Iz_8_1009	DJI_0895	Carex sp.	4	n	1	0	Cyperaceae Juss.
Iz_8_1009	DJI_0895	Centaurea majorovii Dumbadze	0	n	1	0	Asteraceae Bercht. & J.Presl
Iz_8_1009	DJI_0895	Chamaecytisus ruthenicus (Fisch.ex Wol.) Klaskova	0	n	1	0	Fabaceae Lindl.
Iz_8_1009	DJI_0895	Chelidonium majus L.	0	n	1	0	Papaveraceae Juss.
Iz_8_1009	DJI_0895	Chenopodium betaceum Andrz.	0	n	1	0	Amaranthaceae Juss.
Iz_8_1009	DJI_0895	Chenopodium cf. album	0	n	1	0	Amaranthaceae Juss.
Iz_8_1009	DJI_0895	Chenopodium sp.	0		NA	0	Amaranthaceae Juss.
Iz_8_1009	DJI_0895	Chondrilla juncea L.	0	n	1	0	Asteraceae Bercht. & J.Presl

Iz_8_1009	DJI_0895	Cirsium vulgare (Savi) Ten.	0	n	1	0	Asteraceae Bercht. & J.Presl
Iz_8_1009	DJI_0895	Clinopodium acinos (L.) Kuntze	0	n	1	0	Lamiacea Lindl.
Iz_8_1009	DJI_0895	Convallaria majalis L.	0	n	1	0	Asparagaceae Juss.
Iz_8_1009	DJI_0895	Crataegus rhipidophylla Gand.	0	n	1	1	Rosaceae Juss.
Iz_8_1009	DJI_0895	Crepis foetida L.	0	n	1	0	Asteraceae Bercht. & J.Presl
Iz_8_1009	DJI_0895	Dactylis glomerata L.	0	n	1	0	Poaceae Barnhart
Iz_8_1009	DJI_0895	Dianthus campestris M.Bieb.	0	n	1	0	Caryophyllaceae Juss.
Iz_8_1009	DJI_0895	Digitaria sanguinalis (L.) Scop.	0	a	0	0	Poaceae Barnhart
Iz_8_1009	DJI_0895	Elymus repens (L.) Gould	0	n	1	0	Poaceae Barnhart
Iz_8_1009	DJI_0895	Epilobium tetragonum L.	0	n	1	0	Onagraceae Juss.
Iz_8_1009	DJI_0895	Eragrostis minor Host	0	a	0	0	Poaceae Barnhart
Iz_8_1009	DJI_0895	Erigeron canadensis L.	10	a	0	0	Asteraceae Bercht. & J.Presl
Iz_8_1009	DJI_0895	Euphorbia seguieriana Neck.	0	n	1	0	Euphorbiaceae Juss.
Iz_8_1009	DJI_0895	Fallopia dumetorum (L.) Holub	0	n	1	0	Polygonaceae Juss.
Iz_8_1009	DJI_0895	Festuca beckeri (Hack.) Trautv.	0	n	1	0	Poaceae Barnhart
Iz_8_1009	DJI_0895	Fragaria vesca L.	0	n	1	0	Rosaceae Juss.
Iz_8_1009	DJI_0895	Frangula alnus Mill.	0	n	1	1	Rhamnaceae Juss.
Iz_8_1009	DJI_0895	Fraxinus pennsylvanica Marshall	0	a	0	1	Oleaceae Hoffmanns. & Link
Iz_8_1009	DJI_0895	Galeopsis bifida Boenn.	0	n	1	0	Lamiacea Lindl.
Iz_8_1009	DJI_0895	Galium verum L.	0	n	1	0	Rubiaceae Juss.
Iz_8_1009	DJI_0895	Genista tinctoria L.	0	n	1	0	Fabaceae Lindl.
Iz_8_1009	DJI_0895	Geranium robertianum L.	0	n	1	0	Geraniaceae Juss.
Iz_8_1009	DJI_0895	Geranium sanquineum L.	0	n	1	0	Geraniaceae Juss.
Iz_8_1009	DJI_0895	Gypsophila paniculata L.	0	n	1	0	Caryophyllaceae Juss.
Iz_8_1009	DJI_0895	Helichrysum arenarium (L.) Moench	0	n	1	0	Asteraceae Bercht. & J.Presl
Iz_8_1009	DJI_0895	Herniaria polygama J.Gay	0	n	1	0	Caryophyllaceae Juss.
Iz_8_1009	DJI_0895	Hieracium robustum Fr.	0	n	1	0	Asteraceae Bercht. & J.Presl
Iz_8_1009	DJI_0895	Hieracium umbellatum L.	0	n	1	NA	Asteraceae Bercht. & J.Presl
Iz_8_1009	DJI_0895	Hylotelephium maximum (L.) Holub	0	n	1	0	Crassulaceae J.St.-Hil.
Iz_8_1009	DJI_0895	Jacobaea borysthenica (DC.) B.Nord. & Greuter	0	n	1	0	Asteraceae Bercht. & J.Presl
Iz_8_1009	DJI_0895	Jasione montana L.	0	n	1	0	Campanulaceae Juss.
Iz_8_1009	DJI_0895	Jurinea cyanoides (L.) Rchb.s.l.	0	n	1	0	Asteraceae Bercht. & J.Presl
Iz_8_1009	DJI_0895	Koeleria glauca (Spreng.) DC.	0	n	1	0	Poaceae Barnhart
Iz_8_1009	DJI_0895	Lactuca serriola L.	0	a	0	0	Asteraceae Bercht. & J.Presl
Iz_8_1009	DJI_0895	Linaria genistifolia (L.) Mill.	0	n	1	0	Plantaginaceae Juss.
Iz_8_1009	DJI_0895	Lomelosia argentea (L.) Greuter & Burdet	0	n	1	0	Caprifoliaceae Juss.
Iz_8_1009	DJI_0895	Melampyrum pratense L.	0	n	1	0	Orobanchaceae Vent.
Iz_8_1009	DJI_0895	Oenothera biennis L.	0	a	0	0	Onagraceae Juss.
Iz_8_1009	DJI_0895	Pentanema britannica (L.) D.Gut.Larr., Santos-Vicente, Anderb., E.Rico & M.M.Mart.Ort.	0	n	1	0	Asteraceae Bercht. & J.Presl
Iz_8_1009	DJI_0895	Peucedanum oreoselinum (L.) Moench	0	n	1	0	Apiaceae Lindl.
Iz_8_1009	DJI_0895	Peucedanum ruthenicum M.Bieb.	0	n	1	0	Apiaceae Lindl.
Iz_8_1009	DJI_0895	Pilosella echioides (Lumn.) F.W.Schultz & Sch.Bip.	0	n	1	0	Asteraceae Bercht. & J.Presl
Iz_8_1009	DJI_0895	Pilosella officinarum F. Schultz et Sch. Bip.	0	n	1	0	Asteraceae Bercht. & J.Presl
Iz_8_1009	DJI_0895	Pilosella sp.	0		NA	0	Asteraceae Bercht. & J.Presl
Iz_8_1009	DJI_0895	Pinus sylvestris L.	0	n	1	1	Pinaceae Spreng. ex F.Rudolphi
Iz_8_1009	DJI_0895	Poa annua L.	0	n	1	0	Poaceae Barnhart
Iz_8_1009	DJI_0895	Poa nemoralis L.	0	n	1	0	Poaceae Barnhart
Iz_8_1009	DJI_0895	Poa sp.	0		NA	0	Poaceae Barnhart
Iz_8_1009	DJI_0895	Polygonatum odoratum (Mill.) Druce	0	n	1	0	Asparagaceae Juss.
Iz_8_1009	DJI_0895	Polygonum arenarium Waldst. & Kit.	0	n	1	0	Polygonaceae Juss.
Iz_8_1009	DJI_0895	Populus nigra L.	0	n	1	1	Salicaceae Mirb.
Iz_8_1009	DJI_0895	Populus tremula L.	0	n	1	1	Salicaceae Mirb.
Iz_8_1009	DJI_0895	Populus Ã canescens (Aiton) Sm.	0	n	1	1	Salicaceae Mirb.
Iz_8_1009	DJI_0895	Potentilla argentea L.	0	n	1	0	Rosaceae Juss.
Iz_8_1009	DJI_0895	Psammophiliella muralis (L.) Ikonn.	0	n	1	0	Caryophyllaceae Juss.
Iz_8_1009	DJI_0895	Psephellus sumensis (Kalen.) Greuter	0.5	n	1	0	Asteraceae Bercht. & J.Presl
Iz_8_1009	DJI_0895	Pulsatilla pratensis (L.) Mill. s.l.	0	n	1	0	Ranunculaceae Juss.
Iz_8_1009	DJI_0895	Pyrus communis L.	0	a	0	1	Rosaceae Juss.
Iz_8_1009	DJI_0895	Quercus robur L.	0	n	1	1	Fagaceae Dumort.
Iz_8_1009	DJI_0895	Robinia pseudoacacia L.	0	a	0	1	Fabaceae Lindl.
Iz_8_1009	DJI_0895	Rosa sp.	0		NA	1	Rosaceae Juss.
Iz_8_1009	DJI_0895	Rubus idaeus L.	0	n	1	0	Rosaceae Juss.
Iz_8_1009	DJI_0895	Rumex acetosella L.	0	n	1	0	Polygonaceae Juss.
Iz_8_1009	DJI_0895	Rumex thyrsiflorus Fingerh.	0	n	1	0	Polygonaceae Juss.
Iz_8_1009	DJI_0895	Salix alba L.	0	n	1	1	Salicaceae Mirb.
Iz_8_1009	DJI_0895	Sambucus nigra L.	0	n	1	1	Viburnaceae Raf.
Iz_8_1009	DJI_0895	Secale sylvestre Host	0	n	1	0	Poaceae Barnhart
Iz_8_1009	DJI_0895	Sempervivum ruthenicum Schnittsp. & C.B. Lehm.	0	n	1	0	Crassulaceae J.St.-Hil.
Iz_8_1009	DJI_0895	Setaria viridis (L.) P. Beauv.	0	n	1	0	Poaceae Barnhart
Iz_8_1009	DJI_0895	Silene borysthenica (Gruner) Walters	0	n	1	0	Caryophyllaceae Juss.
Iz_8_1009	DJI_0895	Silene latifolia subsp. alba (Mill.) Greuter & Burdet	0	n	1	0	Caryophyllaceae Juss.
Iz_8_1009	DJI_0895	Solidago virgaurea L.	0	n	1	0	Asteraceae Bercht. & J.Presl
Iz_8_1009	DJI_0895	Sorbus aucuparia L.	0	n	1	1	Rosaceae Juss.
Iz_8_1009	DJI_0895	Stachys recta L.	0	n	1	0	Lamiacea Lindl.
Iz_8_1009	DJI_0895	Tanacetum vulgare L.	4	n	1	0	Asteraceae Bercht. & J.Presl
Iz_8_1009	DJI_0895	Thymus pallasianus Heinr.Braun	0	n	1	0	Lamiacea Lindl.
Iz_8_1009	DJI_0895	Tilia cordata Mill.	0	n	1	1	Malvaceae Juss.
Iz_8_1009	DJI_0895	Trifolium alpestre L.	0	n	1	0	Fabaceae Lindl.
Iz_8_1009	DJI_0895	Trifolium arvense L.	0	n	1	0	Fabaceae Lindl.
Iz_8_1009	DJI_0895	Ulmus glabra Huds.	0	n	1	1	Ulmaceae Mirb.
Iz_8_1009	DJI_0895	Verbascum densiflorum Bertol.	0	n	1	0	Scrophulariaceae Juss.
Iz_8_1009	DJI_0895	Veronica austriaca L.	0	n	1	0	Plantaginaceae Juss.
Iz_8_1009	DJI_0895	Veronica incana L.	0	n	1	0	Plantaginaceae Juss.
Iz_8_1009	DJI_0895	Veronica verna L.	0	n	1	0	Plantaginaceae Juss.
Iz_8_1009	DJI_0895	Vincetoxicum hirundinaria Medik	0	n	1	0	Apocynaceae Juss.
Iz_8_1009	DJI_0896	Acer tataricum L.	0	n	1	1	Sapindaceae Juss.
Iz_8_1009	DJI_0896	Achillea millefolium L. s.l.	0	n	1	0	Asteraceae Bercht. & J.Presl
Iz_8_1009	DJI_0896	Allium oleraceum L.	0	n	1	0	Amaryllidaceae J.St.-Hil.
Iz_8_1009	DJI_0896	Ambrosia artemisiifolia L.	0	a	0	0	Asteraceae Bercht. & J.Presl
Iz_8_1009	DJI_0896	Amorpha fruticosa L.	0	a	0	0	Fabaceae Lindl.
Iz_8_1009	DJI_0896	Anthericum ramosum L.	0	n	1	0	Asparagaceae Juss.
Iz_8_1009	DJI_0896	Artemisia marschalliana Spreng.	0	n	1	0	Asteraceae Bercht. & J.Presl
Iz_8_1009	DJI_0896	Asparagus officinalis L.	0	n	1	0	Asparagaceae Juss.
Iz_8_1009	DJI_0896	Astragalus cicer L.	0	n	1	0	Fabaceae Lindl.
Iz_8_1009	DJI_0896	Bassia laniflora (S.G.Gmel.) A.J.Scott	0	n	1	0	Amaranthaceae Juss.
Iz_8_1009	DJI_0896	Berberis vulgaris L.	0	n	1	0	Berberidaceae Juss.
Iz_8_1009	DJI_0896	Betula pendula Roth.	0	n	1	1	Betulaceae Gray
Iz_8_1009	DJI_0896	Betula pendula Roth.	0	n	1	0	Poaceae Barnhart

Iz_8_1009	DJI_0896	Bromus inermis Leyss.	0		NA	NA	
Iz_8_1009	DJI_0896	Bromus squarrosus L.	0	a	0	0	Poaceae Barnhart
Iz_8_1009	DJI_0896	Bromus tectorum L.	0	a	0	0	Poaceae Barnhart
Iz_8_1009	DJI_0896	Calamagrostis epigejos (L.) Roth	20	n	1	0	Poaceae Barnhart
Iz_8_1009	DJI_0896	Carduus acanthoides L.	0	n	1	0	Asteraceae Bercht. & J.Presl
Iz_8_1009	DJI_0896	Carex sp.	0	n	1	0	Cyperaceae Juss.
Iz_8_1009	DJI_0896	Centaurea majorovii Dumbadze	0	n	1	0	Asteraceae Bercht. & J.Presl
Iz_8_1009	DJI_0896	Chamaecytisus ruthenicus (Fisch.ex Wol.) Klaskova	2	n	1	0	Fabaceae Lindl.
Iz_8_1009	DJI_0896	Chelidonium majus L.	0	n	1	0	Papaveraceae Juss.
Iz_8_1009	DJI_0896	Chenopodium betaceum Andrz.	0	n	1	0	Amaranthaceae Juss.
Iz_8_1009	DJI_0896	Chenopodium cf. album	0	n	1	0	Amaranthaceae Juss.
Iz_8_1009	DJI_0896	Chenopodium sp.	2		NA	0	Amaranthaceae Juss.
Iz_8_1009	DJI_0896	Chondrilla juncea L.	0	n	1	0	Asteraceae Bercht. & J.Presl
Iz_8_1009	DJI_0896	Cirsium vulgare (Savi) Ten.	0	n	1	0	Asteraceae Bercht. & J.Presl
Iz_8_1009	DJI_0896	Clinopodium acinos (L.) Kuntze	0	n	1	0	Lamiacea Lindl.
Iz_8_1009	DJI_0896	Convallaria majalis L.	0	n	1	0	Asparagaceae Juss.
Iz_8_1009	DJI_0896	Crataegus rhipidophylla Gand.	0	n	1	1	Rosaceae Juss.
Iz_8_1009	DJI_0896	Crepis foetida L.	0	n	1	0	Asteraceae Bercht. & J.Presl
Iz_8_1009	DJI_0896	Dactylis glomerata L.	0	n	1	0	Poaceae Barnhart
Iz_8_1009	DJI_0896	Dianthus campestris M.Bieb.	0	n	1	0	Caryophyllaceae Juss.
Iz_8_1009	DJI_0896	Digitaria sanguinalis (L.) Scop.	0	a	0	0	Poaceae Barnhart
Iz_8_1009	DJI_0896	Elymus repens (L.) Gould	0	n	1	0	Poaceae Barnhart
Iz_8_1009	DJI_0896	Epilobium tetragonum L.	0	n	1	0	Onagraceae Juss.
Iz_8_1009	DJI_0896	Eragrostis minor Host	0	a	0	0	Poaceae Barnhart
Iz_8_1009	DJI_0896	Erigeron canadensis L.	5	a	0	0	Asteraceae Bercht. & J.Presl
Iz_8_1009	DJI_0896	Euphorbia seguieriana Neck.	0	n	1	0	Euphorbiaceae Juss.
Iz_8_1009	DJI_0896	Fallopia dumetorum (L.) Holub	0	n	1	0	Polygonaceae Juss.
Iz_8_1009	DJI_0896	Festuca beckeri (Hack.) Trautv.	0	n	1	0	Poaceae Barnhart
Iz_8_1009	DJI_0896	Fragaria vesca L.	0	n	1	0	Rosaceae Juss.
Iz_8_1009	DJI_0896	Frangula alnus Mill.	0	n	1	1	Rhamnaceae Juss.
Iz_8_1009	DJI_0896	Fraxinus pennsylvanica Marshall	0	a	0	1	Oleaceae Hoffmanns. & Link
Iz_8_1009	DJI_0896	Galeopsis bifida Boenn.	0	n	1	0	Lamiacea Lindl.
Iz_8_1009	DJI_0896	Galium verum L.	0	n	1	0	Rubiaceae Juss.
Iz_8_1009	DJI_0896	Genista tinctoria L.	0	n	1	0	Fabaceae Lindl.
Iz_8_1009	DJI_0896	Geranium robertianum L.	0	n	1	0	Geraniaceae Juss.
Iz_8_1009	DJI_0896	Geranium sanquineum L.	0	n	1	0	Geraniaceae Juss.
Iz_8_1009	DJI_0896	Gypsophila paniculata L.	0	n	1	0	Caryophyllaceae Juss.
Iz_8_1009	DJI_0896	Helichrysum arenarium (L.) Moench	0	n	1	0	Asteraceae Bercht. & J.Presl
Iz_8_1009	DJI_0896	Herniaria polygama J.Gay	0	n	1	0	Caryophyllaceae Juss.
Iz_8_1009	DJI_0896	Hieracium robustum Fr.	0	n	1	0	Asteraceae Bercht. & J.Presl
Iz_8_1009	DJI_0896	Hieracium umbellatum L.	0	n	1	NA	Asteraceae Bercht. & J.Presl
Iz_8_1009	DJI_0896	Hylotelephium maximum (L.) Holub	0	n	1	0	Crassulaceae J.St.-Hil.
Iz_8_1009	DJI_0896	Jacobaea borysthenica (DC.) B.Nord. & Greuter	0	n	1	0	Asteraceae Bercht. & J.Presl
Iz_8_1009	DJI_0896	Jasione montana L.	0	n	1	0	Campanulaceae Juss.
Iz_8_1009	DJI_0896	Jurinea cyanoides (L.) Rchb.s.l.	0	n	1	0	Asteraceae Bercht. & J.Presl
Iz_8_1009	DJI_0896	Koeleria glauca (Spreng.) DC.	0	n	1	0	Poaceae Barnhart
Iz_8_1009	DJI_0896	Lactuca serriola L.	0	a	0	0	Asteraceae Bercht. & J.Presl
Iz_8_1009	DJI_0896	Linaria genistifolia (L.) Mill.	0	n	1	0	Plantaginaceae Juss.
Iz_8_1009	DJI_0896	Lomelosia argentea (L.) Greuter & Burdet	0	n	1	0	Caprifoliaceae Juss.
Iz_8_1009	DJI_0896	Melampyrum pratense L.	0	n	1	0	Orobanchaceae Vent.
Iz_8_1009	DJI_0896	Oenothera biennis L.	0	a	0	0	Onagraceae Juss.
Iz_8_1009	DJI_0896	Pentanema britannica (L.) D.Gut.Larr., Santos-Vicente, Anderb., E.Rico & M.M.Mart.Ort.	0	n	1	0	Asteraceae Bercht. & J.Presl
Iz_8_1009	DJI_0896	Peucedanum oreoselinum (L.) Moench	0	n	1	0	Apiaceae Lindl.
Iz_8_1009	DJI_0896	Peucedanum ruthenicum M.Bieb.	0	n	1	0	Apiaceae Lindl.
Iz_8_1009	DJI_0896	Pilosella echioides (Lumn.) F.W.Schultz & Sch.Bip.	0	n	1	0	Asteraceae Bercht. & J.Presl
Iz_8_1009	DJI_0896	Pilosella officinarum F. Schultz et Sch. Bip.	0	n	1	0	Asteraceae Bercht. & J.Presl
Iz_8_1009	DJI_0896	Pilosella sp.	0		NA	0	Asteraceae Bercht. & J.Presl
Iz_8_1009	DJI_0896	Pinus sylvestris L.	0	n	1	1	Pinaceae Spreng. ex F.Rudolphi
Iz_8_1009	DJI_0896	Poa annua L.	0	n	1	0	Poaceae Barnhart
Iz_8_1009	DJI_0896	Poa nemoralis L.	0	n	1	0	Poaceae Barnhart
Iz_8_1009	DJI_0896	Poa sp.	0		NA	0	Poaceae Barnhart
Iz_8_1009	DJI_0896	Polygonatum odoratum (Mill.) Druce	0	n	1	0	Asparagaceae Juss.
Iz_8_1009	DJI_0896	Polygonum arenarium Waldst. & Kit.	0	n	1	0	Polygonaceae Juss.
Iz_8_1009	DJI_0896	Populus nigra L.	0	n	1	1	Salicaceae Mirb.
Iz_8_1009	DJI_0896	Populus tremula L.	0	n	1	1	Salicaceae Mirb.
Iz_8_1009	DJI_0896	Populus Ã canescens (Aiton) Sm.	0	n	1	1	Salicaceae Mirb.
Iz_8_1009	DJI_0896	Potentilla argentea L.	0	n	1	0	Rosaceae Juss.
Iz_8_1009	DJI_0896	Psammophiliella muralis (L.) Ikonn.	0	n	1	0	Caryophyllaceae Juss.
Iz_8_1009	DJI_0896	Psephellus sumensis (Kalen.) Greuter	0	n	1	0	Asteraceae Bercht. & J.Presl
Iz_8_1009	DJI_0896	Pulsatilla pratensis (L.) Mill. s.l.	0	n	1	0	Ranunculaceae Juss.
Iz_8_1009	DJI_0896	Pyrus communis L.	0	a	0	1	Rosaceae Juss.
Iz_8_1009	DJI_0896	Quercus robur L.	0	n	1	1	Fagaceae Dumort.
Iz_8_1009	DJI_0896	Robinia pseudoacacia L.	0	a	0	1	Fabaceae Lindl.
Iz_8_1009	DJI_0896	Rosa sp.	0		NA	1	Rosaceae Juss.
Iz_8_1009	DJI_0896	Rubus idaeus L.	0	n	1	0	Rosaceae Juss.
Iz_8_1009	DJI_0896	Rumex acetosella L.	2	n	1	0	Polygonaceae Juss.
Iz_8_1009	DJI_0896	Rumex thyrsiflorus Fingerh.	0	n	1	0	Polygonaceae Juss.
Iz_8_1009	DJI_0896	Salix alba L.	0	n	1	1	Salicaceae Mirb.
Iz_8_1009	DJI_0896	Sambucus nigra L.	0	n	1	1	Viburnaceae Raf.
Iz_8_1009	DJI_0896	Secale sylvestre Host	0	n	1	0	Poaceae Barnhart
Iz_8_1009	DJI_0896	Sempervivum ruthenicum Schnittsp. & C.B. Lehm.	0	n	1	0	Crassulaceae J.St.-Hil.
Iz_8_1009	DJI_0896	Setaria viridis (L.) P. Beauv.	0	n	1	0	Poaceae Barnhart
Iz_8_1009	DJI_0896	Silene borysthenica (Gruner) Walters	0	n	1	0	Caryophyllaceae Juss.
Iz_8_1009	DJI_0896	Silene latifolia subsp. alba (Mill.) Greuter & Burdet	0	n	1	0	Caryophyllaceae Juss.
Iz_8_1009	DJI_0896	Solidago virgaurea L.	0	n	1	0	Asteraceae Bercht. & J.Presl
Iz_8_1009	DJI_0896	Sorbus aucuparia L.	0	n	1	1	Rosaceae Juss.
Iz_8_1009	DJI_0896	Stachys recta L.	0	n	1	0	Lamiacea Lindl.
Iz_8_1009	DJI_0896	Tanacetum vulgare L.	3	n	1	0	Asteraceae Bercht. & J.Presl
Iz_8_1009	DJI_0896	Thymus pallasianus Heinr.Braun	0	n	1	0	Lamiacea Lindl.
Iz_8_1009	DJI_0896	Tilia cordata Mill.	0	n	1	1	Malvaceae Juss.
Iz_8_1009	DJI_0896	Trifolium alpestre L.	0	n	1	0	Fabaceae Lindl.
Iz_8_1009	DJI_0896	Trifolium arvense L.	0	n	1	0	Fabaceae Lindl.
Iz_8_1009	DJI_0896	Ulmus glabra Huds.	0	n	1	1	Ulmaceae Mirb.
Iz_8_1009	DJI_0896	Verbascum densiflorum Bertol.	0	n	1	0	Scrophulariaceae Juss.
Iz_8_1009	DJI_0896	Veronica austriaca L.	0	n	1	0	Plantaginaceae Juss.
Iz_8_1009	DJI_0896	Veronica incana L.	0	n	1	0	Plantaginaceae Juss.
Iz_8_1009	DJI_0896	Veronica verna L.	0	n	1	0	Plantaginaceae Juss.
Iz_8_1009	DJI_0896	Vincetoxicum hirundinaria Medik	0	n	1	0	Apocynaceae Juss.

Iz_8_1009	DJI_0897	Acer tataricum L.	0	n	1	1	Sapindaceae Juss.
Iz_8_1009	DJI_0897	Achillea millefolium L. s.l.	0	n	1	0	Asteraceae Bercht. & J.Presl
Iz_8_1009	DJI_0897	Allium oleraceum L.	0	n	1	0	Amaryllidaceae J.St.-Hil.
Iz_8_1009	DJI_0897	Ambrosia artemisiifolia L.	0	a	0	0	Asteraceae Bercht. & J.Presl
Iz_8_1009	DJI_0897	Amorpha fruticosa L.	0	a	0	0	Fabaceae Lindl.
Iz_8_1009	DJI_0897	Anthericum ramosum L.	0	n	1	0	Asparagaceae Juss.
Iz_8_1009	DJI_0897	Artemisia marschalliana Spreng.	0	n	1	0	Asteraceae Bercht. & J.Presl
Iz_8_1009	DJI_0897	Asparagus officinalis L.	0	n	1	0	Asparagaceae Juss.
Iz_8_1009	DJI_0897	Astragalus cicer L.	0	n	1	0	Fabaceae Lindl.
Iz_8_1009	DJI_0897	Bassia laniflora (S.G.Gmel.) A.J.Scott	0	n	1	0	Amaranthaceae Juss.
Iz_8_1009	DJI_0897	Berberis vulgaris L.	0	n	1	0	Berberidaceae Juss.
Iz_8_1009	DJI_0897	Betula pendula Roth.	0	n	1	1	Betulaceae Gray
Iz_8_1009	DJI_0897	Betula pendula Roth.	0	n	1	0	Poaceae Barnhart
Iz_8_1009	DJI_0897	Bromus inermis Leyss.	0		NA	NA	
Iz_8_1009	DJI_0897	Bromus squarrosus L.	0	a	0	0	Poaceae Barnhart
Iz_8_1009	DJI_0897	Bromus tectorum L.	0	a	0	0	Poaceae Barnhart
Iz_8_1009	DJI_0897	Calamagrostis epigejos (L.) Roth	60	n	1	0	Poaceae Barnhart
Iz_8_1009	DJI_0897	Carduus acanthoides L.	0	n	1	0	Asteraceae Bercht. & J.Presl
Iz_8_1009	DJI_0897	Carex sp.	0	n	1	0	Cyperaceae Juss.
Iz_8_1009	DJI_0897	Centaurea majorovii Dumbadze	0	n	1	0	Asteraceae Bercht. & J.Presl
Iz_8_1009	DJI_0897	Chamaecytisus ruthenicus (Fisch.ex Wol.) Klaskova	0	n	1	0	Fabaceae Lindl.
Iz_8_1009	DJI_0897	Chelidonium majus L.	0	n	1	0	Papaveraceae Juss.
Iz_8_1009	DJI_0897	Chenopodium betaceum Andrz.	0	n	1	0	Amaranthaceae Juss.
Iz_8_1009	DJI_0897	Chenopodium cf. album	0	n	1	0	Amaranthaceae Juss.
Iz_8_1009	DJI_0897	Chenopodium sp.	0		NA	0	Amaranthaceae Juss.
Iz_8_1009	DJI_0897	Chondrilla juncea L.	0	n	1	0	Asteraceae Bercht. & J.Presl
Iz_8_1009	DJI_0897	Cirsium vulgare (Savi) Ten.	0	n	1	0	Asteraceae Bercht. & J.Presl
Iz_8_1009	DJI_0897	Clinopodium acinos (L.) Kuntze	0	n	1	0	Lamiacea Lindl.
Iz_8_1009	DJI_0897	Convallaria majalis L.	0	n	1	0	Asparagaceae Juss.
Iz_8_1009	DJI_0897	Crataegus rhipidophylla Gand.	0	n	1	1	Rosaceae Juss.
Iz_8_1009	DJI_0897	Crepis foetida L.	0	n	1	0	Asteraceae Bercht. & J.Presl
Iz_8_1009	DJI_0897	Dactylis glomerata L.	0	n	1	0	Poaceae Barnhart
Iz_8_1009	DJI_0897	Dianthus campestris M.Bieb.	0	n	1	0	Caryophyllaceae Juss.
Iz_8_1009	DJI_0897	Digitaria sanguinalis (L.) Scop.	0	a	0	0	Poaceae Barnhart
Iz_8_1009	DJI_0897	Elymus repens (L.) Gould	0	n	1	0	Poaceae Barnhart
Iz_8_1009	DJI_0897	Epilobium tetragonum L.	0	n	1	0	Onagraceae Juss.
Iz_8_1009	DJI_0897	Eragrostis minor Host	0	a	0	0	Poaceae Barnhart
Iz_8_1009	DJI_0897	Erigeron canadensis L.	2	a	0	0	Asteraceae Bercht. & J.Presl
Iz_8_1009	DJI_0897	Euphorbia seguieriana Neck.	0	n	1	0	Euphorbiaceae Juss.
Iz_8_1009	DJI_0897	Fallopia dumetorum (L.) Holub	0	n	1	0	Polygonaceae Juss.
Iz_8_1009	DJI_0897	Festuca beckeri (Hack.) Trautv.	0	n	1	0	Poaceae Barnhart
Iz_8_1009	DJI_0897	Fragaria vesca L.	0	n	1	0	Rosaceae Juss.
Iz_8_1009	DJI_0897	Frangula alnus Mill.	0	n	1	1	Rhamnaceae Juss.
Iz_8_1009	DJI_0897	Fraxinus pennsylvanica Marshall	0	a	0	1	Oleaceae Hoffmanns. & Link
Iz_8_1009	DJI_0897	Galeopsis bifida Boenn.	0	n	1	0	Lamiacea Lindl.
Iz_8_1009	DJI_0897	Galium verum L.	0	n	1	0	Rubiaceae Juss.
Iz_8_1009	DJI_0897	Genista tinctoria L.	0	n	1	0	Fabaceae Lindl.
Iz_8_1009	DJI_0897	Geranium robertianum L.	0	n	1	0	Geraniaceae Juss.
Iz_8_1009	DJI_0897	Geranium sanquineum L.	0	n	1	0	Geraniaceae Juss.
Iz_8_1009	DJI_0897	Gypsophila paniculata L.	0	n	1	0	Caryophyllaceae Juss.
Iz_8_1009	DJI_0897	Helichrysum arenarium (L.) Moench	0	n	1	0	Asteraceae Bercht. & J.Presl
Iz_8_1009	DJI_0897	Herniaria polygama J.Gay	0	n	1	0	Caryophyllaceae Juss.
Iz_8_1009	DJI_0897	Hieracium robustum Fr.	0	n	1	0	Asteraceae Bercht. & J.Presl
Iz_8_1009	DJI_0897	Hieracium umbellatum L.	0	n	1	NA	Asteraceae Bercht. & J.Presl
Iz_8_1009	DJI_0897	Hylotelephium maximum (L.) Holub	0	n	1	0	Crassulaceae J.St.-Hil.
Iz_8_1009	DJI_0897	Jacobaea borysthenica (DC.) B.Nord. & Greuter	0	n	1	0	Asteraceae Bercht. & J.Presl
Iz_8_1009	DJI_0897	Jasione montana L.	0	n	1	0	Campanulaceae Juss.
Iz_8_1009	DJI_0897	Jurinea cyanoides (L.) Rchb.s.l.	0	n	1	0	Asteraceae Bercht. & J.Presl
Iz_8_1009	DJI_0897	Koeleria glauca (Spreng.) DC.	0	n	1	0	Poaceae Barnhart
Iz_8_1009	DJI_0897	Lactuca serriola L.	0	a	0	0	Asteraceae Bercht. & J.Presl
Iz_8_1009	DJI_0897	Linaria genistifolia (L.) Mill.	0	n	1	0	Plantaginaceae Juss.
Iz_8_1009	DJI_0897	Lomelosia argentea (L.) Greuter & Burdet	0	n	1	0	Caprifoliaceae Juss.
Iz_8_1009	DJI_0897	Melampyrum pratense L.	0	n	1	0	Orobanchaceae Vent.
Iz_8_1009	DJI_0897	Oenothera biennis L.	0	a	0	0	Onagraceae Juss.
Iz_8_1009	DJI_0897	Pentanema britannica (L.) D.Gut.Larr., Santos-Vicente, Anderb., E.Rico & M.M.Mart.Ort.	0	n	1	0	Asteraceae Bercht. & J.Presl
Iz_8_1009	DJI_0897	Peucedanum oreoselinum (L.) Moench	0	n	1	0	Apiaceae Lindl.
Iz_8_1009	DJI_0897	Peucedanum ruthenicum M.Bieb.	0	n	1	0	Apiaceae Lindl.
Iz_8_1009	DJI_0897	Pilosella echioides (Lumn.) F.W.Schultz & Sch.Bip.	0	n	1	0	Asteraceae Bercht. & J.Presl
Iz_8_1009	DJI_0897	Pilosella officinarum F. Schultz et Sch. Bip.	0	n	1	0	Asteraceae Bercht. & J.Presl
Iz_8_1009	DJI_0897	Pilosella sp.	0		NA	0	Asteraceae Bercht. & J.Presl
Iz_8_1009	DJI_0897	Pinus sylvestris L.	0	n	1	1	Pinaceae Spreng. ex F.Rudolphi
Iz_8_1009	DJI_0897	Poa annua L.	0	n	1	0	Poaceae Barnhart
Iz_8_1009	DJI_0897	Poa nemoralis L.	0	n	1	0	Poaceae Barnhart
Iz_8_1009	DJI_0897	Poa sp.	0		NA	0	Poaceae Barnhart
Iz_8_1009	DJI_0897	Polygonatum odoratum (Mill.) Druce	0	n	1	0	Asparagaceae Juss.
Iz_8_1009	DJI_0897	Polygonum arenarium Waldst. & Kit.	0	n	1	0	Polygonaceae Juss.
Iz_8_1009	DJI_0897	Populus nigra L.	0	n	1	1	Salicaceae Mirb.
Iz_8_1009	DJI_0897	Populus tremula L.	0	n	1	1	Salicaceae Mirb.
Iz_8_1009	DJI_0897	Populus Ã canescens (Aiton) Sm.	0	n	1	1	Salicaceae Mirb.
Iz_8_1009	DJI_0897	Potentilla argentea L.	0	n	1	0	Rosaceae Juss.
Iz_8_1009	DJI_0897	Psammophiliella muralis (L.) Ikonn.	0	n	1	0	Caryophyllaceae Juss.
Iz_8_1009	DJI_0897	Psephellus sumensis (Kalen.) Greuter	0	n	1	0	Asteraceae Bercht. & J.Presl
Iz_8_1009	DJI_0897	Pulsatilla pratensis (L.) Mill. s.l.	0	n	1	0	Ranunculaceae Juss.
Iz_8_1009	DJI_0897	Pyrus communis L.	0	a	0	1	Rosaceae Juss.
Iz_8_1009	DJI_0897	Quercus robur L.	0	n	1	1	Fagaceae Dumort.
Iz_8_1009	DJI_0897	Robinia pseudoacacia L.	0	a	0	1	Fabaceae Lindl.
Iz_8_1009	DJI_0897	Rosa sp.	0		NA	1	Rosaceae Juss.
Iz_8_1009	DJI_0897	Rubus idaeus L.	0	n	1	0	Rosaceae Juss.
Iz_8_1009	DJI_0897	Rumex acetosella L.	0	n	1	0	Polygonaceae Juss.
Iz_8_1009	DJI_0897	Rumex thyrsiflorus Fingerh.	0	n	1	0	Polygonaceae Juss.
Iz_8_1009	DJI_0897	Salix alba L.	0	n	1	1	Salicaceae Mirb.
Iz_8_1009	DJI_0897	Sambucus nigra L.	0	n	1	1	Viburnaceae Raf.
Iz_8_1009	DJI_0897	Secale sylvestre Host	0	n	1	0	Poaceae Barnhart
Iz_8_1009	DJI_0897	Sempervivum ruthenicum Schnittsp. & C.B. Lehm.	0	n	1	0	Crassulaceae J.St.-Hil.
Iz_8_1009	DJI_0897	Setaria viridis (L.) P. Beauv.	0	n	1	0	Poaceae Barnhart
Iz_8_1009	DJI_0897	Silene borysthenica (Gruner) Walters	0	n	1	0	Caryophyllaceae Juss.
Iz_8_1009	DJI_0897	Silene latifolia subsp. alba (Mill.) Greuter & Burdet	0	n	1	0	Caryophyllaceae Juss.
Iz_8_1009	DJI_0897	Solidago virgaurea L.	0	n	1	0	Asteraceae Bercht. & J.Presl

Iz_8_1009	DJI_0897	Sorbus aucuparia L.	0	n	1	1	Rosaceae Juss.
Iz_8_1009	DJI_0897	Stachys recta L.	0	n	1	0	Lamiacea Lindl.
Iz_8_1009	DJI_0897	Tanacetum vulgare L.	0	n	1	0	Asteraceae Bercht. & J.Presl
Iz_8_1009	DJI_0897	Thymus pallasianus Heinr.Braun	0	n	1	0	Lamiacea Lindl.
Iz_8_1009	DJI_0897	Tilia cordata Mill.	0	n	1	1	Malvaceae Juss.
Iz_8_1009	DJI_0897	Trifolium alpestre L.	0	n	1	0	Fabaceae Lindl.
Iz_8_1009	DJI_0897	Trifolium arvense L.	0	n	1	0	Fabaceae Lindl.
Iz_8_1009	DJI_0897	Ulmus glabra Huds.	0	n	1	1	Ulmaceae Mirb.
Iz_8_1009	DJI_0897	Verbascum densiflorum Bertol.	0	n	1	0	Scrophulariaceae Juss.
Iz_8_1009	DJI_0897	Veronica austriaca L.	0	n	1	0	Plantaginaceae Juss.
Iz_8_1009	DJI_0897	Veronica incana L.	0	n	1	0	Plantaginaceae Juss.
Iz_8_1009	DJI_0897	Veronica verna L.	0	n	1	0	Plantaginaceae Juss.
Iz_8_1009	DJI_0897	Vincetoxicum hirundinaria Medik	0	n	1	0	Apocynaceae Juss.
Iz_8_1009	DJI_0898	Acer tataricum L.	0	n	1	1	Sapindaceae Juss.
Iz_8_1009	DJI_0898	Achillea millefolium L. s.l.	0	n	1	0	Asteraceae Bercht. & J.Presl
Iz_8_1009	DJI_0898	Allium oleraceum L.	0	n	1	0	Amaryllidaceae J.St.-Hil.
Iz_8_1009	DJI_0898	Ambrosia artemisiifolia L.	0	a	0	0	Asteraceae Bercht. & J.Presl
Iz_8_1009	DJI_0898	Amorpha fruticosa L.	0	a	0	0	Fabaceae Lindl.
Iz_8_1009	DJI_0898	Anthericum ramosum L.	0	n	1	0	Asparagaceae Juss.
Iz_8_1009	DJI_0898	Artemisia marschalliana Spreng.	0	n	1	0	Asteraceae Bercht. & J.Presl
Iz_8_1009	DJI_0898	Asparagus officinalis L.	0	n	1	0	Asparagaceae Juss.
Iz_8_1009	DJI_0898	Astragalus cicer L.	0	n	1	0	Fabaceae Lindl.
Iz_8_1009	DJI_0898	Bassia laniflora (S.G.Gmel.) A.J.Scott	0	n	1	0	Amaranthaceae Juss.
Iz_8_1009	DJI_0898	Berberis vulgaris L.	0	n	1	0	Berberidaceae Juss.
Iz_8_1009	DJI_0898	Betula pendula Roth.	0	n	1	1	Betulaceae Gray
Iz_8_1009	DJI_0898	Betula pendula Roth.	0	n	1	0	Poaceae Barnhart
Iz_8_1009	DJI_0898	Bromus inermis Leyss.	0		NA	NA	
Iz_8_1009	DJI_0898	Bromus squarrosus L.	0	a	0	0	Poaceae Barnhart
Iz_8_1009	DJI_0898	Bromus tectorum L.	0	a	0	0	Poaceae Barnhart
Iz_8_1009	DJI_0898	Calamagrostis epigejos (L.) Roth	70	n	1	0	Poaceae Barnhart
Iz_8_1009	DJI_0898	Carduus acanthoides L.	0	n	1	0	Asteraceae Bercht. & J.Presl
Iz_8_1009	DJI_0898	Carex sp.	0	n	1	0	Cyperaceae Juss.
Iz_8_1009	DJI_0898	Centaurea majorovii Dumbadze	0	n	1	0	Asteraceae Bercht. & J.Presl
Iz_8_1009	DJI_0898	Chamaecytisus ruthenicus (Fisch.ex Wol.) Klaskova	0	n	1	0	Fabaceae Lindl.
Iz_8_1009	DJI_0898	Chelidonium majus L.	0	n	1	0	Papaveraceae Juss.
Iz_8_1009	DJI_0898	Chenopodium betaceum Andrz.	0	n	1	0	Amaranthaceae Juss.
Iz_8_1009	DJI_0898	Chenopodium cf. album	0	n	1	0	Amaranthaceae Juss.
Iz_8_1009	DJI_0898	Chenopodium sp.	0		NA	0	Amaranthaceae Juss.
Iz_8_1009	DJI_0898	Chondrilla juncea L.	0	n	1	0	Asteraceae Bercht. & J.Presl
Iz_8_1009	DJI_0898	Cirsium vulgare (Savi) Ten.	0	n	1	0	Asteraceae Bercht. & J.Presl
Iz_8_1009	DJI_0898	Clinopodium acinos (L.) Kuntze	0	n	1	0	Lamiacea Lindl.
Iz_8_1009	DJI_0898	Convallaria majalis L.	0	n	1	0	Asparagaceae Juss.
Iz_8_1009	DJI_0898	Crataegus rhipidophylla Gand.	0	n	1	1	Rosaceae Juss.
Iz_8_1009	DJI_0898	Crepis foetida L.	0	n	1	0	Asteraceae Bercht. & J.Presl
Iz_8_1009	DJI_0898	Dactylis glomerata L.	0	n	1	0	Poaceae Barnhart
Iz_8_1009	DJI_0898	Dianthus campestris M.Bieb.	0	n	1	0	Caryophyllaceae Juss.
Iz_8_1009	DJI_0898	Digitaria sanguinalis (L.) Scop.	0	a	0	0	Poaceae Barnhart
Iz_8_1009	DJI_0898	Elymus repens (L.) Gould	0	n	1	0	Poaceae Barnhart
Iz_8_1009	DJI_0898	Epilobium tetragonum L.	0	n	1	0	Onagraceae Juss.
Iz_8_1009	DJI_0898	Eragrostis minor Host	0	a	0	0	Poaceae Barnhart
Iz_8_1009	DJI_0898	Erigeron canadensis L.	0	a	0	0	Asteraceae Bercht. & J.Presl
Iz_8_1009	DJI_0898	Euphorbia seguieriana Neck.	0	n	1	0	Euphorbiaceae Juss.
Iz_8_1009	DJI_0898	Fallopia dumetorum (L.) Holub	0	n	1	0	Polygonaceae Juss.
Iz_8_1009	DJI_0898	Festuca beckeri (Hack.) Trautv.	0	n	1	0	Poaceae Barnhart
Iz_8_1009	DJI_0898	Fragaria vesca L.	0	n	1	0	Rosaceae Juss.
Iz_8_1009	DJI_0898	Frangula alnus Mill.	0	n	1	1	Rhamnaceae Juss.
Iz_8_1009	DJI_0898	Fraxinus pennsylvanica Marshall	0	a	0	1	Oleaceae Hoffmanns. & Link
Iz_8_1009	DJI_0898	Galeopsis bifida Boenn.	0	n	1	0	Lamiacea Lindl.
Iz_8_1009	DJI_0898	Galium verum L.	0	n	1	0	Rubiaceae Juss.
Iz_8_1009	DJI_0898	Genista tinctoria L.	0	n	1	0	Fabaceae Lindl.
Iz_8_1009	DJI_0898	Geranium robertianum L.	0	n	1	0	Geraniaceae Juss.
Iz_8_1009	DJI_0898	Geranium sanquineum L.	0	n	1	0	Geraniaceae Juss.
Iz_8_1009	DJI_0898	Gypsophila paniculata L.	0	n	1	0	Caryophyllaceae Juss.
Iz_8_1009	DJI_0898	Helichrysum arenarium (L.) Moench	0	n	1	0	Asteraceae Bercht. & J.Presl
Iz_8_1009	DJI_0898	Herniaria polygama J.Gay	0	n	1	0	Caryophyllaceae Juss.
Iz_8_1009	DJI_0898	Hieracium robustum Fr.	0	n	1	0	Asteraceae Bercht. & J.Presl
Iz_8_1009	DJI_0898	Hieracium umbellatum L.	0	n	1	NA	Asteraceae Bercht. & J.Presl
Iz_8_1009	DJI_0898	Hylotelephium maximum (L.) Holub	0	n	1	0	Crassulaceae J.St.-Hil.
Iz_8_1009	DJI_0898	Jacobaea borysthenica (DC.) B.Nord. & Greuter	0	n	1	0	Asteraceae Bercht. & J.Presl
Iz_8_1009	DJI_0898	Jasione montana L.	0	n	1	0	Campanulaceae Juss.
Iz_8_1009	DJI_0898	Jurinea cyanoides (L.) Rchb.s.l.	0	n	1	0	Asteraceae Bercht. & J.Presl
Iz_8_1009	DJI_0898	Koeleria glauca (Spreng.) DC.	0	n	1	0	Poaceae Barnhart
Iz_8_1009	DJI_0898	Lactuca serriola L.	0	a	0	0	Asteraceae Bercht. & J.Presl
Iz_8_1009	DJI_0898	Linaria genistifolia (L.) Mill.	0	n	1	0	Plantaginaceae Juss.
Iz_8_1009	DJI_0898	Lomelosia argentea (L.) Greuter & Burdet	0	n	1	0	Caprifoliaceae Juss.
Iz_8_1009	DJI_0898	Melampyrum pratense L.	0	n	1	0	Orobanchaceae Vent.
Iz_8_1009	DJI_0898	Oenothera biennis L.	0	a	0	0	Onagraceae Juss.
Iz_8_1009	DJI_0898	Pentanema britannica (L.) D.Gut.Larr., Santos-Vicente, Anderb., E.Rico & M.M.Mart.Ort.	0	n	1	0	Asteraceae Bercht. & J.Presl
Iz_8_1009	DJI_0898	Peucedanum oreoselinum (L.) Moench	0	n	1	0	Apiaceae Lindl.
Iz_8_1009	DJI_0898	Peucedanum ruthenicum M.Bieb.	0	n	1	0	Apiaceae Lindl.
Iz_8_1009	DJI_0898	Pilosella echioides (Lumn.) F.W.Schultz & Sch.Bip.	0	n	1	0	Asteraceae Bercht. & J.Presl
Iz_8_1009	DJI_0898	Pilosella officinarum F. Schultz et Sch. Bip.	0	n	1	0	Asteraceae Bercht. & J.Presl
Iz_8_1009	DJI_0898	Pilosella sp.	0		NA	0	Asteraceae Bercht. & J.Presl
Iz_8_1009	DJI_0898	Pinus sylvestris L.	0	n	1	1	Pinaceae Spreng. ex F.Rudolphi
Iz_8_1009	DJI_0898	Poa annua L.	0	n	1	0	Poaceae Barnhart
Iz_8_1009	DJI_0898	Poa nemoralis L.	0	n	1	0	Poaceae Barnhart
Iz_8_1009	DJI_0898	Poa sp.	0		NA	0	Poaceae Barnhart
Iz_8_1009	DJI_0898	Polygonatum odoratum (Mill.) Druce	0	n	1	0	Asparagaceae Juss.
Iz_8_1009	DJI_0898	Polygonum arenarium Waldst. & Kit.	0	n	1	0	Polygonaceae Juss.
Iz_8_1009	DJI_0898	Populus nigra L.	0	n	1	1	Salicaceae Mirb.
Iz_8_1009	DJI_0898	Populus tremula L.	10	n	1	1	Salicaceae Mirb.
Iz_8_1009	DJI_0898	Populus Ã canescens (Aiton) Sm.	0	n	1	1	Salicaceae Mirb.
Iz_8_1009	DJI_0898	Potentilla argentea L.	0	n	1	0	Rosaceae Juss.
Iz_8_1009	DJI_0898	Psammophiliella muralis (L.) Ikonn.	0	n	1	0	Caryophyllaceae Juss.
Iz_8_1009	DJI_0898	Psephellus sumensis (Kalen.) Greuter	0	n	1	0	Asteraceae Bercht. & J.Presl
Iz_8_1009	DJI_0898	Pulsatilla pratensis (L.) Mill. s.l.	0	n	1	0	Ranunculaceae Juss.
Iz_8_1009	DJI_0898	Pyrus communis L.	0	a	0	1	Rosaceae Juss.
Iz_8_1009	DJI_0898	Quercus robur L.	0	n	1	1	Fagaceae Dumort.

Iz_8_1009	DJI_0898	Robinia pseudoacacia L.	0	a	0	1	Fabaceae Lindl.
Iz_8_1009	DJI_0898	Rosa sp.	0		NA	1	Rosaceae Juss.
Iz_8_1009	DJI_0898	Rubus idaeus L.	0	n	1	0	Rosaceae Juss.
Iz_8_1009	DJI_0898	Rumex acetosella L.	0	n	1	0	Polygonaceae Juss.
Iz_8_1009	DJI_0898	Rumex thyrsiflorus Fingerh.	0	n	1	0	Polygonaceae Juss.
Iz_8_1009	DJI_0898	Salix alba L.	0	n	1	1	Salicaceae Mirb.
Iz_8_1009	DJI_0898	Sambucus nigra L.	0	n	1	1	Viburnaceae Raf.
Iz_8_1009	DJI_0898	Secale sylvestre Host	0	n	1	0	Poaceae Barnhart
Iz_8_1009	DJI_0898	Sempervivum ruthenicum Schnittsp. & C.B. Lehm.	0	n	1	0	Crassulaceae J.St.-Hil.
Iz_8_1009	DJI_0898	Setaria viridis (L.) P. Beauv.	0	n	1	0	Poaceae Barnhart
Iz_8_1009	DJI_0898	Silene borysthenica (Gruner) Walters	0	n	1	0	Caryophyllaceae Juss.
Iz_8_1009	DJI_0898	Silene latifolia subsp. alba (Mill.) Greuter & Burdet	0	n	1	0	Caryophyllaceae Juss.
Iz_8_1009	DJI_0898	Solidago virgaurea L.	0	n	1	0	Asteraceae Bercht. & J.Presl
Iz_8_1009	DJI_0898	Sorbus aucuparia L.	0	n	1	1	Rosaceae Juss.
Iz_8_1009	DJI_0898	Stachys recta L.	0	n	1	0	Lamiacea Lindl.
Iz_8_1009	DJI_0898	Tanacetum vulgare L.	0	n	1	0	Asteraceae Bercht. & J.Presl
Iz_8_1009	DJI_0898	Thymus pallasianus Heinr.Braun	0	n	1	0	Lamiacea Lindl.
Iz_8_1009	DJI_0898	Tilia cordata Mill.	0	n	1	1	Malvaceae Juss.
Iz_8_1009	DJI_0898	Trifolium alpestre L.	0	n	1	0	Fabaceae Lindl.
Iz_8_1009	DJI_0898	Trifolium arvense L.	0	n	1	0	Fabaceae Lindl.
Iz_8_1009	DJI_0898	Ulmus glabra Huds.	0	n	1	1	Ulmaceae Mirb.
Iz_8_1009	DJI_0898	Verbascum densiflorum Bertol.	0	n	1	0	Scrophulariaceae Juss.
Iz_8_1009	DJI_0898	Veronica austriaca L.	0	n	1	0	Plantaginaceae Juss.
Iz_8_1009	DJI_0898	Veronica incana L.	0	n	1	0	Plantaginaceae Juss.
Iz_8_1009	DJI_0898	Veronica verna L.	0	n	1	0	Plantaginaceae Juss.
Iz_8_1009	DJI_0898	Vincetoxicum hirundinaria Medik	0	n	1	0	Apocynaceae Juss.
Iz_8_1009	DJI_0899	Acer tataricum L.	0	n	1	1	Sapindaceae Juss.
Iz_8_1009	DJI_0899	Achillea millefolium L. s.l.	0	n	1	0	Asteraceae Bercht. & J.Presl
Iz_8_1009	DJI_0899	Allium oleraceum L.	0	n	1	0	Amaryllidaceae J.St.-Hil.
Iz_8_1009	DJI_0899	Ambrosia artemisiifolia L.	0	a	0	0	Asteraceae Bercht. & J.Presl
Iz_8_1009	DJI_0899	Amorpha fruticosa L.	0	a	0	0	Fabaceae Lindl.
Iz_8_1009	DJI_0899	Anthericum ramosum L.	0	n	1	0	Asparagaceae Juss.
Iz_8_1009	DJI_0899	Artemisia marschalliana Spreng.	0	n	1	0	Asteraceae Bercht. & J.Presl
Iz_8_1009	DJI_0899	Asparagus officinalis L.	0	n	1	0	Asparagaceae Juss.
Iz_8_1009	DJI_0899	Astragalus cicer L.	0	n	1	0	Fabaceae Lindl.
Iz_8_1009	DJI_0899	Bassia laniflora (S.G.Gmel.) A.J.Scott	0	n	1	0	Amaranthaceae Juss.
Iz_8_1009	DJI_0899	Berberis vulgaris L.	0	n	1	0	Berberidaceae Juss.
Iz_8_1009	DJI_0899	Betula pendula Roth.	0	n	1	1	Betulaceae Gray
Iz_8_1009	DJI_0899	Betula pendula Roth.	0	n	1	0	Poaceae Barnhart
Iz_8_1009	DJI_0899	Bromus inermis Leyss.	0		NA	NA	
Iz_8_1009	DJI_0899	Bromus squarrosus L.	0	a	0	0	Poaceae Barnhart
Iz_8_1009	DJI_0899	Bromus tectorum L.	0	a	0	0	Poaceae Barnhart
Iz_8_1009	DJI_0899	Calamagrostis epigejos (L.) Roth	40	n	1	0	Poaceae Barnhart
Iz_8_1009	DJI_0899	Carduus acanthoides L.	0	n	1	0	Asteraceae Bercht. & J.Presl
Iz_8_1009	DJI_0899	Carex sp.	0	n	1	0	Cyperaceae Juss.
Iz_8_1009	DJI_0899	Centaurea majorovii Dumbadze	0	n	1	0	Asteraceae Bercht. & J.Presl
Iz_8_1009	DJI_0899	Chamaecytisus ruthenicus (Fisch.ex Wol.) Klaskova	0	n	1	0	Fabaceae Lindl.
Iz_8_1009	DJI_0899	Chelidonium majus L.	0	n	1	0	Papaveraceae Juss.
Iz_8_1009	DJI_0899	Chenopodium betaceum Andrz.	0	n	1	0	Amaranthaceae Juss.
Iz_8_1009	DJI_0899	Chenopodium cf. album	0	n	1	0	Amaranthaceae Juss.
Iz_8_1009	DJI_0899	Chenopodium sp.	0		NA	0	Amaranthaceae Juss.
Iz_8_1009	DJI_0899	Chondrilla juncea L.	0	n	1	0	Asteraceae Bercht. & J.Presl
Iz_8_1009	DJI_0899	Cirsium vulgare (Savi) Ten.	0	n	1	0	Asteraceae Bercht. & J.Presl
Iz_8_1009	DJI_0899	Clinopodium acinos (L.) Kuntze	0	n	1	0	Lamiacea Lindl.
Iz_8_1009	DJI_0899	Convallaria majalis L.	0	n	1	0	Asparagaceae Juss.
Iz_8_1009	DJI_0899	Crataegus rhipidophylla Gand.	0	n	1	1	Rosaceae Juss.
Iz_8_1009	DJI_0899	Crepis foetida L.	0	n	1	0	Asteraceae Bercht. & J.Presl
Iz_8_1009	DJI_0899	Dactylis glomerata L.	0	n	1	0	Poaceae Barnhart
Iz_8_1009	DJI_0899	Dianthus campestris M.Bieb.	0	n	1	0	Caryophyllaceae Juss.
Iz_8_1009	DJI_0899	Digitaria sanguinalis (L.) Scop.	0	a	0	0	Poaceae Barnhart
Iz_8_1009	DJI_0899	Elymus repens (L.) Gould	0	n	1	0	Poaceae Barnhart
Iz_8_1009	DJI_0899	Epilobium tetragonum L.	0	n	1	0	Onagraceae Juss.
Iz_8_1009	DJI_0899	Eragrostis minor Host	0	a	0	0	Poaceae Barnhart
Iz_8_1009	DJI_0899	Erigeron canadensis L.	10	a	0	0	Asteraceae Bercht. & J.Presl
Iz_8_1009	DJI_0899	Euphorbia seguieriana Neck.	0	n	1	0	Euphorbiaceae Juss.
Iz_8_1009	DJI_0899	Fallopia dumetorum (L.) Holub	0	n	1	0	Polygonaceae Juss.
Iz_8_1009	DJI_0899	Festuca beckeri (Hack.) Trautv.	0	n	1	0	Poaceae Barnhart
Iz_8_1009	DJI_0899	Fragaria vesca L.	0	n	1	0	Rosaceae Juss.
Iz_8_1009	DJI_0899	Frangula alnus Mill.	0	n	1	1	Rhamnaceae Juss.
Iz_8_1009	DJI_0899	Fraxinus pennsylvanica Marshall	0	a	0	1	Oleaceae Hoffmanns. & Link
Iz_8_1009	DJI_0899	Galeopsis bifida Boenn.	0	n	1	0	Lamiacea Lindl.
Iz_8_1009	DJI_0899	Galium verum L.	0	n	1	0	Rubiaceae Juss.
Iz_8_1009	DJI_0899	Genista tinctoria L.	0	n	1	0	Fabaceae Lindl.
Iz_8_1009	DJI_0899	Geranium robertianum L.	0	n	1	0	Geraniaceae Juss.
Iz_8_1009	DJI_0899	Geranium sanquineum L.	0	n	1	0	Geraniaceae Juss.
Iz_8_1009	DJI_0899	Gypsophila paniculata L.	0	n	1	0	Caryophyllaceae Juss.
Iz_8_1009	DJI_0899	Helichrysum arenarium (L.) Moench	0	n	1	0	Asteraceae Bercht. & J.Presl
Iz_8_1009	DJI_0899	Herniaria polygama J.Gay	0	n	1	0	Caryophyllaceae Juss.
Iz_8_1009	DJI_0899	Hieracium robustum Fr.	0	n	1	0	Asteraceae Bercht. & J.Presl
Iz_8_1009	DJI_0899	Hieracium umbellatum L.	0	n	1	NA	Asteraceae Bercht. & J.Presl
Iz_8_1009	DJI_0899	Hylotelephium maximum (L.) Holub	0	n	1	0	Crassulaceae J.St.-Hil.
Iz_8_1009	DJI_0899	Jacobaea borysthenica (DC.) B.Nord. & Greuter	0	n	1	0	Asteraceae Bercht. & J.Presl
Iz_8_1009	DJI_0899	Jasione montana L.	0	n	1	0	Campanulaceae Juss.
Iz_8_1009	DJI_0899	Jurinea cyanoides (L.) Rchb.s.l.	0	n	1	0	Asteraceae Bercht. & J.Presl
Iz_8_1009	DJI_0899	Koeleria glauca (Spreng.) DC.	0	n	1	0	Poaceae Barnhart
Iz_8_1009	DJI_0899	Lactuca serriola L.	0	a	0	0	Asteraceae Bercht. & J.Presl
Iz_8_1009	DJI_0899	Linaria genistifolia (L.) Mill.	0	n	1	0	Plantaginaceae Juss.
Iz_8_1009	DJI_0899	Lomelosia argentea (L.) Greuter & Burdet	0	n	1	0	Caprifoliaceae Juss.
Iz_8_1009	DJI_0899	Melampyrum pratense L.	0	n	1	0	Orobanchaceae Vent.
Iz_8_1009	DJI_0899	Oenothera biennis L.	0	a	0	0	Onagraceae Juss.
Iz_8_1009	DJI_0899	Pentanema britannica (L.) D.Gut.Larr., Santos-Vicente, Anderb., E.Rico & M.M.Mart.Ort.	0	n	1	0	Asteraceae Bercht. & J.Presl
Iz_8_1009	DJI_0899	Peucedanum oreoselinum (L.) Moench	0	n	1	0	Apiaceae Lindl.
Iz_8_1009	DJI_0899	Peucedanum ruthenicum M.Bieb.	0	n	1	0	Apiaceae Lindl.
Iz_8_1009	DJI_0899	Pilosella echioides (Lumn.) F.W.Schultz & Sch.Bip.	0	n	1	0	Asteraceae Bercht. & J.Presl
Iz_8_1009	DJI_0899	Pilosella officinarum F. Schultz et Sch. Bip.	0	n	1	0	Asteraceae Bercht. & J.Presl
Iz_8_1009	DJI_0899	Pilosella sp.	0		NA	0	Asteraceae Bercht. & J.Presl
Iz_8_1009	DJI_0899	Pinus sylvestris L.	0	n	1	1	Pinaceae Spreng. ex F.Rudolphi
Iz_8_1009	DJI_0899	Poa annua L.	0	n	1	0	Poaceae Barnhart

Iz_8_1009	DJI_0899	Poa nemoralis L.	0	n	1	0	Poaceae Barnhart
Iz_8_1009	DJI_0899	Poa sp.	0		NA	0	Poaceae Barnhart
Iz_8_1009	DJI_0899	Polygonatum odoratum (Mill.) Druce	0	n	1	0	Asparagaceae Juss.
Iz_8_1009	DJI_0899	Polygonum arenarium Waldst. & Kit.	0	n	1	0	Polygonaceae Juss.
Iz_8_1009	DJI_0899	Populus nigra L.	0	n	1	1	Salicaceae Mirb.
Iz_8_1009	DJI_0899	Populus tremula L.	1	n	1	1	Salicaceae Mirb.
Iz_8_1009	DJI_0899	Populus Ã canescens (Aiton) Sm.	0	n	1	1	Salicaceae Mirb.
Iz_8_1009	DJI_0899	Potentilla argentea L.	0	n	1	0	Rosaceae Juss.
Iz_8_1009	DJI_0899	Psammophiliella muralis (L.) Ikonn.	0	n	1	0	Caryophyllaceae Juss.
Iz_8_1009	DJI_0899	Psephellus sumensis (Kalen.) Greuter	0	n	1	0	Asteraceae Bercht. & J.Presl
Iz_8_1009	DJI_0899	Pulsatilla pratensis (L.) Mill. s.l.	0	n	1	0	Ranunculaceae Juss.
Iz_8_1009	DJI_0899	Pyrus communis L.	0	a	0	1	Rosaceae Juss.
Iz_8_1009	DJI_0899	Quercus robur L.	0	n	1	1	Fagaceae Dumort.
Iz_8_1009	DJI_0899	Robinia pseudoacacia L.	0	a	0	1	Fabaceae Lindl.
Iz_8_1009	DJI_0899	Rosa sp.	0		NA	1	Rosaceae Juss.
Iz_8_1009	DJI_0899	Rubus idaeus L.	0	n	1	0	Rosaceae Juss.
Iz_8_1009	DJI_0899	Rumex acetosella L.	0	n	1	0	Polygonaceae Juss.
Iz_8_1009	DJI_0899	Rumex thyrsiflorus Fingerh.	0	n	1	0	Polygonaceae Juss.
Iz_8_1009	DJI_0899	Salix alba L.	0	n	1	1	Salicaceae Mirb.
Iz_8_1009	DJI_0899	Sambucus nigra L.	0	n	1	1	Viburnaceae Raf.
Iz_8_1009	DJI_0899	Secale sylvestre Host	0	n	1	0	Poaceae Barnhart
Iz_8_1009	DJI_0899	Sempervivum ruthenicum Schnittsp. & C.B. Lehm.	0	n	1	0	Crassulaceae J.St.-Hil.
Iz_8_1009	DJI_0899	Setaria viridis (L.) P. Beauv.	0	n	1	0	Poaceae Barnhart
Iz_8_1009	DJI_0899	Silene borysthenica (Gruner) Walters	0	n	1	0	Caryophyllaceae Juss.
Iz_8_1009	DJI_0899	Silene latifolia subsp. alba (Mill.) Greuter & Burdet	0	n	1	0	Caryophyllaceae Juss.
Iz_8_1009	DJI_0899	Solidago virgaurea L.	0	n	1	0	Asteraceae Bercht. & J.Presl
Iz_8_1009	DJI_0899	Sorbus aucuparia L.	0	n	1	1	Rosaceae Juss.
Iz_8_1009	DJI_0899	Stachys recta L.	0	n	1	0	Lamiacea Lindl.
Iz_8_1009	DJI_0899	Tanacetum vulgare L.	0	n	1	0	Asteraceae Bercht. & J.Presl
Iz_8_1009	DJI_0899	Thymus pallasianus Heinr.Braun	0	n	1	0	Lamiacea Lindl.
Iz_8_1009	DJI_0899	Tilia cordata Mill.	0	n	1	1	Malvaceae Juss.
Iz_8_1009	DJI_0899	Trifolium alpestre L.	0	n	1	0	Fabaceae Lindl.
Iz_8_1009	DJI_0899	Trifolium arvense L.	0	n	1	0	Fabaceae Lindl.
Iz_8_1009	DJI_0899	Ulmus glabra Huds.	0	n	1	1	Ulmaceae Mirb.
Iz_8_1009	DJI_0899	Verbascum densiflorum Bertol.	0	n	1	0	Scrophulariaceae Juss.
Iz_8_1009	DJI_0899	Veronica austriaca L.	0	n	1	0	Plantaginaceae Juss.
Iz_8_1009	DJI_0899	Veronica incana L.	0	n	1	0	Plantaginaceae Juss.
Iz_8_1009	DJI_0899	Veronica verna L.	0	n	1	0	Plantaginaceae Juss.
Iz_8_1009	DJI_0899	Vincetoxicum hirundinaria Medik	0	n	1	0	Apocynaceae Juss.
Iz_8_1009	DJI_0904	Acer tataricum L.	0	n	1	1	Sapindaceae Juss.
Iz_8_1009	DJI_0904	Achillea millefolium L. s.l.	0	n	1	0	Asteraceae Bercht. & J.Presl
Iz_8_1009	DJI_0904	Allium oleraceum L.	0	n	1	0	Amaryllidaceae J.St.-Hil.
Iz_8_1009	DJI_0904	Ambrosia artemisiifolia L.	0	a	0	0	Asteraceae Bercht. & J.Presl
Iz_8_1009	DJI_0904	Amorpha fruticosa L.	0	a	0	0	Fabaceae Lindl.
Iz_8_1009	DJI_0904	Anthericum ramosum L.	0	n	1	0	Asparagaceae Juss.
Iz_8_1009	DJI_0904	Artemisia marschalliana Spreng.	0	n	1	0	Asteraceae Bercht. & J.Presl
Iz_8_1009	DJI_0904	Asparagus officinalis L.	0	n	1	0	Asparagaceae Juss.
Iz_8_1009	DJI_0904	Astragalus cicer L.	0	n	1	0	Fabaceae Lindl.
Iz_8_1009	DJI_0904	Bassia laniflora (S.G.Gmel.) A.J.Scott	0	n	1	0	Amaranthaceae Juss.
Iz_8_1009	DJI_0904	Berberis vulgaris L.	0	n	1	0	Berberidaceae Juss.
Iz_8_1009	DJI_0904	Betula pendula Roth.	0	n	1	1	Betulaceae Gray
Iz_8_1009	DJI_0904	Betula pendula Roth.	0	n	1	0	Poaceae Barnhart
Iz_8_1009	DJI_0904	Bromus inermis Leyss.	0		NA	NA	
Iz_8_1009	DJI_0904	Bromus squarrosus L.	0	a	0	0	Poaceae Barnhart
Iz_8_1009	DJI_0904	Bromus tectorum L.	0	a	0	0	Poaceae Barnhart
Iz_8_1009	DJI_0904	Calamagrostis epigejos (L.) Roth	25	n	1	0	Poaceae Barnhart
Iz_8_1009	DJI_0904	Carduus acanthoides L.	0	n	1	0	Asteraceae Bercht. & J.Presl
Iz_8_1009	DJI_0904	Carex sp.	6	n	1	0	Cyperaceae Juss.
Iz_8_1009	DJI_0904	Centaurea majorovii Dumbadze	0	n	1	0	Asteraceae Bercht. & J.Presl
Iz_8_1009	DJI_0904	Chamaecytisus ruthenicus (Fisch.ex Wol.) Klaskova	0	n	1	0	Fabaceae Lindl.
Iz_8_1009	DJI_0904	Chelidonium majus L.	0	n	1	0	Papaveraceae Juss.
Iz_8_1009	DJI_0904	Chenopodium betaceum Andrz.	0	n	1	0	Amaranthaceae Juss.
Iz_8_1009	DJI_0904	Chenopodium cf. album	0	n	1	0	Amaranthaceae Juss.
Iz_8_1009	DJI_0904	Chenopodium sp.	0		NA	0	Amaranthaceae Juss.
Iz_8_1009	DJI_0904	Chondrilla juncea L.	0	n	1	0	Asteraceae Bercht. & J.Presl
Iz_8_1009	DJI_0904	Cirsium vulgare (Savi) Ten.	0	n	1	0	Asteraceae Bercht. & J.Presl
Iz_8_1009	DJI_0904	Clinopodium acinos (L.) Kuntze	0	n	1	0	Lamiacea Lindl.
Iz_8_1009	DJI_0904	Convallaria majalis L.	0	n	1	0	Asparagaceae Juss.
Iz_8_1009	DJI_0904	Crataegus rhipidophylla Gand.	0	n	1	1	Rosaceae Juss.
Iz_8_1009	DJI_0904	Crepis foetida L.	0	n	1	0	Asteraceae Bercht. & J.Presl
Iz_8_1009	DJI_0904	Dactylis glomerata L.	0	n	1	0	Poaceae Barnhart
Iz_8_1009	DJI_0904	Dianthus campestris M.Bieb.	0	n	1	0	Caryophyllaceae Juss.
Iz_8_1009	DJI_0904	Digitaria sanguinalis (L.) Scop.	0	a	0	0	Poaceae Barnhart
Iz_8_1009	DJI_0904	Elymus repens (L.) Gould	0	n	1	0	Poaceae Barnhart
Iz_8_1009	DJI_0904	Epilobium tetragonum L.	0	n	1	0	Onagraceae Juss.
Iz_8_1009	DJI_0904	Eragrostis minor Host	0	a	0	0	Poaceae Barnhart
Iz_8_1009	DJI_0904	Erigeron canadensis L.	8	a	0	0	Asteraceae Bercht. & J.Presl
Iz_8_1009	DJI_0904	Euphorbia seguieriana Neck.	0	n	1	0	Euphorbiaceae Juss.
Iz_8_1009	DJI_0904	Fallopia dumetorum (L.) Holub	0	n	1	0	Polygonaceae Juss.
Iz_8_1009	DJI_0904	Festuca beckeri (Hack.) Trautv.	0	n	1	0	Poaceae Barnhart
Iz_8_1009	DJI_0904	Fragaria vesca L.	0	n	1	0	Rosaceae Juss.
Iz_8_1009	DJI_0904	Frangula alnus Mill.	0	n	1	1	Rhamnaceae Juss.
Iz_8_1009	DJI_0904	Fraxinus pennsylvanica Marshall	0	a	0	1	Oleaceae Hoffmanns. & Link
Iz_8_1009	DJI_0904	Galeopsis bifida Boenn.	0	n	1	0	Lamiacea Lindl.
Iz_8_1009	DJI_0904	Galium verum L.	0	n	1	0	Rubiaceae Juss.
Iz_8_1009	DJI_0904	Genista tinctoria L.	0	n	1	0	Fabaceae Lindl.
Iz_8_1009	DJI_0904	Geranium robertianum L.	0	n	1	0	Geraniaceae Juss.
Iz_8_1009	DJI_0904	Geranium sanquineum L.	0	n	1	0	Geraniaceae Juss.
Iz_8_1009	DJI_0904	Gypsophila paniculata L.	0	n	1	0	Caryophyllaceae Juss.
Iz_8_1009	DJI_0904	Helichrysum arenarium (L.) Moench	0	n	1	0	Asteraceae Bercht. & J.Presl
Iz_8_1009	DJI_0904	Herniaria polygama J.Gay	0	n	1	0	Caryophyllaceae Juss.
Iz_8_1009	DJI_0904	Hieracium robustum Fr.	0	n	1	0	Asteraceae Bercht. & J.Presl
Iz_8_1009	DJI_0904	Hieracium umbellatum L.	0	n	1	NA	Asteraceae Bercht. & J.Presl
Iz_8_1009	DJI_0904	Hylotelephium maximum (L.) Holub	0	n	1	0	Crassulaceae J.St.-Hil.
Iz_8_1009	DJI_0904	Jacobaea borysthenica (DC.) B.Nord. & Greuter	0	n	1	0	Asteraceae Bercht. & J.Presl
Iz_8_1009	DJI_0904	Jasione montana L.	0	n	1	0	Campanulaceae Juss.
Iz_8_1009	DJI_0904	Jurinea cyanoides (L.) Rchb.s.l.	0	n	1	0	Asteraceae Bercht. & J.Presl
Iz_8_1009	DJI_0904	Koeleria glauca (Spreng.) DC.	0	n	1	0	Poaceae Barnhart

Iz_8_1009	DJI_0904	Lactuca serriola L.	0	a	0	0	Asteraceae Bercht. & J.Presl
Iz_8_1009	DJI_0904	Linaria genistifolia (L.) Mill.	0	n	1	0	Plantaginaceae Juss.
Iz_8_1009	DJI_0904	Lomelosia argentea (L.) Greuter & Burdet	0	n	1	0	Caprifoliaceae Juss.
Iz_8_1009	DJI_0904	Melampyrum pratense L.	0	n	1	0	Orobanchaceae Vent.
Iz_8_1009	DJI_0904	Oenothera biennis L.	0	a	0	0	Onagraceae Juss.
Iz_8_1009	DJI_0904	Pentanema britannica (L.) D.Gut.Larr., Santos-Vicente, Anderb., E.Rico & M.M.Mart.Ort.	0	n	1	0	Asteraceae Bercht. & J.Presl
Iz_8_1009	DJI_0904	Peucedanum oreoselinum (L.) Moench	3	n	1	0	Apiaceae Lindl.
Iz_8_1009	DJI_0904	Peucedanum ruthenicum M.Bieb.	0	n	1	0	Apiaceae Lindl.
Iz_8_1009	DJI_0904	Pilosella echioides (Lumn.) F.W.Schultz & Sch.Bip.	0	n	1	0	Asteraceae Bercht. & J.Presl
Iz_8_1009	DJI_0904	Pilosella officinarum F. Schultz et Sch. Bip.	0	n	1	0	Asteraceae Bercht. & J.Presl
Iz_8_1009	DJI_0904	Pilosella sp.	0		NA	0	Asteraceae Bercht. & J.Presl
Iz_8_1009	DJI_0904	Pinus sylvestris L.	0	n	1	1	Pinaceae Spreng. ex F.Rudolphi
Iz_8_1009	DJI_0904	Poa annua L.	0	n	1	0	Poaceae Barnhart
Iz_8_1009	DJI_0904	Poa nemoralis L.	0	n	1	0	Poaceae Barnhart
Iz_8_1009	DJI_0904	Poa sp.	0		NA	0	Poaceae Barnhart
Iz_8_1009	DJI_0904	Polygonatum odoratum (Mill.) Druce	0	n	1	0	Asparagaceae Juss.
Iz_8_1009	DJI_0904	Polygonum arenarium Waldst. & Kit.	0	n	1	0	Polygonaceae Juss.
Iz_8_1009	DJI_0904	Populus nigra L.	0	n	1	1	Salicaceae Mirb.
Iz_8_1009	DJI_0904	Populus tremula L.	0	n	1	1	Salicaceae Mirb.
Iz_8_1009	DJI_0904	Populus Ã canescens (Aiton) Sm.	0	n	1	1	Salicaceae Mirb.
Iz_8_1009	DJI_0904	Potentilla argentea L.	0	n	1	0	Rosaceae Juss.
Iz_8_1009	DJI_0904	Psammophiliella muralis (L.) Ikonn.	0	n	1	0	Caryophyllaceae Juss.
Iz_8_1009	DJI_0904	Psephellus sumensis (Kalen.) Greuter	0	n	1	0	Asteraceae Bercht. & J.Presl
Iz_8_1009	DJI_0904	Pulsatilla pratensis (L.) Mill. s.l.	0	n	1	0	Ranunculaceae Juss.
Iz_8_1009	DJI_0904	Pyrus communis L.	0	a	0	1	Rosaceae Juss.
Iz_8_1009	DJI_0904	Quercus robur L.	0	n	1	1	Fagaceae Dumort.
Iz_8_1009	DJI_0904	Robinia pseudoacacia L.	0	a	0	1	Fabaceae Lindl.
Iz_8_1009	DJI_0904	Rosa sp.	0		NA	1	Rosaceae Juss.
Iz_8_1009	DJI_0904	Rubus idaeus L.	0	n	1	0	Rosaceae Juss.
Iz_8_1009	DJI_0904	Rumex acetosella L.	0	n	1	0	Polygonaceae Juss.
Iz_8_1009	DJI_0904	Rumex thyrsiflorus Fingerh.	0	n	1	0	Polygonaceae Juss.
Iz_8_1009	DJI_0904	Salix alba L.	0	n	1	1	Salicaceae Mirb.
Iz_8_1009	DJI_0904	Sambucus nigra L.	0	n	1	1	Viburnaceae Raf.
Iz_8_1009	DJI_0904	Secale sylvestre Host	0	n	1	0	Poaceae Barnhart
Iz_8_1009	DJI_0904	Sempervivum ruthenicum Schnittsp. & C.B. Lehm.	0	n	1	0	Crassulaceae J.St.-Hil.
Iz_8_1009	DJI_0904	Setaria viridis (L.) P. Beauv.	0	n	1	0	Poaceae Barnhart
Iz_8_1009	DJI_0904	Silene borysthenica (Gruner) Walters	0	n	1	0	Caryophyllaceae Juss.
Iz_8_1009	DJI_0904	Silene latifolia subsp. alba (Mill.) Greuter & Burdet	0	n	1	0	Caryophyllaceae Juss.
Iz_8_1009	DJI_0904	Solidago virgaurea L.	0	n	1	0	Asteraceae Bercht. & J.Presl
Iz_8_1009	DJI_0904	Sorbus aucuparia L.	0	n	1	1	Rosaceae Juss.
Iz_8_1009	DJI_0904	Stachys recta L.	0	n	1	0	Lamiacea Lindl.
Iz_8_1009	DJI_0904	Tanacetum vulgare L.	0	n	1	0	Asteraceae Bercht. & J.Presl
Iz_8_1009	DJI_0904	Thymus pallasianus Heinr.Braun	0	n	1	0	Lamiacea Lindl.
Iz_8_1009	DJI_0904	Tilia cordata Mill.	0	n	1	1	Malvaceae Juss.
Iz_8_1009	DJI_0904	Trifolium alpestre L.	0	n	1	0	Fabaceae Lindl.
Iz_8_1009	DJI_0904	Trifolium arvense L.	0	n	1	0	Fabaceae Lindl.
Iz_8_1009	DJI_0904	Ulmus glabra Huds.	0	n	1	1	Ulmaceae Mirb.
Iz_8_1009	DJI_0904	Verbascum densiflorum Bertol.	0	n	1	0	Scrophulariaceae Juss.
Iz_8_1009	DJI_0904	Veronica austriaca L.	0	n	1	0	Plantaginaceae Juss.
Iz_8_1009	DJI_0904	Veronica incana L.	0	n	1	0	Plantaginaceae Juss.
Iz_8_1009	DJI_0904	Veronica verna L.	0	n	1	0	Plantaginaceae Juss.
Iz_8_1009	DJI_0904	Vincetoxicum hirundinaria Medik	0	n	1	0	Apocynaceae Juss.
Iz_8_1009	DJI_0906	Acer tataricum L.	0	n	1	1	Sapindaceae Juss.
Iz_8_1009	DJI_0906	Achillea millefolium L. s.l.	0	n	1	0	Asteraceae Bercht. & J.Presl
Iz_8_1009	DJI_0906	Allium oleraceum L.	0	n	1	0	Amaryllidaceae J.St.-Hil.
Iz_8_1009	DJI_0906	Ambrosia artemisiifolia L.	0	a	0	0	Asteraceae Bercht. & J.Presl
Iz_8_1009	DJI_0906	Amorpha fruticosa L.	0	a	0	0	Fabaceae Lindl.
Iz_8_1009	DJI_0906	Anthericum ramosum L.	0	n	1	0	Asparagaceae Juss.
Iz_8_1009	DJI_0906	Artemisia marschalliana Spreng.	0	n	1	0	Asteraceae Bercht. & J.Presl
Iz_8_1009	DJI_0906	Asparagus officinalis L.	0	n	1	0	Asparagaceae Juss.
Iz_8_1009	DJI_0906	Astragalus cicer L.	0	n	1	0	Fabaceae Lindl.
Iz_8_1009	DJI_0906	Bassia laniflora (S.G.Gmel.) A.J.Scott	0	n	1	0	Amaranthaceae Juss.
Iz_8_1009	DJI_0906	Berberis vulgaris L.	0	n	1	0	Berberidaceae Juss.
Iz_8_1009	DJI_0906	Betula pendula Roth.	0	n	1	1	Betulaceae Gray
Iz_8_1009	DJI_0906	Betula pendula Roth.	0	n	1	0	Poaceae Barnhart
Iz_8_1009	DJI_0906	Bromus inermis Leyss.	0		NA	NA	
Iz_8_1009	DJI_0906	Bromus squarrosus L.	0	a	0	0	Poaceae Barnhart
Iz_8_1009	DJI_0906	Bromus tectorum L.	0	a	0	0	Poaceae Barnhart
Iz_8_1009	DJI_0906	Calamagrostis epigejos (L.) Roth	10	n	1	0	Poaceae Barnhart
Iz_8_1009	DJI_0906	Carduus acanthoides L.	0	n	1	0	Asteraceae Bercht. & J.Presl
Iz_8_1009	DJI_0906	Carex sp.	6	n	1	0	Cyperaceae Juss.
Iz_8_1009	DJI_0906	Centaurea majorovii Dumbadze	0	n	1	0	Asteraceae Bercht. & J.Presl
Iz_8_1009	DJI_0906	Chamaecytisus ruthenicus (Fisch.ex Wol.) Klaskova	0	n	1	0	Fabaceae Lindl.
Iz_8_1009	DJI_0906	Chelidonium majus L.	0	n	1	0	Papaveraceae Juss.
Iz_8_1009	DJI_0906	Chenopodium betaceum Andrz.	0	n	1	0	Amaranthaceae Juss.
Iz_8_1009	DJI_0906	Chenopodium cf. album	0	n	1	0	Amaranthaceae Juss.
Iz_8_1009	DJI_0906	Chenopodium sp.	0		NA	0	Amaranthaceae Juss.
Iz_8_1009	DJI_0906	Chondrilla juncea L.	0	n	1	0	Asteraceae Bercht. & J.Presl
Iz_8_1009	DJI_0906	Cirsium vulgare (Savi) Ten.	0	n	1	0	Asteraceae Bercht. & J.Presl
Iz_8_1009	DJI_0906	Clinopodium acinos (L.) Kuntze	0	n	1	0	Lamiacea Lindl.
Iz_8_1009	DJI_0906	Convallaria majalis L.	0	n	1	0	Asparagaceae Juss.
Iz_8_1009	DJI_0906	Crataegus rhipidophylla Gand.	0	n	1	1	Rosaceae Juss.
Iz_8_1009	DJI_0906	Crepis foetida L.	0	n	1	0	Asteraceae Bercht. & J.Presl
Iz_8_1009	DJI_0906	Dactylis glomerata L.	0	n	1	0	Poaceae Barnhart
Iz_8_1009	DJI_0906	Dianthus campestris M.Bieb.	0	n	1	0	Caryophyllaceae Juss.
Iz_8_1009	DJI_0906	Digitaria sanguinalis (L.) Scop.	0	a	0	0	Poaceae Barnhart
Iz_8_1009	DJI_0906	Elymus repens (L.) Gould	0	n	1	0	Poaceae Barnhart
Iz_8_1009	DJI_0906	Epilobium tetragonum L.	0	n	1	0	Onagraceae Juss.
Iz_8_1009	DJI_0906	Eragrostis minor Host	0	a	0	0	Poaceae Barnhart
Iz_8_1009	DJI_0906	Erigeron canadensis L.	5	a	0	0	Asteraceae Bercht. & J.Presl
Iz_8_1009	DJI_0906	Euphorbia seguieriana Neck.	0	n	1	0	Euphorbiaceae Juss.
Iz_8_1009	DJI_0906	Fallopia dumetorum (L.) Holub	1	n	1	0	Polygonaceae Juss.
Iz_8_1009	DJI_0906	Festuca beckeri (Hack.) Trautv.	0	n	1	0	Poaceae Barnhart
Iz_8_1009	DJI_0906	Fragaria vesca L.	0	n	1	0	Rosaceae Juss.
Iz_8_1009	DJI_0906	Frangula alnus Mill.	0	n	1	1	Rhamnaceae Juss.
Iz_8_1009	DJI_0906	Fraxinus pennsylvanica Marshall	0	a	0	1	Oleaceae Hoffmanns. & Link
Iz_8_1009	DJI_0906	Galeopsis bifida Boenn.	0	n	1	0	Lamiacea Lindl.
Iz_8_1009	DJI_0906	Galium verum L.	0	n	1	0	Rubiaceae Juss.

Iz_8_1009	DJI_0906	Genista tinctoria L.	0	n	1	0	Fabaceae Lindl.
Iz_8_1009	DJI_0906	Geranium robertianum L.	0	n	1	0	Geraniaceae Juss.
Iz_8_1009	DJI_0906	Geranium sanquineum L.	0	n	1	0	Geraniaceae Juss.
Iz_8_1009	DJI_0906	Gypsophila paniculata L.	0	n	1	0	Caryophyllaceae Juss.
Iz_8_1009	DJI_0906	Helichrysum arenarium (L.) Moench	1	n	1	0	Asteraceae Bercht. & J.Presl
Iz_8_1009	DJI_0906	Herniaria polygama J.Gay	0	n	1	0	Caryophyllaceae Juss.
Iz_8_1009	DJI_0906	Hieracium robustum Fr.	0	n	1	0	Asteraceae Bercht. & J.Presl
Iz_8_1009	DJI_0906	Hieracium umbellatum L.	0	n	1	NA	Asteraceae Bercht. & J.Presl
Iz_8_1009	DJI_0906	Hylotelephium maximum (L.) Holub	0	n	1	0	Crassulaceae J.St.-Hil.
Iz_8_1009	DJI_0906	Jacobaea borysthenica (DC.) B.Nord. & Greuter	0	n	1	0	Asteraceae Bercht. & J.Presl
Iz_8_1009	DJI_0906	Jasione montana L.	0	n	1	0	Campanulaceae Juss.
Iz_8_1009	DJI_0906	Jurinea cyanoides (L.) Rchb.s.l.	0	n	1	0	Asteraceae Bercht. & J.Presl
Iz_8_1009	DJI_0906	Koeleria glauca (Spreng.) DC.	0	n	1	0	Poaceae Barnhart
Iz_8_1009	DJI_0906	Lactuca serriola L.	0	a	0	0	Asteraceae Bercht. & J.Presl
Iz_8_1009	DJI_0906	Linaria genistifolia (L.) Mill.	0	n	1	0	Plantaginaceae Juss.
Iz_8_1009	DJI_0906	Lomelosia argentea (L.) Greuter & Burdet	0	n	1	0	Caprifoliaceae Juss.
Iz_8_1009	DJI_0906	Melampyrum pratense L.	0	n	1	0	Orobanchaceae Vent.
Iz_8_1009	DJI_0906	Oenothera biennis L.	0	a	0	0	Onagraceae Juss.
Iz_8_1009	DJI_0906	Pentanema britannica (L.) D.Gut.Larr., Santos-Vicente, Anderb., E.Rico & M.M.Mart.Ort.	0	n	1	0	Asteraceae Bercht. & J.Presl
Iz_8_1009	DJI_0906	Peucedanum oreoselinum (L.) Moench	0	n	1	0	Apiaceae Lindl.
Iz_8_1009	DJI_0906	Peucedanum ruthenicum M.Bieb.	0	n	1	0	Apiaceae Lindl.
Iz_8_1009	DJI_0906	Pilosella echioides (Lumn.) F.W.Schultz & Sch.Bip.	0	n	1	0	Asteraceae Bercht. & J.Presl
Iz_8_1009	DJI_0906	Pilosella officinarum F. Schultz et Sch. Bip.	0	n	1	0	Asteraceae Bercht. & J.Presl
Iz_8_1009	DJI_0906	Pilosella sp.	0		NA	0	Asteraceae Bercht. & J.Presl
Iz_8_1009	DJI_0906	Pinus sylvestris L.	0	n	1	1	Pinaceae Spreng. ex F.Rudolphi
Iz_8_1009	DJI_0906	Poa annua L.	0	n	1	0	Poaceae Barnhart
Iz_8_1009	DJI_0906	Poa nemoralis L.	0	n	1	0	Poaceae Barnhart
Iz_8_1009	DJI_0906	Poa sp.	0		NA	0	Poaceae Barnhart
Iz_8_1009	DJI_0906	Polygonatum odoratum (Mill.) Druce	0	n	1	0	Asparagaceae Juss.
Iz_8_1009	DJI_0906	Polygonum arenarium Waldst. & Kit.	0	n	1	0	Polygonaceae Juss.
Iz_8_1009	DJI_0906	Populus nigra L.	0	n	1	1	Salicaceae Mirb.
Iz_8_1009	DJI_0906	Populus tremula L.	0	n	1	1	Salicaceae Mirb.
Iz_8_1009	DJI_0906	Populus Ã canescens (Aiton) Sm.	0	n	1	1	Salicaceae Mirb.
Iz_8_1009	DJI_0906	Potentilla argentea L.	0	n	1	0	Rosaceae Juss.
Iz_8_1009	DJI_0906	Psammophiliella muralis (L.) Ikonn.	0	n	1	0	Caryophyllaceae Juss.
Iz_8_1009	DJI_0906	Psephellus sumensis (Kalen.) Greuter	0	n	1	0	Asteraceae Bercht. & J.Presl
Iz_8_1009	DJI_0906	Pulsatilla pratensis (L.) Mill. s.l.	0	n	1	0	Ranunculaceae Juss.
Iz_8_1009	DJI_0906	Pyrus communis L.	0	a	0	1	Rosaceae Juss.
Iz_8_1009	DJI_0906	Quercus robur L.	0	n	1	1	Fagaceae Dumort.
Iz_8_1009	DJI_0906	Robinia pseudoacacia L.	0	a	0	1	Fabaceae Lindl.
Iz_8_1009	DJI_0906	Rosa sp.	0		NA	1	Rosaceae Juss.
Iz_8_1009	DJI_0906	Rubus idaeus L.	0	n	1	0	Rosaceae Juss.
Iz_8_1009	DJI_0906	Rumex acetosella L.	0	n	1	0	Polygonaceae Juss.
Iz_8_1009	DJI_0906	Rumex thyrsiflorus Fingerh.	0	n	1	0	Polygonaceae Juss.
Iz_8_1009	DJI_0906	Salix alba L.	0	n	1	1	Salicaceae Mirb.
Iz_8_1009	DJI_0906	Sambucus nigra L.	0	n	1	1	Viburnaceae Raf.
Iz_8_1009	DJI_0906	Secale sylvestre Host	0	n	1	0	Poaceae Barnhart
Iz_8_1009	DJI_0906	Sempervivum ruthenicum Schnittsp. & C.B. Lehm.	0	n	1	0	Crassulaceae J.St.-Hil.
Iz_8_1009	DJI_0906	Setaria viridis (L.) P. Beauv.	0	n	1	0	Poaceae Barnhart
Iz_8_1009	DJI_0906	Silene borysthenica (Gruner) Walters	0	n	1	0	Caryophyllaceae Juss.
Iz_8_1009	DJI_0906	Silene latifolia subsp. alba (Mill.) Greuter & Burdet	0	n	1	0	Caryophyllaceae Juss.
Iz_8_1009	DJI_0906	Solidago virgaurea L.	0	n	1	0	Asteraceae Bercht. & J.Presl
Iz_8_1009	DJI_0906	Sorbus aucuparia L.	0	n	1	1	Rosaceae Juss.
Iz_8_1009	DJI_0906	Stachys recta L.	0	n	1	0	Lamiacea Lindl.
Iz_8_1009	DJI_0906	Tanacetum vulgare L.	0	n	1	0	Asteraceae Bercht. & J.Presl
Iz_8_1009	DJI_0906	Thymus pallasianus Heinr.Braun	0	n	1	0	Lamiacea Lindl.
Iz_8_1009	DJI_0906	Tilia cordata Mill.	0	n	1	1	Malvaceae Juss.
Iz_8_1009	DJI_0906	Trifolium alpestre L.	0	n	1	0	Fabaceae Lindl.
Iz_8_1009	DJI_0906	Trifolium arvense L.	0	n	1	0	Fabaceae Lindl.
Iz_8_1009	DJI_0906	Ulmus glabra Huds.	0	n	1	1	Ulmaceae Mirb.
Iz_8_1009	DJI_0906	Verbascum densiflorum Bertol.	0	n	1	0	Scrophulariaceae Juss.
Iz_8_1009	DJI_0906	Veronica austriaca L.	0	n	1	0	Plantaginaceae Juss.
Iz_8_1009	DJI_0906	Veronica incana L.	0	n	1	0	Plantaginaceae Juss.
Iz_8_1009	DJI_0906	Veronica verna L.	0	n	1	0	Plantaginaceae Juss.
Iz_8_1009	DJI_0906	Vincetoxicum hirundinaria Medik	0	n	1	0	Apocynaceae Juss.
Iz_8_1009	DJI_0908	Acer tataricum L.	0	n	1	1	Sapindaceae Juss.
Iz_8_1009	DJI_0908	Achillea millefolium L. s.l.	0	n	1	0	Asteraceae Bercht. & J.Presl
Iz_8_1009	DJI_0908	Allium oleraceum L.	0	n	1	0	Amaryllidaceae J.St.-Hil.
Iz_8_1009	DJI_0908	Ambrosia artemisiifolia L.	0	a	0	0	Asteraceae Bercht. & J.Presl
Iz_8_1009	DJI_0908	Amorpha fruticosa L.	0	a	0	0	Fabaceae Lindl.
Iz_8_1009	DJI_0908	Anthericum ramosum L.	0	n	1	0	Asparagaceae Juss.
Iz_8_1009	DJI_0908	Artemisia marschalliana Spreng.	0	n	1	0	Asteraceae Bercht. & J.Presl
Iz_8_1009	DJI_0908	Asparagus officinalis L.	0	n	1	0	Asparagaceae Juss.
Iz_8_1009	DJI_0908	Astragalus cicer L.	0	n	1	0	Fabaceae Lindl.
Iz_8_1009	DJI_0908	Bassia laniflora (S.G.Gmel.) A.J.Scott	0	n	1	0	Amaranthaceae Juss.
Iz_8_1009	DJI_0908	Berberis vulgaris L.	0	n	1	0	Berberidaceae Juss.
Iz_8_1009	DJI_0908	Betula pendula Roth.	0	n	1	1	Betulaceae Gray
Iz_8_1009	DJI_0908	Betula pendula Roth.	0	n	1	0	Poaceae Barnhart
Iz_8_1009	DJI_0908	Bromus inermis Leyss.	0		NA	NA	
Iz_8_1009	DJI_0908	Bromus squarrosus L.	0	a	0	0	Poaceae Barnhart
Iz_8_1009	DJI_0908	Bromus tectorum L.	0	a	0	0	Poaceae Barnhart
Iz_8_1009	DJI_0908	Calamagrostis epigejos (L.) Roth	5	n	1	0	Poaceae Barnhart
Iz_8_1009	DJI_0908	Carduus acanthoides L.	0	n	1	0	Asteraceae Bercht. & J.Presl
Iz_8_1009	DJI_0908	Carex sp.	0	n	1	0	Cyperaceae Juss.
Iz_8_1009	DJI_0908	Centaurea majorovii Dumbadze	0	n	1	0	Asteraceae Bercht. & J.Presl
Iz_8_1009	DJI_0908	Chamaecytisus ruthenicus (Fisch.ex Wol.) Klaskova	0	n	1	0	Fabaceae Lindl.
Iz_8_1009	DJI_0908	Chelidonium majus L.	0	n	1	0	Papaveraceae Juss.
Iz_8_1009	DJI_0908	Chenopodium betaceum Andrz.	0	n	1	0	Amaranthaceae Juss.
Iz_8_1009	DJI_0908	Chenopodium cf. album	0	n	1	0	Amaranthaceae Juss.
Iz_8_1009	DJI_0908	Chenopodium sp.	0		NA	0	Amaranthaceae Juss.
Iz_8_1009	DJI_0908	Chondrilla juncea L.	0	n	1	0	Asteraceae Bercht. & J.Presl
Iz_8_1009	DJI_0908	Cirsium vulgare (Savi) Ten.	0	n	1	0	Asteraceae Bercht. & J.Presl
Iz_8_1009	DJI_0908	Clinopodium acinos (L.) Kuntze	0	n	1	0	Lamiacea Lindl.
Iz_8_1009	DJI_0908	Convallaria majalis L.	0	n	1	0	Asparagaceae Juss.
Iz_8_1009	DJI_0908	Crataegus rhipidophylla Gand.	0	n	1	1	Rosaceae Juss.
Iz_8_1009	DJI_0908	Crepis foetida L.	0	n	1	0	Asteraceae Bercht. & J.Presl
Iz_8_1009	DJI_0908	Dactylis glomerata L.	0	n	1	0	Poaceae Barnhart
Iz_8_1009	DJI_0908	Dianthus campestris M.Bieb.	0	n	1	0	Caryophyllaceae Juss.

Iz_8_1009	DJI_0908	Digitaria sanguinalis (L.) Scop.	0	a	0	0	Poaceae Barnhart
Iz_8_1009	DJI_0908	Elymus repens (L.) Gould	0	n	1	0	Poaceae Barnhart
Iz_8_1009	DJI_0908	Epilobium tetragonum L.	0	n	1	0	Onagraceae Juss.
Iz_8_1009	DJI_0908	Eragrostis minor Host	0	a	0	0	Poaceae Barnhart
Iz_8_1009	DJI_0908	Erigeron canadensis L.	6	a	0	0	Asteraceae Bercht. & J.Presl
Iz_8_1009	DJI_0908	Euphorbia seguieriana Neck.	0	n	1	0	Euphorbiaceae Juss.
Iz_8_1009	DJI_0908	Fallopia dumetorum (L.) Holub	0	n	1	0	Polygonaceae Juss.
Iz_8_1009	DJI_0908	Festuca beckeri (Hack.) Trautv.	0	n	1	0	Poaceae Barnhart
Iz_8_1009	DJI_0908	Fragaria vesca L.	0	n	1	0	Rosaceae Juss.
Iz_8_1009	DJI_0908	Frangula alnus Mill.	0	n	1	1	Rhamnaceae Juss.
Iz_8_1009	DJI_0908	Fraxinus pennsylvanica Marshall	0	a	0	1	Oleaceae Hoffmanns. & Link
Iz_8_1009	DJI_0908	Galeopsis bifida Boenn.	0	n	1	0	Lamiacea Lindl.
Iz_8_1009	DJI_0908	Galium verum L.	0	n	1	0	Rubiaceae Juss.
Iz_8_1009	DJI_0908	Genista tinctoria L.	0	n	1	0	Fabaceae Lindl.
Iz_8_1009	DJI_0908	Geranium robertianum L.	0	n	1	0	Geraniaceae Juss.
Iz_8_1009	DJI_0908	Geranium sanquineum L.	0	n	1	0	Geraniaceae Juss.
Iz_8_1009	DJI_0908	Gypsophila paniculata L.	0	n	1	0	Caryophyllaceae Juss.
Iz_8_1009	DJI_0908	Helichrysum arenarium (L.) Moench	0	n	1	0	Asteraceae Bercht. & J.Presl
Iz_8_1009	DJI_0908	Herniaria polygama J.Gay	0	n	1	0	Caryophyllaceae Juss.
Iz_8_1009	DJI_0908	Hieracium robustum Fr.	0	n	1	0	Asteraceae Bercht. & J.Presl
Iz_8_1009	DJI_0908	Hieracium umbellatum L.	0	n	1	NA	Asteraceae Bercht. & J.Presl
Iz_8_1009	DJI_0908	Hylotelephium maximum (L.) Holub	0	n	1	0	Crassulaceae J.St.-Hil.
Iz_8_1009	DJI_0908	Jacobaea borysthenica (DC.) B.Nord. & Greuter	0	n	1	0	Asteraceae Bercht. & J.Presl
Iz_8_1009	DJI_0908	Jasione montana L.	0	n	1	0	Campanulaceae Juss.
Iz_8_1009	DJI_0908	Jurinea cyanoides (L.) Rchb.s.l.	0	n	1	0	Asteraceae Bercht. & J.Presl
Iz_8_1009	DJI_0908	Koeleria glauca (Spreng.) DC.	0	n	1	0	Poaceae Barnhart
Iz_8_1009	DJI_0908	Lactuca serriola L.	0	a	0	0	Asteraceae Bercht. & J.Presl
Iz_8_1009	DJI_0908	Linaria genistifolia (L.) Mill.	0	n	1	0	Plantaginaceae Juss.
Iz_8_1009	DJI_0908	Lomelosia argentea (L.) Greuter & Burdet	0	n	1	0	Caprifoliaceae Juss.
Iz_8_1009	DJI_0908	Melampyrum pratense L.	0	n	1	0	Orobanchaceae Vent.
Iz_8_1009	DJI_0908	Oenothera biennis L.	0	a	0	0	Onagraceae Juss.
Iz_8_1009	DJI_0908	Pentanema britannica (L.) D.Gut.Larr., Santos-Vicente, Anderb., E.Rico & M.M.Mart.Ort.	0	n	1	0	Asteraceae Bercht. & J.Presl
Iz_8_1009	DJI_0908	Peucedanum oreoselinum (L.) Moench	0	n	1	0	Apiaceae Lindl.
Iz_8_1009	DJI_0908	Peucedanum ruthenicum M.Bieb.	0	n	1	0	Apiaceae Lindl.
Iz_8_1009	DJI_0908	Pilosella echioides (Lumn.) F.W.Schultz & Sch.Bip.	0	n	1	0	Asteraceae Bercht. & J.Presl
Iz_8_1009	DJI_0908	Pilosella officinarum F. Schultz et Sch. Bip.	0	n	1	0	Asteraceae Bercht. & J.Presl
Iz_8_1009	DJI_0908	Pilosella sp.	0		NA	0	Asteraceae Bercht. & J.Presl
Iz_8_1009	DJI_0908	Pinus sylvestris L.	0	n	1	1	Pinaceae Spreng. ex F.Rudolphi
Iz_8_1009	DJI_0908	Poa annua L.	0	n	1	0	Poaceae Barnhart
Iz_8_1009	DJI_0908	Poa nemoralis L.	0	n	1	0	Poaceae Barnhart
Iz_8_1009	DJI_0908	Poa sp.	0		NA	0	Poaceae Barnhart
Iz_8_1009	DJI_0908	Polygonatum odoratum (Mill.) Druce	0	n	1	0	Asparagaceae Juss.
Iz_8_1009	DJI_0908	Polygonum arenarium Waldst. & Kit.	0	n	1	0	Polygonaceae Juss.
Iz_8_1009	DJI_0908	Populus nigra L.	0	n	1	1	Salicaceae Mirb.
Iz_8_1009	DJI_0908	Populus tremula L.	0	n	1	1	Salicaceae Mirb.
Iz_8_1009	DJI_0908	Populus Ã canescens (Aiton) Sm.	0	n	1	1	Salicaceae Mirb.
Iz_8_1009	DJI_0908	Potentilla argentea L.	0	n	1	0	Rosaceae Juss.
Iz_8_1009	DJI_0908	Psammophiliella muralis (L.) Ikonn.	0	n	1	0	Caryophyllaceae Juss.
Iz_8_1009	DJI_0908	Psephellus sumensis (Kalen.) Greuter	0	n	1	0	Asteraceae Bercht. & J.Presl
Iz_8_1009	DJI_0908	Pulsatilla pratensis (L.) Mill. s.l.	0	n	1	0	Ranunculaceae Juss.
Iz_8_1009	DJI_0908	Pyrus communis L.	0	a	0	1	Rosaceae Juss.
Iz_8_1009	DJI_0908	Quercus robur L.	0	n	1	1	Fagaceae Dumort.
Iz_8_1009	DJI_0908	Robinia pseudoacacia L.	0	a	0	1	Fabaceae Lindl.
Iz_8_1009	DJI_0908	Rosa sp.	0		NA	1	Rosaceae Juss.
Iz_8_1009	DJI_0908	Rubus idaeus L.	0	n	1	0	Rosaceae Juss.
Iz_8_1009	DJI_0908	Rumex acetosella L.	0	n	1	0	Polygonaceae Juss.
Iz_8_1009	DJI_0908	Rumex thyrsiflorus Fingerh.	0	n	1	0	Polygonaceae Juss.
Iz_8_1009	DJI_0908	Salix alba L.	0	n	1	1	Salicaceae Mirb.
Iz_8_1009	DJI_0908	Sambucus nigra L.	0	n	1	1	Viburnaceae Raf.
Iz_8_1009	DJI_0908	Secale sylvestre Host	0	n	1	0	Poaceae Barnhart
Iz_8_1009	DJI_0908	Sempervivum ruthenicum Schnittsp. & C.B. Lehm.	0	n	1	0	Crassulaceae J.St.-Hil.
Iz_8_1009	DJI_0908	Setaria viridis (L.) P. Beauv.	0	n	1	0	Poaceae Barnhart
Iz_8_1009	DJI_0908	Silene borysthenica (Gruner) Walters	0	n	1	0	Caryophyllaceae Juss.
Iz_8_1009	DJI_0908	Silene latifolia subsp. alba (Mill.) Greuter & Burdet	0	n	1	0	Caryophyllaceae Juss.
Iz_8_1009	DJI_0908	Solidago virgaurea L.	0	n	1	0	Asteraceae Bercht. & J.Presl
Iz_8_1009	DJI_0908	Sorbus aucuparia L.	0	n	1	1	Rosaceae Juss.
Iz_8_1009	DJI_0908	Stachys recta L.	0	n	1	0	Lamiacea Lindl.
Iz_8_1009	DJI_0908	Tanacetum vulgare L.	0	n	1	0	Asteraceae Bercht. & J.Presl
Iz_8_1009	DJI_0908	Thymus pallasianus Heinr.Braun	0	n	1	0	Lamiacea Lindl.
Iz_8_1009	DJI_0908	Tilia cordata Mill.	0	n	1	1	Malvaceae Juss.
Iz_8_1009	DJI_0908	Trifolium alpestre L.	0	n	1	0	Fabaceae Lindl.
Iz_8_1009	DJI_0908	Trifolium arvense L.	0	n	1	0	Fabaceae Lindl.
Iz_8_1009	DJI_0908	Ulmus glabra Huds.	0	n	1	1	Ulmaceae Mirb.
Iz_8_1009	DJI_0908	Verbascum densiflorum Bertol.	0	n	1	0	Scrophulariaceae Juss.
Iz_8_1009	DJI_0908	Veronica austriaca L.	0	n	1	0	Plantaginaceae Juss.
Iz_8_1009	DJI_0908	Veronica incana L.	0	n	1	0	Plantaginaceae Juss.
Iz_8_1009	DJI_0908	Veronica verna L.	0	n	1	0	Plantaginaceae Juss.
Iz_8_1009	DJI_0908	Vincetoxicum hirundinaria Medik	0	n	1	0	Apocynaceae Juss.
Iz_8_1009	DJI_0911	Acer tataricum L.	0	n	1	1	Sapindaceae Juss.
Iz_8_1009	DJI_0911	Achillea millefolium L. s.l.	0	n	1	0	Asteraceae Bercht. & J.Presl
Iz_8_1009	DJI_0911	Allium oleraceum L.	0	n	1	0	Amaryllidaceae J.St.-Hil.
Iz_8_1009	DJI_0911	Ambrosia artemisiifolia L.	0	a	0	0	Asteraceae Bercht. & J.Presl
Iz_8_1009	DJI_0911	Amorpha fruticosa L.	0	a	0	0	Fabaceae Lindl.
Iz_8_1009	DJI_0911	Anthericum ramosum L.	0	n	1	0	Asparagaceae Juss.
Iz_8_1009	DJI_0911	Artemisia marschalliana Spreng.	0	n	1	0	Asteraceae Bercht. & J.Presl
Iz_8_1009	DJI_0911	Asparagus officinalis L.	0	n	1	0	Asparagaceae Juss.
Iz_8_1009	DJI_0911	Astragalus cicer L.	0	n	1	0	Fabaceae Lindl.
Iz_8_1009	DJI_0911	Bassia laniflora (S.G.Gmel.) A.J.Scott	0	n	1	0	Amaranthaceae Juss.
Iz_8_1009	DJI_0911	Berberis vulgaris L.	0	n	1	0	Berberidaceae Juss.
Iz_8_1009	DJI_0911	Betula pendula Roth.	0	n	1	1	Betulaceae Gray
Iz_8_1009	DJI_0911	Betula pendula Roth.	0	n	1	0	Poaceae Barnhart
Iz_8_1009	DJI_0911	Bromus inermis Leyss.	0		NA	NA	
Iz_8_1009	DJI_0911	Bromus squarrosus L.	0	a	0	0	Poaceae Barnhart
Iz_8_1009	DJI_0911	Bromus tectorum L.	0	a	0	0	Poaceae Barnhart
Iz_8_1009	DJI_0911	Calamagrostis epigejos (L.) Roth	3	n	1	0	Poaceae Barnhart
Iz_8_1009	DJI_0911	Carduus acanthoides L.	0	n	1	0	Asteraceae Bercht. & J.Presl
Iz_8_1009	DJI_0911	Carex sp.	0	n	1	0	Cyperaceae Juss.
Iz_8_1009	DJI_0911	Centaurea majorovii Dumbadze	0	n	1	0	Asteraceae Bercht. & J.Presl

Iz_8_1009	DJI_0911	Chamaecytisus ruthenicus (Fisch.ex Wol.) Klaskova	0	n	1	0	Fabaceae Lindl.
Iz_8_1009	DJI_0911	Chelidonium majus L.	0	n	1	0	Papaveraceae Juss.
Iz_8_1009	DJI_0911	Chenopodium betaceum Andrz.	0	n	1	0	Amaranthaceae Juss.
Iz_8_1009	DJI_0911	Chenopodium cf. album	0	n	1	0	Amaranthaceae Juss.
Iz_8_1009	DJI_0911	Chenopodium sp.	0		NA	0	Amaranthaceae Juss.
Iz_8_1009	DJI_0911	Chondrilla juncea L.	0	n	1	0	Asteraceae Bercht. & J.Presl
Iz_8_1009	DJI_0911	Cirsium vulgare (Savi) Ten.	0	n	1	0	Asteraceae Bercht. & J.Presl
Iz_8_1009	DJI_0911	Clinopodium acinos (L.) Kuntze	0	n	1	0	Lamiacea Lindl.
Iz_8_1009	DJI_0911	Convallaria majalis L.	0	n	1	0	Asparagaceae Juss.
Iz_8_1009	DJI_0911	Crataegus rhipidophylla Gand.	0	n	1	1	Rosaceae Juss.
Iz_8_1009	DJI_0911	Crepis foetida L.	0	n	1	0	Asteraceae Bercht. & J.Presl
Iz_8_1009	DJI_0911	Dactylis glomerata L.	0	n	1	0	Poaceae Barnhart
Iz_8_1009	DJI_0911	Dianthus campestris M.Bieb.	0	n	1	0	Caryophyllaceae Juss.
Iz_8_1009	DJI_0911	Digitaria sanguinalis (L.) Scop.	0	a	0	0	Poaceae Barnhart
Iz_8_1009	DJI_0911	Elymus repens (L.) Gould	0	n	1	0	Poaceae Barnhart
Iz_8_1009	DJI_0911	Epilobium tetragonum L.	0	n	1	0	Onagraceae Juss.
Iz_8_1009	DJI_0911	Eragrostis minor Host	0	a	0	0	Poaceae Barnhart
Iz_8_1009	DJI_0911	Erigeron canadensis L.	5	a	0	0	Asteraceae Bercht. & J.Presl
Iz_8_1009	DJI_0911	Euphorbia seguieriana Neck.	0	n	1	0	Euphorbiaceae Juss.
Iz_8_1009	DJI_0911	Fallopia dumetorum (L.) Holub	0	n	1	0	Polygonaceae Juss.
Iz_8_1009	DJI_0911	Festuca beckeri (Hack.) Trautv.	0	n	1	0	Poaceae Barnhart
Iz_8_1009	DJI_0911	Fragaria vesca L.	0	n	1	0	Rosaceae Juss.
Iz_8_1009	DJI_0911	Frangula alnus Mill.	0	n	1	1	Rhamnaceae Juss.
Iz_8_1009	DJI_0911	Fraxinus pennsylvanica Marshall	0	a	0	1	Oleaceae Hoffmanns. & Link
Iz_8_1009	DJI_0911	Galeopsis bifida Boenn.	0	n	1	0	Lamiacea Lindl.
Iz_8_1009	DJI_0911	Galium verum L.	0	n	1	0	Rubiaceae Juss.
Iz_8_1009	DJI_0911	Genista tinctoria L.	0	n	1	0	Fabaceae Lindl.
Iz_8_1009	DJI_0911	Geranium robertianum L.	0	n	1	0	Geraniaceae Juss.
Iz_8_1009	DJI_0911	Geranium sanquineum L.	0	n	1	0	Geraniaceae Juss.
Iz_8_1009	DJI_0911	Gypsophila paniculata L.	0	n	1	0	Caryophyllaceae Juss.
Iz_8_1009	DJI_0911	Helichrysum arenarium (L.) Moench	0	n	1	0	Asteraceae Bercht. & J.Presl
Iz_8_1009	DJI_0911	Herniaria polygama J.Gay	0	n	1	0	Caryophyllaceae Juss.
Iz_8_1009	DJI_0911	Hieracium robustum Fr.	0	n	1	0	Asteraceae Bercht. & J.Presl
Iz_8_1009	DJI_0911	Hieracium umbellatum L.	0	n	1	NA	Asteraceae Bercht. & J.Presl
Iz_8_1009	DJI_0911	Hylotelephium maximum (L.) Holub	0	n	1	0	Crassulaceae J.St.-Hil.
Iz_8_1009	DJI_0911	Jacobaea borysthenica (DC.) B.Nord. & Greuter	0	n	1	0	Asteraceae Bercht. & J.Presl
Iz_8_1009	DJI_0911	Jasione montana L.	0	n	1	0	Campanulaceae Juss.
Iz_8_1009	DJI_0911	Jurinea cyanoides (L.) Rchb.s.l.	0	n	1	0	Asteraceae Bercht. & J.Presl
Iz_8_1009	DJI_0911	Koeleria glauca (Spreng.) DC.	0	n	1	0	Poaceae Barnhart
Iz_8_1009	DJI_0911	Lactuca serriola L.	0	a	0	0	Asteraceae Bercht. & J.Presl
Iz_8_1009	DJI_0911	Linaria genistifolia (L.) Mill.	0	n	1	0	Plantaginaceae Juss.
Iz_8_1009	DJI_0911	Lomelosia argentea (L.) Greuter & Burdet	0	n	1	0	Caprifoliaceae Juss.
Iz_8_1009	DJI_0911	Melampyrum pratense L.	0	n	1	0	Orobanchaceae Vent.
Iz_8_1009	DJI_0911	Oenothera biennis L.	0	a	0	0	Onagraceae Juss.
Iz_8_1009	DJI_0911	Pentanema britannica (L.) D.Gut.Larr., Santos-Vicente, Anderb., E.Rico & M.M.Mart.Ort.	0	n	1	0	Asteraceae Bercht. & J.Presl
Iz_8_1009	DJI_0911	Peucedanum oreoselinum (L.) Moench	0	n	1	0	Apiaceae Lindl.
Iz_8_1009	DJI_0911	Peucedanum ruthenicum M.Bieb.	0	n	1	0	Apiaceae Lindl.
Iz_8_1009	DJI_0911	Pilosella echioides (Lumn.) F.W.Schultz & Sch.Bip.	0	n	1	0	Asteraceae Bercht. & J.Presl
Iz_8_1009	DJI_0911	Pilosella officinarum F. Schultz et Sch. Bip.	0	n	1	0	Asteraceae Bercht. & J.Presl
Iz_8_1009	DJI_0911	Pilosella sp.	0		NA	0	Asteraceae Bercht. & J.Presl
Iz_8_1009	DJI_0911	Pinus sylvestris L.	0	n	1	1	Pinaceae Spreng. ex F.Rudolphi
Iz_8_1009	DJI_0911	Poa annua L.	0	n	1	0	Poaceae Barnhart
Iz_8_1009	DJI_0911	Poa nemoralis L.	0	n	1	0	Poaceae Barnhart
Iz_8_1009	DJI_0911	Poa sp.	0		NA	0	Poaceae Barnhart
Iz_8_1009	DJI_0911	Polygonatum odoratum (Mill.) Druce	0	n	1	0	Asparagaceae Juss.
Iz_8_1009	DJI_0911	Polygonum arenarium Waldst. & Kit.	0	n	1	0	Polygonaceae Juss.
Iz_8_1009	DJI_0911	Populus nigra L.	0	n	1	1	Salicaceae Mirb.
Iz_8_1009	DJI_0911	Populus tremula L.	0	n	1	1	Salicaceae Mirb.
Iz_8_1009	DJI_0911	Populus Ã canescens (Aiton) Sm.	0	n	1	1	Salicaceae Mirb.
Iz_8_1009	DJI_0911	Potentilla argentea L.	0	n	1	0	Rosaceae Juss.
Iz_8_1009	DJI_0911	Psammophiliella muralis (L.) Ikonn.	0	n	1	0	Caryophyllaceae Juss.
Iz_8_1009	DJI_0911	Psephellus sumensis (Kalen.) Greuter	0	n	1	0	Asteraceae Bercht. & J.Presl
Iz_8_1009	DJI_0911	Pulsatilla pratensis (L.) Mill. s.l.	0	n	1	0	Ranunculaceae Juss.
Iz_8_1009	DJI_0911	Pyrus communis L.	0	a	0	1	Rosaceae Juss.
Iz_8_1009	DJI_0911	Quercus robur L.	0	n	1	1	Fagaceae Dumort.
Iz_8_1009	DJI_0911	Robinia pseudoacacia L.	0	a	0	1	Fabaceae Lindl.
Iz_8_1009	DJI_0911	Rosa sp.	0		NA	1	Rosaceae Juss.
Iz_8_1009	DJI_0911	Rubus idaeus L.	0	n	1	0	Rosaceae Juss.
Iz_8_1009	DJI_0911	Rumex acetosella L.	0	n	1	0	Polygonaceae Juss.
Iz_8_1009	DJI_0911	Rumex thyrsiflorus Fingerh.	0	n	1	0	Polygonaceae Juss.
Iz_8_1009	DJI_0911	Salix alba L.	0	n	1	1	Salicaceae Mirb.
Iz_8_1009	DJI_0911	Sambucus nigra L.	0	n	1	1	Viburnaceae Raf.
Iz_8_1009	DJI_0911	Secale sylvestre Host	0	n	1	0	Poaceae Barnhart
Iz_8_1009	DJI_0911	Sempervivum ruthenicum Schnittsp. & C.B. Lehm.	0	n	1	0	Crassulaceae J.St.-Hil.
Iz_8_1009	DJI_0911	Setaria viridis (L.) P. Beauv.	0	n	1	0	Poaceae Barnhart
Iz_8_1009	DJI_0911	Silene borysthenica (Gruner) Walters	0	n	1	0	Caryophyllaceae Juss.
Iz_8_1009	DJI_0911	Silene latifolia subsp. alba (Mill.) Greuter & Burdet	0	n	1	0	Caryophyllaceae Juss.
Iz_8_1009	DJI_0911	Solidago virgaurea L.	0	n	1	0	Asteraceae Bercht. & J.Presl
Iz_8_1009	DJI_0911	Sorbus aucuparia L.	0	n	1	1	Rosaceae Juss.
Iz_8_1009	DJI_0911	Stachys recta L.	0	n	1	0	Lamiacea Lindl.
Iz_8_1009	DJI_0911	Tanacetum vulgare L.	0	n	1	0	Asteraceae Bercht. & J.Presl
Iz_8_1009	DJI_0911	Thymus pallasianus Heinr.Braun	0	n	1	0	Lamiacea Lindl.
Iz_8_1009	DJI_0911	Tilia cordata Mill.	0	n	1	1	Malvaceae Juss.
Iz_8_1009	DJI_0911	Trifolium alpestre L.	0	n	1	0	Fabaceae Lindl.
Iz_8_1009	DJI_0911	Trifolium arvense L.	0	n	1	0	Fabaceae Lindl.
Iz_8_1009	DJI_0911	Ulmus glabra Huds.	0	n	1	1	Ulmaceae Mirb.
Iz_8_1009	DJI_0911	Verbascum densiflorum Bertol.	0	n	1	0	Scrophulariaceae Juss.
Iz_8_1009	DJI_0911	Veronica austriaca L.	0	n	1	0	Plantaginaceae Juss.
Iz_8_1009	DJI_0911	Veronica incana L.	0	n	1	0	Plantaginaceae Juss.
Iz_8_1009	DJI_0911	Veronica verna L.	0	n	1	0	Plantaginaceae Juss.
Iz_8_1009	DJI_0911	Vincetoxicum hirundinaria Medik	0	n	1	0	Apocynaceae Juss.
Iz_8_1009	DJI_0912	Acer tataricum L.	0	n	1	1	Sapindaceae Juss.
Iz_8_1009	DJI_0912	Achillea millefolium L. s.l.	0	n	1	0	Asteraceae Bercht. & J.Presl
Iz_8_1009	DJI_0912	Allium oleraceum L.	0	n	1	0	Amaryllidaceae J.St.-Hil.
Iz_8_1009	DJI_0912	Ambrosia artemisiifolia L.	0	a	0	0	Asteraceae Bercht. & J.Presl
Iz_8_1009	DJI_0912	Amorpha fruticosa L.	0	a	0	0	Fabaceae Lindl.
Iz_8_1009	DJI_0912	Anthericum ramosum L.	0	n	1	0	Asparagaceae Juss.
Iz_8_1009	DJI_0912	Artemisia marschalliana Spreng.	0	n	1	0	Asteraceae Bercht. & J.Presl

Iz_8_1009	DJI_0912	Asparagus officinalis L.	0	n	1	0	Asparagaceae Juss.
Iz_8_1009	DJI_0912	Astragalus cicer L.	0	n	1	0	Fabaceae Lindl.
Iz_8_1009	DJI_0912	Bassia laniflora (S.G.Gmel.) A.J.Scott	0	n	1	0	Amaranthaceae Juss.
Iz_8_1009	DJI_0912	Berberis vulgaris L.	0	n	1	0	Berberidaceae Juss.
Iz_8_1009	DJI_0912	Betula pendula Roth.	0	n	1	1	Betulaceae Gray
Iz_8_1009	DJI_0912	Betula pendula Roth.	0	n	1	0	Poaceae Barnhart
Iz_8_1009	DJI_0912	Bromus inermis Leyss.	0		NA	NA	
Iz_8_1009	DJI_0912	Bromus squarrosus L.	0	a	0	0	Poaceae Barnhart
Iz_8_1009	DJI_0912	Bromus tectorum L.	0	a	0	0	Poaceae Barnhart
Iz_8_1009	DJI_0912	Calamagrostis epigejos (L.) Roth	0	n	1	0	Poaceae Barnhart
Iz_8_1009	DJI_0912	Carduus acanthoides L.	0	n	1	0	Asteraceae Bercht. & J.Presl
Iz_8_1009	DJI_0912	Carex sp.	0	n	1	0	Cyperaceae Juss.
Iz_8_1009	DJI_0912	Centaurea majorovii Dumbadze	0	n	1	0	Asteraceae Bercht. & J.Presl
Iz_8_1009	DJI_0912	Chamaecytisus ruthenicus (Fisch.ex Wol.) Klaskova	0	n	1	0	Fabaceae Lindl.
Iz_8_1009	DJI_0912	Chelidonium majus L.	0	n	1	0	Papaveraceae Juss.
Iz_8_1009	DJI_0912	Chenopodium betaceum Andrz.	0	n	1	0	Amaranthaceae Juss.
Iz_8_1009	DJI_0912	Chenopodium cf. album	0	n	1	0	Amaranthaceae Juss.
Iz_8_1009	DJI_0912	Chenopodium sp.	0		NA	0	Amaranthaceae Juss.
Iz_8_1009	DJI_0912	Chondrilla juncea L.	0	n	1	0	Asteraceae Bercht. & J.Presl
Iz_8_1009	DJI_0912	Cirsium vulgare (Savi) Ten.	0	n	1	0	Asteraceae Bercht. & J.Presl
Iz_8_1009	DJI_0912	Clinopodium acinos (L.) Kuntze	0	n	1	0	Lamiacea Lindl.
Iz_8_1009	DJI_0912	Convallaria majalis L.	0	n	1	0	Asparagaceae Juss.
Iz_8_1009	DJI_0912	Crataegus rhipidophylla Gand.	0	n	1	1	Rosaceae Juss.
Iz_8_1009	DJI_0912	Crepis foetida L.	0	n	1	0	Asteraceae Bercht. & J.Presl
Iz_8_1009	DJI_0912	Dactylis glomerata L.	0	n	1	0	Poaceae Barnhart
Iz_8_1009	DJI_0912	Dianthus campestris M.Bieb.	0	n	1	0	Caryophyllaceae Juss.
Iz_8_1009	DJI_0912	Digitaria sanguinalis (L.) Scop.	0	a	0	0	Poaceae Barnhart
Iz_8_1009	DJI_0912	Elymus repens (L.) Gould	0	n	1	0	Poaceae Barnhart
Iz_8_1009	DJI_0912	Epilobium tetragonum L.	0	n	1	0	Onagraceae Juss.
Iz_8_1009	DJI_0912	Eragrostis minor Host	0	a	0	0	Poaceae Barnhart
Iz_8_1009	DJI_0912	Erigeron canadensis L.	5	a	0	0	Asteraceae Bercht. & J.Presl
Iz_8_1009	DJI_0912	Euphorbia seguieriana Neck.	0	n	1	0	Euphorbiaceae Juss.
Iz_8_1009	DJI_0912	Fallopia dumetorum (L.) Holub	0	n	1	0	Polygonaceae Juss.
Iz_8_1009	DJI_0912	Festuca beckeri (Hack.) Trautv.	0	n	1	0	Poaceae Barnhart
Iz_8_1009	DJI_0912	Fragaria vesca L.	0	n	1	0	Rosaceae Juss.
Iz_8_1009	DJI_0912	Frangula alnus Mill.	0	n	1	1	Rhamnaceae Juss.
Iz_8_1009	DJI_0912	Fraxinus pennsylvanica Marshall	0	a	0	1	Oleaceae Hoffmanns. & Link
Iz_8_1009	DJI_0912	Galeopsis bifida Boenn.	0	n	1	0	Lamiacea Lindl.
Iz_8_1009	DJI_0912	Galium verum L.	0	n	1	0	Rubiaceae Juss.
Iz_8_1009	DJI_0912	Genista tinctoria L.	0	n	1	0	Fabaceae Lindl.
Iz_8_1009	DJI_0912	Geranium robertianum L.	0	n	1	0	Geraniaceae Juss.
Iz_8_1009	DJI_0912	Geranium sanquineum L.	0	n	1	0	Geraniaceae Juss.
Iz_8_1009	DJI_0912	Gypsophila paniculata L.	0	n	1	0	Caryophyllaceae Juss.
Iz_8_1009	DJI_0912	Helichrysum arenarium (L.) Moench	0	n	1	0	Asteraceae Bercht. & J.Presl
Iz_8_1009	DJI_0912	Herniaria polygama J.Gay	0	n	1	0	Caryophyllaceae Juss.
Iz_8_1009	DJI_0912	Hieracium robustum Fr.	0	n	1	0	Asteraceae Bercht. & J.Presl
Iz_8_1009	DJI_0912	Hieracium umbellatum L.	0	n	1	NA	Asteraceae Bercht. & J.Presl
Iz_8_1009	DJI_0912	Hylotelephium maximum (L.) Holub	0	n	1	0	Crassulaceae J.St.-Hil.
Iz_8_1009	DJI_0912	Jacobaea borysthenica (DC.) B.Nord. & Greuter	0	n	1	0	Asteraceae Bercht. & J.Presl
Iz_8_1009	DJI_0912	Jasione montana L.	0	n	1	0	Campanulaceae Juss.
Iz_8_1009	DJI_0912	Jurinea cyanoides (L.) Rchb.s.l.	0	n	1	0	Asteraceae Bercht. & J.Presl
Iz_8_1009	DJI_0912	Koeleria glauca (Spreng.) DC.	0	n	1	0	Poaceae Barnhart
Iz_8_1009	DJI_0912	Lactuca serriola L.	0	a	0	0	Asteraceae Bercht. & J.Presl
Iz_8_1009	DJI_0912	Linaria genistifolia (L.) Mill.	0	n	1	0	Plantaginaceae Juss.
Iz_8_1009	DJI_0912	Lomelosia argentea (L.) Greuter & Burdet	0	n	1	0	Caprifoliaceae Juss.
Iz_8_1009	DJI_0912	Melampyrum pratense L.	0	n	1	0	Orobanchaceae Vent.
Iz_8_1009	DJI_0912	Oenothera biennis L.	0	a	0	0	Onagraceae Juss.
Iz_8_1009	DJI_0912	Pentanema britannica (L.) D.Gut.Larr., Santos-Vicente, Anderb., E.Rico & M.M.Mart.Ort.	0	n	1	0	Asteraceae Bercht. & J.Presl
Iz_8_1009	DJI_0912	Peucedanum oreoselinum (L.) Moench	0	n	1	0	Apiaceae Lindl.
Iz_8_1009	DJI_0912	Peucedanum ruthenicum M.Bieb.	0	n	1	0	Apiaceae Lindl.
Iz_8_1009	DJI_0912	Pilosella echioides (Lumn.) F.W.Schultz & Sch.Bip.	0	n	1	0	Asteraceae Bercht. & J.Presl
Iz_8_1009	DJI_0912	Pilosella officinarum F. Schultz et Sch. Bip.	0	n	1	0	Asteraceae Bercht. & J.Presl
Iz_8_1009	DJI_0912	Pilosella sp.	0		NA	0	Asteraceae Bercht. & J.Presl
Iz_8_1009	DJI_0912	Pinus sylvestris L.	0	n	1	1	Pinaceae Spreng. ex F.Rudolphi
Iz_8_1009	DJI_0912	Poa annua L.	0	n	1	0	Poaceae Barnhart
Iz_8_1009	DJI_0912	Poa nemoralis L.	0	n	1	0	Poaceae Barnhart
Iz_8_1009	DJI_0912	Poa sp.	0		NA	0	Poaceae Barnhart
Iz_8_1009	DJI_0912	Polygonatum odoratum (Mill.) Druce	0	n	1	0	Asparagaceae Juss.
Iz_8_1009	DJI_0912	Polygonum arenarium Waldst. & Kit.	0	n	1	0	Polygonaceae Juss.
Iz_8_1009	DJI_0912	Populus nigra L.	0	n	1	1	Salicaceae Mirb.
Iz_8_1009	DJI_0912	Populus tremula L.	0	n	1	1	Salicaceae Mirb.
Iz_8_1009	DJI_0912	Populus Ã canescens (Aiton) Sm.	0	n	1	1	Salicaceae Mirb.
Iz_8_1009	DJI_0912	Potentilla argentea L.	0	n	1	0	Rosaceae Juss.
Iz_8_1009	DJI_0912	Psammophiliella muralis (L.) Ikonn.	0	n	1	0	Caryophyllaceae Juss.
Iz_8_1009	DJI_0912	Psephellus sumensis (Kalen.) Greuter	0	n	1	0	Asteraceae Bercht. & J.Presl
Iz_8_1009	DJI_0912	Pulsatilla pratensis (L.) Mill. s.l.	0	n	1	0	Ranunculaceae Juss.
Iz_8_1009	DJI_0912	Pyrus communis L.	0	a	0	1	Rosaceae Juss.
Iz_8_1009	DJI_0912	Quercus robur L.	0	n	1	1	Fagaceae Dumort.
Iz_8_1009	DJI_0912	Robinia pseudoacacia L.	0	a	0	1	Fabaceae Lindl.
Iz_8_1009	DJI_0912	Rosa sp.	0		NA	1	Rosaceae Juss.
Iz_8_1009	DJI_0912	Rubus idaeus L.	0	n	1	0	Rosaceae Juss.
Iz_8_1009	DJI_0912	Rumex acetosella L.	0	n	1	0	Polygonaceae Juss.
Iz_8_1009	DJI_0912	Rumex thyrsiflorus Fingerh.	0	n	1	0	Polygonaceae Juss.
Iz_8_1009	DJI_0912	Salix alba L.	0	n	1	1	Salicaceae Mirb.
Iz_8_1009	DJI_0912	Sambucus nigra L.	0	n	1	1	Viburnaceae Raf.
Iz_8_1009	DJI_0912	Secale sylvestre Host	0	n	1	0	Poaceae Barnhart
Iz_8_1009	DJI_0912	Sempervivum ruthenicum Schnittsp. & C.B. Lehm.	0	n	1	0	Crassulaceae J.St.-Hil.
Iz_8_1009	DJI_0912	Setaria viridis (L.) P. Beauv.	0	n	1	0	Poaceae Barnhart
Iz_8_1009	DJI_0912	Silene borysthenica (Gruner) Walters	0	n	1	0	Caryophyllaceae Juss.
Iz_8_1009	DJI_0912	Silene latifolia subsp. alba (Mill.) Greuter & Burdet	0	n	1	0	Caryophyllaceae Juss.
Iz_8_1009	DJI_0912	Solidago virgaurea L.	0	n	1	0	Asteraceae Bercht. & J.Presl
Iz_8_1009	DJI_0912	Sorbus aucuparia L.	0	n	1	1	Rosaceae Juss.
Iz_8_1009	DJI_0912	Stachys recta L.	0	n	1	0	Lamiacea Lindl.
Iz_8_1009	DJI_0912	Tanacetum vulgare L.	0	n	1	0	Asteraceae Bercht. & J.Presl
Iz_8_1009	DJI_0912	Thymus pallasianus Heinr.Braun	0	n	1	0	Lamiacea Lindl.
Iz_8_1009	DJI_0912	Tilia cordata Mill.	0	n	1	1	Malvaceae Juss.
Iz_8_1009	DJI_0912	Trifolium alpestre L.	0	n	1	0	Fabaceae Lindl.
Iz_8_1009	DJI_0912	Trifolium arvense L.	0	n	1	0	Fabaceae Lindl.

Iz_8_1009	DJI_0912	Ulmus glabra Huds.	0	n	1	1	Ulmaceae Mirb.
Iz_8_1009	DJI_0912	Verbascum densiflorum Bertol.	0	n	1	0	Scrophulariaceae Juss.
Iz_8_1009	DJI_0912	Veronica austriaca L.	0	n	1	0	Plantaginaceae Juss.
Iz_8_1009	DJI_0912	Veronica incana L.	0	n	1	0	Plantaginaceae Juss.
Iz_8_1009	DJI_0912	Veronica verna L.	0	n	1	0	Plantaginaceae Juss.
Iz_8_1009	DJI_0912	Vincetoxicum hirundinaria Medik	0	n	1	0	Apocynaceae Juss.
Iz_8_1009	DJI_0913	Acer tataricum L.	0	n	1	1	Sapindaceae Juss.
Iz_8_1009	DJI_0913	Achillea millefolium L. s.l.	0	n	1	0	Asteraceae Bercht. & J.Presl
Iz_8_1009	DJI_0913	Allium oleraceum L.	0	n	1	0	Amaryllidaceae J.St.-Hil.
Iz_8_1009	DJI_0913	Ambrosia artemisiifolia L.	0	a	0	0	Asteraceae Bercht. & J.Presl
Iz_8_1009	DJI_0913	Amorpha fruticosa L.	0	a	0	0	Fabaceae Lindl.
Iz_8_1009	DJI_0913	Anthericum ramosum L.	0	n	1	0	Asparagaceae Juss.
Iz_8_1009	DJI_0913	Artemisia marschalliana Spreng.	0	n	1	0	Asteraceae Bercht. & J.Presl
Iz_8_1009	DJI_0913	Asparagus officinalis L.	0	n	1	0	Asparagaceae Juss.
Iz_8_1009	DJI_0913	Astragalus cicer L.	0	n	1	0	Fabaceae Lindl.
Iz_8_1009	DJI_0913	Bassia laniflora (S.G.Gmel.) A.J.Scott	0	n	1	0	Amaranthaceae Juss.
Iz_8_1009	DJI_0913	Berberis vulgaris L.	0	n	1	0	Berberidaceae Juss.
Iz_8_1009	DJI_0913	Betula pendula Roth.	0	n	1	1	Betulaceae Gray
Iz_8_1009	DJI_0913	Betula pendula Roth.	0	n	1	0	Poaceae Barnhart
Iz_8_1009	DJI_0913	Bromus inermis Leyss.	0		NA	NA	
Iz_8_1009	DJI_0913	Bromus squarrosus L.	0	a	0	0	Poaceae Barnhart
Iz_8_1009	DJI_0913	Bromus tectorum L.	0	a	0	0	Poaceae Barnhart
Iz_8_1009	DJI_0913	Calamagrostis epigejos (L.) Roth	5	n	1	0	Poaceae Barnhart
Iz_8_1009	DJI_0913	Carduus acanthoides L.	0	n	1	0	Asteraceae Bercht. & J.Presl
Iz_8_1009	DJI_0913	Carex sp.	0	n	1	0	Cyperaceae Juss.
Iz_8_1009	DJI_0913	Centaurea majorovii Dumbadze	0	n	1	0	Asteraceae Bercht. & J.Presl
Iz_8_1009	DJI_0913	Chamaecytisus ruthenicus (Fisch.ex Wol.) Klaskova	0	n	1	0	Fabaceae Lindl.
Iz_8_1009	DJI_0913	Chelidonium majus L.	0	n	1	0	Papaveraceae Juss.
Iz_8_1009	DJI_0913	Chenopodium betaceum Andrz.	0	n	1	0	Amaranthaceae Juss.
Iz_8_1009	DJI_0913	Chenopodium cf. album	0	n	1	0	Amaranthaceae Juss.
Iz_8_1009	DJI_0913	Chenopodium sp.	0		NA	0	Amaranthaceae Juss.
Iz_8_1009	DJI_0913	Chondrilla juncea L.	0	n	1	0	Asteraceae Bercht. & J.Presl
Iz_8_1009	DJI_0913	Cirsium vulgare (Savi) Ten.	0	n	1	0	Asteraceae Bercht. & J.Presl
Iz_8_1009	DJI_0913	Clinopodium acinos (L.) Kuntze	0	n	1	0	Lamiacea Lindl.
Iz_8_1009	DJI_0913	Convallaria majalis L.	0	n	1	0	Asparagaceae Juss.
Iz_8_1009	DJI_0913	Crataegus rhipidophylla Gand.	0	n	1	1	Rosaceae Juss.
Iz_8_1009	DJI_0913	Crepis foetida L.	0	n	1	0	Asteraceae Bercht. & J.Presl
Iz_8_1009	DJI_0913	Dactylis glomerata L.	0	n	1	0	Poaceae Barnhart
Iz_8_1009	DJI_0913	Dianthus campestris M.Bieb.	0	n	1	0	Caryophyllaceae Juss.
Iz_8_1009	DJI_0913	Digitaria sanguinalis (L.) Scop.	0	a	0	0	Poaceae Barnhart
Iz_8_1009	DJI_0913	Elymus repens (L.) Gould	0	n	1	0	Poaceae Barnhart
Iz_8_1009	DJI_0913	Epilobium tetragonum L.	0	n	1	0	Onagraceae Juss.
Iz_8_1009	DJI_0913	Eragrostis minor Host	0	a	0	0	Poaceae Barnhart
Iz_8_1009	DJI_0913	Erigeron canadensis L.	8	a	0	0	Asteraceae Bercht. & J.Presl
Iz_8_1009	DJI_0913	Euphorbia seguieriana Neck.	0	n	1	0	Euphorbiaceae Juss.
Iz_8_1009	DJI_0913	Fallopia dumetorum (L.) Holub	0	n	1	0	Polygonaceae Juss.
Iz_8_1009	DJI_0913	Festuca beckeri (Hack.) Trautv.	0	n	1	0	Poaceae Barnhart
Iz_8_1009	DJI_0913	Fragaria vesca L.	0	n	1	0	Rosaceae Juss.
Iz_8_1009	DJI_0913	Frangula alnus Mill.	0	n	1	1	Rhamnaceae Juss.
Iz_8_1009	DJI_0913	Fraxinus pennsylvanica Marshall	0	a	0	1	Oleaceae Hoffmanns. & Link
Iz_8_1009	DJI_0913	Galeopsis bifida Boenn.	0	n	1	0	Lamiacea Lindl.
Iz_8_1009	DJI_0913	Galium verum L.	0	n	1	0	Rubiaceae Juss.
Iz_8_1009	DJI_0913	Genista tinctoria L.	0	n	1	0	Fabaceae Lindl.
Iz_8_1009	DJI_0913	Geranium robertianum L.	0	n	1	0	Geraniaceae Juss.
Iz_8_1009	DJI_0913	Geranium sanquineum L.	0	n	1	0	Geraniaceae Juss.
Iz_8_1009	DJI_0913	Gypsophila paniculata L.	0	n	1	0	Caryophyllaceae Juss.
Iz_8_1009	DJI_0913	Helichrysum arenarium (L.) Moench	0	n	1	0	Asteraceae Bercht. & J.Presl
Iz_8_1009	DJI_0913	Herniaria polygama J.Gay	0	n	1	0	Caryophyllaceae Juss.
Iz_8_1009	DJI_0913	Hieracium robustum Fr.	0	n	1	0	Asteraceae Bercht. & J.Presl
Iz_8_1009	DJI_0913	Hieracium umbellatum L.	0	n	1	NA	Asteraceae Bercht. & J.Presl
Iz_8_1009	DJI_0913	Hylotelephium maximum (L.) Holub	0	n	1	0	Crassulaceae J.St.-Hil.
Iz_8_1009	DJI_0913	Jacobaea borysthenica (DC.) B.Nord. & Greuter	0	n	1	0	Asteraceae Bercht. & J.Presl
Iz_8_1009	DJI_0913	Jasione montana L.	0	n	1	0	Campanulaceae Juss.
Iz_8_1009	DJI_0913	Jurinea cyanoides (L.) Rchb.s.l.	0	n	1	0	Asteraceae Bercht. & J.Presl
Iz_8_1009	DJI_0913	Koeleria glauca (Spreng.) DC.	0	n	1	0	Poaceae Barnhart
Iz_8_1009	DJI_0913	Lactuca serriola L.	0	a	0	0	Asteraceae Bercht. & J.Presl
Iz_8_1009	DJI_0913	Linaria genistifolia (L.) Mill.	0	n	1	0	Plantaginaceae Juss.
Iz_8_1009	DJI_0913	Lomelosia argentea (L.) Greuter & Burdet	0	n	1	0	Caprifoliaceae Juss.
Iz_8_1009	DJI_0913	Melampyrum pratense L.	0	n	1	0	Orobanchaceae Vent.
Iz_8_1009	DJI_0913	Oenothera biennis L.	0	a	0	0	Onagraceae Juss.
Iz_8_1009	DJI_0913	Pentanema britannica (L.) D.Gut.Larr., Santos-Vicente, Anderb., E.Rico & M.M.Mart.Ort.	0	n	1	0	Asteraceae Bercht. & J.Presl
Iz_8_1009	DJI_0913	Peucedanum oreoselinum (L.) Moench	0	n	1	0	Apiaceae Lindl.
Iz_8_1009	DJI_0913	Peucedanum ruthenicum M.Bieb.	0	n	1	0	Apiaceae Lindl.
Iz_8_1009	DJI_0913	Pilosella echioides (Lumn.) F.W.Schultz & Sch.Bip.	0	n	1	0	Asteraceae Bercht. & J.Presl
Iz_8_1009	DJI_0913	Pilosella officinarum F. Schultz et Sch. Bip.	0	n	1	0	Asteraceae Bercht. & J.Presl
Iz_8_1009	DJI_0913	Pilosella sp.	0		NA	0	Asteraceae Bercht. & J.Presl
Iz_8_1009	DJI_0913	Pinus sylvestris L.	0	n	1	1	Pinaceae Spreng. ex F.Rudolphi
Iz_8_1009	DJI_0913	Poa annua L.	0	n	1	0	Poaceae Barnhart
Iz_8_1009	DJI_0913	Poa nemoralis L.	0	n	1	0	Poaceae Barnhart
Iz_8_1009	DJI_0913	Poa sp.	0		NA	0	Poaceae Barnhart
Iz_8_1009	DJI_0913	Polygonatum odoratum (Mill.) Druce	0	n	1	0	Asparagaceae Juss.
Iz_8_1009	DJI_0913	Polygonum arenarium Waldst. & Kit.	0	n	1	0	Polygonaceae Juss.
Iz_8_1009	DJI_0913	Populus nigra L.	0	n	1	1	Salicaceae Mirb.
Iz_8_1009	DJI_0913	Populus tremula L.	0	n	1	1	Salicaceae Mirb.
Iz_8_1009	DJI_0913	Populus Ã canescens (Aiton) Sm.	0	n	1	1	Salicaceae Mirb.
Iz_8_1009	DJI_0913	Potentilla argentea L.	0	n	1	0	Rosaceae Juss.
Iz_8_1009	DJI_0913	Psammophiliella muralis (L.) Ikonn.	0	n	1	0	Caryophyllaceae Juss.
Iz_8_1009	DJI_0913	Psephellus sumensis (Kalen.) Greuter	0	n	1	0	Asteraceae Bercht. & J.Presl
Iz_8_1009	DJI_0913	Pulsatilla pratensis (L.) Mill. s.l.	0	n	1	0	Ranunculaceae Juss.
Iz_8_1009	DJI_0913	Pyrus communis L.	0	a	0	1	Rosaceae Juss.
Iz_8_1009	DJI_0913	Quercus robur L.	0	n	1	1	Fagaceae Dumort.
Iz_8_1009	DJI_0913	Robinia pseudoacacia L.	0	a	0	1	Fabaceae Lindl.
Iz_8_1009	DJI_0913	Rosa sp.	0		NA	1	Rosaceae Juss.
Iz_8_1009	DJI_0913	Rubus idaeus L.	0	n	1	0	Rosaceae Juss.
Iz_8_1009	DJI_0913	Rumex acetosella L.	0	n	1	0	Polygonaceae Juss.
Iz_8_1009	DJI_0913	Rumex thyrsiflorus Fingerh.	0	n	1	0	Polygonaceae Juss.
Iz_8_1009	DJI_0913	Salix alba L.	0	n	1	1	Salicaceae Mirb.
Iz_8_1009	DJI_0913	Sambucus nigra L.	0	n	1	1	Viburnaceae Raf.

Iz_8_1009	DJI_0913	Secale sylvestre Host	0	n	1	0	Poaceae Barnhart
Iz_8_1009	DJI_0913	Sempervivum ruthenicum Schnittsp. & C.B. Lehm.	0	n	1	0	Crassulaceae J.St.-Hil.
Iz_8_1009	DJI_0913	Setaria viridis (L.) P. Beauv.	0	n	1	0	Poaceae Barnhart
Iz_8_1009	DJI_0913	Silene borysthenica (Gruner) Walters	0	n	1	0	Caryophyllaceae Juss.
Iz_8_1009	DJI_0913	Silene latifolia subsp. alba (Mill.) Greuter & Burdet	0	n	1	0	Caryophyllaceae Juss.
Iz_8_1009	DJI_0913	Solidago virgaurea L.	0	n	1	0	Asteraceae Bercht. & J.Presl
Iz_8_1009	DJI_0913	Sorbus aucuparia L.	0	n	1	1	Rosaceae Juss.
Iz_8_1009	DJI_0913	Stachys recta L.	0	n	1	0	Lamiacea Lindl.
Iz_8_1009	DJI_0913	Tanacetum vulgare L.	0	n	1	0	Asteraceae Bercht. & J.Presl
Iz_8_1009	DJI_0913	Thymus pallasianus Heinr.Braun	0	n	1	0	Lamiacea Lindl.
Iz_8_1009	DJI_0913	Tilia cordata Mill.	0	n	1	1	Malvaceae Juss.
Iz_8_1009	DJI_0913	Trifolium alpestre L.	0	n	1	0	Fabaceae Lindl.
Iz_8_1009	DJI_0913	Trifolium arvense L.	0	n	1	0	Fabaceae Lindl.
Iz_8_1009	DJI_0913	Ulmus glabra Huds.	0	n	1	1	Ulmaceae Mirb.
Iz_8_1009	DJI_0913	Verbascum densiflorum Bertol.	0	n	1	0	Scrophulariaceae Juss.
Iz_8_1009	DJI_0913	Veronica austriaca L.	0	n	1	0	Plantaginaceae Juss.
Iz_8_1009	DJI_0913	Veronica incana L.	0	n	1	0	Plantaginaceae Juss.
Iz_8_1009	DJI_0913	Veronica verna L.	0	n	1	0	Plantaginaceae Juss.
Iz_8_1009	DJI_0913	Vincetoxicum hirundinaria Medik	0	n	1	0	Apocynaceae Juss.
Iz_8_1009	DJI_0914	Acer tataricum L.	0	n	1	1	Sapindaceae Juss.
Iz_8_1009	DJI_0914	Achillea millefolium L. s.l.	0	n	1	0	Asteraceae Bercht. & J.Presl
Iz_8_1009	DJI_0914	Allium oleraceum L.	0	n	1	0	Amaryllidaceae J.St.-Hil.
Iz_8_1009	DJI_0914	Ambrosia artemisiifolia L.	0	a	0	0	Asteraceae Bercht. & J.Presl
Iz_8_1009	DJI_0914	Amorpha fruticosa L.	0	a	0	0	Fabaceae Lindl.
Iz_8_1009	DJI_0914	Anthericum ramosum L.	0	n	1	0	Asparagaceae Juss.
Iz_8_1009	DJI_0914	Artemisia marschalliana Spreng.	0	n	1	0	Asteraceae Bercht. & J.Presl
Iz_8_1009	DJI_0914	Asparagus officinalis L.	0	n	1	0	Asparagaceae Juss.
Iz_8_1009	DJI_0914	Astragalus cicer L.	0	n	1	0	Fabaceae Lindl.
Iz_8_1009	DJI_0914	Bassia laniflora (S.G.Gmel.) A.J.Scott	0	n	1	0	Amaranthaceae Juss.
Iz_8_1009	DJI_0914	Berberis vulgaris L.	0	n	1	0	Berberidaceae Juss.
Iz_8_1009	DJI_0914	Betula pendula Roth.	0	n	1	1	Betulaceae Gray
Iz_8_1009	DJI_0914	Betula pendula Roth.	0	n	1	0	Poaceae Barnhart
Iz_8_1009	DJI_0914	Bromus inermis Leyss.	0		NA	NA	
Iz_8_1009	DJI_0914	Bromus squarrosus L.	0	a	0	0	Poaceae Barnhart
Iz_8_1009	DJI_0914	Bromus tectorum L.	0	a	0	0	Poaceae Barnhart
Iz_8_1009	DJI_0914	Calamagrostis epigejos (L.) Roth	8	n	1	0	Poaceae Barnhart
Iz_8_1009	DJI_0914	Carduus acanthoides L.	0	n	1	0	Asteraceae Bercht. & J.Presl
Iz_8_1009	DJI_0914	Carex sp.	0	n	1	0	Cyperaceae Juss.
Iz_8_1009	DJI_0914	Centaurea majorovii Dumbadze	0	n	1	0	Asteraceae Bercht. & J.Presl
Iz_8_1009	DJI_0914	Chamaecytisus ruthenicus (Fisch.ex Wol.) Klaskova	0	n	1	0	Fabaceae Lindl.
Iz_8_1009	DJI_0914	Chelidonium majus L.	0	n	1	0	Papaveraceae Juss.
Iz_8_1009	DJI_0914	Chenopodium betaceum Andrz.	0	n	1	0	Amaranthaceae Juss.
Iz_8_1009	DJI_0914	Chenopodium cf. album	0	n	1	0	Amaranthaceae Juss.
Iz_8_1009	DJI_0914	Chenopodium sp.	0		NA	0	Amaranthaceae Juss.
Iz_8_1009	DJI_0914	Chondrilla juncea L.	0	n	1	0	Asteraceae Bercht. & J.Presl
Iz_8_1009	DJI_0914	Cirsium vulgare (Savi) Ten.	0	n	1	0	Asteraceae Bercht. & J.Presl
Iz_8_1009	DJI_0914	Clinopodium acinos (L.) Kuntze	0	n	1	0	Lamiacea Lindl.
Iz_8_1009	DJI_0914	Convallaria majalis L.	0	n	1	0	Asparagaceae Juss.
Iz_8_1009	DJI_0914	Crataegus rhipidophylla Gand.	0	n	1	1	Rosaceae Juss.
Iz_8_1009	DJI_0914	Crepis foetida L.	0	n	1	0	Asteraceae Bercht. & J.Presl
Iz_8_1009	DJI_0914	Dactylis glomerata L.	0	n	1	0	Poaceae Barnhart
Iz_8_1009	DJI_0914	Dianthus campestris M.Bieb.	0	n	1	0	Caryophyllaceae Juss.
Iz_8_1009	DJI_0914	Digitaria sanguinalis (L.) Scop.	0	a	0	0	Poaceae Barnhart
Iz_8_1009	DJI_0914	Elymus repens (L.) Gould	0	n	1	0	Poaceae Barnhart
Iz_8_1009	DJI_0914	Epilobium tetragonum L.	0	n	1	0	Onagraceae Juss.
Iz_8_1009	DJI_0914	Eragrostis minor Host	0	a	0	0	Poaceae Barnhart
Iz_8_1009	DJI_0914	Erigeron canadensis L.	5	a	0	0	Asteraceae Bercht. & J.Presl
Iz_8_1009	DJI_0914	Euphorbia seguieriana Neck.	0	n	1	0	Euphorbiaceae Juss.
Iz_8_1009	DJI_0914	Fallopia dumetorum (L.) Holub	0	n	1	0	Polygonaceae Juss.
Iz_8_1009	DJI_0914	Festuca beckeri (Hack.) Trautv.	0	n	1	0	Poaceae Barnhart
Iz_8_1009	DJI_0914	Fragaria vesca L.	0	n	1	0	Rosaceae Juss.
Iz_8_1009	DJI_0914	Frangula alnus Mill.	0	n	1	1	Rhamnaceae Juss.
Iz_8_1009	DJI_0914	Fraxinus pennsylvanica Marshall	0	a	0	1	Oleaceae Hoffmanns. & Link
Iz_8_1009	DJI_0914	Galeopsis bifida Boenn.	0	n	1	0	Lamiacea Lindl.
Iz_8_1009	DJI_0914	Galium verum L.	0	n	1	0	Rubiaceae Juss.
Iz_8_1009	DJI_0914	Genista tinctoria L.	0	n	1	0	Fabaceae Lindl.
Iz_8_1009	DJI_0914	Geranium robertianum L.	0	n	1	0	Geraniaceae Juss.
Iz_8_1009	DJI_0914	Geranium sanquineum L.	0	n	1	0	Geraniaceae Juss.
Iz_8_1009	DJI_0914	Gypsophila paniculata L.	0	n	1	0	Caryophyllaceae Juss.
Iz_8_1009	DJI_0914	Helichrysum arenarium (L.) Moench	0	n	1	0	Asteraceae Bercht. & J.Presl
Iz_8_1009	DJI_0914	Herniaria polygama J.Gay	0	n	1	0	Caryophyllaceae Juss.
Iz_8_1009	DJI_0914	Hieracium robustum Fr.	0	n	1	0	Asteraceae Bercht. & J.Presl
Iz_8_1009	DJI_0914	Hieracium umbellatum L.	0	n	1	NA	Asteraceae Bercht. & J.Presl
Iz_8_1009	DJI_0914	Hylotelephium maximum (L.) Holub	0	n	1	0	Crassulaceae J.St.-Hil.
Iz_8_1009	DJI_0914	Jacobaea borysthenica (DC.) B.Nord. & Greuter	0	n	1	0	Asteraceae Bercht. & J.Presl
Iz_8_1009	DJI_0914	Jasione montana L.	0	n	1	0	Campanulaceae Juss.
Iz_8_1009	DJI_0914	Jurinea cyanoides (L.) Rchb.s.l.	0	n	1	0	Asteraceae Bercht. & J.Presl
Iz_8_1009	DJI_0914	Koeleria glauca (Spreng.) DC.	0	n	1	0	Poaceae Barnhart
Iz_8_1009	DJI_0914	Lactuca serriola L.	0	a	0	0	Asteraceae Bercht. & J.Presl
Iz_8_1009	DJI_0914	Linaria genistifolia (L.) Mill.	0	n	1	0	Plantaginaceae Juss.
Iz_8_1009	DJI_0914	Lomelosia argentea (L.) Greuter & Burdet	0	n	1	0	Caprifoliaceae Juss.
Iz_8_1009	DJI_0914	Melampyrum pratense L.	0	n	1	0	Orobanchaceae Vent.
Iz_8_1009	DJI_0914	Oenothera biennis L.	0	a	0	0	Onagraceae Juss.
Iz_8_1009	DJI_0914	Pentanema britannica (L.) D.Gut.Larr., Santos-Vicente, Anderb., E.Rico & M.M.Mart.Ort.	0	n	1	0	Asteraceae Bercht. & J.Presl
Iz_8_1009	DJI_0914	Peucedanum oreoselinum (L.) Moench	0	n	1	0	Apiaceae Lindl.
Iz_8_1009	DJI_0914	Peucedanum ruthenicum M.Bieb.	0	n	1	0	Apiaceae Lindl.
Iz_8_1009	DJI_0914	Pilosella echioides (Lumn.) F.W.Schultz & Sch.Bip.	0	n	1	0	Asteraceae Bercht. & J.Presl
Iz_8_1009	DJI_0914	Pilosella officinarum F. Schultz et Sch. Bip.	0	n	1	0	Asteraceae Bercht. & J.Presl
Iz_8_1009	DJI_0914	Pilosella sp.	0		NA	0	Asteraceae Bercht. & J.Presl
Iz_8_1009	DJI_0914	Pinus sylvestris L.	0	n	1	1	Pinaceae Spreng. ex F.Rudolphi
Iz_8_1009	DJI_0914	Poa annua L.	0	n	1	0	Poaceae Barnhart
Iz_8_1009	DJI_0914	Poa nemoralis L.	0	n	1	0	Poaceae Barnhart
Iz_8_1009	DJI_0914	Poa sp.	0		NA	0	Poaceae Barnhart
Iz_8_1009	DJI_0914	Polygonatum odoratum (Mill.) Druce	0	n	1	0	Asparagaceae Juss.
Iz_8_1009	DJI_0914	Polygonum arenarium Waldst. & Kit.	0	n	1	0	Polygonaceae Juss.
Iz_8_1009	DJI_0914	Populus nigra L.	0	n	1	1	Salicaceae Mirb.
Iz_8_1009	DJI_0914	Populus tremula L.	0	n	1	1	Salicaceae Mirb.
Iz_8_1009	DJI_0914	Populus Ã canescens (Aiton) Sm.	0	n	1	1	Salicaceae Mirb.

Iz_8_1009	DJI_0914	Potentilla argentea L.	0	n	1	0	Rosaceae Juss.
Iz_8_1009	DJI_0914	Psammophiliella muralis (L.) Ikonn.	0	n	1	0	Caryophyllaceae Juss.
Iz_8_1009	DJI_0914	Psephellus sumensis (Kalen.) Greuter	0	n	1	0	Asteraceae Bercht. & J.Presl
Iz_8_1009	DJI_0914	Pulsatilla pratensis (L.) Mill. s.l.	0	n	1	0	Ranunculaceae Juss.
Iz_8_1009	DJI_0914	Pyrus communis L.	0	a	0	1	Rosaceae Juss.
Iz_8_1009	DJI_0914	Quercus robur L.	0	n	1	1	Fagaceae Dumort.
Iz_8_1009	DJI_0914	Robinia pseudoacacia L.	0	a	0	1	Fabaceae Lindl.
Iz_8_1009	DJI_0914	Rosa sp.	0		NA	1	Rosaceae Juss.
Iz_8_1009	DJI_0914	Rubus idaeus L.	0	n	1	0	Rosaceae Juss.
Iz_8_1009	DJI_0914	Rumex acetosella L.	0	n	1	0	Polygonaceae Juss.
Iz_8_1009	DJI_0914	Rumex thyrsiflorus Fingerh.	0	n	1	0	Polygonaceae Juss.
Iz_8_1009	DJI_0914	Salix alba L.	0	n	1	1	Salicaceae Mirb.
Iz_8_1009	DJI_0914	Sambucus nigra L.	0	n	1	1	Viburnaceae Raf.
Iz_8_1009	DJI_0914	Secale sylvestre Host	0	n	1	0	Poaceae Barnhart
Iz_8_1009	DJI_0914	Sempervivum ruthenicum Schnittsp. & C.B. Lehm.	0	n	1	0	Crassulaceae J.St.-Hil.
Iz_8_1009	DJI_0914	Setaria viridis (L.) P. Beauv.	0	n	1	0	Poaceae Barnhart
Iz_8_1009	DJI_0914	Silene borysthenica (Gruner) Walters	0	n	1	0	Caryophyllaceae Juss.
Iz_8_1009	DJI_0914	Silene latifolia subsp. alba (Mill.) Greuter & Burdet	0	n	1	0	Caryophyllaceae Juss.
Iz_8_1009	DJI_0914	Solidago virgaurea L.	0	n	1	0	Asteraceae Bercht. & J.Presl
Iz_8_1009	DJI_0914	Sorbus aucuparia L.	0	n	1	1	Rosaceae Juss.
Iz_8_1009	DJI_0914	Stachys recta L.	0	n	1	0	Lamiacea Lindl.
Iz_8_1009	DJI_0914	Tanacetum vulgare L.	0	n	1	0	Asteraceae Bercht. & J.Presl
Iz_8_1009	DJI_0914	Thymus pallasianus Heinr.Braun	0	n	1	0	Lamiacea Lindl.
Iz_8_1009	DJI_0914	Tilia cordata Mill.	0	n	1	1	Malvaceae Juss.
Iz_8_1009	DJI_0914	Trifolium alpestre L.	0	n	1	0	Fabaceae Lindl.
Iz_8_1009	DJI_0914	Trifolium arvense L.	0	n	1	0	Fabaceae Lindl.
Iz_8_1009	DJI_0914	Ulmus glabra Huds.	0	n	1	1	Ulmaceae Mirb.
Iz_8_1009	DJI_0914	Verbascum densiflorum Bertol.	0	n	1	0	Scrophulariaceae Juss.
Iz_8_1009	DJI_0914	Veronica austriaca L.	0	n	1	0	Plantaginaceae Juss.
Iz_8_1009	DJI_0914	Veronica incana L.	0	n	1	0	Plantaginaceae Juss.
Iz_8_1009	DJI_0914	Veronica verna L.	0	n	1	0	Plantaginaceae Juss.
Iz_8_1009	DJI_0914	Vincetoxicum hirundinaria Medik	0	n	1	0	Apocynaceae Juss.
Iz_8_1009	DJI_0916	Acer tataricum L.	0	n	1	1	Sapindaceae Juss.
Iz_8_1009	DJI_0916	Achillea millefolium L. s.l.	0	n	1	0	Asteraceae Bercht. & J.Presl
Iz_8_1009	DJI_0916	Allium oleraceum L.	0	n	1	0	Amaryllidaceae J.St.-Hil.
Iz_8_1009	DJI_0916	Ambrosia artemisiifolia L.	0	a	0	0	Asteraceae Bercht. & J.Presl
Iz_8_1009	DJI_0916	Amorpha fruticosa L.	0	a	0	0	Fabaceae Lindl.
Iz_8_1009	DJI_0916	Anthericum ramosum L.	0	n	1	0	Asparagaceae Juss.
Iz_8_1009	DJI_0916	Artemisia marschalliana Spreng.	0	n	1	0	Asteraceae Bercht. & J.Presl
Iz_8_1009	DJI_0916	Asparagus officinalis L.	0	n	1	0	Asparagaceae Juss.
Iz_8_1009	DJI_0916	Astragalus cicer L.	0	n	1	0	Fabaceae Lindl.
Iz_8_1009	DJI_0916	Bassia laniflora (S.G.Gmel.) A.J.Scott	0	n	1	0	Amaranthaceae Juss.
Iz_8_1009	DJI_0916	Berberis vulgaris L.	0	n	1	0	Berberidaceae Juss.
Iz_8_1009	DJI_0916	Betula pendula Roth.	0	n	1	1	Betulaceae Gray
Iz_8_1009	DJI_0916	Betula pendula Roth.	0	n	1	0	Poaceae Barnhart
Iz_8_1009	DJI_0916	Bromus inermis Leyss.	0		NA	NA	
Iz_8_1009	DJI_0916	Bromus squarrosus L.	0	a	0	0	Poaceae Barnhart
Iz_8_1009	DJI_0916	Bromus tectorum L.	0	a	0	0	Poaceae Barnhart
Iz_8_1009	DJI_0916	Calamagrostis epigejos (L.) Roth	3	n	1	0	Poaceae Barnhart
Iz_8_1009	DJI_0916	Carduus acanthoides L.	0	n	1	0	Asteraceae Bercht. & J.Presl
Iz_8_1009	DJI_0916	Carex sp.	0	n	1	0	Cyperaceae Juss.
Iz_8_1009	DJI_0916	Centaurea majorovii Dumbadze	0	n	1	0	Asteraceae Bercht. & J.Presl
Iz_8_1009	DJI_0916	Chamaecytisus ruthenicus (Fisch.ex Wol.) Klaskova	0	n	1	0	Fabaceae Lindl.
Iz_8_1009	DJI_0916	Chelidonium majus L.	0	n	1	0	Papaveraceae Juss.
Iz_8_1009	DJI_0916	Chenopodium betaceum Andrz.	0	n	1	0	Amaranthaceae Juss.
Iz_8_1009	DJI_0916	Chenopodium cf. album	0	n	1	0	Amaranthaceae Juss.
Iz_8_1009	DJI_0916	Chenopodium sp.	0		NA	0	Amaranthaceae Juss.
Iz_8_1009	DJI_0916	Chondrilla juncea L.	0	n	1	0	Asteraceae Bercht. & J.Presl
Iz_8_1009	DJI_0916	Cirsium vulgare (Savi) Ten.	0	n	1	0	Asteraceae Bercht. & J.Presl
Iz_8_1009	DJI_0916	Clinopodium acinos (L.) Kuntze	0	n	1	0	Lamiacea Lindl.
Iz_8_1009	DJI_0916	Convallaria majalis L.	0	n	1	0	Asparagaceae Juss.
Iz_8_1009	DJI_0916	Crataegus rhipidophylla Gand.	0	n	1	1	Rosaceae Juss.
Iz_8_1009	DJI_0916	Crepis foetida L.	0	n	1	0	Asteraceae Bercht. & J.Presl
Iz_8_1009	DJI_0916	Dactylis glomerata L.	0	n	1	0	Poaceae Barnhart
Iz_8_1009	DJI_0916	Dianthus campestris M.Bieb.	0	n	1	0	Caryophyllaceae Juss.
Iz_8_1009	DJI_0916	Digitaria sanguinalis (L.) Scop.	0	a	0	0	Poaceae Barnhart
Iz_8_1009	DJI_0916	Elymus repens (L.) Gould	0	n	1	0	Poaceae Barnhart
Iz_8_1009	DJI_0916	Epilobium tetragonum L.	0	n	1	0	Onagraceae Juss.
Iz_8_1009	DJI_0916	Eragrostis minor Host	0	a	0	0	Poaceae Barnhart
Iz_8_1009	DJI_0916	Erigeron canadensis L.	5	a	0	0	Asteraceae Bercht. & J.Presl
Iz_8_1009	DJI_0916	Euphorbia seguieriana Neck.	0	n	1	0	Euphorbiaceae Juss.
Iz_8_1009	DJI_0916	Fallopia dumetorum (L.) Holub	0	n	1	0	Polygonaceae Juss.
Iz_8_1009	DJI_0916	Festuca beckeri (Hack.) Trautv.	0	n	1	0	Poaceae Barnhart
Iz_8_1009	DJI_0916	Fragaria vesca L.	0	n	1	0	Rosaceae Juss.
Iz_8_1009	DJI_0916	Frangula alnus Mill.	0	n	1	1	Rhamnaceae Juss.
Iz_8_1009	DJI_0916	Fraxinus pennsylvanica Marshall	0	a	0	1	Oleaceae Hoffmanns. & Link
Iz_8_1009	DJI_0916	Galeopsis bifida Boenn.	0	n	1	0	Lamiacea Lindl.
Iz_8_1009	DJI_0916	Galium verum L.	0	n	1	0	Rubiaceae Juss.
Iz_8_1009	DJI_0916	Genista tinctoria L.	0	n	1	0	Fabaceae Lindl.
Iz_8_1009	DJI_0916	Geranium robertianum L.	0	n	1	0	Geraniaceae Juss.
Iz_8_1009	DJI_0916	Geranium sanquineum L.	0	n	1	0	Geraniaceae Juss.
Iz_8_1009	DJI_0916	Gypsophila paniculata L.	0	n	1	0	Caryophyllaceae Juss.
Iz_8_1009	DJI_0916	Helichrysum arenarium (L.) Moench	0	n	1	0	Asteraceae Bercht. & J.Presl
Iz_8_1009	DJI_0916	Herniaria polygama J.Gay	0	n	1	0	Caryophyllaceae Juss.
Iz_8_1009	DJI_0916	Hieracium robustum Fr.	0	n	1	0	Asteraceae Bercht. & J.Presl
Iz_8_1009	DJI_0916	Hieracium umbellatum L.	0	n	1	NA	Asteraceae Bercht. & J.Presl
Iz_8_1009	DJI_0916	Hylotelephium maximum (L.) Holub	0	n	1	0	Crassulaceae J.St.-Hil.
Iz_8_1009	DJI_0916	Jacobaea borysthenica (DC.) B.Nord. & Greuter	0	n	1	0	Asteraceae Bercht. & J.Presl
Iz_8_1009	DJI_0916	Jasione montana L.	0	n	1	0	Campanulaceae Juss.
Iz_8_1009	DJI_0916	Jurinea cyanoides (L.) Rchb.s.l.	0	n	1	0	Asteraceae Bercht. & J.Presl
Iz_8_1009	DJI_0916	Koeleria glauca (Spreng.) DC.	0	n	1	0	Poaceae Barnhart
Iz_8_1009	DJI_0916	Lactuca serriola L.	0	a	0	0	Asteraceae Bercht. & J.Presl
Iz_8_1009	DJI_0916	Linaria genistifolia (L.) Mill.	0	n	1	0	Plantaginaceae Juss.
Iz_8_1009	DJI_0916	Lomelosia argentea (L.) Greuter & Burdet	0	n	1	0	Caprifoliaceae Juss.
Iz_8_1009	DJI_0916	Melampyrum pratense L.	0	n	1	0	Orobanchaceae Vent.
Iz_8_1009	DJI_0916	Oenothera biennis L.	0	a	0	0	Onagraceae Juss.
Iz_8_1009	DJI_0916	Pentanema britannica (L.) D.Gut.Larr., Santos-Vicente, Anderb., E.Rico & M.M.Mart.Ort.	0	n	1	0	Asteraceae Bercht. & J.Presl
Iz_8_1009	DJI_0916	Peucedanum oreoselinum (L.) Moench	0	n	1	0	Apiaceae Lindl.

Iz_8_1009	DJI_0916	Peucedanum ruthenicum M.Bieb.	0	n	1	0	Apiaceae Lindl.
Iz_8_1009	DJI_0916	Pilosella echioides (Lumn.) F.W.Schultz & Sch.Bip.	0	n	1	0	Asteraceae Bercht. & J.Presl
Iz_8_1009	DJI_0916	Pilosella officinarum F. Schultz et Sch. Bip.	0	n	1	0	Asteraceae Bercht. & J.Presl
Iz_8_1009	DJI_0916	Pilosella sp.	0		NA	0	Asteraceae Bercht. & J.Presl
Iz_8_1009	DJI_0916	Pinus sylvestris L.	0	n	1	1	Pinaceae Spreng. ex F.Rudolphi
Iz_8_1009	DJI_0916	Poa annua L.	0	n	1	0	Poaceae Barnhart
Iz_8_1009	DJI_0916	Poa nemoralis L.	0	n	1	0	Poaceae Barnhart
Iz_8_1009	DJI_0916	Poa sp.	0		NA	0	Poaceae Barnhart
Iz_8_1009	DJI_0916	Polygonatum odoratum (Mill.) Druce	0	n	1	0	Asparagaceae Juss.
Iz_8_1009	DJI_0916	Polygonum arenarium Waldst. & Kit.	0	n	1	0	Polygonaceae Juss.
Iz_8_1009	DJI_0916	Populus nigra L.	0	n	1	1	Salicaceae Mirb.
Iz_8_1009	DJI_0916	Populus tremula L.	0	n	1	1	Salicaceae Mirb.
Iz_8_1009	DJI_0916	Populus Ã canescens (Aiton) Sm.	0	n	1	1	Salicaceae Mirb.
Iz_8_1009	DJI_0916	Potentilla argentea L.	0	n	1	0	Rosaceae Juss.
Iz_8_1009	DJI_0916	Psammophiliella muralis (L.) Ikonn.	0	n	1	0	Caryophyllaceae Juss.
Iz_8_1009	DJI_0916	Psephellus sumensis (Kalen.) Greuter	0	n	1	0	Asteraceae Bercht. & J.Presl
Iz_8_1009	DJI_0916	Pulsatilla pratensis (L.) Mill. s.l.	0	n	1	0	Ranunculaceae Juss.
Iz_8_1009	DJI_0916	Pyrus communis L.	0	a	0	1	Rosaceae Juss.
Iz_8_1009	DJI_0916	Quercus robur L.	0	n	1	1	Fagaceae Dumort.
Iz_8_1009	DJI_0916	Robinia pseudoacacia L.	0	a	0	1	Fabaceae Lindl.
Iz_8_1009	DJI_0916	Rosa sp.	0		NA	1	Rosaceae Juss.
Iz_8_1009	DJI_0916	Rubus idaeus L.	0	n	1	0	Rosaceae Juss.
Iz_8_1009	DJI_0916	Rumex acetosella L.	0	n	1	0	Polygonaceae Juss.
Iz_8_1009	DJI_0916	Rumex thyrsiflorus Fingerh.	0	n	1	0	Polygonaceae Juss.
Iz_8_1009	DJI_0916	Salix alba L.	0	n	1	1	Salicaceae Mirb.
Iz_8_1009	DJI_0916	Sambucus nigra L.	0	n	1	1	Viburnaceae Raf.
Iz_8_1009	DJI_0916	Secale sylvestre Host	0	n	1	0	Poaceae Barnhart
Iz_8_1009	DJI_0916	Sempervivum ruthenicum Schnittsp. & C.B. Lehm.	0	n	1	0	Crassulaceae J.St.-Hil.
Iz_8_1009	DJI_0916	Setaria viridis (L.) P. Beauv.	0	n	1	0	Poaceae Barnhart
Iz_8_1009	DJI_0916	Silene borysthenica (Gruner) Walters	0	n	1	0	Caryophyllaceae Juss.
Iz_8_1009	DJI_0916	Silene latifolia subsp. alba (Mill.) Greuter & Burdet	0	n	1	0	Caryophyllaceae Juss.
Iz_8_1009	DJI_0916	Solidago virgaurea L.	0	n	1	0	Asteraceae Bercht. & J.Presl
Iz_8_1009	DJI_0916	Sorbus aucuparia L.	0	n	1	1	Rosaceae Juss.
Iz_8_1009	DJI_0916	Stachys recta L.	0	n	1	0	Lamiacea Lindl.
Iz_8_1009	DJI_0916	Tanacetum vulgare L.	0	n	1	0	Asteraceae Bercht. & J.Presl
Iz_8_1009	DJI_0916	Thymus pallasianus Heinr.Braun	0	n	1	0	Lamiacea Lindl.
Iz_8_1009	DJI_0916	Tilia cordata Mill.	0	n	1	1	Malvaceae Juss.
Iz_8_1009	DJI_0916	Trifolium alpestre L.	0	n	1	0	Fabaceae Lindl.
Iz_8_1009	DJI_0916	Trifolium arvense L.	0	n	1	0	Fabaceae Lindl.
Iz_8_1009	DJI_0916	Ulmus glabra Huds.	0	n	1	1	Ulmaceae Mirb.
Iz_8_1009	DJI_0916	Verbascum densiflorum Bertol.	0	n	1	0	Scrophulariaceae Juss.
Iz_8_1009	DJI_0916	Veronica austriaca L.	0	n	1	0	Plantaginaceae Juss.
Iz_8_1009	DJI_0916	Veronica incana L.	0	n	1	0	Plantaginaceae Juss.
Iz_8_1009	DJI_0916	Veronica verna L.	0	n	1	0	Plantaginaceae Juss.
Iz_8_1009	DJI_0916	Vincetoxicum hirundinaria Medik	0	n	1	0	Apocynaceae Juss.
Iz_8_1009	DJI_0918	Acer tataricum L.	0	n	1	1	Sapindaceae Juss.
Iz_8_1009	DJI_0918	Achillea millefolium L. s.l.	0	n	1	0	Asteraceae Bercht. & J.Presl
Iz_8_1009	DJI_0918	Allium oleraceum L.	0	n	1	0	Amaryllidaceae J.St.-Hil.
Iz_8_1009	DJI_0918	Ambrosia artemisiifolia L.	0	a	0	0	Asteraceae Bercht. & J.Presl
Iz_8_1009	DJI_0918	Amorpha fruticosa L.	0	a	0	0	Fabaceae Lindl.
Iz_8_1009	DJI_0918	Anthericum ramosum L.	0	n	1	0	Asparagaceae Juss.
Iz_8_1009	DJI_0918	Artemisia marschalliana Spreng.	0	n	1	0	Asteraceae Bercht. & J.Presl
Iz_8_1009	DJI_0918	Asparagus officinalis L.	0	n	1	0	Asparagaceae Juss.
Iz_8_1009	DJI_0918	Astragalus cicer L.	0	n	1	0	Fabaceae Lindl.
Iz_8_1009	DJI_0918	Bassia laniflora (S.G.Gmel.) A.J.Scott	0	n	1	0	Amaranthaceae Juss.
Iz_8_1009	DJI_0918	Berberis vulgaris L.	0	n	1	0	Berberidaceae Juss.
Iz_8_1009	DJI_0918	Betula pendula Roth.	0	n	1	1	Betulaceae Gray
Iz_8_1009	DJI_0918	Betula pendula Roth.	0	n	1	0	Poaceae Barnhart
Iz_8_1009	DJI_0918	Bromus inermis Leyss.	0		NA	NA	
Iz_8_1009	DJI_0918	Bromus squarrosus L.	0	a	0	0	Poaceae Barnhart
Iz_8_1009	DJI_0918	Bromus tectorum L.	0	a	0	0	Poaceae Barnhart
Iz_8_1009	DJI_0918	Calamagrostis epigejos (L.) Roth	15	n	1	0	Poaceae Barnhart
Iz_8_1009	DJI_0918	Carduus acanthoides L.	0	n	1	0	Asteraceae Bercht. & J.Presl
Iz_8_1009	DJI_0918	Carex sp.	0	n	1	0	Cyperaceae Juss.
Iz_8_1009	DJI_0918	Centaurea majorovii Dumbadze	0	n	1	0	Asteraceae Bercht. & J.Presl
Iz_8_1009	DJI_0918	Chamaecytisus ruthenicus (Fisch.ex Wol.) Klaskova	0	n	1	0	Fabaceae Lindl.
Iz_8_1009	DJI_0918	Chelidonium majus L.	0	n	1	0	Papaveraceae Juss.
Iz_8_1009	DJI_0918	Chenopodium betaceum Andrz.	0	n	1	0	Amaranthaceae Juss.
Iz_8_1009	DJI_0918	Chenopodium cf. album	0	n	1	0	Amaranthaceae Juss.
Iz_8_1009	DJI_0918	Chenopodium sp.	0		NA	0	Amaranthaceae Juss.
Iz_8_1009	DJI_0918	Chondrilla juncea L.	0	n	1	0	Asteraceae Bercht. & J.Presl
Iz_8_1009	DJI_0918	Cirsium vulgare (Savi) Ten.	0	n	1	0	Asteraceae Bercht. & J.Presl
Iz_8_1009	DJI_0918	Clinopodium acinos (L.) Kuntze	0	n	1	0	Lamiacea Lindl.
Iz_8_1009	DJI_0918	Convallaria majalis L.	0	n	1	0	Asparagaceae Juss.
Iz_8_1009	DJI_0918	Crataegus rhipidophylla Gand.	0	n	1	1	Rosaceae Juss.
Iz_8_1009	DJI_0918	Crepis foetida L.	0	n	1	0	Asteraceae Bercht. & J.Presl
Iz_8_1009	DJI_0918	Dactylis glomerata L.	0	n	1	0	Poaceae Barnhart
Iz_8_1009	DJI_0918	Dianthus campestris M.Bieb.	0	n	1	0	Caryophyllaceae Juss.
Iz_8_1009	DJI_0918	Digitaria sanguinalis (L.) Scop.	0	a	0	0	Poaceae Barnhart
Iz_8_1009	DJI_0918	Elymus repens (L.) Gould	0	n	1	0	Poaceae Barnhart
Iz_8_1009	DJI_0918	Epilobium tetragonum L.	0	n	1	0	Onagraceae Juss.
Iz_8_1009	DJI_0918	Eragrostis minor Host	0	a	0	0	Poaceae Barnhart
Iz_8_1009	DJI_0918	Erigeron canadensis L.	1	a	0	0	Asteraceae Bercht. & J.Presl
Iz_8_1009	DJI_0918	Euphorbia seguieriana Neck.	0	n	1	0	Euphorbiaceae Juss.
Iz_8_1009	DJI_0918	Fallopia dumetorum (L.) Holub	0	n	1	0	Polygonaceae Juss.
Iz_8_1009	DJI_0918	Festuca beckeri (Hack.) Trautv.	0	n	1	0	Poaceae Barnhart
Iz_8_1009	DJI_0918	Fragaria vesca L.	0	n	1	0	Rosaceae Juss.
Iz_8_1009	DJI_0918	Frangula alnus Mill.	0	n	1	1	Rhamnaceae Juss.
Iz_8_1009	DJI_0918	Fraxinus pennsylvanica Marshall	0	a	0	1	Oleaceae Hoffmanns. & Link
Iz_8_1009	DJI_0918	Galeopsis bifida Boenn.	0	n	1	0	Lamiacea Lindl.
Iz_8_1009	DJI_0918	Galium verum L.	0	n	1	0	Rubiaceae Juss.
Iz_8_1009	DJI_0918	Genista tinctoria L.	0	n	1	0	Fabaceae Lindl.
Iz_8_1009	DJI_0918	Geranium robertianum L.	0	n	1	0	Geraniaceae Juss.
Iz_8_1009	DJI_0918	Geranium sanquineum L.	0	n	1	0	Geraniaceae Juss.
Iz_8_1009	DJI_0918	Gypsophila paniculata L.	0	n	1	0	Caryophyllaceae Juss.
Iz_8_1009	DJI_0918	Helichrysum arenarium (L.) Moench	0	n	1	0	Asteraceae Bercht. & J.Presl
Iz_8_1009	DJI_0918	Herniaria polygama J.Gay	0	n	1	0	Caryophyllaceae Juss.
Iz_8_1009	DJI_0918	Hieracium robustum Fr.	0	n	1	0	Asteraceae Bercht. & J.Presl

Iz_8_1009	DJI_0918	Hieracium umbellatum L.	0	n	1	NA	Asteraceae Bercht. & J.Presl
Iz_8_1009	DJI_0918	Hylotelephium maximum (L.) Holub	0	n	1	0	Crassulaceae J.St.-Hil.
Iz_8_1009	DJI_0918	Jacobaea borysthenica (DC.) B.Nord. & Greuter	0	n	1	0	Asteraceae Bercht. & J.Presl
Iz_8_1009	DJI_0918	Jasione montana L.	0	n	1	0	Campanulaceae Juss.
Iz_8_1009	DJI_0918	Jurinea cyanoides (L.) Rchb.s.l.	0	n	1	0	Asteraceae Bercht. & J.Presl
Iz_8_1009	DJI_0918	Koeleria glauca (Spreng.) DC.	0	n	1	0	Poaceae Barnhart
Iz_8_1009	DJI_0918	Lactuca serriola L.	0	a	0	0	Asteraceae Bercht. & J.Presl
Iz_8_1009	DJI_0918	Linaria genistifolia (L.) Mill.	0	n	1	0	Plantaginaceae Juss.
Iz_8_1009	DJI_0918	Lomelosia argentea (L.) Greuter & Burdet	0	n	1	0	Caprifoliaceae Juss.
Iz_8_1009	DJI_0918	Melampyrum pratense L.	0	n	1	0	Orobanchaceae Vent.
Iz_8_1009	DJI_0918	Oenothera biennis L.	0	a	0	0	Onagraceae Juss.
Iz_8_1009	DJI_0918	Pentanema britannica (L.) D.Gut.Larr., Santos-Vicente, Anderb., E.Rico & M.M.Mart.Ort.	0	n	1	0	Asteraceae Bercht. & J.Presl
Iz_8_1009	DJI_0918	Peucedanum oreoselinum (L.) Moench	0	n	1	0	Apiaceae Lindl.
Iz_8_1009	DJI_0918	Peucedanum ruthenicum M.Bieb.	0	n	1	0	Apiaceae Lindl.
Iz_8_1009	DJI_0918	Pilosella echioides (Lumn.) F.W.Schultz & Sch.Bip.	0	n	1	0	Asteraceae Bercht. & J.Presl
Iz_8_1009	DJI_0918	Pilosella officinarum F. Schultz et Sch. Bip.	0	n	1	0	Asteraceae Bercht. & J.Presl
Iz_8_1009	DJI_0918	Pilosella sp.	0		NA	0	Asteraceae Bercht. & J.Presl
Iz_8_1009	DJI_0918	Pinus sylvestris L.	0	n	1	1	Pinaceae Spreng. ex F.Rudolphi
Iz_8_1009	DJI_0918	Poa annua L.	0	n	1	0	Poaceae Barnhart
Iz_8_1009	DJI_0918	Poa nemoralis L.	0	n	1	0	Poaceae Barnhart
Iz_8_1009	DJI_0918	Poa sp.	0		NA	0	Poaceae Barnhart
Iz_8_1009	DJI_0918	Polygonatum odoratum (Mill.) Druce	0	n	1	0	Asparagaceae Juss.
Iz_8_1009	DJI_0918	Polygonum arenarium Waldst. & Kit.	0	n	1	0	Polygonaceae Juss.
Iz_8_1009	DJI_0918	Populus nigra L.	0	n	1	1	Salicaceae Mirb.
Iz_8_1009	DJI_0918	Populus tremula L.	0	n	1	1	Salicaceae Mirb.
Iz_8_1009	DJI_0918	Populus Ã canescens (Aiton) Sm.	0	n	1	1	Salicaceae Mirb.
Iz_8_1009	DJI_0918	Potentilla argentea L.	0	n	1	0	Rosaceae Juss.
Iz_8_1009	DJI_0918	Psammophiliella muralis (L.) Ikonn.	0	n	1	0	Caryophyllaceae Juss.
Iz_8_1009	DJI_0918	Psephellus sumensis (Kalen.) Greuter	0	n	1	0	Asteraceae Bercht. & J.Presl
Iz_8_1009	DJI_0918	Pulsatilla pratensis (L.) Mill. s.l.	0	n	1	0	Ranunculaceae Juss.
Iz_8_1009	DJI_0918	Pyrus communis L.	0	a	0	1	Rosaceae Juss.
Iz_8_1009	DJI_0918	Quercus robur L.	0	n	1	1	Fagaceae Dumort.
Iz_8_1009	DJI_0918	Robinia pseudoacacia L.	0	a	0	1	Fabaceae Lindl.
Iz_8_1009	DJI_0918	Rosa sp.	0		NA	1	Rosaceae Juss.
Iz_8_1009	DJI_0918	Rubus idaeus L.	0	n	1	0	Rosaceae Juss.
Iz_8_1009	DJI_0918	Rumex acetosella L.	0	n	1	0	Polygonaceae Juss.
Iz_8_1009	DJI_0918	Rumex thyrsiflorus Fingerh.	0	n	1	0	Polygonaceae Juss.
Iz_8_1009	DJI_0918	Salix alba L.	0	n	1	1	Salicaceae Mirb.
Iz_8_1009	DJI_0918	Sambucus nigra L.	0	n	1	1	Viburnaceae Raf.
Iz_8_1009	DJI_0918	Secale sylvestre Host	0	n	1	0	Poaceae Barnhart
Iz_8_1009	DJI_0918	Sempervivum ruthenicum Schnittsp. & C.B. Lehm.	0	n	1	0	Crassulaceae J.St.-Hil.
Iz_8_1009	DJI_0918	Setaria viridis (L.) P. Beauv.	0	n	1	0	Poaceae Barnhart
Iz_8_1009	DJI_0918	Silene borysthenica (Gruner) Walters	0	n	1	0	Caryophyllaceae Juss.
Iz_8_1009	DJI_0918	Silene latifolia subsp. alba (Mill.) Greuter & Burdet	0	n	1	0	Caryophyllaceae Juss.
Iz_8_1009	DJI_0918	Solidago virgaurea L.	0	n	1	0	Asteraceae Bercht. & J.Presl
Iz_8_1009	DJI_0918	Sorbus aucuparia L.	0	n	1	1	Rosaceae Juss.
Iz_8_1009	DJI_0918	Stachys recta L.	0	n	1	0	Lamiacea Lindl.
Iz_8_1009	DJI_0918	Tanacetum vulgare L.	0	n	1	0	Asteraceae Bercht. & J.Presl
Iz_8_1009	DJI_0918	Thymus pallasianus Heinr.Braun	0	n	1	0	Lamiacea Lindl.
Iz_8_1009	DJI_0918	Tilia cordata Mill.	0	n	1	1	Malvaceae Juss.
Iz_8_1009	DJI_0918	Trifolium alpestre L.	0	n	1	0	Fabaceae Lindl.
Iz_8_1009	DJI_0918	Trifolium arvense L.	0	n	1	0	Fabaceae Lindl.
Iz_8_1009	DJI_0918	Ulmus glabra Huds.	0	n	1	1	Ulmaceae Mirb.
Iz_8_1009	DJI_0918	Verbascum densiflorum Bertol.	0	n	1	0	Scrophulariaceae Juss.
Iz_8_1009	DJI_0918	Veronica austriaca L.	0	n	1	0	Plantaginaceae Juss.
Iz_8_1009	DJI_0918	Veronica incana L.	0	n	1	0	Plantaginaceae Juss.
Iz_8_1009	DJI_0918	Veronica verna L.	0	n	1	0	Plantaginaceae Juss.
Iz_8_1009	DJI_0918	Vincetoxicum hirundinaria Medik	0	n	1	0	Apocynaceae Juss.
Iz_8_1009	DJI_0919	Acer tataricum L.	0	n	1	1	Sapindaceae Juss.
Iz_8_1009	DJI_0919	Achillea millefolium L. s.l.	0	n	1	0	Asteraceae Bercht. & J.Presl
Iz_8_1009	DJI_0919	Allium oleraceum L.	0	n	1	0	Amaryllidaceae J.St.-Hil.
Iz_8_1009	DJI_0919	Ambrosia artemisiifolia L.	0	a	0	0	Asteraceae Bercht. & J.Presl
Iz_8_1009	DJI_0919	Amorpha fruticosa L.	0	a	0	0	Fabaceae Lindl.
Iz_8_1009	DJI_0919	Anthericum ramosum L.	0	n	1	0	Asparagaceae Juss.
Iz_8_1009	DJI_0919	Artemisia marschalliana Spreng.	0	n	1	0	Asteraceae Bercht. & J.Presl
Iz_8_1009	DJI_0919	Asparagus officinalis L.	0	n	1	0	Asparagaceae Juss.
Iz_8_1009	DJI_0919	Astragalus cicer L.	0	n	1	0	Fabaceae Lindl.
Iz_8_1009	DJI_0919	Bassia laniflora (S.G.Gmel.) A.J.Scott	0	n	1	0	Amaranthaceae Juss.
Iz_8_1009	DJI_0919	Berberis vulgaris L.	0	n	1	0	Berberidaceae Juss.
Iz_8_1009	DJI_0919	Betula pendula Roth.	0	n	1	1	Betulaceae Gray
Iz_8_1009	DJI_0919	Betula pendula Roth.	0	n	1	0	Poaceae Barnhart
Iz_8_1009	DJI_0919	Bromus inermis Leyss.	0		NA	NA	
Iz_8_1009	DJI_0919	Bromus squarrosus L.	0	a	0	0	Poaceae Barnhart
Iz_8_1009	DJI_0919	Bromus tectorum L.	0	a	0	0	Poaceae Barnhart
Iz_8_1009	DJI_0919	Calamagrostis epigejos (L.) Roth	30	n	1	0	Poaceae Barnhart
Iz_8_1009	DJI_0919	Carduus acanthoides L.	0	n	1	0	Asteraceae Bercht. & J.Presl
Iz_8_1009	DJI_0919	Carex sp.	0	n	1	0	Cyperaceae Juss.
Iz_8_1009	DJI_0919	Centaurea majorovii Dumbadze	0	n	1	0	Asteraceae Bercht. & J.Presl
Iz_8_1009	DJI_0919	Chamaecytisus ruthenicus (Fisch.ex Wol.) Klaskova	0	n	1	0	Fabaceae Lindl.
Iz_8_1009	DJI_0919	Chelidonium majus L.	0	n	1	0	Papaveraceae Juss.
Iz_8_1009	DJI_0919	Chenopodium betaceum Andrz.	0	n	1	0	Amaranthaceae Juss.
Iz_8_1009	DJI_0919	Chenopodium cf. album	0	n	1	0	Amaranthaceae Juss.
Iz_8_1009	DJI_0919	Chenopodium sp.	0		NA	0	Amaranthaceae Juss.
Iz_8_1009	DJI_0919	Chondrilla juncea L.	0	n	1	0	Asteraceae Bercht. & J.Presl
Iz_8_1009	DJI_0919	Cirsium vulgare (Savi) Ten.	0	n	1	0	Asteraceae Bercht. & J.Presl
Iz_8_1009	DJI_0919	Clinopodium acinos (L.) Kuntze	0	n	1	0	Lamiacea Lindl.
Iz_8_1009	DJI_0919	Convallaria majalis L.	0	n	1	0	Asparagaceae Juss.
Iz_8_1009	DJI_0919	Crataegus rhipidophylla Gand.	0	n	1	1	Rosaceae Juss.
Iz_8_1009	DJI_0919	Crepis foetida L.	0	n	1	0	Asteraceae Bercht. & J.Presl
Iz_8_1009	DJI_0919	Dactylis glomerata L.	0	n	1	0	Poaceae Barnhart
Iz_8_1009	DJI_0919	Dianthus campestris M.Bieb.	0	n	1	0	Caryophyllaceae Juss.
Iz_8_1009	DJI_0919	Digitaria sanguinalis (L.) Scop.	0	a	0	0	Poaceae Barnhart
Iz_8_1009	DJI_0919	Elymus repens (L.) Gould	0	n	1	0	Poaceae Barnhart
Iz_8_1009	DJI_0919	Epilobium tetragonum L.	0	n	1	0	Onagraceae Juss.
Iz_8_1009	DJI_0919	Eragrostis minor Host	0	a	0	0	Poaceae Barnhart
Iz_8_1009	DJI_0919	Erigeron canadensis L.	2	a	0	0	Asteraceae Bercht. & J.Presl
Iz_8_1009	DJI_0919	Euphorbia seguieriana Neck.	0	n	1	0	Euphorbiaceae Juss.
Iz_8_1009	DJI_0919	Fallopia dumetorum (L.) Holub	0	n	1	0	Polygonaceae Juss.

Iz_8_1009	DJI_0919	Festuca beckeri (Hack.) Trautv.	0	n	1	0	Poaceae Barnhart
Iz_8_1009	DJI_0919	Fragaria vesca L.	0	n	1	0	Rosaceae Juss.
Iz_8_1009	DJI_0919	Frangula alnus Mill.	0	n	1	1	Rhamnaceae Juss.
Iz_8_1009	DJI_0919	Fraxinus pennsylvanica Marshall	0	a	0	1	Oleaceae Hoffmanns. & Link
Iz_8_1009	DJI_0919	Galeopsis bifida Boenn.	0	n	1	0	Lamiacea Lindl.
Iz_8_1009	DJI_0919	Galium verum L.	0	n	1	0	Rubiaceae Juss.
Iz_8_1009	DJI_0919	Genista tinctoria L.	0	n	1	0	Fabaceae Lindl.
Iz_8_1009	DJI_0919	Geranium robertianum L.	0	n	1	0	Geraniaceae Juss.
Iz_8_1009	DJI_0919	Geranium sanquineum L.	0	n	1	0	Geraniaceae Juss.
Iz_8_1009	DJI_0919	Gypsophila paniculata L.	0	n	1	0	Caryophyllaceae Juss.
Iz_8_1009	DJI_0919	Helichrysum arenarium (L.) Moench	0	n	1	0	Asteraceae Bercht. & J.Presl
Iz_8_1009	DJI_0919	Herniaria polygama J.Gay	0	n	1	0	Caryophyllaceae Juss.
Iz_8_1009	DJI_0919	Hieracium robustum Fr.	0	n	1	0	Asteraceae Bercht. & J.Presl
Iz_8_1009	DJI_0919	Hieracium umbellatum L.	0	n	1	NA	Asteraceae Bercht. & J.Presl
Iz_8_1009	DJI_0919	Hylotelephium maximum (L.) Holub	0	n	1	0	Crassulaceae J.St.-Hil.
Iz_8_1009	DJI_0919	Jacobaea borysthenica (DC.) B.Nord. & Greuter	0	n	1	0	Asteraceae Bercht. & J.Presl
Iz_8_1009	DJI_0919	Jasione montana L.	0	n	1	0	Campanulaceae Juss.
Iz_8_1009	DJI_0919	Jurinea cyanoides (L.) Rchb.s.l.	0	n	1	0	Asteraceae Bercht. & J.Presl
Iz_8_1009	DJI_0919	Koeleria glauca (Spreng.) DC.	0	n	1	0	Poaceae Barnhart
Iz_8_1009	DJI_0919	Lactuca serriola L.	0	a	0	0	Asteraceae Bercht. & J.Presl
Iz_8_1009	DJI_0919	Linaria genistifolia (L.) Mill.	0	n	1	0	Plantaginaceae Juss.
Iz_8_1009	DJI_0919	Lomelosia argentea (L.) Greuter & Burdet	0	n	1	0	Caprifoliaceae Juss.
Iz_8_1009	DJI_0919	Melampyrum pratense L.	0	n	1	0	Orobanchaceae Vent.
Iz_8_1009	DJI_0919	Oenothera biennis L.	0	a	0	0	Onagraceae Juss.
Iz_8_1009	DJI_0919	Pentanema britannica (L.) D.Gut.Larr., Santos-Vicente, Anderb., E.Rico & M.M.Mart.Ort.	0	n	1	0	Asteraceae Bercht. & J.Presl
Iz_8_1009	DJI_0919	Peucedanum oreoselinum (L.) Moench	0	n	1	0	Apiaceae Lindl.
Iz_8_1009	DJI_0919	Peucedanum ruthenicum M.Bieb.	0	n	1	0	Apiaceae Lindl.
Iz_8_1009	DJI_0919	Pilosella echioides (Lumn.) F.W.Schultz & Sch.Bip.	0	n	1	0	Asteraceae Bercht. & J.Presl
Iz_8_1009	DJI_0919	Pilosella officinarum F. Schultz et Sch. Bip.	0	n	1	0	Asteraceae Bercht. & J.Presl
Iz_8_1009	DJI_0919	Pilosella sp.	0		NA	0	Asteraceae Bercht. & J.Presl
Iz_8_1009	DJI_0919	Pinus sylvestris L.	0	n	1	1	Pinaceae Spreng. ex F.Rudolphi
Iz_8_1009	DJI_0919	Poa annua L.	0	n	1	0	Poaceae Barnhart
Iz_8_1009	DJI_0919	Poa nemoralis L.	0	n	1	0	Poaceae Barnhart
Iz_8_1009	DJI_0919	Poa sp.	0		NA	0	Poaceae Barnhart
Iz_8_1009	DJI_0919	Polygonatum odoratum (Mill.) Druce	0	n	1	0	Asparagaceae Juss.
Iz_8_1009	DJI_0919	Polygonum arenarium Waldst. & Kit.	0	n	1	0	Polygonaceae Juss.
Iz_8_1009	DJI_0919	Populus nigra L.	0	n	1	1	Salicaceae Mirb.
Iz_8_1009	DJI_0919	Populus tremula L.	0	n	1	1	Salicaceae Mirb.
Iz_8_1009	DJI_0919	Populus Ã canescens (Aiton) Sm.	0	n	1	1	Salicaceae Mirb.
Iz_8_1009	DJI_0919	Potentilla argentea L.	0	n	1	0	Rosaceae Juss.
Iz_8_1009	DJI_0919	Psammophiliella muralis (L.) Ikonn.	0	n	1	0	Caryophyllaceae Juss.
Iz_8_1009	DJI_0919	Psephellus sumensis (Kalen.) Greuter	0	n	1	0	Asteraceae Bercht. & J.Presl
Iz_8_1009	DJI_0919	Pulsatilla pratensis (L.) Mill. s.l.	0	n	1	0	Ranunculaceae Juss.
Iz_8_1009	DJI_0919	Pyrus communis L.	0	a	0	1	Rosaceae Juss.
Iz_8_1009	DJI_0919	Quercus robur L.	0	n	1	1	Fagaceae Dumort.
Iz_8_1009	DJI_0919	Robinia pseudoacacia L.	0	a	0	1	Fabaceae Lindl.
Iz_8_1009	DJI_0919	Rosa sp.	0		NA	1	Rosaceae Juss.
Iz_8_1009	DJI_0919	Rubus idaeus L.	0	n	1	0	Rosaceae Juss.
Iz_8_1009	DJI_0919	Rumex acetosella L.	0	n	1	0	Polygonaceae Juss.
Iz_8_1009	DJI_0919	Rumex thyrsiflorus Fingerh.	0	n	1	0	Polygonaceae Juss.
Iz_8_1009	DJI_0919	Salix alba L.	0	n	1	1	Salicaceae Mirb.
Iz_8_1009	DJI_0919	Sambucus nigra L.	0	n	1	1	Viburnaceae Raf.
Iz_8_1009	DJI_0919	Secale sylvestre Host	0	n	1	0	Poaceae Barnhart
Iz_8_1009	DJI_0919	Sempervivum ruthenicum Schnittsp. & C.B. Lehm.	0	n	1	0	Crassulaceae J.St.-Hil.
Iz_8_1009	DJI_0919	Setaria viridis (L.) P. Beauv.	0	n	1	0	Poaceae Barnhart
Iz_8_1009	DJI_0919	Silene borysthenica (Gruner) Walters	0	n	1	0	Caryophyllaceae Juss.
Iz_8_1009	DJI_0919	Silene latifolia subsp. alba (Mill.) Greuter & Burdet	0	n	1	0	Caryophyllaceae Juss.
Iz_8_1009	DJI_0919	Solidago virgaurea L.	0	n	1	0	Asteraceae Bercht. & J.Presl
Iz_8_1009	DJI_0919	Sorbus aucuparia L.	0	n	1	1	Rosaceae Juss.
Iz_8_1009	DJI_0919	Stachys recta L.	0	n	1	0	Lamiacea Lindl.
Iz_8_1009	DJI_0919	Tanacetum vulgare L.	0	n	1	0	Asteraceae Bercht. & J.Presl
Iz_8_1009	DJI_0919	Thymus pallasianus Heinr.Braun	0	n	1	0	Lamiacea Lindl.
Iz_8_1009	DJI_0919	Tilia cordata Mill.	0	n	1	1	Malvaceae Juss.
Iz_8_1009	DJI_0919	Trifolium alpestre L.	0	n	1	0	Fabaceae Lindl.
Iz_8_1009	DJI_0919	Trifolium arvense L.	0	n	1	0	Fabaceae Lindl.
Iz_8_1009	DJI_0919	Ulmus glabra Huds.	0	n	1	1	Ulmaceae Mirb.
Iz_8_1009	DJI_0919	Verbascum densiflorum Bertol.	0	n	1	0	Scrophulariaceae Juss.
Iz_8_1009	DJI_0919	Veronica austriaca L.	0	n	1	0	Plantaginaceae Juss.
Iz_8_1009	DJI_0919	Veronica incana L.	0	n	1	0	Plantaginaceae Juss.
Iz_8_1009	DJI_0919	Veronica verna L.	0	n	1	0	Plantaginaceae Juss.
Iz_8_1009	DJI_0919	Vincetoxicum hirundinaria Medik	0	n	1	0	Apocynaceae Juss.
Iz_8_1009	DJI_0922	Acer tataricum L.	0	n	1	1	Sapindaceae Juss.
Iz_8_1009	DJI_0922	Achillea millefolium L. s.l.	0	n	1	0	Asteraceae Bercht. & J.Presl
Iz_8_1009	DJI_0922	Allium oleraceum L.	0	n	1	0	Amaryllidaceae J.St.-Hil.
Iz_8_1009	DJI_0922	Ambrosia artemisiifolia L.	0	a	0	0	Asteraceae Bercht. & J.Presl
Iz_8_1009	DJI_0922	Amorpha fruticosa L.	0	a	0	0	Fabaceae Lindl.
Iz_8_1009	DJI_0922	Anthericum ramosum L.	0	n	1	0	Asparagaceae Juss.
Iz_8_1009	DJI_0922	Artemisia marschalliana Spreng.	0	n	1	0	Asteraceae Bercht. & J.Presl
Iz_8_1009	DJI_0922	Asparagus officinalis L.	0	n	1	0	Asparagaceae Juss.
Iz_8_1009	DJI_0922	Astragalus cicer L.	0	n	1	0	Fabaceae Lindl.
Iz_8_1009	DJI_0922	Bassia laniflora (S.G.Gmel.) A.J.Scott	0	n	1	0	Amaranthaceae Juss.
Iz_8_1009	DJI_0922	Berberis vulgaris L.	0	n	1	0	Berberidaceae Juss.
Iz_8_1009	DJI_0922	Betula pendula Roth.	0	n	1	1	Betulaceae Gray
Iz_8_1009	DJI_0922	Betula pendula Roth.	0	n	1	0	Poaceae Barnhart
Iz_8_1009	DJI_0922	Bromus inermis Leyss.	0		NA	NA	
Iz_8_1009	DJI_0922	Bromus squarrosus L.	0	a	0	0	Poaceae Barnhart
Iz_8_1009	DJI_0922	Bromus tectorum L.	0	a	0	0	Poaceae Barnhart
Iz_8_1009	DJI_0922	Calamagrostis epigejos (L.) Roth	25	n	1	0	Poaceae Barnhart
Iz_8_1009	DJI_0922	Carduus acanthoides L.	0	n	1	0	Asteraceae Bercht. & J.Presl
Iz_8_1009	DJI_0922	Carex sp.	0	n	1	0	Cyperaceae Juss.
Iz_8_1009	DJI_0922	Centaurea majorovii Dumbadze	0	n	1	0	Asteraceae Bercht. & J.Presl
Iz_8_1009	DJI_0922	Chamaecytisus ruthenicus (Fisch.ex Wol.) Klaskova	0	n	1	0	Fabaceae Lindl.
Iz_8_1009	DJI_0922	Chelidonium majus L.	0	n	1	0	Papaveraceae Juss.
Iz_8_1009	DJI_0922	Chenopodium betaceum Andrz.	0	n	1	0	Amaranthaceae Juss.
Iz_8_1009	DJI_0922	Chenopodium cf. album	0	n	1	0	Amaranthaceae Juss.
Iz_8_1009	DJI_0922	Chenopodium sp.	0		NA	0	Amaranthaceae Juss.
Iz_8_1009	DJI_0922	Chondrilla juncea L.	0	n	1	0	Asteraceae Bercht. & J.Presl
Iz_8_1009	DJI_0922	Cirsium vulgare (Savi) Ten.	0	n	1	0	Asteraceae Bercht. & J.Presl

Iz_8_1009	DJI_0922	Clinopodium acinos (L.) Kuntze	0	n	1	0	Lamiacea Lindl.
Iz_8_1009	DJI_0922	Convallaria majalis L.	0	n	1	0	Asparagaceae Juss.
Iz_8_1009	DJI_0922	Crataegus rhipidophylla Gand.	0	n	1	1	Rosaceae Juss.
Iz_8_1009	DJI_0922	Crepis foetida L.	0	n	1	0	Asteraceae Bercht. & J.Presl
Iz_8_1009	DJI_0922	Dactylis glomerata L.	0	n	1	0	Poaceae Barnhart
Iz_8_1009	DJI_0922	Dianthus campestris M.Bieb.	0	n	1	0	Caryophyllaceae Juss.
Iz_8_1009	DJI_0922	Digitaria sanguinalis (L.) Scop.	0	a	0	0	Poaceae Barnhart
Iz_8_1009	DJI_0922	Elymus repens (L.) Gould	0	n	1	0	Poaceae Barnhart
Iz_8_1009	DJI_0922	Epilobium tetragonum L.	0	n	1	0	Onagraceae Juss.
Iz_8_1009	DJI_0922	Eragrostis minor Host	0	a	0	0	Poaceae Barnhart
Iz_8_1009	DJI_0922	Erigeron canadensis L.	30	a	0	0	Asteraceae Bercht. & J.Presl
Iz_8_1009	DJI_0922	Euphorbia seguieriana Neck.	0	n	1	0	Euphorbiaceae Juss.
Iz_8_1009	DJI_0922	Fallopia dumetorum (L.) Holub	0	n	1	0	Polygonaceae Juss.
Iz_8_1009	DJI_0922	Festuca beckeri (Hack.) Trautv.	0	n	1	0	Poaceae Barnhart
Iz_8_1009	DJI_0922	Fragaria vesca L.	0	n	1	0	Rosaceae Juss.
Iz_8_1009	DJI_0922	Frangula alnus Mill.	0	n	1	1	Rhamnaceae Juss.
Iz_8_1009	DJI_0922	Fraxinus pennsylvanica Marshall	0	a	0	1	Oleaceae Hoffmanns. & Link
Iz_8_1009	DJI_0922	Galeopsis bifida Boenn.	0	n	1	0	Lamiacea Lindl.
Iz_8_1009	DJI_0922	Galium verum L.	0	n	1	0	Rubiaceae Juss.
Iz_8_1009	DJI_0922	Genista tinctoria L.	0	n	1	0	Fabaceae Lindl.
Iz_8_1009	DJI_0922	Geranium robertianum L.	0	n	1	0	Geraniaceae Juss.
Iz_8_1009	DJI_0922	Geranium sanquineum L.	0	n	1	0	Geraniaceae Juss.
Iz_8_1009	DJI_0922	Gypsophila paniculata L.	0	n	1	0	Caryophyllaceae Juss.
Iz_8_1009	DJI_0922	Helichrysum arenarium (L.) Moench	0	n	1	0	Asteraceae Bercht. & J.Presl
Iz_8_1009	DJI_0922	Herniaria polygama J.Gay	0	n	1	0	Caryophyllaceae Juss.
Iz_8_1009	DJI_0922	Hieracium robustum Fr.	0	n	1	0	Asteraceae Bercht. & J.Presl
Iz_8_1009	DJI_0922	Hieracium umbellatum L.	0	n	1	NA	Asteraceae Bercht. & J.Presl
Iz_8_1009	DJI_0922	Hylotelephium maximum (L.) Holub	0	n	1	0	Crassulaceae J.St.-Hil.
Iz_8_1009	DJI_0922	Jacobaea borysthenica (DC.) B.Nord. & Greuter	0	n	1	0	Asteraceae Bercht. & J.Presl
Iz_8_1009	DJI_0922	Jasione montana L.	0	n	1	0	Campanulaceae Juss.
Iz_8_1009	DJI_0922	Jurinea cyanoides (L.) Rchb.s.l.	0	n	1	0	Asteraceae Bercht. & J.Presl
Iz_8_1009	DJI_0922	Koeleria glauca (Spreng.) DC.	0	n	1	0	Poaceae Barnhart
Iz_8_1009	DJI_0922	Lactuca serriola L.	0	a	0	0	Asteraceae Bercht. & J.Presl
Iz_8_1009	DJI_0922	Linaria genistifolia (L.) Mill.	0	n	1	0	Plantaginaceae Juss.
Iz_8_1009	DJI_0922	Lomelosia argentea (L.) Greuter & Burdet	0	n	1	0	Caprifoliaceae Juss.
Iz_8_1009	DJI_0922	Melampyrum pratense L.	0	n	1	0	Orobanchaceae Vent.
Iz_8_1009	DJI_0922	Oenothera biennis L.	0	a	0	0	Onagraceae Juss.
Iz_8_1009	DJI_0922	Pentanema britannica (L.) D.Gut.Larr., Santos-Vicente, Anderb., E.Rico & M.M.Mart.Ort.	0	n	1	0	Asteraceae Bercht. & J.Presl
Iz_8_1009	DJI_0922	Peucedanum oreoselinum (L.) Moench	0	n	1	0	Apiaceae Lindl.
Iz_8_1009	DJI_0922	Peucedanum ruthenicum M.Bieb.	0	n	1	0	Apiaceae Lindl.
Iz_8_1009	DJI_0922	Pilosella echioides (Lumn.) F.W.Schultz & Sch.Bip.	0	n	1	0	Asteraceae Bercht. & J.Presl
Iz_8_1009	DJI_0922	Pilosella officinarum F. Schultz et Sch. Bip.	0	n	1	0	Asteraceae Bercht. & J.Presl
Iz_8_1009	DJI_0922	Pilosella sp.	0		NA	0	Asteraceae Bercht. & J.Presl
Iz_8_1009	DJI_0922	Pinus sylvestris L.	0	n	1	1	Pinaceae Spreng. ex F.Rudolphi
Iz_8_1009	DJI_0922	Poa annua L.	0	n	1	0	Poaceae Barnhart
Iz_8_1009	DJI_0922	Poa nemoralis L.	0	n	1	0	Poaceae Barnhart
Iz_8_1009	DJI_0922	Poa sp.	0		NA	0	Poaceae Barnhart
Iz_8_1009	DJI_0922	Polygonatum odoratum (Mill.) Druce	0	n	1	0	Asparagaceae Juss.
Iz_8_1009	DJI_0922	Polygonum arenarium Waldst. & Kit.	0	n	1	0	Polygonaceae Juss.
Iz_8_1009	DJI_0922	Populus nigra L.	0	n	1	1	Salicaceae Mirb.
Iz_8_1009	DJI_0922	Populus tremula L.	0	n	1	1	Salicaceae Mirb.
Iz_8_1009	DJI_0922	Populus Ã canescens (Aiton) Sm.	0	n	1	1	Salicaceae Mirb.
Iz_8_1009	DJI_0922	Potentilla argentea L.	0	n	1	0	Rosaceae Juss.
Iz_8_1009	DJI_0922	Psammophiliella muralis (L.) Ikonn.	0	n	1	0	Caryophyllaceae Juss.
Iz_8_1009	DJI_0922	Psephellus sumensis (Kalen.) Greuter	0	n	1	0	Asteraceae Bercht. & J.Presl
Iz_8_1009	DJI_0922	Pulsatilla pratensis (L.) Mill. s.l.	0	n	1	0	Ranunculaceae Juss.
Iz_8_1009	DJI_0922	Pyrus communis L.	0	a	0	1	Rosaceae Juss.
Iz_8_1009	DJI_0922	Quercus robur L.	0	n	1	1	Fagaceae Dumort.
Iz_8_1009	DJI_0922	Robinia pseudoacacia L.	0	a	0	1	Fabaceae Lindl.
Iz_8_1009	DJI_0922	Rosa sp.	0		NA	1	Rosaceae Juss.
Iz_8_1009	DJI_0922	Rubus idaeus L.	0	n	1	0	Rosaceae Juss.
Iz_8_1009	DJI_0922	Rumex acetosella L.	0	n	1	0	Polygonaceae Juss.
Iz_8_1009	DJI_0922	Rumex thyrsiflorus Fingerh.	0	n	1	0	Polygonaceae Juss.
Iz_8_1009	DJI_0922	Salix alba L.	0	n	1	1	Salicaceae Mirb.
Iz_8_1009	DJI_0922	Sambucus nigra L.	0	n	1	1	Viburnaceae Raf.
Iz_8_1009	DJI_0922	Secale sylvestre Host	0	n	1	0	Poaceae Barnhart
Iz_8_1009	DJI_0922	Sempervivum ruthenicum Schnittsp. & C.B. Lehm.	0	n	1	0	Crassulaceae J.St.-Hil.
Iz_8_1009	DJI_0922	Setaria viridis (L.) P. Beauv.	0	n	1	0	Poaceae Barnhart
Iz_8_1009	DJI_0922	Silene borysthenica (Gruner) Walters	0	n	1	0	Caryophyllaceae Juss.
Iz_8_1009	DJI_0922	Silene latifolia subsp. alba (Mill.) Greuter & Burdet	0	n	1	0	Caryophyllaceae Juss.
Iz_8_1009	DJI_0922	Solidago virgaurea L.	0	n	1	0	Asteraceae Bercht. & J.Presl
Iz_8_1009	DJI_0922	Sorbus aucuparia L.	0	n	1	1	Rosaceae Juss.
Iz_8_1009	DJI_0922	Stachys recta L.	0	n	1	0	Lamiacea Lindl.
Iz_8_1009	DJI_0922	Tanacetum vulgare L.	0	n	1	0	Asteraceae Bercht. & J.Presl
Iz_8_1009	DJI_0922	Thymus pallasianus Heinr.Braun	0	n	1	0	Lamiacea Lindl.
Iz_8_1009	DJI_0922	Tilia cordata Mill.	0	n	1	1	Malvaceae Juss.
Iz_8_1009	DJI_0922	Trifolium alpestre L.	0	n	1	0	Fabaceae Lindl.
Iz_8_1009	DJI_0922	Trifolium arvense L.	0	n	1	0	Fabaceae Lindl.
Iz_8_1009	DJI_0922	Ulmus glabra Huds.	0	n	1	1	Ulmaceae Mirb.
Iz_8_1009	DJI_0922	Verbascum densiflorum Bertol.	0	n	1	0	Scrophulariaceae Juss.
Iz_8_1009	DJI_0922	Veronica austriaca L.	0	n	1	0	Plantaginaceae Juss.
Iz_8_1009	DJI_0922	Veronica incana L.	0	n	1	0	Plantaginaceae Juss.
Iz_8_1009	DJI_0922	Veronica verna L.	0	n	1	0	Plantaginaceae Juss.
Iz_8_1009	DJI_0922	Vincetoxicum hirundinaria Medik	0	n	1	0	Apocynaceae Juss.
Iz_8_1009	DJI_0924	Acer tataricum L.	0	n	1	1	Sapindaceae Juss.
Iz_8_1009	DJI_0924	Achillea millefolium L. s.l.	0	n	1	0	Asteraceae Bercht. & J.Presl
Iz_8_1009	DJI_0924	Allium oleraceum L.	0	n	1	0	Amaryllidaceae J.St.-Hil.
Iz_8_1009	DJI_0924	Ambrosia artemisiifolia L.	0	a	0	0	Asteraceae Bercht. & J.Presl
Iz_8_1009	DJI_0924	Amorpha fruticosa L.	0	a	0	0	Fabaceae Lindl.
Iz_8_1009	DJI_0924	Anthericum ramosum L.	0	n	1	0	Asparagaceae Juss.
Iz_8_1009	DJI_0924	Artemisia marschalliana Spreng.	0	n	1	0	Asteraceae Bercht. & J.Presl
Iz_8_1009	DJI_0924	Asparagus officinalis L.	0	n	1	0	Asparagaceae Juss.
Iz_8_1009	DJI_0924	Astragalus cicer L.	0	n	1	0	Fabaceae Lindl.
Iz_8_1009	DJI_0924	Bassia laniflora (S.G.Gmel.) A.J.Scott	0	n	1	0	Amaranthaceae Juss.
Iz_8_1009	DJI_0924	Berberis vulgaris L.	0	n	1	0	Berberidaceae Juss.
Iz_8_1009	DJI_0924	Betula pendula Roth.	0	n	1	1	Betulaceae Gray
Iz_8_1009	DJI_0924	Betula pendula Roth.	0	n	1	0	Poaceae Barnhart
Iz_8_1009	DJI_0924	Bromus inermis Leyss.	0		NA	NA	

Iz_8_1009	DJI_0924	Bromus squarrosus L.	0	a	0	0	Poaceae Barnhart
Iz_8_1009	DJI_0924	Bromus tectorum L.	0	a	0	0	Poaceae Barnhart
Iz_8_1009	DJI_0924	Calamagrostis epigejos (L.) Roth	0	n	1	0	Poaceae Barnhart
Iz_8_1009	DJI_0924	Carduus acanthoides L.	0	n	1	0	Asteraceae Bercht. & J.Presl
Iz_8_1009	DJI_0924	Carex sp.	0	n	1	0	Cyperaceae Juss.
Iz_8_1009	DJI_0924	Centaurea majorovii Dumbadze	0	n	1	0	Asteraceae Bercht. & J.Presl
Iz_8_1009	DJI_0924	Chamaecytisus ruthenicus (Fisch.ex Wol.) Klaskova	0	n	1	0	Fabaceae Lindl.
Iz_8_1009	DJI_0924	Chelidonium majus L.	0	n	1	0	Papaveraceae Juss.
Iz_8_1009	DJI_0924	Chenopodium betaceum Andrz.	0	n	1	0	Amaranthaceae Juss.
Iz_8_1009	DJI_0924	Chenopodium cf. album	0	n	1	0	Amaranthaceae Juss.
Iz_8_1009	DJI_0924	Chenopodium sp.	0		NA	0	Amaranthaceae Juss.
Iz_8_1009	DJI_0924	Chondrilla juncea L.	0	n	1	0	Asteraceae Bercht. & J.Presl
Iz_8_1009	DJI_0924	Cirsium vulgare (Savi) Ten.	0	n	1	0	Asteraceae Bercht. & J.Presl
Iz_8_1009	DJI_0924	Clinopodium acinos (L.) Kuntze	0	n	1	0	Lamiacea Lindl.
Iz_8_1009	DJI_0924	Convallaria majalis L.	0	n	1	0	Asparagaceae Juss.
Iz_8_1009	DJI_0924	Crataegus rhipidophylla Gand.	0	n	1	1	Rosaceae Juss.
Iz_8_1009	DJI_0924	Crepis foetida L.	0	n	1	0	Asteraceae Bercht. & J.Presl
Iz_8_1009	DJI_0924	Dactylis glomerata L.	0	n	1	0	Poaceae Barnhart
Iz_8_1009	DJI_0924	Dianthus campestris M.Bieb.	0	n	1	0	Caryophyllaceae Juss.
Iz_8_1009	DJI_0924	Digitaria sanguinalis (L.) Scop.	0	a	0	0	Poaceae Barnhart
Iz_8_1009	DJI_0924	Elymus repens (L.) Gould	0	n	1	0	Poaceae Barnhart
Iz_8_1009	DJI_0924	Epilobium tetragonum L.	0	n	1	0	Onagraceae Juss.
Iz_8_1009	DJI_0924	Eragrostis minor Host	0	a	0	0	Poaceae Barnhart
Iz_8_1009	DJI_0924	Erigeron canadensis L.	15	a	0	0	Asteraceae Bercht. & J.Presl
Iz_8_1009	DJI_0924	Euphorbia seguieriana Neck.	0	n	1	0	Euphorbiaceae Juss.
Iz_8_1009	DJI_0924	Fallopia dumetorum (L.) Holub	0	n	1	0	Polygonaceae Juss.
Iz_8_1009	DJI_0924	Festuca beckeri (Hack.) Trautv.	0	n	1	0	Poaceae Barnhart
Iz_8_1009	DJI_0924	Fragaria vesca L.	0	n	1	0	Rosaceae Juss.
Iz_8_1009	DJI_0924	Frangula alnus Mill.	0	n	1	1	Rhamnaceae Juss.
Iz_8_1009	DJI_0924	Fraxinus pennsylvanica Marshall	0	a	0	1	Oleaceae Hoffmanns. & Link
Iz_8_1009	DJI_0924	Galeopsis bifida Boenn.	0	n	1	0	Lamiacea Lindl.
Iz_8_1009	DJI_0924	Galium verum L.	0	n	1	0	Rubiaceae Juss.
Iz_8_1009	DJI_0924	Genista tinctoria L.	0	n	1	0	Fabaceae Lindl.
Iz_8_1009	DJI_0924	Geranium robertianum L.	0	n	1	0	Geraniaceae Juss.
Iz_8_1009	DJI_0924	Geranium sanquineum L.	0	n	1	0	Geraniaceae Juss.
Iz_8_1009	DJI_0924	Gypsophila paniculata L.	0	n	1	0	Caryophyllaceae Juss.
Iz_8_1009	DJI_0924	Helichrysum arenarium (L.) Moench	0	n	1	0	Asteraceae Bercht. & J.Presl
Iz_8_1009	DJI_0924	Herniaria polygama J.Gay	0	n	1	0	Caryophyllaceae Juss.
Iz_8_1009	DJI_0924	Hieracium robustum Fr.	0	n	1	0	Asteraceae Bercht. & J.Presl
Iz_8_1009	DJI_0924	Hieracium umbellatum L.	0	n	1	NA	Asteraceae Bercht. & J.Presl
Iz_8_1009	DJI_0924	Hylotelephium maximum (L.) Holub	0	n	1	0	Crassulaceae J.St.-Hil.
Iz_8_1009	DJI_0924	Jacobaea borysthenica (DC.) B.Nord. & Greuter	0	n	1	0	Asteraceae Bercht. & J.Presl
Iz_8_1009	DJI_0924	Jasione montana L.	0	n	1	0	Campanulaceae Juss.
Iz_8_1009	DJI_0924	Jurinea cyanoides (L.) Rchb.s.l.	0	n	1	0	Asteraceae Bercht. & J.Presl
Iz_8_1009	DJI_0924	Koeleria glauca (Spreng.) DC.	0	n	1	0	Poaceae Barnhart
Iz_8_1009	DJI_0924	Lactuca serriola L.	0	a	0	0	Asteraceae Bercht. & J.Presl
Iz_8_1009	DJI_0924	Linaria genistifolia (L.) Mill.	0	n	1	0	Plantaginaceae Juss.
Iz_8_1009	DJI_0924	Lomelosia argentea (L.) Greuter & Burdet	0	n	1	0	Caprifoliaceae Juss.
Iz_8_1009	DJI_0924	Melampyrum pratense L.	0	n	1	0	Orobanchaceae Vent.
Iz_8_1009	DJI_0924	Oenothera biennis L.	0	a	0	0	Onagraceae Juss.
Iz_8_1009	DJI_0924	Pentanema britannica (L.) D.Gut.Larr., Santos-Vicente, Anderb., E.Rico & M.M.Mart.Ort.	0	n	1	0	Asteraceae Bercht. & J.Presl
Iz_8_1009	DJI_0924	Peucedanum oreoselinum (L.) Moench	0	n	1	0	Apiaceae Lindl.
Iz_8_1009	DJI_0924	Peucedanum ruthenicum M.Bieb.	0	n	1	0	Apiaceae Lindl.
Iz_8_1009	DJI_0924	Pilosella echioides (Lumn.) F.W.Schultz & Sch.Bip.	0	n	1	0	Asteraceae Bercht. & J.Presl
Iz_8_1009	DJI_0924	Pilosella officinarum F. Schultz et Sch. Bip.	0	n	1	0	Asteraceae Bercht. & J.Presl
Iz_8_1009	DJI_0924	Pilosella sp.	0		NA	0	Asteraceae Bercht. & J.Presl
Iz_8_1009	DJI_0924	Pinus sylvestris L.	0	n	1	1	Pinaceae Spreng. ex F.Rudolphi
Iz_8_1009	DJI_0924	Poa annua L.	0	n	1	0	Poaceae Barnhart
Iz_8_1009	DJI_0924	Poa nemoralis L.	0	n	1	0	Poaceae Barnhart
Iz_8_1009	DJI_0924	Poa sp.	0		NA	0	Poaceae Barnhart
Iz_8_1009	DJI_0924	Polygonatum odoratum (Mill.) Druce	0	n	1	0	Asparagaceae Juss.
Iz_8_1009	DJI_0924	Polygonum arenarium Waldst. & Kit.	0	n	1	0	Polygonaceae Juss.
Iz_8_1009	DJI_0924	Populus nigra L.	0	n	1	1	Salicaceae Mirb.
Iz_8_1009	DJI_0924	Populus tremula L.	0	n	1	1	Salicaceae Mirb.
Iz_8_1009	DJI_0924	Populus Ã canescens (Aiton) Sm.	0	n	1	1	Salicaceae Mirb.
Iz_8_1009	DJI_0924	Potentilla argentea L.	0	n	1	0	Rosaceae Juss.
Iz_8_1009	DJI_0924	Psammophiliella muralis (L.) Ikonn.	0	n	1	0	Caryophyllaceae Juss.
Iz_8_1009	DJI_0924	Psephellus sumensis (Kalen.) Greuter	0	n	1	0	Asteraceae Bercht. & J.Presl
Iz_8_1009	DJI_0924	Pulsatilla pratensis (L.) Mill. s.l.	0	n	1	0	Ranunculaceae Juss.
Iz_8_1009	DJI_0924	Pyrus communis L.	0	a	0	1	Rosaceae Juss.
Iz_8_1009	DJI_0924	Quercus robur L.	0	n	1	1	Fagaceae Dumort.
Iz_8_1009	DJI_0924	Robinia pseudoacacia L.	0	a	0	1	Fabaceae Lindl.
Iz_8_1009	DJI_0924	Rosa sp.	0		NA	1	Rosaceae Juss.
Iz_8_1009	DJI_0924	Rubus idaeus L.	0	n	1	0	Rosaceae Juss.
Iz_8_1009	DJI_0924	Rumex acetosella L.	0	n	1	0	Polygonaceae Juss.
Iz_8_1009	DJI_0924	Rumex thyrsiflorus Fingerh.	0	n	1	0	Polygonaceae Juss.
Iz_8_1009	DJI_0924	Salix alba L.	0	n	1	1	Salicaceae Mirb.
Iz_8_1009	DJI_0924	Sambucus nigra L.	0	n	1	1	Viburnaceae Raf.
Iz_8_1009	DJI_0924	Secale sylvestre Host	0	n	1	0	Poaceae Barnhart
Iz_8_1009	DJI_0924	Sempervivum ruthenicum Schnittsp. & C.B. Lehm.	0	n	1	0	Crassulaceae J.St.-Hil.
Iz_8_1009	DJI_0924	Setaria viridis (L.) P. Beauv.	0	n	1	0	Poaceae Barnhart
Iz_8_1009	DJI_0924	Silene borysthenica (Gruner) Walters	0	n	1	0	Caryophyllaceae Juss.
Iz_8_1009	DJI_0924	Silene latifolia subsp. alba (Mill.) Greuter & Burdet	0	n	1	0	Caryophyllaceae Juss.
Iz_8_1009	DJI_0924	Solidago virgaurea L.	0	n	1	0	Asteraceae Bercht. & J.Presl
Iz_8_1009	DJI_0924	Sorbus aucuparia L.	0	n	1	1	Rosaceae Juss.
Iz_8_1009	DJI_0924	Stachys recta L.	0	n	1	0	Lamiacea Lindl.
Iz_8_1009	DJI_0924	Tanacetum vulgare L.	0	n	1	0	Asteraceae Bercht. & J.Presl
Iz_8_1009	DJI_0924	Thymus pallasianus Heinr.Braun	0	n	1	0	Lamiacea Lindl.
Iz_8_1009	DJI_0924	Tilia cordata Mill.	0	n	1	1	Malvaceae Juss.
Iz_8_1009	DJI_0924	Trifolium alpestre L.	0	n	1	0	Fabaceae Lindl.
Iz_8_1009	DJI_0924	Trifolium arvense L.	0	n	1	0	Fabaceae Lindl.
Iz_8_1009	DJI_0924	Ulmus glabra Huds.	0	n	1	1	Ulmaceae Mirb.
Iz_8_1009	DJI_0924	Verbascum densiflorum Bertol.	0	n	1	0	Scrophulariaceae Juss.
Iz_8_1009	DJI_0924	Veronica austriaca L.	0	n	1	0	Plantaginaceae Juss.
Iz_8_1009	DJI_0924	Veronica incana L.	0	n	1	0	Plantaginaceae Juss.
Iz_8_1009	DJI_0924	Veronica verna L.	0	n	1	0	Plantaginaceae Juss.
Iz_8_1009	DJI_0924	Vincetoxicum hirundinaria Medik	0	n	1	0	Apocynaceae Juss.
Iz_8_1009	DJI_0925	Acer tataricum L.	0	n	1	1	Sapindaceae Juss.

Iz_8_1009	DJI_0925	Achillea millefolium L. s.l.	0	n	1	0	Asteraceae Bercht. & J.Presl
Iz_8_1009	DJI_0925	Allium oleraceum L.	0	n	1	0	Amaryllidaceae J.St.-Hil.
Iz_8_1009	DJI_0925	Ambrosia artemisiifolia L.	0	a	0	0	Asteraceae Bercht. & J.Presl
Iz_8_1009	DJI_0925	Amorpha fruticosa L.	0	a	0	0	Fabaceae Lindl.
Iz_8_1009	DJI_0925	Anthericum ramosum L.	0	n	1	0	Asparagaceae Juss.
Iz_8_1009	DJI_0925	Artemisia marschalliana Spreng.	0	n	1	0	Asteraceae Bercht. & J.Presl
Iz_8_1009	DJI_0925	Asparagus officinalis L.	0	n	1	0	Asparagaceae Juss.
Iz_8_1009	DJI_0925	Astragalus cicer L.	0	n	1	0	Fabaceae Lindl.
Iz_8_1009	DJI_0925	Bassia laniflora (S.G.Gmel.) A.J.Scott	0	n	1	0	Amaranthaceae Juss.
Iz_8_1009	DJI_0925	Berberis vulgaris L.	0	n	1	0	Berberidaceae Juss.
Iz_8_1009	DJI_0925	Betula pendula Roth.	0	n	1	1	Betulaceae Gray
Iz_8_1009	DJI_0925	Betula pendula Roth.	0	n	1	0	Poaceae Barnhart
Iz_8_1009	DJI_0925	Bromus inermis Leyss.	0		NA	NA	
Iz_8_1009	DJI_0925	Bromus squarrosus L.	0	a	0	0	Poaceae Barnhart
Iz_8_1009	DJI_0925	Bromus tectorum L.	0	a	0	0	Poaceae Barnhart
Iz_8_1009	DJI_0925	Calamagrostis epigejos (L.) Roth	0	n	1	0	Poaceae Barnhart
Iz_8_1009	DJI_0925	Carduus acanthoides L.	0	n	1	0	Asteraceae Bercht. & J.Presl
Iz_8_1009	DJI_0925	Carex sp.	0	n	1	0	Cyperaceae Juss.
Iz_8_1009	DJI_0925	Centaurea majorovii Dumbadze	0	n	1	0	Asteraceae Bercht. & J.Presl
Iz_8_1009	DJI_0925	Chamaecytisus ruthenicus (Fisch.ex Wol.) Klaskova	0	n	1	0	Fabaceae Lindl.
Iz_8_1009	DJI_0925	Chelidonium majus L.	0	n	1	0	Papaveraceae Juss.
Iz_8_1009	DJI_0925	Chenopodium betaceum Andrz.	0	n	1	0	Amaranthaceae Juss.
Iz_8_1009	DJI_0925	Chenopodium cf. album	0	n	1	0	Amaranthaceae Juss.
Iz_8_1009	DJI_0925	Chenopodium sp.	0		NA	0	Amaranthaceae Juss.
Iz_8_1009	DJI_0925	Chondrilla juncea L.	0	n	1	0	Asteraceae Bercht. & J.Presl
Iz_8_1009	DJI_0925	Cirsium vulgare (Savi) Ten.	0	n	1	0	Asteraceae Bercht. & J.Presl
Iz_8_1009	DJI_0925	Clinopodium acinos (L.) Kuntze	0	n	1	0	Lamiacea Lindl.
Iz_8_1009	DJI_0925	Convallaria majalis L.	0	n	1	0	Asparagaceae Juss.
Iz_8_1009	DJI_0925	Crataegus rhipidophylla Gand.	0	n	1	1	Rosaceae Juss.
Iz_8_1009	DJI_0925	Crepis foetida L.	0	n	1	0	Asteraceae Bercht. & J.Presl
Iz_8_1009	DJI_0925	Dactylis glomerata L.	0	n	1	0	Poaceae Barnhart
Iz_8_1009	DJI_0925	Dianthus campestris M.Bieb.	0	n	1	0	Caryophyllaceae Juss.
Iz_8_1009	DJI_0925	Digitaria sanguinalis (L.) Scop.	0	a	0	0	Poaceae Barnhart
Iz_8_1009	DJI_0925	Elymus repens (L.) Gould	0	n	1	0	Poaceae Barnhart
Iz_8_1009	DJI_0925	Epilobium tetragonum L.	0	n	1	0	Onagraceae Juss.
Iz_8_1009	DJI_0925	Eragrostis minor Host	0	a	0	0	Poaceae Barnhart
Iz_8_1009	DJI_0925	Erigeron canadensis L.	5	a	0	0	Asteraceae Bercht. & J.Presl
Iz_8_1009	DJI_0925	Euphorbia seguieriana Neck.	0	n	1	0	Euphorbiaceae Juss.
Iz_8_1009	DJI_0925	Fallopia dumetorum (L.) Holub	0	n	1	0	Polygonaceae Juss.
Iz_8_1009	DJI_0925	Festuca beckeri (Hack.) Trautv.	0	n	1	0	Poaceae Barnhart
Iz_8_1009	DJI_0925	Fragaria vesca L.	0	n	1	0	Rosaceae Juss.
Iz_8_1009	DJI_0925	Frangula alnus Mill.	0	n	1	1	Rhamnaceae Juss.
Iz_8_1009	DJI_0925	Fraxinus pennsylvanica Marshall	0	a	0	1	Oleaceae Hoffmanns. & Link
Iz_8_1009	DJI_0925	Galeopsis bifida Boenn.	0	n	1	0	Lamiacea Lindl.
Iz_8_1009	DJI_0925	Galium verum L.	0	n	1	0	Rubiaceae Juss.
Iz_8_1009	DJI_0925	Genista tinctoria L.	0	n	1	0	Fabaceae Lindl.
Iz_8_1009	DJI_0925	Geranium robertianum L.	0	n	1	0	Geraniaceae Juss.
Iz_8_1009	DJI_0925	Geranium sanquineum L.	0	n	1	0	Geraniaceae Juss.
Iz_8_1009	DJI_0925	Gypsophila paniculata L.	0	n	1	0	Caryophyllaceae Juss.
Iz_8_1009	DJI_0925	Helichrysum arenarium (L.) Moench	0	n	1	0	Asteraceae Bercht. & J.Presl
Iz_8_1009	DJI_0925	Herniaria polygama J.Gay	0	n	1	0	Caryophyllaceae Juss.
Iz_8_1009	DJI_0925	Hieracium robustum Fr.	0	n	1	0	Asteraceae Bercht. & J.Presl
Iz_8_1009	DJI_0925	Hieracium umbellatum L.	0	n	1	NA	Asteraceae Bercht. & J.Presl
Iz_8_1009	DJI_0925	Hylotelephium maximum (L.) Holub	0	n	1	0	Crassulaceae J.St.-Hil.
Iz_8_1009	DJI_0925	Jacobaea borysthenica (DC.) B.Nord. & Greuter	0	n	1	0	Asteraceae Bercht. & J.Presl
Iz_8_1009	DJI_0925	Jasione montana L.	0	n	1	0	Campanulaceae Juss.
Iz_8_1009	DJI_0925	Jurinea cyanoides (L.) Rchb.s.l.	0	n	1	0	Asteraceae Bercht. & J.Presl
Iz_8_1009	DJI_0925	Koeleria glauca (Spreng.) DC.	0	n	1	0	Poaceae Barnhart
Iz_8_1009	DJI_0925	Lactuca serriola L.	0	a	0	0	Asteraceae Bercht. & J.Presl
Iz_8_1009	DJI_0925	Linaria genistifolia (L.) Mill.	0	n	1	0	Plantaginaceae Juss.
Iz_8_1009	DJI_0925	Lomelosia argentea (L.) Greuter & Burdet	0	n	1	0	Caprifoliaceae Juss.
Iz_8_1009	DJI_0925	Melampyrum pratense L.	0	n	1	0	Orobanchaceae Vent.
Iz_8_1009	DJI_0925	Oenothera biennis L.	0	a	0	0	Onagraceae Juss.
Iz_8_1009	DJI_0925	Pentanema britannica (L.) D.Gut.Larr., Santos-Vicente, Anderb., E.Rico & M.M.Mart.Ort.	0	n	1	0	Asteraceae Bercht. & J.Presl
Iz_8_1009	DJI_0925	Peucedanum oreoselinum (L.) Moench	0	n	1	0	Apiaceae Lindl.
Iz_8_1009	DJI_0925	Peucedanum ruthenicum M.Bieb.	0	n	1	0	Apiaceae Lindl.
Iz_8_1009	DJI_0925	Pilosella echioides (Lumn.) F.W.Schultz & Sch.Bip.	0	n	1	0	Asteraceae Bercht. & J.Presl
Iz_8_1009	DJI_0925	Pilosella officinarum F. Schultz et Sch. Bip.	0	n	1	0	Asteraceae Bercht. & J.Presl
Iz_8_1009	DJI_0925	Pilosella sp.	0		NA	0	Asteraceae Bercht. & J.Presl
Iz_8_1009	DJI_0925	Pinus sylvestris L.	0	n	1	1	Pinaceae Spreng. ex F.Rudolphi
Iz_8_1009	DJI_0925	Poa annua L.	0	n	1	0	Poaceae Barnhart
Iz_8_1009	DJI_0925	Poa nemoralis L.	0	n	1	0	Poaceae Barnhart
Iz_8_1009	DJI_0925	Poa sp.	0		NA	0	Poaceae Barnhart
Iz_8_1009	DJI_0925	Polygonatum odoratum (Mill.) Druce	0	n	1	0	Asparagaceae Juss.
Iz_8_1009	DJI_0925	Polygonum arenarium Waldst. & Kit.	0	n	1	0	Polygonaceae Juss.
Iz_8_1009	DJI_0925	Populus nigra L.	0	n	1	1	Salicaceae Mirb.
Iz_8_1009	DJI_0925	Populus tremula L.	0	n	1	1	Salicaceae Mirb.
Iz_8_1009	DJI_0925	Populus Ã canescens (Aiton) Sm.	0	n	1	1	Salicaceae Mirb.
Iz_8_1009	DJI_0925	Potentilla argentea L.	0	n	1	0	Rosaceae Juss.
Iz_8_1009	DJI_0925	Psammophiliella muralis (L.) Ikonn.	0	n	1	0	Caryophyllaceae Juss.
Iz_8_1009	DJI_0925	Psephellus sumensis (Kalen.) Greuter	0	n	1	0	Asteraceae Bercht. & J.Presl
Iz_8_1009	DJI_0925	Pulsatilla pratensis (L.) Mill. s.l.	0	n	1	0	Ranunculaceae Juss.
Iz_8_1009	DJI_0925	Pyrus communis L.	0	a	0	1	Rosaceae Juss.
Iz_8_1009	DJI_0925	Quercus robur L.	0	n	1	1	Fagaceae Dumort.
Iz_8_1009	DJI_0925	Robinia pseudoacacia L.	0	a	0	1	Fabaceae Lindl.
Iz_8_1009	DJI_0925	Rosa sp.	0		NA	1	Rosaceae Juss.
Iz_8_1009	DJI_0925	Rubus idaeus L.	0	n	1	0	Rosaceae Juss.
Iz_8_1009	DJI_0925	Rumex acetosella L.	0	n	1	0	Polygonaceae Juss.
Iz_8_1009	DJI_0925	Rumex thyrsiflorus Fingerh.	0	n	1	0	Polygonaceae Juss.
Iz_8_1009	DJI_0925	Salix alba L.	0	n	1	1	Salicaceae Mirb.
Iz_8_1009	DJI_0925	Sambucus nigra L.	0	n	1	1	Viburnaceae Raf.
Iz_8_1009	DJI_0925	Secale sylvestre Host	0	n	1	0	Poaceae Barnhart
Iz_8_1009	DJI_0925	Sempervivum ruthenicum Schnittsp. & C.B. Lehm.	0	n	1	0	Crassulaceae J.St.-Hil.
Iz_8_1009	DJI_0925	Setaria viridis (L.) P. Beauv.	0	n	1	0	Poaceae Barnhart
Iz_8_1009	DJI_0925	Silene borysthenica (Gruner) Walters	0	n	1	0	Caryophyllaceae Juss.
Iz_8_1009	DJI_0925	Silene latifolia subsp. alba (Mill.) Greuter & Burdet	0	n	1	0	Caryophyllaceae Juss.
Iz_8_1009	DJI_0925	Solidago virgaurea L.	0	n	1	0	Asteraceae Bercht. & J.Presl
Iz_8_1009	DJI_0925	Sorbus aucuparia L.	0	n	1	1	Rosaceae Juss.

Iz_8_1009	DJI_0925	Stachys recta L.	0	n	1	0	Lamiacea Lindl.
Iz_8_1009	DJI_0925	Tanacetum vulgare L.	0	n	1	0	Asteraceae Bercht. & J.Presl
Iz_8_1009	DJI_0925	Thymus pallasianus Heinr.Braun	0	n	1	0	Lamiacea Lindl.
Iz_8_1009	DJI_0925	Tilia cordata Mill.	0	n	1	1	Malvaceae Juss.
Iz_8_1009	DJI_0925	Trifolium alpestre L.	0	n	1	0	Fabaceae Lindl.
Iz_8_1009	DJI_0925	Trifolium arvense L.	0	n	1	0	Fabaceae Lindl.
Iz_8_1009	DJI_0925	Ulmus glabra Huds.	0	n	1	1	Ulmaceae Mirb.
Iz_8_1009	DJI_0925	Verbascum densiflorum Bertol.	0	n	1	0	Scrophulariaceae Juss.
Iz_8_1009	DJI_0925	Veronica austriaca L.	0	n	1	0	Plantaginaceae Juss.
Iz_8_1009	DJI_0925	Veronica incana L.	0	n	1	0	Plantaginaceae Juss.
Iz_8_1009	DJI_0925	Veronica verna L.	0	n	1	0	Plantaginaceae Juss.
Iz_8_1009	DJI_0925	Vincetoxicum hirundinaria Medik	0	n	1	0	Apocynaceae Juss.
Iz_8_1009	DJI_0927	Acer tataricum L.	0	n	1	1	Sapindaceae Juss.
Iz_8_1009	DJI_0927	Achillea millefolium L. s.l.	0	n	1	0	Asteraceae Bercht. & J.Presl
Iz_8_1009	DJI_0927	Allium oleraceum L.	0	n	1	0	Amaryllidaceae J.St.-Hil.
Iz_8_1009	DJI_0927	Ambrosia artemisiifolia L.	0	a	0	0	Asteraceae Bercht. & J.Presl
Iz_8_1009	DJI_0927	Amorpha fruticosa L.	0	a	0	0	Fabaceae Lindl.
Iz_8_1009	DJI_0927	Anthericum ramosum L.	0	n	1	0	Asparagaceae Juss.
Iz_8_1009	DJI_0927	Artemisia marschalliana Spreng.	0	n	1	0	Asteraceae Bercht. & J.Presl
Iz_8_1009	DJI_0927	Asparagus officinalis L.	0	n	1	0	Asparagaceae Juss.
Iz_8_1009	DJI_0927	Astragalus cicer L.	0	n	1	0	Fabaceae Lindl.
Iz_8_1009	DJI_0927	Bassia laniflora (S.G.Gmel.) A.J.Scott	0	n	1	0	Amaranthaceae Juss.
Iz_8_1009	DJI_0927	Berberis vulgaris L.	0	n	1	0	Berberidaceae Juss.
Iz_8_1009	DJI_0927	Betula pendula Roth.	0	n	1	1	Betulaceae Gray
Iz_8_1009	DJI_0927	Betula pendula Roth.	0	n	1	0	Poaceae Barnhart
Iz_8_1009	DJI_0927	Bromus inermis Leyss.	0		NA	NA	
Iz_8_1009	DJI_0927	Bromus squarrosus L.	0	a	0	0	Poaceae Barnhart
Iz_8_1009	DJI_0927	Bromus tectorum L.	0	a	0	0	Poaceae Barnhart
Iz_8_1009	DJI_0927	Calamagrostis epigejos (L.) Roth	2	n	1	0	Poaceae Barnhart
Iz_8_1009	DJI_0927	Carduus acanthoides L.	0	n	1	0	Asteraceae Bercht. & J.Presl
Iz_8_1009	DJI_0927	Carex sp.	0	n	1	0	Cyperaceae Juss.
Iz_8_1009	DJI_0927	Centaurea majorovii Dumbadze	0	n	1	0	Asteraceae Bercht. & J.Presl
Iz_8_1009	DJI_0927	Chamaecytisus ruthenicus (Fisch.ex Wol.) Klaskova	0	n	1	0	Fabaceae Lindl.
Iz_8_1009	DJI_0927	Chelidonium majus L.	0	n	1	0	Papaveraceae Juss.
Iz_8_1009	DJI_0927	Chenopodium betaceum Andrz.	0	n	1	0	Amaranthaceae Juss.
Iz_8_1009	DJI_0927	Chenopodium cf. album	0	n	1	0	Amaranthaceae Juss.
Iz_8_1009	DJI_0927	Chenopodium sp.	0		NA	0	Amaranthaceae Juss.
Iz_8_1009	DJI_0927	Chondrilla juncea L.	0	n	1	0	Asteraceae Bercht. & J.Presl
Iz_8_1009	DJI_0927	Cirsium vulgare (Savi) Ten.	0	n	1	0	Asteraceae Bercht. & J.Presl
Iz_8_1009	DJI_0927	Clinopodium acinos (L.) Kuntze	0	n	1	0	Lamiacea Lindl.
Iz_8_1009	DJI_0927	Convallaria majalis L.	0	n	1	0	Asparagaceae Juss.
Iz_8_1009	DJI_0927	Crataegus rhipidophylla Gand.	0	n	1	1	Rosaceae Juss.
Iz_8_1009	DJI_0927	Crepis foetida L.	0	n	1	0	Asteraceae Bercht. & J.Presl
Iz_8_1009	DJI_0927	Dactylis glomerata L.	0	n	1	0	Poaceae Barnhart
Iz_8_1009	DJI_0927	Dianthus campestris M.Bieb.	0	n	1	0	Caryophyllaceae Juss.
Iz_8_1009	DJI_0927	Digitaria sanguinalis (L.) Scop.	0	a	0	0	Poaceae Barnhart
Iz_8_1009	DJI_0927	Elymus repens (L.) Gould	0	n	1	0	Poaceae Barnhart
Iz_8_1009	DJI_0927	Epilobium tetragonum L.	0	n	1	0	Onagraceae Juss.
Iz_8_1009	DJI_0927	Eragrostis minor Host	0	a	0	0	Poaceae Barnhart
Iz_8_1009	DJI_0927	Erigeron canadensis L.	4	a	0	0	Asteraceae Bercht. & J.Presl
Iz_8_1009	DJI_0927	Euphorbia seguieriana Neck.	0	n	1	0	Euphorbiaceae Juss.
Iz_8_1009	DJI_0927	Fallopia dumetorum (L.) Holub	0	n	1	0	Polygonaceae Juss.
Iz_8_1009	DJI_0927	Festuca beckeri (Hack.) Trautv.	0	n	1	0	Poaceae Barnhart
Iz_8_1009	DJI_0927	Fragaria vesca L.	0	n	1	0	Rosaceae Juss.
Iz_8_1009	DJI_0927	Frangula alnus Mill.	0	n	1	1	Rhamnaceae Juss.
Iz_8_1009	DJI_0927	Fraxinus pennsylvanica Marshall	0	a	0	1	Oleaceae Hoffmanns. & Link
Iz_8_1009	DJI_0927	Galeopsis bifida Boenn.	0	n	1	0	Lamiacea Lindl.
Iz_8_1009	DJI_0927	Galium verum L.	0	n	1	0	Rubiaceae Juss.
Iz_8_1009	DJI_0927	Genista tinctoria L.	0	n	1	0	Fabaceae Lindl.
Iz_8_1009	DJI_0927	Geranium robertianum L.	0	n	1	0	Geraniaceae Juss.
Iz_8_1009	DJI_0927	Geranium sanquineum L.	0	n	1	0	Geraniaceae Juss.
Iz_8_1009	DJI_0927	Gypsophila paniculata L.	0	n	1	0	Caryophyllaceae Juss.
Iz_8_1009	DJI_0927	Helichrysum arenarium (L.) Moench	0	n	1	0	Asteraceae Bercht. & J.Presl
Iz_8_1009	DJI_0927	Herniaria polygama J.Gay	0	n	1	0	Caryophyllaceae Juss.
Iz_8_1009	DJI_0927	Hieracium robustum Fr.	0	n	1	0	Asteraceae Bercht. & J.Presl
Iz_8_1009	DJI_0927	Hieracium umbellatum L.	0	n	1	NA	Asteraceae Bercht. & J.Presl
Iz_8_1009	DJI_0927	Hylotelephium maximum (L.) Holub	0	n	1	0	Crassulaceae J.St.-Hil.
Iz_8_1009	DJI_0927	Jacobaea borysthenica (DC.) B.Nord. & Greuter	0	n	1	0	Asteraceae Bercht. & J.Presl
Iz_8_1009	DJI_0927	Jasione montana L.	0	n	1	0	Campanulaceae Juss.
Iz_8_1009	DJI_0927	Jurinea cyanoides (L.) Rchb.s.l.	0	n	1	0	Asteraceae Bercht. & J.Presl
Iz_8_1009	DJI_0927	Koeleria glauca (Spreng.) DC.	0	n	1	0	Poaceae Barnhart
Iz_8_1009	DJI_0927	Lactuca serriola L.	0	a	0	0	Asteraceae Bercht. & J.Presl
Iz_8_1009	DJI_0927	Linaria genistifolia (L.) Mill.	0	n	1	0	Plantaginaceae Juss.
Iz_8_1009	DJI_0927	Lomelosia argentea (L.) Greuter & Burdet	0	n	1	0	Caprifoliaceae Juss.
Iz_8_1009	DJI_0927	Melampyrum pratense L.	0	n	1	0	Orobanchaceae Vent.
Iz_8_1009	DJI_0927	Oenothera biennis L.	0	a	0	0	Onagraceae Juss.
Iz_8_1009	DJI_0927	Pentanema britannica (L.) D.Gut.Larr., Santos-Vicente, Anderb., E.Rico & M.M.Mart.Ort.	0	n	1	0	Asteraceae Bercht. & J.Presl
Iz_8_1009	DJI_0927	Peucedanum oreoselinum (L.) Moench	0	n	1	0	Apiaceae Lindl.
Iz_8_1009	DJI_0927	Peucedanum ruthenicum M.Bieb.	0	n	1	0	Apiaceae Lindl.
Iz_8_1009	DJI_0927	Pilosella echioides (Lumn.) F.W.Schultz & Sch.Bip.	0	n	1	0	Asteraceae Bercht. & J.Presl
Iz_8_1009	DJI_0927	Pilosella officinarum F. Schultz et Sch. Bip.	0	n	1	0	Asteraceae Bercht. & J.Presl
Iz_8_1009	DJI_0927	Pilosella sp.	0		NA	0	Asteraceae Bercht. & J.Presl
Iz_8_1009	DJI_0927	Pinus sylvestris L.	0	n	1	1	Pinaceae Spreng. ex F.Rudolphi
Iz_8_1009	DJI_0927	Poa annua L.	0	n	1	0	Poaceae Barnhart
Iz_8_1009	DJI_0927	Poa nemoralis L.	0	n	1	0	Poaceae Barnhart
Iz_8_1009	DJI_0927	Poa sp.	0		NA	0	Poaceae Barnhart
Iz_8_1009	DJI_0927	Polygonatum odoratum (Mill.) Druce	0	n	1	0	Asparagaceae Juss.
Iz_8_1009	DJI_0927	Polygonum arenarium Waldst. & Kit.	0.5	n	1	0	Polygonaceae Juss.
Iz_8_1009	DJI_0927	Populus nigra L.	0	n	1	1	Salicaceae Mirb.
Iz_8_1009	DJI_0927	Populus tremula L.	0	n	1	1	Salicaceae Mirb.
Iz_8_1009	DJI_0927	Populus Ã canescens (Aiton) Sm.	0	n	1	1	Salicaceae Mirb.
Iz_8_1009	DJI_0927	Potentilla argentea L.	0	n	1	0	Rosaceae Juss.
Iz_8_1009	DJI_0927	Psammophiliella muralis (L.) Ikonn.	0	n	1	0	Caryophyllaceae Juss.
Iz_8_1009	DJI_0927	Psephellus sumensis (Kalen.) Greuter	0	n	1	0	Asteraceae Bercht. & J.Presl
Iz_8_1009	DJI_0927	Pulsatilla pratensis (L.) Mill. s.l.	0	n	1	0	Ranunculaceae Juss.
Iz_8_1009	DJI_0927	Pyrus communis L.	0	a	0	1	Rosaceae Juss.
Iz_8_1009	DJI_0927	Quercus robur L.	0	n	1	1	Fagaceae Dumort.
Iz_8_1009	DJI_0927	Robinia pseudoacacia L.	0	a	0	1	Fabaceae Lindl.

Iz_8_1009	DJI_0927	Rosa sp.	0		NA	1	Rosaceae Juss.
Iz_8_1009	DJI_0927	Rubus idaeus L.	0	n	1	0	Rosaceae Juss.
Iz_8_1009	DJI_0927	Rumex acetosella L.	0	n	1	0	Polygonaceae Juss.
Iz_8_1009	DJI_0927	Rumex thyrsiflorus Fingerh.	0	n	1	0	Polygonaceae Juss.
Iz_8_1009	DJI_0927	Salix alba L.	0	n	1	1	Salicaceae Mirb.
Iz_8_1009	DJI_0927	Sambucus nigra L.	0	n	1	1	Viburnaceae Raf.
Iz_8_1009	DJI_0927	Secale sylvestre Host	0	n	1	0	Poaceae Barnhart
Iz_8_1009	DJI_0927	Sempervivum ruthenicum Schnittsp. & C.B. Lehm.	0	n	1	0	Crassulaceae J.St.-Hil.
Iz_8_1009	DJI_0927	Setaria viridis (L.) P. Beauv.	0	n	1	0	Poaceae Barnhart
Iz_8_1009	DJI_0927	Silene borysthenica (Gruner) Walters	0	n	1	0	Caryophyllaceae Juss.
Iz_8_1009	DJI_0927	Silene latifolia subsp. alba (Mill.) Greuter & Burdet	0	n	1	0	Caryophyllaceae Juss.
Iz_8_1009	DJI_0927	Solidago virgaurea L.	0	n	1	0	Asteraceae Bercht. & J.Presl
Iz_8_1009	DJI_0927	Sorbus aucuparia L.	0	n	1	1	Rosaceae Juss.
Iz_8_1009	DJI_0927	Stachys recta L.	0	n	1	0	Lamiacea Lindl.
Iz_8_1009	DJI_0927	Tanacetum vulgare L.	0	n	1	0	Asteraceae Bercht. & J.Presl
Iz_8_1009	DJI_0927	Thymus pallasianus Heinr.Braun	0	n	1	0	Lamiacea Lindl.
Iz_8_1009	DJI_0927	Tilia cordata Mill.	0	n	1	1	Malvaceae Juss.
Iz_8_1009	DJI_0927	Trifolium alpestre L.	0	n	1	0	Fabaceae Lindl.
Iz_8_1009	DJI_0927	Trifolium arvense L.	0	n	1	0	Fabaceae Lindl.
Iz_8_1009	DJI_0927	Ulmus glabra Huds.	0	n	1	1	Ulmaceae Mirb.
Iz_8_1009	DJI_0927	Verbascum densiflorum Bertol.	0	n	1	0	Scrophulariaceae Juss.
Iz_8_1009	DJI_0927	Veronica austriaca L.	0	n	1	0	Plantaginaceae Juss.
Iz_8_1009	DJI_0927	Veronica incana L.	0	n	1	0	Plantaginaceae Juss.
Iz_8_1009	DJI_0927	Veronica verna L.	0	n	1	0	Plantaginaceae Juss.
Iz_8_1009	DJI_0927	Vincetoxicum hirundinaria Medik	0	n	1	0	Apocynaceae Juss.
Iz_8_1009	DJI_0930	Acer tataricum L.	0	n	1	1	Sapindaceae Juss.
Iz_8_1009	DJI_0930	Achillea millefolium L. s.l.	0	n	1	0	Asteraceae Bercht. & J.Presl
Iz_8_1009	DJI_0930	Allium oleraceum L.	0	n	1	0	Amaryllidaceae J.St.-Hil.
Iz_8_1009	DJI_0930	Ambrosia artemisiifolia L.	0	a	0	0	Asteraceae Bercht. & J.Presl
Iz_8_1009	DJI_0930	Amorpha fruticosa L.	0	a	0	0	Fabaceae Lindl.
Iz_8_1009	DJI_0930	Anthericum ramosum L.	0	n	1	0	Asparagaceae Juss.
Iz_8_1009	DJI_0930	Artemisia marschalliana Spreng.	0	n	1	0	Asteraceae Bercht. & J.Presl
Iz_8_1009	DJI_0930	Asparagus officinalis L.	0	n	1	0	Asparagaceae Juss.
Iz_8_1009	DJI_0930	Astragalus cicer L.	0	n	1	0	Fabaceae Lindl.
Iz_8_1009	DJI_0930	Bassia laniflora (S.G.Gmel.) A.J.Scott	0	n	1	0	Amaranthaceae Juss.
Iz_8_1009	DJI_0930	Berberis vulgaris L.	0	n	1	0	Berberidaceae Juss.
Iz_8_1009	DJI_0930	Betula pendula Roth.	0	n	1	1	Betulaceae Gray
Iz_8_1009	DJI_0930	Betula pendula Roth.	0	n	1	0	Poaceae Barnhart
Iz_8_1009	DJI_0930	Bromus inermis Leyss.	0		NA	NA	
Iz_8_1009	DJI_0930	Bromus squarrosus L.	0	a	0	0	Poaceae Barnhart
Iz_8_1009	DJI_0930	Bromus tectorum L.	0	a	0	0	Poaceae Barnhart
Iz_8_1009	DJI_0930	Calamagrostis epigejos (L.) Roth	0.5	n	1	0	Poaceae Barnhart
Iz_8_1009	DJI_0930	Carduus acanthoides L.	0	n	1	0	Asteraceae Bercht. & J.Presl
Iz_8_1009	DJI_0930	Carex sp.	0	n	1	0	Cyperaceae Juss.
Iz_8_1009	DJI_0930	Centaurea majorovii Dumbadze	0	n	1	0	Asteraceae Bercht. & J.Presl
Iz_8_1009	DJI_0930	Chamaecytisus ruthenicus (Fisch.ex Wol.) Klaskova	0	n	1	0	Fabaceae Lindl.
Iz_8_1009	DJI_0930	Chelidonium majus L.	0	n	1	0	Papaveraceae Juss.
Iz_8_1009	DJI_0930	Chenopodium betaceum Andrz.	0	n	1	0	Amaranthaceae Juss.
Iz_8_1009	DJI_0930	Chenopodium cf. album	0	n	1	0	Amaranthaceae Juss.
Iz_8_1009	DJI_0930	Chenopodium sp.	0		NA	0	Amaranthaceae Juss.
Iz_8_1009	DJI_0930	Chondrilla juncea L.	0	n	1	0	Asteraceae Bercht. & J.Presl
Iz_8_1009	DJI_0930	Cirsium vulgare (Savi) Ten.	0	n	1	0	Asteraceae Bercht. & J.Presl
Iz_8_1009	DJI_0930	Clinopodium acinos (L.) Kuntze	0	n	1	0	Lamiacea Lindl.
Iz_8_1009	DJI_0930	Convallaria majalis L.	0	n	1	0	Asparagaceae Juss.
Iz_8_1009	DJI_0930	Crataegus rhipidophylla Gand.	0	n	1	1	Rosaceae Juss.
Iz_8_1009	DJI_0930	Crepis foetida L.	0	n	1	0	Asteraceae Bercht. & J.Presl
Iz_8_1009	DJI_0930	Dactylis glomerata L.	0	n	1	0	Poaceae Barnhart
Iz_8_1009	DJI_0930	Dianthus campestris M.Bieb.	0	n	1	0	Caryophyllaceae Juss.
Iz_8_1009	DJI_0930	Digitaria sanguinalis (L.) Scop.	0	a	0	0	Poaceae Barnhart
Iz_8_1009	DJI_0930	Elymus repens (L.) Gould	0	n	1	0	Poaceae Barnhart
Iz_8_1009	DJI_0930	Epilobium tetragonum L.	0	n	1	0	Onagraceae Juss.
Iz_8_1009	DJI_0930	Eragrostis minor Host	0	a	0	0	Poaceae Barnhart
Iz_8_1009	DJI_0930	Erigeron canadensis L.	5	a	0	0	Asteraceae Bercht. & J.Presl
Iz_8_1009	DJI_0930	Euphorbia seguieriana Neck.	0	n	1	0	Euphorbiaceae Juss.
Iz_8_1009	DJI_0930	Fallopia dumetorum (L.) Holub	0	n	1	0	Polygonaceae Juss.
Iz_8_1009	DJI_0930	Festuca beckeri (Hack.) Trautv.	0	n	1	0	Poaceae Barnhart
Iz_8_1009	DJI_0930	Fragaria vesca L.	0	n	1	0	Rosaceae Juss.
Iz_8_1009	DJI_0930	Frangula alnus Mill.	0	n	1	1	Rhamnaceae Juss.
Iz_8_1009	DJI_0930	Fraxinus pennsylvanica Marshall	0	a	0	1	Oleaceae Hoffmanns. & Link
Iz_8_1009	DJI_0930	Galeopsis bifida Boenn.	0	n	1	0	Lamiacea Lindl.
Iz_8_1009	DJI_0930	Galium verum L.	0	n	1	0	Rubiaceae Juss.
Iz_8_1009	DJI_0930	Genista tinctoria L.	0	n	1	0	Fabaceae Lindl.
Iz_8_1009	DJI_0930	Geranium robertianum L.	0	n	1	0	Geraniaceae Juss.
Iz_8_1009	DJI_0930	Geranium sanquineum L.	0	n	1	0	Geraniaceae Juss.
Iz_8_1009	DJI_0930	Gypsophila paniculata L.	0	n	1	0	Caryophyllaceae Juss.
Iz_8_1009	DJI_0930	Helichrysum arenarium (L.) Moench	0	n	1	0	Asteraceae Bercht. & J.Presl
Iz_8_1009	DJI_0930	Herniaria polygama J.Gay	0	n	1	0	Caryophyllaceae Juss.
Iz_8_1009	DJI_0930	Hieracium robustum Fr.	0	n	1	0	Asteraceae Bercht. & J.Presl
Iz_8_1009	DJI_0930	Hieracium umbellatum L.	0	n	1	NA	Asteraceae Bercht. & J.Presl
Iz_8_1009	DJI_0930	Hylotelephium maximum (L.) Holub	0	n	1	0	Crassulaceae J.St.-Hil.
Iz_8_1009	DJI_0930	Jacobaea borysthenica (DC.) B.Nord. & Greuter	0	n	1	0	Asteraceae Bercht. & J.Presl
Iz_8_1009	DJI_0930	Jasione montana L.	0	n	1	0	Campanulaceae Juss.
Iz_8_1009	DJI_0930	Jurinea cyanoides (L.) Rchb.s.l.	0	n	1	0	Asteraceae Bercht. & J.Presl
Iz_8_1009	DJI_0930	Koeleria glauca (Spreng.) DC.	0	n	1	0	Poaceae Barnhart
Iz_8_1009	DJI_0930	Lactuca serriola L.	0	a	0	0	Asteraceae Bercht. & J.Presl
Iz_8_1009	DJI_0930	Linaria genistifolia (L.) Mill.	0	n	1	0	Plantaginaceae Juss.
Iz_8_1009	DJI_0930	Lomelosia argentea (L.) Greuter & Burdet	0	n	1	0	Caprifoliaceae Juss.
Iz_8_1009	DJI_0930	Melampyrum pratense L.	0	n	1	0	Orobanchaceae Vent.
Iz_8_1009	DJI_0930	Oenothera biennis L.	0	a	0	0	Onagraceae Juss.
Iz_8_1009	DJI_0930	Pentanema britannica (L.) D.Gut.Larr., Santos-Vicente, Anderb., E.Rico & M.M.Mart.Ort.	0	n	1	0	Asteraceae Bercht. & J.Presl
Iz_8_1009	DJI_0930	Peucedanum oreoselinum (L.) Moench	0	n	1	0	Apiaceae Lindl.
Iz_8_1009	DJI_0930	Peucedanum ruthenicum M.Bieb.	0	n	1	0	Apiaceae Lindl.
Iz_8_1009	DJI_0930	Pilosella echioides (Lumn.) F.W.Schultz & Sch.Bip.	0	n	1	0	Asteraceae Bercht. & J.Presl
Iz_8_1009	DJI_0930	Pilosella officinarum F. Schultz et Sch. Bip.	0	n	1	0	Asteraceae Bercht. & J.Presl
Iz_8_1009	DJI_0930	Pilosella sp.	0		NA	0	Asteraceae Bercht. & J.Presl
Iz_8_1009	DJI_0930	Pinus sylvestris L.	0	n	1	1	Pinaceae Spreng. ex F.Rudolphi
Iz_8_1009	DJI_0930	Poa annua L.	0	n	1	0	Poaceae Barnhart
Iz_8_1009	DJI_0930	Poa nemoralis L.	0	n	1	0	Poaceae Barnhart

Iz_8_1009	DJI_0930	Poa sp.	0		NA	0	Poaceae Barnhart
Iz_8_1009	DJI_0930	Polygonatum odoratum (Mill.) Druce	0	n	1	0	Asparagaceae Juss.
Iz_8_1009	DJI_0930	Polygonum arenarium Waldst. & Kit.	0	n	1	0	Polygonaceae Juss.
Iz_8_1009	DJI_0930	Populus nigra L.	0	n	1	1	Salicaceae Mirb.
Iz_8_1009	DJI_0930	Populus tremula L.	0	n	1	1	Salicaceae Mirb.
Iz_8_1009	DJI_0930	Populus Ã canescens (Aiton) Sm.	0	n	1	1	Salicaceae Mirb.
Iz_8_1009	DJI_0930	Potentilla argentea L.	0	n	1	0	Rosaceae Juss.
Iz_8_1009	DJI_0930	Psammophiliella muralis (L.) Ikonn.	0	n	1	0	Caryophyllaceae Juss.
Iz_8_1009	DJI_0930	Psephellus sumensis (Kalen.) Greuter	0	n	1	0	Asteraceae Bercht. & J.Presl
Iz_8_1009	DJI_0930	Pulsatilla pratensis (L.) Mill. s.l.	0	n	1	0	Ranunculaceae Juss.
Iz_8_1009	DJI_0930	Pyrus communis L.	0	a	0	1	Rosaceae Juss.
Iz_8_1009	DJI_0930	Quercus robur L.	0	n	1	1	Fagaceae Dumort.
Iz_8_1009	DJI_0930	Robinia pseudoacacia L.	0	a	0	1	Fabaceae Lindl.
Iz_8_1009	DJI_0930	Rosa sp.	0		NA	1	Rosaceae Juss.
Iz_8_1009	DJI_0930	Rubus idaeus L.	0	n	1	0	Rosaceae Juss.
Iz_8_1009	DJI_0930	Rumex acetosella L.	0	n	1	0	Polygonaceae Juss.
Iz_8_1009	DJI_0930	Rumex thyrsiflorus Fingerh.	0	n	1	0	Polygonaceae Juss.
Iz_8_1009	DJI_0930	Salix alba L.	0	n	1	1	Salicaceae Mirb.
Iz_8_1009	DJI_0930	Sambucus nigra L.	0	n	1	1	Viburnaceae Raf.
Iz_8_1009	DJI_0930	Secale sylvestre Host	0	n	1	0	Poaceae Barnhart
Iz_8_1009	DJI_0930	Sempervivum ruthenicum Schnittsp. & C.B. Lehm.	0	n	1	0	Crassulaceae J.St.-Hil.
Iz_8_1009	DJI_0930	Setaria viridis (L.) P. Beauv.	0	n	1	0	Poaceae Barnhart
Iz_8_1009	DJI_0930	Silene borysthenica (Gruner) Walters	0	n	1	0	Caryophyllaceae Juss.
Iz_8_1009	DJI_0930	Silene latifolia subsp. alba (Mill.) Greuter & Burdet	0	n	1	0	Caryophyllaceae Juss.
Iz_8_1009	DJI_0930	Solidago virgaurea L.	0	n	1	0	Asteraceae Bercht. & J.Presl
Iz_8_1009	DJI_0930	Sorbus aucuparia L.	0	n	1	1	Rosaceae Juss.
Iz_8_1009	DJI_0930	Stachys recta L.	0	n	1	0	Lamiacea Lindl.
Iz_8_1009	DJI_0930	Tanacetum vulgare L.	0	n	1	0	Asteraceae Bercht. & J.Presl
Iz_8_1009	DJI_0930	Thymus pallasianus Heinr.Braun	0	n	1	0	Lamiacea Lindl.
Iz_8_1009	DJI_0930	Tilia cordata Mill.	0	n	1	1	Malvaceae Juss.
Iz_8_1009	DJI_0930	Trifolium alpestre L.	0	n	1	0	Fabaceae Lindl.
Iz_8_1009	DJI_0930	Trifolium arvense L.	0	n	1	0	Fabaceae Lindl.
Iz_8_1009	DJI_0930	Ulmus glabra Huds.	0	n	1	1	Ulmaceae Mirb.
Iz_8_1009	DJI_0930	Verbascum densiflorum Bertol.	0	n	1	0	Scrophulariaceae Juss.
Iz_8_1009	DJI_0930	Veronica austriaca L.	0	n	1	0	Plantaginaceae Juss.
Iz_8_1009	DJI_0930	Veronica incana L.	0	n	1	0	Plantaginaceae Juss.
Iz_8_1009	DJI_0930	Veronica verna L.	0	n	1	0	Plantaginaceae Juss.
Iz_8_1009	DJI_0930	Vincetoxicum hirundinaria Medik	0	n	1	0	Apocynaceae Juss.
Iz_8_1009	DJI_0931	Acer tataricum L.	0	n	1	1	Sapindaceae Juss.
Iz_8_1009	DJI_0931	Achillea millefolium L. s.l.	0	n	1	0	Asteraceae Bercht. & J.Presl
Iz_8_1009	DJI_0931	Allium oleraceum L.	0	n	1	0	Amaryllidaceae J.St.-Hil.
Iz_8_1009	DJI_0931	Ambrosia artemisiifolia L.	0	a	0	0	Asteraceae Bercht. & J.Presl
Iz_8_1009	DJI_0931	Amorpha fruticosa L.	0	a	0	0	Fabaceae Lindl.
Iz_8_1009	DJI_0931	Anthericum ramosum L.	0	n	1	0	Asparagaceae Juss.
Iz_8_1009	DJI_0931	Artemisia marschalliana Spreng.	0	n	1	0	Asteraceae Bercht. & J.Presl
Iz_8_1009	DJI_0931	Asparagus officinalis L.	0	n	1	0	Asparagaceae Juss.
Iz_8_1009	DJI_0931	Astragalus cicer L.	0	n	1	0	Fabaceae Lindl.
Iz_8_1009	DJI_0931	Bassia laniflora (S.G.Gmel.) A.J.Scott	0	n	1	0	Amaranthaceae Juss.
Iz_8_1009	DJI_0931	Berberis vulgaris L.	0	n	1	0	Berberidaceae Juss.
Iz_8_1009	DJI_0931	Betula pendula Roth.	0	n	1	1	Betulaceae Gray
Iz_8_1009	DJI_0931	Betula pendula Roth.	0	n	1	0	Poaceae Barnhart
Iz_8_1009	DJI_0931	Bromus inermis Leyss.	0		NA	NA	
Iz_8_1009	DJI_0931	Bromus squarrosus L.	0	a	0	0	Poaceae Barnhart
Iz_8_1009	DJI_0931	Bromus tectorum L.	0	a	0	0	Poaceae Barnhart
Iz_8_1009	DJI_0931	Calamagrostis epigejos (L.) Roth	0	n	1	0	Poaceae Barnhart
Iz_8_1009	DJI_0931	Carduus acanthoides L.	0	n	1	0	Asteraceae Bercht. & J.Presl
Iz_8_1009	DJI_0931	Carex sp.	0	n	1	0	Cyperaceae Juss.
Iz_8_1009	DJI_0931	Centaurea majorovii Dumbadze	0	n	1	0	Asteraceae Bercht. & J.Presl
Iz_8_1009	DJI_0931	Chamaecytisus ruthenicus (Fisch.ex Wol.) Klaskova	0	n	1	0	Fabaceae Lindl.
Iz_8_1009	DJI_0931	Chelidonium majus L.	0	n	1	0	Papaveraceae Juss.
Iz_8_1009	DJI_0931	Chenopodium betaceum Andrz.	0	n	1	0	Amaranthaceae Juss.
Iz_8_1009	DJI_0931	Chenopodium cf. album	0	n	1	0	Amaranthaceae Juss.
Iz_8_1009	DJI_0931	Chenopodium sp.	0		NA	0	Amaranthaceae Juss.
Iz_8_1009	DJI_0931	Chondrilla juncea L.	0	n	1	0	Asteraceae Bercht. & J.Presl
Iz_8_1009	DJI_0931	Cirsium vulgare (Savi) Ten.	0	n	1	0	Asteraceae Bercht. & J.Presl
Iz_8_1009	DJI_0931	Clinopodium acinos (L.) Kuntze	0	n	1	0	Lamiacea Lindl.
Iz_8_1009	DJI_0931	Convallaria majalis L.	0	n	1	0	Asparagaceae Juss.
Iz_8_1009	DJI_0931	Crataegus rhipidophylla Gand.	0	n	1	1	Rosaceae Juss.
Iz_8_1009	DJI_0931	Crepis foetida L.	0	n	1	0	Asteraceae Bercht. & J.Presl
Iz_8_1009	DJI_0931	Dactylis glomerata L.	0	n	1	0	Poaceae Barnhart
Iz_8_1009	DJI_0931	Dianthus campestris M.Bieb.	0	n	1	0	Caryophyllaceae Juss.
Iz_8_1009	DJI_0931	Digitaria sanguinalis (L.) Scop.	0	a	0	0	Poaceae Barnhart
Iz_8_1009	DJI_0931	Elymus repens (L.) Gould	0	n	1	0	Poaceae Barnhart
Iz_8_1009	DJI_0931	Epilobium tetragonum L.	0	n	1	0	Onagraceae Juss.
Iz_8_1009	DJI_0931	Eragrostis minor Host	0	a	0	0	Poaceae Barnhart
Iz_8_1009	DJI_0931	Erigeron canadensis L.	25	a	0	0	Asteraceae Bercht. & J.Presl
Iz_8_1009	DJI_0931	Euphorbia seguieriana Neck.	0	n	1	0	Euphorbiaceae Juss.
Iz_8_1009	DJI_0931	Fallopia dumetorum (L.) Holub	0	n	1	0	Polygonaceae Juss.
Iz_8_1009	DJI_0931	Festuca beckeri (Hack.) Trautv.	0	n	1	0	Poaceae Barnhart
Iz_8_1009	DJI_0931	Fragaria vesca L.	0	n	1	0	Rosaceae Juss.
Iz_8_1009	DJI_0931	Frangula alnus Mill.	0	n	1	1	Rhamnaceae Juss.
Iz_8_1009	DJI_0931	Fraxinus pennsylvanica Marshall	0	a	0	1	Oleaceae Hoffmanns. & Link
Iz_8_1009	DJI_0931	Galeopsis bifida Boenn.	0	n	1	0	Lamiacea Lindl.
Iz_8_1009	DJI_0931	Galium verum L.	0	n	1	0	Rubiaceae Juss.
Iz_8_1009	DJI_0931	Genista tinctoria L.	0	n	1	0	Fabaceae Lindl.
Iz_8_1009	DJI_0931	Geranium robertianum L.	0	n	1	0	Geraniaceae Juss.
Iz_8_1009	DJI_0931	Geranium sanquineum L.	0	n	1	0	Geraniaceae Juss.
Iz_8_1009	DJI_0931	Gypsophila paniculata L.	0	n	1	0	Caryophyllaceae Juss.
Iz_8_1009	DJI_0931	Helichrysum arenarium (L.) Moench	0	n	1	0	Asteraceae Bercht. & J.Presl
Iz_8_1009	DJI_0931	Herniaria polygama J.Gay	0	n	1	0	Caryophyllaceae Juss.
Iz_8_1009	DJI_0931	Hieracium robustum Fr.	0	n	1	0	Asteraceae Bercht. & J.Presl
Iz_8_1009	DJI_0931	Hieracium umbellatum L.	0	n	1	NA	Asteraceae Bercht. & J.Presl
Iz_8_1009	DJI_0931	Hylotelephium maximum (L.) Holub	0	n	1	0	Crassulaceae J.St.-Hil.
Iz_8_1009	DJI_0931	Jacobaea borysthenica (DC.) B.Nord. & Greuter	0	n	1	0	Asteraceae Bercht. & J.Presl
Iz_8_1009	DJI_0931	Jasione montana L.	0	n	1	0	Campanulaceae Juss.
Iz_8_1009	DJI_0931	Jurinea cyanoides (L.) Rchb.s.l.	0	n	1	0	Asteraceae Bercht. & J.Presl
Iz_8_1009	DJI_0931	Koeleria glauca (Spreng.) DC.	0	n	1	0	Poaceae Barnhart
Iz_8_1009	DJI_0931	Lactuca serriola L.	0	a	0	0	Asteraceae Bercht. & J.Presl

Iz_8_1009	DJI_0931	Linaria genistifolia (L.) Mill.	0	n	1	0	Plantaginaceae Juss.
Iz_8_1009	DJI_0931	Lomelosia argentea (L.) Greuter & Burdet	0	n	1	0	Caprifoliaceae Juss.
Iz_8_1009	DJI_0931	Melampyrum pratense L.	0	n	1	0	Orobanchaceae Vent.
Iz_8_1009	DJI_0931	Oenothera biennis L.	0	a	0	0	Onagraceae Juss.
Iz_8_1009	DJI_0931	Pentanema britannica (L.) D.Gut.Larr., Santos-Vicente, Anderb., E.Rico & M.M.Mart.Ort.	0	n	1	0	Asteraceae Bercht. & J.Presl
Iz_8_1009	DJI_0931	Peucedanum oreoselinum (L.) Moench	0	n	1	0	Apiaceae Lindl.
Iz_8_1009	DJI_0931	Peucedanum ruthenicum M.Bieb.	0	n	1	0	Apiaceae Lindl.
Iz_8_1009	DJI_0931	Pilosella echioides (Lumn.) F.W.Schultz & Sch.Bip.	0	n	1	0	Asteraceae Bercht. & J.Presl
Iz_8_1009	DJI_0931	Pilosella officinarum F. Schultz et Sch. Bip.	0	n	1	0	Asteraceae Bercht. & J.Presl
Iz_8_1009	DJI_0931	Pilosella sp.	0		NA	0	Asteraceae Bercht. & J.Presl
Iz_8_1009	DJI_0931	Pinus sylvestris L.	0	n	1	1	Pinaceae Spreng. ex F.Rudolphi
Iz_8_1009	DJI_0931	Poa annua L.	0	n	1	0	Poaceae Barnhart
Iz_8_1009	DJI_0931	Poa nemoralis L.	0	n	1	0	Poaceae Barnhart
Iz_8_1009	DJI_0931	Poa sp.	0		NA	0	Poaceae Barnhart
Iz_8_1009	DJI_0931	Polygonatum odoratum (Mill.) Druce	0	n	1	0	Asparagaceae Juss.
Iz_8_1009	DJI_0931	Polygonum arenarium Waldst. & Kit.	0	n	1	0	Polygonaceae Juss.
Iz_8_1009	DJI_0931	Populus nigra L.	0	n	1	1	Salicaceae Mirb.
Iz_8_1009	DJI_0931	Populus tremula L.	0	n	1	1	Salicaceae Mirb.
Iz_8_1009	DJI_0931	Populus Ã canescens (Aiton) Sm.	0	n	1	1	Salicaceae Mirb.
Iz_8_1009	DJI_0931	Potentilla argentea L.	0	n	1	0	Rosaceae Juss.
Iz_8_1009	DJI_0931	Psammophiliella muralis (L.) Ikonn.	0	n	1	0	Caryophyllaceae Juss.
Iz_8_1009	DJI_0931	Psephellus sumensis (Kalen.) Greuter	0	n	1	0	Asteraceae Bercht. & J.Presl
Iz_8_1009	DJI_0931	Pulsatilla pratensis (L.) Mill. s.l.	0	n	1	0	Ranunculaceae Juss.
Iz_8_1009	DJI_0931	Pyrus communis L.	0	a	0	1	Rosaceae Juss.
Iz_8_1009	DJI_0931	Quercus robur L.	0	n	1	1	Fagaceae Dumort.
Iz_8_1009	DJI_0931	Robinia pseudoacacia L.	0	a	0	1	Fabaceae Lindl.
Iz_8_1009	DJI_0931	Rosa sp.	0		NA	1	Rosaceae Juss.
Iz_8_1009	DJI_0931	Rubus idaeus L.	0	n	1	0	Rosaceae Juss.
Iz_8_1009	DJI_0931	Rumex acetosella L.	0	n	1	0	Polygonaceae Juss.
Iz_8_1009	DJI_0931	Rumex thyrsiflorus Fingerh.	0	n	1	0	Polygonaceae Juss.
Iz_8_1009	DJI_0931	Salix alba L.	0	n	1	1	Salicaceae Mirb.
Iz_8_1009	DJI_0931	Sambucus nigra L.	0	n	1	1	Viburnaceae Raf.
Iz_8_1009	DJI_0931	Secale sylvestre Host	0	n	1	0	Poaceae Barnhart
Iz_8_1009	DJI_0931	Sempervivum ruthenicum Schnittsp. & C.B. Lehm.	0	n	1	0	Crassulaceae J.St.-Hil.
Iz_8_1009	DJI_0931	Setaria viridis (L.) P. Beauv.	0	n	1	0	Poaceae Barnhart
Iz_8_1009	DJI_0931	Silene borysthenica (Gruner) Walters	0	n	1	0	Caryophyllaceae Juss.
Iz_8_1009	DJI_0931	Silene latifolia subsp. alba (Mill.) Greuter & Burdet	0	n	1	0	Caryophyllaceae Juss.
Iz_8_1009	DJI_0931	Solidago virgaurea L.	0	n	1	0	Asteraceae Bercht. & J.Presl
Iz_8_1009	DJI_0931	Sorbus aucuparia L.	0	n	1	1	Rosaceae Juss.
Iz_8_1009	DJI_0931	Stachys recta L.	0	n	1	0	Lamiacea Lindl.
Iz_8_1009	DJI_0931	Tanacetum vulgare L.	0	n	1	0	Asteraceae Bercht. & J.Presl
Iz_8_1009	DJI_0931	Thymus pallasianus Heinr.Braun	0	n	1	0	Lamiacea Lindl.
Iz_8_1009	DJI_0931	Tilia cordata Mill.	0	n	1	1	Malvaceae Juss.
Iz_8_1009	DJI_0931	Trifolium alpestre L.	0	n	1	0	Fabaceae Lindl.
Iz_8_1009	DJI_0931	Trifolium arvense L.	0	n	1	0	Fabaceae Lindl.
Iz_8_1009	DJI_0931	Ulmus glabra Huds.	0	n	1	1	Ulmaceae Mirb.
Iz_8_1009	DJI_0931	Verbascum densiflorum Bertol.	0	n	1	0	Scrophulariaceae Juss.
Iz_8_1009	DJI_0931	Veronica austriaca L.	0	n	1	0	Plantaginaceae Juss.
Iz_8_1009	DJI_0931	Veronica incana L.	0	n	1	0	Plantaginaceae Juss.
Iz_8_1009	DJI_0931	Veronica verna L.	0	n	1	0	Plantaginaceae Juss.
Iz_8_1009	DJI_0931	Vincetoxicum hirundinaria Medik	0	n	1	0	Apocynaceae Juss.
Iz_8_1009	DJI_0932	Acer tataricum L.	0	n	1	1	Sapindaceae Juss.
Iz_8_1009	DJI_0932	Achillea millefolium L. s.l.	0	n	1	0	Asteraceae Bercht. & J.Presl
Iz_8_1009	DJI_0932	Allium oleraceum L.	0	n	1	0	Amaryllidaceae J.St.-Hil.
Iz_8_1009	DJI_0932	Ambrosia artemisiifolia L.	0	a	0	0	Asteraceae Bercht. & J.Presl
Iz_8_1009	DJI_0932	Amorpha fruticosa L.	0	a	0	0	Fabaceae Lindl.
Iz_8_1009	DJI_0932	Anthericum ramosum L.	0	n	1	0	Asparagaceae Juss.
Iz_8_1009	DJI_0932	Artemisia marschalliana Spreng.	0	n	1	0	Asteraceae Bercht. & J.Presl
Iz_8_1009	DJI_0932	Asparagus officinalis L.	0	n	1	0	Asparagaceae Juss.
Iz_8_1009	DJI_0932	Astragalus cicer L.	0	n	1	0	Fabaceae Lindl.
Iz_8_1009	DJI_0932	Bassia laniflora (S.G.Gmel.) A.J.Scott	0	n	1	0	Amaranthaceae Juss.
Iz_8_1009	DJI_0932	Berberis vulgaris L.	0	n	1	0	Berberidaceae Juss.
Iz_8_1009	DJI_0932	Betula pendula Roth.	0	n	1	1	Betulaceae Gray
Iz_8_1009	DJI_0932	Betula pendula Roth.	0	n	1	0	Poaceae Barnhart
Iz_8_1009	DJI_0932	Bromus inermis Leyss.	0		NA	NA	
Iz_8_1009	DJI_0932	Bromus squarrosus L.	0	a	0	0	Poaceae Barnhart
Iz_8_1009	DJI_0932	Bromus tectorum L.	0	a	0	0	Poaceae Barnhart
Iz_8_1009	DJI_0932	Calamagrostis epigejos (L.) Roth	0	n	1	0	Poaceae Barnhart
Iz_8_1009	DJI_0932	Carduus acanthoides L.	0	n	1	0	Asteraceae Bercht. & J.Presl
Iz_8_1009	DJI_0932	Carex sp.	0	n	1	0	Cyperaceae Juss.
Iz_8_1009	DJI_0932	Centaurea majorovii Dumbadze	0	n	1	0	Asteraceae Bercht. & J.Presl
Iz_8_1009	DJI_0932	Chamaecytisus ruthenicus (Fisch.ex Wol.) Klaskova	0	n	1	0	Fabaceae Lindl.
Iz_8_1009	DJI_0932	Chelidonium majus L.	0	n	1	0	Papaveraceae Juss.
Iz_8_1009	DJI_0932	Chenopodium betaceum Andrz.	0	n	1	0	Amaranthaceae Juss.
Iz_8_1009	DJI_0932	Chenopodium cf. album	0	n	1	0	Amaranthaceae Juss.
Iz_8_1009	DJI_0932	Chenopodium sp.	0		NA	0	Amaranthaceae Juss.
Iz_8_1009	DJI_0932	Chondrilla juncea L.	0	n	1	0	Asteraceae Bercht. & J.Presl
Iz_8_1009	DJI_0932	Cirsium vulgare (Savi) Ten.	0	n	1	0	Asteraceae Bercht. & J.Presl
Iz_8_1009	DJI_0932	Clinopodium acinos (L.) Kuntze	0	n	1	0	Lamiacea Lindl.
Iz_8_1009	DJI_0932	Convallaria majalis L.	0	n	1	0	Asparagaceae Juss.
Iz_8_1009	DJI_0932	Crataegus rhipidophylla Gand.	0	n	1	1	Rosaceae Juss.
Iz_8_1009	DJI_0932	Crepis foetida L.	0	n	1	0	Asteraceae Bercht. & J.Presl
Iz_8_1009	DJI_0932	Dactylis glomerata L.	0	n	1	0	Poaceae Barnhart
Iz_8_1009	DJI_0932	Dianthus campestris M.Bieb.	0	n	1	0	Caryophyllaceae Juss.
Iz_8_1009	DJI_0932	Digitaria sanguinalis (L.) Scop.	0	a	0	0	Poaceae Barnhart
Iz_8_1009	DJI_0932	Elymus repens (L.) Gould	0	n	1	0	Poaceae Barnhart
Iz_8_1009	DJI_0932	Epilobium tetragonum L.	0	n	1	0	Onagraceae Juss.
Iz_8_1009	DJI_0932	Eragrostis minor Host	0	a	0	0	Poaceae Barnhart
Iz_8_1009	DJI_0932	Erigeron canadensis L.	10	a	0	0	Asteraceae Bercht. & J.Presl
Iz_8_1009	DJI_0932	Euphorbia seguieriana Neck.	0	n	1	0	Euphorbiaceae Juss.
Iz_8_1009	DJI_0932	Fallopia dumetorum (L.) Holub	0	n	1	0	Polygonaceae Juss.
Iz_8_1009	DJI_0932	Festuca beckeri (Hack.) Trautv.	0	n	1	0	Poaceae Barnhart
Iz_8_1009	DJI_0932	Fragaria vesca L.	0	n	1	0	Rosaceae Juss.
Iz_8_1009	DJI_0932	Frangula alnus Mill.	0	n	1	1	Rhamnaceae Juss.
Iz_8_1009	DJI_0932	Fraxinus pennsylvanica Marshall	0	a	0	1	Oleaceae Hoffmanns. & Link
Iz_8_1009	DJI_0932	Galeopsis bifida Boenn.	0	n	1	0	Lamiacea Lindl.
Iz_8_1009	DJI_0932	Galium verum L.	0	n	1	0	Rubiaceae Juss.
Iz_8_1009	DJI_0932	Genista tinctoria L.	0	n	1	0	Fabaceae Lindl.

Iz_8_1009	DJI_0932	Geranium robertianum L.	0	n	1	0	Geraniaceae Juss.
Iz_8_1009	DJI_0932	Geranium sanquineum L.	0	n	1	0	Geraniaceae Juss.
Iz_8_1009	DJI_0932	Gypsophila paniculata L.	0	n	1	0	Caryophyllaceae Juss.
Iz_8_1009	DJI_0932	Helichrysum arenarium (L.) Moench	0	n	1	0	Asteraceae Bercht. & J.Presl
Iz_8_1009	DJI_0932	Herniaria polygama J.Gay	0	n	1	0	Caryophyllaceae Juss.
Iz_8_1009	DJI_0932	Hieracium robustum Fr.	0	n	1	0	Asteraceae Bercht. & J.Presl
Iz_8_1009	DJI_0932	Hieracium umbellatum L.	0	n	1	NA	Asteraceae Bercht. & J.Presl
Iz_8_1009	DJI_0932	Hylotelephium maximum (L.) Holub	0	n	1	0	Crassulaceae J.St.-Hil.
Iz_8_1009	DJI_0932	Jacobaea borysthenica (DC.) B.Nord. & Greuter	0	n	1	0	Asteraceae Bercht. & J.Presl
Iz_8_1009	DJI_0932	Jasione montana L.	0	n	1	0	Campanulaceae Juss.
Iz_8_1009	DJI_0932	Jurinea cyanoides (L.) Rchb.s.l.	0	n	1	0	Asteraceae Bercht. & J.Presl
Iz_8_1009	DJI_0932	Koeleria glauca (Spreng.) DC.	0	n	1	0	Poaceae Barnhart
Iz_8_1009	DJI_0932	Lactuca serriola L.	0	a	0	0	Asteraceae Bercht. & J.Presl
Iz_8_1009	DJI_0932	Linaria genistifolia (L.) Mill.	0	n	1	0	Plantaginaceae Juss.
Iz_8_1009	DJI_0932	Lomelosia argentea (L.) Greuter & Burdet	0	n	1	0	Caprifoliaceae Juss.
Iz_8_1009	DJI_0932	Melampyrum pratense L.	0	n	1	0	Orobanchaceae Vent.
Iz_8_1009	DJI_0932	Oenothera biennis L.	0	a	0	0	Onagraceae Juss.
Iz_8_1009	DJI_0932	Pentanema britannica (L.) D.Gut.Larr., Santos-Vicente, Anderb., E.Rico & M.M.Mart.Ort.	0	n	1	0	Asteraceae Bercht. & J.Presl
Iz_8_1009	DJI_0932	Peucedanum oreoselinum (L.) Moench	0	n	1	0	Apiaceae Lindl.
Iz_8_1009	DJI_0932	Peucedanum ruthenicum M.Bieb.	0	n	1	0	Apiaceae Lindl.
Iz_8_1009	DJI_0932	Pilosella echioides (Lumn.) F.W.Schultz & Sch.Bip.	0	n	1	0	Asteraceae Bercht. & J.Presl
Iz_8_1009	DJI_0932	Pilosella officinarum F. Schultz et Sch. Bip.	0	n	1	0	Asteraceae Bercht. & J.Presl
Iz_8_1009	DJI_0932	Pilosella sp.	0		NA	0	Asteraceae Bercht. & J.Presl
Iz_8_1009	DJI_0932	Pinus sylvestris L.	0	n	1	1	Pinaceae Spreng. ex F.Rudolphi
Iz_8_1009	DJI_0932	Poa annua L.	0	n	1	0	Poaceae Barnhart
Iz_8_1009	DJI_0932	Poa nemoralis L.	0	n	1	0	Poaceae Barnhart
Iz_8_1009	DJI_0932	Poa sp.	0		NA	0	Poaceae Barnhart
Iz_8_1009	DJI_0932	Polygonatum odoratum (Mill.) Druce	0	n	1	0	Asparagaceae Juss.
Iz_8_1009	DJI_0932	Polygonum arenarium Waldst. & Kit.	0	n	1	0	Polygonaceae Juss.
Iz_8_1009	DJI_0932	Populus nigra L.	0	n	1	1	Salicaceae Mirb.
Iz_8_1009	DJI_0932	Populus tremula L.	0	n	1	1	Salicaceae Mirb.
Iz_8_1009	DJI_0932	Populus Ã canescens (Aiton) Sm.	0	n	1	1	Salicaceae Mirb.
Iz_8_1009	DJI_0932	Potentilla argentea L.	0	n	1	0	Rosaceae Juss.
Iz_8_1009	DJI_0932	Psammophiliella muralis (L.) Ikonn.	0	n	1	0	Caryophyllaceae Juss.
Iz_8_1009	DJI_0932	Psephellus sumensis (Kalen.) Greuter	0	n	1	0	Asteraceae Bercht. & J.Presl
Iz_8_1009	DJI_0932	Pulsatilla pratensis (L.) Mill. s.l.	0	n	1	0	Ranunculaceae Juss.
Iz_8_1009	DJI_0932	Pyrus communis L.	0	a	0	1	Rosaceae Juss.
Iz_8_1009	DJI_0932	Quercus robur L.	0	n	1	1	Fagaceae Dumort.
Iz_8_1009	DJI_0932	Robinia pseudoacacia L.	0	a	0	1	Fabaceae Lindl.
Iz_8_1009	DJI_0932	Rosa sp.	0		NA	1	Rosaceae Juss.
Iz_8_1009	DJI_0932	Rubus idaeus L.	0	n	1	0	Rosaceae Juss.
Iz_8_1009	DJI_0932	Rumex acetosella L.	0	n	1	0	Polygonaceae Juss.
Iz_8_1009	DJI_0932	Rumex thyrsiflorus Fingerh.	0	n	1	0	Polygonaceae Juss.
Iz_8_1009	DJI_0932	Salix alba L.	0	n	1	1	Salicaceae Mirb.
Iz_8_1009	DJI_0932	Sambucus nigra L.	0	n	1	1	Viburnaceae Raf.
Iz_8_1009	DJI_0932	Secale sylvestre Host	0	n	1	0	Poaceae Barnhart
Iz_8_1009	DJI_0932	Sempervivum ruthenicum Schnittsp. & C.B. Lehm.	0	n	1	0	Crassulaceae J.St.-Hil.
Iz_8_1009	DJI_0932	Setaria viridis (L.) P. Beauv.	0	n	1	0	Poaceae Barnhart
Iz_8_1009	DJI_0932	Silene borysthenica (Gruner) Walters	0	n	1	0	Caryophyllaceae Juss.
Iz_8_1009	DJI_0932	Silene latifolia subsp. alba (Mill.) Greuter & Burdet	0	n	1	0	Caryophyllaceae Juss.
Iz_8_1009	DJI_0932	Solidago virgaurea L.	0	n	1	0	Asteraceae Bercht. & J.Presl
Iz_8_1009	DJI_0932	Sorbus aucuparia L.	0	n	1	1	Rosaceae Juss.
Iz_8_1009	DJI_0932	Stachys recta L.	0	n	1	0	Lamiacea Lindl.
Iz_8_1009	DJI_0932	Tanacetum vulgare L.	0	n	1	0	Asteraceae Bercht. & J.Presl
Iz_8_1009	DJI_0932	Thymus pallasianus Heinr.Braun	0	n	1	0	Lamiacea Lindl.
Iz_8_1009	DJI_0932	Tilia cordata Mill.	0	n	1	1	Malvaceae Juss.
Iz_8_1009	DJI_0932	Trifolium alpestre L.	0	n	1	0	Fabaceae Lindl.
Iz_8_1009	DJI_0932	Trifolium arvense L.	0	n	1	0	Fabaceae Lindl.
Iz_8_1009	DJI_0932	Ulmus glabra Huds.	0	n	1	1	Ulmaceae Mirb.
Iz_8_1009	DJI_0932	Verbascum densiflorum Bertol.	0	n	1	0	Scrophulariaceae Juss.
Iz_8_1009	DJI_0932	Veronica austriaca L.	0	n	1	0	Plantaginaceae Juss.
Iz_8_1009	DJI_0932	Veronica incana L.	0	n	1	0	Plantaginaceae Juss.
Iz_8_1009	DJI_0932	Veronica verna L.	0	n	1	0	Plantaginaceae Juss.
Iz_8_1009	DJI_0932	Vincetoxicum hirundinaria Medik	0	n	1	0	Apocynaceae Juss.
Iz_8_1009	DJI_0933	Acer tataricum L.	0	n	1	1	Sapindaceae Juss.
Iz_8_1009	DJI_0933	Achillea millefolium L. s.l.	0	n	1	0	Asteraceae Bercht. & J.Presl
Iz_8_1009	DJI_0933	Allium oleraceum L.	0	n	1	0	Amaryllidaceae J.St.-Hil.
Iz_8_1009	DJI_0933	Ambrosia artemisiifolia L.	0	a	0	0	Asteraceae Bercht. & J.Presl
Iz_8_1009	DJI_0933	Amorpha fruticosa L.	0	a	0	0	Fabaceae Lindl.
Iz_8_1009	DJI_0933	Anthericum ramosum L.	0	n	1	0	Asparagaceae Juss.
Iz_8_1009	DJI_0933	Artemisia marschalliana Spreng.	0	n	1	0	Asteraceae Bercht. & J.Presl
Iz_8_1009	DJI_0933	Asparagus officinalis L.	0	n	1	0	Asparagaceae Juss.
Iz_8_1009	DJI_0933	Astragalus cicer L.	0	n	1	0	Fabaceae Lindl.
Iz_8_1009	DJI_0933	Bassia laniflora (S.G.Gmel.) A.J.Scott	0	n	1	0	Amaranthaceae Juss.
Iz_8_1009	DJI_0933	Berberis vulgaris L.	0	n	1	0	Berberidaceae Juss.
Iz_8_1009	DJI_0933	Betula pendula Roth.	0	n	1	1	Betulaceae Gray
Iz_8_1009	DJI_0933	Betula pendula Roth.	0	n	1	0	Poaceae Barnhart
Iz_8_1009	DJI_0933	Bromus inermis Leyss.	0		NA	NA	
Iz_8_1009	DJI_0933	Bromus squarrosus L.	0	a	0	0	Poaceae Barnhart
Iz_8_1009	DJI_0933	Bromus tectorum L.	0	a	0	0	Poaceae Barnhart
Iz_8_1009	DJI_0933	Calamagrostis epigejos (L.) Roth	0	n	1	0	Poaceae Barnhart
Iz_8_1009	DJI_0933	Carduus acanthoides L.	0	n	1	0	Asteraceae Bercht. & J.Presl
Iz_8_1009	DJI_0933	Carex sp.	0	n	1	0	Cyperaceae Juss.
Iz_8_1009	DJI_0933	Centaurea majorovii Dumbadze	0	n	1	0	Asteraceae Bercht. & J.Presl
Iz_8_1009	DJI_0933	Chamaecytisus ruthenicus (Fisch.ex Wol.) Klaskova	0	n	1	0	Fabaceae Lindl.
Iz_8_1009	DJI_0933	Chelidonium majus L.	0	n	1	0	Papaveraceae Juss.
Iz_8_1009	DJI_0933	Chenopodium betaceum Andrz.	0	n	1	0	Amaranthaceae Juss.
Iz_8_1009	DJI_0933	Chenopodium cf. album	0	n	1	0	Amaranthaceae Juss.
Iz_8_1009	DJI_0933	Chenopodium sp.	0		NA	0	Amaranthaceae Juss.
Iz_8_1009	DJI_0933	Chondrilla juncea L.	0	n	1	0	Asteraceae Bercht. & J.Presl
Iz_8_1009	DJI_0933	Cirsium vulgare (Savi) Ten.	0	n	1	0	Asteraceae Bercht. & J.Presl
Iz_8_1009	DJI_0933	Clinopodium acinos (L.) Kuntze	0	n	1	0	Lamiacea Lindl.
Iz_8_1009	DJI_0933	Convallaria majalis L.	0	n	1	0	Asparagaceae Juss.
Iz_8_1009	DJI_0933	Crataegus rhipidophylla Gand.	0	n	1	1	Rosaceae Juss.
Iz_8_1009	DJI_0933	Crepis foetida L.	0	n	1	0	Asteraceae Bercht. & J.Presl
Iz_8_1009	DJI_0933	Dactylis glomerata L.	0	n	1	0	Poaceae Barnhart
Iz_8_1009	DJI_0933	Dianthus campestris M.Bieb.	0	n	1	0	Caryophyllaceae Juss.
Iz_8_1009	DJI_0933	Digitaria sanguinalis (L.) Scop.	0	a	0	0	Poaceae Barnhart

Iz_8_1009	DJI_0933	Elymus repens (L.) Gould	0	n	1	0	Poaceae Barnhart
Iz_8_1009	DJI_0933	Epilobium tetragonum L.	0	n	1	0	Onagraceae Juss.
Iz_8_1009	DJI_0933	Eragrostis minor Host	0	a	0	0	Poaceae Barnhart
Iz_8_1009	DJI_0933	Erigeron canadensis L.	15	a	0	0	Asteraceae Bercht. & J.Presl
Iz_8_1009	DJI_0933	Euphorbia seguieriana Neck.	0	n	1	0	Euphorbiaceae Juss.
Iz_8_1009	DJI_0933	Fallopia dumetorum (L.) Holub	0	n	1	0	Polygonaceae Juss.
Iz_8_1009	DJI_0933	Festuca beckeri (Hack.) Trautv.	0	n	1	0	Poaceae Barnhart
Iz_8_1009	DJI_0933	Fragaria vesca L.	0	n	1	0	Rosaceae Juss.
Iz_8_1009	DJI_0933	Frangula alnus Mill.	0	n	1	1	Rhamnaceae Juss.
Iz_8_1009	DJI_0933	Fraxinus pennsylvanica Marshall	0	a	0	1	Oleaceae Hoffmanns. & Link
Iz_8_1009	DJI_0933	Galeopsis bifida Boenn.	0	n	1	0	Lamiacea Lindl.
Iz_8_1009	DJI_0933	Galium verum L.	0	n	1	0	Rubiaceae Juss.
Iz_8_1009	DJI_0933	Genista tinctoria L.	0	n	1	0	Fabaceae Lindl.
Iz_8_1009	DJI_0933	Geranium robertianum L.	0	n	1	0	Geraniaceae Juss.
Iz_8_1009	DJI_0933	Geranium sanquineum L.	0	n	1	0	Geraniaceae Juss.
Iz_8_1009	DJI_0933	Gypsophila paniculata L.	0	n	1	0	Caryophyllaceae Juss.
Iz_8_1009	DJI_0933	Helichrysum arenarium (L.) Moench	0	n	1	0	Asteraceae Bercht. & J.Presl
Iz_8_1009	DJI_0933	Herniaria polygama J.Gay	0	n	1	0	Caryophyllaceae Juss.
Iz_8_1009	DJI_0933	Hieracium robustum Fr.	0	n	1	0	Asteraceae Bercht. & J.Presl
Iz_8_1009	DJI_0933	Hieracium umbellatum L.	0	n	1	NA	Asteraceae Bercht. & J.Presl
Iz_8_1009	DJI_0933	Hylotelephium maximum (L.) Holub	0	n	1	0	Crassulaceae J.St.-Hil.
Iz_8_1009	DJI_0933	Jacobaea borysthenica (DC.) B.Nord. & Greuter	0	n	1	0	Asteraceae Bercht. & J.Presl
Iz_8_1009	DJI_0933	Jasione montana L.	0	n	1	0	Campanulaceae Juss.
Iz_8_1009	DJI_0933	Jurinea cyanoides (L.) Rchb.s.l.	0	n	1	0	Asteraceae Bercht. & J.Presl
Iz_8_1009	DJI_0933	Koeleria glauca (Spreng.) DC.	0	n	1	0	Poaceae Barnhart
Iz_8_1009	DJI_0933	Lactuca serriola L.	0	a	0	0	Asteraceae Bercht. & J.Presl
Iz_8_1009	DJI_0933	Linaria genistifolia (L.) Mill.	0	n	1	0	Plantaginaceae Juss.
Iz_8_1009	DJI_0933	Lomelosia argentea (L.) Greuter & Burdet	0	n	1	0	Caprifoliaceae Juss.
Iz_8_1009	DJI_0933	Melampyrum pratense L.	0	n	1	0	Orobanchaceae Vent.
Iz_8_1009	DJI_0933	Oenothera biennis L.	0	a	0	0	Onagraceae Juss.
Iz_8_1009	DJI_0933	Pentanema britannica (L.) D.Gut.Larr., Santos-Vicente, Anderb., E.Rico & M.M.Mart.Ort.	0	n	1	0	Asteraceae Bercht. & J.Presl
Iz_8_1009	DJI_0933	Peucedanum oreoselinum (L.) Moench	0	n	1	0	Apiaceae Lindl.
Iz_8_1009	DJI_0933	Peucedanum ruthenicum M.Bieb.	0	n	1	0	Apiaceae Lindl.
Iz_8_1009	DJI_0933	Pilosella echioides (Lumn.) F.W.Schultz & Sch.Bip.	0	n	1	0	Asteraceae Bercht. & J.Presl
Iz_8_1009	DJI_0933	Pilosella officinarum F. Schultz et Sch. Bip.	0	n	1	0	Asteraceae Bercht. & J.Presl
Iz_8_1009	DJI_0933	Pilosella sp.	0		NA	0	Asteraceae Bercht. & J.Presl
Iz_8_1009	DJI_0933	Pinus sylvestris L.	0	n	1	1	Pinaceae Spreng. ex F.Rudolphi
Iz_8_1009	DJI_0933	Poa annua L.	0	n	1	0	Poaceae Barnhart
Iz_8_1009	DJI_0933	Poa nemoralis L.	0	n	1	0	Poaceae Barnhart
Iz_8_1009	DJI_0933	Poa sp.	0		NA	0	Poaceae Barnhart
Iz_8_1009	DJI_0933	Polygonatum odoratum (Mill.) Druce	0	n	1	0	Asparagaceae Juss.
Iz_8_1009	DJI_0933	Polygonum arenarium Waldst. & Kit.	0	n	1	0	Polygonaceae Juss.
Iz_8_1009	DJI_0933	Populus nigra L.	0	n	1	1	Salicaceae Mirb.
Iz_8_1009	DJI_0933	Populus tremula L.	0	n	1	1	Salicaceae Mirb.
Iz_8_1009	DJI_0933	Populus Ã canescens (Aiton) Sm.	0	n	1	1	Salicaceae Mirb.
Iz_8_1009	DJI_0933	Potentilla argentea L.	0	n	1	0	Rosaceae Juss.
Iz_8_1009	DJI_0933	Psammophiliella muralis (L.) Ikonn.	0	n	1	0	Caryophyllaceae Juss.
Iz_8_1009	DJI_0933	Psephellus sumensis (Kalen.) Greuter	0	n	1	0	Asteraceae Bercht. & J.Presl
Iz_8_1009	DJI_0933	Pulsatilla pratensis (L.) Mill. s.l.	0	n	1	0	Ranunculaceae Juss.
Iz_8_1009	DJI_0933	Pyrus communis L.	0	a	0	1	Rosaceae Juss.
Iz_8_1009	DJI_0933	Quercus robur L.	0	n	1	1	Fagaceae Dumort.
Iz_8_1009	DJI_0933	Robinia pseudoacacia L.	0	a	0	1	Fabaceae Lindl.
Iz_8_1009	DJI_0933	Rosa sp.	0		NA	1	Rosaceae Juss.
Iz_8_1009	DJI_0933	Rubus idaeus L.	0	n	1	0	Rosaceae Juss.
Iz_8_1009	DJI_0933	Rumex acetosella L.	0	n	1	0	Polygonaceae Juss.
Iz_8_1009	DJI_0933	Rumex thyrsiflorus Fingerh.	0	n	1	0	Polygonaceae Juss.
Iz_8_1009	DJI_0933	Salix alba L.	0	n	1	1	Salicaceae Mirb.
Iz_8_1009	DJI_0933	Sambucus nigra L.	0	n	1	1	Viburnaceae Raf.
Iz_8_1009	DJI_0933	Secale sylvestre Host	0	n	1	0	Poaceae Barnhart
Iz_8_1009	DJI_0933	Sempervivum ruthenicum Schnittsp. & C.B. Lehm.	0	n	1	0	Crassulaceae J.St.-Hil.
Iz_8_1009	DJI_0933	Setaria viridis (L.) P. Beauv.	0	n	1	0	Poaceae Barnhart
Iz_8_1009	DJI_0933	Silene borysthenica (Gruner) Walters	0	n	1	0	Caryophyllaceae Juss.
Iz_8_1009	DJI_0933	Silene latifolia subsp. alba (Mill.) Greuter & Burdet	0	n	1	0	Caryophyllaceae Juss.
Iz_8_1009	DJI_0933	Solidago virgaurea L.	0	n	1	0	Asteraceae Bercht. & J.Presl
Iz_8_1009	DJI_0933	Sorbus aucuparia L.	0	n	1	1	Rosaceae Juss.
Iz_8_1009	DJI_0933	Stachys recta L.	0	n	1	0	Lamiacea Lindl.
Iz_8_1009	DJI_0933	Tanacetum vulgare L.	0	n	1	0	Asteraceae Bercht. & J.Presl
Iz_8_1009	DJI_0933	Thymus pallasianus Heinr.Braun	0	n	1	0	Lamiacea Lindl.
Iz_8_1009	DJI_0933	Tilia cordata Mill.	0	n	1	1	Malvaceae Juss.
Iz_8_1009	DJI_0933	Trifolium alpestre L.	0	n	1	0	Fabaceae Lindl.
Iz_8_1009	DJI_0933	Trifolium arvense L.	0	n	1	0	Fabaceae Lindl.
Iz_8_1009	DJI_0933	Ulmus glabra Huds.	0	n	1	1	Ulmaceae Mirb.
Iz_8_1009	DJI_0933	Verbascum densiflorum Bertol.	0	n	1	0	Scrophulariaceae Juss.
Iz_8_1009	DJI_0933	Veronica austriaca L.	0	n	1	0	Plantaginaceae Juss.
Iz_8_1009	DJI_0933	Veronica incana L.	0	n	1	0	Plantaginaceae Juss.
Iz_8_1009	DJI_0933	Veronica verna L.	0	n	1	0	Plantaginaceae Juss.
Iz_8_1009	DJI_0933	Vincetoxicum hirundinaria Medik	0	n	1	0	Apocynaceae Juss.
Iz_8_1009	DJI_0934	Acer tataricum L.	0	n	1	1	Sapindaceae Juss.
Iz_8_1009	DJI_0934	Achillea millefolium L. s.l.	0	n	1	0	Asteraceae Bercht. & J.Presl
Iz_8_1009	DJI_0934	Allium oleraceum L.	0	n	1	0	Amaryllidaceae J.St.-Hil.
Iz_8_1009	DJI_0934	Ambrosia artemisiifolia L.	0	a	0	0	Asteraceae Bercht. & J.Presl
Iz_8_1009	DJI_0934	Amorpha fruticosa L.	0	a	0	0	Fabaceae Lindl.
Iz_8_1009	DJI_0934	Anthericum ramosum L.	0	n	1	0	Asparagaceae Juss.
Iz_8_1009	DJI_0934	Artemisia marschalliana Spreng.	0	n	1	0	Asteraceae Bercht. & J.Presl
Iz_8_1009	DJI_0934	Asparagus officinalis L.	0	n	1	0	Asparagaceae Juss.
Iz_8_1009	DJI_0934	Astragalus cicer L.	0	n	1	0	Fabaceae Lindl.
Iz_8_1009	DJI_0934	Bassia laniflora (S.G.Gmel.) A.J.Scott	0	n	1	0	Amaranthaceae Juss.
Iz_8_1009	DJI_0934	Berberis vulgaris L.	0	n	1	0	Berberidaceae Juss.
Iz_8_1009	DJI_0934	Betula pendula Roth.	0	n	1	1	Betulaceae Gray
Iz_8_1009	DJI_0934	Betula pendula Roth.	0	n	1	0	Poaceae Barnhart
Iz_8_1009	DJI_0934	Bromus inermis Leyss.	0		NA	NA	
Iz_8_1009	DJI_0934	Bromus squarrosus L.	0	a	0	0	Poaceae Barnhart
Iz_8_1009	DJI_0934	Bromus tectorum L.	0	a	0	0	Poaceae Barnhart
Iz_8_1009	DJI_0934	Calamagrostis epigejos (L.) Roth	0.5	n	1	0	Poaceae Barnhart
Iz_8_1009	DJI_0934	Carduus acanthoides L.	0	n	1	0	Asteraceae Bercht. & J.Presl
Iz_8_1009	DJI_0934	Carex sp.	0	n	1	0	Cyperaceae Juss.
Iz_8_1009	DJI_0934	Centaurea majorovii Dumbadze	0	n	1	0	Asteraceae Bercht. & J.Presl
Iz_8_1009	DJI_0934	Chamaecytisus ruthenicus (Fisch.ex Wol.) Klaskova	0	n	1	0	Fabaceae Lindl.

Iz_8_1009	DJI_0934	Chelidonium majus L.	0	n	1	0	Papaveraceae Juss.
Iz_8_1009	DJI_0934	Chenopodium betaceum Andrz.	0	n	1	0	Amaranthaceae Juss.
Iz_8_1009	DJI_0934	Chenopodium cf. album	0	n	1	0	Amaranthaceae Juss.
Iz_8_1009	DJI_0934	Chenopodium sp.	0		NA	0	Amaranthaceae Juss.
Iz_8_1009	DJI_0934	Chondrilla juncea L.	0	n	1	0	Asteraceae Bercht. & J.Presl
Iz_8_1009	DJI_0934	Cirsium vulgare (Savi) Ten.	0	n	1	0	Asteraceae Bercht. & J.Presl
Iz_8_1009	DJI_0934	Clinopodium acinos (L.) Kuntze	0	n	1	0	Lamiacea Lindl.
Iz_8_1009	DJI_0934	Convallaria majalis L.	0	n	1	0	Asparagaceae Juss.
Iz_8_1009	DJI_0934	Crataegus rhipidophylla Gand.	0	n	1	1	Rosaceae Juss.
Iz_8_1009	DJI_0934	Crepis foetida L.	0	n	1	0	Asteraceae Bercht. & J.Presl
Iz_8_1009	DJI_0934	Dactylis glomerata L.	0	n	1	0	Poaceae Barnhart
Iz_8_1009	DJI_0934	Dianthus campestris M.Bieb.	0	n	1	0	Caryophyllaceae Juss.
Iz_8_1009	DJI_0934	Digitaria sanguinalis (L.) Scop.	0	a	0	0	Poaceae Barnhart
Iz_8_1009	DJI_0934	Elymus repens (L.) Gould	0	n	1	0	Poaceae Barnhart
Iz_8_1009	DJI_0934	Epilobium tetragonum L.	0	n	1	0	Onagraceae Juss.
Iz_8_1009	DJI_0934	Eragrostis minor Host	0	a	0	0	Poaceae Barnhart
Iz_8_1009	DJI_0934	Erigeron canadensis L.	5	a	0	0	Asteraceae Bercht. & J.Presl
Iz_8_1009	DJI_0934	Euphorbia seguieriana Neck.	0	n	1	0	Euphorbiaceae Juss.
Iz_8_1009	DJI_0934	Fallopia dumetorum (L.) Holub	0	n	1	0	Polygonaceae Juss.
Iz_8_1009	DJI_0934	Festuca beckeri (Hack.) Trautv.	0	n	1	0	Poaceae Barnhart
Iz_8_1009	DJI_0934	Fragaria vesca L.	0	n	1	0	Rosaceae Juss.
Iz_8_1009	DJI_0934	Frangula alnus Mill.	0	n	1	1	Rhamnaceae Juss.
Iz_8_1009	DJI_0934	Fraxinus pennsylvanica Marshall	0	a	0	1	Oleaceae Hoffmanns. & Link
Iz_8_1009	DJI_0934	Galeopsis bifida Boenn.	0	n	1	0	Lamiacea Lindl.
Iz_8_1009	DJI_0934	Galium verum L.	0	n	1	0	Rubiaceae Juss.
Iz_8_1009	DJI_0934	Genista tinctoria L.	0	n	1	0	Fabaceae Lindl.
Iz_8_1009	DJI_0934	Geranium robertianum L.	0	n	1	0	Geraniaceae Juss.
Iz_8_1009	DJI_0934	Geranium sanquineum L.	0	n	1	0	Geraniaceae Juss.
Iz_8_1009	DJI_0934	Gypsophila paniculata L.	0	n	1	0	Caryophyllaceae Juss.
Iz_8_1009	DJI_0934	Helichrysum arenarium (L.) Moench	0	n	1	0	Asteraceae Bercht. & J.Presl
Iz_8_1009	DJI_0934	Herniaria polygama J.Gay	0	n	1	0	Caryophyllaceae Juss.
Iz_8_1009	DJI_0934	Hieracium robustum Fr.	0	n	1	0	Asteraceae Bercht. & J.Presl
Iz_8_1009	DJI_0934	Hieracium umbellatum L.	0	n	1	NA	Asteraceae Bercht. & J.Presl
Iz_8_1009	DJI_0934	Hylotelephium maximum (L.) Holub	0	n	1	0	Crassulaceae J.St.-Hil.
Iz_8_1009	DJI_0934	Jacobaea borysthenica (DC.) B.Nord. & Greuter	0	n	1	0	Asteraceae Bercht. & J.Presl
Iz_8_1009	DJI_0934	Jasione montana L.	0	n	1	0	Campanulaceae Juss.
Iz_8_1009	DJI_0934	Jurinea cyanoides (L.) Rchb.s.l.	0	n	1	0	Asteraceae Bercht. & J.Presl
Iz_8_1009	DJI_0934	Koeleria glauca (Spreng.) DC.	0	n	1	0	Poaceae Barnhart
Iz_8_1009	DJI_0934	Lactuca serriola L.	0	a	0	0	Asteraceae Bercht. & J.Presl
Iz_8_1009	DJI_0934	Linaria genistifolia (L.) Mill.	0	n	1	0	Plantaginaceae Juss.
Iz_8_1009	DJI_0934	Lomelosia argentea (L.) Greuter & Burdet	0	n	1	0	Caprifoliaceae Juss.
Iz_8_1009	DJI_0934	Melampyrum pratense L.	0	n	1	0	Orobanchaceae Vent.
Iz_8_1009	DJI_0934	Oenothera biennis L.	0	a	0	0	Onagraceae Juss.
Iz_8_1009	DJI_0934	Pentanema britannica (L.) D.Gut.Larr., Santos-Vicente, Anderb., E.Rico & M.M.Mart.Ort.	0	n	1	0	Asteraceae Bercht. & J.Presl
Iz_8_1009	DJI_0934	Peucedanum oreoselinum (L.) Moench	0	n	1	0	Apiaceae Lindl.
Iz_8_1009	DJI_0934	Peucedanum ruthenicum M.Bieb.	0	n	1	0	Apiaceae Lindl.
Iz_8_1009	DJI_0934	Pilosella echioides (Lumn.) F.W.Schultz & Sch.Bip.	0	n	1	0	Asteraceae Bercht. & J.Presl
Iz_8_1009	DJI_0934	Pilosella officinarum F. Schultz et Sch. Bip.	0	n	1	0	Asteraceae Bercht. & J.Presl
Iz_8_1009	DJI_0934	Pilosella sp.	0		NA	0	Asteraceae Bercht. & J.Presl
Iz_8_1009	DJI_0934	Pinus sylvestris L.	0	n	1	1	Pinaceae Spreng. ex F.Rudolphi
Iz_8_1009	DJI_0934	Poa annua L.	0	n	1	0	Poaceae Barnhart
Iz_8_1009	DJI_0934	Poa nemoralis L.	0	n	1	0	Poaceae Barnhart
Iz_8_1009	DJI_0934	Poa sp.	0		NA	0	Poaceae Barnhart
Iz_8_1009	DJI_0934	Polygonatum odoratum (Mill.) Druce	0	n	1	0	Asparagaceae Juss.
Iz_8_1009	DJI_0934	Polygonum arenarium Waldst. & Kit.	0.2	n	1	0	Polygonaceae Juss.
Iz_8_1009	DJI_0934	Populus nigra L.	0	n	1	1	Salicaceae Mirb.
Iz_8_1009	DJI_0934	Populus tremula L.	0	n	1	1	Salicaceae Mirb.
Iz_8_1009	DJI_0934	Populus Ã canescens (Aiton) Sm.	0	n	1	1	Salicaceae Mirb.
Iz_8_1009	DJI_0934	Potentilla argentea L.	0	n	1	0	Rosaceae Juss.
Iz_8_1009	DJI_0934	Psammophiliella muralis (L.) Ikonn.	0	n	1	0	Caryophyllaceae Juss.
Iz_8_1009	DJI_0934	Psephellus sumensis (Kalen.) Greuter	0	n	1	0	Asteraceae Bercht. & J.Presl
Iz_8_1009	DJI_0934	Pulsatilla pratensis (L.) Mill. s.l.	0	n	1	0	Ranunculaceae Juss.
Iz_8_1009	DJI_0934	Pyrus communis L.	0	a	0	1	Rosaceae Juss.
Iz_8_1009	DJI_0934	Quercus robur L.	0	n	1	1	Fagaceae Dumort.
Iz_8_1009	DJI_0934	Robinia pseudoacacia L.	0	a	0	1	Fabaceae Lindl.
Iz_8_1009	DJI_0934	Rosa sp.	0		NA	1	Rosaceae Juss.
Iz_8_1009	DJI_0934	Rubus idaeus L.	0	n	1	0	Rosaceae Juss.
Iz_8_1009	DJI_0934	Rumex acetosella L.	0	n	1	0	Polygonaceae Juss.
Iz_8_1009	DJI_0934	Rumex thyrsiflorus Fingerh.	0	n	1	0	Polygonaceae Juss.
Iz_8_1009	DJI_0934	Salix alba L.	0	n	1	1	Salicaceae Mirb.
Iz_8_1009	DJI_0934	Sambucus nigra L.	0	n	1	1	Viburnaceae Raf.
Iz_8_1009	DJI_0934	Secale sylvestre Host	0	n	1	0	Poaceae Barnhart
Iz_8_1009	DJI_0934	Sempervivum ruthenicum Schnittsp. & C.B. Lehm.	0	n	1	0	Crassulaceae J.St.-Hil.
Iz_8_1009	DJI_0934	Setaria viridis (L.) P. Beauv.	0	n	1	0	Poaceae Barnhart
Iz_8_1009	DJI_0934	Silene borysthenica (Gruner) Walters	0	n	1	0	Caryophyllaceae Juss.
Iz_8_1009	DJI_0934	Silene latifolia subsp. alba (Mill.) Greuter & Burdet	0	n	1	0	Caryophyllaceae Juss.
Iz_8_1009	DJI_0934	Solidago virgaurea L.	0	n	1	0	Asteraceae Bercht. & J.Presl
Iz_8_1009	DJI_0934	Sorbus aucuparia L.	0	n	1	1	Rosaceae Juss.
Iz_8_1009	DJI_0934	Stachys recta L.	0	n	1	0	Lamiacea Lindl.
Iz_8_1009	DJI_0934	Tanacetum vulgare L.	0	n	1	0	Asteraceae Bercht. & J.Presl
Iz_8_1009	DJI_0934	Thymus pallasianus Heinr.Braun	0	n	1	0	Lamiacea Lindl.
Iz_8_1009	DJI_0934	Tilia cordata Mill.	0	n	1	1	Malvaceae Juss.
Iz_8_1009	DJI_0934	Trifolium alpestre L.	0	n	1	0	Fabaceae Lindl.
Iz_8_1009	DJI_0934	Trifolium arvense L.	0	n	1	0	Fabaceae Lindl.
Iz_8_1009	DJI_0934	Ulmus glabra Huds.	0	n	1	1	Ulmaceae Mirb.
Iz_8_1009	DJI_0934	Verbascum densiflorum Bertol.	0	n	1	0	Scrophulariaceae Juss.
Iz_8_1009	DJI_0934	Veronica austriaca L.	0	n	1	0	Plantaginaceae Juss.
Iz_8_1009	DJI_0934	Veronica incana L.	0	n	1	0	Plantaginaceae Juss.
Iz_8_1009	DJI_0934	Veronica verna L.	0	n	1	0	Plantaginaceae Juss.
Iz_8_1009	DJI_0934	Vincetoxicum hirundinaria Medik	0	n	1	0	Apocynaceae Juss.
Iz_8_1009	DJI_0936	Acer tataricum L.	0	n	1	1	Sapindaceae Juss.
Iz_8_1009	DJI_0936	Achillea millefolium L. s.l.	0	n	1	0	Asteraceae Bercht. & J.Presl
Iz_8_1009	DJI_0936	Allium oleraceum L.	0	n	1	0	Amaryllidaceae J.St.-Hil.
Iz_8_1009	DJI_0936	Ambrosia artemisiifolia L.	0	a	0	0	Asteraceae Bercht. & J.Presl
Iz_8_1009	DJI_0936	Amorpha fruticosa L.	0	a	0	0	Fabaceae Lindl.
Iz_8_1009	DJI_0936	Anthericum ramosum L.	0	n	1	0	Asparagaceae Juss.
Iz_8_1009	DJI_0936	Artemisia marschalliana Spreng.	0	n	1	0	Asteraceae Bercht. & J.Presl
Iz_8_1009	DJI_0936	Asparagus officinalis L.	0	n	1	0	Asparagaceae Juss.

Iz_8_1009	DJI_0936	Astragalus cicer L.	0	n	1	0	Fabaceae Lindl.
Iz_8_1009	DJI_0936	Bassia laniflora (S.G.Gmel.) A.J.Scott	0	n	1	0	Amaranthaceae Juss.
Iz_8_1009	DJI_0936	Berberis vulgaris L.	0	n	1	0	Berberidaceae Juss.
Iz_8_1009	DJI_0936	Betula pendula Roth.	0	n	1	1	Betulaceae Gray
Iz_8_1009	DJI_0936	Betula pendula Roth.	0	n	1	0	Poaceae Barnhart
Iz_8_1009	DJI_0936	Bromus inermis Leyss.	0		NA	NA	
Iz_8_1009	DJI_0936	Bromus squarrosus L.	0	a	0	0	Poaceae Barnhart
Iz_8_1009	DJI_0936	Bromus tectorum L.	0	a	0	0	Poaceae Barnhart
Iz_8_1009	DJI_0936	Calamagrostis epigejos (L.) Roth	0	n	1	0	Poaceae Barnhart
Iz_8_1009	DJI_0936	Carduus acanthoides L.	0	n	1	0	Asteraceae Bercht. & J.Presl
Iz_8_1009	DJI_0936	Carex sp.	0	n	1	0	Cyperaceae Juss.
Iz_8_1009	DJI_0936	Centaurea majorovii Dumbadze	0	n	1	0	Asteraceae Bercht. & J.Presl
Iz_8_1009	DJI_0936	Chamaecytisus ruthenicus (Fisch.ex Wol.) Klaskova	0	n	1	0	Fabaceae Lindl.
Iz_8_1009	DJI_0936	Chelidonium majus L.	0	n	1	0	Papaveraceae Juss.
Iz_8_1009	DJI_0936	Chenopodium betaceum Andrz.	0	n	1	0	Amaranthaceae Juss.
Iz_8_1009	DJI_0936	Chenopodium cf. album	0	n	1	0	Amaranthaceae Juss.
Iz_8_1009	DJI_0936	Chenopodium sp.	0		NA	0	Amaranthaceae Juss.
Iz_8_1009	DJI_0936	Chondrilla juncea L.	0	n	1	0	Asteraceae Bercht. & J.Presl
Iz_8_1009	DJI_0936	Cirsium vulgare (Savi) Ten.	0	n	1	0	Asteraceae Bercht. & J.Presl
Iz_8_1009	DJI_0936	Clinopodium acinos (L.) Kuntze	0	n	1	0	Lamiacea Lindl.
Iz_8_1009	DJI_0936	Convallaria majalis L.	0	n	1	0	Asparagaceae Juss.
Iz_8_1009	DJI_0936	Crataegus rhipidophylla Gand.	0	n	1	1	Rosaceae Juss.
Iz_8_1009	DJI_0936	Crepis foetida L.	0	n	1	0	Asteraceae Bercht. & J.Presl
Iz_8_1009	DJI_0936	Dactylis glomerata L.	0	n	1	0	Poaceae Barnhart
Iz_8_1009	DJI_0936	Dianthus campestris M.Bieb.	0	n	1	0	Caryophyllaceae Juss.
Iz_8_1009	DJI_0936	Digitaria sanguinalis (L.) Scop.	0	a	0	0	Poaceae Barnhart
Iz_8_1009	DJI_0936	Elymus repens (L.) Gould	0	n	1	0	Poaceae Barnhart
Iz_8_1009	DJI_0936	Epilobium tetragonum L.	0	n	1	0	Onagraceae Juss.
Iz_8_1009	DJI_0936	Eragrostis minor Host	0	a	0	0	Poaceae Barnhart
Iz_8_1009	DJI_0936	Erigeron canadensis L.	15	a	0	0	Asteraceae Bercht. & J.Presl
Iz_8_1009	DJI_0936	Euphorbia seguieriana Neck.	0	n	1	0	Euphorbiaceae Juss.
Iz_8_1009	DJI_0936	Fallopia dumetorum (L.) Holub	0	n	1	0	Polygonaceae Juss.
Iz_8_1009	DJI_0936	Festuca beckeri (Hack.) Trautv.	0	n	1	0	Poaceae Barnhart
Iz_8_1009	DJI_0936	Fragaria vesca L.	0	n	1	0	Rosaceae Juss.
Iz_8_1009	DJI_0936	Frangula alnus Mill.	0	n	1	1	Rhamnaceae Juss.
Iz_8_1009	DJI_0936	Fraxinus pennsylvanica Marshall	0	a	0	1	Oleaceae Hoffmanns. & Link
Iz_8_1009	DJI_0936	Galeopsis bifida Boenn.	0	n	1	0	Lamiacea Lindl.
Iz_8_1009	DJI_0936	Galium verum L.	0	n	1	0	Rubiaceae Juss.
Iz_8_1009	DJI_0936	Genista tinctoria L.	0	n	1	0	Fabaceae Lindl.
Iz_8_1009	DJI_0936	Geranium robertianum L.	0	n	1	0	Geraniaceae Juss.
Iz_8_1009	DJI_0936	Geranium sanquineum L.	0	n	1	0	Geraniaceae Juss.
Iz_8_1009	DJI_0936	Gypsophila paniculata L.	0	n	1	0	Caryophyllaceae Juss.
Iz_8_1009	DJI_0936	Helichrysum arenarium (L.) Moench	0	n	1	0	Asteraceae Bercht. & J.Presl
Iz_8_1009	DJI_0936	Herniaria polygama J.Gay	0	n	1	0	Caryophyllaceae Juss.
Iz_8_1009	DJI_0936	Hieracium robustum Fr.	0	n	1	0	Asteraceae Bercht. & J.Presl
Iz_8_1009	DJI_0936	Hieracium umbellatum L.	0	n	1	NA	Asteraceae Bercht. & J.Presl
Iz_8_1009	DJI_0936	Hylotelephium maximum (L.) Holub	0	n	1	0	Crassulaceae J.St.-Hil.
Iz_8_1009	DJI_0936	Jacobaea borysthenica (DC.) B.Nord. & Greuter	0	n	1	0	Asteraceae Bercht. & J.Presl
Iz_8_1009	DJI_0936	Jasione montana L.	0	n	1	0	Campanulaceae Juss.
Iz_8_1009	DJI_0936	Jurinea cyanoides (L.) Rchb.s.l.	0	n	1	0	Asteraceae Bercht. & J.Presl
Iz_8_1009	DJI_0936	Koeleria glauca (Spreng.) DC.	0	n	1	0	Poaceae Barnhart
Iz_8_1009	DJI_0936	Lactuca serriola L.	0	a	0	0	Asteraceae Bercht. & J.Presl
Iz_8_1009	DJI_0936	Linaria genistifolia (L.) Mill.	0	n	1	0	Plantaginaceae Juss.
Iz_8_1009	DJI_0936	Lomelosia argentea (L.) Greuter & Burdet	0	n	1	0	Caprifoliaceae Juss.
Iz_8_1009	DJI_0936	Melampyrum pratense L.	0	n	1	0	Orobanchaceae Vent.
Iz_8_1009	DJI_0936	Oenothera biennis L.	0	a	0	0	Onagraceae Juss.
Iz_8_1009	DJI_0936	Pentanema britannica (L.) D.Gut.Larr., Santos-Vicente, Anderb., E.Rico & M.M.Mart.Ort.	0	n	1	0	Asteraceae Bercht. & J.Presl
Iz_8_1009	DJI_0936	Peucedanum oreoselinum (L.) Moench	0	n	1	0	Apiaceae Lindl.
Iz_8_1009	DJI_0936	Peucedanum ruthenicum M.Bieb.	0	n	1	0	Apiaceae Lindl.
Iz_8_1009	DJI_0936	Pilosella echioides (Lumn.) F.W.Schultz & Sch.Bip.	0	n	1	0	Asteraceae Bercht. & J.Presl
Iz_8_1009	DJI_0936	Pilosella officinarum F. Schultz et Sch. Bip.	0	n	1	0	Asteraceae Bercht. & J.Presl
Iz_8_1009	DJI_0936	Pilosella sp.	0		NA	0	Asteraceae Bercht. & J.Presl
Iz_8_1009	DJI_0936	Pinus sylvestris L.	0	n	1	1	Pinaceae Spreng. ex F.Rudolphi
Iz_8_1009	DJI_0936	Poa annua L.	0	n	1	0	Poaceae Barnhart
Iz_8_1009	DJI_0936	Poa nemoralis L.	0	n	1	0	Poaceae Barnhart
Iz_8_1009	DJI_0936	Poa sp.	0		NA	0	Poaceae Barnhart
Iz_8_1009	DJI_0936	Polygonatum odoratum (Mill.) Druce	0	n	1	0	Asparagaceae Juss.
Iz_8_1009	DJI_0936	Polygonum arenarium Waldst. & Kit.	0	n	1	0	Polygonaceae Juss.
Iz_8_1009	DJI_0936	Populus nigra L.	0	n	1	1	Salicaceae Mirb.
Iz_8_1009	DJI_0936	Populus tremula L.	0	n	1	1	Salicaceae Mirb.
Iz_8_1009	DJI_0936	Populus Ã canescens (Aiton) Sm.	0	n	1	1	Salicaceae Mirb.
Iz_8_1009	DJI_0936	Potentilla argentea L.	0	n	1	0	Rosaceae Juss.
Iz_8_1009	DJI_0936	Psammophiliella muralis (L.) Ikonn.	0	n	1	0	Caryophyllaceae Juss.
Iz_8_1009	DJI_0936	Psephellus sumensis (Kalen.) Greuter	0	n	1	0	Asteraceae Bercht. & J.Presl
Iz_8_1009	DJI_0936	Pulsatilla pratensis (L.) Mill. s.l.	0	n	1	0	Ranunculaceae Juss.
Iz_8_1009	DJI_0936	Pyrus communis L.	0	a	0	1	Rosaceae Juss.
Iz_8_1009	DJI_0936	Quercus robur L.	0	n	1	1	Fagaceae Dumort.
Iz_8_1009	DJI_0936	Robinia pseudoacacia L.	0	a	0	1	Fabaceae Lindl.
Iz_8_1009	DJI_0936	Rosa sp.	0		NA	1	Rosaceae Juss.
Iz_8_1009	DJI_0936	Rubus idaeus L.	0	n	1	0	Rosaceae Juss.
Iz_8_1009	DJI_0936	Rumex acetosella L.	0	n	1	0	Polygonaceae Juss.
Iz_8_1009	DJI_0936	Rumex thyrsiflorus Fingerh.	0	n	1	0	Polygonaceae Juss.
Iz_8_1009	DJI_0936	Salix alba L.	0	n	1	1	Salicaceae Mirb.
Iz_8_1009	DJI_0936	Sambucus nigra L.	0	n	1	1	Viburnaceae Raf.
Iz_8_1009	DJI_0936	Secale sylvestre Host	0	n	1	0	Poaceae Barnhart
Iz_8_1009	DJI_0936	Sempervivum ruthenicum Schnittsp. & C.B. Lehm.	0	n	1	0	Crassulaceae J.St.-Hil.
Iz_8_1009	DJI_0936	Setaria viridis (L.) P. Beauv.	0	n	1	0	Poaceae Barnhart
Iz_8_1009	DJI_0936	Silene borysthenica (Gruner) Walters	0	n	1	0	Caryophyllaceae Juss.
Iz_8_1009	DJI_0936	Silene latifolia subsp. alba (Mill.) Greuter & Burdet	0	n	1	0	Caryophyllaceae Juss.
Iz_8_1009	DJI_0936	Solidago virgaurea L.	0	n	1	0	Asteraceae Bercht. & J.Presl
Iz_8_1009	DJI_0936	Sorbus aucuparia L.	0	n	1	1	Rosaceae Juss.
Iz_8_1009	DJI_0936	Stachys recta L.	0	n	1	0	Lamiacea Lindl.
Iz_8_1009	DJI_0936	Tanacetum vulgare L.	0	n	1	0	Asteraceae Bercht. & J.Presl
Iz_8_1009	DJI_0936	Thymus pallasianus Heinr.Braun	0	n	1	0	Lamiacea Lindl.
Iz_8_1009	DJI_0936	Tilia cordata Mill.	0	n	1	1	Malvaceae Juss.
Iz_8_1009	DJI_0936	Trifolium alpestre L.	0	n	1	0	Fabaceae Lindl.
Iz_8_1009	DJI_0936	Trifolium arvense L.	0	n	1	0	Fabaceae Lindl.
Iz_8_1009	DJI_0936	Ulmus glabra Huds.	0	n	1	1	Ulmaceae Mirb.

Iz_8_1009	DJI_0936	Verbascum densiflorum Bertol.	0	n	1	0	Scrophulariaceae Juss.
Iz_8_1009	DJI_0936	Veronica austriaca L.	0	n	1	0	Plantaginaceae Juss.
Iz_8_1009	DJI_0936	Veronica incana L.	0	n	1	0	Plantaginaceae Juss.
Iz_8_1009	DJI_0936	Veronica verna L.	0	n	1	0	Plantaginaceae Juss.
Iz_8_1009	DJI_0936	Vincetoxicum hirundinaria Medik	0	n	1	0	Apocynaceae Juss.
Iz_8_1009	DJI_0937	Acer tataricum L.	0	n	1	1	Sapindaceae Juss.
Iz_8_1009	DJI_0937	Achillea millefolium L. s.l.	0	n	1	0	Asteraceae Bercht. & J.Presl
Iz_8_1009	DJI_0937	Allium oleraceum L.	0	n	1	0	Amaryllidaceae J.St.-Hil.
Iz_8_1009	DJI_0937	Ambrosia artemisiifolia L.	0	a	0	0	Asteraceae Bercht. & J.Presl
Iz_8_1009	DJI_0937	Amorpha fruticosa L.	0	a	0	0	Fabaceae Lindl.
Iz_8_1009	DJI_0937	Anthericum ramosum L.	0	n	1	0	Asparagaceae Juss.
Iz_8_1009	DJI_0937	Artemisia marschalliana Spreng.	0	n	1	0	Asteraceae Bercht. & J.Presl
Iz_8_1009	DJI_0937	Asparagus officinalis L.	0	n	1	0	Asparagaceae Juss.
Iz_8_1009	DJI_0937	Astragalus cicer L.	0	n	1	0	Fabaceae Lindl.
Iz_8_1009	DJI_0937	Bassia laniflora (S.G.Gmel.) A.J.Scott	0	n	1	0	Amaranthaceae Juss.
Iz_8_1009	DJI_0937	Berberis vulgaris L.	0	n	1	0	Berberidaceae Juss.
Iz_8_1009	DJI_0937	Betula pendula Roth.	0	n	1	1	Betulaceae Gray
Iz_8_1009	DJI_0937	Betula pendula Roth.	0	n	1	0	Poaceae Barnhart
Iz_8_1009	DJI_0937	Bromus inermis Leyss.	0		NA	NA	
Iz_8_1009	DJI_0937	Bromus squarrosus L.	0	a	0	0	Poaceae Barnhart
Iz_8_1009	DJI_0937	Bromus tectorum L.	0	a	0	0	Poaceae Barnhart
Iz_8_1009	DJI_0937	Calamagrostis epigejos (L.) Roth	0	n	1	0	Poaceae Barnhart
Iz_8_1009	DJI_0937	Carduus acanthoides L.	0	n	1	0	Asteraceae Bercht. & J.Presl
Iz_8_1009	DJI_0937	Carex sp.	0	n	1	0	Cyperaceae Juss.
Iz_8_1009	DJI_0937	Centaurea majorovii Dumbadze	0	n	1	0	Asteraceae Bercht. & J.Presl
Iz_8_1009	DJI_0937	Chamaecytisus ruthenicus (Fisch.ex Wol.) Klaskova	0	n	1	0	Fabaceae Lindl.
Iz_8_1009	DJI_0937	Chelidonium majus L.	0	n	1	0	Papaveraceae Juss.
Iz_8_1009	DJI_0937	Chenopodium betaceum Andrz.	0	n	1	0	Amaranthaceae Juss.
Iz_8_1009	DJI_0937	Chenopodium cf. album	0	n	1	0	Amaranthaceae Juss.
Iz_8_1009	DJI_0937	Chenopodium sp.	0		NA	0	Amaranthaceae Juss.
Iz_8_1009	DJI_0937	Chondrilla juncea L.	0	n	1	0	Asteraceae Bercht. & J.Presl
Iz_8_1009	DJI_0937	Cirsium vulgare (Savi) Ten.	0	n	1	0	Asteraceae Bercht. & J.Presl
Iz_8_1009	DJI_0937	Clinopodium acinos (L.) Kuntze	0	n	1	0	Lamiacea Lindl.
Iz_8_1009	DJI_0937	Convallaria majalis L.	0	n	1	0	Asparagaceae Juss.
Iz_8_1009	DJI_0937	Crataegus rhipidophylla Gand.	0	n	1	1	Rosaceae Juss.
Iz_8_1009	DJI_0937	Crepis foetida L.	0	n	1	0	Asteraceae Bercht. & J.Presl
Iz_8_1009	DJI_0937	Dactylis glomerata L.	0	n	1	0	Poaceae Barnhart
Iz_8_1009	DJI_0937	Dianthus campestris M.Bieb.	0	n	1	0	Caryophyllaceae Juss.
Iz_8_1009	DJI_0937	Digitaria sanguinalis (L.) Scop.	0	a	0	0	Poaceae Barnhart
Iz_8_1009	DJI_0937	Elymus repens (L.) Gould	0	n	1	0	Poaceae Barnhart
Iz_8_1009	DJI_0937	Epilobium tetragonum L.	0	n	1	0	Onagraceae Juss.
Iz_8_1009	DJI_0937	Eragrostis minor Host	0	a	0	0	Poaceae Barnhart
Iz_8_1009	DJI_0937	Erigeron canadensis L.	10	a	0	0	Asteraceae Bercht. & J.Presl
Iz_8_1009	DJI_0937	Euphorbia seguieriana Neck.	0	n	1	0	Euphorbiaceae Juss.
Iz_8_1009	DJI_0937	Fallopia dumetorum (L.) Holub	0	n	1	0	Polygonaceae Juss.
Iz_8_1009	DJI_0937	Festuca beckeri (Hack.) Trautv.	0	n	1	0	Poaceae Barnhart
Iz_8_1009	DJI_0937	Fragaria vesca L.	0	n	1	0	Rosaceae Juss.
Iz_8_1009	DJI_0937	Frangula alnus Mill.	0	n	1	1	Rhamnaceae Juss.
Iz_8_1009	DJI_0937	Fraxinus pennsylvanica Marshall	0	a	0	1	Oleaceae Hoffmanns. & Link
Iz_8_1009	DJI_0937	Galeopsis bifida Boenn.	0	n	1	0	Lamiacea Lindl.
Iz_8_1009	DJI_0937	Galium verum L.	0	n	1	0	Rubiaceae Juss.
Iz_8_1009	DJI_0937	Genista tinctoria L.	0	n	1	0	Fabaceae Lindl.
Iz_8_1009	DJI_0937	Geranium robertianum L.	0	n	1	0	Geraniaceae Juss.
Iz_8_1009	DJI_0937	Geranium sanquineum L.	0	n	1	0	Geraniaceae Juss.
Iz_8_1009	DJI_0937	Gypsophila paniculata L.	0	n	1	0	Caryophyllaceae Juss.
Iz_8_1009	DJI_0937	Helichrysum arenarium (L.) Moench	0	n	1	0	Asteraceae Bercht. & J.Presl
Iz_8_1009	DJI_0937	Herniaria polygama J.Gay	0	n	1	0	Caryophyllaceae Juss.
Iz_8_1009	DJI_0937	Hieracium robustum Fr.	0	n	1	0	Asteraceae Bercht. & J.Presl
Iz_8_1009	DJI_0937	Hieracium umbellatum L.	0	n	1	NA	Asteraceae Bercht. & J.Presl
Iz_8_1009	DJI_0937	Hylotelephium maximum (L.) Holub	0	n	1	0	Crassulaceae J.St.-Hil.
Iz_8_1009	DJI_0937	Jacobaea borysthenica (DC.) B.Nord. & Greuter	0	n	1	0	Asteraceae Bercht. & J.Presl
Iz_8_1009	DJI_0937	Jasione montana L.	0	n	1	0	Campanulaceae Juss.
Iz_8_1009	DJI_0937	Jurinea cyanoides (L.) Rchb.s.l.	0	n	1	0	Asteraceae Bercht. & J.Presl
Iz_8_1009	DJI_0937	Koeleria glauca (Spreng.) DC.	0	n	1	0	Poaceae Barnhart
Iz_8_1009	DJI_0937	Lactuca serriola L.	0	a	0	0	Asteraceae Bercht. & J.Presl
Iz_8_1009	DJI_0937	Linaria genistifolia (L.) Mill.	0	n	1	0	Plantaginaceae Juss.
Iz_8_1009	DJI_0937	Lomelosia argentea (L.) Greuter & Burdet	0	n	1	0	Caprifoliaceae Juss.
Iz_8_1009	DJI_0937	Melampyrum pratense L.	0	n	1	0	Orobanchaceae Vent.
Iz_8_1009	DJI_0937	Oenothera biennis L.	0	a	0	0	Onagraceae Juss.
Iz_8_1009	DJI_0937	Pentanema britannica (L.) D.Gut.Larr., Santos-Vicente, Anderb., E.Rico & M.M.Mart.Ort.	0	n	1	0	Asteraceae Bercht. & J.Presl
Iz_8_1009	DJI_0937	Peucedanum oreoselinum (L.) Moench	0	n	1	0	Apiaceae Lindl.
Iz_8_1009	DJI_0937	Peucedanum ruthenicum M.Bieb.	0	n	1	0	Apiaceae Lindl.
Iz_8_1009	DJI_0937	Pilosella echioides (Lumn.) F.W.Schultz & Sch.Bip.	0	n	1	0	Asteraceae Bercht. & J.Presl
Iz_8_1009	DJI_0937	Pilosella officinarum F. Schultz et Sch. Bip.	0	n	1	0	Asteraceae Bercht. & J.Presl
Iz_8_1009	DJI_0937	Pilosella sp.	0		NA	0	Asteraceae Bercht. & J.Presl
Iz_8_1009	DJI_0937	Pinus sylvestris L.	0	n	1	1	Pinaceae Spreng. ex F.Rudolphi
Iz_8_1009	DJI_0937	Poa annua L.	0	n	1	0	Poaceae Barnhart
Iz_8_1009	DJI_0937	Poa nemoralis L.	0	n	1	0	Poaceae Barnhart
Iz_8_1009	DJI_0937	Poa sp.	0		NA	0	Poaceae Barnhart
Iz_8_1009	DJI_0937	Polygonatum odoratum (Mill.) Druce	0	n	1	0	Asparagaceae Juss.
Iz_8_1009	DJI_0937	Polygonum arenarium Waldst. & Kit.	0	n	1	0	Polygonaceae Juss.
Iz_8_1009	DJI_0937	Populus nigra L.	0	n	1	1	Salicaceae Mirb.
Iz_8_1009	DJI_0937	Populus tremula L.	0	n	1	1	Salicaceae Mirb.
Iz_8_1009	DJI_0937	Populus Ã canescens (Aiton) Sm.	0	n	1	1	Salicaceae Mirb.
Iz_8_1009	DJI_0937	Potentilla argentea L.	0	n	1	0	Rosaceae Juss.
Iz_8_1009	DJI_0937	Psammophiliella muralis (L.) Ikonn.	0	n	1	0	Caryophyllaceae Juss.
Iz_8_1009	DJI_0937	Psephellus sumensis (Kalen.) Greuter	0	n	1	0	Asteraceae Bercht. & J.Presl
Iz_8_1009	DJI_0937	Pulsatilla pratensis (L.) Mill. s.l.	0	n	1	0	Ranunculaceae Juss.
Iz_8_1009	DJI_0937	Pyrus communis L.	0	a	0	1	Rosaceae Juss.
Iz_8_1009	DJI_0937	Quercus robur L.	0	n	1	1	Fagaceae Dumort.
Iz_8_1009	DJI_0937	Robinia pseudoacacia L.	0	a	0	1	Fabaceae Lindl.
Iz_8_1009	DJI_0937	Rosa sp.	0		NA	1	Rosaceae Juss.
Iz_8_1009	DJI_0937	Rubus idaeus L.	0	n	1	0	Rosaceae Juss.
Iz_8_1009	DJI_0937	Rumex acetosella L.	0	n	1	0	Polygonaceae Juss.
Iz_8_1009	DJI_0937	Rumex thyrsiflorus Fingerh.	0	n	1	0	Polygonaceae Juss.
Iz_8_1009	DJI_0937	Salix alba L.	0	n	1	1	Salicaceae Mirb.
Iz_8_1009	DJI_0937	Sambucus nigra L.	0	n	1	1	Viburnaceae Raf.
Iz_8_1009	DJI_0937	Secale sylvestre Host	0	n	1	0	Poaceae Barnhart

Iz_8_1009	DJI_0937	Sempervivum ruthenicum Schnittsp. & C.B. Lehm.	0	n	1	0	Crassulaceae J.St.-Hil.
Iz_8_1009	DJI_0937	Setaria viridis (L.) P. Beauv.	0	n	1	0	Poaceae Barnhart
Iz_8_1009	DJI_0937	Silene borysthenica (Gruner) Walters	0	n	1	0	Caryophyllaceae Juss.
Iz_8_1009	DJI_0937	Silene latifolia subsp. alba (Mill.) Greuter & Burdet	0	n	1	0	Caryophyllaceae Juss.
Iz_8_1009	DJI_0937	Solidago virgaurea L.	0	n	1	0	Asteraceae Bercht. & J.Presl
Iz_8_1009	DJI_0937	Sorbus aucuparia L.	0	n	1	1	Rosaceae Juss.
Iz_8_1009	DJI_0937	Stachys recta L.	0	n	1	0	Lamiacea Lindl.
Iz_8_1009	DJI_0937	Tanacetum vulgare L.	0	n	1	0	Asteraceae Bercht. & J.Presl
Iz_8_1009	DJI_0937	Thymus pallasianus Heinr.Braun	0	n	1	0	Lamiacea Lindl.
Iz_8_1009	DJI_0937	Tilia cordata Mill.	0	n	1	1	Malvaceae Juss.
Iz_8_1009	DJI_0937	Trifolium alpestre L.	0	n	1	0	Fabaceae Lindl.
Iz_8_1009	DJI_0937	Trifolium arvense L.	0	n	1	0	Fabaceae Lindl.
Iz_8_1009	DJI_0937	Ulmus glabra Huds.	0	n	1	1	Ulmaceae Mirb.
Iz_8_1009	DJI_0937	Verbascum densiflorum Bertol.	0	n	1	0	Scrophulariaceae Juss.
Iz_8_1009	DJI_0937	Veronica austriaca L.	0	n	1	0	Plantaginaceae Juss.
Iz_8_1009	DJI_0937	Veronica incana L.	0	n	1	0	Plantaginaceae Juss.
Iz_8_1009	DJI_0937	Veronica verna L.	0	n	1	0	Plantaginaceae Juss.
Iz_8_1009	DJI_0937	Vincetoxicum hirundinaria Medik	0	n	1	0	Apocynaceae Juss.
Iz_8_1009	DJI_0939	Acer tataricum L.	0	n	1	1	Sapindaceae Juss.
Iz_8_1009	DJI_0939	Achillea millefolium L. s.l.	0	n	1	0	Asteraceae Bercht. & J.Presl
Iz_8_1009	DJI_0939	Allium oleraceum L.	0	n	1	0	Amaryllidaceae J.St.-Hil.
Iz_8_1009	DJI_0939	Ambrosia artemisiifolia L.	0	a	0	0	Asteraceae Bercht. & J.Presl
Iz_8_1009	DJI_0939	Amorpha fruticosa L.	0	a	0	0	Fabaceae Lindl.
Iz_8_1009	DJI_0939	Anthericum ramosum L.	0	n	1	0	Asparagaceae Juss.
Iz_8_1009	DJI_0939	Artemisia marschalliana Spreng.	0	n	1	0	Asteraceae Bercht. & J.Presl
Iz_8_1009	DJI_0939	Asparagus officinalis L.	0	n	1	0	Asparagaceae Juss.
Iz_8_1009	DJI_0939	Astragalus cicer L.	0	n	1	0	Fabaceae Lindl.
Iz_8_1009	DJI_0939	Bassia laniflora (S.G.Gmel.) A.J.Scott	0	n	1	0	Amaranthaceae Juss.
Iz_8_1009	DJI_0939	Berberis vulgaris L.	0	n	1	0	Berberidaceae Juss.
Iz_8_1009	DJI_0939	Betula pendula Roth.	0	n	1	1	Betulaceae Gray
Iz_8_1009	DJI_0939	Betula pendula Roth.	0	n	1	0	Poaceae Barnhart
Iz_8_1009	DJI_0939	Bromus inermis Leyss.	0		NA	NA	
Iz_8_1009	DJI_0939	Bromus squarrosus L.	0	a	0	0	Poaceae Barnhart
Iz_8_1009	DJI_0939	Bromus tectorum L.	0	a	0	0	Poaceae Barnhart
Iz_8_1009	DJI_0939	Calamagrostis epigejos (L.) Roth	0	n	1	0	Poaceae Barnhart
Iz_8_1009	DJI_0939	Carduus acanthoides L.	0	n	1	0	Asteraceae Bercht. & J.Presl
Iz_8_1009	DJI_0939	Carex sp.	0	n	1	0	Cyperaceae Juss.
Iz_8_1009	DJI_0939	Centaurea majorovii Dumbadze	0	n	1	0	Asteraceae Bercht. & J.Presl
Iz_8_1009	DJI_0939	Chamaecytisus ruthenicus (Fisch.ex Wol.) Klaskova	0	n	1	0	Fabaceae Lindl.
Iz_8_1009	DJI_0939	Chelidonium majus L.	0	n	1	0	Papaveraceae Juss.
Iz_8_1009	DJI_0939	Chenopodium betaceum Andrz.	0	n	1	0	Amaranthaceae Juss.
Iz_8_1009	DJI_0939	Chenopodium cf. album	0	n	1	0	Amaranthaceae Juss.
Iz_8_1009	DJI_0939	Chenopodium sp.	0		NA	0	Amaranthaceae Juss.
Iz_8_1009	DJI_0939	Chondrilla juncea L.	0	n	1	0	Asteraceae Bercht. & J.Presl
Iz_8_1009	DJI_0939	Cirsium vulgare (Savi) Ten.	0	n	1	0	Asteraceae Bercht. & J.Presl
Iz_8_1009	DJI_0939	Clinopodium acinos (L.) Kuntze	0	n	1	0	Lamiacea Lindl.
Iz_8_1009	DJI_0939	Convallaria majalis L.	0	n	1	0	Asparagaceae Juss.
Iz_8_1009	DJI_0939	Crataegus rhipidophylla Gand.	0	n	1	1	Rosaceae Juss.
Iz_8_1009	DJI_0939	Crepis foetida L.	0	n	1	0	Asteraceae Bercht. & J.Presl
Iz_8_1009	DJI_0939	Dactylis glomerata L.	0	n	1	0	Poaceae Barnhart
Iz_8_1009	DJI_0939	Dianthus campestris M.Bieb.	0	n	1	0	Caryophyllaceae Juss.
Iz_8_1009	DJI_0939	Digitaria sanguinalis (L.) Scop.	0	a	0	0	Poaceae Barnhart
Iz_8_1009	DJI_0939	Elymus repens (L.) Gould	0	n	1	0	Poaceae Barnhart
Iz_8_1009	DJI_0939	Epilobium tetragonum L.	0	n	1	0	Onagraceae Juss.
Iz_8_1009	DJI_0939	Eragrostis minor Host	0	a	0	0	Poaceae Barnhart
Iz_8_1009	DJI_0939	Erigeron canadensis L.	15	a	0	0	Asteraceae Bercht. & J.Presl
Iz_8_1009	DJI_0939	Euphorbia seguieriana Neck.	0	n	1	0	Euphorbiaceae Juss.
Iz_8_1009	DJI_0939	Fallopia dumetorum (L.) Holub	0	n	1	0	Polygonaceae Juss.
Iz_8_1009	DJI_0939	Festuca beckeri (Hack.) Trautv.	0	n	1	0	Poaceae Barnhart
Iz_8_1009	DJI_0939	Fragaria vesca L.	0	n	1	0	Rosaceae Juss.
Iz_8_1009	DJI_0939	Frangula alnus Mill.	0	n	1	1	Rhamnaceae Juss.
Iz_8_1009	DJI_0939	Fraxinus pennsylvanica Marshall	0	a	0	1	Oleaceae Hoffmanns. & Link
Iz_8_1009	DJI_0939	Galeopsis bifida Boenn.	0	n	1	0	Lamiacea Lindl.
Iz_8_1009	DJI_0939	Galium verum L.	0	n	1	0	Rubiaceae Juss.
Iz_8_1009	DJI_0939	Genista tinctoria L.	0	n	1	0	Fabaceae Lindl.
Iz_8_1009	DJI_0939	Geranium robertianum L.	0	n	1	0	Geraniaceae Juss.
Iz_8_1009	DJI_0939	Geranium sanquineum L.	0	n	1	0	Geraniaceae Juss.
Iz_8_1009	DJI_0939	Gypsophila paniculata L.	0	n	1	0	Caryophyllaceae Juss.
Iz_8_1009	DJI_0939	Helichrysum arenarium (L.) Moench	0	n	1	0	Asteraceae Bercht. & J.Presl
Iz_8_1009	DJI_0939	Herniaria polygama J.Gay	0	n	1	0	Caryophyllaceae Juss.
Iz_8_1009	DJI_0939	Hieracium robustum Fr.	0	n	1	0	Asteraceae Bercht. & J.Presl
Iz_8_1009	DJI_0939	Hieracium umbellatum L.	0	n	1	NA	Asteraceae Bercht. & J.Presl
Iz_8_1009	DJI_0939	Hylotelephium maximum (L.) Holub	0	n	1	0	Crassulaceae J.St.-Hil.
Iz_8_1009	DJI_0939	Jacobaea borysthenica (DC.) B.Nord. & Greuter	0	n	1	0	Asteraceae Bercht. & J.Presl
Iz_8_1009	DJI_0939	Jasione montana L.	0	n	1	0	Campanulaceae Juss.
Iz_8_1009	DJI_0939	Jurinea cyanoides (L.) Rchb.s.l.	0	n	1	0	Asteraceae Bercht. & J.Presl
Iz_8_1009	DJI_0939	Koeleria glauca (Spreng.) DC.	0	n	1	0	Poaceae Barnhart
Iz_8_1009	DJI_0939	Lactuca serriola L.	0	a	0	0	Asteraceae Bercht. & J.Presl
Iz_8_1009	DJI_0939	Linaria genistifolia (L.) Mill.	0	n	1	0	Plantaginaceae Juss.
Iz_8_1009	DJI_0939	Lomelosia argentea (L.) Greuter & Burdet	0	n	1	0	Caprifoliaceae Juss.
Iz_8_1009	DJI_0939	Melampyrum pratense L.	0	n	1	0	Orobanchaceae Vent.
Iz_8_1009	DJI_0939	Oenothera biennis L.	0	a	0	0	Onagraceae Juss.
Iz_8_1009	DJI_0939	Pentanema britannica (L.) D.Gut.Larr., Santos-Vicente, Anderb., E.Rico & M.M.Mart.Ort.	0	n	1	0	Asteraceae Bercht. & J.Presl
Iz_8_1009	DJI_0939	Peucedanum oreoselinum (L.) Moench	0	n	1	0	Apiaceae Lindl.
Iz_8_1009	DJI_0939	Peucedanum ruthenicum M.Bieb.	0	n	1	0	Apiaceae Lindl.
Iz_8_1009	DJI_0939	Pilosella echioides (Lumn.) F.W.Schultz & Sch.Bip.	0	n	1	0	Asteraceae Bercht. & J.Presl
Iz_8_1009	DJI_0939	Pilosella officinarum F. Schultz et Sch. Bip.	0	n	1	0	Asteraceae Bercht. & J.Presl
Iz_8_1009	DJI_0939	Pilosella sp.	0		NA	0	Asteraceae Bercht. & J.Presl
Iz_8_1009	DJI_0939	Pinus sylvestris L.	0	n	1	1	Pinaceae Spreng. ex F.Rudolphi
Iz_8_1009	DJI_0939	Poa annua L.	0	n	1	0	Poaceae Barnhart
Iz_8_1009	DJI_0939	Poa nemoralis L.	0	n	1	0	Poaceae Barnhart
Iz_8_1009	DJI_0939	Poa sp.	0		NA	0	Poaceae Barnhart
Iz_8_1009	DJI_0939	Polygonatum odoratum (Mill.) Druce	0	n	1	0	Asparagaceae Juss.
Iz_8_1009	DJI_0939	Polygonum arenarium Waldst. & Kit.	0	n	1	0	Polygonaceae Juss.
Iz_8_1009	DJI_0939	Populus nigra L.	0	n	1	1	Salicaceae Mirb.
Iz_8_1009	DJI_0939	Populus tremula L.	0	n	1	1	Salicaceae Mirb.
Iz_8_1009	DJI_0939	Populus Ã canescens (Aiton) Sm.	0	n	1	1	Salicaceae Mirb.
Iz_8_1009	DJI_0939	Potentilla argentea L.	0	n	1	0	Rosaceae Juss.

Iz_8_1009	DJI_0939	Psammophiliella muralis (L.) Ikonn.	0	n	1	0	Caryophyllaceae Juss.
Iz_8_1009	DJI_0939	Psephellus sumensis (Kalen.) Greuter	0	n	1	0	Asteraceae Bercht. & J.Presl
Iz_8_1009	DJI_0939	Pulsatilla pratensis (L.) Mill. s.l.	0	n	1	0	Ranunculaceae Juss.
Iz_8_1009	DJI_0939	Pyrus communis L.	0	a	0	1	Rosaceae Juss.
Iz_8_1009	DJI_0939	Quercus robur L.	0	n	1	1	Fagaceae Dumort.
Iz_8_1009	DJI_0939	Robinia pseudoacacia L.	0	a	0	1	Fabaceae Lindl.
Iz_8_1009	DJI_0939	Rosa sp.	0		NA	1	Rosaceae Juss.
Iz_8_1009	DJI_0939	Rubus idaeus L.	0	n	1	0	Rosaceae Juss.
Iz_8_1009	DJI_0939	Rumex acetosella L.	0	n	1	0	Polygonaceae Juss.
Iz_8_1009	DJI_0939	Rumex thyrsiflorus Fingerh.	0	n	1	0	Polygonaceae Juss.
Iz_8_1009	DJI_0939	Salix alba L.	0	n	1	1	Salicaceae Mirb.
Iz_8_1009	DJI_0939	Sambucus nigra L.	0	n	1	1	Viburnaceae Raf.
Iz_8_1009	DJI_0939	Secale sylvestre Host	0	n	1	0	Poaceae Barnhart
Iz_8_1009	DJI_0939	Sempervivum ruthenicum Schnittsp. & C.B. Lehm.	0	n	1	0	Crassulaceae J.St.-Hil.
Iz_8_1009	DJI_0939	Setaria viridis (L.) P. Beauv.	0	n	1	0	Poaceae Barnhart
Iz_8_1009	DJI_0939	Silene borysthenica (Gruner) Walters	0	n	1	0	Caryophyllaceae Juss.
Iz_8_1009	DJI_0939	Silene latifolia subsp. alba (Mill.) Greuter & Burdet	0	n	1	0	Caryophyllaceae Juss.
Iz_8_1009	DJI_0939	Solidago virgaurea L.	0	n	1	0	Asteraceae Bercht. & J.Presl
Iz_8_1009	DJI_0939	Sorbus aucuparia L.	0	n	1	1	Rosaceae Juss.
Iz_8_1009	DJI_0939	Stachys recta L.	0	n	1	0	Lamiacea Lindl.
Iz_8_1009	DJI_0939	Tanacetum vulgare L.	0	n	1	0	Asteraceae Bercht. & J.Presl
Iz_8_1009	DJI_0939	Thymus pallasianus Heinr.Braun	0	n	1	0	Lamiacea Lindl.
Iz_8_1009	DJI_0939	Tilia cordata Mill.	0	n	1	1	Malvaceae Juss.
Iz_8_1009	DJI_0939	Trifolium alpestre L.	0	n	1	0	Fabaceae Lindl.
Iz_8_1009	DJI_0939	Trifolium arvense L.	0	n	1	0	Fabaceae Lindl.
Iz_8_1009	DJI_0939	Ulmus glabra Huds.	0	n	1	1	Ulmaceae Mirb.
Iz_8_1009	DJI_0939	Verbascum densiflorum Bertol.	0	n	1	0	Scrophulariaceae Juss.
Iz_8_1009	DJI_0939	Veronica austriaca L.	0	n	1	0	Plantaginaceae Juss.
Iz_8_1009	DJI_0939	Veronica incana L.	0	n	1	0	Plantaginaceae Juss.
Iz_8_1009	DJI_0939	Veronica verna L.	0	n	1	0	Plantaginaceae Juss.
Iz_8_1009	DJI_0939	Vincetoxicum hirundinaria Medik	0	n	1	0	Apocynaceae Juss.
Iz_8_1009	DJI_0940	Acer tataricum L.	0	n	1	1	Sapindaceae Juss.
Iz_8_1009	DJI_0940	Achillea millefolium L. s.l.	0	n	1	0	Asteraceae Bercht. & J.Presl
Iz_8_1009	DJI_0940	Allium oleraceum L.	0	n	1	0	Amaryllidaceae J.St.-Hil.
Iz_8_1009	DJI_0940	Ambrosia artemisiifolia L.	0	a	0	0	Asteraceae Bercht. & J.Presl
Iz_8_1009	DJI_0940	Amorpha fruticosa L.	0	a	0	0	Fabaceae Lindl.
Iz_8_1009	DJI_0940	Anthericum ramosum L.	0	n	1	0	Asparagaceae Juss.
Iz_8_1009	DJI_0940	Artemisia marschalliana Spreng.	0	n	1	0	Asteraceae Bercht. & J.Presl
Iz_8_1009	DJI_0940	Asparagus officinalis L.	0	n	1	0	Asparagaceae Juss.
Iz_8_1009	DJI_0940	Astragalus cicer L.	0	n	1	0	Fabaceae Lindl.
Iz_8_1009	DJI_0940	Bassia laniflora (S.G.Gmel.) A.J.Scott	0	n	1	0	Amaranthaceae Juss.
Iz_8_1009	DJI_0940	Berberis vulgaris L.	0	n	1	0	Berberidaceae Juss.
Iz_8_1009	DJI_0940	Betula pendula Roth.	0	n	1	1	Betulaceae Gray
Iz_8_1009	DJI_0940	Betula pendula Roth.	0	n	1	0	Poaceae Barnhart
Iz_8_1009	DJI_0940	Bromus inermis Leyss.	0		NA	NA	
Iz_8_1009	DJI_0940	Bromus squarrosus L.	0	a	0	0	Poaceae Barnhart
Iz_8_1009	DJI_0940	Bromus tectorum L.	0	a	0	0	Poaceae Barnhart
Iz_8_1009	DJI_0940	Calamagrostis epigejos (L.) Roth	15	n	1	0	Poaceae Barnhart
Iz_8_1009	DJI_0940	Carduus acanthoides L.	0	n	1	0	Asteraceae Bercht. & J.Presl
Iz_8_1009	DJI_0940	Carex sp.	0	n	1	0	Cyperaceae Juss.
Iz_8_1009	DJI_0940	Centaurea majorovii Dumbadze	0	n	1	0	Asteraceae Bercht. & J.Presl
Iz_8_1009	DJI_0940	Chamaecytisus ruthenicus (Fisch.ex Wol.) Klaskova	0	n	1	0	Fabaceae Lindl.
Iz_8_1009	DJI_0940	Chelidonium majus L.	0	n	1	0	Papaveraceae Juss.
Iz_8_1009	DJI_0940	Chenopodium betaceum Andrz.	0	n	1	0	Amaranthaceae Juss.
Iz_8_1009	DJI_0940	Chenopodium cf. album	0	n	1	0	Amaranthaceae Juss.
Iz_8_1009	DJI_0940	Chenopodium sp.	0		NA	0	Amaranthaceae Juss.
Iz_8_1009	DJI_0940	Chondrilla juncea L.	0	n	1	0	Asteraceae Bercht. & J.Presl
Iz_8_1009	DJI_0940	Cirsium vulgare (Savi) Ten.	0	n	1	0	Asteraceae Bercht. & J.Presl
Iz_8_1009	DJI_0940	Clinopodium acinos (L.) Kuntze	0	n	1	0	Lamiacea Lindl.
Iz_8_1009	DJI_0940	Convallaria majalis L.	0	n	1	0	Asparagaceae Juss.
Iz_8_1009	DJI_0940	Crataegus rhipidophylla Gand.	0	n	1	1	Rosaceae Juss.
Iz_8_1009	DJI_0940	Crepis foetida L.	0	n	1	0	Asteraceae Bercht. & J.Presl
Iz_8_1009	DJI_0940	Dactylis glomerata L.	0	n	1	0	Poaceae Barnhart
Iz_8_1009	DJI_0940	Dianthus campestris M.Bieb.	0	n	1	0	Caryophyllaceae Juss.
Iz_8_1009	DJI_0940	Digitaria sanguinalis (L.) Scop.	0	a	0	0	Poaceae Barnhart
Iz_8_1009	DJI_0940	Elymus repens (L.) Gould	0	n	1	0	Poaceae Barnhart
Iz_8_1009	DJI_0940	Epilobium tetragonum L.	0	n	1	0	Onagraceae Juss.
Iz_8_1009	DJI_0940	Eragrostis minor Host	0	a	0	0	Poaceae Barnhart
Iz_8_1009	DJI_0940	Erigeron canadensis L.	10	a	0	0	Asteraceae Bercht. & J.Presl
Iz_8_1009	DJI_0940	Euphorbia seguieriana Neck.	0	n	1	0	Euphorbiaceae Juss.
Iz_8_1009	DJI_0940	Fallopia dumetorum (L.) Holub	0	n	1	0	Polygonaceae Juss.
Iz_8_1009	DJI_0940	Festuca beckeri (Hack.) Trautv.	0	n	1	0	Poaceae Barnhart
Iz_8_1009	DJI_0940	Fragaria vesca L.	0	n	1	0	Rosaceae Juss.
Iz_8_1009	DJI_0940	Frangula alnus Mill.	0	n	1	1	Rhamnaceae Juss.
Iz_8_1009	DJI_0940	Fraxinus pennsylvanica Marshall	0	a	0	1	Oleaceae Hoffmanns. & Link
Iz_8_1009	DJI_0940	Galeopsis bifida Boenn.	0	n	1	0	Lamiacea Lindl.
Iz_8_1009	DJI_0940	Galium verum L.	0	n	1	0	Rubiaceae Juss.
Iz_8_1009	DJI_0940	Genista tinctoria L.	0	n	1	0	Fabaceae Lindl.
Iz_8_1009	DJI_0940	Geranium robertianum L.	0	n	1	0	Geraniaceae Juss.
Iz_8_1009	DJI_0940	Geranium sanquineum L.	0	n	1	0	Geraniaceae Juss.
Iz_8_1009	DJI_0940	Gypsophila paniculata L.	0	n	1	0	Caryophyllaceae Juss.
Iz_8_1009	DJI_0940	Helichrysum arenarium (L.) Moench	0	n	1	0	Asteraceae Bercht. & J.Presl
Iz_8_1009	DJI_0940	Herniaria polygama J.Gay	0	n	1	0	Caryophyllaceae Juss.
Iz_8_1009	DJI_0940	Hieracium robustum Fr.	0	n	1	0	Asteraceae Bercht. & J.Presl
Iz_8_1009	DJI_0940	Hieracium umbellatum L.	0	n	1	NA	Asteraceae Bercht. & J.Presl
Iz_8_1009	DJI_0940	Hylotelephium maximum (L.) Holub	0	n	1	0	Crassulaceae J.St.-Hil.
Iz_8_1009	DJI_0940	Jacobaea borysthenica (DC.) B.Nord. & Greuter	0	n	1	0	Asteraceae Bercht. & J.Presl
Iz_8_1009	DJI_0940	Jasione montana L.	0	n	1	0	Campanulaceae Juss.
Iz_8_1009	DJI_0940	Jurinea cyanoides (L.) Rchb.s.l.	0	n	1	0	Asteraceae Bercht. & J.Presl
Iz_8_1009	DJI_0940	Koeleria glauca (Spreng.) DC.	0	n	1	0	Poaceae Barnhart
Iz_8_1009	DJI_0940	Lactuca serriola L.	0	a	0	0	Asteraceae Bercht. & J.Presl
Iz_8_1009	DJI_0940	Linaria genistifolia (L.) Mill.	0	n	1	0	Plantaginaceae Juss.
Iz_8_1009	DJI_0940	Lomelosia argentea (L.) Greuter & Burdet	0	n	1	0	Caprifoliaceae Juss.
Iz_8_1009	DJI_0940	Melampyrum pratense L.	0	n	1	0	Orobanchaceae Vent.
Iz_8_1009	DJI_0940	Oenothera biennis L.	0	a	0	0	Onagraceae Juss.
Iz_8_1009	DJI_0940	Pentanema britannica (L.) D.Gut.Larr., Santos-Vicente, Anderb., E.Rico & M.M.Mart.Ort.	0	n	1	0	Asteraceae Bercht. & J.Presl
Iz_8_1009	DJI_0940	Peucedanum oreoselinum (L.) Moench	0	n	1	0	Apiaceae Lindl.
Iz_8_1009	DJI_0940	Peucedanum ruthenicum M.Bieb.	0	n	1	0	Apiaceae Lindl.

Iz_8_1009	DJI_0940	Pilosella echioides (Lumn.) F.W.Schultz & Sch.Bip.	0	n	1	0	Asteraceae Bercht. & J.Presl
Iz_8_1009	DJI_0940	Pilosella officinarum F. Schultz et Sch. Bip.	0	n	1	0	Asteraceae Bercht. & J.Presl
Iz_8_1009	DJI_0940	Pilosella sp.	0		NA	0	Asteraceae Bercht. & J.Presl
Iz_8_1009	DJI_0940	Pinus sylvestris L.	0	n	1	1	Pinaceae Spreng. ex F.Rudolphi
Iz_8_1009	DJI_0940	Poa annua L.	0	n	1	0	Poaceae Barnhart
Iz_8_1009	DJI_0940	Poa nemoralis L.	0	n	1	0	Poaceae Barnhart
Iz_8_1009	DJI_0940	Poa sp.	0		NA	0	Poaceae Barnhart
Iz_8_1009	DJI_0940	Polygonatum odoratum (Mill.) Druce	0	n	1	0	Asparagaceae Juss.
Iz_8_1009	DJI_0940	Polygonum arenarium Waldst. & Kit.	0	n	1	0	Polygonaceae Juss.
Iz_8_1009	DJI_0940	Populus nigra L.	0	n	1	1	Salicaceae Mirb.
Iz_8_1009	DJI_0940	Populus tremula L.	0	n	1	1	Salicaceae Mirb.
Iz_8_1009	DJI_0940	Populus Ã canescens (Aiton) Sm.	0	n	1	1	Salicaceae Mirb.
Iz_8_1009	DJI_0940	Potentilla argentea L.	0	n	1	0	Rosaceae Juss.
Iz_8_1009	DJI_0940	Psammophiliella muralis (L.) Ikonn.	0	n	1	0	Caryophyllaceae Juss.
Iz_8_1009	DJI_0940	Psephellus sumensis (Kalen.) Greuter	0	n	1	0	Asteraceae Bercht. & J.Presl
Iz_8_1009	DJI_0940	Pulsatilla pratensis (L.) Mill. s.l.	0	n	1	0	Ranunculaceae Juss.
Iz_8_1009	DJI_0940	Pyrus communis L.	0	a	0	1	Rosaceae Juss.
Iz_8_1009	DJI_0940	Quercus robur L.	0	n	1	1	Fagaceae Dumort.
Iz_8_1009	DJI_0940	Robinia pseudoacacia L.	0	a	0	1	Fabaceae Lindl.
Iz_8_1009	DJI_0940	Rosa sp.	0		NA	1	Rosaceae Juss.
Iz_8_1009	DJI_0940	Rubus idaeus L.	0	n	1	0	Rosaceae Juss.
Iz_8_1009	DJI_0940	Rumex acetosella L.	0	n	1	0	Polygonaceae Juss.
Iz_8_1009	DJI_0940	Rumex thyrsiflorus Fingerh.	0	n	1	0	Polygonaceae Juss.
Iz_8_1009	DJI_0940	Salix alba L.	0	n	1	1	Salicaceae Mirb.
Iz_8_1009	DJI_0940	Sambucus nigra L.	0	n	1	1	Viburnaceae Raf.
Iz_8_1009	DJI_0940	Secale sylvestre Host	0	n	1	0	Poaceae Barnhart
Iz_8_1009	DJI_0940	Sempervivum ruthenicum Schnittsp. & C.B. Lehm.	0	n	1	0	Crassulaceae J.St.-Hil.
Iz_8_1009	DJI_0940	Setaria viridis (L.) P. Beauv.	0	n	1	0	Poaceae Barnhart
Iz_8_1009	DJI_0940	Silene borysthenica (Gruner) Walters	0	n	1	0	Caryophyllaceae Juss.
Iz_8_1009	DJI_0940	Silene latifolia subsp. alba (Mill.) Greuter & Burdet	0	n	1	0	Caryophyllaceae Juss.
Iz_8_1009	DJI_0940	Solidago virgaurea L.	0	n	1	0	Asteraceae Bercht. & J.Presl
Iz_8_1009	DJI_0940	Sorbus aucuparia L.	0	n	1	1	Rosaceae Juss.
Iz_8_1009	DJI_0940	Stachys recta L.	0	n	1	0	Lamiacea Lindl.
Iz_8_1009	DJI_0940	Tanacetum vulgare L.	0	n	1	0	Asteraceae Bercht. & J.Presl
Iz_8_1009	DJI_0940	Thymus pallasianus Heinr.Braun	0	n	1	0	Lamiacea Lindl.
Iz_8_1009	DJI_0940	Tilia cordata Mill.	0	n	1	1	Malvaceae Juss.
Iz_8_1009	DJI_0940	Trifolium alpestre L.	0	n	1	0	Fabaceae Lindl.
Iz_8_1009	DJI_0940	Trifolium arvense L.	0	n	1	0	Fabaceae Lindl.
Iz_8_1009	DJI_0940	Ulmus glabra Huds.	0	n	1	1	Ulmaceae Mirb.
Iz_8_1009	DJI_0940	Verbascum densiflorum Bertol.	0	n	1	0	Scrophulariaceae Juss.
Iz_8_1009	DJI_0940	Veronica austriaca L.	0	n	1	0	Plantaginaceae Juss.
Iz_8_1009	DJI_0940	Veronica incana L.	0	n	1	0	Plantaginaceae Juss.
Iz_8_1009	DJI_0940	Veronica verna L.	0	n	1	0	Plantaginaceae Juss.
Iz_8_1009	DJI_0940	Vincetoxicum hirundinaria Medik	0	n	1	0	Apocynaceae Juss.
Iz_8_1009	DJI_0942	Acer tataricum L.	0	n	1	1	Sapindaceae Juss.
Iz_8_1009	DJI_0942	Achillea millefolium L. s.l.	0	n	1	0	Asteraceae Bercht. & J.Presl
Iz_8_1009	DJI_0942	Allium oleraceum L.	0	n	1	0	Amaryllidaceae J.St.-Hil.
Iz_8_1009	DJI_0942	Ambrosia artemisiifolia L.	0	a	0	0	Asteraceae Bercht. & J.Presl
Iz_8_1009	DJI_0942	Amorpha fruticosa L.	0	a	0	0	Fabaceae Lindl.
Iz_8_1009	DJI_0942	Anthericum ramosum L.	0	n	1	0	Asparagaceae Juss.
Iz_8_1009	DJI_0942	Artemisia marschalliana Spreng.	0	n	1	0	Asteraceae Bercht. & J.Presl
Iz_8_1009	DJI_0942	Asparagus officinalis L.	0	n	1	0	Asparagaceae Juss.
Iz_8_1009	DJI_0942	Astragalus cicer L.	0	n	1	0	Fabaceae Lindl.
Iz_8_1009	DJI_0942	Bassia laniflora (S.G.Gmel.) A.J.Scott	0	n	1	0	Amaranthaceae Juss.
Iz_8_1009	DJI_0942	Berberis vulgaris L.	0	n	1	0	Berberidaceae Juss.
Iz_8_1009	DJI_0942	Betula pendula Roth.	0	n	1	1	Betulaceae Gray
Iz_8_1009	DJI_0942	Betula pendula Roth.	0	n	1	0	Poaceae Barnhart
Iz_8_1009	DJI_0942	Bromus inermis Leyss.	0		NA	NA	
Iz_8_1009	DJI_0942	Bromus squarrosus L.	0	a	0	0	Poaceae Barnhart
Iz_8_1009	DJI_0942	Bromus tectorum L.	0	a	0	0	Poaceae Barnhart
Iz_8_1009	DJI_0942	Calamagrostis epigejos (L.) Roth	3	n	1	0	Poaceae Barnhart
Iz_8_1009	DJI_0942	Carduus acanthoides L.	0	n	1	0	Asteraceae Bercht. & J.Presl
Iz_8_1009	DJI_0942	Carex sp.	0	n	1	0	Cyperaceae Juss.
Iz_8_1009	DJI_0942	Centaurea majorovii Dumbadze	0	n	1	0	Asteraceae Bercht. & J.Presl
Iz_8_1009	DJI_0942	Chamaecytisus ruthenicus (Fisch.ex Wol.) Klaskova	0	n	1	0	Fabaceae Lindl.
Iz_8_1009	DJI_0942	Chelidonium majus L.	0	n	1	0	Papaveraceae Juss.
Iz_8_1009	DJI_0942	Chenopodium betaceum Andrz.	0	n	1	0	Amaranthaceae Juss.
Iz_8_1009	DJI_0942	Chenopodium cf. album	0	n	1	0	Amaranthaceae Juss.
Iz_8_1009	DJI_0942	Chenopodium sp.	0		NA	0	Amaranthaceae Juss.
Iz_8_1009	DJI_0942	Chondrilla juncea L.	0	n	1	0	Asteraceae Bercht. & J.Presl
Iz_8_1009	DJI_0942	Cirsium vulgare (Savi) Ten.	0	n	1	0	Asteraceae Bercht. & J.Presl
Iz_8_1009	DJI_0942	Clinopodium acinos (L.) Kuntze	0	n	1	0	Lamiacea Lindl.
Iz_8_1009	DJI_0942	Convallaria majalis L.	0	n	1	0	Asparagaceae Juss.
Iz_8_1009	DJI_0942	Crataegus rhipidophylla Gand.	0	n	1	1	Rosaceae Juss.
Iz_8_1009	DJI_0942	Crepis foetida L.	0	n	1	0	Asteraceae Bercht. & J.Presl
Iz_8_1009	DJI_0942	Dactylis glomerata L.	0	n	1	0	Poaceae Barnhart
Iz_8_1009	DJI_0942	Dianthus campestris M.Bieb.	0	n	1	0	Caryophyllaceae Juss.
Iz_8_1009	DJI_0942	Digitaria sanguinalis (L.) Scop.	0	a	0	0	Poaceae Barnhart
Iz_8_1009	DJI_0942	Elymus repens (L.) Gould	0	n	1	0	Poaceae Barnhart
Iz_8_1009	DJI_0942	Epilobium tetragonum L.	0	n	1	0	Onagraceae Juss.
Iz_8_1009	DJI_0942	Eragrostis minor Host	0	a	0	0	Poaceae Barnhart
Iz_8_1009	DJI_0942	Erigeron canadensis L.	15	a	0	0	Asteraceae Bercht. & J.Presl
Iz_8_1009	DJI_0942	Euphorbia seguieriana Neck.	0	n	1	0	Euphorbiaceae Juss.
Iz_8_1009	DJI_0942	Fallopia dumetorum (L.) Holub	0	n	1	0	Polygonaceae Juss.
Iz_8_1009	DJI_0942	Festuca beckeri (Hack.) Trautv.	0	n	1	0	Poaceae Barnhart
Iz_8_1009	DJI_0942	Fragaria vesca L.	0	n	1	0	Rosaceae Juss.
Iz_8_1009	DJI_0942	Frangula alnus Mill.	0	n	1	1	Rhamnaceae Juss.
Iz_8_1009	DJI_0942	Fraxinus pennsylvanica Marshall	0	a	0	1	Oleaceae Hoffmanns. & Link
Iz_8_1009	DJI_0942	Galeopsis bifida Boenn.	0	n	1	0	Lamiacea Lindl.
Iz_8_1009	DJI_0942	Galium verum L.	0	n	1	0	Rubiaceae Juss.
Iz_8_1009	DJI_0942	Genista tinctoria L.	0	n	1	0	Fabaceae Lindl.
Iz_8_1009	DJI_0942	Geranium robertianum L.	0	n	1	0	Geraniaceae Juss.
Iz_8_1009	DJI_0942	Geranium sanquineum L.	0	n	1	0	Geraniaceae Juss.
Iz_8_1009	DJI_0942	Gypsophila paniculata L.	0	n	1	0	Caryophyllaceae Juss.
Iz_8_1009	DJI_0942	Helichrysum arenarium (L.) Moench	0	n	1	0	Asteraceae Bercht. & J.Presl
Iz_8_1009	DJI_0942	Herniaria polygama J.Gay	0	n	1	0	Caryophyllaceae Juss.
Iz_8_1009	DJI_0942	Hieracium robustum Fr.	0	n	1	0	Asteraceae Bercht. & J.Presl
Iz_8_1009	DJI_0942	Hieracium umbellatum L.	0	n	1	NA	Asteraceae Bercht. & J.Presl

Iz_8_1009	DJI_0942	Hylotelephium maximum (L.) Holub	0	n	1	0	Crassulaceae J.St.-Hil.
Iz_8_1009	DJI_0942	Jacobaea borysthenica (DC.) B.Nord. & Greuter	0	n	1	0	Asteraceae Bercht. & J.Presl
Iz_8_1009	DJI_0942	Jasione montana L.	0	n	1	0	Campanulaceae Juss.
Iz_8_1009	DJI_0942	Jurinea cyanoides (L.) Rchb.s.l.	0	n	1	0	Asteraceae Bercht. & J.Presl
Iz_8_1009	DJI_0942	Koeleria glauca (Spreng.) DC.	0	n	1	0	Poaceae Barnhart
Iz_8_1009	DJI_0942	Lactuca serriola L.	0	a	0	0	Asteraceae Bercht. & J.Presl
Iz_8_1009	DJI_0942	Linaria genistifolia (L.) Mill.	0	n	1	0	Plantaginaceae Juss.
Iz_8_1009	DJI_0942	Lomelosia argentea (L.) Greuter & Burdet	0	n	1	0	Caprifoliaceae Juss.
Iz_8_1009	DJI_0942	Melampyrum pratense L.	0	n	1	0	Orobanchaceae Vent.
Iz_8_1009	DJI_0942	Oenothera biennis L.	0	a	0	0	Onagraceae Juss.
Iz_8_1009	DJI_0942	Pentanema britannica (L.) D.Gut.Larr., Santos-Vicente, Anderb., E.Rico & M.M.Mart.Ort.	0	n	1	0	Asteraceae Bercht. & J.Presl
Iz_8_1009	DJI_0942	Peucedanum oreoselinum (L.) Moench	0	n	1	0	Apiaceae Lindl.
Iz_8_1009	DJI_0942	Peucedanum ruthenicum M.Bieb.	0	n	1	0	Apiaceae Lindl.
Iz_8_1009	DJI_0942	Pilosella echioides (Lumn.) F.W.Schultz & Sch.Bip.	0	n	1	0	Asteraceae Bercht. & J.Presl
Iz_8_1009	DJI_0942	Pilosella officinarum F. Schultz et Sch. Bip.	0	n	1	0	Asteraceae Bercht. & J.Presl
Iz_8_1009	DJI_0942	Pilosella sp.	0		NA	0	Asteraceae Bercht. & J.Presl
Iz_8_1009	DJI_0942	Pinus sylvestris L.	0	n	1	1	Pinaceae Spreng. ex F.Rudolphi
Iz_8_1009	DJI_0942	Poa annua L.	0	n	1	0	Poaceae Barnhart
Iz_8_1009	DJI_0942	Poa nemoralis L.	0	n	1	0	Poaceae Barnhart
Iz_8_1009	DJI_0942	Poa sp.	0		NA	0	Poaceae Barnhart
Iz_8_1009	DJI_0942	Polygonatum odoratum (Mill.) Druce	0	n	1	0	Asparagaceae Juss.
Iz_8_1009	DJI_0942	Polygonum arenarium Waldst. & Kit.	0	n	1	0	Polygonaceae Juss.
Iz_8_1009	DJI_0942	Populus nigra L.	0	n	1	1	Salicaceae Mirb.
Iz_8_1009	DJI_0942	Populus tremula L.	0	n	1	1	Salicaceae Mirb.
Iz_8_1009	DJI_0942	Populus Ã canescens (Aiton) Sm.	0	n	1	1	Salicaceae Mirb.
Iz_8_1009	DJI_0942	Potentilla argentea L.	0	n	1	0	Rosaceae Juss.
Iz_8_1009	DJI_0942	Psammophiliella muralis (L.) Ikonn.	0	n	1	0	Caryophyllaceae Juss.
Iz_8_1009	DJI_0942	Psephellus sumensis (Kalen.) Greuter	0	n	1	0	Asteraceae Bercht. & J.Presl
Iz_8_1009	DJI_0942	Pulsatilla pratensis (L.) Mill. s.l.	0	n	1	0	Ranunculaceae Juss.
Iz_8_1009	DJI_0942	Pyrus communis L.	0	a	0	1	Rosaceae Juss.
Iz_8_1009	DJI_0942	Quercus robur L.	0	n	1	1	Fagaceae Dumort.
Iz_8_1009	DJI_0942	Robinia pseudoacacia L.	0	a	0	1	Fabaceae Lindl.
Iz_8_1009	DJI_0942	Rosa sp.	0		NA	1	Rosaceae Juss.
Iz_8_1009	DJI_0942	Rubus idaeus L.	0	n	1	0	Rosaceae Juss.
Iz_8_1009	DJI_0942	Rumex acetosella L.	0	n	1	0	Polygonaceae Juss.
Iz_8_1009	DJI_0942	Rumex thyrsiflorus Fingerh.	0	n	1	0	Polygonaceae Juss.
Iz_8_1009	DJI_0942	Salix alba L.	0	n	1	1	Salicaceae Mirb.
Iz_8_1009	DJI_0942	Sambucus nigra L.	0	n	1	1	Viburnaceae Raf.
Iz_8_1009	DJI_0942	Secale sylvestre Host	0	n	1	0	Poaceae Barnhart
Iz_8_1009	DJI_0942	Sempervivum ruthenicum Schnittsp. & C.B. Lehm.	0	n	1	0	Crassulaceae J.St.-Hil.
Iz_8_1009	DJI_0942	Setaria viridis (L.) P. Beauv.	0	n	1	0	Poaceae Barnhart
Iz_8_1009	DJI_0942	Silene borysthenica (Gruner) Walters	0	n	1	0	Caryophyllaceae Juss.
Iz_8_1009	DJI_0942	Silene latifolia subsp. alba (Mill.) Greuter & Burdet	0	n	1	0	Caryophyllaceae Juss.
Iz_8_1009	DJI_0942	Solidago virgaurea L.	0	n	1	0	Asteraceae Bercht. & J.Presl
Iz_8_1009	DJI_0942	Sorbus aucuparia L.	0	n	1	1	Rosaceae Juss.
Iz_8_1009	DJI_0942	Stachys recta L.	0	n	1	0	Lamiacea Lindl.
Iz_8_1009	DJI_0942	Tanacetum vulgare L.	0	n	1	0	Asteraceae Bercht. & J.Presl
Iz_8_1009	DJI_0942	Thymus pallasianus Heinr.Braun	0	n	1	0	Lamiacea Lindl.
Iz_8_1009	DJI_0942	Tilia cordata Mill.	0	n	1	1	Malvaceae Juss.
Iz_8_1009	DJI_0942	Trifolium alpestre L.	0	n	1	0	Fabaceae Lindl.
Iz_8_1009	DJI_0942	Trifolium arvense L.	0	n	1	0	Fabaceae Lindl.
Iz_8_1009	DJI_0942	Ulmus glabra Huds.	0	n	1	1	Ulmaceae Mirb.
Iz_8_1009	DJI_0942	Verbascum densiflorum Bertol.	0	n	1	0	Scrophulariaceae Juss.
Iz_8_1009	DJI_0942	Veronica austriaca L.	0	n	1	0	Plantaginaceae Juss.
Iz_8_1009	DJI_0942	Veronica incana L.	0	n	1	0	Plantaginaceae Juss.
Iz_8_1009	DJI_0942	Veronica verna L.	0	n	1	0	Plantaginaceae Juss.
Iz_8_1009	DJI_0942	Vincetoxicum hirundinaria Medik	0	n	1	0	Apocynaceae Juss.
Iz_8_1009	DJI_0943	Acer tataricum L.	0	n	1	1	Sapindaceae Juss.
Iz_8_1009	DJI_0943	Achillea millefolium L. s.l.	0	n	1	0	Asteraceae Bercht. & J.Presl
Iz_8_1009	DJI_0943	Allium oleraceum L.	0	n	1	0	Amaryllidaceae J.St.-Hil.
Iz_8_1009	DJI_0943	Ambrosia artemisiifolia L.	0	a	0	0	Asteraceae Bercht. & J.Presl
Iz_8_1009	DJI_0943	Amorpha fruticosa L.	0	a	0	0	Fabaceae Lindl.
Iz_8_1009	DJI_0943	Anthericum ramosum L.	0	n	1	0	Asparagaceae Juss.
Iz_8_1009	DJI_0943	Artemisia marschalliana Spreng.	0	n	1	0	Asteraceae Bercht. & J.Presl
Iz_8_1009	DJI_0943	Asparagus officinalis L.	0	n	1	0	Asparagaceae Juss.
Iz_8_1009	DJI_0943	Astragalus cicer L.	0	n	1	0	Fabaceae Lindl.
Iz_8_1009	DJI_0943	Bassia laniflora (S.G.Gmel.) A.J.Scott	0	n	1	0	Amaranthaceae Juss.
Iz_8_1009	DJI_0943	Berberis vulgaris L.	0	n	1	0	Berberidaceae Juss.
Iz_8_1009	DJI_0943	Betula pendula Roth.	0	n	1	1	Betulaceae Gray
Iz_8_1009	DJI_0943	Betula pendula Roth.	0	n	1	0	Poaceae Barnhart
Iz_8_1009	DJI_0943	Bromus inermis Leyss.	0		NA	NA	
Iz_8_1009	DJI_0943	Bromus squarrosus L.	0	a	0	0	Poaceae Barnhart
Iz_8_1009	DJI_0943	Bromus tectorum L.	0	a	0	0	Poaceae Barnhart
Iz_8_1009	DJI_0943	Calamagrostis epigejos (L.) Roth	0	n	1	0	Poaceae Barnhart
Iz_8_1009	DJI_0943	Carduus acanthoides L.	0	n	1	0	Asteraceae Bercht. & J.Presl
Iz_8_1009	DJI_0943	Carex sp.	0	n	1	0	Cyperaceae Juss.
Iz_8_1009	DJI_0943	Centaurea majorovii Dumbadze	0	n	1	0	Asteraceae Bercht. & J.Presl
Iz_8_1009	DJI_0943	Chamaecytisus ruthenicus (Fisch.ex Wol.) Klaskova	0	n	1	0	Fabaceae Lindl.
Iz_8_1009	DJI_0943	Chelidonium majus L.	0	n	1	0	Papaveraceae Juss.
Iz_8_1009	DJI_0943	Chenopodium betaceum Andrz.	0	n	1	0	Amaranthaceae Juss.
Iz_8_1009	DJI_0943	Chenopodium cf. album	0	n	1	0	Amaranthaceae Juss.
Iz_8_1009	DJI_0943	Chenopodium sp.	0		NA	0	Amaranthaceae Juss.
Iz_8_1009	DJI_0943	Chondrilla juncea L.	0	n	1	0	Asteraceae Bercht. & J.Presl
Iz_8_1009	DJI_0943	Cirsium vulgare (Savi) Ten.	0	n	1	0	Asteraceae Bercht. & J.Presl
Iz_8_1009	DJI_0943	Clinopodium acinos (L.) Kuntze	0	n	1	0	Lamiacea Lindl.
Iz_8_1009	DJI_0943	Convallaria majalis L.	0	n	1	0	Asparagaceae Juss.
Iz_8_1009	DJI_0943	Crataegus rhipidophylla Gand.	0	n	1	1	Rosaceae Juss.
Iz_8_1009	DJI_0943	Crepis foetida L.	0	n	1	0	Asteraceae Bercht. & J.Presl
Iz_8_1009	DJI_0943	Dactylis glomerata L.	0	n	1	0	Poaceae Barnhart
Iz_8_1009	DJI_0943	Dianthus campestris M.Bieb.	0	n	1	0	Caryophyllaceae Juss.
Iz_8_1009	DJI_0943	Digitaria sanguinalis (L.) Scop.	0	a	0	0	Poaceae Barnhart
Iz_8_1009	DJI_0943	Elymus repens (L.) Gould	0	n	1	0	Poaceae Barnhart
Iz_8_1009	DJI_0943	Epilobium tetragonum L.	0	n	1	0	Onagraceae Juss.
Iz_8_1009	DJI_0943	Eragrostis minor Host	0	a	0	0	Poaceae Barnhart
Iz_8_1009	DJI_0943	Erigeron canadensis L.	35	a	0	0	Asteraceae Bercht. & J.Presl
Iz_8_1009	DJI_0943	Euphorbia seguieriana Neck.	0	n	1	0	Euphorbiaceae Juss.
Iz_8_1009	DJI_0943	Fallopia dumetorum (L.) Holub	0	n	1	0	Polygonaceae Juss.
Iz_8_1009	DJI_0943	Festuca beckeri (Hack.) Trautv.	0	n	1	0	Poaceae Barnhart

Iz_8_1009	DJI_0943	Fragaria vesca L.	0	n	1	0	Rosaceae Juss.
Iz_8_1009	DJI_0943	Frangula alnus Mill.	0	n	1	1	Rhamnaceae Juss.
Iz_8_1009	DJI_0943	Fraxinus pennsylvanica Marshall	0	a	0	1	Oleaceae Hoffmanns. & Link
Iz_8_1009	DJI_0943	Galeopsis bifida Boenn.	0	n	1	0	Lamiacea Lindl.
Iz_8_1009	DJI_0943	Galium verum L.	0	n	1	0	Rubiaceae Juss.
Iz_8_1009	DJI_0943	Genista tinctoria L.	0	n	1	0	Fabaceae Lindl.
Iz_8_1009	DJI_0943	Geranium robertianum L.	0	n	1	0	Geraniaceae Juss.
Iz_8_1009	DJI_0943	Geranium sanquineum L.	0	n	1	0	Geraniaceae Juss.
Iz_8_1009	DJI_0943	Gypsophila paniculata L.	0	n	1	0	Caryophyllaceae Juss.
Iz_8_1009	DJI_0943	Helichrysum arenarium (L.) Moench	0	n	1	0	Asteraceae Bercht. & J.Presl
Iz_8_1009	DJI_0943	Herniaria polygama J.Gay	0	n	1	0	Caryophyllaceae Juss.
Iz_8_1009	DJI_0943	Hieracium robustum Fr.	0	n	1	0	Asteraceae Bercht. & J.Presl
Iz_8_1009	DJI_0943	Hieracium umbellatum L.	0	n	1	NA	Asteraceae Bercht. & J.Presl
Iz_8_1009	DJI_0943	Hylotelephium maximum (L.) Holub	0	n	1	0	Crassulaceae J.St.-Hil.
Iz_8_1009	DJI_0943	Jacobaea borysthenica (DC.) B.Nord. & Greuter	0	n	1	0	Asteraceae Bercht. & J.Presl
Iz_8_1009	DJI_0943	Jasione montana L.	0	n	1	0	Campanulaceae Juss.
Iz_8_1009	DJI_0943	Jurinea cyanoides (L.) Rchb.s.l.	0	n	1	0	Asteraceae Bercht. & J.Presl
Iz_8_1009	DJI_0943	Koeleria glauca (Spreng.) DC.	0	n	1	0	Poaceae Barnhart
Iz_8_1009	DJI_0943	Lactuca serriola L.	0	a	0	0	Asteraceae Bercht. & J.Presl
Iz_8_1009	DJI_0943	Linaria genistifolia (L.) Mill.	0	n	1	0	Plantaginaceae Juss.
Iz_8_1009	DJI_0943	Lomelosia argentea (L.) Greuter & Burdet	0	n	1	0	Caprifoliaceae Juss.
Iz_8_1009	DJI_0943	Melampyrum pratense L.	0	n	1	0	Orobanchaceae Vent.
Iz_8_1009	DJI_0943	Oenothera biennis L.	0	a	0	0	Onagraceae Juss.
Iz_8_1009	DJI_0943	Pentanema britannica (L.) D.Gut.Larr., Santos-Vicente, Anderb., E.Rico & M.M.Mart.Ort.	0	n	1	0	Asteraceae Bercht. & J.Presl
Iz_8_1009	DJI_0943	Peucedanum oreoselinum (L.) Moench	0	n	1	0	Apiaceae Lindl.
Iz_8_1009	DJI_0943	Peucedanum ruthenicum M.Bieb.	0	n	1	0	Apiaceae Lindl.
Iz_8_1009	DJI_0943	Pilosella echioides (Lumn.) F.W.Schultz & Sch.Bip.	0	n	1	0	Asteraceae Bercht. & J.Presl
Iz_8_1009	DJI_0943	Pilosella officinarum F. Schultz et Sch. Bip.	0	n	1	0	Asteraceae Bercht. & J.Presl
Iz_8_1009	DJI_0943	Pilosella sp.	0		NA	0	Asteraceae Bercht. & J.Presl
Iz_8_1009	DJI_0943	Pinus sylvestris L.	0	n	1	1	Pinaceae Spreng. ex F.Rudolphi
Iz_8_1009	DJI_0943	Poa annua L.	0	n	1	0	Poaceae Barnhart
Iz_8_1009	DJI_0943	Poa nemoralis L.	0	n	1	0	Poaceae Barnhart
Iz_8_1009	DJI_0943	Poa sp.	0		NA	0	Poaceae Barnhart
Iz_8_1009	DJI_0943	Polygonatum odoratum (Mill.) Druce	0	n	1	0	Asparagaceae Juss.
Iz_8_1009	DJI_0943	Polygonum arenarium Waldst. & Kit.	0	n	1	0	Polygonaceae Juss.
Iz_8_1009	DJI_0943	Populus nigra L.	0	n	1	1	Salicaceae Mirb.
Iz_8_1009	DJI_0943	Populus tremula L.	0	n	1	1	Salicaceae Mirb.
Iz_8_1009	DJI_0943	Populus Ã canescens (Aiton) Sm.	0	n	1	1	Salicaceae Mirb.
Iz_8_1009	DJI_0943	Potentilla argentea L.	0	n	1	0	Rosaceae Juss.
Iz_8_1009	DJI_0943	Psammophiliella muralis (L.) Ikonn.	0	n	1	0	Caryophyllaceae Juss.
Iz_8_1009	DJI_0943	Psephellus sumensis (Kalen.) Greuter	0	n	1	0	Asteraceae Bercht. & J.Presl
Iz_8_1009	DJI_0943	Pulsatilla pratensis (L.) Mill. s.l.	0	n	1	0	Ranunculaceae Juss.
Iz_8_1009	DJI_0943	Pyrus communis L.	0	a	0	1	Rosaceae Juss.
Iz_8_1009	DJI_0943	Quercus robur L.	0	n	1	1	Fagaceae Dumort.
Iz_8_1009	DJI_0943	Robinia pseudoacacia L.	0	a	0	1	Fabaceae Lindl.
Iz_8_1009	DJI_0943	Rosa sp.	0		NA	1	Rosaceae Juss.
Iz_8_1009	DJI_0943	Rubus idaeus L.	0	n	1	0	Rosaceae Juss.
Iz_8_1009	DJI_0943	Rumex acetosella L.	0	n	1	0	Polygonaceae Juss.
Iz_8_1009	DJI_0943	Rumex thyrsiflorus Fingerh.	0	n	1	0	Polygonaceae Juss.
Iz_8_1009	DJI_0943	Salix alba L.	0	n	1	1	Salicaceae Mirb.
Iz_8_1009	DJI_0943	Sambucus nigra L.	0	n	1	1	Viburnaceae Raf.
Iz_8_1009	DJI_0943	Secale sylvestre Host	0	n	1	0	Poaceae Barnhart
Iz_8_1009	DJI_0943	Sempervivum ruthenicum Schnittsp. & C.B. Lehm.	0	n	1	0	Crassulaceae J.St.-Hil.
Iz_8_1009	DJI_0943	Setaria viridis (L.) P. Beauv.	0	n	1	0	Poaceae Barnhart
Iz_8_1009	DJI_0943	Silene borysthenica (Gruner) Walters	0	n	1	0	Caryophyllaceae Juss.
Iz_8_1009	DJI_0943	Silene latifolia subsp. alba (Mill.) Greuter & Burdet	0	n	1	0	Caryophyllaceae Juss.
Iz_8_1009	DJI_0943	Solidago virgaurea L.	0	n	1	0	Asteraceae Bercht. & J.Presl
Iz_8_1009	DJI_0943	Sorbus aucuparia L.	0	n	1	1	Rosaceae Juss.
Iz_8_1009	DJI_0943	Stachys recta L.	0	n	1	0	Lamiacea Lindl.
Iz_8_1009	DJI_0943	Tanacetum vulgare L.	0	n	1	0	Asteraceae Bercht. & J.Presl
Iz_8_1009	DJI_0943	Thymus pallasianus Heinr.Braun	0	n	1	0	Lamiacea Lindl.
Iz_8_1009	DJI_0943	Tilia cordata Mill.	0	n	1	1	Malvaceae Juss.
Iz_8_1009	DJI_0943	Trifolium alpestre L.	0	n	1	0	Fabaceae Lindl.
Iz_8_1009	DJI_0943	Trifolium arvense L.	0	n	1	0	Fabaceae Lindl.
Iz_8_1009	DJI_0943	Ulmus glabra Huds.	0	n	1	1	Ulmaceae Mirb.
Iz_8_1009	DJI_0943	Verbascum densiflorum Bertol.	0	n	1	0	Scrophulariaceae Juss.
Iz_8_1009	DJI_0943	Veronica austriaca L.	0	n	1	0	Plantaginaceae Juss.
Iz_8_1009	DJI_0943	Veronica incana L.	0	n	1	0	Plantaginaceae Juss.
Iz_8_1009	DJI_0943	Veronica verna L.	0	n	1	0	Plantaginaceae Juss.
Iz_8_1009	DJI_0943	Vincetoxicum hirundinaria Medik	0	n	1	0	Apocynaceae Juss.
Iz_8_1009	DJI_0946	Acer tataricum L.	0	n	1	1	Sapindaceae Juss.
Iz_8_1009	DJI_0946	Achillea millefolium L. s.l.	0	n	1	0	Asteraceae Bercht. & J.Presl
Iz_8_1009	DJI_0946	Allium oleraceum L.	0	n	1	0	Amaryllidaceae J.St.-Hil.
Iz_8_1009	DJI_0946	Ambrosia artemisiifolia L.	0	a	0	0	Asteraceae Bercht. & J.Presl
Iz_8_1009	DJI_0946	Amorpha fruticosa L.	0	a	0	0	Fabaceae Lindl.
Iz_8_1009	DJI_0946	Anthericum ramosum L.	0	n	1	0	Asparagaceae Juss.
Iz_8_1009	DJI_0946	Artemisia marschalliana Spreng.	0	n	1	0	Asteraceae Bercht. & J.Presl
Iz_8_1009	DJI_0946	Asparagus officinalis L.	0	n	1	0	Asparagaceae Juss.
Iz_8_1009	DJI_0946	Astragalus cicer L.	0	n	1	0	Fabaceae Lindl.
Iz_8_1009	DJI_0946	Bassia laniflora (S.G.Gmel.) A.J.Scott	0	n	1	0	Amaranthaceae Juss.
Iz_8_1009	DJI_0946	Berberis vulgaris L.	0	n	1	0	Berberidaceae Juss.
Iz_8_1009	DJI_0946	Betula pendula Roth.	0	n	1	1	Betulaceae Gray
Iz_8_1009	DJI_0946	Betula pendula Roth.	0	n	1	0	Poaceae Barnhart
Iz_8_1009	DJI_0946	Bromus inermis Leyss.	0		NA	NA	
Iz_8_1009	DJI_0946	Bromus squarrosus L.	0	a	0	0	Poaceae Barnhart
Iz_8_1009	DJI_0946	Bromus tectorum L.	0	a	0	0	Poaceae Barnhart
Iz_8_1009	DJI_0946	Calamagrostis epigejos (L.) Roth	3	n	1	0	Poaceae Barnhart
Iz_8_1009	DJI_0946	Carduus acanthoides L.	0	n	1	0	Asteraceae Bercht. & J.Presl
Iz_8_1009	DJI_0946	Carex sp.	0	n	1	0	Cyperaceae Juss.
Iz_8_1009	DJI_0946	Centaurea majorovii Dumbadze	0	n	1	0	Asteraceae Bercht. & J.Presl
Iz_8_1009	DJI_0946	Chamaecytisus ruthenicus (Fisch.ex Wol.) Klaskova	0	n	1	0	Fabaceae Lindl.
Iz_8_1009	DJI_0946	Chelidonium majus L.	0	n	1	0	Papaveraceae Juss.
Iz_8_1009	DJI_0946	Chenopodium betaceum Andrz.	0	n	1	0	Amaranthaceae Juss.
Iz_8_1009	DJI_0946	Chenopodium cf. album	0	n	1	0	Amaranthaceae Juss.
Iz_8_1009	DJI_0946	Chenopodium sp.	0		NA	0	Amaranthaceae Juss.
Iz_8_1009	DJI_0946	Chondrilla juncea L.	0	n	1	0	Asteraceae Bercht. & J.Presl
Iz_8_1009	DJI_0946	Cirsium vulgare (Savi) Ten.	0	n	1	0	Asteraceae Bercht. & J.Presl
Iz_8_1009	DJI_0946	Clinopodium acinos (L.) Kuntze	0	n	1	0	Lamiacea Lindl.

Iz_8_1009	DJI_0946	Convallaria majalis L.	0	n	1	0	Asparagaceae Juss.
Iz_8_1009	DJI_0946	Crataegus rhipidophylla Gand.	0	n	1	1	Rosaceae Juss.
Iz_8_1009	DJI_0946	Crepis foetida L.	0	n	1	0	Asteraceae Bercht. & J.Presl
Iz_8_1009	DJI_0946	Dactylis glomerata L.	0	n	1	0	Poaceae Barnhart
Iz_8_1009	DJI_0946	Dianthus campestris M.Bieb.	0	n	1	0	Caryophyllaceae Juss.
Iz_8_1009	DJI_0946	Digitaria sanguinalis (L.) Scop.	0	a	0	0	Poaceae Barnhart
Iz_8_1009	DJI_0946	Elymus repens (L.) Gould	0	n	1	0	Poaceae Barnhart
Iz_8_1009	DJI_0946	Epilobium tetragonum L.	0	n	1	0	Onagraceae Juss.
Iz_8_1009	DJI_0946	Eragrostis minor Host	0	a	0	0	Poaceae Barnhart
Iz_8_1009	DJI_0946	Erigeron canadensis L.	10	a	0	0	Asteraceae Bercht. & J.Presl
Iz_8_1009	DJI_0946	Euphorbia seguieriana Neck.	0	n	1	0	Euphorbiaceae Juss.
Iz_8_1009	DJI_0946	Fallopia dumetorum (L.) Holub	0	n	1	0	Polygonaceae Juss.
Iz_8_1009	DJI_0946	Festuca beckeri (Hack.) Trautv.	0	n	1	0	Poaceae Barnhart
Iz_8_1009	DJI_0946	Fragaria vesca L.	0	n	1	0	Rosaceae Juss.
Iz_8_1009	DJI_0946	Frangula alnus Mill.	0	n	1	1	Rhamnaceae Juss.
Iz_8_1009	DJI_0946	Fraxinus pennsylvanica Marshall	0	a	0	1	Oleaceae Hoffmanns. & Link
Iz_8_1009	DJI_0946	Galeopsis bifida Boenn.	0	n	1	0	Lamiacea Lindl.
Iz_8_1009	DJI_0946	Galium verum L.	0	n	1	0	Rubiaceae Juss.
Iz_8_1009	DJI_0946	Genista tinctoria L.	0	n	1	0	Fabaceae Lindl.
Iz_8_1009	DJI_0946	Geranium robertianum L.	0	n	1	0	Geraniaceae Juss.
Iz_8_1009	DJI_0946	Geranium sanquineum L.	0	n	1	0	Geraniaceae Juss.
Iz_8_1009	DJI_0946	Gypsophila paniculata L.	0	n	1	0	Caryophyllaceae Juss.
Iz_8_1009	DJI_0946	Helichrysum arenarium (L.) Moench	0	n	1	0	Asteraceae Bercht. & J.Presl
Iz_8_1009	DJI_0946	Herniaria polygama J.Gay	0	n	1	0	Caryophyllaceae Juss.
Iz_8_1009	DJI_0946	Hieracium robustum Fr.	0	n	1	0	Asteraceae Bercht. & J.Presl
Iz_8_1009	DJI_0946	Hieracium umbellatum L.	0	n	1	NA	Asteraceae Bercht. & J.Presl
Iz_8_1009	DJI_0946	Hylotelephium maximum (L.) Holub	0	n	1	0	Crassulaceae J.St.-Hil.
Iz_8_1009	DJI_0946	Jacobaea borysthenica (DC.) B.Nord. & Greuter	0	n	1	0	Asteraceae Bercht. & J.Presl
Iz_8_1009	DJI_0946	Jasione montana L.	0	n	1	0	Campanulaceae Juss.
Iz_8_1009	DJI_0946	Jurinea cyanoides (L.) Rchb.s.l.	0	n	1	0	Asteraceae Bercht. & J.Presl
Iz_8_1009	DJI_0946	Koeleria glauca (Spreng.) DC.	0	n	1	0	Poaceae Barnhart
Iz_8_1009	DJI_0946	Lactuca serriola L.	0	a	0	0	Asteraceae Bercht. & J.Presl
Iz_8_1009	DJI_0946	Linaria genistifolia (L.) Mill.	0	n	1	0	Plantaginaceae Juss.
Iz_8_1009	DJI_0946	Lomelosia argentea (L.) Greuter & Burdet	0	n	1	0	Caprifoliaceae Juss.
Iz_8_1009	DJI_0946	Melampyrum pratense L.	0	n	1	0	Orobanchaceae Vent.
Iz_8_1009	DJI_0946	Oenothera biennis L.	0	a	0	0	Onagraceae Juss.
Iz_8_1009	DJI_0946	Pentanema britannica (L.) D.Gut.Larr., Santos-Vicente, Anderb., E.Rico & M.M.Mart.Ort.	0	n	1	0	Asteraceae Bercht. & J.Presl
Iz_8_1009	DJI_0946	Peucedanum oreoselinum (L.) Moench	0	n	1	0	Apiaceae Lindl.
Iz_8_1009	DJI_0946	Peucedanum ruthenicum M.Bieb.	0	n	1	0	Apiaceae Lindl.
Iz_8_1009	DJI_0946	Pilosella echioides (Lumn.) F.W.Schultz & Sch.Bip.	0	n	1	0	Asteraceae Bercht. & J.Presl
Iz_8_1009	DJI_0946	Pilosella officinarum F. Schultz et Sch. Bip.	0	n	1	0	Asteraceae Bercht. & J.Presl
Iz_8_1009	DJI_0946	Pilosella sp.	0		NA	0	Asteraceae Bercht. & J.Presl
Iz_8_1009	DJI_0946	Pinus sylvestris L.	0	n	1	1	Pinaceae Spreng. ex F.Rudolphi
Iz_8_1009	DJI_0946	Poa annua L.	0	n	1	0	Poaceae Barnhart
Iz_8_1009	DJI_0946	Poa nemoralis L.	0	n	1	0	Poaceae Barnhart
Iz_8_1009	DJI_0946	Poa sp.	0		NA	0	Poaceae Barnhart
Iz_8_1009	DJI_0946	Polygonatum odoratum (Mill.) Druce	0	n	1	0	Asparagaceae Juss.
Iz_8_1009	DJI_0946	Polygonum arenarium Waldst. & Kit.	0	n	1	0	Polygonaceae Juss.
Iz_8_1009	DJI_0946	Populus nigra L.	0	n	1	1	Salicaceae Mirb.
Iz_8_1009	DJI_0946	Populus tremula L.	15	n	1	1	Salicaceae Mirb.
Iz_8_1009	DJI_0946	Populus Ã canescens (Aiton) Sm.	0	n	1	1	Salicaceae Mirb.
Iz_8_1009	DJI_0946	Potentilla argentea L.	0	n	1	0	Rosaceae Juss.
Iz_8_1009	DJI_0946	Psammophiliella muralis (L.) Ikonn.	0	n	1	0	Caryophyllaceae Juss.
Iz_8_1009	DJI_0946	Psephellus sumensis (Kalen.) Greuter	0	n	1	0	Asteraceae Bercht. & J.Presl
Iz_8_1009	DJI_0946	Pulsatilla pratensis (L.) Mill. s.l.	0	n	1	0	Ranunculaceae Juss.
Iz_8_1009	DJI_0946	Pyrus communis L.	0	a	0	1	Rosaceae Juss.
Iz_8_1009	DJI_0946	Quercus robur L.	0	n	1	1	Fagaceae Dumort.
Iz_8_1009	DJI_0946	Robinia pseudoacacia L.	0	a	0	1	Fabaceae Lindl.
Iz_8_1009	DJI_0946	Rosa sp.	0		NA	1	Rosaceae Juss.
Iz_8_1009	DJI_0946	Rubus idaeus L.	0	n	1	0	Rosaceae Juss.
Iz_8_1009	DJI_0946	Rumex acetosella L.	0	n	1	0	Polygonaceae Juss.
Iz_8_1009	DJI_0946	Rumex thyrsiflorus Fingerh.	0	n	1	0	Polygonaceae Juss.
Iz_8_1009	DJI_0946	Salix alba L.	0	n	1	1	Salicaceae Mirb.
Iz_8_1009	DJI_0946	Sambucus nigra L.	0	n	1	1	Viburnaceae Raf.
Iz_8_1009	DJI_0946	Secale sylvestre Host	0	n	1	0	Poaceae Barnhart
Iz_8_1009	DJI_0946	Sempervivum ruthenicum Schnittsp. & C.B. Lehm.	0	n	1	0	Crassulaceae J.St.-Hil.
Iz_8_1009	DJI_0946	Setaria viridis (L.) P. Beauv.	0	n	1	0	Poaceae Barnhart
Iz_8_1009	DJI_0946	Silene borysthenica (Gruner) Walters	0	n	1	0	Caryophyllaceae Juss.
Iz_8_1009	DJI_0946	Silene latifolia subsp. alba (Mill.) Greuter & Burdet	0	n	1	0	Caryophyllaceae Juss.
Iz_8_1009	DJI_0946	Solidago virgaurea L.	1	n	1	0	Asteraceae Bercht. & J.Presl
Iz_8_1009	DJI_0946	Sorbus aucuparia L.	0	n	1	1	Rosaceae Juss.
Iz_8_1009	DJI_0946	Stachys recta L.	0	n	1	0	Lamiacea Lindl.
Iz_8_1009	DJI_0946	Tanacetum vulgare L.	0	n	1	0	Asteraceae Bercht. & J.Presl
Iz_8_1009	DJI_0946	Thymus pallasianus Heinr.Braun	0	n	1	0	Lamiacea Lindl.
Iz_8_1009	DJI_0946	Tilia cordata Mill.	0	n	1	1	Malvaceae Juss.
Iz_8_1009	DJI_0946	Trifolium alpestre L.	0	n	1	0	Fabaceae Lindl.
Iz_8_1009	DJI_0946	Trifolium arvense L.	0	n	1	0	Fabaceae Lindl.
Iz_8_1009	DJI_0946	Ulmus glabra Huds.	0	n	1	1	Ulmaceae Mirb.
Iz_8_1009	DJI_0946	Verbascum densiflorum Bertol.	0	n	1	0	Scrophulariaceae Juss.
Iz_8_1009	DJI_0946	Veronica austriaca L.	0	n	1	0	Plantaginaceae Juss.
Iz_8_1009	DJI_0946	Veronica incana L.	0	n	1	0	Plantaginaceae Juss.
Iz_8_1009	DJI_0946	Veronica verna L.	0	n	1	0	Plantaginaceae Juss.
Iz_8_1009	DJI_0946	Vincetoxicum hirundinaria Medik	0	n	1	0	Apocynaceae Juss.
Iz_8_1009	DJI_0947	Acer tataricum L.	0	n	1	1	Sapindaceae Juss.
Iz_8_1009	DJI_0947	Achillea millefolium L. s.l.	0	n	1	0	Asteraceae Bercht. & J.Presl
Iz_8_1009	DJI_0947	Allium oleraceum L.	0	n	1	0	Amaryllidaceae J.St.-Hil.
Iz_8_1009	DJI_0947	Ambrosia artemisiifolia L.	0	a	0	0	Asteraceae Bercht. & J.Presl
Iz_8_1009	DJI_0947	Amorpha fruticosa L.	0	a	0	0	Fabaceae Lindl.
Iz_8_1009	DJI_0947	Anthericum ramosum L.	0	n	1	0	Asparagaceae Juss.
Iz_8_1009	DJI_0947	Artemisia marschalliana Spreng.	0	n	1	0	Asteraceae Bercht. & J.Presl
Iz_8_1009	DJI_0947	Asparagus officinalis L.	0	n	1	0	Asparagaceae Juss.
Iz_8_1009	DJI_0947	Astragalus cicer L.	0	n	1	0	Fabaceae Lindl.
Iz_8_1009	DJI_0947	Bassia laniflora (S.G.Gmel.) A.J.Scott	0	n	1	0	Amaranthaceae Juss.
Iz_8_1009	DJI_0947	Berberis vulgaris L.	0	n	1	0	Berberidaceae Juss.
Iz_8_1009	DJI_0947	Betula pendula Roth.	0	n	1	1	Betulaceae Gray
Iz_8_1009	DJI_0947	Betula pendula Roth.	0	n	1	0	Poaceae Barnhart
Iz_8_1009	DJI_0947	Bromus inermis Leyss.	0		NA	NA	
Iz_8_1009	DJI_0947	Bromus squarrosus L.	0	a	0	0	Poaceae Barnhart

Iz_8_1009	DJI_0947	Bromus tectorum L.	0	a	0	0	Poaceae Barnhart
Iz_8_1009	DJI_0947	Calamagrostis epigejos (L.) Roth	3	n	1	0	Poaceae Barnhart
Iz_8_1009	DJI_0947	Carduus acanthoides L.	0	n	1	0	Asteraceae Bercht. & J.Presl
Iz_8_1009	DJI_0947	Carex sp.	0	n	1	0	Cyperaceae Juss.
Iz_8_1009	DJI_0947	Centaurea majorovii Dumbadze	0	n	1	0	Asteraceae Bercht. & J.Presl
Iz_8_1009	DJI_0947	Chamaecytisus ruthenicus (Fisch.ex Wol.) Klaskova	0	n	1	0	Fabaceae Lindl.
Iz_8_1009	DJI_0947	Chelidonium majus L.	0	n	1	0	Papaveraceae Juss.
Iz_8_1009	DJI_0947	Chenopodium betaceum Andrz.	0	n	1	0	Amaranthaceae Juss.
Iz_8_1009	DJI_0947	Chenopodium cf. album	0	n	1	0	Amaranthaceae Juss.
Iz_8_1009	DJI_0947	Chenopodium sp.	0		NA	0	Amaranthaceae Juss.
Iz_8_1009	DJI_0947	Chondrilla juncea L.	0	n	1	0	Asteraceae Bercht. & J.Presl
Iz_8_1009	DJI_0947	Cirsium vulgare (Savi) Ten.	0	n	1	0	Asteraceae Bercht. & J.Presl
Iz_8_1009	DJI_0947	Clinopodium acinos (L.) Kuntze	0	n	1	0	Lamiacea Lindl.
Iz_8_1009	DJI_0947	Convallaria majalis L.	0	n	1	0	Asparagaceae Juss.
Iz_8_1009	DJI_0947	Crataegus rhipidophylla Gand.	0	n	1	1	Rosaceae Juss.
Iz_8_1009	DJI_0947	Crepis foetida L.	0	n	1	0	Asteraceae Bercht. & J.Presl
Iz_8_1009	DJI_0947	Dactylis glomerata L.	0	n	1	0	Poaceae Barnhart
Iz_8_1009	DJI_0947	Dianthus campestris M.Bieb.	0	n	1	0	Caryophyllaceae Juss.
Iz_8_1009	DJI_0947	Digitaria sanguinalis (L.) Scop.	0	a	0	0	Poaceae Barnhart
Iz_8_1009	DJI_0947	Elymus repens (L.) Gould	0	n	1	0	Poaceae Barnhart
Iz_8_1009	DJI_0947	Epilobium tetragonum L.	0	n	1	0	Onagraceae Juss.
Iz_8_1009	DJI_0947	Eragrostis minor Host	0	a	0	0	Poaceae Barnhart
Iz_8_1009	DJI_0947	Erigeron canadensis L.	15	a	0	0	Asteraceae Bercht. & J.Presl
Iz_8_1009	DJI_0947	Euphorbia seguieriana Neck.	0	n	1	0	Euphorbiaceae Juss.
Iz_8_1009	DJI_0947	Fallopia dumetorum (L.) Holub	0	n	1	0	Polygonaceae Juss.
Iz_8_1009	DJI_0947	Festuca beckeri (Hack.) Trautv.	0	n	1	0	Poaceae Barnhart
Iz_8_1009	DJI_0947	Fragaria vesca L.	0	n	1	0	Rosaceae Juss.
Iz_8_1009	DJI_0947	Frangula alnus Mill.	0	n	1	1	Rhamnaceae Juss.
Iz_8_1009	DJI_0947	Fraxinus pennsylvanica Marshall	0	a	0	1	Oleaceae Hoffmanns. & Link
Iz_8_1009	DJI_0947	Galeopsis bifida Boenn.	0	n	1	0	Lamiacea Lindl.
Iz_8_1009	DJI_0947	Galium verum L.	0	n	1	0	Rubiaceae Juss.
Iz_8_1009	DJI_0947	Genista tinctoria L.	0	n	1	0	Fabaceae Lindl.
Iz_8_1009	DJI_0947	Geranium robertianum L.	0	n	1	0	Geraniaceae Juss.
Iz_8_1009	DJI_0947	Geranium sanquineum L.	0	n	1	0	Geraniaceae Juss.
Iz_8_1009	DJI_0947	Gypsophila paniculata L.	0	n	1	0	Caryophyllaceae Juss.
Iz_8_1009	DJI_0947	Helichrysum arenarium (L.) Moench	0	n	1	0	Asteraceae Bercht. & J.Presl
Iz_8_1009	DJI_0947	Herniaria polygama J.Gay	0	n	1	0	Caryophyllaceae Juss.
Iz_8_1009	DJI_0947	Hieracium robustum Fr.	0	n	1	0	Asteraceae Bercht. & J.Presl
Iz_8_1009	DJI_0947	Hieracium umbellatum L.	0	n	1	NA	Asteraceae Bercht. & J.Presl
Iz_8_1009	DJI_0947	Hylotelephium maximum (L.) Holub	0	n	1	0	Crassulaceae J.St.-Hil.
Iz_8_1009	DJI_0947	Jacobaea borysthenica (DC.) B.Nord. & Greuter	0	n	1	0	Asteraceae Bercht. & J.Presl
Iz_8_1009	DJI_0947	Jasione montana L.	0	n	1	0	Campanulaceae Juss.
Iz_8_1009	DJI_0947	Jurinea cyanoides (L.) Rchb.s.l.	0	n	1	0	Asteraceae Bercht. & J.Presl
Iz_8_1009	DJI_0947	Koeleria glauca (Spreng.) DC.	0	n	1	0	Poaceae Barnhart
Iz_8_1009	DJI_0947	Lactuca serriola L.	0	a	0	0	Asteraceae Bercht. & J.Presl
Iz_8_1009	DJI_0947	Linaria genistifolia (L.) Mill.	0	n	1	0	Plantaginaceae Juss.
Iz_8_1009	DJI_0947	Lomelosia argentea (L.) Greuter & Burdet	0	n	1	0	Caprifoliaceae Juss.
Iz_8_1009	DJI_0947	Melampyrum pratense L.	0	n	1	0	Orobanchaceae Vent.
Iz_8_1009	DJI_0947	Oenothera biennis L.	0	a	0	0	Onagraceae Juss.
Iz_8_1009	DJI_0947	Pentanema britannica (L.) D.Gut.Larr., Santos-Vicente, Anderb., E.Rico & M.M.Mart.Ort.	0	n	1	0	Asteraceae Bercht. & J.Presl
Iz_8_1009	DJI_0947	Peucedanum oreoselinum (L.) Moench	2	n	1	0	Apiaceae Lindl.
Iz_8_1009	DJI_0947	Peucedanum ruthenicum M.Bieb.	0	n	1	0	Apiaceae Lindl.
Iz_8_1009	DJI_0947	Pilosella echioides (Lumn.) F.W.Schultz & Sch.Bip.	0	n	1	0	Asteraceae Bercht. & J.Presl
Iz_8_1009	DJI_0947	Pilosella officinarum F. Schultz et Sch. Bip.	0	n	1	0	Asteraceae Bercht. & J.Presl
Iz_8_1009	DJI_0947	Pilosella sp.	0		NA	0	Asteraceae Bercht. & J.Presl
Iz_8_1009	DJI_0947	Pinus sylvestris L.	0	n	1	1	Pinaceae Spreng. ex F.Rudolphi
Iz_8_1009	DJI_0947	Poa annua L.	0	n	1	0	Poaceae Barnhart
Iz_8_1009	DJI_0947	Poa nemoralis L.	0	n	1	0	Poaceae Barnhart
Iz_8_1009	DJI_0947	Poa sp.	0		NA	0	Poaceae Barnhart
Iz_8_1009	DJI_0947	Polygonatum odoratum (Mill.) Druce	0	n	1	0	Asparagaceae Juss.
Iz_8_1009	DJI_0947	Polygonum arenarium Waldst. & Kit.	0	n	1	0	Polygonaceae Juss.
Iz_8_1009	DJI_0947	Populus nigra L.	0	n	1	1	Salicaceae Mirb.
Iz_8_1009	DJI_0947	Populus tremula L.	5	n	1	1	Salicaceae Mirb.
Iz_8_1009	DJI_0947	Populus Ã canescens (Aiton) Sm.	0	n	1	1	Salicaceae Mirb.
Iz_8_1009	DJI_0947	Potentilla argentea L.	0	n	1	0	Rosaceae Juss.
Iz_8_1009	DJI_0947	Psammophiliella muralis (L.) Ikonn.	0	n	1	0	Caryophyllaceae Juss.
Iz_8_1009	DJI_0947	Psephellus sumensis (Kalen.) Greuter	0	n	1	0	Asteraceae Bercht. & J.Presl
Iz_8_1009	DJI_0947	Pulsatilla pratensis (L.) Mill. s.l.	0	n	1	0	Ranunculaceae Juss.
Iz_8_1009	DJI_0947	Pyrus communis L.	0	a	0	1	Rosaceae Juss.
Iz_8_1009	DJI_0947	Quercus robur L.	0	n	1	1	Fagaceae Dumort.
Iz_8_1009	DJI_0947	Robinia pseudoacacia L.	0	a	0	1	Fabaceae Lindl.
Iz_8_1009	DJI_0947	Rosa sp.	0		NA	1	Rosaceae Juss.
Iz_8_1009	DJI_0947	Rubus idaeus L.	0	n	1	0	Rosaceae Juss.
Iz_8_1009	DJI_0947	Rumex acetosella L.	0	n	1	0	Polygonaceae Juss.
Iz_8_1009	DJI_0947	Rumex thyrsiflorus Fingerh.	0	n	1	0	Polygonaceae Juss.
Iz_8_1009	DJI_0947	Salix alba L.	0	n	1	1	Salicaceae Mirb.
Iz_8_1009	DJI_0947	Sambucus nigra L.	0	n	1	1	Viburnaceae Raf.
Iz_8_1009	DJI_0947	Secale sylvestre Host	0	n	1	0	Poaceae Barnhart
Iz_8_1009	DJI_0947	Sempervivum ruthenicum Schnittsp. & C.B. Lehm.	0	n	1	0	Crassulaceae J.St.-Hil.
Iz_8_1009	DJI_0947	Setaria viridis (L.) P. Beauv.	0	n	1	0	Poaceae Barnhart
Iz_8_1009	DJI_0947	Silene borysthenica (Gruner) Walters	0	n	1	0	Caryophyllaceae Juss.
Iz_8_1009	DJI_0947	Silene latifolia subsp. alba (Mill.) Greuter & Burdet	0	n	1	0	Caryophyllaceae Juss.
Iz_8_1009	DJI_0947	Solidago virgaurea L.	0	n	1	0	Asteraceae Bercht. & J.Presl
Iz_8_1009	DJI_0947	Sorbus aucuparia L.	0	n	1	1	Rosaceae Juss.
Iz_8_1009	DJI_0947	Stachys recta L.	0	n	1	0	Lamiacea Lindl.
Iz_8_1009	DJI_0947	Tanacetum vulgare L.	0	n	1	0	Asteraceae Bercht. & J.Presl
Iz_8_1009	DJI_0947	Thymus pallasianus Heinr.Braun	0	n	1	0	Lamiacea Lindl.
Iz_8_1009	DJI_0947	Tilia cordata Mill.	0	n	1	1	Malvaceae Juss.
Iz_8_1009	DJI_0947	Trifolium alpestre L.	0	n	1	0	Fabaceae Lindl.
Iz_8_1009	DJI_0947	Trifolium arvense L.	0	n	1	0	Fabaceae Lindl.
Iz_8_1009	DJI_0947	Ulmus glabra Huds.	0	n	1	1	Ulmaceae Mirb.
Iz_8_1009	DJI_0947	Verbascum densiflorum Bertol.	0	n	1	0	Scrophulariaceae Juss.
Iz_8_1009	DJI_0947	Veronica austriaca L.	0	n	1	0	Plantaginaceae Juss.
Iz_8_1009	DJI_0947	Veronica incana L.	0	n	1	0	Plantaginaceae Juss.
Iz_8_1009	DJI_0947	Veronica verna L.	0	n	1	0	Plantaginaceae Juss.
Iz_8_1009	DJI_0947	Vincetoxicum hirundinaria Medik	0	n	1	0	Apocynaceae Juss.
Iz_8_1009	DJI_0953	Acer tataricum L.	0	n	1	1	Sapindaceae Juss.
Iz_8_1009	DJI_0953	Achillea millefolium L. s.l.	0	n	1	0	Asteraceae Bercht. & J.Presl

Iz_8_1009	DJI_0953	Allium oleraceum L.	0	n	1	0	Amaryllidaceae J.St.-Hil.
Iz_8_1009	DJI_0953	Ambrosia artemisiifolia L.	0	a	0	0	Asteraceae Bercht. & J.Presl
Iz_8_1009	DJI_0953	Amorpha fruticosa L.	0	a	0	0	Fabaceae Lindl.
Iz_8_1009	DJI_0953	Anthericum ramosum L.	0	n	1	0	Asparagaceae Juss.
Iz_8_1009	DJI_0953	Artemisia marschalliana Spreng.	0	n	1	0	Asteraceae Bercht. & J.Presl
Iz_8_1009	DJI_0953	Asparagus officinalis L.	0	n	1	0	Asparagaceae Juss.
Iz_8_1009	DJI_0953	Astragalus cicer L.	0	n	1	0	Fabaceae Lindl.
Iz_8_1009	DJI_0953	Bassia laniflora (S.G.Gmel.) A.J.Scott	0	n	1	0	Amaranthaceae Juss.
Iz_8_1009	DJI_0953	Berberis vulgaris L.	0	n	1	0	Berberidaceae Juss.
Iz_8_1009	DJI_0953	Betula pendula Roth.	0	n	1	1	Betulaceae Gray
Iz_8_1009	DJI_0953	Betula pendula Roth.	0	n	1	0	Poaceae Barnhart
Iz_8_1009	DJI_0953	Bromus inermis Leyss.	0		NA	NA	
Iz_8_1009	DJI_0953	Bromus squarrosus L.	0	a	0	0	Poaceae Barnhart
Iz_8_1009	DJI_0953	Bromus tectorum L.	0	a	0	0	Poaceae Barnhart
Iz_8_1009	DJI_0953	Calamagrostis epigejos (L.) Roth	20	n	1	0	Poaceae Barnhart
Iz_8_1009	DJI_0953	Carduus acanthoides L.	0	n	1	0	Asteraceae Bercht. & J.Presl
Iz_8_1009	DJI_0953	Carex sp.	0	n	1	0	Cyperaceae Juss.
Iz_8_1009	DJI_0953	Centaurea majorovii Dumbadze	0	n	1	0	Asteraceae Bercht. & J.Presl
Iz_8_1009	DJI_0953	Chamaecytisus ruthenicus (Fisch.ex Wol.) Klaskova	0	n	1	0	Fabaceae Lindl.
Iz_8_1009	DJI_0953	Chelidonium majus L.	0	n	1	0	Papaveraceae Juss.
Iz_8_1009	DJI_0953	Chenopodium betaceum Andrz.	0	n	1	0	Amaranthaceae Juss.
Iz_8_1009	DJI_0953	Chenopodium cf. album	0	n	1	0	Amaranthaceae Juss.
Iz_8_1009	DJI_0953	Chenopodium sp.	0		NA	0	Amaranthaceae Juss.
Iz_8_1009	DJI_0953	Chondrilla juncea L.	0	n	1	0	Asteraceae Bercht. & J.Presl
Iz_8_1009	DJI_0953	Cirsium vulgare (Savi) Ten.	0	n	1	0	Asteraceae Bercht. & J.Presl
Iz_8_1009	DJI_0953	Clinopodium acinos (L.) Kuntze	0	n	1	0	Lamiacea Lindl.
Iz_8_1009	DJI_0953	Convallaria majalis L.	0	n	1	0	Asparagaceae Juss.
Iz_8_1009	DJI_0953	Crataegus rhipidophylla Gand.	0	n	1	1	Rosaceae Juss.
Iz_8_1009	DJI_0953	Crepis foetida L.	0	n	1	0	Asteraceae Bercht. & J.Presl
Iz_8_1009	DJI_0953	Dactylis glomerata L.	0	n	1	0	Poaceae Barnhart
Iz_8_1009	DJI_0953	Dianthus campestris M.Bieb.	0	n	1	0	Caryophyllaceae Juss.
Iz_8_1009	DJI_0953	Digitaria sanguinalis (L.) Scop.	0	a	0	0	Poaceae Barnhart
Iz_8_1009	DJI_0953	Elymus repens (L.) Gould	0	n	1	0	Poaceae Barnhart
Iz_8_1009	DJI_0953	Epilobium tetragonum L.	0	n	1	0	Onagraceae Juss.
Iz_8_1009	DJI_0953	Eragrostis minor Host	0	a	0	0	Poaceae Barnhart
Iz_8_1009	DJI_0953	Erigeron canadensis L.	0	a	0	0	Asteraceae Bercht. & J.Presl
Iz_8_1009	DJI_0953	Euphorbia seguieriana Neck.	0	n	1	0	Euphorbiaceae Juss.
Iz_8_1009	DJI_0953	Fallopia dumetorum (L.) Holub	0	n	1	0	Polygonaceae Juss.
Iz_8_1009	DJI_0953	Festuca beckeri (Hack.) Trautv.	0	n	1	0	Poaceae Barnhart
Iz_8_1009	DJI_0953	Fragaria vesca L.	0	n	1	0	Rosaceae Juss.
Iz_8_1009	DJI_0953	Frangula alnus Mill.	0	n	1	1	Rhamnaceae Juss.
Iz_8_1009	DJI_0953	Fraxinus pennsylvanica Marshall	0	a	0	1	Oleaceae Hoffmanns. & Link
Iz_8_1009	DJI_0953	Galeopsis bifida Boenn.	0	n	1	0	Lamiacea Lindl.
Iz_8_1009	DJI_0953	Galium verum L.	0	n	1	0	Rubiaceae Juss.
Iz_8_1009	DJI_0953	Genista tinctoria L.	0	n	1	0	Fabaceae Lindl.
Iz_8_1009	DJI_0953	Geranium robertianum L.	0	n	1	0	Geraniaceae Juss.
Iz_8_1009	DJI_0953	Geranium sanquineum L.	0	n	1	0	Geraniaceae Juss.
Iz_8_1009	DJI_0953	Gypsophila paniculata L.	0	n	1	0	Caryophyllaceae Juss.
Iz_8_1009	DJI_0953	Helichrysum arenarium (L.) Moench	0	n	1	0	Asteraceae Bercht. & J.Presl
Iz_8_1009	DJI_0953	Herniaria polygama J.Gay	0	n	1	0	Caryophyllaceae Juss.
Iz_8_1009	DJI_0953	Hieracium robustum Fr.	0	n	1	0	Asteraceae Bercht. & J.Presl
Iz_8_1009	DJI_0953	Hieracium umbellatum L.	0	n	1	NA	Asteraceae Bercht. & J.Presl
Iz_8_1009	DJI_0953	Hylotelephium maximum (L.) Holub	0	n	1	0	Crassulaceae J.St.-Hil.
Iz_8_1009	DJI_0953	Jacobaea borysthenica (DC.) B.Nord. & Greuter	0	n	1	0	Asteraceae Bercht. & J.Presl
Iz_8_1009	DJI_0953	Jasione montana L.	0	n	1	0	Campanulaceae Juss.
Iz_8_1009	DJI_0953	Jurinea cyanoides (L.) Rchb.s.l.	0	n	1	0	Asteraceae Bercht. & J.Presl
Iz_8_1009	DJI_0953	Koeleria glauca (Spreng.) DC.	0	n	1	0	Poaceae Barnhart
Iz_8_1009	DJI_0953	Lactuca serriola L.	0	a	0	0	Asteraceae Bercht. & J.Presl
Iz_8_1009	DJI_0953	Linaria genistifolia (L.) Mill.	0	n	1	0	Plantaginaceae Juss.
Iz_8_1009	DJI_0953	Lomelosia argentea (L.) Greuter & Burdet	0	n	1	0	Caprifoliaceae Juss.
Iz_8_1009	DJI_0953	Melampyrum pratense L.	0	n	1	0	Orobanchaceae Vent.
Iz_8_1009	DJI_0953	Oenothera biennis L.	0	a	0	0	Onagraceae Juss.
Iz_8_1009	DJI_0953	Pentanema britannica (L.) D.Gut.Larr., Santos-Vicente, Anderb., E.Rico & M.M.Mart.Ort.	0	n	1	0	Asteraceae Bercht. & J.Presl
Iz_8_1009	DJI_0953	Peucedanum oreoselinum (L.) Moench	8	n	1	0	Apiaceae Lindl.
Iz_8_1009	DJI_0953	Peucedanum ruthenicum M.Bieb.	0	n	1	0	Apiaceae Lindl.
Iz_8_1009	DJI_0953	Pilosella echioides (Lumn.) F.W.Schultz & Sch.Bip.	0	n	1	0	Asteraceae Bercht. & J.Presl
Iz_8_1009	DJI_0953	Pilosella officinarum F. Schultz et Sch. Bip.	0	n	1	0	Asteraceae Bercht. & J.Presl
Iz_8_1009	DJI_0953	Pilosella sp.	0		NA	0	Asteraceae Bercht. & J.Presl
Iz_8_1009	DJI_0953	Pinus sylvestris L.	0	n	1	1	Pinaceae Spreng. ex F.Rudolphi
Iz_8_1009	DJI_0953	Poa annua L.	0	n	1	0	Poaceae Barnhart
Iz_8_1009	DJI_0953	Poa nemoralis L.	0	n	1	0	Poaceae Barnhart
Iz_8_1009	DJI_0953	Poa sp.	0		NA	0	Poaceae Barnhart
Iz_8_1009	DJI_0953	Polygonatum odoratum (Mill.) Druce	0	n	1	0	Asparagaceae Juss.
Iz_8_1009	DJI_0953	Polygonum arenarium Waldst. & Kit.	0	n	1	0	Polygonaceae Juss.
Iz_8_1009	DJI_0953	Populus nigra L.	0	n	1	1	Salicaceae Mirb.
Iz_8_1009	DJI_0953	Populus tremula L.	0	n	1	1	Salicaceae Mirb.
Iz_8_1009	DJI_0953	Populus Ã canescens (Aiton) Sm.	0	n	1	1	Salicaceae Mirb.
Iz_8_1009	DJI_0953	Potentilla argentea L.	0	n	1	0	Rosaceae Juss.
Iz_8_1009	DJI_0953	Psammophiliella muralis (L.) Ikonn.	0	n	1	0	Caryophyllaceae Juss.
Iz_8_1009	DJI_0953	Psephellus sumensis (Kalen.) Greuter	0	n	1	0	Asteraceae Bercht. & J.Presl
Iz_8_1009	DJI_0953	Pulsatilla pratensis (L.) Mill. s.l.	0	n	1	0	Ranunculaceae Juss.
Iz_8_1009	DJI_0953	Pyrus communis L.	0	a	0	1	Rosaceae Juss.
Iz_8_1009	DJI_0953	Quercus robur L.	0	n	1	1	Fagaceae Dumort.
Iz_8_1009	DJI_0953	Robinia pseudoacacia L.	0	a	0	1	Fabaceae Lindl.
Iz_8_1009	DJI_0953	Rosa sp.	0		NA	1	Rosaceae Juss.
Iz_8_1009	DJI_0953	Rubus idaeus L.	0	n	1	0	Rosaceae Juss.
Iz_8_1009	DJI_0953	Rumex acetosella L.	0	n	1	0	Polygonaceae Juss.
Iz_8_1009	DJI_0953	Rumex thyrsiflorus Fingerh.	0	n	1	0	Polygonaceae Juss.
Iz_8_1009	DJI_0953	Salix alba L.	0	n	1	1	Salicaceae Mirb.
Iz_8_1009	DJI_0953	Sambucus nigra L.	0	n	1	1	Viburnaceae Raf.
Iz_8_1009	DJI_0953	Secale sylvestre Host	0	n	1	0	Poaceae Barnhart
Iz_8_1009	DJI_0953	Sempervivum ruthenicum Schnittsp. & C.B. Lehm.	0	n	1	0	Crassulaceae J.St.-Hil.
Iz_8_1009	DJI_0953	Setaria viridis (L.) P. Beauv.	0	n	1	0	Poaceae Barnhart
Iz_8_1009	DJI_0953	Silene borysthenica (Gruner) Walters	0	n	1	0	Caryophyllaceae Juss.
Iz_8_1009	DJI_0953	Silene latifolia subsp. alba (Mill.) Greuter & Burdet	0	n	1	0	Caryophyllaceae Juss.
Iz_8_1009	DJI_0953	Solidago virgaurea L.	0	n	1	0	Asteraceae Bercht. & J.Presl
Iz_8_1009	DJI_0953	Sorbus aucuparia L.	0	n	1	1	Rosaceae Juss.
Iz_8_1009	DJI_0953	Stachys recta L.	0	n	1	0	Lamiacea Lindl.

Iz_8_1009	DJI_0953	Tanacetum vulgare L.	5	n	1	0	Asteraceae Bercht. & J.Presl
Iz_8_1009	DJI_0953	Thymus pallasianus Heinr.Braun	0	n	1	0	Lamiacea Lindl.
Iz_8_1009	DJI_0953	Tilia cordata Mill.	0	n	1	1	Malvaceae Juss.
Iz_8_1009	DJI_0953	Trifolium alpestre L.	0	n	1	0	Fabaceae Lindl.
Iz_8_1009	DJI_0953	Trifolium arvense L.	0	n	1	0	Fabaceae Lindl.
Iz_8_1009	DJI_0953	Ulmus glabra Huds.	0	n	1	1	Ulmaceae Mirb.
Iz_8_1009	DJI_0953	Verbascum densiflorum Bertol.	0	n	1	0	Scrophulariaceae Juss.
Iz_8_1009	DJI_0953	Veronica austriaca L.	0	n	1	0	Plantaginaceae Juss.
Iz_8_1009	DJI_0953	Veronica incana L.	0	n	1	0	Plantaginaceae Juss.
Iz_8_1009	DJI_0953	Veronica verna L.	0	n	1	0	Plantaginaceae Juss.
Iz_8_1009	DJI_0953	Vincetoxicum hirundinaria Medik	0	n	1	0	Apocynaceae Juss.
Iz_8_1009	DJI_0954	Acer tataricum L.	0	n	1	1	Sapindaceae Juss.
Iz_8_1009	DJI_0954	Achillea millefolium L. s.l.	0	n	1	0	Asteraceae Bercht. & J.Presl
Iz_8_1009	DJI_0954	Allium oleraceum L.	0	n	1	0	Amaryllidaceae J.St.-Hil.
Iz_8_1009	DJI_0954	Ambrosia artemisiifolia L.	0	a	0	0	Asteraceae Bercht. & J.Presl
Iz_8_1009	DJI_0954	Amorpha fruticosa L.	0	a	0	0	Fabaceae Lindl.
Iz_8_1009	DJI_0954	Anthericum ramosum L.	0	n	1	0	Asparagaceae Juss.
Iz_8_1009	DJI_0954	Artemisia marschalliana Spreng.	0	n	1	0	Asteraceae Bercht. & J.Presl
Iz_8_1009	DJI_0954	Asparagus officinalis L.	0	n	1	0	Asparagaceae Juss.
Iz_8_1009	DJI_0954	Astragalus cicer L.	0	n	1	0	Fabaceae Lindl.
Iz_8_1009	DJI_0954	Bassia laniflora (S.G.Gmel.) A.J.Scott	0	n	1	0	Amaranthaceae Juss.
Iz_8_1009	DJI_0954	Berberis vulgaris L.	0	n	1	0	Berberidaceae Juss.
Iz_8_1009	DJI_0954	Betula pendula Roth.	0	n	1	1	Betulaceae Gray
Iz_8_1009	DJI_0954	Betula pendula Roth.	0	n	1	0	Poaceae Barnhart
Iz_8_1009	DJI_0954	Bromus inermis Leyss.	0		NA	NA	
Iz_8_1009	DJI_0954	Bromus squarrosus L.	0	a	0	0	Poaceae Barnhart
Iz_8_1009	DJI_0954	Bromus tectorum L.	0	a	0	0	Poaceae Barnhart
Iz_8_1009	DJI_0954	Calamagrostis epigejos (L.) Roth	5	n	1	0	Poaceae Barnhart
Iz_8_1009	DJI_0954	Carduus acanthoides L.	0	n	1	0	Asteraceae Bercht. & J.Presl
Iz_8_1009	DJI_0954	Carex sp.	0	n	1	0	Cyperaceae Juss.
Iz_8_1009	DJI_0954	Centaurea majorovii Dumbadze	0	n	1	0	Asteraceae Bercht. & J.Presl
Iz_8_1009	DJI_0954	Chamaecytisus ruthenicus (Fisch.ex Wol.) Klaskova	0	n	1	0	Fabaceae Lindl.
Iz_8_1009	DJI_0954	Chelidonium majus L.	0	n	1	0	Papaveraceae Juss.
Iz_8_1009	DJI_0954	Chenopodium betaceum Andrz.	0	n	1	0	Amaranthaceae Juss.
Iz_8_1009	DJI_0954	Chenopodium cf. album	0	n	1	0	Amaranthaceae Juss.
Iz_8_1009	DJI_0954	Chenopodium sp.	0		NA	0	Amaranthaceae Juss.
Iz_8_1009	DJI_0954	Chondrilla juncea L.	10	n	1	0	Asteraceae Bercht. & J.Presl
Iz_8_1009	DJI_0954	Cirsium vulgare (Savi) Ten.	0	n	1	0	Asteraceae Bercht. & J.Presl
Iz_8_1009	DJI_0954	Clinopodium acinos (L.) Kuntze	0	n	1	0	Lamiacea Lindl.
Iz_8_1009	DJI_0954	Convallaria majalis L.	0	n	1	0	Asparagaceae Juss.
Iz_8_1009	DJI_0954	Crataegus rhipidophylla Gand.	0	n	1	1	Rosaceae Juss.
Iz_8_1009	DJI_0954	Crepis foetida L.	0	n	1	0	Asteraceae Bercht. & J.Presl
Iz_8_1009	DJI_0954	Dactylis glomerata L.	0	n	1	0	Poaceae Barnhart
Iz_8_1009	DJI_0954	Dianthus campestris M.Bieb.	0	n	1	0	Caryophyllaceae Juss.
Iz_8_1009	DJI_0954	Digitaria sanguinalis (L.) Scop.	0	a	0	0	Poaceae Barnhart
Iz_8_1009	DJI_0954	Elymus repens (L.) Gould	0	n	1	0	Poaceae Barnhart
Iz_8_1009	DJI_0954	Epilobium tetragonum L.	0	n	1	0	Onagraceae Juss.
Iz_8_1009	DJI_0954	Eragrostis minor Host	0	a	0	0	Poaceae Barnhart
Iz_8_1009	DJI_0954	Erigeron canadensis L.	3	a	0	0	Asteraceae Bercht. & J.Presl
Iz_8_1009	DJI_0954	Euphorbia seguieriana Neck.	0	n	1	0	Euphorbiaceae Juss.
Iz_8_1009	DJI_0954	Fallopia dumetorum (L.) Holub	0	n	1	0	Polygonaceae Juss.
Iz_8_1009	DJI_0954	Festuca beckeri (Hack.) Trautv.	0	n	1	0	Poaceae Barnhart
Iz_8_1009	DJI_0954	Fragaria vesca L.	0	n	1	0	Rosaceae Juss.
Iz_8_1009	DJI_0954	Frangula alnus Mill.	0	n	1	1	Rhamnaceae Juss.
Iz_8_1009	DJI_0954	Fraxinus pennsylvanica Marshall	0	a	0	1	Oleaceae Hoffmanns. & Link
Iz_8_1009	DJI_0954	Galeopsis bifida Boenn.	0	n	1	0	Lamiacea Lindl.
Iz_8_1009	DJI_0954	Galium verum L.	0	n	1	0	Rubiaceae Juss.
Iz_8_1009	DJI_0954	Genista tinctoria L.	0	n	1	0	Fabaceae Lindl.
Iz_8_1009	DJI_0954	Geranium robertianum L.	0	n	1	0	Geraniaceae Juss.
Iz_8_1009	DJI_0954	Geranium sanquineum L.	0	n	1	0	Geraniaceae Juss.
Iz_8_1009	DJI_0954	Gypsophila paniculata L.	0	n	1	0	Caryophyllaceae Juss.
Iz_8_1009	DJI_0954	Helichrysum arenarium (L.) Moench	0	n	1	0	Asteraceae Bercht. & J.Presl
Iz_8_1009	DJI_0954	Herniaria polygama J.Gay	0	n	1	0	Caryophyllaceae Juss.
Iz_8_1009	DJI_0954	Hieracium robustum Fr.	0	n	1	0	Asteraceae Bercht. & J.Presl
Iz_8_1009	DJI_0954	Hieracium umbellatum L.	0	n	1	NA	Asteraceae Bercht. & J.Presl
Iz_8_1009	DJI_0954	Hylotelephium maximum (L.) Holub	0	n	1	0	Crassulaceae J.St.-Hil.
Iz_8_1009	DJI_0954	Jacobaea borysthenica (DC.) B.Nord. & Greuter	0	n	1	0	Asteraceae Bercht. & J.Presl
Iz_8_1009	DJI_0954	Jasione montana L.	0	n	1	0	Campanulaceae Juss.
Iz_8_1009	DJI_0954	Jurinea cyanoides (L.) Rchb.s.l.	0	n	1	0	Asteraceae Bercht. & J.Presl
Iz_8_1009	DJI_0954	Koeleria glauca (Spreng.) DC.	0	n	1	0	Poaceae Barnhart
Iz_8_1009	DJI_0954	Lactuca serriola L.	0	a	0	0	Asteraceae Bercht. & J.Presl
Iz_8_1009	DJI_0954	Linaria genistifolia (L.) Mill.	0	n	1	0	Plantaginaceae Juss.
Iz_8_1009	DJI_0954	Lomelosia argentea (L.) Greuter & Burdet	0	n	1	0	Caprifoliaceae Juss.
Iz_8_1009	DJI_0954	Melampyrum pratense L.	0	n	1	0	Orobanchaceae Vent.
Iz_8_1009	DJI_0954	Oenothera biennis L.	0	a	0	0	Onagraceae Juss.
Iz_8_1009	DJI_0954	Pentanema britannica (L.) D.Gut.Larr., Santos-Vicente, Anderb., E.Rico & M.M.Mart.Ort.	0	n	1	0	Asteraceae Bercht. & J.Presl
Iz_8_1009	DJI_0954	Peucedanum oreoselinum (L.) Moench	2	n	1	0	Apiaceae Lindl.
Iz_8_1009	DJI_0954	Peucedanum ruthenicum M.Bieb.	0	n	1	0	Apiaceae Lindl.
Iz_8_1009	DJI_0954	Pilosella echioides (Lumn.) F.W.Schultz & Sch.Bip.	0	n	1	0	Asteraceae Bercht. & J.Presl
Iz_8_1009	DJI_0954	Pilosella officinarum F. Schultz et Sch. Bip.	0	n	1	0	Asteraceae Bercht. & J.Presl
Iz_8_1009	DJI_0954	Pilosella sp.	0		NA	0	Asteraceae Bercht. & J.Presl
Iz_8_1009	DJI_0954	Pinus sylvestris L.	0	n	1	1	Pinaceae Spreng. ex F.Rudolphi
Iz_8_1009	DJI_0954	Poa annua L.	0	n	1	0	Poaceae Barnhart
Iz_8_1009	DJI_0954	Poa nemoralis L.	0	n	1	0	Poaceae Barnhart
Iz_8_1009	DJI_0954	Poa sp.	0		NA	0	Poaceae Barnhart
Iz_8_1009	DJI_0954	Polygonatum odoratum (Mill.) Druce	0	n	1	0	Asparagaceae Juss.
Iz_8_1009	DJI_0954	Polygonum arenarium Waldst. & Kit.	0	n	1	0	Polygonaceae Juss.
Iz_8_1009	DJI_0954	Populus nigra L.	0	n	1	1	Salicaceae Mirb.
Iz_8_1009	DJI_0954	Populus tremula L.	0	n	1	1	Salicaceae Mirb.
Iz_8_1009	DJI_0954	Populus Ã canescens (Aiton) Sm.	0	n	1	1	Salicaceae Mirb.
Iz_8_1009	DJI_0954	Potentilla argentea L.	0	n	1	0	Rosaceae Juss.
Iz_8_1009	DJI_0954	Psammophiliella muralis (L.) Ikonn.	0	n	1	0	Caryophyllaceae Juss.
Iz_8_1009	DJI_0954	Psephellus sumensis (Kalen.) Greuter	0	n	1	0	Asteraceae Bercht. & J.Presl
Iz_8_1009	DJI_0954	Pulsatilla pratensis (L.) Mill. s.l.	0	n	1	0	Ranunculaceae Juss.
Iz_8_1009	DJI_0954	Pyrus communis L.	0	a	0	1	Rosaceae Juss.
Iz_8_1009	DJI_0954	Quercus robur L.	0	n	1	1	Fagaceae Dumort.
Iz_8_1009	DJI_0954	Robinia pseudoacacia L.	0	a	0	1	Fabaceae Lindl.
Iz_8_1009	DJI_0954	Rosa sp.	0		NA	1	Rosaceae Juss.

Iz_8_1009	DJI_0954	Rubus idaeus L.	0	n	1	0	Rosaceae Juss.
Iz_8_1009	DJI_0954	Rumex acetosella L.	0	n	1	0	Polygonaceae Juss.
Iz_8_1009	DJI_0954	Rumex thyrsiflorus Fingerh.	0	n	1	0	Polygonaceae Juss.
Iz_8_1009	DJI_0954	Salix alba L.	0	n	1	1	Salicaceae Mirb.
Iz_8_1009	DJI_0954	Sambucus nigra L.	0	n	1	1	Viburnaceae Raf.
Iz_8_1009	DJI_0954	Secale sylvestre Host	0	n	1	0	Poaceae Barnhart
Iz_8_1009	DJI_0954	Sempervivum ruthenicum Schnittsp. & C.B. Lehm.	0	n	1	0	Crassulaceae J.St.-Hil.
Iz_8_1009	DJI_0954	Setaria viridis (L.) P. Beauv.	0	n	1	0	Poaceae Barnhart
Iz_8_1009	DJI_0954	Silene borysthenica (Gruner) Walters	0	n	1	0	Caryophyllaceae Juss.
Iz_8_1009	DJI_0954	Silene latifolia subsp. alba (Mill.) Greuter & Burdet	0	n	1	0	Caryophyllaceae Juss.
Iz_8_1009	DJI_0954	Solidago virgaurea L.	0	n	1	0	Asteraceae Bercht. & J.Presl
Iz_8_1009	DJI_0954	Sorbus aucuparia L.	0	n	1	1	Rosaceae Juss.
Iz_8_1009	DJI_0954	Stachys recta L.	0	n	1	0	Lamiacea Lindl.
Iz_8_1009	DJI_0954	Tanacetum vulgare L.	1	n	1	0	Asteraceae Bercht. & J.Presl
Iz_8_1009	DJI_0954	Thymus pallasianus Heinr.Braun	0	n	1	0	Lamiacea Lindl.
Iz_8_1009	DJI_0954	Tilia cordata Mill.	0	n	1	1	Malvaceae Juss.
Iz_8_1009	DJI_0954	Trifolium alpestre L.	0	n	1	0	Fabaceae Lindl.
Iz_8_1009	DJI_0954	Trifolium arvense L.	0	n	1	0	Fabaceae Lindl.
Iz_8_1009	DJI_0954	Ulmus glabra Huds.	0	n	1	1	Ulmaceae Mirb.
Iz_8_1009	DJI_0954	Verbascum densiflorum Bertol.	0	n	1	0	Scrophulariaceae Juss.
Iz_8_1009	DJI_0954	Veronica austriaca L.	0	n	1	0	Plantaginaceae Juss.
Iz_8_1009	DJI_0954	Veronica incana L.	0	n	1	0	Plantaginaceae Juss.
Iz_8_1009	DJI_0954	Veronica verna L.	0	n	1	0	Plantaginaceae Juss.
Iz_8_1009	DJI_0954	Vincetoxicum hirundinaria Medik	0	n	1	0	Apocynaceae Juss.
Iz_8_1009	DJI_0955	Acer tataricum L.	0	n	1	1	Sapindaceae Juss.
Iz_8_1009	DJI_0955	Achillea millefolium L. s.l.	0	n	1	0	Asteraceae Bercht. & J.Presl
Iz_8_1009	DJI_0955	Allium oleraceum L.	0	n	1	0	Amaryllidaceae J.St.-Hil.
Iz_8_1009	DJI_0955	Ambrosia artemisiifolia L.	0	a	0	0	Asteraceae Bercht. & J.Presl
Iz_8_1009	DJI_0955	Amorpha fruticosa L.	0	a	0	0	Fabaceae Lindl.
Iz_8_1009	DJI_0955	Anthericum ramosum L.	0	n	1	0	Asparagaceae Juss.
Iz_8_1009	DJI_0955	Artemisia marschalliana Spreng.	0	n	1	0	Asteraceae Bercht. & J.Presl
Iz_8_1009	DJI_0955	Asparagus officinalis L.	0	n	1	0	Asparagaceae Juss.
Iz_8_1009	DJI_0955	Astragalus cicer L.	0	n	1	0	Fabaceae Lindl.
Iz_8_1009	DJI_0955	Bassia laniflora (S.G.Gmel.) A.J.Scott	0	n	1	0	Amaranthaceae Juss.
Iz_8_1009	DJI_0955	Berberis vulgaris L.	0	n	1	0	Berberidaceae Juss.
Iz_8_1009	DJI_0955	Betula pendula Roth.	0	n	1	1	Betulaceae Gray
Iz_8_1009	DJI_0955	Betula pendula Roth.	0	n	1	0	Poaceae Barnhart
Iz_8_1009	DJI_0955	Bromus inermis Leyss.	0		NA	NA	
Iz_8_1009	DJI_0955	Bromus squarrosus L.	0	a	0	0	Poaceae Barnhart
Iz_8_1009	DJI_0955	Bromus tectorum L.	0	a	0	0	Poaceae Barnhart
Iz_8_1009	DJI_0955	Calamagrostis epigejos (L.) Roth	0.5	n	1	0	Poaceae Barnhart
Iz_8_1009	DJI_0955	Carduus acanthoides L.	0	n	1	0	Asteraceae Bercht. & J.Presl
Iz_8_1009	DJI_0955	Carex sp.	0	n	1	0	Cyperaceae Juss.
Iz_8_1009	DJI_0955	Centaurea majorovii Dumbadze	0	n	1	0	Asteraceae Bercht. & J.Presl
Iz_8_1009	DJI_0955	Chamaecytisus ruthenicus (Fisch.ex Wol.) Klaskova	0	n	1	0	Fabaceae Lindl.
Iz_8_1009	DJI_0955	Chelidonium majus L.	0	n	1	0	Papaveraceae Juss.
Iz_8_1009	DJI_0955	Chenopodium betaceum Andrz.	0	n	1	0	Amaranthaceae Juss.
Iz_8_1009	DJI_0955	Chenopodium cf. album	0	n	1	0	Amaranthaceae Juss.
Iz_8_1009	DJI_0955	Chenopodium sp.	1		NA	0	Amaranthaceae Juss.
Iz_8_1009	DJI_0955	Chondrilla juncea L.	1	n	1	0	Asteraceae Bercht. & J.Presl
Iz_8_1009	DJI_0955	Cirsium vulgare (Savi) Ten.	0	n	1	0	Asteraceae Bercht. & J.Presl
Iz_8_1009	DJI_0955	Clinopodium acinos (L.) Kuntze	0	n	1	0	Lamiacea Lindl.
Iz_8_1009	DJI_0955	Convallaria majalis L.	0	n	1	0	Asparagaceae Juss.
Iz_8_1009	DJI_0955	Crataegus rhipidophylla Gand.	0	n	1	1	Rosaceae Juss.
Iz_8_1009	DJI_0955	Crepis foetida L.	0	n	1	0	Asteraceae Bercht. & J.Presl
Iz_8_1009	DJI_0955	Dactylis glomerata L.	0	n	1	0	Poaceae Barnhart
Iz_8_1009	DJI_0955	Dianthus campestris M.Bieb.	0	n	1	0	Caryophyllaceae Juss.
Iz_8_1009	DJI_0955	Digitaria sanguinalis (L.) Scop.	0	a	0	0	Poaceae Barnhart
Iz_8_1009	DJI_0955	Elymus repens (L.) Gould	0	n	1	0	Poaceae Barnhart
Iz_8_1009	DJI_0955	Epilobium tetragonum L.	0	n	1	0	Onagraceae Juss.
Iz_8_1009	DJI_0955	Eragrostis minor Host	0	a	0	0	Poaceae Barnhart
Iz_8_1009	DJI_0955	Erigeron canadensis L.	5	a	0	0	Asteraceae Bercht. & J.Presl
Iz_8_1009	DJI_0955	Euphorbia seguieriana Neck.	0	n	1	0	Euphorbiaceae Juss.
Iz_8_1009	DJI_0955	Fallopia dumetorum (L.) Holub	0	n	1	0	Polygonaceae Juss.
Iz_8_1009	DJI_0955	Festuca beckeri (Hack.) Trautv.	0	n	1	0	Poaceae Barnhart
Iz_8_1009	DJI_0955	Fragaria vesca L.	0	n	1	0	Rosaceae Juss.
Iz_8_1009	DJI_0955	Frangula alnus Mill.	0	n	1	1	Rhamnaceae Juss.
Iz_8_1009	DJI_0955	Fraxinus pennsylvanica Marshall	0	a	0	1	Oleaceae Hoffmanns. & Link
Iz_8_1009	DJI_0955	Galeopsis bifida Boenn.	0	n	1	0	Lamiacea Lindl.
Iz_8_1009	DJI_0955	Galium verum L.	0	n	1	0	Rubiaceae Juss.
Iz_8_1009	DJI_0955	Genista tinctoria L.	0	n	1	0	Fabaceae Lindl.
Iz_8_1009	DJI_0955	Geranium robertianum L.	0	n	1	0	Geraniaceae Juss.
Iz_8_1009	DJI_0955	Geranium sanquineum L.	0	n	1	0	Geraniaceae Juss.
Iz_8_1009	DJI_0955	Gypsophila paniculata L.	0	n	1	0	Caryophyllaceae Juss.
Iz_8_1009	DJI_0955	Helichrysum arenarium (L.) Moench	0	n	1	0	Asteraceae Bercht. & J.Presl
Iz_8_1009	DJI_0955	Herniaria polygama J.Gay	0	n	1	0	Caryophyllaceae Juss.
Iz_8_1009	DJI_0955	Hieracium robustum Fr.	0	n	1	0	Asteraceae Bercht. & J.Presl
Iz_8_1009	DJI_0955	Hieracium umbellatum L.	0	n	1	NA	Asteraceae Bercht. & J.Presl
Iz_8_1009	DJI_0955	Hylotelephium maximum (L.) Holub	0	n	1	0	Crassulaceae J.St.-Hil.
Iz_8_1009	DJI_0955	Jacobaea borysthenica (DC.) B.Nord. & Greuter	0	n	1	0	Asteraceae Bercht. & J.Presl
Iz_8_1009	DJI_0955	Jasione montana L.	0	n	1	0	Campanulaceae Juss.
Iz_8_1009	DJI_0955	Jurinea cyanoides (L.) Rchb.s.l.	0	n	1	0	Asteraceae Bercht. & J.Presl
Iz_8_1009	DJI_0955	Koeleria glauca (Spreng.) DC.	0	n	1	0	Poaceae Barnhart
Iz_8_1009	DJI_0955	Lactuca serriola L.	0	a	0	0	Asteraceae Bercht. & J.Presl
Iz_8_1009	DJI_0955	Linaria genistifolia (L.) Mill.	0	n	1	0	Plantaginaceae Juss.
Iz_8_1009	DJI_0955	Lomelosia argentea (L.) Greuter & Burdet	0	n	1	0	Caprifoliaceae Juss.
Iz_8_1009	DJI_0955	Melampyrum pratense L.	0	n	1	0	Orobanchaceae Vent.
Iz_8_1009	DJI_0955	Oenothera biennis L.	0	a	0	0	Onagraceae Juss.
Iz_8_1009	DJI_0955	Pentanema britannica (L.) D.Gut.Larr., Santos-Vicente, Anderb., E.Rico & M.M.Mart.Ort.	0	n	1	0	Asteraceae Bercht. & J.Presl
Iz_8_1009	DJI_0955	Peucedanum oreoselinum (L.) Moench	0	n	1	0	Apiaceae Lindl.
Iz_8_1009	DJI_0955	Peucedanum ruthenicum M.Bieb.	0	n	1	0	Apiaceae Lindl.
Iz_8_1009	DJI_0955	Pilosella echioides (Lumn.) F.W.Schultz & Sch.Bip.	0	n	1	0	Asteraceae Bercht. & J.Presl
Iz_8_1009	DJI_0955	Pilosella officinarum F. Schultz et Sch. Bip.	0	n	1	0	Asteraceae Bercht. & J.Presl
Iz_8_1009	DJI_0955	Pilosella sp.	0		NA	0	Asteraceae Bercht. & J.Presl
Iz_8_1009	DJI_0955	Pinus sylvestris L.	0	n	1	1	Pinaceae Spreng. ex F.Rudolphi
Iz_8_1009	DJI_0955	Poa annua L.	0	n	1	0	Poaceae Barnhart
Iz_8_1009	DJI_0955	Poa nemoralis L.	0	n	1	0	Poaceae Barnhart
Iz_8_1009	DJI_0955	Poa sp.	0		NA	0	Poaceae Barnhart

Iz_8_1009	DJI_0955	Polygonatum odoratum (Mill.) Druce	0	n	1	0	Asparagaceae Juss.
Iz_8_1009	DJI_0955	Polygonum arenarium Waldst. & Kit.	0	n	1	0	Polygonaceae Juss.
Iz_8_1009	DJI_0955	Populus nigra L.	0	n	1	1	Salicaceae Mirb.
Iz_8_1009	DJI_0955	Populus tremula L.	0	n	1	1	Salicaceae Mirb.
Iz_8_1009	DJI_0955	Populus Ã canescens (Aiton) Sm.	0	n	1	1	Salicaceae Mirb.
Iz_8_1009	DJI_0955	Potentilla argentea L.	0	n	1	0	Rosaceae Juss.
Iz_8_1009	DJI_0955	Psammophiliella muralis (L.) Ikonn.	0	n	1	0	Caryophyllaceae Juss.
Iz_8_1009	DJI_0955	Psephellus sumensis (Kalen.) Greuter	0	n	1	0	Asteraceae Bercht. & J.Presl
Iz_8_1009	DJI_0955	Pulsatilla pratensis (L.) Mill. s.l.	0	n	1	0	Ranunculaceae Juss.
Iz_8_1009	DJI_0955	Pyrus communis L.	0	a	0	1	Rosaceae Juss.
Iz_8_1009	DJI_0955	Quercus robur L.	0	n	1	1	Fagaceae Dumort.
Iz_8_1009	DJI_0955	Robinia pseudoacacia L.	0	a	0	1	Fabaceae Lindl.
Iz_8_1009	DJI_0955	Rosa sp.	0		NA	1	Rosaceae Juss.
Iz_8_1009	DJI_0955	Rubus idaeus L.	0	n	1	0	Rosaceae Juss.
Iz_8_1009	DJI_0955	Rumex acetosella L.	0	n	1	0	Polygonaceae Juss.
Iz_8_1009	DJI_0955	Rumex thyrsiflorus Fingerh.	0	n	1	0	Polygonaceae Juss.
Iz_8_1009	DJI_0955	Salix alba L.	0	n	1	1	Salicaceae Mirb.
Iz_8_1009	DJI_0955	Sambucus nigra L.	0	n	1	1	Viburnaceae Raf.
Iz_8_1009	DJI_0955	Secale sylvestre Host	0	n	1	0	Poaceae Barnhart
Iz_8_1009	DJI_0955	Sempervivum ruthenicum Schnittsp. & C.B. Lehm.	0	n	1	0	Crassulaceae J.St.-Hil.
Iz_8_1009	DJI_0955	Setaria viridis (L.) P. Beauv.	0	n	1	0	Poaceae Barnhart
Iz_8_1009	DJI_0955	Silene borysthenica (Gruner) Walters	0	n	1	0	Caryophyllaceae Juss.
Iz_8_1009	DJI_0955	Silene latifolia subsp. alba (Mill.) Greuter & Burdet	0	n	1	0	Caryophyllaceae Juss.
Iz_8_1009	DJI_0955	Solidago virgaurea L.	0	n	1	0	Asteraceae Bercht. & J.Presl
Iz_8_1009	DJI_0955	Sorbus aucuparia L.	0	n	1	1	Rosaceae Juss.
Iz_8_1009	DJI_0955	Stachys recta L.	0	n	1	0	Lamiacea Lindl.
Iz_8_1009	DJI_0955	Tanacetum vulgare L.	0	n	1	0	Asteraceae Bercht. & J.Presl
Iz_8_1009	DJI_0955	Thymus pallasianus Heinr.Braun	0	n	1	0	Lamiacea Lindl.
Iz_8_1009	DJI_0955	Tilia cordata Mill.	0	n	1	1	Malvaceae Juss.
Iz_8_1009	DJI_0955	Trifolium alpestre L.	0	n	1	0	Fabaceae Lindl.
Iz_8_1009	DJI_0955	Trifolium arvense L.	0	n	1	0	Fabaceae Lindl.
Iz_8_1009	DJI_0955	Ulmus glabra Huds.	0	n	1	1	Ulmaceae Mirb.
Iz_8_1009	DJI_0955	Verbascum densiflorum Bertol.	0	n	1	0	Scrophulariaceae Juss.
Iz_8_1009	DJI_0955	Veronica austriaca L.	0	n	1	0	Plantaginaceae Juss.
Iz_8_1009	DJI_0955	Veronica incana L.	0	n	1	0	Plantaginaceae Juss.
Iz_8_1009	DJI_0955	Veronica verna L.	0	n	1	0	Plantaginaceae Juss.
Iz_8_1009	DJI_0955	Vincetoxicum hirundinaria Medik	0	n	1	0	Apocynaceae Juss.
Iz_8_1009	DJI_0956	Acer tataricum L.	0	n	1	1	Sapindaceae Juss.
Iz_8_1009	DJI_0956	Achillea millefolium L. s.l.	0	n	1	0	Asteraceae Bercht. & J.Presl
Iz_8_1009	DJI_0956	Allium oleraceum L.	0	n	1	0	Amaryllidaceae J.St.-Hil.
Iz_8_1009	DJI_0956	Ambrosia artemisiifolia L.	0	a	0	0	Asteraceae Bercht. & J.Presl
Iz_8_1009	DJI_0956	Amorpha fruticosa L.	0	a	0	0	Fabaceae Lindl.
Iz_8_1009	DJI_0956	Anthericum ramosum L.	0	n	1	0	Asparagaceae Juss.
Iz_8_1009	DJI_0956	Artemisia marschalliana Spreng.	0	n	1	0	Asteraceae Bercht. & J.Presl
Iz_8_1009	DJI_0956	Asparagus officinalis L.	0	n	1	0	Asparagaceae Juss.
Iz_8_1009	DJI_0956	Astragalus cicer L.	0	n	1	0	Fabaceae Lindl.
Iz_8_1009	DJI_0956	Bassia laniflora (S.G.Gmel.) A.J.Scott	0	n	1	0	Amaranthaceae Juss.
Iz_8_1009	DJI_0956	Berberis vulgaris L.	0	n	1	0	Berberidaceae Juss.
Iz_8_1009	DJI_0956	Betula pendula Roth.	0	n	1	1	Betulaceae Gray
Iz_8_1009	DJI_0956	Betula pendula Roth.	0	n	1	0	Poaceae Barnhart
Iz_8_1009	DJI_0956	Bromus inermis Leyss.	0		NA	NA	
Iz_8_1009	DJI_0956	Bromus squarrosus L.	0	a	0	0	Poaceae Barnhart
Iz_8_1009	DJI_0956	Bromus tectorum L.	0	a	0	0	Poaceae Barnhart
Iz_8_1009	DJI_0956	Calamagrostis epigejos (L.) Roth	0.2	n	1	0	Poaceae Barnhart
Iz_8_1009	DJI_0956	Carduus acanthoides L.	0	n	1	0	Asteraceae Bercht. & J.Presl
Iz_8_1009	DJI_0956	Carex sp.	0	n	1	0	Cyperaceae Juss.
Iz_8_1009	DJI_0956	Centaurea majorovii Dumbadze	0	n	1	0	Asteraceae Bercht. & J.Presl
Iz_8_1009	DJI_0956	Chamaecytisus ruthenicus (Fisch.ex Wol.) Klaskova	0	n	1	0	Fabaceae Lindl.
Iz_8_1009	DJI_0956	Chelidonium majus L.	0	n	1	0	Papaveraceae Juss.
Iz_8_1009	DJI_0956	Chenopodium betaceum Andrz.	0	n	1	0	Amaranthaceae Juss.
Iz_8_1009	DJI_0956	Chenopodium cf. album	0	n	1	0	Amaranthaceae Juss.
Iz_8_1009	DJI_0956	Chenopodium sp.	0		NA	0	Amaranthaceae Juss.
Iz_8_1009	DJI_0956	Chondrilla juncea L.	1	n	1	0	Asteraceae Bercht. & J.Presl
Iz_8_1009	DJI_0956	Cirsium vulgare (Savi) Ten.	0	n	1	0	Asteraceae Bercht. & J.Presl
Iz_8_1009	DJI_0956	Clinopodium acinos (L.) Kuntze	0	n	1	0	Lamiacea Lindl.
Iz_8_1009	DJI_0956	Convallaria majalis L.	0	n	1	0	Asparagaceae Juss.
Iz_8_1009	DJI_0956	Crataegus rhipidophylla Gand.	0	n	1	1	Rosaceae Juss.
Iz_8_1009	DJI_0956	Crepis foetida L.	0	n	1	0	Asteraceae Bercht. & J.Presl
Iz_8_1009	DJI_0956	Dactylis glomerata L.	0	n	1	0	Poaceae Barnhart
Iz_8_1009	DJI_0956	Dianthus campestris M.Bieb.	0	n	1	0	Caryophyllaceae Juss.
Iz_8_1009	DJI_0956	Digitaria sanguinalis (L.) Scop.	0	a	0	0	Poaceae Barnhart
Iz_8_1009	DJI_0956	Elymus repens (L.) Gould	0	n	1	0	Poaceae Barnhart
Iz_8_1009	DJI_0956	Epilobium tetragonum L.	0	n	1	0	Onagraceae Juss.
Iz_8_1009	DJI_0956	Eragrostis minor Host	0	a	0	0	Poaceae Barnhart
Iz_8_1009	DJI_0956	Erigeron canadensis L.	4	a	0	0	Asteraceae Bercht. & J.Presl
Iz_8_1009	DJI_0956	Euphorbia seguieriana Neck.	0	n	1	0	Euphorbiaceae Juss.
Iz_8_1009	DJI_0956	Fallopia dumetorum (L.) Holub	0	n	1	0	Polygonaceae Juss.
Iz_8_1009	DJI_0956	Festuca beckeri (Hack.) Trautv.	0	n	1	0	Poaceae Barnhart
Iz_8_1009	DJI_0956	Fragaria vesca L.	0	n	1	0	Rosaceae Juss.
Iz_8_1009	DJI_0956	Frangula alnus Mill.	0	n	1	1	Rhamnaceae Juss.
Iz_8_1009	DJI_0956	Fraxinus pennsylvanica Marshall	0	a	0	1	Oleaceae Hoffmanns. & Link
Iz_8_1009	DJI_0956	Galeopsis bifida Boenn.	0	n	1	0	Lamiacea Lindl.
Iz_8_1009	DJI_0956	Galium verum L.	0	n	1	0	Rubiaceae Juss.
Iz_8_1009	DJI_0956	Genista tinctoria L.	0	n	1	0	Fabaceae Lindl.
Iz_8_1009	DJI_0956	Geranium robertianum L.	0	n	1	0	Geraniaceae Juss.
Iz_8_1009	DJI_0956	Geranium sanquineum L.	0	n	1	0	Geraniaceae Juss.
Iz_8_1009	DJI_0956	Gypsophila paniculata L.	0	n	1	0	Caryophyllaceae Juss.
Iz_8_1009	DJI_0956	Helichrysum arenarium (L.) Moench	0	n	1	0	Asteraceae Bercht. & J.Presl
Iz_8_1009	DJI_0956	Herniaria polygama J.Gay	0	n	1	0	Caryophyllaceae Juss.
Iz_8_1009	DJI_0956	Hieracium robustum Fr.	0	n	1	0	Asteraceae Bercht. & J.Presl
Iz_8_1009	DJI_0956	Hieracium umbellatum L.	0	n	1	NA	Asteraceae Bercht. & J.Presl
Iz_8_1009	DJI_0956	Hylotelephium maximum (L.) Holub	0	n	1	0	Crassulaceae J.St.-Hil.
Iz_8_1009	DJI_0956	Jacobaea borysthenica (DC.) B.Nord. & Greuter	0	n	1	0	Asteraceae Bercht. & J.Presl
Iz_8_1009	DJI_0956	Jasione montana L.	0	n	1	0	Campanulaceae Juss.
Iz_8_1009	DJI_0956	Jurinea cyanoides (L.) Rchb.s.l.	0	n	1	0	Asteraceae Bercht. & J.Presl
Iz_8_1009	DJI_0956	Koeleria glauca (Spreng.) DC.	0	n	1	0	Poaceae Barnhart
Iz_8_1009	DJI_0956	Lactuca serriola L.	0	a	0	0	Asteraceae Bercht. & J.Presl
Iz_8_1009	DJI_0956	Linaria genistifolia (L.) Mill.	0	n	1	0	Plantaginaceae Juss.

Iz_8_1009	DJI_0956	Lomelosia argentea (L.) Greuter & Burdet	0	n	1	0	Caprifoliaceae Juss.
Iz_8_1009	DJI_0956	Melampyrum pratense L.	0	n	1	0	Orobanchaceae Vent.
Iz_8_1009	DJI_0956	Oenothera biennis L.	0	a	0	0	Onagraceae Juss.
Iz_8_1009	DJI_0956	Pentanema britannica (L.) D.Gut.Larr., Santos-Vicente, Anderb., E.Rico & M.M.Mart.Ort.	0	n	1	0	Asteraceae Bercht. & J.Presl
Iz_8_1009	DJI_0956	Peucedanum oreoselinum (L.) Moench	0	n	1	0	Apiaceae Lindl.
Iz_8_1009	DJI_0956	Peucedanum ruthenicum M.Bieb.	0	n	1	0	Apiaceae Lindl.
Iz_8_1009	DJI_0956	Pilosella echioides (Lumn.) F.W.Schultz & Sch.Bip.	0	n	1	0	Asteraceae Bercht. & J.Presl
Iz_8_1009	DJI_0956	Pilosella officinarum F. Schultz et Sch. Bip.	0	n	1	0	Asteraceae Bercht. & J.Presl
Iz_8_1009	DJI_0956	Pilosella sp.	0		NA	0	Asteraceae Bercht. & J.Presl
Iz_8_1009	DJI_0956	Pinus sylvestris L.	0	n	1	1	Pinaceae Spreng. ex F.Rudolphi
Iz_8_1009	DJI_0956	Poa annua L.	0	n	1	0	Poaceae Barnhart
Iz_8_1009	DJI_0956	Poa nemoralis L.	0	n	1	0	Poaceae Barnhart
Iz_8_1009	DJI_0956	Poa sp.	0		NA	0	Poaceae Barnhart
Iz_8_1009	DJI_0956	Polygonatum odoratum (Mill.) Druce	0	n	1	0	Asparagaceae Juss.
Iz_8_1009	DJI_0956	Polygonum arenarium Waldst. & Kit.	0	n	1	0	Polygonaceae Juss.
Iz_8_1009	DJI_0956	Populus nigra L.	0	n	1	1	Salicaceae Mirb.
Iz_8_1009	DJI_0956	Populus tremula L.	0	n	1	1	Salicaceae Mirb.
Iz_8_1009	DJI_0956	Populus Ã canescens (Aiton) Sm.	0	n	1	1	Salicaceae Mirb.
Iz_8_1009	DJI_0956	Potentilla argentea L.	0	n	1	0	Rosaceae Juss.
Iz_8_1009	DJI_0956	Psammophiliella muralis (L.) Ikonn.	0	n	1	0	Caryophyllaceae Juss.
Iz_8_1009	DJI_0956	Psephellus sumensis (Kalen.) Greuter	0	n	1	0	Asteraceae Bercht. & J.Presl
Iz_8_1009	DJI_0956	Pulsatilla pratensis (L.) Mill. s.l.	0	n	1	0	Ranunculaceae Juss.
Iz_8_1009	DJI_0956	Pyrus communis L.	0	a	0	1	Rosaceae Juss.
Iz_8_1009	DJI_0956	Quercus robur L.	0	n	1	1	Fagaceae Dumort.
Iz_8_1009	DJI_0956	Robinia pseudoacacia L.	0	a	0	1	Fabaceae Lindl.
Iz_8_1009	DJI_0956	Rosa sp.	0		NA	1	Rosaceae Juss.
Iz_8_1009	DJI_0956	Rubus idaeus L.	0	n	1	0	Rosaceae Juss.
Iz_8_1009	DJI_0956	Rumex acetosella L.	0	n	1	0	Polygonaceae Juss.
Iz_8_1009	DJI_0956	Rumex thyrsiflorus Fingerh.	0	n	1	0	Polygonaceae Juss.
Iz_8_1009	DJI_0956	Salix alba L.	0	n	1	1	Salicaceae Mirb.
Iz_8_1009	DJI_0956	Sambucus nigra L.	0	n	1	1	Viburnaceae Raf.
Iz_8_1009	DJI_0956	Secale sylvestre Host	0	n	1	0	Poaceae Barnhart
Iz_8_1009	DJI_0956	Sempervivum ruthenicum Schnittsp. & C.B. Lehm.	0	n	1	0	Crassulaceae J.St.-Hil.
Iz_8_1009	DJI_0956	Setaria viridis (L.) P. Beauv.	0	n	1	0	Poaceae Barnhart
Iz_8_1009	DJI_0956	Silene borysthenica (Gruner) Walters	0	n	1	0	Caryophyllaceae Juss.
Iz_8_1009	DJI_0956	Silene latifolia subsp. alba (Mill.) Greuter & Burdet	0	n	1	0	Caryophyllaceae Juss.
Iz_8_1009	DJI_0956	Solidago virgaurea L.	0	n	1	0	Asteraceae Bercht. & J.Presl
Iz_8_1009	DJI_0956	Sorbus aucuparia L.	0	n	1	1	Rosaceae Juss.
Iz_8_1009	DJI_0956	Stachys recta L.	0	n	1	0	Lamiacea Lindl.
Iz_8_1009	DJI_0956	Tanacetum vulgare L.	0	n	1	0	Asteraceae Bercht. & J.Presl
Iz_8_1009	DJI_0956	Thymus pallasianus Heinr.Braun	0	n	1	0	Lamiacea Lindl.
Iz_8_1009	DJI_0956	Tilia cordata Mill.	0	n	1	1	Malvaceae Juss.
Iz_8_1009	DJI_0956	Trifolium alpestre L.	0	n	1	0	Fabaceae Lindl.
Iz_8_1009	DJI_0956	Trifolium arvense L.	0	n	1	0	Fabaceae Lindl.
Iz_8_1009	DJI_0956	Ulmus glabra Huds.	0	n	1	1	Ulmaceae Mirb.
Iz_8_1009	DJI_0956	Verbascum densiflorum Bertol.	0	n	1	0	Scrophulariaceae Juss.
Iz_8_1009	DJI_0956	Veronica austriaca L.	0	n	1	0	Plantaginaceae Juss.
Iz_8_1009	DJI_0956	Veronica incana L.	0	n	1	0	Plantaginaceae Juss.
Iz_8_1009	DJI_0956	Veronica verna L.	0	n	1	0	Plantaginaceae Juss.
Iz_8_1009	DJI_0956	Vincetoxicum hirundinaria Medik	0	n	1	0	Apocynaceae Juss.
Iz_8_1009	DJI_0963	Acer tataricum L.	0	n	1	1	Sapindaceae Juss.
Iz_8_1009	DJI_0963	Achillea millefolium L. s.l.	0	n	1	0	Asteraceae Bercht. & J.Presl
Iz_8_1009	DJI_0963	Allium oleraceum L.	0	n	1	0	Amaryllidaceae J.St.-Hil.
Iz_8_1009	DJI_0963	Ambrosia artemisiifolia L.	0	a	0	0	Asteraceae Bercht. & J.Presl
Iz_8_1009	DJI_0963	Amorpha fruticosa L.	0	a	0	0	Fabaceae Lindl.
Iz_8_1009	DJI_0963	Anthericum ramosum L.	0	n	1	0	Asparagaceae Juss.
Iz_8_1009	DJI_0963	Artemisia marschalliana Spreng.	0	n	1	0	Asteraceae Bercht. & J.Presl
Iz_8_1009	DJI_0963	Asparagus officinalis L.	0	n	1	0	Asparagaceae Juss.
Iz_8_1009	DJI_0963	Astragalus cicer L.	0	n	1	0	Fabaceae Lindl.
Iz_8_1009	DJI_0963	Bassia laniflora (S.G.Gmel.) A.J.Scott	0	n	1	0	Amaranthaceae Juss.
Iz_8_1009	DJI_0963	Berberis vulgaris L.	0	n	1	0	Berberidaceae Juss.
Iz_8_1009	DJI_0963	Betula pendula Roth.	0	n	1	1	Betulaceae Gray
Iz_8_1009	DJI_0963	Betula pendula Roth.	0	n	1	0	Poaceae Barnhart
Iz_8_1009	DJI_0963	Bromus inermis Leyss.	0		NA	NA	
Iz_8_1009	DJI_0963	Bromus squarrosus L.	0	a	0	0	Poaceae Barnhart
Iz_8_1009	DJI_0963	Bromus tectorum L.	0	a	0	0	Poaceae Barnhart
Iz_8_1009	DJI_0963	Calamagrostis epigejos (L.) Roth	0.5	n	1	0	Poaceae Barnhart
Iz_8_1009	DJI_0963	Carduus acanthoides L.	0	n	1	0	Asteraceae Bercht. & J.Presl
Iz_8_1009	DJI_0963	Carex sp.	0	n	1	0	Cyperaceae Juss.
Iz_8_1009	DJI_0963	Centaurea majorovii Dumbadze	0	n	1	0	Asteraceae Bercht. & J.Presl
Iz_8_1009	DJI_0963	Chamaecytisus ruthenicus (Fisch.ex Wol.) Klaskova	0	n	1	0	Fabaceae Lindl.
Iz_8_1009	DJI_0963	Chelidonium majus L.	0	n	1	0	Papaveraceae Juss.
Iz_8_1009	DJI_0963	Chenopodium betaceum Andrz.	0	n	1	0	Amaranthaceae Juss.
Iz_8_1009	DJI_0963	Chenopodium cf. album	0	n	1	0	Amaranthaceae Juss.
Iz_8_1009	DJI_0963	Chenopodium sp.	0		NA	0	Amaranthaceae Juss.
Iz_8_1009	DJI_0963	Chondrilla juncea L.	0	n	1	0	Asteraceae Bercht. & J.Presl
Iz_8_1009	DJI_0963	Cirsium vulgare (Savi) Ten.	0	n	1	0	Asteraceae Bercht. & J.Presl
Iz_8_1009	DJI_0963	Clinopodium acinos (L.) Kuntze	0	n	1	0	Lamiacea Lindl.
Iz_8_1009	DJI_0963	Convallaria majalis L.	0	n	1	0	Asparagaceae Juss.
Iz_8_1009	DJI_0963	Crataegus rhipidophylla Gand.	0	n	1	1	Rosaceae Juss.
Iz_8_1009	DJI_0963	Crepis foetida L.	0	n	1	0	Asteraceae Bercht. & J.Presl
Iz_8_1009	DJI_0963	Dactylis glomerata L.	0	n	1	0	Poaceae Barnhart
Iz_8_1009	DJI_0963	Dianthus campestris M.Bieb.	0	n	1	0	Caryophyllaceae Juss.
Iz_8_1009	DJI_0963	Digitaria sanguinalis (L.) Scop.	0	a	0	0	Poaceae Barnhart
Iz_8_1009	DJI_0963	Elymus repens (L.) Gould	0	n	1	0	Poaceae Barnhart
Iz_8_1009	DJI_0963	Epilobium tetragonum L.	0	n	1	0	Onagraceae Juss.
Iz_8_1009	DJI_0963	Eragrostis minor Host	0	a	0	0	Poaceae Barnhart
Iz_8_1009	DJI_0963	Erigeron canadensis L.	10	a	0	0	Asteraceae Bercht. & J.Presl
Iz_8_1009	DJI_0963	Euphorbia seguieriana Neck.	0	n	1	0	Euphorbiaceae Juss.
Iz_8_1009	DJI_0963	Fallopia dumetorum (L.) Holub	0	n	1	0	Polygonaceae Juss.
Iz_8_1009	DJI_0963	Festuca beckeri (Hack.) Trautv.	0	n	1	0	Poaceae Barnhart
Iz_8_1009	DJI_0963	Fragaria vesca L.	0	n	1	0	Rosaceae Juss.
Iz_8_1009	DJI_0963	Frangula alnus Mill.	0	n	1	1	Rhamnaceae Juss.
Iz_8_1009	DJI_0963	Fraxinus pennsylvanica Marshall	0	a	0	1	Oleaceae Hoffmanns. & Link
Iz_8_1009	DJI_0963	Galeopsis bifida Boenn.	0	n	1	0	Lamiacea Lindl.
Iz_8_1009	DJI_0963	Galium verum L.	0	n	1	0	Rubiaceae Juss.
Iz_8_1009	DJI_0963	Genista tinctoria L.	0	n	1	0	Fabaceae Lindl.
Iz_8_1009	DJI_0963	Geranium robertianum L.	0	n	1	0	Geraniaceae Juss.

Iz_8_1009	DJI_0963	Geranium sanquineum L.	0	n	1	0	Geraniaceae Juss.
Iz_8_1009	DJI_0963	Gypsophila paniculata L.	0	n	1	0	Caryophyllaceae Juss.
Iz_8_1009	DJI_0963	Helichrysum arenarium (L.) Moench	0	n	1	0	Asteraceae Bercht. & J.Presl
Iz_8_1009	DJI_0963	Herniaria polygama J.Gay	0	n	1	0	Caryophyllaceae Juss.
Iz_8_1009	DJI_0963	Hieracium robustum Fr.	0	n	1	0	Asteraceae Bercht. & J.Presl
Iz_8_1009	DJI_0963	Hieracium umbellatum L.	0	n	1	NA	Asteraceae Bercht. & J.Presl
Iz_8_1009	DJI_0963	Hylotelephium maximum (L.) Holub	0	n	1	0	Crassulaceae J.St.-Hil.
Iz_8_1009	DJI_0963	Jacobaea borysthenica (DC.) B.Nord. & Greuter	0	n	1	0	Asteraceae Bercht. & J.Presl
Iz_8_1009	DJI_0963	Jasione montana L.	0	n	1	0	Campanulaceae Juss.
Iz_8_1009	DJI_0963	Jurinea cyanoides (L.) Rchb.s.l.	0	n	1	0	Asteraceae Bercht. & J.Presl
Iz_8_1009	DJI_0963	Koeleria glauca (Spreng.) DC.	0	n	1	0	Poaceae Barnhart
Iz_8_1009	DJI_0963	Lactuca serriola L.	0	a	0	0	Asteraceae Bercht. & J.Presl
Iz_8_1009	DJI_0963	Linaria genistifolia (L.) Mill.	0	n	1	0	Plantaginaceae Juss.
Iz_8_1009	DJI_0963	Lomelosia argentea (L.) Greuter & Burdet	0	n	1	0	Caprifoliaceae Juss.
Iz_8_1009	DJI_0963	Melampyrum pratense L.	0	n	1	0	Orobanchaceae Vent.
Iz_8_1009	DJI_0963	Oenothera biennis L.	0	a	0	0	Onagraceae Juss.
Iz_8_1009	DJI_0963	Pentanema britannica (L.) D.Gut.Larr., Santos-Vicente, Anderb., E.Rico & M.M.Mart.Ort.	0	n	1	0	Asteraceae Bercht. & J.Presl
Iz_8_1009	DJI_0963	Peucedanum oreoselinum (L.) Moench	0	n	1	0	Apiaceae Lindl.
Iz_8_1009	DJI_0963	Peucedanum ruthenicum M.Bieb.	0	n	1	0	Apiaceae Lindl.
Iz_8_1009	DJI_0963	Pilosella echioides (Lumn.) F.W.Schultz & Sch.Bip.	0	n	1	0	Asteraceae Bercht. & J.Presl
Iz_8_1009	DJI_0963	Pilosella officinarum F. Schultz et Sch. Bip.	0	n	1	0	Asteraceae Bercht. & J.Presl
Iz_8_1009	DJI_0963	Pilosella sp.	0		NA	0	Asteraceae Bercht. & J.Presl
Iz_8_1009	DJI_0963	Pinus sylvestris L.	0	n	1	1	Pinaceae Spreng. ex F.Rudolphi
Iz_8_1009	DJI_0963	Poa annua L.	0	n	1	0	Poaceae Barnhart
Iz_8_1009	DJI_0963	Poa nemoralis L.	0	n	1	0	Poaceae Barnhart
Iz_8_1009	DJI_0963	Poa sp.	0		NA	0	Poaceae Barnhart
Iz_8_1009	DJI_0963	Polygonatum odoratum (Mill.) Druce	0	n	1	0	Asparagaceae Juss.
Iz_8_1009	DJI_0963	Polygonum arenarium Waldst. & Kit.	0	n	1	0	Polygonaceae Juss.
Iz_8_1009	DJI_0963	Populus nigra L.	0	n	1	1	Salicaceae Mirb.
Iz_8_1009	DJI_0963	Populus tremula L.	0	n	1	1	Salicaceae Mirb.
Iz_8_1009	DJI_0963	Populus Ã canescens (Aiton) Sm.	0	n	1	1	Salicaceae Mirb.
Iz_8_1009	DJI_0963	Potentilla argentea L.	0	n	1	0	Rosaceae Juss.
Iz_8_1009	DJI_0963	Psammophiliella muralis (L.) Ikonn.	0	n	1	0	Caryophyllaceae Juss.
Iz_8_1009	DJI_0963	Psephellus sumensis (Kalen.) Greuter	0	n	1	0	Asteraceae Bercht. & J.Presl
Iz_8_1009	DJI_0963	Pulsatilla pratensis (L.) Mill. s.l.	0	n	1	0	Ranunculaceae Juss.
Iz_8_1009	DJI_0963	Pyrus communis L.	0	a	0	1	Rosaceae Juss.
Iz_8_1009	DJI_0963	Quercus robur L.	0	n	1	1	Fagaceae Dumort.
Iz_8_1009	DJI_0963	Robinia pseudoacacia L.	0	a	0	1	Fabaceae Lindl.
Iz_8_1009	DJI_0963	Rosa sp.	0		NA	1	Rosaceae Juss.
Iz_8_1009	DJI_0963	Rubus idaeus L.	0	n	1	0	Rosaceae Juss.
Iz_8_1009	DJI_0963	Rumex acetosella L.	0	n	1	0	Polygonaceae Juss.
Iz_8_1009	DJI_0963	Rumex thyrsiflorus Fingerh.	0	n	1	0	Polygonaceae Juss.
Iz_8_1009	DJI_0963	Salix alba L.	0	n	1	1	Salicaceae Mirb.
Iz_8_1009	DJI_0963	Sambucus nigra L.	0	n	1	1	Viburnaceae Raf.
Iz_8_1009	DJI_0963	Secale sylvestre Host	0	n	1	0	Poaceae Barnhart
Iz_8_1009	DJI_0963	Sempervivum ruthenicum Schnittsp. & C.B. Lehm.	0	n	1	0	Crassulaceae J.St.-Hil.
Iz_8_1009	DJI_0963	Setaria viridis (L.) P. Beauv.	0	n	1	0	Poaceae Barnhart
Iz_8_1009	DJI_0963	Silene borysthenica (Gruner) Walters	0	n	1	0	Caryophyllaceae Juss.
Iz_8_1009	DJI_0963	Silene latifolia subsp. alba (Mill.) Greuter & Burdet	0	n	1	0	Caryophyllaceae Juss.
Iz_8_1009	DJI_0963	Solidago virgaurea L.	0	n	1	0	Asteraceae Bercht. & J.Presl
Iz_8_1009	DJI_0963	Sorbus aucuparia L.	0	n	1	1	Rosaceae Juss.
Iz_8_1009	DJI_0963	Stachys recta L.	0	n	1	0	Lamiacea Lindl.
Iz_8_1009	DJI_0963	Tanacetum vulgare L.	0	n	1	0	Asteraceae Bercht. & J.Presl
Iz_8_1009	DJI_0963	Thymus pallasianus Heinr.Braun	0	n	1	0	Lamiacea Lindl.
Iz_8_1009	DJI_0963	Tilia cordata Mill.	0	n	1	1	Malvaceae Juss.
Iz_8_1009	DJI_0963	Trifolium alpestre L.	0	n	1	0	Fabaceae Lindl.
Iz_8_1009	DJI_0963	Trifolium arvense L.	0	n	1	0	Fabaceae Lindl.
Iz_8_1009	DJI_0963	Ulmus glabra Huds.	0	n	1	1	Ulmaceae Mirb.
Iz_8_1009	DJI_0963	Verbascum densiflorum Bertol.	0	n	1	0	Scrophulariaceae Juss.
Iz_8_1009	DJI_0963	Veronica austriaca L.	0	n	1	0	Plantaginaceae Juss.
Iz_8_1009	DJI_0963	Veronica incana L.	0	n	1	0	Plantaginaceae Juss.
Iz_8_1009	DJI_0963	Veronica verna L.	0	n	1	0	Plantaginaceae Juss.
Iz_8_1009	DJI_0963	Vincetoxicum hirundinaria Medik	0	n	1	0	Apocynaceae Juss.
Iz_8_1009	DJI_0965	Acer tataricum L.	0	n	1	1	Sapindaceae Juss.
Iz_8_1009	DJI_0965	Achillea millefolium L. s.l.	0	n	1	0	Asteraceae Bercht. & J.Presl
Iz_8_1009	DJI_0965	Allium oleraceum L.	0	n	1	0	Amaryllidaceae J.St.-Hil.
Iz_8_1009	DJI_0965	Ambrosia artemisiifolia L.	0	a	0	0	Asteraceae Bercht. & J.Presl
Iz_8_1009	DJI_0965	Amorpha fruticosa L.	0	a	0	0	Fabaceae Lindl.
Iz_8_1009	DJI_0965	Anthericum ramosum L.	0	n	1	0	Asparagaceae Juss.
Iz_8_1009	DJI_0965	Artemisia marschalliana Spreng.	0	n	1	0	Asteraceae Bercht. & J.Presl
Iz_8_1009	DJI_0965	Asparagus officinalis L.	0	n	1	0	Asparagaceae Juss.
Iz_8_1009	DJI_0965	Astragalus cicer L.	0	n	1	0	Fabaceae Lindl.
Iz_8_1009	DJI_0965	Bassia laniflora (S.G.Gmel.) A.J.Scott	0	n	1	0	Amaranthaceae Juss.
Iz_8_1009	DJI_0965	Berberis vulgaris L.	0	n	1	0	Berberidaceae Juss.
Iz_8_1009	DJI_0965	Betula pendula Roth.	0	n	1	1	Betulaceae Gray
Iz_8_1009	DJI_0965	Betula pendula Roth.	0	n	1	0	Poaceae Barnhart
Iz_8_1009	DJI_0965	Bromus inermis Leyss.	0		NA	NA	
Iz_8_1009	DJI_0965	Bromus squarrosus L.	0	a	0	0	Poaceae Barnhart
Iz_8_1009	DJI_0965	Bromus tectorum L.	0	a	0	0	Poaceae Barnhart
Iz_8_1009	DJI_0965	Calamagrostis epigejos (L.) Roth	5	n	1	0	Poaceae Barnhart
Iz_8_1009	DJI_0965	Carduus acanthoides L.	0	n	1	0	Asteraceae Bercht. & J.Presl
Iz_8_1009	DJI_0965	Carex sp.	0	n	1	0	Cyperaceae Juss.
Iz_8_1009	DJI_0965	Centaurea majorovii Dumbadze	0	n	1	0	Asteraceae Bercht. & J.Presl
Iz_8_1009	DJI_0965	Chamaecytisus ruthenicus (Fisch.ex Wol.) Klaskova	0	n	1	0	Fabaceae Lindl.
Iz_8_1009	DJI_0965	Chelidonium majus L.	0	n	1	0	Papaveraceae Juss.
Iz_8_1009	DJI_0965	Chenopodium betaceum Andrz.	0	n	1	0	Amaranthaceae Juss.
Iz_8_1009	DJI_0965	Chenopodium cf. album	0	n	1	0	Amaranthaceae Juss.
Iz_8_1009	DJI_0965	Chenopodium sp.	0		NA	0	Amaranthaceae Juss.
Iz_8_1009	DJI_0965	Chondrilla juncea L.	0	n	1	0	Asteraceae Bercht. & J.Presl
Iz_8_1009	DJI_0965	Cirsium vulgare (Savi) Ten.	0	n	1	0	Asteraceae Bercht. & J.Presl
Iz_8_1009	DJI_0965	Clinopodium acinos (L.) Kuntze	0	n	1	0	Lamiacea Lindl.
Iz_8_1009	DJI_0965	Convallaria majalis L.	0	n	1	0	Asparagaceae Juss.
Iz_8_1009	DJI_0965	Crataegus rhipidophylla Gand.	0	n	1	1	Rosaceae Juss.
Iz_8_1009	DJI_0965	Crepis foetida L.	0	n	1	0	Asteraceae Bercht. & J.Presl
Iz_8_1009	DJI_0965	Dactylis glomerata L.	0	n	1	0	Poaceae Barnhart
Iz_8_1009	DJI_0965	Dianthus campestris M.Bieb.	0	n	1	0	Caryophyllaceae Juss.
Iz_8_1009	DJI_0965	Digitaria sanguinalis (L.) Scop.	0	a	0	0	Poaceae Barnhart
Iz_8_1009	DJI_0965	Elymus repens (L.) Gould	0	n	1	0	Poaceae Barnhart

Iz_8_1009	DJI_0965	Epilobium tetragonum L.	0	n	1	0	Onagraceae Juss.
Iz_8_1009	DJI_0965	Eragrostis minor Host	0	a	0	0	Poaceae Barnhart
Iz_8_1009	DJI_0965	Erigeron canadensis L.	15	a	0	0	Asteraceae Bercht. & J.Presl
Iz_8_1009	DJI_0965	Euphorbia seguieriana Neck.	0	n	1	0	Euphorbiaceae Juss.
Iz_8_1009	DJI_0965	Fallopia dumetorum (L.) Holub	0	n	1	0	Polygonaceae Juss.
Iz_8_1009	DJI_0965	Festuca beckeri (Hack.) Trautv.	0	n	1	0	Poaceae Barnhart
Iz_8_1009	DJI_0965	Fragaria vesca L.	0	n	1	0	Rosaceae Juss.
Iz_8_1009	DJI_0965	Frangula alnus Mill.	0	n	1	1	Rhamnaceae Juss.
Iz_8_1009	DJI_0965	Fraxinus pennsylvanica Marshall	0	a	0	1	Oleaceae Hoffmanns. & Link
Iz_8_1009	DJI_0965	Galeopsis bifida Boenn.	0	n	1	0	Lamiacea Lindl.
Iz_8_1009	DJI_0965	Galium verum L.	0	n	1	0	Rubiaceae Juss.
Iz_8_1009	DJI_0965	Genista tinctoria L.	0	n	1	0	Fabaceae Lindl.
Iz_8_1009	DJI_0965	Geranium robertianum L.	0	n	1	0	Geraniaceae Juss.
Iz_8_1009	DJI_0965	Geranium sanquineum L.	0	n	1	0	Geraniaceae Juss.
Iz_8_1009	DJI_0965	Gypsophila paniculata L.	0	n	1	0	Caryophyllaceae Juss.
Iz_8_1009	DJI_0965	Helichrysum arenarium (L.) Moench	0	n	1	0	Asteraceae Bercht. & J.Presl
Iz_8_1009	DJI_0965	Herniaria polygama J.Gay	0	n	1	0	Caryophyllaceae Juss.
Iz_8_1009	DJI_0965	Hieracium robustum Fr.	0	n	1	0	Asteraceae Bercht. & J.Presl
Iz_8_1009	DJI_0965	Hieracium umbellatum L.	0	n	1	NA	Asteraceae Bercht. & J.Presl
Iz_8_1009	DJI_0965	Hylotelephium maximum (L.) Holub	0	n	1	0	Crassulaceae J.St.-Hil.
Iz_8_1009	DJI_0965	Jacobaea borysthenica (DC.) B.Nord. & Greuter	0	n	1	0	Asteraceae Bercht. & J.Presl
Iz_8_1009	DJI_0965	Jasione montana L.	0	n	1	0	Campanulaceae Juss.
Iz_8_1009	DJI_0965	Jurinea cyanoides (L.) Rchb.s.l.	0	n	1	0	Asteraceae Bercht. & J.Presl
Iz_8_1009	DJI_0965	Koeleria glauca (Spreng.) DC.	0	n	1	0	Poaceae Barnhart
Iz_8_1009	DJI_0965	Lactuca serriola L.	0	a	0	0	Asteraceae Bercht. & J.Presl
Iz_8_1009	DJI_0965	Linaria genistifolia (L.) Mill.	0	n	1	0	Plantaginaceae Juss.
Iz_8_1009	DJI_0965	Lomelosia argentea (L.) Greuter & Burdet	0	n	1	0	Caprifoliaceae Juss.
Iz_8_1009	DJI_0965	Melampyrum pratense L.	0	n	1	0	Orobanchaceae Vent.
Iz_8_1009	DJI_0965	Oenothera biennis L.	0	a	0	0	Onagraceae Juss.
Iz_8_1009	DJI_0965	Pentanema britannica (L.) D.Gut.Larr., Santos-Vicente, Anderb., E.Rico & M.M.Mart.Ort.	0	n	1	0	Asteraceae Bercht. & J.Presl
Iz_8_1009	DJI_0965	Peucedanum oreoselinum (L.) Moench	0	n	1	0	Apiaceae Lindl.
Iz_8_1009	DJI_0965	Peucedanum ruthenicum M.Bieb.	0	n	1	0	Apiaceae Lindl.
Iz_8_1009	DJI_0965	Pilosella echioides (Lumn.) F.W.Schultz & Sch.Bip.	0	n	1	0	Asteraceae Bercht. & J.Presl
Iz_8_1009	DJI_0965	Pilosella officinarum F. Schultz et Sch. Bip.	0	n	1	0	Asteraceae Bercht. & J.Presl
Iz_8_1009	DJI_0965	Pilosella sp.	0		NA	0	Asteraceae Bercht. & J.Presl
Iz_8_1009	DJI_0965	Pinus sylvestris L.	0	n	1	1	Pinaceae Spreng. ex F.Rudolphi
Iz_8_1009	DJI_0965	Poa annua L.	0	n	1	0	Poaceae Barnhart
Iz_8_1009	DJI_0965	Poa nemoralis L.	0	n	1	0	Poaceae Barnhart
Iz_8_1009	DJI_0965	Poa sp.	0		NA	0	Poaceae Barnhart
Iz_8_1009	DJI_0965	Polygonatum odoratum (Mill.) Druce	0	n	1	0	Asparagaceae Juss.
Iz_8_1009	DJI_0965	Polygonum arenarium Waldst. & Kit.	0	n	1	0	Polygonaceae Juss.
Iz_8_1009	DJI_0965	Populus nigra L.	0	n	1	1	Salicaceae Mirb.
Iz_8_1009	DJI_0965	Populus tremula L.	0	n	1	1	Salicaceae Mirb.
Iz_8_1009	DJI_0965	Populus Ã canescens (Aiton) Sm.	0	n	1	1	Salicaceae Mirb.
Iz_8_1009	DJI_0965	Potentilla argentea L.	0	n	1	0	Rosaceae Juss.
Iz_8_1009	DJI_0965	Psammophiliella muralis (L.) Ikonn.	0	n	1	0	Caryophyllaceae Juss.
Iz_8_1009	DJI_0965	Psephellus sumensis (Kalen.) Greuter	0	n	1	0	Asteraceae Bercht. & J.Presl
Iz_8_1009	DJI_0965	Pulsatilla pratensis (L.) Mill. s.l.	0	n	1	0	Ranunculaceae Juss.
Iz_8_1009	DJI_0965	Pyrus communis L.	0	a	0	1	Rosaceae Juss.
Iz_8_1009	DJI_0965	Quercus robur L.	0	n	1	1	Fagaceae Dumort.
Iz_8_1009	DJI_0965	Robinia pseudoacacia L.	0	a	0	1	Fabaceae Lindl.
Iz_8_1009	DJI_0965	Rosa sp.	0		NA	1	Rosaceae Juss.
Iz_8_1009	DJI_0965	Rubus idaeus L.	0	n	1	0	Rosaceae Juss.
Iz_8_1009	DJI_0965	Rumex acetosella L.	0	n	1	0	Polygonaceae Juss.
Iz_8_1009	DJI_0965	Rumex thyrsiflorus Fingerh.	0	n	1	0	Polygonaceae Juss.
Iz_8_1009	DJI_0965	Salix alba L.	0	n	1	1	Salicaceae Mirb.
Iz_8_1009	DJI_0965	Sambucus nigra L.	0	n	1	1	Viburnaceae Raf.
Iz_8_1009	DJI_0965	Secale sylvestre Host	0	n	1	0	Poaceae Barnhart
Iz_8_1009	DJI_0965	Sempervivum ruthenicum Schnittsp. & C.B. Lehm.	0	n	1	0	Crassulaceae J.St.-Hil.
Iz_8_1009	DJI_0965	Setaria viridis (L.) P. Beauv.	0	n	1	0	Poaceae Barnhart
Iz_8_1009	DJI_0965	Silene borysthenica (Gruner) Walters	0	n	1	0	Caryophyllaceae Juss.
Iz_8_1009	DJI_0965	Silene latifolia subsp. alba (Mill.) Greuter & Burdet	0	n	1	0	Caryophyllaceae Juss.
Iz_8_1009	DJI_0965	Solidago virgaurea L.	0	n	1	0	Asteraceae Bercht. & J.Presl
Iz_8_1009	DJI_0965	Sorbus aucuparia L.	0	n	1	1	Rosaceae Juss.
Iz_8_1009	DJI_0965	Stachys recta L.	0	n	1	0	Lamiacea Lindl.
Iz_8_1009	DJI_0965	Tanacetum vulgare L.	0	n	1	0	Asteraceae Bercht. & J.Presl
Iz_8_1009	DJI_0965	Thymus pallasianus Heinr.Braun	0	n	1	0	Lamiacea Lindl.
Iz_8_1009	DJI_0965	Tilia cordata Mill.	0	n	1	1	Malvaceae Juss.
Iz_8_1009	DJI_0965	Trifolium alpestre L.	0	n	1	0	Fabaceae Lindl.
Iz_8_1009	DJI_0965	Trifolium arvense L.	0	n	1	0	Fabaceae Lindl.
Iz_8_1009	DJI_0965	Ulmus glabra Huds.	0	n	1	1	Ulmaceae Mirb.
Iz_8_1009	DJI_0965	Verbascum densiflorum Bertol.	0	n	1	0	Scrophulariaceae Juss.
Iz_8_1009	DJI_0965	Veronica austriaca L.	0	n	1	0	Plantaginaceae Juss.
Iz_8_1009	DJI_0965	Veronica incana L.	0	n	1	0	Plantaginaceae Juss.
Iz_8_1009	DJI_0965	Veronica verna L.	0	n	1	0	Plantaginaceae Juss.
Iz_8_1009	DJI_0965	Vincetoxicum hirundinaria Medik	0	n	1	0	Apocynaceae Juss.
Iz_8_1009	DJI_0966	Acer tataricum L.	0	n	1	1	Sapindaceae Juss.
Iz_8_1009	DJI_0966	Achillea millefolium L. s.l.	0	n	1	0	Asteraceae Bercht. & J.Presl
Iz_8_1009	DJI_0966	Allium oleraceum L.	0	n	1	0	Amaryllidaceae J.St.-Hil.
Iz_8_1009	DJI_0966	Ambrosia artemisiifolia L.	0	a	0	0	Asteraceae Bercht. & J.Presl
Iz_8_1009	DJI_0966	Amorpha fruticosa L.	0	a	0	0	Fabaceae Lindl.
Iz_8_1009	DJI_0966	Anthericum ramosum L.	0	n	1	0	Asparagaceae Juss.
Iz_8_1009	DJI_0966	Artemisia marschalliana Spreng.	0	n	1	0	Asteraceae Bercht. & J.Presl
Iz_8_1009	DJI_0966	Asparagus officinalis L.	0	n	1	0	Asparagaceae Juss.
Iz_8_1009	DJI_0966	Astragalus cicer L.	0	n	1	0	Fabaceae Lindl.
Iz_8_1009	DJI_0966	Bassia laniflora (S.G.Gmel.) A.J.Scott	0	n	1	0	Amaranthaceae Juss.
Iz_8_1009	DJI_0966	Berberis vulgaris L.	0	n	1	0	Berberidaceae Juss.
Iz_8_1009	DJI_0966	Betula pendula Roth.	0	n	1	1	Betulaceae Gray
Iz_8_1009	DJI_0966	Betula pendula Roth.	0	n	1	0	Poaceae Barnhart
Iz_8_1009	DJI_0966	Bromus inermis Leyss.	0		NA	NA	
Iz_8_1009	DJI_0966	Bromus squarrosus L.	0	a	0	0	Poaceae Barnhart
Iz_8_1009	DJI_0966	Bromus tectorum L.	0	a	0	0	Poaceae Barnhart
Iz_8_1009	DJI_0966	Calamagrostis epigejos (L.) Roth	3	n	1	0	Poaceae Barnhart
Iz_8_1009	DJI_0966	Carduus acanthoides L.	0	n	1	0	Asteraceae Bercht. & J.Presl
Iz_8_1009	DJI_0966	Carex sp.	0	n	1	0	Cyperaceae Juss.
Iz_8_1009	DJI_0966	Centaurea majorovii Dumbadze	0	n	1	0	Asteraceae Bercht. & J.Presl
Iz_8_1009	DJI_0966	Chamaecytisus ruthenicus (Fisch.ex Wol.) Klaskova	0	n	1	0	Fabaceae Lindl.
Iz_8_1009	DJI_0966	Chelidonium majus L.	0	n	1	0	Papaveraceae Juss.

Iz_8_1009	DJI_0966	Chenopodium betaceum Andrz.	0	n	1	0	Amaranthaceae Juss.
Iz_8_1009	DJI_0966	Chenopodium cf. album	0	n	1	0	Amaranthaceae Juss.
Iz_8_1009	DJI_0966	Chenopodium sp.	0		NA	0	Amaranthaceae Juss.
Iz_8_1009	DJI_0966	Chondrilla juncea L.	0	n	1	0	Asteraceae Bercht. & J.Presl
Iz_8_1009	DJI_0966	Cirsium vulgare (Savi) Ten.	0	n	1	0	Asteraceae Bercht. & J.Presl
Iz_8_1009	DJI_0966	Clinopodium acinos (L.) Kuntze	0	n	1	0	Lamiacea Lindl.
Iz_8_1009	DJI_0966	Convallaria majalis L.	0	n	1	0	Asparagaceae Juss.
Iz_8_1009	DJI_0966	Crataegus rhipidophylla Gand.	0	n	1	1	Rosaceae Juss.
Iz_8_1009	DJI_0966	Crepis foetida L.	0	n	1	0	Asteraceae Bercht. & J.Presl
Iz_8_1009	DJI_0966	Dactylis glomerata L.	0	n	1	0	Poaceae Barnhart
Iz_8_1009	DJI_0966	Dianthus campestris M.Bieb.	0	n	1	0	Caryophyllaceae Juss.
Iz_8_1009	DJI_0966	Digitaria sanguinalis (L.) Scop.	0	a	0	0	Poaceae Barnhart
Iz_8_1009	DJI_0966	Elymus repens (L.) Gould	0	n	1	0	Poaceae Barnhart
Iz_8_1009	DJI_0966	Epilobium tetragonum L.	0	n	1	0	Onagraceae Juss.
Iz_8_1009	DJI_0966	Eragrostis minor Host	0	a	0	0	Poaceae Barnhart
Iz_8_1009	DJI_0966	Erigeron canadensis L.	4	a	0	0	Asteraceae Bercht. & J.Presl
Iz_8_1009	DJI_0966	Euphorbia seguieriana Neck.	0	n	1	0	Euphorbiaceae Juss.
Iz_8_1009	DJI_0966	Fallopia dumetorum (L.) Holub	0	n	1	0	Polygonaceae Juss.
Iz_8_1009	DJI_0966	Festuca beckeri (Hack.) Trautv.	0	n	1	0	Poaceae Barnhart
Iz_8_1009	DJI_0966	Fragaria vesca L.	0	n	1	0	Rosaceae Juss.
Iz_8_1009	DJI_0966	Frangula alnus Mill.	0	n	1	1	Rhamnaceae Juss.
Iz_8_1009	DJI_0966	Fraxinus pennsylvanica Marshall	0	a	0	1	Oleaceae Hoffmanns. & Link
Iz_8_1009	DJI_0966	Galeopsis bifida Boenn.	0	n	1	0	Lamiacea Lindl.
Iz_8_1009	DJI_0966	Galium verum L.	0	n	1	0	Rubiaceae Juss.
Iz_8_1009	DJI_0966	Genista tinctoria L.	0	n	1	0	Fabaceae Lindl.
Iz_8_1009	DJI_0966	Geranium robertianum L.	0	n	1	0	Geraniaceae Juss.
Iz_8_1009	DJI_0966	Geranium sanquineum L.	0	n	1	0	Geraniaceae Juss.
Iz_8_1009	DJI_0966	Gypsophila paniculata L.	0	n	1	0	Caryophyllaceae Juss.
Iz_8_1009	DJI_0966	Helichrysum arenarium (L.) Moench	0	n	1	0	Asteraceae Bercht. & J.Presl
Iz_8_1009	DJI_0966	Herniaria polygama J.Gay	0	n	1	0	Caryophyllaceae Juss.
Iz_8_1009	DJI_0966	Hieracium robustum Fr.	0	n	1	0	Asteraceae Bercht. & J.Presl
Iz_8_1009	DJI_0966	Hieracium umbellatum L.	0	n	1	NA	Asteraceae Bercht. & J.Presl
Iz_8_1009	DJI_0966	Hylotelephium maximum (L.) Holub	0	n	1	0	Crassulaceae J.St.-Hil.
Iz_8_1009	DJI_0966	Jacobaea borysthenica (DC.) B.Nord. & Greuter	0	n	1	0	Asteraceae Bercht. & J.Presl
Iz_8_1009	DJI_0966	Jasione montana L.	0	n	1	0	Campanulaceae Juss.
Iz_8_1009	DJI_0966	Jurinea cyanoides (L.) Rchb.s.l.	0	n	1	0	Asteraceae Bercht. & J.Presl
Iz_8_1009	DJI_0966	Koeleria glauca (Spreng.) DC.	0	n	1	0	Poaceae Barnhart
Iz_8_1009	DJI_0966	Lactuca serriola L.	0	a	0	0	Asteraceae Bercht. & J.Presl
Iz_8_1009	DJI_0966	Linaria genistifolia (L.) Mill.	0	n	1	0	Plantaginaceae Juss.
Iz_8_1009	DJI_0966	Lomelosia argentea (L.) Greuter & Burdet	0	n	1	0	Caprifoliaceae Juss.
Iz_8_1009	DJI_0966	Melampyrum pratense L.	0	n	1	0	Orobanchaceae Vent.
Iz_8_1009	DJI_0966	Oenothera biennis L.	0	a	0	0	Onagraceae Juss.
Iz_8_1009	DJI_0966	Pentanema britannica (L.) D.Gut.Larr., Santos-Vicente, Anderb., E.Rico & M.M.Mart.Ort.	0	n	1	0	Asteraceae Bercht. & J.Presl
Iz_8_1009	DJI_0966	Peucedanum oreoselinum (L.) Moench	0	n	1	0	Apiaceae Lindl.
Iz_8_1009	DJI_0966	Peucedanum ruthenicum M.Bieb.	0	n	1	0	Apiaceae Lindl.
Iz_8_1009	DJI_0966	Pilosella echioides (Lumn.) F.W.Schultz & Sch.Bip.	0	n	1	0	Asteraceae Bercht. & J.Presl
Iz_8_1009	DJI_0966	Pilosella officinarum F. Schultz et Sch. Bip.	0	n	1	0	Asteraceae Bercht. & J.Presl
Iz_8_1009	DJI_0966	Pilosella sp.	0		NA	0	Asteraceae Bercht. & J.Presl
Iz_8_1009	DJI_0966	Pinus sylvestris L.	0	n	1	1	Pinaceae Spreng. ex F.Rudolphi
Iz_8_1009	DJI_0966	Poa annua L.	0	n	1	0	Poaceae Barnhart
Iz_8_1009	DJI_0966	Poa nemoralis L.	0	n	1	0	Poaceae Barnhart
Iz_8_1009	DJI_0966	Poa sp.	0		NA	0	Poaceae Barnhart
Iz_8_1009	DJI_0966	Polygonatum odoratum (Mill.) Druce	0	n	1	0	Asparagaceae Juss.
Iz_8_1009	DJI_0966	Polygonum arenarium Waldst. & Kit.	0.5	n	1	0	Polygonaceae Juss.
Iz_8_1009	DJI_0966	Populus nigra L.	0	n	1	1	Salicaceae Mirb.
Iz_8_1009	DJI_0966	Populus tremula L.	0	n	1	1	Salicaceae Mirb.
Iz_8_1009	DJI_0966	Populus Ã canescens (Aiton) Sm.	0	n	1	1	Salicaceae Mirb.
Iz_8_1009	DJI_0966	Potentilla argentea L.	0	n	1	0	Rosaceae Juss.
Iz_8_1009	DJI_0966	Psammophiliella muralis (L.) Ikonn.	0	n	1	0	Caryophyllaceae Juss.
Iz_8_1009	DJI_0966	Psephellus sumensis (Kalen.) Greuter	0	n	1	0	Asteraceae Bercht. & J.Presl
Iz_8_1009	DJI_0966	Pulsatilla pratensis (L.) Mill. s.l.	0	n	1	0	Ranunculaceae Juss.
Iz_8_1009	DJI_0966	Pyrus communis L.	0	a	0	1	Rosaceae Juss.
Iz_8_1009	DJI_0966	Quercus robur L.	0	n	1	1	Fagaceae Dumort.
Iz_8_1009	DJI_0966	Robinia pseudoacacia L.	0	a	0	1	Fabaceae Lindl.
Iz_8_1009	DJI_0966	Rosa sp.	0		NA	1	Rosaceae Juss.
Iz_8_1009	DJI_0966	Rubus idaeus L.	0	n	1	0	Rosaceae Juss.
Iz_8_1009	DJI_0966	Rumex acetosella L.	0	n	1	0	Polygonaceae Juss.
Iz_8_1009	DJI_0966	Rumex thyrsiflorus Fingerh.	0	n	1	0	Polygonaceae Juss.
Iz_8_1009	DJI_0966	Salix alba L.	0	n	1	1	Salicaceae Mirb.
Iz_8_1009	DJI_0966	Sambucus nigra L.	0	n	1	1	Viburnaceae Raf.
Iz_8_1009	DJI_0966	Secale sylvestre Host	0	n	1	0	Poaceae Barnhart
Iz_8_1009	DJI_0966	Sempervivum ruthenicum Schnittsp. & C.B. Lehm.	0	n	1	0	Crassulaceae J.St.-Hil.
Iz_8_1009	DJI_0966	Setaria viridis (L.) P. Beauv.	0	n	1	0	Poaceae Barnhart
Iz_8_1009	DJI_0966	Silene borysthenica (Gruner) Walters	0	n	1	0	Caryophyllaceae Juss.
Iz_8_1009	DJI_0966	Silene latifolia subsp. alba (Mill.) Greuter & Burdet	0	n	1	0	Caryophyllaceae Juss.
Iz_8_1009	DJI_0966	Solidago virgaurea L.	0	n	1	0	Asteraceae Bercht. & J.Presl
Iz_8_1009	DJI_0966	Sorbus aucuparia L.	0	n	1	1	Rosaceae Juss.
Iz_8_1009	DJI_0966	Stachys recta L.	0	n	1	0	Lamiacea Lindl.
Iz_8_1009	DJI_0966	Tanacetum vulgare L.	0	n	1	0	Asteraceae Bercht. & J.Presl
Iz_8_1009	DJI_0966	Thymus pallasianus Heinr.Braun	0	n	1	0	Lamiacea Lindl.
Iz_8_1009	DJI_0966	Tilia cordata Mill.	0	n	1	1	Malvaceae Juss.
Iz_8_1009	DJI_0966	Trifolium alpestre L.	0	n	1	0	Fabaceae Lindl.
Iz_8_1009	DJI_0966	Trifolium arvense L.	0	n	1	0	Fabaceae Lindl.
Iz_8_1009	DJI_0966	Ulmus glabra Huds.	0	n	1	1	Ulmaceae Mirb.
Iz_8_1009	DJI_0966	Verbascum densiflorum Bertol.	0	n	1	0	Scrophulariaceae Juss.
Iz_8_1009	DJI_0966	Veronica austriaca L.	0	n	1	0	Plantaginaceae Juss.
Iz_8_1009	DJI_0966	Veronica incana L.	0	n	1	0	Plantaginaceae Juss.
Iz_8_1009	DJI_0966	Veronica verna L.	0	n	1	0	Plantaginaceae Juss.
Iz_8_1009	DJI_0966	Vincetoxicum hirundinaria Medik	0	n	1	0	Apocynaceae Juss.
Iz_8_1009	DJI_0967	Acer tataricum L.	0	n	1	1	Sapindaceae Juss.
Iz_8_1009	DJI_0967	Achillea millefolium L. s.l.	0	n	1	0	Asteraceae Bercht. & J.Presl
Iz_8_1009	DJI_0967	Allium oleraceum L.	0	n	1	0	Amaryllidaceae J.St.-Hil.
Iz_8_1009	DJI_0967	Ambrosia artemisiifolia L.	0	a	0	0	Asteraceae Bercht. & J.Presl
Iz_8_1009	DJI_0967	Amorpha fruticosa L.	0	a	0	0	Fabaceae Lindl.
Iz_8_1009	DJI_0967	Anthericum ramosum L.	0	n	1	0	Asparagaceae Juss.
Iz_8_1009	DJI_0967	Artemisia marschalliana Spreng.	0	n	1	0	Asteraceae Bercht. & J.Presl
Iz_8_1009	DJI_0967	Asparagus officinalis L.	0	n	1	0	Asparagaceae Juss.
Iz_8_1009	DJI_0967	Astragalus cicer L.	0	n	1	0	Fabaceae Lindl.

Iz_8_1009	DJI_0967	Bassia laniflora (S.G.Gmel.) A.J.Scott	0	n	1	0	Amaranthaceae Juss.
Iz_8_1009	DJI_0967	Berberis vulgaris L.	0	n	1	0	Berberidaceae Juss.
Iz_8_1009	DJI_0967	Betula pendula Roth.	0	n	1	1	Betulaceae Gray
Iz_8_1009	DJI_0967	Betula pendula Roth.	0	n	1	0	Poaceae Barnhart
Iz_8_1009	DJI_0967	Bromus inermis Leyss.	0		NA	NA	
Iz_8_1009	DJI_0967	Bromus squarrosus L.	0	a	0	0	Poaceae Barnhart
Iz_8_1009	DJI_0967	Bromus tectorum L.	0	a	0	0	Poaceae Barnhart
Iz_8_1009	DJI_0967	Calamagrostis epigejos (L.) Roth	0.5	n	1	0	Poaceae Barnhart
Iz_8_1009	DJI_0967	Carduus acanthoides L.	0	n	1	0	Asteraceae Bercht. & J.Presl
Iz_8_1009	DJI_0967	Carex sp.	0	n	1	0	Cyperaceae Juss.
Iz_8_1009	DJI_0967	Centaurea majorovii Dumbadze	0	n	1	0	Asteraceae Bercht. & J.Presl
Iz_8_1009	DJI_0967	Chamaecytisus ruthenicus (Fisch.ex Wol.) Klaskova	0	n	1	0	Fabaceae Lindl.
Iz_8_1009	DJI_0967	Chelidonium majus L.	0	n	1	0	Papaveraceae Juss.
Iz_8_1009	DJI_0967	Chenopodium betaceum Andrz.	0	n	1	0	Amaranthaceae Juss.
Iz_8_1009	DJI_0967	Chenopodium cf. album	0	n	1	0	Amaranthaceae Juss.
Iz_8_1009	DJI_0967	Chenopodium sp.	0		NA	0	Amaranthaceae Juss.
Iz_8_1009	DJI_0967	Chondrilla juncea L.	0	n	1	0	Asteraceae Bercht. & J.Presl
Iz_8_1009	DJI_0967	Cirsium vulgare (Savi) Ten.	0	n	1	0	Asteraceae Bercht. & J.Presl
Iz_8_1009	DJI_0967	Clinopodium acinos (L.) Kuntze	0	n	1	0	Lamiacea Lindl.
Iz_8_1009	DJI_0967	Convallaria majalis L.	0	n	1	0	Asparagaceae Juss.
Iz_8_1009	DJI_0967	Crataegus rhipidophylla Gand.	0	n	1	1	Rosaceae Juss.
Iz_8_1009	DJI_0967	Crepis foetida L.	0	n	1	0	Asteraceae Bercht. & J.Presl
Iz_8_1009	DJI_0967	Dactylis glomerata L.	0	n	1	0	Poaceae Barnhart
Iz_8_1009	DJI_0967	Dianthus campestris M.Bieb.	0	n	1	0	Caryophyllaceae Juss.
Iz_8_1009	DJI_0967	Digitaria sanguinalis (L.) Scop.	0	a	0	0	Poaceae Barnhart
Iz_8_1009	DJI_0967	Elymus repens (L.) Gould	0	n	1	0	Poaceae Barnhart
Iz_8_1009	DJI_0967	Epilobium tetragonum L.	0	n	1	0	Onagraceae Juss.
Iz_8_1009	DJI_0967	Eragrostis minor Host	0	a	0	0	Poaceae Barnhart
Iz_8_1009	DJI_0967	Erigeron canadensis L.	7	a	0	0	Asteraceae Bercht. & J.Presl
Iz_8_1009	DJI_0967	Euphorbia seguieriana Neck.	0	n	1	0	Euphorbiaceae Juss.
Iz_8_1009	DJI_0967	Fallopia dumetorum (L.) Holub	0	n	1	0	Polygonaceae Juss.
Iz_8_1009	DJI_0967	Festuca beckeri (Hack.) Trautv.	0	n	1	0	Poaceae Barnhart
Iz_8_1009	DJI_0967	Fragaria vesca L.	0	n	1	0	Rosaceae Juss.
Iz_8_1009	DJI_0967	Frangula alnus Mill.	0	n	1	1	Rhamnaceae Juss.
Iz_8_1009	DJI_0967	Fraxinus pennsylvanica Marshall	0	a	0	1	Oleaceae Hoffmanns. & Link
Iz_8_1009	DJI_0967	Galeopsis bifida Boenn.	0	n	1	0	Lamiacea Lindl.
Iz_8_1009	DJI_0967	Galium verum L.	0	n	1	0	Rubiaceae Juss.
Iz_8_1009	DJI_0967	Genista tinctoria L.	0	n	1	0	Fabaceae Lindl.
Iz_8_1009	DJI_0967	Geranium robertianum L.	0	n	1	0	Geraniaceae Juss.
Iz_8_1009	DJI_0967	Geranium sanquineum L.	0	n	1	0	Geraniaceae Juss.
Iz_8_1009	DJI_0967	Gypsophila paniculata L.	0	n	1	0	Caryophyllaceae Juss.
Iz_8_1009	DJI_0967	Helichrysum arenarium (L.) Moench	0	n	1	0	Asteraceae Bercht. & J.Presl
Iz_8_1009	DJI_0967	Herniaria polygama J.Gay	0	n	1	0	Caryophyllaceae Juss.
Iz_8_1009	DJI_0967	Hieracium robustum Fr.	0	n	1	0	Asteraceae Bercht. & J.Presl
Iz_8_1009	DJI_0967	Hieracium umbellatum L.	0	n	1	NA	Asteraceae Bercht. & J.Presl
Iz_8_1009	DJI_0967	Hylotelephium maximum (L.) Holub	0	n	1	0	Crassulaceae J.St.-Hil.
Iz_8_1009	DJI_0967	Jacobaea borysthenica (DC.) B.Nord. & Greuter	0	n	1	0	Asteraceae Bercht. & J.Presl
Iz_8_1009	DJI_0967	Jasione montana L.	0	n	1	0	Campanulaceae Juss.
Iz_8_1009	DJI_0967	Jurinea cyanoides (L.) Rchb.s.l.	0	n	1	0	Asteraceae Bercht. & J.Presl
Iz_8_1009	DJI_0967	Koeleria glauca (Spreng.) DC.	0	n	1	0	Poaceae Barnhart
Iz_8_1009	DJI_0967	Lactuca serriola L.	0	a	0	0	Asteraceae Bercht. & J.Presl
Iz_8_1009	DJI_0967	Linaria genistifolia (L.) Mill.	0	n	1	0	Plantaginaceae Juss.
Iz_8_1009	DJI_0967	Lomelosia argentea (L.) Greuter & Burdet	0	n	1	0	Caprifoliaceae Juss.
Iz_8_1009	DJI_0967	Melampyrum pratense L.	0	n	1	0	Orobanchaceae Vent.
Iz_8_1009	DJI_0967	Oenothera biennis L.	0	a	0	0	Onagraceae Juss.
Iz_8_1009	DJI_0967	Pentanema britannica (L.) D.Gut.Larr., Santos-Vicente, Anderb., E.Rico & M.M.Mart.Ort.	0	n	1	0	Asteraceae Bercht. & J.Presl
Iz_8_1009	DJI_0967	Peucedanum oreoselinum (L.) Moench	0	n	1	0	Apiaceae Lindl.
Iz_8_1009	DJI_0967	Peucedanum ruthenicum M.Bieb.	0	n	1	0	Apiaceae Lindl.
Iz_8_1009	DJI_0967	Pilosella echioides (Lumn.) F.W.Schultz & Sch.Bip.	0	n	1	0	Asteraceae Bercht. & J.Presl
Iz_8_1009	DJI_0967	Pilosella officinarum F. Schultz et Sch. Bip.	0	n	1	0	Asteraceae Bercht. & J.Presl
Iz_8_1009	DJI_0967	Pilosella sp.	0		NA	0	Asteraceae Bercht. & J.Presl
Iz_8_1009	DJI_0967	Pinus sylvestris L.	0	n	1	1	Pinaceae Spreng. ex F.Rudolphi
Iz_8_1009	DJI_0967	Poa annua L.	0	n	1	0	Poaceae Barnhart
Iz_8_1009	DJI_0967	Poa nemoralis L.	0	n	1	0	Poaceae Barnhart
Iz_8_1009	DJI_0967	Poa sp.	0		NA	0	Poaceae Barnhart
Iz_8_1009	DJI_0967	Polygonatum odoratum (Mill.) Druce	0	n	1	0	Asparagaceae Juss.
Iz_8_1009	DJI_0967	Polygonum arenarium Waldst. & Kit.	0	n	1	0	Polygonaceae Juss.
Iz_8_1009	DJI_0967	Populus nigra L.	0	n	1	1	Salicaceae Mirb.
Iz_8_1009	DJI_0967	Populus tremula L.	0	n	1	1	Salicaceae Mirb.
Iz_8_1009	DJI_0967	Populus Ã canescens (Aiton) Sm.	0	n	1	1	Salicaceae Mirb.
Iz_8_1009	DJI_0967	Potentilla argentea L.	0	n	1	0	Rosaceae Juss.
Iz_8_1009	DJI_0967	Psammophiliella muralis (L.) Ikonn.	0	n	1	0	Caryophyllaceae Juss.
Iz_8_1009	DJI_0967	Psephellus sumensis (Kalen.) Greuter	0	n	1	0	Asteraceae Bercht. & J.Presl
Iz_8_1009	DJI_0967	Pulsatilla pratensis (L.) Mill. s.l.	0	n	1	0	Ranunculaceae Juss.
Iz_8_1009	DJI_0967	Pyrus communis L.	0	a	0	1	Rosaceae Juss.
Iz_8_1009	DJI_0967	Quercus robur L.	0	n	1	1	Fagaceae Dumort.
Iz_8_1009	DJI_0967	Robinia pseudoacacia L.	0	a	0	1	Fabaceae Lindl.
Iz_8_1009	DJI_0967	Rosa sp.	0		NA	1	Rosaceae Juss.
Iz_8_1009	DJI_0967	Rubus idaeus L.	0	n	1	0	Rosaceae Juss.
Iz_8_1009	DJI_0967	Rumex acetosella L.	0	n	1	0	Polygonaceae Juss.
Iz_8_1009	DJI_0967	Rumex thyrsiflorus Fingerh.	0	n	1	0	Polygonaceae Juss.
Iz_8_1009	DJI_0967	Salix alba L.	0	n	1	1	Salicaceae Mirb.
Iz_8_1009	DJI_0967	Sambucus nigra L.	0	n	1	1	Viburnaceae Raf.
Iz_8_1009	DJI_0967	Secale sylvestre Host	0	n	1	0	Poaceae Barnhart
Iz_8_1009	DJI_0967	Sempervivum ruthenicum Schnittsp. & C.B. Lehm.	0	n	1	0	Crassulaceae J.St.-Hil.
Iz_8_1009	DJI_0967	Setaria viridis (L.) P. Beauv.	0	n	1	0	Poaceae Barnhart
Iz_8_1009	DJI_0967	Silene borysthenica (Gruner) Walters	0	n	1	0	Caryophyllaceae Juss.
Iz_8_1009	DJI_0967	Silene latifolia subsp. alba (Mill.) Greuter & Burdet	0	n	1	0	Caryophyllaceae Juss.
Iz_8_1009	DJI_0967	Solidago virgaurea L.	0	n	1	0	Asteraceae Bercht. & J.Presl
Iz_8_1009	DJI_0967	Sorbus aucuparia L.	0	n	1	1	Rosaceae Juss.
Iz_8_1009	DJI_0967	Stachys recta L.	0	n	1	0	Lamiacea Lindl.
Iz_8_1009	DJI_0967	Tanacetum vulgare L.	0	n	1	0	Asteraceae Bercht. & J.Presl
Iz_8_1009	DJI_0967	Thymus pallasianus Heinr.Braun	0	n	1	0	Lamiacea Lindl.
Iz_8_1009	DJI_0967	Tilia cordata Mill.	0	n	1	1	Malvaceae Juss.
Iz_8_1009	DJI_0967	Trifolium alpestre L.	0	n	1	0	Fabaceae Lindl.
Iz_8_1009	DJI_0967	Trifolium arvense L.	0	n	1	0	Fabaceae Lindl.
Iz_8_1009	DJI_0967	Ulmus glabra Huds.	0	n	1	1	Ulmaceae Mirb.
Iz_8_1009	DJI_0967	Verbascum densiflorum Bertol.	0	n	1	0	Scrophulariaceae Juss.

Iz_8_1009	DJI_0967	Veronica austriaca L.	0	n	1	0	Plantaginaceae Juss.
Iz_8_1009	DJI_0967	Veronica incana L.	0	n	1	0	Plantaginaceae Juss.
Iz_8_1009	DJI_0967	Veronica verna L.	0	n	1	0	Plantaginaceae Juss.
Iz_8_1009	DJI_0967	Vincetoxicum hirundinaria Medik	0	n	1	0	Apocynaceae Juss.
Iz_8_1009	DJI_0971	Acer tataricum L.	0	n	1	1	Sapindaceae Juss.
Iz_8_1009	DJI_0971	Achillea millefolium L. s.l.	0	n	1	0	Asteraceae Bercht. & J.Presl
Iz_8_1009	DJI_0971	Allium oleraceum L.	0	n	1	0	Amaryllidaceae J.St.-Hil.
Iz_8_1009	DJI_0971	Ambrosia artemisiifolia L.	0	a	0	0	Asteraceae Bercht. & J.Presl
Iz_8_1009	DJI_0971	Amorpha fruticosa L.	0	a	0	0	Fabaceae Lindl.
Iz_8_1009	DJI_0971	Anthericum ramosum L.	0	n	1	0	Asparagaceae Juss.
Iz_8_1009	DJI_0971	Artemisia marschalliana Spreng.	0	n	1	0	Asteraceae Bercht. & J.Presl
Iz_8_1009	DJI_0971	Asparagus officinalis L.	0	n	1	0	Asparagaceae Juss.
Iz_8_1009	DJI_0971	Astragalus cicer L.	0	n	1	0	Fabaceae Lindl.
Iz_8_1009	DJI_0971	Bassia laniflora (S.G.Gmel.) A.J.Scott	0	n	1	0	Amaranthaceae Juss.
Iz_8_1009	DJI_0971	Berberis vulgaris L.	0	n	1	0	Berberidaceae Juss.
Iz_8_1009	DJI_0971	Betula pendula Roth.	0	n	1	1	Betulaceae Gray
Iz_8_1009	DJI_0971	Betula pendula Roth.	0	n	1	0	Poaceae Barnhart
Iz_8_1009	DJI_0971	Bromus inermis Leyss.	0		NA	NA	
Iz_8_1009	DJI_0971	Bromus squarrosus L.	0	a	0	0	Poaceae Barnhart
Iz_8_1009	DJI_0971	Bromus tectorum L.	0	a	0	0	Poaceae Barnhart
Iz_8_1009	DJI_0971	Calamagrostis epigejos (L.) Roth	0.2	n	1	0	Poaceae Barnhart
Iz_8_1009	DJI_0971	Carduus acanthoides L.	0	n	1	0	Asteraceae Bercht. & J.Presl
Iz_8_1009	DJI_0971	Carex sp.	0	n	1	0	Cyperaceae Juss.
Iz_8_1009	DJI_0971	Centaurea majorovii Dumbadze	0	n	1	0	Asteraceae Bercht. & J.Presl
Iz_8_1009	DJI_0971	Chamaecytisus ruthenicus (Fisch.ex Wol.) Klaskova	0	n	1	0	Fabaceae Lindl.
Iz_8_1009	DJI_0971	Chelidonium majus L.	0	n	1	0	Papaveraceae Juss.
Iz_8_1009	DJI_0971	Chenopodium betaceum Andrz.	0	n	1	0	Amaranthaceae Juss.
Iz_8_1009	DJI_0971	Chenopodium cf. album	0	n	1	0	Amaranthaceae Juss.
Iz_8_1009	DJI_0971	Chenopodium sp.	0		NA	0	Amaranthaceae Juss.
Iz_8_1009	DJI_0971	Chondrilla juncea L.	0	n	1	0	Asteraceae Bercht. & J.Presl
Iz_8_1009	DJI_0971	Cirsium vulgare (Savi) Ten.	0	n	1	0	Asteraceae Bercht. & J.Presl
Iz_8_1009	DJI_0971	Clinopodium acinos (L.) Kuntze	0	n	1	0	Lamiacea Lindl.
Iz_8_1009	DJI_0971	Convallaria majalis L.	0	n	1	0	Asparagaceae Juss.
Iz_8_1009	DJI_0971	Crataegus rhipidophylla Gand.	0	n	1	1	Rosaceae Juss.
Iz_8_1009	DJI_0971	Crepis foetida L.	0	n	1	0	Asteraceae Bercht. & J.Presl
Iz_8_1009	DJI_0971	Dactylis glomerata L.	0	n	1	0	Poaceae Barnhart
Iz_8_1009	DJI_0971	Dianthus campestris M.Bieb.	0	n	1	0	Caryophyllaceae Juss.
Iz_8_1009	DJI_0971	Digitaria sanguinalis (L.) Scop.	0	a	0	0	Poaceae Barnhart
Iz_8_1009	DJI_0971	Elymus repens (L.) Gould	0	n	1	0	Poaceae Barnhart
Iz_8_1009	DJI_0971	Epilobium tetragonum L.	0	n	1	0	Onagraceae Juss.
Iz_8_1009	DJI_0971	Eragrostis minor Host	0	a	0	0	Poaceae Barnhart
Iz_8_1009	DJI_0971	Erigeron canadensis L.	5	a	0	0	Asteraceae Bercht. & J.Presl
Iz_8_1009	DJI_0971	Euphorbia seguieriana Neck.	0	n	1	0	Euphorbiaceae Juss.
Iz_8_1009	DJI_0971	Fallopia dumetorum (L.) Holub	0	n	1	0	Polygonaceae Juss.
Iz_8_1009	DJI_0971	Festuca beckeri (Hack.) Trautv.	0	n	1	0	Poaceae Barnhart
Iz_8_1009	DJI_0971	Fragaria vesca L.	0	n	1	0	Rosaceae Juss.
Iz_8_1009	DJI_0971	Frangula alnus Mill.	0	n	1	1	Rhamnaceae Juss.
Iz_8_1009	DJI_0971	Fraxinus pennsylvanica Marshall	0	a	0	1	Oleaceae Hoffmanns. & Link
Iz_8_1009	DJI_0971	Galeopsis bifida Boenn.	0	n	1	0	Lamiacea Lindl.
Iz_8_1009	DJI_0971	Galium verum L.	0	n	1	0	Rubiaceae Juss.
Iz_8_1009	DJI_0971	Genista tinctoria L.	0	n	1	0	Fabaceae Lindl.
Iz_8_1009	DJI_0971	Geranium robertianum L.	0	n	1	0	Geraniaceae Juss.
Iz_8_1009	DJI_0971	Geranium sanquineum L.	0	n	1	0	Geraniaceae Juss.
Iz_8_1009	DJI_0971	Gypsophila paniculata L.	0	n	1	0	Caryophyllaceae Juss.
Iz_8_1009	DJI_0971	Helichrysum arenarium (L.) Moench	0	n	1	0	Asteraceae Bercht. & J.Presl
Iz_8_1009	DJI_0971	Herniaria polygama J.Gay	0	n	1	0	Caryophyllaceae Juss.
Iz_8_1009	DJI_0971	Hieracium robustum Fr.	0	n	1	0	Asteraceae Bercht. & J.Presl
Iz_8_1009	DJI_0971	Hieracium umbellatum L.	0	n	1	NA	Asteraceae Bercht. & J.Presl
Iz_8_1009	DJI_0971	Hylotelephium maximum (L.) Holub	0	n	1	0	Crassulaceae J.St.-Hil.
Iz_8_1009	DJI_0971	Jacobaea borysthenica (DC.) B.Nord. & Greuter	0	n	1	0	Asteraceae Bercht. & J.Presl
Iz_8_1009	DJI_0971	Jasione montana L.	0	n	1	0	Campanulaceae Juss.
Iz_8_1009	DJI_0971	Jurinea cyanoides (L.) Rchb.s.l.	0	n	1	0	Asteraceae Bercht. & J.Presl
Iz_8_1009	DJI_0971	Koeleria glauca (Spreng.) DC.	0	n	1	0	Poaceae Barnhart
Iz_8_1009	DJI_0971	Lactuca serriola L.	0	a	0	0	Asteraceae Bercht. & J.Presl
Iz_8_1009	DJI_0971	Linaria genistifolia (L.) Mill.	0	n	1	0	Plantaginaceae Juss.
Iz_8_1009	DJI_0971	Lomelosia argentea (L.) Greuter & Burdet	0	n	1	0	Caprifoliaceae Juss.
Iz_8_1009	DJI_0971	Melampyrum pratense L.	0	n	1	0	Orobanchaceae Vent.
Iz_8_1009	DJI_0971	Oenothera biennis L.	0	a	0	0	Onagraceae Juss.
Iz_8_1009	DJI_0971	Pentanema britannica (L.) D.Gut.Larr., Santos-Vicente, Anderb., E.Rico & M.M.Mart.Ort.	0	n	1	0	Asteraceae Bercht. & J.Presl
Iz_8_1009	DJI_0971	Peucedanum oreoselinum (L.) Moench	0	n	1	0	Apiaceae Lindl.
Iz_8_1009	DJI_0971	Peucedanum ruthenicum M.Bieb.	0	n	1	0	Apiaceae Lindl.
Iz_8_1009	DJI_0971	Pilosella echioides (Lumn.) F.W.Schultz & Sch.Bip.	0	n	1	0	Asteraceae Bercht. & J.Presl
Iz_8_1009	DJI_0971	Pilosella officinarum F. Schultz et Sch. Bip.	0	n	1	0	Asteraceae Bercht. & J.Presl
Iz_8_1009	DJI_0971	Pilosella sp.	0		NA	0	Asteraceae Bercht. & J.Presl
Iz_8_1009	DJI_0971	Pinus sylvestris L.	0	n	1	1	Pinaceae Spreng. ex F.Rudolphi
Iz_8_1009	DJI_0971	Poa annua L.	0	n	1	0	Poaceae Barnhart
Iz_8_1009	DJI_0971	Poa nemoralis L.	0	n	1	0	Poaceae Barnhart
Iz_8_1009	DJI_0971	Poa sp.	0		NA	0	Poaceae Barnhart
Iz_8_1009	DJI_0971	Polygonatum odoratum (Mill.) Druce	0	n	1	0	Asparagaceae Juss.
Iz_8_1009	DJI_0971	Polygonum arenarium Waldst. & Kit.	0	n	1	0	Polygonaceae Juss.
Iz_8_1009	DJI_0971	Populus nigra L.	0	n	1	1	Salicaceae Mirb.
Iz_8_1009	DJI_0971	Populus tremula L.	0	n	1	1	Salicaceae Mirb.
Iz_8_1009	DJI_0971	Populus Ã canescens (Aiton) Sm.	0	n	1	1	Salicaceae Mirb.
Iz_8_1009	DJI_0971	Potentilla argentea L.	0	n	1	0	Rosaceae Juss.
Iz_8_1009	DJI_0971	Psammophiliella muralis (L.) Ikonn.	0	n	1	0	Caryophyllaceae Juss.
Iz_8_1009	DJI_0971	Psephellus sumensis (Kalen.) Greuter	0	n	1	0	Asteraceae Bercht. & J.Presl
Iz_8_1009	DJI_0971	Pulsatilla pratensis (L.) Mill. s.l.	0	n	1	0	Ranunculaceae Juss.
Iz_8_1009	DJI_0971	Pyrus communis L.	0	a	0	1	Rosaceae Juss.
Iz_8_1009	DJI_0971	Quercus robur L.	0	n	1	1	Fagaceae Dumort.
Iz_8_1009	DJI_0971	Robinia pseudoacacia L.	0	a	0	1	Fabaceae Lindl.
Iz_8_1009	DJI_0971	Rosa sp.	0		NA	1	Rosaceae Juss.
Iz_8_1009	DJI_0971	Rubus idaeus L.	0	n	1	0	Rosaceae Juss.
Iz_8_1009	DJI_0971	Rumex acetosella L.	0	n	1	0	Polygonaceae Juss.
Iz_8_1009	DJI_0971	Rumex thyrsiflorus Fingerh.	0	n	1	0	Polygonaceae Juss.
Iz_8_1009	DJI_0971	Salix alba L.	0	n	1	1	Salicaceae Mirb.
Iz_8_1009	DJI_0971	Sambucus nigra L.	0	n	1	1	Viburnaceae Raf.
Iz_8_1009	DJI_0971	Secale sylvestre Host	0	n	1	0	Poaceae Barnhart
Iz_8_1009	DJI_0971	Sempervivum ruthenicum Schnittsp. & C.B. Lehm.	0	n	1	0	Crassulaceae J.St.-Hil.

Iz_8_1009	DJI_0971	Setaria viridis (L.) P. Beauv.	0	n	1	0	Poaceae Barnhart
Iz_8_1009	DJI_0971	Silene borysthenica (Gruner) Walters	0	n	1	0	Caryophyllaceae Juss.
Iz_8_1009	DJI_0971	Silene latifolia subsp. alba (Mill.) Greuter & Burdet	0	n	1	0	Caryophyllaceae Juss.
Iz_8_1009	DJI_0971	Solidago virgaurea L.	0	n	1	0	Asteraceae Bercht. & J.Presl
Iz_8_1009	DJI_0971	Sorbus aucuparia L.	0	n	1	1	Rosaceae Juss.
Iz_8_1009	DJI_0971	Stachys recta L.	0	n	1	0	Lamiacea Lindl.
Iz_8_1009	DJI_0971	Tanacetum vulgare L.	0	n	1	0	Asteraceae Bercht. & J.Presl
Iz_8_1009	DJI_0971	Thymus pallasianus Heinr.Braun	0	n	1	0	Lamiacea Lindl.
Iz_8_1009	DJI_0971	Tilia cordata Mill.	0	n	1	1	Malvaceae Juss.
Iz_8_1009	DJI_0971	Trifolium alpestre L.	0	n	1	0	Fabaceae Lindl.
Iz_8_1009	DJI_0971	Trifolium arvense L.	0	n	1	0	Fabaceae Lindl.
Iz_8_1009	DJI_0971	Ulmus glabra Huds.	0	n	1	1	Ulmaceae Mirb.
Iz_8_1009	DJI_0971	Verbascum densiflorum Bertol.	0	n	1	0	Scrophulariaceae Juss.
Iz_8_1009	DJI_0971	Veronica austriaca L.	0	n	1	0	Plantaginaceae Juss.
Iz_8_1009	DJI_0971	Veronica incana L.	0	n	1	0	Plantaginaceae Juss.
Iz_8_1009	DJI_0971	Veronica verna L.	0	n	1	0	Plantaginaceae Juss.
Iz_8_1009	DJI_0971	Vincetoxicum hirundinaria Medik	0	n	1	0	Apocynaceae Juss.
Iz_8_1009	DJI_0972	Acer tataricum L.	0	n	1	1	Sapindaceae Juss.
Iz_8_1009	DJI_0972	Achillea millefolium L. s.l.	0	n	1	0	Asteraceae Bercht. & J.Presl
Iz_8_1009	DJI_0972	Allium oleraceum L.	0	n	1	0	Amaryllidaceae J.St.-Hil.
Iz_8_1009	DJI_0972	Ambrosia artemisiifolia L.	0	a	0	0	Asteraceae Bercht. & J.Presl
Iz_8_1009	DJI_0972	Amorpha fruticosa L.	0	a	0	0	Fabaceae Lindl.
Iz_8_1009	DJI_0972	Anthericum ramosum L.	0	n	1	0	Asparagaceae Juss.
Iz_8_1009	DJI_0972	Artemisia marschalliana Spreng.	0	n	1	0	Asteraceae Bercht. & J.Presl
Iz_8_1009	DJI_0972	Asparagus officinalis L.	0	n	1	0	Asparagaceae Juss.
Iz_8_1009	DJI_0972	Astragalus cicer L.	0	n	1	0	Fabaceae Lindl.
Iz_8_1009	DJI_0972	Bassia laniflora (S.G.Gmel.) A.J.Scott	0	n	1	0	Amaranthaceae Juss.
Iz_8_1009	DJI_0972	Berberis vulgaris L.	0	n	1	0	Berberidaceae Juss.
Iz_8_1009	DJI_0972	Betula pendula Roth.	0	n	1	1	Betulaceae Gray
Iz_8_1009	DJI_0972	Betula pendula Roth.	0	n	1	0	Poaceae Barnhart
Iz_8_1009	DJI_0972	Bromus inermis Leyss.	0		NA	NA	
Iz_8_1009	DJI_0972	Bromus squarrosus L.	0	a	0	0	Poaceae Barnhart
Iz_8_1009	DJI_0972	Bromus tectorum L.	0	a	0	0	Poaceae Barnhart
Iz_8_1009	DJI_0972	Calamagrostis epigejos (L.) Roth	0.2	n	1	0	Poaceae Barnhart
Iz_8_1009	DJI_0972	Carduus acanthoides L.	0	n	1	0	Asteraceae Bercht. & J.Presl
Iz_8_1009	DJI_0972	Carex sp.	0	n	1	0	Cyperaceae Juss.
Iz_8_1009	DJI_0972	Centaurea majorovii Dumbadze	0	n	1	0	Asteraceae Bercht. & J.Presl
Iz_8_1009	DJI_0972	Chamaecytisus ruthenicus (Fisch.ex Wol.) Klaskova	0	n	1	0	Fabaceae Lindl.
Iz_8_1009	DJI_0972	Chelidonium majus L.	0	n	1	0	Papaveraceae Juss.
Iz_8_1009	DJI_0972	Chenopodium betaceum Andrz.	0	n	1	0	Amaranthaceae Juss.
Iz_8_1009	DJI_0972	Chenopodium cf. album	0	n	1	0	Amaranthaceae Juss.
Iz_8_1009	DJI_0972	Chenopodium sp.	0		NA	0	Amaranthaceae Juss.
Iz_8_1009	DJI_0972	Chondrilla juncea L.	0	n	1	0	Asteraceae Bercht. & J.Presl
Iz_8_1009	DJI_0972	Cirsium vulgare (Savi) Ten.	0	n	1	0	Asteraceae Bercht. & J.Presl
Iz_8_1009	DJI_0972	Clinopodium acinos (L.) Kuntze	0	n	1	0	Lamiacea Lindl.
Iz_8_1009	DJI_0972	Convallaria majalis L.	0	n	1	0	Asparagaceae Juss.
Iz_8_1009	DJI_0972	Crataegus rhipidophylla Gand.	0	n	1	1	Rosaceae Juss.
Iz_8_1009	DJI_0972	Crepis foetida L.	0	n	1	0	Asteraceae Bercht. & J.Presl
Iz_8_1009	DJI_0972	Dactylis glomerata L.	0	n	1	0	Poaceae Barnhart
Iz_8_1009	DJI_0972	Dianthus campestris M.Bieb.	0	n	1	0	Caryophyllaceae Juss.
Iz_8_1009	DJI_0972	Digitaria sanguinalis (L.) Scop.	0	a	0	0	Poaceae Barnhart
Iz_8_1009	DJI_0972	Elymus repens (L.) Gould	0	n	1	0	Poaceae Barnhart
Iz_8_1009	DJI_0972	Epilobium tetragonum L.	0	n	1	0	Onagraceae Juss.
Iz_8_1009	DJI_0972	Eragrostis minor Host	0	a	0	0	Poaceae Barnhart
Iz_8_1009	DJI_0972	Erigeron canadensis L.	10	a	0	0	Asteraceae Bercht. & J.Presl
Iz_8_1009	DJI_0972	Euphorbia seguieriana Neck.	0	n	1	0	Euphorbiaceae Juss.
Iz_8_1009	DJI_0972	Fallopia dumetorum (L.) Holub	0	n	1	0	Polygonaceae Juss.
Iz_8_1009	DJI_0972	Festuca beckeri (Hack.) Trautv.	0	n	1	0	Poaceae Barnhart
Iz_8_1009	DJI_0972	Fragaria vesca L.	0	n	1	0	Rosaceae Juss.
Iz_8_1009	DJI_0972	Frangula alnus Mill.	0	n	1	1	Rhamnaceae Juss.
Iz_8_1009	DJI_0972	Fraxinus pennsylvanica Marshall	0	a	0	1	Oleaceae Hoffmanns. & Link
Iz_8_1009	DJI_0972	Galeopsis bifida Boenn.	0	n	1	0	Lamiacea Lindl.
Iz_8_1009	DJI_0972	Galium verum L.	0	n	1	0	Rubiaceae Juss.
Iz_8_1009	DJI_0972	Genista tinctoria L.	0	n	1	0	Fabaceae Lindl.
Iz_8_1009	DJI_0972	Geranium robertianum L.	0	n	1	0	Geraniaceae Juss.
Iz_8_1009	DJI_0972	Geranium sanquineum L.	0	n	1	0	Geraniaceae Juss.
Iz_8_1009	DJI_0972	Gypsophila paniculata L.	0	n	1	0	Caryophyllaceae Juss.
Iz_8_1009	DJI_0972	Helichrysum arenarium (L.) Moench	0	n	1	0	Asteraceae Bercht. & J.Presl
Iz_8_1009	DJI_0972	Herniaria polygama J.Gay	0	n	1	0	Caryophyllaceae Juss.
Iz_8_1009	DJI_0972	Hieracium robustum Fr.	0	n	1	0	Asteraceae Bercht. & J.Presl
Iz_8_1009	DJI_0972	Hieracium umbellatum L.	0	n	1	NA	Asteraceae Bercht. & J.Presl
Iz_8_1009	DJI_0972	Hylotelephium maximum (L.) Holub	0	n	1	0	Crassulaceae J.St.-Hil.
Iz_8_1009	DJI_0972	Jacobaea borysthenica (DC.) B.Nord. & Greuter	0	n	1	0	Asteraceae Bercht. & J.Presl
Iz_8_1009	DJI_0972	Jasione montana L.	0	n	1	0	Campanulaceae Juss.
Iz_8_1009	DJI_0972	Jurinea cyanoides (L.) Rchb.s.l.	0	n	1	0	Asteraceae Bercht. & J.Presl
Iz_8_1009	DJI_0972	Koeleria glauca (Spreng.) DC.	0	n	1	0	Poaceae Barnhart
Iz_8_1009	DJI_0972	Lactuca serriola L.	0	a	0	0	Asteraceae Bercht. & J.Presl
Iz_8_1009	DJI_0972	Linaria genistifolia (L.) Mill.	0	n	1	0	Plantaginaceae Juss.
Iz_8_1009	DJI_0972	Lomelosia argentea (L.) Greuter & Burdet	0	n	1	0	Caprifoliaceae Juss.
Iz_8_1009	DJI_0972	Melampyrum pratense L.	0	n	1	0	Orobanchaceae Vent.
Iz_8_1009	DJI_0972	Oenothera biennis L.	0	a	0	0	Onagraceae Juss.
Iz_8_1009	DJI_0972	Pentanema britannica (L.) D.Gut.Larr., Santos-Vicente, Anderb., E.Rico & M.M.Mart.Ort.	0	n	1	0	Asteraceae Bercht. & J.Presl
Iz_8_1009	DJI_0972	Peucedanum oreoselinum (L.) Moench	0	n	1	0	Apiaceae Lindl.
Iz_8_1009	DJI_0972	Peucedanum ruthenicum M.Bieb.	0	n	1	0	Apiaceae Lindl.
Iz_8_1009	DJI_0972	Pilosella echioides (Lumn.) F.W.Schultz & Sch.Bip.	0	n	1	0	Asteraceae Bercht. & J.Presl
Iz_8_1009	DJI_0972	Pilosella officinarum F. Schultz et Sch. Bip.	0	n	1	0	Asteraceae Bercht. & J.Presl
Iz_8_1009	DJI_0972	Pilosella sp.	0		NA	0	Asteraceae Bercht. & J.Presl
Iz_8_1009	DJI_0972	Pinus sylvestris L.	0	n	1	1	Pinaceae Spreng. ex F.Rudolphi
Iz_8_1009	DJI_0972	Poa annua L.	0	n	1	0	Poaceae Barnhart
Iz_8_1009	DJI_0972	Poa nemoralis L.	0	n	1	0	Poaceae Barnhart
Iz_8_1009	DJI_0972	Poa sp.	0		NA	0	Poaceae Barnhart
Iz_8_1009	DJI_0972	Polygonatum odoratum (Mill.) Druce	0	n	1	0	Asparagaceae Juss.
Iz_8_1009	DJI_0972	Polygonum arenarium Waldst. & Kit.	0	n	1	0	Polygonaceae Juss.
Iz_8_1009	DJI_0972	Populus nigra L.	0	n	1	1	Salicaceae Mirb.
Iz_8_1009	DJI_0972	Populus tremula L.	0	n	1	1	Salicaceae Mirb.
Iz_8_1009	DJI_0972	Populus Ã canescens (Aiton) Sm.	0	n	1	1	Salicaceae Mirb.
Iz_8_1009	DJI_0972	Potentilla argentea L.	0	n	1	0	Rosaceae Juss.
Iz_8_1009	DJI_0972	Psammophiliella muralis (L.) Ikonn.	0	n	1	0	Caryophyllaceae Juss.

Iz_8_1009	DJI_0972	Psephellus sumensis (Kalen.) Greuter	0	n	1	0	Asteraceae Bercht. & J.Presl
Iz_8_1009	DJI_0972	Pulsatilla pratensis (L.) Mill. s.l.	0	n	1	0	Ranunculaceae Juss.
Iz_8_1009	DJI_0972	Pyrus communis L.	0	a	0	1	Rosaceae Juss.
Iz_8_1009	DJI_0972	Quercus robur L.	0	n	1	1	Fagaceae Dumort.
Iz_8_1009	DJI_0972	Robinia pseudoacacia L.	0	a	0	1	Fabaceae Lindl.
Iz_8_1009	DJI_0972	Rosa sp.	0		NA	1	Rosaceae Juss.
Iz_8_1009	DJI_0972	Rubus idaeus L.	0	n	1	0	Rosaceae Juss.
Iz_8_1009	DJI_0972	Rumex acetosella L.	0	n	1	0	Polygonaceae Juss.
Iz_8_1009	DJI_0972	Rumex thyrsiflorus Fingerh.	0	n	1	0	Polygonaceae Juss.
Iz_8_1009	DJI_0972	Salix alba L.	0	n	1	1	Salicaceae Mirb.
Iz_8_1009	DJI_0972	Sambucus nigra L.	0	n	1	1	Viburnaceae Raf.
Iz_8_1009	DJI_0972	Secale sylvestre Host	0	n	1	0	Poaceae Barnhart
Iz_8_1009	DJI_0972	Sempervivum ruthenicum Schnittsp. & C.B. Lehm.	0	n	1	0	Crassulaceae J.St.-Hil.
Iz_8_1009	DJI_0972	Setaria viridis (L.) P. Beauv.	0	n	1	0	Poaceae Barnhart
Iz_8_1009	DJI_0972	Silene borysthenica (Gruner) Walters	0	n	1	0	Caryophyllaceae Juss.
Iz_8_1009	DJI_0972	Silene latifolia subsp. alba (Mill.) Greuter & Burdet	0	n	1	0	Caryophyllaceae Juss.
Iz_8_1009	DJI_0972	Solidago virgaurea L.	0	n	1	0	Asteraceae Bercht. & J.Presl
Iz_8_1009	DJI_0972	Sorbus aucuparia L.	0	n	1	1	Rosaceae Juss.
Iz_8_1009	DJI_0972	Stachys recta L.	0	n	1	0	Lamiacea Lindl.
Iz_8_1009	DJI_0972	Tanacetum vulgare L.	0	n	1	0	Asteraceae Bercht. & J.Presl
Iz_8_1009	DJI_0972	Thymus pallasianus Heinr.Braun	0	n	1	0	Lamiacea Lindl.
Iz_8_1009	DJI_0972	Tilia cordata Mill.	0	n	1	1	Malvaceae Juss.
Iz_8_1009	DJI_0972	Trifolium alpestre L.	0	n	1	0	Fabaceae Lindl.
Iz_8_1009	DJI_0972	Trifolium arvense L.	0	n	1	0	Fabaceae Lindl.
Iz_8_1009	DJI_0972	Ulmus glabra Huds.	0	n	1	1	Ulmaceae Mirb.
Iz_8_1009	DJI_0972	Verbascum densiflorum Bertol.	0	n	1	0	Scrophulariaceae Juss.
Iz_8_1009	DJI_0972	Veronica austriaca L.	0	n	1	0	Plantaginaceae Juss.
Iz_8_1009	DJI_0972	Veronica incana L.	0	n	1	0	Plantaginaceae Juss.
Iz_8_1009	DJI_0972	Veronica verna L.	0	n	1	0	Plantaginaceae Juss.
Iz_8_1009	DJI_0972	Vincetoxicum hirundinaria Medik	0	n	1	0	Apocynaceae Juss.
Iz_8_1009	DJI_0973	Acer tataricum L.	0	n	1	1	Sapindaceae Juss.
Iz_8_1009	DJI_0973	Achillea millefolium L. s.l.	0	n	1	0	Asteraceae Bercht. & J.Presl
Iz_8_1009	DJI_0973	Allium oleraceum L.	0	n	1	0	Amaryllidaceae J.St.-Hil.
Iz_8_1009	DJI_0973	Ambrosia artemisiifolia L.	0	a	0	0	Asteraceae Bercht. & J.Presl
Iz_8_1009	DJI_0973	Amorpha fruticosa L.	0	a	0	0	Fabaceae Lindl.
Iz_8_1009	DJI_0973	Anthericum ramosum L.	0	n	1	0	Asparagaceae Juss.
Iz_8_1009	DJI_0973	Artemisia marschalliana Spreng.	0	n	1	0	Asteraceae Bercht. & J.Presl
Iz_8_1009	DJI_0973	Asparagus officinalis L.	0	n	1	0	Asparagaceae Juss.
Iz_8_1009	DJI_0973	Astragalus cicer L.	0	n	1	0	Fabaceae Lindl.
Iz_8_1009	DJI_0973	Bassia laniflora (S.G.Gmel.) A.J.Scott	0	n	1	0	Amaranthaceae Juss.
Iz_8_1009	DJI_0973	Berberis vulgaris L.	0	n	1	0	Berberidaceae Juss.
Iz_8_1009	DJI_0973	Betula pendula Roth.	0	n	1	1	Betulaceae Gray
Iz_8_1009	DJI_0973	Betula pendula Roth.	0	n	1	0	Poaceae Barnhart
Iz_8_1009	DJI_0973	Bromus inermis Leyss.	0		NA	NA	
Iz_8_1009	DJI_0973	Bromus squarrosus L.	0	a	0	0	Poaceae Barnhart
Iz_8_1009	DJI_0973	Bromus tectorum L.	0	a	0	0	Poaceae Barnhart
Iz_8_1009	DJI_0973	Calamagrostis epigejos (L.) Roth	0.5	n	1	0	Poaceae Barnhart
Iz_8_1009	DJI_0973	Carduus acanthoides L.	0	n	1	0	Asteraceae Bercht. & J.Presl
Iz_8_1009	DJI_0973	Carex sp.	0	n	1	0	Cyperaceae Juss.
Iz_8_1009	DJI_0973	Centaurea majorovii Dumbadze	0	n	1	0	Asteraceae Bercht. & J.Presl
Iz_8_1009	DJI_0973	Chamaecytisus ruthenicus (Fisch.ex Wol.) Klaskova	0	n	1	0	Fabaceae Lindl.
Iz_8_1009	DJI_0973	Chelidonium majus L.	0	n	1	0	Papaveraceae Juss.
Iz_8_1009	DJI_0973	Chenopodium betaceum Andrz.	0	n	1	0	Amaranthaceae Juss.
Iz_8_1009	DJI_0973	Chenopodium cf. album	0	n	1	0	Amaranthaceae Juss.
Iz_8_1009	DJI_0973	Chenopodium sp.	0		NA	0	Amaranthaceae Juss.
Iz_8_1009	DJI_0973	Chondrilla juncea L.	0	n	1	0	Asteraceae Bercht. & J.Presl
Iz_8_1009	DJI_0973	Cirsium vulgare (Savi) Ten.	0	n	1	0	Asteraceae Bercht. & J.Presl
Iz_8_1009	DJI_0973	Clinopodium acinos (L.) Kuntze	0	n	1	0	Lamiacea Lindl.
Iz_8_1009	DJI_0973	Convallaria majalis L.	0	n	1	0	Asparagaceae Juss.
Iz_8_1009	DJI_0973	Crataegus rhipidophylla Gand.	0	n	1	1	Rosaceae Juss.
Iz_8_1009	DJI_0973	Crepis foetida L.	0	n	1	0	Asteraceae Bercht. & J.Presl
Iz_8_1009	DJI_0973	Dactylis glomerata L.	0	n	1	0	Poaceae Barnhart
Iz_8_1009	DJI_0973	Dianthus campestris M.Bieb.	0	n	1	0	Caryophyllaceae Juss.
Iz_8_1009	DJI_0973	Digitaria sanguinalis (L.) Scop.	0	a	0	0	Poaceae Barnhart
Iz_8_1009	DJI_0973	Elymus repens (L.) Gould	0	n	1	0	Poaceae Barnhart
Iz_8_1009	DJI_0973	Epilobium tetragonum L.	0	n	1	0	Onagraceae Juss.
Iz_8_1009	DJI_0973	Eragrostis minor Host	0	a	0	0	Poaceae Barnhart
Iz_8_1009	DJI_0973	Erigeron canadensis L.	10	a	0	0	Asteraceae Bercht. & J.Presl
Iz_8_1009	DJI_0973	Euphorbia seguieriana Neck.	0	n	1	0	Euphorbiaceae Juss.
Iz_8_1009	DJI_0973	Fallopia dumetorum (L.) Holub	0	n	1	0	Polygonaceae Juss.
Iz_8_1009	DJI_0973	Festuca beckeri (Hack.) Trautv.	0	n	1	0	Poaceae Barnhart
Iz_8_1009	DJI_0973	Fragaria vesca L.	0	n	1	0	Rosaceae Juss.
Iz_8_1009	DJI_0973	Frangula alnus Mill.	0	n	1	1	Rhamnaceae Juss.
Iz_8_1009	DJI_0973	Fraxinus pennsylvanica Marshall	0	a	0	1	Oleaceae Hoffmanns. & Link
Iz_8_1009	DJI_0973	Galeopsis bifida Boenn.	0	n	1	0	Lamiacea Lindl.
Iz_8_1009	DJI_0973	Galium verum L.	0	n	1	0	Rubiaceae Juss.
Iz_8_1009	DJI_0973	Genista tinctoria L.	0	n	1	0	Fabaceae Lindl.
Iz_8_1009	DJI_0973	Geranium robertianum L.	0	n	1	0	Geraniaceae Juss.
Iz_8_1009	DJI_0973	Geranium sanquineum L.	0	n	1	0	Geraniaceae Juss.
Iz_8_1009	DJI_0973	Gypsophila paniculata L.	0	n	1	0	Caryophyllaceae Juss.
Iz_8_1009	DJI_0973	Helichrysum arenarium (L.) Moench	0	n	1	0	Asteraceae Bercht. & J.Presl
Iz_8_1009	DJI_0973	Herniaria polygama J.Gay	0	n	1	0	Caryophyllaceae Juss.
Iz_8_1009	DJI_0973	Hieracium robustum Fr.	0	n	1	0	Asteraceae Bercht. & J.Presl
Iz_8_1009	DJI_0973	Hieracium umbellatum L.	0	n	1	NA	Asteraceae Bercht. & J.Presl
Iz_8_1009	DJI_0973	Hylotelephium maximum (L.) Holub	0	n	1	0	Crassulaceae J.St.-Hil.
Iz_8_1009	DJI_0973	Jacobaea borysthenica (DC.) B.Nord. & Greuter	0	n	1	0	Asteraceae Bercht. & J.Presl
Iz_8_1009	DJI_0973	Jasione montana L.	0	n	1	0	Campanulaceae Juss.
Iz_8_1009	DJI_0973	Jurinea cyanoides (L.) Rchb.s.l.	0	n	1	0	Asteraceae Bercht. & J.Presl
Iz_8_1009	DJI_0973	Koeleria glauca (Spreng.) DC.	0	n	1	0	Poaceae Barnhart
Iz_8_1009	DJI_0973	Lactuca serriola L.	0	a	0	0	Asteraceae Bercht. & J.Presl
Iz_8_1009	DJI_0973	Linaria genistifolia (L.) Mill.	0	n	1	0	Plantaginaceae Juss.
Iz_8_1009	DJI_0973	Lomelosia argentea (L.) Greuter & Burdet	0	n	1	0	Caprifoliaceae Juss.
Iz_8_1009	DJI_0973	Melampyrum pratense L.	0	n	1	0	Orobanchaceae Vent.
Iz_8_1009	DJI_0973	Oenothera biennis L.	0	a	0	0	Onagraceae Juss.
Iz_8_1009	DJI_0973	Pentanema britannica (L.) D.Gut.Larr., Santos-Vicente, Anderb., E.Rico & M.M.Mart.Ort.	0	n	1	0	Asteraceae Bercht. & J.Presl
Iz_8_1009	DJI_0973	Peucedanum oreoselinum (L.) Moench	0	n	1	0	Apiaceae Lindl.
Iz_8_1009	DJI_0973	Peucedanum ruthenicum M.Bieb.	0	n	1	0	Apiaceae Lindl.
Iz_8_1009	DJI_0973	Pilosella echioides (Lumn.) F.W.Schultz & Sch.Bip.	0	n	1	0	Asteraceae Bercht. & J.Presl

Iz_8_1009	DJI_0973	Pilosella officinarum F. Schultz et Sch. Bip.	0	n	1	0	Asteraceae Bercht. & J.Presl
Iz_8_1009	DJI_0973	Pilosella sp.	0		NA	0	Asteraceae Bercht. & J.Presl
Iz_8_1009	DJI_0973	Pinus sylvestris L.	0	n	1	1	Pinaceae Spreng. ex F.Rudolphi
Iz_8_1009	DJI_0973	Poa annua L.	0	n	1	0	Poaceae Barnhart
Iz_8_1009	DJI_0973	Poa nemoralis L.	0	n	1	0	Poaceae Barnhart
Iz_8_1009	DJI_0973	Poa sp.	0		NA	0	Poaceae Barnhart
Iz_8_1009	DJI_0973	Polygonatum odoratum (Mill.) Druce	0	n	1	0	Asparagaceae Juss.
Iz_8_1009	DJI_0973	Polygonum arenarium Waldst. & Kit.	0	n	1	0	Polygonaceae Juss.
Iz_8_1009	DJI_0973	Populus nigra L.	0	n	1	1	Salicaceae Mirb.
Iz_8_1009	DJI_0973	Populus tremula L.	0	n	1	1	Salicaceae Mirb.
Iz_8_1009	DJI_0973	Populus Ã canescens (Aiton) Sm.	0	n	1	1	Salicaceae Mirb.
Iz_8_1009	DJI_0973	Potentilla argentea L.	0	n	1	0	Rosaceae Juss.
Iz_8_1009	DJI_0973	Psammophiliella muralis (L.) Ikonn.	0	n	1	0	Caryophyllaceae Juss.
Iz_8_1009	DJI_0973	Psephellus sumensis (Kalen.) Greuter	0	n	1	0	Asteraceae Bercht. & J.Presl
Iz_8_1009	DJI_0973	Pulsatilla pratensis (L.) Mill. s.l.	0	n	1	0	Ranunculaceae Juss.
Iz_8_1009	DJI_0973	Pyrus communis L.	0	a	0	1	Rosaceae Juss.
Iz_8_1009	DJI_0973	Quercus robur L.	0	n	1	1	Fagaceae Dumort.
Iz_8_1009	DJI_0973	Robinia pseudoacacia L.	0	a	0	1	Fabaceae Lindl.
Iz_8_1009	DJI_0973	Rosa sp.	0		NA	1	Rosaceae Juss.
Iz_8_1009	DJI_0973	Rubus idaeus L.	0	n	1	0	Rosaceae Juss.
Iz_8_1009	DJI_0973	Rumex acetosella L.	0	n	1	0	Polygonaceae Juss.
Iz_8_1009	DJI_0973	Rumex thyrsiflorus Fingerh.	0	n	1	0	Polygonaceae Juss.
Iz_8_1009	DJI_0973	Salix alba L.	0	n	1	1	Salicaceae Mirb.
Iz_8_1009	DJI_0973	Sambucus nigra L.	0	n	1	1	Viburnaceae Raf.
Iz_8_1009	DJI_0973	Secale sylvestre Host	0	n	1	0	Poaceae Barnhart
Iz_8_1009	DJI_0973	Sempervivum ruthenicum Schnittsp. & C.B. Lehm.	0	n	1	0	Crassulaceae J.St.-Hil.
Iz_8_1009	DJI_0973	Setaria viridis (L.) P. Beauv.	0	n	1	0	Poaceae Barnhart
Iz_8_1009	DJI_0973	Silene borysthenica (Gruner) Walters	0	n	1	0	Caryophyllaceae Juss.
Iz_8_1009	DJI_0973	Silene latifolia subsp. alba (Mill.) Greuter & Burdet	0	n	1	0	Caryophyllaceae Juss.
Iz_8_1009	DJI_0973	Solidago virgaurea L.	0	n	1	0	Asteraceae Bercht. & J.Presl
Iz_8_1009	DJI_0973	Sorbus aucuparia L.	0	n	1	1	Rosaceae Juss.
Iz_8_1009	DJI_0973	Stachys recta L.	0	n	1	0	Lamiacea Lindl.
Iz_8_1009	DJI_0973	Tanacetum vulgare L.	0	n	1	0	Asteraceae Bercht. & J.Presl
Iz_8_1009	DJI_0973	Thymus pallasianus Heinr.Braun	0	n	1	0	Lamiacea Lindl.
Iz_8_1009	DJI_0973	Tilia cordata Mill.	0	n	1	1	Malvaceae Juss.
Iz_8_1009	DJI_0973	Trifolium alpestre L.	0	n	1	0	Fabaceae Lindl.
Iz_8_1009	DJI_0973	Trifolium arvense L.	0	n	1	0	Fabaceae Lindl.
Iz_8_1009	DJI_0973	Ulmus glabra Huds.	0	n	1	1	Ulmaceae Mirb.
Iz_8_1009	DJI_0973	Verbascum densiflorum Bertol.	0	n	1	0	Scrophulariaceae Juss.
Iz_8_1009	DJI_0973	Veronica austriaca L.	0	n	1	0	Plantaginaceae Juss.
Iz_8_1009	DJI_0973	Veronica incana L.	0	n	1	0	Plantaginaceae Juss.
Iz_8_1009	DJI_0973	Veronica verna L.	0	n	1	0	Plantaginaceae Juss.
Iz_8_1009	DJI_0973	Vincetoxicum hirundinaria Medik	0	n	1	0	Apocynaceae Juss.
Iz_8_1009	DJI_0974	Acer tataricum L.	0	n	1	1	Sapindaceae Juss.
Iz_8_1009	DJI_0974	Achillea millefolium L. s.l.	0	n	1	0	Asteraceae Bercht. & J.Presl
Iz_8_1009	DJI_0974	Allium oleraceum L.	0	n	1	0	Amaryllidaceae J.St.-Hil.
Iz_8_1009	DJI_0974	Ambrosia artemisiifolia L.	0	a	0	0	Asteraceae Bercht. & J.Presl
Iz_8_1009	DJI_0974	Amorpha fruticosa L.	0	a	0	0	Fabaceae Lindl.
Iz_8_1009	DJI_0974	Anthericum ramosum L.	0	n	1	0	Asparagaceae Juss.
Iz_8_1009	DJI_0974	Artemisia marschalliana Spreng.	0	n	1	0	Asteraceae Bercht. & J.Presl
Iz_8_1009	DJI_0974	Asparagus officinalis L.	0	n	1	0	Asparagaceae Juss.
Iz_8_1009	DJI_0974	Astragalus cicer L.	0	n	1	0	Fabaceae Lindl.
Iz_8_1009	DJI_0974	Bassia laniflora (S.G.Gmel.) A.J.Scott	0	n	1	0	Amaranthaceae Juss.
Iz_8_1009	DJI_0974	Berberis vulgaris L.	0	n	1	0	Berberidaceae Juss.
Iz_8_1009	DJI_0974	Betula pendula Roth.	0	n	1	1	Betulaceae Gray
Iz_8_1009	DJI_0974	Betula pendula Roth.	0	n	1	0	Poaceae Barnhart
Iz_8_1009	DJI_0974	Bromus inermis Leyss.	0		NA	NA	
Iz_8_1009	DJI_0974	Bromus squarrosus L.	0	a	0	0	Poaceae Barnhart
Iz_8_1009	DJI_0974	Bromus tectorum L.	0	a	0	0	Poaceae Barnhart
Iz_8_1009	DJI_0974	Calamagrostis epigejos (L.) Roth	2	n	1	0	Poaceae Barnhart
Iz_8_1009	DJI_0974	Carduus acanthoides L.	0	n	1	0	Asteraceae Bercht. & J.Presl
Iz_8_1009	DJI_0974	Carex sp.	0	n	1	0	Cyperaceae Juss.
Iz_8_1009	DJI_0974	Centaurea majorovii Dumbadze	0	n	1	0	Asteraceae Bercht. & J.Presl
Iz_8_1009	DJI_0974	Chamaecytisus ruthenicus (Fisch.ex Wol.) Klaskova	0	n	1	0	Fabaceae Lindl.
Iz_8_1009	DJI_0974	Chelidonium majus L.	0	n	1	0	Papaveraceae Juss.
Iz_8_1009	DJI_0974	Chenopodium betaceum Andrz.	0	n	1	0	Amaranthaceae Juss.
Iz_8_1009	DJI_0974	Chenopodium cf. album	0	n	1	0	Amaranthaceae Juss.
Iz_8_1009	DJI_0974	Chenopodium sp.	0		NA	0	Amaranthaceae Juss.
Iz_8_1009	DJI_0974	Chondrilla juncea L.	0	n	1	0	Asteraceae Bercht. & J.Presl
Iz_8_1009	DJI_0974	Cirsium vulgare (Savi) Ten.	0	n	1	0	Asteraceae Bercht. & J.Presl
Iz_8_1009	DJI_0974	Clinopodium acinos (L.) Kuntze	0	n	1	0	Lamiacea Lindl.
Iz_8_1009	DJI_0974	Convallaria majalis L.	0	n	1	0	Asparagaceae Juss.
Iz_8_1009	DJI_0974	Crataegus rhipidophylla Gand.	0	n	1	1	Rosaceae Juss.
Iz_8_1009	DJI_0974	Crepis foetida L.	0	n	1	0	Asteraceae Bercht. & J.Presl
Iz_8_1009	DJI_0974	Dactylis glomerata L.	0	n	1	0	Poaceae Barnhart
Iz_8_1009	DJI_0974	Dianthus campestris M.Bieb.	0	n	1	0	Caryophyllaceae Juss.
Iz_8_1009	DJI_0974	Digitaria sanguinalis (L.) Scop.	0	a	0	0	Poaceae Barnhart
Iz_8_1009	DJI_0974	Elymus repens (L.) Gould	0	n	1	0	Poaceae Barnhart
Iz_8_1009	DJI_0974	Epilobium tetragonum L.	0	n	1	0	Onagraceae Juss.
Iz_8_1009	DJI_0974	Eragrostis minor Host	0	a	0	0	Poaceae Barnhart
Iz_8_1009	DJI_0974	Erigeron canadensis L.	5	a	0	0	Asteraceae Bercht. & J.Presl
Iz_8_1009	DJI_0974	Euphorbia seguieriana Neck.	0	n	1	0	Euphorbiaceae Juss.
Iz_8_1009	DJI_0974	Fallopia dumetorum (L.) Holub	0	n	1	0	Polygonaceae Juss.
Iz_8_1009	DJI_0974	Festuca beckeri (Hack.) Trautv.	0	n	1	0	Poaceae Barnhart
Iz_8_1009	DJI_0974	Fragaria vesca L.	0	n	1	0	Rosaceae Juss.
Iz_8_1009	DJI_0974	Frangula alnus Mill.	0	n	1	1	Rhamnaceae Juss.
Iz_8_1009	DJI_0974	Fraxinus pennsylvanica Marshall	0	a	0	1	Oleaceae Hoffmanns. & Link
Iz_8_1009	DJI_0974	Galeopsis bifida Boenn.	0	n	1	0	Lamiacea Lindl.
Iz_8_1009	DJI_0974	Galium verum L.	0	n	1	0	Rubiaceae Juss.
Iz_8_1009	DJI_0974	Genista tinctoria L.	0	n	1	0	Fabaceae Lindl.
Iz_8_1009	DJI_0974	Geranium robertianum L.	0	n	1	0	Geraniaceae Juss.
Iz_8_1009	DJI_0974	Geranium sanquineum L.	0	n	1	0	Geraniaceae Juss.
Iz_8_1009	DJI_0974	Gypsophila paniculata L.	0	n	1	0	Caryophyllaceae Juss.
Iz_8_1009	DJI_0974	Helichrysum arenarium (L.) Moench	0	n	1	0	Asteraceae Bercht. & J.Presl
Iz_8_1009	DJI_0974	Herniaria polygama J.Gay	0	n	1	0	Caryophyllaceae Juss.
Iz_8_1009	DJI_0974	Hieracium robustum Fr.	0	n	1	0	Asteraceae Bercht. & J.Presl
Iz_8_1009	DJI_0974	Hieracium umbellatum L.	0	n	1	NA	Asteraceae Bercht. & J.Presl
Iz_8_1009	DJI_0974	Hylotelephium maximum (L.) Holub	0	n	1	0	Crassulaceae J.St.-Hil.

Iz_8_1009	DJI_0974	Jacobaea borysthenica (DC.) B.Nord. & Greuter	0	n	1	0	Asteraceae Bercht. & J.Presl
Iz_8_1009	DJI_0974	Jasione montana L.	0	n	1	0	Campanulaceae Juss.
Iz_8_1009	DJI_0974	Jurinea cyanoides (L.) Rchb.s.l.	0	n	1	0	Asteraceae Bercht. & J.Presl
Iz_8_1009	DJI_0974	Koeleria glauca (Spreng.) DC.	0	n	1	0	Poaceae Barnhart
Iz_8_1009	DJI_0974	Lactuca serriola L.	0	a	0	0	Asteraceae Bercht. & J.Presl
Iz_8_1009	DJI_0974	Linaria genistifolia (L.) Mill.	0	n	1	0	Plantaginaceae Juss.
Iz_8_1009	DJI_0974	Lomelosia argentea (L.) Greuter & Burdet	0	n	1	0	Caprifoliaceae Juss.
Iz_8_1009	DJI_0974	Melampyrum pratense L.	0	n	1	0	Orobanchaceae Vent.
Iz_8_1009	DJI_0974	Oenothera biennis L.	0	a	0	0	Onagraceae Juss.
Iz_8_1009	DJI_0974	Pentanema britannica (L.) D.Gut.Larr., Santos-Vicente, Anderb., E.Rico & M.M.Mart.Ort.	0	n	1	0	Asteraceae Bercht. & J.Presl
Iz_8_1009	DJI_0974	Peucedanum oreoselinum (L.) Moench	0	n	1	0	Apiaceae Lindl.
Iz_8_1009	DJI_0974	Peucedanum ruthenicum M.Bieb.	0	n	1	0	Apiaceae Lindl.
Iz_8_1009	DJI_0974	Pilosella echioides (Lumn.) F.W.Schultz & Sch.Bip.	0	n	1	0	Asteraceae Bercht. & J.Presl
Iz_8_1009	DJI_0974	Pilosella officinarum F. Schultz et Sch. Bip.	0	n	1	0	Asteraceae Bercht. & J.Presl
Iz_8_1009	DJI_0974	Pilosella sp.	0		NA	0	Asteraceae Bercht. & J.Presl
Iz_8_1009	DJI_0974	Pinus sylvestris L.	0	n	1	1	Pinaceae Spreng. ex F.Rudolphi
Iz_8_1009	DJI_0974	Poa annua L.	0	n	1	0	Poaceae Barnhart
Iz_8_1009	DJI_0974	Poa nemoralis L.	0	n	1	0	Poaceae Barnhart
Iz_8_1009	DJI_0974	Poa sp.	0		NA	0	Poaceae Barnhart
Iz_8_1009	DJI_0974	Polygonatum odoratum (Mill.) Druce	0	n	1	0	Asparagaceae Juss.
Iz_8_1009	DJI_0974	Polygonum arenarium Waldst. & Kit.	0	n	1	0	Polygonaceae Juss.
Iz_8_1009	DJI_0974	Populus nigra L.	0	n	1	1	Salicaceae Mirb.
Iz_8_1009	DJI_0974	Populus tremula L.	0	n	1	1	Salicaceae Mirb.
Iz_8_1009	DJI_0974	Populus Ã canescens (Aiton) Sm.	0	n	1	1	Salicaceae Mirb.
Iz_8_1009	DJI_0974	Potentilla argentea L.	0	n	1	0	Rosaceae Juss.
Iz_8_1009	DJI_0974	Psammophiliella muralis (L.) Ikonn.	0	n	1	0	Caryophyllaceae Juss.
Iz_8_1009	DJI_0974	Psephellus sumensis (Kalen.) Greuter	0	n	1	0	Asteraceae Bercht. & J.Presl
Iz_8_1009	DJI_0974	Pulsatilla pratensis (L.) Mill. s.l.	0	n	1	0	Ranunculaceae Juss.
Iz_8_1009	DJI_0974	Pyrus communis L.	0	a	0	1	Rosaceae Juss.
Iz_8_1009	DJI_0974	Quercus robur L.	0	n	1	1	Fagaceae Dumort.
Iz_8_1009	DJI_0974	Robinia pseudoacacia L.	0	a	0	1	Fabaceae Lindl.
Iz_8_1009	DJI_0974	Rosa sp.	0		NA	1	Rosaceae Juss.
Iz_8_1009	DJI_0974	Rubus idaeus L.	0	n	1	0	Rosaceae Juss.
Iz_8_1009	DJI_0974	Rumex acetosella L.	0	n	1	0	Polygonaceae Juss.
Iz_8_1009	DJI_0974	Rumex thyrsiflorus Fingerh.	0	n	1	0	Polygonaceae Juss.
Iz_8_1009	DJI_0974	Salix alba L.	0	n	1	1	Salicaceae Mirb.
Iz_8_1009	DJI_0974	Sambucus nigra L.	0	n	1	1	Viburnaceae Raf.
Iz_8_1009	DJI_0974	Secale sylvestre Host	0	n	1	0	Poaceae Barnhart
Iz_8_1009	DJI_0974	Sempervivum ruthenicum Schnittsp. & C.B. Lehm.	0	n	1	0	Crassulaceae J.St.-Hil.
Iz_8_1009	DJI_0974	Setaria viridis (L.) P. Beauv.	0	n	1	0	Poaceae Barnhart
Iz_8_1009	DJI_0974	Silene borysthenica (Gruner) Walters	0	n	1	0	Caryophyllaceae Juss.
Iz_8_1009	DJI_0974	Silene latifolia subsp. alba (Mill.) Greuter & Burdet	0	n	1	0	Caryophyllaceae Juss.
Iz_8_1009	DJI_0974	Solidago virgaurea L.	0	n	1	0	Asteraceae Bercht. & J.Presl
Iz_8_1009	DJI_0974	Sorbus aucuparia L.	0	n	1	1	Rosaceae Juss.
Iz_8_1009	DJI_0974	Stachys recta L.	0	n	1	0	Lamiacea Lindl.
Iz_8_1009	DJI_0974	Tanacetum vulgare L.	0	n	1	0	Asteraceae Bercht. & J.Presl
Iz_8_1009	DJI_0974	Thymus pallasianus Heinr.Braun	0	n	1	0	Lamiacea Lindl.
Iz_8_1009	DJI_0974	Tilia cordata Mill.	0	n	1	1	Malvaceae Juss.
Iz_8_1009	DJI_0974	Trifolium alpestre L.	0	n	1	0	Fabaceae Lindl.
Iz_8_1009	DJI_0974	Trifolium arvense L.	0	n	1	0	Fabaceae Lindl.
Iz_8_1009	DJI_0974	Ulmus glabra Huds.	0	n	1	1	Ulmaceae Mirb.
Iz_8_1009	DJI_0974	Verbascum densiflorum Bertol.	0	n	1	0	Scrophulariaceae Juss.
Iz_8_1009	DJI_0974	Veronica austriaca L.	0	n	1	0	Plantaginaceae Juss.
Iz_8_1009	DJI_0974	Veronica incana L.	0	n	1	0	Plantaginaceae Juss.
Iz_8_1009	DJI_0974	Veronica verna L.	0	n	1	0	Plantaginaceae Juss.
Iz_8_1009	DJI_0974	Vincetoxicum hirundinaria Medik	0	n	1	0	Apocynaceae Juss.
Iz_8_1209	DJI_0456	Acer tataricum L.	0	n	1	1	Sapindaceae Juss.
Iz_8_1209	DJI_0456	Achillea millefolium L. s.l.	0	n	1	0	Asteraceae Bercht. & J.Presl
Iz_8_1209	DJI_0456	Allium oleraceum L.	0	n	1	0	Amaryllidaceae J.St.-Hil.
Iz_8_1209	DJI_0456	Ambrosia artemisiifolia L.	0	a	0	0	Asteraceae Bercht. & J.Presl
Iz_8_1209	DJI_0456	Amorpha fruticosa L.	0	a	0	0	Fabaceae Lindl.
Iz_8_1209	DJI_0456	Anthericum ramosum L.	0	n	1	0	Asparagaceae Juss.
Iz_8_1209	DJI_0456	Artemisia marschalliana Spreng.	0	n	1	0	Asteraceae Bercht. & J.Presl
Iz_8_1209	DJI_0456	Asparagus officinalis L.	0	n	1	0	Asparagaceae Juss.
Iz_8_1209	DJI_0456	Astragalus cicer L.	0	n	1	0	Fabaceae Lindl.
Iz_8_1209	DJI_0456	Bassia laniflora (S.G.Gmel.) A.J.Scott	0	n	1	0	Amaranthaceae Juss.
Iz_8_1209	DJI_0456	Berberis vulgaris L.	0	n	1	0	Berberidaceae Juss.
Iz_8_1209	DJI_0456	Betula pendula Roth.	0	n	1	1	Betulaceae Gray
Iz_8_1209	DJI_0456	Betula pendula Roth.	0	n	1	0	Poaceae Barnhart
Iz_8_1209	DJI_0456	Bromus inermis Leyss.	0		NA	NA	
Iz_8_1209	DJI_0456	Bromus squarrosus L.	0	a	0	0	Poaceae Barnhart
Iz_8_1209	DJI_0456	Bromus tectorum L.	0	a	0	0	Poaceae Barnhart
Iz_8_1209	DJI_0456	Calamagrostis epigejos (L.) Roth	2	n	1	0	Poaceae Barnhart
Iz_8_1209	DJI_0456	Carduus acanthoides L.	0	n	1	0	Asteraceae Bercht. & J.Presl
Iz_8_1209	DJI_0456	Carex sp.	0	n	1	0	Cyperaceae Juss.
Iz_8_1209	DJI_0456	Centaurea majorovii Dumbadze	0	n	1	0	Asteraceae Bercht. & J.Presl
Iz_8_1209	DJI_0456	Chamaecytisus ruthenicus (Fisch.ex Wol.) Klaskova	0	n	1	0	Fabaceae Lindl.
Iz_8_1209	DJI_0456	Chelidonium majus L.	0	n	1	0	Papaveraceae Juss.
Iz_8_1209	DJI_0456	Chenopodium betaceum Andrz.	0	n	1	0	Amaranthaceae Juss.
Iz_8_1209	DJI_0456	Chenopodium cf. album	0	n	1	0	Amaranthaceae Juss.
Iz_8_1209	DJI_0456	Chenopodium sp.	0		NA	0	Amaranthaceae Juss.
Iz_8_1209	DJI_0456	Chondrilla juncea L.	0	n	1	0	Asteraceae Bercht. & J.Presl
Iz_8_1209	DJI_0456	Cirsium vulgare (Savi) Ten.	0	n	1	0	Asteraceae Bercht. & J.Presl
Iz_8_1209	DJI_0456	Clinopodium acinos (L.) Kuntze	0	n	1	0	Lamiacea Lindl.
Iz_8_1209	DJI_0456	Convallaria majalis L.	0	n	1	0	Asparagaceae Juss.
Iz_8_1209	DJI_0456	Crataegus rhipidophylla Gand.	0	n	1	1	Rosaceae Juss.
Iz_8_1209	DJI_0456	Crepis foetida L.	0	n	1	0	Asteraceae Bercht. & J.Presl
Iz_8_1209	DJI_0456	Dactylis glomerata L.	0	n	1	0	Poaceae Barnhart
Iz_8_1209	DJI_0456	Dianthus campestris M.Bieb.	0	n	1	0	Caryophyllaceae Juss.
Iz_8_1209	DJI_0456	Digitaria sanguinalis (L.) Scop.	0	a	0	0	Poaceae Barnhart
Iz_8_1209	DJI_0456	Elymus repens (L.) Gould	0	n	1	0	Poaceae Barnhart
Iz_8_1209	DJI_0456	Epilobium tetragonum L.	0	n	1	0	Onagraceae Juss.
Iz_8_1209	DJI_0456	Eragrostis minor Host	0	a	0	0	Poaceae Barnhart
Iz_8_1209	DJI_0456	Erigeron canadensis L.	5	a	0	0	Asteraceae Bercht. & J.Presl
Iz_8_1209	DJI_0456	Euphorbia seguieriana Neck.	0	n	1	0	Euphorbiaceae Juss.
Iz_8_1209	DJI_0456	Fallopia dumetorum (L.) Holub	0	n	1	0	Polygonaceae Juss.
Iz_8_1209	DJI_0456	Festuca beckeri (Hack.) Trautv.	0	n	1	0	Poaceae Barnhart
Iz_8_1209	DJI_0456	Fragaria vesca L.	0	n	1	0	Rosaceae Juss.

Iz_8_1209	DJI_0456	Frangula alnus Mill.	0	n	1	1	Rhamnaceae Juss.
Iz_8_1209	DJI_0456	Fraxinus pennsylvanica Marshall	0	a	0	1	Oleaceae Hoffmanns. & Link
Iz_8_1209	DJI_0456	Galeopsis bifida Boenn.	0	n	1	0	Lamiacea Lindl.
Iz_8_1209	DJI_0456	Galium verum L.	0	n	1	0	Rubiaceae Juss.
Iz_8_1209	DJI_0456	Genista tinctoria L.	0	n	1	0	Fabaceae Lindl.
Iz_8_1209	DJI_0456	Geranium robertianum L.	0	n	1	0	Geraniaceae Juss.
Iz_8_1209	DJI_0456	Geranium sanquineum L.	0	n	1	0	Geraniaceae Juss.
Iz_8_1209	DJI_0456	Gypsophila paniculata L.	0	n	1	0	Caryophyllaceae Juss.
Iz_8_1209	DJI_0456	Helichrysum arenarium (L.) Moench	0	n	1	0	Asteraceae Bercht. & J.Presl
Iz_8_1209	DJI_0456	Herniaria polygama J.Gay	0	n	1	0	Caryophyllaceae Juss.
Iz_8_1209	DJI_0456	Hieracium robustum Fr.	0	n	1	0	Asteraceae Bercht. & J.Presl
Iz_8_1209	DJI_0456	Hieracium umbellatum L.	0	n	1	NA	Asteraceae Bercht. & J.Presl
Iz_8_1209	DJI_0456	Hylotelephium maximum (L.) Holub	0	n	1	0	Crassulaceae J.St.-Hil.
Iz_8_1209	DJI_0456	Jacobaea borysthenica (DC.) B.Nord. & Greuter	0	n	1	0	Asteraceae Bercht. & J.Presl
Iz_8_1209	DJI_0456	Jasione montana L.	0	n	1	0	Campanulaceae Juss.
Iz_8_1209	DJI_0456	Jurinea cyanoides (L.) Rchb.s.l.	0	n	1	0	Asteraceae Bercht. & J.Presl
Iz_8_1209	DJI_0456	Koeleria glauca (Spreng.) DC.	0	n	1	0	Poaceae Barnhart
Iz_8_1209	DJI_0456	Lactuca serriola L.	0	a	0	0	Asteraceae Bercht. & J.Presl
Iz_8_1209	DJI_0456	Linaria genistifolia (L.) Mill.	0	n	1	0	Plantaginaceae Juss.
Iz_8_1209	DJI_0456	Lomelosia argentea (L.) Greuter & Burdet	0	n	1	0	Caprifoliaceae Juss.
Iz_8_1209	DJI_0456	Melampyrum pratense L.	0	n	1	0	Orobanchaceae Vent.
Iz_8_1209	DJI_0456	Oenothera biennis L.	0	a	0	0	Onagraceae Juss.
Iz_8_1209	DJI_0456	Pentanema britannica (L.) D.Gut.Larr., Santos-Vicente, Anderb., E.Rico & M.M.Mart.Ort.	0	n	1	0	Asteraceae Bercht. & J.Presl
Iz_8_1209	DJI_0456	Peucedanum oreoselinum (L.) Moench	0	n	1	0	Apiaceae Lindl.
Iz_8_1209	DJI_0456	Peucedanum ruthenicum M.Bieb.	0	n	1	0	Apiaceae Lindl.
Iz_8_1209	DJI_0456	Pilosella echioides (Lumn.) F.W.Schultz & Sch.Bip.	0	n	1	0	Asteraceae Bercht. & J.Presl
Iz_8_1209	DJI_0456	Pilosella officinarum F. Schultz et Sch. Bip.	0	n	1	0	Asteraceae Bercht. & J.Presl
Iz_8_1209	DJI_0456	Pilosella sp.	0		NA	0	Asteraceae Bercht. & J.Presl
Iz_8_1209	DJI_0456	Pinus sylvestris L.	0	n	1	1	Pinaceae Spreng. ex F.Rudolphi
Iz_8_1209	DJI_0456	Poa annua L.	0	n	1	0	Poaceae Barnhart
Iz_8_1209	DJI_0456	Poa nemoralis L.	0	n	1	0	Poaceae Barnhart
Iz_8_1209	DJI_0456	Poa sp.	0		NA	0	Poaceae Barnhart
Iz_8_1209	DJI_0456	Polygonatum odoratum (Mill.) Druce	0	n	1	0	Asparagaceae Juss.
Iz_8_1209	DJI_0456	Polygonum arenarium Waldst. & Kit.	0	n	1	0	Polygonaceae Juss.
Iz_8_1209	DJI_0456	Populus nigra L.	0	n	1	1	Salicaceae Mirb.
Iz_8_1209	DJI_0456	Populus tremula L.	0	n	1	1	Salicaceae Mirb.
Iz_8_1209	DJI_0456	Populus Ã canescens (Aiton) Sm.	0	n	1	1	Salicaceae Mirb.
Iz_8_1209	DJI_0456	Potentilla argentea L.	0	n	1	0	Rosaceae Juss.
Iz_8_1209	DJI_0456	Psammophiliella muralis (L.) Ikonn.	0	n	1	0	Caryophyllaceae Juss.
Iz_8_1209	DJI_0456	Psephellus sumensis (Kalen.) Greuter	0	n	1	0	Asteraceae Bercht. & J.Presl
Iz_8_1209	DJI_0456	Pulsatilla pratensis (L.) Mill. s.l.	0	n	1	0	Ranunculaceae Juss.
Iz_8_1209	DJI_0456	Pyrus communis L.	0	a	0	1	Rosaceae Juss.
Iz_8_1209	DJI_0456	Quercus robur L.	0	n	1	1	Fagaceae Dumort.
Iz_8_1209	DJI_0456	Robinia pseudoacacia L.	0	a	0	1	Fabaceae Lindl.
Iz_8_1209	DJI_0456	Rosa sp.	0		NA	1	Rosaceae Juss.
Iz_8_1209	DJI_0456	Rubus idaeus L.	0	n	1	0	Rosaceae Juss.
Iz_8_1209	DJI_0456	Rumex acetosella L.	0	n	1	0	Polygonaceae Juss.
Iz_8_1209	DJI_0456	Rumex thyrsiflorus Fingerh.	0	n	1	0	Polygonaceae Juss.
Iz_8_1209	DJI_0456	Salix alba L.	0	n	1	1	Salicaceae Mirb.
Iz_8_1209	DJI_0456	Sambucus nigra L.	0	n	1	1	Viburnaceae Raf.
Iz_8_1209	DJI_0456	Secale sylvestre Host	0	n	1	0	Poaceae Barnhart
Iz_8_1209	DJI_0456	Sempervivum ruthenicum Schnittsp. & C.B. Lehm.	0	n	1	0	Crassulaceae J.St.-Hil.
Iz_8_1209	DJI_0456	Setaria viridis (L.) P. Beauv.	0	n	1	0	Poaceae Barnhart
Iz_8_1209	DJI_0456	Silene borysthenica (Gruner) Walters	0	n	1	0	Caryophyllaceae Juss.
Iz_8_1209	DJI_0456	Silene latifolia subsp. alba (Mill.) Greuter & Burdet	0	n	1	0	Caryophyllaceae Juss.
Iz_8_1209	DJI_0456	Solidago virgaurea L.	2	n	1	0	Asteraceae Bercht. & J.Presl
Iz_8_1209	DJI_0456	Sorbus aucuparia L.	0	n	1	1	Rosaceae Juss.
Iz_8_1209	DJI_0456	Stachys recta L.	0	n	1	0	Lamiacea Lindl.
Iz_8_1209	DJI_0456	Tanacetum vulgare L.	0	n	1	0	Asteraceae Bercht. & J.Presl
Iz_8_1209	DJI_0456	Thymus pallasianus Heinr.Braun	0	n	1	0	Lamiacea Lindl.
Iz_8_1209	DJI_0456	Tilia cordata Mill.	0	n	1	1	Malvaceae Juss.
Iz_8_1209	DJI_0456	Trifolium alpestre L.	0	n	1	0	Fabaceae Lindl.
Iz_8_1209	DJI_0456	Trifolium arvense L.	0	n	1	0	Fabaceae Lindl.
Iz_8_1209	DJI_0456	Ulmus glabra Huds.	0	n	1	1	Ulmaceae Mirb.
Iz_8_1209	DJI_0456	Verbascum densiflorum Bertol.	0	n	1	0	Scrophulariaceae Juss.
Iz_8_1209	DJI_0456	Veronica austriaca L.	0	n	1	0	Plantaginaceae Juss.
Iz_8_1209	DJI_0456	Veronica incana L.	0	n	1	0	Plantaginaceae Juss.
Iz_8_1209	DJI_0456	Veronica verna L.	0	n	1	0	Plantaginaceae Juss.
Iz_8_1209	DJI_0456	Vincetoxicum hirundinaria Medik	0	n	1	0	Apocynaceae Juss.
Iz_8_1209	DJI_0460	Acer tataricum L.	0	n	1	1	Sapindaceae Juss.
Iz_8_1209	DJI_0460	Achillea millefolium L. s.l.	0	n	1	0	Asteraceae Bercht. & J.Presl
Iz_8_1209	DJI_0460	Allium oleraceum L.	0	n	1	0	Amaryllidaceae J.St.-Hil.
Iz_8_1209	DJI_0460	Ambrosia artemisiifolia L.	0	a	0	0	Asteraceae Bercht. & J.Presl
Iz_8_1209	DJI_0460	Amorpha fruticosa L.	0	a	0	0	Fabaceae Lindl.
Iz_8_1209	DJI_0460	Anthericum ramosum L.	0	n	1	0	Asparagaceae Juss.
Iz_8_1209	DJI_0460	Artemisia marschalliana Spreng.	0	n	1	0	Asteraceae Bercht. & J.Presl
Iz_8_1209	DJI_0460	Asparagus officinalis L.	0	n	1	0	Asparagaceae Juss.
Iz_8_1209	DJI_0460	Astragalus cicer L.	0	n	1	0	Fabaceae Lindl.
Iz_8_1209	DJI_0460	Bassia laniflora (S.G.Gmel.) A.J.Scott	0	n	1	0	Amaranthaceae Juss.
Iz_8_1209	DJI_0460	Berberis vulgaris L.	0	n	1	0	Berberidaceae Juss.
Iz_8_1209	DJI_0460	Betula pendula Roth.	0	n	1	1	Betulaceae Gray
Iz_8_1209	DJI_0460	Betula pendula Roth.	0	n	1	0	Poaceae Barnhart
Iz_8_1209	DJI_0460	Bromus inermis Leyss.	0		NA	NA	
Iz_8_1209	DJI_0460	Bromus squarrosus L.	0	a	0	0	Poaceae Barnhart
Iz_8_1209	DJI_0460	Bromus tectorum L.	0	a	0	0	Poaceae Barnhart
Iz_8_1209	DJI_0460	Calamagrostis epigejos (L.) Roth	8	n	1	0	Poaceae Barnhart
Iz_8_1209	DJI_0460	Carduus acanthoides L.	0	n	1	0	Asteraceae Bercht. & J.Presl
Iz_8_1209	DJI_0460	Carex sp.	0	n	1	0	Cyperaceae Juss.
Iz_8_1209	DJI_0460	Centaurea majorovii Dumbadze	0	n	1	0	Asteraceae Bercht. & J.Presl
Iz_8_1209	DJI_0460	Chamaecytisus ruthenicus (Fisch.ex Wol.) Klaskova	0	n	1	0	Fabaceae Lindl.
Iz_8_1209	DJI_0460	Chelidonium majus L.	0	n	1	0	Papaveraceae Juss.
Iz_8_1209	DJI_0460	Chenopodium betaceum Andrz.	0	n	1	0	Amaranthaceae Juss.
Iz_8_1209	DJI_0460	Chenopodium cf. album	0	n	1	0	Amaranthaceae Juss.
Iz_8_1209	DJI_0460	Chenopodium sp.	0		NA	0	Amaranthaceae Juss.
Iz_8_1209	DJI_0460	Chondrilla juncea L.	0	n	1	0	Asteraceae Bercht. & J.Presl
Iz_8_1209	DJI_0460	Cirsium vulgare (Savi) Ten.	0	n	1	0	Asteraceae Bercht. & J.Presl
Iz_8_1209	DJI_0460	Clinopodium acinos (L.) Kuntze	0	n	1	0	Lamiacea Lindl.
Iz_8_1209	DJI_0460	Convallaria majalis L.	0	n	1	0	Asparagaceae Juss.

Iz_8_1209	DJI_0460	Crataegus rhipidophylla Gand.	0	n	1	1	Rosaceae Juss.
Iz_8_1209	DJI_0460	Crepis foetida L.	0	n	1	0	Asteraceae Bercht. & J.Presl
Iz_8_1209	DJI_0460	Dactylis glomerata L.	0	n	1	0	Poaceae Barnhart
Iz_8_1209	DJI_0460	Dianthus campestris M.Bieb.	0	n	1	0	Caryophyllaceae Juss.
Iz_8_1209	DJI_0460	Digitaria sanguinalis (L.) Scop.	0	a	0	0	Poaceae Barnhart
Iz_8_1209	DJI_0460	Elymus repens (L.) Gould	0	n	1	0	Poaceae Barnhart
Iz_8_1209	DJI_0460	Epilobium tetragonum L.	0	n	1	0	Onagraceae Juss.
Iz_8_1209	DJI_0460	Eragrostis minor Host	0	a	0	0	Poaceae Barnhart
Iz_8_1209	DJI_0460	Erigeron canadensis L.	6	a	0	0	Asteraceae Bercht. & J.Presl
Iz_8_1209	DJI_0460	Euphorbia seguieriana Neck.	0	n	1	0	Euphorbiaceae Juss.
Iz_8_1209	DJI_0460	Fallopia dumetorum (L.) Holub	0	n	1	0	Polygonaceae Juss.
Iz_8_1209	DJI_0460	Festuca beckeri (Hack.) Trautv.	0	n	1	0	Poaceae Barnhart
Iz_8_1209	DJI_0460	Fragaria vesca L.	0	n	1	0	Rosaceae Juss.
Iz_8_1209	DJI_0460	Frangula alnus Mill.	0	n	1	1	Rhamnaceae Juss.
Iz_8_1209	DJI_0460	Fraxinus pennsylvanica Marshall	0	a	0	1	Oleaceae Hoffmanns. & Link
Iz_8_1209	DJI_0460	Galeopsis bifida Boenn.	0	n	1	0	Lamiacea Lindl.
Iz_8_1209	DJI_0460	Galium verum L.	0	n	1	0	Rubiaceae Juss.
Iz_8_1209	DJI_0460	Genista tinctoria L.	0	n	1	0	Fabaceae Lindl.
Iz_8_1209	DJI_0460	Geranium robertianum L.	0	n	1	0	Geraniaceae Juss.
Iz_8_1209	DJI_0460	Geranium sanquineum L.	0	n	1	0	Geraniaceae Juss.
Iz_8_1209	DJI_0460	Gypsophila paniculata L.	0	n	1	0	Caryophyllaceae Juss.
Iz_8_1209	DJI_0460	Helichrysum arenarium (L.) Moench	0	n	1	0	Asteraceae Bercht. & J.Presl
Iz_8_1209	DJI_0460	Herniaria polygama J.Gay	0	n	1	0	Caryophyllaceae Juss.
Iz_8_1209	DJI_0460	Hieracium robustum Fr.	0	n	1	0	Asteraceae Bercht. & J.Presl
Iz_8_1209	DJI_0460	Hieracium umbellatum L.	0.5	n	1	NA	Asteraceae Bercht. & J.Presl
Iz_8_1209	DJI_0460	Hylotelephium maximum (L.) Holub	0	n	1	0	Crassulaceae J.St.-Hil.
Iz_8_1209	DJI_0460	Jacobaea borysthenica (DC.) B.Nord. & Greuter	0	n	1	0	Asteraceae Bercht. & J.Presl
Iz_8_1209	DJI_0460	Jasione montana L.	0	n	1	0	Campanulaceae Juss.
Iz_8_1209	DJI_0460	Jurinea cyanoides (L.) Rchb.s.l.	0	n	1	0	Asteraceae Bercht. & J.Presl
Iz_8_1209	DJI_0460	Koeleria glauca (Spreng.) DC.	0	n	1	0	Poaceae Barnhart
Iz_8_1209	DJI_0460	Lactuca serriola L.	0	a	0	0	Asteraceae Bercht. & J.Presl
Iz_8_1209	DJI_0460	Linaria genistifolia (L.) Mill.	0	n	1	0	Plantaginaceae Juss.
Iz_8_1209	DJI_0460	Lomelosia argentea (L.) Greuter & Burdet	0	n	1	0	Caprifoliaceae Juss.
Iz_8_1209	DJI_0460	Melampyrum pratense L.	0	n	1	0	Orobanchaceae Vent.
Iz_8_1209	DJI_0460	Oenothera biennis L.	0	a	0	0	Onagraceae Juss.
Iz_8_1209	DJI_0460	Pentanema britannica (L.) D.Gut.Larr., Santos-Vicente, Anderb., E.Rico & M.M.Mart.Ort.	0	n	1	0	Asteraceae Bercht. & J.Presl
Iz_8_1209	DJI_0460	Peucedanum oreoselinum (L.) Moench	0	n	1	0	Apiaceae Lindl.
Iz_8_1209	DJI_0460	Peucedanum ruthenicum M.Bieb.	0	n	1	0	Apiaceae Lindl.
Iz_8_1209	DJI_0460	Pilosella echioides (Lumn.) F.W.Schultz & Sch.Bip.	0	n	1	0	Asteraceae Bercht. & J.Presl
Iz_8_1209	DJI_0460	Pilosella officinarum F. Schultz et Sch. Bip.	0	n	1	0	Asteraceae Bercht. & J.Presl
Iz_8_1209	DJI_0460	Pilosella sp.	0		NA	0	Asteraceae Bercht. & J.Presl
Iz_8_1209	DJI_0460	Pinus sylvestris L.	0	n	1	1	Pinaceae Spreng. ex F.Rudolphi
Iz_8_1209	DJI_0460	Poa annua L.	0	n	1	0	Poaceae Barnhart
Iz_8_1209	DJI_0460	Poa nemoralis L.	0	n	1	0	Poaceae Barnhart
Iz_8_1209	DJI_0460	Poa sp.	0		NA	0	Poaceae Barnhart
Iz_8_1209	DJI_0460	Polygonatum odoratum (Mill.) Druce	0	n	1	0	Asparagaceae Juss.
Iz_8_1209	DJI_0460	Polygonum arenarium Waldst. & Kit.	0	n	1	0	Polygonaceae Juss.
Iz_8_1209	DJI_0460	Populus nigra L.	0	n	1	1	Salicaceae Mirb.
Iz_8_1209	DJI_0460	Populus tremula L.	0	n	1	1	Salicaceae Mirb.
Iz_8_1209	DJI_0460	Populus Ã canescens (Aiton) Sm.	0	n	1	1	Salicaceae Mirb.
Iz_8_1209	DJI_0460	Potentilla argentea L.	0	n	1	0	Rosaceae Juss.
Iz_8_1209	DJI_0460	Psammophiliella muralis (L.) Ikonn.	0	n	1	0	Caryophyllaceae Juss.
Iz_8_1209	DJI_0460	Psephellus sumensis (Kalen.) Greuter	0	n	1	0	Asteraceae Bercht. & J.Presl
Iz_8_1209	DJI_0460	Pulsatilla pratensis (L.) Mill. s.l.	0	n	1	0	Ranunculaceae Juss.
Iz_8_1209	DJI_0460	Pyrus communis L.	0	a	0	1	Rosaceae Juss.
Iz_8_1209	DJI_0460	Quercus robur L.	0	n	1	1	Fagaceae Dumort.
Iz_8_1209	DJI_0460	Robinia pseudoacacia L.	0	a	0	1	Fabaceae Lindl.
Iz_8_1209	DJI_0460	Rosa sp.	0		NA	1	Rosaceae Juss.
Iz_8_1209	DJI_0460	Rubus idaeus L.	0	n	1	0	Rosaceae Juss.
Iz_8_1209	DJI_0460	Rumex acetosella L.	0	n	1	0	Polygonaceae Juss.
Iz_8_1209	DJI_0460	Rumex thyrsiflorus Fingerh.	0	n	1	0	Polygonaceae Juss.
Iz_8_1209	DJI_0460	Salix alba L.	0	n	1	1	Salicaceae Mirb.
Iz_8_1209	DJI_0460	Sambucus nigra L.	0	n	1	1	Viburnaceae Raf.
Iz_8_1209	DJI_0460	Secale sylvestre Host	0	n	1	0	Poaceae Barnhart
Iz_8_1209	DJI_0460	Sempervivum ruthenicum Schnittsp. & C.B. Lehm.	0	n	1	0	Crassulaceae J.St.-Hil.
Iz_8_1209	DJI_0460	Setaria viridis (L.) P. Beauv.	0	n	1	0	Poaceae Barnhart
Iz_8_1209	DJI_0460	Silene borysthenica (Gruner) Walters	0	n	1	0	Caryophyllaceae Juss.
Iz_8_1209	DJI_0460	Silene latifolia subsp. alba (Mill.) Greuter & Burdet	0	n	1	0	Caryophyllaceae Juss.
Iz_8_1209	DJI_0460	Solidago virgaurea L.	0	n	1	0	Asteraceae Bercht. & J.Presl
Iz_8_1209	DJI_0460	Sorbus aucuparia L.	0	n	1	1	Rosaceae Juss.
Iz_8_1209	DJI_0460	Stachys recta L.	0	n	1	0	Lamiacea Lindl.
Iz_8_1209	DJI_0460	Tanacetum vulgare L.	0	n	1	0	Asteraceae Bercht. & J.Presl
Iz_8_1209	DJI_0460	Thymus pallasianus Heinr.Braun	0	n	1	0	Lamiacea Lindl.
Iz_8_1209	DJI_0460	Tilia cordata Mill.	0	n	1	1	Malvaceae Juss.
Iz_8_1209	DJI_0460	Trifolium alpestre L.	0	n	1	0	Fabaceae Lindl.
Iz_8_1209	DJI_0460	Trifolium arvense L.	0	n	1	0	Fabaceae Lindl.
Iz_8_1209	DJI_0460	Ulmus glabra Huds.	0	n	1	1	Ulmaceae Mirb.
Iz_8_1209	DJI_0460	Verbascum densiflorum Bertol.	0	n	1	0	Scrophulariaceae Juss.
Iz_8_1209	DJI_0460	Veronica austriaca L.	0	n	1	0	Plantaginaceae Juss.
Iz_8_1209	DJI_0460	Veronica incana L.	0	n	1	0	Plantaginaceae Juss.
Iz_8_1209	DJI_0460	Veronica verna L.	0	n	1	0	Plantaginaceae Juss.
Iz_8_1209	DJI_0460	Vincetoxicum hirundinaria Medik	0	n	1	0	Apocynaceae Juss.
Iz_8_1209	DJI_0464	Acer tataricum L.	0	n	1	1	Sapindaceae Juss.
Iz_8_1209	DJI_0464	Achillea millefolium L. s.l.	0	n	1	0	Asteraceae Bercht. & J.Presl
Iz_8_1209	DJI_0464	Allium oleraceum L.	0	n	1	0	Amaryllidaceae J.St.-Hil.
Iz_8_1209	DJI_0464	Ambrosia artemisiifolia L.	0	a	0	0	Asteraceae Bercht. & J.Presl
Iz_8_1209	DJI_0464	Amorpha fruticosa L.	0	a	0	0	Fabaceae Lindl.
Iz_8_1209	DJI_0464	Anthericum ramosum L.	0	n	1	0	Asparagaceae Juss.
Iz_8_1209	DJI_0464	Artemisia marschalliana Spreng.	0	n	1	0	Asteraceae Bercht. & J.Presl
Iz_8_1209	DJI_0464	Asparagus officinalis L.	0	n	1	0	Asparagaceae Juss.
Iz_8_1209	DJI_0464	Astragalus cicer L.	0	n	1	0	Fabaceae Lindl.
Iz_8_1209	DJI_0464	Bassia laniflora (S.G.Gmel.) A.J.Scott	0	n	1	0	Amaranthaceae Juss.
Iz_8_1209	DJI_0464	Berberis vulgaris L.	0	n	1	0	Berberidaceae Juss.
Iz_8_1209	DJI_0464	Betula pendula Roth.	0	n	1	1	Betulaceae Gray
Iz_8_1209	DJI_0464	Betula pendula Roth.	0	n	1	0	Poaceae Barnhart
Iz_8_1209	DJI_0464	Bromus inermis Leyss.	0		NA	NA	
Iz_8_1209	DJI_0464	Bromus squarrosus L.	0	a	0	0	Poaceae Barnhart
Iz_8_1209	DJI_0464	Bromus tectorum L.	0	a	0	0	Poaceae Barnhart

Iz_8_1209	DJI_0464	Calamagrostis epigejos (L.) Roth	20	n	1	0	Poaceae Barnhart
Iz_8_1209	DJI_0464	Carduus acanthoides L.	0	n	1	0	Asteraceae Bercht. & J.Presl
Iz_8_1209	DJI_0464	Carex sp.	0	n	1	0	Cyperaceae Juss.
Iz_8_1209	DJI_0464	Centaurea majorovii Dumbadze	0	n	1	0	Asteraceae Bercht. & J.Presl
Iz_8_1209	DJI_0464	Chamaecytisus ruthenicus (Fisch.ex Wol.) Klaskova	0	n	1	0	Fabaceae Lindl.
Iz_8_1209	DJI_0464	Chelidonium majus L.	0	n	1	0	Papaveraceae Juss.
Iz_8_1209	DJI_0464	Chenopodium betaceum Andrz.	0	n	1	0	Amaranthaceae Juss.
Iz_8_1209	DJI_0464	Chenopodium cf. album	0	n	1	0	Amaranthaceae Juss.
Iz_8_1209	DJI_0464	Chenopodium sp.	0		NA	0	Amaranthaceae Juss.
Iz_8_1209	DJI_0464	Chondrilla juncea L.	0	n	1	0	Asteraceae Bercht. & J.Presl
Iz_8_1209	DJI_0464	Cirsium vulgare (Savi) Ten.	0	n	1	0	Asteraceae Bercht. & J.Presl
Iz_8_1209	DJI_0464	Clinopodium acinos (L.) Kuntze	0	n	1	0	Lamiacea Lindl.
Iz_8_1209	DJI_0464	Convallaria majalis L.	0	n	1	0	Asparagaceae Juss.
Iz_8_1209	DJI_0464	Crataegus rhipidophylla Gand.	0	n	1	1	Rosaceae Juss.
Iz_8_1209	DJI_0464	Crepis foetida L.	0	n	1	0	Asteraceae Bercht. & J.Presl
Iz_8_1209	DJI_0464	Dactylis glomerata L.	0	n	1	0	Poaceae Barnhart
Iz_8_1209	DJI_0464	Dianthus campestris M.Bieb.	0	n	1	0	Caryophyllaceae Juss.
Iz_8_1209	DJI_0464	Digitaria sanguinalis (L.) Scop.	0	a	0	0	Poaceae Barnhart
Iz_8_1209	DJI_0464	Elymus repens (L.) Gould	0	n	1	0	Poaceae Barnhart
Iz_8_1209	DJI_0464	Epilobium tetragonum L.	0	n	1	0	Onagraceae Juss.
Iz_8_1209	DJI_0464	Eragrostis minor Host	0	a	0	0	Poaceae Barnhart
Iz_8_1209	DJI_0464	Erigeron canadensis L.	11	a	0	0	Asteraceae Bercht. & J.Presl
Iz_8_1209	DJI_0464	Euphorbia seguieriana Neck.	0	n	1	0	Euphorbiaceae Juss.
Iz_8_1209	DJI_0464	Fallopia dumetorum (L.) Holub	0	n	1	0	Polygonaceae Juss.
Iz_8_1209	DJI_0464	Festuca beckeri (Hack.) Trautv.	0	n	1	0	Poaceae Barnhart
Iz_8_1209	DJI_0464	Fragaria vesca L.	0	n	1	0	Rosaceae Juss.
Iz_8_1209	DJI_0464	Frangula alnus Mill.	0	n	1	1	Rhamnaceae Juss.
Iz_8_1209	DJI_0464	Fraxinus pennsylvanica Marshall	0	a	0	1	Oleaceae Hoffmanns. & Link
Iz_8_1209	DJI_0464	Galeopsis bifida Boenn.	0	n	1	0	Lamiacea Lindl.
Iz_8_1209	DJI_0464	Galium verum L.	0	n	1	0	Rubiaceae Juss.
Iz_8_1209	DJI_0464	Genista tinctoria L.	0	n	1	0	Fabaceae Lindl.
Iz_8_1209	DJI_0464	Geranium robertianum L.	0	n	1	0	Geraniaceae Juss.
Iz_8_1209	DJI_0464	Geranium sanquineum L.	0	n	1	0	Geraniaceae Juss.
Iz_8_1209	DJI_0464	Gypsophila paniculata L.	0	n	1	0	Caryophyllaceae Juss.
Iz_8_1209	DJI_0464	Helichrysum arenarium (L.) Moench	0	n	1	0	Asteraceae Bercht. & J.Presl
Iz_8_1209	DJI_0464	Herniaria polygama J.Gay	0	n	1	0	Caryophyllaceae Juss.
Iz_8_1209	DJI_0464	Hieracium robustum Fr.	0	n	1	0	Asteraceae Bercht. & J.Presl
Iz_8_1209	DJI_0464	Hieracium umbellatum L.	0	n	1	NA	Asteraceae Bercht. & J.Presl
Iz_8_1209	DJI_0464	Hylotelephium maximum (L.) Holub	0	n	1	0	Crassulaceae J.St.-Hil.
Iz_8_1209	DJI_0464	Jacobaea borysthenica (DC.) B.Nord. & Greuter	0	n	1	0	Asteraceae Bercht. & J.Presl
Iz_8_1209	DJI_0464	Jasione montana L.	0	n	1	0	Campanulaceae Juss.
Iz_8_1209	DJI_0464	Jurinea cyanoides (L.) Rchb.s.l.	0	n	1	0	Asteraceae Bercht. & J.Presl
Iz_8_1209	DJI_0464	Koeleria glauca (Spreng.) DC.	0	n	1	0	Poaceae Barnhart
Iz_8_1209	DJI_0464	Lactuca serriola L.	0	a	0	0	Asteraceae Bercht. & J.Presl
Iz_8_1209	DJI_0464	Linaria genistifolia (L.) Mill.	0	n	1	0	Plantaginaceae Juss.
Iz_8_1209	DJI_0464	Lomelosia argentea (L.) Greuter & Burdet	0	n	1	0	Caprifoliaceae Juss.
Iz_8_1209	DJI_0464	Melampyrum pratense L.	0	n	1	0	Orobanchaceae Vent.
Iz_8_1209	DJI_0464	Oenothera biennis L.	0	a	0	0	Onagraceae Juss.
Iz_8_1209	DJI_0464	Pentanema britannica (L.) D.Gut.Larr., Santos-Vicente, Anderb., E.Rico & M.M.Mart.Ort.	0	n	1	0	Asteraceae Bercht. & J.Presl
Iz_8_1209	DJI_0464	Peucedanum oreoselinum (L.) Moench	0	n	1	0	Apiaceae Lindl.
Iz_8_1209	DJI_0464	Peucedanum ruthenicum M.Bieb.	0	n	1	0	Apiaceae Lindl.
Iz_8_1209	DJI_0464	Pilosella echioides (Lumn.) F.W.Schultz & Sch.Bip.	0	n	1	0	Asteraceae Bercht. & J.Presl
Iz_8_1209	DJI_0464	Pilosella officinarum F. Schultz et Sch. Bip.	0	n	1	0	Asteraceae Bercht. & J.Presl
Iz_8_1209	DJI_0464	Pilosella sp.	0		NA	0	Asteraceae Bercht. & J.Presl
Iz_8_1209	DJI_0464	Pinus sylvestris L.	0	n	1	1	Pinaceae Spreng. ex F.Rudolphi
Iz_8_1209	DJI_0464	Poa annua L.	0	n	1	0	Poaceae Barnhart
Iz_8_1209	DJI_0464	Poa nemoralis L.	0	n	1	0	Poaceae Barnhart
Iz_8_1209	DJI_0464	Poa sp.	0		NA	0	Poaceae Barnhart
Iz_8_1209	DJI_0464	Polygonatum odoratum (Mill.) Druce	0	n	1	0	Asparagaceae Juss.
Iz_8_1209	DJI_0464	Polygonum arenarium Waldst. & Kit.	0	n	1	0	Polygonaceae Juss.
Iz_8_1209	DJI_0464	Populus nigra L.	0	n	1	1	Salicaceae Mirb.
Iz_8_1209	DJI_0464	Populus tremula L.	0	n	1	1	Salicaceae Mirb.
Iz_8_1209	DJI_0464	Populus Ã canescens (Aiton) Sm.	0	n	1	1	Salicaceae Mirb.
Iz_8_1209	DJI_0464	Potentilla argentea L.	0	n	1	0	Rosaceae Juss.
Iz_8_1209	DJI_0464	Psammophiliella muralis (L.) Ikonn.	0	n	1	0	Caryophyllaceae Juss.
Iz_8_1209	DJI_0464	Psephellus sumensis (Kalen.) Greuter	0	n	1	0	Asteraceae Bercht. & J.Presl
Iz_8_1209	DJI_0464	Pulsatilla pratensis (L.) Mill. s.l.	0	n	1	0	Ranunculaceae Juss.
Iz_8_1209	DJI_0464	Pyrus communis L.	0	a	0	1	Rosaceae Juss.
Iz_8_1209	DJI_0464	Quercus robur L.	0	n	1	1	Fagaceae Dumort.
Iz_8_1209	DJI_0464	Robinia pseudoacacia L.	0	a	0	1	Fabaceae Lindl.
Iz_8_1209	DJI_0464	Rosa sp.	0		NA	1	Rosaceae Juss.
Iz_8_1209	DJI_0464	Rubus idaeus L.	0	n	1	0	Rosaceae Juss.
Iz_8_1209	DJI_0464	Rumex acetosella L.	0	n	1	0	Polygonaceae Juss.
Iz_8_1209	DJI_0464	Rumex thyrsiflorus Fingerh.	0	n	1	0	Polygonaceae Juss.
Iz_8_1209	DJI_0464	Salix alba L.	0	n	1	1	Salicaceae Mirb.
Iz_8_1209	DJI_0464	Sambucus nigra L.	0	n	1	1	Viburnaceae Raf.
Iz_8_1209	DJI_0464	Secale sylvestre Host	0	n	1	0	Poaceae Barnhart
Iz_8_1209	DJI_0464	Sempervivum ruthenicum Schnittsp. & C.B. Lehm.	0	n	1	0	Crassulaceae J.St.-Hil.
Iz_8_1209	DJI_0464	Setaria viridis (L.) P. Beauv.	0	n	1	0	Poaceae Barnhart
Iz_8_1209	DJI_0464	Silene borysthenica (Gruner) Walters	0	n	1	0	Caryophyllaceae Juss.
Iz_8_1209	DJI_0464	Silene latifolia subsp. alba (Mill.) Greuter & Burdet	0	n	1	0	Caryophyllaceae Juss.
Iz_8_1209	DJI_0464	Solidago virgaurea L.	0	n	1	0	Asteraceae Bercht. & J.Presl
Iz_8_1209	DJI_0464	Sorbus aucuparia L.	0	n	1	1	Rosaceae Juss.
Iz_8_1209	DJI_0464	Stachys recta L.	0	n	1	0	Lamiacea Lindl.
Iz_8_1209	DJI_0464	Tanacetum vulgare L.	0	n	1	0	Asteraceae Bercht. & J.Presl
Iz_8_1209	DJI_0464	Thymus pallasianus Heinr.Braun	0	n	1	0	Lamiacea Lindl.
Iz_8_1209	DJI_0464	Tilia cordata Mill.	0	n	1	1	Malvaceae Juss.
Iz_8_1209	DJI_0464	Trifolium alpestre L.	0	n	1	0	Fabaceae Lindl.
Iz_8_1209	DJI_0464	Trifolium arvense L.	0	n	1	0	Fabaceae Lindl.
Iz_8_1209	DJI_0464	Ulmus glabra Huds.	0	n	1	1	Ulmaceae Mirb.
Iz_8_1209	DJI_0464	Verbascum densiflorum Bertol.	0	n	1	0	Scrophulariaceae Juss.
Iz_8_1209	DJI_0464	Veronica austriaca L.	0	n	1	0	Plantaginaceae Juss.
Iz_8_1209	DJI_0464	Veronica incana L.	0	n	1	0	Plantaginaceae Juss.
Iz_8_1209	DJI_0464	Veronica verna L.	0	n	1	0	Plantaginaceae Juss.
Iz_8_1209	DJI_0464	Vincetoxicum hirundinaria Medik	0	n	1	0	Apocynaceae Juss.
Iz_8_1209	DJI_0467	Acer tataricum L.	0	n	1	1	Sapindaceae Juss.
Iz_8_1209	DJI_0467	Achillea millefolium L. s.l.	0	n	1	0	Asteraceae Bercht. & J.Presl
Iz_8_1209	DJI_0467	Allium oleraceum L.	0	n	1	0	Amaryllidaceae J.St.-Hil.

Iz_8_1209	DJI_0467	Ambrosia artemisiifolia L.	0	a	0	0	Asteraceae Bercht. & J.Presl
Iz_8_1209	DJI_0467	Amorpha fruticosa L.	0	a	0	0	Fabaceae Lindl.
Iz_8_1209	DJI_0467	Anthericum ramosum L.	0	n	1	0	Asparagaceae Juss.
Iz_8_1209	DJI_0467	Artemisia marschalliana Spreng.	0	n	1	0	Asteraceae Bercht. & J.Presl
Iz_8_1209	DJI_0467	Asparagus officinalis L.	0	n	1	0	Asparagaceae Juss.
Iz_8_1209	DJI_0467	Astragalus cicer L.	0	n	1	0	Fabaceae Lindl.
Iz_8_1209	DJI_0467	Bassia laniflora (S.G.Gmel.) A.J.Scott	0	n	1	0	Amaranthaceae Juss.
Iz_8_1209	DJI_0467	Berberis vulgaris L.	0	n	1	0	Berberidaceae Juss.
Iz_8_1209	DJI_0467	Betula pendula Roth.	0	n	1	1	Betulaceae Gray
Iz_8_1209	DJI_0467	Betula pendula Roth.	0	n	1	0	Poaceae Barnhart
Iz_8_1209	DJI_0467	Bromus inermis Leyss.	0		NA	NA	
Iz_8_1209	DJI_0467	Bromus squarrosus L.	0	a	0	0	Poaceae Barnhart
Iz_8_1209	DJI_0467	Bromus tectorum L.	0	a	0	0	Poaceae Barnhart
Iz_8_1209	DJI_0467	Calamagrostis epigejos (L.) Roth	25	n	1	0	Poaceae Barnhart
Iz_8_1209	DJI_0467	Carduus acanthoides L.	0	n	1	0	Asteraceae Bercht. & J.Presl
Iz_8_1209	DJI_0467	Carex sp.	0	n	1	0	Cyperaceae Juss.
Iz_8_1209	DJI_0467	Centaurea majorovii Dumbadze	0	n	1	0	Asteraceae Bercht. & J.Presl
Iz_8_1209	DJI_0467	Chamaecytisus ruthenicus (Fisch.ex Wol.) Klaskova	0	n	1	0	Fabaceae Lindl.
Iz_8_1209	DJI_0467	Chelidonium majus L.	0	n	1	0	Papaveraceae Juss.
Iz_8_1209	DJI_0467	Chenopodium betaceum Andrz.	0	n	1	0	Amaranthaceae Juss.
Iz_8_1209	DJI_0467	Chenopodium cf. album	0	n	1	0	Amaranthaceae Juss.
Iz_8_1209	DJI_0467	Chenopodium sp.	0		NA	0	Amaranthaceae Juss.
Iz_8_1209	DJI_0467	Chondrilla juncea L.	0	n	1	0	Asteraceae Bercht. & J.Presl
Iz_8_1209	DJI_0467	Cirsium vulgare (Savi) Ten.	0	n	1	0	Asteraceae Bercht. & J.Presl
Iz_8_1209	DJI_0467	Clinopodium acinos (L.) Kuntze	0	n	1	0	Lamiacea Lindl.
Iz_8_1209	DJI_0467	Convallaria majalis L.	0	n	1	0	Asparagaceae Juss.
Iz_8_1209	DJI_0467	Crataegus rhipidophylla Gand.	0	n	1	1	Rosaceae Juss.
Iz_8_1209	DJI_0467	Crepis foetida L.	0	n	1	0	Asteraceae Bercht. & J.Presl
Iz_8_1209	DJI_0467	Dactylis glomerata L.	0	n	1	0	Poaceae Barnhart
Iz_8_1209	DJI_0467	Dianthus campestris M.Bieb.	0	n	1	0	Caryophyllaceae Juss.
Iz_8_1209	DJI_0467	Digitaria sanguinalis (L.) Scop.	0	a	0	0	Poaceae Barnhart
Iz_8_1209	DJI_0467	Elymus repens (L.) Gould	0	n	1	0	Poaceae Barnhart
Iz_8_1209	DJI_0467	Epilobium tetragonum L.	0	n	1	0	Onagraceae Juss.
Iz_8_1209	DJI_0467	Eragrostis minor Host	0	a	0	0	Poaceae Barnhart
Iz_8_1209	DJI_0467	Erigeron canadensis L.	3	a	0	0	Asteraceae Bercht. & J.Presl
Iz_8_1209	DJI_0467	Euphorbia seguieriana Neck.	0	n	1	0	Euphorbiaceae Juss.
Iz_8_1209	DJI_0467	Fallopia dumetorum (L.) Holub	0	n	1	0	Polygonaceae Juss.
Iz_8_1209	DJI_0467	Festuca beckeri (Hack.) Trautv.	0	n	1	0	Poaceae Barnhart
Iz_8_1209	DJI_0467	Fragaria vesca L.	0	n	1	0	Rosaceae Juss.
Iz_8_1209	DJI_0467	Frangula alnus Mill.	0	n	1	1	Rhamnaceae Juss.
Iz_8_1209	DJI_0467	Fraxinus pennsylvanica Marshall	0	a	0	1	Oleaceae Hoffmanns. & Link
Iz_8_1209	DJI_0467	Galeopsis bifida Boenn.	0	n	1	0	Lamiacea Lindl.
Iz_8_1209	DJI_0467	Galium verum L.	0	n	1	0	Rubiaceae Juss.
Iz_8_1209	DJI_0467	Genista tinctoria L.	0	n	1	0	Fabaceae Lindl.
Iz_8_1209	DJI_0467	Geranium robertianum L.	0	n	1	0	Geraniaceae Juss.
Iz_8_1209	DJI_0467	Geranium sanquineum L.	0	n	1	0	Geraniaceae Juss.
Iz_8_1209	DJI_0467	Gypsophila paniculata L.	0	n	1	0	Caryophyllaceae Juss.
Iz_8_1209	DJI_0467	Helichrysum arenarium (L.) Moench	0	n	1	0	Asteraceae Bercht. & J.Presl
Iz_8_1209	DJI_0467	Herniaria polygama J.Gay	0	n	1	0	Caryophyllaceae Juss.
Iz_8_1209	DJI_0467	Hieracium robustum Fr.	0	n	1	0	Asteraceae Bercht. & J.Presl
Iz_8_1209	DJI_0467	Hieracium umbellatum L.	2	n	1	NA	Asteraceae Bercht. & J.Presl
Iz_8_1209	DJI_0467	Hylotelephium maximum (L.) Holub	0	n	1	0	Crassulaceae J.St.-Hil.
Iz_8_1209	DJI_0467	Jacobaea borysthenica (DC.) B.Nord. & Greuter	0	n	1	0	Asteraceae Bercht. & J.Presl
Iz_8_1209	DJI_0467	Jasione montana L.	0	n	1	0	Campanulaceae Juss.
Iz_8_1209	DJI_0467	Jurinea cyanoides (L.) Rchb.s.l.	0	n	1	0	Asteraceae Bercht. & J.Presl
Iz_8_1209	DJI_0467	Koeleria glauca (Spreng.) DC.	0	n	1	0	Poaceae Barnhart
Iz_8_1209	DJI_0467	Lactuca serriola L.	0	a	0	0	Asteraceae Bercht. & J.Presl
Iz_8_1209	DJI_0467	Linaria genistifolia (L.) Mill.	0	n	1	0	Plantaginaceae Juss.
Iz_8_1209	DJI_0467	Lomelosia argentea (L.) Greuter & Burdet	0	n	1	0	Caprifoliaceae Juss.
Iz_8_1209	DJI_0467	Melampyrum pratense L.	0	n	1	0	Orobanchaceae Vent.
Iz_8_1209	DJI_0467	Oenothera biennis L.	0	a	0	0	Onagraceae Juss.
Iz_8_1209	DJI_0467	Pentanema britannica (L.) D.Gut.Larr., Santos-Vicente, Anderb., E.Rico & M.M.Mart.Ort.	0	n	1	0	Asteraceae Bercht. & J.Presl
Iz_8_1209	DJI_0467	Peucedanum oreoselinum (L.) Moench	0	n	1	0	Apiaceae Lindl.
Iz_8_1209	DJI_0467	Peucedanum ruthenicum M.Bieb.	0	n	1	0	Apiaceae Lindl.
Iz_8_1209	DJI_0467	Pilosella echioides (Lumn.) F.W.Schultz & Sch.Bip.	0	n	1	0	Asteraceae Bercht. & J.Presl
Iz_8_1209	DJI_0467	Pilosella officinarum F. Schultz et Sch. Bip.	0	n	1	0	Asteraceae Bercht. & J.Presl
Iz_8_1209	DJI_0467	Pilosella sp.	0		NA	0	Asteraceae Bercht. & J.Presl
Iz_8_1209	DJI_0467	Pinus sylvestris L.	0	n	1	1	Pinaceae Spreng. ex F.Rudolphi
Iz_8_1209	DJI_0467	Poa annua L.	0	n	1	0	Poaceae Barnhart
Iz_8_1209	DJI_0467	Poa nemoralis L.	0	n	1	0	Poaceae Barnhart
Iz_8_1209	DJI_0467	Poa sp.	0		NA	0	Poaceae Barnhart
Iz_8_1209	DJI_0467	Polygonatum odoratum (Mill.) Druce	0	n	1	0	Asparagaceae Juss.
Iz_8_1209	DJI_0467	Polygonum arenarium Waldst. & Kit.	0	n	1	0	Polygonaceae Juss.
Iz_8_1209	DJI_0467	Populus nigra L.	0	n	1	1	Salicaceae Mirb.
Iz_8_1209	DJI_0467	Populus tremula L.	0	n	1	1	Salicaceae Mirb.
Iz_8_1209	DJI_0467	Populus Ã canescens (Aiton) Sm.	0	n	1	1	Salicaceae Mirb.
Iz_8_1209	DJI_0467	Potentilla argentea L.	0	n	1	0	Rosaceae Juss.
Iz_8_1209	DJI_0467	Psammophiliella muralis (L.) Ikonn.	0	n	1	0	Caryophyllaceae Juss.
Iz_8_1209	DJI_0467	Psephellus sumensis (Kalen.) Greuter	0	n	1	0	Asteraceae Bercht. & J.Presl
Iz_8_1209	DJI_0467	Pulsatilla pratensis (L.) Mill. s.l.	0	n	1	0	Ranunculaceae Juss.
Iz_8_1209	DJI_0467	Pyrus communis L.	0	a	0	1	Rosaceae Juss.
Iz_8_1209	DJI_0467	Quercus robur L.	0	n	1	1	Fagaceae Dumort.
Iz_8_1209	DJI_0467	Robinia pseudoacacia L.	0	a	0	1	Fabaceae Lindl.
Iz_8_1209	DJI_0467	Rosa sp.	0		NA	1	Rosaceae Juss.
Iz_8_1209	DJI_0467	Rubus idaeus L.	0	n	1	0	Rosaceae Juss.
Iz_8_1209	DJI_0467	Rumex acetosella L.	0	n	1	0	Polygonaceae Juss.
Iz_8_1209	DJI_0467	Rumex thyrsiflorus Fingerh.	0	n	1	0	Polygonaceae Juss.
Iz_8_1209	DJI_0467	Salix alba L.	0	n	1	1	Salicaceae Mirb.
Iz_8_1209	DJI_0467	Sambucus nigra L.	0	n	1	1	Viburnaceae Raf.
Iz_8_1209	DJI_0467	Secale sylvestre Host	0	n	1	0	Poaceae Barnhart
Iz_8_1209	DJI_0467	Sempervivum ruthenicum Schnittsp. & C.B. Lehm.	0	n	1	0	Crassulaceae J.St.-Hil.
Iz_8_1209	DJI_0467	Setaria viridis (L.) P. Beauv.	0	n	1	0	Poaceae Barnhart
Iz_8_1209	DJI_0467	Silene borysthenica (Gruner) Walters	0	n	1	0	Caryophyllaceae Juss.
Iz_8_1209	DJI_0467	Silene latifolia subsp. alba (Mill.) Greuter & Burdet	0	n	1	0	Caryophyllaceae Juss.
Iz_8_1209	DJI_0467	Solidago virgaurea L.	0	n	1	0	Asteraceae Bercht. & J.Presl
Iz_8_1209	DJI_0467	Sorbus aucuparia L.	0	n	1	1	Rosaceae Juss.
Iz_8_1209	DJI_0467	Stachys recta L.	0	n	1	0	Lamiacea Lindl.
Iz_8_1209	DJI_0467	Tanacetum vulgare L.	0	n	1	0	Asteraceae Bercht. & J.Presl

Iz_8_1209	DJI_0467	Thymus pallasianus Heinr.Braun	0	n	1	0	Lamiacea Lindl.
Iz_8_1209	DJI_0467	Tilia cordata Mill.	0	n	1	1	Malvaceae Juss.
Iz_8_1209	DJI_0467	Trifolium alpestre L.	0	n	1	0	Fabaceae Lindl.
Iz_8_1209	DJI_0467	Trifolium arvense L.	0	n	1	0	Fabaceae Lindl.
Iz_8_1209	DJI_0467	Ulmus glabra Huds.	0	n	1	1	Ulmaceae Mirb.
Iz_8_1209	DJI_0467	Verbascum densiflorum Bertol.	0	n	1	0	Scrophulariaceae Juss.
Iz_8_1209	DJI_0467	Veronica austriaca L.	0	n	1	0	Plantaginaceae Juss.
Iz_8_1209	DJI_0467	Veronica incana L.	0	n	1	0	Plantaginaceae Juss.
Iz_8_1209	DJI_0467	Veronica verna L.	0	n	1	0	Plantaginaceae Juss.
Iz_8_1209	DJI_0467	Vincetoxicum hirundinaria Medik	0	n	1	0	Apocynaceae Juss.
Iz_8_1209	DJI_0470	Acer tataricum L.	0	n	1	1	Sapindaceae Juss.
Iz_8_1209	DJI_0470	Achillea millefolium L. s.l.	0	n	1	0	Asteraceae Bercht. & J.Presl
Iz_8_1209	DJI_0470	Allium oleraceum L.	0	n	1	0	Amaryllidaceae J.St.-Hil.
Iz_8_1209	DJI_0470	Ambrosia artemisiifolia L.	0	a	0	0	Asteraceae Bercht. & J.Presl
Iz_8_1209	DJI_0470	Amorpha fruticosa L.	0	a	0	0	Fabaceae Lindl.
Iz_8_1209	DJI_0470	Anthericum ramosum L.	0	n	1	0	Asparagaceae Juss.
Iz_8_1209	DJI_0470	Artemisia marschalliana Spreng.	0	n	1	0	Asteraceae Bercht. & J.Presl
Iz_8_1209	DJI_0470	Asparagus officinalis L.	0	n	1	0	Asparagaceae Juss.
Iz_8_1209	DJI_0470	Astragalus cicer L.	0	n	1	0	Fabaceae Lindl.
Iz_8_1209	DJI_0470	Bassia laniflora (S.G.Gmel.) A.J.Scott	0	n	1	0	Amaranthaceae Juss.
Iz_8_1209	DJI_0470	Berberis vulgaris L.	0	n	1	0	Berberidaceae Juss.
Iz_8_1209	DJI_0470	Betula pendula Roth.	0	n	1	1	Betulaceae Gray
Iz_8_1209	DJI_0470	Betula pendula Roth.	0	n	1	0	Poaceae Barnhart
Iz_8_1209	DJI_0470	Bromus inermis Leyss.	0		NA	NA	
Iz_8_1209	DJI_0470	Bromus squarrosus L.	0	a	0	0	Poaceae Barnhart
Iz_8_1209	DJI_0470	Bromus tectorum L.	0	a	0	0	Poaceae Barnhart
Iz_8_1209	DJI_0470	Calamagrostis epigejos (L.) Roth	4	n	1	0	Poaceae Barnhart
Iz_8_1209	DJI_0470	Carduus acanthoides L.	0	n	1	0	Asteraceae Bercht. & J.Presl
Iz_8_1209	DJI_0470	Carex sp.	0	n	1	0	Cyperaceae Juss.
Iz_8_1209	DJI_0470	Centaurea majorovii Dumbadze	0	n	1	0	Asteraceae Bercht. & J.Presl
Iz_8_1209	DJI_0470	Chamaecytisus ruthenicus (Fisch.ex Wol.) Klaskova	0	n	1	0	Fabaceae Lindl.
Iz_8_1209	DJI_0470	Chelidonium majus L.	0	n	1	0	Papaveraceae Juss.
Iz_8_1209	DJI_0470	Chenopodium betaceum Andrz.	0	n	1	0	Amaranthaceae Juss.
Iz_8_1209	DJI_0470	Chenopodium cf. album	0	n	1	0	Amaranthaceae Juss.
Iz_8_1209	DJI_0470	Chenopodium sp.	0		NA	0	Amaranthaceae Juss.
Iz_8_1209	DJI_0470	Chondrilla juncea L.	0	n	1	0	Asteraceae Bercht. & J.Presl
Iz_8_1209	DJI_0470	Cirsium vulgare (Savi) Ten.	0	n	1	0	Asteraceae Bercht. & J.Presl
Iz_8_1209	DJI_0470	Clinopodium acinos (L.) Kuntze	0	n	1	0	Lamiacea Lindl.
Iz_8_1209	DJI_0470	Convallaria majalis L.	0	n	1	0	Asparagaceae Juss.
Iz_8_1209	DJI_0470	Crataegus rhipidophylla Gand.	0	n	1	1	Rosaceae Juss.
Iz_8_1209	DJI_0470	Crepis foetida L.	0	n	1	0	Asteraceae Bercht. & J.Presl
Iz_8_1209	DJI_0470	Dactylis glomerata L.	0	n	1	0	Poaceae Barnhart
Iz_8_1209	DJI_0470	Dianthus campestris M.Bieb.	0	n	1	0	Caryophyllaceae Juss.
Iz_8_1209	DJI_0470	Digitaria sanguinalis (L.) Scop.	0	a	0	0	Poaceae Barnhart
Iz_8_1209	DJI_0470	Elymus repens (L.) Gould	0	n	1	0	Poaceae Barnhart
Iz_8_1209	DJI_0470	Epilobium tetragonum L.	0	n	1	0	Onagraceae Juss.
Iz_8_1209	DJI_0470	Eragrostis minor Host	0	a	0	0	Poaceae Barnhart
Iz_8_1209	DJI_0470	Erigeron canadensis L.	15	a	0	0	Asteraceae Bercht. & J.Presl
Iz_8_1209	DJI_0470	Euphorbia seguieriana Neck.	0	n	1	0	Euphorbiaceae Juss.
Iz_8_1209	DJI_0470	Fallopia dumetorum (L.) Holub	0	n	1	0	Polygonaceae Juss.
Iz_8_1209	DJI_0470	Festuca beckeri (Hack.) Trautv.	0	n	1	0	Poaceae Barnhart
Iz_8_1209	DJI_0470	Fragaria vesca L.	0	n	1	0	Rosaceae Juss.
Iz_8_1209	DJI_0470	Frangula alnus Mill.	0	n	1	1	Rhamnaceae Juss.
Iz_8_1209	DJI_0470	Fraxinus pennsylvanica Marshall	0	a	0	1	Oleaceae Hoffmanns. & Link
Iz_8_1209	DJI_0470	Galeopsis bifida Boenn.	0	n	1	0	Lamiacea Lindl.
Iz_8_1209	DJI_0470	Galium verum L.	0	n	1	0	Rubiaceae Juss.
Iz_8_1209	DJI_0470	Genista tinctoria L.	0	n	1	0	Fabaceae Lindl.
Iz_8_1209	DJI_0470	Geranium robertianum L.	0	n	1	0	Geraniaceae Juss.
Iz_8_1209	DJI_0470	Geranium sanquineum L.	0	n	1	0	Geraniaceae Juss.
Iz_8_1209	DJI_0470	Gypsophila paniculata L.	0	n	1	0	Caryophyllaceae Juss.
Iz_8_1209	DJI_0470	Helichrysum arenarium (L.) Moench	0	n	1	0	Asteraceae Bercht. & J.Presl
Iz_8_1209	DJI_0470	Herniaria polygama J.Gay	0	n	1	0	Caryophyllaceae Juss.
Iz_8_1209	DJI_0470	Hieracium robustum Fr.	0	n	1	0	Asteraceae Bercht. & J.Presl
Iz_8_1209	DJI_0470	Hieracium umbellatum L.	0	n	1	NA	Asteraceae Bercht. & J.Presl
Iz_8_1209	DJI_0470	Hylotelephium maximum (L.) Holub	0	n	1	0	Crassulaceae J.St.-Hil.
Iz_8_1209	DJI_0470	Jacobaea borysthenica (DC.) B.Nord. & Greuter	0	n	1	0	Asteraceae Bercht. & J.Presl
Iz_8_1209	DJI_0470	Jasione montana L.	0	n	1	0	Campanulaceae Juss.
Iz_8_1209	DJI_0470	Jurinea cyanoides (L.) Rchb.s.l.	0	n	1	0	Asteraceae Bercht. & J.Presl
Iz_8_1209	DJI_0470	Koeleria glauca (Spreng.) DC.	0	n	1	0	Poaceae Barnhart
Iz_8_1209	DJI_0470	Lactuca serriola L.	0	a	0	0	Asteraceae Bercht. & J.Presl
Iz_8_1209	DJI_0470	Linaria genistifolia (L.) Mill.	0	n	1	0	Plantaginaceae Juss.
Iz_8_1209	DJI_0470	Lomelosia argentea (L.) Greuter & Burdet	0	n	1	0	Caprifoliaceae Juss.
Iz_8_1209	DJI_0470	Melampyrum pratense L.	0	n	1	0	Orobanchaceae Vent.
Iz_8_1209	DJI_0470	Oenothera biennis L.	0	a	0	0	Onagraceae Juss.
Iz_8_1209	DJI_0470	Pentanema britannica (L.) D.Gut.Larr., Santos-Vicente, Anderb., E.Rico & M.M.Mart.Ort.	0	n	1	0	Asteraceae Bercht. & J.Presl
Iz_8_1209	DJI_0470	Peucedanum oreoselinum (L.) Moench	0	n	1	0	Apiaceae Lindl.
Iz_8_1209	DJI_0470	Peucedanum ruthenicum M.Bieb.	0	n	1	0	Apiaceae Lindl.
Iz_8_1209	DJI_0470	Pilosella echioides (Lumn.) F.W.Schultz & Sch.Bip.	0	n	1	0	Asteraceae Bercht. & J.Presl
Iz_8_1209	DJI_0470	Pilosella officinarum F. Schultz et Sch. Bip.	0	n	1	0	Asteraceae Bercht. & J.Presl
Iz_8_1209	DJI_0470	Pilosella sp.	0		NA	0	Asteraceae Bercht. & J.Presl
Iz_8_1209	DJI_0470	Pinus sylvestris L.	0	n	1	1	Pinaceae Spreng. ex F.Rudolphi
Iz_8_1209	DJI_0470	Poa annua L.	0	n	1	0	Poaceae Barnhart
Iz_8_1209	DJI_0470	Poa nemoralis L.	0	n	1	0	Poaceae Barnhart
Iz_8_1209	DJI_0470	Poa sp.	0		NA	0	Poaceae Barnhart
Iz_8_1209	DJI_0470	Polygonatum odoratum (Mill.) Druce	0	n	1	0	Asparagaceae Juss.
Iz_8_1209	DJI_0470	Polygonum arenarium Waldst. & Kit.	0	n	1	0	Polygonaceae Juss.
Iz_8_1209	DJI_0470	Populus nigra L.	0	n	1	1	Salicaceae Mirb.
Iz_8_1209	DJI_0470	Populus tremula L.	0	n	1	1	Salicaceae Mirb.
Iz_8_1209	DJI_0470	Populus Ã canescens (Aiton) Sm.	0	n	1	1	Salicaceae Mirb.
Iz_8_1209	DJI_0470	Potentilla argentea L.	0	n	1	0	Rosaceae Juss.
Iz_8_1209	DJI_0470	Psammophiliella muralis (L.) Ikonn.	0	n	1	0	Caryophyllaceae Juss.
Iz_8_1209	DJI_0470	Psephellus sumensis (Kalen.) Greuter	0	n	1	0	Asteraceae Bercht. & J.Presl
Iz_8_1209	DJI_0470	Pulsatilla pratensis (L.) Mill. s.l.	0	n	1	0	Ranunculaceae Juss.
Iz_8_1209	DJI_0470	Pyrus communis L.	0	a	0	1	Rosaceae Juss.
Iz_8_1209	DJI_0470	Quercus robur L.	0	n	1	1	Fagaceae Dumort.
Iz_8_1209	DJI_0470	Robinia pseudoacacia L.	0	a	0	1	Fabaceae Lindl.
Iz_8_1209	DJI_0470	Rosa sp.	0		NA	1	Rosaceae Juss.
Iz_8_1209	DJI_0470	Rubus idaeus L.	0	n	1	0	Rosaceae Juss.

Iz_8_1209	DJI_0470	Rumex acetosella L.	0	n	1	0	Polygonaceae Juss.
Iz_8_1209	DJI_0470	Rumex thyrsiflorus Fingerh.	0	n	1	0	Polygonaceae Juss.
Iz_8_1209	DJI_0470	Salix alba L.	0	n	1	1	Salicaceae Mirb.
Iz_8_1209	DJI_0470	Sambucus nigra L.	0	n	1	1	Viburnaceae Raf.
Iz_8_1209	DJI_0470	Secale sylvestre Host	0	n	1	0	Poaceae Barnhart
Iz_8_1209	DJI_0470	Sempervivum ruthenicum Schnittsp. & C.B. Lehm.	0	n	1	0	Crassulaceae J.St.-Hil.
Iz_8_1209	DJI_0470	Setaria viridis (L.) P. Beauv.	0	n	1	0	Poaceae Barnhart
Iz_8_1209	DJI_0470	Silene borysthenica (Gruner) Walters	0	n	1	0	Caryophyllaceae Juss.
Iz_8_1209	DJI_0470	Silene latifolia subsp. alba (Mill.) Greuter & Burdet	0	n	1	0	Caryophyllaceae Juss.
Iz_8_1209	DJI_0470	Solidago virgaurea L.	0	n	1	0	Asteraceae Bercht. & J.Presl
Iz_8_1209	DJI_0470	Sorbus aucuparia L.	0	n	1	1	Rosaceae Juss.
Iz_8_1209	DJI_0470	Stachys recta L.	0	n	1	0	Lamiacea Lindl.
Iz_8_1209	DJI_0470	Tanacetum vulgare L.	0	n	1	0	Asteraceae Bercht. & J.Presl
Iz_8_1209	DJI_0470	Thymus pallasianus Heinr.Braun	0	n	1	0	Lamiacea Lindl.
Iz_8_1209	DJI_0470	Tilia cordata Mill.	0	n	1	1	Malvaceae Juss.
Iz_8_1209	DJI_0470	Trifolium alpestre L.	0	n	1	0	Fabaceae Lindl.
Iz_8_1209	DJI_0470	Trifolium arvense L.	0	n	1	0	Fabaceae Lindl.
Iz_8_1209	DJI_0470	Ulmus glabra Huds.	0	n	1	1	Ulmaceae Mirb.
Iz_8_1209	DJI_0470	Verbascum densiflorum Bertol.	0	n	1	0	Scrophulariaceae Juss.
Iz_8_1209	DJI_0470	Veronica austriaca L.	0	n	1	0	Plantaginaceae Juss.
Iz_8_1209	DJI_0470	Veronica incana L.	0	n	1	0	Plantaginaceae Juss.
Iz_8_1209	DJI_0470	Veronica verna L.	0	n	1	0	Plantaginaceae Juss.
Iz_8_1209	DJI_0470	Vincetoxicum hirundinaria Medik	0	n	1	0	Apocynaceae Juss.
Iz_8_1209	DJI_0474	Acer tataricum L.	0	n	1	1	Sapindaceae Juss.
Iz_8_1209	DJI_0474	Achillea millefolium L. s.l.	0	n	1	0	Asteraceae Bercht. & J.Presl
Iz_8_1209	DJI_0474	Allium oleraceum L.	0	n	1	0	Amaryllidaceae J.St.-Hil.
Iz_8_1209	DJI_0474	Ambrosia artemisiifolia L.	0	a	0	0	Asteraceae Bercht. & J.Presl
Iz_8_1209	DJI_0474	Amorpha fruticosa L.	0	a	0	0	Fabaceae Lindl.
Iz_8_1209	DJI_0474	Anthericum ramosum L.	0	n	1	0	Asparagaceae Juss.
Iz_8_1209	DJI_0474	Artemisia marschalliana Spreng.	0	n	1	0	Asteraceae Bercht. & J.Presl
Iz_8_1209	DJI_0474	Asparagus officinalis L.	0	n	1	0	Asparagaceae Juss.
Iz_8_1209	DJI_0474	Astragalus cicer L.	0	n	1	0	Fabaceae Lindl.
Iz_8_1209	DJI_0474	Bassia laniflora (S.G.Gmel.) A.J.Scott	0	n	1	0	Amaranthaceae Juss.
Iz_8_1209	DJI_0474	Berberis vulgaris L.	0	n	1	0	Berberidaceae Juss.
Iz_8_1209	DJI_0474	Betula pendula Roth.	0	n	1	1	Betulaceae Gray
Iz_8_1209	DJI_0474	Betula pendula Roth.	0	n	1	0	Poaceae Barnhart
Iz_8_1209	DJI_0474	Bromus inermis Leyss.	0		NA	NA	
Iz_8_1209	DJI_0474	Bromus squarrosus L.	0	a	0	0	Poaceae Barnhart
Iz_8_1209	DJI_0474	Bromus tectorum L.	0	a	0	0	Poaceae Barnhart
Iz_8_1209	DJI_0474	Calamagrostis epigejos (L.) Roth	0	n	1	0	Poaceae Barnhart
Iz_8_1209	DJI_0474	Carduus acanthoides L.	0	n	1	0	Asteraceae Bercht. & J.Presl
Iz_8_1209	DJI_0474	Carex sp.	0	n	1	0	Cyperaceae Juss.
Iz_8_1209	DJI_0474	Centaurea majorovii Dumbadze	0	n	1	0	Asteraceae Bercht. & J.Presl
Iz_8_1209	DJI_0474	Chamaecytisus ruthenicus (Fisch.ex Wol.) Klaskova	0	n	1	0	Fabaceae Lindl.
Iz_8_1209	DJI_0474	Chelidonium majus L.	0	n	1	0	Papaveraceae Juss.
Iz_8_1209	DJI_0474	Chenopodium betaceum Andrz.	0	n	1	0	Amaranthaceae Juss.
Iz_8_1209	DJI_0474	Chenopodium cf. album	0	n	1	0	Amaranthaceae Juss.
Iz_8_1209	DJI_0474	Chenopodium sp.	0		NA	0	Amaranthaceae Juss.
Iz_8_1209	DJI_0474	Chondrilla juncea L.	0	n	1	0	Asteraceae Bercht. & J.Presl
Iz_8_1209	DJI_0474	Cirsium vulgare (Savi) Ten.	0	n	1	0	Asteraceae Bercht. & J.Presl
Iz_8_1209	DJI_0474	Clinopodium acinos (L.) Kuntze	0	n	1	0	Lamiacea Lindl.
Iz_8_1209	DJI_0474	Convallaria majalis L.	0	n	1	0	Asparagaceae Juss.
Iz_8_1209	DJI_0474	Crataegus rhipidophylla Gand.	0	n	1	1	Rosaceae Juss.
Iz_8_1209	DJI_0474	Crepis foetida L.	0	n	1	0	Asteraceae Bercht. & J.Presl
Iz_8_1209	DJI_0474	Dactylis glomerata L.	0	n	1	0	Poaceae Barnhart
Iz_8_1209	DJI_0474	Dianthus campestris M.Bieb.	0	n	1	0	Caryophyllaceae Juss.
Iz_8_1209	DJI_0474	Digitaria sanguinalis (L.) Scop.	0	a	0	0	Poaceae Barnhart
Iz_8_1209	DJI_0474	Elymus repens (L.) Gould	0	n	1	0	Poaceae Barnhart
Iz_8_1209	DJI_0474	Epilobium tetragonum L.	0	n	1	0	Onagraceae Juss.
Iz_8_1209	DJI_0474	Eragrostis minor Host	0	a	0	0	Poaceae Barnhart
Iz_8_1209	DJI_0474	Erigeron canadensis L.	0.2	a	0	0	Asteraceae Bercht. & J.Presl
Iz_8_1209	DJI_0474	Euphorbia seguieriana Neck.	0	n	1	0	Euphorbiaceae Juss.
Iz_8_1209	DJI_0474	Fallopia dumetorum (L.) Holub	0	n	1	0	Polygonaceae Juss.
Iz_8_1209	DJI_0474	Festuca beckeri (Hack.) Trautv.	0	n	1	0	Poaceae Barnhart
Iz_8_1209	DJI_0474	Fragaria vesca L.	0	n	1	0	Rosaceae Juss.
Iz_8_1209	DJI_0474	Frangula alnus Mill.	0	n	1	1	Rhamnaceae Juss.
Iz_8_1209	DJI_0474	Fraxinus pennsylvanica Marshall	0	a	0	1	Oleaceae Hoffmanns. & Link
Iz_8_1209	DJI_0474	Galeopsis bifida Boenn.	0	n	1	0	Lamiacea Lindl.
Iz_8_1209	DJI_0474	Galium verum L.	0	n	1	0	Rubiaceae Juss.
Iz_8_1209	DJI_0474	Genista tinctoria L.	0	n	1	0	Fabaceae Lindl.
Iz_8_1209	DJI_0474	Geranium robertianum L.	0	n	1	0	Geraniaceae Juss.
Iz_8_1209	DJI_0474	Geranium sanquineum L.	0	n	1	0	Geraniaceae Juss.
Iz_8_1209	DJI_0474	Gypsophila paniculata L.	0	n	1	0	Caryophyllaceae Juss.
Iz_8_1209	DJI_0474	Helichrysum arenarium (L.) Moench	0	n	1	0	Asteraceae Bercht. & J.Presl
Iz_8_1209	DJI_0474	Herniaria polygama J.Gay	0	n	1	0	Caryophyllaceae Juss.
Iz_8_1209	DJI_0474	Hieracium robustum Fr.	0	n	1	0	Asteraceae Bercht. & J.Presl
Iz_8_1209	DJI_0474	Hieracium umbellatum L.	0	n	1	NA	Asteraceae Bercht. & J.Presl
Iz_8_1209	DJI_0474	Hylotelephium maximum (L.) Holub	0	n	1	0	Crassulaceae J.St.-Hil.
Iz_8_1209	DJI_0474	Jacobaea borysthenica (DC.) B.Nord. & Greuter	0	n	1	0	Asteraceae Bercht. & J.Presl
Iz_8_1209	DJI_0474	Jasione montana L.	0	n	1	0	Campanulaceae Juss.
Iz_8_1209	DJI_0474	Jurinea cyanoides (L.) Rchb.s.l.	0	n	1	0	Asteraceae Bercht. & J.Presl
Iz_8_1209	DJI_0474	Koeleria glauca (Spreng.) DC.	0	n	1	0	Poaceae Barnhart
Iz_8_1209	DJI_0474	Lactuca serriola L.	0	a	0	0	Asteraceae Bercht. & J.Presl
Iz_8_1209	DJI_0474	Linaria genistifolia (L.) Mill.	0	n	1	0	Plantaginaceae Juss.
Iz_8_1209	DJI_0474	Lomelosia argentea (L.) Greuter & Burdet	0	n	1	0	Caprifoliaceae Juss.
Iz_8_1209	DJI_0474	Melampyrum pratense L.	0	n	1	0	Orobanchaceae Vent.
Iz_8_1209	DJI_0474	Oenothera biennis L.	0	a	0	0	Onagraceae Juss.
Iz_8_1209	DJI_0474	Pentanema britannica (L.) D.Gut.Larr., Santos-Vicente, Anderb., E.Rico & M.M.Mart.Ort.	0	n	1	0	Asteraceae Bercht. & J.Presl
Iz_8_1209	DJI_0474	Peucedanum oreoselinum (L.) Moench	0	n	1	0	Apiaceae Lindl.
Iz_8_1209	DJI_0474	Peucedanum ruthenicum M.Bieb.	0	n	1	0	Apiaceae Lindl.
Iz_8_1209	DJI_0474	Pilosella echioides (Lumn.) F.W.Schultz & Sch.Bip.	0	n	1	0	Asteraceae Bercht. & J.Presl
Iz_8_1209	DJI_0474	Pilosella officinarum F. Schultz et Sch. Bip.	0	n	1	0	Asteraceae Bercht. & J.Presl
Iz_8_1209	DJI_0474	Pilosella sp.	0		NA	0	Asteraceae Bercht. & J.Presl
Iz_8_1209	DJI_0474	Pinus sylvestris L.	0	n	1	1	Pinaceae Spreng. ex F.Rudolphi
Iz_8_1209	DJI_0474	Poa annua L.	0	n	1	0	Poaceae Barnhart
Iz_8_1209	DJI_0474	Poa nemoralis L.	0	n	1	0	Poaceae Barnhart
Iz_8_1209	DJI_0474	Poa sp.	0		NA	0	Poaceae Barnhart
Iz_8_1209	DJI_0474	Polygonatum odoratum (Mill.) Druce	0	n	1	0	Asparagaceae Juss.

Iz_8_1209	DJI_0474	Polygonum arenarium Waldst. & Kit.	0	n	1	0	Polygonaceae Juss.
Iz_8_1209	DJI_0474	Populus nigra L.	0	n	1	1	Salicaceae Mirb.
Iz_8_1209	DJI_0474	Populus tremula L.	0	n	1	1	Salicaceae Mirb.
Iz_8_1209	DJI_0474	Populus Ã canescens (Aiton) Sm.	0	n	1	1	Salicaceae Mirb.
Iz_8_1209	DJI_0474	Potentilla argentea L.	0	n	1	0	Rosaceae Juss.
Iz_8_1209	DJI_0474	Psammophiliella muralis (L.) Ikonn.	0	n	1	0	Caryophyllaceae Juss.
Iz_8_1209	DJI_0474	Psephellus sumensis (Kalen.) Greuter	0	n	1	0	Asteraceae Bercht. & J.Presl
Iz_8_1209	DJI_0474	Pulsatilla pratensis (L.) Mill. s.l.	0	n	1	0	Ranunculaceae Juss.
Iz_8_1209	DJI_0474	Pyrus communis L.	0	a	0	1	Rosaceae Juss.
Iz_8_1209	DJI_0474	Quercus robur L.	0	n	1	1	Fagaceae Dumort.
Iz_8_1209	DJI_0474	Robinia pseudoacacia L.	0	a	0	1	Fabaceae Lindl.
Iz_8_1209	DJI_0474	Rosa sp.	0		NA	1	Rosaceae Juss.
Iz_8_1209	DJI_0474	Rubus idaeus L.	0	n	1	0	Rosaceae Juss.
Iz_8_1209	DJI_0474	Rumex acetosella L.	0	n	1	0	Polygonaceae Juss.
Iz_8_1209	DJI_0474	Rumex thyrsiflorus Fingerh.	0	n	1	0	Polygonaceae Juss.
Iz_8_1209	DJI_0474	Salix alba L.	0	n	1	1	Salicaceae Mirb.
Iz_8_1209	DJI_0474	Sambucus nigra L.	0	n	1	1	Viburnaceae Raf.
Iz_8_1209	DJI_0474	Secale sylvestre Host	0	n	1	0	Poaceae Barnhart
Iz_8_1209	DJI_0474	Sempervivum ruthenicum Schnittsp. & C.B. Lehm.	0	n	1	0	Crassulaceae J.St.-Hil.
Iz_8_1209	DJI_0474	Setaria viridis (L.) P. Beauv.	0	n	1	0	Poaceae Barnhart
Iz_8_1209	DJI_0474	Silene borysthenica (Gruner) Walters	0	n	1	0	Caryophyllaceae Juss.
Iz_8_1209	DJI_0474	Silene latifolia subsp. alba (Mill.) Greuter & Burdet	0	n	1	0	Caryophyllaceae Juss.
Iz_8_1209	DJI_0474	Solidago virgaurea L.	0	n	1	0	Asteraceae Bercht. & J.Presl
Iz_8_1209	DJI_0474	Sorbus aucuparia L.	0	n	1	1	Rosaceae Juss.
Iz_8_1209	DJI_0474	Stachys recta L.	0	n	1	0	Lamiacea Lindl.
Iz_8_1209	DJI_0474	Tanacetum vulgare L.	0	n	1	0	Asteraceae Bercht. & J.Presl
Iz_8_1209	DJI_0474	Thymus pallasianus Heinr.Braun	0	n	1	0	Lamiacea Lindl.
Iz_8_1209	DJI_0474	Tilia cordata Mill.	0	n	1	1	Malvaceae Juss.
Iz_8_1209	DJI_0474	Trifolium alpestre L.	0	n	1	0	Fabaceae Lindl.
Iz_8_1209	DJI_0474	Trifolium arvense L.	0	n	1	0	Fabaceae Lindl.
Iz_8_1209	DJI_0474	Ulmus glabra Huds.	0	n	1	1	Ulmaceae Mirb.
Iz_8_1209	DJI_0474	Verbascum densiflorum Bertol.	0	n	1	0	Scrophulariaceae Juss.
Iz_8_1209	DJI_0474	Veronica austriaca L.	0	n	1	0	Plantaginaceae Juss.
Iz_8_1209	DJI_0474	Veronica incana L.	0	n	1	0	Plantaginaceae Juss.
Iz_8_1209	DJI_0474	Veronica verna L.	0	n	1	0	Plantaginaceae Juss.
Iz_8_1209	DJI_0474	Vincetoxicum hirundinaria Medik	0	n	1	0	Apocynaceae Juss.
Iz_8_1209	DJI_0480	Acer tataricum L.	0	n	1	1	Sapindaceae Juss.
Iz_8_1209	DJI_0480	Achillea millefolium L. s.l.	0	n	1	0	Asteraceae Bercht. & J.Presl
Iz_8_1209	DJI_0480	Allium oleraceum L.	0	n	1	0	Amaryllidaceae J.St.-Hil.
Iz_8_1209	DJI_0480	Ambrosia artemisiifolia L.	0	a	0	0	Asteraceae Bercht. & J.Presl
Iz_8_1209	DJI_0480	Amorpha fruticosa L.	0	a	0	0	Fabaceae Lindl.
Iz_8_1209	DJI_0480	Anthericum ramosum L.	0	n	1	0	Asparagaceae Juss.
Iz_8_1209	DJI_0480	Artemisia marschalliana Spreng.	0	n	1	0	Asteraceae Bercht. & J.Presl
Iz_8_1209	DJI_0480	Asparagus officinalis L.	0	n	1	0	Asparagaceae Juss.
Iz_8_1209	DJI_0480	Astragalus cicer L.	0	n	1	0	Fabaceae Lindl.
Iz_8_1209	DJI_0480	Bassia laniflora (S.G.Gmel.) A.J.Scott	0	n	1	0	Amaranthaceae Juss.
Iz_8_1209	DJI_0480	Berberis vulgaris L.	0	n	1	0	Berberidaceae Juss.
Iz_8_1209	DJI_0480	Betula pendula Roth.	0	n	1	1	Betulaceae Gray
Iz_8_1209	DJI_0480	Betula pendula Roth.	0	n	1	0	Poaceae Barnhart
Iz_8_1209	DJI_0480	Bromus inermis Leyss.	0		NA	NA	
Iz_8_1209	DJI_0480	Bromus squarrosus L.	0	a	0	0	Poaceae Barnhart
Iz_8_1209	DJI_0480	Bromus tectorum L.	0	a	0	0	Poaceae Barnhart
Iz_8_1209	DJI_0480	Calamagrostis epigejos (L.) Roth	0	n	1	0	Poaceae Barnhart
Iz_8_1209	DJI_0480	Carduus acanthoides L.	0	n	1	0	Asteraceae Bercht. & J.Presl
Iz_8_1209	DJI_0480	Carex sp.	0	n	1	0	Cyperaceae Juss.
Iz_8_1209	DJI_0480	Centaurea majorovii Dumbadze	0	n	1	0	Asteraceae Bercht. & J.Presl
Iz_8_1209	DJI_0480	Chamaecytisus ruthenicus (Fisch.ex Wol.) Klaskova	0	n	1	0	Fabaceae Lindl.
Iz_8_1209	DJI_0480	Chelidonium majus L.	0	n	1	0	Papaveraceae Juss.
Iz_8_1209	DJI_0480	Chenopodium betaceum Andrz.	0	n	1	0	Amaranthaceae Juss.
Iz_8_1209	DJI_0480	Chenopodium cf. album	0	n	1	0	Amaranthaceae Juss.
Iz_8_1209	DJI_0480	Chenopodium sp.	0		NA	0	Amaranthaceae Juss.
Iz_8_1209	DJI_0480	Chondrilla juncea L.	0	n	1	0	Asteraceae Bercht. & J.Presl
Iz_8_1209	DJI_0480	Cirsium vulgare (Savi) Ten.	0	n	1	0	Asteraceae Bercht. & J.Presl
Iz_8_1209	DJI_0480	Clinopodium acinos (L.) Kuntze	0	n	1	0	Lamiacea Lindl.
Iz_8_1209	DJI_0480	Convallaria majalis L.	0	n	1	0	Asparagaceae Juss.
Iz_8_1209	DJI_0480	Crataegus rhipidophylla Gand.	0	n	1	1	Rosaceae Juss.
Iz_8_1209	DJI_0480	Crepis foetida L.	0	n	1	0	Asteraceae Bercht. & J.Presl
Iz_8_1209	DJI_0480	Dactylis glomerata L.	0	n	1	0	Poaceae Barnhart
Iz_8_1209	DJI_0480	Dianthus campestris M.Bieb.	0	n	1	0	Caryophyllaceae Juss.
Iz_8_1209	DJI_0480	Digitaria sanguinalis (L.) Scop.	0	a	0	0	Poaceae Barnhart
Iz_8_1209	DJI_0480	Elymus repens (L.) Gould	0	n	1	0	Poaceae Barnhart
Iz_8_1209	DJI_0480	Epilobium tetragonum L.	0	n	1	0	Onagraceae Juss.
Iz_8_1209	DJI_0480	Eragrostis minor Host	0	a	0	0	Poaceae Barnhart
Iz_8_1209	DJI_0480	Erigeron canadensis L.	3	a	0	0	Asteraceae Bercht. & J.Presl
Iz_8_1209	DJI_0480	Euphorbia seguieriana Neck.	0	n	1	0	Euphorbiaceae Juss.
Iz_8_1209	DJI_0480	Fallopia dumetorum (L.) Holub	0	n	1	0	Polygonaceae Juss.
Iz_8_1209	DJI_0480	Festuca beckeri (Hack.) Trautv.	0	n	1	0	Poaceae Barnhart
Iz_8_1209	DJI_0480	Fragaria vesca L.	0	n	1	0	Rosaceae Juss.
Iz_8_1209	DJI_0480	Frangula alnus Mill.	0	n	1	1	Rhamnaceae Juss.
Iz_8_1209	DJI_0480	Fraxinus pennsylvanica Marshall	0	a	0	1	Oleaceae Hoffmanns. & Link
Iz_8_1209	DJI_0480	Galeopsis bifida Boenn.	0	n	1	0	Lamiacea Lindl.
Iz_8_1209	DJI_0480	Galium verum L.	0	n	1	0	Rubiaceae Juss.
Iz_8_1209	DJI_0480	Genista tinctoria L.	0	n	1	0	Fabaceae Lindl.
Iz_8_1209	DJI_0480	Geranium robertianum L.	0	n	1	0	Geraniaceae Juss.
Iz_8_1209	DJI_0480	Geranium sanquineum L.	0	n	1	0	Geraniaceae Juss.
Iz_8_1209	DJI_0480	Gypsophila paniculata L.	0	n	1	0	Caryophyllaceae Juss.
Iz_8_1209	DJI_0480	Helichrysum arenarium (L.) Moench	0	n	1	0	Asteraceae Bercht. & J.Presl
Iz_8_1209	DJI_0480	Herniaria polygama J.Gay	0	n	1	0	Caryophyllaceae Juss.
Iz_8_1209	DJI_0480	Hieracium robustum Fr.	0	n	1	0	Asteraceae Bercht. & J.Presl
Iz_8_1209	DJI_0480	Hieracium umbellatum L.	0	n	1	NA	Asteraceae Bercht. & J.Presl
Iz_8_1209	DJI_0480	Hylotelephium maximum (L.) Holub	0	n	1	0	Crassulaceae J.St.-Hil.
Iz_8_1209	DJI_0480	Jacobaea borysthenica (DC.) B.Nord. & Greuter	0	n	1	0	Asteraceae Bercht. & J.Presl
Iz_8_1209	DJI_0480	Jasione montana L.	0	n	1	0	Campanulaceae Juss.
Iz_8_1209	DJI_0480	Jurinea cyanoides (L.) Rchb.s.l.	0	n	1	0	Asteraceae Bercht. & J.Presl
Iz_8_1209	DJI_0480	Koeleria glauca (Spreng.) DC.	0	n	1	0	Poaceae Barnhart
Iz_8_1209	DJI_0480	Lactuca serriola L.	0	a	0	0	Asteraceae Bercht. & J.Presl
Iz_8_1209	DJI_0480	Linaria genistifolia (L.) Mill.	0	n	1	0	Plantaginaceae Juss.
Iz_8_1209	DJI_0480	Lomelosia argentea (L.) Greuter & Burdet	0	n	1	0	Caprifoliaceae Juss.

Iz_8_1209	DJI_0480	Melampyrum pratense L.	0	n	1	0	Orobanchaceae Vent.
Iz_8_1209	DJI_0480	Oenothera biennis L.	0	a	0	0	Onagraceae Juss.
Iz_8_1209	DJI_0480	Pentanema britannica (L.) D.Gut.Larr., Santos-Vicente, Anderb., E.Rico & M.M.Mart.Ort.	0	n	1	0	Asteraceae Bercht. & J.Presl
Iz_8_1209	DJI_0480	Peucedanum oreoselinum (L.) Moench	0	n	1	0	Apiaceae Lindl.
Iz_8_1209	DJI_0480	Peucedanum ruthenicum M.Bieb.	0	n	1	0	Apiaceae Lindl.
Iz_8_1209	DJI_0480	Pilosella echioides (Lumn.) F.W.Schultz & Sch.Bip.	0	n	1	0	Asteraceae Bercht. & J.Presl
Iz_8_1209	DJI_0480	Pilosella officinarum F. Schultz et Sch. Bip.	0	n	1	0	Asteraceae Bercht. & J.Presl
Iz_8_1209	DJI_0480	Pilosella sp.	0		NA	0	Asteraceae Bercht. & J.Presl
Iz_8_1209	DJI_0480	Pinus sylvestris L.	0	n	1	1	Pinaceae Spreng. ex F.Rudolphi
Iz_8_1209	DJI_0480	Poa annua L.	0	n	1	0	Poaceae Barnhart
Iz_8_1209	DJI_0480	Poa nemoralis L.	0	n	1	0	Poaceae Barnhart
Iz_8_1209	DJI_0480	Poa sp.	0		NA	0	Poaceae Barnhart
Iz_8_1209	DJI_0480	Polygonatum odoratum (Mill.) Druce	0	n	1	0	Asparagaceae Juss.
Iz_8_1209	DJI_0480	Polygonum arenarium Waldst. & Kit.	0	n	1	0	Polygonaceae Juss.
Iz_8_1209	DJI_0480	Populus nigra L.	0	n	1	1	Salicaceae Mirb.
Iz_8_1209	DJI_0480	Populus tremula L.	0	n	1	1	Salicaceae Mirb.
Iz_8_1209	DJI_0480	Populus Ã canescens (Aiton) Sm.	0	n	1	1	Salicaceae Mirb.
Iz_8_1209	DJI_0480	Potentilla argentea L.	0	n	1	0	Rosaceae Juss.
Iz_8_1209	DJI_0480	Psammophiliella muralis (L.) Ikonn.	0	n	1	0	Caryophyllaceae Juss.
Iz_8_1209	DJI_0480	Psephellus sumensis (Kalen.) Greuter	0	n	1	0	Asteraceae Bercht. & J.Presl
Iz_8_1209	DJI_0480	Pulsatilla pratensis (L.) Mill. s.l.	0	n	1	0	Ranunculaceae Juss.
Iz_8_1209	DJI_0480	Pyrus communis L.	0	a	0	1	Rosaceae Juss.
Iz_8_1209	DJI_0480	Quercus robur L.	0	n	1	1	Fagaceae Dumort.
Iz_8_1209	DJI_0480	Robinia pseudoacacia L.	0	a	0	1	Fabaceae Lindl.
Iz_8_1209	DJI_0480	Rosa sp.	0		NA	1	Rosaceae Juss.
Iz_8_1209	DJI_0480	Rubus idaeus L.	0	n	1	0	Rosaceae Juss.
Iz_8_1209	DJI_0480	Rumex acetosella L.	0	n	1	0	Polygonaceae Juss.
Iz_8_1209	DJI_0480	Rumex thyrsiflorus Fingerh.	0	n	1	0	Polygonaceae Juss.
Iz_8_1209	DJI_0480	Salix alba L.	0	n	1	1	Salicaceae Mirb.
Iz_8_1209	DJI_0480	Sambucus nigra L.	0	n	1	1	Viburnaceae Raf.
Iz_8_1209	DJI_0480	Secale sylvestre Host	0	n	1	0	Poaceae Barnhart
Iz_8_1209	DJI_0480	Sempervivum ruthenicum Schnittsp. & C.B. Lehm.	0	n	1	0	Crassulaceae J.St.-Hil.
Iz_8_1209	DJI_0480	Setaria viridis (L.) P. Beauv.	0	n	1	0	Poaceae Barnhart
Iz_8_1209	DJI_0480	Silene borysthenica (Gruner) Walters	0	n	1	0	Caryophyllaceae Juss.
Iz_8_1209	DJI_0480	Silene latifolia subsp. alba (Mill.) Greuter & Burdet	0	n	1	0	Caryophyllaceae Juss.
Iz_8_1209	DJI_0480	Solidago virgaurea L.	0	n	1	0	Asteraceae Bercht. & J.Presl
Iz_8_1209	DJI_0480	Sorbus aucuparia L.	0	n	1	1	Rosaceae Juss.
Iz_8_1209	DJI_0480	Stachys recta L.	0	n	1	0	Lamiacea Lindl.
Iz_8_1209	DJI_0480	Tanacetum vulgare L.	0	n	1	0	Asteraceae Bercht. & J.Presl
Iz_8_1209	DJI_0480	Thymus pallasianus Heinr.Braun	0	n	1	0	Lamiacea Lindl.
Iz_8_1209	DJI_0480	Tilia cordata Mill.	0	n	1	1	Malvaceae Juss.
Iz_8_1209	DJI_0480	Trifolium alpestre L.	0	n	1	0	Fabaceae Lindl.
Iz_8_1209	DJI_0480	Trifolium arvense L.	0	n	1	0	Fabaceae Lindl.
Iz_8_1209	DJI_0480	Ulmus glabra Huds.	0	n	1	1	Ulmaceae Mirb.
Iz_8_1209	DJI_0480	Verbascum densiflorum Bertol.	0	n	1	0	Scrophulariaceae Juss.
Iz_8_1209	DJI_0480	Veronica austriaca L.	0	n	1	0	Plantaginaceae Juss.
Iz_8_1209	DJI_0480	Veronica incana L.	0	n	1	0	Plantaginaceae Juss.
Iz_8_1209	DJI_0480	Veronica verna L.	0	n	1	0	Plantaginaceae Juss.
Iz_8_1209	DJI_0480	Vincetoxicum hirundinaria Medik	0	n	1	0	Apocynaceae Juss.
Iz_8_1209	DJI_0486	Acer tataricum L.	0	n	1	1	Sapindaceae Juss.
Iz_8_1209	DJI_0486	Achillea millefolium L. s.l.	0	n	1	0	Asteraceae Bercht. & J.Presl
Iz_8_1209	DJI_0486	Allium oleraceum L.	0	n	1	0	Amaryllidaceae J.St.-Hil.
Iz_8_1209	DJI_0486	Ambrosia artemisiifolia L.	0	a	0	0	Asteraceae Bercht. & J.Presl
Iz_8_1209	DJI_0486	Amorpha fruticosa L.	0	a	0	0	Fabaceae Lindl.
Iz_8_1209	DJI_0486	Anthericum ramosum L.	0	n	1	0	Asparagaceae Juss.
Iz_8_1209	DJI_0486	Artemisia marschalliana Spreng.	0	n	1	0	Asteraceae Bercht. & J.Presl
Iz_8_1209	DJI_0486	Asparagus officinalis L.	0	n	1	0	Asparagaceae Juss.
Iz_8_1209	DJI_0486	Astragalus cicer L.	0	n	1	0	Fabaceae Lindl.
Iz_8_1209	DJI_0486	Bassia laniflora (S.G.Gmel.) A.J.Scott	0	n	1	0	Amaranthaceae Juss.
Iz_8_1209	DJI_0486	Berberis vulgaris L.	0	n	1	0	Berberidaceae Juss.
Iz_8_1209	DJI_0486	Betula pendula Roth.	0	n	1	1	Betulaceae Gray
Iz_8_1209	DJI_0486	Betula pendula Roth.	0	n	1	0	Poaceae Barnhart
Iz_8_1209	DJI_0486	Bromus inermis Leyss.	0		NA	NA	
Iz_8_1209	DJI_0486	Bromus squarrosus L.	0	a	0	0	Poaceae Barnhart
Iz_8_1209	DJI_0486	Bromus tectorum L.	0	a	0	0	Poaceae Barnhart
Iz_8_1209	DJI_0486	Calamagrostis epigejos (L.) Roth	0	n	1	0	Poaceae Barnhart
Iz_8_1209	DJI_0486	Carduus acanthoides L.	0	n	1	0	Asteraceae Bercht. & J.Presl
Iz_8_1209	DJI_0486	Carex sp.	0	n	1	0	Cyperaceae Juss.
Iz_8_1209	DJI_0486	Centaurea majorovii Dumbadze	0	n	1	0	Asteraceae Bercht. & J.Presl
Iz_8_1209	DJI_0486	Chamaecytisus ruthenicus (Fisch.ex Wol.) Klaskova	0	n	1	0	Fabaceae Lindl.
Iz_8_1209	DJI_0486	Chelidonium majus L.	0	n	1	0	Papaveraceae Juss.
Iz_8_1209	DJI_0486	Chenopodium betaceum Andrz.	0	n	1	0	Amaranthaceae Juss.
Iz_8_1209	DJI_0486	Chenopodium cf. album	0	n	1	0	Amaranthaceae Juss.
Iz_8_1209	DJI_0486	Chenopodium sp.	0		NA	0	Amaranthaceae Juss.
Iz_8_1209	DJI_0486	Chondrilla juncea L.	0	n	1	0	Asteraceae Bercht. & J.Presl
Iz_8_1209	DJI_0486	Cirsium vulgare (Savi) Ten.	0	n	1	0	Asteraceae Bercht. & J.Presl
Iz_8_1209	DJI_0486	Clinopodium acinos (L.) Kuntze	0	n	1	0	Lamiacea Lindl.
Iz_8_1209	DJI_0486	Convallaria majalis L.	0	n	1	0	Asparagaceae Juss.
Iz_8_1209	DJI_0486	Crataegus rhipidophylla Gand.	0	n	1	1	Rosaceae Juss.
Iz_8_1209	DJI_0486	Crepis foetida L.	0	n	1	0	Asteraceae Bercht. & J.Presl
Iz_8_1209	DJI_0486	Dactylis glomerata L.	0	n	1	0	Poaceae Barnhart
Iz_8_1209	DJI_0486	Dianthus campestris M.Bieb.	0	n	1	0	Caryophyllaceae Juss.
Iz_8_1209	DJI_0486	Digitaria sanguinalis (L.) Scop.	0	a	0	0	Poaceae Barnhart
Iz_8_1209	DJI_0486	Elymus repens (L.) Gould	0	n	1	0	Poaceae Barnhart
Iz_8_1209	DJI_0486	Epilobium tetragonum L.	0	n	1	0	Onagraceae Juss.
Iz_8_1209	DJI_0486	Eragrostis minor Host	0	a	0	0	Poaceae Barnhart
Iz_8_1209	DJI_0486	Erigeron canadensis L.	0.3	a	0	0	Asteraceae Bercht. & J.Presl
Iz_8_1209	DJI_0486	Euphorbia seguieriana Neck.	0	n	1	0	Euphorbiaceae Juss.
Iz_8_1209	DJI_0486	Fallopia dumetorum (L.) Holub	0	n	1	0	Polygonaceae Juss.
Iz_8_1209	DJI_0486	Festuca beckeri (Hack.) Trautv.	0	n	1	0	Poaceae Barnhart
Iz_8_1209	DJI_0486	Fragaria vesca L.	0	n	1	0	Rosaceae Juss.
Iz_8_1209	DJI_0486	Frangula alnus Mill.	0	n	1	1	Rhamnaceae Juss.
Iz_8_1209	DJI_0486	Fraxinus pennsylvanica Marshall	0	a	0	1	Oleaceae Hoffmanns. & Link
Iz_8_1209	DJI_0486	Galeopsis bifida Boenn.	0	n	1	0	Lamiacea Lindl.
Iz_8_1209	DJI_0486	Galium verum L.	0	n	1	0	Rubiaceae Juss.
Iz_8_1209	DJI_0486	Genista tinctoria L.	0	n	1	0	Fabaceae Lindl.
Iz_8_1209	DJI_0486	Geranium robertianum L.	0	n	1	0	Geraniaceae Juss.
Iz_8_1209	DJI_0486	Geranium sanquineum L.	0	n	1	0	Geraniaceae Juss.

Iz_8_1209	DJI_0486	Gypsophila paniculata L.	0	n	1	0	Caryophyllaceae Juss.
Iz_8_1209	DJI_0486	Helichrysum arenarium (L.) Moench	0	n	1	0	Asteraceae Bercht. & J.Presl
Iz_8_1209	DJI_0486	Herniaria polygama J.Gay	0	n	1	0	Caryophyllaceae Juss.
Iz_8_1209	DJI_0486	Hieracium robustum Fr.	0	n	1	0	Asteraceae Bercht. & J.Presl
Iz_8_1209	DJI_0486	Hieracium umbellatum L.	0	n	1	NA	Asteraceae Bercht. & J.Presl
Iz_8_1209	DJI_0486	Hylotelephium maximum (L.) Holub	0	n	1	0	Crassulaceae J.St.-Hil.
Iz_8_1209	DJI_0486	Jacobaea borysthenica (DC.) B.Nord. & Greuter	0	n	1	0	Asteraceae Bercht. & J.Presl
Iz_8_1209	DJI_0486	Jasione montana L.	0	n	1	0	Campanulaceae Juss.
Iz_8_1209	DJI_0486	Jurinea cyanoides (L.) Rchb.s.l.	0	n	1	0	Asteraceae Bercht. & J.Presl
Iz_8_1209	DJI_0486	Koeleria glauca (Spreng.) DC.	0	n	1	0	Poaceae Barnhart
Iz_8_1209	DJI_0486	Lactuca serriola L.	0	a	0	0	Asteraceae Bercht. & J.Presl
Iz_8_1209	DJI_0486	Linaria genistifolia (L.) Mill.	0	n	1	0	Plantaginaceae Juss.
Iz_8_1209	DJI_0486	Lomelosia argentea (L.) Greuter & Burdet	0	n	1	0	Caprifoliaceae Juss.
Iz_8_1209	DJI_0486	Melampyrum pratense L.	0	n	1	0	Orobanchaceae Vent.
Iz_8_1209	DJI_0486	Oenothera biennis L.	0	a	0	0	Onagraceae Juss.
Iz_8_1209	DJI_0486	Pentanema britannica (L.) D.Gut.Larr., Santos-Vicente, Anderb., E.Rico & M.M.Mart.Ort.	0	n	1	0	Asteraceae Bercht. & J.Presl
Iz_8_1209	DJI_0486	Peucedanum oreoselinum (L.) Moench	0	n	1	0	Apiaceae Lindl.
Iz_8_1209	DJI_0486	Peucedanum ruthenicum M.Bieb.	0	n	1	0	Apiaceae Lindl.
Iz_8_1209	DJI_0486	Pilosella echioides (Lumn.) F.W.Schultz & Sch.Bip.	0	n	1	0	Asteraceae Bercht. & J.Presl
Iz_8_1209	DJI_0486	Pilosella officinarum F. Schultz et Sch. Bip.	0	n	1	0	Asteraceae Bercht. & J.Presl
Iz_8_1209	DJI_0486	Pilosella sp.	0		NA	0	Asteraceae Bercht. & J.Presl
Iz_8_1209	DJI_0486	Pinus sylvestris L.	0	n	1	1	Pinaceae Spreng. ex F.Rudolphi
Iz_8_1209	DJI_0486	Poa annua L.	0	n	1	0	Poaceae Barnhart
Iz_8_1209	DJI_0486	Poa nemoralis L.	0	n	1	0	Poaceae Barnhart
Iz_8_1209	DJI_0486	Poa sp.	0		NA	0	Poaceae Barnhart
Iz_8_1209	DJI_0486	Polygonatum odoratum (Mill.) Druce	0	n	1	0	Asparagaceae Juss.
Iz_8_1209	DJI_0486	Polygonum arenarium Waldst. & Kit.	0	n	1	0	Polygonaceae Juss.
Iz_8_1209	DJI_0486	Populus nigra L.	0	n	1	1	Salicaceae Mirb.
Iz_8_1209	DJI_0486	Populus tremula L.	0	n	1	1	Salicaceae Mirb.
Iz_8_1209	DJI_0486	Populus Ã canescens (Aiton) Sm.	0	n	1	1	Salicaceae Mirb.
Iz_8_1209	DJI_0486	Potentilla argentea L.	0	n	1	0	Rosaceae Juss.
Iz_8_1209	DJI_0486	Psammophiliella muralis (L.) Ikonn.	0	n	1	0	Caryophyllaceae Juss.
Iz_8_1209	DJI_0486	Psephellus sumensis (Kalen.) Greuter	0	n	1	0	Asteraceae Bercht. & J.Presl
Iz_8_1209	DJI_0486	Pulsatilla pratensis (L.) Mill. s.l.	0	n	1	0	Ranunculaceae Juss.
Iz_8_1209	DJI_0486	Pyrus communis L.	0	a	0	1	Rosaceae Juss.
Iz_8_1209	DJI_0486	Quercus robur L.	0	n	1	1	Fagaceae Dumort.
Iz_8_1209	DJI_0486	Robinia pseudoacacia L.	0	a	0	1	Fabaceae Lindl.
Iz_8_1209	DJI_0486	Rosa sp.	0		NA	1	Rosaceae Juss.
Iz_8_1209	DJI_0486	Rubus idaeus L.	0	n	1	0	Rosaceae Juss.
Iz_8_1209	DJI_0486	Rumex acetosella L.	0	n	1	0	Polygonaceae Juss.
Iz_8_1209	DJI_0486	Rumex thyrsiflorus Fingerh.	0	n	1	0	Polygonaceae Juss.
Iz_8_1209	DJI_0486	Salix alba L.	0	n	1	1	Salicaceae Mirb.
Iz_8_1209	DJI_0486	Sambucus nigra L.	0	n	1	1	Viburnaceae Raf.
Iz_8_1209	DJI_0486	Secale sylvestre Host	0	n	1	0	Poaceae Barnhart
Iz_8_1209	DJI_0486	Sempervivum ruthenicum Schnittsp. & C.B. Lehm.	0	n	1	0	Crassulaceae J.St.-Hil.
Iz_8_1209	DJI_0486	Setaria viridis (L.) P. Beauv.	0	n	1	0	Poaceae Barnhart
Iz_8_1209	DJI_0486	Silene borysthenica (Gruner) Walters	0	n	1	0	Caryophyllaceae Juss.
Iz_8_1209	DJI_0486	Silene latifolia subsp. alba (Mill.) Greuter & Burdet	0	n	1	0	Caryophyllaceae Juss.
Iz_8_1209	DJI_0486	Solidago virgaurea L.	0	n	1	0	Asteraceae Bercht. & J.Presl
Iz_8_1209	DJI_0486	Sorbus aucuparia L.	0	n	1	1	Rosaceae Juss.
Iz_8_1209	DJI_0486	Stachys recta L.	0	n	1	0	Lamiacea Lindl.
Iz_8_1209	DJI_0486	Tanacetum vulgare L.	0	n	1	0	Asteraceae Bercht. & J.Presl
Iz_8_1209	DJI_0486	Thymus pallasianus Heinr.Braun	0	n	1	0	Lamiacea Lindl.
Iz_8_1209	DJI_0486	Tilia cordata Mill.	0	n	1	1	Malvaceae Juss.
Iz_8_1209	DJI_0486	Trifolium alpestre L.	0	n	1	0	Fabaceae Lindl.
Iz_8_1209	DJI_0486	Trifolium arvense L.	0	n	1	0	Fabaceae Lindl.
Iz_8_1209	DJI_0486	Ulmus glabra Huds.	0	n	1	1	Ulmaceae Mirb.
Iz_8_1209	DJI_0486	Verbascum densiflorum Bertol.	0	n	1	0	Scrophulariaceae Juss.
Iz_8_1209	DJI_0486	Veronica austriaca L.	0	n	1	0	Plantaginaceae Juss.
Iz_8_1209	DJI_0486	Veronica incana L.	0	n	1	0	Plantaginaceae Juss.
Iz_8_1209	DJI_0486	Veronica verna L.	0	n	1	0	Plantaginaceae Juss.
Iz_8_1209	DJI_0486	Vincetoxicum hirundinaria Medik	0	n	1	0	Apocynaceae Juss.
Iz_8_1209	DJI_0488	Acer tataricum L.	0	n	1	1	Sapindaceae Juss.
Iz_8_1209	DJI_0488	Achillea millefolium L. s.l.	0	n	1	0	Asteraceae Bercht. & J.Presl
Iz_8_1209	DJI_0488	Allium oleraceum L.	0	n	1	0	Amaryllidaceae J.St.-Hil.
Iz_8_1209	DJI_0488	Ambrosia artemisiifolia L.	0	a	0	0	Asteraceae Bercht. & J.Presl
Iz_8_1209	DJI_0488	Amorpha fruticosa L.	0	a	0	0	Fabaceae Lindl.
Iz_8_1209	DJI_0488	Anthericum ramosum L.	0	n	1	0	Asparagaceae Juss.
Iz_8_1209	DJI_0488	Artemisia marschalliana Spreng.	0	n	1	0	Asteraceae Bercht. & J.Presl
Iz_8_1209	DJI_0488	Asparagus officinalis L.	0	n	1	0	Asparagaceae Juss.
Iz_8_1209	DJI_0488	Astragalus cicer L.	0	n	1	0	Fabaceae Lindl.
Iz_8_1209	DJI_0488	Bassia laniflora (S.G.Gmel.) A.J.Scott	0	n	1	0	Amaranthaceae Juss.
Iz_8_1209	DJI_0488	Berberis vulgaris L.	0	n	1	0	Berberidaceae Juss.
Iz_8_1209	DJI_0488	Betula pendula Roth.	0	n	1	1	Betulaceae Gray
Iz_8_1209	DJI_0488	Betula pendula Roth.	0	n	1	0	Poaceae Barnhart
Iz_8_1209	DJI_0488	Bromus inermis Leyss.	0		NA	NA	
Iz_8_1209	DJI_0488	Bromus squarrosus L.	0	a	0	0	Poaceae Barnhart
Iz_8_1209	DJI_0488	Bromus tectorum L.	0	a	0	0	Poaceae Barnhart
Iz_8_1209	DJI_0488	Calamagrostis epigejos (L.) Roth	0	n	1	0	Poaceae Barnhart
Iz_8_1209	DJI_0488	Carduus acanthoides L.	0	n	1	0	Asteraceae Bercht. & J.Presl
Iz_8_1209	DJI_0488	Carex sp.	0	n	1	0	Cyperaceae Juss.
Iz_8_1209	DJI_0488	Centaurea majorovii Dumbadze	0	n	1	0	Asteraceae Bercht. & J.Presl
Iz_8_1209	DJI_0488	Chamaecytisus ruthenicus (Fisch.ex Wol.) Klaskova	0	n	1	0	Fabaceae Lindl.
Iz_8_1209	DJI_0488	Chelidonium majus L.	0	n	1	0	Papaveraceae Juss.
Iz_8_1209	DJI_0488	Chenopodium betaceum Andrz.	0	n	1	0	Amaranthaceae Juss.
Iz_8_1209	DJI_0488	Chenopodium cf. album	0	n	1	0	Amaranthaceae Juss.
Iz_8_1209	DJI_0488	Chenopodium sp.	0		NA	0	Amaranthaceae Juss.
Iz_8_1209	DJI_0488	Chondrilla juncea L.	0	n	1	0	Asteraceae Bercht. & J.Presl
Iz_8_1209	DJI_0488	Cirsium vulgare (Savi) Ten.	0	n	1	0	Asteraceae Bercht. & J.Presl
Iz_8_1209	DJI_0488	Clinopodium acinos (L.) Kuntze	0	n	1	0	Lamiacea Lindl.
Iz_8_1209	DJI_0488	Convallaria majalis L.	0	n	1	0	Asparagaceae Juss.
Iz_8_1209	DJI_0488	Crataegus rhipidophylla Gand.	0	n	1	1	Rosaceae Juss.
Iz_8_1209	DJI_0488	Crepis foetida L.	0	n	1	0	Asteraceae Bercht. & J.Presl
Iz_8_1209	DJI_0488	Dactylis glomerata L.	0	n	1	0	Poaceae Barnhart
Iz_8_1209	DJI_0488	Dianthus campestris M.Bieb.	0	n	1	0	Caryophyllaceae Juss.
Iz_8_1209	DJI_0488	Digitaria sanguinalis (L.) Scop.	0	a	0	0	Poaceae Barnhart
Iz_8_1209	DJI_0488	Elymus repens (L.) Gould	0	n	1	0	Poaceae Barnhart
Iz_8_1209	DJI_0488	Epilobium tetragonum L.	0	n	1	0	Onagraceae Juss.

Iz_8_1209	DJI_0488	Eragrostis minor Host	0	a	0	0	Poaceae Barnhart
Iz_8_1209	DJI_0488	Erigeron canadensis L.	0	a	0	0	Asteraceae Bercht. & J.Presl
Iz_8_1209	DJI_0488	Euphorbia seguieriana Neck.	0	n	1	0	Euphorbiaceae Juss.
Iz_8_1209	DJI_0488	Fallopia dumetorum (L.) Holub	0	n	1	0	Polygonaceae Juss.
Iz_8_1209	DJI_0488	Festuca beckeri (Hack.) Trautv.	0	n	1	0	Poaceae Barnhart
Iz_8_1209	DJI_0488	Fragaria vesca L.	0	n	1	0	Rosaceae Juss.
Iz_8_1209	DJI_0488	Frangula alnus Mill.	0	n	1	1	Rhamnaceae Juss.
Iz_8_1209	DJI_0488	Fraxinus pennsylvanica Marshall	0	a	0	1	Oleaceae Hoffmanns. & Link
Iz_8_1209	DJI_0488	Galeopsis bifida Boenn.	0	n	1	0	Lamiacea Lindl.
Iz_8_1209	DJI_0488	Galium verum L.	0	n	1	0	Rubiaceae Juss.
Iz_8_1209	DJI_0488	Genista tinctoria L.	0	n	1	0	Fabaceae Lindl.
Iz_8_1209	DJI_0488	Geranium robertianum L.	0	n	1	0	Geraniaceae Juss.
Iz_8_1209	DJI_0488	Geranium sanquineum L.	0	n	1	0	Geraniaceae Juss.
Iz_8_1209	DJI_0488	Gypsophila paniculata L.	0	n	1	0	Caryophyllaceae Juss.
Iz_8_1209	DJI_0488	Helichrysum arenarium (L.) Moench	0	n	1	0	Asteraceae Bercht. & J.Presl
Iz_8_1209	DJI_0488	Herniaria polygama J.Gay	0	n	1	0	Caryophyllaceae Juss.
Iz_8_1209	DJI_0488	Hieracium robustum Fr.	0	n	1	0	Asteraceae Bercht. & J.Presl
Iz_8_1209	DJI_0488	Hieracium umbellatum L.	0	n	1	NA	Asteraceae Bercht. & J.Presl
Iz_8_1209	DJI_0488	Hylotelephium maximum (L.) Holub	0	n	1	0	Crassulaceae J.St.-Hil.
Iz_8_1209	DJI_0488	Jacobaea borysthenica (DC.) B.Nord. & Greuter	0	n	1	0	Asteraceae Bercht. & J.Presl
Iz_8_1209	DJI_0488	Jasione montana L.	0	n	1	0	Campanulaceae Juss.
Iz_8_1209	DJI_0488	Jurinea cyanoides (L.) Rchb.s.l.	0	n	1	0	Asteraceae Bercht. & J.Presl
Iz_8_1209	DJI_0488	Koeleria glauca (Spreng.) DC.	0	n	1	0	Poaceae Barnhart
Iz_8_1209	DJI_0488	Lactuca serriola L.	0	a	0	0	Asteraceae Bercht. & J.Presl
Iz_8_1209	DJI_0488	Linaria genistifolia (L.) Mill.	0	n	1	0	Plantaginaceae Juss.
Iz_8_1209	DJI_0488	Lomelosia argentea (L.) Greuter & Burdet	0	n	1	0	Caprifoliaceae Juss.
Iz_8_1209	DJI_0488	Melampyrum pratense L.	0	n	1	0	Orobanchaceae Vent.
Iz_8_1209	DJI_0488	Oenothera biennis L.	0	a	0	0	Onagraceae Juss.
Iz_8_1209	DJI_0488	Pentanema britannica (L.) D.Gut.Larr., Santos-Vicente, Anderb., E.Rico & M.M.Mart.Ort.	0	n	1	0	Asteraceae Bercht. & J.Presl
Iz_8_1209	DJI_0488	Peucedanum oreoselinum (L.) Moench	0	n	1	0	Apiaceae Lindl.
Iz_8_1209	DJI_0488	Peucedanum ruthenicum M.Bieb.	0	n	1	0	Apiaceae Lindl.
Iz_8_1209	DJI_0488	Pilosella echioides (Lumn.) F.W.Schultz & Sch.Bip.	0	n	1	0	Asteraceae Bercht. & J.Presl
Iz_8_1209	DJI_0488	Pilosella officinarum F. Schultz et Sch. Bip.	0	n	1	0	Asteraceae Bercht. & J.Presl
Iz_8_1209	DJI_0488	Pilosella sp.	0		NA	0	Asteraceae Bercht. & J.Presl
Iz_8_1209	DJI_0488	Pinus sylvestris L.	0	n	1	1	Pinaceae Spreng. ex F.Rudolphi
Iz_8_1209	DJI_0488	Poa annua L.	0	n	1	0	Poaceae Barnhart
Iz_8_1209	DJI_0488	Poa nemoralis L.	0	n	1	0	Poaceae Barnhart
Iz_8_1209	DJI_0488	Poa sp.	0		NA	0	Poaceae Barnhart
Iz_8_1209	DJI_0488	Polygonatum odoratum (Mill.) Druce	0	n	1	0	Asparagaceae Juss.
Iz_8_1209	DJI_0488	Polygonum arenarium Waldst. & Kit.	0	n	1	0	Polygonaceae Juss.
Iz_8_1209	DJI_0488	Populus nigra L.	0	n	1	1	Salicaceae Mirb.
Iz_8_1209	DJI_0488	Populus tremula L.	0	n	1	1	Salicaceae Mirb.
Iz_8_1209	DJI_0488	Populus Ã canescens (Aiton) Sm.	0	n	1	1	Salicaceae Mirb.
Iz_8_1209	DJI_0488	Potentilla argentea L.	0	n	1	0	Rosaceae Juss.
Iz_8_1209	DJI_0488	Psammophiliella muralis (L.) Ikonn.	0	n	1	0	Caryophyllaceae Juss.
Iz_8_1209	DJI_0488	Psephellus sumensis (Kalen.) Greuter	0	n	1	0	Asteraceae Bercht. & J.Presl
Iz_8_1209	DJI_0488	Pulsatilla pratensis (L.) Mill. s.l.	0	n	1	0	Ranunculaceae Juss.
Iz_8_1209	DJI_0488	Pyrus communis L.	0	a	0	1	Rosaceae Juss.
Iz_8_1209	DJI_0488	Quercus robur L.	0	n	1	1	Fagaceae Dumort.
Iz_8_1209	DJI_0488	Robinia pseudoacacia L.	0	a	0	1	Fabaceae Lindl.
Iz_8_1209	DJI_0488	Rosa sp.	0		NA	1	Rosaceae Juss.
Iz_8_1209	DJI_0488	Rubus idaeus L.	0	n	1	0	Rosaceae Juss.
Iz_8_1209	DJI_0488	Rumex acetosella L.	0	n	1	0	Polygonaceae Juss.
Iz_8_1209	DJI_0488	Rumex thyrsiflorus Fingerh.	0	n	1	0	Polygonaceae Juss.
Iz_8_1209	DJI_0488	Salix alba L.	0	n	1	1	Salicaceae Mirb.
Iz_8_1209	DJI_0488	Sambucus nigra L.	0	n	1	1	Viburnaceae Raf.
Iz_8_1209	DJI_0488	Secale sylvestre Host	0	n	1	0	Poaceae Barnhart
Iz_8_1209	DJI_0488	Sempervivum ruthenicum Schnittsp. & C.B. Lehm.	0	n	1	0	Crassulaceae J.St.-Hil.
Iz_8_1209	DJI_0488	Setaria viridis (L.) P. Beauv.	0	n	1	0	Poaceae Barnhart
Iz_8_1209	DJI_0488	Silene borysthenica (Gruner) Walters	0	n	1	0	Caryophyllaceae Juss.
Iz_8_1209	DJI_0488	Silene latifolia subsp. alba (Mill.) Greuter & Burdet	0	n	1	0	Caryophyllaceae Juss.
Iz_8_1209	DJI_0488	Solidago virgaurea L.	0	n	1	0	Asteraceae Bercht. & J.Presl
Iz_8_1209	DJI_0488	Sorbus aucuparia L.	0	n	1	1	Rosaceae Juss.
Iz_8_1209	DJI_0488	Stachys recta L.	0	n	1	0	Lamiacea Lindl.
Iz_8_1209	DJI_0488	Tanacetum vulgare L.	0	n	1	0	Asteraceae Bercht. & J.Presl
Iz_8_1209	DJI_0488	Thymus pallasianus Heinr.Braun	0	n	1	0	Lamiacea Lindl.
Iz_8_1209	DJI_0488	Tilia cordata Mill.	0	n	1	1	Malvaceae Juss.
Iz_8_1209	DJI_0488	Trifolium alpestre L.	0	n	1	0	Fabaceae Lindl.
Iz_8_1209	DJI_0488	Trifolium arvense L.	0	n	1	0	Fabaceae Lindl.
Iz_8_1209	DJI_0488	Ulmus glabra Huds.	0	n	1	1	Ulmaceae Mirb.
Iz_8_1209	DJI_0488	Verbascum densiflorum Bertol.	0	n	1	0	Scrophulariaceae Juss.
Iz_8_1209	DJI_0488	Veronica austriaca L.	0	n	1	0	Plantaginaceae Juss.
Iz_8_1209	DJI_0488	Veronica incana L.	0	n	1	0	Plantaginaceae Juss.
Iz_8_1209	DJI_0488	Veronica verna L.	0	n	1	0	Plantaginaceae Juss.
Iz_8_1209	DJI_0488	Vincetoxicum hirundinaria Medik	0	n	1	0	Apocynaceae Juss.
Iz_8_1209	DJI_0490	Acer tataricum L.	0	n	1	1	Sapindaceae Juss.
Iz_8_1209	DJI_0490	Achillea millefolium L. s.l.	0	n	1	0	Asteraceae Bercht. & J.Presl
Iz_8_1209	DJI_0490	Allium oleraceum L.	0	n	1	0	Amaryllidaceae J.St.-Hil.
Iz_8_1209	DJI_0490	Ambrosia artemisiifolia L.	0	a	0	0	Asteraceae Bercht. & J.Presl
Iz_8_1209	DJI_0490	Amorpha fruticosa L.	0	a	0	0	Fabaceae Lindl.
Iz_8_1209	DJI_0490	Anthericum ramosum L.	0	n	1	0	Asparagaceae Juss.
Iz_8_1209	DJI_0490	Artemisia marschalliana Spreng.	0	n	1	0	Asteraceae Bercht. & J.Presl
Iz_8_1209	DJI_0490	Asparagus officinalis L.	0	n	1	0	Asparagaceae Juss.
Iz_8_1209	DJI_0490	Astragalus cicer L.	0	n	1	0	Fabaceae Lindl.
Iz_8_1209	DJI_0490	Bassia laniflora (S.G.Gmel.) A.J.Scott	0	n	1	0	Amaranthaceae Juss.
Iz_8_1209	DJI_0490	Berberis vulgaris L.	0	n	1	0	Berberidaceae Juss.
Iz_8_1209	DJI_0490	Betula pendula Roth.	0	n	1	1	Betulaceae Gray
Iz_8_1209	DJI_0490	Betula pendula Roth.	0	n	1	0	Poaceae Barnhart
Iz_8_1209	DJI_0490	Bromus inermis Leyss.	0		NA	NA	
Iz_8_1209	DJI_0490	Bromus squarrosus L.	0	a	0	0	Poaceae Barnhart
Iz_8_1209	DJI_0490	Bromus tectorum L.	0	a	0	0	Poaceae Barnhart
Iz_8_1209	DJI_0490	Calamagrostis epigejos (L.) Roth	0	n	1	0	Poaceae Barnhart
Iz_8_1209	DJI_0490	Carduus acanthoides L.	0	n	1	0	Asteraceae Bercht. & J.Presl
Iz_8_1209	DJI_0490	Carex sp.	0	n	1	0	Cyperaceae Juss.
Iz_8_1209	DJI_0490	Centaurea majorovii Dumbadze	0	n	1	0	Asteraceae Bercht. & J.Presl
Iz_8_1209	DJI_0490	Chamaecytisus ruthenicus (Fisch.ex Wol.) Klaskova	0	n	1	0	Fabaceae Lindl.
Iz_8_1209	DJI_0490	Chelidonium majus L.	0	n	1	0	Papaveraceae Juss.
Iz_8_1209	DJI_0490	Chenopodium betaceum Andrz.	0	n	1	0	Amaranthaceae Juss.

Iz_8_1209	DJI_0490	Chenopodium cf. album	0	n	1	0	Amaranthaceae Juss.
Iz_8_1209	DJI_0490	Chenopodium sp.	0		NA	0	Amaranthaceae Juss.
Iz_8_1209	DJI_0490	Chondrilla juncea L.	0	n	1	0	Asteraceae Bercht. & J.Presl
Iz_8_1209	DJI_0490	Cirsium vulgare (Savi) Ten.	0	n	1	0	Asteraceae Bercht. & J.Presl
Iz_8_1209	DJI_0490	Clinopodium acinos (L.) Kuntze	0	n	1	0	Lamiacea Lindl.
Iz_8_1209	DJI_0490	Convallaria majalis L.	0	n	1	0	Asparagaceae Juss.
Iz_8_1209	DJI_0490	Crataegus rhipidophylla Gand.	0	n	1	1	Rosaceae Juss.
Iz_8_1209	DJI_0490	Crepis foetida L.	0	n	1	0	Asteraceae Bercht. & J.Presl
Iz_8_1209	DJI_0490	Dactylis glomerata L.	0	n	1	0	Poaceae Barnhart
Iz_8_1209	DJI_0490	Dianthus campestris M.Bieb.	0	n	1	0	Caryophyllaceae Juss.
Iz_8_1209	DJI_0490	Digitaria sanguinalis (L.) Scop.	0	a	0	0	Poaceae Barnhart
Iz_8_1209	DJI_0490	Elymus repens (L.) Gould	0	n	1	0	Poaceae Barnhart
Iz_8_1209	DJI_0490	Epilobium tetragonum L.	0	n	1	0	Onagraceae Juss.
Iz_8_1209	DJI_0490	Eragrostis minor Host	0	a	0	0	Poaceae Barnhart
Iz_8_1209	DJI_0490	Erigeron canadensis L.	0	a	0	0	Asteraceae Bercht. & J.Presl
Iz_8_1209	DJI_0490	Euphorbia seguieriana Neck.	0	n	1	0	Euphorbiaceae Juss.
Iz_8_1209	DJI_0490	Fallopia dumetorum (L.) Holub	0	n	1	0	Polygonaceae Juss.
Iz_8_1209	DJI_0490	Festuca beckeri (Hack.) Trautv.	0	n	1	0	Poaceae Barnhart
Iz_8_1209	DJI_0490	Fragaria vesca L.	0	n	1	0	Rosaceae Juss.
Iz_8_1209	DJI_0490	Frangula alnus Mill.	0	n	1	1	Rhamnaceae Juss.
Iz_8_1209	DJI_0490	Fraxinus pennsylvanica Marshall	0	a	0	1	Oleaceae Hoffmanns. & Link
Iz_8_1209	DJI_0490	Galeopsis bifida Boenn.	0	n	1	0	Lamiacea Lindl.
Iz_8_1209	DJI_0490	Galium verum L.	0	n	1	0	Rubiaceae Juss.
Iz_8_1209	DJI_0490	Genista tinctoria L.	0	n	1	0	Fabaceae Lindl.
Iz_8_1209	DJI_0490	Geranium robertianum L.	0	n	1	0	Geraniaceae Juss.
Iz_8_1209	DJI_0490	Geranium sanquineum L.	0	n	1	0	Geraniaceae Juss.
Iz_8_1209	DJI_0490	Gypsophila paniculata L.	0	n	1	0	Caryophyllaceae Juss.
Iz_8_1209	DJI_0490	Helichrysum arenarium (L.) Moench	0	n	1	0	Asteraceae Bercht. & J.Presl
Iz_8_1209	DJI_0490	Herniaria polygama J.Gay	0	n	1	0	Caryophyllaceae Juss.
Iz_8_1209	DJI_0490	Hieracium robustum Fr.	0	n	1	0	Asteraceae Bercht. & J.Presl
Iz_8_1209	DJI_0490	Hieracium umbellatum L.	0	n	1	NA	Asteraceae Bercht. & J.Presl
Iz_8_1209	DJI_0490	Hylotelephium maximum (L.) Holub	0	n	1	0	Crassulaceae J.St.-Hil.
Iz_8_1209	DJI_0490	Jacobaea borysthenica (DC.) B.Nord. & Greuter	0	n	1	0	Asteraceae Bercht. & J.Presl
Iz_8_1209	DJI_0490	Jasione montana L.	0	n	1	0	Campanulaceae Juss.
Iz_8_1209	DJI_0490	Jurinea cyanoides (L.) Rchb.s.l.	0	n	1	0	Asteraceae Bercht. & J.Presl
Iz_8_1209	DJI_0490	Koeleria glauca (Spreng.) DC.	0	n	1	0	Poaceae Barnhart
Iz_8_1209	DJI_0490	Lactuca serriola L.	0	a	0	0	Asteraceae Bercht. & J.Presl
Iz_8_1209	DJI_0490	Linaria genistifolia (L.) Mill.	0	n	1	0	Plantaginaceae Juss.
Iz_8_1209	DJI_0490	Lomelosia argentea (L.) Greuter & Burdet	0	n	1	0	Caprifoliaceae Juss.
Iz_8_1209	DJI_0490	Melampyrum pratense L.	0	n	1	0	Orobanchaceae Vent.
Iz_8_1209	DJI_0490	Oenothera biennis L.	0	a	0	0	Onagraceae Juss.
Iz_8_1209	DJI_0490	Pentanema britannica (L.) D.Gut.Larr., Santos-Vicente, Anderb., E.Rico & M.M.Mart.Ort.	0	n	1	0	Asteraceae Bercht. & J.Presl
Iz_8_1209	DJI_0490	Peucedanum oreoselinum (L.) Moench	0	n	1	0	Apiaceae Lindl.
Iz_8_1209	DJI_0490	Peucedanum ruthenicum M.Bieb.	0	n	1	0	Apiaceae Lindl.
Iz_8_1209	DJI_0490	Pilosella echioides (Lumn.) F.W.Schultz & Sch.Bip.	0	n	1	0	Asteraceae Bercht. & J.Presl
Iz_8_1209	DJI_0490	Pilosella officinarum F. Schultz et Sch. Bip.	0	n	1	0	Asteraceae Bercht. & J.Presl
Iz_8_1209	DJI_0490	Pilosella sp.	0		NA	0	Asteraceae Bercht. & J.Presl
Iz_8_1209	DJI_0490	Pinus sylvestris L.	0	n	1	1	Pinaceae Spreng. ex F.Rudolphi
Iz_8_1209	DJI_0490	Poa annua L.	0	n	1	0	Poaceae Barnhart
Iz_8_1209	DJI_0490	Poa nemoralis L.	0	n	1	0	Poaceae Barnhart
Iz_8_1209	DJI_0490	Poa sp.	0		NA	0	Poaceae Barnhart
Iz_8_1209	DJI_0490	Polygonatum odoratum (Mill.) Druce	0	n	1	0	Asparagaceae Juss.
Iz_8_1209	DJI_0490	Polygonum arenarium Waldst. & Kit.	0	n	1	0	Polygonaceae Juss.
Iz_8_1209	DJI_0490	Populus nigra L.	0	n	1	1	Salicaceae Mirb.
Iz_8_1209	DJI_0490	Populus tremula L.	0	n	1	1	Salicaceae Mirb.
Iz_8_1209	DJI_0490	Populus Ã canescens (Aiton) Sm.	0	n	1	1	Salicaceae Mirb.
Iz_8_1209	DJI_0490	Potentilla argentea L.	0	n	1	0	Rosaceae Juss.
Iz_8_1209	DJI_0490	Psammophiliella muralis (L.) Ikonn.	0	n	1	0	Caryophyllaceae Juss.
Iz_8_1209	DJI_0490	Psephellus sumensis (Kalen.) Greuter	0	n	1	0	Asteraceae Bercht. & J.Presl
Iz_8_1209	DJI_0490	Pulsatilla pratensis (L.) Mill. s.l.	0	n	1	0	Ranunculaceae Juss.
Iz_8_1209	DJI_0490	Pyrus communis L.	0	a	0	1	Rosaceae Juss.
Iz_8_1209	DJI_0490	Quercus robur L.	0	n	1	1	Fagaceae Dumort.
Iz_8_1209	DJI_0490	Robinia pseudoacacia L.	0	a	0	1	Fabaceae Lindl.
Iz_8_1209	DJI_0490	Rosa sp.	0		NA	1	Rosaceae Juss.
Iz_8_1209	DJI_0490	Rubus idaeus L.	0	n	1	0	Rosaceae Juss.
Iz_8_1209	DJI_0490	Rumex acetosella L.	0	n	1	0	Polygonaceae Juss.
Iz_8_1209	DJI_0490	Rumex thyrsiflorus Fingerh.	0	n	1	0	Polygonaceae Juss.
Iz_8_1209	DJI_0490	Salix alba L.	0	n	1	1	Salicaceae Mirb.
Iz_8_1209	DJI_0490	Sambucus nigra L.	0	n	1	1	Viburnaceae Raf.
Iz_8_1209	DJI_0490	Secale sylvestre Host	0	n	1	0	Poaceae Barnhart
Iz_8_1209	DJI_0490	Sempervivum ruthenicum Schnittsp. & C.B. Lehm.	0	n	1	0	Crassulaceae J.St.-Hil.
Iz_8_1209	DJI_0490	Setaria viridis (L.) P. Beauv.	0	n	1	0	Poaceae Barnhart
Iz_8_1209	DJI_0490	Silene borysthenica (Gruner) Walters	0	n	1	0	Caryophyllaceae Juss.
Iz_8_1209	DJI_0490	Silene latifolia subsp. alba (Mill.) Greuter & Burdet	0	n	1	0	Caryophyllaceae Juss.
Iz_8_1209	DJI_0490	Solidago virgaurea L.	0	n	1	0	Asteraceae Bercht. & J.Presl
Iz_8_1209	DJI_0490	Sorbus aucuparia L.	0	n	1	1	Rosaceae Juss.
Iz_8_1209	DJI_0490	Stachys recta L.	0	n	1	0	Lamiacea Lindl.
Iz_8_1209	DJI_0490	Tanacetum vulgare L.	0	n	1	0	Asteraceae Bercht. & J.Presl
Iz_8_1209	DJI_0490	Thymus pallasianus Heinr.Braun	0	n	1	0	Lamiacea Lindl.
Iz_8_1209	DJI_0490	Tilia cordata Mill.	0	n	1	1	Malvaceae Juss.
Iz_8_1209	DJI_0490	Trifolium alpestre L.	0	n	1	0	Fabaceae Lindl.
Iz_8_1209	DJI_0490	Trifolium arvense L.	0	n	1	0	Fabaceae Lindl.
Iz_8_1209	DJI_0490	Ulmus glabra Huds.	0	n	1	1	Ulmaceae Mirb.
Iz_8_1209	DJI_0490	Verbascum densiflorum Bertol.	0	n	1	0	Scrophulariaceae Juss.
Iz_8_1209	DJI_0490	Veronica austriaca L.	0	n	1	0	Plantaginaceae Juss.
Iz_8_1209	DJI_0490	Veronica incana L.	0	n	1	0	Plantaginaceae Juss.
Iz_8_1209	DJI_0490	Veronica verna L.	0	n	1	0	Plantaginaceae Juss.
Iz_8_1209	DJI_0490	Vincetoxicum hirundinaria Medik	0	n	1	0	Apocynaceae Juss.
Iz_8_1209	DJI_0493	Acer tataricum L.	0	n	1	1	Sapindaceae Juss.
Iz_8_1209	DJI_0493	Achillea millefolium L. s.l.	0	n	1	0	Asteraceae Bercht. & J.Presl
Iz_8_1209	DJI_0493	Allium oleraceum L.	0	n	1	0	Amaryllidaceae J.St.-Hil.
Iz_8_1209	DJI_0493	Ambrosia artemisiifolia L.	0	a	0	0	Asteraceae Bercht. & J.Presl
Iz_8_1209	DJI_0493	Amorpha fruticosa L.	0	a	0	0	Fabaceae Lindl.
Iz_8_1209	DJI_0493	Anthericum ramosum L.	0	n	1	0	Asparagaceae Juss.
Iz_8_1209	DJI_0493	Artemisia marschalliana Spreng.	0	n	1	0	Asteraceae Bercht. & J.Presl
Iz_8_1209	DJI_0493	Asparagus officinalis L.	0	n	1	0	Asparagaceae Juss.
Iz_8_1209	DJI_0493	Astragalus cicer L.	0	n	1	0	Fabaceae Lindl.
Iz_8_1209	DJI_0493	Bassia laniflora (S.G.Gmel.) A.J.Scott	0	n	1	0	Amaranthaceae Juss.

Iz_8_1209	DJI_0493	Berberis vulgaris L.	0	n	1	0	Berberidaceae Juss.
Iz_8_1209	DJI_0493	Betula pendula Roth.	0	n	1	1	Betulaceae Gray
Iz_8_1209	DJI_0493	Betula pendula Roth.	0	n	1	0	Poaceae Barnhart
Iz_8_1209	DJI_0493	Bromus inermis Leyss.	0		NA	NA	
Iz_8_1209	DJI_0493	Bromus squarrosus L.	0	a	0	0	Poaceae Barnhart
Iz_8_1209	DJI_0493	Bromus tectorum L.	0	a	0	0	Poaceae Barnhart
Iz_8_1209	DJI_0493	Calamagrostis epigejos (L.) Roth	0	n	1	0	Poaceae Barnhart
Iz_8_1209	DJI_0493	Carduus acanthoides L.	0	n	1	0	Asteraceae Bercht. & J.Presl
Iz_8_1209	DJI_0493	Carex sp.	0	n	1	0	Cyperaceae Juss.
Iz_8_1209	DJI_0493	Centaurea majorovii Dumbadze	0	n	1	0	Asteraceae Bercht. & J.Presl
Iz_8_1209	DJI_0493	Chamaecytisus ruthenicus (Fisch.ex Wol.) Klaskova	0	n	1	0	Fabaceae Lindl.
Iz_8_1209	DJI_0493	Chelidonium majus L.	0	n	1	0	Papaveraceae Juss.
Iz_8_1209	DJI_0493	Chenopodium betaceum Andrz.	0	n	1	0	Amaranthaceae Juss.
Iz_8_1209	DJI_0493	Chenopodium cf. album	0	n	1	0	Amaranthaceae Juss.
Iz_8_1209	DJI_0493	Chenopodium sp.	0		NA	0	Amaranthaceae Juss.
Iz_8_1209	DJI_0493	Chondrilla juncea L.	0	n	1	0	Asteraceae Bercht. & J.Presl
Iz_8_1209	DJI_0493	Cirsium vulgare (Savi) Ten.	0	n	1	0	Asteraceae Bercht. & J.Presl
Iz_8_1209	DJI_0493	Clinopodium acinos (L.) Kuntze	0	n	1	0	Lamiacea Lindl.
Iz_8_1209	DJI_0493	Convallaria majalis L.	0	n	1	0	Asparagaceae Juss.
Iz_8_1209	DJI_0493	Crataegus rhipidophylla Gand.	0	n	1	1	Rosaceae Juss.
Iz_8_1209	DJI_0493	Crepis foetida L.	0	n	1	0	Asteraceae Bercht. & J.Presl
Iz_8_1209	DJI_0493	Dactylis glomerata L.	0	n	1	0	Poaceae Barnhart
Iz_8_1209	DJI_0493	Dianthus campestris M.Bieb.	0	n	1	0	Caryophyllaceae Juss.
Iz_8_1209	DJI_0493	Digitaria sanguinalis (L.) Scop.	0	a	0	0	Poaceae Barnhart
Iz_8_1209	DJI_0493	Elymus repens (L.) Gould	0	n	1	0	Poaceae Barnhart
Iz_8_1209	DJI_0493	Epilobium tetragonum L.	0	n	1	0	Onagraceae Juss.
Iz_8_1209	DJI_0493	Eragrostis minor Host	0	a	0	0	Poaceae Barnhart
Iz_8_1209	DJI_0493	Erigeron canadensis L.	0	a	0	0	Asteraceae Bercht. & J.Presl
Iz_8_1209	DJI_0493	Euphorbia seguieriana Neck.	0	n	1	0	Euphorbiaceae Juss.
Iz_8_1209	DJI_0493	Fallopia dumetorum (L.) Holub	0	n	1	0	Polygonaceae Juss.
Iz_8_1209	DJI_0493	Festuca beckeri (Hack.) Trautv.	0	n	1	0	Poaceae Barnhart
Iz_8_1209	DJI_0493	Fragaria vesca L.	0	n	1	0	Rosaceae Juss.
Iz_8_1209	DJI_0493	Frangula alnus Mill.	0	n	1	1	Rhamnaceae Juss.
Iz_8_1209	DJI_0493	Fraxinus pennsylvanica Marshall	0	a	0	1	Oleaceae Hoffmanns. & Link
Iz_8_1209	DJI_0493	Galeopsis bifida Boenn.	0	n	1	0	Lamiacea Lindl.
Iz_8_1209	DJI_0493	Galium verum L.	0	n	1	0	Rubiaceae Juss.
Iz_8_1209	DJI_0493	Genista tinctoria L.	0	n	1	0	Fabaceae Lindl.
Iz_8_1209	DJI_0493	Geranium robertianum L.	0	n	1	0	Geraniaceae Juss.
Iz_8_1209	DJI_0493	Geranium sanquineum L.	0	n	1	0	Geraniaceae Juss.
Iz_8_1209	DJI_0493	Gypsophila paniculata L.	0	n	1	0	Caryophyllaceae Juss.
Iz_8_1209	DJI_0493	Helichrysum arenarium (L.) Moench	0	n	1	0	Asteraceae Bercht. & J.Presl
Iz_8_1209	DJI_0493	Herniaria polygama J.Gay	0	n	1	0	Caryophyllaceae Juss.
Iz_8_1209	DJI_0493	Hieracium robustum Fr.	0	n	1	0	Asteraceae Bercht. & J.Presl
Iz_8_1209	DJI_0493	Hieracium umbellatum L.	0	n	1	NA	Asteraceae Bercht. & J.Presl
Iz_8_1209	DJI_0493	Hylotelephium maximum (L.) Holub	0	n	1	0	Crassulaceae J.St.-Hil.
Iz_8_1209	DJI_0493	Jacobaea borysthenica (DC.) B.Nord. & Greuter	0	n	1	0	Asteraceae Bercht. & J.Presl
Iz_8_1209	DJI_0493	Jasione montana L.	0	n	1	0	Campanulaceae Juss.
Iz_8_1209	DJI_0493	Jurinea cyanoides (L.) Rchb.s.l.	0	n	1	0	Asteraceae Bercht. & J.Presl
Iz_8_1209	DJI_0493	Koeleria glauca (Spreng.) DC.	0	n	1	0	Poaceae Barnhart
Iz_8_1209	DJI_0493	Lactuca serriola L.	0	a	0	0	Asteraceae Bercht. & J.Presl
Iz_8_1209	DJI_0493	Linaria genistifolia (L.) Mill.	0	n	1	0	Plantaginaceae Juss.
Iz_8_1209	DJI_0493	Lomelosia argentea (L.) Greuter & Burdet	0	n	1	0	Caprifoliaceae Juss.
Iz_8_1209	DJI_0493	Melampyrum pratense L.	0	n	1	0	Orobanchaceae Vent.
Iz_8_1209	DJI_0493	Oenothera biennis L.	0	a	0	0	Onagraceae Juss.
Iz_8_1209	DJI_0493	Pentanema britannica (L.) D.Gut.Larr., Santos-Vicente, Anderb., E.Rico & M.M.Mart.Ort.	0	n	1	0	Asteraceae Bercht. & J.Presl
Iz_8_1209	DJI_0493	Peucedanum oreoselinum (L.) Moench	0	n	1	0	Apiaceae Lindl.
Iz_8_1209	DJI_0493	Peucedanum ruthenicum M.Bieb.	0	n	1	0	Apiaceae Lindl.
Iz_8_1209	DJI_0493	Pilosella echioides (Lumn.) F.W.Schultz & Sch.Bip.	0	n	1	0	Asteraceae Bercht. & J.Presl
Iz_8_1209	DJI_0493	Pilosella officinarum F. Schultz et Sch. Bip.	0	n	1	0	Asteraceae Bercht. & J.Presl
Iz_8_1209	DJI_0493	Pilosella sp.	0		NA	0	Asteraceae Bercht. & J.Presl
Iz_8_1209	DJI_0493	Pinus sylvestris L.	0	n	1	1	Pinaceae Spreng. ex F.Rudolphi
Iz_8_1209	DJI_0493	Poa annua L.	0	n	1	0	Poaceae Barnhart
Iz_8_1209	DJI_0493	Poa nemoralis L.	0	n	1	0	Poaceae Barnhart
Iz_8_1209	DJI_0493	Poa sp.	0		NA	0	Poaceae Barnhart
Iz_8_1209	DJI_0493	Polygonatum odoratum (Mill.) Druce	0	n	1	0	Asparagaceae Juss.
Iz_8_1209	DJI_0493	Polygonum arenarium Waldst. & Kit.	0	n	1	0	Polygonaceae Juss.
Iz_8_1209	DJI_0493	Populus nigra L.	0	n	1	1	Salicaceae Mirb.
Iz_8_1209	DJI_0493	Populus tremula L.	0	n	1	1	Salicaceae Mirb.
Iz_8_1209	DJI_0493	Populus Ã canescens (Aiton) Sm.	0	n	1	1	Salicaceae Mirb.
Iz_8_1209	DJI_0493	Potentilla argentea L.	0	n	1	0	Rosaceae Juss.
Iz_8_1209	DJI_0493	Psammophiliella muralis (L.) Ikonn.	0	n	1	0	Caryophyllaceae Juss.
Iz_8_1209	DJI_0493	Psephellus sumensis (Kalen.) Greuter	0	n	1	0	Asteraceae Bercht. & J.Presl
Iz_8_1209	DJI_0493	Pulsatilla pratensis (L.) Mill. s.l.	0	n	1	0	Ranunculaceae Juss.
Iz_8_1209	DJI_0493	Pyrus communis L.	0	a	0	1	Rosaceae Juss.
Iz_8_1209	DJI_0493	Quercus robur L.	0	n	1	1	Fagaceae Dumort.
Iz_8_1209	DJI_0493	Robinia pseudoacacia L.	0	a	0	1	Fabaceae Lindl.
Iz_8_1209	DJI_0493	Rosa sp.	0		NA	1	Rosaceae Juss.
Iz_8_1209	DJI_0493	Rubus idaeus L.	0	n	1	0	Rosaceae Juss.
Iz_8_1209	DJI_0493	Rumex acetosella L.	0	n	1	0	Polygonaceae Juss.
Iz_8_1209	DJI_0493	Rumex thyrsiflorus Fingerh.	0	n	1	0	Polygonaceae Juss.
Iz_8_1209	DJI_0493	Salix alba L.	0	n	1	1	Salicaceae Mirb.
Iz_8_1209	DJI_0493	Sambucus nigra L.	0	n	1	1	Viburnaceae Raf.
Iz_8_1209	DJI_0493	Secale sylvestre Host	0	n	1	0	Poaceae Barnhart
Iz_8_1209	DJI_0493	Sempervivum ruthenicum Schnittsp. & C.B. Lehm.	0	n	1	0	Crassulaceae J.St.-Hil.
Iz_8_1209	DJI_0493	Setaria viridis (L.) P. Beauv.	0	n	1	0	Poaceae Barnhart
Iz_8_1209	DJI_0493	Silene borysthenica (Gruner) Walters	0	n	1	0	Caryophyllaceae Juss.
Iz_8_1209	DJI_0493	Silene latifolia subsp. alba (Mill.) Greuter & Burdet	0	n	1	0	Caryophyllaceae Juss.
Iz_8_1209	DJI_0493	Solidago virgaurea L.	0	n	1	0	Asteraceae Bercht. & J.Presl
Iz_8_1209	DJI_0493	Sorbus aucuparia L.	0	n	1	1	Rosaceae Juss.
Iz_8_1209	DJI_0493	Stachys recta L.	0	n	1	0	Lamiacea Lindl.
Iz_8_1209	DJI_0493	Tanacetum vulgare L.	0	n	1	0	Asteraceae Bercht. & J.Presl
Iz_8_1209	DJI_0493	Thymus pallasianus Heinr.Braun	0	n	1	0	Lamiacea Lindl.
Iz_8_1209	DJI_0493	Tilia cordata Mill.	0	n	1	1	Malvaceae Juss.
Iz_8_1209	DJI_0493	Trifolium alpestre L.	0	n	1	0	Fabaceae Lindl.
Iz_8_1209	DJI_0493	Trifolium arvense L.	0	n	1	0	Fabaceae Lindl.
Iz_8_1209	DJI_0493	Ulmus glabra Huds.	0	n	1	1	Ulmaceae Mirb.
Iz_8_1209	DJI_0493	Verbascum densiflorum Bertol.	0	n	1	0	Scrophulariaceae Juss.
Iz_8_1209	DJI_0493	Veronica austriaca L.	0	n	1	0	Plantaginaceae Juss.

Iz_8_1209	DJI_0493	Veronica incana L.	0	n	1	0	Plantaginaceae Juss.
Iz_8_1209	DJI_0493	Veronica verna L.	0	n	1	0	Plantaginaceae Juss.
Iz_8_1209	DJI_0493	Vincetoxicum hirundinaria Medik	0	n	1	0	Apocynaceae Juss.
Iz_8_1209	DJI_0497	Acer tataricum L.	0	n	1	1	Sapindaceae Juss.
Iz_8_1209	DJI_0497	Achillea millefolium L. s.l.	0	n	1	0	Asteraceae Bercht. & J.Presl
Iz_8_1209	DJI_0497	Allium oleraceum L.	0	n	1	0	Amaryllidaceae J.St.-Hil.
Iz_8_1209	DJI_0497	Ambrosia artemisiifolia L.	0	a	0	0	Asteraceae Bercht. & J.Presl
Iz_8_1209	DJI_0497	Amorpha fruticosa L.	0	a	0	0	Fabaceae Lindl.
Iz_8_1209	DJI_0497	Anthericum ramosum L.	0	n	1	0	Asparagaceae Juss.
Iz_8_1209	DJI_0497	Artemisia marschalliana Spreng.	0	n	1	0	Asteraceae Bercht. & J.Presl
Iz_8_1209	DJI_0497	Asparagus officinalis L.	0	n	1	0	Asparagaceae Juss.
Iz_8_1209	DJI_0497	Astragalus cicer L.	0	n	1	0	Fabaceae Lindl.
Iz_8_1209	DJI_0497	Bassia laniflora (S.G.Gmel.) A.J.Scott	0	n	1	0	Amaranthaceae Juss.
Iz_8_1209	DJI_0497	Berberis vulgaris L.	0	n	1	0	Berberidaceae Juss.
Iz_8_1209	DJI_0497	Betula pendula Roth.	0	n	1	1	Betulaceae Gray
Iz_8_1209	DJI_0497	Betula pendula Roth.	0	n	1	0	Poaceae Barnhart
Iz_8_1209	DJI_0497	Bromus inermis Leyss.	0		NA	NA	
Iz_8_1209	DJI_0497	Bromus squarrosus L.	0	a	0	0	Poaceae Barnhart
Iz_8_1209	DJI_0497	Bromus tectorum L.	0	a	0	0	Poaceae Barnhart
Iz_8_1209	DJI_0497	Calamagrostis epigejos (L.) Roth	0	n	1	0	Poaceae Barnhart
Iz_8_1209	DJI_0497	Carduus acanthoides L.	0	n	1	0	Asteraceae Bercht. & J.Presl
Iz_8_1209	DJI_0497	Carex sp.	0	n	1	0	Cyperaceae Juss.
Iz_8_1209	DJI_0497	Centaurea majorovii Dumbadze	0	n	1	0	Asteraceae Bercht. & J.Presl
Iz_8_1209	DJI_0497	Chamaecytisus ruthenicus (Fisch.ex Wol.) Klaskova	0	n	1	0	Fabaceae Lindl.
Iz_8_1209	DJI_0497	Chelidonium majus L.	0	n	1	0	Papaveraceae Juss.
Iz_8_1209	DJI_0497	Chenopodium betaceum Andrz.	0	n	1	0	Amaranthaceae Juss.
Iz_8_1209	DJI_0497	Chenopodium cf. album	0	n	1	0	Amaranthaceae Juss.
Iz_8_1209	DJI_0497	Chenopodium sp.	0		NA	0	Amaranthaceae Juss.
Iz_8_1209	DJI_0497	Chondrilla juncea L.	0	n	1	0	Asteraceae Bercht. & J.Presl
Iz_8_1209	DJI_0497	Cirsium vulgare (Savi) Ten.	0	n	1	0	Asteraceae Bercht. & J.Presl
Iz_8_1209	DJI_0497	Clinopodium acinos (L.) Kuntze	0	n	1	0	Lamiacea Lindl.
Iz_8_1209	DJI_0497	Convallaria majalis L.	0	n	1	0	Asparagaceae Juss.
Iz_8_1209	DJI_0497	Crataegus rhipidophylla Gand.	0	n	1	1	Rosaceae Juss.
Iz_8_1209	DJI_0497	Crepis foetida L.	0	n	1	0	Asteraceae Bercht. & J.Presl
Iz_8_1209	DJI_0497	Dactylis glomerata L.	0	n	1	0	Poaceae Barnhart
Iz_8_1209	DJI_0497	Dianthus campestris M.Bieb.	0	n	1	0	Caryophyllaceae Juss.
Iz_8_1209	DJI_0497	Digitaria sanguinalis (L.) Scop.	0	a	0	0	Poaceae Barnhart
Iz_8_1209	DJI_0497	Elymus repens (L.) Gould	0	n	1	0	Poaceae Barnhart
Iz_8_1209	DJI_0497	Epilobium tetragonum L.	0	n	1	0	Onagraceae Juss.
Iz_8_1209	DJI_0497	Eragrostis minor Host	0	a	0	0	Poaceae Barnhart
Iz_8_1209	DJI_0497	Erigeron canadensis L.	0	a	0	0	Asteraceae Bercht. & J.Presl
Iz_8_1209	DJI_0497	Euphorbia seguieriana Neck.	0	n	1	0	Euphorbiaceae Juss.
Iz_8_1209	DJI_0497	Fallopia dumetorum (L.) Holub	0	n	1	0	Polygonaceae Juss.
Iz_8_1209	DJI_0497	Festuca beckeri (Hack.) Trautv.	0	n	1	0	Poaceae Barnhart
Iz_8_1209	DJI_0497	Fragaria vesca L.	0	n	1	0	Rosaceae Juss.
Iz_8_1209	DJI_0497	Frangula alnus Mill.	0	n	1	1	Rhamnaceae Juss.
Iz_8_1209	DJI_0497	Fraxinus pennsylvanica Marshall	0	a	0	1	Oleaceae Hoffmanns. & Link
Iz_8_1209	DJI_0497	Galeopsis bifida Boenn.	0	n	1	0	Lamiacea Lindl.
Iz_8_1209	DJI_0497	Galium verum L.	0	n	1	0	Rubiaceae Juss.
Iz_8_1209	DJI_0497	Genista tinctoria L.	0	n	1	0	Fabaceae Lindl.
Iz_8_1209	DJI_0497	Geranium robertianum L.	0	n	1	0	Geraniaceae Juss.
Iz_8_1209	DJI_0497	Geranium sanquineum L.	0	n	1	0	Geraniaceae Juss.
Iz_8_1209	DJI_0497	Gypsophila paniculata L.	0	n	1	0	Caryophyllaceae Juss.
Iz_8_1209	DJI_0497	Helichrysum arenarium (L.) Moench	0	n	1	0	Asteraceae Bercht. & J.Presl
Iz_8_1209	DJI_0497	Herniaria polygama J.Gay	0	n	1	0	Caryophyllaceae Juss.
Iz_8_1209	DJI_0497	Hieracium robustum Fr.	0	n	1	0	Asteraceae Bercht. & J.Presl
Iz_8_1209	DJI_0497	Hieracium umbellatum L.	0	n	1	NA	Asteraceae Bercht. & J.Presl
Iz_8_1209	DJI_0497	Hylotelephium maximum (L.) Holub	0	n	1	0	Crassulaceae J.St.-Hil.
Iz_8_1209	DJI_0497	Jacobaea borysthenica (DC.) B.Nord. & Greuter	0	n	1	0	Asteraceae Bercht. & J.Presl
Iz_8_1209	DJI_0497	Jasione montana L.	0	n	1	0	Campanulaceae Juss.
Iz_8_1209	DJI_0497	Jurinea cyanoides (L.) Rchb.s.l.	0	n	1	0	Asteraceae Bercht. & J.Presl
Iz_8_1209	DJI_0497	Koeleria glauca (Spreng.) DC.	0	n	1	0	Poaceae Barnhart
Iz_8_1209	DJI_0497	Lactuca serriola L.	0	a	0	0	Asteraceae Bercht. & J.Presl
Iz_8_1209	DJI_0497	Linaria genistifolia (L.) Mill.	0	n	1	0	Plantaginaceae Juss.
Iz_8_1209	DJI_0497	Lomelosia argentea (L.) Greuter & Burdet	0	n	1	0	Caprifoliaceae Juss.
Iz_8_1209	DJI_0497	Melampyrum pratense L.	0	n	1	0	Orobanchaceae Vent.
Iz_8_1209	DJI_0497	Oenothera biennis L.	0	a	0	0	Onagraceae Juss.
Iz_8_1209	DJI_0497	Pentanema britannica (L.) D.Gut.Larr., Santos-Vicente, Anderb., E.Rico & M.M.Mart.Ort.	0	n	1	0	Asteraceae Bercht. & J.Presl
Iz_8_1209	DJI_0497	Peucedanum oreoselinum (L.) Moench	0	n	1	0	Apiaceae Lindl.
Iz_8_1209	DJI_0497	Peucedanum ruthenicum M.Bieb.	0	n	1	0	Apiaceae Lindl.
Iz_8_1209	DJI_0497	Pilosella echioides (Lumn.) F.W.Schultz & Sch.Bip.	0	n	1	0	Asteraceae Bercht. & J.Presl
Iz_8_1209	DJI_0497	Pilosella officinarum F. Schultz et Sch. Bip.	0	n	1	0	Asteraceae Bercht. & J.Presl
Iz_8_1209	DJI_0497	Pilosella sp.	0		NA	0	Asteraceae Bercht. & J.Presl
Iz_8_1209	DJI_0497	Pinus sylvestris L.	0	n	1	1	Pinaceae Spreng. ex F.Rudolphi
Iz_8_1209	DJI_0497	Poa annua L.	0	n	1	0	Poaceae Barnhart
Iz_8_1209	DJI_0497	Poa nemoralis L.	0	n	1	0	Poaceae Barnhart
Iz_8_1209	DJI_0497	Poa sp.	0		NA	0	Poaceae Barnhart
Iz_8_1209	DJI_0497	Polygonatum odoratum (Mill.) Druce	0	n	1	0	Asparagaceae Juss.
Iz_8_1209	DJI_0497	Polygonum arenarium Waldst. & Kit.	0	n	1	0	Polygonaceae Juss.
Iz_8_1209	DJI_0497	Populus nigra L.	0	n	1	1	Salicaceae Mirb.
Iz_8_1209	DJI_0497	Populus tremula L.	0	n	1	1	Salicaceae Mirb.
Iz_8_1209	DJI_0497	Populus Ã canescens (Aiton) Sm.	0	n	1	1	Salicaceae Mirb.
Iz_8_1209	DJI_0497	Potentilla argentea L.	0	n	1	0	Rosaceae Juss.
Iz_8_1209	DJI_0497	Psammophiliella muralis (L.) Ikonn.	0	n	1	0	Caryophyllaceae Juss.
Iz_8_1209	DJI_0497	Psephellus sumensis (Kalen.) Greuter	0	n	1	0	Asteraceae Bercht. & J.Presl
Iz_8_1209	DJI_0497	Pulsatilla pratensis (L.) Mill. s.l.	0	n	1	0	Ranunculaceae Juss.
Iz_8_1209	DJI_0497	Pyrus communis L.	0	a	0	1	Rosaceae Juss.
Iz_8_1209	DJI_0497	Quercus robur L.	0	n	1	1	Fagaceae Dumort.
Iz_8_1209	DJI_0497	Robinia pseudoacacia L.	0	a	0	1	Fabaceae Lindl.
Iz_8_1209	DJI_0497	Rosa sp.	0		NA	1	Rosaceae Juss.
Iz_8_1209	DJI_0497	Rubus idaeus L.	0	n	1	0	Rosaceae Juss.
Iz_8_1209	DJI_0497	Rumex acetosella L.	0	n	1	0	Polygonaceae Juss.
Iz_8_1209	DJI_0497	Rumex thyrsiflorus Fingerh.	0	n	1	0	Polygonaceae Juss.
Iz_8_1209	DJI_0497	Salix alba L.	0	n	1	1	Salicaceae Mirb.
Iz_8_1209	DJI_0497	Sambucus nigra L.	0	n	1	1	Viburnaceae Raf.
Iz_8_1209	DJI_0497	Secale sylvestre Host	0	n	1	0	Poaceae Barnhart
Iz_8_1209	DJI_0497	Sempervivum ruthenicum Schnittsp. & C.B. Lehm.	0	n	1	0	Crassulaceae J.St.-Hil.
Iz_8_1209	DJI_0497	Setaria viridis (L.) P. Beauv.	0	n	1	0	Poaceae Barnhart

Iz_8_1209	DJI_0497	Silene borysthenica (Gruner) Walters	0	n	1	0	Caryophyllaceae Juss.
Iz_8_1209	DJI_0497	Silene latifolia subsp. alba (Mill.) Greuter & Burdet	0	n	1	0	Caryophyllaceae Juss.
Iz_8_1209	DJI_0497	Solidago virgaurea L.	0	n	1	0	Asteraceae Bercht. & J.Presl
Iz_8_1209	DJI_0497	Sorbus aucuparia L.	0	n	1	1	Rosaceae Juss.
Iz_8_1209	DJI_0497	Stachys recta L.	0	n	1	0	Lamiacea Lindl.
Iz_8_1209	DJI_0497	Tanacetum vulgare L.	0	n	1	0	Asteraceae Bercht. & J.Presl
Iz_8_1209	DJI_0497	Thymus pallasianus Heinr.Braun	0	n	1	0	Lamiacea Lindl.
Iz_8_1209	DJI_0497	Tilia cordata Mill.	0	n	1	1	Malvaceae Juss.
Iz_8_1209	DJI_0497	Trifolium alpestre L.	0	n	1	0	Fabaceae Lindl.
Iz_8_1209	DJI_0497	Trifolium arvense L.	0	n	1	0	Fabaceae Lindl.
Iz_8_1209	DJI_0497	Ulmus glabra Huds.	0	n	1	1	Ulmaceae Mirb.
Iz_8_1209	DJI_0497	Verbascum densiflorum Bertol.	0	n	1	0	Scrophulariaceae Juss.
Iz_8_1209	DJI_0497	Veronica austriaca L.	0	n	1	0	Plantaginaceae Juss.
Iz_8_1209	DJI_0497	Veronica incana L.	0	n	1	0	Plantaginaceae Juss.
Iz_8_1209	DJI_0497	Veronica verna L.	0	n	1	0	Plantaginaceae Juss.
Iz_8_1209	DJI_0497	Vincetoxicum hirundinaria Medik	0	n	1	0	Apocynaceae Juss.
Iz_8_1209	DJI_0500	Acer tataricum L.	0	n	1	1	Sapindaceae Juss.
Iz_8_1209	DJI_0500	Achillea millefolium L. s.l.	0	n	1	0	Asteraceae Bercht. & J.Presl
Iz_8_1209	DJI_0500	Allium oleraceum L.	0	n	1	0	Amaryllidaceae J.St.-Hil.
Iz_8_1209	DJI_0500	Ambrosia artemisiifolia L.	0	a	0	0	Asteraceae Bercht. & J.Presl
Iz_8_1209	DJI_0500	Amorpha fruticosa L.	0	a	0	0	Fabaceae Lindl.
Iz_8_1209	DJI_0500	Anthericum ramosum L.	0	n	1	0	Asparagaceae Juss.
Iz_8_1209	DJI_0500	Artemisia marschalliana Spreng.	0	n	1	0	Asteraceae Bercht. & J.Presl
Iz_8_1209	DJI_0500	Asparagus officinalis L.	0	n	1	0	Asparagaceae Juss.
Iz_8_1209	DJI_0500	Astragalus cicer L.	0	n	1	0	Fabaceae Lindl.
Iz_8_1209	DJI_0500	Bassia laniflora (S.G.Gmel.) A.J.Scott	0	n	1	0	Amaranthaceae Juss.
Iz_8_1209	DJI_0500	Berberis vulgaris L.	0	n	1	0	Berberidaceae Juss.
Iz_8_1209	DJI_0500	Betula pendula Roth.	0	n	1	1	Betulaceae Gray
Iz_8_1209	DJI_0500	Betula pendula Roth.	0	n	1	0	Poaceae Barnhart
Iz_8_1209	DJI_0500	Bromus inermis Leyss.	0		NA	NA	
Iz_8_1209	DJI_0500	Bromus squarrosus L.	0	a	0	0	Poaceae Barnhart
Iz_8_1209	DJI_0500	Bromus tectorum L.	0	a	0	0	Poaceae Barnhart
Iz_8_1209	DJI_0500	Calamagrostis epigejos (L.) Roth	0	n	1	0	Poaceae Barnhart
Iz_8_1209	DJI_0500	Carduus acanthoides L.	0	n	1	0	Asteraceae Bercht. & J.Presl
Iz_8_1209	DJI_0500	Carex sp.	0	n	1	0	Cyperaceae Juss.
Iz_8_1209	DJI_0500	Centaurea majorovii Dumbadze	0	n	1	0	Asteraceae Bercht. & J.Presl
Iz_8_1209	DJI_0500	Chamaecytisus ruthenicus (Fisch.ex Wol.) Klaskova	0	n	1	0	Fabaceae Lindl.
Iz_8_1209	DJI_0500	Chelidonium majus L.	0	n	1	0	Papaveraceae Juss.
Iz_8_1209	DJI_0500	Chenopodium betaceum Andrz.	0	n	1	0	Amaranthaceae Juss.
Iz_8_1209	DJI_0500	Chenopodium cf. album	0	n	1	0	Amaranthaceae Juss.
Iz_8_1209	DJI_0500	Chenopodium sp.	0		NA	0	Amaranthaceae Juss.
Iz_8_1209	DJI_0500	Chondrilla juncea L.	0	n	1	0	Asteraceae Bercht. & J.Presl
Iz_8_1209	DJI_0500	Cirsium vulgare (Savi) Ten.	0	n	1	0	Asteraceae Bercht. & J.Presl
Iz_8_1209	DJI_0500	Clinopodium acinos (L.) Kuntze	0	n	1	0	Lamiacea Lindl.
Iz_8_1209	DJI_0500	Convallaria majalis L.	0	n	1	0	Asparagaceae Juss.
Iz_8_1209	DJI_0500	Crataegus rhipidophylla Gand.	0	n	1	1	Rosaceae Juss.
Iz_8_1209	DJI_0500	Crepis foetida L.	0	n	1	0	Asteraceae Bercht. & J.Presl
Iz_8_1209	DJI_0500	Dactylis glomerata L.	0	n	1	0	Poaceae Barnhart
Iz_8_1209	DJI_0500	Dianthus campestris M.Bieb.	0	n	1	0	Caryophyllaceae Juss.
Iz_8_1209	DJI_0500	Digitaria sanguinalis (L.) Scop.	0	a	0	0	Poaceae Barnhart
Iz_8_1209	DJI_0500	Elymus repens (L.) Gould	0	n	1	0	Poaceae Barnhart
Iz_8_1209	DJI_0500	Epilobium tetragonum L.	0	n	1	0	Onagraceae Juss.
Iz_8_1209	DJI_0500	Eragrostis minor Host	0	a	0	0	Poaceae Barnhart
Iz_8_1209	DJI_0500	Erigeron canadensis L.	3	a	0	0	Asteraceae Bercht. & J.Presl
Iz_8_1209	DJI_0500	Euphorbia seguieriana Neck.	0	n	1	0	Euphorbiaceae Juss.
Iz_8_1209	DJI_0500	Fallopia dumetorum (L.) Holub	0	n	1	0	Polygonaceae Juss.
Iz_8_1209	DJI_0500	Festuca beckeri (Hack.) Trautv.	0	n	1	0	Poaceae Barnhart
Iz_8_1209	DJI_0500	Fragaria vesca L.	0	n	1	0	Rosaceae Juss.
Iz_8_1209	DJI_0500	Frangula alnus Mill.	0	n	1	1	Rhamnaceae Juss.
Iz_8_1209	DJI_0500	Fraxinus pennsylvanica Marshall	0	a	0	1	Oleaceae Hoffmanns. & Link
Iz_8_1209	DJI_0500	Galeopsis bifida Boenn.	0	n	1	0	Lamiacea Lindl.
Iz_8_1209	DJI_0500	Galium verum L.	0	n	1	0	Rubiaceae Juss.
Iz_8_1209	DJI_0500	Genista tinctoria L.	0	n	1	0	Fabaceae Lindl.
Iz_8_1209	DJI_0500	Geranium robertianum L.	0	n	1	0	Geraniaceae Juss.
Iz_8_1209	DJI_0500	Geranium sanquineum L.	0	n	1	0	Geraniaceae Juss.
Iz_8_1209	DJI_0500	Gypsophila paniculata L.	0	n	1	0	Caryophyllaceae Juss.
Iz_8_1209	DJI_0500	Helichrysum arenarium (L.) Moench	0	n	1	0	Asteraceae Bercht. & J.Presl
Iz_8_1209	DJI_0500	Herniaria polygama J.Gay	0	n	1	0	Caryophyllaceae Juss.
Iz_8_1209	DJI_0500	Hieracium robustum Fr.	0	n	1	0	Asteraceae Bercht. & J.Presl
Iz_8_1209	DJI_0500	Hieracium umbellatum L.	0	n	1	NA	Asteraceae Bercht. & J.Presl
Iz_8_1209	DJI_0500	Hylotelephium maximum (L.) Holub	0	n	1	0	Crassulaceae J.St.-Hil.
Iz_8_1209	DJI_0500	Jacobaea borysthenica (DC.) B.Nord. & Greuter	0	n	1	0	Asteraceae Bercht. & J.Presl
Iz_8_1209	DJI_0500	Jasione montana L.	0	n	1	0	Campanulaceae Juss.
Iz_8_1209	DJI_0500	Jurinea cyanoides (L.) Rchb.s.l.	0	n	1	0	Asteraceae Bercht. & J.Presl
Iz_8_1209	DJI_0500	Koeleria glauca (Spreng.) DC.	0	n	1	0	Poaceae Barnhart
Iz_8_1209	DJI_0500	Lactuca serriola L.	0	a	0	0	Asteraceae Bercht. & J.Presl
Iz_8_1209	DJI_0500	Linaria genistifolia (L.) Mill.	0	n	1	0	Plantaginaceae Juss.
Iz_8_1209	DJI_0500	Lomelosia argentea (L.) Greuter & Burdet	0	n	1	0	Caprifoliaceae Juss.
Iz_8_1209	DJI_0500	Melampyrum pratense L.	0	n	1	0	Orobanchaceae Vent.
Iz_8_1209	DJI_0500	Oenothera biennis L.	0	a	0	0	Onagraceae Juss.
Iz_8_1209	DJI_0500	Pentanema britannica (L.) D.Gut.Larr., Santos-Vicente, Anderb., E.Rico & M.M.Mart.Ort.	0	n	1	0	Asteraceae Bercht. & J.Presl
Iz_8_1209	DJI_0500	Peucedanum oreoselinum (L.) Moench	0	n	1	0	Apiaceae Lindl.
Iz_8_1209	DJI_0500	Peucedanum ruthenicum M.Bieb.	0	n	1	0	Apiaceae Lindl.
Iz_8_1209	DJI_0500	Pilosella echioides (Lumn.) F.W.Schultz & Sch.Bip.	0	n	1	0	Asteraceae Bercht. & J.Presl
Iz_8_1209	DJI_0500	Pilosella officinarum F. Schultz et Sch. Bip.	0	n	1	0	Asteraceae Bercht. & J.Presl
Iz_8_1209	DJI_0500	Pilosella sp.	0		NA	0	Asteraceae Bercht. & J.Presl
Iz_8_1209	DJI_0500	Pinus sylvestris L.	0	n	1	1	Pinaceae Spreng. ex F.Rudolphi
Iz_8_1209	DJI_0500	Poa annua L.	0	n	1	0	Poaceae Barnhart
Iz_8_1209	DJI_0500	Poa nemoralis L.	0	n	1	0	Poaceae Barnhart
Iz_8_1209	DJI_0500	Poa sp.	0		NA	0	Poaceae Barnhart
Iz_8_1209	DJI_0500	Polygonatum odoratum (Mill.) Druce	0	n	1	0	Asparagaceae Juss.
Iz_8_1209	DJI_0500	Polygonum arenarium Waldst. & Kit.	0	n	1	0	Polygonaceae Juss.
Iz_8_1209	DJI_0500	Populus nigra L.	0	n	1	1	Salicaceae Mirb.
Iz_8_1209	DJI_0500	Populus tremula L.	0	n	1	1	Salicaceae Mirb.
Iz_8_1209	DJI_0500	Populus Ã canescens (Aiton) Sm.	0	n	1	1	Salicaceae Mirb.
Iz_8_1209	DJI_0500	Potentilla argentea L.	0	n	1	0	Rosaceae Juss.
Iz_8_1209	DJI_0500	Psammophiliella muralis (L.) Ikonn.	0	n	1	0	Caryophyllaceae Juss.
Iz_8_1209	DJI_0500	Psephellus sumensis (Kalen.) Greuter	0	n	1	0	Asteraceae Bercht. & J.Presl

Iz_8_1209	DJI_0500	Pulsatilla pratensis (L.) Mill. s.l.	0	n	1	0	Ranunculaceae Juss.
Iz_8_1209	DJI_0500	Pyrus communis L.	0	a	0	1	Rosaceae Juss.
Iz_8_1209	DJI_0500	Quercus robur L.	0	n	1	1	Fagaceae Dumort.
Iz_8_1209	DJI_0500	Robinia pseudoacacia L.	0	a	0	1	Fabaceae Lindl.
Iz_8_1209	DJI_0500	Rosa sp.	0		NA	1	Rosaceae Juss.
Iz_8_1209	DJI_0500	Rubus idaeus L.	0	n	1	0	Rosaceae Juss.
Iz_8_1209	DJI_0500	Rumex acetosella L.	0	n	1	0	Polygonaceae Juss.
Iz_8_1209	DJI_0500	Rumex thyrsiflorus Fingerh.	0	n	1	0	Polygonaceae Juss.
Iz_8_1209	DJI_0500	Salix alba L.	0	n	1	1	Salicaceae Mirb.
Iz_8_1209	DJI_0500	Sambucus nigra L.	0	n	1	1	Viburnaceae Raf.
Iz_8_1209	DJI_0500	Secale sylvestre Host	0	n	1	0	Poaceae Barnhart
Iz_8_1209	DJI_0500	Sempervivum ruthenicum Schnittsp. & C.B. Lehm.	0	n	1	0	Crassulaceae J.St.-Hil.
Iz_8_1209	DJI_0500	Setaria viridis (L.) P. Beauv.	0	n	1	0	Poaceae Barnhart
Iz_8_1209	DJI_0500	Silene borysthenica (Gruner) Walters	0	n	1	0	Caryophyllaceae Juss.
Iz_8_1209	DJI_0500	Silene latifolia subsp. alba (Mill.) Greuter & Burdet	0	n	1	0	Caryophyllaceae Juss.
Iz_8_1209	DJI_0500	Solidago virgaurea L.	0	n	1	0	Asteraceae Bercht. & J.Presl
Iz_8_1209	DJI_0500	Sorbus aucuparia L.	0	n	1	1	Rosaceae Juss.
Iz_8_1209	DJI_0500	Stachys recta L.	0	n	1	0	Lamiacea Lindl.
Iz_8_1209	DJI_0500	Tanacetum vulgare L.	0	n	1	0	Asteraceae Bercht. & J.Presl
Iz_8_1209	DJI_0500	Thymus pallasianus Heinr.Braun	0	n	1	0	Lamiacea Lindl.
Iz_8_1209	DJI_0500	Tilia cordata Mill.	0	n	1	1	Malvaceae Juss.
Iz_8_1209	DJI_0500	Trifolium alpestre L.	0	n	1	0	Fabaceae Lindl.
Iz_8_1209	DJI_0500	Trifolium arvense L.	0	n	1	0	Fabaceae Lindl.
Iz_8_1209	DJI_0500	Ulmus glabra Huds.	0	n	1	1	Ulmaceae Mirb.
Iz_8_1209	DJI_0500	Verbascum densiflorum Bertol.	0	n	1	0	Scrophulariaceae Juss.
Iz_8_1209	DJI_0500	Veronica austriaca L.	0	n	1	0	Plantaginaceae Juss.
Iz_8_1209	DJI_0500	Veronica incana L.	0	n	1	0	Plantaginaceae Juss.
Iz_8_1209	DJI_0500	Veronica verna L.	0	n	1	0	Plantaginaceae Juss.
Iz_8_1209	DJI_0500	Vincetoxicum hirundinaria Medik	0	n	1	0	Apocynaceae Juss.
Iz_8_1209	DJI_0505	Acer tataricum L.	0	n	1	1	Sapindaceae Juss.
Iz_8_1209	DJI_0505	Achillea millefolium L. s.l.	0	n	1	0	Asteraceae Bercht. & J.Presl
Iz_8_1209	DJI_0505	Allium oleraceum L.	0	n	1	0	Amaryllidaceae J.St.-Hil.
Iz_8_1209	DJI_0505	Ambrosia artemisiifolia L.	0	a	0	0	Asteraceae Bercht. & J.Presl
Iz_8_1209	DJI_0505	Amorpha fruticosa L.	0	a	0	0	Fabaceae Lindl.
Iz_8_1209	DJI_0505	Anthericum ramosum L.	0	n	1	0	Asparagaceae Juss.
Iz_8_1209	DJI_0505	Artemisia marschalliana Spreng.	0	n	1	0	Asteraceae Bercht. & J.Presl
Iz_8_1209	DJI_0505	Asparagus officinalis L.	0	n	1	0	Asparagaceae Juss.
Iz_8_1209	DJI_0505	Astragalus cicer L.	0	n	1	0	Fabaceae Lindl.
Iz_8_1209	DJI_0505	Bassia laniflora (S.G.Gmel.) A.J.Scott	0	n	1	0	Amaranthaceae Juss.
Iz_8_1209	DJI_0505	Berberis vulgaris L.	0	n	1	0	Berberidaceae Juss.
Iz_8_1209	DJI_0505	Betula pendula Roth.	0	n	1	1	Betulaceae Gray
Iz_8_1209	DJI_0505	Betula pendula Roth.	0	n	1	0	Poaceae Barnhart
Iz_8_1209	DJI_0505	Bromus inermis Leyss.	0		NA	NA	
Iz_8_1209	DJI_0505	Bromus squarrosus L.	0	a	0	0	Poaceae Barnhart
Iz_8_1209	DJI_0505	Bromus tectorum L.	0	a	0	0	Poaceae Barnhart
Iz_8_1209	DJI_0505	Calamagrostis epigejos (L.) Roth	0	n	1	0	Poaceae Barnhart
Iz_8_1209	DJI_0505	Carduus acanthoides L.	0	n	1	0	Asteraceae Bercht. & J.Presl
Iz_8_1209	DJI_0505	Carex sp.	0	n	1	0	Cyperaceae Juss.
Iz_8_1209	DJI_0505	Centaurea majorovii Dumbadze	0	n	1	0	Asteraceae Bercht. & J.Presl
Iz_8_1209	DJI_0505	Chamaecytisus ruthenicus (Fisch.ex Wol.) Klaskova	0	n	1	0	Fabaceae Lindl.
Iz_8_1209	DJI_0505	Chelidonium majus L.	0	n	1	0	Papaveraceae Juss.
Iz_8_1209	DJI_0505	Chenopodium betaceum Andrz.	0	n	1	0	Amaranthaceae Juss.
Iz_8_1209	DJI_0505	Chenopodium cf. album	0	n	1	0	Amaranthaceae Juss.
Iz_8_1209	DJI_0505	Chenopodium sp.	0		NA	0	Amaranthaceae Juss.
Iz_8_1209	DJI_0505	Chondrilla juncea L.	0	n	1	0	Asteraceae Bercht. & J.Presl
Iz_8_1209	DJI_0505	Cirsium vulgare (Savi) Ten.	0	n	1	0	Asteraceae Bercht. & J.Presl
Iz_8_1209	DJI_0505	Clinopodium acinos (L.) Kuntze	0	n	1	0	Lamiacea Lindl.
Iz_8_1209	DJI_0505	Convallaria majalis L.	0	n	1	0	Asparagaceae Juss.
Iz_8_1209	DJI_0505	Crataegus rhipidophylla Gand.	0	n	1	1	Rosaceae Juss.
Iz_8_1209	DJI_0505	Crepis foetida L.	0	n	1	0	Asteraceae Bercht. & J.Presl
Iz_8_1209	DJI_0505	Dactylis glomerata L.	0	n	1	0	Poaceae Barnhart
Iz_8_1209	DJI_0505	Dianthus campestris M.Bieb.	0	n	1	0	Caryophyllaceae Juss.
Iz_8_1209	DJI_0505	Digitaria sanguinalis (L.) Scop.	0	a	0	0	Poaceae Barnhart
Iz_8_1209	DJI_0505	Elymus repens (L.) Gould	0	n	1	0	Poaceae Barnhart
Iz_8_1209	DJI_0505	Epilobium tetragonum L.	0	n	1	0	Onagraceae Juss.
Iz_8_1209	DJI_0505	Eragrostis minor Host	0	a	0	0	Poaceae Barnhart
Iz_8_1209	DJI_0505	Erigeron canadensis L.	0.2	a	0	0	Asteraceae Bercht. & J.Presl
Iz_8_1209	DJI_0505	Euphorbia seguieriana Neck.	0	n	1	0	Euphorbiaceae Juss.
Iz_8_1209	DJI_0505	Fallopia dumetorum (L.) Holub	0	n	1	0	Polygonaceae Juss.
Iz_8_1209	DJI_0505	Festuca beckeri (Hack.) Trautv.	0	n	1	0	Poaceae Barnhart
Iz_8_1209	DJI_0505	Fragaria vesca L.	0	n	1	0	Rosaceae Juss.
Iz_8_1209	DJI_0505	Frangula alnus Mill.	0	n	1	1	Rhamnaceae Juss.
Iz_8_1209	DJI_0505	Fraxinus pennsylvanica Marshall	0	a	0	1	Oleaceae Hoffmanns. & Link
Iz_8_1209	DJI_0505	Galeopsis bifida Boenn.	0	n	1	0	Lamiacea Lindl.
Iz_8_1209	DJI_0505	Galium verum L.	0	n	1	0	Rubiaceae Juss.
Iz_8_1209	DJI_0505	Genista tinctoria L.	0	n	1	0	Fabaceae Lindl.
Iz_8_1209	DJI_0505	Geranium robertianum L.	0	n	1	0	Geraniaceae Juss.
Iz_8_1209	DJI_0505	Geranium sanquineum L.	0	n	1	0	Geraniaceae Juss.
Iz_8_1209	DJI_0505	Gypsophila paniculata L.	0	n	1	0	Caryophyllaceae Juss.
Iz_8_1209	DJI_0505	Helichrysum arenarium (L.) Moench	0	n	1	0	Asteraceae Bercht. & J.Presl
Iz_8_1209	DJI_0505	Herniaria polygama J.Gay	0	n	1	0	Caryophyllaceae Juss.
Iz_8_1209	DJI_0505	Hieracium robustum Fr.	0	n	1	0	Asteraceae Bercht. & J.Presl
Iz_8_1209	DJI_0505	Hieracium umbellatum L.	0	n	1	NA	Asteraceae Bercht. & J.Presl
Iz_8_1209	DJI_0505	Hylotelephium maximum (L.) Holub	0	n	1	0	Crassulaceae J.St.-Hil.
Iz_8_1209	DJI_0505	Jacobaea borysthenica (DC.) B.Nord. & Greuter	0	n	1	0	Asteraceae Bercht. & J.Presl
Iz_8_1209	DJI_0505	Jasione montana L.	0	n	1	0	Campanulaceae Juss.
Iz_8_1209	DJI_0505	Jurinea cyanoides (L.) Rchb.s.l.	0	n	1	0	Asteraceae Bercht. & J.Presl
Iz_8_1209	DJI_0505	Koeleria glauca (Spreng.) DC.	0	n	1	0	Poaceae Barnhart
Iz_8_1209	DJI_0505	Lactuca serriola L.	0	a	0	0	Asteraceae Bercht. & J.Presl
Iz_8_1209	DJI_0505	Linaria genistifolia (L.) Mill.	0	n	1	0	Plantaginaceae Juss.
Iz_8_1209	DJI_0505	Lomelosia argentea (L.) Greuter & Burdet	0	n	1	0	Caprifoliaceae Juss.
Iz_8_1209	DJI_0505	Melampyrum pratense L.	0	n	1	0	Orobanchaceae Vent.
Iz_8_1209	DJI_0505	Oenothera biennis L.	0	a	0	0	Onagraceae Juss.
Iz_8_1209	DJI_0505	Pentanema britannica (L.) D.Gut.Larr., Santos-Vicente, Anderb., E.Rico & M.M.Mart.Ort.	0	n	1	0	Asteraceae Bercht. & J.Presl
Iz_8_1209	DJI_0505	Peucedanum oreoselinum (L.) Moench	0	n	1	0	Apiaceae Lindl.
Iz_8_1209	DJI_0505	Peucedanum ruthenicum M.Bieb.	0	n	1	0	Apiaceae Lindl.
Iz_8_1209	DJI_0505	Pilosella echioides (Lumn.) F.W.Schultz & Sch.Bip.	0	n	1	0	Asteraceae Bercht. & J.Presl
Iz_8_1209	DJI_0505	Pilosella officinarum F. Schultz et Sch. Bip.	0	n	1	0	Asteraceae Bercht. & J.Presl

Iz_8_1209	DJI_0505	Pilosella sp.	0		NA	0	Asteraceae Bercht. & J.Presl
Iz_8_1209	DJI_0505	Pinus sylvestris L.	0	n	1	1	Pinaceae Spreng. ex F.Rudolphi
Iz_8_1209	DJI_0505	Poa annua L.	0	n	1	0	Poaceae Barnhart
Iz_8_1209	DJI_0505	Poa nemoralis L.	0	n	1	0	Poaceae Barnhart
Iz_8_1209	DJI_0505	Poa sp.	0		NA	0	Poaceae Barnhart
Iz_8_1209	DJI_0505	Polygonatum odoratum (Mill.) Druce	0	n	1	0	Asparagaceae Juss.
Iz_8_1209	DJI_0505	Polygonum arenarium Waldst. & Kit.	0	n	1	0	Polygonaceae Juss.
Iz_8_1209	DJI_0505	Populus nigra L.	0	n	1	1	Salicaceae Mirb.
Iz_8_1209	DJI_0505	Populus tremula L.	0	n	1	1	Salicaceae Mirb.
Iz_8_1209	DJI_0505	Populus Ã canescens (Aiton) Sm.	0	n	1	1	Salicaceae Mirb.
Iz_8_1209	DJI_0505	Potentilla argentea L.	0	n	1	0	Rosaceae Juss.
Iz_8_1209	DJI_0505	Psammophiliella muralis (L.) Ikonn.	0	n	1	0	Caryophyllaceae Juss.
Iz_8_1209	DJI_0505	Psephellus sumensis (Kalen.) Greuter	0	n	1	0	Asteraceae Bercht. & J.Presl
Iz_8_1209	DJI_0505	Pulsatilla pratensis (L.) Mill. s.l.	0	n	1	0	Ranunculaceae Juss.
Iz_8_1209	DJI_0505	Pyrus communis L.	0	a	0	1	Rosaceae Juss.
Iz_8_1209	DJI_0505	Quercus robur L.	0	n	1	1	Fagaceae Dumort.
Iz_8_1209	DJI_0505	Robinia pseudoacacia L.	0	a	0	1	Fabaceae Lindl.
Iz_8_1209	DJI_0505	Rosa sp.	0		NA	1	Rosaceae Juss.
Iz_8_1209	DJI_0505	Rubus idaeus L.	0	n	1	0	Rosaceae Juss.
Iz_8_1209	DJI_0505	Rumex acetosella L.	0	n	1	0	Polygonaceae Juss.
Iz_8_1209	DJI_0505	Rumex thyrsiflorus Fingerh.	0	n	1	0	Polygonaceae Juss.
Iz_8_1209	DJI_0505	Salix alba L.	0	n	1	1	Salicaceae Mirb.
Iz_8_1209	DJI_0505	Sambucus nigra L.	0	n	1	1	Viburnaceae Raf.
Iz_8_1209	DJI_0505	Secale sylvestre Host	0	n	1	0	Poaceae Barnhart
Iz_8_1209	DJI_0505	Sempervivum ruthenicum Schnittsp. & C.B. Lehm.	0	n	1	0	Crassulaceae J.St.-Hil.
Iz_8_1209	DJI_0505	Setaria viridis (L.) P. Beauv.	0	n	1	0	Poaceae Barnhart
Iz_8_1209	DJI_0505	Silene borysthenica (Gruner) Walters	0	n	1	0	Caryophyllaceae Juss.
Iz_8_1209	DJI_0505	Silene latifolia subsp. alba (Mill.) Greuter & Burdet	0	n	1	0	Caryophyllaceae Juss.
Iz_8_1209	DJI_0505	Solidago virgaurea L.	0	n	1	0	Asteraceae Bercht. & J.Presl
Iz_8_1209	DJI_0505	Sorbus aucuparia L.	0	n	1	1	Rosaceae Juss.
Iz_8_1209	DJI_0505	Stachys recta L.	0	n	1	0	Lamiacea Lindl.
Iz_8_1209	DJI_0505	Tanacetum vulgare L.	0	n	1	0	Asteraceae Bercht. & J.Presl
Iz_8_1209	DJI_0505	Thymus pallasianus Heinr.Braun	0	n	1	0	Lamiacea Lindl.
Iz_8_1209	DJI_0505	Tilia cordata Mill.	0	n	1	1	Malvaceae Juss.
Iz_8_1209	DJI_0505	Trifolium alpestre L.	0	n	1	0	Fabaceae Lindl.
Iz_8_1209	DJI_0505	Trifolium arvense L.	0	n	1	0	Fabaceae Lindl.
Iz_8_1209	DJI_0505	Ulmus glabra Huds.	0	n	1	1	Ulmaceae Mirb.
Iz_8_1209	DJI_0505	Verbascum densiflorum Bertol.	0	n	1	0	Scrophulariaceae Juss.
Iz_8_1209	DJI_0505	Veronica austriaca L.	0	n	1	0	Plantaginaceae Juss.
Iz_8_1209	DJI_0505	Veronica incana L.	0	n	1	0	Plantaginaceae Juss.
Iz_8_1209	DJI_0505	Veronica verna L.	0	n	1	0	Plantaginaceae Juss.
Iz_8_1209	DJI_0505	Vincetoxicum hirundinaria Medik	0	n	1	0	Apocynaceae Juss.
Iz_9_1209	DJI_0541	Acer tataricum L.	0	n	1	1	Sapindaceae Juss.
Iz_9_1209	DJI_0541	Achillea millefolium L. s.l.	0	n	1	0	Asteraceae Bercht. & J.Presl
Iz_9_1209	DJI_0541	Allium oleraceum L.	0	n	1	0	Amaryllidaceae J.St.-Hil.
Iz_9_1209	DJI_0541	Ambrosia artemisiifolia L.	0	a	0	0	Asteraceae Bercht. & J.Presl
Iz_9_1209	DJI_0541	Amorpha fruticosa L.	0	a	0	0	Fabaceae Lindl.
Iz_9_1209	DJI_0541	Anthericum ramosum L.	0	n	1	0	Asparagaceae Juss.
Iz_9_1209	DJI_0541	Artemisia marschalliana Spreng.	0	n	1	0	Asteraceae Bercht. & J.Presl
Iz_9_1209	DJI_0541	Asparagus officinalis L.	0	n	1	0	Asparagaceae Juss.
Iz_9_1209	DJI_0541	Astragalus cicer L.	0	n	1	0	Fabaceae Lindl.
Iz_9_1209	DJI_0541	Bassia laniflora (S.G.Gmel.) A.J.Scott	0	n	1	0	Amaranthaceae Juss.
Iz_9_1209	DJI_0541	Berberis vulgaris L.	0	n	1	0	Berberidaceae Juss.
Iz_9_1209	DJI_0541	Betula pendula Roth.	0	n	1	1	Betulaceae Gray
Iz_9_1209	DJI_0541	Betula pendula Roth.	0	n	1	0	Poaceae Barnhart
Iz_9_1209	DJI_0541	Bromus inermis Leyss.	0		NA	NA	
Iz_9_1209	DJI_0541	Bromus squarrosus L.	0	a	0	0	Poaceae Barnhart
Iz_9_1209	DJI_0541	Bromus tectorum L.	0	a	0	0	Poaceae Barnhart
Iz_9_1209	DJI_0541	Calamagrostis epigejos (L.) Roth	30	n	1	0	Poaceae Barnhart
Iz_9_1209	DJI_0541	Carduus acanthoides L.	0	n	1	0	Asteraceae Bercht. & J.Presl
Iz_9_1209	DJI_0541	Carex sp.	0	n	1	0	Cyperaceae Juss.
Iz_9_1209	DJI_0541	Centaurea majorovii Dumbadze	0	n	1	0	Asteraceae Bercht. & J.Presl
Iz_9_1209	DJI_0541	Chamaecytisus ruthenicus (Fisch.ex Wol.) Klaskova	0	n	1	0	Fabaceae Lindl.
Iz_9_1209	DJI_0541	Chelidonium majus L.	0	n	1	0	Papaveraceae Juss.
Iz_9_1209	DJI_0541	Chenopodium betaceum Andrz.	0	n	1	0	Amaranthaceae Juss.
Iz_9_1209	DJI_0541	Chenopodium cf. album	0	n	1	0	Amaranthaceae Juss.
Iz_9_1209	DJI_0541	Chenopodium sp.	0		NA	0	Amaranthaceae Juss.
Iz_9_1209	DJI_0541	Chondrilla juncea L.	1	n	1	0	Asteraceae Bercht. & J.Presl
Iz_9_1209	DJI_0541	Cirsium vulgare (Savi) Ten.	2	n	1	0	Asteraceae Bercht. & J.Presl
Iz_9_1209	DJI_0541	Clinopodium acinos (L.) Kuntze	0	n	1	0	Lamiacea Lindl.
Iz_9_1209	DJI_0541	Convallaria majalis L.	0	n	1	0	Asparagaceae Juss.
Iz_9_1209	DJI_0541	Crataegus rhipidophylla Gand.	0	n	1	1	Rosaceae Juss.
Iz_9_1209	DJI_0541	Crepis foetida L.	0	n	1	0	Asteraceae Bercht. & J.Presl
Iz_9_1209	DJI_0541	Dactylis glomerata L.	0	n	1	0	Poaceae Barnhart
Iz_9_1209	DJI_0541	Dianthus campestris M.Bieb.	0	n	1	0	Caryophyllaceae Juss.
Iz_9_1209	DJI_0541	Digitaria sanguinalis (L.) Scop.	0	a	0	0	Poaceae Barnhart
Iz_9_1209	DJI_0541	Elymus repens (L.) Gould	0	n	1	0	Poaceae Barnhart
Iz_9_1209	DJI_0541	Epilobium tetragonum L.	0	n	1	0	Onagraceae Juss.
Iz_9_1209	DJI_0541	Eragrostis minor Host	0	a	0	0	Poaceae Barnhart
Iz_9_1209	DJI_0541	Erigeron canadensis L.	0	a	0	0	Asteraceae Bercht. & J.Presl
Iz_9_1209	DJI_0541	Euphorbia seguieriana Neck.	0	n	1	0	Euphorbiaceae Juss.
Iz_9_1209	DJI_0541	Fallopia dumetorum (L.) Holub	0	n	1	0	Polygonaceae Juss.
Iz_9_1209	DJI_0541	Festuca beckeri (Hack.) Trautv.	0	n	1	0	Poaceae Barnhart
Iz_9_1209	DJI_0541	Fragaria vesca L.	0	n	1	0	Rosaceae Juss.
Iz_9_1209	DJI_0541	Frangula alnus Mill.	0	n	1	1	Rhamnaceae Juss.
Iz_9_1209	DJI_0541	Fraxinus pennsylvanica Marshall	0	a	0	1	Oleaceae Hoffmanns. & Link
Iz_9_1209	DJI_0541	Galeopsis bifida Boenn.	0	n	1	0	Lamiacea Lindl.
Iz_9_1209	DJI_0541	Galium verum L.	0	n	1	0	Rubiaceae Juss.
Iz_9_1209	DJI_0541	Genista tinctoria L.	0	n	1	0	Fabaceae Lindl.
Iz_9_1209	DJI_0541	Geranium robertianum L.	0	n	1	0	Geraniaceae Juss.
Iz_9_1209	DJI_0541	Geranium sanquineum L.	0	n	1	0	Geraniaceae Juss.
Iz_9_1209	DJI_0541	Gypsophila paniculata L.	0	n	1	0	Caryophyllaceae Juss.
Iz_9_1209	DJI_0541	Helichrysum arenarium (L.) Moench	0	n	1	0	Asteraceae Bercht. & J.Presl
Iz_9_1209	DJI_0541	Herniaria polygama J.Gay	0	n	1	0	Caryophyllaceae Juss.
Iz_9_1209	DJI_0541	Hieracium robustum Fr.	0	n	1	0	Asteraceae Bercht. & J.Presl
Iz_9_1209	DJI_0541	Hieracium umbellatum L.	0	n	1	NA	Asteraceae Bercht. & J.Presl
Iz_9_1209	DJI_0541	Hylotelephium maximum (L.) Holub	0	n	1	0	Crassulaceae J.St.-Hil.
Iz_9_1209	DJI_0541	Jacobaea borysthenica (DC.) B.Nord. & Greuter	0	n	1	0	Asteraceae Bercht. & J.Presl

Iz_9_1209	DJI_0541	Jasione montana L.	0	n	1	0	Campanulaceae Juss.
Iz_9_1209	DJI_0541	Jurinea cyanoides (L.) Rchb.s.l.	0	n	1	0	Asteraceae Bercht. & J.Presl
Iz_9_1209	DJI_0541	Koeleria glauca (Spreng.) DC.	0	n	1	0	Poaceae Barnhart
Iz_9_1209	DJI_0541	Lactuca serriola L.	0	a	0	0	Asteraceae Bercht. & J.Presl
Iz_9_1209	DJI_0541	Linaria genistifolia (L.) Mill.	0	n	1	0	Plantaginaceae Juss.
Iz_9_1209	DJI_0541	Lomelosia argentea (L.) Greuter & Burdet	0	n	1	0	Caprifoliaceae Juss.
Iz_9_1209	DJI_0541	Melampyrum pratense L.	0	n	1	0	Orobanchaceae Vent.
Iz_9_1209	DJI_0541	Oenothera biennis L.	0	a	0	0	Onagraceae Juss.
Iz_9_1209	DJI_0541	Pentanema britannica (L.) D.Gut.Larr., Santos-Vicente, Anderb., E.Rico & M.M.Mart.Ort.	0	n	1	0	Asteraceae Bercht. & J.Presl
Iz_9_1209	DJI_0541	Peucedanum oreoselinum (L.) Moench	0	n	1	0	Apiaceae Lindl.
Iz_9_1209	DJI_0541	Peucedanum ruthenicum M.Bieb.	0	n	1	0	Apiaceae Lindl.
Iz_9_1209	DJI_0541	Pilosella echioides (Lumn.) F.W.Schultz & Sch.Bip.	0	n	1	0	Asteraceae Bercht. & J.Presl
Iz_9_1209	DJI_0541	Pilosella officinarum F. Schultz et Sch. Bip.	0	n	1	0	Asteraceae Bercht. & J.Presl
Iz_9_1209	DJI_0541	Pilosella sp.	0		NA	0	Asteraceae Bercht. & J.Presl
Iz_9_1209	DJI_0541	Pinus sylvestris L.	0	n	1	1	Pinaceae Spreng. ex F.Rudolphi
Iz_9_1209	DJI_0541	Poa annua L.	0	n	1	0	Poaceae Barnhart
Iz_9_1209	DJI_0541	Poa nemoralis L.	0	n	1	0	Poaceae Barnhart
Iz_9_1209	DJI_0541	Poa sp.	0		NA	0	Poaceae Barnhart
Iz_9_1209	DJI_0541	Polygonatum odoratum (Mill.) Druce	0	n	1	0	Asparagaceae Juss.
Iz_9_1209	DJI_0541	Polygonum arenarium Waldst. & Kit.	0	n	1	0	Polygonaceae Juss.
Iz_9_1209	DJI_0541	Populus nigra L.	0	n	1	1	Salicaceae Mirb.
Iz_9_1209	DJI_0541	Populus tremula L.	0	n	1	1	Salicaceae Mirb.
Iz_9_1209	DJI_0541	Populus Ã canescens (Aiton) Sm.	0	n	1	1	Salicaceae Mirb.
Iz_9_1209	DJI_0541	Potentilla argentea L.	0	n	1	0	Rosaceae Juss.
Iz_9_1209	DJI_0541	Psammophiliella muralis (L.) Ikonn.	0	n	1	0	Caryophyllaceae Juss.
Iz_9_1209	DJI_0541	Psephellus sumensis (Kalen.) Greuter	0	n	1	0	Asteraceae Bercht. & J.Presl
Iz_9_1209	DJI_0541	Pulsatilla pratensis (L.) Mill. s.l.	0	n	1	0	Ranunculaceae Juss.
Iz_9_1209	DJI_0541	Pyrus communis L.	0	a	0	1	Rosaceae Juss.
Iz_9_1209	DJI_0541	Quercus robur L.	0	n	1	1	Fagaceae Dumort.
Iz_9_1209	DJI_0541	Robinia pseudoacacia L.	0	a	0	1	Fabaceae Lindl.
Iz_9_1209	DJI_0541	Rosa sp.	0		NA	1	Rosaceae Juss.
Iz_9_1209	DJI_0541	Rubus idaeus L.	0	n	1	0	Rosaceae Juss.
Iz_9_1209	DJI_0541	Rumex acetosella L.	0	n	1	0	Polygonaceae Juss.
Iz_9_1209	DJI_0541	Rumex thyrsiflorus Fingerh.	0	n	1	0	Polygonaceae Juss.
Iz_9_1209	DJI_0541	Salix alba L.	0	n	1	1	Salicaceae Mirb.
Iz_9_1209	DJI_0541	Sambucus nigra L.	0	n	1	1	Viburnaceae Raf.
Iz_9_1209	DJI_0541	Secale sylvestre Host	0	n	1	0	Poaceae Barnhart
Iz_9_1209	DJI_0541	Sempervivum ruthenicum Schnittsp. & C.B. Lehm.	0	n	1	0	Crassulaceae J.St.-Hil.
Iz_9_1209	DJI_0541	Setaria viridis (L.) P. Beauv.	0	n	1	0	Poaceae Barnhart
Iz_9_1209	DJI_0541	Silene borysthenica (Gruner) Walters	0	n	1	0	Caryophyllaceae Juss.
Iz_9_1209	DJI_0541	Silene latifolia subsp. alba (Mill.) Greuter & Burdet	0	n	1	0	Caryophyllaceae Juss.
Iz_9_1209	DJI_0541	Solidago virgaurea L.	0	n	1	0	Asteraceae Bercht. & J.Presl
Iz_9_1209	DJI_0541	Sorbus aucuparia L.	0	n	1	1	Rosaceae Juss.
Iz_9_1209	DJI_0541	Species 12.09_9_0601	0		NA	NA	
Iz_9_1209	DJI_0541	Stachys recta L.	0	n	1	0	Lamiacea Lindl.
Iz_9_1209	DJI_0541	Tanacetum vulgare L.	0	n	1	0	Asteraceae Bercht. & J.Presl
Iz_9_1209	DJI_0541	Thymus pallasianus Heinr.Braun	0	n	1	0	Lamiacea Lindl.
Iz_9_1209	DJI_0541	Tilia cordata Mill.	0	n	1	1	Malvaceae Juss.
Iz_9_1209	DJI_0541	Trifolium alpestre L.	0	n	1	0	Fabaceae Lindl.
Iz_9_1209	DJI_0541	Trifolium arvense L.	0	n	1	0	Fabaceae Lindl.
Iz_9_1209	DJI_0541	Ulmus glabra Huds.	0	n	1	1	Ulmaceae Mirb.
Iz_9_1209	DJI_0541	Verbascum densiflorum Bertol.	0	n	1	0	Scrophulariaceae Juss.
Iz_9_1209	DJI_0541	Veronica austriaca L.	0	n	1	0	Plantaginaceae Juss.
Iz_9_1209	DJI_0541	Veronica incana L.	0	n	1	0	Plantaginaceae Juss.
Iz_9_1209	DJI_0541	Veronica verna L.	0	n	1	0	Plantaginaceae Juss.
Iz_9_1209	DJI_0541	Vincetoxicum hirundinaria Medik	0	n	1	0	Apocynaceae Juss.
Iz_9_1209	DJI_0545	Acer tataricum L.	0	n	1	1	Sapindaceae Juss.
Iz_9_1209	DJI_0545	Achillea millefolium L. s.l.	0	n	1	0	Asteraceae Bercht. & J.Presl
Iz_9_1209	DJI_0545	Allium oleraceum L.	0	n	1	0	Amaryllidaceae J.St.-Hil.
Iz_9_1209	DJI_0545	Ambrosia artemisiifolia L.	0	a	0	0	Asteraceae Bercht. & J.Presl
Iz_9_1209	DJI_0545	Amorpha fruticosa L.	0	a	0	0	Fabaceae Lindl.
Iz_9_1209	DJI_0545	Anthericum ramosum L.	0	n	1	0	Asparagaceae Juss.
Iz_9_1209	DJI_0545	Artemisia marschalliana Spreng.	0	n	1	0	Asteraceae Bercht. & J.Presl
Iz_9_1209	DJI_0545	Asparagus officinalis L.	0	n	1	0	Asparagaceae Juss.
Iz_9_1209	DJI_0545	Astragalus cicer L.	0	n	1	0	Fabaceae Lindl.
Iz_9_1209	DJI_0545	Bassia laniflora (S.G.Gmel.) A.J.Scott	0	n	1	0	Amaranthaceae Juss.
Iz_9_1209	DJI_0545	Berberis vulgaris L.	0	n	1	0	Berberidaceae Juss.
Iz_9_1209	DJI_0545	Betula pendula Roth.	0	n	1	1	Betulaceae Gray
Iz_9_1209	DJI_0545	Betula pendula Roth.	0	n	1	0	Poaceae Barnhart
Iz_9_1209	DJI_0545	Bromus inermis Leyss.	0		NA	NA	
Iz_9_1209	DJI_0545	Bromus squarrosus L.	0	a	0	0	Poaceae Barnhart
Iz_9_1209	DJI_0545	Bromus tectorum L.	0	a	0	0	Poaceae Barnhart
Iz_9_1209	DJI_0545	Calamagrostis epigejos (L.) Roth	0.5	n	1	0	Poaceae Barnhart
Iz_9_1209	DJI_0545	Carduus acanthoides L.	0	n	1	0	Asteraceae Bercht. & J.Presl
Iz_9_1209	DJI_0545	Carex sp.	0	n	1	0	Cyperaceae Juss.
Iz_9_1209	DJI_0545	Centaurea majorovii Dumbadze	0	n	1	0	Asteraceae Bercht. & J.Presl
Iz_9_1209	DJI_0545	Chamaecytisus ruthenicus (Fisch.ex Wol.) Klaskova	0	n	1	0	Fabaceae Lindl.
Iz_9_1209	DJI_0545	Chelidonium majus L.	0	n	1	0	Papaveraceae Juss.
Iz_9_1209	DJI_0545	Chenopodium betaceum Andrz.	0	n	1	0	Amaranthaceae Juss.
Iz_9_1209	DJI_0545	Chenopodium cf. album	0	n	1	0	Amaranthaceae Juss.
Iz_9_1209	DJI_0545	Chenopodium sp.	0		NA	0	Amaranthaceae Juss.
Iz_9_1209	DJI_0545	Chondrilla juncea L.	0	n	1	0	Asteraceae Bercht. & J.Presl
Iz_9_1209	DJI_0545	Cirsium vulgare (Savi) Ten.	0	n	1	0	Asteraceae Bercht. & J.Presl
Iz_9_1209	DJI_0545	Clinopodium acinos (L.) Kuntze	0	n	1	0	Lamiacea Lindl.
Iz_9_1209	DJI_0545	Convallaria majalis L.	0	n	1	0	Asparagaceae Juss.
Iz_9_1209	DJI_0545	Crataegus rhipidophylla Gand.	0	n	1	1	Rosaceae Juss.
Iz_9_1209	DJI_0545	Crepis foetida L.	0	n	1	0	Asteraceae Bercht. & J.Presl
Iz_9_1209	DJI_0545	Dactylis glomerata L.	0	n	1	0	Poaceae Barnhart
Iz_9_1209	DJI_0545	Dianthus campestris M.Bieb.	0	n	1	0	Caryophyllaceae Juss.
Iz_9_1209	DJI_0545	Digitaria sanguinalis (L.) Scop.	0	a	0	0	Poaceae Barnhart
Iz_9_1209	DJI_0545	Elymus repens (L.) Gould	0	n	1	0	Poaceae Barnhart
Iz_9_1209	DJI_0545	Epilobium tetragonum L.	0	n	1	0	Onagraceae Juss.
Iz_9_1209	DJI_0545	Eragrostis minor Host	0	a	0	0	Poaceae Barnhart
Iz_9_1209	DJI_0545	Erigeron canadensis L.	0	a	0	0	Asteraceae Bercht. & J.Presl
Iz_9_1209	DJI_0545	Euphorbia seguieriana Neck.	0	n	1	0	Euphorbiaceae Juss.
Iz_9_1209	DJI_0545	Fallopia dumetorum (L.) Holub	0	n	1	0	Polygonaceae Juss.
Iz_9_1209	DJI_0545	Festuca beckeri (Hack.) Trautv.	0	n	1	0	Poaceae Barnhart
Iz_9_1209	DJI_0545	Fragaria vesca L.	0	n	1	0	Rosaceae Juss.

Iz_9_1209	DJI_0545	Frangula alnus Mill.	0	n	1	1	Rhamnaceae Juss.
Iz_9_1209	DJI_0545	Fraxinus pennsylvanica Marshall	0	a	0	1	Oleaceae Hoffmanns. & Link
Iz_9_1209	DJI_0545	Galeopsis bifida Boenn.	0	n	1	0	Lamiacea Lindl.
Iz_9_1209	DJI_0545	Galium verum L.	0	n	1	0	Rubiaceae Juss.
Iz_9_1209	DJI_0545	Genista tinctoria L.	0	n	1	0	Fabaceae Lindl.
Iz_9_1209	DJI_0545	Geranium robertianum L.	0	n	1	0	Geraniaceae Juss.
Iz_9_1209	DJI_0545	Geranium sanquineum L.	0	n	1	0	Geraniaceae Juss.
Iz_9_1209	DJI_0545	Gypsophila paniculata L.	0	n	1	0	Caryophyllaceae Juss.
Iz_9_1209	DJI_0545	Helichrysum arenarium (L.) Moench	0	n	1	0	Asteraceae Bercht. & J.Presl
Iz_9_1209	DJI_0545	Herniaria polygama J.Gay	0	n	1	0	Caryophyllaceae Juss.
Iz_9_1209	DJI_0545	Hieracium robustum Fr.	0	n	1	0	Asteraceae Bercht. & J.Presl
Iz_9_1209	DJI_0545	Hieracium umbellatum L.	0	n	1	NA	Asteraceae Bercht. & J.Presl
Iz_9_1209	DJI_0545	Hylotelephium maximum (L.) Holub	0	n	1	0	Crassulaceae J.St.-Hil.
Iz_9_1209	DJI_0545	Jacobaea borysthenica (DC.) B.Nord. & Greuter	0	n	1	0	Asteraceae Bercht. & J.Presl
Iz_9_1209	DJI_0545	Jasione montana L.	0	n	1	0	Campanulaceae Juss.
Iz_9_1209	DJI_0545	Jurinea cyanoides (L.) Rchb.s.l.	0	n	1	0	Asteraceae Bercht. & J.Presl
Iz_9_1209	DJI_0545	Koeleria glauca (Spreng.) DC.	0	n	1	0	Poaceae Barnhart
Iz_9_1209	DJI_0545	Lactuca serriola L.	0	a	0	0	Asteraceae Bercht. & J.Presl
Iz_9_1209	DJI_0545	Linaria genistifolia (L.) Mill.	0	n	1	0	Plantaginaceae Juss.
Iz_9_1209	DJI_0545	Lomelosia argentea (L.) Greuter & Burdet	0	n	1	0	Caprifoliaceae Juss.
Iz_9_1209	DJI_0545	Melampyrum pratense L.	0	n	1	0	Orobanchaceae Vent.
Iz_9_1209	DJI_0545	Oenothera biennis L.	0	a	0	0	Onagraceae Juss.
Iz_9_1209	DJI_0545	Pentanema britannica (L.) D.Gut.Larr., Santos-Vicente, Anderb., E.Rico & M.M.Mart.Ort.	0	n	1	0	Asteraceae Bercht. & J.Presl
Iz_9_1209	DJI_0545	Peucedanum oreoselinum (L.) Moench	0	n	1	0	Apiaceae Lindl.
Iz_9_1209	DJI_0545	Peucedanum ruthenicum M.Bieb.	0	n	1	0	Apiaceae Lindl.
Iz_9_1209	DJI_0545	Pilosella echioides (Lumn.) F.W.Schultz & Sch.Bip.	0	n	1	0	Asteraceae Bercht. & J.Presl
Iz_9_1209	DJI_0545	Pilosella officinarum F. Schultz et Sch. Bip.	0	n	1	0	Asteraceae Bercht. & J.Presl
Iz_9_1209	DJI_0545	Pilosella sp.	0		NA	0	Asteraceae Bercht. & J.Presl
Iz_9_1209	DJI_0545	Pinus sylvestris L.	0	n	1	1	Pinaceae Spreng. ex F.Rudolphi
Iz_9_1209	DJI_0545	Poa annua L.	0	n	1	0	Poaceae Barnhart
Iz_9_1209	DJI_0545	Poa nemoralis L.	0	n	1	0	Poaceae Barnhart
Iz_9_1209	DJI_0545	Poa sp.	0		NA	0	Poaceae Barnhart
Iz_9_1209	DJI_0545	Polygonatum odoratum (Mill.) Druce	0	n	1	0	Asparagaceae Juss.
Iz_9_1209	DJI_0545	Polygonum arenarium Waldst. & Kit.	0	n	1	0	Polygonaceae Juss.
Iz_9_1209	DJI_0545	Populus nigra L.	0	n	1	1	Salicaceae Mirb.
Iz_9_1209	DJI_0545	Populus tremula L.	0	n	1	1	Salicaceae Mirb.
Iz_9_1209	DJI_0545	Populus Ã canescens (Aiton) Sm.	0	n	1	1	Salicaceae Mirb.
Iz_9_1209	DJI_0545	Potentilla argentea L.	0	n	1	0	Rosaceae Juss.
Iz_9_1209	DJI_0545	Psammophiliella muralis (L.) Ikonn.	0	n	1	0	Caryophyllaceae Juss.
Iz_9_1209	DJI_0545	Psephellus sumensis (Kalen.) Greuter	0	n	1	0	Asteraceae Bercht. & J.Presl
Iz_9_1209	DJI_0545	Pulsatilla pratensis (L.) Mill. s.l.	0	n	1	0	Ranunculaceae Juss.
Iz_9_1209	DJI_0545	Pyrus communis L.	0	a	0	1	Rosaceae Juss.
Iz_9_1209	DJI_0545	Quercus robur L.	0	n	1	1	Fagaceae Dumort.
Iz_9_1209	DJI_0545	Robinia pseudoacacia L.	0	a	0	1	Fabaceae Lindl.
Iz_9_1209	DJI_0545	Rosa sp.	0		NA	1	Rosaceae Juss.
Iz_9_1209	DJI_0545	Rubus idaeus L.	0	n	1	0	Rosaceae Juss.
Iz_9_1209	DJI_0545	Rumex acetosella L.	0	n	1	0	Polygonaceae Juss.
Iz_9_1209	DJI_0545	Rumex thyrsiflorus Fingerh.	0	n	1	0	Polygonaceae Juss.
Iz_9_1209	DJI_0545	Salix alba L.	0	n	1	1	Salicaceae Mirb.
Iz_9_1209	DJI_0545	Sambucus nigra L.	0	n	1	1	Viburnaceae Raf.
Iz_9_1209	DJI_0545	Secale sylvestre Host	0	n	1	0	Poaceae Barnhart
Iz_9_1209	DJI_0545	Sempervivum ruthenicum Schnittsp. & C.B. Lehm.	0	n	1	0	Crassulaceae J.St.-Hil.
Iz_9_1209	DJI_0545	Setaria viridis (L.) P. Beauv.	0	n	1	0	Poaceae Barnhart
Iz_9_1209	DJI_0545	Silene borysthenica (Gruner) Walters	0	n	1	0	Caryophyllaceae Juss.
Iz_9_1209	DJI_0545	Silene latifolia subsp. alba (Mill.) Greuter & Burdet	0	n	1	0	Caryophyllaceae Juss.
Iz_9_1209	DJI_0545	Solidago virgaurea L.	0	n	1	0	Asteraceae Bercht. & J.Presl
Iz_9_1209	DJI_0545	Sorbus aucuparia L.	0	n	1	1	Rosaceae Juss.
Iz_9_1209	DJI_0545	Species 12.09_9_0601	0		NA	NA	
Iz_9_1209	DJI_0545	Stachys recta L.	0	n	1	0	Lamiacea Lindl.
Iz_9_1209	DJI_0545	Tanacetum vulgare L.	0	n	1	0	Asteraceae Bercht. & J.Presl
Iz_9_1209	DJI_0545	Thymus pallasianus Heinr.Braun	0	n	1	0	Lamiacea Lindl.
Iz_9_1209	DJI_0545	Tilia cordata Mill.	0	n	1	1	Malvaceae Juss.
Iz_9_1209	DJI_0545	Trifolium alpestre L.	0	n	1	0	Fabaceae Lindl.
Iz_9_1209	DJI_0545	Trifolium arvense L.	0	n	1	0	Fabaceae Lindl.
Iz_9_1209	DJI_0545	Ulmus glabra Huds.	0	n	1	1	Ulmaceae Mirb.
Iz_9_1209	DJI_0545	Verbascum densiflorum Bertol.	0	n	1	0	Scrophulariaceae Juss.
Iz_9_1209	DJI_0545	Veronica austriaca L.	0	n	1	0	Plantaginaceae Juss.
Iz_9_1209	DJI_0545	Veronica incana L.	0	n	1	0	Plantaginaceae Juss.
Iz_9_1209	DJI_0545	Veronica verna L.	0	n	1	0	Plantaginaceae Juss.
Iz_9_1209	DJI_0545	Vincetoxicum hirundinaria Medik	0	n	1	0	Apocynaceae Juss.
Iz_9_1209	DJI_0564	Acer tataricum L.	0	n	1	1	Sapindaceae Juss.
Iz_9_1209	DJI_0564	Achillea millefolium L. s.l.	0	n	1	0	Asteraceae Bercht. & J.Presl
Iz_9_1209	DJI_0564	Allium oleraceum L.	0	n	1	0	Amaryllidaceae J.St.-Hil.
Iz_9_1209	DJI_0564	Ambrosia artemisiifolia L.	0	a	0	0	Asteraceae Bercht. & J.Presl
Iz_9_1209	DJI_0564	Amorpha fruticosa L.	0	a	0	0	Fabaceae Lindl.
Iz_9_1209	DJI_0564	Anthericum ramosum L.	0	n	1	0	Asparagaceae Juss.
Iz_9_1209	DJI_0564	Artemisia marschalliana Spreng.	0	n	1	0	Asteraceae Bercht. & J.Presl
Iz_9_1209	DJI_0564	Asparagus officinalis L.	0	n	1	0	Asparagaceae Juss.
Iz_9_1209	DJI_0564	Astragalus cicer L.	0	n	1	0	Fabaceae Lindl.
Iz_9_1209	DJI_0564	Bassia laniflora (S.G.Gmel.) A.J.Scott	0	n	1	0	Amaranthaceae Juss.
Iz_9_1209	DJI_0564	Berberis vulgaris L.	0	n	1	0	Berberidaceae Juss.
Iz_9_1209	DJI_0564	Betula pendula Roth.	0	n	1	1	Betulaceae Gray
Iz_9_1209	DJI_0564	Betula pendula Roth.	0	n	1	0	Poaceae Barnhart
Iz_9_1209	DJI_0564	Bromus inermis Leyss.	0		NA	NA	
Iz_9_1209	DJI_0564	Bromus squarrosus L.	0	a	0	0	Poaceae Barnhart
Iz_9_1209	DJI_0564	Bromus tectorum L.	0	a	0	0	Poaceae Barnhart
Iz_9_1209	DJI_0564	Calamagrostis epigejos (L.) Roth	25	n	1	0	Poaceae Barnhart
Iz_9_1209	DJI_0564	Carduus acanthoides L.	0	n	1	0	Asteraceae Bercht. & J.Presl
Iz_9_1209	DJI_0564	Carex sp.	0	n	1	0	Cyperaceae Juss.
Iz_9_1209	DJI_0564	Centaurea majorovii Dumbadze	0	n	1	0	Asteraceae Bercht. & J.Presl
Iz_9_1209	DJI_0564	Chamaecytisus ruthenicus (Fisch.ex Wol.) Klaskova	0	n	1	0	Fabaceae Lindl.
Iz_9_1209	DJI_0564	Chelidonium majus L.	0	n	1	0	Papaveraceae Juss.
Iz_9_1209	DJI_0564	Chenopodium betaceum Andrz.	0	n	1	0	Amaranthaceae Juss.
Iz_9_1209	DJI_0564	Chenopodium cf. album	0	n	1	0	Amaranthaceae Juss.
Iz_9_1209	DJI_0564	Chenopodium sp.	1		NA	0	Amaranthaceae Juss.
Iz_9_1209	DJI_0564	Chondrilla juncea L.	0	n	1	0	Asteraceae Bercht. & J.Presl
Iz_9_1209	DJI_0564	Cirsium vulgare (Savi) Ten.	0	n	1	0	Asteraceae Bercht. & J.Presl
Iz_9_1209	DJI_0564	Clinopodium acinos (L.) Kuntze	0	n	1	0	Lamiacea Lindl.

Iz_9_1209	DJI_0564	Convallaria majalis L.	0	n	1	0	Asparagaceae Juss.
Iz_9_1209	DJI_0564	Crataegus rhipidophylla Gand.	0	n	1	1	Rosaceae Juss.
Iz_9_1209	DJI_0564	Crepis foetida L.	0	n	1	0	Asteraceae Bercht. & J.Presl
Iz_9_1209	DJI_0564	Dactylis glomerata L.	0	n	1	0	Poaceae Barnhart
Iz_9_1209	DJI_0564	Dianthus campestris M.Bieb.	0	n	1	0	Caryophyllaceae Juss.
Iz_9_1209	DJI_0564	Digitaria sanguinalis (L.) Scop.	0	a	0	0	Poaceae Barnhart
Iz_9_1209	DJI_0564	Elymus repens (L.) Gould	0	n	1	0	Poaceae Barnhart
Iz_9_1209	DJI_0564	Epilobium tetragonum L.	0	n	1	0	Onagraceae Juss.
Iz_9_1209	DJI_0564	Eragrostis minor Host	0	a	0	0	Poaceae Barnhart
Iz_9_1209	DJI_0564	Erigeron canadensis L.	2	a	0	0	Asteraceae Bercht. & J.Presl
Iz_9_1209	DJI_0564	Euphorbia seguieriana Neck.	0	n	1	0	Euphorbiaceae Juss.
Iz_9_1209	DJI_0564	Fallopia dumetorum (L.) Holub	0	n	1	0	Polygonaceae Juss.
Iz_9_1209	DJI_0564	Festuca beckeri (Hack.) Trautv.	0	n	1	0	Poaceae Barnhart
Iz_9_1209	DJI_0564	Fragaria vesca L.	0	n	1	0	Rosaceae Juss.
Iz_9_1209	DJI_0564	Frangula alnus Mill.	0	n	1	1	Rhamnaceae Juss.
Iz_9_1209	DJI_0564	Fraxinus pennsylvanica Marshall	0	a	0	1	Oleaceae Hoffmanns. & Link
Iz_9_1209	DJI_0564	Galeopsis bifida Boenn.	0	n	1	0	Lamiacea Lindl.
Iz_9_1209	DJI_0564	Galium verum L.	0	n	1	0	Rubiaceae Juss.
Iz_9_1209	DJI_0564	Genista tinctoria L.	0	n	1	0	Fabaceae Lindl.
Iz_9_1209	DJI_0564	Geranium robertianum L.	0	n	1	0	Geraniaceae Juss.
Iz_9_1209	DJI_0564	Geranium sanquineum L.	0	n	1	0	Geraniaceae Juss.
Iz_9_1209	DJI_0564	Gypsophila paniculata L.	0	n	1	0	Caryophyllaceae Juss.
Iz_9_1209	DJI_0564	Helichrysum arenarium (L.) Moench	0	n	1	0	Asteraceae Bercht. & J.Presl
Iz_9_1209	DJI_0564	Herniaria polygama J.Gay	0	n	1	0	Caryophyllaceae Juss.
Iz_9_1209	DJI_0564	Hieracium robustum Fr.	0	n	1	0	Asteraceae Bercht. & J.Presl
Iz_9_1209	DJI_0564	Hieracium umbellatum L.	1	n	1	NA	Asteraceae Bercht. & J.Presl
Iz_9_1209	DJI_0564	Hylotelephium maximum (L.) Holub	0	n	1	0	Crassulaceae J.St.-Hil.
Iz_9_1209	DJI_0564	Jacobaea borysthenica (DC.) B.Nord. & Greuter	0	n	1	0	Asteraceae Bercht. & J.Presl
Iz_9_1209	DJI_0564	Jasione montana L.	0	n	1	0	Campanulaceae Juss.
Iz_9_1209	DJI_0564	Jurinea cyanoides (L.) Rchb.s.l.	0	n	1	0	Asteraceae Bercht. & J.Presl
Iz_9_1209	DJI_0564	Koeleria glauca (Spreng.) DC.	0	n	1	0	Poaceae Barnhart
Iz_9_1209	DJI_0564	Lactuca serriola L.	0	a	0	0	Asteraceae Bercht. & J.Presl
Iz_9_1209	DJI_0564	Linaria genistifolia (L.) Mill.	0	n	1	0	Plantaginaceae Juss.
Iz_9_1209	DJI_0564	Lomelosia argentea (L.) Greuter & Burdet	0	n	1	0	Caprifoliaceae Juss.
Iz_9_1209	DJI_0564	Melampyrum pratense L.	0	n	1	0	Orobanchaceae Vent.
Iz_9_1209	DJI_0564	Oenothera biennis L.	0	a	0	0	Onagraceae Juss.
Iz_9_1209	DJI_0564	Pentanema britannica (L.) D.Gut.Larr., Santos-Vicente, Anderb., E.Rico & M.M.Mart.Ort.	0	n	1	0	Asteraceae Bercht. & J.Presl
Iz_9_1209	DJI_0564	Peucedanum oreoselinum (L.) Moench	0	n	1	0	Apiaceae Lindl.
Iz_9_1209	DJI_0564	Peucedanum ruthenicum M.Bieb.	0	n	1	0	Apiaceae Lindl.
Iz_9_1209	DJI_0564	Pilosella echioides (Lumn.) F.W.Schultz & Sch.Bip.	0	n	1	0	Asteraceae Bercht. & J.Presl
Iz_9_1209	DJI_0564	Pilosella officinarum F. Schultz et Sch. Bip.	0	n	1	0	Asteraceae Bercht. & J.Presl
Iz_9_1209	DJI_0564	Pilosella sp.	0		NA	0	Asteraceae Bercht. & J.Presl
Iz_9_1209	DJI_0564	Pinus sylvestris L.	0	n	1	1	Pinaceae Spreng. ex F.Rudolphi
Iz_9_1209	DJI_0564	Poa annua L.	0	n	1	0	Poaceae Barnhart
Iz_9_1209	DJI_0564	Poa nemoralis L.	0	n	1	0	Poaceae Barnhart
Iz_9_1209	DJI_0564	Poa sp.	0		NA	0	Poaceae Barnhart
Iz_9_1209	DJI_0564	Polygonatum odoratum (Mill.) Druce	0	n	1	0	Asparagaceae Juss.
Iz_9_1209	DJI_0564	Polygonum arenarium Waldst. & Kit.	0	n	1	0	Polygonaceae Juss.
Iz_9_1209	DJI_0564	Populus nigra L.	0	n	1	1	Salicaceae Mirb.
Iz_9_1209	DJI_0564	Populus tremula L.	0	n	1	1	Salicaceae Mirb.
Iz_9_1209	DJI_0564	Populus Ã canescens (Aiton) Sm.	0	n	1	1	Salicaceae Mirb.
Iz_9_1209	DJI_0564	Potentilla argentea L.	0	n	1	0	Rosaceae Juss.
Iz_9_1209	DJI_0564	Psammophiliella muralis (L.) Ikonn.	0	n	1	0	Caryophyllaceae Juss.
Iz_9_1209	DJI_0564	Psephellus sumensis (Kalen.) Greuter	0	n	1	0	Asteraceae Bercht. & J.Presl
Iz_9_1209	DJI_0564	Pulsatilla pratensis (L.) Mill. s.l.	0	n	1	0	Ranunculaceae Juss.
Iz_9_1209	DJI_0564	Pyrus communis L.	0	a	0	1	Rosaceae Juss.
Iz_9_1209	DJI_0564	Quercus robur L.	0	n	1	1	Fagaceae Dumort.
Iz_9_1209	DJI_0564	Robinia pseudoacacia L.	0	a	0	1	Fabaceae Lindl.
Iz_9_1209	DJI_0564	Rosa sp.	0		NA	1	Rosaceae Juss.
Iz_9_1209	DJI_0564	Rubus idaeus L.	0	n	1	0	Rosaceae Juss.
Iz_9_1209	DJI_0564	Rumex acetosella L.	0	n	1	0	Polygonaceae Juss.
Iz_9_1209	DJI_0564	Rumex thyrsiflorus Fingerh.	0	n	1	0	Polygonaceae Juss.
Iz_9_1209	DJI_0564	Salix alba L.	0	n	1	1	Salicaceae Mirb.
Iz_9_1209	DJI_0564	Sambucus nigra L.	0	n	1	1	Viburnaceae Raf.
Iz_9_1209	DJI_0564	Secale sylvestre Host	0	n	1	0	Poaceae Barnhart
Iz_9_1209	DJI_0564	Sempervivum ruthenicum Schnittsp. & C.B. Lehm.	0	n	1	0	Crassulaceae J.St.-Hil.
Iz_9_1209	DJI_0564	Setaria viridis (L.) P. Beauv.	0	n	1	0	Poaceae Barnhart
Iz_9_1209	DJI_0564	Silene borysthenica (Gruner) Walters	0	n	1	0	Caryophyllaceae Juss.
Iz_9_1209	DJI_0564	Silene latifolia subsp. alba (Mill.) Greuter & Burdet	0	n	1	0	Caryophyllaceae Juss.
Iz_9_1209	DJI_0564	Solidago virgaurea L.	0	n	1	0	Asteraceae Bercht. & J.Presl
Iz_9_1209	DJI_0564	Sorbus aucuparia L.	0	n	1	1	Rosaceae Juss.
Iz_9_1209	DJI_0564	Species 12.09_9_0601	0		NA	NA	
Iz_9_1209	DJI_0564	Stachys recta L.	0	n	1	0	Lamiacea Lindl.
Iz_9_1209	DJI_0564	Tanacetum vulgare L.	0	n	1	0	Asteraceae Bercht. & J.Presl
Iz_9_1209	DJI_0564	Thymus pallasianus Heinr.Braun	0	n	1	0	Lamiacea Lindl.
Iz_9_1209	DJI_0564	Tilia cordata Mill.	0	n	1	1	Malvaceae Juss.
Iz_9_1209	DJI_0564	Trifolium alpestre L.	0	n	1	0	Fabaceae Lindl.
Iz_9_1209	DJI_0564	Trifolium arvense L.	0	n	1	0	Fabaceae Lindl.
Iz_9_1209	DJI_0564	Ulmus glabra Huds.	0	n	1	1	Ulmaceae Mirb.
Iz_9_1209	DJI_0564	Verbascum densiflorum Bertol.	0	n	1	0	Scrophulariaceae Juss.
Iz_9_1209	DJI_0564	Veronica austriaca L.	0	n	1	0	Plantaginaceae Juss.
Iz_9_1209	DJI_0564	Veronica incana L.	0	n	1	0	Plantaginaceae Juss.
Iz_9_1209	DJI_0564	Veronica verna L.	0	n	1	0	Plantaginaceae Juss.
Iz_9_1209	DJI_0564	Vincetoxicum hirundinaria Medik	0	n	1	0	Apocynaceae Juss.
Iz_9_1209	DJI_0567	Acer tataricum L.	0	n	1	1	Sapindaceae Juss.
Iz_9_1209	DJI_0567	Achillea millefolium L. s.l.	0	n	1	0	Asteraceae Bercht. & J.Presl
Iz_9_1209	DJI_0567	Allium oleraceum L.	0	n	1	0	Amaryllidaceae J.St.-Hil.
Iz_9_1209	DJI_0567	Ambrosia artemisiifolia L.	0	a	0	0	Asteraceae Bercht. & J.Presl
Iz_9_1209	DJI_0567	Amorpha fruticosa L.	0	a	0	0	Fabaceae Lindl.
Iz_9_1209	DJI_0567	Anthericum ramosum L.	0	n	1	0	Asparagaceae Juss.
Iz_9_1209	DJI_0567	Artemisia marschalliana Spreng.	0	n	1	0	Asteraceae Bercht. & J.Presl
Iz_9_1209	DJI_0567	Asparagus officinalis L.	0	n	1	0	Asparagaceae Juss.
Iz_9_1209	DJI_0567	Astragalus cicer L.	0	n	1	0	Fabaceae Lindl.
Iz_9_1209	DJI_0567	Bassia laniflora (S.G.Gmel.) A.J.Scott	0	n	1	0	Amaranthaceae Juss.
Iz_9_1209	DJI_0567	Berberis vulgaris L.	0	n	1	0	Berberidaceae Juss.
Iz_9_1209	DJI_0567	Betula pendula Roth.	0	n	1	1	Betulaceae Gray
Iz_9_1209	DJI_0567	Betula pendula Roth.	0	n	1	0	Poaceae Barnhart
Iz_9_1209	DJI_0567	Bromus inermis Leyss.	0		NA	NA	

Iz_9_1209	DJI_0567	Bromus squarrosus L.	0	a	0	0	Poaceae Barnhart
Iz_9_1209	DJI_0567	Bromus tectorum L.	0	a	0	0	Poaceae Barnhart
Iz_9_1209	DJI_0567	Calamagrostis epigejos (L.) Roth	15	n	1	0	Poaceae Barnhart
Iz_9_1209	DJI_0567	Carduus acanthoides L.	0	n	1	0	Asteraceae Bercht. & J.Presl
Iz_9_1209	DJI_0567	Carex sp.	0	n	1	0	Cyperaceae Juss.
Iz_9_1209	DJI_0567	Centaurea majorovii Dumbadze	0	n	1	0	Asteraceae Bercht. & J.Presl
Iz_9_1209	DJI_0567	Chamaecytisus ruthenicus (Fisch.ex Wol.) Klaskova	0	n	1	0	Fabaceae Lindl.
Iz_9_1209	DJI_0567	Chelidonium majus L.	0	n	1	0	Papaveraceae Juss.
Iz_9_1209	DJI_0567	Chenopodium betaceum Andrz.	0	n	1	0	Amaranthaceae Juss.
Iz_9_1209	DJI_0567	Chenopodium cf. album	0	n	1	0	Amaranthaceae Juss.
Iz_9_1209	DJI_0567	Chenopodium sp.	1		NA	0	Amaranthaceae Juss.
Iz_9_1209	DJI_0567	Chondrilla juncea L.	0	n	1	0	Asteraceae Bercht. & J.Presl
Iz_9_1209	DJI_0567	Cirsium vulgare (Savi) Ten.	0	n	1	0	Asteraceae Bercht. & J.Presl
Iz_9_1209	DJI_0567	Clinopodium acinos (L.) Kuntze	0	n	1	0	Lamiacea Lindl.
Iz_9_1209	DJI_0567	Convallaria majalis L.	0	n	1	0	Asparagaceae Juss.
Iz_9_1209	DJI_0567	Crataegus rhipidophylla Gand.	0	n	1	1	Rosaceae Juss.
Iz_9_1209	DJI_0567	Crepis foetida L.	0	n	1	0	Asteraceae Bercht. & J.Presl
Iz_9_1209	DJI_0567	Dactylis glomerata L.	0	n	1	0	Poaceae Barnhart
Iz_9_1209	DJI_0567	Dianthus campestris M.Bieb.	0	n	1	0	Caryophyllaceae Juss.
Iz_9_1209	DJI_0567	Digitaria sanguinalis (L.) Scop.	0	a	0	0	Poaceae Barnhart
Iz_9_1209	DJI_0567	Elymus repens (L.) Gould	0	n	1	0	Poaceae Barnhart
Iz_9_1209	DJI_0567	Epilobium tetragonum L.	0	n	1	0	Onagraceae Juss.
Iz_9_1209	DJI_0567	Eragrostis minor Host	0	a	0	0	Poaceae Barnhart
Iz_9_1209	DJI_0567	Erigeron canadensis L.	2	a	0	0	Asteraceae Bercht. & J.Presl
Iz_9_1209	DJI_0567	Euphorbia seguieriana Neck.	0	n	1	0	Euphorbiaceae Juss.
Iz_9_1209	DJI_0567	Fallopia dumetorum (L.) Holub	0	n	1	0	Polygonaceae Juss.
Iz_9_1209	DJI_0567	Festuca beckeri (Hack.) Trautv.	0	n	1	0	Poaceae Barnhart
Iz_9_1209	DJI_0567	Fragaria vesca L.	0	n	1	0	Rosaceae Juss.
Iz_9_1209	DJI_0567	Frangula alnus Mill.	0	n	1	1	Rhamnaceae Juss.
Iz_9_1209	DJI_0567	Fraxinus pennsylvanica Marshall	0	a	0	1	Oleaceae Hoffmanns. & Link
Iz_9_1209	DJI_0567	Galeopsis bifida Boenn.	0	n	1	0	Lamiacea Lindl.
Iz_9_1209	DJI_0567	Galium verum L.	0	n	1	0	Rubiaceae Juss.
Iz_9_1209	DJI_0567	Genista tinctoria L.	0	n	1	0	Fabaceae Lindl.
Iz_9_1209	DJI_0567	Geranium robertianum L.	0	n	1	0	Geraniaceae Juss.
Iz_9_1209	DJI_0567	Geranium sanquineum L.	0	n	1	0	Geraniaceae Juss.
Iz_9_1209	DJI_0567	Gypsophila paniculata L.	0	n	1	0	Caryophyllaceae Juss.
Iz_9_1209	DJI_0567	Helichrysum arenarium (L.) Moench	0	n	1	0	Asteraceae Bercht. & J.Presl
Iz_9_1209	DJI_0567	Herniaria polygama J.Gay	0	n	1	0	Caryophyllaceae Juss.
Iz_9_1209	DJI_0567	Hieracium robustum Fr.	0	n	1	0	Asteraceae Bercht. & J.Presl
Iz_9_1209	DJI_0567	Hieracium umbellatum L.	0	n	1	NA	Asteraceae Bercht. & J.Presl
Iz_9_1209	DJI_0567	Hylotelephium maximum (L.) Holub	0	n	1	0	Crassulaceae J.St.-Hil.
Iz_9_1209	DJI_0567	Jacobaea borysthenica (DC.) B.Nord. & Greuter	0	n	1	0	Asteraceae Bercht. & J.Presl
Iz_9_1209	DJI_0567	Jasione montana L.	0	n	1	0	Campanulaceae Juss.
Iz_9_1209	DJI_0567	Jurinea cyanoides (L.) Rchb.s.l.	0	n	1	0	Asteraceae Bercht. & J.Presl
Iz_9_1209	DJI_0567	Koeleria glauca (Spreng.) DC.	0	n	1	0	Poaceae Barnhart
Iz_9_1209	DJI_0567	Lactuca serriola L.	0	a	0	0	Asteraceae Bercht. & J.Presl
Iz_9_1209	DJI_0567	Linaria genistifolia (L.) Mill.	0	n	1	0	Plantaginaceae Juss.
Iz_9_1209	DJI_0567	Lomelosia argentea (L.) Greuter & Burdet	0	n	1	0	Caprifoliaceae Juss.
Iz_9_1209	DJI_0567	Melampyrum pratense L.	0	n	1	0	Orobanchaceae Vent.
Iz_9_1209	DJI_0567	Oenothera biennis L.	0	a	0	0	Onagraceae Juss.
Iz_9_1209	DJI_0567	Pentanema britannica (L.) D.Gut.Larr., Santos-Vicente, Anderb., E.Rico & M.M.Mart.Ort.	0	n	1	0	Asteraceae Bercht. & J.Presl
Iz_9_1209	DJI_0567	Peucedanum oreoselinum (L.) Moench	0	n	1	0	Apiaceae Lindl.
Iz_9_1209	DJI_0567	Peucedanum ruthenicum M.Bieb.	0	n	1	0	Apiaceae Lindl.
Iz_9_1209	DJI_0567	Pilosella echioides (Lumn.) F.W.Schultz & Sch.Bip.	0	n	1	0	Asteraceae Bercht. & J.Presl
Iz_9_1209	DJI_0567	Pilosella officinarum F. Schultz et Sch. Bip.	0	n	1	0	Asteraceae Bercht. & J.Presl
Iz_9_1209	DJI_0567	Pilosella sp.	0		NA	0	Asteraceae Bercht. & J.Presl
Iz_9_1209	DJI_0567	Pinus sylvestris L.	0	n	1	1	Pinaceae Spreng. ex F.Rudolphi
Iz_9_1209	DJI_0567	Poa annua L.	0	n	1	0	Poaceae Barnhart
Iz_9_1209	DJI_0567	Poa nemoralis L.	0	n	1	0	Poaceae Barnhart
Iz_9_1209	DJI_0567	Poa sp.	0		NA	0	Poaceae Barnhart
Iz_9_1209	DJI_0567	Polygonatum odoratum (Mill.) Druce	0	n	1	0	Asparagaceae Juss.
Iz_9_1209	DJI_0567	Polygonum arenarium Waldst. & Kit.	0	n	1	0	Polygonaceae Juss.
Iz_9_1209	DJI_0567	Populus nigra L.	0	n	1	1	Salicaceae Mirb.
Iz_9_1209	DJI_0567	Populus tremula L.	0	n	1	1	Salicaceae Mirb.
Iz_9_1209	DJI_0567	Populus Ã canescens (Aiton) Sm.	0	n	1	1	Salicaceae Mirb.
Iz_9_1209	DJI_0567	Potentilla argentea L.	0	n	1	0	Rosaceae Juss.
Iz_9_1209	DJI_0567	Psammophiliella muralis (L.) Ikonn.	0	n	1	0	Caryophyllaceae Juss.
Iz_9_1209	DJI_0567	Psephellus sumensis (Kalen.) Greuter	0	n	1	0	Asteraceae Bercht. & J.Presl
Iz_9_1209	DJI_0567	Pulsatilla pratensis (L.) Mill. s.l.	0	n	1	0	Ranunculaceae Juss.
Iz_9_1209	DJI_0567	Pyrus communis L.	0	a	0	1	Rosaceae Juss.
Iz_9_1209	DJI_0567	Quercus robur L.	0	n	1	1	Fagaceae Dumort.
Iz_9_1209	DJI_0567	Robinia pseudoacacia L.	0	a	0	1	Fabaceae Lindl.
Iz_9_1209	DJI_0567	Rosa sp.	0		NA	1	Rosaceae Juss.
Iz_9_1209	DJI_0567	Rubus idaeus L.	0	n	1	0	Rosaceae Juss.
Iz_9_1209	DJI_0567	Rumex acetosella L.	0	n	1	0	Polygonaceae Juss.
Iz_9_1209	DJI_0567	Rumex thyrsiflorus Fingerh.	0	n	1	0	Polygonaceae Juss.
Iz_9_1209	DJI_0567	Salix alba L.	0	n	1	1	Salicaceae Mirb.
Iz_9_1209	DJI_0567	Sambucus nigra L.	0	n	1	1	Viburnaceae Raf.
Iz_9_1209	DJI_0567	Secale sylvestre Host	0	n	1	0	Poaceae Barnhart
Iz_9_1209	DJI_0567	Sempervivum ruthenicum Schnittsp. & C.B. Lehm.	0	n	1	0	Crassulaceae J.St.-Hil.
Iz_9_1209	DJI_0567	Setaria viridis (L.) P. Beauv.	0	n	1	0	Poaceae Barnhart
Iz_9_1209	DJI_0567	Silene borysthenica (Gruner) Walters	0	n	1	0	Caryophyllaceae Juss.
Iz_9_1209	DJI_0567	Silene latifolia subsp. alba (Mill.) Greuter & Burdet	0	n	1	0	Caryophyllaceae Juss.
Iz_9_1209	DJI_0567	Solidago virgaurea L.	0	n	1	0	Asteraceae Bercht. & J.Presl
Iz_9_1209	DJI_0567	Sorbus aucuparia L.	0	n	1	1	Rosaceae Juss.
Iz_9_1209	DJI_0567	Species 12.09_9_0601	0		NA	NA	
Iz_9_1209	DJI_0567	Stachys recta L.	0	n	1	0	Lamiacea Lindl.
Iz_9_1209	DJI_0567	Tanacetum vulgare L.	0	n	1	0	Asteraceae Bercht. & J.Presl
Iz_9_1209	DJI_0567	Thymus pallasianus Heinr.Braun	0	n	1	0	Lamiacea Lindl.
Iz_9_1209	DJI_0567	Tilia cordata Mill.	0	n	1	1	Malvaceae Juss.
Iz_9_1209	DJI_0567	Trifolium alpestre L.	0	n	1	0	Fabaceae Lindl.
Iz_9_1209	DJI_0567	Trifolium arvense L.	0	n	1	0	Fabaceae Lindl.
Iz_9_1209	DJI_0567	Ulmus glabra Huds.	0	n	1	1	Ulmaceae Mirb.
Iz_9_1209	DJI_0567	Verbascum densiflorum Bertol.	0	n	1	0	Scrophulariaceae Juss.
Iz_9_1209	DJI_0567	Veronica austriaca L.	0	n	1	0	Plantaginaceae Juss.
Iz_9_1209	DJI_0567	Veronica incana L.	0	n	1	0	Plantaginaceae Juss.
Iz_9_1209	DJI_0567	Veronica verna L.	0	n	1	0	Plantaginaceae Juss.
Iz_9_1209	DJI_0567	Vincetoxicum hirundinaria Medik	0	n	1	0	Apocynaceae Juss.

Iz_9_1209	DJI_0569	Acer tataricum L.	0	n	1	1	Sapindaceae Juss.
Iz_9_1209	DJI_0569	Achillea millefolium L. s.l.	0	n	1	0	Asteraceae Bercht. & J.Presl
Iz_9_1209	DJI_0569	Allium oleraceum L.	0	n	1	0	Amaryllidaceae J.St.-Hil.
Iz_9_1209	DJI_0569	Ambrosia artemisiifolia L.	0	a	0	0	Asteraceae Bercht. & J.Presl
Iz_9_1209	DJI_0569	Amorpha fruticosa L.	0	a	0	0	Fabaceae Lindl.
Iz_9_1209	DJI_0569	Anthericum ramosum L.	0	n	1	0	Asparagaceae Juss.
Iz_9_1209	DJI_0569	Artemisia marschalliana Spreng.	0	n	1	0	Asteraceae Bercht. & J.Presl
Iz_9_1209	DJI_0569	Asparagus officinalis L.	0	n	1	0	Asparagaceae Juss.
Iz_9_1209	DJI_0569	Astragalus cicer L.	0	n	1	0	Fabaceae Lindl.
Iz_9_1209	DJI_0569	Bassia laniflora (S.G.Gmel.) A.J.Scott	0	n	1	0	Amaranthaceae Juss.
Iz_9_1209	DJI_0569	Berberis vulgaris L.	0	n	1	0	Berberidaceae Juss.
Iz_9_1209	DJI_0569	Betula pendula Roth.	0	n	1	1	Betulaceae Gray
Iz_9_1209	DJI_0569	Betula pendula Roth.	0	n	1	0	Poaceae Barnhart
Iz_9_1209	DJI_0569	Bromus inermis Leyss.	0		NA	NA	
Iz_9_1209	DJI_0569	Bromus squarrosus L.	0	a	0	0	Poaceae Barnhart
Iz_9_1209	DJI_0569	Bromus tectorum L.	0	a	0	0	Poaceae Barnhart
Iz_9_1209	DJI_0569	Calamagrostis epigejos (L.) Roth	60	n	1	0	Poaceae Barnhart
Iz_9_1209	DJI_0569	Carduus acanthoides L.	0	n	1	0	Asteraceae Bercht. & J.Presl
Iz_9_1209	DJI_0569	Carex sp.	0	n	1	0	Cyperaceae Juss.
Iz_9_1209	DJI_0569	Centaurea majorovii Dumbadze	0	n	1	0	Asteraceae Bercht. & J.Presl
Iz_9_1209	DJI_0569	Chamaecytisus ruthenicus (Fisch.ex Wol.) Klaskova	0	n	1	0	Fabaceae Lindl.
Iz_9_1209	DJI_0569	Chelidonium majus L.	0	n	1	0	Papaveraceae Juss.
Iz_9_1209	DJI_0569	Chenopodium betaceum Andrz.	0	n	1	0	Amaranthaceae Juss.
Iz_9_1209	DJI_0569	Chenopodium cf. album	0	n	1	0	Amaranthaceae Juss.
Iz_9_1209	DJI_0569	Chenopodium sp.	0		NA	0	Amaranthaceae Juss.
Iz_9_1209	DJI_0569	Chondrilla juncea L.	0	n	1	0	Asteraceae Bercht. & J.Presl
Iz_9_1209	DJI_0569	Cirsium vulgare (Savi) Ten.	0	n	1	0	Asteraceae Bercht. & J.Presl
Iz_9_1209	DJI_0569	Clinopodium acinos (L.) Kuntze	0	n	1	0	Lamiacea Lindl.
Iz_9_1209	DJI_0569	Convallaria majalis L.	0	n	1	0	Asparagaceae Juss.
Iz_9_1209	DJI_0569	Crataegus rhipidophylla Gand.	0	n	1	1	Rosaceae Juss.
Iz_9_1209	DJI_0569	Crepis foetida L.	0	n	1	0	Asteraceae Bercht. & J.Presl
Iz_9_1209	DJI_0569	Dactylis glomerata L.	0	n	1	0	Poaceae Barnhart
Iz_9_1209	DJI_0569	Dianthus campestris M.Bieb.	0	n	1	0	Caryophyllaceae Juss.
Iz_9_1209	DJI_0569	Digitaria sanguinalis (L.) Scop.	0	a	0	0	Poaceae Barnhart
Iz_9_1209	DJI_0569	Elymus repens (L.) Gould	0	n	1	0	Poaceae Barnhart
Iz_9_1209	DJI_0569	Epilobium tetragonum L.	0	n	1	0	Onagraceae Juss.
Iz_9_1209	DJI_0569	Eragrostis minor Host	0	a	0	0	Poaceae Barnhart
Iz_9_1209	DJI_0569	Erigeron canadensis L.	0	a	0	0	Asteraceae Bercht. & J.Presl
Iz_9_1209	DJI_0569	Euphorbia seguieriana Neck.	0	n	1	0	Euphorbiaceae Juss.
Iz_9_1209	DJI_0569	Fallopia dumetorum (L.) Holub	0	n	1	0	Polygonaceae Juss.
Iz_9_1209	DJI_0569	Festuca beckeri (Hack.) Trautv.	0	n	1	0	Poaceae Barnhart
Iz_9_1209	DJI_0569	Fragaria vesca L.	0	n	1	0	Rosaceae Juss.
Iz_9_1209	DJI_0569	Frangula alnus Mill.	0	n	1	1	Rhamnaceae Juss.
Iz_9_1209	DJI_0569	Fraxinus pennsylvanica Marshall	0	a	0	1	Oleaceae Hoffmanns. & Link
Iz_9_1209	DJI_0569	Galeopsis bifida Boenn.	0	n	1	0	Lamiacea Lindl.
Iz_9_1209	DJI_0569	Galium verum L.	0	n	1	0	Rubiaceae Juss.
Iz_9_1209	DJI_0569	Genista tinctoria L.	0	n	1	0	Fabaceae Lindl.
Iz_9_1209	DJI_0569	Geranium robertianum L.	0	n	1	0	Geraniaceae Juss.
Iz_9_1209	DJI_0569	Geranium sanquineum L.	0	n	1	0	Geraniaceae Juss.
Iz_9_1209	DJI_0569	Gypsophila paniculata L.	0	n	1	0	Caryophyllaceae Juss.
Iz_9_1209	DJI_0569	Helichrysum arenarium (L.) Moench	0	n	1	0	Asteraceae Bercht. & J.Presl
Iz_9_1209	DJI_0569	Herniaria polygama J.Gay	0	n	1	0	Caryophyllaceae Juss.
Iz_9_1209	DJI_0569	Hieracium robustum Fr.	0	n	1	0	Asteraceae Bercht. & J.Presl
Iz_9_1209	DJI_0569	Hieracium umbellatum L.	0	n	1	NA	Asteraceae Bercht. & J.Presl
Iz_9_1209	DJI_0569	Hylotelephium maximum (L.) Holub	0	n	1	0	Crassulaceae J.St.-Hil.
Iz_9_1209	DJI_0569	Jacobaea borysthenica (DC.) B.Nord. & Greuter	0	n	1	0	Asteraceae Bercht. & J.Presl
Iz_9_1209	DJI_0569	Jasione montana L.	0	n	1	0	Campanulaceae Juss.
Iz_9_1209	DJI_0569	Jurinea cyanoides (L.) Rchb.s.l.	0	n	1	0	Asteraceae Bercht. & J.Presl
Iz_9_1209	DJI_0569	Koeleria glauca (Spreng.) DC.	0	n	1	0	Poaceae Barnhart
Iz_9_1209	DJI_0569	Lactuca serriola L.	0	a	0	0	Asteraceae Bercht. & J.Presl
Iz_9_1209	DJI_0569	Linaria genistifolia (L.) Mill.	0	n	1	0	Plantaginaceae Juss.
Iz_9_1209	DJI_0569	Lomelosia argentea (L.) Greuter & Burdet	0	n	1	0	Caprifoliaceae Juss.
Iz_9_1209	DJI_0569	Melampyrum pratense L.	0	n	1	0	Orobanchaceae Vent.
Iz_9_1209	DJI_0569	Oenothera biennis L.	0	a	0	0	Onagraceae Juss.
Iz_9_1209	DJI_0569	Pentanema britannica (L.) D.Gut.Larr., Santos-Vicente, Anderb., E.Rico & M.M.Mart.Ort.	0	n	1	0	Asteraceae Bercht. & J.Presl
Iz_9_1209	DJI_0569	Peucedanum oreoselinum (L.) Moench	0	n	1	0	Apiaceae Lindl.
Iz_9_1209	DJI_0569	Peucedanum ruthenicum M.Bieb.	0	n	1	0	Apiaceae Lindl.
Iz_9_1209	DJI_0569	Pilosella echioides (Lumn.) F.W.Schultz & Sch.Bip.	0	n	1	0	Asteraceae Bercht. & J.Presl
Iz_9_1209	DJI_0569	Pilosella officinarum F. Schultz et Sch. Bip.	0	n	1	0	Asteraceae Bercht. & J.Presl
Iz_9_1209	DJI_0569	Pilosella sp.	0		NA	0	Asteraceae Bercht. & J.Presl
Iz_9_1209	DJI_0569	Pinus sylvestris L.	0	n	1	1	Pinaceae Spreng. ex F.Rudolphi
Iz_9_1209	DJI_0569	Poa annua L.	0	n	1	0	Poaceae Barnhart
Iz_9_1209	DJI_0569	Poa nemoralis L.	0	n	1	0	Poaceae Barnhart
Iz_9_1209	DJI_0569	Poa sp.	0		NA	0	Poaceae Barnhart
Iz_9_1209	DJI_0569	Polygonatum odoratum (Mill.) Druce	0	n	1	0	Asparagaceae Juss.
Iz_9_1209	DJI_0569	Polygonum arenarium Waldst. & Kit.	0	n	1	0	Polygonaceae Juss.
Iz_9_1209	DJI_0569	Populus nigra L.	0	n	1	1	Salicaceae Mirb.
Iz_9_1209	DJI_0569	Populus tremula L.	0	n	1	1	Salicaceae Mirb.
Iz_9_1209	DJI_0569	Populus Ã canescens (Aiton) Sm.	0	n	1	1	Salicaceae Mirb.
Iz_9_1209	DJI_0569	Potentilla argentea L.	0	n	1	0	Rosaceae Juss.
Iz_9_1209	DJI_0569	Psammophiliella muralis (L.) Ikonn.	0	n	1	0	Caryophyllaceae Juss.
Iz_9_1209	DJI_0569	Psephellus sumensis (Kalen.) Greuter	0	n	1	0	Asteraceae Bercht. & J.Presl
Iz_9_1209	DJI_0569	Pulsatilla pratensis (L.) Mill. s.l.	0	n	1	0	Ranunculaceae Juss.
Iz_9_1209	DJI_0569	Pyrus communis L.	0	a	0	1	Rosaceae Juss.
Iz_9_1209	DJI_0569	Quercus robur L.	0	n	1	1	Fagaceae Dumort.
Iz_9_1209	DJI_0569	Robinia pseudoacacia L.	0	a	0	1	Fabaceae Lindl.
Iz_9_1209	DJI_0569	Rosa sp.	0		NA	1	Rosaceae Juss.
Iz_9_1209	DJI_0569	Rubus idaeus L.	0	n	1	0	Rosaceae Juss.
Iz_9_1209	DJI_0569	Rumex acetosella L.	0	n	1	0	Polygonaceae Juss.
Iz_9_1209	DJI_0569	Rumex thyrsiflorus Fingerh.	0	n	1	0	Polygonaceae Juss.
Iz_9_1209	DJI_0569	Salix alba L.	0	n	1	1	Salicaceae Mirb.
Iz_9_1209	DJI_0569	Sambucus nigra L.	0	n	1	1	Viburnaceae Raf.
Iz_9_1209	DJI_0569	Secale sylvestre Host	0	n	1	0	Poaceae Barnhart
Iz_9_1209	DJI_0569	Sempervivum ruthenicum Schnittsp. & C.B. Lehm.	0	n	1	0	Crassulaceae J.St.-Hil.
Iz_9_1209	DJI_0569	Setaria viridis (L.) P. Beauv.	0	n	1	0	Poaceae Barnhart
Iz_9_1209	DJI_0569	Silene borysthenica (Gruner) Walters	0	n	1	0	Caryophyllaceae Juss.
Iz_9_1209	DJI_0569	Silene latifolia subsp. alba (Mill.) Greuter & Burdet	0	n	1	0	Caryophyllaceae Juss.
Iz_9_1209	DJI_0569	Solidago virgaurea L.	0	n	1	0	Asteraceae Bercht. & J.Presl

Iz_9_1209	DJI_0569	Sorbus aucuparia L.	0	n	1	1	Rosaceae Juss.
Iz_9_1209	DJI_0569	Species 12.09_9_0601	0		NA	NA	
Iz_9_1209	DJI_0569	Stachys recta L.	0	n	1	0	Lamiacea Lindl.
Iz_9_1209	DJI_0569	Tanacetum vulgare L.	0	n	1	0	Asteraceae Bercht. & J.Presl
Iz_9_1209	DJI_0569	Thymus pallasianus Heinr.Braun	0	n	1	0	Lamiacea Lindl.
Iz_9_1209	DJI_0569	Tilia cordata Mill.	0	n	1	1	Malvaceae Juss.
Iz_9_1209	DJI_0569	Trifolium alpestre L.	0	n	1	0	Fabaceae Lindl.
Iz_9_1209	DJI_0569	Trifolium arvense L.	0	n	1	0	Fabaceae Lindl.
Iz_9_1209	DJI_0569	Ulmus glabra Huds.	0	n	1	1	Ulmaceae Mirb.
Iz_9_1209	DJI_0569	Verbascum densiflorum Bertol.	0	n	1	0	Scrophulariaceae Juss.
Iz_9_1209	DJI_0569	Veronica austriaca L.	0	n	1	0	Plantaginaceae Juss.
Iz_9_1209	DJI_0569	Veronica incana L.	0	n	1	0	Plantaginaceae Juss.
Iz_9_1209	DJI_0569	Veronica verna L.	0	n	1	0	Plantaginaceae Juss.
Iz_9_1209	DJI_0569	Vincetoxicum hirundinaria Medik	0	n	1	0	Apocynaceae Juss.
Iz_9_1209	DJI_0572	Acer tataricum L.	0	n	1	1	Sapindaceae Juss.
Iz_9_1209	DJI_0572	Achillea millefolium L. s.l.	0	n	1	0	Asteraceae Bercht. & J.Presl
Iz_9_1209	DJI_0572	Allium oleraceum L.	0	n	1	0	Amaryllidaceae J.St.-Hil.
Iz_9_1209	DJI_0572	Ambrosia artemisiifolia L.	0	a	0	0	Asteraceae Bercht. & J.Presl
Iz_9_1209	DJI_0572	Amorpha fruticosa L.	0	a	0	0	Fabaceae Lindl.
Iz_9_1209	DJI_0572	Anthericum ramosum L.	0	n	1	0	Asparagaceae Juss.
Iz_9_1209	DJI_0572	Artemisia marschalliana Spreng.	0	n	1	0	Asteraceae Bercht. & J.Presl
Iz_9_1209	DJI_0572	Asparagus officinalis L.	0	n	1	0	Asparagaceae Juss.
Iz_9_1209	DJI_0572	Astragalus cicer L.	0	n	1	0	Fabaceae Lindl.
Iz_9_1209	DJI_0572	Bassia laniflora (S.G.Gmel.) A.J.Scott	0	n	1	0	Amaranthaceae Juss.
Iz_9_1209	DJI_0572	Berberis vulgaris L.	0	n	1	0	Berberidaceae Juss.
Iz_9_1209	DJI_0572	Betula pendula Roth.	0	n	1	1	Betulaceae Gray
Iz_9_1209	DJI_0572	Betula pendula Roth.	0	n	1	0	Poaceae Barnhart
Iz_9_1209	DJI_0572	Bromus inermis Leyss.	0		NA	NA	
Iz_9_1209	DJI_0572	Bromus squarrosus L.	0	a	0	0	Poaceae Barnhart
Iz_9_1209	DJI_0572	Bromus tectorum L.	0	a	0	0	Poaceae Barnhart
Iz_9_1209	DJI_0572	Calamagrostis epigejos (L.) Roth	20	n	1	0	Poaceae Barnhart
Iz_9_1209	DJI_0572	Carduus acanthoides L.	0	n	1	0	Asteraceae Bercht. & J.Presl
Iz_9_1209	DJI_0572	Carex sp.	0	n	1	0	Cyperaceae Juss.
Iz_9_1209	DJI_0572	Centaurea majorovii Dumbadze	0	n	1	0	Asteraceae Bercht. & J.Presl
Iz_9_1209	DJI_0572	Chamaecytisus ruthenicus (Fisch.ex Wol.) Klaskova	0	n	1	0	Fabaceae Lindl.
Iz_9_1209	DJI_0572	Chelidonium majus L.	0	n	1	0	Papaveraceae Juss.
Iz_9_1209	DJI_0572	Chenopodium betaceum Andrz.	0	n	1	0	Amaranthaceae Juss.
Iz_9_1209	DJI_0572	Chenopodium cf. album	0	n	1	0	Amaranthaceae Juss.
Iz_9_1209	DJI_0572	Chenopodium sp.	0		NA	0	Amaranthaceae Juss.
Iz_9_1209	DJI_0572	Chondrilla juncea L.	0	n	1	0	Asteraceae Bercht. & J.Presl
Iz_9_1209	DJI_0572	Cirsium vulgare (Savi) Ten.	0	n	1	0	Asteraceae Bercht. & J.Presl
Iz_9_1209	DJI_0572	Clinopodium acinos (L.) Kuntze	0	n	1	0	Lamiacea Lindl.
Iz_9_1209	DJI_0572	Convallaria majalis L.	0	n	1	0	Asparagaceae Juss.
Iz_9_1209	DJI_0572	Crataegus rhipidophylla Gand.	0	n	1	1	Rosaceae Juss.
Iz_9_1209	DJI_0572	Crepis foetida L.	0	n	1	0	Asteraceae Bercht. & J.Presl
Iz_9_1209	DJI_0572	Dactylis glomerata L.	0	n	1	0	Poaceae Barnhart
Iz_9_1209	DJI_0572	Dianthus campestris M.Bieb.	0	n	1	0	Caryophyllaceae Juss.
Iz_9_1209	DJI_0572	Digitaria sanguinalis (L.) Scop.	0	a	0	0	Poaceae Barnhart
Iz_9_1209	DJI_0572	Elymus repens (L.) Gould	0	n	1	0	Poaceae Barnhart
Iz_9_1209	DJI_0572	Epilobium tetragonum L.	0	n	1	0	Onagraceae Juss.
Iz_9_1209	DJI_0572	Eragrostis minor Host	0	a	0	0	Poaceae Barnhart
Iz_9_1209	DJI_0572	Erigeron canadensis L.	3	a	0	0	Asteraceae Bercht. & J.Presl
Iz_9_1209	DJI_0572	Euphorbia seguieriana Neck.	0	n	1	0	Euphorbiaceae Juss.
Iz_9_1209	DJI_0572	Fallopia dumetorum (L.) Holub	0	n	1	0	Polygonaceae Juss.
Iz_9_1209	DJI_0572	Festuca beckeri (Hack.) Trautv.	0	n	1	0	Poaceae Barnhart
Iz_9_1209	DJI_0572	Fragaria vesca L.	0	n	1	0	Rosaceae Juss.
Iz_9_1209	DJI_0572	Frangula alnus Mill.	0	n	1	1	Rhamnaceae Juss.
Iz_9_1209	DJI_0572	Fraxinus pennsylvanica Marshall	0	a	0	1	Oleaceae Hoffmanns. & Link
Iz_9_1209	DJI_0572	Galeopsis bifida Boenn.	0	n	1	0	Lamiacea Lindl.
Iz_9_1209	DJI_0572	Galium verum L.	0	n	1	0	Rubiaceae Juss.
Iz_9_1209	DJI_0572	Genista tinctoria L.	0	n	1	0	Fabaceae Lindl.
Iz_9_1209	DJI_0572	Geranium robertianum L.	0	n	1	0	Geraniaceae Juss.
Iz_9_1209	DJI_0572	Geranium sanquineum L.	0	n	1	0	Geraniaceae Juss.
Iz_9_1209	DJI_0572	Gypsophila paniculata L.	0	n	1	0	Caryophyllaceae Juss.
Iz_9_1209	DJI_0572	Helichrysum arenarium (L.) Moench	0	n	1	0	Asteraceae Bercht. & J.Presl
Iz_9_1209	DJI_0572	Herniaria polygama J.Gay	0	n	1	0	Caryophyllaceae Juss.
Iz_9_1209	DJI_0572	Hieracium robustum Fr.	0	n	1	0	Asteraceae Bercht. & J.Presl
Iz_9_1209	DJI_0572	Hieracium umbellatum L.	0	n	1	NA	Asteraceae Bercht. & J.Presl
Iz_9_1209	DJI_0572	Hylotelephium maximum (L.) Holub	0	n	1	0	Crassulaceae J.St.-Hil.
Iz_9_1209	DJI_0572	Jacobaea borysthenica (DC.) B.Nord. & Greuter	0	n	1	0	Asteraceae Bercht. & J.Presl
Iz_9_1209	DJI_0572	Jasione montana L.	0	n	1	0	Campanulaceae Juss.
Iz_9_1209	DJI_0572	Jurinea cyanoides (L.) Rchb.s.l.	0	n	1	0	Asteraceae Bercht. & J.Presl
Iz_9_1209	DJI_0572	Koeleria glauca (Spreng.) DC.	0	n	1	0	Poaceae Barnhart
Iz_9_1209	DJI_0572	Lactuca serriola L.	0	a	0	0	Asteraceae Bercht. & J.Presl
Iz_9_1209	DJI_0572	Linaria genistifolia (L.) Mill.	0	n	1	0	Plantaginaceae Juss.
Iz_9_1209	DJI_0572	Lomelosia argentea (L.) Greuter & Burdet	0	n	1	0	Caprifoliaceae Juss.
Iz_9_1209	DJI_0572	Melampyrum pratense L.	0	n	1	0	Orobanchaceae Vent.
Iz_9_1209	DJI_0572	Oenothera biennis L.	0	a	0	0	Onagraceae Juss.
Iz_9_1209	DJI_0572	Pentanema britannica (L.) D.Gut.Larr., Santos-Vicente, Anderb., E.Rico & M.M.Mart.Ort.	0	n	1	0	Asteraceae Bercht. & J.Presl
Iz_9_1209	DJI_0572	Peucedanum oreoselinum (L.) Moench	0	n	1	0	Apiaceae Lindl.
Iz_9_1209	DJI_0572	Peucedanum ruthenicum M.Bieb.	0	n	1	0	Apiaceae Lindl.
Iz_9_1209	DJI_0572	Pilosella echioides (Lumn.) F.W.Schultz & Sch.Bip.	0	n	1	0	Asteraceae Bercht. & J.Presl
Iz_9_1209	DJI_0572	Pilosella officinarum F. Schultz et Sch. Bip.	0	n	1	0	Asteraceae Bercht. & J.Presl
Iz_9_1209	DJI_0572	Pilosella sp.	0		NA	0	Asteraceae Bercht. & J.Presl
Iz_9_1209	DJI_0572	Pinus sylvestris L.	0	n	1	1	Pinaceae Spreng. ex F.Rudolphi
Iz_9_1209	DJI_0572	Poa annua L.	0	n	1	0	Poaceae Barnhart
Iz_9_1209	DJI_0572	Poa nemoralis L.	0	n	1	0	Poaceae Barnhart
Iz_9_1209	DJI_0572	Poa sp.	0		NA	0	Poaceae Barnhart
Iz_9_1209	DJI_0572	Polygonatum odoratum (Mill.) Druce	0	n	1	0	Asparagaceae Juss.
Iz_9_1209	DJI_0572	Polygonum arenarium Waldst. & Kit.	0	n	1	0	Polygonaceae Juss.
Iz_9_1209	DJI_0572	Populus nigra L.	0	n	1	1	Salicaceae Mirb.
Iz_9_1209	DJI_0572	Populus tremula L.	0	n	1	1	Salicaceae Mirb.
Iz_9_1209	DJI_0572	Populus Ã canescens (Aiton) Sm.	0	n	1	1	Salicaceae Mirb.
Iz_9_1209	DJI_0572	Potentilla argentea L.	0	n	1	0	Rosaceae Juss.
Iz_9_1209	DJI_0572	Psammophiliella muralis (L.) Ikonn.	0	n	1	0	Caryophyllaceae Juss.
Iz_9_1209	DJI_0572	Psephellus sumensis (Kalen.) Greuter	0	n	1	0	Asteraceae Bercht. & J.Presl
Iz_9_1209	DJI_0572	Pulsatilla pratensis (L.) Mill. s.l.	0	n	1	0	Ranunculaceae Juss.
Iz_9_1209	DJI_0572	Pyrus communis L.	0	a	0	1	Rosaceae Juss.

Iz_9_1209	DJI_0572	Quercus robur L.	0	n	1	1	Fagaceae Dumort.
Iz_9_1209	DJI_0572	Robinia pseudoacacia L.	0	a	0	1	Fabaceae Lindl.
Iz_9_1209	DJI_0572	Rosa sp.	0		NA	1	Rosaceae Juss.
Iz_9_1209	DJI_0572	Rubus idaeus L.	0	n	1	0	Rosaceae Juss.
Iz_9_1209	DJI_0572	Rumex acetosella L.	0	n	1	0	Polygonaceae Juss.
Iz_9_1209	DJI_0572	Rumex thyrsiflorus Fingerh.	0	n	1	0	Polygonaceae Juss.
Iz_9_1209	DJI_0572	Salix alba L.	0	n	1	1	Salicaceae Mirb.
Iz_9_1209	DJI_0572	Sambucus nigra L.	0	n	1	1	Viburnaceae Raf.
Iz_9_1209	DJI_0572	Secale sylvestre Host	0	n	1	0	Poaceae Barnhart
Iz_9_1209	DJI_0572	Sempervivum ruthenicum Schnittsp. & C.B. Lehm.	0	n	1	0	Crassulaceae J.St.-Hil.
Iz_9_1209	DJI_0572	Setaria viridis (L.) P. Beauv.	0	n	1	0	Poaceae Barnhart
Iz_9_1209	DJI_0572	Silene borysthenica (Gruner) Walters	0	n	1	0	Caryophyllaceae Juss.
Iz_9_1209	DJI_0572	Silene latifolia subsp. alba (Mill.) Greuter & Burdet	0	n	1	0	Caryophyllaceae Juss.
Iz_9_1209	DJI_0572	Solidago virgaurea L.	0	n	1	0	Asteraceae Bercht. & J.Presl
Iz_9_1209	DJI_0572	Sorbus aucuparia L.	0	n	1	1	Rosaceae Juss.
Iz_9_1209	DJI_0572	Species 12.09_9_0601	0		NA	NA	
Iz_9_1209	DJI_0572	Stachys recta L.	0	n	1	0	Lamiacea Lindl.
Iz_9_1209	DJI_0572	Tanacetum vulgare L.	0	n	1	0	Asteraceae Bercht. & J.Presl
Iz_9_1209	DJI_0572	Thymus pallasianus Heinr.Braun	0	n	1	0	Lamiacea Lindl.
Iz_9_1209	DJI_0572	Tilia cordata Mill.	0	n	1	1	Malvaceae Juss.
Iz_9_1209	DJI_0572	Trifolium alpestre L.	0	n	1	0	Fabaceae Lindl.
Iz_9_1209	DJI_0572	Trifolium arvense L.	0	n	1	0	Fabaceae Lindl.
Iz_9_1209	DJI_0572	Ulmus glabra Huds.	0	n	1	1	Ulmaceae Mirb.
Iz_9_1209	DJI_0572	Verbascum densiflorum Bertol.	0	n	1	0	Scrophulariaceae Juss.
Iz_9_1209	DJI_0572	Veronica austriaca L.	0	n	1	0	Plantaginaceae Juss.
Iz_9_1209	DJI_0572	Veronica incana L.	0	n	1	0	Plantaginaceae Juss.
Iz_9_1209	DJI_0572	Veronica verna L.	0	n	1	0	Plantaginaceae Juss.
Iz_9_1209	DJI_0572	Vincetoxicum hirundinaria Medik	0	n	1	0	Apocynaceae Juss.
Iz_9_1209	DJI_0575	Acer tataricum L.	0	n	1	1	Sapindaceae Juss.
Iz_9_1209	DJI_0575	Achillea millefolium L. s.l.	0	n	1	0	Asteraceae Bercht. & J.Presl
Iz_9_1209	DJI_0575	Allium oleraceum L.	0	n	1	0	Amaryllidaceae J.St.-Hil.
Iz_9_1209	DJI_0575	Ambrosia artemisiifolia L.	0	a	0	0	Asteraceae Bercht. & J.Presl
Iz_9_1209	DJI_0575	Amorpha fruticosa L.	0	a	0	0	Fabaceae Lindl.
Iz_9_1209	DJI_0575	Anthericum ramosum L.	0	n	1	0	Asparagaceae Juss.
Iz_9_1209	DJI_0575	Artemisia marschalliana Spreng.	0	n	1	0	Asteraceae Bercht. & J.Presl
Iz_9_1209	DJI_0575	Asparagus officinalis L.	0	n	1	0	Asparagaceae Juss.
Iz_9_1209	DJI_0575	Astragalus cicer L.	0	n	1	0	Fabaceae Lindl.
Iz_9_1209	DJI_0575	Bassia laniflora (S.G.Gmel.) A.J.Scott	0	n	1	0	Amaranthaceae Juss.
Iz_9_1209	DJI_0575	Berberis vulgaris L.	0	n	1	0	Berberidaceae Juss.
Iz_9_1209	DJI_0575	Betula pendula Roth.	0	n	1	1	Betulaceae Gray
Iz_9_1209	DJI_0575	Betula pendula Roth.	0	n	1	0	Poaceae Barnhart
Iz_9_1209	DJI_0575	Bromus inermis Leyss.	0		NA	NA	
Iz_9_1209	DJI_0575	Bromus squarrosus L.	0	a	0	0	Poaceae Barnhart
Iz_9_1209	DJI_0575	Bromus tectorum L.	0	a	0	0	Poaceae Barnhart
Iz_9_1209	DJI_0575	Calamagrostis epigejos (L.) Roth	25	n	1	0	Poaceae Barnhart
Iz_9_1209	DJI_0575	Carduus acanthoides L.	0	n	1	0	Asteraceae Bercht. & J.Presl
Iz_9_1209	DJI_0575	Carex sp.	0	n	1	0	Cyperaceae Juss.
Iz_9_1209	DJI_0575	Centaurea majorovii Dumbadze	0	n	1	0	Asteraceae Bercht. & J.Presl
Iz_9_1209	DJI_0575	Chamaecytisus ruthenicus (Fisch.ex Wol.) Klaskova	0	n	1	0	Fabaceae Lindl.
Iz_9_1209	DJI_0575	Chelidonium majus L.	0	n	1	0	Papaveraceae Juss.
Iz_9_1209	DJI_0575	Chenopodium betaceum Andrz.	0	n	1	0	Amaranthaceae Juss.
Iz_9_1209	DJI_0575	Chenopodium cf. album	0	n	1	0	Amaranthaceae Juss.
Iz_9_1209	DJI_0575	Chenopodium sp.	0		NA	0	Amaranthaceae Juss.
Iz_9_1209	DJI_0575	Chondrilla juncea L.	0	n	1	0	Asteraceae Bercht. & J.Presl
Iz_9_1209	DJI_0575	Cirsium vulgare (Savi) Ten.	0	n	1	0	Asteraceae Bercht. & J.Presl
Iz_9_1209	DJI_0575	Clinopodium acinos (L.) Kuntze	0	n	1	0	Lamiacea Lindl.
Iz_9_1209	DJI_0575	Convallaria majalis L.	0	n	1	0	Asparagaceae Juss.
Iz_9_1209	DJI_0575	Crataegus rhipidophylla Gand.	0	n	1	1	Rosaceae Juss.
Iz_9_1209	DJI_0575	Crepis foetida L.	0	n	1	0	Asteraceae Bercht. & J.Presl
Iz_9_1209	DJI_0575	Dactylis glomerata L.	0	n	1	0	Poaceae Barnhart
Iz_9_1209	DJI_0575	Dianthus campestris M.Bieb.	0	n	1	0	Caryophyllaceae Juss.
Iz_9_1209	DJI_0575	Digitaria sanguinalis (L.) Scop.	0	a	0	0	Poaceae Barnhart
Iz_9_1209	DJI_0575	Elymus repens (L.) Gould	0	n	1	0	Poaceae Barnhart
Iz_9_1209	DJI_0575	Epilobium tetragonum L.	0	n	1	0	Onagraceae Juss.
Iz_9_1209	DJI_0575	Eragrostis minor Host	0	a	0	0	Poaceae Barnhart
Iz_9_1209	DJI_0575	Erigeron canadensis L.	2	a	0	0	Asteraceae Bercht. & J.Presl
Iz_9_1209	DJI_0575	Euphorbia seguieriana Neck.	0	n	1	0	Euphorbiaceae Juss.
Iz_9_1209	DJI_0575	Fallopia dumetorum (L.) Holub	0	n	1	0	Polygonaceae Juss.
Iz_9_1209	DJI_0575	Festuca beckeri (Hack.) Trautv.	0	n	1	0	Poaceae Barnhart
Iz_9_1209	DJI_0575	Fragaria vesca L.	0	n	1	0	Rosaceae Juss.
Iz_9_1209	DJI_0575	Frangula alnus Mill.	0	n	1	1	Rhamnaceae Juss.
Iz_9_1209	DJI_0575	Fraxinus pennsylvanica Marshall	0	a	0	1	Oleaceae Hoffmanns. & Link
Iz_9_1209	DJI_0575	Galeopsis bifida Boenn.	0	n	1	0	Lamiacea Lindl.
Iz_9_1209	DJI_0575	Galium verum L.	0	n	1	0	Rubiaceae Juss.
Iz_9_1209	DJI_0575	Genista tinctoria L.	0	n	1	0	Fabaceae Lindl.
Iz_9_1209	DJI_0575	Geranium robertianum L.	0	n	1	0	Geraniaceae Juss.
Iz_9_1209	DJI_0575	Geranium sanquineum L.	0	n	1	0	Geraniaceae Juss.
Iz_9_1209	DJI_0575	Gypsophila paniculata L.	0	n	1	0	Caryophyllaceae Juss.
Iz_9_1209	DJI_0575	Helichrysum arenarium (L.) Moench	0	n	1	0	Asteraceae Bercht. & J.Presl
Iz_9_1209	DJI_0575	Herniaria polygama J.Gay	0	n	1	0	Caryophyllaceae Juss.
Iz_9_1209	DJI_0575	Hieracium robustum Fr.	0	n	1	0	Asteraceae Bercht. & J.Presl
Iz_9_1209	DJI_0575	Hieracium umbellatum L.	0	n	1	NA	Asteraceae Bercht. & J.Presl
Iz_9_1209	DJI_0575	Hylotelephium maximum (L.) Holub	0	n	1	0	Crassulaceae J.St.-Hil.
Iz_9_1209	DJI_0575	Jacobaea borysthenica (DC.) B.Nord. & Greuter	0	n	1	0	Asteraceae Bercht. & J.Presl
Iz_9_1209	DJI_0575	Jasione montana L.	0	n	1	0	Campanulaceae Juss.
Iz_9_1209	DJI_0575	Jurinea cyanoides (L.) Rchb.s.l.	0	n	1	0	Asteraceae Bercht. & J.Presl
Iz_9_1209	DJI_0575	Koeleria glauca (Spreng.) DC.	0	n	1	0	Poaceae Barnhart
Iz_9_1209	DJI_0575	Lactuca serriola L.	0	a	0	0	Asteraceae Bercht. & J.Presl
Iz_9_1209	DJI_0575	Linaria genistifolia (L.) Mill.	0	n	1	0	Plantaginaceae Juss.
Iz_9_1209	DJI_0575	Lomelosia argentea (L.) Greuter & Burdet	0	n	1	0	Caprifoliaceae Juss.
Iz_9_1209	DJI_0575	Melampyrum pratense L.	0	n	1	0	Orobanchaceae Vent.
Iz_9_1209	DJI_0575	Oenothera biennis L.	0	a	0	0	Onagraceae Juss.
Iz_9_1209	DJI_0575	Pentanema britannica (L.) D.Gut.Larr., Santos-Vicente, Anderb., E.Rico & M.M.Mart.Ort.	0	n	1	0	Asteraceae Bercht. & J.Presl
Iz_9_1209	DJI_0575	Peucedanum oreoselinum (L.) Moench	0.5	n	1	0	Apiaceae Lindl.
Iz_9_1209	DJI_0575	Peucedanum ruthenicum M.Bieb.	0	n	1	0	Apiaceae Lindl.
Iz_9_1209	DJI_0575	Pilosella echioides (Lumn.) F.W.Schultz & Sch.Bip.	0	n	1	0	Asteraceae Bercht. & J.Presl
Iz_9_1209	DJI_0575	Pilosella officinarum F. Schultz et Sch. Bip.	0	n	1	0	Asteraceae Bercht. & J.Presl
Iz_9_1209	DJI_0575	Pilosella sp.	0		NA	0	Asteraceae Bercht. & J.Presl

Iz_9_1209	DJI_0575	Pinus sylvestris L.	0	n	1	1	Pinaceae Spreng. ex F.Rudolphi
Iz_9_1209	DJI_0575	Poa annua L.	0	n	1	0	Poaceae Barnhart
Iz_9_1209	DJI_0575	Poa nemoralis L.	0	n	1	0	Poaceae Barnhart
Iz_9_1209	DJI_0575	Poa sp.	0		NA	0	Poaceae Barnhart
Iz_9_1209	DJI_0575	Polygonatum odoratum (Mill.) Druce	0	n	1	0	Asparagaceae Juss.
Iz_9_1209	DJI_0575	Polygonum arenarium Waldst. & Kit.	0	n	1	0	Polygonaceae Juss.
Iz_9_1209	DJI_0575	Populus nigra L.	0	n	1	1	Salicaceae Mirb.
Iz_9_1209	DJI_0575	Populus tremula L.	0	n	1	1	Salicaceae Mirb.
Iz_9_1209	DJI_0575	Populus Ã canescens (Aiton) Sm.	0	n	1	1	Salicaceae Mirb.
Iz_9_1209	DJI_0575	Potentilla argentea L.	0	n	1	0	Rosaceae Juss.
Iz_9_1209	DJI_0575	Psammophiliella muralis (L.) Ikonn.	0	n	1	0	Caryophyllaceae Juss.
Iz_9_1209	DJI_0575	Psephellus sumensis (Kalen.) Greuter	0	n	1	0	Asteraceae Bercht. & J.Presl
Iz_9_1209	DJI_0575	Pulsatilla pratensis (L.) Mill. s.l.	0	n	1	0	Ranunculaceae Juss.
Iz_9_1209	DJI_0575	Pyrus communis L.	0	a	0	1	Rosaceae Juss.
Iz_9_1209	DJI_0575	Quercus robur L.	0	n	1	1	Fagaceae Dumort.
Iz_9_1209	DJI_0575	Robinia pseudoacacia L.	0	a	0	1	Fabaceae Lindl.
Iz_9_1209	DJI_0575	Rosa sp.	0		NA	1	Rosaceae Juss.
Iz_9_1209	DJI_0575	Rubus idaeus L.	0	n	1	0	Rosaceae Juss.
Iz_9_1209	DJI_0575	Rumex acetosella L.	0	n	1	0	Polygonaceae Juss.
Iz_9_1209	DJI_0575	Rumex thyrsiflorus Fingerh.	0	n	1	0	Polygonaceae Juss.
Iz_9_1209	DJI_0575	Salix alba L.	0	n	1	1	Salicaceae Mirb.
Iz_9_1209	DJI_0575	Sambucus nigra L.	0	n	1	1	Viburnaceae Raf.
Iz_9_1209	DJI_0575	Secale sylvestre Host	0	n	1	0	Poaceae Barnhart
Iz_9_1209	DJI_0575	Sempervivum ruthenicum Schnittsp. & C.B. Lehm.	0	n	1	0	Crassulaceae J.St.-Hil.
Iz_9_1209	DJI_0575	Setaria viridis (L.) P. Beauv.	0	n	1	0	Poaceae Barnhart
Iz_9_1209	DJI_0575	Silene borysthenica (Gruner) Walters	0	n	1	0	Caryophyllaceae Juss.
Iz_9_1209	DJI_0575	Silene latifolia subsp. alba (Mill.) Greuter & Burdet	0	n	1	0	Caryophyllaceae Juss.
Iz_9_1209	DJI_0575	Solidago virgaurea L.	0	n	1	0	Asteraceae Bercht. & J.Presl
Iz_9_1209	DJI_0575	Sorbus aucuparia L.	0	n	1	1	Rosaceae Juss.
Iz_9_1209	DJI_0575	Species 12.09_9_0601	0		NA	NA	
Iz_9_1209	DJI_0575	Stachys recta L.	0	n	1	0	Lamiacea Lindl.
Iz_9_1209	DJI_0575	Tanacetum vulgare L.	0	n	1	0	Asteraceae Bercht. & J.Presl
Iz_9_1209	DJI_0575	Thymus pallasianus Heinr.Braun	0	n	1	0	Lamiacea Lindl.
Iz_9_1209	DJI_0575	Tilia cordata Mill.	0	n	1	1	Malvaceae Juss.
Iz_9_1209	DJI_0575	Trifolium alpestre L.	0	n	1	0	Fabaceae Lindl.
Iz_9_1209	DJI_0575	Trifolium arvense L.	0	n	1	0	Fabaceae Lindl.
Iz_9_1209	DJI_0575	Ulmus glabra Huds.	0	n	1	1	Ulmaceae Mirb.
Iz_9_1209	DJI_0575	Verbascum densiflorum Bertol.	0	n	1	0	Scrophulariaceae Juss.
Iz_9_1209	DJI_0575	Veronica austriaca L.	0	n	1	0	Plantaginaceae Juss.
Iz_9_1209	DJI_0575	Veronica incana L.	0	n	1	0	Plantaginaceae Juss.
Iz_9_1209	DJI_0575	Veronica verna L.	0	n	1	0	Plantaginaceae Juss.
Iz_9_1209	DJI_0575	Vincetoxicum hirundinaria Medik	0	n	1	0	Apocynaceae Juss.
Iz_9_1209	DJI_0579	Acer tataricum L.	0	n	1	1	Sapindaceae Juss.
Iz_9_1209	DJI_0579	Achillea millefolium L. s.l.	0	n	1	0	Asteraceae Bercht. & J.Presl
Iz_9_1209	DJI_0579	Allium oleraceum L.	0	n	1	0	Amaryllidaceae J.St.-Hil.
Iz_9_1209	DJI_0579	Ambrosia artemisiifolia L.	0	a	0	0	Asteraceae Bercht. & J.Presl
Iz_9_1209	DJI_0579	Amorpha fruticosa L.	0	a	0	0	Fabaceae Lindl.
Iz_9_1209	DJI_0579	Anthericum ramosum L.	0	n	1	0	Asparagaceae Juss.
Iz_9_1209	DJI_0579	Artemisia marschalliana Spreng.	0	n	1	0	Asteraceae Bercht. & J.Presl
Iz_9_1209	DJI_0579	Asparagus officinalis L.	0	n	1	0	Asparagaceae Juss.
Iz_9_1209	DJI_0579	Astragalus cicer L.	0	n	1	0	Fabaceae Lindl.
Iz_9_1209	DJI_0579	Bassia laniflora (S.G.Gmel.) A.J.Scott	0	n	1	0	Amaranthaceae Juss.
Iz_9_1209	DJI_0579	Berberis vulgaris L.	0	n	1	0	Berberidaceae Juss.
Iz_9_1209	DJI_0579	Betula pendula Roth.	0	n	1	1	Betulaceae Gray
Iz_9_1209	DJI_0579	Betula pendula Roth.	0	n	1	0	Poaceae Barnhart
Iz_9_1209	DJI_0579	Bromus inermis Leyss.	0		NA	NA	
Iz_9_1209	DJI_0579	Bromus squarrosus L.	0	a	0	0	Poaceae Barnhart
Iz_9_1209	DJI_0579	Bromus tectorum L.	0	a	0	0	Poaceae Barnhart
Iz_9_1209	DJI_0579	Calamagrostis epigejos (L.) Roth	30	n	1	0	Poaceae Barnhart
Iz_9_1209	DJI_0579	Carduus acanthoides L.	0	n	1	0	Asteraceae Bercht. & J.Presl
Iz_9_1209	DJI_0579	Carex sp.	0	n	1	0	Cyperaceae Juss.
Iz_9_1209	DJI_0579	Centaurea majorovii Dumbadze	0	n	1	0	Asteraceae Bercht. & J.Presl
Iz_9_1209	DJI_0579	Chamaecytisus ruthenicus (Fisch.ex Wol.) Klaskova	0	n	1	0	Fabaceae Lindl.
Iz_9_1209	DJI_0579	Chelidonium majus L.	0	n	1	0	Papaveraceae Juss.
Iz_9_1209	DJI_0579	Chenopodium betaceum Andrz.	0	n	1	0	Amaranthaceae Juss.
Iz_9_1209	DJI_0579	Chenopodium cf. album	0	n	1	0	Amaranthaceae Juss.
Iz_9_1209	DJI_0579	Chenopodium sp.	0		NA	0	Amaranthaceae Juss.
Iz_9_1209	DJI_0579	Chondrilla juncea L.	0	n	1	0	Asteraceae Bercht. & J.Presl
Iz_9_1209	DJI_0579	Cirsium vulgare (Savi) Ten.	0	n	1	0	Asteraceae Bercht. & J.Presl
Iz_9_1209	DJI_0579	Clinopodium acinos (L.) Kuntze	0	n	1	0	Lamiacea Lindl.
Iz_9_1209	DJI_0579	Convallaria majalis L.	0	n	1	0	Asparagaceae Juss.
Iz_9_1209	DJI_0579	Crataegus rhipidophylla Gand.	0	n	1	1	Rosaceae Juss.
Iz_9_1209	DJI_0579	Crepis foetida L.	0	n	1	0	Asteraceae Bercht. & J.Presl
Iz_9_1209	DJI_0579	Dactylis glomerata L.	0	n	1	0	Poaceae Barnhart
Iz_9_1209	DJI_0579	Dianthus campestris M.Bieb.	0	n	1	0	Caryophyllaceae Juss.
Iz_9_1209	DJI_0579	Digitaria sanguinalis (L.) Scop.	0	a	0	0	Poaceae Barnhart
Iz_9_1209	DJI_0579	Elymus repens (L.) Gould	0	n	1	0	Poaceae Barnhart
Iz_9_1209	DJI_0579	Epilobium tetragonum L.	0	n	1	0	Onagraceae Juss.
Iz_9_1209	DJI_0579	Eragrostis minor Host	0	a	0	0	Poaceae Barnhart
Iz_9_1209	DJI_0579	Erigeron canadensis L.	0	a	0	0	Asteraceae Bercht. & J.Presl
Iz_9_1209	DJI_0579	Euphorbia seguieriana Neck.	0	n	1	0	Euphorbiaceae Juss.
Iz_9_1209	DJI_0579	Fallopia dumetorum (L.) Holub	0	n	1	0	Polygonaceae Juss.
Iz_9_1209	DJI_0579	Festuca beckeri (Hack.) Trautv.	0	n	1	0	Poaceae Barnhart
Iz_9_1209	DJI_0579	Fragaria vesca L.	0	n	1	0	Rosaceae Juss.
Iz_9_1209	DJI_0579	Frangula alnus Mill.	0	n	1	1	Rhamnaceae Juss.
Iz_9_1209	DJI_0579	Fraxinus pennsylvanica Marshall	0	a	0	1	Oleaceae Hoffmanns. & Link
Iz_9_1209	DJI_0579	Galeopsis bifida Boenn.	0	n	1	0	Lamiacea Lindl.
Iz_9_1209	DJI_0579	Galium verum L.	0	n	1	0	Rubiaceae Juss.
Iz_9_1209	DJI_0579	Genista tinctoria L.	0	n	1	0	Fabaceae Lindl.
Iz_9_1209	DJI_0579	Geranium robertianum L.	0	n	1	0	Geraniaceae Juss.
Iz_9_1209	DJI_0579	Geranium sanquineum L.	0	n	1	0	Geraniaceae Juss.
Iz_9_1209	DJI_0579	Gypsophila paniculata L.	0	n	1	0	Caryophyllaceae Juss.
Iz_9_1209	DJI_0579	Helichrysum arenarium (L.) Moench	0	n	1	0	Asteraceae Bercht. & J.Presl
Iz_9_1209	DJI_0579	Herniaria polygama J.Gay	0	n	1	0	Caryophyllaceae Juss.
Iz_9_1209	DJI_0579	Hieracium robustum Fr.	0	n	1	0	Asteraceae Bercht. & J.Presl
Iz_9_1209	DJI_0579	Hieracium umbellatum L.	0	n	1	NA	Asteraceae Bercht. & J.Presl
Iz_9_1209	DJI_0579	Hylotelephium maximum (L.) Holub	0	n	1	0	Crassulaceae J.St.-Hil.
Iz_9_1209	DJI_0579	Jacobaea borysthenica (DC.) B.Nord. & Greuter	0	n	1	0	Asteraceae Bercht. & J.Presl

Iz_9_1209	DJI_0579	Jasione montana L.	0	n	1	0	Campanulaceae Juss.
Iz_9_1209	DJI_0579	Jurinea cyanoides (L.) Rchb.s.l.	0	n	1	0	Asteraceae Bercht. & J.Presl
Iz_9_1209	DJI_0579	Koeleria glauca (Spreng.) DC.	0	n	1	0	Poaceae Barnhart
Iz_9_1209	DJI_0579	Lactuca serriola L.	0	a	0	0	Asteraceae Bercht. & J.Presl
Iz_9_1209	DJI_0579	Linaria genistifolia (L.) Mill.	0	n	1	0	Plantaginaceae Juss.
Iz_9_1209	DJI_0579	Lomelosia argentea (L.) Greuter & Burdet	0	n	1	0	Caprifoliaceae Juss.
Iz_9_1209	DJI_0579	Melampyrum pratense L.	0	n	1	0	Orobanchaceae Vent.
Iz_9_1209	DJI_0579	Oenothera biennis L.	0	a	0	0	Onagraceae Juss.
Iz_9_1209	DJI_0579	Pentanema britannica (L.) D.Gut.Larr., Santos-Vicente, Anderb., E.Rico & M.M.Mart.Ort.	0	n	1	0	Asteraceae Bercht. & J.Presl
Iz_9_1209	DJI_0579	Peucedanum oreoselinum (L.) Moench	2	n	1	0	Apiaceae Lindl.
Iz_9_1209	DJI_0579	Peucedanum ruthenicum M.Bieb.	0	n	1	0	Apiaceae Lindl.
Iz_9_1209	DJI_0579	Pilosella echioides (Lumn.) F.W.Schultz & Sch.Bip.	0	n	1	0	Asteraceae Bercht. & J.Presl
Iz_9_1209	DJI_0579	Pilosella officinarum F. Schultz et Sch. Bip.	0	n	1	0	Asteraceae Bercht. & J.Presl
Iz_9_1209	DJI_0579	Pilosella sp.	0		NA	0	Asteraceae Bercht. & J.Presl
Iz_9_1209	DJI_0579	Pinus sylvestris L.	0	n	1	1	Pinaceae Spreng. ex F.Rudolphi
Iz_9_1209	DJI_0579	Poa annua L.	0	n	1	0	Poaceae Barnhart
Iz_9_1209	DJI_0579	Poa nemoralis L.	0	n	1	0	Poaceae Barnhart
Iz_9_1209	DJI_0579	Poa sp.	0		NA	0	Poaceae Barnhart
Iz_9_1209	DJI_0579	Polygonatum odoratum (Mill.) Druce	0	n	1	0	Asparagaceae Juss.
Iz_9_1209	DJI_0579	Polygonum arenarium Waldst. & Kit.	0	n	1	0	Polygonaceae Juss.
Iz_9_1209	DJI_0579	Populus nigra L.	0	n	1	1	Salicaceae Mirb.
Iz_9_1209	DJI_0579	Populus tremula L.	0	n	1	1	Salicaceae Mirb.
Iz_9_1209	DJI_0579	Populus Ã canescens (Aiton) Sm.	0	n	1	1	Salicaceae Mirb.
Iz_9_1209	DJI_0579	Potentilla argentea L.	0	n	1	0	Rosaceae Juss.
Iz_9_1209	DJI_0579	Psammophiliella muralis (L.) Ikonn.	0	n	1	0	Caryophyllaceae Juss.
Iz_9_1209	DJI_0579	Psephellus sumensis (Kalen.) Greuter	0	n	1	0	Asteraceae Bercht. & J.Presl
Iz_9_1209	DJI_0579	Pulsatilla pratensis (L.) Mill. s.l.	0	n	1	0	Ranunculaceae Juss.
Iz_9_1209	DJI_0579	Pyrus communis L.	0	a	0	1	Rosaceae Juss.
Iz_9_1209	DJI_0579	Quercus robur L.	0	n	1	1	Fagaceae Dumort.
Iz_9_1209	DJI_0579	Robinia pseudoacacia L.	0	a	0	1	Fabaceae Lindl.
Iz_9_1209	DJI_0579	Rosa sp.	0		NA	1	Rosaceae Juss.
Iz_9_1209	DJI_0579	Rubus idaeus L.	0	n	1	0	Rosaceae Juss.
Iz_9_1209	DJI_0579	Rumex acetosella L.	0	n	1	0	Polygonaceae Juss.
Iz_9_1209	DJI_0579	Rumex thyrsiflorus Fingerh.	0	n	1	0	Polygonaceae Juss.
Iz_9_1209	DJI_0579	Salix alba L.	0	n	1	1	Salicaceae Mirb.
Iz_9_1209	DJI_0579	Sambucus nigra L.	0	n	1	1	Viburnaceae Raf.
Iz_9_1209	DJI_0579	Secale sylvestre Host	0	n	1	0	Poaceae Barnhart
Iz_9_1209	DJI_0579	Sempervivum ruthenicum Schnittsp. & C.B. Lehm.	0	n	1	0	Crassulaceae J.St.-Hil.
Iz_9_1209	DJI_0579	Setaria viridis (L.) P. Beauv.	0	n	1	0	Poaceae Barnhart
Iz_9_1209	DJI_0579	Silene borysthenica (Gruner) Walters	0	n	1	0	Caryophyllaceae Juss.
Iz_9_1209	DJI_0579	Silene latifolia subsp. alba (Mill.) Greuter & Burdet	0	n	1	0	Caryophyllaceae Juss.
Iz_9_1209	DJI_0579	Solidago virgaurea L.	0.5	n	1	0	Asteraceae Bercht. & J.Presl
Iz_9_1209	DJI_0579	Sorbus aucuparia L.	0	n	1	1	Rosaceae Juss.
Iz_9_1209	DJI_0579	Species 12.09_9_0601	0		NA	NA	
Iz_9_1209	DJI_0579	Stachys recta L.	0	n	1	0	Lamiacea Lindl.
Iz_9_1209	DJI_0579	Tanacetum vulgare L.	0	n	1	0	Asteraceae Bercht. & J.Presl
Iz_9_1209	DJI_0579	Thymus pallasianus Heinr.Braun	0	n	1	0	Lamiacea Lindl.
Iz_9_1209	DJI_0579	Tilia cordata Mill.	0	n	1	1	Malvaceae Juss.
Iz_9_1209	DJI_0579	Trifolium alpestre L.	0	n	1	0	Fabaceae Lindl.
Iz_9_1209	DJI_0579	Trifolium arvense L.	0	n	1	0	Fabaceae Lindl.
Iz_9_1209	DJI_0579	Ulmus glabra Huds.	0	n	1	1	Ulmaceae Mirb.
Iz_9_1209	DJI_0579	Verbascum densiflorum Bertol.	0	n	1	0	Scrophulariaceae Juss.
Iz_9_1209	DJI_0579	Veronica austriaca L.	0	n	1	0	Plantaginaceae Juss.
Iz_9_1209	DJI_0579	Veronica incana L.	0	n	1	0	Plantaginaceae Juss.
Iz_9_1209	DJI_0579	Veronica verna L.	0	n	1	0	Plantaginaceae Juss.
Iz_9_1209	DJI_0579	Vincetoxicum hirundinaria Medik	0	n	1	0	Apocynaceae Juss.
Iz_9_1209	DJI_0582	Acer tataricum L.	0	n	1	1	Sapindaceae Juss.
Iz_9_1209	DJI_0582	Achillea millefolium L. s.l.	0	n	1	0	Asteraceae Bercht. & J.Presl
Iz_9_1209	DJI_0582	Allium oleraceum L.	0	n	1	0	Amaryllidaceae J.St.-Hil.
Iz_9_1209	DJI_0582	Ambrosia artemisiifolia L.	0	a	0	0	Asteraceae Bercht. & J.Presl
Iz_9_1209	DJI_0582	Amorpha fruticosa L.	0	a	0	0	Fabaceae Lindl.
Iz_9_1209	DJI_0582	Anthericum ramosum L.	0	n	1	0	Asparagaceae Juss.
Iz_9_1209	DJI_0582	Artemisia marschalliana Spreng.	0	n	1	0	Asteraceae Bercht. & J.Presl
Iz_9_1209	DJI_0582	Asparagus officinalis L.	0	n	1	0	Asparagaceae Juss.
Iz_9_1209	DJI_0582	Astragalus cicer L.	0	n	1	0	Fabaceae Lindl.
Iz_9_1209	DJI_0582	Bassia laniflora (S.G.Gmel.) A.J.Scott	0	n	1	0	Amaranthaceae Juss.
Iz_9_1209	DJI_0582	Berberis vulgaris L.	0	n	1	0	Berberidaceae Juss.
Iz_9_1209	DJI_0582	Betula pendula Roth.	0	n	1	1	Betulaceae Gray
Iz_9_1209	DJI_0582	Betula pendula Roth.	0	n	1	0	Poaceae Barnhart
Iz_9_1209	DJI_0582	Bromus inermis Leyss.	0		NA	NA	
Iz_9_1209	DJI_0582	Bromus squarrosus L.	0	a	0	0	Poaceae Barnhart
Iz_9_1209	DJI_0582	Bromus tectorum L.	0	a	0	0	Poaceae Barnhart
Iz_9_1209	DJI_0582	Calamagrostis epigejos (L.) Roth	65	n	1	0	Poaceae Barnhart
Iz_9_1209	DJI_0582	Carduus acanthoides L.	0	n	1	0	Asteraceae Bercht. & J.Presl
Iz_9_1209	DJI_0582	Carex sp.	0	n	1	0	Cyperaceae Juss.
Iz_9_1209	DJI_0582	Centaurea majorovii Dumbadze	0	n	1	0	Asteraceae Bercht. & J.Presl
Iz_9_1209	DJI_0582	Chamaecytisus ruthenicus (Fisch.ex Wol.) Klaskova	0	n	1	0	Fabaceae Lindl.
Iz_9_1209	DJI_0582	Chelidonium majus L.	0	n	1	0	Papaveraceae Juss.
Iz_9_1209	DJI_0582	Chenopodium betaceum Andrz.	0	n	1	0	Amaranthaceae Juss.
Iz_9_1209	DJI_0582	Chenopodium cf. album	0	n	1	0	Amaranthaceae Juss.
Iz_9_1209	DJI_0582	Chenopodium sp.	0		NA	0	Amaranthaceae Juss.
Iz_9_1209	DJI_0582	Chondrilla juncea L.	0	n	1	0	Asteraceae Bercht. & J.Presl
Iz_9_1209	DJI_0582	Cirsium vulgare (Savi) Ten.	0	n	1	0	Asteraceae Bercht. & J.Presl
Iz_9_1209	DJI_0582	Clinopodium acinos (L.) Kuntze	0	n	1	0	Lamiacea Lindl.
Iz_9_1209	DJI_0582	Convallaria majalis L.	0	n	1	0	Asparagaceae Juss.
Iz_9_1209	DJI_0582	Crataegus rhipidophylla Gand.	0	n	1	1	Rosaceae Juss.
Iz_9_1209	DJI_0582	Crepis foetida L.	0	n	1	0	Asteraceae Bercht. & J.Presl
Iz_9_1209	DJI_0582	Dactylis glomerata L.	0	n	1	0	Poaceae Barnhart
Iz_9_1209	DJI_0582	Dianthus campestris M.Bieb.	0	n	1	0	Caryophyllaceae Juss.
Iz_9_1209	DJI_0582	Digitaria sanguinalis (L.) Scop.	0	a	0	0	Poaceae Barnhart
Iz_9_1209	DJI_0582	Elymus repens (L.) Gould	0	n	1	0	Poaceae Barnhart
Iz_9_1209	DJI_0582	Epilobium tetragonum L.	0	n	1	0	Onagraceae Juss.
Iz_9_1209	DJI_0582	Eragrostis minor Host	0	a	0	0	Poaceae Barnhart
Iz_9_1209	DJI_0582	Erigeron canadensis L.	0	a	0	0	Asteraceae Bercht. & J.Presl
Iz_9_1209	DJI_0582	Euphorbia seguieriana Neck.	0	n	1	0	Euphorbiaceae Juss.
Iz_9_1209	DJI_0582	Fallopia dumetorum (L.) Holub	0	n	1	0	Polygonaceae Juss.
Iz_9_1209	DJI_0582	Festuca beckeri (Hack.) Trautv.	0	n	1	0	Poaceae Barnhart
Iz_9_1209	DJI_0582	Fragaria vesca L.	0	n	1	0	Rosaceae Juss.

Iz_9_1209	DJI_0582	Frangula alnus Mill.	0	n	1	1	Rhamnaceae Juss.
Iz_9_1209	DJI_0582	Fraxinus pennsylvanica Marshall	0	a	0	1	Oleaceae Hoffmanns. & Link
Iz_9_1209	DJI_0582	Galeopsis bifida Boenn.	0	n	1	0	Lamiacea Lindl.
Iz_9_1209	DJI_0582	Galium verum L.	0	n	1	0	Rubiaceae Juss.
Iz_9_1209	DJI_0582	Genista tinctoria L.	0	n	1	0	Fabaceae Lindl.
Iz_9_1209	DJI_0582	Geranium robertianum L.	0	n	1	0	Geraniaceae Juss.
Iz_9_1209	DJI_0582	Geranium sanquineum L.	0	n	1	0	Geraniaceae Juss.
Iz_9_1209	DJI_0582	Gypsophila paniculata L.	0	n	1	0	Caryophyllaceae Juss.
Iz_9_1209	DJI_0582	Helichrysum arenarium (L.) Moench	0	n	1	0	Asteraceae Bercht. & J.Presl
Iz_9_1209	DJI_0582	Herniaria polygama J.Gay	0	n	1	0	Caryophyllaceae Juss.
Iz_9_1209	DJI_0582	Hieracium robustum Fr.	0	n	1	0	Asteraceae Bercht. & J.Presl
Iz_9_1209	DJI_0582	Hieracium umbellatum L.	0	n	1	NA	Asteraceae Bercht. & J.Presl
Iz_9_1209	DJI_0582	Hylotelephium maximum (L.) Holub	0	n	1	0	Crassulaceae J.St.-Hil.
Iz_9_1209	DJI_0582	Jacobaea borysthenica (DC.) B.Nord. & Greuter	0	n	1	0	Asteraceae Bercht. & J.Presl
Iz_9_1209	DJI_0582	Jasione montana L.	0	n	1	0	Campanulaceae Juss.
Iz_9_1209	DJI_0582	Jurinea cyanoides (L.) Rchb.s.l.	0	n	1	0	Asteraceae Bercht. & J.Presl
Iz_9_1209	DJI_0582	Koeleria glauca (Spreng.) DC.	0	n	1	0	Poaceae Barnhart
Iz_9_1209	DJI_0582	Lactuca serriola L.	0.2	a	0	0	Asteraceae Bercht. & J.Presl
Iz_9_1209	DJI_0582	Linaria genistifolia (L.) Mill.	0	n	1	0	Plantaginaceae Juss.
Iz_9_1209	DJI_0582	Lomelosia argentea (L.) Greuter & Burdet	0	n	1	0	Caprifoliaceae Juss.
Iz_9_1209	DJI_0582	Melampyrum pratense L.	0	n	1	0	Orobanchaceae Vent.
Iz_9_1209	DJI_0582	Oenothera biennis L.	0	a	0	0	Onagraceae Juss.
Iz_9_1209	DJI_0582	Pentanema britannica (L.) D.Gut.Larr., Santos-Vicente, Anderb., E.Rico & M.M.Mart.Ort.	0	n	1	0	Asteraceae Bercht. & J.Presl
Iz_9_1209	DJI_0582	Peucedanum oreoselinum (L.) Moench	0	n	1	0	Apiaceae Lindl.
Iz_9_1209	DJI_0582	Peucedanum ruthenicum M.Bieb.	0	n	1	0	Apiaceae Lindl.
Iz_9_1209	DJI_0582	Pilosella echioides (Lumn.) F.W.Schultz & Sch.Bip.	0	n	1	0	Asteraceae Bercht. & J.Presl
Iz_9_1209	DJI_0582	Pilosella officinarum F. Schultz et Sch. Bip.	0	n	1	0	Asteraceae Bercht. & J.Presl
Iz_9_1209	DJI_0582	Pilosella sp.	0		NA	0	Asteraceae Bercht. & J.Presl
Iz_9_1209	DJI_0582	Pinus sylvestris L.	0	n	1	1	Pinaceae Spreng. ex F.Rudolphi
Iz_9_1209	DJI_0582	Poa annua L.	0	n	1	0	Poaceae Barnhart
Iz_9_1209	DJI_0582	Poa nemoralis L.	0	n	1	0	Poaceae Barnhart
Iz_9_1209	DJI_0582	Poa sp.	0		NA	0	Poaceae Barnhart
Iz_9_1209	DJI_0582	Polygonatum odoratum (Mill.) Druce	0	n	1	0	Asparagaceae Juss.
Iz_9_1209	DJI_0582	Polygonum arenarium Waldst. & Kit.	0	n	1	0	Polygonaceae Juss.
Iz_9_1209	DJI_0582	Populus nigra L.	0	n	1	1	Salicaceae Mirb.
Iz_9_1209	DJI_0582	Populus tremula L.	0	n	1	1	Salicaceae Mirb.
Iz_9_1209	DJI_0582	Populus Ã canescens (Aiton) Sm.	0	n	1	1	Salicaceae Mirb.
Iz_9_1209	DJI_0582	Potentilla argentea L.	0	n	1	0	Rosaceae Juss.
Iz_9_1209	DJI_0582	Psammophiliella muralis (L.) Ikonn.	0	n	1	0	Caryophyllaceae Juss.
Iz_9_1209	DJI_0582	Psephellus sumensis (Kalen.) Greuter	0	n	1	0	Asteraceae Bercht. & J.Presl
Iz_9_1209	DJI_0582	Pulsatilla pratensis (L.) Mill. s.l.	0	n	1	0	Ranunculaceae Juss.
Iz_9_1209	DJI_0582	Pyrus communis L.	0	a	0	1	Rosaceae Juss.
Iz_9_1209	DJI_0582	Quercus robur L.	0	n	1	1	Fagaceae Dumort.
Iz_9_1209	DJI_0582	Robinia pseudoacacia L.	0	a	0	1	Fabaceae Lindl.
Iz_9_1209	DJI_0582	Rosa sp.	0		NA	1	Rosaceae Juss.
Iz_9_1209	DJI_0582	Rubus idaeus L.	0	n	1	0	Rosaceae Juss.
Iz_9_1209	DJI_0582	Rumex acetosella L.	0	n	1	0	Polygonaceae Juss.
Iz_9_1209	DJI_0582	Rumex thyrsiflorus Fingerh.	0	n	1	0	Polygonaceae Juss.
Iz_9_1209	DJI_0582	Salix alba L.	0	n	1	1	Salicaceae Mirb.
Iz_9_1209	DJI_0582	Sambucus nigra L.	0	n	1	1	Viburnaceae Raf.
Iz_9_1209	DJI_0582	Secale sylvestre Host	0	n	1	0	Poaceae Barnhart
Iz_9_1209	DJI_0582	Sempervivum ruthenicum Schnittsp. & C.B. Lehm.	0	n	1	0	Crassulaceae J.St.-Hil.
Iz_9_1209	DJI_0582	Setaria viridis (L.) P. Beauv.	0	n	1	0	Poaceae Barnhart
Iz_9_1209	DJI_0582	Silene borysthenica (Gruner) Walters	0	n	1	0	Caryophyllaceae Juss.
Iz_9_1209	DJI_0582	Silene latifolia subsp. alba (Mill.) Greuter & Burdet	0	n	1	0	Caryophyllaceae Juss.
Iz_9_1209	DJI_0582	Solidago virgaurea L.	0	n	1	0	Asteraceae Bercht. & J.Presl
Iz_9_1209	DJI_0582	Sorbus aucuparia L.	0	n	1	1	Rosaceae Juss.
Iz_9_1209	DJI_0582	Species 12.09_9_0601	0		NA	NA	
Iz_9_1209	DJI_0582	Stachys recta L.	0	n	1	0	Lamiacea Lindl.
Iz_9_1209	DJI_0582	Tanacetum vulgare L.	0	n	1	0	Asteraceae Bercht. & J.Presl
Iz_9_1209	DJI_0582	Thymus pallasianus Heinr.Braun	0	n	1	0	Lamiacea Lindl.
Iz_9_1209	DJI_0582	Tilia cordata Mill.	0	n	1	1	Malvaceae Juss.
Iz_9_1209	DJI_0582	Trifolium alpestre L.	0	n	1	0	Fabaceae Lindl.
Iz_9_1209	DJI_0582	Trifolium arvense L.	0	n	1	0	Fabaceae Lindl.
Iz_9_1209	DJI_0582	Ulmus glabra Huds.	0	n	1	1	Ulmaceae Mirb.
Iz_9_1209	DJI_0582	Verbascum densiflorum Bertol.	0	n	1	0	Scrophulariaceae Juss.
Iz_9_1209	DJI_0582	Veronica austriaca L.	0	n	1	0	Plantaginaceae Juss.
Iz_9_1209	DJI_0582	Veronica incana L.	0	n	1	0	Plantaginaceae Juss.
Iz_9_1209	DJI_0582	Veronica verna L.	0	n	1	0	Plantaginaceae Juss.
Iz_9_1209	DJI_0582	Vincetoxicum hirundinaria Medik	0	n	1	0	Apocynaceae Juss.
Iz_9_1209	DJI_0588	Acer tataricum L.	0	n	1	1	Sapindaceae Juss.
Iz_9_1209	DJI_0588	Achillea millefolium L. s.l.	0	n	1	0	Asteraceae Bercht. & J.Presl
Iz_9_1209	DJI_0588	Allium oleraceum L.	0	n	1	0	Amaryllidaceae J.St.-Hil.
Iz_9_1209	DJI_0588	Ambrosia artemisiifolia L.	0	a	0	0	Asteraceae Bercht. & J.Presl
Iz_9_1209	DJI_0588	Amorpha fruticosa L.	0	a	0	0	Fabaceae Lindl.
Iz_9_1209	DJI_0588	Anthericum ramosum L.	0	n	1	0	Asparagaceae Juss.
Iz_9_1209	DJI_0588	Artemisia marschalliana Spreng.	0	n	1	0	Asteraceae Bercht. & J.Presl
Iz_9_1209	DJI_0588	Asparagus officinalis L.	0	n	1	0	Asparagaceae Juss.
Iz_9_1209	DJI_0588	Astragalus cicer L.	0	n	1	0	Fabaceae Lindl.
Iz_9_1209	DJI_0588	Bassia laniflora (S.G.Gmel.) A.J.Scott	0	n	1	0	Amaranthaceae Juss.
Iz_9_1209	DJI_0588	Berberis vulgaris L.	0	n	1	0	Berberidaceae Juss.
Iz_9_1209	DJI_0588	Betula pendula Roth.	0	n	1	1	Betulaceae Gray
Iz_9_1209	DJI_0588	Betula pendula Roth.	0	n	1	0	Poaceae Barnhart
Iz_9_1209	DJI_0588	Bromus inermis Leyss.	0		NA	NA	
Iz_9_1209	DJI_0588	Bromus squarrosus L.	0	a	0	0	Poaceae Barnhart
Iz_9_1209	DJI_0588	Bromus tectorum L.	0	a	0	0	Poaceae Barnhart
Iz_9_1209	DJI_0588	Calamagrostis epigejos (L.) Roth	30	n	1	0	Poaceae Barnhart
Iz_9_1209	DJI_0588	Carduus acanthoides L.	0	n	1	0	Asteraceae Bercht. & J.Presl
Iz_9_1209	DJI_0588	Carex sp.	0	n	1	0	Cyperaceae Juss.
Iz_9_1209	DJI_0588	Centaurea majorovii Dumbadze	0	n	1	0	Asteraceae Bercht. & J.Presl
Iz_9_1209	DJI_0588	Chamaecytisus ruthenicus (Fisch.ex Wol.) Klaskova	0	n	1	0	Fabaceae Lindl.
Iz_9_1209	DJI_0588	Chelidonium majus L.	0	n	1	0	Papaveraceae Juss.
Iz_9_1209	DJI_0588	Chenopodium betaceum Andrz.	0	n	1	0	Amaranthaceae Juss.
Iz_9_1209	DJI_0588	Chenopodium cf. album	0	n	1	0	Amaranthaceae Juss.
Iz_9_1209	DJI_0588	Chenopodium sp.	0		NA	0	Amaranthaceae Juss.
Iz_9_1209	DJI_0588	Chondrilla juncea L.	0	n	1	0	Asteraceae Bercht. & J.Presl
Iz_9_1209	DJI_0588	Cirsium vulgare (Savi) Ten.	0	n	1	0	Asteraceae Bercht. & J.Presl
Iz_9_1209	DJI_0588	Clinopodium acinos (L.) Kuntze	0	n	1	0	Lamiacea Lindl.

Iz_9_1209	DJI_0588	Convallaria majalis L.	0	n	1	0	Asparagaceae Juss.
Iz_9_1209	DJI_0588	Crataegus rhipidophylla Gand.	0	n	1	1	Rosaceae Juss.
Iz_9_1209	DJI_0588	Crepis foetida L.	0	n	1	0	Asteraceae Bercht. & J.Presl
Iz_9_1209	DJI_0588	Dactylis glomerata L.	0	n	1	0	Poaceae Barnhart
Iz_9_1209	DJI_0588	Dianthus campestris M.Bieb.	0	n	1	0	Caryophyllaceae Juss.
Iz_9_1209	DJI_0588	Digitaria sanguinalis (L.) Scop.	0	a	0	0	Poaceae Barnhart
Iz_9_1209	DJI_0588	Elymus repens (L.) Gould	0	n	1	0	Poaceae Barnhart
Iz_9_1209	DJI_0588	Epilobium tetragonum L.	0	n	1	0	Onagraceae Juss.
Iz_9_1209	DJI_0588	Eragrostis minor Host	0	a	0	0	Poaceae Barnhart
Iz_9_1209	DJI_0588	Erigeron canadensis L.	0	a	0	0	Asteraceae Bercht. & J.Presl
Iz_9_1209	DJI_0588	Euphorbia seguieriana Neck.	0	n	1	0	Euphorbiaceae Juss.
Iz_9_1209	DJI_0588	Fallopia dumetorum (L.) Holub	0	n	1	0	Polygonaceae Juss.
Iz_9_1209	DJI_0588	Festuca beckeri (Hack.) Trautv.	0	n	1	0	Poaceae Barnhart
Iz_9_1209	DJI_0588	Fragaria vesca L.	0	n	1	0	Rosaceae Juss.
Iz_9_1209	DJI_0588	Frangula alnus Mill.	0	n	1	1	Rhamnaceae Juss.
Iz_9_1209	DJI_0588	Fraxinus pennsylvanica Marshall	0	a	0	1	Oleaceae Hoffmanns. & Link
Iz_9_1209	DJI_0588	Galeopsis bifida Boenn.	0	n	1	0	Lamiacea Lindl.
Iz_9_1209	DJI_0588	Galium verum L.	0	n	1	0	Rubiaceae Juss.
Iz_9_1209	DJI_0588	Genista tinctoria L.	0	n	1	0	Fabaceae Lindl.
Iz_9_1209	DJI_0588	Geranium robertianum L.	0	n	1	0	Geraniaceae Juss.
Iz_9_1209	DJI_0588	Geranium sanquineum L.	0	n	1	0	Geraniaceae Juss.
Iz_9_1209	DJI_0588	Gypsophila paniculata L.	0	n	1	0	Caryophyllaceae Juss.
Iz_9_1209	DJI_0588	Helichrysum arenarium (L.) Moench	0	n	1	0	Asteraceae Bercht. & J.Presl
Iz_9_1209	DJI_0588	Herniaria polygama J.Gay	0	n	1	0	Caryophyllaceae Juss.
Iz_9_1209	DJI_0588	Hieracium robustum Fr.	0	n	1	0	Asteraceae Bercht. & J.Presl
Iz_9_1209	DJI_0588	Hieracium umbellatum L.	0	n	1	NA	Asteraceae Bercht. & J.Presl
Iz_9_1209	DJI_0588	Hylotelephium maximum (L.) Holub	0	n	1	0	Crassulaceae J.St.-Hil.
Iz_9_1209	DJI_0588	Jacobaea borysthenica (DC.) B.Nord. & Greuter	0	n	1	0	Asteraceae Bercht. & J.Presl
Iz_9_1209	DJI_0588	Jasione montana L.	0	n	1	0	Campanulaceae Juss.
Iz_9_1209	DJI_0588	Jurinea cyanoides (L.) Rchb.s.l.	0	n	1	0	Asteraceae Bercht. & J.Presl
Iz_9_1209	DJI_0588	Koeleria glauca (Spreng.) DC.	0	n	1	0	Poaceae Barnhart
Iz_9_1209	DJI_0588	Lactuca serriola L.	0	a	0	0	Asteraceae Bercht. & J.Presl
Iz_9_1209	DJI_0588	Linaria genistifolia (L.) Mill.	0	n	1	0	Plantaginaceae Juss.
Iz_9_1209	DJI_0588	Lomelosia argentea (L.) Greuter & Burdet	0	n	1	0	Caprifoliaceae Juss.
Iz_9_1209	DJI_0588	Melampyrum pratense L.	0	n	1	0	Orobanchaceae Vent.
Iz_9_1209	DJI_0588	Oenothera biennis L.	0	a	0	0	Onagraceae Juss.
Iz_9_1209	DJI_0588	Pentanema britannica (L.) D.Gut.Larr., Santos-Vicente, Anderb., E.Rico & M.M.Mart.Ort.	0	n	1	0	Asteraceae Bercht. & J.Presl
Iz_9_1209	DJI_0588	Peucedanum oreoselinum (L.) Moench	0	n	1	0	Apiaceae Lindl.
Iz_9_1209	DJI_0588	Peucedanum ruthenicum M.Bieb.	0	n	1	0	Apiaceae Lindl.
Iz_9_1209	DJI_0588	Pilosella echioides (Lumn.) F.W.Schultz & Sch.Bip.	0	n	1	0	Asteraceae Bercht. & J.Presl
Iz_9_1209	DJI_0588	Pilosella officinarum F. Schultz et Sch. Bip.	0	n	1	0	Asteraceae Bercht. & J.Presl
Iz_9_1209	DJI_0588	Pilosella sp.	0		NA	0	Asteraceae Bercht. & J.Presl
Iz_9_1209	DJI_0588	Pinus sylvestris L.	0	n	1	1	Pinaceae Spreng. ex F.Rudolphi
Iz_9_1209	DJI_0588	Poa annua L.	0	n	1	0	Poaceae Barnhart
Iz_9_1209	DJI_0588	Poa nemoralis L.	0	n	1	0	Poaceae Barnhart
Iz_9_1209	DJI_0588	Poa sp.	0		NA	0	Poaceae Barnhart
Iz_9_1209	DJI_0588	Polygonatum odoratum (Mill.) Druce	0	n	1	0	Asparagaceae Juss.
Iz_9_1209	DJI_0588	Polygonum arenarium Waldst. & Kit.	0	n	1	0	Polygonaceae Juss.
Iz_9_1209	DJI_0588	Populus nigra L.	0	n	1	1	Salicaceae Mirb.
Iz_9_1209	DJI_0588	Populus tremula L.	0	n	1	1	Salicaceae Mirb.
Iz_9_1209	DJI_0588	Populus Ã canescens (Aiton) Sm.	0	n	1	1	Salicaceae Mirb.
Iz_9_1209	DJI_0588	Potentilla argentea L.	0	n	1	0	Rosaceae Juss.
Iz_9_1209	DJI_0588	Psammophiliella muralis (L.) Ikonn.	0	n	1	0	Caryophyllaceae Juss.
Iz_9_1209	DJI_0588	Psephellus sumensis (Kalen.) Greuter	0	n	1	0	Asteraceae Bercht. & J.Presl
Iz_9_1209	DJI_0588	Pulsatilla pratensis (L.) Mill. s.l.	0	n	1	0	Ranunculaceae Juss.
Iz_9_1209	DJI_0588	Pyrus communis L.	0	a	0	1	Rosaceae Juss.
Iz_9_1209	DJI_0588	Quercus robur L.	0	n	1	1	Fagaceae Dumort.
Iz_9_1209	DJI_0588	Robinia pseudoacacia L.	0	a	0	1	Fabaceae Lindl.
Iz_9_1209	DJI_0588	Rosa sp.	0		NA	1	Rosaceae Juss.
Iz_9_1209	DJI_0588	Rubus idaeus L.	0	n	1	0	Rosaceae Juss.
Iz_9_1209	DJI_0588	Rumex acetosella L.	0	n	1	0	Polygonaceae Juss.
Iz_9_1209	DJI_0588	Rumex thyrsiflorus Fingerh.	0	n	1	0	Polygonaceae Juss.
Iz_9_1209	DJI_0588	Salix alba L.	0	n	1	1	Salicaceae Mirb.
Iz_9_1209	DJI_0588	Sambucus nigra L.	0	n	1	1	Viburnaceae Raf.
Iz_9_1209	DJI_0588	Secale sylvestre Host	0	n	1	0	Poaceae Barnhart
Iz_9_1209	DJI_0588	Sempervivum ruthenicum Schnittsp. & C.B. Lehm.	0	n	1	0	Crassulaceae J.St.-Hil.
Iz_9_1209	DJI_0588	Setaria viridis (L.) P. Beauv.	0	n	1	0	Poaceae Barnhart
Iz_9_1209	DJI_0588	Silene borysthenica (Gruner) Walters	0	n	1	0	Caryophyllaceae Juss.
Iz_9_1209	DJI_0588	Silene latifolia subsp. alba (Mill.) Greuter & Burdet	0	n	1	0	Caryophyllaceae Juss.
Iz_9_1209	DJI_0588	Solidago virgaurea L.	0.2	n	1	0	Asteraceae Bercht. & J.Presl
Iz_9_1209	DJI_0588	Sorbus aucuparia L.	0	n	1	1	Rosaceae Juss.
Iz_9_1209	DJI_0588	Species 12.09_9_0601	0		NA	NA	
Iz_9_1209	DJI_0588	Stachys recta L.	0	n	1	0	Lamiacea Lindl.
Iz_9_1209	DJI_0588	Tanacetum vulgare L.	0	n	1	0	Asteraceae Bercht. & J.Presl
Iz_9_1209	DJI_0588	Thymus pallasianus Heinr.Braun	0	n	1	0	Lamiacea Lindl.
Iz_9_1209	DJI_0588	Tilia cordata Mill.	0	n	1	1	Malvaceae Juss.
Iz_9_1209	DJI_0588	Trifolium alpestre L.	0	n	1	0	Fabaceae Lindl.
Iz_9_1209	DJI_0588	Trifolium arvense L.	0	n	1	0	Fabaceae Lindl.
Iz_9_1209	DJI_0588	Ulmus glabra Huds.	0	n	1	1	Ulmaceae Mirb.
Iz_9_1209	DJI_0588	Verbascum densiflorum Bertol.	0	n	1	0	Scrophulariaceae Juss.
Iz_9_1209	DJI_0588	Veronica austriaca L.	0	n	1	0	Plantaginaceae Juss.
Iz_9_1209	DJI_0588	Veronica incana L.	0	n	1	0	Plantaginaceae Juss.
Iz_9_1209	DJI_0588	Veronica verna L.	0	n	1	0	Plantaginaceae Juss.
Iz_9_1209	DJI_0588	Vincetoxicum hirundinaria Medik	0	n	1	0	Apocynaceae Juss.
Iz_9_1209	DJI_0601	Acer tataricum L.	0	n	1	1	Sapindaceae Juss.
Iz_9_1209	DJI_0601	Achillea millefolium L. s.l.	0	n	1	0	Asteraceae Bercht. & J.Presl
Iz_9_1209	DJI_0601	Allium oleraceum L.	0	n	1	0	Amaryllidaceae J.St.-Hil.
Iz_9_1209	DJI_0601	Ambrosia artemisiifolia L.	0	a	0	0	Asteraceae Bercht. & J.Presl
Iz_9_1209	DJI_0601	Amorpha fruticosa L.	0	a	0	0	Fabaceae Lindl.
Iz_9_1209	DJI_0601	Anthericum ramosum L.	0	n	1	0	Asparagaceae Juss.
Iz_9_1209	DJI_0601	Artemisia marschalliana Spreng.	0	n	1	0	Asteraceae Bercht. & J.Presl
Iz_9_1209	DJI_0601	Asparagus officinalis L.	0	n	1	0	Asparagaceae Juss.
Iz_9_1209	DJI_0601	Astragalus cicer L.	0	n	1	0	Fabaceae Lindl.
Iz_9_1209	DJI_0601	Bassia laniflora (S.G.Gmel.) A.J.Scott	0	n	1	0	Amaranthaceae Juss.
Iz_9_1209	DJI_0601	Berberis vulgaris L.	0	n	1	0	Berberidaceae Juss.
Iz_9_1209	DJI_0601	Betula pendula Roth.	0	n	1	1	Betulaceae Gray
Iz_9_1209	DJI_0601	Betula pendula Roth.	0	n	1	0	Poaceae Barnhart
Iz_9_1209	DJI_0601	Bromus inermis Leyss.	0		NA	NA	

Iz_9_1209	DJI_0601	Bromus squarrosus L.	0	a	0	0	Poaceae Barnhart
Iz_9_1209	DJI_0601	Bromus tectorum L.	0	a	0	0	Poaceae Barnhart
Iz_9_1209	DJI_0601	Calamagrostis epigejos (L.) Roth	3	n	1	0	Poaceae Barnhart
Iz_9_1209	DJI_0601	Carduus acanthoides L.	0	n	1	0	Asteraceae Bercht. & J.Presl
Iz_9_1209	DJI_0601	Carex sp.	0	n	1	0	Cyperaceae Juss.
Iz_9_1209	DJI_0601	Centaurea majorovii Dumbadze	0	n	1	0	Asteraceae Bercht. & J.Presl
Iz_9_1209	DJI_0601	Chamaecytisus ruthenicus (Fisch.ex Wol.) Klaskova	0	n	1	0	Fabaceae Lindl.
Iz_9_1209	DJI_0601	Chelidonium majus L.	0	n	1	0	Papaveraceae Juss.
Iz_9_1209	DJI_0601	Chenopodium betaceum Andrz.	0	n	1	0	Amaranthaceae Juss.
Iz_9_1209	DJI_0601	Chenopodium cf. album	0	n	1	0	Amaranthaceae Juss.
Iz_9_1209	DJI_0601	Chenopodium sp.	0		NA	0	Amaranthaceae Juss.
Iz_9_1209	DJI_0601	Chondrilla juncea L.	2	n	1	0	Asteraceae Bercht. & J.Presl
Iz_9_1209	DJI_0601	Cirsium vulgare (Savi) Ten.	0	n	1	0	Asteraceae Bercht. & J.Presl
Iz_9_1209	DJI_0601	Clinopodium acinos (L.) Kuntze	0	n	1	0	Lamiacea Lindl.
Iz_9_1209	DJI_0601	Convallaria majalis L.	0	n	1	0	Asparagaceae Juss.
Iz_9_1209	DJI_0601	Crataegus rhipidophylla Gand.	0	n	1	1	Rosaceae Juss.
Iz_9_1209	DJI_0601	Crepis foetida L.	0	n	1	0	Asteraceae Bercht. & J.Presl
Iz_9_1209	DJI_0601	Dactylis glomerata L.	0	n	1	0	Poaceae Barnhart
Iz_9_1209	DJI_0601	Dianthus campestris M.Bieb.	0	n	1	0	Caryophyllaceae Juss.
Iz_9_1209	DJI_0601	Digitaria sanguinalis (L.) Scop.	0	a	0	0	Poaceae Barnhart
Iz_9_1209	DJI_0601	Elymus repens (L.) Gould	0	n	1	0	Poaceae Barnhart
Iz_9_1209	DJI_0601	Epilobium tetragonum L.	0	n	1	0	Onagraceae Juss.
Iz_9_1209	DJI_0601	Eragrostis minor Host	0	a	0	0	Poaceae Barnhart
Iz_9_1209	DJI_0601	Erigeron canadensis L.	10	a	0	0	Asteraceae Bercht. & J.Presl
Iz_9_1209	DJI_0601	Euphorbia seguieriana Neck.	0	n	1	0	Euphorbiaceae Juss.
Iz_9_1209	DJI_0601	Fallopia dumetorum (L.) Holub	0	n	1	0	Polygonaceae Juss.
Iz_9_1209	DJI_0601	Festuca beckeri (Hack.) Trautv.	0	n	1	0	Poaceae Barnhart
Iz_9_1209	DJI_0601	Fragaria vesca L.	0	n	1	0	Rosaceae Juss.
Iz_9_1209	DJI_0601	Frangula alnus Mill.	0	n	1	1	Rhamnaceae Juss.
Iz_9_1209	DJI_0601	Fraxinus pennsylvanica Marshall	0	a	0	1	Oleaceae Hoffmanns. & Link
Iz_9_1209	DJI_0601	Galeopsis bifida Boenn.	0	n	1	0	Lamiacea Lindl.
Iz_9_1209	DJI_0601	Galium verum L.	0	n	1	0	Rubiaceae Juss.
Iz_9_1209	DJI_0601	Genista tinctoria L.	0	n	1	0	Fabaceae Lindl.
Iz_9_1209	DJI_0601	Geranium robertianum L.	0	n	1	0	Geraniaceae Juss.
Iz_9_1209	DJI_0601	Geranium sanquineum L.	0	n	1	0	Geraniaceae Juss.
Iz_9_1209	DJI_0601	Gypsophila paniculata L.	0	n	1	0	Caryophyllaceae Juss.
Iz_9_1209	DJI_0601	Helichrysum arenarium (L.) Moench	0	n	1	0	Asteraceae Bercht. & J.Presl
Iz_9_1209	DJI_0601	Herniaria polygama J.Gay	0	n	1	0	Caryophyllaceae Juss.
Iz_9_1209	DJI_0601	Hieracium robustum Fr.	0	n	1	0	Asteraceae Bercht. & J.Presl
Iz_9_1209	DJI_0601	Hieracium umbellatum L.	0	n	1	NA	Asteraceae Bercht. & J.Presl
Iz_9_1209	DJI_0601	Hylotelephium maximum (L.) Holub	0	n	1	0	Crassulaceae J.St.-Hil.
Iz_9_1209	DJI_0601	Jacobaea borysthenica (DC.) B.Nord. & Greuter	0	n	1	0	Asteraceae Bercht. & J.Presl
Iz_9_1209	DJI_0601	Jasione montana L.	0	n	1	0	Campanulaceae Juss.
Iz_9_1209	DJI_0601	Jurinea cyanoides (L.) Rchb.s.l.	0	n	1	0	Asteraceae Bercht. & J.Presl
Iz_9_1209	DJI_0601	Koeleria glauca (Spreng.) DC.	0	n	1	0	Poaceae Barnhart
Iz_9_1209	DJI_0601	Lactuca serriola L.	0	a	0	0	Asteraceae Bercht. & J.Presl
Iz_9_1209	DJI_0601	Linaria genistifolia (L.) Mill.	0	n	1	0	Plantaginaceae Juss.
Iz_9_1209	DJI_0601	Lomelosia argentea (L.) Greuter & Burdet	0	n	1	0	Caprifoliaceae Juss.
Iz_9_1209	DJI_0601	Melampyrum pratense L.	0	n	1	0	Orobanchaceae Vent.
Iz_9_1209	DJI_0601	Oenothera biennis L.	0	a	0	0	Onagraceae Juss.
Iz_9_1209	DJI_0601	Pentanema britannica (L.) D.Gut.Larr., Santos-Vicente, Anderb., E.Rico & M.M.Mart.Ort.	0	n	1	0	Asteraceae Bercht. & J.Presl
Iz_9_1209	DJI_0601	Peucedanum oreoselinum (L.) Moench	0	n	1	0	Apiaceae Lindl.
Iz_9_1209	DJI_0601	Peucedanum ruthenicum M.Bieb.	0	n	1	0	Apiaceae Lindl.
Iz_9_1209	DJI_0601	Pilosella echioides (Lumn.) F.W.Schultz & Sch.Bip.	0	n	1	0	Asteraceae Bercht. & J.Presl
Iz_9_1209	DJI_0601	Pilosella officinarum F. Schultz et Sch. Bip.	0	n	1	0	Asteraceae Bercht. & J.Presl
Iz_9_1209	DJI_0601	Pilosella sp.	0		NA	0	Asteraceae Bercht. & J.Presl
Iz_9_1209	DJI_0601	Pinus sylvestris L.	0	n	1	1	Pinaceae Spreng. ex F.Rudolphi
Iz_9_1209	DJI_0601	Poa annua L.	0	n	1	0	Poaceae Barnhart
Iz_9_1209	DJI_0601	Poa nemoralis L.	0	n	1	0	Poaceae Barnhart
Iz_9_1209	DJI_0601	Poa sp.	0		NA	0	Poaceae Barnhart
Iz_9_1209	DJI_0601	Polygonatum odoratum (Mill.) Druce	0	n	1	0	Asparagaceae Juss.
Iz_9_1209	DJI_0601	Polygonum arenarium Waldst. & Kit.	0	n	1	0	Polygonaceae Juss.
Iz_9_1209	DJI_0601	Populus nigra L.	0	n	1	1	Salicaceae Mirb.
Iz_9_1209	DJI_0601	Populus tremula L.	0	n	1	1	Salicaceae Mirb.
Iz_9_1209	DJI_0601	Populus Ã canescens (Aiton) Sm.	0	n	1	1	Salicaceae Mirb.
Iz_9_1209	DJI_0601	Potentilla argentea L.	0	n	1	0	Rosaceae Juss.
Iz_9_1209	DJI_0601	Psammophiliella muralis (L.) Ikonn.	0	n	1	0	Caryophyllaceae Juss.
Iz_9_1209	DJI_0601	Psephellus sumensis (Kalen.) Greuter	0	n	1	0	Asteraceae Bercht. & J.Presl
Iz_9_1209	DJI_0601	Pulsatilla pratensis (L.) Mill. s.l.	0	n	1	0	Ranunculaceae Juss.
Iz_9_1209	DJI_0601	Pyrus communis L.	0	a	0	1	Rosaceae Juss.
Iz_9_1209	DJI_0601	Quercus robur L.	0	n	1	1	Fagaceae Dumort.
Iz_9_1209	DJI_0601	Robinia pseudoacacia L.	0	a	0	1	Fabaceae Lindl.
Iz_9_1209	DJI_0601	Rosa sp.	0		NA	1	Rosaceae Juss.
Iz_9_1209	DJI_0601	Rubus idaeus L.	0	n	1	0	Rosaceae Juss.
Iz_9_1209	DJI_0601	Rumex acetosella L.	0	n	1	0	Polygonaceae Juss.
Iz_9_1209	DJI_0601	Rumex thyrsiflorus Fingerh.	0	n	1	0	Polygonaceae Juss.
Iz_9_1209	DJI_0601	Salix alba L.	0	n	1	1	Salicaceae Mirb.
Iz_9_1209	DJI_0601	Sambucus nigra L.	0	n	1	1	Viburnaceae Raf.
Iz_9_1209	DJI_0601	Secale sylvestre Host	0	n	1	0	Poaceae Barnhart
Iz_9_1209	DJI_0601	Sempervivum ruthenicum Schnittsp. & C.B. Lehm.	0	n	1	0	Crassulaceae J.St.-Hil.
Iz_9_1209	DJI_0601	Setaria viridis (L.) P. Beauv.	0	n	1	0	Poaceae Barnhart
Iz_9_1209	DJI_0601	Silene borysthenica (Gruner) Walters	0	n	1	0	Caryophyllaceae Juss.
Iz_9_1209	DJI_0601	Silene latifolia subsp. alba (Mill.) Greuter & Burdet	0	n	1	0	Caryophyllaceae Juss.
Iz_9_1209	DJI_0601	Solidago virgaurea L.	0	n	1	0	Asteraceae Bercht. & J.Presl
Iz_9_1209	DJI_0601	Sorbus aucuparia L.	0	n	1	1	Rosaceae Juss.
Iz_9_1209	DJI_0601	Species 12.09_9_0601	0.2		NA	NA	
Iz_9_1209	DJI_0601	Stachys recta L.	0	n	1	0	Lamiacea Lindl.
Iz_9_1209	DJI_0601	Tanacetum vulgare L.	0	n	1	0	Asteraceae Bercht. & J.Presl
Iz_9_1209	DJI_0601	Thymus pallasianus Heinr.Braun	0	n	1	0	Lamiacea Lindl.
Iz_9_1209	DJI_0601	Tilia cordata Mill.	0	n	1	1	Malvaceae Juss.
Iz_9_1209	DJI_0601	Trifolium alpestre L.	0	n	1	0	Fabaceae Lindl.
Iz_9_1209	DJI_0601	Trifolium arvense L.	0	n	1	0	Fabaceae Lindl.
Iz_9_1209	DJI_0601	Ulmus glabra Huds.	0	n	1	1	Ulmaceae Mirb.
Iz_9_1209	DJI_0601	Verbascum densiflorum Bertol.	0	n	1	0	Scrophulariaceae Juss.
Iz_9_1209	DJI_0601	Veronica austriaca L.	0	n	1	0	Plantaginaceae Juss.
Iz_9_1209	DJI_0601	Veronica incana L.	0	n	1	0	Plantaginaceae Juss.
Iz_9_1209	DJI_0601	Veronica verna L.	0	n	1	0	Plantaginaceae Juss.
Iz_9_1209	DJI_0601	Vincetoxicum hirundinaria Medik	0	n	1	0	Apocynaceae Juss.

Plot_ID	Photo_ID	Species	n_trees	family_name	native_alien	native_alien_mod
Iz_1_1009	DJI_0212	Acer tataricum L.	0	Sapindaceae Juss.	n	1
Iz_1_1009	DJI_0212	Betula pendula Roth.	0	Betulaceae Gray	n	1
Iz_1_1009	DJI_0212	Crataegus rhipidophylla Gand.	0	Rosaceae Juss.	n	1
Iz_1_1009	DJI_0212	Frangula alnus Mill.	0	Rhamnaceae Juss.	n	1
Iz_1_1009	DJI_0212	Fraxinus pennsylvanica Marshall	0	Oleaceae Hoffmanns. & Link	a	0
Iz_1_1009	DJI_0212	Pinus sylvestris L.	0	Pinaceae Spreng. ex F.Rudolphi	n	1
Iz_1_1009	DJI_0212	Populus × canescens (Aiton) Sm.	0	Salicaceae Mirb.	n	1
Iz_1_1009	DJI_0212	Populus tremula L.	0	Salicaceae Mirb.	n	1
Iz_1_1009	DJI_0212	Populus nigra L.	0	Salicaceae Mirb.	n	1
Iz_1_1009	DJI_0212	Pyrus communis L.	0	Rosaceae Juss.	a	0
Iz_1_1009	DJI_0212	Quercus robur L.	0	Fagaceae Dumort.	n	1
Iz_1_1009	DJI_0212	Robinia pseudoacacia L.	0	Fabaceae Lindl.	a	0
Iz_1_1009	DJI_0212	Rosa sp.	0	Rosaceae Juss.		
Iz_1_1009	DJI_0212	Salix alba L.	0	Salicaceae Mirb.	n	1
Iz_1_1009	DJI_0212	Sambucus nigra L.	0	Viburnaceae Raf.	n	1
Iz_1_1009	DJI_0212	Sorbus aucuparia L.	0	Rosaceae Juss.	n	1
Iz_1_1009	DJI_0212	Tilia cordata Mill.	0	Malvaceae Juss.	n	1
Iz_1_1009	DJI_0212	Ulmus glabra Huds.	0	Ulmaceae Mirb.	n	1
Iz_1_1009	DJI_0213	Acer tataricum L.	0	Sapindaceae Juss.	n	1
Iz_1_1009	DJI_0213	Betula pendula Roth.	0	Betulaceae Gray	n	1
Iz_1_1009	DJI_0213	Crataegus rhipidophylla Gand.	0	Rosaceae Juss.	n	1
Iz_1_1009	DJI_0213	Frangula alnus Mill.	0	Rhamnaceae Juss.	n	1
Iz_1_1009	DJI_0213	Fraxinus pennsylvanica Marshall	0	Oleaceae Hoffmanns. & Link	a	0
Iz_1_1009	DJI_0213	Pinus sylvestris L.	0	Pinaceae Spreng. ex F.Rudolphi	n	1
Iz_1_1009	DJI_0213	Populus × canescens (Aiton) Sm.	0	Salicaceae Mirb.	n	1
Iz_1_1009	DJI_0213	Populus tremula L.	0	Salicaceae Mirb.	n	1
Iz_1_1009	DJI_0213	Populus nigra L.	0	Salicaceae Mirb.	n	1
Iz_1_1009	DJI_0213	Pyrus communis L.	0	Rosaceae Juss.	a	0
Iz_1_1009	DJI_0213	Quercus robur L.	0	Fagaceae Dumort.	n	1
Iz_1_1009	DJI_0213	Robinia pseudoacacia L.	0	Fabaceae Lindl.	a	0
Iz_1_1009	DJI_0213	Rosa sp.	0	Rosaceae Juss.		
Iz_1_1009	DJI_0213	Salix alba L.	0	Salicaceae Mirb.	n	1
Iz_1_1009	DJI_0213	Sambucus nigra L.	0	Viburnaceae Raf.	n	1
Iz_1_1009	DJI_0213	Sorbus aucuparia L.	0	Rosaceae Juss.	n	1
Iz_1_1009	DJI_0213	Tilia cordata Mill.	0	Malvaceae Juss.	n	1
Iz_1_1009	DJI_0213	Ulmus glabra Huds.	0	Ulmaceae Mirb.	n	1
Iz_1_1009	DJI_0215	Acer tataricum L.	0	Sapindaceae Juss.	n	1
Iz_1_1009	DJI_0215	Betula pendula Roth.	0	Betulaceae Gray	n	1
Iz_1_1009	DJI_0215	Crataegus rhipidophylla Gand.	0	Rosaceae Juss.	n	1
Iz_1_1009	DJI_0215	Frangula alnus Mill.	0	Rhamnaceae Juss.	n	1
Iz_1_1009	DJI_0215	Fraxinus pennsylvanica Marshall	0	Oleaceae Hoffmanns. & Link	a	0
Iz_1_1009	DJI_0215	Pinus sylvestris L.	0	Pinaceae Spreng. ex F.Rudolphi	n	1
Iz_1_1009	DJI_0215	Populus × canescens (Aiton) Sm.	0	Salicaceae Mirb.	n	1
Iz_1_1009	DJI_0215	Populus tremula L.	0	Salicaceae Mirb.	n	1
Iz_1_1009	DJI_0215	Populus nigra L.	0	Salicaceae Mirb.	n	1
Iz_1_1009	DJI_0215	Pyrus communis L.	0	Rosaceae Juss.	a	0
Iz_1_1009	DJI_0215	Quercus robur L.	0	Fagaceae Dumort.	n	1
Iz_1_1009	DJI_0215	Robinia pseudoacacia L.	0	Fabaceae Lindl.	a	0
Iz_1_1009	DJI_0215	Rosa sp.	0	Rosaceae Juss.		
Iz_1_1009	DJI_0215	Salix alba L.	0	Salicaceae Mirb.	n	1
Iz_1_1009	DJI_0215	Sambucus nigra L.	0	Viburnaceae Raf.	n	1
Iz_1_1009	DJI_0215	Sorbus aucuparia L.	0	Rosaceae Juss.	n	1
Iz_1_1009	DJI_0215	Tilia cordata Mill.	0	Malvaceae Juss.	n	1
Iz_1_1009	DJI_0215	Ulmus glabra Huds.	0	Ulmaceae Mirb.	n	1
Iz_1_1009	DJI_0217	Acer tataricum L.	0	Sapindaceae Juss.	n	1
Iz_1_1009	DJI_0217	Betula pendula Roth.	0	Betulaceae Gray	n	1
Iz_1_1009	DJI_0217	Crataegus rhipidophylla Gand.	0	Rosaceae Juss.	n	1
Iz_1_1009	DJI_0217	Frangula alnus Mill.	0	Rhamnaceae Juss.	n	1
Iz_1_1009	DJI_0217	Fraxinus pennsylvanica Marshall	0	Oleaceae Hoffmanns. & Link	a	0
Iz_1_1009	DJI_0217	Pinus sylvestris L.	0	Pinaceae Spreng. ex F.Rudolphi	n	1
Iz_1_1009	DJI_0217	Populus × canescens (Aiton) Sm.	0	Salicaceae Mirb.	n	1
Iz_1_1009	DJI_0217	Populus tremula L.	0	Salicaceae Mirb.	n	1
Iz_1_1009	DJI_0217	Populus nigra L.	0	Salicaceae Mirb.	n	1

Iz_1_1009	DJI_0217	Pyrus communis L.	0	Rosaceae Juss.	a	0
Iz_1_1009	DJI_0217	Quercus robur L.	0	Fagaceae Dumort.	n	1
Iz_1_1009	DJI_0217	Robinia pseudoacacia L.	0	Fabaceae Lindl.	a	0
Iz_1_1009	DJI_0217	Rosa sp.	0	Rosaceae Juss.		
Iz_1_1009	DJI_0217	Salix alba L.	0	Salicaceae Mirb.	n	1
Iz_1_1009	DJI_0217	Sambucus nigra L.	0	Viburnaceae Raf.	n	1
Iz_1_1009	DJI_0217	Sorbus aucuparia L.	0	Rosaceae Juss.	n	1
Iz_1_1009	DJI_0217	Tilia cordata Mill.	0	Malvaceae Juss.	n	1
Iz_1_1009	DJI_0217	Ulmus glabra Huds.	0	Ulmaceae Mirb.	n	1
Iz_1_1009	DJI_0218	Acer tataricum L.	0	Sapindaceae Juss.	n	1
Iz_1_1009	DJI_0218	Betula pendula Roth.	0	Betulaceae Gray	n	1
Iz_1_1009	DJI_0218	Crataegus rhipidophylla Gand.	0	Rosaceae Juss.	n	1
Iz_1_1009	DJI_0218	Frangula alnus Mill.	0	Rhamnaceae Juss.	n	1
Iz_1_1009	DJI_0218	Fraxinus pennsylvanica Marshall	0	Oleaceae Hoffmanns. & Link	a	0
Iz_1_1009	DJI_0218	Pinus sylvestris L.	0	Pinaceae Spreng. ex F.Rudolphi	n	1
Iz_1_1009	DJI_0218	Populus × canescens (Aiton) Sm.	0	Salicaceae Mirb.	n	1
Iz_1_1009	DJI_0218	Populus tremula L.	0	Salicaceae Mirb.	n	1
Iz_1_1009	DJI_0218	Populus nigra L.	0	Salicaceae Mirb.	n	1
Iz_1_1009	DJI_0218	Pyrus communis L.	0	Rosaceae Juss.	a	0
Iz_1_1009	DJI_0218	Quercus robur L.	0	Fagaceae Dumort.	n	1
Iz_1_1009	DJI_0218	Robinia pseudoacacia L.	0	Fabaceae Lindl.	a	0
Iz_1_1009	DJI_0218	Rosa sp.	0	Rosaceae Juss.		
Iz_1_1009	DJI_0218	Salix alba L.	0	Salicaceae Mirb.	n	1
Iz_1_1009	DJI_0218	Sambucus nigra L.	0	Viburnaceae Raf.	n	1
Iz_1_1009	DJI_0218	Sorbus aucuparia L.	0	Rosaceae Juss.	n	1
Iz_1_1009	DJI_0218	Tilia cordata Mill.	0	Malvaceae Juss.	n	1
Iz_1_1009	DJI_0218	Ulmus glabra Huds.	0	Ulmaceae Mirb.	n	1
Iz_1_1009	DJI_0220	Acer tataricum L.	0	Sapindaceae Juss.	n	1
Iz_1_1009	DJI_0220	Betula pendula Roth.	0	Betulaceae Gray	n	1
Iz_1_1009	DJI_0220	Crataegus rhipidophylla Gand.	0	Rosaceae Juss.	n	1
Iz_1_1009	DJI_0220	Frangula alnus Mill.	0	Rhamnaceae Juss.	n	1
Iz_1_1009	DJI_0220	Fraxinus pennsylvanica Marshall	0	Oleaceae Hoffmanns. & Link	a	0
Iz_1_1009	DJI_0220	Pinus sylvestris L.	0	Pinaceae Spreng. ex F.Rudolphi	n	1
Iz_1_1009	DJI_0220	Populus × canescens (Aiton) Sm.	0	Salicaceae Mirb.	n	1
Iz_1_1009	DJI_0220	Populus tremula L.	0	Salicaceae Mirb.	n	1
Iz_1_1009	DJI_0220	Populus nigra L.	0	Salicaceae Mirb.	n	1
Iz_1_1009	DJI_0220	Pyrus communis L.	0	Rosaceae Juss.	a	0
Iz_1_1009	DJI_0220	Quercus robur L.	0	Fagaceae Dumort.	n	1
Iz_1_1009	DJI_0220	Robinia pseudoacacia L.	0	Fabaceae Lindl.	a	0
Iz_1_1009	DJI_0220	Rosa sp.	0	Rosaceae Juss.		
Iz_1_1009	DJI_0220	Salix alba L.	0	Salicaceae Mirb.	n	1
Iz_1_1009	DJI_0220	Sambucus nigra L.	0	Viburnaceae Raf.	n	1
Iz_1_1009	DJI_0220	Sorbus aucuparia L.	0	Rosaceae Juss.	n	1
Iz_1_1009	DJI_0220	Tilia cordata Mill.	0	Malvaceae Juss.	n	1
Iz_1_1009	DJI_0220	Ulmus glabra Huds.	0	Ulmaceae Mirb.	n	1
Iz_1_1009	DJI_0229	Acer tataricum L.	0	Sapindaceae Juss.	n	1
Iz_1_1009	DJI_0229	Betula pendula Roth.	0	Betulaceae Gray	n	1
Iz_1_1009	DJI_0229	Crataegus rhipidophylla Gand.	0	Rosaceae Juss.	n	1
Iz_1_1009	DJI_0229	Frangula alnus Mill.	0	Rhamnaceae Juss.	n	1
Iz_1_1009	DJI_0229	Fraxinus pennsylvanica Marshall	0	Oleaceae Hoffmanns. & Link	a	0
Iz_1_1009	DJI_0229	Pinus sylvestris L.	0	Pinaceae Spreng. ex F.Rudolphi	n	1
Iz_1_1009	DJI_0229	Populus × canescens (Aiton) Sm.	0	Salicaceae Mirb.	n	1
Iz_1_1009	DJI_0229	Populus tremula L.	0	Salicaceae Mirb.	n	1
Iz_1_1009	DJI_0229	Populus nigra L.	0	Salicaceae Mirb.	n	1
Iz_1_1009	DJI_0229	Pyrus communis L.	0	Rosaceae Juss.	a	0
Iz_1_1009	DJI_0229	Quercus robur L.	0	Fagaceae Dumort.	n	1
Iz_1_1009	DJI_0229	Robinia pseudoacacia L.	0	Fabaceae Lindl.	a	0
Iz_1_1009	DJI_0229	Rosa sp.	0	Rosaceae Juss.		
Iz_1_1009	DJI_0229	Salix alba L.	0	Salicaceae Mirb.	n	1
Iz_1_1009	DJI_0229	Sambucus nigra L.	0	Viburnaceae Raf.	n	1
Iz_1_1009	DJI_0229	Sorbus aucuparia L.	0	Rosaceae Juss.	n	1
Iz_1_1009	DJI_0229	Tilia cordata Mill.	0	Malvaceae Juss.	n	1
Iz_1_1009	DJI_0229	Ulmus glabra Huds.	0	Ulmaceae Mirb.	n	1
Iz_1_1009	DJI_0230	Acer tataricum L.	0	Sapindaceae Juss.	n	1

Iz_1_1009	DJI_0230	Betula pendula Roth.	0	Betulaceae Gray	n	1
Iz_1_1009	DJI_0230	Crataegus rhipidophylla Gand.	0	Rosaceae Juss.	n	1
Iz_1_1009	DJI_0230	Frangula alnus Mill.	0	Rhamnaceae Juss.	n	1
Iz_1_1009	DJI_0230	Fraxinus pennsylvanica Marshall	0	Oleaceae Hoffmanns. & Link	a	0
Iz_1_1009	DJI_0230	Pinus sylvestris L.	0	Pinaceae Spreng. ex F.Rudolphi	n	1
Iz_1_1009	DJI_0230	Populus × canescens (Aiton) Sm.	0	Salicaceae Mirb.	n	1
Iz_1_1009	DJI_0230	Populus tremula L.	0	Salicaceae Mirb.	n	1
Iz_1_1009	DJI_0230	Populus nigra L.	0	Salicaceae Mirb.	n	1
Iz_1_1009	DJI_0230	Pyrus communis L.	0	Rosaceae Juss.	a	0
Iz_1_1009	DJI_0230	Quercus robur L.	0	Fagaceae Dumort.	n	1
Iz_1_1009	DJI_0230	Robinia pseudoacacia L.	0	Fabaceae Lindl.	a	0
Iz_1_1009	DJI_0230	Rosa sp.	0	Rosaceae Juss.		
Iz_1_1009	DJI_0230	Salix alba L.	0	Salicaceae Mirb.	n	1
Iz_1_1009	DJI_0230	Sambucus nigra L.	0	Viburnaceae Raf.	n	1
Iz_1_1009	DJI_0230	Sorbus aucuparia L.	0	Rosaceae Juss.	n	1
Iz_1_1009	DJI_0230	Tilia cordata Mill.	0	Malvaceae Juss.	n	1
Iz_1_1009	DJI_0230	Ulmus glabra Huds.	0	Ulmaceae Mirb.	n	1
Iz_1_1009	DJI_0231	Acer tataricum L.	0	Sapindaceae Juss.	n	1
Iz_1_1009	DJI_0231	Betula pendula Roth.	0	Betulaceae Gray	n	1
Iz_1_1009	DJI_0231	Crataegus rhipidophylla Gand.	0	Rosaceae Juss.	n	1
Iz_1_1009	DJI_0231	Frangula alnus Mill.	0	Rhamnaceae Juss.	n	1
Iz_1_1009	DJI_0231	Fraxinus pennsylvanica Marshall	0	Oleaceae Hoffmanns. & Link	a	0
Iz_1_1009	DJI_0231	Pinus sylvestris L.	0	Pinaceae Spreng. ex F.Rudolphi	n	1
Iz_1_1009	DJI_0231	Populus × canescens (Aiton) Sm.	0	Salicaceae Mirb.	n	1
Iz_1_1009	DJI_0231	Populus tremula L.	0	Salicaceae Mirb.	n	1
Iz_1_1009	DJI_0231	Populus nigra L.	0	Salicaceae Mirb.	n	1
Iz_1_1009	DJI_0231	Pyrus communis L.	0	Rosaceae Juss.	a	0
Iz_1_1009	DJI_0231	Quercus robur L.	0	Fagaceae Dumort.	n	1
Iz_1_1009	DJI_0231	Robinia pseudoacacia L.	0	Fabaceae Lindl.	a	0
Iz_1_1009	DJI_0231	Rosa sp.	0	Rosaceae Juss.		
Iz_1_1009	DJI_0231	Salix alba L.	0	Salicaceae Mirb.	n	1
Iz_1_1009	DJI_0231	Sambucus nigra L.	0	Viburnaceae Raf.	n	1
Iz_1_1009	DJI_0231	Sorbus aucuparia L.	0	Rosaceae Juss.	n	1
Iz_1_1009	DJI_0231	Tilia cordata Mill.	0	Malvaceae Juss.	n	1
Iz_1_1009	DJI_0231	Ulmus glabra Huds.	0	Ulmaceae Mirb.	n	1
Iz_1_1009	DJI_0234	Acer tataricum L.	0	Sapindaceae Juss.	n	1
Iz_1_1009	DJI_0234	Betula pendula Roth.	0	Betulaceae Gray	n	1
Iz_1_1009	DJI_0234	Crataegus rhipidophylla Gand.	0	Rosaceae Juss.	n	1
Iz_1_1009	DJI_0234	Frangula alnus Mill.	0	Rhamnaceae Juss.	n	1
Iz_1_1009	DJI_0234	Fraxinus pennsylvanica Marshall	0	Oleaceae Hoffmanns. & Link	a	0
Iz_1_1009	DJI_0234	Pinus sylvestris L.	0	Pinaceae Spreng. ex F.Rudolphi	n	1
Iz_1_1009	DJI_0234	Populus × canescens (Aiton) Sm.	0	Salicaceae Mirb.	n	1
Iz_1_1009	DJI_0234	Populus tremula L.	0	Salicaceae Mirb.	n	1
Iz_1_1009	DJI_0234	Populus nigra L.	0	Salicaceae Mirb.	n	1
Iz_1_1009	DJI_0234	Pyrus communis L.	0	Rosaceae Juss.	a	0
Iz_1_1009	DJI_0234	Quercus robur L.	0	Fagaceae Dumort.	n	1
Iz_1_1009	DJI_0234	Robinia pseudoacacia L.	0	Fabaceae Lindl.	a	0
Iz_1_1009	DJI_0234	Rosa sp.	0	Rosaceae Juss.		
Iz_1_1009	DJI_0234	Salix alba L.	0	Salicaceae Mirb.	n	1
Iz_1_1009	DJI_0234	Sambucus nigra L.	0	Viburnaceae Raf.	n	1
Iz_1_1009	DJI_0234	Sorbus aucuparia L.	0	Rosaceae Juss.	n	1
Iz_1_1009	DJI_0234	Tilia cordata Mill.	0	Malvaceae Juss.	n	1
Iz_1_1009	DJI_0234	Ulmus glabra Huds.	0	Ulmaceae Mirb.	n	1
Iz_1_1009	DJI_0238	Acer tataricum L.	0	Sapindaceae Juss.	n	1
Iz_1_1009	DJI_0238	Betula pendula Roth.	0	Betulaceae Gray	n	1
Iz_1_1009	DJI_0238	Crataegus rhipidophylla Gand.	0	Rosaceae Juss.	n	1
Iz_1_1009	DJI_0238	Frangula alnus Mill.	0	Rhamnaceae Juss.	n	1
Iz_1_1009	DJI_0238	Fraxinus pennsylvanica Marshall	0	Oleaceae Hoffmanns. & Link	a	0
Iz_1_1009	DJI_0238	Pinus sylvestris L.	0	Pinaceae Spreng. ex F.Rudolphi	n	1
Iz_1_1009	DJI_0238	Populus × canescens (Aiton) Sm.	0	Salicaceae Mirb.	n	1
Iz_1_1009	DJI_0238	Populus tremula L.	0	Salicaceae Mirb.	n	1
Iz_1_1009	DJI_0238	Populus nigra L.	0	Salicaceae Mirb.	n	1
Iz_1_1009	DJI_0238	Pyrus communis L.	0	Rosaceae Juss.	a	0
Iz_1_1009	DJI_0238	Quercus robur L.	0	Fagaceae Dumort.	n	1

Iz_1_1009	DJI_0238	Robinia pseudoacacia L.	0	Fabaceae Lindl.	a	0
Iz_1_1009	DJI_0238	Rosa sp.	0	Rosaceae Juss.		
Iz_1_1009	DJI_0238	Salix alba L.	0	Salicaceae Mirb.	n	1
Iz_1_1009	DJI_0238	Sambucus nigra L.	0	Viburnaceae Raf.	n	1
Iz_1_1009	DJI_0238	Sorbus aucuparia L.	0	Rosaceae Juss.	n	1
Iz_1_1009	DJI_0238	Tilia cordata Mill.	0	Malvaceae Juss.	n	1
Iz_1_1009	DJI_0238	Ulmus glabra Huds.	0	Ulmaceae Mirb.	n	1
Iz_1_1009	DJI_0239	Acer tataricum L.	0	Sapindaceae Juss.	n	1
Iz_1_1009	DJI_0239	Betula pendula Roth.	0	Betulaceae Gray	n	1
Iz_1_1009	DJI_0239	Crataegus rhipidophylla Gand.	0	Rosaceae Juss.	n	1
Iz_1_1009	DJI_0239	Frangula alnus Mill.	0	Rhamnaceae Juss.	n	1
Iz_1_1009	DJI_0239	Fraxinus pennsylvanica Marshall	0	Oleaceae Hoffmanns. & Link	a	0
Iz_1_1009	DJI_0239	Pinus sylvestris L.	0	Pinaceae Spreng. ex F.Rudolphi	n	1
Iz_1_1009	DJI_0239	Populus × canescens (Aiton) Sm.	0	Salicaceae Mirb.	n	1
Iz_1_1009	DJI_0239	Populus tremula L.	0	Salicaceae Mirb.	n	1
Iz_1_1009	DJI_0239	Populus nigra L.	0	Salicaceae Mirb.	n	1
Iz_1_1009	DJI_0239	Pyrus communis L.	0	Rosaceae Juss.	a	0
Iz_1_1009	DJI_0239	Quercus robur L.	0	Fagaceae Dumort.	n	1
Iz_1_1009	DJI_0239	Robinia pseudoacacia L.	0	Fabaceae Lindl.	a	0
Iz_1_1009	DJI_0239	Rosa sp.	0	Rosaceae Juss.		
Iz_1_1009	DJI_0239	Salix alba L.	0	Salicaceae Mirb.	n	1
Iz_1_1009	DJI_0239	Sambucus nigra L.	0	Viburnaceae Raf.	n	1
Iz_1_1009	DJI_0239	Sorbus aucuparia L.	0	Rosaceae Juss.	n	1
Iz_1_1009	DJI_0239	Tilia cordata Mill.	0	Malvaceae Juss.	n	1
Iz_1_1009	DJI_0239	Ulmus glabra Huds.	0	Ulmaceae Mirb.	n	1
Iz_1_1009	DJI_0242	Acer tataricum L.	0	Sapindaceae Juss.	n	1
Iz_1_1009	DJI_0242	Betula pendula Roth.	0	Betulaceae Gray	n	1
Iz_1_1009	DJI_0242	Crataegus rhipidophylla Gand.	0	Rosaceae Juss.	n	1
Iz_1_1009	DJI_0242	Frangula alnus Mill.	0	Rhamnaceae Juss.	n	1
Iz_1_1009	DJI_0242	Fraxinus pennsylvanica Marshall	0	Oleaceae Hoffmanns. & Link	a	0
Iz_1_1009	DJI_0242	Pinus sylvestris L.	0	Pinaceae Spreng. ex F.Rudolphi	n	1
Iz_1_1009	DJI_0242	Populus × canescens (Aiton) Sm.	0	Salicaceae Mirb.	n	1
Iz_1_1009	DJI_0242	Populus tremula L.	0	Salicaceae Mirb.	n	1
Iz_1_1009	DJI_0242	Populus nigra L.	0	Salicaceae Mirb.	n	1
Iz_1_1009	DJI_0242	Pyrus communis L.	0	Rosaceae Juss.	a	0
Iz_1_1009	DJI_0242	Quercus robur L.	0	Fagaceae Dumort.	n	1
Iz_1_1009	DJI_0242	Robinia pseudoacacia L.	0	Fabaceae Lindl.	a	0
Iz_1_1009	DJI_0242	Rosa sp.	0	Rosaceae Juss.		
Iz_1_1009	DJI_0242	Salix alba L.	0	Salicaceae Mirb.	n	1
Iz_1_1009	DJI_0242	Sambucus nigra L.	0	Viburnaceae Raf.	n	1
Iz_1_1009	DJI_0242	Sorbus aucuparia L.	0	Rosaceae Juss.	n	1
Iz_1_1009	DJI_0242	Tilia cordata Mill.	0	Malvaceae Juss.	n	1
Iz_1_1009	DJI_0242	Ulmus glabra Huds.	0	Ulmaceae Mirb.	n	1
Iz_1_1009	DJI_0250	Acer tataricum L.	0	Sapindaceae Juss.	n	1
Iz_1_1009	DJI_0250	Betula pendula Roth.	0	Betulaceae Gray	n	1
Iz_1_1009	DJI_0250	Crataegus rhipidophylla Gand.	0	Rosaceae Juss.	n	1
Iz_1_1009	DJI_0250	Frangula alnus Mill.	0	Rhamnaceae Juss.	n	1
Iz_1_1009	DJI_0250	Fraxinus pennsylvanica Marshall	0	Oleaceae Hoffmanns. & Link	a	0
Iz_1_1009	DJI_0250	Pinus sylvestris L.	0	Pinaceae Spreng. ex F.Rudolphi	n	1
Iz_1_1009	DJI_0250	Populus × canescens (Aiton) Sm.	0	Salicaceae Mirb.	n	1
Iz_1_1009	DJI_0250	Populus tremula L.	0	Salicaceae Mirb.	n	1
Iz_1_1009	DJI_0250	Populus nigra L.	0	Salicaceae Mirb.	n	1
Iz_1_1009	DJI_0250	Pyrus communis L.	0	Rosaceae Juss.	a	0
Iz_1_1009	DJI_0250	Quercus robur L.	0	Fagaceae Dumort.	n	1
Iz_1_1009	DJI_0250	Robinia pseudoacacia L.	0	Fabaceae Lindl.	a	0
Iz_1_1009	DJI_0250	Rosa sp.	0	Rosaceae Juss.		
Iz_1_1009	DJI_0250	Salix alba L.	0	Salicaceae Mirb.	n	1
Iz_1_1009	DJI_0250	Sambucus nigra L.	0	Viburnaceae Raf.	n	1
Iz_1_1009	DJI_0250	Sorbus aucuparia L.	0	Rosaceae Juss.	n	1
Iz_1_1009	DJI_0250	Tilia cordata Mill.	0	Malvaceae Juss.	n	1
Iz_1_1009	DJI_0250	Ulmus glabra Huds.	0	Ulmaceae Mirb.	n	1
Iz_1_1009	DJI_0256	Acer tataricum L.	0	Sapindaceae Juss.	n	1
Iz_1_1009	DJI_0256	Betula pendula Roth.	0	Betulaceae Gray	n	1
Iz_1_1009	DJI_0256	Crataegus rhipidophylla Gand.	0	Rosaceae Juss.	n	1

Iz_1_1009	DJI_0256	Frangula alnus Mill.	0	Rhamnaceae Juss.	n	1
Iz_1_1009	DJI_0256	Fraxinus pennsylvanica Marshall	0	Oleaceae Hoffmanns. & Link	a	0
Iz_1_1009	DJI_0256	Pinus sylvestris L.	0	Pinaceae Spreng. ex F.Rudolphi	n	1
Iz_1_1009	DJI_0256	Populus × canescens (Aiton) Sm.	0	Salicaceae Mirb.	n	1
Iz_1_1009	DJI_0256	Populus tremula L.	0	Salicaceae Mirb.	n	1
Iz_1_1009	DJI_0256	Populus nigra L.	0	Salicaceae Mirb.	n	1
Iz_1_1009	DJI_0256	Pyrus communis L.	0	Rosaceae Juss.	a	0
Iz_1_1009	DJI_0256	Quercus robur L.	0	Fagaceae Dumort.	n	1
Iz_1_1009	DJI_0256	Robinia pseudoacacia L.	0	Fabaceae Lindl.	a	0
Iz_1_1009	DJI_0256	Rosa sp.	0	Rosaceae Juss.		
Iz_1_1009	DJI_0256	Salix alba L.	0	Salicaceae Mirb.	n	1
Iz_1_1009	DJI_0256	Sambucus nigra L.	0	Viburnaceae Raf.	n	1
Iz_1_1009	DJI_0256	Sorbus aucuparia L.	0	Rosaceae Juss.	n	1
Iz_1_1009	DJI_0256	Tilia cordata Mill.	0	Malvaceae Juss.	n	1
Iz_1_1009	DJI_0256	Ulmus glabra Huds.	0	Ulmaceae Mirb.	n	1
Iz_2_1009	DJI_0437	Acer tataricum L.	0	Sapindaceae Juss.	n	1
Iz_2_1009	DJI_0437	Betula pendula Roth.	0	Betulaceae Gray	n	1
Iz_2_1009	DJI_0437	Crataegus rhipidophylla Gand.	0	Rosaceae Juss.	n	1
Iz_2_1009	DJI_0437	Frangula alnus Mill.	0	Rhamnaceae Juss.	n	1
Iz_2_1009	DJI_0437	Fraxinus pennsylvanica Marshall	0	Oleaceae Hoffmanns. & Link	a	0
Iz_2_1009	DJI_0437	Pinus sylvestris L.	0	Pinaceae Spreng. ex F.Rudolphi	n	1
Iz_2_1009	DJI_0437	Populus × canescens (Aiton) Sm.	0	Salicaceae Mirb.	n	1
Iz_2_1009	DJI_0437	Populus tremula L.	0	Salicaceae Mirb.	n	1
Iz_2_1009	DJI_0437	Populus nigra L.	0	Salicaceae Mirb.	n	1
Iz_2_1009	DJI_0437	Pyrus communis L.	0	Rosaceae Juss.	a	0
Iz_2_1009	DJI_0437	Quercus robur L.	0	Fagaceae Dumort.	n	1
Iz_2_1009	DJI_0437	Robinia pseudoacacia L.	0	Fabaceae Lindl.	a	0
Iz_2_1009	DJI_0437	Rosa sp.	0	Rosaceae Juss.		
Iz_2_1009	DJI_0437	Salix alba L.	0	Salicaceae Mirb.	n	1
Iz_2_1009	DJI_0437	Sambucus nigra L.	0	Viburnaceae Raf.	n	1
Iz_2_1009	DJI_0437	Sorbus aucuparia L.	0	Rosaceae Juss.	n	1
Iz_2_1009	DJI_0437	Tilia cordata Mill.	0	Malvaceae Juss.	n	1
Iz_2_1009	DJI_0437	Ulmus glabra Huds.	0	Ulmaceae Mirb.	n	1
Iz_2_1009	DJI_0438	Acer tataricum L.	0	Sapindaceae Juss.	n	1
Iz_2_1009	DJI_0438	Betula pendula Roth.	0	Betulaceae Gray	n	1
Iz_2_1009	DJI_0438	Crataegus rhipidophylla Gand.	0	Rosaceae Juss.	n	1
Iz_2_1009	DJI_0438	Frangula alnus Mill.	0	Rhamnaceae Juss.	n	1
Iz_2_1009	DJI_0438	Fraxinus pennsylvanica Marshall	0	Oleaceae Hoffmanns. & Link	a	0
Iz_2_1009	DJI_0438	Pinus sylvestris L.	0	Pinaceae Spreng. ex F.Rudolphi	n	1
Iz_2_1009	DJI_0438	Populus × canescens (Aiton) Sm.	0	Salicaceae Mirb.	n	1
Iz_2_1009	DJI_0438	Populus tremula L.	0	Salicaceae Mirb.	n	1
Iz_2_1009	DJI_0438	Populus nigra L.	0	Salicaceae Mirb.	n	1
Iz_2_1009	DJI_0438	Pyrus communis L.	0	Rosaceae Juss.	a	0
Iz_2_1009	DJI_0438	Quercus robur L.	0	Fagaceae Dumort.	n	1
Iz_2_1009	DJI_0438	Robinia pseudoacacia L.	0	Fabaceae Lindl.	a	0
Iz_2_1009	DJI_0438	Rosa sp.	0	Rosaceae Juss.		
Iz_2_1009	DJI_0438	Salix alba L.	0	Salicaceae Mirb.	n	1
Iz_2_1009	DJI_0438	Sambucus nigra L.	0	Viburnaceae Raf.	n	1
Iz_2_1009	DJI_0438	Sorbus aucuparia L.	0	Rosaceae Juss.	n	1
Iz_2_1009	DJI_0438	Tilia cordata Mill.	0	Malvaceae Juss.	n	1
Iz_2_1009	DJI_0438	Ulmus glabra Huds.	0	Ulmaceae Mirb.	n	1
Iz_2_1009	DJI_0439	Acer tataricum L.	0	Sapindaceae Juss.	n	1
Iz_2_1009	DJI_0439	Betula pendula Roth.	0	Betulaceae Gray	n	1
Iz_2_1009	DJI_0439	Crataegus rhipidophylla Gand.	0	Rosaceae Juss.	n	1
Iz_2_1009	DJI_0439	Frangula alnus Mill.	0	Rhamnaceae Juss.	n	1
Iz_2_1009	DJI_0439	Fraxinus pennsylvanica Marshall	0	Oleaceae Hoffmanns. & Link	a	0
Iz_2_1009	DJI_0439	Pinus sylvestris L.	0	Pinaceae Spreng. ex F.Rudolphi	n	1
Iz_2_1009	DJI_0439	Populus × canescens (Aiton) Sm.	0	Salicaceae Mirb.	n	1
Iz_2_1009	DJI_0439	Populus tremula L.	0	Salicaceae Mirb.	n	1
Iz_2_1009	DJI_0439	Populus nigra L.	0	Salicaceae Mirb.	n	1
Iz_2_1009	DJI_0439	Pyrus communis L.	0	Rosaceae Juss.	a	0
Iz_2_1009	DJI_0439	Quercus robur L.	0	Fagaceae Dumort.	n	1
Iz_2_1009	DJI_0439	Robinia pseudoacacia L.	0	Fabaceae Lindl.	a	0
Iz_2_1009	DJI_0439	Rosa sp.	0	Rosaceae Juss.		

Iz_2_1009	DJI_0439	Salix alba L.	0	Salicaceae Mirb.	n	1
Iz_2_1009	DJI_0439	Sambucus nigra L.	0	Viburnaceae Raf.	n	1
Iz_2_1009	DJI_0439	Sorbus aucuparia L.	0	Rosaceae Juss.	n	1
Iz_2_1009	DJI_0439	Tilia cordata Mill.	0	Malvaceae Juss.	n	1
Iz_2_1009	DJI_0439	Ulmus glabra Huds.	0	Ulmaceae Mirb.	n	1
Iz_2_1009	DJI_0440	Acer tataricum L.	0	Sapindaceae Juss.	n	1
Iz_2_1009	DJI_0440	Betula pendula Roth.	0	Betulaceae Gray	n	1
Iz_2_1009	DJI_0440	Crataegus rhipidophylla Gand.	0	Rosaceae Juss.	n	1
Iz_2_1009	DJI_0440	Frangula alnus Mill.	0	Rhamnaceae Juss.	n	1
Iz_2_1009	DJI_0440	Fraxinus pennsylvanica Marshall	0	Oleaceae Hoffmanns. & Link	a	0
Iz_2_1009	DJI_0440	Pinus sylvestris L.	0	Pinaceae Spreng. ex F.Rudolphi	n	1
Iz_2_1009	DJI_0440	Populus × canescens (Aiton) Sm.	0	Salicaceae Mirb.	n	1
Iz_2_1009	DJI_0440	Populus tremula L.	0	Salicaceae Mirb.	n	1
Iz_2_1009	DJI_0440	Populus nigra L.	0	Salicaceae Mirb.	n	1
Iz_2_1009	DJI_0440	Pyrus communis L.	0	Rosaceae Juss.	a	0
Iz_2_1009	DJI_0440	Quercus robur L.	0	Fagaceae Dumort.	n	1
Iz_2_1009	DJI_0440	Robinia pseudoacacia L.	0	Fabaceae Lindl.	a	0
Iz_2_1009	DJI_0440	Rosa sp.	0	Rosaceae Juss.		
Iz_2_1009	DJI_0440	Salix alba L.	0	Salicaceae Mirb.	n	1
Iz_2_1009	DJI_0440	Sambucus nigra L.	0	Viburnaceae Raf.	n	1
Iz_2_1009	DJI_0440	Sorbus aucuparia L.	0	Rosaceae Juss.	n	1
Iz_2_1009	DJI_0440	Tilia cordata Mill.	0	Malvaceae Juss.	n	1
Iz_2_1009	DJI_0440	Ulmus glabra Huds.	0	Ulmaceae Mirb.	n	1
Iz_2_1009	DJI_0443	Acer tataricum L.	0	Sapindaceae Juss.	n	1
Iz_2_1009	DJI_0443	Betula pendula Roth.	0	Betulaceae Gray	n	1
Iz_2_1009	DJI_0443	Crataegus rhipidophylla Gand.	0	Rosaceae Juss.	n	1
Iz_2_1009	DJI_0443	Frangula alnus Mill.	0	Rhamnaceae Juss.	n	1
Iz_2_1009	DJI_0443	Fraxinus pennsylvanica Marshall	0	Oleaceae Hoffmanns. & Link	a	0
Iz_2_1009	DJI_0443	Pinus sylvestris L.	0	Pinaceae Spreng. ex F.Rudolphi	n	1
Iz_2_1009	DJI_0443	Populus × canescens (Aiton) Sm.	0	Salicaceae Mirb.	n	1
Iz_2_1009	DJI_0443	Populus tremula L.	0	Salicaceae Mirb.	n	1
Iz_2_1009	DJI_0443	Populus nigra L.	0	Salicaceae Mirb.	n	1
Iz_2_1009	DJI_0443	Pyrus communis L.	0	Rosaceae Juss.	a	0
Iz_2_1009	DJI_0443	Quercus robur L.	0	Fagaceae Dumort.	n	1
Iz_2_1009	DJI_0443	Robinia pseudoacacia L.	0	Fabaceae Lindl.	a	0
Iz_2_1009	DJI_0443	Rosa sp.	0	Rosaceae Juss.		
Iz_2_1009	DJI_0443	Salix alba L.	0	Salicaceae Mirb.	n	1
Iz_2_1009	DJI_0443	Sambucus nigra L.	0	Viburnaceae Raf.	n	1
Iz_2_1009	DJI_0443	Sorbus aucuparia L.	0	Rosaceae Juss.	n	1
Iz_2_1009	DJI_0443	Tilia cordata Mill.	0	Malvaceae Juss.	n	1
Iz_2_1009	DJI_0443	Ulmus glabra Huds.	0	Ulmaceae Mirb.	n	1
Iz_2_1009	DJI_0444	Acer tataricum L.	0	Sapindaceae Juss.	n	1
Iz_2_1009	DJI_0444	Betula pendula Roth.	0	Betulaceae Gray	n	1
Iz_2_1009	DJI_0444	Crataegus rhipidophylla Gand.	0	Rosaceae Juss.	n	1
Iz_2_1009	DJI_0444	Frangula alnus Mill.	0	Rhamnaceae Juss.	n	1
Iz_2_1009	DJI_0444	Fraxinus pennsylvanica Marshall	0	Oleaceae Hoffmanns. & Link	a	0
Iz_2_1009	DJI_0444	Pinus sylvestris L.	0	Pinaceae Spreng. ex F.Rudolphi	n	1
Iz_2_1009	DJI_0444	Populus × canescens (Aiton) Sm.	0	Salicaceae Mirb.	n	1
Iz_2_1009	DJI_0444	Populus tremula L.	0	Salicaceae Mirb.	n	1
Iz_2_1009	DJI_0444	Populus nigra L.	0	Salicaceae Mirb.	n	1
Iz_2_1009	DJI_0444	Pyrus communis L.	0	Rosaceae Juss.	a	0
Iz_2_1009	DJI_0444	Quercus robur L.	0	Fagaceae Dumort.	n	1
Iz_2_1009	DJI_0444	Robinia pseudoacacia L.	0	Fabaceae Lindl.	a	0
Iz_2_1009	DJI_0444	Rosa sp.	0	Rosaceae Juss.		
Iz_2_1009	DJI_0444	Salix alba L.	0	Salicaceae Mirb.	n	1
Iz_2_1009	DJI_0444	Sambucus nigra L.	0	Viburnaceae Raf.	n	1
Iz_2_1009	DJI_0444	Sorbus aucuparia L.	0	Rosaceae Juss.	n	1
Iz_2_1009	DJI_0444	Tilia cordata Mill.	0	Malvaceae Juss.	n	1
Iz_2_1009	DJI_0444	Ulmus glabra Huds.	0	Ulmaceae Mirb.	n	1
Iz_2_1009	DJI_0445	Acer tataricum L.	0	Sapindaceae Juss.	n	1
Iz_2_1009	DJI_0445	Betula pendula Roth.	0	Betulaceae Gray	n	1
Iz_2_1009	DJI_0445	Crataegus rhipidophylla Gand.	0	Rosaceae Juss.	n	1
Iz_2_1009	DJI_0445	Frangula alnus Mill.	0	Rhamnaceae Juss.	n	1
Iz_2_1009	DJI_0445	Fraxinus pennsylvanica Marshall	0	Oleaceae Hoffmanns. & Link	a	0

Iz_2_1009	DJI_0445	Pinus sylvestris L.	0	Pinaceae Spreng. ex F.Rudolphi	n	1
Iz_2_1009	DJI_0445	Populus × canescens (Aiton) Sm.	0	Salicaceae Mirb.	n	1
Iz_2_1009	DJI_0445	Populus tremula L.	0	Salicaceae Mirb.	n	1
Iz_2_1009	DJI_0445	Populus nigra L.	0	Salicaceae Mirb.	n	1
Iz_2_1009	DJI_0445	Pyrus communis L.	0	Rosaceae Juss.	a	0
Iz_2_1009	DJI_0445	Quercus robur L.	0	Fagaceae Dumort.	n	1
Iz_2_1009	DJI_0445	Robinia pseudoacacia L.	0	Fabaceae Lindl.	a	0
Iz_2_1009	DJI_0445	Rosa sp.	0	Rosaceae Juss.		
Iz_2_1009	DJI_0445	Salix alba L.	0	Salicaceae Mirb.	n	1
Iz_2_1009	DJI_0445	Sambucus nigra L.	0	Viburnaceae Raf.	n	1
Iz_2_1009	DJI_0445	Sorbus aucuparia L.	0	Rosaceae Juss.	n	1
Iz_2_1009	DJI_0445	Tilia cordata Mill.	0	Malvaceae Juss.	n	1
Iz_2_1009	DJI_0445	Ulmus glabra Huds.	0	Ulmaceae Mirb.	n	1
Iz_2_1009	DJI_0446	Acer tataricum L.	0	Sapindaceae Juss.	n	1
Iz_2_1009	DJI_0446	Betula pendula Roth.	0	Betulaceae Gray	n	1
Iz_2_1009	DJI_0446	Crataegus rhipidophylla Gand.	0	Rosaceae Juss.	n	1
Iz_2_1009	DJI_0446	Frangula alnus Mill.	0	Rhamnaceae Juss.	n	1
Iz_2_1009	DJI_0446	Fraxinus pennsylvanica Marshall	0	Oleaceae Hoffmanns. & Link	a	0
Iz_2_1009	DJI_0446	Pinus sylvestris L.	0	Pinaceae Spreng. ex F.Rudolphi	n	1
Iz_2_1009	DJI_0446	Populus × canescens (Aiton) Sm.	0	Salicaceae Mirb.	n	1
Iz_2_1009	DJI_0446	Populus tremula L.	0	Salicaceae Mirb.	n	1
Iz_2_1009	DJI_0446	Populus nigra L.	0	Salicaceae Mirb.	n	1
Iz_2_1009	DJI_0446	Pyrus communis L.	0	Rosaceae Juss.	a	0
Iz_2_1009	DJI_0446	Quercus robur L.	0	Fagaceae Dumort.	n	1
Iz_2_1009	DJI_0446	Robinia pseudoacacia L.	0	Fabaceae Lindl.	a	0
Iz_2_1009	DJI_0446	Rosa sp.	0	Rosaceae Juss.		
Iz_2_1009	DJI_0446	Salix alba L.	0	Salicaceae Mirb.	n	1
Iz_2_1009	DJI_0446	Sambucus nigra L.	0	Viburnaceae Raf.	n	1
Iz_2_1009	DJI_0446	Sorbus aucuparia L.	0	Rosaceae Juss.	n	1
Iz_2_1009	DJI_0446	Tilia cordata Mill.	0	Malvaceae Juss.	n	1
Iz_2_1009	DJI_0446	Ulmus glabra Huds.	0	Ulmaceae Mirb.	n	1
Iz_2_1009	DJI_0447	Acer tataricum L.	0	Sapindaceae Juss.	n	1
Iz_2_1009	DJI_0447	Betula pendula Roth.	0	Betulaceae Gray	n	1
Iz_2_1009	DJI_0447	Crataegus rhipidophylla Gand.	0	Rosaceae Juss.	n	1
Iz_2_1009	DJI_0447	Frangula alnus Mill.	0	Rhamnaceae Juss.	n	1
Iz_2_1009	DJI_0447	Fraxinus pennsylvanica Marshall	0	Oleaceae Hoffmanns. & Link	a	0
Iz_2_1009	DJI_0447	Pinus sylvestris L.	0	Pinaceae Spreng. ex F.Rudolphi	n	1
Iz_2_1009	DJI_0447	Populus × canescens (Aiton) Sm.	0	Salicaceae Mirb.	n	1
Iz_2_1009	DJI_0447	Populus tremula L.	0	Salicaceae Mirb.	n	1
Iz_2_1009	DJI_0447	Populus nigra L.	0	Salicaceae Mirb.	n	1
Iz_2_1009	DJI_0447	Pyrus communis L.	0	Rosaceae Juss.	a	0
Iz_2_1009	DJI_0447	Quercus robur L.	0	Fagaceae Dumort.	n	1
Iz_2_1009	DJI_0447	Robinia pseudoacacia L.	0	Fabaceae Lindl.	a	0
Iz_2_1009	DJI_0447	Rosa sp.	0	Rosaceae Juss.		
Iz_2_1009	DJI_0447	Salix alba L.	0	Salicaceae Mirb.	n	1
Iz_2_1009	DJI_0447	Sambucus nigra L.	0	Viburnaceae Raf.	n	1
Iz_2_1009	DJI_0447	Sorbus aucuparia L.	0	Rosaceae Juss.	n	1
Iz_2_1009	DJI_0447	Tilia cordata Mill.	0	Malvaceae Juss.	n	1
Iz_2_1009	DJI_0447	Ulmus glabra Huds.	0	Ulmaceae Mirb.	n	1
Iz_2_1009	DJI_0448	Acer tataricum L.	0	Sapindaceae Juss.	n	1
Iz_2_1009	DJI_0448	Betula pendula Roth.	0	Betulaceae Gray	n	1
Iz_2_1009	DJI_0448	Crataegus rhipidophylla Gand.	0	Rosaceae Juss.	n	1
Iz_2_1009	DJI_0448	Frangula alnus Mill.	0	Rhamnaceae Juss.	n	1
Iz_2_1009	DJI_0448	Fraxinus pennsylvanica Marshall	0	Oleaceae Hoffmanns. & Link	a	0
Iz_2_1009	DJI_0448	Pinus sylvestris L.	0	Pinaceae Spreng. ex F.Rudolphi	n	1
Iz_2_1009	DJI_0448	Populus × canescens (Aiton) Sm.	0	Salicaceae Mirb.	n	1
Iz_2_1009	DJI_0448	Populus tremula L.	0	Salicaceae Mirb.	n	1
Iz_2_1009	DJI_0448	Populus nigra L.	0	Salicaceae Mirb.	n	1
Iz_2_1009	DJI_0448	Pyrus communis L.	0	Rosaceae Juss.	a	0
Iz_2_1009	DJI_0448	Quercus robur L.	0	Fagaceae Dumort.	n	1
Iz_2_1009	DJI_0448	Robinia pseudoacacia L.	0	Fabaceae Lindl.	a	0
Iz_2_1009	DJI_0448	Rosa sp.	0	Rosaceae Juss.		
Iz_2_1009	DJI_0448	Salix alba L.	0	Salicaceae Mirb.	n	1
Iz_2_1009	DJI_0448	Sambucus nigra L.	0	Viburnaceae Raf.	n	1

Iz_2_1009	DJI_0448	Sorbus aucuparia L.	0	Rosaceae Juss.	n	1
Iz_2_1009	DJI_0448	Tilia cordata Mill.	0	Malvaceae Juss.	n	1
Iz_2_1009	DJI_0448	Ulmus glabra Huds.	0	Ulmaceae Mirb.	n	1
Iz_2_1009	DJI_0449	Acer tataricum L.	0	Sapindaceae Juss.	n	1
Iz_2_1009	DJI_0449	Betula pendula Roth.	0	Betulaceae Gray	n	1
Iz_2_1009	DJI_0449	Crataegus rhipidophylla Gand.	0	Rosaceae Juss.	n	1
Iz_2_1009	DJI_0449	Frangula alnus Mill.	0	Rhamnaceae Juss.	n	1
Iz_2_1009	DJI_0449	Fraxinus pennsylvanica Marshall	0	Oleaceae Hoffmanns. & Link	a	0
Iz_2_1009	DJI_0449	Pinus sylvestris L.	0	Pinaceae Spreng. ex F.Rudolphi	n	1
Iz_2_1009	DJI_0449	Populus × canescens (Aiton) Sm.	0	Salicaceae Mirb.	n	1
Iz_2_1009	DJI_0449	Populus tremula L.	0	Salicaceae Mirb.	n	1
Iz_2_1009	DJI_0449	Populus nigra L.	0	Salicaceae Mirb.	n	1
Iz_2_1009	DJI_0449	Pyrus communis L.	0	Rosaceae Juss.	a	0
Iz_2_1009	DJI_0449	Quercus robur L.	0	Fagaceae Dumort.	n	1
Iz_2_1009	DJI_0449	Robinia pseudoacacia L.	0	Fabaceae Lindl.	a	0
Iz_2_1009	DJI_0449	Rosa sp.	0	Rosaceae Juss.		
Iz_2_1009	DJI_0449	Salix alba L.	0	Salicaceae Mirb.	n	1
Iz_2_1009	DJI_0449	Sambucus nigra L.	0	Viburnaceae Raf.	n	1
Iz_2_1009	DJI_0449	Sorbus aucuparia L.	0	Rosaceae Juss.	n	1
Iz_2_1009	DJI_0449	Tilia cordata Mill.	0	Malvaceae Juss.	n	1
Iz_2_1009	DJI_0449	Ulmus glabra Huds.	0	Ulmaceae Mirb.	n	1
Iz_2_1009	DJI_0450	Acer tataricum L.	0	Sapindaceae Juss.	n	1
Iz_2_1009	DJI_0450	Betula pendula Roth.	0	Betulaceae Gray	n	1
Iz_2_1009	DJI_0450	Crataegus rhipidophylla Gand.	0	Rosaceae Juss.	n	1
Iz_2_1009	DJI_0450	Frangula alnus Mill.	0	Rhamnaceae Juss.	n	1
Iz_2_1009	DJI_0450	Fraxinus pennsylvanica Marshall	0	Oleaceae Hoffmanns. & Link	a	0
Iz_2_1009	DJI_0450	Pinus sylvestris L.	0	Pinaceae Spreng. ex F.Rudolphi	n	1
Iz_2_1009	DJI_0450	Populus × canescens (Aiton) Sm.	0	Salicaceae Mirb.	n	1
Iz_2_1009	DJI_0450	Populus tremula L.	0	Salicaceae Mirb.	n	1
Iz_2_1009	DJI_0450	Populus nigra L.	0	Salicaceae Mirb.	n	1
Iz_2_1009	DJI_0450	Pyrus communis L.	0	Rosaceae Juss.	a	0
Iz_2_1009	DJI_0450	Quercus robur L.	0	Fagaceae Dumort.	n	1
Iz_2_1009	DJI_0450	Robinia pseudoacacia L.	0	Fabaceae Lindl.	a	0
Iz_2_1009	DJI_0450	Rosa sp.	0	Rosaceae Juss.		
Iz_2_1009	DJI_0450	Salix alba L.	0	Salicaceae Mirb.	n	1
Iz_2_1009	DJI_0450	Sambucus nigra L.	0	Viburnaceae Raf.	n	1
Iz_2_1009	DJI_0450	Sorbus aucuparia L.	0	Rosaceae Juss.	n	1
Iz_2_1009	DJI_0450	Tilia cordata Mill.	0	Malvaceae Juss.	n	1
Iz_2_1009	DJI_0450	Ulmus glabra Huds.	0	Ulmaceae Mirb.	n	1
Iz_2_1009	DJI_0453	Acer tataricum L.	0	Sapindaceae Juss.	n	1
Iz_2_1009	DJI_0453	Betula pendula Roth.	0	Betulaceae Gray	n	1
Iz_2_1009	DJI_0453	Crataegus rhipidophylla Gand.	0	Rosaceae Juss.	n	1
Iz_2_1009	DJI_0453	Frangula alnus Mill.	0	Rhamnaceae Juss.	n	1
Iz_2_1009	DJI_0453	Fraxinus pennsylvanica Marshall	0	Oleaceae Hoffmanns. & Link	a	0
Iz_2_1009	DJI_0453	Pinus sylvestris L.	0	Pinaceae Spreng. ex F.Rudolphi	n	1
Iz_2_1009	DJI_0453	Populus × canescens (Aiton) Sm.	0	Salicaceae Mirb.	n	1
Iz_2_1009	DJI_0453	Populus tremula L.	0	Salicaceae Mirb.	n	1
Iz_2_1009	DJI_0453	Populus nigra L.	0	Salicaceae Mirb.	n	1
Iz_2_1009	DJI_0453	Pyrus communis L.	0	Rosaceae Juss.	a	0
Iz_2_1009	DJI_0453	Quercus robur L.	0	Fagaceae Dumort.	n	1
Iz_2_1009	DJI_0453	Robinia pseudoacacia L.	0	Fabaceae Lindl.	a	0
Iz_2_1009	DJI_0453	Rosa sp.	0	Rosaceae Juss.		
Iz_2_1009	DJI_0453	Salix alba L.	0	Salicaceae Mirb.	n	1
Iz_2_1009	DJI_0453	Sambucus nigra L.	0	Viburnaceae Raf.	n	1
Iz_2_1009	DJI_0453	Sorbus aucuparia L.	0	Rosaceae Juss.	n	1
Iz_2_1009	DJI_0453	Tilia cordata Mill.	0	Malvaceae Juss.	n	1
Iz_2_1009	DJI_0453	Ulmus glabra Huds.	0	Ulmaceae Mirb.	n	1
Iz_2_1009	DJI_0454	Acer tataricum L.	0	Sapindaceae Juss.	n	1
Iz_2_1009	DJI_0454	Betula pendula Roth.	0	Betulaceae Gray	n	1
Iz_2_1009	DJI_0454	Crataegus rhipidophylla Gand.	0	Rosaceae Juss.	n	1
Iz_2_1009	DJI_0454	Frangula alnus Mill.	0	Rhamnaceae Juss.	n	1
Iz_2_1009	DJI_0454	Fraxinus pennsylvanica Marshall	0	Oleaceae Hoffmanns. & Link	a	0
Iz_2_1009	DJI_0454	Pinus sylvestris L.	0	Pinaceae Spreng. ex F.Rudolphi	n	1
Iz_2_1009	DJI_0454	Populus × canescens (Aiton) Sm.	0	Salicaceae Mirb.	n	1

Iz_2_1009	DJI_0454	Populus tremula L.	0	Salicaceae Mirb.	n	1
Iz_2_1009	DJI_0454	Populus nigra L.	0	Salicaceae Mirb.	n	1
Iz_2_1009	DJI_0454	Pyrus communis L.	0	Rosaceae Juss.	a	0
Iz_2_1009	DJI_0454	Quercus robur L.	0	Fagaceae Dumort.	n	1
Iz_2_1009	DJI_0454	Robinia pseudoacacia L.	0	Fabaceae Lindl.	a	0
Iz_2_1009	DJI_0454	Rosa sp.	0	Rosaceae Juss.		
Iz_2_1009	DJI_0454	Salix alba L.	0	Salicaceae Mirb.	n	1
Iz_2_1009	DJI_0454	Sambucus nigra L.	0	Viburnaceae Raf.	n	1
Iz_2_1009	DJI_0454	Sorbus aucuparia L.	0	Rosaceae Juss.	n	1
Iz_2_1009	DJI_0454	Tilia cordata Mill.	0	Malvaceae Juss.	n	1
Iz_2_1009	DJI_0454	Ulmus glabra Huds.	0	Ulmaceae Mirb.	n	1
Iz_2_1009	DJI_0459	Acer tataricum L.	0	Sapindaceae Juss.	n	1
Iz_2_1009	DJI_0459	Betula pendula Roth.	0	Betulaceae Gray	n	1
Iz_2_1009	DJI_0459	Crataegus rhipidophylla Gand.	0	Rosaceae Juss.	n	1
Iz_2_1009	DJI_0459	Frangula alnus Mill.	0	Rhamnaceae Juss.	n	1
Iz_2_1009	DJI_0459	Fraxinus pennsylvanica Marshall	0	Oleaceae Hoffmanns. & Link	a	0
Iz_2_1009	DJI_0459	Pinus sylvestris L.	0	Pinaceae Spreng. ex F.Rudolphi	n	1
Iz_2_1009	DJI_0459	Populus × canescens (Aiton) Sm.	0	Salicaceae Mirb.	n	1
Iz_2_1009	DJI_0459	Populus tremula L.	0	Salicaceae Mirb.	n	1
Iz_2_1009	DJI_0459	Populus nigra L.	0	Salicaceae Mirb.	n	1
Iz_2_1009	DJI_0459	Pyrus communis L.	0	Rosaceae Juss.	a	0
Iz_2_1009	DJI_0459	Quercus robur L.	0	Fagaceae Dumort.	n	1
Iz_2_1009	DJI_0459	Robinia pseudoacacia L.	0	Fabaceae Lindl.	a	0
Iz_2_1009	DJI_0459	Rosa sp.	0	Rosaceae Juss.		
Iz_2_1009	DJI_0459	Salix alba L.	0	Salicaceae Mirb.	n	1
Iz_2_1009	DJI_0459	Sambucus nigra L.	0	Viburnaceae Raf.	n	1
Iz_2_1009	DJI_0459	Sorbus aucuparia L.	0	Rosaceae Juss.	n	1
Iz_2_1009	DJI_0459	Tilia cordata Mill.	0	Malvaceae Juss.	n	1
Iz_2_1009	DJI_0459	Ulmus glabra Huds.	0	Ulmaceae Mirb.	n	1
Iz_2_1009	DJI_0461	Acer tataricum L.	0	Sapindaceae Juss.	n	1
Iz_2_1009	DJI_0461	Betula pendula Roth.	0	Betulaceae Gray	n	1
Iz_2_1009	DJI_0461	Crataegus rhipidophylla Gand.	0	Rosaceae Juss.	n	1
Iz_2_1009	DJI_0461	Frangula alnus Mill.	0	Rhamnaceae Juss.	n	1
Iz_2_1009	DJI_0461	Fraxinus pennsylvanica Marshall	0	Oleaceae Hoffmanns. & Link	a	0
Iz_2_1009	DJI_0461	Pinus sylvestris L.	0	Pinaceae Spreng. ex F.Rudolphi	n	1
Iz_2_1009	DJI_0461	Populus × canescens (Aiton) Sm.	0	Salicaceae Mirb.	n	1
Iz_2_1009	DJI_0461	Populus tremula L.	0	Salicaceae Mirb.	n	1
Iz_2_1009	DJI_0461	Populus nigra L.	0	Salicaceae Mirb.	n	1
Iz_2_1009	DJI_0461	Pyrus communis L.	0	Rosaceae Juss.	a	0
Iz_2_1009	DJI_0461	Quercus robur L.	0	Fagaceae Dumort.	n	1
Iz_2_1009	DJI_0461	Robinia pseudoacacia L.	0	Fabaceae Lindl.	a	0
Iz_2_1009	DJI_0461	Rosa sp.	0	Rosaceae Juss.		
Iz_2_1009	DJI_0461	Salix alba L.	0	Salicaceae Mirb.	n	1
Iz_2_1009	DJI_0461	Sambucus nigra L.	0	Viburnaceae Raf.	n	1
Iz_2_1009	DJI_0461	Sorbus aucuparia L.	0	Rosaceae Juss.	n	1
Iz_2_1009	DJI_0461	Tilia cordata Mill.	0	Malvaceae Juss.	n	1
Iz_2_1009	DJI_0461	Ulmus glabra Huds.	0	Ulmaceae Mirb.	n	1
Iz_2_1009	DJI_0462	Acer tataricum L.	0	Sapindaceae Juss.	n	1
Iz_2_1009	DJI_0462	Betula pendula Roth.	0	Betulaceae Gray	n	1
Iz_2_1009	DJI_0462	Crataegus rhipidophylla Gand.	0	Rosaceae Juss.	n	1
Iz_2_1009	DJI_0462	Frangula alnus Mill.	0	Rhamnaceae Juss.	n	1
Iz_2_1009	DJI_0462	Fraxinus pennsylvanica Marshall	0	Oleaceae Hoffmanns. & Link	a	0
Iz_2_1009	DJI_0462	Pinus sylvestris L.	0	Pinaceae Spreng. ex F.Rudolphi	n	1
Iz_2_1009	DJI_0462	Populus × canescens (Aiton) Sm.	0	Salicaceae Mirb.	n	1
Iz_2_1009	DJI_0462	Populus tremula L.	0	Salicaceae Mirb.	n	1
Iz_2_1009	DJI_0462	Populus nigra L.	0	Salicaceae Mirb.	n	1
Iz_2_1009	DJI_0462	Pyrus communis L.	0	Rosaceae Juss.	a	0
Iz_2_1009	DJI_0462	Quercus robur L.	0	Fagaceae Dumort.	n	1
Iz_2_1009	DJI_0462	Robinia pseudoacacia L.	0	Fabaceae Lindl.	a	0
Iz_2_1009	DJI_0462	Rosa sp.	0	Rosaceae Juss.		
Iz_2_1009	DJI_0462	Salix alba L.	0	Salicaceae Mirb.	n	1
Iz_2_1009	DJI_0462	Sambucus nigra L.	0	Viburnaceae Raf.	n	1
Iz_2_1009	DJI_0462	Sorbus aucuparia L.	0	Rosaceae Juss.	n	1
Iz_2_1009	DJI_0462	Tilia cordata Mill.	0	Malvaceae Juss.	n	1

Iz_2_1009	DJI_0462	Ulmus glabra Huds.	0	Ulmaceae Mirb.	n	1
Iz_2_1009	DJI_0463	Acer tataricum L.	0	Sapindaceae Juss.	n	1
Iz_2_1009	DJI_0463	Betula pendula Roth.	0	Betulaceae Gray	n	1
Iz_2_1009	DJI_0463	Crataegus rhipidophylla Gand.	0	Rosaceae Juss.	n	1
Iz_2_1009	DJI_0463	Frangula alnus Mill.	0	Rhamnaceae Juss.	n	1
Iz_2_1009	DJI_0463	Fraxinus pennsylvanica Marshall	0	Oleaceae Hoffmanns. & Link	a	0
Iz_2_1009	DJI_0463	Pinus sylvestris L.	0	Pinaceae Spreng. ex F.Rudolphi	n	1
Iz_2_1009	DJI_0463	Populus × canescens (Aiton) Sm.	0	Salicaceae Mirb.	n	1
Iz_2_1009	DJI_0463	Populus tremula L.	0	Salicaceae Mirb.	n	1
Iz_2_1009	DJI_0463	Populus nigra L.	0	Salicaceae Mirb.	n	1
Iz_2_1009	DJI_0463	Pyrus communis L.	0	Rosaceae Juss.	a	0
Iz_2_1009	DJI_0463	Quercus robur L.	0	Fagaceae Dumort.	n	1
Iz_2_1009	DJI_0463	Robinia pseudoacacia L.	0	Fabaceae Lindl.	a	0
Iz_2_1009	DJI_0463	Rosa sp.	0	Rosaceae Juss.		
Iz_2_1009	DJI_0463	Salix alba L.	0	Salicaceae Mirb.	n	1
Iz_2_1009	DJI_0463	Sambucus nigra L.	0	Viburnaceae Raf.	n	1
Iz_2_1009	DJI_0463	Sorbus aucuparia L.	0	Rosaceae Juss.	n	1
Iz_2_1009	DJI_0463	Tilia cordata Mill.	0	Malvaceae Juss.	n	1
Iz_2_1009	DJI_0463	Ulmus glabra Huds.	0	Ulmaceae Mirb.	n	1
Iz_2_1009	DJI_0469	Acer tataricum L.	0	Sapindaceae Juss.	n	1
Iz_2_1009	DJI_0469	Betula pendula Roth.	0	Betulaceae Gray	n	1
Iz_2_1009	DJI_0469	Crataegus rhipidophylla Gand.	0	Rosaceae Juss.	n	1
Iz_2_1009	DJI_0469	Frangula alnus Mill.	0	Rhamnaceae Juss.	n	1
Iz_2_1009	DJI_0469	Fraxinus pennsylvanica Marshall	0	Oleaceae Hoffmanns. & Link	a	0
Iz_2_1009	DJI_0469	Pinus sylvestris L.	0	Pinaceae Spreng. ex F.Rudolphi	n	1
Iz_2_1009	DJI_0469	Populus × canescens (Aiton) Sm.	0	Salicaceae Mirb.	n	1
Iz_2_1009	DJI_0469	Populus tremula L.	0	Salicaceae Mirb.	n	1
Iz_2_1009	DJI_0469	Populus nigra L.	0	Salicaceae Mirb.	n	1
Iz_2_1009	DJI_0469	Pyrus communis L.	0	Rosaceae Juss.	a	0
Iz_2_1009	DJI_0469	Quercus robur L.	0	Fagaceae Dumort.	n	1
Iz_2_1009	DJI_0469	Robinia pseudoacacia L.	0	Fabaceae Lindl.	a	0
Iz_2_1009	DJI_0469	Rosa sp.	0	Rosaceae Juss.		
Iz_2_1009	DJI_0469	Salix alba L.	0	Salicaceae Mirb.	n	1
Iz_2_1009	DJI_0469	Sambucus nigra L.	0	Viburnaceae Raf.	n	1
Iz_2_1009	DJI_0469	Sorbus aucuparia L.	0	Rosaceae Juss.	n	1
Iz_2_1009	DJI_0469	Tilia cordata Mill.	0	Malvaceae Juss.	n	1
Iz_2_1009	DJI_0469	Ulmus glabra Huds.	0	Ulmaceae Mirb.	n	1
Iz_2_1009	DJI_0471	Acer tataricum L.	0	Sapindaceae Juss.	n	1
Iz_2_1009	DJI_0471	Betula pendula Roth.	0	Betulaceae Gray	n	1
Iz_2_1009	DJI_0471	Crataegus rhipidophylla Gand.	0	Rosaceae Juss.	n	1
Iz_2_1009	DJI_0471	Frangula alnus Mill.	0	Rhamnaceae Juss.	n	1
Iz_2_1009	DJI_0471	Fraxinus pennsylvanica Marshall	0	Oleaceae Hoffmanns. & Link	a	0
Iz_2_1009	DJI_0471	Pinus sylvestris L.	0	Pinaceae Spreng. ex F.Rudolphi	n	1
Iz_2_1009	DJI_0471	Populus × canescens (Aiton) Sm.	0	Salicaceae Mirb.	n	1
Iz_2_1009	DJI_0471	Populus tremula L.	0	Salicaceae Mirb.	n	1
Iz_2_1009	DJI_0471	Populus nigra L.	0	Salicaceae Mirb.	n	1
Iz_2_1009	DJI_0471	Pyrus communis L.	0	Rosaceae Juss.	a	0
Iz_2_1009	DJI_0471	Quercus robur L.	0	Fagaceae Dumort.	n	1
Iz_2_1009	DJI_0471	Robinia pseudoacacia L.	0	Fabaceae Lindl.	a	0
Iz_2_1009	DJI_0471	Rosa sp.	0	Rosaceae Juss.		
Iz_2_1009	DJI_0471	Salix alba L.	0	Salicaceae Mirb.	n	1
Iz_2_1009	DJI_0471	Sambucus nigra L.	0	Viburnaceae Raf.	n	1
Iz_2_1009	DJI_0471	Sorbus aucuparia L.	0	Rosaceae Juss.	n	1
Iz_2_1009	DJI_0471	Tilia cordata Mill.	0	Malvaceae Juss.	n	1
Iz_2_1009	DJI_0471	Ulmus glabra Huds.	0	Ulmaceae Mirb.	n	1
Iz_2_1009	DJI_0472	Acer tataricum L.	0	Sapindaceae Juss.	n	1
Iz_2_1009	DJI_0472	Betula pendula Roth.	0	Betulaceae Gray	n	1
Iz_2_1009	DJI_0472	Crataegus rhipidophylla Gand.	0	Rosaceae Juss.	n	1
Iz_2_1009	DJI_0472	Frangula alnus Mill.	0	Rhamnaceae Juss.	n	1
Iz_2_1009	DJI_0472	Fraxinus pennsylvanica Marshall	0	Oleaceae Hoffmanns. & Link	a	0
Iz_2_1009	DJI_0472	Pinus sylvestris L.	0	Pinaceae Spreng. ex F.Rudolphi	n	1
Iz_2_1009	DJI_0472	Populus × canescens (Aiton) Sm.	0	Salicaceae Mirb.	n	1
Iz_2_1009	DJI_0472	Populus tremula L.	0	Salicaceae Mirb.	n	1
Iz_2_1009	DJI_0472	Populus nigra L.	0	Salicaceae Mirb.	n	1

Iz_2_1009	DJI_0472	Pyrus communis L.	0	Rosaceae Juss.	a	0
Iz_2_1009	DJI_0472	Quercus robur L.	0	Fagaceae Dumort.	n	1
Iz_2_1009	DJI_0472	Robinia pseudoacacia L.	0	Fabaceae Lindl.	a	0
Iz_2_1009	DJI_0472	Rosa sp.	0	Rosaceae Juss.		
Iz_2_1009	DJI_0472	Salix alba L.	0	Salicaceae Mirb.	n	1
Iz_2_1009	DJI_0472	Sambucus nigra L.	0	Viburnaceae Raf.	n	1
Iz_2_1009	DJI_0472	Sorbus aucuparia L.	0	Rosaceae Juss.	n	1
Iz_2_1009	DJI_0472	Tilia cordata Mill.	0	Malvaceae Juss.	n	1
Iz_2_1009	DJI_0472	Ulmus glabra Huds.	0	Ulmaceae Mirb.	n	1
Iz_2_1009	DJI_0473	Acer tataricum L.	0	Sapindaceae Juss.	n	1
Iz_2_1009	DJI_0473	Betula pendula Roth.	0	Betulaceae Gray	n	1
Iz_2_1009	DJI_0473	Crataegus rhipidophylla Gand.	0	Rosaceae Juss.	n	1
Iz_2_1009	DJI_0473	Frangula alnus Mill.	0	Rhamnaceae Juss.	n	1
Iz_2_1009	DJI_0473	Fraxinus pennsylvanica Marshall	0	Oleaceae Hoffmanns. & Link	a	0
Iz_2_1009	DJI_0473	Pinus sylvestris L.	0	Pinaceae Spreng. ex F.Rudolphi	n	1
Iz_2_1009	DJI_0473	Populus × canescens (Aiton) Sm.	0	Salicaceae Mirb.	n	1
Iz_2_1009	DJI_0473	Populus tremula L.	0	Salicaceae Mirb.	n	1
Iz_2_1009	DJI_0473	Populus nigra L.	0	Salicaceae Mirb.	n	1
Iz_2_1009	DJI_0473	Pyrus communis L.	0	Rosaceae Juss.	a	0
Iz_2_1009	DJI_0473	Quercus robur L.	0	Fagaceae Dumort.	n	1
Iz_2_1009	DJI_0473	Robinia pseudoacacia L.	0	Fabaceae Lindl.	a	0
Iz_2_1009	DJI_0473	Rosa sp.	0	Rosaceae Juss.		
Iz_2_1009	DJI_0473	Salix alba L.	0	Salicaceae Mirb.	n	1
Iz_2_1009	DJI_0473	Sambucus nigra L.	0	Viburnaceae Raf.	n	1
Iz_2_1009	DJI_0473	Sorbus aucuparia L.	0	Rosaceae Juss.	n	1
Iz_2_1009	DJI_0473	Tilia cordata Mill.	0	Malvaceae Juss.	n	1
Iz_2_1009	DJI_0473	Ulmus glabra Huds.	0	Ulmaceae Mirb.	n	1
Iz_2_1009	DJI_0475	Acer tataricum L.	0	Sapindaceae Juss.	n	1
Iz_2_1009	DJI_0475	Betula pendula Roth.	0	Betulaceae Gray	n	1
Iz_2_1009	DJI_0475	Crataegus rhipidophylla Gand.	0	Rosaceae Juss.	n	1
Iz_2_1009	DJI_0475	Frangula alnus Mill.	0	Rhamnaceae Juss.	n	1
Iz_2_1009	DJI_0475	Fraxinus pennsylvanica Marshall	0	Oleaceae Hoffmanns. & Link	a	0
Iz_2_1009	DJI_0475	Pinus sylvestris L.	0	Pinaceae Spreng. ex F.Rudolphi	n	1
Iz_2_1009	DJI_0475	Populus × canescens (Aiton) Sm.	0	Salicaceae Mirb.	n	1
Iz_2_1009	DJI_0475	Populus tremula L.	0	Salicaceae Mirb.	n	1
Iz_2_1009	DJI_0475	Populus nigra L.	0	Salicaceae Mirb.	n	1
Iz_2_1009	DJI_0475	Pyrus communis L.	0	Rosaceae Juss.	a	0
Iz_2_1009	DJI_0475	Quercus robur L.	0	Fagaceae Dumort.	n	1
Iz_2_1009	DJI_0475	Robinia pseudoacacia L.	0	Fabaceae Lindl.	a	0
Iz_2_1009	DJI_0475	Rosa sp.	0	Rosaceae Juss.		
Iz_2_1009	DJI_0475	Salix alba L.	0	Salicaceae Mirb.	n	1
Iz_2_1009	DJI_0475	Sambucus nigra L.	0	Viburnaceae Raf.	n	1
Iz_2_1009	DJI_0475	Sorbus aucuparia L.	0	Rosaceae Juss.	n	1
Iz_2_1009	DJI_0475	Tilia cordata Mill.	0	Malvaceae Juss.	n	1
Iz_2_1009	DJI_0475	Ulmus glabra Huds.	0	Ulmaceae Mirb.	n	1
Iz_2_1009	DJI_0478	Acer tataricum L.	0	Sapindaceae Juss.	n	1
Iz_2_1009	DJI_0478	Betula pendula Roth.	0	Betulaceae Gray	n	1
Iz_2_1009	DJI_0478	Crataegus rhipidophylla Gand.	0	Rosaceae Juss.	n	1
Iz_2_1009	DJI_0478	Frangula alnus Mill.	0	Rhamnaceae Juss.	n	1
Iz_2_1009	DJI_0478	Fraxinus pennsylvanica Marshall	0	Oleaceae Hoffmanns. & Link	a	0
Iz_2_1009	DJI_0478	Pinus sylvestris L.	0	Pinaceae Spreng. ex F.Rudolphi	n	1
Iz_2_1009	DJI_0478	Populus × canescens (Aiton) Sm.	0	Salicaceae Mirb.	n	1
Iz_2_1009	DJI_0478	Populus tremula L.	0	Salicaceae Mirb.	n	1
Iz_2_1009	DJI_0478	Populus nigra L.	0	Salicaceae Mirb.	n	1
Iz_2_1009	DJI_0478	Pyrus communis L.	0	Rosaceae Juss.	a	0
Iz_2_1009	DJI_0478	Quercus robur L.	0	Fagaceae Dumort.	n	1
Iz_2_1009	DJI_0478	Robinia pseudoacacia L.	0	Fabaceae Lindl.	a	0
Iz_2_1009	DJI_0478	Rosa sp.	0	Rosaceae Juss.		
Iz_2_1009	DJI_0478	Salix alba L.	0	Salicaceae Mirb.	n	1
Iz_2_1009	DJI_0478	Sambucus nigra L.	0	Viburnaceae Raf.	n	1
Iz_2_1009	DJI_0478	Sorbus aucuparia L.	0	Rosaceae Juss.	n	1
Iz_2_1009	DJI_0478	Tilia cordata Mill.	0	Malvaceae Juss.	n	1
Iz_2_1009	DJI_0478	Ulmus glabra Huds.	0	Ulmaceae Mirb.	n	1
Iz_2_1009	DJI_0481	Acer tataricum L.	0	Sapindaceae Juss.	n	1

Iz_2_1009	DJI_0481	Betula pendula Roth.	0	Betulaceae Gray	n	1
Iz_2_1009	DJI_0481	Crataegus rhipidophylla Gand.	0	Rosaceae Juss.	n	1
Iz_2_1009	DJI_0481	Frangula alnus Mill.	0	Rhamnaceae Juss.	n	1
Iz_2_1009	DJI_0481	Fraxinus pennsylvanica Marshall	0	Oleaceae Hoffmanns. & Link	a	0
Iz_2_1009	DJI_0481	Pinus sylvestris L.	0	Pinaceae Spreng. ex F.Rudolphi	n	1
Iz_2_1009	DJI_0481	Populus × canescens (Aiton) Sm.	0	Salicaceae Mirb.	n	1
Iz_2_1009	DJI_0481	Populus tremula L.	0	Salicaceae Mirb.	n	1
Iz_2_1009	DJI_0481	Populus nigra L.	0	Salicaceae Mirb.	n	1
Iz_2_1009	DJI_0481	Pyrus communis L.	0	Rosaceae Juss.	a	0
Iz_2_1009	DJI_0481	Quercus robur L.	1	Fagaceae Dumort.	n	1
Iz_2_1009	DJI_0481	Robinia pseudoacacia L.	0	Fabaceae Lindl.	a	0
Iz_2_1009	DJI_0481	Rosa sp.	0	Rosaceae Juss.		
Iz_2_1009	DJI_0481	Salix alba L.	0	Salicaceae Mirb.	n	1
Iz_2_1009	DJI_0481	Sambucus nigra L.	0	Viburnaceae Raf.	n	1
Iz_2_1009	DJI_0481	Sorbus aucuparia L.	0	Rosaceae Juss.	n	1
Iz_2_1009	DJI_0481	Tilia cordata Mill.	0	Malvaceae Juss.	n	1
Iz_2_1009	DJI_0481	Ulmus glabra Huds.	0	Ulmaceae Mirb.	n	1
Iz_2_1009	DJI_0491	Acer tataricum L.	0	Sapindaceae Juss.	n	1
Iz_2_1009	DJI_0491	Betula pendula Roth.	0	Betulaceae Gray	n	1
Iz_2_1009	DJI_0491	Crataegus rhipidophylla Gand.	0	Rosaceae Juss.	n	1
Iz_2_1009	DJI_0491	Frangula alnus Mill.	0	Rhamnaceae Juss.	n	1
Iz_2_1009	DJI_0491	Fraxinus pennsylvanica Marshall	0	Oleaceae Hoffmanns. & Link	a	0
Iz_2_1009	DJI_0491	Pinus sylvestris L.	0	Pinaceae Spreng. ex F.Rudolphi	n	1
Iz_2_1009	DJI_0491	Populus × canescens (Aiton) Sm.	0	Salicaceae Mirb.	n	1
Iz_2_1009	DJI_0491	Populus tremula L.	0	Salicaceae Mirb.	n	1
Iz_2_1009	DJI_0491	Populus nigra L.	0	Salicaceae Mirb.	n	1
Iz_2_1009	DJI_0491	Pyrus communis L.	0	Rosaceae Juss.	a	0
Iz_2_1009	DJI_0491	Quercus robur L.	0	Fagaceae Dumort.	n	1
Iz_2_1009	DJI_0491	Robinia pseudoacacia L.	0	Fabaceae Lindl.	a	0
Iz_2_1009	DJI_0491	Rosa sp.	0	Rosaceae Juss.		
Iz_2_1009	DJI_0491	Salix alba L.	0	Salicaceae Mirb.	n	1
Iz_2_1009	DJI_0491	Sambucus nigra L.	0	Viburnaceae Raf.	n	1
Iz_2_1009	DJI_0491	Sorbus aucuparia L.	0	Rosaceae Juss.	n	1
Iz_2_1009	DJI_0491	Tilia cordata Mill.	0	Malvaceae Juss.	n	1
Iz_2_1009	DJI_0491	Ulmus glabra Huds.	0	Ulmaceae Mirb.	n	1
Iz_2_1009	DJI_0494	Acer tataricum L.	0	Sapindaceae Juss.	n	1
Iz_2_1009	DJI_0494	Betula pendula Roth.	0	Betulaceae Gray	n	1
Iz_2_1009	DJI_0494	Crataegus rhipidophylla Gand.	0	Rosaceae Juss.	n	1
Iz_2_1009	DJI_0494	Frangula alnus Mill.	0	Rhamnaceae Juss.	n	1
Iz_2_1009	DJI_0494	Fraxinus pennsylvanica Marshall	0	Oleaceae Hoffmanns. & Link	a	0
Iz_2_1009	DJI_0494	Pinus sylvestris L.	0	Pinaceae Spreng. ex F.Rudolphi	n	1
Iz_2_1009	DJI_0494	Populus × canescens (Aiton) Sm.	0	Salicaceae Mirb.	n	1
Iz_2_1009	DJI_0494	Populus tremula L.	0	Salicaceae Mirb.	n	1
Iz_2_1009	DJI_0494	Populus nigra L.	0	Salicaceae Mirb.	n	1
Iz_2_1009	DJI_0494	Pyrus communis L.	0	Rosaceae Juss.	a	0
Iz_2_1009	DJI_0494	Quercus robur L.	0	Fagaceae Dumort.	n	1
Iz_2_1009	DJI_0494	Robinia pseudoacacia L.	0	Fabaceae Lindl.	a	0
Iz_2_1009	DJI_0494	Rosa sp.	0	Rosaceae Juss.		
Iz_2_1009	DJI_0494	Salix alba L.	0	Salicaceae Mirb.	n	1
Iz_2_1009	DJI_0494	Sambucus nigra L.	0	Viburnaceae Raf.	n	1
Iz_2_1009	DJI_0494	Sorbus aucuparia L.	0	Rosaceae Juss.	n	1
Iz_2_1009	DJI_0494	Tilia cordata Mill.	0	Malvaceae Juss.	n	1
Iz_2_1009	DJI_0494	Ulmus glabra Huds.	0	Ulmaceae Mirb.	n	1
Iz_2_1009	DJI_0497	Acer tataricum L.	0	Sapindaceae Juss.	n	1
Iz_2_1009	DJI_0497	Betula pendula Roth.	0	Betulaceae Gray	n	1
Iz_2_1009	DJI_0497	Crataegus rhipidophylla Gand.	0	Rosaceae Juss.	n	1
Iz_2_1009	DJI_0497	Frangula alnus Mill.	0	Rhamnaceae Juss.	n	1
Iz_2_1009	DJI_0497	Fraxinus pennsylvanica Marshall	0	Oleaceae Hoffmanns. & Link	a	0
Iz_2_1009	DJI_0497	Pinus sylvestris L.	0	Pinaceae Spreng. ex F.Rudolphi	n	1
Iz_2_1009	DJI_0497	Populus × canescens (Aiton) Sm.	0	Salicaceae Mirb.	n	1
Iz_2_1009	DJI_0497	Populus tremula L.	0	Salicaceae Mirb.	n	1
Iz_2_1009	DJI_0497	Populus nigra L.	0	Salicaceae Mirb.	n	1
Iz_2_1009	DJI_0497	Pyrus communis L.	0	Rosaceae Juss.	a	0
Iz_2_1009	DJI_0497	Quercus robur L.	0	Fagaceae Dumort.	n	1

Iz_2_1009	DJI_0497	Robinia pseudoacacia L.	0	Fabaceae Lindl.	a	0
Iz_2_1009	DJI_0497	Rosa sp.	0	Rosaceae Juss.		
Iz_2_1009	DJI_0497	Salix alba L.	0	Salicaceae Mirb.	n	1
Iz_2_1009	DJI_0497	Sambucus nigra L.	0	Viburnaceae Raf.	n	1
Iz_2_1009	DJI_0497	Sorbus aucuparia L.	0	Rosaceae Juss.	n	1
Iz_2_1009	DJI_0497	Tilia cordata Mill.	0	Malvaceae Juss.	n	1
Iz_2_1009	DJI_0497	Ulmus glabra Huds.	0	Ulmaceae Mirb.	n	1
Iz_2_1009	DJI_0498	Acer tataricum L.	0	Sapindaceae Juss.	n	1
Iz_2_1009	DJI_0498	Betula pendula Roth.	0	Betulaceae Gray	n	1
Iz_2_1009	DJI_0498	Crataegus rhipidophylla Gand.	0	Rosaceae Juss.	n	1
Iz_2_1009	DJI_0498	Frangula alnus Mill.	0	Rhamnaceae Juss.	n	1
Iz_2_1009	DJI_0498	Fraxinus pennsylvanica Marshall	0	Oleaceae Hoffmanns. & Link	a	0
Iz_2_1009	DJI_0498	Pinus sylvestris L.	0	Pinaceae Spreng. ex F.Rudolphi	n	1
Iz_2_1009	DJI_0498	Populus × canescens (Aiton) Sm.	0	Salicaceae Mirb.	n	1
Iz_2_1009	DJI_0498	Populus tremula L.	0	Salicaceae Mirb.	n	1
Iz_2_1009	DJI_0498	Populus nigra L.	0	Salicaceae Mirb.	n	1
Iz_2_1009	DJI_0498	Pyrus communis L.	0	Rosaceae Juss.	a	0
Iz_2_1009	DJI_0498	Quercus robur L.	3	Fagaceae Dumort.	n	1
Iz_2_1009	DJI_0498	Robinia pseudoacacia L.	0	Fabaceae Lindl.	a	0
Iz_2_1009	DJI_0498	Rosa sp.	0	Rosaceae Juss.		
Iz_2_1009	DJI_0498	Salix alba L.	0	Salicaceae Mirb.	n	1
Iz_2_1009	DJI_0498	Sambucus nigra L.	0	Viburnaceae Raf.	n	1
Iz_2_1009	DJI_0498	Sorbus aucuparia L.	0	Rosaceae Juss.	n	1
Iz_2_1009	DJI_0498	Tilia cordata Mill.	0	Malvaceae Juss.	n	1
Iz_2_1009	DJI_0498	Ulmus glabra Huds.	0	Ulmaceae Mirb.	n	1
Iz_2_1009	DJI_0500	Acer tataricum L.	0	Sapindaceae Juss.	n	1
Iz_2_1009	DJI_0500	Betula pendula Roth.	0	Betulaceae Gray	n	1
Iz_2_1009	DJI_0500	Crataegus rhipidophylla Gand.	0	Rosaceae Juss.	n	1
Iz_2_1009	DJI_0500	Frangula alnus Mill.	0	Rhamnaceae Juss.	n	1
Iz_2_1009	DJI_0500	Fraxinus pennsylvanica Marshall	0	Oleaceae Hoffmanns. & Link	a	0
Iz_2_1009	DJI_0500	Pinus sylvestris L.	0	Pinaceae Spreng. ex F.Rudolphi	n	1
Iz_2_1009	DJI_0500	Populus × canescens (Aiton) Sm.	0	Salicaceae Mirb.	n	1
Iz_2_1009	DJI_0500	Populus tremula L.	0	Salicaceae Mirb.	n	1
Iz_2_1009	DJI_0500	Populus nigra L.	0	Salicaceae Mirb.	n	1
Iz_2_1009	DJI_0500	Pyrus communis L.	0	Rosaceae Juss.	a	0
Iz_2_1009	DJI_0500	Quercus robur L.	1	Fagaceae Dumort.	n	1
Iz_2_1009	DJI_0500	Robinia pseudoacacia L.	0	Fabaceae Lindl.	a	0
Iz_2_1009	DJI_0500	Rosa sp.	0	Rosaceae Juss.		
Iz_2_1009	DJI_0500	Salix alba L.	0	Salicaceae Mirb.	n	1
Iz_2_1009	DJI_0500	Sambucus nigra L.	0	Viburnaceae Raf.	n	1
Iz_2_1009	DJI_0500	Sorbus aucuparia L.	0	Rosaceae Juss.	n	1
Iz_2_1009	DJI_0500	Tilia cordata Mill.	0	Malvaceae Juss.	n	1
Iz_2_1009	DJI_0500	Ulmus glabra Huds.	0	Ulmaceae Mirb.	n	1
Iz_2_1009	DJI_0504	Acer tataricum L.	0	Sapindaceae Juss.	n	1
Iz_2_1009	DJI_0504	Betula pendula Roth.	0	Betulaceae Gray	n	1
Iz_2_1009	DJI_0504	Crataegus rhipidophylla Gand.	0	Rosaceae Juss.	n	1
Iz_2_1009	DJI_0504	Frangula alnus Mill.	0	Rhamnaceae Juss.	n	1
Iz_2_1009	DJI_0504	Fraxinus pennsylvanica Marshall	0	Oleaceae Hoffmanns. & Link	a	0
Iz_2_1009	DJI_0504	Pinus sylvestris L.	0	Pinaceae Spreng. ex F.Rudolphi	n	1
Iz_2_1009	DJI_0504	Populus × canescens (Aiton) Sm.	0	Salicaceae Mirb.	n	1
Iz_2_1009	DJI_0504	Populus tremula L.	0	Salicaceae Mirb.	n	1
Iz_2_1009	DJI_0504	Populus nigra L.	0	Salicaceae Mirb.	n	1
Iz_2_1009	DJI_0504	Pyrus communis L.	0	Rosaceae Juss.	a	0
Iz_2_1009	DJI_0504	Quercus robur L.	3	Fagaceae Dumort.	n	1
Iz_2_1009	DJI_0504	Robinia pseudoacacia L.	0	Fabaceae Lindl.	a	0
Iz_2_1009	DJI_0504	Rosa sp.	0	Rosaceae Juss.		
Iz_2_1009	DJI_0504	Salix alba L.	0	Salicaceae Mirb.	n	1
Iz_2_1009	DJI_0504	Sambucus nigra L.	0	Viburnaceae Raf.	n	1
Iz_2_1009	DJI_0504	Sorbus aucuparia L.	0	Rosaceae Juss.	n	1
Iz_2_1009	DJI_0504	Tilia cordata Mill.	0	Malvaceae Juss.	n	1
Iz_2_1009	DJI_0504	Ulmus glabra Huds.	0	Ulmaceae Mirb.	n	1
Iz_2_1009	DJI_0507	Acer tataricum L.	0	Sapindaceae Juss.	n	1
Iz_2_1009	DJI_0507	Betula pendula Roth.	0	Betulaceae Gray	n	1
Iz_2_1009	DJI_0507	Crataegus rhipidophylla Gand.	0	Rosaceae Juss.	n	1

Iz_2_1009	DJI_0507	Frangula alnus Mill.	0	Rhamnaceae Juss.	n	1
Iz_2_1009	DJI_0507	Fraxinus pennsylvanica Marshall	0	Oleaceae Hoffmanns. & Link	a	0
Iz_2_1009	DJI_0507	Pinus sylvestris L.	0	Pinaceae Spreng. ex F.Rudolphi	n	1
Iz_2_1009	DJI_0507	Populus × canescens (Aiton) Sm.	0	Salicaceae Mirb.	n	1
Iz_2_1009	DJI_0507	Populus tremula L.	0	Salicaceae Mirb.	n	1
Iz_2_1009	DJI_0507	Populus nigra L.	0	Salicaceae Mirb.	n	1
Iz_2_1009	DJI_0507	Pyrus communis L.	0	Rosaceae Juss.	a	0
Iz_2_1009	DJI_0507	Quercus robur L.	0	Fagaceae Dumort.	n	1
Iz_2_1009	DJI_0507	Robinia pseudoacacia L.	0	Fabaceae Lindl.	a	0
Iz_2_1009	DJI_0507	Rosa sp.	0	Rosaceae Juss.		
Iz_2_1009	DJI_0507	Salix alba L.	0	Salicaceae Mirb.	n	1
Iz_2_1009	DJI_0507	Sambucus nigra L.	0	Viburnaceae Raf.	n	1
Iz_2_1009	DJI_0507	Sorbus aucuparia L.	0	Rosaceae Juss.	n	1
Iz_2_1009	DJI_0507	Tilia cordata Mill.	0	Malvaceae Juss.	n	1
Iz_2_1009	DJI_0507	Ulmus glabra Huds.	0	Ulmaceae Mirb.	n	1
Iz_2_1009	DJI_0508	Acer tataricum L.	0	Sapindaceae Juss.	n	1
Iz_2_1009	DJI_0508	Betula pendula Roth.	0	Betulaceae Gray	n	1
Iz_2_1009	DJI_0508	Crataegus rhipidophylla Gand.	0	Rosaceae Juss.	n	1
Iz_2_1009	DJI_0508	Frangula alnus Mill.	0	Rhamnaceae Juss.	n	1
Iz_2_1009	DJI_0508	Fraxinus pennsylvanica Marshall	0	Oleaceae Hoffmanns. & Link	a	0
Iz_2_1009	DJI_0508	Pinus sylvestris L.	0	Pinaceae Spreng. ex F.Rudolphi	n	1
Iz_2_1009	DJI_0508	Populus × canescens (Aiton) Sm.	0	Salicaceae Mirb.	n	1
Iz_2_1009	DJI_0508	Populus tremula L.	0	Salicaceae Mirb.	n	1
Iz_2_1009	DJI_0508	Populus nigra L.	0	Salicaceae Mirb.	n	1
Iz_2_1009	DJI_0508	Pyrus communis L.	0	Rosaceae Juss.	a	0
Iz_2_1009	DJI_0508	Quercus robur L.	0	Fagaceae Dumort.	n	1
Iz_2_1009	DJI_0508	Robinia pseudoacacia L.	0	Fabaceae Lindl.	a	0
Iz_2_1009	DJI_0508	Rosa sp.	0	Rosaceae Juss.		
Iz_2_1009	DJI_0508	Salix alba L.	0	Salicaceae Mirb.	n	1
Iz_2_1009	DJI_0508	Sambucus nigra L.	0	Viburnaceae Raf.	n	1
Iz_2_1009	DJI_0508	Sorbus aucuparia L.	0	Rosaceae Juss.	n	1
Iz_2_1009	DJI_0508	Tilia cordata Mill.	0	Malvaceae Juss.	n	1
Iz_2_1009	DJI_0508	Ulmus glabra Huds.	0	Ulmaceae Mirb.	n	1
Iz_2_1009	DJI_0511	Acer tataricum L.	0	Sapindaceae Juss.	n	1
Iz_2_1009	DJI_0511	Betula pendula Roth.	0	Betulaceae Gray	n	1
Iz_2_1009	DJI_0511	Crataegus rhipidophylla Gand.	0	Rosaceae Juss.	n	1
Iz_2_1009	DJI_0511	Frangula alnus Mill.	0	Rhamnaceae Juss.	n	1
Iz_2_1009	DJI_0511	Fraxinus pennsylvanica Marshall	0	Oleaceae Hoffmanns. & Link	a	0
Iz_2_1009	DJI_0511	Pinus sylvestris L.	0	Pinaceae Spreng. ex F.Rudolphi	n	1
Iz_2_1009	DJI_0511	Populus × canescens (Aiton) Sm.	0	Salicaceae Mirb.	n	1
Iz_2_1009	DJI_0511	Populus tremula L.	0	Salicaceae Mirb.	n	1
Iz_2_1009	DJI_0511	Populus nigra L.	0	Salicaceae Mirb.	n	1
Iz_2_1009	DJI_0511	Pyrus communis L.	0	Rosaceae Juss.	a	0
Iz_2_1009	DJI_0511	Quercus robur L.	0	Fagaceae Dumort.	n	1
Iz_2_1009	DJI_0511	Robinia pseudoacacia L.	0	Fabaceae Lindl.	a	0
Iz_2_1009	DJI_0511	Rosa sp.	0	Rosaceae Juss.		
Iz_2_1009	DJI_0511	Salix alba L.	0	Salicaceae Mirb.	n	1
Iz_2_1009	DJI_0511	Sambucus nigra L.	0	Viburnaceae Raf.	n	1
Iz_2_1009	DJI_0511	Sorbus aucuparia L.	0	Rosaceae Juss.	n	1
Iz_2_1009	DJI_0511	Tilia cordata Mill.	0	Malvaceae Juss.	n	1
Iz_2_1009	DJI_0511	Ulmus glabra Huds.	0	Ulmaceae Mirb.	n	1
Iz_2_1009	DJI_0513	Acer tataricum L.	0	Sapindaceae Juss.	n	1
Iz_2_1009	DJI_0513	Betula pendula Roth.	0	Betulaceae Gray	n	1
Iz_2_1009	DJI_0513	Crataegus rhipidophylla Gand.	0	Rosaceae Juss.	n	1
Iz_2_1009	DJI_0513	Frangula alnus Mill.	0	Rhamnaceae Juss.	n	1
Iz_2_1009	DJI_0513	Fraxinus pennsylvanica Marshall	0	Oleaceae Hoffmanns. & Link	a	0
Iz_2_1009	DJI_0513	Pinus sylvestris L.	0	Pinaceae Spreng. ex F.Rudolphi	n	1
Iz_2_1009	DJI_0513	Populus × canescens (Aiton) Sm.	0	Salicaceae Mirb.	n	1
Iz_2_1009	DJI_0513	Populus tremula L.	0	Salicaceae Mirb.	n	1
Iz_2_1009	DJI_0513	Populus nigra L.	0	Salicaceae Mirb.	n	1
Iz_2_1009	DJI_0513	Pyrus communis L.	0	Rosaceae Juss.	a	0
Iz_2_1009	DJI_0513	Quercus robur L.	0	Fagaceae Dumort.	n	1
Iz_2_1009	DJI_0513	Robinia pseudoacacia L.	0	Fabaceae Lindl.	a	0
Iz_2_1009	DJI_0513	Rosa sp.	0	Rosaceae Juss.		

Iz_2_1009	DJI_0513	Salix alba L.	0	Salicaceae Mirb.	n	1
Iz_2_1009	DJI_0513	Sambucus nigra L.	0	Viburnaceae Raf.	n	1
Iz_2_1009	DJI_0513	Sorbus aucuparia L.	0	Rosaceae Juss.	n	1
Iz_2_1009	DJI_0513	Tilia cordata Mill.	0	Malvaceae Juss.	n	1
Iz_2_1009	DJI_0513	Ulmus glabra Huds.	0	Ulmaceae Mirb.	n	1
Iz_2_1009	DJI_0515	Acer tataricum L.	0	Sapindaceae Juss.	n	1
Iz_2_1009	DJI_0515	Betula pendula Roth.	0	Betulaceae Gray	n	1
Iz_2_1009	DJI_0515	Crataegus rhipidophylla Gand.	0	Rosaceae Juss.	n	1
Iz_2_1009	DJI_0515	Frangula alnus Mill.	0	Rhamnaceae Juss.	n	1
Iz_2_1009	DJI_0515	Fraxinus pennsylvanica Marshall	0	Oleaceae Hoffmanns. & Link	a	0
Iz_2_1009	DJI_0515	Pinus sylvestris L.	0	Pinaceae Spreng. ex F.Rudolphi	n	1
Iz_2_1009	DJI_0515	Populus × canescens (Aiton) Sm.	0	Salicaceae Mirb.	n	1
Iz_2_1009	DJI_0515	Populus tremula L.	0	Salicaceae Mirb.	n	1
Iz_2_1009	DJI_0515	Populus nigra L.	0	Salicaceae Mirb.	n	1
Iz_2_1009	DJI_0515	Pyrus communis L.	0	Rosaceae Juss.	a	0
Iz_2_1009	DJI_0515	Quercus robur L.	0	Fagaceae Dumort.	n	1
Iz_2_1009	DJI_0515	Robinia pseudoacacia L.	0	Fabaceae Lindl.	a	0
Iz_2_1009	DJI_0515	Rosa sp.	0	Rosaceae Juss.		
Iz_2_1009	DJI_0515	Salix alba L.	0	Salicaceae Mirb.	n	1
Iz_2_1009	DJI_0515	Sambucus nigra L.	0	Viburnaceae Raf.	n	1
Iz_2_1009	DJI_0515	Sorbus aucuparia L.	0	Rosaceae Juss.	n	1
Iz_2_1009	DJI_0515	Tilia cordata Mill.	0	Malvaceae Juss.	n	1
Iz_2_1009	DJI_0515	Ulmus glabra Huds.	0	Ulmaceae Mirb.	n	1
Iz_2_1009	DJI_0524	Acer tataricum L.	0	Sapindaceae Juss.	n	1
Iz_2_1009	DJI_0524	Betula pendula Roth.	0	Betulaceae Gray	n	1
Iz_2_1009	DJI_0524	Crataegus rhipidophylla Gand.	0	Rosaceae Juss.	n	1
Iz_2_1009	DJI_0524	Frangula alnus Mill.	0	Rhamnaceae Juss.	n	1
Iz_2_1009	DJI_0524	Fraxinus pennsylvanica Marshall	0	Oleaceae Hoffmanns. & Link	a	0
Iz_2_1009	DJI_0524	Pinus sylvestris L.	0	Pinaceae Spreng. ex F.Rudolphi	n	1
Iz_2_1009	DJI_0524	Populus × canescens (Aiton) Sm.	0	Salicaceae Mirb.	n	1
Iz_2_1009	DJI_0524	Populus tremula L.	0	Salicaceae Mirb.	n	1
Iz_2_1009	DJI_0524	Populus nigra L.	0	Salicaceae Mirb.	n	1
Iz_2_1009	DJI_0524	Pyrus communis L.	0	Rosaceae Juss.	a	0
Iz_2_1009	DJI_0524	Quercus robur L.	0	Fagaceae Dumort.	n	1
Iz_2_1009	DJI_0524	Robinia pseudoacacia L.	0	Fabaceae Lindl.	a	0
Iz_2_1009	DJI_0524	Rosa sp.	0	Rosaceae Juss.		
Iz_2_1009	DJI_0524	Salix alba L.	0	Salicaceae Mirb.	n	1
Iz_2_1009	DJI_0524	Sambucus nigra L.	0	Viburnaceae Raf.	n	1
Iz_2_1009	DJI_0524	Sorbus aucuparia L.	0	Rosaceae Juss.	n	1
Iz_2_1009	DJI_0524	Tilia cordata Mill.	0	Malvaceae Juss.	n	1
Iz_2_1009	DJI_0524	Ulmus glabra Huds.	0	Ulmaceae Mirb.	n	1
Iz_2_1009	DJI_0526	Acer tataricum L.	0	Sapindaceae Juss.	n	1
Iz_2_1009	DJI_0526	Betula pendula Roth.	0	Betulaceae Gray	n	1
Iz_2_1009	DJI_0526	Crataegus rhipidophylla Gand.	0	Rosaceae Juss.	n	1
Iz_2_1009	DJI_0526	Frangula alnus Mill.	0	Rhamnaceae Juss.	n	1
Iz_2_1009	DJI_0526	Fraxinus pennsylvanica Marshall	0	Oleaceae Hoffmanns. & Link	a	0
Iz_2_1009	DJI_0526	Pinus sylvestris L.	0	Pinaceae Spreng. ex F.Rudolphi	n	1
Iz_2_1009	DJI_0526	Populus × canescens (Aiton) Sm.	0	Salicaceae Mirb.	n	1
Iz_2_1009	DJI_0526	Populus tremula L.	0	Salicaceae Mirb.	n	1
Iz_2_1009	DJI_0526	Populus nigra L.	0	Salicaceae Mirb.	n	1
Iz_2_1009	DJI_0526	Pyrus communis L.	0	Rosaceae Juss.	a	0
Iz_2_1009	DJI_0526	Quercus robur L.	0	Fagaceae Dumort.	n	1
Iz_2_1009	DJI_0526	Robinia pseudoacacia L.	0	Fabaceae Lindl.	a	0
Iz_2_1009	DJI_0526	Rosa sp.	0	Rosaceae Juss.		
Iz_2_1009	DJI_0526	Salix alba L.	0	Salicaceae Mirb.	n	1
Iz_2_1009	DJI_0526	Sambucus nigra L.	0	Viburnaceae Raf.	n	1
Iz_2_1009	DJI_0526	Sorbus aucuparia L.	0	Rosaceae Juss.	n	1
Iz_2_1009	DJI_0526	Tilia cordata Mill.	0	Malvaceae Juss.	n	1
Iz_2_1009	DJI_0526	Ulmus glabra Huds.	0	Ulmaceae Mirb.	n	1
Iz_2_1009	DJI_0527	Acer tataricum L.	0	Sapindaceae Juss.	n	1
Iz_2_1009	DJI_0527	Betula pendula Roth.	0	Betulaceae Gray	n	1
Iz_2_1009	DJI_0527	Crataegus rhipidophylla Gand.	0	Rosaceae Juss.	n	1
Iz_2_1009	DJI_0527	Frangula alnus Mill.	0	Rhamnaceae Juss.	n	1
Iz_2_1009	DJI_0527	Fraxinus pennsylvanica Marshall	0	Oleaceae Hoffmanns. & Link	a	0

Iz_2_1009	DJI_0527	Pinus sylvestris L.	0	Pinaceae Spreng. ex F.Rudolphi	n	1
Iz_2_1009	DJI_0527	Populus × canescens (Aiton) Sm.	0	Salicaceae Mirb.	n	1
Iz_2_1009	DJI_0527	Populus tremula L.	0	Salicaceae Mirb.	n	1
Iz_2_1009	DJI_0527	Populus nigra L.	0	Salicaceae Mirb.	n	1
Iz_2_1009	DJI_0527	Pyrus communis L.	0	Rosaceae Juss.	a	0
Iz_2_1009	DJI_0527	Quercus robur L.	0	Fagaceae Dumort.	n	1
Iz_2_1009	DJI_0527	Robinia pseudoacacia L.	0	Fabaceae Lindl.	a	0
Iz_2_1009	DJI_0527	Rosa sp.	0	Rosaceae Juss.		
Iz_2_1009	DJI_0527	Salix alba L.	0	Salicaceae Mirb.	n	1
Iz_2_1009	DJI_0527	Sambucus nigra L.	0	Viburnaceae Raf.	n	1
Iz_2_1009	DJI_0527	Sorbus aucuparia L.	0	Rosaceae Juss.	n	1
Iz_2_1009	DJI_0527	Tilia cordata Mill.	0	Malvaceae Juss.	n	1
Iz_2_1009	DJI_0527	Ulmus glabra Huds.	0	Ulmaceae Mirb.	n	1
Iz_2_1009	DJI_0529	Acer tataricum L.	0	Sapindaceae Juss.	n	1
Iz_2_1009	DJI_0529	Betula pendula Roth.	0	Betulaceae Gray	n	1
Iz_2_1009	DJI_0529	Crataegus rhipidophylla Gand.	0	Rosaceae Juss.	n	1
Iz_2_1009	DJI_0529	Frangula alnus Mill.	0	Rhamnaceae Juss.	n	1
Iz_2_1009	DJI_0529	Fraxinus pennsylvanica Marshall	0	Oleaceae Hoffmanns. & Link	a	0
Iz_2_1009	DJI_0529	Pinus sylvestris L.	0	Pinaceae Spreng. ex F.Rudolphi	n	1
Iz_2_1009	DJI_0529	Populus × canescens (Aiton) Sm.	0	Salicaceae Mirb.	n	1
Iz_2_1009	DJI_0529	Populus tremula L.	0	Salicaceae Mirb.	n	1
Iz_2_1009	DJI_0529	Populus nigra L.	0	Salicaceae Mirb.	n	1
Iz_2_1009	DJI_0529	Pyrus communis L.	0	Rosaceae Juss.	a	0
Iz_2_1009	DJI_0529	Quercus robur L.	0	Fagaceae Dumort.	n	1
Iz_2_1009	DJI_0529	Robinia pseudoacacia L.	0	Fabaceae Lindl.	a	0
Iz_2_1009	DJI_0529	Rosa sp.	0	Rosaceae Juss.		
Iz_2_1009	DJI_0529	Salix alba L.	0	Salicaceae Mirb.	n	1
Iz_2_1009	DJI_0529	Sambucus nigra L.	0	Viburnaceae Raf.	n	1
Iz_2_1009	DJI_0529	Sorbus aucuparia L.	0	Rosaceae Juss.	n	1
Iz_2_1009	DJI_0529	Tilia cordata Mill.	0	Malvaceae Juss.	n	1
Iz_2_1009	DJI_0529	Ulmus glabra Huds.	0	Ulmaceae Mirb.	n	1
Iz_2_1009	DJI_0530	Acer tataricum L.	0	Sapindaceae Juss.	n	1
Iz_2_1009	DJI_0530	Betula pendula Roth.	0	Betulaceae Gray	n	1
Iz_2_1009	DJI_0530	Crataegus rhipidophylla Gand.	0	Rosaceae Juss.	n	1
Iz_2_1009	DJI_0530	Frangula alnus Mill.	0	Rhamnaceae Juss.	n	1
Iz_2_1009	DJI_0530	Fraxinus pennsylvanica Marshall	0	Oleaceae Hoffmanns. & Link	a	0
Iz_2_1009	DJI_0530	Pinus sylvestris L.	0	Pinaceae Spreng. ex F.Rudolphi	n	1
Iz_2_1009	DJI_0530	Populus × canescens (Aiton) Sm.	0	Salicaceae Mirb.	n	1
Iz_2_1009	DJI_0530	Populus tremula L.	0	Salicaceae Mirb.	n	1
Iz_2_1009	DJI_0530	Populus nigra L.	0	Salicaceae Mirb.	n	1
Iz_2_1009	DJI_0530	Pyrus communis L.	0	Rosaceae Juss.	a	0
Iz_2_1009	DJI_0530	Quercus robur L.	0	Fagaceae Dumort.	n	1
Iz_2_1009	DJI_0530	Robinia pseudoacacia L.	0	Fabaceae Lindl.	a	0
Iz_2_1009	DJI_0530	Rosa sp.	0	Rosaceae Juss.		
Iz_2_1009	DJI_0530	Salix alba L.	0	Salicaceae Mirb.	n	1
Iz_2_1009	DJI_0530	Sambucus nigra L.	0	Viburnaceae Raf.	n	1
Iz_2_1009	DJI_0530	Sorbus aucuparia L.	0	Rosaceae Juss.	n	1
Iz_2_1009	DJI_0530	Tilia cordata Mill.	0	Malvaceae Juss.	n	1
Iz_2_1009	DJI_0530	Ulmus glabra Huds.	0	Ulmaceae Mirb.	n	1
Iz_2_1009	DJI_0533	Acer tataricum L.	0	Sapindaceae Juss.	n	1
Iz_2_1009	DJI_0533	Betula pendula Roth.	0	Betulaceae Gray	n	1
Iz_2_1009	DJI_0533	Crataegus rhipidophylla Gand.	0	Rosaceae Juss.	n	1
Iz_2_1009	DJI_0533	Frangula alnus Mill.	0	Rhamnaceae Juss.	n	1
Iz_2_1009	DJI_0533	Fraxinus pennsylvanica Marshall	0	Oleaceae Hoffmanns. & Link	a	0
Iz_2_1009	DJI_0533	Pinus sylvestris L.	0	Pinaceae Spreng. ex F.Rudolphi	n	1
Iz_2_1009	DJI_0533	Populus × canescens (Aiton) Sm.	0	Salicaceae Mirb.	n	1
Iz_2_1009	DJI_0533	Populus tremula L.	0	Salicaceae Mirb.	n	1
Iz_2_1009	DJI_0533	Populus nigra L.	0	Salicaceae Mirb.	n	1
Iz_2_1009	DJI_0533	Pyrus communis L.	0	Rosaceae Juss.	a	0
Iz_2_1009	DJI_0533	Quercus robur L.	0	Fagaceae Dumort.	n	1
Iz_2_1009	DJI_0533	Robinia pseudoacacia L.	0	Fabaceae Lindl.	a	0
Iz_2_1009	DJI_0533	Rosa sp.	0	Rosaceae Juss.		
Iz_2_1009	DJI_0533	Salix alba L.	0	Salicaceae Mirb.	n	1
Iz_2_1009	DJI_0533	Sambucus nigra L.	0	Viburnaceae Raf.	n	1

Iz_2_1009	DJI_0533	Sorbus aucuparia L.	0	Rosaceae Juss.	n	1
Iz_2_1009	DJI_0533	Tilia cordata Mill.	0	Malvaceae Juss.	n	1
Iz_2_1009	DJI_0533	Ulmus glabra Huds.	0	Ulmaceae Mirb.	n	1
Iz_2_1009	DJI_0534	Acer tataricum L.	0	Sapindaceae Juss.	n	1
Iz_2_1009	DJI_0534	Betula pendula Roth.	0	Betulaceae Gray	n	1
Iz_2_1009	DJI_0534	Crataegus rhipidophylla Gand.	0	Rosaceae Juss.	n	1
Iz_2_1009	DJI_0534	Frangula alnus Mill.	0	Rhamnaceae Juss.	n	1
Iz_2_1009	DJI_0534	Fraxinus pennsylvanica Marshall	0	Oleaceae Hoffmanns. & Link	a	0
Iz_2_1009	DJI_0534	Pinus sylvestris L.	0	Pinaceae Spreng. ex F.Rudolphi	n	1
Iz_2_1009	DJI_0534	Populus × canescens (Aiton) Sm.	0	Salicaceae Mirb.	n	1
Iz_2_1009	DJI_0534	Populus tremula L.	0	Salicaceae Mirb.	n	1
Iz_2_1009	DJI_0534	Populus nigra L.	0	Salicaceae Mirb.	n	1
Iz_2_1009	DJI_0534	Pyrus communis L.	0	Rosaceae Juss.	a	0
Iz_2_1009	DJI_0534	Quercus robur L.	0	Fagaceae Dumort.	n	1
Iz_2_1009	DJI_0534	Robinia pseudoacacia L.	0	Fabaceae Lindl.	a	0
Iz_2_1009	DJI_0534	Rosa sp.	0	Rosaceae Juss.		
Iz_2_1009	DJI_0534	Salix alba L.	0	Salicaceae Mirb.	n	1
Iz_2_1009	DJI_0534	Sambucus nigra L.	0	Viburnaceae Raf.	n	1
Iz_2_1009	DJI_0534	Sorbus aucuparia L.	0	Rosaceae Juss.	n	1
Iz_2_1009	DJI_0534	Tilia cordata Mill.	0	Malvaceae Juss.	n	1
Iz_2_1009	DJI_0534	Ulmus glabra Huds.	0	Ulmaceae Mirb.	n	1
Iz_2_1009	DJI_0536	Acer tataricum L.	0	Sapindaceae Juss.	n	1
Iz_2_1009	DJI_0536	Betula pendula Roth.	0	Betulaceae Gray	n	1
Iz_2_1009	DJI_0536	Crataegus rhipidophylla Gand.	0	Rosaceae Juss.	n	1
Iz_2_1009	DJI_0536	Frangula alnus Mill.	0	Rhamnaceae Juss.	n	1
Iz_2_1009	DJI_0536	Fraxinus pennsylvanica Marshall	0	Oleaceae Hoffmanns. & Link	a	0
Iz_2_1009	DJI_0536	Pinus sylvestris L.	0	Pinaceae Spreng. ex F.Rudolphi	n	1
Iz_2_1009	DJI_0536	Populus × canescens (Aiton) Sm.	0	Salicaceae Mirb.	n	1
Iz_2_1009	DJI_0536	Populus tremula L.	0	Salicaceae Mirb.	n	1
Iz_2_1009	DJI_0536	Populus nigra L.	0	Salicaceae Mirb.	n	1
Iz_2_1009	DJI_0536	Pyrus communis L.	0	Rosaceae Juss.	a	0
Iz_2_1009	DJI_0536	Quercus robur L.	0	Fagaceae Dumort.	n	1
Iz_2_1009	DJI_0536	Robinia pseudoacacia L.	0	Fabaceae Lindl.	a	0
Iz_2_1009	DJI_0536	Rosa sp.	0	Rosaceae Juss.		
Iz_2_1009	DJI_0536	Salix alba L.	0	Salicaceae Mirb.	n	1
Iz_2_1009	DJI_0536	Sambucus nigra L.	0	Viburnaceae Raf.	n	1
Iz_2_1009	DJI_0536	Sorbus aucuparia L.	0	Rosaceae Juss.	n	1
Iz_2_1009	DJI_0536	Tilia cordata Mill.	0	Malvaceae Juss.	n	1
Iz_2_1009	DJI_0536	Ulmus glabra Huds.	0	Ulmaceae Mirb.	n	1
Iz_2_1009	DJI_0537	Acer tataricum L.	0	Sapindaceae Juss.	n	1
Iz_2_1009	DJI_0537	Betula pendula Roth.	0	Betulaceae Gray	n	1
Iz_2_1009	DJI_0537	Crataegus rhipidophylla Gand.	0	Rosaceae Juss.	n	1
Iz_2_1009	DJI_0537	Frangula alnus Mill.	0	Rhamnaceae Juss.	n	1
Iz_2_1009	DJI_0537	Fraxinus pennsylvanica Marshall	0	Oleaceae Hoffmanns. & Link	a	0
Iz_2_1009	DJI_0537	Pinus sylvestris L.	0	Pinaceae Spreng. ex F.Rudolphi	n	1
Iz_2_1009	DJI_0537	Populus × canescens (Aiton) Sm.	0	Salicaceae Mirb.	n	1
Iz_2_1009	DJI_0537	Populus tremula L.	0	Salicaceae Mirb.	n	1
Iz_2_1009	DJI_0537	Populus nigra L.	0	Salicaceae Mirb.	n	1
Iz_2_1009	DJI_0537	Pyrus communis L.	0	Rosaceae Juss.	a	0
Iz_2_1009	DJI_0537	Quercus robur L.	0	Fagaceae Dumort.	n	1
Iz_2_1009	DJI_0537	Robinia pseudoacacia L.	0	Fabaceae Lindl.	a	0
Iz_2_1009	DJI_0537	Rosa sp.	0	Rosaceae Juss.		
Iz_2_1009	DJI_0537	Salix alba L.	0	Salicaceae Mirb.	n	1
Iz_2_1009	DJI_0537	Sambucus nigra L.	0	Viburnaceae Raf.	n	1
Iz_2_1009	DJI_0537	Sorbus aucuparia L.	0	Rosaceae Juss.	n	1
Iz_2_1009	DJI_0537	Tilia cordata Mill.	0	Malvaceae Juss.	n	1
Iz_2_1009	DJI_0537	Ulmus glabra Huds.	0	Ulmaceae Mirb.	n	1
Iz_2_1009	DJI_0539	Acer tataricum L.	0	Sapindaceae Juss.	n	1
Iz_2_1009	DJI_0539	Betula pendula Roth.	0	Betulaceae Gray	n	1
Iz_2_1009	DJI_0539	Crataegus rhipidophylla Gand.	0	Rosaceae Juss.	n	1
Iz_2_1009	DJI_0539	Frangula alnus Mill.	0	Rhamnaceae Juss.	n	1
Iz_2_1009	DJI_0539	Fraxinus pennsylvanica Marshall	0	Oleaceae Hoffmanns. & Link	a	0
Iz_2_1009	DJI_0539	Pinus sylvestris L.	0	Pinaceae Spreng. ex F.Rudolphi	n	1
Iz_2_1009	DJI_0539	Populus × canescens (Aiton) Sm.	0	Salicaceae Mirb.	n	1

Iz_2_1009	DJI_0539	Populus tremula L.	0	Salicaceae Mirb.	n	1
Iz_2_1009	DJI_0539	Populus nigra L.	0	Salicaceae Mirb.	n	1
Iz_2_1009	DJI_0539	Pyrus communis L.	0	Rosaceae Juss.	a	0
Iz_2_1009	DJI_0539	Quercus robur L.	0	Fagaceae Dumort.	n	1
Iz_2_1009	DJI_0539	Robinia pseudoacacia L.	0	Fabaceae Lindl.	a	0
Iz_2_1009	DJI_0539	Rosa sp.	0	Rosaceae Juss.		
Iz_2_1009	DJI_0539	Salix alba L.	0	Salicaceae Mirb.	n	1
Iz_2_1009	DJI_0539	Sambucus nigra L.	0	Viburnaceae Raf.	n	1
Iz_2_1009	DJI_0539	Sorbus aucuparia L.	0	Rosaceae Juss.	n	1
Iz_2_1009	DJI_0539	Tilia cordata Mill.	0	Malvaceae Juss.	n	1
Iz_2_1009	DJI_0539	Ulmus glabra Huds.	0	Ulmaceae Mirb.	n	1
Iz_2_1009	DJI_0540	Acer tataricum L.	0	Sapindaceae Juss.	n	1
Iz_2_1009	DJI_0540	Betula pendula Roth.	0	Betulaceae Gray	n	1
Iz_2_1009	DJI_0540	Crataegus rhipidophylla Gand.	0	Rosaceae Juss.	n	1
Iz_2_1009	DJI_0540	Frangula alnus Mill.	0	Rhamnaceae Juss.	n	1
Iz_2_1009	DJI_0540	Fraxinus pennsylvanica Marshall	0	Oleaceae Hoffmanns. & Link	a	0
Iz_2_1009	DJI_0540	Pinus sylvestris L.	0	Pinaceae Spreng. ex F.Rudolphi	n	1
Iz_2_1009	DJI_0540	Populus × canescens (Aiton) Sm.	0	Salicaceae Mirb.	n	1
Iz_2_1009	DJI_0540	Populus tremula L.	0	Salicaceae Mirb.	n	1
Iz_2_1009	DJI_0540	Populus nigra L.	0	Salicaceae Mirb.	n	1
Iz_2_1009	DJI_0540	Pyrus communis L.	0	Rosaceae Juss.	a	0
Iz_2_1009	DJI_0540	Quercus robur L.	0	Fagaceae Dumort.	n	1
Iz_2_1009	DJI_0540	Robinia pseudoacacia L.	0	Fabaceae Lindl.	a	0
Iz_2_1009	DJI_0540	Rosa sp.	0	Rosaceae Juss.		
Iz_2_1009	DJI_0540	Salix alba L.	0	Salicaceae Mirb.	n	1
Iz_2_1009	DJI_0540	Sambucus nigra L.	0	Viburnaceae Raf.	n	1
Iz_2_1009	DJI_0540	Sorbus aucuparia L.	0	Rosaceae Juss.	n	1
Iz_2_1009	DJI_0540	Tilia cordata Mill.	0	Malvaceae Juss.	n	1
Iz_2_1009	DJI_0540	Ulmus glabra Huds.	0	Ulmaceae Mirb.	n	1
Iz_2_1009	DJI_0542	Acer tataricum L.	0	Sapindaceae Juss.	n	1
Iz_2_1009	DJI_0542	Betula pendula Roth.	0	Betulaceae Gray	n	1
Iz_2_1009	DJI_0542	Crataegus rhipidophylla Gand.	0	Rosaceae Juss.	n	1
Iz_2_1009	DJI_0542	Frangula alnus Mill.	0	Rhamnaceae Juss.	n	1
Iz_2_1009	DJI_0542	Fraxinus pennsylvanica Marshall	0	Oleaceae Hoffmanns. & Link	a	0
Iz_2_1009	DJI_0542	Pinus sylvestris L.	0	Pinaceae Spreng. ex F.Rudolphi	n	1
Iz_2_1009	DJI_0542	Populus × canescens (Aiton) Sm.	0	Salicaceae Mirb.	n	1
Iz_2_1009	DJI_0542	Populus tremula L.	0	Salicaceae Mirb.	n	1
Iz_2_1009	DJI_0542	Populus nigra L.	0	Salicaceae Mirb.	n	1
Iz_2_1009	DJI_0542	Pyrus communis L.	0	Rosaceae Juss.	a	0
Iz_2_1009	DJI_0542	Quercus robur L.	0	Fagaceae Dumort.	n	1
Iz_2_1009	DJI_0542	Robinia pseudoacacia L.	0	Fabaceae Lindl.	a	0
Iz_2_1009	DJI_0542	Rosa sp.	0	Rosaceae Juss.		
Iz_2_1009	DJI_0542	Salix alba L.	0	Salicaceae Mirb.	n	1
Iz_2_1009	DJI_0542	Sambucus nigra L.	0	Viburnaceae Raf.	n	1
Iz_2_1009	DJI_0542	Sorbus aucuparia L.	0	Rosaceae Juss.	n	1
Iz_2_1009	DJI_0542	Tilia cordata Mill.	0	Malvaceae Juss.	n	1
Iz_2_1009	DJI_0542	Ulmus glabra Huds.	0	Ulmaceae Mirb.	n	1
Iz_2_1009	DJI_0545	Acer tataricum L.	0	Sapindaceae Juss.	n	1
Iz_2_1009	DJI_0545	Betula pendula Roth.	0	Betulaceae Gray	n	1
Iz_2_1009	DJI_0545	Crataegus rhipidophylla Gand.	0	Rosaceae Juss.	n	1
Iz_2_1009	DJI_0545	Frangula alnus Mill.	0	Rhamnaceae Juss.	n	1
Iz_2_1009	DJI_0545	Fraxinus pennsylvanica Marshall	0	Oleaceae Hoffmanns. & Link	a	0
Iz_2_1009	DJI_0545	Pinus sylvestris L.	0	Pinaceae Spreng. ex F.Rudolphi	n	1
Iz_2_1009	DJI_0545	Populus × canescens (Aiton) Sm.	0	Salicaceae Mirb.	n	1
Iz_2_1009	DJI_0545	Populus tremula L.	0	Salicaceae Mirb.	n	1
Iz_2_1009	DJI_0545	Populus nigra L.	0	Salicaceae Mirb.	n	1
Iz_2_1009	DJI_0545	Pyrus communis L.	0	Rosaceae Juss.	a	0
Iz_2_1009	DJI_0545	Quercus robur L.	0	Fagaceae Dumort.	n	1
Iz_2_1009	DJI_0545	Robinia pseudoacacia L.	0	Fabaceae Lindl.	a	0
Iz_2_1009	DJI_0545	Rosa sp.	0	Rosaceae Juss.		
Iz_2_1009	DJI_0545	Salix alba L.	0	Salicaceae Mirb.	n	1
Iz_2_1009	DJI_0545	Sambucus nigra L.	0	Viburnaceae Raf.	n	1
Iz_2_1009	DJI_0545	Sorbus aucuparia L.	0	Rosaceae Juss.	n	1
Iz_2_1009	DJI_0545	Tilia cordata Mill.	0	Malvaceae Juss.	n	1

Iz_2_1009	DJI_0545	Ulmus glabra Huds.	0	Ulmaceae Mirb.	n	1
Iz_2_1009	DJI_0547	Acer tataricum L.	0	Sapindaceae Juss.	n	1
Iz_2_1009	DJI_0547	Betula pendula Roth.	0	Betulaceae Gray	n	1
Iz_2_1009	DJI_0547	Crataegus rhipidophylla Gand.	0	Rosaceae Juss.	n	1
Iz_2_1009	DJI_0547	Frangula alnus Mill.	0	Rhamnaceae Juss.	n	1
Iz_2_1009	DJI_0547	Fraxinus pennsylvanica Marshall	0	Oleaceae Hoffmanns. & Link	a	0
Iz_2_1009	DJI_0547	Pinus sylvestris L.	0	Pinaceae Spreng. ex F.Rudolphi	n	1
Iz_2_1009	DJI_0547	Populus × canescens (Aiton) Sm.	0	Salicaceae Mirb.	n	1
Iz_2_1009	DJI_0547	Populus tremula L.	0	Salicaceae Mirb.	n	1
Iz_2_1009	DJI_0547	Populus nigra L.	0	Salicaceae Mirb.	n	1
Iz_2_1009	DJI_0547	Pyrus communis L.	0	Rosaceae Juss.	a	0
Iz_2_1009	DJI_0547	Quercus robur L.	0	Fagaceae Dumort.	n	1
Iz_2_1009	DJI_0547	Robinia pseudoacacia L.	0	Fabaceae Lindl.	a	0
Iz_2_1009	DJI_0547	Rosa sp.	0	Rosaceae Juss.		
Iz_2_1009	DJI_0547	Salix alba L.	0	Salicaceae Mirb.	n	1
Iz_2_1009	DJI_0547	Sambucus nigra L.	0	Viburnaceae Raf.	n	1
Iz_2_1009	DJI_0547	Sorbus aucuparia L.	0	Rosaceae Juss.	n	1
Iz_2_1009	DJI_0547	Tilia cordata Mill.	0	Malvaceae Juss.	n	1
Iz_2_1009	DJI_0547	Ulmus glabra Huds.	0	Ulmaceae Mirb.	n	1
Iz_2_1009	DJI_0549	Acer tataricum L.	0	Sapindaceae Juss.	n	1
Iz_2_1009	DJI_0549	Betula pendula Roth.	0	Betulaceae Gray	n	1
Iz_2_1009	DJI_0549	Crataegus rhipidophylla Gand.	0	Rosaceae Juss.	n	1
Iz_2_1009	DJI_0549	Frangula alnus Mill.	0	Rhamnaceae Juss.	n	1
Iz_2_1009	DJI_0549	Fraxinus pennsylvanica Marshall	0	Oleaceae Hoffmanns. & Link	a	0
Iz_2_1009	DJI_0549	Pinus sylvestris L.	0	Pinaceae Spreng. ex F.Rudolphi	n	1
Iz_2_1009	DJI_0549	Populus × canescens (Aiton) Sm.	0	Salicaceae Mirb.	n	1
Iz_2_1009	DJI_0549	Populus tremula L.	0	Salicaceae Mirb.	n	1
Iz_2_1009	DJI_0549	Populus nigra L.	0	Salicaceae Mirb.	n	1
Iz_2_1009	DJI_0549	Pyrus communis L.	0	Rosaceae Juss.	a	0
Iz_2_1009	DJI_0549	Quercus robur L.	0	Fagaceae Dumort.	n	1
Iz_2_1009	DJI_0549	Robinia pseudoacacia L.	0	Fabaceae Lindl.	a	0
Iz_2_1009	DJI_0549	Rosa sp.	0	Rosaceae Juss.		
Iz_2_1009	DJI_0549	Salix alba L.	0	Salicaceae Mirb.	n	1
Iz_2_1009	DJI_0549	Sambucus nigra L.	0	Viburnaceae Raf.	n	1
Iz_2_1009	DJI_0549	Sorbus aucuparia L.	0	Rosaceae Juss.	n	1
Iz_2_1009	DJI_0549	Tilia cordata Mill.	0	Malvaceae Juss.	n	1
Iz_2_1009	DJI_0549	Ulmus glabra Huds.	0	Ulmaceae Mirb.	n	1
Iz_2_1009	DJI_0551	Acer tataricum L.	0	Sapindaceae Juss.	n	1
Iz_2_1009	DJI_0551	Betula pendula Roth.	0	Betulaceae Gray	n	1
Iz_2_1009	DJI_0551	Crataegus rhipidophylla Gand.	0	Rosaceae Juss.	n	1
Iz_2_1009	DJI_0551	Frangula alnus Mill.	0	Rhamnaceae Juss.	n	1
Iz_2_1009	DJI_0551	Fraxinus pennsylvanica Marshall	0	Oleaceae Hoffmanns. & Link	a	0
Iz_2_1009	DJI_0551	Pinus sylvestris L.	0	Pinaceae Spreng. ex F.Rudolphi	n	1
Iz_2_1009	DJI_0551	Populus × canescens (Aiton) Sm.	0	Salicaceae Mirb.	n	1
Iz_2_1009	DJI_0551	Populus tremula L.	0	Salicaceae Mirb.	n	1
Iz_2_1009	DJI_0551	Populus nigra L.	0	Salicaceae Mirb.	n	1
Iz_2_1009	DJI_0551	Pyrus communis L.	0	Rosaceae Juss.	a	0
Iz_2_1009	DJI_0551	Quercus robur L.	0	Fagaceae Dumort.	n	1
Iz_2_1009	DJI_0551	Robinia pseudoacacia L.	0	Fabaceae Lindl.	a	0
Iz_2_1009	DJI_0551	Rosa sp.	0	Rosaceae Juss.		
Iz_2_1009	DJI_0551	Salix alba L.	0	Salicaceae Mirb.	n	1
Iz_2_1009	DJI_0551	Sambucus nigra L.	0	Viburnaceae Raf.	n	1
Iz_2_1009	DJI_0551	Sorbus aucuparia L.	0	Rosaceae Juss.	n	1
Iz_2_1009	DJI_0551	Tilia cordata Mill.	0	Malvaceae Juss.	n	1
Iz_2_1009	DJI_0551	Ulmus glabra Huds.	0	Ulmaceae Mirb.	n	1
Iz_2_1009	DJI_0553	Acer tataricum L.	0	Sapindaceae Juss.	n	1
Iz_2_1009	DJI_0553	Betula pendula Roth.	0	Betulaceae Gray	n	1
Iz_2_1009	DJI_0553	Crataegus rhipidophylla Gand.	0	Rosaceae Juss.	n	1
Iz_2_1009	DJI_0553	Frangula alnus Mill.	0	Rhamnaceae Juss.	n	1
Iz_2_1009	DJI_0553	Fraxinus pennsylvanica Marshall	0	Oleaceae Hoffmanns. & Link	a	0
Iz_2_1009	DJI_0553	Pinus sylvestris L.	0	Pinaceae Spreng. ex F.Rudolphi	n	1
Iz_2_1009	DJI_0553	Populus × canescens (Aiton) Sm.	0	Salicaceae Mirb.	n	1
Iz_2_1009	DJI_0553	Populus tremula L.	0	Salicaceae Mirb.	n	1
Iz_2_1009	DJI_0553	Populus nigra L.	0	Salicaceae Mirb.	n	1

Iz_2_1009	DJI_0553	Pyrus communis L.	0	Rosaceae Juss.	a	0
Iz_2_1009	DJI_0553	Quercus robur L.	0	Fagaceae Dumort.	n	1
Iz_2_1009	DJI_0553	Robinia pseudoacacia L.	0	Fabaceae Lindl.	a	0
Iz_2_1009	DJI_0553	Rosa sp.	0	Rosaceae Juss.		
Iz_2_1009	DJI_0553	Salix alba L.	0	Salicaceae Mirb.	n	1
Iz_2_1009	DJI_0553	Sambucus nigra L.	0	Viburnaceae Raf.	n	1
Iz_2_1009	DJI_0553	Sorbus aucuparia L.	0	Rosaceae Juss.	n	1
Iz_2_1009	DJI_0553	Tilia cordata Mill.	0	Malvaceae Juss.	n	1
Iz_2_1009	DJI_0553	Ulmus glabra Huds.	0	Ulmaceae Mirb.	n	1
Iz_2_1009	DJI_0558	Acer tataricum L.	0	Sapindaceae Juss.	n	1
Iz_2_1009	DJI_0558	Betula pendula Roth.	0	Betulaceae Gray	n	1
Iz_2_1009	DJI_0558	Crataegus rhipidophylla Gand.	0	Rosaceae Juss.	n	1
Iz_2_1009	DJI_0558	Frangula alnus Mill.	0	Rhamnaceae Juss.	n	1
Iz_2_1009	DJI_0558	Fraxinus pennsylvanica Marshall	0	Oleaceae Hoffmanns. & Link	a	0
Iz_2_1009	DJI_0558	Pinus sylvestris L.	0	Pinaceae Spreng. ex F.Rudolphi	n	1
Iz_2_1009	DJI_0558	Populus × canescens (Aiton) Sm.	0	Salicaceae Mirb.	n	1
Iz_2_1009	DJI_0558	Populus tremula L.	0	Salicaceae Mirb.	n	1
Iz_2_1009	DJI_0558	Populus nigra L.	0	Salicaceae Mirb.	n	1
Iz_2_1009	DJI_0558	Pyrus communis L.	0	Rosaceae Juss.	a	0
Iz_2_1009	DJI_0558	Quercus robur L.	0	Fagaceae Dumort.	n	1
Iz_2_1009	DJI_0558	Robinia pseudoacacia L.	0	Fabaceae Lindl.	a	0
Iz_2_1009	DJI_0558	Rosa sp.	0	Rosaceae Juss.		
Iz_2_1009	DJI_0558	Salix alba L.	0	Salicaceae Mirb.	n	1
Iz_2_1009	DJI_0558	Sambucus nigra L.	0	Viburnaceae Raf.	n	1
Iz_2_1009	DJI_0558	Sorbus aucuparia L.	0	Rosaceae Juss.	n	1
Iz_2_1009	DJI_0558	Tilia cordata Mill.	0	Malvaceae Juss.	n	1
Iz_2_1009	DJI_0558	Ulmus glabra Huds.	0	Ulmaceae Mirb.	n	1
Iz_2_1009	DJI_0560	Acer tataricum L.	0	Sapindaceae Juss.	n	1
Iz_2_1009	DJI_0560	Betula pendula Roth.	0	Betulaceae Gray	n	1
Iz_2_1009	DJI_0560	Crataegus rhipidophylla Gand.	0	Rosaceae Juss.	n	1
Iz_2_1009	DJI_0560	Frangula alnus Mill.	0	Rhamnaceae Juss.	n	1
Iz_2_1009	DJI_0560	Fraxinus pennsylvanica Marshall	0	Oleaceae Hoffmanns. & Link	a	0
Iz_2_1009	DJI_0560	Pinus sylvestris L.	0	Pinaceae Spreng. ex F.Rudolphi	n	1
Iz_2_1009	DJI_0560	Populus × canescens (Aiton) Sm.	0	Salicaceae Mirb.	n	1
Iz_2_1009	DJI_0560	Populus tremula L.	0	Salicaceae Mirb.	n	1
Iz_2_1009	DJI_0560	Populus nigra L.	0	Salicaceae Mirb.	n	1
Iz_2_1009	DJI_0560	Pyrus communis L.	0	Rosaceae Juss.	a	0
Iz_2_1009	DJI_0560	Quercus robur L.	0	Fagaceae Dumort.	n	1
Iz_2_1009	DJI_0560	Robinia pseudoacacia L.	0	Fabaceae Lindl.	a	0
Iz_2_1009	DJI_0560	Rosa sp.	0	Rosaceae Juss.		
Iz_2_1009	DJI_0560	Salix alba L.	0	Salicaceae Mirb.	n	1
Iz_2_1009	DJI_0560	Sambucus nigra L.	0	Viburnaceae Raf.	n	1
Iz_2_1009	DJI_0560	Sorbus aucuparia L.	0	Rosaceae Juss.	n	1
Iz_2_1009	DJI_0560	Tilia cordata Mill.	0	Malvaceae Juss.	n	1
Iz_2_1009	DJI_0560	Ulmus glabra Huds.	0	Ulmaceae Mirb.	n	1
Iz_2_1009	DJI_0562	Acer tataricum L.	0	Sapindaceae Juss.	n	1
Iz_2_1009	DJI_0562	Betula pendula Roth.	0	Betulaceae Gray	n	1
Iz_2_1009	DJI_0562	Crataegus rhipidophylla Gand.	0	Rosaceae Juss.	n	1
Iz_2_1009	DJI_0562	Frangula alnus Mill.	0	Rhamnaceae Juss.	n	1
Iz_2_1009	DJI_0562	Fraxinus pennsylvanica Marshall	0	Oleaceae Hoffmanns. & Link	a	0
Iz_2_1009	DJI_0562	Pinus sylvestris L.	0	Pinaceae Spreng. ex F.Rudolphi	n	1
Iz_2_1009	DJI_0562	Populus × canescens (Aiton) Sm.	0	Salicaceae Mirb.	n	1
Iz_2_1009	DJI_0562	Populus tremula L.	0	Salicaceae Mirb.	n	1
Iz_2_1009	DJI_0562	Populus nigra L.	0	Salicaceae Mirb.	n	1
Iz_2_1009	DJI_0562	Pyrus communis L.	0	Rosaceae Juss.	a	0
Iz_2_1009	DJI_0562	Quercus robur L.	0	Fagaceae Dumort.	n	1
Iz_2_1009	DJI_0562	Robinia pseudoacacia L.	0	Fabaceae Lindl.	a	0
Iz_2_1009	DJI_0562	Rosa sp.	0	Rosaceae Juss.		
Iz_2_1009	DJI_0562	Salix alba L.	0	Salicaceae Mirb.	n	1
Iz_2_1009	DJI_0562	Sambucus nigra L.	0	Viburnaceae Raf.	n	1
Iz_2_1009	DJI_0562	Sorbus aucuparia L.	0	Rosaceae Juss.	n	1
Iz_2_1009	DJI_0562	Tilia cordata Mill.	0	Malvaceae Juss.	n	1
Iz_2_1009	DJI_0562	Ulmus glabra Huds.	0	Ulmaceae Mirb.	n	1
Iz_2_1009	DJI_0569	Acer tataricum L.	0	Sapindaceae Juss.	n	1

Iz_2_1009	DJI_0569	Betula pendula Roth.	0	Betulaceae Gray	n	1
Iz_2_1009	DJI_0569	Crataegus rhipidophylla Gand.	0	Rosaceae Juss.	n	1
Iz_2_1009	DJI_0569	Frangula alnus Mill.	0	Rhamnaceae Juss.	n	1
Iz_2_1009	DJI_0569	Fraxinus pennsylvanica Marshall	0	Oleaceae Hoffmanns. & Link	a	0
Iz_2_1009	DJI_0569	Pinus sylvestris L.	0	Pinaceae Spreng. ex F.Rudolphi	n	1
Iz_2_1009	DJI_0569	Populus × canescens (Aiton) Sm.	0	Salicaceae Mirb.	n	1
Iz_2_1009	DJI_0569	Populus tremula L.	0	Salicaceae Mirb.	n	1
Iz_2_1009	DJI_0569	Populus nigra L.	0	Salicaceae Mirb.	n	1
Iz_2_1009	DJI_0569	Pyrus communis L.	0	Rosaceae Juss.	a	0
Iz_2_1009	DJI_0569	Quercus robur L.	0	Fagaceae Dumort.	n	1
Iz_2_1009	DJI_0569	Robinia pseudoacacia L.	0	Fabaceae Lindl.	a	0
Iz_2_1009	DJI_0569	Rosa sp.	0	Rosaceae Juss.		
Iz_2_1009	DJI_0569	Salix alba L.	0	Salicaceae Mirb.	n	1
Iz_2_1009	DJI_0569	Sambucus nigra L.	0	Viburnaceae Raf.	n	1
Iz_2_1009	DJI_0569	Sorbus aucuparia L.	0	Rosaceae Juss.	n	1
Iz_2_1009	DJI_0569	Tilia cordata Mill.	0	Malvaceae Juss.	n	1
Iz_2_1009	DJI_0569	Ulmus glabra Huds.	0	Ulmaceae Mirb.	n	1
Iz_2_1009	DJI_0572	Acer tataricum L.	0	Sapindaceae Juss.	n	1
Iz_2_1009	DJI_0572	Betula pendula Roth.	0	Betulaceae Gray	n	1
Iz_2_1009	DJI_0572	Crataegus rhipidophylla Gand.	0	Rosaceae Juss.	n	1
Iz_2_1009	DJI_0572	Frangula alnus Mill.	0	Rhamnaceae Juss.	n	1
Iz_2_1009	DJI_0572	Fraxinus pennsylvanica Marshall	0	Oleaceae Hoffmanns. & Link	a	0
Iz_2_1009	DJI_0572	Pinus sylvestris L.	0	Pinaceae Spreng. ex F.Rudolphi	n	1
Iz_2_1009	DJI_0572	Populus × canescens (Aiton) Sm.	0	Salicaceae Mirb.	n	1
Iz_2_1009	DJI_0572	Populus tremula L.	0	Salicaceae Mirb.	n	1
Iz_2_1009	DJI_0572	Populus nigra L.	0	Salicaceae Mirb.	n	1
Iz_2_1009	DJI_0572	Pyrus communis L.	0	Rosaceae Juss.	a	0
Iz_2_1009	DJI_0572	Quercus robur L.	0	Fagaceae Dumort.	n	1
Iz_2_1009	DJI_0572	Robinia pseudoacacia L.	0	Fabaceae Lindl.	a	0
Iz_2_1009	DJI_0572	Rosa sp.	0	Rosaceae Juss.		
Iz_2_1009	DJI_0572	Salix alba L.	0	Salicaceae Mirb.	n	1
Iz_2_1009	DJI_0572	Sambucus nigra L.	0	Viburnaceae Raf.	n	1
Iz_2_1009	DJI_0572	Sorbus aucuparia L.	0	Rosaceae Juss.	n	1
Iz_2_1009	DJI_0572	Tilia cordata Mill.	0	Malvaceae Juss.	n	1
Iz_2_1009	DJI_0572	Ulmus glabra Huds.	0	Ulmaceae Mirb.	n	1
Iz_3_1009	DJI_0831	Acer tataricum L.	0	Sapindaceae Juss.	n	1
Iz_3_1009	DJI_0831	Betula pendula Roth.	0	Betulaceae Gray	n	1
Iz_3_1009	DJI_0831	Crataegus rhipidophylla Gand.	0	Rosaceae Juss.	n	1
Iz_3_1009	DJI_0831	Frangula alnus Mill.	0	Rhamnaceae Juss.	n	1
Iz_3_1009	DJI_0831	Fraxinus pennsylvanica Marshall	0	Oleaceae Hoffmanns. & Link	a	0
Iz_3_1009	DJI_0831	Pinus sylvestris L.	0	Pinaceae Spreng. ex F.Rudolphi	n	1
Iz_3_1009	DJI_0831	Populus × canescens (Aiton) Sm.	0	Salicaceae Mirb.	n	1
Iz_3_1009	DJI_0831	Populus tremula L.	0	Salicaceae Mirb.	n	1
Iz_3_1009	DJI_0831	Populus nigra L.	0	Salicaceae Mirb.	n	1
Iz_3_1009	DJI_0831	Pyrus communis L.	0	Rosaceae Juss.	a	0
Iz_3_1009	DJI_0831	Quercus robur L.	2	Fagaceae Dumort.	n	1
Iz_3_1009	DJI_0831	Robinia pseudoacacia L.	0	Fabaceae Lindl.	a	0
Iz_3_1009	DJI_0831	Rosa sp.	0	Rosaceae Juss.		
Iz_3_1009	DJI_0831	Salix alba L.	0	Salicaceae Mirb.	n	1
Iz_3_1009	DJI_0831	Sambucus nigra L.	0	Viburnaceae Raf.	n	1
Iz_3_1009	DJI_0831	Sorbus aucuparia L.	0	Rosaceae Juss.	n	1
Iz_3_1009	DJI_0831	Tilia cordata Mill.	0	Malvaceae Juss.	n	1
Iz_3_1009	DJI_0831	Ulmus glabra Huds.	0	Ulmaceae Mirb.	n	1
Iz_3_1009	DJI_0832	Acer tataricum L.	0	Sapindaceae Juss.	n	1
Iz_3_1009	DJI_0832	Betula pendula Roth.	0	Betulaceae Gray	n	1
Iz_3_1009	DJI_0832	Crataegus rhipidophylla Gand.	0	Rosaceae Juss.	n	1
Iz_3_1009	DJI_0832	Frangula alnus Mill.	0	Rhamnaceae Juss.	n	1
Iz_3_1009	DJI_0832	Fraxinus pennsylvanica Marshall	0	Oleaceae Hoffmanns. & Link	a	0
Iz_3_1009	DJI_0832	Pinus sylvestris L.	0	Pinaceae Spreng. ex F.Rudolphi	n	1
Iz_3_1009	DJI_0832	Populus × canescens (Aiton) Sm.	0	Salicaceae Mirb.	n	1
Iz_3_1009	DJI_0832	Populus tremula L.	0	Salicaceae Mirb.	n	1
Iz_3_1009	DJI_0832	Populus nigra L.	0	Salicaceae Mirb.	n	1
Iz_3_1009	DJI_0832	Pyrus communis L.	0	Rosaceae Juss.	a	0
Iz_3_1009	DJI_0832	Quercus robur L.	0	Fagaceae Dumort.	n	1

Iz_3_1009	DJI_0832	Robinia pseudoacacia L.	0	Fabaceae Lindl.	a	0
Iz_3_1009	DJI_0832	Rosa sp.	0	Rosaceae Juss.		
Iz_3_1009	DJI_0832	Salix alba L.	0	Salicaceae Mirb.	n	1
Iz_3_1009	DJI_0832	Sambucus nigra L.	0	Viburnaceae Raf.	n	1
Iz_3_1009	DJI_0832	Sorbus aucuparia L.	0	Rosaceae Juss.	n	1
Iz_3_1009	DJI_0832	Tilia cordata Mill.	0	Malvaceae Juss.	n	1
Iz_3_1009	DJI_0832	Ulmus glabra Huds.	0	Ulmaceae Mirb.	n	1
Iz_3_1009	DJI_0833	Acer tataricum L.	0	Sapindaceae Juss.	n	1
Iz_3_1009	DJI_0833	Betula pendula Roth.	0	Betulaceae Gray	n	1
Iz_3_1009	DJI_0833	Crataegus rhipidophylla Gand.	0	Rosaceae Juss.	n	1
Iz_3_1009	DJI_0833	Frangula alnus Mill.	0	Rhamnaceae Juss.	n	1
Iz_3_1009	DJI_0833	Fraxinus pennsylvanica Marshall	0	Oleaceae Hoffmanns. & Link	a	0
Iz_3_1009	DJI_0833	Pinus sylvestris L.	0	Pinaceae Spreng. ex F.Rudolphi	n	1
Iz_3_1009	DJI_0833	Populus × canescens (Aiton) Sm.	0	Salicaceae Mirb.	n	1
Iz_3_1009	DJI_0833	Populus tremula L.	0	Salicaceae Mirb.	n	1
Iz_3_1009	DJI_0833	Populus nigra L.	0	Salicaceae Mirb.	n	1
Iz_3_1009	DJI_0833	Pyrus communis L.	0	Rosaceae Juss.	a	0
Iz_3_1009	DJI_0833	Quercus robur L.	0	Fagaceae Dumort.	n	1
Iz_3_1009	DJI_0833	Robinia pseudoacacia L.	0	Fabaceae Lindl.	a	0
Iz_3_1009	DJI_0833	Rosa sp.	0	Rosaceae Juss.		
Iz_3_1009	DJI_0833	Salix alba L.	0	Salicaceae Mirb.	n	1
Iz_3_1009	DJI_0833	Sambucus nigra L.	0	Viburnaceae Raf.	n	1
Iz_3_1009	DJI_0833	Sorbus aucuparia L.	0	Rosaceae Juss.	n	1
Iz_3_1009	DJI_0833	Tilia cordata Mill.	0	Malvaceae Juss.	n	1
Iz_3_1009	DJI_0833	Ulmus glabra Huds.	0	Ulmaceae Mirb.	n	1
Iz_3_1009	DJI_0837	Acer tataricum L.	0	Sapindaceae Juss.	n	1
Iz_3_1009	DJI_0837	Betula pendula Roth.	0	Betulaceae Gray	n	1
Iz_3_1009	DJI_0837	Crataegus rhipidophylla Gand.	0	Rosaceae Juss.	n	1
Iz_3_1009	DJI_0837	Frangula alnus Mill.	0	Rhamnaceae Juss.	n	1
Iz_3_1009	DJI_0837	Fraxinus pennsylvanica Marshall	0	Oleaceae Hoffmanns. & Link	a	0
Iz_3_1009	DJI_0837	Pinus sylvestris L.	0	Pinaceae Spreng. ex F.Rudolphi	n	1
Iz_3_1009	DJI_0837	Populus × canescens (Aiton) Sm.	0	Salicaceae Mirb.	n	1
Iz_3_1009	DJI_0837	Populus tremula L.	0	Salicaceae Mirb.	n	1
Iz_3_1009	DJI_0837	Populus nigra L.	0	Salicaceae Mirb.	n	1
Iz_3_1009	DJI_0837	Pyrus communis L.	0	Rosaceae Juss.	a	0
Iz_3_1009	DJI_0837	Quercus robur L.	0	Fagaceae Dumort.	n	1
Iz_3_1009	DJI_0837	Robinia pseudoacacia L.	0	Fabaceae Lindl.	a	0
Iz_3_1009	DJI_0837	Rosa sp.	0	Rosaceae Juss.		
Iz_3_1009	DJI_0837	Salix alba L.	0	Salicaceae Mirb.	n	1
Iz_3_1009	DJI_0837	Sambucus nigra L.	0	Viburnaceae Raf.	n	1
Iz_3_1009	DJI_0837	Sorbus aucuparia L.	0	Rosaceae Juss.	n	1
Iz_3_1009	DJI_0837	Tilia cordata Mill.	0	Malvaceae Juss.	n	1
Iz_3_1009	DJI_0837	Ulmus glabra Huds.	0	Ulmaceae Mirb.	n	1
Iz_3_1009	DJI_0838	Acer tataricum L.	0	Sapindaceae Juss.	n	1
Iz_3_1009	DJI_0838	Betula pendula Roth.	0	Betulaceae Gray	n	1
Iz_3_1009	DJI_0838	Crataegus rhipidophylla Gand.	0	Rosaceae Juss.	n	1
Iz_3_1009	DJI_0838	Frangula alnus Mill.	0	Rhamnaceae Juss.	n	1
Iz_3_1009	DJI_0838	Fraxinus pennsylvanica Marshall	0	Oleaceae Hoffmanns. & Link	a	0
Iz_3_1009	DJI_0838	Pinus sylvestris L.	0	Pinaceae Spreng. ex F.Rudolphi	n	1
Iz_3_1009	DJI_0838	Populus × canescens (Aiton) Sm.	0	Salicaceae Mirb.	n	1
Iz_3_1009	DJI_0838	Populus tremula L.	0	Salicaceae Mirb.	n	1
Iz_3_1009	DJI_0838	Populus nigra L.	0	Salicaceae Mirb.	n	1
Iz_3_1009	DJI_0838	Pyrus communis L.	0	Rosaceae Juss.	a	0
Iz_3_1009	DJI_0838	Quercus robur L.	0	Fagaceae Dumort.	n	1
Iz_3_1009	DJI_0838	Robinia pseudoacacia L.	0	Fabaceae Lindl.	a	0
Iz_3_1009	DJI_0838	Rosa sp.	0	Rosaceae Juss.		
Iz_3_1009	DJI_0838	Salix alba L.	0	Salicaceae Mirb.	n	1
Iz_3_1009	DJI_0838	Sambucus nigra L.	0	Viburnaceae Raf.	n	1
Iz_3_1009	DJI_0838	Sorbus aucuparia L.	0	Rosaceae Juss.	n	1
Iz_3_1009	DJI_0838	Tilia cordata Mill.	0	Malvaceae Juss.	n	1
Iz_3_1009	DJI_0838	Ulmus glabra Huds.	0	Ulmaceae Mirb.	n	1
Iz_3_1009	DJI_0842	Acer tataricum L.	0	Sapindaceae Juss.	n	1
Iz_3_1009	DJI_0842	Betula pendula Roth.	0	Betulaceae Gray	n	1
Iz_3_1009	DJI_0842	Crataegus rhipidophylla Gand.	0	Rosaceae Juss.	n	1

Iz_3_1009	DJI_0842	Frangula alnus Mill.	0	Rhamnaceae Juss.	n	1
Iz_3_1009	DJI_0842	Fraxinus pennsylvanica Marshall	0	Oleaceae Hoffmanns. & Link	a	0
Iz_3_1009	DJI_0842	Pinus sylvestris L.	0	Pinaceae Spreng. ex F.Rudolphi	n	1
Iz_3_1009	DJI_0842	Populus × canescens (Aiton) Sm.	0	Salicaceae Mirb.	n	1
Iz_3_1009	DJI_0842	Populus tremula L.	0	Salicaceae Mirb.	n	1
Iz_3_1009	DJI_0842	Populus nigra L.	0	Salicaceae Mirb.	n	1
Iz_3_1009	DJI_0842	Pyrus communis L.	0	Rosaceae Juss.	a	0
Iz_3_1009	DJI_0842	Quercus robur L.	0	Fagaceae Dumort.	n	1
Iz_3_1009	DJI_0842	Robinia pseudoacacia L.	0	Fabaceae Lindl.	a	0
Iz_3_1009	DJI_0842	Rosa sp.	0	Rosaceae Juss.		
Iz_3_1009	DJI_0842	Salix alba L.	0	Salicaceae Mirb.	n	1
Iz_3_1009	DJI_0842	Sambucus nigra L.	0	Viburnaceae Raf.	n	1
Iz_3_1009	DJI_0842	Sorbus aucuparia L.	0	Rosaceae Juss.	n	1
Iz_3_1009	DJI_0842	Tilia cordata Mill.	0	Malvaceae Juss.	n	1
Iz_3_1009	DJI_0842	Ulmus glabra Huds.	0	Ulmaceae Mirb.	n	1
Iz_3_1009	DJI_0843	Acer tataricum L.	0	Sapindaceae Juss.	n	1
Iz_3_1009	DJI_0843	Betula pendula Roth.	0	Betulaceae Gray	n	1
Iz_3_1009	DJI_0843	Crataegus rhipidophylla Gand.	0	Rosaceae Juss.	n	1
Iz_3_1009	DJI_0843	Frangula alnus Mill.	0	Rhamnaceae Juss.	n	1
Iz_3_1009	DJI_0843	Fraxinus pennsylvanica Marshall	0	Oleaceae Hoffmanns. & Link	a	0
Iz_3_1009	DJI_0843	Pinus sylvestris L.	0	Pinaceae Spreng. ex F.Rudolphi	n	1
Iz_3_1009	DJI_0843	Populus × canescens (Aiton) Sm.	0	Salicaceae Mirb.	n	1
Iz_3_1009	DJI_0843	Populus tremula L.	0	Salicaceae Mirb.	n	1
Iz_3_1009	DJI_0843	Populus nigra L.	0	Salicaceae Mirb.	n	1
Iz_3_1009	DJI_0843	Pyrus communis L.	0	Rosaceae Juss.	a	0
Iz_3_1009	DJI_0843	Quercus robur L.	0	Fagaceae Dumort.	n	1
Iz_3_1009	DJI_0843	Robinia pseudoacacia L.	0	Fabaceae Lindl.	a	0
Iz_3_1009	DJI_0843	Rosa sp.	0	Rosaceae Juss.		
Iz_3_1009	DJI_0843	Salix alba L.	0	Salicaceae Mirb.	n	1
Iz_3_1009	DJI_0843	Sambucus nigra L.	0	Viburnaceae Raf.	n	1
Iz_3_1009	DJI_0843	Sorbus aucuparia L.	0	Rosaceae Juss.	n	1
Iz_3_1009	DJI_0843	Tilia cordata Mill.	0	Malvaceae Juss.	n	1
Iz_3_1009	DJI_0843	Ulmus glabra Huds.	0	Ulmaceae Mirb.	n	1
Iz_3_1009	DJI_0845	Acer tataricum L.	0	Sapindaceae Juss.	n	1
Iz_3_1009	DJI_0845	Betula pendula Roth.	0	Betulaceae Gray	n	1
Iz_3_1009	DJI_0845	Crataegus rhipidophylla Gand.	0	Rosaceae Juss.	n	1
Iz_3_1009	DJI_0845	Frangula alnus Mill.	0	Rhamnaceae Juss.	n	1
Iz_3_1009	DJI_0845	Fraxinus pennsylvanica Marshall	0	Oleaceae Hoffmanns. & Link	a	0
Iz_3_1009	DJI_0845	Pinus sylvestris L.	0	Pinaceae Spreng. ex F.Rudolphi	n	1
Iz_3_1009	DJI_0845	Populus × canescens (Aiton) Sm.	0	Salicaceae Mirb.	n	1
Iz_3_1009	DJI_0845	Populus tremula L.	0	Salicaceae Mirb.	n	1
Iz_3_1009	DJI_0845	Populus nigra L.	0	Salicaceae Mirb.	n	1
Iz_3_1009	DJI_0845	Pyrus communis L.	0	Rosaceae Juss.	a	0
Iz_3_1009	DJI_0845	Quercus robur L.	0	Fagaceae Dumort.	n	1
Iz_3_1009	DJI_0845	Robinia pseudoacacia L.	0	Fabaceae Lindl.	a	0
Iz_3_1009	DJI_0845	Rosa sp.	0	Rosaceae Juss.		
Iz_3_1009	DJI_0845	Salix alba L.	0	Salicaceae Mirb.	n	1
Iz_3_1009	DJI_0845	Sambucus nigra L.	0	Viburnaceae Raf.	n	1
Iz_3_1009	DJI_0845	Sorbus aucuparia L.	0	Rosaceae Juss.	n	1
Iz_3_1009	DJI_0845	Tilia cordata Mill.	0	Malvaceae Juss.	n	1
Iz_3_1009	DJI_0845	Ulmus glabra Huds.	0	Ulmaceae Mirb.	n	1
Iz_3_1009	DJI_0850	Acer tataricum L.	0	Sapindaceae Juss.	n	1
Iz_3_1009	DJI_0850	Betula pendula Roth.	0	Betulaceae Gray	n	1
Iz_3_1009	DJI_0850	Crataegus rhipidophylla Gand.	0	Rosaceae Juss.	n	1
Iz_3_1009	DJI_0850	Frangula alnus Mill.	0	Rhamnaceae Juss.	n	1
Iz_3_1009	DJI_0850	Fraxinus pennsylvanica Marshall	0	Oleaceae Hoffmanns. & Link	a	0
Iz_3_1009	DJI_0850	Pinus sylvestris L.	0	Pinaceae Spreng. ex F.Rudolphi	n	1
Iz_3_1009	DJI_0850	Populus × canescens (Aiton) Sm.	0	Salicaceae Mirb.	n	1
Iz_3_1009	DJI_0850	Populus tremula L.	0	Salicaceae Mirb.	n	1
Iz_3_1009	DJI_0850	Populus nigra L.	0	Salicaceae Mirb.	n	1
Iz_3_1009	DJI_0850	Pyrus communis L.	0	Rosaceae Juss.	a	0
Iz_3_1009	DJI_0850	Quercus robur L.	0	Fagaceae Dumort.	n	1
Iz_3_1009	DJI_0850	Robinia pseudoacacia L.	0	Fabaceae Lindl.	a	0
Iz_3_1009	DJI_0850	Rosa sp.	0	Rosaceae Juss.		

Iz_3_1009	DJI_0850	Salix alba L.	0	Salicaceae Mirb.	n	1
Iz_3_1009	DJI_0850	Sambucus nigra L.	0	Viburnaceae Raf.	n	1
Iz_3_1009	DJI_0850	Sorbus aucuparia L.	0	Rosaceae Juss.	n	1
Iz_3_1009	DJI_0850	Tilia cordata Mill.	0	Malvaceae Juss.	n	1
Iz_3_1009	DJI_0850	Ulmus glabra Huds.	0	Ulmaceae Mirb.	n	1
Iz_3_1009	DJI_0852	Acer tataricum L.	0	Sapindaceae Juss.	n	1
Iz_3_1009	DJI_0852	Betula pendula Roth.	0	Betulaceae Gray	n	1
Iz_3_1009	DJI_0852	Crataegus rhipidophylla Gand.	0	Rosaceae Juss.	n	1
Iz_3_1009	DJI_0852	Frangula alnus Mill.	0	Rhamnaceae Juss.	n	1
Iz_3_1009	DJI_0852	Fraxinus pennsylvanica Marshall	0	Oleaceae Hoffmanns. & Link	a	0
Iz_3_1009	DJI_0852	Pinus sylvestris L.	0	Pinaceae Spreng. ex F.Rudolphi	n	1
Iz_3_1009	DJI_0852	Populus × canescens (Aiton) Sm.	0	Salicaceae Mirb.	n	1
Iz_3_1009	DJI_0852	Populus tremula L.	0	Salicaceae Mirb.	n	1
Iz_3_1009	DJI_0852	Populus nigra L.	0	Salicaceae Mirb.	n	1
Iz_3_1009	DJI_0852	Pyrus communis L.	0	Rosaceae Juss.	a	0
Iz_3_1009	DJI_0852	Quercus robur L.	0	Fagaceae Dumort.	n	1
Iz_3_1009	DJI_0852	Robinia pseudoacacia L.	0	Fabaceae Lindl.	a	0
Iz_3_1009	DJI_0852	Rosa sp.	0	Rosaceae Juss.		
Iz_3_1009	DJI_0852	Salix alba L.	0	Salicaceae Mirb.	n	1
Iz_3_1009	DJI_0852	Sambucus nigra L.	0	Viburnaceae Raf.	n	1
Iz_3_1009	DJI_0852	Sorbus aucuparia L.	0	Rosaceae Juss.	n	1
Iz_3_1009	DJI_0852	Tilia cordata Mill.	0	Malvaceae Juss.	n	1
Iz_3_1009	DJI_0852	Ulmus glabra Huds.	0	Ulmaceae Mirb.	n	1
Iz_3_1009	DJI_0853	Acer tataricum L.	0	Sapindaceae Juss.	n	1
Iz_3_1009	DJI_0853	Betula pendula Roth.	1	Betulaceae Gray	n	1
Iz_3_1009	DJI_0853	Crataegus rhipidophylla Gand.	0	Rosaceae Juss.	n	1
Iz_3_1009	DJI_0853	Frangula alnus Mill.	0	Rhamnaceae Juss.	n	1
Iz_3_1009	DJI_0853	Fraxinus pennsylvanica Marshall	0	Oleaceae Hoffmanns. & Link	a	0
Iz_3_1009	DJI_0853	Pinus sylvestris L.	0	Pinaceae Spreng. ex F.Rudolphi	n	1
Iz_3_1009	DJI_0853	Populus × canescens (Aiton) Sm.	0	Salicaceae Mirb.	n	1
Iz_3_1009	DJI_0853	Populus tremula L.	0	Salicaceae Mirb.	n	1
Iz_3_1009	DJI_0853	Populus nigra L.	0	Salicaceae Mirb.	n	1
Iz_3_1009	DJI_0853	Pyrus communis L.	0	Rosaceae Juss.	a	0
Iz_3_1009	DJI_0853	Quercus robur L.	4	Fagaceae Dumort.	n	1
Iz_3_1009	DJI_0853	Robinia pseudoacacia L.	0	Fabaceae Lindl.	a	0
Iz_3_1009	DJI_0853	Rosa sp.	0	Rosaceae Juss.		
Iz_3_1009	DJI_0853	Salix alba L.	0	Salicaceae Mirb.	n	1
Iz_3_1009	DJI_0853	Sambucus nigra L.	0	Viburnaceae Raf.	n	1
Iz_3_1009	DJI_0853	Sorbus aucuparia L.	0	Rosaceae Juss.	n	1
Iz_3_1009	DJI_0853	Tilia cordata Mill.	0	Malvaceae Juss.	n	1
Iz_3_1009	DJI_0853	Ulmus glabra Huds.	0	Ulmaceae Mirb.	n	1
Iz_3_1009	DJI_0855	Acer tataricum L.	0	Sapindaceae Juss.	n	1
Iz_3_1009	DJI_0855	Betula pendula Roth.	0	Betulaceae Gray	n	1
Iz_3_1009	DJI_0855	Crataegus rhipidophylla Gand.	0	Rosaceae Juss.	n	1
Iz_3_1009	DJI_0855	Frangula alnus Mill.	0	Rhamnaceae Juss.	n	1
Iz_3_1009	DJI_0855	Fraxinus pennsylvanica Marshall	0	Oleaceae Hoffmanns. & Link	a	0
Iz_3_1009	DJI_0855	Pinus sylvestris L.	0	Pinaceae Spreng. ex F.Rudolphi	n	1
Iz_3_1009	DJI_0855	Populus × canescens (Aiton) Sm.	0	Salicaceae Mirb.	n	1
Iz_3_1009	DJI_0855	Populus tremula L.	0	Salicaceae Mirb.	n	1
Iz_3_1009	DJI_0855	Populus nigra L.	0	Salicaceae Mirb.	n	1
Iz_3_1009	DJI_0855	Pyrus communis L.	0	Rosaceae Juss.	a	0
Iz_3_1009	DJI_0855	Quercus robur L.	0	Fagaceae Dumort.	n	1
Iz_3_1009	DJI_0855	Robinia pseudoacacia L.	0	Fabaceae Lindl.	a	0
Iz_3_1009	DJI_0855	Rosa sp.	0	Rosaceae Juss.		
Iz_3_1009	DJI_0855	Salix alba L.	0	Salicaceae Mirb.	n	1
Iz_3_1009	DJI_0855	Sambucus nigra L.	0	Viburnaceae Raf.	n	1
Iz_3_1009	DJI_0855	Sorbus aucuparia L.	0	Rosaceae Juss.	n	1
Iz_3_1009	DJI_0855	Tilia cordata Mill.	0	Malvaceae Juss.	n	1
Iz_3_1009	DJI_0855	Ulmus glabra Huds.	0	Ulmaceae Mirb.	n	1
Iz_3_1009	DJI_0857	Acer tataricum L.	0	Sapindaceae Juss.	n	1
Iz_3_1009	DJI_0857	Betula pendula Roth.	0	Betulaceae Gray	n	1
Iz_3_1009	DJI_0857	Crataegus rhipidophylla Gand.	0	Rosaceae Juss.	n	1
Iz_3_1009	DJI_0857	Frangula alnus Mill.	0	Rhamnaceae Juss.	n	1
Iz_3_1009	DJI_0857	Fraxinus pennsylvanica Marshall	0	Oleaceae Hoffmanns. & Link	a	0

Iz_3_1009	DJI_0857	Pinus sylvestris L.	0	Pinaceae Spreng. ex F.Rudolphi	n	1
Iz_3_1009	DJI_0857	Populus × canescens (Aiton) Sm.	0	Salicaceae Mirb.	n	1
Iz_3_1009	DJI_0857	Populus tremula L.	0	Salicaceae Mirb.	n	1
Iz_3_1009	DJI_0857	Populus nigra L.	0	Salicaceae Mirb.	n	1
Iz_3_1009	DJI_0857	Pyrus communis L.	0	Rosaceae Juss.	a	0
Iz_3_1009	DJI_0857	Quercus robur L.	0	Fagaceae Dumort.	n	1
Iz_3_1009	DJI_0857	Robinia pseudoacacia L.	0	Fabaceae Lindl.	a	0
Iz_3_1009	DJI_0857	Rosa sp.	0	Rosaceae Juss.		
Iz_3_1009	DJI_0857	Salix alba L.	0	Salicaceae Mirb.	n	1
Iz_3_1009	DJI_0857	Sambucus nigra L.	0	Viburnaceae Raf.	n	1
Iz_3_1009	DJI_0857	Sorbus aucuparia L.	0	Rosaceae Juss.	n	1
Iz_3_1009	DJI_0857	Tilia cordata Mill.	0	Malvaceae Juss.	n	1
Iz_3_1009	DJI_0857	Ulmus glabra Huds.	0	Ulmaceae Mirb.	n	1
Iz_3_1009	DJI_0858	Acer tataricum L.	0	Sapindaceae Juss.	n	1
Iz_3_1009	DJI_0858	Betula pendula Roth.	0	Betulaceae Gray	n	1
Iz_3_1009	DJI_0858	Crataegus rhipidophylla Gand.	0	Rosaceae Juss.	n	1
Iz_3_1009	DJI_0858	Frangula alnus Mill.	0	Rhamnaceae Juss.	n	1
Iz_3_1009	DJI_0858	Fraxinus pennsylvanica Marshall	0	Oleaceae Hoffmanns. & Link	a	0
Iz_3_1009	DJI_0858	Pinus sylvestris L.	0	Pinaceae Spreng. ex F.Rudolphi	n	1
Iz_3_1009	DJI_0858	Populus × canescens (Aiton) Sm.	0	Salicaceae Mirb.	n	1
Iz_3_1009	DJI_0858	Populus tremula L.	0	Salicaceae Mirb.	n	1
Iz_3_1009	DJI_0858	Populus nigra L.	0	Salicaceae Mirb.	n	1
Iz_3_1009	DJI_0858	Pyrus communis L.	0	Rosaceae Juss.	a	0
Iz_3_1009	DJI_0858	Quercus robur L.	0	Fagaceae Dumort.	n	1
Iz_3_1009	DJI_0858	Robinia pseudoacacia L.	0	Fabaceae Lindl.	a	0
Iz_3_1009	DJI_0858	Rosa sp.	0	Rosaceae Juss.		
Iz_3_1009	DJI_0858	Salix alba L.	0	Salicaceae Mirb.	n	1
Iz_3_1009	DJI_0858	Sambucus nigra L.	0	Viburnaceae Raf.	n	1
Iz_3_1009	DJI_0858	Sorbus aucuparia L.	0	Rosaceae Juss.	n	1
Iz_3_1009	DJI_0858	Tilia cordata Mill.	0	Malvaceae Juss.	n	1
Iz_3_1009	DJI_0858	Ulmus glabra Huds.	0	Ulmaceae Mirb.	n	1
Iz_3_1009	DJI_0859	Acer tataricum L.	0	Sapindaceae Juss.	n	1
Iz_3_1009	DJI_0859	Betula pendula Roth.	0	Betulaceae Gray	n	1
Iz_3_1009	DJI_0859	Crataegus rhipidophylla Gand.	0	Rosaceae Juss.	n	1
Iz_3_1009	DJI_0859	Frangula alnus Mill.	0	Rhamnaceae Juss.	n	1
Iz_3_1009	DJI_0859	Fraxinus pennsylvanica Marshall	0	Oleaceae Hoffmanns. & Link	a	0
Iz_3_1009	DJI_0859	Pinus sylvestris L.	0	Pinaceae Spreng. ex F.Rudolphi	n	1
Iz_3_1009	DJI_0859	Populus × canescens (Aiton) Sm.	0	Salicaceae Mirb.	n	1
Iz_3_1009	DJI_0859	Populus tremula L.	0	Salicaceae Mirb.	n	1
Iz_3_1009	DJI_0859	Populus nigra L.	0	Salicaceae Mirb.	n	1
Iz_3_1009	DJI_0859	Pyrus communis L.	0	Rosaceae Juss.	a	0
Iz_3_1009	DJI_0859	Quercus robur L.	0	Fagaceae Dumort.	n	1
Iz_3_1009	DJI_0859	Robinia pseudoacacia L.	0	Fabaceae Lindl.	a	0
Iz_3_1009	DJI_0859	Rosa sp.	0	Rosaceae Juss.		
Iz_3_1009	DJI_0859	Salix alba L.	0	Salicaceae Mirb.	n	1
Iz_3_1009	DJI_0859	Sambucus nigra L.	0	Viburnaceae Raf.	n	1
Iz_3_1009	DJI_0859	Sorbus aucuparia L.	0	Rosaceae Juss.	n	1
Iz_3_1009	DJI_0859	Tilia cordata Mill.	0	Malvaceae Juss.	n	1
Iz_3_1009	DJI_0859	Ulmus glabra Huds.	0	Ulmaceae Mirb.	n	1
Iz_3_1009	DJI_0860	Acer tataricum L.	0	Sapindaceae Juss.	n	1
Iz_3_1009	DJI_0860	Betula pendula Roth.	0	Betulaceae Gray	n	1
Iz_3_1009	DJI_0860	Crataegus rhipidophylla Gand.	0	Rosaceae Juss.	n	1
Iz_3_1009	DJI_0860	Frangula alnus Mill.	0	Rhamnaceae Juss.	n	1
Iz_3_1009	DJI_0860	Fraxinus pennsylvanica Marshall	0	Oleaceae Hoffmanns. & Link	a	0
Iz_3_1009	DJI_0860	Pinus sylvestris L.	0	Pinaceae Spreng. ex F.Rudolphi	n	1
Iz_3_1009	DJI_0860	Populus × canescens (Aiton) Sm.	0	Salicaceae Mirb.	n	1
Iz_3_1009	DJI_0860	Populus tremula L.	0	Salicaceae Mirb.	n	1
Iz_3_1009	DJI_0860	Populus nigra L.	0	Salicaceae Mirb.	n	1
Iz_3_1009	DJI_0860	Pyrus communis L.	0	Rosaceae Juss.	a	0
Iz_3_1009	DJI_0860	Quercus robur L.	0	Fagaceae Dumort.	n	1
Iz_3_1009	DJI_0860	Robinia pseudoacacia L.	0	Fabaceae Lindl.	a	0
Iz_3_1009	DJI_0860	Rosa sp.	0	Rosaceae Juss.		
Iz_3_1009	DJI_0860	Salix alba L.	0	Salicaceae Mirb.	n	1
Iz_3_1009	DJI_0860	Sambucus nigra L.	0	Viburnaceae Raf.	n	1

Iz_3_1009	DJI_0860	Sorbus aucuparia L.	0	Rosaceae Juss.	n	1
Iz_3_1009	DJI_0860	Tilia cordata Mill.	0	Malvaceae Juss.	n	1
Iz_3_1009	DJI_0860	Ulmus glabra Huds.	0	Ulmaceae Mirb.	n	1
Iz_3_1009	DJI_0861	Acer tataricum L.	0	Sapindaceae Juss.	n	1
Iz_3_1009	DJI_0861	Betula pendula Roth.	0	Betulaceae Gray	n	1
Iz_3_1009	DJI_0861	Crataegus rhipidophylla Gand.	0	Rosaceae Juss.	n	1
Iz_3_1009	DJI_0861	Frangula alnus Mill.	0	Rhamnaceae Juss.	n	1
Iz_3_1009	DJI_0861	Fraxinus pennsylvanica Marshall	0	Oleaceae Hoffmanns. & Link	a	0
Iz_3_1009	DJI_0861	Pinus sylvestris L.	0	Pinaceae Spreng. ex F.Rudolphi	n	1
Iz_3_1009	DJI_0861	Populus × canescens (Aiton) Sm.	0	Salicaceae Mirb.	n	1
Iz_3_1009	DJI_0861	Populus tremula L.	0	Salicaceae Mirb.	n	1
Iz_3_1009	DJI_0861	Populus nigra L.	0	Salicaceae Mirb.	n	1
Iz_3_1009	DJI_0861	Pyrus communis L.	0	Rosaceae Juss.	a	0
Iz_3_1009	DJI_0861	Quercus robur L.	0	Fagaceae Dumort.	n	1
Iz_3_1009	DJI_0861	Robinia pseudoacacia L.	0	Fabaceae Lindl.	a	0
Iz_3_1009	DJI_0861	Rosa sp.	0	Rosaceae Juss.		
Iz_3_1009	DJI_0861	Salix alba L.	0	Salicaceae Mirb.	n	1
Iz_3_1009	DJI_0861	Sambucus nigra L.	0	Viburnaceae Raf.	n	1
Iz_3_1009	DJI_0861	Sorbus aucuparia L.	0	Rosaceae Juss.	n	1
Iz_3_1009	DJI_0861	Tilia cordata Mill.	0	Malvaceae Juss.	n	1
Iz_3_1009	DJI_0861	Ulmus glabra Huds.	0	Ulmaceae Mirb.	n	1
Iz_3_1009	DJI_0862	Acer tataricum L.	0	Sapindaceae Juss.	n	1
Iz_3_1009	DJI_0862	Betula pendula Roth.	0	Betulaceae Gray	n	1
Iz_3_1009	DJI_0862	Crataegus rhipidophylla Gand.	0	Rosaceae Juss.	n	1
Iz_3_1009	DJI_0862	Frangula alnus Mill.	0	Rhamnaceae Juss.	n	1
Iz_3_1009	DJI_0862	Fraxinus pennsylvanica Marshall	0	Oleaceae Hoffmanns. & Link	a	0
Iz_3_1009	DJI_0862	Pinus sylvestris L.	0	Pinaceae Spreng. ex F.Rudolphi	n	1
Iz_3_1009	DJI_0862	Populus × canescens (Aiton) Sm.	0	Salicaceae Mirb.	n	1
Iz_3_1009	DJI_0862	Populus tremula L.	0	Salicaceae Mirb.	n	1
Iz_3_1009	DJI_0862	Populus nigra L.	0	Salicaceae Mirb.	n	1
Iz_3_1009	DJI_0862	Pyrus communis L.	0	Rosaceae Juss.	a	0
Iz_3_1009	DJI_0862	Quercus robur L.	0	Fagaceae Dumort.	n	1
Iz_3_1009	DJI_0862	Robinia pseudoacacia L.	0	Fabaceae Lindl.	a	0
Iz_3_1009	DJI_0862	Rosa sp.	0	Rosaceae Juss.		
Iz_3_1009	DJI_0862	Salix alba L.	0	Salicaceae Mirb.	n	1
Iz_3_1009	DJI_0862	Sambucus nigra L.	0	Viburnaceae Raf.	n	1
Iz_3_1009	DJI_0862	Sorbus aucuparia L.	0	Rosaceae Juss.	n	1
Iz_3_1009	DJI_0862	Tilia cordata Mill.	0	Malvaceae Juss.	n	1
Iz_3_1009	DJI_0862	Ulmus glabra Huds.	0	Ulmaceae Mirb.	n	1
Iz_3_1009	DJI_0863	Acer tataricum L.	0	Sapindaceae Juss.	n	1
Iz_3_1009	DJI_0863	Betula pendula Roth.	0	Betulaceae Gray	n	1
Iz_3_1009	DJI_0863	Crataegus rhipidophylla Gand.	0	Rosaceae Juss.	n	1
Iz_3_1009	DJI_0863	Frangula alnus Mill.	0	Rhamnaceae Juss.	n	1
Iz_3_1009	DJI_0863	Fraxinus pennsylvanica Marshall	0	Oleaceae Hoffmanns. & Link	a	0
Iz_3_1009	DJI_0863	Pinus sylvestris L.	0	Pinaceae Spreng. ex F.Rudolphi	n	1
Iz_3_1009	DJI_0863	Populus × canescens (Aiton) Sm.	0	Salicaceae Mirb.	n	1
Iz_3_1009	DJI_0863	Populus tremula L.	0	Salicaceae Mirb.	n	1
Iz_3_1009	DJI_0863	Populus nigra L.	0	Salicaceae Mirb.	n	1
Iz_3_1009	DJI_0863	Pyrus communis L.	0	Rosaceae Juss.	a	0
Iz_3_1009	DJI_0863	Quercus robur L.	0	Fagaceae Dumort.	n	1
Iz_3_1009	DJI_0863	Robinia pseudoacacia L.	0	Fabaceae Lindl.	a	0
Iz_3_1009	DJI_0863	Rosa sp.	0	Rosaceae Juss.		
Iz_3_1009	DJI_0863	Salix alba L.	0	Salicaceae Mirb.	n	1
Iz_3_1009	DJI_0863	Sambucus nigra L.	0	Viburnaceae Raf.	n	1
Iz_3_1009	DJI_0863	Sorbus aucuparia L.	0	Rosaceae Juss.	n	1
Iz_3_1009	DJI_0863	Tilia cordata Mill.	0	Malvaceae Juss.	n	1
Iz_3_1009	DJI_0863	Ulmus glabra Huds.	0	Ulmaceae Mirb.	n	1
Iz_3_1009	DJI_0864	Acer tataricum L.	0	Sapindaceae Juss.	n	1
Iz_3_1009	DJI_0864	Betula pendula Roth.	0	Betulaceae Gray	n	1
Iz_3_1009	DJI_0864	Crataegus rhipidophylla Gand.	0	Rosaceae Juss.	n	1
Iz_3_1009	DJI_0864	Frangula alnus Mill.	0	Rhamnaceae Juss.	n	1
Iz_3_1009	DJI_0864	Fraxinus pennsylvanica Marshall	0	Oleaceae Hoffmanns. & Link	a	0
Iz_3_1009	DJI_0864	Pinus sylvestris L.	0	Pinaceae Spreng. ex F.Rudolphi	n	1
Iz_3_1009	DJI_0864	Populus × canescens (Aiton) Sm.	0	Salicaceae Mirb.	n	1

Iz_3_1009	DJI_0864	Populus tremula L.	0	Salicaceae Mirb.	n	1
Iz_3_1009	DJI_0864	Populus nigra L.	0	Salicaceae Mirb.	n	1
Iz_3_1009	DJI_0864	Pyrus communis L.	0	Rosaceae Juss.	a	0
Iz_3_1009	DJI_0864	Quercus robur L.	0	Fagaceae Dumort.	n	1
Iz_3_1009	DJI_0864	Robinia pseudoacacia L.	0	Fabaceae Lindl.	a	0
Iz_3_1009	DJI_0864	Rosa sp.	0	Rosaceae Juss.		
Iz_3_1009	DJI_0864	Salix alba L.	0	Salicaceae Mirb.	n	1
Iz_3_1009	DJI_0864	Sambucus nigra L.	0	Viburnaceae Raf.	n	1
Iz_3_1009	DJI_0864	Sorbus aucuparia L.	0	Rosaceae Juss.	n	1
Iz_3_1009	DJI_0864	Tilia cordata Mill.	0	Malvaceae Juss.	n	1
Iz_3_1009	DJI_0864	Ulmus glabra Huds.	0	Ulmaceae Mirb.	n	1
Iz_3_1009	DJI_0865	Acer tataricum L.	0	Sapindaceae Juss.	n	1
Iz_3_1009	DJI_0865	Betula pendula Roth.	0	Betulaceae Gray	n	1
Iz_3_1009	DJI_0865	Crataegus rhipidophylla Gand.	0	Rosaceae Juss.	n	1
Iz_3_1009	DJI_0865	Frangula alnus Mill.	0	Rhamnaceae Juss.	n	1
Iz_3_1009	DJI_0865	Fraxinus pennsylvanica Marshall	0	Oleaceae Hoffmanns. & Link	a	0
Iz_3_1009	DJI_0865	Pinus sylvestris L.	0	Pinaceae Spreng. ex F.Rudolphi	n	1
Iz_3_1009	DJI_0865	Populus × canescens (Aiton) Sm.	0	Salicaceae Mirb.	n	1
Iz_3_1009	DJI_0865	Populus tremula L.	0	Salicaceae Mirb.	n	1
Iz_3_1009	DJI_0865	Populus nigra L.	0	Salicaceae Mirb.	n	1
Iz_3_1009	DJI_0865	Pyrus communis L.	0	Rosaceae Juss.	a	0
Iz_3_1009	DJI_0865	Quercus robur L.	0	Fagaceae Dumort.	n	1
Iz_3_1009	DJI_0865	Robinia pseudoacacia L.	0	Fabaceae Lindl.	a	0
Iz_3_1009	DJI_0865	Rosa sp.	0	Rosaceae Juss.		
Iz_3_1009	DJI_0865	Salix alba L.	0	Salicaceae Mirb.	n	1
Iz_3_1009	DJI_0865	Sambucus nigra L.	0	Viburnaceae Raf.	n	1
Iz_3_1009	DJI_0865	Sorbus aucuparia L.	0	Rosaceae Juss.	n	1
Iz_3_1009	DJI_0865	Tilia cordata Mill.	0	Malvaceae Juss.	n	1
Iz_3_1009	DJI_0865	Ulmus glabra Huds.	0	Ulmaceae Mirb.	n	1
Iz_3_1009	DJI_0866	Acer tataricum L.	0	Sapindaceae Juss.	n	1
Iz_3_1009	DJI_0866	Betula pendula Roth.	0	Betulaceae Gray	n	1
Iz_3_1009	DJI_0866	Crataegus rhipidophylla Gand.	0	Rosaceae Juss.	n	1
Iz_3_1009	DJI_0866	Frangula alnus Mill.	0	Rhamnaceae Juss.	n	1
Iz_3_1009	DJI_0866	Fraxinus pennsylvanica Marshall	0	Oleaceae Hoffmanns. & Link	a	0
Iz_3_1009	DJI_0866	Pinus sylvestris L.	0	Pinaceae Spreng. ex F.Rudolphi	n	1
Iz_3_1009	DJI_0866	Populus × canescens (Aiton) Sm.	0	Salicaceae Mirb.	n	1
Iz_3_1009	DJI_0866	Populus tremula L.	0	Salicaceae Mirb.	n	1
Iz_3_1009	DJI_0866	Populus nigra L.	0	Salicaceae Mirb.	n	1
Iz_3_1009	DJI_0866	Pyrus communis L.	0	Rosaceae Juss.	a	0
Iz_3_1009	DJI_0866	Quercus robur L.	0	Fagaceae Dumort.	n	1
Iz_3_1009	DJI_0866	Robinia pseudoacacia L.	0	Fabaceae Lindl.	a	0
Iz_3_1009	DJI_0866	Rosa sp.	0	Rosaceae Juss.		
Iz_3_1009	DJI_0866	Salix alba L.	0	Salicaceae Mirb.	n	1
Iz_3_1009	DJI_0866	Sambucus nigra L.	0	Viburnaceae Raf.	n	1
Iz_3_1009	DJI_0866	Sorbus aucuparia L.	0	Rosaceae Juss.	n	1
Iz_3_1009	DJI_0866	Tilia cordata Mill.	0	Malvaceae Juss.	n	1
Iz_3_1009	DJI_0866	Ulmus glabra Huds.	0	Ulmaceae Mirb.	n	1
Iz_3_1009	DJI_0867	Acer tataricum L.	0	Sapindaceae Juss.	n	1
Iz_3_1009	DJI_0867	Betula pendula Roth.	0	Betulaceae Gray	n	1
Iz_3_1009	DJI_0867	Crataegus rhipidophylla Gand.	0	Rosaceae Juss.	n	1
Iz_3_1009	DJI_0867	Frangula alnus Mill.	0	Rhamnaceae Juss.	n	1
Iz_3_1009	DJI_0867	Fraxinus pennsylvanica Marshall	0	Oleaceae Hoffmanns. & Link	a	0
Iz_3_1009	DJI_0867	Pinus sylvestris L.	0	Pinaceae Spreng. ex F.Rudolphi	n	1
Iz_3_1009	DJI_0867	Populus × canescens (Aiton) Sm.	0	Salicaceae Mirb.	n	1
Iz_3_1009	DJI_0867	Populus tremula L.	0	Salicaceae Mirb.	n	1
Iz_3_1009	DJI_0867	Populus nigra L.	0	Salicaceae Mirb.	n	1
Iz_3_1009	DJI_0867	Pyrus communis L.	0	Rosaceae Juss.	a	0
Iz_3_1009	DJI_0867	Quercus robur L.	0	Fagaceae Dumort.	n	1
Iz_3_1009	DJI_0867	Robinia pseudoacacia L.	0	Fabaceae Lindl.	a	0
Iz_3_1009	DJI_0867	Rosa sp.	0	Rosaceae Juss.		
Iz_3_1009	DJI_0867	Salix alba L.	0	Salicaceae Mirb.	n	1
Iz_3_1009	DJI_0867	Sambucus nigra L.	0	Viburnaceae Raf.	n	1
Iz_3_1009	DJI_0867	Sorbus aucuparia L.	0	Rosaceae Juss.	n	1
Iz_3_1009	DJI_0867	Tilia cordata Mill.	0	Malvaceae Juss.	n	1

Iz_3_1009	DJI_0867	Ulmus glabra Huds.	0	Ulmaceae Mirb.	n	1
Iz_3_1009	DJI_0868	Acer tataricum L.	0	Sapindaceae Juss.	n	1
Iz_3_1009	DJI_0868	Betula pendula Roth.	0	Betulaceae Gray	n	1
Iz_3_1009	DJI_0868	Crataegus rhipidophylla Gand.	0	Rosaceae Juss.	n	1
Iz_3_1009	DJI_0868	Frangula alnus Mill.	0	Rhamnaceae Juss.	n	1
Iz_3_1009	DJI_0868	Fraxinus pennsylvanica Marshall	0	Oleaceae Hoffmanns. & Link	a	0
Iz_3_1009	DJI_0868	Pinus sylvestris L.	0	Pinaceae Spreng. ex F.Rudolphi	n	1
Iz_3_1009	DJI_0868	Populus × canescens (Aiton) Sm.	0	Salicaceae Mirb.	n	1
Iz_3_1009	DJI_0868	Populus tremula L.	0	Salicaceae Mirb.	n	1
Iz_3_1009	DJI_0868	Populus nigra L.	0	Salicaceae Mirb.	n	1
Iz_3_1009	DJI_0868	Pyrus communis L.	0	Rosaceae Juss.	a	0
Iz_3_1009	DJI_0868	Quercus robur L.	2	Fagaceae Dumort.	n	1
Iz_3_1009	DJI_0868	Robinia pseudoacacia L.	0	Fabaceae Lindl.	a	0
Iz_3_1009	DJI_0868	Rosa sp.	0	Rosaceae Juss.		
Iz_3_1009	DJI_0868	Salix alba L.	0	Salicaceae Mirb.	n	1
Iz_3_1009	DJI_0868	Sambucus nigra L.	0	Viburnaceae Raf.	n	1
Iz_3_1009	DJI_0868	Sorbus aucuparia L.	0	Rosaceae Juss.	n	1
Iz_3_1009	DJI_0868	Tilia cordata Mill.	0	Malvaceae Juss.	n	1
Iz_3_1009	DJI_0868	Ulmus glabra Huds.	0	Ulmaceae Mirb.	n	1
Iz_3_1009	DJI_0869	Acer tataricum L.	0	Sapindaceae Juss.	n	1
Iz_3_1009	DJI_0869	Betula pendula Roth.	0	Betulaceae Gray	n	1
Iz_3_1009	DJI_0869	Crataegus rhipidophylla Gand.	0	Rosaceae Juss.	n	1
Iz_3_1009	DJI_0869	Frangula alnus Mill.	0	Rhamnaceae Juss.	n	1
Iz_3_1009	DJI_0869	Fraxinus pennsylvanica Marshall	0	Oleaceae Hoffmanns. & Link	a	0
Iz_3_1009	DJI_0869	Pinus sylvestris L.	0	Pinaceae Spreng. ex F.Rudolphi	n	1
Iz_3_1009	DJI_0869	Populus × canescens (Aiton) Sm.	0	Salicaceae Mirb.	n	1
Iz_3_1009	DJI_0869	Populus tremula L.	0	Salicaceae Mirb.	n	1
Iz_3_1009	DJI_0869	Populus nigra L.	0	Salicaceae Mirb.	n	1
Iz_3_1009	DJI_0869	Pyrus communis L.	0	Rosaceae Juss.	a	0
Iz_3_1009	DJI_0869	Quercus robur L.	0	Fagaceae Dumort.	n	1
Iz_3_1009	DJI_0869	Robinia pseudoacacia L.	0	Fabaceae Lindl.	a	0
Iz_3_1009	DJI_0869	Rosa sp.	0	Rosaceae Juss.		
Iz_3_1009	DJI_0869	Salix alba L.	0	Salicaceae Mirb.	n	1
Iz_3_1009	DJI_0869	Sambucus nigra L.	0	Viburnaceae Raf.	n	1
Iz_3_1009	DJI_0869	Sorbus aucuparia L.	0	Rosaceae Juss.	n	1
Iz_3_1009	DJI_0869	Tilia cordata Mill.	0	Malvaceae Juss.	n	1
Iz_3_1009	DJI_0869	Ulmus glabra Huds.	0	Ulmaceae Mirb.	n	1
Iz_3_1009	DJI_0870	Acer tataricum L.	0	Sapindaceae Juss.	n	1
Iz_3_1009	DJI_0870	Betula pendula Roth.	0	Betulaceae Gray	n	1
Iz_3_1009	DJI_0870	Crataegus rhipidophylla Gand.	0	Rosaceae Juss.	n	1
Iz_3_1009	DJI_0870	Frangula alnus Mill.	0	Rhamnaceae Juss.	n	1
Iz_3_1009	DJI_0870	Fraxinus pennsylvanica Marshall	0	Oleaceae Hoffmanns. & Link	a	0
Iz_3_1009	DJI_0870	Pinus sylvestris L.	0	Pinaceae Spreng. ex F.Rudolphi	n	1
Iz_3_1009	DJI_0870	Populus × canescens (Aiton) Sm.	0	Salicaceae Mirb.	n	1
Iz_3_1009	DJI_0870	Populus tremula L.	0	Salicaceae Mirb.	n	1
Iz_3_1009	DJI_0870	Populus nigra L.	0	Salicaceae Mirb.	n	1
Iz_3_1009	DJI_0870	Pyrus communis L.	0	Rosaceae Juss.	a	0
Iz_3_1009	DJI_0870	Quercus robur L.	3	Fagaceae Dumort.	n	1
Iz_3_1009	DJI_0870	Robinia pseudoacacia L.	0	Fabaceae Lindl.	a	0
Iz_3_1009	DJI_0870	Rosa sp.	0	Rosaceae Juss.		
Iz_3_1009	DJI_0870	Salix alba L.	0	Salicaceae Mirb.	n	1
Iz_3_1009	DJI_0870	Sambucus nigra L.	0	Viburnaceae Raf.	n	1
Iz_3_1009	DJI_0870	Sorbus aucuparia L.	0	Rosaceae Juss.	n	1
Iz_3_1009	DJI_0870	Tilia cordata Mill.	0	Malvaceae Juss.	n	1
Iz_3_1009	DJI_0870	Ulmus glabra Huds.	0	Ulmaceae Mirb.	n	1
Iz_3_1009	DJI_0871	Acer tataricum L.	0	Sapindaceae Juss.	n	1
Iz_3_1009	DJI_0871	Betula pendula Roth.	0	Betulaceae Gray	n	1
Iz_3_1009	DJI_0871	Crataegus rhipidophylla Gand.	0	Rosaceae Juss.	n	1
Iz_3_1009	DJI_0871	Frangula alnus Mill.	0	Rhamnaceae Juss.	n	1
Iz_3_1009	DJI_0871	Fraxinus pennsylvanica Marshall	0	Oleaceae Hoffmanns. & Link	a	0
Iz_3_1009	DJI_0871	Pinus sylvestris L.	0	Pinaceae Spreng. ex F.Rudolphi	n	1
Iz_3_1009	DJI_0871	Populus × canescens (Aiton) Sm.	0	Salicaceae Mirb.	n	1
Iz_3_1009	DJI_0871	Populus tremula L.	0	Salicaceae Mirb.	n	1
Iz_3_1009	DJI_0871	Populus nigra L.	0	Salicaceae Mirb.	n	1

Iz_3_1009	DJI_0871	Pyrus communis L.	0	Rosaceae Juss.	a	0
Iz_3_1009	DJI_0871	Quercus robur L.	7	Fagaceae Dumort.	n	1
Iz_3_1009	DJI_0871	Robinia pseudoacacia L.	0	Fabaceae Lindl.	a	0
Iz_3_1009	DJI_0871	Rosa sp.	0	Rosaceae Juss.		
Iz_3_1009	DJI_0871	Salix alba L.	0	Salicaceae Mirb.	n	1
Iz_3_1009	DJI_0871	Sambucus nigra L.	0	Viburnaceae Raf.	n	1
Iz_3_1009	DJI_0871	Sorbus aucuparia L.	0	Rosaceae Juss.	n	1
Iz_3_1009	DJI_0871	Tilia cordata Mill.	0	Malvaceae Juss.	n	1
Iz_3_1009	DJI_0871	Ulmus glabra Huds.	0	Ulmaceae Mirb.	n	1
Iz_3_1009	DJI_0874	Acer tataricum L.	0	Sapindaceae Juss.	n	1
Iz_3_1009	DJI_0874	Betula pendula Roth.	0	Betulaceae Gray	n	1
Iz_3_1009	DJI_0874	Crataegus rhipidophylla Gand.	0	Rosaceae Juss.	n	1
Iz_3_1009	DJI_0874	Frangula alnus Mill.	0	Rhamnaceae Juss.	n	1
Iz_3_1009	DJI_0874	Fraxinus pennsylvanica Marshall	0	Oleaceae Hoffmanns. & Link	a	0
Iz_3_1009	DJI_0874	Pinus sylvestris L.	0	Pinaceae Spreng. ex F.Rudolphi	n	1
Iz_3_1009	DJI_0874	Populus × canescens (Aiton) Sm.	0	Salicaceae Mirb.	n	1
Iz_3_1009	DJI_0874	Populus tremula L.	0	Salicaceae Mirb.	n	1
Iz_3_1009	DJI_0874	Populus nigra L.	0	Salicaceae Mirb.	n	1
Iz_3_1009	DJI_0874	Pyrus communis L.	0	Rosaceae Juss.	a	0
Iz_3_1009	DJI_0874	Quercus robur L.	0	Fagaceae Dumort.	n	1
Iz_3_1009	DJI_0874	Robinia pseudoacacia L.	0	Fabaceae Lindl.	a	0
Iz_3_1009	DJI_0874	Rosa sp.	0	Rosaceae Juss.		
Iz_3_1009	DJI_0874	Salix alba L.	0	Salicaceae Mirb.	n	1
Iz_3_1009	DJI_0874	Sambucus nigra L.	0	Viburnaceae Raf.	n	1
Iz_3_1009	DJI_0874	Sorbus aucuparia L.	0	Rosaceae Juss.	n	1
Iz_3_1009	DJI_0874	Tilia cordata Mill.	0	Malvaceae Juss.	n	1
Iz_3_1009	DJI_0874	Ulmus glabra Huds.	0	Ulmaceae Mirb.	n	1
Iz_3_1009	DJI_0875	Acer tataricum L.	0	Sapindaceae Juss.	n	1
Iz_3_1009	DJI_0875	Betula pendula Roth.	0	Betulaceae Gray	n	1
Iz_3_1009	DJI_0875	Crataegus rhipidophylla Gand.	0	Rosaceae Juss.	n	1
Iz_3_1009	DJI_0875	Frangula alnus Mill.	0	Rhamnaceae Juss.	n	1
Iz_3_1009	DJI_0875	Fraxinus pennsylvanica Marshall	0	Oleaceae Hoffmanns. & Link	a	0
Iz_3_1009	DJI_0875	Pinus sylvestris L.	0	Pinaceae Spreng. ex F.Rudolphi	n	1
Iz_3_1009	DJI_0875	Populus × canescens (Aiton) Sm.	0	Salicaceae Mirb.	n	1
Iz_3_1009	DJI_0875	Populus tremula L.	1	Salicaceae Mirb.	n	1
Iz_3_1009	DJI_0875	Populus nigra L.	0	Salicaceae Mirb.	n	1
Iz_3_1009	DJI_0875	Pyrus communis L.	0	Rosaceae Juss.	a	0
Iz_3_1009	DJI_0875	Quercus robur L.	0	Fagaceae Dumort.	n	1
Iz_3_1009	DJI_0875	Robinia pseudoacacia L.	0	Fabaceae Lindl.	a	0
Iz_3_1009	DJI_0875	Rosa sp.	0	Rosaceae Juss.		
Iz_3_1009	DJI_0875	Salix alba L.	0	Salicaceae Mirb.	n	1
Iz_3_1009	DJI_0875	Sambucus nigra L.	0	Viburnaceae Raf.	n	1
Iz_3_1009	DJI_0875	Sorbus aucuparia L.	0	Rosaceae Juss.	n	1
Iz_3_1009	DJI_0875	Tilia cordata Mill.	0	Malvaceae Juss.	n	1
Iz_3_1009	DJI_0875	Ulmus glabra Huds.	0	Ulmaceae Mirb.	n	1
Iz_3_1009	DJI_0876	Acer tataricum L.	2	Sapindaceae Juss.	n	1
Iz_3_1009	DJI_0876	Betula pendula Roth.	0	Betulaceae Gray	n	1
Iz_3_1009	DJI_0876	Crataegus rhipidophylla Gand.	0	Rosaceae Juss.	n	1
Iz_3_1009	DJI_0876	Frangula alnus Mill.	0	Rhamnaceae Juss.	n	1
Iz_3_1009	DJI_0876	Fraxinus pennsylvanica Marshall	0	Oleaceae Hoffmanns. & Link	a	0
Iz_3_1009	DJI_0876	Pinus sylvestris L.	0	Pinaceae Spreng. ex F.Rudolphi	n	1
Iz_3_1009	DJI_0876	Populus × canescens (Aiton) Sm.	0	Salicaceae Mirb.	n	1
Iz_3_1009	DJI_0876	Populus tremula L.	0	Salicaceae Mirb.	n	1
Iz_3_1009	DJI_0876	Populus nigra L.	0	Salicaceae Mirb.	n	1
Iz_3_1009	DJI_0876	Pyrus communis L.	0	Rosaceae Juss.	a	0
Iz_3_1009	DJI_0876	Quercus robur L.	0	Fagaceae Dumort.	n	1
Iz_3_1009	DJI_0876	Robinia pseudoacacia L.	0	Fabaceae Lindl.	a	0
Iz_3_1009	DJI_0876	Rosa sp.	0	Rosaceae Juss.		
Iz_3_1009	DJI_0876	Salix alba L.	0	Salicaceae Mirb.	n	1
Iz_3_1009	DJI_0876	Sambucus nigra L.	0	Viburnaceae Raf.	n	1
Iz_3_1009	DJI_0876	Sorbus aucuparia L.	0	Rosaceae Juss.	n	1
Iz_3_1009	DJI_0876	Tilia cordata Mill.	0	Malvaceae Juss.	n	1
Iz_3_1009	DJI_0876	Ulmus glabra Huds.	0	Ulmaceae Mirb.	n	1
Iz_3_1009	DJI_0880	Acer tataricum L.	5	Sapindaceae Juss.	n	1

Iz_3_1009	DJI_0880	Betula pendula Roth.	0	Betulaceae Gray	n	1
Iz_3_1009	DJI_0880	Crataegus rhipidophylla Gand.	0	Rosaceae Juss.	n	1
Iz_3_1009	DJI_0880	Frangula alnus Mill.	0	Rhamnaceae Juss.	n	1
Iz_3_1009	DJI_0880	Fraxinus pennsylvanica Marshall	0	Oleaceae Hoffmanns. & Link	a	0
Iz_3_1009	DJI_0880	Pinus sylvestris L.	0	Pinaceae Spreng. ex F.Rudolphi	n	1
Iz_3_1009	DJI_0880	Populus × canescens (Aiton) Sm.	0	Salicaceae Mirb.	n	1
Iz_3_1009	DJI_0880	Populus tremula L.	0	Salicaceae Mirb.	n	1
Iz_3_1009	DJI_0880	Populus nigra L.	0	Salicaceae Mirb.	n	1
Iz_3_1009	DJI_0880	Pyrus communis L.	0	Rosaceae Juss.	a	0
Iz_3_1009	DJI_0880	Quercus robur L.	0	Fagaceae Dumort.	n	1
Iz_3_1009	DJI_0880	Robinia pseudoacacia L.	0	Fabaceae Lindl.	a	0
Iz_3_1009	DJI_0880	Rosa sp.	0	Rosaceae Juss.		
Iz_3_1009	DJI_0880	Salix alba L.	0	Salicaceae Mirb.	n	1
Iz_3_1009	DJI_0880	Sambucus nigra L.	0	Viburnaceae Raf.	n	1
Iz_3_1009	DJI_0880	Sorbus aucuparia L.	0	Rosaceae Juss.	n	1
Iz_3_1009	DJI_0880	Tilia cordata Mill.	0	Malvaceae Juss.	n	1
Iz_3_1009	DJI_0880	Ulmus glabra Huds.	0	Ulmaceae Mirb.	n	1
Iz_3_1009	DJI_0887	Acer tataricum L.	0	Sapindaceae Juss.	n	1
Iz_3_1009	DJI_0887	Betula pendula Roth.	0	Betulaceae Gray	n	1
Iz_3_1009	DJI_0887	Crataegus rhipidophylla Gand.	0	Rosaceae Juss.	n	1
Iz_3_1009	DJI_0887	Frangula alnus Mill.	0	Rhamnaceae Juss.	n	1
Iz_3_1009	DJI_0887	Fraxinus pennsylvanica Marshall	0	Oleaceae Hoffmanns. & Link	a	0
Iz_3_1009	DJI_0887	Pinus sylvestris L.	0	Pinaceae Spreng. ex F.Rudolphi	n	1
Iz_3_1009	DJI_0887	Populus × canescens (Aiton) Sm.	0	Salicaceae Mirb.	n	1
Iz_3_1009	DJI_0887	Populus tremula L.	0	Salicaceae Mirb.	n	1
Iz_3_1009	DJI_0887	Populus nigra L.	0	Salicaceae Mirb.	n	1
Iz_3_1009	DJI_0887	Pyrus communis L.	0	Rosaceae Juss.	a	0
Iz_3_1009	DJI_0887	Quercus robur L.	0	Fagaceae Dumort.	n	1
Iz_3_1009	DJI_0887	Robinia pseudoacacia L.	0	Fabaceae Lindl.	a	0
Iz_3_1009	DJI_0887	Rosa sp.	0	Rosaceae Juss.		
Iz_3_1009	DJI_0887	Salix alba L.	0	Salicaceae Mirb.	n	1
Iz_3_1009	DJI_0887	Sambucus nigra L.	0	Viburnaceae Raf.	n	1
Iz_3_1009	DJI_0887	Sorbus aucuparia L.	0	Rosaceae Juss.	n	1
Iz_3_1009	DJI_0887	Tilia cordata Mill.	0	Malvaceae Juss.	n	1
Iz_3_1009	DJI_0887	Ulmus glabra Huds.	0	Ulmaceae Mirb.	n	1
Iz_3_1009	DJI_0888	Acer tataricum L.	0	Sapindaceae Juss.	n	1
Iz_3_1009	DJI_0888	Betula pendula Roth.	0	Betulaceae Gray	n	1
Iz_3_1009	DJI_0888	Crataegus rhipidophylla Gand.	0	Rosaceae Juss.	n	1
Iz_3_1009	DJI_0888	Frangula alnus Mill.	0	Rhamnaceae Juss.	n	1
Iz_3_1009	DJI_0888	Fraxinus pennsylvanica Marshall	0	Oleaceae Hoffmanns. & Link	a	0
Iz_3_1009	DJI_0888	Pinus sylvestris L.	0	Pinaceae Spreng. ex F.Rudolphi	n	1
Iz_3_1009	DJI_0888	Populus × canescens (Aiton) Sm.	0	Salicaceae Mirb.	n	1
Iz_3_1009	DJI_0888	Populus tremula L.	0	Salicaceae Mirb.	n	1
Iz_3_1009	DJI_0888	Populus nigra L.	0	Salicaceae Mirb.	n	1
Iz_3_1009	DJI_0888	Pyrus communis L.	0	Rosaceae Juss.	a	0
Iz_3_1009	DJI_0888	Quercus robur L.	12	Fagaceae Dumort.	n	1
Iz_3_1009	DJI_0888	Robinia pseudoacacia L.	0	Fabaceae Lindl.	a	0
Iz_3_1009	DJI_0888	Rosa sp.	0	Rosaceae Juss.		
Iz_3_1009	DJI_0888	Salix alba L.	0	Salicaceae Mirb.	n	1
Iz_3_1009	DJI_0888	Sambucus nigra L.	0	Viburnaceae Raf.	n	1
Iz_3_1009	DJI_0888	Sorbus aucuparia L.	0	Rosaceae Juss.	n	1
Iz_3_1009	DJI_0888	Tilia cordata Mill.	0	Malvaceae Juss.	n	1
Iz_3_1009	DJI_0888	Ulmus glabra Huds.	0	Ulmaceae Mirb.	n	1
Iz_3_1009	DJI_0893	Acer tataricum L.	0	Sapindaceae Juss.	n	1
Iz_3_1009	DJI_0893	Betula pendula Roth.	0	Betulaceae Gray	n	1
Iz_3_1009	DJI_0893	Crataegus rhipidophylla Gand.	0	Rosaceae Juss.	n	1
Iz_3_1009	DJI_0893	Frangula alnus Mill.	0	Rhamnaceae Juss.	n	1
Iz_3_1009	DJI_0893	Fraxinus pennsylvanica Marshall	0	Oleaceae Hoffmanns. & Link	a	0
Iz_3_1009	DJI_0893	Pinus sylvestris L.	0	Pinaceae Spreng. ex F.Rudolphi	n	1
Iz_3_1009	DJI_0893	Populus × canescens (Aiton) Sm.	0	Salicaceae Mirb.	n	1
Iz_3_1009	DJI_0893	Populus tremula L.	0	Salicaceae Mirb.	n	1
Iz_3_1009	DJI_0893	Populus nigra L.	0	Salicaceae Mirb.	n	1
Iz_3_1009	DJI_0893	Pyrus communis L.	0	Rosaceae Juss.	a	0
Iz_3_1009	DJI_0893	Quercus robur L.	0	Fagaceae Dumort.	n	1

Iz_3_1009	DJI_0893	Robinia pseudoacacia L.	0	Fabaceae Lindl.	a	0
Iz_3_1009	DJI_0893	Rosa sp.	0	Rosaceae Juss.		
Iz_3_1009	DJI_0893	Salix alba L.	0	Salicaceae Mirb.	n	1
Iz_3_1009	DJI_0893	Sambucus nigra L.	0	Viburnaceae Raf.	n	1
Iz_3_1009	DJI_0893	Sorbus aucuparia L.	0	Rosaceae Juss.	n	1
Iz_3_1009	DJI_0893	Tilia cordata Mill.	0	Malvaceae Juss.	n	1
Iz_3_1009	DJI_0893	Ulmus glabra Huds.	0	Ulmaceae Mirb.	n	1
Iz_3_1009	DJI_0895	Acer tataricum L.	0	Sapindaceae Juss.	n	1
Iz_3_1009	DJI_0895	Betula pendula Roth.	0	Betulaceae Gray	n	1
Iz_3_1009	DJI_0895	Crataegus rhipidophylla Gand.	0	Rosaceae Juss.	n	1
Iz_3_1009	DJI_0895	Frangula alnus Mill.	0	Rhamnaceae Juss.	n	1
Iz_3_1009	DJI_0895	Fraxinus pennsylvanica Marshall	0	Oleaceae Hoffmanns. & Link	a	0
Iz_3_1009	DJI_0895	Pinus sylvestris L.	0	Pinaceae Spreng. ex F.Rudolphi	n	1
Iz_3_1009	DJI_0895	Populus × canescens (Aiton) Sm.	0	Salicaceae Mirb.	n	1
Iz_3_1009	DJI_0895	Populus tremula L.	0	Salicaceae Mirb.	n	1
Iz_3_1009	DJI_0895	Populus nigra L.	0	Salicaceae Mirb.	n	1
Iz_3_1009	DJI_0895	Pyrus communis L.	0	Rosaceae Juss.	a	0
Iz_3_1009	DJI_0895	Quercus robur L.	0	Fagaceae Dumort.	n	1
Iz_3_1009	DJI_0895	Robinia pseudoacacia L.	0	Fabaceae Lindl.	a	0
Iz_3_1009	DJI_0895	Rosa sp.	0	Rosaceae Juss.		
Iz_3_1009	DJI_0895	Salix alba L.	0	Salicaceae Mirb.	n	1
Iz_3_1009	DJI_0895	Sambucus nigra L.	0	Viburnaceae Raf.	n	1
Iz_3_1009	DJI_0895	Sorbus aucuparia L.	0	Rosaceae Juss.	n	1
Iz_3_1009	DJI_0895	Tilia cordata Mill.	0	Malvaceae Juss.	n	1
Iz_3_1009	DJI_0895	Ulmus glabra Huds.	0	Ulmaceae Mirb.	n	1
Iz_3_1009	DJI_0896	Acer tataricum L.	0	Sapindaceae Juss.	n	1
Iz_3_1009	DJI_0896	Betula pendula Roth.	0	Betulaceae Gray	n	1
Iz_3_1009	DJI_0896	Crataegus rhipidophylla Gand.	0	Rosaceae Juss.	n	1
Iz_3_1009	DJI_0896	Frangula alnus Mill.	0	Rhamnaceae Juss.	n	1
Iz_3_1009	DJI_0896	Fraxinus pennsylvanica Marshall	0	Oleaceae Hoffmanns. & Link	a	0
Iz_3_1009	DJI_0896	Pinus sylvestris L.	0	Pinaceae Spreng. ex F.Rudolphi	n	1
Iz_3_1009	DJI_0896	Populus × canescens (Aiton) Sm.	0	Salicaceae Mirb.	n	1
Iz_3_1009	DJI_0896	Populus tremula L.	0	Salicaceae Mirb.	n	1
Iz_3_1009	DJI_0896	Populus nigra L.	0	Salicaceae Mirb.	n	1
Iz_3_1009	DJI_0896	Pyrus communis L.	0	Rosaceae Juss.	a	0
Iz_3_1009	DJI_0896	Quercus robur L.	3	Fagaceae Dumort.	n	1
Iz_3_1009	DJI_0896	Robinia pseudoacacia L.	0	Fabaceae Lindl.	a	0
Iz_3_1009	DJI_0896	Rosa sp.	0	Rosaceae Juss.		
Iz_3_1009	DJI_0896	Salix alba L.	0	Salicaceae Mirb.	n	1
Iz_3_1009	DJI_0896	Sambucus nigra L.	0	Viburnaceae Raf.	n	1
Iz_3_1009	DJI_0896	Sorbus aucuparia L.	0	Rosaceae Juss.	n	1
Iz_3_1009	DJI_0896	Tilia cordata Mill.	0	Malvaceae Juss.	n	1
Iz_3_1009	DJI_0896	Ulmus glabra Huds.	0	Ulmaceae Mirb.	n	1
Iz_3_1009	DJI_0897	Acer tataricum L.	0	Sapindaceae Juss.	n	1
Iz_3_1009	DJI_0897	Betula pendula Roth.	0	Betulaceae Gray	n	1
Iz_3_1009	DJI_0897	Crataegus rhipidophylla Gand.	0	Rosaceae Juss.	n	1
Iz_3_1009	DJI_0897	Frangula alnus Mill.	0	Rhamnaceae Juss.	n	1
Iz_3_1009	DJI_0897	Fraxinus pennsylvanica Marshall	0	Oleaceae Hoffmanns. & Link	a	0
Iz_3_1009	DJI_0897	Pinus sylvestris L.	0	Pinaceae Spreng. ex F.Rudolphi	n	1
Iz_3_1009	DJI_0897	Populus × canescens (Aiton) Sm.	0	Salicaceae Mirb.	n	1
Iz_3_1009	DJI_0897	Populus tremula L.	0	Salicaceae Mirb.	n	1
Iz_3_1009	DJI_0897	Populus nigra L.	0	Salicaceae Mirb.	n	1
Iz_3_1009	DJI_0897	Pyrus communis L.	0	Rosaceae Juss.	a	0
Iz_3_1009	DJI_0897	Quercus robur L.	0	Fagaceae Dumort.	n	1
Iz_3_1009	DJI_0897	Robinia pseudoacacia L.	0	Fabaceae Lindl.	a	0
Iz_3_1009	DJI_0897	Rosa sp.	0	Rosaceae Juss.		
Iz_3_1009	DJI_0897	Salix alba L.	0	Salicaceae Mirb.	n	1
Iz_3_1009	DJI_0897	Sambucus nigra L.	0	Viburnaceae Raf.	n	1
Iz_3_1009	DJI_0897	Sorbus aucuparia L.	0	Rosaceae Juss.	n	1
Iz_3_1009	DJI_0897	Tilia cordata Mill.	0	Malvaceae Juss.	n	1
Iz_3_1009	DJI_0897	Ulmus glabra Huds.	0	Ulmaceae Mirb.	n	1
Iz_3_1009	DJI_0900	Acer tataricum L.	0	Sapindaceae Juss.	n	1
Iz_3_1009	DJI_0900	Betula pendula Roth.	0	Betulaceae Gray	n	1
Iz_3_1009	DJI_0900	Crataegus rhipidophylla Gand.	0	Rosaceae Juss.	n	1

Iz_3_1009	DJI_0900	Frangula alnus Mill.	0	Rhamnaceae Juss.	n	1
Iz_3_1009	DJI_0900	Fraxinus pennsylvanica Marshall	0	Oleaceae Hoffmanns. & Link	a	0
Iz_3_1009	DJI_0900	Pinus sylvestris L.	0	Pinaceae Spreng. ex F.Rudolphi	n	1
Iz_3_1009	DJI_0900	Populus × canescens (Aiton) Sm.	0	Salicaceae Mirb.	n	1
Iz_3_1009	DJI_0900	Populus tremula L.	0	Salicaceae Mirb.	n	1
Iz_3_1009	DJI_0900	Populus nigra L.	0	Salicaceae Mirb.	n	1
Iz_3_1009	DJI_0900	Pyrus communis L.	0	Rosaceae Juss.	a	0
Iz_3_1009	DJI_0900	Quercus robur L.	0	Fagaceae Dumort.	n	1
Iz_3_1009	DJI_0900	Robinia pseudoacacia L.	0	Fabaceae Lindl.	a	0
Iz_3_1009	DJI_0900	Rosa sp.	0	Rosaceae Juss.		
Iz_3_1009	DJI_0900	Salix alba L.	0	Salicaceae Mirb.	n	1
Iz_3_1009	DJI_0900	Sambucus nigra L.	0	Viburnaceae Raf.	n	1
Iz_3_1009	DJI_0900	Sorbus aucuparia L.	0	Rosaceae Juss.	n	1
Iz_3_1009	DJI_0900	Tilia cordata Mill.	0	Malvaceae Juss.	n	1
Iz_3_1009	DJI_0900	Ulmus glabra Huds.	0	Ulmaceae Mirb.	n	1
Iz_3_1009	DJI_0901	Acer tataricum L.	0	Sapindaceae Juss.	n	1
Iz_3_1009	DJI_0901	Betula pendula Roth.	0	Betulaceae Gray	n	1
Iz_3_1009	DJI_0901	Crataegus rhipidophylla Gand.	0	Rosaceae Juss.	n	1
Iz_3_1009	DJI_0901	Frangula alnus Mill.	0	Rhamnaceae Juss.	n	1
Iz_3_1009	DJI_0901	Fraxinus pennsylvanica Marshall	0	Oleaceae Hoffmanns. & Link	a	0
Iz_3_1009	DJI_0901	Pinus sylvestris L.	0	Pinaceae Spreng. ex F.Rudolphi	n	1
Iz_3_1009	DJI_0901	Populus × canescens (Aiton) Sm.	0	Salicaceae Mirb.	n	1
Iz_3_1009	DJI_0901	Populus tremula L.	0	Salicaceae Mirb.	n	1
Iz_3_1009	DJI_0901	Populus nigra L.	0	Salicaceae Mirb.	n	1
Iz_3_1009	DJI_0901	Pyrus communis L.	0	Rosaceae Juss.	a	0
Iz_3_1009	DJI_0901	Quercus robur L.	0	Fagaceae Dumort.	n	1
Iz_3_1009	DJI_0901	Robinia pseudoacacia L.	0	Fabaceae Lindl.	a	0
Iz_3_1009	DJI_0901	Rosa sp.	0	Rosaceae Juss.		
Iz_3_1009	DJI_0901	Salix alba L.	0	Salicaceae Mirb.	n	1
Iz_3_1009	DJI_0901	Sambucus nigra L.	0	Viburnaceae Raf.	n	1
Iz_3_1009	DJI_0901	Sorbus aucuparia L.	0	Rosaceae Juss.	n	1
Iz_3_1009	DJI_0901	Tilia cordata Mill.	0	Malvaceae Juss.	n	1
Iz_3_1009	DJI_0901	Ulmus glabra Huds.	0	Ulmaceae Mirb.	n	1
Iz_3_1009	DJI_0902	Acer tataricum L.	0	Sapindaceae Juss.	n	1
Iz_3_1009	DJI_0902	Betula pendula Roth.	0	Betulaceae Gray	n	1
Iz_3_1009	DJI_0902	Crataegus rhipidophylla Gand.	0	Rosaceae Juss.	n	1
Iz_3_1009	DJI_0902	Frangula alnus Mill.	0	Rhamnaceae Juss.	n	1
Iz_3_1009	DJI_0902	Fraxinus pennsylvanica Marshall	0	Oleaceae Hoffmanns. & Link	a	0
Iz_3_1009	DJI_0902	Pinus sylvestris L.	0	Pinaceae Spreng. ex F.Rudolphi	n	1
Iz_3_1009	DJI_0902	Populus × canescens (Aiton) Sm.	0	Salicaceae Mirb.	n	1
Iz_3_1009	DJI_0902	Populus tremula L.	0	Salicaceae Mirb.	n	1
Iz_3_1009	DJI_0902	Populus nigra L.	0	Salicaceae Mirb.	n	1
Iz_3_1009	DJI_0902	Pyrus communis L.	0	Rosaceae Juss.	a	0
Iz_3_1009	DJI_0902	Quercus robur L.	0	Fagaceae Dumort.	n	1
Iz_3_1009	DJI_0902	Robinia pseudoacacia L.	0	Fabaceae Lindl.	a	0
Iz_3_1009	DJI_0902	Rosa sp.	0	Rosaceae Juss.		
Iz_3_1009	DJI_0902	Salix alba L.	0	Salicaceae Mirb.	n	1
Iz_3_1009	DJI_0902	Sambucus nigra L.	0	Viburnaceae Raf.	n	1
Iz_3_1009	DJI_0902	Sorbus aucuparia L.	0	Rosaceae Juss.	n	1
Iz_3_1009	DJI_0902	Tilia cordata Mill.	0	Malvaceae Juss.	n	1
Iz_3_1009	DJI_0902	Ulmus glabra Huds.	0	Ulmaceae Mirb.	n	1
Iz_3_1009	DJI_0903	Acer tataricum L.	0	Sapindaceae Juss.	n	1
Iz_3_1009	DJI_0903	Betula pendula Roth.	0	Betulaceae Gray	n	1
Iz_3_1009	DJI_0903	Crataegus rhipidophylla Gand.	0	Rosaceae Juss.	n	1
Iz_3_1009	DJI_0903	Frangula alnus Mill.	0	Rhamnaceae Juss.	n	1
Iz_3_1009	DJI_0903	Fraxinus pennsylvanica Marshall	0	Oleaceae Hoffmanns. & Link	a	0
Iz_3_1009	DJI_0903	Pinus sylvestris L.	0	Pinaceae Spreng. ex F.Rudolphi	n	1
Iz_3_1009	DJI_0903	Populus × canescens (Aiton) Sm.	0	Salicaceae Mirb.	n	1
Iz_3_1009	DJI_0903	Populus tremula L.	0	Salicaceae Mirb.	n	1
Iz_3_1009	DJI_0903	Populus nigra L.	0	Salicaceae Mirb.	n	1
Iz_3_1009	DJI_0903	Pyrus communis L.	0	Rosaceae Juss.	a	0
Iz_3_1009	DJI_0903	Quercus robur L.	0	Fagaceae Dumort.	n	1
Iz_3_1009	DJI_0903	Robinia pseudoacacia L.	0	Fabaceae Lindl.	a	0
Iz_3_1009	DJI_0903	Rosa sp.	0	Rosaceae Juss.		

Iz_3_1009	DJI_0903	Salix alba L.	0	Salicaceae Mirb.	n	1
Iz_3_1009	DJI_0903	Sambucus nigra L.	0	Viburnaceae Raf.	n	1
Iz_3_1009	DJI_0903	Sorbus aucuparia L.	0	Rosaceae Juss.	n	1
Iz_3_1009	DJI_0903	Tilia cordata Mill.	0	Malvaceae Juss.	n	1
Iz_3_1009	DJI_0903	Ulmus glabra Huds.	0	Ulmaceae Mirb.	n	1
Iz_3_1009	DJI_0904	Acer tataricum L.	0	Sapindaceae Juss.	n	1
Iz_3_1009	DJI_0904	Betula pendula Roth.	0	Betulaceae Gray	n	1
Iz_3_1009	DJI_0904	Crataegus rhipidophylla Gand.	0	Rosaceae Juss.	n	1
Iz_3_1009	DJI_0904	Frangula alnus Mill.	0	Rhamnaceae Juss.	n	1
Iz_3_1009	DJI_0904	Fraxinus pennsylvanica Marshall	0	Oleaceae Hoffmanns. & Link	a	0
Iz_3_1009	DJI_0904	Pinus sylvestris L.	0	Pinaceae Spreng. ex F.Rudolphi	n	1
Iz_3_1009	DJI_0904	Populus × canescens (Aiton) Sm.	0	Salicaceae Mirb.	n	1
Iz_3_1009	DJI_0904	Populus tremula L.	0	Salicaceae Mirb.	n	1
Iz_3_1009	DJI_0904	Populus nigra L.	0	Salicaceae Mirb.	n	1
Iz_3_1009	DJI_0904	Pyrus communis L.	0	Rosaceae Juss.	a	0
Iz_3_1009	DJI_0904	Quercus robur L.	0	Fagaceae Dumort.	n	1
Iz_3_1009	DJI_0904	Robinia pseudoacacia L.	0	Fabaceae Lindl.	a	0
Iz_3_1009	DJI_0904	Rosa sp.	0	Rosaceae Juss.		
Iz_3_1009	DJI_0904	Salix alba L.	0	Salicaceae Mirb.	n	1
Iz_3_1009	DJI_0904	Sambucus nigra L.	0	Viburnaceae Raf.	n	1
Iz_3_1009	DJI_0904	Sorbus aucuparia L.	0	Rosaceae Juss.	n	1
Iz_3_1009	DJI_0904	Tilia cordata Mill.	0	Malvaceae Juss.	n	1
Iz_3_1009	DJI_0904	Ulmus glabra Huds.	0	Ulmaceae Mirb.	n	1
Iz_3_1009	DJI_0908	Acer tataricum L.	0	Sapindaceae Juss.	n	1
Iz_3_1009	DJI_0908	Betula pendula Roth.	0	Betulaceae Gray	n	1
Iz_3_1009	DJI_0908	Crataegus rhipidophylla Gand.	0	Rosaceae Juss.	n	1
Iz_3_1009	DJI_0908	Frangula alnus Mill.	0	Rhamnaceae Juss.	n	1
Iz_3_1009	DJI_0908	Fraxinus pennsylvanica Marshall	0	Oleaceae Hoffmanns. & Link	a	0
Iz_3_1009	DJI_0908	Pinus sylvestris L.	0	Pinaceae Spreng. ex F.Rudolphi	n	1
Iz_3_1009	DJI_0908	Populus × canescens (Aiton) Sm.	0	Salicaceae Mirb.	n	1
Iz_3_1009	DJI_0908	Populus tremula L.	0	Salicaceae Mirb.	n	1
Iz_3_1009	DJI_0908	Populus nigra L.	0	Salicaceae Mirb.	n	1
Iz_3_1009	DJI_0908	Pyrus communis L.	0	Rosaceae Juss.	a	0
Iz_3_1009	DJI_0908	Quercus robur L.	0	Fagaceae Dumort.	n	1
Iz_3_1009	DJI_0908	Robinia pseudoacacia L.	0	Fabaceae Lindl.	a	0
Iz_3_1009	DJI_0908	Rosa sp.	0	Rosaceae Juss.		
Iz_3_1009	DJI_0908	Salix alba L.	0	Salicaceae Mirb.	n	1
Iz_3_1009	DJI_0908	Sambucus nigra L.	0	Viburnaceae Raf.	n	1
Iz_3_1009	DJI_0908	Sorbus aucuparia L.	0	Rosaceae Juss.	n	1
Iz_3_1009	DJI_0908	Tilia cordata Mill.	0	Malvaceae Juss.	n	1
Iz_3_1009	DJI_0908	Ulmus glabra Huds.	0	Ulmaceae Mirb.	n	1
Iz_3_1009	DJI_0911	Acer tataricum L.	0	Sapindaceae Juss.	n	1
Iz_3_1009	DJI_0911	Betula pendula Roth.	0	Betulaceae Gray	n	1
Iz_3_1009	DJI_0911	Crataegus rhipidophylla Gand.	0	Rosaceae Juss.	n	1
Iz_3_1009	DJI_0911	Frangula alnus Mill.	0	Rhamnaceae Juss.	n	1
Iz_3_1009	DJI_0911	Fraxinus pennsylvanica Marshall	0	Oleaceae Hoffmanns. & Link	a	0
Iz_3_1009	DJI_0911	Pinus sylvestris L.	0	Pinaceae Spreng. ex F.Rudolphi	n	1
Iz_3_1009	DJI_0911	Populus × canescens (Aiton) Sm.	0	Salicaceae Mirb.	n	1
Iz_3_1009	DJI_0911	Populus tremula L.	0	Salicaceae Mirb.	n	1
Iz_3_1009	DJI_0911	Populus nigra L.	0	Salicaceae Mirb.	n	1
Iz_3_1009	DJI_0911	Pyrus communis L.	0	Rosaceae Juss.	a	0
Iz_3_1009	DJI_0911	Quercus robur L.	0	Fagaceae Dumort.	n	1
Iz_3_1009	DJI_0911	Robinia pseudoacacia L.	0	Fabaceae Lindl.	a	0
Iz_3_1009	DJI_0911	Rosa sp.	0	Rosaceae Juss.		
Iz_3_1009	DJI_0911	Salix alba L.	0	Salicaceae Mirb.	n	1
Iz_3_1009	DJI_0911	Sambucus nigra L.	0	Viburnaceae Raf.	n	1
Iz_3_1009	DJI_0911	Sorbus aucuparia L.	0	Rosaceae Juss.	n	1
Iz_3_1009	DJI_0911	Tilia cordata Mill.	0	Malvaceae Juss.	n	1
Iz_3_1009	DJI_0911	Ulmus glabra Huds.	0	Ulmaceae Mirb.	n	1
Iz_3_1009	DJI_0926	Acer tataricum L.	0	Sapindaceae Juss.	n	1
Iz_3_1009	DJI_0926	Betula pendula Roth.	0	Betulaceae Gray	n	1
Iz_3_1009	DJI_0926	Crataegus rhipidophylla Gand.	0	Rosaceae Juss.	n	1
Iz_3_1009	DJI_0926	Frangula alnus Mill.	0	Rhamnaceae Juss.	n	1
Iz_3_1009	DJI_0926	Fraxinus pennsylvanica Marshall	0	Oleaceae Hoffmanns. & Link	a	0

Iz_3_1009	DJI_0926	Pinus sylvestris L.	0	Pinaceae Spreng. ex F.Rudolphi	n	1
Iz_3_1009	DJI_0926	Populus × canescens (Aiton) Sm.	0	Salicaceae Mirb.	n	1
Iz_3_1009	DJI_0926	Populus tremula L.	0	Salicaceae Mirb.	n	1
Iz_3_1009	DJI_0926	Populus nigra L.	0	Salicaceae Mirb.	n	1
Iz_3_1009	DJI_0926	Pyrus communis L.	0	Rosaceae Juss.	a	0
Iz_3_1009	DJI_0926	Quercus robur L.	0	Fagaceae Dumort.	n	1
Iz_3_1009	DJI_0926	Robinia pseudoacacia L.	0	Fabaceae Lindl.	a	0
Iz_3_1009	DJI_0926	Rosa sp.	0	Rosaceae Juss.		
Iz_3_1009	DJI_0926	Salix alba L.	0	Salicaceae Mirb.	n	1
Iz_3_1009	DJI_0926	Sambucus nigra L.	0	Viburnaceae Raf.	n	1
Iz_3_1009	DJI_0926	Sorbus aucuparia L.	0	Rosaceae Juss.	n	1
Iz_3_1009	DJI_0926	Tilia cordata Mill.	0	Malvaceae Juss.	n	1
Iz_3_1009	DJI_0926	Ulmus glabra Huds.	0	Ulmaceae Mirb.	n	1
Iz_3_1009	DJI_0929	Acer tataricum L.	0	Sapindaceae Juss.	n	1
Iz_3_1009	DJI_0929	Betula pendula Roth.	0	Betulaceae Gray	n	1
Iz_3_1009	DJI_0929	Crataegus rhipidophylla Gand.	0	Rosaceae Juss.	n	1
Iz_3_1009	DJI_0929	Frangula alnus Mill.	0	Rhamnaceae Juss.	n	1
Iz_3_1009	DJI_0929	Fraxinus pennsylvanica Marshall	0	Oleaceae Hoffmanns. & Link	a	0
Iz_3_1009	DJI_0929	Pinus sylvestris L.	0	Pinaceae Spreng. ex F.Rudolphi	n	1
Iz_3_1009	DJI_0929	Populus × canescens (Aiton) Sm.	0	Salicaceae Mirb.	n	1
Iz_3_1009	DJI_0929	Populus tremula L.	0	Salicaceae Mirb.	n	1
Iz_3_1009	DJI_0929	Populus nigra L.	0	Salicaceae Mirb.	n	1
Iz_3_1009	DJI_0929	Pyrus communis L.	0	Rosaceae Juss.	a	0
Iz_3_1009	DJI_0929	Quercus robur L.	1	Fagaceae Dumort.	n	1
Iz_3_1009	DJI_0929	Robinia pseudoacacia L.	0	Fabaceae Lindl.	a	0
Iz_3_1009	DJI_0929	Rosa sp.	0	Rosaceae Juss.		
Iz_3_1009	DJI_0929	Salix alba L.	0	Salicaceae Mirb.	n	1
Iz_3_1009	DJI_0929	Sambucus nigra L.	0	Viburnaceae Raf.	n	1
Iz_3_1009	DJI_0929	Sorbus aucuparia L.	0	Rosaceae Juss.	n	1
Iz_3_1009	DJI_0929	Tilia cordata Mill.	0	Malvaceae Juss.	n	1
Iz_3_1009	DJI_0929	Ulmus glabra Huds.	0	Ulmaceae Mirb.	n	1
Iz_3_1009	DJI_0931	Acer tataricum L.	0	Sapindaceae Juss.	n	1
Iz_3_1009	DJI_0931	Betula pendula Roth.	0	Betulaceae Gray	n	1
Iz_3_1009	DJI_0931	Crataegus rhipidophylla Gand.	0	Rosaceae Juss.	n	1
Iz_3_1009	DJI_0931	Frangula alnus Mill.	0	Rhamnaceae Juss.	n	1
Iz_3_1009	DJI_0931	Fraxinus pennsylvanica Marshall	0	Oleaceae Hoffmanns. & Link	a	0
Iz_3_1009	DJI_0931	Pinus sylvestris L.	0	Pinaceae Spreng. ex F.Rudolphi	n	1
Iz_3_1009	DJI_0931	Populus × canescens (Aiton) Sm.	0	Salicaceae Mirb.	n	1
Iz_3_1009	DJI_0931	Populus tremula L.	0	Salicaceae Mirb.	n	1
Iz_3_1009	DJI_0931	Populus nigra L.	0	Salicaceae Mirb.	n	1
Iz_3_1009	DJI_0931	Pyrus communis L.	0	Rosaceae Juss.	a	0
Iz_3_1009	DJI_0931	Quercus robur L.	1	Fagaceae Dumort.	n	1
Iz_3_1009	DJI_0931	Robinia pseudoacacia L.	0	Fabaceae Lindl.	a	0
Iz_3_1009	DJI_0931	Rosa sp.	0	Rosaceae Juss.		
Iz_3_1009	DJI_0931	Salix alba L.	0	Salicaceae Mirb.	n	1
Iz_3_1009	DJI_0931	Sambucus nigra L.	0	Viburnaceae Raf.	n	1
Iz_3_1009	DJI_0931	Sorbus aucuparia L.	0	Rosaceae Juss.	n	1
Iz_3_1009	DJI_0931	Tilia cordata Mill.	0	Malvaceae Juss.	n	1
Iz_3_1009	DJI_0931	Ulmus glabra Huds.	0	Ulmaceae Mirb.	n	1
Iz_3_1009	DJI_0933	Acer tataricum L.	0	Sapindaceae Juss.	n	1
Iz_3_1009	DJI_0933	Betula pendula Roth.	0	Betulaceae Gray	n	1
Iz_3_1009	DJI_0933	Crataegus rhipidophylla Gand.	0	Rosaceae Juss.	n	1
Iz_3_1009	DJI_0933	Frangula alnus Mill.	0	Rhamnaceae Juss.	n	1
Iz_3_1009	DJI_0933	Fraxinus pennsylvanica Marshall	0	Oleaceae Hoffmanns. & Link	a	0
Iz_3_1009	DJI_0933	Pinus sylvestris L.	0	Pinaceae Spreng. ex F.Rudolphi	n	1
Iz_3_1009	DJI_0933	Populus × canescens (Aiton) Sm.	0	Salicaceae Mirb.	n	1
Iz_3_1009	DJI_0933	Populus tremula L.	0	Salicaceae Mirb.	n	1
Iz_3_1009	DJI_0933	Populus nigra L.	0	Salicaceae Mirb.	n	1
Iz_3_1009	DJI_0933	Pyrus communis L.	0	Rosaceae Juss.	a	0
Iz_3_1009	DJI_0933	Quercus robur L.	0	Fagaceae Dumort.	n	1
Iz_3_1009	DJI_0933	Robinia pseudoacacia L.	0	Fabaceae Lindl.	a	0
Iz_3_1009	DJI_0933	Rosa sp.	0	Rosaceae Juss.		
Iz_3_1009	DJI_0933	Salix alba L.	0	Salicaceae Mirb.	n	1
Iz_3_1009	DJI_0933	Sambucus nigra L.	0	Viburnaceae Raf.	n	1

Iz_3_1009	DJI_0933	Sorbus aucuparia L.	0	Rosaceae Juss.	n	1
Iz_3_1009	DJI_0933	Tilia cordata Mill.	0	Malvaceae Juss.	n	1
Iz_3_1009	DJI_0933	Ulmus glabra Huds.	0	Ulmaceae Mirb.	n	1
Iz_3_1009	DJI_0935	Acer tataricum L.	0	Sapindaceae Juss.	n	1
Iz_3_1009	DJI_0935	Betula pendula Roth.	0	Betulaceae Gray	n	1
Iz_3_1009	DJI_0935	Crataegus rhipidophylla Gand.	0	Rosaceae Juss.	n	1
Iz_3_1009	DJI_0935	Frangula alnus Mill.	0	Rhamnaceae Juss.	n	1
Iz_3_1009	DJI_0935	Fraxinus pennsylvanica Marshall	0	Oleaceae Hoffmanns. & Link	a	0
Iz_3_1009	DJI_0935	Pinus sylvestris L.	0	Pinaceae Spreng. ex F.Rudolphi	n	1
Iz_3_1009	DJI_0935	Populus × canescens (Aiton) Sm.	0	Salicaceae Mirb.	n	1
Iz_3_1009	DJI_0935	Populus tremula L.	0	Salicaceae Mirb.	n	1
Iz_3_1009	DJI_0935	Populus nigra L.	0	Salicaceae Mirb.	n	1
Iz_3_1009	DJI_0935	Pyrus communis L.	0	Rosaceae Juss.	a	0
Iz_3_1009	DJI_0935	Quercus robur L.	5	Fagaceae Dumort.	n	1
Iz_3_1009	DJI_0935	Robinia pseudoacacia L.	0	Fabaceae Lindl.	a	0
Iz_3_1009	DJI_0935	Rosa sp.	0	Rosaceae Juss.		
Iz_3_1009	DJI_0935	Salix alba L.	0	Salicaceae Mirb.	n	1
Iz_3_1009	DJI_0935	Sambucus nigra L.	0	Viburnaceae Raf.	n	1
Iz_3_1009	DJI_0935	Sorbus aucuparia L.	0	Rosaceae Juss.	n	1
Iz_3_1009	DJI_0935	Tilia cordata Mill.	0	Malvaceae Juss.	n	1
Iz_3_1009	DJI_0935	Ulmus glabra Huds.	0	Ulmaceae Mirb.	n	1
Iz_3_1009	DJI_0938	Acer tataricum L.	0	Sapindaceae Juss.	n	1
Iz_3_1009	DJI_0938	Betula pendula Roth.	0	Betulaceae Gray	n	1
Iz_3_1009	DJI_0938	Crataegus rhipidophylla Gand.	0	Rosaceae Juss.	n	1
Iz_3_1009	DJI_0938	Frangula alnus Mill.	0	Rhamnaceae Juss.	n	1
Iz_3_1009	DJI_0938	Fraxinus pennsylvanica Marshall	0	Oleaceae Hoffmanns. & Link	a	0
Iz_3_1009	DJI_0938	Pinus sylvestris L.	0	Pinaceae Spreng. ex F.Rudolphi	n	1
Iz_3_1009	DJI_0938	Populus × canescens (Aiton) Sm.	0	Salicaceae Mirb.	n	1
Iz_3_1009	DJI_0938	Populus tremula L.	1	Salicaceae Mirb.	n	1
Iz_3_1009	DJI_0938	Populus nigra L.	0	Salicaceae Mirb.	n	1
Iz_3_1009	DJI_0938	Pyrus communis L.	0	Rosaceae Juss.	a	0
Iz_3_1009	DJI_0938	Quercus robur L.	0	Fagaceae Dumort.	n	1
Iz_3_1009	DJI_0938	Robinia pseudoacacia L.	0	Fabaceae Lindl.	a	0
Iz_3_1009	DJI_0938	Rosa sp.	0	Rosaceae Juss.		
Iz_3_1009	DJI_0938	Salix alba L.	0	Salicaceae Mirb.	n	1
Iz_3_1009	DJI_0938	Sambucus nigra L.	0	Viburnaceae Raf.	n	1
Iz_3_1009	DJI_0938	Sorbus aucuparia L.	0	Rosaceae Juss.	n	1
Iz_3_1009	DJI_0938	Tilia cordata Mill.	0	Malvaceae Juss.	n	1
Iz_3_1009	DJI_0938	Ulmus glabra Huds.	0	Ulmaceae Mirb.	n	1
Iz_3_1009	DJI_0945	Acer tataricum L.	0	Sapindaceae Juss.	n	1
Iz_3_1009	DJI_0945	Betula pendula Roth.	0	Betulaceae Gray	n	1
Iz_3_1009	DJI_0945	Crataegus rhipidophylla Gand.	0	Rosaceae Juss.	n	1
Iz_3_1009	DJI_0945	Frangula alnus Mill.	0	Rhamnaceae Juss.	n	1
Iz_3_1009	DJI_0945	Fraxinus pennsylvanica Marshall	0	Oleaceae Hoffmanns. & Link	a	0
Iz_3_1009	DJI_0945	Pinus sylvestris L.	0	Pinaceae Spreng. ex F.Rudolphi	n	1
Iz_3_1009	DJI_0945	Populus × canescens (Aiton) Sm.	0	Salicaceae Mirb.	n	1
Iz_3_1009	DJI_0945	Populus tremula L.	0	Salicaceae Mirb.	n	1
Iz_3_1009	DJI_0945	Populus nigra L.	0	Salicaceae Mirb.	n	1
Iz_3_1009	DJI_0945	Pyrus communis L.	0	Rosaceae Juss.	a	0
Iz_3_1009	DJI_0945	Quercus robur L.	0	Fagaceae Dumort.	n	1
Iz_3_1009	DJI_0945	Robinia pseudoacacia L.	0	Fabaceae Lindl.	a	0
Iz_3_1009	DJI_0945	Rosa sp.	0	Rosaceae Juss.		
Iz_3_1009	DJI_0945	Salix alba L.	0	Salicaceae Mirb.	n	1
Iz_3_1009	DJI_0945	Sambucus nigra L.	0	Viburnaceae Raf.	n	1
Iz_3_1009	DJI_0945	Sorbus aucuparia L.	0	Rosaceae Juss.	n	1
Iz_3_1009	DJI_0945	Tilia cordata Mill.	0	Malvaceae Juss.	n	1
Iz_3_1009	DJI_0945	Ulmus glabra Huds.	0	Ulmaceae Mirb.	n	1
Iz_3_1009	DJI_0951	Acer tataricum L.	0	Sapindaceae Juss.	n	1
Iz_3_1009	DJI_0951	Betula pendula Roth.	0	Betulaceae Gray	n	1
Iz_3_1009	DJI_0951	Crataegus rhipidophylla Gand.	0	Rosaceae Juss.	n	1
Iz_3_1009	DJI_0951	Frangula alnus Mill.	0	Rhamnaceae Juss.	n	1
Iz_3_1009	DJI_0951	Fraxinus pennsylvanica Marshall	0	Oleaceae Hoffmanns. & Link	a	0
Iz_3_1009	DJI_0951	Pinus sylvestris L.	0	Pinaceae Spreng. ex F.Rudolphi	n	1
Iz_3_1009	DJI_0951	Populus × canescens (Aiton) Sm.	0	Salicaceae Mirb.	n	1

Iz_3_1009	DJI_0951	Populus tremula L.	0	Salicaceae Mirb.	n	1
Iz_3_1009	DJI_0951	Populus nigra L.	0	Salicaceae Mirb.	n	1
Iz_3_1009	DJI_0951	Pyrus communis L.	0	Rosaceae Juss.	a	0
Iz_3_1009	DJI_0951	Quercus robur L.	0	Fagaceae Dumort.	n	1
Iz_3_1009	DJI_0951	Robinia pseudoacacia L.	0	Fabaceae Lindl.	a	0
Iz_3_1009	DJI_0951	Rosa sp.	0	Rosaceae Juss.		
Iz_3_1009	DJI_0951	Salix alba L.	0	Salicaceae Mirb.	n	1
Iz_3_1009	DJI_0951	Sambucus nigra L.	0	Viburnaceae Raf.	n	1
Iz_3_1009	DJI_0951	Sorbus aucuparia L.	0	Rosaceae Juss.	n	1
Iz_3_1009	DJI_0951	Tilia cordata Mill.	0	Malvaceae Juss.	n	1
Iz_3_1009	DJI_0951	Ulmus glabra Huds.	0	Ulmaceae Mirb.	n	1
Iz_3_1009	DJI_0954	Acer tataricum L.	0	Sapindaceae Juss.	n	1
Iz_3_1009	DJI_0954	Betula pendula Roth.	0	Betulaceae Gray	n	1
Iz_3_1009	DJI_0954	Crataegus rhipidophylla Gand.	0	Rosaceae Juss.	n	1
Iz_3_1009	DJI_0954	Frangula alnus Mill.	0	Rhamnaceae Juss.	n	1
Iz_3_1009	DJI_0954	Fraxinus pennsylvanica Marshall	0	Oleaceae Hoffmanns. & Link	a	0
Iz_3_1009	DJI_0954	Pinus sylvestris L.	0	Pinaceae Spreng. ex F.Rudolphi	n	1
Iz_3_1009	DJI_0954	Populus × canescens (Aiton) Sm.	0	Salicaceae Mirb.	n	1
Iz_3_1009	DJI_0954	Populus tremula L.	0	Salicaceae Mirb.	n	1
Iz_3_1009	DJI_0954	Populus nigra L.	0	Salicaceae Mirb.	n	1
Iz_3_1009	DJI_0954	Pyrus communis L.	0	Rosaceae Juss.	a	0
Iz_3_1009	DJI_0954	Quercus robur L.	0	Fagaceae Dumort.	n	1
Iz_3_1009	DJI_0954	Robinia pseudoacacia L.	0	Fabaceae Lindl.	a	0
Iz_3_1009	DJI_0954	Rosa sp.	0	Rosaceae Juss.		
Iz_3_1009	DJI_0954	Salix alba L.	0	Salicaceae Mirb.	n	1
Iz_3_1009	DJI_0954	Sambucus nigra L.	0	Viburnaceae Raf.	n	1
Iz_3_1009	DJI_0954	Sorbus aucuparia L.	0	Rosaceae Juss.	n	1
Iz_3_1009	DJI_0954	Tilia cordata Mill.	0	Malvaceae Juss.	n	1
Iz_3_1009	DJI_0954	Ulmus glabra Huds.	0	Ulmaceae Mirb.	n	1
Iz_3_1009	DJI_0956	Acer tataricum L.	0	Sapindaceae Juss.	n	1
Iz_3_1009	DJI_0956	Betula pendula Roth.	0	Betulaceae Gray	n	1
Iz_3_1009	DJI_0956	Crataegus rhipidophylla Gand.	0	Rosaceae Juss.	n	1
Iz_3_1009	DJI_0956	Frangula alnus Mill.	0	Rhamnaceae Juss.	n	1
Iz_3_1009	DJI_0956	Fraxinus pennsylvanica Marshall	0	Oleaceae Hoffmanns. & Link	a	0
Iz_3_1009	DJI_0956	Pinus sylvestris L.	0	Pinaceae Spreng. ex F.Rudolphi	n	1
Iz_3_1009	DJI_0956	Populus × canescens (Aiton) Sm.	0	Salicaceae Mirb.	n	1
Iz_3_1009	DJI_0956	Populus tremula L.	0	Salicaceae Mirb.	n	1
Iz_3_1009	DJI_0956	Populus nigra L.	0	Salicaceae Mirb.	n	1
Iz_3_1009	DJI_0956	Pyrus communis L.	0	Rosaceae Juss.	a	0
Iz_3_1009	DJI_0956	Quercus robur L.	0	Fagaceae Dumort.	n	1
Iz_3_1009	DJI_0956	Robinia pseudoacacia L.	0	Fabaceae Lindl.	a	0
Iz_3_1009	DJI_0956	Rosa sp.	0	Rosaceae Juss.		
Iz_3_1009	DJI_0956	Salix alba L.	0	Salicaceae Mirb.	n	1
Iz_3_1009	DJI_0956	Sambucus nigra L.	0	Viburnaceae Raf.	n	1
Iz_3_1009	DJI_0956	Sorbus aucuparia L.	0	Rosaceae Juss.	n	1
Iz_3_1009	DJI_0956	Tilia cordata Mill.	0	Malvaceae Juss.	n	1
Iz_3_1009	DJI_0956	Ulmus glabra Huds.	0	Ulmaceae Mirb.	n	1
Iz_3_1009	DJI_0966	Acer tataricum L.	0	Sapindaceae Juss.	n	1
Iz_3_1009	DJI_0966	Betula pendula Roth.	0	Betulaceae Gray	n	1
Iz_3_1009	DJI_0966	Crataegus rhipidophylla Gand.	0	Rosaceae Juss.	n	1
Iz_3_1009	DJI_0966	Frangula alnus Mill.	0	Rhamnaceae Juss.	n	1
Iz_3_1009	DJI_0966	Fraxinus pennsylvanica Marshall	0	Oleaceae Hoffmanns. & Link	a	0
Iz_3_1009	DJI_0966	Pinus sylvestris L.	0	Pinaceae Spreng. ex F.Rudolphi	n	1
Iz_3_1009	DJI_0966	Populus × canescens (Aiton) Sm.	0	Salicaceae Mirb.	n	1
Iz_3_1009	DJI_0966	Populus tremula L.	0	Salicaceae Mirb.	n	1
Iz_3_1009	DJI_0966	Populus nigra L.	0	Salicaceae Mirb.	n	1
Iz_3_1009	DJI_0966	Pyrus communis L.	0	Rosaceae Juss.	a	0
Iz_3_1009	DJI_0966	Quercus robur L.	0	Fagaceae Dumort.	n	1
Iz_3_1009	DJI_0966	Robinia pseudoacacia L.	0	Fabaceae Lindl.	a	0
Iz_3_1009	DJI_0966	Rosa sp.	0	Rosaceae Juss.		
Iz_3_1009	DJI_0966	Salix alba L.	0	Salicaceae Mirb.	n	1
Iz_3_1009	DJI_0966	Sambucus nigra L.	0	Viburnaceae Raf.	n	1
Iz_3_1009	DJI_0966	Sorbus aucuparia L.	0	Rosaceae Juss.	n	1
Iz_3_1009	DJI_0966	Tilia cordata Mill.	0	Malvaceae Juss.	n	1

Iz_3_1009	DJI_0966	Ulmus glabra Huds.	0	Ulmaceae Mirb.	n	1
Iz_3_1009	DJI_0969	Acer tataricum L.	0	Sapindaceae Juss.	n	1
Iz_3_1009	DJI_0969	Betula pendula Roth.	0	Betulaceae Gray	n	1
Iz_3_1009	DJI_0969	Crataegus rhipidophylla Gand.	0	Rosaceae Juss.	n	1
Iz_3_1009	DJI_0969	Frangula alnus Mill.	0	Rhamnaceae Juss.	n	1
Iz_3_1009	DJI_0969	Fraxinus pennsylvanica Marshall	0	Oleaceae Hoffmanns. & Link	a	0
Iz_3_1009	DJI_0969	Pinus sylvestris L.	0	Pinaceae Spreng. ex F.Rudolphi	n	1
Iz_3_1009	DJI_0969	Populus × canescens (Aiton) Sm.	0	Salicaceae Mirb.	n	1
Iz_3_1009	DJI_0969	Populus tremula L.	0	Salicaceae Mirb.	n	1
Iz_3_1009	DJI_0969	Populus nigra L.	0	Salicaceae Mirb.	n	1
Iz_3_1009	DJI_0969	Pyrus communis L.	0	Rosaceae Juss.	a	0
Iz_3_1009	DJI_0969	Quercus robur L.	45	Fagaceae Dumort.	n	1
Iz_3_1009	DJI_0969	Robinia pseudoacacia L.	0	Fabaceae Lindl.	a	0
Iz_3_1009	DJI_0969	Rosa sp.	0	Rosaceae Juss.		
Iz_3_1009	DJI_0969	Salix alba L.	0	Salicaceae Mirb.	n	1
Iz_3_1009	DJI_0969	Sambucus nigra L.	0	Viburnaceae Raf.	n	1
Iz_3_1009	DJI_0969	Sorbus aucuparia L.	0	Rosaceae Juss.	n	1
Iz_3_1009	DJI_0969	Tilia cordata Mill.	0	Malvaceae Juss.	n	1
Iz_3_1009	DJI_0969	Ulmus glabra Huds.	0	Ulmaceae Mirb.	n	1
Iz_3_1009	DJI_0977	Acer tataricum L.	0	Sapindaceae Juss.	n	1
Iz_3_1009	DJI_0977	Betula pendula Roth.	0	Betulaceae Gray	n	1
Iz_3_1009	DJI_0977	Crataegus rhipidophylla Gand.	0	Rosaceae Juss.	n	1
Iz_3_1009	DJI_0977	Frangula alnus Mill.	0	Rhamnaceae Juss.	n	1
Iz_3_1009	DJI_0977	Fraxinus pennsylvanica Marshall	0	Oleaceae Hoffmanns. & Link	a	0
Iz_3_1009	DJI_0977	Pinus sylvestris L.	0	Pinaceae Spreng. ex F.Rudolphi	n	1
Iz_3_1009	DJI_0977	Populus × canescens (Aiton) Sm.	0	Salicaceae Mirb.	n	1
Iz_3_1009	DJI_0977	Populus tremula L.	0	Salicaceae Mirb.	n	1
Iz_3_1009	DJI_0977	Populus nigra L.	0	Salicaceae Mirb.	n	1
Iz_3_1009	DJI_0977	Pyrus communis L.	0	Rosaceae Juss.	a	0
Iz_3_1009	DJI_0977	Quercus robur L.	0	Fagaceae Dumort.	n	1
Iz_3_1009	DJI_0977	Robinia pseudoacacia L.	0	Fabaceae Lindl.	a	0
Iz_3_1009	DJI_0977	Rosa sp.	0	Rosaceae Juss.		
Iz_3_1009	DJI_0977	Salix alba L.	0	Salicaceae Mirb.	n	1
Iz_3_1009	DJI_0977	Sambucus nigra L.	0	Viburnaceae Raf.	n	1
Iz_3_1009	DJI_0977	Sorbus aucuparia L.	0	Rosaceae Juss.	n	1
Iz_3_1009	DJI_0977	Tilia cordata Mill.	0	Malvaceae Juss.	n	1
Iz_3_1009	DJI_0977	Ulmus glabra Huds.	0	Ulmaceae Mirb.	n	1
Iz_3_1009	DJI_0978	Acer tataricum L.	0	Sapindaceae Juss.	n	1
Iz_3_1009	DJI_0978	Betula pendula Roth.	0	Betulaceae Gray	n	1
Iz_3_1009	DJI_0978	Crataegus rhipidophylla Gand.	0	Rosaceae Juss.	n	1
Iz_3_1009	DJI_0978	Frangula alnus Mill.	0	Rhamnaceae Juss.	n	1
Iz_3_1009	DJI_0978	Fraxinus pennsylvanica Marshall	0	Oleaceae Hoffmanns. & Link	a	0
Iz_3_1009	DJI_0978	Pinus sylvestris L.	0	Pinaceae Spreng. ex F.Rudolphi	n	1
Iz_3_1009	DJI_0978	Populus × canescens (Aiton) Sm.	0	Salicaceae Mirb.	n	1
Iz_3_1009	DJI_0978	Populus tremula L.	0	Salicaceae Mirb.	n	1
Iz_3_1009	DJI_0978	Populus nigra L.	0	Salicaceae Mirb.	n	1
Iz_3_1009	DJI_0978	Pyrus communis L.	0	Rosaceae Juss.	a	0
Iz_3_1009	DJI_0978	Quercus robur L.	6	Fagaceae Dumort.	n	1
Iz_3_1009	DJI_0978	Robinia pseudoacacia L.	0	Fabaceae Lindl.	a	0
Iz_3_1009	DJI_0978	Rosa sp.	0	Rosaceae Juss.		
Iz_3_1009	DJI_0978	Salix alba L.	0	Salicaceae Mirb.	n	1
Iz_3_1009	DJI_0978	Sambucus nigra L.	0	Viburnaceae Raf.	n	1
Iz_3_1009	DJI_0978	Sorbus aucuparia L.	0	Rosaceae Juss.	n	1
Iz_3_1009	DJI_0978	Tilia cordata Mill.	0	Malvaceae Juss.	n	1
Iz_3_1009	DJI_0978	Ulmus glabra Huds.	0	Ulmaceae Mirb.	n	1
Iz_3_1009	DJI_0979	Acer tataricum L.	0	Sapindaceae Juss.	n	1
Iz_3_1009	DJI_0979	Betula pendula Roth.	0	Betulaceae Gray	n	1
Iz_3_1009	DJI_0979	Crataegus rhipidophylla Gand.	0	Rosaceae Juss.	n	1
Iz_3_1009	DJI_0979	Frangula alnus Mill.	0	Rhamnaceae Juss.	n	1
Iz_3_1009	DJI_0979	Fraxinus pennsylvanica Marshall	0	Oleaceae Hoffmanns. & Link	a	0
Iz_3_1009	DJI_0979	Pinus sylvestris L.	0	Pinaceae Spreng. ex F.Rudolphi	n	1
Iz_3_1009	DJI_0979	Populus × canescens (Aiton) Sm.	0	Salicaceae Mirb.	n	1
Iz_3_1009	DJI_0979	Populus tremula L.	0	Salicaceae Mirb.	n	1
Iz_3_1009	DJI_0979	Populus nigra L.	0	Salicaceae Mirb.	n	1

Iz_3_1009	DJI_0979	Pyrus communis L.	0	Rosaceae Juss.	a	0
Iz_3_1009	DJI_0979	Quercus robur L.	0	Fagaceae Dumort.	n	1
Iz_3_1009	DJI_0979	Robinia pseudoacacia L.	0	Fabaceae Lindl.	a	0
Iz_3_1009	DJI_0979	Rosa sp.	0	Rosaceae Juss.		
Iz_3_1009	DJI_0979	Salix alba L.	0	Salicaceae Mirb.	n	1
Iz_3_1009	DJI_0979	Sambucus nigra L.	0	Viburnaceae Raf.	n	1
Iz_3_1009	DJI_0979	Sorbus aucuparia L.	0	Rosaceae Juss.	n	1
Iz_3_1009	DJI_0979	Tilia cordata Mill.	0	Malvaceae Juss.	n	1
Iz_3_1009	DJI_0979	Ulmus glabra Huds.	0	Ulmaceae Mirb.	n	1
Iz_3_1009	DJI_0991	Acer tataricum L.	0	Sapindaceae Juss.	n	1
Iz_3_1009	DJI_0991	Betula pendula Roth.	0	Betulaceae Gray	n	1
Iz_3_1009	DJI_0991	Crataegus rhipidophylla Gand.	0	Rosaceae Juss.	n	1
Iz_3_1009	DJI_0991	Frangula alnus Mill.	0	Rhamnaceae Juss.	n	1
Iz_3_1009	DJI_0991	Fraxinus pennsylvanica Marshall	0	Oleaceae Hoffmanns. & Link	a	0
Iz_3_1009	DJI_0991	Pinus sylvestris L.	0	Pinaceae Spreng. ex F.Rudolphi	n	1
Iz_3_1009	DJI_0991	Populus × canescens (Aiton) Sm.	0	Salicaceae Mirb.	n	1
Iz_3_1009	DJI_0991	Populus tremula L.	0	Salicaceae Mirb.	n	1
Iz_3_1009	DJI_0991	Populus nigra L.	0	Salicaceae Mirb.	n	1
Iz_3_1009	DJI_0991	Pyrus communis L.	0	Rosaceae Juss.	a	0
Iz_3_1009	DJI_0991	Quercus robur L.	5	Fagaceae Dumort.	n	1
Iz_3_1009	DJI_0991	Robinia pseudoacacia L.	0	Fabaceae Lindl.	a	0
Iz_3_1009	DJI_0991	Rosa sp.	0	Rosaceae Juss.		
Iz_3_1009	DJI_0991	Salix alba L.	0	Salicaceae Mirb.	n	1
Iz_3_1009	DJI_0991	Sambucus nigra L.	0	Viburnaceae Raf.	n	1
Iz_3_1009	DJI_0991	Sorbus aucuparia L.	0	Rosaceae Juss.	n	1
Iz_3_1009	DJI_0991	Tilia cordata Mill.	0	Malvaceae Juss.	n	1
Iz_3_1009	DJI_0991	Ulmus glabra Huds.	0	Ulmaceae Mirb.	n	1
Iz_3_1009	DJI_0001	Acer tataricum L.	0	Sapindaceae Juss.	n	1
Iz_3_1009	DJI_0001	Betula pendula Roth.	0	Betulaceae Gray	n	1
Iz_3_1009	DJI_0001	Crataegus rhipidophylla Gand.	0	Rosaceae Juss.	n	1
Iz_3_1009	DJI_0001	Frangula alnus Mill.	0	Rhamnaceae Juss.	n	1
Iz_3_1009	DJI_0001	Fraxinus pennsylvanica Marshall	0	Oleaceae Hoffmanns. & Link	a	0
Iz_3_1009	DJI_0001	Pinus sylvestris L.	0	Pinaceae Spreng. ex F.Rudolphi	n	1
Iz_3_1009	DJI_0001	Populus × canescens (Aiton) Sm.	0	Salicaceae Mirb.	n	1
Iz_3_1009	DJI_0001	Populus tremula L.	0	Salicaceae Mirb.	n	1
Iz_3_1009	DJI_0001	Populus nigra L.	0	Salicaceae Mirb.	n	1
Iz_3_1009	DJI_0001	Pyrus communis L.	0	Rosaceae Juss.	a	0
Iz_3_1009	DJI_0001	Quercus robur L.	0	Fagaceae Dumort.	n	1
Iz_3_1009	DJI_0001	Robinia pseudoacacia L.	0	Fabaceae Lindl.	a	0
Iz_3_1009	DJI_0001	Rosa sp.	0	Rosaceae Juss.		
Iz_3_1009	DJI_0001	Salix alba L.	0	Salicaceae Mirb.	n	1
Iz_3_1009	DJI_0001	Sambucus nigra L.	0	Viburnaceae Raf.	n	1
Iz_3_1009	DJI_0001	Sorbus aucuparia L.	0	Rosaceae Juss.	n	1
Iz_3_1009	DJI_0001	Tilia cordata Mill.	0	Malvaceae Juss.	n	1
Iz_3_1009	DJI_0001	Ulmus glabra Huds.	0	Ulmaceae Mirb.	n	1
Iz_3_1009	DJI_0002	Acer tataricum L.	0	Sapindaceae Juss.	n	1
Iz_3_1009	DJI_0002	Betula pendula Roth.	0	Betulaceae Gray	n	1
Iz_3_1009	DJI_0002	Crataegus rhipidophylla Gand.	0	Rosaceae Juss.	n	1
Iz_3_1009	DJI_0002	Frangula alnus Mill.	0	Rhamnaceae Juss.	n	1
Iz_3_1009	DJI_0002	Fraxinus pennsylvanica Marshall	0	Oleaceae Hoffmanns. & Link	a	0
Iz_3_1009	DJI_0002	Pinus sylvestris L.	0	Pinaceae Spreng. ex F.Rudolphi	n	1
Iz_3_1009	DJI_0002	Populus × canescens (Aiton) Sm.	0	Salicaceae Mirb.	n	1
Iz_3_1009	DJI_0002	Populus tremula L.	0	Salicaceae Mirb.	n	1
Iz_3_1009	DJI_0002	Populus nigra L.	0	Salicaceae Mirb.	n	1
Iz_3_1009	DJI_0002	Pyrus communis L.	0	Rosaceae Juss.	a	0
Iz_3_1009	DJI_0002	Quercus robur L.	0	Fagaceae Dumort.	n	1
Iz_3_1009	DJI_0002	Robinia pseudoacacia L.	0	Fabaceae Lindl.	a	0
Iz_3_1009	DJI_0002	Rosa sp.	0	Rosaceae Juss.		
Iz_3_1009	DJI_0002	Salix alba L.	0	Salicaceae Mirb.	n	1
Iz_3_1009	DJI_0002	Sambucus nigra L.	0	Viburnaceae Raf.	n	1
Iz_3_1009	DJI_0002	Sorbus aucuparia L.	0	Rosaceae Juss.	n	1
Iz_3_1009	DJI_0002	Tilia cordata Mill.	0	Malvaceae Juss.	n	1
Iz_3_1009	DJI_0002	Ulmus glabra Huds.	0	Ulmaceae Mirb.	n	1
Iz_3_1009	DJI_0004	Acer tataricum L.	0	Sapindaceae Juss.	n	1

Iz_3_1009	DJI_0004	Betula pendula Roth.	0	Betulaceae Gray	n	1
Iz_3_1009	DJI_0004	Crataegus rhipidophylla Gand.	0	Rosaceae Juss.	n	1
Iz_3_1009	DJI_0004	Frangula alnus Mill.	0	Rhamnaceae Juss.	n	1
Iz_3_1009	DJI_0004	Fraxinus pennsylvanica Marshall	0	Oleaceae Hoffmanns. & Link	a	0
Iz_3_1009	DJI_0004	Pinus sylvestris L.	0	Pinaceae Spreng. ex F.Rudolphi	n	1
Iz_3_1009	DJI_0004	Populus × canescens (Aiton) Sm.	0	Salicaceae Mirb.	n	1
Iz_3_1009	DJI_0004	Populus tremula L.	0	Salicaceae Mirb.	n	1
Iz_3_1009	DJI_0004	Populus nigra L.	0	Salicaceae Mirb.	n	1
Iz_3_1009	DJI_0004	Pyrus communis L.	0	Rosaceae Juss.	a	0
Iz_3_1009	DJI_0004	Quercus robur L.	0	Fagaceae Dumort.	n	1
Iz_3_1009	DJI_0004	Robinia pseudoacacia L.	0	Fabaceae Lindl.	a	0
Iz_3_1009	DJI_0004	Rosa sp.	0	Rosaceae Juss.		
Iz_3_1009	DJI_0004	Salix alba L.	0	Salicaceae Mirb.	n	1
Iz_3_1009	DJI_0004	Sambucus nigra L.	0	Viburnaceae Raf.	n	1
Iz_3_1009	DJI_0004	Sorbus aucuparia L.	0	Rosaceae Juss.	n	1
Iz_3_1009	DJI_0004	Tilia cordata Mill.	0	Malvaceae Juss.	n	1
Iz_3_1009	DJI_0004	Ulmus glabra Huds.	0	Ulmaceae Mirb.	n	1
Iz_4_1009	DJI_0287	Acer tataricum L.	0	Sapindaceae Juss.	n	1
Iz_4_1009	DJI_0287	Betula pendula Roth.	0	Betulaceae Gray	n	1
Iz_4_1009	DJI_0287	Crataegus rhipidophylla Gand.	0	Rosaceae Juss.	n	1
Iz_4_1009	DJI_0287	Frangula alnus Mill.	0	Rhamnaceae Juss.	n	1
Iz_4_1009	DJI_0287	Fraxinus pennsylvanica Marshall	0	Oleaceae Hoffmanns. & Link	a	0
Iz_4_1009	DJI_0287	Pinus sylvestris L.	0	Pinaceae Spreng. ex F.Rudolphi	n	1
Iz_4_1009	DJI_0287	Populus × canescens (Aiton) Sm.	0	Salicaceae Mirb.	n	1
Iz_4_1009	DJI_0287	Populus tremula L.	0	Salicaceae Mirb.	n	1
Iz_4_1009	DJI_0287	Populus nigra L.	0	Salicaceae Mirb.	n	1
Iz_4_1009	DJI_0287	Pyrus communis L.	0	Rosaceae Juss.	a	0
Iz_4_1009	DJI_0287	Quercus robur L.	0	Fagaceae Dumort.	n	1
Iz_4_1009	DJI_0287	Robinia pseudoacacia L.	0	Fabaceae Lindl.	a	0
Iz_4_1009	DJI_0287	Rosa sp.	0	Rosaceae Juss.		
Iz_4_1009	DJI_0287	Salix alba L.	0	Salicaceae Mirb.	n	1
Iz_4_1009	DJI_0287	Sambucus nigra L.	0	Viburnaceae Raf.	n	1
Iz_4_1009	DJI_0287	Sorbus aucuparia L.	0	Rosaceae Juss.	n	1
Iz_4_1009	DJI_0287	Tilia cordata Mill.	0	Malvaceae Juss.	n	1
Iz_4_1009	DJI_0287	Ulmus glabra Huds.	0	Ulmaceae Mirb.	n	1
Iz_4_1009	DJI_0288	Acer tataricum L.	0	Sapindaceae Juss.	n	1
Iz_4_1009	DJI_0288	Betula pendula Roth.	0	Betulaceae Gray	n	1
Iz_4_1009	DJI_0288	Crataegus rhipidophylla Gand.	0	Rosaceae Juss.	n	1
Iz_4_1009	DJI_0288	Frangula alnus Mill.	0	Rhamnaceae Juss.	n	1
Iz_4_1009	DJI_0288	Fraxinus pennsylvanica Marshall	0	Oleaceae Hoffmanns. & Link	a	0
Iz_4_1009	DJI_0288	Pinus sylvestris L.	0	Pinaceae Spreng. ex F.Rudolphi	n	1
Iz_4_1009	DJI_0288	Populus × canescens (Aiton) Sm.	0	Salicaceae Mirb.	n	1
Iz_4_1009	DJI_0288	Populus tremula L.	0	Salicaceae Mirb.	n	1
Iz_4_1009	DJI_0288	Populus nigra L.	0	Salicaceae Mirb.	n	1
Iz_4_1009	DJI_0288	Pyrus communis L.	0	Rosaceae Juss.	a	0
Iz_4_1009	DJI_0288	Quercus robur L.	0	Fagaceae Dumort.	n	1
Iz_4_1009	DJI_0288	Robinia pseudoacacia L.	0	Fabaceae Lindl.	a	0
Iz_4_1009	DJI_0288	Rosa sp.	0	Rosaceae Juss.		
Iz_4_1009	DJI_0288	Salix alba L.	0	Salicaceae Mirb.	n	1
Iz_4_1009	DJI_0288	Sambucus nigra L.	0	Viburnaceae Raf.	n	1
Iz_4_1009	DJI_0288	Sorbus aucuparia L.	0	Rosaceae Juss.	n	1
Iz_4_1009	DJI_0288	Tilia cordata Mill.	0	Malvaceae Juss.	n	1
Iz_4_1009	DJI_0288	Ulmus glabra Huds.	0	Ulmaceae Mirb.	n	1
Iz_4_1009	DJI_0292	Acer tataricum L.	0	Sapindaceae Juss.	n	1
Iz_4_1009	DJI_0292	Betula pendula Roth.	0	Betulaceae Gray	n	1
Iz_4_1009	DJI_0292	Crataegus rhipidophylla Gand.	0	Rosaceae Juss.	n	1
Iz_4_1009	DJI_0292	Frangula alnus Mill.	0	Rhamnaceae Juss.	n	1
Iz_4_1009	DJI_0292	Fraxinus pennsylvanica Marshall	0	Oleaceae Hoffmanns. & Link	a	0
Iz_4_1009	DJI_0292	Pinus sylvestris L.	0	Pinaceae Spreng. ex F.Rudolphi	n	1
Iz_4_1009	DJI_0292	Populus × canescens (Aiton) Sm.	0	Salicaceae Mirb.	n	1
Iz_4_1009	DJI_0292	Populus tremula L.	0	Salicaceae Mirb.	n	1
Iz_4_1009	DJI_0292	Populus nigra L.	0	Salicaceae Mirb.	n	1
Iz_4_1009	DJI_0292	Pyrus communis L.	0	Rosaceae Juss.	a	0
Iz_4_1009	DJI_0292	Quercus robur L.	0	Fagaceae Dumort.	n	1

Iz_4_1009	DJI_0292	Robinia pseudoacacia L.	0	Fabaceae Lindl.	a	0
Iz_4_1009	DJI_0292	Rosa sp.	0	Rosaceae Juss.		
Iz_4_1009	DJI_0292	Salix alba L.	0	Salicaceae Mirb.	n	1
Iz_4_1009	DJI_0292	Sambucus nigra L.	0	Viburnaceae Raf.	n	1
Iz_4_1009	DJI_0292	Sorbus aucuparia L.	0	Rosaceae Juss.	n	1
Iz_4_1009	DJI_0292	Tilia cordata Mill.	0	Malvaceae Juss.	n	1
Iz_4_1009	DJI_0292	Ulmus glabra Huds.	0	Ulmaceae Mirb.	n	1
Iz_4_1009	DJI_0297	Acer tataricum L.	0	Sapindaceae Juss.	n	1
Iz_4_1009	DJI_0297	Betula pendula Roth.	0	Betulaceae Gray	n	1
Iz_4_1009	DJI_0297	Crataegus rhipidophylla Gand.	0	Rosaceae Juss.	n	1
Iz_4_1009	DJI_0297	Frangula alnus Mill.	0	Rhamnaceae Juss.	n	1
Iz_4_1009	DJI_0297	Fraxinus pennsylvanica Marshall	0	Oleaceae Hoffmanns. & Link	a	0
Iz_4_1009	DJI_0297	Pinus sylvestris L.	0	Pinaceae Spreng. ex F.Rudolphi	n	1
Iz_4_1009	DJI_0297	Populus × canescens (Aiton) Sm.	0	Salicaceae Mirb.	n	1
Iz_4_1009	DJI_0297	Populus tremula L.	0	Salicaceae Mirb.	n	1
Iz_4_1009	DJI_0297	Populus nigra L.	0	Salicaceae Mirb.	n	1
Iz_4_1009	DJI_0297	Pyrus communis L.	0	Rosaceae Juss.	a	0
Iz_4_1009	DJI_0297	Quercus robur L.	0	Fagaceae Dumort.	n	1
Iz_4_1009	DJI_0297	Robinia pseudoacacia L.	0	Fabaceae Lindl.	a	0
Iz_4_1009	DJI_0297	Rosa sp.	0	Rosaceae Juss.		
Iz_4_1009	DJI_0297	Salix alba L.	0	Salicaceae Mirb.	n	1
Iz_4_1009	DJI_0297	Sambucus nigra L.	0	Viburnaceae Raf.	n	1
Iz_4_1009	DJI_0297	Sorbus aucuparia L.	0	Rosaceae Juss.	n	1
Iz_4_1009	DJI_0297	Tilia cordata Mill.	0	Malvaceae Juss.	n	1
Iz_4_1009	DJI_0297	Ulmus glabra Huds.	0	Ulmaceae Mirb.	n	1
Iz_4_1009	DJI_0299	Acer tataricum L.	0	Sapindaceae Juss.	n	1
Iz_4_1009	DJI_0299	Betula pendula Roth.	0	Betulaceae Gray	n	1
Iz_4_1009	DJI_0299	Crataegus rhipidophylla Gand.	0	Rosaceae Juss.	n	1
Iz_4_1009	DJI_0299	Frangula alnus Mill.	0	Rhamnaceae Juss.	n	1
Iz_4_1009	DJI_0299	Fraxinus pennsylvanica Marshall	0	Oleaceae Hoffmanns. & Link	a	0
Iz_4_1009	DJI_0299	Pinus sylvestris L.	0	Pinaceae Spreng. ex F.Rudolphi	n	1
Iz_4_1009	DJI_0299	Populus × canescens (Aiton) Sm.	0	Salicaceae Mirb.	n	1
Iz_4_1009	DJI_0299	Populus tremula L.	0	Salicaceae Mirb.	n	1
Iz_4_1009	DJI_0299	Populus nigra L.	0	Salicaceae Mirb.	n	1
Iz_4_1009	DJI_0299	Pyrus communis L.	0	Rosaceae Juss.	a	0
Iz_4_1009	DJI_0299	Quercus robur L.	0	Fagaceae Dumort.	n	1
Iz_4_1009	DJI_0299	Robinia pseudoacacia L.	0	Fabaceae Lindl.	a	0
Iz_4_1009	DJI_0299	Rosa sp.	0	Rosaceae Juss.		
Iz_4_1009	DJI_0299	Salix alba L.	0	Salicaceae Mirb.	n	1
Iz_4_1009	DJI_0299	Sambucus nigra L.	0	Viburnaceae Raf.	n	1
Iz_4_1009	DJI_0299	Sorbus aucuparia L.	0	Rosaceae Juss.	n	1
Iz_4_1009	DJI_0299	Tilia cordata Mill.	0	Malvaceae Juss.	n	1
Iz_4_1009	DJI_0299	Ulmus glabra Huds.	0	Ulmaceae Mirb.	n	1
Iz_4_1009	DJI_0300	Acer tataricum L.	0	Sapindaceae Juss.	n	1
Iz_4_1009	DJI_0300	Betula pendula Roth.	0	Betulaceae Gray	n	1
Iz_4_1009	DJI_0300	Crataegus rhipidophylla Gand.	0	Rosaceae Juss.	n	1
Iz_4_1009	DJI_0300	Frangula alnus Mill.	0	Rhamnaceae Juss.	n	1
Iz_4_1009	DJI_0300	Fraxinus pennsylvanica Marshall	0	Oleaceae Hoffmanns. & Link	a	0
Iz_4_1009	DJI_0300	Pinus sylvestris L.	0	Pinaceae Spreng. ex F.Rudolphi	n	1
Iz_4_1009	DJI_0300	Populus × canescens (Aiton) Sm.	0	Salicaceae Mirb.	n	1
Iz_4_1009	DJI_0300	Populus tremula L.	0	Salicaceae Mirb.	n	1
Iz_4_1009	DJI_0300	Populus nigra L.	0	Salicaceae Mirb.	n	1
Iz_4_1009	DJI_0300	Pyrus communis L.	0	Rosaceae Juss.	a	0
Iz_4_1009	DJI_0300	Quercus robur L.	0	Fagaceae Dumort.	n	1
Iz_4_1009	DJI_0300	Robinia pseudoacacia L.	0	Fabaceae Lindl.	a	0
Iz_4_1009	DJI_0300	Rosa sp.	0	Rosaceae Juss.		
Iz_4_1009	DJI_0300	Salix alba L.	0	Salicaceae Mirb.	n	1
Iz_4_1009	DJI_0300	Sambucus nigra L.	0	Viburnaceae Raf.	n	1
Iz_4_1009	DJI_0300	Sorbus aucuparia L.	0	Rosaceae Juss.	n	1
Iz_4_1009	DJI_0300	Tilia cordata Mill.	0	Malvaceae Juss.	n	1
Iz_4_1009	DJI_0300	Ulmus glabra Huds.	0	Ulmaceae Mirb.	n	1
Iz_4_1009	DJI_0303	Acer tataricum L.	0	Sapindaceae Juss.	n	1
Iz_4_1009	DJI_0303	Betula pendula Roth.	0	Betulaceae Gray	n	1
Iz_4_1009	DJI_0303	Crataegus rhipidophylla Gand.	0	Rosaceae Juss.	n	1

Iz_4_1009	DJI_0303	Frangula alnus Mill.	0	Rhamnaceae Juss.	n	1
Iz_4_1009	DJI_0303	Fraxinus pennsylvanica Marshall	0	Oleaceae Hoffmanns. & Link	a	0
Iz_4_1009	DJI_0303	Pinus sylvestris L.	0	Pinaceae Spreng. ex F.Rudolphi	n	1
Iz_4_1009	DJI_0303	Populus × canescens (Aiton) Sm.	0	Salicaceae Mirb.	n	1
Iz_4_1009	DJI_0303	Populus tremula L.	0	Salicaceae Mirb.	n	1
Iz_4_1009	DJI_0303	Populus nigra L.	0	Salicaceae Mirb.	n	1
Iz_4_1009	DJI_0303	Pyrus communis L.	0	Rosaceae Juss.	a	0
Iz_4_1009	DJI_0303	Quercus robur L.	0	Fagaceae Dumort.	n	1
Iz_4_1009	DJI_0303	Robinia pseudoacacia L.	0	Fabaceae Lindl.	a	0
Iz_4_1009	DJI_0303	Rosa sp.	0	Rosaceae Juss.		
Iz_4_1009	DJI_0303	Salix alba L.	0	Salicaceae Mirb.	n	1
Iz_4_1009	DJI_0303	Sambucus nigra L.	0	Viburnaceae Raf.	n	1
Iz_4_1009	DJI_0303	Sorbus aucuparia L.	0	Rosaceae Juss.	n	1
Iz_4_1009	DJI_0303	Tilia cordata Mill.	0	Malvaceae Juss.	n	1
Iz_4_1009	DJI_0303	Ulmus glabra Huds.	0	Ulmaceae Mirb.	n	1
Iz_4_1009	DJI_0309	Acer tataricum L.	0	Sapindaceae Juss.	n	1
Iz_4_1009	DJI_0309	Betula pendula Roth.	0	Betulaceae Gray	n	1
Iz_4_1009	DJI_0309	Crataegus rhipidophylla Gand.	0	Rosaceae Juss.	n	1
Iz_4_1009	DJI_0309	Frangula alnus Mill.	0	Rhamnaceae Juss.	n	1
Iz_4_1009	DJI_0309	Fraxinus pennsylvanica Marshall	0	Oleaceae Hoffmanns. & Link	a	0
Iz_4_1009	DJI_0309	Pinus sylvestris L.	0	Pinaceae Spreng. ex F.Rudolphi	n	1
Iz_4_1009	DJI_0309	Populus × canescens (Aiton) Sm.	0	Salicaceae Mirb.	n	1
Iz_4_1009	DJI_0309	Populus tremula L.	0	Salicaceae Mirb.	n	1
Iz_4_1009	DJI_0309	Populus nigra L.	0	Salicaceae Mirb.	n	1
Iz_4_1009	DJI_0309	Pyrus communis L.	0	Rosaceae Juss.	a	0
Iz_4_1009	DJI_0309	Quercus robur L.	0	Fagaceae Dumort.	n	1
Iz_4_1009	DJI_0309	Robinia pseudoacacia L.	0	Fabaceae Lindl.	a	0
Iz_4_1009	DJI_0309	Rosa sp.	0	Rosaceae Juss.		
Iz_4_1009	DJI_0309	Salix alba L.	0	Salicaceae Mirb.	n	1
Iz_4_1009	DJI_0309	Sambucus nigra L.	0	Viburnaceae Raf.	n	1
Iz_4_1009	DJI_0309	Sorbus aucuparia L.	0	Rosaceae Juss.	n	1
Iz_4_1009	DJI_0309	Tilia cordata Mill.	0	Malvaceae Juss.	n	1
Iz_4_1009	DJI_0309	Ulmus glabra Huds.	0	Ulmaceae Mirb.	n	1
Iz_4_1009	DJI_0314	Acer tataricum L.	0	Sapindaceae Juss.	n	1
Iz_4_1009	DJI_0314	Betula pendula Roth.	0	Betulaceae Gray	n	1
Iz_4_1009	DJI_0314	Crataegus rhipidophylla Gand.	0	Rosaceae Juss.	n	1
Iz_4_1009	DJI_0314	Frangula alnus Mill.	0	Rhamnaceae Juss.	n	1
Iz_4_1009	DJI_0314	Fraxinus pennsylvanica Marshall	0	Oleaceae Hoffmanns. & Link	a	0
Iz_4_1009	DJI_0314	Pinus sylvestris L.	0	Pinaceae Spreng. ex F.Rudolphi	n	1
Iz_4_1009	DJI_0314	Populus × canescens (Aiton) Sm.	0	Salicaceae Mirb.	n	1
Iz_4_1009	DJI_0314	Populus tremula L.	0	Salicaceae Mirb.	n	1
Iz_4_1009	DJI_0314	Populus nigra L.	0	Salicaceae Mirb.	n	1
Iz_4_1009	DJI_0314	Pyrus communis L.	0	Rosaceae Juss.	a	0
Iz_4_1009	DJI_0314	Quercus robur L.	0	Fagaceae Dumort.	n	1
Iz_4_1009	DJI_0314	Robinia pseudoacacia L.	0	Fabaceae Lindl.	a	0
Iz_4_1009	DJI_0314	Rosa sp.	0	Rosaceae Juss.		
Iz_4_1009	DJI_0314	Salix alba L.	0	Salicaceae Mirb.	n	1
Iz_4_1009	DJI_0314	Sambucus nigra L.	0	Viburnaceae Raf.	n	1
Iz_4_1009	DJI_0314	Sorbus aucuparia L.	0	Rosaceae Juss.	n	1
Iz_4_1009	DJI_0314	Tilia cordata Mill.	0	Malvaceae Juss.	n	1
Iz_4_1009	DJI_0314	Ulmus glabra Huds.	0	Ulmaceae Mirb.	n	1
Iz_4_1009	DJI_0316	Acer tataricum L.	0	Sapindaceae Juss.	n	1
Iz_4_1009	DJI_0316	Betula pendula Roth.	0	Betulaceae Gray	n	1
Iz_4_1009	DJI_0316	Crataegus rhipidophylla Gand.	0	Rosaceae Juss.	n	1
Iz_4_1009	DJI_0316	Frangula alnus Mill.	0	Rhamnaceae Juss.	n	1
Iz_4_1009	DJI_0316	Fraxinus pennsylvanica Marshall	0	Oleaceae Hoffmanns. & Link	a	0
Iz_4_1009	DJI_0316	Pinus sylvestris L.	0	Pinaceae Spreng. ex F.Rudolphi	n	1
Iz_4_1009	DJI_0316	Populus × canescens (Aiton) Sm.	0	Salicaceae Mirb.	n	1
Iz_4_1009	DJI_0316	Populus tremula L.	0	Salicaceae Mirb.	n	1
Iz_4_1009	DJI_0316	Populus nigra L.	0	Salicaceae Mirb.	n	1
Iz_4_1009	DJI_0316	Pyrus communis L.	0	Rosaceae Juss.	a	0
Iz_4_1009	DJI_0316	Quercus robur L.	0	Fagaceae Dumort.	n	1
Iz_4_1009	DJI_0316	Robinia pseudoacacia L.	0	Fabaceae Lindl.	a	0
Iz_4_1009	DJI_0316	Rosa sp.	0	Rosaceae Juss.		

Iz_4_1009	DJI_0316	Salix alba L.	0	Salicaceae Mirb.	n	1
Iz_4_1009	DJI_0316	Sambucus nigra L.	0	Viburnaceae Raf.	n	1
Iz_4_1009	DJI_0316	Sorbus aucuparia L.	0	Rosaceae Juss.	n	1
Iz_4_1009	DJI_0316	Tilia cordata Mill.	0	Malvaceae Juss.	n	1
Iz_4_1009	DJI_0316	Ulmus glabra Huds.	0	Ulmaceae Mirb.	n	1
Iz_4_1009	DJI_0319	Acer tataricum L.	0	Sapindaceae Juss.	n	1
Iz_4_1009	DJI_0319	Betula pendula Roth.	0	Betulaceae Gray	n	1
Iz_4_1009	DJI_0319	Crataegus rhipidophylla Gand.	0	Rosaceae Juss.	n	1
Iz_4_1009	DJI_0319	Frangula alnus Mill.	0	Rhamnaceae Juss.	n	1
Iz_4_1009	DJI_0319	Fraxinus pennsylvanica Marshall	0	Oleaceae Hoffmanns. & Link	a	0
Iz_4_1009	DJI_0319	Pinus sylvestris L.	0	Pinaceae Spreng. ex F.Rudolphi	n	1
Iz_4_1009	DJI_0319	Populus × canescens (Aiton) Sm.	0	Salicaceae Mirb.	n	1
Iz_4_1009	DJI_0319	Populus tremula L.	0	Salicaceae Mirb.	n	1
Iz_4_1009	DJI_0319	Populus nigra L.	0	Salicaceae Mirb.	n	1
Iz_4_1009	DJI_0319	Pyrus communis L.	0	Rosaceae Juss.	a	0
Iz_4_1009	DJI_0319	Quercus robur L.	0	Fagaceae Dumort.	n	1
Iz_4_1009	DJI_0319	Robinia pseudoacacia L.	0	Fabaceae Lindl.	a	0
Iz_4_1009	DJI_0319	Rosa sp.	0	Rosaceae Juss.		
Iz_4_1009	DJI_0319	Salix alba L.	0	Salicaceae Mirb.	n	1
Iz_4_1009	DJI_0319	Sambucus nigra L.	0	Viburnaceae Raf.	n	1
Iz_4_1009	DJI_0319	Sorbus aucuparia L.	0	Rosaceae Juss.	n	1
Iz_4_1009	DJI_0319	Tilia cordata Mill.	0	Malvaceae Juss.	n	1
Iz_4_1009	DJI_0319	Ulmus glabra Huds.	0	Ulmaceae Mirb.	n	1
Iz_4_1009	DJI_0323	Acer tataricum L.	0	Sapindaceae Juss.	n	1
Iz_4_1009	DJI_0323	Betula pendula Roth.	0	Betulaceae Gray	n	1
Iz_4_1009	DJI_0323	Crataegus rhipidophylla Gand.	0	Rosaceae Juss.	n	1
Iz_4_1009	DJI_0323	Frangula alnus Mill.	0	Rhamnaceae Juss.	n	1
Iz_4_1009	DJI_0323	Fraxinus pennsylvanica Marshall	0	Oleaceae Hoffmanns. & Link	a	0
Iz_4_1009	DJI_0323	Pinus sylvestris L.	0	Pinaceae Spreng. ex F.Rudolphi	n	1
Iz_4_1009	DJI_0323	Populus × canescens (Aiton) Sm.	0	Salicaceae Mirb.	n	1
Iz_4_1009	DJI_0323	Populus tremula L.	0	Salicaceae Mirb.	n	1
Iz_4_1009	DJI_0323	Populus nigra L.	0	Salicaceae Mirb.	n	1
Iz_4_1009	DJI_0323	Pyrus communis L.	0	Rosaceae Juss.	a	0
Iz_4_1009	DJI_0323	Quercus robur L.	0	Fagaceae Dumort.	n	1
Iz_4_1009	DJI_0323	Robinia pseudoacacia L.	0	Fabaceae Lindl.	a	0
Iz_4_1009	DJI_0323	Rosa sp.	0	Rosaceae Juss.		
Iz_4_1009	DJI_0323	Salix alba L.	0	Salicaceae Mirb.	n	1
Iz_4_1009	DJI_0323	Sambucus nigra L.	0	Viburnaceae Raf.	n	1
Iz_4_1009	DJI_0323	Sorbus aucuparia L.	0	Rosaceae Juss.	n	1
Iz_4_1009	DJI_0323	Tilia cordata Mill.	0	Malvaceae Juss.	n	1
Iz_4_1009	DJI_0323	Ulmus glabra Huds.	0	Ulmaceae Mirb.	n	1
Iz_4_1009	DJI_0325	Acer tataricum L.	0	Sapindaceae Juss.	n	1
Iz_4_1009	DJI_0325	Betula pendula Roth.	0	Betulaceae Gray	n	1
Iz_4_1009	DJI_0325	Crataegus rhipidophylla Gand.	0	Rosaceae Juss.	n	1
Iz_4_1009	DJI_0325	Frangula alnus Mill.	0	Rhamnaceae Juss.	n	1
Iz_4_1009	DJI_0325	Fraxinus pennsylvanica Marshall	0	Oleaceae Hoffmanns. & Link	a	0
Iz_4_1009	DJI_0325	Pinus sylvestris L.	0	Pinaceae Spreng. ex F.Rudolphi	n	1
Iz_4_1009	DJI_0325	Populus × canescens (Aiton) Sm.	0	Salicaceae Mirb.	n	1
Iz_4_1009	DJI_0325	Populus tremula L.	0	Salicaceae Mirb.	n	1
Iz_4_1009	DJI_0325	Populus nigra L.	0	Salicaceae Mirb.	n	1
Iz_4_1009	DJI_0325	Pyrus communis L.	0	Rosaceae Juss.	a	0
Iz_4_1009	DJI_0325	Quercus robur L.	0	Fagaceae Dumort.	n	1
Iz_4_1009	DJI_0325	Robinia pseudoacacia L.	0	Fabaceae Lindl.	a	0
Iz_4_1009	DJI_0325	Rosa sp.	0	Rosaceae Juss.		
Iz_4_1009	DJI_0325	Salix alba L.	0	Salicaceae Mirb.	n	1
Iz_4_1009	DJI_0325	Sambucus nigra L.	0	Viburnaceae Raf.	n	1
Iz_4_1009	DJI_0325	Sorbus aucuparia L.	0	Rosaceae Juss.	n	1
Iz_4_1009	DJI_0325	Tilia cordata Mill.	0	Malvaceae Juss.	n	1
Iz_4_1009	DJI_0325	Ulmus glabra Huds.	0	Ulmaceae Mirb.	n	1
Iz_4_1009	DJI_0327	Acer tataricum L.	0	Sapindaceae Juss.	n	1
Iz_4_1009	DJI_0327	Betula pendula Roth.	0	Betulaceae Gray	n	1
Iz_4_1009	DJI_0327	Crataegus rhipidophylla Gand.	0	Rosaceae Juss.	n	1
Iz_4_1009	DJI_0327	Frangula alnus Mill.	0	Rhamnaceae Juss.	n	1
Iz_4_1009	DJI_0327	Fraxinus pennsylvanica Marshall	0	Oleaceae Hoffmanns. & Link	a	0

Iz_4_1009	DJI_0327	Pinus sylvestris L.	0	Pinaceae Spreng. ex F.Rudolphi	n	1
Iz_4_1009	DJI_0327	Populus × canescens (Aiton) Sm.	0	Salicaceae Mirb.	n	1
Iz_4_1009	DJI_0327	Populus tremula L.	0	Salicaceae Mirb.	n	1
Iz_4_1009	DJI_0327	Populus nigra L.	0	Salicaceae Mirb.	n	1
Iz_4_1009	DJI_0327	Pyrus communis L.	0	Rosaceae Juss.	a	0
Iz_4_1009	DJI_0327	Quercus robur L.	0	Fagaceae Dumort.	n	1
Iz_4_1009	DJI_0327	Robinia pseudoacacia L.	0	Fabaceae Lindl.	a	0
Iz_4_1009	DJI_0327	Rosa sp.	0	Rosaceae Juss.		
Iz_4_1009	DJI_0327	Salix alba L.	0	Salicaceae Mirb.	n	1
Iz_4_1009	DJI_0327	Sambucus nigra L.	0	Viburnaceae Raf.	n	1
Iz_4_1009	DJI_0327	Sorbus aucuparia L.	0	Rosaceae Juss.	n	1
Iz_4_1009	DJI_0327	Tilia cordata Mill.	0	Malvaceae Juss.	n	1
Iz_4_1009	DJI_0327	Ulmus glabra Huds.	0	Ulmaceae Mirb.	n	1
Iz_4_1009	DJI_0330	Acer tataricum L.	0	Sapindaceae Juss.	n	1
Iz_4_1009	DJI_0330	Betula pendula Roth.	0	Betulaceae Gray	n	1
Iz_4_1009	DJI_0330	Crataegus rhipidophylla Gand.	0	Rosaceae Juss.	n	1
Iz_4_1009	DJI_0330	Frangula alnus Mill.	0	Rhamnaceae Juss.	n	1
Iz_4_1009	DJI_0330	Fraxinus pennsylvanica Marshall	0	Oleaceae Hoffmanns. & Link	a	0
Iz_4_1009	DJI_0330	Pinus sylvestris L.	0	Pinaceae Spreng. ex F.Rudolphi	n	1
Iz_4_1009	DJI_0330	Populus × canescens (Aiton) Sm.	0	Salicaceae Mirb.	n	1
Iz_4_1009	DJI_0330	Populus tremula L.	0	Salicaceae Mirb.	n	1
Iz_4_1009	DJI_0330	Populus nigra L.	0	Salicaceae Mirb.	n	1
Iz_4_1009	DJI_0330	Pyrus communis L.	0	Rosaceae Juss.	a	0
Iz_4_1009	DJI_0330	Quercus robur L.	0	Fagaceae Dumort.	n	1
Iz_4_1009	DJI_0330	Robinia pseudoacacia L.	0	Fabaceae Lindl.	a	0
Iz_4_1009	DJI_0330	Rosa sp.	0	Rosaceae Juss.		
Iz_4_1009	DJI_0330	Salix alba L.	0	Salicaceae Mirb.	n	1
Iz_4_1009	DJI_0330	Sambucus nigra L.	0	Viburnaceae Raf.	n	1
Iz_4_1009	DJI_0330	Sorbus aucuparia L.	0	Rosaceae Juss.	n	1
Iz_4_1009	DJI_0330	Tilia cordata Mill.	0	Malvaceae Juss.	n	1
Iz_4_1009	DJI_0330	Ulmus glabra Huds.	0	Ulmaceae Mirb.	n	1
Iz_4_1009	DJI_0333	Acer tataricum L.	0	Sapindaceae Juss.	n	1
Iz_4_1009	DJI_0333	Betula pendula Roth.	0	Betulaceae Gray	n	1
Iz_4_1009	DJI_0333	Crataegus rhipidophylla Gand.	0	Rosaceae Juss.	n	1
Iz_4_1009	DJI_0333	Frangula alnus Mill.	0	Rhamnaceae Juss.	n	1
Iz_4_1009	DJI_0333	Fraxinus pennsylvanica Marshall	0	Oleaceae Hoffmanns. & Link	a	0
Iz_4_1009	DJI_0333	Pinus sylvestris L.	0	Pinaceae Spreng. ex F.Rudolphi	n	1
Iz_4_1009	DJI_0333	Populus × canescens (Aiton) Sm.	0	Salicaceae Mirb.	n	1
Iz_4_1009	DJI_0333	Populus tremula L.	0	Salicaceae Mirb.	n	1
Iz_4_1009	DJI_0333	Populus nigra L.	0	Salicaceae Mirb.	n	1
Iz_4_1009	DJI_0333	Pyrus communis L.	0	Rosaceae Juss.	a	0
Iz_4_1009	DJI_0333	Quercus robur L.	0	Fagaceae Dumort.	n	1
Iz_4_1009	DJI_0333	Robinia pseudoacacia L.	0	Fabaceae Lindl.	a	0
Iz_4_1009	DJI_0333	Rosa sp.	0	Rosaceae Juss.		
Iz_4_1009	DJI_0333	Salix alba L.	0	Salicaceae Mirb.	n	1
Iz_4_1009	DJI_0333	Sambucus nigra L.	0	Viburnaceae Raf.	n	1
Iz_4_1009	DJI_0333	Sorbus aucuparia L.	0	Rosaceae Juss.	n	1
Iz_4_1009	DJI_0333	Tilia cordata Mill.	0	Malvaceae Juss.	n	1
Iz_4_1009	DJI_0333	Ulmus glabra Huds.	0	Ulmaceae Mirb.	n	1
Iz_4_1009	DJI_0335	Acer tataricum L.	0	Sapindaceae Juss.	n	1
Iz_4_1009	DJI_0335	Betula pendula Roth.	0	Betulaceae Gray	n	1
Iz_4_1009	DJI_0335	Crataegus rhipidophylla Gand.	0	Rosaceae Juss.	n	1
Iz_4_1009	DJI_0335	Frangula alnus Mill.	0	Rhamnaceae Juss.	n	1
Iz_4_1009	DJI_0335	Fraxinus pennsylvanica Marshall	0	Oleaceae Hoffmanns. & Link	a	0
Iz_4_1009	DJI_0335	Pinus sylvestris L.	0	Pinaceae Spreng. ex F.Rudolphi	n	1
Iz_4_1009	DJI_0335	Populus × canescens (Aiton) Sm.	0	Salicaceae Mirb.	n	1
Iz_4_1009	DJI_0335	Populus tremula L.	0	Salicaceae Mirb.	n	1
Iz_4_1009	DJI_0335	Populus nigra L.	0	Salicaceae Mirb.	n	1
Iz_4_1009	DJI_0335	Pyrus communis L.	0	Rosaceae Juss.	a	0
Iz_4_1009	DJI_0335	Quercus robur L.	0	Fagaceae Dumort.	n	1
Iz_4_1009	DJI_0335	Robinia pseudoacacia L.	0	Fabaceae Lindl.	a	0
Iz_4_1009	DJI_0335	Rosa sp.	0	Rosaceae Juss.		
Iz_4_1009	DJI_0335	Salix alba L.	0	Salicaceae Mirb.	n	1
Iz_4_1009	DJI_0335	Sambucus nigra L.	0	Viburnaceae Raf.	n	1

Iz_4_1009	DJI_0335	Sorbus aucuparia L.	0	Rosaceae Juss.	n	1
Iz_4_1009	DJI_0335	Tilia cordata Mill.	0	Malvaceae Juss.	n	1
Iz_4_1009	DJI_0335	Ulmus glabra Huds.	0	Ulmaceae Mirb.	n	1
Iz_4_1009	DJI_0336	Acer tataricum L.	0	Sapindaceae Juss.	n	1
Iz_4_1009	DJI_0336	Betula pendula Roth.	0	Betulaceae Gray	n	1
Iz_4_1009	DJI_0336	Crataegus rhipidophylla Gand.	0	Rosaceae Juss.	n	1
Iz_4_1009	DJI_0336	Frangula alnus Mill.	0	Rhamnaceae Juss.	n	1
Iz_4_1009	DJI_0336	Fraxinus pennsylvanica Marshall	0	Oleaceae Hoffmanns. & Link	a	0
Iz_4_1009	DJI_0336	Pinus sylvestris L.	0	Pinaceae Spreng. ex F.Rudolphi	n	1
Iz_4_1009	DJI_0336	Populus × canescens (Aiton) Sm.	0	Salicaceae Mirb.	n	1
Iz_4_1009	DJI_0336	Populus tremula L.	0	Salicaceae Mirb.	n	1
Iz_4_1009	DJI_0336	Populus nigra L.	0	Salicaceae Mirb.	n	1
Iz_4_1009	DJI_0336	Pyrus communis L.	0	Rosaceae Juss.	a	0
Iz_4_1009	DJI_0336	Quercus robur L.	0	Fagaceae Dumort.	n	1
Iz_4_1009	DJI_0336	Robinia pseudoacacia L.	0	Fabaceae Lindl.	a	0
Iz_4_1009	DJI_0336	Rosa sp.	0	Rosaceae Juss.		
Iz_4_1009	DJI_0336	Salix alba L.	0	Salicaceae Mirb.	n	1
Iz_4_1009	DJI_0336	Sambucus nigra L.	0	Viburnaceae Raf.	n	1
Iz_4_1009	DJI_0336	Sorbus aucuparia L.	0	Rosaceae Juss.	n	1
Iz_4_1009	DJI_0336	Tilia cordata Mill.	0	Malvaceae Juss.	n	1
Iz_4_1009	DJI_0336	Ulmus glabra Huds.	0	Ulmaceae Mirb.	n	1
Iz_4_1009	DJI_0339	Acer tataricum L.	0	Sapindaceae Juss.	n	1
Iz_4_1009	DJI_0339	Betula pendula Roth.	0	Betulaceae Gray	n	1
Iz_4_1009	DJI_0339	Crataegus rhipidophylla Gand.	0	Rosaceae Juss.	n	1
Iz_4_1009	DJI_0339	Frangula alnus Mill.	0	Rhamnaceae Juss.	n	1
Iz_4_1009	DJI_0339	Fraxinus pennsylvanica Marshall	0	Oleaceae Hoffmanns. & Link	a	0
Iz_4_1009	DJI_0339	Pinus sylvestris L.	0	Pinaceae Spreng. ex F.Rudolphi	n	1
Iz_4_1009	DJI_0339	Populus × canescens (Aiton) Sm.	0	Salicaceae Mirb.	n	1
Iz_4_1009	DJI_0339	Populus tremula L.	0	Salicaceae Mirb.	n	1
Iz_4_1009	DJI_0339	Populus nigra L.	0	Salicaceae Mirb.	n	1
Iz_4_1009	DJI_0339	Pyrus communis L.	0	Rosaceae Juss.	a	0
Iz_4_1009	DJI_0339	Quercus robur L.	0	Fagaceae Dumort.	n	1
Iz_4_1009	DJI_0339	Robinia pseudoacacia L.	0	Fabaceae Lindl.	a	0
Iz_4_1009	DJI_0339	Rosa sp.	0	Rosaceae Juss.		
Iz_4_1009	DJI_0339	Salix alba L.	0	Salicaceae Mirb.	n	1
Iz_4_1009	DJI_0339	Sambucus nigra L.	0	Viburnaceae Raf.	n	1
Iz_4_1009	DJI_0339	Sorbus aucuparia L.	0	Rosaceae Juss.	n	1
Iz_4_1009	DJI_0339	Tilia cordata Mill.	0	Malvaceae Juss.	n	1
Iz_4_1009	DJI_0339	Ulmus glabra Huds.	0	Ulmaceae Mirb.	n	1
Iz_4_1009	DJI_0342	Acer tataricum L.	0	Sapindaceae Juss.	n	1
Iz_4_1009	DJI_0342	Betula pendula Roth.	0	Betulaceae Gray	n	1
Iz_4_1009	DJI_0342	Crataegus rhipidophylla Gand.	0	Rosaceae Juss.	n	1
Iz_4_1009	DJI_0342	Frangula alnus Mill.	0	Rhamnaceae Juss.	n	1
Iz_4_1009	DJI_0342	Fraxinus pennsylvanica Marshall	0	Oleaceae Hoffmanns. & Link	a	0
Iz_4_1009	DJI_0342	Pinus sylvestris L.	0	Pinaceae Spreng. ex F.Rudolphi	n	1
Iz_4_1009	DJI_0342	Populus × canescens (Aiton) Sm.	0	Salicaceae Mirb.	n	1
Iz_4_1009	DJI_0342	Populus tremula L.	0	Salicaceae Mirb.	n	1
Iz_4_1009	DJI_0342	Populus nigra L.	0	Salicaceae Mirb.	n	1
Iz_4_1009	DJI_0342	Pyrus communis L.	0	Rosaceae Juss.	a	0
Iz_4_1009	DJI_0342	Quercus robur L.	0	Fagaceae Dumort.	n	1
Iz_4_1009	DJI_0342	Robinia pseudoacacia L.	0	Fabaceae Lindl.	a	0
Iz_4_1009	DJI_0342	Rosa sp.	0	Rosaceae Juss.		
Iz_4_1009	DJI_0342	Salix alba L.	0	Salicaceae Mirb.	n	1
Iz_4_1009	DJI_0342	Sambucus nigra L.	0	Viburnaceae Raf.	n	1
Iz_4_1009	DJI_0342	Sorbus aucuparia L.	0	Rosaceae Juss.	n	1
Iz_4_1009	DJI_0342	Tilia cordata Mill.	0	Malvaceae Juss.	n	1
Iz_4_1009	DJI_0342	Ulmus glabra Huds.	0	Ulmaceae Mirb.	n	1
Iz_4_1009	DJI_0343	Acer tataricum L.	0	Sapindaceae Juss.	n	1
Iz_4_1009	DJI_0343	Betula pendula Roth.	0	Betulaceae Gray	n	1
Iz_4_1009	DJI_0343	Crataegus rhipidophylla Gand.	0	Rosaceae Juss.	n	1
Iz_4_1009	DJI_0343	Frangula alnus Mill.	0	Rhamnaceae Juss.	n	1
Iz_4_1009	DJI_0343	Fraxinus pennsylvanica Marshall	0	Oleaceae Hoffmanns. & Link	a	0
Iz_4_1009	DJI_0343	Pinus sylvestris L.	0	Pinaceae Spreng. ex F.Rudolphi	n	1
Iz_4_1009	DJI_0343	Populus × canescens (Aiton) Sm.	0	Salicaceae Mirb.	n	1

Iz_4_1009	DJI_0343	Populus tremula L.	0	Salicaceae Mirb.	n	1
Iz_4_1009	DJI_0343	Populus nigra L.	0	Salicaceae Mirb.	n	1
Iz_4_1009	DJI_0343	Pyrus communis L.	0	Rosaceae Juss.	a	0
Iz_4_1009	DJI_0343	Quercus robur L.	0	Fagaceae Dumort.	n	1
Iz_4_1009	DJI_0343	Robinia pseudoacacia L.	0	Fabaceae Lindl.	a	0
Iz_4_1009	DJI_0343	Rosa sp.	0	Rosaceae Juss.		
Iz_4_1009	DJI_0343	Salix alba L.	0	Salicaceae Mirb.	n	1
Iz_4_1009	DJI_0343	Sambucus nigra L.	0	Viburnaceae Raf.	n	1
Iz_4_1009	DJI_0343	Sorbus aucuparia L.	0	Rosaceae Juss.	n	1
Iz_4_1009	DJI_0343	Tilia cordata Mill.	0	Malvaceae Juss.	n	1
Iz_4_1009	DJI_0343	Ulmus glabra Huds.	0	Ulmaceae Mirb.	n	1
Iz_4_1009	DJI_0344	Acer tataricum L.	0	Sapindaceae Juss.	n	1
Iz_4_1009	DJI_0344	Betula pendula Roth.	0	Betulaceae Gray	n	1
Iz_4_1009	DJI_0344	Crataegus rhipidophylla Gand.	0	Rosaceae Juss.	n	1
Iz_4_1009	DJI_0344	Frangula alnus Mill.	0	Rhamnaceae Juss.	n	1
Iz_4_1009	DJI_0344	Fraxinus pennsylvanica Marshall	0	Oleaceae Hoffmanns. & Link	a	0
Iz_4_1009	DJI_0344	Pinus sylvestris L.	0	Pinaceae Spreng. ex F.Rudolphi	n	1
Iz_4_1009	DJI_0344	Populus × canescens (Aiton) Sm.	0	Salicaceae Mirb.	n	1
Iz_4_1009	DJI_0344	Populus tremula L.	0	Salicaceae Mirb.	n	1
Iz_4_1009	DJI_0344	Populus nigra L.	0	Salicaceae Mirb.	n	1
Iz_4_1009	DJI_0344	Pyrus communis L.	0	Rosaceae Juss.	a	0
Iz_4_1009	DJI_0344	Quercus robur L.	0	Fagaceae Dumort.	n	1
Iz_4_1009	DJI_0344	Robinia pseudoacacia L.	0	Fabaceae Lindl.	a	0
Iz_4_1009	DJI_0344	Rosa sp.	0	Rosaceae Juss.		
Iz_4_1009	DJI_0344	Salix alba L.	0	Salicaceae Mirb.	n	1
Iz_4_1009	DJI_0344	Sambucus nigra L.	0	Viburnaceae Raf.	n	1
Iz_4_1009	DJI_0344	Sorbus aucuparia L.	0	Rosaceae Juss.	n	1
Iz_4_1009	DJI_0344	Tilia cordata Mill.	0	Malvaceae Juss.	n	1
Iz_4_1009	DJI_0344	Ulmus glabra Huds.	0	Ulmaceae Mirb.	n	1
Iz_4_1009	DJI_0346	Acer tataricum L.	0	Sapindaceae Juss.	n	1
Iz_4_1009	DJI_0346	Betula pendula Roth.	0	Betulaceae Gray	n	1
Iz_4_1009	DJI_0346	Crataegus rhipidophylla Gand.	0	Rosaceae Juss.	n	1
Iz_4_1009	DJI_0346	Frangula alnus Mill.	0	Rhamnaceae Juss.	n	1
Iz_4_1009	DJI_0346	Fraxinus pennsylvanica Marshall	0	Oleaceae Hoffmanns. & Link	a	0
Iz_4_1009	DJI_0346	Pinus sylvestris L.	0	Pinaceae Spreng. ex F.Rudolphi	n	1
Iz_4_1009	DJI_0346	Populus × canescens (Aiton) Sm.	0	Salicaceae Mirb.	n	1
Iz_4_1009	DJI_0346	Populus tremula L.	0	Salicaceae Mirb.	n	1
Iz_4_1009	DJI_0346	Populus nigra L.	0	Salicaceae Mirb.	n	1
Iz_4_1009	DJI_0346	Pyrus communis L.	0	Rosaceae Juss.	a	0
Iz_4_1009	DJI_0346	Quercus robur L.	0	Fagaceae Dumort.	n	1
Iz_4_1009	DJI_0346	Robinia pseudoacacia L.	0	Fabaceae Lindl.	a	0
Iz_4_1009	DJI_0346	Rosa sp.	0	Rosaceae Juss.		
Iz_4_1009	DJI_0346	Salix alba L.	0	Salicaceae Mirb.	n	1
Iz_4_1009	DJI_0346	Sambucus nigra L.	0	Viburnaceae Raf.	n	1
Iz_4_1009	DJI_0346	Sorbus aucuparia L.	0	Rosaceae Juss.	n	1
Iz_4_1009	DJI_0346	Tilia cordata Mill.	0	Malvaceae Juss.	n	1
Iz_4_1009	DJI_0346	Ulmus glabra Huds.	0	Ulmaceae Mirb.	n	1
Iz_4_1009	DJI_0348	Acer tataricum L.	0	Sapindaceae Juss.	n	1
Iz_4_1009	DJI_0348	Betula pendula Roth.	0	Betulaceae Gray	n	1
Iz_4_1009	DJI_0348	Crataegus rhipidophylla Gand.	0	Rosaceae Juss.	n	1
Iz_4_1009	DJI_0348	Frangula alnus Mill.	0	Rhamnaceae Juss.	n	1
Iz_4_1009	DJI_0348	Fraxinus pennsylvanica Marshall	0	Oleaceae Hoffmanns. & Link	a	0
Iz_4_1009	DJI_0348	Pinus sylvestris L.	0	Pinaceae Spreng. ex F.Rudolphi	n	1
Iz_4_1009	DJI_0348	Populus × canescens (Aiton) Sm.	0	Salicaceae Mirb.	n	1
Iz_4_1009	DJI_0348	Populus tremula L.	0	Salicaceae Mirb.	n	1
Iz_4_1009	DJI_0348	Populus nigra L.	0	Salicaceae Mirb.	n	1
Iz_4_1009	DJI_0348	Pyrus communis L.	0	Rosaceae Juss.	a	0
Iz_4_1009	DJI_0348	Quercus robur L.	0	Fagaceae Dumort.	n	1
Iz_4_1009	DJI_0348	Robinia pseudoacacia L.	0	Fabaceae Lindl.	a	0
Iz_4_1009	DJI_0348	Rosa sp.	0	Rosaceae Juss.		
Iz_4_1009	DJI_0348	Salix alba L.	0	Salicaceae Mirb.	n	1
Iz_4_1009	DJI_0348	Sambucus nigra L.	0	Viburnaceae Raf.	n	1
Iz_4_1009	DJI_0348	Sorbus aucuparia L.	0	Rosaceae Juss.	n	1
Iz_4_1009	DJI_0348	Tilia cordata Mill.	0	Malvaceae Juss.	n	1

Iz_4_1009	DJI_0348	Ulmus glabra Huds.	0	Ulmaceae Mirb.	n	1
Iz_4_1009	DJI_0351	Acer tataricum L.	0	Sapindaceae Juss.	n	1
Iz_4_1009	DJI_0351	Betula pendula Roth.	0	Betulaceae Gray	n	1
Iz_4_1009	DJI_0351	Crataegus rhipidophylla Gand.	0	Rosaceae Juss.	n	1
Iz_4_1009	DJI_0351	Frangula alnus Mill.	0	Rhamnaceae Juss.	n	1
Iz_4_1009	DJI_0351	Fraxinus pennsylvanica Marshall	0	Oleaceae Hoffmanns. & Link	a	0
Iz_4_1009	DJI_0351	Pinus sylvestris L.	0	Pinaceae Spreng. ex F.Rudolphi	n	1
Iz_4_1009	DJI_0351	Populus × canescens (Aiton) Sm.	0	Salicaceae Mirb.	n	1
Iz_4_1009	DJI_0351	Populus tremula L.	0	Salicaceae Mirb.	n	1
Iz_4_1009	DJI_0351	Populus nigra L.	0	Salicaceae Mirb.	n	1
Iz_4_1009	DJI_0351	Pyrus communis L.	0	Rosaceae Juss.	a	0
Iz_4_1009	DJI_0351	Quercus robur L.	0	Fagaceae Dumort.	n	1
Iz_4_1009	DJI_0351	Robinia pseudoacacia L.	0	Fabaceae Lindl.	a	0
Iz_4_1009	DJI_0351	Rosa sp.	0	Rosaceae Juss.		
Iz_4_1009	DJI_0351	Salix alba L.	0	Salicaceae Mirb.	n	1
Iz_4_1009	DJI_0351	Sambucus nigra L.	0	Viburnaceae Raf.	n	1
Iz_4_1009	DJI_0351	Sorbus aucuparia L.	0	Rosaceae Juss.	n	1
Iz_4_1009	DJI_0351	Tilia cordata Mill.	0	Malvaceae Juss.	n	1
Iz_4_1009	DJI_0351	Ulmus glabra Huds.	0	Ulmaceae Mirb.	n	1
Iz_4_1009	DJI_0352	Acer tataricum L.	0	Sapindaceae Juss.	n	1
Iz_4_1009	DJI_0352	Betula pendula Roth.	0	Betulaceae Gray	n	1
Iz_4_1009	DJI_0352	Crataegus rhipidophylla Gand.	0	Rosaceae Juss.	n	1
Iz_4_1009	DJI_0352	Frangula alnus Mill.	0	Rhamnaceae Juss.	n	1
Iz_4_1009	DJI_0352	Fraxinus pennsylvanica Marshall	0	Oleaceae Hoffmanns. & Link	a	0
Iz_4_1009	DJI_0352	Pinus sylvestris L.	0	Pinaceae Spreng. ex F.Rudolphi	n	1
Iz_4_1009	DJI_0352	Populus × canescens (Aiton) Sm.	0	Salicaceae Mirb.	n	1
Iz_4_1009	DJI_0352	Populus tremula L.	0	Salicaceae Mirb.	n	1
Iz_4_1009	DJI_0352	Populus nigra L.	0	Salicaceae Mirb.	n	1
Iz_4_1009	DJI_0352	Pyrus communis L.	0	Rosaceae Juss.	a	0
Iz_4_1009	DJI_0352	Quercus robur L.	0	Fagaceae Dumort.	n	1
Iz_4_1009	DJI_0352	Robinia pseudoacacia L.	0	Fabaceae Lindl.	a	0
Iz_4_1009	DJI_0352	Rosa sp.	0	Rosaceae Juss.		
Iz_4_1009	DJI_0352	Salix alba L.	0	Salicaceae Mirb.	n	1
Iz_4_1009	DJI_0352	Sambucus nigra L.	0	Viburnaceae Raf.	n	1
Iz_4_1009	DJI_0352	Sorbus aucuparia L.	0	Rosaceae Juss.	n	1
Iz_4_1009	DJI_0352	Tilia cordata Mill.	0	Malvaceae Juss.	n	1
Iz_4_1009	DJI_0352	Ulmus glabra Huds.	0	Ulmaceae Mirb.	n	1
Iz_4_1009	DJI_0355	Acer tataricum L.	0	Sapindaceae Juss.	n	1
Iz_4_1009	DJI_0355	Betula pendula Roth.	0	Betulaceae Gray	n	1
Iz_4_1009	DJI_0355	Crataegus rhipidophylla Gand.	0	Rosaceae Juss.	n	1
Iz_4_1009	DJI_0355	Frangula alnus Mill.	0	Rhamnaceae Juss.	n	1
Iz_4_1009	DJI_0355	Fraxinus pennsylvanica Marshall	0	Oleaceae Hoffmanns. & Link	a	0
Iz_4_1009	DJI_0355	Pinus sylvestris L.	0	Pinaceae Spreng. ex F.Rudolphi	n	1
Iz_4_1009	DJI_0355	Populus × canescens (Aiton) Sm.	0	Salicaceae Mirb.	n	1
Iz_4_1009	DJI_0355	Populus tremula L.	0	Salicaceae Mirb.	n	1
Iz_4_1009	DJI_0355	Populus nigra L.	0	Salicaceae Mirb.	n	1
Iz_4_1009	DJI_0355	Pyrus communis L.	0	Rosaceae Juss.	a	0
Iz_4_1009	DJI_0355	Quercus robur L.	0	Fagaceae Dumort.	n	1
Iz_4_1009	DJI_0355	Robinia pseudoacacia L.	0	Fabaceae Lindl.	a	0
Iz_4_1009	DJI_0355	Rosa sp.	0	Rosaceae Juss.		
Iz_4_1009	DJI_0355	Salix alba L.	0	Salicaceae Mirb.	n	1
Iz_4_1009	DJI_0355	Sambucus nigra L.	0	Viburnaceae Raf.	n	1
Iz_4_1009	DJI_0355	Sorbus aucuparia L.	0	Rosaceae Juss.	n	1
Iz_4_1009	DJI_0355	Tilia cordata Mill.	0	Malvaceae Juss.	n	1
Iz_4_1009	DJI_0355	Ulmus glabra Huds.	0	Ulmaceae Mirb.	n	1
Iz_4_1009	DJI_0356	Acer tataricum L.	0	Sapindaceae Juss.	n	1
Iz_4_1009	DJI_0356	Betula pendula Roth.	0	Betulaceae Gray	n	1
Iz_4_1009	DJI_0356	Crataegus rhipidophylla Gand.	0	Rosaceae Juss.	n	1
Iz_4_1009	DJI_0356	Frangula alnus Mill.	0	Rhamnaceae Juss.	n	1
Iz_4_1009	DJI_0356	Fraxinus pennsylvanica Marshall	0	Oleaceae Hoffmanns. & Link	a	0
Iz_4_1009	DJI_0356	Pinus sylvestris L.	0	Pinaceae Spreng. ex F.Rudolphi	n	1
Iz_4_1009	DJI_0356	Populus × canescens (Aiton) Sm.	0	Salicaceae Mirb.	n	1
Iz_4_1009	DJI_0356	Populus tremula L.	0	Salicaceae Mirb.	n	1
Iz_4_1009	DJI_0356	Populus nigra L.	0	Salicaceae Mirb.	n	1

Iz_4_1009	DJI_0356	Pyrus communis L.	0	Rosaceae Juss.	a	0
Iz_4_1009	DJI_0356	Quercus robur L.	0	Fagaceae Dumort.	n	1
Iz_4_1009	DJI_0356	Robinia pseudoacacia L.	0	Fabaceae Lindl.	a	0
Iz_4_1009	DJI_0356	Rosa sp.	0	Rosaceae Juss.		
Iz_4_1009	DJI_0356	Salix alba L.	0	Salicaceae Mirb.	n	1
Iz_4_1009	DJI_0356	Sambucus nigra L.	0	Viburnaceae Raf.	n	1
Iz_4_1009	DJI_0356	Sorbus aucuparia L.	0	Rosaceae Juss.	n	1
Iz_4_1009	DJI_0356	Tilia cordata Mill.	0	Malvaceae Juss.	n	1
Iz_4_1009	DJI_0356	Ulmus glabra Huds.	0	Ulmaceae Mirb.	n	1
Iz_4_1009	DJI_0357	Acer tataricum L.	0	Sapindaceae Juss.	n	1
Iz_4_1009	DJI_0357	Betula pendula Roth.	0	Betulaceae Gray	n	1
Iz_4_1009	DJI_0357	Crataegus rhipidophylla Gand.	0	Rosaceae Juss.	n	1
Iz_4_1009	DJI_0357	Frangula alnus Mill.	0	Rhamnaceae Juss.	n	1
Iz_4_1009	DJI_0357	Fraxinus pennsylvanica Marshall	0	Oleaceae Hoffmanns. & Link	a	0
Iz_4_1009	DJI_0357	Pinus sylvestris L.	0	Pinaceae Spreng. ex F.Rudolphi	n	1
Iz_4_1009	DJI_0357	Populus × canescens (Aiton) Sm.	0	Salicaceae Mirb.	n	1
Iz_4_1009	DJI_0357	Populus tremula L.	0	Salicaceae Mirb.	n	1
Iz_4_1009	DJI_0357	Populus nigra L.	0	Salicaceae Mirb.	n	1
Iz_4_1009	DJI_0357	Pyrus communis L.	0	Rosaceae Juss.	a	0
Iz_4_1009	DJI_0357	Quercus robur L.	0	Fagaceae Dumort.	n	1
Iz_4_1009	DJI_0357	Robinia pseudoacacia L.	0	Fabaceae Lindl.	a	0
Iz_4_1009	DJI_0357	Rosa sp.	0	Rosaceae Juss.		
Iz_4_1009	DJI_0357	Salix alba L.	0	Salicaceae Mirb.	n	1
Iz_4_1009	DJI_0357	Sambucus nigra L.	0	Viburnaceae Raf.	n	1
Iz_4_1009	DJI_0357	Sorbus aucuparia L.	0	Rosaceae Juss.	n	1
Iz_4_1009	DJI_0357	Tilia cordata Mill.	0	Malvaceae Juss.	n	1
Iz_4_1009	DJI_0357	Ulmus glabra Huds.	0	Ulmaceae Mirb.	n	1
Iz_4_1009	DJI_0358	Acer tataricum L.	0	Sapindaceae Juss.	n	1
Iz_4_1009	DJI_0358	Betula pendula Roth.	0	Betulaceae Gray	n	1
Iz_4_1009	DJI_0358	Crataegus rhipidophylla Gand.	0	Rosaceae Juss.	n	1
Iz_4_1009	DJI_0358	Frangula alnus Mill.	0	Rhamnaceae Juss.	n	1
Iz_4_1009	DJI_0358	Fraxinus pennsylvanica Marshall	0	Oleaceae Hoffmanns. & Link	a	0
Iz_4_1009	DJI_0358	Pinus sylvestris L.	0	Pinaceae Spreng. ex F.Rudolphi	n	1
Iz_4_1009	DJI_0358	Populus × canescens (Aiton) Sm.	0	Salicaceae Mirb.	n	1
Iz_4_1009	DJI_0358	Populus tremula L.	0	Salicaceae Mirb.	n	1
Iz_4_1009	DJI_0358	Populus nigra L.	0	Salicaceae Mirb.	n	1
Iz_4_1009	DJI_0358	Pyrus communis L.	0	Rosaceae Juss.	a	0
Iz_4_1009	DJI_0358	Quercus robur L.	0	Fagaceae Dumort.	n	1
Iz_4_1009	DJI_0358	Robinia pseudoacacia L.	0	Fabaceae Lindl.	a	0
Iz_4_1009	DJI_0358	Rosa sp.	0	Rosaceae Juss.		
Iz_4_1009	DJI_0358	Salix alba L.	0	Salicaceae Mirb.	n	1
Iz_4_1009	DJI_0358	Sambucus nigra L.	0	Viburnaceae Raf.	n	1
Iz_4_1009	DJI_0358	Sorbus aucuparia L.	0	Rosaceae Juss.	n	1
Iz_4_1009	DJI_0358	Tilia cordata Mill.	0	Malvaceae Juss.	n	1
Iz_4_1009	DJI_0358	Ulmus glabra Huds.	0	Ulmaceae Mirb.	n	1
Iz_4_1009	DJI_0359	Acer tataricum L.	0	Sapindaceae Juss.	n	1
Iz_4_1009	DJI_0359	Betula pendula Roth.	0	Betulaceae Gray	n	1
Iz_4_1009	DJI_0359	Crataegus rhipidophylla Gand.	0	Rosaceae Juss.	n	1
Iz_4_1009	DJI_0359	Frangula alnus Mill.	0	Rhamnaceae Juss.	n	1
Iz_4_1009	DJI_0359	Fraxinus pennsylvanica Marshall	0	Oleaceae Hoffmanns. & Link	a	0
Iz_4_1009	DJI_0359	Pinus sylvestris L.	0	Pinaceae Spreng. ex F.Rudolphi	n	1
Iz_4_1009	DJI_0359	Populus × canescens (Aiton) Sm.	0	Salicaceae Mirb.	n	1
Iz_4_1009	DJI_0359	Populus tremula L.	0	Salicaceae Mirb.	n	1
Iz_4_1009	DJI_0359	Populus nigra L.	0	Salicaceae Mirb.	n	1
Iz_4_1009	DJI_0359	Pyrus communis L.	0	Rosaceae Juss.	a	0
Iz_4_1009	DJI_0359	Quercus robur L.	0	Fagaceae Dumort.	n	1
Iz_4_1009	DJI_0359	Robinia pseudoacacia L.	0	Fabaceae Lindl.	a	0
Iz_4_1009	DJI_0359	Rosa sp.	0	Rosaceae Juss.		
Iz_4_1009	DJI_0359	Salix alba L.	0	Salicaceae Mirb.	n	1
Iz_4_1009	DJI_0359	Sambucus nigra L.	0	Viburnaceae Raf.	n	1
Iz_4_1009	DJI_0359	Sorbus aucuparia L.	0	Rosaceae Juss.	n	1
Iz_4_1009	DJI_0359	Tilia cordata Mill.	0	Malvaceae Juss.	n	1
Iz_4_1009	DJI_0359	Ulmus glabra Huds.	0	Ulmaceae Mirb.	n	1
Iz_4_1009	DJI_0360	Acer tataricum L.	0	Sapindaceae Juss.	n	1

Iz_4_1009	DJI_0360	Betula pendula Roth.	0	Betulaceae Gray	n	1
Iz_4_1009	DJI_0360	Crataegus rhipidophylla Gand.	0	Rosaceae Juss.	n	1
Iz_4_1009	DJI_0360	Frangula alnus Mill.	0	Rhamnaceae Juss.	n	1
Iz_4_1009	DJI_0360	Fraxinus pennsylvanica Marshall	0	Oleaceae Hoffmanns. & Link	a	0
Iz_4_1009	DJI_0360	Pinus sylvestris L.	0	Pinaceae Spreng. ex F.Rudolphi	n	1
Iz_4_1009	DJI_0360	Populus × canescens (Aiton) Sm.	0	Salicaceae Mirb.	n	1
Iz_4_1009	DJI_0360	Populus tremula L.	0	Salicaceae Mirb.	n	1
Iz_4_1009	DJI_0360	Populus nigra L.	0	Salicaceae Mirb.	n	1
Iz_4_1009	DJI_0360	Pyrus communis L.	0	Rosaceae Juss.	a	0
Iz_4_1009	DJI_0360	Quercus robur L.	0	Fagaceae Dumort.	n	1
Iz_4_1009	DJI_0360	Robinia pseudoacacia L.	0	Fabaceae Lindl.	a	0
Iz_4_1009	DJI_0360	Rosa sp.	0	Rosaceae Juss.		
Iz_4_1009	DJI_0360	Salix alba L.	0	Salicaceae Mirb.	n	1
Iz_4_1009	DJI_0360	Sambucus nigra L.	0	Viburnaceae Raf.	n	1
Iz_4_1009	DJI_0360	Sorbus aucuparia L.	0	Rosaceae Juss.	n	1
Iz_4_1009	DJI_0360	Tilia cordata Mill.	0	Malvaceae Juss.	n	1
Iz_4_1009	DJI_0360	Ulmus glabra Huds.	0	Ulmaceae Mirb.	n	1
Iz_4_1009	DJI_0361	Acer tataricum L.	0	Sapindaceae Juss.	n	1
Iz_4_1009	DJI_0361	Betula pendula Roth.	0	Betulaceae Gray	n	1
Iz_4_1009	DJI_0361	Crataegus rhipidophylla Gand.	0	Rosaceae Juss.	n	1
Iz_4_1009	DJI_0361	Frangula alnus Mill.	0	Rhamnaceae Juss.	n	1
Iz_4_1009	DJI_0361	Fraxinus pennsylvanica Marshall	0	Oleaceae Hoffmanns. & Link	a	0
Iz_4_1009	DJI_0361	Pinus sylvestris L.	0	Pinaceae Spreng. ex F.Rudolphi	n	1
Iz_4_1009	DJI_0361	Populus × canescens (Aiton) Sm.	0	Salicaceae Mirb.	n	1
Iz_4_1009	DJI_0361	Populus tremula L.	0	Salicaceae Mirb.	n	1
Iz_4_1009	DJI_0361	Populus nigra L.	0	Salicaceae Mirb.	n	1
Iz_4_1009	DJI_0361	Pyrus communis L.	0	Rosaceae Juss.	a	0
Iz_4_1009	DJI_0361	Quercus robur L.	0	Fagaceae Dumort.	n	1
Iz_4_1009	DJI_0361	Robinia pseudoacacia L.	0	Fabaceae Lindl.	a	0
Iz_4_1009	DJI_0361	Rosa sp.	0	Rosaceae Juss.		
Iz_4_1009	DJI_0361	Salix alba L.	0	Salicaceae Mirb.	n	1
Iz_4_1009	DJI_0361	Sambucus nigra L.	0	Viburnaceae Raf.	n	1
Iz_4_1009	DJI_0361	Sorbus aucuparia L.	0	Rosaceae Juss.	n	1
Iz_4_1009	DJI_0361	Tilia cordata Mill.	0	Malvaceae Juss.	n	1
Iz_4_1009	DJI_0361	Ulmus glabra Huds.	0	Ulmaceae Mirb.	n	1
Iz_4_1009	DJI_0362	Acer tataricum L.	0	Sapindaceae Juss.	n	1
Iz_4_1009	DJI_0362	Betula pendula Roth.	0	Betulaceae Gray	n	1
Iz_4_1009	DJI_0362	Crataegus rhipidophylla Gand.	0	Rosaceae Juss.	n	1
Iz_4_1009	DJI_0362	Frangula alnus Mill.	0	Rhamnaceae Juss.	n	1
Iz_4_1009	DJI_0362	Fraxinus pennsylvanica Marshall	0	Oleaceae Hoffmanns. & Link	a	0
Iz_4_1009	DJI_0362	Pinus sylvestris L.	0	Pinaceae Spreng. ex F.Rudolphi	n	1
Iz_4_1009	DJI_0362	Populus × canescens (Aiton) Sm.	0	Salicaceae Mirb.	n	1
Iz_4_1009	DJI_0362	Populus tremula L.	0	Salicaceae Mirb.	n	1
Iz_4_1009	DJI_0362	Populus nigra L.	0	Salicaceae Mirb.	n	1
Iz_4_1009	DJI_0362	Pyrus communis L.	0	Rosaceae Juss.	a	0
Iz_4_1009	DJI_0362	Quercus robur L.	0	Fagaceae Dumort.	n	1
Iz_4_1009	DJI_0362	Robinia pseudoacacia L.	0	Fabaceae Lindl.	a	0
Iz_4_1009	DJI_0362	Rosa sp.	0	Rosaceae Juss.		
Iz_4_1009	DJI_0362	Salix alba L.	0	Salicaceae Mirb.	n	1
Iz_4_1009	DJI_0362	Sambucus nigra L.	0	Viburnaceae Raf.	n	1
Iz_4_1009	DJI_0362	Sorbus aucuparia L.	0	Rosaceae Juss.	n	1
Iz_4_1009	DJI_0362	Tilia cordata Mill.	0	Malvaceae Juss.	n	1
Iz_4_1009	DJI_0362	Ulmus glabra Huds.	0	Ulmaceae Mirb.	n	1
Iz_4_1009	DJI_0363	Acer tataricum L.	0	Sapindaceae Juss.	n	1
Iz_4_1009	DJI_0363	Betula pendula Roth.	0	Betulaceae Gray	n	1
Iz_4_1009	DJI_0363	Crataegus rhipidophylla Gand.	0	Rosaceae Juss.	n	1
Iz_4_1009	DJI_0363	Frangula alnus Mill.	0	Rhamnaceae Juss.	n	1
Iz_4_1009	DJI_0363	Fraxinus pennsylvanica Marshall	0	Oleaceae Hoffmanns. & Link	a	0
Iz_4_1009	DJI_0363	Pinus sylvestris L.	0	Pinaceae Spreng. ex F.Rudolphi	n	1
Iz_4_1009	DJI_0363	Populus × canescens (Aiton) Sm.	0	Salicaceae Mirb.	n	1
Iz_4_1009	DJI_0363	Populus tremula L.	0	Salicaceae Mirb.	n	1
Iz_4_1009	DJI_0363	Populus nigra L.	0	Salicaceae Mirb.	n	1
Iz_4_1009	DJI_0363	Pyrus communis L.	0	Rosaceae Juss.	a	0
Iz_4_1009	DJI_0363	Quercus robur L.	0	Fagaceae Dumort.	n	1

Iz_4_1009	DJI_0363	Robinia pseudoacacia L.	0	Fabaceae Lindl.	a	0
Iz_4_1009	DJI_0363	Rosa sp.	0	Rosaceae Juss.		
Iz_4_1009	DJI_0363	Salix alba L.	0	Salicaceae Mirb.	n	1
Iz_4_1009	DJI_0363	Sambucus nigra L.	0	Viburnaceae Raf.	n	1
Iz_4_1009	DJI_0363	Sorbus aucuparia L.	0	Rosaceae Juss.	n	1
Iz_4_1009	DJI_0363	Tilia cordata Mill.	0	Malvaceae Juss.	n	1
Iz_4_1009	DJI_0363	Ulmus glabra Huds.	0	Ulmaceae Mirb.	n	1
Iz_4_1009	DJI_0364	Acer tataricum L.	0	Sapindaceae Juss.	n	1
Iz_4_1009	DJI_0364	Betula pendula Roth.	0	Betulaceae Gray	n	1
Iz_4_1009	DJI_0364	Crataegus rhipidophylla Gand.	0	Rosaceae Juss.	n	1
Iz_4_1009	DJI_0364	Frangula alnus Mill.	0	Rhamnaceae Juss.	n	1
Iz_4_1009	DJI_0364	Fraxinus pennsylvanica Marshall	0	Oleaceae Hoffmanns. & Link	a	0
Iz_4_1009	DJI_0364	Pinus sylvestris L.	0	Pinaceae Spreng. ex F.Rudolphi	n	1
Iz_4_1009	DJI_0364	Populus × canescens (Aiton) Sm.	0	Salicaceae Mirb.	n	1
Iz_4_1009	DJI_0364	Populus tremula L.	0	Salicaceae Mirb.	n	1
Iz_4_1009	DJI_0364	Populus nigra L.	0	Salicaceae Mirb.	n	1
Iz_4_1009	DJI_0364	Pyrus communis L.	0	Rosaceae Juss.	a	0
Iz_4_1009	DJI_0364	Quercus robur L.	0	Fagaceae Dumort.	n	1
Iz_4_1009	DJI_0364	Robinia pseudoacacia L.	0	Fabaceae Lindl.	a	0
Iz_4_1009	DJI_0364	Rosa sp.	0	Rosaceae Juss.		
Iz_4_1009	DJI_0364	Salix alba L.	0	Salicaceae Mirb.	n	1
Iz_4_1009	DJI_0364	Sambucus nigra L.	0	Viburnaceae Raf.	n	1
Iz_4_1009	DJI_0364	Sorbus aucuparia L.	0	Rosaceae Juss.	n	1
Iz_4_1009	DJI_0364	Tilia cordata Mill.	0	Malvaceae Juss.	n	1
Iz_4_1009	DJI_0364	Ulmus glabra Huds.	0	Ulmaceae Mirb.	n	1
Iz_4_1009	DJI_0365	Acer tataricum L.	0	Sapindaceae Juss.	n	1
Iz_4_1009	DJI_0365	Betula pendula Roth.	0	Betulaceae Gray	n	1
Iz_4_1009	DJI_0365	Crataegus rhipidophylla Gand.	0	Rosaceae Juss.	n	1
Iz_4_1009	DJI_0365	Frangula alnus Mill.	0	Rhamnaceae Juss.	n	1
Iz_4_1009	DJI_0365	Fraxinus pennsylvanica Marshall	0	Oleaceae Hoffmanns. & Link	a	0
Iz_4_1009	DJI_0365	Pinus sylvestris L.	0	Pinaceae Spreng. ex F.Rudolphi	n	1
Iz_4_1009	DJI_0365	Populus × canescens (Aiton) Sm.	0	Salicaceae Mirb.	n	1
Iz_4_1009	DJI_0365	Populus tremula L.	0	Salicaceae Mirb.	n	1
Iz_4_1009	DJI_0365	Populus nigra L.	0	Salicaceae Mirb.	n	1
Iz_4_1009	DJI_0365	Pyrus communis L.	0	Rosaceae Juss.	a	0
Iz_4_1009	DJI_0365	Quercus robur L.	0	Fagaceae Dumort.	n	1
Iz_4_1009	DJI_0365	Robinia pseudoacacia L.	0	Fabaceae Lindl.	a	0
Iz_4_1009	DJI_0365	Rosa sp.	0	Rosaceae Juss.		
Iz_4_1009	DJI_0365	Salix alba L.	0	Salicaceae Mirb.	n	1
Iz_4_1009	DJI_0365	Sambucus nigra L.	0	Viburnaceae Raf.	n	1
Iz_4_1009	DJI_0365	Sorbus aucuparia L.	0	Rosaceae Juss.	n	1
Iz_4_1009	DJI_0365	Tilia cordata Mill.	0	Malvaceae Juss.	n	1
Iz_4_1009	DJI_0365	Ulmus glabra Huds.	0	Ulmaceae Mirb.	n	1
Iz_4_1009	DJI_0366	Acer tataricum L.	0	Sapindaceae Juss.	n	1
Iz_4_1009	DJI_0366	Betula pendula Roth.	0	Betulaceae Gray	n	1
Iz_4_1009	DJI_0366	Crataegus rhipidophylla Gand.	0	Rosaceae Juss.	n	1
Iz_4_1009	DJI_0366	Frangula alnus Mill.	0	Rhamnaceae Juss.	n	1
Iz_4_1009	DJI_0366	Fraxinus pennsylvanica Marshall	0	Oleaceae Hoffmanns. & Link	a	0
Iz_4_1009	DJI_0366	Pinus sylvestris L.	0	Pinaceae Spreng. ex F.Rudolphi	n	1
Iz_4_1009	DJI_0366	Populus × canescens (Aiton) Sm.	0	Salicaceae Mirb.	n	1
Iz_4_1009	DJI_0366	Populus tremula L.	0	Salicaceae Mirb.	n	1
Iz_4_1009	DJI_0366	Populus nigra L.	0	Salicaceae Mirb.	n	1
Iz_4_1009	DJI_0366	Pyrus communis L.	0	Rosaceae Juss.	a	0
Iz_4_1009	DJI_0366	Quercus robur L.	0	Fagaceae Dumort.	n	1
Iz_4_1009	DJI_0366	Robinia pseudoacacia L.	0	Fabaceae Lindl.	a	0
Iz_4_1009	DJI_0366	Rosa sp.	0	Rosaceae Juss.		
Iz_4_1009	DJI_0366	Salix alba L.	0	Salicaceae Mirb.	n	1
Iz_4_1009	DJI_0366	Sambucus nigra L.	0	Viburnaceae Raf.	n	1
Iz_4_1009	DJI_0366	Sorbus aucuparia L.	0	Rosaceae Juss.	n	1
Iz_4_1009	DJI_0366	Tilia cordata Mill.	0	Malvaceae Juss.	n	1
Iz_4_1009	DJI_0366	Ulmus glabra Huds.	0	Ulmaceae Mirb.	n	1
Iz_4_1009	DJI_0367	Acer tataricum L.	0	Sapindaceae Juss.	n	1
Iz_4_1009	DJI_0367	Betula pendula Roth.	0	Betulaceae Gray	n	1
Iz_4_1009	DJI_0367	Crataegus rhipidophylla Gand.	0	Rosaceae Juss.	n	1

Iz_4_1009	DJI_0367	Frangula alnus Mill.	0	Rhamnaceae Juss.	n	1
Iz_4_1009	DJI_0367	Fraxinus pennsylvanica Marshall	0	Oleaceae Hoffmanns. & Link	a	0
Iz_4_1009	DJI_0367	Pinus sylvestris L.	0	Pinaceae Spreng. ex F.Rudolphi	n	1
Iz_4_1009	DJI_0367	Populus × canescens (Aiton) Sm.	0	Salicaceae Mirb.	n	1
Iz_4_1009	DJI_0367	Populus tremula L.	0	Salicaceae Mirb.	n	1
Iz_4_1009	DJI_0367	Populus nigra L.	0	Salicaceae Mirb.	n	1
Iz_4_1009	DJI_0367	Pyrus communis L.	0	Rosaceae Juss.	a	0
Iz_4_1009	DJI_0367	Quercus robur L.	0	Fagaceae Dumort.	n	1
Iz_4_1009	DJI_0367	Robinia pseudoacacia L.	0	Fabaceae Lindl.	a	0
Iz_4_1009	DJI_0367	Rosa sp.	0	Rosaceae Juss.		
Iz_4_1009	DJI_0367	Salix alba L.	0	Salicaceae Mirb.	n	1
Iz_4_1009	DJI_0367	Sambucus nigra L.	0	Viburnaceae Raf.	n	1
Iz_4_1009	DJI_0367	Sorbus aucuparia L.	0	Rosaceae Juss.	n	1
Iz_4_1009	DJI_0367	Tilia cordata Mill.	0	Malvaceae Juss.	n	1
Iz_4_1009	DJI_0367	Ulmus glabra Huds.	0	Ulmaceae Mirb.	n	1
Iz_4_1009	DJI_0372	Acer tataricum L.	0	Sapindaceae Juss.	n	1
Iz_4_1009	DJI_0372	Betula pendula Roth.	0	Betulaceae Gray	n	1
Iz_4_1009	DJI_0372	Crataegus rhipidophylla Gand.	0	Rosaceae Juss.	n	1
Iz_4_1009	DJI_0372	Frangula alnus Mill.	0	Rhamnaceae Juss.	n	1
Iz_4_1009	DJI_0372	Fraxinus pennsylvanica Marshall	0	Oleaceae Hoffmanns. & Link	a	0
Iz_4_1009	DJI_0372	Pinus sylvestris L.	0	Pinaceae Spreng. ex F.Rudolphi	n	1
Iz_4_1009	DJI_0372	Populus × canescens (Aiton) Sm.	0	Salicaceae Mirb.	n	1
Iz_4_1009	DJI_0372	Populus tremula L.	0	Salicaceae Mirb.	n	1
Iz_4_1009	DJI_0372	Populus nigra L.	0	Salicaceae Mirb.	n	1
Iz_4_1009	DJI_0372	Pyrus communis L.	0	Rosaceae Juss.	a	0
Iz_4_1009	DJI_0372	Quercus robur L.	0	Fagaceae Dumort.	n	1
Iz_4_1009	DJI_0372	Robinia pseudoacacia L.	0	Fabaceae Lindl.	a	0
Iz_4_1009	DJI_0372	Rosa sp.	0	Rosaceae Juss.		
Iz_4_1009	DJI_0372	Salix alba L.	0	Salicaceae Mirb.	n	1
Iz_4_1009	DJI_0372	Sambucus nigra L.	0	Viburnaceae Raf.	n	1
Iz_4_1009	DJI_0372	Sorbus aucuparia L.	0	Rosaceae Juss.	n	1
Iz_4_1009	DJI_0372	Tilia cordata Mill.	0	Malvaceae Juss.	n	1
Iz_4_1009	DJI_0372	Ulmus glabra Huds.	0	Ulmaceae Mirb.	n	1
Iz_4_1009	DJI_0373	Acer tataricum L.	0	Sapindaceae Juss.	n	1
Iz_4_1009	DJI_0373	Betula pendula Roth.	0	Betulaceae Gray	n	1
Iz_4_1009	DJI_0373	Crataegus rhipidophylla Gand.	0	Rosaceae Juss.	n	1
Iz_4_1009	DJI_0373	Frangula alnus Mill.	0	Rhamnaceae Juss.	n	1
Iz_4_1009	DJI_0373	Fraxinus pennsylvanica Marshall	0	Oleaceae Hoffmanns. & Link	a	0
Iz_4_1009	DJI_0373	Pinus sylvestris L.	0	Pinaceae Spreng. ex F.Rudolphi	n	1
Iz_4_1009	DJI_0373	Populus × canescens (Aiton) Sm.	0	Salicaceae Mirb.	n	1
Iz_4_1009	DJI_0373	Populus tremula L.	0	Salicaceae Mirb.	n	1
Iz_4_1009	DJI_0373	Populus nigra L.	0	Salicaceae Mirb.	n	1
Iz_4_1009	DJI_0373	Pyrus communis L.	0	Rosaceae Juss.	a	0
Iz_4_1009	DJI_0373	Quercus robur L.	0	Fagaceae Dumort.	n	1
Iz_4_1009	DJI_0373	Robinia pseudoacacia L.	0	Fabaceae Lindl.	a	0
Iz_4_1009	DJI_0373	Rosa sp.	0	Rosaceae Juss.		
Iz_4_1009	DJI_0373	Salix alba L.	0	Salicaceae Mirb.	n	1
Iz_4_1009	DJI_0373	Sambucus nigra L.	0	Viburnaceae Raf.	n	1
Iz_4_1009	DJI_0373	Sorbus aucuparia L.	0	Rosaceae Juss.	n	1
Iz_4_1009	DJI_0373	Tilia cordata Mill.	0	Malvaceae Juss.	n	1
Iz_4_1009	DJI_0373	Ulmus glabra Huds.	0	Ulmaceae Mirb.	n	1
Iz_4_1009	DJI_0375	Acer tataricum L.	0	Sapindaceae Juss.	n	1
Iz_4_1009	DJI_0375	Betula pendula Roth.	0	Betulaceae Gray	n	1
Iz_4_1009	DJI_0375	Crataegus rhipidophylla Gand.	0	Rosaceae Juss.	n	1
Iz_4_1009	DJI_0375	Frangula alnus Mill.	0	Rhamnaceae Juss.	n	1
Iz_4_1009	DJI_0375	Fraxinus pennsylvanica Marshall	0	Oleaceae Hoffmanns. & Link	a	0
Iz_4_1009	DJI_0375	Pinus sylvestris L.	0	Pinaceae Spreng. ex F.Rudolphi	n	1
Iz_4_1009	DJI_0375	Populus × canescens (Aiton) Sm.	0	Salicaceae Mirb.	n	1
Iz_4_1009	DJI_0375	Populus tremula L.	0	Salicaceae Mirb.	n	1
Iz_4_1009	DJI_0375	Populus nigra L.	0	Salicaceae Mirb.	n	1
Iz_4_1009	DJI_0375	Pyrus communis L.	0	Rosaceae Juss.	a	0
Iz_4_1009	DJI_0375	Quercus robur L.	0	Fagaceae Dumort.	n	1
Iz_4_1009	DJI_0375	Robinia pseudoacacia L.	0	Fabaceae Lindl.	a	0
Iz_4_1009	DJI_0375	Rosa sp.	0	Rosaceae Juss.		

Iz_4_1009	DJI_0375	Salix alba L.	0	Salicaceae Mirb.	n	1
Iz_4_1009	DJI_0375	Sambucus nigra L.	0	Viburnaceae Raf.	n	1
Iz_4_1009	DJI_0375	Sorbus aucuparia L.	0	Rosaceae Juss.	n	1
Iz_4_1009	DJI_0375	Tilia cordata Mill.	0	Malvaceae Juss.	n	1
Iz_4_1009	DJI_0375	Ulmus glabra Huds.	0	Ulmaceae Mirb.	n	1
Iz_4_1009	DJI_0377	Acer tataricum L.	0	Sapindaceae Juss.	n	1
Iz_4_1009	DJI_0377	Betula pendula Roth.	0	Betulaceae Gray	n	1
Iz_4_1009	DJI_0377	Crataegus rhipidophylla Gand.	0	Rosaceae Juss.	n	1
Iz_4_1009	DJI_0377	Frangula alnus Mill.	0	Rhamnaceae Juss.	n	1
Iz_4_1009	DJI_0377	Fraxinus pennsylvanica Marshall	0	Oleaceae Hoffmanns. & Link	a	0
Iz_4_1009	DJI_0377	Pinus sylvestris L.	0	Pinaceae Spreng. ex F.Rudolphi	n	1
Iz_4_1009	DJI_0377	Populus × canescens (Aiton) Sm.	0	Salicaceae Mirb.	n	1
Iz_4_1009	DJI_0377	Populus tremula L.	0	Salicaceae Mirb.	n	1
Iz_4_1009	DJI_0377	Populus nigra L.	0	Salicaceae Mirb.	n	1
Iz_4_1009	DJI_0377	Pyrus communis L.	0	Rosaceae Juss.	a	0
Iz_4_1009	DJI_0377	Quercus robur L.	0	Fagaceae Dumort.	n	1
Iz_4_1009	DJI_0377	Robinia pseudoacacia L.	0	Fabaceae Lindl.	a	0
Iz_4_1009	DJI_0377	Rosa sp.	0	Rosaceae Juss.		
Iz_4_1009	DJI_0377	Salix alba L.	0	Salicaceae Mirb.	n	1
Iz_4_1009	DJI_0377	Sambucus nigra L.	0	Viburnaceae Raf.	n	1
Iz_4_1009	DJI_0377	Sorbus aucuparia L.	0	Rosaceae Juss.	n	1
Iz_4_1009	DJI_0377	Tilia cordata Mill.	0	Malvaceae Juss.	n	1
Iz_4_1009	DJI_0377	Ulmus glabra Huds.	0	Ulmaceae Mirb.	n	1
Iz_4_1009	DJI_0378	Acer tataricum L.	0	Sapindaceae Juss.	n	1
Iz_4_1009	DJI_0378	Betula pendula Roth.	0	Betulaceae Gray	n	1
Iz_4_1009	DJI_0378	Crataegus rhipidophylla Gand.	0	Rosaceae Juss.	n	1
Iz_4_1009	DJI_0378	Frangula alnus Mill.	0	Rhamnaceae Juss.	n	1
Iz_4_1009	DJI_0378	Fraxinus pennsylvanica Marshall	0	Oleaceae Hoffmanns. & Link	a	0
Iz_4_1009	DJI_0378	Pinus sylvestris L.	0	Pinaceae Spreng. ex F.Rudolphi	n	1
Iz_4_1009	DJI_0378	Populus × canescens (Aiton) Sm.	0	Salicaceae Mirb.	n	1
Iz_4_1009	DJI_0378	Populus tremula L.	0	Salicaceae Mirb.	n	1
Iz_4_1009	DJI_0378	Populus nigra L.	0	Salicaceae Mirb.	n	1
Iz_4_1009	DJI_0378	Pyrus communis L.	0	Rosaceae Juss.	a	0
Iz_4_1009	DJI_0378	Quercus robur L.	0	Fagaceae Dumort.	n	1
Iz_4_1009	DJI_0378	Robinia pseudoacacia L.	0	Fabaceae Lindl.	a	0
Iz_4_1009	DJI_0378	Rosa sp.	0	Rosaceae Juss.		
Iz_4_1009	DJI_0378	Salix alba L.	0	Salicaceae Mirb.	n	1
Iz_4_1009	DJI_0378	Sambucus nigra L.	0	Viburnaceae Raf.	n	1
Iz_4_1009	DJI_0378	Sorbus aucuparia L.	0	Rosaceae Juss.	n	1
Iz_4_1009	DJI_0378	Tilia cordata Mill.	0	Malvaceae Juss.	n	1
Iz_4_1009	DJI_0378	Ulmus glabra Huds.	0	Ulmaceae Mirb.	n	1
Iz_4_1009	DJI_0381	Acer tataricum L.	0	Sapindaceae Juss.	n	1
Iz_4_1009	DJI_0381	Betula pendula Roth.	0	Betulaceae Gray	n	1
Iz_4_1009	DJI_0381	Crataegus rhipidophylla Gand.	0	Rosaceae Juss.	n	1
Iz_4_1009	DJI_0381	Frangula alnus Mill.	0	Rhamnaceae Juss.	n	1
Iz_4_1009	DJI_0381	Fraxinus pennsylvanica Marshall	0	Oleaceae Hoffmanns. & Link	a	0
Iz_4_1009	DJI_0381	Pinus sylvestris L.	0	Pinaceae Spreng. ex F.Rudolphi	n	1
Iz_4_1009	DJI_0381	Populus × canescens (Aiton) Sm.	0	Salicaceae Mirb.	n	1
Iz_4_1009	DJI_0381	Populus tremula L.	0	Salicaceae Mirb.	n	1
Iz_4_1009	DJI_0381	Populus nigra L.	0	Salicaceae Mirb.	n	1
Iz_4_1009	DJI_0381	Pyrus communis L.	0	Rosaceae Juss.	a	0
Iz_4_1009	DJI_0381	Quercus robur L.	0	Fagaceae Dumort.	n	1
Iz_4_1009	DJI_0381	Robinia pseudoacacia L.	0	Fabaceae Lindl.	a	0
Iz_4_1009	DJI_0381	Rosa sp.	0	Rosaceae Juss.		
Iz_4_1009	DJI_0381	Salix alba L.	0	Salicaceae Mirb.	n	1
Iz_4_1009	DJI_0381	Sambucus nigra L.	0	Viburnaceae Raf.	n	1
Iz_4_1009	DJI_0381	Sorbus aucuparia L.	0	Rosaceae Juss.	n	1
Iz_4_1009	DJI_0381	Tilia cordata Mill.	0	Malvaceae Juss.	n	1
Iz_4_1009	DJI_0381	Ulmus glabra Huds.	0	Ulmaceae Mirb.	n	1
Iz_4_1009	DJI_0383	Acer tataricum L.	0	Sapindaceae Juss.	n	1
Iz_4_1009	DJI_0383	Betula pendula Roth.	0	Betulaceae Gray	n	1
Iz_4_1009	DJI_0383	Crataegus rhipidophylla Gand.	0	Rosaceae Juss.	n	1
Iz_4_1009	DJI_0383	Frangula alnus Mill.	0	Rhamnaceae Juss.	n	1
Iz_4_1009	DJI_0383	Fraxinus pennsylvanica Marshall	0	Oleaceae Hoffmanns. & Link	a	0

Iz_4_1009	DJI_0383	Pinus sylvestris L.	0	Pinaceae Spreng. ex F.Rudolphi	n	1
Iz_4_1009	DJI_0383	Populus × canescens (Aiton) Sm.	0	Salicaceae Mirb.	n	1
Iz_4_1009	DJI_0383	Populus tremula L.	0	Salicaceae Mirb.	n	1
Iz_4_1009	DJI_0383	Populus nigra L.	0	Salicaceae Mirb.	n	1
Iz_4_1009	DJI_0383	Pyrus communis L.	0	Rosaceae Juss.	a	0
Iz_4_1009	DJI_0383	Quercus robur L.	0	Fagaceae Dumort.	n	1
Iz_4_1009	DJI_0383	Robinia pseudoacacia L.	0	Fabaceae Lindl.	a	0
Iz_4_1009	DJI_0383	Rosa sp.	0	Rosaceae Juss.		
Iz_4_1009	DJI_0383	Salix alba L.	0	Salicaceae Mirb.	n	1
Iz_4_1009	DJI_0383	Sambucus nigra L.	0	Viburnaceae Raf.	n	1
Iz_4_1009	DJI_0383	Sorbus aucuparia L.	0	Rosaceae Juss.	n	1
Iz_4_1009	DJI_0383	Tilia cordata Mill.	0	Malvaceae Juss.	n	1
Iz_4_1009	DJI_0383	Ulmus glabra Huds.	0	Ulmaceae Mirb.	n	1
Iz_4_1009	DJI_0385	Acer tataricum L.	0	Sapindaceae Juss.	n	1
Iz_4_1009	DJI_0385	Betula pendula Roth.	0	Betulaceae Gray	n	1
Iz_4_1009	DJI_0385	Crataegus rhipidophylla Gand.	0	Rosaceae Juss.	n	1
Iz_4_1009	DJI_0385	Frangula alnus Mill.	0	Rhamnaceae Juss.	n	1
Iz_4_1009	DJI_0385	Fraxinus pennsylvanica Marshall	0	Oleaceae Hoffmanns. & Link	a	0
Iz_4_1009	DJI_0385	Pinus sylvestris L.	0	Pinaceae Spreng. ex F.Rudolphi	n	1
Iz_4_1009	DJI_0385	Populus × canescens (Aiton) Sm.	0	Salicaceae Mirb.	n	1
Iz_4_1009	DJI_0385	Populus tremula L.	0	Salicaceae Mirb.	n	1
Iz_4_1009	DJI_0385	Populus nigra L.	0	Salicaceae Mirb.	n	1
Iz_4_1009	DJI_0385	Pyrus communis L.	0	Rosaceae Juss.	a	0
Iz_4_1009	DJI_0385	Quercus robur L.	0	Fagaceae Dumort.	n	1
Iz_4_1009	DJI_0385	Robinia pseudoacacia L.	0	Fabaceae Lindl.	a	0
Iz_4_1009	DJI_0385	Rosa sp.	0	Rosaceae Juss.		
Iz_4_1009	DJI_0385	Salix alba L.	0	Salicaceae Mirb.	n	1
Iz_4_1009	DJI_0385	Sambucus nigra L.	0	Viburnaceae Raf.	n	1
Iz_4_1009	DJI_0385	Sorbus aucuparia L.	0	Rosaceae Juss.	n	1
Iz_4_1009	DJI_0385	Tilia cordata Mill.	0	Malvaceae Juss.	n	1
Iz_4_1009	DJI_0385	Ulmus glabra Huds.	0	Ulmaceae Mirb.	n	1
Iz_4_1009	DJI_0386	Acer tataricum L.	0	Sapindaceae Juss.	n	1
Iz_4_1009	DJI_0386	Betula pendula Roth.	0	Betulaceae Gray	n	1
Iz_4_1009	DJI_0386	Crataegus rhipidophylla Gand.	0	Rosaceae Juss.	n	1
Iz_4_1009	DJI_0386	Frangula alnus Mill.	0	Rhamnaceae Juss.	n	1
Iz_4_1009	DJI_0386	Fraxinus pennsylvanica Marshall	0	Oleaceae Hoffmanns. & Link	a	0
Iz_4_1009	DJI_0386	Pinus sylvestris L.	0	Pinaceae Spreng. ex F.Rudolphi	n	1
Iz_4_1009	DJI_0386	Populus × canescens (Aiton) Sm.	0	Salicaceae Mirb.	n	1
Iz_4_1009	DJI_0386	Populus tremula L.	0	Salicaceae Mirb.	n	1
Iz_4_1009	DJI_0386	Populus nigra L.	0	Salicaceae Mirb.	n	1
Iz_4_1009	DJI_0386	Pyrus communis L.	0	Rosaceae Juss.	a	0
Iz_4_1009	DJI_0386	Quercus robur L.	0	Fagaceae Dumort.	n	1
Iz_4_1009	DJI_0386	Robinia pseudoacacia L.	0	Fabaceae Lindl.	a	0
Iz_4_1009	DJI_0386	Rosa sp.	0	Rosaceae Juss.		
Iz_4_1009	DJI_0386	Salix alba L.	0	Salicaceae Mirb.	n	1
Iz_4_1009	DJI_0386	Sambucus nigra L.	0	Viburnaceae Raf.	n	1
Iz_4_1009	DJI_0386	Sorbus aucuparia L.	0	Rosaceae Juss.	n	1
Iz_4_1009	DJI_0386	Tilia cordata Mill.	0	Malvaceae Juss.	n	1
Iz_4_1009	DJI_0386	Ulmus glabra Huds.	0	Ulmaceae Mirb.	n	1
Iz_4_1009	DJI_0387	Acer tataricum L.	0	Sapindaceae Juss.	n	1
Iz_4_1009	DJI_0387	Betula pendula Roth.	0	Betulaceae Gray	n	1
Iz_4_1009	DJI_0387	Crataegus rhipidophylla Gand.	0	Rosaceae Juss.	n	1
Iz_4_1009	DJI_0387	Frangula alnus Mill.	0	Rhamnaceae Juss.	n	1
Iz_4_1009	DJI_0387	Fraxinus pennsylvanica Marshall	0	Oleaceae Hoffmanns. & Link	a	0
Iz_4_1009	DJI_0387	Pinus sylvestris L.	0	Pinaceae Spreng. ex F.Rudolphi	n	1
Iz_4_1009	DJI_0387	Populus × canescens (Aiton) Sm.	0	Salicaceae Mirb.	n	1
Iz_4_1009	DJI_0387	Populus tremula L.	0	Salicaceae Mirb.	n	1
Iz_4_1009	DJI_0387	Populus nigra L.	0	Salicaceae Mirb.	n	1
Iz_4_1009	DJI_0387	Pyrus communis L.	0	Rosaceae Juss.	a	0
Iz_4_1009	DJI_0387	Quercus robur L.	0	Fagaceae Dumort.	n	1
Iz_4_1009	DJI_0387	Robinia pseudoacacia L.	0	Fabaceae Lindl.	a	0
Iz_4_1009	DJI_0387	Rosa sp.	0	Rosaceae Juss.		
Iz_4_1009	DJI_0387	Salix alba L.	0	Salicaceae Mirb.	n	1
Iz_4_1009	DJI_0387	Sambucus nigra L.	0	Viburnaceae Raf.	n	1

Iz_4_1009	DJI_0387	Sorbus aucuparia L.	0	Rosaceae Juss.	n	1
Iz_4_1009	DJI_0387	Tilia cordata Mill.	0	Malvaceae Juss.	n	1
Iz_4_1009	DJI_0387	Ulmus glabra Huds.	0	Ulmaceae Mirb.	n	1
Iz_4_1009	DJI_0388	Acer tataricum L.	0	Sapindaceae Juss.	n	1
Iz_4_1009	DJI_0388	Betula pendula Roth.	0	Betulaceae Gray	n	1
Iz_4_1009	DJI_0388	Crataegus rhipidophylla Gand.	0	Rosaceae Juss.	n	1
Iz_4_1009	DJI_0388	Frangula alnus Mill.	0	Rhamnaceae Juss.	n	1
Iz_4_1009	DJI_0388	Fraxinus pennsylvanica Marshall	0	Oleaceae Hoffmanns. & Link	a	0
Iz_4_1009	DJI_0388	Pinus sylvestris L.	0	Pinaceae Spreng. ex F.Rudolphi	n	1
Iz_4_1009	DJI_0388	Populus × canescens (Aiton) Sm.	0	Salicaceae Mirb.	n	1
Iz_4_1009	DJI_0388	Populus tremula L.	0	Salicaceae Mirb.	n	1
Iz_4_1009	DJI_0388	Populus nigra L.	0	Salicaceae Mirb.	n	1
Iz_4_1009	DJI_0388	Pyrus communis L.	0	Rosaceae Juss.	a	0
Iz_4_1009	DJI_0388	Quercus robur L.	0	Fagaceae Dumort.	n	1
Iz_4_1009	DJI_0388	Robinia pseudoacacia L.	0	Fabaceae Lindl.	a	0
Iz_4_1009	DJI_0388	Rosa sp.	0	Rosaceae Juss.		
Iz_4_1009	DJI_0388	Salix alba L.	0	Salicaceae Mirb.	n	1
Iz_4_1009	DJI_0388	Sambucus nigra L.	0	Viburnaceae Raf.	n	1
Iz_4_1009	DJI_0388	Sorbus aucuparia L.	0	Rosaceae Juss.	n	1
Iz_4_1009	DJI_0388	Tilia cordata Mill.	0	Malvaceae Juss.	n	1
Iz_4_1009	DJI_0388	Ulmus glabra Huds.	0	Ulmaceae Mirb.	n	1
Iz_4_1009	DJI_0389	Acer tataricum L.	0	Sapindaceae Juss.	n	1
Iz_4_1009	DJI_0389	Betula pendula Roth.	0	Betulaceae Gray	n	1
Iz_4_1009	DJI_0389	Crataegus rhipidophylla Gand.	0	Rosaceae Juss.	n	1
Iz_4_1009	DJI_0389	Frangula alnus Mill.	0	Rhamnaceae Juss.	n	1
Iz_4_1009	DJI_0389	Fraxinus pennsylvanica Marshall	0	Oleaceae Hoffmanns. & Link	a	0
Iz_4_1009	DJI_0389	Pinus sylvestris L.	0	Pinaceae Spreng. ex F.Rudolphi	n	1
Iz_4_1009	DJI_0389	Populus × canescens (Aiton) Sm.	0	Salicaceae Mirb.	n	1
Iz_4_1009	DJI_0389	Populus tremula L.	0	Salicaceae Mirb.	n	1
Iz_4_1009	DJI_0389	Populus nigra L.	0	Salicaceae Mirb.	n	1
Iz_4_1009	DJI_0389	Pyrus communis L.	0	Rosaceae Juss.	a	0
Iz_4_1009	DJI_0389	Quercus robur L.	0	Fagaceae Dumort.	n	1
Iz_4_1009	DJI_0389	Robinia pseudoacacia L.	0	Fabaceae Lindl.	a	0
Iz_4_1009	DJI_0389	Rosa sp.	0	Rosaceae Juss.		
Iz_4_1009	DJI_0389	Salix alba L.	0	Salicaceae Mirb.	n	1
Iz_4_1009	DJI_0389	Sambucus nigra L.	0	Viburnaceae Raf.	n	1
Iz_4_1009	DJI_0389	Sorbus aucuparia L.	0	Rosaceae Juss.	n	1
Iz_4_1009	DJI_0389	Tilia cordata Mill.	0	Malvaceae Juss.	n	1
Iz_4_1009	DJI_0389	Ulmus glabra Huds.	0	Ulmaceae Mirb.	n	1
Iz_4_1009	DJI_0392	Acer tataricum L.	0	Sapindaceae Juss.	n	1
Iz_4_1009	DJI_0392	Betula pendula Roth.	0	Betulaceae Gray	n	1
Iz_4_1009	DJI_0392	Crataegus rhipidophylla Gand.	0	Rosaceae Juss.	n	1
Iz_4_1009	DJI_0392	Frangula alnus Mill.	0	Rhamnaceae Juss.	n	1
Iz_4_1009	DJI_0392	Fraxinus pennsylvanica Marshall	0	Oleaceae Hoffmanns. & Link	a	0
Iz_4_1009	DJI_0392	Pinus sylvestris L.	0	Pinaceae Spreng. ex F.Rudolphi	n	1
Iz_4_1009	DJI_0392	Populus × canescens (Aiton) Sm.	0	Salicaceae Mirb.	n	1
Iz_4_1009	DJI_0392	Populus tremula L.	0	Salicaceae Mirb.	n	1
Iz_4_1009	DJI_0392	Populus nigra L.	0	Salicaceae Mirb.	n	1
Iz_4_1009	DJI_0392	Pyrus communis L.	0	Rosaceae Juss.	a	0
Iz_4_1009	DJI_0392	Quercus robur L.	0	Fagaceae Dumort.	n	1
Iz_4_1009	DJI_0392	Robinia pseudoacacia L.	0	Fabaceae Lindl.	a	0
Iz_4_1009	DJI_0392	Rosa sp.	0	Rosaceae Juss.		
Iz_4_1009	DJI_0392	Salix alba L.	0	Salicaceae Mirb.	n	1
Iz_4_1009	DJI_0392	Sambucus nigra L.	0	Viburnaceae Raf.	n	1
Iz_4_1009	DJI_0392	Sorbus aucuparia L.	0	Rosaceae Juss.	n	1
Iz_4_1009	DJI_0392	Tilia cordata Mill.	0	Malvaceae Juss.	n	1
Iz_4_1009	DJI_0392	Ulmus glabra Huds.	0	Ulmaceae Mirb.	n	1
Iz_4_1009	DJI_0395	Acer tataricum L.	0	Sapindaceae Juss.	n	1
Iz_4_1009	DJI_0395	Betula pendula Roth.	0	Betulaceae Gray	n	1
Iz_4_1009	DJI_0395	Crataegus rhipidophylla Gand.	0	Rosaceae Juss.	n	1
Iz_4_1009	DJI_0395	Frangula alnus Mill.	0	Rhamnaceae Juss.	n	1
Iz_4_1009	DJI_0395	Fraxinus pennsylvanica Marshall	0	Oleaceae Hoffmanns. & Link	a	0
Iz_4_1009	DJI_0395	Pinus sylvestris L.	0	Pinaceae Spreng. ex F.Rudolphi	n	1
Iz_4_1009	DJI_0395	Populus × canescens (Aiton) Sm.	0	Salicaceae Mirb.	n	1

Iz_4_1009	DJI_0395	Populus tremula L.	0	Salicaceae Mirb.	n	1
Iz_4_1009	DJI_0395	Populus nigra L.	0	Salicaceae Mirb.	n	1
Iz_4_1009	DJI_0395	Pyrus communis L.	0	Rosaceae Juss.	a	0
Iz_4_1009	DJI_0395	Quercus robur L.	0	Fagaceae Dumort.	n	1
Iz_4_1009	DJI_0395	Robinia pseudoacacia L.	0	Fabaceae Lindl.	a	0
Iz_4_1009	DJI_0395	Rosa sp.	0	Rosaceae Juss.		
Iz_4_1009	DJI_0395	Salix alba L.	0	Salicaceae Mirb.	n	1
Iz_4_1009	DJI_0395	Sambucus nigra L.	0	Viburnaceae Raf.	n	1
Iz_4_1009	DJI_0395	Sorbus aucuparia L.	0	Rosaceae Juss.	n	1
Iz_4_1009	DJI_0395	Tilia cordata Mill.	0	Malvaceae Juss.	n	1
Iz_4_1009	DJI_0395	Ulmus glabra Huds.	0	Ulmaceae Mirb.	n	1
Iz_4_1009	DJI_0397	Acer tataricum L.	0	Sapindaceae Juss.	n	1
Iz_4_1009	DJI_0397	Betula pendula Roth.	0	Betulaceae Gray	n	1
Iz_4_1009	DJI_0397	Crataegus rhipidophylla Gand.	0	Rosaceae Juss.	n	1
Iz_4_1009	DJI_0397	Frangula alnus Mill.	0	Rhamnaceae Juss.	n	1
Iz_4_1009	DJI_0397	Fraxinus pennsylvanica Marshall	0	Oleaceae Hoffmanns. & Link	a	0
Iz_4_1009	DJI_0397	Pinus sylvestris L.	0	Pinaceae Spreng. ex F.Rudolphi	n	1
Iz_4_1009	DJI_0397	Populus × canescens (Aiton) Sm.	0	Salicaceae Mirb.	n	1
Iz_4_1009	DJI_0397	Populus tremula L.	0	Salicaceae Mirb.	n	1
Iz_4_1009	DJI_0397	Populus nigra L.	0	Salicaceae Mirb.	n	1
Iz_4_1009	DJI_0397	Pyrus communis L.	0	Rosaceae Juss.	a	0
Iz_4_1009	DJI_0397	Quercus robur L.	0	Fagaceae Dumort.	n	1
Iz_4_1009	DJI_0397	Robinia pseudoacacia L.	0	Fabaceae Lindl.	a	0
Iz_4_1009	DJI_0397	Rosa sp.	0	Rosaceae Juss.		
Iz_4_1009	DJI_0397	Salix alba L.	0	Salicaceae Mirb.	n	1
Iz_4_1009	DJI_0397	Sambucus nigra L.	0	Viburnaceae Raf.	n	1
Iz_4_1009	DJI_0397	Sorbus aucuparia L.	0	Rosaceae Juss.	n	1
Iz_4_1009	DJI_0397	Tilia cordata Mill.	0	Malvaceae Juss.	n	1
Iz_4_1009	DJI_0397	Ulmus glabra Huds.	0	Ulmaceae Mirb.	n	1
Iz_4_1009	DJI_0399	Acer tataricum L.	0	Sapindaceae Juss.	n	1
Iz_4_1009	DJI_0399	Betula pendula Roth.	0	Betulaceae Gray	n	1
Iz_4_1009	DJI_0399	Crataegus rhipidophylla Gand.	0	Rosaceae Juss.	n	1
Iz_4_1009	DJI_0399	Frangula alnus Mill.	0	Rhamnaceae Juss.	n	1
Iz_4_1009	DJI_0399	Fraxinus pennsylvanica Marshall	0	Oleaceae Hoffmanns. & Link	a	0
Iz_4_1009	DJI_0399	Pinus sylvestris L.	0	Pinaceae Spreng. ex F.Rudolphi	n	1
Iz_4_1009	DJI_0399	Populus × canescens (Aiton) Sm.	0	Salicaceae Mirb.	n	1
Iz_4_1009	DJI_0399	Populus tremula L.	0	Salicaceae Mirb.	n	1
Iz_4_1009	DJI_0399	Populus nigra L.	0	Salicaceae Mirb.	n	1
Iz_4_1009	DJI_0399	Pyrus communis L.	0	Rosaceae Juss.	a	0
Iz_4_1009	DJI_0399	Quercus robur L.	0	Fagaceae Dumort.	n	1
Iz_4_1009	DJI_0399	Robinia pseudoacacia L.	0	Fabaceae Lindl.	a	0
Iz_4_1009	DJI_0399	Rosa sp.	0	Rosaceae Juss.		
Iz_4_1009	DJI_0399	Salix alba L.	0	Salicaceae Mirb.	n	1
Iz_4_1009	DJI_0399	Sambucus nigra L.	0	Viburnaceae Raf.	n	1
Iz_4_1009	DJI_0399	Sorbus aucuparia L.	0	Rosaceae Juss.	n	1
Iz_4_1009	DJI_0399	Tilia cordata Mill.	0	Malvaceae Juss.	n	1
Iz_4_1009	DJI_0399	Ulmus glabra Huds.	0	Ulmaceae Mirb.	n	1
Iz_4_1009	DJI_0405	Acer tataricum L.	0	Sapindaceae Juss.	n	1
Iz_4_1009	DJI_0405	Betula pendula Roth.	0	Betulaceae Gray	n	1
Iz_4_1009	DJI_0405	Crataegus rhipidophylla Gand.	0	Rosaceae Juss.	n	1
Iz_4_1009	DJI_0405	Frangula alnus Mill.	0	Rhamnaceae Juss.	n	1
Iz_4_1009	DJI_0405	Fraxinus pennsylvanica Marshall	0	Oleaceae Hoffmanns. & Link	a	0
Iz_4_1009	DJI_0405	Pinus sylvestris L.	0	Pinaceae Spreng. ex F.Rudolphi	n	1
Iz_4_1009	DJI_0405	Populus × canescens (Aiton) Sm.	0	Salicaceae Mirb.	n	1
Iz_4_1009	DJI_0405	Populus tremula L.	0	Salicaceae Mirb.	n	1
Iz_4_1009	DJI_0405	Populus nigra L.	0	Salicaceae Mirb.	n	1
Iz_4_1009	DJI_0405	Pyrus communis L.	0	Rosaceae Juss.	a	0
Iz_4_1009	DJI_0405	Quercus robur L.	0	Fagaceae Dumort.	n	1
Iz_4_1009	DJI_0405	Robinia pseudoacacia L.	0	Fabaceae Lindl.	a	0
Iz_4_1009	DJI_0405	Rosa sp.	0	Rosaceae Juss.		
Iz_4_1009	DJI_0405	Salix alba L.	0	Salicaceae Mirb.	n	1
Iz_4_1009	DJI_0405	Sambucus nigra L.	0	Viburnaceae Raf.	n	1
Iz_4_1009	DJI_0405	Sorbus aucuparia L.	0	Rosaceae Juss.	n	1
Iz_4_1009	DJI_0405	Tilia cordata Mill.	0	Malvaceae Juss.	n	1

Iz_4_1009	DJI_0405	Ulmus glabra Huds.	0	Ulmaceae Mirb.	n	1
Iz_4_1009	DJI_0406	Acer tataricum L.	0	Sapindaceae Juss.	n	1
Iz_4_1009	DJI_0406	Betula pendula Roth.	0	Betulaceae Gray	n	1
Iz_4_1009	DJI_0406	Crataegus rhipidophylla Gand.	0	Rosaceae Juss.	n	1
Iz_4_1009	DJI_0406	Frangula alnus Mill.	0	Rhamnaceae Juss.	n	1
Iz_4_1009	DJI_0406	Fraxinus pennsylvanica Marshall	0	Oleaceae Hoffmanns. & Link	a	0
Iz_4_1009	DJI_0406	Pinus sylvestris L.	0	Pinaceae Spreng. ex F.Rudolphi	n	1
Iz_4_1009	DJI_0406	Populus × canescens (Aiton) Sm.	0	Salicaceae Mirb.	n	1
Iz_4_1009	DJI_0406	Populus tremula L.	0	Salicaceae Mirb.	n	1
Iz_4_1009	DJI_0406	Populus nigra L.	0	Salicaceae Mirb.	n	1
Iz_4_1009	DJI_0406	Pyrus communis L.	0	Rosaceae Juss.	a	0
Iz_4_1009	DJI_0406	Quercus robur L.	0	Fagaceae Dumort.	n	1
Iz_4_1009	DJI_0406	Robinia pseudoacacia L.	0	Fabaceae Lindl.	a	0
Iz_4_1009	DJI_0406	Rosa sp.	0	Rosaceae Juss.		
Iz_4_1009	DJI_0406	Salix alba L.	0	Salicaceae Mirb.	n	1
Iz_4_1009	DJI_0406	Sambucus nigra L.	0	Viburnaceae Raf.	n	1
Iz_4_1009	DJI_0406	Sorbus aucuparia L.	0	Rosaceae Juss.	n	1
Iz_4_1009	DJI_0406	Tilia cordata Mill.	0	Malvaceae Juss.	n	1
Iz_4_1009	DJI_0406	Ulmus glabra Huds.	0	Ulmaceae Mirb.	n	1
Iz_4_1009	DJI_0407	Acer tataricum L.	0	Sapindaceae Juss.	n	1
Iz_4_1009	DJI_0407	Betula pendula Roth.	0	Betulaceae Gray	n	1
Iz_4_1009	DJI_0407	Crataegus rhipidophylla Gand.	0	Rosaceae Juss.	n	1
Iz_4_1009	DJI_0407	Frangula alnus Mill.	0	Rhamnaceae Juss.	n	1
Iz_4_1009	DJI_0407	Fraxinus pennsylvanica Marshall	0	Oleaceae Hoffmanns. & Link	a	0
Iz_4_1009	DJI_0407	Pinus sylvestris L.	0	Pinaceae Spreng. ex F.Rudolphi	n	1
Iz_4_1009	DJI_0407	Populus × canescens (Aiton) Sm.	0	Salicaceae Mirb.	n	1
Iz_4_1009	DJI_0407	Populus tremula L.	0	Salicaceae Mirb.	n	1
Iz_4_1009	DJI_0407	Populus nigra L.	0	Salicaceae Mirb.	n	1
Iz_4_1009	DJI_0407	Pyrus communis L.	0	Rosaceae Juss.	a	0
Iz_4_1009	DJI_0407	Quercus robur L.	0	Fagaceae Dumort.	n	1
Iz_4_1009	DJI_0407	Robinia pseudoacacia L.	0	Fabaceae Lindl.	a	0
Iz_4_1009	DJI_0407	Rosa sp.	0	Rosaceae Juss.		
Iz_4_1009	DJI_0407	Salix alba L.	0	Salicaceae Mirb.	n	1
Iz_4_1009	DJI_0407	Sambucus nigra L.	0	Viburnaceae Raf.	n	1
Iz_4_1009	DJI_0407	Sorbus aucuparia L.	0	Rosaceae Juss.	n	1
Iz_4_1009	DJI_0407	Tilia cordata Mill.	0	Malvaceae Juss.	n	1
Iz_4_1009	DJI_0407	Ulmus glabra Huds.	0	Ulmaceae Mirb.	n	1
Iz_4_1009	DJI_0408	Acer tataricum L.	0	Sapindaceae Juss.	n	1
Iz_4_1009	DJI_0408	Betula pendula Roth.	0	Betulaceae Gray	n	1
Iz_4_1009	DJI_0408	Crataegus rhipidophylla Gand.	0	Rosaceae Juss.	n	1
Iz_4_1009	DJI_0408	Frangula alnus Mill.	0	Rhamnaceae Juss.	n	1
Iz_4_1009	DJI_0408	Fraxinus pennsylvanica Marshall	0	Oleaceae Hoffmanns. & Link	a	0
Iz_4_1009	DJI_0408	Pinus sylvestris L.	0	Pinaceae Spreng. ex F.Rudolphi	n	1
Iz_4_1009	DJI_0408	Populus × canescens (Aiton) Sm.	0	Salicaceae Mirb.	n	1
Iz_4_1009	DJI_0408	Populus tremula L.	0	Salicaceae Mirb.	n	1
Iz_4_1009	DJI_0408	Populus nigra L.	0	Salicaceae Mirb.	n	1
Iz_4_1009	DJI_0408	Pyrus communis L.	0	Rosaceae Juss.	a	0
Iz_4_1009	DJI_0408	Quercus robur L.	0	Fagaceae Dumort.	n	1
Iz_4_1009	DJI_0408	Robinia pseudoacacia L.	0	Fabaceae Lindl.	a	0
Iz_4_1009	DJI_0408	Rosa sp.	0	Rosaceae Juss.		
Iz_4_1009	DJI_0408	Salix alba L.	0	Salicaceae Mirb.	n	1
Iz_4_1009	DJI_0408	Sambucus nigra L.	0	Viburnaceae Raf.	n	1
Iz_4_1009	DJI_0408	Sorbus aucuparia L.	0	Rosaceae Juss.	n	1
Iz_4_1009	DJI_0408	Tilia cordata Mill.	0	Malvaceae Juss.	n	1
Iz_4_1009	DJI_0408	Ulmus glabra Huds.	0	Ulmaceae Mirb.	n	1
Iz_4_1009	DJI_0411	Acer tataricum L.	0	Sapindaceae Juss.	n	1
Iz_4_1009	DJI_0411	Betula pendula Roth.	0	Betulaceae Gray	n	1
Iz_4_1009	DJI_0411	Crataegus rhipidophylla Gand.	0	Rosaceae Juss.	n	1
Iz_4_1009	DJI_0411	Frangula alnus Mill.	0	Rhamnaceae Juss.	n	1
Iz_4_1009	DJI_0411	Fraxinus pennsylvanica Marshall	0	Oleaceae Hoffmanns. & Link	a	0
Iz_4_1009	DJI_0411	Pinus sylvestris L.	0	Pinaceae Spreng. ex F.Rudolphi	n	1
Iz_4_1009	DJI_0411	Populus × canescens (Aiton) Sm.	0	Salicaceae Mirb.	n	1
Iz_4_1009	DJI_0411	Populus tremula L.	0	Salicaceae Mirb.	n	1
Iz_4_1009	DJI_0411	Populus nigra L.	0	Salicaceae Mirb.	n	1

Iz_4_1009	DJI_0411	Pyrus communis L.	0	Rosaceae Juss.	a	0
Iz_4_1009	DJI_0411	Quercus robur L.	0	Fagaceae Dumort.	n	1
Iz_4_1009	DJI_0411	Robinia pseudoacacia L.	0	Fabaceae Lindl.	a	0
Iz_4_1009	DJI_0411	Rosa sp.	0	Rosaceae Juss.		
Iz_4_1009	DJI_0411	Salix alba L.	0	Salicaceae Mirb.	n	1
Iz_4_1009	DJI_0411	Sambucus nigra L.	0	Viburnaceae Raf.	n	1
Iz_4_1009	DJI_0411	Sorbus aucuparia L.	0	Rosaceae Juss.	n	1
Iz_4_1009	DJI_0411	Tilia cordata Mill.	0	Malvaceae Juss.	n	1
Iz_4_1009	DJI_0411	Ulmus glabra Huds.	0	Ulmaceae Mirb.	n	1
Iz_5_1009	DJI_0671	Acer tataricum L.	0	Sapindaceae Juss.	n	1
Iz_5_1009	DJI_0671	Betula pendula Roth.	0	Betulaceae Gray	n	1
Iz_5_1009	DJI_0671	Crataegus rhipidophylla Gand.	0	Rosaceae Juss.	n	1
Iz_5_1009	DJI_0671	Frangula alnus Mill.	0	Rhamnaceae Juss.	n	1
Iz_5_1009	DJI_0671	Fraxinus pennsylvanica Marshall	0	Oleaceae Hoffmanns. & Link	a	0
Iz_5_1009	DJI_0671	Pinus sylvestris L.	0	Pinaceae Spreng. ex F.Rudolphi	n	1
Iz_5_1009	DJI_0671	Populus × canescens (Aiton) Sm.	0	Salicaceae Mirb.	n	1
Iz_5_1009	DJI_0671	Populus tremula L.	0	Salicaceae Mirb.	n	1
Iz_5_1009	DJI_0671	Populus nigra L.	0	Salicaceae Mirb.	n	1
Iz_5_1009	DJI_0671	Populus sp.	0	Salicaceae Mirb.	n	1
Iz_5_1009	DJI_0671	Pyrus communis L.	0	Rosaceae Juss.	a	0
Iz_5_1009	DJI_0671	Quercus robur L.	0	Fagaceae Dumort.	n	1
Iz_5_1009	DJI_0671	Robinia pseudoacacia L.	0	Fabaceae Lindl.	a	0
Iz_5_1009	DJI_0671	Rosa sp.	0	Rosaceae Juss.		
Iz_5_1009	DJI_0671	Salix alba L.	0	Salicaceae Mirb.	n	1
Iz_5_1009	DJI_0671	Sambucus nigra L.	0	Viburnaceae Raf.	n	1
Iz_5_1009	DJI_0671	Sorbus aucuparia L.	0	Rosaceae Juss.	n	1
Iz_5_1009	DJI_0671	Tilia cordata Mill.	0	Malvaceae Juss.	n	1
Iz_5_1009	DJI_0671	Ulmus glabra Huds.	0	Ulmaceae Mirb.	n	1
Iz_5_1009	DJI_0672	Acer tataricum L.	0	Sapindaceae Juss.	n	1
Iz_5_1009	DJI_0672	Betula pendula Roth.	0	Betulaceae Gray	n	1
Iz_5_1009	DJI_0672	Crataegus rhipidophylla Gand.	0	Rosaceae Juss.	n	1
Iz_5_1009	DJI_0672	Frangula alnus Mill.	0	Rhamnaceae Juss.	n	1
Iz_5_1009	DJI_0672	Fraxinus pennsylvanica Marshall	0	Oleaceae Hoffmanns. & Link	a	0
Iz_5_1009	DJI_0672	Pinus sylvestris L.	0	Pinaceae Spreng. ex F.Rudolphi	n	1
Iz_5_1009	DJI_0672	Populus × canescens (Aiton) Sm.	0	Salicaceae Mirb.	n	1
Iz_5_1009	DJI_0672	Populus tremula L.	2	Salicaceae Mirb.	n	1
Iz_5_1009	DJI_0672	Populus nigra L.	0	Salicaceae Mirb.	n	1
Iz_5_1009	DJI_0672	Populus sp.	0	Salicaceae Mirb.	n	1
Iz_5_1009	DJI_0672	Pyrus communis L.	0	Rosaceae Juss.	a	0
Iz_5_1009	DJI_0672	Quercus robur L.	0	Fagaceae Dumort.	n	1
Iz_5_1009	DJI_0672	Robinia pseudoacacia L.	0	Fabaceae Lindl.	a	0
Iz_5_1009	DJI_0672	Rosa sp.	0	Rosaceae Juss.		
Iz_5_1009	DJI_0672	Salix alba L.	0	Salicaceae Mirb.	n	1
Iz_5_1009	DJI_0672	Sambucus nigra L.	0	Viburnaceae Raf.	n	1
Iz_5_1009	DJI_0672	Sorbus aucuparia L.	0	Rosaceae Juss.	n	1
Iz_5_1009	DJI_0672	Tilia cordata Mill.	0	Malvaceae Juss.	n	1
Iz_5_1009	DJI_0672	Ulmus glabra Huds.	0	Ulmaceae Mirb.	n	1
Iz_5_1009	DJI_0673	Acer tataricum L.	0	Sapindaceae Juss.	n	1
Iz_5_1009	DJI_0673	Betula pendula Roth.	0	Betulaceae Gray	n	1
Iz_5_1009	DJI_0673	Crataegus rhipidophylla Gand.	0	Rosaceae Juss.	n	1
Iz_5_1009	DJI_0673	Frangula alnus Mill.	0	Rhamnaceae Juss.	n	1
Iz_5_1009	DJI_0673	Fraxinus pennsylvanica Marshall	0	Oleaceae Hoffmanns. & Link	a	0
Iz_5_1009	DJI_0673	Pinus sylvestris L.	0	Pinaceae Spreng. ex F.Rudolphi	n	1
Iz_5_1009	DJI_0673	Populus × canescens (Aiton) Sm.	0	Salicaceae Mirb.	n	1
Iz_5_1009	DJI_0673	Populus tremula L.	2	Salicaceae Mirb.	n	1
Iz_5_1009	DJI_0673	Populus nigra L.	0	Salicaceae Mirb.	n	1
Iz_5_1009	DJI_0673	Populus sp.	0	Salicaceae Mirb.	n	1
Iz_5_1009	DJI_0673	Pyrus communis L.	0	Rosaceae Juss.	a	0
Iz_5_1009	DJI_0673	Quercus robur L.	0	Fagaceae Dumort.	n	1
Iz_5_1009	DJI_0673	Robinia pseudoacacia L.	0	Fabaceae Lindl.	a	0
Iz_5_1009	DJI_0673	Rosa sp.	0	Rosaceae Juss.		
Iz_5_1009	DJI_0673	Salix alba L.	0	Salicaceae Mirb.	n	1
Iz_5_1009	DJI_0673	Sambucus nigra L.	0	Viburnaceae Raf.	n	1
Iz_5_1009	DJI_0673	Sorbus aucuparia L.	0	Rosaceae Juss.	n	1

Iz_5_1009	DJI_0673	Tilia cordata Mill.	0	Malvaceae Juss.	n	1
Iz_5_1009	DJI_0673	Ulmus glabra Huds.	0	Ulmaceae Mirb.	n	1
Iz_5_1009	DJI_0674	Acer tataricum L.	0	Sapindaceae Juss.	n	1
Iz_5_1009	DJI_0674	Betula pendula Roth.	0	Betulaceae Gray	n	1
Iz_5_1009	DJI_0674	Crataegus rhipidophylla Gand.	0	Rosaceae Juss.	n	1
Iz_5_1009	DJI_0674	Frangula alnus Mill.	0	Rhamnaceae Juss.	n	1
Iz_5_1009	DJI_0674	Fraxinus pennsylvanica Marshall	0	Oleaceae Hoffmanns. & Link	a	0
Iz_5_1009	DJI_0674	Pinus sylvestris L.	0	Pinaceae Spreng. ex F.Rudolphi	n	1
Iz_5_1009	DJI_0674	Populus × canescens (Aiton) Sm.	0	Salicaceae Mirb.	n	1
Iz_5_1009	DJI_0674	Populus tremula L.	0	Salicaceae Mirb.	n	1
Iz_5_1009	DJI_0674	Populus nigra L.	0	Salicaceae Mirb.	n	1
Iz_5_1009	DJI_0674	Populus sp.	0	Salicaceae Mirb.	n	1
Iz_5_1009	DJI_0674	Pyrus communis L.	0	Rosaceae Juss.	a	0
Iz_5_1009	DJI_0674	Quercus robur L.	0	Fagaceae Dumort.	n	1
Iz_5_1009	DJI_0674	Robinia pseudoacacia L.	0	Fabaceae Lindl.	a	0
Iz_5_1009	DJI_0674	Rosa sp.	0	Rosaceae Juss.		
Iz_5_1009	DJI_0674	Salix alba L.	0	Salicaceae Mirb.	n	1
Iz_5_1009	DJI_0674	Sambucus nigra L.	0	Viburnaceae Raf.	n	1
Iz_5_1009	DJI_0674	Sorbus aucuparia L.	0	Rosaceae Juss.	n	1
Iz_5_1009	DJI_0674	Tilia cordata Mill.	0	Malvaceae Juss.	n	1
Iz_5_1009	DJI_0674	Ulmus glabra Huds.	0	Ulmaceae Mirb.	n	1
Iz_5_1009	DJI_0676	Acer tataricum L.	0	Sapindaceae Juss.	n	1
Iz_5_1009	DJI_0676	Betula pendula Roth.	0	Betulaceae Gray	n	1
Iz_5_1009	DJI_0676	Crataegus rhipidophylla Gand.	0	Rosaceae Juss.	n	1
Iz_5_1009	DJI_0676	Frangula alnus Mill.	0	Rhamnaceae Juss.	n	1
Iz_5_1009	DJI_0676	Fraxinus pennsylvanica Marshall	0	Oleaceae Hoffmanns. & Link	a	0
Iz_5_1009	DJI_0676	Pinus sylvestris L.	0	Pinaceae Spreng. ex F.Rudolphi	n	1
Iz_5_1009	DJI_0676	Populus × canescens (Aiton) Sm.	0	Salicaceae Mirb.	n	1
Iz_5_1009	DJI_0676	Populus tremula L.	0	Salicaceae Mirb.	n	1
Iz_5_1009	DJI_0676	Populus nigra L.	0	Salicaceae Mirb.	n	1
Iz_5_1009	DJI_0676	Populus sp.	0	Salicaceae Mirb.	n	1
Iz_5_1009	DJI_0676	Pyrus communis L.	0	Rosaceae Juss.	a	0
Iz_5_1009	DJI_0676	Quercus robur L.	0	Fagaceae Dumort.	n	1
Iz_5_1009	DJI_0676	Robinia pseudoacacia L.	0	Fabaceae Lindl.	a	0
Iz_5_1009	DJI_0676	Rosa sp.	0	Rosaceae Juss.		
Iz_5_1009	DJI_0676	Salix alba L.	0	Salicaceae Mirb.	n	1
Iz_5_1009	DJI_0676	Sambucus nigra L.	0	Viburnaceae Raf.	n	1
Iz_5_1009	DJI_0676	Sorbus aucuparia L.	0	Rosaceae Juss.	n	1
Iz_5_1009	DJI_0676	Tilia cordata Mill.	0	Malvaceae Juss.	n	1
Iz_5_1009	DJI_0676	Ulmus glabra Huds.	0	Ulmaceae Mirb.	n	1
Iz_5_1009	DJI_0677	Acer tataricum L.	0	Sapindaceae Juss.	n	1
Iz_5_1009	DJI_0677	Betula pendula Roth.	0	Betulaceae Gray	n	1
Iz_5_1009	DJI_0677	Crataegus rhipidophylla Gand.	0	Rosaceae Juss.	n	1
Iz_5_1009	DJI_0677	Frangula alnus Mill.	0	Rhamnaceae Juss.	n	1
Iz_5_1009	DJI_0677	Fraxinus pennsylvanica Marshall	0	Oleaceae Hoffmanns. & Link	a	0
Iz_5_1009	DJI_0677	Pinus sylvestris L.	0	Pinaceae Spreng. ex F.Rudolphi	n	1
Iz_5_1009	DJI_0677	Populus × canescens (Aiton) Sm.	0	Salicaceae Mirb.	n	1
Iz_5_1009	DJI_0677	Populus tremula L.	0	Salicaceae Mirb.	n	1
Iz_5_1009	DJI_0677	Populus nigra L.	0	Salicaceae Mirb.	n	1
Iz_5_1009	DJI_0677	Populus sp.	0	Salicaceae Mirb.	n	1
Iz_5_1009	DJI_0677	Pyrus communis L.	0	Rosaceae Juss.	a	0
Iz_5_1009	DJI_0677	Quercus robur L.	0	Fagaceae Dumort.	n	1
Iz_5_1009	DJI_0677	Robinia pseudoacacia L.	0	Fabaceae Lindl.	a	0
Iz_5_1009	DJI_0677	Rosa sp.	0	Rosaceae Juss.		
Iz_5_1009	DJI_0677	Salix alba L.	0	Salicaceae Mirb.	n	1
Iz_5_1009	DJI_0677	Sambucus nigra L.	0	Viburnaceae Raf.	n	1
Iz_5_1009	DJI_0677	Sorbus aucuparia L.	0	Rosaceae Juss.	n	1
Iz_5_1009	DJI_0677	Tilia cordata Mill.	0	Malvaceae Juss.	n	1
Iz_5_1009	DJI_0677	Ulmus glabra Huds.	0	Ulmaceae Mirb.	n	1
Iz_5_1009	DJI_0679	Acer tataricum L.	0	Sapindaceae Juss.	n	1
Iz_5_1009	DJI_0679	Betula pendula Roth.	0	Betulaceae Gray	n	1
Iz_5_1009	DJI_0679	Crataegus rhipidophylla Gand.	0	Rosaceae Juss.	n	1
Iz_5_1009	DJI_0679	Frangula alnus Mill.	0	Rhamnaceae Juss.	n	1
Iz_5_1009	DJI_0679	Fraxinus pennsylvanica Marshall	0	Oleaceae Hoffmanns. & Link	a	0

Iz_5_1009	DJI_0679	Pinus sylvestris L.	0	Pinaceae Spreng. ex F.Rudolphi	n	1
Iz_5_1009	DJI_0679	Populus × canescens (Aiton) Sm.	0	Salicaceae Mirb.	n	1
Iz_5_1009	DJI_0679	Populus tremula L.	2	Salicaceae Mirb.	n	1
Iz_5_1009	DJI_0679	Populus nigra L.	0	Salicaceae Mirb.	n	1
Iz_5_1009	DJI_0679	Populus sp.	0	Salicaceae Mirb.	n	1
Iz_5_1009	DJI_0679	Pyrus communis L.	0	Rosaceae Juss.	a	0
Iz_5_1009	DJI_0679	Quercus robur L.	0	Fagaceae Dumort.	n	1
Iz_5_1009	DJI_0679	Robinia pseudoacacia L.	0	Fabaceae Lindl.	a	0
Iz_5_1009	DJI_0679	Rosa sp.	0	Rosaceae Juss.		
Iz_5_1009	DJI_0679	Salix alba L.	0	Salicaceae Mirb.	n	1
Iz_5_1009	DJI_0679	Sambucus nigra L.	0	Viburnaceae Raf.	n	1
Iz_5_1009	DJI_0679	Sorbus aucuparia L.	0	Rosaceae Juss.	n	1
Iz_5_1009	DJI_0679	Tilia cordata Mill.	0	Malvaceae Juss.	n	1
Iz_5_1009	DJI_0679	Ulmus glabra Huds.	0	Ulmaceae Mirb.	n	1
Iz_5_1009	DJI_0685	Acer tataricum L.	0	Sapindaceae Juss.	n	1
Iz_5_1009	DJI_0685	Betula pendula Roth.	0	Betulaceae Gray	n	1
Iz_5_1009	DJI_0685	Crataegus rhipidophylla Gand.	0	Rosaceae Juss.	n	1
Iz_5_1009	DJI_0685	Frangula alnus Mill.	0	Rhamnaceae Juss.	n	1
Iz_5_1009	DJI_0685	Fraxinus pennsylvanica Marshall	0	Oleaceae Hoffmanns. & Link	a	0
Iz_5_1009	DJI_0685	Pinus sylvestris L.	0	Pinaceae Spreng. ex F.Rudolphi	n	1
Iz_5_1009	DJI_0685	Populus × canescens (Aiton) Sm.	0	Salicaceae Mirb.	n	1
Iz_5_1009	DJI_0685	Populus tremula L.	0	Salicaceae Mirb.	n	1
Iz_5_1009	DJI_0685	Populus nigra L.	0	Salicaceae Mirb.	n	1
Iz_5_1009	DJI_0685	Populus sp.	0	Salicaceae Mirb.	n	1
Iz_5_1009	DJI_0685	Pyrus communis L.	0	Rosaceae Juss.	a	0
Iz_5_1009	DJI_0685	Quercus robur L.	0	Fagaceae Dumort.	n	1
Iz_5_1009	DJI_0685	Robinia pseudoacacia L.	0	Fabaceae Lindl.	a	0
Iz_5_1009	DJI_0685	Rosa sp.	0	Rosaceae Juss.		
Iz_5_1009	DJI_0685	Salix alba L.	0	Salicaceae Mirb.	n	1
Iz_5_1009	DJI_0685	Sambucus nigra L.	0	Viburnaceae Raf.	n	1
Iz_5_1009	DJI_0685	Sorbus aucuparia L.	0	Rosaceae Juss.	n	1
Iz_5_1009	DJI_0685	Tilia cordata Mill.	0	Malvaceae Juss.	n	1
Iz_5_1009	DJI_0685	Ulmus glabra Huds.	0	Ulmaceae Mirb.	n	1
Iz_5_1009	DJI_0686	Acer tataricum L.	0	Sapindaceae Juss.	n	1
Iz_5_1009	DJI_0686	Betula pendula Roth.	0	Betulaceae Gray	n	1
Iz_5_1009	DJI_0686	Crataegus rhipidophylla Gand.	0	Rosaceae Juss.	n	1
Iz_5_1009	DJI_0686	Frangula alnus Mill.	0	Rhamnaceae Juss.	n	1
Iz_5_1009	DJI_0686	Fraxinus pennsylvanica Marshall	0	Oleaceae Hoffmanns. & Link	a	0
Iz_5_1009	DJI_0686	Pinus sylvestris L.	0	Pinaceae Spreng. ex F.Rudolphi	n	1
Iz_5_1009	DJI_0686	Populus × canescens (Aiton) Sm.	0	Salicaceae Mirb.	n	1
Iz_5_1009	DJI_0686	Populus tremula L.	0	Salicaceae Mirb.	n	1
Iz_5_1009	DJI_0686	Populus nigra L.	0	Salicaceae Mirb.	n	1
Iz_5_1009	DJI_0686	Populus sp.	0	Salicaceae Mirb.	n	1
Iz_5_1009	DJI_0686	Pyrus communis L.	0	Rosaceae Juss.	a	0
Iz_5_1009	DJI_0686	Quercus robur L.	0	Fagaceae Dumort.	n	1
Iz_5_1009	DJI_0686	Robinia pseudoacacia L.	0	Fabaceae Lindl.	a	0
Iz_5_1009	DJI_0686	Rosa sp.	0	Rosaceae Juss.		
Iz_5_1009	DJI_0686	Salix alba L.	0	Salicaceae Mirb.	n	1
Iz_5_1009	DJI_0686	Sambucus nigra L.	0	Viburnaceae Raf.	n	1
Iz_5_1009	DJI_0686	Sorbus aucuparia L.	0	Rosaceae Juss.	n	1
Iz_5_1009	DJI_0686	Tilia cordata Mill.	0	Malvaceae Juss.	n	1
Iz_5_1009	DJI_0686	Ulmus glabra Huds.	0	Ulmaceae Mirb.	n	1
Iz_5_1009	DJI_0689	Acer tataricum L.	0	Sapindaceae Juss.	n	1
Iz_5_1009	DJI_0689	Betula pendula Roth.	0	Betulaceae Gray	n	1
Iz_5_1009	DJI_0689	Crataegus rhipidophylla Gand.	0	Rosaceae Juss.	n	1
Iz_5_1009	DJI_0689	Frangula alnus Mill.	0	Rhamnaceae Juss.	n	1
Iz_5_1009	DJI_0689	Fraxinus pennsylvanica Marshall	0	Oleaceae Hoffmanns. & Link	a	0
Iz_5_1009	DJI_0689	Pinus sylvestris L.	0	Pinaceae Spreng. ex F.Rudolphi	n	1
Iz_5_1009	DJI_0689	Populus × canescens (Aiton) Sm.	0	Salicaceae Mirb.	n	1
Iz_5_1009	DJI_0689	Populus tremula L.	0	Salicaceae Mirb.	n	1
Iz_5_1009	DJI_0689	Populus nigra L.	0	Salicaceae Mirb.	n	1
Iz_5_1009	DJI_0689	Populus sp.	0	Salicaceae Mirb.	n	1
Iz_5_1009	DJI_0689	Pyrus communis L.	0	Rosaceae Juss.	a	0
Iz_5_1009	DJI_0689	Quercus robur L.	0	Fagaceae Dumort.	n	1

Iz_5_1009	DJI_0689	Robinia pseudoacacia L.	0	Fabaceae Lindl.	a	0
Iz_5_1009	DJI_0689	Rosa sp.	0	Rosaceae Juss.		
Iz_5_1009	DJI_0689	Salix alba L.	0	Salicaceae Mirb.	n	1
Iz_5_1009	DJI_0689	Sambucus nigra L.	0	Viburnaceae Raf.	n	1
Iz_5_1009	DJI_0689	Sorbus aucuparia L.	0	Rosaceae Juss.	n	1
Iz_5_1009	DJI_0689	Tilia cordata Mill.	0	Malvaceae Juss.	n	1
Iz_5_1009	DJI_0689	Ulmus glabra Huds.	0	Ulmaceae Mirb.	n	1
Iz_5_1009	DJI_0691	Acer tataricum L.	0	Sapindaceae Juss.	n	1
Iz_5_1009	DJI_0691	Betula pendula Roth.	0	Betulaceae Gray	n	1
Iz_5_1009	DJI_0691	Crataegus rhipidophylla Gand.	0	Rosaceae Juss.	n	1
Iz_5_1009	DJI_0691	Frangula alnus Mill.	0	Rhamnaceae Juss.	n	1
Iz_5_1009	DJI_0691	Fraxinus pennsylvanica Marshall	0	Oleaceae Hoffmanns. & Link	a	0
Iz_5_1009	DJI_0691	Pinus sylvestris L.	0	Pinaceae Spreng. ex F.Rudolphi	n	1
Iz_5_1009	DJI_0691	Populus × canescens (Aiton) Sm.	0	Salicaceae Mirb.	n	1
Iz_5_1009	DJI_0691	Populus tremula L.	0	Salicaceae Mirb.	n	1
Iz_5_1009	DJI_0691	Populus nigra L.	0	Salicaceae Mirb.	n	1
Iz_5_1009	DJI_0691	Populus sp.	0	Salicaceae Mirb.	n	1
Iz_5_1009	DJI_0691	Pyrus communis L.	0	Rosaceae Juss.	a	0
Iz_5_1009	DJI_0691	Quercus robur L.	0	Fagaceae Dumort.	n	1
Iz_5_1009	DJI_0691	Robinia pseudoacacia L.	0	Fabaceae Lindl.	a	0
Iz_5_1009	DJI_0691	Rosa sp.	0	Rosaceae Juss.		
Iz_5_1009	DJI_0691	Salix alba L.	0	Salicaceae Mirb.	n	1
Iz_5_1009	DJI_0691	Sambucus nigra L.	0	Viburnaceae Raf.	n	1
Iz_5_1009	DJI_0691	Sorbus aucuparia L.	0	Rosaceae Juss.	n	1
Iz_5_1009	DJI_0691	Tilia cordata Mill.	0	Malvaceae Juss.	n	1
Iz_5_1009	DJI_0691	Ulmus glabra Huds.	0	Ulmaceae Mirb.	n	1
Iz_5_1009	DJI_0693	Acer tataricum L.	0	Sapindaceae Juss.	n	1
Iz_5_1009	DJI_0693	Betula pendula Roth.	0	Betulaceae Gray	n	1
Iz_5_1009	DJI_0693	Crataegus rhipidophylla Gand.	0	Rosaceae Juss.	n	1
Iz_5_1009	DJI_0693	Frangula alnus Mill.	0	Rhamnaceae Juss.	n	1
Iz_5_1009	DJI_0693	Fraxinus pennsylvanica Marshall	0	Oleaceae Hoffmanns. & Link	a	0
Iz_5_1009	DJI_0693	Pinus sylvestris L.	0	Pinaceae Spreng. ex F.Rudolphi	n	1
Iz_5_1009	DJI_0693	Populus × canescens (Aiton) Sm.	0	Salicaceae Mirb.	n	1
Iz_5_1009	DJI_0693	Populus tremula L.	1	Salicaceae Mirb.	n	1
Iz_5_1009	DJI_0693	Populus nigra L.	0	Salicaceae Mirb.	n	1
Iz_5_1009	DJI_0693	Populus sp.	0	Salicaceae Mirb.	n	1
Iz_5_1009	DJI_0693	Pyrus communis L.	0	Rosaceae Juss.	a	0
Iz_5_1009	DJI_0693	Quercus robur L.	0	Fagaceae Dumort.	n	1
Iz_5_1009	DJI_0693	Robinia pseudoacacia L.	0	Fabaceae Lindl.	a	0
Iz_5_1009	DJI_0693	Rosa sp.	0	Rosaceae Juss.		
Iz_5_1009	DJI_0693	Salix alba L.	0	Salicaceae Mirb.	n	1
Iz_5_1009	DJI_0693	Sambucus nigra L.	0	Viburnaceae Raf.	n	1
Iz_5_1009	DJI_0693	Sorbus aucuparia L.	0	Rosaceae Juss.	n	1
Iz_5_1009	DJI_0693	Tilia cordata Mill.	0	Malvaceae Juss.	n	1
Iz_5_1009	DJI_0693	Ulmus glabra Huds.	0	Ulmaceae Mirb.	n	1
Iz_5_1009	DJI_0695	Acer tataricum L.	0	Sapindaceae Juss.	n	1
Iz_5_1009	DJI_0695	Betula pendula Roth.	0	Betulaceae Gray	n	1
Iz_5_1009	DJI_0695	Crataegus rhipidophylla Gand.	0	Rosaceae Juss.	n	1
Iz_5_1009	DJI_0695	Frangula alnus Mill.	0	Rhamnaceae Juss.	n	1
Iz_5_1009	DJI_0695	Fraxinus pennsylvanica Marshall	0	Oleaceae Hoffmanns. & Link	a	0
Iz_5_1009	DJI_0695	Pinus sylvestris L.	0	Pinaceae Spreng. ex F.Rudolphi	n	1
Iz_5_1009	DJI_0695	Populus × canescens (Aiton) Sm.	0	Salicaceae Mirb.	n	1
Iz_5_1009	DJI_0695	Populus tremula L.	0	Salicaceae Mirb.	n	1
Iz_5_1009	DJI_0695	Populus nigra L.	0	Salicaceae Mirb.	n	1
Iz_5_1009	DJI_0695	Populus sp.	0	Salicaceae Mirb.	n	1
Iz_5_1009	DJI_0695	Pyrus communis L.	0	Rosaceae Juss.	a	0
Iz_5_1009	DJI_0695	Quercus robur L.	0	Fagaceae Dumort.	n	1
Iz_5_1009	DJI_0695	Robinia pseudoacacia L.	0	Fabaceae Lindl.	a	0
Iz_5_1009	DJI_0695	Rosa sp.	0	Rosaceae Juss.		
Iz_5_1009	DJI_0695	Salix alba L.	0	Salicaceae Mirb.	n	1
Iz_5_1009	DJI_0695	Sambucus nigra L.	0	Viburnaceae Raf.	n	1
Iz_5_1009	DJI_0695	Sorbus aucuparia L.	0	Rosaceae Juss.	n	1
Iz_5_1009	DJI_0695	Tilia cordata Mill.	0	Malvaceae Juss.	n	1
Iz_5_1009	DJI_0695	Ulmus glabra Huds.	0	Ulmaceae Mirb.	n	1

Iz_5_1009	DJI_0697	Acer tataricum L.	0	Sapindaceae Juss.	n	1
Iz_5_1009	DJI_0697	Betula pendula Roth.	0	Betulaceae Gray	n	1
Iz_5_1009	DJI_0697	Crataegus rhipidophylla Gand.	0	Rosaceae Juss.	n	1
Iz_5_1009	DJI_0697	Frangula alnus Mill.	0	Rhamnaceae Juss.	n	1
Iz_5_1009	DJI_0697	Fraxinus pennsylvanica Marshall	0	Oleaceae Hoffmanns. & Link	a	0
Iz_5_1009	DJI_0697	Pinus sylvestris L.	0	Pinaceae Spreng. ex F.Rudolphi	n	1
Iz_5_1009	DJI_0697	Populus × canescens (Aiton) Sm.	0	Salicaceae Mirb.	n	1
Iz_5_1009	DJI_0697	Populus tremula L.	0	Salicaceae Mirb.	n	1
Iz_5_1009	DJI_0697	Populus nigra L.	0	Salicaceae Mirb.	n	1
Iz_5_1009	DJI_0697	Populus sp.	0	Salicaceae Mirb.	n	1
Iz_5_1009	DJI_0697	Pyrus communis L.	0	Rosaceae Juss.	a	0
Iz_5_1009	DJI_0697	Quercus robur L.	0	Fagaceae Dumort.	n	1
Iz_5_1009	DJI_0697	Robinia pseudoacacia L.	0	Fabaceae Lindl.	a	0
Iz_5_1009	DJI_0697	Rosa sp.	0	Rosaceae Juss.		
Iz_5_1009	DJI_0697	Salix alba L.	0	Salicaceae Mirb.	n	1
Iz_5_1009	DJI_0697	Sambucus nigra L.	0	Viburnaceae Raf.	n	1
Iz_5_1009	DJI_0697	Sorbus aucuparia L.	0	Rosaceae Juss.	n	1
Iz_5_1009	DJI_0697	Tilia cordata Mill.	0	Malvaceae Juss.	n	1
Iz_5_1009	DJI_0697	Ulmus glabra Huds.	0	Ulmaceae Mirb.	n	1
Iz_5_1009	DJI_0701	Acer tataricum L.	0	Sapindaceae Juss.	n	1
Iz_5_1009	DJI_0701	Betula pendula Roth.	0	Betulaceae Gray	n	1
Iz_5_1009	DJI_0701	Crataegus rhipidophylla Gand.	0	Rosaceae Juss.	n	1
Iz_5_1009	DJI_0701	Frangula alnus Mill.	0	Rhamnaceae Juss.	n	1
Iz_5_1009	DJI_0701	Fraxinus pennsylvanica Marshall	0	Oleaceae Hoffmanns. & Link	a	0
Iz_5_1009	DJI_0701	Pinus sylvestris L.	0	Pinaceae Spreng. ex F.Rudolphi	n	1
Iz_5_1009	DJI_0701	Populus × canescens (Aiton) Sm.	0	Salicaceae Mirb.	n	1
Iz_5_1009	DJI_0701	Populus tremula L.	0	Salicaceae Mirb.	n	1
Iz_5_1009	DJI_0701	Populus nigra L.	0	Salicaceae Mirb.	n	1
Iz_5_1009	DJI_0701	Populus sp.	0	Salicaceae Mirb.	n	1
Iz_5_1009	DJI_0701	Pyrus communis L.	0	Rosaceae Juss.	a	0
Iz_5_1009	DJI_0701	Quercus robur L.	0	Fagaceae Dumort.	n	1
Iz_5_1009	DJI_0701	Robinia pseudoacacia L.	0	Fabaceae Lindl.	a	0
Iz_5_1009	DJI_0701	Rosa sp.	0	Rosaceae Juss.		
Iz_5_1009	DJI_0701	Salix alba L.	0	Salicaceae Mirb.	n	1
Iz_5_1009	DJI_0701	Sambucus nigra L.	0	Viburnaceae Raf.	n	1
Iz_5_1009	DJI_0701	Sorbus aucuparia L.	0	Rosaceae Juss.	n	1
Iz_5_1009	DJI_0701	Tilia cordata Mill.	0	Malvaceae Juss.	n	1
Iz_5_1009	DJI_0701	Ulmus glabra Huds.	0	Ulmaceae Mirb.	n	1
Iz_5_1009	DJI_0703	Acer tataricum L.	0	Sapindaceae Juss.	n	1
Iz_5_1009	DJI_0703	Betula pendula Roth.	0	Betulaceae Gray	n	1
Iz_5_1009	DJI_0703	Crataegus rhipidophylla Gand.	0	Rosaceae Juss.	n	1
Iz_5_1009	DJI_0703	Frangula alnus Mill.	0	Rhamnaceae Juss.	n	1
Iz_5_1009	DJI_0703	Fraxinus pennsylvanica Marshall	0	Oleaceae Hoffmanns. & Link	a	0
Iz_5_1009	DJI_0703	Pinus sylvestris L.	0	Pinaceae Spreng. ex F.Rudolphi	n	1
Iz_5_1009	DJI_0703	Populus × canescens (Aiton) Sm.	0	Salicaceae Mirb.	n	1
Iz_5_1009	DJI_0703	Populus tremula L.	4	Salicaceae Mirb.	n	1
Iz_5_1009	DJI_0703	Populus nigra L.	0	Salicaceae Mirb.	n	1
Iz_5_1009	DJI_0703	Populus sp.	0	Salicaceae Mirb.	n	1
Iz_5_1009	DJI_0703	Pyrus communis L.	0	Rosaceae Juss.	a	0
Iz_5_1009	DJI_0703	Quercus robur L.	0	Fagaceae Dumort.	n	1
Iz_5_1009	DJI_0703	Robinia pseudoacacia L.	0	Fabaceae Lindl.	a	0
Iz_5_1009	DJI_0703	Rosa sp.	0	Rosaceae Juss.		
Iz_5_1009	DJI_0703	Salix alba L.	0	Salicaceae Mirb.	n	1
Iz_5_1009	DJI_0703	Sambucus nigra L.	0	Viburnaceae Raf.	n	1
Iz_5_1009	DJI_0703	Sorbus aucuparia L.	0	Rosaceae Juss.	n	1
Iz_5_1009	DJI_0703	Tilia cordata Mill.	0	Malvaceae Juss.	n	1
Iz_5_1009	DJI_0703	Ulmus glabra Huds.	0	Ulmaceae Mirb.	n	1
Iz_5_1009	DJI_0707	Acer tataricum L.	0	Sapindaceae Juss.	n	1
Iz_5_1009	DJI_0707	Betula pendula Roth.	0	Betulaceae Gray	n	1
Iz_5_1009	DJI_0707	Crataegus rhipidophylla Gand.	0	Rosaceae Juss.	n	1
Iz_5_1009	DJI_0707	Frangula alnus Mill.	0	Rhamnaceae Juss.	n	1
Iz_5_1009	DJI_0707	Fraxinus pennsylvanica Marshall	0	Oleaceae Hoffmanns. & Link	a	0
Iz_5_1009	DJI_0707	Pinus sylvestris L.	0	Pinaceae Spreng. ex F.Rudolphi	n	1
Iz_5_1009	DJI_0707	Populus × canescens (Aiton) Sm.	0	Salicaceae Mirb.	n	1

Iz_5_1009	DJI_0707	Populus tremula L.	0	Salicaceae Mirb.	n	1
Iz_5_1009	DJI_0707	Populus nigra L.	0	Salicaceae Mirb.	n	1
Iz_5_1009	DJI_0707	Populus sp.	0	Salicaceae Mirb.	n	1
Iz_5_1009	DJI_0707	Pyrus communis L.	0	Rosaceae Juss.	a	0
Iz_5_1009	DJI_0707	Quercus robur L.	0	Fagaceae Dumort.	n	1
Iz_5_1009	DJI_0707	Robinia pseudoacacia L.	0	Fabaceae Lindl.	a	0
Iz_5_1009	DJI_0707	Rosa sp.	0	Rosaceae Juss.		
Iz_5_1009	DJI_0707	Salix alba L.	0	Salicaceae Mirb.	n	1
Iz_5_1009	DJI_0707	Sambucus nigra L.	0	Viburnaceae Raf.	n	1
Iz_5_1009	DJI_0707	Sorbus aucuparia L.	0	Rosaceae Juss.	n	1
Iz_5_1009	DJI_0707	Tilia cordata Mill.	0	Malvaceae Juss.	n	1
Iz_5_1009	DJI_0707	Ulmus glabra Huds.	0	Ulmaceae Mirb.	n	1
Iz_5_1009	DJI_0710	Acer tataricum L.	0	Sapindaceae Juss.	n	1
Iz_5_1009	DJI_0710	Betula pendula Roth.	0	Betulaceae Gray	n	1
Iz_5_1009	DJI_0710	Crataegus rhipidophylla Gand.	0	Rosaceae Juss.	n	1
Iz_5_1009	DJI_0710	Frangula alnus Mill.	0	Rhamnaceae Juss.	n	1
Iz_5_1009	DJI_0710	Fraxinus pennsylvanica Marshall	0	Oleaceae Hoffmanns. & Link	a	0
Iz_5_1009	DJI_0710	Pinus sylvestris L.	0	Pinaceae Spreng. ex F.Rudolphi	n	1
Iz_5_1009	DJI_0710	Populus × canescens (Aiton) Sm.	0	Salicaceae Mirb.	n	1
Iz_5_1009	DJI_0710	Populus tremula L.	0	Salicaceae Mirb.	n	1
Iz_5_1009	DJI_0710	Populus nigra L.	0	Salicaceae Mirb.	n	1
Iz_5_1009	DJI_0710	Populus sp.	0	Salicaceae Mirb.	n	1
Iz_5_1009	DJI_0710	Pyrus communis L.	0	Rosaceae Juss.	a	0
Iz_5_1009	DJI_0710	Quercus robur L.	0	Fagaceae Dumort.	n	1
Iz_5_1009	DJI_0710	Robinia pseudoacacia L.	0	Fabaceae Lindl.	a	0
Iz_5_1009	DJI_0710	Rosa sp.	0	Rosaceae Juss.		
Iz_5_1009	DJI_0710	Salix alba L.	0	Salicaceae Mirb.	n	1
Iz_5_1009	DJI_0710	Sambucus nigra L.	0	Viburnaceae Raf.	n	1
Iz_5_1009	DJI_0710	Sorbus aucuparia L.	0	Rosaceae Juss.	n	1
Iz_5_1009	DJI_0710	Tilia cordata Mill.	0	Malvaceae Juss.	n	1
Iz_5_1009	DJI_0710	Ulmus glabra Huds.	0	Ulmaceae Mirb.	n	1
Iz_5_1009	DJI_0713	Acer tataricum L.	0	Sapindaceae Juss.	n	1
Iz_5_1009	DJI_0713	Betula pendula Roth.	0	Betulaceae Gray	n	1
Iz_5_1009	DJI_0713	Crataegus rhipidophylla Gand.	0	Rosaceae Juss.	n	1
Iz_5_1009	DJI_0713	Frangula alnus Mill.	0	Rhamnaceae Juss.	n	1
Iz_5_1009	DJI_0713	Fraxinus pennsylvanica Marshall	0	Oleaceae Hoffmanns. & Link	a	0
Iz_5_1009	DJI_0713	Pinus sylvestris L.	0	Pinaceae Spreng. ex F.Rudolphi	n	1
Iz_5_1009	DJI_0713	Populus × canescens (Aiton) Sm.	0	Salicaceae Mirb.	n	1
Iz_5_1009	DJI_0713	Populus tremula L.	0	Salicaceae Mirb.	n	1
Iz_5_1009	DJI_0713	Populus nigra L.	0	Salicaceae Mirb.	n	1
Iz_5_1009	DJI_0713	Populus sp.	0	Salicaceae Mirb.	n	1
Iz_5_1009	DJI_0713	Pyrus communis L.	0	Rosaceae Juss.	a	0
Iz_5_1009	DJI_0713	Quercus robur L.	0	Fagaceae Dumort.	n	1
Iz_5_1009	DJI_0713	Robinia pseudoacacia L.	0	Fabaceae Lindl.	a	0
Iz_5_1009	DJI_0713	Rosa sp.	0	Rosaceae Juss.		
Iz_5_1009	DJI_0713	Salix alba L.	0	Salicaceae Mirb.	n	1
Iz_5_1009	DJI_0713	Sambucus nigra L.	0	Viburnaceae Raf.	n	1
Iz_5_1009	DJI_0713	Sorbus aucuparia L.	0	Rosaceae Juss.	n	1
Iz_5_1009	DJI_0713	Tilia cordata Mill.	0	Malvaceae Juss.	n	1
Iz_5_1009	DJI_0713	Ulmus glabra Huds.	0	Ulmaceae Mirb.	n	1
Iz_5_1009	DJI_0715	Acer tataricum L.	0	Sapindaceae Juss.	n	1
Iz_5_1009	DJI_0715	Betula pendula Roth.	0	Betulaceae Gray	n	1
Iz_5_1009	DJI_0715	Crataegus rhipidophylla Gand.	0	Rosaceae Juss.	n	1
Iz_5_1009	DJI_0715	Frangula alnus Mill.	0	Rhamnaceae Juss.	n	1
Iz_5_1009	DJI_0715	Fraxinus pennsylvanica Marshall	0	Oleaceae Hoffmanns. & Link	a	0
Iz_5_1009	DJI_0715	Pinus sylvestris L.	0	Pinaceae Spreng. ex F.Rudolphi	n	1
Iz_5_1009	DJI_0715	Populus × canescens (Aiton) Sm.	0	Salicaceae Mirb.	n	1
Iz_5_1009	DJI_0715	Populus tremula L.	0	Salicaceae Mirb.	n	1
Iz_5_1009	DJI_0715	Populus nigra L.	0	Salicaceae Mirb.	n	1
Iz_5_1009	DJI_0715	Populus sp.	0	Salicaceae Mirb.	n	1
Iz_5_1009	DJI_0715	Pyrus communis L.	0	Rosaceae Juss.	a	0
Iz_5_1009	DJI_0715	Quercus robur L.	0	Fagaceae Dumort.	n	1
Iz_5_1009	DJI_0715	Robinia pseudoacacia L.	0	Fabaceae Lindl.	a	0
Iz_5_1009	DJI_0715	Rosa sp.	0	Rosaceae Juss.		

Iz_5_1009	DJI_0715	Salix alba L.	0	Salicaceae Mirb.	n	1
Iz_5_1009	DJI_0715	Sambucus nigra L.	0	Viburnaceae Raf.	n	1
Iz_5_1009	DJI_0715	Sorbus aucuparia L.	0	Rosaceae Juss.	n	1
Iz_5_1009	DJI_0715	Tilia cordata Mill.	0	Malvaceae Juss.	n	1
Iz_5_1009	DJI_0715	Ulmus glabra Huds.	0	Ulmaceae Mirb.	n	1
Iz_5_1009	DJI_0717	Acer tataricum L.	0	Sapindaceae Juss.	n	1
Iz_5_1009	DJI_0717	Betula pendula Roth.	0	Betulaceae Gray	n	1
Iz_5_1009	DJI_0717	Crataegus rhipidophylla Gand.	0	Rosaceae Juss.	n	1
Iz_5_1009	DJI_0717	Frangula alnus Mill.	0	Rhamnaceae Juss.	n	1
Iz_5_1009	DJI_0717	Fraxinus pennsylvanica Marshall	0	Oleaceae Hoffmanns. & Link	a	0
Iz_5_1009	DJI_0717	Pinus sylvestris L.	0	Pinaceae Spreng. ex F.Rudolphi	n	1
Iz_5_1009	DJI_0717	Populus × canescens (Aiton) Sm.	0	Salicaceae Mirb.	n	1
Iz_5_1009	DJI_0717	Populus tremula L.	0	Salicaceae Mirb.	n	1
Iz_5_1009	DJI_0717	Populus nigra L.	0	Salicaceae Mirb.	n	1
Iz_5_1009	DJI_0717	Populus sp.	0	Salicaceae Mirb.	n	1
Iz_5_1009	DJI_0717	Pyrus communis L.	0	Rosaceae Juss.	a	0
Iz_5_1009	DJI_0717	Quercus robur L.	0	Fagaceae Dumort.	n	1
Iz_5_1009	DJI_0717	Robinia pseudoacacia L.	0	Fabaceae Lindl.	a	0
Iz_5_1009	DJI_0717	Rosa sp.	0	Rosaceae Juss.		
Iz_5_1009	DJI_0717	Salix alba L.	0	Salicaceae Mirb.	n	1
Iz_5_1009	DJI_0717	Sambucus nigra L.	0	Viburnaceae Raf.	n	1
Iz_5_1009	DJI_0717	Sorbus aucuparia L.	0	Rosaceae Juss.	n	1
Iz_5_1009	DJI_0717	Tilia cordata Mill.	0	Malvaceae Juss.	n	1
Iz_5_1009	DJI_0717	Ulmus glabra Huds.	0	Ulmaceae Mirb.	n	1
Iz_5_1009	DJI_0725	Acer tataricum L.	0	Sapindaceae Juss.	n	1
Iz_5_1009	DJI_0725	Betula pendula Roth.	0	Betulaceae Gray	n	1
Iz_5_1009	DJI_0725	Crataegus rhipidophylla Gand.	0	Rosaceae Juss.	n	1
Iz_5_1009	DJI_0725	Frangula alnus Mill.	0	Rhamnaceae Juss.	n	1
Iz_5_1009	DJI_0725	Fraxinus pennsylvanica Marshall	0	Oleaceae Hoffmanns. & Link	a	0
Iz_5_1009	DJI_0725	Pinus sylvestris L.	0	Pinaceae Spreng. ex F.Rudolphi	n	1
Iz_5_1009	DJI_0725	Populus × canescens (Aiton) Sm.	0	Salicaceae Mirb.	n	1
Iz_5_1009	DJI_0725	Populus tremula L.	0	Salicaceae Mirb.	n	1
Iz_5_1009	DJI_0725	Populus nigra L.	0	Salicaceae Mirb.	n	1
Iz_5_1009	DJI_0725	Populus sp.	0	Salicaceae Mirb.	n	1
Iz_5_1009	DJI_0725	Pyrus communis L.	0	Rosaceae Juss.	a	0
Iz_5_1009	DJI_0725	Quercus robur L.	0	Fagaceae Dumort.	n	1
Iz_5_1009	DJI_0725	Robinia pseudoacacia L.	0	Fabaceae Lindl.	a	0
Iz_5_1009	DJI_0725	Rosa sp.	0	Rosaceae Juss.		
Iz_5_1009	DJI_0725	Salix alba L.	0	Salicaceae Mirb.	n	1
Iz_5_1009	DJI_0725	Sambucus nigra L.	0	Viburnaceae Raf.	n	1
Iz_5_1009	DJI_0725	Sorbus aucuparia L.	0	Rosaceae Juss.	n	1
Iz_5_1009	DJI_0725	Tilia cordata Mill.	0	Malvaceae Juss.	n	1
Iz_5_1009	DJI_0725	Ulmus glabra Huds.	0	Ulmaceae Mirb.	n	1
Iz_5_1009	DJI_0731	Acer tataricum L.	0	Sapindaceae Juss.	n	1
Iz_5_1009	DJI_0731	Betula pendula Roth.	0	Betulaceae Gray	n	1
Iz_5_1009	DJI_0731	Crataegus rhipidophylla Gand.	0	Rosaceae Juss.	n	1
Iz_5_1009	DJI_0731	Frangula alnus Mill.	0	Rhamnaceae Juss.	n	1
Iz_5_1009	DJI_0731	Fraxinus pennsylvanica Marshall	0	Oleaceae Hoffmanns. & Link	a	0
Iz_5_1009	DJI_0731	Pinus sylvestris L.	0	Pinaceae Spreng. ex F.Rudolphi	n	1
Iz_5_1009	DJI_0731	Populus × canescens (Aiton) Sm.	0	Salicaceae Mirb.	n	1
Iz_5_1009	DJI_0731	Populus tremula L.	0	Salicaceae Mirb.	n	1
Iz_5_1009	DJI_0731	Populus nigra L.	0	Salicaceae Mirb.	n	1
Iz_5_1009	DJI_0731	Populus sp.	0	Salicaceae Mirb.	n	1
Iz_5_1009	DJI_0731	Pyrus communis L.	0	Rosaceae Juss.	a	0
Iz_5_1009	DJI_0731	Quercus robur L.	0	Fagaceae Dumort.	n	1
Iz_5_1009	DJI_0731	Robinia pseudoacacia L.	0	Fabaceae Lindl.	a	0
Iz_5_1009	DJI_0731	Rosa sp.	0	Rosaceae Juss.		
Iz_5_1009	DJI_0731	Salix alba L.	0	Salicaceae Mirb.	n	1
Iz_5_1009	DJI_0731	Sambucus nigra L.	0	Viburnaceae Raf.	n	1
Iz_5_1009	DJI_0731	Sorbus aucuparia L.	0	Rosaceae Juss.	n	1
Iz_5_1009	DJI_0731	Tilia cordata Mill.	0	Malvaceae Juss.	n	1
Iz_5_1009	DJI_0731	Ulmus glabra Huds.	0	Ulmaceae Mirb.	n	1
Iz_5_1009	DJI_0735	Acer tataricum L.	0	Sapindaceae Juss.	n	1
Iz_5_1009	DJI_0735	Betula pendula Roth.	0	Betulaceae Gray	n	1

Iz_5_1009	DJI_0735	Crataegus rhipidophylla Gand.	0	Rosaceae Juss.	n	1
Iz_5_1009	DJI_0735	Frangula alnus Mill.	0	Rhamnaceae Juss.	n	1
Iz_5_1009	DJI_0735	Fraxinus pennsylvanica Marshall	0	Oleaceae Hoffmanns. & Link	a	0
Iz_5_1009	DJI_0735	Pinus sylvestris L.	0	Pinaceae Spreng. ex F.Rudolphi	n	1
Iz_5_1009	DJI_0735	Populus × canescens (Aiton) Sm.	0	Salicaceae Mirb.	n	1
Iz_5_1009	DJI_0735	Populus tremula L.	0	Salicaceae Mirb.	n	1
Iz_5_1009	DJI_0735	Populus nigra L.	0	Salicaceae Mirb.	n	1
Iz_5_1009	DJI_0735	Populus sp.	0	Salicaceae Mirb.	n	1
Iz_5_1009	DJI_0735	Pyrus communis L.	0	Rosaceae Juss.	a	0
Iz_5_1009	DJI_0735	Quercus robur L.	0	Fagaceae Dumort.	n	1
Iz_5_1009	DJI_0735	Robinia pseudoacacia L.	0	Fabaceae Lindl.	a	0
Iz_5_1009	DJI_0735	Rosa sp.	0	Rosaceae Juss.		
Iz_5_1009	DJI_0735	Salix alba L.	0	Salicaceae Mirb.	n	1
Iz_5_1009	DJI_0735	Sambucus nigra L.	0	Viburnaceae Raf.	n	1
Iz_5_1009	DJI_0735	Sorbus aucuparia L.	0	Rosaceae Juss.	n	1
Iz_5_1009	DJI_0735	Tilia cordata Mill.	0	Malvaceae Juss.	n	1
Iz_5_1009	DJI_0735	Ulmus glabra Huds.	0	Ulmaceae Mirb.	n	1
Iz_5_1009	DJI_0756	Acer tataricum L.	0	Sapindaceae Juss.	n	1
Iz_5_1009	DJI_0756	Betula pendula Roth.	0	Betulaceae Gray	n	1
Iz_5_1009	DJI_0756	Crataegus rhipidophylla Gand.	0	Rosaceae Juss.	n	1
Iz_5_1009	DJI_0756	Frangula alnus Mill.	0	Rhamnaceae Juss.	n	1
Iz_5_1009	DJI_0756	Fraxinus pennsylvanica Marshall	0	Oleaceae Hoffmanns. & Link	a	0
Iz_5_1009	DJI_0756	Pinus sylvestris L.	0	Pinaceae Spreng. ex F.Rudolphi	n	1
Iz_5_1009	DJI_0756	Populus × canescens (Aiton) Sm.	0	Salicaceae Mirb.	n	1
Iz_5_1009	DJI_0756	Populus tremula L.	0	Salicaceae Mirb.	n	1
Iz_5_1009	DJI_0756	Populus nigra L.	0	Salicaceae Mirb.	n	1
Iz_5_1009	DJI_0756	Populus sp.	0	Salicaceae Mirb.	n	1
Iz_5_1009	DJI_0756	Pyrus communis L.	0	Rosaceae Juss.	a	0
Iz_5_1009	DJI_0756	Quercus robur L.	0	Fagaceae Dumort.	n	1
Iz_5_1009	DJI_0756	Robinia pseudoacacia L.	0	Fabaceae Lindl.	a	0
Iz_5_1009	DJI_0756	Rosa sp.	0	Rosaceae Juss.		
Iz_5_1009	DJI_0756	Salix alba L.	0	Salicaceae Mirb.	n	1
Iz_5_1009	DJI_0756	Sambucus nigra L.	0	Viburnaceae Raf.	n	1
Iz_5_1009	DJI_0756	Sorbus aucuparia L.	0	Rosaceae Juss.	n	1
Iz_5_1009	DJI_0756	Tilia cordata Mill.	0	Malvaceae Juss.	n	1
Iz_5_1009	DJI_0756	Ulmus glabra Huds.	0	Ulmaceae Mirb.	n	1
Iz_5_1009	DJI_0761	Acer tataricum L.	0	Sapindaceae Juss.	n	1
Iz_5_1009	DJI_0761	Betula pendula Roth.	0	Betulaceae Gray	n	1
Iz_5_1009	DJI_0761	Crataegus rhipidophylla Gand.	0	Rosaceae Juss.	n	1
Iz_5_1009	DJI_0761	Frangula alnus Mill.	0	Rhamnaceae Juss.	n	1
Iz_5_1009	DJI_0761	Fraxinus pennsylvanica Marshall	0	Oleaceae Hoffmanns. & Link	a	0
Iz_5_1009	DJI_0761	Pinus sylvestris L.	0	Pinaceae Spreng. ex F.Rudolphi	n	1
Iz_5_1009	DJI_0761	Populus × canescens (Aiton) Sm.	0	Salicaceae Mirb.	n	1
Iz_5_1009	DJI_0761	Populus tremula L.	0	Salicaceae Mirb.	n	1
Iz_5_1009	DJI_0761	Populus nigra L.	0	Salicaceae Mirb.	n	1
Iz_5_1009	DJI_0761	Populus sp.	0	Salicaceae Mirb.	n	1
Iz_5_1009	DJI_0761	Pyrus communis L.	0	Rosaceae Juss.	a	0
Iz_5_1009	DJI_0761	Quercus robur L.	0	Fagaceae Dumort.	n	1
Iz_5_1009	DJI_0761	Robinia pseudoacacia L.	0	Fabaceae Lindl.	a	0
Iz_5_1009	DJI_0761	Rosa sp.	0	Rosaceae Juss.		
Iz_5_1009	DJI_0761	Salix alba L.	0	Salicaceae Mirb.	n	1
Iz_5_1009	DJI_0761	Sambucus nigra L.	0	Viburnaceae Raf.	n	1
Iz_5_1009	DJI_0761	Sorbus aucuparia L.	0	Rosaceae Juss.	n	1
Iz_5_1009	DJI_0761	Tilia cordata Mill.	0	Malvaceae Juss.	n	1
Iz_5_1009	DJI_0761	Ulmus glabra Huds.	0	Ulmaceae Mirb.	n	1
Iz_5_1009	DJI_0765	Acer tataricum L.	0	Sapindaceae Juss.	n	1
Iz_5_1009	DJI_0765	Betula pendula Roth.	0	Betulaceae Gray	n	1
Iz_5_1009	DJI_0765	Crataegus rhipidophylla Gand.	0	Rosaceae Juss.	n	1
Iz_5_1009	DJI_0765	Frangula alnus Mill.	0	Rhamnaceae Juss.	n	1
Iz_5_1009	DJI_0765	Fraxinus pennsylvanica Marshall	0	Oleaceae Hoffmanns. & Link	a	0
Iz_5_1009	DJI_0765	Pinus sylvestris L.	0	Pinaceae Spreng. ex F.Rudolphi	n	1
Iz_5_1009	DJI_0765	Populus × canescens (Aiton) Sm.	0	Salicaceae Mirb.	n	1
Iz_5_1009	DJI_0765	Populus tremula L.	1	Salicaceae Mirb.	n	1
Iz_5_1009	DJI_0765	Populus nigra L.	0	Salicaceae Mirb.	n	1

Iz_5_1009	DJI_0765	Populus sp.	0	Salicaceae Mirb.	n	1
Iz_5_1009	DJI_0765	Pyrus communis L.	0	Rosaceae Juss.	a	0
Iz_5_1009	DJI_0765	Quercus robur L.	0	Fagaceae Dumort.	n	1
Iz_5_1009	DJI_0765	Robinia pseudoacacia L.	0	Fabaceae Lindl.	a	0
Iz_5_1009	DJI_0765	Rosa sp.	0	Rosaceae Juss.		
Iz_5_1009	DJI_0765	Salix alba L.	0	Salicaceae Mirb.	n	1
Iz_5_1009	DJI_0765	Sambucus nigra L.	0	Viburnaceae Raf.	n	1
Iz_5_1009	DJI_0765	Sorbus aucuparia L.	0	Rosaceae Juss.	n	1
Iz_5_1009	DJI_0765	Tilia cordata Mill.	0	Malvaceae Juss.	n	1
Iz_5_1009	DJI_0765	Ulmus glabra Huds.	0	Ulmaceae Mirb.	n	1
Iz_5_1009	DJI_0772	Acer tataricum L.	0	Sapindaceae Juss.	n	1
Iz_5_1009	DJI_0772	Betula pendula Roth.	0	Betulaceae Gray	n	1
Iz_5_1009	DJI_0772	Crataegus rhipidophylla Gand.	0	Rosaceae Juss.	n	1
Iz_5_1009	DJI_0772	Frangula alnus Mill.	0	Rhamnaceae Juss.	n	1
Iz_5_1009	DJI_0772	Fraxinus pennsylvanica Marshall	0	Oleaceae Hoffmanns. & Link	a	0
Iz_5_1009	DJI_0772	Pinus sylvestris L.	0	Pinaceae Spreng. ex F.Rudolphi	n	1
Iz_5_1009	DJI_0772	Populus × canescens (Aiton) Sm.	0	Salicaceae Mirb.	n	1
Iz_5_1009	DJI_0772	Populus tremula L.	0	Salicaceae Mirb.	n	1
Iz_5_1009	DJI_0772	Populus nigra L.	0	Salicaceae Mirb.	n	1
Iz_5_1009	DJI_0772	Populus sp.	0	Salicaceae Mirb.	n	1
Iz_5_1009	DJI_0772	Pyrus communis L.	0	Rosaceae Juss.	a	0
Iz_5_1009	DJI_0772	Quercus robur L.	0	Fagaceae Dumort.	n	1
Iz_5_1009	DJI_0772	Robinia pseudoacacia L.	0	Fabaceae Lindl.	a	0
Iz_5_1009	DJI_0772	Rosa sp.	0	Rosaceae Juss.		
Iz_5_1009	DJI_0772	Salix alba L.	0	Salicaceae Mirb.	n	1
Iz_5_1009	DJI_0772	Sambucus nigra L.	0	Viburnaceae Raf.	n	1
Iz_5_1009	DJI_0772	Sorbus aucuparia L.	0	Rosaceae Juss.	n	1
Iz_5_1009	DJI_0772	Tilia cordata Mill.	0	Malvaceae Juss.	n	1
Iz_5_1009	DJI_0772	Ulmus glabra Huds.	0	Ulmaceae Mirb.	n	1
Iz_5_1009	DJI_0774	Acer tataricum L.	0	Sapindaceae Juss.	n	1
Iz_5_1009	DJI_0774	Betula pendula Roth.	0	Betulaceae Gray	n	1
Iz_5_1009	DJI_0774	Crataegus rhipidophylla Gand.	0	Rosaceae Juss.	n	1
Iz_5_1009	DJI_0774	Frangula alnus Mill.	0	Rhamnaceae Juss.	n	1
Iz_5_1009	DJI_0774	Fraxinus pennsylvanica Marshall	0	Oleaceae Hoffmanns. & Link	a	0
Iz_5_1009	DJI_0774	Pinus sylvestris L.	0	Pinaceae Spreng. ex F.Rudolphi	n	1
Iz_5_1009	DJI_0774	Populus × canescens (Aiton) Sm.	0	Salicaceae Mirb.	n	1
Iz_5_1009	DJI_0774	Populus tremula L.	0	Salicaceae Mirb.	n	1
Iz_5_1009	DJI_0774	Populus nigra L.	0	Salicaceae Mirb.	n	1
Iz_5_1009	DJI_0774	Populus sp.	0	Salicaceae Mirb.	n	1
Iz_5_1009	DJI_0774	Pyrus communis L.	0	Rosaceae Juss.	a	0
Iz_5_1009	DJI_0774	Quercus robur L.	0	Fagaceae Dumort.	n	1
Iz_5_1009	DJI_0774	Robinia pseudoacacia L.	0	Fabaceae Lindl.	a	0
Iz_5_1009	DJI_0774	Rosa sp.	0	Rosaceae Juss.		
Iz_5_1009	DJI_0774	Salix alba L.	0	Salicaceae Mirb.	n	1
Iz_5_1009	DJI_0774	Sambucus nigra L.	0	Viburnaceae Raf.	n	1
Iz_5_1009	DJI_0774	Sorbus aucuparia L.	0	Rosaceae Juss.	n	1
Iz_5_1009	DJI_0774	Tilia cordata Mill.	0	Malvaceae Juss.	n	1
Iz_5_1009	DJI_0774	Ulmus glabra Huds.	0	Ulmaceae Mirb.	n	1
Iz_5_1009	DJI_0776	Acer tataricum L.	0	Sapindaceae Juss.	n	1
Iz_5_1009	DJI_0776	Betula pendula Roth.	0	Betulaceae Gray	n	1
Iz_5_1009	DJI_0776	Crataegus rhipidophylla Gand.	0	Rosaceae Juss.	n	1
Iz_5_1009	DJI_0776	Frangula alnus Mill.	0	Rhamnaceae Juss.	n	1
Iz_5_1009	DJI_0776	Fraxinus pennsylvanica Marshall	0	Oleaceae Hoffmanns. & Link	a	0
Iz_5_1009	DJI_0776	Pinus sylvestris L.	0	Pinaceae Spreng. ex F.Rudolphi	n	1
Iz_5_1009	DJI_0776	Populus × canescens (Aiton) Sm.	0	Salicaceae Mirb.	n	1
Iz_5_1009	DJI_0776	Populus tremula L.	0	Salicaceae Mirb.	n	1
Iz_5_1009	DJI_0776	Populus nigra L.	0	Salicaceae Mirb.	n	1
Iz_5_1009	DJI_0776	Populus sp.	0	Salicaceae Mirb.	n	1
Iz_5_1009	DJI_0776	Pyrus communis L.	0	Rosaceae Juss.	a	0
Iz_5_1009	DJI_0776	Quercus robur L.	0	Fagaceae Dumort.	n	1
Iz_5_1009	DJI_0776	Robinia pseudoacacia L.	0	Fabaceae Lindl.	a	0
Iz_5_1009	DJI_0776	Rosa sp.	0	Rosaceae Juss.		
Iz_5_1009	DJI_0776	Salix alba L.	0	Salicaceae Mirb.	n	1
Iz_5_1009	DJI_0776	Sambucus nigra L.	0	Viburnaceae Raf.	n	1

Iz_5_1009	DJI_0776	Sorbus aucuparia L.	0	Rosaceae Juss.	n	1
Iz_5_1009	DJI_0776	Tilia cordata Mill.	0	Malvaceae Juss.	n	1
Iz_5_1009	DJI_0776	Ulmus glabra Huds.	0	Ulmaceae Mirb.	n	1
Iz_5_1009	DJI_0777	Acer tataricum L.	0	Sapindaceae Juss.	n	1
Iz_5_1009	DJI_0777	Betula pendula Roth.	0	Betulaceae Gray	n	1
Iz_5_1009	DJI_0777	Crataegus rhipidophylla Gand.	0	Rosaceae Juss.	n	1
Iz_5_1009	DJI_0777	Frangula alnus Mill.	0	Rhamnaceae Juss.	n	1
Iz_5_1009	DJI_0777	Fraxinus pennsylvanica Marshall	0	Oleaceae Hoffmanns. & Link	a	0
Iz_5_1009	DJI_0777	Pinus sylvestris L.	0	Pinaceae Spreng. ex F.Rudolphi	n	1
Iz_5_1009	DJI_0777	Populus × canescens (Aiton) Sm.	0	Salicaceae Mirb.	n	1
Iz_5_1009	DJI_0777	Populus tremula L.	0	Salicaceae Mirb.	n	1
Iz_5_1009	DJI_0777	Populus nigra L.	0	Salicaceae Mirb.	n	1
Iz_5_1009	DJI_0777	Populus sp.	0	Salicaceae Mirb.	n	1
Iz_5_1009	DJI_0777	Pyrus communis L.	0	Rosaceae Juss.	a	0
Iz_5_1009	DJI_0777	Quercus robur L.	0	Fagaceae Dumort.	n	1
Iz_5_1009	DJI_0777	Robinia pseudoacacia L.	0	Fabaceae Lindl.	a	0
Iz_5_1009	DJI_0777	Rosa sp.	0	Rosaceae Juss.		
Iz_5_1009	DJI_0777	Salix alba L.	0	Salicaceae Mirb.	n	1
Iz_5_1009	DJI_0777	Sambucus nigra L.	0	Viburnaceae Raf.	n	1
Iz_5_1009	DJI_0777	Sorbus aucuparia L.	0	Rosaceae Juss.	n	1
Iz_5_1009	DJI_0777	Tilia cordata Mill.	0	Malvaceae Juss.	n	1
Iz_5_1009	DJI_0777	Ulmus glabra Huds.	0	Ulmaceae Mirb.	n	1
Iz_5_1009	DJI_0781	Acer tataricum L.	0	Sapindaceae Juss.	n	1
Iz_5_1009	DJI_0781	Betula pendula Roth.	0	Betulaceae Gray	n	1
Iz_5_1009	DJI_0781	Crataegus rhipidophylla Gand.	0	Rosaceae Juss.	n	1
Iz_5_1009	DJI_0781	Frangula alnus Mill.	0	Rhamnaceae Juss.	n	1
Iz_5_1009	DJI_0781	Fraxinus pennsylvanica Marshall	0	Oleaceae Hoffmanns. & Link	a	0
Iz_5_1009	DJI_0781	Pinus sylvestris L.	0	Pinaceae Spreng. ex F.Rudolphi	n	1
Iz_5_1009	DJI_0781	Populus × canescens (Aiton) Sm.	0	Salicaceae Mirb.	n	1
Iz_5_1009	DJI_0781	Populus tremula L.	0	Salicaceae Mirb.	n	1
Iz_5_1009	DJI_0781	Populus nigra L.	0	Salicaceae Mirb.	n	1
Iz_5_1009	DJI_0781	Populus sp.	0	Salicaceae Mirb.	n	1
Iz_5_1009	DJI_0781	Pyrus communis L.	0	Rosaceae Juss.	a	0
Iz_5_1009	DJI_0781	Quercus robur L.	0	Fagaceae Dumort.	n	1
Iz_5_1009	DJI_0781	Robinia pseudoacacia L.	0	Fabaceae Lindl.	a	0
Iz_5_1009	DJI_0781	Rosa sp.	0	Rosaceae Juss.		
Iz_5_1009	DJI_0781	Salix alba L.	0	Salicaceae Mirb.	n	1
Iz_5_1009	DJI_0781	Sambucus nigra L.	0	Viburnaceae Raf.	n	1
Iz_5_1009	DJI_0781	Sorbus aucuparia L.	0	Rosaceae Juss.	n	1
Iz_5_1009	DJI_0781	Tilia cordata Mill.	0	Malvaceae Juss.	n	1
Iz_5_1009	DJI_0781	Ulmus glabra Huds.	0	Ulmaceae Mirb.	n	1
Iz_5_1009	DJI_0783	Acer tataricum L.	0	Sapindaceae Juss.	n	1
Iz_5_1009	DJI_0783	Betula pendula Roth.	0	Betulaceae Gray	n	1
Iz_5_1009	DJI_0783	Crataegus rhipidophylla Gand.	0	Rosaceae Juss.	n	1
Iz_5_1009	DJI_0783	Frangula alnus Mill.	0	Rhamnaceae Juss.	n	1
Iz_5_1009	DJI_0783	Fraxinus pennsylvanica Marshall	0	Oleaceae Hoffmanns. & Link	a	0
Iz_5_1009	DJI_0783	Pinus sylvestris L.	0	Pinaceae Spreng. ex F.Rudolphi	n	1
Iz_5_1009	DJI_0783	Populus × canescens (Aiton) Sm.	0	Salicaceae Mirb.	n	1
Iz_5_1009	DJI_0783	Populus tremula L.	0	Salicaceae Mirb.	n	1
Iz_5_1009	DJI_0783	Populus nigra L.	0	Salicaceae Mirb.	n	1
Iz_5_1009	DJI_0783	Populus sp.	0	Salicaceae Mirb.	n	1
Iz_5_1009	DJI_0783	Pyrus communis L.	0	Rosaceae Juss.	a	0
Iz_5_1009	DJI_0783	Quercus robur L.	0	Fagaceae Dumort.	n	1
Iz_5_1009	DJI_0783	Robinia pseudoacacia L.	0	Fabaceae Lindl.	a	0
Iz_5_1009	DJI_0783	Rosa sp.	0	Rosaceae Juss.		
Iz_5_1009	DJI_0783	Salix alba L.	0	Salicaceae Mirb.	n	1
Iz_5_1009	DJI_0783	Sambucus nigra L.	0	Viburnaceae Raf.	n	1
Iz_5_1009	DJI_0783	Sorbus aucuparia L.	0	Rosaceae Juss.	n	1
Iz_5_1009	DJI_0783	Tilia cordata Mill.	0	Malvaceae Juss.	n	1
Iz_5_1009	DJI_0783	Ulmus glabra Huds.	0	Ulmaceae Mirb.	n	1
Iz_5_1009	DJI_0785	Acer tataricum L.	0	Sapindaceae Juss.	n	1
Iz_5_1009	DJI_0785	Betula pendula Roth.	0	Betulaceae Gray	n	1
Iz_5_1009	DJI_0785	Crataegus rhipidophylla Gand.	0	Rosaceae Juss.	n	1
Iz_5_1009	DJI_0785	Frangula alnus Mill.	0	Rhamnaceae Juss.	n	1

Iz_5_1009	DJI_0785	Fraxinus pennsylvanica Marshall	0	Oleaceae Hoffmanns. & Link	a	0
Iz_5_1009	DJI_0785	Pinus sylvestris L.	0	Pinaceae Spreng. ex F.Rudolphi	n	1
Iz_5_1009	DJI_0785	Populus × canescens (Aiton) Sm.	0	Salicaceae Mirb.	n	1
Iz_5_1009	DJI_0785	Populus tremula L.	0	Salicaceae Mirb.	n	1
Iz_5_1009	DJI_0785	Populus nigra L.	0	Salicaceae Mirb.	n	1
Iz_5_1009	DJI_0785	Populus sp.	0	Salicaceae Mirb.	n	1
Iz_5_1009	DJI_0785	Pyrus communis L.	0	Rosaceae Juss.	a	0
Iz_5_1009	DJI_0785	Quercus robur L.	0	Fagaceae Dumort.	n	1
Iz_5_1009	DJI_0785	Robinia pseudoacacia L.	0	Fabaceae Lindl.	a	0
Iz_5_1009	DJI_0785	Rosa sp.	0	Rosaceae Juss.		
Iz_5_1009	DJI_0785	Salix alba L.	0	Salicaceae Mirb.	n	1
Iz_5_1009	DJI_0785	Sambucus nigra L.	0	Viburnaceae Raf.	n	1
Iz_5_1009	DJI_0785	Sorbus aucuparia L.	0	Rosaceae Juss.	n	1
Iz_5_1009	DJI_0785	Tilia cordata Mill.	0	Malvaceae Juss.	n	1
Iz_5_1009	DJI_0785	Ulmus glabra Huds.	0	Ulmaceae Mirb.	n	1
Iz_5_1009	DJI_0789	Acer tataricum L.	0	Sapindaceae Juss.	n	1
Iz_5_1009	DJI_0789	Betula pendula Roth.	0	Betulaceae Gray	n	1
Iz_5_1009	DJI_0789	Crataegus rhipidophylla Gand.	0	Rosaceae Juss.	n	1
Iz_5_1009	DJI_0789	Frangula alnus Mill.	0	Rhamnaceae Juss.	n	1
Iz_5_1009	DJI_0789	Fraxinus pennsylvanica Marshall	0	Oleaceae Hoffmanns. & Link	a	0
Iz_5_1009	DJI_0789	Pinus sylvestris L.	0	Pinaceae Spreng. ex F.Rudolphi	n	1
Iz_5_1009	DJI_0789	Populus × canescens (Aiton) Sm.	0	Salicaceae Mirb.	n	1
Iz_5_1009	DJI_0789	Populus tremula L.	0	Salicaceae Mirb.	n	1
Iz_5_1009	DJI_0789	Populus nigra L.	0	Salicaceae Mirb.	n	1
Iz_5_1009	DJI_0789	Populus sp.	0	Salicaceae Mirb.	n	1
Iz_5_1009	DJI_0789	Pyrus communis L.	0	Rosaceae Juss.	a	0
Iz_5_1009	DJI_0789	Quercus robur L.	0	Fagaceae Dumort.	n	1
Iz_5_1009	DJI_0789	Robinia pseudoacacia L.	0	Fabaceae Lindl.	a	0
Iz_5_1009	DJI_0789	Rosa sp.	0	Rosaceae Juss.		
Iz_5_1009	DJI_0789	Salix alba L.	0	Salicaceae Mirb.	n	1
Iz_5_1009	DJI_0789	Sambucus nigra L.	0	Viburnaceae Raf.	n	1
Iz_5_1009	DJI_0789	Sorbus aucuparia L.	0	Rosaceae Juss.	n	1
Iz_5_1009	DJI_0789	Tilia cordata Mill.	0	Malvaceae Juss.	n	1
Iz_5_1009	DJI_0789	Ulmus glabra Huds.	0	Ulmaceae Mirb.	n	1
Iz_5_1009	DJI_0793	Acer tataricum L.	0	Sapindaceae Juss.	n	1
Iz_5_1009	DJI_0793	Betula pendula Roth.	0	Betulaceae Gray	n	1
Iz_5_1009	DJI_0793	Crataegus rhipidophylla Gand.	0	Rosaceae Juss.	n	1
Iz_5_1009	DJI_0793	Frangula alnus Mill.	0	Rhamnaceae Juss.	n	1
Iz_5_1009	DJI_0793	Fraxinus pennsylvanica Marshall	0	Oleaceae Hoffmanns. & Link	a	0
Iz_5_1009	DJI_0793	Pinus sylvestris L.	0	Pinaceae Spreng. ex F.Rudolphi	n	1
Iz_5_1009	DJI_0793	Populus × canescens (Aiton) Sm.	0	Salicaceae Mirb.	n	1
Iz_5_1009	DJI_0793	Populus tremula L.	0	Salicaceae Mirb.	n	1
Iz_5_1009	DJI_0793	Populus nigra L.	0	Salicaceae Mirb.	n	1
Iz_5_1009	DJI_0793	Populus sp.	0	Salicaceae Mirb.	n	1
Iz_5_1009	DJI_0793	Pyrus communis L.	0	Rosaceae Juss.	a	0
Iz_5_1009	DJI_0793	Quercus robur L.	0	Fagaceae Dumort.	n	1
Iz_5_1009	DJI_0793	Robinia pseudoacacia L.	0	Fabaceae Lindl.	a	0
Iz_5_1009	DJI_0793	Rosa sp.	0	Rosaceae Juss.		
Iz_5_1009	DJI_0793	Salix alba L.	0	Salicaceae Mirb.	n	1
Iz_5_1009	DJI_0793	Sambucus nigra L.	0	Viburnaceae Raf.	n	1
Iz_5_1009	DJI_0793	Sorbus aucuparia L.	0	Rosaceae Juss.	n	1
Iz_5_1009	DJI_0793	Tilia cordata Mill.	0	Malvaceae Juss.	n	1
Iz_5_1009	DJI_0793	Ulmus glabra Huds.	0	Ulmaceae Mirb.	n	1
Iz_5_1009	DJI_0795	Acer tataricum L.	0	Sapindaceae Juss.	n	1
Iz_5_1009	DJI_0795	Betula pendula Roth.	0	Betulaceae Gray	n	1
Iz_5_1009	DJI_0795	Crataegus rhipidophylla Gand.	0	Rosaceae Juss.	n	1
Iz_5_1009	DJI_0795	Frangula alnus Mill.	0	Rhamnaceae Juss.	n	1
Iz_5_1009	DJI_0795	Fraxinus pennsylvanica Marshall	0	Oleaceae Hoffmanns. & Link	a	0
Iz_5_1009	DJI_0795	Pinus sylvestris L.	0	Pinaceae Spreng. ex F.Rudolphi	n	1
Iz_5_1009	DJI_0795	Populus × canescens (Aiton) Sm.	0	Salicaceae Mirb.	n	1
Iz_5_1009	DJI_0795	Populus tremula L.	0	Salicaceae Mirb.	n	1
Iz_5_1009	DJI_0795	Populus nigra L.	0	Salicaceae Mirb.	n	1
Iz_5_1009	DJI_0795	Populus sp.	0	Salicaceae Mirb.	n	1
Iz_5_1009	DJI_0795	Pyrus communis L.	0	Rosaceae Juss.	a	0

Iz_5_1009	DJI_0795	Quercus robur L.	0	Fagaceae Dumort.	n	1
Iz_5_1009	DJI_0795	Robinia pseudoacacia L.	0	Fabaceae Lindl.	a	0
Iz_5_1009	DJI_0795	Rosa sp.	0	Rosaceae Juss.		
Iz_5_1009	DJI_0795	Salix alba L.	0	Salicaceae Mirb.	n	1
Iz_5_1009	DJI_0795	Sambucus nigra L.	0	Viburnaceae Raf.	n	1
Iz_5_1009	DJI_0795	Sorbus aucuparia L.	0	Rosaceae Juss.	n	1
Iz_5_1009	DJI_0795	Tilia cordata Mill.	0	Malvaceae Juss.	n	1
Iz_5_1009	DJI_0795	Ulmus glabra Huds.	0	Ulmaceae Mirb.	n	1
Iz_5_1009	DJI_0800	Acer tataricum L.	0	Sapindaceae Juss.	n	1
Iz_5_1009	DJI_0800	Betula pendula Roth.	0	Betulaceae Gray	n	1
Iz_5_1009	DJI_0800	Crataegus rhipidophylla Gand.	0	Rosaceae Juss.	n	1
Iz_5_1009	DJI_0800	Frangula alnus Mill.	0	Rhamnaceae Juss.	n	1
Iz_5_1009	DJI_0800	Fraxinus pennsylvanica Marshall	0	Oleaceae Hoffmanns. & Link	a	0
Iz_5_1009	DJI_0800	Pinus sylvestris L.	0	Pinaceae Spreng. ex F.Rudolphi	n	1
Iz_5_1009	DJI_0800	Populus × canescens (Aiton) Sm.	0	Salicaceae Mirb.	n	1
Iz_5_1009	DJI_0800	Populus tremula L.	0	Salicaceae Mirb.	n	1
Iz_5_1009	DJI_0800	Populus nigra L.	0	Salicaceae Mirb.	n	1
Iz_5_1009	DJI_0800	Populus sp.	0	Salicaceae Mirb.	n	1
Iz_5_1009	DJI_0800	Pyrus communis L.	0	Rosaceae Juss.	a	0
Iz_5_1009	DJI_0800	Quercus robur L.	0	Fagaceae Dumort.	n	1
Iz_5_1009	DJI_0800	Robinia pseudoacacia L.	0	Fabaceae Lindl.	a	0
Iz_5_1009	DJI_0800	Rosa sp.	0	Rosaceae Juss.		
Iz_5_1009	DJI_0800	Salix alba L.	0	Salicaceae Mirb.	n	1
Iz_5_1009	DJI_0800	Sambucus nigra L.	0	Viburnaceae Raf.	n	1
Iz_5_1009	DJI_0800	Sorbus aucuparia L.	0	Rosaceae Juss.	n	1
Iz_5_1009	DJI_0800	Tilia cordata Mill.	0	Malvaceae Juss.	n	1
Iz_5_1009	DJI_0800	Ulmus glabra Huds.	0	Ulmaceae Mirb.	n	1
Iz_5_1009	DJI_0803	Acer tataricum L.	0	Sapindaceae Juss.	n	1
Iz_5_1009	DJI_0803	Betula pendula Roth.	0	Betulaceae Gray	n	1
Iz_5_1009	DJI_0803	Crataegus rhipidophylla Gand.	0	Rosaceae Juss.	n	1
Iz_5_1009	DJI_0803	Frangula alnus Mill.	0	Rhamnaceae Juss.	n	1
Iz_5_1009	DJI_0803	Fraxinus pennsylvanica Marshall	0	Oleaceae Hoffmanns. & Link	a	0
Iz_5_1009	DJI_0803	Pinus sylvestris L.	0	Pinaceae Spreng. ex F.Rudolphi	n	1
Iz_5_1009	DJI_0803	Populus × canescens (Aiton) Sm.	0	Salicaceae Mirb.	n	1
Iz_5_1009	DJI_0803	Populus tremula L.	0	Salicaceae Mirb.	n	1
Iz_5_1009	DJI_0803	Populus nigra L.	0	Salicaceae Mirb.	n	1
Iz_5_1009	DJI_0803	Populus sp.	0	Salicaceae Mirb.	n	1
Iz_5_1009	DJI_0803	Pyrus communis L.	0	Rosaceae Juss.	a	0
Iz_5_1009	DJI_0803	Quercus robur L.	0	Fagaceae Dumort.	n	1
Iz_5_1009	DJI_0803	Robinia pseudoacacia L.	0	Fabaceae Lindl.	a	0
Iz_5_1009	DJI_0803	Rosa sp.	0	Rosaceae Juss.		
Iz_5_1009	DJI_0803	Salix alba L.	0	Salicaceae Mirb.	n	1
Iz_5_1009	DJI_0803	Sambucus nigra L.	0	Viburnaceae Raf.	n	1
Iz_5_1009	DJI_0803	Sorbus aucuparia L.	0	Rosaceae Juss.	n	1
Iz_5_1009	DJI_0803	Tilia cordata Mill.	0	Malvaceae Juss.	n	1
Iz_5_1009	DJI_0803	Ulmus glabra Huds.	0	Ulmaceae Mirb.	n	1
Iz_5_1009	DJI_0811	Acer tataricum L.	0	Sapindaceae Juss.	n	1
Iz_5_1009	DJI_0811	Betula pendula Roth.	0	Betulaceae Gray	n	1
Iz_5_1009	DJI_0811	Crataegus rhipidophylla Gand.	0	Rosaceae Juss.	n	1
Iz_5_1009	DJI_0811	Frangula alnus Mill.	0	Rhamnaceae Juss.	n	1
Iz_5_1009	DJI_0811	Fraxinus pennsylvanica Marshall	0	Oleaceae Hoffmanns. & Link	a	0
Iz_5_1009	DJI_0811	Pinus sylvestris L.	0	Pinaceae Spreng. ex F.Rudolphi	n	1
Iz_5_1009	DJI_0811	Populus × canescens (Aiton) Sm.	0	Salicaceae Mirb.	n	1
Iz_5_1009	DJI_0811	Populus tremula L.	0	Salicaceae Mirb.	n	1
Iz_5_1009	DJI_0811	Populus nigra L.	0	Salicaceae Mirb.	n	1
Iz_5_1009	DJI_0811	Populus sp.	0	Salicaceae Mirb.	n	1
Iz_5_1009	DJI_0811	Pyrus communis L.	0	Rosaceae Juss.	a	0
Iz_5_1009	DJI_0811	Quercus robur L.	0	Fagaceae Dumort.	n	1
Iz_5_1009	DJI_0811	Robinia pseudoacacia L.	0	Fabaceae Lindl.	a	0
Iz_5_1009	DJI_0811	Rosa sp.	0	Rosaceae Juss.		
Iz_5_1009	DJI_0811	Salix alba L.	0	Salicaceae Mirb.	n	1
Iz_5_1009	DJI_0811	Sambucus nigra L.	0	Viburnaceae Raf.	n	1
Iz_5_1009	DJI_0811	Sorbus aucuparia L.	0	Rosaceae Juss.	n	1
Iz_5_1009	DJI_0811	Tilia cordata Mill.	0	Malvaceae Juss.	n	1

Iz_5_1009	DJI_0811	Ulmus glabra Huds.	0	Ulmaceae Mirb.	n	1
Iz_5_1009	DJI_0814	Acer tataricum L.	0	Sapindaceae Juss.	n	1
Iz_5_1009	DJI_0814	Betula pendula Roth.	0	Betulaceae Gray	n	1
Iz_5_1009	DJI_0814	Crataegus rhipidophylla Gand.	0	Rosaceae Juss.	n	1
Iz_5_1009	DJI_0814	Frangula alnus Mill.	0	Rhamnaceae Juss.	n	1
Iz_5_1009	DJI_0814	Fraxinus pennsylvanica Marshall	0	Oleaceae Hoffmanns. & Link	a	0
Iz_5_1009	DJI_0814	Pinus sylvestris L.	0	Pinaceae Spreng. ex F.Rudolphi	n	1
Iz_5_1009	DJI_0814	Populus × canescens (Aiton) Sm.	0	Salicaceae Mirb.	n	1
Iz_5_1009	DJI_0814	Populus tremula L.	0	Salicaceae Mirb.	n	1
Iz_5_1009	DJI_0814	Populus nigra L.	0	Salicaceae Mirb.	n	1
Iz_5_1009	DJI_0814	Populus sp.	0	Salicaceae Mirb.	n	1
Iz_5_1009	DJI_0814	Pyrus communis L.	0	Rosaceae Juss.	a	0
Iz_5_1009	DJI_0814	Quercus robur L.	0	Fagaceae Dumort.	n	1
Iz_5_1009	DJI_0814	Robinia pseudoacacia L.	0	Fabaceae Lindl.	a	0
Iz_5_1009	DJI_0814	Rosa sp.	0	Rosaceae Juss.		
Iz_5_1009	DJI_0814	Salix alba L.	0	Salicaceae Mirb.	n	1
Iz_5_1009	DJI_0814	Sambucus nigra L.	0	Viburnaceae Raf.	n	1
Iz_5_1009	DJI_0814	Sorbus aucuparia L.	0	Rosaceae Juss.	n	1
Iz_5_1009	DJI_0814	Tilia cordata Mill.	0	Malvaceae Juss.	n	1
Iz_5_1009	DJI_0814	Ulmus glabra Huds.	0	Ulmaceae Mirb.	n	1
Iz_5_1009	DJI_0816	Acer tataricum L.	0	Sapindaceae Juss.	n	1
Iz_5_1009	DJI_0816	Betula pendula Roth.	0	Betulaceae Gray	n	1
Iz_5_1009	DJI_0816	Crataegus rhipidophylla Gand.	0	Rosaceae Juss.	n	1
Iz_5_1009	DJI_0816	Frangula alnus Mill.	0	Rhamnaceae Juss.	n	1
Iz_5_1009	DJI_0816	Fraxinus pennsylvanica Marshall	0	Oleaceae Hoffmanns. & Link	a	0
Iz_5_1009	DJI_0816	Pinus sylvestris L.	0	Pinaceae Spreng. ex F.Rudolphi	n	1
Iz_5_1009	DJI_0816	Populus × canescens (Aiton) Sm.	0	Salicaceae Mirb.	n	1
Iz_5_1009	DJI_0816	Populus tremula L.	0	Salicaceae Mirb.	n	1
Iz_5_1009	DJI_0816	Populus nigra L.	0	Salicaceae Mirb.	n	1
Iz_5_1009	DJI_0816	Populus sp.	0	Salicaceae Mirb.	n	1
Iz_5_1009	DJI_0816	Pyrus communis L.	0	Rosaceae Juss.	a	0
Iz_5_1009	DJI_0816	Quercus robur L.	0	Fagaceae Dumort.	n	1
Iz_5_1009	DJI_0816	Robinia pseudoacacia L.	0	Fabaceae Lindl.	a	0
Iz_5_1009	DJI_0816	Rosa sp.	0	Rosaceae Juss.		
Iz_5_1009	DJI_0816	Salix alba L.	0	Salicaceae Mirb.	n	1
Iz_5_1009	DJI_0816	Sambucus nigra L.	0	Viburnaceae Raf.	n	1
Iz_5_1009	DJI_0816	Sorbus aucuparia L.	0	Rosaceae Juss.	n	1
Iz_5_1009	DJI_0816	Tilia cordata Mill.	0	Malvaceae Juss.	n	1
Iz_5_1009	DJI_0816	Ulmus glabra Huds.	0	Ulmaceae Mirb.	n	1
Iz_5_1009	DJI_0819	Acer tataricum L.	0	Sapindaceae Juss.	n	1
Iz_5_1009	DJI_0819	Betula pendula Roth.	0	Betulaceae Gray	n	1
Iz_5_1009	DJI_0819	Crataegus rhipidophylla Gand.	0	Rosaceae Juss.	n	1
Iz_5_1009	DJI_0819	Frangula alnus Mill.	0	Rhamnaceae Juss.	n	1
Iz_5_1009	DJI_0819	Fraxinus pennsylvanica Marshall	0	Oleaceae Hoffmanns. & Link	a	0
Iz_5_1009	DJI_0819	Pinus sylvestris L.	0	Pinaceae Spreng. ex F.Rudolphi	n	1
Iz_5_1009	DJI_0819	Populus × canescens (Aiton) Sm.	0	Salicaceae Mirb.	n	1
Iz_5_1009	DJI_0819	Populus tremula L.	0	Salicaceae Mirb.	n	1
Iz_5_1009	DJI_0819	Populus nigra L.	0	Salicaceae Mirb.	n	1
Iz_5_1009	DJI_0819	Populus sp.	0	Salicaceae Mirb.	n	1
Iz_5_1009	DJI_0819	Pyrus communis L.	0	Rosaceae Juss.	a	0
Iz_5_1009	DJI_0819	Quercus robur L.	0	Fagaceae Dumort.	n	1
Iz_5_1009	DJI_0819	Robinia pseudoacacia L.	0	Fabaceae Lindl.	a	0
Iz_5_1009	DJI_0819	Rosa sp.	0	Rosaceae Juss.		
Iz_5_1009	DJI_0819	Salix alba L.	0	Salicaceae Mirb.	n	1
Iz_5_1009	DJI_0819	Sambucus nigra L.	0	Viburnaceae Raf.	n	1
Iz_5_1009	DJI_0819	Sorbus aucuparia L.	0	Rosaceae Juss.	n	1
Iz_5_1009	DJI_0819	Tilia cordata Mill.	0	Malvaceae Juss.	n	1
Iz_5_1009	DJI_0819	Ulmus glabra Huds.	0	Ulmaceae Mirb.	n	1
Iz_5_1009	DJI_0820	Acer tataricum L.	0	Sapindaceae Juss.	n	1
Iz_5_1009	DJI_0820	Betula pendula Roth.	0	Betulaceae Gray	n	1
Iz_5_1009	DJI_0820	Crataegus rhipidophylla Gand.	0	Rosaceae Juss.	n	1
Iz_5_1009	DJI_0820	Frangula alnus Mill.	0	Rhamnaceae Juss.	n	1
Iz_5_1009	DJI_0820	Fraxinus pennsylvanica Marshall	0	Oleaceae Hoffmanns. & Link	a	0
Iz_5_1009	DJI_0820	Pinus sylvestris L.	0	Pinaceae Spreng. ex F.Rudolphi	n	1

Iz_5_1009	DJI_0820	Populus × canescens (Aiton) Sm.	0	Salicaceae Mirb.	n	1
Iz_5_1009	DJI_0820	Populus tremula L.	0	Salicaceae Mirb.	n	1
Iz_5_1009	DJI_0820	Populus nigra L.	0	Salicaceae Mirb.	n	1
Iz_5_1009	DJI_0820	Populus sp.	0	Salicaceae Mirb.	n	1
Iz_5_1009	DJI_0820	Pyrus communis L.	0	Rosaceae Juss.	a	0
Iz_5_1009	DJI_0820	Quercus robur L.	0	Fagaceae Dumort.	n	1
Iz_5_1009	DJI_0820	Robinia pseudoacacia L.	0	Fabaceae Lindl.	a	0
Iz_5_1009	DJI_0820	Rosa sp.	0	Rosaceae Juss.		
Iz_5_1009	DJI_0820	Salix alba L.	0	Salicaceae Mirb.	n	1
Iz_5_1009	DJI_0820	Sambucus nigra L.	0	Viburnaceae Raf.	n	1
Iz_5_1009	DJI_0820	Sorbus aucuparia L.	0	Rosaceae Juss.	n	1
Iz_5_1009	DJI_0820	Tilia cordata Mill.	0	Malvaceae Juss.	n	1
Iz_5_1009	DJI_0820	Ulmus glabra Huds.	0	Ulmaceae Mirb.	n	1
Iz_5_1009	DJI_0822	Acer tataricum L.	0	Sapindaceae Juss.	n	1
Iz_5_1009	DJI_0822	Betula pendula Roth.	0	Betulaceae Gray	n	1
Iz_5_1009	DJI_0822	Crataegus rhipidophylla Gand.	0	Rosaceae Juss.	n	1
Iz_5_1009	DJI_0822	Frangula alnus Mill.	0	Rhamnaceae Juss.	n	1
Iz_5_1009	DJI_0822	Fraxinus pennsylvanica Marshall	0	Oleaceae Hoffmanns. & Link	a	0
Iz_5_1009	DJI_0822	Pinus sylvestris L.	0	Pinaceae Spreng. ex F.Rudolphi	n	1
Iz_5_1009	DJI_0822	Populus × canescens (Aiton) Sm.	0	Salicaceae Mirb.	n	1
Iz_5_1009	DJI_0822	Populus tremula L.	0	Salicaceae Mirb.	n	1
Iz_5_1009	DJI_0822	Populus nigra L.	0	Salicaceae Mirb.	n	1
Iz_5_1009	DJI_0822	Populus sp.	0	Salicaceae Mirb.	n	1
Iz_5_1009	DJI_0822	Pyrus communis L.	0	Rosaceae Juss.	a	0
Iz_5_1009	DJI_0822	Quercus robur L.	0	Fagaceae Dumort.	n	1
Iz_5_1009	DJI_0822	Robinia pseudoacacia L.	0	Fabaceae Lindl.	a	0
Iz_5_1009	DJI_0822	Rosa sp.	0	Rosaceae Juss.		
Iz_5_1009	DJI_0822	Salix alba L.	0	Salicaceae Mirb.	n	1
Iz_5_1009	DJI_0822	Sambucus nigra L.	0	Viburnaceae Raf.	n	1
Iz_5_1009	DJI_0822	Sorbus aucuparia L.	0	Rosaceae Juss.	n	1
Iz_5_1009	DJI_0822	Tilia cordata Mill.	0	Malvaceae Juss.	n	1
Iz_5_1009	DJI_0822	Ulmus glabra Huds.	0	Ulmaceae Mirb.	n	1
Iz_5_1009	DJI_0823	Acer tataricum L.	0	Sapindaceae Juss.	n	1
Iz_5_1009	DJI_0823	Betula pendula Roth.	0	Betulaceae Gray	n	1
Iz_5_1009	DJI_0823	Crataegus rhipidophylla Gand.	0	Rosaceae Juss.	n	1
Iz_5_1009	DJI_0823	Frangula alnus Mill.	0	Rhamnaceae Juss.	n	1
Iz_5_1009	DJI_0823	Fraxinus pennsylvanica Marshall	0	Oleaceae Hoffmanns. & Link	a	0
Iz_5_1009	DJI_0823	Pinus sylvestris L.	0	Pinaceae Spreng. ex F.Rudolphi	n	1
Iz_5_1009	DJI_0823	Populus × canescens (Aiton) Sm.	0	Salicaceae Mirb.	n	1
Iz_5_1009	DJI_0823	Populus tremula L.	0	Salicaceae Mirb.	n	1
Iz_5_1009	DJI_0823	Populus nigra L.	0	Salicaceae Mirb.	n	1
Iz_5_1009	DJI_0823	Populus sp.	0	Salicaceae Mirb.	n	1
Iz_5_1009	DJI_0823	Pyrus communis L.	0	Rosaceae Juss.	a	0
Iz_5_1009	DJI_0823	Quercus robur L.	0	Fagaceae Dumort.	n	1
Iz_5_1009	DJI_0823	Robinia pseudoacacia L.	0	Fabaceae Lindl.	a	0
Iz_5_1009	DJI_0823	Rosa sp.	0	Rosaceae Juss.		
Iz_5_1009	DJI_0823	Salix alba L.	0	Salicaceae Mirb.	n	1
Iz_5_1009	DJI_0823	Sambucus nigra L.	0	Viburnaceae Raf.	n	1
Iz_5_1009	DJI_0823	Sorbus aucuparia L.	0	Rosaceae Juss.	n	1
Iz_5_1009	DJI_0823	Tilia cordata Mill.	0	Malvaceae Juss.	n	1
Iz_5_1009	DJI_0823	Ulmus glabra Huds.	0	Ulmaceae Mirb.	n	1
Iz_5_1009	DJI_0832	Acer tataricum L.	0	Sapindaceae Juss.	n	1
Iz_5_1009	DJI_0832	Betula pendula Roth.	0	Betulaceae Gray	n	1
Iz_5_1009	DJI_0832	Crataegus rhipidophylla Gand.	0	Rosaceae Juss.	n	1
Iz_5_1009	DJI_0832	Frangula alnus Mill.	0	Rhamnaceae Juss.	n	1
Iz_5_1009	DJI_0832	Fraxinus pennsylvanica Marshall	0	Oleaceae Hoffmanns. & Link	a	0
Iz_5_1009	DJI_0832	Pinus sylvestris L.	0	Pinaceae Spreng. ex F.Rudolphi	n	1
Iz_5_1009	DJI_0832	Populus × canescens (Aiton) Sm.	0	Salicaceae Mirb.	n	1
Iz_5_1009	DJI_0832	Populus tremula L.	0	Salicaceae Mirb.	n	1
Iz_5_1009	DJI_0832	Populus nigra L.	0	Salicaceae Mirb.	n	1
Iz_5_1009	DJI_0832	Populus sp.	0	Salicaceae Mirb.	n	1
Iz_5_1009	DJI_0832	Pyrus communis L.	0	Rosaceae Juss.	a	0
Iz_5_1009	DJI_0832	Quercus robur L.	0	Fagaceae Dumort.	n	1
Iz_5_1009	DJI_0832	Robinia pseudoacacia L.	0	Fabaceae Lindl.	a	0

Iz_5_1009	DJI_0832	Rosa sp.	0	Rosaceae Juss.		
Iz_5_1009	DJI_0832	Salix alba L.	0	Salicaceae Mirb.	n	1
Iz_5_1009	DJI_0832	Sambucus nigra L.	0	Viburnaceae Raf.	n	1
Iz_5_1009	DJI_0832	Sorbus aucuparia L.	0	Rosaceae Juss.	n	1
Iz_5_1009	DJI_0832	Tilia cordata Mill.	0	Malvaceae Juss.	n	1
Iz_5_1009	DJI_0832	Ulmus glabra Huds.	0	Ulmaceae Mirb.	n	1
Iz_5_1009	DJI_0838	Acer tataricum L.	0	Sapindaceae Juss.	n	1
Iz_5_1009	DJI_0838	Betula pendula Roth.	0	Betulaceae Gray	n	1
Iz_5_1009	DJI_0838	Crataegus rhipidophylla Gand.	0	Rosaceae Juss.	n	1
Iz_5_1009	DJI_0838	Frangula alnus Mill.	0	Rhamnaceae Juss.	n	1
Iz_5_1009	DJI_0838	Fraxinus pennsylvanica Marshall	0	Oleaceae Hoffmanns. & Link	a	0
Iz_5_1009	DJI_0838	Pinus sylvestris L.	0	Pinaceae Spreng. ex F.Rudolphi	n	1
Iz_5_1009	DJI_0838	Populus × canescens (Aiton) Sm.	0	Salicaceae Mirb.	n	1
Iz_5_1009	DJI_0838	Populus tremula L.	0	Salicaceae Mirb.	n	1
Iz_5_1009	DJI_0838	Populus nigra L.	0	Salicaceae Mirb.	n	1
Iz_5_1009	DJI_0838	Populus sp.	0	Salicaceae Mirb.	n	1
Iz_5_1009	DJI_0838	Pyrus communis L.	0	Rosaceae Juss.	a	0
Iz_5_1009	DJI_0838	Quercus robur L.	0	Fagaceae Dumort.	n	1
Iz_5_1009	DJI_0838	Robinia pseudoacacia L.	0	Fabaceae Lindl.	a	0
Iz_5_1009	DJI_0838	Rosa sp.	0	Rosaceae Juss.		
Iz_5_1009	DJI_0838	Salix alba L.	0	Salicaceae Mirb.	n	1
Iz_5_1009	DJI_0838	Sambucus nigra L.	0	Viburnaceae Raf.	n	1
Iz_5_1009	DJI_0838	Sorbus aucuparia L.	0	Rosaceae Juss.	n	1
Iz_5_1009	DJI_0838	Tilia cordata Mill.	0	Malvaceae Juss.	n	1
Iz_5_1009	DJI_0838	Ulmus glabra Huds.	0	Ulmaceae Mirb.	n	1
Iz_5_1009	DJI_0841	Acer tataricum L.	0	Sapindaceae Juss.	n	1
Iz_5_1009	DJI_0841	Betula pendula Roth.	0	Betulaceae Gray	n	1
Iz_5_1009	DJI_0841	Crataegus rhipidophylla Gand.	0	Rosaceae Juss.	n	1
Iz_5_1009	DJI_0841	Frangula alnus Mill.	0	Rhamnaceae Juss.	n	1
Iz_5_1009	DJI_0841	Fraxinus pennsylvanica Marshall	0	Oleaceae Hoffmanns. & Link	a	0
Iz_5_1009	DJI_0841	Pinus sylvestris L.	0	Pinaceae Spreng. ex F.Rudolphi	n	1
Iz_5_1009	DJI_0841	Populus × canescens (Aiton) Sm.	0	Salicaceae Mirb.	n	1
Iz_5_1009	DJI_0841	Populus tremula L.	0	Salicaceae Mirb.	n	1
Iz_5_1009	DJI_0841	Populus nigra L.	0	Salicaceae Mirb.	n	1
Iz_5_1009	DJI_0841	Populus sp.	0	Salicaceae Mirb.	n	1
Iz_5_1009	DJI_0841	Pyrus communis L.	0	Rosaceae Juss.	a	0
Iz_5_1009	DJI_0841	Quercus robur L.	0	Fagaceae Dumort.	n	1
Iz_5_1009	DJI_0841	Robinia pseudoacacia L.	0	Fabaceae Lindl.	a	0
Iz_5_1009	DJI_0841	Rosa sp.	0	Rosaceae Juss.		
Iz_5_1009	DJI_0841	Salix alba L.	0	Salicaceae Mirb.	n	1
Iz_5_1009	DJI_0841	Sambucus nigra L.	0	Viburnaceae Raf.	n	1
Iz_5_1009	DJI_0841	Sorbus aucuparia L.	0	Rosaceae Juss.	n	1
Iz_5_1009	DJI_0841	Tilia cordata Mill.	0	Malvaceae Juss.	n	1
Iz_5_1009	DJI_0841	Ulmus glabra Huds.	0	Ulmaceae Mirb.	n	1
Iz_5_1009	DJI_0843	Acer tataricum L.	0	Sapindaceae Juss.	n	1
Iz_5_1009	DJI_0843	Betula pendula Roth.	0	Betulaceae Gray	n	1
Iz_5_1009	DJI_0843	Crataegus rhipidophylla Gand.	0	Rosaceae Juss.	n	1
Iz_5_1009	DJI_0843	Frangula alnus Mill.	0	Rhamnaceae Juss.	n	1
Iz_5_1009	DJI_0843	Fraxinus pennsylvanica Marshall	0	Oleaceae Hoffmanns. & Link	a	0
Iz_5_1009	DJI_0843	Pinus sylvestris L.	0	Pinaceae Spreng. ex F.Rudolphi	n	1
Iz_5_1009	DJI_0843	Populus × canescens (Aiton) Sm.	0	Salicaceae Mirb.	n	1
Iz_5_1009	DJI_0843	Populus tremula L.	0	Salicaceae Mirb.	n	1
Iz_5_1009	DJI_0843	Populus nigra L.	0	Salicaceae Mirb.	n	1
Iz_5_1009	DJI_0843	Populus sp.	0	Salicaceae Mirb.	n	1
Iz_5_1009	DJI_0843	Pyrus communis L.	0	Rosaceae Juss.	a	0
Iz_5_1009	DJI_0843	Quercus robur L.	0	Fagaceae Dumort.	n	1
Iz_5_1009	DJI_0843	Robinia pseudoacacia L.	0	Fabaceae Lindl.	a	0
Iz_5_1009	DJI_0843	Rosa sp.	0	Rosaceae Juss.		
Iz_5_1009	DJI_0843	Salix alba L.	0	Salicaceae Mirb.	n	1
Iz_5_1009	DJI_0843	Sambucus nigra L.	0	Viburnaceae Raf.	n	1
Iz_5_1009	DJI_0843	Sorbus aucuparia L.	0	Rosaceae Juss.	n	1
Iz_5_1009	DJI_0843	Tilia cordata Mill.	0	Malvaceae Juss.	n	1
Iz_5_1009	DJI_0843	Ulmus glabra Huds.	0	Ulmaceae Mirb.	n	1
Iz_5_1009	DJI_0851	Acer tataricum L.	0	Sapindaceae Juss.	n	1

Iz_5_1009	DJI_0851	Betula pendula Roth.	0	Betulaceae Gray	n	1
Iz_5_1009	DJI_0851	Crataegus rhipidophylla Gand.	0	Rosaceae Juss.	n	1
Iz_5_1009	DJI_0851	Frangula alnus Mill.	0	Rhamnaceae Juss.	n	1
Iz_5_1009	DJI_0851	Fraxinus pennsylvanica Marshall	0	Oleaceae Hoffmanns. & Link	a	0
Iz_5_1009	DJI_0851	Pinus sylvestris L.	0	Pinaceae Spreng. ex F.Rudolphi	n	1
Iz_5_1009	DJI_0851	Populus × canescens (Aiton) Sm.	0	Salicaceae Mirb.	n	1
Iz_5_1009	DJI_0851	Populus tremula L.	0	Salicaceae Mirb.	n	1
Iz_5_1009	DJI_0851	Populus nigra L.	0	Salicaceae Mirb.	n	1
Iz_5_1009	DJI_0851	Populus sp.	0	Salicaceae Mirb.	n	1
Iz_5_1009	DJI_0851	Pyrus communis L.	0	Rosaceae Juss.	a	0
Iz_5_1009	DJI_0851	Quercus robur L.	0	Fagaceae Dumort.	n	1
Iz_5_1009	DJI_0851	Robinia pseudoacacia L.	0	Fabaceae Lindl.	a	0
Iz_5_1009	DJI_0851	Rosa sp.	0	Rosaceae Juss.		
Iz_5_1009	DJI_0851	Salix alba L.	0	Salicaceae Mirb.	n	1
Iz_5_1009	DJI_0851	Sambucus nigra L.	0	Viburnaceae Raf.	n	1
Iz_5_1009	DJI_0851	Sorbus aucuparia L.	0	Rosaceae Juss.	n	1
Iz_5_1009	DJI_0851	Tilia cordata Mill.	0	Malvaceae Juss.	n	1
Iz_5_1009	DJI_0851	Ulmus glabra Huds.	0	Ulmaceae Mirb.	n	1
Iz_5_1009	DJI_0858	Acer tataricum L.	0	Sapindaceae Juss.	n	1
Iz_5_1009	DJI_0858	Betula pendula Roth.	0	Betulaceae Gray	n	1
Iz_5_1009	DJI_0858	Crataegus rhipidophylla Gand.	0	Rosaceae Juss.	n	1
Iz_5_1009	DJI_0858	Frangula alnus Mill.	0	Rhamnaceae Juss.	n	1
Iz_5_1009	DJI_0858	Fraxinus pennsylvanica Marshall	0	Oleaceae Hoffmanns. & Link	a	0
Iz_5_1009	DJI_0858	Pinus sylvestris L.	0	Pinaceae Spreng. ex F.Rudolphi	n	1
Iz_5_1009	DJI_0858	Populus × canescens (Aiton) Sm.	0	Salicaceae Mirb.	n	1
Iz_5_1009	DJI_0858	Populus tremula L.	0	Salicaceae Mirb.	n	1
Iz_5_1009	DJI_0858	Populus nigra L.	0	Salicaceae Mirb.	n	1
Iz_5_1009	DJI_0858	Populus sp.	0	Salicaceae Mirb.	n	1
Iz_5_1009	DJI_0858	Pyrus communis L.	0	Rosaceae Juss.	a	0
Iz_5_1009	DJI_0858	Quercus robur L.	0	Fagaceae Dumort.	n	1
Iz_5_1009	DJI_0858	Robinia pseudoacacia L.	0	Fabaceae Lindl.	a	0
Iz_5_1009	DJI_0858	Rosa sp.	0	Rosaceae Juss.		
Iz_5_1009	DJI_0858	Salix alba L.	0	Salicaceae Mirb.	n	1
Iz_5_1009	DJI_0858	Sambucus nigra L.	0	Viburnaceae Raf.	n	1
Iz_5_1009	DJI_0858	Sorbus aucuparia L.	0	Rosaceae Juss.	n	1
Iz_5_1009	DJI_0858	Tilia cordata Mill.	0	Malvaceae Juss.	n	1
Iz_5_1009	DJI_0858	Ulmus glabra Huds.	0	Ulmaceae Mirb.	n	1
Iz_5_1009	DJI_0859	Acer tataricum L.	0	Sapindaceae Juss.	n	1
Iz_5_1009	DJI_0859	Betula pendula Roth.	0	Betulaceae Gray	n	1
Iz_5_1009	DJI_0859	Crataegus rhipidophylla Gand.	0	Rosaceae Juss.	n	1
Iz_5_1009	DJI_0859	Frangula alnus Mill.	0	Rhamnaceae Juss.	n	1
Iz_5_1009	DJI_0859	Fraxinus pennsylvanica Marshall	0	Oleaceae Hoffmanns. & Link	a	0
Iz_5_1009	DJI_0859	Pinus sylvestris L.	0	Pinaceae Spreng. ex F.Rudolphi	n	1
Iz_5_1009	DJI_0859	Populus × canescens (Aiton) Sm.	0	Salicaceae Mirb.	n	1
Iz_5_1009	DJI_0859	Populus tremula L.	0	Salicaceae Mirb.	n	1
Iz_5_1009	DJI_0859	Populus nigra L.	0	Salicaceae Mirb.	n	1
Iz_5_1009	DJI_0859	Populus sp.	0	Salicaceae Mirb.	n	1
Iz_5_1009	DJI_0859	Pyrus communis L.	0	Rosaceae Juss.	a	0
Iz_5_1009	DJI_0859	Quercus robur L.	0	Fagaceae Dumort.	n	1
Iz_5_1009	DJI_0859	Robinia pseudoacacia L.	0	Fabaceae Lindl.	a	0
Iz_5_1009	DJI_0859	Rosa sp.	0	Rosaceae Juss.		
Iz_5_1009	DJI_0859	Salix alba L.	0	Salicaceae Mirb.	n	1
Iz_5_1009	DJI_0859	Sambucus nigra L.	0	Viburnaceae Raf.	n	1
Iz_5_1009	DJI_0859	Sorbus aucuparia L.	0	Rosaceae Juss.	n	1
Iz_5_1009	DJI_0859	Tilia cordata Mill.	0	Malvaceae Juss.	n	1
Iz_5_1009	DJI_0859	Ulmus glabra Huds.	0	Ulmaceae Mirb.	n	1
Iz_5_1009	DJI_0860	Acer tataricum L.	0	Sapindaceae Juss.	n	1
Iz_5_1009	DJI_0860	Betula pendula Roth.	0	Betulaceae Gray	n	1
Iz_5_1009	DJI_0860	Crataegus rhipidophylla Gand.	0	Rosaceae Juss.	n	1
Iz_5_1009	DJI_0860	Frangula alnus Mill.	0	Rhamnaceae Juss.	n	1
Iz_5_1009	DJI_0860	Fraxinus pennsylvanica Marshall	0	Oleaceae Hoffmanns. & Link	a	0
Iz_5_1009	DJI_0860	Pinus sylvestris L.	0	Pinaceae Spreng. ex F.Rudolphi	n	1
Iz_5_1009	DJI_0860	Populus × canescens (Aiton) Sm.	0	Salicaceae Mirb.	n	1
Iz_5_1009	DJI_0860	Populus tremula L.	0	Salicaceae Mirb.	n	1

Iz_5_1009	DJI_0860	Populus nigra L.	0	Salicaceae Mirb.	n	1
Iz_5_1009	DJI_0860	Populus sp.	0	Salicaceae Mirb.	n	1
Iz_5_1009	DJI_0860	Pyrus communis L.	0	Rosaceae Juss.	a	0
Iz_5_1009	DJI_0860	Quercus robur L.	0	Fagaceae Dumort.	n	1
Iz_5_1009	DJI_0860	Robinia pseudoacacia L.	0	Fabaceae Lindl.	a	0
Iz_5_1009	DJI_0860	Rosa sp.	0	Rosaceae Juss.		
Iz_5_1009	DJI_0860	Salix alba L.	0	Salicaceae Mirb.	n	1
Iz_5_1009	DJI_0860	Sambucus nigra L.	0	Viburnaceae Raf.	n	1
Iz_5_1009	DJI_0860	Sorbus aucuparia L.	0	Rosaceae Juss.	n	1
Iz_5_1009	DJI_0860	Tilia cordata Mill.	0	Malvaceae Juss.	n	1
Iz_5_1009	DJI_0860	Ulmus glabra Huds.	0	Ulmaceae Mirb.	n	1
Iz_5_1009	DJI_0864	Acer tataricum L.	0	Sapindaceae Juss.	n	1
Iz_5_1009	DJI_0864	Betula pendula Roth.	0	Betulaceae Gray	n	1
Iz_5_1009	DJI_0864	Crataegus rhipidophylla Gand.	0	Rosaceae Juss.	n	1
Iz_5_1009	DJI_0864	Frangula alnus Mill.	0	Rhamnaceae Juss.	n	1
Iz_5_1009	DJI_0864	Fraxinus pennsylvanica Marshall	0	Oleaceae Hoffmanns. & Link	a	0
Iz_5_1009	DJI_0864	Pinus sylvestris L.	0	Pinaceae Spreng. ex F.Rudolphi	n	1
Iz_5_1009	DJI_0864	Populus × canescens (Aiton) Sm.	0	Salicaceae Mirb.	n	1
Iz_5_1009	DJI_0864	Populus tremula L.	0	Salicaceae Mirb.	n	1
Iz_5_1009	DJI_0864	Populus nigra L.	0	Salicaceae Mirb.	n	1
Iz_5_1009	DJI_0864	Populus sp.	0	Salicaceae Mirb.	n	1
Iz_5_1009	DJI_0864	Pyrus communis L.	0	Rosaceae Juss.	a	0
Iz_5_1009	DJI_0864	Quercus robur L.	0	Fagaceae Dumort.	n	1
Iz_5_1009	DJI_0864	Robinia pseudoacacia L.	0	Fabaceae Lindl.	a	0
Iz_5_1009	DJI_0864	Rosa sp.	0	Rosaceae Juss.		
Iz_5_1009	DJI_0864	Salix alba L.	0	Salicaceae Mirb.	n	1
Iz_5_1009	DJI_0864	Sambucus nigra L.	0	Viburnaceae Raf.	n	1
Iz_5_1009	DJI_0864	Sorbus aucuparia L.	0	Rosaceae Juss.	n	1
Iz_5_1009	DJI_0864	Tilia cordata Mill.	0	Malvaceae Juss.	n	1
Iz_5_1009	DJI_0864	Ulmus glabra Huds.	0	Ulmaceae Mirb.	n	1
Iz_6_1009	DJI_0303	Acer tataricum L.	0	Sapindaceae Juss.	n	1
Iz_6_1009	DJI_0303	Betula pendula Roth.	0	Betulaceae Gray	n	1
Iz_6_1009	DJI_0303	Crataegus rhipidophylla Gand.	0	Rosaceae Juss.	n	1
Iz_6_1009	DJI_0303	Frangula alnus Mill.	0	Rhamnaceae Juss.	n	1
Iz_6_1009	DJI_0303	Fraxinus pennsylvanica Marshall	0	Oleaceae Hoffmanns. & Link	a	0
Iz_6_1009	DJI_0303	Pinus sylvestris L.	0	Pinaceae Spreng. ex F.Rudolphi	n	1
Iz_6_1009	DJI_0303	Populus × canescens (Aiton) Sm.	0	Salicaceae Mirb.	n	1
Iz_6_1009	DJI_0303	Populus tremula L.	0	Salicaceae Mirb.	n	1
Iz_6_1009	DJI_0303	Populus nigra L.	0	Salicaceae Mirb.	n	1
Iz_6_1009	DJI_0303	Pyrus communis L.	0	Rosaceae Juss.	a	0
Iz_6_1009	DJI_0303	Quercus robur L.	0	Fagaceae Dumort.	n	1
Iz_6_1009	DJI_0303	Robinia pseudoacacia L.	0	Fabaceae Lindl.	a	0
Iz_6_1009	DJI_0303	Rosa sp.	0	Rosaceae Juss.		
Iz_6_1009	DJI_0303	Salix alba L.	0	Salicaceae Mirb.	n	1
Iz_6_1009	DJI_0303	Sambucus nigra L.	0	Viburnaceae Raf.	n	1
Iz_6_1009	DJI_0303	Sorbus aucuparia L.	0	Rosaceae Juss.	n	1
Iz_6_1009	DJI_0303	Tilia cordata Mill.	0	Malvaceae Juss.	n	1
Iz_6_1009	DJI_0303	Ulmus glabra Huds.	0	Ulmaceae Mirb.	n	1
Iz_6_1009	DJI_0307	Acer tataricum L.	0	Sapindaceae Juss.	n	1
Iz_6_1009	DJI_0307	Betula pendula Roth.	0	Betulaceae Gray	n	1
Iz_6_1009	DJI_0307	Crataegus rhipidophylla Gand.	0	Rosaceae Juss.	n	1
Iz_6_1009	DJI_0307	Frangula alnus Mill.	0	Rhamnaceae Juss.	n	1
Iz_6_1009	DJI_0307	Fraxinus pennsylvanica Marshall	0	Oleaceae Hoffmanns. & Link	a	0
Iz_6_1009	DJI_0307	Pinus sylvestris L.	0	Pinaceae Spreng. ex F.Rudolphi	n	1
Iz_6_1009	DJI_0307	Populus × canescens (Aiton) Sm.	0	Salicaceae Mirb.	n	1
Iz_6_1009	DJI_0307	Populus tremula L.	0	Salicaceae Mirb.	n	1
Iz_6_1009	DJI_0307	Populus nigra L.	0	Salicaceae Mirb.	n	1
Iz_6_1009	DJI_0307	Pyrus communis L.	0	Rosaceae Juss.	a	0
Iz_6_1009	DJI_0307	Quercus robur L.	0	Fagaceae Dumort.	n	1
Iz_6_1009	DJI_0307	Robinia pseudoacacia L.	0	Fabaceae Lindl.	a	0
Iz_6_1009	DJI_0307	Rosa sp.	0	Rosaceae Juss.		
Iz_6_1009	DJI_0307	Salix alba L.	0	Salicaceae Mirb.	n	1
Iz_6_1009	DJI_0307	Sambucus nigra L.	0	Viburnaceae Raf.	n	1
Iz_6_1009	DJI_0307	Sorbus aucuparia L.	0	Rosaceae Juss.	n	1

Iz_6_1009	DJI_0307	Tilia cordata Mill.	0	Malvaceae Juss.	n	1
Iz_6_1009	DJI_0307	Ulmus glabra Huds.	0	Ulmaceae Mirb.	n	1
Iz_6_1009	DJI_0310	Acer tataricum L.	0	Sapindaceae Juss.	n	1
Iz_6_1009	DJI_0310	Betula pendula Roth.	0	Betulaceae Gray	n	1
Iz_6_1009	DJI_0310	Crataegus rhipidophylla Gand.	0	Rosaceae Juss.	n	1
Iz_6_1009	DJI_0310	Frangula alnus Mill.	0	Rhamnaceae Juss.	n	1
Iz_6_1009	DJI_0310	Fraxinus pennsylvanica Marshall	0	Oleaceae Hoffmanns. & Link	a	0
Iz_6_1009	DJI_0310	Pinus sylvestris L.	0	Pinaceae Spreng. ex F.Rudolphi	n	1
Iz_6_1009	DJI_0310	Populus × canescens (Aiton) Sm.	0	Salicaceae Mirb.	n	1
Iz_6_1009	DJI_0310	Populus tremula L.	0	Salicaceae Mirb.	n	1
Iz_6_1009	DJI_0310	Populus nigra L.	0	Salicaceae Mirb.	n	1
Iz_6_1009	DJI_0310	Pyrus communis L.	0	Rosaceae Juss.	a	0
Iz_6_1009	DJI_0310	Quercus robur L.	0	Fagaceae Dumort.	n	1
Iz_6_1009	DJI_0310	Robinia pseudoacacia L.	0	Fabaceae Lindl.	a	0
Iz_6_1009	DJI_0310	Rosa sp.	0	Rosaceae Juss.		
Iz_6_1009	DJI_0310	Salix alba L.	0	Salicaceae Mirb.	n	1
Iz_6_1009	DJI_0310	Sambucus nigra L.	0	Viburnaceae Raf.	n	1
Iz_6_1009	DJI_0310	Sorbus aucuparia L.	0	Rosaceae Juss.	n	1
Iz_6_1009	DJI_0310	Tilia cordata Mill.	0	Malvaceae Juss.	n	1
Iz_6_1009	DJI_0310	Ulmus glabra Huds.	0	Ulmaceae Mirb.	n	1
Iz_6_1009	DJI_0311	Acer tataricum L.	0	Sapindaceae Juss.	n	1
Iz_6_1009	DJI_0311	Betula pendula Roth.	0	Betulaceae Gray	n	1
Iz_6_1009	DJI_0311	Crataegus rhipidophylla Gand.	0	Rosaceae Juss.	n	1
Iz_6_1009	DJI_0311	Frangula alnus Mill.	0	Rhamnaceae Juss.	n	1
Iz_6_1009	DJI_0311	Fraxinus pennsylvanica Marshall	0	Oleaceae Hoffmanns. & Link	a	0
Iz_6_1009	DJI_0311	Pinus sylvestris L.	0	Pinaceae Spreng. ex F.Rudolphi	n	1
Iz_6_1009	DJI_0311	Populus × canescens (Aiton) Sm.	0	Salicaceae Mirb.	n	1
Iz_6_1009	DJI_0311	Populus tremula L.	0	Salicaceae Mirb.	n	1
Iz_6_1009	DJI_0311	Populus nigra L.	0	Salicaceae Mirb.	n	1
Iz_6_1009	DJI_0311	Pyrus communis L.	0	Rosaceae Juss.	a	0
Iz_6_1009	DJI_0311	Quercus robur L.	0	Fagaceae Dumort.	n	1
Iz_6_1009	DJI_0311	Robinia pseudoacacia L.	0	Fabaceae Lindl.	a	0
Iz_6_1009	DJI_0311	Rosa sp.	0	Rosaceae Juss.		
Iz_6_1009	DJI_0311	Salix alba L.	0	Salicaceae Mirb.	n	1
Iz_6_1009	DJI_0311	Sambucus nigra L.	0	Viburnaceae Raf.	n	1
Iz_6_1009	DJI_0311	Sorbus aucuparia L.	0	Rosaceae Juss.	n	1
Iz_6_1009	DJI_0311	Tilia cordata Mill.	0	Malvaceae Juss.	n	1
Iz_6_1009	DJI_0311	Ulmus glabra Huds.	0	Ulmaceae Mirb.	n	1
Iz_6_1009	DJI_0312	Acer tataricum L.	0	Sapindaceae Juss.	n	1
Iz_6_1009	DJI_0312	Betula pendula Roth.	0	Betulaceae Gray	n	1
Iz_6_1009	DJI_0312	Crataegus rhipidophylla Gand.	0	Rosaceae Juss.	n	1
Iz_6_1009	DJI_0312	Frangula alnus Mill.	0	Rhamnaceae Juss.	n	1
Iz_6_1009	DJI_0312	Fraxinus pennsylvanica Marshall	0	Oleaceae Hoffmanns. & Link	a	0
Iz_6_1009	DJI_0312	Pinus sylvestris L.	0	Pinaceae Spreng. ex F.Rudolphi	n	1
Iz_6_1009	DJI_0312	Populus × canescens (Aiton) Sm.	0	Salicaceae Mirb.	n	1
Iz_6_1009	DJI_0312	Populus tremula L.	0	Salicaceae Mirb.	n	1
Iz_6_1009	DJI_0312	Populus nigra L.	0	Salicaceae Mirb.	n	1
Iz_6_1009	DJI_0312	Pyrus communis L.	0	Rosaceae Juss.	a	0
Iz_6_1009	DJI_0312	Quercus robur L.	0	Fagaceae Dumort.	n	1
Iz_6_1009	DJI_0312	Robinia pseudoacacia L.	0	Fabaceae Lindl.	a	0
Iz_6_1009	DJI_0312	Rosa sp.	0	Rosaceae Juss.		
Iz_6_1009	DJI_0312	Salix alba L.	0	Salicaceae Mirb.	n	1
Iz_6_1009	DJI_0312	Sambucus nigra L.	0	Viburnaceae Raf.	n	1
Iz_6_1009	DJI_0312	Sorbus aucuparia L.	0	Rosaceae Juss.	n	1
Iz_6_1009	DJI_0312	Tilia cordata Mill.	0	Malvaceae Juss.	n	1
Iz_6_1009	DJI_0312	Ulmus glabra Huds.	0	Ulmaceae Mirb.	n	1
Iz_6_1009	DJI_0314	Acer tataricum L.	0	Sapindaceae Juss.	n	1
Iz_6_1009	DJI_0314	Betula pendula Roth.	0	Betulaceae Gray	n	1
Iz_6_1009	DJI_0314	Crataegus rhipidophylla Gand.	0	Rosaceae Juss.	n	1
Iz_6_1009	DJI_0314	Frangula alnus Mill.	0	Rhamnaceae Juss.	n	1
Iz_6_1009	DJI_0314	Fraxinus pennsylvanica Marshall	0	Oleaceae Hoffmanns. & Link	a	0
Iz_6_1009	DJI_0314	Pinus sylvestris L.	0	Pinaceae Spreng. ex F.Rudolphi	n	1
Iz_6_1009	DJI_0314	Populus × canescens (Aiton) Sm.	0	Salicaceae Mirb.	n	1
Iz_6_1009	DJI_0314	Populus tremula L.	0	Salicaceae Mirb.	n	1

Iz_6_1009	DJI_0314	Populus nigra L.	0	Salicaceae Mirb.	n	1
Iz_6_1009	DJI_0314	Pyrus communis L.	0	Rosaceae Juss.	a	0
Iz_6_1009	DJI_0314	Quercus robur L.	0	Fagaceae Dumort.	n	1
Iz_6_1009	DJI_0314	Robinia pseudoacacia L.	0	Fabaceae Lindl.	a	0
Iz_6_1009	DJI_0314	Rosa sp.	0	Rosaceae Juss.		
Iz_6_1009	DJI_0314	Salix alba L.	0	Salicaceae Mirb.	n	1
Iz_6_1009	DJI_0314	Sambucus nigra L.	0	Viburnaceae Raf.	n	1
Iz_6_1009	DJI_0314	Sorbus aucuparia L.	0	Rosaceae Juss.	n	1
Iz_6_1009	DJI_0314	Tilia cordata Mill.	0	Malvaceae Juss.	n	1
Iz_6_1009	DJI_0314	Ulmus glabra Huds.	0	Ulmaceae Mirb.	n	1
Iz_6_1009	DJI_0317	Acer tataricum L.	0	Sapindaceae Juss.	n	1
Iz_6_1009	DJI_0317	Betula pendula Roth.	0	Betulaceae Gray	n	1
Iz_6_1009	DJI_0317	Crataegus rhipidophylla Gand.	0	Rosaceae Juss.	n	1
Iz_6_1009	DJI_0317	Frangula alnus Mill.	0	Rhamnaceae Juss.	n	1
Iz_6_1009	DJI_0317	Fraxinus pennsylvanica Marshall	0	Oleaceae Hoffmanns. & Link	a	0
Iz_6_1009	DJI_0317	Pinus sylvestris L.	0	Pinaceae Spreng. ex F.Rudolphi	n	1
Iz_6_1009	DJI_0317	Populus × canescens (Aiton) Sm.	0	Salicaceae Mirb.	n	1
Iz_6_1009	DJI_0317	Populus tremula L.	0	Salicaceae Mirb.	n	1
Iz_6_1009	DJI_0317	Populus nigra L.	0	Salicaceae Mirb.	n	1
Iz_6_1009	DJI_0317	Pyrus communis L.	0	Rosaceae Juss.	a	0
Iz_6_1009	DJI_0317	Quercus robur L.	0	Fagaceae Dumort.	n	1
Iz_6_1009	DJI_0317	Robinia pseudoacacia L.	0	Fabaceae Lindl.	a	0
Iz_6_1009	DJI_0317	Rosa sp.	0	Rosaceae Juss.		
Iz_6_1009	DJI_0317	Salix alba L.	0	Salicaceae Mirb.	n	1
Iz_6_1009	DJI_0317	Sambucus nigra L.	0	Viburnaceae Raf.	n	1
Iz_6_1009	DJI_0317	Sorbus aucuparia L.	0	Rosaceae Juss.	n	1
Iz_6_1009	DJI_0317	Tilia cordata Mill.	0	Malvaceae Juss.	n	1
Iz_6_1009	DJI_0317	Ulmus glabra Huds.	0	Ulmaceae Mirb.	n	1
Iz_6_1009	DJI_0320	Acer tataricum L.	0	Sapindaceae Juss.	n	1
Iz_6_1009	DJI_0320	Betula pendula Roth.	0	Betulaceae Gray	n	1
Iz_6_1009	DJI_0320	Crataegus rhipidophylla Gand.	0	Rosaceae Juss.	n	1
Iz_6_1009	DJI_0320	Frangula alnus Mill.	0	Rhamnaceae Juss.	n	1
Iz_6_1009	DJI_0320	Fraxinus pennsylvanica Marshall	0	Oleaceae Hoffmanns. & Link	a	0
Iz_6_1009	DJI_0320	Pinus sylvestris L.	0	Pinaceae Spreng. ex F.Rudolphi	n	1
Iz_6_1009	DJI_0320	Populus × canescens (Aiton) Sm.	0	Salicaceae Mirb.	n	1
Iz_6_1009	DJI_0320	Populus tremula L.	0	Salicaceae Mirb.	n	1
Iz_6_1009	DJI_0320	Populus nigra L.	0	Salicaceae Mirb.	n	1
Iz_6_1009	DJI_0320	Pyrus communis L.	0	Rosaceae Juss.	a	0
Iz_6_1009	DJI_0320	Quercus robur L.	0	Fagaceae Dumort.	n	1
Iz_6_1009	DJI_0320	Robinia pseudoacacia L.	0	Fabaceae Lindl.	a	0
Iz_6_1009	DJI_0320	Rosa sp.	0	Rosaceae Juss.		
Iz_6_1009	DJI_0320	Salix alba L.	0	Salicaceae Mirb.	n	1
Iz_6_1009	DJI_0320	Sambucus nigra L.	0	Viburnaceae Raf.	n	1
Iz_6_1009	DJI_0320	Sorbus aucuparia L.	0	Rosaceae Juss.	n	1
Iz_6_1009	DJI_0320	Tilia cordata Mill.	0	Malvaceae Juss.	n	1
Iz_6_1009	DJI_0320	Ulmus glabra Huds.	0	Ulmaceae Mirb.	n	1
Iz_6_1009	DJI_0324	Acer tataricum L.	0	Sapindaceae Juss.	n	1
Iz_6_1009	DJI_0324	Betula pendula Roth.	0	Betulaceae Gray	n	1
Iz_6_1009	DJI_0324	Crataegus rhipidophylla Gand.	0	Rosaceae Juss.	n	1
Iz_6_1009	DJI_0324	Frangula alnus Mill.	0	Rhamnaceae Juss.	n	1
Iz_6_1009	DJI_0324	Fraxinus pennsylvanica Marshall	0	Oleaceae Hoffmanns. & Link	a	0
Iz_6_1009	DJI_0324	Pinus sylvestris L.	0	Pinaceae Spreng. ex F.Rudolphi	n	1
Iz_6_1009	DJI_0324	Populus × canescens (Aiton) Sm.	0	Salicaceae Mirb.	n	1
Iz_6_1009	DJI_0324	Populus tremula L.	0	Salicaceae Mirb.	n	1
Iz_6_1009	DJI_0324	Populus nigra L.	0	Salicaceae Mirb.	n	1
Iz_6_1009	DJI_0324	Pyrus communis L.	0	Rosaceae Juss.	a	0
Iz_6_1009	DJI_0324	Quercus robur L.	0	Fagaceae Dumort.	n	1
Iz_6_1009	DJI_0324	Robinia pseudoacacia L.	0	Fabaceae Lindl.	a	0
Iz_6_1009	DJI_0324	Rosa sp.	0	Rosaceae Juss.		
Iz_6_1009	DJI_0324	Salix alba L.	0	Salicaceae Mirb.	n	1
Iz_6_1009	DJI_0324	Sambucus nigra L.	0	Viburnaceae Raf.	n	1
Iz_6_1009	DJI_0324	Sorbus aucuparia L.	0	Rosaceae Juss.	n	1
Iz_6_1009	DJI_0324	Tilia cordata Mill.	0	Malvaceae Juss.	n	1
Iz_6_1009	DJI_0324	Ulmus glabra Huds.	0	Ulmaceae Mirb.	n	1

Iz_6_1009	DJI_0326	Acer tataricum L.	0	Sapindaceae Juss.	n	1
Iz_6_1009	DJI_0326	Betula pendula Roth.	0	Betulaceae Gray	n	1
Iz_6_1009	DJI_0326	Crataegus rhipidophylla Gand.	0	Rosaceae Juss.	n	1
Iz_6_1009	DJI_0326	Frangula alnus Mill.	0	Rhamnaceae Juss.	n	1
Iz_6_1009	DJI_0326	Fraxinus pennsylvanica Marshall	0	Oleaceae Hoffmanns. & Link	a	0
Iz_6_1009	DJI_0326	Pinus sylvestris L.	0	Pinaceae Spreng. ex F.Rudolphi	n	1
Iz_6_1009	DJI_0326	Populus × canescens (Aiton) Sm.	0	Salicaceae Mirb.	n	1
Iz_6_1009	DJI_0326	Populus tremula L.	0	Salicaceae Mirb.	n	1
Iz_6_1009	DJI_0326	Populus nigra L.	0	Salicaceae Mirb.	n	1
Iz_6_1009	DJI_0326	Pyrus communis L.	0	Rosaceae Juss.	a	0
Iz_6_1009	DJI_0326	Quercus robur L.	0	Fagaceae Dumort.	n	1
Iz_6_1009	DJI_0326	Robinia pseudoacacia L.	0	Fabaceae Lindl.	a	0
Iz_6_1009	DJI_0326	Rosa sp.	0	Rosaceae Juss.		
Iz_6_1009	DJI_0326	Salix alba L.	0	Salicaceae Mirb.	n	1
Iz_6_1009	DJI_0326	Sambucus nigra L.	0	Viburnaceae Raf.	n	1
Iz_6_1009	DJI_0326	Sorbus aucuparia L.	0	Rosaceae Juss.	n	1
Iz_6_1009	DJI_0326	Tilia cordata Mill.	0	Malvaceae Juss.	n	1
Iz_6_1009	DJI_0326	Ulmus glabra Huds.	0	Ulmaceae Mirb.	n	1
Iz_6_1009	DJI_0335	Acer tataricum L.	0	Sapindaceae Juss.	n	1
Iz_6_1009	DJI_0335	Betula pendula Roth.	0	Betulaceae Gray	n	1
Iz_6_1009	DJI_0335	Crataegus rhipidophylla Gand.	0	Rosaceae Juss.	n	1
Iz_6_1009	DJI_0335	Frangula alnus Mill.	0	Rhamnaceae Juss.	n	1
Iz_6_1009	DJI_0335	Fraxinus pennsylvanica Marshall	0	Oleaceae Hoffmanns. & Link	a	0
Iz_6_1009	DJI_0335	Pinus sylvestris L.	0	Pinaceae Spreng. ex F.Rudolphi	n	1
Iz_6_1009	DJI_0335	Populus × canescens (Aiton) Sm.	0	Salicaceae Mirb.	n	1
Iz_6_1009	DJI_0335	Populus tremula L.	0	Salicaceae Mirb.	n	1
Iz_6_1009	DJI_0335	Populus nigra L.	0	Salicaceae Mirb.	n	1
Iz_6_1009	DJI_0335	Pyrus communis L.	0	Rosaceae Juss.	a	0
Iz_6_1009	DJI_0335	Quercus robur L.	0	Fagaceae Dumort.	n	1
Iz_6_1009	DJI_0335	Robinia pseudoacacia L.	0	Fabaceae Lindl.	a	0
Iz_6_1009	DJI_0335	Rosa sp.	0	Rosaceae Juss.		
Iz_6_1009	DJI_0335	Salix alba L.	0	Salicaceae Mirb.	n	1
Iz_6_1009	DJI_0335	Sambucus nigra L.	0	Viburnaceae Raf.	n	1
Iz_6_1009	DJI_0335	Sorbus aucuparia L.	0	Rosaceae Juss.	n	1
Iz_6_1009	DJI_0335	Tilia cordata Mill.	0	Malvaceae Juss.	n	1
Iz_6_1009	DJI_0335	Ulmus glabra Huds.	0	Ulmaceae Mirb.	n	1
Iz_6_1009	DJI_0338	Acer tataricum L.	0	Sapindaceae Juss.	n	1
Iz_6_1009	DJI_0338	Betula pendula Roth.	0	Betulaceae Gray	n	1
Iz_6_1009	DJI_0338	Crataegus rhipidophylla Gand.	0	Rosaceae Juss.	n	1
Iz_6_1009	DJI_0338	Frangula alnus Mill.	0	Rhamnaceae Juss.	n	1
Iz_6_1009	DJI_0338	Fraxinus pennsylvanica Marshall	0	Oleaceae Hoffmanns. & Link	a	0
Iz_6_1009	DJI_0338	Pinus sylvestris L.	0	Pinaceae Spreng. ex F.Rudolphi	n	1
Iz_6_1009	DJI_0338	Populus × canescens (Aiton) Sm.	0	Salicaceae Mirb.	n	1
Iz_6_1009	DJI_0338	Populus tremula L.	0	Salicaceae Mirb.	n	1
Iz_6_1009	DJI_0338	Populus nigra L.	0	Salicaceae Mirb.	n	1
Iz_6_1009	DJI_0338	Pyrus communis L.	0	Rosaceae Juss.	a	0
Iz_6_1009	DJI_0338	Quercus robur L.	0	Fagaceae Dumort.	n	1
Iz_6_1009	DJI_0338	Robinia pseudoacacia L.	0	Fabaceae Lindl.	a	0
Iz_6_1009	DJI_0338	Rosa sp.	0	Rosaceae Juss.		
Iz_6_1009	DJI_0338	Salix alba L.	0	Salicaceae Mirb.	n	1
Iz_6_1009	DJI_0338	Sambucus nigra L.	0	Viburnaceae Raf.	n	1
Iz_6_1009	DJI_0338	Sorbus aucuparia L.	0	Rosaceae Juss.	n	1
Iz_6_1009	DJI_0338	Tilia cordata Mill.	0	Malvaceae Juss.	n	1
Iz_6_1009	DJI_0338	Ulmus glabra Huds.	0	Ulmaceae Mirb.	n	1
Iz_6_1009	DJI_0341	Acer tataricum L.	0	Sapindaceae Juss.	n	1
Iz_6_1009	DJI_0341	Betula pendula Roth.	0	Betulaceae Gray	n	1
Iz_6_1009	DJI_0341	Crataegus rhipidophylla Gand.	0	Rosaceae Juss.	n	1
Iz_6_1009	DJI_0341	Frangula alnus Mill.	0	Rhamnaceae Juss.	n	1
Iz_6_1009	DJI_0341	Fraxinus pennsylvanica Marshall	0	Oleaceae Hoffmanns. & Link	a	0
Iz_6_1009	DJI_0341	Pinus sylvestris L.	0	Pinaceae Spreng. ex F.Rudolphi	n	1
Iz_6_1009	DJI_0341	Populus × canescens (Aiton) Sm.	0	Salicaceae Mirb.	n	1
Iz_6_1009	DJI_0341	Populus tremula L.	0	Salicaceae Mirb.	n	1
Iz_6_1009	DJI_0341	Populus nigra L.	0	Salicaceae Mirb.	n	1
Iz_6_1009	DJI_0341	Pyrus communis L.	0	Rosaceae Juss.	a	0

Iz_6_1009	DJI_0341	Quercus robur L.	0	Fagaceae Dumort.	n	1
Iz_6_1009	DJI_0341	Robinia pseudoacacia L.	0	Fabaceae Lindl.	a	0
Iz_6_1009	DJI_0341	Rosa sp.	0	Rosaceae Juss.		
Iz_6_1009	DJI_0341	Salix alba L.	0	Salicaceae Mirb.	n	1
Iz_6_1009	DJI_0341	Sambucus nigra L.	0	Viburnaceae Raf.	n	1
Iz_6_1009	DJI_0341	Sorbus aucuparia L.	0	Rosaceae Juss.	n	1
Iz_6_1009	DJI_0341	Tilia cordata Mill.	0	Malvaceae Juss.	n	1
Iz_6_1009	DJI_0341	Ulmus glabra Huds.	0	Ulmaceae Mirb.	n	1
Iz_6_1009	DJI_0343	Acer tataricum L.	0	Sapindaceae Juss.	n	1
Iz_6_1009	DJI_0343	Betula pendula Roth.	0	Betulaceae Gray	n	1
Iz_6_1009	DJI_0343	Crataegus rhipidophylla Gand.	0	Rosaceae Juss.	n	1
Iz_6_1009	DJI_0343	Frangula alnus Mill.	0	Rhamnaceae Juss.	n	1
Iz_6_1009	DJI_0343	Fraxinus pennsylvanica Marshall	0	Oleaceae Hoffmanns. & Link	a	0
Iz_6_1009	DJI_0343	Pinus sylvestris L.	0	Pinaceae Spreng. ex F.Rudolphi	n	1
Iz_6_1009	DJI_0343	Populus × canescens (Aiton) Sm.	0	Salicaceae Mirb.	n	1
Iz_6_1009	DJI_0343	Populus tremula L.	0	Salicaceae Mirb.	n	1
Iz_6_1009	DJI_0343	Populus nigra L.	0	Salicaceae Mirb.	n	1
Iz_6_1009	DJI_0343	Pyrus communis L.	0	Rosaceae Juss.	a	0
Iz_6_1009	DJI_0343	Quercus robur L.	0	Fagaceae Dumort.	n	1
Iz_6_1009	DJI_0343	Robinia pseudoacacia L.	0	Fabaceae Lindl.	a	0
Iz_6_1009	DJI_0343	Rosa sp.	0	Rosaceae Juss.		
Iz_6_1009	DJI_0343	Salix alba L.	0	Salicaceae Mirb.	n	1
Iz_6_1009	DJI_0343	Sambucus nigra L.	0	Viburnaceae Raf.	n	1
Iz_6_1009	DJI_0343	Sorbus aucuparia L.	0	Rosaceae Juss.	n	1
Iz_6_1009	DJI_0343	Tilia cordata Mill.	0	Malvaceae Juss.	n	1
Iz_6_1009	DJI_0343	Ulmus glabra Huds.	0	Ulmaceae Mirb.	n	1
Iz_6_1009	DJI_0345	Acer tataricum L.	0	Sapindaceae Juss.	n	1
Iz_6_1009	DJI_0345	Betula pendula Roth.	0	Betulaceae Gray	n	1
Iz_6_1009	DJI_0345	Crataegus rhipidophylla Gand.	0	Rosaceae Juss.	n	1
Iz_6_1009	DJI_0345	Frangula alnus Mill.	0	Rhamnaceae Juss.	n	1
Iz_6_1009	DJI_0345	Fraxinus pennsylvanica Marshall	0	Oleaceae Hoffmanns. & Link	a	0
Iz_6_1009	DJI_0345	Pinus sylvestris L.	0	Pinaceae Spreng. ex F.Rudolphi	n	1
Iz_6_1009	DJI_0345	Populus × canescens (Aiton) Sm.	0	Salicaceae Mirb.	n	1
Iz_6_1009	DJI_0345	Populus tremula L.	0	Salicaceae Mirb.	n	1
Iz_6_1009	DJI_0345	Populus nigra L.	0	Salicaceae Mirb.	n	1
Iz_6_1009	DJI_0345	Pyrus communis L.	0	Rosaceae Juss.	a	0
Iz_6_1009	DJI_0345	Quercus robur L.	0	Fagaceae Dumort.	n	1
Iz_6_1009	DJI_0345	Robinia pseudoacacia L.	0	Fabaceae Lindl.	a	0
Iz_6_1009	DJI_0345	Rosa sp.	0	Rosaceae Juss.		
Iz_6_1009	DJI_0345	Salix alba L.	0	Salicaceae Mirb.	n	1
Iz_6_1009	DJI_0345	Sambucus nigra L.	0	Viburnaceae Raf.	n	1
Iz_6_1009	DJI_0345	Sorbus aucuparia L.	0	Rosaceae Juss.	n	1
Iz_6_1009	DJI_0345	Tilia cordata Mill.	0	Malvaceae Juss.	n	1
Iz_6_1009	DJI_0345	Ulmus glabra Huds.	0	Ulmaceae Mirb.	n	1
Iz_6_1009	DJI_0347	Acer tataricum L.	0	Sapindaceae Juss.	n	1
Iz_6_1009	DJI_0347	Betula pendula Roth.	0	Betulaceae Gray	n	1
Iz_6_1009	DJI_0347	Crataegus rhipidophylla Gand.	0	Rosaceae Juss.	n	1
Iz_6_1009	DJI_0347	Frangula alnus Mill.	0	Rhamnaceae Juss.	n	1
Iz_6_1009	DJI_0347	Fraxinus pennsylvanica Marshall	0	Oleaceae Hoffmanns. & Link	a	0
Iz_6_1009	DJI_0347	Pinus sylvestris L.	0	Pinaceae Spreng. ex F.Rudolphi	n	1
Iz_6_1009	DJI_0347	Populus × canescens (Aiton) Sm.	0	Salicaceae Mirb.	n	1
Iz_6_1009	DJI_0347	Populus tremula L.	0	Salicaceae Mirb.	n	1
Iz_6_1009	DJI_0347	Populus nigra L.	0	Salicaceae Mirb.	n	1
Iz_6_1009	DJI_0347	Pyrus communis L.	0	Rosaceae Juss.	a	0
Iz_6_1009	DJI_0347	Quercus robur L.	0	Fagaceae Dumort.	n	1
Iz_6_1009	DJI_0347	Robinia pseudoacacia L.	0	Fabaceae Lindl.	a	0
Iz_6_1009	DJI_0347	Rosa sp.	0	Rosaceae Juss.		
Iz_6_1009	DJI_0347	Salix alba L.	0	Salicaceae Mirb.	n	1
Iz_6_1009	DJI_0347	Sambucus nigra L.	0	Viburnaceae Raf.	n	1
Iz_6_1009	DJI_0347	Sorbus aucuparia L.	0	Rosaceae Juss.	n	1
Iz_6_1009	DJI_0347	Tilia cordata Mill.	0	Malvaceae Juss.	n	1
Iz_6_1009	DJI_0347	Ulmus glabra Huds.	0	Ulmaceae Mirb.	n	1
Iz_6_1009	DJI_0349	Acer tataricum L.	0	Sapindaceae Juss.	n	1
Iz_6_1009	DJI_0349	Betula pendula Roth.	0	Betulaceae Gray	n	1

Iz_6_1009	DJI_0349	Crataegus rhipidophylla Gand.	0	Rosaceae Juss.	n	1
Iz_6_1009	DJI_0349	Frangula alnus Mill.	0	Rhamnaceae Juss.	n	1
Iz_6_1009	DJI_0349	Fraxinus pennsylvanica Marshall	0	Oleaceae Hoffmanns. & Link	a	0
Iz_6_1009	DJI_0349	Pinus sylvestris L.	0	Pinaceae Spreng. ex F.Rudolphi	n	1
Iz_6_1009	DJI_0349	Populus × canescens (Aiton) Sm.	0	Salicaceae Mirb.	n	1
Iz_6_1009	DJI_0349	Populus tremula L.	0	Salicaceae Mirb.	n	1
Iz_6_1009	DJI_0349	Populus nigra L.	0	Salicaceae Mirb.	n	1
Iz_6_1009	DJI_0349	Pyrus communis L.	0	Rosaceae Juss.	a	0
Iz_6_1009	DJI_0349	Quercus robur L.	0	Fagaceae Dumort.	n	1
Iz_6_1009	DJI_0349	Robinia pseudoacacia L.	0	Fabaceae Lindl.	a	0
Iz_6_1009	DJI_0349	Rosa sp.	0	Rosaceae Juss.		
Iz_6_1009	DJI_0349	Salix alba L.	0	Salicaceae Mirb.	n	1
Iz_6_1009	DJI_0349	Sambucus nigra L.	0	Viburnaceae Raf.	n	1
Iz_6_1009	DJI_0349	Sorbus aucuparia L.	0	Rosaceae Juss.	n	1
Iz_6_1009	DJI_0349	Tilia cordata Mill.	0	Malvaceae Juss.	n	1
Iz_6_1009	DJI_0349	Ulmus glabra Huds.	0	Ulmaceae Mirb.	n	1
Iz_6_1009	DJI_0355	Acer tataricum L.	0	Sapindaceae Juss.	n	1
Iz_6_1009	DJI_0355	Betula pendula Roth.	0	Betulaceae Gray	n	1
Iz_6_1009	DJI_0355	Crataegus rhipidophylla Gand.	0	Rosaceae Juss.	n	1
Iz_6_1009	DJI_0355	Frangula alnus Mill.	0	Rhamnaceae Juss.	n	1
Iz_6_1009	DJI_0355	Fraxinus pennsylvanica Marshall	0	Oleaceae Hoffmanns. & Link	a	0
Iz_6_1009	DJI_0355	Pinus sylvestris L.	0	Pinaceae Spreng. ex F.Rudolphi	n	1
Iz_6_1009	DJI_0355	Populus × canescens (Aiton) Sm.	0	Salicaceae Mirb.	n	1
Iz_6_1009	DJI_0355	Populus tremula L.	0	Salicaceae Mirb.	n	1
Iz_6_1009	DJI_0355	Populus nigra L.	0	Salicaceae Mirb.	n	1
Iz_6_1009	DJI_0355	Pyrus communis L.	0	Rosaceae Juss.	a	0
Iz_6_1009	DJI_0355	Quercus robur L.	0	Fagaceae Dumort.	n	1
Iz_6_1009	DJI_0355	Robinia pseudoacacia L.	0	Fabaceae Lindl.	a	0
Iz_6_1009	DJI_0355	Rosa sp.	0	Rosaceae Juss.		
Iz_6_1009	DJI_0355	Salix alba L.	0	Salicaceae Mirb.	n	1
Iz_6_1009	DJI_0355	Sambucus nigra L.	0	Viburnaceae Raf.	n	1
Iz_6_1009	DJI_0355	Sorbus aucuparia L.	0	Rosaceae Juss.	n	1
Iz_6_1009	DJI_0355	Tilia cordata Mill.	0	Malvaceae Juss.	n	1
Iz_6_1009	DJI_0355	Ulmus glabra Huds.	0	Ulmaceae Mirb.	n	1
Iz_6_1009	DJI_0358	Acer tataricum L.	0	Sapindaceae Juss.	n	1
Iz_6_1009	DJI_0358	Betula pendula Roth.	0	Betulaceae Gray	n	1
Iz_6_1009	DJI_0358	Crataegus rhipidophylla Gand.	0	Rosaceae Juss.	n	1
Iz_6_1009	DJI_0358	Frangula alnus Mill.	0	Rhamnaceae Juss.	n	1
Iz_6_1009	DJI_0358	Fraxinus pennsylvanica Marshall	0	Oleaceae Hoffmanns. & Link	a	0
Iz_6_1009	DJI_0358	Pinus sylvestris L.	0	Pinaceae Spreng. ex F.Rudolphi	n	1
Iz_6_1009	DJI_0358	Populus × canescens (Aiton) Sm.	0	Salicaceae Mirb.	n	1
Iz_6_1009	DJI_0358	Populus tremula L.	0	Salicaceae Mirb.	n	1
Iz_6_1009	DJI_0358	Populus nigra L.	0	Salicaceae Mirb.	n	1
Iz_6_1009	DJI_0358	Pyrus communis L.	0	Rosaceae Juss.	a	0
Iz_6_1009	DJI_0358	Quercus robur L.	0	Fagaceae Dumort.	n	1
Iz_6_1009	DJI_0358	Robinia pseudoacacia L.	0	Fabaceae Lindl.	a	0
Iz_6_1009	DJI_0358	Rosa sp.	0	Rosaceae Juss.		
Iz_6_1009	DJI_0358	Salix alba L.	0	Salicaceae Mirb.	n	1
Iz_6_1009	DJI_0358	Sambucus nigra L.	0	Viburnaceae Raf.	n	1
Iz_6_1009	DJI_0358	Sorbus aucuparia L.	0	Rosaceae Juss.	n	1
Iz_6_1009	DJI_0358	Tilia cordata Mill.	0	Malvaceae Juss.	n	1
Iz_6_1009	DJI_0358	Ulmus glabra Huds.	0	Ulmaceae Mirb.	n	1
Iz_6_1009	DJI_0361	Acer tataricum L.	0	Sapindaceae Juss.	n	1
Iz_6_1009	DJI_0361	Betula pendula Roth.	0	Betulaceae Gray	n	1
Iz_6_1009	DJI_0361	Crataegus rhipidophylla Gand.	0	Rosaceae Juss.	n	1
Iz_6_1009	DJI_0361	Frangula alnus Mill.	0	Rhamnaceae Juss.	n	1
Iz_6_1009	DJI_0361	Fraxinus pennsylvanica Marshall	0	Oleaceae Hoffmanns. & Link	a	0
Iz_6_1009	DJI_0361	Pinus sylvestris L.	0	Pinaceae Spreng. ex F.Rudolphi	n	1
Iz_6_1009	DJI_0361	Populus × canescens (Aiton) Sm.	0	Salicaceae Mirb.	n	1
Iz_6_1009	DJI_0361	Populus tremula L.	0	Salicaceae Mirb.	n	1
Iz_6_1009	DJI_0361	Populus nigra L.	0	Salicaceae Mirb.	n	1
Iz_6_1009	DJI_0361	Pyrus communis L.	0	Rosaceae Juss.	a	0
Iz_6_1009	DJI_0361	Quercus robur L.	0	Fagaceae Dumort.	n	1
Iz_6_1009	DJI_0361	Robinia pseudoacacia L.	0	Fabaceae Lindl.	a	0

Iz_6_1009	DJI_0361	Rosa sp.	0	Rosaceae Juss.		
Iz_6_1009	DJI_0361	Salix alba L.	0	Salicaceae Mirb.	n	1
Iz_6_1009	DJI_0361	Sambucus nigra L.	0	Viburnaceae Raf.	n	1
Iz_6_1009	DJI_0361	Sorbus aucuparia L.	0	Rosaceae Juss.	n	1
Iz_6_1009	DJI_0361	Tilia cordata Mill.	0	Malvaceae Juss.	n	1
Iz_6_1009	DJI_0361	Ulmus glabra Huds.	0	Ulmaceae Mirb.	n	1
Iz_6_1009	DJI_0367	Acer tataricum L.	0	Sapindaceae Juss.	n	1
Iz_6_1009	DJI_0367	Betula pendula Roth.	0	Betulaceae Gray	n	1
Iz_6_1009	DJI_0367	Crataegus rhipidophylla Gand.	0	Rosaceae Juss.	n	1
Iz_6_1009	DJI_0367	Frangula alnus Mill.	0	Rhamnaceae Juss.	n	1
Iz_6_1009	DJI_0367	Fraxinus pennsylvanica Marshall	0	Oleaceae Hoffmanns. & Link	a	0
Iz_6_1009	DJI_0367	Pinus sylvestris L.	0	Pinaceae Spreng. ex F.Rudolphi	n	1
Iz_6_1009	DJI_0367	Populus × canescens (Aiton) Sm.	0	Salicaceae Mirb.	n	1
Iz_6_1009	DJI_0367	Populus tremula L.	0	Salicaceae Mirb.	n	1
Iz_6_1009	DJI_0367	Populus nigra L.	0	Salicaceae Mirb.	n	1
Iz_6_1009	DJI_0367	Pyrus communis L.	0	Rosaceae Juss.	a	0
Iz_6_1009	DJI_0367	Quercus robur L.	0	Fagaceae Dumort.	n	1
Iz_6_1009	DJI_0367	Robinia pseudoacacia L.	0	Fabaceae Lindl.	a	0
Iz_6_1009	DJI_0367	Rosa sp.	0	Rosaceae Juss.		
Iz_6_1009	DJI_0367	Salix alba L.	0	Salicaceae Mirb.	n	1
Iz_6_1009	DJI_0367	Sambucus nigra L.	0	Viburnaceae Raf.	n	1
Iz_6_1009	DJI_0367	Sorbus aucuparia L.	0	Rosaceae Juss.	n	1
Iz_6_1009	DJI_0367	Tilia cordata Mill.	0	Malvaceae Juss.	n	1
Iz_6_1009	DJI_0367	Ulmus glabra Huds.	0	Ulmaceae Mirb.	n	1
Iz_6_1009	DJI_0372	Acer tataricum L.	0	Sapindaceae Juss.	n	1
Iz_6_1009	DJI_0372	Betula pendula Roth.	0	Betulaceae Gray	n	1
Iz_6_1009	DJI_0372	Crataegus rhipidophylla Gand.	0	Rosaceae Juss.	n	1
Iz_6_1009	DJI_0372	Frangula alnus Mill.	0	Rhamnaceae Juss.	n	1
Iz_6_1009	DJI_0372	Fraxinus pennsylvanica Marshall	0	Oleaceae Hoffmanns. & Link	a	0
Iz_6_1009	DJI_0372	Pinus sylvestris L.	0	Pinaceae Spreng. ex F.Rudolphi	n	1
Iz_6_1009	DJI_0372	Populus × canescens (Aiton) Sm.	0	Salicaceae Mirb.	n	1
Iz_6_1009	DJI_0372	Populus tremula L.	0	Salicaceae Mirb.	n	1
Iz_6_1009	DJI_0372	Populus nigra L.	0	Salicaceae Mirb.	n	1
Iz_6_1009	DJI_0372	Pyrus communis L.	0	Rosaceae Juss.	a	0
Iz_6_1009	DJI_0372	Quercus robur L.	0	Fagaceae Dumort.	n	1
Iz_6_1009	DJI_0372	Robinia pseudoacacia L.	0	Fabaceae Lindl.	a	0
Iz_6_1009	DJI_0372	Rosa sp.	0	Rosaceae Juss.		
Iz_6_1009	DJI_0372	Salix alba L.	0	Salicaceae Mirb.	n	1
Iz_6_1009	DJI_0372	Sambucus nigra L.	0	Viburnaceae Raf.	n	1
Iz_6_1009	DJI_0372	Sorbus aucuparia L.	0	Rosaceae Juss.	n	1
Iz_6_1009	DJI_0372	Tilia cordata Mill.	0	Malvaceae Juss.	n	1
Iz_6_1009	DJI_0372	Ulmus glabra Huds.	0	Ulmaceae Mirb.	n	1
Iz_6_1009	DJI_0374	Acer tataricum L.	0	Sapindaceae Juss.	n	1
Iz_6_1009	DJI_0374	Betula pendula Roth.	0	Betulaceae Gray	n	1
Iz_6_1009	DJI_0374	Crataegus rhipidophylla Gand.	0	Rosaceae Juss.	n	1
Iz_6_1009	DJI_0374	Frangula alnus Mill.	0	Rhamnaceae Juss.	n	1
Iz_6_1009	DJI_0374	Fraxinus pennsylvanica Marshall	0	Oleaceae Hoffmanns. & Link	a	0
Iz_6_1009	DJI_0374	Pinus sylvestris L.	0	Pinaceae Spreng. ex F.Rudolphi	n	1
Iz_6_1009	DJI_0374	Populus × canescens (Aiton) Sm.	0	Salicaceae Mirb.	n	1
Iz_6_1009	DJI_0374	Populus tremula L.	0	Salicaceae Mirb.	n	1
Iz_6_1009	DJI_0374	Populus nigra L.	0	Salicaceae Mirb.	n	1
Iz_6_1009	DJI_0374	Pyrus communis L.	0	Rosaceae Juss.	a	0
Iz_6_1009	DJI_0374	Quercus robur L.	0	Fagaceae Dumort.	n	1
Iz_6_1009	DJI_0374	Robinia pseudoacacia L.	0	Fabaceae Lindl.	a	0
Iz_6_1009	DJI_0374	Rosa sp.	0	Rosaceae Juss.		
Iz_6_1009	DJI_0374	Salix alba L.	0	Salicaceae Mirb.	n	1
Iz_6_1009	DJI_0374	Sambucus nigra L.	0	Viburnaceae Raf.	n	1
Iz_6_1009	DJI_0374	Sorbus aucuparia L.	0	Rosaceae Juss.	n	1
Iz_6_1009	DJI_0374	Tilia cordata Mill.	0	Malvaceae Juss.	n	1
Iz_6_1009	DJI_0374	Ulmus glabra Huds.	0	Ulmaceae Mirb.	n	1
Iz_6_1009	DJI_0382	Acer tataricum L.	0	Sapindaceae Juss.	n	1
Iz_6_1009	DJI_0382	Betula pendula Roth.	0	Betulaceae Gray	n	1
Iz_6_1009	DJI_0382	Crataegus rhipidophylla Gand.	0	Rosaceae Juss.	n	1
Iz_6_1009	DJI_0382	Frangula alnus Mill.	0	Rhamnaceae Juss.	n	1

Iz_6_1009	DJI_0382	Fraxinus pennsylvanica Marshall	0	Oleaceae Hoffmanns. & Link	a	0
Iz_6_1009	DJI_0382	Pinus sylvestris L.	0	Pinaceae Spreng. ex F.Rudolphi	n	1
Iz_6_1009	DJI_0382	Populus × canescens (Aiton) Sm.	0	Salicaceae Mirb.	n	1
Iz_6_1009	DJI_0382	Populus tremula L.	0	Salicaceae Mirb.	n	1
Iz_6_1009	DJI_0382	Populus nigra L.	0	Salicaceae Mirb.	n	1
Iz_6_1009	DJI_0382	Pyrus communis L.	0	Rosaceae Juss.	a	0
Iz_6_1009	DJI_0382	Quercus robur L.	0	Fagaceae Dumort.	n	1
Iz_6_1009	DJI_0382	Robinia pseudoacacia L.	0	Fabaceae Lindl.	a	0
Iz_6_1009	DJI_0382	Rosa sp.	0	Rosaceae Juss.		
Iz_6_1009	DJI_0382	Salix alba L.	0	Salicaceae Mirb.	n	1
Iz_6_1009	DJI_0382	Sambucus nigra L.	0	Viburnaceae Raf.	n	1
Iz_6_1009	DJI_0382	Sorbus aucuparia L.	0	Rosaceae Juss.	n	1
Iz_6_1009	DJI_0382	Tilia cordata Mill.	0	Malvaceae Juss.	n	1
Iz_6_1009	DJI_0382	Ulmus glabra Huds.	0	Ulmaceae Mirb.	n	1
Iz_6_1009	DJI_0385	Acer tataricum L.	0	Sapindaceae Juss.	n	1
Iz_6_1009	DJI_0385	Betula pendula Roth.	0	Betulaceae Gray	n	1
Iz_6_1009	DJI_0385	Crataegus rhipidophylla Gand.	0	Rosaceae Juss.	n	1
Iz_6_1009	DJI_0385	Frangula alnus Mill.	0	Rhamnaceae Juss.	n	1
Iz_6_1009	DJI_0385	Fraxinus pennsylvanica Marshall	0	Oleaceae Hoffmanns. & Link	a	0
Iz_6_1009	DJI_0385	Pinus sylvestris L.	0	Pinaceae Spreng. ex F.Rudolphi	n	1
Iz_6_1009	DJI_0385	Populus × canescens (Aiton) Sm.	0	Salicaceae Mirb.	n	1
Iz_6_1009	DJI_0385	Populus tremula L.	0	Salicaceae Mirb.	n	1
Iz_6_1009	DJI_0385	Populus nigra L.	0	Salicaceae Mirb.	n	1
Iz_6_1009	DJI_0385	Pyrus communis L.	0	Rosaceae Juss.	a	0
Iz_6_1009	DJI_0385	Quercus robur L.	0	Fagaceae Dumort.	n	1
Iz_6_1009	DJI_0385	Robinia pseudoacacia L.	0	Fabaceae Lindl.	a	0
Iz_6_1009	DJI_0385	Rosa sp.	0	Rosaceae Juss.		
Iz_6_1009	DJI_0385	Salix alba L.	0	Salicaceae Mirb.	n	1
Iz_6_1009	DJI_0385	Sambucus nigra L.	0	Viburnaceae Raf.	n	1
Iz_6_1009	DJI_0385	Sorbus aucuparia L.	0	Rosaceae Juss.	n	1
Iz_6_1009	DJI_0385	Tilia cordata Mill.	0	Malvaceae Juss.	n	1
Iz_6_1009	DJI_0385	Ulmus glabra Huds.	0	Ulmaceae Mirb.	n	1
Iz_6_1009	DJI_0395	Acer tataricum L.	0	Sapindaceae Juss.	n	1
Iz_6_1009	DJI_0395	Betula pendula Roth.	0	Betulaceae Gray	n	1
Iz_6_1009	DJI_0395	Crataegus rhipidophylla Gand.	0	Rosaceae Juss.	n	1
Iz_6_1009	DJI_0395	Frangula alnus Mill.	0	Rhamnaceae Juss.	n	1
Iz_6_1009	DJI_0395	Fraxinus pennsylvanica Marshall	0	Oleaceae Hoffmanns. & Link	a	0
Iz_6_1009	DJI_0395	Pinus sylvestris L.	0	Pinaceae Spreng. ex F.Rudolphi	n	1
Iz_6_1009	DJI_0395	Populus × canescens (Aiton) Sm.	0	Salicaceae Mirb.	n	1
Iz_6_1009	DJI_0395	Populus tremula L.	0	Salicaceae Mirb.	n	1
Iz_6_1009	DJI_0395	Populus nigra L.	0	Salicaceae Mirb.	n	1
Iz_6_1009	DJI_0395	Pyrus communis L.	0	Rosaceae Juss.	a	0
Iz_6_1009	DJI_0395	Quercus robur L.	0	Fagaceae Dumort.	n	1
Iz_6_1009	DJI_0395	Robinia pseudoacacia L.	0	Fabaceae Lindl.	a	0
Iz_6_1009	DJI_0395	Rosa sp.	0	Rosaceae Juss.		
Iz_6_1009	DJI_0395	Salix alba L.	0	Salicaceae Mirb.	n	1
Iz_6_1009	DJI_0395	Sambucus nigra L.	0	Viburnaceae Raf.	n	1
Iz_6_1009	DJI_0395	Sorbus aucuparia L.	0	Rosaceae Juss.	n	1
Iz_6_1009	DJI_0395	Tilia cordata Mill.	0	Malvaceae Juss.	n	1
Iz_6_1009	DJI_0395	Ulmus glabra Huds.	0	Ulmaceae Mirb.	n	1
Iz_6_1009	DJI_0406	Acer tataricum L.	0	Sapindaceae Juss.	n	1
Iz_6_1009	DJI_0406	Betula pendula Roth.	0	Betulaceae Gray	n	1
Iz_6_1009	DJI_0406	Crataegus rhipidophylla Gand.	0	Rosaceae Juss.	n	1
Iz_6_1009	DJI_0406	Frangula alnus Mill.	0	Rhamnaceae Juss.	n	1
Iz_6_1009	DJI_0406	Fraxinus pennsylvanica Marshall	0	Oleaceae Hoffmanns. & Link	a	0
Iz_6_1009	DJI_0406	Pinus sylvestris L.	0	Pinaceae Spreng. ex F.Rudolphi	n	1
Iz_6_1009	DJI_0406	Populus × canescens (Aiton) Sm.	0	Salicaceae Mirb.	n	1
Iz_6_1009	DJI_0406	Populus tremula L.	0	Salicaceae Mirb.	n	1
Iz_6_1009	DJI_0406	Populus nigra L.	0	Salicaceae Mirb.	n	1
Iz_6_1009	DJI_0406	Pyrus communis L.	0	Rosaceae Juss.	a	0
Iz_6_1009	DJI_0406	Quercus robur L.	8	Fagaceae Dumort.	n	1
Iz_6_1009	DJI_0406	Robinia pseudoacacia L.	0	Fabaceae Lindl.	a	0
Iz_6_1009	DJI_0406	Rosa sp.	0	Rosaceae Juss.		
Iz_6_1009	DJI_0406	Salix alba L.	0	Salicaceae Mirb.	n	1

Iz_6_1009	DJI_0406	Sambucus nigra L.	0	Viburnaceae Raf.	n	1
Iz_6_1009	DJI_0406	Sorbus aucuparia L.	0	Rosaceae Juss.	n	1
Iz_6_1009	DJI_0406	Tilia cordata Mill.	0	Malvaceae Juss.	n	1
Iz_6_1009	DJI_0406	Ulmus glabra Huds.	0	Ulmaceae Mirb.	n	1
Iz_7_1009	DJI_0438	Acer tataricum L.	0	Sapindaceae Juss.	n	1
Iz_7_1009	DJI_0438	Betula pendula Roth.	0	Betulaceae Gray	n	1
Iz_7_1009	DJI_0438	Crataegus rhipidophylla Gand.	0	Rosaceae Juss.	n	1
Iz_7_1009	DJI_0438	Frangula alnus Mill.	0	Rhamnaceae Juss.	n	1
Iz_7_1009	DJI_0438	Fraxinus pennsylvanica Marshall	0	Oleaceae Hoffmanns. & Link	a	0
Iz_7_1009	DJI_0438	Pinus sylvestris L.	0	Pinaceae Spreng. ex F.Rudolphi	n	1
Iz_7_1009	DJI_0438	Populus × canescens (Aiton) Sm.	0	Salicaceae Mirb.	n	1
Iz_7_1009	DJI_0438	Populus tremula L.	0	Salicaceae Mirb.	n	1
Iz_7_1009	DJI_0438	Populus nigra L.	0	Salicaceae Mirb.	n	1
Iz_7_1009	DJI_0438	Pyrus communis L.	0	Rosaceae Juss.	a	0
Iz_7_1009	DJI_0438	Quercus robur L.	0	Fagaceae Dumort.	n	1
Iz_7_1009	DJI_0438	Robinia pseudoacacia L.	0	Fabaceae Lindl.	a	0
Iz_7_1009	DJI_0438	Rosa sp.	0	Rosaceae Juss.		
Iz_7_1009	DJI_0438	Salix alba L.	0	Salicaceae Mirb.	n	1
Iz_7_1009	DJI_0438	Sambucus nigra L.	0	Viburnaceae Raf.	n	1
Iz_7_1009	DJI_0438	Sorbus aucuparia L.	0	Rosaceae Juss.	n	1
Iz_7_1009	DJI_0438	Tilia cordata Mill.	0	Malvaceae Juss.	n	1
Iz_7_1009	DJI_0438	Ulmus glabra Huds.	0	Ulmaceae Mirb.	n	1
Iz_7_1009	DJI_0447	Acer tataricum L.	0	Sapindaceae Juss.	n	1
Iz_7_1009	DJI_0447	Betula pendula Roth.	0	Betulaceae Gray	n	1
Iz_7_1009	DJI_0447	Crataegus rhipidophylla Gand.	0	Rosaceae Juss.	n	1
Iz_7_1009	DJI_0447	Frangula alnus Mill.	0	Rhamnaceae Juss.	n	1
Iz_7_1009	DJI_0447	Fraxinus pennsylvanica Marshall	0	Oleaceae Hoffmanns. & Link	a	0
Iz_7_1009	DJI_0447	Pinus sylvestris L.	0	Pinaceae Spreng. ex F.Rudolphi	n	1
Iz_7_1009	DJI_0447	Populus × canescens (Aiton) Sm.	0	Salicaceae Mirb.	n	1
Iz_7_1009	DJI_0447	Populus tremula L.	0	Salicaceae Mirb.	n	1
Iz_7_1009	DJI_0447	Populus nigra L.	0	Salicaceae Mirb.	n	1
Iz_7_1009	DJI_0447	Pyrus communis L.	0	Rosaceae Juss.	a	0
Iz_7_1009	DJI_0447	Quercus robur L.	0	Fagaceae Dumort.	n	1
Iz_7_1009	DJI_0447	Robinia pseudoacacia L.	0	Fabaceae Lindl.	a	0
Iz_7_1009	DJI_0447	Rosa sp.	0	Rosaceae Juss.		
Iz_7_1009	DJI_0447	Salix alba L.	0	Salicaceae Mirb.	n	1
Iz_7_1009	DJI_0447	Sambucus nigra L.	0	Viburnaceae Raf.	n	1
Iz_7_1009	DJI_0447	Sorbus aucuparia L.	0	Rosaceae Juss.	n	1
Iz_7_1009	DJI_0447	Tilia cordata Mill.	0	Malvaceae Juss.	n	1
Iz_7_1009	DJI_0447	Ulmus glabra Huds.	0	Ulmaceae Mirb.	n	1
Iz_7_1009	DJI_0448	Acer tataricum L.	0	Sapindaceae Juss.	n	1
Iz_7_1009	DJI_0448	Betula pendula Roth.	0	Betulaceae Gray	n	1
Iz_7_1009	DJI_0448	Crataegus rhipidophylla Gand.	0	Rosaceae Juss.	n	1
Iz_7_1009	DJI_0448	Frangula alnus Mill.	0	Rhamnaceae Juss.	n	1
Iz_7_1009	DJI_0448	Fraxinus pennsylvanica Marshall	0	Oleaceae Hoffmanns. & Link	a	0
Iz_7_1009	DJI_0448	Pinus sylvestris L.	0	Pinaceae Spreng. ex F.Rudolphi	n	1
Iz_7_1009	DJI_0448	Populus × canescens (Aiton) Sm.	0	Salicaceae Mirb.	n	1
Iz_7_1009	DJI_0448	Populus tremula L.	0	Salicaceae Mirb.	n	1
Iz_7_1009	DJI_0448	Populus nigra L.	0	Salicaceae Mirb.	n	1
Iz_7_1009	DJI_0448	Pyrus communis L.	0	Rosaceae Juss.	a	0
Iz_7_1009	DJI_0448	Quercus robur L.	0	Fagaceae Dumort.	n	1
Iz_7_1009	DJI_0448	Robinia pseudoacacia L.	0	Fabaceae Lindl.	a	0
Iz_7_1009	DJI_0448	Rosa sp.	0	Rosaceae Juss.		
Iz_7_1009	DJI_0448	Salix alba L.	0	Salicaceae Mirb.	n	1
Iz_7_1009	DJI_0448	Sambucus nigra L.	0	Viburnaceae Raf.	n	1
Iz_7_1009	DJI_0448	Sorbus aucuparia L.	0	Rosaceae Juss.	n	1
Iz_7_1009	DJI_0448	Tilia cordata Mill.	0	Malvaceae Juss.	n	1
Iz_7_1009	DJI_0448	Ulmus glabra Huds.	0	Ulmaceae Mirb.	n	1
Iz_7_1009	DJI_0450	Acer tataricum L.	0	Sapindaceae Juss.	n	1
Iz_7_1009	DJI_0450	Betula pendula Roth.	0	Betulaceae Gray	n	1
Iz_7_1009	DJI_0450	Crataegus rhipidophylla Gand.	0	Rosaceae Juss.	n	1
Iz_7_1009	DJI_0450	Frangula alnus Mill.	0	Rhamnaceae Juss.	n	1
Iz_7_1009	DJI_0450	Fraxinus pennsylvanica Marshall	0	Oleaceae Hoffmanns. & Link	a	0
Iz_7_1009	DJI_0450	Pinus sylvestris L.	0	Pinaceae Spreng. ex F.Rudolphi	n	1

Iz_7_1009	DJI_0450	Populus × canescens (Aiton) Sm.	0	Salicaceae Mirb.	n	1
Iz_7_1009	DJI_0450	Populus tremula L.	0	Salicaceae Mirb.	n	1
Iz_7_1009	DJI_0450	Populus nigra L.	0	Salicaceae Mirb.	n	1
Iz_7_1009	DJI_0450	Pyrus communis L.	0	Rosaceae Juss.	a	0
Iz_7_1009	DJI_0450	Quercus robur L.	0	Fagaceae Dumort.	n	1
Iz_7_1009	DJI_0450	Robinia pseudoacacia L.	0	Fabaceae Lindl.	a	0
Iz_7_1009	DJI_0450	Rosa sp.	0	Rosaceae Juss.		
Iz_7_1009	DJI_0450	Salix alba L.	0	Salicaceae Mirb.	n	1
Iz_7_1009	DJI_0450	Sambucus nigra L.	0	Viburnaceae Raf.	n	1
Iz_7_1009	DJI_0450	Sorbus aucuparia L.	0	Rosaceae Juss.	n	1
Iz_7_1009	DJI_0450	Tilia cordata Mill.	0	Malvaceae Juss.	n	1
Iz_7_1009	DJI_0450	Ulmus glabra Huds.	0	Ulmaceae Mirb.	n	1
Iz_7_1009	DJI_0452	Acer tataricum L.	0	Sapindaceae Juss.	n	1
Iz_7_1009	DJI_0452	Betula pendula Roth.	0	Betulaceae Gray	n	1
Iz_7_1009	DJI_0452	Crataegus rhipidophylla Gand.	0	Rosaceae Juss.	n	1
Iz_7_1009	DJI_0452	Frangula alnus Mill.	0	Rhamnaceae Juss.	n	1
Iz_7_1009	DJI_0452	Fraxinus pennsylvanica Marshall	0	Oleaceae Hoffmanns. & Link	a	0
Iz_7_1009	DJI_0452	Pinus sylvestris L.	0	Pinaceae Spreng. ex F.Rudolphi	n	1
Iz_7_1009	DJI_0452	Populus × canescens (Aiton) Sm.	0	Salicaceae Mirb.	n	1
Iz_7_1009	DJI_0452	Populus tremula L.	0	Salicaceae Mirb.	n	1
Iz_7_1009	DJI_0452	Populus nigra L.	0	Salicaceae Mirb.	n	1
Iz_7_1009	DJI_0452	Pyrus communis L.	0	Rosaceae Juss.	a	0
Iz_7_1009	DJI_0452	Quercus robur L.	0	Fagaceae Dumort.	n	1
Iz_7_1009	DJI_0452	Robinia pseudoacacia L.	0	Fabaceae Lindl.	a	0
Iz_7_1009	DJI_0452	Rosa sp.	0	Rosaceae Juss.		
Iz_7_1009	DJI_0452	Salix alba L.	0	Salicaceae Mirb.	n	1
Iz_7_1009	DJI_0452	Sambucus nigra L.	0	Viburnaceae Raf.	n	1
Iz_7_1009	DJI_0452	Sorbus aucuparia L.	0	Rosaceae Juss.	n	1
Iz_7_1009	DJI_0452	Tilia cordata Mill.	0	Malvaceae Juss.	n	1
Iz_7_1009	DJI_0452	Ulmus glabra Huds.	0	Ulmaceae Mirb.	n	1
Iz_7_1009	DJI_0455	Acer tataricum L.	0	Sapindaceae Juss.	n	1
Iz_7_1009	DJI_0455	Betula pendula Roth.	0	Betulaceae Gray	n	1
Iz_7_1009	DJI_0455	Crataegus rhipidophylla Gand.	0	Rosaceae Juss.	n	1
Iz_7_1009	DJI_0455	Frangula alnus Mill.	0	Rhamnaceae Juss.	n	1
Iz_7_1009	DJI_0455	Fraxinus pennsylvanica Marshall	0	Oleaceae Hoffmanns. & Link	a	0
Iz_7_1009	DJI_0455	Pinus sylvestris L.	0	Pinaceae Spreng. ex F.Rudolphi	n	1
Iz_7_1009	DJI_0455	Populus × canescens (Aiton) Sm.	0	Salicaceae Mirb.	n	1
Iz_7_1009	DJI_0455	Populus tremula L.	0	Salicaceae Mirb.	n	1
Iz_7_1009	DJI_0455	Populus nigra L.	0	Salicaceae Mirb.	n	1
Iz_7_1009	DJI_0455	Pyrus communis L.	0	Rosaceae Juss.	a	0
Iz_7_1009	DJI_0455	Quercus robur L.	0	Fagaceae Dumort.	n	1
Iz_7_1009	DJI_0455	Robinia pseudoacacia L.	0	Fabaceae Lindl.	a	0
Iz_7_1009	DJI_0455	Rosa sp.	0	Rosaceae Juss.		
Iz_7_1009	DJI_0455	Salix alba L.	0	Salicaceae Mirb.	n	1
Iz_7_1009	DJI_0455	Sambucus nigra L.	0	Viburnaceae Raf.	n	1
Iz_7_1009	DJI_0455	Sorbus aucuparia L.	0	Rosaceae Juss.	n	1
Iz_7_1009	DJI_0455	Tilia cordata Mill.	0	Malvaceae Juss.	n	1
Iz_7_1009	DJI_0455	Ulmus glabra Huds.	0	Ulmaceae Mirb.	n	1
Iz_7_1009	DJI_0458	Acer tataricum L.	0	Sapindaceae Juss.	n	1
Iz_7_1009	DJI_0458	Betula pendula Roth.	0	Betulaceae Gray	n	1
Iz_7_1009	DJI_0458	Crataegus rhipidophylla Gand.	0	Rosaceae Juss.	n	1
Iz_7_1009	DJI_0458	Frangula alnus Mill.	0	Rhamnaceae Juss.	n	1
Iz_7_1009	DJI_0458	Fraxinus pennsylvanica Marshall	0	Oleaceae Hoffmanns. & Link	a	0
Iz_7_1009	DJI_0458	Pinus sylvestris L.	0	Pinaceae Spreng. ex F.Rudolphi	n	1
Iz_7_1009	DJI_0458	Populus × canescens (Aiton) Sm.	0	Salicaceae Mirb.	n	1
Iz_7_1009	DJI_0458	Populus tremula L.	0	Salicaceae Mirb.	n	1
Iz_7_1009	DJI_0458	Populus nigra L.	0	Salicaceae Mirb.	n	1
Iz_7_1009	DJI_0458	Pyrus communis L.	0	Rosaceae Juss.	a	0
Iz_7_1009	DJI_0458	Quercus robur L.	0	Fagaceae Dumort.	n	1
Iz_7_1009	DJI_0458	Robinia pseudoacacia L.	0	Fabaceae Lindl.	a	0
Iz_7_1009	DJI_0458	Rosa sp.	0	Rosaceae Juss.		
Iz_7_1009	DJI_0458	Salix alba L.	0	Salicaceae Mirb.	n	1
Iz_7_1009	DJI_0458	Sambucus nigra L.	0	Viburnaceae Raf.	n	1
Iz_7_1009	DJI_0458	Sorbus aucuparia L.	0	Rosaceae Juss.	n	1

Iz_7_1009	DJI_0458	Tilia cordata Mill.	0	Malvaceae Juss.	n	1
Iz_7_1009	DJI_0458	Ulmus glabra Huds.	0	Ulmaceae Mirb.	n	1
Iz_7_1009	DJI_0462	Acer tataricum L.	0	Sapindaceae Juss.	n	1
Iz_7_1009	DJI_0462	Betula pendula Roth.	0	Betulaceae Gray	n	1
Iz_7_1009	DJI_0462	Crataegus rhipidophylla Gand.	0	Rosaceae Juss.	n	1
Iz_7_1009	DJI_0462	Frangula alnus Mill.	0	Rhamnaceae Juss.	n	1
Iz_7_1009	DJI_0462	Fraxinus pennsylvanica Marshall	0	Oleaceae Hoffmanns. & Link	a	0
Iz_7_1009	DJI_0462	Pinus sylvestris L.	0	Pinaceae Spreng. ex F.Rudolphi	n	1
Iz_7_1009	DJI_0462	Populus × canescens (Aiton) Sm.	0	Salicaceae Mirb.	n	1
Iz_7_1009	DJI_0462	Populus tremula L.	0	Salicaceae Mirb.	n	1
Iz_7_1009	DJI_0462	Populus nigra L.	0	Salicaceae Mirb.	n	1
Iz_7_1009	DJI_0462	Pyrus communis L.	0	Rosaceae Juss.	a	0
Iz_7_1009	DJI_0462	Quercus robur L.	0	Fagaceae Dumort.	n	1
Iz_7_1009	DJI_0462	Robinia pseudoacacia L.	0	Fabaceae Lindl.	a	0
Iz_7_1009	DJI_0462	Rosa sp.	0	Rosaceae Juss.		
Iz_7_1009	DJI_0462	Salix alba L.	0	Salicaceae Mirb.	n	1
Iz_7_1009	DJI_0462	Sambucus nigra L.	0	Viburnaceae Raf.	n	1
Iz_7_1009	DJI_0462	Sorbus aucuparia L.	0	Rosaceae Juss.	n	1
Iz_7_1009	DJI_0462	Tilia cordata Mill.	0	Malvaceae Juss.	n	1
Iz_7_1009	DJI_0462	Ulmus glabra Huds.	0	Ulmaceae Mirb.	n	1
Iz_7_1009	DJI_0466	Acer tataricum L.	0	Sapindaceae Juss.	n	1
Iz_7_1009	DJI_0466	Betula pendula Roth.	0	Betulaceae Gray	n	1
Iz_7_1009	DJI_0466	Crataegus rhipidophylla Gand.	0	Rosaceae Juss.	n	1
Iz_7_1009	DJI_0466	Frangula alnus Mill.	0	Rhamnaceae Juss.	n	1
Iz_7_1009	DJI_0466	Fraxinus pennsylvanica Marshall	0	Oleaceae Hoffmanns. & Link	a	0
Iz_7_1009	DJI_0466	Pinus sylvestris L.	0	Pinaceae Spreng. ex F.Rudolphi	n	1
Iz_7_1009	DJI_0466	Populus × canescens (Aiton) Sm.	0	Salicaceae Mirb.	n	1
Iz_7_1009	DJI_0466	Populus tremula L.	0	Salicaceae Mirb.	n	1
Iz_7_1009	DJI_0466	Populus nigra L.	0	Salicaceae Mirb.	n	1
Iz_7_1009	DJI_0466	Pyrus communis L.	0	Rosaceae Juss.	a	0
Iz_7_1009	DJI_0466	Quercus robur L.	0	Fagaceae Dumort.	n	1
Iz_7_1009	DJI_0466	Robinia pseudoacacia L.	0	Fabaceae Lindl.	a	0
Iz_7_1009	DJI_0466	Rosa sp.	0	Rosaceae Juss.		
Iz_7_1009	DJI_0466	Salix alba L.	0	Salicaceae Mirb.	n	1
Iz_7_1009	DJI_0466	Sambucus nigra L.	0	Viburnaceae Raf.	n	1
Iz_7_1009	DJI_0466	Sorbus aucuparia L.	0	Rosaceae Juss.	n	1
Iz_7_1009	DJI_0466	Tilia cordata Mill.	0	Malvaceae Juss.	n	1
Iz_7_1009	DJI_0466	Ulmus glabra Huds.	0	Ulmaceae Mirb.	n	1
Iz_7_1009	DJI_0470	Acer tataricum L.	0	Sapindaceae Juss.	n	1
Iz_7_1009	DJI_0470	Betula pendula Roth.	0	Betulaceae Gray	n	1
Iz_7_1009	DJI_0470	Crataegus rhipidophylla Gand.	0	Rosaceae Juss.	n	1
Iz_7_1009	DJI_0470	Frangula alnus Mill.	0	Rhamnaceae Juss.	n	1
Iz_7_1009	DJI_0470	Fraxinus pennsylvanica Marshall	0	Oleaceae Hoffmanns. & Link	a	0
Iz_7_1009	DJI_0470	Pinus sylvestris L.	0	Pinaceae Spreng. ex F.Rudolphi	n	1
Iz_7_1009	DJI_0470	Populus × canescens (Aiton) Sm.	0	Salicaceae Mirb.	n	1
Iz_7_1009	DJI_0470	Populus tremula L.	0	Salicaceae Mirb.	n	1
Iz_7_1009	DJI_0470	Populus nigra L.	0	Salicaceae Mirb.	n	1
Iz_7_1009	DJI_0470	Pyrus communis L.	0	Rosaceae Juss.	a	0
Iz_7_1009	DJI_0470	Quercus robur L.	0	Fagaceae Dumort.	n	1
Iz_7_1009	DJI_0470	Robinia pseudoacacia L.	0	Fabaceae Lindl.	a	0
Iz_7_1009	DJI_0470	Rosa sp.	0	Rosaceae Juss.		
Iz_7_1009	DJI_0470	Salix alba L.	0	Salicaceae Mirb.	n	1
Iz_7_1009	DJI_0470	Sambucus nigra L.	0	Viburnaceae Raf.	n	1
Iz_7_1009	DJI_0470	Sorbus aucuparia L.	0	Rosaceae Juss.	n	1
Iz_7_1009	DJI_0470	Tilia cordata Mill.	0	Malvaceae Juss.	n	1
Iz_7_1009	DJI_0470	Ulmus glabra Huds.	0	Ulmaceae Mirb.	n	1
Iz_7_1009	DJI_0479	Acer tataricum L.	0	Sapindaceae Juss.	n	1
Iz_7_1009	DJI_0479	Betula pendula Roth.	0	Betulaceae Gray	n	1
Iz_7_1009	DJI_0479	Crataegus rhipidophylla Gand.	0	Rosaceae Juss.	n	1
Iz_7_1009	DJI_0479	Frangula alnus Mill.	0	Rhamnaceae Juss.	n	1
Iz_7_1009	DJI_0479	Fraxinus pennsylvanica Marshall	0	Oleaceae Hoffmanns. & Link	a	0
Iz_7_1009	DJI_0479	Pinus sylvestris L.	0	Pinaceae Spreng. ex F.Rudolphi	n	1
Iz_7_1009	DJI_0479	Populus × canescens (Aiton) Sm.	0	Salicaceae Mirb.	n	1
Iz_7_1009	DJI_0479	Populus tremula L.	0	Salicaceae Mirb.	n	1

Iz_7_1009	DJI_0479	Populus nigra L.	0	Salicaceae Mirb.	n	1
Iz_7_1009	DJI_0479	Pyrus communis L.	0	Rosaceae Juss.	a	0
Iz_7_1009	DJI_0479	Quercus robur L.	0	Fagaceae Dumort.	n	1
Iz_7_1009	DJI_0479	Robinia pseudoacacia L.	0	Fabaceae Lindl.	a	0
Iz_7_1009	DJI_0479	Rosa sp.	0	Rosaceae Juss.		
Iz_7_1009	DJI_0479	Salix alba L.	0	Salicaceae Mirb.	n	1
Iz_7_1009	DJI_0479	Sambucus nigra L.	0	Viburnaceae Raf.	n	1
Iz_7_1009	DJI_0479	Sorbus aucuparia L.	0	Rosaceae Juss.	n	1
Iz_7_1009	DJI_0479	Tilia cordata Mill.	0	Malvaceae Juss.	n	1
Iz_7_1009	DJI_0479	Ulmus glabra Huds.	0	Ulmaceae Mirb.	n	1
Iz_7_1009	DJI_0485	Acer tataricum L.	0	Sapindaceae Juss.	n	1
Iz_7_1009	DJI_0485	Betula pendula Roth.	0	Betulaceae Gray	n	1
Iz_7_1009	DJI_0485	Crataegus rhipidophylla Gand.	0	Rosaceae Juss.	n	1
Iz_7_1009	DJI_0485	Frangula alnus Mill.	0	Rhamnaceae Juss.	n	1
Iz_7_1009	DJI_0485	Fraxinus pennsylvanica Marshall	0	Oleaceae Hoffmanns. & Link	a	0
Iz_7_1009	DJI_0485	Pinus sylvestris L.	0	Pinaceae Spreng. ex F.Rudolphi	n	1
Iz_7_1009	DJI_0485	Populus × canescens (Aiton) Sm.	0	Salicaceae Mirb.	n	1
Iz_7_1009	DJI_0485	Populus tremula L.	0	Salicaceae Mirb.	n	1
Iz_7_1009	DJI_0485	Populus nigra L.	0	Salicaceae Mirb.	n	1
Iz_7_1009	DJI_0485	Pyrus communis L.	0	Rosaceae Juss.	a	0
Iz_7_1009	DJI_0485	Quercus robur L.	0	Fagaceae Dumort.	n	1
Iz_7_1009	DJI_0485	Robinia pseudoacacia L.	0	Fabaceae Lindl.	a	0
Iz_7_1009	DJI_0485	Rosa sp.	0	Rosaceae Juss.		
Iz_7_1009	DJI_0485	Salix alba L.	0	Salicaceae Mirb.	n	1
Iz_7_1009	DJI_0485	Sambucus nigra L.	0	Viburnaceae Raf.	n	1
Iz_7_1009	DJI_0485	Sorbus aucuparia L.	0	Rosaceae Juss.	n	1
Iz_7_1009	DJI_0485	Tilia cordata Mill.	0	Malvaceae Juss.	n	1
Iz_7_1009	DJI_0485	Ulmus glabra Huds.	0	Ulmaceae Mirb.	n	1
Iz_7_1009	DJI_0489	Acer tataricum L.	0	Sapindaceae Juss.	n	1
Iz_7_1009	DJI_0489	Betula pendula Roth.	0	Betulaceae Gray	n	1
Iz_7_1009	DJI_0489	Crataegus rhipidophylla Gand.	0	Rosaceae Juss.	n	1
Iz_7_1009	DJI_0489	Frangula alnus Mill.	0	Rhamnaceae Juss.	n	1
Iz_7_1009	DJI_0489	Fraxinus pennsylvanica Marshall	0	Oleaceae Hoffmanns. & Link	a	0
Iz_7_1009	DJI_0489	Pinus sylvestris L.	0	Pinaceae Spreng. ex F.Rudolphi	n	1
Iz_7_1009	DJI_0489	Populus × canescens (Aiton) Sm.	0	Salicaceae Mirb.	n	1
Iz_7_1009	DJI_0489	Populus tremula L.	0	Salicaceae Mirb.	n	1
Iz_7_1009	DJI_0489	Populus nigra L.	0	Salicaceae Mirb.	n	1
Iz_7_1009	DJI_0489	Pyrus communis L.	0	Rosaceae Juss.	a	0
Iz_7_1009	DJI_0489	Quercus robur L.	0	Fagaceae Dumort.	n	1
Iz_7_1009	DJI_0489	Robinia pseudoacacia L.	0	Fabaceae Lindl.	a	0
Iz_7_1009	DJI_0489	Rosa sp.	0	Rosaceae Juss.		
Iz_7_1009	DJI_0489	Salix alba L.	0	Salicaceae Mirb.	n	1
Iz_7_1009	DJI_0489	Sambucus nigra L.	0	Viburnaceae Raf.	n	1
Iz_7_1009	DJI_0489	Sorbus aucuparia L.	0	Rosaceae Juss.	n	1
Iz_7_1009	DJI_0489	Tilia cordata Mill.	0	Malvaceae Juss.	n	1
Iz_7_1009	DJI_0489	Ulmus glabra Huds.	0	Ulmaceae Mirb.	n	1
Iz_7_1009	DJI_0497	Acer tataricum L.	0	Sapindaceae Juss.	n	1
Iz_7_1009	DJI_0497	Betula pendula Roth.	0	Betulaceae Gray	n	1
Iz_7_1009	DJI_0497	Crataegus rhipidophylla Gand.	0	Rosaceae Juss.	n	1
Iz_7_1009	DJI_0497	Frangula alnus Mill.	0	Rhamnaceae Juss.	n	1
Iz_7_1009	DJI_0497	Fraxinus pennsylvanica Marshall	0	Oleaceae Hoffmanns. & Link	a	0
Iz_7_1009	DJI_0497	Pinus sylvestris L.	0	Pinaceae Spreng. ex F.Rudolphi	n	1
Iz_7_1009	DJI_0497	Populus × canescens (Aiton) Sm.	0	Salicaceae Mirb.	n	1
Iz_7_1009	DJI_0497	Populus tremula L.	0	Salicaceae Mirb.	n	1
Iz_7_1009	DJI_0497	Populus nigra L.	0	Salicaceae Mirb.	n	1
Iz_7_1009	DJI_0497	Pyrus communis L.	0	Rosaceae Juss.	a	0
Iz_7_1009	DJI_0497	Quercus robur L.	0	Fagaceae Dumort.	n	1
Iz_7_1009	DJI_0497	Robinia pseudoacacia L.	0	Fabaceae Lindl.	a	0
Iz_7_1009	DJI_0497	Rosa sp.	0	Rosaceae Juss.		
Iz_7_1009	DJI_0497	Salix alba L.	0	Salicaceae Mirb.	n	1
Iz_7_1009	DJI_0497	Sambucus nigra L.	0	Viburnaceae Raf.	n	1
Iz_7_1009	DJI_0497	Sorbus aucuparia L.	0	Rosaceae Juss.	n	1
Iz_7_1009	DJI_0497	Tilia cordata Mill.	0	Malvaceae Juss.	n	1
Iz_7_1009	DJI_0497	Ulmus glabra Huds.	0	Ulmaceae Mirb.	n	1

Iz_7_1009	DJI_0499	Acer tataricum L.	0	Sapindaceae Juss.	n	1
Iz_7_1009	DJI_0499	Betula pendula Roth.	0	Betulaceae Gray	n	1
Iz_7_1009	DJI_0499	Crataegus rhipidophylla Gand.	0	Rosaceae Juss.	n	1
Iz_7_1009	DJI_0499	Frangula alnus Mill.	0	Rhamnaceae Juss.	n	1
Iz_7_1009	DJI_0499	Fraxinus pennsylvanica Marshall	0	Oleaceae Hoffmanns. & Link	a	0
Iz_7_1009	DJI_0499	Pinus sylvestris L.	0	Pinaceae Spreng. ex F.Rudolphi	n	1
Iz_7_1009	DJI_0499	Populus × canescens (Aiton) Sm.	0	Salicaceae Mirb.	n	1
Iz_7_1009	DJI_0499	Populus tremula L.	0	Salicaceae Mirb.	n	1
Iz_7_1009	DJI_0499	Populus nigra L.	0	Salicaceae Mirb.	n	1
Iz_7_1009	DJI_0499	Pyrus communis L.	0	Rosaceae Juss.	a	0
Iz_7_1009	DJI_0499	Quercus robur L.	0	Fagaceae Dumort.	n	1
Iz_7_1009	DJI_0499	Robinia pseudoacacia L.	0	Fabaceae Lindl.	a	0
Iz_7_1009	DJI_0499	Rosa sp.	0	Rosaceae Juss.		
Iz_7_1009	DJI_0499	Salix alba L.	0	Salicaceae Mirb.	n	1
Iz_7_1009	DJI_0499	Sambucus nigra L.	0	Viburnaceae Raf.	n	1
Iz_7_1009	DJI_0499	Sorbus aucuparia L.	0	Rosaceae Juss.	n	1
Iz_7_1009	DJI_0499	Tilia cordata Mill.	0	Malvaceae Juss.	n	1
Iz_7_1009	DJI_0499	Ulmus glabra Huds.	0	Ulmaceae Mirb.	n	1
Iz_7_1009	DJI_0507	Acer tataricum L.	0	Sapindaceae Juss.	n	1
Iz_7_1009	DJI_0507	Betula pendula Roth.	0	Betulaceae Gray	n	1
Iz_7_1009	DJI_0507	Crataegus rhipidophylla Gand.	0	Rosaceae Juss.	n	1
Iz_7_1009	DJI_0507	Frangula alnus Mill.	0	Rhamnaceae Juss.	n	1
Iz_7_1009	DJI_0507	Fraxinus pennsylvanica Marshall	0	Oleaceae Hoffmanns. & Link	a	0
Iz_7_1009	DJI_0507	Pinus sylvestris L.	0	Pinaceae Spreng. ex F.Rudolphi	n	1
Iz_7_1009	DJI_0507	Populus × canescens (Aiton) Sm.	0	Salicaceae Mirb.	n	1
Iz_7_1009	DJI_0507	Populus tremula L.	0	Salicaceae Mirb.	n	1
Iz_7_1009	DJI_0507	Populus nigra L.	0	Salicaceae Mirb.	n	1
Iz_7_1009	DJI_0507	Pyrus communis L.	0	Rosaceae Juss.	a	0
Iz_7_1009	DJI_0507	Quercus robur L.	0	Fagaceae Dumort.	n	1
Iz_7_1009	DJI_0507	Robinia pseudoacacia L.	0	Fabaceae Lindl.	a	0
Iz_7_1009	DJI_0507	Rosa sp.	0	Rosaceae Juss.		
Iz_7_1009	DJI_0507	Salix alba L.	0	Salicaceae Mirb.	n	1
Iz_7_1009	DJI_0507	Sambucus nigra L.	0	Viburnaceae Raf.	n	1
Iz_7_1009	DJI_0507	Sorbus aucuparia L.	0	Rosaceae Juss.	n	1
Iz_7_1009	DJI_0507	Tilia cordata Mill.	0	Malvaceae Juss.	n	1
Iz_7_1009	DJI_0507	Ulmus glabra Huds.	0	Ulmaceae Mirb.	n	1
Iz_8_1009	DJI_0894	Acer tataricum L.	0	Sapindaceae Juss.	n	1
Iz_8_1009	DJI_0894	Betula pendula Roth.	0	Betulaceae Gray	n	1
Iz_8_1009	DJI_0894	Crataegus rhipidophylla Gand.	0	Rosaceae Juss.	n	1
Iz_8_1009	DJI_0894	Frangula alnus Mill.	0	Rhamnaceae Juss.	n	1
Iz_8_1009	DJI_0894	Fraxinus pennsylvanica Marshall	0	Oleaceae Hoffmanns. & Link	a	0
Iz_8_1009	DJI_0894	Pinus sylvestris L.	0	Pinaceae Spreng. ex F.Rudolphi	n	1
Iz_8_1009	DJI_0894	Populus × canescens (Aiton) Sm.	0	Salicaceae Mirb.	n	1
Iz_8_1009	DJI_0894	Populus tremula L.	0	Salicaceae Mirb.	n	1
Iz_8_1009	DJI_0894	Populus nigra L.	0	Salicaceae Mirb.	n	1
Iz_8_1009	DJI_0894	Pyrus communis L.	0	Rosaceae Juss.	a	0
Iz_8_1009	DJI_0894	Quercus robur L.	0	Fagaceae Dumort.	n	1
Iz_8_1009	DJI_0894	Robinia pseudoacacia L.	0	Fabaceae Lindl.	a	0
Iz_8_1009	DJI_0894	Rosa sp.	0	Rosaceae Juss.		
Iz_8_1009	DJI_0894	Salix alba L.	0	Salicaceae Mirb.	n	1
Iz_8_1009	DJI_0894	Sambucus nigra L.	0	Viburnaceae Raf.	n	1
Iz_8_1009	DJI_0894	Sorbus aucuparia L.	0	Rosaceae Juss.	n	1
Iz_8_1009	DJI_0894	Tilia cordata Mill.	0	Malvaceae Juss.	n	1
Iz_8_1009	DJI_0894	Ulmus glabra Huds.	0	Ulmaceae Mirb.	n	1
Iz_8_1009	DJI_0895	Acer tataricum L.	0	Sapindaceae Juss.	n	1
Iz_8_1009	DJI_0895	Betula pendula Roth.	0	Betulaceae Gray	n	1
Iz_8_1009	DJI_0895	Crataegus rhipidophylla Gand.	0	Rosaceae Juss.	n	1
Iz_8_1009	DJI_0895	Frangula alnus Mill.	0	Rhamnaceae Juss.	n	1
Iz_8_1009	DJI_0895	Fraxinus pennsylvanica Marshall	0	Oleaceae Hoffmanns. & Link	a	0
Iz_8_1009	DJI_0895	Pinus sylvestris L.	0	Pinaceae Spreng. ex F.Rudolphi	n	1
Iz_8_1009	DJI_0895	Populus × canescens (Aiton) Sm.	0	Salicaceae Mirb.	n	1
Iz_8_1009	DJI_0895	Populus tremula L.	0	Salicaceae Mirb.	n	1
Iz_8_1009	DJI_0895	Populus nigra L.	0	Salicaceae Mirb.	n	1
Iz_8_1009	DJI_0895	Pyrus communis L.	0	Rosaceae Juss.	a	0

Iz_8_1009	DJI_0895	Quercus robur L.	0	Fagaceae Dumort.	n	1
Iz_8_1009	DJI_0895	Robinia pseudoacacia L.	0	Fabaceae Lindl.	a	0
Iz_8_1009	DJI_0895	Rosa sp.	0	Rosaceae Juss.		
Iz_8_1009	DJI_0895	Salix alba L.	0	Salicaceae Mirb.	n	1
Iz_8_1009	DJI_0895	Sambucus nigra L.	0	Viburnaceae Raf.	n	1
Iz_8_1009	DJI_0895	Sorbus aucuparia L.	0	Rosaceae Juss.	n	1
Iz_8_1009	DJI_0895	Tilia cordata Mill.	0	Malvaceae Juss.	n	1
Iz_8_1009	DJI_0895	Ulmus glabra Huds.	0	Ulmaceae Mirb.	n	1
Iz_8_1009	DJI_0896	Acer tataricum L.	0	Sapindaceae Juss.	n	1
Iz_8_1009	DJI_0896	Betula pendula Roth.	0	Betulaceae Gray	n	1
Iz_8_1009	DJI_0896	Crataegus rhipidophylla Gand.	0	Rosaceae Juss.	n	1
Iz_8_1009	DJI_0896	Frangula alnus Mill.	0	Rhamnaceae Juss.	n	1
Iz_8_1009	DJI_0896	Fraxinus pennsylvanica Marshall	0	Oleaceae Hoffmanns. & Link	a	0
Iz_8_1009	DJI_0896	Pinus sylvestris L.	0	Pinaceae Spreng. ex F.Rudolphi	n	1
Iz_8_1009	DJI_0896	Populus × canescens (Aiton) Sm.	0	Salicaceae Mirb.	n	1
Iz_8_1009	DJI_0896	Populus tremula L.	0	Salicaceae Mirb.	n	1
Iz_8_1009	DJI_0896	Populus nigra L.	0	Salicaceae Mirb.	n	1
Iz_8_1009	DJI_0896	Pyrus communis L.	0	Rosaceae Juss.	a	0
Iz_8_1009	DJI_0896	Quercus robur L.	0	Fagaceae Dumort.	n	1
Iz_8_1009	DJI_0896	Robinia pseudoacacia L.	0	Fabaceae Lindl.	a	0
Iz_8_1009	DJI_0896	Rosa sp.	0	Rosaceae Juss.		
Iz_8_1009	DJI_0896	Salix alba L.	0	Salicaceae Mirb.	n	1
Iz_8_1009	DJI_0896	Sambucus nigra L.	0	Viburnaceae Raf.	n	1
Iz_8_1009	DJI_0896	Sorbus aucuparia L.	0	Rosaceae Juss.	n	1
Iz_8_1009	DJI_0896	Tilia cordata Mill.	0	Malvaceae Juss.	n	1
Iz_8_1009	DJI_0896	Ulmus glabra Huds.	0	Ulmaceae Mirb.	n	1
Iz_8_1009	DJI_0897	Acer tataricum L.	0	Sapindaceae Juss.	n	1
Iz_8_1009	DJI_0897	Betula pendula Roth.	0	Betulaceae Gray	n	1
Iz_8_1009	DJI_0897	Crataegus rhipidophylla Gand.	0	Rosaceae Juss.	n	1
Iz_8_1009	DJI_0897	Frangula alnus Mill.	0	Rhamnaceae Juss.	n	1
Iz_8_1009	DJI_0897	Fraxinus pennsylvanica Marshall	0	Oleaceae Hoffmanns. & Link	a	0
Iz_8_1009	DJI_0897	Pinus sylvestris L.	0	Pinaceae Spreng. ex F.Rudolphi	n	1
Iz_8_1009	DJI_0897	Populus × canescens (Aiton) Sm.	0	Salicaceae Mirb.	n	1
Iz_8_1009	DJI_0897	Populus tremula L.	0	Salicaceae Mirb.	n	1
Iz_8_1009	DJI_0897	Populus nigra L.	0	Salicaceae Mirb.	n	1
Iz_8_1009	DJI_0897	Pyrus communis L.	0	Rosaceae Juss.	a	0
Iz_8_1009	DJI_0897	Quercus robur L.	0	Fagaceae Dumort.	n	1
Iz_8_1009	DJI_0897	Robinia pseudoacacia L.	0	Fabaceae Lindl.	a	0
Iz_8_1009	DJI_0897	Rosa sp.	0	Rosaceae Juss.		
Iz_8_1009	DJI_0897	Salix alba L.	0	Salicaceae Mirb.	n	1
Iz_8_1009	DJI_0897	Sambucus nigra L.	0	Viburnaceae Raf.	n	1
Iz_8_1009	DJI_0897	Sorbus aucuparia L.	0	Rosaceae Juss.	n	1
Iz_8_1009	DJI_0897	Tilia cordata Mill.	0	Malvaceae Juss.	n	1
Iz_8_1009	DJI_0897	Ulmus glabra Huds.	0	Ulmaceae Mirb.	n	1
Iz_8_1009	DJI_0898	Acer tataricum L.	0	Sapindaceae Juss.	n	1
Iz_8_1009	DJI_0898	Betula pendula Roth.	0	Betulaceae Gray	n	1
Iz_8_1009	DJI_0898	Crataegus rhipidophylla Gand.	0	Rosaceae Juss.	n	1
Iz_8_1009	DJI_0898	Frangula alnus Mill.	0	Rhamnaceae Juss.	n	1
Iz_8_1009	DJI_0898	Fraxinus pennsylvanica Marshall	0	Oleaceae Hoffmanns. & Link	a	0
Iz_8_1009	DJI_0898	Pinus sylvestris L.	0	Pinaceae Spreng. ex F.Rudolphi	n	1
Iz_8_1009	DJI_0898	Populus × canescens (Aiton) Sm.	0	Salicaceae Mirb.	n	1
Iz_8_1009	DJI_0898	Populus tremula L.	8	Salicaceae Mirb.	n	1
Iz_8_1009	DJI_0898	Populus nigra L.	0	Salicaceae Mirb.	n	1
Iz_8_1009	DJI_0898	Pyrus communis L.	0	Rosaceae Juss.	a	0
Iz_8_1009	DJI_0898	Quercus robur L.	0	Fagaceae Dumort.	n	1
Iz_8_1009	DJI_0898	Robinia pseudoacacia L.	0	Fabaceae Lindl.	a	0
Iz_8_1009	DJI_0898	Rosa sp.	0	Rosaceae Juss.		
Iz_8_1009	DJI_0898	Salix alba L.	0	Salicaceae Mirb.	n	1
Iz_8_1009	DJI_0898	Sambucus nigra L.	0	Viburnaceae Raf.	n	1
Iz_8_1009	DJI_0898	Sorbus aucuparia L.	0	Rosaceae Juss.	n	1
Iz_8_1009	DJI_0898	Tilia cordata Mill.	0	Malvaceae Juss.	n	1
Iz_8_1009	DJI_0898	Ulmus glabra Huds.	0	Ulmaceae Mirb.	n	1
Iz_8_1009	DJI_0899	Acer tataricum L.	0	Sapindaceae Juss.	n	1
Iz_8_1009	DJI_0899	Betula pendula Roth.	0	Betulaceae Gray	n	1

Iz_8_1009	DJI_0899	Crataegus rhipidophylla Gand.	0	Rosaceae Juss.	n	1
Iz_8_1009	DJI_0899	Frangula alnus Mill.	0	Rhamnaceae Juss.	n	1
Iz_8_1009	DJI_0899	Fraxinus pennsylvanica Marshall	0	Oleaceae Hoffmanns. & Link	a	0
Iz_8_1009	DJI_0899	Pinus sylvestris L.	0	Pinaceae Spreng. ex F.Rudolphi	n	1
Iz_8_1009	DJI_0899	Populus × canescens (Aiton) Sm.	0	Salicaceae Mirb.	n	1
Iz_8_1009	DJI_0899	Populus tremula L.	1	Salicaceae Mirb.	n	1
Iz_8_1009	DJI_0899	Populus nigra L.	0	Salicaceae Mirb.	n	1
Iz_8_1009	DJI_0899	Pyrus communis L.	0	Rosaceae Juss.	a	0
Iz_8_1009	DJI_0899	Quercus robur L.	0	Fagaceae Dumort.	n	1
Iz_8_1009	DJI_0899	Robinia pseudoacacia L.	0	Fabaceae Lindl.	a	0
Iz_8_1009	DJI_0899	Rosa sp.	0	Rosaceae Juss.		
Iz_8_1009	DJI_0899	Salix alba L.	0	Salicaceae Mirb.	n	1
Iz_8_1009	DJI_0899	Sambucus nigra L.	0	Viburnaceae Raf.	n	1
Iz_8_1009	DJI_0899	Sorbus aucuparia L.	0	Rosaceae Juss.	n	1
Iz_8_1009	DJI_0899	Tilia cordata Mill.	0	Malvaceae Juss.	n	1
Iz_8_1009	DJI_0899	Ulmus glabra Huds.	0	Ulmaceae Mirb.	n	1
Iz_8_1009	DJI_0904	Acer tataricum L.	0	Sapindaceae Juss.	n	1
Iz_8_1009	DJI_0904	Betula pendula Roth.	0	Betulaceae Gray	n	1
Iz_8_1009	DJI_0904	Crataegus rhipidophylla Gand.	0	Rosaceae Juss.	n	1
Iz_8_1009	DJI_0904	Frangula alnus Mill.	0	Rhamnaceae Juss.	n	1
Iz_8_1009	DJI_0904	Fraxinus pennsylvanica Marshall	0	Oleaceae Hoffmanns. & Link	a	0
Iz_8_1009	DJI_0904	Pinus sylvestris L.	0	Pinaceae Spreng. ex F.Rudolphi	n	1
Iz_8_1009	DJI_0904	Populus × canescens (Aiton) Sm.	0	Salicaceae Mirb.	n	1
Iz_8_1009	DJI_0904	Populus tremula L.	0	Salicaceae Mirb.	n	1
Iz_8_1009	DJI_0904	Populus nigra L.	0	Salicaceae Mirb.	n	1
Iz_8_1009	DJI_0904	Pyrus communis L.	0	Rosaceae Juss.	a	0
Iz_8_1009	DJI_0904	Quercus robur L.	0	Fagaceae Dumort.	n	1
Iz_8_1009	DJI_0904	Robinia pseudoacacia L.	0	Fabaceae Lindl.	a	0
Iz_8_1009	DJI_0904	Rosa sp.	0	Rosaceae Juss.		
Iz_8_1009	DJI_0904	Salix alba L.	0	Salicaceae Mirb.	n	1
Iz_8_1009	DJI_0904	Sambucus nigra L.	0	Viburnaceae Raf.	n	1
Iz_8_1009	DJI_0904	Sorbus aucuparia L.	0	Rosaceae Juss.	n	1
Iz_8_1009	DJI_0904	Tilia cordata Mill.	0	Malvaceae Juss.	n	1
Iz_8_1009	DJI_0904	Ulmus glabra Huds.	0	Ulmaceae Mirb.	n	1
Iz_8_1009	DJI_0906	Acer tataricum L.	0	Sapindaceae Juss.	n	1
Iz_8_1009	DJI_0906	Betula pendula Roth.	0	Betulaceae Gray	n	1
Iz_8_1009	DJI_0906	Crataegus rhipidophylla Gand.	0	Rosaceae Juss.	n	1
Iz_8_1009	DJI_0906	Frangula alnus Mill.	0	Rhamnaceae Juss.	n	1
Iz_8_1009	DJI_0906	Fraxinus pennsylvanica Marshall	0	Oleaceae Hoffmanns. & Link	a	0
Iz_8_1009	DJI_0906	Pinus sylvestris L.	0	Pinaceae Spreng. ex F.Rudolphi	n	1
Iz_8_1009	DJI_0906	Populus × canescens (Aiton) Sm.	0	Salicaceae Mirb.	n	1
Iz_8_1009	DJI_0906	Populus tremula L.	0	Salicaceae Mirb.	n	1
Iz_8_1009	DJI_0906	Populus nigra L.	0	Salicaceae Mirb.	n	1
Iz_8_1009	DJI_0906	Pyrus communis L.	0	Rosaceae Juss.	a	0
Iz_8_1009	DJI_0906	Quercus robur L.	0	Fagaceae Dumort.	n	1
Iz_8_1009	DJI_0906	Robinia pseudoacacia L.	0	Fabaceae Lindl.	a	0
Iz_8_1009	DJI_0906	Rosa sp.	0	Rosaceae Juss.		
Iz_8_1009	DJI_0906	Salix alba L.	0	Salicaceae Mirb.	n	1
Iz_8_1009	DJI_0906	Sambucus nigra L.	0	Viburnaceae Raf.	n	1
Iz_8_1009	DJI_0906	Sorbus aucuparia L.	0	Rosaceae Juss.	n	1
Iz_8_1009	DJI_0906	Tilia cordata Mill.	0	Malvaceae Juss.	n	1
Iz_8_1009	DJI_0906	Ulmus glabra Huds.	0	Ulmaceae Mirb.	n	1
Iz_8_1009	DJI_0908	Acer tataricum L.	0	Sapindaceae Juss.	n	1
Iz_8_1009	DJI_0908	Betula pendula Roth.	0	Betulaceae Gray	n	1
Iz_8_1009	DJI_0908	Crataegus rhipidophylla Gand.	0	Rosaceae Juss.	n	1
Iz_8_1009	DJI_0908	Frangula alnus Mill.	0	Rhamnaceae Juss.	n	1
Iz_8_1009	DJI_0908	Fraxinus pennsylvanica Marshall	0	Oleaceae Hoffmanns. & Link	a	0
Iz_8_1009	DJI_0908	Pinus sylvestris L.	0	Pinaceae Spreng. ex F.Rudolphi	n	1
Iz_8_1009	DJI_0908	Populus × canescens (Aiton) Sm.	0	Salicaceae Mirb.	n	1
Iz_8_1009	DJI_0908	Populus tremula L.	0	Salicaceae Mirb.	n	1
Iz_8_1009	DJI_0908	Populus nigra L.	0	Salicaceae Mirb.	n	1
Iz_8_1009	DJI_0908	Pyrus communis L.	0	Rosaceae Juss.	a	0
Iz_8_1009	DJI_0908	Quercus robur L.	0	Fagaceae Dumort.	n	1
Iz_8_1009	DJI_0908	Robinia pseudoacacia L.	0	Fabaceae Lindl.	a	0

Iz_8_1009	DJI_0908	Rosa sp.	0	Rosaceae Juss.		
Iz_8_1009	DJI_0908	Salix alba L.	0	Salicaceae Mirb.	n	1
Iz_8_1009	DJI_0908	Sambucus nigra L.	0	Viburnaceae Raf.	n	1
Iz_8_1009	DJI_0908	Sorbus aucuparia L.	0	Rosaceae Juss.	n	1
Iz_8_1009	DJI_0908	Tilia cordata Mill.	0	Malvaceae Juss.	n	1
Iz_8_1009	DJI_0908	Ulmus glabra Huds.	0	Ulmaceae Mirb.	n	1
Iz_8_1009	DJI_0911	Acer tataricum L.	0	Sapindaceae Juss.	n	1
Iz_8_1009	DJI_0911	Betula pendula Roth.	0	Betulaceae Gray	n	1
Iz_8_1009	DJI_0911	Crataegus rhipidophylla Gand.	0	Rosaceae Juss.	n	1
Iz_8_1009	DJI_0911	Frangula alnus Mill.	0	Rhamnaceae Juss.	n	1
Iz_8_1009	DJI_0911	Fraxinus pennsylvanica Marshall	0	Oleaceae Hoffmanns. & Link	a	0
Iz_8_1009	DJI_0911	Pinus sylvestris L.	0	Pinaceae Spreng. ex F.Rudolphi	n	1
Iz_8_1009	DJI_0911	Populus × canescens (Aiton) Sm.	0	Salicaceae Mirb.	n	1
Iz_8_1009	DJI_0911	Populus tremula L.	0	Salicaceae Mirb.	n	1
Iz_8_1009	DJI_0911	Populus nigra L.	0	Salicaceae Mirb.	n	1
Iz_8_1009	DJI_0911	Pyrus communis L.	0	Rosaceae Juss.	a	0
Iz_8_1009	DJI_0911	Quercus robur L.	0	Fagaceae Dumort.	n	1
Iz_8_1009	DJI_0911	Robinia pseudoacacia L.	0	Fabaceae Lindl.	a	0
Iz_8_1009	DJI_0911	Rosa sp.	0	Rosaceae Juss.		
Iz_8_1009	DJI_0911	Salix alba L.	0	Salicaceae Mirb.	n	1
Iz_8_1009	DJI_0911	Sambucus nigra L.	0	Viburnaceae Raf.	n	1
Iz_8_1009	DJI_0911	Sorbus aucuparia L.	0	Rosaceae Juss.	n	1
Iz_8_1009	DJI_0911	Tilia cordata Mill.	0	Malvaceae Juss.	n	1
Iz_8_1009	DJI_0911	Ulmus glabra Huds.	0	Ulmaceae Mirb.	n	1
Iz_8_1009	DJI_0912	Acer tataricum L.	0	Sapindaceae Juss.	n	1
Iz_8_1009	DJI_0912	Betula pendula Roth.	0	Betulaceae Gray	n	1
Iz_8_1009	DJI_0912	Crataegus rhipidophylla Gand.	0	Rosaceae Juss.	n	1
Iz_8_1009	DJI_0912	Frangula alnus Mill.	0	Rhamnaceae Juss.	n	1
Iz_8_1009	DJI_0912	Fraxinus pennsylvanica Marshall	0	Oleaceae Hoffmanns. & Link	a	0
Iz_8_1009	DJI_0912	Pinus sylvestris L.	0	Pinaceae Spreng. ex F.Rudolphi	n	1
Iz_8_1009	DJI_0912	Populus × canescens (Aiton) Sm.	0	Salicaceae Mirb.	n	1
Iz_8_1009	DJI_0912	Populus tremula L.	0	Salicaceae Mirb.	n	1
Iz_8_1009	DJI_0912	Populus nigra L.	0	Salicaceae Mirb.	n	1
Iz_8_1009	DJI_0912	Pyrus communis L.	0	Rosaceae Juss.	a	0
Iz_8_1009	DJI_0912	Quercus robur L.	0	Fagaceae Dumort.	n	1
Iz_8_1009	DJI_0912	Robinia pseudoacacia L.	0	Fabaceae Lindl.	a	0
Iz_8_1009	DJI_0912	Rosa sp.	0	Rosaceae Juss.		
Iz_8_1009	DJI_0912	Salix alba L.	0	Salicaceae Mirb.	n	1
Iz_8_1009	DJI_0912	Sambucus nigra L.	0	Viburnaceae Raf.	n	1
Iz_8_1009	DJI_0912	Sorbus aucuparia L.	0	Rosaceae Juss.	n	1
Iz_8_1009	DJI_0912	Tilia cordata Mill.	0	Malvaceae Juss.	n	1
Iz_8_1009	DJI_0912	Ulmus glabra Huds.	0	Ulmaceae Mirb.	n	1
Iz_8_1009	DJI_0913	Acer tataricum L.	0	Sapindaceae Juss.	n	1
Iz_8_1009	DJI_0913	Betula pendula Roth.	0	Betulaceae Gray	n	1
Iz_8_1009	DJI_0913	Crataegus rhipidophylla Gand.	0	Rosaceae Juss.	n	1
Iz_8_1009	DJI_0913	Frangula alnus Mill.	0	Rhamnaceae Juss.	n	1
Iz_8_1009	DJI_0913	Fraxinus pennsylvanica Marshall	0	Oleaceae Hoffmanns. & Link	a	0
Iz_8_1009	DJI_0913	Pinus sylvestris L.	0	Pinaceae Spreng. ex F.Rudolphi	n	1
Iz_8_1009	DJI_0913	Populus × canescens (Aiton) Sm.	0	Salicaceae Mirb.	n	1
Iz_8_1009	DJI_0913	Populus tremula L.	0	Salicaceae Mirb.	n	1
Iz_8_1009	DJI_0913	Populus nigra L.	0	Salicaceae Mirb.	n	1
Iz_8_1009	DJI_0913	Pyrus communis L.	0	Rosaceae Juss.	a	0
Iz_8_1009	DJI_0913	Quercus robur L.	0	Fagaceae Dumort.	n	1
Iz_8_1009	DJI_0913	Robinia pseudoacacia L.	0	Fabaceae Lindl.	a	0
Iz_8_1009	DJI_0913	Rosa sp.	0	Rosaceae Juss.		
Iz_8_1009	DJI_0913	Salix alba L.	0	Salicaceae Mirb.	n	1
Iz_8_1009	DJI_0913	Sambucus nigra L.	0	Viburnaceae Raf.	n	1
Iz_8_1009	DJI_0913	Sorbus aucuparia L.	0	Rosaceae Juss.	n	1
Iz_8_1009	DJI_0913	Tilia cordata Mill.	0	Malvaceae Juss.	n	1
Iz_8_1009	DJI_0913	Ulmus glabra Huds.	0	Ulmaceae Mirb.	n	1
Iz_8_1009	DJI_0914	Acer tataricum L.	0	Sapindaceae Juss.	n	1
Iz_8_1009	DJI_0914	Betula pendula Roth.	0	Betulaceae Gray	n	1
Iz_8_1009	DJI_0914	Crataegus rhipidophylla Gand.	0	Rosaceae Juss.	n	1
Iz_8_1009	DJI_0914	Frangula alnus Mill.	0	Rhamnaceae Juss.	n	1

Iz_8_1009	DJI_0914	Fraxinus pennsylvanica Marshall	0	Oleaceae Hoffmanns. & Link	a	0
Iz_8_1009	DJI_0914	Pinus sylvestris L.	0	Pinaceae Spreng. ex F.Rudolphi	n	1
Iz_8_1009	DJI_0914	Populus × canescens (Aiton) Sm.	0	Salicaceae Mirb.	n	1
Iz_8_1009	DJI_0914	Populus tremula L.	0	Salicaceae Mirb.	n	1
Iz_8_1009	DJI_0914	Populus nigra L.	0	Salicaceae Mirb.	n	1
Iz_8_1009	DJI_0914	Pyrus communis L.	0	Rosaceae Juss.	a	0
Iz_8_1009	DJI_0914	Quercus robur L.	0	Fagaceae Dumort.	n	1
Iz_8_1009	DJI_0914	Robinia pseudoacacia L.	0	Fabaceae Lindl.	a	0
Iz_8_1009	DJI_0914	Rosa sp.	0	Rosaceae Juss.		
Iz_8_1009	DJI_0914	Salix alba L.	0	Salicaceae Mirb.	n	1
Iz_8_1009	DJI_0914	Sambucus nigra L.	0	Viburnaceae Raf.	n	1
Iz_8_1009	DJI_0914	Sorbus aucuparia L.	0	Rosaceae Juss.	n	1
Iz_8_1009	DJI_0914	Tilia cordata Mill.	0	Malvaceae Juss.	n	1
Iz_8_1009	DJI_0914	Ulmus glabra Huds.	0	Ulmaceae Mirb.	n	1
Iz_8_1009	DJI_0916	Acer tataricum L.	0	Sapindaceae Juss.	n	1
Iz_8_1009	DJI_0916	Betula pendula Roth.	0	Betulaceae Gray	n	1
Iz_8_1009	DJI_0916	Crataegus rhipidophylla Gand.	0	Rosaceae Juss.	n	1
Iz_8_1009	DJI_0916	Frangula alnus Mill.	0	Rhamnaceae Juss.	n	1
Iz_8_1009	DJI_0916	Fraxinus pennsylvanica Marshall	0	Oleaceae Hoffmanns. & Link	a	0
Iz_8_1009	DJI_0916	Pinus sylvestris L.	0	Pinaceae Spreng. ex F.Rudolphi	n	1
Iz_8_1009	DJI_0916	Populus × canescens (Aiton) Sm.	0	Salicaceae Mirb.	n	1
Iz_8_1009	DJI_0916	Populus tremula L.	0	Salicaceae Mirb.	n	1
Iz_8_1009	DJI_0916	Populus nigra L.	0	Salicaceae Mirb.	n	1
Iz_8_1009	DJI_0916	Pyrus communis L.	0	Rosaceae Juss.	a	0
Iz_8_1009	DJI_0916	Quercus robur L.	0	Fagaceae Dumort.	n	1
Iz_8_1009	DJI_0916	Robinia pseudoacacia L.	0	Fabaceae Lindl.	a	0
Iz_8_1009	DJI_0916	Rosa sp.	0	Rosaceae Juss.		
Iz_8_1009	DJI_0916	Salix alba L.	0	Salicaceae Mirb.	n	1
Iz_8_1009	DJI_0916	Sambucus nigra L.	0	Viburnaceae Raf.	n	1
Iz_8_1009	DJI_0916	Sorbus aucuparia L.	0	Rosaceae Juss.	n	1
Iz_8_1009	DJI_0916	Tilia cordata Mill.	0	Malvaceae Juss.	n	1
Iz_8_1009	DJI_0916	Ulmus glabra Huds.	0	Ulmaceae Mirb.	n	1
Iz_8_1009	DJI_0918	Acer tataricum L.	0	Sapindaceae Juss.	n	1
Iz_8_1009	DJI_0918	Betula pendula Roth.	0	Betulaceae Gray	n	1
Iz_8_1009	DJI_0918	Crataegus rhipidophylla Gand.	0	Rosaceae Juss.	n	1
Iz_8_1009	DJI_0918	Frangula alnus Mill.	0	Rhamnaceae Juss.	n	1
Iz_8_1009	DJI_0918	Fraxinus pennsylvanica Marshall	0	Oleaceae Hoffmanns. & Link	a	0
Iz_8_1009	DJI_0918	Pinus sylvestris L.	0	Pinaceae Spreng. ex F.Rudolphi	n	1
Iz_8_1009	DJI_0918	Populus × canescens (Aiton) Sm.	0	Salicaceae Mirb.	n	1
Iz_8_1009	DJI_0918	Populus tremula L.	0	Salicaceae Mirb.	n	1
Iz_8_1009	DJI_0918	Populus nigra L.	0	Salicaceae Mirb.	n	1
Iz_8_1009	DJI_0918	Pyrus communis L.	0	Rosaceae Juss.	a	0
Iz_8_1009	DJI_0918	Quercus robur L.	0	Fagaceae Dumort.	n	1
Iz_8_1009	DJI_0918	Robinia pseudoacacia L.	0	Fabaceae Lindl.	a	0
Iz_8_1009	DJI_0918	Rosa sp.	0	Rosaceae Juss.		
Iz_8_1009	DJI_0918	Salix alba L.	0	Salicaceae Mirb.	n	1
Iz_8_1009	DJI_0918	Sambucus nigra L.	0	Viburnaceae Raf.	n	1
Iz_8_1009	DJI_0918	Sorbus aucuparia L.	0	Rosaceae Juss.	n	1
Iz_8_1009	DJI_0918	Tilia cordata Mill.	0	Malvaceae Juss.	n	1
Iz_8_1009	DJI_0918	Ulmus glabra Huds.	0	Ulmaceae Mirb.	n	1
Iz_8_1009	DJI_0919	Acer tataricum L.	0	Sapindaceae Juss.	n	1
Iz_8_1009	DJI_0919	Betula pendula Roth.	0	Betulaceae Gray	n	1
Iz_8_1009	DJI_0919	Crataegus rhipidophylla Gand.	0	Rosaceae Juss.	n	1
Iz_8_1009	DJI_0919	Frangula alnus Mill.	0	Rhamnaceae Juss.	n	1
Iz_8_1009	DJI_0919	Fraxinus pennsylvanica Marshall	0	Oleaceae Hoffmanns. & Link	a	0
Iz_8_1009	DJI_0919	Pinus sylvestris L.	0	Pinaceae Spreng. ex F.Rudolphi	n	1
Iz_8_1009	DJI_0919	Populus × canescens (Aiton) Sm.	0	Salicaceae Mirb.	n	1
Iz_8_1009	DJI_0919	Populus tremula L.	0	Salicaceae Mirb.	n	1
Iz_8_1009	DJI_0919	Populus nigra L.	0	Salicaceae Mirb.	n	1
Iz_8_1009	DJI_0919	Pyrus communis L.	0	Rosaceae Juss.	a	0
Iz_8_1009	DJI_0919	Quercus robur L.	0	Fagaceae Dumort.	n	1
Iz_8_1009	DJI_0919	Robinia pseudoacacia L.	0	Fabaceae Lindl.	a	0
Iz_8_1009	DJI_0919	Rosa sp.	0	Rosaceae Juss.		
Iz_8_1009	DJI_0919	Salix alba L.	0	Salicaceae Mirb.	n	1

Iz_8_1009	DJI_0919	Sambucus nigra L.	0	Viburnaceae Raf.	n	1
Iz_8_1009	DJI_0919	Sorbus aucuparia L.	0	Rosaceae Juss.	n	1
Iz_8_1009	DJI_0919	Tilia cordata Mill.	0	Malvaceae Juss.	n	1
Iz_8_1009	DJI_0919	Ulmus glabra Huds.	0	Ulmaceae Mirb.	n	1
Iz_8_1009	DJI_0922	Acer tataricum L.	0	Sapindaceae Juss.	n	1
Iz_8_1009	DJI_0922	Betula pendula Roth.	0	Betulaceae Gray	n	1
Iz_8_1009	DJI_0922	Crataegus rhipidophylla Gand.	0	Rosaceae Juss.	n	1
Iz_8_1009	DJI_0922	Frangula alnus Mill.	0	Rhamnaceae Juss.	n	1
Iz_8_1009	DJI_0922	Fraxinus pennsylvanica Marshall	0	Oleaceae Hoffmanns. & Link	a	0
Iz_8_1009	DJI_0922	Pinus sylvestris L.	0	Pinaceae Spreng. ex F.Rudolphi	n	1
Iz_8_1009	DJI_0922	Populus × canescens (Aiton) Sm.	0	Salicaceae Mirb.	n	1
Iz_8_1009	DJI_0922	Populus tremula L.	0	Salicaceae Mirb.	n	1
Iz_8_1009	DJI_0922	Populus nigra L.	0	Salicaceae Mirb.	n	1
Iz_8_1009	DJI_0922	Pyrus communis L.	0	Rosaceae Juss.	a	0
Iz_8_1009	DJI_0922	Quercus robur L.	0	Fagaceae Dumort.	n	1
Iz_8_1009	DJI_0922	Robinia pseudoacacia L.	0	Fabaceae Lindl.	a	0
Iz_8_1009	DJI_0922	Rosa sp.	0	Rosaceae Juss.		
Iz_8_1009	DJI_0922	Salix alba L.	0	Salicaceae Mirb.	n	1
Iz_8_1009	DJI_0922	Sambucus nigra L.	0	Viburnaceae Raf.	n	1
Iz_8_1009	DJI_0922	Sorbus aucuparia L.	0	Rosaceae Juss.	n	1
Iz_8_1009	DJI_0922	Tilia cordata Mill.	0	Malvaceae Juss.	n	1
Iz_8_1009	DJI_0922	Ulmus glabra Huds.	0	Ulmaceae Mirb.	n	1
Iz_8_1009	DJI_0924	Acer tataricum L.	0	Sapindaceae Juss.	n	1
Iz_8_1009	DJI_0924	Betula pendula Roth.	0	Betulaceae Gray	n	1
Iz_8_1009	DJI_0924	Crataegus rhipidophylla Gand.	0	Rosaceae Juss.	n	1
Iz_8_1009	DJI_0924	Frangula alnus Mill.	0	Rhamnaceae Juss.	n	1
Iz_8_1009	DJI_0924	Fraxinus pennsylvanica Marshall	0	Oleaceae Hoffmanns. & Link	a	0
Iz_8_1009	DJI_0924	Pinus sylvestris L.	0	Pinaceae Spreng. ex F.Rudolphi	n	1
Iz_8_1009	DJI_0924	Populus × canescens (Aiton) Sm.	0	Salicaceae Mirb.	n	1
Iz_8_1009	DJI_0924	Populus tremula L.	0	Salicaceae Mirb.	n	1
Iz_8_1009	DJI_0924	Populus nigra L.	0	Salicaceae Mirb.	n	1
Iz_8_1009	DJI_0924	Pyrus communis L.	0	Rosaceae Juss.	a	0
Iz_8_1009	DJI_0924	Quercus robur L.	0	Fagaceae Dumort.	n	1
Iz_8_1009	DJI_0924	Robinia pseudoacacia L.	0	Fabaceae Lindl.	a	0
Iz_8_1009	DJI_0924	Rosa sp.	0	Rosaceae Juss.		
Iz_8_1009	DJI_0924	Salix alba L.	0	Salicaceae Mirb.	n	1
Iz_8_1009	DJI_0924	Sambucus nigra L.	0	Viburnaceae Raf.	n	1
Iz_8_1009	DJI_0924	Sorbus aucuparia L.	0	Rosaceae Juss.	n	1
Iz_8_1009	DJI_0924	Tilia cordata Mill.	0	Malvaceae Juss.	n	1
Iz_8_1009	DJI_0924	Ulmus glabra Huds.	0	Ulmaceae Mirb.	n	1
Iz_8_1009	DJI_0925	Acer tataricum L.	0	Sapindaceae Juss.	n	1
Iz_8_1009	DJI_0925	Betula pendula Roth.	0	Betulaceae Gray	n	1
Iz_8_1009	DJI_0925	Crataegus rhipidophylla Gand.	0	Rosaceae Juss.	n	1
Iz_8_1009	DJI_0925	Frangula alnus Mill.	0	Rhamnaceae Juss.	n	1
Iz_8_1009	DJI_0925	Fraxinus pennsylvanica Marshall	0	Oleaceae Hoffmanns. & Link	a	0
Iz_8_1009	DJI_0925	Pinus sylvestris L.	0	Pinaceae Spreng. ex F.Rudolphi	n	1
Iz_8_1009	DJI_0925	Populus × canescens (Aiton) Sm.	0	Salicaceae Mirb.	n	1
Iz_8_1009	DJI_0925	Populus tremula L.	0	Salicaceae Mirb.	n	1
Iz_8_1009	DJI_0925	Populus nigra L.	0	Salicaceae Mirb.	n	1
Iz_8_1009	DJI_0925	Pyrus communis L.	0	Rosaceae Juss.	a	0
Iz_8_1009	DJI_0925	Quercus robur L.	0	Fagaceae Dumort.	n	1
Iz_8_1009	DJI_0925	Robinia pseudoacacia L.	0	Fabaceae Lindl.	a	0
Iz_8_1009	DJI_0925	Rosa sp.	0	Rosaceae Juss.		
Iz_8_1009	DJI_0925	Salix alba L.	0	Salicaceae Mirb.	n	1
Iz_8_1009	DJI_0925	Sambucus nigra L.	0	Viburnaceae Raf.	n	1
Iz_8_1009	DJI_0925	Sorbus aucuparia L.	0	Rosaceae Juss.	n	1
Iz_8_1009	DJI_0925	Tilia cordata Mill.	0	Malvaceae Juss.	n	1
Iz_8_1009	DJI_0925	Ulmus glabra Huds.	0	Ulmaceae Mirb.	n	1
Iz_8_1009	DJI_0927	Acer tataricum L.	0	Sapindaceae Juss.	n	1
Iz_8_1009	DJI_0927	Betula pendula Roth.	0	Betulaceae Gray	n	1
Iz_8_1009	DJI_0927	Crataegus rhipidophylla Gand.	0	Rosaceae Juss.	n	1
Iz_8_1009	DJI_0927	Frangula alnus Mill.	0	Rhamnaceae Juss.	n	1
Iz_8_1009	DJI_0927	Fraxinus pennsylvanica Marshall	0	Oleaceae Hoffmanns. & Link	a	0
Iz_8_1009	DJI_0927	Pinus sylvestris L.	0	Pinaceae Spreng. ex F.Rudolphi	n	1

Iz_8_1009	DJI_0927	Populus × canescens (Aiton) Sm.	0	Salicaceae Mirb.	n	1
Iz_8_1009	DJI_0927	Populus tremula L.	0	Salicaceae Mirb.	n	1
Iz_8_1009	DJI_0927	Populus nigra L.	0	Salicaceae Mirb.	n	1
Iz_8_1009	DJI_0927	Pyrus communis L.	0	Rosaceae Juss.	a	0
Iz_8_1009	DJI_0927	Quercus robur L.	0	Fagaceae Dumort.	n	1
Iz_8_1009	DJI_0927	Robinia pseudoacacia L.	0	Fabaceae Lindl.	a	0
Iz_8_1009	DJI_0927	Rosa sp.	0	Rosaceae Juss.		
Iz_8_1009	DJI_0927	Salix alba L.	0	Salicaceae Mirb.	n	1
Iz_8_1009	DJI_0927	Sambucus nigra L.	0	Viburnaceae Raf.	n	1
Iz_8_1009	DJI_0927	Sorbus aucuparia L.	0	Rosaceae Juss.	n	1
Iz_8_1009	DJI_0927	Tilia cordata Mill.	0	Malvaceae Juss.	n	1
Iz_8_1009	DJI_0927	Ulmus glabra Huds.	0	Ulmaceae Mirb.	n	1
Iz_8_1009	DJI_0930	Acer tataricum L.	0	Sapindaceae Juss.	n	1
Iz_8_1009	DJI_0930	Betula pendula Roth.	0	Betulaceae Gray	n	1
Iz_8_1009	DJI_0930	Crataegus rhipidophylla Gand.	0	Rosaceae Juss.	n	1
Iz_8_1009	DJI_0930	Frangula alnus Mill.	0	Rhamnaceae Juss.	n	1
Iz_8_1009	DJI_0930	Fraxinus pennsylvanica Marshall	0	Oleaceae Hoffmanns. & Link	a	0
Iz_8_1009	DJI_0930	Pinus sylvestris L.	0	Pinaceae Spreng. ex F.Rudolphi	n	1
Iz_8_1009	DJI_0930	Populus × canescens (Aiton) Sm.	0	Salicaceae Mirb.	n	1
Iz_8_1009	DJI_0930	Populus tremula L.	0	Salicaceae Mirb.	n	1
Iz_8_1009	DJI_0930	Populus nigra L.	0	Salicaceae Mirb.	n	1
Iz_8_1009	DJI_0930	Pyrus communis L.	0	Rosaceae Juss.	a	0
Iz_8_1009	DJI_0930	Quercus robur L.	0	Fagaceae Dumort.	n	1
Iz_8_1009	DJI_0930	Robinia pseudoacacia L.	0	Fabaceae Lindl.	a	0
Iz_8_1009	DJI_0930	Rosa sp.	0	Rosaceae Juss.		
Iz_8_1009	DJI_0930	Salix alba L.	0	Salicaceae Mirb.	n	1
Iz_8_1009	DJI_0930	Sambucus nigra L.	0	Viburnaceae Raf.	n	1
Iz_8_1009	DJI_0930	Sorbus aucuparia L.	0	Rosaceae Juss.	n	1
Iz_8_1009	DJI_0930	Tilia cordata Mill.	0	Malvaceae Juss.	n	1
Iz_8_1009	DJI_0930	Ulmus glabra Huds.	0	Ulmaceae Mirb.	n	1
Iz_8_1009	DJI_0931	Acer tataricum L.	0	Sapindaceae Juss.	n	1
Iz_8_1009	DJI_0931	Betula pendula Roth.	0	Betulaceae Gray	n	1
Iz_8_1009	DJI_0931	Crataegus rhipidophylla Gand.	0	Rosaceae Juss.	n	1
Iz_8_1009	DJI_0931	Frangula alnus Mill.	0	Rhamnaceae Juss.	n	1
Iz_8_1009	DJI_0931	Fraxinus pennsylvanica Marshall	0	Oleaceae Hoffmanns. & Link	a	0
Iz_8_1009	DJI_0931	Pinus sylvestris L.	0	Pinaceae Spreng. ex F.Rudolphi	n	1
Iz_8_1009	DJI_0931	Populus × canescens (Aiton) Sm.	0	Salicaceae Mirb.	n	1
Iz_8_1009	DJI_0931	Populus tremula L.	0	Salicaceae Mirb.	n	1
Iz_8_1009	DJI_0931	Populus nigra L.	0	Salicaceae Mirb.	n	1
Iz_8_1009	DJI_0931	Pyrus communis L.	0	Rosaceae Juss.	a	0
Iz_8_1009	DJI_0931	Quercus robur L.	0	Fagaceae Dumort.	n	1
Iz_8_1009	DJI_0931	Robinia pseudoacacia L.	0	Fabaceae Lindl.	a	0
Iz_8_1009	DJI_0931	Rosa sp.	0	Rosaceae Juss.		
Iz_8_1009	DJI_0931	Salix alba L.	0	Salicaceae Mirb.	n	1
Iz_8_1009	DJI_0931	Sambucus nigra L.	0	Viburnaceae Raf.	n	1
Iz_8_1009	DJI_0931	Sorbus aucuparia L.	0	Rosaceae Juss.	n	1
Iz_8_1009	DJI_0931	Tilia cordata Mill.	0	Malvaceae Juss.	n	1
Iz_8_1009	DJI_0931	Ulmus glabra Huds.	0	Ulmaceae Mirb.	n	1
Iz_8_1009	DJI_0932	Acer tataricum L.	0	Sapindaceae Juss.	n	1
Iz_8_1009	DJI_0932	Betula pendula Roth.	0	Betulaceae Gray	n	1
Iz_8_1009	DJI_0932	Crataegus rhipidophylla Gand.	0	Rosaceae Juss.	n	1
Iz_8_1009	DJI_0932	Frangula alnus Mill.	0	Rhamnaceae Juss.	n	1
Iz_8_1009	DJI_0932	Fraxinus pennsylvanica Marshall	0	Oleaceae Hoffmanns. & Link	a	0
Iz_8_1009	DJI_0932	Pinus sylvestris L.	0	Pinaceae Spreng. ex F.Rudolphi	n	1
Iz_8_1009	DJI_0932	Populus × canescens (Aiton) Sm.	0	Salicaceae Mirb.	n	1
Iz_8_1009	DJI_0932	Populus tremula L.	0	Salicaceae Mirb.	n	1
Iz_8_1009	DJI_0932	Populus nigra L.	0	Salicaceae Mirb.	n	1
Iz_8_1009	DJI_0932	Pyrus communis L.	0	Rosaceae Juss.	a	0
Iz_8_1009	DJI_0932	Quercus robur L.	0	Fagaceae Dumort.	n	1
Iz_8_1009	DJI_0932	Robinia pseudoacacia L.	0	Fabaceae Lindl.	a	0
Iz_8_1009	DJI_0932	Rosa sp.	0	Rosaceae Juss.		
Iz_8_1009	DJI_0932	Salix alba L.	0	Salicaceae Mirb.	n	1
Iz_8_1009	DJI_0932	Sambucus nigra L.	0	Viburnaceae Raf.	n	1
Iz_8_1009	DJI_0932	Sorbus aucuparia L.	0	Rosaceae Juss.	n	1

Iz_8_1009	DJI_0932	Tilia cordata Mill.	0	Malvaceae Juss.	n	1
Iz_8_1009	DJI_0932	Ulmus glabra Huds.	0	Ulmaceae Mirb.	n	1
Iz_8_1009	DJI_0933	Acer tataricum L.	0	Sapindaceae Juss.	n	1
Iz_8_1009	DJI_0933	Betula pendula Roth.	0	Betulaceae Gray	n	1
Iz_8_1009	DJI_0933	Crataegus rhipidophylla Gand.	0	Rosaceae Juss.	n	1
Iz_8_1009	DJI_0933	Frangula alnus Mill.	0	Rhamnaceae Juss.	n	1
Iz_8_1009	DJI_0933	Fraxinus pennsylvanica Marshall	0	Oleaceae Hoffmanns. & Link	a	0
Iz_8_1009	DJI_0933	Pinus sylvestris L.	0	Pinaceae Spreng. ex F.Rudolphi	n	1
Iz_8_1009	DJI_0933	Populus × canescens (Aiton) Sm.	0	Salicaceae Mirb.	n	1
Iz_8_1009	DJI_0933	Populus tremula L.	0	Salicaceae Mirb.	n	1
Iz_8_1009	DJI_0933	Populus nigra L.	0	Salicaceae Mirb.	n	1
Iz_8_1009	DJI_0933	Pyrus communis L.	0	Rosaceae Juss.	a	0
Iz_8_1009	DJI_0933	Quercus robur L.	0	Fagaceae Dumort.	n	1
Iz_8_1009	DJI_0933	Robinia pseudoacacia L.	0	Fabaceae Lindl.	a	0
Iz_8_1009	DJI_0933	Rosa sp.	0	Rosaceae Juss.		
Iz_8_1009	DJI_0933	Salix alba L.	0	Salicaceae Mirb.	n	1
Iz_8_1009	DJI_0933	Sambucus nigra L.	0	Viburnaceae Raf.	n	1
Iz_8_1009	DJI_0933	Sorbus aucuparia L.	0	Rosaceae Juss.	n	1
Iz_8_1009	DJI_0933	Tilia cordata Mill.	0	Malvaceae Juss.	n	1
Iz_8_1009	DJI_0933	Ulmus glabra Huds.	0	Ulmaceae Mirb.	n	1
Iz_8_1009	DJI_0934	Acer tataricum L.	0	Sapindaceae Juss.	n	1
Iz_8_1009	DJI_0934	Betula pendula Roth.	0	Betulaceae Gray	n	1
Iz_8_1009	DJI_0934	Crataegus rhipidophylla Gand.	0	Rosaceae Juss.	n	1
Iz_8_1009	DJI_0934	Frangula alnus Mill.	0	Rhamnaceae Juss.	n	1
Iz_8_1009	DJI_0934	Fraxinus pennsylvanica Marshall	0	Oleaceae Hoffmanns. & Link	a	0
Iz_8_1009	DJI_0934	Pinus sylvestris L.	0	Pinaceae Spreng. ex F.Rudolphi	n	1
Iz_8_1009	DJI_0934	Populus × canescens (Aiton) Sm.	0	Salicaceae Mirb.	n	1
Iz_8_1009	DJI_0934	Populus tremula L.	0	Salicaceae Mirb.	n	1
Iz_8_1009	DJI_0934	Populus nigra L.	0	Salicaceae Mirb.	n	1
Iz_8_1009	DJI_0934	Pyrus communis L.	0	Rosaceae Juss.	a	0
Iz_8_1009	DJI_0934	Quercus robur L.	0	Fagaceae Dumort.	n	1
Iz_8_1009	DJI_0934	Robinia pseudoacacia L.	0	Fabaceae Lindl.	a	0
Iz_8_1009	DJI_0934	Rosa sp.	0	Rosaceae Juss.		
Iz_8_1009	DJI_0934	Salix alba L.	0	Salicaceae Mirb.	n	1
Iz_8_1009	DJI_0934	Sambucus nigra L.	0	Viburnaceae Raf.	n	1
Iz_8_1009	DJI_0934	Sorbus aucuparia L.	0	Rosaceae Juss.	n	1
Iz_8_1009	DJI_0934	Tilia cordata Mill.	0	Malvaceae Juss.	n	1
Iz_8_1009	DJI_0934	Ulmus glabra Huds.	0	Ulmaceae Mirb.	n	1
Iz_8_1009	DJI_0936	Acer tataricum L.	0	Sapindaceae Juss.	n	1
Iz_8_1009	DJI_0936	Betula pendula Roth.	0	Betulaceae Gray	n	1
Iz_8_1009	DJI_0936	Crataegus rhipidophylla Gand.	0	Rosaceae Juss.	n	1
Iz_8_1009	DJI_0936	Frangula alnus Mill.	0	Rhamnaceae Juss.	n	1
Iz_8_1009	DJI_0936	Fraxinus pennsylvanica Marshall	0	Oleaceae Hoffmanns. & Link	a	0
Iz_8_1009	DJI_0936	Pinus sylvestris L.	0	Pinaceae Spreng. ex F.Rudolphi	n	1
Iz_8_1009	DJI_0936	Populus × canescens (Aiton) Sm.	0	Salicaceae Mirb.	n	1
Iz_8_1009	DJI_0936	Populus tremula L.	0	Salicaceae Mirb.	n	1
Iz_8_1009	DJI_0936	Populus nigra L.	0	Salicaceae Mirb.	n	1
Iz_8_1009	DJI_0936	Pyrus communis L.	0	Rosaceae Juss.	a	0
Iz_8_1009	DJI_0936	Quercus robur L.	0	Fagaceae Dumort.	n	1
Iz_8_1009	DJI_0936	Robinia pseudoacacia L.	0	Fabaceae Lindl.	a	0
Iz_8_1009	DJI_0936	Rosa sp.	0	Rosaceae Juss.		
Iz_8_1009	DJI_0936	Salix alba L.	0	Salicaceae Mirb.	n	1
Iz_8_1009	DJI_0936	Sambucus nigra L.	0	Viburnaceae Raf.	n	1
Iz_8_1009	DJI_0936	Sorbus aucuparia L.	0	Rosaceae Juss.	n	1
Iz_8_1009	DJI_0936	Tilia cordata Mill.	0	Malvaceae Juss.	n	1
Iz_8_1009	DJI_0936	Ulmus glabra Huds.	0	Ulmaceae Mirb.	n	1
Iz_8_1009	DJI_0937	Acer tataricum L.	0	Sapindaceae Juss.	n	1
Iz_8_1009	DJI_0937	Betula pendula Roth.	0	Betulaceae Gray	n	1
Iz_8_1009	DJI_0937	Crataegus rhipidophylla Gand.	0	Rosaceae Juss.	n	1
Iz_8_1009	DJI_0937	Frangula alnus Mill.	0	Rhamnaceae Juss.	n	1
Iz_8_1009	DJI_0937	Fraxinus pennsylvanica Marshall	0	Oleaceae Hoffmanns. & Link	a	0
Iz_8_1009	DJI_0937	Pinus sylvestris L.	0	Pinaceae Spreng. ex F.Rudolphi	n	1
Iz_8_1009	DJI_0937	Populus × canescens (Aiton) Sm.	0	Salicaceae Mirb.	n	1
Iz_8_1009	DJI_0937	Populus tremula L.	0	Salicaceae Mirb.	n	1

Iz_8_1009	DJI_0937	Populus nigra L.	0	Salicaceae Mirb.	n	1
Iz_8_1009	DJI_0937	Pyrus communis L.	0	Rosaceae Juss.	a	0
Iz_8_1009	DJI_0937	Quercus robur L.	0	Fagaceae Dumort.	n	1
Iz_8_1009	DJI_0937	Robinia pseudoacacia L.	0	Fabaceae Lindl.	a	0
Iz_8_1009	DJI_0937	Rosa sp.	0	Rosaceae Juss.		
Iz_8_1009	DJI_0937	Salix alba L.	0	Salicaceae Mirb.	n	1
Iz_8_1009	DJI_0937	Sambucus nigra L.	0	Viburnaceae Raf.	n	1
Iz_8_1009	DJI_0937	Sorbus aucuparia L.	0	Rosaceae Juss.	n	1
Iz_8_1009	DJI_0937	Tilia cordata Mill.	0	Malvaceae Juss.	n	1
Iz_8_1009	DJI_0937	Ulmus glabra Huds.	0	Ulmaceae Mirb.	n	1
Iz_8_1009	DJI_0939	Acer tataricum L.	0	Sapindaceae Juss.	n	1
Iz_8_1009	DJI_0939	Betula pendula Roth.	0	Betulaceae Gray	n	1
Iz_8_1009	DJI_0939	Crataegus rhipidophylla Gand.	0	Rosaceae Juss.	n	1
Iz_8_1009	DJI_0939	Frangula alnus Mill.	0	Rhamnaceae Juss.	n	1
Iz_8_1009	DJI_0939	Fraxinus pennsylvanica Marshall	0	Oleaceae Hoffmanns. & Link	a	0
Iz_8_1009	DJI_0939	Pinus sylvestris L.	0	Pinaceae Spreng. ex F.Rudolphi	n	1
Iz_8_1009	DJI_0939	Populus × canescens (Aiton) Sm.	0	Salicaceae Mirb.	n	1
Iz_8_1009	DJI_0939	Populus tremula L.	0	Salicaceae Mirb.	n	1
Iz_8_1009	DJI_0939	Populus nigra L.	0	Salicaceae Mirb.	n	1
Iz_8_1009	DJI_0939	Pyrus communis L.	0	Rosaceae Juss.	a	0
Iz_8_1009	DJI_0939	Quercus robur L.	0	Fagaceae Dumort.	n	1
Iz_8_1009	DJI_0939	Robinia pseudoacacia L.	0	Fabaceae Lindl.	a	0
Iz_8_1009	DJI_0939	Rosa sp.	0	Rosaceae Juss.		
Iz_8_1009	DJI_0939	Salix alba L.	0	Salicaceae Mirb.	n	1
Iz_8_1009	DJI_0939	Sambucus nigra L.	0	Viburnaceae Raf.	n	1
Iz_8_1009	DJI_0939	Sorbus aucuparia L.	0	Rosaceae Juss.	n	1
Iz_8_1009	DJI_0939	Tilia cordata Mill.	0	Malvaceae Juss.	n	1
Iz_8_1009	DJI_0939	Ulmus glabra Huds.	0	Ulmaceae Mirb.	n	1
Iz_8_1009	DJI_0940	Acer tataricum L.	0	Sapindaceae Juss.	n	1
Iz_8_1009	DJI_0940	Betula pendula Roth.	0	Betulaceae Gray	n	1
Iz_8_1009	DJI_0940	Crataegus rhipidophylla Gand.	0	Rosaceae Juss.	n	1
Iz_8_1009	DJI_0940	Frangula alnus Mill.	0	Rhamnaceae Juss.	n	1
Iz_8_1009	DJI_0940	Fraxinus pennsylvanica Marshall	0	Oleaceae Hoffmanns. & Link	a	0
Iz_8_1009	DJI_0940	Pinus sylvestris L.	0	Pinaceae Spreng. ex F.Rudolphi	n	1
Iz_8_1009	DJI_0940	Populus × canescens (Aiton) Sm.	0	Salicaceae Mirb.	n	1
Iz_8_1009	DJI_0940	Populus tremula L.	0	Salicaceae Mirb.	n	1
Iz_8_1009	DJI_0940	Populus nigra L.	0	Salicaceae Mirb.	n	1
Iz_8_1009	DJI_0940	Pyrus communis L.	0	Rosaceae Juss.	a	0
Iz_8_1009	DJI_0940	Quercus robur L.	0	Fagaceae Dumort.	n	1
Iz_8_1009	DJI_0940	Robinia pseudoacacia L.	0	Fabaceae Lindl.	a	0
Iz_8_1009	DJI_0940	Rosa sp.	0	Rosaceae Juss.		
Iz_8_1009	DJI_0940	Salix alba L.	0	Salicaceae Mirb.	n	1
Iz_8_1009	DJI_0940	Sambucus nigra L.	0	Viburnaceae Raf.	n	1
Iz_8_1009	DJI_0940	Sorbus aucuparia L.	0	Rosaceae Juss.	n	1
Iz_8_1009	DJI_0940	Tilia cordata Mill.	0	Malvaceae Juss.	n	1
Iz_8_1009	DJI_0940	Ulmus glabra Huds.	0	Ulmaceae Mirb.	n	1
Iz_8_1009	DJI_0942	Acer tataricum L.	0	Sapindaceae Juss.	n	1
Iz_8_1009	DJI_0942	Betula pendula Roth.	0	Betulaceae Gray	n	1
Iz_8_1009	DJI_0942	Crataegus rhipidophylla Gand.	0	Rosaceae Juss.	n	1
Iz_8_1009	DJI_0942	Frangula alnus Mill.	0	Rhamnaceae Juss.	n	1
Iz_8_1009	DJI_0942	Fraxinus pennsylvanica Marshall	0	Oleaceae Hoffmanns. & Link	a	0
Iz_8_1009	DJI_0942	Pinus sylvestris L.	0	Pinaceae Spreng. ex F.Rudolphi	n	1
Iz_8_1009	DJI_0942	Populus × canescens (Aiton) Sm.	0	Salicaceae Mirb.	n	1
Iz_8_1009	DJI_0942	Populus tremula L.	0	Salicaceae Mirb.	n	1
Iz_8_1009	DJI_0942	Populus nigra L.	0	Salicaceae Mirb.	n	1
Iz_8_1009	DJI_0942	Pyrus communis L.	0	Rosaceae Juss.	a	0
Iz_8_1009	DJI_0942	Quercus robur L.	0	Fagaceae Dumort.	n	1
Iz_8_1009	DJI_0942	Robinia pseudoacacia L.	0	Fabaceae Lindl.	a	0
Iz_8_1009	DJI_0942	Rosa sp.	0	Rosaceae Juss.		
Iz_8_1009	DJI_0942	Salix alba L.	0	Salicaceae Mirb.	n	1
Iz_8_1009	DJI_0942	Sambucus nigra L.	0	Viburnaceae Raf.	n	1
Iz_8_1009	DJI_0942	Sorbus aucuparia L.	0	Rosaceae Juss.	n	1
Iz_8_1009	DJI_0942	Tilia cordata Mill.	0	Malvaceae Juss.	n	1
Iz_8_1009	DJI_0942	Ulmus glabra Huds.	0	Ulmaceae Mirb.	n	1

Iz_8_1009	DJI_0943	Acer tataricum L.	0	Sapindaceae Juss.	n	1
Iz_8_1009	DJI_0943	Betula pendula Roth.	0	Betulaceae Gray	n	1
Iz_8_1009	DJI_0943	Crataegus rhipidophylla Gand.	0	Rosaceae Juss.	n	1
Iz_8_1009	DJI_0943	Frangula alnus Mill.	0	Rhamnaceae Juss.	n	1
Iz_8_1009	DJI_0943	Fraxinus pennsylvanica Marshall	0	Oleaceae Hoffmanns. & Link	a	0
Iz_8_1009	DJI_0943	Pinus sylvestris L.	0	Pinaceae Spreng. ex F.Rudolphi	n	1
Iz_8_1009	DJI_0943	Populus × canescens (Aiton) Sm.	0	Salicaceae Mirb.	n	1
Iz_8_1009	DJI_0943	Populus tremula L.	0	Salicaceae Mirb.	n	1
Iz_8_1009	DJI_0943	Populus nigra L.	0	Salicaceae Mirb.	n	1
Iz_8_1009	DJI_0943	Pyrus communis L.	0	Rosaceae Juss.	a	0
Iz_8_1009	DJI_0943	Quercus robur L.	0	Fagaceae Dumort.	n	1
Iz_8_1009	DJI_0943	Robinia pseudoacacia L.	0	Fabaceae Lindl.	a	0
Iz_8_1009	DJI_0943	Rosa sp.	0	Rosaceae Juss.		
Iz_8_1009	DJI_0943	Salix alba L.	0	Salicaceae Mirb.	n	1
Iz_8_1009	DJI_0943	Sambucus nigra L.	0	Viburnaceae Raf.	n	1
Iz_8_1009	DJI_0943	Sorbus aucuparia L.	0	Rosaceae Juss.	n	1
Iz_8_1009	DJI_0943	Tilia cordata Mill.	0	Malvaceae Juss.	n	1
Iz_8_1009	DJI_0943	Ulmus glabra Huds.	0	Ulmaceae Mirb.	n	1
Iz_8_1009	DJI_0946	Acer tataricum L.	0	Sapindaceae Juss.	n	1
Iz_8_1009	DJI_0946	Betula pendula Roth.	0	Betulaceae Gray	n	1
Iz_8_1009	DJI_0946	Crataegus rhipidophylla Gand.	0	Rosaceae Juss.	n	1
Iz_8_1009	DJI_0946	Frangula alnus Mill.	0	Rhamnaceae Juss.	n	1
Iz_8_1009	DJI_0946	Fraxinus pennsylvanica Marshall	0	Oleaceae Hoffmanns. & Link	a	0
Iz_8_1009	DJI_0946	Pinus sylvestris L.	0	Pinaceae Spreng. ex F.Rudolphi	n	1
Iz_8_1009	DJI_0946	Populus × canescens (Aiton) Sm.	0	Salicaceae Mirb.	n	1
Iz_8_1009	DJI_0946	Populus tremula L.	25	Salicaceae Mirb.	n	1
Iz_8_1009	DJI_0946	Populus nigra L.	0	Salicaceae Mirb.	n	1
Iz_8_1009	DJI_0946	Pyrus communis L.	0	Rosaceae Juss.	a	0
Iz_8_1009	DJI_0946	Quercus robur L.	0	Fagaceae Dumort.	n	1
Iz_8_1009	DJI_0946	Robinia pseudoacacia L.	0	Fabaceae Lindl.	a	0
Iz_8_1009	DJI_0946	Rosa sp.	0	Rosaceae Juss.		
Iz_8_1009	DJI_0946	Salix alba L.	0	Salicaceae Mirb.	n	1
Iz_8_1009	DJI_0946	Sambucus nigra L.	0	Viburnaceae Raf.	n	1
Iz_8_1009	DJI_0946	Sorbus aucuparia L.	0	Rosaceae Juss.	n	1
Iz_8_1009	DJI_0946	Tilia cordata Mill.	0	Malvaceae Juss.	n	1
Iz_8_1009	DJI_0946	Ulmus glabra Huds.	0	Ulmaceae Mirb.	n	1
Iz_8_1009	DJI_0947	Acer tataricum L.	0	Sapindaceae Juss.	n	1
Iz_8_1009	DJI_0947	Betula pendula Roth.	0	Betulaceae Gray	n	1
Iz_8_1009	DJI_0947	Crataegus rhipidophylla Gand.	0	Rosaceae Juss.	n	1
Iz_8_1009	DJI_0947	Frangula alnus Mill.	0	Rhamnaceae Juss.	n	1
Iz_8_1009	DJI_0947	Fraxinus pennsylvanica Marshall	0	Oleaceae Hoffmanns. & Link	a	0
Iz_8_1009	DJI_0947	Pinus sylvestris L.	0	Pinaceae Spreng. ex F.Rudolphi	n	1
Iz_8_1009	DJI_0947	Populus × canescens (Aiton) Sm.	0	Salicaceae Mirb.	n	1
Iz_8_1009	DJI_0947	Populus tremula L.	5	Salicaceae Mirb.	n	1
Iz_8_1009	DJI_0947	Populus nigra L.	0	Salicaceae Mirb.	n	1
Iz_8_1009	DJI_0947	Pyrus communis L.	0	Rosaceae Juss.	a	0
Iz_8_1009	DJI_0947	Quercus robur L.	0	Fagaceae Dumort.	n	1
Iz_8_1009	DJI_0947	Robinia pseudoacacia L.	0	Fabaceae Lindl.	a	0
Iz_8_1009	DJI_0947	Rosa sp.	0	Rosaceae Juss.		
Iz_8_1009	DJI_0947	Salix alba L.	0	Salicaceae Mirb.	n	1
Iz_8_1009	DJI_0947	Sambucus nigra L.	0	Viburnaceae Raf.	n	1
Iz_8_1009	DJI_0947	Sorbus aucuparia L.	0	Rosaceae Juss.	n	1
Iz_8_1009	DJI_0947	Tilia cordata Mill.	0	Malvaceae Juss.	n	1
Iz_8_1009	DJI_0947	Ulmus glabra Huds.	0	Ulmaceae Mirb.	n	1
Iz_8_1009	DJI_0953	Acer tataricum L.	0	Sapindaceae Juss.	n	1
Iz_8_1009	DJI_0953	Betula pendula Roth.	0	Betulaceae Gray	n	1
Iz_8_1009	DJI_0953	Crataegus rhipidophylla Gand.	0	Rosaceae Juss.	n	1
Iz_8_1009	DJI_0953	Frangula alnus Mill.	0	Rhamnaceae Juss.	n	1
Iz_8_1009	DJI_0953	Fraxinus pennsylvanica Marshall	0	Oleaceae Hoffmanns. & Link	a	0
Iz_8_1009	DJI_0953	Pinus sylvestris L.	0	Pinaceae Spreng. ex F.Rudolphi	n	1
Iz_8_1009	DJI_0953	Populus × canescens (Aiton) Sm.	0	Salicaceae Mirb.	n	1
Iz_8_1009	DJI_0953	Populus tremula L.	0	Salicaceae Mirb.	n	1
Iz_8_1009	DJI_0953	Populus nigra L.	0	Salicaceae Mirb.	n	1
Iz_8_1009	DJI_0953	Pyrus communis L.	0	Rosaceae Juss.	a	0

Iz_8_1009	DJI_0953	Quercus robur L.	0	Fagaceae Dumort.	n	1
Iz_8_1009	DJI_0953	Robinia pseudoacacia L.	0	Fabaceae Lindl.	a	0
Iz_8_1009	DJI_0953	Rosa sp.	0	Rosaceae Juss.		
Iz_8_1009	DJI_0953	Salix alba L.	0	Salicaceae Mirb.	n	1
Iz_8_1009	DJI_0953	Sambucus nigra L.	0	Viburnaceae Raf.	n	1
Iz_8_1009	DJI_0953	Sorbus aucuparia L.	0	Rosaceae Juss.	n	1
Iz_8_1009	DJI_0953	Tilia cordata Mill.	0	Malvaceae Juss.	n	1
Iz_8_1009	DJI_0953	Ulmus glabra Huds.	0	Ulmaceae Mirb.	n	1
Iz_8_1009	DJI_0954	Acer tataricum L.	0	Sapindaceae Juss.	n	1
Iz_8_1009	DJI_0954	Betula pendula Roth.	0	Betulaceae Gray	n	1
Iz_8_1009	DJI_0954	Crataegus rhipidophylla Gand.	0	Rosaceae Juss.	n	1
Iz_8_1009	DJI_0954	Frangula alnus Mill.	0	Rhamnaceae Juss.	n	1
Iz_8_1009	DJI_0954	Fraxinus pennsylvanica Marshall	0	Oleaceae Hoffmanns. & Link	a	0
Iz_8_1009	DJI_0954	Pinus sylvestris L.	0	Pinaceae Spreng. ex F.Rudolphi	n	1
Iz_8_1009	DJI_0954	Populus × canescens (Aiton) Sm.	0	Salicaceae Mirb.	n	1
Iz_8_1009	DJI_0954	Populus tremula L.	0	Salicaceae Mirb.	n	1
Iz_8_1009	DJI_0954	Populus nigra L.	0	Salicaceae Mirb.	n	1
Iz_8_1009	DJI_0954	Pyrus communis L.	0	Rosaceae Juss.	a	0
Iz_8_1009	DJI_0954	Quercus robur L.	0	Fagaceae Dumort.	n	1
Iz_8_1009	DJI_0954	Robinia pseudoacacia L.	0	Fabaceae Lindl.	a	0
Iz_8_1009	DJI_0954	Rosa sp.	0	Rosaceae Juss.		
Iz_8_1009	DJI_0954	Salix alba L.	0	Salicaceae Mirb.	n	1
Iz_8_1009	DJI_0954	Sambucus nigra L.	0	Viburnaceae Raf.	n	1
Iz_8_1009	DJI_0954	Sorbus aucuparia L.	0	Rosaceae Juss.	n	1
Iz_8_1009	DJI_0954	Tilia cordata Mill.	0	Malvaceae Juss.	n	1
Iz_8_1009	DJI_0954	Ulmus glabra Huds.	0	Ulmaceae Mirb.	n	1
Iz_8_1009	DJI_0955	Acer tataricum L.	0	Sapindaceae Juss.	n	1
Iz_8_1009	DJI_0955	Betula pendula Roth.	0	Betulaceae Gray	n	1
Iz_8_1009	DJI_0955	Crataegus rhipidophylla Gand.	0	Rosaceae Juss.	n	1
Iz_8_1009	DJI_0955	Frangula alnus Mill.	0	Rhamnaceae Juss.	n	1
Iz_8_1009	DJI_0955	Fraxinus pennsylvanica Marshall	0	Oleaceae Hoffmanns. & Link	a	0
Iz_8_1009	DJI_0955	Pinus sylvestris L.	0	Pinaceae Spreng. ex F.Rudolphi	n	1
Iz_8_1009	DJI_0955	Populus × canescens (Aiton) Sm.	0	Salicaceae Mirb.	n	1
Iz_8_1009	DJI_0955	Populus tremula L.	0	Salicaceae Mirb.	n	1
Iz_8_1009	DJI_0955	Populus nigra L.	0	Salicaceae Mirb.	n	1
Iz_8_1009	DJI_0955	Pyrus communis L.	0	Rosaceae Juss.	a	0
Iz_8_1009	DJI_0955	Quercus robur L.	0	Fagaceae Dumort.	n	1
Iz_8_1009	DJI_0955	Robinia pseudoacacia L.	0	Fabaceae Lindl.	a	0
Iz_8_1009	DJI_0955	Rosa sp.	0	Rosaceae Juss.		
Iz_8_1009	DJI_0955	Salix alba L.	0	Salicaceae Mirb.	n	1
Iz_8_1009	DJI_0955	Sambucus nigra L.	0	Viburnaceae Raf.	n	1
Iz_8_1009	DJI_0955	Sorbus aucuparia L.	0	Rosaceae Juss.	n	1
Iz_8_1009	DJI_0955	Tilia cordata Mill.	0	Malvaceae Juss.	n	1
Iz_8_1009	DJI_0955	Ulmus glabra Huds.	0	Ulmaceae Mirb.	n	1
Iz_8_1009	DJI_0956	Acer tataricum L.	0	Sapindaceae Juss.	n	1
Iz_8_1009	DJI_0956	Betula pendula Roth.	0	Betulaceae Gray	n	1
Iz_8_1009	DJI_0956	Crataegus rhipidophylla Gand.	0	Rosaceae Juss.	n	1
Iz_8_1009	DJI_0956	Frangula alnus Mill.	0	Rhamnaceae Juss.	n	1
Iz_8_1009	DJI_0956	Fraxinus pennsylvanica Marshall	0	Oleaceae Hoffmanns. & Link	a	0
Iz_8_1009	DJI_0956	Pinus sylvestris L.	0	Pinaceae Spreng. ex F.Rudolphi	n	1
Iz_8_1009	DJI_0956	Populus × canescens (Aiton) Sm.	0	Salicaceae Mirb.	n	1
Iz_8_1009	DJI_0956	Populus tremula L.	0	Salicaceae Mirb.	n	1
Iz_8_1009	DJI_0956	Populus nigra L.	0	Salicaceae Mirb.	n	1
Iz_8_1009	DJI_0956	Pyrus communis L.	0	Rosaceae Juss.	a	0
Iz_8_1009	DJI_0956	Quercus robur L.	0	Fagaceae Dumort.	n	1
Iz_8_1009	DJI_0956	Robinia pseudoacacia L.	0	Fabaceae Lindl.	a	0
Iz_8_1009	DJI_0956	Rosa sp.	0	Rosaceae Juss.		
Iz_8_1009	DJI_0956	Salix alba L.	0	Salicaceae Mirb.	n	1
Iz_8_1009	DJI_0956	Sambucus nigra L.	0	Viburnaceae Raf.	n	1
Iz_8_1009	DJI_0956	Sorbus aucuparia L.	0	Rosaceae Juss.	n	1
Iz_8_1009	DJI_0956	Tilia cordata Mill.	0	Malvaceae Juss.	n	1
Iz_8_1009	DJI_0956	Ulmus glabra Huds.	0	Ulmaceae Mirb.	n	1
Iz_8_1009	DJI_0963	Acer tataricum L.	0	Sapindaceae Juss.	n	1
Iz_8_1009	DJI_0963	Betula pendula Roth.	0	Betulaceae Gray	n	1

Iz_8_1009	DJI_0963	Crataegus rhipidophylla Gand.	0	Rosaceae Juss.	n	1
Iz_8_1009	DJI_0963	Frangula alnus Mill.	0	Rhamnaceae Juss.	n	1
Iz_8_1009	DJI_0963	Fraxinus pennsylvanica Marshall	0	Oleaceae Hoffmanns. & Link	a	0
Iz_8_1009	DJI_0963	Pinus sylvestris L.	0	Pinaceae Spreng. ex F.Rudolphi	n	1
Iz_8_1009	DJI_0963	Populus × canescens (Aiton) Sm.	0	Salicaceae Mirb.	n	1
Iz_8_1009	DJI_0963	Populus tremula L.	0	Salicaceae Mirb.	n	1
Iz_8_1009	DJI_0963	Populus nigra L.	0	Salicaceae Mirb.	n	1
Iz_8_1009	DJI_0963	Pyrus communis L.	0	Rosaceae Juss.	a	0
Iz_8_1009	DJI_0963	Quercus robur L.	0	Fagaceae Dumort.	n	1
Iz_8_1009	DJI_0963	Robinia pseudoacacia L.	0	Fabaceae Lindl.	a	0
Iz_8_1009	DJI_0963	Rosa sp.	0	Rosaceae Juss.		
Iz_8_1009	DJI_0963	Salix alba L.	0	Salicaceae Mirb.	n	1
Iz_8_1009	DJI_0963	Sambucus nigra L.	0	Viburnaceae Raf.	n	1
Iz_8_1009	DJI_0963	Sorbus aucuparia L.	0	Rosaceae Juss.	n	1
Iz_8_1009	DJI_0963	Tilia cordata Mill.	0	Malvaceae Juss.	n	1
Iz_8_1009	DJI_0963	Ulmus glabra Huds.	0	Ulmaceae Mirb.	n	1
Iz_8_1009	DJI_0965	Acer tataricum L.	0	Sapindaceae Juss.	n	1
Iz_8_1009	DJI_0965	Betula pendula Roth.	0	Betulaceae Gray	n	1
Iz_8_1009	DJI_0965	Crataegus rhipidophylla Gand.	0	Rosaceae Juss.	n	1
Iz_8_1009	DJI_0965	Frangula alnus Mill.	0	Rhamnaceae Juss.	n	1
Iz_8_1009	DJI_0965	Fraxinus pennsylvanica Marshall	0	Oleaceae Hoffmanns. & Link	a	0
Iz_8_1009	DJI_0965	Pinus sylvestris L.	0	Pinaceae Spreng. ex F.Rudolphi	n	1
Iz_8_1009	DJI_0965	Populus × canescens (Aiton) Sm.	0	Salicaceae Mirb.	n	1
Iz_8_1009	DJI_0965	Populus tremula L.	0	Salicaceae Mirb.	n	1
Iz_8_1009	DJI_0965	Populus nigra L.	0	Salicaceae Mirb.	n	1
Iz_8_1009	DJI_0965	Pyrus communis L.	0	Rosaceae Juss.	a	0
Iz_8_1009	DJI_0965	Quercus robur L.	0	Fagaceae Dumort.	n	1
Iz_8_1009	DJI_0965	Robinia pseudoacacia L.	0	Fabaceae Lindl.	a	0
Iz_8_1009	DJI_0965	Rosa sp.	0	Rosaceae Juss.		
Iz_8_1009	DJI_0965	Salix alba L.	0	Salicaceae Mirb.	n	1
Iz_8_1009	DJI_0965	Sambucus nigra L.	0	Viburnaceae Raf.	n	1
Iz_8_1009	DJI_0965	Sorbus aucuparia L.	0	Rosaceae Juss.	n	1
Iz_8_1009	DJI_0965	Tilia cordata Mill.	0	Malvaceae Juss.	n	1
Iz_8_1009	DJI_0965	Ulmus glabra Huds.	0	Ulmaceae Mirb.	n	1
Iz_8_1009	DJI_0966	Acer tataricum L.	0	Sapindaceae Juss.	n	1
Iz_8_1009	DJI_0966	Betula pendula Roth.	0	Betulaceae Gray	n	1
Iz_8_1009	DJI_0966	Crataegus rhipidophylla Gand.	0	Rosaceae Juss.	n	1
Iz_8_1009	DJI_0966	Frangula alnus Mill.	0	Rhamnaceae Juss.	n	1
Iz_8_1009	DJI_0966	Fraxinus pennsylvanica Marshall	0	Oleaceae Hoffmanns. & Link	a	0
Iz_8_1009	DJI_0966	Pinus sylvestris L.	0	Pinaceae Spreng. ex F.Rudolphi	n	1
Iz_8_1009	DJI_0966	Populus × canescens (Aiton) Sm.	0	Salicaceae Mirb.	n	1
Iz_8_1009	DJI_0966	Populus tremula L.	0	Salicaceae Mirb.	n	1
Iz_8_1009	DJI_0966	Populus nigra L.	0	Salicaceae Mirb.	n	1
Iz_8_1009	DJI_0966	Pyrus communis L.	0	Rosaceae Juss.	a	0
Iz_8_1009	DJI_0966	Quercus robur L.	0	Fagaceae Dumort.	n	1
Iz_8_1009	DJI_0966	Robinia pseudoacacia L.	0	Fabaceae Lindl.	a	0
Iz_8_1009	DJI_0966	Rosa sp.	0	Rosaceae Juss.		
Iz_8_1009	DJI_0966	Salix alba L.	0	Salicaceae Mirb.	n	1
Iz_8_1009	DJI_0966	Sambucus nigra L.	0	Viburnaceae Raf.	n	1
Iz_8_1009	DJI_0966	Sorbus aucuparia L.	0	Rosaceae Juss.	n	1
Iz_8_1009	DJI_0966	Tilia cordata Mill.	0	Malvaceae Juss.	n	1
Iz_8_1009	DJI_0966	Ulmus glabra Huds.	0	Ulmaceae Mirb.	n	1
Iz_8_1009	DJI_0967	Acer tataricum L.	0	Sapindaceae Juss.	n	1
Iz_8_1009	DJI_0967	Betula pendula Roth.	0	Betulaceae Gray	n	1
Iz_8_1009	DJI_0967	Crataegus rhipidophylla Gand.	0	Rosaceae Juss.	n	1
Iz_8_1009	DJI_0967	Frangula alnus Mill.	0	Rhamnaceae Juss.	n	1
Iz_8_1009	DJI_0967	Fraxinus pennsylvanica Marshall	0	Oleaceae Hoffmanns. & Link	a	0
Iz_8_1009	DJI_0967	Pinus sylvestris L.	0	Pinaceae Spreng. ex F.Rudolphi	n	1
Iz_8_1009	DJI_0967	Populus × canescens (Aiton) Sm.	0	Salicaceae Mirb.	n	1
Iz_8_1009	DJI_0967	Populus tremula L.	0	Salicaceae Mirb.	n	1
Iz_8_1009	DJI_0967	Populus nigra L.	0	Salicaceae Mirb.	n	1
Iz_8_1009	DJI_0967	Pyrus communis L.	0	Rosaceae Juss.	a	0
Iz_8_1009	DJI_0967	Quercus robur L.	0	Fagaceae Dumort.	n	1
Iz_8_1009	DJI_0967	Robinia pseudoacacia L.	0	Fabaceae Lindl.	a	0

Iz_8_1009	DJI_0967	Rosa sp.	0	Rosaceae Juss.		
Iz_8_1009	DJI_0967	Salix alba L.	0	Salicaceae Mirb.	n	1
Iz_8_1009	DJI_0967	Sambucus nigra L.	0	Viburnaceae Raf.	n	1
Iz_8_1009	DJI_0967	Sorbus aucuparia L.	0	Rosaceae Juss.	n	1
Iz_8_1009	DJI_0967	Tilia cordata Mill.	0	Malvaceae Juss.	n	1
Iz_8_1009	DJI_0967	Ulmus glabra Huds.	0	Ulmaceae Mirb.	n	1
Iz_8_1009	DJI_0971	Acer tataricum L.	0	Sapindaceae Juss.	n	1
Iz_8_1009	DJI_0971	Betula pendula Roth.	0	Betulaceae Gray	n	1
Iz_8_1009	DJI_0971	Crataegus rhipidophylla Gand.	0	Rosaceae Juss.	n	1
Iz_8_1009	DJI_0971	Frangula alnus Mill.	0	Rhamnaceae Juss.	n	1
Iz_8_1009	DJI_0971	Fraxinus pennsylvanica Marshall	0	Oleaceae Hoffmanns. & Link	a	0
Iz_8_1009	DJI_0971	Pinus sylvestris L.	0	Pinaceae Spreng. ex F.Rudolphi	n	1
Iz_8_1009	DJI_0971	Populus × canescens (Aiton) Sm.	0	Salicaceae Mirb.	n	1
Iz_8_1009	DJI_0971	Populus tremula L.	0	Salicaceae Mirb.	n	1
Iz_8_1009	DJI_0971	Populus nigra L.	0	Salicaceae Mirb.	n	1
Iz_8_1009	DJI_0971	Pyrus communis L.	0	Rosaceae Juss.	a	0
Iz_8_1009	DJI_0971	Quercus robur L.	0	Fagaceae Dumort.	n	1
Iz_8_1009	DJI_0971	Robinia pseudoacacia L.	0	Fabaceae Lindl.	a	0
Iz_8_1009	DJI_0971	Rosa sp.	0	Rosaceae Juss.		
Iz_8_1009	DJI_0971	Salix alba L.	0	Salicaceae Mirb.	n	1
Iz_8_1009	DJI_0971	Sambucus nigra L.	0	Viburnaceae Raf.	n	1
Iz_8_1009	DJI_0971	Sorbus aucuparia L.	0	Rosaceae Juss.	n	1
Iz_8_1009	DJI_0971	Tilia cordata Mill.	0	Malvaceae Juss.	n	1
Iz_8_1009	DJI_0971	Ulmus glabra Huds.	0	Ulmaceae Mirb.	n	1
Iz_8_1009	DJI_0972	Acer tataricum L.	0	Sapindaceae Juss.	n	1
Iz_8_1009	DJI_0972	Betula pendula Roth.	0	Betulaceae Gray	n	1
Iz_8_1009	DJI_0972	Crataegus rhipidophylla Gand.	0	Rosaceae Juss.	n	1
Iz_8_1009	DJI_0972	Frangula alnus Mill.	0	Rhamnaceae Juss.	n	1
Iz_8_1009	DJI_0972	Fraxinus pennsylvanica Marshall	0	Oleaceae Hoffmanns. & Link	a	0
Iz_8_1009	DJI_0972	Pinus sylvestris L.	0	Pinaceae Spreng. ex F.Rudolphi	n	1
Iz_8_1009	DJI_0972	Populus × canescens (Aiton) Sm.	0	Salicaceae Mirb.	n	1
Iz_8_1009	DJI_0972	Populus tremula L.	0	Salicaceae Mirb.	n	1
Iz_8_1009	DJI_0972	Populus nigra L.	0	Salicaceae Mirb.	n	1
Iz_8_1009	DJI_0972	Pyrus communis L.	0	Rosaceae Juss.	a	0
Iz_8_1009	DJI_0972	Quercus robur L.	0	Fagaceae Dumort.	n	1
Iz_8_1009	DJI_0972	Robinia pseudoacacia L.	0	Fabaceae Lindl.	a	0
Iz_8_1009	DJI_0972	Rosa sp.	0	Rosaceae Juss.		
Iz_8_1009	DJI_0972	Salix alba L.	0	Salicaceae Mirb.	n	1
Iz_8_1009	DJI_0972	Sambucus nigra L.	0	Viburnaceae Raf.	n	1
Iz_8_1009	DJI_0972	Sorbus aucuparia L.	0	Rosaceae Juss.	n	1
Iz_8_1009	DJI_0972	Tilia cordata Mill.	0	Malvaceae Juss.	n	1
Iz_8_1009	DJI_0972	Ulmus glabra Huds.	0	Ulmaceae Mirb.	n	1
Iz_8_1009	DJI_0973	Acer tataricum L.	0	Sapindaceae Juss.	n	1
Iz_8_1009	DJI_0973	Betula pendula Roth.	0	Betulaceae Gray	n	1
Iz_8_1009	DJI_0973	Crataegus rhipidophylla Gand.	0	Rosaceae Juss.	n	1
Iz_8_1009	DJI_0973	Frangula alnus Mill.	0	Rhamnaceae Juss.	n	1
Iz_8_1009	DJI_0973	Fraxinus pennsylvanica Marshall	0	Oleaceae Hoffmanns. & Link	a	0
Iz_8_1009	DJI_0973	Pinus sylvestris L.	0	Pinaceae Spreng. ex F.Rudolphi	n	1
Iz_8_1009	DJI_0973	Populus × canescens (Aiton) Sm.	0	Salicaceae Mirb.	n	1
Iz_8_1009	DJI_0973	Populus tremula L.	0	Salicaceae Mirb.	n	1
Iz_8_1009	DJI_0973	Populus nigra L.	0	Salicaceae Mirb.	n	1
Iz_8_1009	DJI_0973	Pyrus communis L.	0	Rosaceae Juss.	a	0
Iz_8_1009	DJI_0973	Quercus robur L.	0	Fagaceae Dumort.	n	1
Iz_8_1009	DJI_0973	Robinia pseudoacacia L.	0	Fabaceae Lindl.	a	0
Iz_8_1009	DJI_0973	Rosa sp.	0	Rosaceae Juss.		
Iz_8_1009	DJI_0973	Salix alba L.	0	Salicaceae Mirb.	n	1
Iz_8_1009	DJI_0973	Sambucus nigra L.	0	Viburnaceae Raf.	n	1
Iz_8_1009	DJI_0973	Sorbus aucuparia L.	0	Rosaceae Juss.	n	1
Iz_8_1009	DJI_0973	Tilia cordata Mill.	0	Malvaceae Juss.	n	1
Iz_8_1009	DJI_0973	Ulmus glabra Huds.	0	Ulmaceae Mirb.	n	1
Iz_8_1009	DJI_0974	Acer tataricum L.	0	Sapindaceae Juss.	n	1
Iz_8_1009	DJI_0974	Betula pendula Roth.	0	Betulaceae Gray	n	1
Iz_8_1009	DJI_0974	Crataegus rhipidophylla Gand.	0	Rosaceae Juss.	n	1
Iz_8_1009	DJI_0974	Frangula alnus Mill.	0	Rhamnaceae Juss.	n	1

Iz_8_1009	DJI_0974	Fraxinus pennsylvanica Marshall	0	Oleaceae Hoffmanns. & Link	a	0
Iz_8_1009	DJI_0974	Pinus sylvestris L.	0	Pinaceae Spreng. ex F.Rudolphi	n	1
Iz_8_1009	DJI_0974	Populus × canescens (Aiton) Sm.	0	Salicaceae Mirb.	n	1
Iz_8_1009	DJI_0974	Populus tremula L.	0	Salicaceae Mirb.	n	1
Iz_8_1009	DJI_0974	Populus nigra L.	0	Salicaceae Mirb.	n	1
Iz_8_1009	DJI_0974	Pyrus communis L.	0	Rosaceae Juss.	a	0
Iz_8_1009	DJI_0974	Quercus robur L.	0	Fagaceae Dumort.	n	1
Iz_8_1009	DJI_0974	Robinia pseudoacacia L.	0	Fabaceae Lindl.	a	0
Iz_8_1009	DJI_0974	Rosa sp.	0	Rosaceae Juss.		
Iz_8_1009	DJI_0974	Salix alba L.	0	Salicaceae Mirb.	n	1
Iz_8_1009	DJI_0974	Sambucus nigra L.	0	Viburnaceae Raf.	n	1
Iz_8_1009	DJI_0974	Sorbus aucuparia L.	0	Rosaceae Juss.	n	1
Iz_8_1009	DJI_0974	Tilia cordata Mill.	0	Malvaceae Juss.	n	1
Iz_8_1009	DJI_0974	Ulmus glabra Huds.	0	Ulmaceae Mirb.	n	1
Iz_1_1109	DJI_0102	Acer tataricum L.	0	Sapindaceae Juss.	n	1
Iz_1_1109	DJI_0102	Betula pendula Roth.	0	Betulaceae Gray	n	1
Iz_1_1109	DJI_0102	Crataegus rhipidophylla Gand.	0	Rosaceae Juss.	n	1
Iz_1_1109	DJI_0102	Frangula alnus Mill.	0	Rhamnaceae Juss.	n	1
Iz_1_1109	DJI_0102	Fraxinus pennsylvanica Marshall	0	Oleaceae Hoffmanns. & Link	a	0
Iz_1_1109	DJI_0102	Pinus sylvestris L.	0	Pinaceae Spreng. ex F.Rudolphi	n	1
Iz_1_1109	DJI_0102	Populus × canescens (Aiton) Sm.	0	Salicaceae Mirb.	n	1
Iz_1_1109	DJI_0102	Populus tremula L.	0	Salicaceae Mirb.	n	1
Iz_1_1109	DJI_0102	Populus nigra L.	0	Salicaceae Mirb.	n	1
Iz_1_1109	DJI_0102	Populus sp.	0	Salicaceae Mirb.	n	1
Iz_1_1109	DJI_0102	Pyrus communis L.	0	Rosaceae Juss.	a	0
Iz_1_1109	DJI_0102	Quercus robur L.	0	Fagaceae Dumort.	n	1
Iz_1_1109	DJI_0102	Robinia pseudoacacia L.	0	Fabaceae Lindl.	a	0
Iz_1_1109	DJI_0102	Rosa sp.	0	Rosaceae Juss.		
Iz_1_1109	DJI_0102	Salix alba L.	0	Salicaceae Mirb.	n	1
Iz_1_1109	DJI_0102	Sambucus nigra L.	0	Viburnaceae Raf.	n	1
Iz_1_1109	DJI_0102	Sorbus aucuparia L.	0	Rosaceae Juss.	n	1
Iz_1_1109	DJI_0102	Tilia cordata Mill.	0	Malvaceae Juss.	n	1
Iz_1_1109	DJI_0102	Ulmus glabra Huds.	0	Ulmaceae Mirb.	n	1
Iz_1_1109	DJI_0098	Acer tataricum L.	0	Sapindaceae Juss.	n	1
Iz_1_1109	DJI_0098	Betula pendula Roth.	0	Betulaceae Gray	n	1
Iz_1_1109	DJI_0098	Crataegus rhipidophylla Gand.	0	Rosaceae Juss.	n	1
Iz_1_1109	DJI_0098	Frangula alnus Mill.	0	Rhamnaceae Juss.	n	1
Iz_1_1109	DJI_0098	Fraxinus pennsylvanica Marshall	0	Oleaceae Hoffmanns. & Link	a	0
Iz_1_1109	DJI_0098	Pinus sylvestris L.	0	Pinaceae Spreng. ex F.Rudolphi	n	1
Iz_1_1109	DJI_0098	Populus × canescens (Aiton) Sm.	0	Salicaceae Mirb.	n	1
Iz_1_1109	DJI_0098	Populus tremula L.	0	Salicaceae Mirb.	n	1
Iz_1_1109	DJI_0098	Populus nigra L.	0	Salicaceae Mirb.	n	1
Iz_1_1109	DJI_0098	Populus sp.	0	Salicaceae Mirb.	n	1
Iz_1_1109	DJI_0098	Pyrus communis L.	0	Rosaceae Juss.	a	0
Iz_1_1109	DJI_0098	Quercus robur L.	0	Fagaceae Dumort.	n	1
Iz_1_1109	DJI_0098	Robinia pseudoacacia L.	0	Fabaceae Lindl.	a	0
Iz_1_1109	DJI_0098	Rosa sp.	0	Rosaceae Juss.		
Iz_1_1109	DJI_0098	Salix alba L.	0	Salicaceae Mirb.	n	1
Iz_1_1109	DJI_0098	Sambucus nigra L.	0	Viburnaceae Raf.	n	1
Iz_1_1109	DJI_0098	Sorbus aucuparia L.	0	Rosaceae Juss.	n	1
Iz_1_1109	DJI_0098	Tilia cordata Mill.	0	Malvaceae Juss.	n	1
Iz_1_1109	DJI_0098	Ulmus glabra Huds.	0	Ulmaceae Mirb.	n	1
Iz_1_1109	DJI_0090	Acer tataricum L.	0	Sapindaceae Juss.	n	1
Iz_1_1109	DJI_0090	Betula pendula Roth.	0	Betulaceae Gray	n	1
Iz_1_1109	DJI_0090	Crataegus rhipidophylla Gand.	0	Rosaceae Juss.	n	1
Iz_1_1109	DJI_0090	Frangula alnus Mill.	0	Rhamnaceae Juss.	n	1
Iz_1_1109	DJI_0090	Fraxinus pennsylvanica Marshall	0	Oleaceae Hoffmanns. & Link	a	0
Iz_1_1109	DJI_0090	Pinus sylvestris L.	0	Pinaceae Spreng. ex F.Rudolphi	n	1
Iz_1_1109	DJI_0090	Populus × canescens (Aiton) Sm.	0	Salicaceae Mirb.	n	1
Iz_1_1109	DJI_0090	Populus tremula L.	0	Salicaceae Mirb.	n	1
Iz_1_1109	DJI_0090	Populus nigra L.	0	Salicaceae Mirb.	n	1
Iz_1_1109	DJI_0090	Populus sp.	0	Salicaceae Mirb.	n	1
Iz_1_1109	DJI_0090	Pyrus communis L.	0	Rosaceae Juss.	a	0
Iz_1_1109	DJI_0090	Quercus robur L.	0	Fagaceae Dumort.	n	1

Iz_1_1109	DJI_0090	Robinia pseudoacacia L.	0	Fabaceae Lindl.	a	0
Iz_1_1109	DJI_0090	Rosa sp.	0	Rosaceae Juss.		
Iz_1_1109	DJI_0090	Salix alba L.	0	Salicaceae Mirb.	n	1
Iz_1_1109	DJI_0090	Sambucus nigra L.	0	Viburnaceae Raf.	n	1
Iz_1_1109	DJI_0090	Sorbus aucuparia L.	0	Rosaceae Juss.	n	1
Iz_1_1109	DJI_0090	Tilia cordata Mill.	0	Malvaceae Juss.	n	1
Iz_1_1109	DJI_0090	Ulmus glabra Huds.	0	Ulmaceae Mirb.	n	1
Iz_1_1109	DJI_0083	Acer tataricum L.	0	Sapindaceae Juss.	n	1
Iz_1_1109	DJI_0083	Betula pendula Roth.	0	Betulaceae Gray	n	1
Iz_1_1109	DJI_0083	Crataegus rhipidophylla Gand.	0	Rosaceae Juss.	n	1
Iz_1_1109	DJI_0083	Frangula alnus Mill.	0	Rhamnaceae Juss.	n	1
Iz_1_1109	DJI_0083	Fraxinus pennsylvanica Marshall	0	Oleaceae Hoffmanns. & Link	a	0
Iz_1_1109	DJI_0083	Pinus sylvestris L.	0	Pinaceae Spreng. ex F.Rudolphi	n	1
Iz_1_1109	DJI_0083	Populus × canescens (Aiton) Sm.	0	Salicaceae Mirb.	n	1
Iz_1_1109	DJI_0083	Populus tremula L.	0	Salicaceae Mirb.	n	1
Iz_1_1109	DJI_0083	Populus nigra L.	0	Salicaceae Mirb.	n	1
Iz_1_1109	DJI_0083	Populus sp.	0	Salicaceae Mirb.	n	1
Iz_1_1109	DJI_0083	Pyrus communis L.	0	Rosaceae Juss.	a	0
Iz_1_1109	DJI_0083	Quercus robur L.	0	Fagaceae Dumort.	n	1
Iz_1_1109	DJI_0083	Robinia pseudoacacia L.	0	Fabaceae Lindl.	a	0
Iz_1_1109	DJI_0083	Rosa sp.	0	Rosaceae Juss.		
Iz_1_1109	DJI_0083	Salix alba L.	0	Salicaceae Mirb.	n	1
Iz_1_1109	DJI_0083	Sambucus nigra L.	0	Viburnaceae Raf.	n	1
Iz_1_1109	DJI_0083	Sorbus aucuparia L.	0	Rosaceae Juss.	n	1
Iz_1_1109	DJI_0083	Tilia cordata Mill.	0	Malvaceae Juss.	n	1
Iz_1_1109	DJI_0083	Ulmus glabra Huds.	0	Ulmaceae Mirb.	n	1
Iz_1_1109	DJI_0080	Acer tataricum L.	0	Sapindaceae Juss.	n	1
Iz_1_1109	DJI_0080	Betula pendula Roth.	0	Betulaceae Gray	n	1
Iz_1_1109	DJI_0080	Crataegus rhipidophylla Gand.	0	Rosaceae Juss.	n	1
Iz_1_1109	DJI_0080	Frangula alnus Mill.	0	Rhamnaceae Juss.	n	1
Iz_1_1109	DJI_0080	Fraxinus pennsylvanica Marshall	0	Oleaceae Hoffmanns. & Link	a	0
Iz_1_1109	DJI_0080	Pinus sylvestris L.	0	Pinaceae Spreng. ex F.Rudolphi	n	1
Iz_1_1109	DJI_0080	Populus × canescens (Aiton) Sm.	0	Salicaceae Mirb.	n	1
Iz_1_1109	DJI_0080	Populus tremula L.	0	Salicaceae Mirb.	n	1
Iz_1_1109	DJI_0080	Populus nigra L.	0	Salicaceae Mirb.	n	1
Iz_1_1109	DJI_0080	Populus sp.	0	Salicaceae Mirb.	n	1
Iz_1_1109	DJI_0080	Pyrus communis L.	0	Rosaceae Juss.	a	0
Iz_1_1109	DJI_0080	Quercus robur L.	0	Fagaceae Dumort.	n	1
Iz_1_1109	DJI_0080	Robinia pseudoacacia L.	0	Fabaceae Lindl.	a	0
Iz_1_1109	DJI_0080	Rosa sp.	0	Rosaceae Juss.		
Iz_1_1109	DJI_0080	Salix alba L.	0	Salicaceae Mirb.	n	1
Iz_1_1109	DJI_0080	Sambucus nigra L.	0	Viburnaceae Raf.	n	1
Iz_1_1109	DJI_0080	Sorbus aucuparia L.	0	Rosaceae Juss.	n	1
Iz_1_1109	DJI_0080	Tilia cordata Mill.	0	Malvaceae Juss.	n	1
Iz_1_1109	DJI_0080	Ulmus glabra Huds.	0	Ulmaceae Mirb.	n	1
Iz_1_1109	DJI_0076	Acer tataricum L.	0	Sapindaceae Juss.	n	1
Iz_1_1109	DJI_0076	Betula pendula Roth.	0	Betulaceae Gray	n	1
Iz_1_1109	DJI_0076	Crataegus rhipidophylla Gand.	0	Rosaceae Juss.	n	1
Iz_1_1109	DJI_0076	Frangula alnus Mill.	0	Rhamnaceae Juss.	n	1
Iz_1_1109	DJI_0076	Fraxinus pennsylvanica Marshall	0	Oleaceae Hoffmanns. & Link	a	0
Iz_1_1109	DJI_0076	Pinus sylvestris L.	0	Pinaceae Spreng. ex F.Rudolphi	n	1
Iz_1_1109	DJI_0076	Populus × canescens (Aiton) Sm.	0	Salicaceae Mirb.	n	1
Iz_1_1109	DJI_0076	Populus tremula L.	0	Salicaceae Mirb.	n	1
Iz_1_1109	DJI_0076	Populus nigra L.	0	Salicaceae Mirb.	n	1
Iz_1_1109	DJI_0076	Populus sp.	0	Salicaceae Mirb.	n	1
Iz_1_1109	DJI_0076	Pyrus communis L.	0	Rosaceae Juss.	a	0
Iz_1_1109	DJI_0076	Quercus robur L.	0	Fagaceae Dumort.	n	1
Iz_1_1109	DJI_0076	Robinia pseudoacacia L.	0	Fabaceae Lindl.	a	0
Iz_1_1109	DJI_0076	Rosa sp.	0	Rosaceae Juss.		
Iz_1_1109	DJI_0076	Salix alba L.	0	Salicaceae Mirb.	n	1
Iz_1_1109	DJI_0076	Sambucus nigra L.	0	Viburnaceae Raf.	n	1
Iz_1_1109	DJI_0076	Sorbus aucuparia L.	0	Rosaceae Juss.	n	1
Iz_1_1109	DJI_0076	Tilia cordata Mill.	0	Malvaceae Juss.	n	1
Iz_1_1109	DJI_0076	Ulmus glabra Huds.	0	Ulmaceae Mirb.	n	1

Iz_1_1109	DJI_0074	Acer tataricum L.	0	Sapindaceae Juss.	n	1
Iz_1_1109	DJI_0074	Betula pendula Roth.	0	Betulaceae Gray	n	1
Iz_1_1109	DJI_0074	Crataegus rhipidophylla Gand.	0	Rosaceae Juss.	n	1
Iz_1_1109	DJI_0074	Frangula alnus Mill.	0	Rhamnaceae Juss.	n	1
Iz_1_1109	DJI_0074	Fraxinus pennsylvanica Marshall	0	Oleaceae Hoffmanns. & Link	a	0
Iz_1_1109	DJI_0074	Pinus sylvestris L.	0	Pinaceae Spreng. ex F.Rudolphi	n	1
Iz_1_1109	DJI_0074	Populus × canescens (Aiton) Sm.	0	Salicaceae Mirb.	n	1
Iz_1_1109	DJI_0074	Populus tremula L.	0	Salicaceae Mirb.	n	1
Iz_1_1109	DJI_0074	Populus nigra L.	0	Salicaceae Mirb.	n	1
Iz_1_1109	DJI_0074	Populus sp.	0	Salicaceae Mirb.	n	1
Iz_1_1109	DJI_0074	Pyrus communis L.	0	Rosaceae Juss.	a	0
Iz_1_1109	DJI_0074	Quercus robur L.	0	Fagaceae Dumort.	n	1
Iz_1_1109	DJI_0074	Robinia pseudoacacia L.	0	Fabaceae Lindl.	a	0
Iz_1_1109	DJI_0074	Rosa sp.	0	Rosaceae Juss.		
Iz_1_1109	DJI_0074	Salix alba L.	0	Salicaceae Mirb.	n	1
Iz_1_1109	DJI_0074	Sambucus nigra L.	0	Viburnaceae Raf.	n	1
Iz_1_1109	DJI_0074	Sorbus aucuparia L.	0	Rosaceae Juss.	n	1
Iz_1_1109	DJI_0074	Tilia cordata Mill.	0	Malvaceae Juss.	n	1
Iz_1_1109	DJI_0074	Ulmus glabra Huds.	0	Ulmaceae Mirb.	n	1
Iz_1_1109	DJI_0071	Acer tataricum L.	0	Sapindaceae Juss.	n	1
Iz_1_1109	DJI_0071	Betula pendula Roth.	0	Betulaceae Gray	n	1
Iz_1_1109	DJI_0071	Crataegus rhipidophylla Gand.	0	Rosaceae Juss.	n	1
Iz_1_1109	DJI_0071	Frangula alnus Mill.	0	Rhamnaceae Juss.	n	1
Iz_1_1109	DJI_0071	Fraxinus pennsylvanica Marshall	0	Oleaceae Hoffmanns. & Link	a	0
Iz_1_1109	DJI_0071	Pinus sylvestris L.	0	Pinaceae Spreng. ex F.Rudolphi	n	1
Iz_1_1109	DJI_0071	Populus × canescens (Aiton) Sm.	0	Salicaceae Mirb.	n	1
Iz_1_1109	DJI_0071	Populus tremula L.	0	Salicaceae Mirb.	n	1
Iz_1_1109	DJI_0071	Populus nigra L.	0	Salicaceae Mirb.	n	1
Iz_1_1109	DJI_0071	Populus sp.	0	Salicaceae Mirb.	n	1
Iz_1_1109	DJI_0071	Pyrus communis L.	0	Rosaceae Juss.	a	0
Iz_1_1109	DJI_0071	Quercus robur L.	0	Fagaceae Dumort.	n	1
Iz_1_1109	DJI_0071	Robinia pseudoacacia L.	0	Fabaceae Lindl.	a	0
Iz_1_1109	DJI_0071	Rosa sp.	0	Rosaceae Juss.		
Iz_1_1109	DJI_0071	Salix alba L.	0	Salicaceae Mirb.	n	1
Iz_1_1109	DJI_0071	Sambucus nigra L.	0	Viburnaceae Raf.	n	1
Iz_1_1109	DJI_0071	Sorbus aucuparia L.	0	Rosaceae Juss.	n	1
Iz_1_1109	DJI_0071	Tilia cordata Mill.	0	Malvaceae Juss.	n	1
Iz_1_1109	DJI_0071	Ulmus glabra Huds.	0	Ulmaceae Mirb.	n	1
Iz_1_1109	DJI_0068	Acer tataricum L.	0	Sapindaceae Juss.	n	1
Iz_1_1109	DJI_0068	Betula pendula Roth.	0	Betulaceae Gray	n	1
Iz_1_1109	DJI_0068	Crataegus rhipidophylla Gand.	0	Rosaceae Juss.	n	1
Iz_1_1109	DJI_0068	Frangula alnus Mill.	0	Rhamnaceae Juss.	n	1
Iz_1_1109	DJI_0068	Fraxinus pennsylvanica Marshall	0	Oleaceae Hoffmanns. & Link	a	0
Iz_1_1109	DJI_0068	Pinus sylvestris L.	0	Pinaceae Spreng. ex F.Rudolphi	n	1
Iz_1_1109	DJI_0068	Populus × canescens (Aiton) Sm.	0	Salicaceae Mirb.	n	1
Iz_1_1109	DJI_0068	Populus tremula L.	0	Salicaceae Mirb.	n	1
Iz_1_1109	DJI_0068	Populus nigra L.	0	Salicaceae Mirb.	n	1
Iz_1_1109	DJI_0068	Populus sp.	0	Salicaceae Mirb.	n	1
Iz_1_1109	DJI_0068	Pyrus communis L.	0	Rosaceae Juss.	a	0
Iz_1_1109	DJI_0068	Quercus robur L.	0	Fagaceae Dumort.	n	1
Iz_1_1109	DJI_0068	Robinia pseudoacacia L.	0	Fabaceae Lindl.	a	0
Iz_1_1109	DJI_0068	Rosa sp.	0	Rosaceae Juss.		
Iz_1_1109	DJI_0068	Salix alba L.	0	Salicaceae Mirb.	n	1
Iz_1_1109	DJI_0068	Sambucus nigra L.	0	Viburnaceae Raf.	n	1
Iz_1_1109	DJI_0068	Sorbus aucuparia L.	0	Rosaceae Juss.	n	1
Iz_1_1109	DJI_0068	Tilia cordata Mill.	0	Malvaceae Juss.	n	1
Iz_1_1109	DJI_0068	Ulmus glabra Huds.	0	Ulmaceae Mirb.	n	1
Iz_1_1109	DJI_0066	Acer tataricum L.	0	Sapindaceae Juss.	n	1
Iz_1_1109	DJI_0066	Betula pendula Roth.	0	Betulaceae Gray	n	1
Iz_1_1109	DJI_0066	Crataegus rhipidophylla Gand.	0	Rosaceae Juss.	n	1
Iz_1_1109	DJI_0066	Frangula alnus Mill.	0	Rhamnaceae Juss.	n	1
Iz_1_1109	DJI_0066	Fraxinus pennsylvanica Marshall	0	Oleaceae Hoffmanns. & Link	a	0
Iz_1_1109	DJI_0066	Pinus sylvestris L.	0	Pinaceae Spreng. ex F.Rudolphi	n	1
Iz_1_1109	DJI_0066	Populus × canescens (Aiton) Sm.	0	Salicaceae Mirb.	n	1

Iz_1_1109	DJI_0066	Populus tremula L.	0	Salicaceae Mirb.	n	1
Iz_1_1109	DJI_0066	Populus nigra L.	0	Salicaceae Mirb.	n	1
Iz_1_1109	DJI_0066	Populus sp.	0	Salicaceae Mirb.	n	1
Iz_1_1109	DJI_0066	Pyrus communis L.	0	Rosaceae Juss.	a	0
Iz_1_1109	DJI_0066	Quercus robur L.	0	Fagaceae Dumort.	n	1
Iz_1_1109	DJI_0066	Robinia pseudoacacia L.	0	Fabaceae Lindl.	a	0
Iz_1_1109	DJI_0066	Rosa sp.	0	Rosaceae Juss.		
Iz_1_1109	DJI_0066	Salix alba L.	0	Salicaceae Mirb.	n	1
Iz_1_1109	DJI_0066	Sambucus nigra L.	0	Viburnaceae Raf.	n	1
Iz_1_1109	DJI_0066	Sorbus aucuparia L.	0	Rosaceae Juss.	n	1
Iz_1_1109	DJI_0066	Tilia cordata Mill.	0	Malvaceae Juss.	n	1
Iz_1_1109	DJI_0066	Ulmus glabra Huds.	0	Ulmaceae Mirb.	n	1
Iz_1_1109	DJI_0064	Acer tataricum L.	0	Sapindaceae Juss.	n	1
Iz_1_1109	DJI_0064	Betula pendula Roth.	0	Betulaceae Gray	n	1
Iz_1_1109	DJI_0064	Crataegus rhipidophylla Gand.	0	Rosaceae Juss.	n	1
Iz_1_1109	DJI_0064	Frangula alnus Mill.	0	Rhamnaceae Juss.	n	1
Iz_1_1109	DJI_0064	Fraxinus pennsylvanica Marshall	0	Oleaceae Hoffmanns. & Link	a	0
Iz_1_1109	DJI_0064	Pinus sylvestris L.	0	Pinaceae Spreng. ex F.Rudolphi	n	1
Iz_1_1109	DJI_0064	Populus × canescens (Aiton) Sm.	0	Salicaceae Mirb.	n	1
Iz_1_1109	DJI_0064	Populus tremula L.	0	Salicaceae Mirb.	n	1
Iz_1_1109	DJI_0064	Populus nigra L.	0	Salicaceae Mirb.	n	1
Iz_1_1109	DJI_0064	Populus sp.	0	Salicaceae Mirb.	n	1
Iz_1_1109	DJI_0064	Pyrus communis L.	0	Rosaceae Juss.	a	0
Iz_1_1109	DJI_0064	Quercus robur L.	0	Fagaceae Dumort.	n	1
Iz_1_1109	DJI_0064	Robinia pseudoacacia L.	0	Fabaceae Lindl.	a	0
Iz_1_1109	DJI_0064	Rosa sp.	0	Rosaceae Juss.		
Iz_1_1109	DJI_0064	Salix alba L.	0	Salicaceae Mirb.	n	1
Iz_1_1109	DJI_0064	Sambucus nigra L.	0	Viburnaceae Raf.	n	1
Iz_1_1109	DJI_0064	Sorbus aucuparia L.	0	Rosaceae Juss.	n	1
Iz_1_1109	DJI_0064	Tilia cordata Mill.	0	Malvaceae Juss.	n	1
Iz_1_1109	DJI_0064	Ulmus glabra Huds.	0	Ulmaceae Mirb.	n	1
Iz_1_1109	DJI_0062	Acer tataricum L.	0	Sapindaceae Juss.	n	1
Iz_1_1109	DJI_0062	Betula pendula Roth.	0	Betulaceae Gray	n	1
Iz_1_1109	DJI_0062	Crataegus rhipidophylla Gand.	0	Rosaceae Juss.	n	1
Iz_1_1109	DJI_0062	Frangula alnus Mill.	0	Rhamnaceae Juss.	n	1
Iz_1_1109	DJI_0062	Fraxinus pennsylvanica Marshall	0	Oleaceae Hoffmanns. & Link	a	0
Iz_1_1109	DJI_0062	Pinus sylvestris L.	0	Pinaceae Spreng. ex F.Rudolphi	n	1
Iz_1_1109	DJI_0062	Populus × canescens (Aiton) Sm.	0	Salicaceae Mirb.	n	1
Iz_1_1109	DJI_0062	Populus tremula L.	0	Salicaceae Mirb.	n	1
Iz_1_1109	DJI_0062	Populus nigra L.	0	Salicaceae Mirb.	n	1
Iz_1_1109	DJI_0062	Populus sp.	0	Salicaceae Mirb.	n	1
Iz_1_1109	DJI_0062	Pyrus communis L.	0	Rosaceae Juss.	a	0
Iz_1_1109	DJI_0062	Quercus robur L.	0	Fagaceae Dumort.	n	1
Iz_1_1109	DJI_0062	Robinia pseudoacacia L.	0	Fabaceae Lindl.	a	0
Iz_1_1109	DJI_0062	Rosa sp.	0	Rosaceae Juss.		
Iz_1_1109	DJI_0062	Salix alba L.	0	Salicaceae Mirb.	n	1
Iz_1_1109	DJI_0062	Sambucus nigra L.	0	Viburnaceae Raf.	n	1
Iz_1_1109	DJI_0062	Sorbus aucuparia L.	0	Rosaceae Juss.	n	1
Iz_1_1109	DJI_0062	Tilia cordata Mill.	0	Malvaceae Juss.	n	1
Iz_1_1109	DJI_0062	Ulmus glabra Huds.	0	Ulmaceae Mirb.	n	1
Iz_1_1109	DJI_0060	Acer tataricum L.	0	Sapindaceae Juss.	n	1
Iz_1_1109	DJI_0060	Betula pendula Roth.	0	Betulaceae Gray	n	1
Iz_1_1109	DJI_0060	Crataegus rhipidophylla Gand.	0	Rosaceae Juss.	n	1
Iz_1_1109	DJI_0060	Frangula alnus Mill.	0	Rhamnaceae Juss.	n	1
Iz_1_1109	DJI_0060	Fraxinus pennsylvanica Marshall	0	Oleaceae Hoffmanns. & Link	a	0
Iz_1_1109	DJI_0060	Pinus sylvestris L.	0	Pinaceae Spreng. ex F.Rudolphi	n	1
Iz_1_1109	DJI_0060	Populus × canescens (Aiton) Sm.	0	Salicaceae Mirb.	n	1
Iz_1_1109	DJI_0060	Populus tremula L.	0	Salicaceae Mirb.	n	1
Iz_1_1109	DJI_0060	Populus nigra L.	0	Salicaceae Mirb.	n	1
Iz_1_1109	DJI_0060	Populus sp.	0	Salicaceae Mirb.	n	1
Iz_1_1109	DJI_0060	Pyrus communis L.	0	Rosaceae Juss.	a	0
Iz_1_1109	DJI_0060	Quercus robur L.	0	Fagaceae Dumort.	n	1
Iz_1_1109	DJI_0060	Robinia pseudoacacia L.	0	Fabaceae Lindl.	a	0
Iz_1_1109	DJI_0060	Rosa sp.	0	Rosaceae Juss.		

Iz_1_1109	DJI_0060	Salix alba L.	0	Salicaceae Mirb.	n	1
Iz_1_1109	DJI_0060	Sambucus nigra L.	0	Viburnaceae Raf.	n	1
Iz_1_1109	DJI_0060	Sorbus aucuparia L.	0	Rosaceae Juss.	n	1
Iz_1_1109	DJI_0060	Tilia cordata Mill.	0	Malvaceae Juss.	n	1
Iz_1_1109	DJI_0060	Ulmus glabra Huds.	0	Ulmaceae Mirb.	n	1
Iz_1_1109	DJI_0058	Acer tataricum L.	0	Sapindaceae Juss.	n	1
Iz_1_1109	DJI_0058	Betula pendula Roth.	0	Betulaceae Gray	n	1
Iz_1_1109	DJI_0058	Crataegus rhipidophylla Gand.	0	Rosaceae Juss.	n	1
Iz_1_1109	DJI_0058	Frangula alnus Mill.	0	Rhamnaceae Juss.	n	1
Iz_1_1109	DJI_0058	Fraxinus pennsylvanica Marshall	0	Oleaceae Hoffmanns. & Link	a	0
Iz_1_1109	DJI_0058	Pinus sylvestris L.	0	Pinaceae Spreng. ex F.Rudolphi	n	1
Iz_1_1109	DJI_0058	Populus × canescens (Aiton) Sm.	0	Salicaceae Mirb.	n	1
Iz_1_1109	DJI_0058	Populus tremula L.	0	Salicaceae Mirb.	n	1
Iz_1_1109	DJI_0058	Populus nigra L.	0	Salicaceae Mirb.	n	1
Iz_1_1109	DJI_0058	Populus sp.	0	Salicaceae Mirb.	n	1
Iz_1_1109	DJI_0058	Pyrus communis L.	0	Rosaceae Juss.	a	0
Iz_1_1109	DJI_0058	Quercus robur L.	0	Fagaceae Dumort.	n	1
Iz_1_1109	DJI_0058	Robinia pseudoacacia L.	0	Fabaceae Lindl.	a	0
Iz_1_1109	DJI_0058	Rosa sp.	0	Rosaceae Juss.		
Iz_1_1109	DJI_0058	Salix alba L.	0	Salicaceae Mirb.	n	1
Iz_1_1109	DJI_0058	Sambucus nigra L.	0	Viburnaceae Raf.	n	1
Iz_1_1109	DJI_0058	Sorbus aucuparia L.	0	Rosaceae Juss.	n	1
Iz_1_1109	DJI_0058	Tilia cordata Mill.	0	Malvaceae Juss.	n	1
Iz_1_1109	DJI_0058	Ulmus glabra Huds.	0	Ulmaceae Mirb.	n	1
Iz_1_1109	DJI_0055	Acer tataricum L.	0	Sapindaceae Juss.	n	1
Iz_1_1109	DJI_0055	Betula pendula Roth.	0	Betulaceae Gray	n	1
Iz_1_1109	DJI_0055	Crataegus rhipidophylla Gand.	0	Rosaceae Juss.	n	1
Iz_1_1109	DJI_0055	Frangula alnus Mill.	0	Rhamnaceae Juss.	n	1
Iz_1_1109	DJI_0055	Fraxinus pennsylvanica Marshall	0	Oleaceae Hoffmanns. & Link	a	0
Iz_1_1109	DJI_0055	Pinus sylvestris L.	0	Pinaceae Spreng. ex F.Rudolphi	n	1
Iz_1_1109	DJI_0055	Populus × canescens (Aiton) Sm.	0	Salicaceae Mirb.	n	1
Iz_1_1109	DJI_0055	Populus tremula L.	1	Salicaceae Mirb.	n	1
Iz_1_1109	DJI_0055	Populus nigra L.	0	Salicaceae Mirb.	n	1
Iz_1_1109	DJI_0055	Populus sp.	0	Salicaceae Mirb.	n	1
Iz_1_1109	DJI_0055	Pyrus communis L.	0	Rosaceae Juss.	a	0
Iz_1_1109	DJI_0055	Quercus robur L.	0	Fagaceae Dumort.	n	1
Iz_1_1109	DJI_0055	Robinia pseudoacacia L.	0	Fabaceae Lindl.	a	0
Iz_1_1109	DJI_0055	Rosa sp.	0	Rosaceae Juss.		
Iz_1_1109	DJI_0055	Salix alba L.	0	Salicaceae Mirb.	n	1
Iz_1_1109	DJI_0055	Sambucus nigra L.	0	Viburnaceae Raf.	n	1
Iz_1_1109	DJI_0055	Sorbus aucuparia L.	0	Rosaceae Juss.	n	1
Iz_1_1109	DJI_0055	Tilia cordata Mill.	0	Malvaceae Juss.	n	1
Iz_1_1109	DJI_0055	Ulmus glabra Huds.	0	Ulmaceae Mirb.	n	1
Iz_1_1109	DJI_0051	Acer tataricum L.	0	Sapindaceae Juss.	n	1
Iz_1_1109	DJI_0051	Betula pendula Roth.	0	Betulaceae Gray	n	1
Iz_1_1109	DJI_0051	Crataegus rhipidophylla Gand.	0	Rosaceae Juss.	n	1
Iz_1_1109	DJI_0051	Frangula alnus Mill.	0	Rhamnaceae Juss.	n	1
Iz_1_1109	DJI_0051	Fraxinus pennsylvanica Marshall	0	Oleaceae Hoffmanns. & Link	a	0
Iz_1_1109	DJI_0051	Pinus sylvestris L.	0	Pinaceae Spreng. ex F.Rudolphi	n	1
Iz_1_1109	DJI_0051	Populus × canescens (Aiton) Sm.	0	Salicaceae Mirb.	n	1
Iz_1_1109	DJI_0051	Populus tremula L.	0	Salicaceae Mirb.	n	1
Iz_1_1109	DJI_0051	Populus nigra L.	0	Salicaceae Mirb.	n	1
Iz_1_1109	DJI_0051	Populus sp.	0	Salicaceae Mirb.	n	1
Iz_1_1109	DJI_0051	Pyrus communis L.	0	Rosaceae Juss.	a	0
Iz_1_1109	DJI_0051	Quercus robur L.	0	Fagaceae Dumort.	n	1
Iz_1_1109	DJI_0051	Robinia pseudoacacia L.	0	Fabaceae Lindl.	a	0
Iz_1_1109	DJI_0051	Rosa sp.	0	Rosaceae Juss.		
Iz_1_1109	DJI_0051	Salix alba L.	0	Salicaceae Mirb.	n	1
Iz_1_1109	DJI_0051	Sambucus nigra L.	0	Viburnaceae Raf.	n	1
Iz_1_1109	DJI_0051	Sorbus aucuparia L.	0	Rosaceae Juss.	n	1
Iz_1_1109	DJI_0051	Tilia cordata Mill.	0	Malvaceae Juss.	n	1
Iz_1_1109	DJI_0051	Ulmus glabra Huds.	0	Ulmaceae Mirb.	n	1
Iz_1_1109	DJI_0048	Acer tataricum L.	0	Sapindaceae Juss.	n	1
Iz_1_1109	DJI_0048	Betula pendula Roth.	0	Betulaceae Gray	n	1

Iz_1_1109	DJI_0048	Crataegus rhipidophylla Gand.	0	Rosaceae Juss.	n	1
Iz_1_1109	DJI_0048	Frangula alnus Mill.	0	Rhamnaceae Juss.	n	1
Iz_1_1109	DJI_0048	Fraxinus pennsylvanica Marshall	0	Oleaceae Hoffmanns. & Link	a	0
Iz_1_1109	DJI_0048	Pinus sylvestris L.	0	Pinaceae Spreng. ex F.Rudolphi	n	1
Iz_1_1109	DJI_0048	Populus × canescens (Aiton) Sm.	0	Salicaceae Mirb.	n	1
Iz_1_1109	DJI_0048	Populus tremula L.	0	Salicaceae Mirb.	n	1
Iz_1_1109	DJI_0048	Populus nigra L.	0	Salicaceae Mirb.	n	1
Iz_1_1109	DJI_0048	Populus sp.	0	Salicaceae Mirb.	n	1
Iz_1_1109	DJI_0048	Pyrus communis L.	0	Rosaceae Juss.	a	0
Iz_1_1109	DJI_0048	Quercus robur L.	0	Fagaceae Dumort.	n	1
Iz_1_1109	DJI_0048	Robinia pseudoacacia L.	0	Fabaceae Lindl.	a	0
Iz_1_1109	DJI_0048	Rosa sp.	0	Rosaceae Juss.		
Iz_1_1109	DJI_0048	Salix alba L.	0	Salicaceae Mirb.	n	1
Iz_1_1109	DJI_0048	Sambucus nigra L.	0	Viburnaceae Raf.	n	1
Iz_1_1109	DJI_0048	Sorbus aucuparia L.	0	Rosaceae Juss.	n	1
Iz_1_1109	DJI_0048	Tilia cordata Mill.	0	Malvaceae Juss.	n	1
Iz_1_1109	DJI_0048	Ulmus glabra Huds.	0	Ulmaceae Mirb.	n	1
Iz_1_1109	DJI_0046	Acer tataricum L.	0	Sapindaceae Juss.	n	1
Iz_1_1109	DJI_0046	Betula pendula Roth.	0	Betulaceae Gray	n	1
Iz_1_1109	DJI_0046	Crataegus rhipidophylla Gand.	0	Rosaceae Juss.	n	1
Iz_1_1109	DJI_0046	Frangula alnus Mill.	0	Rhamnaceae Juss.	n	1
Iz_1_1109	DJI_0046	Fraxinus pennsylvanica Marshall	0	Oleaceae Hoffmanns. & Link	a	0
Iz_1_1109	DJI_0046	Pinus sylvestris L.	0	Pinaceae Spreng. ex F.Rudolphi	n	1
Iz_1_1109	DJI_0046	Populus × canescens (Aiton) Sm.	0	Salicaceae Mirb.	n	1
Iz_1_1109	DJI_0046	Populus tremula L.	0	Salicaceae Mirb.	n	1
Iz_1_1109	DJI_0046	Populus nigra L.	0	Salicaceae Mirb.	n	1
Iz_1_1109	DJI_0046	Populus sp.	0	Salicaceae Mirb.	n	1
Iz_1_1109	DJI_0046	Pyrus communis L.	0	Rosaceae Juss.	a	0
Iz_1_1109	DJI_0046	Quercus robur L.	0	Fagaceae Dumort.	n	1
Iz_1_1109	DJI_0046	Robinia pseudoacacia L.	0	Fabaceae Lindl.	a	0
Iz_1_1109	DJI_0046	Rosa sp.	0	Rosaceae Juss.		
Iz_1_1109	DJI_0046	Salix alba L.	0	Salicaceae Mirb.	n	1
Iz_1_1109	DJI_0046	Sambucus nigra L.	0	Viburnaceae Raf.	n	1
Iz_1_1109	DJI_0046	Sorbus aucuparia L.	0	Rosaceae Juss.	n	1
Iz_1_1109	DJI_0046	Tilia cordata Mill.	0	Malvaceae Juss.	n	1
Iz_1_1109	DJI_0046	Ulmus glabra Huds.	0	Ulmaceae Mirb.	n	1
Iz_1_1109	DJI_0044	Acer tataricum L.	0	Sapindaceae Juss.	n	1
Iz_1_1109	DJI_0044	Betula pendula Roth.	0	Betulaceae Gray	n	1
Iz_1_1109	DJI_0044	Crataegus rhipidophylla Gand.	0	Rosaceae Juss.	n	1
Iz_1_1109	DJI_0044	Frangula alnus Mill.	0	Rhamnaceae Juss.	n	1
Iz_1_1109	DJI_0044	Fraxinus pennsylvanica Marshall	0	Oleaceae Hoffmanns. & Link	a	0
Iz_1_1109	DJI_0044	Pinus sylvestris L.	0	Pinaceae Spreng. ex F.Rudolphi	n	1
Iz_1_1109	DJI_0044	Populus × canescens (Aiton) Sm.	0	Salicaceae Mirb.	n	1
Iz_1_1109	DJI_0044	Populus tremula L.	0	Salicaceae Mirb.	n	1
Iz_1_1109	DJI_0044	Populus nigra L.	0	Salicaceae Mirb.	n	1
Iz_1_1109	DJI_0044	Populus sp.	0	Salicaceae Mirb.	n	1
Iz_1_1109	DJI_0044	Pyrus communis L.	0	Rosaceae Juss.	a	0
Iz_1_1109	DJI_0044	Quercus robur L.	0	Fagaceae Dumort.	n	1
Iz_1_1109	DJI_0044	Robinia pseudoacacia L.	0	Fabaceae Lindl.	a	0
Iz_1_1109	DJI_0044	Rosa sp.	0	Rosaceae Juss.		
Iz_1_1109	DJI_0044	Salix alba L.	0	Salicaceae Mirb.	n	1
Iz_1_1109	DJI_0044	Sambucus nigra L.	0	Viburnaceae Raf.	n	1
Iz_1_1109	DJI_0044	Sorbus aucuparia L.	0	Rosaceae Juss.	n	1
Iz_1_1109	DJI_0044	Tilia cordata Mill.	0	Malvaceae Juss.	n	1
Iz_1_1109	DJI_0044	Ulmus glabra Huds.	0	Ulmaceae Mirb.	n	1
Iz_1_1109	DJI_0041	Acer tataricum L.	0	Sapindaceae Juss.	n	1
Iz_1_1109	DJI_0041	Betula pendula Roth.	0	Betulaceae Gray	n	1
Iz_1_1109	DJI_0041	Crataegus rhipidophylla Gand.	0	Rosaceae Juss.	n	1
Iz_1_1109	DJI_0041	Frangula alnus Mill.	0	Rhamnaceae Juss.	n	1
Iz_1_1109	DJI_0041	Fraxinus pennsylvanica Marshall	0	Oleaceae Hoffmanns. & Link	a	0
Iz_1_1109	DJI_0041	Pinus sylvestris L.	0	Pinaceae Spreng. ex F.Rudolphi	n	1
Iz_1_1109	DJI_0041	Populus × canescens (Aiton) Sm.	0	Salicaceae Mirb.	n	1
Iz_1_1109	DJI_0041	Populus tremula L.	0	Salicaceae Mirb.	n	1
Iz_1_1109	DJI_0041	Populus nigra L.	0	Salicaceae Mirb.	n	1

Iz_1_1109	DJI_0041	Populus sp.	0	Salicaceae Mirb.	n	1
Iz_1_1109	DJI_0041	Pyrus communis L.	0	Rosaceae Juss.	a	0
Iz_1_1109	DJI_0041	Quercus robur L.	0	Fagaceae Dumort.	n	1
Iz_1_1109	DJI_0041	Robinia pseudoacacia L.	0	Fabaceae Lindl.	a	0
Iz_1_1109	DJI_0041	Rosa sp.	0	Rosaceae Juss.		
Iz_1_1109	DJI_0041	Salix alba L.	0	Salicaceae Mirb.	n	1
Iz_1_1109	DJI_0041	Sambucus nigra L.	0	Viburnaceae Raf.	n	1
Iz_1_1109	DJI_0041	Sorbus aucuparia L.	0	Rosaceae Juss.	n	1
Iz_1_1109	DJI_0041	Tilia cordata Mill.	0	Malvaceae Juss.	n	1
Iz_1_1109	DJI_0041	Ulmus glabra Huds.	0	Ulmaceae Mirb.	n	1
Iz_1_1109	DJI_0037	Acer tataricum L.	0	Sapindaceae Juss.	n	1
Iz_1_1109	DJI_0037	Betula pendula Roth.	0	Betulaceae Gray	n	1
Iz_1_1109	DJI_0037	Crataegus rhipidophylla Gand.	0	Rosaceae Juss.	n	1
Iz_1_1109	DJI_0037	Frangula alnus Mill.	0	Rhamnaceae Juss.	n	1
Iz_1_1109	DJI_0037	Fraxinus pennsylvanica Marshall	0	Oleaceae Hoffmanns. & Link	a	0
Iz_1_1109	DJI_0037	Pinus sylvestris L.	0	Pinaceae Spreng. ex F.Rudolphi	n	1
Iz_1_1109	DJI_0037	Populus × canescens (Aiton) Sm.	0	Salicaceae Mirb.	n	1
Iz_1_1109	DJI_0037	Populus tremula L.	0	Salicaceae Mirb.	n	1
Iz_1_1109	DJI_0037	Populus nigra L.	0	Salicaceae Mirb.	n	1
Iz_1_1109	DJI_0037	Populus sp.	0	Salicaceae Mirb.	n	1
Iz_1_1109	DJI_0037	Pyrus communis L.	0	Rosaceae Juss.	a	0
Iz_1_1109	DJI_0037	Quercus robur L.	0	Fagaceae Dumort.	n	1
Iz_1_1109	DJI_0037	Robinia pseudoacacia L.	0	Fabaceae Lindl.	a	0
Iz_1_1109	DJI_0037	Rosa sp.	0	Rosaceae Juss.		
Iz_1_1109	DJI_0037	Salix alba L.	0	Salicaceae Mirb.	n	1
Iz_1_1109	DJI_0037	Sambucus nigra L.	0	Viburnaceae Raf.	n	1
Iz_1_1109	DJI_0037	Sorbus aucuparia L.	0	Rosaceae Juss.	n	1
Iz_1_1109	DJI_0037	Tilia cordata Mill.	0	Malvaceae Juss.	n	1
Iz_1_1109	DJI_0037	Ulmus glabra Huds.	0	Ulmaceae Mirb.	n	1
Iz_1_1109	DJI_0034	Acer tataricum L.	0	Sapindaceae Juss.	n	1
Iz_1_1109	DJI_0034	Betula pendula Roth.	0	Betulaceae Gray	n	1
Iz_1_1109	DJI_0034	Crataegus rhipidophylla Gand.	0	Rosaceae Juss.	n	1
Iz_1_1109	DJI_0034	Frangula alnus Mill.	0	Rhamnaceae Juss.	n	1
Iz_1_1109	DJI_0034	Fraxinus pennsylvanica Marshall	0	Oleaceae Hoffmanns. & Link	a	0
Iz_1_1109	DJI_0034	Pinus sylvestris L.	0	Pinaceae Spreng. ex F.Rudolphi	n	1
Iz_1_1109	DJI_0034	Populus × canescens (Aiton) Sm.	0	Salicaceae Mirb.	n	1
Iz_1_1109	DJI_0034	Populus tremula L.	0	Salicaceae Mirb.	n	1
Iz_1_1109	DJI_0034	Populus nigra L.	0	Salicaceae Mirb.	n	1
Iz_1_1109	DJI_0034	Populus sp.	0	Salicaceae Mirb.	n	1
Iz_1_1109	DJI_0034	Pyrus communis L.	0	Rosaceae Juss.	a	0
Iz_1_1109	DJI_0034	Quercus robur L.	0	Fagaceae Dumort.	n	1
Iz_1_1109	DJI_0034	Robinia pseudoacacia L.	0	Fabaceae Lindl.	a	0
Iz_1_1109	DJI_0034	Rosa sp.	0	Rosaceae Juss.		
Iz_1_1109	DJI_0034	Salix alba L.	0	Salicaceae Mirb.	n	1
Iz_1_1109	DJI_0034	Sambucus nigra L.	0	Viburnaceae Raf.	n	1
Iz_1_1109	DJI_0034	Sorbus aucuparia L.	0	Rosaceae Juss.	n	1
Iz_1_1109	DJI_0034	Tilia cordata Mill.	0	Malvaceae Juss.	n	1
Iz_1_1109	DJI_0034	Ulmus glabra Huds.	0	Ulmaceae Mirb.	n	1
Iz_1_1109	DJI_0032	Acer tataricum L.	0	Sapindaceae Juss.	n	1
Iz_1_1109	DJI_0032	Betula pendula Roth.	0	Betulaceae Gray	n	1
Iz_1_1109	DJI_0032	Crataegus rhipidophylla Gand.	0	Rosaceae Juss.	n	1
Iz_1_1109	DJI_0032	Frangula alnus Mill.	0	Rhamnaceae Juss.	n	1
Iz_1_1109	DJI_0032	Fraxinus pennsylvanica Marshall	0	Oleaceae Hoffmanns. & Link	a	0
Iz_1_1109	DJI_0032	Pinus sylvestris L.	0	Pinaceae Spreng. ex F.Rudolphi	n	1
Iz_1_1109	DJI_0032	Populus × canescens (Aiton) Sm.	0	Salicaceae Mirb.	n	1
Iz_1_1109	DJI_0032	Populus tremula L.	0	Salicaceae Mirb.	n	1
Iz_1_1109	DJI_0032	Populus nigra L.	0	Salicaceae Mirb.	n	1
Iz_1_1109	DJI_0032	Populus sp.	0	Salicaceae Mirb.	n	1
Iz_1_1109	DJI_0032	Pyrus communis L.	0	Rosaceae Juss.	a	0
Iz_1_1109	DJI_0032	Quercus robur L.	0	Fagaceae Dumort.	n	1
Iz_1_1109	DJI_0032	Robinia pseudoacacia L.	0	Fabaceae Lindl.	a	0
Iz_1_1109	DJI_0032	Rosa sp.	0	Rosaceae Juss.		
Iz_1_1109	DJI_0032	Salix alba L.	0	Salicaceae Mirb.	n	1
Iz_1_1109	DJI_0032	Sambucus nigra L.	0	Viburnaceae Raf.	n	1

Iz_1_1109	DJI_0032	Sorbus aucuparia L.	0	Rosaceae Juss.	n	1
Iz_1_1109	DJI_0032	Tilia cordata Mill.	0	Malvaceae Juss.	n	1
Iz_1_1109	DJI_0032	Ulmus glabra Huds.	0	Ulmaceae Mirb.	n	1
Iz_1_1109	DJI_0029	Acer tataricum L.	0	Sapindaceae Juss.	n	1
Iz_1_1109	DJI_0029	Betula pendula Roth.	0	Betulaceae Gray	n	1
Iz_1_1109	DJI_0029	Crataegus rhipidophylla Gand.	0	Rosaceae Juss.	n	1
Iz_1_1109	DJI_0029	Frangula alnus Mill.	0	Rhamnaceae Juss.	n	1
Iz_1_1109	DJI_0029	Fraxinus pennsylvanica Marshall	0	Oleaceae Hoffmanns. & Link	a	0
Iz_1_1109	DJI_0029	Pinus sylvestris L.	0	Pinaceae Spreng. ex F.Rudolphi	n	1
Iz_1_1109	DJI_0029	Populus × canescens (Aiton) Sm.	0	Salicaceae Mirb.	n	1
Iz_1_1109	DJI_0029	Populus tremula L.	0	Salicaceae Mirb.	n	1
Iz_1_1109	DJI_0029	Populus nigra L.	0	Salicaceae Mirb.	n	1
Iz_1_1109	DJI_0029	Populus sp.	0	Salicaceae Mirb.	n	1
Iz_1_1109	DJI_0029	Pyrus communis L.	0	Rosaceae Juss.	a	0
Iz_1_1109	DJI_0029	Quercus robur L.	0	Fagaceae Dumort.	n	1
Iz_1_1109	DJI_0029	Robinia pseudoacacia L.	0	Fabaceae Lindl.	a	0
Iz_1_1109	DJI_0029	Rosa sp.	0	Rosaceae Juss.		
Iz_1_1109	DJI_0029	Salix alba L.	0	Salicaceae Mirb.	n	1
Iz_1_1109	DJI_0029	Sambucus nigra L.	0	Viburnaceae Raf.	n	1
Iz_1_1109	DJI_0029	Sorbus aucuparia L.	0	Rosaceae Juss.	n	1
Iz_1_1109	DJI_0029	Tilia cordata Mill.	0	Malvaceae Juss.	n	1
Iz_1_1109	DJI_0029	Ulmus glabra Huds.	0	Ulmaceae Mirb.	n	1
Iz_2_1109	DJI_0255	Acer tataricum L.	0	Sapindaceae Juss.	n	1
Iz_2_1109	DJI_0255	Betula pendula Roth.	0	Betulaceae Gray	n	1
Iz_2_1109	DJI_0255	Crataegus rhipidophylla Gand.	0	Rosaceae Juss.	n	1
Iz_2_1109	DJI_0255	Frangula alnus Mill.	0	Rhamnaceae Juss.	n	1
Iz_2_1109	DJI_0255	Fraxinus pennsylvanica Marshall	0	Oleaceae Hoffmanns. & Link	a	0
Iz_2_1109	DJI_0255	Pinus sylvestris L.	0	Pinaceae Spreng. ex F.Rudolphi	n	1
Iz_2_1109	DJI_0255	Populus × canescens (Aiton) Sm.	0	Salicaceae Mirb.	n	1
Iz_2_1109	DJI_0255	Populus tremula L.	0	Salicaceae Mirb.	n	1
Iz_2_1109	DJI_0255	Populus nigra L.	0	Salicaceae Mirb.	n	1
Iz_2_1109	DJI_0255	Pyrus communis L.	0	Rosaceae Juss.	a	0
Iz_2_1109	DJI_0255	Quercus robur L.	0	Fagaceae Dumort.	n	1
Iz_2_1109	DJI_0255	Robinia pseudoacacia L.	0	Fabaceae Lindl.	a	0
Iz_2_1109	DJI_0255	Rosa sp.	0	Rosaceae Juss.		
Iz_2_1109	DJI_0255	Salix alba L.	0	Salicaceae Mirb.	n	1
Iz_2_1109	DJI_0255	Sambucus nigra L.	0	Viburnaceae Raf.	n	1
Iz_2_1109	DJI_0255	Sorbus aucuparia L.	0	Rosaceae Juss.	n	1
Iz_2_1109	DJI_0255	Tilia cordata Mill.	0	Malvaceae Juss.	n	1
Iz_2_1109	DJI_0255	Ulmus glabra Huds.	0	Ulmaceae Mirb.	n	1
Iz_2_1109	DJI_0250	Acer tataricum L.	0	Sapindaceae Juss.	n	1
Iz_2_1109	DJI_0250	Betula pendula Roth.	0	Betulaceae Gray	n	1
Iz_2_1109	DJI_0250	Crataegus rhipidophylla Gand.	0	Rosaceae Juss.	n	1
Iz_2_1109	DJI_0250	Frangula alnus Mill.	0	Rhamnaceae Juss.	n	1
Iz_2_1109	DJI_0250	Fraxinus pennsylvanica Marshall	0	Oleaceae Hoffmanns. & Link	a	0
Iz_2_1109	DJI_0250	Pinus sylvestris L.	0	Pinaceae Spreng. ex F.Rudolphi	n	1
Iz_2_1109	DJI_0250	Populus × canescens (Aiton) Sm.	0	Salicaceae Mirb.	n	1
Iz_2_1109	DJI_0250	Populus tremula L.	0	Salicaceae Mirb.	n	1
Iz_2_1109	DJI_0250	Populus nigra L.	0	Salicaceae Mirb.	n	1
Iz_2_1109	DJI_0250	Pyrus communis L.	0	Rosaceae Juss.	a	0
Iz_2_1109	DJI_0250	Quercus robur L.	0	Fagaceae Dumort.	n	1
Iz_2_1109	DJI_0250	Robinia pseudoacacia L.	0	Fabaceae Lindl.	a	0
Iz_2_1109	DJI_0250	Rosa sp.	0	Rosaceae Juss.		
Iz_2_1109	DJI_0250	Salix alba L.	0	Salicaceae Mirb.	n	1
Iz_2_1109	DJI_0250	Sambucus nigra L.	0	Viburnaceae Raf.	n	1
Iz_2_1109	DJI_0250	Sorbus aucuparia L.	0	Rosaceae Juss.	n	1
Iz_2_1109	DJI_0250	Tilia cordata Mill.	0	Malvaceae Juss.	n	1
Iz_2_1109	DJI_0250	Ulmus glabra Huds.	0	Ulmaceae Mirb.	n	1
Iz_2_1109	DJI_0245	Acer tataricum L.	0	Sapindaceae Juss.	n	1
Iz_2_1109	DJI_0245	Betula pendula Roth.	0	Betulaceae Gray	n	1
Iz_2_1109	DJI_0245	Crataegus rhipidophylla Gand.	0	Rosaceae Juss.	n	1
Iz_2_1109	DJI_0245	Frangula alnus Mill.	0	Rhamnaceae Juss.	n	1
Iz_2_1109	DJI_0245	Fraxinus pennsylvanica Marshall	0	Oleaceae Hoffmanns. & Link	a	0
Iz_2_1109	DJI_0245	Pinus sylvestris L.	0	Pinaceae Spreng. ex F.Rudolphi	n	1

Iz_2_1109	DJI_0245	Populus × canescens (Aiton) Sm.	0	Salicaceae Mirb.	n	1
Iz_2_1109	DJI_0245	Populus tremula L.	0	Salicaceae Mirb.	n	1
Iz_2_1109	DJI_0245	Populus nigra L.	0	Salicaceae Mirb.	n	1
Iz_2_1109	DJI_0245	Pyrus communis L.	0	Rosaceae Juss.	a	0
Iz_2_1109	DJI_0245	Quercus robur L.	0	Fagaceae Dumort.	n	1
Iz_2_1109	DJI_0245	Robinia pseudoacacia L.	0	Fabaceae Lindl.	a	0
Iz_2_1109	DJI_0245	Rosa sp.	0	Rosaceae Juss.		
Iz_2_1109	DJI_0245	Salix alba L.	0	Salicaceae Mirb.	n	1
Iz_2_1109	DJI_0245	Sambucus nigra L.	0	Viburnaceae Raf.	n	1
Iz_2_1109	DJI_0245	Sorbus aucuparia L.	0	Rosaceae Juss.	n	1
Iz_2_1109	DJI_0245	Tilia cordata Mill.	0	Malvaceae Juss.	n	1
Iz_2_1109	DJI_0245	Ulmus glabra Huds.	0	Ulmaceae Mirb.	n	1
Iz_2_1109	DJI_0238	Acer tataricum L.	0	Sapindaceae Juss.	n	1
Iz_2_1109	DJI_0238	Betula pendula Roth.	0	Betulaceae Gray	n	1
Iz_2_1109	DJI_0238	Crataegus rhipidophylla Gand.	0	Rosaceae Juss.	n	1
Iz_2_1109	DJI_0238	Frangula alnus Mill.	0	Rhamnaceae Juss.	n	1
Iz_2_1109	DJI_0238	Fraxinus pennsylvanica Marshall	0	Oleaceae Hoffmanns. & Link	a	0
Iz_2_1109	DJI_0238	Pinus sylvestris L.	0	Pinaceae Spreng. ex F.Rudolphi	n	1
Iz_2_1109	DJI_0238	Populus × canescens (Aiton) Sm.	0	Salicaceae Mirb.	n	1
Iz_2_1109	DJI_0238	Populus tremula L.	0	Salicaceae Mirb.	n	1
Iz_2_1109	DJI_0238	Populus nigra L.	0	Salicaceae Mirb.	n	1
Iz_2_1109	DJI_0238	Pyrus communis L.	0	Rosaceae Juss.	a	0
Iz_2_1109	DJI_0238	Quercus robur L.	0	Fagaceae Dumort.	n	1
Iz_2_1109	DJI_0238	Robinia pseudoacacia L.	0	Fabaceae Lindl.	a	0
Iz_2_1109	DJI_0238	Rosa sp.	0	Rosaceae Juss.		
Iz_2_1109	DJI_0238	Salix alba L.	0	Salicaceae Mirb.	n	1
Iz_2_1109	DJI_0238	Sambucus nigra L.	0	Viburnaceae Raf.	n	1
Iz_2_1109	DJI_0238	Sorbus aucuparia L.	0	Rosaceae Juss.	n	1
Iz_2_1109	DJI_0238	Tilia cordata Mill.	0	Malvaceae Juss.	n	1
Iz_2_1109	DJI_0238	Ulmus glabra Huds.	0	Ulmaceae Mirb.	n	1
Iz_2_1109	DJI_00237	Acer tataricum L.	0	Sapindaceae Juss.	n	1
Iz_2_1109	DJI_00237	Betula pendula Roth.	0	Betulaceae Gray	n	1
Iz_2_1109	DJI_00237	Crataegus rhipidophylla Gand.	0	Rosaceae Juss.	n	1
Iz_2_1109	DJI_00237	Frangula alnus Mill.	0	Rhamnaceae Juss.	n	1
Iz_2_1109	DJI_00237	Fraxinus pennsylvanica Marshall	0	Oleaceae Hoffmanns. & Link	a	0
Iz_2_1109	DJI_00237	Pinus sylvestris L.	0	Pinaceae Spreng. ex F.Rudolphi	n	1
Iz_2_1109	DJI_00237	Populus × canescens (Aiton) Sm.	0	Salicaceae Mirb.	n	1
Iz_2_1109	DJI_00237	Populus tremula L.	0	Salicaceae Mirb.	n	1
Iz_2_1109	DJI_00237	Populus nigra L.	0	Salicaceae Mirb.	n	1
Iz_2_1109	DJI_00237	Pyrus communis L.	0	Rosaceae Juss.	a	0
Iz_2_1109	DJI_00237	Quercus robur L.	0	Fagaceae Dumort.	n	1
Iz_2_1109	DJI_00237	Robinia pseudoacacia L.	0	Fabaceae Lindl.	a	0
Iz_2_1109	DJI_00237	Rosa sp.	0	Rosaceae Juss.		
Iz_2_1109	DJI_00237	Salix alba L.	0	Salicaceae Mirb.	n	1
Iz_2_1109	DJI_00237	Sambucus nigra L.	0	Viburnaceae Raf.	n	1
Iz_2_1109	DJI_00237	Sorbus aucuparia L.	0	Rosaceae Juss.	n	1
Iz_2_1109	DJI_00237	Tilia cordata Mill.	0	Malvaceae Juss.	n	1
Iz_2_1109	DJI_00237	Ulmus glabra Huds.	0	Ulmaceae Mirb.	n	1
Iz_2_1109	DJI_0231	Acer tataricum L.	0	Sapindaceae Juss.	n	1
Iz_2_1109	DJI_0231	Betula pendula Roth.	0	Betulaceae Gray	n	1
Iz_2_1109	DJI_0231	Crataegus rhipidophylla Gand.	0	Rosaceae Juss.	n	1
Iz_2_1109	DJI_0231	Frangula alnus Mill.	0	Rhamnaceae Juss.	n	1
Iz_2_1109	DJI_0231	Fraxinus pennsylvanica Marshall	0	Oleaceae Hoffmanns. & Link	a	0
Iz_2_1109	DJI_0231	Pinus sylvestris L.	0	Pinaceae Spreng. ex F.Rudolphi	n	1
Iz_2_1109	DJI_0231	Populus × canescens (Aiton) Sm.	0	Salicaceae Mirb.	n	1
Iz_2_1109	DJI_0231	Populus tremula L.	2	Salicaceae Mirb.	n	1
Iz_2_1109	DJI_0231	Populus nigra L.	0	Salicaceae Mirb.	n	1
Iz_2_1109	DJI_0231	Pyrus communis L.	0	Rosaceae Juss.	a	0
Iz_2_1109	DJI_0231	Quercus robur L.	0	Fagaceae Dumort.	n	1
Iz_2_1109	DJI_0231	Robinia pseudoacacia L.	0	Fabaceae Lindl.	a	0
Iz_2_1109	DJI_0231	Rosa sp.	0	Rosaceae Juss.		
Iz_2_1109	DJI_0231	Salix alba L.	0	Salicaceae Mirb.	n	1
Iz_2_1109	DJI_0231	Sambucus nigra L.	0	Viburnaceae Raf.	n	1
Iz_2_1109	DJI_0231	Sorbus aucuparia L.	0	Rosaceae Juss.	n	1

Iz_2_1109	DJI_0231	Tilia cordata Mill.	0	Malvaceae Juss.	n	1
Iz_2_1109	DJI_0231	Ulmus glabra Huds.	0	Ulmaceae Mirb.	n	1
Iz_2_1109	DJI_0229	Acer tataricum L.	0	Sapindaceae Juss.	n	1
Iz_2_1109	DJI_0229	Betula pendula Roth.	0	Betulaceae Gray	n	1
Iz_2_1109	DJI_0229	Crataegus rhipidophylla Gand.	0	Rosaceae Juss.	n	1
Iz_2_1109	DJI_0229	Frangula alnus Mill.	0	Rhamnaceae Juss.	n	1
Iz_2_1109	DJI_0229	Fraxinus pennsylvanica Marshall	0	Oleaceae Hoffmanns. & Link	a	0
Iz_2_1109	DJI_0229	Pinus sylvestris L.	0	Pinaceae Spreng. ex F.Rudolphi	n	1
Iz_2_1109	DJI_0229	Populus × canescens (Aiton) Sm.	0	Salicaceae Mirb.	n	1
Iz_2_1109	DJI_0229	Populus tremula L.	0	Salicaceae Mirb.	n	1
Iz_2_1109	DJI_0229	Populus nigra L.	0	Salicaceae Mirb.	n	1
Iz_2_1109	DJI_0229	Pyrus communis L.	0	Rosaceae Juss.	a	0
Iz_2_1109	DJI_0229	Quercus robur L.	0	Fagaceae Dumort.	n	1
Iz_2_1109	DJI_0229	Robinia pseudoacacia L.	0	Fabaceae Lindl.	a	0
Iz_2_1109	DJI_0229	Rosa sp.	0	Rosaceae Juss.		
Iz_2_1109	DJI_0229	Salix alba L.	0	Salicaceae Mirb.	n	1
Iz_2_1109	DJI_0229	Sambucus nigra L.	0	Viburnaceae Raf.	n	1
Iz_2_1109	DJI_0229	Sorbus aucuparia L.	0	Rosaceae Juss.	n	1
Iz_2_1109	DJI_0229	Tilia cordata Mill.	0	Malvaceae Juss.	n	1
Iz_2_1109	DJI_0229	Ulmus glabra Huds.	0	Ulmaceae Mirb.	n	1
Iz_2_1109	DJI_0225	Acer tataricum L.	0	Sapindaceae Juss.	n	1
Iz_2_1109	DJI_0225	Betula pendula Roth.	0	Betulaceae Gray	n	1
Iz_2_1109	DJI_0225	Crataegus rhipidophylla Gand.	0	Rosaceae Juss.	n	1
Iz_2_1109	DJI_0225	Frangula alnus Mill.	0	Rhamnaceae Juss.	n	1
Iz_2_1109	DJI_0225	Fraxinus pennsylvanica Marshall	0	Oleaceae Hoffmanns. & Link	a	0
Iz_2_1109	DJI_0225	Pinus sylvestris L.	0	Pinaceae Spreng. ex F.Rudolphi	n	1
Iz_2_1109	DJI_0225	Populus × canescens (Aiton) Sm.	0	Salicaceae Mirb.	n	1
Iz_2_1109	DJI_0225	Populus tremula L.	0	Salicaceae Mirb.	n	1
Iz_2_1109	DJI_0225	Populus nigra L.	0	Salicaceae Mirb.	n	1
Iz_2_1109	DJI_0225	Pyrus communis L.	0	Rosaceae Juss.	a	0
Iz_2_1109	DJI_0225	Quercus robur L.	0	Fagaceae Dumort.	n	1
Iz_2_1109	DJI_0225	Robinia pseudoacacia L.	0	Fabaceae Lindl.	a	0
Iz_2_1109	DJI_0225	Rosa sp.	0	Rosaceae Juss.		
Iz_2_1109	DJI_0225	Salix alba L.	0	Salicaceae Mirb.	n	1
Iz_2_1109	DJI_0225	Sambucus nigra L.	0	Viburnaceae Raf.	n	1
Iz_2_1109	DJI_0225	Sorbus aucuparia L.	0	Rosaceae Juss.	n	1
Iz_2_1109	DJI_0225	Tilia cordata Mill.	0	Malvaceae Juss.	n	1
Iz_2_1109	DJI_0225	Ulmus glabra Huds.	0	Ulmaceae Mirb.	n	1
Iz_2_1109	DJI_0222	Acer tataricum L.	0	Sapindaceae Juss.	n	1
Iz_2_1109	DJI_0222	Betula pendula Roth.	0	Betulaceae Gray	n	1
Iz_2_1109	DJI_0222	Crataegus rhipidophylla Gand.	0	Rosaceae Juss.	n	1
Iz_2_1109	DJI_0222	Frangula alnus Mill.	0	Rhamnaceae Juss.	n	1
Iz_2_1109	DJI_0222	Fraxinus pennsylvanica Marshall	0	Oleaceae Hoffmanns. & Link	a	0
Iz_2_1109	DJI_0222	Pinus sylvestris L.	0	Pinaceae Spreng. ex F.Rudolphi	n	1
Iz_2_1109	DJI_0222	Populus × canescens (Aiton) Sm.	0	Salicaceae Mirb.	n	1
Iz_2_1109	DJI_0222	Populus tremula L.	0	Salicaceae Mirb.	n	1
Iz_2_1109	DJI_0222	Populus nigra L.	0	Salicaceae Mirb.	n	1
Iz_2_1109	DJI_0222	Pyrus communis L.	0	Rosaceae Juss.	a	0
Iz_2_1109	DJI_0222	Quercus robur L.	0	Fagaceae Dumort.	n	1
Iz_2_1109	DJI_0222	Robinia pseudoacacia L.	0	Fabaceae Lindl.	a	0
Iz_2_1109	DJI_0222	Rosa sp.	0	Rosaceae Juss.		
Iz_2_1109	DJI_0222	Salix alba L.	0	Salicaceae Mirb.	n	1
Iz_2_1109	DJI_0222	Sambucus nigra L.	0	Viburnaceae Raf.	n	1
Iz_2_1109	DJI_0222	Sorbus aucuparia L.	0	Rosaceae Juss.	n	1
Iz_2_1109	DJI_0222	Tilia cordata Mill.	0	Malvaceae Juss.	n	1
Iz_2_1109	DJI_0222	Ulmus glabra Huds.	0	Ulmaceae Mirb.	n	1
Iz_3_1109	DJI_0140	Acer tataricum L.	0	Sapindaceae Juss.	n	1
Iz_3_1109	DJI_0140	Betula pendula Roth.	0	Betulaceae Gray	n	1
Iz_3_1109	DJI_0140	Crataegus rhipidophylla Gand.	0	Rosaceae Juss.	n	1
Iz_3_1109	DJI_0140	Frangula alnus Mill.	0	Rhamnaceae Juss.	n	1
Iz_3_1109	DJI_0140	Fraxinus pennsylvanica Marshall	0	Oleaceae Hoffmanns. & Link	a	0
Iz_3_1109	DJI_0140	Pinus sylvestris L.	0	Pinaceae Spreng. ex F.Rudolphi	n	1
Iz_3_1109	DJI_0140	Populus × canescens (Aiton) Sm.	0	Salicaceae Mirb.	n	1
Iz_3_1109	DJI_0140	Populus tremula L.	0	Salicaceae Mirb.	n	1

Iz_3_1109	DJI_0140	Populus nigra L.	0	Salicaceae Mirb.	n	1
Iz_3_1109	DJI_0140	Pyrus communis L.	0	Rosaceae Juss.	a	0
Iz_3_1109	DJI_0140	Quercus robur L.	0	Fagaceae Dumort.	n	1
Iz_3_1109	DJI_0140	Robinia pseudoacacia L.	0	Fabaceae Lindl.	a	0
Iz_3_1109	DJI_0140	Rosa sp.	0	Rosaceae Juss.		
Iz_3_1109	DJI_0140	Salix alba L.	0	Salicaceae Mirb.	n	1
Iz_3_1109	DJI_0140	Sambucus nigra L.	0	Viburnaceae Raf.	n	1
Iz_3_1109	DJI_0140	Sorbus aucuparia L.	0	Rosaceae Juss.	n	1
Iz_3_1109	DJI_0140	Tilia cordata Mill.	0	Malvaceae Juss.	n	1
Iz_3_1109	DJI_0140	Ulmus glabra Huds.	0	Ulmaceae Mirb.	n	1
Iz_3_1109	DJI_0139	Acer tataricum L.	0	Sapindaceae Juss.	n	1
Iz_3_1109	DJI_0139	Betula pendula Roth.	0	Betulaceae Gray	n	1
Iz_3_1109	DJI_0139	Crataegus rhipidophylla Gand.	0	Rosaceae Juss.	n	1
Iz_3_1109	DJI_0139	Frangula alnus Mill.	0	Rhamnaceae Juss.	n	1
Iz_3_1109	DJI_0139	Fraxinus pennsylvanica Marshall	0	Oleaceae Hoffmanns. & Link	a	0
Iz_3_1109	DJI_0139	Pinus sylvestris L.	0	Pinaceae Spreng. ex F.Rudolphi	n	1
Iz_3_1109	DJI_0139	Populus × canescens (Aiton) Sm.	0	Salicaceae Mirb.	n	1
Iz_3_1109	DJI_0139	Populus tremula L.	0	Salicaceae Mirb.	n	1
Iz_3_1109	DJI_0139	Populus nigra L.	0	Salicaceae Mirb.	n	1
Iz_3_1109	DJI_0139	Pyrus communis L.	0	Rosaceae Juss.	a	0
Iz_3_1109	DJI_0139	Quercus robur L.	0	Fagaceae Dumort.	n	1
Iz_3_1109	DJI_0139	Robinia pseudoacacia L.	0	Fabaceae Lindl.	a	0
Iz_3_1109	DJI_0139	Rosa sp.	0	Rosaceae Juss.		
Iz_3_1109	DJI_0139	Salix alba L.	0	Salicaceae Mirb.	n	1
Iz_3_1109	DJI_0139	Sambucus nigra L.	0	Viburnaceae Raf.	n	1
Iz_3_1109	DJI_0139	Sorbus aucuparia L.	0	Rosaceae Juss.	n	1
Iz_3_1109	DJI_0139	Tilia cordata Mill.	0	Malvaceae Juss.	n	1
Iz_3_1109	DJI_0139	Ulmus glabra Huds.	0	Ulmaceae Mirb.	n	1
Iz_3_1109	DJI_0137	Acer tataricum L.	0	Sapindaceae Juss.	n	1
Iz_3_1109	DJI_0137	Betula pendula Roth.	0	Betulaceae Gray	n	1
Iz_3_1109	DJI_0137	Crataegus rhipidophylla Gand.	0	Rosaceae Juss.	n	1
Iz_3_1109	DJI_0137	Frangula alnus Mill.	0	Rhamnaceae Juss.	n	1
Iz_3_1109	DJI_0137	Fraxinus pennsylvanica Marshall	0	Oleaceae Hoffmanns. & Link	a	0
Iz_3_1109	DJI_0137	Pinus sylvestris L.	0	Pinaceae Spreng. ex F.Rudolphi	n	1
Iz_3_1109	DJI_0137	Populus × canescens (Aiton) Sm.	0	Salicaceae Mirb.	n	1
Iz_3_1109	DJI_0137	Populus tremula L.	0	Salicaceae Mirb.	n	1
Iz_3_1109	DJI_0137	Populus nigra L.	0	Salicaceae Mirb.	n	1
Iz_3_1109	DJI_0137	Pyrus communis L.	0	Rosaceae Juss.	a	0
Iz_3_1109	DJI_0137	Quercus robur L.	0	Fagaceae Dumort.	n	1
Iz_3_1109	DJI_0137	Robinia pseudoacacia L.	0	Fabaceae Lindl.	a	0
Iz_3_1109	DJI_0137	Rosa sp.	0	Rosaceae Juss.		
Iz_3_1109	DJI_0137	Salix alba L.	0	Salicaceae Mirb.	n	1
Iz_3_1109	DJI_0137	Sambucus nigra L.	0	Viburnaceae Raf.	n	1
Iz_3_1109	DJI_0137	Sorbus aucuparia L.	0	Rosaceae Juss.	n	1
Iz_3_1109	DJI_0137	Tilia cordata Mill.	0	Malvaceae Juss.	n	1
Iz_3_1109	DJI_0137	Ulmus glabra Huds.	0	Ulmaceae Mirb.	n	1
Iz_3_1109	DJI_0135	Acer tataricum L.	0	Sapindaceae Juss.	n	1
Iz_3_1109	DJI_0135	Betula pendula Roth.	0	Betulaceae Gray	n	1
Iz_3_1109	DJI_0135	Crataegus rhipidophylla Gand.	0	Rosaceae Juss.	n	1
Iz_3_1109	DJI_0135	Frangula alnus Mill.	0	Rhamnaceae Juss.	n	1
Iz_3_1109	DJI_0135	Fraxinus pennsylvanica Marshall	0	Oleaceae Hoffmanns. & Link	a	0
Iz_3_1109	DJI_0135	Pinus sylvestris L.	0	Pinaceae Spreng. ex F.Rudolphi	n	1
Iz_3_1109	DJI_0135	Populus × canescens (Aiton) Sm.	0	Salicaceae Mirb.	n	1
Iz_3_1109	DJI_0135	Populus tremula L.	0	Salicaceae Mirb.	n	1
Iz_3_1109	DJI_0135	Populus nigra L.	0	Salicaceae Mirb.	n	1
Iz_3_1109	DJI_0135	Pyrus communis L.	0	Rosaceae Juss.	a	0
Iz_3_1109	DJI_0135	Quercus robur L.	0	Fagaceae Dumort.	n	1
Iz_3_1109	DJI_0135	Robinia pseudoacacia L.	0	Fabaceae Lindl.	a	0
Iz_3_1109	DJI_0135	Rosa sp.	0	Rosaceae Juss.		
Iz_3_1109	DJI_0135	Salix alba L.	0	Salicaceae Mirb.	n	1
Iz_3_1109	DJI_0135	Sambucus nigra L.	0	Viburnaceae Raf.	n	1
Iz_3_1109	DJI_0135	Sorbus aucuparia L.	0	Rosaceae Juss.	n	1
Iz_3_1109	DJI_0135	Tilia cordata Mill.	0	Malvaceae Juss.	n	1
Iz_3_1109	DJI_0135	Ulmus glabra Huds.	0	Ulmaceae Mirb.	n	1

Iz_3_1109	DJI_0132	Acer tataricum L.	0	Sapindaceae Juss.	n	1
Iz_3_1109	DJI_0132	Betula pendula Roth.	0	Betulaceae Gray	n	1
Iz_3_1109	DJI_0132	Crataegus rhipidophylla Gand.	0	Rosaceae Juss.	n	1
Iz_3_1109	DJI_0132	Frangula alnus Mill.	0	Rhamnaceae Juss.	n	1
Iz_3_1109	DJI_0132	Fraxinus pennsylvanica Marshall	0	Oleaceae Hoffmanns. & Link	a	0
Iz_3_1109	DJI_0132	Pinus sylvestris L.	0	Pinaceae Spreng. ex F.Rudolphi	n	1
Iz_3_1109	DJI_0132	Populus × canescens (Aiton) Sm.	0	Salicaceae Mirb.	n	1
Iz_3_1109	DJI_0132	Populus tremula L.	0	Salicaceae Mirb.	n	1
Iz_3_1109	DJI_0132	Populus nigra L.	0	Salicaceae Mirb.	n	1
Iz_3_1109	DJI_0132	Pyrus communis L.	0	Rosaceae Juss.	a	0
Iz_3_1109	DJI_0132	Quercus robur L.	0	Fagaceae Dumort.	n	1
Iz_3_1109	DJI_0132	Robinia pseudoacacia L.	0	Fabaceae Lindl.	a	0
Iz_3_1109	DJI_0132	Rosa sp.	0	Rosaceae Juss.		
Iz_3_1109	DJI_0132	Salix alba L.	0	Salicaceae Mirb.	n	1
Iz_3_1109	DJI_0132	Sambucus nigra L.	0	Viburnaceae Raf.	n	1
Iz_3_1109	DJI_0132	Sorbus aucuparia L.	0	Rosaceae Juss.	n	1
Iz_3_1109	DJI_0132	Tilia cordata Mill.	0	Malvaceae Juss.	n	1
Iz_3_1109	DJI_0132	Ulmus glabra Huds.	0	Ulmaceae Mirb.	n	1
Iz_3_1109	DJI_0128	Acer tataricum L.	0	Sapindaceae Juss.	n	1
Iz_3_1109	DJI_0128	Betula pendula Roth.	0	Betulaceae Gray	n	1
Iz_3_1109	DJI_0128	Crataegus rhipidophylla Gand.	0	Rosaceae Juss.	n	1
Iz_3_1109	DJI_0128	Frangula alnus Mill.	0	Rhamnaceae Juss.	n	1
Iz_3_1109	DJI_0128	Fraxinus pennsylvanica Marshall	0	Oleaceae Hoffmanns. & Link	a	0
Iz_3_1109	DJI_0128	Pinus sylvestris L.	0	Pinaceae Spreng. ex F.Rudolphi	n	1
Iz_3_1109	DJI_0128	Populus × canescens (Aiton) Sm.	0	Salicaceae Mirb.	n	1
Iz_3_1109	DJI_0128	Populus tremula L.	0	Salicaceae Mirb.	n	1
Iz_3_1109	DJI_0128	Populus nigra L.	0	Salicaceae Mirb.	n	1
Iz_3_1109	DJI_0128	Pyrus communis L.	0	Rosaceae Juss.	a	0
Iz_3_1109	DJI_0128	Quercus robur L.	0	Fagaceae Dumort.	n	1
Iz_3_1109	DJI_0128	Robinia pseudoacacia L.	0	Fabaceae Lindl.	a	0
Iz_3_1109	DJI_0128	Rosa sp.	0	Rosaceae Juss.		
Iz_3_1109	DJI_0128	Salix alba L.	0	Salicaceae Mirb.	n	1
Iz_3_1109	DJI_0128	Sambucus nigra L.	0	Viburnaceae Raf.	n	1
Iz_3_1109	DJI_0128	Sorbus aucuparia L.	0	Rosaceae Juss.	n	1
Iz_3_1109	DJI_0128	Tilia cordata Mill.	0	Malvaceae Juss.	n	1
Iz_3_1109	DJI_0128	Ulmus glabra Huds.	0	Ulmaceae Mirb.	n	1
Iz_3_1109	DJI_0126	Acer tataricum L.	0	Sapindaceae Juss.	n	1
Iz_3_1109	DJI_0126	Betula pendula Roth.	0	Betulaceae Gray	n	1
Iz_3_1109	DJI_0126	Crataegus rhipidophylla Gand.	0	Rosaceae Juss.	n	1
Iz_3_1109	DJI_0126	Frangula alnus Mill.	0	Rhamnaceae Juss.	n	1
Iz_3_1109	DJI_0126	Fraxinus pennsylvanica Marshall	0	Oleaceae Hoffmanns. & Link	a	0
Iz_3_1109	DJI_0126	Pinus sylvestris L.	0	Pinaceae Spreng. ex F.Rudolphi	n	1
Iz_3_1109	DJI_0126	Populus × canescens (Aiton) Sm.	0	Salicaceae Mirb.	n	1
Iz_3_1109	DJI_0126	Populus tremula L.	0	Salicaceae Mirb.	n	1
Iz_3_1109	DJI_0126	Populus nigra L.	0	Salicaceae Mirb.	n	1
Iz_3_1109	DJI_0126	Pyrus communis L.	0	Rosaceae Juss.	a	0
Iz_3_1109	DJI_0126	Quercus robur L.	0	Fagaceae Dumort.	n	1
Iz_3_1109	DJI_0126	Robinia pseudoacacia L.	0	Fabaceae Lindl.	a	0
Iz_3_1109	DJI_0126	Rosa sp.	0	Rosaceae Juss.		
Iz_3_1109	DJI_0126	Salix alba L.	0	Salicaceae Mirb.	n	1
Iz_3_1109	DJI_0126	Sambucus nigra L.	0	Viburnaceae Raf.	n	1
Iz_3_1109	DJI_0126	Sorbus aucuparia L.	0	Rosaceae Juss.	n	1
Iz_3_1109	DJI_0126	Tilia cordata Mill.	0	Malvaceae Juss.	n	1
Iz_3_1109	DJI_0126	Ulmus glabra Huds.	0	Ulmaceae Mirb.	n	1
Iz_3_1109	DJI_0123	Acer tataricum L.	0	Sapindaceae Juss.	n	1
Iz_3_1109	DJI_0123	Betula pendula Roth.	0	Betulaceae Gray	n	1
Iz_3_1109	DJI_0123	Crataegus rhipidophylla Gand.	0	Rosaceae Juss.	n	1
Iz_3_1109	DJI_0123	Frangula alnus Mill.	0	Rhamnaceae Juss.	n	1
Iz_3_1109	DJI_0123	Fraxinus pennsylvanica Marshall	0	Oleaceae Hoffmanns. & Link	a	0
Iz_3_1109	DJI_0123	Pinus sylvestris L.	0	Pinaceae Spreng. ex F.Rudolphi	n	1
Iz_3_1109	DJI_0123	Populus × canescens (Aiton) Sm.	0	Salicaceae Mirb.	n	1
Iz_3_1109	DJI_0123	Populus tremula L.	0	Salicaceae Mirb.	n	1
Iz_3_1109	DJI_0123	Populus nigra L.	0	Salicaceae Mirb.	n	1
Iz_3_1109	DJI_0123	Pyrus communis L.	0	Rosaceae Juss.	a	0

Iz_3_1109	DJI_0123	Quercus robur L.	0	Fagaceae Dumort.	n	1
Iz_3_1109	DJI_0123	Robinia pseudoacacia L.	0	Fabaceae Lindl.	a	0
Iz_3_1109	DJI_0123	Rosa sp.	0	Rosaceae Juss.		
Iz_3_1109	DJI_0123	Salix alba L.	0	Salicaceae Mirb.	n	1
Iz_3_1109	DJI_0123	Sambucus nigra L.	0	Viburnaceae Raf.	n	1
Iz_3_1109	DJI_0123	Sorbus aucuparia L.	0	Rosaceae Juss.	n	1
Iz_3_1109	DJI_0123	Tilia cordata Mill.	0	Malvaceae Juss.	n	1
Iz_3_1109	DJI_0123	Ulmus glabra Huds.	0	Ulmaceae Mirb.	n	1
Iz_3_1109	DJI_0120	Acer tataricum L.	0	Sapindaceae Juss.	n	1
Iz_3_1109	DJI_0120	Betula pendula Roth.	0	Betulaceae Gray	n	1
Iz_3_1109	DJI_0120	Crataegus rhipidophylla Gand.	0	Rosaceae Juss.	n	1
Iz_3_1109	DJI_0120	Frangula alnus Mill.	0	Rhamnaceae Juss.	n	1
Iz_3_1109	DJI_0120	Fraxinus pennsylvanica Marshall	0	Oleaceae Hoffmanns. & Link	a	0
Iz_3_1109	DJI_0120	Pinus sylvestris L.	0	Pinaceae Spreng. ex F.Rudolphi	n	1
Iz_3_1109	DJI_0120	Populus × canescens (Aiton) Sm.	0	Salicaceae Mirb.	n	1
Iz_3_1109	DJI_0120	Populus tremula L.	0	Salicaceae Mirb.	n	1
Iz_3_1109	DJI_0120	Populus nigra L.	0	Salicaceae Mirb.	n	1
Iz_3_1109	DJI_0120	Pyrus communis L.	0	Rosaceae Juss.	a	0
Iz_3_1109	DJI_0120	Quercus robur L.	0	Fagaceae Dumort.	n	1
Iz_3_1109	DJI_0120	Robinia pseudoacacia L.	0	Fabaceae Lindl.	a	0
Iz_3_1109	DJI_0120	Rosa sp.	0	Rosaceae Juss.		
Iz_3_1109	DJI_0120	Salix alba L.	0	Salicaceae Mirb.	n	1
Iz_3_1109	DJI_0120	Sambucus nigra L.	0	Viburnaceae Raf.	n	1
Iz_3_1109	DJI_0120	Sorbus aucuparia L.	0	Rosaceae Juss.	n	1
Iz_3_1109	DJI_0120	Tilia cordata Mill.	0	Malvaceae Juss.	n	1
Iz_3_1109	DJI_0120	Ulmus glabra Huds.	0	Ulmaceae Mirb.	n	1
Iz_3_1109	DJI_0118	Acer tataricum L.	0	Sapindaceae Juss.	n	1
Iz_3_1109	DJI_0118	Betula pendula Roth.	0	Betulaceae Gray	n	1
Iz_3_1109	DJI_0118	Crataegus rhipidophylla Gand.	0	Rosaceae Juss.	n	1
Iz_3_1109	DJI_0118	Frangula alnus Mill.	0	Rhamnaceae Juss.	n	1
Iz_3_1109	DJI_0118	Fraxinus pennsylvanica Marshall	0	Oleaceae Hoffmanns. & Link	a	0
Iz_3_1109	DJI_0118	Pinus sylvestris L.	0	Pinaceae Spreng. ex F.Rudolphi	n	1
Iz_3_1109	DJI_0118	Populus × canescens (Aiton) Sm.	0	Salicaceae Mirb.	n	1
Iz_3_1109	DJI_0118	Populus tremula L.	0	Salicaceae Mirb.	n	1
Iz_3_1109	DJI_0118	Populus nigra L.	0	Salicaceae Mirb.	n	1
Iz_3_1109	DJI_0118	Pyrus communis L.	0	Rosaceae Juss.	a	0
Iz_3_1109	DJI_0118	Quercus robur L.	0	Fagaceae Dumort.	n	1
Iz_3_1109	DJI_0118	Robinia pseudoacacia L.	0	Fabaceae Lindl.	a	0
Iz_3_1109	DJI_0118	Rosa sp.	0	Rosaceae Juss.		
Iz_3_1109	DJI_0118	Salix alba L.	0	Salicaceae Mirb.	n	1
Iz_3_1109	DJI_0118	Sambucus nigra L.	0	Viburnaceae Raf.	n	1
Iz_3_1109	DJI_0118	Sorbus aucuparia L.	0	Rosaceae Juss.	n	1
Iz_3_1109	DJI_0118	Tilia cordata Mill.	0	Malvaceae Juss.	n	1
Iz_3_1109	DJI_0118	Ulmus glabra Huds.	0	Ulmaceae Mirb.	n	1
Iz_3_1109	DJI_0116	Acer tataricum L.	0	Sapindaceae Juss.	n	1
Iz_3_1109	DJI_0116	Betula pendula Roth.	0	Betulaceae Gray	n	1
Iz_3_1109	DJI_0116	Crataegus rhipidophylla Gand.	0	Rosaceae Juss.	n	1
Iz_3_1109	DJI_0116	Frangula alnus Mill.	0	Rhamnaceae Juss.	n	1
Iz_3_1109	DJI_0116	Fraxinus pennsylvanica Marshall	0	Oleaceae Hoffmanns. & Link	a	0
Iz_3_1109	DJI_0116	Pinus sylvestris L.	0	Pinaceae Spreng. ex F.Rudolphi	n	1
Iz_3_1109	DJI_0116	Populus × canescens (Aiton) Sm.	0	Salicaceae Mirb.	n	1
Iz_3_1109	DJI_0116	Populus tremula L.	0	Salicaceae Mirb.	n	1
Iz_3_1109	DJI_0116	Populus nigra L.	0	Salicaceae Mirb.	n	1
Iz_3_1109	DJI_0116	Pyrus communis L.	0	Rosaceae Juss.	a	0
Iz_3_1109	DJI_0116	Quercus robur L.	0	Fagaceae Dumort.	n	1
Iz_3_1109	DJI_0116	Robinia pseudoacacia L.	0	Fabaceae Lindl.	a	0
Iz_3_1109	DJI_0116	Rosa sp.	0	Rosaceae Juss.		
Iz_3_1109	DJI_0116	Salix alba L.	0	Salicaceae Mirb.	n	1
Iz_3_1109	DJI_0116	Sambucus nigra L.	0	Viburnaceae Raf.	n	1
Iz_3_1109	DJI_0116	Sorbus aucuparia L.	0	Rosaceae Juss.	n	1
Iz_3_1109	DJI_0116	Tilia cordata Mill.	0	Malvaceae Juss.	n	1
Iz_3_1109	DJI_0116	Ulmus glabra Huds.	0	Ulmaceae Mirb.	n	1
Iz_3_1109	DJI_0114	Acer tataricum L.	0	Sapindaceae Juss.	n	1
Iz_3_1109	DJI_0114	Betula pendula Roth.	0	Betulaceae Gray	n	1

Iz_3_1109	DJI_0114	Crataegus rhipidophylla Gand.	0	Rosaceae Juss.	n	1
Iz_3_1109	DJI_0114	Frangula alnus Mill.	0	Rhamnaceae Juss.	n	1
Iz_3_1109	DJI_0114	Fraxinus pennsylvanica Marshall	0	Oleaceae Hoffmanns. & Link	a	0
Iz_3_1109	DJI_0114	Pinus sylvestris L.	0	Pinaceae Spreng. ex F.Rudolphi	n	1
Iz_3_1109	DJI_0114	Populus × canescens (Aiton) Sm.	0	Salicaceae Mirb.	n	1
Iz_3_1109	DJI_0114	Populus tremula L.	0	Salicaceae Mirb.	n	1
Iz_3_1109	DJI_0114	Populus nigra L.	0	Salicaceae Mirb.	n	1
Iz_3_1109	DJI_0114	Pyrus communis L.	0	Rosaceae Juss.	a	0
Iz_3_1109	DJI_0114	Quercus robur L.	0	Fagaceae Dumort.	n	1
Iz_3_1109	DJI_0114	Robinia pseudoacacia L.	0	Fabaceae Lindl.	a	0
Iz_3_1109	DJI_0114	Rosa sp.	0	Rosaceae Juss.		
Iz_3_1109	DJI_0114	Salix alba L.	0	Salicaceae Mirb.	n	1
Iz_3_1109	DJI_0114	Sambucus nigra L.	0	Viburnaceae Raf.	n	1
Iz_3_1109	DJI_0114	Sorbus aucuparia L.	0	Rosaceae Juss.	n	1
Iz_3_1109	DJI_0114	Tilia cordata Mill.	0	Malvaceae Juss.	n	1
Iz_3_1109	DJI_0114	Ulmus glabra Huds.	0	Ulmaceae Mirb.	n	1
Iz_3_1109	DJI_0110	Acer tataricum L.	0	Sapindaceae Juss.	n	1
Iz_3_1109	DJI_0110	Betula pendula Roth.	0	Betulaceae Gray	n	1
Iz_3_1109	DJI_0110	Crataegus rhipidophylla Gand.	0	Rosaceae Juss.	n	1
Iz_3_1109	DJI_0110	Frangula alnus Mill.	0	Rhamnaceae Juss.	n	1
Iz_3_1109	DJI_0110	Fraxinus pennsylvanica Marshall	0	Oleaceae Hoffmanns. & Link	a	0
Iz_3_1109	DJI_0110	Pinus sylvestris L.	0	Pinaceae Spreng. ex F.Rudolphi	n	1
Iz_3_1109	DJI_0110	Populus × canescens (Aiton) Sm.	0	Salicaceae Mirb.	n	1
Iz_3_1109	DJI_0110	Populus tremula L.	0	Salicaceae Mirb.	n	1
Iz_3_1109	DJI_0110	Populus nigra L.	0	Salicaceae Mirb.	n	1
Iz_3_1109	DJI_0110	Pyrus communis L.	0	Rosaceae Juss.	a	0
Iz_3_1109	DJI_0110	Quercus robur L.	0	Fagaceae Dumort.	n	1
Iz_3_1109	DJI_0110	Robinia pseudoacacia L.	0	Fabaceae Lindl.	a	0
Iz_3_1109	DJI_0110	Rosa sp.	0	Rosaceae Juss.		
Iz_3_1109	DJI_0110	Salix alba L.	0	Salicaceae Mirb.	n	1
Iz_3_1109	DJI_0110	Sambucus nigra L.	0	Viburnaceae Raf.	n	1
Iz_3_1109	DJI_0110	Sorbus aucuparia L.	0	Rosaceae Juss.	n	1
Iz_3_1109	DJI_0110	Tilia cordata Mill.	0	Malvaceae Juss.	n	1
Iz_3_1109	DJI_0110	Ulmus glabra Huds.	0	Ulmaceae Mirb.	n	1
Iz_3_1109	DJI_0107	Acer tataricum L.	0	Sapindaceae Juss.	n	1
Iz_3_1109	DJI_0107	Betula pendula Roth.	0	Betulaceae Gray	n	1
Iz_3_1109	DJI_0107	Crataegus rhipidophylla Gand.	0	Rosaceae Juss.	n	1
Iz_3_1109	DJI_0107	Frangula alnus Mill.	0	Rhamnaceae Juss.	n	1
Iz_3_1109	DJI_0107	Fraxinus pennsylvanica Marshall	0	Oleaceae Hoffmanns. & Link	a	0
Iz_3_1109	DJI_0107	Pinus sylvestris L.	0	Pinaceae Spreng. ex F.Rudolphi	n	1
Iz_3_1109	DJI_0107	Populus × canescens (Aiton) Sm.	0	Salicaceae Mirb.	n	1
Iz_3_1109	DJI_0107	Populus tremula L.	0	Salicaceae Mirb.	n	1
Iz_3_1109	DJI_0107	Populus nigra L.	0	Salicaceae Mirb.	n	1
Iz_3_1109	DJI_0107	Pyrus communis L.	0	Rosaceae Juss.	a	0
Iz_3_1109	DJI_0107	Quercus robur L.	0	Fagaceae Dumort.	n	1
Iz_3_1109	DJI_0107	Robinia pseudoacacia L.	0	Fabaceae Lindl.	a	0
Iz_3_1109	DJI_0107	Rosa sp.	0	Rosaceae Juss.		
Iz_3_1109	DJI_0107	Salix alba L.	0	Salicaceae Mirb.	n	1
Iz_3_1109	DJI_0107	Sambucus nigra L.	0	Viburnaceae Raf.	n	1
Iz_3_1109	DJI_0107	Sorbus aucuparia L.	0	Rosaceae Juss.	n	1
Iz_3_1109	DJI_0107	Tilia cordata Mill.	0	Malvaceae Juss.	n	1
Iz_3_1109	DJI_0107	Ulmus glabra Huds.	0	Ulmaceae Mirb.	n	1
Iz_3_1109	DJI_0104	Acer tataricum L.	0	Sapindaceae Juss.	n	1
Iz_3_1109	DJI_0104	Betula pendula Roth.	0	Betulaceae Gray	n	1
Iz_3_1109	DJI_0104	Crataegus rhipidophylla Gand.	0	Rosaceae Juss.	n	1
Iz_3_1109	DJI_0104	Frangula alnus Mill.	0	Rhamnaceae Juss.	n	1
Iz_3_1109	DJI_0104	Fraxinus pennsylvanica Marshall	0	Oleaceae Hoffmanns. & Link	a	0
Iz_3_1109	DJI_0104	Pinus sylvestris L.	0	Pinaceae Spreng. ex F.Rudolphi	n	1
Iz_3_1109	DJI_0104	Populus × canescens (Aiton) Sm.	0	Salicaceae Mirb.	n	1
Iz_3_1109	DJI_0104	Populus tremula L.	0	Salicaceae Mirb.	n	1
Iz_3_1109	DJI_0104	Populus nigra L.	0	Salicaceae Mirb.	n	1
Iz_3_1109	DJI_0104	Pyrus communis L.	0	Rosaceae Juss.	a	0
Iz_3_1109	DJI_0104	Quercus robur L.	0	Fagaceae Dumort.	n	1
Iz_3_1109	DJI_0104	Robinia pseudoacacia L.	0	Fabaceae Lindl.	a	0

Iz_3_1109	DJI_0104	Rosa sp.	0	Rosaceae Juss.		
Iz_3_1109	DJI_0104	Salix alba L.	0	Salicaceae Mirb.	n	1
Iz_3_1109	DJI_0104	Sambucus nigra L.	0	Viburnaceae Raf.	n	1
Iz_3_1109	DJI_0104	Sorbus aucuparia L.	0	Rosaceae Juss.	n	1
Iz_3_1109	DJI_0104	Tilia cordata Mill.	0	Malvaceae Juss.	n	1
Iz_3_1109	DJI_0104	Ulmus glabra Huds.	0	Ulmaceae Mirb.	n	1
Iz_3_1109	DJI_0101	Acer tataricum L.	0	Sapindaceae Juss.	n	1
Iz_3_1109	DJI_0101	Betula pendula Roth.	0	Betulaceae Gray	n	1
Iz_3_1109	DJI_0101	Crataegus rhipidophylla Gand.	0	Rosaceae Juss.	n	1
Iz_3_1109	DJI_0101	Frangula alnus Mill.	0	Rhamnaceae Juss.	n	1
Iz_3_1109	DJI_0101	Fraxinus pennsylvanica Marshall	0	Oleaceae Hoffmanns. & Link	a	0
Iz_3_1109	DJI_0101	Pinus sylvestris L.	0	Pinaceae Spreng. ex F.Rudolphi	n	1
Iz_3_1109	DJI_0101	Populus × canescens (Aiton) Sm.	0	Salicaceae Mirb.	n	1
Iz_3_1109	DJI_0101	Populus tremula L.	0	Salicaceae Mirb.	n	1
Iz_3_1109	DJI_0101	Populus nigra L.	0	Salicaceae Mirb.	n	1
Iz_3_1109	DJI_0101	Pyrus communis L.	0	Rosaceae Juss.	a	0
Iz_3_1109	DJI_0101	Quercus robur L.	0	Fagaceae Dumort.	n	1
Iz_3_1109	DJI_0101	Robinia pseudoacacia L.	0	Fabaceae Lindl.	a	0
Iz_3_1109	DJI_0101	Rosa sp.	0	Rosaceae Juss.		
Iz_3_1109	DJI_0101	Salix alba L.	0	Salicaceae Mirb.	n	1
Iz_3_1109	DJI_0101	Sambucus nigra L.	0	Viburnaceae Raf.	n	1
Iz_3_1109	DJI_0101	Sorbus aucuparia L.	0	Rosaceae Juss.	n	1
Iz_3_1109	DJI_0101	Tilia cordata Mill.	0	Malvaceae Juss.	n	1
Iz_3_1109	DJI_0101	Ulmus glabra Huds.	0	Ulmaceae Mirb.	n	1
Iz_3_1109	DJI_0099	Acer tataricum L.	0	Sapindaceae Juss.	n	1
Iz_3_1109	DJI_0099	Betula pendula Roth.	0	Betulaceae Gray	n	1
Iz_3_1109	DJI_0099	Crataegus rhipidophylla Gand.	0	Rosaceae Juss.	n	1
Iz_3_1109	DJI_0099	Frangula alnus Mill.	0	Rhamnaceae Juss.	n	1
Iz_3_1109	DJI_0099	Fraxinus pennsylvanica Marshall	0	Oleaceae Hoffmanns. & Link	a	0
Iz_3_1109	DJI_0099	Pinus sylvestris L.	0	Pinaceae Spreng. ex F.Rudolphi	n	1
Iz_3_1109	DJI_0099	Populus × canescens (Aiton) Sm.	0	Salicaceae Mirb.	n	1
Iz_3_1109	DJI_0099	Populus tremula L.	0	Salicaceae Mirb.	n	1
Iz_3_1109	DJI_0099	Populus nigra L.	0	Salicaceae Mirb.	n	1
Iz_3_1109	DJI_0099	Pyrus communis L.	0	Rosaceae Juss.	a	0
Iz_3_1109	DJI_0099	Quercus robur L.	0	Fagaceae Dumort.	n	1
Iz_3_1109	DJI_0099	Robinia pseudoacacia L.	0	Fabaceae Lindl.	a	0
Iz_3_1109	DJI_0099	Rosa sp.	0	Rosaceae Juss.		
Iz_3_1109	DJI_0099	Salix alba L.	0	Salicaceae Mirb.	n	1
Iz_3_1109	DJI_0099	Sambucus nigra L.	0	Viburnaceae Raf.	n	1
Iz_3_1109	DJI_0099	Sorbus aucuparia L.	0	Rosaceae Juss.	n	1
Iz_3_1109	DJI_0099	Tilia cordata Mill.	0	Malvaceae Juss.	n	1
Iz_3_1109	DJI_0099	Ulmus glabra Huds.	0	Ulmaceae Mirb.	n	1
Iz_3_1109	DJI_0097	Acer tataricum L.	0	Sapindaceae Juss.	n	1
Iz_3_1109	DJI_0097	Betula pendula Roth.	0	Betulaceae Gray	n	1
Iz_3_1109	DJI_0097	Crataegus rhipidophylla Gand.	0	Rosaceae Juss.	n	1
Iz_3_1109	DJI_0097	Frangula alnus Mill.	0	Rhamnaceae Juss.	n	1
Iz_3_1109	DJI_0097	Fraxinus pennsylvanica Marshall	0	Oleaceae Hoffmanns. & Link	a	0
Iz_3_1109	DJI_0097	Pinus sylvestris L.	0	Pinaceae Spreng. ex F.Rudolphi	n	1
Iz_3_1109	DJI_0097	Populus × canescens (Aiton) Sm.	0	Salicaceae Mirb.	n	1
Iz_3_1109	DJI_0097	Populus tremula L.	0	Salicaceae Mirb.	n	1
Iz_3_1109	DJI_0097	Populus nigra L.	0	Salicaceae Mirb.	n	1
Iz_3_1109	DJI_0097	Pyrus communis L.	0	Rosaceae Juss.	a	0
Iz_3_1109	DJI_0097	Quercus robur L.	0	Fagaceae Dumort.	n	1
Iz_3_1109	DJI_0097	Robinia pseudoacacia L.	0	Fabaceae Lindl.	a	0
Iz_3_1109	DJI_0097	Rosa sp.	0	Rosaceae Juss.		
Iz_3_1109	DJI_0097	Salix alba L.	0	Salicaceae Mirb.	n	1
Iz_3_1109	DJI_0097	Sambucus nigra L.	0	Viburnaceae Raf.	n	1
Iz_3_1109	DJI_0097	Sorbus aucuparia L.	0	Rosaceae Juss.	n	1
Iz_3_1109	DJI_0097	Tilia cordata Mill.	0	Malvaceae Juss.	n	1
Iz_3_1109	DJI_0097	Ulmus glabra Huds.	0	Ulmaceae Mirb.	n	1
Iz_3_1109	DJI_0095	Acer tataricum L.	0	Sapindaceae Juss.	n	1
Iz_3_1109	DJI_0095	Betula pendula Roth.	0	Betulaceae Gray	n	1
Iz_3_1109	DJI_0095	Crataegus rhipidophylla Gand.	0	Rosaceae Juss.	n	1
Iz_3_1109	DJI_0095	Frangula alnus Mill.	0	Rhamnaceae Juss.	n	1

Iz_3_1109	DJI_0095	Fraxinus pennsylvanica Marshall	0	Oleaceae Hoffmanns. & Link	a	0
Iz_3_1109	DJI_0095	Pinus sylvestris L.	0	Pinaceae Spreng. ex F.Rudolphi	n	1
Iz_3_1109	DJI_0095	Populus × canescens (Aiton) Sm.	0	Salicaceae Mirb.	n	1
Iz_3_1109	DJI_0095	Populus tremula L.	0	Salicaceae Mirb.	n	1
Iz_3_1109	DJI_0095	Populus nigra L.	0	Salicaceae Mirb.	n	1
Iz_3_1109	DJI_0095	Pyrus communis L.	0	Rosaceae Juss.	a	0
Iz_3_1109	DJI_0095	Quercus robur L.	0	Fagaceae Dumort.	n	1
Iz_3_1109	DJI_0095	Robinia pseudoacacia L.	0	Fabaceae Lindl.	a	0
Iz_3_1109	DJI_0095	Rosa sp.	0	Rosaceae Juss.		
Iz_3_1109	DJI_0095	Salix alba L.	0	Salicaceae Mirb.	n	1
Iz_3_1109	DJI_0095	Sambucus nigra L.	0	Viburnaceae Raf.	n	1
Iz_3_1109	DJI_0095	Sorbus aucuparia L.	0	Rosaceae Juss.	n	1
Iz_3_1109	DJI_0095	Tilia cordata Mill.	0	Malvaceae Juss.	n	1
Iz_3_1109	DJI_0095	Ulmus glabra Huds.	0	Ulmaceae Mirb.	n	1
Iz_3_1109	DJI_0075	Acer tataricum L.	0	Sapindaceae Juss.	n	1
Iz_3_1109	DJI_0075	Betula pendula Roth.	0	Betulaceae Gray	n	1
Iz_3_1109	DJI_0075	Crataegus rhipidophylla Gand.	0	Rosaceae Juss.	n	1
Iz_3_1109	DJI_0075	Frangula alnus Mill.	0	Rhamnaceae Juss.	n	1
Iz_3_1109	DJI_0075	Fraxinus pennsylvanica Marshall	0	Oleaceae Hoffmanns. & Link	a	0
Iz_3_1109	DJI_0075	Pinus sylvestris L.	0	Pinaceae Spreng. ex F.Rudolphi	n	1
Iz_3_1109	DJI_0075	Populus × canescens (Aiton) Sm.	0	Salicaceae Mirb.	n	1
Iz_3_1109	DJI_0075	Populus tremula L.	0	Salicaceae Mirb.	n	1
Iz_3_1109	DJI_0075	Populus nigra L.	0	Salicaceae Mirb.	n	1
Iz_3_1109	DJI_0075	Pyrus communis L.	0	Rosaceae Juss.	a	0
Iz_3_1109	DJI_0075	Quercus robur L.	0	Fagaceae Dumort.	n	1
Iz_3_1109	DJI_0075	Robinia pseudoacacia L.	0	Fabaceae Lindl.	a	0
Iz_3_1109	DJI_0075	Rosa sp.	0	Rosaceae Juss.		
Iz_3_1109	DJI_0075	Salix alba L.	0	Salicaceae Mirb.	n	1
Iz_3_1109	DJI_0075	Sambucus nigra L.	0	Viburnaceae Raf.	n	1
Iz_3_1109	DJI_0075	Sorbus aucuparia L.	0	Rosaceae Juss.	n	1
Iz_3_1109	DJI_0075	Tilia cordata Mill.	0	Malvaceae Juss.	n	1
Iz_3_1109	DJI_0075	Ulmus glabra Huds.	0	Ulmaceae Mirb.	n	1
Iz_3_1109	DJI_0073	Acer tataricum L.	0	Sapindaceae Juss.	n	1
Iz_3_1109	DJI_0073	Betula pendula Roth.	0	Betulaceae Gray	n	1
Iz_3_1109	DJI_0073	Crataegus rhipidophylla Gand.	0	Rosaceae Juss.	n	1
Iz_3_1109	DJI_0073	Frangula alnus Mill.	0	Rhamnaceae Juss.	n	1
Iz_3_1109	DJI_0073	Fraxinus pennsylvanica Marshall	0	Oleaceae Hoffmanns. & Link	a	0
Iz_3_1109	DJI_0073	Pinus sylvestris L.	0	Pinaceae Spreng. ex F.Rudolphi	n	1
Iz_3_1109	DJI_0073	Populus × canescens (Aiton) Sm.	0	Salicaceae Mirb.	n	1
Iz_3_1109	DJI_0073	Populus tremula L.	0	Salicaceae Mirb.	n	1
Iz_3_1109	DJI_0073	Populus nigra L.	0	Salicaceae Mirb.	n	1
Iz_3_1109	DJI_0073	Pyrus communis L.	0	Rosaceae Juss.	a	0
Iz_3_1109	DJI_0073	Quercus robur L.	0	Fagaceae Dumort.	n	1
Iz_3_1109	DJI_0073	Robinia pseudoacacia L.	0	Fabaceae Lindl.	a	0
Iz_3_1109	DJI_0073	Rosa sp.	0	Rosaceae Juss.		
Iz_3_1109	DJI_0073	Salix alba L.	0	Salicaceae Mirb.	n	1
Iz_3_1109	DJI_0073	Sambucus nigra L.	0	Viburnaceae Raf.	n	1
Iz_3_1109	DJI_0073	Sorbus aucuparia L.	0	Rosaceae Juss.	n	1
Iz_3_1109	DJI_0073	Tilia cordata Mill.	0	Malvaceae Juss.	n	1
Iz_3_1109	DJI_0073	Ulmus glabra Huds.	0	Ulmaceae Mirb.	n	1
Iz_3_1109	DJI_0070	Acer tataricum L.	0	Sapindaceae Juss.	n	1
Iz_3_1109	DJI_0070	Betula pendula Roth.	0	Betulaceae Gray	n	1
Iz_3_1109	DJI_0070	Crataegus rhipidophylla Gand.	0	Rosaceae Juss.	n	1
Iz_3_1109	DJI_0070	Frangula alnus Mill.	0	Rhamnaceae Juss.	n	1
Iz_3_1109	DJI_0070	Fraxinus pennsylvanica Marshall	0	Oleaceae Hoffmanns. & Link	a	0
Iz_3_1109	DJI_0070	Pinus sylvestris L.	0	Pinaceae Spreng. ex F.Rudolphi	n	1
Iz_3_1109	DJI_0070	Populus × canescens (Aiton) Sm.	0	Salicaceae Mirb.	n	1
Iz_3_1109	DJI_0070	Populus tremula L.	0	Salicaceae Mirb.	n	1
Iz_3_1109	DJI_0070	Populus nigra L.	0	Salicaceae Mirb.	n	1
Iz_3_1109	DJI_0070	Pyrus communis L.	0	Rosaceae Juss.	a	0
Iz_3_1109	DJI_0070	Quercus robur L.	0	Fagaceae Dumort.	n	1
Iz_3_1109	DJI_0070	Robinia pseudoacacia L.	0	Fabaceae Lindl.	a	0
Iz_3_1109	DJI_0070	Rosa sp.	0	Rosaceae Juss.		
Iz_3_1109	DJI_0070	Salix alba L.	0	Salicaceae Mirb.	n	1

Iz_3_1109	DJI_0070	Sambucus nigra L.	0	Viburnaceae Raf.	n	1
Iz_3_1109	DJI_0070	Sorbus aucuparia L.	0	Rosaceae Juss.	n	1
Iz_3_1109	DJI_0070	Tilia cordata Mill.	0	Malvaceae Juss.	n	1
Iz_3_1109	DJI_0070	Ulmus glabra Huds.	0	Ulmaceae Mirb.	n	1
Iz_3_1109	DJI_0063	Acer tataricum L.	0	Sapindaceae Juss.	n	1
Iz_3_1109	DJI_0063	Betula pendula Roth.	0	Betulaceae Gray	n	1
Iz_3_1109	DJI_0063	Crataegus rhipidophylla Gand.	0	Rosaceae Juss.	n	1
Iz_3_1109	DJI_0063	Frangula alnus Mill.	0	Rhamnaceae Juss.	n	1
Iz_3_1109	DJI_0063	Fraxinus pennsylvanica Marshall	0	Oleaceae Hoffmanns. & Link	a	0
Iz_3_1109	DJI_0063	Pinus sylvestris L.	0	Pinaceae Spreng. ex F.Rudolphi	n	1
Iz_3_1109	DJI_0063	Populus × canescens (Aiton) Sm.	0	Salicaceae Mirb.	n	1
Iz_3_1109	DJI_0063	Populus tremula L.	0	Salicaceae Mirb.	n	1
Iz_3_1109	DJI_0063	Populus nigra L.	0	Salicaceae Mirb.	n	1
Iz_3_1109	DJI_0063	Pyrus communis L.	0	Rosaceae Juss.	a	0
Iz_3_1109	DJI_0063	Quercus robur L.	0	Fagaceae Dumort.	n	1
Iz_3_1109	DJI_0063	Robinia pseudoacacia L.	0	Fabaceae Lindl.	a	0
Iz_3_1109	DJI_0063	Rosa sp.	0	Rosaceae Juss.		
Iz_3_1109	DJI_0063	Salix alba L.	0	Salicaceae Mirb.	n	1
Iz_3_1109	DJI_0063	Sambucus nigra L.	0	Viburnaceae Raf.	n	1
Iz_3_1109	DJI_0063	Sorbus aucuparia L.	0	Rosaceae Juss.	n	1
Iz_3_1109	DJI_0063	Tilia cordata Mill.	0	Malvaceae Juss.	n	1
Iz_3_1109	DJI_0063	Ulmus glabra Huds.	0	Ulmaceae Mirb.	n	1
Iz_3_1109	DJI_0058	Acer tataricum L.	0	Sapindaceae Juss.	n	1
Iz_3_1109	DJI_0058	Betula pendula Roth.	0	Betulaceae Gray	n	1
Iz_3_1109	DJI_0058	Crataegus rhipidophylla Gand.	0	Rosaceae Juss.	n	1
Iz_3_1109	DJI_0058	Frangula alnus Mill.	0	Rhamnaceae Juss.	n	1
Iz_3_1109	DJI_0058	Fraxinus pennsylvanica Marshall	0	Oleaceae Hoffmanns. & Link	a	0
Iz_3_1109	DJI_0058	Pinus sylvestris L.	0	Pinaceae Spreng. ex F.Rudolphi	n	1
Iz_3_1109	DJI_0058	Populus × canescens (Aiton) Sm.	0	Salicaceae Mirb.	n	1
Iz_3_1109	DJI_0058	Populus tremula L.	0	Salicaceae Mirb.	n	1
Iz_3_1109	DJI_0058	Populus nigra L.	0	Salicaceae Mirb.	n	1
Iz_3_1109	DJI_0058	Pyrus communis L.	0	Rosaceae Juss.	a	0
Iz_3_1109	DJI_0058	Quercus robur L.	0	Fagaceae Dumort.	n	1
Iz_3_1109	DJI_0058	Robinia pseudoacacia L.	0	Fabaceae Lindl.	a	0
Iz_3_1109	DJI_0058	Rosa sp.	0	Rosaceae Juss.		
Iz_3_1109	DJI_0058	Salix alba L.	0	Salicaceae Mirb.	n	1
Iz_3_1109	DJI_0058	Sambucus nigra L.	0	Viburnaceae Raf.	n	1
Iz_3_1109	DJI_0058	Sorbus aucuparia L.	0	Rosaceae Juss.	n	1
Iz_3_1109	DJI_0058	Tilia cordata Mill.	0	Malvaceae Juss.	n	1
Iz_3_1109	DJI_0058	Ulmus glabra Huds.	0	Ulmaceae Mirb.	n	1
Iz_3_1109	DJI_0055	Acer tataricum L.	0	Sapindaceae Juss.	n	1
Iz_3_1109	DJI_0055	Betula pendula Roth.	0	Betulaceae Gray	n	1
Iz_3_1109	DJI_0055	Crataegus rhipidophylla Gand.	0	Rosaceae Juss.	n	1
Iz_3_1109	DJI_0055	Frangula alnus Mill.	0	Rhamnaceae Juss.	n	1
Iz_3_1109	DJI_0055	Fraxinus pennsylvanica Marshall	0	Oleaceae Hoffmanns. & Link	a	0
Iz_3_1109	DJI_0055	Pinus sylvestris L.	0	Pinaceae Spreng. ex F.Rudolphi	n	1
Iz_3_1109	DJI_0055	Populus × canescens (Aiton) Sm.	0	Salicaceae Mirb.	n	1
Iz_3_1109	DJI_0055	Populus tremula L.	0	Salicaceae Mirb.	n	1
Iz_3_1109	DJI_0055	Populus nigra L.	0	Salicaceae Mirb.	n	1
Iz_3_1109	DJI_0055	Pyrus communis L.	0	Rosaceae Juss.	a	0
Iz_3_1109	DJI_0055	Quercus robur L.	0	Fagaceae Dumort.	n	1
Iz_3_1109	DJI_0055	Robinia pseudoacacia L.	0	Fabaceae Lindl.	a	0
Iz_3_1109	DJI_0055	Rosa sp.	0	Rosaceae Juss.		
Iz_3_1109	DJI_0055	Salix alba L.	0	Salicaceae Mirb.	n	1
Iz_3_1109	DJI_0055	Sambucus nigra L.	0	Viburnaceae Raf.	n	1
Iz_3_1109	DJI_0055	Sorbus aucuparia L.	0	Rosaceae Juss.	n	1
Iz_3_1109	DJI_0055	Tilia cordata Mill.	0	Malvaceae Juss.	n	1
Iz_3_1109	DJI_0055	Ulmus glabra Huds.	0	Ulmaceae Mirb.	n	1
Iz_3_1109	DJI_0051	Acer tataricum L.	0	Sapindaceae Juss.	n	1
Iz_3_1109	DJI_0051	Betula pendula Roth.	0	Betulaceae Gray	n	1
Iz_3_1109	DJI_0051	Crataegus rhipidophylla Gand.	0	Rosaceae Juss.	n	1
Iz_3_1109	DJI_0051	Frangula alnus Mill.	0	Rhamnaceae Juss.	n	1
Iz_3_1109	DJI_0051	Fraxinus pennsylvanica Marshall	0	Oleaceae Hoffmanns. & Link	a	0
Iz_3_1109	DJI_0051	Pinus sylvestris L.	0	Pinaceae Spreng. ex F.Rudolphi	n	1

Iz_3_1109	DJI_0051	Populus × canescens (Aiton) Sm.	0	Salicaceae Mirb.	n	1
Iz_3_1109	DJI_0051	Populus tremula L.	0	Salicaceae Mirb.	n	1
Iz_3_1109	DJI_0051	Populus nigra L.	0	Salicaceae Mirb.	n	1
Iz_3_1109	DJI_0051	Pyrus communis L.	0	Rosaceae Juss.	a	0
Iz_3_1109	DJI_0051	Quercus robur L.	0	Fagaceae Dumort.	n	1
Iz_3_1109	DJI_0051	Robinia pseudoacacia L.	0	Fabaceae Lindl.	a	0
Iz_3_1109	DJI_0051	Rosa sp.	0	Rosaceae Juss.		
Iz_3_1109	DJI_0051	Salix alba L.	0	Salicaceae Mirb.	n	1
Iz_3_1109	DJI_0051	Sambucus nigra L.	0	Viburnaceae Raf.	n	1
Iz_3_1109	DJI_0051	Sorbus aucuparia L.	0	Rosaceae Juss.	n	1
Iz_3_1109	DJI_0051	Tilia cordata Mill.	0	Malvaceae Juss.	n	1
Iz_3_1109	DJI_0051	Ulmus glabra Huds.	0	Ulmaceae Mirb.	n	1
Iz_3_1109	DJI_0048	Acer tataricum L.	0	Sapindaceae Juss.	n	1
Iz_3_1109	DJI_0048	Betula pendula Roth.	0	Betulaceae Gray	n	1
Iz_3_1109	DJI_0048	Crataegus rhipidophylla Gand.	0	Rosaceae Juss.	n	1
Iz_3_1109	DJI_0048	Frangula alnus Mill.	0	Rhamnaceae Juss.	n	1
Iz_3_1109	DJI_0048	Fraxinus pennsylvanica Marshall	0	Oleaceae Hoffmanns. & Link	a	0
Iz_3_1109	DJI_0048	Pinus sylvestris L.	0	Pinaceae Spreng. ex F.Rudolphi	n	1
Iz_3_1109	DJI_0048	Populus × canescens (Aiton) Sm.	0	Salicaceae Mirb.	n	1
Iz_3_1109	DJI_0048	Populus tremula L.	0	Salicaceae Mirb.	n	1
Iz_3_1109	DJI_0048	Populus nigra L.	0	Salicaceae Mirb.	n	1
Iz_3_1109	DJI_0048	Pyrus communis L.	0	Rosaceae Juss.	a	0
Iz_3_1109	DJI_0048	Quercus robur L.	0	Fagaceae Dumort.	n	1
Iz_3_1109	DJI_0048	Robinia pseudoacacia L.	0	Fabaceae Lindl.	a	0
Iz_3_1109	DJI_0048	Rosa sp.	0	Rosaceae Juss.		
Iz_3_1109	DJI_0048	Salix alba L.	0	Salicaceae Mirb.	n	1
Iz_3_1109	DJI_0048	Sambucus nigra L.	0	Viburnaceae Raf.	n	1
Iz_3_1109	DJI_0048	Sorbus aucuparia L.	0	Rosaceae Juss.	n	1
Iz_3_1109	DJI_0048	Tilia cordata Mill.	0	Malvaceae Juss.	n	1
Iz_3_1109	DJI_0048	Ulmus glabra Huds.	0	Ulmaceae Mirb.	n	1
Iz_3_1109	DJI_0045	Acer tataricum L.	0	Sapindaceae Juss.	n	1
Iz_3_1109	DJI_0045	Betula pendula Roth.	0	Betulaceae Gray	n	1
Iz_3_1109	DJI_0045	Crataegus rhipidophylla Gand.	0	Rosaceae Juss.	n	1
Iz_3_1109	DJI_0045	Frangula alnus Mill.	0	Rhamnaceae Juss.	n	1
Iz_3_1109	DJI_0045	Fraxinus pennsylvanica Marshall	0	Oleaceae Hoffmanns. & Link	a	0
Iz_3_1109	DJI_0045	Pinus sylvestris L.	0	Pinaceae Spreng. ex F.Rudolphi	n	1
Iz_3_1109	DJI_0045	Populus × canescens (Aiton) Sm.	0	Salicaceae Mirb.	n	1
Iz_3_1109	DJI_0045	Populus tremula L.	2	Salicaceae Mirb.	n	1
Iz_3_1109	DJI_0045	Populus nigra L.	0	Salicaceae Mirb.	n	1
Iz_3_1109	DJI_0045	Pyrus communis L.	0	Rosaceae Juss.	a	0
Iz_3_1109	DJI_0045	Quercus robur L.	0	Fagaceae Dumort.	n	1
Iz_3_1109	DJI_0045	Robinia pseudoacacia L.	0	Fabaceae Lindl.	a	0
Iz_3_1109	DJI_0045	Rosa sp.	0	Rosaceae Juss.		
Iz_3_1109	DJI_0045	Salix alba L.	0	Salicaceae Mirb.	n	1
Iz_3_1109	DJI_0045	Sambucus nigra L.	0	Viburnaceae Raf.	n	1
Iz_3_1109	DJI_0045	Sorbus aucuparia L.	0	Rosaceae Juss.	n	1
Iz_3_1109	DJI_0045	Tilia cordata Mill.	0	Malvaceae Juss.	n	1
Iz_3_1109	DJI_0045	Ulmus glabra Huds.	0	Ulmaceae Mirb.	n	1
Iz_3_1109	DJI_0042	Acer tataricum L.	0	Sapindaceae Juss.	n	1
Iz_3_1109	DJI_0042	Betula pendula Roth.	0	Betulaceae Gray	n	1
Iz_3_1109	DJI_0042	Crataegus rhipidophylla Gand.	0	Rosaceae Juss.	n	1
Iz_3_1109	DJI_0042	Frangula alnus Mill.	0	Rhamnaceae Juss.	n	1
Iz_3_1109	DJI_0042	Fraxinus pennsylvanica Marshall	0	Oleaceae Hoffmanns. & Link	a	0
Iz_3_1109	DJI_0042	Pinus sylvestris L.	0	Pinaceae Spreng. ex F.Rudolphi	n	1
Iz_3_1109	DJI_0042	Populus × canescens (Aiton) Sm.	0	Salicaceae Mirb.	n	1
Iz_3_1109	DJI_0042	Populus tremula L.	0	Salicaceae Mirb.	n	1
Iz_3_1109	DJI_0042	Populus nigra L.	0	Salicaceae Mirb.	n	1
Iz_3_1109	DJI_0042	Pyrus communis L.	0	Rosaceae Juss.	a	0
Iz_3_1109	DJI_0042	Quercus robur L.	0	Fagaceae Dumort.	n	1
Iz_3_1109	DJI_0042	Robinia pseudoacacia L.	0	Fabaceae Lindl.	a	0
Iz_3_1109	DJI_0042	Rosa sp.	0	Rosaceae Juss.		
Iz_3_1109	DJI_0042	Salix alba L.	0	Salicaceae Mirb.	n	1
Iz_3_1109	DJI_0042	Sambucus nigra L.	0	Viburnaceae Raf.	n	1
Iz_3_1109	DJI_0042	Sorbus aucuparia L.	0	Rosaceae Juss.	n	1

Iz_3_1109	DJI_0042	Tilia cordata Mill.	0	Malvaceae Juss.	n	1
Iz_3_1109	DJI_0042	Ulmus glabra Huds.	0	Ulmaceae Mirb.	n	1
Iz_3_1109	DJI_0038	Acer tataricum L.	0	Sapindaceae Juss.	n	1
Iz_3_1109	DJI_0038	Betula pendula Roth.	0	Betulaceae Gray	n	1
Iz_3_1109	DJI_0038	Crataegus rhipidophylla Gand.	0	Rosaceae Juss.	n	1
Iz_3_1109	DJI_0038	Frangula alnus Mill.	0	Rhamnaceae Juss.	n	1
Iz_3_1109	DJI_0038	Fraxinus pennsylvanica Marshall	0	Oleaceae Hoffmanns. & Link	a	0
Iz_3_1109	DJI_0038	Pinus sylvestris L.	0	Pinaceae Spreng. ex F.Rudolphi	n	1
Iz_3_1109	DJI_0038	Populus × canescens (Aiton) Sm.	0	Salicaceae Mirb.	n	1
Iz_3_1109	DJI_0038	Populus tremula L.	0	Salicaceae Mirb.	n	1
Iz_3_1109	DJI_0038	Populus nigra L.	0	Salicaceae Mirb.	n	1
Iz_3_1109	DJI_0038	Pyrus communis L.	0	Rosaceae Juss.	a	0
Iz_3_1109	DJI_0038	Quercus robur L.	0	Fagaceae Dumort.	n	1
Iz_3_1109	DJI_0038	Robinia pseudoacacia L.	0	Fabaceae Lindl.	a	0
Iz_3_1109	DJI_0038	Rosa sp.	0	Rosaceae Juss.		
Iz_3_1109	DJI_0038	Salix alba L.	0	Salicaceae Mirb.	n	1
Iz_3_1109	DJI_0038	Sambucus nigra L.	0	Viburnaceae Raf.	n	1
Iz_3_1109	DJI_0038	Sorbus aucuparia L.	0	Rosaceae Juss.	n	1
Iz_3_1109	DJI_0038	Tilia cordata Mill.	0	Malvaceae Juss.	n	1
Iz_3_1109	DJI_0038	Ulmus glabra Huds.	0	Ulmaceae Mirb.	n	1
Iz_3_1109	DJI_0035	Acer tataricum L.	0	Sapindaceae Juss.	n	1
Iz_3_1109	DJI_0035	Betula pendula Roth.	0	Betulaceae Gray	n	1
Iz_3_1109	DJI_0035	Crataegus rhipidophylla Gand.	0	Rosaceae Juss.	n	1
Iz_3_1109	DJI_0035	Frangula alnus Mill.	0	Rhamnaceae Juss.	n	1
Iz_3_1109	DJI_0035	Fraxinus pennsylvanica Marshall	0	Oleaceae Hoffmanns. & Link	a	0
Iz_3_1109	DJI_0035	Pinus sylvestris L.	0	Pinaceae Spreng. ex F.Rudolphi	n	1
Iz_3_1109	DJI_0035	Populus × canescens (Aiton) Sm.	0	Salicaceae Mirb.	n	1
Iz_3_1109	DJI_0035	Populus tremula L.	0	Salicaceae Mirb.	n	1
Iz_3_1109	DJI_0035	Populus nigra L.	0	Salicaceae Mirb.	n	1
Iz_3_1109	DJI_0035	Pyrus communis L.	0	Rosaceae Juss.	a	0
Iz_3_1109	DJI_0035	Quercus robur L.	0	Fagaceae Dumort.	n	1
Iz_3_1109	DJI_0035	Robinia pseudoacacia L.	0	Fabaceae Lindl.	a	0
Iz_3_1109	DJI_0035	Rosa sp.	0	Rosaceae Juss.		
Iz_3_1109	DJI_0035	Salix alba L.	0	Salicaceae Mirb.	n	1
Iz_3_1109	DJI_0035	Sambucus nigra L.	0	Viburnaceae Raf.	n	1
Iz_3_1109	DJI_0035	Sorbus aucuparia L.	0	Rosaceae Juss.	n	1
Iz_3_1109	DJI_0035	Tilia cordata Mill.	0	Malvaceae Juss.	n	1
Iz_3_1109	DJI_0035	Ulmus glabra Huds.	0	Ulmaceae Mirb.	n	1
Iz_3_1109	DJI_0033	Acer tataricum L.	0	Sapindaceae Juss.	n	1
Iz_3_1109	DJI_0033	Betula pendula Roth.	0	Betulaceae Gray	n	1
Iz_3_1109	DJI_0033	Crataegus rhipidophylla Gand.	0	Rosaceae Juss.	n	1
Iz_3_1109	DJI_0033	Frangula alnus Mill.	0	Rhamnaceae Juss.	n	1
Iz_3_1109	DJI_0033	Fraxinus pennsylvanica Marshall	0	Oleaceae Hoffmanns. & Link	a	0
Iz_3_1109	DJI_0033	Pinus sylvestris L.	0	Pinaceae Spreng. ex F.Rudolphi	n	1
Iz_3_1109	DJI_0033	Populus × canescens (Aiton) Sm.	0	Salicaceae Mirb.	n	1
Iz_3_1109	DJI_0033	Populus tremula L.	0	Salicaceae Mirb.	n	1
Iz_3_1109	DJI_0033	Populus nigra L.	0	Salicaceae Mirb.	n	1
Iz_3_1109	DJI_0033	Pyrus communis L.	0	Rosaceae Juss.	a	0
Iz_3_1109	DJI_0033	Quercus robur L.	0	Fagaceae Dumort.	n	1
Iz_3_1109	DJI_0033	Robinia pseudoacacia L.	0	Fabaceae Lindl.	a	0
Iz_3_1109	DJI_0033	Rosa sp.	0	Rosaceae Juss.		
Iz_3_1109	DJI_0033	Salix alba L.	0	Salicaceae Mirb.	n	1
Iz_3_1109	DJI_0033	Sambucus nigra L.	0	Viburnaceae Raf.	n	1
Iz_3_1109	DJI_0033	Sorbus aucuparia L.	0	Rosaceae Juss.	n	1
Iz_3_1109	DJI_0033	Tilia cordata Mill.	0	Malvaceae Juss.	n	1
Iz_3_1109	DJI_0033	Ulmus glabra Huds.	0	Ulmaceae Mirb.	n	1
Iz_3_1109	DJI_0031	Acer tataricum L.	0	Sapindaceae Juss.	n	1
Iz_3_1109	DJI_0031	Betula pendula Roth.	0	Betulaceae Gray	n	1
Iz_3_1109	DJI_0031	Crataegus rhipidophylla Gand.	0	Rosaceae Juss.	n	1
Iz_3_1109	DJI_0031	Frangula alnus Mill.	0	Rhamnaceae Juss.	n	1
Iz_3_1109	DJI_0031	Fraxinus pennsylvanica Marshall	0	Oleaceae Hoffmanns. & Link	a	0
Iz_3_1109	DJI_0031	Pinus sylvestris L.	0	Pinaceae Spreng. ex F.Rudolphi	n	1
Iz_3_1109	DJI_0031	Populus × canescens (Aiton) Sm.	0	Salicaceae Mirb.	n	1
Iz_3_1109	DJI_0031	Populus tremula L.	0	Salicaceae Mirb.	n	1

Iz_3_1109	DJI_0031	Populus nigra L.	0	Salicaceae Mirb.	n	1
Iz_3_1109	DJI_0031	Pyrus communis L.	0	Rosaceae Juss.	a	0
Iz_3_1109	DJI_0031	Quercus robur L.	0	Fagaceae Dumort.	n	1
Iz_3_1109	DJI_0031	Robinia pseudoacacia L.	0	Fabaceae Lindl.	a	0
Iz_3_1109	DJI_0031	Rosa sp.	0	Rosaceae Juss.		
Iz_3_1109	DJI_0031	Salix alba L.	0	Salicaceae Mirb.	n	1
Iz_3_1109	DJI_0031	Sambucus nigra L.	0	Viburnaceae Raf.	n	1
Iz_3_1109	DJI_0031	Sorbus aucuparia L.	0	Rosaceae Juss.	n	1
Iz_3_1109	DJI_0031	Tilia cordata Mill.	0	Malvaceae Juss.	n	1
Iz_3_1109	DJI_0031	Ulmus glabra Huds.	0	Ulmaceae Mirb.	n	1
Iz_3_1109	DJI_0027	Acer tataricum L.	0	Sapindaceae Juss.	n	1
Iz_3_1109	DJI_0027	Betula pendula Roth.	0	Betulaceae Gray	n	1
Iz_3_1109	DJI_0027	Crataegus rhipidophylla Gand.	0	Rosaceae Juss.	n	1
Iz_3_1109	DJI_0027	Frangula alnus Mill.	0	Rhamnaceae Juss.	n	1
Iz_3_1109	DJI_0027	Fraxinus pennsylvanica Marshall	0	Oleaceae Hoffmanns. & Link	a	0
Iz_3_1109	DJI_0027	Pinus sylvestris L.	0	Pinaceae Spreng. ex F.Rudolphi	n	1
Iz_3_1109	DJI_0027	Populus × canescens (Aiton) Sm.	0	Salicaceae Mirb.	n	1
Iz_3_1109	DJI_0027	Populus tremula L.	0	Salicaceae Mirb.	n	1
Iz_3_1109	DJI_0027	Populus nigra L.	0	Salicaceae Mirb.	n	1
Iz_3_1109	DJI_0027	Pyrus communis L.	0	Rosaceae Juss.	a	0
Iz_3_1109	DJI_0027	Quercus robur L.	0	Fagaceae Dumort.	n	1
Iz_3_1109	DJI_0027	Robinia pseudoacacia L.	0	Fabaceae Lindl.	a	0
Iz_3_1109	DJI_0027	Rosa sp.	0	Rosaceae Juss.		
Iz_3_1109	DJI_0027	Salix alba L.	0	Salicaceae Mirb.	n	1
Iz_3_1109	DJI_0027	Sambucus nigra L.	0	Viburnaceae Raf.	n	1
Iz_3_1109	DJI_0027	Sorbus aucuparia L.	0	Rosaceae Juss.	n	1
Iz_3_1109	DJI_0027	Tilia cordata Mill.	0	Malvaceae Juss.	n	1
Iz_3_1109	DJI_0027	Ulmus glabra Huds.	0	Ulmaceae Mirb.	n	1
Iz_3_1109	DJI_0023	Acer tataricum L.	0	Sapindaceae Juss.	n	1
Iz_3_1109	DJI_0023	Betula pendula Roth.	0	Betulaceae Gray	n	1
Iz_3_1109	DJI_0023	Crataegus rhipidophylla Gand.	0	Rosaceae Juss.	n	1
Iz_3_1109	DJI_0023	Frangula alnus Mill.	0	Rhamnaceae Juss.	n	1
Iz_3_1109	DJI_0023	Fraxinus pennsylvanica Marshall	0	Oleaceae Hoffmanns. & Link	a	0
Iz_3_1109	DJI_0023	Pinus sylvestris L.	0	Pinaceae Spreng. ex F.Rudolphi	n	1
Iz_3_1109	DJI_0023	Populus × canescens (Aiton) Sm.	0	Salicaceae Mirb.	n	1
Iz_3_1109	DJI_0023	Populus tremula L.	0	Salicaceae Mirb.	n	1
Iz_3_1109	DJI_0023	Populus nigra L.	0	Salicaceae Mirb.	n	1
Iz_3_1109	DJI_0023	Pyrus communis L.	0	Rosaceae Juss.	a	0
Iz_3_1109	DJI_0023	Quercus robur L.	0	Fagaceae Dumort.	n	1
Iz_3_1109	DJI_0023	Robinia pseudoacacia L.	0	Fabaceae Lindl.	a	0
Iz_3_1109	DJI_0023	Rosa sp.	0	Rosaceae Juss.		
Iz_3_1109	DJI_0023	Salix alba L.	0	Salicaceae Mirb.	n	1
Iz_3_1109	DJI_0023	Sambucus nigra L.	0	Viburnaceae Raf.	n	1
Iz_3_1109	DJI_0023	Sorbus aucuparia L.	0	Rosaceae Juss.	n	1
Iz_3_1109	DJI_0023	Tilia cordata Mill.	0	Malvaceae Juss.	n	1
Iz_3_1109	DJI_0023	Ulmus glabra Huds.	0	Ulmaceae Mirb.	n	1
Iz_3_1109	DJI_0020	Acer tataricum L.	0	Sapindaceae Juss.	n	1
Iz_3_1109	DJI_0020	Betula pendula Roth.	0	Betulaceae Gray	n	1
Iz_3_1109	DJI_0020	Crataegus rhipidophylla Gand.	0	Rosaceae Juss.	n	1
Iz_3_1109	DJI_0020	Frangula alnus Mill.	0	Rhamnaceae Juss.	n	1
Iz_3_1109	DJI_0020	Fraxinus pennsylvanica Marshall	0	Oleaceae Hoffmanns. & Link	a	0
Iz_3_1109	DJI_0020	Pinus sylvestris L.	0	Pinaceae Spreng. ex F.Rudolphi	n	1
Iz_3_1109	DJI_0020	Populus × canescens (Aiton) Sm.	0	Salicaceae Mirb.	n	1
Iz_3_1109	DJI_0020	Populus tremula L.	0	Salicaceae Mirb.	n	1
Iz_3_1109	DJI_0020	Populus nigra L.	0	Salicaceae Mirb.	n	1
Iz_3_1109	DJI_0020	Pyrus communis L.	0	Rosaceae Juss.	a	0
Iz_3_1109	DJI_0020	Quercus robur L.	0	Fagaceae Dumort.	n	1
Iz_3_1109	DJI_0020	Robinia pseudoacacia L.	0	Fabaceae Lindl.	a	0
Iz_3_1109	DJI_0020	Rosa sp.	0	Rosaceae Juss.		
Iz_3_1109	DJI_0020	Salix alba L.	0	Salicaceae Mirb.	n	1
Iz_3_1109	DJI_0020	Sambucus nigra L.	0	Viburnaceae Raf.	n	1
Iz_3_1109	DJI_0020	Sorbus aucuparia L.	0	Rosaceae Juss.	n	1
Iz_3_1109	DJI_0020	Tilia cordata Mill.	0	Malvaceae Juss.	n	1
Iz_3_1109	DJI_0020	Ulmus glabra Huds.	0	Ulmaceae Mirb.	n	1

Iz_3_1109	DJI_0016	Acer tataricum L.	0	Sapindaceae Juss.	n	1
Iz_3_1109	DJI_0016	Betula pendula Roth.	0	Betulaceae Gray	n	1
Iz_3_1109	DJI_0016	Crataegus rhipidophylla Gand.	0	Rosaceae Juss.	n	1
Iz_3_1109	DJI_0016	Frangula alnus Mill.	0	Rhamnaceae Juss.	n	1
Iz_3_1109	DJI_0016	Fraxinus pennsylvanica Marshall	0	Oleaceae Hoffmanns. & Link	a	0
Iz_3_1109	DJI_0016	Pinus sylvestris L.	0	Pinaceae Spreng. ex F.Rudolphi	n	1
Iz_3_1109	DJI_0016	Populus × canescens (Aiton) Sm.	0	Salicaceae Mirb.	n	1
Iz_3_1109	DJI_0016	Populus tremula L.	0	Salicaceae Mirb.	n	1
Iz_3_1109	DJI_0016	Populus nigra L.	0	Salicaceae Mirb.	n	1
Iz_3_1109	DJI_0016	Pyrus communis L.	0	Rosaceae Juss.	a	0
Iz_3_1109	DJI_0016	Quercus robur L.	0	Fagaceae Dumort.	n	1
Iz_3_1109	DJI_0016	Robinia pseudoacacia L.	0	Fabaceae Lindl.	a	0
Iz_3_1109	DJI_0016	Rosa sp.	0	Rosaceae Juss.		
Iz_3_1109	DJI_0016	Salix alba L.	0	Salicaceae Mirb.	n	1
Iz_3_1109	DJI_0016	Sambucus nigra L.	0	Viburnaceae Raf.	n	1
Iz_3_1109	DJI_0016	Sorbus aucuparia L.	0	Rosaceae Juss.	n	1
Iz_3_1109	DJI_0016	Tilia cordata Mill.	0	Malvaceae Juss.	n	1
Iz_3_1109	DJI_0016	Ulmus glabra Huds.	0	Ulmaceae Mirb.	n	1
Iz_3_1109	DJI_0014	Acer tataricum L.	0	Sapindaceae Juss.	n	1
Iz_3_1109	DJI_0014	Betula pendula Roth.	0	Betulaceae Gray	n	1
Iz_3_1109	DJI_0014	Crataegus rhipidophylla Gand.	0	Rosaceae Juss.	n	1
Iz_3_1109	DJI_0014	Frangula alnus Mill.	0	Rhamnaceae Juss.	n	1
Iz_3_1109	DJI_0014	Fraxinus pennsylvanica Marshall	0	Oleaceae Hoffmanns. & Link	a	0
Iz_3_1109	DJI_0014	Pinus sylvestris L.	0	Pinaceae Spreng. ex F.Rudolphi	n	1
Iz_3_1109	DJI_0014	Populus × canescens (Aiton) Sm.	0	Salicaceae Mirb.	n	1
Iz_3_1109	DJI_0014	Populus tremula L.	0	Salicaceae Mirb.	n	1
Iz_3_1109	DJI_0014	Populus nigra L.	0	Salicaceae Mirb.	n	1
Iz_3_1109	DJI_0014	Pyrus communis L.	0	Rosaceae Juss.	a	0
Iz_3_1109	DJI_0014	Quercus robur L.	0	Fagaceae Dumort.	n	1
Iz_3_1109	DJI_0014	Robinia pseudoacacia L.	0	Fabaceae Lindl.	a	0
Iz_3_1109	DJI_0014	Rosa sp.	0	Rosaceae Juss.		
Iz_3_1109	DJI_0014	Salix alba L.	0	Salicaceae Mirb.	n	1
Iz_3_1109	DJI_0014	Sambucus nigra L.	0	Viburnaceae Raf.	n	1
Iz_3_1109	DJI_0014	Sorbus aucuparia L.	0	Rosaceae Juss.	n	1
Iz_3_1109	DJI_0014	Tilia cordata Mill.	0	Malvaceae Juss.	n	1
Iz_3_1109	DJI_0014	Ulmus glabra Huds.	0	Ulmaceae Mirb.	n	1
Iz_3_1109	DJI_0009	Acer tataricum L.	0	Sapindaceae Juss.	n	1
Iz_3_1109	DJI_0009	Betula pendula Roth.	0	Betulaceae Gray	n	1
Iz_3_1109	DJI_0009	Crataegus rhipidophylla Gand.	0	Rosaceae Juss.	n	1
Iz_3_1109	DJI_0009	Frangula alnus Mill.	0	Rhamnaceae Juss.	n	1
Iz_3_1109	DJI_0009	Fraxinus pennsylvanica Marshall	0	Oleaceae Hoffmanns. & Link	a	0
Iz_3_1109	DJI_0009	Pinus sylvestris L.	0	Pinaceae Spreng. ex F.Rudolphi	n	1
Iz_3_1109	DJI_0009	Populus × canescens (Aiton) Sm.	0	Salicaceae Mirb.	n	1
Iz_3_1109	DJI_0009	Populus tremula L.	0	Salicaceae Mirb.	n	1
Iz_3_1109	DJI_0009	Populus nigra L.	0	Salicaceae Mirb.	n	1
Iz_3_1109	DJI_0009	Pyrus communis L.	0	Rosaceae Juss.	a	0
Iz_3_1109	DJI_0009	Quercus robur L.	0	Fagaceae Dumort.	n	1
Iz_3_1109	DJI_0009	Robinia pseudoacacia L.	0	Fabaceae Lindl.	a	0
Iz_3_1109	DJI_0009	Rosa sp.	0	Rosaceae Juss.		
Iz_3_1109	DJI_0009	Salix alba L.	0	Salicaceae Mirb.	n	1
Iz_3_1109	DJI_0009	Sambucus nigra L.	0	Viburnaceae Raf.	n	1
Iz_3_1109	DJI_0009	Sorbus aucuparia L.	0	Rosaceae Juss.	n	1
Iz_3_1109	DJI_0009	Tilia cordata Mill.	0	Malvaceae Juss.	n	1
Iz_3_1109	DJI_0009	Ulmus glabra Huds.	0	Ulmaceae Mirb.	n	1
Iz_4_1109	DJI_0111	Acer tataricum L.	0	Sapindaceae Juss.	n	1
Iz_4_1109	DJI_0111	Betula pendula Roth.	0	Betulaceae Gray	n	1
Iz_4_1109	DJI_0111	Crataegus rhipidophylla Gand.	0	Rosaceae Juss.	n	1
Iz_4_1109	DJI_0111	Frangula alnus Mill.	0	Rhamnaceae Juss.	n	1
Iz_4_1109	DJI_0111	Fraxinus pennsylvanica Marshall	0	Oleaceae Hoffmanns. & Link	a	0
Iz_4_1109	DJI_0111	Pinus sylvestris L.	0	Pinaceae Spreng. ex F.Rudolphi	n	1
Iz_4_1109	DJI_0111	Populus × canescens (Aiton) Sm.	0	Salicaceae Mirb.	n	1
Iz_4_1109	DJI_0111	Populus tremula L.	0	Salicaceae Mirb.	n	1
Iz_4_1109	DJI_0111	Populus nigra L.	0	Salicaceae Mirb.	n	1
Iz_4_1109	DJI_0111	Pyrus communis L.	0	Rosaceae Juss.	a	0

Iz_4_1109	DJI_0111	Quercus robur L.	0	Fagaceae Dumort.	n	1
Iz_4_1109	DJI_0111	Robinia pseudoacacia L.	0	Fabaceae Lindl.	a	0
Iz_4_1109	DJI_0111	Rosa sp.	0	Rosaceae Juss.		
Iz_4_1109	DJI_0111	Salix alba L.	0	Salicaceae Mirb.	n	1
Iz_4_1109	DJI_0111	Sambucus nigra L.	0	Viburnaceae Raf.	n	1
Iz_4_1109	DJI_0111	Sorbus aucuparia L.	0	Rosaceae Juss.	n	1
Iz_4_1109	DJI_0111	Tilia cordata Mill.	0	Malvaceae Juss.	n	1
Iz_4_1109	DJI_0111	Ulmus glabra Huds.	0	Ulmaceae Mirb.	n	1
Iz_4_1109	DJI_0103	Acer tataricum L.	0	Sapindaceae Juss.	n	1
Iz_4_1109	DJI_0103	Betula pendula Roth.	0	Betulaceae Gray	n	1
Iz_4_1109	DJI_0103	Crataegus rhipidophylla Gand.	0	Rosaceae Juss.	n	1
Iz_4_1109	DJI_0103	Frangula alnus Mill.	0	Rhamnaceae Juss.	n	1
Iz_4_1109	DJI_0103	Fraxinus pennsylvanica Marshall	0	Oleaceae Hoffmanns. & Link	a	0
Iz_4_1109	DJI_0103	Pinus sylvestris L.	0	Pinaceae Spreng. ex F.Rudolphi	n	1
Iz_4_1109	DJI_0103	Populus × canescens (Aiton) Sm.	0	Salicaceae Mirb.	n	1
Iz_4_1109	DJI_0103	Populus tremula L.	0	Salicaceae Mirb.	n	1
Iz_4_1109	DJI_0103	Populus nigra L.	0	Salicaceae Mirb.	n	1
Iz_4_1109	DJI_0103	Pyrus communis L.	0	Rosaceae Juss.	a	0
Iz_4_1109	DJI_0103	Quercus robur L.	0	Fagaceae Dumort.	n	1
Iz_4_1109	DJI_0103	Robinia pseudoacacia L.	0	Fabaceae Lindl.	a	0
Iz_4_1109	DJI_0103	Rosa sp.	0	Rosaceae Juss.		
Iz_4_1109	DJI_0103	Salix alba L.	0	Salicaceae Mirb.	n	1
Iz_4_1109	DJI_0103	Sambucus nigra L.	0	Viburnaceae Raf.	n	1
Iz_4_1109	DJI_0103	Sorbus aucuparia L.	0	Rosaceae Juss.	n	1
Iz_4_1109	DJI_0103	Tilia cordata Mill.	0	Malvaceae Juss.	n	1
Iz_4_1109	DJI_0103	Ulmus glabra Huds.	0	Ulmaceae Mirb.	n	1
Iz_4_1109	DJI_0096	Acer tataricum L.	0	Sapindaceae Juss.	n	1
Iz_4_1109	DJI_0096	Betula pendula Roth.	0	Betulaceae Gray	n	1
Iz_4_1109	DJI_0096	Crataegus rhipidophylla Gand.	0	Rosaceae Juss.	n	1
Iz_4_1109	DJI_0096	Frangula alnus Mill.	0	Rhamnaceae Juss.	n	1
Iz_4_1109	DJI_0096	Fraxinus pennsylvanica Marshall	0	Oleaceae Hoffmanns. & Link	a	0
Iz_4_1109	DJI_0096	Pinus sylvestris L.	0	Pinaceae Spreng. ex F.Rudolphi	n	1
Iz_4_1109	DJI_0096	Populus × canescens (Aiton) Sm.	0	Salicaceae Mirb.	n	1
Iz_4_1109	DJI_0096	Populus tremula L.	0	Salicaceae Mirb.	n	1
Iz_4_1109	DJI_0096	Populus nigra L.	0	Salicaceae Mirb.	n	1
Iz_4_1109	DJI_0096	Pyrus communis L.	0	Rosaceae Juss.	a	0
Iz_4_1109	DJI_0096	Quercus robur L.	0	Fagaceae Dumort.	n	1
Iz_4_1109	DJI_0096	Robinia pseudoacacia L.	0	Fabaceae Lindl.	a	0
Iz_4_1109	DJI_0096	Rosa sp.	0	Rosaceae Juss.		
Iz_4_1109	DJI_0096	Salix alba L.	0	Salicaceae Mirb.	n	1
Iz_4_1109	DJI_0096	Sambucus nigra L.	0	Viburnaceae Raf.	n	1
Iz_4_1109	DJI_0096	Sorbus aucuparia L.	0	Rosaceae Juss.	n	1
Iz_4_1109	DJI_0096	Tilia cordata Mill.	0	Malvaceae Juss.	n	1
Iz_4_1109	DJI_0096	Ulmus glabra Huds.	0	Ulmaceae Mirb.	n	1
Iz_4_1109	DJI_0091	Acer tataricum L.	0	Sapindaceae Juss.	n	1
Iz_4_1109	DJI_0091	Betula pendula Roth.	0	Betulaceae Gray	n	1
Iz_4_1109	DJI_0091	Crataegus rhipidophylla Gand.	0	Rosaceae Juss.	n	1
Iz_4_1109	DJI_0091	Frangula alnus Mill.	0	Rhamnaceae Juss.	n	1
Iz_4_1109	DJI_0091	Fraxinus pennsylvanica Marshall	0	Oleaceae Hoffmanns. & Link	a	0
Iz_4_1109	DJI_0091	Pinus sylvestris L.	0	Pinaceae Spreng. ex F.Rudolphi	n	1
Iz_4_1109	DJI_0091	Populus × canescens (Aiton) Sm.	0	Salicaceae Mirb.	n	1
Iz_4_1109	DJI_0091	Populus tremula L.	0	Salicaceae Mirb.	n	1
Iz_4_1109	DJI_0091	Populus nigra L.	0	Salicaceae Mirb.	n	1
Iz_4_1109	DJI_0091	Pyrus communis L.	0	Rosaceae Juss.	a	0
Iz_4_1109	DJI_0091	Quercus robur L.	0	Fagaceae Dumort.	n	1
Iz_4_1109	DJI_0091	Robinia pseudoacacia L.	0	Fabaceae Lindl.	a	0
Iz_4_1109	DJI_0091	Rosa sp.	0	Rosaceae Juss.		
Iz_4_1109	DJI_0091	Salix alba L.	0	Salicaceae Mirb.	n	1
Iz_4_1109	DJI_0091	Sambucus nigra L.	0	Viburnaceae Raf.	n	1
Iz_4_1109	DJI_0091	Sorbus aucuparia L.	0	Rosaceae Juss.	n	1
Iz_4_1109	DJI_0091	Tilia cordata Mill.	0	Malvaceae Juss.	n	1
Iz_4_1109	DJI_0091	Ulmus glabra Huds.	0	Ulmaceae Mirb.	n	1
Iz_4_1109	DJI_0090	Acer tataricum L.	0	Sapindaceae Juss.	n	1
Iz_4_1109	DJI_0090	Betula pendula Roth.	0	Betulaceae Gray	n	1

Iz_4_1109	DJI_0090	Crataegus rhipidophylla Gand.	0	Rosaceae Juss.	n	1
Iz_4_1109	DJI_0090	Frangula alnus Mill.	0	Rhamnaceae Juss.	n	1
Iz_4_1109	DJI_0090	Fraxinus pennsylvanica Marshall	0	Oleaceae Hoffmanns. & Link	a	0
Iz_4_1109	DJI_0090	Pinus sylvestris L.	0	Pinaceae Spreng. ex F.Rudolphi	n	1
Iz_4_1109	DJI_0090	Populus × canescens (Aiton) Sm.	0	Salicaceae Mirb.	n	1
Iz_4_1109	DJI_0090	Populus tremula L.	0	Salicaceae Mirb.	n	1
Iz_4_1109	DJI_0090	Populus nigra L.	0	Salicaceae Mirb.	n	1
Iz_4_1109	DJI_0090	Pyrus communis L.	0	Rosaceae Juss.	a	0
Iz_4_1109	DJI_0090	Quercus robur L.	0	Fagaceae Dumort.	n	1
Iz_4_1109	DJI_0090	Robinia pseudoacacia L.	0	Fabaceae Lindl.	a	0
Iz_4_1109	DJI_0090	Rosa sp.	0	Rosaceae Juss.		
Iz_4_1109	DJI_0090	Salix alba L.	0	Salicaceae Mirb.	n	1
Iz_4_1109	DJI_0090	Sambucus nigra L.	0	Viburnaceae Raf.	n	1
Iz_4_1109	DJI_0090	Sorbus aucuparia L.	0	Rosaceae Juss.	n	1
Iz_4_1109	DJI_0090	Tilia cordata Mill.	0	Malvaceae Juss.	n	1
Iz_4_1109	DJI_0090	Ulmus glabra Huds.	0	Ulmaceae Mirb.	n	1
Iz_4_1109	DJI_0087	Acer tataricum L.	0	Sapindaceae Juss.	n	1
Iz_4_1109	DJI_0087	Betula pendula Roth.	0	Betulaceae Gray	n	1
Iz_4_1109	DJI_0087	Crataegus rhipidophylla Gand.	0	Rosaceae Juss.	n	1
Iz_4_1109	DJI_0087	Frangula alnus Mill.	0	Rhamnaceae Juss.	n	1
Iz_4_1109	DJI_0087	Fraxinus pennsylvanica Marshall	0	Oleaceae Hoffmanns. & Link	a	0
Iz_4_1109	DJI_0087	Pinus sylvestris L.	0	Pinaceae Spreng. ex F.Rudolphi	n	1
Iz_4_1109	DJI_0087	Populus × canescens (Aiton) Sm.	0	Salicaceae Mirb.	n	1
Iz_4_1109	DJI_0087	Populus tremula L.	0	Salicaceae Mirb.	n	1
Iz_4_1109	DJI_0087	Populus nigra L.	0	Salicaceae Mirb.	n	1
Iz_4_1109	DJI_0087	Pyrus communis L.	0	Rosaceae Juss.	a	0
Iz_4_1109	DJI_0087	Quercus robur L.	1	Fagaceae Dumort.	n	1
Iz_4_1109	DJI_0087	Robinia pseudoacacia L.	0	Fabaceae Lindl.	a	0
Iz_4_1109	DJI_0087	Rosa sp.	0	Rosaceae Juss.		
Iz_4_1109	DJI_0087	Salix alba L.	0	Salicaceae Mirb.	n	1
Iz_4_1109	DJI_0087	Sambucus nigra L.	0	Viburnaceae Raf.	n	1
Iz_4_1109	DJI_0087	Sorbus aucuparia L.	0	Rosaceae Juss.	n	1
Iz_4_1109	DJI_0087	Tilia cordata Mill.	0	Malvaceae Juss.	n	1
Iz_4_1109	DJI_0087	Ulmus glabra Huds.	0	Ulmaceae Mirb.	n	1
Iz_4_1109	DJI_0084	Acer tataricum L.	0	Sapindaceae Juss.	n	1
Iz_4_1109	DJI_0084	Betula pendula Roth.	0	Betulaceae Gray	n	1
Iz_4_1109	DJI_0084	Crataegus rhipidophylla Gand.	0	Rosaceae Juss.	n	1
Iz_4_1109	DJI_0084	Frangula alnus Mill.	0	Rhamnaceae Juss.	n	1
Iz_4_1109	DJI_0084	Fraxinus pennsylvanica Marshall	0	Oleaceae Hoffmanns. & Link	a	0
Iz_4_1109	DJI_0084	Pinus sylvestris L.	1	Pinaceae Spreng. ex F.Rudolphi	n	1
Iz_4_1109	DJI_0084	Populus × canescens (Aiton) Sm.	0	Salicaceae Mirb.	n	1
Iz_4_1109	DJI_0084	Populus tremula L.	0	Salicaceae Mirb.	n	1
Iz_4_1109	DJI_0084	Populus nigra L.	0	Salicaceae Mirb.	n	1
Iz_4_1109	DJI_0084	Pyrus communis L.	0	Rosaceae Juss.	a	0
Iz_4_1109	DJI_0084	Quercus robur L.	0	Fagaceae Dumort.	n	1
Iz_4_1109	DJI_0084	Robinia pseudoacacia L.	0	Fabaceae Lindl.	a	0
Iz_4_1109	DJI_0084	Rosa sp.	0	Rosaceae Juss.		
Iz_4_1109	DJI_0084	Salix alba L.	0	Salicaceae Mirb.	n	1
Iz_4_1109	DJI_0084	Sambucus nigra L.	0	Viburnaceae Raf.	n	1
Iz_4_1109	DJI_0084	Sorbus aucuparia L.	0	Rosaceae Juss.	n	1
Iz_4_1109	DJI_0084	Tilia cordata Mill.	0	Malvaceae Juss.	n	1
Iz_4_1109	DJI_0084	Ulmus glabra Huds.	0	Ulmaceae Mirb.	n	1
Iz_4_1109	DJI_0079	Acer tataricum L.	0	Sapindaceae Juss.	n	1
Iz_4_1109	DJI_0079	Betula pendula Roth.	0	Betulaceae Gray	n	1
Iz_4_1109	DJI_0079	Crataegus rhipidophylla Gand.	0	Rosaceae Juss.	n	1
Iz_4_1109	DJI_0079	Frangula alnus Mill.	0	Rhamnaceae Juss.	n	1
Iz_4_1109	DJI_0079	Fraxinus pennsylvanica Marshall	0	Oleaceae Hoffmanns. & Link	a	0
Iz_4_1109	DJI_0079	Pinus sylvestris L.	0	Pinaceae Spreng. ex F.Rudolphi	n	1
Iz_4_1109	DJI_0079	Populus × canescens (Aiton) Sm.	0	Salicaceae Mirb.	n	1
Iz_4_1109	DJI_0079	Populus tremula L.	0	Salicaceae Mirb.	n	1
Iz_4_1109	DJI_0079	Populus nigra L.	0	Salicaceae Mirb.	n	1
Iz_4_1109	DJI_0079	Pyrus communis L.	0	Rosaceae Juss.	a	0
Iz_4_1109	DJI_0079	Quercus robur L.	0	Fagaceae Dumort.	n	1
Iz_4_1109	DJI_0079	Robinia pseudoacacia L.	0	Fabaceae Lindl.	a	0

Iz_4_1109	DJI_0079	Rosa sp.	0	Rosaceae Juss.		
Iz_4_1109	DJI_0079	Salix alba L.	0	Salicaceae Mirb.	n	1
Iz_4_1109	DJI_0079	Sambucus nigra L.	0	Viburnaceae Raf.	n	1
Iz_4_1109	DJI_0079	Sorbus aucuparia L.	0	Rosaceae Juss.	n	1
Iz_4_1109	DJI_0079	Tilia cordata Mill.	0	Malvaceae Juss.	n	1
Iz_4_1109	DJI_0079	Ulmus glabra Huds.	0	Ulmaceae Mirb.	n	1
Iz_4_1109	DJI_0069	Acer tataricum L.	0	Sapindaceae Juss.	n	1
Iz_4_1109	DJI_0069	Betula pendula Roth.	0	Betulaceae Gray	n	1
Iz_4_1109	DJI_0069	Crataegus rhipidophylla Gand.	0	Rosaceae Juss.	n	1
Iz_4_1109	DJI_0069	Frangula alnus Mill.	0	Rhamnaceae Juss.	n	1
Iz_4_1109	DJI_0069	Fraxinus pennsylvanica Marshall	0	Oleaceae Hoffmanns. & Link	a	0
Iz_4_1109	DJI_0069	Pinus sylvestris L.	0	Pinaceae Spreng. ex F.Rudolphi	n	1
Iz_4_1109	DJI_0069	Populus × canescens (Aiton) Sm.	0	Salicaceae Mirb.	n	1
Iz_4_1109	DJI_0069	Populus tremula L.	0	Salicaceae Mirb.	n	1
Iz_4_1109	DJI_0069	Populus nigra L.	0	Salicaceae Mirb.	n	1
Iz_4_1109	DJI_0069	Pyrus communis L.	0	Rosaceae Juss.	a	0
Iz_4_1109	DJI_0069	Quercus robur L.	0	Fagaceae Dumort.	n	1
Iz_4_1109	DJI_0069	Robinia pseudoacacia L.	0	Fabaceae Lindl.	a	0
Iz_4_1109	DJI_0069	Rosa sp.	0	Rosaceae Juss.		
Iz_4_1109	DJI_0069	Salix alba L.	0	Salicaceae Mirb.	n	1
Iz_4_1109	DJI_0069	Sambucus nigra L.	0	Viburnaceae Raf.	n	1
Iz_4_1109	DJI_0069	Sorbus aucuparia L.	0	Rosaceae Juss.	n	1
Iz_4_1109	DJI_0069	Tilia cordata Mill.	0	Malvaceae Juss.	n	1
Iz_4_1109	DJI_0069	Ulmus glabra Huds.	0	Ulmaceae Mirb.	n	1
Iz_4_1109	DJI_0065	Acer tataricum L.	0	Sapindaceae Juss.	n	1
Iz_4_1109	DJI_0065	Betula pendula Roth.	0	Betulaceae Gray	n	1
Iz_4_1109	DJI_0065	Crataegus rhipidophylla Gand.	0	Rosaceae Juss.	n	1
Iz_4_1109	DJI_0065	Frangula alnus Mill.	0	Rhamnaceae Juss.	n	1
Iz_4_1109	DJI_0065	Fraxinus pennsylvanica Marshall	0	Oleaceae Hoffmanns. & Link	a	0
Iz_4_1109	DJI_0065	Pinus sylvestris L.	0	Pinaceae Spreng. ex F.Rudolphi	n	1
Iz_4_1109	DJI_0065	Populus × canescens (Aiton) Sm.	0	Salicaceae Mirb.	n	1
Iz_4_1109	DJI_0065	Populus tremula L.	0	Salicaceae Mirb.	n	1
Iz_4_1109	DJI_0065	Populus nigra L.	0	Salicaceae Mirb.	n	1
Iz_4_1109	DJI_0065	Pyrus communis L.	0	Rosaceae Juss.	a	0
Iz_4_1109	DJI_0065	Quercus robur L.	0	Fagaceae Dumort.	n	1
Iz_4_1109	DJI_0065	Robinia pseudoacacia L.	0	Fabaceae Lindl.	a	0
Iz_4_1109	DJI_0065	Rosa sp.	0	Rosaceae Juss.		
Iz_4_1109	DJI_0065	Salix alba L.	0	Salicaceae Mirb.	n	1
Iz_4_1109	DJI_0065	Sambucus nigra L.	0	Viburnaceae Raf.	n	1
Iz_4_1109	DJI_0065	Sorbus aucuparia L.	0	Rosaceae Juss.	n	1
Iz_4_1109	DJI_0065	Tilia cordata Mill.	0	Malvaceae Juss.	n	1
Iz_4_1109	DJI_0065	Ulmus glabra Huds.	0	Ulmaceae Mirb.	n	1
Iz_4_1109	DJI_0063	Acer tataricum L.	0	Sapindaceae Juss.	n	1
Iz_4_1109	DJI_0063	Betula pendula Roth.	0	Betulaceae Gray	n	1
Iz_4_1109	DJI_0063	Crataegus rhipidophylla Gand.	0	Rosaceae Juss.	n	1
Iz_4_1109	DJI_0063	Frangula alnus Mill.	0	Rhamnaceae Juss.	n	1
Iz_4_1109	DJI_0063	Fraxinus pennsylvanica Marshall	0	Oleaceae Hoffmanns. & Link	a	0
Iz_4_1109	DJI_0063	Pinus sylvestris L.	1	Pinaceae Spreng. ex F.Rudolphi	n	1
Iz_4_1109	DJI_0063	Populus × canescens (Aiton) Sm.	0	Salicaceae Mirb.	n	1
Iz_4_1109	DJI_0063	Populus tremula L.	0	Salicaceae Mirb.	n	1
Iz_4_1109	DJI_0063	Populus nigra L.	0	Salicaceae Mirb.	n	1
Iz_4_1109	DJI_0063	Pyrus communis L.	0	Rosaceae Juss.	a	0
Iz_4_1109	DJI_0063	Quercus robur L.	0	Fagaceae Dumort.	n	1
Iz_4_1109	DJI_0063	Robinia pseudoacacia L.	0	Fabaceae Lindl.	a	0
Iz_4_1109	DJI_0063	Rosa sp.	0	Rosaceae Juss.		
Iz_4_1109	DJI_0063	Salix alba L.	0	Salicaceae Mirb.	n	1
Iz_4_1109	DJI_0063	Sambucus nigra L.	0	Viburnaceae Raf.	n	1
Iz_4_1109	DJI_0063	Sorbus aucuparia L.	0	Rosaceae Juss.	n	1
Iz_4_1109	DJI_0063	Tilia cordata Mill.	0	Malvaceae Juss.	n	1
Iz_4_1109	DJI_0063	Ulmus glabra Huds.	0	Ulmaceae Mirb.	n	1
Iz_4_1109	DJI_0060	Acer tataricum L.	0	Sapindaceae Juss.	n	1
Iz_4_1109	DJI_0060	Betula pendula Roth.	0	Betulaceae Gray	n	1
Iz_4_1109	DJI_0060	Crataegus rhipidophylla Gand.	0	Rosaceae Juss.	n	1
Iz_4_1109	DJI_0060	Frangula alnus Mill.	0	Rhamnaceae Juss.	n	1

Iz_4_1109	DJI_0060	Fraxinus pennsylvanica Marshall	0	Oleaceae Hoffmanns. & Link	a	0
Iz_4_1109	DJI_0060	Pinus sylvestris L.	0	Pinaceae Spreng. ex F.Rudolphi	n	1
Iz_4_1109	DJI_0060	Populus × canescens (Aiton) Sm.	0	Salicaceae Mirb.	n	1
Iz_4_1109	DJI_0060	Populus tremula L.	0	Salicaceae Mirb.	n	1
Iz_4_1109	DJI_0060	Populus nigra L.	0	Salicaceae Mirb.	n	1
Iz_4_1109	DJI_0060	Pyrus communis L.	0	Rosaceae Juss.	a	0
Iz_4_1109	DJI_0060	Quercus robur L.	0	Fagaceae Dumort.	n	1
Iz_4_1109	DJI_0060	Robinia pseudoacacia L.	0	Fabaceae Lindl.	a	0
Iz_4_1109	DJI_0060	Rosa sp.	0	Rosaceae Juss.		
Iz_4_1109	DJI_0060	Salix alba L.	0	Salicaceae Mirb.	n	1
Iz_4_1109	DJI_0060	Sambucus nigra L.	0	Viburnaceae Raf.	n	1
Iz_4_1109	DJI_0060	Sorbus aucuparia L.	0	Rosaceae Juss.	n	1
Iz_4_1109	DJI_0060	Tilia cordata Mill.	0	Malvaceae Juss.	n	1
Iz_4_1109	DJI_0060	Ulmus glabra Huds.	0	Ulmaceae Mirb.	n	1
Iz_4_1109	DJI_0058	Acer tataricum L.	0	Sapindaceae Juss.	n	1
Iz_4_1109	DJI_0058	Betula pendula Roth.	0	Betulaceae Gray	n	1
Iz_4_1109	DJI_0058	Crataegus rhipidophylla Gand.	0	Rosaceae Juss.	n	1
Iz_4_1109	DJI_0058	Frangula alnus Mill.	0	Rhamnaceae Juss.	n	1
Iz_4_1109	DJI_0058	Fraxinus pennsylvanica Marshall	0	Oleaceae Hoffmanns. & Link	a	0
Iz_4_1109	DJI_0058	Pinus sylvestris L.	0	Pinaceae Spreng. ex F.Rudolphi	n	1
Iz_4_1109	DJI_0058	Populus × canescens (Aiton) Sm.	0	Salicaceae Mirb.	n	1
Iz_4_1109	DJI_0058	Populus tremula L.	0	Salicaceae Mirb.	n	1
Iz_4_1109	DJI_0058	Populus nigra L.	0	Salicaceae Mirb.	n	1
Iz_4_1109	DJI_0058	Pyrus communis L.	0	Rosaceae Juss.	a	0
Iz_4_1109	DJI_0058	Quercus robur L.	0	Fagaceae Dumort.	n	1
Iz_4_1109	DJI_0058	Robinia pseudoacacia L.	0	Fabaceae Lindl.	a	0
Iz_4_1109	DJI_0058	Rosa sp.	0	Rosaceae Juss.		
Iz_4_1109	DJI_0058	Salix alba L.	0	Salicaceae Mirb.	n	1
Iz_4_1109	DJI_0058	Sambucus nigra L.	0	Viburnaceae Raf.	n	1
Iz_4_1109	DJI_0058	Sorbus aucuparia L.	0	Rosaceae Juss.	n	1
Iz_4_1109	DJI_0058	Tilia cordata Mill.	0	Malvaceae Juss.	n	1
Iz_4_1109	DJI_0058	Ulmus glabra Huds.	0	Ulmaceae Mirb.	n	1
Iz_4_1109	DJI_0053	Acer tataricum L.	0	Sapindaceae Juss.	n	1
Iz_4_1109	DJI_0053	Betula pendula Roth.	0	Betulaceae Gray	n	1
Iz_4_1109	DJI_0053	Crataegus rhipidophylla Gand.	0	Rosaceae Juss.	n	1
Iz_4_1109	DJI_0053	Frangula alnus Mill.	0	Rhamnaceae Juss.	n	1
Iz_4_1109	DJI_0053	Fraxinus pennsylvanica Marshall	0	Oleaceae Hoffmanns. & Link	a	0
Iz_4_1109	DJI_0053	Pinus sylvestris L.	0	Pinaceae Spreng. ex F.Rudolphi	n	1
Iz_4_1109	DJI_0053	Populus × canescens (Aiton) Sm.	0	Salicaceae Mirb.	n	1
Iz_4_1109	DJI_0053	Populus tremula L.	0	Salicaceae Mirb.	n	1
Iz_4_1109	DJI_0053	Populus nigra L.	0	Salicaceae Mirb.	n	1
Iz_4_1109	DJI_0053	Pyrus communis L.	0	Rosaceae Juss.	a	0
Iz_4_1109	DJI_0053	Quercus robur L.	0	Fagaceae Dumort.	n	1
Iz_4_1109	DJI_0053	Robinia pseudoacacia L.	0	Fabaceae Lindl.	a	0
Iz_4_1109	DJI_0053	Rosa sp.	0	Rosaceae Juss.		
Iz_4_1109	DJI_0053	Salix alba L.	0	Salicaceae Mirb.	n	1
Iz_4_1109	DJI_0053	Sambucus nigra L.	0	Viburnaceae Raf.	n	1
Iz_4_1109	DJI_0053	Sorbus aucuparia L.	0	Rosaceae Juss.	n	1
Iz_4_1109	DJI_0053	Tilia cordata Mill.	0	Malvaceae Juss.	n	1
Iz_4_1109	DJI_0053	Ulmus glabra Huds.	1	Ulmaceae Mirb.	n	1
Iz_4_1109	DJI_0050	Acer tataricum L.	0	Sapindaceae Juss.	n	1
Iz_4_1109	DJI_0050	Betula pendula Roth.	0	Betulaceae Gray	n	1
Iz_4_1109	DJI_0050	Crataegus rhipidophylla Gand.	0	Rosaceae Juss.	n	1
Iz_4_1109	DJI_0050	Frangula alnus Mill.	0	Rhamnaceae Juss.	n	1
Iz_4_1109	DJI_0050	Fraxinus pennsylvanica Marshall	0	Oleaceae Hoffmanns. & Link	a	0
Iz_4_1109	DJI_0050	Pinus sylvestris L.	0	Pinaceae Spreng. ex F.Rudolphi	n	1
Iz_4_1109	DJI_0050	Populus × canescens (Aiton) Sm.	0	Salicaceae Mirb.	n	1
Iz_4_1109	DJI_0050	Populus tremula L.	0	Salicaceae Mirb.	n	1
Iz_4_1109	DJI_0050	Populus nigra L.	0	Salicaceae Mirb.	n	1
Iz_4_1109	DJI_0050	Pyrus communis L.	2	Rosaceae Juss.	a	0
Iz_4_1109	DJI_0050	Quercus robur L.	0	Fagaceae Dumort.	n	1
Iz_4_1109	DJI_0050	Robinia pseudoacacia L.	0	Fabaceae Lindl.	a	0
Iz_4_1109	DJI_0050	Rosa sp.	0	Rosaceae Juss.		
Iz_4_1109	DJI_0050	Salix alba L.	0	Salicaceae Mirb.	n	1

Iz_4_1109	DJI_0050	Sambucus nigra L.	0	Viburnaceae Raf.	n	1
Iz_4_1109	DJI_0050	Sorbus aucuparia L.	0	Rosaceae Juss.	n	1
Iz_4_1109	DJI_0050	Tilia cordata Mill.	0	Malvaceae Juss.	n	1
Iz_4_1109	DJI_0050	Ulmus glabra Huds.	0	Ulmaceae Mirb.	n	1
Iz_4_1109	DJI_0043	Acer tataricum L.	0	Sapindaceae Juss.	n	1
Iz_4_1109	DJI_0043	Betula pendula Roth.	0	Betulaceae Gray	n	1
Iz_4_1109	DJI_0043	Crataegus rhipidophylla Gand.	0	Rosaceae Juss.	n	1
Iz_4_1109	DJI_0043	Frangula alnus Mill.	0	Rhamnaceae Juss.	n	1
Iz_4_1109	DJI_0043	Fraxinus pennsylvanica Marshall	0	Oleaceae Hoffmanns. & Link	a	0
Iz_4_1109	DJI_0043	Pinus sylvestris L.	0	Pinaceae Spreng. ex F.Rudolphi	n	1
Iz_4_1109	DJI_0043	Populus × canescens (Aiton) Sm.	0	Salicaceae Mirb.	n	1
Iz_4_1109	DJI_0043	Populus tremula L.	0	Salicaceae Mirb.	n	1
Iz_4_1109	DJI_0043	Populus nigra L.	0	Salicaceae Mirb.	n	1
Iz_4_1109	DJI_0043	Pyrus communis L.	0	Rosaceae Juss.	a	0
Iz_4_1109	DJI_0043	Quercus robur L.	0	Fagaceae Dumort.	n	1
Iz_4_1109	DJI_0043	Robinia pseudoacacia L.	0	Fabaceae Lindl.	a	0
Iz_4_1109	DJI_0043	Rosa sp.	0	Rosaceae Juss.		
Iz_4_1109	DJI_0043	Salix alba L.	0	Salicaceae Mirb.	n	1
Iz_4_1109	DJI_0043	Sambucus nigra L.	0	Viburnaceae Raf.	n	1
Iz_4_1109	DJI_0043	Sorbus aucuparia L.	0	Rosaceae Juss.	n	1
Iz_4_1109	DJI_0043	Tilia cordata Mill.	0	Malvaceae Juss.	n	1
Iz_4_1109	DJI_0043	Ulmus glabra Huds.	0	Ulmaceae Mirb.	n	1
Iz_4_1109	DJI_0030	Acer tataricum L.	0	Sapindaceae Juss.	n	1
Iz_4_1109	DJI_0030	Betula pendula Roth.	0	Betulaceae Gray	n	1
Iz_4_1109	DJI_0030	Crataegus rhipidophylla Gand.	0	Rosaceae Juss.	n	1
Iz_4_1109	DJI_0030	Frangula alnus Mill.	0	Rhamnaceae Juss.	n	1
Iz_4_1109	DJI_0030	Fraxinus pennsylvanica Marshall	0	Oleaceae Hoffmanns. & Link	a	0
Iz_4_1109	DJI_0030	Pinus sylvestris L.	0	Pinaceae Spreng. ex F.Rudolphi	n	1
Iz_4_1109	DJI_0030	Populus × canescens (Aiton) Sm.	0	Salicaceae Mirb.	n	1
Iz_4_1109	DJI_0030	Populus tremula L.	0	Salicaceae Mirb.	n	1
Iz_4_1109	DJI_0030	Populus nigra L.	0	Salicaceae Mirb.	n	1
Iz_4_1109	DJI_0030	Pyrus communis L.	0	Rosaceae Juss.	a	0
Iz_4_1109	DJI_0030	Quercus robur L.	0	Fagaceae Dumort.	n	1
Iz_4_1109	DJI_0030	Robinia pseudoacacia L.	0	Fabaceae Lindl.	a	0
Iz_4_1109	DJI_0030	Rosa sp.	0	Rosaceae Juss.		
Iz_4_1109	DJI_0030	Salix alba L.	0	Salicaceae Mirb.	n	1
Iz_4_1109	DJI_0030	Sambucus nigra L.	0	Viburnaceae Raf.	n	1
Iz_4_1109	DJI_0030	Sorbus aucuparia L.	0	Rosaceae Juss.	n	1
Iz_4_1109	DJI_0030	Tilia cordata Mill.	0	Malvaceae Juss.	n	1
Iz_4_1109	DJI_0030	Ulmus glabra Huds.	0	Ulmaceae Mirb.	n	1
Iz_4_1109	DJI_0025	Acer tataricum L.	0	Sapindaceae Juss.	n	1
Iz_4_1109	DJI_0025	Betula pendula Roth.	0	Betulaceae Gray	n	1
Iz_4_1109	DJI_0025	Crataegus rhipidophylla Gand.	0	Rosaceae Juss.	n	1
Iz_4_1109	DJI_0025	Frangula alnus Mill.	0	Rhamnaceae Juss.	n	1
Iz_4_1109	DJI_0025	Fraxinus pennsylvanica Marshall	0	Oleaceae Hoffmanns. & Link	a	0
Iz_4_1109	DJI_0025	Pinus sylvestris L.	0	Pinaceae Spreng. ex F.Rudolphi	n	1
Iz_4_1109	DJI_0025	Populus × canescens (Aiton) Sm.	0	Salicaceae Mirb.	n	1
Iz_4_1109	DJI_0025	Populus tremula L.	0	Salicaceae Mirb.	n	1
Iz_4_1109	DJI_0025	Populus nigra L.	0	Salicaceae Mirb.	n	1
Iz_4_1109	DJI_0025	Pyrus communis L.	0	Rosaceae Juss.	a	0
Iz_4_1109	DJI_0025	Quercus robur L.	0	Fagaceae Dumort.	n	1
Iz_4_1109	DJI_0025	Robinia pseudoacacia L.	0	Fabaceae Lindl.	a	0
Iz_4_1109	DJI_0025	Rosa sp.	0	Rosaceae Juss.		
Iz_4_1109	DJI_0025	Salix alba L.	0	Salicaceae Mirb.	n	1
Iz_4_1109	DJI_0025	Sambucus nigra L.	0	Viburnaceae Raf.	n	1
Iz_4_1109	DJI_0025	Sorbus aucuparia L.	0	Rosaceae Juss.	n	1
Iz_4_1109	DJI_0025	Tilia cordata Mill.	0	Malvaceae Juss.	n	1
Iz_4_1109	DJI_0025	Ulmus glabra Huds.	0	Ulmaceae Mirb.	n	1
Iz_4_1109	DJI_0016	Acer tataricum L.	0	Sapindaceae Juss.	n	1
Iz_4_1109	DJI_0016	Betula pendula Roth.	0	Betulaceae Gray	n	1
Iz_4_1109	DJI_0016	Crataegus rhipidophylla Gand.	0	Rosaceae Juss.	n	1
Iz_4_1109	DJI_0016	Frangula alnus Mill.	0	Rhamnaceae Juss.	n	1
Iz_4_1109	DJI_0016	Fraxinus pennsylvanica Marshall	0	Oleaceae Hoffmanns. & Link	a	0
Iz_4_1109	DJI_0016	Pinus sylvestris L.	0	Pinaceae Spreng. ex F.Rudolphi	n	1

Iz_4_1109	DJI_0016	Populus × canescens (Aiton) Sm.	0	Salicaceae Mirb.	n	1
Iz_4_1109	DJI_0016	Populus tremula L.	0	Salicaceae Mirb.	n	1
Iz_4_1109	DJI_0016	Populus nigra L.	0	Salicaceae Mirb.	n	1
Iz_4_1109	DJI_0016	Pyrus communis L.	0	Rosaceae Juss.	a	0
Iz_4_1109	DJI_0016	Quercus robur L.	0	Fagaceae Dumort.	n	1
Iz_4_1109	DJI_0016	Robinia pseudoacacia L.	0	Fabaceae Lindl.	a	0
Iz_4_1109	DJI_0016	Rosa sp.	0	Rosaceae Juss.		
Iz_4_1109	DJI_0016	Salix alba L.	0	Salicaceae Mirb.	n	1
Iz_4_1109	DJI_0016	Sambucus nigra L.	0	Viburnaceae Raf.	n	1
Iz_4_1109	DJI_0016	Sorbus aucuparia L.	0	Rosaceae Juss.	n	1
Iz_4_1109	DJI_0016	Tilia cordata Mill.	0	Malvaceae Juss.	n	1
Iz_4_1109	DJI_0016	Ulmus glabra Huds.	0	Ulmaceae Mirb.	n	1
Iz_4_1109	DJI_0012	Acer tataricum L.	0	Sapindaceae Juss.	n	1
Iz_4_1109	DJI_0012	Betula pendula Roth.	0	Betulaceae Gray	n	1
Iz_4_1109	DJI_0012	Crataegus rhipidophylla Gand.	0	Rosaceae Juss.	n	1
Iz_4_1109	DJI_0012	Frangula alnus Mill.	0	Rhamnaceae Juss.	n	1
Iz_4_1109	DJI_0012	Fraxinus pennsylvanica Marshall	0	Oleaceae Hoffmanns. & Link	a	0
Iz_4_1109	DJI_0012	Pinus sylvestris L.	0	Pinaceae Spreng. ex F.Rudolphi	n	1
Iz_4_1109	DJI_0012	Populus × canescens (Aiton) Sm.	0	Salicaceae Mirb.	n	1
Iz_4_1109	DJI_0012	Populus tremula L.	0	Salicaceae Mirb.	n	1
Iz_4_1109	DJI_0012	Populus nigra L.	0	Salicaceae Mirb.	n	1
Iz_4_1109	DJI_0012	Pyrus communis L.	0	Rosaceae Juss.	a	0
Iz_4_1109	DJI_0012	Quercus robur L.	0	Fagaceae Dumort.	n	1
Iz_4_1109	DJI_0012	Robinia pseudoacacia L.	0	Fabaceae Lindl.	a	0
Iz_4_1109	DJI_0012	Rosa sp.	0	Rosaceae Juss.		
Iz_4_1109	DJI_0012	Salix alba L.	0	Salicaceae Mirb.	n	1
Iz_4_1109	DJI_0012	Sambucus nigra L.	0	Viburnaceae Raf.	n	1
Iz_4_1109	DJI_0012	Sorbus aucuparia L.	0	Rosaceae Juss.	n	1
Iz_4_1109	DJI_0012	Tilia cordata Mill.	0	Malvaceae Juss.	n	1
Iz_4_1109	DJI_0012	Ulmus glabra Huds.	0	Ulmaceae Mirb.	n	1
Iz_4_1109	DJI_0011	Acer tataricum L.	0	Sapindaceae Juss.	n	1
Iz_4_1109	DJI_0011	Betula pendula Roth.	0	Betulaceae Gray	n	1
Iz_4_1109	DJI_0011	Crataegus rhipidophylla Gand.	0	Rosaceae Juss.	n	1
Iz_4_1109	DJI_0011	Frangula alnus Mill.	0	Rhamnaceae Juss.	n	1
Iz_4_1109	DJI_0011	Fraxinus pennsylvanica Marshall	0	Oleaceae Hoffmanns. & Link	a	0
Iz_4_1109	DJI_0011	Pinus sylvestris L.	0	Pinaceae Spreng. ex F.Rudolphi	n	1
Iz_4_1109	DJI_0011	Populus × canescens (Aiton) Sm.	0	Salicaceae Mirb.	n	1
Iz_4_1109	DJI_0011	Populus tremula L.	0	Salicaceae Mirb.	n	1
Iz_4_1109	DJI_0011	Populus nigra L.	0	Salicaceae Mirb.	n	1
Iz_4_1109	DJI_0011	Pyrus communis L.	0	Rosaceae Juss.	a	0
Iz_4_1109	DJI_0011	Quercus robur L.	0	Fagaceae Dumort.	n	1
Iz_4_1109	DJI_0011	Robinia pseudoacacia L.	0	Fabaceae Lindl.	a	0
Iz_4_1109	DJI_0011	Rosa sp.	0	Rosaceae Juss.		
Iz_4_1109	DJI_0011	Salix alba L.	0	Salicaceae Mirb.	n	1
Iz_4_1109	DJI_0011	Sambucus nigra L.	0	Viburnaceae Raf.	n	1
Iz_4_1109	DJI_0011	Sorbus aucuparia L.	0	Rosaceae Juss.	n	1
Iz_4_1109	DJI_0011	Tilia cordata Mill.	0	Malvaceae Juss.	n	1
Iz_4_1109	DJI_0011	Ulmus glabra Huds.	0	Ulmaceae Mirb.	n	1
Iz_4_1109	DJI_009	Acer tataricum L.	0	Sapindaceae Juss.	n	1
Iz_4_1109	DJI_009	Betula pendula Roth.	0	Betulaceae Gray	n	1
Iz_4_1109	DJI_009	Crataegus rhipidophylla Gand.	0	Rosaceae Juss.	n	1
Iz_4_1109	DJI_009	Frangula alnus Mill.	0	Rhamnaceae Juss.	n	1
Iz_4_1109	DJI_009	Fraxinus pennsylvanica Marshall	0	Oleaceae Hoffmanns. & Link	a	0
Iz_4_1109	DJI_009	Pinus sylvestris L.	0	Pinaceae Spreng. ex F.Rudolphi	n	1
Iz_4_1109	DJI_009	Populus × canescens (Aiton) Sm.	0	Salicaceae Mirb.	n	1
Iz_4_1109	DJI_009	Populus tremula L.	0	Salicaceae Mirb.	n	1
Iz_4_1109	DJI_009	Populus nigra L.	0	Salicaceae Mirb.	n	1
Iz_4_1109	DJI_009	Pyrus communis L.	0	Rosaceae Juss.	a	0
Iz_4_1109	DJI_009	Quercus robur L.	0	Fagaceae Dumort.	n	1
Iz_4_1109	DJI_009	Robinia pseudoacacia L.	0	Fabaceae Lindl.	a	0
Iz_4_1109	DJI_009	Rosa sp.	0	Rosaceae Juss.		
Iz_4_1109	DJI_009	Salix alba L.	0	Salicaceae Mirb.	n	1
Iz_4_1109	DJI_009	Sambucus nigra L.	0	Viburnaceae Raf.	n	1
Iz_4_1109	DJI_009	Sorbus aucuparia L.	0	Rosaceae Juss.	n	1

Iz_4_1109	DJI_009	Tilia cordata Mill.	0	Malvaceae Juss.	n	1
Iz_4_1109	DJI_009	Ulmus glabra Huds.	0	Ulmaceae Mirb.	n	1
Iz_4_1109	DJI_006	Acer tataricum L.	0	Sapindaceae Juss.	n	1
Iz_4_1109	DJI_006	Betula pendula Roth.	0	Betulaceae Gray	n	1
Iz_4_1109	DJI_006	Crataegus rhipidophylla Gand.	0	Rosaceae Juss.	n	1
Iz_4_1109	DJI_006	Frangula alnus Mill.	0	Rhamnaceae Juss.	n	1
Iz_4_1109	DJI_006	Fraxinus pennsylvanica Marshall	0	Oleaceae Hoffmanns. & Link	a	0
Iz_4_1109	DJI_006	Pinus sylvestris L.	0	Pinaceae Spreng. ex F.Rudolphi	n	1
Iz_4_1109	DJI_006	Populus × canescens (Aiton) Sm.	0	Salicaceae Mirb.	n	1
Iz_4_1109	DJI_006	Populus tremula L.	0	Salicaceae Mirb.	n	1
Iz_4_1109	DJI_006	Populus nigra L.	0	Salicaceae Mirb.	n	1
Iz_4_1109	DJI_006	Pyrus communis L.	0	Rosaceae Juss.	a	0
Iz_4_1109	DJI_006	Quercus robur L.	0	Fagaceae Dumort.	n	1
Iz_4_1109	DJI_006	Robinia pseudoacacia L.	0	Fabaceae Lindl.	a	0
Iz_4_1109	DJI_006	Rosa sp.	0	Rosaceae Juss.		
Iz_4_1109	DJI_006	Salix alba L.	0	Salicaceae Mirb.	n	1
Iz_4_1109	DJI_006	Sambucus nigra L.	0	Viburnaceae Raf.	n	1
Iz_4_1109	DJI_006	Sorbus aucuparia L.	0	Rosaceae Juss.	n	1
Iz_4_1109	DJI_006	Tilia cordata Mill.	0	Malvaceae Juss.	n	1
Iz_4_1109	DJI_006	Ulmus glabra Huds.	0	Ulmaceae Mirb.	n	1
Iz_5_1109	DJI_0223	Acer tataricum L.	0	Sapindaceae Juss.	n	1
Iz_5_1109	DJI_0223	Betula pendula Roth.	0	Betulaceae Gray	n	1
Iz_5_1109	DJI_0223	Crataegus rhipidophylla Gand.	0	Rosaceae Juss.	n	1
Iz_5_1109	DJI_0223	Frangula alnus Mill.	0	Rhamnaceae Juss.	n	1
Iz_5_1109	DJI_0223	Fraxinus pennsylvanica Marshall	0	Oleaceae Hoffmanns. & Link	a	0
Iz_5_1109	DJI_0223	Pinus sylvestris L.	0	Pinaceae Spreng. ex F.Rudolphi	n	1
Iz_5_1109	DJI_0223	Populus × canescens (Aiton) Sm.	0	Salicaceae Mirb.	n	1
Iz_5_1109	DJI_0223	Populus tremula L.	0	Salicaceae Mirb.	n	1
Iz_5_1109	DJI_0223	Populus nigra L.	0	Salicaceae Mirb.	n	1
Iz_5_1109	DJI_0223	Pyrus communis L.	0	Rosaceae Juss.	a	0
Iz_5_1109	DJI_0223	Quercus robur L.	0	Fagaceae Dumort.	n	1
Iz_5_1109	DJI_0223	Robinia pseudoacacia L.	0	Fabaceae Lindl.	a	0
Iz_5_1109	DJI_0223	Rosa sp.	0	Rosaceae Juss.		
Iz_5_1109	DJI_0223	Salix alba L.	0	Salicaceae Mirb.	n	1
Iz_5_1109	DJI_0223	Sambucus nigra L.	0	Viburnaceae Raf.	n	1
Iz_5_1109	DJI_0223	Sorbus aucuparia L.	0	Rosaceae Juss.	n	1
Iz_5_1109	DJI_0223	Tilia cordata Mill.	0	Malvaceae Juss.	n	1
Iz_5_1109	DJI_0223	Ulmus glabra Huds.	0	Ulmaceae Mirb.	n	1
Iz_5_1109	DJI_0220	Acer tataricum L.	0	Sapindaceae Juss.	n	1
Iz_5_1109	DJI_0220	Betula pendula Roth.	0	Betulaceae Gray	n	1
Iz_5_1109	DJI_0220	Crataegus rhipidophylla Gand.	0	Rosaceae Juss.	n	1
Iz_5_1109	DJI_0220	Frangula alnus Mill.	0	Rhamnaceae Juss.	n	1
Iz_5_1109	DJI_0220	Fraxinus pennsylvanica Marshall	0	Oleaceae Hoffmanns. & Link	a	0
Iz_5_1109	DJI_0220	Pinus sylvestris L.	0	Pinaceae Spreng. ex F.Rudolphi	n	1
Iz_5_1109	DJI_0220	Populus × canescens (Aiton) Sm.	0	Salicaceae Mirb.	n	1
Iz_5_1109	DJI_0220	Populus tremula L.	0	Salicaceae Mirb.	n	1
Iz_5_1109	DJI_0220	Populus nigra L.	0	Salicaceae Mirb.	n	1
Iz_5_1109	DJI_0220	Pyrus communis L.	0	Rosaceae Juss.	a	0
Iz_5_1109	DJI_0220	Quercus robur L.	0	Fagaceae Dumort.	n	1
Iz_5_1109	DJI_0220	Robinia pseudoacacia L.	0	Fabaceae Lindl.	a	0
Iz_5_1109	DJI_0220	Rosa sp.	0	Rosaceae Juss.		
Iz_5_1109	DJI_0220	Salix alba L.	0	Salicaceae Mirb.	n	1
Iz_5_1109	DJI_0220	Sambucus nigra L.	0	Viburnaceae Raf.	n	1
Iz_5_1109	DJI_0220	Sorbus aucuparia L.	0	Rosaceae Juss.	n	1
Iz_5_1109	DJI_0220	Tilia cordata Mill.	0	Malvaceae Juss.	n	1
Iz_5_1109	DJI_0220	Ulmus glabra Huds.	0	Ulmaceae Mirb.	n	1
Iz_5_1109	DJI_0217	Acer tataricum L.	0	Sapindaceae Juss.	n	1
Iz_5_1109	DJI_0217	Betula pendula Roth.	0	Betulaceae Gray	n	1
Iz_5_1109	DJI_0217	Crataegus rhipidophylla Gand.	0	Rosaceae Juss.	n	1
Iz_5_1109	DJI_0217	Frangula alnus Mill.	0	Rhamnaceae Juss.	n	1
Iz_5_1109	DJI_0217	Fraxinus pennsylvanica Marshall	0	Oleaceae Hoffmanns. & Link	a	0
Iz_5_1109	DJI_0217	Pinus sylvestris L.	0	Pinaceae Spreng. ex F.Rudolphi	n	1
Iz_5_1109	DJI_0217	Populus × canescens (Aiton) Sm.	0	Salicaceae Mirb.	n	1
Iz_5_1109	DJI_0217	Populus tremula L.	0	Salicaceae Mirb.	n	1

Iz_5_1109	DJI_0217	Populus nigra L.	0	Salicaceae Mirb.	n	1
Iz_5_1109	DJI_0217	Pyrus communis L.	0	Rosaceae Juss.	a	0
Iz_5_1109	DJI_0217	Quercus robur L.	0	Fagaceae Dumort.	n	1
Iz_5_1109	DJI_0217	Robinia pseudoacacia L.	0	Fabaceae Lindl.	a	0
Iz_5_1109	DJI_0217	Rosa sp.	0	Rosaceae Juss.		
Iz_5_1109	DJI_0217	Salix alba L.	0	Salicaceae Mirb.	n	1
Iz_5_1109	DJI_0217	Sambucus nigra L.	0	Viburnaceae Raf.	n	1
Iz_5_1109	DJI_0217	Sorbus aucuparia L.	0	Rosaceae Juss.	n	1
Iz_5_1109	DJI_0217	Tilia cordata Mill.	0	Malvaceae Juss.	n	1
Iz_5_1109	DJI_0217	Ulmus glabra Huds.	0	Ulmaceae Mirb.	n	1
Iz_5_1109	DJI_0213	Acer tataricum L.	0	Sapindaceae Juss.	n	1
Iz_5_1109	DJI_0213	Betula pendula Roth.	0	Betulaceae Gray	n	1
Iz_5_1109	DJI_0213	Crataegus rhipidophylla Gand.	0	Rosaceae Juss.	n	1
Iz_5_1109	DJI_0213	Frangula alnus Mill.	0	Rhamnaceae Juss.	n	1
Iz_5_1109	DJI_0213	Fraxinus pennsylvanica Marshall	0	Oleaceae Hoffmanns. & Link	a	0
Iz_5_1109	DJI_0213	Pinus sylvestris L.	0	Pinaceae Spreng. ex F.Rudolphi	n	1
Iz_5_1109	DJI_0213	Populus × canescens (Aiton) Sm.	0	Salicaceae Mirb.	n	1
Iz_5_1109	DJI_0213	Populus tremula L.	0	Salicaceae Mirb.	n	1
Iz_5_1109	DJI_0213	Populus nigra L.	0	Salicaceae Mirb.	n	1
Iz_5_1109	DJI_0213	Pyrus communis L.	0	Rosaceae Juss.	a	0
Iz_5_1109	DJI_0213	Quercus robur L.	0	Fagaceae Dumort.	n	1
Iz_5_1109	DJI_0213	Robinia pseudoacacia L.	0	Fabaceae Lindl.	a	0
Iz_5_1109	DJI_0213	Rosa sp.	0	Rosaceae Juss.		
Iz_5_1109	DJI_0213	Salix alba L.	0	Salicaceae Mirb.	n	1
Iz_5_1109	DJI_0213	Sambucus nigra L.	0	Viburnaceae Raf.	n	1
Iz_5_1109	DJI_0213	Sorbus aucuparia L.	0	Rosaceae Juss.	n	1
Iz_5_1109	DJI_0213	Tilia cordata Mill.	0	Malvaceae Juss.	n	1
Iz_5_1109	DJI_0213	Ulmus glabra Huds.	0	Ulmaceae Mirb.	n	1
Iz_5_1109	DJI_0210	Acer tataricum L.	0	Sapindaceae Juss.	n	1
Iz_5_1109	DJI_0210	Betula pendula Roth.	0	Betulaceae Gray	n	1
Iz_5_1109	DJI_0210	Crataegus rhipidophylla Gand.	0	Rosaceae Juss.	n	1
Iz_5_1109	DJI_0210	Frangula alnus Mill.	0	Rhamnaceae Juss.	n	1
Iz_5_1109	DJI_0210	Fraxinus pennsylvanica Marshall	0	Oleaceae Hoffmanns. & Link	a	0
Iz_5_1109	DJI_0210	Pinus sylvestris L.	0	Pinaceae Spreng. ex F.Rudolphi	n	1
Iz_5_1109	DJI_0210	Populus × canescens (Aiton) Sm.	0	Salicaceae Mirb.	n	1
Iz_5_1109	DJI_0210	Populus tremula L.	0	Salicaceae Mirb.	n	1
Iz_5_1109	DJI_0210	Populus nigra L.	0	Salicaceae Mirb.	n	1
Iz_5_1109	DJI_0210	Pyrus communis L.	0	Rosaceae Juss.	a	0
Iz_5_1109	DJI_0210	Quercus robur L.	0	Fagaceae Dumort.	n	1
Iz_5_1109	DJI_0210	Robinia pseudoacacia L.	0	Fabaceae Lindl.	a	0
Iz_5_1109	DJI_0210	Rosa sp.	0	Rosaceae Juss.		
Iz_5_1109	DJI_0210	Salix alba L.	0	Salicaceae Mirb.	n	1
Iz_5_1109	DJI_0210	Sambucus nigra L.	0	Viburnaceae Raf.	n	1
Iz_5_1109	DJI_0210	Sorbus aucuparia L.	0	Rosaceae Juss.	n	1
Iz_5_1109	DJI_0210	Tilia cordata Mill.	0	Malvaceae Juss.	n	1
Iz_5_1109	DJI_0210	Ulmus glabra Huds.	0	Ulmaceae Mirb.	n	1
Iz_5_1109	DJI_0207	Acer tataricum L.	0	Sapindaceae Juss.	n	1
Iz_5_1109	DJI_0207	Betula pendula Roth.	0	Betulaceae Gray	n	1
Iz_5_1109	DJI_0207	Crataegus rhipidophylla Gand.	0	Rosaceae Juss.	n	1
Iz_5_1109	DJI_0207	Frangula alnus Mill.	0	Rhamnaceae Juss.	n	1
Iz_5_1109	DJI_0207	Fraxinus pennsylvanica Marshall	0	Oleaceae Hoffmanns. & Link	a	0
Iz_5_1109	DJI_0207	Pinus sylvestris L.	0	Pinaceae Spreng. ex F.Rudolphi	n	1
Iz_5_1109	DJI_0207	Populus × canescens (Aiton) Sm.	0	Salicaceae Mirb.	n	1
Iz_5_1109	DJI_0207	Populus tremula L.	0	Salicaceae Mirb.	n	1
Iz_5_1109	DJI_0207	Populus nigra L.	0	Salicaceae Mirb.	n	1
Iz_5_1109	DJI_0207	Pyrus communis L.	0	Rosaceae Juss.	a	0
Iz_5_1109	DJI_0207	Quercus robur L.	0	Fagaceae Dumort.	n	1
Iz_5_1109	DJI_0207	Robinia pseudoacacia L.	0	Fabaceae Lindl.	a	0
Iz_5_1109	DJI_0207	Rosa sp.	0	Rosaceae Juss.		
Iz_5_1109	DJI_0207	Salix alba L.	0	Salicaceae Mirb.	n	1
Iz_5_1109	DJI_0207	Sambucus nigra L.	0	Viburnaceae Raf.	n	1
Iz_5_1109	DJI_0207	Sorbus aucuparia L.	0	Rosaceae Juss.	n	1
Iz_5_1109	DJI_0207	Tilia cordata Mill.	0	Malvaceae Juss.	n	1
Iz_5_1109	DJI_0207	Ulmus glabra Huds.	0	Ulmaceae Mirb.	n	1

Iz_5_1109	DJI_0204	Acer tataricum L.	0	Sapindaceae Juss.	n	1
Iz_5_1109	DJI_0204	Betula pendula Roth.	0	Betulaceae Gray	n	1
Iz_5_1109	DJI_0204	Crataegus rhipidophylla Gand.	0	Rosaceae Juss.	n	1
Iz_5_1109	DJI_0204	Frangula alnus Mill.	0	Rhamnaceae Juss.	n	1
Iz_5_1109	DJI_0204	Fraxinus pennsylvanica Marshall	0	Oleaceae Hoffmanns. & Link	a	0
Iz_5_1109	DJI_0204	Pinus sylvestris L.	0	Pinaceae Spreng. ex F.Rudolphi	n	1
Iz_5_1109	DJI_0204	Populus × canescens (Aiton) Sm.	0	Salicaceae Mirb.	n	1
Iz_5_1109	DJI_0204	Populus tremula L.	0	Salicaceae Mirb.	n	1
Iz_5_1109	DJI_0204	Populus nigra L.	0	Salicaceae Mirb.	n	1
Iz_5_1109	DJI_0204	Pyrus communis L.	0	Rosaceae Juss.	a	0
Iz_5_1109	DJI_0204	Quercus robur L.	0	Fagaceae Dumort.	n	1
Iz_5_1109	DJI_0204	Robinia pseudoacacia L.	0	Fabaceae Lindl.	a	0
Iz_5_1109	DJI_0204	Rosa sp.	0	Rosaceae Juss.		
Iz_5_1109	DJI_0204	Salix alba L.	0	Salicaceae Mirb.	n	1
Iz_5_1109	DJI_0204	Sambucus nigra L.	0	Viburnaceae Raf.	n	1
Iz_5_1109	DJI_0204	Sorbus aucuparia L.	0	Rosaceae Juss.	n	1
Iz_5_1109	DJI_0204	Tilia cordata Mill.	0	Malvaceae Juss.	n	1
Iz_5_1109	DJI_0204	Ulmus glabra Huds.	0	Ulmaceae Mirb.	n	1
Iz_5_1109	DJI_0199	Acer tataricum L.	0	Sapindaceae Juss.	n	1
Iz_5_1109	DJI_0199	Betula pendula Roth.	0	Betulaceae Gray	n	1
Iz_5_1109	DJI_0199	Crataegus rhipidophylla Gand.	0	Rosaceae Juss.	n	1
Iz_5_1109	DJI_0199	Frangula alnus Mill.	0	Rhamnaceae Juss.	n	1
Iz_5_1109	DJI_0199	Fraxinus pennsylvanica Marshall	0	Oleaceae Hoffmanns. & Link	a	0
Iz_5_1109	DJI_0199	Pinus sylvestris L.	0	Pinaceae Spreng. ex F.Rudolphi	n	1
Iz_5_1109	DJI_0199	Populus × canescens (Aiton) Sm.	0	Salicaceae Mirb.	n	1
Iz_5_1109	DJI_0199	Populus tremula L.	0	Salicaceae Mirb.	n	1
Iz_5_1109	DJI_0199	Populus nigra L.	0	Salicaceae Mirb.	n	1
Iz_5_1109	DJI_0199	Pyrus communis L.	0	Rosaceae Juss.	a	0
Iz_5_1109	DJI_0199	Quercus robur L.	0	Fagaceae Dumort.	n	1
Iz_5_1109	DJI_0199	Robinia pseudoacacia L.	0	Fabaceae Lindl.	a	0
Iz_5_1109	DJI_0199	Rosa sp.	0	Rosaceae Juss.		
Iz_5_1109	DJI_0199	Salix alba L.	0	Salicaceae Mirb.	n	1
Iz_5_1109	DJI_0199	Sambucus nigra L.	0	Viburnaceae Raf.	n	1
Iz_5_1109	DJI_0199	Sorbus aucuparia L.	0	Rosaceae Juss.	n	1
Iz_5_1109	DJI_0199	Tilia cordata Mill.	0	Malvaceae Juss.	n	1
Iz_5_1109	DJI_0199	Ulmus glabra Huds.	0	Ulmaceae Mirb.	n	1
Iz_5_1109	DJI_0195	Acer tataricum L.	0	Sapindaceae Juss.	n	1
Iz_5_1109	DJI_0195	Betula pendula Roth.	0	Betulaceae Gray	n	1
Iz_5_1109	DJI_0195	Crataegus rhipidophylla Gand.	0	Rosaceae Juss.	n	1
Iz_5_1109	DJI_0195	Frangula alnus Mill.	0	Rhamnaceae Juss.	n	1
Iz_5_1109	DJI_0195	Fraxinus pennsylvanica Marshall	0	Oleaceae Hoffmanns. & Link	a	0
Iz_5_1109	DJI_0195	Pinus sylvestris L.	0	Pinaceae Spreng. ex F.Rudolphi	n	1
Iz_5_1109	DJI_0195	Populus × canescens (Aiton) Sm.	0	Salicaceae Mirb.	n	1
Iz_5_1109	DJI_0195	Populus tremula L.	0	Salicaceae Mirb.	n	1
Iz_5_1109	DJI_0195	Populus nigra L.	0	Salicaceae Mirb.	n	1
Iz_5_1109	DJI_0195	Pyrus communis L.	0	Rosaceae Juss.	a	0
Iz_5_1109	DJI_0195	Quercus robur L.	0	Fagaceae Dumort.	n	1
Iz_5_1109	DJI_0195	Robinia pseudoacacia L.	0	Fabaceae Lindl.	a	0
Iz_5_1109	DJI_0195	Rosa sp.	0	Rosaceae Juss.		
Iz_5_1109	DJI_0195	Salix alba L.	0	Salicaceae Mirb.	n	1
Iz_5_1109	DJI_0195	Sambucus nigra L.	0	Viburnaceae Raf.	n	1
Iz_5_1109	DJI_0195	Sorbus aucuparia L.	0	Rosaceae Juss.	n	1
Iz_5_1109	DJI_0195	Tilia cordata Mill.	0	Malvaceae Juss.	n	1
Iz_5_1109	DJI_0195	Ulmus glabra Huds.	0	Ulmaceae Mirb.	n	1
Iz_5_1109	DJI_0193	Acer tataricum L.	0	Sapindaceae Juss.	n	1
Iz_5_1109	DJI_0193	Betula pendula Roth.	0	Betulaceae Gray	n	1
Iz_5_1109	DJI_0193	Crataegus rhipidophylla Gand.	0	Rosaceae Juss.	n	1
Iz_5_1109	DJI_0193	Frangula alnus Mill.	0	Rhamnaceae Juss.	n	1
Iz_5_1109	DJI_0193	Fraxinus pennsylvanica Marshall	0	Oleaceae Hoffmanns. & Link	a	0
Iz_5_1109	DJI_0193	Pinus sylvestris L.	0	Pinaceae Spreng. ex F.Rudolphi	n	1
Iz_5_1109	DJI_0193	Populus × canescens (Aiton) Sm.	0	Salicaceae Mirb.	n	1
Iz_5_1109	DJI_0193	Populus tremula L.	0	Salicaceae Mirb.	n	1
Iz_5_1109	DJI_0193	Populus nigra L.	0	Salicaceae Mirb.	n	1
Iz_5_1109	DJI_0193	Pyrus communis L.	0	Rosaceae Juss.	a	0

Iz_5_1109	DJI_0193	Quercus robur L.	0	Fagaceae Dumort.	n	1
Iz_5_1109	DJI_0193	Robinia pseudoacacia L.	0	Fabaceae Lindl.	a	0
Iz_5_1109	DJI_0193	Rosa sp.	0	Rosaceae Juss.		
Iz_5_1109	DJI_0193	Salix alba L.	0	Salicaceae Mirb.	n	1
Iz_5_1109	DJI_0193	Sambucus nigra L.	0	Viburnaceae Raf.	n	1
Iz_5_1109	DJI_0193	Sorbus aucuparia L.	0	Rosaceae Juss.	n	1
Iz_5_1109	DJI_0193	Tilia cordata Mill.	0	Malvaceae Juss.	n	1
Iz_5_1109	DJI_0193	Ulmus glabra Huds.	0	Ulmaceae Mirb.	n	1
Iz_5_1109	DJI_0192	Acer tataricum L.	0	Sapindaceae Juss.	n	1
Iz_5_1109	DJI_0192	Betula pendula Roth.	0	Betulaceae Gray	n	1
Iz_5_1109	DJI_0192	Crataegus rhipidophylla Gand.	0	Rosaceae Juss.	n	1
Iz_5_1109	DJI_0192	Frangula alnus Mill.	0	Rhamnaceae Juss.	n	1
Iz_5_1109	DJI_0192	Fraxinus pennsylvanica Marshall	0	Oleaceae Hoffmanns. & Link	a	0
Iz_5_1109	DJI_0192	Pinus sylvestris L.	0	Pinaceae Spreng. ex F.Rudolphi	n	1
Iz_5_1109	DJI_0192	Populus × canescens (Aiton) Sm.	0	Salicaceae Mirb.	n	1
Iz_5_1109	DJI_0192	Populus tremula L.	0	Salicaceae Mirb.	n	1
Iz_5_1109	DJI_0192	Populus nigra L.	0	Salicaceae Mirb.	n	1
Iz_5_1109	DJI_0192	Pyrus communis L.	0	Rosaceae Juss.	a	0
Iz_5_1109	DJI_0192	Quercus robur L.	0	Fagaceae Dumort.	n	1
Iz_5_1109	DJI_0192	Robinia pseudoacacia L.	0	Fabaceae Lindl.	a	0
Iz_5_1109	DJI_0192	Rosa sp.	0	Rosaceae Juss.		
Iz_5_1109	DJI_0192	Salix alba L.	0	Salicaceae Mirb.	n	1
Iz_5_1109	DJI_0192	Sambucus nigra L.	0	Viburnaceae Raf.	n	1
Iz_5_1109	DJI_0192	Sorbus aucuparia L.	0	Rosaceae Juss.	n	1
Iz_5_1109	DJI_0192	Tilia cordata Mill.	0	Malvaceae Juss.	n	1
Iz_5_1109	DJI_0192	Ulmus glabra Huds.	0	Ulmaceae Mirb.	n	1
Iz_5_1109	DJI_0191	Acer tataricum L.	0	Sapindaceae Juss.	n	1
Iz_5_1109	DJI_0191	Betula pendula Roth.	0	Betulaceae Gray	n	1
Iz_5_1109	DJI_0191	Crataegus rhipidophylla Gand.	0	Rosaceae Juss.	n	1
Iz_5_1109	DJI_0191	Frangula alnus Mill.	0	Rhamnaceae Juss.	n	1
Iz_5_1109	DJI_0191	Fraxinus pennsylvanica Marshall	0	Oleaceae Hoffmanns. & Link	a	0
Iz_5_1109	DJI_0191	Pinus sylvestris L.	0	Pinaceae Spreng. ex F.Rudolphi	n	1
Iz_5_1109	DJI_0191	Populus × canescens (Aiton) Sm.	0	Salicaceae Mirb.	n	1
Iz_5_1109	DJI_0191	Populus tremula L.	0	Salicaceae Mirb.	n	1
Iz_5_1109	DJI_0191	Populus nigra L.	0	Salicaceae Mirb.	n	1
Iz_5_1109	DJI_0191	Pyrus communis L.	0	Rosaceae Juss.	a	0
Iz_5_1109	DJI_0191	Quercus robur L.	0	Fagaceae Dumort.	n	1
Iz_5_1109	DJI_0191	Robinia pseudoacacia L.	0	Fabaceae Lindl.	a	0
Iz_5_1109	DJI_0191	Rosa sp.	0	Rosaceae Juss.		
Iz_5_1109	DJI_0191	Salix alba L.	0	Salicaceae Mirb.	n	1
Iz_5_1109	DJI_0191	Sambucus nigra L.	0	Viburnaceae Raf.	n	1
Iz_5_1109	DJI_0191	Sorbus aucuparia L.	0	Rosaceae Juss.	n	1
Iz_5_1109	DJI_0191	Tilia cordata Mill.	0	Malvaceae Juss.	n	1
Iz_5_1109	DJI_0191	Ulmus glabra Huds.	0	Ulmaceae Mirb.	n	1
Iz_5_1109	DJI_0190	Acer tataricum L.	0	Sapindaceae Juss.	n	1
Iz_5_1109	DJI_0190	Betula pendula Roth.	0	Betulaceae Gray	n	1
Iz_5_1109	DJI_0190	Crataegus rhipidophylla Gand.	0	Rosaceae Juss.	n	1
Iz_5_1109	DJI_0190	Frangula alnus Mill.	0	Rhamnaceae Juss.	n	1
Iz_5_1109	DJI_0190	Fraxinus pennsylvanica Marshall	0	Oleaceae Hoffmanns. & Link	a	0
Iz_5_1109	DJI_0190	Pinus sylvestris L.	0	Pinaceae Spreng. ex F.Rudolphi	n	1
Iz_5_1109	DJI_0190	Populus × canescens (Aiton) Sm.	0	Salicaceae Mirb.	n	1
Iz_5_1109	DJI_0190	Populus tremula L.	0	Salicaceae Mirb.	n	1
Iz_5_1109	DJI_0190	Populus nigra L.	0	Salicaceae Mirb.	n	1
Iz_5_1109	DJI_0190	Pyrus communis L.	0	Rosaceae Juss.	a	0
Iz_5_1109	DJI_0190	Quercus robur L.	0	Fagaceae Dumort.	n	1
Iz_5_1109	DJI_0190	Robinia pseudoacacia L.	0	Fabaceae Lindl.	a	0
Iz_5_1109	DJI_0190	Rosa sp.	0	Rosaceae Juss.		
Iz_5_1109	DJI_0190	Salix alba L.	0	Salicaceae Mirb.	n	1
Iz_5_1109	DJI_0190	Sambucus nigra L.	0	Viburnaceae Raf.	n	1
Iz_5_1109	DJI_0190	Sorbus aucuparia L.	0	Rosaceae Juss.	n	1
Iz_5_1109	DJI_0190	Tilia cordata Mill.	0	Malvaceae Juss.	n	1
Iz_5_1109	DJI_0190	Ulmus glabra Huds.	0	Ulmaceae Mirb.	n	1
Iz_5_1109	DJI_0167	Acer tataricum L.	0	Sapindaceae Juss.	n	1
Iz_5_1109	DJI_0167	Betula pendula Roth.	0	Betulaceae Gray	n	1

Iz_5_1109	DJI_0167	Crataegus rhipidophylla Gand.	0	Rosaceae Juss.	n	1
Iz_5_1109	DJI_0167	Frangula alnus Mill.	0	Rhamnaceae Juss.	n	1
Iz_5_1109	DJI_0167	Fraxinus pennsylvanica Marshall	0	Oleaceae Hoffmanns. & Link	a	0
Iz_5_1109	DJI_0167	Pinus sylvestris L.	0	Pinaceae Spreng. ex F.Rudolphi	n	1
Iz_5_1109	DJI_0167	Populus × canescens (Aiton) Sm.	0	Salicaceae Mirb.	n	1
Iz_5_1109	DJI_0167	Populus tremula L.	0	Salicaceae Mirb.	n	1
Iz_5_1109	DJI_0167	Populus nigra L.	0	Salicaceae Mirb.	n	1
Iz_5_1109	DJI_0167	Pyrus communis L.	0	Rosaceae Juss.	a	0
Iz_5_1109	DJI_0167	Quercus robur L.	1	Fagaceae Dumort.	n	1
Iz_5_1109	DJI_0167	Robinia pseudoacacia L.	0	Fabaceae Lindl.	a	0
Iz_5_1109	DJI_0167	Rosa sp.	0	Rosaceae Juss.		
Iz_5_1109	DJI_0167	Salix alba L.	0	Salicaceae Mirb.	n	1
Iz_5_1109	DJI_0167	Sambucus nigra L.	0	Viburnaceae Raf.	n	1
Iz_5_1109	DJI_0167	Sorbus aucuparia L.	0	Rosaceae Juss.	n	1
Iz_5_1109	DJI_0167	Tilia cordata Mill.	0	Malvaceae Juss.	n	1
Iz_5_1109	DJI_0167	Ulmus glabra Huds.	0	Ulmaceae Mirb.	n	1
Iz_5_1109	DJI_0162	Acer tataricum L.	0	Sapindaceae Juss.	n	1
Iz_5_1109	DJI_0162	Betula pendula Roth.	0	Betulaceae Gray	n	1
Iz_5_1109	DJI_0162	Crataegus rhipidophylla Gand.	0	Rosaceae Juss.	n	1
Iz_5_1109	DJI_0162	Frangula alnus Mill.	0	Rhamnaceae Juss.	n	1
Iz_5_1109	DJI_0162	Fraxinus pennsylvanica Marshall	0	Oleaceae Hoffmanns. & Link	a	0
Iz_5_1109	DJI_0162	Pinus sylvestris L.	0	Pinaceae Spreng. ex F.Rudolphi	n	1
Iz_5_1109	DJI_0162	Populus × canescens (Aiton) Sm.	0	Salicaceae Mirb.	n	1
Iz_5_1109	DJI_0162	Populus tremula L.	0	Salicaceae Mirb.	n	1
Iz_5_1109	DJI_0162	Populus nigra L.	0	Salicaceae Mirb.	n	1
Iz_5_1109	DJI_0162	Pyrus communis L.	0	Rosaceae Juss.	a	0
Iz_5_1109	DJI_0162	Quercus robur L.	1	Fagaceae Dumort.	n	1
Iz_5_1109	DJI_0162	Robinia pseudoacacia L.	0	Fabaceae Lindl.	a	0
Iz_5_1109	DJI_0162	Rosa sp.	0	Rosaceae Juss.		
Iz_5_1109	DJI_0162	Salix alba L.	0	Salicaceae Mirb.	n	1
Iz_5_1109	DJI_0162	Sambucus nigra L.	0	Viburnaceae Raf.	n	1
Iz_5_1109	DJI_0162	Sorbus aucuparia L.	0	Rosaceae Juss.	n	1
Iz_5_1109	DJI_0162	Tilia cordata Mill.	0	Malvaceae Juss.	n	1
Iz_5_1109	DJI_0162	Ulmus glabra Huds.	0	Ulmaceae Mirb.	n	1
Iz_5_1109	DJI_0156	Acer tataricum L.	0	Sapindaceae Juss.	n	1
Iz_5_1109	DJI_0156	Betula pendula Roth.	0	Betulaceae Gray	n	1
Iz_5_1109	DJI_0156	Crataegus rhipidophylla Gand.	0	Rosaceae Juss.	n	1
Iz_5_1109	DJI_0156	Frangula alnus Mill.	0	Rhamnaceae Juss.	n	1
Iz_5_1109	DJI_0156	Fraxinus pennsylvanica Marshall	0	Oleaceae Hoffmanns. & Link	a	0
Iz_5_1109	DJI_0156	Pinus sylvestris L.	0	Pinaceae Spreng. ex F.Rudolphi	n	1
Iz_5_1109	DJI_0156	Populus × canescens (Aiton) Sm.	0	Salicaceae Mirb.	n	1
Iz_5_1109	DJI_0156	Populus tremula L.	0	Salicaceae Mirb.	n	1
Iz_5_1109	DJI_0156	Populus nigra L.	0	Salicaceae Mirb.	n	1
Iz_5_1109	DJI_0156	Pyrus communis L.	0	Rosaceae Juss.	a	0
Iz_5_1109	DJI_0156	Quercus robur L.	0	Fagaceae Dumort.	n	1
Iz_5_1109	DJI_0156	Robinia pseudoacacia L.	0	Fabaceae Lindl.	a	0
Iz_5_1109	DJI_0156	Rosa sp.	0	Rosaceae Juss.		
Iz_5_1109	DJI_0156	Salix alba L.	0	Salicaceae Mirb.	n	1
Iz_5_1109	DJI_0156	Sambucus nigra L.	0	Viburnaceae Raf.	n	1
Iz_5_1109	DJI_0156	Sorbus aucuparia L.	0	Rosaceae Juss.	n	1
Iz_5_1109	DJI_0156	Tilia cordata Mill.	0	Malvaceae Juss.	n	1
Iz_5_1109	DJI_0156	Ulmus glabra Huds.	0	Ulmaceae Mirb.	n	1
Iz_5_1109	DJI_0154	Acer tataricum L.	0	Sapindaceae Juss.	n	1
Iz_5_1109	DJI_0154	Betula pendula Roth.	0	Betulaceae Gray	n	1
Iz_5_1109	DJI_0154	Crataegus rhipidophylla Gand.	0	Rosaceae Juss.	n	1
Iz_5_1109	DJI_0154	Frangula alnus Mill.	0	Rhamnaceae Juss.	n	1
Iz_5_1109	DJI_0154	Fraxinus pennsylvanica Marshall	0	Oleaceae Hoffmanns. & Link	a	0
Iz_5_1109	DJI_0154	Pinus sylvestris L.	0	Pinaceae Spreng. ex F.Rudolphi	n	1
Iz_5_1109	DJI_0154	Populus × canescens (Aiton) Sm.	0	Salicaceae Mirb.	n	1
Iz_5_1109	DJI_0154	Populus tremula L.	0	Salicaceae Mirb.	n	1
Iz_5_1109	DJI_0154	Populus nigra L.	0	Salicaceae Mirb.	n	1
Iz_5_1109	DJI_0154	Pyrus communis L.	0	Rosaceae Juss.	a	0
Iz_5_1109	DJI_0154	Quercus robur L.	0	Fagaceae Dumort.	n	1
Iz_5_1109	DJI_0154	Robinia pseudoacacia L.	0	Fabaceae Lindl.	a	0

Iz_5_1109	DJI_0154	Rosa sp.	0	Rosaceae Juss.		
Iz_5_1109	DJI_0154	Salix alba L.	0	Salicaceae Mirb.	n	1
Iz_5_1109	DJI_0154	Sambucus nigra L.	0	Viburnaceae Raf.	n	1
Iz_5_1109	DJI_0154	Sorbus aucuparia L.	0	Rosaceae Juss.	n	1
Iz_5_1109	DJI_0154	Tilia cordata Mill.	0	Malvaceae Juss.	n	1
Iz_5_1109	DJI_0154	Ulmus glabra Huds.	0	Ulmaceae Mirb.	n	1
Iz_5_1109	DJI_0152	Acer tataricum L.	0	Sapindaceae Juss.	n	1
Iz_5_1109	DJI_0152	Betula pendula Roth.	0	Betulaceae Gray	n	1
Iz_5_1109	DJI_0152	Crataegus rhipidophylla Gand.	0	Rosaceae Juss.	n	1
Iz_5_1109	DJI_0152	Frangula alnus Mill.	0	Rhamnaceae Juss.	n	1
Iz_5_1109	DJI_0152	Fraxinus pennsylvanica Marshall	0	Oleaceae Hoffmanns. & Link	a	0
Iz_5_1109	DJI_0152	Pinus sylvestris L.	1	Pinaceae Spreng. ex F.Rudolphi	n	1
Iz_5_1109	DJI_0152	Populus × canescens (Aiton) Sm.	0	Salicaceae Mirb.	n	1
Iz_5_1109	DJI_0152	Populus tremula L.	0	Salicaceae Mirb.	n	1
Iz_5_1109	DJI_0152	Populus nigra L.	0	Salicaceae Mirb.	n	1
Iz_5_1109	DJI_0152	Pyrus communis L.	0	Rosaceae Juss.	a	0
Iz_5_1109	DJI_0152	Quercus robur L.	1	Fagaceae Dumort.	n	1
Iz_5_1109	DJI_0152	Robinia pseudoacacia L.	25	Fabaceae Lindl.	a	0
Iz_5_1109	DJI_0152	Rosa sp.	0	Rosaceae Juss.		
Iz_5_1109	DJI_0152	Salix alba L.	0	Salicaceae Mirb.	n	1
Iz_5_1109	DJI_0152	Sambucus nigra L.	0	Viburnaceae Raf.	n	1
Iz_5_1109	DJI_0152	Sorbus aucuparia L.	0	Rosaceae Juss.	n	1
Iz_5_1109	DJI_0152	Tilia cordata Mill.	0	Malvaceae Juss.	n	1
Iz_5_1109	DJI_0152	Ulmus glabra Huds.	0	Ulmaceae Mirb.	n	1
Iz_5_1109	DJI_0148	Acer tataricum L.	0	Sapindaceae Juss.	n	1
Iz_5_1109	DJI_0148	Betula pendula Roth.	0	Betulaceae Gray	n	1
Iz_5_1109	DJI_0148	Crataegus rhipidophylla Gand.	0	Rosaceae Juss.	n	1
Iz_5_1109	DJI_0148	Frangula alnus Mill.	0	Rhamnaceae Juss.	n	1
Iz_5_1109	DJI_0148	Fraxinus pennsylvanica Marshall	0	Oleaceae Hoffmanns. & Link	a	0
Iz_5_1109	DJI_0148	Pinus sylvestris L.	0	Pinaceae Spreng. ex F.Rudolphi	n	1
Iz_5_1109	DJI_0148	Populus × canescens (Aiton) Sm.	0	Salicaceae Mirb.	n	1
Iz_5_1109	DJI_0148	Populus tremula L.	0	Salicaceae Mirb.	n	1
Iz_5_1109	DJI_0148	Populus nigra L.	0	Salicaceae Mirb.	n	1
Iz_5_1109	DJI_0148	Pyrus communis L.	0	Rosaceae Juss.	a	0
Iz_5_1109	DJI_0148	Quercus robur L.	0	Fagaceae Dumort.	n	1
Iz_5_1109	DJI_0148	Robinia pseudoacacia L.	0	Fabaceae Lindl.	a	0
Iz_5_1109	DJI_0148	Rosa sp.	0	Rosaceae Juss.		
Iz_5_1109	DJI_0148	Salix alba L.	0	Salicaceae Mirb.	n	1
Iz_5_1109	DJI_0148	Sambucus nigra L.	0	Viburnaceae Raf.	n	1
Iz_5_1109	DJI_0148	Sorbus aucuparia L.	0	Rosaceae Juss.	n	1
Iz_5_1109	DJI_0148	Tilia cordata Mill.	0	Malvaceae Juss.	n	1
Iz_5_1109	DJI_0148	Ulmus glabra Huds.	0	Ulmaceae Mirb.	n	1
Iz_5_1109	DJI_0146	Acer tataricum L.	0	Sapindaceae Juss.	n	1
Iz_5_1109	DJI_0146	Betula pendula Roth.	0	Betulaceae Gray	n	1
Iz_5_1109	DJI_0146	Crataegus rhipidophylla Gand.	0	Rosaceae Juss.	n	1
Iz_5_1109	DJI_0146	Frangula alnus Mill.	0	Rhamnaceae Juss.	n	1
Iz_5_1109	DJI_0146	Fraxinus pennsylvanica Marshall	0	Oleaceae Hoffmanns. & Link	a	0
Iz_5_1109	DJI_0146	Pinus sylvestris L.	0	Pinaceae Spreng. ex F.Rudolphi	n	1
Iz_5_1109	DJI_0146	Populus × canescens (Aiton) Sm.	0	Salicaceae Mirb.	n	1
Iz_5_1109	DJI_0146	Populus tremula L.	0	Salicaceae Mirb.	n	1
Iz_5_1109	DJI_0146	Populus nigra L.	0	Salicaceae Mirb.	n	1
Iz_5_1109	DJI_0146	Pyrus communis L.	0	Rosaceae Juss.	a	0
Iz_5_1109	DJI_0146	Quercus robur L.	0	Fagaceae Dumort.	n	1
Iz_5_1109	DJI_0146	Robinia pseudoacacia L.	0	Fabaceae Lindl.	a	0
Iz_5_1109	DJI_0146	Rosa sp.	0	Rosaceae Juss.		
Iz_5_1109	DJI_0146	Salix alba L.	0	Salicaceae Mirb.	n	1
Iz_5_1109	DJI_0146	Sambucus nigra L.	0	Viburnaceae Raf.	n	1
Iz_5_1109	DJI_0146	Sorbus aucuparia L.	0	Rosaceae Juss.	n	1
Iz_5_1109	DJI_0146	Tilia cordata Mill.	0	Malvaceae Juss.	n	1
Iz_5_1109	DJI_0146	Ulmus glabra Huds.	0	Ulmaceae Mirb.	n	1
Iz_5_1109	DJI_0143	Acer tataricum L.	0	Sapindaceae Juss.	n	1
Iz_5_1109	DJI_0143	Betula pendula Roth.	0	Betulaceae Gray	n	1
Iz_5_1109	DJI_0143	Crataegus rhipidophylla Gand.	0	Rosaceae Juss.	n	1
Iz_5_1109	DJI_0143	Frangula alnus Mill.	0	Rhamnaceae Juss.	n	1

Iz_5_1109	DJI_0143	Fraxinus pennsylvanica Marshall	0	Oleaceae Hoffmanns. & Link	a	0
Iz_5_1109	DJI_0143	Pinus sylvestris L.	0	Pinaceae Spreng. ex F.Rudolphi	n	1
Iz_5_1109	DJI_0143	Populus × canescens (Aiton) Sm.	0	Salicaceae Mirb.	n	1
Iz_5_1109	DJI_0143	Populus tremula L.	0	Salicaceae Mirb.	n	1
Iz_5_1109	DJI_0143	Populus nigra L.	0	Salicaceae Mirb.	n	1
Iz_5_1109	DJI_0143	Pyrus communis L.	0	Rosaceae Juss.	a	0
Iz_5_1109	DJI_0143	Quercus robur L.	0	Fagaceae Dumort.	n	1
Iz_5_1109	DJI_0143	Robinia pseudoacacia L.	0	Fabaceae Lindl.	a	0
Iz_5_1109	DJI_0143	Rosa sp.	0	Rosaceae Juss.		
Iz_5_1109	DJI_0143	Salix alba L.	0	Salicaceae Mirb.	n	1
Iz_5_1109	DJI_0143	Sambucus nigra L.	0	Viburnaceae Raf.	n	1
Iz_5_1109	DJI_0143	Sorbus aucuparia L.	0	Rosaceae Juss.	n	1
Iz_5_1109	DJI_0143	Tilia cordata Mill.	0	Malvaceae Juss.	n	1
Iz_5_1109	DJI_0143	Ulmus glabra Huds.	0	Ulmaceae Mirb.	n	1
Iz_5_1109	DJI_0140	Acer tataricum L.	0	Sapindaceae Juss.	n	1
Iz_5_1109	DJI_0140	Betula pendula Roth.	0	Betulaceae Gray	n	1
Iz_5_1109	DJI_0140	Crataegus rhipidophylla Gand.	0	Rosaceae Juss.	n	1
Iz_5_1109	DJI_0140	Frangula alnus Mill.	0	Rhamnaceae Juss.	n	1
Iz_5_1109	DJI_0140	Fraxinus pennsylvanica Marshall	0	Oleaceae Hoffmanns. & Link	a	0
Iz_5_1109	DJI_0140	Pinus sylvestris L.	0	Pinaceae Spreng. ex F.Rudolphi	n	1
Iz_5_1109	DJI_0140	Populus × canescens (Aiton) Sm.	0	Salicaceae Mirb.	n	1
Iz_5_1109	DJI_0140	Populus tremula L.	0	Salicaceae Mirb.	n	1
Iz_5_1109	DJI_0140	Populus nigra L.	0	Salicaceae Mirb.	n	1
Iz_5_1109	DJI_0140	Pyrus communis L.	0	Rosaceae Juss.	a	0
Iz_5_1109	DJI_0140	Quercus robur L.	0	Fagaceae Dumort.	n	1
Iz_5_1109	DJI_0140	Robinia pseudoacacia L.	0	Fabaceae Lindl.	a	0
Iz_5_1109	DJI_0140	Rosa sp.	0	Rosaceae Juss.		
Iz_5_1109	DJI_0140	Salix alba L.	0	Salicaceae Mirb.	n	1
Iz_5_1109	DJI_0140	Sambucus nigra L.	0	Viburnaceae Raf.	n	1
Iz_5_1109	DJI_0140	Sorbus aucuparia L.	0	Rosaceae Juss.	n	1
Iz_5_1109	DJI_0140	Tilia cordata Mill.	0	Malvaceae Juss.	n	1
Iz_5_1109	DJI_0140	Ulmus glabra Huds.	0	Ulmaceae Mirb.	n	1
Iz_5_1109	DJI_0138	Acer tataricum L.	0	Sapindaceae Juss.	n	1
Iz_5_1109	DJI_0138	Betula pendula Roth.	0	Betulaceae Gray	n	1
Iz_5_1109	DJI_0138	Crataegus rhipidophylla Gand.	0	Rosaceae Juss.	n	1
Iz_5_1109	DJI_0138	Frangula alnus Mill.	0	Rhamnaceae Juss.	n	1
Iz_5_1109	DJI_0138	Fraxinus pennsylvanica Marshall	0	Oleaceae Hoffmanns. & Link	a	0
Iz_5_1109	DJI_0138	Pinus sylvestris L.	0	Pinaceae Spreng. ex F.Rudolphi	n	1
Iz_5_1109	DJI_0138	Populus × canescens (Aiton) Sm.	0	Salicaceae Mirb.	n	1
Iz_5_1109	DJI_0138	Populus tremula L.	0	Salicaceae Mirb.	n	1
Iz_5_1109	DJI_0138	Populus nigra L.	0	Salicaceae Mirb.	n	1
Iz_5_1109	DJI_0138	Pyrus communis L.	0	Rosaceae Juss.	a	0
Iz_5_1109	DJI_0138	Quercus robur L.	0	Fagaceae Dumort.	n	1
Iz_5_1109	DJI_0138	Robinia pseudoacacia L.	0	Fabaceae Lindl.	a	0
Iz_5_1109	DJI_0138	Rosa sp.	0	Rosaceae Juss.		
Iz_5_1109	DJI_0138	Salix alba L.	0	Salicaceae Mirb.	n	1
Iz_5_1109	DJI_0138	Sambucus nigra L.	0	Viburnaceae Raf.	n	1
Iz_5_1109	DJI_0138	Sorbus aucuparia L.	0	Rosaceae Juss.	n	1
Iz_5_1109	DJI_0138	Tilia cordata Mill.	0	Malvaceae Juss.	n	1
Iz_5_1109	DJI_0138	Ulmus glabra Huds.	0	Ulmaceae Mirb.	n	1
Iz_5_1109	DJI_0136	Acer tataricum L.	0	Sapindaceae Juss.	n	1
Iz_5_1109	DJI_0136	Betula pendula Roth.	0	Betulaceae Gray	n	1
Iz_5_1109	DJI_0136	Crataegus rhipidophylla Gand.	0	Rosaceae Juss.	n	1
Iz_5_1109	DJI_0136	Frangula alnus Mill.	0	Rhamnaceae Juss.	n	1
Iz_5_1109	DJI_0136	Fraxinus pennsylvanica Marshall	0	Oleaceae Hoffmanns. & Link	a	0
Iz_5_1109	DJI_0136	Pinus sylvestris L.	0	Pinaceae Spreng. ex F.Rudolphi	n	1
Iz_5_1109	DJI_0136	Populus × canescens (Aiton) Sm.	0	Salicaceae Mirb.	n	1
Iz_5_1109	DJI_0136	Populus tremula L.	0	Salicaceae Mirb.	n	1
Iz_5_1109	DJI_0136	Populus nigra L.	0	Salicaceae Mirb.	n	1
Iz_5_1109	DJI_0136	Pyrus communis L.	0	Rosaceae Juss.	a	0
Iz_5_1109	DJI_0136	Quercus robur L.	0	Fagaceae Dumort.	n	1
Iz_5_1109	DJI_0136	Robinia pseudoacacia L.	0	Fabaceae Lindl.	a	0
Iz_5_1109	DJI_0136	Rosa sp.	0	Rosaceae Juss.		
Iz_5_1109	DJI_0136	Salix alba L.	0	Salicaceae Mirb.	n	1

Iz_5_1109	DJI_0136	Sambucus nigra L.	0	Viburnaceae Raf.	n	1
Iz_5_1109	DJI_0136	Sorbus aucuparia L.	0	Rosaceae Juss.	n	1
Iz_5_1109	DJI_0136	Tilia cordata Mill.	0	Malvaceae Juss.	n	1
Iz_5_1109	DJI_0136	Ulmus glabra Huds.	0	Ulmaceae Mirb.	n	1
Iz_5_1109	DJI_0134	Acer tataricum L.	0	Sapindaceae Juss.	n	1
Iz_5_1109	DJI_0134	Betula pendula Roth.	0	Betulaceae Gray	n	1
Iz_5_1109	DJI_0134	Crataegus rhipidophylla Gand.	0	Rosaceae Juss.	n	1
Iz_5_1109	DJI_0134	Frangula alnus Mill.	0	Rhamnaceae Juss.	n	1
Iz_5_1109	DJI_0134	Fraxinus pennsylvanica Marshall	0	Oleaceae Hoffmanns. & Link	a	0
Iz_5_1109	DJI_0134	Pinus sylvestris L.	0	Pinaceae Spreng. ex F.Rudolphi	n	1
Iz_5_1109	DJI_0134	Populus × canescens (Aiton) Sm.	0	Salicaceae Mirb.	n	1
Iz_5_1109	DJI_0134	Populus tremula L.	0	Salicaceae Mirb.	n	1
Iz_5_1109	DJI_0134	Populus nigra L.	0	Salicaceae Mirb.	n	1
Iz_5_1109	DJI_0134	Pyrus communis L.	0	Rosaceae Juss.	a	0
Iz_5_1109	DJI_0134	Quercus robur L.	0	Fagaceae Dumort.	n	1
Iz_5_1109	DJI_0134	Robinia pseudoacacia L.	0	Fabaceae Lindl.	a	0
Iz_5_1109	DJI_0134	Rosa sp.	0	Rosaceae Juss.		
Iz_5_1109	DJI_0134	Salix alba L.	0	Salicaceae Mirb.	n	1
Iz_5_1109	DJI_0134	Sambucus nigra L.	0	Viburnaceae Raf.	n	1
Iz_5_1109	DJI_0134	Sorbus aucuparia L.	0	Rosaceae Juss.	n	1
Iz_5_1109	DJI_0134	Tilia cordata Mill.	0	Malvaceae Juss.	n	1
Iz_5_1109	DJI_0134	Ulmus glabra Huds.	0	Ulmaceae Mirb.	n	1
Iz_5_1109	DJI_0132	Acer tataricum L.	0	Sapindaceae Juss.	n	1
Iz_5_1109	DJI_0132	Betula pendula Roth.	0	Betulaceae Gray	n	1
Iz_5_1109	DJI_0132	Crataegus rhipidophylla Gand.	0	Rosaceae Juss.	n	1
Iz_5_1109	DJI_0132	Frangula alnus Mill.	0	Rhamnaceae Juss.	n	1
Iz_5_1109	DJI_0132	Fraxinus pennsylvanica Marshall	0	Oleaceae Hoffmanns. & Link	a	0
Iz_5_1109	DJI_0132	Pinus sylvestris L.	0	Pinaceae Spreng. ex F.Rudolphi	n	1
Iz_5_1109	DJI_0132	Populus × canescens (Aiton) Sm.	0	Salicaceae Mirb.	n	1
Iz_5_1109	DJI_0132	Populus tremula L.	0	Salicaceae Mirb.	n	1
Iz_5_1109	DJI_0132	Populus nigra L.	0	Salicaceae Mirb.	n	1
Iz_5_1109	DJI_0132	Pyrus communis L.	0	Rosaceae Juss.	a	0
Iz_5_1109	DJI_0132	Quercus robur L.	0	Fagaceae Dumort.	n	1
Iz_5_1109	DJI_0132	Robinia pseudoacacia L.	0	Fabaceae Lindl.	a	0
Iz_5_1109	DJI_0132	Rosa sp.	0	Rosaceae Juss.		
Iz_5_1109	DJI_0132	Salix alba L.	0	Salicaceae Mirb.	n	1
Iz_5_1109	DJI_0132	Sambucus nigra L.	0	Viburnaceae Raf.	n	1
Iz_5_1109	DJI_0132	Sorbus aucuparia L.	0	Rosaceae Juss.	n	1
Iz_5_1109	DJI_0132	Tilia cordata Mill.	0	Malvaceae Juss.	n	1
Iz_5_1109	DJI_0132	Ulmus glabra Huds.	0	Ulmaceae Mirb.	n	1
Iz_5_1109	DJI_0130	Acer tataricum L.	0	Sapindaceae Juss.	n	1
Iz_5_1109	DJI_0130	Betula pendula Roth.	0	Betulaceae Gray	n	1
Iz_5_1109	DJI_0130	Crataegus rhipidophylla Gand.	0	Rosaceae Juss.	n	1
Iz_5_1109	DJI_0130	Frangula alnus Mill.	0	Rhamnaceae Juss.	n	1
Iz_5_1109	DJI_0130	Fraxinus pennsylvanica Marshall	0	Oleaceae Hoffmanns. & Link	a	0
Iz_5_1109	DJI_0130	Pinus sylvestris L.	0	Pinaceae Spreng. ex F.Rudolphi	n	1
Iz_5_1109	DJI_0130	Populus × canescens (Aiton) Sm.	0	Salicaceae Mirb.	n	1
Iz_5_1109	DJI_0130	Populus tremula L.	0	Salicaceae Mirb.	n	1
Iz_5_1109	DJI_0130	Populus nigra L.	0	Salicaceae Mirb.	n	1
Iz_5_1109	DJI_0130	Pyrus communis L.	0	Rosaceae Juss.	a	0
Iz_5_1109	DJI_0130	Quercus robur L.	0	Fagaceae Dumort.	n	1
Iz_5_1109	DJI_0130	Robinia pseudoacacia L.	0	Fabaceae Lindl.	a	0
Iz_5_1109	DJI_0130	Rosa sp.	0	Rosaceae Juss.		
Iz_5_1109	DJI_0130	Salix alba L.	0	Salicaceae Mirb.	n	1
Iz_5_1109	DJI_0130	Sambucus nigra L.	0	Viburnaceae Raf.	n	1
Iz_5_1109	DJI_0130	Sorbus aucuparia L.	0	Rosaceae Juss.	n	1
Iz_5_1109	DJI_0130	Tilia cordata Mill.	0	Malvaceae Juss.	n	1
Iz_5_1109	DJI_0130	Ulmus glabra Huds.	0	Ulmaceae Mirb.	n	1
Iz_5_1109	DJI_0127	Acer tataricum L.	0	Sapindaceae Juss.	n	1
Iz_5_1109	DJI_0127	Betula pendula Roth.	0	Betulaceae Gray	n	1
Iz_5_1109	DJI_0127	Crataegus rhipidophylla Gand.	0	Rosaceae Juss.	n	1
Iz_5_1109	DJI_0127	Frangula alnus Mill.	0	Rhamnaceae Juss.	n	1
Iz_5_1109	DJI_0127	Fraxinus pennsylvanica Marshall	0	Oleaceae Hoffmanns. & Link	a	0
Iz_5_1109	DJI_0127	Pinus sylvestris L.	0	Pinaceae Spreng. ex F.Rudolphi	n	1

Iz_5_1109	DJI_0127	Populus × canescens (Aiton) Sm.	0	Salicaceae Mirb.	n	1
Iz_5_1109	DJI_0127	Populus tremula L.	0	Salicaceae Mirb.	n	1
Iz_5_1109	DJI_0127	Populus nigra L.	0	Salicaceae Mirb.	n	1
Iz_5_1109	DJI_0127	Pyrus communis L.	0	Rosaceae Juss.	a	0
Iz_5_1109	DJI_0127	Quercus robur L.	0	Fagaceae Dumort.	n	1
Iz_5_1109	DJI_0127	Robinia pseudoacacia L.	0	Fabaceae Lindl.	a	0
Iz_5_1109	DJI_0127	Rosa sp.	0	Rosaceae Juss.		
Iz_5_1109	DJI_0127	Salix alba L.	0	Salicaceae Mirb.	n	1
Iz_5_1109	DJI_0127	Sambucus nigra L.	0	Viburnaceae Raf.	n	1
Iz_5_1109	DJI_0127	Sorbus aucuparia L.	0	Rosaceae Juss.	n	1
Iz_5_1109	DJI_0127	Tilia cordata Mill.	0	Malvaceae Juss.	n	1
Iz_5_1109	DJI_0127	Ulmus glabra Huds.	0	Ulmaceae Mirb.	n	1
Iz_5_1109	DJI_0126	Acer tataricum L.	0	Sapindaceae Juss.	n	1
Iz_5_1109	DJI_0126	Betula pendula Roth.	0	Betulaceae Gray	n	1
Iz_5_1109	DJI_0126	Crataegus rhipidophylla Gand.	0	Rosaceae Juss.	n	1
Iz_5_1109	DJI_0126	Frangula alnus Mill.	0	Rhamnaceae Juss.	n	1
Iz_5_1109	DJI_0126	Fraxinus pennsylvanica Marshall	0	Oleaceae Hoffmanns. & Link	a	0
Iz_5_1109	DJI_0126	Pinus sylvestris L.	0	Pinaceae Spreng. ex F.Rudolphi	n	1
Iz_5_1109	DJI_0126	Populus × canescens (Aiton) Sm.	0	Salicaceae Mirb.	n	1
Iz_5_1109	DJI_0126	Populus tremula L.	0	Salicaceae Mirb.	n	1
Iz_5_1109	DJI_0126	Populus nigra L.	0	Salicaceae Mirb.	n	1
Iz_5_1109	DJI_0126	Pyrus communis L.	0	Rosaceae Juss.	a	0
Iz_5_1109	DJI_0126	Quercus robur L.	0	Fagaceae Dumort.	n	1
Iz_5_1109	DJI_0126	Robinia pseudoacacia L.	0	Fabaceae Lindl.	a	0
Iz_5_1109	DJI_0126	Rosa sp.	0	Rosaceae Juss.		
Iz_5_1109	DJI_0126	Salix alba L.	0	Salicaceae Mirb.	n	1
Iz_5_1109	DJI_0126	Sambucus nigra L.	0	Viburnaceae Raf.	n	1
Iz_5_1109	DJI_0126	Sorbus aucuparia L.	0	Rosaceae Juss.	n	1
Iz_5_1109	DJI_0126	Tilia cordata Mill.	0	Malvaceae Juss.	n	1
Iz_5_1109	DJI_0126	Ulmus glabra Huds.	0	Ulmaceae Mirb.	n	1
Iz_5_1109	DJI_0125	Acer tataricum L.	0	Sapindaceae Juss.	n	1
Iz_5_1109	DJI_0125	Betula pendula Roth.	0	Betulaceae Gray	n	1
Iz_5_1109	DJI_0125	Crataegus rhipidophylla Gand.	0	Rosaceae Juss.	n	1
Iz_5_1109	DJI_0125	Frangula alnus Mill.	0	Rhamnaceae Juss.	n	1
Iz_5_1109	DJI_0125	Fraxinus pennsylvanica Marshall	0	Oleaceae Hoffmanns. & Link	a	0
Iz_5_1109	DJI_0125	Pinus sylvestris L.	0	Pinaceae Spreng. ex F.Rudolphi	n	1
Iz_5_1109	DJI_0125	Populus × canescens (Aiton) Sm.	0	Salicaceae Mirb.	n	1
Iz_5_1109	DJI_0125	Populus tremula L.	0	Salicaceae Mirb.	n	1
Iz_5_1109	DJI_0125	Populus nigra L.	0	Salicaceae Mirb.	n	1
Iz_5_1109	DJI_0125	Pyrus communis L.	0	Rosaceae Juss.	a	0
Iz_5_1109	DJI_0125	Quercus robur L.	0	Fagaceae Dumort.	n	1
Iz_5_1109	DJI_0125	Robinia pseudoacacia L.	0	Fabaceae Lindl.	a	0
Iz_5_1109	DJI_0125	Rosa sp.	0	Rosaceae Juss.		
Iz_5_1109	DJI_0125	Salix alba L.	0	Salicaceae Mirb.	n	1
Iz_5_1109	DJI_0125	Sambucus nigra L.	0	Viburnaceae Raf.	n	1
Iz_5_1109	DJI_0125	Sorbus aucuparia L.	0	Rosaceae Juss.	n	1
Iz_5_1109	DJI_0125	Tilia cordata Mill.	0	Malvaceae Juss.	n	1
Iz_5_1109	DJI_0125	Ulmus glabra Huds.	0	Ulmaceae Mirb.	n	1
Iz_6_1109	DJI_0292	Acer tataricum L.	0	Sapindaceae Juss.	n	1
Iz_6_1109	DJI_0292	Betula pendula Roth.	0	Betulaceae Gray	n	1
Iz_6_1109	DJI_0292	Crataegus rhipidophylla Gand.	0	Rosaceae Juss.	n	1
Iz_6_1109	DJI_0292	Frangula alnus Mill.	0	Rhamnaceae Juss.	n	1
Iz_6_1109	DJI_0292	Fraxinus pennsylvanica Marshall	0	Oleaceae Hoffmanns. & Link	a	0
Iz_6_1109	DJI_0292	Pinus sylvestris L.	0	Pinaceae Spreng. ex F.Rudolphi	n	1
Iz_6_1109	DJI_0292	Populus × canescens (Aiton) Sm.	0	Salicaceae Mirb.	n	1
Iz_6_1109	DJI_0292	Populus tremula L.	0	Salicaceae Mirb.	n	1
Iz_6_1109	DJI_0292	Populus nigra L.	0	Salicaceae Mirb.	n	1
Iz_6_1109	DJI_0292	Pyrus communis L.	0	Rosaceae Juss.	a	0
Iz_6_1109	DJI_0292	Quercus robur L.	0	Fagaceae Dumort.	n	1
Iz_6_1109	DJI_0292	Robinia pseudoacacia L.	0	Fabaceae Lindl.	a	0
Iz_6_1109	DJI_0292	Rosa sp.	0	Rosaceae Juss.		
Iz_6_1109	DJI_0292	Salix alba L.	0	Salicaceae Mirb.	n	1
Iz_6_1109	DJI_0292	Sambucus nigra L.	0	Viburnaceae Raf.	n	1
Iz_6_1109	DJI_0292	Sorbus aucuparia L.	0	Rosaceae Juss.	n	1

Iz_6_1109	DJI_0292	Tilia cordata Mill.	0	Malvaceae Juss.	n	1
Iz_6_1109	DJI_0292	Ulmus glabra Huds.	0	Ulmaceae Mirb.	n	1
Iz_6_1109	DJI_0289	Acer tataricum L.	0	Sapindaceae Juss.	n	1
Iz_6_1109	DJI_0289	Betula pendula Roth.	0	Betulaceae Gray	n	1
Iz_6_1109	DJI_0289	Crataegus rhipidophylla Gand.	0	Rosaceae Juss.	n	1
Iz_6_1109	DJI_0289	Frangula alnus Mill.	0	Rhamnaceae Juss.	n	1
Iz_6_1109	DJI_0289	Fraxinus pennsylvanica Marshall	0	Oleaceae Hoffmanns. & Link	a	0
Iz_6_1109	DJI_0289	Pinus sylvestris L.	0	Pinaceae Spreng. ex F.Rudolphi	n	1
Iz_6_1109	DJI_0289	Populus × canescens (Aiton) Sm.	0	Salicaceae Mirb.	n	1
Iz_6_1109	DJI_0289	Populus tremula L.	0	Salicaceae Mirb.	n	1
Iz_6_1109	DJI_0289	Populus nigra L.	0	Salicaceae Mirb.	n	1
Iz_6_1109	DJI_0289	Pyrus communis L.	0	Rosaceae Juss.	a	0
Iz_6_1109	DJI_0289	Quercus robur L.	0	Fagaceae Dumort.	n	1
Iz_6_1109	DJI_0289	Robinia pseudoacacia L.	0	Fabaceae Lindl.	a	0
Iz_6_1109	DJI_0289	Rosa sp.	0	Rosaceae Juss.		
Iz_6_1109	DJI_0289	Salix alba L.	0	Salicaceae Mirb.	n	1
Iz_6_1109	DJI_0289	Sambucus nigra L.	0	Viburnaceae Raf.	n	1
Iz_6_1109	DJI_0289	Sorbus aucuparia L.	0	Rosaceae Juss.	n	1
Iz_6_1109	DJI_0289	Tilia cordata Mill.	0	Malvaceae Juss.	n	1
Iz_6_1109	DJI_0289	Ulmus glabra Huds.	0	Ulmaceae Mirb.	n	1
Iz_6_1109	DJI_0286	Acer tataricum L.	0	Sapindaceae Juss.	n	1
Iz_6_1109	DJI_0286	Betula pendula Roth.	0	Betulaceae Gray	n	1
Iz_6_1109	DJI_0286	Crataegus rhipidophylla Gand.	0	Rosaceae Juss.	n	1
Iz_6_1109	DJI_0286	Frangula alnus Mill.	0	Rhamnaceae Juss.	n	1
Iz_6_1109	DJI_0286	Fraxinus pennsylvanica Marshall	0	Oleaceae Hoffmanns. & Link	a	0
Iz_6_1109	DJI_0286	Pinus sylvestris L.	0	Pinaceae Spreng. ex F.Rudolphi	n	1
Iz_6_1109	DJI_0286	Populus × canescens (Aiton) Sm.	0	Salicaceae Mirb.	n	1
Iz_6_1109	DJI_0286	Populus tremula L.	0	Salicaceae Mirb.	n	1
Iz_6_1109	DJI_0286	Populus nigra L.	0	Salicaceae Mirb.	n	1
Iz_6_1109	DJI_0286	Pyrus communis L.	0	Rosaceae Juss.	a	0
Iz_6_1109	DJI_0286	Quercus robur L.	0	Fagaceae Dumort.	n	1
Iz_6_1109	DJI_0286	Robinia pseudoacacia L.	0	Fabaceae Lindl.	a	0
Iz_6_1109	DJI_0286	Rosa sp.	0	Rosaceae Juss.		
Iz_6_1109	DJI_0286	Salix alba L.	0	Salicaceae Mirb.	n	1
Iz_6_1109	DJI_0286	Sambucus nigra L.	0	Viburnaceae Raf.	n	1
Iz_6_1109	DJI_0286	Sorbus aucuparia L.	0	Rosaceae Juss.	n	1
Iz_6_1109	DJI_0286	Tilia cordata Mill.	0	Malvaceae Juss.	n	1
Iz_6_1109	DJI_0286	Ulmus glabra Huds.	0	Ulmaceae Mirb.	n	1
Iz_6_1109	DJI_0283	Acer tataricum L.	0	Sapindaceae Juss.	n	1
Iz_6_1109	DJI_0283	Betula pendula Roth.	0	Betulaceae Gray	n	1
Iz_6_1109	DJI_0283	Crataegus rhipidophylla Gand.	0	Rosaceae Juss.	n	1
Iz_6_1109	DJI_0283	Frangula alnus Mill.	0	Rhamnaceae Juss.	n	1
Iz_6_1109	DJI_0283	Fraxinus pennsylvanica Marshall	0	Oleaceae Hoffmanns. & Link	a	0
Iz_6_1109	DJI_0283	Pinus sylvestris L.	0	Pinaceae Spreng. ex F.Rudolphi	n	1
Iz_6_1109	DJI_0283	Populus × canescens (Aiton) Sm.	0	Salicaceae Mirb.	n	1
Iz_6_1109	DJI_0283	Populus tremula L.	0	Salicaceae Mirb.	n	1
Iz_6_1109	DJI_0283	Populus nigra L.	0	Salicaceae Mirb.	n	1
Iz_6_1109	DJI_0283	Pyrus communis L.	0	Rosaceae Juss.	a	0
Iz_6_1109	DJI_0283	Quercus robur L.	0	Fagaceae Dumort.	n	1
Iz_6_1109	DJI_0283	Robinia pseudoacacia L.	0	Fabaceae Lindl.	a	0
Iz_6_1109	DJI_0283	Rosa sp.	0	Rosaceae Juss.		
Iz_6_1109	DJI_0283	Salix alba L.	0	Salicaceae Mirb.	n	1
Iz_6_1109	DJI_0283	Sambucus nigra L.	0	Viburnaceae Raf.	n	1
Iz_6_1109	DJI_0283	Sorbus aucuparia L.	0	Rosaceae Juss.	n	1
Iz_6_1109	DJI_0283	Tilia cordata Mill.	0	Malvaceae Juss.	n	1
Iz_6_1109	DJI_0283	Ulmus glabra Huds.	0	Ulmaceae Mirb.	n	1
Iz_6_1109	DJI_0280	Acer tataricum L.	0	Sapindaceae Juss.	n	1
Iz_6_1109	DJI_0280	Betula pendula Roth.	0	Betulaceae Gray	n	1
Iz_6_1109	DJI_0280	Crataegus rhipidophylla Gand.	0	Rosaceae Juss.	n	1
Iz_6_1109	DJI_0280	Frangula alnus Mill.	0	Rhamnaceae Juss.	n	1
Iz_6_1109	DJI_0280	Fraxinus pennsylvanica Marshall	0	Oleaceae Hoffmanns. & Link	a	0
Iz_6_1109	DJI_0280	Pinus sylvestris L.	0	Pinaceae Spreng. ex F.Rudolphi	n	1
Iz_6_1109	DJI_0280	Populus × canescens (Aiton) Sm.	0	Salicaceae Mirb.	n	1
Iz_6_1109	DJI_0280	Populus tremula L.	0	Salicaceae Mirb.	n	1

Iz_6_1109	DJI_0280	Populus nigra L.	0	Salicaceae Mirb.	n	1
Iz_6_1109	DJI_0280	Pyrus communis L.	0	Rosaceae Juss.	a	0
Iz_6_1109	DJI_0280	Quercus robur L.	0	Fagaceae Dumort.	n	1
Iz_6_1109	DJI_0280	Robinia pseudoacacia L.	0	Fabaceae Lindl.	a	0
Iz_6_1109	DJI_0280	Rosa sp.	0	Rosaceae Juss.		
Iz_6_1109	DJI_0280	Salix alba L.	0	Salicaceae Mirb.	n	1
Iz_6_1109	DJI_0280	Sambucus nigra L.	0	Viburnaceae Raf.	n	1
Iz_6_1109	DJI_0280	Sorbus aucuparia L.	0	Rosaceae Juss.	n	1
Iz_6_1109	DJI_0280	Tilia cordata Mill.	0	Malvaceae Juss.	n	1
Iz_6_1109	DJI_0280	Ulmus glabra Huds.	0	Ulmaceae Mirb.	n	1
Iz_6_1109	DJI_0278	Acer tataricum L.	0	Sapindaceae Juss.	n	1
Iz_6_1109	DJI_0278	Betula pendula Roth.	0	Betulaceae Gray	n	1
Iz_6_1109	DJI_0278	Crataegus rhipidophylla Gand.	0	Rosaceae Juss.	n	1
Iz_6_1109	DJI_0278	Frangula alnus Mill.	0	Rhamnaceae Juss.	n	1
Iz_6_1109	DJI_0278	Fraxinus pennsylvanica Marshall	0	Oleaceae Hoffmanns. & Link	a	0
Iz_6_1109	DJI_0278	Pinus sylvestris L.	0	Pinaceae Spreng. ex F.Rudolphi	n	1
Iz_6_1109	DJI_0278	Populus × canescens (Aiton) Sm.	0	Salicaceae Mirb.	n	1
Iz_6_1109	DJI_0278	Populus tremula L.	0	Salicaceae Mirb.	n	1
Iz_6_1109	DJI_0278	Populus nigra L.	0	Salicaceae Mirb.	n	1
Iz_6_1109	DJI_0278	Pyrus communis L.	0	Rosaceae Juss.	a	0
Iz_6_1109	DJI_0278	Quercus robur L.	0	Fagaceae Dumort.	n	1
Iz_6_1109	DJI_0278	Robinia pseudoacacia L.	0	Fabaceae Lindl.	a	0
Iz_6_1109	DJI_0278	Rosa sp.	0	Rosaceae Juss.		
Iz_6_1109	DJI_0278	Salix alba L.	0	Salicaceae Mirb.	n	1
Iz_6_1109	DJI_0278	Sambucus nigra L.	0	Viburnaceae Raf.	n	1
Iz_6_1109	DJI_0278	Sorbus aucuparia L.	0	Rosaceae Juss.	n	1
Iz_6_1109	DJI_0278	Tilia cordata Mill.	0	Malvaceae Juss.	n	1
Iz_6_1109	DJI_0278	Ulmus glabra Huds.	0	Ulmaceae Mirb.	n	1
Iz_6_1109	DJI_0275	Acer tataricum L.	0	Sapindaceae Juss.	n	1
Iz_6_1109	DJI_0275	Betula pendula Roth.	0	Betulaceae Gray	n	1
Iz_6_1109	DJI_0275	Crataegus rhipidophylla Gand.	0	Rosaceae Juss.	n	1
Iz_6_1109	DJI_0275	Frangula alnus Mill.	0	Rhamnaceae Juss.	n	1
Iz_6_1109	DJI_0275	Fraxinus pennsylvanica Marshall	0	Oleaceae Hoffmanns. & Link	a	0
Iz_6_1109	DJI_0275	Pinus sylvestris L.	0	Pinaceae Spreng. ex F.Rudolphi	n	1
Iz_6_1109	DJI_0275	Populus × canescens (Aiton) Sm.	0	Salicaceae Mirb.	n	1
Iz_6_1109	DJI_0275	Populus tremula L.	0	Salicaceae Mirb.	n	1
Iz_6_1109	DJI_0275	Populus nigra L.	0	Salicaceae Mirb.	n	1
Iz_6_1109	DJI_0275	Pyrus communis L.	0	Rosaceae Juss.	a	0
Iz_6_1109	DJI_0275	Quercus robur L.	0	Fagaceae Dumort.	n	1
Iz_6_1109	DJI_0275	Robinia pseudoacacia L.	0	Fabaceae Lindl.	a	0
Iz_6_1109	DJI_0275	Rosa sp.	0	Rosaceae Juss.		
Iz_6_1109	DJI_0275	Salix alba L.	0	Salicaceae Mirb.	n	1
Iz_6_1109	DJI_0275	Sambucus nigra L.	0	Viburnaceae Raf.	n	1
Iz_6_1109	DJI_0275	Sorbus aucuparia L.	0	Rosaceae Juss.	n	1
Iz_6_1109	DJI_0275	Tilia cordata Mill.	0	Malvaceae Juss.	n	1
Iz_6_1109	DJI_0275	Ulmus glabra Huds.	0	Ulmaceae Mirb.	n	1
Iz_6_1109	DJI_0272	Acer tataricum L.	0	Sapindaceae Juss.	n	1
Iz_6_1109	DJI_0272	Betula pendula Roth.	0	Betulaceae Gray	n	1
Iz_6_1109	DJI_0272	Crataegus rhipidophylla Gand.	0	Rosaceae Juss.	n	1
Iz_6_1109	DJI_0272	Frangula alnus Mill.	0	Rhamnaceae Juss.	n	1
Iz_6_1109	DJI_0272	Fraxinus pennsylvanica Marshall	0	Oleaceae Hoffmanns. & Link	a	0
Iz_6_1109	DJI_0272	Pinus sylvestris L.	0	Pinaceae Spreng. ex F.Rudolphi	n	1
Iz_6_1109	DJI_0272	Populus × canescens (Aiton) Sm.	0	Salicaceae Mirb.	n	1
Iz_6_1109	DJI_0272	Populus tremula L.	0	Salicaceae Mirb.	n	1
Iz_6_1109	DJI_0272	Populus nigra L.	0	Salicaceae Mirb.	n	1
Iz_6_1109	DJI_0272	Pyrus communis L.	0	Rosaceae Juss.	a	0
Iz_6_1109	DJI_0272	Quercus robur L.	0	Fagaceae Dumort.	n	1
Iz_6_1109	DJI_0272	Robinia pseudoacacia L.	0	Fabaceae Lindl.	a	0
Iz_6_1109	DJI_0272	Rosa sp.	0	Rosaceae Juss.		
Iz_6_1109	DJI_0272	Salix alba L.	0	Salicaceae Mirb.	n	1
Iz_6_1109	DJI_0272	Sambucus nigra L.	0	Viburnaceae Raf.	n	1
Iz_6_1109	DJI_0272	Sorbus aucuparia L.	0	Rosaceae Juss.	n	1
Iz_6_1109	DJI_0272	Tilia cordata Mill.	0	Malvaceae Juss.	n	1
Iz_6_1109	DJI_0272	Ulmus glabra Huds.	0	Ulmaceae Mirb.	n	1

Iz_6_1109	DJI_0267	Acer tataricum L.	0	Sapindaceae Juss.	n	1
Iz_6_1109	DJI_0267	Betula pendula Roth.	0	Betulaceae Gray	n	1
Iz_6_1109	DJI_0267	Crataegus rhipidophylla Gand.	0	Rosaceae Juss.	n	1
Iz_6_1109	DJI_0267	Frangula alnus Mill.	0	Rhamnaceae Juss.	n	1
Iz_6_1109	DJI_0267	Fraxinus pennsylvanica Marshall	0	Oleaceae Hoffmanns. & Link	a	0
Iz_6_1109	DJI_0267	Pinus sylvestris L.	0	Pinaceae Spreng. ex F.Rudolphi	n	1
Iz_6_1109	DJI_0267	Populus × canescens (Aiton) Sm.	0	Salicaceae Mirb.	n	1
Iz_6_1109	DJI_0267	Populus tremula L.	0	Salicaceae Mirb.	n	1
Iz_6_1109	DJI_0267	Populus nigra L.	0	Salicaceae Mirb.	n	1
Iz_6_1109	DJI_0267	Pyrus communis L.	0	Rosaceae Juss.	a	0
Iz_6_1109	DJI_0267	Quercus robur L.	0	Fagaceae Dumort.	n	1
Iz_6_1109	DJI_0267	Robinia pseudoacacia L.	0	Fabaceae Lindl.	a	0
Iz_6_1109	DJI_0267	Rosa sp.	0	Rosaceae Juss.		
Iz_6_1109	DJI_0267	Salix alba L.	0	Salicaceae Mirb.	n	1
Iz_6_1109	DJI_0267	Sambucus nigra L.	0	Viburnaceae Raf.	n	1
Iz_6_1109	DJI_0267	Sorbus aucuparia L.	0	Rosaceae Juss.	n	1
Iz_6_1109	DJI_0267	Tilia cordata Mill.	0	Malvaceae Juss.	n	1
Iz_6_1109	DJI_0267	Ulmus glabra Huds.	0	Ulmaceae Mirb.	n	1
Iz_6_1109	DJI_0261	Acer tataricum L.	0	Sapindaceae Juss.	n	1
Iz_6_1109	DJI_0261	Betula pendula Roth.	0	Betulaceae Gray	n	1
Iz_6_1109	DJI_0261	Crataegus rhipidophylla Gand.	0	Rosaceae Juss.	n	1
Iz_6_1109	DJI_0261	Frangula alnus Mill.	0	Rhamnaceae Juss.	n	1
Iz_6_1109	DJI_0261	Fraxinus pennsylvanica Marshall	0	Oleaceae Hoffmanns. & Link	a	0
Iz_6_1109	DJI_0261	Pinus sylvestris L.	0	Pinaceae Spreng. ex F.Rudolphi	n	1
Iz_6_1109	DJI_0261	Populus × canescens (Aiton) Sm.	0	Salicaceae Mirb.	n	1
Iz_6_1109	DJI_0261	Populus tremula L.	0	Salicaceae Mirb.	n	1
Iz_6_1109	DJI_0261	Populus nigra L.	0	Salicaceae Mirb.	n	1
Iz_6_1109	DJI_0261	Pyrus communis L.	0	Rosaceae Juss.	a	0
Iz_6_1109	DJI_0261	Quercus robur L.	0	Fagaceae Dumort.	n	1
Iz_6_1109	DJI_0261	Robinia pseudoacacia L.	0	Fabaceae Lindl.	a	0
Iz_6_1109	DJI_0261	Rosa sp.	0	Rosaceae Juss.		
Iz_6_1109	DJI_0261	Salix alba L.	0	Salicaceae Mirb.	n	1
Iz_6_1109	DJI_0261	Sambucus nigra L.	0	Viburnaceae Raf.	n	1
Iz_6_1109	DJI_0261	Sorbus aucuparia L.	0	Rosaceae Juss.	n	1
Iz_6_1109	DJI_0261	Tilia cordata Mill.	0	Malvaceae Juss.	n	1
Iz_6_1109	DJI_0261	Ulmus glabra Huds.	0	Ulmaceae Mirb.	n	1
Iz_6_1109	DJI_0259	Acer tataricum L.	0	Sapindaceae Juss.	n	1
Iz_6_1109	DJI_0259	Betula pendula Roth.	0	Betulaceae Gray	n	1
Iz_6_1109	DJI_0259	Crataegus rhipidophylla Gand.	0	Rosaceae Juss.	n	1
Iz_6_1109	DJI_0259	Frangula alnus Mill.	0	Rhamnaceae Juss.	n	1
Iz_6_1109	DJI_0259	Fraxinus pennsylvanica Marshall	0	Oleaceae Hoffmanns. & Link	a	0
Iz_6_1109	DJI_0259	Pinus sylvestris L.	0	Pinaceae Spreng. ex F.Rudolphi	n	1
Iz_6_1109	DJI_0259	Populus × canescens (Aiton) Sm.	0	Salicaceae Mirb.	n	1
Iz_6_1109	DJI_0259	Populus tremula L.	1	Salicaceae Mirb.	n	1
Iz_6_1109	DJI_0259	Populus nigra L.	0	Salicaceae Mirb.	n	1
Iz_6_1109	DJI_0259	Pyrus communis L.	0	Rosaceae Juss.	a	0
Iz_6_1109	DJI_0259	Quercus robur L.	0	Fagaceae Dumort.	n	1
Iz_6_1109	DJI_0259	Robinia pseudoacacia L.	0	Fabaceae Lindl.	a	0
Iz_6_1109	DJI_0259	Rosa sp.	0	Rosaceae Juss.		
Iz_6_1109	DJI_0259	Salix alba L.	0	Salicaceae Mirb.	n	1
Iz_6_1109	DJI_0259	Sambucus nigra L.	0	Viburnaceae Raf.	n	1
Iz_6_1109	DJI_0259	Sorbus aucuparia L.	0	Rosaceae Juss.	n	1
Iz_6_1109	DJI_0259	Tilia cordata Mill.	0	Malvaceae Juss.	n	1
Iz_6_1109	DJI_0259	Ulmus glabra Huds.	0	Ulmaceae Mirb.	n	1
Iz_6_1109	DJI_0256	Acer tataricum L.	0	Sapindaceae Juss.	n	1
Iz_6_1109	DJI_0256	Betula pendula Roth.	0	Betulaceae Gray	n	1
Iz_6_1109	DJI_0256	Crataegus rhipidophylla Gand.	0	Rosaceae Juss.	n	1
Iz_6_1109	DJI_0256	Frangula alnus Mill.	0	Rhamnaceae Juss.	n	1
Iz_6_1109	DJI_0256	Fraxinus pennsylvanica Marshall	0	Oleaceae Hoffmanns. & Link	a	0
Iz_6_1109	DJI_0256	Pinus sylvestris L.	0	Pinaceae Spreng. ex F.Rudolphi	n	1
Iz_6_1109	DJI_0256	Populus × canescens (Aiton) Sm.	0	Salicaceae Mirb.	n	1
Iz_6_1109	DJI_0256	Populus tremula L.	0	Salicaceae Mirb.	n	1
Iz_6_1109	DJI_0256	Populus nigra L.	0	Salicaceae Mirb.	n	1
Iz_6_1109	DJI_0256	Pyrus communis L.	0	Rosaceae Juss.	a	0

Iz_6_1109	DJI_0256	Quercus robur L.	0	Fagaceae Dumort.	n	1
Iz_6_1109	DJI_0256	Robinia pseudoacacia L.	0	Fabaceae Lindl.	a	0
Iz_6_1109	DJI_0256	Rosa sp.	0	Rosaceae Juss.		
Iz_6_1109	DJI_0256	Salix alba L.	0	Salicaceae Mirb.	n	1
Iz_6_1109	DJI_0256	Sambucus nigra L.	0	Viburnaceae Raf.	n	1
Iz_6_1109	DJI_0256	Sorbus aucuparia L.	0	Rosaceae Juss.	n	1
Iz_6_1109	DJI_0256	Tilia cordata Mill.	0	Malvaceae Juss.	n	1
Iz_6_1109	DJI_0256	Ulmus glabra Huds.	0	Ulmaceae Mirb.	n	1
Iz_6_1109	DJI_0254	Acer tataricum L.	0	Sapindaceae Juss.	n	1
Iz_6_1109	DJI_0254	Betula pendula Roth.	0	Betulaceae Gray	n	1
Iz_6_1109	DJI_0254	Crataegus rhipidophylla Gand.	0	Rosaceae Juss.	n	1
Iz_6_1109	DJI_0254	Frangula alnus Mill.	0	Rhamnaceae Juss.	n	1
Iz_6_1109	DJI_0254	Fraxinus pennsylvanica Marshall	0	Oleaceae Hoffmanns. & Link	a	0
Iz_6_1109	DJI_0254	Pinus sylvestris L.	0	Pinaceae Spreng. ex F.Rudolphi	n	1
Iz_6_1109	DJI_0254	Populus × canescens (Aiton) Sm.	0	Salicaceae Mirb.	n	1
Iz_6_1109	DJI_0254	Populus tremula L.	0	Salicaceae Mirb.	n	1
Iz_6_1109	DJI_0254	Populus nigra L.	0	Salicaceae Mirb.	n	1
Iz_6_1109	DJI_0254	Pyrus communis L.	0	Rosaceae Juss.	a	0
Iz_6_1109	DJI_0254	Quercus robur L.	0	Fagaceae Dumort.	n	1
Iz_6_1109	DJI_0254	Robinia pseudoacacia L.	0	Fabaceae Lindl.	a	0
Iz_6_1109	DJI_0254	Rosa sp.	0	Rosaceae Juss.		
Iz_6_1109	DJI_0254	Salix alba L.	0	Salicaceae Mirb.	n	1
Iz_6_1109	DJI_0254	Sambucus nigra L.	0	Viburnaceae Raf.	n	1
Iz_6_1109	DJI_0254	Sorbus aucuparia L.	0	Rosaceae Juss.	n	1
Iz_6_1109	DJI_0254	Tilia cordata Mill.	0	Malvaceae Juss.	n	1
Iz_6_1109	DJI_0254	Ulmus glabra Huds.	0	Ulmaceae Mirb.	n	1
Iz_6_1109	DJI_0252	Acer tataricum L.	0	Sapindaceae Juss.	n	1
Iz_6_1109	DJI_0252	Betula pendula Roth.	0	Betulaceae Gray	n	1
Iz_6_1109	DJI_0252	Crataegus rhipidophylla Gand.	0	Rosaceae Juss.	n	1
Iz_6_1109	DJI_0252	Frangula alnus Mill.	0	Rhamnaceae Juss.	n	1
Iz_6_1109	DJI_0252	Fraxinus pennsylvanica Marshall	0	Oleaceae Hoffmanns. & Link	a	0
Iz_6_1109	DJI_0252	Pinus sylvestris L.	0	Pinaceae Spreng. ex F.Rudolphi	n	1
Iz_6_1109	DJI_0252	Populus × canescens (Aiton) Sm.	0	Salicaceae Mirb.	n	1
Iz_6_1109	DJI_0252	Populus tremula L.	0	Salicaceae Mirb.	n	1
Iz_6_1109	DJI_0252	Populus nigra L.	0	Salicaceae Mirb.	n	1
Iz_6_1109	DJI_0252	Pyrus communis L.	0	Rosaceae Juss.	a	0
Iz_6_1109	DJI_0252	Quercus robur L.	0	Fagaceae Dumort.	n	1
Iz_6_1109	DJI_0252	Robinia pseudoacacia L.	0	Fabaceae Lindl.	a	0
Iz_6_1109	DJI_0252	Rosa sp.	0	Rosaceae Juss.		
Iz_6_1109	DJI_0252	Salix alba L.	0	Salicaceae Mirb.	n	1
Iz_6_1109	DJI_0252	Sambucus nigra L.	0	Viburnaceae Raf.	n	1
Iz_6_1109	DJI_0252	Sorbus aucuparia L.	0	Rosaceae Juss.	n	1
Iz_6_1109	DJI_0252	Tilia cordata Mill.	0	Malvaceae Juss.	n	1
Iz_6_1109	DJI_0252	Ulmus glabra Huds.	0	Ulmaceae Mirb.	n	1
Iz_6_1109	DJI_0250	Acer tataricum L.	0	Sapindaceae Juss.	n	1
Iz_6_1109	DJI_0250	Betula pendula Roth.	0	Betulaceae Gray	n	1
Iz_6_1109	DJI_0250	Crataegus rhipidophylla Gand.	0	Rosaceae Juss.	n	1
Iz_6_1109	DJI_0250	Frangula alnus Mill.	0	Rhamnaceae Juss.	n	1
Iz_6_1109	DJI_0250	Fraxinus pennsylvanica Marshall	0	Oleaceae Hoffmanns. & Link	a	0
Iz_6_1109	DJI_0250	Pinus sylvestris L.	0	Pinaceae Spreng. ex F.Rudolphi	n	1
Iz_6_1109	DJI_0250	Populus × canescens (Aiton) Sm.	0	Salicaceae Mirb.	n	1
Iz_6_1109	DJI_0250	Populus tremula L.	0	Salicaceae Mirb.	n	1
Iz_6_1109	DJI_0250	Populus nigra L.	0	Salicaceae Mirb.	n	1
Iz_6_1109	DJI_0250	Pyrus communis L.	0	Rosaceae Juss.	a	0
Iz_6_1109	DJI_0250	Quercus robur L.	0	Fagaceae Dumort.	n	1
Iz_6_1109	DJI_0250	Robinia pseudoacacia L.	0	Fabaceae Lindl.	a	0
Iz_6_1109	DJI_0250	Rosa sp.	0	Rosaceae Juss.		
Iz_6_1109	DJI_0250	Salix alba L.	0	Salicaceae Mirb.	n	1
Iz_6_1109	DJI_0250	Sambucus nigra L.	0	Viburnaceae Raf.	n	1
Iz_6_1109	DJI_0250	Sorbus aucuparia L.	0	Rosaceae Juss.	n	1
Iz_6_1109	DJI_0250	Tilia cordata Mill.	0	Malvaceae Juss.	n	1
Iz_6_1109	DJI_0250	Ulmus glabra Huds.	0	Ulmaceae Mirb.	n	1
Iz_6_1109	DJI_0248	Acer tataricum L.	0	Sapindaceae Juss.	n	1
Iz_6_1109	DJI_0248	Betula pendula Roth.	0	Betulaceae Gray	n	1

Iz_6_1109	DJI_0248	Crataegus rhipidophylla Gand.	0	Rosaceae Juss.	n	1
Iz_6_1109	DJI_0248	Frangula alnus Mill.	0	Rhamnaceae Juss.	n	1
Iz_6_1109	DJI_0248	Fraxinus pennsylvanica Marshall	0	Oleaceae Hoffmanns. & Link	a	0
Iz_6_1109	DJI_0248	Pinus sylvestris L.	0	Pinaceae Spreng. ex F.Rudolphi	n	1
Iz_6_1109	DJI_0248	Populus × canescens (Aiton) Sm.	0	Salicaceae Mirb.	n	1
Iz_6_1109	DJI_0248	Populus tremula L.	0	Salicaceae Mirb.	n	1
Iz_6_1109	DJI_0248	Populus nigra L.	0	Salicaceae Mirb.	n	1
Iz_6_1109	DJI_0248	Pyrus communis L.	0	Rosaceae Juss.	a	0
Iz_6_1109	DJI_0248	Quercus robur L.	0	Fagaceae Dumort.	n	1
Iz_6_1109	DJI_0248	Robinia pseudoacacia L.	0	Fabaceae Lindl.	a	0
Iz_6_1109	DJI_0248	Rosa sp.	0	Rosaceae Juss.		
Iz_6_1109	DJI_0248	Salix alba L.	0	Salicaceae Mirb.	n	1
Iz_6_1109	DJI_0248	Sambucus nigra L.	0	Viburnaceae Raf.	n	1
Iz_6_1109	DJI_0248	Sorbus aucuparia L.	0	Rosaceae Juss.	n	1
Iz_6_1109	DJI_0248	Tilia cordata Mill.	0	Malvaceae Juss.	n	1
Iz_6_1109	DJI_0248	Ulmus glabra Huds.	0	Ulmaceae Mirb.	n	1
Iz_6_1109	DJI_0247	Acer tataricum L.	0	Sapindaceae Juss.	n	1
Iz_6_1109	DJI_0247	Betula pendula Roth.	0	Betulaceae Gray	n	1
Iz_6_1109	DJI_0247	Crataegus rhipidophylla Gand.	0	Rosaceae Juss.	n	1
Iz_6_1109	DJI_0247	Frangula alnus Mill.	0	Rhamnaceae Juss.	n	1
Iz_6_1109	DJI_0247	Fraxinus pennsylvanica Marshall	0	Oleaceae Hoffmanns. & Link	a	0
Iz_6_1109	DJI_0247	Pinus sylvestris L.	0	Pinaceae Spreng. ex F.Rudolphi	n	1
Iz_6_1109	DJI_0247	Populus × canescens (Aiton) Sm.	0	Salicaceae Mirb.	n	1
Iz_6_1109	DJI_0247	Populus tremula L.	0	Salicaceae Mirb.	n	1
Iz_6_1109	DJI_0247	Populus nigra L.	0	Salicaceae Mirb.	n	1
Iz_6_1109	DJI_0247	Pyrus communis L.	0	Rosaceae Juss.	a	0
Iz_6_1109	DJI_0247	Quercus robur L.	0	Fagaceae Dumort.	n	1
Iz_6_1109	DJI_0247	Robinia pseudoacacia L.	0	Fabaceae Lindl.	a	0
Iz_6_1109	DJI_0247	Rosa sp.	0	Rosaceae Juss.		
Iz_6_1109	DJI_0247	Salix alba L.	0	Salicaceae Mirb.	n	1
Iz_6_1109	DJI_0247	Sambucus nigra L.	0	Viburnaceae Raf.	n	1
Iz_6_1109	DJI_0247	Sorbus aucuparia L.	0	Rosaceae Juss.	n	1
Iz_6_1109	DJI_0247	Tilia cordata Mill.	0	Malvaceae Juss.	n	1
Iz_6_1109	DJI_0247	Ulmus glabra Huds.	0	Ulmaceae Mirb.	n	1
Iz_6_1109	DJI_0244	Acer tataricum L.	0	Sapindaceae Juss.	n	1
Iz_6_1109	DJI_0244	Betula pendula Roth.	0	Betulaceae Gray	n	1
Iz_6_1109	DJI_0244	Crataegus rhipidophylla Gand.	0	Rosaceae Juss.	n	1
Iz_6_1109	DJI_0244	Frangula alnus Mill.	0	Rhamnaceae Juss.	n	1
Iz_6_1109	DJI_0244	Fraxinus pennsylvanica Marshall	0	Oleaceae Hoffmanns. & Link	a	0
Iz_6_1109	DJI_0244	Pinus sylvestris L.	0	Pinaceae Spreng. ex F.Rudolphi	n	1
Iz_6_1109	DJI_0244	Populus × canescens (Aiton) Sm.	0	Salicaceae Mirb.	n	1
Iz_6_1109	DJI_0244	Populus tremula L.	0	Salicaceae Mirb.	n	1
Iz_6_1109	DJI_0244	Populus nigra L.	0	Salicaceae Mirb.	n	1
Iz_6_1109	DJI_0244	Pyrus communis L.	0	Rosaceae Juss.	a	0
Iz_6_1109	DJI_0244	Quercus robur L.	0	Fagaceae Dumort.	n	1
Iz_6_1109	DJI_0244	Robinia pseudoacacia L.	0	Fabaceae Lindl.	a	0
Iz_6_1109	DJI_0244	Rosa sp.	0	Rosaceae Juss.		
Iz_6_1109	DJI_0244	Salix alba L.	0	Salicaceae Mirb.	n	1
Iz_6_1109	DJI_0244	Sambucus nigra L.	0	Viburnaceae Raf.	n	1
Iz_6_1109	DJI_0244	Sorbus aucuparia L.	0	Rosaceae Juss.	n	1
Iz_6_1109	DJI_0244	Tilia cordata Mill.	0	Malvaceae Juss.	n	1
Iz_6_1109	DJI_0244	Ulmus glabra Huds.	0	Ulmaceae Mirb.	n	1
Iz_6_1109	DJI_0242	Acer tataricum L.	0	Sapindaceae Juss.	n	1
Iz_6_1109	DJI_0242	Betula pendula Roth.	0	Betulaceae Gray	n	1
Iz_6_1109	DJI_0242	Crataegus rhipidophylla Gand.	0	Rosaceae Juss.	n	1
Iz_6_1109	DJI_0242	Frangula alnus Mill.	0	Rhamnaceae Juss.	n	1
Iz_6_1109	DJI_0242	Fraxinus pennsylvanica Marshall	0	Oleaceae Hoffmanns. & Link	a	0
Iz_6_1109	DJI_0242	Pinus sylvestris L.	0	Pinaceae Spreng. ex F.Rudolphi	n	1
Iz_6_1109	DJI_0242	Populus × canescens (Aiton) Sm.	0	Salicaceae Mirb.	n	1
Iz_6_1109	DJI_0242	Populus tremula L.	0	Salicaceae Mirb.	n	1
Iz_6_1109	DJI_0242	Populus nigra L.	0	Salicaceae Mirb.	n	1
Iz_6_1109	DJI_0242	Pyrus communis L.	0	Rosaceae Juss.	a	0
Iz_6_1109	DJI_0242	Quercus robur L.	0	Fagaceae Dumort.	n	1
Iz_6_1109	DJI_0242	Robinia pseudoacacia L.	0	Fabaceae Lindl.	a	0

Iz_6_1109	DJI_0242	Rosa sp.	0	Rosaceae Juss.		
Iz_6_1109	DJI_0242	Salix alba L.	0	Salicaceae Mirb.	n	1
Iz_6_1109	DJI_0242	Sambucus nigra L.	0	Viburnaceae Raf.	n	1
Iz_6_1109	DJI_0242	Sorbus aucuparia L.	0	Rosaceae Juss.	n	1
Iz_6_1109	DJI_0242	Tilia cordata Mill.	0	Malvaceae Juss.	n	1
Iz_6_1109	DJI_0242	Ulmus glabra Huds.	0	Ulmaceae Mirb.	n	1
Iz_6_1109	DJI_0239	Acer tataricum L.	0	Sapindaceae Juss.	n	1
Iz_6_1109	DJI_0239	Betula pendula Roth.	0	Betulaceae Gray	n	1
Iz_6_1109	DJI_0239	Crataegus rhipidophylla Gand.	0	Rosaceae Juss.	n	1
Iz_6_1109	DJI_0239	Frangula alnus Mill.	0	Rhamnaceae Juss.	n	1
Iz_6_1109	DJI_0239	Fraxinus pennsylvanica Marshall	0	Oleaceae Hoffmanns. & Link	a	0
Iz_6_1109	DJI_0239	Pinus sylvestris L.	0	Pinaceae Spreng. ex F.Rudolphi	n	1
Iz_6_1109	DJI_0239	Populus × canescens (Aiton) Sm.	0	Salicaceae Mirb.	n	1
Iz_6_1109	DJI_0239	Populus tremula L.	0	Salicaceae Mirb.	n	1
Iz_6_1109	DJI_0239	Populus nigra L.	0	Salicaceae Mirb.	n	1
Iz_6_1109	DJI_0239	Pyrus communis L.	0	Rosaceae Juss.	a	0
Iz_6_1109	DJI_0239	Quercus robur L.	0	Fagaceae Dumort.	n	1
Iz_6_1109	DJI_0239	Robinia pseudoacacia L.	0	Fabaceae Lindl.	a	0
Iz_6_1109	DJI_0239	Rosa sp.	0	Rosaceae Juss.		
Iz_6_1109	DJI_0239	Salix alba L.	0	Salicaceae Mirb.	n	1
Iz_6_1109	DJI_0239	Sambucus nigra L.	0	Viburnaceae Raf.	n	1
Iz_6_1109	DJI_0239	Sorbus aucuparia L.	0	Rosaceae Juss.	n	1
Iz_6_1109	DJI_0239	Tilia cordata Mill.	0	Malvaceae Juss.	n	1
Iz_6_1109	DJI_0239	Ulmus glabra Huds.	0	Ulmaceae Mirb.	n	1
Iz_6_1109	DJI_0238	Acer tataricum L.	0	Sapindaceae Juss.	n	1
Iz_6_1109	DJI_0238	Betula pendula Roth.	0	Betulaceae Gray	n	1
Iz_6_1109	DJI_0238	Crataegus rhipidophylla Gand.	0	Rosaceae Juss.	n	1
Iz_6_1109	DJI_0238	Frangula alnus Mill.	0	Rhamnaceae Juss.	n	1
Iz_6_1109	DJI_0238	Fraxinus pennsylvanica Marshall	0	Oleaceae Hoffmanns. & Link	a	0
Iz_6_1109	DJI_0238	Pinus sylvestris L.	0	Pinaceae Spreng. ex F.Rudolphi	n	1
Iz_6_1109	DJI_0238	Populus × canescens (Aiton) Sm.	0	Salicaceae Mirb.	n	1
Iz_6_1109	DJI_0238	Populus tremula L.	0	Salicaceae Mirb.	n	1
Iz_6_1109	DJI_0238	Populus nigra L.	0	Salicaceae Mirb.	n	1
Iz_6_1109	DJI_0238	Pyrus communis L.	0	Rosaceae Juss.	a	0
Iz_6_1109	DJI_0238	Quercus robur L.	0	Fagaceae Dumort.	n	1
Iz_6_1109	DJI_0238	Robinia pseudoacacia L.	0	Fabaceae Lindl.	a	0
Iz_6_1109	DJI_0238	Rosa sp.	0	Rosaceae Juss.		
Iz_6_1109	DJI_0238	Salix alba L.	0	Salicaceae Mirb.	n	1
Iz_6_1109	DJI_0238	Sambucus nigra L.	0	Viburnaceae Raf.	n	1
Iz_6_1109	DJI_0238	Sorbus aucuparia L.	0	Rosaceae Juss.	n	1
Iz_6_1109	DJI_0238	Tilia cordata Mill.	0	Malvaceae Juss.	n	1
Iz_6_1109	DJI_0238	Ulmus glabra Huds.	0	Ulmaceae Mirb.	n	1
Iz_6_1109	DJI_0234	Acer tataricum L.	0	Sapindaceae Juss.	n	1
Iz_6_1109	DJI_0234	Betula pendula Roth.	0	Betulaceae Gray	n	1
Iz_6_1109	DJI_0234	Crataegus rhipidophylla Gand.	0	Rosaceae Juss.	n	1
Iz_6_1109	DJI_0234	Frangula alnus Mill.	0	Rhamnaceae Juss.	n	1
Iz_6_1109	DJI_0234	Fraxinus pennsylvanica Marshall	0	Oleaceae Hoffmanns. & Link	a	0
Iz_6_1109	DJI_0234	Pinus sylvestris L.	0	Pinaceae Spreng. ex F.Rudolphi	n	1
Iz_6_1109	DJI_0234	Populus × canescens (Aiton) Sm.	0	Salicaceae Mirb.	n	1
Iz_6_1109	DJI_0234	Populus tremula L.	0	Salicaceae Mirb.	n	1
Iz_6_1109	DJI_0234	Populus nigra L.	0	Salicaceae Mirb.	n	1
Iz_6_1109	DJI_0234	Pyrus communis L.	0	Rosaceae Juss.	a	0
Iz_6_1109	DJI_0234	Quercus robur L.	0	Fagaceae Dumort.	n	1
Iz_6_1109	DJI_0234	Robinia pseudoacacia L.	0	Fabaceae Lindl.	a	0
Iz_6_1109	DJI_0234	Rosa sp.	0	Rosaceae Juss.		
Iz_6_1109	DJI_0234	Salix alba L.	0	Salicaceae Mirb.	n	1
Iz_6_1109	DJI_0234	Sambucus nigra L.	0	Viburnaceae Raf.	n	1
Iz_6_1109	DJI_0234	Sorbus aucuparia L.	0	Rosaceae Juss.	n	1
Iz_6_1109	DJI_0234	Tilia cordata Mill.	0	Malvaceae Juss.	n	1
Iz_6_1109	DJI_0234	Ulmus glabra Huds.	0	Ulmaceae Mirb.	n	1
Iz_7_1109	DJI_0359	Acer tataricum L.	0	Sapindaceae Juss.	n	1
Iz_7_1109	DJI_0359	Betula pendula Roth.	0	Betulaceae Gray	n	1
Iz_7_1109	DJI_0359	Crataegus rhipidophylla Gand.	0	Rosaceae Juss.	n	1
Iz_7_1109	DJI_0359	Frangula alnus Mill.	0	Rhamnaceae Juss.	n	1

Iz_7_1109	DJI_0359	Fraxinus pennsylvanica Marshall	0	Oleaceae Hoffmanns. & Link	a	0
Iz_7_1109	DJI_0359	Pinus sylvestris L.	0	Pinaceae Spreng. ex F.Rudolphi	n	1
Iz_7_1109	DJI_0359	Populus × canescens (Aiton) Sm.	0	Salicaceae Mirb.	n	1
Iz_7_1109	DJI_0359	Populus tremula L.	0	Salicaceae Mirb.	n	1
Iz_7_1109	DJI_0359	Populus nigra L.	0	Salicaceae Mirb.	n	1
Iz_7_1109	DJI_0359	Pyrus communis L.	0	Rosaceae Juss.	a	0
Iz_7_1109	DJI_0359	Quercus robur L.	0	Fagaceae Dumort.	n	1
Iz_7_1109	DJI_0359	Robinia pseudoacacia L.	0	Fabaceae Lindl.	a	0
Iz_7_1109	DJI_0359	Rosa sp.	0	Rosaceae Juss.		
Iz_7_1109	DJI_0359	Salix alba L.	0	Salicaceae Mirb.	n	1
Iz_7_1109	DJI_0359	Sambucus nigra L.	0	Viburnaceae Raf.	n	1
Iz_7_1109	DJI_0359	Sorbus aucuparia L.	0	Rosaceae Juss.	n	1
Iz_7_1109	DJI_0359	Tilia cordata Mill.	0	Malvaceae Juss.	n	1
Iz_7_1109	DJI_0359	Ulmus glabra Huds.	0	Ulmaceae Mirb.	n	1
Iz_7_1109	DJI_0356	Acer tataricum L.	0	Sapindaceae Juss.	n	1
Iz_7_1109	DJI_0356	Betula pendula Roth.	0	Betulaceae Gray	n	1
Iz_7_1109	DJI_0356	Crataegus rhipidophylla Gand.	0	Rosaceae Juss.	n	1
Iz_7_1109	DJI_0356	Frangula alnus Mill.	0	Rhamnaceae Juss.	n	1
Iz_7_1109	DJI_0356	Fraxinus pennsylvanica Marshall	0	Oleaceae Hoffmanns. & Link	a	0
Iz_7_1109	DJI_0356	Pinus sylvestris L.	0	Pinaceae Spreng. ex F.Rudolphi	n	1
Iz_7_1109	DJI_0356	Populus × canescens (Aiton) Sm.	0	Salicaceae Mirb.	n	1
Iz_7_1109	DJI_0356	Populus tremula L.	0	Salicaceae Mirb.	n	1
Iz_7_1109	DJI_0356	Populus nigra L.	0	Salicaceae Mirb.	n	1
Iz_7_1109	DJI_0356	Pyrus communis L.	0	Rosaceae Juss.	a	0
Iz_7_1109	DJI_0356	Quercus robur L.	0	Fagaceae Dumort.	n	1
Iz_7_1109	DJI_0356	Robinia pseudoacacia L.	0	Fabaceae Lindl.	a	0
Iz_7_1109	DJI_0356	Rosa sp.	0	Rosaceae Juss.		
Iz_7_1109	DJI_0356	Salix alba L.	0	Salicaceae Mirb.	n	1
Iz_7_1109	DJI_0356	Sambucus nigra L.	0	Viburnaceae Raf.	n	1
Iz_7_1109	DJI_0356	Sorbus aucuparia L.	0	Rosaceae Juss.	n	1
Iz_7_1109	DJI_0356	Tilia cordata Mill.	0	Malvaceae Juss.	n	1
Iz_7_1109	DJI_0356	Ulmus glabra Huds.	0	Ulmaceae Mirb.	n	1
Iz_7_1109	DJI_0352	Acer tataricum L.	0	Sapindaceae Juss.	n	1
Iz_7_1109	DJI_0352	Betula pendula Roth.	0	Betulaceae Gray	n	1
Iz_7_1109	DJI_0352	Crataegus rhipidophylla Gand.	0	Rosaceae Juss.	n	1
Iz_7_1109	DJI_0352	Frangula alnus Mill.	0	Rhamnaceae Juss.	n	1
Iz_7_1109	DJI_0352	Fraxinus pennsylvanica Marshall	0	Oleaceae Hoffmanns. & Link	a	0
Iz_7_1109	DJI_0352	Pinus sylvestris L.	0	Pinaceae Spreng. ex F.Rudolphi	n	1
Iz_7_1109	DJI_0352	Populus × canescens (Aiton) Sm.	0	Salicaceae Mirb.	n	1
Iz_7_1109	DJI_0352	Populus tremula L.	0	Salicaceae Mirb.	n	1
Iz_7_1109	DJI_0352	Populus nigra L.	0	Salicaceae Mirb.	n	1
Iz_7_1109	DJI_0352	Pyrus communis L.	0	Rosaceae Juss.	a	0
Iz_7_1109	DJI_0352	Quercus robur L.	0	Fagaceae Dumort.	n	1
Iz_7_1109	DJI_0352	Robinia pseudoacacia L.	0	Fabaceae Lindl.	a	0
Iz_7_1109	DJI_0352	Rosa sp.	0	Rosaceae Juss.		
Iz_7_1109	DJI_0352	Salix alba L.	0	Salicaceae Mirb.	n	1
Iz_7_1109	DJI_0352	Sambucus nigra L.	0	Viburnaceae Raf.	n	1
Iz_7_1109	DJI_0352	Sorbus aucuparia L.	0	Rosaceae Juss.	n	1
Iz_7_1109	DJI_0352	Tilia cordata Mill.	0	Malvaceae Juss.	n	1
Iz_7_1109	DJI_0352	Ulmus glabra Huds.	0	Ulmaceae Mirb.	n	1
Iz_7_1109	DJI_0349	Acer tataricum L.	0	Sapindaceae Juss.	n	1
Iz_7_1109	DJI_0349	Betula pendula Roth.	0	Betulaceae Gray	n	1
Iz_7_1109	DJI_0349	Crataegus rhipidophylla Gand.	0	Rosaceae Juss.	n	1
Iz_7_1109	DJI_0349	Frangula alnus Mill.	0	Rhamnaceae Juss.	n	1
Iz_7_1109	DJI_0349	Fraxinus pennsylvanica Marshall	0	Oleaceae Hoffmanns. & Link	a	0
Iz_7_1109	DJI_0349	Pinus sylvestris L.	0	Pinaceae Spreng. ex F.Rudolphi	n	1
Iz_7_1109	DJI_0349	Populus × canescens (Aiton) Sm.	0	Salicaceae Mirb.	n	1
Iz_7_1109	DJI_0349	Populus tremula L.	0	Salicaceae Mirb.	n	1
Iz_7_1109	DJI_0349	Populus nigra L.	0	Salicaceae Mirb.	n	1
Iz_7_1109	DJI_0349	Pyrus communis L.	0	Rosaceae Juss.	a	0
Iz_7_1109	DJI_0349	Quercus robur L.	0	Fagaceae Dumort.	n	1
Iz_7_1109	DJI_0349	Robinia pseudoacacia L.	0	Fabaceae Lindl.	a	0
Iz_7_1109	DJI_0349	Rosa sp.	0	Rosaceae Juss.		
Iz_7_1109	DJI_0349	Salix alba L.	0	Salicaceae Mirb.	n	1

Iz_7_1109	DJI_0349	Sambucus nigra L.	0	Viburnaceae Raf.	n	1
Iz_7_1109	DJI_0349	Sorbus aucuparia L.	0	Rosaceae Juss.	n	1
Iz_7_1109	DJI_0349	Tilia cordata Mill.	0	Malvaceae Juss.	n	1
Iz_7_1109	DJI_0349	Ulmus glabra Huds.	0	Ulmaceae Mirb.	n	1
Iz_7_1109	DJI_0345	Acer tataricum L.	0	Sapindaceae Juss.	n	1
Iz_7_1109	DJI_0345	Betula pendula Roth.	0	Betulaceae Gray	n	1
Iz_7_1109	DJI_0345	Crataegus rhipidophylla Gand.	0	Rosaceae Juss.	n	1
Iz_7_1109	DJI_0345	Frangula alnus Mill.	0	Rhamnaceae Juss.	n	1
Iz_7_1109	DJI_0345	Fraxinus pennsylvanica Marshall	0	Oleaceae Hoffmanns. & Link	a	0
Iz_7_1109	DJI_0345	Pinus sylvestris L.	0	Pinaceae Spreng. ex F.Rudolphi	n	1
Iz_7_1109	DJI_0345	Populus × canescens (Aiton) Sm.	0	Salicaceae Mirb.	n	1
Iz_7_1109	DJI_0345	Populus tremula L.	0	Salicaceae Mirb.	n	1
Iz_7_1109	DJI_0345	Populus nigra L.	0	Salicaceae Mirb.	n	1
Iz_7_1109	DJI_0345	Pyrus communis L.	0	Rosaceae Juss.	a	0
Iz_7_1109	DJI_0345	Quercus robur L.	0	Fagaceae Dumort.	n	1
Iz_7_1109	DJI_0345	Robinia pseudoacacia L.	0	Fabaceae Lindl.	a	0
Iz_7_1109	DJI_0345	Rosa sp.	0	Rosaceae Juss.		
Iz_7_1109	DJI_0345	Salix alba L.	0	Salicaceae Mirb.	n	1
Iz_7_1109	DJI_0345	Sambucus nigra L.	0	Viburnaceae Raf.	n	1
Iz_7_1109	DJI_0345	Sorbus aucuparia L.	0	Rosaceae Juss.	n	1
Iz_7_1109	DJI_0345	Tilia cordata Mill.	0	Malvaceae Juss.	n	1
Iz_7_1109	DJI_0345	Ulmus glabra Huds.	0	Ulmaceae Mirb.	n	1
Iz_7_1109	DJI_0341	Acer tataricum L.	0	Sapindaceae Juss.	n	1
Iz_7_1109	DJI_0341	Betula pendula Roth.	0	Betulaceae Gray	n	1
Iz_7_1109	DJI_0341	Crataegus rhipidophylla Gand.	0	Rosaceae Juss.	n	1
Iz_7_1109	DJI_0341	Frangula alnus Mill.	0	Rhamnaceae Juss.	n	1
Iz_7_1109	DJI_0341	Fraxinus pennsylvanica Marshall	0	Oleaceae Hoffmanns. & Link	a	0
Iz_7_1109	DJI_0341	Pinus sylvestris L.	0	Pinaceae Spreng. ex F.Rudolphi	n	1
Iz_7_1109	DJI_0341	Populus × canescens (Aiton) Sm.	0	Salicaceae Mirb.	n	1
Iz_7_1109	DJI_0341	Populus tremula L.	0	Salicaceae Mirb.	n	1
Iz_7_1109	DJI_0341	Populus nigra L.	0	Salicaceae Mirb.	n	1
Iz_7_1109	DJI_0341	Pyrus communis L.	0	Rosaceae Juss.	a	0
Iz_7_1109	DJI_0341	Quercus robur L.	0	Fagaceae Dumort.	n	1
Iz_7_1109	DJI_0341	Robinia pseudoacacia L.	0	Fabaceae Lindl.	a	0
Iz_7_1109	DJI_0341	Rosa sp.	0	Rosaceae Juss.		
Iz_7_1109	DJI_0341	Salix alba L.	0	Salicaceae Mirb.	n	1
Iz_7_1109	DJI_0341	Sambucus nigra L.	0	Viburnaceae Raf.	n	1
Iz_7_1109	DJI_0341	Sorbus aucuparia L.	0	Rosaceae Juss.	n	1
Iz_7_1109	DJI_0341	Tilia cordata Mill.	0	Malvaceae Juss.	n	1
Iz_7_1109	DJI_0341	Ulmus glabra Huds.	0	Ulmaceae Mirb.	n	1
Iz_7_1109	DJI_0359.1	Acer tataricum L.	0	Sapindaceae Juss.	n	1
Iz_7_1109	DJI_0359.1	Betula pendula Roth.	0	Betulaceae Gray	n	1
Iz_7_1109	DJI_0359.1	Crataegus rhipidophylla Gand.	0	Rosaceae Juss.	n	1
Iz_7_1109	DJI_0359.1	Frangula alnus Mill.	0	Rhamnaceae Juss.	n	1
Iz_7_1109	DJI_0359.1	Fraxinus pennsylvanica Marshall	0	Oleaceae Hoffmanns. & Link	a	0
Iz_7_1109	DJI_0359.1	Pinus sylvestris L.	0	Pinaceae Spreng. ex F.Rudolphi	n	1
Iz_7_1109	DJI_0359.1	Populus × canescens (Aiton) Sm.	0	Salicaceae Mirb.	n	1
Iz_7_1109	DJI_0359.1	Populus tremula L.	0	Salicaceae Mirb.	n	1
Iz_7_1109	DJI_0359.1	Populus nigra L.	0	Salicaceae Mirb.	n	1
Iz_7_1109	DJI_0359.1	Pyrus communis L.	0	Rosaceae Juss.	a	0
Iz_7_1109	DJI_0359.1	Quercus robur L.	0	Fagaceae Dumort.	n	1
Iz_7_1109	DJI_0359.1	Robinia pseudoacacia L.	0	Fabaceae Lindl.	a	0
Iz_7_1109	DJI_0359.1	Rosa sp.	0	Rosaceae Juss.		
Iz_7_1109	DJI_0359.1	Salix alba L.	0	Salicaceae Mirb.	n	1
Iz_7_1109	DJI_0359.1	Sambucus nigra L.	0	Viburnaceae Raf.	n	1
Iz_7_1109	DJI_0359.1	Sorbus aucuparia L.	0	Rosaceae Juss.	n	1
Iz_7_1109	DJI_0359.1	Tilia cordata Mill.	0	Malvaceae Juss.	n	1
Iz_7_1109	DJI_0359.1	Ulmus glabra Huds.	0	Ulmaceae Mirb.	n	1
Iz_7_1109	DJI_0334	Acer tataricum L.	0	Sapindaceae Juss.	n	1
Iz_7_1109	DJI_0334	Betula pendula Roth.	0	Betulaceae Gray	n	1
Iz_7_1109	DJI_0334	Crataegus rhipidophylla Gand.	0	Rosaceae Juss.	n	1
Iz_7_1109	DJI_0334	Frangula alnus Mill.	0	Rhamnaceae Juss.	n	1
Iz_7_1109	DJI_0334	Fraxinus pennsylvanica Marshall	0	Oleaceae Hoffmanns. & Link	a	0
Iz_7_1109	DJI_0334	Pinus sylvestris L.	0	Pinaceae Spreng. ex F.Rudolphi	n	1

Iz_7_1109	DJI_0334	Populus × canescens (Aiton) Sm.	0	Salicaceae Mirb.	n	1
Iz_7_1109	DJI_0334	Populus tremula L.	0	Salicaceae Mirb.	n	1
Iz_7_1109	DJI_0334	Populus nigra L.	0	Salicaceae Mirb.	n	1
Iz_7_1109	DJI_0334	Pyrus communis L.	0	Rosaceae Juss.	a	0
Iz_7_1109	DJI_0334	Quercus robur L.	0	Fagaceae Dumort.	n	1
Iz_7_1109	DJI_0334	Robinia pseudoacacia L.	0	Fabaceae Lindl.	a	0
Iz_7_1109	DJI_0334	Rosa sp.	0	Rosaceae Juss.		
Iz_7_1109	DJI_0334	Salix alba L.	0	Salicaceae Mirb.	n	1
Iz_7_1109	DJI_0334	Sambucus nigra L.	0	Viburnaceae Raf.	n	1
Iz_7_1109	DJI_0334	Sorbus aucuparia L.	0	Rosaceae Juss.	n	1
Iz_7_1109	DJI_0334	Tilia cordata Mill.	0	Malvaceae Juss.	n	1
Iz_7_1109	DJI_0334	Ulmus glabra Huds.	0	Ulmaceae Mirb.	n	1
Iz_7_1109	DJI_0331	Acer tataricum L.	0	Sapindaceae Juss.	n	1
Iz_7_1109	DJI_0331	Betula pendula Roth.	0	Betulaceae Gray	n	1
Iz_7_1109	DJI_0331	Crataegus rhipidophylla Gand.	0	Rosaceae Juss.	n	1
Iz_7_1109	DJI_0331	Frangula alnus Mill.	0	Rhamnaceae Juss.	n	1
Iz_7_1109	DJI_0331	Fraxinus pennsylvanica Marshall	0	Oleaceae Hoffmanns. & Link	a	0
Iz_7_1109	DJI_0331	Pinus sylvestris L.	0	Pinaceae Spreng. ex F.Rudolphi	n	1
Iz_7_1109	DJI_0331	Populus × canescens (Aiton) Sm.	0	Salicaceae Mirb.	n	1
Iz_7_1109	DJI_0331	Populus tremula L.	0	Salicaceae Mirb.	n	1
Iz_7_1109	DJI_0331	Populus nigra L.	0	Salicaceae Mirb.	n	1
Iz_7_1109	DJI_0331	Pyrus communis L.	0	Rosaceae Juss.	a	0
Iz_7_1109	DJI_0331	Quercus robur L.	0	Fagaceae Dumort.	n	1
Iz_7_1109	DJI_0331	Robinia pseudoacacia L.	0	Fabaceae Lindl.	a	0
Iz_7_1109	DJI_0331	Rosa sp.	0	Rosaceae Juss.		
Iz_7_1109	DJI_0331	Salix alba L.	0	Salicaceae Mirb.	n	1
Iz_7_1109	DJI_0331	Sambucus nigra L.	0	Viburnaceae Raf.	n	1
Iz_7_1109	DJI_0331	Sorbus aucuparia L.	0	Rosaceae Juss.	n	1
Iz_7_1109	DJI_0331	Tilia cordata Mill.	0	Malvaceae Juss.	n	1
Iz_7_1109	DJI_0331	Ulmus glabra Huds.	0	Ulmaceae Mirb.	n	1
Iz_7_1109	DJI_0328	Acer tataricum L.	0	Sapindaceae Juss.	n	1
Iz_7_1109	DJI_0328	Betula pendula Roth.	0	Betulaceae Gray	n	1
Iz_7_1109	DJI_0328	Crataegus rhipidophylla Gand.	0	Rosaceae Juss.	n	1
Iz_7_1109	DJI_0328	Frangula alnus Mill.	0	Rhamnaceae Juss.	n	1
Iz_7_1109	DJI_0328	Fraxinus pennsylvanica Marshall	0	Oleaceae Hoffmanns. & Link	a	0
Iz_7_1109	DJI_0328	Pinus sylvestris L.	0	Pinaceae Spreng. ex F.Rudolphi	n	1
Iz_7_1109	DJI_0328	Populus × canescens (Aiton) Sm.	0	Salicaceae Mirb.	n	1
Iz_7_1109	DJI_0328	Populus tremula L.	1	Salicaceae Mirb.	n	1
Iz_7_1109	DJI_0328	Populus nigra L.	0	Salicaceae Mirb.	n	1
Iz_7_1109	DJI_0328	Pyrus communis L.	0	Rosaceae Juss.	a	0
Iz_7_1109	DJI_0328	Quercus robur L.	0	Fagaceae Dumort.	n	1
Iz_7_1109	DJI_0328	Robinia pseudoacacia L.	0	Fabaceae Lindl.	a	0
Iz_7_1109	DJI_0328	Rosa sp.	0	Rosaceae Juss.		
Iz_7_1109	DJI_0328	Salix alba L.	0	Salicaceae Mirb.	n	1
Iz_7_1109	DJI_0328	Sambucus nigra L.	0	Viburnaceae Raf.	n	1
Iz_7_1109	DJI_0328	Sorbus aucuparia L.	0	Rosaceae Juss.	n	1
Iz_7_1109	DJI_0328	Tilia cordata Mill.	0	Malvaceae Juss.	n	1
Iz_7_1109	DJI_0328	Ulmus glabra Huds.	0	Ulmaceae Mirb.	n	1
Iz_7_1109	DJI_0325	Acer tataricum L.	0	Sapindaceae Juss.	n	1
Iz_7_1109	DJI_0325	Betula pendula Roth.	0	Betulaceae Gray	n	1
Iz_7_1109	DJI_0325	Crataegus rhipidophylla Gand.	0	Rosaceae Juss.	n	1
Iz_7_1109	DJI_0325	Frangula alnus Mill.	0	Rhamnaceae Juss.	n	1
Iz_7_1109	DJI_0325	Fraxinus pennsylvanica Marshall	0	Oleaceae Hoffmanns. & Link	a	0
Iz_7_1109	DJI_0325	Pinus sylvestris L.	0	Pinaceae Spreng. ex F.Rudolphi	n	1
Iz_7_1109	DJI_0325	Populus × canescens (Aiton) Sm.	0	Salicaceae Mirb.	n	1
Iz_7_1109	DJI_0325	Populus tremula L.	0	Salicaceae Mirb.	n	1
Iz_7_1109	DJI_0325	Populus nigra L.	0	Salicaceae Mirb.	n	1
Iz_7_1109	DJI_0325	Pyrus communis L.	0	Rosaceae Juss.	a	0
Iz_7_1109	DJI_0325	Quercus robur L.	0	Fagaceae Dumort.	n	1
Iz_7_1109	DJI_0325	Robinia pseudoacacia L.	0	Fabaceae Lindl.	a	0
Iz_7_1109	DJI_0325	Rosa sp.	0	Rosaceae Juss.		
Iz_7_1109	DJI_0325	Salix alba L.	0	Salicaceae Mirb.	n	1
Iz_7_1109	DJI_0325	Sambucus nigra L.	0	Viburnaceae Raf.	n	1
Iz_7_1109	DJI_0325	Sorbus aucuparia L.	0	Rosaceae Juss.	n	1

Iz_7_1109	DJI_0325	Tilia cordata Mill.	0	Malvaceae Juss.	n	1
Iz_7_1109	DJI_0325	Ulmus glabra Huds.	0	Ulmaceae Mirb.	n	1
Iz_7_1109	DJI_0321	Acer tataricum L.	0	Sapindaceae Juss.	n	1
Iz_7_1109	DJI_0321	Betula pendula Roth.	0	Betulaceae Gray	n	1
Iz_7_1109	DJI_0321	Crataegus rhipidophylla Gand.	0	Rosaceae Juss.	n	1
Iz_7_1109	DJI_0321	Frangula alnus Mill.	0	Rhamnaceae Juss.	n	1
Iz_7_1109	DJI_0321	Fraxinus pennsylvanica Marshall	0	Oleaceae Hoffmanns. & Link	a	0
Iz_7_1109	DJI_0321	Pinus sylvestris L.	0	Pinaceae Spreng. ex F.Rudolphi	n	1
Iz_7_1109	DJI_0321	Populus × canescens (Aiton) Sm.	0	Salicaceae Mirb.	n	1
Iz_7_1109	DJI_0321	Populus tremula L.	0	Salicaceae Mirb.	n	1
Iz_7_1109	DJI_0321	Populus nigra L.	0	Salicaceae Mirb.	n	1
Iz_7_1109	DJI_0321	Pyrus communis L.	0	Rosaceae Juss.	a	0
Iz_7_1109	DJI_0321	Quercus robur L.	0	Fagaceae Dumort.	n	1
Iz_7_1109	DJI_0321	Robinia pseudoacacia L.	0	Fabaceae Lindl.	a	0
Iz_7_1109	DJI_0321	Rosa sp.	0	Rosaceae Juss.		
Iz_7_1109	DJI_0321	Salix alba L.	0	Salicaceae Mirb.	n	1
Iz_7_1109	DJI_0321	Sambucus nigra L.	0	Viburnaceae Raf.	n	1
Iz_7_1109	DJI_0321	Sorbus aucuparia L.	0	Rosaceae Juss.	n	1
Iz_7_1109	DJI_0321	Tilia cordata Mill.	0	Malvaceae Juss.	n	1
Iz_7_1109	DJI_0321	Ulmus glabra Huds.	0	Ulmaceae Mirb.	n	1
Iz_7_1109	DJI_0315	Acer tataricum L.	0	Sapindaceae Juss.	n	1
Iz_7_1109	DJI_0315	Betula pendula Roth.	0	Betulaceae Gray	n	1
Iz_7_1109	DJI_0315	Crataegus rhipidophylla Gand.	0	Rosaceae Juss.	n	1
Iz_7_1109	DJI_0315	Frangula alnus Mill.	0	Rhamnaceae Juss.	n	1
Iz_7_1109	DJI_0315	Fraxinus pennsylvanica Marshall	0	Oleaceae Hoffmanns. & Link	a	0
Iz_7_1109	DJI_0315	Pinus sylvestris L.	0	Pinaceae Spreng. ex F.Rudolphi	n	1
Iz_7_1109	DJI_0315	Populus × canescens (Aiton) Sm.	0	Salicaceae Mirb.	n	1
Iz_7_1109	DJI_0315	Populus tremula L.	0	Salicaceae Mirb.	n	1
Iz_7_1109	DJI_0315	Populus nigra L.	0	Salicaceae Mirb.	n	1
Iz_7_1109	DJI_0315	Pyrus communis L.	0	Rosaceae Juss.	a	0
Iz_7_1109	DJI_0315	Quercus robur L.	0	Fagaceae Dumort.	n	1
Iz_7_1109	DJI_0315	Robinia pseudoacacia L.	0	Fabaceae Lindl.	a	0
Iz_7_1109	DJI_0315	Rosa sp.	0	Rosaceae Juss.		
Iz_7_1109	DJI_0315	Salix alba L.	0	Salicaceae Mirb.	n	1
Iz_7_1109	DJI_0315	Sambucus nigra L.	0	Viburnaceae Raf.	n	1
Iz_7_1109	DJI_0315	Sorbus aucuparia L.	0	Rosaceae Juss.	n	1
Iz_7_1109	DJI_0315	Tilia cordata Mill.	0	Malvaceae Juss.	n	1
Iz_7_1109	DJI_0315	Ulmus glabra Huds.	0	Ulmaceae Mirb.	n	1
Iz_1_1209	DJI_0035	Acer tataricum L.	0	Sapindaceae Juss.	n	1
Iz_1_1209	DJI_0035	Betula pendula Roth.	0	Betulaceae Gray	n	1
Iz_1_1209	DJI_0035	Crataegus rhipidophylla Gand.	0	Rosaceae Juss.	n	1
Iz_1_1209	DJI_0035	Frangula alnus Mill.	0	Rhamnaceae Juss.	n	1
Iz_1_1209	DJI_0035	Fraxinus pennsylvanica Marshall	0	Oleaceae Hoffmanns. & Link	a	0
Iz_1_1209	DJI_0035	Pinus sylvestris L.	0	Pinaceae Spreng. ex F.Rudolphi	n	1
Iz_1_1209	DJI_0035	Populus × canescens (Aiton) Sm.	0	Salicaceae Mirb.	n	1
Iz_1_1209	DJI_0035	Populus tremula L.	0	Salicaceae Mirb.	n	1
Iz_1_1209	DJI_0035	Populus nigra L.	0	Salicaceae Mirb.	n	1
Iz_1_1209	DJI_0035	Pyrus communis L.	0	Rosaceae Juss.	a	0
Iz_1_1209	DJI_0035	Quercus robur L.	0	Fagaceae Dumort.	n	1
Iz_1_1209	DJI_0035	Robinia pseudoacacia L.	0	Fabaceae Lindl.	a	0
Iz_1_1209	DJI_0035	Rosa sp.	0	Rosaceae Juss.		
Iz_1_1209	DJI_0035	Salix alba L.	0	Salicaceae Mirb.	n	1
Iz_1_1209	DJI_0035	Sambucus nigra L.	0	Viburnaceae Raf.	n	1
Iz_1_1209	DJI_0035	Sorbus aucuparia L.	0	Rosaceae Juss.	n	1
Iz_1_1209	DJI_0035	Tilia cordata Mill.	0	Malvaceae Juss.	n	1
Iz_1_1209	DJI_0035	Ulmus glabra Huds.	0	Ulmaceae Mirb.	n	1
Iz_1_1209	DJI_0032	Acer tataricum L.	0	Sapindaceae Juss.	n	1
Iz_1_1209	DJI_0032	Betula pendula Roth.	0	Betulaceae Gray	n	1
Iz_1_1209	DJI_0032	Crataegus rhipidophylla Gand.	0	Rosaceae Juss.	n	1
Iz_1_1209	DJI_0032	Frangula alnus Mill.	0	Rhamnaceae Juss.	n	1
Iz_1_1209	DJI_0032	Fraxinus pennsylvanica Marshall	0	Oleaceae Hoffmanns. & Link	a	0
Iz_1_1209	DJI_0032	Pinus sylvestris L.	0	Pinaceae Spreng. ex F.Rudolphi	n	1
Iz_1_1209	DJI_0032	Populus × canescens (Aiton) Sm.	0	Salicaceae Mirb.	n	1
Iz_1_1209	DJI_0032	Populus tremula L.	0	Salicaceae Mirb.	n	1

Iz_1_1209	DJI_0032	Populus nigra L.	0	Salicaceae Mirb.	n	1
Iz_1_1209	DJI_0032	Pyrus communis L.	0	Rosaceae Juss.	a	0
Iz_1_1209	DJI_0032	Quercus robur L.	0	Fagaceae Dumort.	n	1
Iz_1_1209	DJI_0032	Robinia pseudoacacia L.	0	Fabaceae Lindl.	a	0
Iz_1_1209	DJI_0032	Rosa sp.	0	Rosaceae Juss.		
Iz_1_1209	DJI_0032	Salix alba L.	0	Salicaceae Mirb.	n	1
Iz_1_1209	DJI_0032	Sambucus nigra L.	0	Viburnaceae Raf.	n	1
Iz_1_1209	DJI_0032	Sorbus aucuparia L.	0	Rosaceae Juss.	n	1
Iz_1_1209	DJI_0032	Tilia cordata Mill.	0	Malvaceae Juss.	n	1
Iz_1_1209	DJI_0032	Ulmus glabra Huds.	0	Ulmaceae Mirb.	n	1
Iz_1_1209	DJI_0029	Acer tataricum L.	0	Sapindaceae Juss.	n	1
Iz_1_1209	DJI_0029	Betula pendula Roth.	0	Betulaceae Gray	n	1
Iz_1_1209	DJI_0029	Crataegus rhipidophylla Gand.	0	Rosaceae Juss.	n	1
Iz_1_1209	DJI_0029	Frangula alnus Mill.	0	Rhamnaceae Juss.	n	1
Iz_1_1209	DJI_0029	Fraxinus pennsylvanica Marshall	0	Oleaceae Hoffmanns. & Link	a	0
Iz_1_1209	DJI_0029	Pinus sylvestris L.	0	Pinaceae Spreng. ex F.Rudolphi	n	1
Iz_1_1209	DJI_0029	Populus × canescens (Aiton) Sm.	0	Salicaceae Mirb.	n	1
Iz_1_1209	DJI_0029	Populus tremula L.	0	Salicaceae Mirb.	n	1
Iz_1_1209	DJI_0029	Populus nigra L.	0	Salicaceae Mirb.	n	1
Iz_1_1209	DJI_0029	Pyrus communis L.	0	Rosaceae Juss.	a	0
Iz_1_1209	DJI_0029	Quercus robur L.	0	Fagaceae Dumort.	n	1
Iz_1_1209	DJI_0029	Robinia pseudoacacia L.	0	Fabaceae Lindl.	a	0
Iz_1_1209	DJI_0029	Rosa sp.	0	Rosaceae Juss.		
Iz_1_1209	DJI_0029	Salix alba L.	0	Salicaceae Mirb.	n	1
Iz_1_1209	DJI_0029	Sambucus nigra L.	0	Viburnaceae Raf.	n	1
Iz_1_1209	DJI_0029	Sorbus aucuparia L.	0	Rosaceae Juss.	n	1
Iz_1_1209	DJI_0029	Tilia cordata Mill.	0	Malvaceae Juss.	n	1
Iz_1_1209	DJI_0029	Ulmus glabra Huds.	0	Ulmaceae Mirb.	n	1
Iz_1_1209	DJI_0026	Acer tataricum L.	0	Sapindaceae Juss.	n	1
Iz_1_1209	DJI_0026	Betula pendula Roth.	0	Betulaceae Gray	n	1
Iz_1_1209	DJI_0026	Crataegus rhipidophylla Gand.	0	Rosaceae Juss.	n	1
Iz_1_1209	DJI_0026	Frangula alnus Mill.	0	Rhamnaceae Juss.	n	1
Iz_1_1209	DJI_0026	Fraxinus pennsylvanica Marshall	0	Oleaceae Hoffmanns. & Link	a	0
Iz_1_1209	DJI_0026	Pinus sylvestris L.	0	Pinaceae Spreng. ex F.Rudolphi	n	1
Iz_1_1209	DJI_0026	Populus × canescens (Aiton) Sm.	0	Salicaceae Mirb.	n	1
Iz_1_1209	DJI_0026	Populus tremula L.	0	Salicaceae Mirb.	n	1
Iz_1_1209	DJI_0026	Populus nigra L.	0	Salicaceae Mirb.	n	1
Iz_1_1209	DJI_0026	Pyrus communis L.	0	Rosaceae Juss.	a	0
Iz_1_1209	DJI_0026	Quercus robur L.	0	Fagaceae Dumort.	n	1
Iz_1_1209	DJI_0026	Robinia pseudoacacia L.	0	Fabaceae Lindl.	a	0
Iz_1_1209	DJI_0026	Rosa sp.	0	Rosaceae Juss.		
Iz_1_1209	DJI_0026	Salix alba L.	0	Salicaceae Mirb.	n	1
Iz_1_1209	DJI_0026	Sambucus nigra L.	0	Viburnaceae Raf.	n	1
Iz_1_1209	DJI_0026	Sorbus aucuparia L.	0	Rosaceae Juss.	n	1
Iz_1_1209	DJI_0026	Tilia cordata Mill.	0	Malvaceae Juss.	n	1
Iz_1_1209	DJI_0026	Ulmus glabra Huds.	0	Ulmaceae Mirb.	n	1
Iz_1_1209	DJI_0023	Acer tataricum L.	0	Sapindaceae Juss.	n	1
Iz_1_1209	DJI_0023	Betula pendula Roth.	0	Betulaceae Gray	n	1
Iz_1_1209	DJI_0023	Crataegus rhipidophylla Gand.	0	Rosaceae Juss.	n	1
Iz_1_1209	DJI_0023	Frangula alnus Mill.	0	Rhamnaceae Juss.	n	1
Iz_1_1209	DJI_0023	Fraxinus pennsylvanica Marshall	0	Oleaceae Hoffmanns. & Link	a	0
Iz_1_1209	DJI_0023	Pinus sylvestris L.	0	Pinaceae Spreng. ex F.Rudolphi	n	1
Iz_1_1209	DJI_0023	Populus × canescens (Aiton) Sm.	0	Salicaceae Mirb.	n	1
Iz_1_1209	DJI_0023	Populus tremula L.	0	Salicaceae Mirb.	n	1
Iz_1_1209	DJI_0023	Populus nigra L.	0	Salicaceae Mirb.	n	1
Iz_1_1209	DJI_0023	Pyrus communis L.	0	Rosaceae Juss.	a	0
Iz_1_1209	DJI_0023	Quercus robur L.	0	Fagaceae Dumort.	n	1
Iz_1_1209	DJI_0023	Robinia pseudoacacia L.	0	Fabaceae Lindl.	a	0
Iz_1_1209	DJI_0023	Rosa sp.	0	Rosaceae Juss.		
Iz_1_1209	DJI_0023	Salix alba L.	0	Salicaceae Mirb.	n	1
Iz_1_1209	DJI_0023	Sambucus nigra L.	0	Viburnaceae Raf.	n	1
Iz_1_1209	DJI_0023	Sorbus aucuparia L.	0	Rosaceae Juss.	n	1
Iz_1_1209	DJI_0023	Tilia cordata Mill.	0	Malvaceae Juss.	n	1
Iz_1_1209	DJI_0023	Ulmus glabra Huds.	0	Ulmaceae Mirb.	n	1

Iz_1_1209	DJI_0020	Acer tataricum L.	0	Sapindaceae Juss.	n	1
Iz_1_1209	DJI_0020	Betula pendula Roth.	0	Betulaceae Gray	n	1
Iz_1_1209	DJI_0020	Crataegus rhipidophylla Gand.	0	Rosaceae Juss.	n	1
Iz_1_1209	DJI_0020	Frangula alnus Mill.	0	Rhamnaceae Juss.	n	1
Iz_1_1209	DJI_0020	Fraxinus pennsylvanica Marshall	0	Oleaceae Hoffmanns. & Link	a	0
Iz_1_1209	DJI_0020	Pinus sylvestris L.	0	Pinaceae Spreng. ex F.Rudolphi	n	1
Iz_1_1209	DJI_0020	Populus × canescens (Aiton) Sm.	0	Salicaceae Mirb.	n	1
Iz_1_1209	DJI_0020	Populus tremula L.	0	Salicaceae Mirb.	n	1
Iz_1_1209	DJI_0020	Populus nigra L.	0	Salicaceae Mirb.	n	1
Iz_1_1209	DJI_0020	Pyrus communis L.	0	Rosaceae Juss.	a	0
Iz_1_1209	DJI_0020	Quercus robur L.	0	Fagaceae Dumort.	n	1
Iz_1_1209	DJI_0020	Robinia pseudoacacia L.	0	Fabaceae Lindl.	a	0
Iz_1_1209	DJI_0020	Rosa sp.	0	Rosaceae Juss.		
Iz_1_1209	DJI_0020	Salix alba L.	0	Salicaceae Mirb.	n	1
Iz_1_1209	DJI_0020	Sambucus nigra L.	0	Viburnaceae Raf.	n	1
Iz_1_1209	DJI_0020	Sorbus aucuparia L.	0	Rosaceae Juss.	n	1
Iz_1_1209	DJI_0020	Tilia cordata Mill.	0	Malvaceae Juss.	n	1
Iz_1_1209	DJI_0020	Ulmus glabra Huds.	0	Ulmaceae Mirb.	n	1
Iz_1_1209	DJI_0016	Acer tataricum L.	0	Sapindaceae Juss.	n	1
Iz_1_1209	DJI_0016	Betula pendula Roth.	0	Betulaceae Gray	n	1
Iz_1_1209	DJI_0016	Crataegus rhipidophylla Gand.	0	Rosaceae Juss.	n	1
Iz_1_1209	DJI_0016	Frangula alnus Mill.	0	Rhamnaceae Juss.	n	1
Iz_1_1209	DJI_0016	Fraxinus pennsylvanica Marshall	0	Oleaceae Hoffmanns. & Link	a	0
Iz_1_1209	DJI_0016	Pinus sylvestris L.	0	Pinaceae Spreng. ex F.Rudolphi	n	1
Iz_1_1209	DJI_0016	Populus × canescens (Aiton) Sm.	0	Salicaceae Mirb.	n	1
Iz_1_1209	DJI_0016	Populus tremula L.	0	Salicaceae Mirb.	n	1
Iz_1_1209	DJI_0016	Populus nigra L.	0	Salicaceae Mirb.	n	1
Iz_1_1209	DJI_0016	Pyrus communis L.	0	Rosaceae Juss.	a	0
Iz_1_1209	DJI_0016	Quercus robur L.	0	Fagaceae Dumort.	n	1
Iz_1_1209	DJI_0016	Robinia pseudoacacia L.	0	Fabaceae Lindl.	a	0
Iz_1_1209	DJI_0016	Rosa sp.	0	Rosaceae Juss.		
Iz_1_1209	DJI_0016	Salix alba L.	0	Salicaceae Mirb.	n	1
Iz_1_1209	DJI_0016	Sambucus nigra L.	0	Viburnaceae Raf.	n	1
Iz_1_1209	DJI_0016	Sorbus aucuparia L.	0	Rosaceae Juss.	n	1
Iz_1_1209	DJI_0016	Tilia cordata Mill.	0	Malvaceae Juss.	n	1
Iz_1_1209	DJI_0016	Ulmus glabra Huds.	0	Ulmaceae Mirb.	n	1
Iz_1_1209	DJI_0013	Acer tataricum L.	0	Sapindaceae Juss.	n	1
Iz_1_1209	DJI_0013	Betula pendula Roth.	0	Betulaceae Gray	n	1
Iz_1_1209	DJI_0013	Crataegus rhipidophylla Gand.	0	Rosaceae Juss.	n	1
Iz_1_1209	DJI_0013	Frangula alnus Mill.	0	Rhamnaceae Juss.	n	1
Iz_1_1209	DJI_0013	Fraxinus pennsylvanica Marshall	0	Oleaceae Hoffmanns. & Link	a	0
Iz_1_1209	DJI_0013	Pinus sylvestris L.	0	Pinaceae Spreng. ex F.Rudolphi	n	1
Iz_1_1209	DJI_0013	Populus × canescens (Aiton) Sm.	0	Salicaceae Mirb.	n	1
Iz_1_1209	DJI_0013	Populus tremula L.	0	Salicaceae Mirb.	n	1
Iz_1_1209	DJI_0013	Populus nigra L.	0	Salicaceae Mirb.	n	1
Iz_1_1209	DJI_0013	Pyrus communis L.	0	Rosaceae Juss.	a	0
Iz_1_1209	DJI_0013	Quercus robur L.	0	Fagaceae Dumort.	n	1
Iz_1_1209	DJI_0013	Robinia pseudoacacia L.	0	Fabaceae Lindl.	a	0
Iz_1_1209	DJI_0013	Rosa sp.	0	Rosaceae Juss.		
Iz_1_1209	DJI_0013	Salix alba L.	0	Salicaceae Mirb.	n	1
Iz_1_1209	DJI_0013	Sambucus nigra L.	0	Viburnaceae Raf.	n	1
Iz_1_1209	DJI_0013	Sorbus aucuparia L.	0	Rosaceae Juss.	n	1
Iz_1_1209	DJI_0013	Tilia cordata Mill.	0	Malvaceae Juss.	n	1
Iz_1_1209	DJI_0013	Ulmus glabra Huds.	0	Ulmaceae Mirb.	n	1
Iz_1_1209	DJI_0009	Acer tataricum L.	0	Sapindaceae Juss.	n	1
Iz_1_1209	DJI_0009	Betula pendula Roth.	1	Betulaceae Gray	n	1
Iz_1_1209	DJI_0009	Crataegus rhipidophylla Gand.	0	Rosaceae Juss.	n	1
Iz_1_1209	DJI_0009	Frangula alnus Mill.	0	Rhamnaceae Juss.	n	1
Iz_1_1209	DJI_0009	Fraxinus pennsylvanica Marshall	0	Oleaceae Hoffmanns. & Link	a	0
Iz_1_1209	DJI_0009	Pinus sylvestris L.	0	Pinaceae Spreng. ex F.Rudolphi	n	1
Iz_1_1209	DJI_0009	Populus × canescens (Aiton) Sm.	0	Salicaceae Mirb.	n	1
Iz_1_1209	DJI_0009	Populus tremula L.	0	Salicaceae Mirb.	n	1
Iz_1_1209	DJI_0009	Populus nigra L.	0	Salicaceae Mirb.	n	1
Iz_1_1209	DJI_0009	Pyrus communis L.	0	Rosaceae Juss.	a	0

Iz_1_1209	DJI_0009	Quercus robur L.	0	Fagaceae Dumort.	n	1
Iz_1_1209	DJI_0009	Robinia pseudoacacia L.	0	Fabaceae Lindl.	a	0
Iz_1_1209	DJI_0009	Rosa sp.	0	Rosaceae Juss.		
Iz_1_1209	DJI_0009	Salix alba L.	0	Salicaceae Mirb.	n	1
Iz_1_1209	DJI_0009	Sambucus nigra L.	0	Viburnaceae Raf.	n	1
Iz_1_1209	DJI_0009	Sorbus aucuparia L.	0	Rosaceae Juss.	n	1
Iz_1_1209	DJI_0009	Tilia cordata Mill.	0	Malvaceae Juss.	n	1
Iz_1_1209	DJI_0009	Ulmus glabra Huds.	0	Ulmaceae Mirb.	n	1
Iz_1_1209	DJI_0005	Acer tataricum L.	0	Sapindaceae Juss.	n	1
Iz_1_1209	DJI_0005	Betula pendula Roth.	0	Betulaceae Gray	n	1
Iz_1_1209	DJI_0005	Crataegus rhipidophylla Gand.	0	Rosaceae Juss.	n	1
Iz_1_1209	DJI_0005	Frangula alnus Mill.	0	Rhamnaceae Juss.	n	1
Iz_1_1209	DJI_0005	Fraxinus pennsylvanica Marshall	0	Oleaceae Hoffmanns. & Link	a	0
Iz_1_1209	DJI_0005	Pinus sylvestris L.	0	Pinaceae Spreng. ex F.Rudolphi	n	1
Iz_1_1209	DJI_0005	Populus × canescens (Aiton) Sm.	0	Salicaceae Mirb.	n	1
Iz_1_1209	DJI_0005	Populus tremula L.	0	Salicaceae Mirb.	n	1
Iz_1_1209	DJI_0005	Populus nigra L.	0	Salicaceae Mirb.	n	1
Iz_1_1209	DJI_0005	Pyrus communis L.	0	Rosaceae Juss.	a	0
Iz_1_1209	DJI_0005	Quercus robur L.	0	Fagaceae Dumort.	n	1
Iz_1_1209	DJI_0005	Robinia pseudoacacia L.	0	Fabaceae Lindl.	a	0
Iz_1_1209	DJI_0005	Rosa sp.	0	Rosaceae Juss.		
Iz_1_1209	DJI_0005	Salix alba L.	0	Salicaceae Mirb.	n	1
Iz_1_1209	DJI_0005	Sambucus nigra L.	0	Viburnaceae Raf.	n	1
Iz_1_1209	DJI_0005	Sorbus aucuparia L.	0	Rosaceae Juss.	n	1
Iz_1_1209	DJI_0005	Tilia cordata Mill.	0	Malvaceae Juss.	n	1
Iz_1_1209	DJI_0005	Ulmus glabra Huds.	0	Ulmaceae Mirb.	n	1
Iz_2_1209	DJI_0043	Acer tataricum L.	0	Sapindaceae Juss.	n	1
Iz_2_1209	DJI_0043	Betula pendula Roth.	0	Betulaceae Gray	n	1
Iz_2_1209	DJI_0043	Crataegus rhipidophylla Gand.	0	Rosaceae Juss.	n	1
Iz_2_1209	DJI_0043	Frangula alnus Mill.	0	Rhamnaceae Juss.	n	1
Iz_2_1209	DJI_0043	Fraxinus pennsylvanica Marshall	0	Oleaceae Hoffmanns. & Link	a	0
Iz_2_1209	DJI_0043	Pinus sylvestris L.	0	Pinaceae Spreng. ex F.Rudolphi	n	1
Iz_2_1209	DJI_0043	Populus × canescens (Aiton) Sm.	0	Salicaceae Mirb.	n	1
Iz_2_1209	DJI_0043	Populus tremula L.	0	Salicaceae Mirb.	n	1
Iz_2_1209	DJI_0043	Populus nigra L.	0	Salicaceae Mirb.	n	1
Iz_2_1209	DJI_0043	Pyrus communis L.	0	Rosaceae Juss.	a	0
Iz_2_1209	DJI_0043	Quercus robur L.	0	Fagaceae Dumort.	n	1
Iz_2_1209	DJI_0043	Robinia pseudoacacia L.	0	Fabaceae Lindl.	a	0
Iz_2_1209	DJI_0043	Rosa sp.	0	Rosaceae Juss.		
Iz_2_1209	DJI_0043	Salix alba L.	0	Salicaceae Mirb.	n	1
Iz_2_1209	DJI_0043	Sambucus nigra L.	0	Viburnaceae Raf.	n	1
Iz_2_1209	DJI_0043	Sorbus aucuparia L.	0	Rosaceae Juss.	n	1
Iz_2_1209	DJI_0043	Tilia cordata Mill.	0	Malvaceae Juss.	n	1
Iz_2_1209	DJI_0043	Ulmus glabra Huds.	0	Ulmaceae Mirb.	n	1
Iz_2_1209	DJI_0046	Acer tataricum L.	0	Sapindaceae Juss.	n	1
Iz_2_1209	DJI_0046	Betula pendula Roth.	0	Betulaceae Gray	n	1
Iz_2_1209	DJI_0046	Crataegus rhipidophylla Gand.	0	Rosaceae Juss.	n	1
Iz_2_1209	DJI_0046	Frangula alnus Mill.	0	Rhamnaceae Juss.	n	1
Iz_2_1209	DJI_0046	Fraxinus pennsylvanica Marshall	0	Oleaceae Hoffmanns. & Link	a	0
Iz_2_1209	DJI_0046	Pinus sylvestris L.	0	Pinaceae Spreng. ex F.Rudolphi	n	1
Iz_2_1209	DJI_0046	Populus × canescens (Aiton) Sm.	0	Salicaceae Mirb.	n	1
Iz_2_1209	DJI_0046	Populus tremula L.	0	Salicaceae Mirb.	n	1
Iz_2_1209	DJI_0046	Populus nigra L.	0	Salicaceae Mirb.	n	1
Iz_2_1209	DJI_0046	Pyrus communis L.	0	Rosaceae Juss.	a	0
Iz_2_1209	DJI_0046	Quercus robur L.	0	Fagaceae Dumort.	n	1
Iz_2_1209	DJI_0046	Robinia pseudoacacia L.	0	Fabaceae Lindl.	a	0
Iz_2_1209	DJI_0046	Rosa sp.	0	Rosaceae Juss.		
Iz_2_1209	DJI_0046	Salix alba L.	0	Salicaceae Mirb.	n	1
Iz_2_1209	DJI_0046	Sambucus nigra L.	0	Viburnaceae Raf.	n	1
Iz_2_1209	DJI_0046	Sorbus aucuparia L.	0	Rosaceae Juss.	n	1
Iz_2_1209	DJI_0046	Tilia cordata Mill.	0	Malvaceae Juss.	n	1
Iz_2_1209	DJI_0046	Ulmus glabra Huds.	0	Ulmaceae Mirb.	n	1
Iz_2_1209	DJI_0050	Acer tataricum L.	0	Sapindaceae Juss.	n	1
Iz_2_1209	DJI_0050	Betula pendula Roth.	0	Betulaceae Gray	n	1

Iz_2_1209	DJI_0050	Crataegus rhipidophylla Gand.	0	Rosaceae Juss.	n	1
Iz_2_1209	DJI_0050	Frangula alnus Mill.	0	Rhamnaceae Juss.	n	1
Iz_2_1209	DJI_0050	Fraxinus pennsylvanica Marshall	0	Oleaceae Hoffmanns. & Link	a	0
Iz_2_1209	DJI_0050	Pinus sylvestris L.	0	Pinaceae Spreng. ex F.Rudolphi	n	1
Iz_2_1209	DJI_0050	Populus × canescens (Aiton) Sm.	0	Salicaceae Mirb.	n	1
Iz_2_1209	DJI_0050	Populus tremula L.	0	Salicaceae Mirb.	n	1
Iz_2_1209	DJI_0050	Populus nigra L.	0	Salicaceae Mirb.	n	1
Iz_2_1209	DJI_0050	Pyrus communis L.	0	Rosaceae Juss.	a	0
Iz_2_1209	DJI_0050	Quercus robur L.	0	Fagaceae Dumort.	n	1
Iz_2_1209	DJI_0050	Robinia pseudoacacia L.	0	Fabaceae Lindl.	a	0
Iz_2_1209	DJI_0050	Rosa sp.	0	Rosaceae Juss.		
Iz_2_1209	DJI_0050	Salix alba L.	0	Salicaceae Mirb.	n	1
Iz_2_1209	DJI_0050	Sambucus nigra L.	0	Viburnaceae Raf.	n	1
Iz_2_1209	DJI_0050	Sorbus aucuparia L.	0	Rosaceae Juss.	n	1
Iz_2_1209	DJI_0050	Tilia cordata Mill.	0	Malvaceae Juss.	n	1
Iz_2_1209	DJI_0050	Ulmus glabra Huds.	0	Ulmaceae Mirb.	n	1
Iz_2_1209	DJI_0053	Acer tataricum L.	0	Sapindaceae Juss.	n	1
Iz_2_1209	DJI_0053	Betula pendula Roth.	0	Betulaceae Gray	n	1
Iz_2_1209	DJI_0053	Crataegus rhipidophylla Gand.	0	Rosaceae Juss.	n	1
Iz_2_1209	DJI_0053	Frangula alnus Mill.	0	Rhamnaceae Juss.	n	1
Iz_2_1209	DJI_0053	Fraxinus pennsylvanica Marshall	0	Oleaceae Hoffmanns. & Link	a	0
Iz_2_1209	DJI_0053	Pinus sylvestris L.	0	Pinaceae Spreng. ex F.Rudolphi	n	1
Iz_2_1209	DJI_0053	Populus × canescens (Aiton) Sm.	0	Salicaceae Mirb.	n	1
Iz_2_1209	DJI_0053	Populus tremula L.	0	Salicaceae Mirb.	n	1
Iz_2_1209	DJI_0053	Populus nigra L.	0	Salicaceae Mirb.	n	1
Iz_2_1209	DJI_0053	Pyrus communis L.	0	Rosaceae Juss.	a	0
Iz_2_1209	DJI_0053	Quercus robur L.	0	Fagaceae Dumort.	n	1
Iz_2_1209	DJI_0053	Robinia pseudoacacia L.	0	Fabaceae Lindl.	a	0
Iz_2_1209	DJI_0053	Rosa sp.	0	Rosaceae Juss.		
Iz_2_1209	DJI_0053	Salix alba L.	0	Salicaceae Mirb.	n	1
Iz_2_1209	DJI_0053	Sambucus nigra L.	0	Viburnaceae Raf.	n	1
Iz_2_1209	DJI_0053	Sorbus aucuparia L.	0	Rosaceae Juss.	n	1
Iz_2_1209	DJI_0053	Tilia cordata Mill.	0	Malvaceae Juss.	n	1
Iz_2_1209	DJI_0053	Ulmus glabra Huds.	0	Ulmaceae Mirb.	n	1
Iz_2_1209	DJI_0056	Acer tataricum L.	0	Sapindaceae Juss.	n	1
Iz_2_1209	DJI_0056	Betula pendula Roth.	0	Betulaceae Gray	n	1
Iz_2_1209	DJI_0056	Crataegus rhipidophylla Gand.	0	Rosaceae Juss.	n	1
Iz_2_1209	DJI_0056	Frangula alnus Mill.	0	Rhamnaceae Juss.	n	1
Iz_2_1209	DJI_0056	Fraxinus pennsylvanica Marshall	0	Oleaceae Hoffmanns. & Link	a	0
Iz_2_1209	DJI_0056	Pinus sylvestris L.	0	Pinaceae Spreng. ex F.Rudolphi	n	1
Iz_2_1209	DJI_0056	Populus × canescens (Aiton) Sm.	0	Salicaceae Mirb.	n	1
Iz_2_1209	DJI_0056	Populus tremula L.	0	Salicaceae Mirb.	n	1
Iz_2_1209	DJI_0056	Populus nigra L.	0	Salicaceae Mirb.	n	1
Iz_2_1209	DJI_0056	Pyrus communis L.	0	Rosaceae Juss.	a	0
Iz_2_1209	DJI_0056	Quercus robur L.	0	Fagaceae Dumort.	n	1
Iz_2_1209	DJI_0056	Robinia pseudoacacia L.	0	Fabaceae Lindl.	a	0
Iz_2_1209	DJI_0056	Rosa sp.	0	Rosaceae Juss.		
Iz_2_1209	DJI_0056	Salix alba L.	0	Salicaceae Mirb.	n	1
Iz_2_1209	DJI_0056	Sambucus nigra L.	0	Viburnaceae Raf.	n	1
Iz_2_1209	DJI_0056	Sorbus aucuparia L.	0	Rosaceae Juss.	n	1
Iz_2_1209	DJI_0056	Tilia cordata Mill.	0	Malvaceae Juss.	n	1
Iz_2_1209	DJI_0056	Ulmus glabra Huds.	0	Ulmaceae Mirb.	n	1
Iz_2_1209	DJI_0059	Acer tataricum L.	0	Sapindaceae Juss.	n	1
Iz_2_1209	DJI_0059	Betula pendula Roth.	0	Betulaceae Gray	n	1
Iz_2_1209	DJI_0059	Crataegus rhipidophylla Gand.	0	Rosaceae Juss.	n	1
Iz_2_1209	DJI_0059	Frangula alnus Mill.	0	Rhamnaceae Juss.	n	1
Iz_2_1209	DJI_0059	Fraxinus pennsylvanica Marshall	0	Oleaceae Hoffmanns. & Link	a	0
Iz_2_1209	DJI_0059	Pinus sylvestris L.	0	Pinaceae Spreng. ex F.Rudolphi	n	1
Iz_2_1209	DJI_0059	Populus × canescens (Aiton) Sm.	0	Salicaceae Mirb.	n	1
Iz_2_1209	DJI_0059	Populus tremula L.	0	Salicaceae Mirb.	n	1
Iz_2_1209	DJI_0059	Populus nigra L.	0	Salicaceae Mirb.	n	1
Iz_2_1209	DJI_0059	Pyrus communis L.	0	Rosaceae Juss.	a	0
Iz_2_1209	DJI_0059	Quercus robur L.	0	Fagaceae Dumort.	n	1
Iz_2_1209	DJI_0059	Robinia pseudoacacia L.	0	Fabaceae Lindl.	a	0

Iz_2_1209	DJI_0059	Rosa sp.	0	Rosaceae Juss.		
Iz_2_1209	DJI_0059	Salix alba L.	0	Salicaceae Mirb.	n	1
Iz_2_1209	DJI_0059	Sambucus nigra L.	0	Viburnaceae Raf.	n	1
Iz_2_1209	DJI_0059	Sorbus aucuparia L.	0	Rosaceae Juss.	n	1
Iz_2_1209	DJI_0059	Tilia cordata Mill.	0	Malvaceae Juss.	n	1
Iz_2_1209	DJI_0059	Ulmus glabra Huds.	0	Ulmaceae Mirb.	n	1
Iz_2_1209	DJI_0064	Acer tataricum L.	0	Sapindaceae Juss.	n	1
Iz_2_1209	DJI_0064	Betula pendula Roth.	0	Betulaceae Gray	n	1
Iz_2_1209	DJI_0064	Crataegus rhipidophylla Gand.	0	Rosaceae Juss.	n	1
Iz_2_1209	DJI_0064	Frangula alnus Mill.	0	Rhamnaceae Juss.	n	1
Iz_2_1209	DJI_0064	Fraxinus pennsylvanica Marshall	0	Oleaceae Hoffmanns. & Link	a	0
Iz_2_1209	DJI_0064	Pinus sylvestris L.	0	Pinaceae Spreng. ex F.Rudolphi	n	1
Iz_2_1209	DJI_0064	Populus × canescens (Aiton) Sm.	0	Salicaceae Mirb.	n	1
Iz_2_1209	DJI_0064	Populus tremula L.	0	Salicaceae Mirb.	n	1
Iz_2_1209	DJI_0064	Populus nigra L.	0	Salicaceae Mirb.	n	1
Iz_2_1209	DJI_0064	Pyrus communis L.	0	Rosaceae Juss.	a	0
Iz_2_1209	DJI_0064	Quercus robur L.	0	Fagaceae Dumort.	n	1
Iz_2_1209	DJI_0064	Robinia pseudoacacia L.	0	Fabaceae Lindl.	a	0
Iz_2_1209	DJI_0064	Rosa sp.	0	Rosaceae Juss.		
Iz_2_1209	DJI_0064	Salix alba L.	0	Salicaceae Mirb.	n	1
Iz_2_1209	DJI_0064	Sambucus nigra L.	0	Viburnaceae Raf.	n	1
Iz_2_1209	DJI_0064	Sorbus aucuparia L.	0	Rosaceae Juss.	n	1
Iz_2_1209	DJI_0064	Tilia cordata Mill.	0	Malvaceae Juss.	n	1
Iz_2_1209	DJI_0064	Ulmus glabra Huds.	0	Ulmaceae Mirb.	n	1
Iz_2_1209	DJI_0067	Acer tataricum L.	0	Sapindaceae Juss.	n	1
Iz_2_1209	DJI_0067	Betula pendula Roth.	0	Betulaceae Gray	n	1
Iz_2_1209	DJI_0067	Crataegus rhipidophylla Gand.	0	Rosaceae Juss.	n	1
Iz_2_1209	DJI_0067	Frangula alnus Mill.	0	Rhamnaceae Juss.	n	1
Iz_2_1209	DJI_0067	Fraxinus pennsylvanica Marshall	0	Oleaceae Hoffmanns. & Link	a	0
Iz_2_1209	DJI_0067	Pinus sylvestris L.	0	Pinaceae Spreng. ex F.Rudolphi	n	1
Iz_2_1209	DJI_0067	Populus × canescens (Aiton) Sm.	0	Salicaceae Mirb.	n	1
Iz_2_1209	DJI_0067	Populus tremula L.	0	Salicaceae Mirb.	n	1
Iz_2_1209	DJI_0067	Populus nigra L.	0	Salicaceae Mirb.	n	1
Iz_2_1209	DJI_0067	Pyrus communis L.	0	Rosaceae Juss.	a	0
Iz_2_1209	DJI_0067	Quercus robur L.	0	Fagaceae Dumort.	n	1
Iz_2_1209	DJI_0067	Robinia pseudoacacia L.	0	Fabaceae Lindl.	a	0
Iz_2_1209	DJI_0067	Rosa sp.	0	Rosaceae Juss.		
Iz_2_1209	DJI_0067	Salix alba L.	0	Salicaceae Mirb.	n	1
Iz_2_1209	DJI_0067	Sambucus nigra L.	0	Viburnaceae Raf.	n	1
Iz_2_1209	DJI_0067	Sorbus aucuparia L.	0	Rosaceae Juss.	n	1
Iz_2_1209	DJI_0067	Tilia cordata Mill.	0	Malvaceae Juss.	n	1
Iz_2_1209	DJI_0067	Ulmus glabra Huds.	0	Ulmaceae Mirb.	n	1
Iz_3_1209	DJI_0132	Acer tataricum L.	0	Sapindaceae Juss.	n	1
Iz_3_1209	DJI_0132	Betula pendula Roth.	0	Betulaceae Gray	n	1
Iz_3_1209	DJI_0132	Crataegus rhipidophylla Gand.	0	Rosaceae Juss.	n	1
Iz_3_1209	DJI_0132	Frangula alnus Mill.	0	Rhamnaceae Juss.	n	1
Iz_3_1209	DJI_0132	Fraxinus pennsylvanica Marshall	0	Oleaceae Hoffmanns. & Link	a	0
Iz_3_1209	DJI_0132	Pinus sylvestris L.	0	Pinaceae Spreng. ex F.Rudolphi	n	1
Iz_3_1209	DJI_0132	Populus × canescens (Aiton) Sm.	0	Salicaceae Mirb.	n	1
Iz_3_1209	DJI_0132	Populus tremula L.	0	Salicaceae Mirb.	n	1
Iz_3_1209	DJI_0132	Populus nigra L.	0	Salicaceae Mirb.	n	1
Iz_3_1209	DJI_0132	Pyrus communis L.	0	Rosaceae Juss.	a	0
Iz_3_1209	DJI_0132	Quercus robur L.	0	Fagaceae Dumort.	n	1
Iz_3_1209	DJI_0132	Robinia pseudoacacia L.	0	Fabaceae Lindl.	a	0
Iz_3_1209	DJI_0132	Rosa sp.	0	Rosaceae Juss.		
Iz_3_1209	DJI_0132	Salix alba L.	0	Salicaceae Mirb.	n	1
Iz_3_1209	DJI_0132	Sambucus nigra L.	0	Viburnaceae Raf.	n	1
Iz_3_1209	DJI_0132	Sorbus aucuparia L.	0	Rosaceae Juss.	n	1
Iz_3_1209	DJI_0132	Tilia cordata Mill.	0	Malvaceae Juss.	n	1
Iz_3_1209	DJI_0132	Ulmus glabra Huds.	0	Ulmaceae Mirb.	n	1
Iz_3_1209	DJI_0128	Acer tataricum L.	0	Sapindaceae Juss.	n	1
Iz_3_1209	DJI_0128	Betula pendula Roth.	0	Betulaceae Gray	n	1
Iz_3_1209	DJI_0128	Crataegus rhipidophylla Gand.	0	Rosaceae Juss.	n	1
Iz_3_1209	DJI_0128	Frangula alnus Mill.	0	Rhamnaceae Juss.	n	1

Iz_3_1209	DJI_0128	Fraxinus pennsylvanica Marshall	0	Oleaceae Hoffmanns. & Link	a	0
Iz_3_1209	DJI_0128	Pinus sylvestris L.	0	Pinaceae Spreng. ex F.Rudolphi	n	1
Iz_3_1209	DJI_0128	Populus × canescens (Aiton) Sm.	0	Salicaceae Mirb.	n	1
Iz_3_1209	DJI_0128	Populus tremula L.	0	Salicaceae Mirb.	n	1
Iz_3_1209	DJI_0128	Populus nigra L.	0	Salicaceae Mirb.	n	1
Iz_3_1209	DJI_0128	Pyrus communis L.	0	Rosaceae Juss.	a	0
Iz_3_1209	DJI_0128	Quercus robur L.	1	Fagaceae Dumort.	n	1
Iz_3_1209	DJI_0128	Robinia pseudoacacia L.	0	Fabaceae Lindl.	a	0
Iz_3_1209	DJI_0128	Rosa sp.	0	Rosaceae Juss.		
Iz_3_1209	DJI_0128	Salix alba L.	0	Salicaceae Mirb.	n	1
Iz_3_1209	DJI_0128	Sambucus nigra L.	0	Viburnaceae Raf.	n	1
Iz_3_1209	DJI_0128	Sorbus aucuparia L.	0	Rosaceae Juss.	n	1
Iz_3_1209	DJI_0128	Tilia cordata Mill.	0	Malvaceae Juss.	n	1
Iz_3_1209	DJI_0128	Ulmus glabra Huds.	0	Ulmaceae Mirb.	n	1
Iz_3_1209	DJI_0125	Acer tataricum L.	0	Sapindaceae Juss.	n	1
Iz_3_1209	DJI_0125	Betula pendula Roth.	0	Betulaceae Gray	n	1
Iz_3_1209	DJI_0125	Crataegus rhipidophylla Gand.	0	Rosaceae Juss.	n	1
Iz_3_1209	DJI_0125	Frangula alnus Mill.	0	Rhamnaceae Juss.	n	1
Iz_3_1209	DJI_0125	Fraxinus pennsylvanica Marshall	0	Oleaceae Hoffmanns. & Link	a	0
Iz_3_1209	DJI_0125	Pinus sylvestris L.	0	Pinaceae Spreng. ex F.Rudolphi	n	1
Iz_3_1209	DJI_0125	Populus × canescens (Aiton) Sm.	0	Salicaceae Mirb.	n	1
Iz_3_1209	DJI_0125	Populus tremula L.	0	Salicaceae Mirb.	n	1
Iz_3_1209	DJI_0125	Populus nigra L.	0	Salicaceae Mirb.	n	1
Iz_3_1209	DJI_0125	Pyrus communis L.	0	Rosaceae Juss.	a	0
Iz_3_1209	DJI_0125	Quercus robur L.	0	Fagaceae Dumort.	n	1
Iz_3_1209	DJI_0125	Robinia pseudoacacia L.	0	Fabaceae Lindl.	a	0
Iz_3_1209	DJI_0125	Rosa sp.	0	Rosaceae Juss.		
Iz_3_1209	DJI_0125	Salix alba L.	0	Salicaceae Mirb.	n	1
Iz_3_1209	DJI_0125	Sambucus nigra L.	0	Viburnaceae Raf.	n	1
Iz_3_1209	DJI_0125	Sorbus aucuparia L.	0	Rosaceae Juss.	n	1
Iz_3_1209	DJI_0125	Tilia cordata Mill.	0	Malvaceae Juss.	n	1
Iz_3_1209	DJI_0125	Ulmus glabra Huds.	0	Ulmaceae Mirb.	n	1
Iz_3_1209	DJI_0122	Acer tataricum L.	0	Sapindaceae Juss.	n	1
Iz_3_1209	DJI_0122	Betula pendula Roth.	0	Betulaceae Gray	n	1
Iz_3_1209	DJI_0122	Crataegus rhipidophylla Gand.	0	Rosaceae Juss.	n	1
Iz_3_1209	DJI_0122	Frangula alnus Mill.	0	Rhamnaceae Juss.	n	1
Iz_3_1209	DJI_0122	Fraxinus pennsylvanica Marshall	0	Oleaceae Hoffmanns. & Link	a	0
Iz_3_1209	DJI_0122	Pinus sylvestris L.	0	Pinaceae Spreng. ex F.Rudolphi	n	1
Iz_3_1209	DJI_0122	Populus × canescens (Aiton) Sm.	0	Salicaceae Mirb.	n	1
Iz_3_1209	DJI_0122	Populus tremula L.	0	Salicaceae Mirb.	n	1
Iz_3_1209	DJI_0122	Populus nigra L.	0	Salicaceae Mirb.	n	1
Iz_3_1209	DJI_0122	Pyrus communis L.	0	Rosaceae Juss.	a	0
Iz_3_1209	DJI_0122	Quercus robur L.	0	Fagaceae Dumort.	n	1
Iz_3_1209	DJI_0122	Robinia pseudoacacia L.	0	Fabaceae Lindl.	a	0
Iz_3_1209	DJI_0122	Rosa sp.	0	Rosaceae Juss.		
Iz_3_1209	DJI_0122	Salix alba L.	0	Salicaceae Mirb.	n	1
Iz_3_1209	DJI_0122	Sambucus nigra L.	0	Viburnaceae Raf.	n	1
Iz_3_1209	DJI_0122	Sorbus aucuparia L.	0	Rosaceae Juss.	n	1
Iz_3_1209	DJI_0122	Tilia cordata Mill.	0	Malvaceae Juss.	n	1
Iz_3_1209	DJI_0122	Ulmus glabra Huds.	0	Ulmaceae Mirb.	n	1
Iz_3_1209	DJI_0119	Acer tataricum L.	0	Sapindaceae Juss.	n	1
Iz_3_1209	DJI_0119	Betula pendula Roth.	0	Betulaceae Gray	n	1
Iz_3_1209	DJI_0119	Crataegus rhipidophylla Gand.	0	Rosaceae Juss.	n	1
Iz_3_1209	DJI_0119	Frangula alnus Mill.	0	Rhamnaceae Juss.	n	1
Iz_3_1209	DJI_0119	Fraxinus pennsylvanica Marshall	0	Oleaceae Hoffmanns. & Link	a	0
Iz_3_1209	DJI_0119	Pinus sylvestris L.	0	Pinaceae Spreng. ex F.Rudolphi	n	1
Iz_3_1209	DJI_0119	Populus × canescens (Aiton) Sm.	0	Salicaceae Mirb.	n	1
Iz_3_1209	DJI_0119	Populus tremula L.	0	Salicaceae Mirb.	n	1
Iz_3_1209	DJI_0119	Populus nigra L.	0	Salicaceae Mirb.	n	1
Iz_3_1209	DJI_0119	Pyrus communis L.	0	Rosaceae Juss.	a	0
Iz_3_1209	DJI_0119	Quercus robur L.	0	Fagaceae Dumort.	n	1
Iz_3_1209	DJI_0119	Robinia pseudoacacia L.	0	Fabaceae Lindl.	a	0
Iz_3_1209	DJI_0119	Rosa sp.	0	Rosaceae Juss.		
Iz_3_1209	DJI_0119	Salix alba L.	0	Salicaceae Mirb.	n	1

Iz_3_1209	DJI_0119	Sambucus nigra L.	0	Viburnaceae Raf.	n	1
Iz_3_1209	DJI_0119	Sorbus aucuparia L.	0	Rosaceae Juss.	n	1
Iz_3_1209	DJI_0119	Tilia cordata Mill.	0	Malvaceae Juss.	n	1
Iz_3_1209	DJI_0119	Ulmus glabra Huds.	0	Ulmaceae Mirb.	n	1
Iz_3_1209	DJI_0116	Acer tataricum L.	0	Sapindaceae Juss.	n	1
Iz_3_1209	DJI_0116	Betula pendula Roth.	0	Betulaceae Gray	n	1
Iz_3_1209	DJI_0116	Crataegus rhipidophylla Gand.	0	Rosaceae Juss.	n	1
Iz_3_1209	DJI_0116	Frangula alnus Mill.	0	Rhamnaceae Juss.	n	1
Iz_3_1209	DJI_0116	Fraxinus pennsylvanica Marshall	0	Oleaceae Hoffmanns. & Link	a	0
Iz_3_1209	DJI_0116	Pinus sylvestris L.	0	Pinaceae Spreng. ex F.Rudolphi	n	1
Iz_3_1209	DJI_0116	Populus × canescens (Aiton) Sm.	0	Salicaceae Mirb.	n	1
Iz_3_1209	DJI_0116	Populus tremula L.	0	Salicaceae Mirb.	n	1
Iz_3_1209	DJI_0116	Populus nigra L.	0	Salicaceae Mirb.	n	1
Iz_3_1209	DJI_0116	Pyrus communis L.	0	Rosaceae Juss.	a	0
Iz_3_1209	DJI_0116	Quercus robur L.	0	Fagaceae Dumort.	n	1
Iz_3_1209	DJI_0116	Robinia pseudoacacia L.	0	Fabaceae Lindl.	a	0
Iz_3_1209	DJI_0116	Rosa sp.	0	Rosaceae Juss.		
Iz_3_1209	DJI_0116	Salix alba L.	0	Salicaceae Mirb.	n	1
Iz_3_1209	DJI_0116	Sambucus nigra L.	0	Viburnaceae Raf.	n	1
Iz_3_1209	DJI_0116	Sorbus aucuparia L.	0	Rosaceae Juss.	n	1
Iz_3_1209	DJI_0116	Tilia cordata Mill.	0	Malvaceae Juss.	n	1
Iz_3_1209	DJI_0116	Ulmus glabra Huds.	0	Ulmaceae Mirb.	n	1
Iz_3_1209	DJI_0113	Acer tataricum L.	0	Sapindaceae Juss.	n	1
Iz_3_1209	DJI_0113	Betula pendula Roth.	0	Betulaceae Gray	n	1
Iz_3_1209	DJI_0113	Crataegus rhipidophylla Gand.	0	Rosaceae Juss.	n	1
Iz_3_1209	DJI_0113	Frangula alnus Mill.	0	Rhamnaceae Juss.	n	1
Iz_3_1209	DJI_0113	Fraxinus pennsylvanica Marshall	0	Oleaceae Hoffmanns. & Link	a	0
Iz_3_1209	DJI_0113	Pinus sylvestris L.	0	Pinaceae Spreng. ex F.Rudolphi	n	1
Iz_3_1209	DJI_0113	Populus × canescens (Aiton) Sm.	0	Salicaceae Mirb.	n	1
Iz_3_1209	DJI_0113	Populus tremula L.	0	Salicaceae Mirb.	n	1
Iz_3_1209	DJI_0113	Populus nigra L.	0	Salicaceae Mirb.	n	1
Iz_3_1209	DJI_0113	Pyrus communis L.	0	Rosaceae Juss.	a	0
Iz_3_1209	DJI_0113	Quercus robur L.	0	Fagaceae Dumort.	n	1
Iz_3_1209	DJI_0113	Robinia pseudoacacia L.	0	Fabaceae Lindl.	a	0
Iz_3_1209	DJI_0113	Rosa sp.	0	Rosaceae Juss.		
Iz_3_1209	DJI_0113	Salix alba L.	0	Salicaceae Mirb.	n	1
Iz_3_1209	DJI_0113	Sambucus nigra L.	0	Viburnaceae Raf.	n	1
Iz_3_1209	DJI_0113	Sorbus aucuparia L.	0	Rosaceae Juss.	n	1
Iz_3_1209	DJI_0113	Tilia cordata Mill.	0	Malvaceae Juss.	n	1
Iz_3_1209	DJI_0113	Ulmus glabra Huds.	0	Ulmaceae Mirb.	n	1
Iz_3_1209	DJI_0109	Acer tataricum L.	0	Sapindaceae Juss.	n	1
Iz_3_1209	DJI_0109	Betula pendula Roth.	0	Betulaceae Gray	n	1
Iz_3_1209	DJI_0109	Crataegus rhipidophylla Gand.	0	Rosaceae Juss.	n	1
Iz_3_1209	DJI_0109	Frangula alnus Mill.	0	Rhamnaceae Juss.	n	1
Iz_3_1209	DJI_0109	Fraxinus pennsylvanica Marshall	0	Oleaceae Hoffmanns. & Link	a	0
Iz_3_1209	DJI_0109	Pinus sylvestris L.	0	Pinaceae Spreng. ex F.Rudolphi	n	1
Iz_3_1209	DJI_0109	Populus × canescens (Aiton) Sm.	0	Salicaceae Mirb.	n	1
Iz_3_1209	DJI_0109	Populus tremula L.	0	Salicaceae Mirb.	n	1
Iz_3_1209	DJI_0109	Populus nigra L.	0	Salicaceae Mirb.	n	1
Iz_3_1209	DJI_0109	Pyrus communis L.	0	Rosaceae Juss.	a	0
Iz_3_1209	DJI_0109	Quercus robur L.	0	Fagaceae Dumort.	n	1
Iz_3_1209	DJI_0109	Robinia pseudoacacia L.	0	Fabaceae Lindl.	a	0
Iz_3_1209	DJI_0109	Rosa sp.	0	Rosaceae Juss.		
Iz_3_1209	DJI_0109	Salix alba L.	0	Salicaceae Mirb.	n	1
Iz_3_1209	DJI_0109	Sambucus nigra L.	0	Viburnaceae Raf.	n	1
Iz_3_1209	DJI_0109	Sorbus aucuparia L.	0	Rosaceae Juss.	n	1
Iz_3_1209	DJI_0109	Tilia cordata Mill.	0	Malvaceae Juss.	n	1
Iz_3_1209	DJI_0109	Ulmus glabra Huds.	0	Ulmaceae Mirb.	n	1
Iz_3_1209	DJI_0106	Acer tataricum L.	0	Sapindaceae Juss.	n	1
Iz_3_1209	DJI_0106	Betula pendula Roth.	0	Betulaceae Gray	n	1
Iz_3_1209	DJI_0106	Crataegus rhipidophylla Gand.	0	Rosaceae Juss.	n	1
Iz_3_1209	DJI_0106	Frangula alnus Mill.	0	Rhamnaceae Juss.	n	1
Iz_3_1209	DJI_0106	Fraxinus pennsylvanica Marshall	0	Oleaceae Hoffmanns. & Link	a	0
Iz_3_1209	DJI_0106	Pinus sylvestris L.	0	Pinaceae Spreng. ex F.Rudolphi	n	1

Iz_3_1209	DJI_0106	Populus × canescens (Aiton) Sm.	0	Salicaceae Mirb.	n	1
Iz_3_1209	DJI_0106	Populus tremula L.	0	Salicaceae Mirb.	n	1
Iz_3_1209	DJI_0106	Populus nigra L.	0	Salicaceae Mirb.	n	1
Iz_3_1209	DJI_0106	Pyrus communis L.	0	Rosaceae Juss.	a	0
Iz_3_1209	DJI_0106	Quercus robur L.	0	Fagaceae Dumort.	n	1
Iz_3_1209	DJI_0106	Robinia pseudoacacia L.	0	Fabaceae Lindl.	a	0
Iz_3_1209	DJI_0106	Rosa sp.	0	Rosaceae Juss.		
Iz_3_1209	DJI_0106	Salix alba L.	0	Salicaceae Mirb.	n	1
Iz_3_1209	DJI_0106	Sambucus nigra L.	0	Viburnaceae Raf.	n	1
Iz_3_1209	DJI_0106	Sorbus aucuparia L.	0	Rosaceae Juss.	n	1
Iz_3_1209	DJI_0106	Tilia cordata Mill.	0	Malvaceae Juss.	n	1
Iz_3_1209	DJI_0106	Ulmus glabra Huds.	0	Ulmaceae Mirb.	n	1
Iz_3_1209	DJI_0103	Acer tataricum L.	0	Sapindaceae Juss.	n	1
Iz_3_1209	DJI_0103	Betula pendula Roth.	0	Betulaceae Gray	n	1
Iz_3_1209	DJI_0103	Crataegus rhipidophylla Gand.	0	Rosaceae Juss.	n	1
Iz_3_1209	DJI_0103	Frangula alnus Mill.	0	Rhamnaceae Juss.	n	1
Iz_3_1209	DJI_0103	Fraxinus pennsylvanica Marshall	0	Oleaceae Hoffmanns. & Link	a	0
Iz_3_1209	DJI_0103	Pinus sylvestris L.	0	Pinaceae Spreng. ex F.Rudolphi	n	1
Iz_3_1209	DJI_0103	Populus × canescens (Aiton) Sm.	0	Salicaceae Mirb.	n	1
Iz_3_1209	DJI_0103	Populus tremula L.	0	Salicaceae Mirb.	n	1
Iz_3_1209	DJI_0103	Populus nigra L.	0	Salicaceae Mirb.	n	1
Iz_3_1209	DJI_0103	Pyrus communis L.	0	Rosaceae Juss.	a	0
Iz_3_1209	DJI_0103	Quercus robur L.	0	Fagaceae Dumort.	n	1
Iz_3_1209	DJI_0103	Robinia pseudoacacia L.	0	Fabaceae Lindl.	a	0
Iz_3_1209	DJI_0103	Rosa sp.	0	Rosaceae Juss.		
Iz_3_1209	DJI_0103	Salix alba L.	0	Salicaceae Mirb.	n	1
Iz_3_1209	DJI_0103	Sambucus nigra L.	0	Viburnaceae Raf.	n	1
Iz_3_1209	DJI_0103	Sorbus aucuparia L.	0	Rosaceae Juss.	n	1
Iz_3_1209	DJI_0103	Tilia cordata Mill.	0	Malvaceae Juss.	n	1
Iz_3_1209	DJI_0103	Ulmus glabra Huds.	0	Ulmaceae Mirb.	n	1
Iz_3_1209	DJI_0100	Acer tataricum L.	0	Sapindaceae Juss.	n	1
Iz_3_1209	DJI_0100	Betula pendula Roth.	0	Betulaceae Gray	n	1
Iz_3_1209	DJI_0100	Crataegus rhipidophylla Gand.	0	Rosaceae Juss.	n	1
Iz_3_1209	DJI_0100	Frangula alnus Mill.	0	Rhamnaceae Juss.	n	1
Iz_3_1209	DJI_0100	Fraxinus pennsylvanica Marshall	0	Oleaceae Hoffmanns. & Link	a	0
Iz_3_1209	DJI_0100	Pinus sylvestris L.	0	Pinaceae Spreng. ex F.Rudolphi	n	1
Iz_3_1209	DJI_0100	Populus × canescens (Aiton) Sm.	0	Salicaceae Mirb.	n	1
Iz_3_1209	DJI_0100	Populus tremula L.	0	Salicaceae Mirb.	n	1
Iz_3_1209	DJI_0100	Populus nigra L.	0	Salicaceae Mirb.	n	1
Iz_3_1209	DJI_0100	Pyrus communis L.	0	Rosaceae Juss.	a	0
Iz_3_1209	DJI_0100	Quercus robur L.	0	Fagaceae Dumort.	n	1
Iz_3_1209	DJI_0100	Robinia pseudoacacia L.	0	Fabaceae Lindl.	a	0
Iz_3_1209	DJI_0100	Rosa sp.	0	Rosaceae Juss.		
Iz_3_1209	DJI_0100	Salix alba L.	0	Salicaceae Mirb.	n	1
Iz_3_1209	DJI_0100	Sambucus nigra L.	0	Viburnaceae Raf.	n	1
Iz_3_1209	DJI_0100	Sorbus aucuparia L.	0	Rosaceae Juss.	n	1
Iz_3_1209	DJI_0100	Tilia cordata Mill.	0	Malvaceae Juss.	n	1
Iz_3_1209	DJI_0100	Ulmus glabra Huds.	0	Ulmaceae Mirb.	n	1
Iz_3_1209	DJI_0097	Acer tataricum L.	0	Sapindaceae Juss.	n	1
Iz_3_1209	DJI_0097	Betula pendula Roth.	0	Betulaceae Gray	n	1
Iz_3_1209	DJI_0097	Crataegus rhipidophylla Gand.	0	Rosaceae Juss.	n	1
Iz_3_1209	DJI_0097	Frangula alnus Mill.	0	Rhamnaceae Juss.	n	1
Iz_3_1209	DJI_0097	Fraxinus pennsylvanica Marshall	0	Oleaceae Hoffmanns. & Link	a	0
Iz_3_1209	DJI_0097	Pinus sylvestris L.	0	Pinaceae Spreng. ex F.Rudolphi	n	1
Iz_3_1209	DJI_0097	Populus × canescens (Aiton) Sm.	0	Salicaceae Mirb.	n	1
Iz_3_1209	DJI_0097	Populus tremula L.	0	Salicaceae Mirb.	n	1
Iz_3_1209	DJI_0097	Populus nigra L.	0	Salicaceae Mirb.	n	1
Iz_3_1209	DJI_0097	Pyrus communis L.	0	Rosaceae Juss.	a	0
Iz_3_1209	DJI_0097	Quercus robur L.	0	Fagaceae Dumort.	n	1
Iz_3_1209	DJI_0097	Robinia pseudoacacia L.	0	Fabaceae Lindl.	a	0
Iz_3_1209	DJI_0097	Rosa sp.	0	Rosaceae Juss.		
Iz_3_1209	DJI_0097	Salix alba L.	0	Salicaceae Mirb.	n	1
Iz_3_1209	DJI_0097	Sambucus nigra L.	0	Viburnaceae Raf.	n	1
Iz_3_1209	DJI_0097	Sorbus aucuparia L.	0	Rosaceae Juss.	n	1

Iz_3_1209	DJI_0097	Tilia cordata Mill.	0	Malvaceae Juss.	n	1
Iz_3_1209	DJI_0097	Ulmus glabra Huds.	0	Ulmaceae Mirb.	n	1
Iz_4_1209	DJI_0210	Acer tataricum L.	0	Sapindaceae Juss.	n	1
Iz_4_1209	DJI_0210	Betula pendula Roth.	0	Betulaceae Gray	n	1
Iz_4_1209	DJI_0210	Crataegus rhipidophylla Gand.	0	Rosaceae Juss.	n	1
Iz_4_1209	DJI_0210	Frangula alnus Mill.	0	Rhamnaceae Juss.	n	1
Iz_4_1209	DJI_0210	Fraxinus pennsylvanica Marshall	0	Oleaceae Hoffmanns. & Link	a	0
Iz_4_1209	DJI_0210	Pinus sylvestris L.	0	Pinaceae Spreng. ex F.Rudolphi	n	1
Iz_4_1209	DJI_0210	Populus × canescens (Aiton) Sm.	0	Salicaceae Mirb.	n	1
Iz_4_1209	DJI_0210	Populus tremula L.	0	Salicaceae Mirb.	n	1
Iz_4_1209	DJI_0210	Populus nigra L.	0	Salicaceae Mirb.	n	1
Iz_4_1209	DJI_0210	Pyrus communis L.	0	Rosaceae Juss.	a	0
Iz_4_1209	DJI_0210	Quercus robur L.	0	Fagaceae Dumort.	n	1
Iz_4_1209	DJI_0210	Robinia pseudoacacia L.	0	Fabaceae Lindl.	a	0
Iz_4_1209	DJI_0210	Rosa sp.	0	Rosaceae Juss.		
Iz_4_1209	DJI_0210	Salix alba L.	0	Salicaceae Mirb.	n	1
Iz_4_1209	DJI_0210	Sambucus nigra L.	0	Viburnaceae Raf.	n	1
Iz_4_1209	DJI_0210	Sorbus aucuparia L.	0	Rosaceae Juss.	n	1
Iz_4_1209	DJI_0210	Tilia cordata Mill.	0	Malvaceae Juss.	n	1
Iz_4_1209	DJI_0210	Ulmus glabra Huds.	0	Ulmaceae Mirb.	n	1
Iz_4_1209	DJI_0207	Acer tataricum L.	0	Sapindaceae Juss.	n	1
Iz_4_1209	DJI_0207	Betula pendula Roth.	0	Betulaceae Gray	n	1
Iz_4_1209	DJI_0207	Crataegus rhipidophylla Gand.	0	Rosaceae Juss.	n	1
Iz_4_1209	DJI_0207	Frangula alnus Mill.	0	Rhamnaceae Juss.	n	1
Iz_4_1209	DJI_0207	Fraxinus pennsylvanica Marshall	0	Oleaceae Hoffmanns. & Link	a	0
Iz_4_1209	DJI_0207	Pinus sylvestris L.	0	Pinaceae Spreng. ex F.Rudolphi	n	1
Iz_4_1209	DJI_0207	Populus × canescens (Aiton) Sm.	0	Salicaceae Mirb.	n	1
Iz_4_1209	DJI_0207	Populus tremula L.	0	Salicaceae Mirb.	n	1
Iz_4_1209	DJI_0207	Populus nigra L.	0	Salicaceae Mirb.	n	1
Iz_4_1209	DJI_0207	Pyrus communis L.	0	Rosaceae Juss.	a	0
Iz_4_1209	DJI_0207	Quercus robur L.	0	Fagaceae Dumort.	n	1
Iz_4_1209	DJI_0207	Robinia pseudoacacia L.	0	Fabaceae Lindl.	a	0
Iz_4_1209	DJI_0207	Rosa sp.	0	Rosaceae Juss.		
Iz_4_1209	DJI_0207	Salix alba L.	0	Salicaceae Mirb.	n	1
Iz_4_1209	DJI_0207	Sambucus nigra L.	0	Viburnaceae Raf.	n	1
Iz_4_1209	DJI_0207	Sorbus aucuparia L.	0	Rosaceae Juss.	n	1
Iz_4_1209	DJI_0207	Tilia cordata Mill.	0	Malvaceae Juss.	n	1
Iz_4_1209	DJI_0207	Ulmus glabra Huds.	0	Ulmaceae Mirb.	n	1
Iz_4_1209	DJI_0203	Acer tataricum L.	0	Sapindaceae Juss.	n	1
Iz_4_1209	DJI_0203	Betula pendula Roth.	0	Betulaceae Gray	n	1
Iz_4_1209	DJI_0203	Crataegus rhipidophylla Gand.	0	Rosaceae Juss.	n	1
Iz_4_1209	DJI_0203	Frangula alnus Mill.	0	Rhamnaceae Juss.	n	1
Iz_4_1209	DJI_0203	Fraxinus pennsylvanica Marshall	0	Oleaceae Hoffmanns. & Link	a	0
Iz_4_1209	DJI_0203	Pinus sylvestris L.	0	Pinaceae Spreng. ex F.Rudolphi	n	1
Iz_4_1209	DJI_0203	Populus × canescens (Aiton) Sm.	0	Salicaceae Mirb.	n	1
Iz_4_1209	DJI_0203	Populus tremula L.	0	Salicaceae Mirb.	n	1
Iz_4_1209	DJI_0203	Populus nigra L.	0	Salicaceae Mirb.	n	1
Iz_4_1209	DJI_0203	Pyrus communis L.	0	Rosaceae Juss.	a	0
Iz_4_1209	DJI_0203	Quercus robur L.	0	Fagaceae Dumort.	n	1
Iz_4_1209	DJI_0203	Robinia pseudoacacia L.	0	Fabaceae Lindl.	a	0
Iz_4_1209	DJI_0203	Rosa sp.	0	Rosaceae Juss.		
Iz_4_1209	DJI_0203	Salix alba L.	0	Salicaceae Mirb.	n	1
Iz_4_1209	DJI_0203	Sambucus nigra L.	0	Viburnaceae Raf.	n	1
Iz_4_1209	DJI_0203	Sorbus aucuparia L.	0	Rosaceae Juss.	n	1
Iz_4_1209	DJI_0203	Tilia cordata Mill.	0	Malvaceae Juss.	n	1
Iz_4_1209	DJI_0203	Ulmus glabra Huds.	0	Ulmaceae Mirb.	n	1
Iz_4_1209	DJI_0199	Acer tataricum L.	0	Sapindaceae Juss.	n	1
Iz_4_1209	DJI_0199	Betula pendula Roth.	0	Betulaceae Gray	n	1
Iz_4_1209	DJI_0199	Crataegus rhipidophylla Gand.	0	Rosaceae Juss.	n	1
Iz_4_1209	DJI_0199	Frangula alnus Mill.	0	Rhamnaceae Juss.	n	1
Iz_4_1209	DJI_0199	Fraxinus pennsylvanica Marshall	0	Oleaceae Hoffmanns. & Link	a	0
Iz_4_1209	DJI_0199	Pinus sylvestris L.	0	Pinaceae Spreng. ex F.Rudolphi	n	1
Iz_4_1209	DJI_0199	Populus × canescens (Aiton) Sm.	0	Salicaceae Mirb.	n	1
Iz_4_1209	DJI_0199	Populus tremula L.	0	Salicaceae Mirb.	n	1

Iz_4_1209	DJI_0199	Populus nigra L.	0	Salicaceae Mirb.	n	1
Iz_4_1209	DJI_0199	Pyrus communis L.	0	Rosaceae Juss.	a	0
Iz_4_1209	DJI_0199	Quercus robur L.	0	Fagaceae Dumort.	n	1
Iz_4_1209	DJI_0199	Robinia pseudoacacia L.	0	Fabaceae Lindl.	a	0
Iz_4_1209	DJI_0199	Rosa sp.	0	Rosaceae Juss.		
Iz_4_1209	DJI_0199	Salix alba L.	0	Salicaceae Mirb.	n	1
Iz_4_1209	DJI_0199	Sambucus nigra L.	0	Viburnaceae Raf.	n	1
Iz_4_1209	DJI_0199	Sorbus aucuparia L.	0	Rosaceae Juss.	n	1
Iz_4_1209	DJI_0199	Tilia cordata Mill.	0	Malvaceae Juss.	n	1
Iz_4_1209	DJI_0199	Ulmus glabra Huds.	0	Ulmaceae Mirb.	n	1
Iz_4_1209	DJI_0195	Acer tataricum L.	0	Sapindaceae Juss.	n	1
Iz_4_1209	DJI_0195	Betula pendula Roth.	0	Betulaceae Gray	n	1
Iz_4_1209	DJI_0195	Crataegus rhipidophylla Gand.	0	Rosaceae Juss.	n	1
Iz_4_1209	DJI_0195	Frangula alnus Mill.	0	Rhamnaceae Juss.	n	1
Iz_4_1209	DJI_0195	Fraxinus pennsylvanica Marshall	0	Oleaceae Hoffmanns. & Link	a	0
Iz_4_1209	DJI_0195	Pinus sylvestris L.	0	Pinaceae Spreng. ex F.Rudolphi	n	1
Iz_4_1209	DJI_0195	Populus × canescens (Aiton) Sm.	0	Salicaceae Mirb.	n	1
Iz_4_1209	DJI_0195	Populus tremula L.	0	Salicaceae Mirb.	n	1
Iz_4_1209	DJI_0195	Populus nigra L.	0	Salicaceae Mirb.	n	1
Iz_4_1209	DJI_0195	Pyrus communis L.	0	Rosaceae Juss.	a	0
Iz_4_1209	DJI_0195	Quercus robur L.	0	Fagaceae Dumort.	n	1
Iz_4_1209	DJI_0195	Robinia pseudoacacia L.	0	Fabaceae Lindl.	a	0
Iz_4_1209	DJI_0195	Rosa sp.	0	Rosaceae Juss.		
Iz_4_1209	DJI_0195	Salix alba L.	0	Salicaceae Mirb.	n	1
Iz_4_1209	DJI_0195	Sambucus nigra L.	0	Viburnaceae Raf.	n	1
Iz_4_1209	DJI_0195	Sorbus aucuparia L.	0	Rosaceae Juss.	n	1
Iz_4_1209	DJI_0195	Tilia cordata Mill.	0	Malvaceae Juss.	n	1
Iz_4_1209	DJI_0195	Ulmus glabra Huds.	0	Ulmaceae Mirb.	n	1
Iz_4_1209	DJI_0191	Acer tataricum L.	0	Sapindaceae Juss.	n	1
Iz_4_1209	DJI_0191	Betula pendula Roth.	0	Betulaceae Gray	n	1
Iz_4_1209	DJI_0191	Crataegus rhipidophylla Gand.	0	Rosaceae Juss.	n	1
Iz_4_1209	DJI_0191	Frangula alnus Mill.	0	Rhamnaceae Juss.	n	1
Iz_4_1209	DJI_0191	Fraxinus pennsylvanica Marshall	0	Oleaceae Hoffmanns. & Link	a	0
Iz_4_1209	DJI_0191	Pinus sylvestris L.	0	Pinaceae Spreng. ex F.Rudolphi	n	1
Iz_4_1209	DJI_0191	Populus × canescens (Aiton) Sm.	0	Salicaceae Mirb.	n	1
Iz_4_1209	DJI_0191	Populus tremula L.	0	Salicaceae Mirb.	n	1
Iz_4_1209	DJI_0191	Populus nigra L.	0	Salicaceae Mirb.	n	1
Iz_4_1209	DJI_0191	Pyrus communis L.	0	Rosaceae Juss.	a	0
Iz_4_1209	DJI_0191	Quercus robur L.	0	Fagaceae Dumort.	n	1
Iz_4_1209	DJI_0191	Robinia pseudoacacia L.	0	Fabaceae Lindl.	a	0
Iz_4_1209	DJI_0191	Rosa sp.	0	Rosaceae Juss.		
Iz_4_1209	DJI_0191	Salix alba L.	0	Salicaceae Mirb.	n	1
Iz_4_1209	DJI_0191	Sambucus nigra L.	0	Viburnaceae Raf.	n	1
Iz_4_1209	DJI_0191	Sorbus aucuparia L.	0	Rosaceae Juss.	n	1
Iz_4_1209	DJI_0191	Tilia cordata Mill.	0	Malvaceae Juss.	n	1
Iz_4_1209	DJI_0191	Ulmus glabra Huds.	0	Ulmaceae Mirb.	n	1
Iz_4_1209	DJI_0188	Acer tataricum L.	0	Sapindaceae Juss.	n	1
Iz_4_1209	DJI_0188	Betula pendula Roth.	0	Betulaceae Gray	n	1
Iz_4_1209	DJI_0188	Crataegus rhipidophylla Gand.	0	Rosaceae Juss.	n	1
Iz_4_1209	DJI_0188	Frangula alnus Mill.	0	Rhamnaceae Juss.	n	1
Iz_4_1209	DJI_0188	Fraxinus pennsylvanica Marshall	0	Oleaceae Hoffmanns. & Link	a	0
Iz_4_1209	DJI_0188	Pinus sylvestris L.	0	Pinaceae Spreng. ex F.Rudolphi	n	1
Iz_4_1209	DJI_0188	Populus × canescens (Aiton) Sm.	0	Salicaceae Mirb.	n	1
Iz_4_1209	DJI_0188	Populus tremula L.	0	Salicaceae Mirb.	n	1
Iz_4_1209	DJI_0188	Populus nigra L.	0	Salicaceae Mirb.	n	1
Iz_4_1209	DJI_0188	Pyrus communis L.	0	Rosaceae Juss.	a	0
Iz_4_1209	DJI_0188	Quercus robur L.	0	Fagaceae Dumort.	n	1
Iz_4_1209	DJI_0188	Robinia pseudoacacia L.	0	Fabaceae Lindl.	a	0
Iz_4_1209	DJI_0188	Rosa sp.	0	Rosaceae Juss.		
Iz_4_1209	DJI_0188	Salix alba L.	0	Salicaceae Mirb.	n	1
Iz_4_1209	DJI_0188	Sambucus nigra L.	0	Viburnaceae Raf.	n	1
Iz_4_1209	DJI_0188	Sorbus aucuparia L.	0	Rosaceae Juss.	n	1
Iz_4_1209	DJI_0188	Tilia cordata Mill.	0	Malvaceae Juss.	n	1
Iz_4_1209	DJI_0188	Ulmus glabra Huds.	0	Ulmaceae Mirb.	n	1

Iz_4_1209	DJI_0185	Acer tataricum L.	0	Sapindaceae Juss.	n	1
Iz_4_1209	DJI_0185	Betula pendula Roth.	0	Betulaceae Gray	n	1
Iz_4_1209	DJI_0185	Crataegus rhipidophylla Gand.	0	Rosaceae Juss.	n	1
Iz_4_1209	DJI_0185	Frangula alnus Mill.	0	Rhamnaceae Juss.	n	1
Iz_4_1209	DJI_0185	Fraxinus pennsylvanica Marshall	0	Oleaceae Hoffmanns. & Link	a	0
Iz_4_1209	DJI_0185	Pinus sylvestris L.	0	Pinaceae Spreng. ex F.Rudolphi	n	1
Iz_4_1209	DJI_0185	Populus × canescens (Aiton) Sm.	0	Salicaceae Mirb.	n	1
Iz_4_1209	DJI_0185	Populus tremula L.	0	Salicaceae Mirb.	n	1
Iz_4_1209	DJI_0185	Populus nigra L.	0	Salicaceae Mirb.	n	1
Iz_4_1209	DJI_0185	Pyrus communis L.	0	Rosaceae Juss.	a	0
Iz_4_1209	DJI_0185	Quercus robur L.	0	Fagaceae Dumort.	n	1
Iz_4_1209	DJI_0185	Robinia pseudoacacia L.	0	Fabaceae Lindl.	a	0
Iz_4_1209	DJI_0185	Rosa sp.	0	Rosaceae Juss.		
Iz_4_1209	DJI_0185	Salix alba L.	0	Salicaceae Mirb.	n	1
Iz_4_1209	DJI_0185	Sambucus nigra L.	0	Viburnaceae Raf.	n	1
Iz_4_1209	DJI_0185	Sorbus aucuparia L.	0	Rosaceae Juss.	n	1
Iz_4_1209	DJI_0185	Tilia cordata Mill.	0	Malvaceae Juss.	n	1
Iz_4_1209	DJI_0185	Ulmus glabra Huds.	0	Ulmaceae Mirb.	n	1
Iz_4_1209	DJI_0181	Acer tataricum L.	0	Sapindaceae Juss.	n	1
Iz_4_1209	DJI_0181	Betula pendula Roth.	0	Betulaceae Gray	n	1
Iz_4_1209	DJI_0181	Crataegus rhipidophylla Gand.	0	Rosaceae Juss.	n	1
Iz_4_1209	DJI_0181	Frangula alnus Mill.	0	Rhamnaceae Juss.	n	1
Iz_4_1209	DJI_0181	Fraxinus pennsylvanica Marshall	0	Oleaceae Hoffmanns. & Link	a	0
Iz_4_1209	DJI_0181	Pinus sylvestris L.	0	Pinaceae Spreng. ex F.Rudolphi	n	1
Iz_4_1209	DJI_0181	Populus × canescens (Aiton) Sm.	0	Salicaceae Mirb.	n	1
Iz_4_1209	DJI_0181	Populus tremula L.	0	Salicaceae Mirb.	n	1
Iz_4_1209	DJI_0181	Populus nigra L.	0	Salicaceae Mirb.	n	1
Iz_4_1209	DJI_0181	Pyrus communis L.	0	Rosaceae Juss.	a	0
Iz_4_1209	DJI_0181	Quercus robur L.	0	Fagaceae Dumort.	n	1
Iz_4_1209	DJI_0181	Robinia pseudoacacia L.	0	Fabaceae Lindl.	a	0
Iz_4_1209	DJI_0181	Rosa sp.	0	Rosaceae Juss.		
Iz_4_1209	DJI_0181	Salix alba L.	0	Salicaceae Mirb.	n	1
Iz_4_1209	DJI_0181	Sambucus nigra L.	0	Viburnaceae Raf.	n	1
Iz_4_1209	DJI_0181	Sorbus aucuparia L.	0	Rosaceae Juss.	n	1
Iz_4_1209	DJI_0181	Tilia cordata Mill.	0	Malvaceae Juss.	n	1
Iz_4_1209	DJI_0181	Ulmus glabra Huds.	0	Ulmaceae Mirb.	n	1
Iz_4_1209	DJI_0178	Acer tataricum L.	0	Sapindaceae Juss.	n	1
Iz_4_1209	DJI_0178	Betula pendula Roth.	0	Betulaceae Gray	n	1
Iz_4_1209	DJI_0178	Crataegus rhipidophylla Gand.	0	Rosaceae Juss.	n	1
Iz_4_1209	DJI_0178	Frangula alnus Mill.	0	Rhamnaceae Juss.	n	1
Iz_4_1209	DJI_0178	Fraxinus pennsylvanica Marshall	0	Oleaceae Hoffmanns. & Link	a	0
Iz_4_1209	DJI_0178	Pinus sylvestris L.	0	Pinaceae Spreng. ex F.Rudolphi	n	1
Iz_4_1209	DJI_0178	Populus × canescens (Aiton) Sm.	0	Salicaceae Mirb.	n	1
Iz_4_1209	DJI_0178	Populus tremula L.	0	Salicaceae Mirb.	n	1
Iz_4_1209	DJI_0178	Populus nigra L.	0	Salicaceae Mirb.	n	1
Iz_4_1209	DJI_0178	Pyrus communis L.	0	Rosaceae Juss.	a	0
Iz_4_1209	DJI_0178	Quercus robur L.	0	Fagaceae Dumort.	n	1
Iz_4_1209	DJI_0178	Robinia pseudoacacia L.	0	Fabaceae Lindl.	a	0
Iz_4_1209	DJI_0178	Rosa sp.	0	Rosaceae Juss.		
Iz_4_1209	DJI_0178	Salix alba L.	0	Salicaceae Mirb.	n	1
Iz_4_1209	DJI_0178	Sambucus nigra L.	0	Viburnaceae Raf.	n	1
Iz_4_1209	DJI_0178	Sorbus aucuparia L.	0	Rosaceae Juss.	n	1
Iz_4_1209	DJI_0178	Tilia cordata Mill.	0	Malvaceae Juss.	n	1
Iz_4_1209	DJI_0178	Ulmus glabra Huds.	0	Ulmaceae Mirb.	n	1
Iz_4_1209	DJI_0174	Acer tataricum L.	0	Sapindaceae Juss.	n	1
Iz_4_1209	DJI_0174	Betula pendula Roth.	0	Betulaceae Gray	n	1
Iz_4_1209	DJI_0174	Crataegus rhipidophylla Gand.	0	Rosaceae Juss.	n	1
Iz_4_1209	DJI_0174	Frangula alnus Mill.	0	Rhamnaceae Juss.	n	1
Iz_4_1209	DJI_0174	Fraxinus pennsylvanica Marshall	0	Oleaceae Hoffmanns. & Link	a	0
Iz_4_1209	DJI_0174	Pinus sylvestris L.	0	Pinaceae Spreng. ex F.Rudolphi	n	1
Iz_4_1209	DJI_0174	Populus × canescens (Aiton) Sm.	0	Salicaceae Mirb.	n	1
Iz_4_1209	DJI_0174	Populus tremula L.	0	Salicaceae Mirb.	n	1
Iz_4_1209	DJI_0174	Populus nigra L.	0	Salicaceae Mirb.	n	1
Iz_4_1209	DJI_0174	Pyrus communis L.	0	Rosaceae Juss.	a	0

Iz_4_1209	DJI_0174	Quercus robur L.	0	Fagaceae Dumort.	n	1
Iz_4_1209	DJI_0174	Robinia pseudoacacia L.	0	Fabaceae Lindl.	a	0
Iz_4_1209	DJI_0174	Rosa sp.	0	Rosaceae Juss.		
Iz_4_1209	DJI_0174	Salix alba L.	0	Salicaceae Mirb.	n	1
Iz_4_1209	DJI_0174	Sambucus nigra L.	0	Viburnaceae Raf.	n	1
Iz_4_1209	DJI_0174	Sorbus aucuparia L.	0	Rosaceae Juss.	n	1
Iz_4_1209	DJI_0174	Tilia cordata Mill.	0	Malvaceae Juss.	n	1
Iz_4_1209	DJI_0174	Ulmus glabra Huds.	0	Ulmaceae Mirb.	n	1
Iz_4_1209	DJI_0171	Acer tataricum L.	0	Sapindaceae Juss.	n	1
Iz_4_1209	DJI_0171	Betula pendula Roth.	0	Betulaceae Gray	n	1
Iz_4_1209	DJI_0171	Crataegus rhipidophylla Gand.	0	Rosaceae Juss.	n	1
Iz_4_1209	DJI_0171	Frangula alnus Mill.	0	Rhamnaceae Juss.	n	1
Iz_4_1209	DJI_0171	Fraxinus pennsylvanica Marshall	0	Oleaceae Hoffmanns. & Link	a	0
Iz_4_1209	DJI_0171	Pinus sylvestris L.	0	Pinaceae Spreng. ex F.Rudolphi	n	1
Iz_4_1209	DJI_0171	Populus × canescens (Aiton) Sm.	0	Salicaceae Mirb.	n	1
Iz_4_1209	DJI_0171	Populus tremula L.	0	Salicaceae Mirb.	n	1
Iz_4_1209	DJI_0171	Populus nigra L.	0	Salicaceae Mirb.	n	1
Iz_4_1209	DJI_0171	Pyrus communis L.	0	Rosaceae Juss.	a	0
Iz_4_1209	DJI_0171	Quercus robur L.	0	Fagaceae Dumort.	n	1
Iz_4_1209	DJI_0171	Robinia pseudoacacia L.	0	Fabaceae Lindl.	a	0
Iz_4_1209	DJI_0171	Rosa sp.	0	Rosaceae Juss.		
Iz_4_1209	DJI_0171	Salix alba L.	0	Salicaceae Mirb.	n	1
Iz_4_1209	DJI_0171	Sambucus nigra L.	0	Viburnaceae Raf.	n	1
Iz_4_1209	DJI_0171	Sorbus aucuparia L.	0	Rosaceae Juss.	n	1
Iz_4_1209	DJI_0171	Tilia cordata Mill.	0	Malvaceae Juss.	n	1
Iz_4_1209	DJI_0171	Ulmus glabra Huds.	0	Ulmaceae Mirb.	n	1
Iz_5_1209	DJI_0291	Acer tataricum L.	0	Sapindaceae Juss.	n	1
Iz_5_1209	DJI_0291	Betula pendula Roth.	0	Betulaceae Gray	n	1
Iz_5_1209	DJI_0291	Crataegus rhipidophylla Gand.	0	Rosaceae Juss.	n	1
Iz_5_1209	DJI_0291	Frangula alnus Mill.	0	Rhamnaceae Juss.	n	1
Iz_5_1209	DJI_0291	Fraxinus pennsylvanica Marshall	0	Oleaceae Hoffmanns. & Link	a	0
Iz_5_1209	DJI_0291	Pinus sylvestris L.	0	Pinaceae Spreng. ex F.Rudolphi	n	1
Iz_5_1209	DJI_0291	Populus × canescens (Aiton) Sm.	0	Salicaceae Mirb.	n	1
Iz_5_1209	DJI_0291	Populus tremula L.	0	Salicaceae Mirb.	n	1
Iz_5_1209	DJI_0291	Populus nigra L.	0	Salicaceae Mirb.	n	1
Iz_5_1209	DJI_0291	Pyrus communis L.	1	Rosaceae Juss.	a	0
Iz_5_1209	DJI_0291	Quercus robur L.	0	Fagaceae Dumort.	n	1
Iz_5_1209	DJI_0291	Robinia pseudoacacia L.	0	Fabaceae Lindl.	a	0
Iz_5_1209	DJI_0291	Rosa sp.	0	Rosaceae Juss.		
Iz_5_1209	DJI_0291	Salix alba L.	0	Salicaceae Mirb.	n	1
Iz_5_1209	DJI_0291	Sambucus nigra L.	0	Viburnaceae Raf.	n	1
Iz_5_1209	DJI_0291	Sorbus aucuparia L.	0	Rosaceae Juss.	n	1
Iz_5_1209	DJI_0291	Tilia cordata Mill.	0	Malvaceae Juss.	n	1
Iz_5_1209	DJI_0291	Ulmus glabra Huds.	0	Ulmaceae Mirb.	n	1
Iz_5_1209	DJI_0289	Acer tataricum L.	0	Sapindaceae Juss.	n	1
Iz_5_1209	DJI_0289	Betula pendula Roth.	0	Betulaceae Gray	n	1
Iz_5_1209	DJI_0289	Crataegus rhipidophylla Gand.	0	Rosaceae Juss.	n	1
Iz_5_1209	DJI_0289	Frangula alnus Mill.	0	Rhamnaceae Juss.	n	1
Iz_5_1209	DJI_0289	Fraxinus pennsylvanica Marshall	0	Oleaceae Hoffmanns. & Link	a	0
Iz_5_1209	DJI_0289	Pinus sylvestris L.	0	Pinaceae Spreng. ex F.Rudolphi	n	1
Iz_5_1209	DJI_0289	Populus × canescens (Aiton) Sm.	0	Salicaceae Mirb.	n	1
Iz_5_1209	DJI_0289	Populus tremula L.	0	Salicaceae Mirb.	n	1
Iz_5_1209	DJI_0289	Populus nigra L.	0	Salicaceae Mirb.	n	1
Iz_5_1209	DJI_0289	Pyrus communis L.	1	Rosaceae Juss.	a	0
Iz_5_1209	DJI_0289	Quercus robur L.	0	Fagaceae Dumort.	n	1
Iz_5_1209	DJI_0289	Robinia pseudoacacia L.	0	Fabaceae Lindl.	a	0
Iz_5_1209	DJI_0289	Rosa sp.	0	Rosaceae Juss.		
Iz_5_1209	DJI_0289	Salix alba L.	0	Salicaceae Mirb.	n	1
Iz_5_1209	DJI_0289	Sambucus nigra L.	0	Viburnaceae Raf.	n	1
Iz_5_1209	DJI_0289	Sorbus aucuparia L.	0	Rosaceae Juss.	n	1
Iz_5_1209	DJI_0289	Tilia cordata Mill.	0	Malvaceae Juss.	n	1
Iz_5_1209	DJI_0289	Ulmus glabra Huds.	0	Ulmaceae Mirb.	n	1
Iz_5_1209	DJI_0286	Acer tataricum L.	0	Sapindaceae Juss.	n	1
Iz_5_1209	DJI_0286	Betula pendula Roth.	0	Betulaceae Gray	n	1

Iz_5_1209	DJI_0286	Crataegus rhipidophylla Gand.	0	Rosaceae Juss.	n	1
Iz_5_1209	DJI_0286	Frangula alnus Mill.	0	Rhamnaceae Juss.	n	1
Iz_5_1209	DJI_0286	Fraxinus pennsylvanica Marshall	0	Oleaceae Hoffmanns. & Link	a	0
Iz_5_1209	DJI_0286	Pinus sylvestris L.	0	Pinaceae Spreng. ex F.Rudolphi	n	1
Iz_5_1209	DJI_0286	Populus × canescens (Aiton) Sm.	0	Salicaceae Mirb.	n	1
Iz_5_1209	DJI_0286	Populus tremula L.	0	Salicaceae Mirb.	n	1
Iz_5_1209	DJI_0286	Populus nigra L.	0	Salicaceae Mirb.	n	1
Iz_5_1209	DJI_0286	Pyrus communis L.	0	Rosaceae Juss.	a	0
Iz_5_1209	DJI_0286	Quercus robur L.	0	Fagaceae Dumort.	n	1
Iz_5_1209	DJI_0286	Robinia pseudoacacia L.	0	Fabaceae Lindl.	a	0
Iz_5_1209	DJI_0286	Rosa sp.	0	Rosaceae Juss.		
Iz_5_1209	DJI_0286	Salix alba L.	0	Salicaceae Mirb.	n	1
Iz_5_1209	DJI_0286	Sambucus nigra L.	0	Viburnaceae Raf.	n	1
Iz_5_1209	DJI_0286	Sorbus aucuparia L.	0	Rosaceae Juss.	n	1
Iz_5_1209	DJI_0286	Tilia cordata Mill.	0	Malvaceae Juss.	n	1
Iz_5_1209	DJI_0286	Ulmus glabra Huds.	0	Ulmaceae Mirb.	n	1
Iz_5_1209	DJI_0284	Acer tataricum L.	0	Sapindaceae Juss.	n	1
Iz_5_1209	DJI_0284	Betula pendula Roth.	0	Betulaceae Gray	n	1
Iz_5_1209	DJI_0284	Crataegus rhipidophylla Gand.	0	Rosaceae Juss.	n	1
Iz_5_1209	DJI_0284	Frangula alnus Mill.	0	Rhamnaceae Juss.	n	1
Iz_5_1209	DJI_0284	Fraxinus pennsylvanica Marshall	0	Oleaceae Hoffmanns. & Link	a	0
Iz_5_1209	DJI_0284	Pinus sylvestris L.	0	Pinaceae Spreng. ex F.Rudolphi	n	1
Iz_5_1209	DJI_0284	Populus × canescens (Aiton) Sm.	0	Salicaceae Mirb.	n	1
Iz_5_1209	DJI_0284	Populus tremula L.	0	Salicaceae Mirb.	n	1
Iz_5_1209	DJI_0284	Populus nigra L.	0	Salicaceae Mirb.	n	1
Iz_5_1209	DJI_0284	Pyrus communis L.	0	Rosaceae Juss.	a	0
Iz_5_1209	DJI_0284	Quercus robur L.	0	Fagaceae Dumort.	n	1
Iz_5_1209	DJI_0284	Robinia pseudoacacia L.	0	Fabaceae Lindl.	a	0
Iz_5_1209	DJI_0284	Rosa sp.	0	Rosaceae Juss.		
Iz_5_1209	DJI_0284	Salix alba L.	0	Salicaceae Mirb.	n	1
Iz_5_1209	DJI_0284	Sambucus nigra L.	0	Viburnaceae Raf.	n	1
Iz_5_1209	DJI_0284	Sorbus aucuparia L.	0	Rosaceae Juss.	n	1
Iz_5_1209	DJI_0284	Tilia cordata Mill.	0	Malvaceae Juss.	n	1
Iz_5_1209	DJI_0284	Ulmus glabra Huds.	0	Ulmaceae Mirb.	n	1
Iz_5_1209	DJI_0281	Acer tataricum L.	0	Sapindaceae Juss.	n	1
Iz_5_1209	DJI_0281	Betula pendula Roth.	0	Betulaceae Gray	n	1
Iz_5_1209	DJI_0281	Crataegus rhipidophylla Gand.	0	Rosaceae Juss.	n	1
Iz_5_1209	DJI_0281	Frangula alnus Mill.	0	Rhamnaceae Juss.	n	1
Iz_5_1209	DJI_0281	Fraxinus pennsylvanica Marshall	0	Oleaceae Hoffmanns. & Link	a	0
Iz_5_1209	DJI_0281	Pinus sylvestris L.	0	Pinaceae Spreng. ex F.Rudolphi	n	1
Iz_5_1209	DJI_0281	Populus × canescens (Aiton) Sm.	0	Salicaceae Mirb.	n	1
Iz_5_1209	DJI_0281	Populus tremula L.	0	Salicaceae Mirb.	n	1
Iz_5_1209	DJI_0281	Populus nigra L.	0	Salicaceae Mirb.	n	1
Iz_5_1209	DJI_0281	Pyrus communis L.	0	Rosaceae Juss.	a	0
Iz_5_1209	DJI_0281	Quercus robur L.	0	Fagaceae Dumort.	n	1
Iz_5_1209	DJI_0281	Robinia pseudoacacia L.	0	Fabaceae Lindl.	a	0
Iz_5_1209	DJI_0281	Rosa sp.	0	Rosaceae Juss.		
Iz_5_1209	DJI_0281	Salix alba L.	0	Salicaceae Mirb.	n	1
Iz_5_1209	DJI_0281	Sambucus nigra L.	0	Viburnaceae Raf.	n	1
Iz_5_1209	DJI_0281	Sorbus aucuparia L.	0	Rosaceae Juss.	n	1
Iz_5_1209	DJI_0281	Tilia cordata Mill.	0	Malvaceae Juss.	n	1
Iz_5_1209	DJI_0281	Ulmus glabra Huds.	0	Ulmaceae Mirb.	n	1
Iz_5_1209	DJI_0276	Acer tataricum L.	0	Sapindaceae Juss.	n	1
Iz_5_1209	DJI_0276	Betula pendula Roth.	0	Betulaceae Gray	n	1
Iz_5_1209	DJI_0276	Crataegus rhipidophylla Gand.	0	Rosaceae Juss.	n	1
Iz_5_1209	DJI_0276	Frangula alnus Mill.	0	Rhamnaceae Juss.	n	1
Iz_5_1209	DJI_0276	Fraxinus pennsylvanica Marshall	0	Oleaceae Hoffmanns. & Link	a	0
Iz_5_1209	DJI_0276	Pinus sylvestris L.	0	Pinaceae Spreng. ex F.Rudolphi	n	1
Iz_5_1209	DJI_0276	Populus × canescens (Aiton) Sm.	0	Salicaceae Mirb.	n	1
Iz_5_1209	DJI_0276	Populus tremula L.	1	Salicaceae Mirb.	n	1
Iz_5_1209	DJI_0276	Populus nigra L.	0	Salicaceae Mirb.	n	1
Iz_5_1209	DJI_0276	Pyrus communis L.	0	Rosaceae Juss.	a	0
Iz_5_1209	DJI_0276	Quercus robur L.	0	Fagaceae Dumort.	n	1
Iz_5_1209	DJI_0276	Robinia pseudoacacia L.	0	Fabaceae Lindl.	a	0

Iz_5_1209	DJI_0276	Rosa sp.	0	Rosaceae Juss.		
Iz_5_1209	DJI_0276	Salix alba L.	0	Salicaceae Mirb.	n	1
Iz_5_1209	DJI_0276	Sambucus nigra L.	0	Viburnaceae Raf.	n	1
Iz_5_1209	DJI_0276	Sorbus aucuparia L.	0	Rosaceae Juss.	n	1
Iz_5_1209	DJI_0276	Tilia cordata Mill.	0	Malvaceae Juss.	n	1
Iz_5_1209	DJI_0276	Ulmus glabra Huds.	0	Ulmaceae Mirb.	n	1
Iz_5_1209	DJI_0272	Acer tataricum L.	0	Sapindaceae Juss.	n	1
Iz_5_1209	DJI_0272	Betula pendula Roth.	0	Betulaceae Gray	n	1
Iz_5_1209	DJI_0272	Crataegus rhipidophylla Gand.	0	Rosaceae Juss.	n	1
Iz_5_1209	DJI_0272	Frangula alnus Mill.	0	Rhamnaceae Juss.	n	1
Iz_5_1209	DJI_0272	Fraxinus pennsylvanica Marshall	0	Oleaceae Hoffmanns. & Link	a	0
Iz_5_1209	DJI_0272	Pinus sylvestris L.	0	Pinaceae Spreng. ex F.Rudolphi	n	1
Iz_5_1209	DJI_0272	Populus × canescens (Aiton) Sm.	0	Salicaceae Mirb.	n	1
Iz_5_1209	DJI_0272	Populus tremula L.	0	Salicaceae Mirb.	n	1
Iz_5_1209	DJI_0272	Populus nigra L.	0	Salicaceae Mirb.	n	1
Iz_5_1209	DJI_0272	Pyrus communis L.	0	Rosaceae Juss.	a	0
Iz_5_1209	DJI_0272	Quercus robur L.	0	Fagaceae Dumort.	n	1
Iz_5_1209	DJI_0272	Robinia pseudoacacia L.	0	Fabaceae Lindl.	a	0
Iz_5_1209	DJI_0272	Rosa sp.	0	Rosaceae Juss.		
Iz_5_1209	DJI_0272	Salix alba L.	0	Salicaceae Mirb.	n	1
Iz_5_1209	DJI_0272	Sambucus nigra L.	0	Viburnaceae Raf.	n	1
Iz_5_1209	DJI_0272	Sorbus aucuparia L.	0	Rosaceae Juss.	n	1
Iz_5_1209	DJI_0272	Tilia cordata Mill.	0	Malvaceae Juss.	n	1
Iz_5_1209	DJI_0272	Ulmus glabra Huds.	0	Ulmaceae Mirb.	n	1
Iz_5_1209	DJI_0270	Acer tataricum L.	0	Sapindaceae Juss.	n	1
Iz_5_1209	DJI_0270	Betula pendula Roth.	0	Betulaceae Gray	n	1
Iz_5_1209	DJI_0270	Crataegus rhipidophylla Gand.	0	Rosaceae Juss.	n	1
Iz_5_1209	DJI_0270	Frangula alnus Mill.	0	Rhamnaceae Juss.	n	1
Iz_5_1209	DJI_0270	Fraxinus pennsylvanica Marshall	0	Oleaceae Hoffmanns. & Link	a	0
Iz_5_1209	DJI_0270	Pinus sylvestris L.	0	Pinaceae Spreng. ex F.Rudolphi	n	1
Iz_5_1209	DJI_0270	Populus × canescens (Aiton) Sm.	0	Salicaceae Mirb.	n	1
Iz_5_1209	DJI_0270	Populus tremula L.	0	Salicaceae Mirb.	n	1
Iz_5_1209	DJI_0270	Populus nigra L.	0	Salicaceae Mirb.	n	1
Iz_5_1209	DJI_0270	Pyrus communis L.	0	Rosaceae Juss.	a	0
Iz_5_1209	DJI_0270	Quercus robur L.	0	Fagaceae Dumort.	n	1
Iz_5_1209	DJI_0270	Robinia pseudoacacia L.	0	Fabaceae Lindl.	a	0
Iz_5_1209	DJI_0270	Rosa sp.	0	Rosaceae Juss.		
Iz_5_1209	DJI_0270	Salix alba L.	0	Salicaceae Mirb.	n	1
Iz_5_1209	DJI_0270	Sambucus nigra L.	0	Viburnaceae Raf.	n	1
Iz_5_1209	DJI_0270	Sorbus aucuparia L.	0	Rosaceae Juss.	n	1
Iz_5_1209	DJI_0270	Tilia cordata Mill.	0	Malvaceae Juss.	n	1
Iz_5_1209	DJI_0270	Ulmus glabra Huds.	0	Ulmaceae Mirb.	n	1
Iz_5_1209	DJI_0265	Acer tataricum L.	0	Sapindaceae Juss.	n	1
Iz_5_1209	DJI_0265	Betula pendula Roth.	0	Betulaceae Gray	n	1
Iz_5_1209	DJI_0265	Crataegus rhipidophylla Gand.	0	Rosaceae Juss.	n	1
Iz_5_1209	DJI_0265	Frangula alnus Mill.	0	Rhamnaceae Juss.	n	1
Iz_5_1209	DJI_0265	Fraxinus pennsylvanica Marshall	0	Oleaceae Hoffmanns. & Link	a	0
Iz_5_1209	DJI_0265	Pinus sylvestris L.	0	Pinaceae Spreng. ex F.Rudolphi	n	1
Iz_5_1209	DJI_0265	Populus × canescens (Aiton) Sm.	0	Salicaceae Mirb.	n	1
Iz_5_1209	DJI_0265	Populus tremula L.	0	Salicaceae Mirb.	n	1
Iz_5_1209	DJI_0265	Populus nigra L.	0	Salicaceae Mirb.	n	1
Iz_5_1209	DJI_0265	Pyrus communis L.	0	Rosaceae Juss.	a	0
Iz_5_1209	DJI_0265	Quercus robur L.	0	Fagaceae Dumort.	n	1
Iz_5_1209	DJI_0265	Robinia pseudoacacia L.	0	Fabaceae Lindl.	a	0
Iz_5_1209	DJI_0265	Rosa sp.	0	Rosaceae Juss.		
Iz_5_1209	DJI_0265	Salix alba L.	0	Salicaceae Mirb.	n	1
Iz_5_1209	DJI_0265	Sambucus nigra L.	0	Viburnaceae Raf.	n	1
Iz_5_1209	DJI_0265	Sorbus aucuparia L.	0	Rosaceae Juss.	n	1
Iz_5_1209	DJI_0265	Tilia cordata Mill.	0	Malvaceae Juss.	n	1
Iz_5_1209	DJI_0265	Ulmus glabra Huds.	0	Ulmaceae Mirb.	n	1
Iz_5_1209	DJI_0262	Acer tataricum L.	0	Sapindaceae Juss.	n	1
Iz_5_1209	DJI_0262	Betula pendula Roth.	0	Betulaceae Gray	n	1
Iz_5_1209	DJI_0262	Crataegus rhipidophylla Gand.	0	Rosaceae Juss.	n	1
Iz_5_1209	DJI_0262	Frangula alnus Mill.	0	Rhamnaceae Juss.	n	1

Iz_5_1209	DJI_0262	Fraxinus pennsylvanica Marshall	0	Oleaceae Hoffmanns. & Link	a	0
Iz_5_1209	DJI_0262	Pinus sylvestris L.	0	Pinaceae Spreng. ex F.Rudolphi	n	1
Iz_5_1209	DJI_0262	Populus × canescens (Aiton) Sm.	0	Salicaceae Mirb.	n	1
Iz_5_1209	DJI_0262	Populus tremula L.	0	Salicaceae Mirb.	n	1
Iz_5_1209	DJI_0262	Populus nigra L.	0	Salicaceae Mirb.	n	1
Iz_5_1209	DJI_0262	Pyrus communis L.	0	Rosaceae Juss.	a	0
Iz_5_1209	DJI_0262	Quercus robur L.	0	Fagaceae Dumort.	n	1
Iz_5_1209	DJI_0262	Robinia pseudoacacia L.	0	Fabaceae Lindl.	a	0
Iz_5_1209	DJI_0262	Rosa sp.	0	Rosaceae Juss.		
Iz_5_1209	DJI_0262	Salix alba L.	0	Salicaceae Mirb.	n	1
Iz_5_1209	DJI_0262	Sambucus nigra L.	0	Viburnaceae Raf.	n	1
Iz_5_1209	DJI_0262	Sorbus aucuparia L.	0	Rosaceae Juss.	n	1
Iz_5_1209	DJI_0262	Tilia cordata Mill.	0	Malvaceae Juss.	n	1
Iz_5_1209	DJI_0262	Ulmus glabra Huds.	0	Ulmaceae Mirb.	n	1
Iz_5_1209	DJI_0259	Acer tataricum L.	0	Sapindaceae Juss.	n	1
Iz_5_1209	DJI_0259	Betula pendula Roth.	0	Betulaceae Gray	n	1
Iz_5_1209	DJI_0259	Crataegus rhipidophylla Gand.	0	Rosaceae Juss.	n	1
Iz_5_1209	DJI_0259	Frangula alnus Mill.	0	Rhamnaceae Juss.	n	1
Iz_5_1209	DJI_0259	Fraxinus pennsylvanica Marshall	0	Oleaceae Hoffmanns. & Link	a	0
Iz_5_1209	DJI_0259	Pinus sylvestris L.	0	Pinaceae Spreng. ex F.Rudolphi	n	1
Iz_5_1209	DJI_0259	Populus × canescens (Aiton) Sm.	0	Salicaceae Mirb.	n	1
Iz_5_1209	DJI_0259	Populus tremula L.	0	Salicaceae Mirb.	n	1
Iz_5_1209	DJI_0259	Populus nigra L.	0	Salicaceae Mirb.	n	1
Iz_5_1209	DJI_0259	Pyrus communis L.	0	Rosaceae Juss.	a	0
Iz_5_1209	DJI_0259	Quercus robur L.	0	Fagaceae Dumort.	n	1
Iz_5_1209	DJI_0259	Robinia pseudoacacia L.	0	Fabaceae Lindl.	a	0
Iz_5_1209	DJI_0259	Rosa sp.	0	Rosaceae Juss.		
Iz_5_1209	DJI_0259	Salix alba L.	0	Salicaceae Mirb.	n	1
Iz_5_1209	DJI_0259	Sambucus nigra L.	0	Viburnaceae Raf.	n	1
Iz_5_1209	DJI_0259	Sorbus aucuparia L.	0	Rosaceae Juss.	n	1
Iz_5_1209	DJI_0259	Tilia cordata Mill.	0	Malvaceae Juss.	n	1
Iz_5_1209	DJI_0259	Ulmus glabra Huds.	0	Ulmaceae Mirb.	n	1
Iz_5_1209	DJI_0256	Acer tataricum L.	0	Sapindaceae Juss.	n	1
Iz_5_1209	DJI_0256	Betula pendula Roth.	0	Betulaceae Gray	n	1
Iz_5_1209	DJI_0256	Crataegus rhipidophylla Gand.	0	Rosaceae Juss.	n	1
Iz_5_1209	DJI_0256	Frangula alnus Mill.	0	Rhamnaceae Juss.	n	1
Iz_5_1209	DJI_0256	Fraxinus pennsylvanica Marshall	0	Oleaceae Hoffmanns. & Link	a	0
Iz_5_1209	DJI_0256	Pinus sylvestris L.	0	Pinaceae Spreng. ex F.Rudolphi	n	1
Iz_5_1209	DJI_0256	Populus × canescens (Aiton) Sm.	0	Salicaceae Mirb.	n	1
Iz_5_1209	DJI_0256	Populus tremula L.	0	Salicaceae Mirb.	n	1
Iz_5_1209	DJI_0256	Populus nigra L.	0	Salicaceae Mirb.	n	1
Iz_5_1209	DJI_0256	Pyrus communis L.	0	Rosaceae Juss.	a	0
Iz_5_1209	DJI_0256	Quercus robur L.	0	Fagaceae Dumort.	n	1
Iz_5_1209	DJI_0256	Robinia pseudoacacia L.	0	Fabaceae Lindl.	a	0
Iz_5_1209	DJI_0256	Rosa sp.	0	Rosaceae Juss.		
Iz_5_1209	DJI_0256	Salix alba L.	0	Salicaceae Mirb.	n	1
Iz_5_1209	DJI_0256	Sambucus nigra L.	0	Viburnaceae Raf.	n	1
Iz_5_1209	DJI_0256	Sorbus aucuparia L.	0	Rosaceae Juss.	n	1
Iz_5_1209	DJI_0256	Tilia cordata Mill.	0	Malvaceae Juss.	n	1
Iz_5_1209	DJI_0256	Ulmus glabra Huds.	0	Ulmaceae Mirb.	n	1
Iz_5_1209	DJI_0253	Acer tataricum L.	0	Sapindaceae Juss.	n	1
Iz_5_1209	DJI_0253	Betula pendula Roth.	0	Betulaceae Gray	n	1
Iz_5_1209	DJI_0253	Crataegus rhipidophylla Gand.	0	Rosaceae Juss.	n	1
Iz_5_1209	DJI_0253	Frangula alnus Mill.	0	Rhamnaceae Juss.	n	1
Iz_5_1209	DJI_0253	Fraxinus pennsylvanica Marshall	0	Oleaceae Hoffmanns. & Link	a	0
Iz_5_1209	DJI_0253	Pinus sylvestris L.	0	Pinaceae Spreng. ex F.Rudolphi	n	1
Iz_5_1209	DJI_0253	Populus × canescens (Aiton) Sm.	0	Salicaceae Mirb.	n	1
Iz_5_1209	DJI_0253	Populus tremula L.	0	Salicaceae Mirb.	n	1
Iz_5_1209	DJI_0253	Populus nigra L.	0	Salicaceae Mirb.	n	1
Iz_5_1209	DJI_0253	Pyrus communis L.	0	Rosaceae Juss.	a	0
Iz_5_1209	DJI_0253	Quercus robur L.	0	Fagaceae Dumort.	n	1
Iz_5_1209	DJI_0253	Robinia pseudoacacia L.	0	Fabaceae Lindl.	a	0
Iz_5_1209	DJI_0253	Rosa sp.	0	Rosaceae Juss.		
Iz_5_1209	DJI_0253	Salix alba L.	0	Salicaceae Mirb.	n	1

Iz_5_1209	DJI_0253	Sambucus nigra L.	0	Viburnaceae Raf.	n	1
Iz_5_1209	DJI_0253	Sorbus aucuparia L.	0	Rosaceae Juss.	n	1
Iz_5_1209	DJI_0253	Tilia cordata Mill.	0	Malvaceae Juss.	n	1
Iz_5_1209	DJI_0253	Ulmus glabra Huds.	0	Ulmaceae Mirb.	n	1
Iz_5_1209	DJI_0249	Acer tataricum L.	0	Sapindaceae Juss.	n	1
Iz_5_1209	DJI_0249	Betula pendula Roth.	0	Betulaceae Gray	n	1
Iz_5_1209	DJI_0249	Crataegus rhipidophylla Gand.	0	Rosaceae Juss.	n	1
Iz_5_1209	DJI_0249	Frangula alnus Mill.	0	Rhamnaceae Juss.	n	1
Iz_5_1209	DJI_0249	Fraxinus pennsylvanica Marshall	0	Oleaceae Hoffmanns. & Link	a	0
Iz_5_1209	DJI_0249	Pinus sylvestris L.	0	Pinaceae Spreng. ex F.Rudolphi	n	1
Iz_5_1209	DJI_0249	Populus × canescens (Aiton) Sm.	0	Salicaceae Mirb.	n	1
Iz_5_1209	DJI_0249	Populus tremula L.	0	Salicaceae Mirb.	n	1
Iz_5_1209	DJI_0249	Populus nigra L.	0	Salicaceae Mirb.	n	1
Iz_5_1209	DJI_0249	Pyrus communis L.	0	Rosaceae Juss.	a	0
Iz_5_1209	DJI_0249	Quercus robur L.	0	Fagaceae Dumort.	n	1
Iz_5_1209	DJI_0249	Robinia pseudoacacia L.	0	Fabaceae Lindl.	a	0
Iz_5_1209	DJI_0249	Rosa sp.	0	Rosaceae Juss.		
Iz_5_1209	DJI_0249	Salix alba L.	0	Salicaceae Mirb.	n	1
Iz_5_1209	DJI_0249	Sambucus nigra L.	0	Viburnaceae Raf.	n	1
Iz_5_1209	DJI_0249	Sorbus aucuparia L.	0	Rosaceae Juss.	n	1
Iz_5_1209	DJI_0249	Tilia cordata Mill.	0	Malvaceae Juss.	n	1
Iz_5_1209	DJI_0249	Ulmus glabra Huds.	0	Ulmaceae Mirb.	n	1
Iz_5_1209	DJI_0245	Acer tataricum L.	0	Sapindaceae Juss.	n	1
Iz_5_1209	DJI_0245	Betula pendula Roth.	0	Betulaceae Gray	n	1
Iz_5_1209	DJI_0245	Crataegus rhipidophylla Gand.	0	Rosaceae Juss.	n	1
Iz_5_1209	DJI_0245	Frangula alnus Mill.	0	Rhamnaceae Juss.	n	1
Iz_5_1209	DJI_0245	Fraxinus pennsylvanica Marshall	0	Oleaceae Hoffmanns. & Link	a	0
Iz_5_1209	DJI_0245	Pinus sylvestris L.	0	Pinaceae Spreng. ex F.Rudolphi	n	1
Iz_5_1209	DJI_0245	Populus × canescens (Aiton) Sm.	0	Salicaceae Mirb.	n	1
Iz_5_1209	DJI_0245	Populus tremula L.	0	Salicaceae Mirb.	n	1
Iz_5_1209	DJI_0245	Populus nigra L.	0	Salicaceae Mirb.	n	1
Iz_5_1209	DJI_0245	Pyrus communis L.	0	Rosaceae Juss.	a	0
Iz_5_1209	DJI_0245	Quercus robur L.	0	Fagaceae Dumort.	n	1
Iz_5_1209	DJI_0245	Robinia pseudoacacia L.	0	Fabaceae Lindl.	a	0
Iz_5_1209	DJI_0245	Rosa sp.	0	Rosaceae Juss.		
Iz_5_1209	DJI_0245	Salix alba L.	0	Salicaceae Mirb.	n	1
Iz_5_1209	DJI_0245	Sambucus nigra L.	0	Viburnaceae Raf.	n	1
Iz_5_1209	DJI_0245	Sorbus aucuparia L.	0	Rosaceae Juss.	n	1
Iz_5_1209	DJI_0245	Tilia cordata Mill.	0	Malvaceae Juss.	n	1
Iz_5_1209	DJI_0245	Ulmus glabra Huds.	0	Ulmaceae Mirb.	n	1
Iz_5_1209	DJI_0242	Acer tataricum L.	0	Sapindaceae Juss.	n	1
Iz_5_1209	DJI_0242	Betula pendula Roth.	0	Betulaceae Gray	n	1
Iz_5_1209	DJI_0242	Crataegus rhipidophylla Gand.	0	Rosaceae Juss.	n	1
Iz_5_1209	DJI_0242	Frangula alnus Mill.	0	Rhamnaceae Juss.	n	1
Iz_5_1209	DJI_0242	Fraxinus pennsylvanica Marshall	0	Oleaceae Hoffmanns. & Link	a	0
Iz_5_1209	DJI_0242	Pinus sylvestris L.	0	Pinaceae Spreng. ex F.Rudolphi	n	1
Iz_5_1209	DJI_0242	Populus × canescens (Aiton) Sm.	0	Salicaceae Mirb.	n	1
Iz_5_1209	DJI_0242	Populus tremula L.	0	Salicaceae Mirb.	n	1
Iz_5_1209	DJI_0242	Populus nigra L.	0	Salicaceae Mirb.	n	1
Iz_5_1209	DJI_0242	Pyrus communis L.	0	Rosaceae Juss.	a	0
Iz_5_1209	DJI_0242	Quercus robur L.	0	Fagaceae Dumort.	n	1
Iz_5_1209	DJI_0242	Robinia pseudoacacia L.	0	Fabaceae Lindl.	a	0
Iz_5_1209	DJI_0242	Rosa sp.	0	Rosaceae Juss.		
Iz_5_1209	DJI_0242	Salix alba L.	0	Salicaceae Mirb.	n	1
Iz_5_1209	DJI_0242	Sambucus nigra L.	0	Viburnaceae Raf.	n	1
Iz_5_1209	DJI_0242	Sorbus aucuparia L.	0	Rosaceae Juss.	n	1
Iz_5_1209	DJI_0242	Tilia cordata Mill.	0	Malvaceae Juss.	n	1
Iz_5_1209	DJI_0242	Ulmus glabra Huds.	0	Ulmaceae Mirb.	n	1
Iz_6_1209	DJI_0358	Acer tataricum L.	0	Sapindaceae Juss.	n	1
Iz_6_1209	DJI_0358	Betula pendula Roth.	0	Betulaceae Gray	n	1
Iz_6_1209	DJI_0358	Crataegus rhipidophylla Gand.	0	Rosaceae Juss.	n	1
Iz_6_1209	DJI_0358	Frangula alnus Mill.	0	Rhamnaceae Juss.	n	1
Iz_6_1209	DJI_0358	Fraxinus pennsylvanica Marshall	0	Oleaceae Hoffmanns. & Link	a	0
Iz_6_1209	DJI_0358	Pinus sylvestris L.	0	Pinaceae Spreng. ex F.Rudolphi	n	1

Iz_6_1209	DJI_0358	Populus × canescens (Aiton) Sm.	0	Salicaceae Mirb.	n	1
Iz_6_1209	DJI_0358	Populus tremula L.	0	Salicaceae Mirb.	n	1
Iz_6_1209	DJI_0358	Populus nigra L.	0	Salicaceae Mirb.	n	1
Iz_6_1209	DJI_0358	Pyrus communis L.	0	Rosaceae Juss.	a	0
Iz_6_1209	DJI_0358	Quercus robur L.	0	Fagaceae Dumort.	n	1
Iz_6_1209	DJI_0358	Robinia pseudoacacia L.	0	Fabaceae Lindl.	a	0
Iz_6_1209	DJI_0358	Rosa sp.	0	Rosaceae Juss.		
Iz_6_1209	DJI_0358	Salix alba L.	0	Salicaceae Mirb.	n	1
Iz_6_1209	DJI_0358	Sambucus nigra L.	0	Viburnaceae Raf.	n	1
Iz_6_1209	DJI_0358	Sorbus aucuparia L.	0	Rosaceae Juss.	n	1
Iz_6_1209	DJI_0358	Tilia cordata Mill.	0	Malvaceae Juss.	n	1
Iz_6_1209	DJI_0358	Ulmus glabra Huds.	0	Ulmaceae Mirb.	n	1
Iz_6_1209	DJI_0355	Acer tataricum L.	0	Sapindaceae Juss.	n	1
Iz_6_1209	DJI_0355	Betula pendula Roth.	0	Betulaceae Gray	n	1
Iz_6_1209	DJI_0355	Crataegus rhipidophylla Gand.	0	Rosaceae Juss.	n	1
Iz_6_1209	DJI_0355	Frangula alnus Mill.	0	Rhamnaceae Juss.	n	1
Iz_6_1209	DJI_0355	Fraxinus pennsylvanica Marshall	0	Oleaceae Hoffmanns. & Link	a	0
Iz_6_1209	DJI_0355	Pinus sylvestris L.	0	Pinaceae Spreng. ex F.Rudolphi	n	1
Iz_6_1209	DJI_0355	Populus × canescens (Aiton) Sm.	0	Salicaceae Mirb.	n	1
Iz_6_1209	DJI_0355	Populus tremula L.	0	Salicaceae Mirb.	n	1
Iz_6_1209	DJI_0355	Populus nigra L.	0	Salicaceae Mirb.	n	1
Iz_6_1209	DJI_0355	Pyrus communis L.	0	Rosaceae Juss.	a	0
Iz_6_1209	DJI_0355	Quercus robur L.	0	Fagaceae Dumort.	n	1
Iz_6_1209	DJI_0355	Robinia pseudoacacia L.	0	Fabaceae Lindl.	a	0
Iz_6_1209	DJI_0355	Rosa sp.	0	Rosaceae Juss.		
Iz_6_1209	DJI_0355	Salix alba L.	0	Salicaceae Mirb.	n	1
Iz_6_1209	DJI_0355	Sambucus nigra L.	0	Viburnaceae Raf.	n	1
Iz_6_1209	DJI_0355	Sorbus aucuparia L.	0	Rosaceae Juss.	n	1
Iz_6_1209	DJI_0355	Tilia cordata Mill.	0	Malvaceae Juss.	n	1
Iz_6_1209	DJI_0355	Ulmus glabra Huds.	0	Ulmaceae Mirb.	n	1
Iz_6_1209	DJI_0352	Acer tataricum L.	0	Sapindaceae Juss.	n	1
Iz_6_1209	DJI_0352	Betula pendula Roth.	0	Betulaceae Gray	n	1
Iz_6_1209	DJI_0352	Crataegus rhipidophylla Gand.	0	Rosaceae Juss.	n	1
Iz_6_1209	DJI_0352	Frangula alnus Mill.	0	Rhamnaceae Juss.	n	1
Iz_6_1209	DJI_0352	Fraxinus pennsylvanica Marshall	0	Oleaceae Hoffmanns. & Link	a	0
Iz_6_1209	DJI_0352	Pinus sylvestris L.	0	Pinaceae Spreng. ex F.Rudolphi	n	1
Iz_6_1209	DJI_0352	Populus × canescens (Aiton) Sm.	0	Salicaceae Mirb.	n	1
Iz_6_1209	DJI_0352	Populus tremula L.	0	Salicaceae Mirb.	n	1
Iz_6_1209	DJI_0352	Populus nigra L.	0	Salicaceae Mirb.	n	1
Iz_6_1209	DJI_0352	Pyrus communis L.	0	Rosaceae Juss.	a	0
Iz_6_1209	DJI_0352	Quercus robur L.	0	Fagaceae Dumort.	n	1
Iz_6_1209	DJI_0352	Robinia pseudoacacia L.	0	Fabaceae Lindl.	a	0
Iz_6_1209	DJI_0352	Rosa sp.	0	Rosaceae Juss.		
Iz_6_1209	DJI_0352	Salix alba L.	0	Salicaceae Mirb.	n	1
Iz_6_1209	DJI_0352	Sambucus nigra L.	0	Viburnaceae Raf.	n	1
Iz_6_1209	DJI_0352	Sorbus aucuparia L.	0	Rosaceae Juss.	n	1
Iz_6_1209	DJI_0352	Tilia cordata Mill.	0	Malvaceae Juss.	n	1
Iz_6_1209	DJI_0352	Ulmus glabra Huds.	0	Ulmaceae Mirb.	n	1
Iz_6_1209	DJI_0349	Acer tataricum L.	0	Sapindaceae Juss.	n	1
Iz_6_1209	DJI_0349	Betula pendula Roth.	0	Betulaceae Gray	n	1
Iz_6_1209	DJI_0349	Crataegus rhipidophylla Gand.	0	Rosaceae Juss.	n	1
Iz_6_1209	DJI_0349	Frangula alnus Mill.	0	Rhamnaceae Juss.	n	1
Iz_6_1209	DJI_0349	Fraxinus pennsylvanica Marshall	0	Oleaceae Hoffmanns. & Link	a	0
Iz_6_1209	DJI_0349	Pinus sylvestris L.	0	Pinaceae Spreng. ex F.Rudolphi	n	1
Iz_6_1209	DJI_0349	Populus × canescens (Aiton) Sm.	0	Salicaceae Mirb.	n	1
Iz_6_1209	DJI_0349	Populus tremula L.	0	Salicaceae Mirb.	n	1
Iz_6_1209	DJI_0349	Populus nigra L.	0	Salicaceae Mirb.	n	1
Iz_6_1209	DJI_0349	Pyrus communis L.	0	Rosaceae Juss.	a	0
Iz_6_1209	DJI_0349	Quercus robur L.	0	Fagaceae Dumort.	n	1
Iz_6_1209	DJI_0349	Robinia pseudoacacia L.	0	Fabaceae Lindl.	a	0
Iz_6_1209	DJI_0349	Rosa sp.	0	Rosaceae Juss.		
Iz_6_1209	DJI_0349	Salix alba L.	0	Salicaceae Mirb.	n	1
Iz_6_1209	DJI_0349	Sambucus nigra L.	0	Viburnaceae Raf.	n	1
Iz_6_1209	DJI_0349	Sorbus aucuparia L.	0	Rosaceae Juss.	n	1

Iz_6_1209	DJI_0349	Tilia cordata Mill.	0	Malvaceae Juss.	n	1
Iz_6_1209	DJI_0349	Ulmus glabra Huds.	0	Ulmaceae Mirb.	n	1
Iz_6_1209	DJI_0346	Acer tataricum L.	0	Sapindaceae Juss.	n	1
Iz_6_1209	DJI_0346	Betula pendula Roth.	0	Betulaceae Gray	n	1
Iz_6_1209	DJI_0346	Crataegus rhipidophylla Gand.	0	Rosaceae Juss.	n	1
Iz_6_1209	DJI_0346	Frangula alnus Mill.	0	Rhamnaceae Juss.	n	1
Iz_6_1209	DJI_0346	Fraxinus pennsylvanica Marshall	0	Oleaceae Hoffmanns. & Link	a	0
Iz_6_1209	DJI_0346	Pinus sylvestris L.	0	Pinaceae Spreng. ex F.Rudolphi	n	1
Iz_6_1209	DJI_0346	Populus × canescens (Aiton) Sm.	0	Salicaceae Mirb.	n	1
Iz_6_1209	DJI_0346	Populus tremula L.	0	Salicaceae Mirb.	n	1
Iz_6_1209	DJI_0346	Populus nigra L.	0	Salicaceae Mirb.	n	1
Iz_6_1209	DJI_0346	Pyrus communis L.	0	Rosaceae Juss.	a	0
Iz_6_1209	DJI_0346	Quercus robur L.	0	Fagaceae Dumort.	n	1
Iz_6_1209	DJI_0346	Robinia pseudoacacia L.	0	Fabaceae Lindl.	a	0
Iz_6_1209	DJI_0346	Rosa sp.	0	Rosaceae Juss.		
Iz_6_1209	DJI_0346	Salix alba L.	0	Salicaceae Mirb.	n	1
Iz_6_1209	DJI_0346	Sambucus nigra L.	0	Viburnaceae Raf.	n	1
Iz_6_1209	DJI_0346	Sorbus aucuparia L.	0	Rosaceae Juss.	n	1
Iz_6_1209	DJI_0346	Tilia cordata Mill.	0	Malvaceae Juss.	n	1
Iz_6_1209	DJI_0346	Ulmus glabra Huds.	0	Ulmaceae Mirb.	n	1
Iz_6_1209	DJI_0341	Acer tataricum L.	0	Sapindaceae Juss.	n	1
Iz_6_1209	DJI_0341	Betula pendula Roth.	0	Betulaceae Gray	n	1
Iz_6_1209	DJI_0341	Crataegus rhipidophylla Gand.	0	Rosaceae Juss.	n	1
Iz_6_1209	DJI_0341	Frangula alnus Mill.	0	Rhamnaceae Juss.	n	1
Iz_6_1209	DJI_0341	Fraxinus pennsylvanica Marshall	0	Oleaceae Hoffmanns. & Link	a	0
Iz_6_1209	DJI_0341	Pinus sylvestris L.	0	Pinaceae Spreng. ex F.Rudolphi	n	1
Iz_6_1209	DJI_0341	Populus × canescens (Aiton) Sm.	0	Salicaceae Mirb.	n	1
Iz_6_1209	DJI_0341	Populus tremula L.	1	Salicaceae Mirb.	n	1
Iz_6_1209	DJI_0341	Populus nigra L.	0	Salicaceae Mirb.	n	1
Iz_6_1209	DJI_0341	Pyrus communis L.	0	Rosaceae Juss.	a	0
Iz_6_1209	DJI_0341	Quercus robur L.	0	Fagaceae Dumort.	n	1
Iz_6_1209	DJI_0341	Robinia pseudoacacia L.	0	Fabaceae Lindl.	a	0
Iz_6_1209	DJI_0341	Rosa sp.	0	Rosaceae Juss.		
Iz_6_1209	DJI_0341	Salix alba L.	0	Salicaceae Mirb.	n	1
Iz_6_1209	DJI_0341	Sambucus nigra L.	0	Viburnaceae Raf.	n	1
Iz_6_1209	DJI_0341	Sorbus aucuparia L.	0	Rosaceae Juss.	n	1
Iz_6_1209	DJI_0341	Tilia cordata Mill.	0	Malvaceae Juss.	n	1
Iz_6_1209	DJI_0341	Ulmus glabra Huds.	0	Ulmaceae Mirb.	n	1
Iz_6_1209	DJI_0336	Acer tataricum L.	0	Sapindaceae Juss.	n	1
Iz_6_1209	DJI_0336	Betula pendula Roth.	0	Betulaceae Gray	n	1
Iz_6_1209	DJI_0336	Crataegus rhipidophylla Gand.	0	Rosaceae Juss.	n	1
Iz_6_1209	DJI_0336	Frangula alnus Mill.	0	Rhamnaceae Juss.	n	1
Iz_6_1209	DJI_0336	Fraxinus pennsylvanica Marshall	0	Oleaceae Hoffmanns. & Link	a	0
Iz_6_1209	DJI_0336	Pinus sylvestris L.	0	Pinaceae Spreng. ex F.Rudolphi	n	1
Iz_6_1209	DJI_0336	Populus × canescens (Aiton) Sm.	0	Salicaceae Mirb.	n	1
Iz_6_1209	DJI_0336	Populus tremula L.	0	Salicaceae Mirb.	n	1
Iz_6_1209	DJI_0336	Populus nigra L.	0	Salicaceae Mirb.	n	1
Iz_6_1209	DJI_0336	Pyrus communis L.	0	Rosaceae Juss.	a	0
Iz_6_1209	DJI_0336	Quercus robur L.	0	Fagaceae Dumort.	n	1
Iz_6_1209	DJI_0336	Robinia pseudoacacia L.	0	Fabaceae Lindl.	a	0
Iz_6_1209	DJI_0336	Rosa sp.	0	Rosaceae Juss.		
Iz_6_1209	DJI_0336	Salix alba L.	0	Salicaceae Mirb.	n	1
Iz_6_1209	DJI_0336	Sambucus nigra L.	0	Viburnaceae Raf.	n	1
Iz_6_1209	DJI_0336	Sorbus aucuparia L.	0	Rosaceae Juss.	n	1
Iz_6_1209	DJI_0336	Tilia cordata Mill.	0	Malvaceae Juss.	n	1
Iz_6_1209	DJI_0336	Ulmus glabra Huds.	0	Ulmaceae Mirb.	n	1
Iz_6_1209	DJI_0333	Acer tataricum L.	0	Sapindaceae Juss.	n	1
Iz_6_1209	DJI_0333	Betula pendula Roth.	0	Betulaceae Gray	n	1
Iz_6_1209	DJI_0333	Crataegus rhipidophylla Gand.	0	Rosaceae Juss.	n	1
Iz_6_1209	DJI_0333	Frangula alnus Mill.	0	Rhamnaceae Juss.	n	1
Iz_6_1209	DJI_0333	Fraxinus pennsylvanica Marshall	0	Oleaceae Hoffmanns. & Link	a	0
Iz_6_1209	DJI_0333	Pinus sylvestris L.	0	Pinaceae Spreng. ex F.Rudolphi	n	1
Iz_6_1209	DJI_0333	Populus × canescens (Aiton) Sm.	0	Salicaceae Mirb.	n	1
Iz_6_1209	DJI_0333	Populus tremula L.	0	Salicaceae Mirb.	n	1

Iz_6_1209	DJI_0333	Populus nigra L.	0	Salicaceae Mirb.	n	1
Iz_6_1209	DJI_0333	Pyrus communis L.	0	Rosaceae Juss.	a	0
Iz_6_1209	DJI_0333	Quercus robur L.	0	Fagaceae Dumort.	n	1
Iz_6_1209	DJI_0333	Robinia pseudoacacia L.	0	Fabaceae Lindl.	a	0
Iz_6_1209	DJI_0333	Rosa sp.	0	Rosaceae Juss.		
Iz_6_1209	DJI_0333	Salix alba L.	0	Salicaceae Mirb.	n	1
Iz_6_1209	DJI_0333	Sambucus nigra L.	0	Viburnaceae Raf.	n	1
Iz_6_1209	DJI_0333	Sorbus aucuparia L.	0	Rosaceae Juss.	n	1
Iz_6_1209	DJI_0333	Tilia cordata Mill.	0	Malvaceae Juss.	n	1
Iz_6_1209	DJI_0333	Ulmus glabra Huds.	0	Ulmaceae Mirb.	n	1
Iz_6_1209	DJI_0330	Acer tataricum L.	0	Sapindaceae Juss.	n	1
Iz_6_1209	DJI_0330	Betula pendula Roth.	0	Betulaceae Gray	n	1
Iz_6_1209	DJI_0330	Crataegus rhipidophylla Gand.	0	Rosaceae Juss.	n	1
Iz_6_1209	DJI_0330	Frangula alnus Mill.	0	Rhamnaceae Juss.	n	1
Iz_6_1209	DJI_0330	Fraxinus pennsylvanica Marshall	0	Oleaceae Hoffmanns. & Link	a	0
Iz_6_1209	DJI_0330	Pinus sylvestris L.	0	Pinaceae Spreng. ex F.Rudolphi	n	1
Iz_6_1209	DJI_0330	Populus × canescens (Aiton) Sm.	0	Salicaceae Mirb.	n	1
Iz_6_1209	DJI_0330	Populus tremula L.	0	Salicaceae Mirb.	n	1
Iz_6_1209	DJI_0330	Populus nigra L.	0	Salicaceae Mirb.	n	1
Iz_6_1209	DJI_0330	Pyrus communis L.	0	Rosaceae Juss.	a	0
Iz_6_1209	DJI_0330	Quercus robur L.	0	Fagaceae Dumort.	n	1
Iz_6_1209	DJI_0330	Robinia pseudoacacia L.	0	Fabaceae Lindl.	a	0
Iz_6_1209	DJI_0330	Rosa sp.	0	Rosaceae Juss.		
Iz_6_1209	DJI_0330	Salix alba L.	0	Salicaceae Mirb.	n	1
Iz_6_1209	DJI_0330	Sambucus nigra L.	0	Viburnaceae Raf.	n	1
Iz_6_1209	DJI_0330	Sorbus aucuparia L.	0	Rosaceae Juss.	n	1
Iz_6_1209	DJI_0330	Tilia cordata Mill.	0	Malvaceae Juss.	n	1
Iz_6_1209	DJI_0330	Ulmus glabra Huds.	0	Ulmaceae Mirb.	n	1
Iz_6_1209	DJI_0327	Acer tataricum L.	0	Sapindaceae Juss.	n	1
Iz_6_1209	DJI_0327	Betula pendula Roth.	0	Betulaceae Gray	n	1
Iz_6_1209	DJI_0327	Crataegus rhipidophylla Gand.	0	Rosaceae Juss.	n	1
Iz_6_1209	DJI_0327	Frangula alnus Mill.	0	Rhamnaceae Juss.	n	1
Iz_6_1209	DJI_0327	Fraxinus pennsylvanica Marshall	0	Oleaceae Hoffmanns. & Link	a	0
Iz_6_1209	DJI_0327	Pinus sylvestris L.	0	Pinaceae Spreng. ex F.Rudolphi	n	1
Iz_6_1209	DJI_0327	Populus × canescens (Aiton) Sm.	0	Salicaceae Mirb.	n	1
Iz_6_1209	DJI_0327	Populus tremula L.	6	Salicaceae Mirb.	n	1
Iz_6_1209	DJI_0327	Populus nigra L.	0	Salicaceae Mirb.	n	1
Iz_6_1209	DJI_0327	Pyrus communis L.	0	Rosaceae Juss.	a	0
Iz_6_1209	DJI_0327	Quercus robur L.	0	Fagaceae Dumort.	n	1
Iz_6_1209	DJI_0327	Robinia pseudoacacia L.	0	Fabaceae Lindl.	a	0
Iz_6_1209	DJI_0327	Rosa sp.	0	Rosaceae Juss.		
Iz_6_1209	DJI_0327	Salix alba L.	0	Salicaceae Mirb.	n	1
Iz_6_1209	DJI_0327	Sambucus nigra L.	0	Viburnaceae Raf.	n	1
Iz_6_1209	DJI_0327	Sorbus aucuparia L.	0	Rosaceae Juss.	n	1
Iz_6_1209	DJI_0327	Tilia cordata Mill.	0	Malvaceae Juss.	n	1
Iz_6_1209	DJI_0327	Ulmus glabra Huds.	0	Ulmaceae Mirb.	n	1
Iz_6_1209	DJI_0311	Acer tataricum L.	0	Sapindaceae Juss.	n	1
Iz_6_1209	DJI_0311	Betula pendula Roth.	0	Betulaceae Gray	n	1
Iz_6_1209	DJI_0311	Crataegus rhipidophylla Gand.	0	Rosaceae Juss.	n	1
Iz_6_1209	DJI_0311	Frangula alnus Mill.	0	Rhamnaceae Juss.	n	1
Iz_6_1209	DJI_0311	Fraxinus pennsylvanica Marshall	0	Oleaceae Hoffmanns. & Link	a	0
Iz_6_1209	DJI_0311	Pinus sylvestris L.	0	Pinaceae Spreng. ex F.Rudolphi	n	1
Iz_6_1209	DJI_0311	Populus × canescens (Aiton) Sm.	0	Salicaceae Mirb.	n	1
Iz_6_1209	DJI_0311	Populus tremula L.	4	Salicaceae Mirb.	n	1
Iz_6_1209	DJI_0311	Populus nigra L.	0	Salicaceae Mirb.	n	1
Iz_6_1209	DJI_0311	Pyrus communis L.	0	Rosaceae Juss.	a	0
Iz_6_1209	DJI_0311	Quercus robur L.	0	Fagaceae Dumort.	n	1
Iz_6_1209	DJI_0311	Robinia pseudoacacia L.	0	Fabaceae Lindl.	a	0
Iz_6_1209	DJI_0311	Rosa sp.	0	Rosaceae Juss.		
Iz_6_1209	DJI_0311	Salix alba L.	0	Salicaceae Mirb.	n	1
Iz_6_1209	DJI_0311	Sambucus nigra L.	0	Viburnaceae Raf.	n	1
Iz_6_1209	DJI_0311	Sorbus aucuparia L.	0	Rosaceae Juss.	n	1
Iz_6_1209	DJI_0311	Tilia cordata Mill.	0	Malvaceae Juss.	n	1
Iz_6_1209	DJI_0311	Ulmus glabra Huds.	0	Ulmaceae Mirb.	n	1

Iz_6_1209	DJI_0309	Acer tataricum L.	0	Sapindaceae Juss.	n	1
Iz_6_1209	DJI_0309	Betula pendula Roth.	0	Betulaceae Gray	n	1
Iz_6_1209	DJI_0309	Crataegus rhipidophylla Gand.	0	Rosaceae Juss.	n	1
Iz_6_1209	DJI_0309	Frangula alnus Mill.	0	Rhamnaceae Juss.	n	1
Iz_6_1209	DJI_0309	Fraxinus pennsylvanica Marshall	0	Oleaceae Hoffmanns. & Link	a	0
Iz_6_1209	DJI_0309	Pinus sylvestris L.	0	Pinaceae Spreng. ex F.Rudolphi	n	1
Iz_6_1209	DJI_0309	Populus × canescens (Aiton) Sm.	0	Salicaceae Mirb.	n	1
Iz_6_1209	DJI_0309	Populus tremula L.	0	Salicaceae Mirb.	n	1
Iz_6_1209	DJI_0309	Populus nigra L.	0	Salicaceae Mirb.	n	1
Iz_6_1209	DJI_0309	Pyrus communis L.	0	Rosaceae Juss.	a	0
Iz_6_1209	DJI_0309	Quercus robur L.	0	Fagaceae Dumort.	n	1
Iz_6_1209	DJI_0309	Robinia pseudoacacia L.	0	Fabaceae Lindl.	a	0
Iz_6_1209	DJI_0309	Rosa sp.	0	Rosaceae Juss.		
Iz_6_1209	DJI_0309	Salix alba L.	0	Salicaceae Mirb.	n	1
Iz_6_1209	DJI_0309	Sambucus nigra L.	0	Viburnaceae Raf.	n	1
Iz_6_1209	DJI_0309	Sorbus aucuparia L.	0	Rosaceae Juss.	n	1
Iz_6_1209	DJI_0309	Tilia cordata Mill.	0	Malvaceae Juss.	n	1
Iz_6_1209	DJI_0309	Ulmus glabra Huds.	0	Ulmaceae Mirb.	n	1
Iz_6_1209	DJI_0306	Acer tataricum L.	0	Sapindaceae Juss.	n	1
Iz_6_1209	DJI_0306	Betula pendula Roth.	0	Betulaceae Gray	n	1
Iz_6_1209	DJI_0306	Crataegus rhipidophylla Gand.	0	Rosaceae Juss.	n	1
Iz_6_1209	DJI_0306	Frangula alnus Mill.	0	Rhamnaceae Juss.	n	1
Iz_6_1209	DJI_0306	Fraxinus pennsylvanica Marshall	0	Oleaceae Hoffmanns. & Link	a	0
Iz_6_1209	DJI_0306	Pinus sylvestris L.	0	Pinaceae Spreng. ex F.Rudolphi	n	1
Iz_6_1209	DJI_0306	Populus × canescens (Aiton) Sm.	0	Salicaceae Mirb.	n	1
Iz_6_1209	DJI_0306	Populus tremula L.	0	Salicaceae Mirb.	n	1
Iz_6_1209	DJI_0306	Populus nigra L.	0	Salicaceae Mirb.	n	1
Iz_6_1209	DJI_0306	Pyrus communis L.	0	Rosaceae Juss.	a	0
Iz_6_1209	DJI_0306	Quercus robur L.	0	Fagaceae Dumort.	n	1
Iz_6_1209	DJI_0306	Robinia pseudoacacia L.	0	Fabaceae Lindl.	a	0
Iz_6_1209	DJI_0306	Rosa sp.	0	Rosaceae Juss.		
Iz_6_1209	DJI_0306	Salix alba L.	0	Salicaceae Mirb.	n	1
Iz_6_1209	DJI_0306	Sambucus nigra L.	0	Viburnaceae Raf.	n	1
Iz_6_1209	DJI_0306	Sorbus aucuparia L.	0	Rosaceae Juss.	n	1
Iz_6_1209	DJI_0306	Tilia cordata Mill.	0	Malvaceae Juss.	n	1
Iz_6_1209	DJI_0306	Ulmus glabra Huds.	0	Ulmaceae Mirb.	n	1
Iz_6_1209	DJI_0303	Acer tataricum L.	0	Sapindaceae Juss.	n	1
Iz_6_1209	DJI_0303	Betula pendula Roth.	0	Betulaceae Gray	n	1
Iz_6_1209	DJI_0303	Crataegus rhipidophylla Gand.	0	Rosaceae Juss.	n	1
Iz_6_1209	DJI_0303	Frangula alnus Mill.	0	Rhamnaceae Juss.	n	1
Iz_6_1209	DJI_0303	Fraxinus pennsylvanica Marshall	0	Oleaceae Hoffmanns. & Link	a	0
Iz_6_1209	DJI_0303	Pinus sylvestris L.	0	Pinaceae Spreng. ex F.Rudolphi	n	1
Iz_6_1209	DJI_0303	Populus × canescens (Aiton) Sm.	0	Salicaceae Mirb.	n	1
Iz_6_1209	DJI_0303	Populus tremula L.	1	Salicaceae Mirb.	n	1
Iz_6_1209	DJI_0303	Populus nigra L.	0	Salicaceae Mirb.	n	1
Iz_6_1209	DJI_0303	Pyrus communis L.	0	Rosaceae Juss.	a	0
Iz_6_1209	DJI_0303	Quercus robur L.	0	Fagaceae Dumort.	n	1
Iz_6_1209	DJI_0303	Robinia pseudoacacia L.	0	Fabaceae Lindl.	a	0
Iz_6_1209	DJI_0303	Rosa sp.	0	Rosaceae Juss.		
Iz_6_1209	DJI_0303	Salix alba L.	0	Salicaceae Mirb.	n	1
Iz_6_1209	DJI_0303	Sambucus nigra L.	0	Viburnaceae Raf.	n	1
Iz_6_1209	DJI_0303	Sorbus aucuparia L.	0	Rosaceae Juss.	n	1
Iz_6_1209	DJI_0303	Tilia cordata Mill.	0	Malvaceae Juss.	n	1
Iz_6_1209	DJI_0303	Ulmus glabra Huds.	0	Ulmaceae Mirb.	n	1
Iz_6_1209	DJI_0300	Acer tataricum L.	0	Sapindaceae Juss.	n	1
Iz_6_1209	DJI_0300	Betula pendula Roth.	0	Betulaceae Gray	n	1
Iz_6_1209	DJI_0300	Crataegus rhipidophylla Gand.	0	Rosaceae Juss.	n	1
Iz_6_1209	DJI_0300	Frangula alnus Mill.	0	Rhamnaceae Juss.	n	1
Iz_6_1209	DJI_0300	Fraxinus pennsylvanica Marshall	0	Oleaceae Hoffmanns. & Link	a	0
Iz_6_1209	DJI_0300	Pinus sylvestris L.	0	Pinaceae Spreng. ex F.Rudolphi	n	1
Iz_6_1209	DJI_0300	Populus × canescens (Aiton) Sm.	0	Salicaceae Mirb.	n	1
Iz_6_1209	DJI_0300	Populus tremula L.	0	Salicaceae Mirb.	n	1
Iz_6_1209	DJI_0300	Populus nigra L.	0	Salicaceae Mirb.	n	1
Iz_6_1209	DJI_0300	Pyrus communis L.	0	Rosaceae Juss.	a	0

Iz_6_1209	DJI_0300	Quercus robur L.	0	Fagaceae Dumort.	n	1
Iz_6_1209	DJI_0300	Robinia pseudoacacia L.	0	Fabaceae Lindl.	a	0
Iz_6_1209	DJI_0300	Rosa sp.	0	Rosaceae Juss.		
Iz_6_1209	DJI_0300	Salix alba L.	0	Salicaceae Mirb.	n	1
Iz_6_1209	DJI_0300	Sambucus nigra L.	0	Viburnaceae Raf.	n	1
Iz_6_1209	DJI_0300	Sorbus aucuparia L.	0	Rosaceae Juss.	n	1
Iz_6_1209	DJI_0300	Tilia cordata Mill.	0	Malvaceae Juss.	n	1
Iz_6_1209	DJI_0300	Ulmus glabra Huds.	0	Ulmaceae Mirb.	n	1
Iz_7_1209	DJI_0442	Acer tataricum L.	0	Sapindaceae Juss.	n	1
Iz_7_1209	DJI_0442	Betula pendula Roth.	0	Betulaceae Gray	n	1
Iz_7_1209	DJI_0442	Crataegus rhipidophylla Gand.	0	Rosaceae Juss.	n	1
Iz_7_1209	DJI_0442	Frangula alnus Mill.	0	Rhamnaceae Juss.	n	1
Iz_7_1209	DJI_0442	Fraxinus pennsylvanica Marshall	0	Oleaceae Hoffmanns. & Link	a	0
Iz_7_1209	DJI_0442	Pinus sylvestris L.	0	Pinaceae Spreng. ex F.Rudolphi	n	1
Iz_7_1209	DJI_0442	Populus × canescens (Aiton) Sm.	0	Salicaceae Mirb.	n	1
Iz_7_1209	DJI_0442	Populus tremula L.	0	Salicaceae Mirb.	n	1
Iz_7_1209	DJI_0442	Populus nigra L.	0	Salicaceae Mirb.	n	1
Iz_7_1209	DJI_0442	Pyrus communis L.	0	Rosaceae Juss.	a	0
Iz_7_1209	DJI_0442	Quercus robur L.	0	Fagaceae Dumort.	n	1
Iz_7_1209	DJI_0442	Robinia pseudoacacia L.	0	Fabaceae Lindl.	a	0
Iz_7_1209	DJI_0442	Rosa sp.	0	Rosaceae Juss.		
Iz_7_1209	DJI_0442	Salix alba L.	0	Salicaceae Mirb.	n	1
Iz_7_1209	DJI_0442	Sambucus nigra L.	0	Viburnaceae Raf.	n	1
Iz_7_1209	DJI_0442	Sorbus aucuparia L.	0	Rosaceae Juss.	n	1
Iz_7_1209	DJI_0442	Tilia cordata Mill.	0	Malvaceae Juss.	n	1
Iz_7_1209	DJI_0442	Ulmus glabra Huds.	0	Ulmaceae Mirb.	n	1
Iz_7_1209	DJI_0439	Acer tataricum L.	0	Sapindaceae Juss.	n	1
Iz_7_1209	DJI_0439	Betula pendula Roth.	0	Betulaceae Gray	n	1
Iz_7_1209	DJI_0439	Crataegus rhipidophylla Gand.	0	Rosaceae Juss.	n	1
Iz_7_1209	DJI_0439	Frangula alnus Mill.	0	Rhamnaceae Juss.	n	1
Iz_7_1209	DJI_0439	Fraxinus pennsylvanica Marshall	0	Oleaceae Hoffmanns. & Link	a	0
Iz_7_1209	DJI_0439	Pinus sylvestris L.	0	Pinaceae Spreng. ex F.Rudolphi	n	1
Iz_7_1209	DJI_0439	Populus × canescens (Aiton) Sm.	0	Salicaceae Mirb.	n	1
Iz_7_1209	DJI_0439	Populus tremula L.	0	Salicaceae Mirb.	n	1
Iz_7_1209	DJI_0439	Populus nigra L.	0	Salicaceae Mirb.	n	1
Iz_7_1209	DJI_0439	Pyrus communis L.	0	Rosaceae Juss.	a	0
Iz_7_1209	DJI_0439	Quercus robur L.	0	Fagaceae Dumort.	n	1
Iz_7_1209	DJI_0439	Robinia pseudoacacia L.	0	Fabaceae Lindl.	a	0
Iz_7_1209	DJI_0439	Rosa sp.	0	Rosaceae Juss.		
Iz_7_1209	DJI_0439	Salix alba L.	0	Salicaceae Mirb.	n	1
Iz_7_1209	DJI_0439	Sambucus nigra L.	0	Viburnaceae Raf.	n	1
Iz_7_1209	DJI_0439	Sorbus aucuparia L.	0	Rosaceae Juss.	n	1
Iz_7_1209	DJI_0439	Tilia cordata Mill.	0	Malvaceae Juss.	n	1
Iz_7_1209	DJI_0439	Ulmus glabra Huds.	0	Ulmaceae Mirb.	n	1
Iz_7_1209	DJI_0431	Acer tataricum L.	0	Sapindaceae Juss.	n	1
Iz_7_1209	DJI_0431	Betula pendula Roth.	0	Betulaceae Gray	n	1
Iz_7_1209	DJI_0431	Crataegus rhipidophylla Gand.	0	Rosaceae Juss.	n	1
Iz_7_1209	DJI_0431	Frangula alnus Mill.	0	Rhamnaceae Juss.	n	1
Iz_7_1209	DJI_0431	Fraxinus pennsylvanica Marshall	0	Oleaceae Hoffmanns. & Link	a	0
Iz_7_1209	DJI_0431	Pinus sylvestris L.	0	Pinaceae Spreng. ex F.Rudolphi	n	1
Iz_7_1209	DJI_0431	Populus × canescens (Aiton) Sm.	0	Salicaceae Mirb.	n	1
Iz_7_1209	DJI_0431	Populus tremula L.	1	Salicaceae Mirb.	n	1
Iz_7_1209	DJI_0431	Populus nigra L.	0	Salicaceae Mirb.	n	1
Iz_7_1209	DJI_0431	Pyrus communis L.	0	Rosaceae Juss.	a	0
Iz_7_1209	DJI_0431	Quercus robur L.	0	Fagaceae Dumort.	n	1
Iz_7_1209	DJI_0431	Robinia pseudoacacia L.	0	Fabaceae Lindl.	a	0
Iz_7_1209	DJI_0431	Rosa sp.	0	Rosaceae Juss.		
Iz_7_1209	DJI_0431	Salix alba L.	0	Salicaceae Mirb.	n	1
Iz_7_1209	DJI_0431	Sambucus nigra L.	0	Viburnaceae Raf.	n	1
Iz_7_1209	DJI_0431	Sorbus aucuparia L.	0	Rosaceae Juss.	n	1
Iz_7_1209	DJI_0431	Tilia cordata Mill.	0	Malvaceae Juss.	n	1
Iz_7_1209	DJI_0431	Ulmus glabra Huds.	0	Ulmaceae Mirb.	n	1
Iz_7_1209	DJI_0427	Acer tataricum L.	0	Sapindaceae Juss.	n	1
Iz_7_1209	DJI_0427	Betula pendula Roth.	0	Betulaceae Gray	n	1

Iz_7_1209	DJI_0427	Crataegus rhipidophylla Gand.	0	Rosaceae Juss.	n	1
Iz_7_1209	DJI_0427	Frangula alnus Mill.	0	Rhamnaceae Juss.	n	1
Iz_7_1209	DJI_0427	Fraxinus pennsylvanica Marshall	0	Oleaceae Hoffmanns. & Link	a	0
Iz_7_1209	DJI_0427	Pinus sylvestris L.	0	Pinaceae Spreng. ex F.Rudolphi	n	1
Iz_7_1209	DJI_0427	Populus × canescens (Aiton) Sm.	0	Salicaceae Mirb.	n	1
Iz_7_1209	DJI_0427	Populus tremula L.	0	Salicaceae Mirb.	n	1
Iz_7_1209	DJI_0427	Populus nigra L.	0	Salicaceae Mirb.	n	1
Iz_7_1209	DJI_0427	Pyrus communis L.	0	Rosaceae Juss.	a	0
Iz_7_1209	DJI_0427	Quercus robur L.	0	Fagaceae Dumort.	n	1
Iz_7_1209	DJI_0427	Robinia pseudoacacia L.	0	Fabaceae Lindl.	a	0
Iz_7_1209	DJI_0427	Rosa sp.	0	Rosaceae Juss.		
Iz_7_1209	DJI_0427	Salix alba L.	0	Salicaceae Mirb.	n	1
Iz_7_1209	DJI_0427	Sambucus nigra L.	0	Viburnaceae Raf.	n	1
Iz_7_1209	DJI_0427	Sorbus aucuparia L.	0	Rosaceae Juss.	n	1
Iz_7_1209	DJI_0427	Tilia cordata Mill.	0	Malvaceae Juss.	n	1
Iz_7_1209	DJI_0427	Ulmus glabra Huds.	0	Ulmaceae Mirb.	n	1
Iz_7_1209	DJI_0424	Acer tataricum L.	0	Sapindaceae Juss.	n	1
Iz_7_1209	DJI_0424	Betula pendula Roth.	0	Betulaceae Gray	n	1
Iz_7_1209	DJI_0424	Crataegus rhipidophylla Gand.	0	Rosaceae Juss.	n	1
Iz_7_1209	DJI_0424	Frangula alnus Mill.	0	Rhamnaceae Juss.	n	1
Iz_7_1209	DJI_0424	Fraxinus pennsylvanica Marshall	0	Oleaceae Hoffmanns. & Link	a	0
Iz_7_1209	DJI_0424	Pinus sylvestris L.	0	Pinaceae Spreng. ex F.Rudolphi	n	1
Iz_7_1209	DJI_0424	Populus × canescens (Aiton) Sm.	0	Salicaceae Mirb.	n	1
Iz_7_1209	DJI_0424	Populus tremula L.	0	Salicaceae Mirb.	n	1
Iz_7_1209	DJI_0424	Populus nigra L.	0	Salicaceae Mirb.	n	1
Iz_7_1209	DJI_0424	Pyrus communis L.	0	Rosaceae Juss.	a	0
Iz_7_1209	DJI_0424	Quercus robur L.	0	Fagaceae Dumort.	n	1
Iz_7_1209	DJI_0424	Robinia pseudoacacia L.	0	Fabaceae Lindl.	a	0
Iz_7_1209	DJI_0424	Rosa sp.	0	Rosaceae Juss.		
Iz_7_1209	DJI_0424	Salix alba L.	0	Salicaceae Mirb.	n	1
Iz_7_1209	DJI_0424	Sambucus nigra L.	0	Viburnaceae Raf.	n	1
Iz_7_1209	DJI_0424	Sorbus aucuparia L.	0	Rosaceae Juss.	n	1
Iz_7_1209	DJI_0424	Tilia cordata Mill.	0	Malvaceae Juss.	n	1
Iz_7_1209	DJI_0424	Ulmus glabra Huds.	0	Ulmaceae Mirb.	n	1
Iz_7_1209	DJI_0419	Acer tataricum L.	0	Sapindaceae Juss.	n	1
Iz_7_1209	DJI_0419	Betula pendula Roth.	0	Betulaceae Gray	n	1
Iz_7_1209	DJI_0419	Crataegus rhipidophylla Gand.	0	Rosaceae Juss.	n	1
Iz_7_1209	DJI_0419	Frangula alnus Mill.	0	Rhamnaceae Juss.	n	1
Iz_7_1209	DJI_0419	Fraxinus pennsylvanica Marshall	0	Oleaceae Hoffmanns. & Link	a	0
Iz_7_1209	DJI_0419	Pinus sylvestris L.	0	Pinaceae Spreng. ex F.Rudolphi	n	1
Iz_7_1209	DJI_0419	Populus × canescens (Aiton) Sm.	0	Salicaceae Mirb.	n	1
Iz_7_1209	DJI_0419	Populus tremula L.	0	Salicaceae Mirb.	n	1
Iz_7_1209	DJI_0419	Populus nigra L.	0	Salicaceae Mirb.	n	1
Iz_7_1209	DJI_0419	Pyrus communis L.	0	Rosaceae Juss.	a	0
Iz_7_1209	DJI_0419	Quercus robur L.	0	Fagaceae Dumort.	n	1
Iz_7_1209	DJI_0419	Robinia pseudoacacia L.	0	Fabaceae Lindl.	a	0
Iz_7_1209	DJI_0419	Rosa sp.	0	Rosaceae Juss.		
Iz_7_1209	DJI_0419	Salix alba L.	0	Salicaceae Mirb.	n	1
Iz_7_1209	DJI_0419	Sambucus nigra L.	0	Viburnaceae Raf.	n	1
Iz_7_1209	DJI_0419	Sorbus aucuparia L.	0	Rosaceae Juss.	n	1
Iz_7_1209	DJI_0419	Tilia cordata Mill.	0	Malvaceae Juss.	n	1
Iz_7_1209	DJI_0419	Ulmus glabra Huds.	0	Ulmaceae Mirb.	n	1
Iz_7_1209	DJI_0416	Acer tataricum L.	0	Sapindaceae Juss.	n	1
Iz_7_1209	DJI_0416	Betula pendula Roth.	0	Betulaceae Gray	n	1
Iz_7_1209	DJI_0416	Crataegus rhipidophylla Gand.	0	Rosaceae Juss.	n	1
Iz_7_1209	DJI_0416	Frangula alnus Mill.	0	Rhamnaceae Juss.	n	1
Iz_7_1209	DJI_0416	Fraxinus pennsylvanica Marshall	0	Oleaceae Hoffmanns. & Link	a	0
Iz_7_1209	DJI_0416	Pinus sylvestris L.	1	Pinaceae Spreng. ex F.Rudolphi	n	1
Iz_7_1209	DJI_0416	Populus × canescens (Aiton) Sm.	0	Salicaceae Mirb.	n	1
Iz_7_1209	DJI_0416	Populus tremula L.	0	Salicaceae Mirb.	n	1
Iz_7_1209	DJI_0416	Populus nigra L.	0	Salicaceae Mirb.	n	1
Iz_7_1209	DJI_0416	Pyrus communis L.	0	Rosaceae Juss.	a	0
Iz_7_1209	DJI_0416	Quercus robur L.	0	Fagaceae Dumort.	n	1
Iz_7_1209	DJI_0416	Robinia pseudoacacia L.	0	Fabaceae Lindl.	a	0

Iz_7_1209	DJI_0416	Rosa sp.	0	Rosaceae Juss.		
Iz_7_1209	DJI_0416	Salix alba L.	0	Salicaceae Mirb.	n	1
Iz_7_1209	DJI_0416	Sambucus nigra L.	0	Viburnaceae Raf.	n	1
Iz_7_1209	DJI_0416	Sorbus aucuparia L.	0	Rosaceae Juss.	n	1
Iz_7_1209	DJI_0416	Tilia cordata Mill.	0	Malvaceae Juss.	n	1
Iz_7_1209	DJI_0416	Ulmus glabra Huds.	0	Ulmaceae Mirb.	n	1
Iz_7_1209	DJI_0413	Acer tataricum L.	0	Sapindaceae Juss.	n	1
Iz_7_1209	DJI_0413	Betula pendula Roth.	0	Betulaceae Gray	n	1
Iz_7_1209	DJI_0413	Crataegus rhipidophylla Gand.	0	Rosaceae Juss.	n	1
Iz_7_1209	DJI_0413	Frangula alnus Mill.	0	Rhamnaceae Juss.	n	1
Iz_7_1209	DJI_0413	Fraxinus pennsylvanica Marshall	0	Oleaceae Hoffmanns. & Link	a	0
Iz_7_1209	DJI_0413	Pinus sylvestris L.	0	Pinaceae Spreng. ex F.Rudolphi	n	1
Iz_7_1209	DJI_0413	Populus × canescens (Aiton) Sm.	0	Salicaceae Mirb.	n	1
Iz_7_1209	DJI_0413	Populus tremula L.	0	Salicaceae Mirb.	n	1
Iz_7_1209	DJI_0413	Populus nigra L.	0	Salicaceae Mirb.	n	1
Iz_7_1209	DJI_0413	Pyrus communis L.	0	Rosaceae Juss.	a	0
Iz_7_1209	DJI_0413	Quercus robur L.	0	Fagaceae Dumort.	n	1
Iz_7_1209	DJI_0413	Robinia pseudoacacia L.	0	Fabaceae Lindl.	a	0
Iz_7_1209	DJI_0413	Rosa sp.	0	Rosaceae Juss.		
Iz_7_1209	DJI_0413	Salix alba L.	0	Salicaceae Mirb.	n	1
Iz_7_1209	DJI_0413	Sambucus nigra L.	0	Viburnaceae Raf.	n	1
Iz_7_1209	DJI_0413	Sorbus aucuparia L.	0	Rosaceae Juss.	n	1
Iz_7_1209	DJI_0413	Tilia cordata Mill.	0	Malvaceae Juss.	n	1
Iz_7_1209	DJI_0413	Ulmus glabra Huds.	0	Ulmaceae Mirb.	n	1
Iz_7_1209	DJI_0409	Acer tataricum L.	0	Sapindaceae Juss.	n	1
Iz_7_1209	DJI_0409	Betula pendula Roth.	0	Betulaceae Gray	n	1
Iz_7_1209	DJI_0409	Crataegus rhipidophylla Gand.	0	Rosaceae Juss.	n	1
Iz_7_1209	DJI_0409	Frangula alnus Mill.	0	Rhamnaceae Juss.	n	1
Iz_7_1209	DJI_0409	Fraxinus pennsylvanica Marshall	0	Oleaceae Hoffmanns. & Link	a	0
Iz_7_1209	DJI_0409	Pinus sylvestris L.	0	Pinaceae Spreng. ex F.Rudolphi	n	1
Iz_7_1209	DJI_0409	Populus × canescens (Aiton) Sm.	0	Salicaceae Mirb.	n	1
Iz_7_1209	DJI_0409	Populus tremula L.	0	Salicaceae Mirb.	n	1
Iz_7_1209	DJI_0409	Populus nigra L.	0	Salicaceae Mirb.	n	1
Iz_7_1209	DJI_0409	Pyrus communis L.	0	Rosaceae Juss.	a	0
Iz_7_1209	DJI_0409	Quercus robur L.	0	Fagaceae Dumort.	n	1
Iz_7_1209	DJI_0409	Robinia pseudoacacia L.	0	Fabaceae Lindl.	a	0
Iz_7_1209	DJI_0409	Rosa sp.	0	Rosaceae Juss.		
Iz_7_1209	DJI_0409	Salix alba L.	0	Salicaceae Mirb.	n	1
Iz_7_1209	DJI_0409	Sambucus nigra L.	0	Viburnaceae Raf.	n	1
Iz_7_1209	DJI_0409	Sorbus aucuparia L.	0	Rosaceae Juss.	n	1
Iz_7_1209	DJI_0409	Tilia cordata Mill.	0	Malvaceae Juss.	n	1
Iz_7_1209	DJI_0409	Ulmus glabra Huds.	0	Ulmaceae Mirb.	n	1
Iz_7_1209	DJI_0405	Acer tataricum L.	0	Sapindaceae Juss.	n	1
Iz_7_1209	DJI_0405	Betula pendula Roth.	0	Betulaceae Gray	n	1
Iz_7_1209	DJI_0405	Crataegus rhipidophylla Gand.	0	Rosaceae Juss.	n	1
Iz_7_1209	DJI_0405	Frangula alnus Mill.	0	Rhamnaceae Juss.	n	1
Iz_7_1209	DJI_0405	Fraxinus pennsylvanica Marshall	0	Oleaceae Hoffmanns. & Link	a	0
Iz_7_1209	DJI_0405	Pinus sylvestris L.	0	Pinaceae Spreng. ex F.Rudolphi	n	1
Iz_7_1209	DJI_0405	Populus × canescens (Aiton) Sm.	0	Salicaceae Mirb.	n	1
Iz_7_1209	DJI_0405	Populus tremula L.	0	Salicaceae Mirb.	n	1
Iz_7_1209	DJI_0405	Populus nigra L.	0	Salicaceae Mirb.	n	1
Iz_7_1209	DJI_0405	Pyrus communis L.	0	Rosaceae Juss.	a	0
Iz_7_1209	DJI_0405	Quercus robur L.	0	Fagaceae Dumort.	n	1
Iz_7_1209	DJI_0405	Robinia pseudoacacia L.	0	Fabaceae Lindl.	a	0
Iz_7_1209	DJI_0405	Rosa sp.	0	Rosaceae Juss.		
Iz_7_1209	DJI_0405	Salix alba L.	0	Salicaceae Mirb.	n	1
Iz_7_1209	DJI_0405	Sambucus nigra L.	0	Viburnaceae Raf.	n	1
Iz_7_1209	DJI_0405	Sorbus aucuparia L.	0	Rosaceae Juss.	n	1
Iz_7_1209	DJI_0405	Tilia cordata Mill.	0	Malvaceae Juss.	n	1
Iz_7_1209	DJI_0405	Ulmus glabra Huds.	0	Ulmaceae Mirb.	n	1
Iz_7_1209	DJI_0398	Acer tataricum L.	0	Sapindaceae Juss.	n	1
Iz_7_1209	DJI_0398	Betula pendula Roth.	0	Betulaceae Gray	n	1
Iz_7_1209	DJI_0398	Crataegus rhipidophylla Gand.	0	Rosaceae Juss.	n	1
Iz_7_1209	DJI_0398	Frangula alnus Mill.	0	Rhamnaceae Juss.	n	1

Iz_7_1209	DJI_0398	Fraxinus pennsylvanica Marshall	0	Oleaceae Hoffmanns. & Link	a	0
Iz_7_1209	DJI_0398	Pinus sylvestris L.	0	Pinaceae Spreng. ex F.Rudolphi	n	1
Iz_7_1209	DJI_0398	Populus × canescens (Aiton) Sm.	3	Salicaceae Mirb.	n	1
Iz_7_1209	DJI_0398	Populus tremula L.	0	Salicaceae Mirb.	n	1
Iz_7_1209	DJI_0398	Populus nigra L.	0	Salicaceae Mirb.	n	1
Iz_7_1209	DJI_0398	Pyrus communis L.	0	Rosaceae Juss.	a	0
Iz_7_1209	DJI_0398	Quercus robur L.	0	Fagaceae Dumort.	n	1
Iz_7_1209	DJI_0398	Robinia pseudoacacia L.	0	Fabaceae Lindl.	a	0
Iz_7_1209	DJI_0398	Rosa sp.	0	Rosaceae Juss.		
Iz_7_1209	DJI_0398	Salix alba L.	0	Salicaceae Mirb.	n	1
Iz_7_1209	DJI_0398	Sambucus nigra L.	0	Viburnaceae Raf.	n	1
Iz_7_1209	DJI_0398	Sorbus aucuparia L.	0	Rosaceae Juss.	n	1
Iz_7_1209	DJI_0398	Tilia cordata Mill.	0	Malvaceae Juss.	n	1
Iz_7_1209	DJI_0398	Ulmus glabra Huds.	0	Ulmaceae Mirb.	n	1
Iz_7_1209	DJI_0395	Acer tataricum L.	0	Sapindaceae Juss.	n	1
Iz_7_1209	DJI_0395	Betula pendula Roth.	0	Betulaceae Gray	n	1
Iz_7_1209	DJI_0395	Crataegus rhipidophylla Gand.	0	Rosaceae Juss.	n	1
Iz_7_1209	DJI_0395	Frangula alnus Mill.	0	Rhamnaceae Juss.	n	1
Iz_7_1209	DJI_0395	Fraxinus pennsylvanica Marshall	0	Oleaceae Hoffmanns. & Link	a	0
Iz_7_1209	DJI_0395	Pinus sylvestris L.	0	Pinaceae Spreng. ex F.Rudolphi	n	1
Iz_7_1209	DJI_0395	Populus × canescens (Aiton) Sm.	2	Salicaceae Mirb.	n	1
Iz_7_1209	DJI_0395	Populus tremula L.	0	Salicaceae Mirb.	n	1
Iz_7_1209	DJI_0395	Populus nigra L.	0	Salicaceae Mirb.	n	1
Iz_7_1209	DJI_0395	Pyrus communis L.	0	Rosaceae Juss.	a	0
Iz_7_1209	DJI_0395	Quercus robur L.	0	Fagaceae Dumort.	n	1
Iz_7_1209	DJI_0395	Robinia pseudoacacia L.	0	Fabaceae Lindl.	a	0
Iz_7_1209	DJI_0395	Rosa sp.	0	Rosaceae Juss.		
Iz_7_1209	DJI_0395	Salix alba L.	0	Salicaceae Mirb.	n	1
Iz_7_1209	DJI_0395	Sambucus nigra L.	0	Viburnaceae Raf.	n	1
Iz_7_1209	DJI_0395	Sorbus aucuparia L.	0	Rosaceae Juss.	n	1
Iz_7_1209	DJI_0395	Tilia cordata Mill.	0	Malvaceae Juss.	n	1
Iz_7_1209	DJI_0395	Ulmus glabra Huds.	0	Ulmaceae Mirb.	n	1
Iz_7_1209	DJI_0392	Acer tataricum L.	0	Sapindaceae Juss.	n	1
Iz_7_1209	DJI_0392	Betula pendula Roth.	0	Betulaceae Gray	n	1
Iz_7_1209	DJI_0392	Crataegus rhipidophylla Gand.	0	Rosaceae Juss.	n	1
Iz_7_1209	DJI_0392	Frangula alnus Mill.	0	Rhamnaceae Juss.	n	1
Iz_7_1209	DJI_0392	Fraxinus pennsylvanica Marshall	0	Oleaceae Hoffmanns. & Link	a	0
Iz_7_1209	DJI_0392	Pinus sylvestris L.	0	Pinaceae Spreng. ex F.Rudolphi	n	1
Iz_7_1209	DJI_0392	Populus × canescens (Aiton) Sm.	0	Salicaceae Mirb.	n	1
Iz_7_1209	DJI_0392	Populus tremula L.	0	Salicaceae Mirb.	n	1
Iz_7_1209	DJI_0392	Populus nigra L.	0	Salicaceae Mirb.	n	1
Iz_7_1209	DJI_0392	Pyrus communis L.	0	Rosaceae Juss.	a	0
Iz_7_1209	DJI_0392	Quercus robur L.	0	Fagaceae Dumort.	n	1
Iz_7_1209	DJI_0392	Robinia pseudoacacia L.	0	Fabaceae Lindl.	a	0
Iz_7_1209	DJI_0392	Rosa sp.	0	Rosaceae Juss.		
Iz_7_1209	DJI_0392	Salix alba L.	0	Salicaceae Mirb.	n	1
Iz_7_1209	DJI_0392	Sambucus nigra L.	0	Viburnaceae Raf.	n	1
Iz_7_1209	DJI_0392	Sorbus aucuparia L.	0	Rosaceae Juss.	n	1
Iz_7_1209	DJI_0392	Tilia cordata Mill.	0	Malvaceae Juss.	n	1
Iz_7_1209	DJI_0392	Ulmus glabra Huds.	0	Ulmaceae Mirb.	n	1
Iz_8_1209	DJI_0505	Acer tataricum L.	0	Sapindaceae Juss.	n	1
Iz_8_1209	DJI_0505	Betula pendula Roth.	0	Betulaceae Gray	n	1
Iz_8_1209	DJI_0505	Crataegus rhipidophylla Gand.	0	Rosaceae Juss.	n	1
Iz_8_1209	DJI_0505	Frangula alnus Mill.	0	Rhamnaceae Juss.	n	1
Iz_8_1209	DJI_0505	Fraxinus pennsylvanica Marshall	0	Oleaceae Hoffmanns. & Link	a	0
Iz_8_1209	DJI_0505	Pinus sylvestris L.	0	Pinaceae Spreng. ex F.Rudolphi	n	1
Iz_8_1209	DJI_0505	Populus × canescens (Aiton) Sm.	0	Salicaceae Mirb.	n	1
Iz_8_1209	DJI_0505	Populus tremula L.	0	Salicaceae Mirb.	n	1
Iz_8_1209	DJI_0505	Populus nigra L.	0	Salicaceae Mirb.	n	1
Iz_8_1209	DJI_0505	Pyrus communis L.	0	Rosaceae Juss.	a	0
Iz_8_1209	DJI_0505	Quercus robur L.	0	Fagaceae Dumort.	n	1
Iz_8_1209	DJI_0505	Robinia pseudoacacia L.	0	Fabaceae Lindl.	a	0
Iz_8_1209	DJI_0505	Rosa sp.	0	Rosaceae Juss.		
Iz_8_1209	DJI_0505	Salix alba L.	0	Salicaceae Mirb.	n	1

Iz_8_1209	DJI_0505	Sambucus nigra L.	0	Viburnaceae Raf.	n	1
Iz_8_1209	DJI_0505	Sorbus aucuparia L.	0	Rosaceae Juss.	n	1
Iz_8_1209	DJI_0505	Tilia cordata Mill.	0	Malvaceae Juss.	n	1
Iz_8_1209	DJI_0505	Ulmus glabra Huds.	0	Ulmaceae Mirb.	n	1
Iz_8_1209	DJI_0500	Acer tataricum L.	0	Sapindaceae Juss.	n	1
Iz_8_1209	DJI_0500	Betula pendula Roth.	0	Betulaceae Gray	n	1
Iz_8_1209	DJI_0500	Crataegus rhipidophylla Gand.	0	Rosaceae Juss.	n	1
Iz_8_1209	DJI_0500	Frangula alnus Mill.	0	Rhamnaceae Juss.	n	1
Iz_8_1209	DJI_0500	Fraxinus pennsylvanica Marshall	0	Oleaceae Hoffmanns. & Link	a	0
Iz_8_1209	DJI_0500	Pinus sylvestris L.	0	Pinaceae Spreng. ex F.Rudolphi	n	1
Iz_8_1209	DJI_0500	Populus × canescens (Aiton) Sm.	0	Salicaceae Mirb.	n	1
Iz_8_1209	DJI_0500	Populus tremula L.	0	Salicaceae Mirb.	n	1
Iz_8_1209	DJI_0500	Populus nigra L.	0	Salicaceae Mirb.	n	1
Iz_8_1209	DJI_0500	Pyrus communis L.	0	Rosaceae Juss.	a	0
Iz_8_1209	DJI_0500	Quercus robur L.	0	Fagaceae Dumort.	n	1
Iz_8_1209	DJI_0500	Robinia pseudoacacia L.	0	Fabaceae Lindl.	a	0
Iz_8_1209	DJI_0500	Rosa sp.	0	Rosaceae Juss.		
Iz_8_1209	DJI_0500	Salix alba L.	0	Salicaceae Mirb.	n	1
Iz_8_1209	DJI_0500	Sambucus nigra L.	0	Viburnaceae Raf.	n	1
Iz_8_1209	DJI_0500	Sorbus aucuparia L.	0	Rosaceae Juss.	n	1
Iz_8_1209	DJI_0500	Tilia cordata Mill.	0	Malvaceae Juss.	n	1
Iz_8_1209	DJI_0500	Ulmus glabra Huds.	0	Ulmaceae Mirb.	n	1
Iz_8_1209	DJI_0497	Acer tataricum L.	0	Sapindaceae Juss.	n	1
Iz_8_1209	DJI_0497	Betula pendula Roth.	0	Betulaceae Gray	n	1
Iz_8_1209	DJI_0497	Crataegus rhipidophylla Gand.	0	Rosaceae Juss.	n	1
Iz_8_1209	DJI_0497	Frangula alnus Mill.	0	Rhamnaceae Juss.	n	1
Iz_8_1209	DJI_0497	Fraxinus pennsylvanica Marshall	0	Oleaceae Hoffmanns. & Link	a	0
Iz_8_1209	DJI_0497	Pinus sylvestris L.	0	Pinaceae Spreng. ex F.Rudolphi	n	1
Iz_8_1209	DJI_0497	Populus × canescens (Aiton) Sm.	0	Salicaceae Mirb.	n	1
Iz_8_1209	DJI_0497	Populus tremula L.	0	Salicaceae Mirb.	n	1
Iz_8_1209	DJI_0497	Populus nigra L.	0	Salicaceae Mirb.	n	1
Iz_8_1209	DJI_0497	Pyrus communis L.	0	Rosaceae Juss.	a	0
Iz_8_1209	DJI_0497	Quercus robur L.	0	Fagaceae Dumort.	n	1
Iz_8_1209	DJI_0497	Robinia pseudoacacia L.	0	Fabaceae Lindl.	a	0
Iz_8_1209	DJI_0497	Rosa sp.	0	Rosaceae Juss.		
Iz_8_1209	DJI_0497	Salix alba L.	0	Salicaceae Mirb.	n	1
Iz_8_1209	DJI_0497	Sambucus nigra L.	0	Viburnaceae Raf.	n	1
Iz_8_1209	DJI_0497	Sorbus aucuparia L.	0	Rosaceae Juss.	n	1
Iz_8_1209	DJI_0497	Tilia cordata Mill.	0	Malvaceae Juss.	n	1
Iz_8_1209	DJI_0497	Ulmus glabra Huds.	0	Ulmaceae Mirb.	n	1
Iz_8_1209	DJI_0493	Acer tataricum L.	0	Sapindaceae Juss.	n	1
Iz_8_1209	DJI_0493	Betula pendula Roth.	0	Betulaceae Gray	n	1
Iz_8_1209	DJI_0493	Crataegus rhipidophylla Gand.	0	Rosaceae Juss.	n	1
Iz_8_1209	DJI_0493	Frangula alnus Mill.	0	Rhamnaceae Juss.	n	1
Iz_8_1209	DJI_0493	Fraxinus pennsylvanica Marshall	0	Oleaceae Hoffmanns. & Link	a	0
Iz_8_1209	DJI_0493	Pinus sylvestris L.	0	Pinaceae Spreng. ex F.Rudolphi	n	1
Iz_8_1209	DJI_0493	Populus × canescens (Aiton) Sm.	0	Salicaceae Mirb.	n	1
Iz_8_1209	DJI_0493	Populus tremula L.	0	Salicaceae Mirb.	n	1
Iz_8_1209	DJI_0493	Populus nigra L.	0	Salicaceae Mirb.	n	1
Iz_8_1209	DJI_0493	Pyrus communis L.	0	Rosaceae Juss.	a	0
Iz_8_1209	DJI_0493	Quercus robur L.	0	Fagaceae Dumort.	n	1
Iz_8_1209	DJI_0493	Robinia pseudoacacia L.	0	Fabaceae Lindl.	a	0
Iz_8_1209	DJI_0493	Rosa sp.	0	Rosaceae Juss.		
Iz_8_1209	DJI_0493	Salix alba L.	0	Salicaceae Mirb.	n	1
Iz_8_1209	DJI_0493	Sambucus nigra L.	0	Viburnaceae Raf.	n	1
Iz_8_1209	DJI_0493	Sorbus aucuparia L.	0	Rosaceae Juss.	n	1
Iz_8_1209	DJI_0493	Tilia cordata Mill.	0	Malvaceae Juss.	n	1
Iz_8_1209	DJI_0493	Ulmus glabra Huds.	0	Ulmaceae Mirb.	n	1
Iz_8_1209	DJI_0490	Acer tataricum L.	0	Sapindaceae Juss.	n	1
Iz_8_1209	DJI_0490	Betula pendula Roth.	0	Betulaceae Gray	n	1
Iz_8_1209	DJI_0490	Crataegus rhipidophylla Gand.	0	Rosaceae Juss.	n	1
Iz_8_1209	DJI_0490	Frangula alnus Mill.	0	Rhamnaceae Juss.	n	1
Iz_8_1209	DJI_0490	Fraxinus pennsylvanica Marshall	0	Oleaceae Hoffmanns. & Link	a	0
Iz_8_1209	DJI_0490	Pinus sylvestris L.	0	Pinaceae Spreng. ex F.Rudolphi	n	1

Iz_8_1209	DJI_0490	Populus × canescens (Aiton) Sm.	0	Salicaceae Mirb.	n	1
Iz_8_1209	DJI_0490	Populus tremula L.	0	Salicaceae Mirb.	n	1
Iz_8_1209	DJI_0490	Populus nigra L.	0	Salicaceae Mirb.	n	1
Iz_8_1209	DJI_0490	Pyrus communis L.	0	Rosaceae Juss.	a	0
Iz_8_1209	DJI_0490	Quercus robur L.	0	Fagaceae Dumort.	n	1
Iz_8_1209	DJI_0490	Robinia pseudoacacia L.	0	Fabaceae Lindl.	a	0
Iz_8_1209	DJI_0490	Rosa sp.	0	Rosaceae Juss.		
Iz_8_1209	DJI_0490	Salix alba L.	0	Salicaceae Mirb.	n	1
Iz_8_1209	DJI_0490	Sambucus nigra L.	0	Viburnaceae Raf.	n	1
Iz_8_1209	DJI_0490	Sorbus aucuparia L.	0	Rosaceae Juss.	n	1
Iz_8_1209	DJI_0490	Tilia cordata Mill.	0	Malvaceae Juss.	n	1
Iz_8_1209	DJI_0490	Ulmus glabra Huds.	0	Ulmaceae Mirb.	n	1
Iz_8_1209	DJI_0486	Acer tataricum L.	0	Sapindaceae Juss.	n	1
Iz_8_1209	DJI_0486	Betula pendula Roth.	0	Betulaceae Gray	n	1
Iz_8_1209	DJI_0486	Crataegus rhipidophylla Gand.	0	Rosaceae Juss.	n	1
Iz_8_1209	DJI_0486	Frangula alnus Mill.	0	Rhamnaceae Juss.	n	1
Iz_8_1209	DJI_0486	Fraxinus pennsylvanica Marshall	0	Oleaceae Hoffmanns. & Link	a	0
Iz_8_1209	DJI_0486	Pinus sylvestris L.	0	Pinaceae Spreng. ex F.Rudolphi	n	1
Iz_8_1209	DJI_0486	Populus × canescens (Aiton) Sm.	0	Salicaceae Mirb.	n	1
Iz_8_1209	DJI_0486	Populus tremula L.	0	Salicaceae Mirb.	n	1
Iz_8_1209	DJI_0486	Populus nigra L.	0	Salicaceae Mirb.	n	1
Iz_8_1209	DJI_0486	Pyrus communis L.	0	Rosaceae Juss.	a	0
Iz_8_1209	DJI_0486	Quercus robur L.	0	Fagaceae Dumort.	n	1
Iz_8_1209	DJI_0486	Robinia pseudoacacia L.	0	Fabaceae Lindl.	a	0
Iz_8_1209	DJI_0486	Rosa sp.	0	Rosaceae Juss.		
Iz_8_1209	DJI_0486	Salix alba L.	0	Salicaceae Mirb.	n	1
Iz_8_1209	DJI_0486	Sambucus nigra L.	0	Viburnaceae Raf.	n	1
Iz_8_1209	DJI_0486	Sorbus aucuparia L.	0	Rosaceae Juss.	n	1
Iz_8_1209	DJI_0486	Tilia cordata Mill.	0	Malvaceae Juss.	n	1
Iz_8_1209	DJI_0486	Ulmus glabra Huds.	0	Ulmaceae Mirb.	n	1
Iz_8_1209	DJI_0480	Acer tataricum L.	0	Sapindaceae Juss.	n	1
Iz_8_1209	DJI_0480	Betula pendula Roth.	0	Betulaceae Gray	n	1
Iz_8_1209	DJI_0480	Crataegus rhipidophylla Gand.	0	Rosaceae Juss.	n	1
Iz_8_1209	DJI_0480	Frangula alnus Mill.	0	Rhamnaceae Juss.	n	1
Iz_8_1209	DJI_0480	Fraxinus pennsylvanica Marshall	0	Oleaceae Hoffmanns. & Link	a	0
Iz_8_1209	DJI_0480	Pinus sylvestris L.	0	Pinaceae Spreng. ex F.Rudolphi	n	1
Iz_8_1209	DJI_0480	Populus × canescens (Aiton) Sm.	0	Salicaceae Mirb.	n	1
Iz_8_1209	DJI_0480	Populus tremula L.	0	Salicaceae Mirb.	n	1
Iz_8_1209	DJI_0480	Populus nigra L.	0	Salicaceae Mirb.	n	1
Iz_8_1209	DJI_0480	Pyrus communis L.	0	Rosaceae Juss.	a	0
Iz_8_1209	DJI_0480	Quercus robur L.	0	Fagaceae Dumort.	n	1
Iz_8_1209	DJI_0480	Robinia pseudoacacia L.	0	Fabaceae Lindl.	a	0
Iz_8_1209	DJI_0480	Rosa sp.	0	Rosaceae Juss.		
Iz_8_1209	DJI_0480	Salix alba L.	0	Salicaceae Mirb.	n	1
Iz_8_1209	DJI_0480	Sambucus nigra L.	0	Viburnaceae Raf.	n	1
Iz_8_1209	DJI_0480	Sorbus aucuparia L.	0	Rosaceae Juss.	n	1
Iz_8_1209	DJI_0480	Tilia cordata Mill.	0	Malvaceae Juss.	n	1
Iz_8_1209	DJI_0480	Ulmus glabra Huds.	0	Ulmaceae Mirb.	n	1
Iz_8_1209	DJI_0488	Acer tataricum L.	0	Sapindaceae Juss.	n	1
Iz_8_1209	DJI_0488	Betula pendula Roth.	0	Betulaceae Gray	n	1
Iz_8_1209	DJI_0488	Crataegus rhipidophylla Gand.	0	Rosaceae Juss.	n	1
Iz_8_1209	DJI_0488	Frangula alnus Mill.	0	Rhamnaceae Juss.	n	1
Iz_8_1209	DJI_0488	Fraxinus pennsylvanica Marshall	0	Oleaceae Hoffmanns. & Link	a	0
Iz_8_1209	DJI_0488	Pinus sylvestris L.	0	Pinaceae Spreng. ex F.Rudolphi	n	1
Iz_8_1209	DJI_0488	Populus × canescens (Aiton) Sm.	0	Salicaceae Mirb.	n	1
Iz_8_1209	DJI_0488	Populus tremula L.	0	Salicaceae Mirb.	n	1
Iz_8_1209	DJI_0488	Populus nigra L.	0	Salicaceae Mirb.	n	1
Iz_8_1209	DJI_0488	Pyrus communis L.	0	Rosaceae Juss.	a	0
Iz_8_1209	DJI_0488	Quercus robur L.	0	Fagaceae Dumort.	n	1
Iz_8_1209	DJI_0488	Robinia pseudoacacia L.	0	Fabaceae Lindl.	a	0
Iz_8_1209	DJI_0488	Rosa sp.	0	Rosaceae Juss.		
Iz_8_1209	DJI_0488	Salix alba L.	0	Salicaceae Mirb.	n	1
Iz_8_1209	DJI_0488	Sambucus nigra L.	0	Viburnaceae Raf.	n	1
Iz_8_1209	DJI_0488	Sorbus aucuparia L.	0	Rosaceae Juss.	n	1

Iz_8_1209	DJI_0488	Tilia cordata Mill.	0	Malvaceae Juss.	n	1
Iz_8_1209	DJI_0488	Ulmus glabra Huds.	0	Ulmaceae Mirb.	n	1
Iz_8_1209	DJI_0474	Acer tataricum L.	0	Sapindaceae Juss.	n	1
Iz_8_1209	DJI_0474	Betula pendula Roth.	0	Betulaceae Gray	n	1
Iz_8_1209	DJI_0474	Crataegus rhipidophylla Gand.	0	Rosaceae Juss.	n	1
Iz_8_1209	DJI_0474	Frangula alnus Mill.	0	Rhamnaceae Juss.	n	1
Iz_8_1209	DJI_0474	Fraxinus pennsylvanica Marshall	0	Oleaceae Hoffmanns. & Link	a	0
Iz_8_1209	DJI_0474	Pinus sylvestris L.	0	Pinaceae Spreng. ex F.Rudolphi	n	1
Iz_8_1209	DJI_0474	Populus × canescens (Aiton) Sm.	0	Salicaceae Mirb.	n	1
Iz_8_1209	DJI_0474	Populus tremula L.	0	Salicaceae Mirb.	n	1
Iz_8_1209	DJI_0474	Populus nigra L.	0	Salicaceae Mirb.	n	1
Iz_8_1209	DJI_0474	Pyrus communis L.	0	Rosaceae Juss.	a	0
Iz_8_1209	DJI_0474	Quercus robur L.	0	Fagaceae Dumort.	n	1
Iz_8_1209	DJI_0474	Robinia pseudoacacia L.	0	Fabaceae Lindl.	a	0
Iz_8_1209	DJI_0474	Rosa sp.	0	Rosaceae Juss.		
Iz_8_1209	DJI_0474	Salix alba L.	0	Salicaceae Mirb.	n	1
Iz_8_1209	DJI_0474	Sambucus nigra L.	0	Viburnaceae Raf.	n	1
Iz_8_1209	DJI_0474	Sorbus aucuparia L.	0	Rosaceae Juss.	n	1
Iz_8_1209	DJI_0474	Tilia cordata Mill.	0	Malvaceae Juss.	n	1
Iz_8_1209	DJI_0474	Ulmus glabra Huds.	0	Ulmaceae Mirb.	n	1
Iz_8_1209	DJI_0470	Acer tataricum L.	0	Sapindaceae Juss.	n	1
Iz_8_1209	DJI_0470	Betula pendula Roth.	0	Betulaceae Gray	n	1
Iz_8_1209	DJI_0470	Crataegus rhipidophylla Gand.	0	Rosaceae Juss.	n	1
Iz_8_1209	DJI_0470	Frangula alnus Mill.	0	Rhamnaceae Juss.	n	1
Iz_8_1209	DJI_0470	Fraxinus pennsylvanica Marshall	0	Oleaceae Hoffmanns. & Link	a	0
Iz_8_1209	DJI_0470	Pinus sylvestris L.	0	Pinaceae Spreng. ex F.Rudolphi	n	1
Iz_8_1209	DJI_0470	Populus × canescens (Aiton) Sm.	0	Salicaceae Mirb.	n	1
Iz_8_1209	DJI_0470	Populus tremula L.	0	Salicaceae Mirb.	n	1
Iz_8_1209	DJI_0470	Populus nigra L.	0	Salicaceae Mirb.	n	1
Iz_8_1209	DJI_0470	Pyrus communis L.	0	Rosaceae Juss.	a	0
Iz_8_1209	DJI_0470	Quercus robur L.	0	Fagaceae Dumort.	n	1
Iz_8_1209	DJI_0470	Robinia pseudoacacia L.	0	Fabaceae Lindl.	a	0
Iz_8_1209	DJI_0470	Rosa sp.	0	Rosaceae Juss.		
Iz_8_1209	DJI_0470	Salix alba L.	0	Salicaceae Mirb.	n	1
Iz_8_1209	DJI_0470	Sambucus nigra L.	0	Viburnaceae Raf.	n	1
Iz_8_1209	DJI_0470	Sorbus aucuparia L.	0	Rosaceae Juss.	n	1
Iz_8_1209	DJI_0470	Tilia cordata Mill.	0	Malvaceae Juss.	n	1
Iz_8_1209	DJI_0470	Ulmus glabra Huds.	0	Ulmaceae Mirb.	n	1
Iz_8_1209	DJI_0467	Acer tataricum L.	0	Sapindaceae Juss.	n	1
Iz_8_1209	DJI_0467	Betula pendula Roth.	0	Betulaceae Gray	n	1
Iz_8_1209	DJI_0467	Crataegus rhipidophylla Gand.	0	Rosaceae Juss.	n	1
Iz_8_1209	DJI_0467	Frangula alnus Mill.	0	Rhamnaceae Juss.	n	1
Iz_8_1209	DJI_0467	Fraxinus pennsylvanica Marshall	0	Oleaceae Hoffmanns. & Link	a	0
Iz_8_1209	DJI_0467	Pinus sylvestris L.	0	Pinaceae Spreng. ex F.Rudolphi	n	1
Iz_8_1209	DJI_0467	Populus × canescens (Aiton) Sm.	0	Salicaceae Mirb.	n	1
Iz_8_1209	DJI_0467	Populus tremula L.	0	Salicaceae Mirb.	n	1
Iz_8_1209	DJI_0467	Populus nigra L.	0	Salicaceae Mirb.	n	1
Iz_8_1209	DJI_0467	Pyrus communis L.	0	Rosaceae Juss.	a	0
Iz_8_1209	DJI_0467	Quercus robur L.	0	Fagaceae Dumort.	n	1
Iz_8_1209	DJI_0467	Robinia pseudoacacia L.	0	Fabaceae Lindl.	a	0
Iz_8_1209	DJI_0467	Rosa sp.	0	Rosaceae Juss.		
Iz_8_1209	DJI_0467	Salix alba L.	0	Salicaceae Mirb.	n	1
Iz_8_1209	DJI_0467	Sambucus nigra L.	0	Viburnaceae Raf.	n	1
Iz_8_1209	DJI_0467	Sorbus aucuparia L.	0	Rosaceae Juss.	n	1
Iz_8_1209	DJI_0467	Tilia cordata Mill.	0	Malvaceae Juss.	n	1
Iz_8_1209	DJI_0467	Ulmus glabra Huds.	0	Ulmaceae Mirb.	n	1
Iz_8_1209	DJI_0464	Acer tataricum L.	0	Sapindaceae Juss.	n	1
Iz_8_1209	DJI_0464	Betula pendula Roth.	0	Betulaceae Gray	n	1
Iz_8_1209	DJI_0464	Crataegus rhipidophylla Gand.	0	Rosaceae Juss.	n	1
Iz_8_1209	DJI_0464	Frangula alnus Mill.	0	Rhamnaceae Juss.	n	1
Iz_8_1209	DJI_0464	Fraxinus pennsylvanica Marshall	0	Oleaceae Hoffmanns. & Link	a	0
Iz_8_1209	DJI_0464	Pinus sylvestris L.	0	Pinaceae Spreng. ex F.Rudolphi	n	1
Iz_8_1209	DJI_0464	Populus × canescens (Aiton) Sm.	0	Salicaceae Mirb.	n	1
Iz_8_1209	DJI_0464	Populus tremula L.	0	Salicaceae Mirb.	n	1

Iz_8_1209	DJI_0464	Populus nigra L.	0	Salicaceae Mirb.	n	1
Iz_8_1209	DJI_0464	Pyrus communis L.	0	Rosaceae Juss.	a	0
Iz_8_1209	DJI_0464	Quercus robur L.	0	Fagaceae Dumort.	n	1
Iz_8_1209	DJI_0464	Robinia pseudoacacia L.	0	Fabaceae Lindl.	a	0
Iz_8_1209	DJI_0464	Rosa sp.	0	Rosaceae Juss.		
Iz_8_1209	DJI_0464	Salix alba L.	0	Salicaceae Mirb.	n	1
Iz_8_1209	DJI_0464	Sambucus nigra L.	0	Viburnaceae Raf.	n	1
Iz_8_1209	DJI_0464	Sorbus aucuparia L.	0	Rosaceae Juss.	n	1
Iz_8_1209	DJI_0464	Tilia cordata Mill.	0	Malvaceae Juss.	n	1
Iz_8_1209	DJI_0464	Ulmus glabra Huds.	0	Ulmaceae Mirb.	n	1
Iz_8_1209	DJI_0460	Acer tataricum L.	0	Sapindaceae Juss.	n	1
Iz_8_1209	DJI_0460	Betula pendula Roth.	0	Betulaceae Gray	n	1
Iz_8_1209	DJI_0460	Crataegus rhipidophylla Gand.	0	Rosaceae Juss.	n	1
Iz_8_1209	DJI_0460	Frangula alnus Mill.	0	Rhamnaceae Juss.	n	1
Iz_8_1209	DJI_0460	Fraxinus pennsylvanica Marshall	0	Oleaceae Hoffmanns. & Link	a	0
Iz_8_1209	DJI_0460	Pinus sylvestris L.	0	Pinaceae Spreng. ex F.Rudolphi	n	1
Iz_8_1209	DJI_0460	Populus × canescens (Aiton) Sm.	0	Salicaceae Mirb.	n	1
Iz_8_1209	DJI_0460	Populus tremula L.	0	Salicaceae Mirb.	n	1
Iz_8_1209	DJI_0460	Populus nigra L.	0	Salicaceae Mirb.	n	1
Iz_8_1209	DJI_0460	Pyrus communis L.	0	Rosaceae Juss.	a	0
Iz_8_1209	DJI_0460	Quercus robur L.	0	Fagaceae Dumort.	n	1
Iz_8_1209	DJI_0460	Robinia pseudoacacia L.	0	Fabaceae Lindl.	a	0
Iz_8_1209	DJI_0460	Rosa sp.	0	Rosaceae Juss.		
Iz_8_1209	DJI_0460	Salix alba L.	0	Salicaceae Mirb.	n	1
Iz_8_1209	DJI_0460	Sambucus nigra L.	0	Viburnaceae Raf.	n	1
Iz_8_1209	DJI_0460	Sorbus aucuparia L.	0	Rosaceae Juss.	n	1
Iz_8_1209	DJI_0460	Tilia cordata Mill.	0	Malvaceae Juss.	n	1
Iz_8_1209	DJI_0460	Ulmus glabra Huds.	0	Ulmaceae Mirb.	n	1
Iz_8_1209	DJI_0456	Acer tataricum L.	0	Sapindaceae Juss.	n	1
Iz_8_1209	DJI_0456	Betula pendula Roth.	0	Betulaceae Gray	n	1
Iz_8_1209	DJI_0456	Crataegus rhipidophylla Gand.	0	Rosaceae Juss.	n	1
Iz_8_1209	DJI_0456	Frangula alnus Mill.	0	Rhamnaceae Juss.	n	1
Iz_8_1209	DJI_0456	Fraxinus pennsylvanica Marshall	0	Oleaceae Hoffmanns. & Link	a	0
Iz_8_1209	DJI_0456	Pinus sylvestris L.	0	Pinaceae Spreng. ex F.Rudolphi	n	1
Iz_8_1209	DJI_0456	Populus × canescens (Aiton) Sm.	0	Salicaceae Mirb.	n	1
Iz_8_1209	DJI_0456	Populus tremula L.	0	Salicaceae Mirb.	n	1
Iz_8_1209	DJI_0456	Populus nigra L.	0	Salicaceae Mirb.	n	1
Iz_8_1209	DJI_0456	Pyrus communis L.	0	Rosaceae Juss.	a	0
Iz_8_1209	DJI_0456	Quercus robur L.	0	Fagaceae Dumort.	n	1
Iz_8_1209	DJI_0456	Robinia pseudoacacia L.	0	Fabaceae Lindl.	a	0
Iz_8_1209	DJI_0456	Rosa sp.	0	Rosaceae Juss.		
Iz_8_1209	DJI_0456	Salix alba L.	0	Salicaceae Mirb.	n	1
Iz_8_1209	DJI_0456	Sambucus nigra L.	0	Viburnaceae Raf.	n	1
Iz_8_1209	DJI_0456	Sorbus aucuparia L.	0	Rosaceae Juss.	n	1
Iz_8_1209	DJI_0456	Tilia cordata Mill.	0	Malvaceae Juss.	n	1
Iz_8_1209	DJI_0456	Ulmus glabra Huds.	0	Ulmaceae Mirb.	n	1
Iz_9_1209	DJI_0601	Acer tataricum L.	0	Sapindaceae Juss.	n	1
Iz_9_1209	DJI_0601	Betula pendula Roth.	0	Betulaceae Gray	n	1
Iz_9_1209	DJI_0601	Crataegus rhipidophylla Gand.	0	Rosaceae Juss.	n	1
Iz_9_1209	DJI_0601	Frangula alnus Mill.	0	Rhamnaceae Juss.	n	1
Iz_9_1209	DJI_0601	Fraxinus pennsylvanica Marshall	0	Oleaceae Hoffmanns. & Link	a	0
Iz_9_1209	DJI_0601	Pinus sylvestris L.	0	Pinaceae Spreng. ex F.Rudolphi	n	1
Iz_9_1209	DJI_0601	Populus × canescens (Aiton) Sm.	0	Salicaceae Mirb.	n	1
Iz_9_1209	DJI_0601	Populus tremula L.	0	Salicaceae Mirb.	n	1
Iz_9_1209	DJI_0601	Populus nigra L.	0	Salicaceae Mirb.	n	1
Iz_9_1209	DJI_0601	Pyrus communis L.	0	Rosaceae Juss.	a	0
Iz_9_1209	DJI_0601	Quercus robur L.	0	Fagaceae Dumort.	n	1
Iz_9_1209	DJI_0601	Robinia pseudoacacia L.	0	Fabaceae Lindl.	a	0
Iz_9_1209	DJI_0601	Rosa sp.	0	Rosaceae Juss.		
Iz_9_1209	DJI_0601	Salix alba L.	0	Salicaceae Mirb.	n	1
Iz_9_1209	DJI_0601	Sambucus nigra L.	0	Viburnaceae Raf.	n	1
Iz_9_1209	DJI_0601	Sorbus aucuparia L.	0	Rosaceae Juss.	n	1
Iz_9_1209	DJI_0601	Tilia cordata Mill.	0	Malvaceae Juss.	n	1
Iz_9_1209	DJI_0601	Ulmus glabra Huds.	0	Ulmaceae Mirb.	n	1

Iz_9_1209	DJI_0582	Acer tataricum L.	0	Sapindaceae Juss.	n	1
Iz_9_1209	DJI_0582	Betula pendula Roth.	0	Betulaceae Gray	n	1
Iz_9_1209	DJI_0582	Crataegus rhipidophylla Gand.	0	Rosaceae Juss.	n	1
Iz_9_1209	DJI_0582	Frangula alnus Mill.	0	Rhamnaceae Juss.	n	1
Iz_9_1209	DJI_0582	Fraxinus pennsylvanica Marshall	0	Oleaceae Hoffmanns. & Link	a	0
Iz_9_1209	DJI_0582	Pinus sylvestris L.	0	Pinaceae Spreng. ex F.Rudolphi	n	1
Iz_9_1209	DJI_0582	Populus × canescens (Aiton) Sm.	0	Salicaceae Mirb.	n	1
Iz_9_1209	DJI_0582	Populus tremula L.	0	Salicaceae Mirb.	n	1
Iz_9_1209	DJI_0582	Populus nigra L.	0	Salicaceae Mirb.	n	1
Iz_9_1209	DJI_0582	Pyrus communis L.	0	Rosaceae Juss.	a	0
Iz_9_1209	DJI_0582	Quercus robur L.	0	Fagaceae Dumort.	n	1
Iz_9_1209	DJI_0582	Robinia pseudoacacia L.	0	Fabaceae Lindl.	a	0
Iz_9_1209	DJI_0582	Rosa sp.	0	Rosaceae Juss.		
Iz_9_1209	DJI_0582	Salix alba L.	0	Salicaceae Mirb.	n	1
Iz_9_1209	DJI_0582	Sambucus nigra L.	0	Viburnaceae Raf.	n	1
Iz_9_1209	DJI_0582	Sorbus aucuparia L.	0	Rosaceae Juss.	n	1
Iz_9_1209	DJI_0582	Tilia cordata Mill.	0	Malvaceae Juss.	n	1
Iz_9_1209	DJI_0582	Ulmus glabra Huds.	0	Ulmaceae Mirb.	n	1
Iz_9_1209	DJI_0588	Acer tataricum L.	0	Sapindaceae Juss.	n	1
Iz_9_1209	DJI_0588	Betula pendula Roth.	0	Betulaceae Gray	n	1
Iz_9_1209	DJI_0588	Crataegus rhipidophylla Gand.	0	Rosaceae Juss.	n	1
Iz_9_1209	DJI_0588	Frangula alnus Mill.	0	Rhamnaceae Juss.	n	1
Iz_9_1209	DJI_0588	Fraxinus pennsylvanica Marshall	0	Oleaceae Hoffmanns. & Link	a	0
Iz_9_1209	DJI_0588	Pinus sylvestris L.	0	Pinaceae Spreng. ex F.Rudolphi	n	1
Iz_9_1209	DJI_0588	Populus × canescens (Aiton) Sm.	0	Salicaceae Mirb.	n	1
Iz_9_1209	DJI_0588	Populus tremula L.	0	Salicaceae Mirb.	n	1
Iz_9_1209	DJI_0588	Populus nigra L.	0	Salicaceae Mirb.	n	1
Iz_9_1209	DJI_0588	Pyrus communis L.	0	Rosaceae Juss.	a	0
Iz_9_1209	DJI_0588	Quercus robur L.	0	Fagaceae Dumort.	n	1
Iz_9_1209	DJI_0588	Robinia pseudoacacia L.	0	Fabaceae Lindl.	a	0
Iz_9_1209	DJI_0588	Rosa sp.	0	Rosaceae Juss.		
Iz_9_1209	DJI_0588	Salix alba L.	0	Salicaceae Mirb.	n	1
Iz_9_1209	DJI_0588	Sambucus nigra L.	0	Viburnaceae Raf.	n	1
Iz_9_1209	DJI_0588	Sorbus aucuparia L.	0	Rosaceae Juss.	n	1
Iz_9_1209	DJI_0588	Tilia cordata Mill.	0	Malvaceae Juss.	n	1
Iz_9_1209	DJI_0588	Ulmus glabra Huds.	0	Ulmaceae Mirb.	n	1
Iz_9_1209	DJI_0579	Acer tataricum L.	0	Sapindaceae Juss.	n	1
Iz_9_1209	DJI_0579	Betula pendula Roth.	0	Betulaceae Gray	n	1
Iz_9_1209	DJI_0579	Crataegus rhipidophylla Gand.	0	Rosaceae Juss.	n	1
Iz_9_1209	DJI_0579	Frangula alnus Mill.	0	Rhamnaceae Juss.	n	1
Iz_9_1209	DJI_0579	Fraxinus pennsylvanica Marshall	0	Oleaceae Hoffmanns. & Link	a	0
Iz_9_1209	DJI_0579	Pinus sylvestris L.	0	Pinaceae Spreng. ex F.Rudolphi	n	1
Iz_9_1209	DJI_0579	Populus × canescens (Aiton) Sm.	0	Salicaceae Mirb.	n	1
Iz_9_1209	DJI_0579	Populus tremula L.	0	Salicaceae Mirb.	n	1
Iz_9_1209	DJI_0579	Populus nigra L.	0	Salicaceae Mirb.	n	1
Iz_9_1209	DJI_0579	Pyrus communis L.	0	Rosaceae Juss.	a	0
Iz_9_1209	DJI_0579	Quercus robur L.	0	Fagaceae Dumort.	n	1
Iz_9_1209	DJI_0579	Robinia pseudoacacia L.	0	Fabaceae Lindl.	a	0
Iz_9_1209	DJI_0579	Rosa sp.	0	Rosaceae Juss.		
Iz_9_1209	DJI_0579	Salix alba L.	0	Salicaceae Mirb.	n	1
Iz_9_1209	DJI_0579	Sambucus nigra L.	0	Viburnaceae Raf.	n	1
Iz_9_1209	DJI_0579	Sorbus aucuparia L.	0	Rosaceae Juss.	n	1
Iz_9_1209	DJI_0579	Tilia cordata Mill.	0	Malvaceae Juss.	n	1
Iz_9_1209	DJI_0579	Ulmus glabra Huds.	0	Ulmaceae Mirb.	n	1
Iz_9_1209	DJI_0575	Acer tataricum L.	0	Sapindaceae Juss.	n	1
Iz_9_1209	DJI_0575	Betula pendula Roth.	0	Betulaceae Gray	n	1
Iz_9_1209	DJI_0575	Crataegus rhipidophylla Gand.	0	Rosaceae Juss.	n	1
Iz_9_1209	DJI_0575	Frangula alnus Mill.	0	Rhamnaceae Juss.	n	1
Iz_9_1209	DJI_0575	Fraxinus pennsylvanica Marshall	0	Oleaceae Hoffmanns. & Link	a	0
Iz_9_1209	DJI_0575	Pinus sylvestris L.	0	Pinaceae Spreng. ex F.Rudolphi	n	1
Iz_9_1209	DJI_0575	Populus × canescens (Aiton) Sm.	0	Salicaceae Mirb.	n	1
Iz_9_1209	DJI_0575	Populus tremula L.	0	Salicaceae Mirb.	n	1
Iz_9_1209	DJI_0575	Populus nigra L.	0	Salicaceae Mirb.	n	1
Iz_9_1209	DJI_0575	Pyrus communis L.	0	Rosaceae Juss.	a	0

Iz_9_1209	DJI_0575	Quercus robur L.	0	Fagaceae Dumort.	n	1
Iz_9_1209	DJI_0575	Robinia pseudoacacia L.	0	Fabaceae Lindl.	a	0
Iz_9_1209	DJI_0575	Rosa sp.	0	Rosaceae Juss.		
Iz_9_1209	DJI_0575	Salix alba L.	0	Salicaceae Mirb.	n	1
Iz_9_1209	DJI_0575	Sambucus nigra L.	0	Viburnaceae Raf.	n	1
Iz_9_1209	DJI_0575	Sorbus aucuparia L.	0	Rosaceae Juss.	n	1
Iz_9_1209	DJI_0575	Tilia cordata Mill.	0	Malvaceae Juss.	n	1
Iz_9_1209	DJI_0575	Ulmus glabra Huds.	0	Ulmaceae Mirb.	n	1
Iz_9_1209	DJI_0572	Acer tataricum L.	0	Sapindaceae Juss.	n	1
Iz_9_1209	DJI_0572	Betula pendula Roth.	0	Betulaceae Gray	n	1
Iz_9_1209	DJI_0572	Crataegus rhipidophylla Gand.	0	Rosaceae Juss.	n	1
Iz_9_1209	DJI_0572	Frangula alnus Mill.	0	Rhamnaceae Juss.	n	1
Iz_9_1209	DJI_0572	Fraxinus pennsylvanica Marshall	0	Oleaceae Hoffmanns. & Link	a	0
Iz_9_1209	DJI_0572	Pinus sylvestris L.	0	Pinaceae Spreng. ex F.Rudolphi	n	1
Iz_9_1209	DJI_0572	Populus × canescens (Aiton) Sm.	0	Salicaceae Mirb.	n	1
Iz_9_1209	DJI_0572	Populus tremula L.	0	Salicaceae Mirb.	n	1
Iz_9_1209	DJI_0572	Populus nigra L.	0	Salicaceae Mirb.	n	1
Iz_9_1209	DJI_0572	Pyrus communis L.	0	Rosaceae Juss.	a	0
Iz_9_1209	DJI_0572	Quercus robur L.	0	Fagaceae Dumort.	n	1
Iz_9_1209	DJI_0572	Robinia pseudoacacia L.	0	Fabaceae Lindl.	a	0
Iz_9_1209	DJI_0572	Rosa sp.	0	Rosaceae Juss.		
Iz_9_1209	DJI_0572	Salix alba L.	0	Salicaceae Mirb.	n	1
Iz_9_1209	DJI_0572	Sambucus nigra L.	0	Viburnaceae Raf.	n	1
Iz_9_1209	DJI_0572	Sorbus aucuparia L.	0	Rosaceae Juss.	n	1
Iz_9_1209	DJI_0572	Tilia cordata Mill.	0	Malvaceae Juss.	n	1
Iz_9_1209	DJI_0572	Ulmus glabra Huds.	0	Ulmaceae Mirb.	n	1
Iz_9_1209	DJI_0569	Acer tataricum L.	0	Sapindaceae Juss.	n	1
Iz_9_1209	DJI_0569	Betula pendula Roth.	0	Betulaceae Gray	n	1
Iz_9_1209	DJI_0569	Crataegus rhipidophylla Gand.	0	Rosaceae Juss.	n	1
Iz_9_1209	DJI_0569	Frangula alnus Mill.	0	Rhamnaceae Juss.	n	1
Iz_9_1209	DJI_0569	Fraxinus pennsylvanica Marshall	0	Oleaceae Hoffmanns. & Link	a	0
Iz_9_1209	DJI_0569	Pinus sylvestris L.	0	Pinaceae Spreng. ex F.Rudolphi	n	1
Iz_9_1209	DJI_0569	Populus × canescens (Aiton) Sm.	0	Salicaceae Mirb.	n	1
Iz_9_1209	DJI_0569	Populus tremula L.	0	Salicaceae Mirb.	n	1
Iz_9_1209	DJI_0569	Populus nigra L.	0	Salicaceae Mirb.	n	1
Iz_9_1209	DJI_0569	Pyrus communis L.	0	Rosaceae Juss.	a	0
Iz_9_1209	DJI_0569	Quercus robur L.	0	Fagaceae Dumort.	n	1
Iz_9_1209	DJI_0569	Robinia pseudoacacia L.	0	Fabaceae Lindl.	a	0
Iz_9_1209	DJI_0569	Rosa sp.	0	Rosaceae Juss.		
Iz_9_1209	DJI_0569	Salix alba L.	0	Salicaceae Mirb.	n	1
Iz_9_1209	DJI_0569	Sambucus nigra L.	0	Viburnaceae Raf.	n	1
Iz_9_1209	DJI_0569	Sorbus aucuparia L.	0	Rosaceae Juss.	n	1
Iz_9_1209	DJI_0569	Tilia cordata Mill.	0	Malvaceae Juss.	n	1
Iz_9_1209	DJI_0569	Ulmus glabra Huds.	0	Ulmaceae Mirb.	n	1
Iz_9_1209	DJI_0567	Acer tataricum L.	0	Sapindaceae Juss.	n	1
Iz_9_1209	DJI_0567	Betula pendula Roth.	0	Betulaceae Gray	n	1
Iz_9_1209	DJI_0567	Crataegus rhipidophylla Gand.	0	Rosaceae Juss.	n	1
Iz_9_1209	DJI_0567	Frangula alnus Mill.	0	Rhamnaceae Juss.	n	1
Iz_9_1209	DJI_0567	Fraxinus pennsylvanica Marshall	0	Oleaceae Hoffmanns. & Link	a	0
Iz_9_1209	DJI_0567	Pinus sylvestris L.	0	Pinaceae Spreng. ex F.Rudolphi	n	1
Iz_9_1209	DJI_0567	Populus × canescens (Aiton) Sm.	0	Salicaceae Mirb.	n	1
Iz_9_1209	DJI_0567	Populus tremula L.	0	Salicaceae Mirb.	n	1
Iz_9_1209	DJI_0567	Populus nigra L.	0	Salicaceae Mirb.	n	1
Iz_9_1209	DJI_0567	Pyrus communis L.	0	Rosaceae Juss.	a	0
Iz_9_1209	DJI_0567	Quercus robur L.	0	Fagaceae Dumort.	n	1
Iz_9_1209	DJI_0567	Robinia pseudoacacia L.	0	Fabaceae Lindl.	a	0
Iz_9_1209	DJI_0567	Rosa sp.	0	Rosaceae Juss.		
Iz_9_1209	DJI_0567	Salix alba L.	0	Salicaceae Mirb.	n	1
Iz_9_1209	DJI_0567	Sambucus nigra L.	0	Viburnaceae Raf.	n	1
Iz_9_1209	DJI_0567	Sorbus aucuparia L.	0	Rosaceae Juss.	n	1
Iz_9_1209	DJI_0567	Tilia cordata Mill.	0	Malvaceae Juss.	n	1
Iz_9_1209	DJI_0567	Ulmus glabra Huds.	0	Ulmaceae Mirb.	n	1
Iz_9_1209	DJI_0564	Acer tataricum L.	0	Sapindaceae Juss.	n	1
Iz_9_1209	DJI_0564	Betula pendula Roth.	0	Betulaceae Gray	n	1

Iz_9_1209	DJI_0564	Crataegus rhipidophylla Gand.	0	Rosaceae Juss.	n	1
Iz_9_1209	DJI_0564	Frangula alnus Mill.	0	Rhamnaceae Juss.	n	1
Iz_9_1209	DJI_0564	Fraxinus pennsylvanica Marshall	0	Oleaceae Hoffmanns. & Link	a	0
Iz_9_1209	DJI_0564	Pinus sylvestris L.	0	Pinaceae Spreng. ex F.Rudolphi	n	1
Iz_9_1209	DJI_0564	Populus × canescens (Aiton) Sm.	0	Salicaceae Mirb.	n	1
Iz_9_1209	DJI_0564	Populus tremula L.	0	Salicaceae Mirb.	n	1
Iz_9_1209	DJI_0564	Populus nigra L.	0	Salicaceae Mirb.	n	1
Iz_9_1209	DJI_0564	Pyrus communis L.	0	Rosaceae Juss.	a	0
Iz_9_1209	DJI_0564	Quercus robur L.	0	Fagaceae Dumort.	n	1
Iz_9_1209	DJI_0564	Robinia pseudoacacia L.	0	Fabaceae Lindl.	a	0
Iz_9_1209	DJI_0564	Rosa sp.	0	Rosaceae Juss.		
Iz_9_1209	DJI_0564	Salix alba L.	0	Salicaceae Mirb.	n	1
Iz_9_1209	DJI_0564	Sambucus nigra L.	0	Viburnaceae Raf.	n	1
Iz_9_1209	DJI_0564	Sorbus aucuparia L.	0	Rosaceae Juss.	n	1
Iz_9_1209	DJI_0564	Tilia cordata Mill.	0	Malvaceae Juss.	n	1
Iz_9_1209	DJI_0564	Ulmus glabra Huds.	0	Ulmaceae Mirb.	n	1
Iz_9_1209	DJI_0545	Acer tataricum L.	0	Sapindaceae Juss.	n	1
Iz_9_1209	DJI_0545	Betula pendula Roth.	0	Betulaceae Gray	n	1
Iz_9_1209	DJI_0545	Crataegus rhipidophylla Gand.	0	Rosaceae Juss.	n	1
Iz_9_1209	DJI_0545	Frangula alnus Mill.	0	Rhamnaceae Juss.	n	1
Iz_9_1209	DJI_0545	Fraxinus pennsylvanica Marshall	0	Oleaceae Hoffmanns. & Link	a	0
Iz_9_1209	DJI_0545	Pinus sylvestris L.	0	Pinaceae Spreng. ex F.Rudolphi	n	1
Iz_9_1209	DJI_0545	Populus × canescens (Aiton) Sm.	0	Salicaceae Mirb.	n	1
Iz_9_1209	DJI_0545	Populus tremula L.	0	Salicaceae Mirb.	n	1
Iz_9_1209	DJI_0545	Populus nigra L.	0	Salicaceae Mirb.	n	1
Iz_9_1209	DJI_0545	Pyrus communis L.	0	Rosaceae Juss.	a	0
Iz_9_1209	DJI_0545	Quercus robur L.	0	Fagaceae Dumort.	n	1
Iz_9_1209	DJI_0545	Robinia pseudoacacia L.	0	Fabaceae Lindl.	a	0
Iz_9_1209	DJI_0545	Rosa sp.	0	Rosaceae Juss.		
Iz_9_1209	DJI_0545	Salix alba L.	0	Salicaceae Mirb.	n	1
Iz_9_1209	DJI_0545	Sambucus nigra L.	0	Viburnaceae Raf.	n	1
Iz_9_1209	DJI_0545	Sorbus aucuparia L.	0	Rosaceae Juss.	n	1
Iz_9_1209	DJI_0545	Tilia cordata Mill.	0	Malvaceae Juss.	n	1
Iz_9_1209	DJI_0545	Ulmus glabra Huds.	0	Ulmaceae Mirb.	n	1
Iz_9_1209	DJI_0541	Acer tataricum L.	0	Sapindaceae Juss.	n	1
Iz_9_1209	DJI_0541	Betula pendula Roth.	0	Betulaceae Gray	n	1
Iz_9_1209	DJI_0541	Crataegus rhipidophylla Gand.	0	Rosaceae Juss.	n	1
Iz_9_1209	DJI_0541	Frangula alnus Mill.	0	Rhamnaceae Juss.	n	1
Iz_9_1209	DJI_0541	Fraxinus pennsylvanica Marshall	0	Oleaceae Hoffmanns. & Link	a	0
Iz_9_1209	DJI_0541	Pinus sylvestris L.	0	Pinaceae Spreng. ex F.Rudolphi	n	1
Iz_9_1209	DJI_0541	Populus × canescens (Aiton) Sm.	0	Salicaceae Mirb.	n	1
Iz_9_1209	DJI_0541	Populus tremula L.	0	Salicaceae Mirb.	n	1
Iz_9_1209	DJI_0541	Populus nigra L.	0	Salicaceae Mirb.	n	1
Iz_9_1209	DJI_0541	Pyrus communis L.	0	Rosaceae Juss.	a	0
Iz_9_1209	DJI_0541	Quercus robur L.	0	Fagaceae Dumort.	n	1
Iz_9_1209	DJI_0541	Robinia pseudoacacia L.	0	Fabaceae Lindl.	a	0
Iz_9_1209	DJI_0541	Rosa sp.	0	Rosaceae Juss.		
Iz_9_1209	DJI_0541	Salix alba L.	0	Salicaceae Mirb.	n	1
Iz_9_1209	DJI_0541	Sambucus nigra L.	0	Viburnaceae Raf.	n	1
Iz_9_1209	DJI_0541	Sorbus aucuparia L.	0	Rosaceae Juss.	n	1
Iz_9_1209	DJI_0541	Tilia cordata Mill.	0	Malvaceae Juss.	n	1
Iz_9_1209	DJI_0541	Ulmus glabra Huds.	0	Ulmaceae Mirb.	n	1
Iz_10_1209	DJI_0691	Acer tataricum L.	0	Sapindaceae Juss.	n	1
Iz_10_1209	DJI_0691	Betula pendula Roth.	0	Betulaceae Gray	n	1
Iz_10_1209	DJI_0691	Crataegus rhipidophylla Gand.	0	Rosaceae Juss.	n	1
Iz_10_1209	DJI_0691	Frangula alnus Mill.	0	Rhamnaceae Juss.	n	1
Iz_10_1209	DJI_0691	Fraxinus pennsylvanica Marshall	0	Oleaceae Hoffmanns. & Link	a	0
Iz_10_1209	DJI_0691	Pinus sylvestris L.	0	Pinaceae Spreng. ex F.Rudolphi	n	1
Iz_10_1209	DJI_0691	Populus × canescens (Aiton) Sm.	0	Salicaceae Mirb.	n	1
Iz_10_1209	DJI_0691	Populus tremula L.	0	Salicaceae Mirb.	n	1
Iz_10_1209	DJI_0691	Populus nigra L.	0	Salicaceae Mirb.	n	1
Iz_10_1209	DJI_0691	Pyrus communis L.	0	Rosaceae Juss.	a	0
Iz_10_1209	DJI_0691	Quercus robur L.	0	Fagaceae Dumort.	n	1
Iz_10_1209	DJI_0691	Robinia pseudoacacia L.	0	Fabaceae Lindl.	a	0

Iz_10_1209	DJI_0691	Rosa sp.	0	Rosaceae Juss.		
Iz_10_1209	DJI_0691	Salix alba L.	0	Salicaceae Mirb.	n	1
Iz_10_1209	DJI_0691	Sambucus nigra L.	0	Viburnaceae Raf.	n	1
Iz_10_1209	DJI_0691	Sorbus aucuparia L.	0	Rosaceae Juss.	n	1
Iz_10_1209	DJI_0691	Tilia cordata Mill.	0	Malvaceae Juss.	n	1
Iz_10_1209	DJI_0691	Ulmus glabra Huds.	0	Ulmaceae Mirb.	n	1
Iz_10_1209	DJI_0687	Acer tataricum L.	0	Sapindaceae Juss.	n	1
Iz_10_1209	DJI_0687	Betula pendula Roth.	0	Betulaceae Gray	n	1
Iz_10_1209	DJI_0687	Crataegus rhipidophylla Gand.	0	Rosaceae Juss.	n	1
Iz_10_1209	DJI_0687	Frangula alnus Mill.	0	Rhamnaceae Juss.	n	1
Iz_10_1209	DJI_0687	Fraxinus pennsylvanica Marshall	0	Oleaceae Hoffmanns. & Link	a	0
Iz_10_1209	DJI_0687	Pinus sylvestris L.	0	Pinaceae Spreng. ex F.Rudolphi	n	1
Iz_10_1209	DJI_0687	Populus × canescens (Aiton) Sm.	0	Salicaceae Mirb.	n	1
Iz_10_1209	DJI_0687	Populus tremula L.	0	Salicaceae Mirb.	n	1
Iz_10_1209	DJI_0687	Populus nigra L.	0	Salicaceae Mirb.	n	1
Iz_10_1209	DJI_0687	Pyrus communis L.	0	Rosaceae Juss.	a	0
Iz_10_1209	DJI_0687	Quercus robur L.	0	Fagaceae Dumort.	n	1
Iz_10_1209	DJI_0687	Robinia pseudoacacia L.	0	Fabaceae Lindl.	a	0
Iz_10_1209	DJI_0687	Rosa sp.	0	Rosaceae Juss.		
Iz_10_1209	DJI_0687	Salix alba L.	0	Salicaceae Mirb.	n	1
Iz_10_1209	DJI_0687	Sambucus nigra L.	0	Viburnaceae Raf.	n	1
Iz_10_1209	DJI_0687	Sorbus aucuparia L.	0	Rosaceae Juss.	n	1
Iz_10_1209	DJI_0687	Tilia cordata Mill.	0	Malvaceae Juss.	n	1
Iz_10_1209	DJI_0687	Ulmus glabra Huds.	0	Ulmaceae Mirb.	n	1
Iz_10_1209	DJI_0683	Acer tataricum L.	0	Sapindaceae Juss.	n	1
Iz_10_1209	DJI_0683	Betula pendula Roth.	0	Betulaceae Gray	n	1
Iz_10_1209	DJI_0683	Crataegus rhipidophylla Gand.	0	Rosaceae Juss.	n	1
Iz_10_1209	DJI_0683	Frangula alnus Mill.	0	Rhamnaceae Juss.	n	1
Iz_10_1209	DJI_0683	Fraxinus pennsylvanica Marshall	0	Oleaceae Hoffmanns. & Link	a	0
Iz_10_1209	DJI_0683	Pinus sylvestris L.	0	Pinaceae Spreng. ex F.Rudolphi	n	1
Iz_10_1209	DJI_0683	Populus × canescens (Aiton) Sm.	0	Salicaceae Mirb.	n	1
Iz_10_1209	DJI_0683	Populus tremula L.	0	Salicaceae Mirb.	n	1
Iz_10_1209	DJI_0683	Populus nigra L.	0	Salicaceae Mirb.	n	1
Iz_10_1209	DJI_0683	Pyrus communis L.	0	Rosaceae Juss.	a	0
Iz_10_1209	DJI_0683	Quercus robur L.	0	Fagaceae Dumort.	n	1
Iz_10_1209	DJI_0683	Robinia pseudoacacia L.	0	Fabaceae Lindl.	a	0
Iz_10_1209	DJI_0683	Rosa sp.	0	Rosaceae Juss.		
Iz_10_1209	DJI_0683	Salix alba L.	0	Salicaceae Mirb.	n	1
Iz_10_1209	DJI_0683	Sambucus nigra L.	0	Viburnaceae Raf.	n	1
Iz_10_1209	DJI_0683	Sorbus aucuparia L.	0	Rosaceae Juss.	n	1
Iz_10_1209	DJI_0683	Tilia cordata Mill.	0	Malvaceae Juss.	n	1
Iz_10_1209	DJI_0683	Ulmus glabra Huds.	0	Ulmaceae Mirb.	n	1
Iz_10_1209	DJI_0681	Acer tataricum L.	0	Sapindaceae Juss.	n	1
Iz_10_1209	DJI_0681	Betula pendula Roth.	0	Betulaceae Gray	n	1
Iz_10_1209	DJI_0681	Crataegus rhipidophylla Gand.	0	Rosaceae Juss.	n	1
Iz_10_1209	DJI_0681	Frangula alnus Mill.	0	Rhamnaceae Juss.	n	1
Iz_10_1209	DJI_0681	Fraxinus pennsylvanica Marshall	0	Oleaceae Hoffmanns. & Link	a	0
Iz_10_1209	DJI_0681	Pinus sylvestris L.	0	Pinaceae Spreng. ex F.Rudolphi	n	1
Iz_10_1209	DJI_0681	Populus × canescens (Aiton) Sm.	0	Salicaceae Mirb.	n	1
Iz_10_1209	DJI_0681	Populus tremula L.	0	Salicaceae Mirb.	n	1
Iz_10_1209	DJI_0681	Populus nigra L.	0	Salicaceae Mirb.	n	1
Iz_10_1209	DJI_0681	Pyrus communis L.	0	Rosaceae Juss.	a	0
Iz_10_1209	DJI_0681	Quercus robur L.	0	Fagaceae Dumort.	n	1
Iz_10_1209	DJI_0681	Robinia pseudoacacia L.	0	Fabaceae Lindl.	a	0
Iz_10_1209	DJI_0681	Rosa sp.	0	Rosaceae Juss.		
Iz_10_1209	DJI_0681	Salix alba L.	0	Salicaceae Mirb.	n	1
Iz_10_1209	DJI_0681	Sambucus nigra L.	0	Viburnaceae Raf.	n	1
Iz_10_1209	DJI_0681	Sorbus aucuparia L.	0	Rosaceae Juss.	n	1
Iz_10_1209	DJI_0681	Tilia cordata Mill.	0	Malvaceae Juss.	n	1
Iz_10_1209	DJI_0681	Ulmus glabra Huds.	0	Ulmaceae Mirb.	n	1
Iz_10_1209	DJI_0678	Acer tataricum L.	0	Sapindaceae Juss.	n	1
Iz_10_1209	DJI_0678	Betula pendula Roth.	0	Betulaceae Gray	n	1
Iz_10_1209	DJI_0678	Crataegus rhipidophylla Gand.	0	Rosaceae Juss.	n	1
Iz_10_1209	DJI_0678	Frangula alnus Mill.	0	Rhamnaceae Juss.	n	1

Iz_10_1209	DJI_0678	Fraxinus pennsylvanica Marshall	0	Oleaceae Hoffmanns. & Link	a	0
Iz_10_1209	DJI_0678	Pinus sylvestris L.	0	Pinaceae Spreng. ex F.Rudolphi	n	1
Iz_10_1209	DJI_0678	Populus × canescens (Aiton) Sm.	0	Salicaceae Mirb.	n	1
Iz_10_1209	DJI_0678	Populus tremula L.	0	Salicaceae Mirb.	n	1
Iz_10_1209	DJI_0678	Populus nigra L.	0	Salicaceae Mirb.	n	1
Iz_10_1209	DJI_0678	Pyrus communis L.	0	Rosaceae Juss.	a	0
Iz_10_1209	DJI_0678	Quercus robur L.	0	Fagaceae Dumort.	n	1
Iz_10_1209	DJI_0678	Robinia pseudoacacia L.	0	Fabaceae Lindl.	a	0
Iz_10_1209	DJI_0678	Rosa sp.	0	Rosaceae Juss.		
Iz_10_1209	DJI_0678	Salix alba L.	0	Salicaceae Mirb.	n	1
Iz_10_1209	DJI_0678	Sambucus nigra L.	0	Viburnaceae Raf.	n	1
Iz_10_1209	DJI_0678	Sorbus aucuparia L.	0	Rosaceae Juss.	n	1
Iz_10_1209	DJI_0678	Tilia cordata Mill.	0	Malvaceae Juss.	n	1
Iz_10_1209	DJI_0678	Ulmus glabra Huds.	0	Ulmaceae Mirb.	n	1
Iz_10_1209	DJI_0675	Acer tataricum L.	0	Sapindaceae Juss.	n	1
Iz_10_1209	DJI_0675	Betula pendula Roth.	0	Betulaceae Gray	n	1
Iz_10_1209	DJI_0675	Crataegus rhipidophylla Gand.	0	Rosaceae Juss.	n	1
Iz_10_1209	DJI_0675	Frangula alnus Mill.	0	Rhamnaceae Juss.	n	1
Iz_10_1209	DJI_0675	Fraxinus pennsylvanica Marshall	0	Oleaceae Hoffmanns. & Link	a	0
Iz_10_1209	DJI_0675	Pinus sylvestris L.	0	Pinaceae Spreng. ex F.Rudolphi	n	1
Iz_10_1209	DJI_0675	Populus × canescens (Aiton) Sm.	0	Salicaceae Mirb.	n	1
Iz_10_1209	DJI_0675	Populus tremula L.	2	Salicaceae Mirb.	n	1
Iz_10_1209	DJI_0675	Populus nigra L.	0	Salicaceae Mirb.	n	1
Iz_10_1209	DJI_0675	Pyrus communis L.	0	Rosaceae Juss.	a	0
Iz_10_1209	DJI_0675	Quercus robur L.	0	Fagaceae Dumort.	n	1
Iz_10_1209	DJI_0675	Robinia pseudoacacia L.	0	Fabaceae Lindl.	a	0
Iz_10_1209	DJI_0675	Rosa sp.	0	Rosaceae Juss.		
Iz_10_1209	DJI_0675	Salix alba L.	0	Salicaceae Mirb.	n	1
Iz_10_1209	DJI_0675	Sambucus nigra L.	0	Viburnaceae Raf.	n	1
Iz_10_1209	DJI_0675	Sorbus aucuparia L.	0	Rosaceae Juss.	n	1
Iz_10_1209	DJI_0675	Tilia cordata Mill.	0	Malvaceae Juss.	n	1
Iz_10_1209	DJI_0675	Ulmus glabra Huds.	0	Ulmaceae Mirb.	n	1
Iz_10_1209	DJI_0672	Acer tataricum L.	0	Sapindaceae Juss.	n	1
Iz_10_1209	DJI_0672	Betula pendula Roth.	0	Betulaceae Gray	n	1
Iz_10_1209	DJI_0672	Crataegus rhipidophylla Gand.	0	Rosaceae Juss.	n	1
Iz_10_1209	DJI_0672	Frangula alnus Mill.	0	Rhamnaceae Juss.	n	1
Iz_10_1209	DJI_0672	Fraxinus pennsylvanica Marshall	0	Oleaceae Hoffmanns. & Link	a	0
Iz_10_1209	DJI_0672	Pinus sylvestris L.	0	Pinaceae Spreng. ex F.Rudolphi	n	1
Iz_10_1209	DJI_0672	Populus × canescens (Aiton) Sm.	0	Salicaceae Mirb.	n	1
Iz_10_1209	DJI_0672	Populus tremula L.	1	Salicaceae Mirb.	n	1
Iz_10_1209	DJI_0672	Populus nigra L.	0	Salicaceae Mirb.	n	1
Iz_10_1209	DJI_0672	Pyrus communis L.	0	Rosaceae Juss.	a	0
Iz_10_1209	DJI_0672	Quercus robur L.	0	Fagaceae Dumort.	n	1
Iz_10_1209	DJI_0672	Robinia pseudoacacia L.	0	Fabaceae Lindl.	a	0
Iz_10_1209	DJI_0672	Rosa sp.	0	Rosaceae Juss.		
Iz_10_1209	DJI_0672	Salix alba L.	0	Salicaceae Mirb.	n	1
Iz_10_1209	DJI_0672	Sambucus nigra L.	0	Viburnaceae Raf.	n	1
Iz_10_1209	DJI_0672	Sorbus aucuparia L.	0	Rosaceae Juss.	n	1
Iz_10_1209	DJI_0672	Tilia cordata Mill.	0	Malvaceae Juss.	n	1
Iz_10_1209	DJI_0672	Ulmus glabra Huds.	0	Ulmaceae Mirb.	n	1
Iz_10_1209	DJI_0669	Acer tataricum L.	0	Sapindaceae Juss.	n	1
Iz_10_1209	DJI_0669	Betula pendula Roth.	0	Betulaceae Gray	n	1
Iz_10_1209	DJI_0669	Crataegus rhipidophylla Gand.	0	Rosaceae Juss.	n	1
Iz_10_1209	DJI_0669	Frangula alnus Mill.	0	Rhamnaceae Juss.	n	1
Iz_10_1209	DJI_0669	Fraxinus pennsylvanica Marshall	0	Oleaceae Hoffmanns. & Link	a	0
Iz_10_1209	DJI_0669	Pinus sylvestris L.	0	Pinaceae Spreng. ex F.Rudolphi	n	1
Iz_10_1209	DJI_0669	Populus × canescens (Aiton) Sm.	0	Salicaceae Mirb.	n	1
Iz_10_1209	DJI_0669	Populus tremula L.	0	Salicaceae Mirb.	n	1
Iz_10_1209	DJI_0669	Populus nigra L.	0	Salicaceae Mirb.	n	1
Iz_10_1209	DJI_0669	Pyrus communis L.	0	Rosaceae Juss.	a	0
Iz_10_1209	DJI_0669	Quercus robur L.	0	Fagaceae Dumort.	n	1
Iz_10_1209	DJI_0669	Robinia pseudoacacia L.	0	Fabaceae Lindl.	a	0
Iz_10_1209	DJI_0669	Rosa sp.	0	Rosaceae Juss.		
Iz_10_1209	DJI_0669	Salix alba L.	0	Salicaceae Mirb.	n	1

Iz_10_1209	DJI_0669	Sambucus nigra L.	0	Viburnaceae Raf.	n	1
Iz_10_1209	DJI_0669	Sorbus aucuparia L.	0	Rosaceae Juss.	n	1
Iz_10_1209	DJI_0669	Tilia cordata Mill.	0	Malvaceae Juss.	n	1
Iz_10_1209	DJI_0669	Ulmus glabra Huds.	0	Ulmaceae Mirb.	n	1
Iz_10_1209	DJI_0666	Acer tataricum L.	0	Sapindaceae Juss.	n	1
Iz_10_1209	DJI_0666	Betula pendula Roth.	0	Betulaceae Gray	n	1
Iz_10_1209	DJI_0666	Crataegus rhipidophylla Gand.	0	Rosaceae Juss.	n	1
Iz_10_1209	DJI_0666	Frangula alnus Mill.	0	Rhamnaceae Juss.	n	1
Iz_10_1209	DJI_0666	Fraxinus pennsylvanica Marshall	0	Oleaceae Hoffmanns. & Link	a	0
Iz_10_1209	DJI_0666	Pinus sylvestris L.	0	Pinaceae Spreng. ex F.Rudolphi	n	1
Iz_10_1209	DJI_0666	Populus × canescens (Aiton) Sm.	0	Salicaceae Mirb.	n	1
Iz_10_1209	DJI_0666	Populus tremula L.	0	Salicaceae Mirb.	n	1
Iz_10_1209	DJI_0666	Populus nigra L.	0	Salicaceae Mirb.	n	1
Iz_10_1209	DJI_0666	Pyrus communis L.	0	Rosaceae Juss.	a	0
Iz_10_1209	DJI_0666	Quercus robur L.	0	Fagaceae Dumort.	n	1
Iz_10_1209	DJI_0666	Robinia pseudoacacia L.	0	Fabaceae Lindl.	a	0
Iz_10_1209	DJI_0666	Rosa sp.	0	Rosaceae Juss.		
Iz_10_1209	DJI_0666	Salix alba L.	0	Salicaceae Mirb.	n	1
Iz_10_1209	DJI_0666	Sambucus nigra L.	0	Viburnaceae Raf.	n	1
Iz_10_1209	DJI_0666	Sorbus aucuparia L.	0	Rosaceae Juss.	n	1
Iz_10_1209	DJI_0666	Tilia cordata Mill.	0	Malvaceae Juss.	n	1
Iz_10_1209	DJI_0666	Ulmus glabra Huds.	0	Ulmaceae Mirb.	n	1
Iz_10_1209	DJI_0662	Acer tataricum L.	0	Sapindaceae Juss.	n	1
Iz_10_1209	DJI_0662	Betula pendula Roth.	0	Betulaceae Gray	n	1
Iz_10_1209	DJI_0662	Crataegus rhipidophylla Gand.	0	Rosaceae Juss.	n	1
Iz_10_1209	DJI_0662	Frangula alnus Mill.	0	Rhamnaceae Juss.	n	1
Iz_10_1209	DJI_0662	Fraxinus pennsylvanica Marshall	0	Oleaceae Hoffmanns. & Link	a	0
Iz_10_1209	DJI_0662	Pinus sylvestris L.	0	Pinaceae Spreng. ex F.Rudolphi	n	1
Iz_10_1209	DJI_0662	Populus × canescens (Aiton) Sm.	0	Salicaceae Mirb.	n	1
Iz_10_1209	DJI_0662	Populus tremula L.	0	Salicaceae Mirb.	n	1
Iz_10_1209	DJI_0662	Populus nigra L.	0	Salicaceae Mirb.	n	1
Iz_10_1209	DJI_0662	Pyrus communis L.	0	Rosaceae Juss.	a	0
Iz_10_1209	DJI_0662	Quercus robur L.	0	Fagaceae Dumort.	n	1
Iz_10_1209	DJI_0662	Robinia pseudoacacia L.	0	Fabaceae Lindl.	a	0
Iz_10_1209	DJI_0662	Rosa sp.	0	Rosaceae Juss.		
Iz_10_1209	DJI_0662	Salix alba L.	0	Salicaceae Mirb.	n	1
Iz_10_1209	DJI_0662	Sambucus nigra L.	0	Viburnaceae Raf.	n	1
Iz_10_1209	DJI_0662	Sorbus aucuparia L.	0	Rosaceae Juss.	n	1
Iz_10_1209	DJI_0662	Tilia cordata Mill.	0	Malvaceae Juss.	n	1
Iz_10_1209	DJI_0662	Ulmus glabra Huds.	0	Ulmaceae Mirb.	n	1
Iz_10_1209	DJI_0658	Acer tataricum L.	0	Sapindaceae Juss.	n	1
Iz_10_1209	DJI_0658	Betula pendula Roth.	0	Betulaceae Gray	n	1
Iz_10_1209	DJI_0658	Crataegus rhipidophylla Gand.	0	Rosaceae Juss.	n	1
Iz_10_1209	DJI_0658	Frangula alnus Mill.	0	Rhamnaceae Juss.	n	1
Iz_10_1209	DJI_0658	Fraxinus pennsylvanica Marshall	0	Oleaceae Hoffmanns. & Link	a	0
Iz_10_1209	DJI_0658	Pinus sylvestris L.	0	Pinaceae Spreng. ex F.Rudolphi	n	1
Iz_10_1209	DJI_0658	Populus × canescens (Aiton) Sm.	0	Salicaceae Mirb.	n	1
Iz_10_1209	DJI_0658	Populus tremula L.	1	Salicaceae Mirb.	n	1
Iz_10_1209	DJI_0658	Populus nigra L.	0	Salicaceae Mirb.	n	1
Iz_10_1209	DJI_0658	Pyrus communis L.	0	Rosaceae Juss.	a	0
Iz_10_1209	DJI_0658	Quercus robur L.	0	Fagaceae Dumort.	n	1
Iz_10_1209	DJI_0658	Robinia pseudoacacia L.	0	Fabaceae Lindl.	a	0
Iz_10_1209	DJI_0658	Rosa sp.	0	Rosaceae Juss.		
Iz_10_1209	DJI_0658	Salix alba L.	0	Salicaceae Mirb.	n	1
Iz_10_1209	DJI_0658	Sambucus nigra L.	0	Viburnaceae Raf.	n	1
Iz_10_1209	DJI_0658	Sorbus aucuparia L.	0	Rosaceae Juss.	n	1
Iz_10_1209	DJI_0658	Tilia cordata Mill.	0	Malvaceae Juss.	n	1
Iz_10_1209	DJI_0658	Ulmus glabra Huds.	0	Ulmaceae Mirb.	n	1
Iz_10_1209	DJI_0654	Acer tataricum L.	0	Sapindaceae Juss.	n	1
Iz_10_1209	DJI_0654	Betula pendula Roth.	0	Betulaceae Gray	n	1
Iz_10_1209	DJI_0654	Crataegus rhipidophylla Gand.	0	Rosaceae Juss.	n	1
Iz_10_1209	DJI_0654	Frangula alnus Mill.	0	Rhamnaceae Juss.	n	1
Iz_10_1209	DJI_0654	Fraxinus pennsylvanica Marshall	0	Oleaceae Hoffmanns. & Link	a	0
Iz_10_1209	DJI_0654	Pinus sylvestris L.	0	Pinaceae Spreng. ex F.Rudolphi	n	1

Iz_10_1209	DJI_0654	Populus × canescens (Aiton) Sm.	0	Salicaceae Mirb.	n	1
Iz_10_1209	DJI_0654	Populus tremula L.	0	Salicaceae Mirb.	n	1
Iz_10_1209	DJI_0654	Populus nigra L.	0	Salicaceae Mirb.	n	1
Iz_10_1209	DJI_0654	Pyrus communis L.	0	Rosaceae Juss.	a	0
Iz_10_1209	DJI_0654	Quercus robur L.	0	Fagaceae Dumort.	n	1
Iz_10_1209	DJI_0654	Robinia pseudoacacia L.	0	Fabaceae Lindl.	a	0
Iz_10_1209	DJI_0654	Rosa sp.	0	Rosaceae Juss.		
Iz_10_1209	DJI_0654	Salix alba L.	0	Salicaceae Mirb.	n	1
Iz_10_1209	DJI_0654	Sambucus nigra L.	0	Viburnaceae Raf.	n	1
Iz_10_1209	DJI_0654	Sorbus aucuparia L.	0	Rosaceae Juss.	n	1
Iz_10_1209	DJI_0654	Tilia cordata Mill.	0	Malvaceae Juss.	n	1
Iz_10_1209	DJI_0654	Ulmus glabra Huds.	0	Ulmaceae Mirb.	n	1
Iz_10_1209	DJI_0649	Acer tataricum L.	0	Sapindaceae Juss.	n	1
Iz_10_1209	DJI_0649	Betula pendula Roth.	0	Betulaceae Gray	n	1
Iz_10_1209	DJI_0649	Crataegus rhipidophylla Gand.	0	Rosaceae Juss.	n	1
Iz_10_1209	DJI_0649	Frangula alnus Mill.	0	Rhamnaceae Juss.	n	1
Iz_10_1209	DJI_0649	Fraxinus pennsylvanica Marshall	0	Oleaceae Hoffmanns. & Link	a	0
Iz_10_1209	DJI_0649	Pinus sylvestris L.	0	Pinaceae Spreng. ex F.Rudolphi	n	1
Iz_10_1209	DJI_0649	Populus × canescens (Aiton) Sm.	0	Salicaceae Mirb.	n	1
Iz_10_1209	DJI_0649	Populus tremula L.	0	Salicaceae Mirb.	n	1
Iz_10_1209	DJI_0649	Populus nigra L.	0	Salicaceae Mirb.	n	1
Iz_10_1209	DJI_0649	Pyrus communis L.	0	Rosaceae Juss.	a	0
Iz_10_1209	DJI_0649	Quercus robur L.	0	Fagaceae Dumort.	n	1
Iz_10_1209	DJI_0649	Robinia pseudoacacia L.	0	Fabaceae Lindl.	a	0
Iz_10_1209	DJI_0649	Rosa sp.	0	Rosaceae Juss.		
Iz_10_1209	DJI_0649	Salix alba L.	0	Salicaceae Mirb.	n	1
Iz_10_1209	DJI_0649	Sambucus nigra L.	0	Viburnaceae Raf.	n	1
Iz_10_1209	DJI_0649	Sorbus aucuparia L.	0	Rosaceae Juss.	n	1
Iz_10_1209	DJI_0649	Tilia cordata Mill.	0	Malvaceae Juss.	n	1
Iz_10_1209	DJI_0649	Ulmus glabra Huds.	0	Ulmaceae Mirb.	n	1
Iz_10_1209	DJI_0643	Acer tataricum L.	0	Sapindaceae Juss.	n	1
Iz_10_1209	DJI_0643	Betula pendula Roth.	0	Betulaceae Gray	n	1
Iz_10_1209	DJI_0643	Crataegus rhipidophylla Gand.	0	Rosaceae Juss.	n	1
Iz_10_1209	DJI_0643	Frangula alnus Mill.	0	Rhamnaceae Juss.	n	1
Iz_10_1209	DJI_0643	Fraxinus pennsylvanica Marshall	0	Oleaceae Hoffmanns. & Link	a	0
Iz_10_1209	DJI_0643	Pinus sylvestris L.	0	Pinaceae Spreng. ex F.Rudolphi	n	1
Iz_10_1209	DJI_0643	Populus × canescens (Aiton) Sm.	0	Salicaceae Mirb.	n	1
Iz_10_1209	DJI_0643	Populus tremula L.	0	Salicaceae Mirb.	n	1
Iz_10_1209	DJI_0643	Populus nigra L.	0	Salicaceae Mirb.	n	1
Iz_10_1209	DJI_0643	Pyrus communis L.	0	Rosaceae Juss.	a	0
Iz_10_1209	DJI_0643	Quercus robur L.	0	Fagaceae Dumort.	n	1
Iz_10_1209	DJI_0643	Robinia pseudoacacia L.	0	Fabaceae Lindl.	a	0
Iz_10_1209	DJI_0643	Rosa sp.	0	Rosaceae Juss.		
Iz_10_1209	DJI_0643	Salix alba L.	0	Salicaceae Mirb.	n	1
Iz_10_1209	DJI_0643	Sambucus nigra L.	0	Viburnaceae Raf.	n	1
Iz_10_1209	DJI_0643	Sorbus aucuparia L.	0	Rosaceae Juss.	n	1
Iz_10_1209	DJI_0643	Tilia cordata Mill.	0	Malvaceae Juss.	n	1
Iz_10_1209	DJI_0643	Ulmus glabra Huds.	0	Ulmaceae Mirb.	n	1
Iz_10_1209	DJI_0637	Acer tataricum L.	0	Sapindaceae Juss.	n	1
Iz_10_1209	DJI_0637	Betula pendula Roth.	0	Betulaceae Gray	n	1
Iz_10_1209	DJI_0637	Crataegus rhipidophylla Gand.	0	Rosaceae Juss.	n	1
Iz_10_1209	DJI_0637	Frangula alnus Mill.	0	Rhamnaceae Juss.	n	1
Iz_10_1209	DJI_0637	Fraxinus pennsylvanica Marshall	0	Oleaceae Hoffmanns. & Link	a	0
Iz_10_1209	DJI_0637	Pinus sylvestris L.	0	Pinaceae Spreng. ex F.Rudolphi	n	1
Iz_10_1209	DJI_0637	Populus × canescens (Aiton) Sm.	0	Salicaceae Mirb.	n	1
Iz_10_1209	DJI_0637	Populus tremula L.	1	Salicaceae Mirb.	n	1
Iz_10_1209	DJI_0637	Populus nigra L.	0	Salicaceae Mirb.	n	1
Iz_10_1209	DJI_0637	Pyrus communis L.	0	Rosaceae Juss.	a	0
Iz_10_1209	DJI_0637	Quercus robur L.	0	Fagaceae Dumort.	n	1
Iz_10_1209	DJI_0637	Robinia pseudoacacia L.	0	Fabaceae Lindl.	a	0
Iz_10_1209	DJI_0637	Rosa sp.	0	Rosaceae Juss.		
Iz_10_1209	DJI_0637	Salix alba L.	0	Salicaceae Mirb.	n	1
Iz_10_1209	DJI_0637	Sambucus nigra L.	0	Viburnaceae Raf.	n	1
Iz_10_1209	DJI_0637	Sorbus aucuparia L.	0	Rosaceae Juss.	n	1

Iz_10_1209	DJI_0637	Tilia cordata Mill.	0	Malvaceae Juss.	n	1
Iz_10_1209	DJI_0637	Ulmus glabra Huds.	0	Ulmaceae Mirb.	n	1
Iz_11_1209	DJI_0771	Acer tataricum L.	0	Sapindaceae Juss.	n	1
Iz_11_1209	DJI_0771	Betula pendula Roth.	0	Betulaceae Gray	n	1
Iz_11_1209	DJI_0771	Crataegus rhipidophylla Gand.	0	Rosaceae Juss.	n	1
Iz_11_1209	DJI_0771	Frangula alnus Mill.	0	Rhamnaceae Juss.	n	1
Iz_11_1209	DJI_0771	Fraxinus pennsylvanica Marshall	0	Oleaceae Hoffmanns. & Link	a	0
Iz_11_1209	DJI_0771	Pinus sylvestris L.	0	Pinaceae Spreng. ex F.Rudolphi	n	1
Iz_11_1209	DJI_0771	Populus × canescens (Aiton) Sm.	0	Salicaceae Mirb.	n	1
Iz_11_1209	DJI_0771	Populus tremula L.	0	Salicaceae Mirb.	n	1
Iz_11_1209	DJI_0771	Populus nigra L.	0	Salicaceae Mirb.	n	1
Iz_11_1209	DJI_0771	Pyrus communis L.	0	Rosaceae Juss.	a	0
Iz_11_1209	DJI_0771	Quercus robur L.	0	Fagaceae Dumort.	n	1
Iz_11_1209	DJI_0771	Robinia pseudoacacia L.	0	Fabaceae Lindl.	a	0
Iz_11_1209	DJI_0771	Rosa sp.	0	Rosaceae Juss.		
Iz_11_1209	DJI_0771	Salix alba L.	0	Salicaceae Mirb.	n	1
Iz_11_1209	DJI_0771	Sambucus nigra L.	0	Viburnaceae Raf.	n	1
Iz_11_1209	DJI_0771	Sorbus aucuparia L.	0	Rosaceae Juss.	n	1
Iz_11_1209	DJI_0771	Tilia cordata Mill.	0	Malvaceae Juss.	n	1
Iz_11_1209	DJI_0771	Ulmus glabra Huds.	0	Ulmaceae Mirb.	n	1
Iz_11_1209	DJI_0765	Acer tataricum L.	0	Sapindaceae Juss.	n	1
Iz_11_1209	DJI_0765	Betula pendula Roth.	0	Betulaceae Gray	n	1
Iz_11_1209	DJI_0765	Crataegus rhipidophylla Gand.	0	Rosaceae Juss.	n	1
Iz_11_1209	DJI_0765	Frangula alnus Mill.	0	Rhamnaceae Juss.	n	1
Iz_11_1209	DJI_0765	Fraxinus pennsylvanica Marshall	0	Oleaceae Hoffmanns. & Link	a	0
Iz_11_1209	DJI_0765	Pinus sylvestris L.	0	Pinaceae Spreng. ex F.Rudolphi	n	1
Iz_11_1209	DJI_0765	Populus × canescens (Aiton) Sm.	0	Salicaceae Mirb.	n	1
Iz_11_1209	DJI_0765	Populus tremula L.	0	Salicaceae Mirb.	n	1
Iz_11_1209	DJI_0765	Populus nigra L.	0	Salicaceae Mirb.	n	1
Iz_11_1209	DJI_0765	Pyrus communis L.	0	Rosaceae Juss.	a	0
Iz_11_1209	DJI_0765	Quercus robur L.	0	Fagaceae Dumort.	n	1
Iz_11_1209	DJI_0765	Robinia pseudoacacia L.	0	Fabaceae Lindl.	a	0
Iz_11_1209	DJI_0765	Rosa sp.	0	Rosaceae Juss.		
Iz_11_1209	DJI_0765	Salix alba L.	0	Salicaceae Mirb.	n	1
Iz_11_1209	DJI_0765	Sambucus nigra L.	0	Viburnaceae Raf.	n	1
Iz_11_1209	DJI_0765	Sorbus aucuparia L.	0	Rosaceae Juss.	n	1
Iz_11_1209	DJI_0765	Tilia cordata Mill.	0	Malvaceae Juss.	n	1
Iz_11_1209	DJI_0765	Ulmus glabra Huds.	0	Ulmaceae Mirb.	n	1
Iz_11_1209	DJI_0761	Acer tataricum L.	0	Sapindaceae Juss.	n	1
Iz_11_1209	DJI_0761	Betula pendula Roth.	0	Betulaceae Gray	n	1
Iz_11_1209	DJI_0761	Crataegus rhipidophylla Gand.	0	Rosaceae Juss.	n	1
Iz_11_1209	DJI_0761	Frangula alnus Mill.	0	Rhamnaceae Juss.	n	1
Iz_11_1209	DJI_0761	Fraxinus pennsylvanica Marshall	0	Oleaceae Hoffmanns. & Link	a	0
Iz_11_1209	DJI_0761	Pinus sylvestris L.	0	Pinaceae Spreng. ex F.Rudolphi	n	1
Iz_11_1209	DJI_0761	Populus × canescens (Aiton) Sm.	0	Salicaceae Mirb.	n	1
Iz_11_1209	DJI_0761	Populus tremula L.	0	Salicaceae Mirb.	n	1
Iz_11_1209	DJI_0761	Populus nigra L.	0	Salicaceae Mirb.	n	1
Iz_11_1209	DJI_0761	Pyrus communis L.	0	Rosaceae Juss.	a	0
Iz_11_1209	DJI_0761	Quercus robur L.	0	Fagaceae Dumort.	n	1
Iz_11_1209	DJI_0761	Robinia pseudoacacia L.	0	Fabaceae Lindl.	a	0
Iz_11_1209	DJI_0761	Rosa sp.	0	Rosaceae Juss.		
Iz_11_1209	DJI_0761	Salix alba L.	0	Salicaceae Mirb.	n	1
Iz_11_1209	DJI_0761	Sambucus nigra L.	0	Viburnaceae Raf.	n	1
Iz_11_1209	DJI_0761	Sorbus aucuparia L.	0	Rosaceae Juss.	n	1
Iz_11_1209	DJI_0761	Tilia cordata Mill.	0	Malvaceae Juss.	n	1
Iz_11_1209	DJI_0761	Ulmus glabra Huds.	0	Ulmaceae Mirb.	n	1
Iz_11_1209	DJI_0757	Acer tataricum L.	0	Sapindaceae Juss.	n	1
Iz_11_1209	DJI_0757	Betula pendula Roth.	0	Betulaceae Gray	n	1
Iz_11_1209	DJI_0757	Crataegus rhipidophylla Gand.	0	Rosaceae Juss.	n	1
Iz_11_1209	DJI_0757	Frangula alnus Mill.	0	Rhamnaceae Juss.	n	1
Iz_11_1209	DJI_0757	Fraxinus pennsylvanica Marshall	0	Oleaceae Hoffmanns. & Link	a	0
Iz_11_1209	DJI_0757	Pinus sylvestris L.	0	Pinaceae Spreng. ex F.Rudolphi	n	1
Iz_11_1209	DJI_0757	Populus × canescens (Aiton) Sm.	0	Salicaceae Mirb.	n	1
Iz_11_1209	DJI_0757	Populus tremula L.	0	Salicaceae Mirb.	n	1

Iz_11_1209	DJI_0757	Populus nigra L.	0	Salicaceae Mirb.	n	1
Iz_11_1209	DJI_0757	Pyrus communis L.	0	Rosaceae Juss.	a	0
Iz_11_1209	DJI_0757	Quercus robur L.	0	Fagaceae Dumort.	n	1
Iz_11_1209	DJI_0757	Robinia pseudoacacia L.	0	Fabaceae Lindl.	a	0
Iz_11_1209	DJI_0757	Rosa sp.	0	Rosaceae Juss.		
Iz_11_1209	DJI_0757	Salix alba L.	0	Salicaceae Mirb.	n	1
Iz_11_1209	DJI_0757	Sambucus nigra L.	0	Viburnaceae Raf.	n	1
Iz_11_1209	DJI_0757	Sorbus aucuparia L.	0	Rosaceae Juss.	n	1
Iz_11_1209	DJI_0757	Tilia cordata Mill.	0	Malvaceae Juss.	n	1
Iz_11_1209	DJI_0757	Ulmus glabra Huds.	0	Ulmaceae Mirb.	n	1
Iz_11_1209	DJI_0751	Acer tataricum L.	0	Sapindaceae Juss.	n	1
Iz_11_1209	DJI_0751	Betula pendula Roth.	0	Betulaceae Gray	n	1
Iz_11_1209	DJI_0751	Crataegus rhipidophylla Gand.	0	Rosaceae Juss.	n	1
Iz_11_1209	DJI_0751	Frangula alnus Mill.	0	Rhamnaceae Juss.	n	1
Iz_11_1209	DJI_0751	Fraxinus pennsylvanica Marshall	0	Oleaceae Hoffmanns. & Link	a	0
Iz_11_1209	DJI_0751	Pinus sylvestris L.	0	Pinaceae Spreng. ex F.Rudolphi	n	1
Iz_11_1209	DJI_0751	Populus × canescens (Aiton) Sm.	0	Salicaceae Mirb.	n	1
Iz_11_1209	DJI_0751	Populus tremula L.	2	Salicaceae Mirb.	n	1
Iz_11_1209	DJI_0751	Populus nigra L.	0	Salicaceae Mirb.	n	1
Iz_11_1209	DJI_0751	Pyrus communis L.	0	Rosaceae Juss.	a	0
Iz_11_1209	DJI_0751	Quercus robur L.	0	Fagaceae Dumort.	n	1
Iz_11_1209	DJI_0751	Robinia pseudoacacia L.	0	Fabaceae Lindl.	a	0
Iz_11_1209	DJI_0751	Rosa sp.	0	Rosaceae Juss.		
Iz_11_1209	DJI_0751	Salix alba L.	0	Salicaceae Mirb.	n	1
Iz_11_1209	DJI_0751	Sambucus nigra L.	0	Viburnaceae Raf.	n	1
Iz_11_1209	DJI_0751	Sorbus aucuparia L.	0	Rosaceae Juss.	n	1
Iz_11_1209	DJI_0751	Tilia cordata Mill.	0	Malvaceae Juss.	n	1
Iz_11_1209	DJI_0751	Ulmus glabra Huds.	0	Ulmaceae Mirb.	n	1
Iz_11_1209	DJI_0749	Acer tataricum L.	0	Sapindaceae Juss.	n	1
Iz_11_1209	DJI_0749	Betula pendula Roth.	0	Betulaceae Gray	n	1
Iz_11_1209	DJI_0749	Crataegus rhipidophylla Gand.	0	Rosaceae Juss.	n	1
Iz_11_1209	DJI_0749	Frangula alnus Mill.	0	Rhamnaceae Juss.	n	1
Iz_11_1209	DJI_0749	Fraxinus pennsylvanica Marshall	0	Oleaceae Hoffmanns. & Link	a	0
Iz_11_1209	DJI_0749	Pinus sylvestris L.	0	Pinaceae Spreng. ex F.Rudolphi	n	1
Iz_11_1209	DJI_0749	Populus × canescens (Aiton) Sm.	0	Salicaceae Mirb.	n	1
Iz_11_1209	DJI_0749	Populus tremula L.	0	Salicaceae Mirb.	n	1
Iz_11_1209	DJI_0749	Populus nigra L.	0	Salicaceae Mirb.	n	1
Iz_11_1209	DJI_0749	Pyrus communis L.	0	Rosaceae Juss.	a	0
Iz_11_1209	DJI_0749	Quercus robur L.	0	Fagaceae Dumort.	n	1
Iz_11_1209	DJI_0749	Robinia pseudoacacia L.	0	Fabaceae Lindl.	a	0
Iz_11_1209	DJI_0749	Rosa sp.	0	Rosaceae Juss.		
Iz_11_1209	DJI_0749	Salix alba L.	0	Salicaceae Mirb.	n	1
Iz_11_1209	DJI_0749	Sambucus nigra L.	0	Viburnaceae Raf.	n	1
Iz_11_1209	DJI_0749	Sorbus aucuparia L.	0	Rosaceae Juss.	n	1
Iz_11_1209	DJI_0749	Tilia cordata Mill.	0	Malvaceae Juss.	n	1
Iz_11_1209	DJI_0749	Ulmus glabra Huds.	0	Ulmaceae Mirb.	n	1
Iz_11_1209	DJI_0746	Acer tataricum L.	0	Sapindaceae Juss.	n	1
Iz_11_1209	DJI_0746	Betula pendula Roth.	0	Betulaceae Gray	n	1
Iz_11_1209	DJI_0746	Crataegus rhipidophylla Gand.	0	Rosaceae Juss.	n	1
Iz_11_1209	DJI_0746	Frangula alnus Mill.	0	Rhamnaceae Juss.	n	1
Iz_11_1209	DJI_0746	Fraxinus pennsylvanica Marshall	0	Oleaceae Hoffmanns. & Link	a	0
Iz_11_1209	DJI_0746	Pinus sylvestris L.	0	Pinaceae Spreng. ex F.Rudolphi	n	1
Iz_11_1209	DJI_0746	Populus × canescens (Aiton) Sm.	0	Salicaceae Mirb.	n	1
Iz_11_1209	DJI_0746	Populus tremula L.	0	Salicaceae Mirb.	n	1
Iz_11_1209	DJI_0746	Populus nigra L.	0	Salicaceae Mirb.	n	1
Iz_11_1209	DJI_0746	Pyrus communis L.	0	Rosaceae Juss.	a	0
Iz_11_1209	DJI_0746	Quercus robur L.	0	Fagaceae Dumort.	n	1
Iz_11_1209	DJI_0746	Robinia pseudoacacia L.	0	Fabaceae Lindl.	a	0
Iz_11_1209	DJI_0746	Rosa sp.	0	Rosaceae Juss.		
Iz_11_1209	DJI_0746	Salix alba L.	0	Salicaceae Mirb.	n	1
Iz_11_1209	DJI_0746	Sambucus nigra L.	0	Viburnaceae Raf.	n	1
Iz_11_1209	DJI_0746	Sorbus aucuparia L.	0	Rosaceae Juss.	n	1
Iz_11_1209	DJI_0746	Tilia cordata Mill.	0	Malvaceae Juss.	n	1
Iz_11_1209	DJI_0746	Ulmus glabra Huds.	0	Ulmaceae Mirb.	n	1

Iz_11_1209	DJI_0743	Acer tataricum L.	0	Sapindaceae Juss.	n	1
Iz_11_1209	DJI_0743	Betula pendula Roth.	0	Betulaceae Gray	n	1
Iz_11_1209	DJI_0743	Crataegus rhipidophylla Gand.	0	Rosaceae Juss.	n	1
Iz_11_1209	DJI_0743	Frangula alnus Mill.	0	Rhamnaceae Juss.	n	1
Iz_11_1209	DJI_0743	Fraxinus pennsylvanica Marshall	0	Oleaceae Hoffmanns. & Link	a	0
Iz_11_1209	DJI_0743	Pinus sylvestris L.	0	Pinaceae Spreng. ex F.Rudolphi	n	1
Iz_11_1209	DJI_0743	Populus × canescens (Aiton) Sm.	0	Salicaceae Mirb.	n	1
Iz_11_1209	DJI_0743	Populus tremula L.	0	Salicaceae Mirb.	n	1
Iz_11_1209	DJI_0743	Populus nigra L.	0	Salicaceae Mirb.	n	1
Iz_11_1209	DJI_0743	Pyrus communis L.	0	Rosaceae Juss.	a	0
Iz_11_1209	DJI_0743	Quercus robur L.	0	Fagaceae Dumort.	n	1
Iz_11_1209	DJI_0743	Robinia pseudoacacia L.	0	Fabaceae Lindl.	a	0
Iz_11_1209	DJI_0743	Rosa sp.	0	Rosaceae Juss.		
Iz_11_1209	DJI_0743	Salix alba L.	0	Salicaceae Mirb.	n	1
Iz_11_1209	DJI_0743	Sambucus nigra L.	0	Viburnaceae Raf.	n	1
Iz_11_1209	DJI_0743	Sorbus aucuparia L.	0	Rosaceae Juss.	n	1
Iz_11_1209	DJI_0743	Tilia cordata Mill.	0	Malvaceae Juss.	n	1
Iz_11_1209	DJI_0743	Ulmus glabra Huds.	0	Ulmaceae Mirb.	n	1
Iz_11_1209	DJI_0740	Acer tataricum L.	0	Sapindaceae Juss.	n	1
Iz_11_1209	DJI_0740	Betula pendula Roth.	0	Betulaceae Gray	n	1
Iz_11_1209	DJI_0740	Crataegus rhipidophylla Gand.	0	Rosaceae Juss.	n	1
Iz_11_1209	DJI_0740	Frangula alnus Mill.	0	Rhamnaceae Juss.	n	1
Iz_11_1209	DJI_0740	Fraxinus pennsylvanica Marshall	0	Oleaceae Hoffmanns. & Link	a	0
Iz_11_1209	DJI_0740	Pinus sylvestris L.	0	Pinaceae Spreng. ex F.Rudolphi	n	1
Iz_11_1209	DJI_0740	Populus × canescens (Aiton) Sm.	0	Salicaceae Mirb.	n	1
Iz_11_1209	DJI_0740	Populus tremula L.	0	Salicaceae Mirb.	n	1
Iz_11_1209	DJI_0740	Populus nigra L.	0	Salicaceae Mirb.	n	1
Iz_11_1209	DJI_0740	Pyrus communis L.	0	Rosaceae Juss.	a	0
Iz_11_1209	DJI_0740	Quercus robur L.	0	Fagaceae Dumort.	n	1
Iz_11_1209	DJI_0740	Robinia pseudoacacia L.	0	Fabaceae Lindl.	a	0
Iz_11_1209	DJI_0740	Rosa sp.	0	Rosaceae Juss.		
Iz_11_1209	DJI_0740	Salix alba L.	0	Salicaceae Mirb.	n	1
Iz_11_1209	DJI_0740	Sambucus nigra L.	0	Viburnaceae Raf.	n	1
Iz_11_1209	DJI_0740	Sorbus aucuparia L.	0	Rosaceae Juss.	n	1
Iz_11_1209	DJI_0740	Tilia cordata Mill.	0	Malvaceae Juss.	n	1
Iz_11_1209	DJI_0740	Ulmus glabra Huds.	0	Ulmaceae Mirb.	n	1
Iz_11_1209	DJI_0737	Acer tataricum L.	0	Sapindaceae Juss.	n	1
Iz_11_1209	DJI_0737	Betula pendula Roth.	0	Betulaceae Gray	n	1
Iz_11_1209	DJI_0737	Crataegus rhipidophylla Gand.	0	Rosaceae Juss.	n	1
Iz_11_1209	DJI_0737	Frangula alnus Mill.	0	Rhamnaceae Juss.	n	1
Iz_11_1209	DJI_0737	Fraxinus pennsylvanica Marshall	0	Oleaceae Hoffmanns. & Link	a	0
Iz_11_1209	DJI_0737	Pinus sylvestris L.	0	Pinaceae Spreng. ex F.Rudolphi	n	1
Iz_11_1209	DJI_0737	Populus × canescens (Aiton) Sm.	0	Salicaceae Mirb.	n	1
Iz_11_1209	DJI_0737	Populus tremula L.	0	Salicaceae Mirb.	n	1
Iz_11_1209	DJI_0737	Populus nigra L.	0	Salicaceae Mirb.	n	1
Iz_11_1209	DJI_0737	Pyrus communis L.	0	Rosaceae Juss.	a	0
Iz_11_1209	DJI_0737	Quercus robur L.	0	Fagaceae Dumort.	n	1
Iz_11_1209	DJI_0737	Robinia pseudoacacia L.	0	Fabaceae Lindl.	a	0
Iz_11_1209	DJI_0737	Rosa sp.	0	Rosaceae Juss.		
Iz_11_1209	DJI_0737	Salix alba L.	0	Salicaceae Mirb.	n	1
Iz_11_1209	DJI_0737	Sambucus nigra L.	0	Viburnaceae Raf.	n	1
Iz_11_1209	DJI_0737	Sorbus aucuparia L.	0	Rosaceae Juss.	n	1
Iz_11_1209	DJI_0737	Tilia cordata Mill.	0	Malvaceae Juss.	n	1
Iz_11_1209	DJI_0737	Ulmus glabra Huds.	0	Ulmaceae Mirb.	n	1
Iz_11_1209	DJI_0733	Acer tataricum L.	0	Sapindaceae Juss.	n	1
Iz_11_1209	DJI_0733	Betula pendula Roth.	0	Betulaceae Gray	n	1
Iz_11_1209	DJI_0733	Crataegus rhipidophylla Gand.	0	Rosaceae Juss.	n	1
Iz_11_1209	DJI_0733	Frangula alnus Mill.	0	Rhamnaceae Juss.	n	1
Iz_11_1209	DJI_0733	Fraxinus pennsylvanica Marshall	0	Oleaceae Hoffmanns. & Link	a	0
Iz_11_1209	DJI_0733	Pinus sylvestris L.	0	Pinaceae Spreng. ex F.Rudolphi	n	1
Iz_11_1209	DJI_0733	Populus × canescens (Aiton) Sm.	0	Salicaceae Mirb.	n	1
Iz_11_1209	DJI_0733	Populus tremula L.	0	Salicaceae Mirb.	n	1
Iz_11_1209	DJI_0733	Populus nigra L.	0	Salicaceae Mirb.	n	1
Iz_11_1209	DJI_0733	Pyrus communis L.	0	Rosaceae Juss.	a	0

Iz_11_1209	DJI_0733	Quercus robur L.	0	Fagaceae Dumort.	n	1
Iz_11_1209	DJI_0733	Robinia pseudoacacia L.	0	Fabaceae Lindl.	a	0
Iz_11_1209	DJI_0733	Rosa sp.	0	Rosaceae Juss.		
Iz_11_1209	DJI_0733	Salix alba L.	0	Salicaceae Mirb.	n	1
Iz_11_1209	DJI_0733	Sambucus nigra L.	0	Viburnaceae Raf.	n	1
Iz_11_1209	DJI_0733	Sorbus aucuparia L.	0	Rosaceae Juss.	n	1
Iz_11_1209	DJI_0733	Tilia cordata Mill.	0	Malvaceae Juss.	n	1
Iz_11_1209	DJI_0733	Ulmus glabra Huds.	0	Ulmaceae Mirb.	n	1
Iz_11_1209	DJI_0728	Acer tataricum L.	0	Sapindaceae Juss.	n	1
Iz_11_1209	DJI_0728	Betula pendula Roth.	0	Betulaceae Gray	n	1
Iz_11_1209	DJI_0728	Crataegus rhipidophylla Gand.	0	Rosaceae Juss.	n	1
Iz_11_1209	DJI_0728	Frangula alnus Mill.	0	Rhamnaceae Juss.	n	1
Iz_11_1209	DJI_0728	Fraxinus pennsylvanica Marshall	0	Oleaceae Hoffmanns. & Link	a	0
Iz_11_1209	DJI_0728	Pinus sylvestris L.	0	Pinaceae Spreng. ex F.Rudolphi	n	1
Iz_11_1209	DJI_0728	Populus × canescens (Aiton) Sm.	0	Salicaceae Mirb.	n	1
Iz_11_1209	DJI_0728	Populus tremula L.	0	Salicaceae Mirb.	n	1
Iz_11_1209	DJI_0728	Populus nigra L.	0	Salicaceae Mirb.	n	1
Iz_11_1209	DJI_0728	Pyrus communis L.	0	Rosaceae Juss.	a	0
Iz_11_1209	DJI_0728	Quercus robur L.	0	Fagaceae Dumort.	n	1
Iz_11_1209	DJI_0728	Robinia pseudoacacia L.	0	Fabaceae Lindl.	a	0
Iz_11_1209	DJI_0728	Rosa sp.	0	Rosaceae Juss.		
Iz_11_1209	DJI_0728	Salix alba L.	0	Salicaceae Mirb.	n	1
Iz_11_1209	DJI_0728	Sambucus nigra L.	0	Viburnaceae Raf.	n	1
Iz_11_1209	DJI_0728	Sorbus aucuparia L.	0	Rosaceae Juss.	n	1
Iz_11_1209	DJI_0728	Tilia cordata Mill.	0	Malvaceae Juss.	n	1
Iz_11_1209	DJI_0728	Ulmus glabra Huds.	0	Ulmaceae Mirb.	n	1
Iz_11_1209	DJI_0725	Acer tataricum L.	0	Sapindaceae Juss.	n	1
Iz_11_1209	DJI_0725	Betula pendula Roth.	0	Betulaceae Gray	n	1
Iz_11_1209	DJI_0725	Crataegus rhipidophylla Gand.	0	Rosaceae Juss.	n	1
Iz_11_1209	DJI_0725	Frangula alnus Mill.	0	Rhamnaceae Juss.	n	1
Iz_11_1209	DJI_0725	Fraxinus pennsylvanica Marshall	0	Oleaceae Hoffmanns. & Link	a	0
Iz_11_1209	DJI_0725	Pinus sylvestris L.	0	Pinaceae Spreng. ex F.Rudolphi	n	1
Iz_11_1209	DJI_0725	Populus × canescens (Aiton) Sm.	0	Salicaceae Mirb.	n	1
Iz_11_1209	DJI_0725	Populus tremula L.	0	Salicaceae Mirb.	n	1
Iz_11_1209	DJI_0725	Populus nigra L.	0	Salicaceae Mirb.	n	1
Iz_11_1209	DJI_0725	Pyrus communis L.	0	Rosaceae Juss.	a	0
Iz_11_1209	DJI_0725	Quercus robur L.	0	Fagaceae Dumort.	n	1
Iz_11_1209	DJI_0725	Robinia pseudoacacia L.	0	Fabaceae Lindl.	a	0
Iz_11_1209	DJI_0725	Rosa sp.	0	Rosaceae Juss.		
Iz_11_1209	DJI_0725	Salix alba L.	0	Salicaceae Mirb.	n	1
Iz_11_1209	DJI_0725	Sambucus nigra L.	0	Viburnaceae Raf.	n	1
Iz_11_1209	DJI_0725	Sorbus aucuparia L.	0	Rosaceae Juss.	n	1
Iz_11_1209	DJI_0725	Tilia cordata Mill.	0	Malvaceae Juss.	n	1
Iz_11_1209	DJI_0725	Ulmus glabra Huds.	0	Ulmaceae Mirb.	n	1
Iz_11_1209	DJI_0722	Acer tataricum L.	0	Sapindaceae Juss.	n	1
Iz_11_1209	DJI_0722	Betula pendula Roth.	0	Betulaceae Gray	n	1
Iz_11_1209	DJI_0722	Crataegus rhipidophylla Gand.	0	Rosaceae Juss.	n	1
Iz_11_1209	DJI_0722	Frangula alnus Mill.	0	Rhamnaceae Juss.	n	1
Iz_11_1209	DJI_0722	Fraxinus pennsylvanica Marshall	0	Oleaceae Hoffmanns. & Link	a	0
Iz_11_1209	DJI_0722	Pinus sylvestris L.	0	Pinaceae Spreng. ex F.Rudolphi	n	1
Iz_11_1209	DJI_0722	Populus × canescens (Aiton) Sm.	0	Salicaceae Mirb.	n	1
Iz_11_1209	DJI_0722	Populus tremula L.	0	Salicaceae Mirb.	n	1
Iz_11_1209	DJI_0722	Populus nigra L.	0	Salicaceae Mirb.	n	1
Iz_11_1209	DJI_0722	Pyrus communis L.	0	Rosaceae Juss.	a	0
Iz_11_1209	DJI_0722	Quercus robur L.	0	Fagaceae Dumort.	n	1
Iz_11_1209	DJI_0722	Robinia pseudoacacia L.	0	Fabaceae Lindl.	a	0
Iz_11_1209	DJI_0722	Rosa sp.	0	Rosaceae Juss.		
Iz_11_1209	DJI_0722	Salix alba L.	0	Salicaceae Mirb.	n	1
Iz_11_1209	DJI_0722	Sambucus nigra L.	0	Viburnaceae Raf.	n	1
Iz_11_1209	DJI_0722	Sorbus aucuparia L.	0	Rosaceae Juss.	n	1
Iz_11_1209	DJI_0722	Tilia cordata Mill.	0	Malvaceae Juss.	n	1
Iz_11_1209	DJI_0722	Ulmus glabra Huds.	0	Ulmaceae Mirb.	n	1
Iz_11_1209	DJI_0719	Acer tataricum L.	0	Sapindaceae Juss.	n	1
Iz_11_1209	DJI_0719	Betula pendula Roth.	0	Betulaceae Gray	n	1

Iz_11_1209	DJI_0719	Crataegus rhipidophylla Gand.	0	Rosaceae Juss.	n	1
Iz_11_1209	DJI_0719	Frangula alnus Mill.	0	Rhamnaceae Juss.	n	1
Iz_11_1209	DJI_0719	Fraxinus pennsylvanica Marshall	0	Oleaceae Hoffmanns. & Link	a	0
Iz_11_1209	DJI_0719	Pinus sylvestris L.	0	Pinaceae Spreng. ex F.Rudolphi	n	1
Iz_11_1209	DJI_0719	Populus × canescens (Aiton) Sm.	0	Salicaceae Mirb.	n	1
Iz_11_1209	DJI_0719	Populus tremula L.	0	Salicaceae Mirb.	n	1
Iz_11_1209	DJI_0719	Populus nigra L.	0	Salicaceae Mirb.	n	1
Iz_11_1209	DJI_0719	Pyrus communis L.	0	Rosaceae Juss.	a	0
Iz_11_1209	DJI_0719	Quercus robur L.	0	Fagaceae Dumort.	n	1
Iz_11_1209	DJI_0719	Robinia pseudoacacia L.	0	Fabaceae Lindl.	a	0
Iz_11_1209	DJI_0719	Rosa sp.	0	Rosaceae Juss.		
Iz_11_1209	DJI_0719	Salix alba L.	0	Salicaceae Mirb.	n	1
Iz_11_1209	DJI_0719	Sambucus nigra L.	0	Viburnaceae Raf.	n	1
Iz_11_1209	DJI_0719	Sorbus aucuparia L.	0	Rosaceae Juss.	n	1
Iz_11_1209	DJI_0719	Tilia cordata Mill.	0	Malvaceae Juss.	n	1
Iz_11_1209	DJI_0719	Ulmus glabra Huds.	0	Ulmaceae Mirb.	n	1
Iz_12_1209	DJI_0831	Acer tataricum L.	0	Sapindaceae Juss.	n	1
Iz_12_1209	DJI_0831	Betula pendula Roth.	0	Betulaceae Gray	n	1
Iz_12_1209	DJI_0831	Crataegus rhipidophylla Gand.	0	Rosaceae Juss.	n	1
Iz_12_1209	DJI_0831	Frangula alnus Mill.	0	Rhamnaceae Juss.	n	1
Iz_12_1209	DJI_0831	Fraxinus pennsylvanica Marshall	0	Oleaceae Hoffmanns. & Link	a	0
Iz_12_1209	DJI_0831	Pinus sylvestris L.	0	Pinaceae Spreng. ex F.Rudolphi	n	1
Iz_12_1209	DJI_0831	Populus × canescens (Aiton) Sm.	0	Salicaceae Mirb.	n	1
Iz_12_1209	DJI_0831	Populus tremula L.	4	Salicaceae Mirb.	n	1
Iz_12_1209	DJI_0831	Populus nigra L.	0	Salicaceae Mirb.	n	1
Iz_12_1209	DJI_0831	Pyrus communis L.	0	Rosaceae Juss.	a	0
Iz_12_1209	DJI_0831	Quercus robur L.	0	Fagaceae Dumort.	n	1
Iz_12_1209	DJI_0831	Robinia pseudoacacia L.	0	Fabaceae Lindl.	a	0
Iz_12_1209	DJI_0831	Rosa sp.	0	Rosaceae Juss.		
Iz_12_1209	DJI_0831	Salix sp.	0			
Iz_12_1209	DJI_0831	Salix alba L.	0	Salicaceae Mirb.	n	1
Iz_12_1209	DJI_0831	Sambucus nigra L.	0	Viburnaceae Raf.	n	1
Iz_12_1209	DJI_0831	Sorbus aucuparia L.	0	Rosaceae Juss.	n	1
Iz_12_1209	DJI_0831	Tilia cordata Mill.	0	Malvaceae Juss.	n	1
Iz_12_1209	DJI_0831	Ulmus glabra Huds.	0	Ulmaceae Mirb.	n	1
Iz_12_1209	DJI_0828	Acer tataricum L.	0	Sapindaceae Juss.	n	1
Iz_12_1209	DJI_0828	Betula pendula Roth.	0	Betulaceae Gray	n	1
Iz_12_1209	DJI_0828	Crataegus rhipidophylla Gand.	0	Rosaceae Juss.	n	1
Iz_12_1209	DJI_0828	Frangula alnus Mill.	0	Rhamnaceae Juss.	n	1
Iz_12_1209	DJI_0828	Fraxinus pennsylvanica Marshall	0	Oleaceae Hoffmanns. & Link	a	0
Iz_12_1209	DJI_0828	Pinus sylvestris L.	0	Pinaceae Spreng. ex F.Rudolphi	n	1
Iz_12_1209	DJI_0828	Populus × canescens (Aiton) Sm.	0	Salicaceae Mirb.	n	1
Iz_12_1209	DJI_0828	Populus tremula L.	0	Salicaceae Mirb.	n	1
Iz_12_1209	DJI_0828	Populus nigra L.	0	Salicaceae Mirb.	n	1
Iz_12_1209	DJI_0828	Pyrus communis L.	0	Rosaceae Juss.	a	0
Iz_12_1209	DJI_0828	Quercus robur L.	0	Fagaceae Dumort.	n	1
Iz_12_1209	DJI_0828	Robinia pseudoacacia L.	0	Fabaceae Lindl.	a	0
Iz_12_1209	DJI_0828	Rosa sp.	0	Rosaceae Juss.		
Iz_12_1209	DJI_0828	Salix sp.	0			
Iz_12_1209	DJI_0828	Salix alba L.	0	Salicaceae Mirb.	n	1
Iz_12_1209	DJI_0828	Sambucus nigra L.	0	Viburnaceae Raf.	n	1
Iz_12_1209	DJI_0828	Sorbus aucuparia L.	0	Rosaceae Juss.	n	1
Iz_12_1209	DJI_0828	Tilia cordata Mill.	0	Malvaceae Juss.	n	1
Iz_12_1209	DJI_0828	Ulmus glabra Huds.	0	Ulmaceae Mirb.	n	1
Iz_12_1209	DJI_0825	Acer tataricum L.	0	Sapindaceae Juss.	n	1
Iz_12_1209	DJI_0825	Betula pendula Roth.	0	Betulaceae Gray	n	1
Iz_12_1209	DJI_0825	Crataegus rhipidophylla Gand.	0	Rosaceae Juss.	n	1
Iz_12_1209	DJI_0825	Frangula alnus Mill.	0	Rhamnaceae Juss.	n	1
Iz_12_1209	DJI_0825	Fraxinus pennsylvanica Marshall	0	Oleaceae Hoffmanns. & Link	a	0
Iz_12_1209	DJI_0825	Pinus sylvestris L.	0	Pinaceae Spreng. ex F.Rudolphi	n	1
Iz_12_1209	DJI_0825	Populus × canescens (Aiton) Sm.	0	Salicaceae Mirb.	n	1
Iz_12_1209	DJI_0825	Populus tremula L.	0	Salicaceae Mirb.	n	1
Iz_12_1209	DJI_0825	Populus nigra L.	0	Salicaceae Mirb.	n	1
Iz_12_1209	DJI_0825	Pyrus communis L.	0	Rosaceae Juss.	a	0

Iz_12_1209	DJI_0825	Quercus robur L.	0	Fagaceae Dumort.	n	1
Iz_12_1209	DJI_0825	Robinia pseudoacacia L.	0	Fabaceae Lindl.	a	0
Iz_12_1209	DJI_0825	Rosa sp.	0	Rosaceae Juss.		
Iz_12_1209	DJI_0825	Salix sp.	0			
Iz_12_1209	DJI_0825	Salix alba L.	0	Salicaceae Mirb.	n	1
Iz_12_1209	DJI_0825	Sambucus nigra L.	0	Viburnaceae Raf.	n	1
Iz_12_1209	DJI_0825	Sorbus aucuparia L.	0	Rosaceae Juss.	n	1
Iz_12_1209	DJI_0825	Tilia cordata Mill.	0	Malvaceae Juss.	n	1
Iz_12_1209	DJI_0825	Ulmus glabra Huds.	0	Ulmaceae Mirb.	n	1
Iz_12_1209	DJI_0817	Acer tataricum L.	0	Sapindaceae Juss.	n	1
Iz_12_1209	DJI_0817	Betula pendula Roth.	0	Betulaceae Gray	n	1
Iz_12_1209	DJI_0817	Crataegus rhipidophylla Gand.	0	Rosaceae Juss.	n	1
Iz_12_1209	DJI_0817	Frangula alnus Mill.	0	Rhamnaceae Juss.	n	1
Iz_12_1209	DJI_0817	Fraxinus pennsylvanica Marshall	0	Oleaceae Hoffmanns. & Link	a	0
Iz_12_1209	DJI_0817	Pinus sylvestris L.	0	Pinaceae Spreng. ex F.Rudolphi	n	1
Iz_12_1209	DJI_0817	Populus × canescens (Aiton) Sm.	0	Salicaceae Mirb.	n	1
Iz_12_1209	DJI_0817	Populus tremula L.	0	Salicaceae Mirb.	n	1
Iz_12_1209	DJI_0817	Populus nigra L.	0	Salicaceae Mirb.	n	1
Iz_12_1209	DJI_0817	Pyrus communis L.	0	Rosaceae Juss.	a	0
Iz_12_1209	DJI_0817	Quercus robur L.	0	Fagaceae Dumort.	n	1
Iz_12_1209	DJI_0817	Robinia pseudoacacia L.	0	Fabaceae Lindl.	a	0
Iz_12_1209	DJI_0817	Rosa sp.	0	Rosaceae Juss.		
Iz_12_1209	DJI_0817	Salix sp.	0			
Iz_12_1209	DJI_0817	Salix alba L.	0	Salicaceae Mirb.	n	1
Iz_12_1209	DJI_0817	Sambucus nigra L.	0	Viburnaceae Raf.	n	1
Iz_12_1209	DJI_0817	Sorbus aucuparia L.	0	Rosaceae Juss.	n	1
Iz_12_1209	DJI_0817	Tilia cordata Mill.	0	Malvaceae Juss.	n	1
Iz_12_1209	DJI_0817	Ulmus glabra Huds.	0	Ulmaceae Mirb.	n	1
Iz_12_1209	DJI_0814	Acer tataricum L.	0	Sapindaceae Juss.	n	1
Iz_12_1209	DJI_0814	Betula pendula Roth.	28	Betulaceae Gray	n	1
Iz_12_1209	DJI_0814	Crataegus rhipidophylla Gand.	0	Rosaceae Juss.	n	1
Iz_12_1209	DJI_0814	Frangula alnus Mill.	0	Rhamnaceae Juss.	n	1
Iz_12_1209	DJI_0814	Fraxinus pennsylvanica Marshall	0	Oleaceae Hoffmanns. & Link	a	0
Iz_12_1209	DJI_0814	Pinus sylvestris L.	0	Pinaceae Spreng. ex F.Rudolphi	n	1
Iz_12_1209	DJI_0814	Populus × canescens (Aiton) Sm.	0	Salicaceae Mirb.	n	1
Iz_12_1209	DJI_0814	Populus tremula L.	4	Salicaceae Mirb.	n	1
Iz_12_1209	DJI_0814	Populus nigra L.	0	Salicaceae Mirb.	n	1
Iz_12_1209	DJI_0814	Pyrus communis L.	0	Rosaceae Juss.	a	0
Iz_12_1209	DJI_0814	Quercus robur L.	0	Fagaceae Dumort.	n	1
Iz_12_1209	DJI_0814	Robinia pseudoacacia L.	0	Fabaceae Lindl.	a	0
Iz_12_1209	DJI_0814	Rosa sp.	0	Rosaceae Juss.		
Iz_12_1209	DJI_0814	Salix sp.	0			
Iz_12_1209	DJI_0814	Salix alba L.	0	Salicaceae Mirb.	n	1
Iz_12_1209	DJI_0814	Sambucus nigra L.	0	Viburnaceae Raf.	n	1
Iz_12_1209	DJI_0814	Sorbus aucuparia L.	0	Rosaceae Juss.	n	1
Iz_12_1209	DJI_0814	Tilia cordata Mill.	0	Malvaceae Juss.	n	1
Iz_12_1209	DJI_0814	Ulmus glabra Huds.	0	Ulmaceae Mirb.	n	1
Iz_12_1209	DJI_0810	Acer tataricum L.	0	Sapindaceae Juss.	n	1
Iz_12_1209	DJI_0810	Betula pendula Roth.	25	Betulaceae Gray	n	1
Iz_12_1209	DJI_0810	Crataegus rhipidophylla Gand.	0	Rosaceae Juss.	n	1
Iz_12_1209	DJI_0810	Frangula alnus Mill.	0	Rhamnaceae Juss.	n	1
Iz_12_1209	DJI_0810	Fraxinus pennsylvanica Marshall	0	Oleaceae Hoffmanns. & Link	a	0
Iz_12_1209	DJI_0810	Pinus sylvestris L.	0	Pinaceae Spreng. ex F.Rudolphi	n	1
Iz_12_1209	DJI_0810	Populus × canescens (Aiton) Sm.	0	Salicaceae Mirb.	n	1
Iz_12_1209	DJI_0810	Populus tremula L.	0	Salicaceae Mirb.	n	1
Iz_12_1209	DJI_0810	Populus nigra L.	0	Salicaceae Mirb.	n	1
Iz_12_1209	DJI_0810	Pyrus communis L.	0	Rosaceae Juss.	a	0
Iz_12_1209	DJI_0810	Quercus robur L.	0	Fagaceae Dumort.	n	1
Iz_12_1209	DJI_0810	Robinia pseudoacacia L.	0	Fabaceae Lindl.	a	0
Iz_12_1209	DJI_0810	Rosa sp.	0	Rosaceae Juss.		
Iz_12_1209	DJI_0810	Salix sp.	0			
Iz_12_1209	DJI_0810	Salix alba L.	0	Salicaceae Mirb.	n	1
Iz_12_1209	DJI_0810	Sambucus nigra L.	0	Viburnaceae Raf.	n	1
Iz_12_1209	DJI_0810	Sorbus aucuparia L.	0	Rosaceae Juss.	n	1

Iz_12_1209	DJI_0810	Tilia cordata Mill.	0	Malvaceae Juss.	n	1
Iz_12_1209	DJI_0810	Ulmus glabra Huds.	0	Ulmaceae Mirb.	n	1
Iz_12_1209	DJI_0805	Acer tataricum L.	0	Sapindaceae Juss.	n	1
Iz_12_1209	DJI_0805	Betula pendula Roth.	2	Betulaceae Gray	n	1
Iz_12_1209	DJI_0805	Crataegus rhipidophylla Gand.	0	Rosaceae Juss.	n	1
Iz_12_1209	DJI_0805	Frangula alnus Mill.	0	Rhamnaceae Juss.	n	1
Iz_12_1209	DJI_0805	Fraxinus pennsylvanica Marshall	0	Oleaceae Hoffmanns. & Link	a	0
Iz_12_1209	DJI_0805	Pinus sylvestris L.	0	Pinaceae Spreng. ex F.Rudolphi	n	1
Iz_12_1209	DJI_0805	Populus × canescens (Aiton) Sm.	0	Salicaceae Mirb.	n	1
Iz_12_1209	DJI_0805	Populus tremula L.	0	Salicaceae Mirb.	n	1
Iz_12_1209	DJI_0805	Populus nigra L.	0	Salicaceae Mirb.	n	1
Iz_12_1209	DJI_0805	Pyrus communis L.	0	Rosaceae Juss.	a	0
Iz_12_1209	DJI_0805	Quercus robur L.	0	Fagaceae Dumort.	n	1
Iz_12_1209	DJI_0805	Robinia pseudoacacia L.	0	Fabaceae Lindl.	a	0
Iz_12_1209	DJI_0805	Rosa sp.	0	Rosaceae Juss.		
Iz_12_1209	DJI_0805	Salix sp.	0			
Iz_12_1209	DJI_0805	Salix alba L.	0	Salicaceae Mirb.	n	1
Iz_12_1209	DJI_0805	Sambucus nigra L.	0	Viburnaceae Raf.	n	1
Iz_12_1209	DJI_0805	Sorbus aucuparia L.	0	Rosaceae Juss.	n	1
Iz_12_1209	DJI_0805	Tilia cordata Mill.	0	Malvaceae Juss.	n	1
Iz_12_1209	DJI_0805	Ulmus glabra Huds.	0	Ulmaceae Mirb.	n	1
Iz_12_1209	DJI_0801	Acer tataricum L.	0	Sapindaceae Juss.	n	1
Iz_12_1209	DJI_0801	Betula pendula Roth.	7	Betulaceae Gray	n	1
Iz_12_1209	DJI_0801	Crataegus rhipidophylla Gand.	0	Rosaceae Juss.	n	1
Iz_12_1209	DJI_0801	Frangula alnus Mill.	0	Rhamnaceae Juss.	n	1
Iz_12_1209	DJI_0801	Fraxinus pennsylvanica Marshall	0	Oleaceae Hoffmanns. & Link	a	0
Iz_12_1209	DJI_0801	Pinus sylvestris L.	0	Pinaceae Spreng. ex F.Rudolphi	n	1
Iz_12_1209	DJI_0801	Populus × canescens (Aiton) Sm.	0	Salicaceae Mirb.	n	1
Iz_12_1209	DJI_0801	Populus tremula L.	0	Salicaceae Mirb.	n	1
Iz_12_1209	DJI_0801	Populus nigra L.	0	Salicaceae Mirb.	n	1
Iz_12_1209	DJI_0801	Pyrus communis L.	0	Rosaceae Juss.	a	0
Iz_12_1209	DJI_0801	Quercus robur L.	0	Fagaceae Dumort.	n	1
Iz_12_1209	DJI_0801	Robinia pseudoacacia L.	0	Fabaceae Lindl.	a	0
Iz_12_1209	DJI_0801	Rosa sp.	0	Rosaceae Juss.		
Iz_12_1209	DJI_0801	Salix sp.	0			
Iz_12_1209	DJI_0801	Salix alba L.	0	Salicaceae Mirb.	n	1
Iz_12_1209	DJI_0801	Sambucus nigra L.	0	Viburnaceae Raf.	n	1
Iz_12_1209	DJI_0801	Sorbus aucuparia L.	0	Rosaceae Juss.	n	1
Iz_12_1209	DJI_0801	Tilia cordata Mill.	0	Malvaceae Juss.	n	1
Iz_12_1209	DJI_0801	Ulmus glabra Huds.	0	Ulmaceae Mirb.	n	1
Iz_12_1209	DJI_0798	Acer tataricum L.	0	Sapindaceae Juss.	n	1
Iz_12_1209	DJI_0798	Betula pendula Roth.	13	Betulaceae Gray	n	1
Iz_12_1209	DJI_0798	Crataegus rhipidophylla Gand.	0	Rosaceae Juss.	n	1
Iz_12_1209	DJI_0798	Frangula alnus Mill.	0	Rhamnaceae Juss.	n	1
Iz_12_1209	DJI_0798	Fraxinus pennsylvanica Marshall	0	Oleaceae Hoffmanns. & Link	a	0
Iz_12_1209	DJI_0798	Pinus sylvestris L.	0	Pinaceae Spreng. ex F.Rudolphi	n	1
Iz_12_1209	DJI_0798	Populus × canescens (Aiton) Sm.	0	Salicaceae Mirb.	n	1
Iz_12_1209	DJI_0798	Populus tremula L.	4	Salicaceae Mirb.	n	1
Iz_12_1209	DJI_0798	Populus nigra L.	0	Salicaceae Mirb.	n	1
Iz_12_1209	DJI_0798	Pyrus communis L.	0	Rosaceae Juss.	a	0
Iz_12_1209	DJI_0798	Quercus robur L.	0	Fagaceae Dumort.	n	1
Iz_12_1209	DJI_0798	Robinia pseudoacacia L.	0	Fabaceae Lindl.	a	0
Iz_12_1209	DJI_0798	Rosa sp.	0	Rosaceae Juss.		
Iz_12_1209	DJI_0798	Salix sp.	0			
Iz_12_1209	DJI_0798	Salix alba L.	0	Salicaceae Mirb.	n	1
Iz_12_1209	DJI_0798	Sambucus nigra L.	0	Viburnaceae Raf.	n	1
Iz_12_1209	DJI_0798	Sorbus aucuparia L.	0	Rosaceae Juss.	n	1
Iz_12_1209	DJI_0798	Tilia cordata Mill.	0	Malvaceae Juss.	n	1
Iz_12_1209	DJI_0798	Ulmus glabra Huds.	0	Ulmaceae Mirb.	n	1
Iz_12_1209	DJI_0794	Acer tataricum L.	0	Sapindaceae Juss.	n	1
Iz_12_1209	DJI_0794	Betula pendula Roth.	2	Betulaceae Gray	n	1
Iz_12_1209	DJI_0794	Crataegus rhipidophylla Gand.	0	Rosaceae Juss.	n	1
Iz_12_1209	DJI_0794	Frangula alnus Mill.	0	Rhamnaceae Juss.	n	1
Iz_12_1209	DJI_0794	Fraxinus pennsylvanica Marshall	0	Oleaceae Hoffmanns. & Link	a	0

Iz_12_1209	DJI_0794	Pinus sylvestris L.	0	Pinaceae Spreng. ex F.Rudolphi	n	1
Iz_12_1209	DJI_0794	Populus × canescens (Aiton) Sm.	0	Salicaceae Mirb.	n	1
Iz_12_1209	DJI_0794	Populus tremula L.	0	Salicaceae Mirb.	n	1
Iz_12_1209	DJI_0794	Populus nigra L.	0	Salicaceae Mirb.	n	1
Iz_12_1209	DJI_0794	Pyrus communis L.	0	Rosaceae Juss.	a	0
Iz_12_1209	DJI_0794	Quercus robur L.	0	Fagaceae Dumort.	n	1
Iz_12_1209	DJI_0794	Robinia pseudoacacia L.	0	Fabaceae Lindl.	a	0
Iz_12_1209	DJI_0794	Rosa sp.	0	Rosaceae Juss.		
Iz_12_1209	DJI_0794	Salix sp.	0			
Iz_12_1209	DJI_0794	Salix alba L.	0	Salicaceae Mirb.	n	1
Iz_12_1209	DJI_0794	Sambucus nigra L.	0	Viburnaceae Raf.	n	1
Iz_12_1209	DJI_0794	Sorbus aucuparia L.	0	Rosaceae Juss.	n	1
Iz_12_1209	DJI_0794	Tilia cordata Mill.	0	Malvaceae Juss.	n	1
Iz_12_1209	DJI_0794	Ulmus glabra Huds.	0	Ulmaceae Mirb.	n	1
Iz_12_1209	DJI_0791	Acer tataricum L.	0	Sapindaceae Juss.	n	1
Iz_12_1209	DJI_0791	Betula pendula Roth.	0	Betulaceae Gray	n	1
Iz_12_1209	DJI_0791	Crataegus rhipidophylla Gand.	0	Rosaceae Juss.	n	1
Iz_12_1209	DJI_0791	Frangula alnus Mill.	0	Rhamnaceae Juss.	n	1
Iz_12_1209	DJI_0791	Fraxinus pennsylvanica Marshall	0	Oleaceae Hoffmanns. & Link	a	0
Iz_12_1209	DJI_0791	Pinus sylvestris L.	0	Pinaceae Spreng. ex F.Rudolphi	n	1
Iz_12_1209	DJI_0791	Populus × canescens (Aiton) Sm.	0	Salicaceae Mirb.	n	1
Iz_12_1209	DJI_0791	Populus tremula L.	0	Salicaceae Mirb.	n	1
Iz_12_1209	DJI_0791	Populus nigra L.	0	Salicaceae Mirb.	n	1
Iz_12_1209	DJI_0791	Pyrus communis L.	0	Rosaceae Juss.	a	0
Iz_12_1209	DJI_0791	Quercus robur L.	0	Fagaceae Dumort.	n	1
Iz_12_1209	DJI_0791	Robinia pseudoacacia L.	0	Fabaceae Lindl.	a	0
Iz_12_1209	DJI_0791	Rosa sp.	0	Rosaceae Juss.		
Iz_12_1209	DJI_0791	Salix sp.	0			
Iz_12_1209	DJI_0791	Salix alba L.	0	Salicaceae Mirb.	n	1
Iz_12_1209	DJI_0791	Sambucus nigra L.	0	Viburnaceae Raf.	n	1
Iz_12_1209	DJI_0791	Sorbus aucuparia L.	0	Rosaceae Juss.	n	1
Iz_12_1209	DJI_0791	Tilia cordata Mill.	0	Malvaceae Juss.	n	1
Iz_12_1209	DJI_0791	Ulmus glabra Huds.	0	Ulmaceae Mirb.	n	1
Iz_12_1209	DJI_0788	Acer tataricum L.	0	Sapindaceae Juss.	n	1
Iz_12_1209	DJI_0788	Betula pendula Roth.	0	Betulaceae Gray	n	1
Iz_12_1209	DJI_0788	Crataegus rhipidophylla Gand.	0	Rosaceae Juss.	n	1
Iz_12_1209	DJI_0788	Frangula alnus Mill.	0	Rhamnaceae Juss.	n	1
Iz_12_1209	DJI_0788	Fraxinus pennsylvanica Marshall	0	Oleaceae Hoffmanns. & Link	a	0
Iz_12_1209	DJI_0788	Pinus sylvestris L.	0	Pinaceae Spreng. ex F.Rudolphi	n	1
Iz_12_1209	DJI_0788	Populus × canescens (Aiton) Sm.	0	Salicaceae Mirb.	n	1
Iz_12_1209	DJI_0788	Populus tremula L.	0	Salicaceae Mirb.	n	1
Iz_12_1209	DJI_0788	Populus nigra L.	0	Salicaceae Mirb.	n	1
Iz_12_1209	DJI_0788	Pyrus communis L.	0	Rosaceae Juss.	a	0
Iz_12_1209	DJI_0788	Quercus robur L.	0	Fagaceae Dumort.	n	1
Iz_12_1209	DJI_0788	Robinia pseudoacacia L.	0	Fabaceae Lindl.	a	0
Iz_12_1209	DJI_0788	Rosa sp.	0	Rosaceae Juss.		
Iz_12_1209	DJI_0788	Salix sp.	0			
Iz_12_1209	DJI_0788	Salix alba L.	0	Salicaceae Mirb.	n	1
Iz_12_1209	DJI_0788	Sambucus nigra L.	0	Viburnaceae Raf.	n	1
Iz_12_1209	DJI_0788	Sorbus aucuparia L.	0	Rosaceae Juss.	n	1
Iz_12_1209	DJI_0788	Tilia cordata Mill.	0	Malvaceae Juss.	n	1
Iz_12_1209	DJI_0788	Ulmus glabra Huds.	0	Ulmaceae Mirb.	n	1
Iz_12_1209	DJI_0785	Acer tataricum L.	0	Sapindaceae Juss.	n	1
Iz_12_1209	DJI_0785	Betula pendula Roth.	0	Betulaceae Gray	n	1
Iz_12_1209	DJI_0785	Crataegus rhipidophylla Gand.	0	Rosaceae Juss.	n	1
Iz_12_1209	DJI_0785	Frangula alnus Mill.	0	Rhamnaceae Juss.	n	1
Iz_12_1209	DJI_0785	Fraxinus pennsylvanica Marshall	0	Oleaceae Hoffmanns. & Link	a	0
Iz_12_1209	DJI_0785	Pinus sylvestris L.	5	Pinaceae Spreng. ex F.Rudolphi	n	1
Iz_12_1209	DJI_0785	Populus × canescens (Aiton) Sm.	1	Salicaceae Mirb.	n	1
Iz_12_1209	DJI_0785	Populus tremula L.	0	Salicaceae Mirb.	n	1
Iz_12_1209	DJI_0785	Populus nigra L.	0	Salicaceae Mirb.	n	1
Iz_12_1209	DJI_0785	Pyrus communis L.	0	Rosaceae Juss.	a	0
Iz_12_1209	DJI_0785	Quercus robur L.	3	Fagaceae Dumort.	n	1
Iz_12_1209	DJI_0785	Robinia pseudoacacia L.	0	Fabaceae Lindl.	a	0

Iz_12_1209	DJI_0785	Rosa sp.	0	Rosaceae Juss.		
Iz_12_1209	DJI_0785	Salix sp.	1			
Iz_12_1209	DJI_0785	Salix alba L.	0	Salicaceae Mirb.	n	1
Iz_12_1209	DJI_0785	Sambucus nigra L.	0	Viburnaceae Raf.	n	1
Iz_12_1209	DJI_0785	Sorbus aucuparia L.	0	Rosaceae Juss.	n	1
Iz_12_1209	DJI_0785	Tilia cordata Mill.	0	Malvaceae Juss.	n	1
Iz_12_1209	DJI_0785	Ulmus glabra Huds.	0	Ulmaceae Mirb.	n	1
Iz_13_1209	DJI_0913	Acer tataricum L.	0	Sapindaceae Juss.	n	1
Iz_13_1209	DJI_0913	Betula pendula Roth.	0	Betulaceae Gray	n	1
Iz_13_1209	DJI_0913	Crataegus rhipidophylla Gand.	0	Rosaceae Juss.	n	1
Iz_13_1209	DJI_0913	Frangula alnus Mill.	0	Rhamnaceae Juss.	n	1
Iz_13_1209	DJI_0913	Fraxinus pennsylvanica Marshall	0	Oleaceae Hoffmanns. & Link	a	0
Iz_13_1209	DJI_0913	Pinus sylvestris L.	0	Pinaceae Spreng. ex F.Rudolphi	n	1
Iz_13_1209	DJI_0913	Populus × canescens (Aiton) Sm.	0	Salicaceae Mirb.	n	1
Iz_13_1209	DJI_0913	Populus tremula L.	1	Salicaceae Mirb.	n	1
Iz_13_1209	DJI_0913	Populus nigra L.	0	Salicaceae Mirb.	n	1
Iz_13_1209	DJI_0913	Populus sp.	0	Salicaceae Mirb.	n	1
Iz_13_1209	DJI_0913	Pyrus communis L.	0	Rosaceae Juss.	a	0
Iz_13_1209	DJI_0913	Quercus robur L.	0	Fagaceae Dumort.	n	1
Iz_13_1209	DJI_0913	Robinia pseudoacacia L.	0	Fabaceae Lindl.	a	0
Iz_13_1209	DJI_0913	Rosa sp.	0	Rosaceae Juss.		
Iz_13_1209	DJI_0913	Salix alba L.	0	Salicaceae Mirb.	n	1
Iz_13_1209	DJI_0913	Sambucus nigra L.	0	Viburnaceae Raf.	n	1
Iz_13_1209	DJI_0913	Sorbus aucuparia L.	0	Rosaceae Juss.	n	1
Iz_13_1209	DJI_0913	Tilia cordata Mill.	0	Malvaceae Juss.	n	1
Iz_13_1209	DJI_0913	Ulmus glabra Huds.	0	Ulmaceae Mirb.	n	1
Iz_13_1209	DJI_0908	Acer tataricum L.	0	Sapindaceae Juss.	n	1
Iz_13_1209	DJI_0908	Betula pendula Roth.	0	Betulaceae Gray	n	1
Iz_13_1209	DJI_0908	Crataegus rhipidophylla Gand.	0	Rosaceae Juss.	n	1
Iz_13_1209	DJI_0908	Frangula alnus Mill.	0	Rhamnaceae Juss.	n	1
Iz_13_1209	DJI_0908	Fraxinus pennsylvanica Marshall	0	Oleaceae Hoffmanns. & Link	a	0
Iz_13_1209	DJI_0908	Pinus sylvestris L.	0	Pinaceae Spreng. ex F.Rudolphi	n	1
Iz_13_1209	DJI_0908	Populus × canescens (Aiton) Sm.	0	Salicaceae Mirb.	n	1
Iz_13_1209	DJI_0908	Populus tremula L.	3	Salicaceae Mirb.	n	1
Iz_13_1209	DJI_0908	Populus nigra L.	0	Salicaceae Mirb.	n	1
Iz_13_1209	DJI_0908	Populus sp.	0	Salicaceae Mirb.	n	1
Iz_13_1209	DJI_0908	Pyrus communis L.	0	Rosaceae Juss.	a	0
Iz_13_1209	DJI_0908	Quercus robur L.	0	Fagaceae Dumort.	n	1
Iz_13_1209	DJI_0908	Robinia pseudoacacia L.	0	Fabaceae Lindl.	a	0
Iz_13_1209	DJI_0908	Rosa sp.	0	Rosaceae Juss.		
Iz_13_1209	DJI_0908	Salix alba L.	0	Salicaceae Mirb.	n	1
Iz_13_1209	DJI_0908	Sambucus nigra L.	0	Viburnaceae Raf.	n	1
Iz_13_1209	DJI_0908	Sorbus aucuparia L.	0	Rosaceae Juss.	n	1
Iz_13_1209	DJI_0908	Tilia cordata Mill.	0	Malvaceae Juss.	n	1
Iz_13_1209	DJI_0908	Ulmus glabra Huds.	0	Ulmaceae Mirb.	n	1
Iz_13_1209	DJI_0905	Acer tataricum L.	0	Sapindaceae Juss.	n	1
Iz_13_1209	DJI_0905	Betula pendula Roth.	0	Betulaceae Gray	n	1
Iz_13_1209	DJI_0905	Crataegus rhipidophylla Gand.	0	Rosaceae Juss.	n	1
Iz_13_1209	DJI_0905	Frangula alnus Mill.	0	Rhamnaceae Juss.	n	1
Iz_13_1209	DJI_0905	Fraxinus pennsylvanica Marshall	0	Oleaceae Hoffmanns. & Link	a	0
Iz_13_1209	DJI_0905	Pinus sylvestris L.	0	Pinaceae Spreng. ex F.Rudolphi	n	1
Iz_13_1209	DJI_0905	Populus × canescens (Aiton) Sm.	0	Salicaceae Mirb.	n	1
Iz_13_1209	DJI_0905	Populus tremula L.	0	Salicaceae Mirb.	n	1
Iz_13_1209	DJI_0905	Populus nigra L.	0	Salicaceae Mirb.	n	1
Iz_13_1209	DJI_0905	Populus sp.	0	Salicaceae Mirb.	n	1
Iz_13_1209	DJI_0905	Pyrus communis L.	0	Rosaceae Juss.	a	0
Iz_13_1209	DJI_0905	Quercus robur L.	0	Fagaceae Dumort.	n	1
Iz_13_1209	DJI_0905	Robinia pseudoacacia L.	0	Fabaceae Lindl.	a	0
Iz_13_1209	DJI_0905	Rosa sp.	0	Rosaceae Juss.		
Iz_13_1209	DJI_0905	Salix alba L.	0	Salicaceae Mirb.	n	1
Iz_13_1209	DJI_0905	Sambucus nigra L.	0	Viburnaceae Raf.	n	1
Iz_13_1209	DJI_0905	Sorbus aucuparia L.	0	Rosaceae Juss.	n	1
Iz_13_1209	DJI_0905	Tilia cordata Mill.	0	Malvaceae Juss.	n	1
Iz_13_1209	DJI_0905	Ulmus glabra Huds.	0	Ulmaceae Mirb.	n	1

Iz_13_1209	DJI_0901	Acer tataricum L.	0	Sapindaceae Juss.	n	1
Iz_13_1209	DJI_0901	Betula pendula Roth.	0	Betulaceae Gray	n	1
Iz_13_1209	DJI_0901	Crataegus rhipidophylla Gand.	0	Rosaceae Juss.	n	1
Iz_13_1209	DJI_0901	Frangula alnus Mill.	0	Rhamnaceae Juss.	n	1
Iz_13_1209	DJI_0901	Fraxinus pennsylvanica Marshall	0	Oleaceae Hoffmanns. & Link	a	0
Iz_13_1209	DJI_0901	Pinus sylvestris L.	0	Pinaceae Spreng. ex F.Rudolphi	n	1
Iz_13_1209	DJI_0901	Populus × canescens (Aiton) Sm.	0	Salicaceae Mirb.	n	1
Iz_13_1209	DJI_0901	Populus tremula L.	0	Salicaceae Mirb.	n	1
Iz_13_1209	DJI_0901	Populus nigra L.	0	Salicaceae Mirb.	n	1
Iz_13_1209	DJI_0901	Populus sp.	0	Salicaceae Mirb.	n	1
Iz_13_1209	DJI_0901	Pyrus communis L.	0	Rosaceae Juss.	a	0
Iz_13_1209	DJI_0901	Quercus robur L.	0	Fagaceae Dumort.	n	1
Iz_13_1209	DJI_0901	Robinia pseudoacacia L.	0	Fabaceae Lindl.	a	0
Iz_13_1209	DJI_0901	Rosa sp.	0	Rosaceae Juss.		
Iz_13_1209	DJI_0901	Salix alba L.	0	Salicaceae Mirb.	n	1
Iz_13_1209	DJI_0901	Sambucus nigra L.	0	Viburnaceae Raf.	n	1
Iz_13_1209	DJI_0901	Sorbus aucuparia L.	0	Rosaceae Juss.	n	1
Iz_13_1209	DJI_0901	Tilia cordata Mill.	0	Malvaceae Juss.	n	1
Iz_13_1209	DJI_0901	Ulmus glabra Huds.	0	Ulmaceae Mirb.	n	1
Iz_13_1209	DJI_0897	Acer tataricum L.	0	Sapindaceae Juss.	n	1
Iz_13_1209	DJI_0897	Betula pendula Roth.	0	Betulaceae Gray	n	1
Iz_13_1209	DJI_0897	Crataegus rhipidophylla Gand.	0	Rosaceae Juss.	n	1
Iz_13_1209	DJI_0897	Frangula alnus Mill.	0	Rhamnaceae Juss.	n	1
Iz_13_1209	DJI_0897	Fraxinus pennsylvanica Marshall	0	Oleaceae Hoffmanns. & Link	a	0
Iz_13_1209	DJI_0897	Pinus sylvestris L.	0	Pinaceae Spreng. ex F.Rudolphi	n	1
Iz_13_1209	DJI_0897	Populus × canescens (Aiton) Sm.	0	Salicaceae Mirb.	n	1
Iz_13_1209	DJI_0897	Populus tremula L.	0	Salicaceae Mirb.	n	1
Iz_13_1209	DJI_0897	Populus nigra L.	0	Salicaceae Mirb.	n	1
Iz_13_1209	DJI_0897	Populus sp.	0	Salicaceae Mirb.	n	1
Iz_13_1209	DJI_0897	Pyrus communis L.	0	Rosaceae Juss.	a	0
Iz_13_1209	DJI_0897	Quercus robur L.	0	Fagaceae Dumort.	n	1
Iz_13_1209	DJI_0897	Robinia pseudoacacia L.	0	Fabaceae Lindl.	a	0
Iz_13_1209	DJI_0897	Rosa sp.	0	Rosaceae Juss.		
Iz_13_1209	DJI_0897	Salix alba L.	0	Salicaceae Mirb.	n	1
Iz_13_1209	DJI_0897	Sambucus nigra L.	0	Viburnaceae Raf.	n	1
Iz_13_1209	DJI_0897	Sorbus aucuparia L.	0	Rosaceae Juss.	n	1
Iz_13_1209	DJI_0897	Tilia cordata Mill.	0	Malvaceae Juss.	n	1
Iz_13_1209	DJI_0897	Ulmus glabra Huds.	0	Ulmaceae Mirb.	n	1
Iz_13_1209	DJI_0893	Acer tataricum L.	0	Sapindaceae Juss.	n	1
Iz_13_1209	DJI_0893	Betula pendula Roth.	0	Betulaceae Gray	n	1
Iz_13_1209	DJI_0893	Crataegus rhipidophylla Gand.	0	Rosaceae Juss.	n	1
Iz_13_1209	DJI_0893	Frangula alnus Mill.	0	Rhamnaceae Juss.	n	1
Iz_13_1209	DJI_0893	Fraxinus pennsylvanica Marshall	0	Oleaceae Hoffmanns. & Link	a	0
Iz_13_1209	DJI_0893	Pinus sylvestris L.	0	Pinaceae Spreng. ex F.Rudolphi	n	1
Iz_13_1209	DJI_0893	Populus × canescens (Aiton) Sm.	0	Salicaceae Mirb.	n	1
Iz_13_1209	DJI_0893	Populus tremula L.	0	Salicaceae Mirb.	n	1
Iz_13_1209	DJI_0893	Populus nigra L.	0	Salicaceae Mirb.	n	1
Iz_13_1209	DJI_0893	Populus sp.	0	Salicaceae Mirb.	n	1
Iz_13_1209	DJI_0893	Pyrus communis L.	0	Rosaceae Juss.	a	0
Iz_13_1209	DJI_0893	Quercus robur L.	0	Fagaceae Dumort.	n	1
Iz_13_1209	DJI_0893	Robinia pseudoacacia L.	0	Fabaceae Lindl.	a	0
Iz_13_1209	DJI_0893	Rosa sp.	0	Rosaceae Juss.		
Iz_13_1209	DJI_0893	Salix alba L.	0	Salicaceae Mirb.	n	1
Iz_13_1209	DJI_0893	Sambucus nigra L.	0	Viburnaceae Raf.	n	1
Iz_13_1209	DJI_0893	Sorbus aucuparia L.	0	Rosaceae Juss.	n	1
Iz_13_1209	DJI_0893	Tilia cordata Mill.	0	Malvaceae Juss.	n	1
Iz_13_1209	DJI_0893	Ulmus glabra Huds.	0	Ulmaceae Mirb.	n	1
Iz_13_1209	DJI_0890	Acer tataricum L.	0	Sapindaceae Juss.	n	1
Iz_13_1209	DJI_0890	Betula pendula Roth.	0	Betulaceae Gray	n	1
Iz_13_1209	DJI_0890	Crataegus rhipidophylla Gand.	0	Rosaceae Juss.	n	1
Iz_13_1209	DJI_0890	Frangula alnus Mill.	0	Rhamnaceae Juss.	n	1
Iz_13_1209	DJI_0890	Fraxinus pennsylvanica Marshall	0	Oleaceae Hoffmanns. & Link	a	0
Iz_13_1209	DJI_0890	Pinus sylvestris L.	0	Pinaceae Spreng. ex F.Rudolphi	n	1
Iz_13_1209	DJI_0890	Populus × canescens (Aiton) Sm.	0	Salicaceae Mirb.	n	1

Iz_13_1209	DJI_0890	Populus tremula L.	0	Salicaceae Mirb.	n	1
Iz_13_1209	DJI_0890	Populus nigra L.	0	Salicaceae Mirb.	n	1
Iz_13_1209	DJI_0890	Populus sp.	0	Salicaceae Mirb.	n	1
Iz_13_1209	DJI_0890	Pyrus communis L.	0	Rosaceae Juss.	a	0
Iz_13_1209	DJI_0890	Quercus robur L.	0	Fagaceae Dumort.	n	1
Iz_13_1209	DJI_0890	Robinia pseudoacacia L.	0	Fabaceae Lindl.	a	0
Iz_13_1209	DJI_0890	Rosa sp.	0	Rosaceae Juss.		
Iz_13_1209	DJI_0890	Salix alba L.	0	Salicaceae Mirb.	n	1
Iz_13_1209	DJI_0890	Sambucus nigra L.	0	Viburnaceae Raf.	n	1
Iz_13_1209	DJI_0890	Sorbus aucuparia L.	0	Rosaceae Juss.	n	1
Iz_13_1209	DJI_0890	Tilia cordata Mill.	0	Malvaceae Juss.	n	1
Iz_13_1209	DJI_0890	Ulmus glabra Huds.	0	Ulmaceae Mirb.	n	1
Iz_13_1209	DJI_0887	Acer tataricum L.	0	Sapindaceae Juss.	n	1
Iz_13_1209	DJI_0887	Betula pendula Roth.	0	Betulaceae Gray	n	1
Iz_13_1209	DJI_0887	Crataegus rhipidophylla Gand.	0	Rosaceae Juss.	n	1
Iz_13_1209	DJI_0887	Frangula alnus Mill.	0	Rhamnaceae Juss.	n	1
Iz_13_1209	DJI_0887	Fraxinus pennsylvanica Marshall	0	Oleaceae Hoffmanns. & Link	a	0
Iz_13_1209	DJI_0887	Pinus sylvestris L.	0	Pinaceae Spreng. ex F.Rudolphi	n	1
Iz_13_1209	DJI_0887	Populus × canescens (Aiton) Sm.	0	Salicaceae Mirb.	n	1
Iz_13_1209	DJI_0887	Populus tremula L.	0	Salicaceae Mirb.	n	1
Iz_13_1209	DJI_0887	Populus nigra L.	0	Salicaceae Mirb.	n	1
Iz_13_1209	DJI_0887	Populus sp.	0	Salicaceae Mirb.	n	1
Iz_13_1209	DJI_0887	Pyrus communis L.	0	Rosaceae Juss.	a	0
Iz_13_1209	DJI_0887	Quercus robur L.	0	Fagaceae Dumort.	n	1
Iz_13_1209	DJI_0887	Robinia pseudoacacia L.	0	Fabaceae Lindl.	a	0
Iz_13_1209	DJI_0887	Rosa sp.	0	Rosaceae Juss.		
Iz_13_1209	DJI_0887	Salix alba L.	0	Salicaceae Mirb.	n	1
Iz_13_1209	DJI_0887	Sambucus nigra L.	0	Viburnaceae Raf.	n	1
Iz_13_1209	DJI_0887	Sorbus aucuparia L.	0	Rosaceae Juss.	n	1
Iz_13_1209	DJI_0887	Tilia cordata Mill.	0	Malvaceae Juss.	n	1
Iz_13_1209	DJI_0887	Ulmus glabra Huds.	0	Ulmaceae Mirb.	n	1
Iz_13_1209	DJI_0883	Acer tataricum L.	0	Sapindaceae Juss.	n	1
Iz_13_1209	DJI_0883	Betula pendula Roth.	0	Betulaceae Gray	n	1
Iz_13_1209	DJI_0883	Crataegus rhipidophylla Gand.	0	Rosaceae Juss.	n	1
Iz_13_1209	DJI_0883	Frangula alnus Mill.	0	Rhamnaceae Juss.	n	1
Iz_13_1209	DJI_0883	Fraxinus pennsylvanica Marshall	0	Oleaceae Hoffmanns. & Link	a	0
Iz_13_1209	DJI_0883	Pinus sylvestris L.	0	Pinaceae Spreng. ex F.Rudolphi	n	1
Iz_13_1209	DJI_0883	Populus × canescens (Aiton) Sm.	0	Salicaceae Mirb.	n	1
Iz_13_1209	DJI_0883	Populus tremula L.	0	Salicaceae Mirb.	n	1
Iz_13_1209	DJI_0883	Populus nigra L.	0	Salicaceae Mirb.	n	1
Iz_13_1209	DJI_0883	Populus sp.	0	Salicaceae Mirb.	n	1
Iz_13_1209	DJI_0883	Pyrus communis L.	0	Rosaceae Juss.	a	0
Iz_13_1209	DJI_0883	Quercus robur L.	0	Fagaceae Dumort.	n	1
Iz_13_1209	DJI_0883	Robinia pseudoacacia L.	0	Fabaceae Lindl.	a	0
Iz_13_1209	DJI_0883	Rosa sp.	0	Rosaceae Juss.		
Iz_13_1209	DJI_0883	Salix alba L.	0	Salicaceae Mirb.	n	1
Iz_13_1209	DJI_0883	Sambucus nigra L.	0	Viburnaceae Raf.	n	1
Iz_13_1209	DJI_0883	Sorbus aucuparia L.	0	Rosaceae Juss.	n	1
Iz_13_1209	DJI_0883	Tilia cordata Mill.	0	Malvaceae Juss.	n	1
Iz_13_1209	DJI_0883	Ulmus glabra Huds.	0	Ulmaceae Mirb.	n	1
Iz_13_1209	DJI_0878	Acer tataricum L.	0	Sapindaceae Juss.	n	1
Iz_13_1209	DJI_0878	Betula pendula Roth.	0	Betulaceae Gray	n	1
Iz_13_1209	DJI_0878	Crataegus rhipidophylla Gand.	0	Rosaceae Juss.	n	1
Iz_13_1209	DJI_0878	Frangula alnus Mill.	0	Rhamnaceae Juss.	n	1
Iz_13_1209	DJI_0878	Fraxinus pennsylvanica Marshall	0	Oleaceae Hoffmanns. & Link	a	0
Iz_13_1209	DJI_0878	Pinus sylvestris L.	0	Pinaceae Spreng. ex F.Rudolphi	n	1
Iz_13_1209	DJI_0878	Populus × canescens (Aiton) Sm.	0	Salicaceae Mirb.	n	1
Iz_13_1209	DJI_0878	Populus tremula L.	0	Salicaceae Mirb.	n	1
Iz_13_1209	DJI_0878	Populus nigra L.	0	Salicaceae Mirb.	n	1
Iz_13_1209	DJI_0878	Populus sp.	0	Salicaceae Mirb.	n	1
Iz_13_1209	DJI_0878	Pyrus communis L.	0	Rosaceae Juss.	a	0
Iz_13_1209	DJI_0878	Quercus robur L.	0	Fagaceae Dumort.	n	1
Iz_13_1209	DJI_0878	Robinia pseudoacacia L.	0	Fabaceae Lindl.	a	0
Iz_13_1209	DJI_0878	Rosa sp.	0	Rosaceae Juss.		

Iz_13_1209	DJI_0878	Salix alba L.	0	Salicaceae Mirb.	n	1
Iz_13_1209	DJI_0878	Sambucus nigra L.	0	Viburnaceae Raf.	n	1
Iz_13_1209	DJI_0878	Sorbus aucuparia L.	0	Rosaceae Juss.	n	1
Iz_13_1209	DJI_0878	Tilia cordata Mill.	0	Malvaceae Juss.	n	1
Iz_13_1209	DJI_0878	Ulmus glabra Huds.	0	Ulmaceae Mirb.	n	1
Iz_13_1209	DJI_0875	Acer tataricum L.	0	Sapindaceae Juss.	n	1
Iz_13_1209	DJI_0875	Betula pendula Roth.	0	Betulaceae Gray	n	1
Iz_13_1209	DJI_0875	Crataegus rhipidophylla Gand.	0	Rosaceae Juss.	n	1
Iz_13_1209	DJI_0875	Frangula alnus Mill.	0	Rhamnaceae Juss.	n	1
Iz_13_1209	DJI_0875	Fraxinus pennsylvanica Marshall	0	Oleaceae Hoffmanns. & Link	a	0
Iz_13_1209	DJI_0875	Pinus sylvestris L.	0	Pinaceae Spreng. ex F.Rudolphi	n	1
Iz_13_1209	DJI_0875	Populus × canescens (Aiton) Sm.	0	Salicaceae Mirb.	n	1
Iz_13_1209	DJI_0875	Populus tremula L.	0	Salicaceae Mirb.	n	1
Iz_13_1209	DJI_0875	Populus nigra L.	0	Salicaceae Mirb.	n	1
Iz_13_1209	DJI_0875	Populus sp.	0	Salicaceae Mirb.	n	1
Iz_13_1209	DJI_0875	Pyrus communis L.	0	Rosaceae Juss.	a	0
Iz_13_1209	DJI_0875	Quercus robur L.	0	Fagaceae Dumort.	n	1
Iz_13_1209	DJI_0875	Robinia pseudoacacia L.	0	Fabaceae Lindl.	a	0
Iz_13_1209	DJI_0875	Rosa sp.	0	Rosaceae Juss.		
Iz_13_1209	DJI_0875	Salix alba L.	0	Salicaceae Mirb.	n	1
Iz_13_1209	DJI_0875	Sambucus nigra L.	0	Viburnaceae Raf.	n	1
Iz_13_1209	DJI_0875	Sorbus aucuparia L.	0	Rosaceae Juss.	n	1
Iz_13_1209	DJI_0875	Tilia cordata Mill.	0	Malvaceae Juss.	n	1
Iz_13_1209	DJI_0875	Ulmus glabra Huds.	0	Ulmaceae Mirb.	n	1
Iz_13_1209	DJI_0873	Acer tataricum L.	0	Sapindaceae Juss.	n	1
Iz_13_1209	DJI_0873	Betula pendula Roth.	0	Betulaceae Gray	n	1
Iz_13_1209	DJI_0873	Crataegus rhipidophylla Gand.	0	Rosaceae Juss.	n	1
Iz_13_1209	DJI_0873	Frangula alnus Mill.	0	Rhamnaceae Juss.	n	1
Iz_13_1209	DJI_0873	Fraxinus pennsylvanica Marshall	0	Oleaceae Hoffmanns. & Link	a	0
Iz_13_1209	DJI_0873	Pinus sylvestris L.	0	Pinaceae Spreng. ex F.Rudolphi	n	1
Iz_13_1209	DJI_0873	Populus × canescens (Aiton) Sm.	0	Salicaceae Mirb.	n	1
Iz_13_1209	DJI_0873	Populus tremula L.	0	Salicaceae Mirb.	n	1
Iz_13_1209	DJI_0873	Populus nigra L.	0	Salicaceae Mirb.	n	1
Iz_13_1209	DJI_0873	Populus sp.	0	Salicaceae Mirb.	n	1
Iz_13_1209	DJI_0873	Pyrus communis L.	0	Rosaceae Juss.	a	0
Iz_13_1209	DJI_0873	Quercus robur L.	0	Fagaceae Dumort.	n	1
Iz_13_1209	DJI_0873	Robinia pseudoacacia L.	0	Fabaceae Lindl.	a	0
Iz_13_1209	DJI_0873	Rosa sp.	0	Rosaceae Juss.		
Iz_13_1209	DJI_0873	Salix alba L.	0	Salicaceae Mirb.	n	1
Iz_13_1209	DJI_0873	Sambucus nigra L.	0	Viburnaceae Raf.	n	1
Iz_13_1209	DJI_0873	Sorbus aucuparia L.	0	Rosaceae Juss.	n	1
Iz_13_1209	DJI_0873	Tilia cordata Mill.	0	Malvaceae Juss.	n	1
Iz_13_1209	DJI_0873	Ulmus glabra Huds.	0	Ulmaceae Mirb.	n	1
Iz_13_1209	DJI_0869	Acer tataricum L.	0	Sapindaceae Juss.	n	1
Iz_13_1209	DJI_0869	Betula pendula Roth.	0	Betulaceae Gray	n	1
Iz_13_1209	DJI_0869	Crataegus rhipidophylla Gand.	0	Rosaceae Juss.	n	1
Iz_13_1209	DJI_0869	Frangula alnus Mill.	0	Rhamnaceae Juss.	n	1
Iz_13_1209	DJI_0869	Fraxinus pennsylvanica Marshall	0	Oleaceae Hoffmanns. & Link	a	0
Iz_13_1209	DJI_0869	Pinus sylvestris L.	0	Pinaceae Spreng. ex F.Rudolphi	n	1
Iz_13_1209	DJI_0869	Populus × canescens (Aiton) Sm.	0	Salicaceae Mirb.	n	1
Iz_13_1209	DJI_0869	Populus tremula L.	0	Salicaceae Mirb.	n	1
Iz_13_1209	DJI_0869	Populus nigra L.	0	Salicaceae Mirb.	n	1
Iz_13_1209	DJI_0869	Populus sp.	0	Salicaceae Mirb.	n	1
Iz_13_1209	DJI_0869	Pyrus communis L.	0	Rosaceae Juss.	a	0
Iz_13_1209	DJI_0869	Quercus robur L.	0	Fagaceae Dumort.	n	1
Iz_13_1209	DJI_0869	Robinia pseudoacacia L.	0	Fabaceae Lindl.	a	0
Iz_13_1209	DJI_0869	Rosa sp.	0	Rosaceae Juss.		
Iz_13_1209	DJI_0869	Salix alba L.	0	Salicaceae Mirb.	n	1
Iz_13_1209	DJI_0869	Sambucus nigra L.	0	Viburnaceae Raf.	n	1
Iz_13_1209	DJI_0869	Sorbus aucuparia L.	0	Rosaceae Juss.	n	1
Iz_13_1209	DJI_0869	Tilia cordata Mill.	0	Malvaceae Juss.	n	1
Iz_13_1209	DJI_0869	Ulmus glabra Huds.	0	Ulmaceae Mirb.	n	1
Iz_13_1209	DJI_0863	Acer tataricum L.	0	Sapindaceae Juss.	n	1
Iz_13_1209	DJI_0863	Betula pendula Roth.	0	Betulaceae Gray	n	1

Iz_13_1209	DJI_0863	Crataegus rhipidophylla Gand.	0	Rosaceae Juss.	n	1
Iz_13_1209	DJI_0863	Frangula alnus Mill.	0	Rhamnaceae Juss.	n	1
Iz_13_1209	DJI_0863	Fraxinus pennsylvanica Marshall	0	Oleaceae Hoffmanns. & Link	a	0
Iz_13_1209	DJI_0863	Pinus sylvestris L.	0	Pinaceae Spreng. ex F.Rudolphi	n	1
Iz_13_1209	DJI_0863	Populus × canescens (Aiton) Sm.	0	Salicaceae Mirb.	n	1
Iz_13_1209	DJI_0863	Populus tremula L.	0	Salicaceae Mirb.	n	1
Iz_13_1209	DJI_0863	Populus nigra L.	0	Salicaceae Mirb.	n	1
Iz_13_1209	DJI_0863	Populus sp.	0	Salicaceae Mirb.	n	1
Iz_13_1209	DJI_0863	Pyrus communis L.	0	Rosaceae Juss.	a	0
Iz_13_1209	DJI_0863	Quercus robur L.	0	Fagaceae Dumort.	n	1
Iz_13_1209	DJI_0863	Robinia pseudoacacia L.	0	Fabaceae Lindl.	a	0
Iz_13_1209	DJI_0863	Rosa sp.	0	Rosaceae Juss.		
Iz_13_1209	DJI_0863	Salix alba L.	0	Salicaceae Mirb.	n	1
Iz_13_1209	DJI_0863	Sambucus nigra L.	0	Viburnaceae Raf.	n	1
Iz_13_1209	DJI_0863	Sorbus aucuparia L.	0	Rosaceae Juss.	n	1
Iz_13_1209	DJI_0863	Tilia cordata Mill.	0	Malvaceae Juss.	n	1
Iz_13_1209	DJI_0863	Ulmus glabra Huds.	0	Ulmaceae Mirb.	n	1
Iz_14_1209	DJI_0982	Acer tataricum L.	0	Sapindaceae Juss.	n	1
Iz_14_1209	DJI_0982	Betula pendula Roth.	0	Betulaceae Gray	n	1
Iz_14_1209	DJI_0982	Crataegus rhipidophylla Gand.	0	Rosaceae Juss.	n	1
Iz_14_1209	DJI_0982	Frangula alnus Mill.	0	Rhamnaceae Juss.	n	1
Iz_14_1209	DJI_0982	Fraxinus pennsylvanica Marshall	0	Oleaceae Hoffmanns. & Link	a	0
Iz_14_1209	DJI_0982	Pinus sylvestris L.	0	Pinaceae Spreng. ex F.Rudolphi	n	1
Iz_14_1209	DJI_0982	Populus × canescens (Aiton) Sm.	0	Salicaceae Mirb.	n	1
Iz_14_1209	DJI_0982	Populus tremula L.	0	Salicaceae Mirb.	n	1
Iz_14_1209	DJI_0982	Populus nigra L.	0	Salicaceae Mirb.	n	1
Iz_14_1209	DJI_0982	Pyrus communis L.	0	Rosaceae Juss.	a	0
Iz_14_1209	DJI_0982	Quercus robur L.	0	Fagaceae Dumort.	n	1
Iz_14_1209	DJI_0982	Robinia pseudoacacia L.	0	Fabaceae Lindl.	a	0
Iz_14_1209	DJI_0982	Rosa sp.	0	Rosaceae Juss.		
Iz_14_1209	DJI_0982	Salix alba L.	0	Salicaceae Mirb.	n	1
Iz_14_1209	DJI_0982	Sambucus nigra L.	0	Viburnaceae Raf.	n	1
Iz_14_1209	DJI_0982	Sorbus aucuparia L.	0	Rosaceae Juss.	n	1
Iz_14_1209	DJI_0982	Tilia cordata Mill.	0	Malvaceae Juss.	n	1
Iz_14_1209	DJI_0982	Ulmus glabra Huds.	0	Ulmaceae Mirb.	n	1
Iz_14_1209	DJI_0979	Acer tataricum L.	0	Sapindaceae Juss.	n	1
Iz_14_1209	DJI_0979	Betula pendula Roth.	0	Betulaceae Gray	n	1
Iz_14_1209	DJI_0979	Crataegus rhipidophylla Gand.	0	Rosaceae Juss.	n	1
Iz_14_1209	DJI_0979	Frangula alnus Mill.	0	Rhamnaceae Juss.	n	1
Iz_14_1209	DJI_0979	Fraxinus pennsylvanica Marshall	0	Oleaceae Hoffmanns. & Link	a	0
Iz_14_1209	DJI_0979	Pinus sylvestris L.	0	Pinaceae Spreng. ex F.Rudolphi	n	1
Iz_14_1209	DJI_0979	Populus × canescens (Aiton) Sm.	0	Salicaceae Mirb.	n	1
Iz_14_1209	DJI_0979	Populus tremula L.	0	Salicaceae Mirb.	n	1
Iz_14_1209	DJI_0979	Populus nigra L.	0	Salicaceae Mirb.	n	1
Iz_14_1209	DJI_0979	Pyrus communis L.	0	Rosaceae Juss.	a	0
Iz_14_1209	DJI_0979	Quercus robur L.	0	Fagaceae Dumort.	n	1
Iz_14_1209	DJI_0979	Robinia pseudoacacia L.	0	Fabaceae Lindl.	a	0
Iz_14_1209	DJI_0979	Rosa sp.	0	Rosaceae Juss.		
Iz_14_1209	DJI_0979	Salix alba L.	0	Salicaceae Mirb.	n	1
Iz_14_1209	DJI_0979	Sambucus nigra L.	0	Viburnaceae Raf.	n	1
Iz_14_1209	DJI_0979	Sorbus aucuparia L.	0	Rosaceae Juss.	n	1
Iz_14_1209	DJI_0979	Tilia cordata Mill.	0	Malvaceae Juss.	n	1
Iz_14_1209	DJI_0979	Ulmus glabra Huds.	0	Ulmaceae Mirb.	n	1
Iz_14_1209	DJI_0976	Acer tataricum L.	0	Sapindaceae Juss.	n	1
Iz_14_1209	DJI_0976	Betula pendula Roth.	0	Betulaceae Gray	n	1
Iz_14_1209	DJI_0976	Crataegus rhipidophylla Gand.	0	Rosaceae Juss.	n	1
Iz_14_1209	DJI_0976	Frangula alnus Mill.	0	Rhamnaceae Juss.	n	1
Iz_14_1209	DJI_0976	Fraxinus pennsylvanica Marshall	0	Oleaceae Hoffmanns. & Link	a	0
Iz_14_1209	DJI_0976	Pinus sylvestris L.	0	Pinaceae Spreng. ex F.Rudolphi	n	1
Iz_14_1209	DJI_0976	Populus × canescens (Aiton) Sm.	0	Salicaceae Mirb.	n	1
Iz_14_1209	DJI_0976	Populus tremula L.	0	Salicaceae Mirb.	n	1
Iz_14_1209	DJI_0976	Populus nigra L.	0	Salicaceae Mirb.	n	1
Iz_14_1209	DJI_0976	Pyrus communis L.	0	Rosaceae Juss.	a	0
Iz_14_1209	DJI_0976	Quercus robur L.	0	Fagaceae Dumort.	n	1

Iz_14_1209	DJI_0976	Robinia pseudoacacia L.	0	Fabaceae Lindl.	a	0
Iz_14_1209	DJI_0976	Rosa sp.	0	Rosaceae Juss.		
Iz_14_1209	DJI_0976	Salix alba L.	0	Salicaceae Mirb.	n	1
Iz_14_1209	DJI_0976	Sambucus nigra L.	0	Viburnaceae Raf.	n	1
Iz_14_1209	DJI_0976	Sorbus aucuparia L.	0	Rosaceae Juss.	n	1
Iz_14_1209	DJI_0976	Tilia cordata Mill.	0	Malvaceae Juss.	n	1
Iz_14_1209	DJI_0976	Ulmus glabra Huds.	0	Ulmaceae Mirb.	n	1
Iz_14_1209	DJI_0972	Acer tataricum L.	0	Sapindaceae Juss.	n	1
Iz_14_1209	DJI_0972	Betula pendula Roth.	0	Betulaceae Gray	n	1
Iz_14_1209	DJI_0972	Crataegus rhipidophylla Gand.	0	Rosaceae Juss.	n	1
Iz_14_1209	DJI_0972	Frangula alnus Mill.	0	Rhamnaceae Juss.	n	1
Iz_14_1209	DJI_0972	Fraxinus pennsylvanica Marshall	0	Oleaceae Hoffmanns. & Link	a	0
Iz_14_1209	DJI_0972	Pinus sylvestris L.	0	Pinaceae Spreng. ex F.Rudolphi	n	1
Iz_14_1209	DJI_0972	Populus × canescens (Aiton) Sm.	0	Salicaceae Mirb.	n	1
Iz_14_1209	DJI_0972	Populus tremula L.	0	Salicaceae Mirb.	n	1
Iz_14_1209	DJI_0972	Populus nigra L.	0	Salicaceae Mirb.	n	1
Iz_14_1209	DJI_0972	Pyrus communis L.	0	Rosaceae Juss.	a	0
Iz_14_1209	DJI_0972	Quercus robur L.	0	Fagaceae Dumort.	n	1
Iz_14_1209	DJI_0972	Robinia pseudoacacia L.	0	Fabaceae Lindl.	a	0
Iz_14_1209	DJI_0972	Rosa sp.	0	Rosaceae Juss.		
Iz_14_1209	DJI_0972	Salix alba L.	0	Salicaceae Mirb.	n	1
Iz_14_1209	DJI_0972	Sambucus nigra L.	0	Viburnaceae Raf.	n	1
Iz_14_1209	DJI_0972	Sorbus aucuparia L.	0	Rosaceae Juss.	n	1
Iz_14_1209	DJI_0972	Tilia cordata Mill.	0	Malvaceae Juss.	n	1
Iz_14_1209	DJI_0972	Ulmus glabra Huds.	2	Ulmaceae Mirb.	n	1
Iz_14_1209	DJI_0966	Acer tataricum L.	0	Sapindaceae Juss.	n	1
Iz_14_1209	DJI_0966	Betula pendula Roth.	0	Betulaceae Gray	n	1
Iz_14_1209	DJI_0966	Crataegus rhipidophylla Gand.	0	Rosaceae Juss.	n	1
Iz_14_1209	DJI_0966	Frangula alnus Mill.	0	Rhamnaceae Juss.	n	1
Iz_14_1209	DJI_0966	Fraxinus pennsylvanica Marshall	0	Oleaceae Hoffmanns. & Link	a	0
Iz_14_1209	DJI_0966	Pinus sylvestris L.	0	Pinaceae Spreng. ex F.Rudolphi	n	1
Iz_14_1209	DJI_0966	Populus × canescens (Aiton) Sm.	0	Salicaceae Mirb.	n	1
Iz_14_1209	DJI_0966	Populus tremula L.	0	Salicaceae Mirb.	n	1
Iz_14_1209	DJI_0966	Populus nigra L.	0	Salicaceae Mirb.	n	1
Iz_14_1209	DJI_0966	Pyrus communis L.	0	Rosaceae Juss.	a	0
Iz_14_1209	DJI_0966	Quercus robur L.	0	Fagaceae Dumort.	n	1
Iz_14_1209	DJI_0966	Robinia pseudoacacia L.	0	Fabaceae Lindl.	a	0
Iz_14_1209	DJI_0966	Rosa sp.	0	Rosaceae Juss.		
Iz_14_1209	DJI_0966	Salix alba L.	0	Salicaceae Mirb.	n	1
Iz_14_1209	DJI_0966	Sambucus nigra L.	0	Viburnaceae Raf.	n	1
Iz_14_1209	DJI_0966	Sorbus aucuparia L.	0	Rosaceae Juss.	n	1
Iz_14_1209	DJI_0966	Tilia cordata Mill.	0	Malvaceae Juss.	n	1
Iz_14_1209	DJI_0966	Ulmus glabra Huds.	0	Ulmaceae Mirb.	n	1
Iz_14_1209	DJI_0960	Acer tataricum L.	0	Sapindaceae Juss.	n	1
Iz_14_1209	DJI_0960	Betula pendula Roth.	0	Betulaceae Gray	n	1
Iz_14_1209	DJI_0960	Crataegus rhipidophylla Gand.	0	Rosaceae Juss.	n	1
Iz_14_1209	DJI_0960	Frangula alnus Mill.	0	Rhamnaceae Juss.	n	1
Iz_14_1209	DJI_0960	Fraxinus pennsylvanica Marshall	0	Oleaceae Hoffmanns. & Link	a	0
Iz_14_1209	DJI_0960	Pinus sylvestris L.	0	Pinaceae Spreng. ex F.Rudolphi	n	1
Iz_14_1209	DJI_0960	Populus × canescens (Aiton) Sm.	0	Salicaceae Mirb.	n	1
Iz_14_1209	DJI_0960	Populus tremula L.	0	Salicaceae Mirb.	n	1
Iz_14_1209	DJI_0960	Populus nigra L.	0	Salicaceae Mirb.	n	1
Iz_14_1209	DJI_0960	Pyrus communis L.	0	Rosaceae Juss.	a	0
Iz_14_1209	DJI_0960	Quercus robur L.	0	Fagaceae Dumort.	n	1
Iz_14_1209	DJI_0960	Robinia pseudoacacia L.	0	Fabaceae Lindl.	a	0
Iz_14_1209	DJI_0960	Rosa sp.	0	Rosaceae Juss.		
Iz_14_1209	DJI_0960	Salix alba L.	0	Salicaceae Mirb.	n	1
Iz_14_1209	DJI_0960	Sambucus nigra L.	0	Viburnaceae Raf.	n	1
Iz_14_1209	DJI_0960	Sorbus aucuparia L.	0	Rosaceae Juss.	n	1
Iz_14_1209	DJI_0960	Tilia cordata Mill.	0	Malvaceae Juss.	n	1
Iz_14_1209	DJI_0960	Ulmus glabra Huds.	0	Ulmaceae Mirb.	n	1
Iz_14_1209	DJI_0956	Acer tataricum L.	0	Sapindaceae Juss.	n	1
Iz_14_1209	DJI_0956	Betula pendula Roth.	0	Betulaceae Gray	n	1
Iz_14_1209	DJI_0956	Crataegus rhipidophylla Gand.	0	Rosaceae Juss.	n	1

Iz_14_1209	DJI_0956	Frangula alnus Mill.	0	Rhamnaceae Juss.	n	1
Iz_14_1209	DJI_0956	Fraxinus pennsylvanica Marshall	0	Oleaceae Hoffmanns. & Link	a	0
Iz_14_1209	DJI_0956	Pinus sylvestris L.	0	Pinaceae Spreng. ex F.Rudolphi	n	1
Iz_14_1209	DJI_0956	Populus × canescens (Aiton) Sm.	0	Salicaceae Mirb.	n	1
Iz_14_1209	DJI_0956	Populus tremula L.	0	Salicaceae Mirb.	n	1
Iz_14_1209	DJI_0956	Populus nigra L.	0	Salicaceae Mirb.	n	1
Iz_14_1209	DJI_0956	Pyrus communis L.	0	Rosaceae Juss.	a	0
Iz_14_1209	DJI_0956	Quercus robur L.	1	Fagaceae Dumort.	n	1
Iz_14_1209	DJI_0956	Robinia pseudoacacia L.	0	Fabaceae Lindl.	a	0
Iz_14_1209	DJI_0956	Rosa sp.	0	Rosaceae Juss.		
Iz_14_1209	DJI_0956	Salix alba L.	0	Salicaceae Mirb.	n	1
Iz_14_1209	DJI_0956	Sambucus nigra L.	0	Viburnaceae Raf.	n	1
Iz_14_1209	DJI_0956	Sorbus aucuparia L.	0	Rosaceae Juss.	n	1
Iz_14_1209	DJI_0956	Tilia cordata Mill.	0	Malvaceae Juss.	n	1
Iz_14_1209	DJI_0956	Ulmus glabra Huds.	0	Ulmaceae Mirb.	n	1
Iz_14_1209	DJI_0953	Acer tataricum L.	0	Sapindaceae Juss.	n	1
Iz_14_1209	DJI_0953	Betula pendula Roth.	0	Betulaceae Gray	n	1
Iz_14_1209	DJI_0953	Crataegus rhipidophylla Gand.	0	Rosaceae Juss.	n	1
Iz_14_1209	DJI_0953	Frangula alnus Mill.	0	Rhamnaceae Juss.	n	1
Iz_14_1209	DJI_0953	Fraxinus pennsylvanica Marshall	0	Oleaceae Hoffmanns. & Link	a	0
Iz_14_1209	DJI_0953	Pinus sylvestris L.	0	Pinaceae Spreng. ex F.Rudolphi	n	1
Iz_14_1209	DJI_0953	Populus × canescens (Aiton) Sm.	0	Salicaceae Mirb.	n	1
Iz_14_1209	DJI_0953	Populus tremula L.	0	Salicaceae Mirb.	n	1
Iz_14_1209	DJI_0953	Populus nigra L.	1	Salicaceae Mirb.	n	1
Iz_14_1209	DJI_0953	Pyrus communis L.	0	Rosaceae Juss.	a	0
Iz_14_1209	DJI_0953	Quercus robur L.	1	Fagaceae Dumort.	n	1
Iz_14_1209	DJI_0953	Robinia pseudoacacia L.	0	Fabaceae Lindl.	a	0
Iz_14_1209	DJI_0953	Rosa sp.	0	Rosaceae Juss.		
Iz_14_1209	DJI_0953	Salix alba L.	0	Salicaceae Mirb.	n	1
Iz_14_1209	DJI_0953	Sambucus nigra L.	0	Viburnaceae Raf.	n	1
Iz_14_1209	DJI_0953	Sorbus aucuparia L.	0	Rosaceae Juss.	n	1
Iz_14_1209	DJI_0953	Tilia cordata Mill.	0	Malvaceae Juss.	n	1
Iz_14_1209	DJI_0953	Ulmus glabra Huds.	0	Ulmaceae Mirb.	n	1
Iz_14_1209	DJI_0950	Acer tataricum L.	0	Sapindaceae Juss.	n	1
Iz_14_1209	DJI_0950	Betula pendula Roth.	0	Betulaceae Gray	n	1
Iz_14_1209	DJI_0950	Crataegus rhipidophylla Gand.	0	Rosaceae Juss.	n	1
Iz_14_1209	DJI_0950	Frangula alnus Mill.	0	Rhamnaceae Juss.	n	1
Iz_14_1209	DJI_0950	Fraxinus pennsylvanica Marshall	0	Oleaceae Hoffmanns. & Link	a	0
Iz_14_1209	DJI_0950	Pinus sylvestris L.	0	Pinaceae Spreng. ex F.Rudolphi	n	1
Iz_14_1209	DJI_0950	Populus × canescens (Aiton) Sm.	0	Salicaceae Mirb.	n	1
Iz_14_1209	DJI_0950	Populus tremula L.	0	Salicaceae Mirb.	n	1
Iz_14_1209	DJI_0950	Populus nigra L.	0	Salicaceae Mirb.	n	1
Iz_14_1209	DJI_0950	Pyrus communis L.	0	Rosaceae Juss.	a	0
Iz_14_1209	DJI_0950	Quercus robur L.	0	Fagaceae Dumort.	n	1
Iz_14_1209	DJI_0950	Robinia pseudoacacia L.	0	Fabaceae Lindl.	a	0
Iz_14_1209	DJI_0950	Rosa sp.	0	Rosaceae Juss.		
Iz_14_1209	DJI_0950	Salix alba L.	0	Salicaceae Mirb.	n	1
Iz_14_1209	DJI_0950	Sambucus nigra L.	0	Viburnaceae Raf.	n	1
Iz_14_1209	DJI_0950	Sorbus aucuparia L.	0	Rosaceae Juss.	n	1
Iz_14_1209	DJI_0950	Tilia cordata Mill.	0	Malvaceae Juss.	n	1
Iz_14_1209	DJI_0950	Ulmus glabra Huds.	0	Ulmaceae Mirb.	n	1
Iz_14_1209	DJI_0942	Acer tataricum L.	0	Sapindaceae Juss.	n	1
Iz_14_1209	DJI_0942	Betula pendula Roth.	0	Betulaceae Gray	n	1
Iz_14_1209	DJI_0942	Crataegus rhipidophylla Gand.	0	Rosaceae Juss.	n	1
Iz_14_1209	DJI_0942	Frangula alnus Mill.	0	Rhamnaceae Juss.	n	1
Iz_14_1209	DJI_0942	Fraxinus pennsylvanica Marshall	0	Oleaceae Hoffmanns. & Link	a	0
Iz_14_1209	DJI_0942	Pinus sylvestris L.	0	Pinaceae Spreng. ex F.Rudolphi	n	1
Iz_14_1209	DJI_0942	Populus × canescens (Aiton) Sm.	0	Salicaceae Mirb.	n	1
Iz_14_1209	DJI_0942	Populus tremula L.	0	Salicaceae Mirb.	n	1
Iz_14_1209	DJI_0942	Populus nigra L.	0	Salicaceae Mirb.	n	1
Iz_14_1209	DJI_0942	Pyrus communis L.	0	Rosaceae Juss.	a	0
Iz_14_1209	DJI_0942	Quercus robur L.	0	Fagaceae Dumort.	n	1
Iz_14_1209	DJI_0942	Robinia pseudoacacia L.	0	Fabaceae Lindl.	a	0
Iz_14_1209	DJI_0942	Rosa sp.	0	Rosaceae Juss.		

Iz_14_1209	DJI_0942	Salix alba L.	0	Salicaceae Mirb.	n	1
Iz_14_1209	DJI_0942	Sambucus nigra L.	0	Viburnaceae Raf.	n	1
Iz_14_1209	DJI_0942	Sorbus aucuparia L.	0	Rosaceae Juss.	n	1
Iz_14_1209	DJI_0942	Tilia cordata Mill.	0	Malvaceae Juss.	n	1
Iz_14_1209	DJI_0942	Ulmus glabra Huds.	0	Ulmaceae Mirb.	n	1
Iz_14_1209	DJI_0939	Acer tataricum L.	0	Sapindaceae Juss.	n	1
Iz_14_1209	DJI_0939	Betula pendula Roth.	0	Betulaceae Gray	n	1
Iz_14_1209	DJI_0939	Crataegus rhipidophylla Gand.	0	Rosaceae Juss.	n	1
Iz_14_1209	DJI_0939	Frangula alnus Mill.	0	Rhamnaceae Juss.	n	1
Iz_14_1209	DJI_0939	Fraxinus pennsylvanica Marshall	0	Oleaceae Hoffmanns. & Link	a	0
Iz_14_1209	DJI_0939	Pinus sylvestris L.	0	Pinaceae Spreng. ex F.Rudolphi	n	1
Iz_14_1209	DJI_0939	Populus × canescens (Aiton) Sm.	0	Salicaceae Mirb.	n	1
Iz_14_1209	DJI_0939	Populus tremula L.	0	Salicaceae Mirb.	n	1
Iz_14_1209	DJI_0939	Populus nigra L.	0	Salicaceae Mirb.	n	1
Iz_14_1209	DJI_0939	Pyrus communis L.	0	Rosaceae Juss.	a	0
Iz_14_1209	DJI_0939	Quercus robur L.	0	Fagaceae Dumort.	n	1
Iz_14_1209	DJI_0939	Robinia pseudoacacia L.	0	Fabaceae Lindl.	a	0
Iz_14_1209	DJI_0939	Rosa sp.	0	Rosaceae Juss.		
Iz_14_1209	DJI_0939	Salix alba L.	0	Salicaceae Mirb.	n	1
Iz_14_1209	DJI_0939	Sambucus nigra L.	0	Viburnaceae Raf.	n	1
Iz_14_1209	DJI_0939	Sorbus aucuparia L.	0	Rosaceae Juss.	n	1
Iz_14_1209	DJI_0939	Tilia cordata Mill.	0	Malvaceae Juss.	n	1
Iz_14_1209	DJI_0939	Ulmus glabra Huds.	0	Ulmaceae Mirb.	n	1
Iz_15_1209	DJI_0083	Acer tataricum L.	0	Sapindaceae Juss.	n	1
Iz_15_1209	DJI_0083	Betula pendula Roth.	0	Betulaceae Gray	n	1
Iz_15_1209	DJI_0083	Crataegus rhipidophylla Gand.	0	Rosaceae Juss.	n	1
Iz_15_1209	DJI_0083	Frangula alnus Mill.	0	Rhamnaceae Juss.	n	1
Iz_15_1209	DJI_0083	Fraxinus pennsylvanica Marshall	0	Oleaceae Hoffmanns. & Link	a	0
Iz_15_1209	DJI_0083	Pinus sylvestris L.	0	Pinaceae Spreng. ex F.Rudolphi	n	1
Iz_15_1209	DJI_0083	Populus × canescens (Aiton) Sm.	0	Salicaceae Mirb.	n	1
Iz_15_1209	DJI_0083	Populus tremula L.	0	Salicaceae Mirb.	n	1
Iz_15_1209	DJI_0083	Populus nigra L.	0	Salicaceae Mirb.	n	1
Iz_15_1209	DJI_0083	Pyrus communis L.	0	Rosaceae Juss.	a	0
Iz_15_1209	DJI_0083	Quercus robur L.	0	Fagaceae Dumort.	n	1
Iz_15_1209	DJI_0083	Robinia pseudoacacia L.	0	Fabaceae Lindl.	a	0
Iz_15_1209	DJI_0083	Rosa sp.	0	Rosaceae Juss.		
Iz_15_1209	DJI_0083	Salix alba L.	0	Salicaceae Mirb.	n	1
Iz_15_1209	DJI_0083	Sambucus nigra L.	0	Viburnaceae Raf.	n	1
Iz_15_1209	DJI_0083	Sorbus aucuparia L.	0	Rosaceae Juss.	n	1
Iz_15_1209	DJI_0083	Tilia cordata Mill.	0	Malvaceae Juss.	n	1
Iz_15_1209	DJI_0083	Ulmus glabra Huds.	0	Ulmaceae Mirb.	n	1
Iz_15_1209	DJI_0080	Acer tataricum L.	0	Sapindaceae Juss.	n	1
Iz_15_1209	DJI_0080	Betula pendula Roth.	0	Betulaceae Gray	n	1
Iz_15_1209	DJI_0080	Crataegus rhipidophylla Gand.	0	Rosaceae Juss.	n	1
Iz_15_1209	DJI_0080	Frangula alnus Mill.	0	Rhamnaceae Juss.	n	1
Iz_15_1209	DJI_0080	Fraxinus pennsylvanica Marshall	0	Oleaceae Hoffmanns. & Link	a	0
Iz_15_1209	DJI_0080	Pinus sylvestris L.	0	Pinaceae Spreng. ex F.Rudolphi	n	1
Iz_15_1209	DJI_0080	Populus × canescens (Aiton) Sm.	0	Salicaceae Mirb.	n	1
Iz_15_1209	DJI_0080	Populus tremula L.	0	Salicaceae Mirb.	n	1
Iz_15_1209	DJI_0080	Populus nigra L.	0	Salicaceae Mirb.	n	1
Iz_15_1209	DJI_0080	Pyrus communis L.	0	Rosaceae Juss.	a	0
Iz_15_1209	DJI_0080	Quercus robur L.	0	Fagaceae Dumort.	n	1
Iz_15_1209	DJI_0080	Robinia pseudoacacia L.	0	Fabaceae Lindl.	a	0
Iz_15_1209	DJI_0080	Rosa sp.	0	Rosaceae Juss.		
Iz_15_1209	DJI_0080	Salix alba L.	0	Salicaceae Mirb.	n	1
Iz_15_1209	DJI_0080	Sambucus nigra L.	0	Viburnaceae Raf.	n	1
Iz_15_1209	DJI_0080	Sorbus aucuparia L.	0	Rosaceae Juss.	n	1
Iz_15_1209	DJI_0080	Tilia cordata Mill.	0	Malvaceae Juss.	n	1
Iz_15_1209	DJI_0080	Ulmus glabra Huds.	0	Ulmaceae Mirb.	n	1
Iz_15_1209	DJI_0077	Acer tataricum L.	0	Sapindaceae Juss.	n	1
Iz_15_1209	DJI_0077	Betula pendula Roth.	0	Betulaceae Gray	n	1
Iz_15_1209	DJI_0077	Crataegus rhipidophylla Gand.	0	Rosaceae Juss.	n	1
Iz_15_1209	DJI_0077	Frangula alnus Mill.	0	Rhamnaceae Juss.	n	1
Iz_15_1209	DJI_0077	Fraxinus pennsylvanica Marshall	0	Oleaceae Hoffmanns. & Link	a	0

Iz_15_1209	DJI_0077	Pinus sylvestris L.	0	Pinaceae Spreng. ex F.Rudolphi	n	1
Iz_15_1209	DJI_0077	Populus × canescens (Aiton) Sm.	0	Salicaceae Mirb.	n	1
Iz_15_1209	DJI_0077	Populus tremula L.	0	Salicaceae Mirb.	n	1
Iz_15_1209	DJI_0077	Populus nigra L.	0	Salicaceae Mirb.	n	1
Iz_15_1209	DJI_0077	Pyrus communis L.	0	Rosaceae Juss.	a	0
Iz_15_1209	DJI_0077	Quercus robur L.	0	Fagaceae Dumort.	n	1
Iz_15_1209	DJI_0077	Robinia pseudoacacia L.	0	Fabaceae Lindl.	a	0
Iz_15_1209	DJI_0077	Rosa sp.	0	Rosaceae Juss.		
Iz_15_1209	DJI_0077	Salix alba L.	0	Salicaceae Mirb.	n	1
Iz_15_1209	DJI_0077	Sambucus nigra L.	0	Viburnaceae Raf.	n	1
Iz_15_1209	DJI_0077	Sorbus aucuparia L.	0	Rosaceae Juss.	n	1
Iz_15_1209	DJI_0077	Tilia cordata Mill.	0	Malvaceae Juss.	n	1
Iz_15_1209	DJI_0077	Ulmus glabra Huds.	0	Ulmaceae Mirb.	n	1
Iz_15_1209	DJI_0072	Acer tataricum L.	0	Sapindaceae Juss.	n	1
Iz_15_1209	DJI_0072	Betula pendula Roth.	0	Betulaceae Gray	n	1
Iz_15_1209	DJI_0072	Crataegus rhipidophylla Gand.	0	Rosaceae Juss.	n	1
Iz_15_1209	DJI_0072	Frangula alnus Mill.	0	Rhamnaceae Juss.	n	1
Iz_15_1209	DJI_0072	Fraxinus pennsylvanica Marshall	0	Oleaceae Hoffmanns. & Link	a	0
Iz_15_1209	DJI_0072	Pinus sylvestris L.	0	Pinaceae Spreng. ex F.Rudolphi	n	1
Iz_15_1209	DJI_0072	Populus × canescens (Aiton) Sm.	0	Salicaceae Mirb.	n	1
Iz_15_1209	DJI_0072	Populus tremula L.	0	Salicaceae Mirb.	n	1
Iz_15_1209	DJI_0072	Populus nigra L.	0	Salicaceae Mirb.	n	1
Iz_15_1209	DJI_0072	Pyrus communis L.	0	Rosaceae Juss.	a	0
Iz_15_1209	DJI_0072	Quercus robur L.	0	Fagaceae Dumort.	n	1
Iz_15_1209	DJI_0072	Robinia pseudoacacia L.	0	Fabaceae Lindl.	a	0
Iz_15_1209	DJI_0072	Rosa sp.	0	Rosaceae Juss.		
Iz_15_1209	DJI_0072	Salix alba L.	0	Salicaceae Mirb.	n	1
Iz_15_1209	DJI_0072	Sambucus nigra L.	0	Viburnaceae Raf.	n	1
Iz_15_1209	DJI_0072	Sorbus aucuparia L.	0	Rosaceae Juss.	n	1
Iz_15_1209	DJI_0072	Tilia cordata Mill.	0	Malvaceae Juss.	n	1
Iz_15_1209	DJI_0072	Ulmus glabra Huds.	0	Ulmaceae Mirb.	n	1
Iz_15_1209	DJI_0069	Acer tataricum L.	0	Sapindaceae Juss.	n	1
Iz_15_1209	DJI_0069	Betula pendula Roth.	0	Betulaceae Gray	n	1
Iz_15_1209	DJI_0069	Crataegus rhipidophylla Gand.	0	Rosaceae Juss.	n	1
Iz_15_1209	DJI_0069	Frangula alnus Mill.	0	Rhamnaceae Juss.	n	1
Iz_15_1209	DJI_0069	Fraxinus pennsylvanica Marshall	0	Oleaceae Hoffmanns. & Link	a	0
Iz_15_1209	DJI_0069	Pinus sylvestris L.	0	Pinaceae Spreng. ex F.Rudolphi	n	1
Iz_15_1209	DJI_0069	Populus × canescens (Aiton) Sm.	0	Salicaceae Mirb.	n	1
Iz_15_1209	DJI_0069	Populus tremula L.	0	Salicaceae Mirb.	n	1
Iz_15_1209	DJI_0069	Populus nigra L.	0	Salicaceae Mirb.	n	1
Iz_15_1209	DJI_0069	Pyrus communis L.	0	Rosaceae Juss.	a	0
Iz_15_1209	DJI_0069	Quercus robur L.	0	Fagaceae Dumort.	n	1
Iz_15_1209	DJI_0069	Robinia pseudoacacia L.	0	Fabaceae Lindl.	a	0
Iz_15_1209	DJI_0069	Rosa sp.	0	Rosaceae Juss.		
Iz_15_1209	DJI_0069	Salix alba L.	0	Salicaceae Mirb.	n	1
Iz_15_1209	DJI_0069	Sambucus nigra L.	0	Viburnaceae Raf.	n	1
Iz_15_1209	DJI_0069	Sorbus aucuparia L.	0	Rosaceae Juss.	n	1
Iz_15_1209	DJI_0069	Tilia cordata Mill.	0	Malvaceae Juss.	n	1
Iz_15_1209	DJI_0069	Ulmus glabra Huds.	0	Ulmaceae Mirb.	n	1
Iz_15_1209	DJI_0065	Acer tataricum L.	0	Sapindaceae Juss.	n	1
Iz_15_1209	DJI_0065	Betula pendula Roth.	0	Betulaceae Gray	n	1
Iz_15_1209	DJI_0065	Crataegus rhipidophylla Gand.	0	Rosaceae Juss.	n	1
Iz_15_1209	DJI_0065	Frangula alnus Mill.	0	Rhamnaceae Juss.	n	1
Iz_15_1209	DJI_0065	Fraxinus pennsylvanica Marshall	0	Oleaceae Hoffmanns. & Link	a	0
Iz_15_1209	DJI_0065	Pinus sylvestris L.	0	Pinaceae Spreng. ex F.Rudolphi	n	1
Iz_15_1209	DJI_0065	Populus × canescens (Aiton) Sm.	0	Salicaceae Mirb.	n	1
Iz_15_1209	DJI_0065	Populus tremula L.	0	Salicaceae Mirb.	n	1
Iz_15_1209	DJI_0065	Populus nigra L.	0	Salicaceae Mirb.	n	1
Iz_15_1209	DJI_0065	Pyrus communis L.	0	Rosaceae Juss.	a	0
Iz_15_1209	DJI_0065	Quercus robur L.	0	Fagaceae Dumort.	n	1
Iz_15_1209	DJI_0065	Robinia pseudoacacia L.	0	Fabaceae Lindl.	a	0
Iz_15_1209	DJI_0065	Rosa sp.	0	Rosaceae Juss.		
Iz_15_1209	DJI_0065	Salix alba L.	0	Salicaceae Mirb.	n	1
Iz_15_1209	DJI_0065	Sambucus nigra L.	0	Viburnaceae Raf.	n	1

Iz_15_1209	DJI_0065	Sorbus aucuparia L.	0	Rosaceae Juss.	n	1
Iz_15_1209	DJI_0065	Tilia cordata Mill.	0	Malvaceae Juss.	n	1
Iz_15_1209	DJI_0065	Ulmus glabra Huds.	0	Ulmaceae Mirb.	n	1
Iz_15_1209	DJI_0056	Acer tataricum L.	0	Sapindaceae Juss.	n	1
Iz_15_1209	DJI_0056	Betula pendula Roth.	0	Betulaceae Gray	n	1
Iz_15_1209	DJI_0056	Crataegus rhipidophylla Gand.	0	Rosaceae Juss.	n	1
Iz_15_1209	DJI_0056	Frangula alnus Mill.	0	Rhamnaceae Juss.	n	1
Iz_15_1209	DJI_0056	Fraxinus pennsylvanica Marshall	0	Oleaceae Hoffmanns. & Link	a	0
Iz_15_1209	DJI_0056	Pinus sylvestris L.	0	Pinaceae Spreng. ex F.Rudolphi	n	1
Iz_15_1209	DJI_0056	Populus × canescens (Aiton) Sm.	0	Salicaceae Mirb.	n	1
Iz_15_1209	DJI_0056	Populus tremula L.	0	Salicaceae Mirb.	n	1
Iz_15_1209	DJI_0056	Populus nigra L.	0	Salicaceae Mirb.	n	1
Iz_15_1209	DJI_0056	Pyrus communis L.	0	Rosaceae Juss.	a	0
Iz_15_1209	DJI_0056	Quercus robur L.	0	Fagaceae Dumort.	n	1
Iz_15_1209	DJI_0056	Robinia pseudoacacia L.	0	Fabaceae Lindl.	a	0
Iz_15_1209	DJI_0056	Rosa sp.	0	Rosaceae Juss.		
Iz_15_1209	DJI_0056	Salix alba L.	0	Salicaceae Mirb.	n	1
Iz_15_1209	DJI_0056	Sambucus nigra L.	0	Viburnaceae Raf.	n	1
Iz_15_1209	DJI_0056	Sorbus aucuparia L.	0	Rosaceae Juss.	n	1
Iz_15_1209	DJI_0056	Tilia cordata Mill.	0	Malvaceae Juss.	n	1
Iz_15_1209	DJI_0056	Ulmus glabra Huds.	0	Ulmaceae Mirb.	n	1
Iz_15_1209	DJI_0053	Acer tataricum L.	0	Sapindaceae Juss.	n	1
Iz_15_1209	DJI_0053	Betula pendula Roth.	0	Betulaceae Gray	n	1
Iz_15_1209	DJI_0053	Crataegus rhipidophylla Gand.	0	Rosaceae Juss.	n	1
Iz_15_1209	DJI_0053	Frangula alnus Mill.	0	Rhamnaceae Juss.	n	1
Iz_15_1209	DJI_0053	Fraxinus pennsylvanica Marshall	0	Oleaceae Hoffmanns. & Link	a	0
Iz_15_1209	DJI_0053	Pinus sylvestris L.	0	Pinaceae Spreng. ex F.Rudolphi	n	1
Iz_15_1209	DJI_0053	Populus × canescens (Aiton) Sm.	0	Salicaceae Mirb.	n	1
Iz_15_1209	DJI_0053	Populus tremula L.	0	Salicaceae Mirb.	n	1
Iz_15_1209	DJI_0053	Populus nigra L.	0	Salicaceae Mirb.	n	1
Iz_15_1209	DJI_0053	Pyrus communis L.	0	Rosaceae Juss.	a	0
Iz_15_1209	DJI_0053	Quercus robur L.	0	Fagaceae Dumort.	n	1
Iz_15_1209	DJI_0053	Robinia pseudoacacia L.	0	Fabaceae Lindl.	a	0
Iz_15_1209	DJI_0053	Rosa sp.	0	Rosaceae Juss.		
Iz_15_1209	DJI_0053	Salix alba L.	0	Salicaceae Mirb.	n	1
Iz_15_1209	DJI_0053	Sambucus nigra L.	0	Viburnaceae Raf.	n	1
Iz_15_1209	DJI_0053	Sorbus aucuparia L.	0	Rosaceae Juss.	n	1
Iz_15_1209	DJI_0053	Tilia cordata Mill.	0	Malvaceae Juss.	n	1
Iz_15_1209	DJI_0053	Ulmus glabra Huds.	0	Ulmaceae Mirb.	n	1
Iz_15_1209	DJI_0050	Acer tataricum L.	0	Sapindaceae Juss.	n	1
Iz_15_1209	DJI_0050	Betula pendula Roth.	0	Betulaceae Gray	n	1
Iz_15_1209	DJI_0050	Crataegus rhipidophylla Gand.	0	Rosaceae Juss.	n	1
Iz_15_1209	DJI_0050	Frangula alnus Mill.	0	Rhamnaceae Juss.	n	1
Iz_15_1209	DJI_0050	Fraxinus pennsylvanica Marshall	0	Oleaceae Hoffmanns. & Link	a	0
Iz_15_1209	DJI_0050	Pinus sylvestris L.	0	Pinaceae Spreng. ex F.Rudolphi	n	1
Iz_15_1209	DJI_0050	Populus × canescens (Aiton) Sm.	0	Salicaceae Mirb.	n	1
Iz_15_1209	DJI_0050	Populus tremula L.	0	Salicaceae Mirb.	n	1
Iz_15_1209	DJI_0050	Populus nigra L.	0	Salicaceae Mirb.	n	1
Iz_15_1209	DJI_0050	Pyrus communis L.	0	Rosaceae Juss.	a	0
Iz_15_1209	DJI_0050	Quercus robur L.	0	Fagaceae Dumort.	n	1
Iz_15_1209	DJI_0050	Robinia pseudoacacia L.	0	Fabaceae Lindl.	a	0
Iz_15_1209	DJI_0050	Rosa sp.	0	Rosaceae Juss.		
Iz_15_1209	DJI_0050	Salix alba L.	0	Salicaceae Mirb.	n	1
Iz_15_1209	DJI_0050	Sambucus nigra L.	0	Viburnaceae Raf.	n	1
Iz_15_1209	DJI_0050	Sorbus aucuparia L.	0	Rosaceae Juss.	n	1
Iz_15_1209	DJI_0050	Tilia cordata Mill.	0	Malvaceae Juss.	n	1
Iz_15_1209	DJI_0050	Ulmus glabra Huds.	0	Ulmaceae Mirb.	n	1
Iz_15_1209	DJI_0046	Acer tataricum L.	0	Sapindaceae Juss.	n	1
Iz_15_1209	DJI_0046	Betula pendula Roth.	0	Betulaceae Gray	n	1
Iz_15_1209	DJI_0046	Crataegus rhipidophylla Gand.	0	Rosaceae Juss.	n	1
Iz_15_1209	DJI_0046	Frangula alnus Mill.	0	Rhamnaceae Juss.	n	1
Iz_15_1209	DJI_0046	Fraxinus pennsylvanica Marshall	0	Oleaceae Hoffmanns. & Link	a	0
Iz_15_1209	DJI_0046	Pinus sylvestris L.	0	Pinaceae Spreng. ex F.Rudolphi	n	1
Iz_15_1209	DJI_0046	Populus × canescens (Aiton) Sm.	0	Salicaceae Mirb.	n	1

Iz_15_1209	DJI_0046	Populus tremula L.	0	Salicaceae Mirb.	n	1
Iz_15_1209	DJI_0046	Populus nigra L.	0	Salicaceae Mirb.	n	1
Iz_15_1209	DJI_0046	Pyrus communis L.	0	Rosaceae Juss.	a	0
Iz_15_1209	DJI_0046	Quercus robur L.	0	Fagaceae Dumort.	n	1
Iz_15_1209	DJI_0046	Robinia pseudoacacia L.	0	Fabaceae Lindl.	a	0
Iz_15_1209	DJI_0046	Rosa sp.	0	Rosaceae Juss.		
Iz_15_1209	DJI_0046	Salix alba L.	0	Salicaceae Mirb.	n	1
Iz_15_1209	DJI_0046	Sambucus nigra L.	0	Viburnaceae Raf.	n	1
Iz_15_1209	DJI_0046	Sorbus aucuparia L.	0	Rosaceae Juss.	n	1
Iz_15_1209	DJI_0046	Tilia cordata Mill.	0	Malvaceae Juss.	n	1
Iz_15_1209	DJI_0046	Ulmus glabra Huds.	0	Ulmaceae Mirb.	n	1
Iz_16_1209	DJI_0157	Acer tataricum L.	0	Sapindaceae Juss.	n	1
Iz_16_1209	DJI_0157	Betula pendula Roth.	0	Betulaceae Gray	n	1
Iz_16_1209	DJI_0157	Crataegus rhipidophylla Gand.	0	Rosaceae Juss.	n	1
Iz_16_1209	DJI_0157	Frangula alnus Mill.	0	Rhamnaceae Juss.	n	1
Iz_16_1209	DJI_0157	Fraxinus pennsylvanica Marshall	0	Oleaceae Hoffmanns. & Link	a	0
Iz_16_1209	DJI_0157	Pinus sylvestris L.	0	Pinaceae Spreng. ex F.Rudolphi	n	1
Iz_16_1209	DJI_0157	Populus × canescens (Aiton) Sm.	0	Salicaceae Mirb.	n	1
Iz_16_1209	DJI_0157	Populus tremula L.	0	Salicaceae Mirb.	n	1
Iz_16_1209	DJI_0157	Populus nigra L.	0	Salicaceae Mirb.	n	1
Iz_16_1209	DJI_0157	Pyrus communis L.	0	Rosaceae Juss.	a	0
Iz_16_1209	DJI_0157	Quercus robur L.	0	Fagaceae Dumort.	n	1
Iz_16_1209	DJI_0157	Robinia pseudoacacia L.	0	Fabaceae Lindl.	a	0
Iz_16_1209	DJI_0157	Rosa sp.	0	Rosaceae Juss.		
Iz_16_1209	DJI_0157	Salix alba L.	0	Salicaceae Mirb.	n	1
Iz_16_1209	DJI_0157	Sambucus nigra L.	0	Viburnaceae Raf.	n	1
Iz_16_1209	DJI_0157	Sorbus aucuparia L.	0	Rosaceae Juss.	n	1
Iz_16_1209	DJI_0157	Tilia cordata Mill.	0	Malvaceae Juss.	n	1
Iz_16_1209	DJI_0157	Ulmus glabra Huds.	0	Ulmaceae Mirb.	n	1
Iz_16_1209	DJI_0150	Acer tataricum L.	0	Sapindaceae Juss.	n	1
Iz_16_1209	DJI_0150	Betula pendula Roth.	0	Betulaceae Gray	n	1
Iz_16_1209	DJI_0150	Crataegus rhipidophylla Gand.	0	Rosaceae Juss.	n	1
Iz_16_1209	DJI_0150	Frangula alnus Mill.	0	Rhamnaceae Juss.	n	1
Iz_16_1209	DJI_0150	Fraxinus pennsylvanica Marshall	0	Oleaceae Hoffmanns. & Link	a	0
Iz_16_1209	DJI_0150	Pinus sylvestris L.	0	Pinaceae Spreng. ex F.Rudolphi	n	1
Iz_16_1209	DJI_0150	Populus × canescens (Aiton) Sm.	0	Salicaceae Mirb.	n	1
Iz_16_1209	DJI_0150	Populus tremula L.	0	Salicaceae Mirb.	n	1
Iz_16_1209	DJI_0150	Populus nigra L.	0	Salicaceae Mirb.	n	1
Iz_16_1209	DJI_0150	Pyrus communis L.	0	Rosaceae Juss.	a	0
Iz_16_1209	DJI_0150	Quercus robur L.	0	Fagaceae Dumort.	n	1
Iz_16_1209	DJI_0150	Robinia pseudoacacia L.	0	Fabaceae Lindl.	a	0
Iz_16_1209	DJI_0150	Rosa sp.	0	Rosaceae Juss.		
Iz_16_1209	DJI_0150	Salix alba L.	0	Salicaceae Mirb.	n	1
Iz_16_1209	DJI_0150	Sambucus nigra L.	0	Viburnaceae Raf.	n	1
Iz_16_1209	DJI_0150	Sorbus aucuparia L.	0	Rosaceae Juss.	n	1
Iz_16_1209	DJI_0150	Tilia cordata Mill.	0	Malvaceae Juss.	n	1
Iz_16_1209	DJI_0150	Ulmus glabra Huds.	0	Ulmaceae Mirb.	n	1
Iz_16_1209	DJI_0146	Acer tataricum L.	0	Sapindaceae Juss.	n	1
Iz_16_1209	DJI_0146	Betula pendula Roth.	0	Betulaceae Gray	n	1
Iz_16_1209	DJI_0146	Crataegus rhipidophylla Gand.	0	Rosaceae Juss.	n	1
Iz_16_1209	DJI_0146	Frangula alnus Mill.	0	Rhamnaceae Juss.	n	1
Iz_16_1209	DJI_0146	Fraxinus pennsylvanica Marshall	0	Oleaceae Hoffmanns. & Link	a	0
Iz_16_1209	DJI_0146	Pinus sylvestris L.	0	Pinaceae Spreng. ex F.Rudolphi	n	1
Iz_16_1209	DJI_0146	Populus × canescens (Aiton) Sm.	0	Salicaceae Mirb.	n	1
Iz_16_1209	DJI_0146	Populus tremula L.	0	Salicaceae Mirb.	n	1
Iz_16_1209	DJI_0146	Populus nigra L.	0	Salicaceae Mirb.	n	1
Iz_16_1209	DJI_0146	Pyrus communis L.	0	Rosaceae Juss.	a	0
Iz_16_1209	DJI_0146	Quercus robur L.	0	Fagaceae Dumort.	n	1
Iz_16_1209	DJI_0146	Robinia pseudoacacia L.	0	Fabaceae Lindl.	a	0
Iz_16_1209	DJI_0146	Rosa sp.	0	Rosaceae Juss.		
Iz_16_1209	DJI_0146	Salix alba L.	0	Salicaceae Mirb.	n	1
Iz_16_1209	DJI_0146	Sambucus nigra L.	0	Viburnaceae Raf.	n	1
Iz_16_1209	DJI_0146	Sorbus aucuparia L.	0	Rosaceae Juss.	n	1
Iz_16_1209	DJI_0146	Tilia cordata Mill.	0	Malvaceae Juss.	n	1

Iz_16_1209	DJI_0146	Ulmus glabra Huds.	0	Ulmaceae Mirb.	n	1
Iz_16_1209	DJI_0135	Acer tataricum L.	0	Sapindaceae Juss.	n	1
Iz_16_1209	DJI_0135	Betula pendula Roth.	0	Betulaceae Gray	n	1
Iz_16_1209	DJI_0135	Crataegus rhipidophylla Gand.	0	Rosaceae Juss.	n	1
Iz_16_1209	DJI_0135	Frangula alnus Mill.	0	Rhamnaceae Juss.	n	1
Iz_16_1209	DJI_0135	Fraxinus pennsylvanica Marshall	0	Oleaceae Hoffmanns. & Link	a	0
Iz_16_1209	DJI_0135	Pinus sylvestris L.	0	Pinaceae Spreng. ex F.Rudolphi	n	1
Iz_16_1209	DJI_0135	Populus × canescens (Aiton) Sm.	0	Salicaceae Mirb.	n	1
Iz_16_1209	DJI_0135	Populus tremula L.	0	Salicaceae Mirb.	n	1
Iz_16_1209	DJI_0135	Populus nigra L.	0	Salicaceae Mirb.	n	1
Iz_16_1209	DJI_0135	Pyrus communis L.	0	Rosaceae Juss.	a	0
Iz_16_1209	DJI_0135	Quercus robur L.	0	Fagaceae Dumort.	n	1
Iz_16_1209	DJI_0135	Robinia pseudoacacia L.	0	Fabaceae Lindl.	a	0
Iz_16_1209	DJI_0135	Rosa sp.	0	Rosaceae Juss.		
Iz_16_1209	DJI_0135	Salix alba L.	0	Salicaceae Mirb.	n	1
Iz_16_1209	DJI_0135	Sambucus nigra L.	0	Viburnaceae Raf.	n	1
Iz_16_1209	DJI_0135	Sorbus aucuparia L.	0	Rosaceae Juss.	n	1
Iz_16_1209	DJI_0135	Tilia cordata Mill.	0	Malvaceae Juss.	n	1
Iz_16_1209	DJI_0135	Ulmus glabra Huds.	0	Ulmaceae Mirb.	n	1
Iz_16_1209	DJI_0130	Acer tataricum L.	0	Sapindaceae Juss.	n	1
Iz_16_1209	DJI_0130	Betula pendula Roth.	0	Betulaceae Gray	n	1
Iz_16_1209	DJI_0130	Crataegus rhipidophylla Gand.	0	Rosaceae Juss.	n	1
Iz_16_1209	DJI_0130	Frangula alnus Mill.	0	Rhamnaceae Juss.	n	1
Iz_16_1209	DJI_0130	Fraxinus pennsylvanica Marshall	0	Oleaceae Hoffmanns. & Link	a	0
Iz_16_1209	DJI_0130	Pinus sylvestris L.	0	Pinaceae Spreng. ex F.Rudolphi	n	1
Iz_16_1209	DJI_0130	Populus × canescens (Aiton) Sm.	0	Salicaceae Mirb.	n	1
Iz_16_1209	DJI_0130	Populus tremula L.	0	Salicaceae Mirb.	n	1
Iz_16_1209	DJI_0130	Populus nigra L.	0	Salicaceae Mirb.	n	1
Iz_16_1209	DJI_0130	Pyrus communis L.	0	Rosaceae Juss.	a	0
Iz_16_1209	DJI_0130	Quercus robur L.	0	Fagaceae Dumort.	n	1
Iz_16_1209	DJI_0130	Robinia pseudoacacia L.	0	Fabaceae Lindl.	a	0
Iz_16_1209	DJI_0130	Rosa sp.	0	Rosaceae Juss.		
Iz_16_1209	DJI_0130	Salix alba L.	0	Salicaceae Mirb.	n	1
Iz_16_1209	DJI_0130	Sambucus nigra L.	0	Viburnaceae Raf.	n	1
Iz_16_1209	DJI_0130	Sorbus aucuparia L.	0	Rosaceae Juss.	n	1
Iz_16_1209	DJI_0130	Tilia cordata Mill.	0	Malvaceae Juss.	n	1
Iz_16_1209	DJI_0130	Ulmus glabra Huds.	0	Ulmaceae Mirb.	n	1
Iz_16_1209	DJI_0127	Acer tataricum L.	0	Sapindaceae Juss.	n	1
Iz_16_1209	DJI_0127	Betula pendula Roth.	0	Betulaceae Gray	n	1
Iz_16_1209	DJI_0127	Crataegus rhipidophylla Gand.	0	Rosaceae Juss.	n	1
Iz_16_1209	DJI_0127	Frangula alnus Mill.	0	Rhamnaceae Juss.	n	1
Iz_16_1209	DJI_0127	Fraxinus pennsylvanica Marshall	0	Oleaceae Hoffmanns. & Link	a	0
Iz_16_1209	DJI_0127	Pinus sylvestris L.	0	Pinaceae Spreng. ex F.Rudolphi	n	1
Iz_16_1209	DJI_0127	Populus × canescens (Aiton) Sm.	0	Salicaceae Mirb.	n	1
Iz_16_1209	DJI_0127	Populus tremula L.	0	Salicaceae Mirb.	n	1
Iz_16_1209	DJI_0127	Populus nigra L.	0	Salicaceae Mirb.	n	1
Iz_16_1209	DJI_0127	Pyrus communis L.	0	Rosaceae Juss.	a	0
Iz_16_1209	DJI_0127	Quercus robur L.	0	Fagaceae Dumort.	n	1
Iz_16_1209	DJI_0127	Robinia pseudoacacia L.	0	Fabaceae Lindl.	a	0
Iz_16_1209	DJI_0127	Rosa sp.	0	Rosaceae Juss.		
Iz_16_1209	DJI_0127	Salix alba L.	0	Salicaceae Mirb.	n	1
Iz_16_1209	DJI_0127	Sambucus nigra L.	0	Viburnaceae Raf.	n	1
Iz_16_1209	DJI_0127	Sorbus aucuparia L.	0	Rosaceae Juss.	n	1
Iz_16_1209	DJI_0127	Tilia cordata Mill.	0	Malvaceae Juss.	n	1
Iz_16_1209	DJI_0127	Ulmus glabra Huds.	0	Ulmaceae Mirb.	n	1
Iz_16_1209	DJI_0122	Acer tataricum L.	0	Sapindaceae Juss.	n	1
Iz_16_1209	DJI_0122	Betula pendula Roth.	0	Betulaceae Gray	n	1
Iz_16_1209	DJI_0122	Crataegus rhipidophylla Gand.	0	Rosaceae Juss.	n	1
Iz_16_1209	DJI_0122	Frangula alnus Mill.	0	Rhamnaceae Juss.	n	1
Iz_16_1209	DJI_0122	Fraxinus pennsylvanica Marshall	0	Oleaceae Hoffmanns. & Link	a	0
Iz_16_1209	DJI_0122	Pinus sylvestris L.	0	Pinaceae Spreng. ex F.Rudolphi	n	1
Iz_16_1209	DJI_0122	Populus × canescens (Aiton) Sm.	0	Salicaceae Mirb.	n	1
Iz_16_1209	DJI_0122	Populus tremula L.	0	Salicaceae Mirb.	n	1
Iz_16_1209	DJI_0122	Populus nigra L.	0	Salicaceae Mirb.	n	1

Iz_16_1209	DJI_0122	Pyrus communis L.	0	Rosaceae Juss.	a	0
Iz_16_1209	DJI_0122	Quercus robur L.	0	Fagaceae Dumort.	n	1
Iz_16_1209	DJI_0122	Robinia pseudoacacia L.	0	Fabaceae Lindl.	a	0
Iz_16_1209	DJI_0122	Rosa sp.	0	Rosaceae Juss.		
Iz_16_1209	DJI_0122	Salix alba L.	0	Salicaceae Mirb.	n	1
Iz_16_1209	DJI_0122	Sambucus nigra L.	0	Viburnaceae Raf.	n	1
Iz_16_1209	DJI_0122	Sorbus aucuparia L.	0	Rosaceae Juss.	n	1
Iz_16_1209	DJI_0122	Tilia cordata Mill.	0	Malvaceae Juss.	n	1
Iz_16_1209	DJI_0122	Ulmus glabra Huds.	0	Ulmaceae Mirb.	n	1
Iz_16_1209	DJI_0119	Acer tataricum L.	0	Sapindaceae Juss.	n	1
Iz_16_1209	DJI_0119	Betula pendula Roth.	0	Betulaceae Gray	n	1
Iz_16_1209	DJI_0119	Crataegus rhipidophylla Gand.	0	Rosaceae Juss.	n	1
Iz_16_1209	DJI_0119	Frangula alnus Mill.	0	Rhamnaceae Juss.	n	1
Iz_16_1209	DJI_0119	Fraxinus pennsylvanica Marshall	0	Oleaceae Hoffmanns. & Link	a	0
Iz_16_1209	DJI_0119	Pinus sylvestris L.	0	Pinaceae Spreng. ex F.Rudolphi	n	1
Iz_16_1209	DJI_0119	Populus × canescens (Aiton) Sm.	0	Salicaceae Mirb.	n	1
Iz_16_1209	DJI_0119	Populus tremula L.	0	Salicaceae Mirb.	n	1
Iz_16_1209	DJI_0119	Populus nigra L.	0	Salicaceae Mirb.	n	1
Iz_16_1209	DJI_0119	Pyrus communis L.	0	Rosaceae Juss.	a	0
Iz_16_1209	DJI_0119	Quercus robur L.	0	Fagaceae Dumort.	n	1
Iz_16_1209	DJI_0119	Robinia pseudoacacia L.	0	Fabaceae Lindl.	a	0
Iz_16_1209	DJI_0119	Rosa sp.	0	Rosaceae Juss.		
Iz_16_1209	DJI_0119	Salix alba L.	0	Salicaceae Mirb.	n	1
Iz_16_1209	DJI_0119	Sambucus nigra L.	0	Viburnaceae Raf.	n	1
Iz_16_1209	DJI_0119	Sorbus aucuparia L.	0	Rosaceae Juss.	n	1
Iz_16_1209	DJI_0119	Tilia cordata Mill.	0	Malvaceae Juss.	n	1
Iz_16_1209	DJI_0119	Ulmus glabra Huds.	0	Ulmaceae Mirb.	n	1
Iz_16_1209	DJI_0114	Acer tataricum L.	0	Sapindaceae Juss.	n	1
Iz_16_1209	DJI_0114	Betula pendula Roth.	0	Betulaceae Gray	n	1
Iz_16_1209	DJI_0114	Crataegus rhipidophylla Gand.	0	Rosaceae Juss.	n	1
Iz_16_1209	DJI_0114	Frangula alnus Mill.	0	Rhamnaceae Juss.	n	1
Iz_16_1209	DJI_0114	Fraxinus pennsylvanica Marshall	0	Oleaceae Hoffmanns. & Link	a	0
Iz_16_1209	DJI_0114	Pinus sylvestris L.	0	Pinaceae Spreng. ex F.Rudolphi	n	1
Iz_16_1209	DJI_0114	Populus × canescens (Aiton) Sm.	0	Salicaceae Mirb.	n	1
Iz_16_1209	DJI_0114	Populus tremula L.	0	Salicaceae Mirb.	n	1
Iz_16_1209	DJI_0114	Populus nigra L.	0	Salicaceae Mirb.	n	1
Iz_16_1209	DJI_0114	Pyrus communis L.	0	Rosaceae Juss.	a	0
Iz_16_1209	DJI_0114	Quercus robur L.	0	Fagaceae Dumort.	n	1
Iz_16_1209	DJI_0114	Robinia pseudoacacia L.	0	Fabaceae Lindl.	a	0
Iz_16_1209	DJI_0114	Rosa sp.	0	Rosaceae Juss.		
Iz_16_1209	DJI_0114	Salix alba L.	0	Salicaceae Mirb.	n	1
Iz_16_1209	DJI_0114	Sambucus nigra L.	0	Viburnaceae Raf.	n	1
Iz_16_1209	DJI_0114	Sorbus aucuparia L.	0	Rosaceae Juss.	n	1
Iz_16_1209	DJI_0114	Tilia cordata Mill.	0	Malvaceae Juss.	n	1
Iz_16_1209	DJI_0114	Ulmus glabra Huds.	0	Ulmaceae Mirb.	n	1
Iz_16_1209	DJI_0109	Acer tataricum L.	0	Sapindaceae Juss.	n	1
Iz_16_1209	DJI_0109	Betula pendula Roth.	0	Betulaceae Gray	n	1
Iz_16_1209	DJI_0109	Crataegus rhipidophylla Gand.	0	Rosaceae Juss.	n	1
Iz_16_1209	DJI_0109	Frangula alnus Mill.	0	Rhamnaceae Juss.	n	1
Iz_16_1209	DJI_0109	Fraxinus pennsylvanica Marshall	0	Oleaceae Hoffmanns. & Link	a	0
Iz_16_1209	DJI_0109	Pinus sylvestris L.	0	Pinaceae Spreng. ex F.Rudolphi	n	1
Iz_16_1209	DJI_0109	Populus × canescens (Aiton) Sm.	0	Salicaceae Mirb.	n	1
Iz_16_1209	DJI_0109	Populus tremula L.	0	Salicaceae Mirb.	n	1
Iz_16_1209	DJI_0109	Populus nigra L.	0	Salicaceae Mirb.	n	1
Iz_16_1209	DJI_0109	Pyrus communis L.	0	Rosaceae Juss.	a	0
Iz_16_1209	DJI_0109	Quercus robur L.	0	Fagaceae Dumort.	n	1
Iz_16_1209	DJI_0109	Robinia pseudoacacia L.	0	Fabaceae Lindl.	a	0
Iz_16_1209	DJI_0109	Rosa sp.	0	Rosaceae Juss.		
Iz_16_1209	DJI_0109	Salix alba L.	0	Salicaceae Mirb.	n	1
Iz_16_1209	DJI_0109	Sambucus nigra L.	0	Viburnaceae Raf.	n	1
Iz_16_1209	DJI_0109	Sorbus aucuparia L.	0	Rosaceae Juss.	n	1
Iz_16_1209	DJI_0109	Tilia cordata Mill.	0	Malvaceae Juss.	n	1
Iz_16_1209	DJI_0109	Ulmus glabra Huds.	0	Ulmaceae Mirb.	n	1
Iz_16_1209	DJI_0106	Acer tataricum L.	0	Sapindaceae Juss.	n	1

Iz_16_1209	DJI_0106	Betula pendula Roth.	0	Betulaceae Gray	n	1
Iz_16_1209	DJI_0106	Crataegus rhipidophylla Gand.	0	Rosaceae Juss.	n	1
Iz_16_1209	DJI_0106	Frangula alnus Mill.	0	Rhamnaceae Juss.	n	1
Iz_16_1209	DJI_0106	Fraxinus pennsylvanica Marshall	0	Oleaceae Hoffmanns. & Link	a	0
Iz_16_1209	DJI_0106	Pinus sylvestris L.	0	Pinaceae Spreng. ex F.Rudolphi	n	1
Iz_16_1209	DJI_0106	Populus × canescens (Aiton) Sm.	0	Salicaceae Mirb.	n	1
Iz_16_1209	DJI_0106	Populus tremula L.	0	Salicaceae Mirb.	n	1
Iz_16_1209	DJI_0106	Populus nigra L.	0	Salicaceae Mirb.	n	1
Iz_16_1209	DJI_0106	Pyrus communis L.	0	Rosaceae Juss.	a	0
Iz_16_1209	DJI_0106	Quercus robur L.	1	Fagaceae Dumort.	n	1
Iz_16_1209	DJI_0106	Robinia pseudoacacia L.	1	Fabaceae Lindl.	a	0
Iz_16_1209	DJI_0106	Rosa sp.	0	Rosaceae Juss.		
Iz_16_1209	DJI_0106	Salix alba L.	0	Salicaceae Mirb.	n	1
Iz_16_1209	DJI_0106	Sambucus nigra L.	0	Viburnaceae Raf.	n	1
Iz_16_1209	DJI_0106	Sorbus aucuparia L.	0	Rosaceae Juss.	n	1
Iz_16_1209	DJI_0106	Tilia cordata Mill.	0	Malvaceae Juss.	n	1
Iz_16_1209	DJI_0106	Ulmus glabra Huds.	0	Ulmaceae Mirb.	n	1
Iz_16_1209	DJI_0103	Acer tataricum L.	0	Sapindaceae Juss.	n	1
Iz_16_1209	DJI_0103	Betula pendula Roth.	0	Betulaceae Gray	n	1
Iz_16_1209	DJI_0103	Crataegus rhipidophylla Gand.	0	Rosaceae Juss.	n	1
Iz_16_1209	DJI_0103	Frangula alnus Mill.	0	Rhamnaceae Juss.	n	1
Iz_16_1209	DJI_0103	Fraxinus pennsylvanica Marshall	0	Oleaceae Hoffmanns. & Link	a	0
Iz_16_1209	DJI_0103	Pinus sylvestris L.	0	Pinaceae Spreng. ex F.Rudolphi	n	1
Iz_16_1209	DJI_0103	Populus × canescens (Aiton) Sm.	0	Salicaceae Mirb.	n	1
Iz_16_1209	DJI_0103	Populus tremula L.	0	Salicaceae Mirb.	n	1
Iz_16_1209	DJI_0103	Populus nigra L.	0	Salicaceae Mirb.	n	1
Iz_16_1209	DJI_0103	Pyrus communis L.	0	Rosaceae Juss.	a	0
Iz_16_1209	DJI_0103	Quercus robur L.	0	Fagaceae Dumort.	n	1
Iz_16_1209	DJI_0103	Robinia pseudoacacia L.	0	Fabaceae Lindl.	a	0
Iz_16_1209	DJI_0103	Rosa sp.	0	Rosaceae Juss.		
Iz_16_1209	DJI_0103	Salix alba L.	0	Salicaceae Mirb.	n	1
Iz_16_1209	DJI_0103	Sambucus nigra L.	0	Viburnaceae Raf.	n	1
Iz_16_1209	DJI_0103	Sorbus aucuparia L.	0	Rosaceae Juss.	n	1
Iz_16_1209	DJI_0103	Tilia cordata Mill.	0	Malvaceae Juss.	n	1
Iz_16_1209	DJI_0103	Ulmus glabra Huds.	0	Ulmaceae Mirb.	n	1
